# Identifying factors associated with the direction and significance of microRNA tumor-normal expression differences in colorectal cancer

**DOI:** 10.1186/s12885-017-3690-x

**Published:** 2017-10-30

**Authors:** John R. Stevens, Jennifer S. Herrick, Roger K. Wolff, Martha L. Slattery

**Affiliations:** 1 Department of Mathematics and Statistics, Utah State University, Logan, USA; 2 Division of Epidemiology, Department of Internal Medicine, University of Utah, Logan, USA

**Keywords:** microRNA, Colorectal cancer, Epigenetics, Differential expression

## Abstract

**Background:**

microRNAs are small non-protein-coding RNA molecules that regulate gene expression, and have a potential epigenetic role in disease progression and survival of colorectal cancer. In terms of tumor-normal expression differences, many microRNAs exhibit evidence of being up-regulated in some subjects but down-regulated in others, or are dysregulated only for a subset of the population. We present and implement an approach to identify factors (lifestyle, tumor molecular phenotype, and survival-related) that are associated with the direction and/or significance of these microRNAs’ tumor-normal expression differences in colorectal cancer.

**Methods:**

Using expression data for 1394 microRNAs and 1836 colorectal cancer subjects (each with both tumor and normal samples), we perform a dip test to identify microRNAs with multimodal distributions of tumor-normal expression differences. For proximal, distal, and rectal tumor sites separately, these microRNAs are tested for tumor-normal differential expression using a signed rank test, both overall and within levels of each lifestyle, tumor molecular phenotype, and survival-related factor. Appropriate adjustments are made to control the overall FDR.

**Results:**

We identify hundreds of microRNAs whose direction and/or significance of tumor-normal differential expression is associated with one or more lifestyle, tumor molecular phenotype, or survival-related factors.

**Conclusions:**

The results of this study demonstrate the benefit to colorectal cancer researchers to consider multiple subject-level factors when studying dysregulation of microRNAs, whose tumor-related changes in expression can be associated with multiple factors. Our results will serve as a publicly-available resource to provide clarifying information about various factors associated with the direction and significance of tumor-normal differential expression of microRNAs in colorectal cancer.

**Electronic supplementary material:**

The online version of this article (10.1186/s12885-017-3690-x) contains supplementary material, which is available to authorized users.

## Background

Dysregulation of microRNAs, which are small non-protein-coding RNA molecules that regulate gene expression [1–3], has been of interest in colorectal cancer patients [4–6] due to the potential epigenetic role of microRNAs in disease progression and survival. Within the context of colorectal cancer patients, we have previously reported on the prognostic role of various microRNAs in disease stage and colorectal cancer-specific mortality [7], on differential expression of microRNAs between tumor and normal samples [8, 9], on predictive microRNAs for differentiating carcinoma from normal mucosa [10], on site-specific associations of microRNAs and survival [11], and on associations of microRNA expression with cigarette smoking [12] and other diet and lifestyle factors [13].

In this study we focus on microRNAs that, in terms of tumor-normal expression differences, exhibit evidence of being up-regulated in some subjects but down-regulated in others, or that are dysregulated only for a subset of the population. We present and implement an approach to identify factors (lifestyle, tumor molecular phenotype, and survival-related) that are associated with the direction and/or significance of these microRNAs’ tumor-normal expression differences. It is important to note that our interest here does not lie simply in identifying microRNAs that are differentially expressed between tumor and normal tissues. Rather, our interest lies in identifying factors that are associated with the *direction* and/or *significance* of microRNA differential expression. Considering additional factors beyond the tumor/normal distinction allows for greater specificity in conclusions regarding differential expression, as microRNA expression seems to be quite dynamic. For example, rather than simply concluding that a given microRNA is significantly dysregulated in tumor compared to normal tissue, we can identify sub-groups of subjects (corresponding to levels of a particular factor) where the dysregulation is no longer significant or even changes direction – with the microRNA tending to be up-regulated in one sub-group but down-regulated in another. This work has the goal of identifying such cases where factors of interest are associated with the direction and significance of microRNA tumor-normal dysregulation in colorectal cancer subjects.

## Methods

### Study design

Data for this study come from two population-based case-control studies. Colon and rectal cancer patients between 30 and 79 years of age at diagnosis were recruited from the Wasatch Front in Utah and the Kaiser Permanente Medical Care Program (KPMCP) in Northern California. Cancer cases had a primary adenocarcinoma diagnosed between October 1991 and September 1994 for colon, and between June 1997 and May 2001 for rectal. This population-based Diet, Activity, and Lifestyle study was approved by the Institutional Review Board at the University of Utah, with study participants signing informed consent. Additional study details have been described previously [7].

### MicroRNA processing

RNA was extracted from formalin-fixed paraffin embedded tissues and processed as previously described [7], using both carcinoma tissue and normal mucosa adjacent to the carcinoma tissue. The Agilent Human miRNA Microarray V19.0 was used given the high number (2006) of microRNAs, its high level of reliability (coefficient 0.98 in our data), amount of RNA needed to run the platform, and good agreement with both NanoString [6] and qRT-PCR [10]. 100 ng total RNA was labeled with Cy3 and hybridized to the microarray and were scanned on an Agilent SureScan microarray scanner model G2600D using Agilent Feature Extract software v.11.5.1.1. Stringent QC parameters established by Agilent were applied to the data, including tests for excessive background fluorescence, excessive variation among probe sequence replicates on the array, and measures of the total gene signal on the array to assess low signal. Samples failing to meet these quality standards were repeated, and if a sample failed QC assessment a second time, it was deemed to be of poor quality and excluded from subsequent analysis. Total gene signal was normalized (adopting GeneSpring’s “scale” option) by multiplying each sample’s expression values by a scaling factor which was the median of the 75th percentiles of all the samples divided by the 75th percentile of the individual sample [14]; this scaling factor normalization was implemented with SAS 9.4.

### Subject-level factors: Lifestyle, tumor phenotype, and survival data

As part of the Diet, Activity, and Lifestyle study, data were collected by trained, certified interviewers using laptop computers. All interviews were audio-taped as previously described and reviewed for quality control purposes [15]. The referent period for the study was two years prior to diagnosis. As part of the study questionnaire (Additional file 1), information was collected on type, amount, and duration of alcohol use, past and current smoking status, and estrogen exposure. Body size information, including height (measured at time of interview) and weight (recalled for referent period) was also recorded.

Alcohol use was defined in terms of liquor (including whiskey, rum, gin, vodka, tequila, liqueurs, etc.), beer (including malt liquor), and wine (including champagne, sherry and wine cooler beverages). Alcohol consumed was measured in number of drinks consumed, as measured by 12-oz (oz.) for beer, 4 oz. for wine, and 1.5 oz. for liquor, per week or month during the reference year, and respondents must have consumed on average at least one beverage a month to be considered a consumer. Subjects reporting having smoked at least 100 cigarettes in their lifetime were considered to have been a cigarette smoker. Cigarette smokers who reported having not smoked during the referent period were considered former smokers. Assessment of subjects’ MSI, CIMP, *BRAF*, *TP53*, and *KRAS* tumor mutation statuses was performed as described previously [16].

Because study participants were from Utah and California, and both states are members of the National Cancer Institute funded Surveillance, Epidemiology, and End Results (SEER) Program, follow-up data were available on all study participants, including SEER summary and AJCC severity stages of tumors, as well as degree of colon tumor differentiation. In addition, the SEER Program provided follow-up data on all participants (through 2006) of total number of months survived, date of death (or date of last follow-up), and cause of death.

Table 1 summarizes the subject-level factors considered in this study. All factors were coded 0/1 in the statistical analysis.

**Table 1:** Summary of subject-level factors considered for association with direction of tumor-normal microRNA differential expression

	Proximal (*N* = 567)			Distal (*N* = 550)			Rectal (*N* = 719)		
Factor: Interpretation	N0	N1	N_miss_	N0	N1	N_miss_	N0	N1	N_miss_
MSI: MSI (0 = stable / MSS, 1 = unstable / MSI)	428	128	11	508	23	19	699	16	4
CIMP: CIMP status (0 = low, 1 = high)	280	204	83	403	63	84	599	76	44
*BRAF: BRAF* mutation status (0 = none, 1 = mutation)	391	73	103	433	15	102	685	19	15
*TP53: TP53* mutation status (0 = none, 1 = mutation)	325	222	20	255	265	30	344	353	22
*KRAS: KRAS* mutation status (0 = none, 1 = mutation)	341	198	28	364	141	45	502	212	5
STAGE_D: SEER summary stage distant (0 = no, 1 = yes)	475	92	0	459	91	0	632	87	0
STAGE_L: SEER summary stage local (0 = no, 1 = yes)	417	150	0	365	185	0	393	326	0
STAGE_R: SEER summary stage regional (0 = no, 1 = yes)	252	315	0	293	257	0	431	288	0
AJCC_1: AJCC stage 1 (0 = no, 1 = yes)	479	88	0	404	146	0	450	269	0
AJCC_2: AJCC stage 1 or 2 (0 = no, 1 = yes)	285	282	0	254	296	0	313	406	0
AJCC_3: AJCC stage 1, 2, or 3 (0 = no [stage 4], 1 = yes)	102	465	0	106	444	0	111	608	0
SEX: 1 = male, 0 = female	286	281	0	300	250	0	409	310	0
DIFF_NA: Tumor differentiation n/a (0 = no, 1 = yes)	242	24	301	508	40	2	0	719	0
DIFF_WELL: Tumor differentiation well (0 = no, 1 = yes)	516	50	1	491	57	2	719	0	0
DIFF_MOD: Tumor differentiation moderate (0 = no, 1 = yes)	199	367	1	161	387	2	719	0	0
DIFF_POOR: Tumor differentiation poor (0 = no, 1 = yes)	441	125	1	484	64	2	719	0	0
VITAL_ALIVE: Vital status at last follow-up (0 = dead, 1 = alive)	281	285	1	261	288	1	341	378	0
SURV5YRS: Survival at least 60 months after sample taken (0 = no, 1 = yes)	257	309	1	233	316	1	297	422	0
COD_CRC: Cause of death CRC (0 = no, 1 = yes)	81	179	307	70	160	320	112	229	378
ALCOHOL_reg: Referent year alcohol consumption at least 1.0 g/day (0 = no, 1 = yes)	247	198	122	243	175	132	292	246	181
WINE_any: More than 0 4 oz. glasses wine per day (0 = no, 1 = yes) during referent year	303	142	122	284	134	132	385	153	181
LIQUOR_any: More than 0 servings liquor per day (0 = no, 1 = yes) during referent year	326	119	122	301	117	132	421	117	181
BEER_any: More than 0 servings beer per day (0 = no, 1 = yes) during referent year	327	118	122	317	101	132	374	164	181
CIG_ever: Ever smoked cigarettes (0 = no, 1 = yes)	180	264	123	183	234	133	239	299	181
CIG_current: Current smoker (0 = no, 1 = yes)	377	67	123	360	57	133	448	90	181
CIG_former: Former smoker (0 = no, 1 = yes)	247	197	123	240	177	133	341	197	181
ESTROGEN: Estrogen exposure within past 2 years (0 = no, 1 = yes; missing for all males)	133	69	365	112	72	366	119	108	492
BMI_normal: BMI [“for analysis 2 years ago”] less than 25 (0 = no, 1 = yes)	299	144	124	270	143	137	355	179	185
BMI_overweight: BMI at least 25 and less than 30 (0 = no, 1 = yes)	249	194	124	258	155	137	344	190	185
BMI_obese: BMI at least 30 and less than 40 (0 = no, 1 = yes)	355	88	124	310	103	137	390	144	185
BMI_extreme: BMI at least 40 (0 = no, 1 = yes)	426	17	124	401	12	137	513	21	185

All factors were coded 0/1, and corresponding sample sizes in proximal, distal, and rectal sites are indicated by N0 and N1. Missing values in some factors result in differences (N_miss_) between overall subject totals (N) and the sum of N0 + N1

### Statistical analysis

For each microRNA, and within each tumor site (proximal colon, distal colon, rectal) separately, the tumor-normal expression difference was calculated using the matched pairs of tumor and normal samples from each subject. We note that the paired nature of our study design provides the advantage that the tumor-normal difference effectively removes the effects of potentially confounding factors (such as age) that could affect any microRNA’s expression in both normal and tumor separately. Because our interest lies in microRNAs that are up-regulated in some subjects but down-regulated in others, or that are dysregulated only for a subset of the population, we focus on first identifying microRNAs whose tumor-normal expression difference distribution has multiple modes – such as a positive mode (representing up-regulation) for some subjects, a negative mode (representing down-regulation) for others, and possibly a third mode centered at zero (representing no dysregulation). Each microRNA’s tumor-normal expression difference distribution was therefore tested for unimodality using Hartigan’s dip test statistic [17]. After using Hommel’s method [18] to control the family-wise error rate at 0.05, only those microRNAs exhibiting significant multimodality were considered further.

For each tumor site separately, each microRNA was tested for overall differential expression (between tumor and normal) using a nonparametric Wilcoxon Signed Rank test [19] on the tumor-normal expression difference. Because this nonparametric test drops data values of zero, the effective sample size for each microRNA depended on its number of observed nonzero tumor-normal expression differences. In our microRNA data, tumor-normal expression differences of zero result from non-expression in both tumor and normal. For each of the factors in Table 1, each microRNA was also tested (using the Wilcoxon test) for differential expression within each factor level whenever the sample size in both factor levels was at least 10. (While a linear mixed model approach would have allowed a direct statistical interaction test of whether the tumor-normal expression difference depended on a factor’s level, such an approach would require unrealistic distributional assumptions for our microRNA data. Specifically, even rough normality could not be achieved using reasonably interpretable transformations such as the log. Instead, the Wilcoxon Signed Rank test was used because of its nonparametric nature.) The resulting *p*-values were adjusted (to control the false discovery rate [20] at 0.05) for each site separately, and for each test (overall, at factor levels 0, at factor levels 1) separately. Each resulting microRNA was classified as significantly down-regulated (“Down”), not significantly differentially expressed (“NS”), or significantly up-regulated (“Up”) in each test. The one-sided alternative was employed in the Wilcoxon test, with (one-sided FDR-adjusted) *p*-value thresholds .05/2 for down-regulation and 1–.05/2 for up-regulation. No significance was called for (adjusted) *p*-values between .15/2 and 1–.15/2. Adjusted p-values between .05/2 and .15/2, or between 1–.15/2 and 1–.05/2 were considered inconclusive and not classified.

## Results

**Fig. 1:**
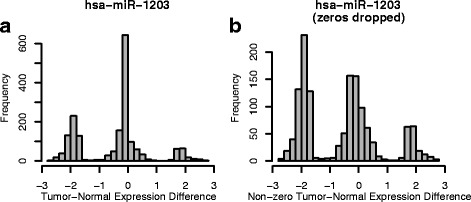
Example of a microRNA with a multimodal expression difference, with (**a**) and without (**b**) values of 0 included

Using expression data for 1394 microRNAs and 1836 colorectal cancer subjects (each with both tumor and normal samples), many microRNAs exhibited multimodal tumor-normal expression differences, as in Fig. 1. The left mode (near −2 in Fig. 1) corresponds to subjects in which the microRNA is down-regulated in tumor, while the right mode (near +2 in Fig. 1) corresponds to subjects in which the microRNA is up-regulated in tumor. The center mode (near 0) can actually be considered two components – one in which the expression differences are exactly 0 (the tall spike in the left panel of Fig. 1), and another in which expression differences are spread around 0 (more easily seen in the right panel of Fig. 1, where expression differences of 0 have been dropped). These center components correspond to subjects in which the microRNA is (either exactly or essentially) not dysregulated in tumor.

When Hartigan’s dip statistic was used to test each microRNA’s tumor-normal difference distribution for unimodality, and the family-wise error rate was controlled at 0.05, this resulted in 122, 123, and 276 microRNAs identified as having multimodal distributions in proximal, distal, and rectal tumor sites, respectively. There were 66 microRNAs exhibiting multimodality in all three tumor sites. After subsequent application of the Wilcoxon Signed Rank test and classification of each microRNA as “Up”, “Down”, or “NS” as described in the “Statistical Analysis” section above, Table 2 summarizes the resulting numbers of microRNAs classified to each outcome (“Up”, “Down”, “NS”) overall and within each factor level, across all site / factor combinations.

**Table 2:** Numbers of microRNAs classified as up-regulated, down-regulated, or not significantly differentially expressed (NS) in tumor relative to normal, at various site / factor level combinations

Factor Level		Overall			
0	1	Down	NS	Up	Superscripts (and totals) – colors named here are used in later tables, figures, and additional files ^b^ (“blue”) overall significance, with agreement in one factor level and NS in the other (2759) ^g^ (“green”) overall NS, but significant in only one factor level (142) ^o^ (“orange”) overall NS, and significant in opposite directions for factor levels (8) ^p^ (“purple”) agreement in both factor levels (direction or NS), but different from overall (NS or direction) (223) ^y^ (“yellow”) overall significance, with agreement in one factor level but opposite direction in the other factor level (10)
Down	Down	4782	1 ^p^	^p^	
Down	NS	1758 ^b^	44 ^g^		
Down	Up	2 ^y^	6 ^o^	^y^	
NS	Down	387 ^b^	40 ^g^		
NS	NS	126 ^p^	1730	96 ^p^	
NS	Up		34 ^g^	157 ^b^	
Up	Down	^y^	2 ^o^	8 ^y^	
Up	NS		24 ^g^	457 ^b^	
Up	Up	^p^	^p^	2217	

**Fig. 2:**
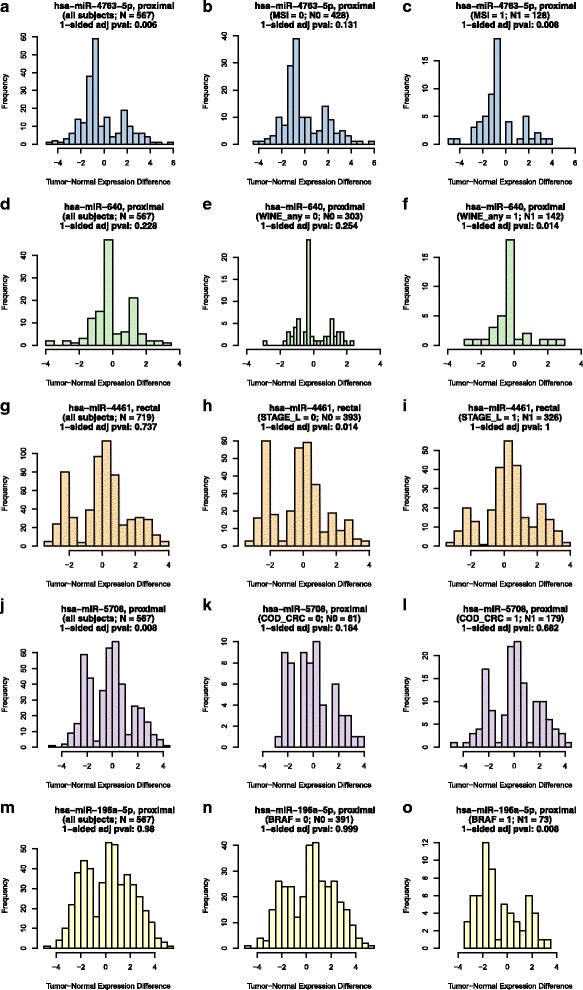
Representative results for outcomes of interest – overall significance, with agreement in one factor level and NS in the other (**a**-**c**; blue); overall NS, but significant in only one factor level (**d**-**f**; green); overall NS, and significant in opposite directions for factor levels (**g**-**i**; orange); agreement in both factor levels (direction or NS), but different from overall (NS or direction) (**j**-**l**; purple); and overall significance, with agreement in one factor level but opposite direction in the other factor level (**m**-**o**; yellow)

For convenience in summarizing results, outcomes of interest in Table 2 are given superscripts corresponding to color names, as reported in Table 2. Representative results for each of these colors (i.e., outcomes of interest) are given in Fig. 2; full results for all colors (i.e., outcomes of interest) are given in Additional files 2, 3, 4, 5 and 6. Each row of plots in Fig. 2 (and each page of plots in Additional files 2, 3, 4, 5 and 6) has the same format, which can be summarized as follows, using the yellow row of Fig. 2 (plots m-o) as an example. The plot titles indicate which microRNA (miR-196a-5p) and site (proximal) are considered, and the left plot is a histogram of the tumor-normal expression difference of the indicated microRNA at the indicated site, using data from all subjects (with sample size reported in the second row of the title). The one-sided *p*-value from the Wilcoxon Rank Sum test for differential expression, after adjustment to control the false discovery rate, is reported in the third row of the plot’s title. Adjusted one-sided *p*-values close to 0 (less than 0.025) suggest down-regulation in tumor relative to normal, while those close to 1 (greater than 0.975) suggest up-regulation. The second row of the titles of the center and right plots indicate which factor levels are considered, with histograms representing tumor-normal expression differences for the same indicated microRNA in corresponding subsets of the data. Subset sample sizes and significance test results are reported in the second and third rows of the plot titles, respectively. Taken together, the row of yellow plots in Fig. 2 (plots m-o) indicate significant overall up-regulation of miR-196a-5p in 567 proximal colon cancer patients (left plot), similarly significant up-regulation of the same microRNA in 391 proximal colon cancer patients whose tumors lack the *BRAF* mutation (center plot), but significant down-regulation of the same microRNA in 73 proximal colon cancer patients whose tumors have the *BRAF* mutation.

In Fig. 2a-c (and Additional file 2; the “blue” outcomes of interest), the left plot indicates an overall tendency of significant differential expression, while the center and right plots disagree on the statistical significance. The same direction and statistical significance of the left plot (the overall test) is reflected in only one of the factor level subsets (as in Fig. 2c). For some microRNA / factor / site combinations this may be due to a smaller effective sample size (and consequent loss of statistical power) in one of the factor levels, particularly for factors whose levels are greatly unbalanced (such as BMI_extreme; see Table 1). However, for most microRNA / factor / site combinations, this outcome can be seen in the shapes of the tumor-normal expression difference distributions – such as the more pronounced negative mode in Fig. 2c resulting in a statistical conclusion of down-regulation, but the more balanced (if not entirely symmetric) modes in Fig. 2b failing to provide overwhelming evidence of any differential expression. Such a “blue” outcome can generally be interpreted as a microRNA that is overall significantly dysregulated in tumor vs. normal, but only for one of the factor’s levels.

In Fig. 2d-f (and Additional file 3; the “green” outcomes of interest), the left plot indicates a lack of evidence of differential expression, usually due to a relative balance between the numbers of negative and positive tumor-normal expression differences (as in Fig. 2d). Such a balance (and corresponding lack of statistical significance) is also seen in one of the factor levels (as in Fig. 2e), but not in the other factor level which has a more pronounced mode on one side or the other (as in the negative mode of Fig. 2f). This is indicative of a microRNA (such as Fig. 2d-f miR-640 in proximal tumor) that is significantly dysregulated in only one of the factor’s levels (here, down-regulated in subjects who regularly consume any wine).

Figure 2g-i (and Additional file 4; the “orange” outcomes of interest) present an interesting scenario where a microRNA is overall not significantly dysregulated in tumor vs. normal, but upon consideration of subject sub-groups it is determined that the microRNA (miR-4461 in Fig. 2g-i) tends to be significantly down-regulated in one factor level (distant or regional SEER summary stage rectal tumors) but significantly up-regulated in the other factor level (local SEER summary stage rectal tumors). Such outcomes are rare (see Table 2), but interesting.

In Fig. 2 (and Additional files 2, 3, 4, 5 and 6), the sample sizes of the subsets (center and right plots) within each row do not necessarily add up to the total sample size (left plot). This occurs here because of missing values in some factors defined in Table 1. In the overall test of differential expression (left plots in Fig. 2 and Additional files 2, 3, 4, 5 and 6), all subjects (with tumors in the indicated site) are used in the test of differential expression, and this sample size is reported in the second row of the plot title. In the tests of differential expression within factor level subsets (center and right plots in Fig. 2 and Additional files 2, 3, 4, 5 and 6), only subjects with recorded values for the indicated factor (and with tumors in the indicated site) were used in the test of differential expression, and these sample sizes are reported in the second row of the plot title. The widespread presence of missing values in several factors here contributes to an effective loss of statistical power for many of these subset tests of differential expression, which is the most likely explanation for the “purple” outcomes of Fig. 2 and Table 2, where all but one such outcome involved a microRNA being significantly dysregulated overall with a larger sample size, but not significantly dysregulated in either factor level subset (where the sample size was much smaller). Consequently, the “purple” outcomes are of lesser interest than the others, which are summarized in greater detail for specific factors by site in Table 3. All outcomes of interest from Tables 2 and 3 are summarized in greater detail in Additional file 7.

**Table 3:** Numbers of microRNAs (out of the indicated numbers considered multimodal at each site) classified with respect to the tumor-normal test of differential expression as: (b, “blue”) overall significant, with significant directional agreement in one factor level and NS in the other; (g, “green”) overall NS, but significant in only one factor level; (o, “orange”) overall NS, and significant in opposite directions for factor levels; and (y, “yellow”) overall significant, with significant directional agreement in one factor level but significant in the opposite direction in the other factor level

	Proximal (of 122)				Distal (of 123)				Rectal (of 276)			
Factor	b	g	o	y	b	g	o	y	b	g	o	y
MSI	27			1	62			1	186			4
CIMP	19			1	29				64	1		
*BRAF*	13	3		1	58				176			
*TP53*	16				10				28	5		
*KRAS*	21	2			14				29	3	1	
STAGE_D	32	1			12	1			53	2		
STAGE_L	23				10				19	3	1	
STAGE_R	19				15				21	1		
AJCC_1	40	2			7	1			19	3		
AJCC_2	18				10				16	2	1	
AJCC_3	28	2			8	1			41	6		
SEX	23	3			6				12	1		
DIFF_NA	55				42							
DIFF_WELL	37				30							
DIFF_MOD	13				16							
DIFF_POOR	21	1			27							
VITAL_ALIVE	16				15	5			27	1		
SURV5YRS	17				21	4	2		18	1		
COD_CRC	19	1			3	6			67	9		
ALCOHOL_reg	11				7				51	5		
WINE_any	9	1			12	1			78	8	1	1
LIQUOR_any	13	1			14	3			68	5	1	1
BEER_any	16	2			11				56	2		
CIG_ever	24	5			4				36	5		
CIG_current	23				17	1			69	8	1	
CIG_former	19	4			5				28	1		
ESTROGEN	12	1			11				46	4		
BMI_normal	9				10	2			33	4		
BMI_overweight	17	4			10				30	1		
BMI_obese	18				22				68	2		
BMI_extreme	66				58				150	1		

In presenting these results, we report all subject-level factors that we considered, acknowledging that some overlap, redundancy, or even superiority between factors may be possible. For example, while survival at five years (SURV5YRS) may be a better indicator for overall survival, there may also be additional value to some researchers in considering the status of the patient at last follow-up (VITAL_ALIVE), so the results for both factors are reported here. Also for example, the degree of concordance between SEER and AJCC staging is approximately reflected in the results – for example, AJCC_3 = 0 and STAGE_D = 1 both refer to patients with distant metastasis (Table 1); of the 28 (AJCC_3) and 32 (STAGE_D) “blue” outcomes in proximal colon reported for these factors in Table 3, an examination of Additional file 7 reveals that 26 microRNA outcomes are in common. (These two factors’ results are not identical because the original data are actually slightly different – of the 102 AJCC_3 = 0 proximal colon patients in Table 1, only 92 were STAGE_D = 1.) Although such overlap, redundancy, or even superiority between factors reported here may be noted by some researchers, we have chosen to be broad in the reporting of our results, in the interest of providing more information.

## Discussion

A disproportionate number of outcomes of interest in Table 3 occur for the rectal site, particularly for the “blue” outcomes. In other words, while there are many microRNAs that are significantly differentially expressed in the tumor vs. normal comparison, but that are only differentially expressed in one of the levels of some factor of interest, such outcomes are especially common in rectal site comparisons. Additionally, more than half of the microRNAs with multimodal tumor-normal expression differences in rectal cancer have their significance associated with MSI (186 of 276) or *BRAF* tumor status (176 of 276).

**Fig. 3:**
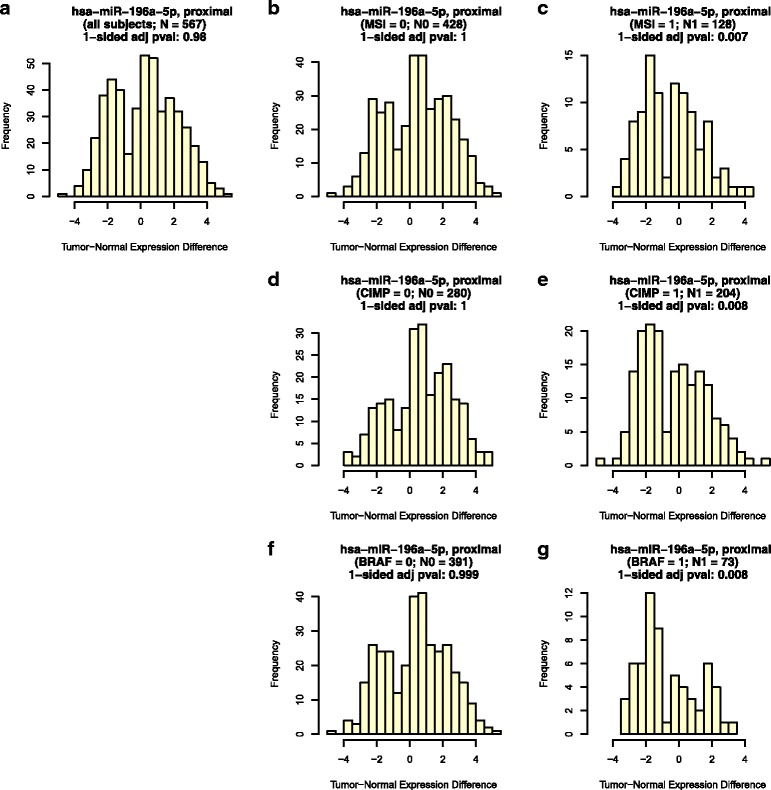
Results for a microRNA with three factors (MSI, CIMP, and *BRAF*) simultaneously associated with the direction and significance of its tumor-normal dysregulation

Differential expression of microRNAs in colorectal cancer is a multi-faceted phenomenon, with multiple factors sometimes being associated with the direction and significance of differential expression of the same microRNA at a given site. For example, Table 3 reports that for each of the factors MSI, CIMP, and *BRAF*, there is one microRNA that is significantly dysregulated in proximal colon tumor relative to proximal colon normal mucosa, but that is significantly dysregulated in the opposite direction for one of the factors’ levels (i.e., a “yellow” outcome). In fact, this is the same microRNA for all three factors, as represented in Fig. 3. Figure 3a indicates that overall, microRNA miR-196a-5p tends to be significantly up-regulated in tumor vs. normal. Figure 3b, d, and f demonstrate that in the absence of MSI (i.e., for MSS), for CIMP status low, or in *BRAF*-mutated tumors, respectively, (i.e., at factor levels 0) this microRNA tends to be significantly up-regulated, with a bimodal tumor-normal expression difference distribution with major node favoring positive values. However, Fig. 3c, e, and g show that in the presence of MSI, for CIMP status high, or in *BRAF*-mutated tumors, respectively, (i.e., at factor levels 1) this microRNA tends to be significantly down-regulated, with bimodal distributions whose modes corresponding to negative values are more pronounced.

It is important to note that all of the conclusions of this study (and resulting classifications of outcomes in interest in Tables 2 and 3) are reached after controlling the overall false discovery rate at 0.05. This means that only as much as 5% of the significant findings in this paper can be expected to be false positives. While alternative error rate thresholds could have been selected, it is encouraging to have so many significant results after controlling for multiple comparisons across so many microRNAs, sites, and factors of interest.

While the results reported in Table 3 (and full results in Additional file 7) involve too many microRNAs to discuss at length individually in this manuscript, we can demonstrate the potential clarifying utility of these results (particularly Additional file 7) by referring to the following few representative examples.

MicroRNAs miR-1266 and miR-4727-3p were classified as the two “orange” outcomes for distal colon tumors in Table 3, being not significantly differentially expressed overall, but differentially expressed in different directions for levels of the SURV5YRS factor. Additional file 7 shows that miR-1266 and miR-4727-3p did not show strong evidence of overall tumor-normal differential expression (respective one-sided FDR-adjusted *p*-values 0.8111 and 0.6329), but were significantly down-regulated (one-sided FDR-adjusted p-values 0.0077 and 0.0153) in subjects that did not survive five years beyond diagnosis, and were significantly up-regulated (one-sided FDR-adjusted *p*-values 0.9996 and 0.981) in subjects that survived beyond five years. These findings are consistent with those previously reported in the literature. miR-1266 has been shown to be significantly down-regulated in gastric cancer tissues [21], with higher expression values correlating with longer patient survival times [22]. miR-4727-3p has been shown to bind with the *BUB1* gene [23], lower expression levels of which have previously been shown to be associated with shorter relapse-free survival after surgery for colon carcinoma [24].

For several years miR-145 has been of interest in rectal cancers as a possible tumor-suppressor [25, 26], being significantly down-regulated in colorectal carcinoma (with up-regulation in response to neoadjuvant chemotherapy) [27]. Our results are consistent with this literature – miR-145-3p was found to be significantly down-regulated in rectal tumors (one-sided FDR-adjusted p-value <0.0001). However, our results (Additional file 7) provide additional insight, as miR-145-3p was classified as a “blue” outcome in rectal tumors in Table 3, exhibiting significant overall down-regulation in rectal tumor, for MSS subjects (one-sided FDR-adjusted *p*-values <0.0001 for MSS vs. 0.2267 for MSI subjects), for non-*BRAF*-mutated tumors (one-sided FDR-adjusted *p*-values <0.0001 for non-*BRAF*-mutated vs. 0.5 for *BRAF*-mutated tumors), for non-wine-drinking subjects (one-sided FDR-adjusted *p*-values <0.0001 for non-wine-drinking vs. 0.1657 for wine-drinking subjects), and for non-extremely-obese subjects (one-sided FDR-adjusted *p*-values <0.0001 for non-extremely-obese vs. 0.2267 for extremely-obese subjects). The public availability of Additional file 7 makes such clarifying insights widely available for colorectal cancer researchers.

Previously, miR-130a has been shown to play a complex role in tumorigenesis, being down-regulated in chronic lymphocytic leukemia [28] but up-regulated in nonsmall cell lung cancer and chronic myeloid leukemia [29, 30], and also up-regulated in colon cancer (compared to paired adjacent normal mucosa) [31]. In rectal cancer, up-regulation of miR-130a-3p is significantly associated with better survival [11]. Additional file 7 shows that in rectal cancer, miR-130a-3p is classified as a “blue” outcome, being significantly up-regulated, but only for low-CIMP subjects (one-sided FDR-adjusted *p*-values 0.999 for low-CIMP vs. 0.6341 for high-CIMP subjects), non-*BRAF*-mutated tumors (one-sided FDR-adjusted p-values 0.9987 for non-*BRAF*-mutated vs. 0.7154 for *BRAF*-mutated tumors), *TP53*-mutated tumors (one-sided FDR-adjusted p-values 0.9987 for *TP53*-mutated vs. 0.8521 for non-*TP53*-mutated tumors), non-*KRAS*-mutated tumors (one-sided FDR-adjusted p-values 0.9992 for non-*KRAS*-mutated vs. 0.7466 for *KRAS*-mutated tumors), non-distant SEER summary stage (one-sided FDR-adjusted p-values 0.9993 for non-distant vs. 0.6568 for distant subjects), subjects with AJCC stage less than 4 (one-sided FDR-adjusted *p*-values 0.9998 for subjects with AJCC stage less than 4 vs. 0.2258 for subjects with AJCC stage 4), subjects alive at last follow-up (one-sided FDR-adjusted p-values 0.9994 for subjects alive at last follow-up vs. 0.8333 for subjects not alive at last follow-up), subjects reaching at least five years survival (one-sided FDR-adjusted *p*-values 0.9984 for five-year survival vs. 0.8875 for subjects surviving less than five years), non-current smokers (one-sided FDR-adjusted p-values >0.9999 for non-current smokers vs. 0.4375 for current smokers), subjects with BMI outside normal range (one-sided FDR-adjusted p-values 0.9998 for non-normal BMI range vs. 0.9136 for normal BMI range subjects), or subjects with non-extreme BMI (one-sided FDR-adjusted p-values 0.9994 for non-extreme BMI vs. 0.3929 for extreme BMI subjects). In addition, Additional file 7 shows miR-130a-3p as a “yellow” outcome in rectal cancer cases, being up-regulated in MSS tumors (one-sided FDR-adjusted *p*-value 0.9998) but down-regulated in MSI tumors (one-sided FDR-adjusted p-value 0.0134). At a minimum, this all suggests the need to account for some of these factors when considering the prognostic role of miR-130a-3p in rectal cancer subjects. It also raises questions regarding the potential roles these factors could play in affecting the survival of rectal cancer subjects.

## Conclusions

The results of this study demonstrate the benefit to colorectal cancer researchers to consider multiple subject-level factors when studying dysregulation of microRNAs, whose tumor-related changes in expression can be associated with multiple factors. In instances where microRNAs can be both up and down regulated, depending on specific factor levels, the consequences of not accounting for these factors would most likely be failure to detect any association with the microRNA. In other instances, failure to consider subject-level factors most likely would result in underestimation of the significance of the association. Since direction of regulation could be an important component when considering functionality of microRNAs, this information is important from a potential translational perspective. Our Additional file 7 will serve as a publicly-available resource to provide clarifying information about various factors associated with the direction and significance of tumor-normal differential expression of microRNAs in colorectal cancer.

## Additional files

Additional file 1: (AF1_ColonStudyQuestionnaire.pdf) Copy of the questionnaire used in the study. (PDF 1180 kb)

Additional file 2: (AF2_blue.pdf) Visualizations of “blue” outcomes of interest – microRNAs that are overall significant in the tumor-normal test of differential expression, with significant directional agreement in one factor level and NS in the other. Each page of this file is in the same format as explained for each row in Fig. 2. (PDF 4490 kb)

Additional file 3: (AF3_green.pdf) Visualizations of “green” outcomes of interest – microRNAs that are overall not significant in the tumor-normal test of differential expression, but significant in only one factor level. Each page of this file is in the same format as explained for each row in Fig. 2. (PDF 243 kb)

Additional file 4: (AF4_orange.pdf) Visualizations of “orange” outcomes of interest – microRNAs that are overall not significant in the tumor-normal test of differential expression, but significant in opposite directions for factor levels. Each page of this file is in the same format as explained for each row in Fig. 2. (PDF 16 kb)

Additional file 5: (AF5_purple.pdf) Visualizations of “purple” outcomes of (lesser) interest – microRNAs that agree in the direction or non-significance of the tumor-normal test of differential expression in each factor level, but different from the overall test’s direction or significance. Each page of this file is in the same format as explained for each row in Fig. 2. (PDF 382 kb)

Additional file 6: (AF6_yellow.pdf) Visualizations of “yellow” outcomes of interest – microRNAs that are overall significant in the tumor-normal test of differential expression, with agreement in one factor level but opposite direction in the other factor level. Each page of this file is in the same format as explained for each row in Fig. 2. (PDF 20 kb)

Additional file 7: (AF7_results.csv) Spreadsheet file for all outcomes of interest, including the FDR-adjusted *p*-values and sample sizes for all site / factor / microRNA combinations that were classified to one of the five categories (colors) in Table 2. (CSV 199 kb)

## Abbreviations

*BRAF*: Human gene encoding a protein called B-Raf
CIMP: CpG island methylator phenotype
FDR: False discovery rate
KPMCP: Kaiser Permanente Medical Care Program
*KRAS*: Human gene encoding a protein called K-Ras
microRNA / miRNA: Small non-protein-coding RNA molecules that regulate gene expression
MSI / MSS: Microsatellite instable / microsatellite stabile
NS: Not significantly differentially expressed (NS) in tumor relative to normal
QC: Quality control
RNA: Ribonucleic acid
SAS: Software developed by SAS Institute; formerly “statistical analysis system”
SEER: Surveillance, Epidemiology, and End Results Program
*TP53*: Human gene encoding a protein called tumor protein p53
