# Supplementary material for: Identifying factors associated with the direction and significance of microRNA tumor-normal expression differences in colorectal cancer
Source: BMC Cancer. 2017 Oct 30;17:707. doi: 10.1186/s12885-017-3690-x (PMC5663119; doi:10.1186/s12885-017-3690-x)
Supplement: Supplementary file 2 — (AF2_blue.pdf) Visualizations of “blue” outcomes of interest – microRNAs that are overall significant in the tumor-normal test of differential expression, with significant directional agreement in one factor level and NS in the other. Each page of this file is in the same format as explained for each row in Fig. 2. (PDF 4490 kb) [file 12885_2017_3690_MOESM1_ESM.pdf]

DIET AND PHYSICAL ACTIVITY RISK FACTOR STUDY

ID #:

RESPONDENT'S REFERENCE DATE:

RESPONDENT'S DATE OF BIRTH:

NAME OF INTERVIEWER:

INTERVIEWER ID#:

DATE OF INTERVIEW:

ENTER TIME:

Thank you for agreeing to participate in our study. As you may recall from the introductory letter, the purpose of this study is to examine factors related to the health of adults in the U.S. We will be discussing a variety of topics, including your past health, your past diet and other health habits. I want to remind you that your participation is voluntary and that all the information collected will be kept completely confidential. Neither your name nor any identifying information will appear on any report of the study. If there is any question you do not wish to answer, just let me know and I will go on to the next question. Unless you have any questions, I will begin the interview now.

ADMINISTER CONSENT FORM.

9 R [SORY]

SECTION A

DEMOGRAPHICS

ATX1Since many people have never been in an interview exactly like this, let me start by reading you a paragraph that tells a little bit about how it works. I am going to read you a set of questions exactly as they are worded so that every respondent in the survey is answering the same questions. You'll be asked to answer two kinds of questions. In some cases, you'll be asked to answer in your own words. For those questions , I will have to write down your answer word for word. In other cases, you will be given a list of answers and asked to choose the one that fits best. If at any time during the interview you are not clear about what is wanted, be sure to ask me. Also, please feel free to take as much time as you need to answer the questions.

A001 I'd like to begin by asking you some questions about your background. What is your date of birth?

MONTH	DAY	YEAR

98 DK

COMPLETE REFERENT WORKSHEET

(CALCULATE AGE AT REFERENT DATE. IF <30 OR >80 EXIT INTERVIEW)

A002What is the highest grade or year of school or college that you have completed?

1 EIGHTH GRADE OR LESS
2 TRADE SCHOOL OR BUSINESS SCHOOL INSTEAD OF HIGH SCHOOL
3 SOME HIGH SCHOOL
4 HIGH SCHOOL GRADUATE OR GED
5 TRADE SCHOOL OR BUSINESS SCHOOL AFTER GRADUATING FROM HIGH SCHOOL
6 SOME COLLEGE INCLUDING 2 YEAR DEGREES
7 RECEIVED BACHELOR'S DEGREE
8 GRADUATE OR PROFESSIONAL EDUCATION BEYOND THE BACHELOR'S DEGREE
9 GRADUATE OR PROFESSIONAL DEGREE
98 DK

A003Please look at this card **(SHOW CARD)** and tell me what is your race?

1 WHITE, NOT OF HISPANIC ORIGIN
2 WHITE, HISPANIC
3 BLACK, NOT OF HISPANIC ORIGIN
4 BLACK, HISPANIC
5 ASIAN OR PACIFIC ISLANDER **(SORY)**
6 AMERICAN INDIAN OR ALASKA NATIVE **(SORY)**
7 OTHER **(SPECIFY)** ______________________
8 DK

A004Are you currently married, living as married, widowed, divorced, separated, or never married?

1 MARRIED
2 LIVING AS MARRIED
3 WIDOWED
4 DIVORCED
5 SEPARATED
6 NEVER MARRIED
8 DK

A005Whether or not you attend church regularly, what is your religious preference? Are you:

(INTV: READ RESPONSE STEMS)

1 Catholic,
2 Protestant,
3 LDS,
4 Jewish,
5 Seventh-day Adventist,
6 Some other religion or
7 Have no religious preference?
8 DK

A006During the last five years, which of the following statements best describes your attendance at religious services or meetings? Do you:

(INTV: READ RESPONSES)

1 Never attend,
2 Attend less than one service or meeting per month,
3 Attend one to three services or meetings per month or
4 Attend one or more services or meetings per week?
8 DK

A007INTV: ENTER SEX

1 MALE
2 FEMALE

GO TO SECTION B, MEDICAL HISTORY

SECTION B

MEDICAL HISTORY

BTX1The next part of the questionnaire asks questions about your health and medical history.

Did a doctor ever tell you that you have, or have had (CONDITION)?	In what year were you first told by a doctor that you had (CONDITION)?	Was it treated with surgery?
B001 familial polyposis an inherited condition in which you and other family members have had numerous colon polyps<br>1 YES (B002)<br>3 NO (B004)<br>8 DK (B004)	B002 familial polyposis<br>__ YEAR<br>98 DK	B003<br>1 YES<br>3 NO<br>8 DK
B004 a colon or rectal polyp (excluding familial polyposis)<br>1 YES (B005)<br>3 NO (B007)<br>8 DK (B007)	B005 a colon or rectal polyp<br>__ YEAR<br>98 DK	B006<br>1 YES<br>3 NO<br>8 DK
B007 ulcerative colitis<br>1 YES (B008)<br>3 NO (B010)<br>8 DK (B010)	B008 ulcerative colitis<br>__ YEAR<br>98 DK	B009<br>1 YES<br>3 NO<br>8 DK
B010 any other diseases of the colon or large bowel<br>1 YES (B011)<br>3 NO (B013)<br>8 DK (B013)	B011 other diseases of the colon or large bowel<br>__ YEAR<br>98 DK	B012<br>1 YES<br>3 NO<br>8 DK
B013 lactose intolerance or the inability to properly digest milk<br>1 YES (B014)<br>3 NO (B015)<br>8 DK (B015)	B014 lactose intolerance<br>__ YEAR<br>98 DK	

B015Before (REFERENT DATE), on the average, how many bowel movements did you have per week?

___ ENTER NUMBER
998 DK

B016Before (REFERENT DATE) were you ever diagnosed as having any type of cancer?

1 YES (B017)
3 NO (B025)
8 DK (B025)

	FIRST CANCER	SECOND CANCER	THIRD CANCER
What was the first/next type of cancer you had?	B017<br>01 SKIN (NOT MELANOMA)<br>03 BREAST<br>04 LUNG<br>05 COLON<br>06 CORPUS (UTERINE)<br>07 RECTUM<br>08 BLADDER<br>09 OVARY<br>10 MELANOMA<br>11 CERVIX<br>12 CANCER OF FEMALE REPRODUCTIVE ORGANS, SITE UNKNOWN<br>13 OTHER CANCER (SPECIFY)<br>14 RESPONDENT HAD CANCER, SITE UNKNOWN<br>15 UNKNOWN IF RESPONDENT HAD CANCER<br>16 CANCER OF THE LARGE BOWEL (COLON/RECTUM), SITE UNKNOWN	B020<br>01 SKIN (NOT MELANOMA)<br>03 BREAST<br>04 LUNG<br>05 COLON<br>06 CORPUS (UTERINE)<br>07 RECTUM<br>08 BLADDER<br>09 OVARY<br>10 MELANOMA<br>11 CERVIX<br>12 CANCER OF FEMALE REPRODUCTIVE ORGAN, SITE UNKNOWN<br>13 OTHER CANCER (SPECIFY)<br>14 RESPONDENT HAD CANCER, SITE UNKNOWN<br>15 UNKNOWN IF RESPONDENT HAD CANCER<br>16 CANCER OF THE LARGE BOWEL (COLON/RECTUM), SITE	B023<br>01 SKIN (NOT MELANOMA)<br>03 BREAST<br>04 LUNG<br>05 COLON<br>06 CORPUS (UTERINE)<br>07 RECTUM<br>08 BLADDER<br>09 OVARY<br>10 MELANOMA<br>11 CERVIX<br>12 CANCER OF FEMALE REPRODUCTIVE ORGAN, SITE UNKNOWN<br>13 OTHER CANCER (SPECIFY)<br>14 RESPONDENT HAD CANCER, SITE UNKNOWN<br>15 UNKNOWN IF RESPONDENT HAD CANCER<br>16 CANCER OF THE LARGE BOWEL (COLON/RECTUM), SITE
How old were you when this cancer was diagnosed?	B018<br>__ ENTER AGE<br>998 DK	B021<br>__ ENTER AGE<br>998 DK	B024<br>__ ENTER AGE<br>998 DK
Before (REFERENT DATE) did you have any other cancer?	B019<br>1 YES (B020)<br>3 NO (B025)<br>8 DK (B020)	B022<br>1 YES (B023)<br>3 NO (B025)<br>8 DK (B023)	

Before (REFERENT DATE) have you every had surgery for removal of all or part of your (PART)?	In what year was the surgery performed?
B025 stomach<br>1 YES (B026)<br>3 NO (B027)<br>8 DK (B027)	B026 _____ YEAR<br>98 DK
B027 intestines or colon<br>1 YES (B028)<br>3 NO (B029)<br>8 DK (B029)	B028 _____ YEAR<br>98 DK
B029 gall bladder<br>1 YES (B030)<br>3 NO (B031)<br>8 DK (B031)	B030 _____ YEAR<br>98 DK

Have you ever taken (MEDICINE) regularly? By regularly, I mean at least 3 times a week for at least 1 month.	At about what age did you start taking (MEDICINE)	About how long did you take (MEDICINE)	Are you presently taking (MEDICINE)
B031 medicine for asthma in the form of tablets or capsules<br>1 YES (B032)<br>3 NO (B035)<br>8 DK (B035)	B032 medicine for asthma<br>__ ENTER AGE<br>98 DK	B033 __ YEARS<br>OR<br>__ MONTHS<br>98 DK	B034<br>1 YES<br>3 NO<br>8 DK
B035 valium, also know as diazepam<br>1 YES (B036)<br>3 NO (B039)<br>8 DK (B039)	B036 valium<br>__ ENTER AGE<br>98 DK	B037 __ YEARS<br>OR<br>__ MONTHS<br>98 DK	B038<br>1 YES<br>3 NO<br>8 DK
B039 digoxin or other digitalis medicine<br>1 YES (B040)<br>3 NO (B043)<br>8 DK (B043)	B040 digoxin<br>__ ENTER AGE<br>98 DK	B041 __ YEARS<br>OR<br>__ MONTHS<br>98 DK	B042<br>1 YES<br>3 NO<br>8 DK
B043 DBI or phenformin, which is one particular type of pill that used to be given for diabetes<br>1 YES (B044)<br>3 NO (B047)<br>8 DK (B047)	B044 DBI or phenformin<br>__ ENTER AGE<br>98 DK	B045 __ YEARS<br>OR<br>__ MONTHS<br>98 DK	B046<br>1 YES<br>3 NO<br>8 DK
B047 preludin or phenmetrazine, which is one particular kind of reducing pill to cut down your appetite<br>1 YES (B048)<br>3 NO (B051)<br>8 DK (B051)	B048 preludin or phenmetrazine<br>__ ENTER AGE<br>98 DK	B049 __ YEARS<br>OR<br>__ MONTHS<br>98 DK	B050<br>1 YES<br>3 NO<br>8 DK
B051 aspirin<br>1 YES (B052)<br>3 NO (B055)<br>8 DK (B055)	B052 aspirin<br>__ ENTER AGE<br>98 DK	B053 __ YEARS<br>OR<br>__ MONTHS<br>98 DK	B054<br>1 YES<br>3 NO<br>8 DK
B055 other nonsteroidal anti-inflammatory drugs or arthritis medicines such as Motrin, Clinoril, Naprosyn, or Feldene<br>1 YES (B056)<br>3 NO (B059)<br>8 DK (B059)	B056 other nonsteroidal drugs<br>__ ENTER AGE<br>98 DK	B057 __ YEARS<br>OR<br>__ MONTHS<br>98 DK	B058<br>1 YES<br>3 NO<br>8 DK

B059In your whole life, have you ever taken penicillin for more than 30 days?

1 YES
3 NO
8 DK

SECTION C

PHYSICAL ACTIVITY

CTX1Now, I am going to ask about your level of physical activity. I will ask about your level of physical activity at work and then at leisure and at home.

C001Between (FILL REFERENT PERIOD) were you employed at a job for pay, either full-time or part-time including self employment?

1 YES
3 NO (C039)
8 DK (C039)
9 R (C039)

C002What was your complete job title?

____
8 DK
9 R

C003In what kind of business or industry did you work; that is, what product was made or what service was given?

____
8 DK
9 R

C004What were your main duties on that job?

____
____
8 DK
9 R

C005In what month and year did you start working at this job?

ENTER MONTH AND YEAR: __MONTH __YR
8 DK
9 R

C006In what month and year did you stop working at this job?

ENTER MONTH AND YEAR: __MONTH __YR
6 STILL WORKING
8 DK
9 R

C007Please look at this card and tell me, between (FILL REFERENT PERIOD), did you perform any moderate activities such as standing or walking not carrying heavy objects at this job?

1 YES
3 NO (C010)
8 DK (C010)
9 R (C010)

C008 Between (FILL REFERENT PERIOD), in how many months did you perform moderate activity at this job?

__ ENTER NUMBER OF MONTHS
98 DK
99 R

C009 Between (FILL REFERENT PERIOD), on average, how many hours per week did you perform moderate activity at this job?

___ ENTER NUMBER OF HOURS
998 DK
999 R

C010 Please look at this card and tell me, between (FILL REFERENT PERIOD), did you perform any vigorous activities such as construction, shoveling dirt or other heavy labor at this job?

1 YES
3 NO (C013)
8 DK (C013)
9 R (C013)

C011 Between (FILL REFERENT PERIOD), in how many months did you perform vigorous activity at this job?

__ ENTER NUMBER OF MONTHS
98 DK
99 R

C012 Between (FILL REFERENT PERIOD), on average, how many hours per week did you perform vigorous activity at this job?

___ ENTER NUMBER OF HOURS
998 DK
999 R

C013 Between (FILL REFERENT PERIOD) were you employed at any other job for pay, either full-time or part-time including self employment?

1 YES
3 NO (C039)
8 DK (C039)
9 R (C039)

C014 What was your complete job title?

____
8 DK
9 R

C015 In what kind of business or industry did you work; that is, what product was made or what service was given?

____
8 DK
9 R

C016What were your main duties on that job?

____

____

8 DK
9 R

C017In what month and year did you start working at this job?

ENTER MONTH AND YEAR: __MONTH __YR
8 DK
9 R

C018In what month and year did you stop working at this job?

ENTER MONTH AND YEAR: __MONTH __YR
6 STILL WORKING
8 DK
9 R

SECTION C

PHYSICAL ACTIVITY

CTX1Now, I am going to ask about your level of physical activity. I will ask about your level of physical activity at work and then at leisure and at home.

C001Between (FILL REFERENT PERIOD) were you employed at a job for pay, either full-time or part-time including self employment?

1 YES
3 NO (C039)
8 DK (C039)
9 R (C039)

C002What was your complete job title?

____

8 DK
9 R

C003In what kind of business or industry did you work; that is, what product was made or what service was given?

____

8 DK
9 R

C004What were your main duties on that job?

____

____

8 DK
9 R

C005In what month and year did you start working at this job?

ENTER MONTH AND YEAR: __MONTH __YR

8 DK
9 R

C006 In what month and year did you stop working at this job?

ENTER MONTH AND YEAR: __MONTH __YR
6 STILL WORKING
8 DK
9 R

C007 Please look at this card and tell me, between (FILL REFERENT PERIOD), did you perform any moderate activities such as standing or walking not carrying heavy objects at this job?

1 YES
3 NO (C010)
8 DK (C010)
9 R (C010)

C008 Between (FILL REFERENT PERIOD), in how many months did you perform moderate activity at this job?

__ ENTER NUMBER OF MONTHS
98 DK
99 R

C009 Between (FILL REFERENT PERIOD), on average, how many hours per week did you perform moderate activity at this job?

___ ENTER NUMBER OF HOURS
998 DK
999 R

C010 Please look at this card and tell me, between (FILL REFERENT PERIOD), did you perform any vigorous activities such as construction, shoveling dirt or other heavy labor at this job?

1 YES
3 NO (C013)
8 DK (C013)
9 R (C013)

C011 Between (FILL REFERENT PERIOD), in how many months did you perform vigorous activity at this job?

__ ENTER NUMBER OF MONTHS
98 DK
99 R

C012 Between (FILL REFERENT PERIOD), on average, how many hours per week did you perform vigorous activity at this job?

___ ENTER NUMBER OF HOURS
998 DK
999 R

C013 Between (FILL REFERENT PERIOD) were you employed at any other job for pay, either full-time or part-time including self employment?

1 YES
3 NO (C039)
8 DK (C039)
9 R (C039)

C014 What was your complete job title?

____

8 DK
9 R

C015 In what kind of business or industry did you work; that is, what product was made or what service was given?

____

8 DK
9 R

C016 What were your main duties on that job?

____

____

8 DK
9 R

C017 In what month and year did you start working at this job?

ENTER MONTH AND YEAR: __MONTH __YR
8 DK
9 R

C018 In what month and year did you stop working at this job?

ENTER MONTH AND YEAR: __MONTH __YR
6 STILL WORKING
8 DK
9 R

C019 Please look at this card and tell me, <u>between (FILL REFERENT PERIOD)</u>, did you perform any moderate activities such as standing or walking not carrying heavy objects at this job?

1 YES
3 NO (C022)
8 DK (C022)
9 R (C022)

C020 <u>Between (FILL REFERENT PERIOD)</u>, in how many <u>months</u> did you perform moderate activity at this job?

__ ENTER NUMBER OF MONTHS
98 DK
99 R

C021 <u>Between (FILL REFERENT PERIOD)</u>, on average, how many <u>hours per week</u> did you perform moderate activity at this job?

___ ENTER NUMBER OF HOURS
998 DK
999 R

C022 Please look at this card and tell me, between (FILL REFERENT PERIOD), did you perform any vigorous activities such as construction, shoveling dirt or other heavy labor at this job?

1 YES
3 NO (C025)
8 DK (C025)
9 R (C025)

C023 Between (FILL REFERENT PERIOD), in how many months did you perform vigorous activity at this job?

__ ENTER NUMBER OF MONTHS
98 DK
99 R

C024 Between (FILL REFERENT PERIOD), on average, how many hours per week did you perform vigorous activity at this job?

___ ENTER NUMBER OF HOURS
998 DK
999 R

C025 Between (FILL REFERENT PERIOD) were you employed at any other job for pay, either full-time or part-time including self employment?

1 YES
3 NO (C039)
8 DK (C039)
9 R (C039)

C026 What was your complete job title?

____

8 DK
9 R

C027 In what kind of business or industry did you work; that is, what product was made or what service was given?

____

8 DK
9 R

C028 What were your main duties on that job?

____

____

8 DK
9 R

C029 In what month and year did you start working at this job?

ENTER MONTH AND YEAR: __MONTH __YR

8 DK
9 R

C030 In what month and year did you stop working at this job?

ENTER MONTH AND YEAR: __MONTH __YR
6 STILL WORKING
8 DK
9 R

C031 Please look at this card and tell me, between (FILL REFERENT PERIOD), did you perform any moderate activities such as standing or walking not carrying heavy objects at this job?

1 YES
3 NO (C034)
8 DK (C034)
9 R (C034)

C032 Between (FILL REFERENT PERIOD), in how many months did you perform moderate activity at this job?

__ ENTER NUMBER OF MONTHS
98 DK
99 R

C033 Between (FILL REFERENT PERIOD), on average, how many hours per week did you perform moderate activity at this job?

___ ENTER NUMBER OF HOURS
998 DK
999 R

C034 Please look at this card and tell me, between (FILL REFERENT PERIOD), did you perform any vigorous activities such as construction, shoveling dirt or other heavy labor at this job?

1 YES
3 NO (C037)
8 DK (C037)
9 R (C037)

C035 Between (FILL REFERENT PERIOD), in how many months did you perform vigorous activity at this job?

__ ENTER NUMBER OF MONTHS
98 DK
99 R

C036 Between (FILL REFERENT PERIOD), on average, how many hours per week did you perform vigorous activity at this job?

___ ENTER NUMBER OF HOURS
998 DK
999 R

C037 Between (FILL REFERENT PERIOD) were you working either fewer hours or at a lower physical effort because of injury, a recent illness or a long-term disease?

1 YES, INJURY (C038)
3 YES, RECENT ILLNESS (C038)
5 YES, LONG TERM ILLNESS (C038)
7 NO (C075)
8 DK (C075)
9 R (C075)

C038In what year did this change occur?

__ ENTER YEAR
98 DK
99 R
(C075)

C039Before (FILL REFERENT DATE), since age 18, have you ever been employed at a job for pay, either full-time or part-time including self employment?

1 YES
3 NO (C081)
8 DK (C081)
9 R (C081)

C040What was your complete job title for the last job you held?

____
8 DK
9 R

C041In what kind of business or industry did you work; that is, what product was made or what service was given?

____
8 DK
9 R

C042What were your main duties on that job?

____
____
8 DK
9 R

C043In what month and year did you start working at this job?

ENTER MONTH AND YEAR: __MONTH __YR
8 DK
9 R

C044In what month and year did you stop working at this job?

ENTER MONTH AND YEAR: __MONTH __YR
8 DK
9 R

C045Please look at this card and tell me, between (FILL LAST YEAR EMPLOYED) did you perform any moderate activities such as standing or walking not carrying

heavy objects at this job?

1 YES
3 NO (C048)
8 DK (C048)
9 R (C048)

C046 Between (FILL LAST YEAR EMPLOYED), in how many months did you perform moderate activity at this job?

__ ENTER NUMBER OF MONTHS
98 DK
99 R

C047 Between (FILL LAST YEAR EMPLOYED), on average, how many hours per week did you perform moderate activity at this job?

___ ENTER NUMBER OF HOURS
998 DK
999 R

C048 Please look at this card and tell me, between (FILL LAST YEAR EMPLOYED) did you perform any vigorous activities such as construction, shoveling dirt or other heavy labor at this job?

1 YES
3 NO (C051)
8 DK (C051)
9 R (C051)

C049 Between (FILL LAST YEAR EMPLOYED), in how many months did you perform vigorous activity at this job?

__ ENTER NUMBER OF MONTHS
98 DK
99 R

C050Between (FILL LAST YEAR EMPLOYED), on average, how many hours per week did you perform vigorous activity at this job?

___ ENTER NUMBER OF HOURS
998 DK
999 R

C051Between (FILL LAST YEAR EMPLOYED) were you employed at any other job for pay, either full-time or part-time including self employment?

1 YES
3 NO (C075)
8 DK (C075)
9 R (C075)

C052What was your complete job title?

____
8 DK
9 R

C053In what kind of business or industry did you work; that is, what product was made or what service was given?

____
8 DK
9 R

C054What were your main duties on that job?

____
____
8 DK
9 R

C055In what month and year did you start working at this job?

ENTER MONTH AND YEAR: __MONTH __YR
8 DK
9 R

C056In what month and year did you stop working at this job?

ENTER MONTH AND YEAR: __MONTH __YR
8 DK
9 R

C057Please look at this card and tell me, between (FILL LAST YEAR EMPLOYED) did you perform any moderate activities such as standing or walking not carrying heavy objects at this job?

1 YES
3 NO (C060)
8 DK (C060)
9 R (C060)

C058Between (FILL LAST YEAR EMPLOYED), in how many months did you perform moderate activity at this job?

__ ENTER NUMBER OF MONTHS
98 DK
99 R

C059Between (FILL LAST YEAR EMPLOYED), on average, how many hours per week did you perform moderate activity at this job?

___ ENTER NUMBER OF HOURS
998 DK
999 R

C060Please look at this card and tell me, between (FILL LAST YEAR EMPLOYED) did you perform any vigorous activities such as construction, shoveling dirt or other heavy labor at this job?

1 YES
3 NO (C063)
8 DK (C063)
9 R (C063)

C061Between (FILL LAST YEAR EMPLOYED), in how many months did you perform vigorous activity at this job?

__ ENTER NUMBER OF MONTHS
98 DK
99 R

C062Between (FILL LAST YEAR EMPLOYED), on average, how many hours per week did you perform vigorous activity at this job?

___ ENTER NUMBER OF HOURS
998 DK
999 R

C063Between (FILL LAST YEAR EMPLOYED) were you employed at any other job for pay, either full-time or part-time including self employment?

1 YES
3 NO (C075)
8 DK (C075)
9 R (C075)

C064What was your complete job title?

____

8 DK
9 R

C065In what kind of business or industry did you work; that is, what product was made or what service was given?

____

8 DK
9 R

C066What were your main duties on that job?

____

____

8 DK
9 R

C067In what month and year did you start working at this job?

ENTER MONTH AND YEAR: __MONTH __YR
8 DK
9 R

C068In what month and year did you stop working at this job?

ENTER MONTH AND YEAR: __MONTH __YR
8 DK
9 R

C069Please look at this card and tell me, <u>between (FILL LAST YEAR EMPLOYED)</u> did you perform any moderate activities such as standing or walking not carrying heavy objects at this job?

1 YES
3 NO (C072)
8 DK (C072)
9 R (C072)

C070<u>Between (FILL LAST YEAR EMPLOYED)</u>, in how many <u>months</u> did you perform moderate activity at this job?

__ ENTER NUMBER OF MONTHS
98 DK
99 R

C071<u>Between (FILL LAST YEAR EMPLOYED)</u>, on average, how many <u>hours per week</u> did you perform moderate activity at this job?
___ ENTER NUMBER OF HOURS
998 DK
999 R

C072 Please look at this card and tell me, between (FILL LAST YEAR EMPLOYED) did you perform any vigorous activities such as construction, shoveling dirt or other heavy labor at this job?

1 YES
3 NO (C075)
8 DK (C075)
9 R (C075)

C073 Between (FILL LAST YEAR EMPLOYED), in how many months did you perform vigorous activity at this job?

__ ENTER NUMBER OF MONTHS
98 DK
99 R

C074 Between (FILL LAST YEAR EMPLOYED), on average, how many hours per week did you perform vigorous activity at this job?

___ ENTER NUMBER OF HOURS
998 DK
999 R

(SCREEN ALL EVER EMPLOYED SINCE AGE 18 FOR AGE NOW--IF EMPLOYED DURING THE RP THEN DO NOT ASK ABOUT 60 IF 62 OR LESS NOW, DO NOT ASK 45 IF 47 OR LESS NOW AND 30 IF 32 OR LESS NOW. IF LAST YR EMPLOYED IS OUTSIDE OF THE RP, THE R WILL SKIP THE QUESTION IF AGE AT THE BEGINNING THE LAST YEAR EMPLOYED IS LESS THAN OR EQUAL TO THE AGE WE ARE ASKING ABOUT)

C075 Compared to your level of physical activity at your job/s between (FILL EITHER REFERENT PERIOD OR LAST YEAR WORKED), how would you describe your level of physical activity at work when you were 60 years old. Would it be: less, about the same or more physical activity?

1 LESS PHYSICAL ACTIVITY
3 ABOUT THE SAME AMOUNT OF PHYSICAL ACTIVITY OR (C077)
5 MORE PHYSICAL ACTIVITY
7 NOT WORKING AT 60 (C077)
8 DK (C077)
9 R (C077)

C076 What per cent (FILL MORE OR LESS) would that be?

__ ENTER PER CENT
98 DK
99 R

C077Compared to your level of physical activity at your job/s between (FILL EITHER REFERENT PERIOD OR LAST YEAR WORKED), how would you describe your level of physical activity at work when you were 45 years old. Would it be: less, about the same or more physical activity?

1 LESS PHYSICAL ACTIVITY
3 ABOUT THE SAME AMOUNT OF PHYSICAL ACTIVITY OR (C079)
5 MORE PHYSICAL ACTIVITY
7 NOT WORKING AT 45 (C079)
8 DK (C079)
9 R (C079)

C078What per cent (FILL MORE OR LESS) would that be?

__ ENTER PER CENT
98 DK
99 R

C079Compared to your level of physical activity at your job/s between (FILL EITHER REFERENT PERIOD OR LAST YEAR WORKED), how would you describe your level of physical activity at work when you were 30 years old. Would it be: less, about the same or more physical activity?

1 LESS PHYSICAL ACTIVITY
3 ABOUT THE SAME AMOUNT OF PHYSICAL ACTIVITY OR (C081)
5 MORE PHYSICAL ACTIVITY
7 NOT WORKING AT 30 (C081)
8 DK (C081)
9 R (C081)

C080What per cent (FILL MORE OR LESS) would that be?

__ ENTER PER CENT
98 DK
99 R

C081Between (FILL REFERENT PERIOD), how many flights of stairs per week did you climb? A flight of stairs is ten steps.

___ ENTER NUMBER
998 DK
999 R

C082Between (FILL REFERENT PERIOD), how many hours of sleep did you get on an average day?

__ ENTER NUMBER
98 DK
99 R

CTX2The next questions ask about your level of physical activity at leisure and around the house between (FILL REFERENT PERIOD). Do not include any occupational activities which you just mentioned.

For each activity indicate whether you performed the activity between (FILL REFERENT PERIOD). Answer "yes" only for those activities performed for an hour or more during any month. Consider the time spent in the activity only. For example, riding a chair lift skiing is not considered time skiing; sitting by a swimming pool should not be included in time spent swimming.

CTX3(INTV: GIVE RESPONDENT MODERATE ACTIVITY CUE CARD) Did you perform any of the following activities at a MODERATE PACE for at least one hour total time in any month between (FILL REFERENT PERIOD)? Moderate activities are those which are done at a more moderate pace than are more strenuous activities, or those which make you sweat or get out of breathe.

C083Did you perform sports at a moderate pace such as softball, shooting baskets, swimming, skiing, bowling or golfing?

1 YES
3 NO (C087)
8 DK (C087)
9 R (C087)

C084Between (FILL REFERENT PERIOD), in how many months was this activity performed?

(ENTER NUMBER)
98 DK
99 R

C085What was the average time per session you performed this activity?

(ENTER NUMBER)
98 DK
99 R

085CINTV: ENTER MINUTES/HOURS

1 MINUTES
3 HOURS
8 DK
9 R

C086What was the average number of days per week or month you performed this activity?

(ENTER NUMBER)
98 DK
99 R

086CINTV: ENTER WEEK/MONTH

1 WEEK
3 MONTH
8 DK
9 R

C087Did you perform moderate paced walks or hikes?

1 YES
3 NO (C091)
8 DK (C091)
9 R (C091)

C088Between (FILL REFERENT PERIOD), in how many months was this activity performed?

(ENTER NUMBER)
98 DK
99 R

C089What was the average time per session you performed this activity?

(ENTER NUMBER)
98 DK
99 R

089CINTV: ENTER MINUTES/HOURS

1 MINUTES
3 HOURS
8 DK
9 R

C090What was the average number of days per week or month you performed this activity?

(ENTER NUMBER)
98 DK
99 R

090CINTV: ENTER WEEK/MONTH

1 WEEK
3 MONTH
8 DK
9 R

C091Did you perform moderate paced home exercise, calisthenics or dancing?

1 YES
3 NO (C095)
8 DK (C095)
9 R (C095)

C092Between (FILL REFERENT PERIOD), in how many months was this activity performed?

(ENTER NUMBER)
98 DK
99 R

C093What was the average time per session you performed this activity?

(ENTER NUMBER)
98 DK
99 R

093CINTV: ENTER MINUTES/HOURS

1 MINUTES
3 HOURS
8 DK
9 R

C094What was the average number of days per week or month you performed this activity?

(ENTER NUMBER)
98 DK
99 R

094CINTV: ENTER WEEK/MONTH

1 WEEK
3 MONTH
8 DK
9 R

C095Did you perform moderate paced home maintenance, gardening, painting, raking, sweeping, mowing with a power mower, cleaning or vacuuming?

1 YES
3 NO (C099)
8 DK (C099)
9 R (C099)

C096Between (FILL REFERENT PERIOD), in how many months was this activity performed?

(ENTER NUMBER)
98 DK
99 R

C097What was the average time per session you performed this activity?

(ENTER NUMBER)
98 DK
99 R

097CINTV: ENTER MINUTES/HOURS

1 MINUTES
3 HOURS
8 DK
9 R

C098What was the average number of days per week or month you performed this activity?

(ENTER NUMBER)
98 DK
99 R

098CINTV: ENTER WEEK/MONTH

1 WEEK

3 MONTH
8 DK
9 R

(INTV: GIVE RESPONDENT VIGOROUS ACTIVITY CUE CARD)

Of the following activities, which ones did you perform at a STRENUOUS OR VIGOROUS PACE for at least one hour total time in any month between (FILL REFERENT PERIOD):	Between (FILL REFERENT PERIOD), in how many months was this activity performed?	What was the average time per session you performed this activity?	What was the average number of days per week or month you performed this activity?
C099 Did you perform vigorous racket sports and other strenuous sports such as tennis, squash or basketball?<br>1 YES<br>3 NO (C103)<br>8 DK (C103)<br>9 R (C103)	C100<br>(ENTER NUMBER)<br>98 DK<br>99 R	C101<br>__ MINUTES<br>**OR**<br>___ HOURS<br>98 DK<br>99 R	C102<br>___ PER WEEK<br>**OR**<br>___ PER MONTH<br>98 DK<br>99 R
C103 Did you perform jogging, running or biking?<br>1 YES<br>3 NO (C107)<br>8 DK (C107)<br>9 R (C107)	C104<br>(ENTER NUMBER)<br>98 DK<br>99 R	C105<br>__ MINUTES<br>**OR**<br>___ HOURS<br>98 DK<br>99 R	C106<br>___ PER WEEK<br>**OR**<br>___ PER MONTH<br>98 DK<br>99 R
C107 Did you perform vigorous exercise class or vigorous dance<br>1 YES<br>3 NO (C111)<br>8 DK (C111)<br>9 R (C111)	C108<br>(ENTER NUMBER)<br>98 DK<br>99 R	C109<br>__ MINUTES<br>**OR**<br>___ HOURS<br>98 DK<br>99 R	C110<br>___ PER WEEK<br>**OR**<br>___ PER MONTH<br>98 DK<br>99 R
C111 Did you perform weightlifting or use of exercise machines?<br>1 YES<br>3 NO (C115)<br>8 DK (C115)<br>9 R (C115)	C112<br>(ENTER NUMBER)<br>98 DK<br>99 R	C113<br>__ MINUTES<br>**OR**<br>___ HOURS<br>98 DK<br>99 R	C114<br>___ PER WEEK<br>**OR**<br>___ PER MONTH<br>98 DK<br>99 R
C115 Did you perform hiking uphill or backpacking?<br>1 YES<br>3 NO (C119)<br>8 DK (C119)<br>9 R (C119)	C116<br>(ENTER NUMBER)<br>98 DK<br>99 R	C117<br>__ MINUTES<br>**OR**<br>___ HOURS<br>98 DK<br>99 R	C118<br>___ PER WEEK<br>**OR**<br>___ PER MONTH<br>98 DK<br>99 R
C119 Did you perform vigorous swimming?<br>1 YES<br>3 NO (C123)<br>8 DK (C123)<br>9 R (C123)	C120<br>(ENTER NUMBER)<br>98 DK<br>99 R	C121<br>__ MINUTES<br>**OR**<br>___ HOURS<br>98 DK<br>99 R	C122<br>___ PER WEEK<br>**OR**<br>___ PER MONTH<br>98 DK<br>99 R
C123 Did you perform scrubbing floors or mowing lawn with a non-power mower?<br>1 YES<br>3 NO (C127)<br>8 DK (C127)	C124<br>(ENTER NUMBER)<br>98 DK<br>99 R	C125<br>__ MINUTES<br>**OR**<br>___ HOURS<br>98 DK	C126<br>___ PER WEEK<br>**OR**<br>___ PER MONTH<br>98 DK

9 R (C127)		99 R	99 R
C127 Did you perform chopping or splitting wood, gardening with heavy tools or other heavy labor such as shoveling dirt?<br>1 YES<br>3 NO (C131)<br>8 DK (C131)<br>9 R (C131)	C128<br>(ENTER NUMBER)<br>98 DK<br>99 R	C129<br>__ MINUTES<br>**OR**<br>___ HOURS<br>98 DK<br>99 R	C130<br>___ PER WEEK<br>**OR**<br>___ PER MONTH<br>98 DK<br>99 R

(SCREEN FOR AGE OF RESPONDENT AT BEGINNING OF REFERENT PERIOD)

C131Compared to your level of physical activity at leisure and at home between (FILL REFERENT PERIOD) how would you describe your leisure time and home activity when you were 60 years old. Would it be: less, about the same or more physical activity?

1 LESS PHYSICAL ACTIVITY
3 ABOUT THE SAME AMOUNT OF PHYSICAL ACTIVITY OR (C133)
5 MORE PHYSICAL ACTIVITY
8 DK (C133)
9 R (C133)

C132What per cent (FILL MORE OR LESS) would that be?

__ ENTER PER CENT
98 DK
99 R

C133Compared to your level of physical activity at leisure and at home between (FILL REFERENT PERIOD), how would you describe your leisure time and home activity when you were 45 years old. Would it be: less, about the same or more physical activity?

1 LESS PHYSICAL ACTIVITY
3 ABOUT THE SAME AMOUNT OF PHYSICAL ACTIVITY OR (C135)
5 MORE PHYSICAL ACTIVITY
8 DK (C135)
9 R (C135)

C134What per cent (FILL MORE OR LESS) would that be?

__ ENTER PER CENT
98 DK
99 R

C135Compared to your level of physical activity at leisure and at home between (FILL REFERENT PERIOD), how would you describe your leisure time and home activity when you were 30 years old. Would it be: less, about the same or more physical activity?

1 LESS PHYSICAL ACTIVITY
3 ABOUT THE SAME AMOUNT OF PHYSICAL ACTIVITY OR (NEXT SECTION)
5 MORE PHYSICAL ACTIVITY
8 DK (NEXT SECTION)
9 R (NEXT SECTION)

C136What per cent (FILL MORE OR LESS) would that be?

__ ENTER PER CENT
98 DK
99 R(ALL RESPONDENTS GO TO SECTION D: DIET HISTORY WHICH IS NOT BEING PROGRAMMED ON CASES)

C019Please look at this card and tell me, between (FILL REFERENT PERIOD), did you perform any moderate activities such as standing or walking not carrying heavy objects at this job?

1 YES

3 NO (C022)
8 DK (C022)
9 R (C022)

C020 Between (FILL REFERENT PERIOD), in how many months did you perform moderate activity at this job?

__ ENTER NUMBER OF MONTHS
98 DK
99 R

C021 Between (FILL REFERENT PERIOD), on average, how many hours per week did you perform moderate activity at this job?

___ ENTER NUMBER OF HOURS
998 DK
999 R

C022 Please look at this card and tell me, between (FILL REFERENT PERIOD), did you perform any vigorous activities such as construction, shoveling dirt or other heavy labor at this job?

1 YES
3 NO (C025)
8 DK (C025)
9 R (C025)

C023 Between (FILL REFERENT PERIOD), in how many months did you perform vigorous activity at this job?

__ ENTER NUMBER OF MONTHS
98 DK
99 R

C024 Between (FILL REFERENT PERIOD), on average, how many hours per week did you perform vigorous activity at this job?

___ ENTER NUMBER OF HOURS
998 DK
999 R

C025 Between (FILL REFERENT PERIOD) were you employed at any other job for pay, either full-time or part-time including self employment?

1 YES
3 NO (C039)
8 DK (C039)
9 R (C039)

C026 What was your complete job title?

____
8 DK
9 R

C027 In what kind of business or industry did you work; that is, what product was made or what service was given?

____

8 DK
9 R

C028What were your main duties on that job?

____

____

8 DK
9 R

C029In what month and year did you start working at this job?

ENTER MONTH AND YEAR: __MONTH __YR
8 DK
9 R

C030In what month and year did you stop working at this job?

ENTER MONTH AND YEAR: __MONTH __YR
6 STILL WORKING
8 DK
9 R

C031Please look at this card and tell me, between (FILL REFERENT PERIOD), did you perform any moderate activities such as standing or walking not carrying heavy objects at this job?

1 YES
3 NO (C034)
8 DK (C034)
9 R (C034)

C032Between (FILL REFERENT PERIOD), in how many months did you perform moderate activity at this job?

__ ENTER NUMBER OF MONTHS
98 DK
99 R

C033Between (FILL REFERENT PERIOD), on average, how many hours per week did you perform moderate activity at this job?

___ ENTER NUMBER OF HOURS
998 DK
999 R

C034Please look at this card and tell me, between (FILL REFERENT PERIOD), did you perform any vigorous activities such as construction, shoveling dirt or other heavy labor at this job?

1 YES
3 NO (C037)
8 DK (C037)
9 R (C037)

C035Between (FILL REFERENT PERIOD), in how many months did you perform vigorous activity at this job?

___ ENTER NUMBER OF MONTHS
98 DK
99 R

C036 Between (FILL REFERENT PERIOD), on average, how many hours per week did you perform vigorous activity at this job?

___ ENTER NUMBER OF HOURS
998 DK
999 R

C037 Between (FILL REFERENT PERIOD) were you working either fewer hours or at a lower physical effort because of injury, a recent illness or a long-term disease?

1 YES, INJURY (C038)
3 YES, RECENT ILLNESS (C038)
5 YES, LONG TERM ILLNESS (C038)
7 NO (C075)
8 DK (C075)
9 R (C075)

C038 In what year did this change occur?

___ ENTER YEAR
98 DK
99 R
(C075)

C039 Before (FILL REFERENT DATE), since age 18, have you ever been employed at a job for pay, either full-time or part-time including self employment?

1 YES
3 NO (C081)
8 DK (C081)
9 R (C081)

C040 What was your complete job title for the last job you held?

____
8 DK
9 R

C041 In what kind of business or industry did you work; that is, what product was made or what service was given?

____
8 DK
9 R

C042 What were your main duties on that job?

____
____
8 DK
9 R

C043 In what month and year did you start working at this job?

ENTER MONTH AND YEAR: __MONTH __YR
8 DK
9 R

C044 In what month and year did you stop working at this job?

ENTER MONTH AND YEAR: __MONTH __YR
8 DK
9 R

C045 Please look at this card and tell me, between (FILL LAST YEAR EMPLOYED) did you perform any moderate activities such as standing or walking not carrying heavy objects at this job?

1 YES
3 NO (C048)
8 DK (C048)
9 R (C048)

C046 Between (FILL LAST YEAR EMPLOYED), in how many months did you perform moderate activity at this job?

__ ENTER NUMBER OF MONTHS
98 DK
99 R

C047 Between (FILL LAST YEAR EMPLOYED), on average, how many hours per week did you perform moderate activity at this job?
___ ENTER NUMBER OF HOURS
998 DK
999 R

C048 Please look at this card and tell me, between (FILL LAST YEAR EMPLOYED) did you perform any vigorous activities such as construction, shoveling dirt or other heavy labor at this job?

1 YES
3 NO (C051)
8 DK (C051)
9 R (C051)

C049 Between (FILL LAST YEAR EMPLOYED), in how many months did you perform vigorous activity at this job?

__ ENTER NUMBER OF MONTHS
98 DK
99 R

C050Between (FILL LAST YEAR EMPLOYED), on average, how many hours per week did you perform vigorous activity at this job?

___ ENTER NUMBER OF HOURS
998 DK
999 R

C051Between (FILL LAST YEAR EMPLOYED) were you employed at any other job for pay, either full-time or part-time including self employment?

1 YES
3 NO (C075)
8 DK (C075)
9 R (C075)

C052What was your complete job title?

____

8 DK
9 R

C053In what kind of business or industry did you work; that is, what product was made or what service was given?

____

8 DK
9 R

C054What were your main duties on that job?

____

____

8 DK
9 R

C055In what month and year did you start working at this job?

ENTER MONTH AND YEAR: __MONTH __YR
8 DK
9 R

C056In what month and year did you stop working at this job?

ENTER MONTH AND YEAR: __MONTH __YR
8 DK
9 R

C057Please look at this card and tell me, between (FILL LAST YEAR EMPLOYED) did you perform any moderate activities such as standing or walking not carrying heavy objects at this job?

1 YES
3 NO (C060)
8 DK (C060)
9 R (C060)

C058Between (FILL LAST YEAR EMPLOYED), in how many months did you perform moderate activity at this job?

__ ENTER NUMBER OF MONTHS
98 DK
99 R

C059Between (FILL LAST YEAR EMPLOYED), on average, how many hours per week did you perform moderate activity at this job?
___ ENTER NUMBER OF HOURS
998 DK
999 R

C060Please look at this card and tell me, between (FILL LAST YEAR EMPLOYED) did you perform any vigorous activities such as construction, shoveling dirt or other heavy labor at this job?

1 YES
3 NO (C063)
8 DK (C063)
9 R (C063)

C061Between (FILL LAST YEAR EMPLOYED), in how many months did you perform vigorous activity at this job?

__ ENTER NUMBER OF MONTHS
98 DK
99 R

C062Between (FILL LAST YEAR EMPLOYED), on average, how many hours per week did you perform vigorous activity at this job?

___ ENTER NUMBER OF HOURS
998 DK
999 R

C063Between (FILL LAST YEAR EMPLOYED) were you employed at any other job for pay, either full-time or part-time including self employment?

1 YES
3 NO (C075)
8 DK (C075)
9 R (C075)

C064 What was your complete job title?

____

8 DK
9 R

C065 In what kind of business or industry did you work; that is, what product was made or what service was given?

____

8 DK
9 R

C066 What were your main duties on that job?

____

____

8 DK
9 R

C067 In what month and year did you start working at this job?

ENTER MONTH AND YEAR: __MONTH __YR
8 DK
9 R

C068 In what month and year did you stop working at this job?

ENTER MONTH AND YEAR: __MONTH __YR
8 DK
9 R

C069 Please look at this card and tell me, between (FILL LAST YEAR EMPLOYED) did you perform any moderate activities such as standing or walking not carrying heavy objects at this job?

1 YES
3 NO (C072)
8 DK (C072)
9 R (C072)

C070 Between (FILL LAST YEAR EMPLOYED), in how many months did you perform moderate activity at this job?

__ ENTER NUMBER OF MONTHS
98 DK
99 R

C071 Between (FILL LAST YEAR EMPLOYED), on average, how many hours per week did you perform moderate activity at this job?
___ ENTER NUMBER OF HOURS
998 DK
999 R

C072Please look at this card and tell me, between (FILL LAST YEAR EMPLOYED) did you perform any vigorous activities such as construction, shoveling dirt or other heavy labor at this job?

1 YES
3 NO (C075)
8 DK (C075)
9 R (C075)

C073Between (FILL LAST YEAR EMPLOYED), in how many months did you perform vigorous activity at this job?

__ ENTER NUMBER OF MONTHS
98 DK
99 R

C074Between (FILL LAST YEAR EMPLOYED), on average, how many hours per week did you perform vigorous activity at this job?

___ ENTER NUMBER OF HOURS
998 DK
999 R

(SCREEN ALL EVER EMPLOYED SINCE AGE 18 FOR AGE NOW--IF EMPLOYED DURING THE RP THEN DO NOT ASK ABOUT 60 IF 62 OR LESS NOW, DO NOT ASK 45 IF 47 OR LESS NOW AND 30 IF 32 OR LESS NOW. IF LAST YR EMPLOYED IS OUTSIDE OF THE RP, THE R WILL SKIP THE QUESTION IF AGE AT THE BEGINNING THE LAST YEAR EMPLOYED IS LESS THAN OR EQUAL TO THE AGE WE ARE ASKING ABOUT)

C075Compared to your level of physical activity at your job/s between (FILL EITHER REFERENT PERIOD OR LAST YEAR WORKED), how would you describe your level of physical activity at work when you were 60 years old. Would it be: less, about the same or more physical activity?

1 LESS PHYSICAL ACTIVITY
3 ABOUT THE SAME AMOUNT OF PHYSICAL ACTIVITY OR (C077)
5 MORE PHYSICAL ACTIVITY
7 NOT WORKING AT 60 (C077)
8 DK (C077)
9 R (C077)

C076What per cent (FILL MORE OR LESS) would that be?

__ ENTER PER CENT
98 DK
99 R

C077Compared to your level of physical activity at your job/s between (FILL EITHER REFERENT PERIOD OR LAST YEAR WORKED), how would you describe your level of physical activity at work when you were 45 years old. Would it be: less, about the same or more physical activity?

1 LESS PHYSICAL ACTIVITY
3 ABOUT THE SAME AMOUNT OF PHYSICAL ACTIVITY OR (C079)
5 MORE PHYSICAL ACTIVITY
7 NOT WORKING AT 45 (C079)
8 DK (C079)
9 R (C079)

C078What per cent (FILL MORE OR LESS) would that be?

__ ENTER PER CENT
98 DK
99 R

C079Compared to your level of physical activity at your job/s between (FILL EITHER REFERENT PERIOD OR LAST YEAR WORKED), how would you describe your level of physical activity at work when you were 30 years old. Would it be: less, about the same or more physical activity?

1 LESS PHYSICAL ACTIVITY
3 ABOUT THE SAME AMOUNT OF PHYSICAL ACTIVITY OR (C081)
5 MORE PHYSICAL ACTIVITY
7 NOT WORKING AT 30 (C081)
8 DK (C081)
9 R (C081)

C080What per cent (FILL MORE OR LESS) would that be?

__ ENTER PER CENT
98 DK
99 R

C081Between (FILL REFERENT PERIOD), how many flights of stairs per week did you climb? A flight of stairs is ten steps.

___ ENTER NUMBER
998 DK
999 R

C082Between (FILL REFERENT PERIOD), how many hours of sleep did you get on an average day?

__ ENTER NUMBER
98 DK
99 R

CTX2The next questions ask about your level of physical activity at leisure and around the house between (FILL REFERENT PERIOD). Do not include any occupational activities which you just mentioned.

For each activity indicate whether you performed the activity between (FILL REFERENT PERIOD). Answer "yes" only for those activities performed for an hour or more during any month. Consider the time spent in the activity only. For example, riding a chair lift skiing is not considered time skiing; sitting by a swimming pool should not be included in time spent swimming.

CTX3(INTV: GIVE RESPONDENT MODERATE ACTIVITY CUE CARD) Did you perform any of the following activities at a MODERATE PACE for at least one hour total time in any month between (FILL REFERENT PERIOD)? Moderate activities are those which are done at a more moderate pace than are more strenuous activities, or those which make you sweat or get out of breathe.

C083Did you perform sports at a moderate pace such as softball, shooting baskets, swimming, skiing, bowling or golfing?

1 YES
3 NO (C087)
8 DK (C087)
9 R (C087)

C084Between (FILL REFERENT PERIOD), in how many months was this activity performed?

(ENTER NUMBER)
98 DK
99 R

C085What was the average time per session you performed this activity?

(ENTER NUMBER)
98 DK
99 R

085CINTV: ENTER MINUTES/HOURS

1 MINUTES
3 HOURS
8 DK
9 R

C086What was the average number of days per week or month you performed this activity?

(ENTER NUMBER)
98 DK
99 R

086CINTV: ENTER WEEK/MONTH

1 WEEK
3 MONTH
8 DK
9 R

C087Did you perform moderate paced walks or hikes?

1 YES
3 NO (C091)
8 DK (C091)
9 R (C091)

C088Between (FILL REFERENT PERIOD), in how many months was this activity performed?

(ENTER NUMBER)
98 DK
99 R

C089What was the average time per session you performed this activity?

(ENTER NUMBER)
98 DK
99 R

089CINTV: ENTER MINUTES/HOURS

1 MINUTES
3 HOURS
8 DK
9 R

C090What was the average number of days per week or month you performed this activity?

(ENTER NUMBER)
98 DK
99 R

090CINTV: ENTER WEEK/MONTH

1 WEEK
3 MONTH
8 DK
9 R

C091Did you perform moderate paced home exercise, calisthenics or dancing?

1 YES
3 NO (C095)
8 DK (C095)
9 R (C095)

C092Between (FILL REFERENT PERIOD), in how many months was this activity performed?

(ENTER NUMBER)
98 DK
99 R

C093What was the average time per session you performed this activity?

(ENTER NUMBER)
98 DK
99 R

093CINTV: ENTER MINUTES/HOURS

1 MINUTES
3 HOURS
8 DK
9 R

C094What was the average number of days per week or month you performed this activity?

(ENTER NUMBER)
98 DK
99 R

094CINTV: ENTER WEEK/MONTH

1 WEEK
3 MONTH
8 DK
9 R

C095Did you perform moderate paced home maintenance, gardening, painting, raking, sweeping, mowing with a power mower, cleaning or vacuuming?

1 YES
3 NO (C099)
8 DK (C099)
9 R (C099)

C096Between (FILL REFERENT PERIOD), in how many months was this activity performed?

(ENTER NUMBER)
98 DK
99 R

C097What was the average time per session you performed this activity?

(ENTER NUMBER)
98 DK
99 R

097CINTV: ENTER MINUTES/HOURS

1 MINUTES
3 HOURS
8 DK
9 R

C098What was the average number of days per week or month you performed this activity?

(ENTER NUMBER)
98 DK
99 R

098CINTV: ENTER WEEK/MONTH

1 WEEK

3 MONTH
8 DK
9 R

(INTV: GIVE RESPONDENT VIGOROUS ACTIVITY CUE CARD)

Of the following activities, which ones did you perform at a STRENUOUS OR VIGOROUS PACE for at least one hour total time in any month between (FILL REFERENT PERIOD):	Between (FILL REFERENT PERIOD), in how many months was this activity performed?	What was the average time per session you performed this activity?	What was the average number of days per week or month you performed this activity?
C099 Did you perform vigorous racket sports and other strenuous sports such as tennis, squash or basketball?<br>1 YES<br>3 NO (C103)<br>8 DK (C103)<br>9 R (C103)	C100<br>(ENTER NUMBER)<br>98 DK<br>99 R	C101<br>__ MINUTES<br>**OR**<br>___ HOURS<br>98 DK<br>99 R	C102<br>___ PER WEEK<br>**OR**<br>___ PER MONTH<br>98 DK<br>99 R
C103 Did you perform jogging, running or biking?<br>1 YES<br>3 NO (C107)<br>8 DK (C107)<br>9 R (C107)	C104<br>(ENTER NUMBER)<br>98 DK<br>99 R	C105<br>__ MINUTES<br>**OR**<br>___ HOURS<br>98 DK<br>99 R	C106<br>___ PER WEEK<br>**OR**<br>___ PER MONTH<br>98 DK<br>99 R
C107 Did you perform vigorous exercise class or vigorous dance<br>1 YES<br>3 NO (C111)<br>8 DK (C111)<br>9 R (C111)	C108<br>(ENTER NUMBER)<br>98 DK<br>99 R	C109<br>__ MINUTES<br>**OR**<br>___ HOURS<br>98 DK<br>99 R	C110<br>___ PER WEEK<br>**OR**<br>___ PER MONTH<br>98 DK<br>99 R
C111 Did you perform weightlifting or use of exercise machines?<br>1 YES<br>3 NO (C115)<br>8 DK (C115)<br>9 R (C115)	C112<br>(ENTER NUMBER)<br>98 DK<br>99 R	C113<br>__ MINUTES<br>**OR**<br>___ HOURS<br>98 DK<br>99 R	C114<br>___ PER WEEK<br>**OR**<br>___ PER MONTH<br>98 DK<br>99 R
C115 Did you perform hiking uphill or backpacking?<br>1 YES<br>3 NO (C119)<br>8 DK (C119)<br>9 R (C119)	C116<br>(ENTER NUMBER)<br>98 DK<br>99 R	C117<br>__ MINUTES<br>**OR**<br>___ HOURS<br>98 DK<br>99 R	C118<br>___ PER WEEK<br>**OR**<br>___ PER MONTH<br>98 DK<br>99 R
C119 Did you perform vigorous swimming?<br>1 YES<br>3 NO (C123)<br>8 DK (C123)<br>9 R (C123)	C120<br>(ENTER NUMBER)<br>98 DK<br>99 R	C121<br>__ MINUTES<br>**OR**<br>___ HOURS<br>98 DK<br>99 R	C122<br>___ PER WEEK<br>**OR**<br>___ PER MONTH<br>98 DK<br>99 R
C123 Did you perform scrubbing floors or mowing lawn with a non-power mower?<br>1 YES<br>3 NO (C127)<br>8 DK (C127)	C124<br>(ENTER NUMBER)<br>98 DK<br>99 R	C125<br>__ MINUTES<br>**OR**<br>___ HOURS<br>98 DK	C126<br>___ PER WEEK<br>**OR**<br>___ PER MONTH<br>98 DK

9 R (C127)		99 R	99 R
C127 Did you perform chopping or splitting wood, gardening with heavy tools or other heavy labor such as shoveling dirt?<br>1 YES<br>3 NO (C131)<br>8 DK (C131)<br>9 R (C131)	C128<br>(ENTER NUMBER)<br>98 DK<br>99 R	C129<br>__ MINUTES<br>**OR**<br>___ HOURS<br>98 DK<br>99 R	C130<br>___ PER WEEK<br>**OR**<br>___ PER MONTH<br>98 DK<br>99 R

(SCREEN FOR AGE OF RESPONDENT AT BEGINNING OF REFERENT PERIOD)

C131 Compared to your level of physical activity at leisure and at home between (FILL REFERENT PERIOD) how would you describe your leisure time and home activity when you were 60 years old. Would it be: less, about the same or more physical activity?

1 LESS PHYSICAL ACTIVITY
3 ABOUT THE SAME AMOUNT OF PHYSICAL ACTIVITY OR (C133)
5 MORE PHYSICAL ACTIVITY
8 DK (C133)
9 R (C133)

C132 What per cent (FILL MORE OR LESS) would that be?

__ ENTER PER CENT
98 DK
99 R

C133 Compared to your level of physical activity at leisure and at home between (FILL REFERENT PERIOD), how would you describe your leisure time and home activity when you were 45 years old. Would it be: less, about the same or more physical activity?

1 LESS PHYSICAL ACTIVITY
3 ABOUT THE SAME AMOUNT OF PHYSICAL ACTIVITY OR (C135)
5 MORE PHYSICAL ACTIVITY
8 DK (C135)
9 R (C135)

C134 What per cent (FILL MORE OR LESS) would that be?

__ ENTER PER CENT
98 DK
99 R

C135 Compared to your level of physical activity at leisure and at home between (FILL REFERENT PERIOD), how would you describe your leisure time and home activity when you were 30 years old. Would it be: less, about the same or more physical activity?

1 LESS PHYSICAL ACTIVITY
3 ABOUT THE SAME AMOUNT OF PHYSICAL ACTIVITY OR (NEXT SECTION)
5 MORE PHYSICAL ACTIVITY
8 DK (NEXT SECTION)
9 R (NEXT SECTION)

C136 What per cent (FILL MORE OR LESS) would that be?

__ ENTER PER CENT
98 DK
99 R (ALL RESPONDENTS GO TO SECTION D: DIET HISTORY WHICH IS NOT BEING PROGRAMMED ON CASES)

SECTION C
PHYSICAL ACTIVITY

CTX1Now, I am going to ask about your level of physical activity. I will ask about your level of physical activity at work and then at leisure and at home.

	FIRST JOB	SECOND JOB	THIRD JOB
Between (FILL REFERENT PERIOD) were you employed at a job for pay, either full-time or part-time including self employment?	C001<br>1 YES (C002)<br>3 NO (C039)<br>8 DK (C039)	C013<br>1 YES (C014)<br>3 NO (C039)<br>8 DK (C039)	C025<br>1 YES (C026)<br>3 NO (C039)<br>8 DK (C039)
What was your complete job title?	C002<br>____________________<br>__8 DK	C014<br>_____________________<br>_<br>8 DK	C026<br>___________________<br>8 DK
In what kind of business or industry did you work; that is, what product was made or what service was given?	C003<br>____________________<br>__8 DK	C015<br>_____________________<br>_<br>8 DK	C027<br>___________________<br>8 DK
What were your main duties on that job?	C004<br>____________________<br>__<br>____________________<br>__<br>8 DK	C016<br>_____________________<br>_<br>_____________________<br>_<br>8 DK	C028<br>___________________<br>___________________<br>___8 DK
In what month and year did you start working at this job? ENTER MONTH AND YEAR	C005<br>__ MONTH __ YEAR<br>8 DK	C017<br>__ MONTH __ YEAR<br>8 DK	C029<br>__ MONTH __ YEAR<br>8 DK
In what month and year did you stop working at this job?	C006<br>__ MONTH __ YEAR<br>6 STILL WORKING<br>8 DK	C018<br>__ MONTH __ YEAR<br>6 STILL WORKING<br>8 DK	C030<br>__ MONTH __ YEAR<br>6 STILL WORKING<br>8 DK
Please look at this card and tell me, between [FILL REFERENT PERIOD], did you perform any moderate activities such as standing or walking not carrying heavy objects at this job?	C007<br>1 YES (C008)<br>3 NO (C010)<br>8 DK (C010)	C019<br>1 YES (C020)<br>3 NO (C022)<br>8 DK (C022)	C031<br>1 YES (C032)<br>3 NO (C034)<br>8 DK (C034)
Between [FILL REFERENT PERIOD], in how many months did you perform moderate activity at this job?	C008<br>___ ENTER # OF MONTHS<br>98 DK	C020<br>___ ENTER # OF MONTHS<br>98 DK	C032<br>___ ENTER # OF MONTHS<br>98 DK
Between [FILL REFERENT PERIOD], on average, how many hours per week did you perform moderate activity at this job?	C009<br>___ ENTER # OF HOURS<br>998 DK	C021<br>___ ENTER # OF HOURS<br>998 DK	C033<br>___ ENTER # OF HOURS<br>998 DK
Please look at this card and tell me, between [FILL REFERENT PERIOD], did you perform any vigorous activities such as construction, shoveling dirt or other heavy labor at this job?	C010<br>1 YES (C011)<br>3 NO (C013)<br>8 DK (C013)	C022<br>1 YES (C023)<br>3 NO (C025)<br>8 DK (C025)	C034<br>1 YES (C035)<br>3 NO (C037)<br>8 DK (C037)
Between [FILL REFERENT PERIOD], in how many	C011	C023	C035

months did you perform vigorous activity at this job?	___ ENTER # OF MONTHS<br>98 DK	___ ENTER # OF MONTHS<br>98 DK	___ ENTER # OF MONTHS<br>98 DK
Between [FILL REFERENT PERIOD], on average, how many hours per week did you perform vigorous activity at this job?	C012<br>___ ENTER # 0F HOURS<br>998 DK	C024<br>___ ENTER # 0F HOURS<br>998 DK	C036<br>___ ENTER # 0F HOURS<br>998 DK

C037Between (FILL REFERENT PERIOD) were you working either fewer hours or at a lower physical effort because of injury, a recent illness or a long-term disease?

1 YES, INJURY (C038)
3 YES, RECENT ILLNESS (C038)
5 YES, LONG TERM ILLNESS (C038)
7 NO (C075)
8 DK (C075)

C038In what year did this change occur?

__ ENTER YEAR
98 DK

	LAST JOB HELD	OTHER JOB	OTHER JOB
Before/between (FILL REFERENT DATE/FILL LAST YEAR EMPLOYED) since age 18, have/were you ever been employed at a job for pay, either full-time or part-time including self employment?	C039<br>1 YES (C040)<br>3 NO (C081)<br>8 DK (C081)	C051<br>1 YES (C052)<br>3 NO (C075)<br>8 DK (C075)	C063<br>1 YES (C064)<br>3 NO (C075)<br>8 DK (C075)
What was your complete job title?	C040<br>____________________<br>__8 DK	C052<br>_____________________<br>_<br>8 DK	C064<br>____________________<br>8 DK
In what kind of business or industry did you work; that is, what product was made or what service was given?	C041<br>____________________<br>__8 DK	C053<br>_____________________<br>_<br>8 DK	C065<br>____________________<br>8 DK
What were your main duties on that job?	C042<br>____________________<br>__<br>____________________<br>__<br>8 DK	C054<br>_____________________<br>_<br>_____________________<br>_<br>8 DK	C066<br>____________________<br>____________________<br>___8 DK
In what month and year did you start working at this job? ENTER MONTH AND YEAR	C043<br>__ MONTH __ YEAR<br>8 DK	C055<br>__ MONTH __ YEAR<br>8 DK	C067<br>__ MONTH __ YEAR<br>8 DK
In what month and year did you start working at this job?	C044<br>__ MONTH __ YEAR<br>8 DK	C056<br>__ MONTH __ YEAR<br>8 DK	C068<br>__ MONTH __ YEAR<br>8 DK
Please look at this card and tell me, between [FILL LAST YEAR EMPLOYED] did you perform any moderate activities such as standing or walking not carrying heavy objects at this job?	C045<br>1 YES (C046)<br>3 NO (C048)<br>8 DK (C048)	C057<br>1 YES (C058)<br>3 NO (C060)<br>8 DK (C060)	C069<br>1 YES (C070)<br>3 NO (C072)<br>8 DK (C072)

Between [FILL LAST YEAR EMPLOYED] in how many months did you perform moderate activity at this job?	C046<br>___ ENTER #<br>OF MONTHS<br>98 DK	C058<br>___ ENTER #<br>OF MONTHS<br>98 DK	C070<br>___ ENTER #<br>OF MONTHS<br>98 DK
Between [FILL LAST YEAR EMPLOYED] on average, how many hours per week did you perform moderate activity at this job?	C047<br>___ ENTER #<br>OF HOURS<br>998 DK	C059<br>___ ENTER #<br>OF HOURS<br>998 DK	C071<br>___ ENTER #<br>OF HOURS<br>998 DK
Please look at this card and tell me, between [FILL LAST YEAR EMPLOYED] did you perform any vigorous activities such as construction, shoveling dirt or other heavy labor at this job?	C048<br>1 YES (C049)<br>3 NO (C051)<br>8 DK (C051)	C060<br>1 YES (C061)<br>3 NO (C063)<br>8 DK (C063)	C072<br>1 YES (C073)<br>3 NO (C075)<br>8 DK (C075)

	LAST JOB HELD	OTHER JOB	OTHER JOB
Between [FILL LAST YEAR EMPLOYED] in how many months did you perform vigorous activity at this job?	C049<br>___ ENTER #<br>OF MONTHS<br>98 DK	C061<br>___ ENTER #<br>OF MONTHS<br>98 DK	C073<br>___ ENTER #<br>OF MONTHS<br>98 DK
Between [FILL LAST YEAR EMPLOYED] on average, how many hours per week did you perform vigorous activity at this job?	C050<br>___ ENTER #<br>0F HOURS<br>998 DK	C062<br>___ ENTER #<br>0F HOURS<br>998 DK	C074<br>___ ENTER #<br>0F HOURS<br>998 DK

(SCREEN ALL EVER EMPLOYED SINCE AGE 18 FOR AGE NOW--IF EMPLOYED DURING THE RP THEN DO NOT ASK ABOUT 60 IF 62 OR LESS NOW, DO NOT ASK 45 IF 47 OR LESS NOW AND 30 IF 32 OR LESS NOW. IF LAST YR EMPLOYED IS OUTSIDE OF THE RP, THE R WILL SKIP THE QUESTION IF AGE AT THE BEGINNING THE LAST YEAR EMPLOYED IS LESS THAN OR EQUAL TO THE AGE WE ARE ASKING ABOUT)

	60 YEARS OLD	45 YEARS OLD	30 YEARS OLD
Compared to your level of physical activity at your job/s between (FILL EITHER REFERENT PERIOD OR LAST YEAR WORKED), how would you describe your level of physical activity at work when you were (FILL AGE). Would it be: less, about the same or more physical activity?	C075<br>1 LESS PHYSICAL ACTIVITY<br>3 ABOUT THE SAME AMOUNT OF PHYSICAL ACTIVITY OR (C077)<br>5 MORE PHYSICAL ACTIVITY<br>7 NOT WORKING AT 60 (C077)<br>8 DK (C077)	C077<br>1 LESS PHYSICAL ACTIVITY (C078)<br>3 ABOUT THE SAME AMOUNT OF PHYSICAL ACTIVITY OR (C079)<br>5 MORE PHYSICAL ACTIVITY (C078)<br>7 NOT WORKING AT 45 (C079)<br>8 DK (C079)	C079<br>1 LESS PHYSICAL ACTIVITY (C080)<br>3 ABOUT THE SAME AMOUNT OF PHYSICAL ACTIVITY OR (C081)<br>5 MORE PHYSICAL ACTIVITY (C080)<br>7 NOT WORKING AT 60 (C081)<br>8 DK (C077)
What per cent (FILL MORE OR LESS) would that be?	C076<br>_ ENTER PER CENT<br>98 DK	C078<br>_ ENTER PER CENT<br>98 DK	C080<br>_ ENTER PER CENT<br>98 DK

C081Between (FILL REFERENT PERIOD), how many flights of stairs per week did you climb? A flight of stairs is ten steps.

__ ENTER NUMBER
998 DK

C082Between (FILL REFERENT PERIOD), how many hours of sleep did you get on an average day?

__ ENTER NUMBER
98 DK

CTX2The next questions ask about your level of physical activity at leisure and around the house between (FILL REFERENT PERIOD). Do not include any occupational activities which you just mentioned.

For each activity indicate whether you performed the activity between (FILL REFERENT PERIOD). Answer "yes" only for those activities performed for an hour or more during any month. Consider the time spent in the activity only. For example, riding a chair lift skiing is not considered time skiing; sitting by a swimming pool should not be included in time spent swimming.

CTX3(INTV: GIVE RESPONDENT MODERATE ACTIVITY CUE CARD)

Did you perform any of the following activities at a MODERATE PACE for at least one hour total time in any month between (FILL REFERENT PERIOD)? Moderate activities are those which are done at a more moderate pace than are more strenuous activities, or those which make you sweat or get out of breathe.	Between (FILL REFERENT PERIOD), in how many months was this activity performed?	What was the average time per session you performed this activity?	What was the average number of days per week or month you performed this activity?
C083<br>Did you perform sports at a moderate pace such as softball, shooting baskets, swimming, skiing, bowling or golfing?<br>1 YES (C084)<br>3 NO (C087)<br>8 DK (C087)	C084<br>(ENTER NUMBER)<br>98 DK	C085<br>1 MINUTES ___<br>3 HOURS ___<br>8 DK	C086<br>1 WEEK ___<br>3 MONTH ___<br>8 DK
C087<br>Did you perform moderate paced walks or hikes?<br>1 YES (C088)<br>3 NO (C091)<br>8 DK (C091)	C088<br>(ENTER NUMBER)<br>98 DK	C089<br>1 MINUTES ___<br>3 HOURS ___<br>8 DK	C090<br>1 WEEK ___<br>3 MONTH ___<br>8 DK
C091<br>Did you perform moderate paced home exercise, calisthenics or dancing?<br>1 YES (C092)<br>3 NO (C095)<br>8 DK (C095)	C092<br>(ENTER NUMBER)<br>98 DK	C093<br>1 MINUTES ___<br>3 HOURS ___<br>8 DK	C094<br>1 WEEK ___<br>3 MONTH ___<br>8 DK
C095<br>Did you perform moderate paced home maintenance, gardening, painting, raking, sweeping, mowing with a power mower, cleaning or vacuuming?<br>1 YES (C096)<br>3 NO (C099)<br>8 DK (C099)	C096<br>(ENTER NUMBER)<br>98 DK	C097<br>1 MINUTES ___<br>3 HOURS ___<br>8 DK	C098<br>1 WEEK ___<br>3 MONTH ___<br>8 DK

(INTV: GIVE RESPONDENT VIGOROUS ACTIVITY CUE CARD)

Of the following activities, which ones did you perform at a STRENUOUS OR VIGOROUS PACE for at least one hour total time in any month between (FILL REFERENT PERIOD).	Between (FILL REFERENT PERIOD), in how many months was this activity performed?	What was the average time per session you performed this activity?	What was the average number of days per week or month you performed this activity?
C099 Did you perform vigorous racket sports and other strenuous sports such as tennis, squash or basketball?<br>1 YES (C100)<br>3 NO (C103)<br>8 DK (C103)	C100<br>(ENTER NUMBER)<br>98 DK	C101<br>1 MINUTES ___<br>3 HOURS ___<br>8 DK	C102<br>1 WEEK ___<br>3 MONTH ___<br>8 DK
C103 Did you perform jogging, running or biking?<br>1 YES (C104)<br>3 NO (C107)<br>8 DK (C107)	C104<br>(ENTER NUMBER)<br>98 DK	C105<br>1 MINUTES ___<br>3 HOURS ___<br>8 DK	C106<br>1 WEEK ___<br>3 MONTH ___<br>8 DK
C107 Did you perform vigorous exercise class or vigorous dance<br>1 YES (C108)	C108<br>(ENTER	C109<br>1 MINUTES ___	C110<br>1 WEEK ___

3 NO (C111)<br>8 DK (C111)	NUMBER)<br>98 DK	3 HOURS ___<br>8 DK	3 MONTH ___<br>8 DK

Of the following activities, which ones did you perform at a STRENUOUS OR VIGOROUS PACE for at least one hour total time in any month between (FILL REFERENT PERIOD).	Between (FILL REFERENT PERIOD), in how many months was this activity performed?	What was the average time per session you performed this activity?	What was the average number of days per week or month you performed this activity?
C111 Did you perform weightlifting or use of exercise machines?<br>1 YES (C112)<br>3 NO (C115)<br>8 DK (C115)	C112<br>(ENTER NUMBER)<br>98 DK	C113<br>1 MINUTES ___<br>3 HOURS ___<br>8 DK	C114<br>1 WEEK ___<br>3 MONTH ___<br>8 DK
C115<br>Did you perform hiking uphill or backpacking?<br>1 YES (C116)<br>3 NO (GOTO C119)<br>8 DK (GOTO C119)	C116<br>(ENTER NUMBER)<br>98 DK	C117<br>1 MINUTES ___<br>3 HOURS ___<br>8 DK	C118<br>1 WEEK ___<br>3 MONTH ___<br>8 DK
C119<br>Did you perform vigorous swimming?<br>1 YES (C129)<br>3 NO (GOTO C123)<br>8 DK (GOTO C123)	C120<br>(ENTER NUMBER)<br>98 DK	C121<br>1 MINUTES ___<br>3 HOURS ___<br>8 DK	C122<br>1 WEEK ___<br>3 MONTH ___<br>8 DK
C123<br>Did you perform scrubbing floors or mowing lawn with a non-power mower?<br>1 YES (C124)<br>3 NO (GOTO C127)<br>8 DK (GOTO C127)	C124<br>(ENTER NUMBER)<br>98 DK	C125<br>1 MINUTES ___<br>3 HOURS ___<br>8 DK	C126<br>1 WEEK ___<br>3 MONTH ___<br>8 DK
C127<br>Did you perform chopping or splitting wood, gardening with heavy tools or other heavy labor such as shoveling dirt?<br>1 YES (C128)<br>3 NO (GOTO C131)<br>8 DK (GOTO C131)	C128<br>(ENTER NUMBER)<br>98 DK	C129<br>1 MINUTES ___<br>3 HOURS ___<br>8 DK	C130<br>1 WEEK ___<br>3 MONTH ___<br>8 DK

[SCREEN FOR AGE OF RESPONDENT AT BEGINNING OF REFERENT PERIOD]

	60 YEARS OLD	45 YEARS OLD	30 YEARS OLD
Compared to your level of physical activity at leisure and at home between [FILL REFERENT PERIOD] how would you describe your leisure time and home activity when you were (ENTER AGE). Would it be: less, about the same or more physical activity?	C131<br>1 LESS PHYSICAL ACTIVITY (C132)<br>3 ABOUT THE SAME AMOUNT OF PHYSICAL ACTIVITY OR (C133)<br>5 MORE PHYSICAL ACTIVITY (C132)<br>8 DK (C133)	C133<br>1 LESS PHYSICAL ACTIVITY (C134)<br>3 ABOUT THE SAME AMOUNT OF PHYSICAL ACTIVITY OR (C135)<br>5 MORE PHYSICAL ACTIVITY (C134)<br>8 DK (C135)	C135<br>1 LESS PHYSICAL ACTIVITY (C136)<br>3 ABOUT THE SAME AMOUNT OF PHYSICAL ACTIVITY OR (NEXT SECTION)<br>5 MORE PHYSICAL ACTIVITY (C136)<br>8 DK (NEXT SECTION)
What per cent (FILL MORE OR LESS) would	C132	C134	C136

that be?	__ ENTER PER CENT<br>98 DK	__ ENTER PER CENT<br>98 DK	__ ENTER PER CENT<br>98 DK

(ALL RESPONDENTS GO TO SECTION D: DIET HISTORY WHICH IS NOT BEGIN PROGRAMMED ON CASES)

SECTION D

DIETARY HISTORY

DTX1Now, I would like to ask about any special diets you were on between (REFERENT PERIOD).

Are you on a (FILL TYPE) diet?	D001 Weight loss	D002 Weight gain	D003 Low salt	D004 Diabetic	D005 Cholesterol lowering	D006 Diet for any other medical condition	D007 Vegetarian	D008 Any other special
	1 YES<br>3 NO<br>8 DK	1 YES<br>3 NO<br>8 DK	1 YES<br>3 NO<br>8 DK	1 YES<br>3 NO<br>8 DK	1 YES<br>3 NO<br>8 DK	1 YES<br>3 NO<br>8 DK	1 YES<br>3 NO<br>8 DK	1 YES (SPECIFY) ___________<br>__3 NO<br>8 DK

DTX2The next questions ask about when you ate and drank during a usual day between (FILL REFERENT PERIOD) and what kinds of food or drink you typically consumed. Include those times when you only had something to drink such as coffee, juice, soda pop or an alcoholic beverage as well as those times when you ate and drank.

(INTV: GIVE RESPONDENT FOOD GROUP CUE CARD)
(IT MIGHT BE HELPFUL IF THE PREVIOUS ANSWERS COULD BE SHOWN ON THESE SERIES OF QUESTIONS)

Between (FILL REFERENT PERIOD), did you eat and/or drink (FILL TIME PERIOD)?	Please look at this cue card and tell me the main foods or drink you typically consumed (FILL TIME PERIOD)?
D009 before breakfast<br>1 YES (D010)<br>3 NO (D011)<br>8 DK (D011)	D010 before breakfast<br>(ENTER FOOD GROUP NUMBERS-ALLOW FOR 15)<br>☐☐☐☐☐☐☐☐☐☐☐☐☐☐☐<br>6 OTHER (SPECIFY)<br>8 DK
D011 at breakfast<br>1 YES (D012)<br>3 NO (D013)<br>8 DK (D013)	D012 at breakfast<br>(ENTER FOOD GROUP NUMBERS-ALLOW FOR 15)<br>☐☐☐☐☐☐☐☐☐☐☐☐☐☐☐<br>6 OTHER (SPECIFY)<br>8 DK
D013 between breakfast and lunch<br>1 YES (D014)<br>3 NO (D015)<br>8 DK (D015)	D014 between breakfast and lunch<br>(ENTER FOOD GROUP NUMBERS-ALLOW FOR 15)<br>☐☐☐☐☐☐☐☐☐☐☐☐☐☐☐<br>6 OTHER (SPECIFY)<br>8 DK

1 COFFEE OR TEA
2 JUICE
3 MILK
4 SODA OR POP
5 BEER, WINE OR MIXED DRINKS
6 OTHER (SPECIFY)
7 FRUIT
8 BREAD, ROLL, TOAST, BAGEL OR MUFFIN
9 CEREAL
10 PANCAKES OR WAFFLES
11 CHEESE
12 ICE CREAM
18 BACON SAUSAGE OR HAM
19 FISH
20 MEAT (BEEF, PORK, CHICKEN)
21 CASSEROLE
22 PASTA AND RICE DISHES
23 PIZZA
24 SANDWICH WITH MEAT INCLUDING HAMBURGERS
25 SANDWICH WITHOUT MEAT INCLUDING PEANUT BUTTER AND CHEESE
26 CAKES, COOKIES, PIES OR OTHER SIMILAR DESSERTS
27 PASTRIES AND DOUGHNUTS
28 CANDY

13 YOGURT
14 SALAD
15 VEGETABLES INCLUDING RAW VEGETABLES AND POTATOES
16 SOUP
17 EGGS OR OMELETTES

29 CHIPS, FRIES OR OTHER SALTY SNACKS
30 CRACKERS
31 NUTS
32 POPCORN
97 NO OTHERS
98 DK

Between (FILL REFERENT PERIOD), did you eat and/or drink (FILL TIME PERIOD)?	Please look at this cue card and tell me the main foods or drink you typically consumed (FILL TIME PERIOD)?
D015 at lunch<br>1 YES (D016)<br>3 NO (D017)<br>8 DK (D017)	D016 at lunch<br>(ENTER FOOD GROUP NUMBERS-ALLOW FOR 15)<br>☐☐☐☐☐☐☐☐☐☐☐☐☐☐☐<br>6 OTHER (SPECIFY)<br>8 DK
D017 between lunch and your evening meal<br>1 YES (D018)<br>3 NO (D019)<br>8 DK (D019)	D018 between lunch and your evening meal<br>(ENTER FOOD GROUP NUMBERS-ALLOW FOR 15)<br>☐☐☐☐☐☐☐☐☐☐☐☐☐☐☐<br>8 DK
D019 at your evening meal<br>1 YES (D020)<br>3 NO (D021)<br>8 DK (D021)	D020 at your evening meal<br>(ENTER FOOD GROUP NUMBERS-ALLOW FOR 15)<br>☐☐☐☐☐☐☐☐☐☐☐☐☐☐☐<br>8 DK
D021 between your evening meal and bedtime<br>1 YES (D022)<br>3 NO (D023)<br>8 DK (D023)	D022<br>(ENTER FOOD GROUP NUMBERS-ALLOW FOR 15)<br>☐☐☐☐☐☐☐☐☐☐☐☐☐☐☐<br>8 DK
D023 after going to bed<br>1 YES (D024)<br>3 NO (DTX3)<br>8 DK (DTX3)	D024<br>(ENTER FOOD GROUP NUMBERS-ALLOW FOR 15)<br>☐☐☐☐☐☐☐☐☐☐☐☐☐☐☐<br>8 DK

1 COFFEE OR TEA
2 JUICE
3 MILK
4 SODA OR POP
5 BEER, WINE OR MIXED DRINKS
6 OTHER (SPECIFY)
7 FRUIT
8 BREAD, ROLL, TOAST, BAGEL OR MUFFIN
9 CEREAL
10 PANCAKES OR WAFFLES
11 CHEESE
12 ICE CREAM
13 YOGURT
14 SALAD
15 VEGETABLES INCLUDING RAW VEGETABLES AND POTATOES
16 SOUP
17 EGGS OR OMELETTES

18 BACON SAUSAGE OR HAM
19 FISH
20 MEAT (BEEF, PORK, CHICKEN)
21 CASSEROLE
22 PASTA AND RICE DISHES
23 PIZZA
24 SANDWICH WITH MEAT INCLUDING HAMBURGERS
25 SANDWICH WITHOUT MEAT INCLUDING PEANUT BUTTER AND CHEESE
26 CAKES, COOKIES, PIES OR OTHER SIMILAR DESSERTS
27 PASTRIES AND DOUGHNUTS
28 CANDY
29 CHIPS, FRIES OR OTHER SALTY SNACKS
30 CRACKERS
31 NUTS
32 POPCORN
97 NO OTHERS
98 DK

GO TO QUANTITATED FOOD QUESTIONNAIRE

DTX3Now, I am going to ask about changes in your diet. (SCREEN RESPONDENT'S FOR AGE AT REFERENT PERIOD)

	60 YEARS OLD	
Did you eat/drink (FILL FOOD OR BEVERAGE) when you were (FILL AGE)?	How often did you usually have it? INTV: ENTER PER DAY/WEEK MONTH	How much did you usually have?
D025 Whole milk<br>1 YES (D026)<br>3 NO (D028)<br>8 DK (D028)	D026<br>1 DAY<br>2 WEEK<br>5 MONTH<br>8 DK	D027<br>______ OZ<br>98 DK
D028 2% or skim milk<br>1 YES (D029)<br>3 NO (D031)<br>8 DK (D031)	D029<br>1 DAY<br>2 WEEK<br>5 MONTH<br>8 DK	D030<br>______ OZ<br>98 DK
D031 Cheese<br>1 YES (D032)<br>3 NO (D034)<br>8 DK (D034)	D032<br>1 DAY<br>2 WEEK<br>5 MONTH<br>8 DK	D033<br>______ OZ<br>98 DK
D034 Fish<br>1 YES (D035)<br>3 NO (D037)<br>8 DK (D037)	D035<br>1 DAY<br>2 WEEK<br>5 MONTH<br>8 DK	D036<br>______ SERVINGS<br>98 DK
D037 Poultry<br>1 YES (D038)<br>3 NO (D040)<br>8 DK (D040)	D038<br>1 DAY<br>2 WEEK<br>5 MONTH<br>8 DK	D039<br>______ SERVINGS<br>98 DK
D040 Red meat such as beef, lamb or pork<br>1 YES (D041)<br>3 NO (D043)<br>8 DK (D043)	D041<br>1 DAY<br>2 WEEK<br>5 MONTH<br>8 DK	D042<br>______ SERVINGS<br>98 DK
D043 Fruits<br>1 YES (D044)<br>3 NO (D046)<br>8 DK (D046)	D044<br>1 DAY<br>2 WEEK<br>5 MONTH<br>8 DK	D045<br>1 ______ PIECES<br>3 ______ CUPS<br>8 DK
D046 Vegetables<br>1 YES (D047)<br>3 NO (D049)<br>8 DK (D049)	D047<br>1 DAY<br>2 WEEK<br>5 MONTH<br>8 DK	D048<br>______ CUPS<br>98 DK
D049 Whole wheat or high fiber bread<br>1 YES (D050)<br>3 NO (D052)<br>8 DK (D052)	D050<br>1 DAY<br>2 WEEK<br>5 MONTH<br>8 DK	D051<br>______ SLICES<br>98 DK

	45 YEARS OLD	
Did you eat/drink (FILL FOOD OR BEVERAGE) when you were (FILL AGE)?	How often did you usually have it? INTV: ENTER PER DAY/WEEK MONTH	How much did you usually have?
D052 Whole milk<br>1 YES (D053)<br>3 NO (D055)<br>8 DK (D055)	D053<br>1 DAY<br>2 WEEK<br>5 MONTH<br>8 DK	D054<br>______ OZ<br>98 DK
D055 2% or skim milk<br>1 YES (D056)<br>3 NO (D058)<br>8 DK (D058)	D056<br>1 DAY<br>2 WEEK<br>5 MONTH<br>8 DK	D057<br>______ OZ<br>98 DK
D058 Cheese<br>1 YES (D059)<br>3 NO (D061)<br>8 DK (D061)	D059<br>1 DAY<br>2 WEEK<br>5 MONTH<br>8 DK	D060<br>______ OZ<br>98 DK
D061 Fish<br>1 YES (D062)<br>3 NO (D064)<br>8 DK (D064)	D062<br>1 DAY<br>2 WEEK<br>5 MONTH<br>8 DK	D063<br>______ SERVINGS<br>98 DK
D064 Poultry<br>1 YES (D065)<br>3 NO (D067)<br>8 DK (D067)	D065<br>1 DAY<br>2 WEEK<br>5 MONTH<br>8 DK	D066<br>______ SERVINGS<br>98 DK
D067 Red meat such as beef, lamb or pork<br>1 YES (D068)<br>3 NO (D070)<br>8 DK (D070)	D068<br>1 DAY<br>2 WEEK<br>5 MONTH<br>8 DK	D069<br>______ SERVINGS<br>98 DK
D070 Fruits<br>1 YES (D071)<br>3 NO (D073)<br>8 DK (D073)	D071<br>1 DAY<br>2 WEEK<br>5 MONTH<br>8 DK	D072<br>1 ______ PIECES<br>3 ______ CUPS<br>8 DK
D073 Vegetables<br>1 YES (D074)<br>3 NO (D076)<br>8 DK (D076)	D074<br>1 DAY<br>2 WEEK<br>5 MONTH<br>8 DK	D075<br>______ CUPS<br>98 DK
D076 Whole wheat or high fiber bread<br>1 YES (D077)<br>3 NO (D079)<br>8 DK (D079)	D077<br>1 DAY<br>2 WEEK<br>5 MONTH<br>8 DK	D078<br>______ SLICES<br>98 DK

	30 YEARS OLD	
Did you eat/drink (FILL FOOD OR BEVERAGE) when you were (FILL AGE)?	How often did you usually have it? INTV: ENTER PER DAY/WEEK MONTH	How much did you usually have?
D079 Whole milk<br>1 YES (D080)<br>3 NO (D082)<br>8 DK (D082)	D080<br>1 DAY<br>2 WEEK<br>5 MONTH<br>8 DK	D081<br>______ OZ<br>98 DK
D082 2% or skim milk<br>1 YES (D083)<br>3 NO (D085)<br>8 DK (D085)	D083<br>1 DAY<br>2 WEEK<br>5 MONTH<br>8 DK	D084<br>______ OZ<br>98 DK
D085 Cheese<br>1 YES (D086)<br>3 NO (D088)<br>8 DK (D088)	D086<br>1 DAY<br>2 WEEK<br>5 MONTH<br>8 DK	D087<br>______ OZ<br>98 DK
D088 Fish<br>1 YES (D089)<br>3 NO (D091)<br>8 DK (D091)	D089<br>1 DAY<br>2 WEEK<br>5 MONTH<br>8 DK	D090<br>______ SERVINGS<br>98 DK
D091 Poultry<br>1 YES (D092)<br>3 NO (D094)<br>8 DK (D094)	D092<br>1 DAY<br>2 WEEK<br>5 MONTH<br>8 DK	D093<br>______ SERVINGS<br>98 DK
D094 Red meat such as beef, lamb or pork<br>1 YES (D095)<br>3 NO (D097)<br>8 DK (D097)	D095<br>1 DAY<br>2 WEEK<br>5 MONTH<br>8 DK	D096<br>______ SERVINGS<br>98 DK
D097 Fruits<br>1 YES (D098)<br>3 NO (E100)<br>8 DK (E100)	D098<br>1 DAY<br>2 WEEK<br>5 MONTH<br>8 DK	D099<br>1 ______ PIECES<br>3 ______ CUPS<br>8 DK
E100 Vegetables<br>1 YES (E101)<br>3 NO (E103)<br>8 DK (E103)	E101<br>1 DAY<br>2 WEEK<br>5 MONTH<br>8 DK	E102<br>______ CUPS<br>98 DK
E103 Whole wheat or high fiber bread<br>1 YES (E104)<br>3 NO (SECTION E)<br>8 DK (SECTION E)	E104<br>1 DAY<br>2 WEEK<br>5 MONTH<br>8 DK	E105<br>______ SLICES<br>98 DK

(GOTO SECTION E)

QUANTITATED FOOD HISTORY

EGGS

NHLBI Code	Se-lect	Item Description	Comments	Serving Size Amount	Frequency D	W	M	Prep Code	Fat Code

1. Q. Do you eat eggs or quiche? ______ NO
Q. How many do you usually have? ______ egg(s)
Q. How are they usually prepared?
Q. How often do you have them?

		Without fat:							
25015		Hard, soft, poached<br>(1 of each = 1 egg)		SM MD LG*<br>XL JM					
		With fat:							
25338		Scrambled, omelet, fried<br>(1 of each = 2 eggs)		SM MD LG*<br>XL JM				APFC	BCOM
25448		Quiche<br>(1 MOD=1/7 of 9" pie)		SI MOD					

Note: Eggs without fat: 1 egg = 1 SV.
Note: Eggs with fat: 2 eggs = 1 SV.

2. Q. Do you eat Egg McMuffins or similar type breakfast sandwiches? ______ NO
Q. How many do you usually have? ______
Q. How often do you have them? ______

25457		Egg McMuffin<br>(1 PC = 1 Egg McMuffin)		PC					

3. Q. Do you eat egg salad? ______ NO
Q. How much do you usually have? ______ CP
Q. How often do you have it?

25411		Egg salad:<br>Mayo type dressing		CP					

DAIRY

NHLBI Code	Se-lect	Item Description	Comments	Serving Size Amount	Frequency D	W	M	Prep Code	Fat Code

4. Q. Do you drink milk as a beverage? _____ NO

Q. How much do you usually have? ______ FO or ______ CP

Q. What kind do you usually have?

Q. How often do you usually have it?

		MILK:							
34256		Milk, % fat unknown		FO CP					
34215		Whole milk		FO CP					
32227		2% milk		FO CP					
32219		1% milk		FO CP					
30221		Skim milk/nonfat liq.		FO CP					
30239		Evap. skim (undil.)		FO CP					
34207		Evap. whole (undil.)		FO CP					
		Other:							
		BUTTERMILK:							
34249		% fat uknown		FO CP					
34231		Whole		FO CP					
32201		2%		FO CP					
30205		Skim		FO CP					
		CHOCOLATE MILK, COMMERCIAL							
33076		% fat unknown		FO CP					
35014		Whole		FO CP					
33019		2%		FO CP					
33160		Skim		FO CP					

5. Q. Do you add anything to your milk? ______ NO
Q. What do you add?
Q. How often?

59139		ADDITIONS TO MILK:<br>Chocolate syrup							
59196		Cocoa powder w/sugar							
59780		Cocoa powder w/o sugar,<br>or w/artific. sweetener							
31160		Instant breakfast							
30213		Non-fat dry milk powder							
59584		Protein powder							
		Other							

DAIRY CONTINUED

NHLBI Code	Se-lect	Item Description	Comments	Serving Size Amount	Frequency D ¦ W ¦ M	Prep Code	Fat Code

6. Q. Do you usually eat cheese? Include hard cheese and processed cheese. ______ NO

Q. How much do you usually have? ______ OZ

Q. How often do you usually have it?

36020		Cheese (natural):<br>Mozzarella and other part skim (1 SL=0.75 oz)		SL OZ					
38026		All other types (1 SL=0.75 oz)		SL OZ					
38083		Processed: American,<br>Swiss (1 SL=0.75 oz)		SL OZ					
34033		Diet (1 SL=0.75 oz)		SL OZ					

Note: Code cream cheese in bread section
Note: Code imitation cheese as diet, regular sodium

7. Q. Do you eat cottage cheese or ricotta cheese? ______ NO

Q. How much do you usually have? ______ CP or ______ OZ

Q. What kind do you usually have?

Q. How often do you usually have it?

34041		Cottage:<br>% fat unknown		OZ CP					
34017		Regular (4% fat)		OZ CP					
32037		Low fat		OZ CP					
34066		Ricotta:<br>% fat unknown		OZ CP					
36038		Regular		OZ CP					
34025		Part skim		OZ CP					

NHLBI Code	Se-lect	Item Description	Comments	Serving Size Amount	Frequency D	W	M	Prep Code	Fat Code

8. Q. Do you usually eat yogurt? ______ NO

Q. How much do you have? ______ OZ or ______ CP

Q. What kind do you have?

Q. How often do you usually have it?

33068		Yogurt (flavored): % fat unknown		OZ CP					
35055		Whole		OZ CP					
33050		Lowfat		OZ CP					
35220		Nonfat		OZ CP					
33170		Sweetened with aspartame		OZ CP					
32250		Unflavored: % fat unknown		OZ CP					
34504		Whole		OZ CP					
32235		Lowfat		OZ CP					
33142		Nonfat		OZ CP					

9. Q. Do you usually eat ice cream or frozen desserts?
Include ice cream snacks, frozen yogurt, sherbet or milkshakes. ______ NO

Q. What kind do you usually have?

Q. How much do you usually have?
(Ascertain serving size for each item mentioned)

Q. How often do you have it?

37101		Ice cream: % fat unknown		CP					
31054		Very low fat (e.g. Weight Watchers)		CP					
35030		Soft serve or ice milk		CP					
37010		Regular fat		CP					
37028		Very rich		CP					
32300		Frozen yogurt		OZ CP					
31021		Sherbet		CP					
35097		Milk shake		FO CP					
37093		Ice Cream Snacks: Sandwiches, bars, Drumsticks (1 bar=1 each)		Bar					
91041		Popsicles (1 PC=1 single stick) (1 DB=1 double stick)		PC DB					

DAIRY CONTINUED

NHLBI Code	Se-lect	Item Description	Comments	Serving Size Amount	Frequency D ¦ W ¦ M	Prep Code	Fat Code

10. **Q. Do you add anything to your ice cream or frozen desserts? ______ NO**

Q. How often?

59121		**ADDITIONS TO ICE CREAM/FROZEN YOGURT**<br>Chocolate sauce							
59618		Caramel topping							
39024		Whipped cream:<br>Dairy							
28415		Nondairy							
45435		Nuts							
81281		Fresh fruit							
59139		Chocolate syrup							
		Other							

GRAINS/CEREALS

NHLBI Code	Se- lect	Item Description	Comments	Serving Size Amount	Frequency D	W	M	Prep Code	Fat Code

11. Q. Do you usually eat bread? ______ NO
Q. What kind?
Q. How often?

60061		BREADS: White (1 SL=approx 4"x4"x1/2", 1 oz)		SL					
60095		Whole wheat/mixed grains (1 SL=approx 4"x4"x1/2", 1 oz)		SL					
60251		High fiber breads (1 SL=approx 4"x4"x1/2", 1 oz)		SL					
60087		French/Italian (1 SL=oval 4-1/4"x2-3/4"x1", 1 oz)		CI SL					
60053		Rye, Pumpernickel (1 SL=1 oz)		SL					
60459		Diet w/fiber added (eg Less, Taystee Lite)(1 SL=0.8 oz)		SL					
60464		White, thin sliced (eg Hollywood, Weight Watchers) (1 SL=0.6 oz)		SL					
60442		Wheat, thin sliced (eg Hollywood, Weight Watchers) (1 SL=0.6 oz)		SL					

12. Q. Do you usually add anything to your bread? ______ NO
Q. To what breads do you add these items?

47035		**ADDITIONS TO BREAD:** Butter							
49030		Margarine: Regular							
49060		Spread							
48918		Diet							
90100		Syrup: Regular							
90258		Reduced calorie							
90050		Molasses							
90035		Jelly/jam: Regular							
90320		Reduced sugar							
45013		Peanut butter							
90027		Honey							
44206		Mayonnaise							
44362		Mayo-type dressing							
44214		Diet mayo-type dressing							
06320		Mustard							
38042		Cream cheese							
06031		Catsup							
		Other:							

GRAINS/CEREALS Continued

NHLBI Code	Se-lect	Item Description	Comments	Serving Size Amount	Frequency D ¦ W ¦ M	Prep Code	Fat Code

13. **Q. Do you eat rolls, including any of these? ______ NO**
Q. What kind?
Q. How often?

60137		Rolls:<br>Hamburger (SM=3" diam; MD=3.5" diam, 8/pound; LG=4" diam)<br>Hot dog (SM=3.75"; MD=5.25", LG= "foot long")		SM MD* LG					
60277		Kaiser/Hard (SM=Kaiser 2.5" diam; MD=Kaiser 3.75" diam, Hard 2.5" diam x 2"; LG=Kaiser 4" diam)		SM MD* LG					
60293		Submarine (SM=5.5"; MD=8.5"; LG=11.5")		SM MD* LG					
54361		Dinner roll (SM=2.25" sq; MD=2.5" sq; LG=3.5"x2.5")		SM MD* LG					
54395		Croissant (SM=1 oz; MD=5"-6", 2 oz)		SM MD*				APFI	BCOM
54403		Crescent		MD					
60152		English muffin		MD					
54015		Biscuit (SM=2" diam; MD=2.33" diam, 1.33 oz; LG=3" diam)		SM MD* LG				APFI	BCOM
60012		Bagel, all kinds (SM=2.33" diam; MD=3" diam, 2 oz; LG=3.5" diam)		SM MD* LG					
60194		Pita (SM=4" diam; MD=5.25" diam; LG=6.5" diam, 3 oz)		SM MD LG*					

Note: In this section, exclude all hamburger buns eaten in a fast food restaurant

GRAINS/CEREALS Continued

14. Q. Do you usually add anything to your rolls? ______ NO
Q. To what rolls do you add these items?

NHLBI Code	Se-lect	Item Description	Comments	Serving Size Amount	Frequency D	W	M	Prep Code	Fat Code
47035		**ADDITIONS TO ROLLS:**<br>Butter							
49030		Margarine: Regular							
49060		Spread							
48918		Diet							
90100		Syrup: Regular							
90258		Reduced calorie							
90050		Molasses							
90035		Jelly/jam: Regular							
90320		Reduced sugar							
45013		Peanut butter							
90027		Honey							
44206		Mayonnaise							
44362		Mayo-type dressing							
44214		Diet mayo-type dressing							
06320		Mustard							
38042		Cream cheese							
06031		Catsup							
		Other:							

GRAINS/CEREALS Continued

NHLBI Code	Se-lect	Item Description	Comments	Serving Size Amount	Frequency D ¦ W ¦ M	Prep Code	Fat Code

15. Q. Do you usually eat pancakes, french toast, coffee cakes, other quick breads or muffins? ______ NO
Q. How much do you usually have?
(Ascertain serving size for each item mentioned)
Q. How often?
Q. How is it usually prepared?

54730		Quick breads: Pancakes (1 SM=3" diam; MD=4" diam; LG=6.5" diam)		SM MD* LG					
54750		Waffles (1 MD=3.5"x2.75"; Lg=4.5"x3.75")		MD LG* XL					
25346		French toast		SL				APFC	BCOM
54528		Nut, banana & other similar bread (1 SL=4 1/2"x1/2")		SL				APFI	BCOM
54536		Coffee cake (1 PC=3"x3")		PC				APFI	BCOM
67370		Muffins: Blueberry (SM=2" diam x 1.5"; LG=2 oz)		SM* LG*					
54262		Muffins: Bran (MD=2.5" diam x 1.5"; LG=2.5 oz)		SM MD* LG*				APFI	BCOM
54023		Cornbread (1 PC=3" square)		PC				APFI	BCOM

16. Q. Do you usually add anything to these foods? ______ NO
Q. To what foods do you usually add these items?

47035		**ADDITIONS:** Butter							
49030		Margarine: Regular							
49060		Spread							
48918		Diet							
90100		Syrup: Regular							
90258		Reduced calorie							
90050		Molasses							
90035		Jelly/jam: Regular							
90320		Reduced sugar							
45013		Peanut butter							
38042		Cream cheese							
90027		Honey							
		Other:							

GRAINS/CEREALS Continued

NHLBI Code	Se-lect	Item Description	Comments	Serving Size Amount	Frequency D	W	M	Prep Code	Fat Code

17. Q. Do you usually eat doughnuts or sweet rolls? ______ NO
Q. How often?

NHLBI Code	Se-lect	Item Description	Comments	Serving Size Amount	Frequency D	W	M	Prep Code	Fat Code
54551		Doughnuts all types (MD=yeast 4"; cake 3.5")		SM MD* LG				APFC	BCOM
54577		Sweet roll		SM MD* LG				APFI	BCOM

18. Q. Do you usually eat pasta, including macaroni, macaroni and cheese, spaghetti, noodles or pasta salads?
Include ravioli and lasagna. ______ NO
Q. How much do you usually have?
Q. How often?

NHLBI Code	Se-lect	Item Description	Comments	Serving Size Amount	Frequency D	W	M	Prep Code	Fat Code
63016		Pasta: Macaroni, spaghetti		CP				SES	BCOM HOME
63024		Egg noodles		CP				SES	
38737		Macaroni & cheese		CP				APFI	BCOM
65410		Pasta salad: Pasta w/ vinaigrette dressing		CP				APFI	BCOM
65400		Macaroni salad		CP					
38661		Ravioli/Pasta: With cheese		CP				APFI	BCOM
14142		With meat		CP				APFI	BCOM
38760		Lasagna: 1 PC = 4" SQ Meatless, spinach		PC				APFI	BCOM
14134		Beef and pork		PC					
38707		Frozen pasta entree, regular		PC					
38708		Frozen pasta entree, low calorie		PC					

GRAINS/CEREALS Continued

NHLBI Code	Se- lect	Item Description	Comments	Serving Size Amount	Frequency D ¦ W ¦ M	Prep Code	Fat Code

19. **Q. Do you add anything to the pasta that you eat? ______ NO**
Q. To which foods do you add these items?

38745		ADDITIONS: Cheese sauce						APFI	BCOM
51797		Spaghetti sauce: meatless							
14118		Spaghetti sauce: with meat						APFI	BCOM
51599		White sauce/Alfredo						APFI	BCOM
47035		Butter							
49030		Margarine: Regular							
49060		Spread							
48918		Diet							
38059		Parmesan cheese							
51940		Pesto						APFI	BCOM
51664		Gravy						APFI	BCOM
		Other:							

20. **Q. Do you usually eat rice or other whole grains? Include rice mixes, fried rice or fried noodles. ______ NO**
Q. How much do you usually have? ______ CP
Q. How often?

61168		Rice: Plain, white		CP				SES	BCOMH OME
61226		Brown rice		CP				SES	BCOM HOME
61406		Rice mixes, pilaf		CP				APFC	BCOM
63120		Fried noodles		CP				APFC	BCOM
61177		Fried rice		CP				APFC	BCOM
61390		Kasha/Buckwheat, cooked		CP				SES	BCOM HOME

21. **Q. Do you add anything to the rice or whole grains that you eat? ______ NO**
Q. To which foods do you add these items?

47035		**ADDITIONS FOR RICE:** Butter							
49030		Margarine: Regular							
49060		Spread							
48918		Diet							
38745		Cheese sauce						APFI	BCOM
06429		Soy sauce							
51789		Sweet & sour sauce							
51664		Gravy						APFI	BCOM
		Other:							

GRAINS/CEREALS Continued

NHLBI Code	Se-lect	Item Description	Comments	Serving Size Amount	Frequency D	W	M	Prep Code	Fat Code

22. **Q. Do you usually eat hot or cold cereal? ______ NO**
Q. How much do you usually have? ______ CP
Q. What kind do you usually have?
Q. How often?

		Hot cereals:							
61143		Oatmeal		CP				SES	BCOM HOME
61890		Farina, Cream of Wheat		CP				SES	
61101		Corn grits		CP				SES	
		Cold Cereals:							
		*Brand/type:							

GRAINS/CEREALS Continued

NHLBI Code	Se-lect	Item Description	Comments	Serving Size Amount	Frequency D	W	M	Prep Code	Fat Code

23. Q. Do you usually add anything to your cereals? Include any unprocessed bran or wheat germ. ______ NO

Q. To which cereals do you add these items?

		**ADDITIONS TO CEREALS:**<br>*Milk, type:							
47035		Butter							
49030		Margarine: Regular							
49060		Spread							
48918		Diet							
28100		Nondairy liquid creamer							
90084		Sugar: White							
90068		Brown							
90027		Honey							
90274		Artificial sweetener:<br>Nutrasweet (Equal)							
90266		Saccharin or unknown							
61713		Unprocessed bran							
61184		Wheat germ							
		Other:							

24. Q. Do you usually eat crackers? ______ NO
Q. How many do you usually have?
Q. What kind or brand?
Q. How often?

		* Crackers - Brand/type:							

GRAINS/CEREALS Continued

NHLBI Code	Se-lect	Item Description	Comments	Serving Size Amount	Frequency D ¦ W ¦ M	Prep Code	Fat Code

25. Q. Do you usually add anything to your crackers? ______ NO
Q. To which crackers do you usually add these items?

45013		**ADDITIONS TO CRACKERS:** Peanut butter							
		*Cheese							
47035		Butter							
49030		Margarine: Regular							
49060		Spread							
48918		Diet							
		Other:							

FRUIT JUICES

NHLBI Code	Se-lect	Item Description	Comments	Serving Size Amount	Frequency D ¦ W ¦ M	Prep Code	Fat Code

26. Q. Do you usually drink any fruit or vegetable juices? ______ NO

Q. How much do you usually have? ______ FO or ______ CP

Q. How often?

		Known:<br>Apple juice or cider:		FO CP					
82016		Unsweetened		FO CP					
84558		Sweetened		FO CP					
80531		Cranberry juice: Low calorie		FO CP					
84509		Sweetened		FO CP					
84517		Grape juice: Sweetened		FO CP					
80606		Grapefruit juice:<br>Unsweetened		FO CP					
84525		Sweetened		FO CP					
80523		Orange juice: Unsweetened		FO CP					
84541		Sweetened		FO CP					
80614		Papaya juice		FO CP					
82032		Pineapple juice: Unsweetened		FO CP					
82040		Prune juice		FO CP					
84533		Nectars, all types: Apricot		FO CP					
84574		Pear, peach		FO CP					
		Other							
		Vegetable Juices: Regular							
70276		Tomato		FO CP					
70615		Mixed, V-8 type		FO CP					

FRUITS

NHLBI Code	Se-lect	Item Description	Comments	Serving Size Amount	Frequency D	W	M	Prep Code	Fat Code

27. Q. Do you eat fresh fruit? ______ NO

Q. Looking at this list, which ones do you eat and how often?

81018		**FRESH FRUITS:**<br>Apple		SM MD* LG					
81026		Apricot		MD					
81034		Banana		LI SM MD* LG					
81059		Berries (all other than strawberries)		PC CP					
81075		Cherries		PC CP					
81232		Figs, fresh		SM MD* LG<br>CP					
80010		Grapefruit		SM MD* LG					
81083		Grapes (15 = 1 CP)		PC CP					
		Melons:							
81067		Cantaloupe		WDG*<br>SM MD LG					
81133		Honeydew		WDG*<br>SM MD LG					
81331		Watermelon		SL CP					
81307		Papaya		SM MD* LG CP					
81091		Mango		MD CP					
81109		Nectarine		SM MD* LG CP					
80028		Orange		SM MD* LG					
81117		Peach		SM MD* LG					
81125		Pear		SM MD* LG					
81141		Pineapple:<br>Chunks (20 chunks = 1 CP)		CK CP					
81141		Slices		SL					
81158		Plum		SM MD* LG					
81174		Strawberries (15 = 1 CP)		CP					
80036		Tangerine		SM MD* LG					
81356		Kiwi fruit		MD CP					

FRUITS Continued

28. Q. Do you eat canned, cooked or frozen fruit? ______ NO

Q. Looking at this list, which ones do you eat and how often?

Q. What is your usual serving size for canned or frozen fruit? _______ CP

NHLBI Code	Se-lect	Item Description	Comments	Serving Size Amount	Frequency D	W	M	Prep Code	Fat Code
		**CND/FRZN/CKD FRUITS:** Applesauce:							
84012		Sweetened		CP					
82057		Unsweetened		CP					
		Apricots, canned (1 PC = 1 half)							
81026		Unsweetened		PC CP					
84020		Sweetened		PC CP					
84103		Berries (all other than strawberries)		CP					
		Cherries:							
81075		Unsweetened/frozen		PC CP					
84038		Canned/frozen/sweetened		CP					
90142		Cranberry sauce		TB CP					
84178		Figs, canned, sweetened (1 PC = 1 fig)		PC CP					
82081		Fruit cocktail: Unsweetened		CP					
84046		Sweetened		CP					
80028		Grapefruit/orange sections: Unsweetened		CP					
84186		Sweetened		CP					
84145		Orange, mandarin		CP					
81117		Peaches: Cnd/frzn, unswt (2 halves=1 md)		MD CP					
84053		Frzn/cnd, swt (1 PC = 1/2)		PC CP					
81125		Pears: Cnd/frzn, unswt (2 halves=1 md)		MD CP					
84061		Frzn/cnd, swt (1 PC=1 half)		PC CP					
81257		Pineapple: Frzn/cnd, unswt (SL = 1 slice)		CK SL CP					
84079		Canned, sweetened		CK SL CP					
81158		Plums: Cnd, unsweetened		SM MD* LG CP					
84087		Cnd, swt (1 pc = 1 plum)		PC CP					

FRUITS Continued

NHLBI Code	Se-lect	Item Description	Comments	Serving Size Amount	Frequency D	W	M	Prep Code	Fat Code
82099		Cnd/Frzn/Ckd fruits:<br>Rhubarb: Unsweetened		CP					
84111		Sweetened		CP					
81174		Strawberries: Frzn, unswt		CP					
84095		Frzn, swt		CP					
		Other:							

29. Q. Do you usually eat dried fruits? ______ NO

Q. How much do you usually have?

(Ascertain serving size for each item mentioned)

Q. How often?

83014		Dried Fruits (uncooked):<br>Apples		RG CP					
83022		Apricots (1 pc=one half)<br>(1 CP = 9 halves)		PC CP					
83030		Dates (1 PC = 1)		PC CP					
83048		Figs (1 PC = 1)		PC CP					
83055		Peaches, pears & similar<br>(1 PC=one half)		PC CP					
83063		Prunes (1 PC = 1)		PC CP					
83071		Raisins		TB CP					
84210		Fruit leather or rolls<br>(MD=1 rollup)		MD OZ					

30. Q. Do you usually add anything to your fruits? ______ NO

Q. To which fruits do you add these items?

		**ADDITIONS TO FRUITS**:<br>Sugar							
90084		White							
90068		Brown							
90027		Honey							
		Artificial sweetener							
90274		Nutrasweet (Equal)							
90266		Saccharin							
		*Milk, type:							
36202		Cream/half & half							
28100		Nondairy creamer							
39024		Whipped cream: Dairy							
28415		Nondairy							
		Other:							

ETHNIC

NHLBI Code	Se-lect	Item Description	Comments	Serving Size Amount	Frequency D	W	M	Prep Code	Fat Code

31. Q. Do you usually eat pizza? ______ NO
Q. How much do you usually have? ______ slices
Q. What kind?
Q. How often?

38780		Pizza: Cheese, reg. (MOD = 1/8 14" diam; SM=9" diam; MD=12" diam)		MOD SM MD					
38729		Cheese pizza, deep dish (1 SL= 1/8 14" diam)		SL				APFI	BCOM
15470		Meat pizza, reg. (MOD = 1/8 14" diam; SM=9" diam; MD=12" diam)		MOD SM MD					
38735		Meat pizza, deep dish		SL				APFI	BCOM

Note: Disregard any vegetables on pizza.

32. Q. Do you eat Oriental foods (include fortune cookies)? ______ NO
Q. What is your usual serving size? ______ CP or ______ SV
Q. What kind?
Q. How often?

14850		Chicken & Oriental veg.		CP				APFC	BCOM
14851		Beef & Oriental veg.		CP				APFC	BCOM
14852		Pork & Oriental veg.		CP				APFC	BCOM
14853		Seafood & Oriental veg.		CP				APFC	BCOM
70375		Oriental veg. (plain)		CP				SES	BCOM
71021		Tofu & oriental veg.		CP				APFC	BCOM
14854		Sweet and sour dishes		CP				PAN	BCOM
25379		Egg roll (1 PC = 1)		PC				APFC	BCOM
25361		Egg foo yung (1 PC = 1 pancake)		PC				APFC	BCOM
93120		Fortune cookie (1 cookie=0.5 PC)		PC					
06429		**ADDITIONS**: Soy sauce							
51789		Sweet and sour sauce							
		Other:							

ETHNIC Continued

33. **Q. Do you usually eat Mexican food? ______ NO**
Q. What kind?
Q. How often?

NHLBI Code	Se-lect	Item Description	Comments	Serving Size Amount	Frequency D	W	M	Prep Code	Fat Code
14229		Mexican:<br>Enchilada:<br>(1 PC = 1 enchilada)<br>Cheese		PC					
14227		Chicken		PC					
14820		Beef		PC				APFC	BCOM
73635		Burrito: Bean		MD LG XL*					
14704		Beef		MD LG* XL					
14696		Taco, beef		MD					
14223		Tostada: Beef		MD					
73643		Bean		MD				APFC	BCOM
73544		Refried beans		CP				APFC	BCOM
10512		Chicken fajitas - meat		OZ				PAN	BCOM
10017		Beef fajitas - meat		OZ				PAN	BCOM
65144		Spanish rice		CP				APFI	BCOM
60285		Tortilla: Flour (6" diam):<br>Plain		SM MD LG*<br>XL JM					
53108		Fried		SM MD LG*<br>XL JM				APFC	BCOM
60301		Corn (5 1/2" diam):<br>Plain		MD					
53173		Fried		MD				APFC	BCOM
		**ADDITIONS:**							
51888		Salsa, taco sauce							
38208		Sour cream							
46078		Guacamole							
38026		Cheese							

Note: Use tortilla codes if eaten plain or separate from Mexican foods.
Code other ingredients of fajitas besides meat separately.
Code tortilla chips in salty snack section.

SOUPS

34. **Q. Do you eat soups? Include stew, chowder and chili. ______ NO**

Q. How much do you usually have? ______ FO or ______ CP

Q. What kind?

Q. How often?

NHLBI Code	Select	Item Description	Comments	Serving Size Amount	Frequency D	Frequency W	Frequency M	Prep Code	Fat Code
51102		Water based: Vegetable, Chicken, Beef Noodle/Rice (RTS)		FO CP					
51094		Milk or cream based: Tomato (undil)		FO CP					
51086		All others (undil)		FO CP					
		*Add type of milk:		FO CP					
06098		Instant soups: Dry onion mix		PKG TB					
51037		Bouillon (RTS)		FO CP					
23275		Fish chowders: New England (cream, undil)		FO CP					
23267		Manhattan (tomato, undil)		FO CP					
23150		Gumbo (RTS)		CP					
51045		Hearty soups: Bean soups (RTS)		FO CP					
14035		Beef vegetable stew		CP				APFI	BCOM
14050		Chili w/meat & beans		CP					
14060		Chili w/o meat, w/beans		CP					
51177		Chunky soups: w/meat (RTS)		FO CP					
51185		w/o meat (RTS)		FO CP					

Note: Ascertain type of milk used in cream based soups.
Note: 1 cup of cream based soup = 1/2 cup soup concentrate + 1/2 cup milk (if used). If other soup noted as undiluted, code 1 cp soup = 1/2 cp soup concentrate
Note: Code 1 tablespoon of dehydrated instant soup per serving for soup used in casseroles and other recipes.

CONVENIENCE FOODS

35. Q. Do you usually eat frozen entrees, including TV dinners or pot pies? ______ NO

Q. What kind do you usually have?

Q. How often?

NHLBI Code	Se-lect	Item Description	Comments	Serving Size Amount	Frequency D	W	M	Prep Code	Fat Code
14121		Frozen Entrees - Regular<br>Meat (1 PC=1 entree)		PC					
10509		Poultry		PC					
23109		Fish		PC					
14037		Pot pies: Meat (1 PC=1 pie)		PC				APFI	BCOM
15160		Poultry		PC				APFI	BCOM
14032		T.V. Dinners (1 PC=1 entree)<br>Meat		PC					
15162		Poultry		PC					
23115		Fish		PC					
14129		Frozen entree:<br>(1 PC=1 entree)<br>Low calorie:<br>Meat		PC					
15179		Poultry		PC					
23237		Fish		PC					

MEATS

NHLBI Code	Se-lect	Item Description	Comments	Serving Size Amount	Frequency D ¦ W ¦ M	Prep Code	Fat Code

36. Q. Do you eat any fast food hamburgers or cheeseburgers? ______ NO

Q. How many do you usually have? ______

Q. What kind?

Q. How often?

		*Fast food hamburgers<br>Restaurant known:							
14659		Hamburger, reg.		SW					
14660		Hamburger, 1/4 lb, unk		SW					
14665		Cheeseburger, reg.		SW					
14666		Cheeseburger, 1/4 lb, unk		SW					

37. Q. Do you eat ground beef including other hamburgers, meatloaf or meatballs? ______ NO

Q. What kind of beef?

Q. How much do you usually have? ______ OZ

Q. How is it usually prepared?

13350		Ground beef:<br>Regular hamburger, 23% fat		OZ				PAN BST<br>PFN BDF	BCOM<br>HOME
13011		Chuck, lean, 20% fat		OZ					
12013		Round, sirloin, extra lean, 15% fat		OZ					
11015		< 15% fat		OZ					
13086		% fat unknown or restaurant, other than fast food		OZ					

MEATS Continued

NHLBI Code	Se-lect	Item Description	Comments	Serving Size Amount	Frequency D ¦ W ¦ M	Prep Code	Fat Code

38. Q. Do you usually add any of these items to your hamburgers, meatloaf, or meatballs? ______ NO
Q. To which foods do you add these items?

06031		**ADDITIONS TO GROUND BEEF**<br>Catsup, chili sauce, cocktail sauce							
06551		Steak sauce							
06429		Soy sauce							
06460		Worcestershire sauce							
06619		Teriyaki sauce							
06320		Mustards, prepared							
51990		BBQ sauce							
51664		Gravy						APFI	BCOM
		Other:							

39. Q. Do you eat any ground beef casseroles or Hamburger Helper? ______ NO
Q. How much do you usually have?
Q. How often?

14373		Hamburger Helper		CP					
14415		Ground beef & rice casserole w/tomato sauce		CP				APFI	BCOM
15390		Casserole w/meat, pasta and vegetables		CP					

40. Q. Do you eat any other beef such as pot roast or steaks? ______ NO
Q. Do you eat it trimmed or untrimmed?
Q. How much do you usually have? ______ OZ
Q. How often?
Q. How is it usually prepared?

11015		*Beef (9% fat)		OZ				PAN BST<br>PFN BDF	BCOM<br>HOME
11015				OZ					
12013		*Beef (15% fat)		OZ					
12013				OZ					

MEATS Continued

NHLBI Code	Se-lect	Item Description	Comments	Serving Size Amount	Frequency D	W	M	Prep Code	Fat Code
		Untrimmed beef:		OZ				PAN BST PFN BDF	BCOM HOME
13011		*Beef (20% fat)		OZ					
13011				OZ					
13011				OZ					

41. Q. Do you usually add any of these items to beef? ______ NO

Q. To which foods do you add these items?

06031		**ADDITIONS TO BEEF:** Catsup, chili sauce, cocktail sauce							
06551		Steak sauce							
06429		Soy sauce							
06460		Worcestershire sauce							
06619		Teriyaki sauce							
06320		Mustards, prepared							
51990		BBQ sauce							
51664		Gravy						APFI	BCOM
		Other:							

42. Q. Do you eat ham or ham hocks? ______ NO

Q. How much do you usually have? ______ OZ

Q. How often?

		Ham							
11213		Ham, smoked		OZ				PAN BST PFN BDF	BCOM HOME
		Fresh ham (leg):							
11106		Trimmed		OZ					
13177		Untrimmed		OZ					
13243		Ham hocks		OZ					

Note: Deli ham coded with cold cuts

MEATS Continued

NHLBI Code	Select	Item Description	Comments	Serving Size Amount	Frequency D ¦ W ¦ M	Prep Code	Fat Code

43. Q. Do you usually add any of these items to ham?

Q. To which foods do you add these items?

NHLBI Code	Select	Item Description	Comments	Serving Size Amount	D	W	M	Prep Code	Fat Code
06031		**ADDITIONS TO HAM:**<br>Catsup, chili sauce, cocktail sauce							
06551		Steak sauce							
06429		Soy sauce							
06460		Worcestershire sauce							
06619		Teriyaki sauce							
06320		Mustards, prepared							
51990		BBQ sauce							
51664		Gravy						APFI	BCOM
		Other:							

44. Q. Do you usually eat any bacon, breakfast sausages, or corned beef hash? ______ NO

Q. How often?

NHLBI Code	Select	Item Description	Comments	Serving Size Amount	D	W	M	Prep Code	Fat Code
13227		Breakfast Pork:<br>Bacon, regular		SL TKS OZ					
12203		Canadian bacon		SL OZ				PAN BST<br>PFN BDF	BCOM<br>HOME
13540		Breakfast sausage:<br>patties or links		LK SMP<br>PY OZ					
14068		Corned beef hash		CP				APFC	BCOM

MEATS Continued

NHLBI Code	Se- lect	Item Description	Comments	Serving Size Amount	Frequency D ¦ W ¦ M	Prep Code	Fat Code

45. **Q. Do you eat any other pork, including pork chops and ribs? ______ NO**

Q. Do you eat it trimmed or untrimmed?

Q. How much do you usually have? ______ OZ

Q. How often?

Q. How is it usually prepared?

12104		Trimmed pork: Chops, steak		OZ				PAN BST<br>PFN BDF	BCOM<br>HOME
12104				OZ					
13102		Untrimmed pork:<br>Chops, steak		OZ					
13102				OZ					
13102		Spareribs, Back ribs		OZ					

46. **Q. Do you eat veal or lamb? ______ NO**

Q. Do you eat it trimmed or untrimmed?

Q. How much do you usually have? ______ OZ

Q. How often?

Q. How is it usually prepared?

10041		Trimmed: Veal		OZ				PAN BST<br>PFN BDF	BCOM<br>HOME
10041				OZ					
10033		Lamb		OZ					
10033				OZ					
12021		Untrimmed: Veal		OZ					
12021				OZ					
13320		Lamb		OZ					
13320				OZ					

MEATS Continued

NHLBI Code	Select	Item Description	Comments	Serving Size Amount	Frequency D	W	M	Prep Code	Fat Code

47. Q. Do you usually add any of these items to your pork, veal or lamb? ______ NO
Q. To which foods do you add these items?

06031		**ADDITIONS:** Catsup, chili sauce, cocktail sauce							
06551		Steak sauce							
06429		Soy sauce							
06460		Worcestershire sauce							
06619		Teriyaki sauce							
06320		Mustards, prepared							
51990		BBQ sauce							
51664		Gravy						APFI	BCOM
		Other:							

48. Q. Do you usually eat any of these cold cuts or sausage? ______ NO
Q. How much do you usually have? ______ OZ
Q. How often?

13508		Cold cuts & sausage: Bologna		OZ					
12229		Deli ham		OZ				PAN BST PFN BDF	BCOM HOME
13466		Luncheon meat		OZ					
13771		Salami		OZ					
10025		Dried chipped beef		OZ					
13532		Liverwurst		OZ					
13599		Braunschweiger, liver sausage		OZ					
13573		Canned meats (Spam)		OZ					
13516		Frankfurters, hot dog: Regular (1 = 1 SV)		REG* JM OZ					
13201		Kosher beef		REG* JM OZ					
13755		Turkey or chicken roll		OZ				PAN BST PFN BDF	BCOM HOME
13730		Turkey/chicken franks		REG JM OZ					
13748		Turkey bologna/salami		OZ					
13557		Sausage: Unknown type/Polish		LK OZ					
13623		Knockwurst		LK OZ					
13631		Italian		LK OZ					
13565		Vienna		LK OZ					

MEATS Continued

NHLBI Code	Se- lect	Item Description	Comments	Serving Size Amount	Frequency D	W	M	Prep Code	Fat Code

49. Q. Do you add any of thse items to your cold cuts or sausages?

Q. To which foods do you add these items?

06031		**ADDITIONS:** Catsup, chili sauce, cocktail sauce							
06551		Steak sauce							
06429		Soy sauce							
06460		Worcestershire sauce							
06619		Teriyaki sauce							
06320		Mustards, prepared							
51990		BBQ sauce							
		Other:							

50. Q. Do you usually eat any liver, kidney, heart, or other organ meats?

Q. How much do you usually have? ______ OZ
(Ascertain serving size for each item mentioned)

Q. How often?

Q. How is it usually prepared?

16055		Organ Meats: Liver, beef		OZ				PAN BST PFN BDF	BCOM HOME
16212		Liver, chicken, giblets		PC OZ				PAN BST PFN BDF	
16105		Liver, calves'		OZ				PAN BST PFN BDF	
16220		Pate		TB OZ				APFI	BCOM
		*Other organ meats							

MEATS Continued

NHLBI Code	Se-lect	Item Description	Comments	Serving Size Amount	Frequency D ¦ W ¦ M	Prep Code	Fat Code

51. Q. Do you eat game, including venison, rabbit or duck? ______ NO
Q. How much do you usually have? ______ OZ
Q. With skin or without skin?
Q. How often?
Q. How is it usually prepared?

		Game							
10538		Guinea hen, pheasant, wild duck		OZ				PAN BST PFN BDF	BCOM HOME
10058		All other game: Venison, rabbit, etc.		OZ				PAN BST PFN BDF	

POULTRY

52. Q. Do you usually eat chicken? Include any chicken sandwiches or chicken eaten at places like Kentucky Fried Chicken, Wendy's or McDonald's. ______ NO

Q. How much do you usually have? ____________________________________
Q. Light meat or dark meat?
Q. With skin or without skin?
Q. How often?
Q. How is it usually prepared?

11726		Chicken: Unknown type: w/skin		OZ				PAN BST PFN BDF	BCOM HOME
				OZ					
11734		Unknown type: w/o skin		OZ				PAN BST PFN BDF	
10504		Light meat w/skin		OZ				PAN BST PFN BDF	
				OZ					
10512		Light meat w/o skin		OZ				PAN BST PFN BDF	
11700		Dark meat w/skin		OZ				PAN BST PFN BDF	
				OZ					
10520		Dark meat w/o skin		OZ				PAN BST PFN BDF	
		*Fast food chicken		OZ				PAN BST PFN BDF	
				OZ					

Note: Record weight without bone. See guide to ascertain weight of each part.
Note: Ascertain fast food vs. home preparation

POULTRY Continued

NHLBI Code	Se-lect	Item Description	Comments	Serving Size Amount	Frequency D	W	M	Prep Code	Fat Code

53. Q. Do you eat turkey, cornish hens, duck or goose? ______ NO
Q. How much do you usually have? ______
Q. With skin or without skin?
Q. How often?
Q. How is it usually prepared?

11726		Turkey/Cornish hens<br>Unknown type: w/skin		OZ				PAN BST<br>PFN BDF	BCOM<br>HOME
				OZ					
11734		Unknown type: w/o skin		OZ				PAN BST<br>PFN BDF	
10504		Light meat: w/skin		OZ				PAN BST<br>PFN BDF	
				OZ					
10512		Light meat: w/o skin		OZ				PAN BST<br>PFN BDF	
				OZ					
11700		Dark meat: w/skin		OZ				PAN BST<br>PFN BDF	
				OZ					
10520		Dark meat: w/o skin		OZ				PAN BST<br>PFN BDF	
13805		Duck w/skin		OZ				PAN BST<br>PFN BDF	
13813		Goose w/skin		OZ				PAN BST<br>PFN BDF	
11718		Duck, goose w/o skin		OZ				PAN BST<br>PFN BDF	

54. Q. Do you usually add any of these items to chicken, turkey, duck or goose? ______ NO
Q. To which foods do you add these items?

06031		**ADDITIONS:** Catsup, chili sauce, cocktail sauce							
06551		Steak sauce							
06429		Soy sauce							
06460		Worcestershire sauce							
06619		Teriyaki sauce							
06320		Mustards, prepared							
51990		BBQ sauce							
51664		Gravy						APFI	BCOM
90027		Honey							
		Other:							

POULTRY Continued

NHLBI Code	Select	Item Description	Comments	Serving Size Amount	Frequency D	W	M	Prep Code	Fat Code

55. **Q. Do you eat chicken or turkey salad? ______ NO**

Q. How much do you usually have? ______ CP

Q. How often?

14548		Chicken/turkey salad<br>Mayo-type dressing		CP					

56. **Q. Do you usually eat stuffing or dressing? ______ NO**

Q. How much do you usually have? ______ CP

Q. How often?

54106		Stuffing:<br>Bread type		CP				APFI	BCOM
54130		Cornbread type		CP				APFI	BCOM

FISH/SHELLFISH

NHLBI Code	Se- lect	Item Description	Comments	Serving Size Amount	Frequency D ¦ W ¦ M	Prep Code	Fat Code

57. Q. Do you usually eat fresh, frozen or smoked fish? Include any fish sandwiches or fish eaten at places such as McDonald's, Burger King, Wendy's, Captain D's, or Arthur Treachers. ______ NO
Q. How much do you usually have? ______ OZ
Q. How often?
Q. How is it usually prepared?

20016		* Lean fish (2% fat)		OZ				PAN BST PFN BDF	BCOM HOME
				OZ					
				OZ					
20024		* Medium fat fish (6%)		OZ					
				OZ					
				OZ					
21014		* High fat fish (12% fat)		OZ					
				OZ					
				OZ					
		*Fast food fish		OZ				PAN BST PFN BDF	BCOM
				OZ					
				OZ					
21469		Lox		OZ					
21520		Pickled herring (1 pc=0.5 oz)		OZ					
21501		Sushi or sashimi (no rice)		OZ					
20016		Fish sticks and breaded fish, frozen		OZ				BDF	BCOM

NOTE: Code rice for sushi separately

58. Q. Do you usually add any of these items to the fish you eat? ______
Q. To which foods do you add these items?

47035		**ADDITIONS TO FISH:** Butter							
49030		Margarine: Regular							
49060		Spread							
48918		Diet							
06031		Cocktail sauce							
06031		Catsup							
44321		Tartar sauce							
80515		Lemon juice							
		Other:							

FISH/SHELLFISH Continued

NHLBI Code	Se-lect	Item Description	Comments	Serving Size Amount	Frequency D ¦ W ¦ M	Prep Code	Fat Code

59. Q. Do you usually eat any fresh or frozen shellfish, octopus or squid? ______ NO

Q. How much do you usually have?

(Ascertain serving size for each item mentioned)

Q. How often?

Q. How is it usually prepared?

22236		Shellfish:<br>Lobster		SML MDL* LGL				PAN BST<br>PFN BDF	BCOM<br>HOME
22236		Lobster tail		SM MD* LG					
22269		Shrimp		SM MD* LG<br>OZ					
22210		Clams, cooked		SM MD LG					
22020		Oysters, raw (E=Eastern)<br>(P=Pacific)		MDE* MDP					
22251		Scallops		SM LG* OZ					
22202		Abalone (1 PC=1 abalone)		PC OZ					
22301		Crab, hard shell<br>(1 PC=1 crab)		PC OZ					
22285		Squid, octopus		OZ					

60. Q. Do you usually add any of these items to the shellfish, squid or octopus? ______ NO

Q. To which foods do you add these items?

47035		**ADDITIONS:**<br>Butter							
49030		Margarine: Regular							
49060		Spread							
48918		Diet							
06031		Cocktail sauce							
06031		Catsup							
44321		Tartar sauce							
80515		Lemon juice							
		Other:							

61. Q. Do you usually eat tuna salad? ______ NO

Q. How much do you usually have? ______ CP

Q. How often?

23360		Tuna salad:<br>Mayo-type dressing		CP					

FISH/SHELLFISH Continued

NHLBI Code	Se-lect	Item Description	Comments	Serving Size Amount	Frequency D	W	M	Prep Code	Fat Code

62. Q. Do you usually eat any other canned fish including tuna, sardines, herring or salmon? ______ NO

Q. How much do you usually have?
(Ascertain serving size for each item separately)

Q. How often?

21436		Canned fish: Sardines		SM MD* LG OZ				PAN BST PFN BDF	BCOM HOME
21410		Herring		SM MD* LG OZ					
22228		Crabmeat		CP OZ					
21428		Salmon		CP OZ					
22806		Caviar		TB OZ					
21402		Anchovies (1 PC=1 fillet)		PC OZ					
21444		Tuna: Oil pack		CP OZ					
20040		Water pack		CP OZ					

LEGUMES

NHLBI Code	Se-lect	Item Description	Comments	Serving Size Amount	Frequency D	W	M	Prep Code	Fat Code

63. Q. Do you eat dried peas or beans? Include canned beans and baked beans. ______ NO

Q. How much do you usually have? ______ CP

Q. How often?

Q. How are they usually prepared?

73790		Navy beans		CP				SES	BCOM HOME
73627		Blackeye peas		CP					
73593		Split peas		CP					
73536		Baked beans		CP					
73800		Pinto beans		CP					
73780		Kidney, red beans		CP					
73577		Northern beans		CP					
73585		Chick peas		CP					
73820		Lentils		CP					
73601		Soybeans		CP					

64. Q. Do you eat tofu or Textured Vegetable Proteins (TVP)? ______ NO

Q. How much do you usually have? ______ CP

Q. How often?

Q. How is it usually prepared?

50054		Soy Products or TVP: Tofu, soybean curd (1 PC=1 piece)		CP OZ				SES	BCOM HOME
50039		TVP: Dehydrated		CP OZ					
50096		Ham substitute		SL OZ				SES	BCOM HOME
50070		Sausage substitute, link		LK OZ					
50088		Sausage substitute, patty		SM MD* LG OZ					
50013		Bacon substitute		SL					
50130		Bacon-like bits		TB					
50104		Frankfurter substitute		LK OZ					

VEGETABLES

NHLBI Code	Se-lect	Item Description	Comments	Serving Size Amount	Frequency D ¦ W ¦ M	Prep Code	Fat Code

65. Q. Do you usually eat lettuce or green leafy salads? _______ NO

Q. How much do you usually have? ______ CP

Q. What kind?

Q. How often?

72074		Salad greens:<br>Iceberg or unknown		CP					
72058		Endive, escarole		CP					
72090		Romaine		CP					
72231		Watercress		CP					
72207		Spinach, raw		CP					

66. Q. What kind of salad dressing do you use on your salads? ______ NO

Q. How much? ______ TS or ______ TB

Q. How often?

44107		Salad dressing:<br>Regular: Blue cheese and all other cheeses		TS TB CP					
44460		Ranch		TS TB CP					
44123		French		TS TB CP					
44156		Italian		TS TB CP					
44180		Russian		TS TB CP					
44289		Thousand Island		TS TB CP					
44099		Yogurt based		TS TB CP					
44123		All other creamy types		TS TB CP					
44156		All other clear types		TS TB CP					
44529		Homemade/Mixes:<br>(Fat source known)<br>Creamy type		TS TB CP				APFI	HOME
44024		Clear type (oil & vinegar)		TS TB CP				APFI	HOME
44131		Oil free: clear		TS TB CP					
44390		Oil free: creamy		TS TB CP					

VEGETABLES Continued

NHLBI Code	Se-lect	Item Description	Comments	Serving Size Amount	Frequency D	W	M	Prep Code	Fat Code
44370		Low calorie: Blue cheese, Roquefort		TS TB CP					
44388		French		TS TB CP					
44172		Italian		TS TB CP					
44198		Russian		TS TB CP					
44297		Thousand Island		TS TB CP					
44480		Reduced calorie ranch, buttermilk		TS TB CP					
44362		Mayonnaise: Mayo or mayo-type, unknown		TS TB CP					
44206		Real mayonnaise		TS TB CP					
44362		Mayo-type salad dressing		TS TB CP					
44410		Diet mayo/mayo-type		TS TB CP					

67. Q. Do you add bacos, croutons, lemon juice or other items to your salads? ______ NO

Q. How often?

50130		**ADDITIONS:** Baco's							
60327		Croutons							
80515		Lemon juice							
		Other:							

FRESH/FROZEN, CANNED VEGETABLES

68. Q. Do you eat vegetables? ______ NO
Q. How much do you usually have? ______ CP or ______ OZ
Q. Looking at this list, which of these vegetables do you eat and how often?
Q. How are they usually prepared?

		Vegetables:							
70011		Artichokes: Fresh (1 PC=1 heart)		PC SM MD* LG CP				SES	BCOM HOME
70029		Asparagus: Fresh/frozen		SM MD* LG CP					
70458		Canned		SM MD* LG CP					
46086		Avocado		SM MD* LG CP					

VEGETABLES Continued

NHLBI Code	Se-lect	Item Description	Comments	Serving Size Amount	Frequency D	W	M	Prep Code	Fat Code
70037		Beans - Green: Fresh/frozen		CP				SES	BCOM<br>HOME
70474		Canned		CP					
70045		Lima: Fresh/frozen, canned		CP					
70052		Wax: Fresh/frozen		CP					
70656		Canned		CP					
70060		Beets: Fresh		CP					
70466		Canned		CP					
72256		Broccoli: Raw, fresh<br>(SP = 1 spear)		SP CP					
70524		Cooked, fresh/frozen<br>(SP = 1 spear)		SP CP				SES	BCOM<br>HOME
70086		Brussel sprouts: fresh/frozen<br>(PC = 1 sprout)		PC CP				SES	
72017		Cabbage: Raw		CP					
70292		Cooked		CP				SES	BCOM<br>HOME
79020		Coleslaw		CP					
72025		Carrots: Raw (1 PC=1 stick)		SM MD* LG<br>PC CP					
70094		Cooked, fresh/frozen		CP				SES	BCOM<br>HOME
70672		Canned		CP				SES	
72264		Cauliflower: Raw<br>(PC = 1 flowerette)		PC CP					
70102		Fresh/frozen<br>(PC = 1 flowerette)		PC CP				SES	BCOM<br>HOME
72033		Celery: Raw (1 PC=1 stick)		SM MD* LG<br>PC CP					
70110		Cooked		CP				SES	BCOM<br>HOME
64014		Corn: Cob		SM MD* LG XL					
64022		Kernel, fresh/frozen		CP				SES	BCOM<br>HOME
70482		Kernel, canned		CP				SES	
72041		Cucumber		SM MD* LG<br>CP					
70128		Eggplant, cooked		CP				SES	BCOM<br>HOME
70540		Greens (collard, kale)		CP				SES	
70557		Greens (turnips, mustard,<br>beet greens):<br>Fresh/frozen		CP				SES	

VEGETABLES Continued

NHLBI Code	Select	Item Description	Comments	Serving Size Amount	Frequency D	W	M	Prep Code	Fat Code
70770		Mixed vegetables (broccoli, cauliflower, carrots		CP				SES	BCOM HOME
70144		Mixed vegetables: Fresh/frozen corn, lima beans, peas, carrots		CP					
70532		Canned		CP					
72199		Mushrooms: Fresh, raw		SM MD* LG CP					
70623		Cooked, fresh		CP				SES	BCOM HOME
70151		Canned		CP					
70169		Okra, cooked		CP					
46045		Olive: Black		SM MD* LG XL CP					
46052		Green		SM MD* LG XL CP					
72132		Onions, fresh: Raw, chopped, sliced		SLM CP					
70177		Cooked		CP				SES	BCOM HOME
72108		Scallion		SM MD* LG CP					
70342		Oriental vegetables: Bamboo shoots: Canned, cooked		CP				SES	BCOM HOME
70375		Mixed Chinese veg: canned		CP					
70326		Water chestnuts: canned		CP					
64030		Parsnips: Fresh/frozen		CP					
70193		Peas, green: Fresh/frozen		CP					
70490		Canned		CP					
70391		Pea pods: Fresh/frozen		CP					
70441		Pea/carrots: Fresh/frozen		CP					
70649		Canned		CP					
72066		Peppers, green or red, fresh: Raw		SM MD* LG CP					
70516		Cooked		SM MD* LG SP CP				SES	BCOM HOME
06064		Pickles: Dill		SM MD* LG CP					
06072		Sweet		SM MD* LG CP					
06080		Relish		TB CP					
70219		Rutabagas, fresh		CP				SES	BCOM HOME
70227		Sauerkraut, canned		CP				SES	BCOM HOME

NHLBI Code	Se-lect	Item Description	Comments	Serving Size Amount	Frequency D	W	M	Prep Code	Fat Code
72249		Seaweed, dried (1 PC=1 sheet)		PC					
70367		Sprouts, fresh: Alfalfa		CP					
72116		Soybean		CP					
70235		Spinach: Fresh/frozen, Cooked		CP				SES	BCOM HOME
70508		Canned		CP				SES	
70243		Squash: Summer, fresh/frozen		CP				SES	
70250		Winter, fresh/frozen		CP				SES	
64055		Sweet Potatoes: Fresh/frozen		SM MD* LG CP				SES	
65460		Canned		CP				SES	
72124		Tomatoes: Raw (SLM=1 slice)		SLM SM MD* LG CP					
70268		Canned, regular		CP				SES	BCOM HOME
70359		Tomato sauce		CP					
70318		Tomato paste		CP					
70284		Turnips, cooked		CP				SES	BCOM HOME

Note: For vegetables frozen in sauce use SES BUTR.

Note: Vegetable combinations coded as the prominent vegetable or as mixed vegetable if equal amounts of vegetables in mixture.

VEGETABLES Continued

NHLBI Code	Se-lect	Item Description	Comments	Serving Size Amount	Frequency D	W	M	Prep Code	Fat Code

69. Q. Do you usually add any of these items to your vegetables? ______ NO

Q. To which foods do you add these items?

47035		**ADDITIONS TO VEGETABLES:**<br>Butter							
49030		Margarine: Regular							
49060		Spread							
48918		Diet							
06031		Catsup							
06429		Soy Sauce							
50130		Baco's							
51771		Hollandaise							
51664		Gravy						APFI	BCOM
38745		Cheese sauce						APFI	BCOM
51599		White sauces						APFI	BCOM
		Other:							

70. Q. Do you usually eat potatoes? Include any french fries or baked potatoes eaten at places such as McDonald's, Wendy's or Arby's. ______ NO

Q. What kind do you usually have?

Q. How much do you have? ______ CP

Q. How often?

64063		Potatoes: Baked, boiled<br>w/skin		SM MD* LG<br>XL CP				SES	BCOM<br>HOME
64048		w/o skin		SM MD* LG<br>XL CP				SES	BCOM<br>HOME
65029		French fried (REG=1 fry)<br>(1 CP=14 reg.)		REG CP				APFC	BCOM
65037		Hash brown		CP				APFC	BCOM
65060		Other pan fried		CP				APFC	BCOM
65052		Mashed		CP				APFI	BCOM
65243		Potato salad<br>(mayo-type dressing)		CP					
65094		Potato salad (oil-type<br>dressing		CP				APFI	BCOM

VEGETABLES Continued

71. Q. Looking at this list, do you add any of these items to your potatoes? ______ NO

Q. To which potatoes do you add these items?

NHLBI Code	Se- lect	Item Description	Comments	Serving Size Amount	Frequency D	W	M	Prep Code	Fat Code
47035		**ADDITIONS FOR POTATOES** Butter							
49030		Margarine: Regular							
49060		Spread							
48918		Diet							
38208		Sour cream							
32235		Yogurt							
06031		Catsup							
06429		Soy sauce							
50130		Baco's							
51771		Hollandaise							
51664		Gravy						APFI	BCOM
38745		Cheese sauce						APFI	BCOM
51599		White sauce						APFI	BCOM
		Other							

DESSERTS

NHLBI Code	Se-lect	Item Description	Comments	Serving Size Amount	Frequency D	W	M	Prep Code	Fat Code

72. Q. Do you eat cake, pie, poptarts or snack cakes? ______ NO
Q. What kind?
Q. How often?

56176		Cakes:<br>Chocolate		ci					
56192		Yellow/White/Unknown		ci					
56432		Pound cake		ci				APFI	BCOM
93013		Angel food		ci					
56408		Cheesecake, cream		ci					
56424		Fruitcake		ci				APFI	BCOM
56283		Carrot		ci				APFI	BCOM

Note: Code crust of cheesecake separately.
Note: Code sponge cake as yellow/white cake.
Note: 1 slice of two layer wedge: 8.4 ci
1 slice of loaf cake: 5.63 ci (1/2 inch thick)
1 slice of sheet cake: 9.0 ci (3 x 3 x 1 inch)
1 cupcake: 4.71 ci (2 x 1 inch)

		Pies: Fillings							
57463		Pumpkin, sweet potato		MOD					
91140		All fruit pies/tarts		MOD					
57372		All cream pies/tarts		MOD					
57067		Crusts: 1 crust		MOD				APFI	BCOM
57059		2 crust		MOD				APFI	BCOM
57042		Graham cracker		MOD				APFI	BCOM
57620		Turnovers/fried pies<br>(1 PC=1 turnover)		PC				APFC	BCOM

Note: Code filling and crust for each serving of pie.

93054		Poptarts/Snack cakes:<br>Poptarts<br>(1 PC=1 Poptart)		PC					
56440		Snack cakes:<br>Ring Ding, Ding Dong<br>(similar)(1 PC=1 RD or DD)		PC					
56750		Twinkie (similar)<br>(1 PC=1 Twinkie)		PC					

73. Q. Are your cakes usually frosted? ______ NO
Q. Which ones are frosted?

56663		Frosting		2 TB				APFI	BCOM

DESSERTS Continued

NHLBI Code	Se-lect	Item Description	Comments	Serving Size Amount	Frequency D ¦ W ¦ M	Prep Code	Fat Code

74. Q. Do you eat jello or pudding? ______ NO

Q. How much do you usually have? ______ CP

Q. How often?

91025		Jello: Regular		CP					
90310		Low calorie		CP					
58099		Puddings:<br>Ingredients unknown:<br>Chocolate		CP					
58123		All other flavors		CP					
		Artificially sweetened,<br>with skim milk							
91180		Chocolate		CP					
91170		All other flavors		CP					

75. Q. Do you usually add any toppings to your desserts? ______ NO

Q. To which desserts do you add these items?

		**ADDITIONS TO DESSERTS:**<br>Whipped cream:							
39024		Dairy							
28415		Nondairy							
		Other:							

DESSERTS Continued

NHLBI Code	Se-lect	Item Description	Comments	Serving Size Amount	Frequency D ¦ W ¦ M	Prep Code	Fat Code

76. **Q. Do you eat cookies or brownies? ______ NO**

Q. How many do you usually have?

Q. How often?

		Brand/type:							

NUTS & SEEDS

NHLBI Code	Se- lect	Item Description	Comments	Serving Size Amount	Frequency D ¦ W ¦ M			Prep Code	Fat Code

77. **Q. Do you usually eat nuts or seeds? Include any trail mix, nuts or peanut butter that you have not reported. ______ NO**

Q. How much do you usually have? ______ OZ or ______ TB or ______ CP

Q. How often?

45328		Almonds		OZ TB CP					
45336		Cashews		OZ TB CP					
45252		Mixed nuts		OZ CP					
45344		Peanuts		OZ TB CP					
45450		Pecans		OZ CP					
73110		Roasted soynuts		OZ CP					
45351		Sunflower seeds		OZ TB CP					
45260		Pumpkin seeds		OZ TB CP					
45377		Trail mix		OZ CP					
45013		Peanut butter		TS TB CP					

Note: Sunflower seeds in shell 1 cp = 1/3 cp
Peanuts in shell 1 cp = 1/4 cp

SALTY SNACKS

NHLBI Code	Se- lect	Item Description	Comments	Serving Size Amount	Frequency D ¦ W ¦ M	Prep Code	Fat Code

78. **Q. Do you usually eat chips or pretzels? Include potato, corn or tortilla chips, pork skins and nachos. ______ NO**

Q. How many do you usually have? ______ OZ or ______ CP

Q. How often?

53470		Chips: Regular		PC OZ CP				APF	BCOM
53095		Nachos w/cheese sauce		CP					
60525		Pretzels		MD OZ CP					

79. **Q. Do you usually add dips to these snacks? ______ NO**

Q. How often?

36210		ADDITIONS:<br>Dips: Sour cream based							
73072		Bean							
36012		Cream cheese based							
32250		Yogurt based							
46078		Guacamole							
51888		Salsa							

80. **Q. Do you eat popcorn? _______ NO**

Q. How much do you usually have? ______ CP

Q. How often?

Q. How is it usually prepared?

61150		Popcorn: Popped w/fat:<br>Salted		CP				APFC	BCOM
61358		Plain, w/o fat:		CP					
59147		Caramel & similar		CP				APFI	BCOM

SALTY SNACKS Continued

NHLBI Code	Se- lect	Item Description	Comments	Serving Size Amount	Frequency D ¦ W ¦ M			Prep Code	Fat Code

81. Q. Do you usually add butter, margarine or some other item to your popcorn? ______ NO

47035		**ADDITIONS TO POPCORN:** Butter							
49030		Margarine: Regular							
49060		Spread							
48918		Diet							
		Other:							

82. Q. Do you usually eat candy? ______ NO
Q. What kind?
Q. How much do you usually have? ______
Q. How often?

		*Brand/type:							

BEVERAGES

83. Q. Do you drink hot chocolate? ______ NO
Q. How much do you usually have? ______
Q. What kind?
Q. How often?

35280		Cocoa: skim milk or pkg. mix		FO CP					
33126		Cocoa, sugar free		FO CP					
35022		Cocoa, whole milk		FO CP					

BEVERAGES Continued

NHLBI Code	Se-lect	Item Description	Comments	Serving Size Amount	Frequency D	W	M	Prep Code	Fat Code

84. Q. Do you usually drink coffee, including cappuccino and flavored types? ______ NO
Q. How much do you usually have ______ FO or ______ CP?
Q. How often?

NHLBI Code	Se-lect	Item Description	Comments	Serving Size Amount	D	W	M	Prep Code	Fat Code
03012		Coffee: Brewed, regular		FO CP					
03046		Instant, regular, prepared		FO CP					
03624		Decaffeinated, all types		FO CP					
03038		Cafe au lait and similar types		FO CP					
03616		Postum, coffee substitute		FO CP					
03707		Cafe au lait & similar types, artificially sweetened		FO CP					

85. Q. Do you usually drink any tea, iced or hot? _____ NO
Q. How much do you usually have ______ FO or ______ CP?
Q. How often?

NHLBI Code	Se-lect	Item Description	Comments	Serving Size Amount	D	W	M	Prep Code	Fat Code
03327		Tea: Hot or iced, unswt		FO CP					
03319		Hot or iced, swt		FO CP					
03699		Hot or iced, swt w/Aspartame		FO CP					
03343		Decaffeinated, herbal		FO CP					

86. Q. Do you add anything to your coffee or tea? ______ NO
Q. To which beverages do you add these items?

NHLBI Code	Se-lect	Item Description	Comments	Serving Size Amount	D	W	M	Prep Code	Fat Code
28100		**ADDITIONS TO BEVERAGES:** Coffee creamers, liquid							
28118		Coffee creamer: powder							
36202		Half & half							
90084		Sugar							
90274		Nutrasweet (Equal)							
90266		Saccharin (Sweet n' Low)							
80515		Lemon juice							
90027		Honey							
		*Milk, type:							
		*Other:							

BEVERAGES Continued

NHLBI Code	Se- lect	Item Description	Comments	Serving Size Amount	Frequency D ¦ W ¦ M	Prep Code	Fat Code

87. Q. Do you usually drink coke, soda or pop? ______ NO

Q. How much do you usually have? ______ FO or ______ CP

Q. How often?

04010		Colas: Regular (include Mello Yello, Mountain Dew)		FO CP					
04523		Low calorie		FO CP					
01040		Caffeine-free colas: Regular		FO CP					
01131		Low calorie		FO CP					
01040		Noncolas: Regular, citrus, tonic water, ginger-ale type, root beer, etc.		FO CP					
01131		Low calorie		FO CP					
01099		Club soda, seltzer		FO CP					
01016		Fruit-flavored		FO CP					

88. Q. Do you drink mineral water? ______ NO

Q. How much do you usually have? ______ FO or ______ CP

Q. How often?

01099		Mineral water		FO CP					
01098		Mineral water & fruit juice		FO CP					

BEVERAGES Continued

NHLBI Code	Se-lect	Item Description	Comments	Serving Size Amount	Frequency D ¦ W ¦ M	Prep Code	Fat Code

89. Q. Do you drink Hi-C, Kool-Aid, lemonade or similar types of beverages? ______ NO

Q. How much do you usually have? ______ FO or ______ CP

Q. How often?

85110		Fruit flavored drink (Hi-C)		FO CP					
85076		Mix, swt, reconst. (Kool Aid)		FO CP					
85092		Mix, swt w/Aspartame, reconst.		FO CP					
85019		Lemonade, sweetened		FO CP					
85050		Electrolyte drinks (Gatorade)		FO CP					

90. Q. Do you drink beer? ______ NO

Q. How much do you usually have? ______ FO

Q. How often?

Q. How often during a month do you drink more than this amount? _______

How much more? ______ FO

02006		Beer: Regular		FO					
02022		Low calorie		FO					
01057		Nonalcoholic		FO					

BEVERAGES Continued

NHLBI Code	Se-lect	Item Description	Comments	Serving Size Amount	Frequency D ¦ W ¦ M	Prep Code	Fat Code

91. Q. Do you drink wine? ______

Q. How much do you usually have? ______ FO

Q. How often?

02113		Wine: Table		FO					
02105		Sweet or dessert		FO					

Note: Code low calorie wine as 02113, 1/2 the amount reported.

92. Q. Do you drink liqueurs, mixed drinks, cocktails or liquor? ______ NO

Q. How much? ______ FO

Q. How often?

02824		Liqueurs/cordials: fruit flavored		FO					
02857		Coffee, chocolate		FO					
		*Mixed drinks/cocktails							
02303		Liquor: Vodka		FO					
02303		Gin		FO					
02303		Rum		FO					
02303		Whiskey/bourbon		FO					
		*Mixers:							

Note: Ascertain any mixers used in drinks

MINERAL/VITAMIN SUPPLEMENTS

NHLBI Code	Se- lect	Item Description	Comments	Serving Size Amount	Frequency D ¦ W ¦ M	Prep Code	Fat Code

93. Q. Do you usually take a vitamin or mineral supplement? ______ NO

Q. What kind?

Q. How many? ______ DS

Q. How often?

		Vitamin/Mineral Supplement: *Brand known	(See screen)						
		Brand unknown:							
07410		Multivitamin w/minerals		DS					
07360		w/o minerals or unknown		DS					
07440		w/iron		DS					

94. Q. Is there anything that I haven't mentioned that you eat on a regular basis? ______ NO

POST INTERVIEW-
Interviewer: How well do you think participant was able to recall dietary information?
(a) Excellent(3) (b) Good(4) (c) Fair(5) (d) Poor(6)

When asked, the participant was able to provide:
a) general information (3)
b) specific information (4)

Interviewer's code number ______________ **THIS FORM IS:** **______ COMPLETE (3)**
Cross checked by _______________________ **______ INCOMPLETE (4)**

SECTION E

BODY SIZE

E001What was your usual adult height? **(ENTER INCHES TO THE NEAREST QUARTER)**

___ FEET ___ INCHES
98 DK

What was your usual weight (ENTER REFERENT DATE)?	E002 12 months ago<br>____ LBS<br>8 DK	E003 2 years ago<br>____ LBS<br>8 DK	E004 5 years ago<br>____ LBS<br>8 DK

E005ENTER HEIGHT: **(ENTER INCHES TO THE NEAREST QUARTER INCH)**

FOR EXAMPLE:
FOR 6 FEET 3 1/2 INCHES ENTER 350
FOR 5 FEET 10 1/4 INCHES ENTER 1025

____
998 DK

E006ENTER WEIGHT:

____
998 DK

	READING #1	READING #2
ENTER WAIST CIRCUMFERENCE	E007<br>_____<br>8 DK	E008<br>_____<br>8 DK
ENTER HIP CIRCUMFERENCE	E009<br>_____<br>8 DK	E010<br>_____<br>8 DK

E011ENTER THIGH CIRCUMFERENCE:

_____
98 DK

E012 SECOND THIGH READING:

_____
98 DK

SECTION G

FAMILY HISTORY

In this section of the questionnaire I would like to ask you about the health history of your immediate blood relatives. This would include your mother, father, sisters, brothers and children. I am interested in living and deceased members of your family, but I'm interested only in your full blood relatives not half or adopted relatives.

I'm going to start with your parents.

G001Is your mother still living?

1 YES
3 NO [GOTO G003]
8 DK [GOTO G015]
9 R [GOTO G015]

G002How old is she?

___ ENTER AGE [GOTO G004]
998 DK [GOTO G004]
999 R [GOTO G004]

G003How old was she when she died?

___ ENTER AGE
998 DK
999 R

G004Did she ever have a colon or rectal polyp?

1 YES
3 NO [GOTO G006]
8 DK [GOTO G006]
9 R [GOTO G006]

G005How old was she when the polyp was first diagnosed?

___ ENTER AGE
998 DK
999 R

G006Was your mother ever diagnosed as having any type of cancer?

1 YES
3 NO [GOTO G015]
8 DK
9 R [GOTO G015]

G007What was the first type of cancer your mother had?

01 SKIN (NOT MELANOMA)
03 BREAST
04 LUNG
05 COLON

06 CORPUS (UTERINE)

07 RECTUM

08 BLADDER

09 OVARY

10 MELANOMA

11 CERVIX

12 CANCER OF FEMALE REPRODUCTIVE ORGAN, SITE UNKNOWN

13 OTHER CANCER [SPECIFY]

14 RELATIVE HAD CANCER, SITE UNKNOWN

15 UNKNOWN IF RELATIVE HAD CANCER

99 R

G008How old was she when this cancer was diagnosed?

___ ENTER AGE

998 DK

999 R

G009Did your mother have any other cancer?

1 YES
3 NO [GOTO G015]
8 DK
9 R [GOTO G015]

G010What was the next type of cancer your mother had?

01 SKIN (NOT MELANOMA)
03 BREAST
04 LUNG
05 COLON
06 CORPUS (UTERINE)
07 RECTUM
08 BLADDER
09 OVARY
10 MELANOMA
11 CERVIX
12 CANCER OF FEMALE REPRODUCTIVE ORGAN, SITE UNKNOWN
13 OTHER CANCER [SPECIFY]
14 RELATIVE HAD CANCER, SITE UNKNOWN
15 UNKNOWN IF RELATIVE HAD CANCER
99 R

G011How old was she when this cancer was diagnosed?

___ ENTER AGE
998 DK
999 R

G012Did your mother have any other cancer?

1 YES
3 NO [GOTO G015]
8 DK
9 R [GOTO G015]

G013What was the next type of cancer your mother had?

01 SKIN (NOT MELANOMA)
03 BREAST
04 LUNG
05 COLON
06 CORPUS (UTERINE)
07 RECTUM
08 BLADDER
09 OVARY
10 MELANOMA
11 CERVIX
12 CANCER OF FEMALE REPRODUCTIVE ORGAN, SITE UNKNOWN
13 OTHER CANCER [SPECIFY]
14 RELATIVE HAD CANCER, SITE UNKNOWN
15 UNKNOWN IF RELATIVE HAD CANCER
99 R

G014How old was she when this cancer was diagnosed?

___ ENTER AGE
998 DK
999 R

G015Is you father still living?

1 YES
3 NO [GOTO G017]
8 DK [GOTO GTX1]
9 R [GOTO GTX1]

G016How old is he?

___ ENTER AGE [GOTO G018]
998 DK [GOTO G018]
999 R [GOTO G018]

G017How old was he when he died?

___ ENTER AGE
998 DK
999 R

G018Did he ever have a colon or rectal polyp?

1 YES
3 NO [GOTO G020]
8 DK [GOTO G020]
9 R [GOTO G020]

G019How old was he when the polyp was first diagnosed?

___ ENTER AGE
998 DK
999 R

G020Was your father ever diagnosed as having any type of cancer?

1 YES
3 NO [GOTO GTX1]
8 DK
9 R [GOTO GTX1]

G021What was the first type of cancer your father had?

01 SKIN (NOT MELANOMA)
02 PROSTATE
04 LUNG
05 COLON
07 RECTUM
08 BLADDER
10 MELANOMA
13 OTHER CANCER [SPECIFY]
14 RELATIVE HAD CANCER, SITE UNKNOWN
15 UNKNOWN IF RELATIVE HAD CANCER
99 R

G022How old was he when this cancer was diagnosed?

___ ENTER AGE
998 DK
999 R

G023Did your father have any other cancer?

1 YES
3 NO [GOTO GTX1]
8 DK
9 R [GOTO GTX1]

G024What was the next type of cancer your father had?

01 SKIN (NOT MELANOMA)
02 PROSTATE
04 LUNG
05 COLON
07 RECTUM
08 BLADDER
10 MELANOMA
13 OTHER CANCER [SPECIFY]
14 RELATIVE HAD CANCER, SITE UNKNOWN
15 UNKNOWN IF RELATIVE HAD CANCER
99 R

G025How old was he when this cancer was diagnosed?

___ ENTER AGE
998 DK
999 R

G026Did your father have any other cancer?

1 YES
3 NO [GOTO GTX1]
8 DK
9 R [GOTO GTX1]

G027What was the next type of cancer your father had?

01 SKIN (NOT MELANOMA)
02 PROSTATE
04 LUNG
05 COLON
07 RECTUM
08 BLADDER
10 MELANOMA
13 OTHER CANCER [SPECIFY]
14 RELATIVE HAD CANCER, SITE UNKNOWN
15 UNKNOWN IF RELATIVE HAD CANCER
99 R

G028How old was he when this cancer was diagnosed?

___ ENTER AGE
998 DK
999 R

GTX1Now I would like to ask you the same questions about your full brothers and sisters. Please include those who are living or deceased, but do not include adopted, foster, half or step brothers and sisters.

G029How many full brothers do you have? Again, please include any who may have died.

__ ENTER NUMBER
00 NONE
98 DK
99 R

G030How many full sisters do you have? Again, please include any who may have died.

__ ENTER NUMBER
00 NONE
98 DK
99 R

[IF G029=0, 98 OR 99 AND 1030=0, 98 OR 99 THEN GOTO GTX2]

G031What is the name of your oldest brother or sister? They will not be contacted. This is a simple way to help you complete this part of the questionnaire.

____________________ ENTER NAME
98 DK
99 R

G032What is [FILL NAME]'s sex?

1 MALE
2 FEMALE
8 DK
9 R

G033Is [FILL NAME] still living?

1 YES
3 NO [GOTO G035]
8 DK [GOTO G036]
9 R [GOTO G036]

G034How old is [FILL NAME]?

___ ENTER AGE [GOTO G036]
998 DK [GOTO G036]
999 R [GOTO G036]

G035How old was [FILL NAME] when s/he died?

___ ENTER AGE
998 DK
999 R

G036Did [FILL NAME] ever have a colon or rectal polyp?

1 YES
3 NO [GOTO G038]
8 DK [GOTO G038]
9 R [GOTO G038]

G037How old was s/he when the polyp was first diagnosed?

___ ENTER AGE
998 DK
999 R

G038Was [FILL NAME] ever diagnosed as having any type of cancer?

1 YES
3 NO [GOTO G047]
8 DK
9 R [GOTO G047]

G039What was the first type of cancer [FILL NAME] had?

01 SKIN (NOT MELANOMA)
02 PROSTATE
03 BREAST
04 LUNG
05 COLON
06 CORPUS (UTERINE)
07 RECTUM
08 BLADDER
09 OVARY
10 MELANOMA
11 CERVIX
12 CANCER OF FEMALE REPRODUCTIVE ORGAN, SITE UNKNOWN
13 OTHER CANCER [SPECIFY]
14 RELATIVE HAD CANCER, SITE UNKNOWN
15 UNKNOWN IF RELATIVE HAD CANCER
99 R

G040How old was s/he when this cancer was diagnosed?

___ ENTER AGE
998 DK
999 R

G041Did [FILL NAME] have any other cancer?

1 YES
3 NO [GOTO G047]

8 DK

9 R [GOTO G047]

G042What was the next type of cancer [FILL NAME] had?

01 SKIN (NOT MELANOMA)
02 PROSTATE
03 BREAST
04 LUNG
05 COLON
06 CORPUS (UTERINE)
07 RECTUM
08 BLADDER
09 OVARY
10 MELANOMA
11 CERVIX
12 CANCER OF FEMALE REPRODUCTIVE ORGAN, SITE UNKNOWN
13 OTHER CANCER [SPECIFY]
14 RELATIVE HAD CANCER, SITE UNKNOWN
15 UNKNOWN IF RELATIVE HAD CANCER
99 R

G043How old was s/he when this cancer was diagnosed?

___ ENTER AGE
998 DK
999 R

G044Did [FILL NAME] have any other cancer?

1 YES
3 NO [GOTO G047]
8 DK
9 R [GOTO G047]

G045What was the next type of cancer [FILL NAME] had?

01 SKIN (NOT MELANOMA)
02 PROSTATE
03 BREAST
04 LUNG
05 COLON
06 CORPUS (UTERINE)
07 RECTUM
08 BLADDER
09 OVARY
10 MELANOMA
11 CERVIX
12 CANCER OF FEMALE REPRODUCTIVE ORGAN, SITE UNKNOWN
13 OTHER CANCER [SPECIFY]
14 RELATIVE HAD CANCER, SITE UNKNOWN
15 UNKNOWN IF RELATIVE HAD CANCER
99 R

G046How old was s/he when this cancer was diagnosed?

___ ENTER AGE
998 DK

999 R

G047What is the name of your next brother or sister? (They will not be contacted. This is a simple way to help you complete this part of the questionnaire.)

____________________ ENTER NAME

00 NO MORE BROTHERS OR SISTERS [GTX2]

98 DK [GOTO GTX2]

99 R [GOTO GTX2]

G048What is [FILL NAME]'s sex?

1 MALE

2 FEMALE

8 DK

9 R

G049Is [FILL NAME] still living?

1 YES
3 NO [GOTO G051]
8 DK [GOTO G052]
9 R [GOTO G052]

G050How old is [FILL NAME]?

___ ENTER AGE [GOTO G052]
998 DK [GOTO G052]
999 R [GOTO G052]

G051How old was [FILL NAME] when s/he died?

___ ENTER AGE
998 DK
999 R

G052Did [FILL NAME] ever have a colon or rectal polyp?

1 YES
3 NO [GOTO G054]
8 DK [GOTO G054]
9 R [GOTO G054]

G053How old was s/he when the polyp was first diagnosed?

___ ENTER AGE
998 DK
999 R

G054Was [FILL NAME] ever diagnosed as having any type of cancer?

1 YES
3 NO [GOTO G063]
8 DK
9 R [GOTO G063]

G055What was the first type of cancer [FILL NAME] had?

01 SKIN (NOT MELANOMA)
02 PROSTATE
03 BREAST
04 LUNG
05 COLON
06 CORPUS (UTERINE)
07 RECTUM
08 BLADDER
09 OVARY
10 MELANOMA
11 CERVIX
12 CANCER OF FEMALE REPRODUCTIVE ORGAN, SITE UNKNOWN
13 OTHER CANCER [SPECIFY]
14 RELATIVE HAD CANCER, SITE UNKNOWN

15 UNKNOWN IF RELATIVE HAD CANCER
99 R

G056How old was s/he when this cancer was diagnosed?

___ ENTER AGE
998 DK
999 R

G057Did [FILL NAME] have any other cancer?

1 YES
3 NO [GOTO G063]
8 DK
9 R [GOTO G063]

G058What was the next type of cancer [FILL NAME] had?

01 SKIN (NOT MELANOMA)
02 PROSTATE
03 BREAST
04 LUNG
05 COLON
06 CORPUS (UTERINE)
07 RECTUM
08 BLADDER
09 OVARY
10 MELANOMA
11 CERVIX
12 CANCER OF FEMALE REPRODUCTIVE ORGAN, SITE UNKNOWN
13 OTHER CANCER [SPECIFY]
14 RELATIVE HAD CANCER, SITE UNKNOWN
15 UNKNOWN IF RELATIVE HAD CANCER
99 R

G059How old was s/he when this cancer was diagnosed?

___ ENTER AGE
998 DK
999 R

G060Did [FILL NAME] have any other cancer?

1 YES
3 NO [GOTO G063]
8 DK
9 R [GOTO G063]

G061What was the next type of cancer [FILL NAME] had?

01 SKIN (NOT MELANOMA)
02 PROSTATE
03 BREAST
04 LUNG
05 COLON
06 CORPUS (UTERINE)
07 RECTUM
08 BLADDER
09 OVARY
10 MELANOMA
11 CERVIX
12 CANCER OF FEMALE REPRODUCTIVE ORGAN, SITE UNKNOWN
13 OTHER CANCER [SPECIFY]
14 RELATIVE HAD CANCER, SITE UNKNOWN
15 UNKNOWN IF RELATIVE HAD CANCER
99 R

G062How old was s/he when this cancer was diagnosed?

___ ENTER AGE
998 DK

999 R

G063What is the name of your next brother or sister? (They will not be contacted. This is a simple way to help you complete this part of the questionnaire.)

____________________ ENTER NAME

00 NO MORE BROTHERS OR SISTERS [GOTO GTX2]

98 DK [GOTO GTX2]

99 R [GOTO GTX2]

G064What is [FILL NAME]'s sex?

1 MALE

2 FEMALE

8 DK

9 R

G065Is [FILL NAME] still living?

1 YES
3 NO [GOTO G067]
8 DK [GOTO G068]
9 R [GOTO G068]

G066How old is [FILL NAME]?

___ ENTER AGE [GOTO G068]
998 DK [GOTO G068]
999 R [GOTO G068]

G067How old was [FILL NAME] when s/he died?

___ ENTER AGE
998 DK
999 R

G068Did [FILL NAME] ever have a colon or rectal polyp?

1 YES
3 NO [GOTO G070]
8 DK [GOTO G070]
9 R [GOTO G070]

G069How old was s/he when the polyp was first diagnosed?

___ ENTER AGE
998 DK
999 R

G070Was [FILL NAME] ever diagnosed as having any type of cancer?

1 YES
3 NO [GOTO G079]
8 DK
9 R [GOTO G079]

G071What was the first type of cancer [FILL NAME] had?

01 SKIN (NOT MELANOMA)
02 PROSTATE
03 BREAST
04 LUNG
05 COLON
06 CORPUS (UTERINE)
07 RECTUM
08 BLADDER
09 OVARY
10 MELANOMA
11 CERVIX
12 CANCER OF FEMALE REPRODUCTIVE ORGAN, SITE UNKNOWN
13 OTHER CANCER [SPECIFY]
14 RELATIVE HAD CANCER, SITE UNKNOWN

15 UNKNOWN IF RELATIVE HAD CANCER
99 R

G072How old was s/he when this cancer was diagnosed?

___ ENTER AGE
998 DK
999 R

G073Did [FILL NAME] have any other cancer?

1 YES
3 NO [GOTO G079]
8 DK
9 R [GOTO G079]

G074What was the next type of cancer [FILL NAME] had?

01 SKIN (NOT MELANOMA)
02 PROSTATE
03 BREAST
04 LUNG
05 COLON
06 CORPUS (UTERINE)
07 RECTUM
08 BLADDER
09 OVARY
10 MELANOMA
11 CERVIX
12 CANCER OF FEMALE REPRODUCTIVE ORGAN, SITE UNKNOWN
13 OTHER CANCER [SPECIFY]
14 RELATIVE HAD CANCER, SITE UNKNOWN
15 UNKNOWN IF RELATIVE HAD CANCER
99 R

G075How old was s/he when this cancer was diagnosed?

___ ENTER AGE
998 DK
999 R

G076Did [FILL NAME] have any other cancer?

1 YES
3 NO [GOTO G079]
8 DK
9 R [GOTO G079]

G077What was the next type of cancer [FILL NAME] had?

01 SKIN (NOT MELANOMA)
02 PROSTATE
03 BREAST
04 LUNG
05 COLON
06 CORPUS (UTERINE)
07 RECTUM
08 BLADDER
09 OVARY
10 MELANOMA
11 CERVIX
12 CANCER OF FEMALE REPRODUCTIVE ORGAN, SITE UNKNOWN
13 OTHER CANCER [SPECIFY]
14 RELATIVE HAD CANCER, SITE UNKNOWN
15 UNKNOWN IF RELATIVE HAD CANCER
99 R

G078How old was s/he when this cancer was diagnosed?

___ ENTER AGE
998 DK

999 R

G079What is the name of your next brother or sister? (They will not be contacted. This is a simple way to help you complete this part of the questionnaire.)

____________________ ENTER NAME

00 NO MORE BROTHERS OR SISTERS [GOTO GTX2]

98 DK [GOTO GTX2]

99 R [GOTO GTX2]

G080What is [FILL NAME]'s sex?

1 MALE

2 FEMALE

8 DK

9 R

G081Is [FILL NAME] still living?

1 YES
3 NO [GOTO G083]
8 DK [GOTO G084]
9 R [GOTO G084]

G082How old is [FILL NAME]?

___ ENTER AGE [GOTO G084]
998 DK [GOTO G084]
999 R [GOTO G084]

G083How old was [FILL NAME] when s/he died?

___ ENTER AGE
998 DK
999 R

G084Did [FILL NAME] ever have a colon or rectal polyp?

1 YES
3 NO [GOTO G086]
8 DK [GOTO G086]
9 R [GOTO G086]

G085How old was s/he when the polyp was first diagnosed?

___ ENTER AGE
998 DK
999 R

G086Was [FILL NAME] ever diagnosed as having any type of cancer?

1 YES
3 NO [GOTO G095]
8 DK
9 R [GOTO G095]

G087What was the first type of cancer [FILL NAME] had?

01 SKIN (NOT MELANOMA)
02 PROSTATE
03 BREAST
04 LUNG
05 COLON
06 CORPUS (UTERINE)
07 RECTUM
08 BLADDER
09 OVARY
10 MELANOMA
11 CERVIX
12 CANCER OF FEMALE REPRODUCTIVE ORGAN, SITE UNKNOWN
13 OTHER CANCER [SPECIFY]
14 RELATIVE HAD CANCER, SITE UNKNOWN

15 UNKNOWN IF RELATIVE HAD CANCER
99 R

G088How old was s/he when this cancer was diagnosed?

___ ENTER AGE
998 DK
999 R

G089Did [FILL NAME] have any other cancer?

1 YES
3 NO [GOTO G095]
8 DK
9 R [GOTO G095]

G090What was the next type of cancer [FILL NAME] had?

01 SKIN (NOT MELANOMA)
02 PROSTATE
03 BREAST
04 LUNG
05 COLON
06 CORPUS (UTERINE)
07 RECTUM
08 BLADDER
09 OVARY
10 MELANOMA
11 CERVIX
12 CANCER OF FEMALE REPRODUCTIVE ORGAN, SITE UNKNOWN
13 OTHER CANCER [SPECIFY]
14 RELATIVE HAD CANCER, SITE UNKNOWN
15 UNKNOWN IF RELATIVE HAD CANCER
99 R

G091How old was s/he when this cancer was diagnosed?

___ ENTER AGE
998 DK
999 R

G092Did [FILL NAME] have any other cancer?

1 YES
3 NO [GOTO G095]
8 DK
9 R [GOTO G095]

G093What was the next type of cancer [FILL NAME] had?

01 SKIN (NOT MELANOMA)
02 PROSTATE
03 BREAST
04 LUNG
05 COLON
06 CORPUS (UTERINE)
07 RECTUM
08 BLADDER
09 OVARY
10 MELANOMA
11 CERVIX
12 CANCER OF FEMALE REPRODUCTIVE ORGAN, SITE UNKNOWN
13 OTHER CANCER [SPECIFY]
14 RELATIVE HAD CANCER, SITE UNKNOWN
15 UNKNOWN IF RELATIVE HAD CANCER
99 R

G094How old was s/he when this cancer was diagnosed?

___ ENTER AGE
998 DK

999 R

G095What is the name of your next brother or sister? (They will not be contacted. This is a simple way to help you complete this part of the questionnaire.)

____________________ ENTER NAME

00 NO MORE BROTHERS OR SISTERS [GOTO GTX2]

98 DK [GOTO GTX2]

99 R [GOTO GTX2]

G096What is [FILL NAME]'s sex?

1 MALE

2 FEMALE

8 DK

9 R

G097Is [FILL NAME] still living?

1 YES
3 NO [GOTO G099]
8 DK [GOTO G100]
9 R [GOTO G100]

G098How old is [FILL NAME]?

___ ENTER AGE [GOTO G100]
998 DK [GOTO G100]
999 R [GOTO G100]

G099How old was [FILL NAME] when s/he died?

___ ENTER AGE
998 DK
999 R

G100Did [FILL NAME] ever have a colon or rectal polyp?

1 YES
3 NO [GOTO G102]
8 DK [GOTO G102]
9 R [GOTO G102]

G101How old was s/he when the polyp was first diagnosed?

___ ENTER AGE
998 DK
999 R

G102Was [FILL NAME] ever diagnosed as having any type of cancer?

1 YES
3 NO [GOTO G111]
8 DK
9 R [GOTO G111]

G103What was the first type of cancer [FILL NAME] had?

01 SKIN (NOT MELANOMA)
02 PROSTATE
03 BREAST
04 LUNG
05 COLON
06 CORPUS (UTERINE)
07 RECTUM
08 BLADDER
09 OVARY
10 MELANOMA
11 CERVIX
12 CANCER OF FEMALE REPRODUCTIVE ORGAN, SITE UNKNOWN
13 OTHER CANCER [SPECIFY]
14 RELATIVE HAD CANCER, SITE UNKNOWN

15 UNKNOWN IF RELATIVE HAD CANCER
99 R

G104How old was s/he when this cancer was diagnosed?

___ ENTER AGE
998 DK
999 R

G105Did [FILL NAME] have any other cancer?

1 YES
3 NO [GOTO G111]
8 DK
9 R [GOTO G111]

G106What was the next type of cancer [FILL NAME] had?

01 SKIN (NOT MELANOMA)
02 PROSTATE
03 BREAST
04 LUNG
05 COLON
06 CORPUS (UTERINE)
07 RECTUM
08 BLADDER
09 OVARY
10 MELANOMA
11 CERVIX
12 CANCER OF FEMALE REPRODUCTIVE ORGAN, SITE UNKNOWN
13 OTHER CANCER [SPECIFY]
14 RELATIVE HAD CANCER, SITE UNKNOWN
15 UNKNOWN IF RELATIVE HAD CANCER
99 R

G107How old was s/he when this cancer was diagnosed?

___ ENTER AGE
998 DK
999 R

G108Did [FILL NAME] have any other cancer?

1 YES
3 NO [GOTO G111]
8 DK
9 R [GOTO G111]

G109What was the next type of cancer [FILL NAME] had?

01 SKIN (NOT MELANOMA)
02 PROSTATE
03 BREAST
04 LUNG
05 COLON
06 CORPUS (UTERINE)
07 RECTUM
08 BLADDER
09 OVARY
10 MELANOMA
11 CERVIX
12 CANCER OF FEMALE REPRODUCTIVE ORGAN, SITE UNKNOWN
13 OTHER CANCER [SPECIFY]
14 RELATIVE HAD CANCER, SITE UNKNOWN
15 UNKNOWN IF RELATIVE HAD CANCER
99 R

G110How old was s/he when this cancer was diagnosed?

___ ENTER AGE
998 DK

999 R

G111What is the name of your next brother or sister? (They will not be contacted. This is a simple way to help you complete this part of the questionnaire.)

____________________ ENTER NAME

00 NO MORE BROTHERS OR SISTERS [GOTO GTX2]

98 DK [GOTO GTX2]

99 R [GOTO GTX2]

G112What is [FILL NAME]'s sex?

1 MALE

2 FEMALE

8 DK

9 R

G113Is [FILL NAME] still living?

1 YES
3 NO [GOTO G115]
8 DK [GOTO G116]
9 R [GOTO G116]

G114How old is [FILL NAME]?

___ ENTER AGE [GOTO G116]
998 DK [GOTO G116]
999 R [GOTO G116]

G115How old was [FILL NAME] when s/he died?

___ ENTER AGE
998 DK
999 R

G116Did [FILL NAME] ever have a colon or rectal polyp?

1 YES
3 NO [GOTO G118]
8 DK [GOTO G118]
9 R [GOTO G118]

G117How old was s/he when the polyp was first diagnosed?

___ ENTER AGE
998 DK
999 R

G118Was [FILL NAME] ever diagnosed as having any type of cancer?

1 YES
3 NO [GOTO G127]
8 DK
9 R [GOTO G127]

G119What was the first type of cancer [FILL NAME] had?

01 SKIN (NOT MELANOMA)
02 PROSTATE
03 BREAST
04 LUNG
05 COLON
06 CORPUS (UTERINE)
07 RECTUM
08 BLADDER
09 OVARY
10 MELANOMA
11 CERVIX
12 CANCER OF FEMALE REPRODUCTIVE ORGAN, SITE UNKNOWN
13 OTHER CANCER [SPECIFY]
14 RELATIVE HAD CANCER, SITE UNKNOWN

15 UNKNOWN IF RELATIVE HAD CANCER
99 R

G120How old was s/he when this cancer was diagnosed?

___ ENTER AGE
998 DK
999 R

G121Did [FILL NAME] have any other cancer?

1 YES
3 NO [GOTO G127]
8 DK
9 R [GOTO G127]

G122What was the next type of cancer [FILL NAME] had?

01 SKIN (NOT MELANOMA)
02 PROSTATE
03 BREAST
04 LUNG
05 COLON
06 CORPUS (UTERINE)
07 RECTUM
08 BLADDER
09 OVARY
10 MELANOMA
11 CERVIX
12 CANCER OF FEMALE REPRODUCTIVE ORGAN, SITE UNKNOWN
13 OTHER CANCER [SPECIFY]
14 RELATIVE HAD CANCER, SITE UNKNOWN
15 UNKNOWN IF RELATIVE HAD CANCER
99 R

G123How old was s/he when this cancer was diagnosed?

___ ENTER AGE
998 DK
999 R

G124Did [FILL NAME] have any other cancer?

1 YES
3 NO [GOTO G127]
8 DK
9 R [GOTO G127]

G125What was the next type of cancer [FILL NAME] had?

01 SKIN (NOT MELANOMA)
02 PROSTATE
03 BREAST
04 LUNG
05 COLON
06 CORPUS (UTERINE)
07 RECTUM
08 BLADDER
09 OVARY
10 MELANOMA
11 CERVIX
12 CANCER OF FEMALE REPRODUCTIVE ORGAN, SITE UNKNOWN
13 OTHER CANCER [SPECIFY]
14 RELATIVE HAD CANCER, SITE UNKNOWN
15 UNKNOWN IF RELATIVE HAD CANCER
99 R

G126How old was s/he when this cancer was diagnosed?

___ ENTER AGE
998 DK

999 R

G127What is the name of your next brother or sister? (They will not be contacted. This is a simple way to help you complete this part of the questionnaire.)

____________________ ENTER NAME

00 NO MORE BROTHERS OR SISTERS [GOTO GTX2]

98 DK [GOTO GTX2]

99 R [GOTO GTX2]

G128What is [FILL NAME]'s sex?

1 MALE

2 FEMALE

8 DK

9 R

G129Is [FILL NAME] still living?

1 YES
3 NO [GOTO G131]
8 DK [GOTO G132]
9 R [GOTO G132]

G130How old is [FILL NAME]?

___ ENTER AGE [GOTO G132]
998 DK [GOTO G132]
999 R [GOTO G132]

G131How old was [FILL NAME] when s/he died?

___ ENTER AGE
998 DK
999 R

G132Did [FILL NAME] ever have a colon or rectal polyp?

1 YES
3 NO [GOTO G134]
8 DK [GOTO G134]
9 R [GOTO G134]

G133How old was s/he when the polyp was first diagnosed?

___ ENTER AGE
998 DK
999 R

G134Was [FILL NAME] ever diagnosed as having any type of cancer?

1 YES
3 NO [GOTO G143]
8 DK
9 R [GOTO G143]

G135What was the first type of cancer [FILL NAME] had?

01 SKIN (NOT MELANOMA)
02 PROSTATE
03 BREAST
04 LUNG
05 COLON
06 CORPUS (UTERINE)
07 RECTUM
08 BLADDER
09 OVARY
10 MELANOMA
11 CERVIX
12 CANCER OF FEMALE REPRODUCTIVE ORGAN, SITE UNKNOWN
13 OTHER CANCER [SPECIFY]
14 RELATIVE HAD CANCER, SITE UNKNOWN

15 UNKNOWN IF RELATIVE HAD CANCER
99 R

G136How old was s/he when this cancer was diagnosed?

___ ENTER AGE
998 DK
999 R

G137Did [FILL NAME] have any other cancer?

1 YES
3 NO [GOTO G143]
8 DK
9 R [GOTO G143]

G138What was the next type of cancer [FILL NAME] had?

01 SKIN (NOT MELANOMA)
02 PROSTATE
03 BREAST
04 LUNG
05 COLON
06 CORPUS (UTERINE)
07 RECTUM
08 BLADDER
09 OVARY
10 MELANOMA
11 CERVIX
12 CANCER OF FEMALE REPRODUCTIVE ORGAN, SITE UNKNOWN
13 OTHER CANCER [SPECIFY]
14 RELATIVE HAD CANCER, SITE UNKNOWN
15 UNKNOWN IF RELATIVE HAD CANCER
99 R

G139How old was s/he when this cancer was diagnosed?

___ ENTER AGE
998 DK
999 R

G140Did [FILL NAME] have any other cancer?

1 YES
3 NO [GOTO G143]
8 DK
9 R [GOTO G143]

G141What was the next type of cancer [FILL NAME] had?

01 SKIN (NOT MELANOMA)
02 PROSTATE
03 BREAST
04 LUNG
05 COLON
06 CORPUS (UTERINE)
07 RECTUM
08 BLADDER
09 OVARY
10 MELANOMA
11 CERVIX
12 CANCER OF FEMALE REPRODUCTIVE ORGAN, SITE UNKNOWN
13 OTHER CANCER [SPECIFY]
14 RELATIVE HAD CANCER, SITE UNKNOWN
15 UNKNOWN IF RELATIVE HAD CANCER
99 R

G142How old was s/he when this cancer was diagnosed?

___ ENTER AGE
998 DK

999 R

G143What is the name of your next brother or sister? (They will not be contacted. This is a simple way to help you complete this part of the questionnaire.)

____________________ ENTER NAME

00 NO MORE BROTHERS OR SISTERS [GOTO GTX2]

98 DK [GOTO GTX2]

99 R [GOTO GTX2]

G144What is [FILL NAME]'s sex?

1 MALE

2 FEMALE

8 DK

9 R

G145Is [FILL NAME] still living?

1 YES
3 NO [GOTO G147]
8 DK [GOTO G148
9 R [GOTO G148]

G146How old is [FILL NAME]?

___ ENTER AGE [GOTO G148]
998 DK [GOTO G148]
999 R [GOTO G148]

G147How old was [FILL NAME] when s/he died?

___ ENTER AGE
998 DK
999 R

G148Did [FILL NAME] ever have a colon or rectal polyp?

1 YES
3 NO [GOTO G150]
8 DK [GOTO G150]
9 R [GOTO G150]

G149How old was s/he when the polyp was first diagnosed?

___ ENTER AGE
998 DK
999 R

G150Was [FILL NAME] ever diagnosed as having any type of cancer?

1 YES
3 NO [GOTO G159]
8 DK
9 R [GOTO G159]

G151What was the first type of cancer [FILL NAME] had?

01 SKIN (NOT MELANOMA)
02 PROSTATE
03 BREAST
04 LUNG
05 COLON
06 CORPUS (UTERINE)
07 RECTUM
08 BLADDER
09 OVARY
10 MELANOMA
11 CERVIX
12 CANCER OF FEMALE REPRODUCTIVE ORGAN, SITE UNKNOWN
13 OTHER CANCER [SPECIFY]
14 RELATIVE HAD CANCER, SITE UNKNOWN

15 UNKNOWN IF RELATIVE HAD CANCER
99 R

G152How old was s/he when this cancer was diagnosed?

___ ENTER AGE
998 DK
999 R

G153Did [FILL NAME] have any other cancer?

1 YES
3 NO [GOTO G159]
8 DK
9 R [GOTO G159]

G154What was the next type of cancer [FILL NAME] had?

01 SKIN (NOT MELANOMA)
02 PROSTATE
03 BREAST
04 LUNG
05 COLON
06 CORPUS (UTERINE)
07 RECTUM
08 BLADDER
09 OVARY
10 MELANOMA
11 CERVIX
12 CANCER OF FEMALE REPRODUCTIVE ORGAN, SITE UNKNOWN
13 OTHER CANCER [SPECIFY]
14 RELATIVE HAD CANCER, SITE UNKNOWN
15 UNKNOWN IF RELATIVE HAD CANCER
99 R

G155How old was s/he when this cancer was diagnosed?

___ ENTER AGE
998 DK
999 R

G156Did [FILL NAME] have any other cancer?

1 YES
3 NO [GOTO G159]
8 DK
9 R [GOTO G159]

G157What was the next type of cancer [FILL NAME] had?

01 SKIN (NOT MELANOMA)
02 PROSTATE
03 BREAST
04 LUNG
05 COLON
06 CORPUS (UTERINE)
07 RECTUM
08 BLADDER
09 OVARY
10 MELANOMA
11 CERVIX
12 CANCER OF FEMALE REPRODUCTIVE ORGAN, SITE UNKNOWN
13 OTHER CANCER [SPECIFY]
14 RELATIVE HAD CANCER, SITE UNKNOWN
15 UNKNOWN IF RELATIVE HAD CANCER
99 R

G158How old was s/he when this cancer was diagnosed?

___ ENTER AGE
998 DK

999 R

G159What is the name of your next brother or sister? (They will not be contacted. This is a simple way to help you complete this part of the questionnaire.)

____________________ ENTER NAME

00 NO MORE BROTHERS OR SISTERS [GOTO GTX2]

98 DK [GOTO GTX2]

99 R [GOTO GTX2]

G160What is [FILL NAME]'s sex?

1 MALE

2 FEMALE

8 DK

9 R

G161Is [FILL NAME] still living?

1 YES
3 NO [GOTO G163]
8 DK [GOTO G164]
9 R [GOTO G164]

G162How old is [FILL NAME]?

___ ENTER AGE [GOTO G164]
998 DK [GOTO G164]
999 R [GOTO G164]

G163How old was [FILL NAME] when s/he died?

___ ENTER AGE
998 DK
999 R

G164Did [FILL NAME] ever have a colon or rectal polyp?

1 YES
3 NO [GOTO G166]
8 DK [GOTO G166]
9 R [GOTO G166]

G165How old was s/he when the polyp was first diagnosed?

___ ENTER AGE
998 DK
999 R

G166Was [FILL NAME] ever diagnosed as having any type of cancer?

1 YES
3 NO [GOTO G175]
8 DK
9 R [GOTO G175]

G167What was the first type of cancer [FILL NAME] had?

01 SKIN (NOT MELANOMA)
02 PROSTATE
03 BREAST
04 LUNG
05 COLON
06 CORPUS (UTERINE)
07 RECTUM
08 BLADDER
09 OVARY
10 MELANOMA
11 CERVIX
12 CANCER OF FEMALE REPRODUCTIVE ORGAN, SITE UNKNOWN
13 OTHER CANCER [SPECIFY]
14 RELATIVE HAD CANCER, SITE UNKNOWN

15 UNKNOWN IF RELATIVE HAD CANCER
99 R

G168How old was s/he when this cancer was diagnosed?

___ ENTER AGE
998 DK
999 R

G169Did [FILL NAME] have any other cancer?

1 YES
3 NO [GOTO G175]
8 DK
9 R [GOTO G175]

G170What was the next type of cancer [FILL NAME] had?

01 SKIN (NOT MELANOMA)
02 PROSTATE
03 BREAST
04 LUNG
05 COLON
06 CORPUS (UTERINE)
07 RECTUM
08 BLADDER
09 OVARY
10 MELANOMA
11 CERVIX
12 CANCER OF FEMALE REPRODUCTIVE ORGAN, SITE UNKNOWN
13 OTHER CANCER [SPECIFY]
14 RELATIVE HAD CANCER, SITE UNKNOWN
15 UNKNOWN IF RELATIVE HAD CANCER
99 R

G171How old was s/he when this cancer was diagnosed?

___ ENTER AGE
998 DK
999 R

G172Did [FILL NAME] have any other cancer?

1 YES
3 NO [GOTO G175]
8 DK
9 R [GOTO G175]

G173What was the next type of cancer [FILL NAME] had?

01 SKIN (NOT MELANOMA)
02 PROSTATE
03 BREAST
04 LUNG
05 COLON
06 CORPUS (UTERINE)
07 RECTUM
08 BLADDER
09 OVARY
10 MELANOMA
11 CERVIX
12 CANCER OF FEMALE REPRODUCTIVE ORGAN, SITE UNKNOWN
13 OTHER CANCER [SPECIFY]
14 RELATIVE HAD CANCER, SITE UNKNOWN
15 UNKNOWN IF RELATIVE HAD CANCER
99 R

G174How old was s/he when this cancer was diagnosed?

___ ENTER AGE
998 DK

999 R

G175What is the name of your next brother or sister? (They will not be contacted. This is a simple way to help you complete this part of the questionnaire.)

____________________ ENTER NAME

00 NO MORE BROTHERS OR SISTERS [GOTO GTX2]

98 DK [GOTO GTX2]

99 R [GOTO GTX2]

G176What is [FILL NAME]'s sex?

1 MALE

2 FEMALE

8 DK

9 R

G177Is [FILL NAME] still living?

1 YES
3 NO [GOTO G179]
8 DK [GOTO G180]
9 R [GOTO G180]

G178How old is [FILL NAME]?

___ ENTER AGE [GOTO G180]
998 DK [GOTO G180]
999 R [GOTO G180]

G179How old was [FILL NAME] when s/he died?

___ ENTER AGE
998 DK
999 R

G180Did [FILL NAME] ever have a colon or rectal polyp?

1 YES
3 NO [GOTO G182]
8 DK [GOTO G182]
9 R [GOTO G182]

G181How old was s/he when the polyp was first diagnosed?

___ ENTER AGE
998 DK
999 R

G182Was [FILL NAME] ever diagnosed as having any type of cancer?

1 YES
3 NO [GOTO G191]
8 DK
9 R [GOTO G191]

G183What was the first type of cancer [FILL NAME] had?

01 SKIN (NOT MELANOMA)
02 PROSTATE
03 BREAST
04 LUNG
05 COLON
06 CORPUS (UTERINE)
07 RECTUM
08 BLADDER
09 OVARY
10 MELANOMA
11 CERVIX
12 CANCER OF FEMALE REPRODUCTIVE ORGAN, SITE UNKNOWN
13 OTHER CANCER [SPECIFY]
14 RELATIVE HAD CANCER, SITE UNKNOWN

15 UNKNOWN IF RELATIVE HAD CANCER
99 R

G184How old was s/he when this cancer was diagnosed?

___ ENTER AGE
998 DK
999 R

G185Did [FILL NAME] have any other cancer?

1 YES
3 NO [GOTO G191]
8 DK
9 R [GOTO G191]

G186What was the next type of cancer [FILL NAME] had?

01 SKIN (NOT MELANOMA)
02 PROSTATE
03 BREAST
04 LUNG
05 COLON
06 CORPUS (UTERINE)
07 RECTUM
08 BLADDER
09 OVARY
10 MELANOMA
11 CERVIX
12 CANCER OF FEMALE REPRODUCTIVE ORGAN, SITE UNKNOWN
13 OTHER CANCER [SPECIFY]
14 RELATIVE HAD CANCER, SITE UNKNOWN
15 UNKNOWN IF RELATIVE HAD CANCER
99 R

G187How old was s/he when this cancer was diagnosed?

___ ENTER AGE
998 DK
999 R

G188Did [FILL NAME] have any other cancer?

1 YES
3 NO [GOTO G191]
8 DK
9 R [GOTO G191]

G189What was the next type of cancer [FILL NAME] had?

01 SKIN (NOT MELANOMA)
02 PROSTATE
03 BREAST
04 LUNG
05 COLON
06 CORPUS (UTERINE)
07 RECTUM
08 BLADDER
09 OVARY
10 MELANOMA
11 CERVIX
12 CANCER OF FEMALE REPRODUCTIVE ORGAN, SITE UNKNOWN
13 OTHER CANCER [SPECIFY]
14 RELATIVE HAD CANCER, SITE UNKNOWN
15 UNKNOWN IF RELATIVE HAD CANCER
99 R

G190How old was s/he when this cancer was diagnosed?

___ ENTER AGE
998 DK

999 R

G191What is the name of your next brother or sister? (They will not be contacted. This is a simple way to help you complete this part of the questionnaire.)

____________________ ENTER NAME

00 NO MORE BROTHERS OR SISTERS [GOTO GTX2]

98 DK [GOTO GTX2]

99 R [GOTO GTX2]

G192What is [FILL NAME]'s sex?

1 MALE

2 FEMALE

8 DK

9 R

G193Is [FILL NAME] still living?

1 YES
3 NO [GOTO G195]
8 DK [GOTO G196]
9 R [GOTO G196]

G194How old is [FILL NAME]?

___ ENTER AGE [GOTO G196]
998 DK [GOTO G196]
999 R [GOTO G196]

G195How old was [FILL NAME] when s/he died?

___ ENTER AGE
998 DK
999 R

G196Did [FILL NAME] ever have a colon or rectal polyp?

1 YES
3 NO [GOTO G198]
8 DK [GOTO G198]
9 R [GOTO G198]

G197How old was s/he when the polyp was first diagnosed?

___ ENTER AGE
998 DK
999 R

G198Was [FILL NAME] ever diagnosed as having any type of cancer?

1 YES
3 NO [GOTO G207]
8 DK
9 R [GOTO G207]

G199What was the first type of cancer [FILL NAME] had?

01 SKIN (NOT MELANOMA)
02 PROSTATE
03 BREAST
04 LUNG
05 COLON
06 CORPUS (UTERINE)
07 RECTUM
08 BLADDER
09 OVARY
10 MELANOMA
11 CERVIX
12 CANCER OF FEMALE REPRODUCTIVE ORGAN, SITE UNKNOWN
13 OTHER CANCER [SPECIFY]

14 RELATIVE HAD CANCER, SITE UNKNOWN
15 UNKNOWN IF RELATIVE HAD CANCER
99 R

G200How old was s/he when this cancer was diagnosed?

___ ENTER AGE
998 DK
999 R

G201Did [FILL NAME] have any other cancer?

1 YES
3 NO [GOTO G207]
8 DK
9 R [GOTO G207]

G202What was the next type of cancer [FILL NAME] had?

01 SKIN (NOT MELANOMA)
02 PROSTATE
03 BREAST
04 LUNG
05 COLON
06 CORPUS (UTERINE)
07 RECTUM
08 BLADDER
09 OVARY
10 MELANOMA
11 CERVIX
12 CANCER OF FEMALE REPRODUCTIVE ORGAN, SITE UNKNOWN
13 OTHER CANCER [SPECIFY]
14 RELATIVE HAD CANCER, SITE UNKNOWN
15 UNKNOWN IF RELATIVE HAD CANCER
99 R

G203How old was s/he when this cancer was diagnosed?

___ ENTER AGE
998 DK
999 R

G204Did [FILL NAME] have any other cancer?

1 YES
3 NO [GOTO G207]
8 DK
9 R [GOTO G207]

G205What was the next type of cancer [FILL NAME] had?

01 SKIN (NOT MELANOMA)
02 PROSTATE
03 BREAST
04 LUNG
05 COLON
06 CORPUS (UTERINE)
07 RECTUM
08 BLADDER
09 OVARY
10 MELANOMA
11 CERVIX
12 CANCER OF FEMALE REPRODUCTIVE ORGAN, SITE UNKNOWN
13 OTHER CANCER [SPECIFY]
14 RELATIVE HAD CANCER, SITE UNKNOWN
15 UNKNOWN IF RELATIVE HAD CANCER
99 R

G206How old was s/he when this cancer was diagnosed?

___ ENTER AGE
998 DK

999 R

G207What is the name of your next brother or sister? (They will not be contacted. This is a simple way to help you complete this part of the questionnaire.)

____________________ ENTER NAME

00 NO MORE BROTHERS OR SISTERS [GOTO GTX2]

98 DK [GOTO GTX2]

99 R [GOTO GTX2]

G208What is [FILL NAME]'s sex?

1 MALE

2 FEMALE

8 DK

9 R

G209Is [FILL NAME] still living?

1 YES
3 NO [GOTO G211]
8 DK [GOTO G212]
9 R [GOTO G212]

G210How old is [FILL NAME]?

___ ENTER AGE [GOTO G212]
998 DK [GOTO G212]
999 R [GOTO G212]

G211How old was [FILL NAME] when s/he died?

___ ENTER AGE
998 DK
999 R

G212Did [FILL NAME] ever have a colon or rectal polyp?

1 YES
3 NO [GOTO G214]
8 DK [GOTO G214]
9 R [GOTO G214]

G213How old was s/he when the polyp was first diagnosed?

___ ENTER AGE
998 DK
999 R

G214Was [FILL NAME] ever diagnosed as having any type of cancer?

1 YES
3 NO [GOTO G223]
8 DK [GOTO G223]
9 R [GOTO G223]

G215What was the first type of cancer [FILL NAME] had?

01 SKIN (NOT MELANOMA)
02 PROSTATE
03 BREAST
04 LUNG
05 COLON
06 CORPUS (UTERINE)
07 RECTUM
08 BLADDER
09 OVARY
10 MELANOMA
11 CERVIX
12 CANCER OF FEMALE REPRODUCTIVE ORGAN, SITE UNKNOWN
13 OTHER CANCER [SPECIFY]
14 RELATIVE HAD CANCER, SITE UNKNOWN

15 UNKNOWN IF RELATIVE HAD CANCER

99 R

G216How old was s/he when this cancer was diagnosed?

___ ENTER AGE

998 DK

999 R

G217Did [FILL NAME] have any other cancer?

1 YES

3 NO [GOTO G223]

8 DK

9 R [GOTO G223]

G218What was the next type of cancer [FILL NAME] had?

01 SKIN (NOT MELANOMA)
02 PROSTATE
03 BREAST
04 LUNG
05 COLON
06 CORPUS (UTERINE)
07 RECTUM
08 BLADDER
09 OVARY
10 MELANOMA
11 CERVIX
12 CANCER OF FEMALE REPRODUCTIVE ORGAN, SITE UNKNOWN
13 OTHER CANCER [SPECIFY]
14 RELATIVE HAD CANCER, SITE UNKNOWN
15 UNKNOWN IF RELATIVE HAD CANCER
99 R

G219How old was s/he when this cancer was diagnosed?

___ ENTER AGE
998 DK
999 R

G220Did [FILL NAME] have any other cancer?

1 YES
3 NO [GOTO G223]
8 DK
9 R [GOTO G223]

G221What was the next type of cancer [FILL NAME] had?

01 SKIN (NOT MELANOMA)
02 PROSTATE
03 BREAST
04 LUNG
05 COLON
06 CORPUS (UTERINE)
07 RECTUM
08 BLADDER
09 OVARY
10 MELANOMA
11 CERVIX
12 CANCER OF FEMALE REPRODUCTIVE ORGAN, SITE UNKNOWN
13 OTHER CANCER [SPECIFY]
14 RELATIVE HAD CANCER, SITE UNKNOWN
15 UNKNOWN IF RELATIVE HAD CANCER
99 R

G222How old was s/he when this cancer was diagnosed?

___ ENTER AGE
998 DK

999 R

G223What is the name of your next brother or sister? (They will not be contacted. This is a simple way to help you complete this part of the questionnaire.)

____________________ ENTER NAME

00 NO MORE BROTHERS OR SISTERS [GOTO GTX2]

98 DK [GOTO GTX2]

99 R [GOTO GTX2]

G224What is [FILL NAME]'s sex?

1 MALE

2 FEMALE

8 DK

9 R

G225Is [FILL NAME] still living?

1 YES
3 NO [GOTO G227]
8 DK [GOTO G228]
9 R [GOTO G228]

G226How old is [FILL NAME]?

___ ENTER AGE [GOTO G228]
998 DK [GOTO G228]
999 R [GOTO G228]

G227How old was [FILL NAME] when s/he died?

___ ENTER AGE
998 DK
999 R

G228Did [FILL NAME] ever have a colon or rectal polyp?

1 YES
3 NO [GOTO G230]
8 DK [GOTO G230]
9 R [GOTO G230]

G229How old was s/he when the polyp was first diagnosed?

___ ENTER AGE
998 DK
999 R

G230Was [FILL NAME] ever diagnosed as having any type of cancer?

1 YES
3 NO [GOTO G239]
8 DK
9 R [GOTO G239]

G231What was the first type of cancer [FILL NAME] had?

01 SKIN (NOT MELANOMA)
02 PROSTATE
03 BREAST
04 LUNG
05 COLON
06 CORPUS (UTERINE)
07 RECTUM
08 BLADDER
09 OVARY
10 MELANOMA
11 CERVIX
12 CANCER OF FEMALE REPRODUCTIVE ORGAN, SITE UNKNOWN
13 OTHER CANCER [SPECIFY]
14 RELATIVE HAD CANCER, SITE UNKNOWN

15 UNKNOWN IF RELATIVE HAD CANCER
99 R

G232How old was s/he when this cancer was diagnosed?

___ ENTER AGE
998 DK
999 R

G233Did [FILL NAME] have any other cancer?

1 YES
3 NO [GOTO G239]
8 DK
9 R [GOTO G239]

G234What was the next type of cancer [FILL NAME] had?

01 SKIN (NOT MELANOMA)
02 PROSTATE
03 BREAST
04 LUNG
05 COLON
06 CORPUS (UTERINE)
07 RECTUM
08 BLADDER
09 OVARY
10 MELANOMA
11 CERVIX
12 CANCER OF FEMALE REPRODUCTIVE ORGAN, SITE UNKNOWN
13 OTHER CANCER [SPECIFY]
14 RELATIVE HAD CANCER, SITE UNKNOWN
15 UNKNOWN IF RELATIVE HAD CANCER
99 R

G235How old was s/he when this cancer was diagnosed?

___ ENTER AGE
998 DK
999 R

G236Did [FILL NAME] have any other cancer?

1 YES
3 NO [GOTO G239]
8 DK
9 R [GOTO G239]

G237What was the next type of cancer [FILL NAME] had?

01 SKIN (NOT MELANOMA)
02 PROSTATE
03 BREAST
04 LUNG
05 COLON
06 CORPUS (UTERINE)
07 RECTUM
08 BLADDER
09 OVARY
10 MELANOMA
11 CERVIX
12 CANCER OF FEMALE REPRODUCTIVE ORGAN, SITE UNKNOWN
13 OTHER CANCER [SPECIFY]
14 RELATIVE HAD CANCER, SITE UNKNOWN
15 UNKNOWN IF RELATIVE HAD CANCER
99 R

G238How old was s/he when this cancer was diagnosed?

___ ENTER AGE
998 DK

999 R

G239What is the name of your next brother or sister? (They will not be contacted. This is a simple way to help you complete this part of the questionnaire.)

____________________ ENTER NAME

00 NO MORE BROTHERS OR SISTERS [GOTO GTX2]

98 DK [GOTO GTX2]

99 R [GOTO GTX2]

G240What is [FILL NAME]'s sex?

1 MALE

2 FEMALE

8 DK

9 R

G241Is [FILL NAME] still living?

1 YES
3 NO [GOTO G243]
8 DK [GOTO G244]
9 R [GOTO G244]

G242How old is [FILL NAME]?

___ ENTER AGE [GOTO G244]
998 DK [GOTO G244]
999 R [GOTO G244]

G243How old was [FILL NAME] when s/he died?

___ ENTER AGE
998 DK
999 R

G244Did [FILL NAME] ever have a colon or rectal polyp?

1 YES
3 NO [GOTO G246]
8 DK [GOTO G246]
9 R [GOTO G246]

G245How old was s/he when the polyp was first diagnosed?

___ ENTER AGE
998 DK
999 R

G246Was [FILL NAME] ever diagnosed as having any type of cancer?

1 YES
3 NO [GOTO G255]
8 DK [GOTO G255]
9 R [GOTO G255]

G247What was the first type of cancer [FILL NAME] had?

01 SKIN (NOT MELANOMA)
02 PROSTATE
03 BREAST
04 LUNG
05 COLON
06 CORPUS (UTERINE)
07 RECTUM
08 BLADDER
09 OVARY
10 MELANOMA
11 CERVIX
12 CANCER OF FEMALE REPRODUCTIVE ORGAN, SITE UNKNOWN
13 OTHER CANCER [SPECIFY]
14 RELATIVE HAD CANCER, SITE UNKNOWN

15 UNKNOWN IF RELATIVE HAD CANCER
99 R

G248How old was s/he when this cancer was diagnosed?

___ ENTER AGE
998 DK
999 R

G249Did [FILL NAME] have any other cancer?

1 YES
3 NO [GOTO G255]
8 DK
9 R [GOTO G255]

G250What was the next type of cancer [FILL NAME] had?

01 SKIN (NOT MELANOMA)
02 PROSTATE
03 BREAST
04 LUNG
05 COLON
06 CORPUS (UTERINE)
07 RECTUM
08 BLADDER
09 OVARY
10 MELANOMA
11 CERVIX
12 CANCER OF FEMALE REPRODUCTIVE ORGAN, SITE UNKNOWN
13 OTHER CANCER [SPECIFY]
14 RELATIVE HAD CANCER, SITE UNKNOWN
15 UNKNOWN IF RELATIVE HAD CANCER
99 R

G251How old was s/he when this cancer was diagnosed?

___ ENTER AGE
998 DK
999 R

G252Did [FILL NAME] have any other cancer?

1 YES
3 NO [GOTO G255]
8 DK
9 R [GOTO G255]

G253What was the next type of cancer [FILL NAME] had?

01 SKIN (NOT MELANOMA)
02 PROSTATE
03 BREAST
04 LUNG
05 COLON
06 CORPUS (UTERINE)
07 RECTUM
08 BLADDER
09 OVARY
10 MELANOMA
11 CERVIX
12 CANCER OF FEMALE REPRODUCTIVE ORGAN, SITE UNKNOWN
13 OTHER CANCER [SPECIFY]
14 RELATIVE HAD CANCER, SITE UNKNOWN
15 UNKNOWN IF RELATIVE HAD CANCER
99 R

G254How old was s/he when this cancer was diagnosed?

___ ENTER AGE
998 DK

999 R

G255What is the name of your next brother or sister? (They will not be contacted. This is a simple way to help you complete this part of the questionnaire.)

____________________ ENTER NAME
00 NO MORE BROTHERS OR SISTERS [GOTO GTX2]
98 DK [GOTO GTX2]
99 R [GOTO GTX2]

G256What is [FILL NAME]'s sex?

1 MALE
2 FEMALE
8 DK
9 R

G257Is [FILL NAME] still living?

1 YES
3 NO [GOTO G259]
8 DK [GOTO G260]
9 R [GOTO G260]

G258How old is [FILL NAME]?

___ ENTER AGE [GOTO G260]
998 DK [GOTO G260]
999 R [GOTO G260]

G259How old was [FILL NAME] when s/he died?

___ ENTER AGE
998 DK
999 R

G260Did [FILL NAME] ever have a colon or rectal polyp?

1 YES
3 NO [GOTO G262]
8 DK [GOTO G262]
9 R [GOTO G262]

G261How old was s/he when the polyp was first diagnosed?

___ ENTER AGE
998 DK
999 R

G262Was [FILL NAME] ever diagnosed as having any type of cancer?

1 YES
3 NO [GOTO GTX2]
8 DK
9 R [GOTO GTX2]

G263What was the first type of cancer [FILL NAME] had?

01 SKIN (NOT MELANOMA)
02 PROSTATE
03 BREAST
04 LUNG
05 COLON
06 CORPUS (UTERINE)
07 RECTUM
08 BLADDER
09 OVARY
10 MELANOMA
11 CERVIX
12 CANCER OF FEMALE REPRODUCTIVE ORGAN, SITE UNKNOWN
13 OTHER CANCER [SPECIFY]
14 RELATIVE HAD CANCER, SITE UNKNOWN

15 UNKNOWN IF RELATIVE HAD CANCER
99 R

G264How old was s/he when this cancer was diagnosed?

___ ENTER AGE
998 DK
999 R

G265Did [FILL NAME] have any other cancer?

1 YES
3 NO [GOTO GTX2]
8 DK
9 R [GOTO GTX2]

G266What was the next type of cancer [FILL NAME] had?

01 SKIN (NOT MELANOMA)
02 PROSTATE
03 BREAST
04 LUNG
05 COLON
06 CORPUS (UTERINE)
07 RECTUM
08 BLADDER
09 OVARY
10 MELANOMA
11 CERVIX
12 CANCER OF FEMALE REPRODUCTIVE ORGAN, SITE UNKNOWN
13 OTHER CANCER [SPECIFY]
14 RELATIVE HAD CANCER, SITE UNKNOWN
15 UNKNOWN IF RELATIVE HAD CANCER
99 R

G267How old was s/he when this cancer was diagnosed?

___ ENTER AGE
998 DK
999 R

G268Did [FILL NAME] have any other cancer?

1 YES
3 NO [GOTO GTX2]
8 DK
9 R [GOTO GTX2]

G269What was the next type of cancer [FILL NAME] had?

01 SKIN (NOT MELANOMA)
02 PROSTATE
03 BREAST
04 LUNG
05 COLON
06 CORPUS (UTERINE)
07 RECTUM
08 BLADDER
09 OVARY
10 MELANOMA
11 CERVIX
12 CANCER OF FEMALE REPRODUCTIVE ORGAN, SITE UNKNOWN
13 OTHER CANCER [SPECIFY]
14 RELATIVE HAD CANCER, SITE UNKNOWN
15 UNKNOWN IF RELATIVE HAD CANCER
99 R

G270How old was s/he when this cancer was diagnosed?

___ ENTER AGE
998 DK

999 R

GTX2I would like to ask you the same questions about your children. Again, include those who are living or deceased, but do not include adopted, foster, or step children.

G271How many children do you have?

__ ENTER NUMBER
00 NONE [GOTO ITX1]
98 DK [GOTO ITX1]
99 R [GOTO ITX1]

G272What is the name of your oldest child? They will not be contacted. This is a simple way to help you complete this part of the questionnaire.

____________________ ENTER NAME
98 DK
99 R

G273What is [FILL NAME]'s sex?

1 MALE
2 FEMALE
8 DK
9 R

G274Is [FILL NAME] still living?

1 YES
3 NO [GOTO G276]
8 DK [GOTO G277]
9 R [GOTO G277]

G275How old is [FILL NAME]?

___ ENTER AGE [GOTO G277]
998 DK [GOTO G277]
999 R [GOTO G277]

G276How old was [FILL NAME] when s/he died?

___ ENTER AGE
998 DK
999 R

G277Did [FILL NAME] ever have a colon or rectal polyp?

1 YES
3 NO [GOTO G279]
8 DK [GOTO G279]
9 R [GOTO G279]

G278How old was s/he when the polyp was first diagnosed?

___ ENTER AGE
998 DK
999 R

G279Was [FILL NAME] ever diagnosed as having any type of cancer?

1 YES
3 NO [GOTO G288]
8 DK
9 R [GOTO G288]

G280What was the first type of cancer [FILL NAME] had?

01 SKIN (NOT MELANOMA)
02 PROSTATE
03 BREAST
04 LUNG
05 COLON
06 CORPUS (UTERINE)
07 RECTUM
08 BLADDER
09 OVARY
10 MELANOMA
11 CERVIX
12 CANCER OF FEMALE REPRODUCTIVE ORGAN, SITE UNKNOWN
13 OTHER CANCER [SPECIFY]
14 RELATIVE HAD CANCER, SITE UNKNOWN
15 UNKNOWN IF RELATIVE HAD CANCER
99 R

G281How old was s/he when this cancer was diagnosed?

___ ENTER AGE
998 DK
999 R

G282Did [FILL NAME] have any other cancer?

1 YES
3 NO [GOTO G288]
8 DK
9 R [GOTO G288]

G283What was the next type of cancer [FILL NAME] had?

01 SKIN (NOT MELANOMA)
02 PROSTATE
03 BREAST
04 LUNG
05 COLON
06 CORPUS (UTERINE)
07 RECTUM
08 BLADDER
09 OVARY
10 MELANOMA
11 CERVIX
12 CANCER OF FEMALE REPRODUCTIVE ORGAN, SITE UNKNOWN
13 OTHER CANCER [SPECIFY]
14 RELATIVE HAD CANCER, SITE UNKNOWN
15 UNKNOWN IF RELATIVE HAD CANCER
99 R

G284How old was s/he when this cancer was diagnosed?

___ ENTER AGE
998 DK
999 R

G285Did [FILL NAME] have any other cancer?

1 YES
3 NO [GOTO G228]
8 DK
9 R [GOTO G228]

G286What was the next type of cancer [FILL NAME] had?

01 SKIN (NOT MELANOMA)
02 PROSTATE
03 BREAST
04 LUNG
05 COLON
06 CORPUS (UTERINE)
07 RECTUM
08 BLADDER

09 OVARY
10 MELANOMA
11 CERVIX
12 CANCER OF FEMALE REPRODUCTIVE ORGAN, SITE UNKNOWN
13 OTHER CANCER [SPECIFY]
14 RELATIVE HAD CANCER, SITE UNKNOWN
15 UNKNOWN IF RELATIVE HAD CANCER
99 R

G287How old was s/he when this cancer was diagnosed?

___ ENTER AGE
998 DK
999 R

G288What is the name of your next child? (They will not be contacted. This is a simple way to help you complete this part of the questionnaire.)

____________________ ENTER NAME
00 NO MORE CHILDREN [GOTO ITX1]
98 DK [GOTO ITX1]
99 R [GOTO ITX1]

G289What is [FILL NAME]'s sex?

1 MALE
2 FEMALE
8 DK
9 R

G290Is [FILL NAME] still living?

1 YES
3 NO [GOTO G292]
8 DK [GOTO G293]
9 R [GOTO G293]

G291How old is [FILL NAME]?

___ ENTER AGE [GOTO G293]
998 DK [GOTO G293]
999 R [GOTO G293]

G292How old was [FILL NAME] when s/he died?

___ ENTER AGE
998 DK
999 R

G293Did [FILL NAME] ever have a colon or rectal polyp?

1 YES
3 NO [GOTO G295]
8 DK [GOTO G295]
9 R [GOTO G295]

G294How old was s/he when the polyp was first diagnosed?

___ ENTER AGE
998 DK
999 R

G295Was [FILL NAME] ever diagnosed as having any type of cancer?

1 YES
3 NO [GOTO G304]
8 DK
9 R [GOTO G304]

G296What was the first type of cancer [FILL NAME] had?

01 SKIN (NOT MELANOMA)
02 PROSTATE
03 BREAST
04 LUNG
05 COLON
06 CORPUS (UTERINE)
07 RECTUM
08 BLADDER
09 OVARY
10 MELANOMA
11 CERVIX
12 CANCER OF FEMALE REPRODUCTIVE ORGAN, SITE UNKNOWN
13 OTHER CANCER [SPECIFY]
14 RELATIVE HAD CANCER, SITE UNKNOWN
15 UNKNOWN IF RELATIVE HAD CANCER
99 R

G297How old was s/he when this cancer was diagnosed?

___ ENTER AGE
998 DK
999 R

G298Did [FILL NAME] have any other cancer?

1 YES
3 NO [GOTO G304]
8 DK
9 R [GOTO G304]

G299What was the next type of cancer [FILL NAME] had?

01 SKIN (NOT MELANOMA)
02 PROSTATE
03 BREAST
04 LUNG
05 COLON
06 CORPUS (UTERINE)
07 RECTUM
08 BLADDER
09 OVARY
10 MELANOMA
11 CERVIX
12 CANCER OF FEMALE REPRODUCTIVE ORGAN, SITE UNKNOWN
13 OTHER CANCER [SPECIFY]
14 RELATIVE HAD CANCER, SITE UNKNOWN
15 UNKNOWN IF RELATIVE HAD CANCER
99 R

G300How old was s/he when this cancer was diagnosed?

___ ENTER AGE
998 DK
999 R

G301Did [FILL NAME] have any other cancer?

1 YES
3 NO [GOTO G304]
8 DK
9 R [GOTO G304]

G302What was the next type of cancer [FILL NAME] had?

01 SKIN (NOT MELANOMA)
02 PROSTATE
03 BREAST
04 LUNG
05 COLON
06 CORPUS (UTERINE)
07 RECTUM
08 BLADDER

09 OVARY
10 MELANOMA
11 CERVIX
12 CANCER OF FEMALE REPRODUCTIVE ORGAN, SITE UNKNOWN
13 OTHER CANCER [SPECIFY]
14 RELATIVE HAD CANCER, SITE UNKNOWN
15 UNKNOWN IF RELATIVE HAD CANCER
99 R

G303How old was s/he when this cancer was diagnosed?

___ ENTER AGE
998 DK
999 R

G304What is the name of your next child? (They will not be contacted. This is a simple way to help you complete this part of the questionnaire.)

____________________ ENTER NAME

00 NO MORE CHILDREN [GOTO ITX1]

98 DK [GOTO ITX1]

99 R [GOTO ITX1]

G305What is [FILL NAME]'s sex?

1 MALE

2 FEMALE

8 DK

9 R

G306Is [FILL NAME] still living?

1 YES

3 NO [GOTO G308]

8 DK [GOTO G309]

9 R [GOTO G309]

G307How old is [FILL NAME]?

___ ENTER AGE [GOTO G309]

998 DK [GOTO G309]

999 R [GOTO G309]

G308How old was [FILL NAME] when s/he died?

___ ENTER AGE

998 DK

999 R

G309Did [FILL NAME] ever have a colon or rectal polyp?

1 YES

3 NO [GOTO G311]

8 DK [GOTO G311]

9 R [GOTO G311]

G310How old was s/he when the polyp was first diagnosed?

___ ENTER AGE

998 DK

999 R

G311Was [FILL NAME] ever diagnosed as having any type of cancer?

1 YES

3 NO [GOTO G320]

8 DK

9 R [GOTO G320]

G312What was the first type of cancer [FILL NAME] had?

01 SKIN (NOT MELANOMA)
02 PROSTATE
03 BREAST
04 LUNG
05 COLON
06 CORPUS (UTERINE)
07 RECTUM
08 BLADDER
09 OVARY
10 MELANOMA
11 CERVIX
12 CANCER OF FEMALE REPRODUCTIVE ORGAN, SITE UNKNOWN
13 OTHER CANCER [SPECIFY]
14 RELATIVE HAD CANCER, SITE UNKNOWN
15 UNKNOWN IF RELATIVE HAD CANCER
99 R

G313How old was s/he when this cancer was diagnosed?

___ ENTER AGE
998 DK
999 R

G314Did [FILL NAME] have any other cancer?

1 YES
3 NO [GOTO G320]
8 DK
9 R [GOTO G320]

G315What was the next type of cancer [FILL NAME] had?

01 SKIN (NOT MELANOMA)
02 PROSTATE
03 BREAST
04 LUNG
05 COLON
06 CORPUS (UTERINE)
07 RECTUM
08 BLADDER
09 OVARY
10 MELANOMA
11 CERVIX
12 CANCER OF FEMALE REPRODUCTIVE ORGAN, SITE UNKNOWN
13 OTHER CANCER [SPECIFY]
14 RELATIVE HAD CANCER, SITE UNKNOWN
15 UNKNOWN IF RELATIVE HAD CANCER
99 R

G316How old was s/he when this cancer was diagnosed?

___ ENTER AGE
998 DK
999 R

G317Did [FILL NAME] have any other cancer?

1 YES
3 NO [GOTO G320]
8 DK
9 R [GOTO G320]

G318What was the next type of cancer [FILL NAME] had?

01 SKIN (NOT MELANOMA)
02 PROSTATE
03 BREAST
04 LUNG
05 COLON
06 CORPUS (UTERINE)
07 RECTUM
08 BLADDER

09 OVARY
10 MELANOMA
11 CERVIX
12 CANCER OF FEMALE REPRODUCTIVE ORGAN, SITE UNKNOWN
13 OTHER CANCER [SPECIFY]
14 RELATIVE HAD CANCER, SITE UNKNOWN
15 UNKNOWN IF RELATIVE HAD CANCER
99 R

G319How old was s/he when this cancer was diagnosed?

___ ENTER AGE
998 DK
999 R

G320What is the name of your next child? (They will not be contacted. This is a simple way to help you complete this part of the questionnaire.)

____________________ ENTER NAME
00 NO MORE CHILDREN [GOTO ITX1]
98 DK [GOTO ITX1]
99 R [GOTO ITX1]

G321What is [FILL NAME]'s sex?

1 MALE
2 FEMALE
8 DK
9 R

G322Is [FILL NAME] still living?

1 YES
3 NO [GOTO G324]
8 DK [GOTO G325]
9 R [GOTO G325]

G323How old is [FILL NAME]?

___ ENTER AGE [GOTO G325]
998 DK [GOTO G325]
999 R [GOTO G325]

G324How old was [FILL NAME] when s/he died?

___ ENTER AGE
998 DK
999 R

G325Did [FILL NAME] ever have a colon or rectal polyp?

1 YES
3 NO [GOTO G327]
8 DK [GOTO G327]
9 R [GOTO G327]

G326How old was s/he when the polyp was first diagnosed?

___ ENTER AGE
998 DK
999 R

G327Was [FILL NAME] ever diagnosed as having any type of cancer?

1 YES
3 NO [GOTO G336]
8 DK
9 R [GOTO G336]

G328What was the first type of cancer [FILL NAME] had?

01 SKIN (NOT MELANOMA)
02 PROSTATE
03 BREAST
04 LUNG
05 COLON
06 CORPUS (UTERINE)
07 RECTUM
08 BLADDER
09 OVARY
10 MELANOMA
11 CERVIX
12 CANCER OF FEMALE REPRODUCTIVE ORGAN, SITE UNKNOWN
13 OTHER CANCER [SPECIFY]
14 RELATIVE HAD CANCER, SITE UNKNOWN
15 UNKNOWN IF RELATIVE HAD CANCER
99 R

G329How old was s/he when this cancer was diagnosed?

___ ENTER AGE
998 DK
999 R

G330Did [FILL NAME] have any other cancer?

1 YES
3 NO [GOTO G336]
8 DK
9 R [GOTO G336]

G331What was the next type of cancer [FILL NAME] had?

01 SKIN (NOT MELANOMA)
02 PROSTATE
03 BREAST
04 LUNG
05 COLON
06 CORPUS (UTERINE)
07 RECTUM
08 BLADDER
09 OVARY
10 MELANOMA
11 CERVIX
12 CANCER OF FEMALE REPRODUCTIVE ORGAN, SITE UNKNOWN
13 OTHER CANCER [SPECIFY]
14 RELATIVE HAD CANCER, SITE UNKNOWN
15 UNKNOWN IF RELATIVE HAD CANCER
99 R

G332How old was s/he when this cancer was diagnosed?

___ ENTER AGE
998 DK
999 R

G333Did [FILL NAME] have any other cancer?

1 YES
3 NO [GOTO G336]
8 DK
9 R [GOTO G336]

G334What was the next type of cancer [FILL NAME] had?

01 SKIN (NOT MELANOMA)
02 PROSTATE
03 BREAST
04 LUNG
05 COLON
06 CORPUS (UTERINE)
07 RECTUM
08 BLADDER

09 OVARY
10 MELANOMA
11 CERVIX
12 CANCER OF FEMALE REPRODUCTIVE ORGAN, SITE UNKNOWN
13 OTHER CANCER [SPECIFY]
14 RELATIVE HAD CANCER, SITE UNKNOWN
15 UNKNOWN IF RELATIVE HAD CANCER
99 R

G335How old was s/he when this cancer was diagnosed?

___ ENTER AGE
998 DK
999 R

G336What is the name of your next child? (They will not be contacted. This is a simple way to help you complete this part of the questionnaire.)

____________________ ENTER NAME
00 NO MORE CHILDREN [GOTO ITX1]
98 DK [GOTO ITX1]
99 R [GOTO ITX1]

G337What is [FILL NAME]'s sex?

1 MALE
2 FEMALE
8 DK
9 R

G338Is [FILL NAME] still living?

1 YES
3 NO [GOTO G340]
8 DK [GOTO G341]
9 R [GOTO G341]

G339How old is [FILL NAME]?

___ ENTER AGE [GOTO G341]
998 DK [GOTO G341]
999 R [GOTO G341]

G340How old was [FILL NAME] when s/he died?

___ ENTER AGE
998 DK
999 R

G341Did [FILL NAME] ever have a colon or rectal polyp?

1 YES
3 NO [GOTO G343]
8 DK [GOTO G343]
9 R [GOTO G343]

G342How old was s/he when the polyp was first diagnosed?

___ ENTER AGE
998 DK
999 R

G343Was [FILL NAME] ever diagnosed as having any type of cancer?

1 YES
3 NO [GOTO G352]
8 DK
9 R [GOTO G352]

G344What was the first type of cancer [FILL NAME] had?

01 SKIN (NOT MELANOMA)
02 PROSTATE
03 BREAST
04 LUNG
05 COLON
06 CORPUS (UTERINE)
07 RECTUM
08 BLADDER
09 OVARY
10 MELANOMA
11 CERVIX
12 CANCER OF FEMALE REPRODUCTIVE ORGAN, SITE UNKNOWN
13 OTHER CANCER [SPECIFY]
14 RELATIVE HAD CANCER, SITE UNKNOWN
15 UNKNOWN IF RELATIVE HAD CANCER
99 R

G345How old was s/he when this cancer was diagnosed?

___ ENTER AGE
998 DK
999 R

G346Did [FILL NAME] have any other cancer?

1 YES
3 NO [GOTO G352]
8 DK
9 R [GOTO G352]

G347What was the next type of cancer [FILL NAME] had?

01 SKIN (NOT MELANOMA)
02 PROSTATE
03 BREAST
04 LUNG
05 COLON
06 CORPUS (UTERINE)
07 RECTUM
08 BLADDER
09 OVARY
10 MELANOMA
11 CERVIX
12 CANCER OF FEMALE REPRODUCTIVE ORGAN, SITE UNKNOWN
13 OTHER CANCER [SPECIFY]
14 RELATIVE HAD CANCER, SITE UNKNOWN
15 UNKNOWN IF RELATIVE HAD CANCER
99 R

G348How old was s/he when this cancer was diagnosed?

___ ENTER AGE
998 DK
999 R

G349Did [FILL NAME] have any other cancer?

1 YES
3 NO [GOTO G352]
8 DK
9 R [GOTO G352]

G350What was the next type of cancer [FILL NAME] had?

01 SKIN (NOT MELANOMA)
02 PROSTATE
03 BREAST
04 LUNG
05 COLON
06 CORPUS (UTERINE)
07 RECTUM
08 BLADDER

09 OVARY
10 MELANOMA
11 CERVIX
12 CANCER OF FEMALE REPRODUCTIVE ORGAN, SITE UNKNOWN
13 OTHER CANCER [SPECIFY]
14 RELATIVE HAD CANCER, SITE UNKNOWN
15 UNKNOWN IF RELATIVE HAD CANCER
99 R

G351How old was s/he when this cancer was diagnosed?

___ ENTER AGE
998 DK
999 R

G352What is the name of your next child? (They will not be contacted. This is a simple way to help you complete this part of the questionnaire.)

____________________ ENTER NAME

00 NO MORE CHILDREN [GOTO ITX1]

98 DK [GOTO ITX1]

99 R [GOTO ITX1]

G353What is [FILL NAME]'s sex?

1 MALE

2 FEMALE

8 DK

9 R

G354Is [FILL NAME] still living?

1 YES

3 NO [GOTO G356]

8 DK [GOTO G357]

9 R [GOTO G357]

G355How old is [FILL NAME]?

___ ENTER AGE [GOTO G357]

998 DK [GOTO G357]

999 R [GOTO G357]

G356How old was [FILL NAME] when s/he died?

___ ENTER AGE

998 DK

999 R

G357Did [FILL NAME] ever have a colon or rectal polyp?

1 YES

3 NO [GOTO G359]

8 DK [GOTO G359]

9 R [GOTO G359]

G358How old was s/he when the polyp was first diagnosed?

___ ENTER AGE

998 DK

999 R

G359Was [FILL NAME] ever diagnosed as having any type of cancer?

1 YES

3 NO [GOTO G368]

8 DK

9 R [GOTO G368]

G360What was the first type of cancer [FILL NAME] had?

01 SKIN (NOT MELANOMA)
02 PROSTATE
03 BREAST
04 LUNG
05 COLON
06 CORPUS (UTERINE)
07 RECTUM
08 BLADDER
09 OVARY
10 MELANOMA
11 CERVIX
12 CANCER OF FEMALE REPRODUCTIVE ORGAN, SITE UNKNOWN
13 OTHER CANCER [SPECIFY]
14 RELATIVE HAD CANCER, SITE UNKNOWN
15 UNKNOWN IF RELATIVE HAD CANCER
99 R

G361How old was s/he when this cancer was diagnosed?

___ ENTER AGE
998 DK
999 R

G362Did [FILL NAME] have any other cancer?

1 YES
3 NO [GOTO G368]
8 DK
9 R [GOTO G368]

G363What was the next type of cancer [FILL NAME] had?

01 SKIN (NOT MELANOMA)
02 PROSTATE
03 BREAST
04 LUNG
05 COLON
06 CORPUS (UTERINE)
07 RECTUM
08 BLADDER
09 OVARY
10 MELANOMA
11 CERVIX
12 CANCER OF FEMALE REPRODUCTIVE ORGAN, SITE UNKNOWN
13 OTHER CANCER [SPECIFY]
14 RELATIVE HAD CANCER, SITE UNKNOWN
15 UNKNOWN IF RELATIVE HAD CANCER
99 R

G364How old was s/he when this cancer was diagnosed?

___ ENTER AGE
998 DK
999 R

G365Did [FILL NAME] have any other cancer?

1 YES
3 NO [GOTO G368]
8 DK
9 R [GOTO G368]

G366What was the next type of cancer [FILL NAME] had?

01 SKIN (NOT MELANOMA)
02 PROSTATE
03 BREAST
04 LUNG
05 COLON
06 CORPUS (UTERINE)
07 RECTUM
08 BLADDER

09 OVARY
10 MELANOMA
11 CERVIX
12 CANCER OF FEMALE REPRODUCTIVE ORGAN, SITE UNKNOWN
13 OTHER CANCER [SPECIFY]
14 RELATIVE HAD CANCER, SITE UNKNOWN
15 UNKNOWN IF RELATIVE HAD CANCER
99 R

G367How old was s/he when this cancer was diagnosed?

___ ENTER AGE
998 DK
999 R

G368What is the name of your next child? (They will not be contacted. This is a simple way to help you complete this part of the questionnaire.)

____________________ ENTER NAME
00 NO MORE CHILDREN [GOTO ITX1]
98 DK
99 R [GOTO ITX1]

G369What is [FILL NAME]'s sex?

1 MALE
2 FEMALE
8 DK
9 R

G370Is [FILL NAME] still living?

1 YES
3 NO [GOTO G372]
8 DK [GOTO G373]
9 R [GOTO G373]

G371How old is [FILL NAME]?

___ ENTER AGE [GOTO G373]
998 DK [GOTO G373]
999 R [GOTO G373]

G372How old was [FILL NAME] when s/he died?

___ ENTER AGE
998 DK
999 R

G373Did [FILL NAME] ever have a colon or rectal polyp?

1 YES
3 NO [GOTO G375]
8 DK [GOTO G375]
9 R [GOTO G375]

G374How old was s/he when the polyp was first diagnosed?

___ ENTER AGE
998 DK
999 R

G375Was [FILL NAME] ever diagnosed as having any type of cancer?

1 YES
3 NO [GOTO G384]
8 DK
9 R [GOTO G384]

G376What was the first type of cancer [FILL NAME] had?

01 SKIN (NOT MELANOMA)
02 PROSTATE
03 BREAST
04 LUNG
05 COLON
06 CORPUS (UTERINE)
07 RECTUM
08 BLADDER
09 OVARY
10 MELANOMA
11 CERVIX
12 CANCER OF FEMALE REPRODUCTIVE ORGAN, SITE UNKNOWN
13 OTHER CANCER [SPECIFY]
14 RELATIVE HAD CANCER, SITE UNKNOWN
15 UNKNOWN IF RELATIVE HAD CANCER
99 R

G377How old was s/he when this cancer was diagnosed?

___ ENTER AGE
998 DK
999 R

G378Did [FILL NAME] have any other cancer?

1 YES
3 NO [GOTO G384]
8 DK
9 R [GOTO G384]

G379What was the next type of cancer [FILL NAME] had?

01 SKIN (NOT MELANOMA)
02 PROSTATE
03 BREAST
04 LUNG
05 COLON
06 CORPUS (UTERINE)
07 RECTUM
08 BLADDER
09 OVARY
10 MELANOMA
11 CERVIX
12 CANCER OF FEMALE REPRODUCTIVE ORGAN, SITE UNKNOWN
13 OTHER CANCER [SPECIFY]
14 RELATIVE HAD CANCER, SITE UNKNOWN
15 UNKNOWN IF RELATIVE HAD CANCER
99 R

G380How old was s/he when this cancer was diagnosed?

___ ENTER AGE
998 DK
999 R

G381Did [FILL NAME] have any other cancer?

1 YES
3 NO [GOTO G384]
8 DK
9 R [GOTO G384]

G382What was the next type of cancer [FILL NAME] had?

01 SKIN (NOT MELANOMA)
02 PROSTATE
03 BREAST
04 LUNG
05 COLON
06 CORPUS (UTERINE)
07 RECTUM
08 BLADDER

09 OVARY

10 MELANOMA

11 CERVIX

12 CANCER OF FEMALE REPRODUCTIVE ORGAN, SITE UNKNOWN

13 OTHER CANCER [SPECIFY]

14 RELATIVE HAD CANCER, SITE UNKNOWN

15 UNKNOWN IF RELATIVE HAD CANCER

99 R

G383How old was s/he when this cancer was diagnosed?

___ ENTER AGE

998 DK

999 R

G384What is the name of your next child? (They will not be contacted. This is a simple way to help you complete this part of the questionnaire.)

____________________ ENTER NAME
00 NO MORE CHILDREN [GOTO ITX1]
98 DK [GOTO ITX1]
99 R [GOTO ITX1]

G385What is [FILL NAME]'s sex?

1 MALE
2 FEMALE
8 DK
9 R

G386Is [FILL NAME] still living?

1 YES
3 NO [GOTO G388]
8 DK [GOTO G389]
9 R [GOTO G389]

G387How old is [FILL NAME]?

___ ENTER AGE [GOTO G389]
998 DK [GOTO G389]
999 R [GOTO G389]

G388How old was [FILL NAME] when s/he died?

___ ENTER AGE
998 DK
999 R

G389Did [FILL NAME] ever have a colon or rectal polyp?

1 YES
3 NO [GOTO G391]
8 DK [GOTO G391]
9 R [GOTO G391]

G390How old was s/he when the polyp was first diagnosed?

___ ENTER AGE
998 DK
999 R

G391Was [FILL NAME] ever diagnosed as having any type of cancer?

1 YES
3 NO [GOTO G400]
8 DK
9 R [GOTO G400]

G392What was the first type of cancer [FILL NAME] had?

01 SKIN (NOT MELANOMA)
02 PROSTATE
03 BREAST
04 LUNG
05 COLON
06 CORPUS (UTERINE)
07 RECTUM
08 BLADDER
09 OVARY
10 MELANOMA
11 CERVIX
12 CANCER OF FEMALE REPRODUCTIVE ORGAN, SITE UNKNOWN
13 OTHER CANCER [SPECIFY]
14 RELATIVE HAD CANCER, SITE UNKNOWN
15 UNKNOWN IF RELATIVE HAD CANCER
99 R

G393How old was s/he when this cancer was diagnosed?

___ ENTER AGE
998 DK
999 R

G394Did [FILL NAME] have any other cancer?

1 YES
3 NO [GOTO H400]
8 DK
9 R [GOTO H400]

G395What was the next type of cancer [FILL NAME] had?

01 SKIN (NOT MELANOMA)
02 PROSTATE
03 BREAST
04 LUNG
05 COLON
06 CORPUS (UTERINE)
07 RECTUM
08 BLADDER
09 OVARY
10 MELANOMA
11 CERVIX
12 CANCER OF FEMALE REPRODUCTIVE ORGAN, SITE UNKNOWN
13 OTHER CANCER [SPECIFY]
14 RELATIVE HAD CANCER, SITE UNKNOWN
15 UNKNOWN IF RELATIVE HAD CANCER
99 R

G396How old was s/he when this cancer was diagnosed?

___ ENTER AGE
998 DK
999 R

G397Did [FILL NAME] have any other cancer?

1 YES
3 NO [GOTO G400]
8 DK
9 R [GOTO G400]

G398What was the next type of cancer [FILL NAME] had?

01 SKIN (NOT MELANOMA)
02 PROSTATE
03 BREAST
04 LUNG
05 COLON
06 CORPUS (UTERINE)
07 RECTUM
08 BLADDER

09 OVARY
10 MELANOMA
11 CERVIX
12 CANCER OF FEMALE REPRODUCTIVE ORGAN, SITE UNKNOWN
13 OTHER CANCER [SPECIFY]
14 RELATIVE HAD CANCER, SITE UNKNOWN
15 UNKNOWN IF RELATIVE HAD CANCER
99 R

G399How old was s/he when this cancer was diagnosed?

___ ENTER AGE
998 DK
999 R

G400What is the name of your next child? (They will not be contacted. This is a simple way to help you complete this part of the questionnaire.)

____________________ ENTER NAME
00 NO MORE CHILDREN [GOTO ITX1]
98 DK [GOTO ITX1]
99 R [GOTO ITX1]

G401What is [FILL NAME]'s sex?

1 MALE
2 FEMALE
8 DK
9 R

G402Is [FILL NAME] still living?

1 YES
3 NO [GOTO G404]
8 DK [GOTO G405]
9 R [GOTO G405]

G403How old is [FILL NAME]?

___ ENTER AGE [GOTO G405]
998 DK [GOTO G405]
999 R [GOTO G405]

G404How old was [FILL NAME] when s/he died?

___ ENTER AGE
998 DK
999 R

G405Did [FILL NAME] ever have a colon or rectal polyp?

1 YES
3 NO [GOTO G407]
8 DK [GOTO G407]
9 R [GOTO G407]

G406How old was s/he when the polyp was first diagnosed?

___ ENTER AGE
998 DK
999 R

G407Was [FILL NAME] ever diagnosed as having any type of cancer?

1 YES
3 NO [GOTO G416]
8 DK
9 R [GOTO G416]

G408What was the first type of cancer [FILL NAME] had?

01 SKIN (NOT MELANOMA)
02 PROSTATE
03 BREAST
04 LUNG
05 COLON
06 CORPUS (UTERINE)
07 RECTUM
08 BLADDER
09 OVARY
10 MELANOMA
11 CERVIX
12 CANCER OF FEMALE REPRODUCTIVE ORGAN, SITE UNKNOWN
13 OTHER CANCER [SPECIFY]
14 RELATIVE HAD CANCER, SITE UNKNOWN
15 UNKNOWN IF RELATIVE HAD CANCER
99 R

G409How old was s/he when this cancer was diagnosed?

___ ENTER AGE
998 DK
999 R

G410Did [FILL NAME] have any other cancer?

1 YES
3 NO [GOTO G416]
8 DK
9 R [GOTO G416]

G411What was the next type of cancer [FILL NAME] had?

01 SKIN (NOT MELANOMA)
02 PROSTATE
03 BREAST
04 LUNG
05 COLON
06 CORPUS (UTERINE)
07 RECTUM
08 BLADDER
09 OVARY
10 MELANOMA
11 CERVIX
12 CANCER OF FEMALE REPRODUCTIVE ORGAN, SITE UNKNOWN
13 OTHER CANCER [SPECIFY]
14 RELATIVE HAD CANCER, SITE UNKNOWN
15 UNKNOWN IF RELATIVE HAD CANCER
99 R

G412How old was s/he when this cancer was diagnosed?

___ ENTER AGE
998 DK
999 R

G413Did [FILL NAME] have any other cancer?

1 YES
3 NO [GOTO G416]
8 DK
9 R [GOTO G416]

G414What was the next type of cancer [FILL NAME] had?

01 SKIN (NOT MELANOMA)
02 PROSTATE
03 BREAST
04 LUNG
05 COLON
06 CORPUS (UTERINE)
07 RECTUM
08 BLADDER

09 OVARY

10 MELANOMA

11 CERVIX

12 CANCER OF FEMALE REPRODUCTIVE ORGAN, SITE UNKNOWN

13 OTHER CANCER [SPECIFY]

14 RELATIVE HAD CANCER, SITE UNKNOWN

15 UNKNOWN IF RELATIVE HAD CANCER

99 R

G415How old was s/he when this cancer was diagnosed?

___ ENTER AGE

998 DK

999 R

G416What is the name of your next child? (They will not be contacted. This is a simple way to help you complete this part of the questionnaire.)

____________________ ENTER NAME
00 NO MORE CHILDREN [GOTO ITX1]
98 DK [GOTO ITX1]
99 R [GOTO ITX1]

G417What is [FILL NAME]'s sex?

1 MALE
2 FEMALE
8 DK
9 R

G418Is [FILL NAME] still living?

1 YES
3 NO [GOTO G420]
8 DK [GOTO G421]
9 R [GOTO G421]

G419How old is [FILL NAME]?

___ ENTER AGE [GOTO G421]
998 DK [GOTO G421]
999 R [GOTO G421]

G420How old was [FILL NAME] when s/he died?

___ ENTER AGE
998 DK
999 R

G421Did [FILL NAME] ever have a colon or rectal polyp?

1 YES
3 NO [GOTO G423]
8 DK [GOTO G423]
9 R [GOTO G423]

G422How old was s/he when the polyp was first diagnosed?

___ ENTER AGE
998 DK
999 R

G423Was [FILL NAME] ever diagnosed as having any type of cancer?

1 YES
3 NO [GOTO G432]
8 DK
9 R [GOTO G432]

G424What was the first type of cancer [FILL NAME] had?

01 SKIN (NOT MELANOMA)
02 PROSTATE
03 BREAST
04 LUNG
05 COLON
06 CORPUS (UTERINE)
07 RECTUM
08 BLADDER
09 OVARY
10 MELANOMA
11 CERVIX
12 CANCER OF FEMALE REPRODUCTIVE ORGAN, SITE UNKNOWN
13 OTHER CANCER [SPECIFY]
14 RELATIVE HAD CANCER, SITE UNKNOWN
15 UNKNOWN IF RELATIVE HAD CANCER
99 R

G425How old was s/he when this cancer was diagnosed?

___ ENTER AGE
998 DK
999 R

G426Did [FILL NAME] have any other cancer?

1 YES
3 NO [GOTO G432]
8 DK
9 R [GOTO G432]

G427What was the next type of cancer [FILL NAME] had?

01 SKIN (NOT MELANOMA)
02 PROSTATE
03 BREAST
04 LUNG
05 COLON
06 CORPUS (UTERINE)
07 RECTUM
08 BLADDER
09 OVARY
10 MELANOMA
11 CERVIX
12 CANCER OF FEMALE REPRODUCTIVE ORGAN, SITE UNKNOWN
13 OTHER CANCER [SPECIFY]
14 RELATIVE HAD CANCER, SITE UNKNOWN
15 UNKNOWN IF RELATIVE HAD CANCER
99 R

G428How old was s/he when this cancer was diagnosed?

___ ENTER AGE
998 DK
999 R

G429Did [FILL NAME] have any other cancer?

1 YES
3 NO [GOTO G432]
8 DK
9 R [GOTO G432]

G430What was the next type of cancer [FILL NAME] had?

01 SKIN (NOT MELANOMA)
02 PROSTATE
03 BREAST
04 LUNG
05 COLON
06 CORPUS (UTERINE)
07 RECTUM
08 BLADDER

09 OVARY

10 MELANOMA

11 CERVIX

12 CANCER OF FEMALE REPRODUCTIVE ORGAN, SITE UNKNOWN

13 OTHER CANCER [SPECIFY]

14 RELATIVE HAD CANCER, SITE UNKNOWN

15 UNKNOWN IF RELATIVE HAD CANCER

99 R

G431How old was s/he when this cancer was diagnosed?

___ ENTER AGE

998 DK

999 R

G432What is the name of your next child? (They will not be contacted. This is a simple way to help you complete this part of the questionnaire.)

____________________ ENTER NAME
00 NO MORE CHILDREN [GOTO ITX1]
98 DK [GOTO ITX1]
99 R [GOTO ITX1]

G433What is [FILL NAME]'s sex?

1 MALE
2 FEMALE
8 DK
9 R

G434Is [FILL NAME] still living?

1 YES
3 NO [GOTO G436]
8 DK [GOTO G437]
9 R [GOTO G437]

G435How old is [FILL NAME]?

___ ENTER AGE [GOTO G437]
998 DK [GOTO G437]
999 R [GOTO G437]

G436How old was [FILL NAME] when s/he died?

___ ENTER AGE
998 DK
999 R

G437Did [FILL NAME] ever have a colon or rectal polyp?

1 YES
3 NO [GOTO G439]
8 DK [GOTO G439]
9 R [GOTO G439]

G438How old was s/he when the polyp was first diagnosed?

___ ENTER AGE
998 DK
999 R

G439Was [FILL NAME] ever diagnosed as having any type of cancer?

1 YES
3 NO [GOTO G448]
8 DK
9 R [GOTO G448]

G440What was the first type of cancer [FILL NAME] had?

01 SKIN (NOT MELANOMA)
02 PROSTATE
03 BREAST
04 LUNG
05 COLON
06 CORPUS (UTERINE)
07 RECTUM
08 BLADDER
09 OVARY
10 MELANOMA
11 CERVIX
12 CANCER OF FEMALE REPRODUCTIVE ORGAN, SITE UNKNOWN
13 OTHER CANCER [SPECIFY]
14 RELATIVE HAD CANCER, SITE UNKNOWN
15 UNKNOWN IF RELATIVE HAD CANCER
99 R

G441How old was s/he when this cancer was diagnosed?

___ ENTER AGE
998 DK
999 R

G442Did [FILL NAME] have any other cancer?

1 YES
3 NO [GOTO G448]
8 DK
9 R [GOTO G448]

G443What was the next type of cancer [FILL NAME] had?

01 SKIN (NOT MELANOMA)
02 PROSTATE
03 BREAST
04 LUNG
05 COLON
06 CORPUS (UTERINE)
07 RECTUM
08 BLADDER
09 OVARY
10 MELANOMA
11 CERVIX
12 CANCER OF FEMALE REPRODUCTIVE ORGAN, SITE UNKNOWN
13 OTHER CANCER [SPECIFY]
14 RELATIVE HAD CANCER, SITE UNKNOWN
15 UNKNOWN IF RELATIVE HAD CANCER
99 R

G444How old was s/he when this cancer was diagnosed?

___ ENTER AGE
998 DK
999 R

G445Did [FILL NAME] have any other cancer?

1 YES
3 NO [GOTO G448]
8 DK
9 R [GOTO G448]

G446What was the next type of cancer [FILL NAME] had?

01 SKIN (NOT MELANOMA)
02 PROSTATE
03 BREAST
04 LUNG
05 COLON
06 CORPUS (UTERINE)
07 RECTUM
08 BLADDER

09 OVARY

10 MELANOMA

11 CERVIX

12 CANCER OF FEMALE REPRODUCTIVE ORGAN, SITE UNKNOWN

13 OTHER CANCER [SPECIFY]

14 RELATIVE HAD CANCER, SITE UNKNOWN

15 UNKNOWN IF RELATIVE HAD CANCER

99 R

G447How old was s/he when this cancer was diagnosed?

___ ENTER AGE

998 DK

999 R

G448What is the name of your next child? (They will not be contacted. This is a simple way to help you complete this part of the questionnaire.)

____________________ ENTER NAME
00 NO MORE CHILDREN [GOTO ITX1]
98 DK [GOTO ITX1]
99 R [GOTO ITX1]

G449What is [FILL NAME]'s sex?

1 MALE
2 FEMALE
8 DK
9 R

G450Is [FILL NAME] still living?

1 YES
3 NO [GOTO G452]
8 DK [GOTO G453]
9 R [GOTO G453]

G451How old is [FILL NAME]?

___ ENTER AGE [GOTO G453]
998 DK [GOTO G453]
999 R [GOTO G453]

G452How old was [FILL NAME] when s/he died?

___ ENTER AGE
998 DK
999 R

G453Did [FILL NAME] ever have a colon or rectal polyp?

1 YES
3 NO [GOTO G455]
8 DK [GOTO G455]
9 R [GOTO G455]

G454How old was s/he when the polyp was first diagnosed?

___ ENTER AGE
998 DK
999 R

G455Was [FILL NAME] ever diagnosed as having any type of cancer?

1 YES
3 NO [GOTO G464]
8 DK
9 R [GOTO G464]

G456What was the first type of cancer [FILL NAME] had?

01 SKIN (NOT MELANOMA)
02 PROSTATE
03 BREAST
04 LUNG
05 COLON
06 CORPUS (UTERINE)
07 RECTUM
08 BLADDER
09 OVARY
10 MELANOMA
11 CERVIX
12 CANCER OF FEMALE REPRODUCTIVE ORGAN, SITE UNKNOWN
13 OTHER CANCER [SPECIFY]
14 RELATIVE HAD CANCER, SITE UNKNOWN
15 UNKNOWN IF RELATIVE HAD CANCER
99 R

G457How old was s/he when this cancer was diagnosed?

___ ENTER AGE
998 DK
999 R

G458Did [FILL NAME] have any other cancer?

1 YES
3 NO [GOTO G464]
8 DK
9 R [GOTO G464]

G459What was the next type of cancer [FILL NAME] had?

01 SKIN (NOT MELANOMA)
02 PROSTATE
03 BREAST
04 LUNG
05 COLON
06 CORPUS (UTERINE)
07 RECTUM
08 BLADDER
09 OVARY
10 MELANOMA
11 CERVIX
12 CANCER OF FEMALE REPRODUCTIVE ORGAN, SITE UNKNOWN
13 OTHER CANCER [SPECIFY]
14 RELATIVE HAD CANCER, SITE UNKNOWN
15 UNKNOWN IF RELATIVE HAD CANCER
99 R

G460How old was s/he when this cancer was diagnosed?

___ ENTER AGE
998 DK
999 R

G461Did [FILL NAME] have any other cancer?

1 YES
3 NO [GOTO G464]
8 DK
9 R [GOTO H64]

G462What was the next type of cancer [FILL NAME] had?

01 SKIN (NOT MELANOMA)
02 PROSTATE
03 BREAST
04 LUNG
05 COLON
06 CORPUS (UTERINE)
07 RECTUM
08 BLADDER

09 OVARY
10 MELANOMA
11 CERVIX
12 CANCER OF FEMALE REPRODUCTIVE ORGAN, SITE UNKNOWN
13 OTHER CANCER [SPECIFY]
14 RELATIVE HAD CANCER, SITE UNKNOWN
15 UNKNOWN IF RELATIVE HAD CANCER
99 R

G463How old was s/he when this cancer was diagnosed?

___ ENTER AGE
998 DK
999 R

G464What is the name of your next child? (They will not be contacted. This is a simple way to help you complete this part of the questionnaire.)

____________________ ENTER NAME
00 NO MORE CHILDREN [GOTO ITX1]
98 DK [GOTO ITX1]
99 R [GOTO ITX1]

G465What is [FILL NAME]'s sex?

1 MALE
2 FEMALE
8 DK
9 R

G466Is [FILL NAME] still living?

1 YES
3 NO [GOTO G468]
8 DK [GOTO G469]
9 R [GOTO G469]

G467How old is [FILL NAME]?

___ ENTER AGE [GOTO G469]
998 DK [GOTO G469]
999 R [GOTO G469]

G468How old was [FILL NAME] when s/he died?

___ ENTER AGE
998 DK
999 R

G469Did [FILL NAME] ever have a colon or rectal polyp?

1 YES
3 NO [GOTO G471]
8 DK [GOTO G471]
9 R [GOTO G471]

G470How old was s/he when the polyp was first diagnosed?

___ ENTER AGE
998 DK
999 R

G471Was [FILL NAME] ever diagnosed as having any type of cancer?

1 YES
3 NO [GOTO G480]
8 DK
9 R [GOTO G480]

G472What was the first type of cancer [FILL NAME] had?

01 SKIN (NOT MELANOMA)
02 PROSTATE
03 BREAST
04 LUNG
05 COLON
06 CORPUS (UTERINE)
07 RECTUM
08 BLADDER
09 OVARY
10 MELANOMA
11 CERVIX
12 CANCER OF FEMALE REPRODUCTIVE ORGAN, SITE UNKNOWN
13 OTHER CANCER [SPECIFY]
14 RELATIVE HAD CANCER, SITE UNKNOWN
15 UNKNOWN IF RELATIVE HAD CANCER
99 R

G473How old was s/he when this cancer was diagnosed?

___ ENTER AGE
998 DK
999 R

G474Did [FILL NAME] have any other cancer?

1 YES
3 NO [GOTO G480]
8 DK
9 R [GOTO G480]

G475What was the next type of cancer [FILL NAME] had?

01 SKIN (NOT MELANOMA)
02 PROSTATE
03 BREAST
04 LUNG
05 COLON
06 CORPUS (UTERINE)
07 RECTUM
08 BLADDER
09 OVARY
10 MELANOMA
11 CERVIX
12 CANCER OF FEMALE REPRODUCTIVE ORGAN, SITE UNKNOWN
13 OTHER CANCER [SPECIFY]
14 RELATIVE HAD CANCER, SITE UNKNOWN
15 UNKNOWN IF RELATIVE HAD CANCER
99 R

G476How old was s/he when this cancer was diagnosed?

___ ENTER AGE
998 DK
999 R

G477Did [FILL NAME] have any other cancer?

1 YES
3 NO [GOTO G480]
8 DK
9 R [GOTO G480]

G478What was the next type of cancer [FILL NAME] had?

01 SKIN (NOT MELANOMA)
02 PROSTATE
03 BREAST
04 LUNG
05 COLON
06 CORPUS (UTERINE)
07 RECTUM
08 BLADDER

09 OVARY
10 MELANOMA
11 CERVIX
12 CANCER OF FEMALE REPRODUCTIVE ORGAN, SITE UNKNOWN
13 OTHER CANCER [SPECIFY]
14 RELATIVE HAD CANCER, SITE UNKNOWN
15 UNKNOWN IF RELATIVE HAD CANCER
99 R

G479How old was s/he when this cancer was diagnosed?

___ ENTER AGE
998 DK
999 R

G480What is the name of your next child? (They will not be contacted. This is a simple way to help you complete this part of the questionnaire.)

____________________ ENTER NAME
00 NO MORE CHILDREN [GOTO ITX1]
98 DK [GOTO ITX1]
99 R [GOTO ITX1]

G481What is [FILL NAME]'s sex?

1 MALE
2 FEMALE
8 DK
9 R

G482Is [FILL NAME] still living?

1 YES
3 NO [GOTO G484]
8 DK [GOTO G485]
9 R [GOTO G485]

G483How old is [FILL NAME]?

___ ENTER AGE [GOTO G485]
998 DK [GOTO G485]
999 R [GOTO G485]

G484How old was [FILL NAME] when s/he died?

___ ENTER AGE
998 DK
999 R

G485Did [FILL NAME] ever have a colon or rectal polyp?

1 YES
3 NO [GOTO G487]
8 DK [GOTO G487]
9 R [GOTO G487]

G486How old was s/he when the polyp was first diagnosed?

___ ENTER AGE
998 DK
999 R

G487Was [FILL NAME] ever diagnosed as having any type of cancer?

1 YES
3 NO [GOTO G496]
8 DK
9 R [GOTO G496]

G488What was the first type of cancer [FILL NAME] had?

01 SKIN (NOT MELANOMA)
02 PROSTATE
03 BREAST
04 LUNG
05 COLON
06 CORPUS (UTERINE)
07 RECTUM
08 BLADDER
09 OVARY
10 MELANOMA
11 CERVIX
12 CANCER OF FEMALE REPRODUCTIVE ORGAN, SITE UNKNOWN
13 OTHER CANCER [SPECIFY]
14 RELATIVE HAD CANCER, SITE UNKNOWN
15 UNKNOWN IF RELATIVE HAD CANCER
99 R

G489How old was s/he when this cancer was diagnosed?

___ ENTER AGE
998 DK
999 R

G490Did [FILL NAME] have any other cancer?

1 YES
3 NO [GOTO G496]
8 DK
9 R [GOTO G496]

G491What was the next type of cancer [FILL NAME] had?

01 SKIN (NOT MELANOMA)
02 PROSTATE
03 BREAST
04 LUNG
05 COLON
06 CORPUS (UTERINE)
07 RECTUM
08 BLADDER
09 OVARY
10 MELANOMA
11 CERVIX
12 CANCER OF FEMALE REPRODUCTIVE ORGAN, SITE UNKNOWN
13 OTHER CANCER [SPECIFY]
14 RELATIVE HAD CANCER, SITE UNKNOWN
15 UNKNOWN IF RELATIVE HAD CANCER
99 R

G492How old was s/he when this cancer was diagnosed?

___ ENTER AGE
998 DK
999 R

G493Did [FILL NAME] have any other cancer?

1 YES
3 NO [GOTO G496]
8 DK
9 R [GOTO G496]

G494What was the next type of cancer [FILL NAME] had?

01 SKIN (NOT MELANOMA)
02 PROSTATE
03 BREAST
04 LUNG
05 COLON
06 CORPUS (UTERINE)
07 RECTUM
08 BLADDER

09 OVARY

10 MELANOMA

11 CERVIX

12 CANCER OF FEMALE REPRODUCTIVE ORGAN, SITE UNKNOWN

13 OTHER CANCER [SPECIFY]

14 RELATIVE HAD CANCER, SITE UNKNOWN

15 UNKNOWN IF RELATIVE HAD CANCER

99 R

G495How old was s/he when this cancer was diagnosed?

___ ENTER AGE

998 DK

999 R

G496What is the name of your next child? (They will not be contacted. This is a simple way to help you complete this part of the questionnaire.)

____________________ ENTER NAME
00 NO MORE CHILDREN [GOTO ITX1]
98 DK [GOTO ITX1]
99 R [GOTO ITX1]

G497What is [FILL NAME]'s sex?

1 MALE
2 FEMALE
8 DK
9 R

G498Is [FILL NAME] still living?

1 YES
3 NO [GOTO G500]
8 DK [GOTO G501]
9 R [GOTO G501]

G499How old is [FILL NAME]?

___ ENTER AGE [GOTO G501]
998 DK [GOTO G501]
999 R [GOTO G501]

G500How old was [FILL NAME] when s/he died?

___ ENTER AGE
998 DK
999 R

G501Did [FILL NAME] ever have a colon or rectal polyp?

1 YES
3 NO [GOTO G503]
8 DK [GOTO G503]
9 R [GOTO G503]

G502How old was s/he when the polyp was first diagnosed?

___ ENTER AGE
998 DK
999 R

G503Was [FILL NAME] ever diagnosed as having any type of cancer?

1 YES
3 NO [GOTO ITX1]
8 DK
9 R [GOTO ITX1]

G504What was the first type of cancer [FILL NAME] had?

01 SKIN (NOT MELANOMA)
02 PROSTATE
03 BREAST
04 LUNG
05 COLON
06 CORPUS (UTERINE)
07 RECTUM
08 BLADDER
09 OVARY
10 MELANOMA
11 CERVIX
12 CANCER OF FEMALE REPRODUCTIVE ORGAN, SITE UNKNOWN
13 OTHER CANCER [SPECIFY]
14 RELATIVE HAD CANCER, SITE UNKNOWN
15 UNKNOWN IF RELATIVE HAD CANCER
99 R

G505How old was s/he when this cancer was diagnosed?

___ ENTER AGE
998 DK
999 R

G506Did [FILL NAME] have any other cancer?

1 YES
3 NO [GOTO ITX1]
8 DK
9 R [GOTO ITX1]

G507What was the next type of cancer [FILL NAME] had?

01 SKIN (NOT MELANOMA)
02 PROSTATE
03 BREAST
04 LUNG
05 COLON
06 CORPUS (UTERINE)
07 RECTUM
08 BLADDER
09 OVARY
10 MELANOMA
11 CERVIX
12 CANCER OF FEMALE REPRODUCTIVE ORGAN, SITE UNKNOWN
13 OTHER CANCER [SPECIFY]
14 RELATIVE HAD CANCER, SITE UNKNOWN
15 UNKNOWN IF RELATIVE HAD CANCER
99 R

G508How old was s/he when this cancer was diagnosed?

___ ENTER AGE
998 DK
999 R

G509Did [FILL NAME] have any other cancer?

1 YES
3 NO [GOTO ITX1]
8 DK
9 R [GOTO ITX1]

G510What was the next type of cancer [FILL NAME] had?

01 SKIN (NOT MELANOMA)
02 PROSTATE
03 BREAST
04 LUNG
05 COLON
06 CORPUS (UTERINE)
07 RECTUM
08 BLADDER

09 OVARY
10 MELANOMA
11 CERVIX
12 CANCER OF FEMALE REPRODUCTIVE ORGAN, SITE UNKNOWN
13 OTHER CANCER [SPECIFY]
14 RELATIVE HAD CANCER, SITE UNKNOWN
15 UNKNOWN IF RELATIVE HAD CANCER
99 R

G511How old was s/he when this cancer was diagnosed?

___ ENTER AGE
998 DK
999 R

SECTION G

GYNECOLOGICAL AND REPRODUCTIVE HISTORY

(SCREEN FOR FEMALE RESPONDENTS)

HTX1This next section of the interview includes questions about your menstrual history as well as any pregnancies you might have had.

H001How old were you when you menstruated for the first time, that is, had your first period?

__ ENTER AGE
97 NEVER MENSTRATED (H004)
98 DK

H002Which of the statements on this card best describes your current menstrual status? (INTV: READ RESPONSES)

1I am still having periods (H004)
2I have had an operation which stopped my periods
3My periods stopped by themselves
4I am taking medication that stopped my periods
5I am presently pregnant; or my pregnancy ended within the past 2 months; or I am nursing. (THNX)
6OTHER (SPECIFY) (H004)
8DK (H004)

H003How old were you when your periods stopped completely?

__ ENTER AGE
98 DK

H004Have you ever had a D & C, that is, a "scraping" or "cleaning out" of your uterus or womb?

1 YES (H005)
3 NO (H006)
8 DK (H006)

H005How many times have you had a D & C?

__ ENTER NUMBER
98 DK

H006Has your uterus or womb been surgically removed?

1 YES (H007)
3 NO (H007)
8 DK

H007Have one or both of your ovaries been surgically removed?

1 YES ONE OVARY
2 YES BOTH OVARIES
3 NO
8 DK

H008How many times in all have you been pregnant? Please count all live births, stillbirths, miscarriages, ectopic or tubal pregnancies, and induced abortions.

__ ENTER NUMBER
00 NONE (H109)
98 DK (H109)

PREGNANCY NUMBER	Starting with your first pregnancy, what was your age at the beginning of the pregnancy?	How many weeks or months did this pregnancy last?	What was the outcome of this pregnancy? (INTV: SHOW CARD)	Did you breastfeed this child?	How many weeks or months did you breastfeed this child?
1	H009<br>__ ENTER AGE<br>98 DK	H010<br>__ MONTHS OR<br>__ WEEKS<br>98 DK	H011<br>1 LIVE BIRTH (H012)<br>2 STILLBIRTH<br>3 MISCARRIAGE<br>4 ECTOPIC/TUBAL<br>5 INDUCED ABORTION<br>8 DK<br>(OTHERS H014)	H012<br>1 YES (H013)<br>3 NO (H014)<br>8 DK (H014)	H013<br>__ MONTHS OR<br>__ WEEKS<br>98 DK
2	H014<br>__ ENTER AGE<br>98 DK	H015<br>__ MONTHS OR<br>__ WEEKS<br>98 DK	H016<br>1 LIVE BIRTH (H017)<br>2 STILLBIRTH<br>3 MISCARRIAGE<br>4 ECTOPIC/TUBAL<br>5 INDUCED ABORTION<br>8 DK<br>(OTHERS H019)	H017<br>1 YES (H018)<br>3 NO (H019)<br>8 DK (H019)	H018<br>__ MONTHS OR<br>__ WEEKS<br>98 DK
3	H019<br>__ ENTER AGE<br>98 DK	H020<br>__ MONTHS OR<br>__ WEEKS<br>98 DK	H021<br>1 LIVE BIRTH (H022)<br>2 STILLBIRTH<br>3 MISCARRIAGE<br>4 ECTOPIC/TUBAL<br>5 INDUCED ABORTION<br>8 DK<br>(OTHERS H024)	H022<br>1 YES (H023)<br>3 NO (H024)<br>8 DK (H024)	H023<br>__ MONTHS OR<br>__ WEEKS<br>98 DK
4	H024<br>__ ENTER AGE<br>98 DK	H025<br>__ MONTHS OR<br>__ WEEKS<br>98 DK	H026<br>1 LIVE BIRTH (H027)<br>2 STILLBIRTH<br>3 MISCARRIAGE<br>4 ECTOPIC/TUBAL<br>5 INDUCED ABORTION<br>8 DK<br>(OTHERS H029)	H027<br>1 YES (H028)<br>3 NO (H029)<br>8 DK (H029)	H028<br>__ MONTHS OR<br>__ WEEKS<br>98 DK

PREGNANCY NUMBER	Starting with your first pregnancy, what was your age at the beginning of the pregnancy?	How many weeks or months did this pregnancy last?	What was the outcome of this pregnancy? (INTV: SHOW CARD)	Did you breastfeed this child?	How many weeks or months did you breastfeed this child?
5	H029<br>__ ENTER AGE<br>98 DK	H030<br>__ MONTHS OR<br>__ WEEKS<br>98 DK	H031<br>1 LIVE BIRTH (H032)<br>2 STILLBIRTH<br>3 MISCARRIAGE<br>4 ECTOPIC/TUBAL<br>5 INDUCED ABORTION<br>8 DK<br>(OTHERS H034)	H032<br>1 YES (H033)<br>3 NO (H034)<br>8 DK (H034)	H033<br>__ MONTHS OR<br>__ WEEKS<br>98 DK
6	H034<br>__ ENTER AGE<br>98 DK	H035<br>__ MONTHS OR<br>__ WEEKS<br>98 DK	H036<br>1 LIVE BIRTH (H037)<br>2 STILLBIRTH<br>3 MISCARRIAGE<br>4 ECTOPIC/TUBAL<br>5 INDUCED ABORTION<br>8 DK<br>(OTHERS H039)	H037<br>1 YES (H038)<br>3 NO (H039)<br>8 DK (H039)	H038<br>__ MONTHS OR<br>__ WEEKS<br>98 DK
7	H039<br>__ ENTER AGE<br>98 DK	H040<br>__ MONTHS OR<br>__ WEEKS<br>98 DK	H041<br>1 LIVE BIRTH (H042)<br>2 STILLBIRTH<br>3 MISCARRIAGE<br>4 ECTOPIC/TUBAL<br>5 INDUCED ABORTION<br>8 DK<br>(OTHERS H044)	H042<br>1 YES (H043)<br>3 NO (H044)<br>8 DK (H044)	H043<br>__ MONTHS OR<br>__ WEEKS<br>98 DK
8	H044<br>__ ENTER AGE<br>98 DK	H045<br>__ MONTHS OR<br>__ WEEKS<br>98 DK	H046<br>1 LIVE BIRTH (H047)<br>2 STILLBIRTH<br>3 MISCARRIAGE<br>4 ECTOPIC/TUBAL<br>5 INDUCED ABORTION<br>8 DK<br>(OTHERS H049)	H047<br>1 YES (H048)<br>3 NO (H049)<br>8 DK (H049)	H048<br>__ MONTHS OR<br>__ WEEKS<br>98 DK
9	H049<br>__ ENTER AGE<br>98 DK	H050<br>__ MONTHS OR<br>__ WEEKS<br>98 DK	H051<br>1 LIVE BIRTH (H052)<br>2 STILLBIRTH<br>3 MISCARRIAGE<br>4 ECTOPIC/TUBAL<br>5 INDUCED ABORTION<br>8 DK<br>(OTHERS H054)	H052<br>1 YES (H053)<br>3 NO (H054)<br>8 DK (H054)	H053<br>__ MONTHS OR<br>__ WEEKS<br>98 DK
10	H054<br>__ ENTER AGE<br>98 DK	H055<br>__ MONTHS OR<br>__ WEEKS<br>98 DK	H056<br>1 LIVE BIRTH (H057)<br>2 STILLBIRTH<br>3 MISCARRIAGE<br>4 ECTOPIC/TUBAL<br>5 INDUCED ABORTION<br>8 DK<br>(OTHERS H059)	H057<br>1 YES (H058)<br>3 NO (H059)<br>8 DK (H059)	H058<br>__ MONTHS OR<br>__ WEEKS<br>98 DK

PREGNANCY NUMBER	Starting with your first pregnancy, what was your age at the beginning of the pregnancy?	How many weeks or months did this pregnancy last?	What was the outcome of this pregnancy? (INTV: SHOW CARD)	Did you breastfeed this child?	How many weeks or months did you breastfeed this child?
11	H059<br>__ ENTER AGE<br>98 DK	H060<br>__ MONTHS OR<br>__ WEEKS<br>98 DK	H061<br>1 LIVE BIRTH (H062)<br>2 STILLBIRTH<br>3 MISCARRIAGE<br>4 ECTOPIC/TUBAL<br>5 INDUCED ABORTION<br>8 DK<br>(OTHERS H064)	H062<br>1 YES<br>3 NO (H064)<br>8 DK (H064)	H063<br>__ MONTHS OR<br>__ WEEKS<br>98 DK
12	H064<br>__ ENTER AGE<br>98 DK	H065<br>__ MONTHS OR<br>__ WEEKS<br>98 DK	H066<br>1 LIVE BIRTH (H067)<br>2 STILLBIRTH<br>3 MISCARRIAGE<br>4 ECTOPIC/TUBAL<br>5 INDUCED ABORTION<br>8 DK<br>(OTHERS H069)	H067<br>1 YES (H068)<br>3 NO (H069)<br>8 DK (H069)	H068<br>__ MONTHS OR<br>__ WEEKS<br>98 DK
13	H069<br>__ ENTER AGE<br>98 DK	H070<br>__ MONTHS OR<br>__ WEEKS<br>98 DK	H071<br>1 LIVE BIRTH (H072)<br>2 STILLBIRTH<br>3 MISCARRIAGE<br>4 ECTOPIC/TUBAL<br>5 INDUCED ABORTION<br>8 DK<br>(OTHERS H074)	H072<br>1 YES (H073)<br>3 NO (H074)<br>8 DK (H074)	H073<br>__ MONTHS OR<br>__ WEEKS<br>98 DK
14	H074<br>__ ENTER AGE<br>98 DK	H075<br>__ MONTHS OR<br>__ WEEKS<br>98 DK	H076<br>1 LIVE BIRTH (H077)<br>2 STILLBIRTH<br>3 MISCARRIAGE<br>4 ECTOPIC/TUBAL<br>5 INDUCED ABORTION<br>8 DK<br>(OTHERS H079)	H077<br>1 YES<br>3 NO (H079)<br>8 DK (H079)	H078<br>__ MONTHS OR<br>__ WEEKS<br>98 DK
15	H079<br>__ ENTER AGE<br>98 DK	H080<br>__ MONTHS OR<br>__ WEEKS<br>98 DK	H081<br>1 LIVE BIRTH (H082)<br>2 STILLBIRTH<br>3 MISCARRIAGE<br>4 ECTOPIC/TUBAL<br>5 INDUCED ABORTION<br>8 DK<br>(OTHERS H084)	H082<br>1 YES (H083)<br>3 NO (H084)<br>8 DK (H084)	H083<br>__ MONTHS OR<br>__ WEEKS<br>98 DK
16	H084<br>__ ENTER AGE<br>98 DK	H085<br>__ MONTHS OR<br>__ WEEKS<br>98 DK	H086<br>1 LIVE BIRTH (H087)<br>2 STILLBIRTH<br>3 MISCARRIAGE<br>4 ECTOPIC/TUBAL<br>5 INDUCED ABORTION<br>8 DK<br>(OTHERS H089)	H087<br>1 YES (H088)<br>3 NO (H089)<br>8 DK (H089)	H088<br>__ MONTHS OR<br>__ WEEKS<br>98 DK

PREGNANCY NUMBER	Starting with your first pregnancy, what was your age at the beginning of the pregnancy?	How many weeks or months did this pregnancy last?	What was the outcome of this pregnancy? (INTV: SHOW CARD)	Did you breastfeed this child?	How many weeks or months did you breastfeed this child?
17	H089<br>__ ENTER AGE<br>98 DK	H090<br>__ MONTHS OR<br>__ WEEKS<br>98 DK	H091<br>1 LIVE BIRTH (H092)<br>2 STILLBIRTH<br>3 MISCARRIAGE<br>4 ECTOPIC/TUBAL<br>5 INDUCED ABORTION<br>8 DK<br>(OTHERS H094)	H092<br>1 YES (H093)<br>3 NO (H094)<br>8 DK (H094)	H093<br>__ MONTHS OR<br>__ WEEKS<br>98 DK
18	H094<br>__ ENTER AGE<br>98 DK	H095<br>__ MONTHS OR<br>__ WEEKS<br>98 DK	H096<br>1 LIVE BIRTH (H097)<br>2 STILLBIRTH<br>3 MISCARRIAGE<br>4 ECTOPIC/TUBAL<br>5 INDUCED ABORTION<br>8 DK<br>(OTHERS H099)	H097<br>1 YES (H098)<br>3 NO (H099)<br>8 DK (H099)	H098<br>__ MONTHS OR<br>__ WEEKS<br>98 DK
19	H099<br>__ ENTER AGE<br>98 DK	H100<br>__ MONTHS OR<br>__ WEEKS<br>98 DK	H101<br>1 LIVE BIRTH (H102)<br>2 STILLBIRTH<br>3 MISCARRIAGE<br>4 ECTOPIC/TUBAL<br>5 INDUCED ABORTION<br>8 DK<br>(OTHERS H104)	H102<br>1 YES (H103)<br>3 NO (H104)<br>8 DK (H104)	H103<br>__ MONTHS OR<br>__ WEEKS<br>98 DK
20	H104<br>__ ENTER AGE<br>98 DK	H105<br>__ MONTHS OR<br>__ WEEKS<br>98 DK	H106<br>1 LIVE BIRTH (H107)<br>2 STILLBIRTH<br>3 MISCARRIAGE<br>4 ECTOPIC/TUBAL<br>5 INDUCED ABORTION<br>8 DK<br>(OTHERS H109)	H107<br>1 YES<br>3 NO (H109)<br>8 DK (H109)	H108<br>__ MONTHS OR<br>__ WEEKS<br>98 DK

H109Did you ever try for one straight year or more to become pregnant and, during that time, not become pregnant?

1 YES (H110)
3 NO (H112)
8 DK (H112)

H110Did you or your partner ever visit a doctor, clinic, or hospital because you had trouble getting pregnant?

1 YES (H111)
3 NO (H112)
8 DK (H112)

H111What was the reason you had a problem getting pregnant? (INTV: READ RESPONSES)

1 a problem with your ovaries or hormones,
2 a problem with your fallopian tubes,
3 a problem with your uterus or cervix,
4 your partner had fertility problems,
5 other fertility problem or
6 no problem was found
8 DK

H112Have you ever taken oral contraceptives that is birth control pills?

1 YES (H113)
3 NO (H116)
8 DK (H116)

H113In what year did you begin taking them?

__ ENTER YEAR
98 DK

H114In what year did you last take them?

__ ENTER YEAR
00 STILL TAKING
98 DK

H115For many women, taking oral contraceptives involves starting and stopping several times. How long, altogether (have you been/were you) actually taking the pills?

__ MONTHS OR __ YEARS
98 DK

H116Have you ever used estrogen, progestin, or other female hormones for any reason? The preparation may be pills, shots, skin patches, vaginal creams, or vaginal suppositories?

1 YES (H117)
3 NO (ITX1)
8 DK (ITX1)

H117In what year did you begin taking them?

__ ENTER YEAR
98 DK

H118In what year did you last take them?

__ ENTER YEAR
00 STILL TAKING
98 DK

H119For many women, taking hormones involves starting and stopping several times. How long, altogether (have you been/were you) actually taking the pills, shots or using skin patches, creams or suppositories?

_ MONTHS _ YEARS
98 DK

SECTION H

LIFESTYLE

HTX1This last section discusses lifestyle and personal habits.

	CIGARETTES	CIGARS	PIPES
	H001<br>Have you smoked at least 100 cigarettes in your lifetime?<br>1 YES (H002)<br>3 NO (H007)<br>8 DK (H007)	H007<br>Have you ever smoked cigars on a regular basis in your lifetime, that is for a period of at least one year?<br>1 YES (H008)<br>3 NO (H013)<br>8 DK (H013)	H013<br>Have you ever smoked pipes on a regular basis in your lifetime, that is for a period of at least one year?<br>1 YES (H014)<br>3 NO (H019)<br>8 DK (H019)
How old were you when you first started smoking **(TYPE TOBACCO)** on a regular basis?	H002<br>_____ AGE (H003)<br>98 DK (H003)	H008<br>_____ AGE (H009)<br>98 DK (H009)	H014<br>_____ AGE (H015)<br>98 DK (H015)
Do you smoke **(TYPE TOBACCO)** now?	H003<br>1 YES (H004)<br>3 NO (H005)<br>8 DK (H005)	H009<br>1 YES (H010)<br>3 NO (H011)<br>8 DK (H011)	H015<br>1 YES (H016)<br>3 NO (H017)<br>8 DK (H017)
On average, about how many **(TYPE TOBACCO)** do you smoke per day?	H004<br>_ _ _ NUMBER (H007)<br>998 DK (H007)	H010<br>_ _ _ NUMBER (H013)<br>998 DK (H013)	H016<br>_ _ _ NUMBER (H019)<br>998 DK (H019)
How old were you when you stopped smoking **(TYPE TOBACCO)**?	H005<br>_____ AGE (H006)<br>98 DK (H006)	H011<br>_____ AGE (H012)<br>98 DK (H012)	H017<br>_____ AGE (H018)<br>98 DK (H018)
On average, about how many **(TYPE TOBACCO)** did you smoke per day before you quit?	H006<br>_ _ _ NUMBER (H007)<br>998 DK (H007)	H012<br>_ _ _ NUMBER (H013)<br>998 DK (H013)	H018<br>_ _ _ NUMBER (H019)<br>998 DK (H019)

H019Which statement best describes your use of alcoholic beverages that is beer, wine and hard liquor including alcoholic cocktails, whiskey, gin, vodka, scotch, bourbon or rum. **(INTV: READ RESPONSES)**

1 I have never consumed alcoholic beverages, (HTX3)
3 I used to consume alcoholic beverages, but I don't anymore or (H020)
5 I currently consume some alcoholic beverages (HTX2)
8 DK (H020)

H020In what month and year did you stop drinking alcoholic beverages?

_ MONTH _ YEAR
8 DK

(SCREEN FOR DRINKING DURING REFERENT PERIOD - ONLY THOSE THAT DRANK DURING RP ASKED H021)

HTX2I am going to ask you about your use of alcoholic beverages between **(REFERENT PERIOD)**.

H021Between **(REFERENT PERIOD)**, did you drink an average of one or more alcoholic beverages a week?

1 YES (H022)
3 NO (H043)
8 DK (H022)

Between **(REFERENT PERIOD)** did you typically consume any alcoholic beverages on **(DAY)**?	Between **(REFERENT PERIOD)** how many 12-ounce cans or bottles of beer did you usually drink on **(DAY)**?	Between **(REFERENT PERIOD)** how many 4-ounce glasses of wine did you usually drink on **(DAY)**?	Between **(REFERENT PERIOD)** how many 1 1/2-ounce shots of hard liquor did you usually drink on **(DAY)**?
H022 Monday<br>1 YES (H023)<br>3 NO (H026)<br>8 DK (H026)	H023<br>__ NUMBER<br>00 NONE<br>98 DK	H024<br>__ NUMBER<br>00 NONE<br>98 DK	H025<br>__ NUMBER<br>00 NONE<br>98 DK
H026 Tuesday<br>1 YES (H027)<br>3 NO (H030)<br>8 DK (H030)	H027<br>__ NUMBER<br>00 NONE<br>98 DK	H028<br>__ NUMBER<br>00 NONE<br>98 DK	H029<br>__ NUMBER<br>00 NONE<br>98 DK
H030 Wednesday<br>1 YES (H031)<br>3 NO (H034)<br>8 DK (H034)	H031<br>__ NUMBER<br>00 NONE<br>98 DK	H032<br>__ NUMBER<br>00 NONE<br>98 DK	H033<br>__ NUMBER<br>00 NONE<br>98 DK
H034 Thursday<br>1 YES (H035)<br>3 NO (H038)<br>8 DK (H038)	H035<br>__ NUMBER<br>00 NONE<br>98 DK	H036<br>__ NUMBER<br>00 NONE<br>98 DK	H037<br>__ NUMBER<br>00 NONE<br>98 DK
H038 Friday<br>1 YES (H039)<br>3 NO (H042)<br>8 DK (H042)	H039<br>__ NUMBER<br>00 NONE<br>98 DK	H040<br>__ NUMBER<br>00 NONE<br>98 DK	H041<br>__ NUMBER<br>00 NONE<br>98 DK
H042 Saturday<br>1 YES (H043)<br>3 NO (H046)<br>8 DK (H046)	H043<br>__ NUMBER<br>00 NONE<br>98 DK	H044<br>__ NUMBER<br>00 NONE<br>98 DK	H045<br>__ NUMBER<br>00 NONE<br>98 DK
H046 Sunday<br>1 YES (H047)<br>3 NO (H050)<br>8 DK (H050)	H047<br>__ NUMBER<br>00 NONE<br>98 DK	H048<br>__ NUMBER<br>00 NONE<br>98 DK	H049<br>__ NUMBER<br>00 NONE<br>98 DK

(SCREEN FOR RESPONDENT AGE AND YEAR QUIT DRINKING)

	60 YEARS OLD	45 YEARS OLD	30 YEARS OLD
Did you drink alcoholic beverages when you were **(AGE)** years old?	H050<br>1 YES (H051)<br>3 NO (H054)<br>8 DK (H054)	H054<br>1 YES (H055)<br>3 NO (H058)<br>8 DK (H058)	H058<br>1 YES (H059)<br>3 NO (HTX3)<br>8 DK (HTX3)
How many 12-ounce cans or bottles of beer did you usually drink per week?	H051<br>__ NUMBER<br>000 NONE<br>998 DK	H055<br>__ NUMBER<br>000 NONE<br>998 DK	H059<br>__ NUMBER<br>000 NONE<br>998 DK
How many 4-ounce glasses of wine did you usually drink per week?	H052<br>__ NUMBER<br>000 NONE<br>998 DK	H056<br>__ NUMBER<br>000 NONE<br>998 DK	H060<br>__ NUMBER<br>000 NONE<br>998 DK
How many 1 1/2-ounce shots of hard liquor did you usually drink per week?	H053<br>__ NUMBER<br>000 NONE<br>998 DK	H057<br>__ NUMBER<br>000 NONE<br>998 DK	H061<br>__ NUMBER<br>000 NONE<br>998 DK

HTX3These next questions ask about how much time you spent out of doors.

	SUMMER	FALL	WINTER	SPRING
Between **(REFERENT PERIOD)**, how many hours per week, on average, did you spend outside during the **(SEASON)**?	H062<br>__ NUMBER (H063)<br>000 NONE (H065)<br>998 DK (H063)	H065<br>__ NUMBER (H066)<br>000 NONE (H068)<br>998 DK (H066)	H068<br>__ NUMBER (H069)<br>000 NONE (H071)<br>998 DK (H069)	H071<br>__ NUMBER (H072)<br>000 NONE (HTX4)<br>998 DK
Approximately what percentage of your body was exposed to the sun?	H063<br>1 very little,<br>(less than 10%)<br>2 some,<br>(10% to 30%)<br>3 a lot,<br>(31% to 50%) or<br>4 most of it?<br>(over 50%)<br>8 DK	H066<br>1 very little,<br>(less than 10%)<br>2some,<br>(10% to 30%)<br>3a lot,<br>(31% to 50%) or<br>4most of it?<br>(over 50%)<br>8DK	H069<br>1 very little,<br>(less than 10%)<br>2 some,<br>(10% to 30%)<br>3 a lot,<br>(31% to 50%) or<br>4 most of it?<br>(over 50%)<br>8 DK	H072<br>1 very little,<br>(less than 10%)<br>2 some,<br>(10% to 30%)<br>3 a lot,<br>(31% to 50%) or<br>4 most of it?<br>(over 50%)<br>8 DK
Did you use sun screen?	H064<br>1 YES (H065)<br>3 NO (H065)<br>8 DK (H065)	H067<br>1 YES (H068)<br>3 NO (H068)<br>8 DK (H068)	H070<br>1 YES (H071)<br>3 NO (H071)<br>8 DK (H071)	H073<br>1 YES (HTX4)<br>3 NO (HTX4)<br>8 DK (HTX4)

HTX4Now, I am going to read you a list of materials you may have handled or been exposed to on a job, at home or as a hobby before **(REFERENT PERIOD)**. You should answer yes if you handled or were exposed to any material at least once a week for six months or longer or had a very heavy exposure for a briefer period of time on a job, at home or as a hobby.

Did you ever handle, or were you exposed to, **(MATERIAL)**?	Was this at work, outside of your job, or both?	What was the first year you handled or were exposed to it/them?	What was the last year you handled, or were exposed to, it/them?	What was the total number of years you handled, or were exposed to, it/them?
H074 asbestos<br>1 YES (H075)<br>3 NO (H079)<br>8 DK (H079)	H075<br>1 WORK<br>3 HOME/HOBBY<br>5 BOTH<br>8 DK	H076 19__<br>8 DK	H077<br>19__<br>8 DK	H078<br>__ NUMBER<br>8 DK
H079 wood or sawdust<br>1 YES (H080)<br>3 NO (H084)<br>8 DK (H084)	H080<br>1 WORK<br>3 HOME/HOBBY<br>5 BOTH<br>8 DK	H081 19__<br>8 DK	H082<br>19__<br>8 DK	H083<br>__ NUMBER<br>8 DK
H084 solvents<br>1 YES (H085)<br>3 NO (H089)<br>8 DK (H089)	H085<br>1 WORK<br>3 HOME/HOBBY<br>5 BOTH<br>8 DK	H086 19__<br>8 DK	H087<br>19__<br>8 DK	H088<br>__ NUMBER<br>8 DK
H089 fiberglass<br>1 YES (H090)<br>3 NO (H094)<br>8 DK (H094)	H090<br>1 WORK<br>3 HOME/HOBBY<br>5 BOTH<br>8 DK	H091 19__<br>8 DK	H092<br>19__<br>8 DK	H093<br>__ NUMBER<br>8 DK
H094 grinding or sand blasting abrasives<br>1 YES (H095)<br>3 NO (H099)<br>8 DK (H099)	H095<br>1 WORK<br>3 HOME/HOBBY<br>5 BOTH<br>8 DK	H096 19__<br>8 DK	HO97<br>19__<br>8 DK	H098<br>__ NUMBER<br>8 DK
H099 paint or varnish<br>1 YES<br>3 NO (H104)<br>8 DK (H104)	H100<br>1 WORK<br>3 HOME/HOBBY<br>5 BOTH<br>8 DK	H101 19__<br>8 DK	H102<br>19__<br>8 DK	H103<br>__ NUMBER<br>8 DK
H104 weed killers, insect killers or other pesticides<br>1 YES (H105)<br>3 NO (H109)<br>8 DK (H109)	H105<br>1 WORK<br>3 HOME/HOBBY<br>5 BOTH<br>8 DK	H106 19__<br>8 DK	H107<br>19__<br>8 DK	H108<br>__ NUMBER<br>8 DK
H109 petroleum or petroleum based products<br>1 YES (H110)<br>3 NO (ITX5)<br>8 DK (ITX5)	H110<br>1 WORK<br>3 HOME/HOBBY<br>5 BOTH<br>8 DK	H111 19__<br>8 DK	H112<br>19__<br>8 DK	H113<br>__ NUMBER<br>8 DK

HTX5These final questions are for statistical purposes only.

H114This question asks about your household last year. Was your total family income last year before deductions and taxes more than or less than 35,000?

1 OVER 35,000 (H115)
3 UNDER 35,000 (H116)
8 DK (H117)

H115I am going to mention several income categories. When I mention the category which describes your total family income last year please stop me.

01 Between 35 and 40 thousand
02 Between 40 and 45 thousand
03 Between 45 and 50 thousand
04 Between 50 and 55 thousand
05 Between 55 and 60 thousand
06 60,000 or more
98 DK
(H117)

H116I am going to mention several income categories. When I mention the category which describes your total family income last year please stop me.

01 Less than 5,000
02 Between 5 and 10 thousand
03 Between 10 and 15 thousand
04 Between 15 and 20 thousand
05 Between 20 and 30 thousand
06 Between 30 and 35 thousand
98 DK
(H117)

H117What was the total number of persons in your household last year, including yourself?

__ NUMBER
998 DK

HTX6If we need to contact you in the future, it is helpful to know the name of an individual outside your household who will always know your whereabouts. What is the name, address, and phone number of a close friend or relative who does not live with you?

1 CONTINUE
8 DK (H123)

H118NAME: **(LAST NAME FIRST)**

H119STREET ADDRESS:

H120CITY, STATE:

H121TELEPHONE:

H122What is **(NAME)'s** relationship to you?

1 MOTHER
2 FATHER
3 SON
4 DAUGHTER
5 OTHER RELATIVE **(SPECIFY)**
6 FRIEND
8 DK

H123Do you have a social security number?

1 YES (H124)
3 NO (H125)
8 DK (H125)

H124 To help identify participants in our study, we are asking for your social security number. Your providing this information to us is voluntary. Your answer or refusal to answer will have no effect in any way on your social security benefits. What is your social security number?

__ __ __ - __ __ - __ __ __ __ NUMBER
8 DK

H125Do you currently have a valid **(STATE)** driver's license?

1 YES
3 NO
8 DK

H126**(IF 65 OR OVER)** Are you enrolled in Medicare?

1 YES
3 NO
8 DK

THNXThat completes the interview. You have been very helpful and I appreciate your time and cooperation.

GO TO SECTION I
