# Supplementary material for: Identifying factors associated with the direction and significance of microRNA tumor-normal expression differences in colorectal cancer
Source: BMC Cancer. 2017 Oct 30;17:707. doi: 10.1186/s12885-017-3690-x (PMC5663119; doi:10.1186/s12885-017-3690-x)
Supplement: Supplementary file 4 — (AF4_orange.pdf) Visualizations of “orange” outcomes of interest – microRNAs that are overall not significant in the tumor-normal test of differential expression, but significant in opposite directions for factor levels. Each page of this file is in the same format as explained for each row in Fig. 2. (PDF 16 kb) [file 12885_2017_3690_MOESM3_ESM.pdf]

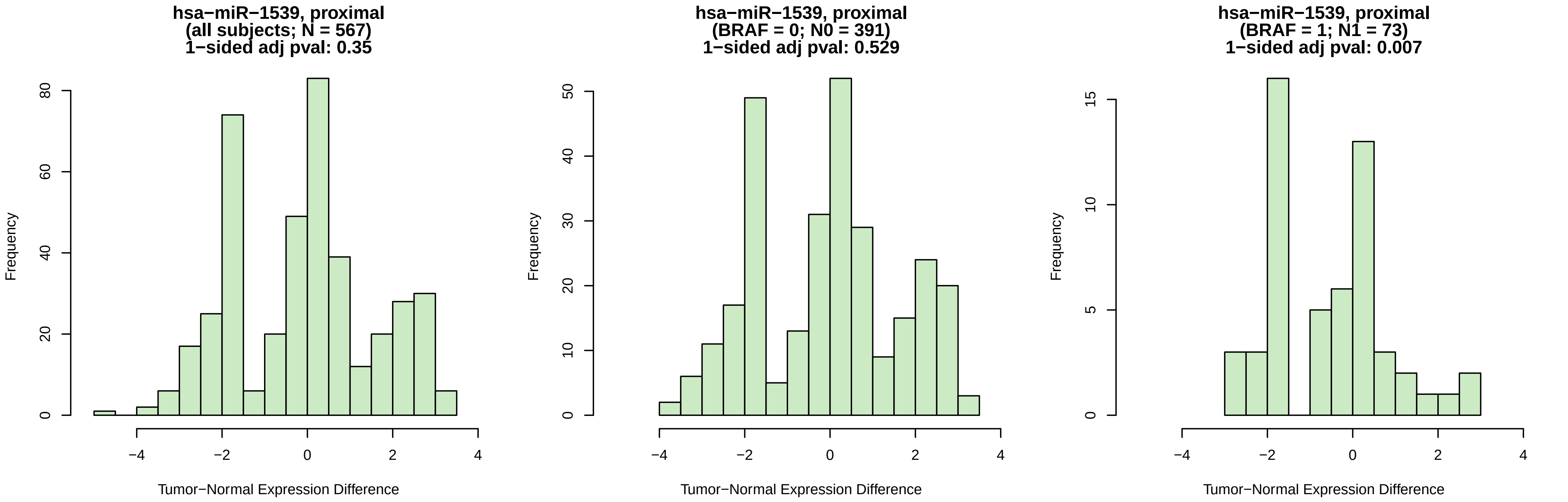
hsa−miR−1539, proximal
(all subjects; N = 567)
1−sided adj pval: 0.35
hsa−miR−1539, proximal
(BRAF = 0; N0 = 391)
1−sided adj pval: 0.529
hsa−miR−1539, proximal
(BRAF = 1; N1 = 73)
1−sided adj pval: 0.007
Frequency
Tumor−Normal Expression Difference

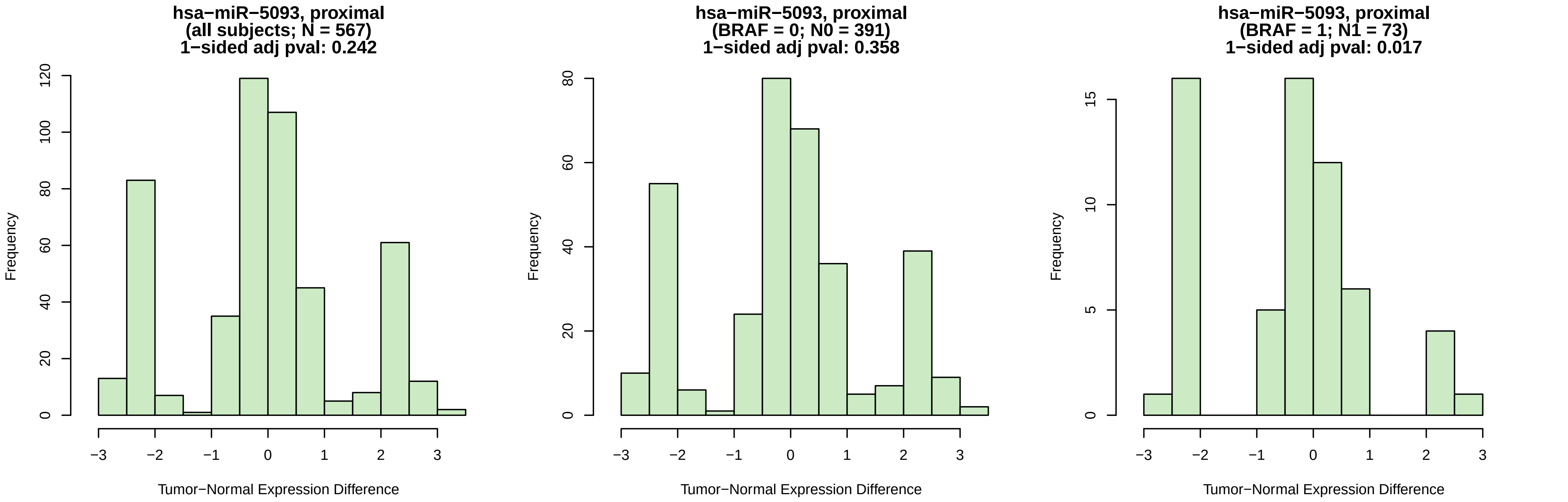

hsa−miR−5093, proximal
(all subjects; N = 567)
1−sided adj pval: 0.242
Frequency
Tumor−Normal Expression Difference
hsa−miR−5093, proximal
(BRAF = 0; N0 = 391)
1−sided adj pval: 0.358
Frequency
Tumor−Normal Expression Difference
hsa−miR−5093, proximal
(BRAF = 1; N1 = 73)
1−sided adj pval: 0.017
Frequency
Tumor−Normal Expression Difference

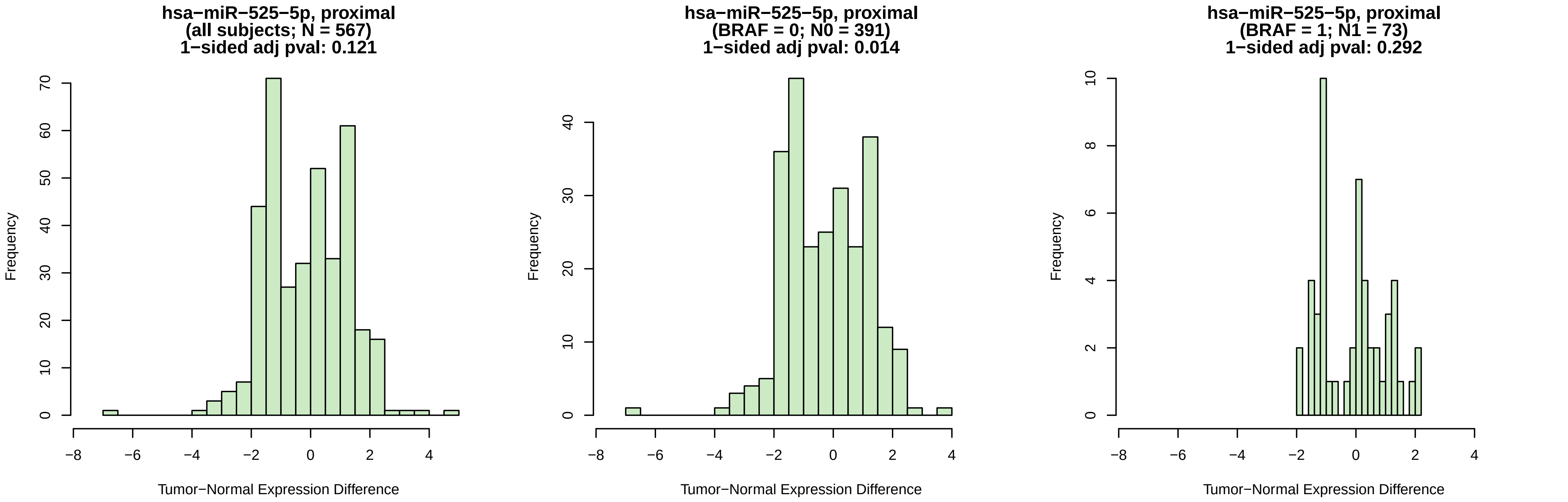
hsa−miR−525−5p, proximal
(all subjects; N = 567)
1−sided adj pval: 0.121
Frequency
Tumor−Normal Expression Difference
hsa−miR−525−5p, proximal
(BRAF = 0; N0 = 391)
1−sided adj pval: 0.014
Frequency
Tumor−Normal Expression Difference
hsa−miR−525−5p, proximal
(BRAF = 1; N1 = 73)
1−sided adj pval: 0.292
Frequency
Tumor−Normal Expression Difference

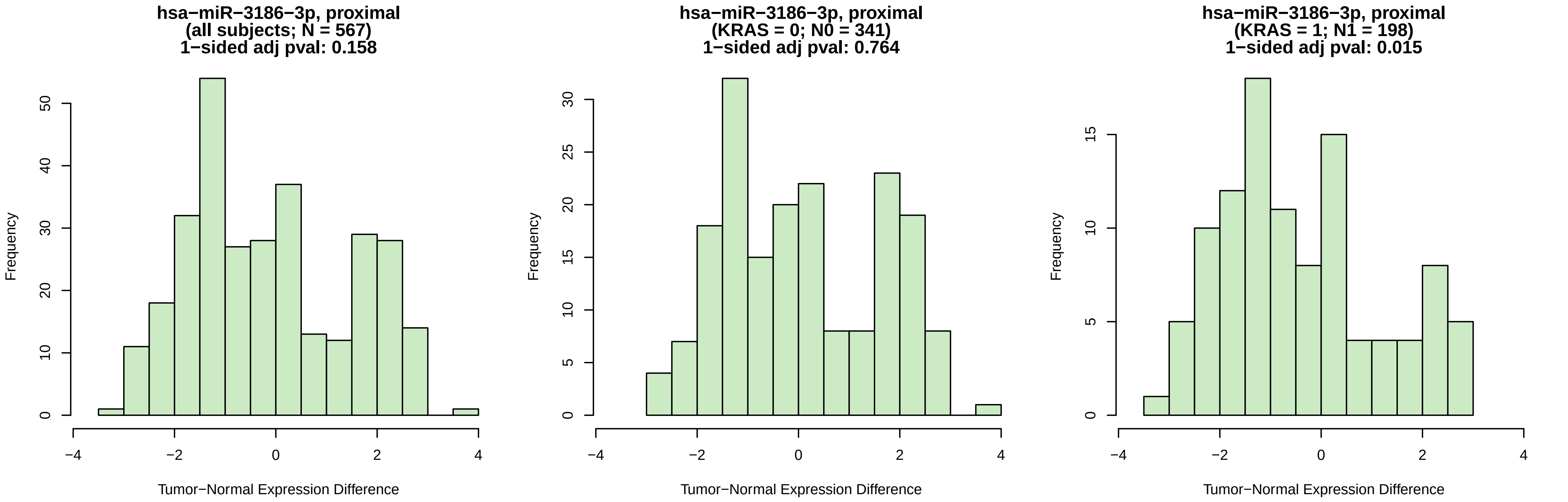

hsa−miR−3186−3p, proximal
(all subjects; N = 567)
1−sided adj pval: 0.158
Frequency
Tumor−Normal Expression Difference
hsa−miR−3186−3p, proximal
(KRAS = 0; N0 = 341)
1−sided adj pval: 0.764
Frequency
Tumor−Normal Expression Difference
hsa−miR−3186−3p, proximal
(KRAS = 1; N1 = 198)
1−sided adj pval: 0.015
Frequency
Tumor−Normal Expression Difference

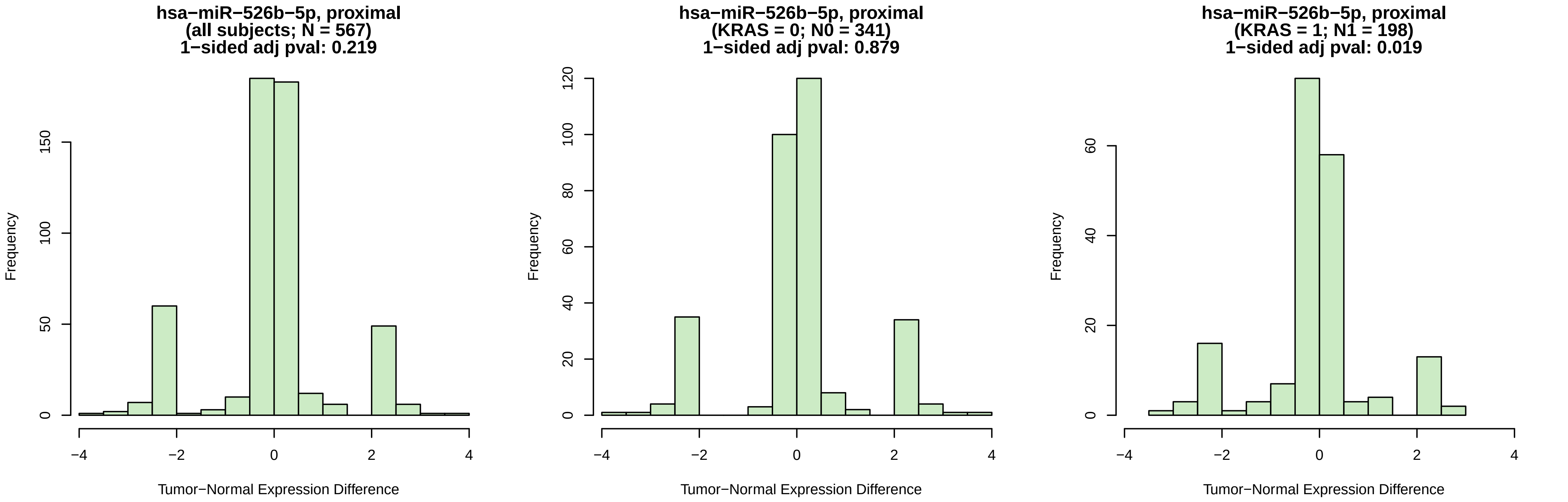
hsa−miR−526b−5p, proximal
(all subjects; N = 567)
1−sided adj pval: 0.219
Frequency
0
50
100
150
−4
−2
0
2
4
Tumor−Normal Expression Difference
hsa−miR−526b−5p, proximal
(KRAS = 0; N0 = 341)
1−sided adj pval: 0.879
Frequency
0
20
40
60
80
100
120
−4
−2
0
2
4
Tumor−Normal Expression Difference
hsa−miR−526b−5p, proximal
(KRAS = 1; N1 = 198)
1−sided adj pval: 0.019
Frequency
0
20
40
60
−4
−2
0
2
4
Tumor−Normal Expression Difference

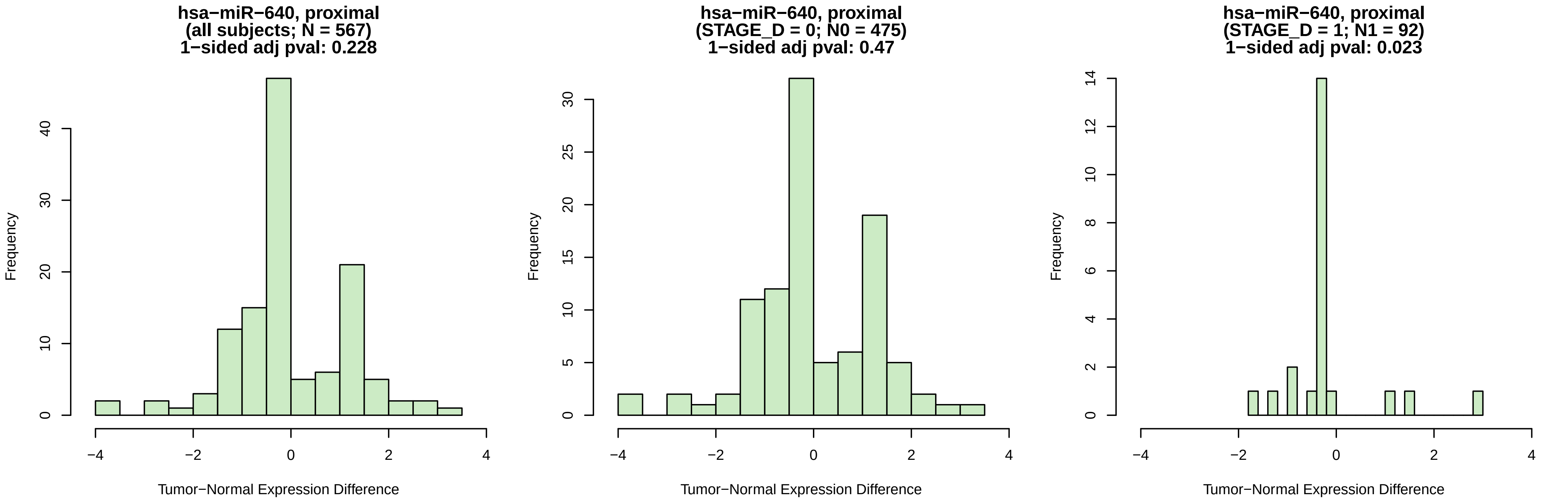
hsa−miR−640, proximal
(all subjects; N = 567)
1−sided adj pval: 0.228
Frequency
Tumor−Normal Expression Difference
hsa−miR−640, proximal
(STAGE_D = 0; N0 = 475)
1−sided adj pval: 0.47
Frequency
Tumor−Normal Expression Difference
hsa−miR−640, proximal
(STAGE_D = 1; N1 = 92)
1−sided adj pval: 0.023
Frequency
Tumor−Normal Expression Difference

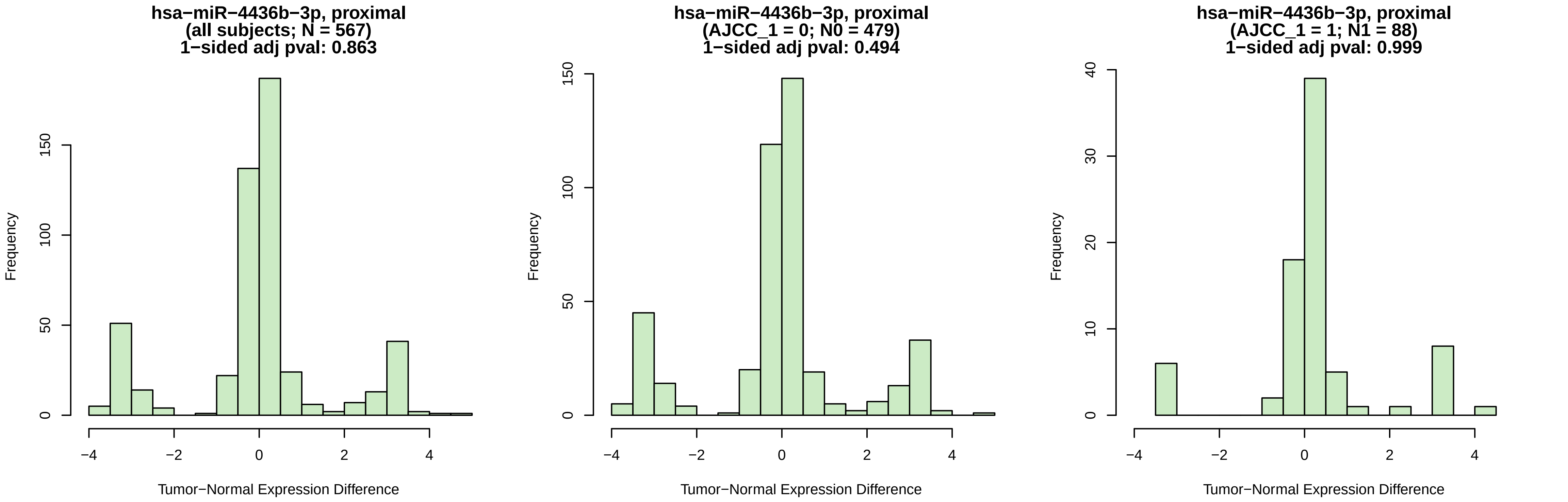
hsa−miR−4436b−3p, proximal
(all subjects; N = 567)
1−sided adj pval: 0.863
Frequency
Tumor−Normal Expression Difference
hsa−miR−4436b−3p, proximal
(AJCC_1 = 0; N0 = 479)
1−sided adj pval: 0.494
Frequency
Tumor−Normal Expression Difference
hsa−miR−4436b−3p, proximal
(AJCC_1 = 1; N1 = 88)
1−sided adj pval: 0.999
Frequency
Tumor−Normal Expression Difference

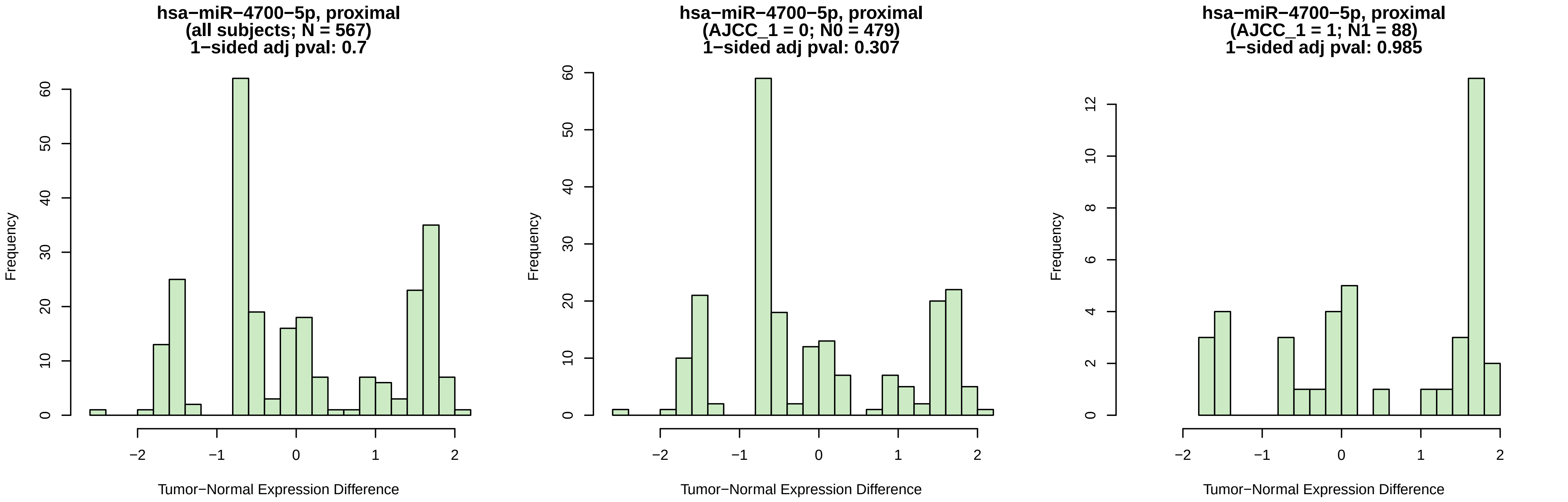

hsa−miR−4700−5p, proximal
(all subjects; N = 567)
1−sided adj pval: 0.7
Frequency
Tumor−Normal Expression Difference
hsa−miR−4700−5p, proximal
(AJCC_1 = 0; N0 = 479)
1−sided adj pval: 0.307
Frequency
Tumor−Normal Expression Difference
hsa−miR−4700−5p, proximal
(AJCC_1 = 1; N1 = 88)
1−sided adj pval: 0.985
Frequency
Tumor−Normal Expression Difference

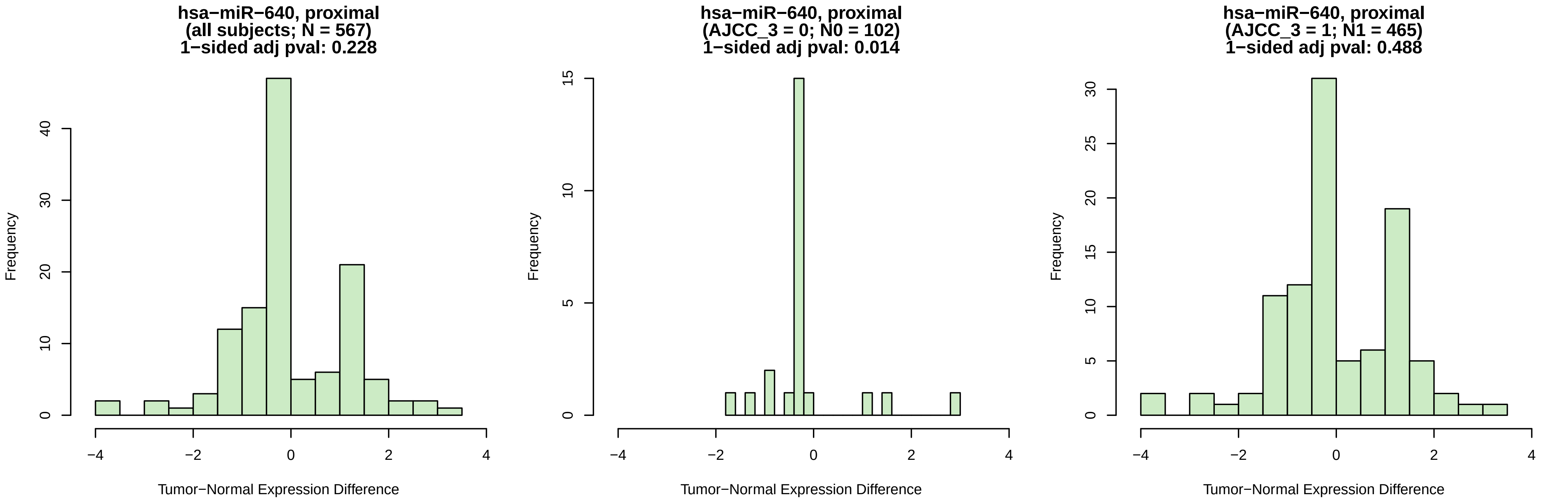

hsa−miR−640, proximal
(all subjects; N = 567)
1−sided adj pval: 0.228
hsa−miR−640, proximal
(AJCC_3 = 0; N0 = 102)
1−sided adj pval: 0.014
hsa−miR−640, proximal
(AJCC_3 = 1; N1 = 465)
1−sided adj pval: 0.488
Frequency
Tumor−Normal Expression Difference

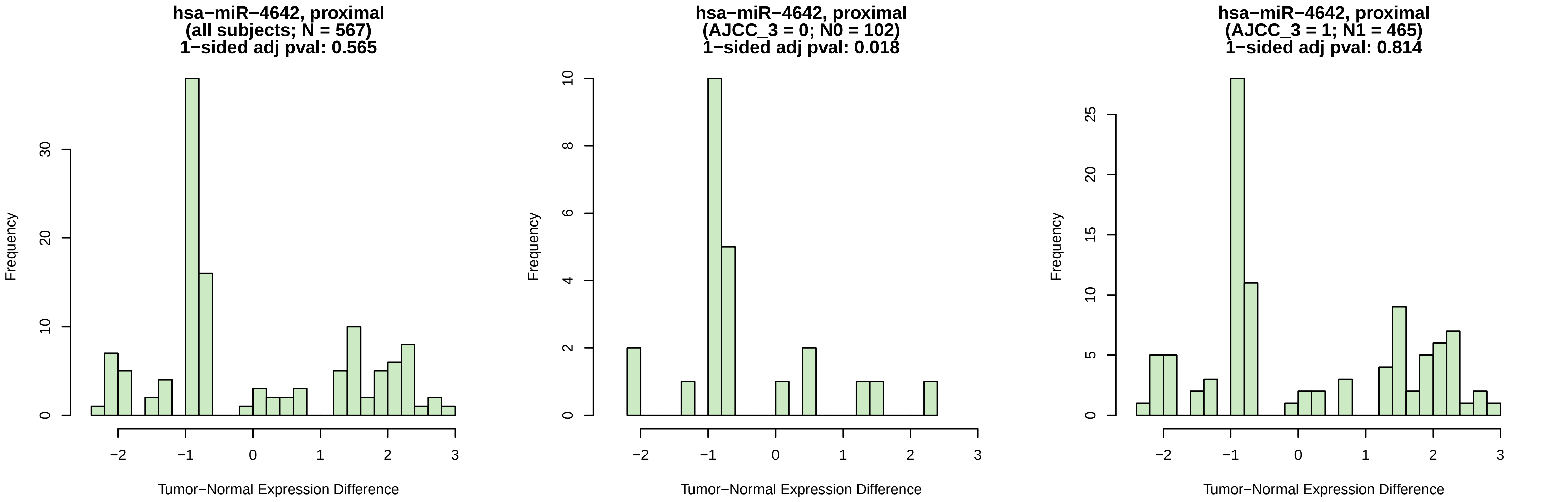
hsa−miR−4642, proximal
(all subjects; N = 567)
1−sided adj pval: 0.565
Frequency
Tumor−Normal Expression Difference
hsa−miR−4642, proximal
(AJCC_3 = 0; N0 = 102)
1−sided adj pval: 0.018
Frequency
Tumor−Normal Expression Difference
hsa−miR−4642, proximal
(AJCC_3 = 1; N1 = 465)
1−sided adj pval: 0.814
Frequency
Tumor−Normal Expression Difference

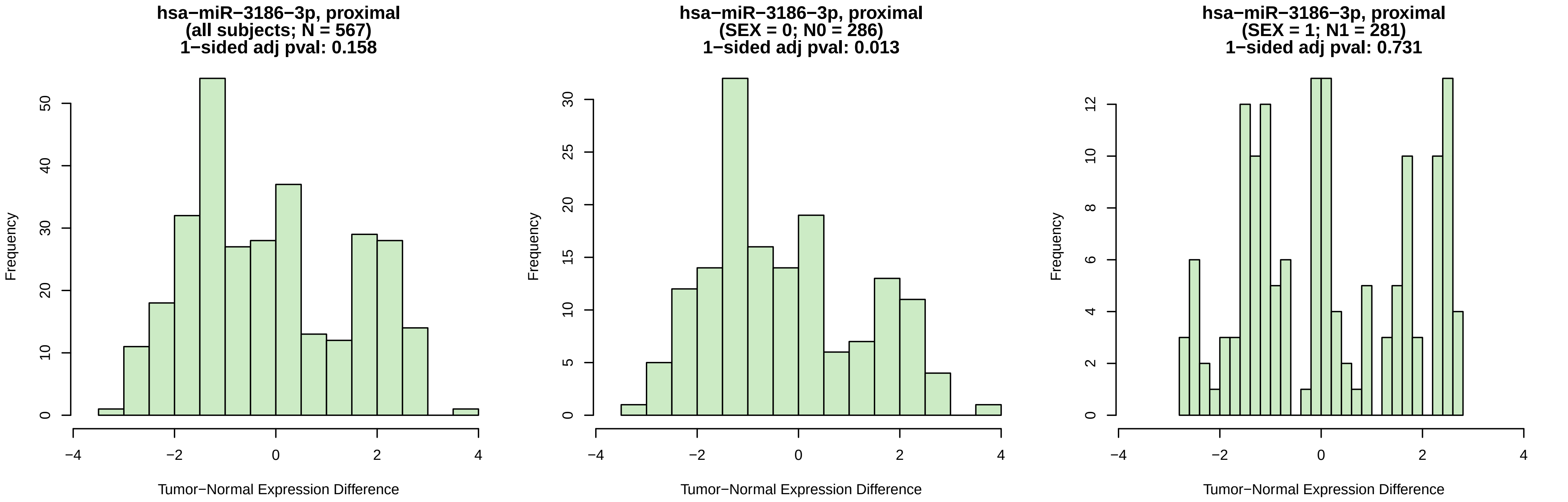
hsa−miR−3186−3p, proximal
(all subjects; N = 567)
1−sided adj pval: 0.158
Frequency
Tumor−Normal Expression Difference
hsa−miR−3186−3p, proximal
(SEX = 0; N0 = 286)
1−sided adj pval: 0.013
Frequency
Tumor−Normal Expression Difference
hsa−miR−3186−3p, proximal
(SEX = 1; N1 = 281)
1−sided adj pval: 0.731
Frequency
Tumor−Normal Expression Difference

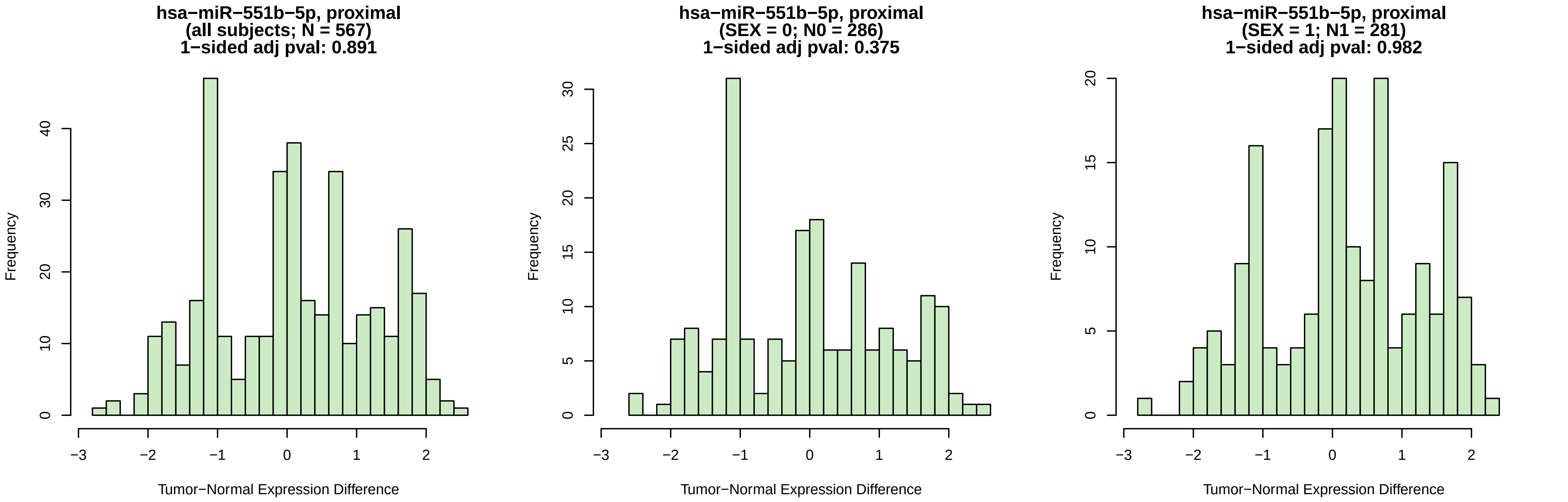

hsa−miR−551b−5p, proximal
(all subjects; N = 567)
1−sided adj pval: 0.891
hsa−miR−551b−5p, proximal
(SEX = 0; N0 = 286)
1−sided adj pval: 0.375
hsa−miR−551b−5p, proximal
(SEX = 1; N1 = 281)
1−sided adj pval: 0.982
Frequency
Tumor−Normal Expression Difference

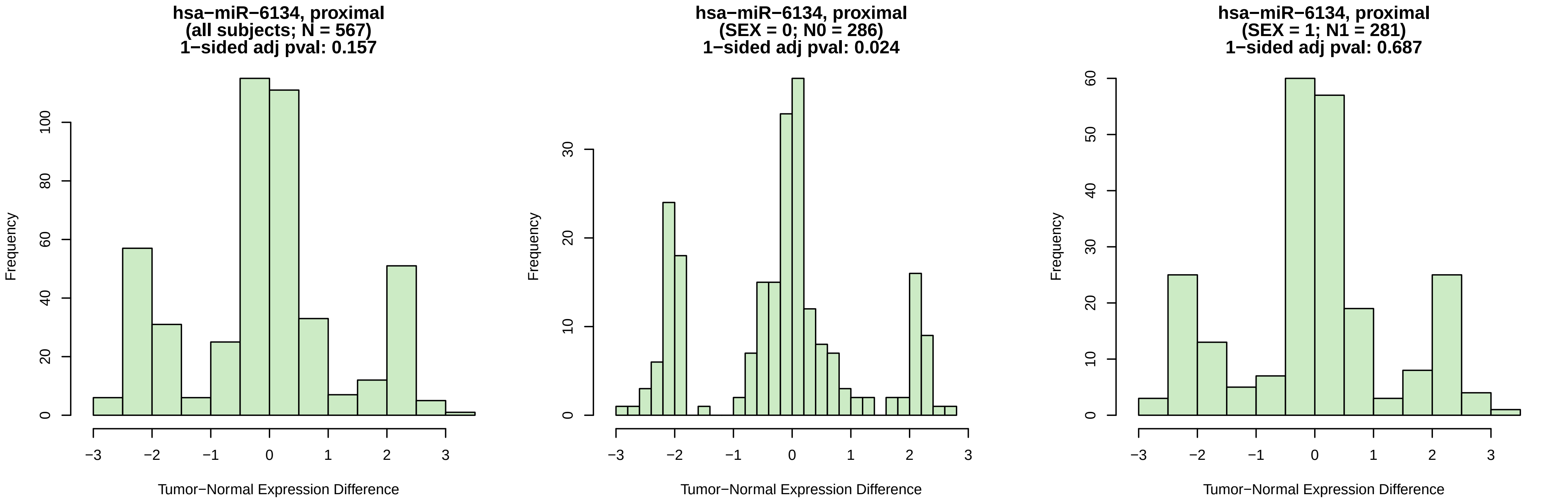
hsa−miR−6134, proximal
(all subjects; N = 567)
1−sided adj pval: 0.157
Frequency
Tumor−Normal Expression Difference
hsa−miR−6134, proximal
(SEX = 0; N0 = 286)
1−sided adj pval: 0.024
Frequency
Tumor−Normal Expression Difference
hsa−miR−6134, proximal
(SEX = 1; N1 = 281)
1−sided adj pval: 0.687
Frequency
Tumor−Normal Expression Difference

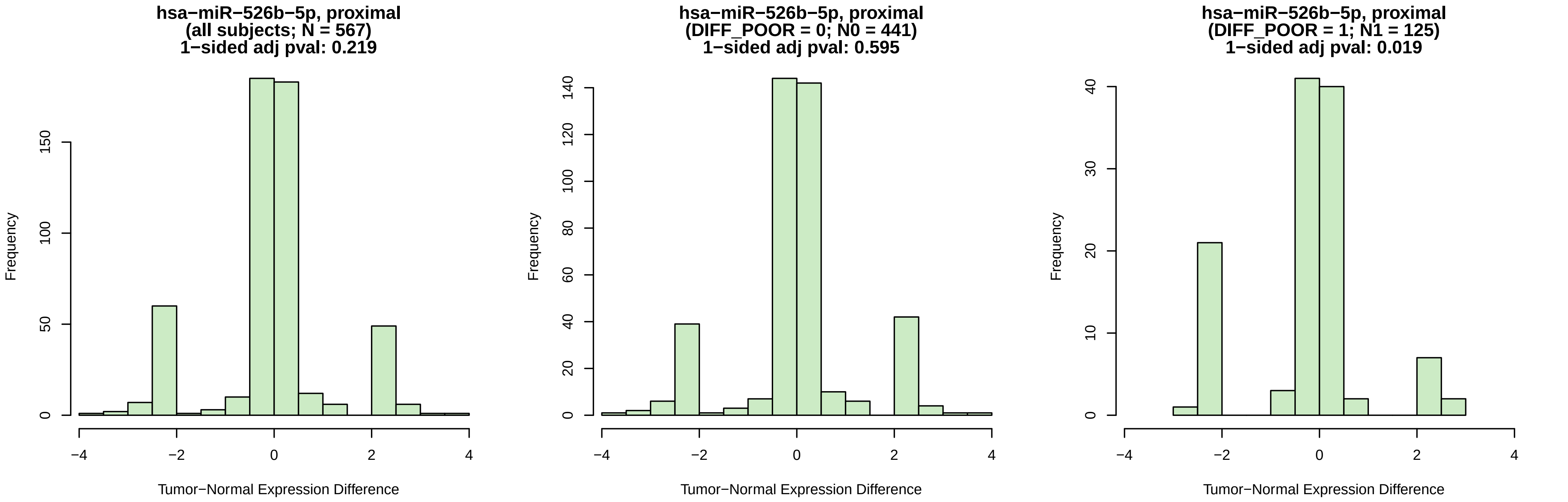
hsa−miR−526b−5p, proximal
(all subjects; N = 567)
1−sided adj pval: 0.219
Frequency
0
50
100
150
−4
−2
0
2
4
Tumor−Normal Expression Difference
hsa−miR−526b−5p, proximal
(DIFF_POOR = 0; N0 = 441)
1−sided adj pval: 0.595
Frequency
0
20
40
60
80
100
120
140
−4
−2
0
2
4
Tumor−Normal Expression Difference
hsa−miR−526b−5p, proximal
(DIFF_POOR = 1; N1 = 125)
1−sided adj pval: 0.019
Frequency
0
10
20
30
40
−4
−2
0
2
4
Tumor−Normal Expression Difference

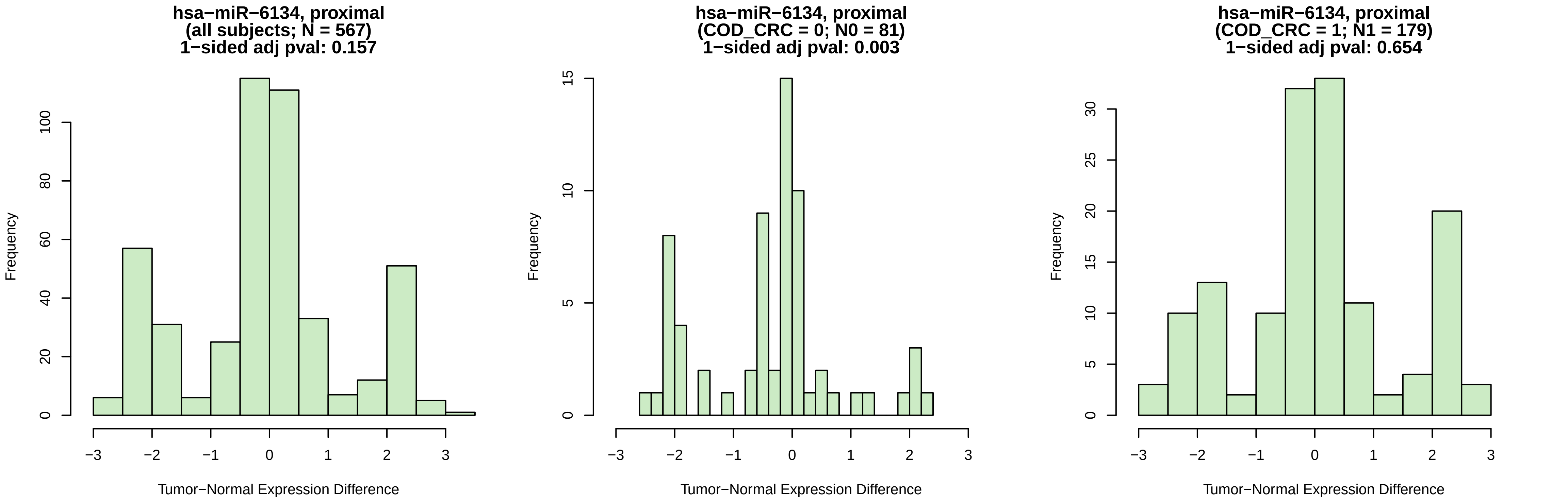
hsa−miR−6134, proximal
(all subjects; N = 567)
1−sided adj pval: 0.157
Frequency
0
20
40
60
80
100
−3
−2
−1
0
1
2
3
Tumor−Normal Expression Difference
hsa−miR−6134, proximal
(COD_CRC = 0; N0 = 81)
1−sided adj pval: 0.003
Frequency
0
5
10
15
−3
−2
−1
0
1
2
3
Tumor−Normal Expression Difference
hsa−miR−6134, proximal
(COD_CRC = 1; N1 = 179)
1−sided adj pval: 0.654
Frequency
0
5
10
15
20
25
30
−3
−2
−1
0
1
2
3
Tumor−Normal Expression Difference

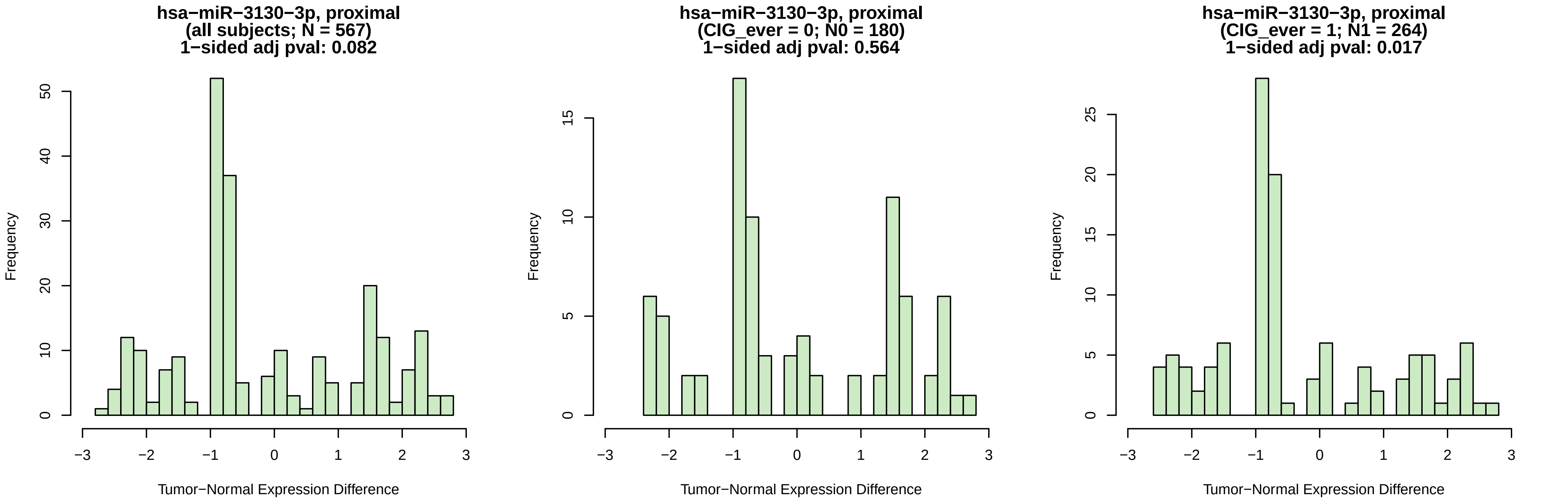
hsa−miR−3130−3p, proximal
(all subjects; N = 567)
1−sided adj pval: 0.082
Frequency
0
10
20
30
40
50
−3
−2
−1
0
1
2
3
Tumor−Normal Expression Difference
hsa−miR−3130−3p, proximal
(CIG_ever = 0; N0 = 180)
1−sided adj pval: 0.564
Frequency
0
5
10
15
−3
−2
−1
0
1
2
3
Tumor−Normal Expression Difference
hsa−miR−3130−3p, proximal
(CIG_ever = 1; N1 = 264)
1−sided adj pval: 0.017
Frequency
0
5
10
15
20
25
−3
−2
−1
0
1
2
3
Tumor−Normal Expression Difference

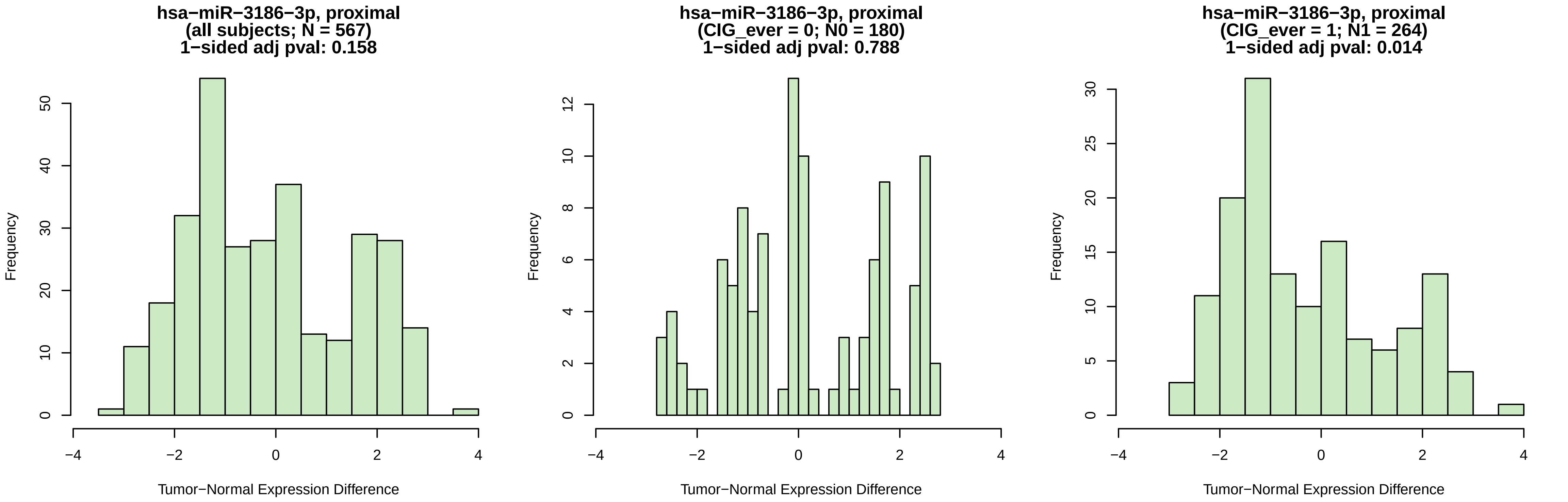

hsa−miR−3186−3p, proximal
(all subjects; N = 567)
1−sided adj pval: 0.158
Frequency
Tumor−Normal Expression Difference
hsa−miR−3186−3p, proximal
(CIG_ever = 0; N0 = 180)
1−sided adj pval: 0.788
Frequency
Tumor−Normal Expression Difference
hsa−miR−3186−3p, proximal
(CIG_ever = 1; N1 = 264)
1−sided adj pval: 0.014
Frequency
Tumor−Normal Expression Difference

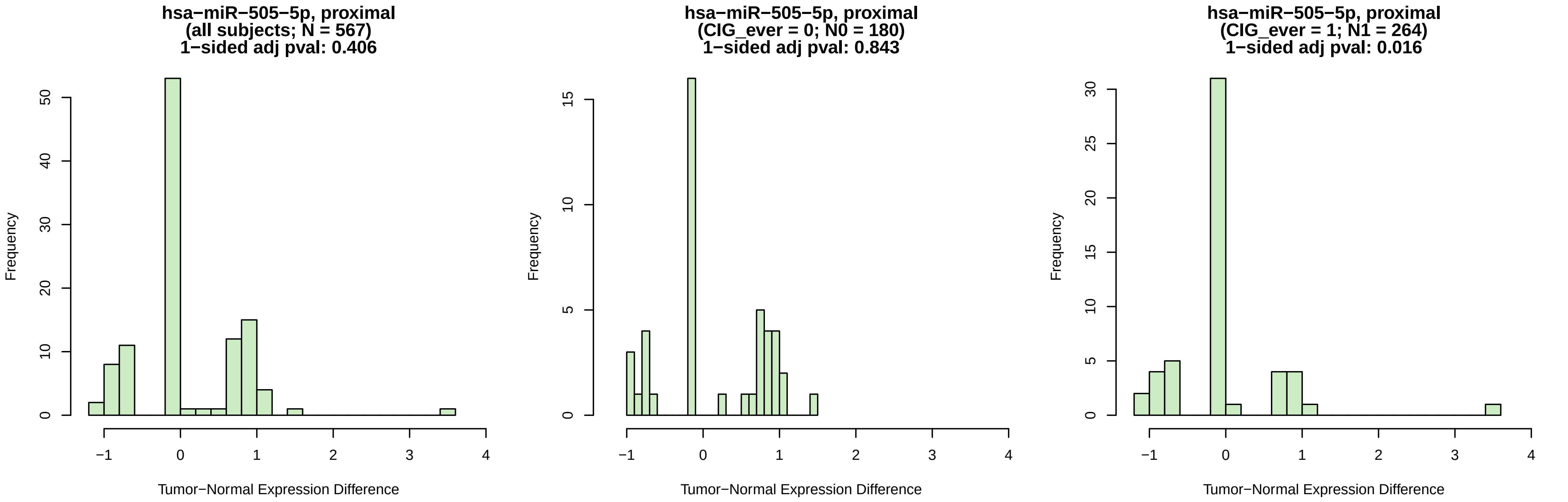

hsa−miR−505−5p, proximal
(all subjects; N = 567)
1−sided adj pval: 0.406
Frequency
Tumor−Normal Expression Difference
hsa−miR−505−5p, proximal
(CIG_ever = 0; N0 = 180)
1−sided adj pval: 0.843
Frequency
Tumor−Normal Expression Difference
hsa−miR−505−5p, proximal
(CIG_ever = 1; N1 = 264)
1−sided adj pval: 0.016
Frequency
Tumor−Normal Expression Difference

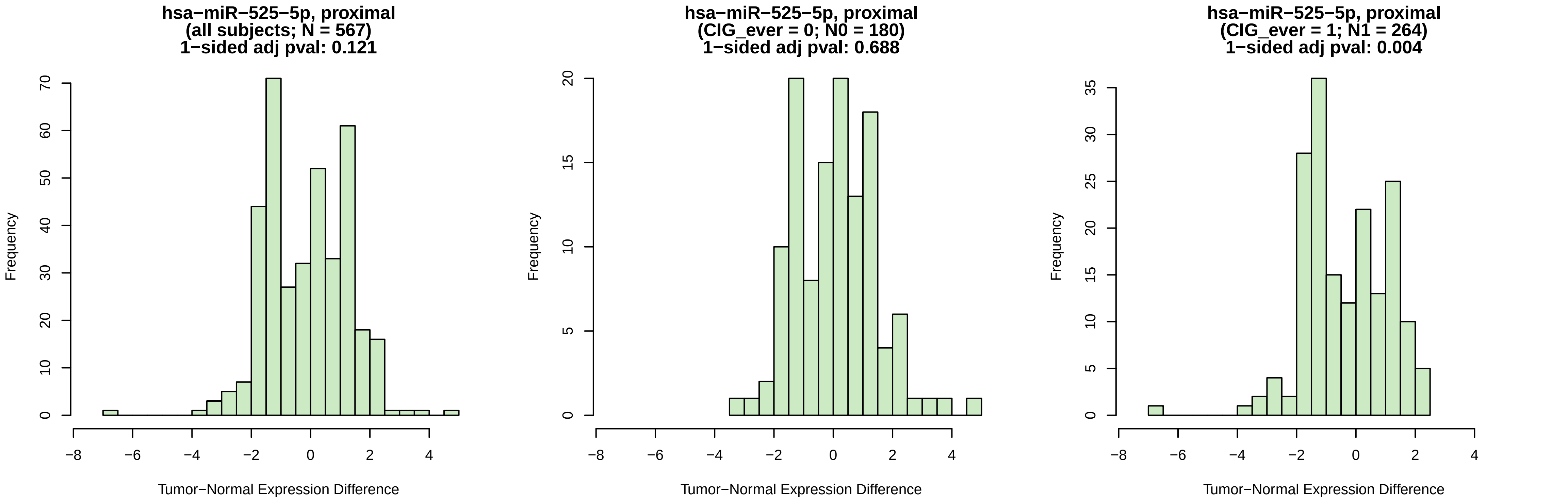
hsa−miR−525−5p, proximal
(all subjects; N = 567)
1−sided adj pval: 0.121
Frequency
Tumor−Normal Expression Difference
hsa−miR−525−5p, proximal
(CIG_ever = 0; N0 = 180)
1−sided adj pval: 0.688
Frequency
Tumor−Normal Expression Difference
hsa−miR−525−5p, proximal
(CIG_ever = 1; N1 = 264)
1−sided adj pval: 0.004
Frequency
Tumor−Normal Expression Difference

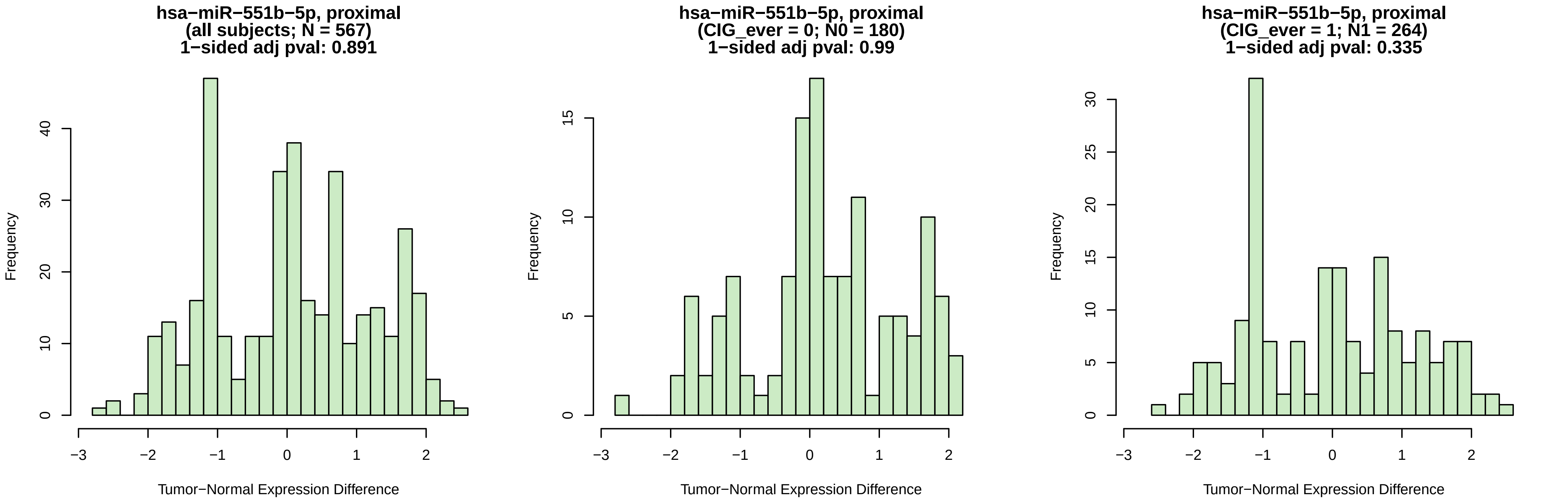

hsa−miR−551b−5p, proximal
(all subjects; N = 567)
1−sided adj pval: 0.891
Frequency
0
10
20
30
40
−3
−2
−1
0
1
2
Tumor−Normal Expression Difference
hsa−miR−551b−5p, proximal
(CIG_ever = 0; N0 = 180)
1−sided adj pval: 0.99
Frequency
0
5
10
15
−3
−2
−1
0
1
2
Tumor−Normal Expression Difference
hsa−miR−551b−5p, proximal
(CIG_ever = 1; N1 = 264)
1−sided adj pval: 0.335
Frequency
0
5
10
15
20
25
30
−3
−2
−1
0
1
2
Tumor−Normal Expression Difference

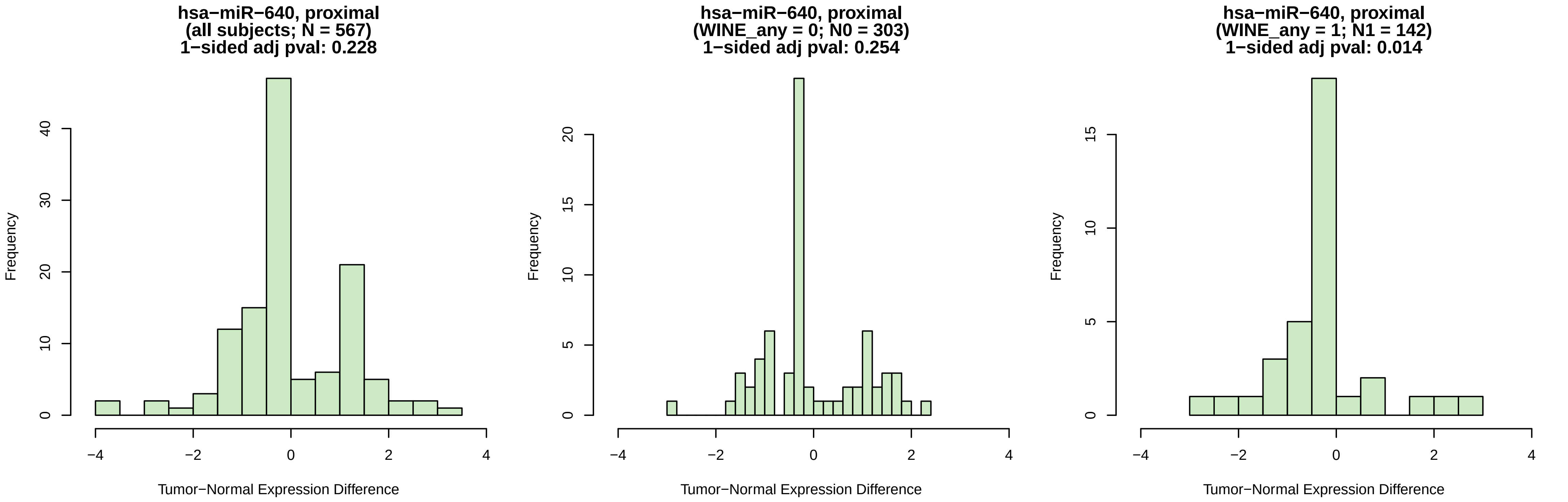
hsa−miR−640, proximal
(all subjects; N = 567)
1−sided adj pval: 0.228
Frequency
Tumor−Normal Expression Difference
hsa−miR−640, proximal
(WINE_any = 0; N0 = 303)
1−sided adj pval: 0.254
Frequency
Tumor−Normal Expression Difference
hsa−miR−640, proximal
(WINE_any = 1; N1 = 142)
1−sided adj pval: 0.014
Frequency
Tumor−Normal Expression Difference

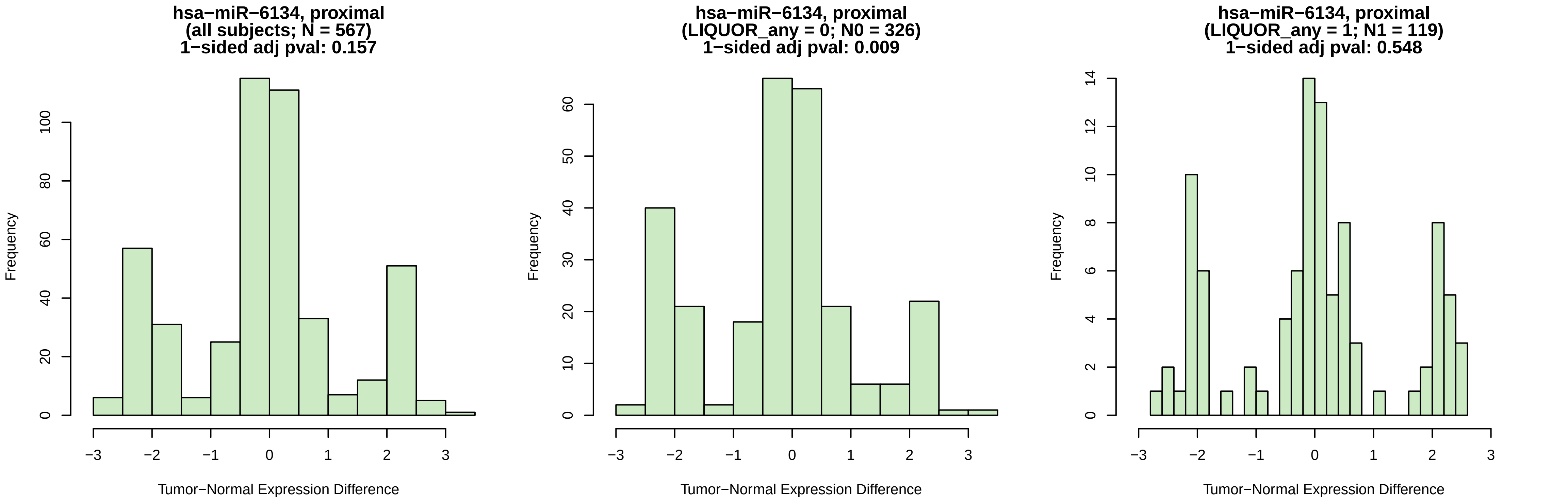

hsa−miR−6134, proximal
(all subjects; N = 567)
1−sided adj pval: 0.157
Frequency
Tumor−Normal Expression Difference
hsa−miR−6134, proximal
(LIQUOR_any = 0; N0 = 326)
1−sided adj pval: 0.009
Frequency
Tumor−Normal Expression Difference
hsa−miR−6134, proximal
(LIQUOR_any = 1; N1 = 119)
1−sided adj pval: 0.548
Frequency
Tumor−Normal Expression Difference

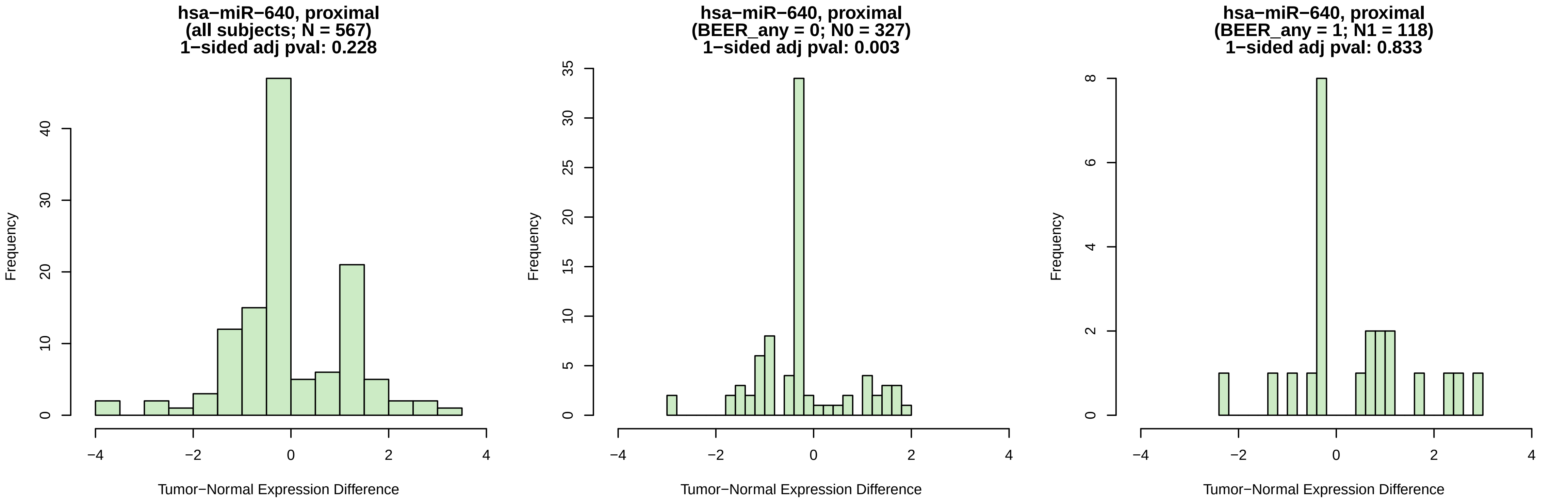

hsa−miR−640, proximal
(all subjects; N = 567)
1−sided adj pval: 0.228
Frequency
Tumor−Normal Expression Difference
hsa−miR−640, proximal
(BEER_any = 0; N0 = 327)
1−sided adj pval: 0.003
Frequency
Tumor−Normal Expression Difference
hsa−miR−640, proximal
(BEER_any = 1; N1 = 118)
1−sided adj pval: 0.833
Frequency
Tumor−Normal Expression Difference

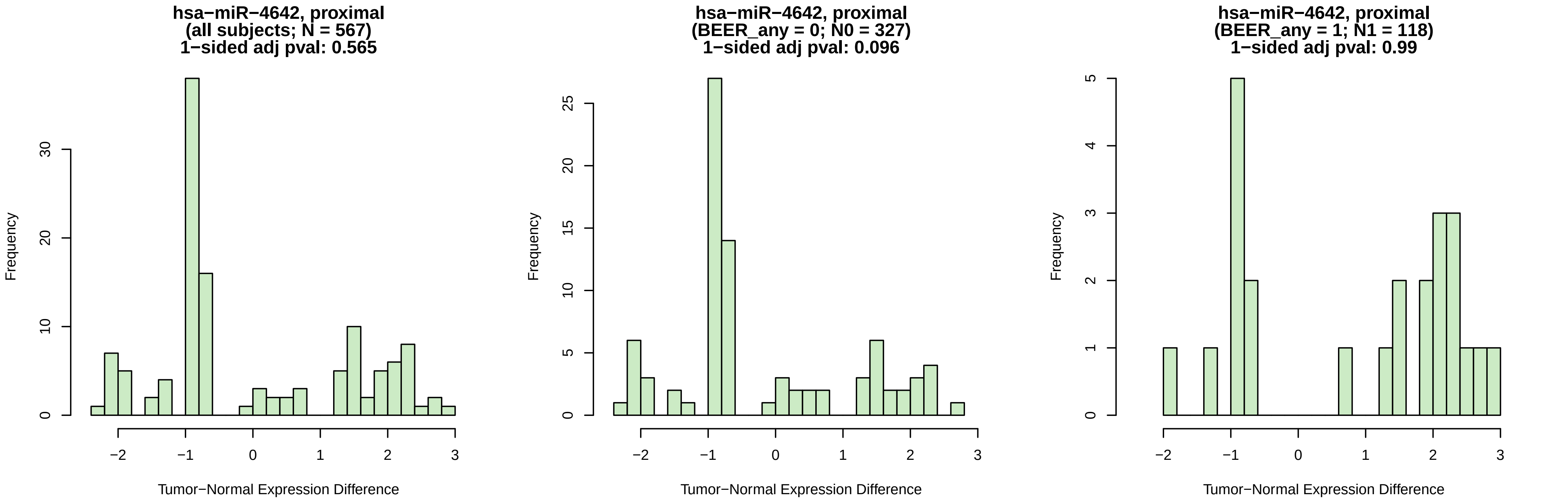

hsa−miR−4642, proximal
(all subjects; N = 567)
1−sided adj pval: 0.565
Frequency
Tumor−Normal Expression Difference
hsa−miR−4642, proximal
(BEER_any = 0; N0 = 327)
1−sided adj pval: 0.096
Frequency
Tumor−Normal Expression Difference
hsa−miR−4642, proximal
(BEER_any = 1; N1 = 118)
1−sided adj pval: 0.99
Frequency
Tumor−Normal Expression Difference

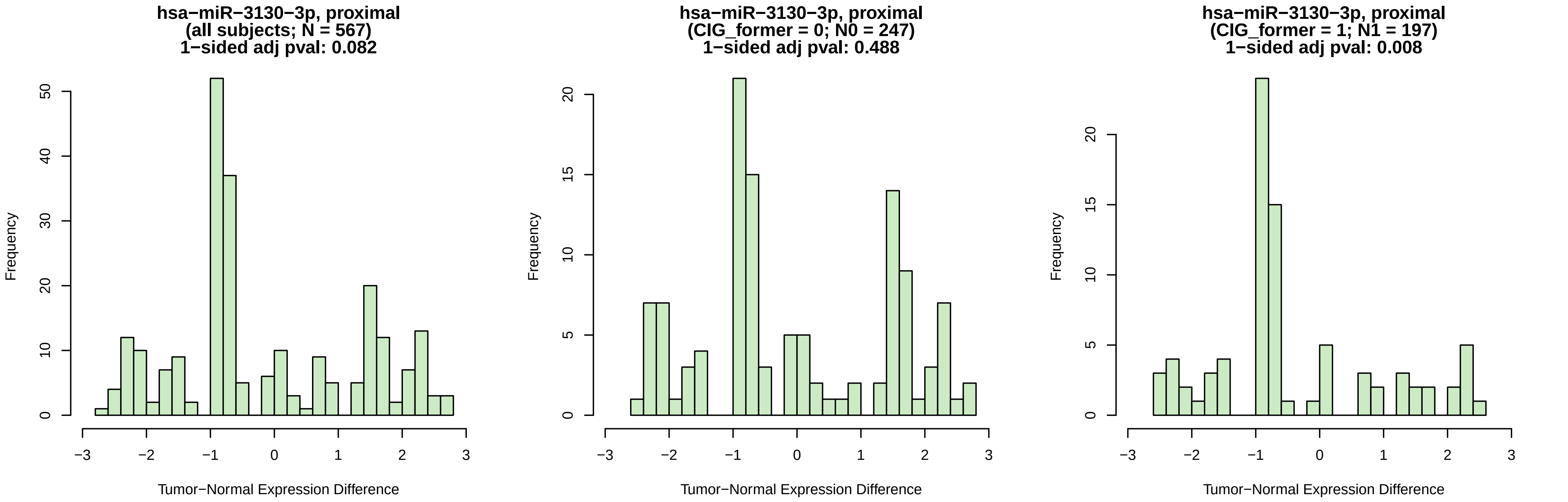
hsa−miR−3130−3p, proximal
(all subjects; N = 567)
1−sided adj pval: 0.082
Frequency
Tumor−Normal Expression Difference
hsa−miR−3130−3p, proximal
(CIG_former = 0; N0 = 247)
1−sided adj pval: 0.488
Frequency
Tumor−Normal Expression Difference
hsa−miR−3130−3p, proximal
(CIG_former = 1; N1 = 197)
1−sided adj pval: 0.008
Frequency
Tumor−Normal Expression Difference

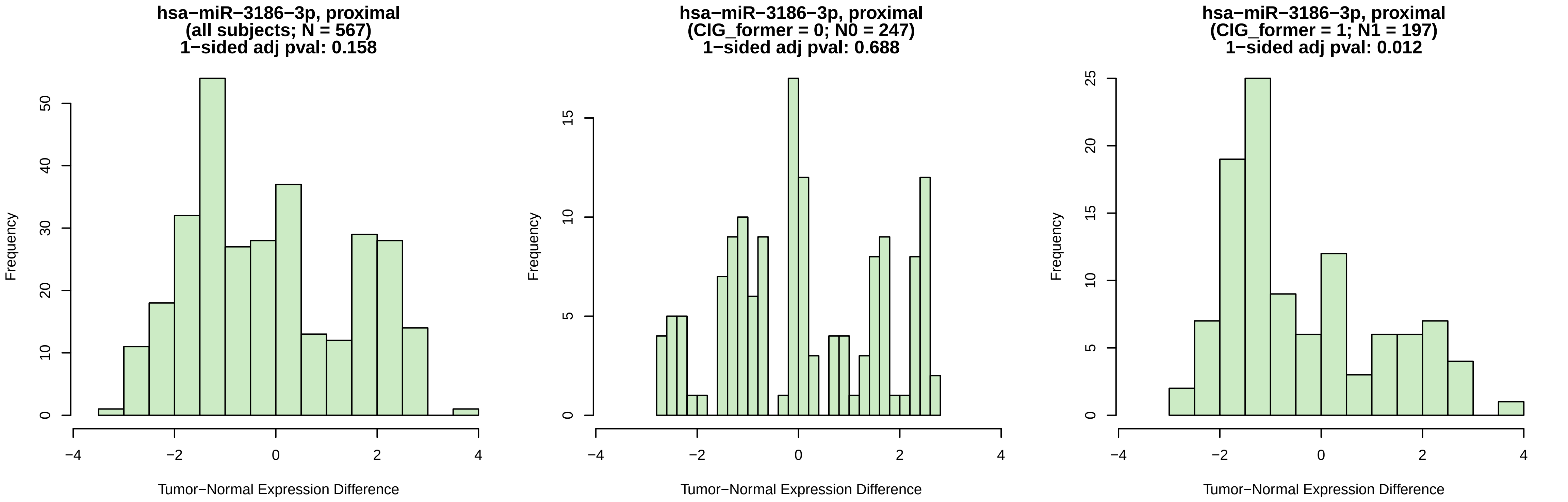
hsa−miR−3186−3p, proximal
(all subjects; N = 567)
1−sided adj pval: 0.158
Frequency
Tumor−Normal Expression Difference
hsa−miR−3186−3p, proximal
(CIG_former = 0; N0 = 247)
1−sided adj pval: 0.688
Frequency
Tumor−Normal Expression Difference
hsa−miR−3186−3p, proximal
(CIG_former = 1; N1 = 197)
1−sided adj pval: 0.012
Frequency
Tumor−Normal Expression Difference

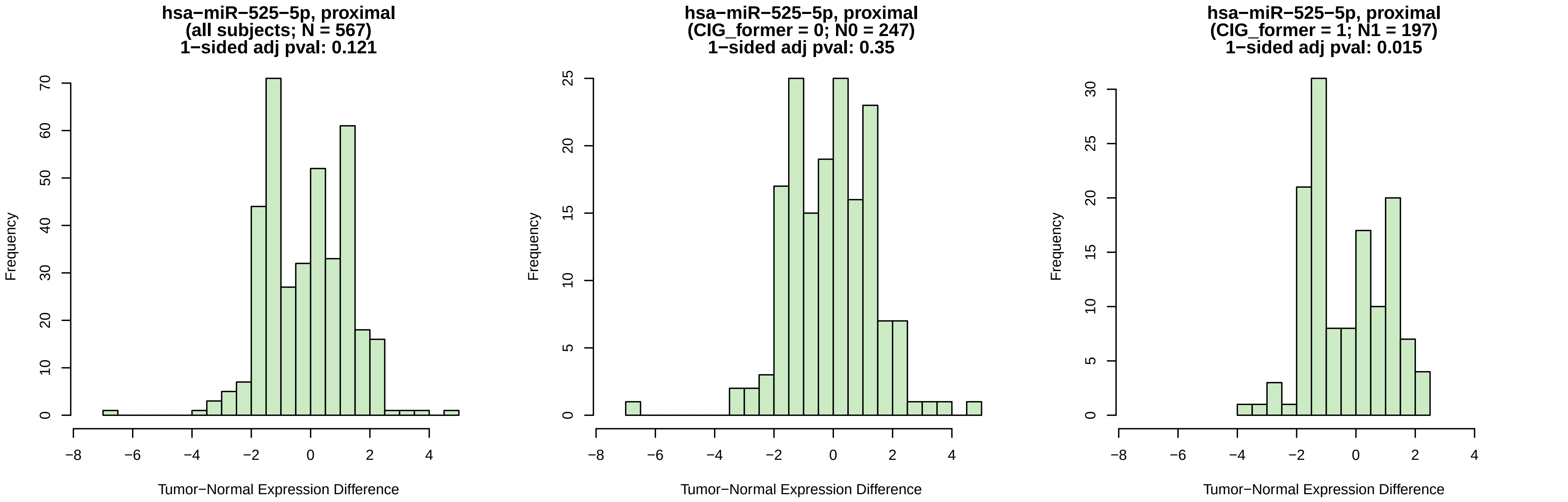
hsa−miR−525−5p, proximal
(all subjects; N = 567)
1−sided adj pval: 0.121
Frequency
Tumor−Normal Expression Difference
hsa−miR−525−5p, proximal
(CIG_former = 0; N0 = 247)
1−sided adj pval: 0.35
Frequency
Tumor−Normal Expression Difference
hsa−miR−525−5p, proximal
(CIG_former = 1; N1 = 197)
1−sided adj pval: 0.015
Frequency
Tumor−Normal Expression Difference

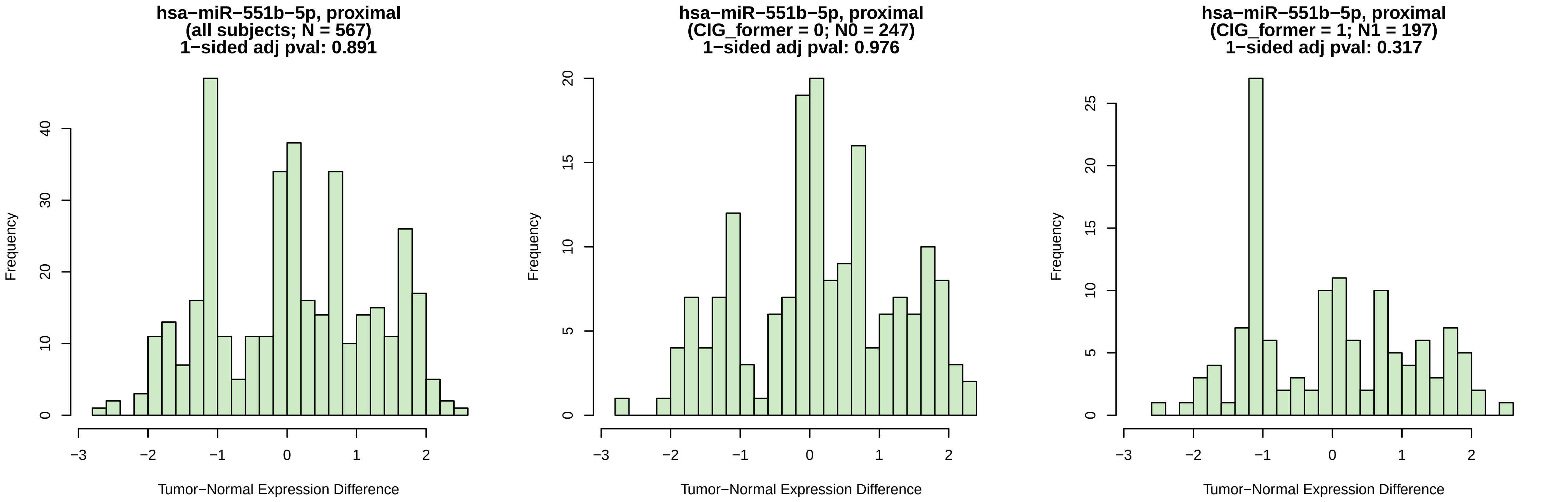
hsa−miR−551b−5p, proximal
(all subjects; N = 567)
1−sided adj pval: 0.891
hsa−miR−551b−5p, proximal
(CIG_former = 0; N0 = 247)
1−sided adj pval: 0.976
hsa−miR−551b−5p, proximal
(CIG_former = 1; N1 = 197)
1−sided adj pval: 0.317
Frequency
Tumor−Normal Expression Difference

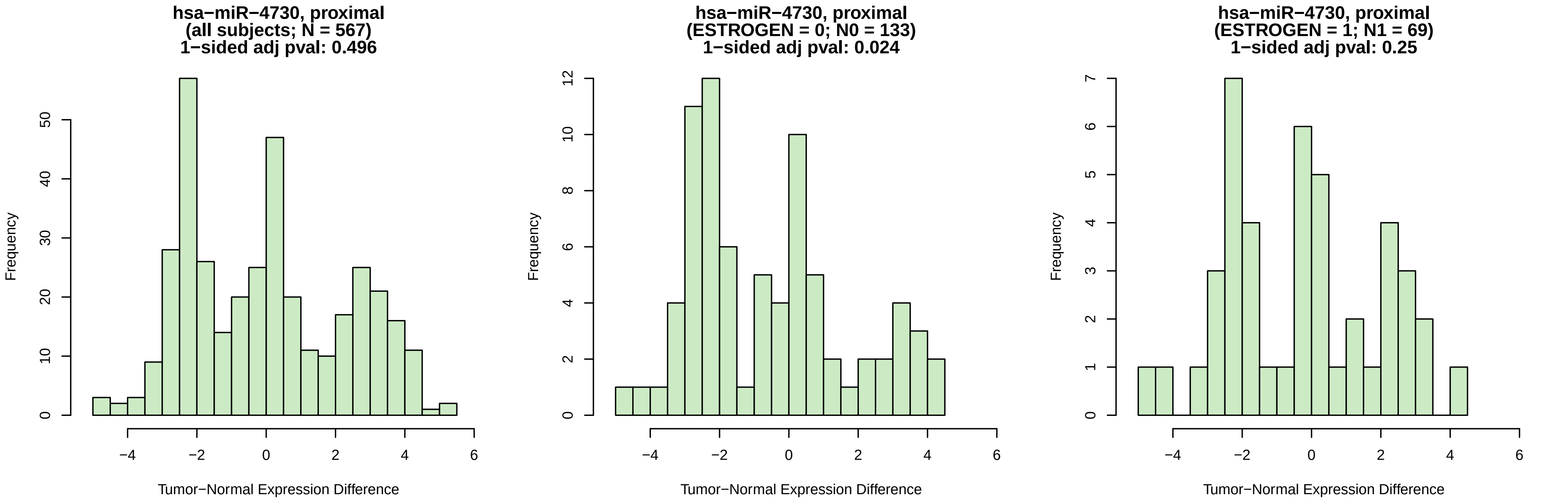
hsa−miR−4730, proximal
(all subjects; N = 567)
1−sided adj pval: 0.496
Frequency
Tumor−Normal Expression Difference
hsa−miR−4730, proximal
(ESTROGEN = 0; N0 = 133)
1−sided adj pval: 0.024
Frequency
Tumor−Normal Expression Difference
hsa−miR−4730, proximal
(ESTROGEN = 1; N1 = 69)
1−sided adj pval: 0.25
Frequency
Tumor−Normal Expression Difference

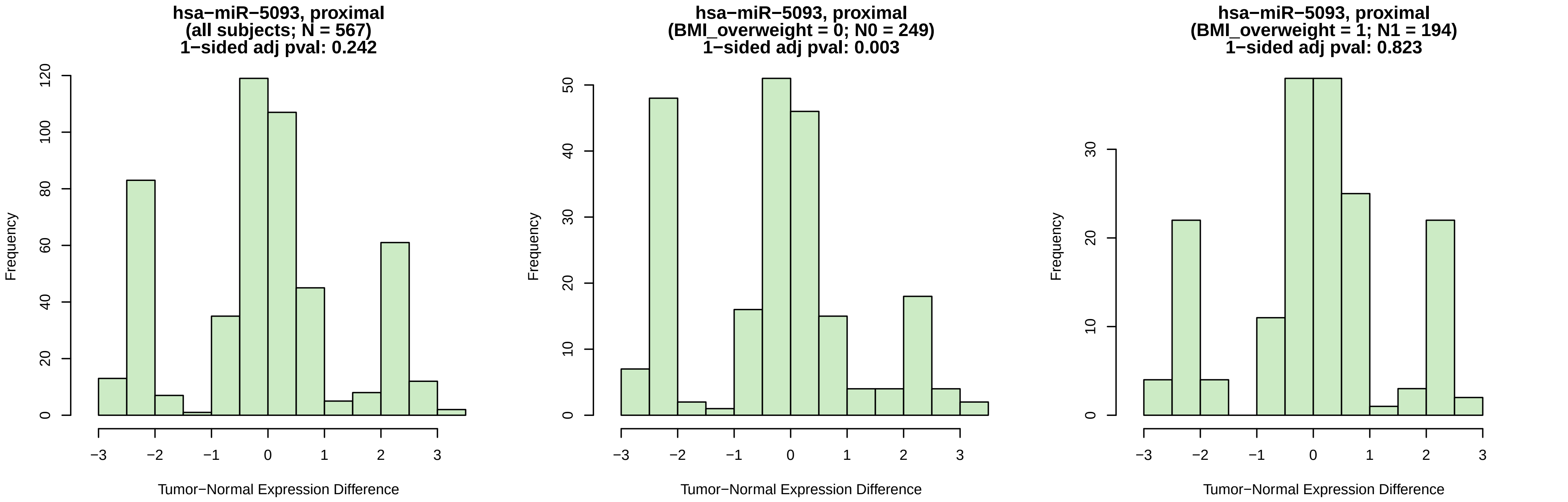
hsa−miR−5093, proximal
(all subjects; N = 567)
1−sided adj pval: 0.242
Frequency
Tumor−Normal Expression Difference
hsa−miR−5093, proximal
(BMI_overweight = 0; N0 = 249)
1−sided adj pval: 0.003
Frequency
Tumor−Normal Expression Difference
hsa−miR−5093, proximal
(BMI_overweight = 1; N1 = 194)
1−sided adj pval: 0.823
Frequency
Tumor−Normal Expression Difference

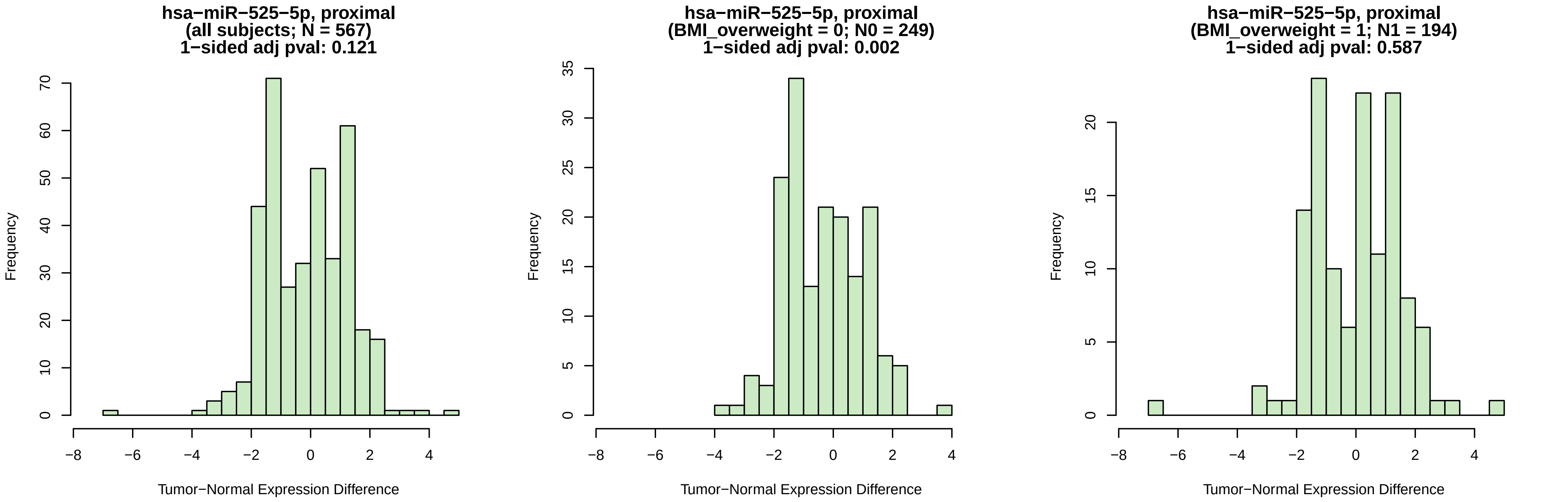
hsa−miR−525−5p, proximal
(all subjects; N = 567)
1−sided adj pval: 0.121
Frequency
Tumor−Normal Expression Difference
hsa−miR−525−5p, proximal
(BMI_overweight = 0; N0 = 249)
1−sided adj pval: 0.002
Frequency
Tumor−Normal Expression Difference
hsa−miR−525−5p, proximal
(BMI_overweight = 1; N1 = 194)
1−sided adj pval: 0.587
Frequency
Tumor−Normal Expression Difference

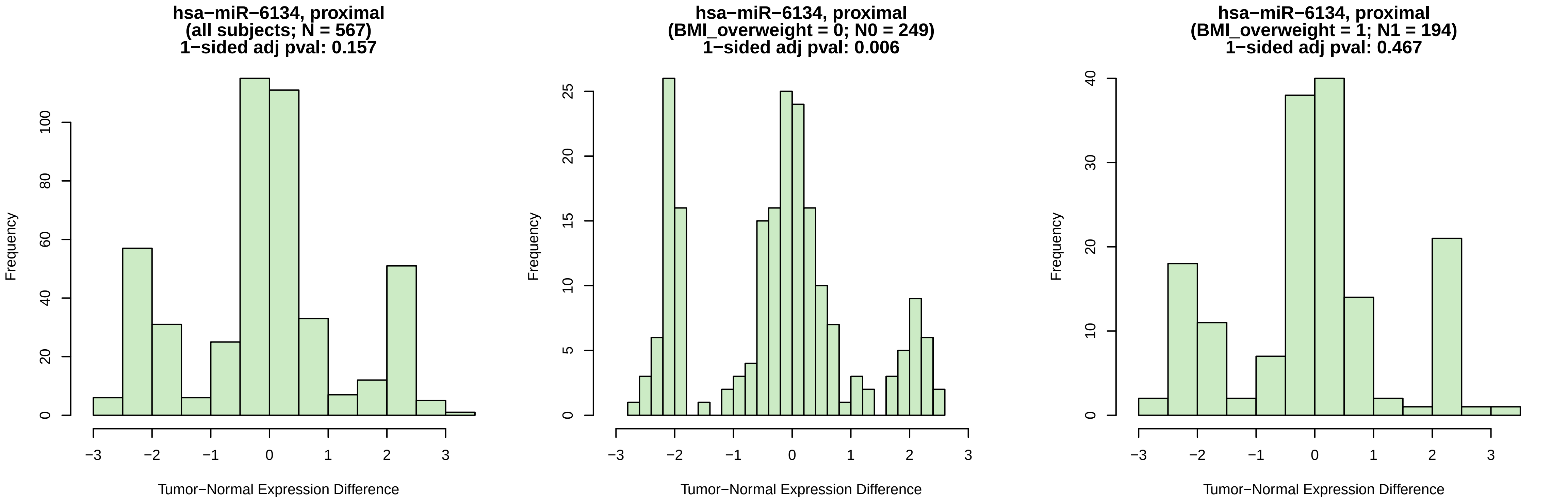
hsa−miR−6134, proximal
(all subjects; N = 567)
1−sided adj pval: 0.157
hsa−miR−6134, proximal
(BMI_overweight = 0; N0 = 249)
1−sided adj pval: 0.006
hsa−miR−6134, proximal
(BMI_overweight = 1; N1 = 194)
1−sided adj pval: 0.467
Frequency
Tumor−Normal Expression Difference

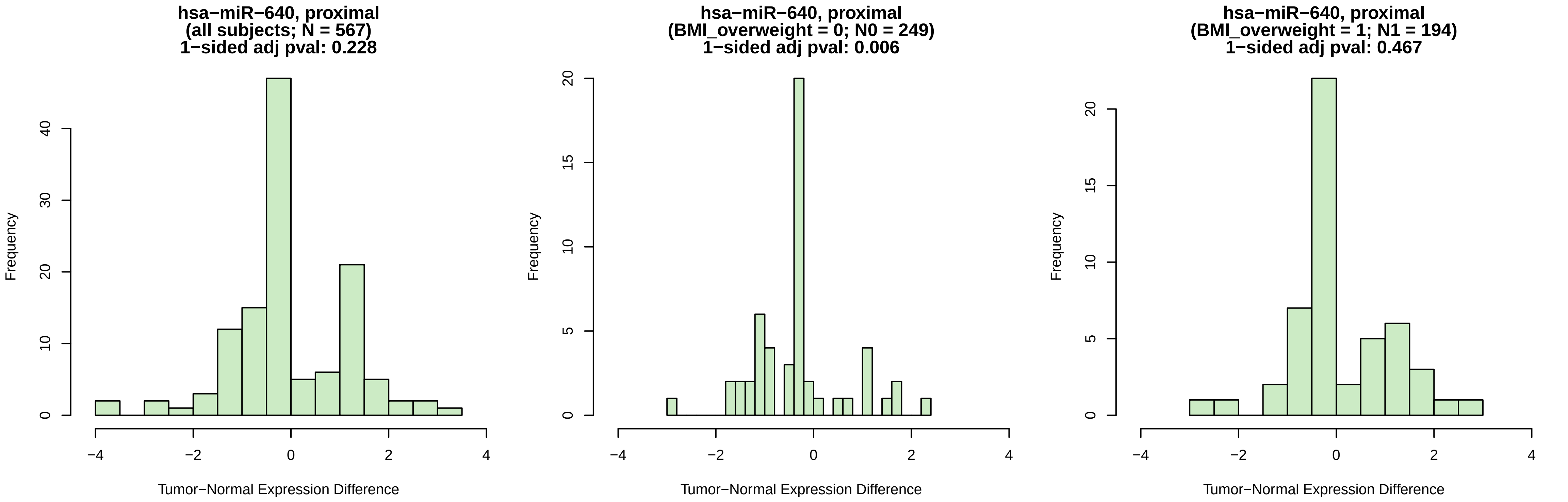
hsa−miR−640, proximal
(all subjects; N = 567)
1−sided adj pval: 0.228
hsa−miR−640, proximal
(BMI_overweight = 0; N0 = 249)
1−sided adj pval: 0.006
hsa−miR−640, proximal
(BMI_overweight = 1; N1 = 194)
1−sided adj pval: 0.467
Frequency
Tumor−Normal Expression Difference
−4
−2
0
2
4
0
10
20
30
40
5
15

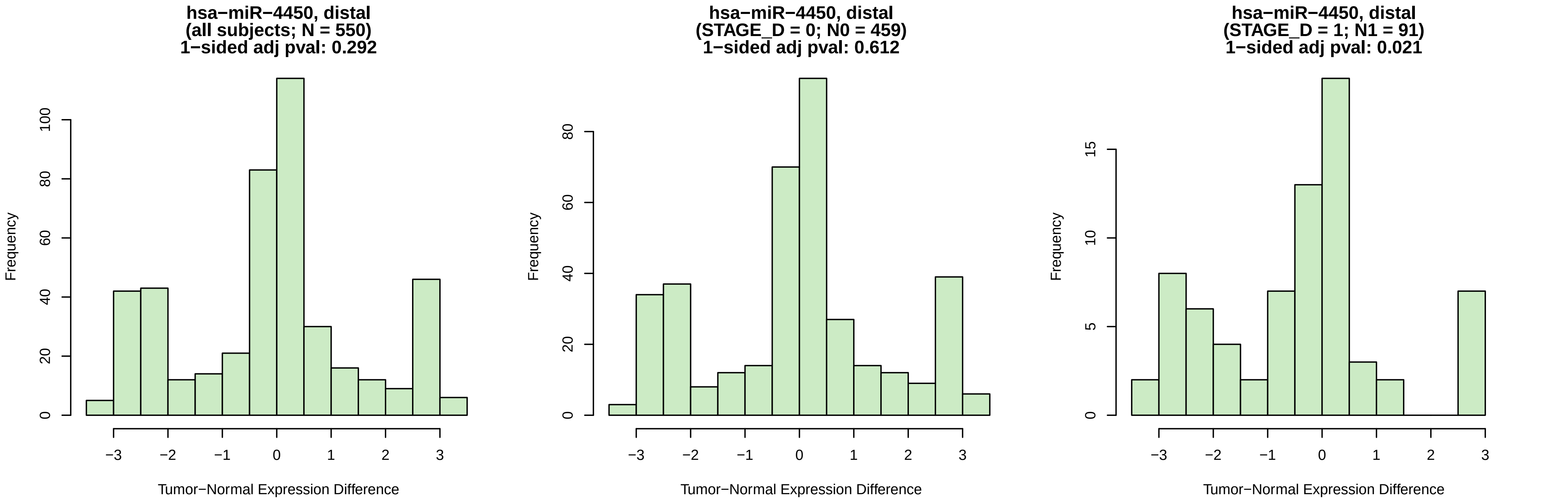
hsa−miR−4450, distal
(all subjects; N = 550)
1−sided adj pval: 0.292
Frequency
0
20
40
60
80
100
−3
−2
−1
0
1
2
3
Tumor−Normal Expression Difference
hsa−miR−4450, distal
(STAGE_D = 0; N0 = 459)
1−sided adj pval: 0.612
Frequency
0
20
40
60
80
−3
−2
−1
0
1
2
3
Tumor−Normal Expression Difference
hsa−miR−4450, distal
(STAGE_D = 1; N1 = 91)
1−sided adj pval: 0.021
Frequency
0
5
10
15
−3
−2
−1
0
1
2
3
Tumor−Normal Expression Difference

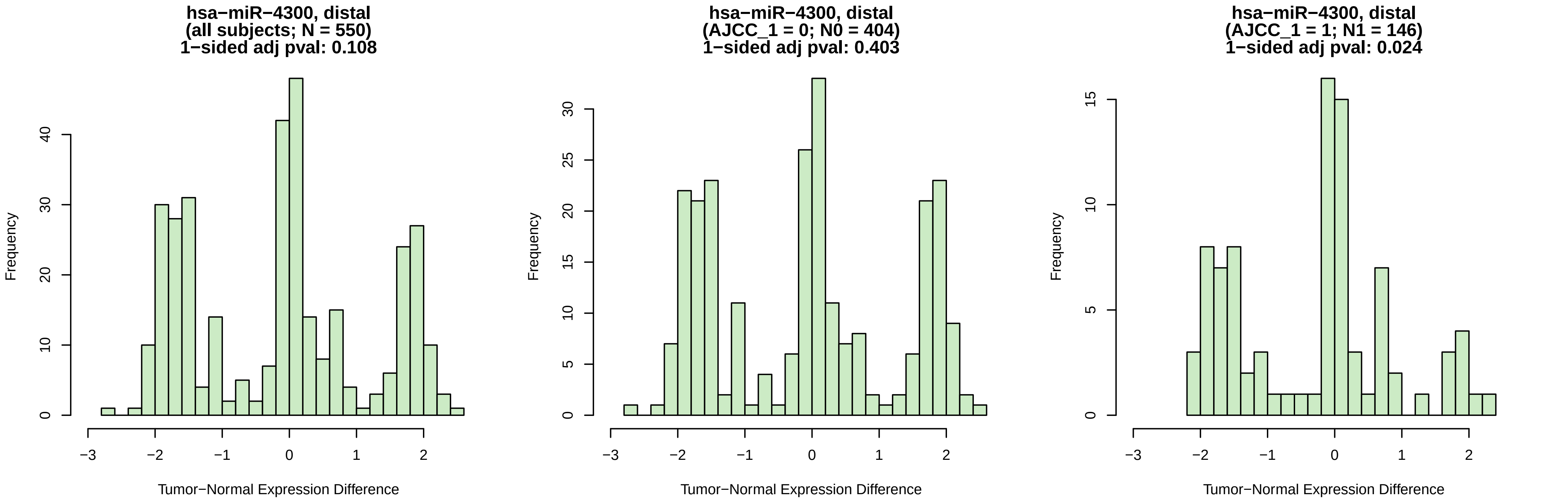

hsa−miR−4300, distal
(all subjects; N = 550)
1−sided adj pval: 0.108
hsa−miR−4300, distal
(AJCC_1 = 0; N0 = 404)
1−sided adj pval: 0.403
hsa−miR−4300, distal
(AJCC_1 = 1; N1 = 146)
1−sided adj pval: 0.024
Frequency
Tumor−Normal Expression Difference

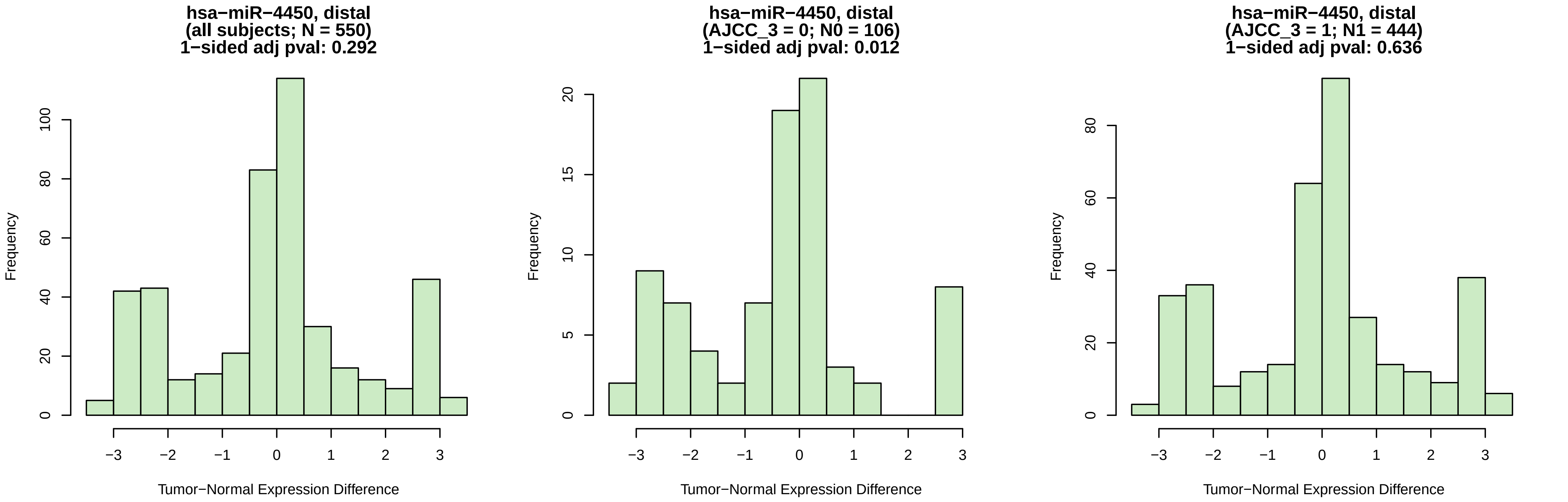

hsa−miR−4450, distal
(all subjects; N = 550)
1−sided adj pval: 0.292
hsa−miR−4450, distal
(AJCC_3 = 0; N0 = 106)
1−sided adj pval: 0.012
hsa−miR−4450, distal
(AJCC_3 = 1; N1 = 444)
1−sided adj pval: 0.636
Frequency
Tumor−Normal Expression Difference

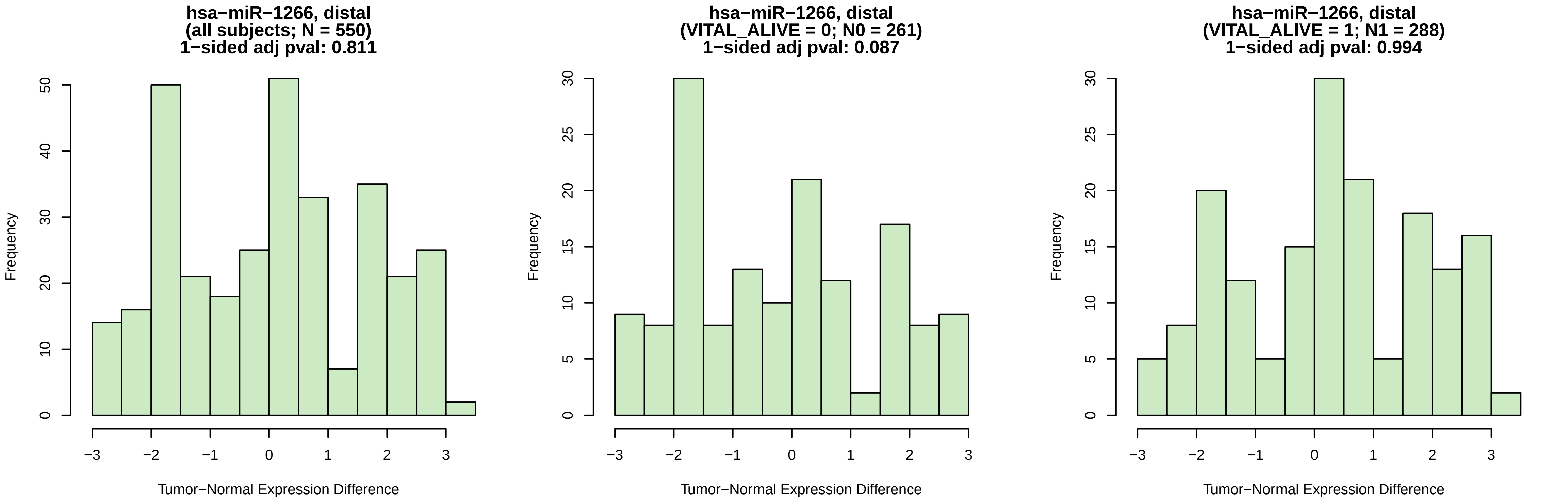
hsa−miR−1266, distal
(all subjects; N = 550)
1−sided adj pval: 0.811
Frequency
Tumor−Normal Expression Difference
hsa−miR−1266, distal
(VITAL_ALIVE = 0; N0 = 261)
1−sided adj pval: 0.087
Frequency
Tumor−Normal Expression Difference
hsa−miR−1266, distal
(VITAL_ALIVE = 1; N1 = 288)
1−sided adj pval: 0.994
Frequency
Tumor−Normal Expression Difference

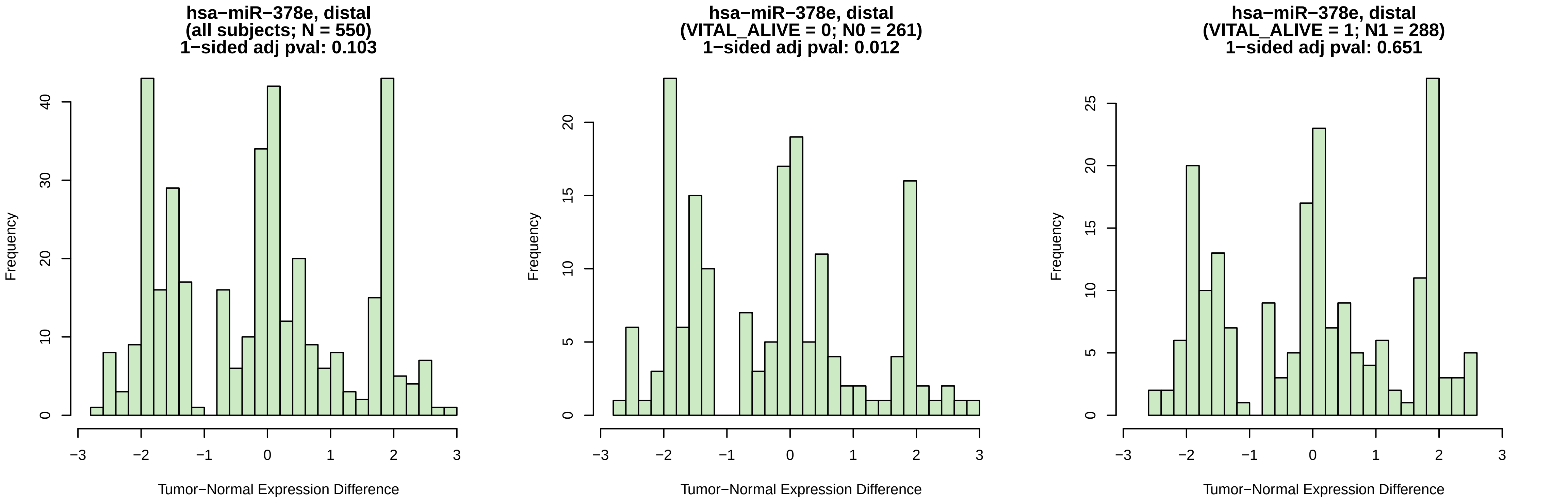

hsa−miR−378e, distal
(all subjects; N = 550)
1−sided adj pval: 0.103
Frequency
Tumor−Normal Expression Difference
hsa−miR−378e, distal
(VITAL_ALIVE = 0; N0 = 261)
1−sided adj pval: 0.012
Frequency
Tumor−Normal Expression Difference
hsa−miR−378e, distal
(VITAL_ALIVE = 1; N1 = 288)
1−sided adj pval: 0.651
Frequency
Tumor−Normal Expression Difference

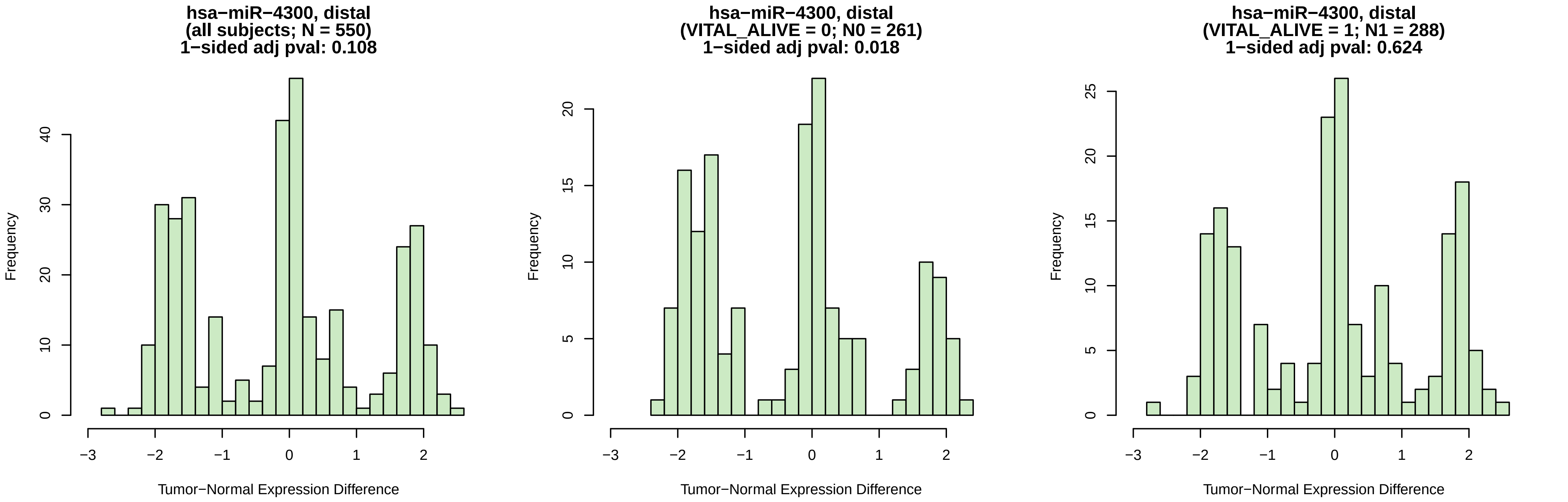

hsa−miR−4300, distal
(all subjects; N = 550)
1−sided adj pval: 0.108
Frequency
Tumor−Normal Expression Difference
hsa−miR−4300, distal
(VITAL_ALIVE = 0; N0 = 261)
1−sided adj pval: 0.018
Frequency
Tumor−Normal Expression Difference
hsa−miR−4300, distal
(VITAL_ALIVE = 1; N1 = 288)
1−sided adj pval: 0.624
Frequency
Tumor−Normal Expression Difference

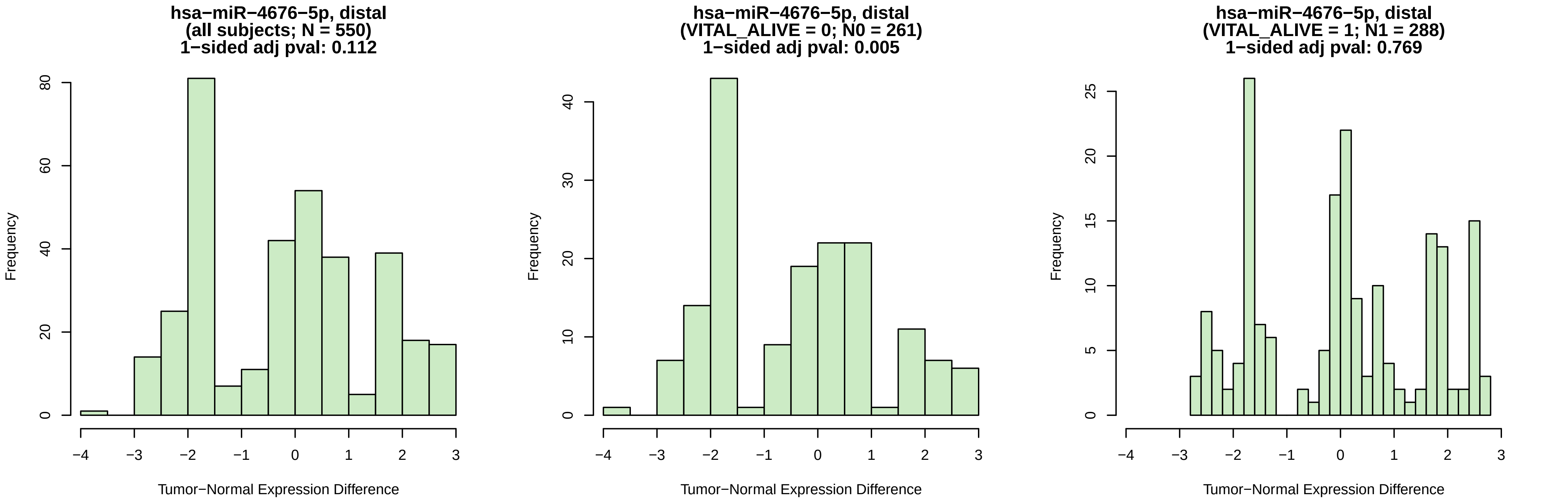
hsa−miR−4676−5p, distal
(all subjects; N = 550)
1−sided adj pval: 0.112
Frequency
Tumor−Normal Expression Difference
hsa−miR−4676−5p, distal
(VITAL_ALIVE = 0; N0 = 261)
1−sided adj pval: 0.005
Frequency
Tumor−Normal Expression Difference
hsa−miR−4676−5p, distal
(VITAL_ALIVE = 1; N1 = 288)
1−sided adj pval: 0.769
Frequency
Tumor−Normal Expression Difference

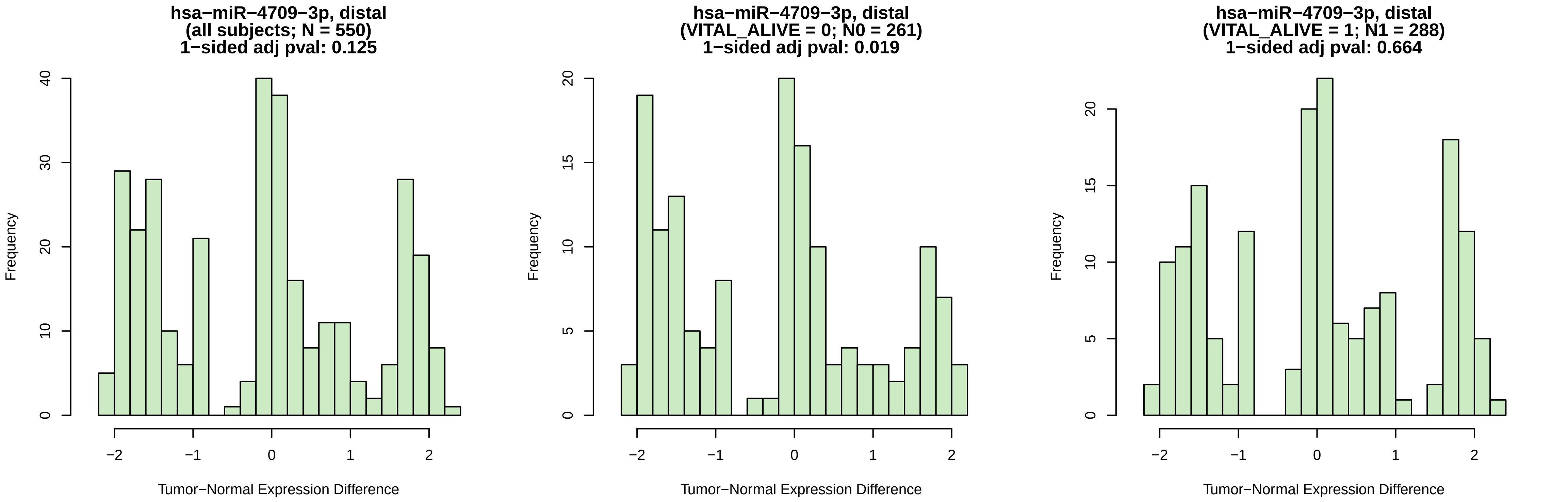

hsa−miR−4709−3p, distal
(all subjects; N = 550)
1−sided adj pval: 0.125
hsa−miR−4709−3p, distal
(VITAL_ALIVE = 0; N0 = 261)
1−sided adj pval: 0.019
hsa−miR−4709−3p, distal
(VITAL_ALIVE = 1; N1 = 288)
1−sided adj pval: 0.664
Frequency
Tumor−Normal Expression Difference

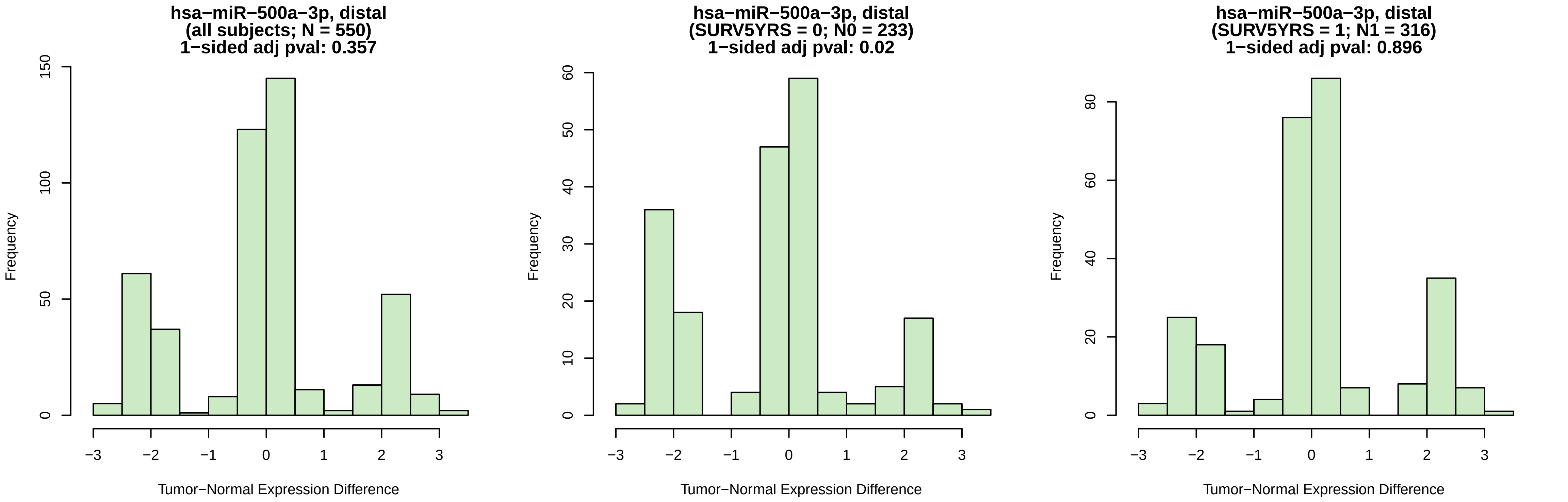
hsa−miR−500a−3p, distal
(all subjects; N = 550)
1−sided adj pval: 0.357
hsa−miR−500a−3p, distal
(SURV5YRS = 0; N0 = 233)
1−sided adj pval: 0.02
hsa−miR−500a−3p, distal
(SURV5YRS = 1; N1 = 316)
1−sided adj pval: 0.896
Frequency
Tumor−Normal Expression Difference

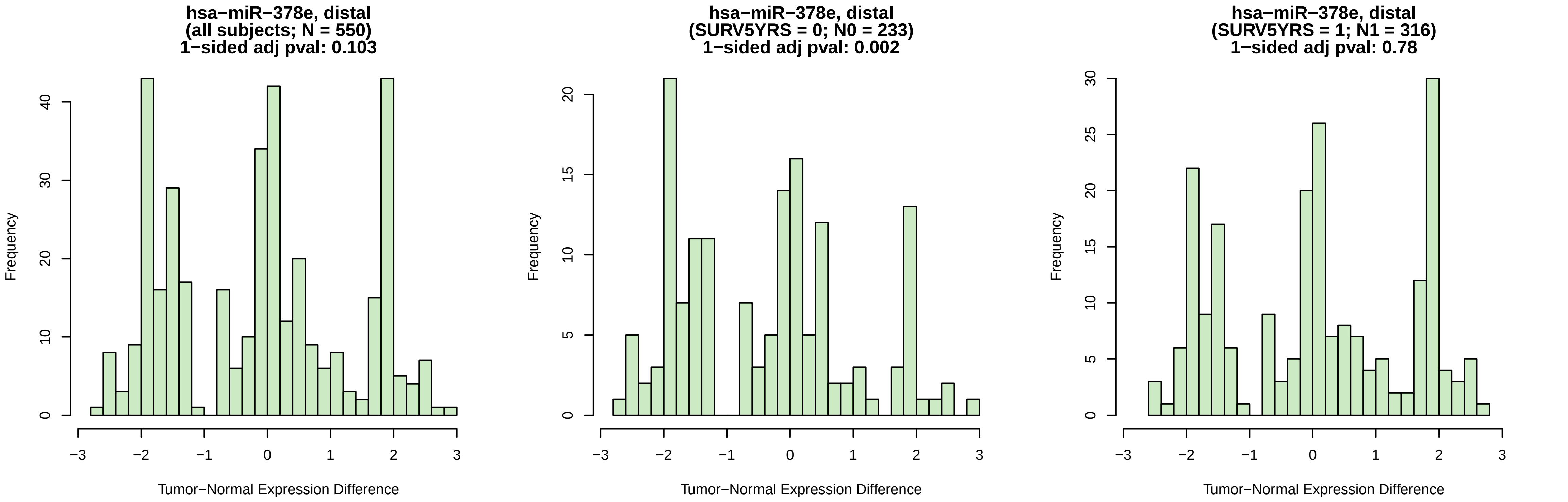

hsa−miR−378e, distal
(all subjects; N = 550)
1−sided adj pval: 0.103
hsa−miR−378e, distal
(SURV5YRS = 0; N0 = 233)
1−sided adj pval: 0.002
hsa−miR−378e, distal
(SURV5YRS = 1; N1 = 316)
1−sided adj pval: 0.78
Frequency
Tumor−Normal Expression Difference

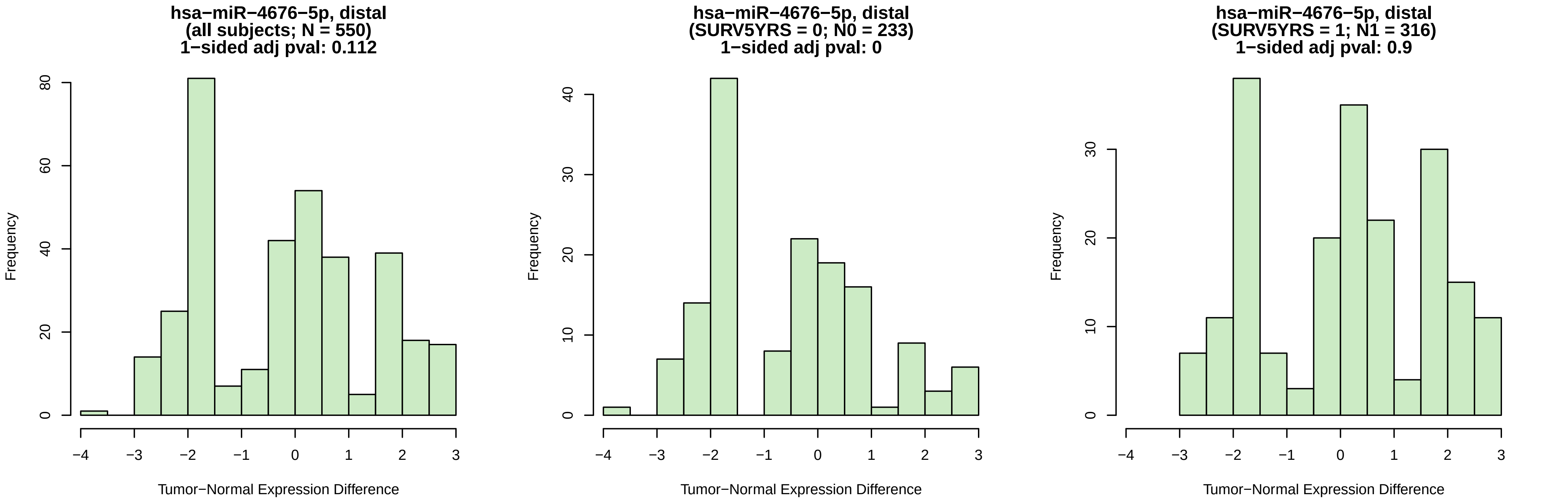

hsa−miR−4676−5p, distal
(all subjects; N = 550)
1−sided adj pval: 0.112
Frequency
0
20
40
60
80
−4
−3
−2
−1
0
1
2
3
Tumor−Normal Expression Difference
hsa−miR−4676−5p, distal
(SURV5YRS = 0; N0 = 233)
1−sided adj pval: 0
Frequency
0
10
20
30
40
−4
−3
−2
−1
0
1
2
3
Tumor−Normal Expression Difference
hsa−miR−4676−5p, distal
(SURV5YRS = 1; N1 = 316)
1−sided adj pval: 0.9
Frequency
0
10
20
30
−4
−3
−2
−1
0
1
2
3
Tumor−Normal Expression Difference

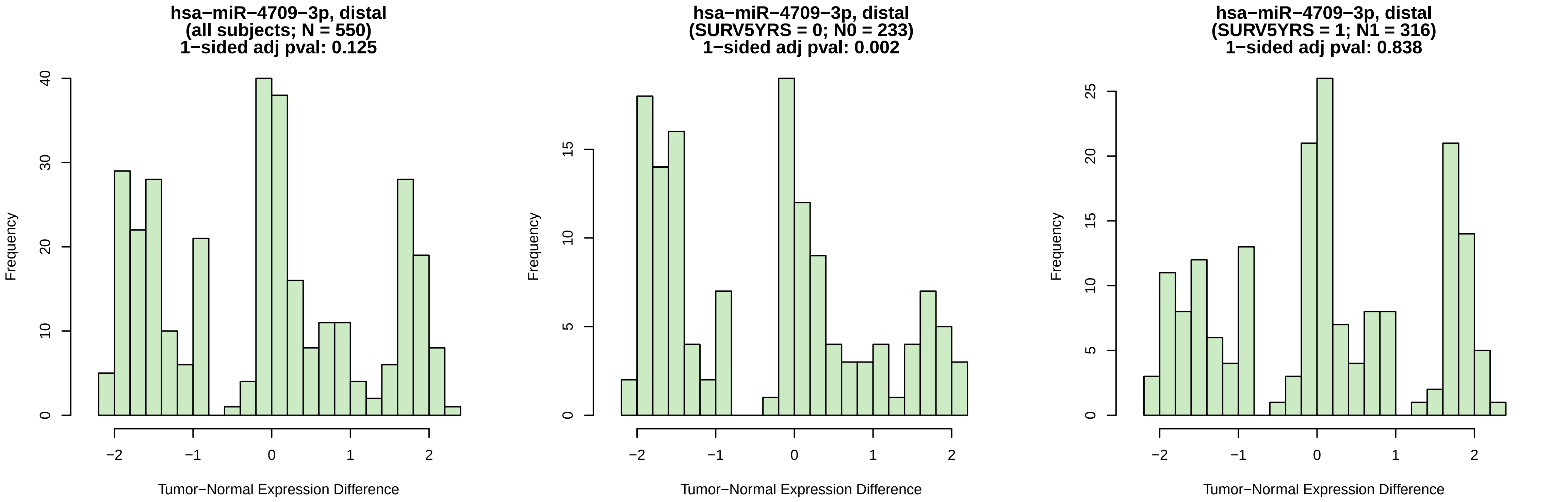
hsa−miR−4709−3p, distal
(all subjects; N = 550)
1−sided adj pval: 0.125
Frequency
Tumor−Normal Expression Difference
hsa−miR−4709−3p, distal
(SURV5YRS = 0; N0 = 233)
1−sided adj pval: 0.002
Frequency
Tumor−Normal Expression Difference
hsa−miR−4709−3p, distal
(SURV5YRS = 1; N1 = 316)
1−sided adj pval: 0.838
Frequency
Tumor−Normal Expression Difference

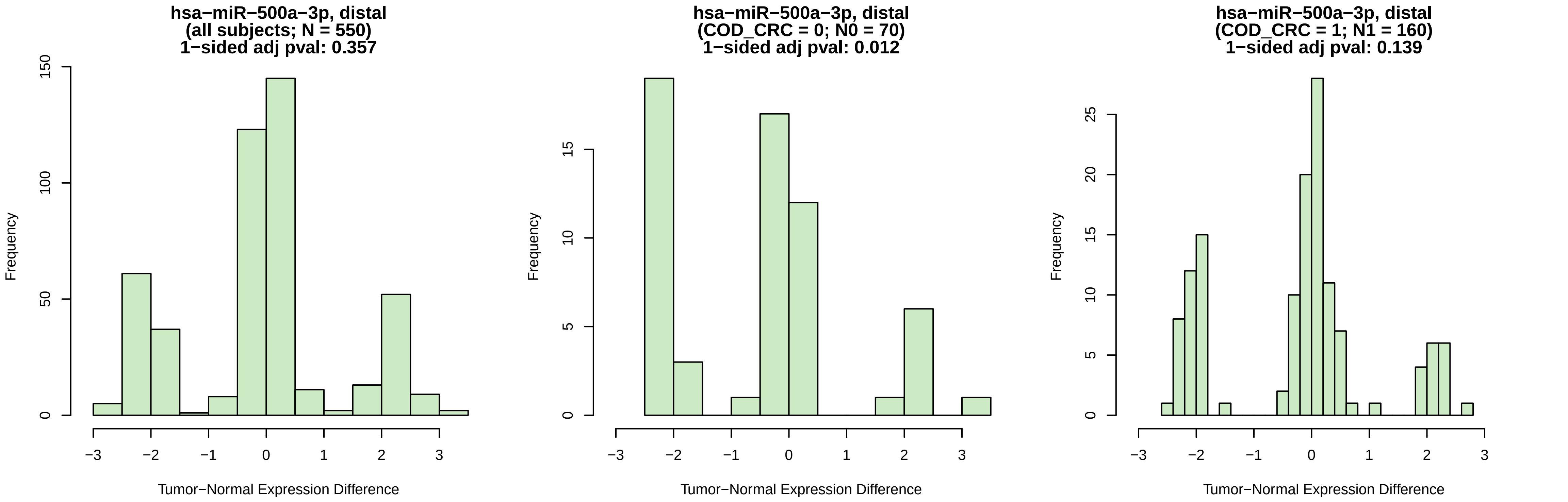
hsa−miR−500a−3p, distal
(all subjects; N = 550)
1−sided adj pval: 0.357
Frequency
Tumor−Normal Expression Difference
hsa−miR−500a−3p, distal
(COD_CRC = 0; N0 = 70)
1−sided adj pval: 0.012
Frequency
Tumor−Normal Expression Difference
hsa−miR−500a−3p, distal
(COD_CRC = 1; N1 = 160)
1−sided adj pval: 0.139
Frequency
Tumor−Normal Expression Difference

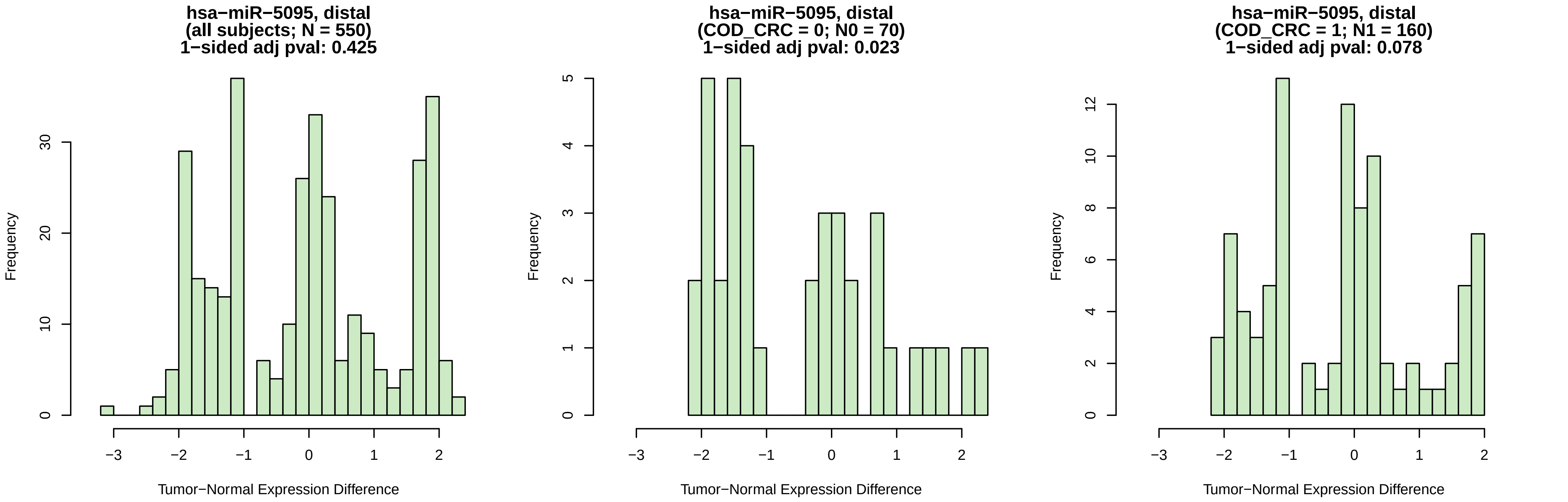
hsa−miR−5095, distal
(all subjects; N = 550)
1−sided adj pval: 0.425
Frequency
0
10
20
30
−3
−2
−1
0
1
2
Tumor−Normal Expression Difference
hsa−miR−5095, distal
(COD_CRC = 0; N0 = 70)
1−sided adj pval: 0.023
Frequency
0
1
2
3
4
5
−3
−2
−1
0
1
2
Tumor−Normal Expression Difference
hsa−miR−5095, distal
(COD_CRC = 1; N1 = 160)
1−sided adj pval: 0.078
Frequency
0
2
4
6
8
10
12
−3
−2
−1
0
1
2
Tumor−Normal Expression Difference

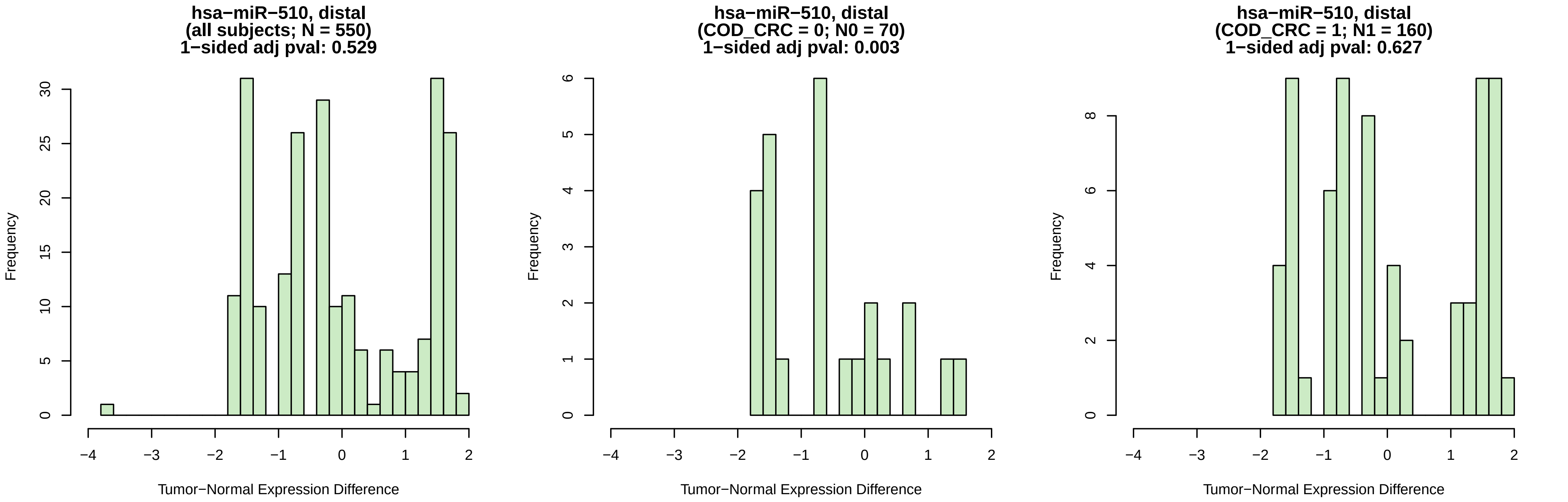
hsa−miR−510, distal
(all subjects; N = 550)
1−sided adj pval: 0.529
hsa−miR−510, distal
(COD_CRC = 0; N0 = 70)
1−sided adj pval: 0.003
hsa−miR−510, distal
(COD_CRC = 1; N1 = 160)
1−sided adj pval: 0.627
Frequency
Tumor−Normal Expression Difference

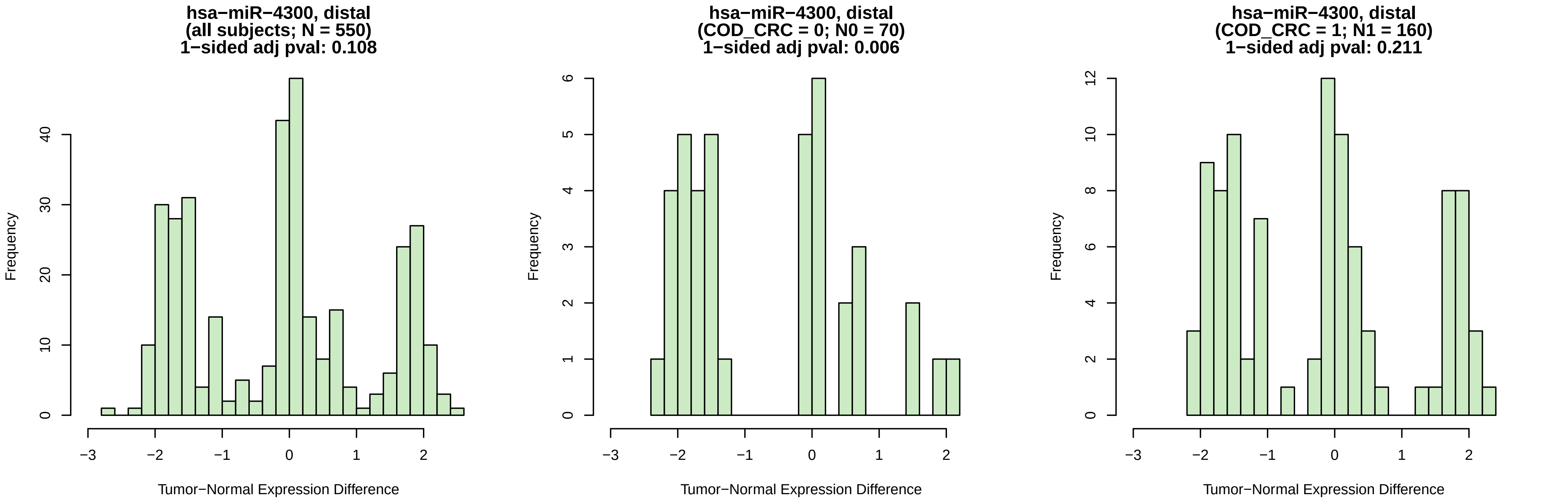
hsa−miR−4300, distal
(all subjects; N = 550)
1−sided adj pval: 0.108
Frequency
0
10
20
30
40
−3
−2
−1
0
1
2
Tumor−Normal Expression Difference
hsa−miR−4300, distal
(COD_CRC = 0; N0 = 70)
1−sided adj pval: 0.006
Frequency
0
1
2
3
4
5
6
−3
−2
−1
0
1
2
Tumor−Normal Expression Difference
hsa−miR−4300, distal
(COD_CRC = 1; N1 = 160)
1−sided adj pval: 0.211
Frequency
0
2
4
6
8
10
12
−3
−2
−1
0
1
2
Tumor−Normal Expression Difference

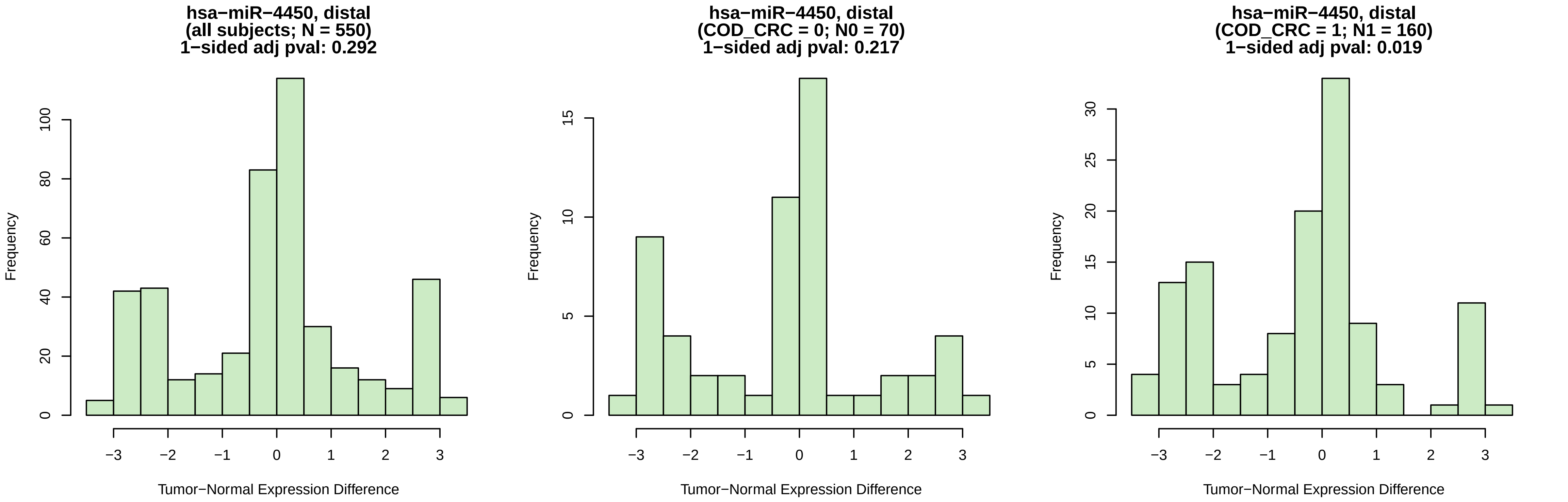
hsa−miR−4450, distal
(all subjects; N = 550)
1−sided adj pval: 0.292
Frequency
0
20
40
60
80
100
−3
−2
−1
0
1
2
3
Tumor−Normal Expression Difference
hsa−miR−4450, distal
(COD_CRC = 0; N0 = 70)
1−sided adj pval: 0.217
Frequency
0
5
10
15
−3
−2
−1
0
1
2
3
Tumor−Normal Expression Difference
hsa−miR−4450, distal
(COD_CRC = 1; N1 = 160)
1−sided adj pval: 0.019
Frequency
0
5
10
15
20
25
30
−3
−2
−1
0
1
2
3
Tumor−Normal Expression Difference

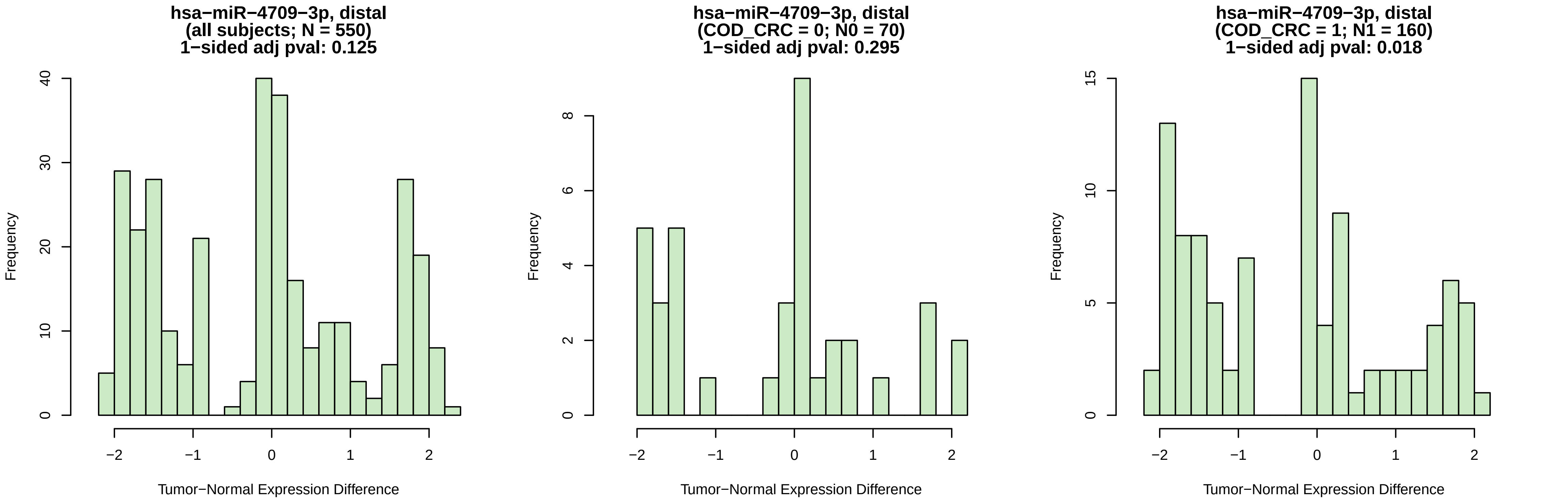
hsa−miR−4709−3p, distal
(all subjects; N = 550)
1−sided adj pval: 0.125
Frequency
Tumor−Normal Expression Difference
hsa−miR−4709−3p, distal
(COD_CRC = 0; N0 = 70)
1−sided adj pval: 0.295
Frequency
Tumor−Normal Expression Difference
hsa−miR−4709−3p, distal
(COD_CRC = 1; N1 = 160)
1−sided adj pval: 0.018
Frequency
Tumor−Normal Expression Difference

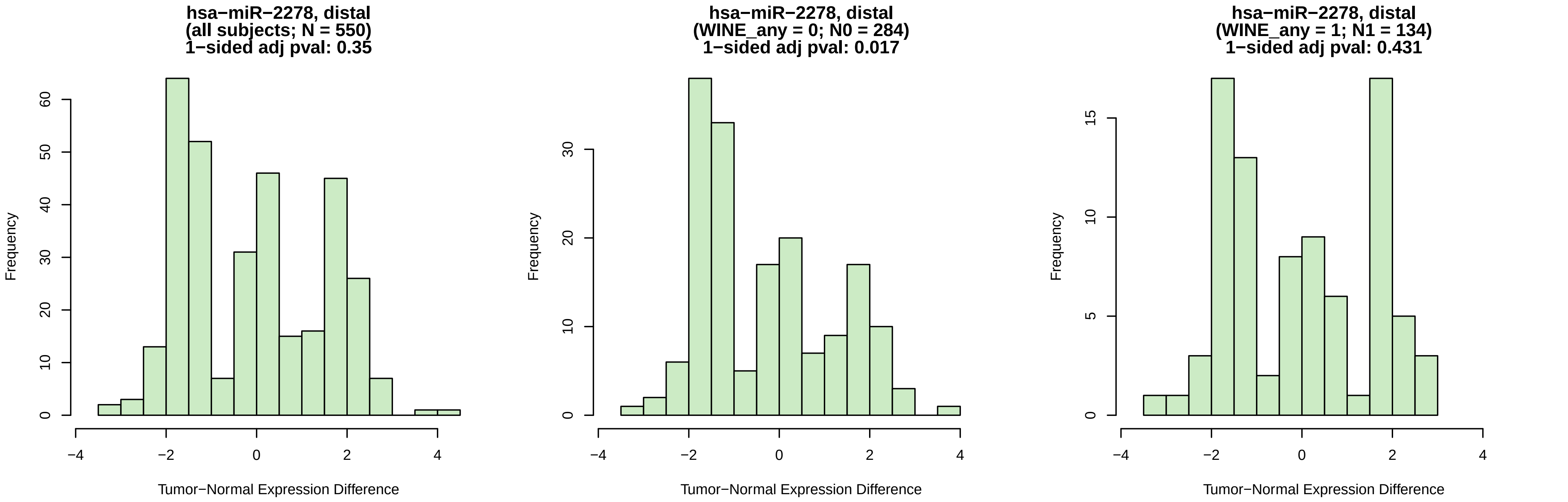

hsa−miR−2278, distal
(all subjects; N = 550)
1−sided adj pval: 0.35
Frequency
Tumor−Normal Expression Difference
hsa−miR−2278, distal
(WINE_any = 0; N0 = 284)
1−sided adj pval: 0.017
Frequency
Tumor−Normal Expression Difference
hsa−miR−2278, distal
(WINE_any = 1; N1 = 134)
1−sided adj pval: 0.431
Frequency
Tumor−Normal Expression Difference

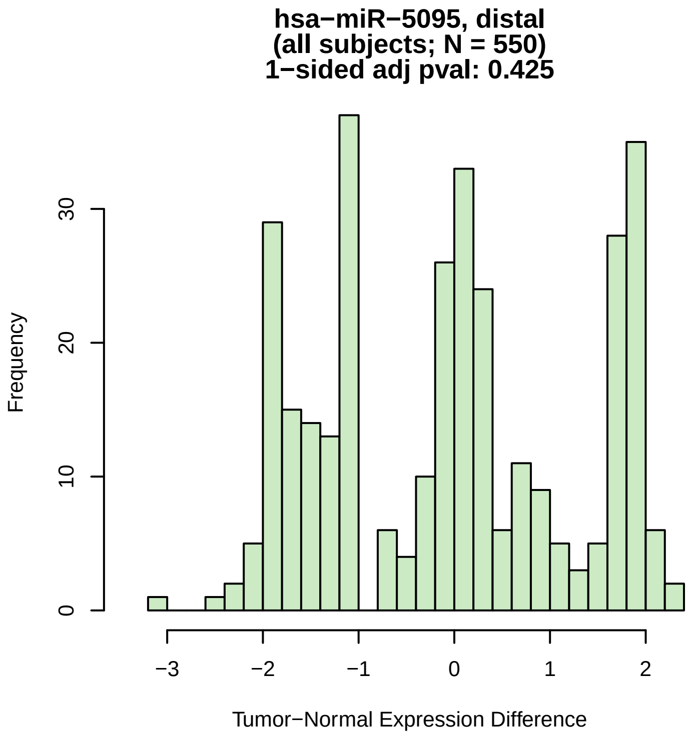
hsa−miR−5095, distal
(all subjects; N = 550)
1−sided adj pval: 0.425
Frequency
Tumor−Normal Expression Difference

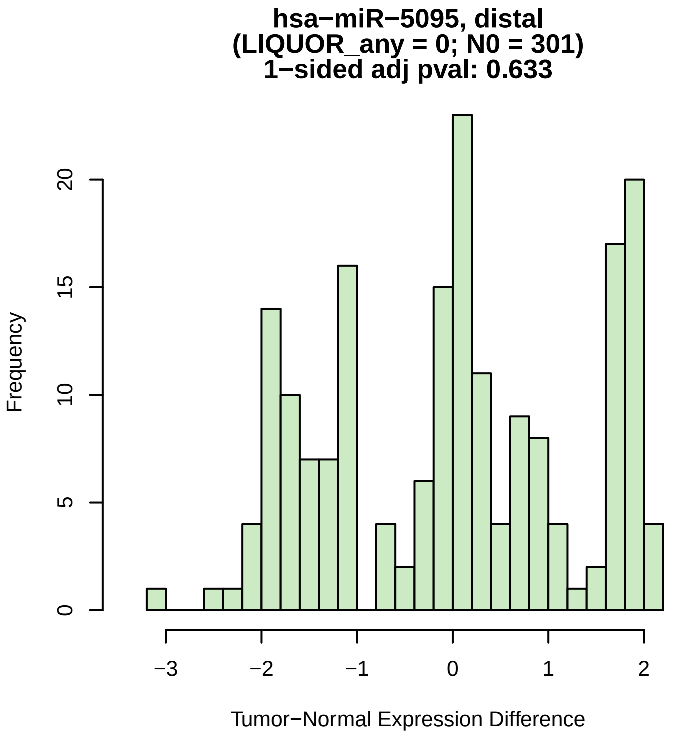
hsa−miR−5095, distal
(LIQUOR_any = 0; N0 = 301)
1−sided adj pval: 0.633
Frequency
Tumor−Normal Expression Difference

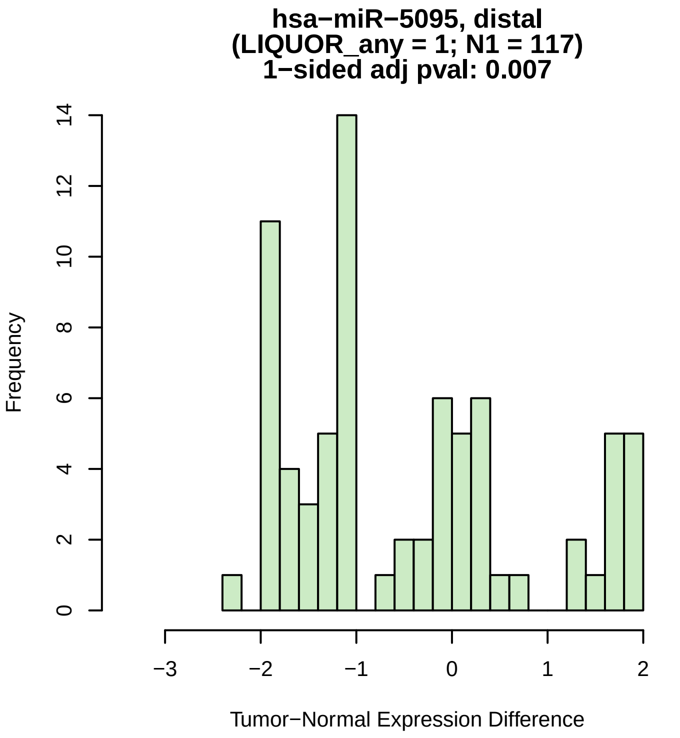
hsa−miR−5095, distal
(LIQUOR_any = 1; N1 = 117)
1−sided adj pval: 0.007
Frequency
Tumor−Normal Expression Difference

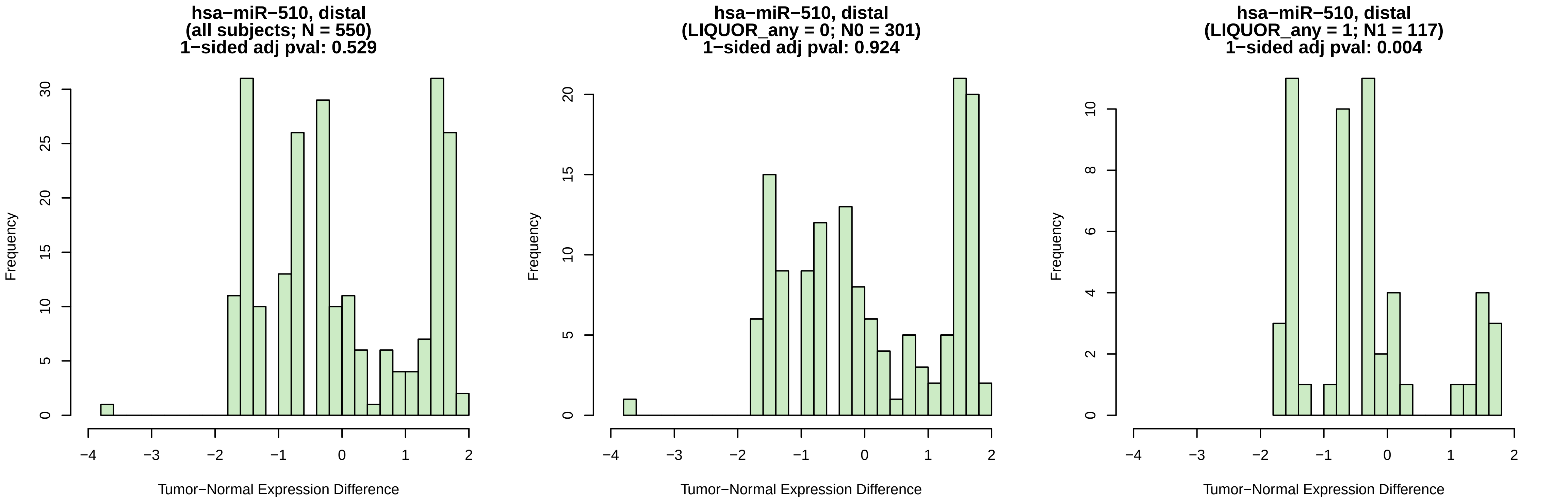
hsa−miR−510, distal
(all subjects; N = 550)
1−sided adj pval: 0.529
Frequency
Tumor−Normal Expression Difference
hsa−miR−510, distal
(LIQUOR_any = 0; N0 = 301)
1−sided adj pval: 0.924
Frequency
Tumor−Normal Expression Difference
hsa−miR−510, distal
(LIQUOR_any = 1; N1 = 117)
1−sided adj pval: 0.004
Frequency
Tumor−Normal Expression Difference

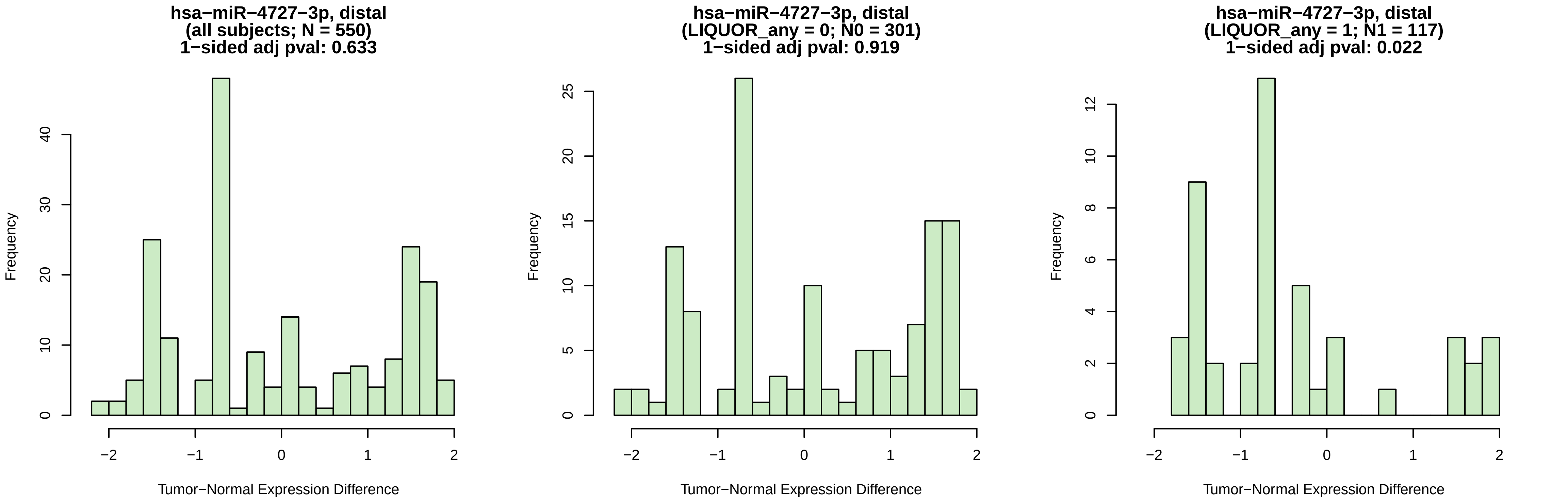

hsa−miR−4727−3p, distal
(all subjects; N = 550)
1−sided adj pval: 0.633
Frequency
0
10
20
30
40
−2
−1
0
1
2
Tumor−Normal Expression Difference
hsa−miR−4727−3p, distal
(LIQUOR_any = 0; N0 = 301)
1−sided adj pval: 0.919
Frequency
0
5
10
15
20
25
−2
−1
0
1
2
Tumor−Normal Expression Difference
hsa−miR−4727−3p, distal
(LIQUOR_any = 1; N1 = 117)
1−sided adj pval: 0.022
Frequency
0
2
4
6
8
10
12
−2
−1
0
1
2
Tumor−Normal Expression Difference

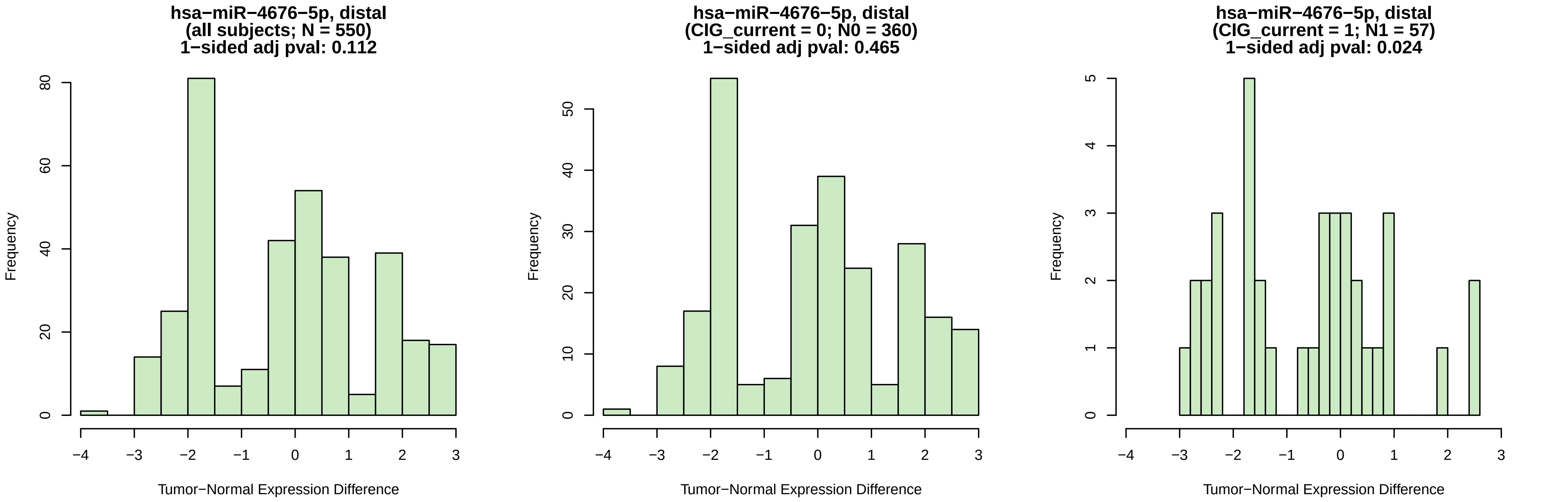

hsa−miR−4676−5p, distal
(all subjects; N = 550)
1−sided adj pval: 0.112
Frequency
Tumor−Normal Expression Difference
0
20
40
60
80
−4
−3
−2
−1
0
1
2
3
hsa−miR−4676−5p, distal
(CIG_current = 0; N0 = 360)
1−sided adj pval: 0.465
Frequency
Tumor−Normal Expression Difference
0
10
20
30
40
50
hsa−miR−4676−5p, distal
(CIG_current = 1; N1 = 57)
1−sided adj pval: 0.024
Frequency
Tumor−Normal Expression Difference
0
1
2
3
4
5

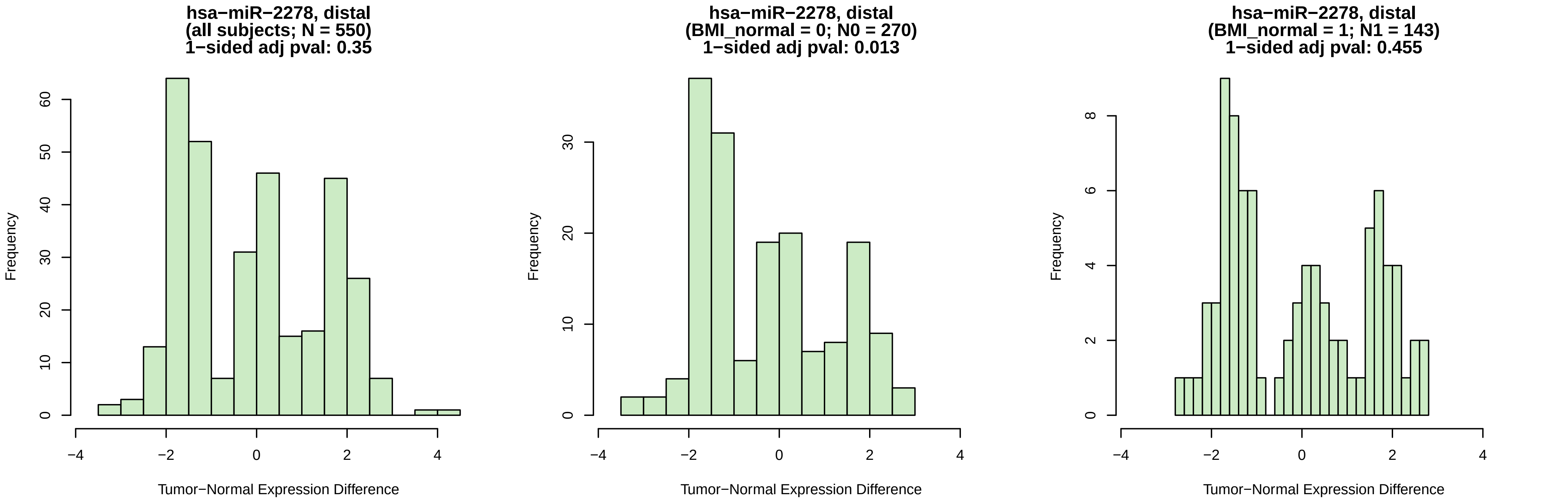
hsa−miR−2278, distal
(all subjects; N = 550)
1−sided adj pval: 0.35
Frequency
Tumor−Normal Expression Difference
hsa−miR−2278, distal
(BMI_normal = 0; N0 = 270)
1−sided adj pval: 0.013
Frequency
Tumor−Normal Expression Difference
hsa−miR−2278, distal
(BMI_normal = 1; N1 = 143)
1−sided adj pval: 0.455
Frequency
Tumor−Normal Expression Difference

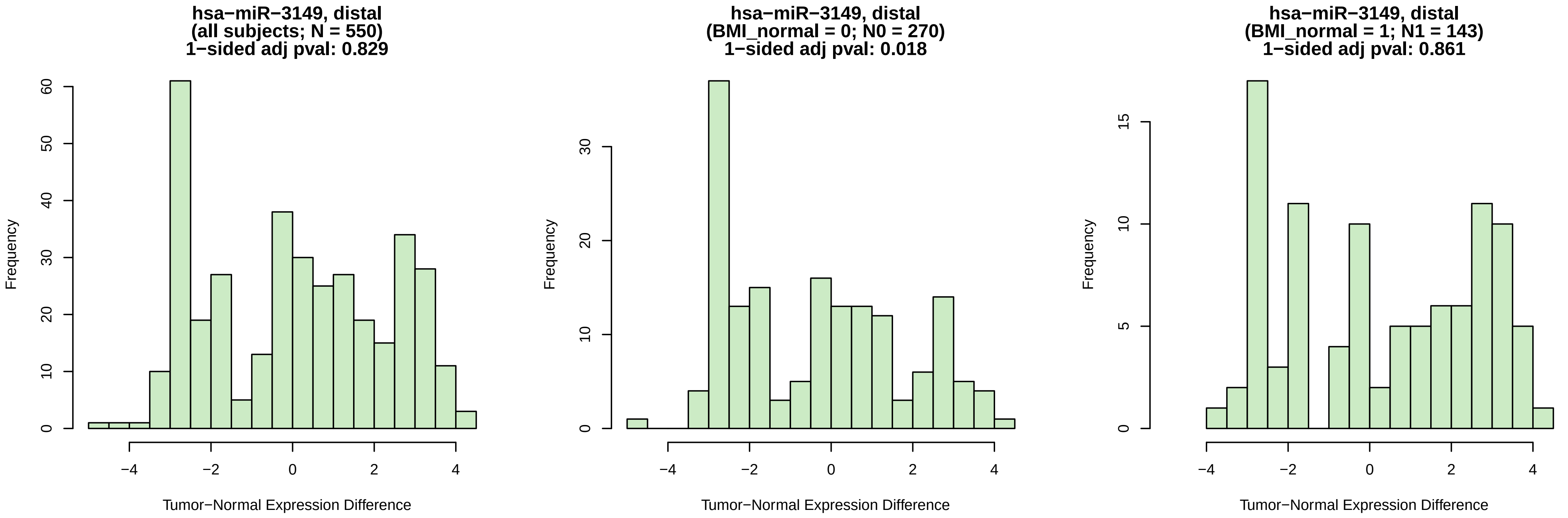

hsa−miR−3149, distal
(all subjects; N = 550)
1−sided adj pval: 0.829
Frequency
0
10
20
30
40
50
60
−4
−2
0
2
4
Tumor−Normal Expression Difference
hsa−miR−3149, distal
(BMI_normal = 0; N0 = 270)
1−sided adj pval: 0.018
Frequency
0
10
20
30
−4
−2
0
2
4
Tumor−Normal Expression Difference
hsa−miR−3149, distal
(BMI_normal = 1; N1 = 143)
1−sided adj pval: 0.861
Frequency
0
5
10
15
−4
−2
0
2
4
Tumor−Normal Expression Difference

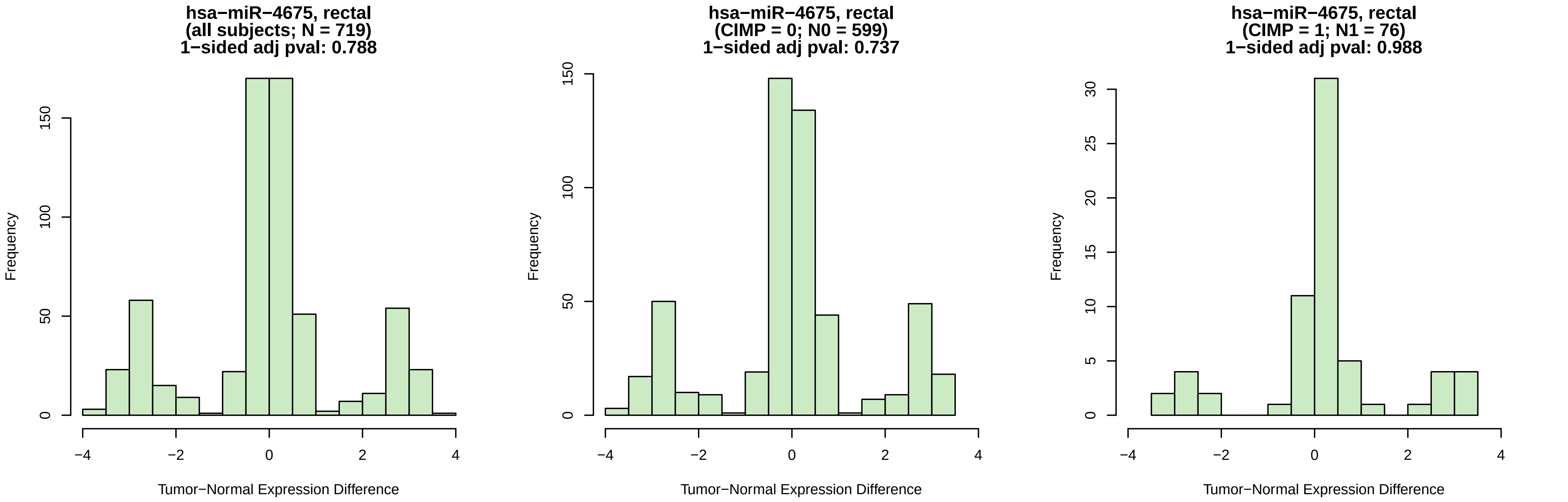

hsa−miR−4675, rectal
(all subjects; N = 719)
1−sided adj pval: 0.788
Frequency
Tumor−Normal Expression Difference
hsa−miR−4675, rectal
(CIMP = 0; N0 = 599)
1−sided adj pval: 0.737
Frequency
Tumor−Normal Expression Difference
hsa−miR−4675, rectal
(CIMP = 1; N1 = 76)
1−sided adj pval: 0.988
Frequency
Tumor−Normal Expression Difference

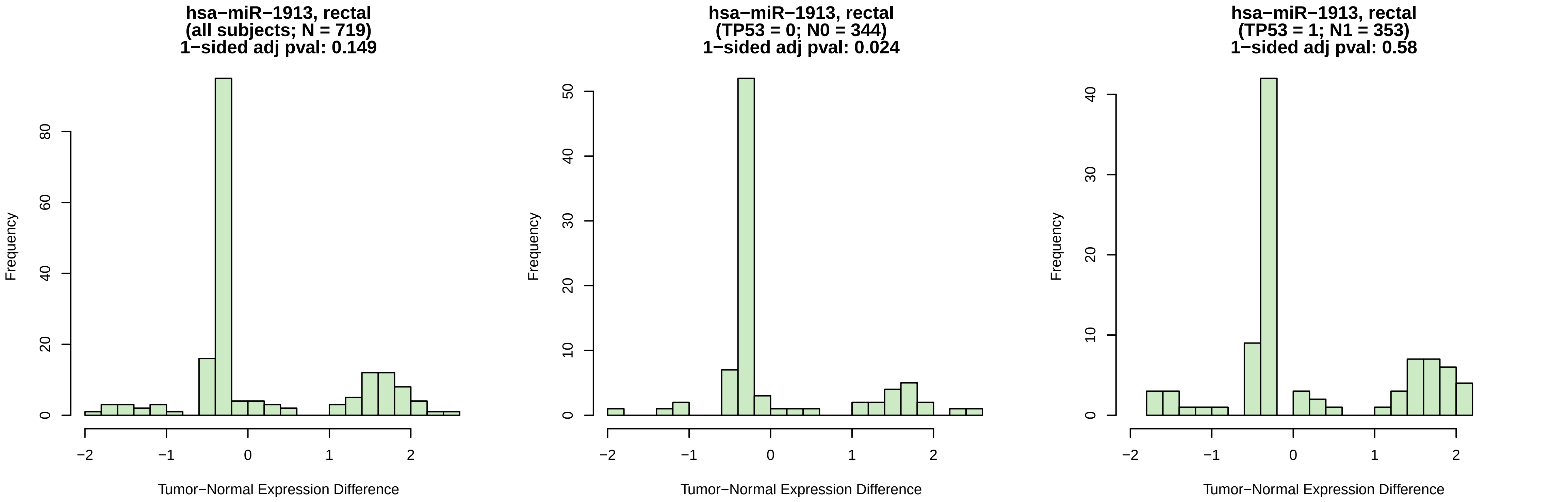

hsa−miR−1913, rectal
(all subjects; N = 719)
1−sided adj pval: 0.149
Frequency
Tumor−Normal Expression Difference
hsa−miR−1913, rectal
(TP53 = 0; N0 = 344)
1−sided adj pval: 0.024
Frequency
Tumor−Normal Expression Difference
hsa−miR−1913, rectal
(TP53 = 1; N1 = 353)
1−sided adj pval: 0.58
Frequency
Tumor−Normal Expression Difference

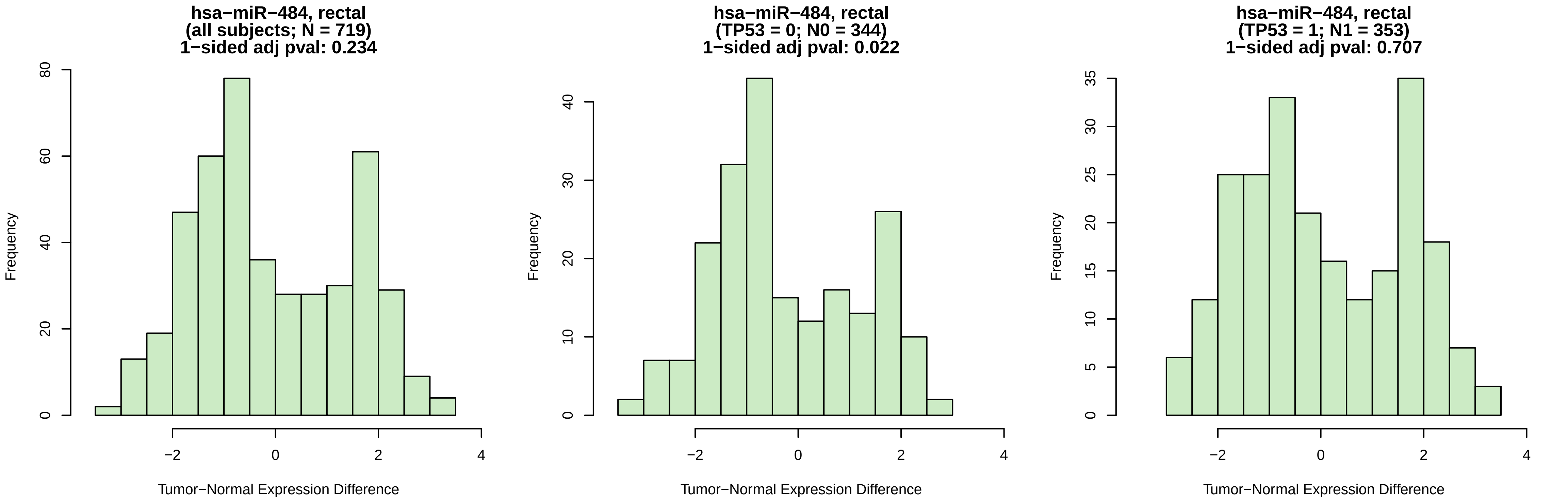

hsa−miR−484, rectal
(all subjects; N = 719)
1−sided adj pval: 0.234
Frequency
0
20
40
60
80
−2
0
2
4
Tumor−Normal Expression Difference
hsa−miR−484, rectal
(TP53 = 0; N0 = 344)
1−sided adj pval: 0.022
Frequency
0
10
20
30
40
−2
0
2
4
Tumor−Normal Expression Difference
hsa−miR−484, rectal
(TP53 = 1; N1 = 353)
1−sided adj pval: 0.707
Frequency
0
5
10
15
20
25
30
35
−2
0
2
4
Tumor−Normal Expression Difference

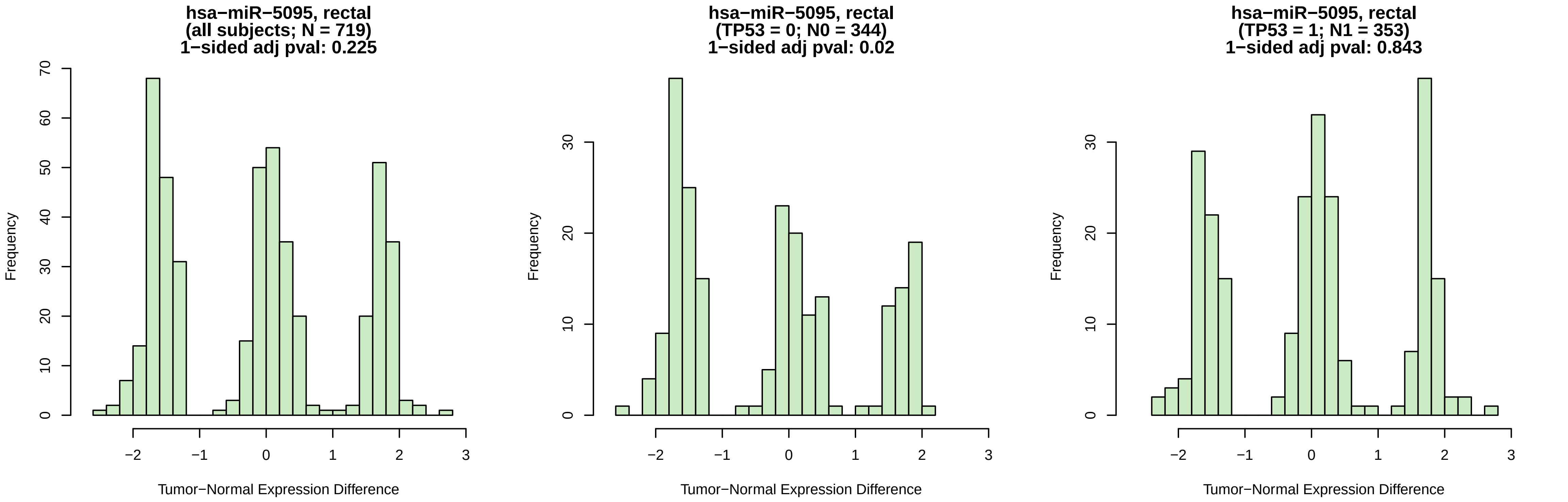

hsa−miR−5095, rectal
(all subjects; N = 719)
1−sided adj pval: 0.225
Frequency
0
10
20
30
40
50
60
70
−2
−1
0
1
2
3
Tumor−Normal Expression Difference
hsa−miR−5095, rectal
(TP53 = 0; N0 = 344)
1−sided adj pval: 0.02
Frequency
0
10
20
30
−2
−1
0
1
2
3
Tumor−Normal Expression Difference
hsa−miR−5095, rectal
(TP53 = 1; N1 = 353)
1−sided adj pval: 0.843
Frequency
0
10
20
30
−2
−1
0
1
2
3
Tumor−Normal Expression Difference

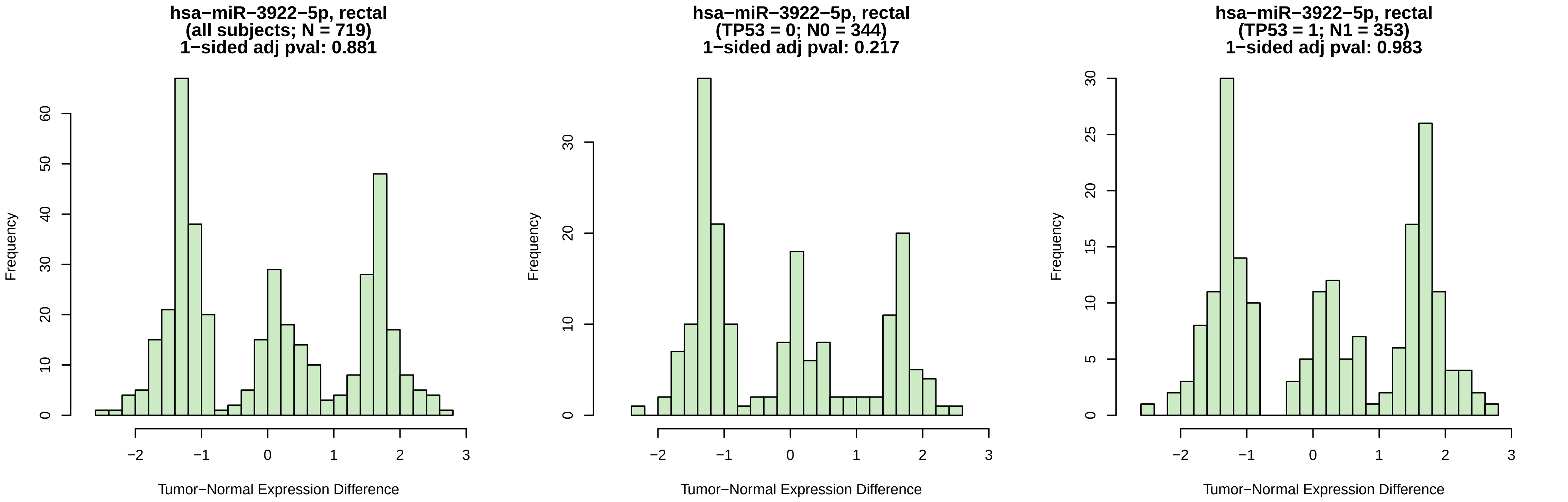

hsa−miR−3922−5p, rectal
(all subjects; N = 719)
1−sided adj pval: 0.881
Frequency
Tumor−Normal Expression Difference
hsa−miR−3922−5p, rectal
(TP53 = 0; N0 = 344)
1−sided adj pval: 0.217
Frequency
Tumor−Normal Expression Difference
hsa−miR−3922−5p, rectal
(TP53 = 1; N1 = 353)
1−sided adj pval: 0.983
Frequency
Tumor−Normal Expression Difference

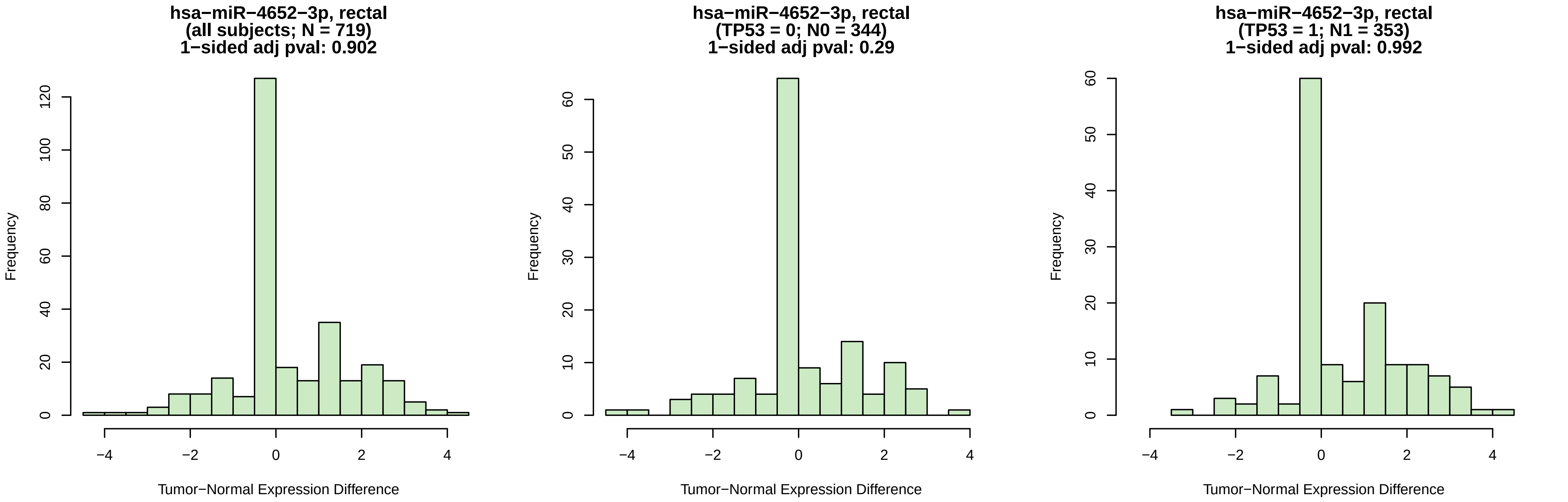

hsa−miR−4652−3p, rectal
(all subjects; N = 719)
1−sided adj pval: 0.902
Frequency
Tumor−Normal Expression Difference
hsa−miR−4652−3p, rectal
(TP53 = 0; N0 = 344)
1−sided adj pval: 0.29
Frequency
Tumor−Normal Expression Difference
hsa−miR−4652−3p, rectal
(TP53 = 1; N1 = 353)
1−sided adj pval: 0.992
Frequency
Tumor−Normal Expression Difference

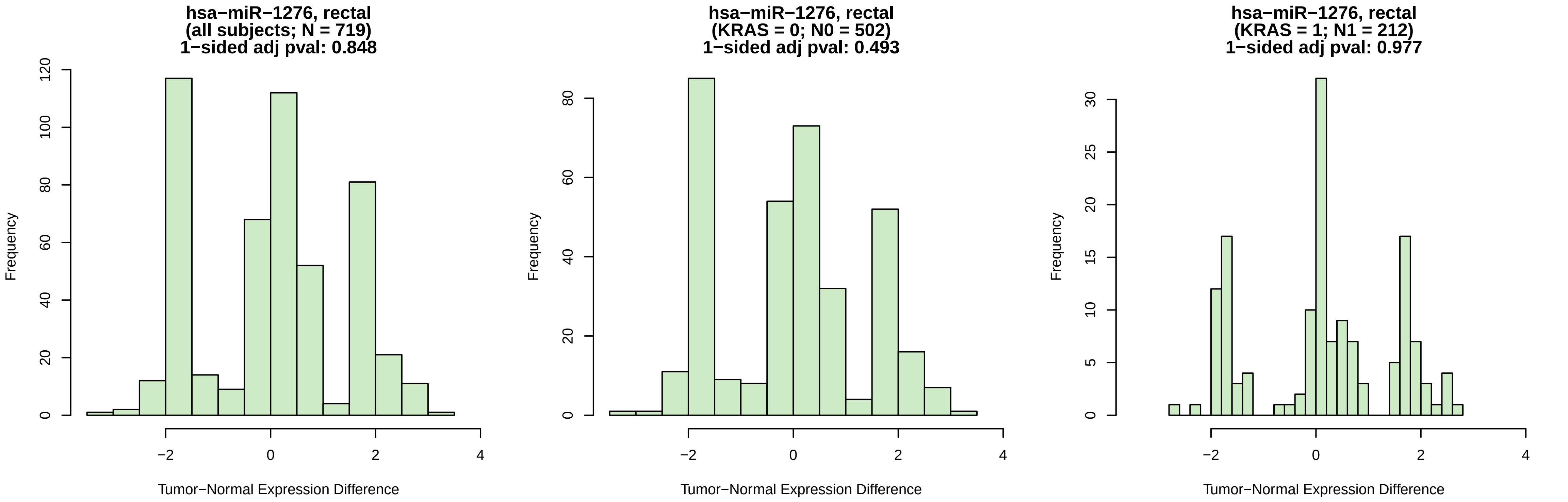
hsa−miR−1276, rectal
(all subjects; N = 719)
1−sided adj pval: 0.848
Frequency
Tumor−Normal Expression Difference
hsa−miR−1276, rectal
(KRAS = 0; N0 = 502)
1−sided adj pval: 0.493
Frequency
Tumor−Normal Expression Difference
hsa−miR−1276, rectal
(KRAS = 1; N1 = 212)
1−sided adj pval: 0.977
Frequency
Tumor−Normal Expression Difference

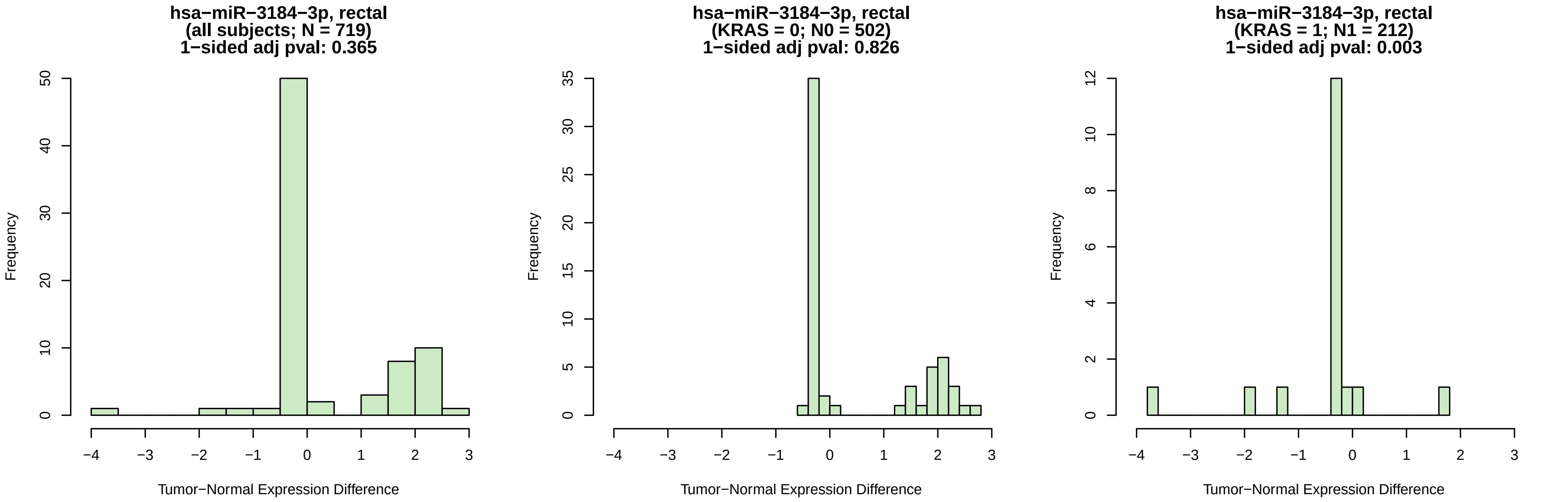
hsa−miR−3184−3p, rectal
(all subjects; N = 719)
1−sided adj pval: 0.365
Frequency
Tumor−Normal Expression Difference
hsa−miR−3184−3p, rectal
(KRAS = 0; N0 = 502)
1−sided adj pval: 0.826
Frequency
Tumor−Normal Expression Difference
hsa−miR−3184−3p, rectal
(KRAS = 1; N1 = 212)
1−sided adj pval: 0.003
Frequency
Tumor−Normal Expression Difference

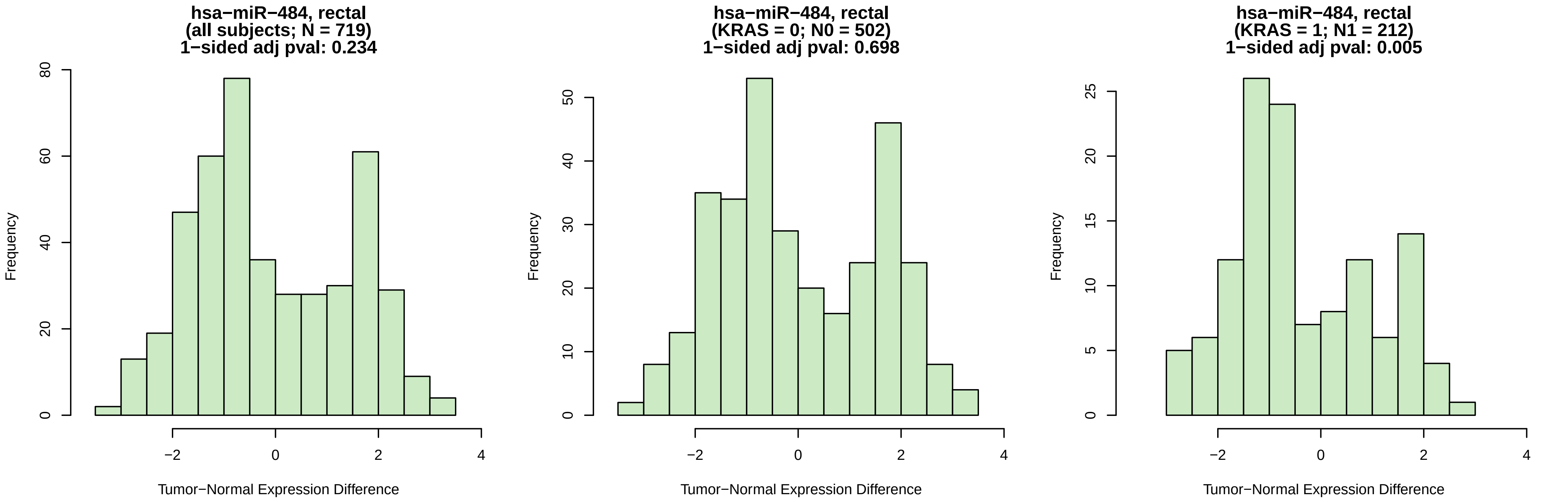
hsa−miR−484, rectal
(all subjects; N = 719)
1−sided adj pval: 0.234
Frequency
Tumor−Normal Expression Difference
hsa−miR−484, rectal
(KRAS = 0; N0 = 502)
1−sided adj pval: 0.698
Frequency
Tumor−Normal Expression Difference
hsa−miR−484, rectal
(KRAS = 1; N1 = 212)
1−sided adj pval: 0.005
Frequency
Tumor−Normal Expression Difference

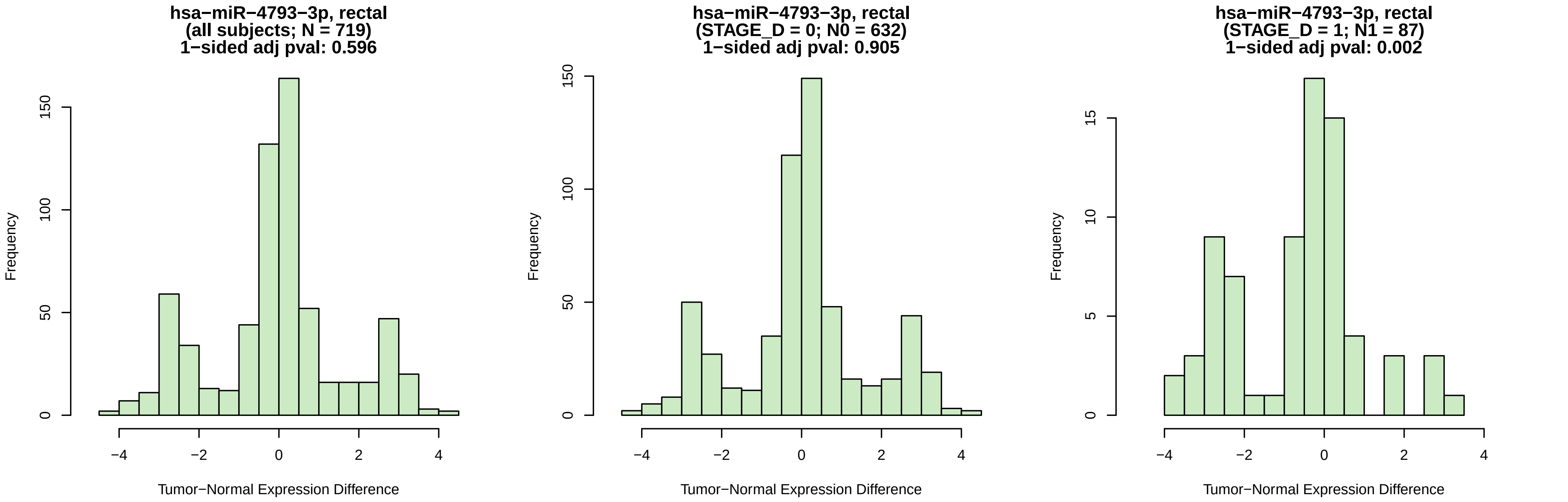
hsa−miR−4793−3p, rectal
(all subjects; N = 719)
1−sided adj pval: 0.596
Frequency
Tumor−Normal Expression Difference
hsa−miR−4793−3p, rectal
(STAGE_D = 0; N0 = 632)
1−sided adj pval: 0.905
Frequency
Tumor−Normal Expression Difference
hsa−miR−4793−3p, rectal
(STAGE_D = 1; N1 = 87)
1−sided adj pval: 0.002
Frequency
Tumor−Normal Expression Difference

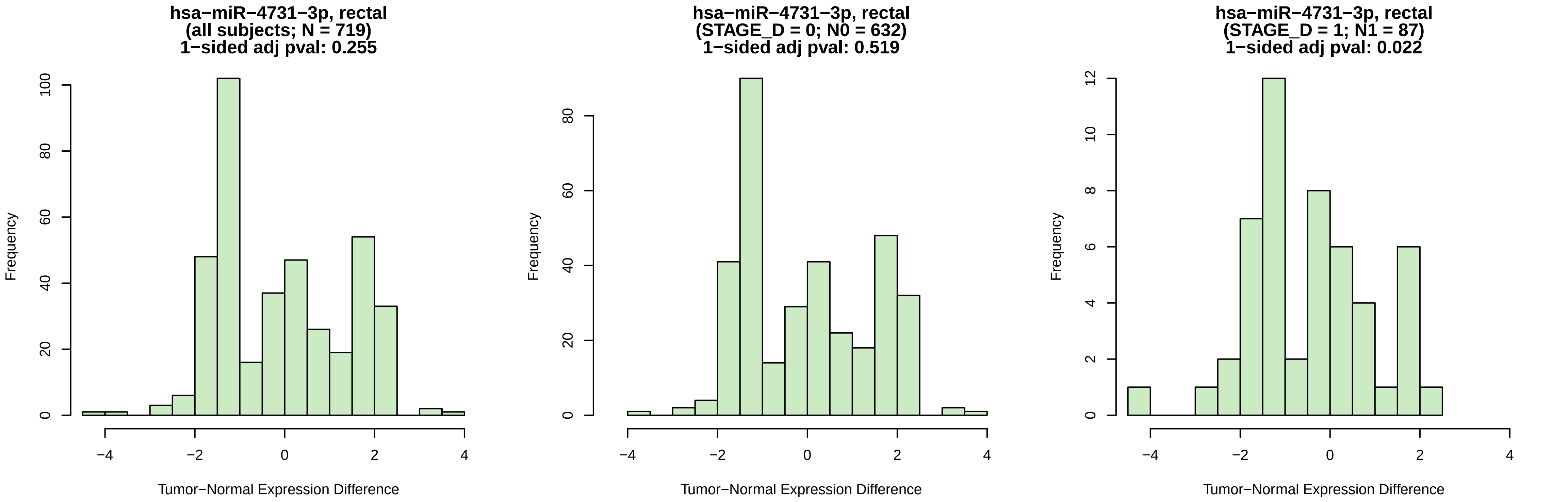
hsa−miR−4731−3p, rectal
(all subjects; N = 719)
1−sided adj pval: 0.255
Frequency
Tumor−Normal Expression Difference
hsa−miR−4731−3p, rectal
(STAGE_D = 0; N0 = 632)
1−sided adj pval: 0.519
Frequency
Tumor−Normal Expression Difference
hsa−miR−4731−3p, rectal
(STAGE_D = 1; N1 = 87)
1−sided adj pval: 0.022
Frequency
Tumor−Normal Expression Difference

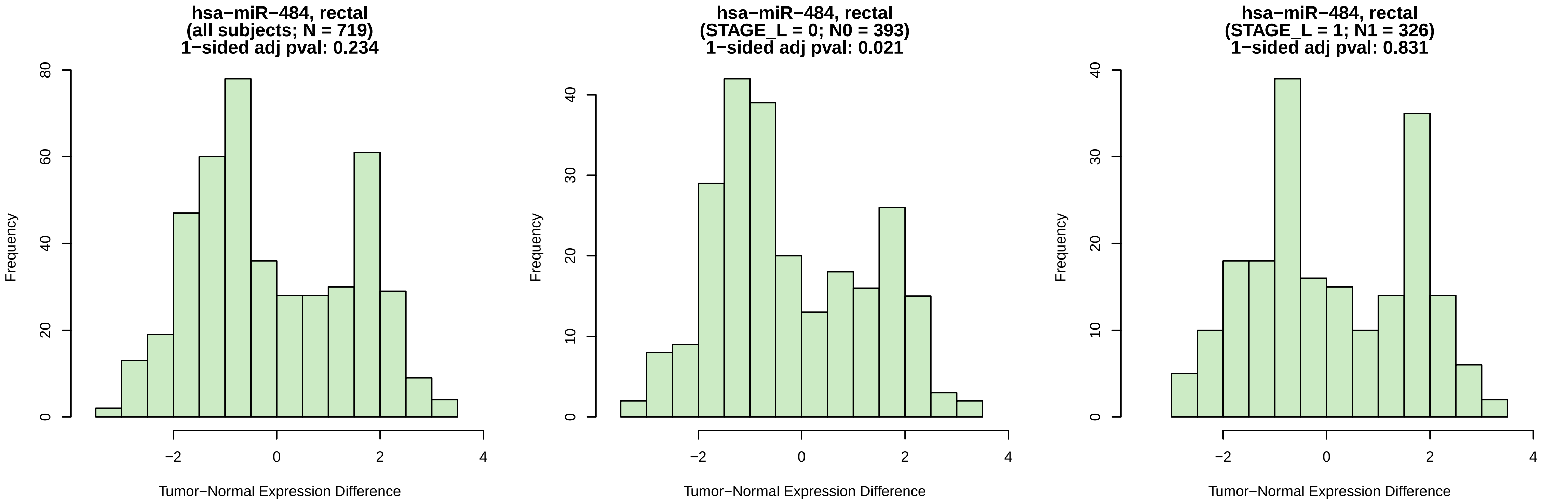
hsa−miR−484, rectal
(all subjects; N = 719)
1−sided adj pval: 0.234
Frequency
Tumor−Normal Expression Difference
hsa−miR−484, rectal
(STAGE_L = 0; N0 = 393)
1−sided adj pval: 0.021
Frequency
Tumor−Normal Expression Difference
hsa−miR−484, rectal
(STAGE_L = 1; N1 = 326)
1−sided adj pval: 0.831
Frequency
Tumor−Normal Expression Difference

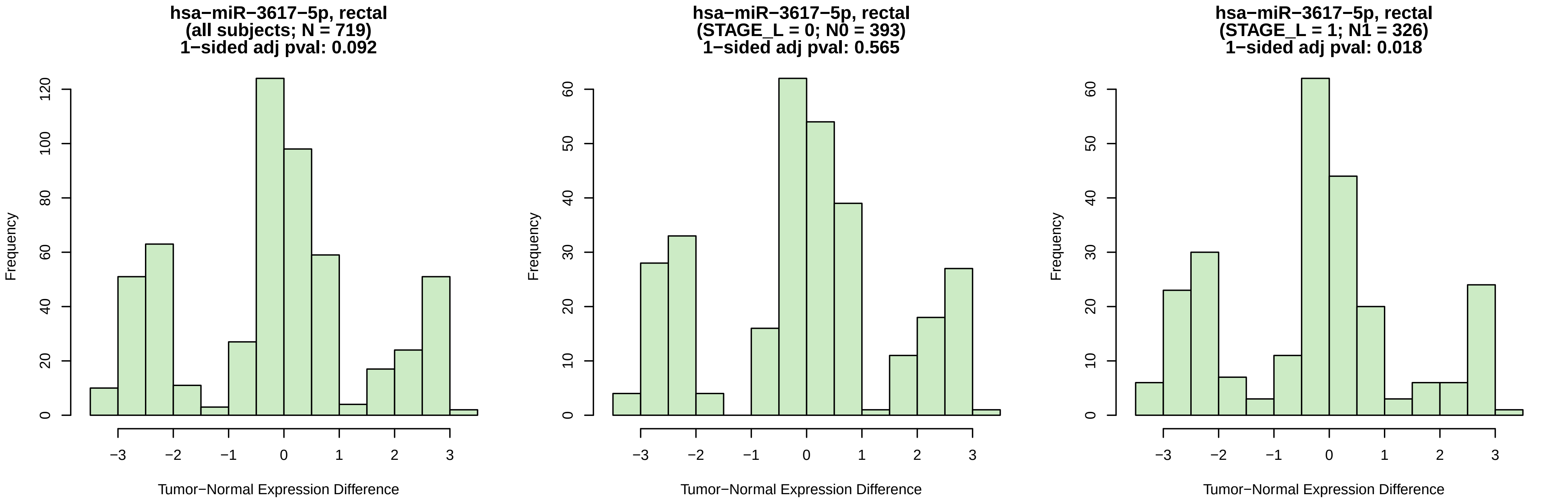

hsa−miR−3617−5p, rectal
(all subjects; N = 719)
1−sided adj pval: 0.092
hsa−miR−3617−5p, rectal
(STAGE_L = 0; N0 = 393)
1−sided adj pval: 0.565
hsa−miR−3617−5p, rectal
(STAGE_L = 1; N1 = 326)
1−sided adj pval: 0.018
Frequency
Tumor−Normal Expression Difference

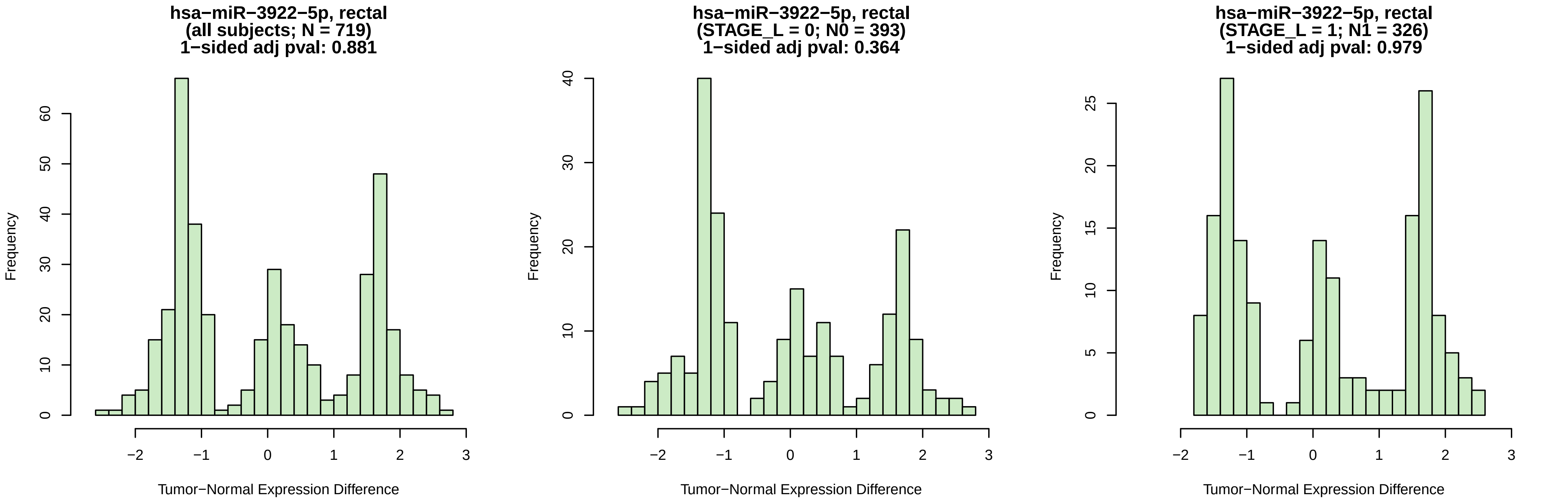
hsa−miR−3922−5p, rectal
(all subjects; N = 719)
1−sided adj pval: 0.881
Frequency
0
10
20
30
40
50
60
−2
−1
0
1
2
3
Tumor−Normal Expression Difference
hsa−miR−3922−5p, rectal
(STAGE_L = 0; N0 = 393)
1−sided adj pval: 0.364
Frequency
0
10
20
30
40
−2
−1
0
1
2
3
Tumor−Normal Expression Difference
hsa−miR−3922−5p, rectal
(STAGE_L = 1; N1 = 326)
1−sided adj pval: 0.979
Frequency
0
5
10
15
20
25
−2
−1
0
1
2
3
Tumor−Normal Expression Difference

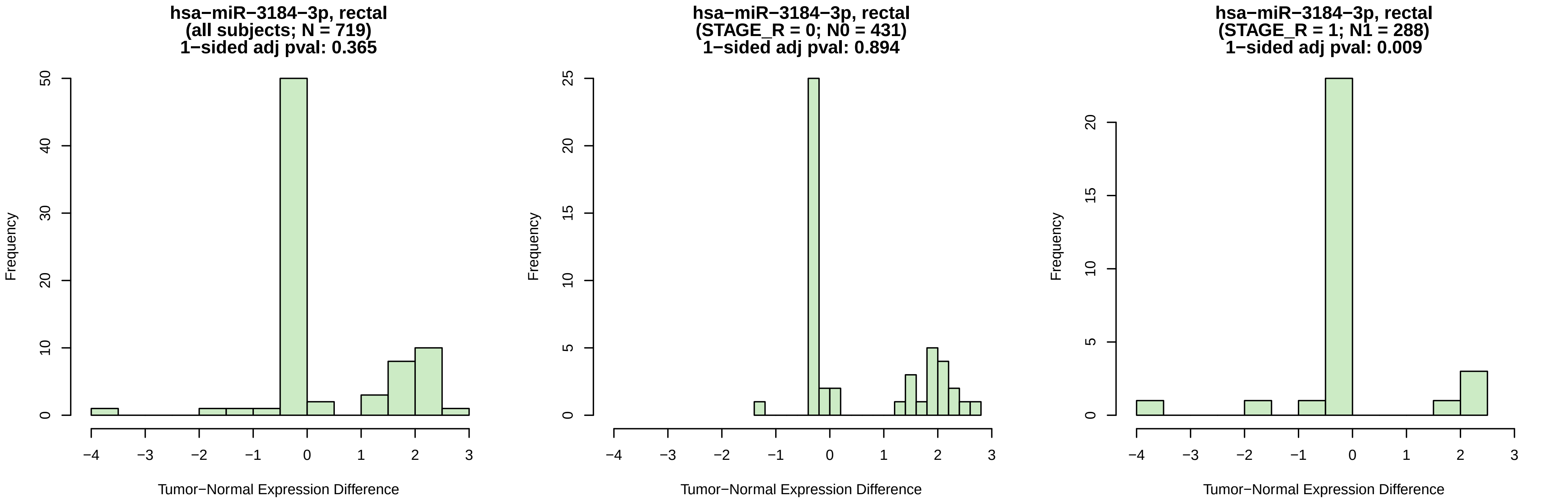
hsa−miR−3184−3p, rectal
(all subjects; N = 719)
1−sided adj pval: 0.365
Frequency
Tumor−Normal Expression Difference
hsa−miR−3184−3p, rectal
(STAGE_R = 0; N0 = 431)
1−sided adj pval: 0.894
Frequency
Tumor−Normal Expression Difference
hsa−miR−3184−3p, rectal
(STAGE_R = 1; N1 = 288)
1−sided adj pval: 0.009
Frequency
Tumor−Normal Expression Difference

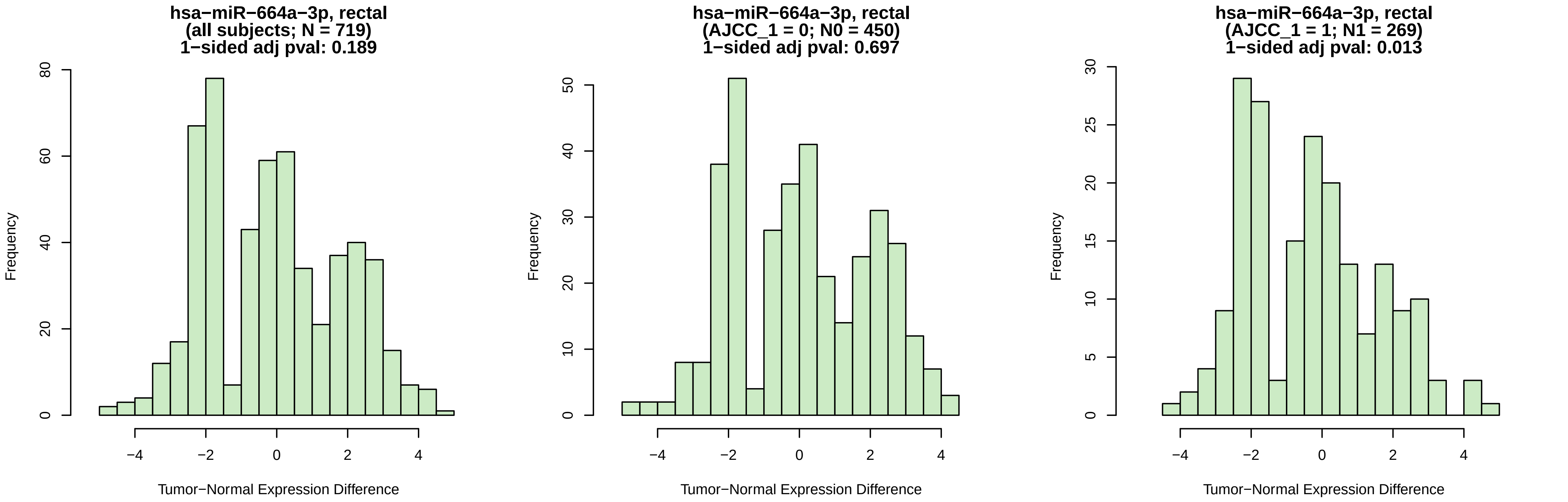
hsa−miR−664a−3p, rectal
(all subjects; N = 719)
1−sided adj pval: 0.189
Frequency
Tumor−Normal Expression Difference
hsa−miR−664a−3p, rectal
(AJCC_1 = 0; N0 = 450)
1−sided adj pval: 0.697
Frequency
Tumor−Normal Expression Difference
hsa−miR−664a−3p, rectal
(AJCC_1 = 1; N1 = 269)
1−sided adj pval: 0.013
Frequency
Tumor−Normal Expression Difference

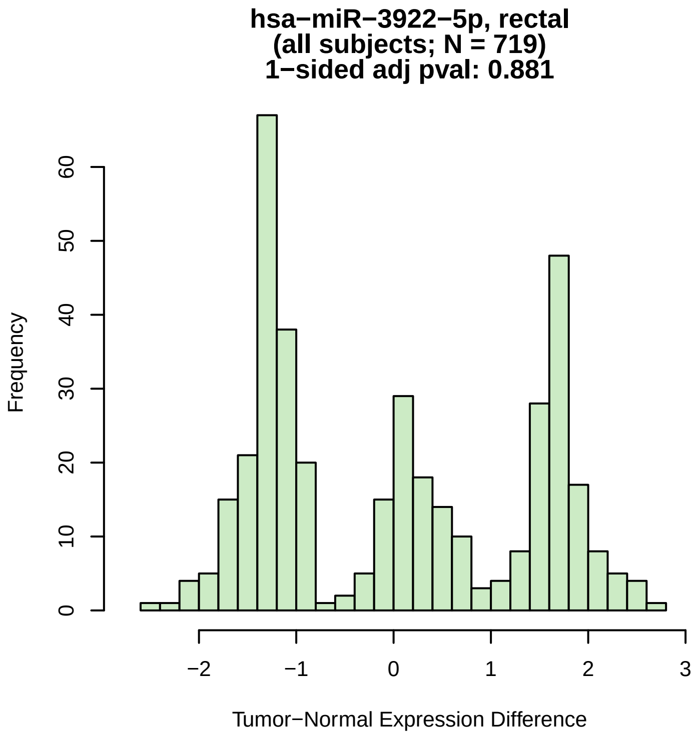
hsa−miR−3922−5p, rectal
(all subjects; N = 719)
1−sided adj pval: 0.881
Frequency
Tumor−Normal Expression Difference

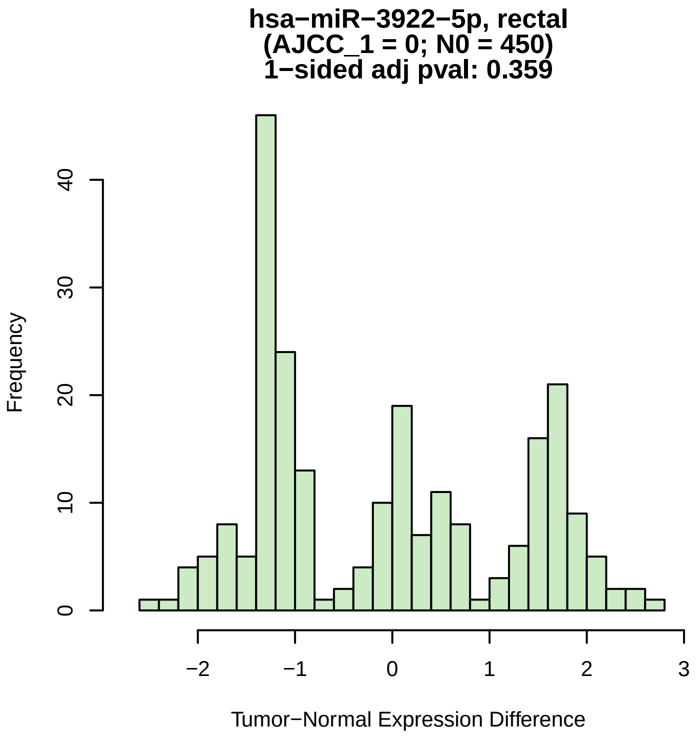
hsa−miR−3922−5p, rectal
(AJCC_1 = 0; N0 = 450)
1−sided adj pval: 0.359
Frequency
Tumor−Normal Expression Difference

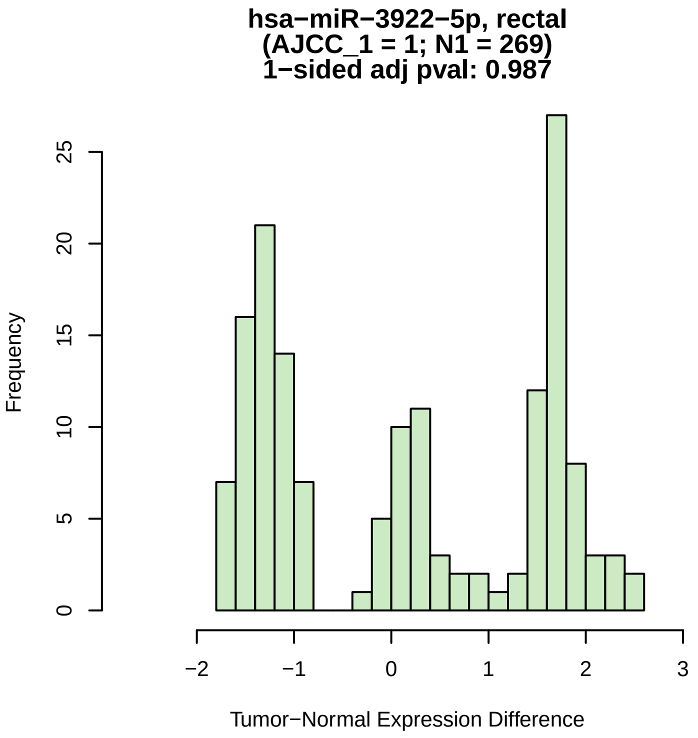
hsa−miR−3922−5p, rectal
(AJCC_1 = 1; N1 = 269)
1−sided adj pval: 0.987
Frequency
Tumor−Normal Expression Difference

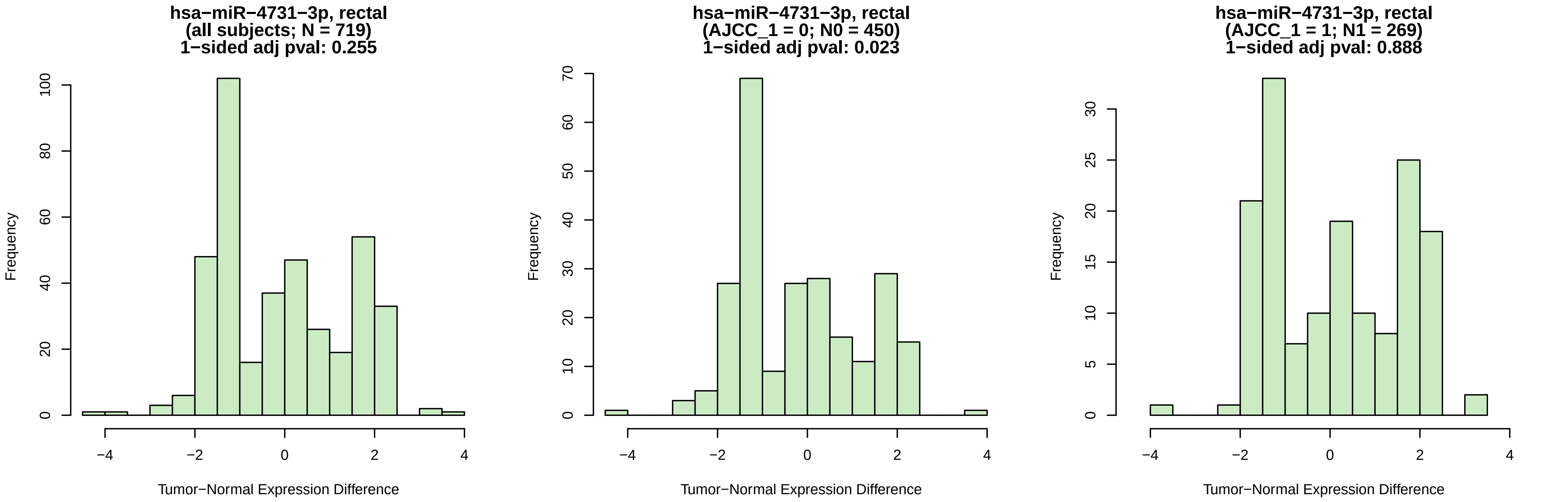

hsa−miR−4731−3p, rectal
(all subjects; N = 719)
1−sided adj pval: 0.255
Frequency
0
20
40
60
80
100
−4
−2
0
2
4
Tumor−Normal Expression Difference
hsa−miR−4731−3p, rectal
(AJCC_1 = 0; N0 = 450)
1−sided adj pval: 0.023
Frequency
0
10
20
30
40
50
60
70
−4
−2
0
2
4
Tumor−Normal Expression Difference
hsa−miR−4731−3p, rectal
(AJCC_1 = 1; N1 = 269)
1−sided adj pval: 0.888
Frequency
0
5
10
15
20
25
30
−4
−2
0
2
4
Tumor−Normal Expression Difference

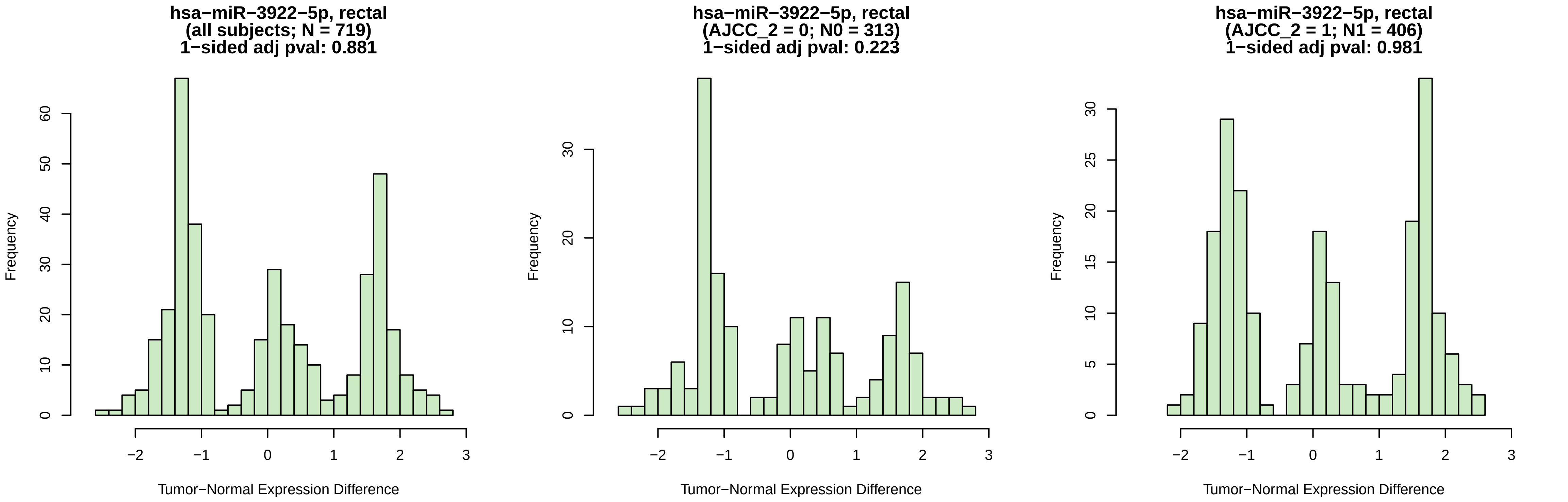

hsa−miR−3922−5p, rectal
(all subjects; N = 719)
1−sided adj pval: 0.881
Frequency
Tumor−Normal Expression Difference
hsa−miR−3922−5p, rectal
(AJCC_2 = 0; N0 = 313)
1−sided adj pval: 0.223
Frequency
Tumor−Normal Expression Difference
hsa−miR−3922−5p, rectal
(AJCC_2 = 1; N1 = 406)
1−sided adj pval: 0.981
Frequency
Tumor−Normal Expression Difference

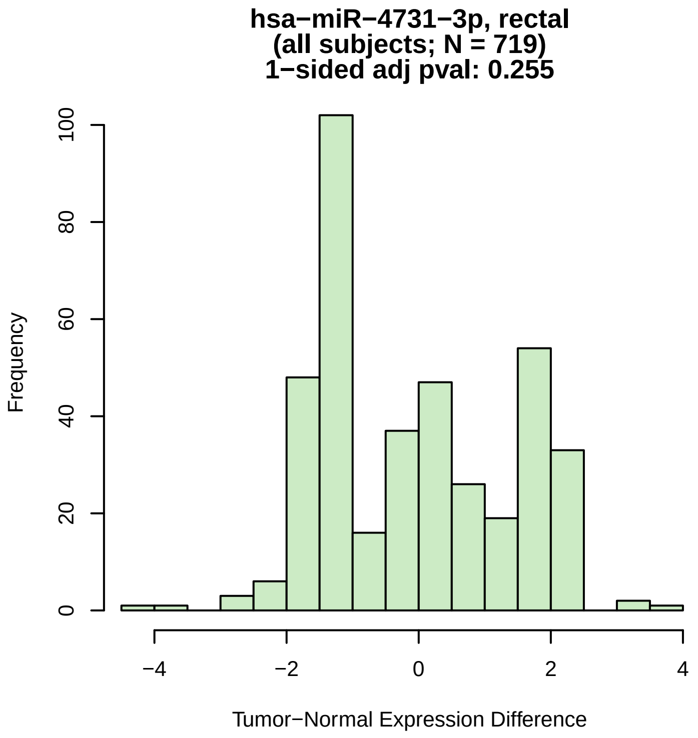
hsa−miR−4731−3p, rectal
(all subjects; N = 719)
1−sided adj pval: 0.255
Frequency
Tumor−Normal Expression Difference

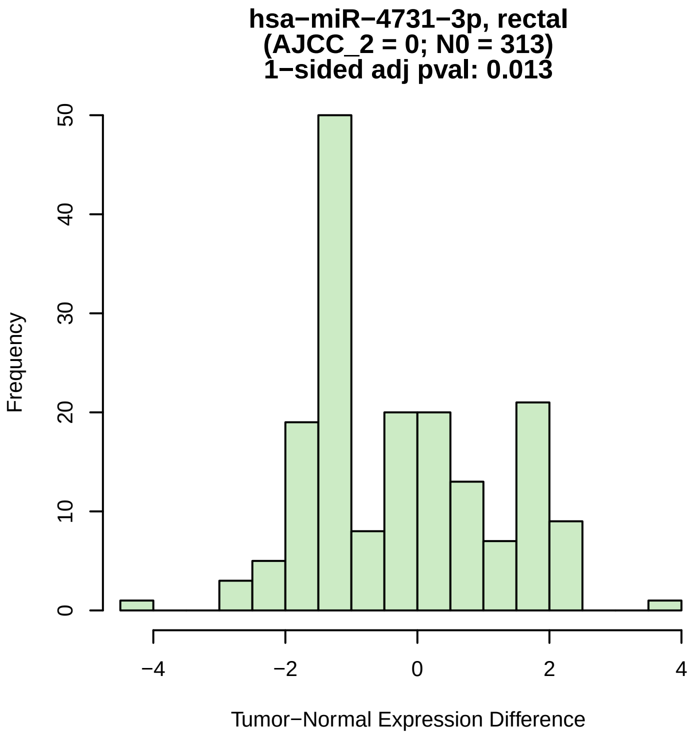
hsa−miR−4731−3p, rectal
(AJCC_2 = 0; N0 = 313)
1−sided adj pval: 0.013
Frequency
Tumor−Normal Expression Difference

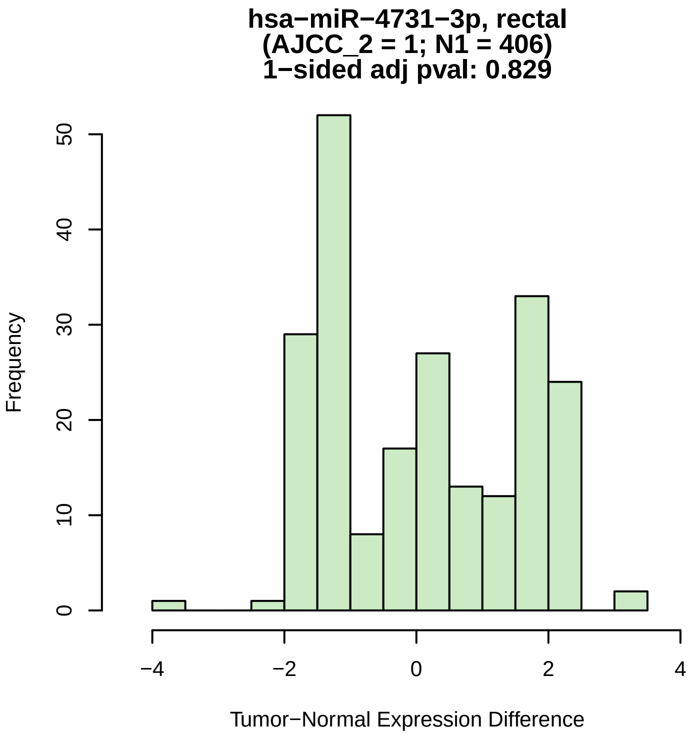
hsa−miR−4731−3p, rectal
(AJCC_2 = 1; N1 = 406)
1−sided adj pval: 0.829
Frequency
Tumor−Normal Expression Difference

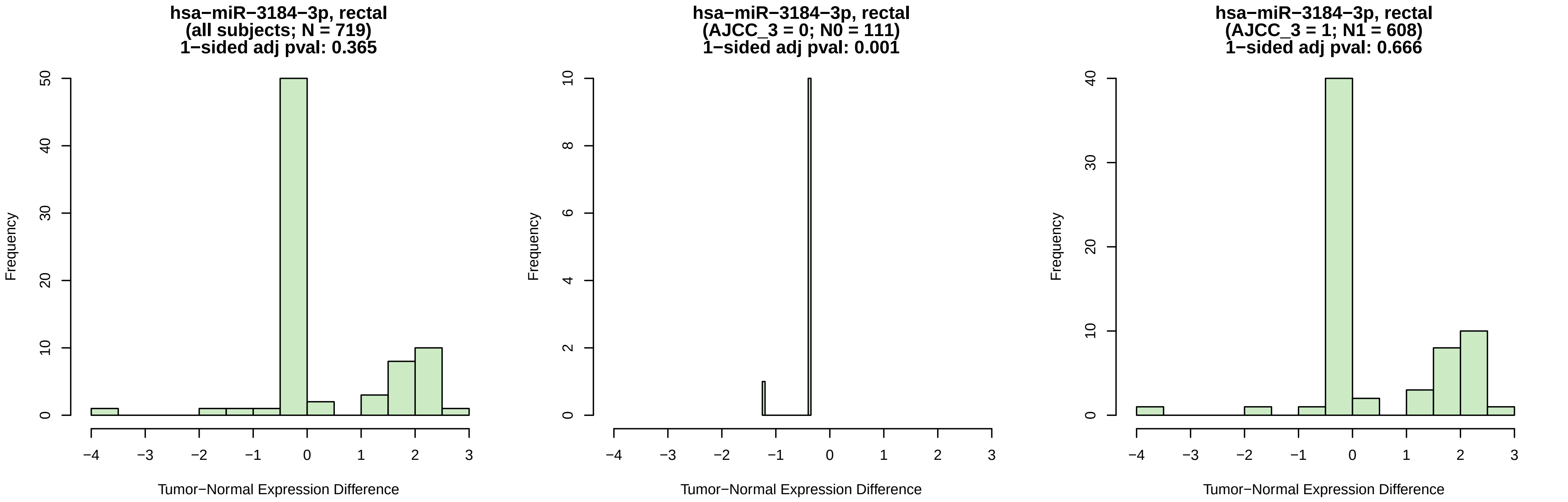

hsa−miR−3184−3p, rectal
(all subjects; N = 719)
1−sided adj pval: 0.365
Frequency
Tumor−Normal Expression Difference
hsa−miR−3184−3p, rectal
(AJCC_3 = 0; N0 = 111)
1−sided adj pval: 0.001
Frequency
Tumor−Normal Expression Difference
hsa−miR−3184−3p, rectal
(AJCC_3 = 1; N1 = 608)
1−sided adj pval: 0.666
Frequency
Tumor−Normal Expression Difference

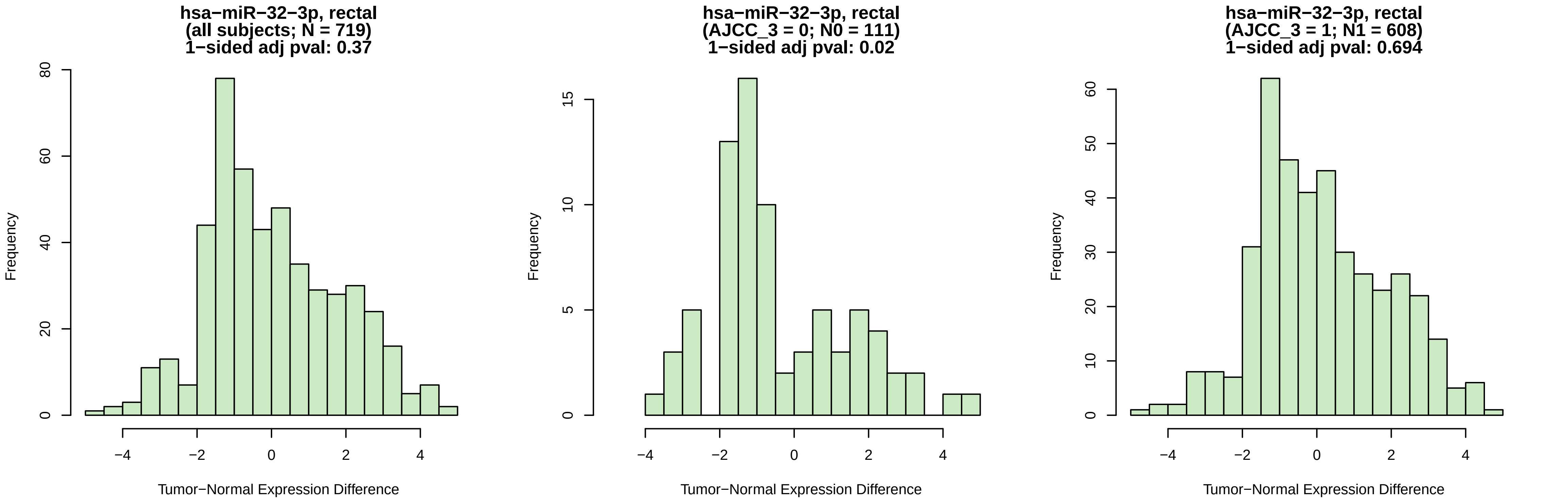
hsa−miR−32−3p, rectal
(all subjects; N = 719)
1−sided adj pval: 0.37
Frequency
Tumor−Normal Expression Difference
hsa−miR−32−3p, rectal
(AJCC_3 = 0; N0 = 111)
1−sided adj pval: 0.02
Frequency
Tumor−Normal Expression Difference
hsa−miR−32−3p, rectal
(AJCC_3 = 1; N1 = 608)
1−sided adj pval: 0.694
Frequency
Tumor−Normal Expression Difference

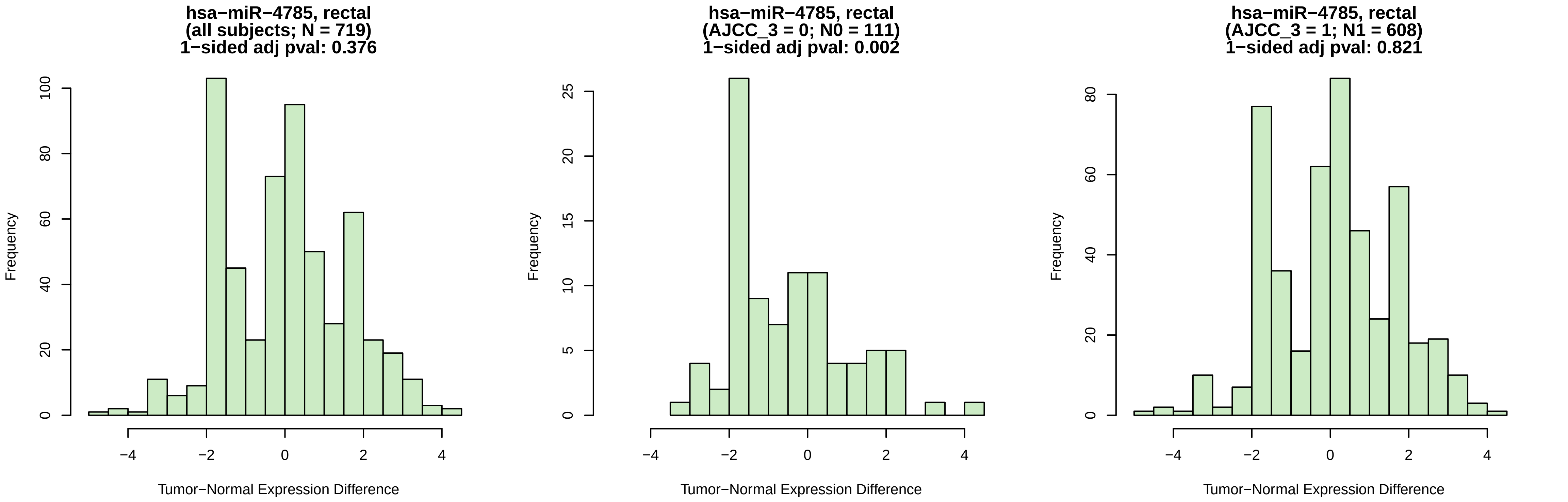
hsa−miR−4785, rectal
(all subjects; N = 719)
1−sided adj pval: 0.376
Frequency
Tumor−Normal Expression Difference
hsa−miR−4785, rectal
(AJCC_3 = 0; N0 = 111)
1−sided adj pval: 0.002
Frequency
Tumor−Normal Expression Difference
hsa−miR−4785, rectal
(AJCC_3 = 1; N1 = 608)
1−sided adj pval: 0.821
Frequency
Tumor−Normal Expression Difference

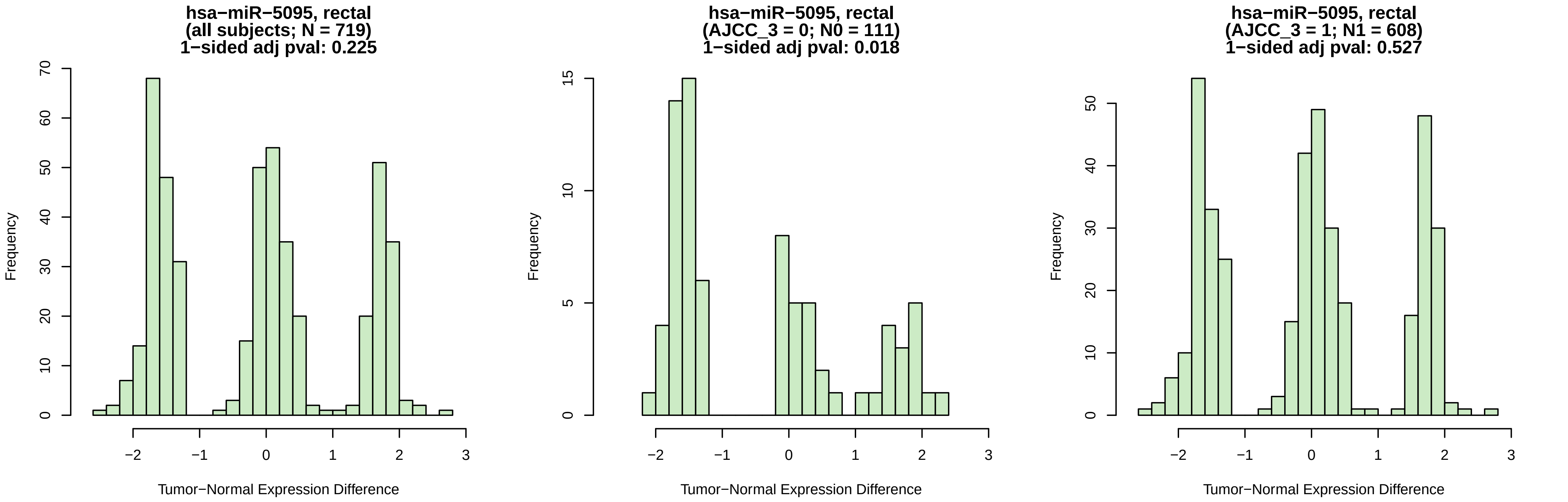

hsa−miR−5095, rectal
(all subjects; N = 719)
1−sided adj pval: 0.225
Frequency
Tumor−Normal Expression Difference
hsa−miR−5095, rectal
(AJCC_3 = 0; N0 = 111)
1−sided adj pval: 0.018
Frequency
Tumor−Normal Expression Difference
hsa−miR−5095, rectal
(AJCC_3 = 1; N1 = 608)
1−sided adj pval: 0.527
Frequency
Tumor−Normal Expression Difference

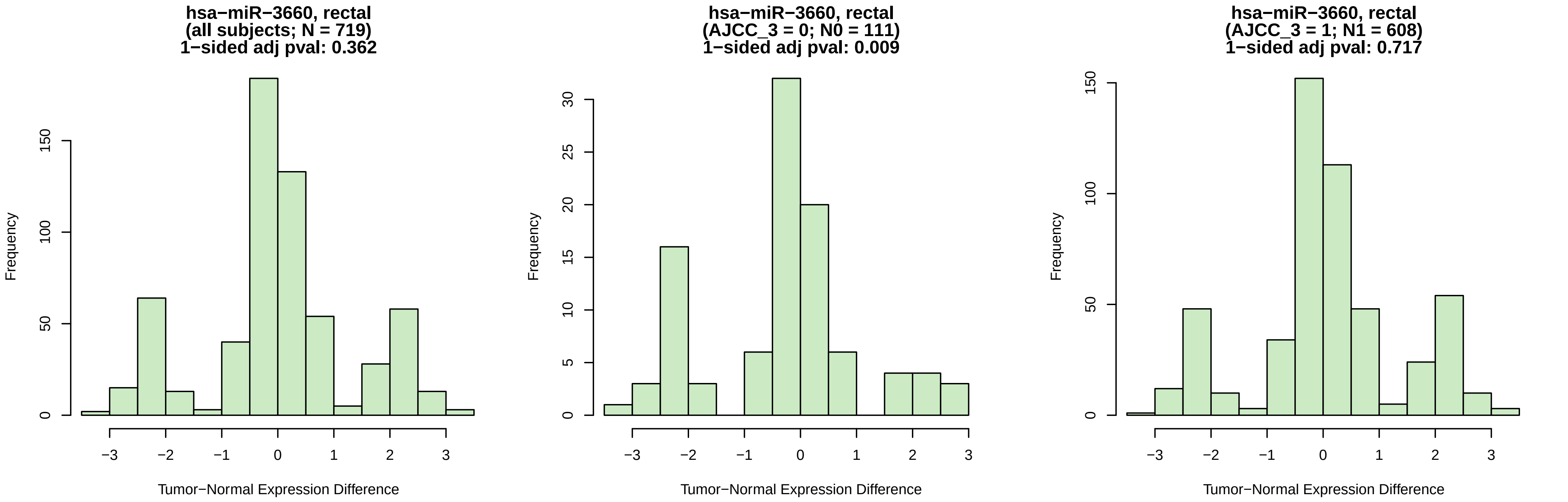

hsa−miR−3660, rectal
(all subjects; N = 719)
1−sided adj pval: 0.362
Frequency
0
50
100
150
−3
−2
−1
0
1
2
3
Tumor−Normal Expression Difference
hsa−miR−3660, rectal
(AJCC_3 = 0; N0 = 111)
1−sided adj pval: 0.009
Frequency
0
5
10
15
20
25
30
−3
−2
−1
0
1
2
3
Tumor−Normal Expression Difference
hsa−miR−3660, rectal
(AJCC_3 = 1; N1 = 608)
1−sided adj pval: 0.717
Frequency
0
50
100
150
−3
−2
−1
0
1
2
3
Tumor−Normal Expression Difference

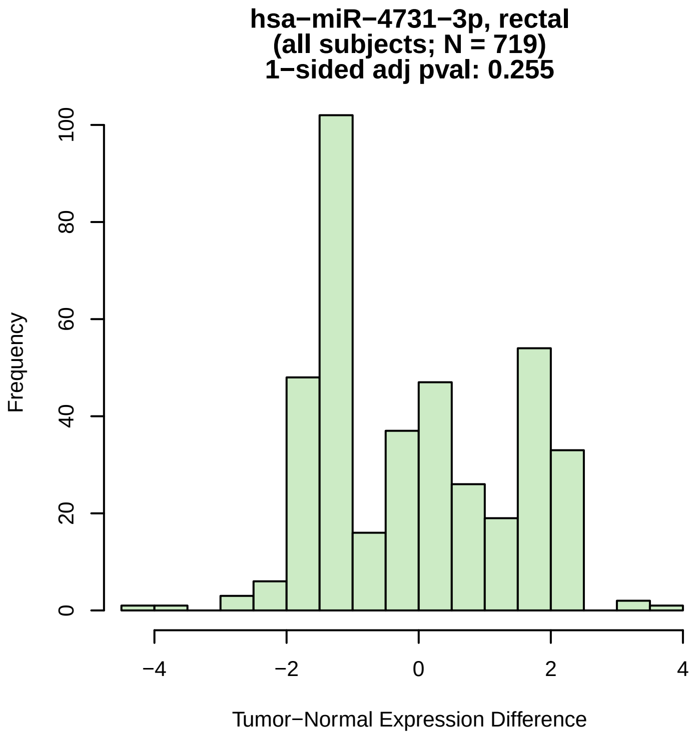

hsa−miR−4731−3p, rectal
(all subjects; N = 719)
1−sided adj pval: 0.255
Frequency
0
20
40
60
80
100
−4
−2
0
2
4
Tumor−Normal Expression Difference

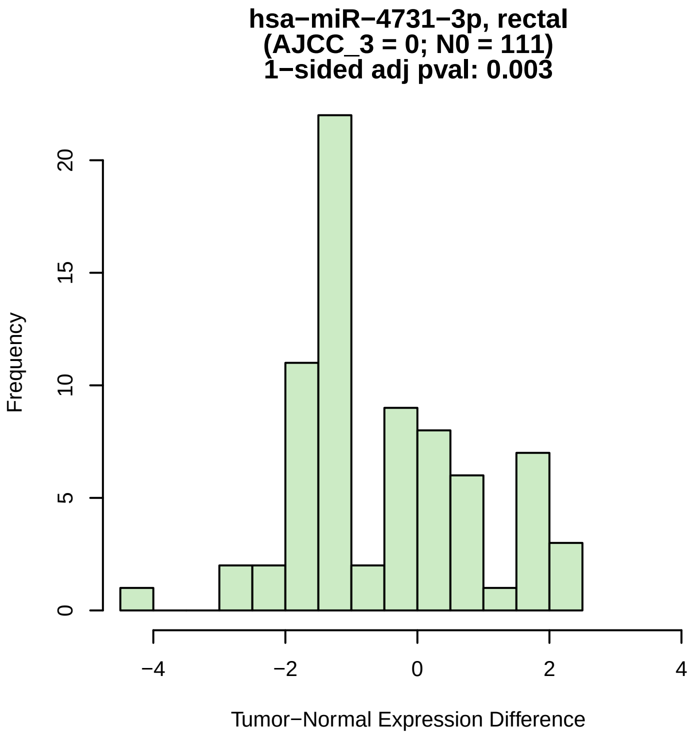

hsa−miR−4731−3p, rectal
(AJCC_3 = 0; N0 = 111)
1−sided adj pval: 0.003
Frequency
0
5
10
15
20
−4
−2
0
2
4
Tumor−Normal Expression Difference

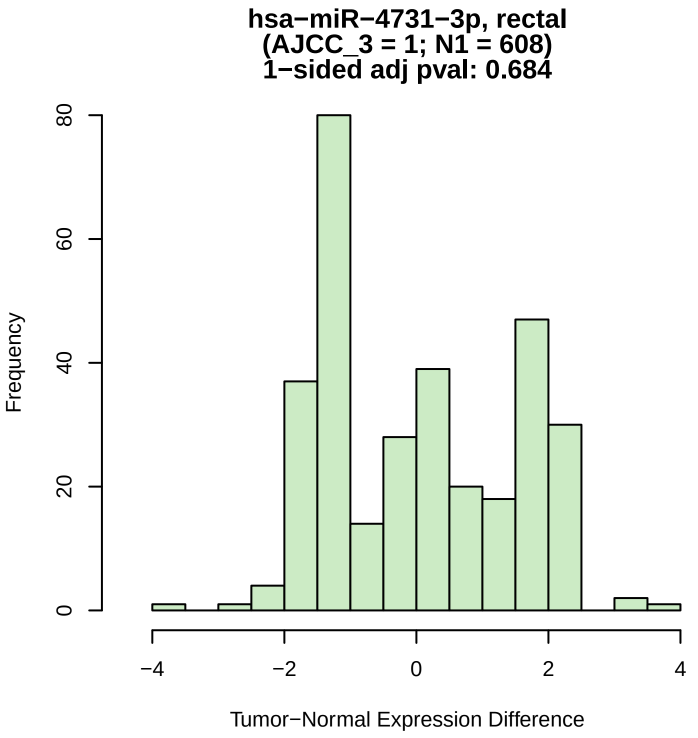

hsa−miR−4731−3p, rectal
(AJCC_3 = 1; N1 = 608)
1−sided adj pval: 0.684
Frequency
0
20
40
60
80
−4
−2
0
2
4
Tumor−Normal Expression Difference

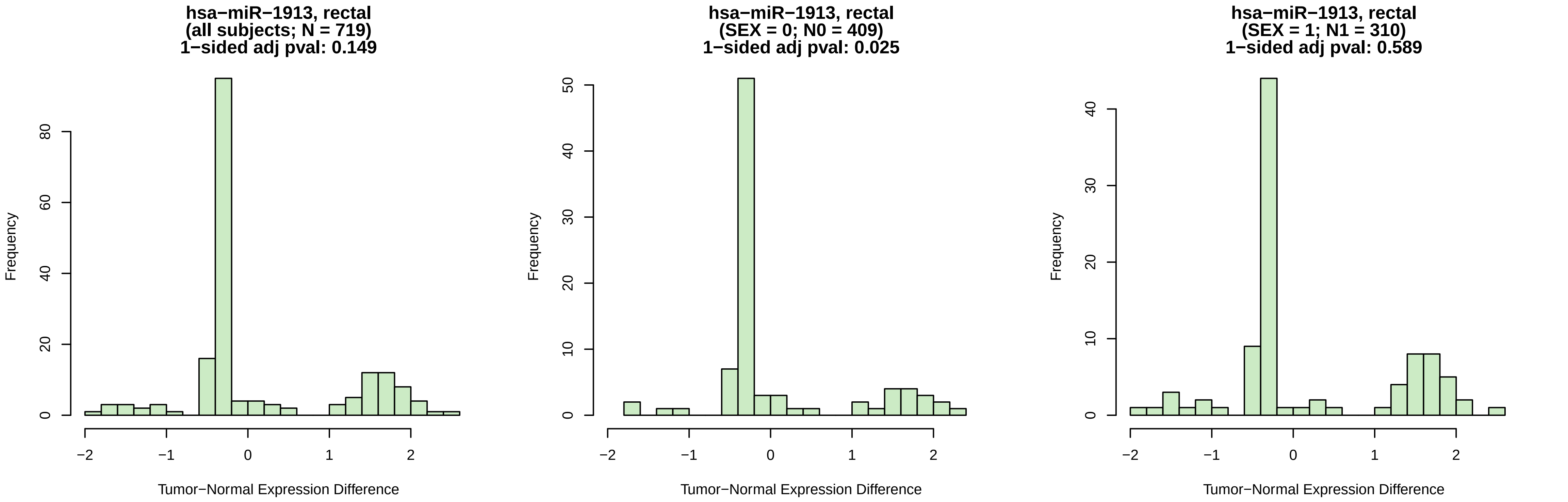

hsa−miR−1913, rectal
(all subjects; N = 719)
1−sided adj pval: 0.149
Frequency
0
20
40
60
80
−2
−1
0
1
2
Tumor−Normal Expression Difference
hsa−miR−1913, rectal
(SEX = 0; N0 = 409)
1−sided adj pval: 0.025
Frequency
0
10
20
30
40
50
−2
−1
0
1
2
Tumor−Normal Expression Difference
hsa−miR−1913, rectal
(SEX = 1; N1 = 310)
1−sided adj pval: 0.589
Frequency
0
10
20
30
40
−2
−1
0
1
2
Tumor−Normal Expression Difference

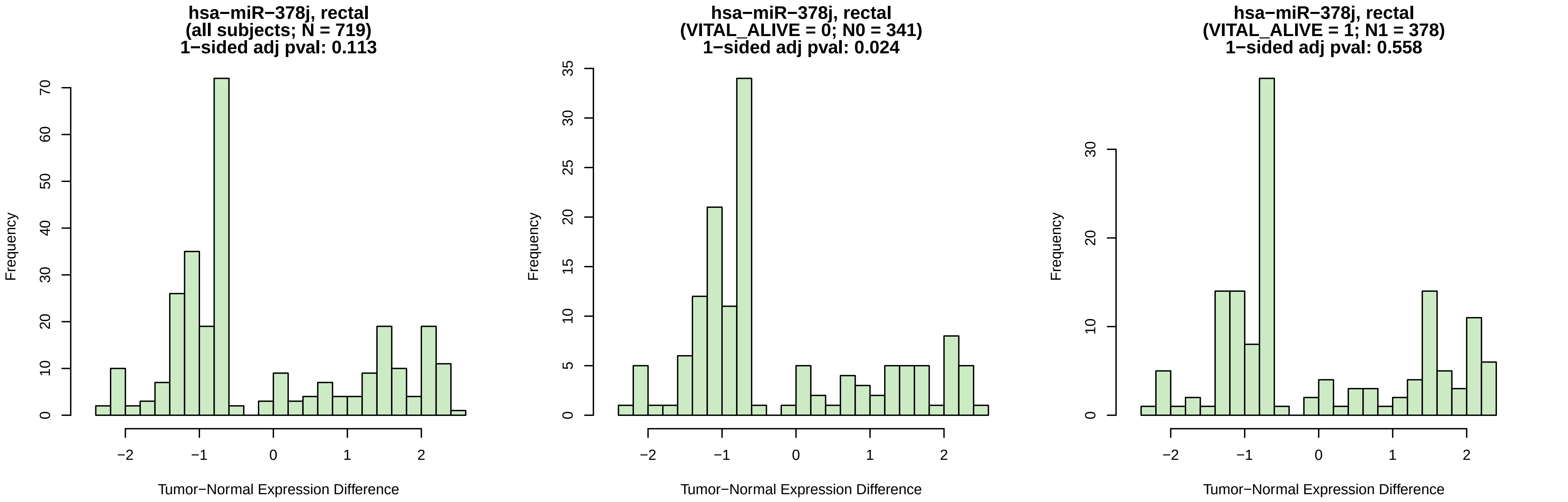

hsa−miR−378j, rectal
(all subjects; N = 719)
1−sided adj pval: 0.113
Frequency
Tumor−Normal Expression Difference
hsa−miR−378j, rectal
(VITAL_ALIVE = 0; N0 = 341)
1−sided adj pval: 0.024
Frequency
Tumor−Normal Expression Difference
hsa−miR−378j, rectal
(VITAL_ALIVE = 1; N1 = 378)
1−sided adj pval: 0.558
Frequency
Tumor−Normal Expression Difference

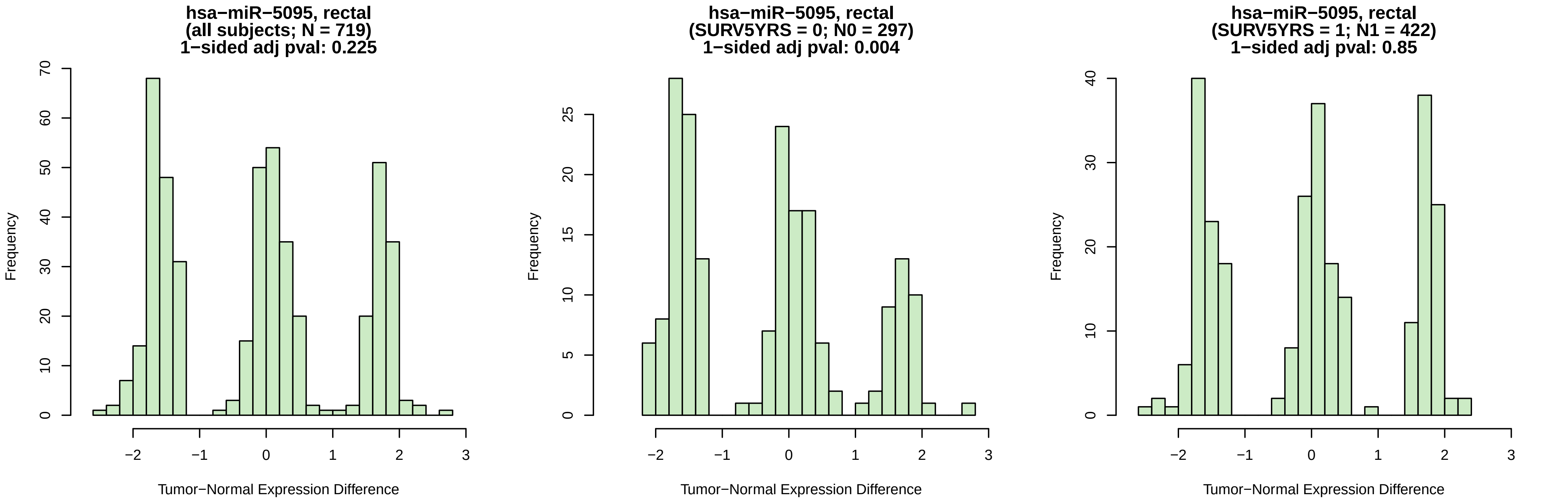
hsa−miR−5095, rectal
(all subjects; N = 719)
1−sided adj pval: 0.225
hsa−miR−5095, rectal
(SURV5YRS = 0; N0 = 297)
1−sided adj pval: 0.004
hsa−miR−5095, rectal
(SURV5YRS = 1; N1 = 422)
1−sided adj pval: 0.85
Frequency
Tumor−Normal Expression Difference
0
10
20
30
40
50
60
70
5
15
25
−2
−1
1
2
3

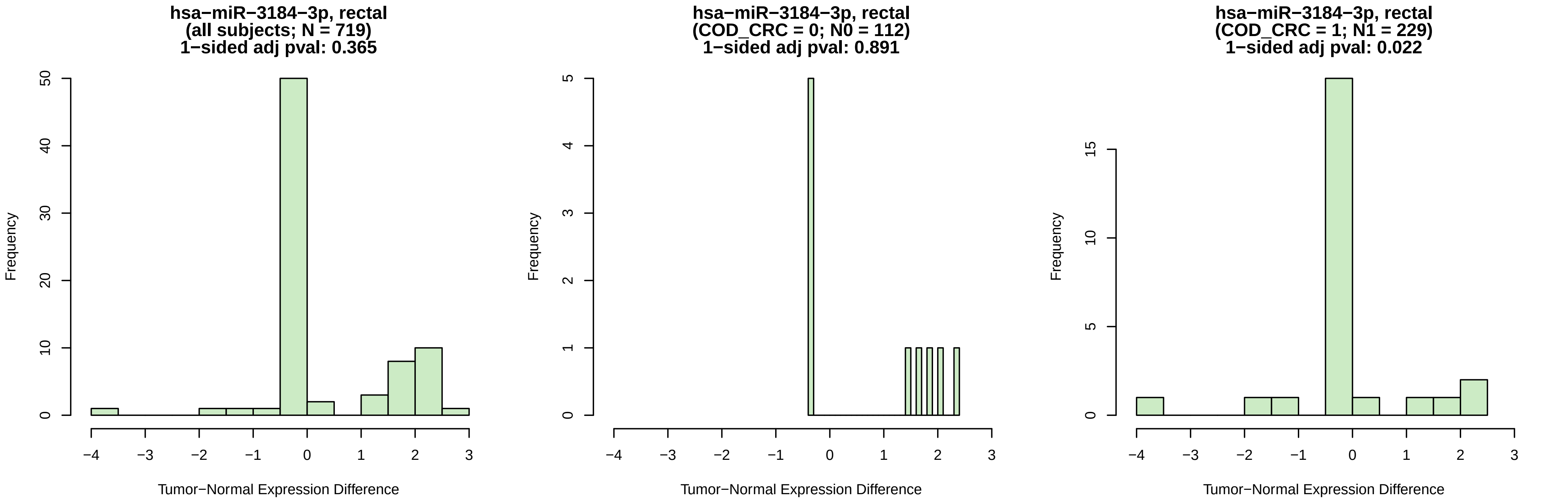

hsa−miR−3184−3p, rectal
(all subjects; N = 719)
1−sided adj pval: 0.365
Frequency
0
10
20
30
40
50
−4
−3
−2
−1
0
1
2
3
Tumor−Normal Expression Difference
hsa−miR−3184−3p, rectal
(COD_CRC = 0; N0 = 112)
1−sided adj pval: 0.891
Frequency
0
1
2
3
4
5
−4
−3
−2
−1
0
1
2
3
Tumor−Normal Expression Difference
hsa−miR−3184−3p, rectal
(COD_CRC = 1; N1 = 229)
1−sided adj pval: 0.022
Frequency
0
5
10
15
−4
−3
−2
−1
0
1
2
3
Tumor−Normal Expression Difference

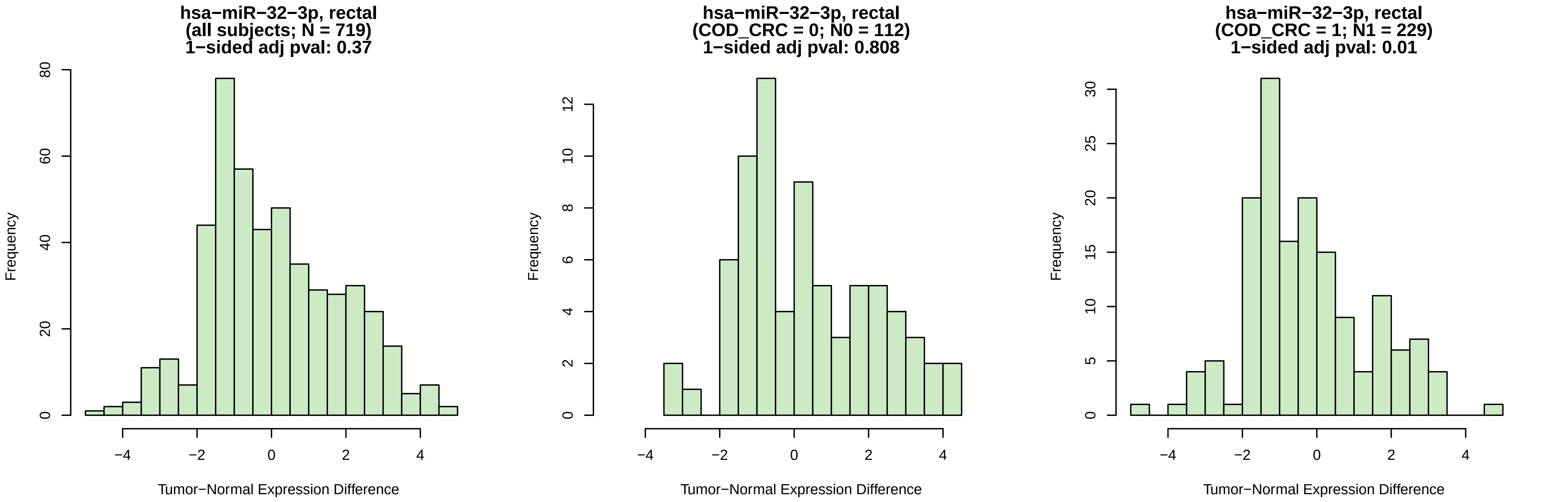

hsa−miR−32−3p, rectal
(all subjects; N = 719)
1−sided adj pval: 0.37
Frequency
Tumor−Normal Expression Difference
hsa−miR−32−3p, rectal
(COD_CRC = 0; N0 = 112)
1−sided adj pval: 0.808
Frequency
Tumor−Normal Expression Difference
hsa−miR−32−3p, rectal
(COD_CRC = 1; N1 = 229)
1−sided adj pval: 0.01
Frequency
Tumor−Normal Expression Difference

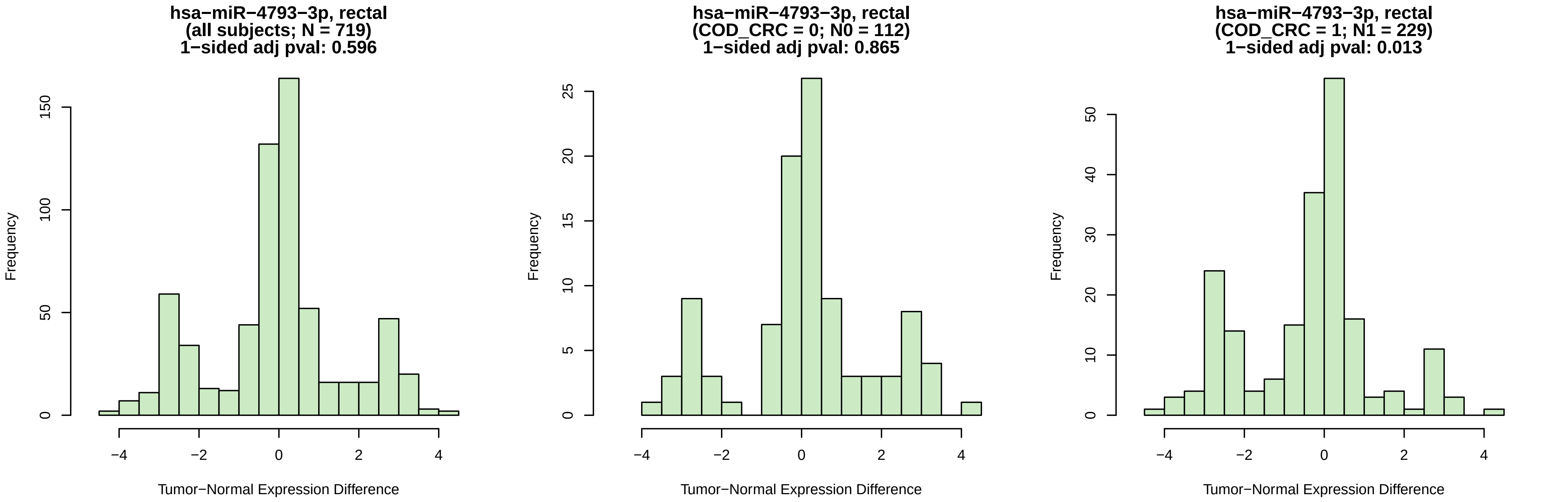
hsa−miR−4793−3p, rectal
(all subjects; N = 719)
1−sided adj pval: 0.596
Frequency
Tumor−Normal Expression Difference
hsa−miR−4793−3p, rectal
(COD_CRC = 0; N0 = 112)
1−sided adj pval: 0.865
Frequency
Tumor−Normal Expression Difference
hsa−miR−4793−3p, rectal
(COD_CRC = 1; N1 = 229)
1−sided adj pval: 0.013
Frequency
Tumor−Normal Expression Difference

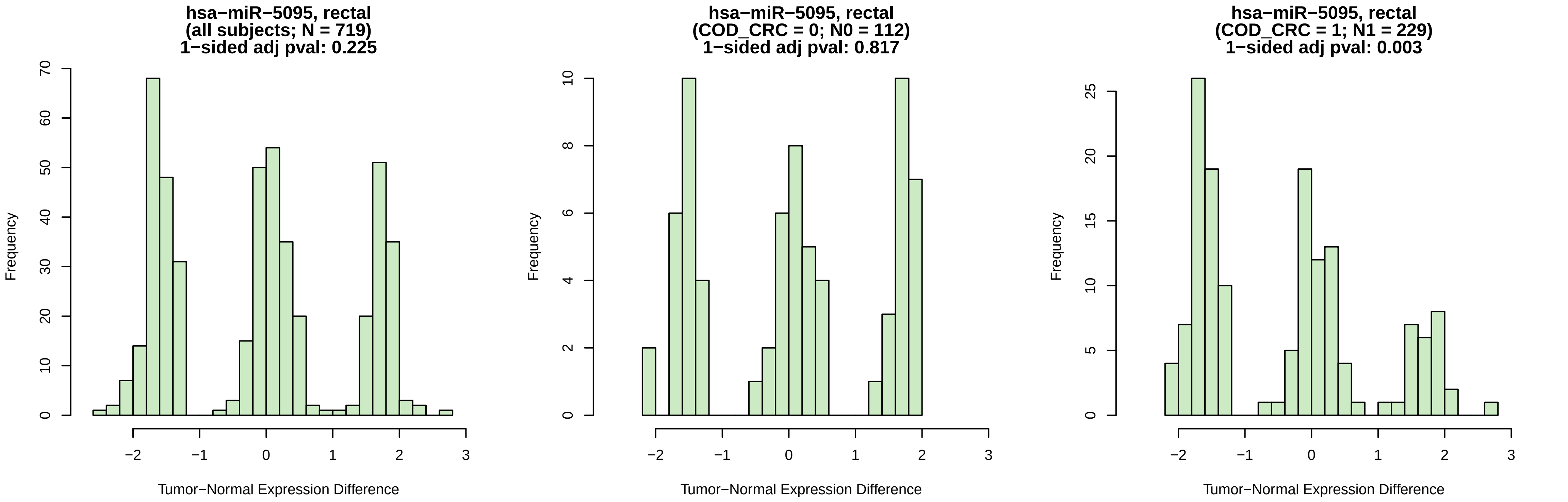
hsa−miR−5095, rectal
(all subjects; N = 719)
1−sided adj pval: 0.225
Frequency
Tumor−Normal Expression Difference
hsa−miR−5095, rectal
(COD_CRC = 0; N0 = 112)
1−sided adj pval: 0.817
Frequency
Tumor−Normal Expression Difference
hsa−miR−5095, rectal
(COD_CRC = 1; N1 = 229)
1−sided adj pval: 0.003
Frequency
Tumor−Normal Expression Difference

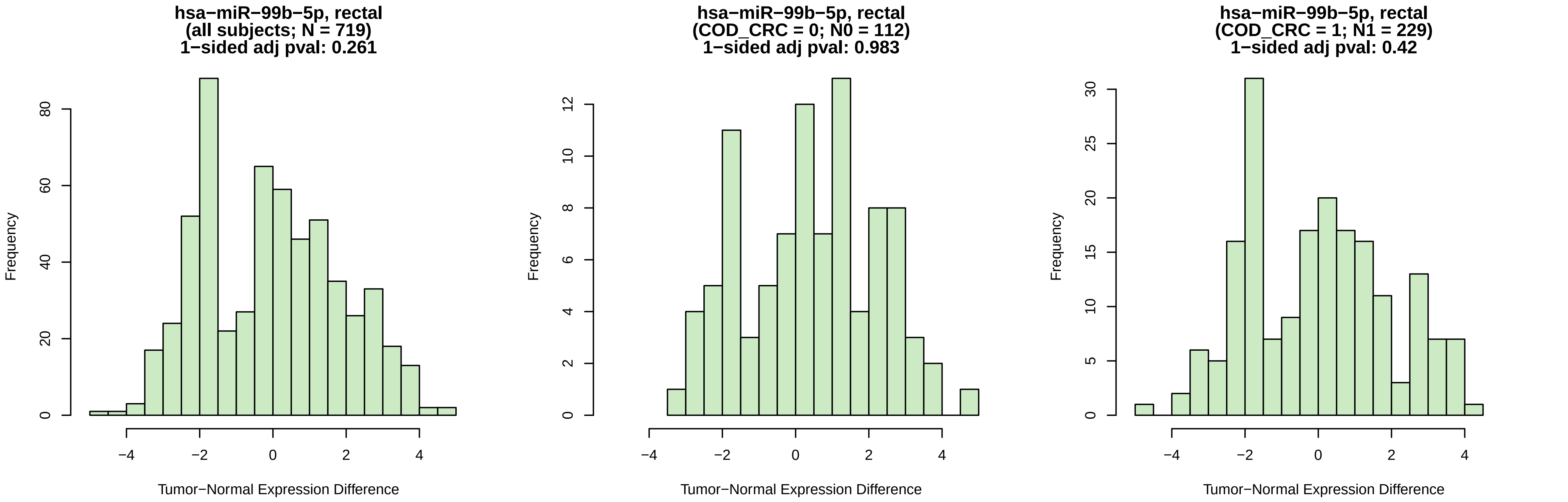
hsa−miR−99b−5p, rectal
(all subjects; N = 719)
1−sided adj pval: 0.261
Frequency
Tumor−Normal Expression Difference
hsa−miR−99b−5p, rectal
(COD_CRC = 0; N0 = 112)
1−sided adj pval: 0.983
Frequency
Tumor−Normal Expression Difference
hsa−miR−99b−5p, rectal
(COD_CRC = 1; N1 = 229)
1−sided adj pval: 0.42
Frequency
Tumor−Normal Expression Difference

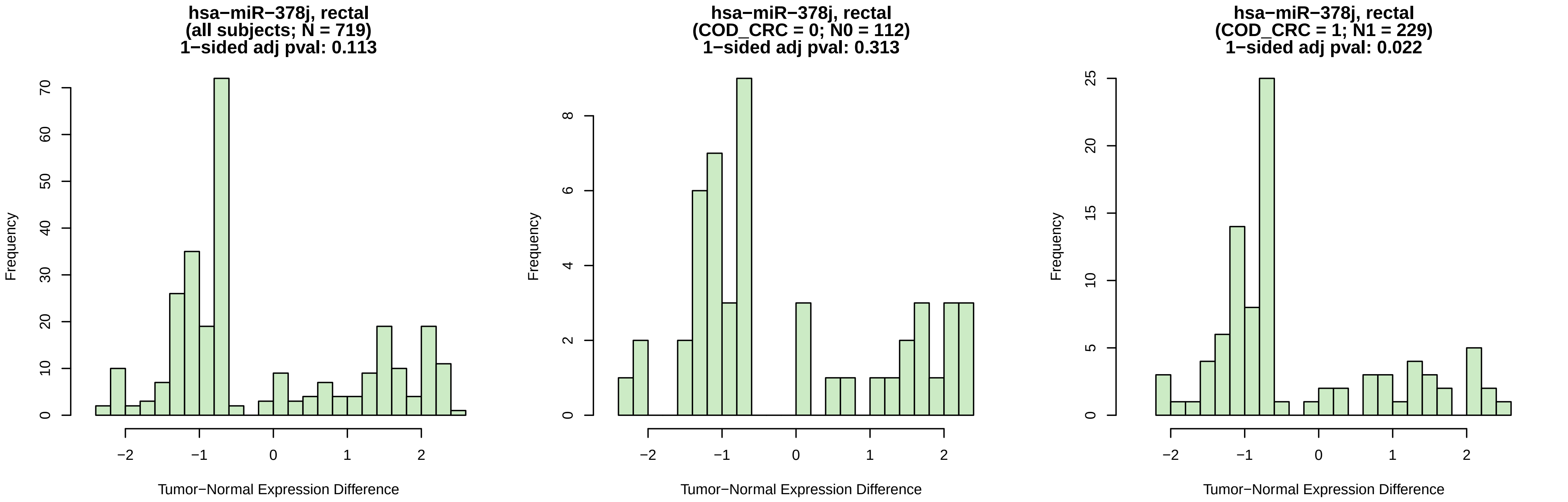
hsa−miR−378j, rectal
(all subjects; N = 719)
1−sided adj pval: 0.113
Frequency
0
10
20
30
40
50
60
70
−2
−1
0
1
2
Tumor−Normal Expression Difference
hsa−miR−378j, rectal
(COD_CRC = 0; N0 = 112)
1−sided adj pval: 0.313
Frequency
0
2
4
6
8
−2
−1
0
1
2
Tumor−Normal Expression Difference
hsa−miR−378j, rectal
(COD_CRC = 1; N1 = 229)
1−sided adj pval: 0.022
Frequency
0
5
10
15
20
25
−2
−1
0
1
2
Tumor−Normal Expression Difference

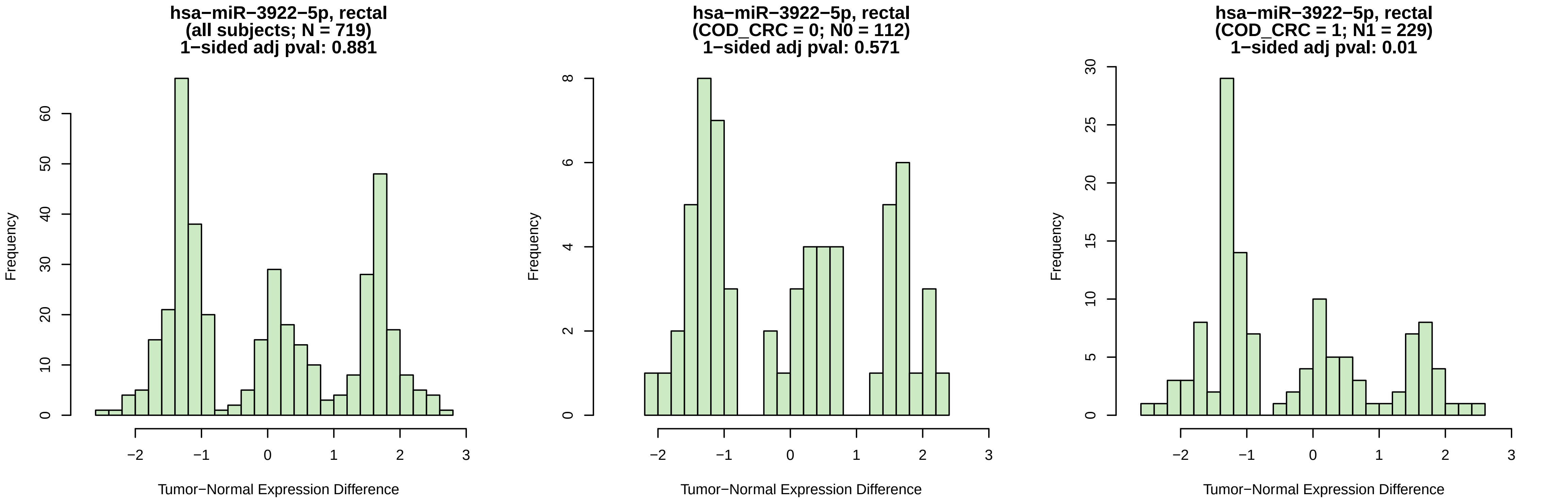
hsa−miR−3922−5p, rectal
(all subjects; N = 719)
1−sided adj pval: 0.881
Frequency
Tumor−Normal Expression Difference
hsa−miR−3922−5p, rectal
(COD_CRC = 0; N0 = 112)
1−sided adj pval: 0.571
Frequency
Tumor−Normal Expression Difference
hsa−miR−3922−5p, rectal
(COD_CRC = 1; N1 = 229)
1−sided adj pval: 0.01
Frequency
Tumor−Normal Expression Difference

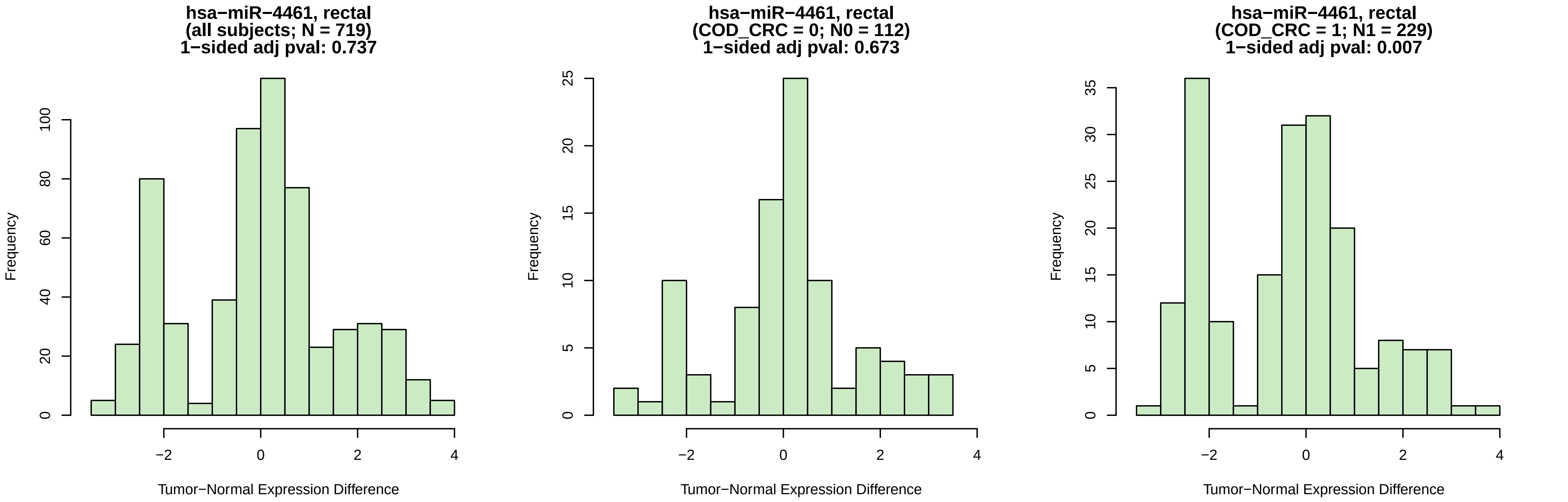
hsa−miR−4461, rectal
(all subjects; N = 719)
1−sided adj pval: 0.737
hsa−miR−4461, rectal
(COD_CRC = 0; N0 = 112)
1−sided adj pval: 0.673
hsa−miR−4461, rectal
(COD_CRC = 1; N1 = 229)
1−sided adj pval: 0.007
Frequency
Tumor−Normal Expression Difference
0
20
40
60
80
100
5
10
15
20
25
30
35
−2
0
2
4

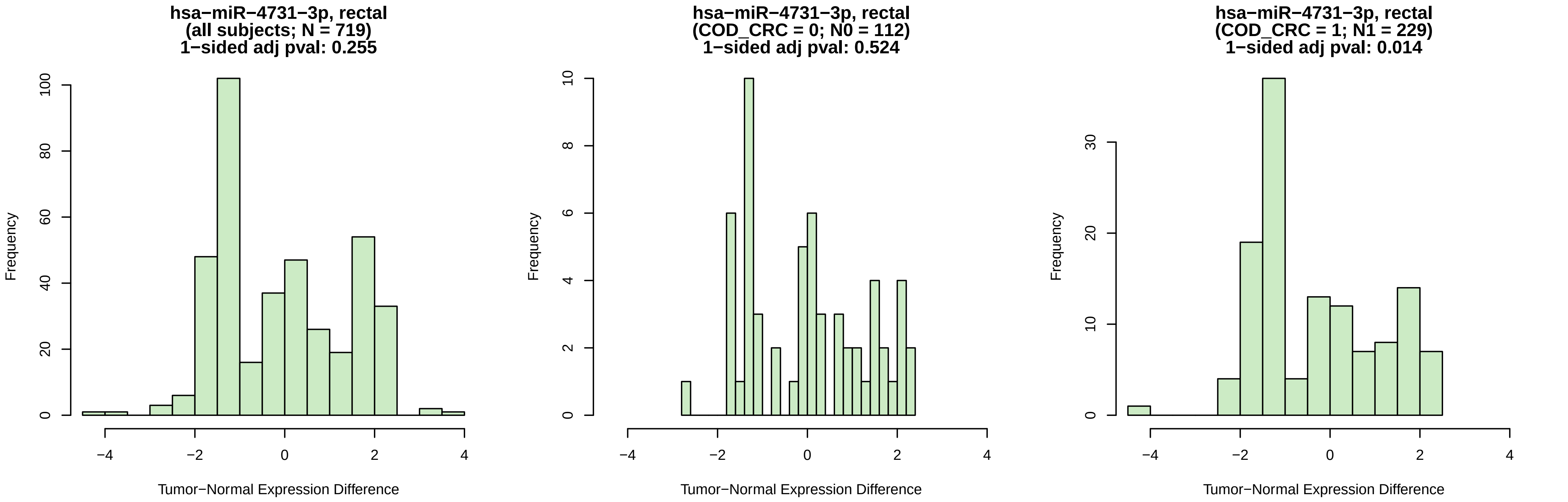
hsa−miR−4731−3p, rectal
(all subjects; N = 719)
1−sided adj pval: 0.255
Frequency
Tumor−Normal Expression Difference
0
20
40
60
80
100
−4
−2
0
2
4
hsa−miR−4731−3p, rectal
(COD_CRC = 0; N0 = 112)
1−sided adj pval: 0.524
Frequency
Tumor−Normal Expression Difference
0
2
4
6
8
10
−4
−2
0
2
4
hsa−miR−4731−3p, rectal
(COD_CRC = 1; N1 = 229)
1−sided adj pval: 0.014
Frequency
Tumor−Normal Expression Difference
0
10
20
30
−4
−2
0
2
4

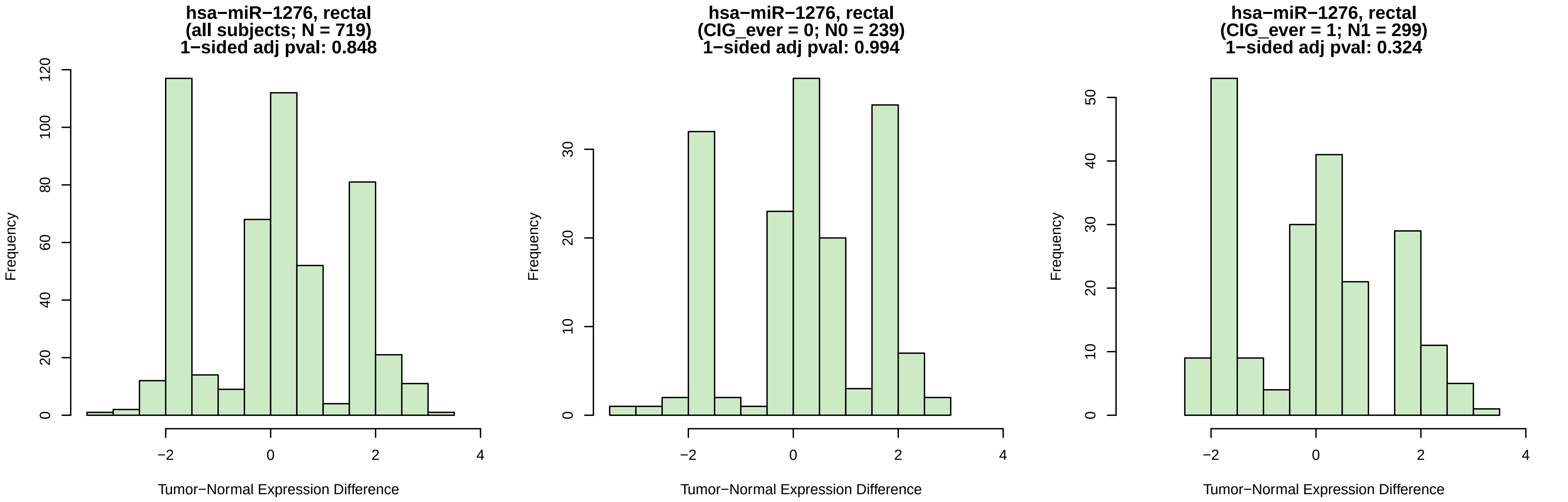
hsa−miR−1276, rectal
(all subjects; N = 719)
1−sided adj pval: 0.848
Frequency
0
20
40
60
80
100
120
−2
0
2
4
Tumor−Normal Expression Difference
hsa−miR−1276, rectal
(CIG_ever = 0; N0 = 239)
1−sided adj pval: 0.994
Frequency
0
10
20
30
−2
0
2
4
Tumor−Normal Expression Difference
hsa−miR−1276, rectal
(CIG_ever = 1; N1 = 299)
1−sided adj pval: 0.324
Frequency
0
10
20
30
40
50
−2
0
2
4
Tumor−Normal Expression Difference

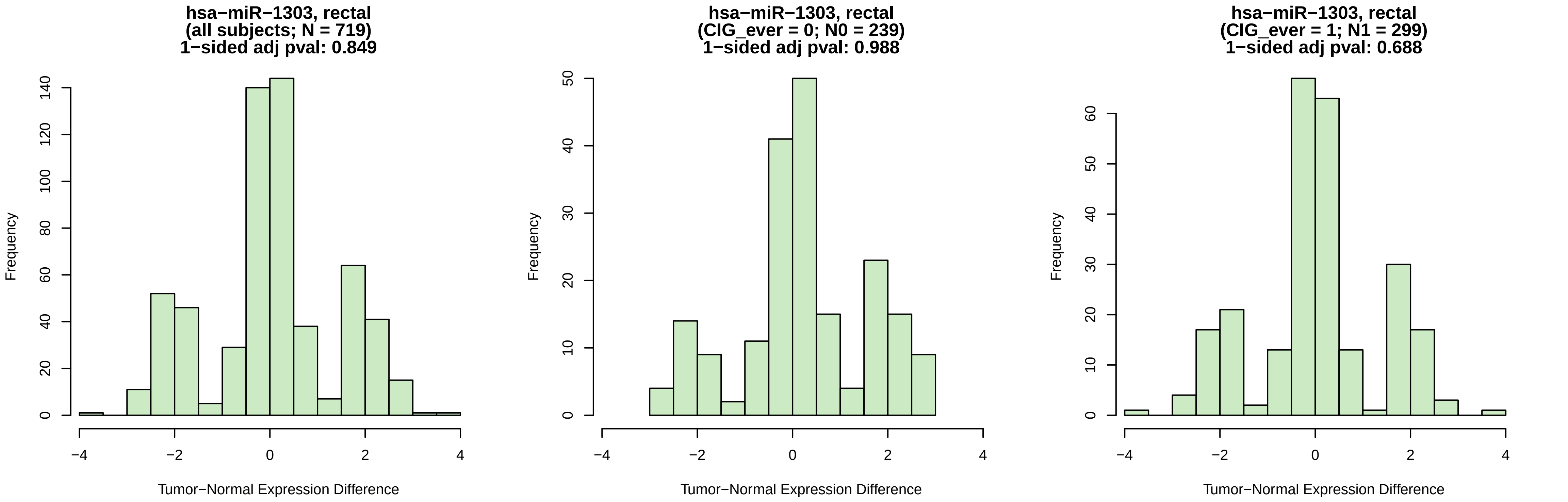

hsa−miR−1303, rectal
(all subjects; N = 719)
1−sided adj pval: 0.849
Frequency
Tumor−Normal Expression Difference
hsa−miR−1303, rectal
(CIG_ever = 0; N0 = 239)
1−sided adj pval: 0.988
Frequency
Tumor−Normal Expression Difference
hsa−miR−1303, rectal
(CIG_ever = 1; N1 = 299)
1−sided adj pval: 0.688
Frequency
Tumor−Normal Expression Difference

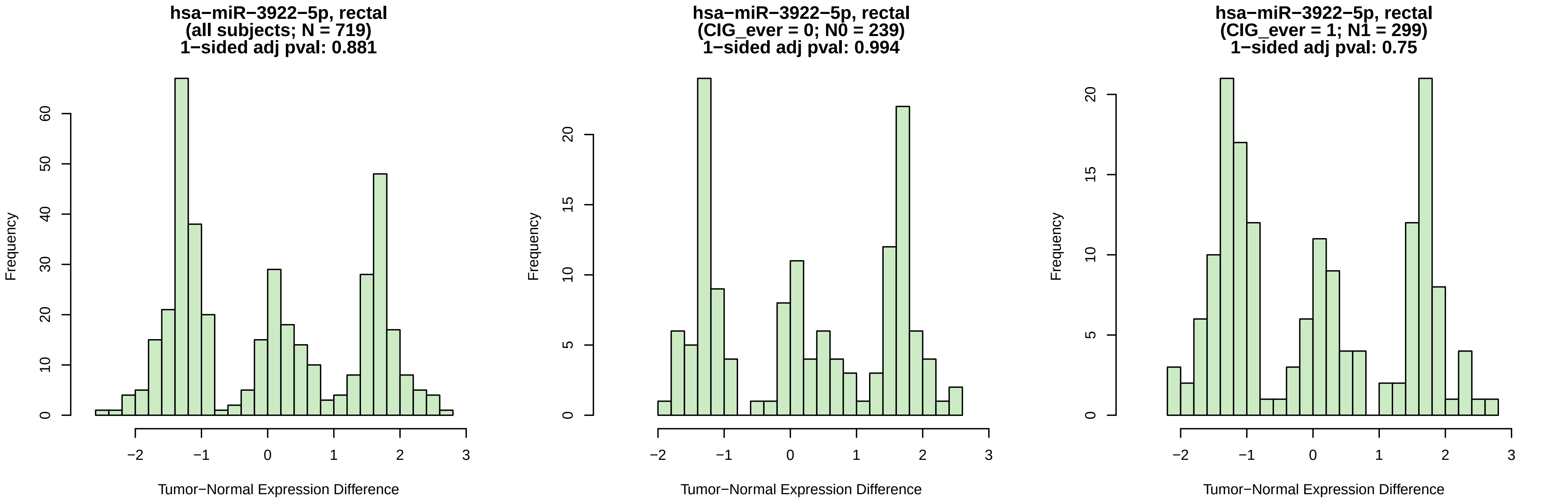
hsa−miR−3922−5p, rectal
(all subjects; N = 719)
1−sided adj pval: 0.881
hsa−miR−3922−5p, rectal
(CIG_ever = 0; N0 = 239)
1−sided adj pval: 0.994
hsa−miR−3922−5p, rectal
(CIG_ever = 1; N1 = 299)
1−sided adj pval: 0.75
Frequency
Tumor−Normal Expression Difference

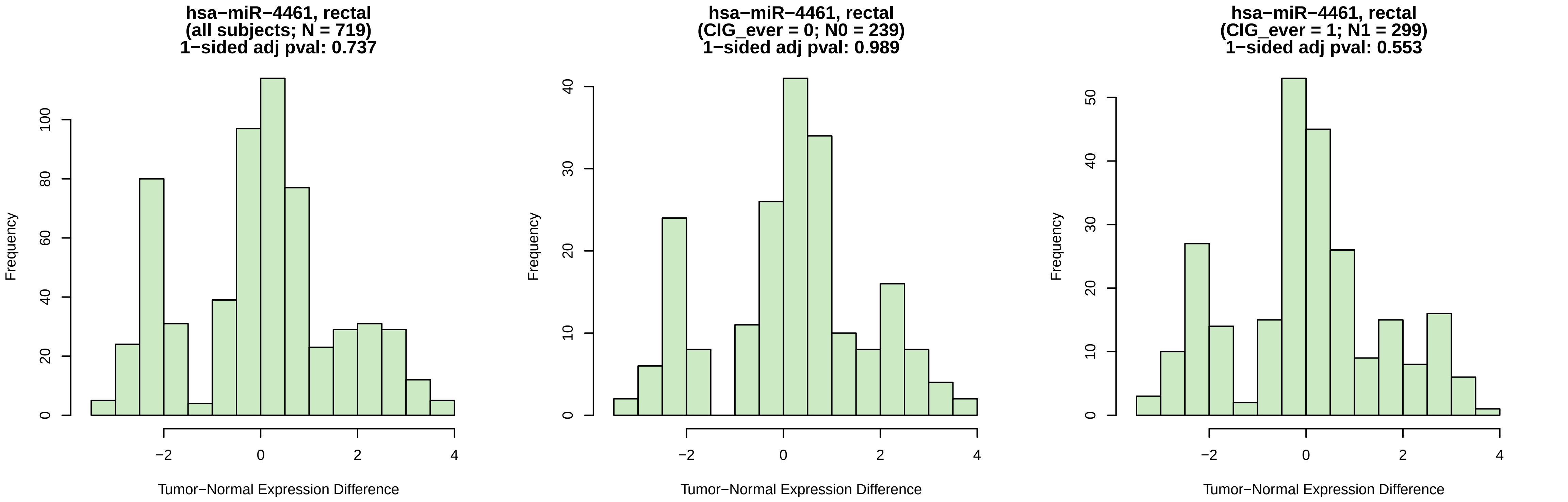
hsa−miR−4461, rectal
(all subjects; N = 719)
1−sided adj pval: 0.737
Frequency
0
20
40
60
80
100
−2
0
2
4
Tumor−Normal Expression Difference
hsa−miR−4461, rectal
(CIG_ever = 0; N0 = 239)
1−sided adj pval: 0.989
Frequency
0
10
20
30
40
−2
0
2
4
Tumor−Normal Expression Difference
hsa−miR−4461, rectal
(CIG_ever = 1; N1 = 299)
1−sided adj pval: 0.553
Frequency
0
10
20
30
40
50
−2
0
2
4
Tumor−Normal Expression Difference

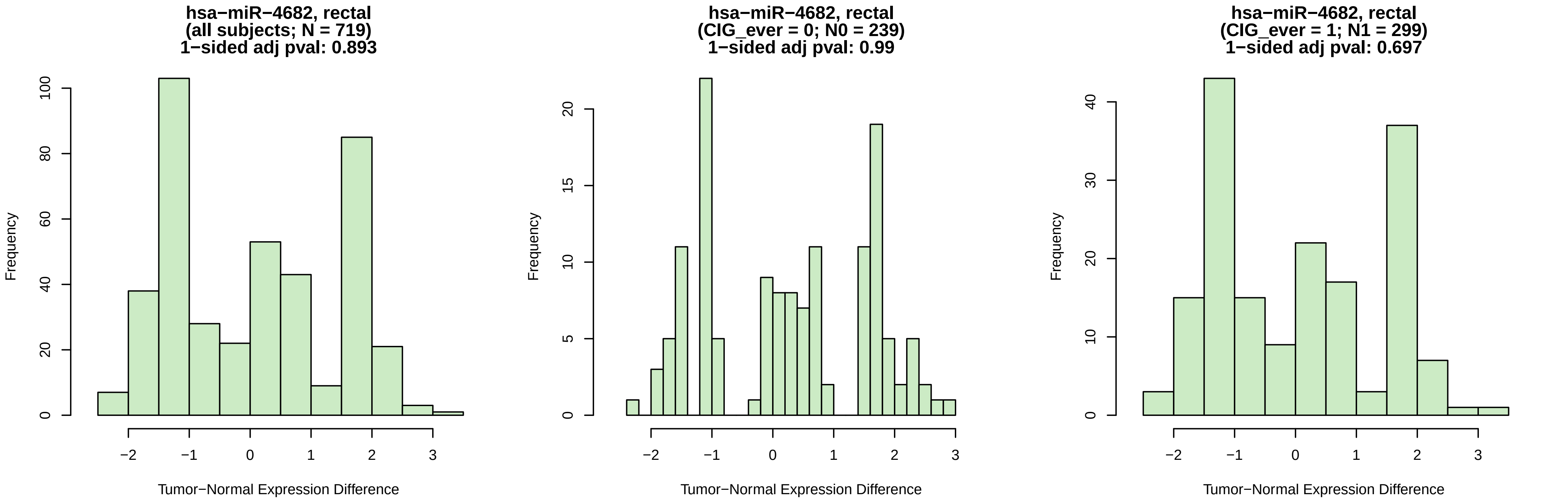
hsa−miR−4682, rectal
(all subjects; N = 719)
1−sided adj pval: 0.893
Frequency
0
20
40
60
80
100
−2
−1
0
1
2
3
Tumor−Normal Expression Difference
hsa−miR−4682, rectal
(CIG_ever = 0; N0 = 239)
1−sided adj pval: 0.99
Frequency
0
5
10
15
20
−2
−1
0
1
2
3
Tumor−Normal Expression Difference
hsa−miR−4682, rectal
(CIG_ever = 1; N1 = 299)
1−sided adj pval: 0.697
Frequency
0
10
20
30
40
−2
−1
0
1
2
3
Tumor−Normal Expression Difference

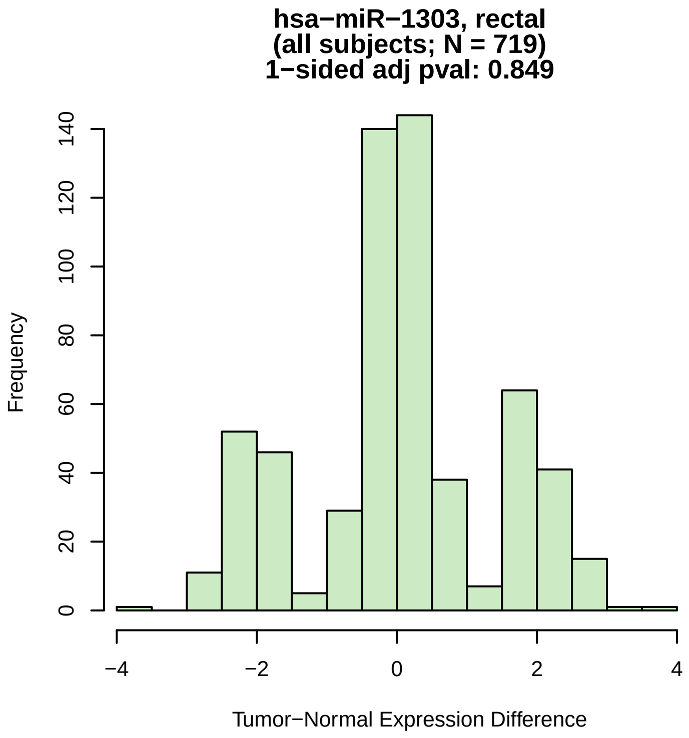
hsa−miR−1303, rectal
(all subjects; N = 719)
1−sided adj pval: 0.849
Frequency
Tumor−Normal Expression Difference

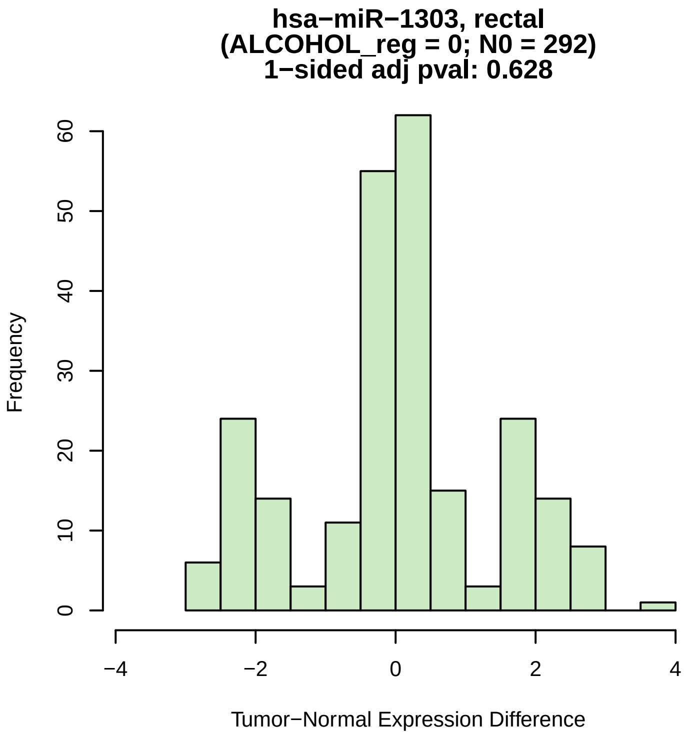
hsa−miR−1303, rectal
(ALCOHOL_reg = 0; N0 = 292)
1−sided adj pval: 0.628
Frequency
Tumor−Normal Expression Difference

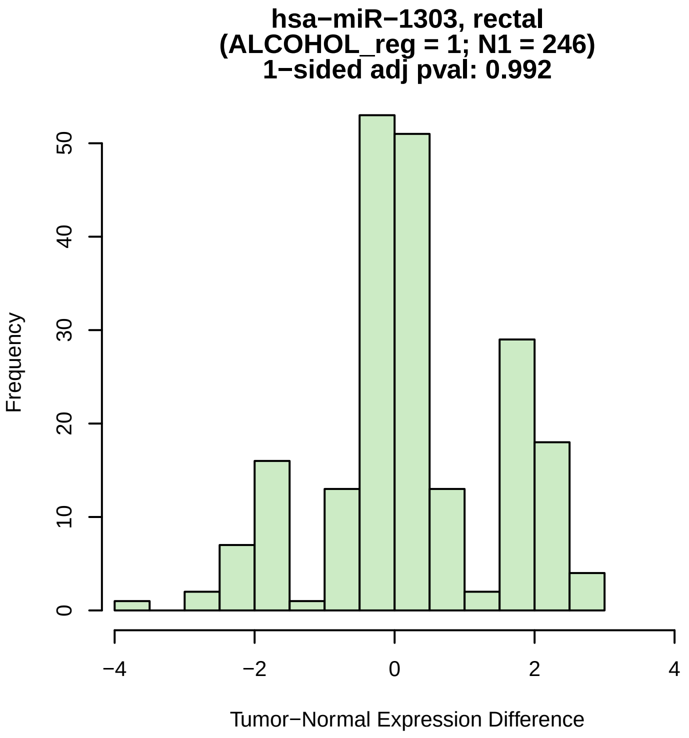
hsa−miR−1303, rectal
(ALCOHOL_reg = 1; N1 = 246)
1−sided adj pval: 0.992
Frequency
Tumor−Normal Expression Difference

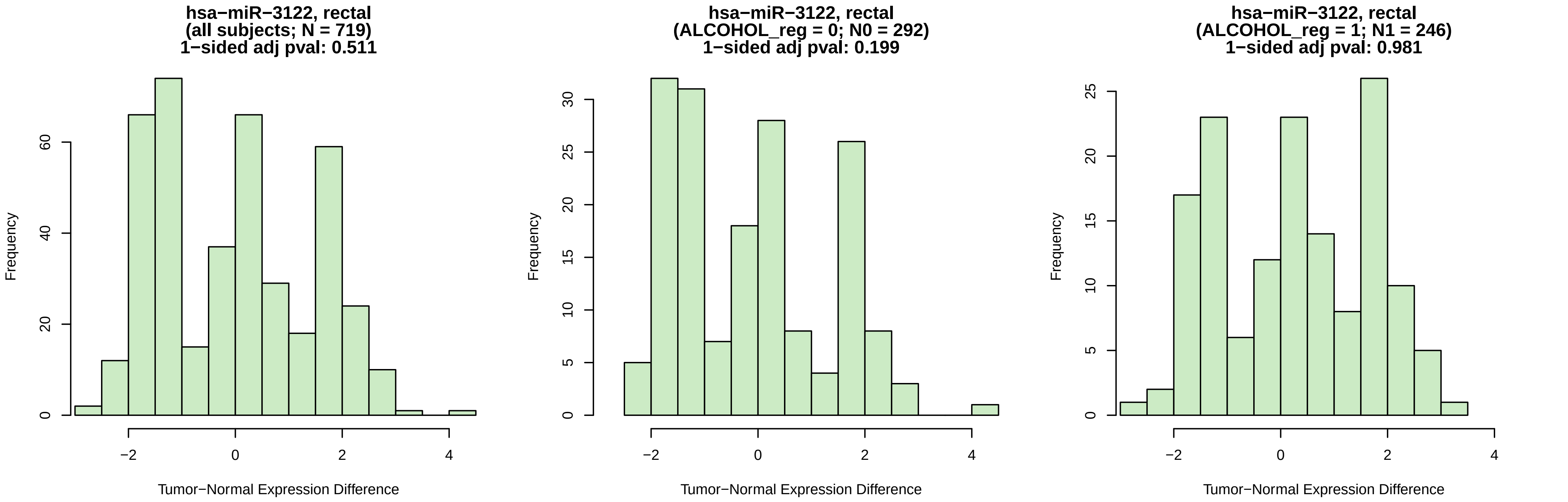
hsa−miR−3122, rectal
(all subjects; N = 719)
1−sided adj pval: 0.511
Frequency
Tumor−Normal Expression Difference
hsa−miR−3122, rectal
(ALCOHOL_reg = 0; N0 = 292)
1−sided adj pval: 0.199
Frequency
Tumor−Normal Expression Difference
hsa−miR−3122, rectal
(ALCOHOL_reg = 1; N1 = 246)
1−sided adj pval: 0.981
Frequency
Tumor−Normal Expression Difference

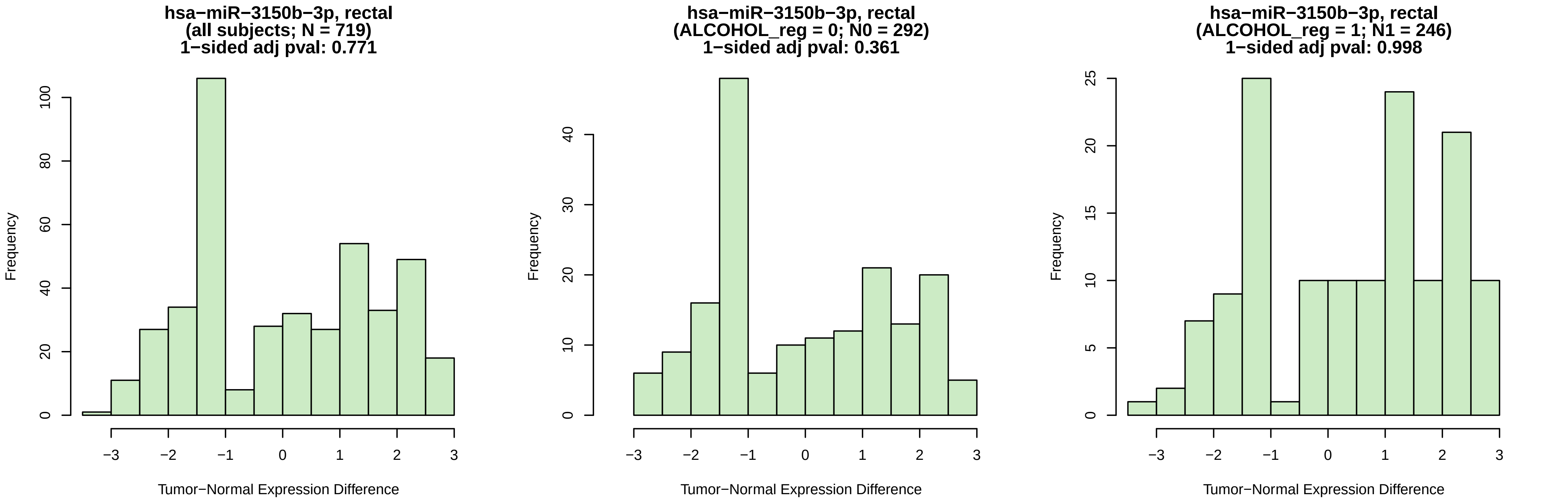

hsa−miR−3150b−3p, rectal
(all subjects; N = 719)
1−sided adj pval: 0.771
hsa−miR−3150b−3p, rectal
(ALCOHOL_reg = 0; N0 = 292)
1−sided adj pval: 0.361
hsa−miR−3150b−3p, rectal
(ALCOHOL_reg = 1; N1 = 246)
1−sided adj pval: 0.998
Frequency
Tumor−Normal Expression Difference

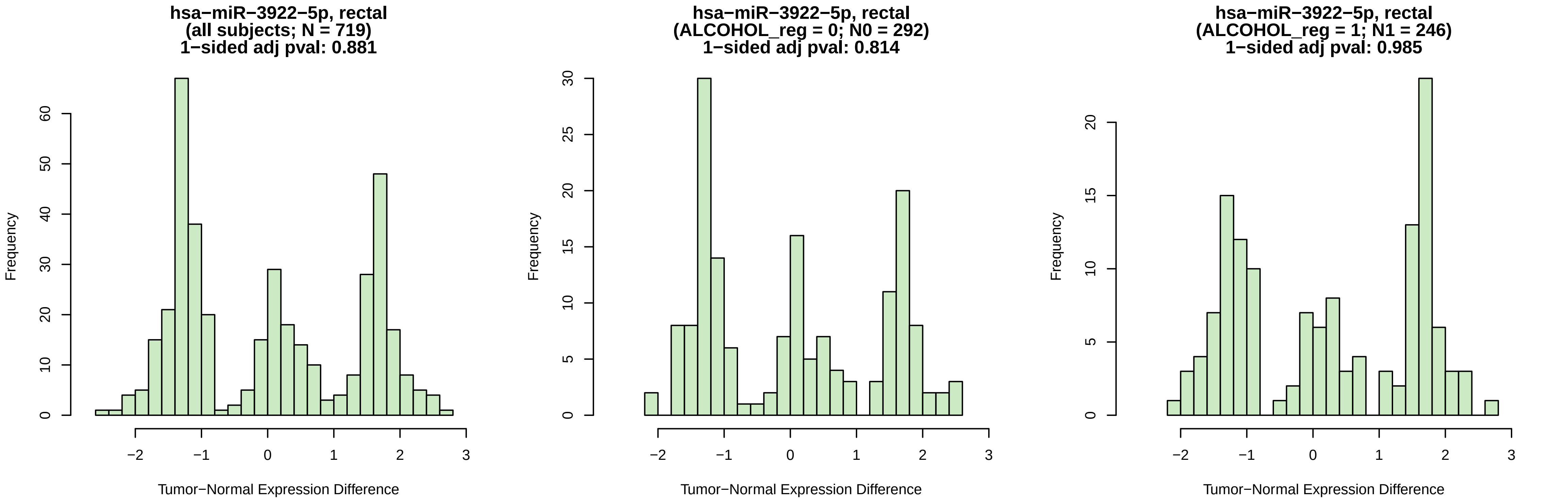
hsa−miR−3922−5p, rectal
(all subjects; N = 719)
1−sided adj pval: 0.881
Frequency
Tumor−Normal Expression Difference
hsa−miR−3922−5p, rectal
(ALCOHOL_reg = 0; N0 = 292)
1−sided adj pval: 0.814
Frequency
Tumor−Normal Expression Difference
hsa−miR−3922−5p, rectal
(ALCOHOL_reg = 1; N1 = 246)
1−sided adj pval: 0.985
Frequency
Tumor−Normal Expression Difference

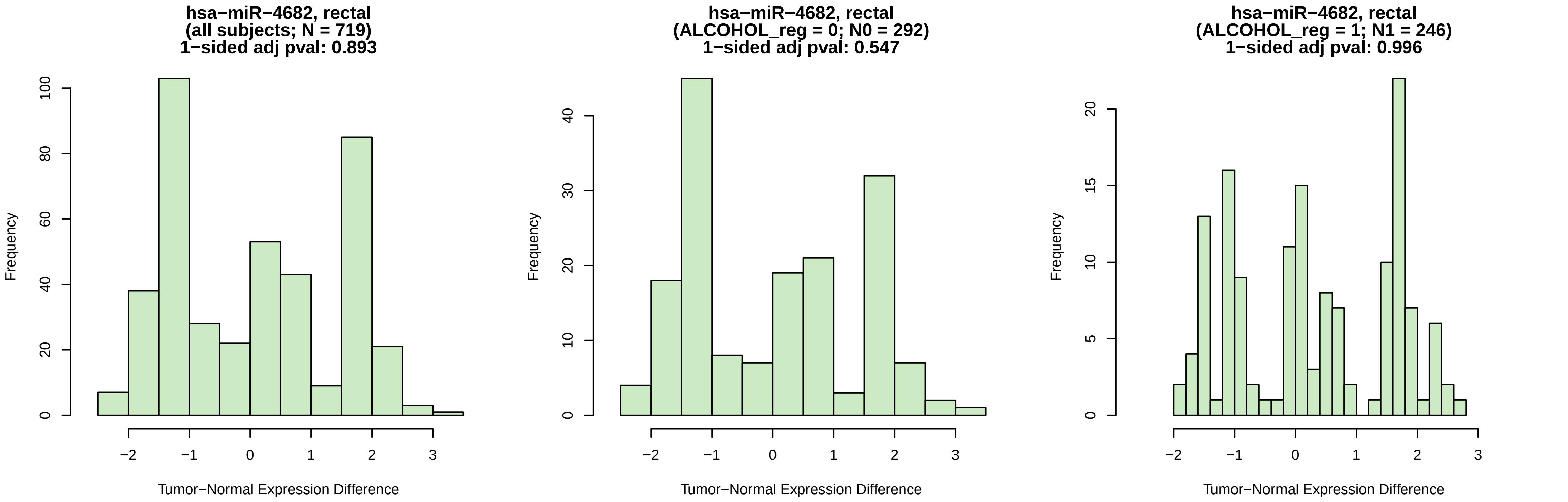

hsa−miR−4682, rectal
(all subjects; N = 719)
1−sided adj pval: 0.893
Frequency
Tumor−Normal Expression Difference
hsa−miR−4682, rectal
(ALCOHOL_reg = 0; N0 = 292)
1−sided adj pval: 0.547
Frequency
Tumor−Normal Expression Difference
hsa−miR−4682, rectal
(ALCOHOL_reg = 1; N1 = 246)
1−sided adj pval: 0.996
Frequency
Tumor−Normal Expression Difference

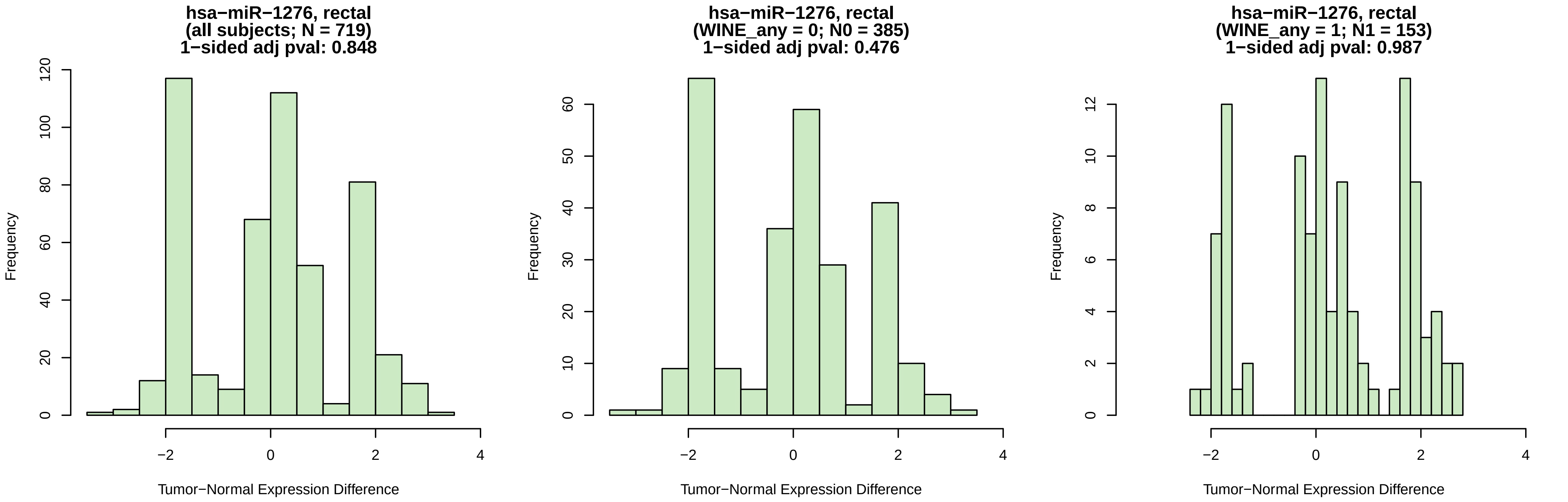
hsa−miR−1276, rectal
(all subjects; N = 719)
1−sided adj pval: 0.848
Frequency
Tumor−Normal Expression Difference
hsa−miR−1276, rectal
(WINE_any = 0; N0 = 385)
1−sided adj pval: 0.476
Frequency
Tumor−Normal Expression Difference
hsa−miR−1276, rectal
(WINE_any = 1; N1 = 153)
1−sided adj pval: 0.987
Frequency
Tumor−Normal Expression Difference

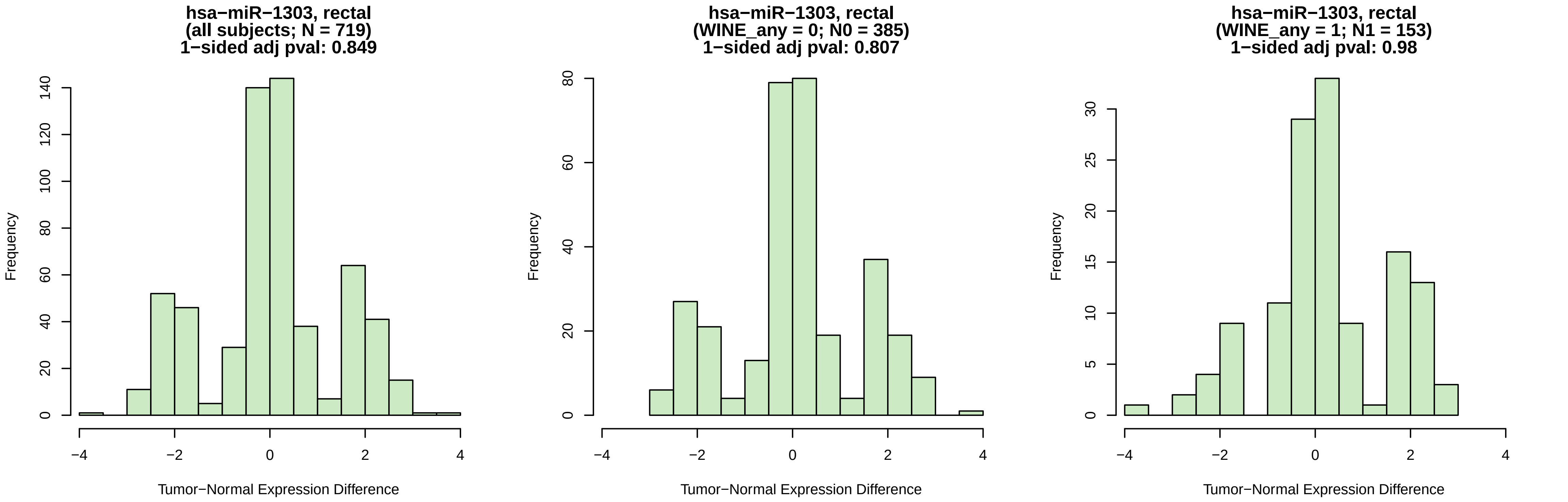

hsa−miR−1303, rectal
(all subjects; N = 719)
1−sided adj pval: 0.849
Frequency
Tumor−Normal Expression Difference
hsa−miR−1303, rectal
(WINE_any = 0; N0 = 385)
1−sided adj pval: 0.807
Frequency
Tumor−Normal Expression Difference
hsa−miR−1303, rectal
(WINE_any = 1; N1 = 153)
1−sided adj pval: 0.98
Frequency
Tumor−Normal Expression Difference

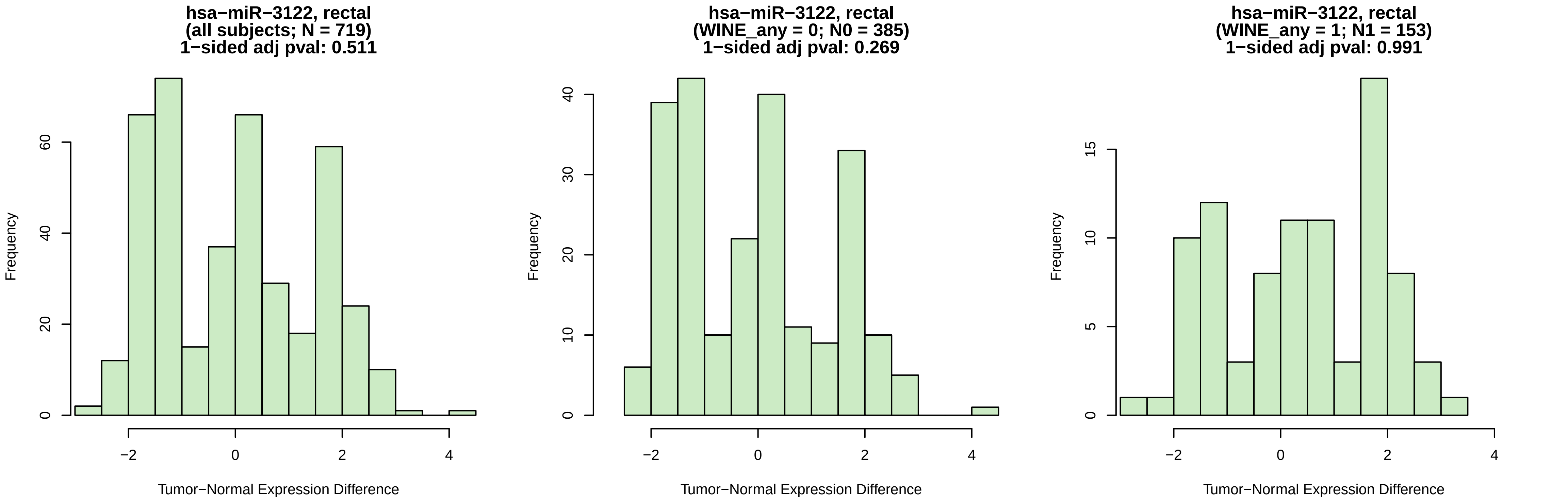

hsa−miR−3122, rectal
(all subjects; N = 719)
1−sided adj pval: 0.511
Frequency
0
20
40
60
−2
0
2
4
Tumor−Normal Expression Difference
hsa−miR−3122, rectal
(WINE_any = 0; N0 = 385)
1−sided adj pval: 0.269
Frequency
0
10
20
30
40
−2
0
2
4
Tumor−Normal Expression Difference
hsa−miR−3122, rectal
(WINE_any = 1; N1 = 153)
1−sided adj pval: 0.991
Frequency
0
5
10
15
−2
0
2
4
Tumor−Normal Expression Difference

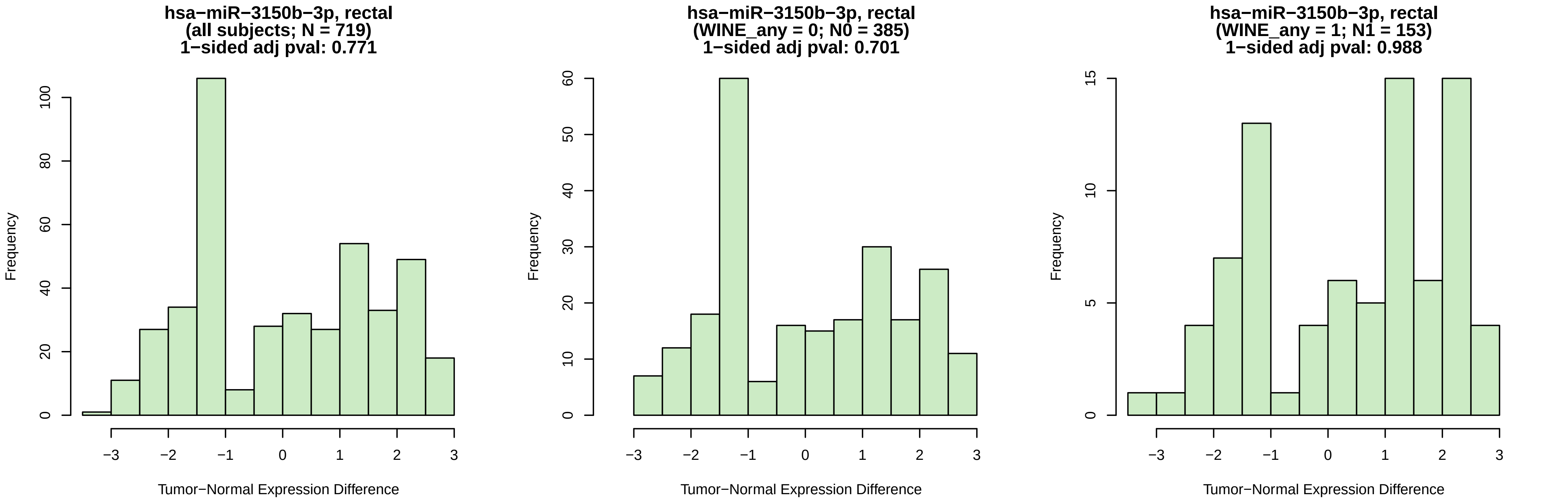
hsa−miR−3150b−3p, rectal
(all subjects; N = 719)
1−sided adj pval: 0.771
Frequency
Tumor−Normal Expression Difference
hsa−miR−3150b−3p, rectal
(WINE_any = 0; N0 = 385)
1−sided adj pval: 0.701
Frequency
Tumor−Normal Expression Difference
hsa−miR−3150b−3p, rectal
(WINE_any = 1; N1 = 153)
1−sided adj pval: 0.988
Frequency
Tumor−Normal Expression Difference

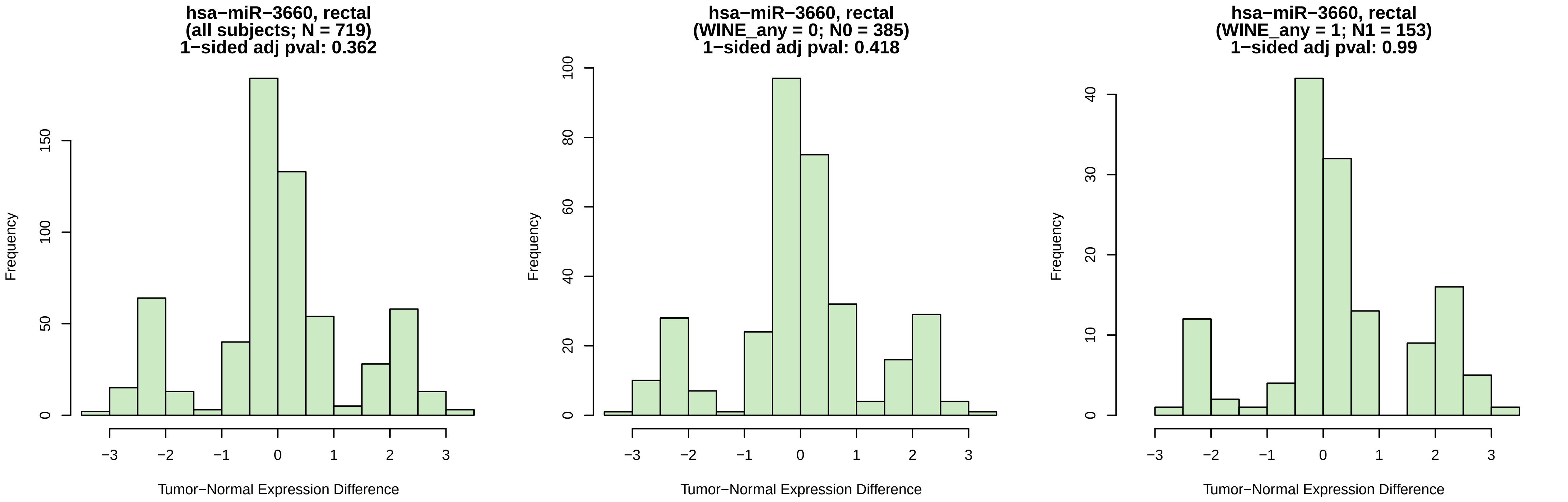

hsa−miR−3660, rectal
(all subjects; N = 719)
1−sided adj pval: 0.362
Frequency
0
50
100
150
−3
−2
−1
0
1
2
3
Tumor−Normal Expression Difference
hsa−miR−3660, rectal
(WINE_any = 0; N0 = 385)
1−sided adj pval: 0.418
Frequency
0
20
40
60
80
100
−3
−2
−1
0
1
2
3
Tumor−Normal Expression Difference
hsa−miR−3660, rectal
(WINE_any = 1; N1 = 153)
1−sided adj pval: 0.99
Frequency
0
10
20
30
40
−3
−2
−1
0
1
2
3
Tumor−Normal Expression Difference

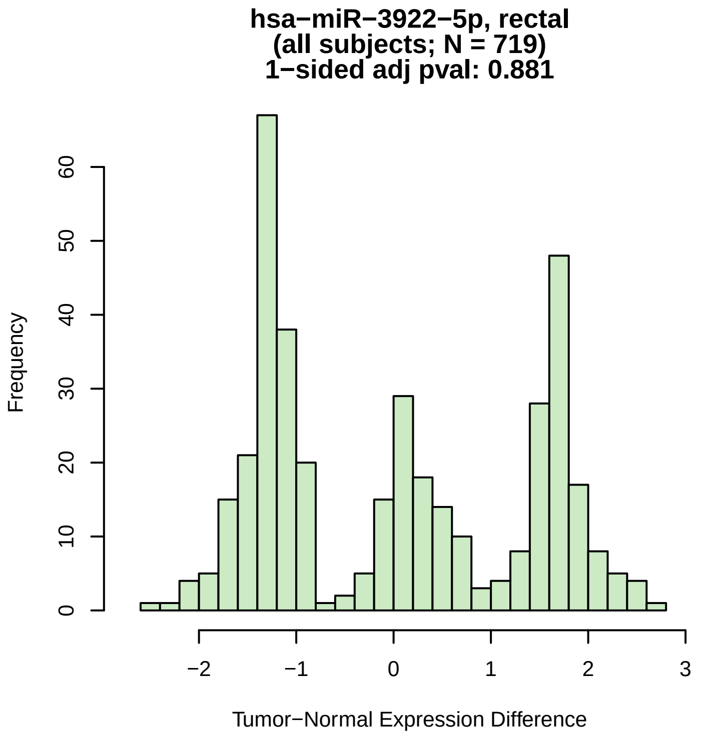

hsa−miR−3922−5p, rectal
(all subjects; N = 719)
1−sided adj pval: 0.881
Frequency
Tumor−Normal Expression Difference

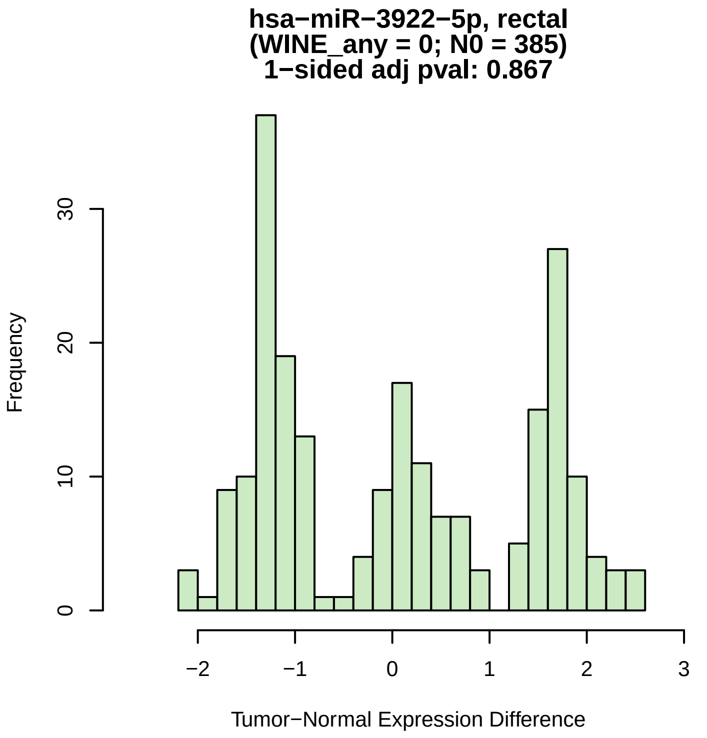

hsa−miR−3922−5p, rectal
(WINE_any = 0; N0 = 385)
1−sided adj pval: 0.867
Frequency
Tumor−Normal Expression Difference

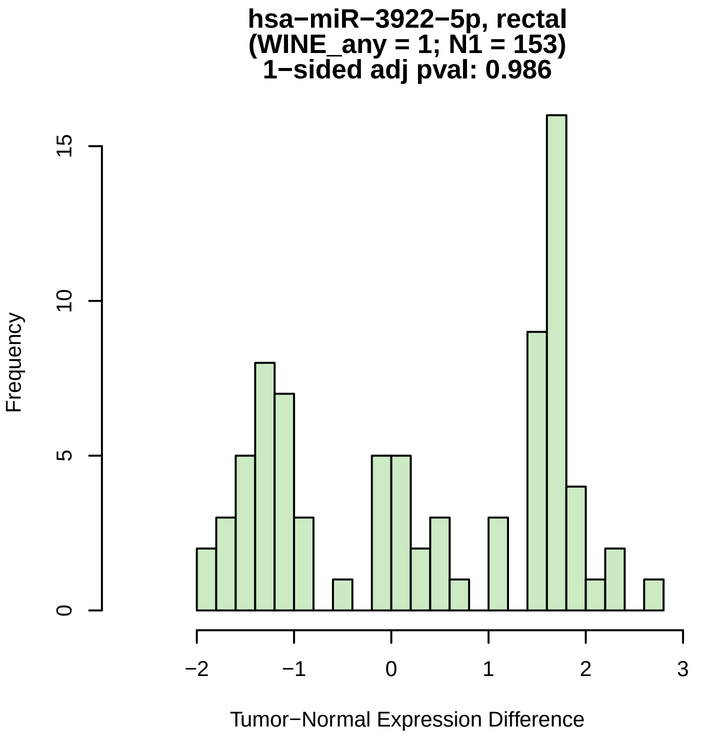

hsa−miR−3922−5p, rectal
(WINE_any = 1; N1 = 153)
1−sided adj pval: 0.986
Frequency
Tumor−Normal Expression Difference

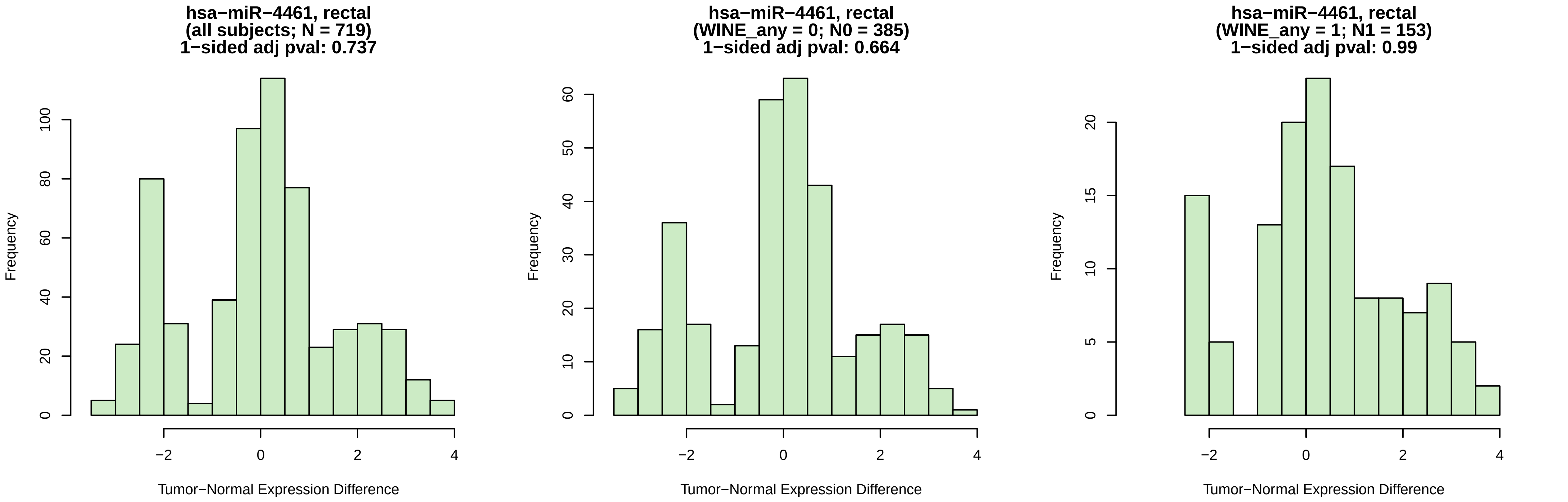

hsa−miR−4461, rectal
(all subjects; N = 719)
1−sided adj pval: 0.737
Frequency
Tumor−Normal Expression Difference
hsa−miR−4461, rectal
(WINE_any = 0; N0 = 385)
1−sided adj pval: 0.664
Frequency
Tumor−Normal Expression Difference
hsa−miR−4461, rectal
(WINE_any = 1; N1 = 153)
1−sided adj pval: 0.99
Frequency
Tumor−Normal Expression Difference

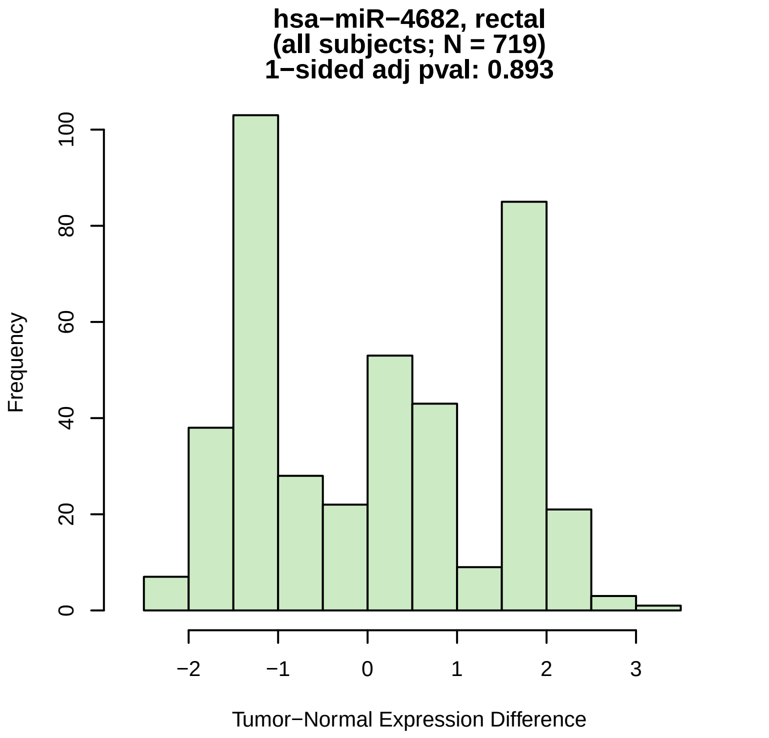
hsa−miR−4682, rectal
(all subjects; N = 719)
1−sided adj pval: 0.893
Frequency
Tumor−Normal Expression Difference

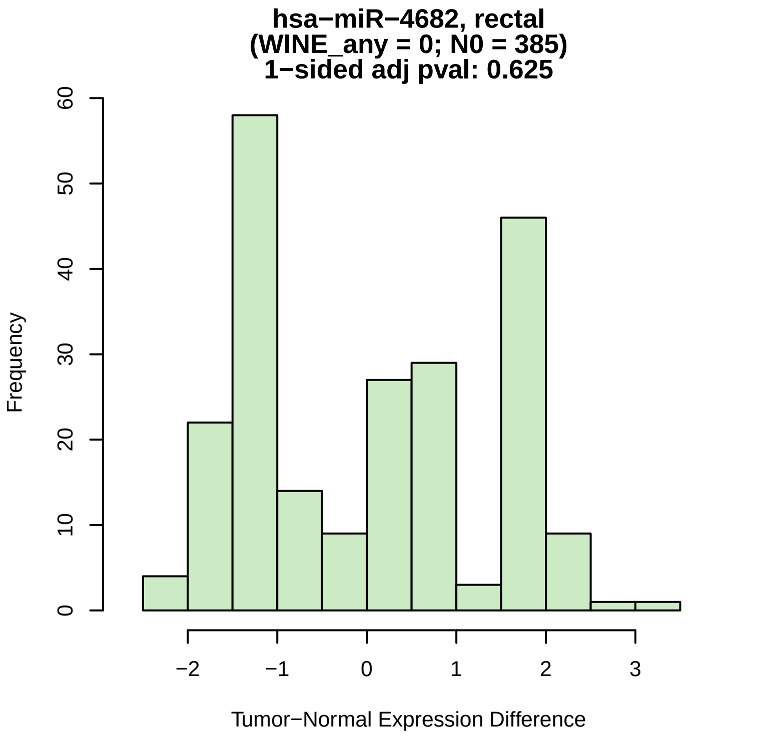
hsa−miR−4682, rectal
(WINE_any = 0; N0 = 385)
1−sided adj pval: 0.625
Frequency
Tumor−Normal Expression Difference

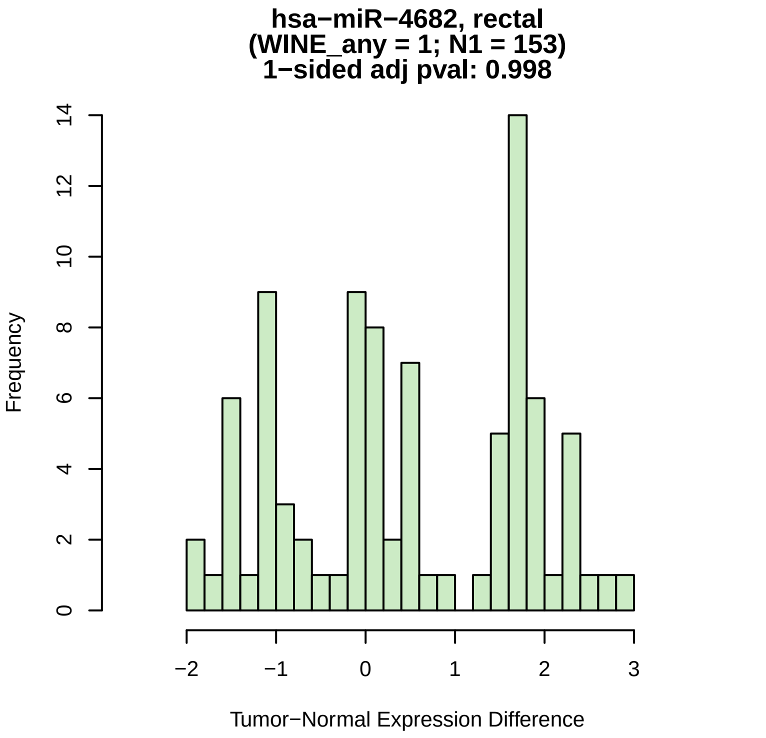
hsa−miR−4682, rectal
(WINE_any = 1; N1 = 153)
1−sided adj pval: 0.998
Frequency
Tumor−Normal Expression Difference

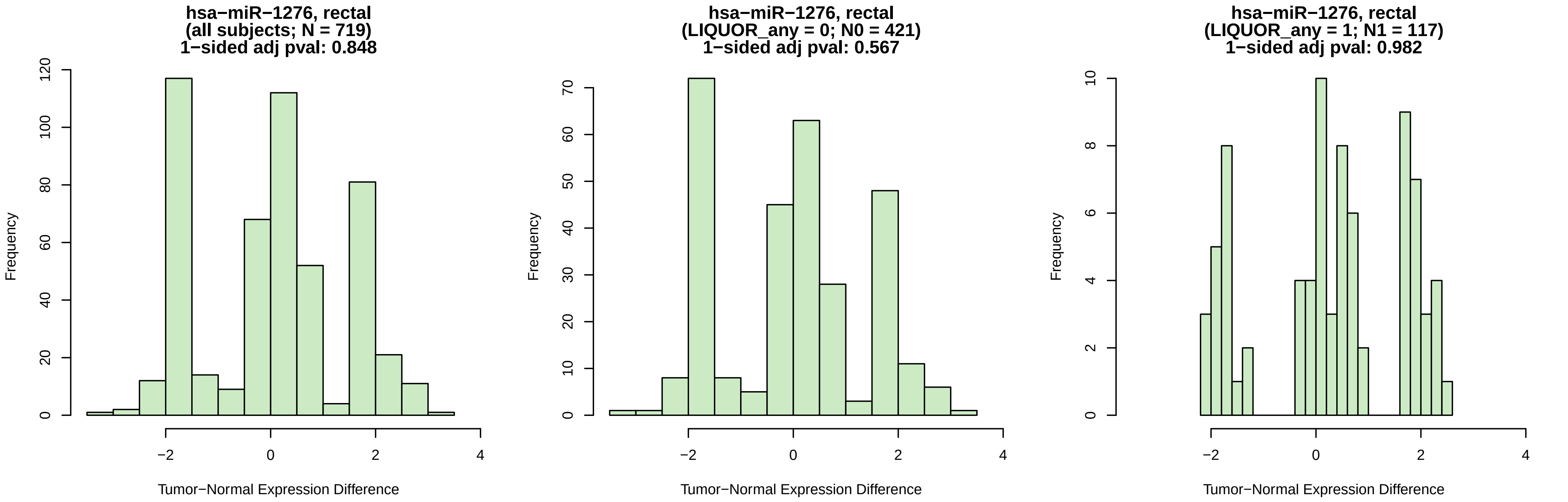
hsa−miR−1276, rectal
(all subjects; N = 719)
1−sided adj pval: 0.848
Frequency
Tumor−Normal Expression Difference
hsa−miR−1276, rectal
(LIQUOR_any = 0; N0 = 421)
1−sided adj pval: 0.567
Frequency
Tumor−Normal Expression Difference
hsa−miR−1276, rectal
(LIQUOR_any = 1; N1 = 117)
1−sided adj pval: 0.982
Frequency
Tumor−Normal Expression Difference

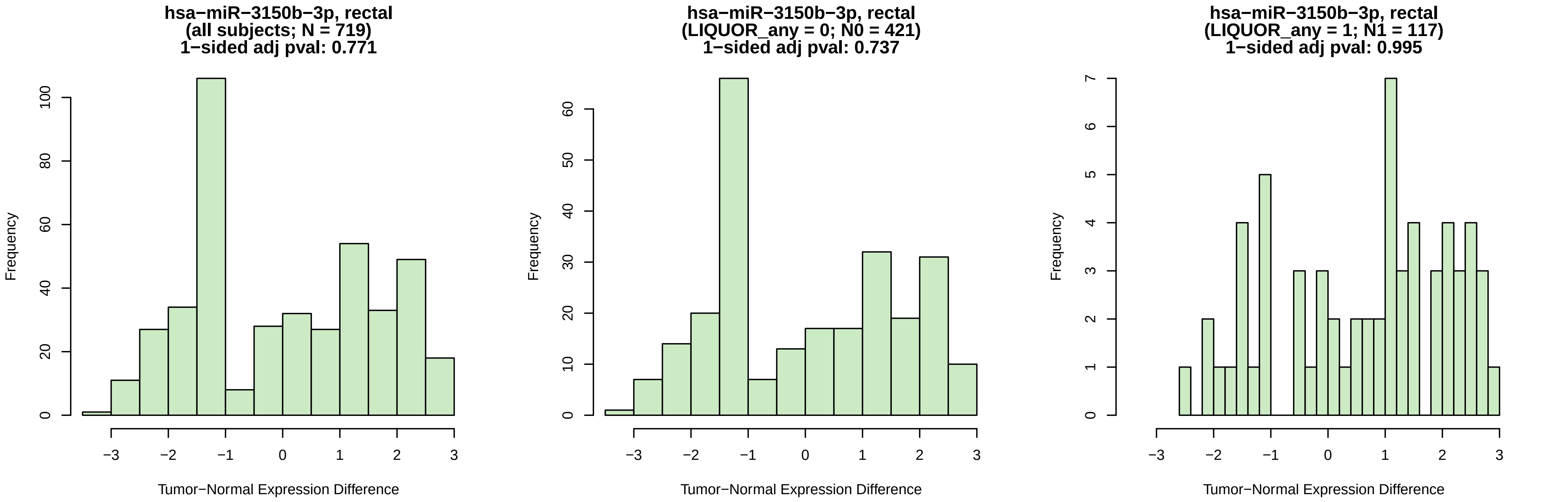

hsa−miR−3150b−3p, rectal
(all subjects; N = 719)
1−sided adj pval: 0.771
Frequency
0
20
40
60
80
100
−3
−2
−1
0
1
2
3
Tumor−Normal Expression Difference
hsa−miR−3150b−3p, rectal
(LIQUOR_any = 0; N0 = 421)
1−sided adj pval: 0.737
Frequency
0
10
20
30
40
50
60
−3
−2
−1
0
1
2
3
Tumor−Normal Expression Difference
hsa−miR−3150b−3p, rectal
(LIQUOR_any = 1; N1 = 117)
1−sided adj pval: 0.995
Frequency
0
1
2
3
4
5
6
7
−3
−2
−1
0
1
2
3
Tumor−Normal Expression Difference

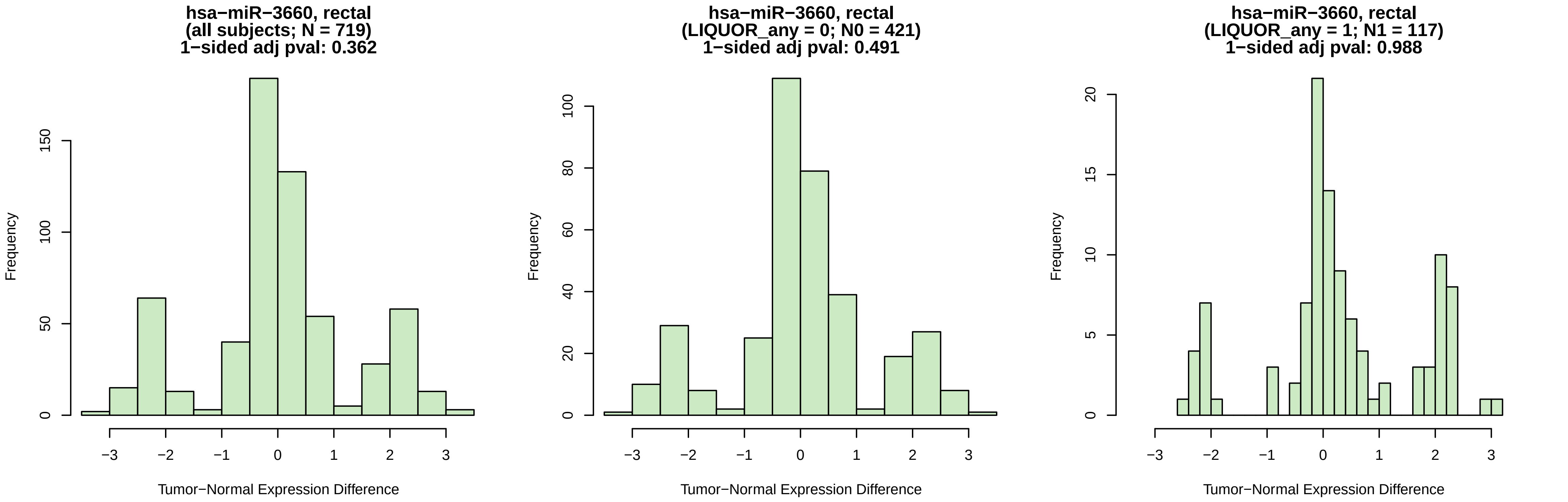

hsa−miR−3660, rectal
(all subjects; N = 719)
1−sided adj pval: 0.362
Frequency
0
50
100
150
−3
−2
−1
0
1
2
3
Tumor−Normal Expression Difference
hsa−miR−3660, rectal
(LIQUOR_any = 0; N0 = 421)
1−sided adj pval: 0.491
Frequency
0
20
40
60
80
100
−3
−2
−1
0
1
2
3
Tumor−Normal Expression Difference
hsa−miR−3660, rectal
(LIQUOR_any = 1; N1 = 117)
1−sided adj pval: 0.988
Frequency
0
5
10
15
20
−3
−2
−1
0
1
2
3
Tumor−Normal Expression Difference

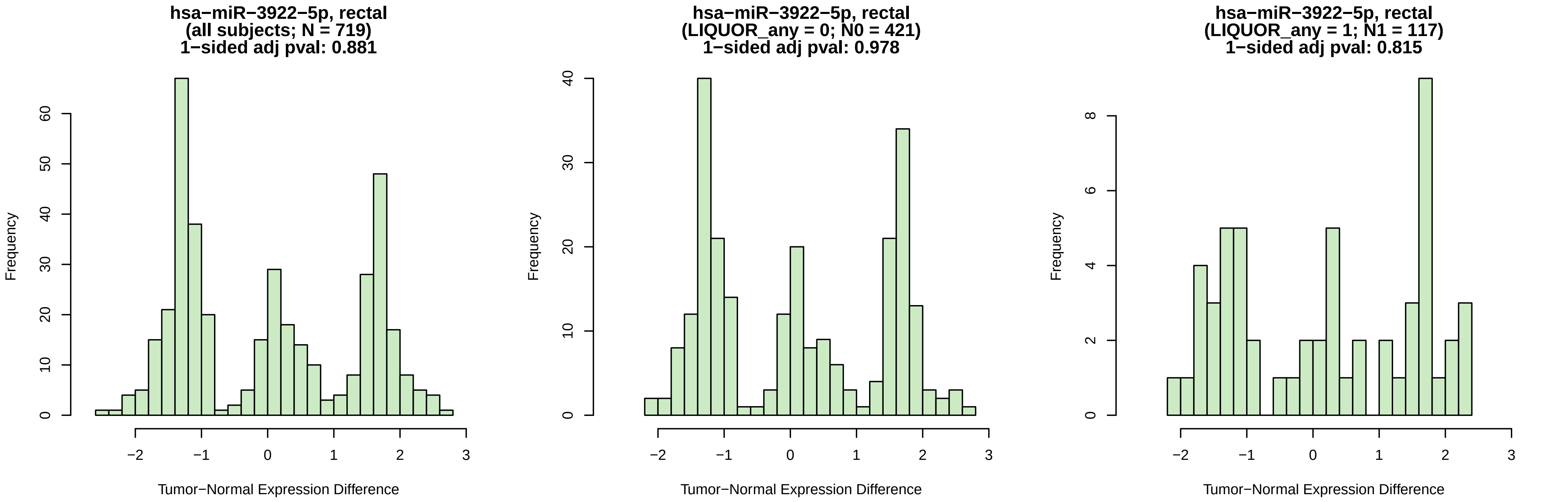

hsa−miR−3922−5p, rectal
(all subjects; N = 719)
1−sided adj pval: 0.881
hsa−miR−3922−5p, rectal
(LIQUOR_any = 0; N0 = 421)
1−sided adj pval: 0.978
hsa−miR−3922−5p, rectal
(LIQUOR_any = 1; N1 = 117)
1−sided adj pval: 0.815
Frequency
Tumor−Normal Expression Difference

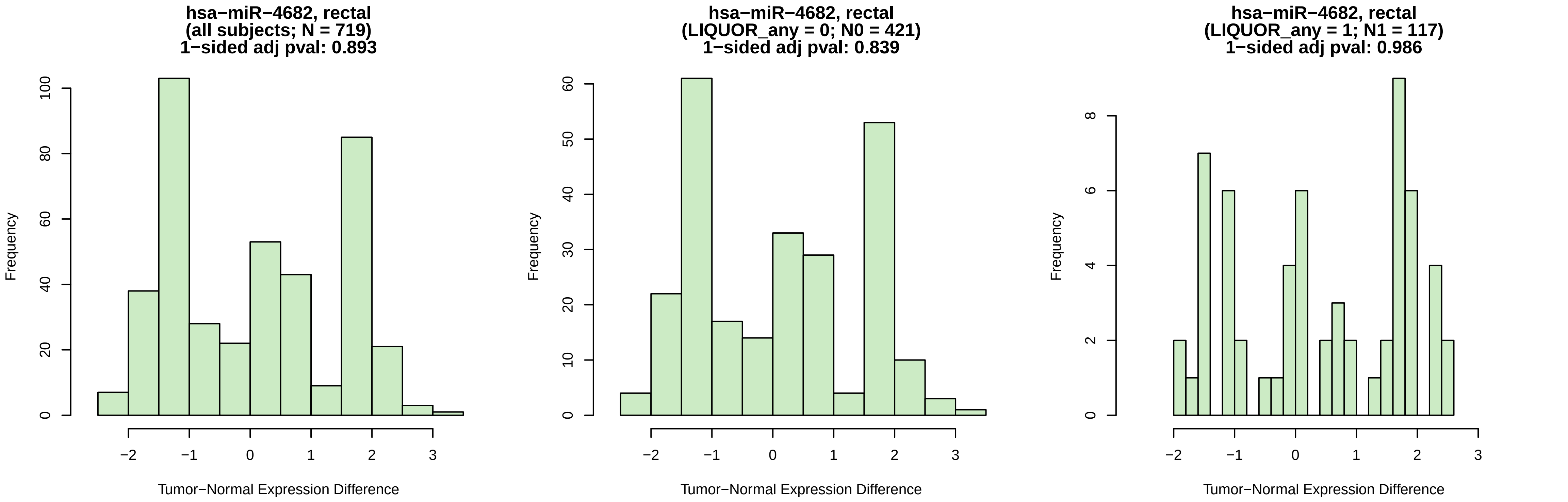

hsa−miR−4682, rectal
(all subjects; N = 719)
1−sided adj pval: 0.893
Frequency
Tumor−Normal Expression Difference
hsa−miR−4682, rectal
(LIQUOR_any = 0; N0 = 421)
1−sided adj pval: 0.839
Frequency
Tumor−Normal Expression Difference
hsa−miR−4682, rectal
(LIQUOR_any = 1; N1 = 117)
1−sided adj pval: 0.986
Frequency
Tumor−Normal Expression Difference

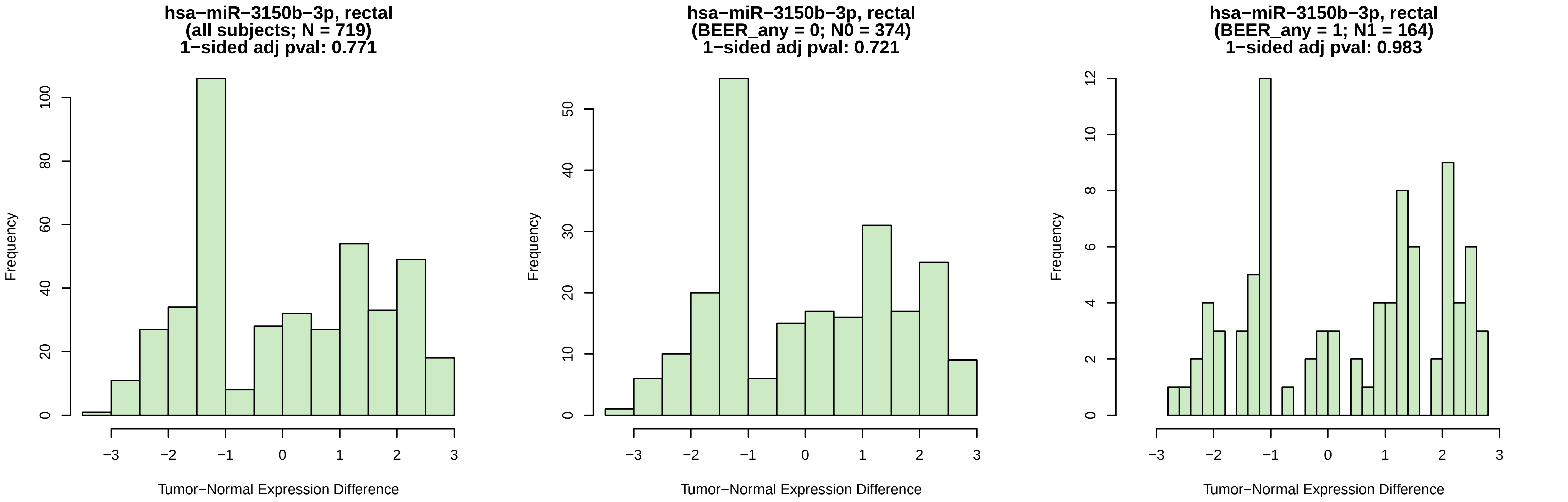
hsa−miR−3150b−3p, rectal
(all subjects; N = 719)
1−sided adj pval: 0.771
Frequency
Tumor−Normal Expression Difference
hsa−miR−3150b−3p, rectal
(BEER_any = 0; N0 = 374)
1−sided adj pval: 0.721
Frequency
Tumor−Normal Expression Difference
hsa−miR−3150b−3p, rectal
(BEER_any = 1; N1 = 164)
1−sided adj pval: 0.983
Frequency
Tumor−Normal Expression Difference

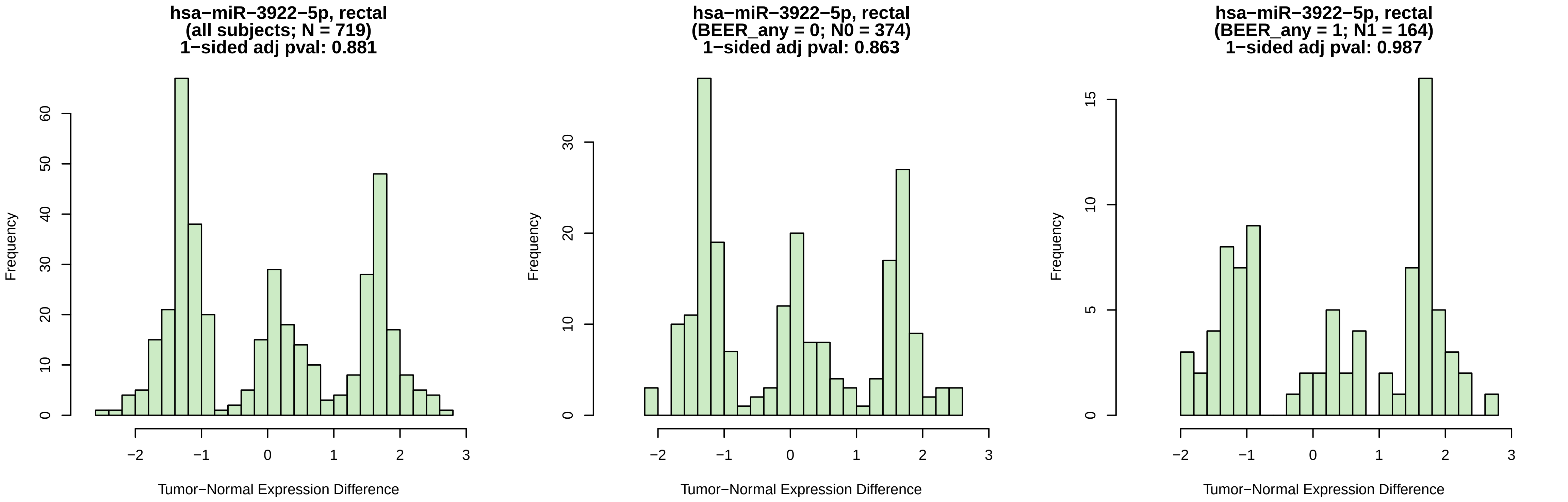
hsa−miR−3922−5p, rectal
(all subjects; N = 719)
1−sided adj pval: 0.881
Frequency
Tumor−Normal Expression Difference
hsa−miR−3922−5p, rectal
(BEER_any = 0; N0 = 374)
1−sided adj pval: 0.863
Frequency
Tumor−Normal Expression Difference
hsa−miR−3922−5p, rectal
(BEER_any = 1; N1 = 164)
1−sided adj pval: 0.987
Frequency
Tumor−Normal Expression Difference

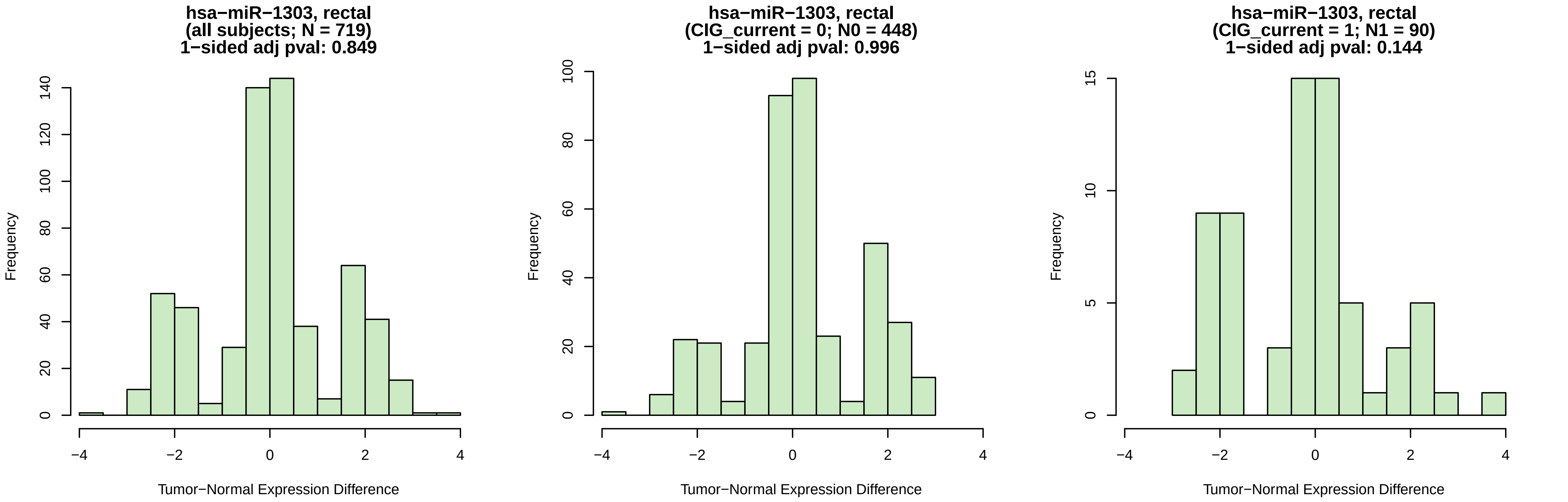

hsa−miR−1303, rectal
(all subjects; N = 719)
1−sided adj pval: 0.849
Frequency
Tumor−Normal Expression Difference
hsa−miR−1303, rectal
(CIG_current = 0; N0 = 448)
1−sided adj pval: 0.996
Frequency
Tumor−Normal Expression Difference
hsa−miR−1303, rectal
(CIG_current = 1; N1 = 90)
1−sided adj pval: 0.144
Frequency
Tumor−Normal Expression Difference

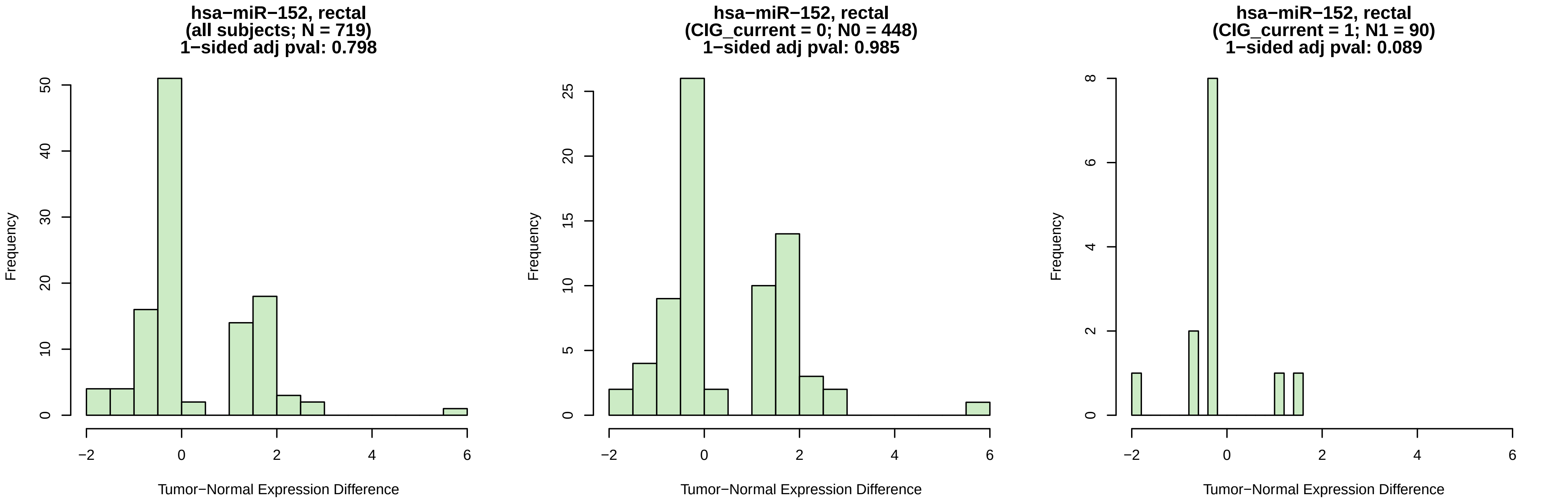

hsa−miR−152, rectal
(all subjects; N = 719)
1−sided adj pval: 0.798
Frequency
Tumor−Normal Expression Difference
hsa−miR−152, rectal
(CIG_current = 0; N0 = 448)
1−sided adj pval: 0.985
Frequency
Tumor−Normal Expression Difference
hsa−miR−152, rectal
(CIG_current = 1; N1 = 90)
1−sided adj pval: 0.089
Frequency
Tumor−Normal Expression Difference

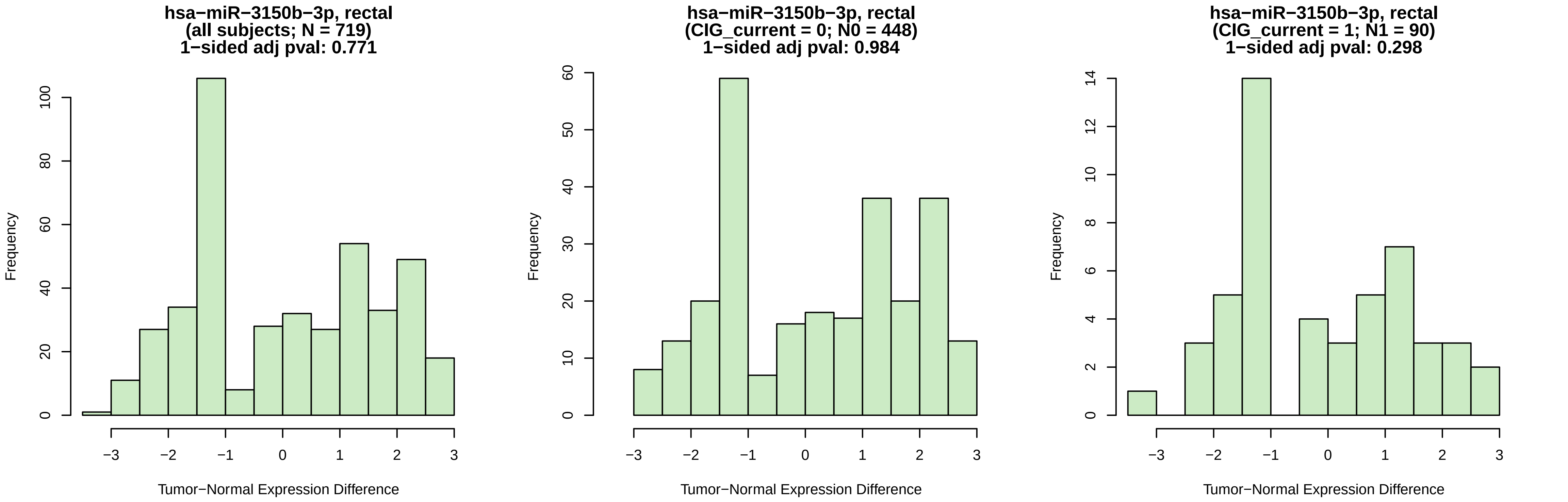
hsa−miR−3150b−3p, rectal
(all subjects; N = 719)
1−sided adj pval: 0.771
Frequency
Tumor−Normal Expression Difference
hsa−miR−3150b−3p, rectal
(CIG_current = 0; N0 = 448)
1−sided adj pval: 0.984
Frequency
Tumor−Normal Expression Difference
hsa−miR−3150b−3p, rectal
(CIG_current = 1; N1 = 90)
1−sided adj pval: 0.298
Frequency
Tumor−Normal Expression Difference

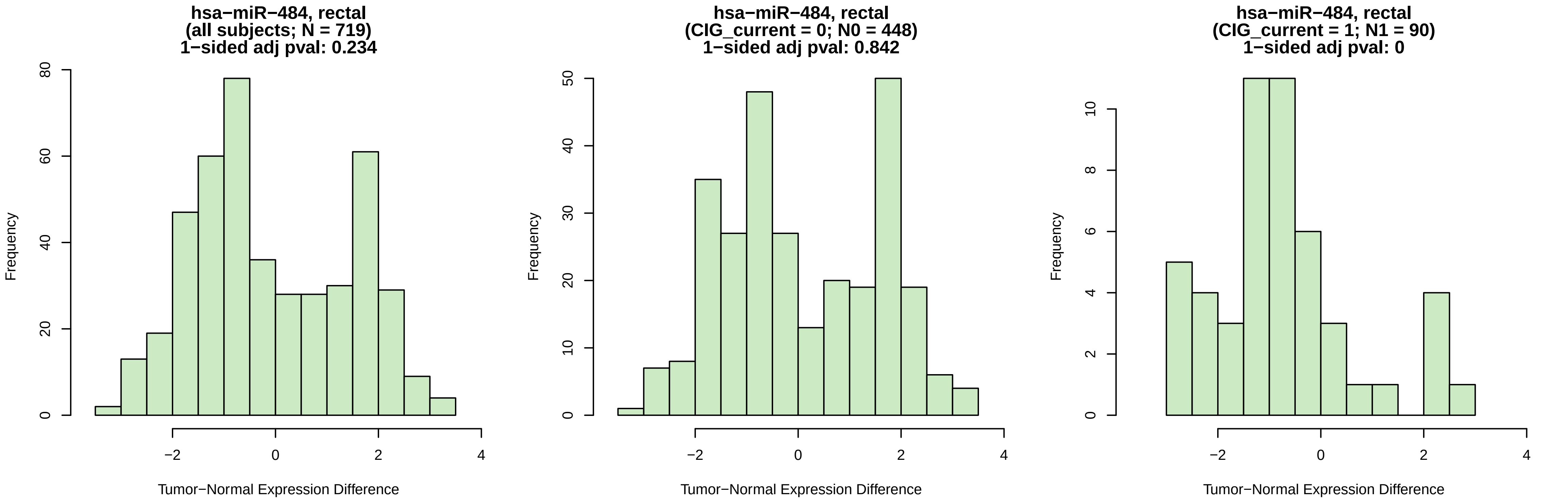
hsa−miR−484, rectal
(all subjects; N = 719)
1−sided adj pval: 0.234
Frequency
Tumor−Normal Expression Difference
hsa−miR−484, rectal
(CIG_current = 0; N0 = 448)
1−sided adj pval: 0.842
Frequency
Tumor−Normal Expression Difference
hsa−miR−484, rectal
(CIG_current = 1; N1 = 90)
1−sided adj pval: 0
Frequency
Tumor−Normal Expression Difference

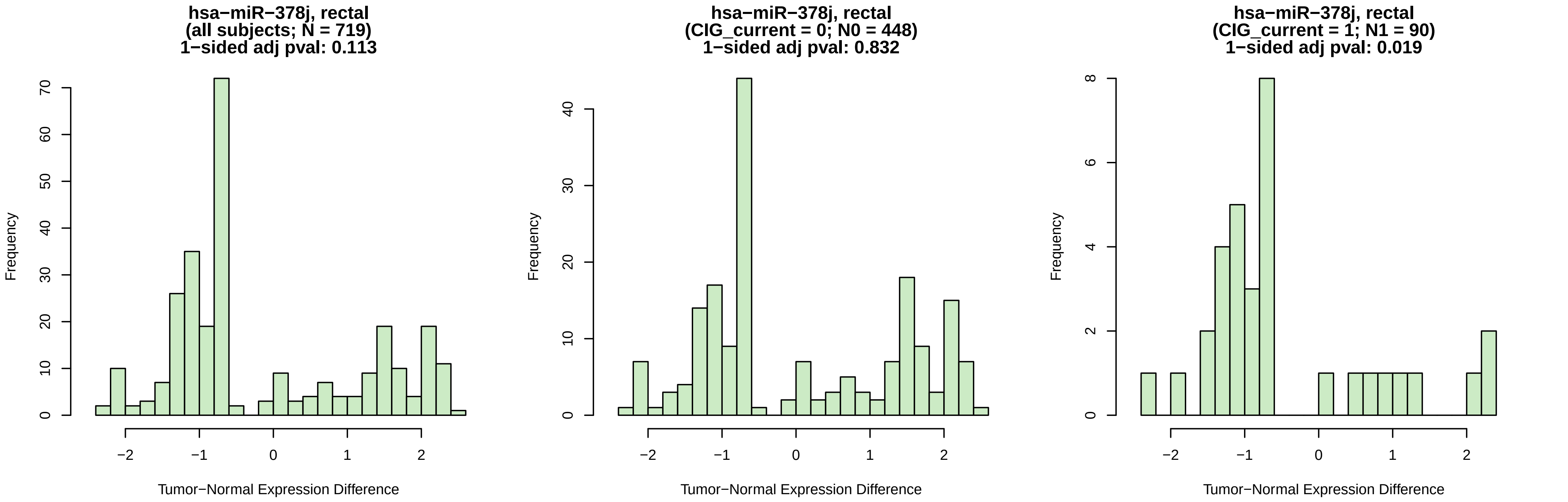
hsa−miR−378j, rectal
(all subjects; N = 719)
1−sided adj pval: 0.113
Frequency
Tumor−Normal Expression Difference
hsa−miR−378j, rectal
(CIG_current = 0; N0 = 448)
1−sided adj pval: 0.832
Frequency
Tumor−Normal Expression Difference
hsa−miR−378j, rectal
(CIG_current = 1; N1 = 90)
1−sided adj pval: 0.019
Frequency
Tumor−Normal Expression Difference

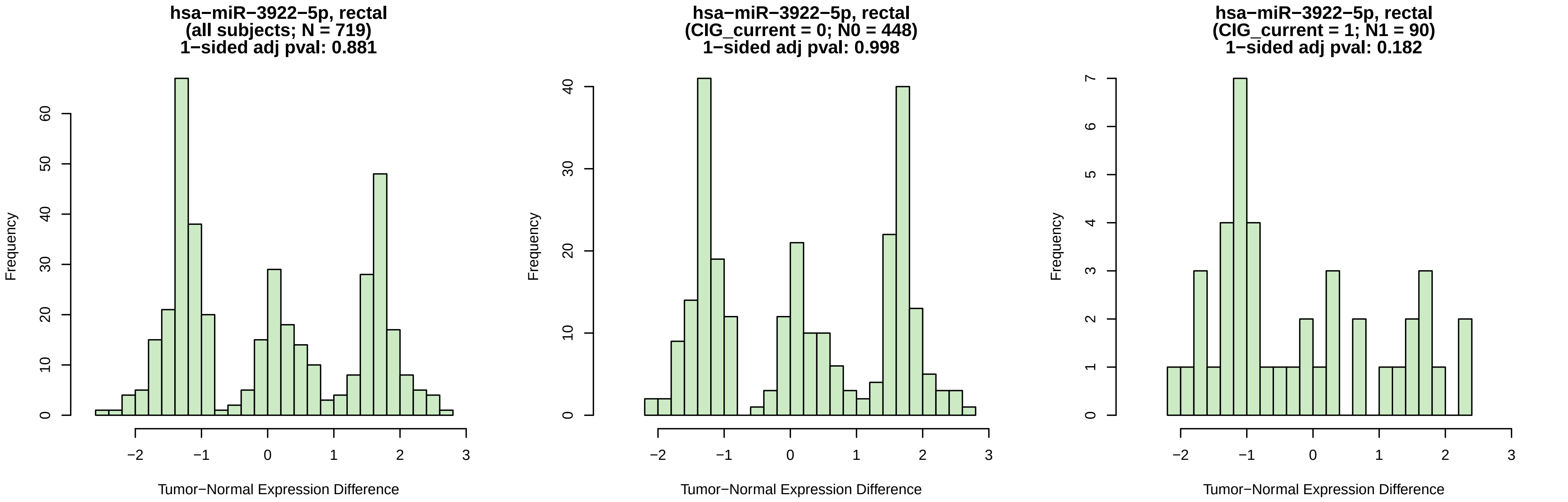
hsa−miR−3922−5p, rectal
(all subjects; N = 719)
1−sided adj pval: 0.881
hsa−miR−3922−5p, rectal
(CIG_current = 0; N0 = 448)
1−sided adj pval: 0.998
hsa−miR−3922−5p, rectal
(CIG_current = 1; N1 = 90)
1−sided adj pval: 0.182
Frequency
Tumor−Normal Expression Difference

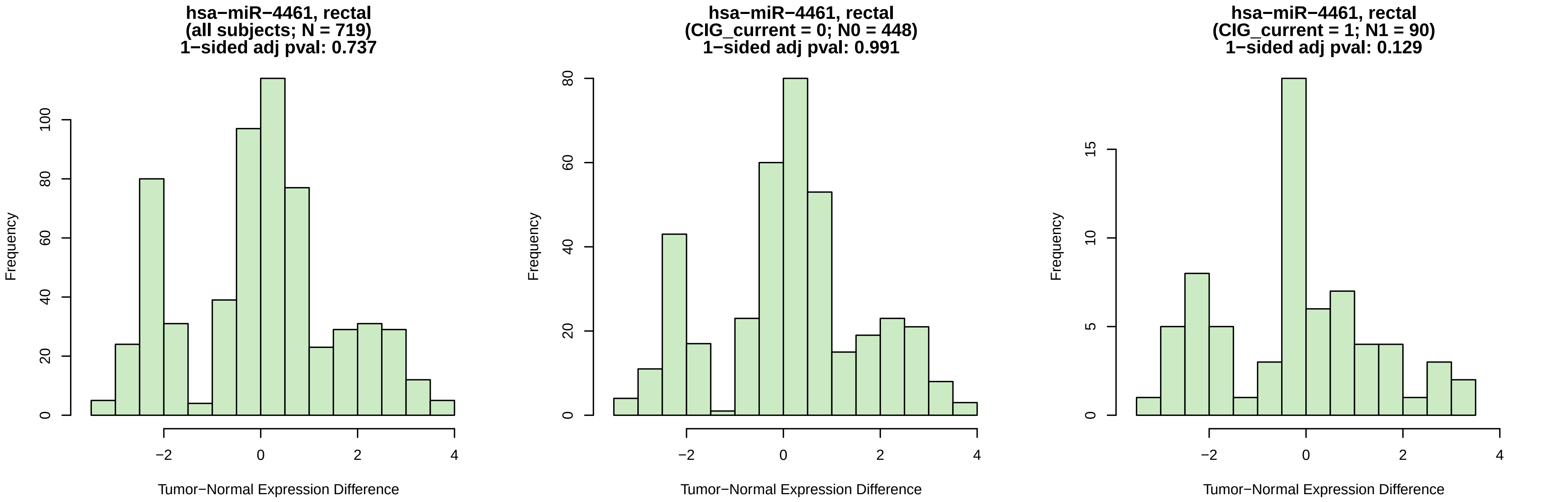
hsa−miR−4461, rectal
(all subjects; N = 719)
1−sided adj pval: 0.737
Frequency
Tumor−Normal Expression Difference
hsa−miR−4461, rectal
(CIG_current = 0; N0 = 448)
1−sided adj pval: 0.991
Frequency
Tumor−Normal Expression Difference
hsa−miR−4461, rectal
(CIG_current = 1; N1 = 90)
1−sided adj pval: 0.129
Frequency
Tumor−Normal Expression Difference

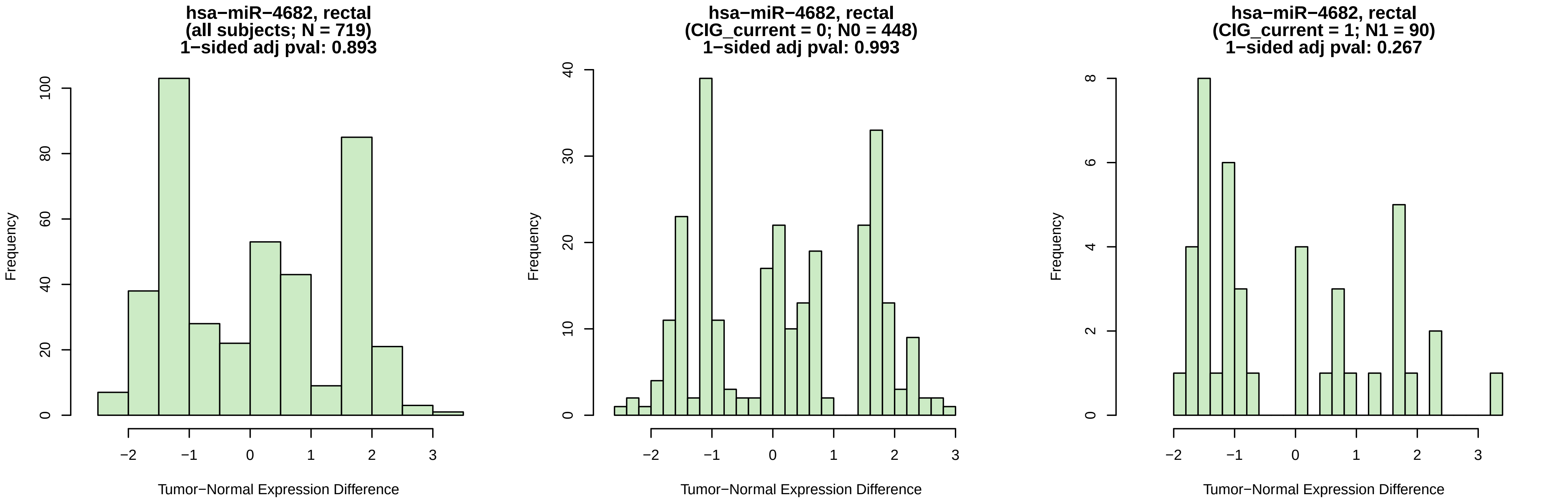

hsa−miR−4682, rectal
(all subjects; N = 719)
1−sided adj pval: 0.893
Frequency
Tumor−Normal Expression Difference
hsa−miR−4682, rectal
(CIG_current = 0; N0 = 448)
1−sided adj pval: 0.993
Frequency
Tumor−Normal Expression Difference
hsa−miR−4682, rectal
(CIG_current = 1; N1 = 90)
1−sided adj pval: 0.267
Frequency
Tumor−Normal Expression Difference

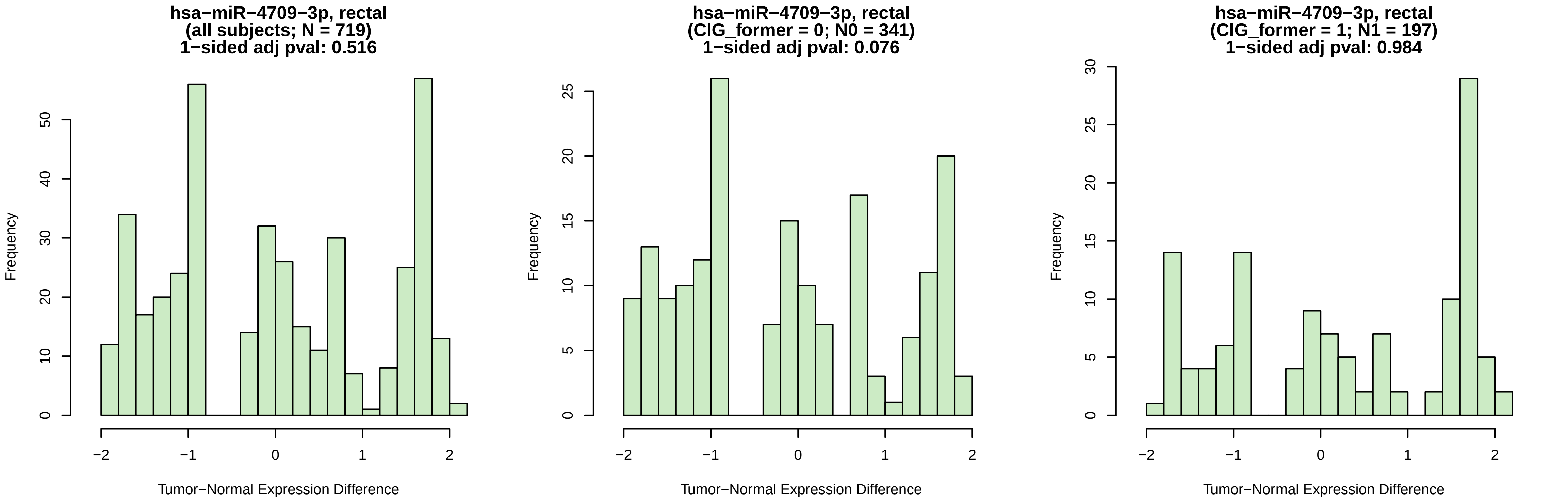

hsa−miR−4709−3p, rectal
(all subjects; N = 719)
1−sided adj pval: 0.516
Frequency
Tumor−Normal Expression Difference
hsa−miR−4709−3p, rectal
(CIG_former = 0; N0 = 341)
1−sided adj pval: 0.076
Frequency
Tumor−Normal Expression Difference
hsa−miR−4709−3p, rectal
(CIG_former = 1; N1 = 197)
1−sided adj pval: 0.984
Frequency
Tumor−Normal Expression Difference

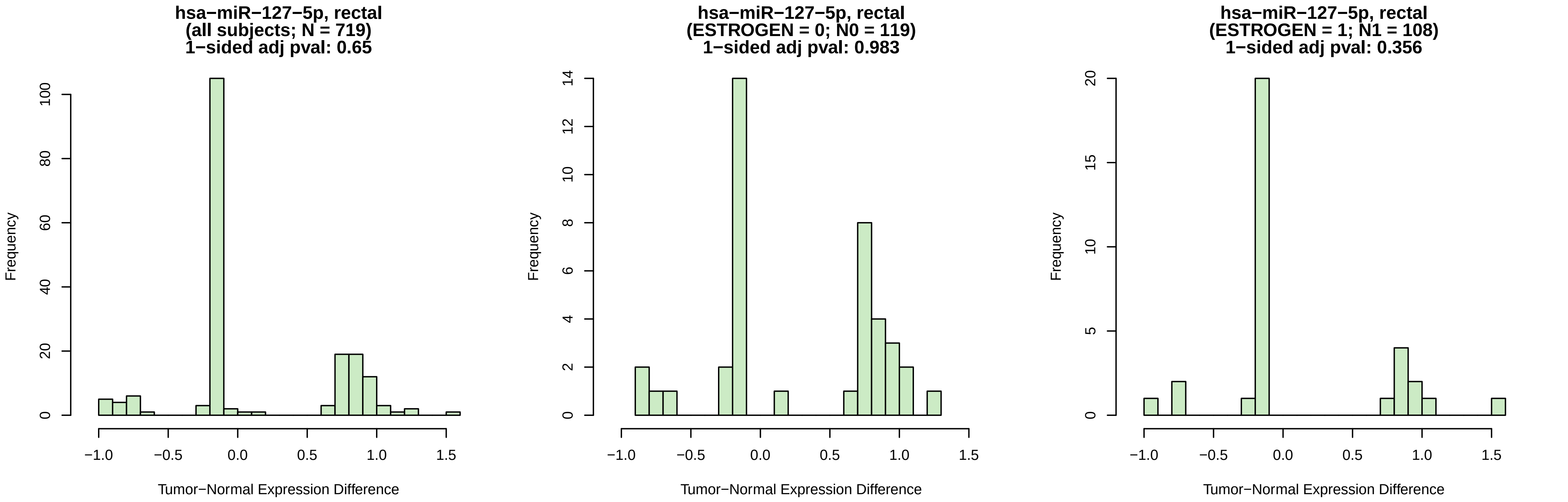

hsa−miR−127−5p, rectal
(all subjects; N = 719)
1−sided adj pval: 0.65
Frequency
Tumor−Normal Expression Difference
hsa−miR−127−5p, rectal
(ESTROGEN = 0; N0 = 119)
1−sided adj pval: 0.983
Frequency
Tumor−Normal Expression Difference
hsa−miR−127−5p, rectal
(ESTROGEN = 1; N1 = 108)
1−sided adj pval: 0.356
Frequency
Tumor−Normal Expression Difference

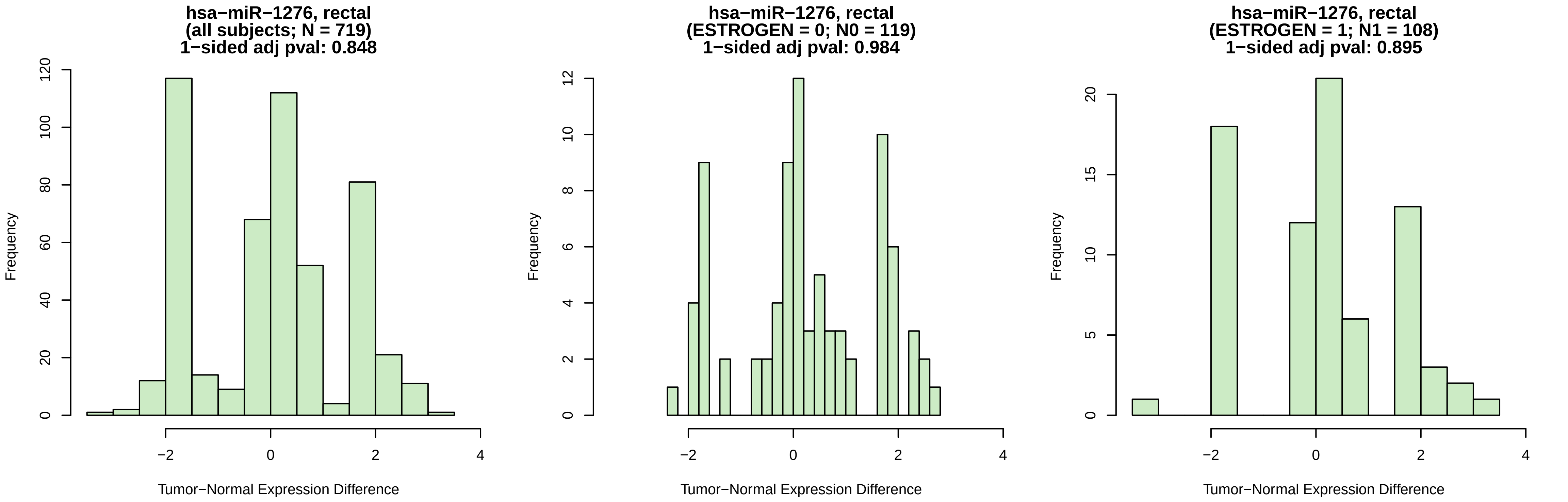
hsa−miR−1276, rectal
(all subjects; N = 719)
1−sided adj pval: 0.848
Frequency
Tumor−Normal Expression Difference
hsa−miR−1276, rectal
(ESTROGEN = 0; N0 = 119)
1−sided adj pval: 0.984
Frequency
Tumor−Normal Expression Difference
hsa−miR−1276, rectal
(ESTROGEN = 1; N1 = 108)
1−sided adj pval: 0.895
Frequency
Tumor−Normal Expression Difference

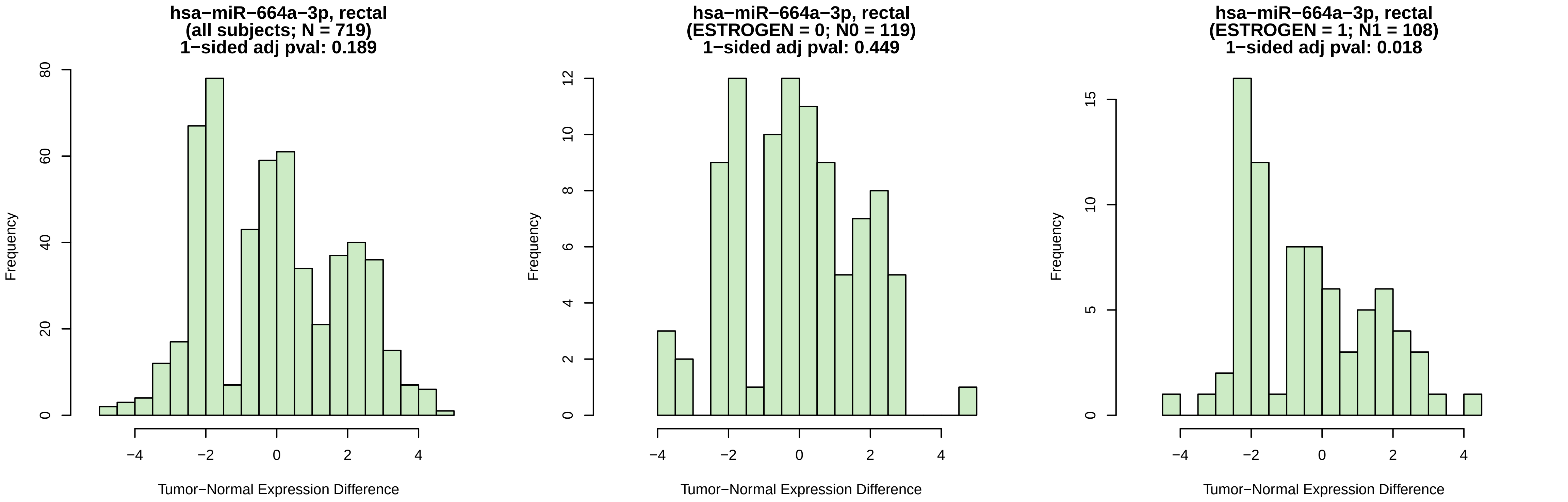

hsa−miR−664a−3p, rectal
(all subjects; N = 719)
1−sided adj pval: 0.189
Frequency
Tumor−Normal Expression Difference
hsa−miR−664a−3p, rectal
(ESTROGEN = 0; N0 = 119)
1−sided adj pval: 0.449
Frequency
Tumor−Normal Expression Difference
hsa−miR−664a−3p, rectal
(ESTROGEN = 1; N1 = 108)
1−sided adj pval: 0.018
Frequency
Tumor−Normal Expression Difference

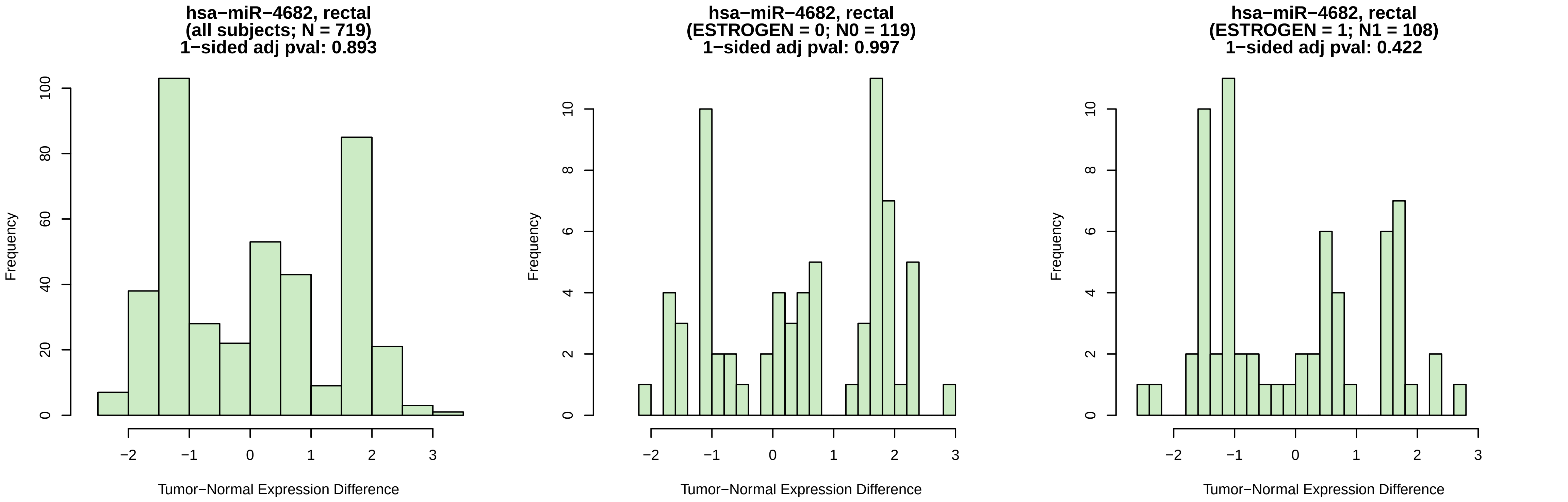

hsa−miR−4682, rectal
(all subjects; N = 719)
1−sided adj pval: 0.893
Frequency
Tumor−Normal Expression Difference
hsa−miR−4682, rectal
(ESTROGEN = 0; N0 = 119)
1−sided adj pval: 0.997
Frequency
Tumor−Normal Expression Difference
hsa−miR−4682, rectal
(ESTROGEN = 1; N1 = 108)
1−sided adj pval: 0.422
Frequency
Tumor−Normal Expression Difference

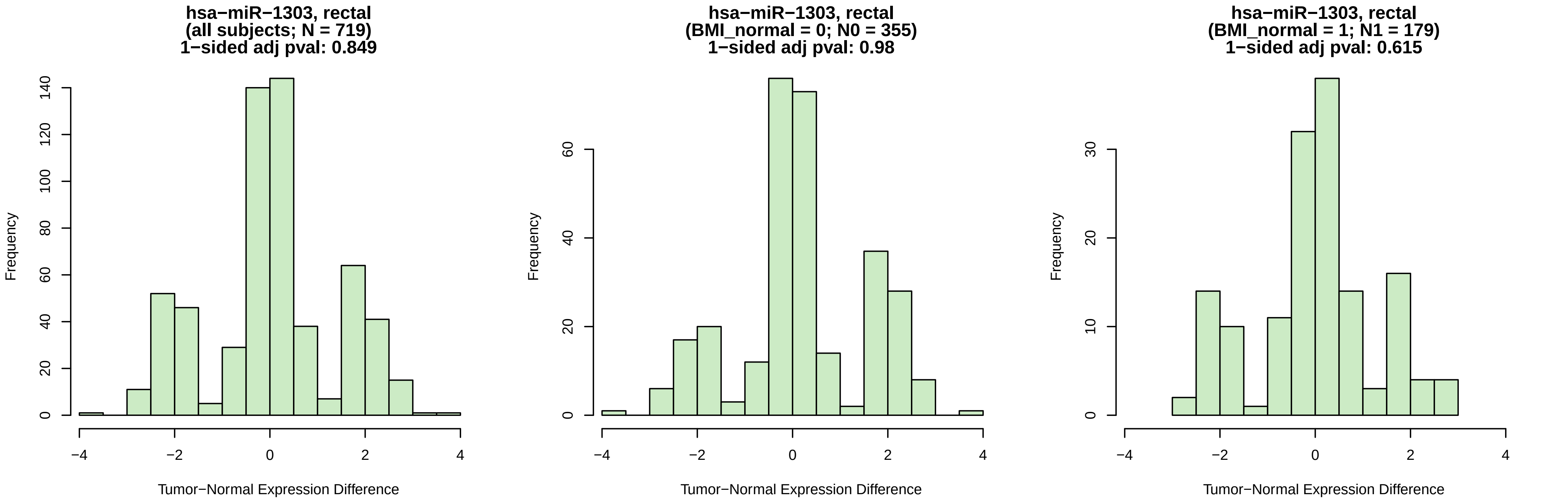
hsa−miR−1303, rectal
(all subjects; N = 719)
1−sided adj pval: 0.849
Frequency
Tumor−Normal Expression Difference
hsa−miR−1303, rectal
(BMI_normal = 0; N0 = 355)
1−sided adj pval: 0.98
Frequency
Tumor−Normal Expression Difference
hsa−miR−1303, rectal
(BMI_normal = 1; N1 = 179)
1−sided adj pval: 0.615
Frequency
Tumor−Normal Expression Difference

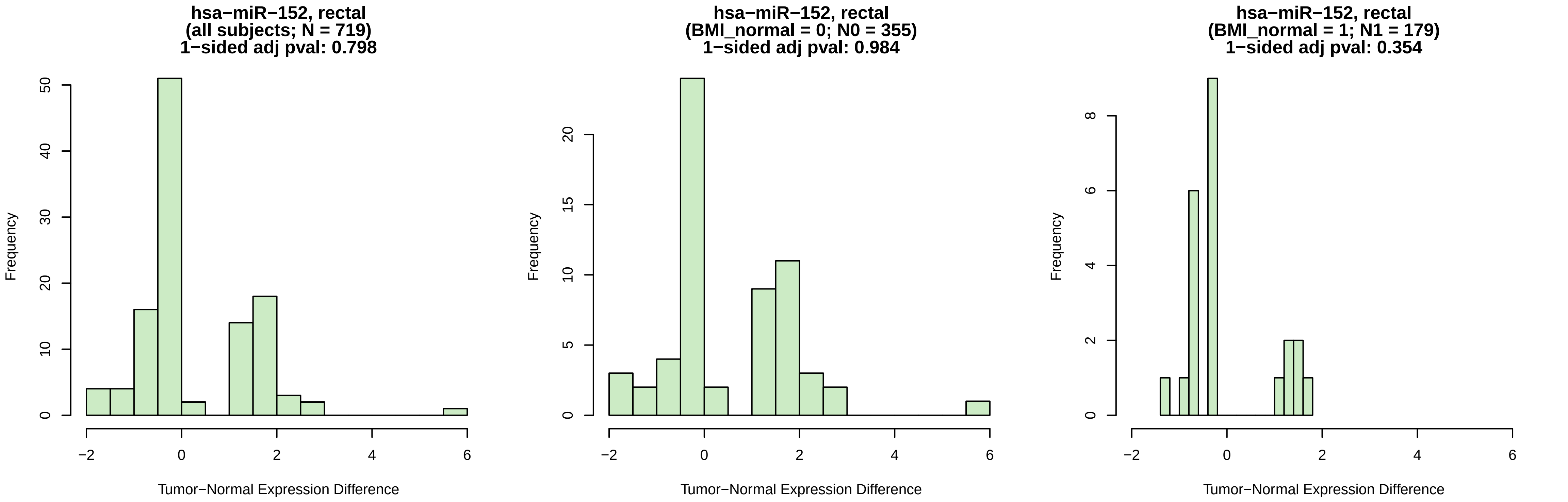
hsa−miR−152, rectal
(all subjects; N = 719)
1−sided adj pval: 0.798
Frequency
Tumor−Normal Expression Difference
hsa−miR−152, rectal
(BMI_normal = 0; N0 = 355)
1−sided adj pval: 0.984
Frequency
Tumor−Normal Expression Difference
hsa−miR−152, rectal
(BMI_normal = 1; N1 = 179)
1−sided adj pval: 0.354
Frequency
Tumor−Normal Expression Difference

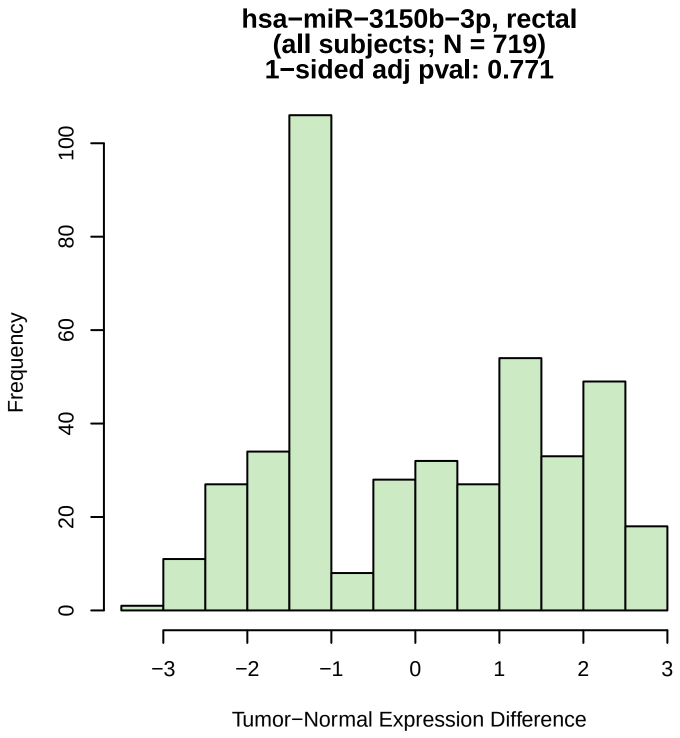
hsa−miR−3150b−3p, rectal
(all subjects; N = 719)
1−sided adj pval: 0.771
Frequency
Tumor−Normal Expression Difference

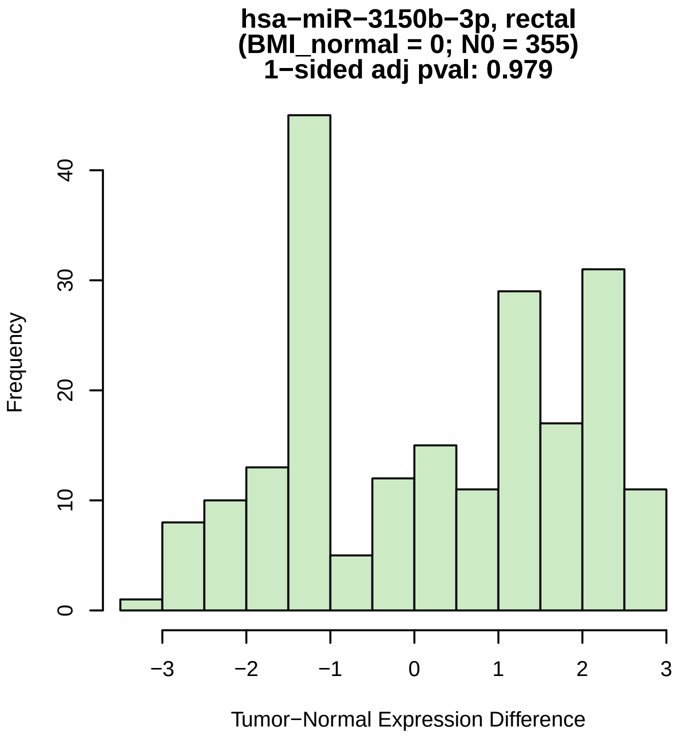
hsa−miR−3150b−3p, rectal
(BMI_normal = 0; N0 = 355)
1−sided adj pval: 0.979
Frequency
Tumor−Normal Expression Difference

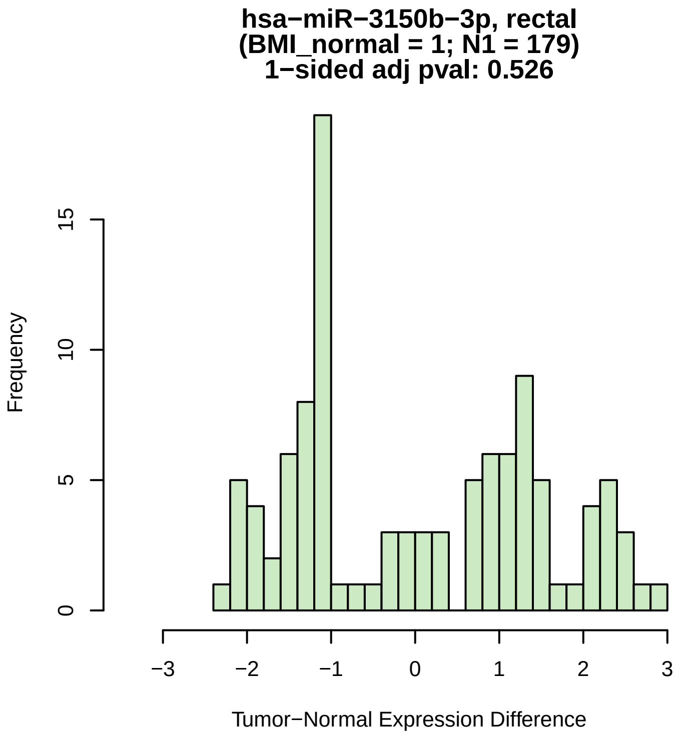
hsa−miR−3150b−3p, rectal
(BMI_normal = 1; N1 = 179)
1−sided adj pval: 0.526
Frequency
Tumor−Normal Expression Difference

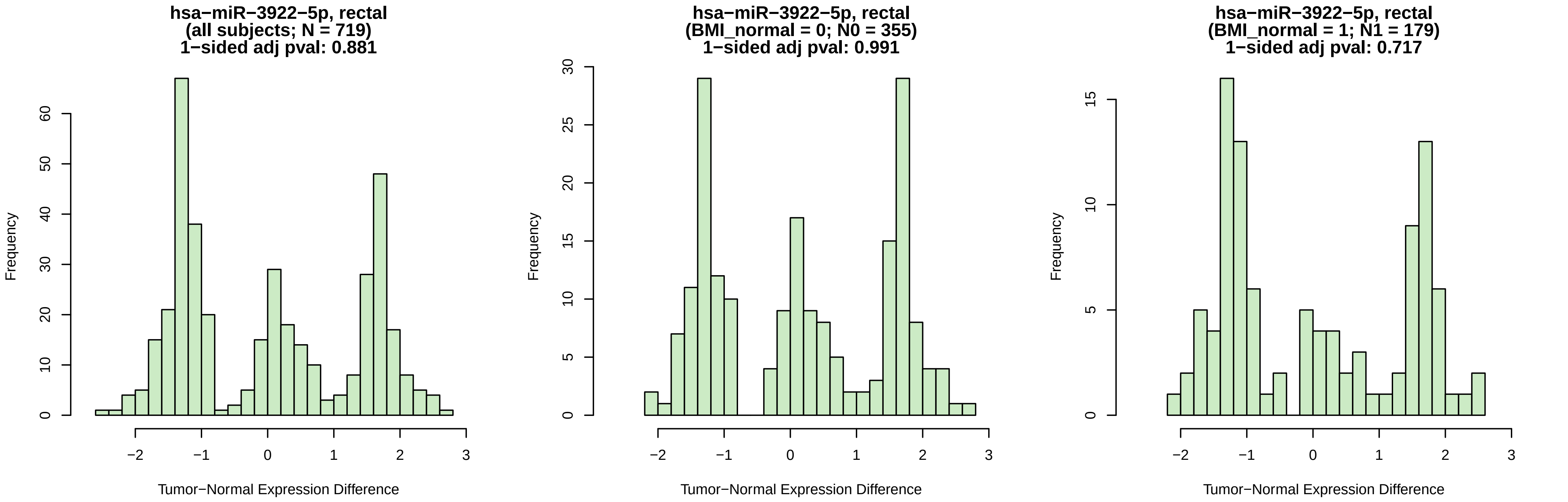
hsa−miR−3922−5p, rectal
(all subjects; N = 719)
1−sided adj pval: 0.881
Frequency
Tumor−Normal Expression Difference
hsa−miR−3922−5p, rectal
(BMI_normal = 0; N0 = 355)
1−sided adj pval: 0.991
Frequency
Tumor−Normal Expression Difference
hsa−miR−3922−5p, rectal
(BMI_normal = 1; N1 = 179)
1−sided adj pval: 0.717
Frequency
Tumor−Normal Expression Difference

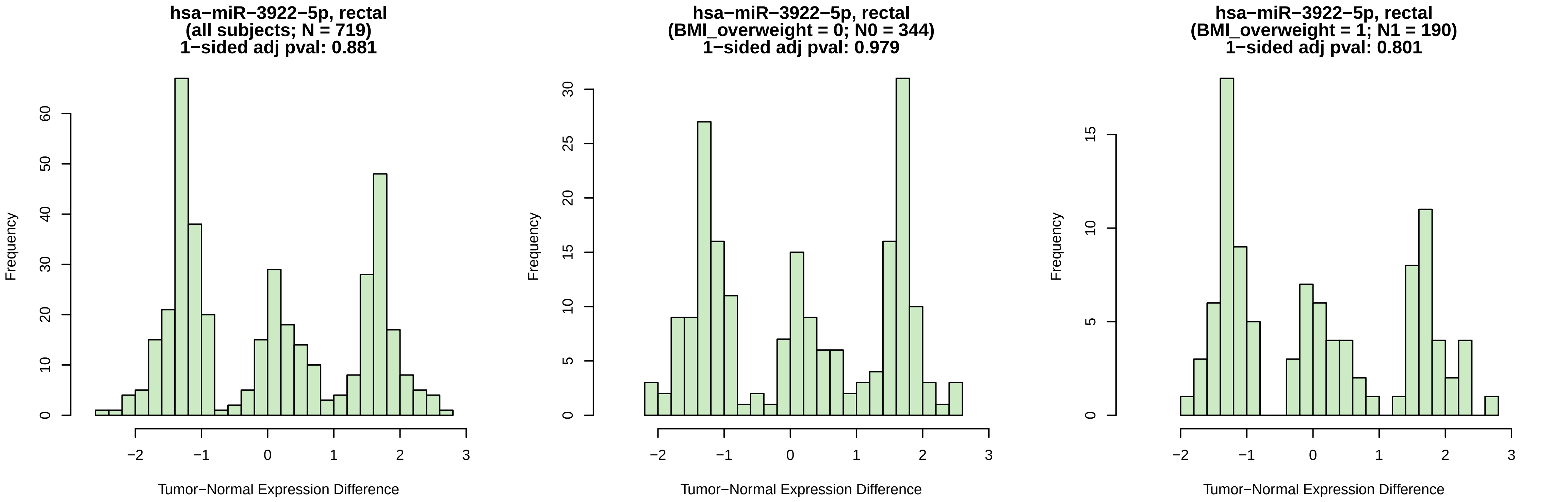

hsa−miR−3922−5p, rectal
(all subjects; N = 719)
1−sided adj pval: 0.881
Frequency
0
10
20
30
40
50
60
−2
−1
0
1
2
3
Tumor−Normal Expression Difference
hsa−miR−3922−5p, rectal
(BMI_overweight = 0; N0 = 344)
1−sided adj pval: 0.979
Frequency
0
5
10
15
20
25
30
−2
−1
0
1
2
3
Tumor−Normal Expression Difference
hsa−miR−3922−5p, rectal
(BMI_overweight = 1; N1 = 190)
1−sided adj pval: 0.801
Frequency
0
5
10
15
−2
−1
0
1
2
3
Tumor−Normal Expression Difference

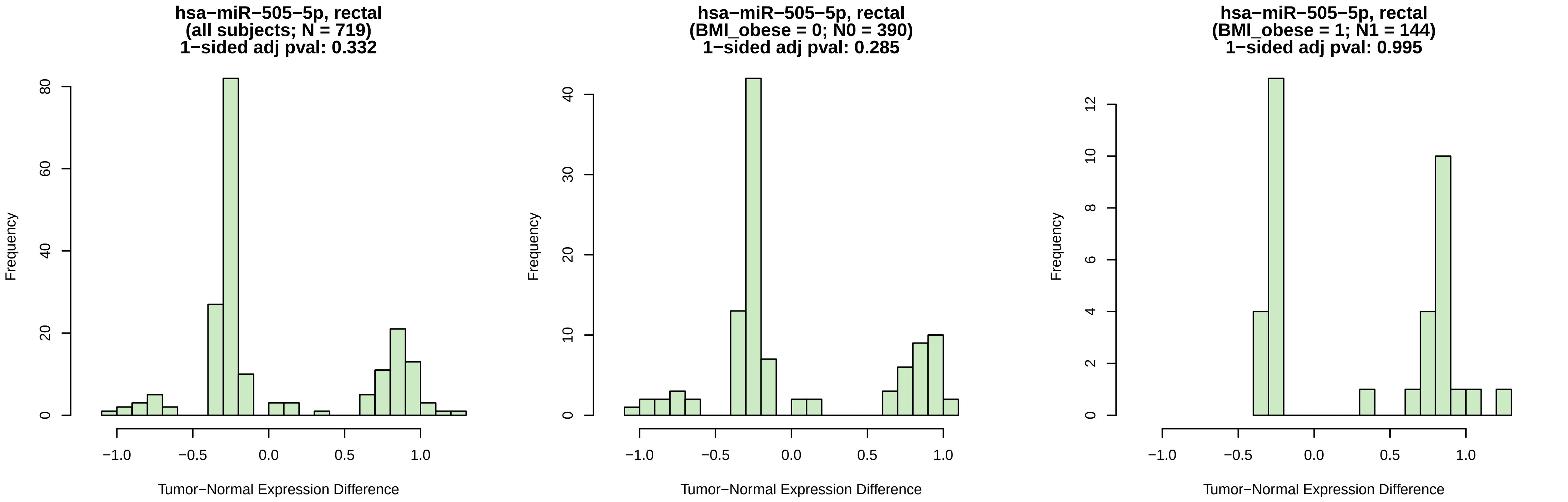
hsa−miR−505−5p, rectal
(all subjects; N = 719)
1−sided adj pval: 0.332
Frequency
Tumor−Normal Expression Difference
hsa−miR−505−5p, rectal
(BMI_obese = 0; N0 = 390)
1−sided adj pval: 0.285
Frequency
Tumor−Normal Expression Difference
hsa−miR−505−5p, rectal
(BMI_obese = 1; N1 = 144)
1−sided adj pval: 0.995
Frequency
Tumor−Normal Expression Difference

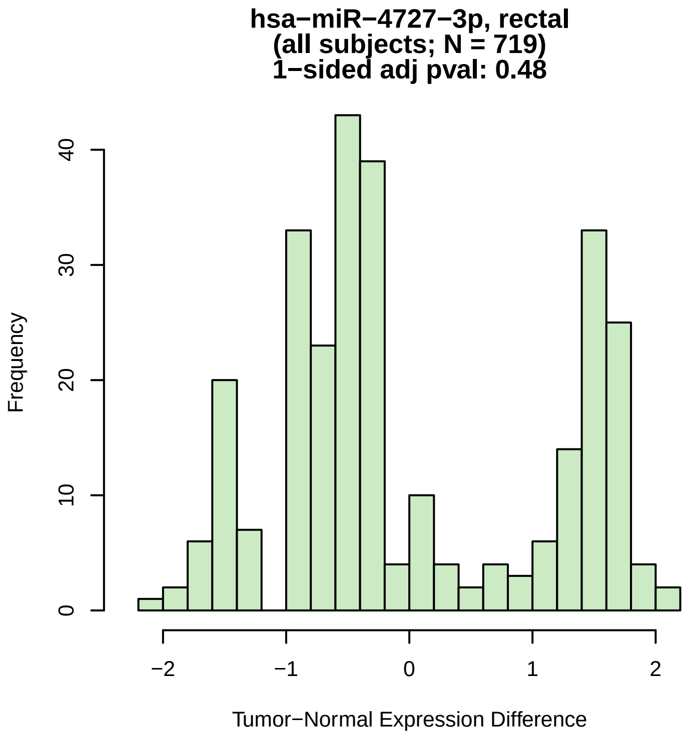
hsa−miR−4727−3p, rectal
(all subjects; N = 719)
1−sided adj pval: 0.48
Frequency
Tumor−Normal Expression Difference
0
10
20
30
40
−2
−1
0
1
2
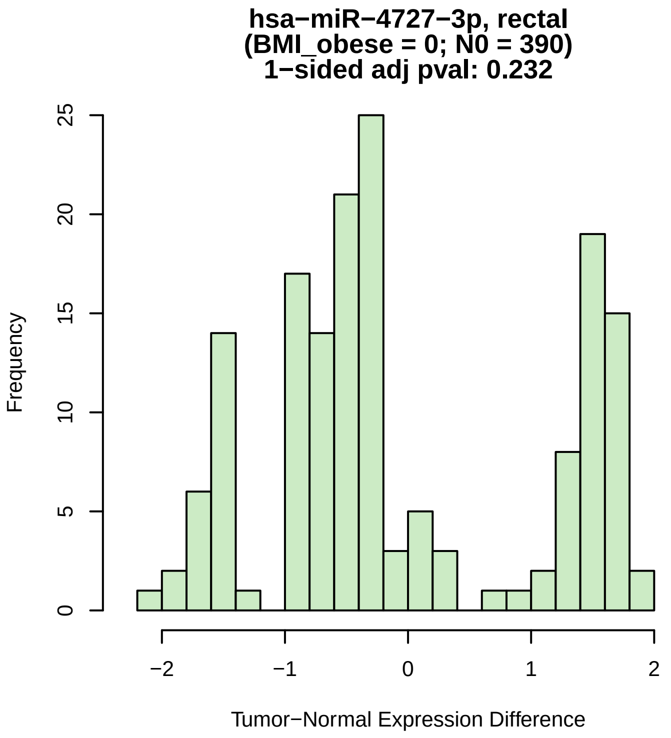
hsa−miR−4727−3p, rectal
(BMI_obese = 0; N0 = 390)
1−sided adj pval: 0.232
Frequency
Tumor−Normal Expression Difference
0
5
10
15
20
25
−2
−1
0
1
2
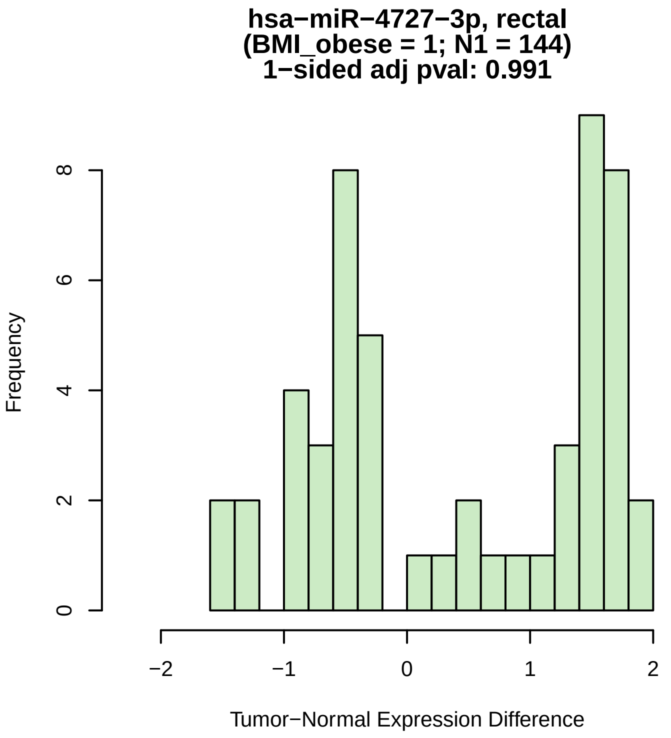
hsa−miR−4727−3p, rectal
(BMI_obese = 1; N1 = 144)
1−sided adj pval: 0.991
Frequency
Tumor−Normal Expression Difference
0
2
4
6
8
−2
−1
0
1
2

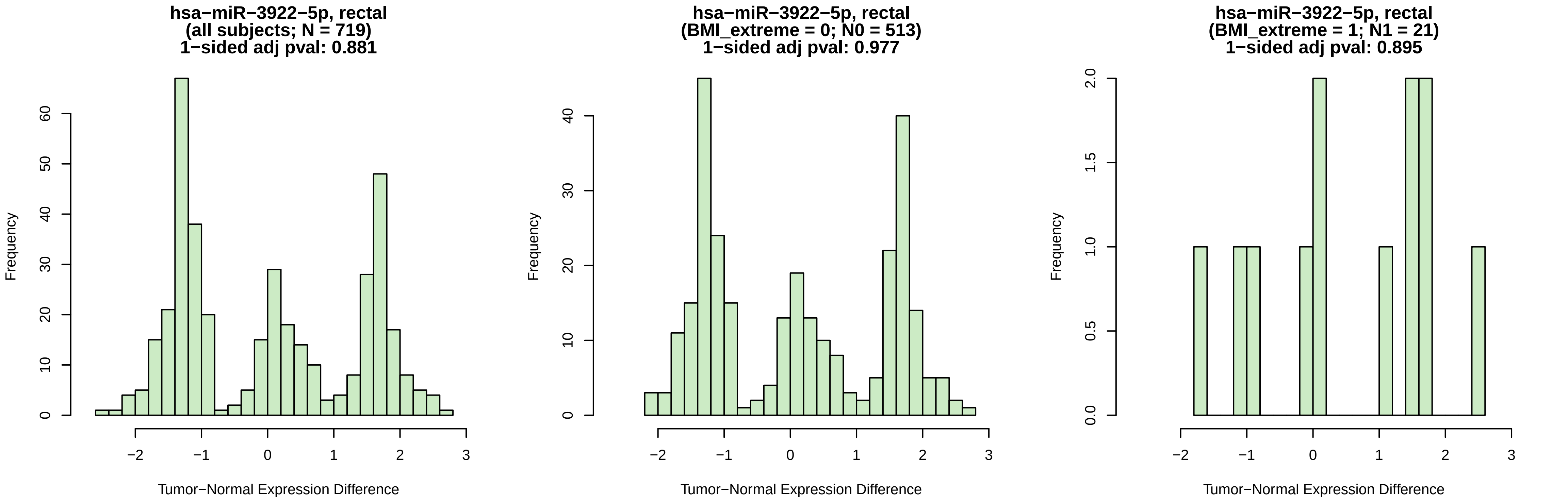

hsa−miR−3922−5p, rectal
(all subjects; N = 719)
1−sided adj pval: 0.881
Frequency
Tumor−Normal Expression Difference
hsa−miR−3922−5p, rectal
(BMI_extreme = 0; N0 = 513)
1−sided adj pval: 0.977
Frequency
Tumor−Normal Expression Difference
hsa−miR−3922−5p, rectal
(BMI_extreme = 1; N1 = 21)
1−sided adj pval: 0.895
Frequency
Tumor−Normal Expression Difference
