# Supplementary material for: Identifying factors associated with the direction and significance of microRNA tumor-normal expression differences in colorectal cancer
Source: BMC Cancer. 2017 Oct 30;17:707. doi: 10.1186/s12885-017-3690-x (PMC5663119; doi:10.1186/s12885-017-3690-x)
Supplement: Supplementary file 5 — (AF5_purple.pdf) Visualizations of “purple” outcomes of (lesser) interest – microRNAs that agree in the direction or non-significance of the tumor-normal test of differential expression in each factor level, but different from the overall test’s direction or significance. Each page of this file is in the same format as explained for each row in Fig. 2. (PDF 382 kb) [file 12885_2017_3690_MOESM4_ESM.pdf]

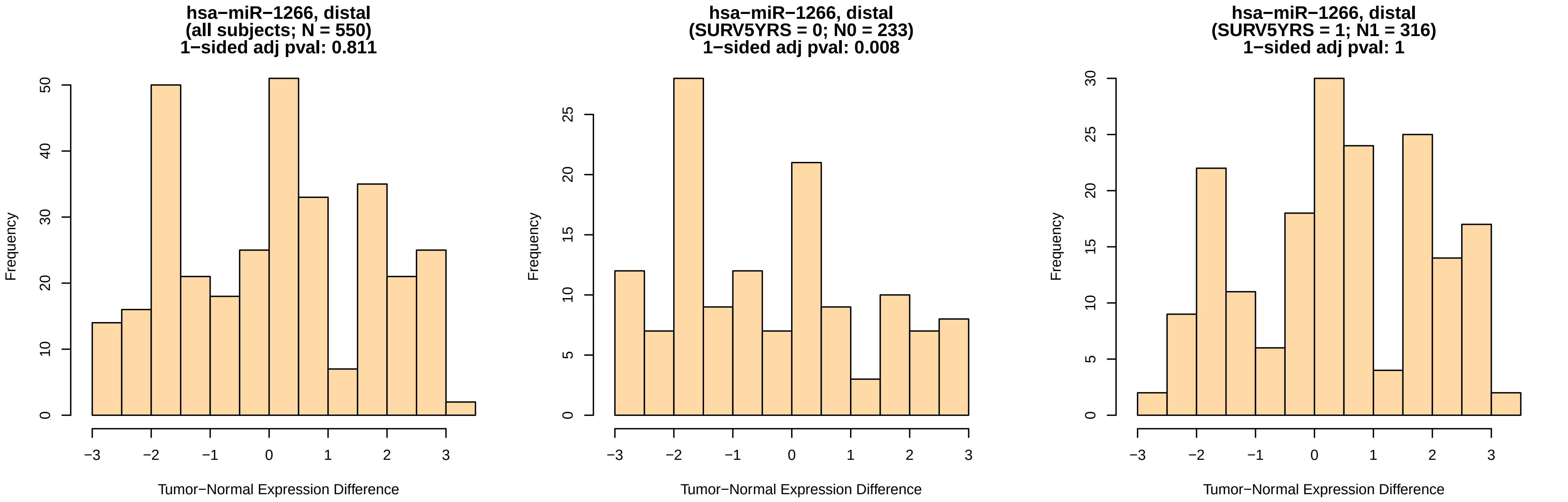
hsa−miR−1266, distal
(all subjects; N = 550)
1−sided adj pval: 0.811
Frequency
Tumor−Normal Expression Difference
hsa−miR−1266, distal
(SURV5YRS = 0; N0 = 233)
1−sided adj pval: 0.008
Frequency
Tumor−Normal Expression Difference
hsa−miR−1266, distal
(SURV5YRS = 1; N1 = 316)
1−sided adj pval: 1
Frequency
Tumor−Normal Expression Difference

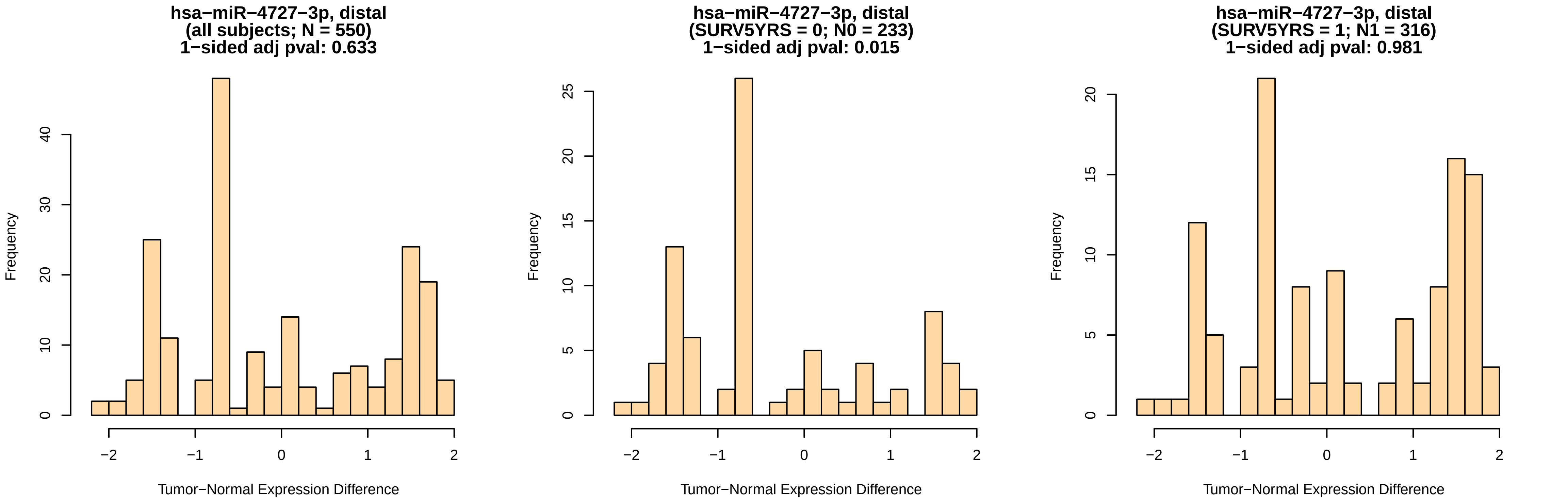
hsa−miR−4727−3p, distal
(all subjects; N = 550)
1−sided adj pval: 0.633
Frequency
Tumor−Normal Expression Difference
hsa−miR−4727−3p, distal
(SURV5YRS = 0; N0 = 233)
1−sided adj pval: 0.015
Frequency
Tumor−Normal Expression Difference
hsa−miR−4727−3p, distal
(SURV5YRS = 1; N1 = 316)
1−sided adj pval: 0.981
Frequency
Tumor−Normal Expression Difference

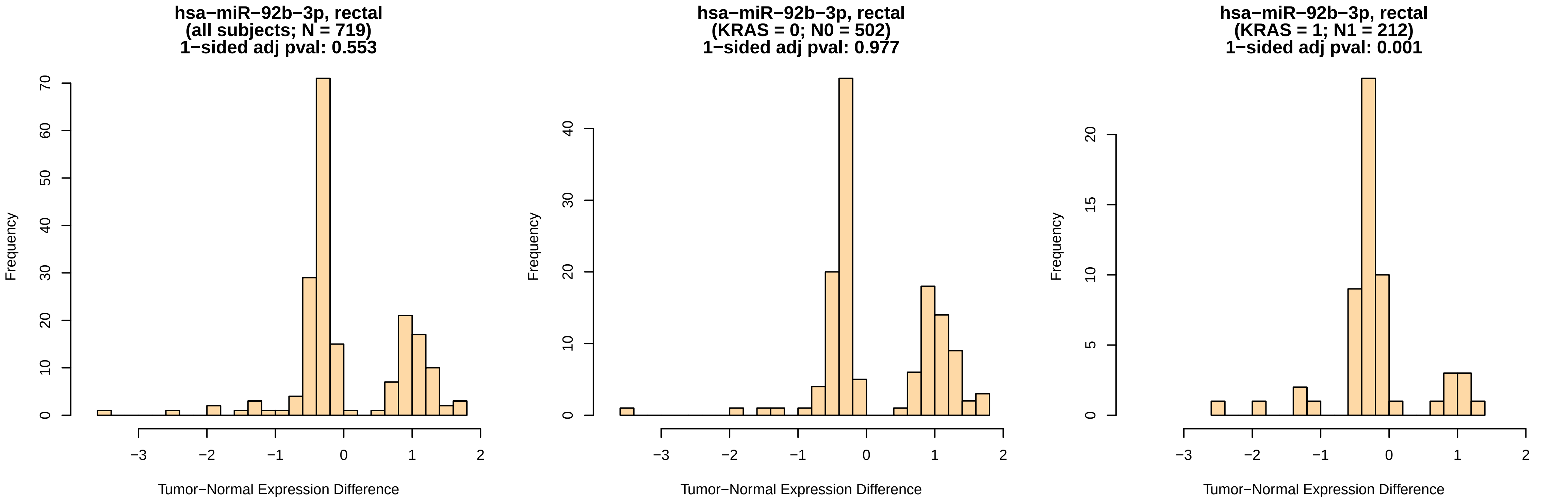

hsa−miR−92b−3p, rectal
(all subjects; N = 719)
1−sided adj pval: 0.553
Frequency
Tumor−Normal Expression Difference
hsa−miR−92b−3p, rectal
(KRAS = 0; N0 = 502)
1−sided adj pval: 0.977
Frequency
Tumor−Normal Expression Difference
hsa−miR−92b−3p, rectal
(KRAS = 1; N1 = 212)
1−sided adj pval: 0.001
Frequency
Tumor−Normal Expression Difference

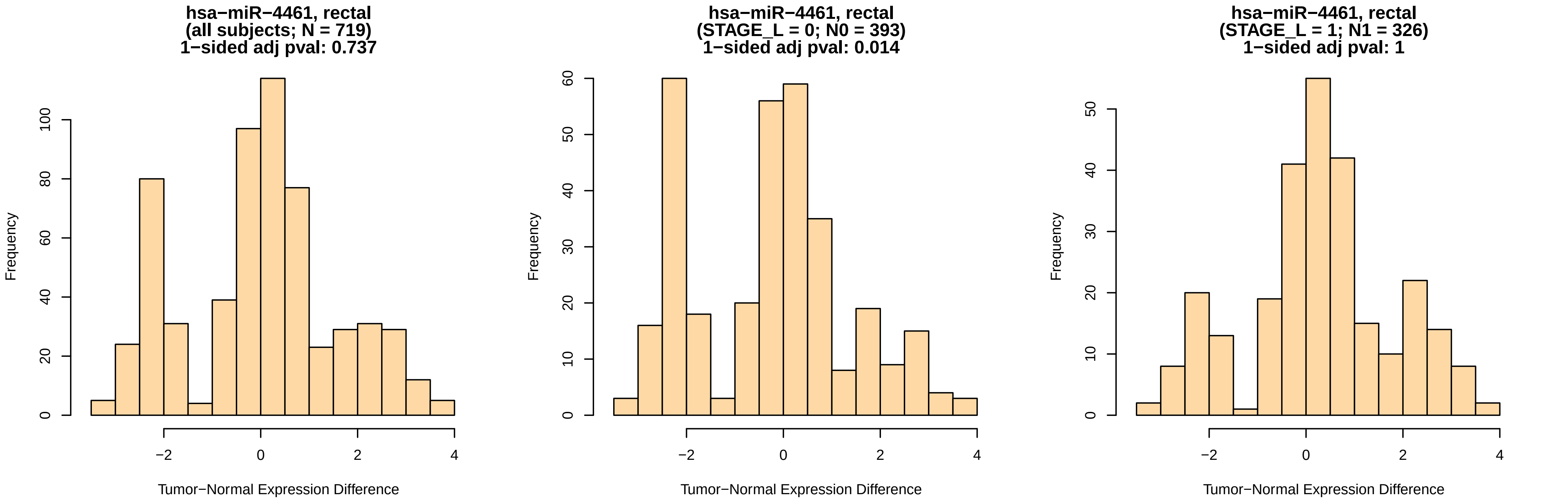

hsa−miR−4461, rectal
(all subjects; N = 719)
1−sided adj pval: 0.737
Frequency
0
20
40
60
80
100
−2
0
2
4
Tumor−Normal Expression Difference
hsa−miR−4461, rectal
(STAGE_L = 0; N0 = 393)
1−sided adj pval: 0.014
Frequency
0
10
20
30
40
50
60
−2
0
2
4
Tumor−Normal Expression Difference
hsa−miR−4461, rectal
(STAGE_L = 1; N1 = 326)
1−sided adj pval: 1
Frequency
0
10
20
30
40
50
−2
0
2
4
Tumor−Normal Expression Difference

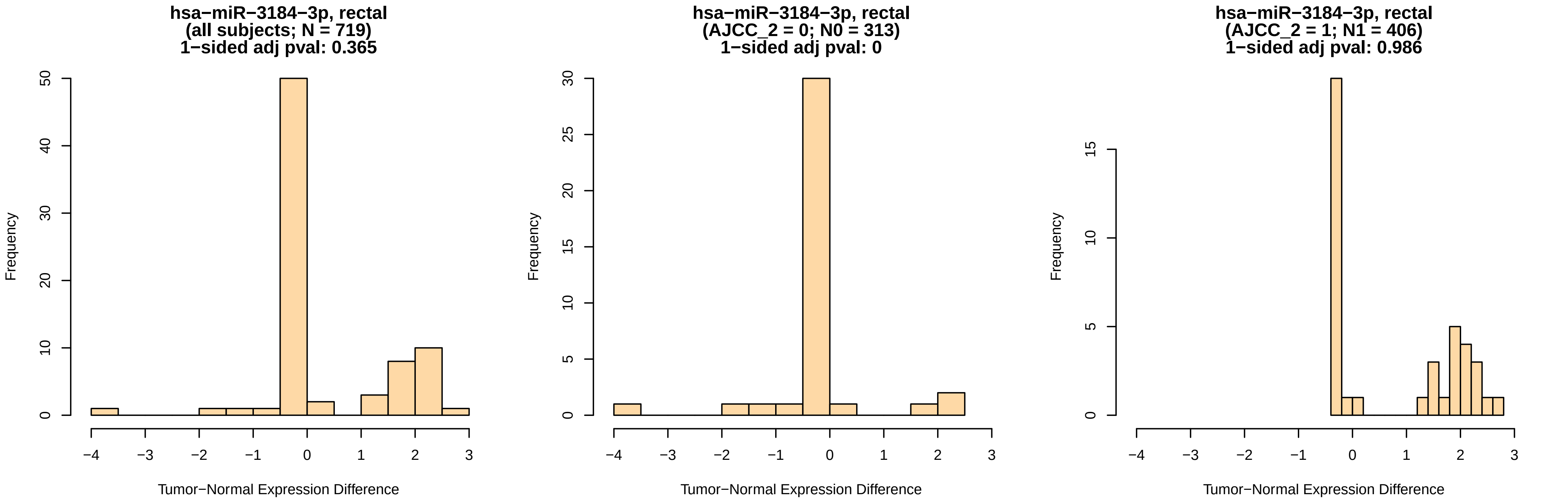
hsa−miR−3184−3p, rectal
(all subjects; N = 719)
1−sided adj pval: 0.365
hsa−miR−3184−3p, rectal
(AJCC_2 = 0; N0 = 313)
1−sided adj pval: 0
hsa−miR−3184−3p, rectal
(AJCC_2 = 1; N1 = 406)
1−sided adj pval: 0.986
Frequency
Tumor−Normal Expression Difference

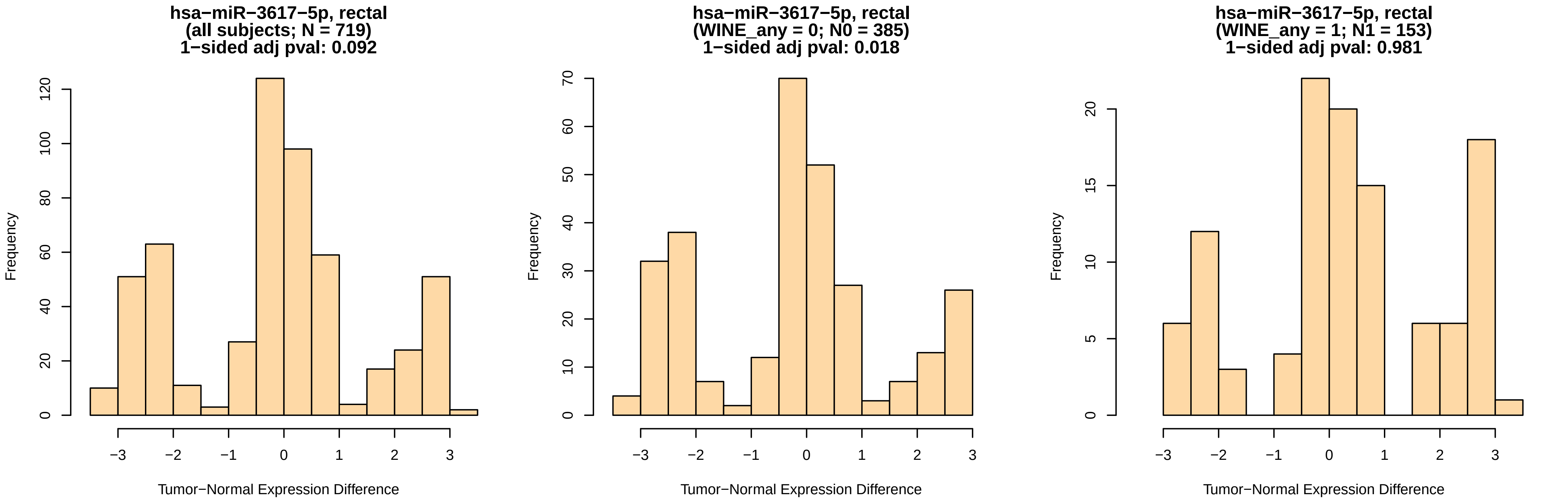
hsa−miR−3617−5p, rectal
(all subjects; N = 719)
1−sided adj pval: 0.092
Frequency
Tumor−Normal Expression Difference
hsa−miR−3617−5p, rectal
(WINE_any = 0; N0 = 385)
1−sided adj pval: 0.018
Frequency
Tumor−Normal Expression Difference
hsa−miR−3617−5p, rectal
(WINE_any = 1; N1 = 153)
1−sided adj pval: 0.981
Frequency
Tumor−Normal Expression Difference

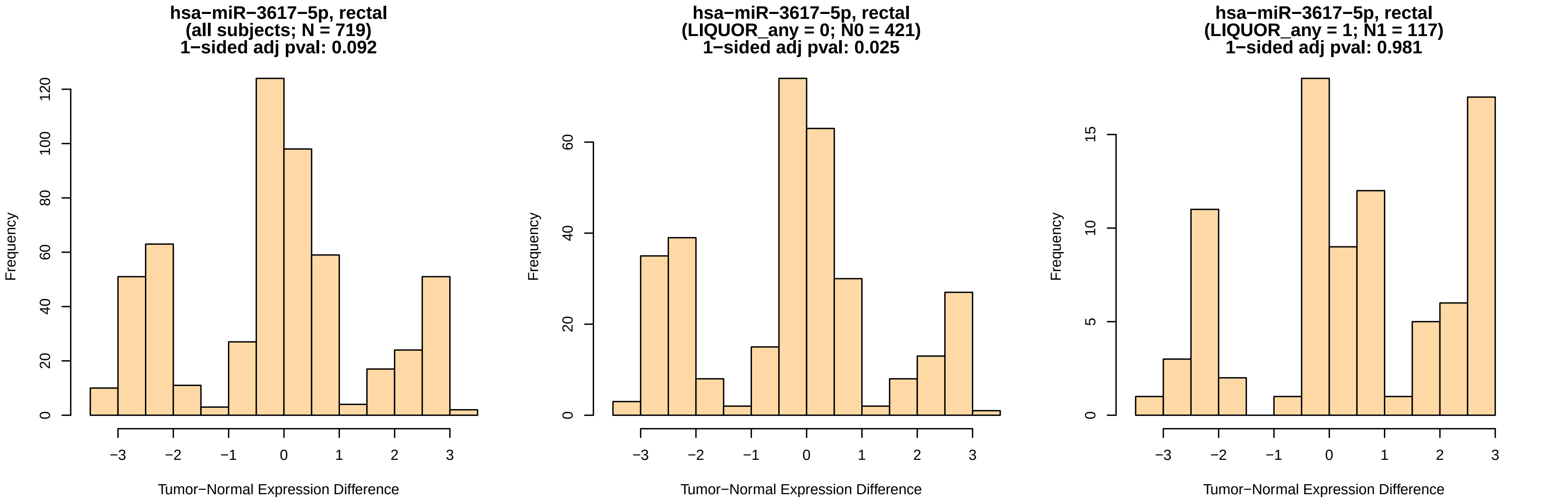
hsa−miR−3617−5p, rectal
(all subjects; N = 719)
1−sided adj pval: 0.092
hsa−miR−3617−5p, rectal
(LIQUOR_any = 0; N0 = 421)
1−sided adj pval: 0.025
hsa−miR−3617−5p, rectal
(LIQUOR_any = 1; N1 = 117)
1−sided adj pval: 0.981
Frequency
Tumor−Normal Expression Difference

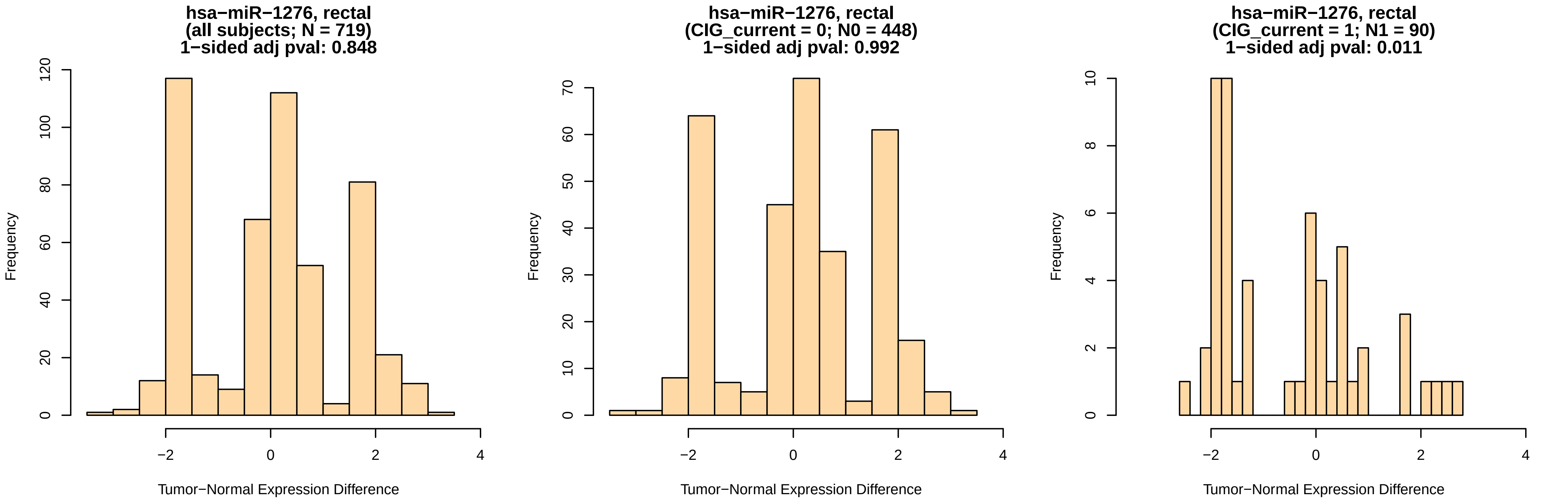

hsa−miR−1276, rectal
(all subjects; N = 719)
1−sided adj pval: 0.848
Frequency
0
20
40
60
80
100
120
−2
0
2
4
Tumor−Normal Expression Difference
hsa−miR−1276, rectal
(CIG_current = 0; N0 = 448)
1−sided adj pval: 0.992
Frequency
0
10
20
30
40
50
60
70
−2
0
2
4
Tumor−Normal Expression Difference
hsa−miR−1276, rectal
(CIG_current = 1; N1 = 90)
1−sided adj pval: 0.011
Frequency
0
2
4
6
8
10
−2
0
2
4
Tumor−Normal Expression Difference
