# Supplementary material for: Identifying factors associated with the direction and significance of microRNA tumor-normal expression differences in colorectal cancer
Source: BMC Cancer. 2017 Oct 30;17:707. doi: 10.1186/s12885-017-3690-x (PMC5663119; doi:10.1186/s12885-017-3690-x)
Supplement: Supplementary file 6 — (AF6_yellow.pdf) Visualizations of “yellow” outcomes of interest – microRNAs that are overall significant in the tumor-normal test of differential expression, with agreement in one factor level but opposite direction in the other factor level. Each page of this file is in the same format as explained for each row in Fig. 2. (PDF 20 kb) [file 12885_2017_3690_MOESM5_ESM.pdf]

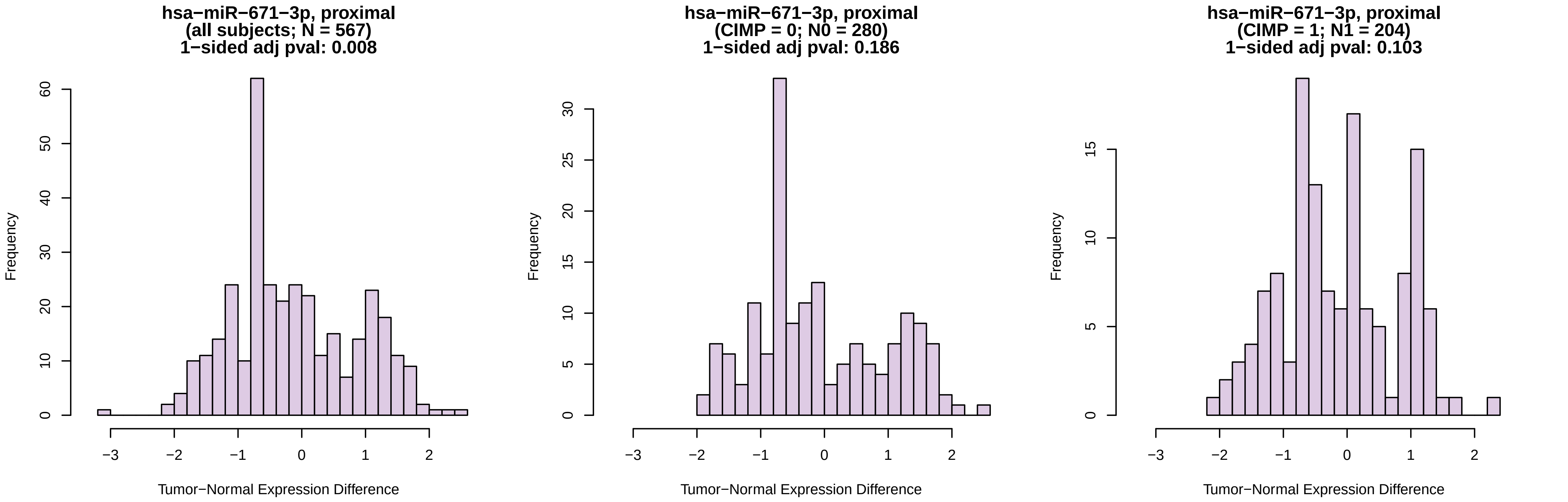
hsa−miR−671−3p, proximal
(all subjects; N = 567)
1−sided adj pval: 0.008
Frequency
Tumor−Normal Expression Difference
hsa−miR−671−3p, proximal
(CIMP = 0; N0 = 280)
1−sided adj pval: 0.186
Frequency
Tumor−Normal Expression Difference
hsa−miR−671−3p, proximal
(CIMP = 1; N1 = 204)
1−sided adj pval: 0.103
Frequency
Tumor−Normal Expression Difference

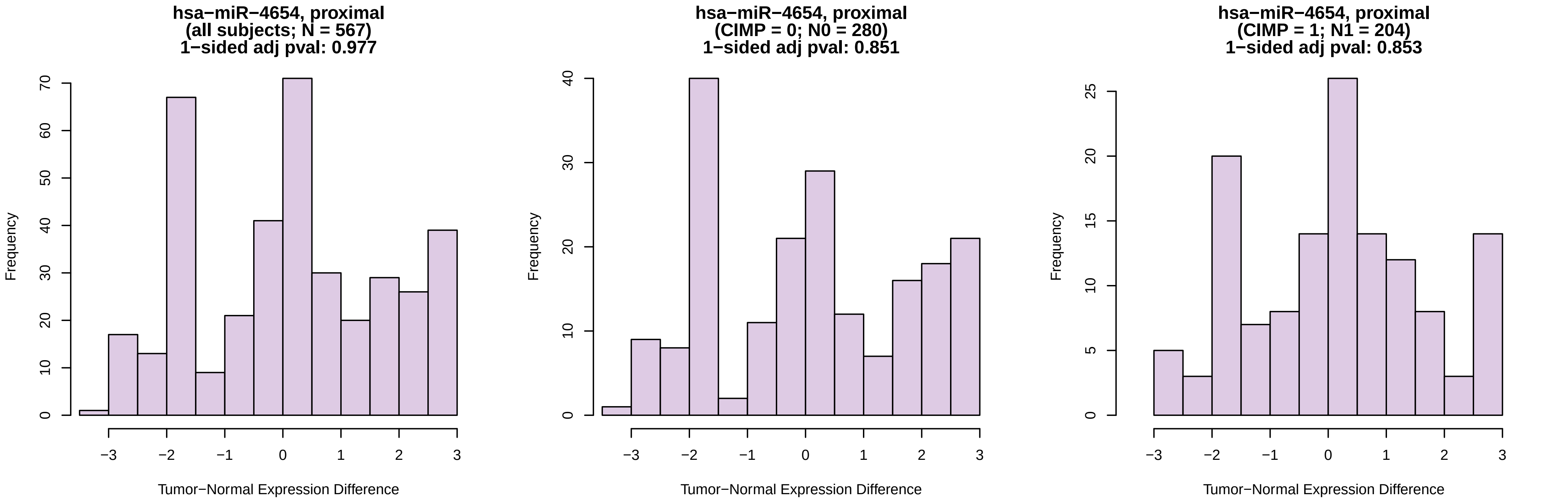
hsa−miR−4654, proximal
(all subjects; N = 567)
1−sided adj pval: 0.977
Frequency
Tumor−Normal Expression Difference
hsa−miR−4654, proximal
(CIMP = 0; N0 = 280)
1−sided adj pval: 0.851
Frequency
Tumor−Normal Expression Difference
hsa−miR−4654, proximal
(CIMP = 1; N1 = 204)
1−sided adj pval: 0.853
Frequency
Tumor−Normal Expression Difference

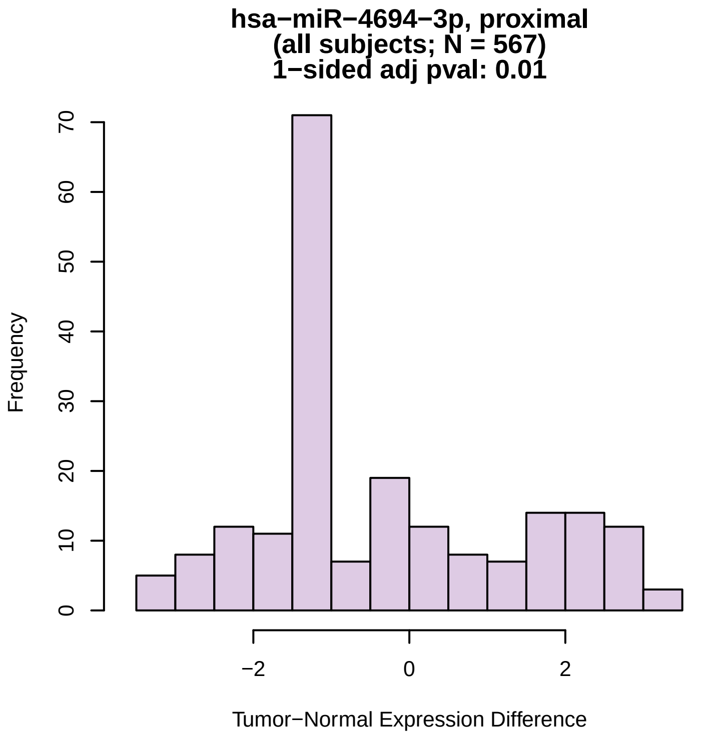
hsa−miR−4694−3p, proximal
(all subjects; N = 567)
1−sided adj pval: 0.01
Frequency
0
10
20
30
40
50
60
70
−2
0
2
Tumor−Normal Expression Difference

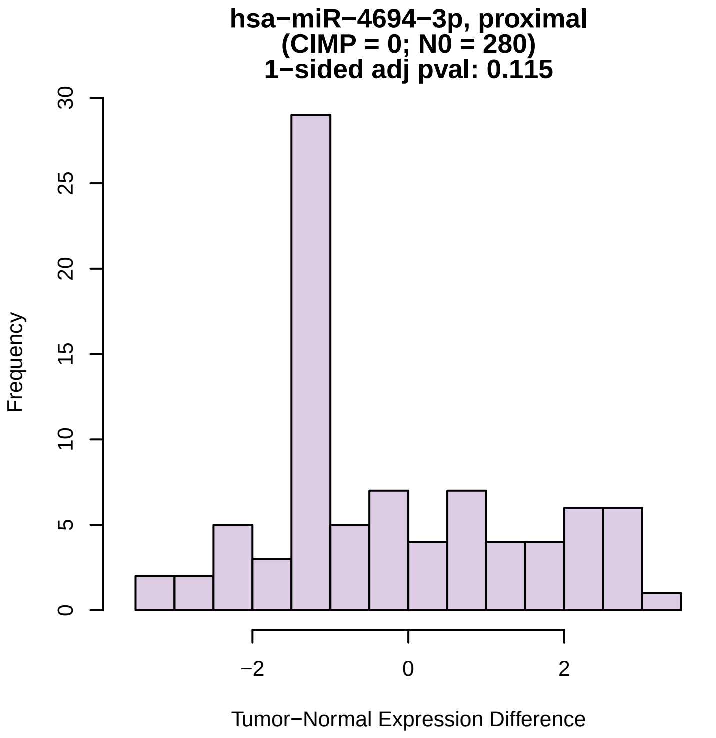
hsa−miR−4694−3p, proximal
(CIMP = 0; N0 = 280)
1−sided adj pval: 0.115
Frequency
0
5
10
15
20
25
30
−2
0
2
Tumor−Normal Expression Difference

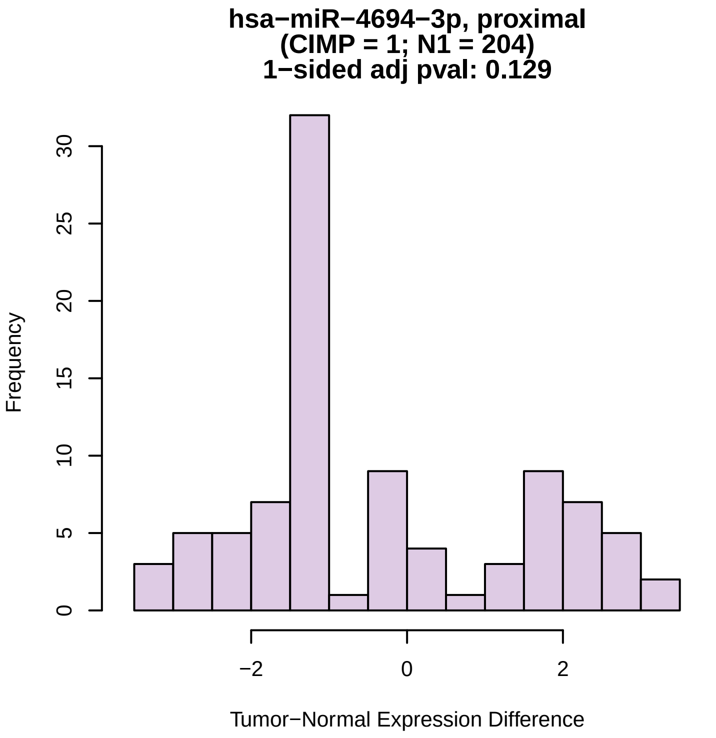
hsa−miR−4694−3p, proximal
(CIMP = 1; N1 = 204)
1−sided adj pval: 0.129
Frequency
0
5
10
15
20
25
30
−2
0
2
Tumor−Normal Expression Difference

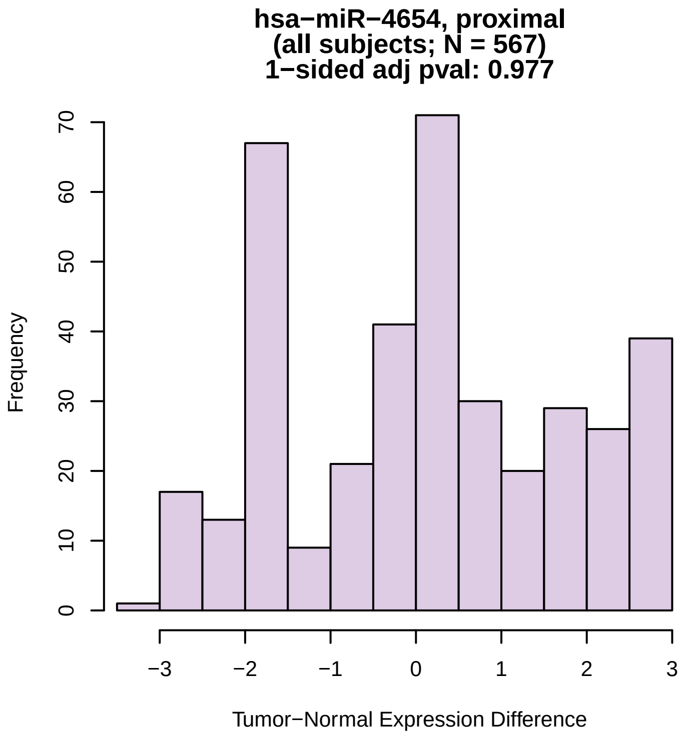

hsa−miR−4654, proximal
(all subjects; N = 567)
1−sided adj pval: 0.977
Frequency
Tumor−Normal Expression Difference

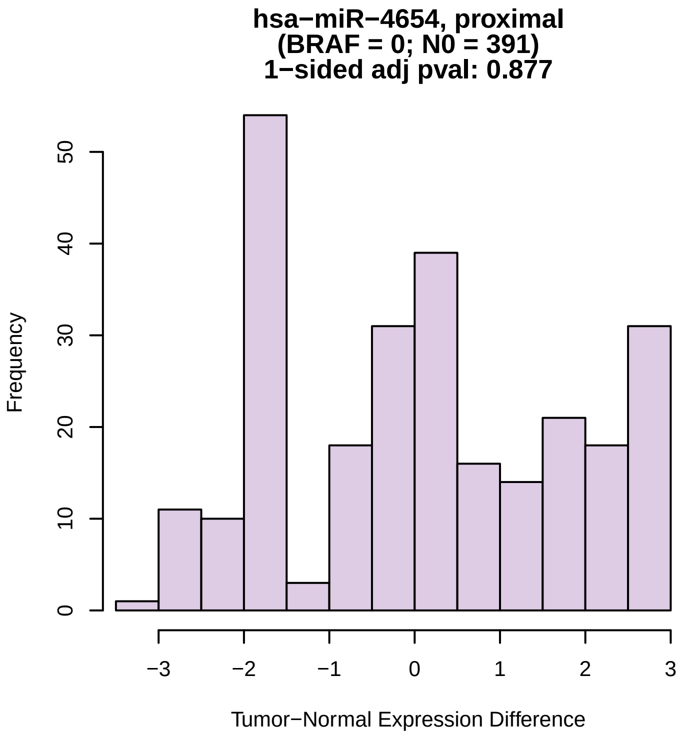

hsa−miR−4654, proximal
(BRAF = 0; N0 = 391)
1−sided adj pval: 0.877
Frequency
Tumor−Normal Expression Difference

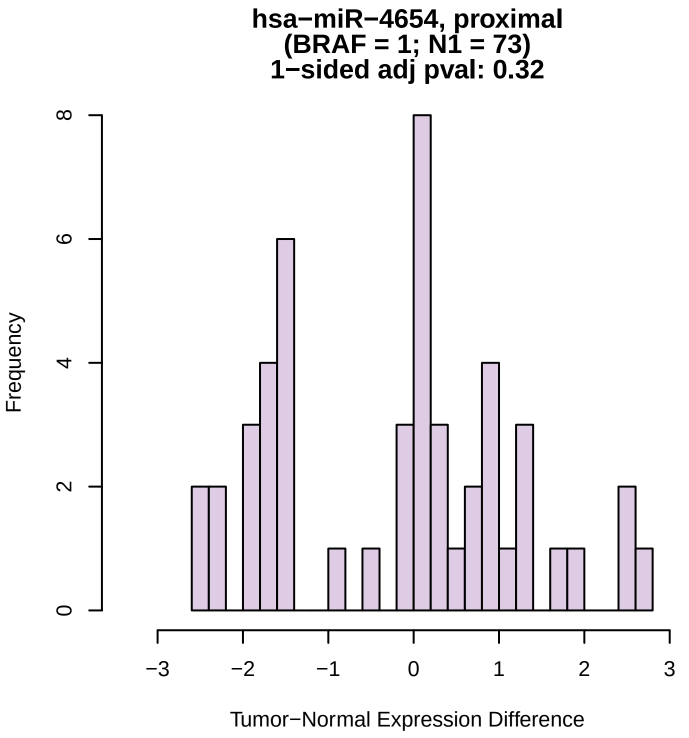

hsa−miR−4654, proximal
(BRAF = 1; N1 = 73)
1−sided adj pval: 0.32
Frequency
Tumor−Normal Expression Difference

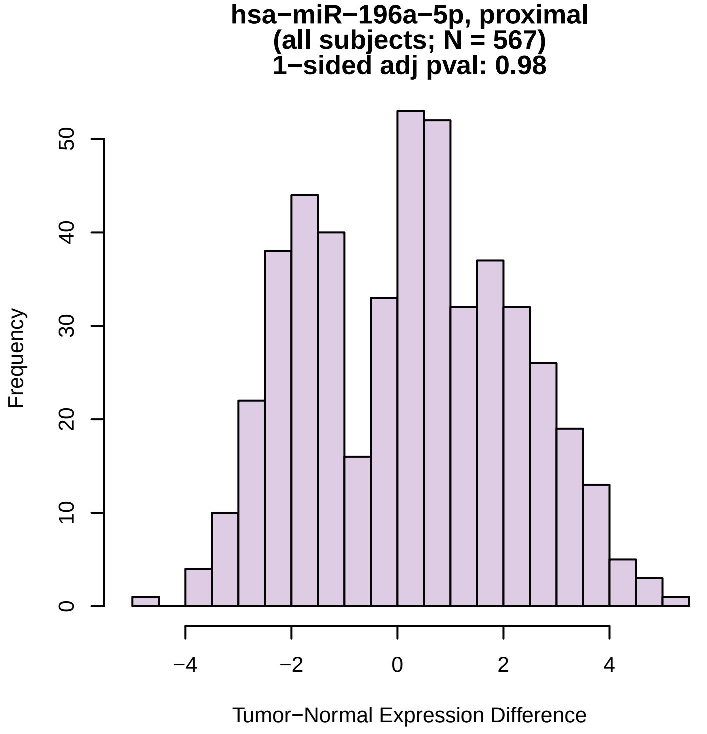
hsa−miR−196a−5p, proximal
(all subjects; N = 567)
1−sided adj pval: 0.98
Frequency
Tumor−Normal Expression Difference

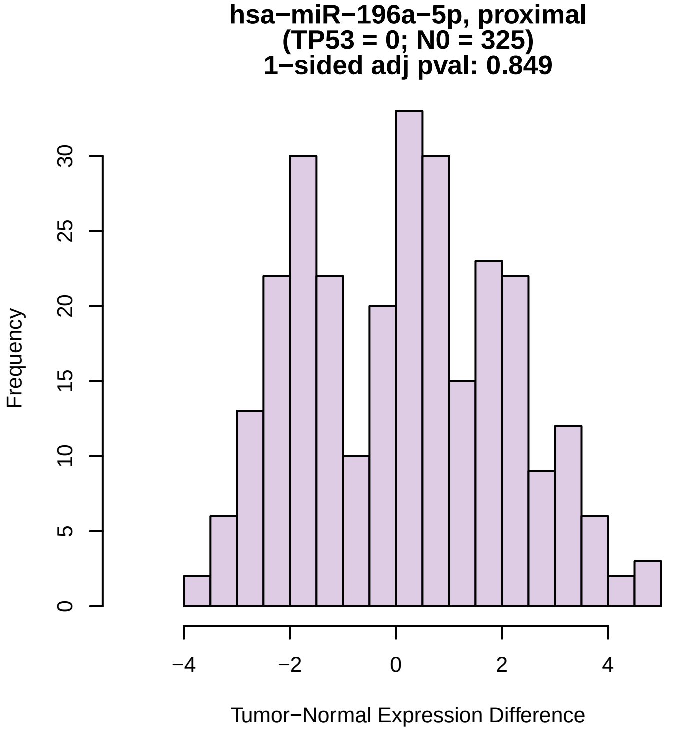
hsa−miR−196a−5p, proximal
(TP53 = 0; N0 = 325)
1−sided adj pval: 0.849
Frequency
Tumor−Normal Expression Difference

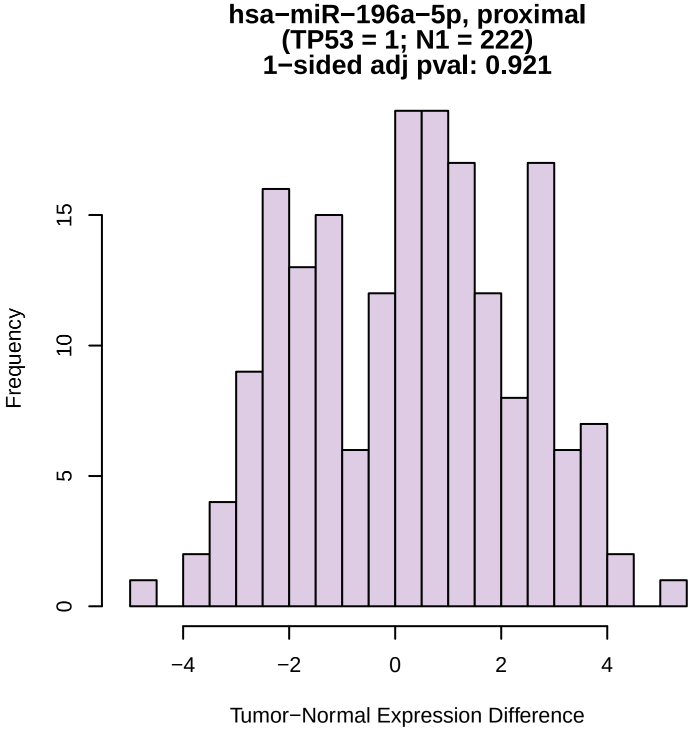
hsa−miR−196a−5p, proximal
(TP53 = 1; N1 = 222)
1−sided adj pval: 0.921
Frequency
Tumor−Normal Expression Difference

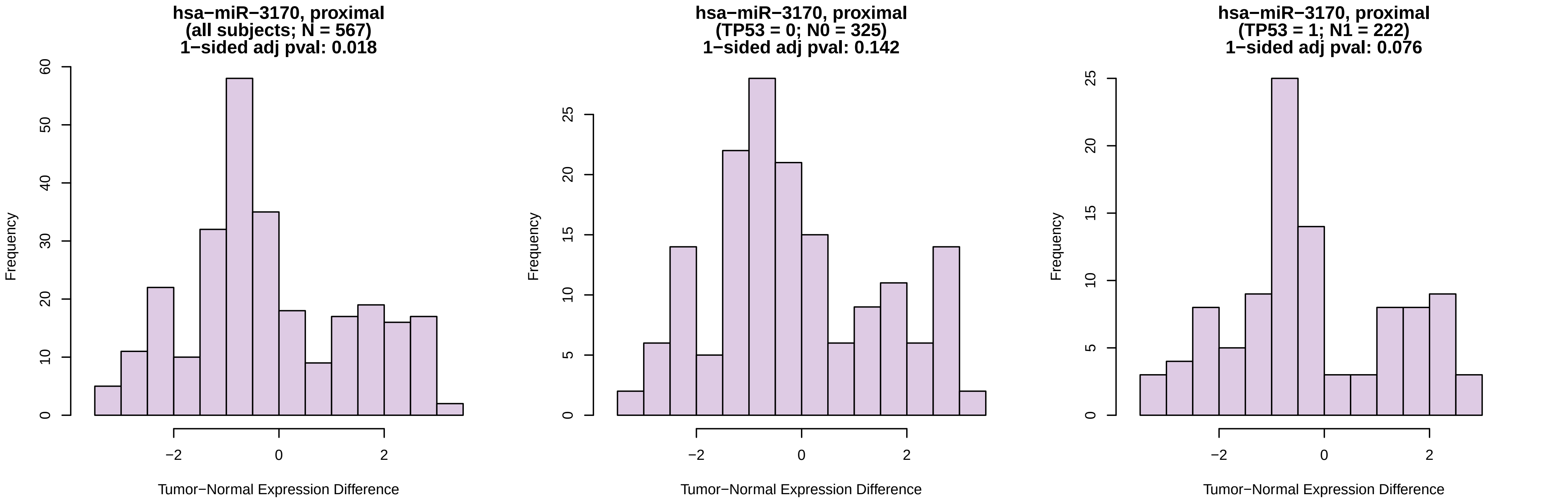
hsa−miR−3170, proximal
(all subjects; N = 567)
1−sided adj pval: 0.018
Frequency
Tumor−Normal Expression Difference
hsa−miR−3170, proximal
(TP53 = 0; N0 = 325)
1−sided adj pval: 0.142
Frequency
Tumor−Normal Expression Difference
hsa−miR−3170, proximal
(TP53 = 1; N1 = 222)
1−sided adj pval: 0.076
Frequency
Tumor−Normal Expression Difference

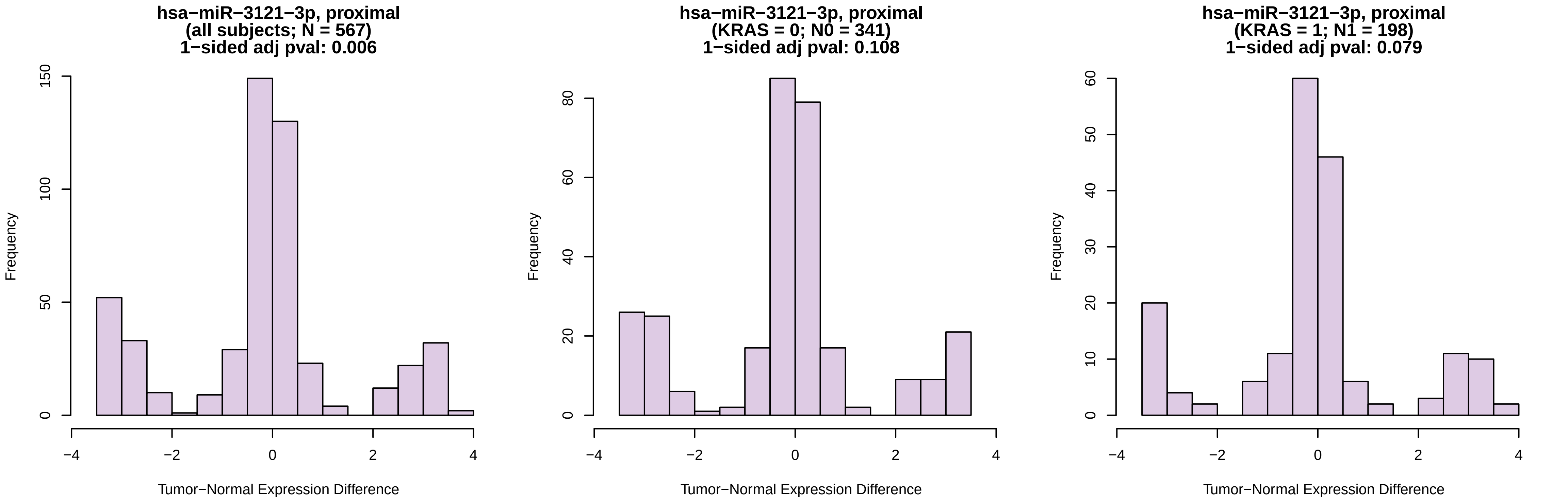

hsa−miR−3121−3p, proximal
(all subjects; N = 567)
1−sided adj pval: 0.006
Frequency
0
50
100
150
−4
−2
0
2
4
Tumor−Normal Expression Difference
hsa−miR−3121−3p, proximal
(KRAS = 0; N0 = 341)
1−sided adj pval: 0.108
Frequency
0
20
40
60
80
−4
−2
0
2
4
Tumor−Normal Expression Difference
hsa−miR−3121−3p, proximal
(KRAS = 1; N1 = 198)
1−sided adj pval: 0.079
Frequency
0
10
20
30
40
50
60
−4
−2
0
2
4
Tumor−Normal Expression Difference

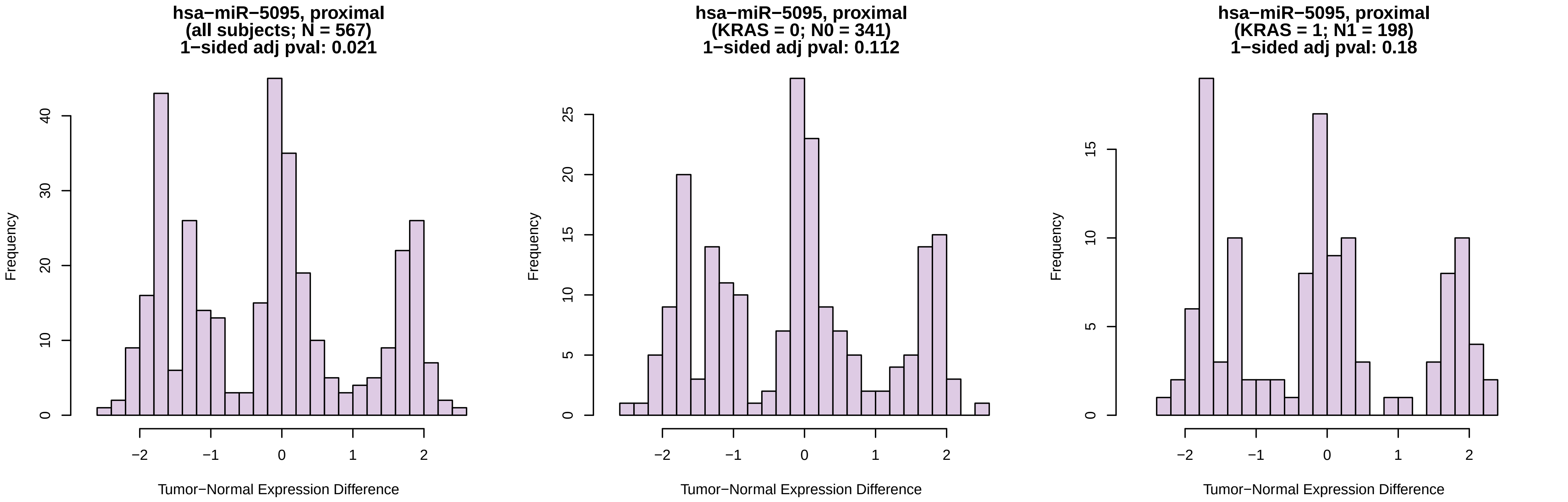
hsa−miR−5095, proximal
(all subjects; N = 567)
1−sided adj pval: 0.021
Frequency
0
10
20
30
40
−2
−1
0
1
2
Tumor−Normal Expression Difference
hsa−miR−5095, proximal
(KRAS = 0; N0 = 341)
1−sided adj pval: 0.112
Frequency
0
5
10
15
20
25
−2
−1
0
1
2
Tumor−Normal Expression Difference
hsa−miR−5095, proximal
(KRAS = 1; N1 = 198)
1−sided adj pval: 0.18
Frequency
0
5
10
15
−2
−1
0
1
2
Tumor−Normal Expression Difference

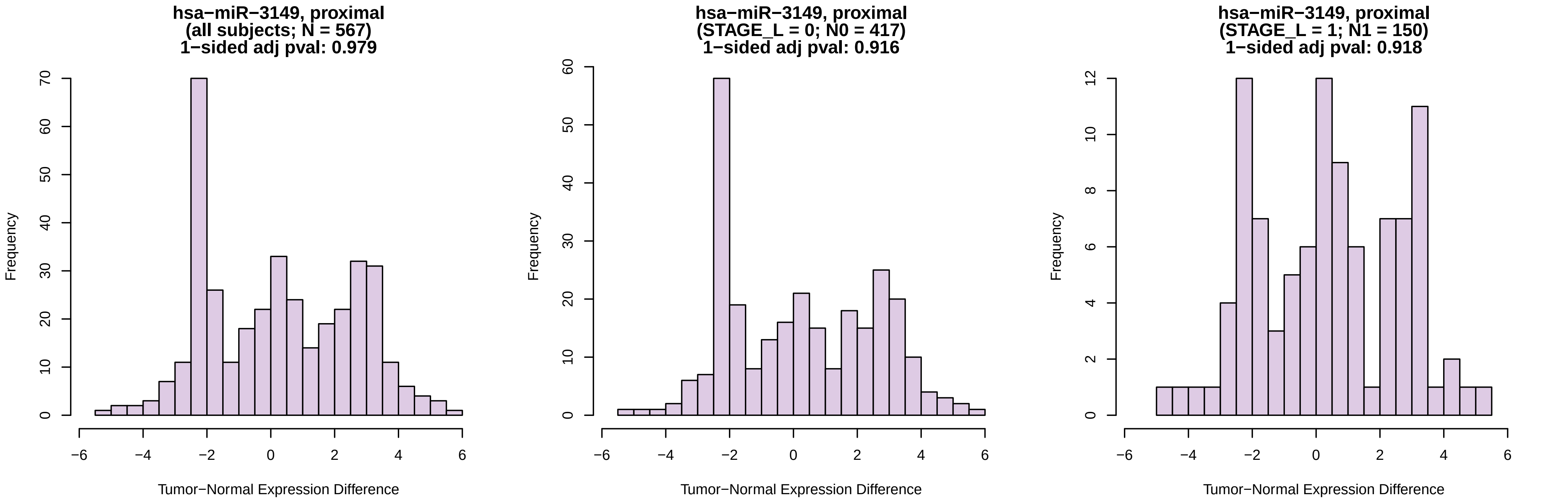

hsa−miR−3149, proximal
(all subjects; N = 567)
1−sided adj pval: 0.979
Frequency
Tumor−Normal Expression Difference
hsa−miR−3149, proximal
(STAGE_L = 0; N0 = 417)
1−sided adj pval: 0.916
Frequency
Tumor−Normal Expression Difference
hsa−miR−3149, proximal
(STAGE_L = 1; N1 = 150)
1−sided adj pval: 0.918
Frequency
Tumor−Normal Expression Difference

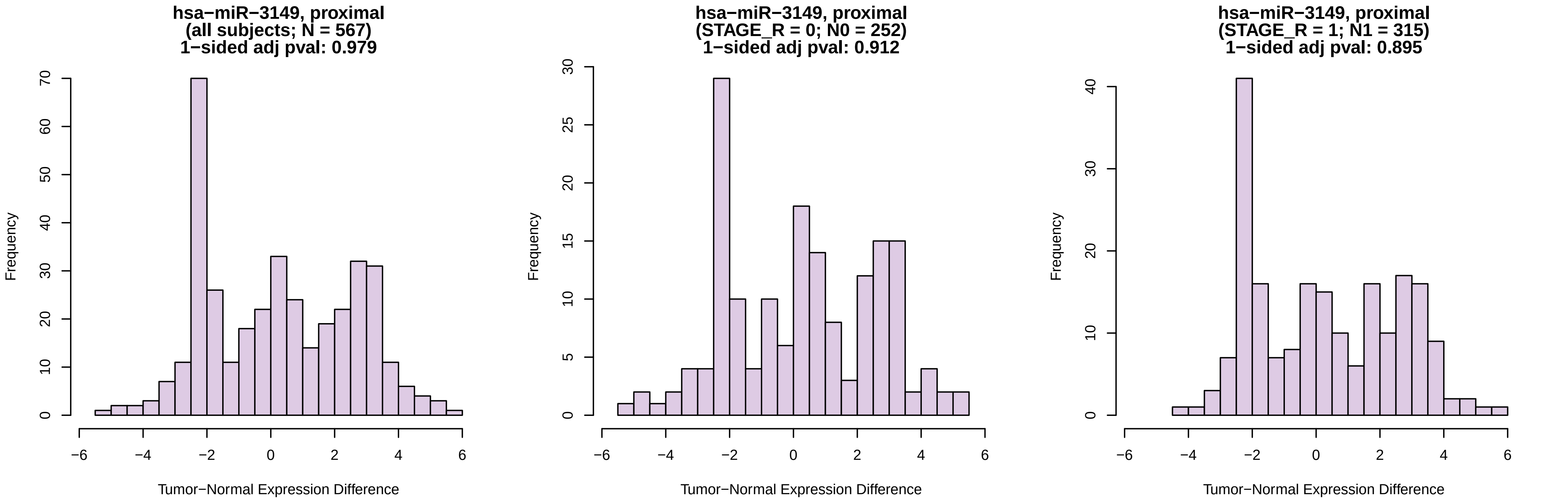

hsa−miR−3149, proximal
(all subjects; N = 567)
1−sided adj pval: 0.979
Frequency
Tumor−Normal Expression Difference
hsa−miR−3149, proximal
(STAGE_R = 0; N0 = 252)
1−sided adj pval: 0.912
Frequency
Tumor−Normal Expression Difference
hsa−miR−3149, proximal
(STAGE_R = 1; N1 = 315)
1−sided adj pval: 0.895
Frequency
Tumor−Normal Expression Difference

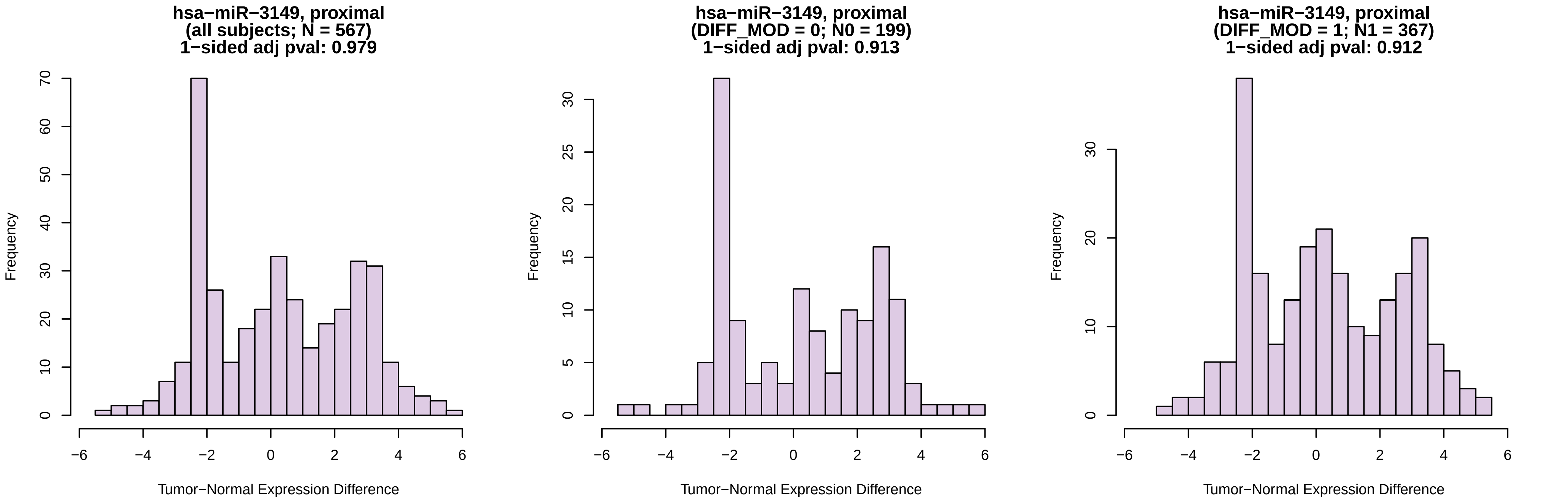
hsa−miR−3149, proximal
(all subjects; N = 567)
1−sided adj pval: 0.979
Frequency
Tumor−Normal Expression Difference
hsa−miR−3149, proximal
(DIFF_MOD = 0; N0 = 199)
1−sided adj pval: 0.913
Frequency
Tumor−Normal Expression Difference
hsa−miR−3149, proximal
(DIFF_MOD = 1; N1 = 367)
1−sided adj pval: 0.912
Frequency
Tumor−Normal Expression Difference

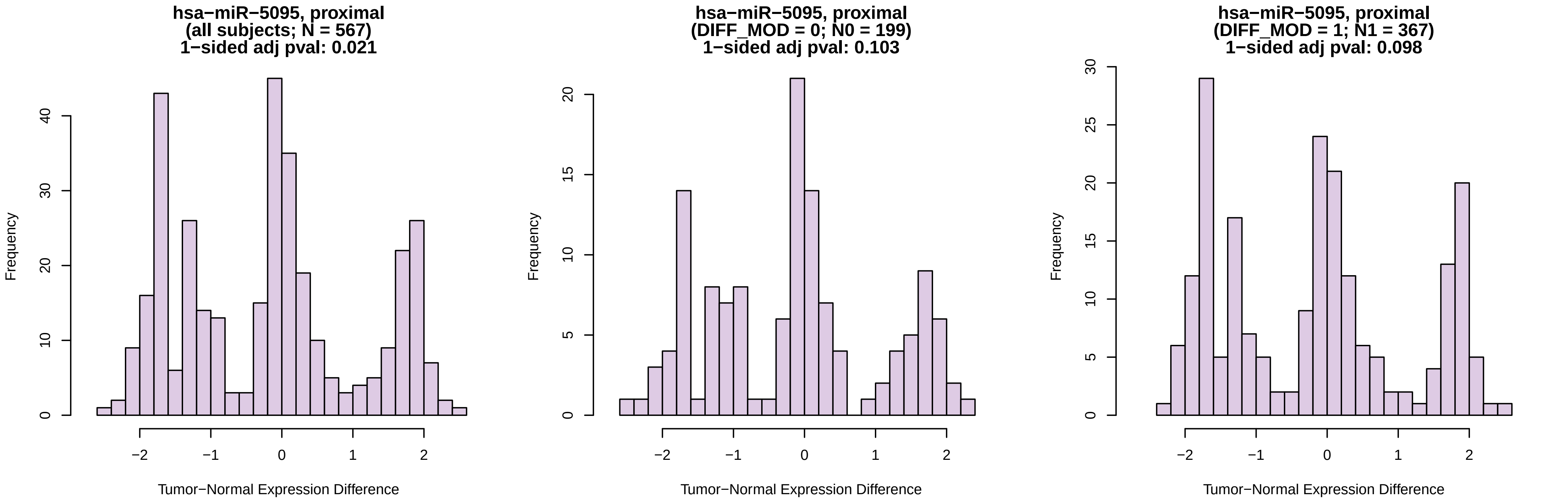

hsa−miR−5095, proximal
(all subjects; N = 567)
1−sided adj pval: 0.021
Frequency
Tumor−Normal Expression Difference
hsa−miR−5095, proximal
(DIFF_MOD = 0; N0 = 199)
1−sided adj pval: 0.103
Frequency
Tumor−Normal Expression Difference
hsa−miR−5095, proximal
(DIFF_MOD = 1; N1 = 367)
1−sided adj pval: 0.098
Frequency
Tumor−Normal Expression Difference

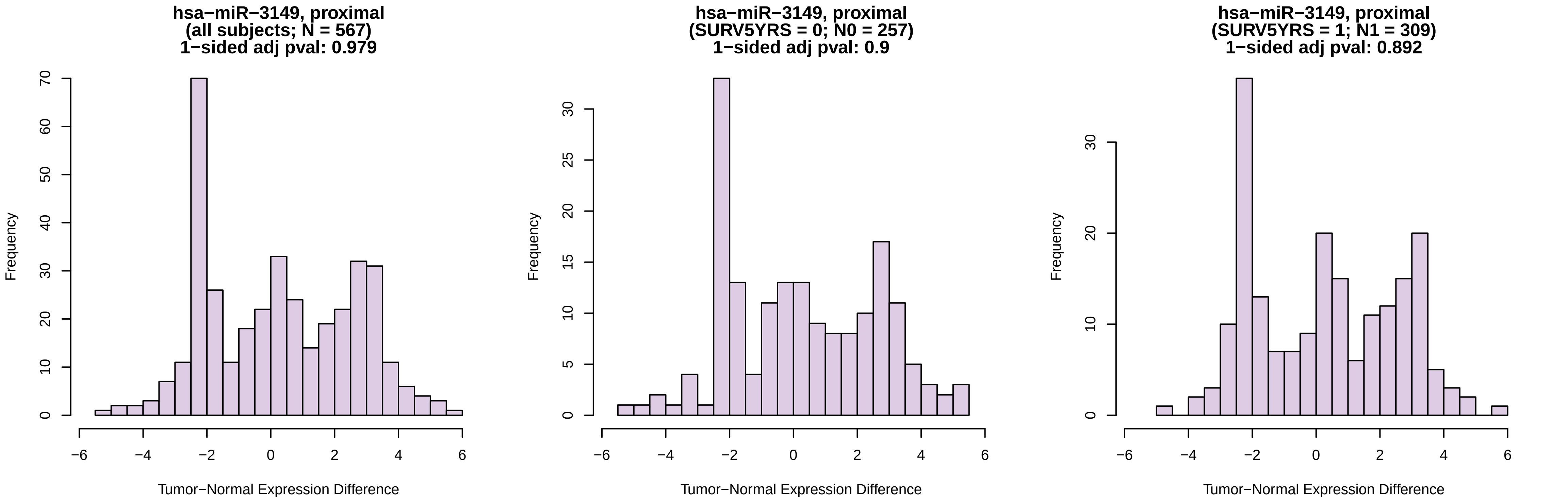
hsa−miR−3149, proximal
(all subjects; N = 567)
1−sided adj pval: 0.979
Frequency
Tumor−Normal Expression Difference
hsa−miR−3149, proximal
(SURV5YRS = 0; N0 = 257)
1−sided adj pval: 0.9
Frequency
Tumor−Normal Expression Difference
hsa−miR−3149, proximal
(SURV5YRS = 1; N1 = 309)
1−sided adj pval: 0.892
Frequency
Tumor−Normal Expression Difference

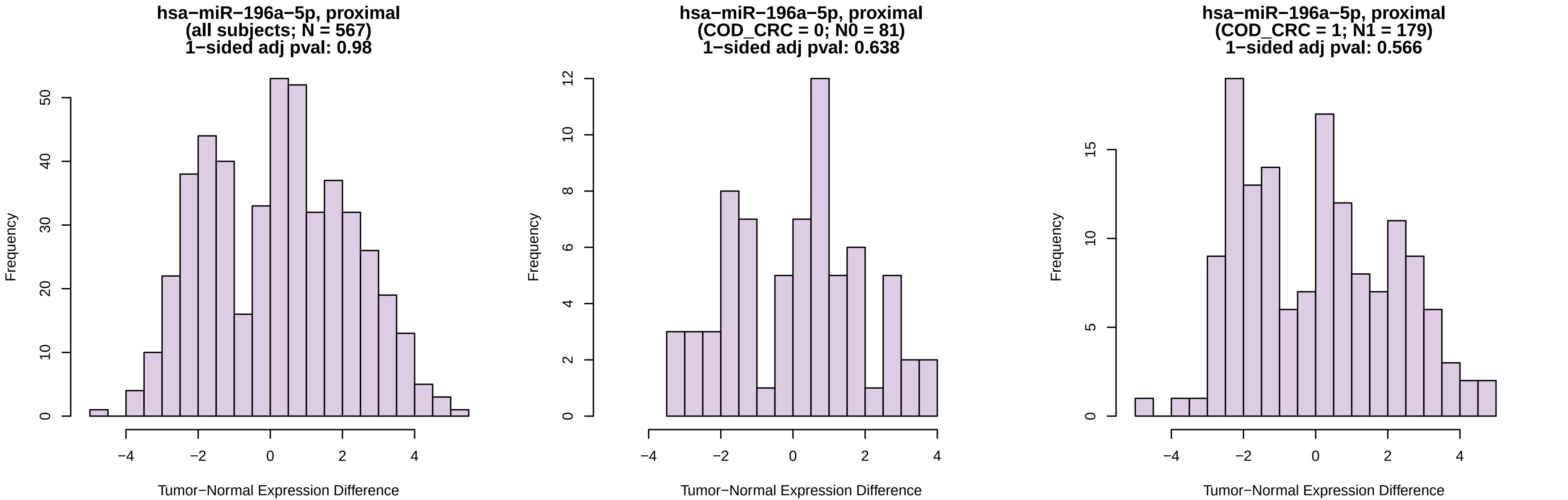
hsa−miR−196a−5p, proximal
(all subjects; N = 567)
1−sided adj pval: 0.98
Frequency
Tumor−Normal Expression Difference
hsa−miR−196a−5p, proximal
(COD_CRC = 0; N0 = 81)
1−sided adj pval: 0.638
Frequency
Tumor−Normal Expression Difference
hsa−miR−196a−5p, proximal
(COD_CRC = 1; N1 = 179)
1−sided adj pval: 0.566
Frequency
Tumor−Normal Expression Difference

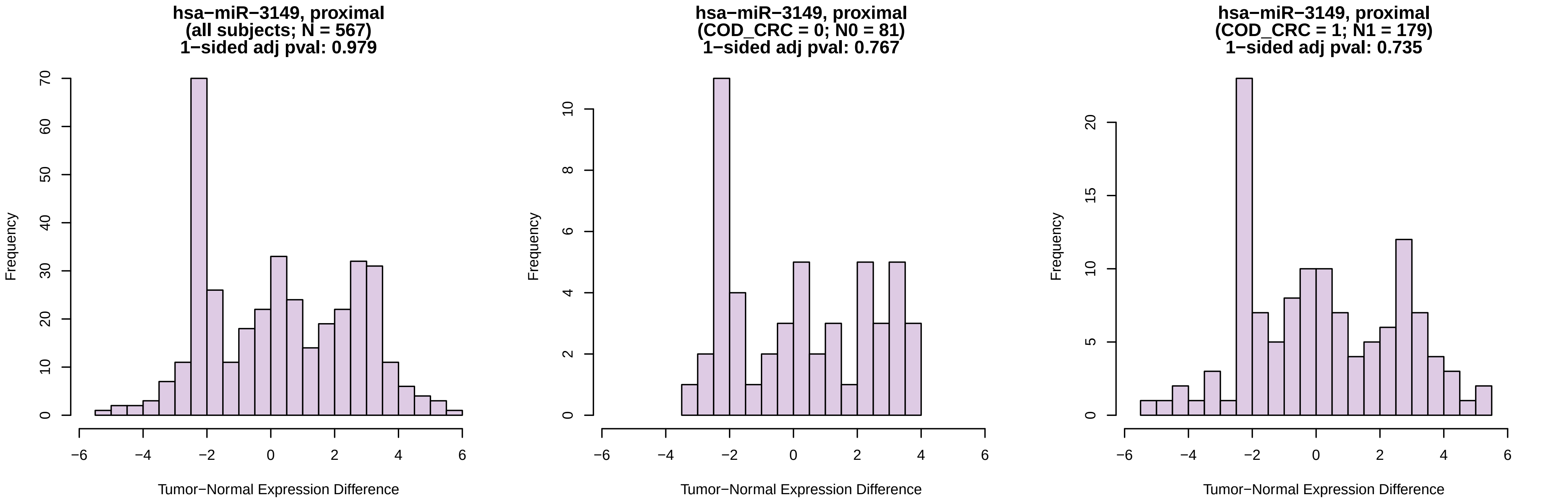
hsa−miR−3149, proximal
(all subjects; N = 567)
1−sided adj pval: 0.979
Frequency
Tumor−Normal Expression Difference
hsa−miR−3149, proximal
(COD_CRC = 0; N0 = 81)
1−sided adj pval: 0.767
Frequency
Tumor−Normal Expression Difference
hsa−miR−3149, proximal
(COD_CRC = 1; N1 = 179)
1−sided adj pval: 0.735
Frequency
Tumor−Normal Expression Difference

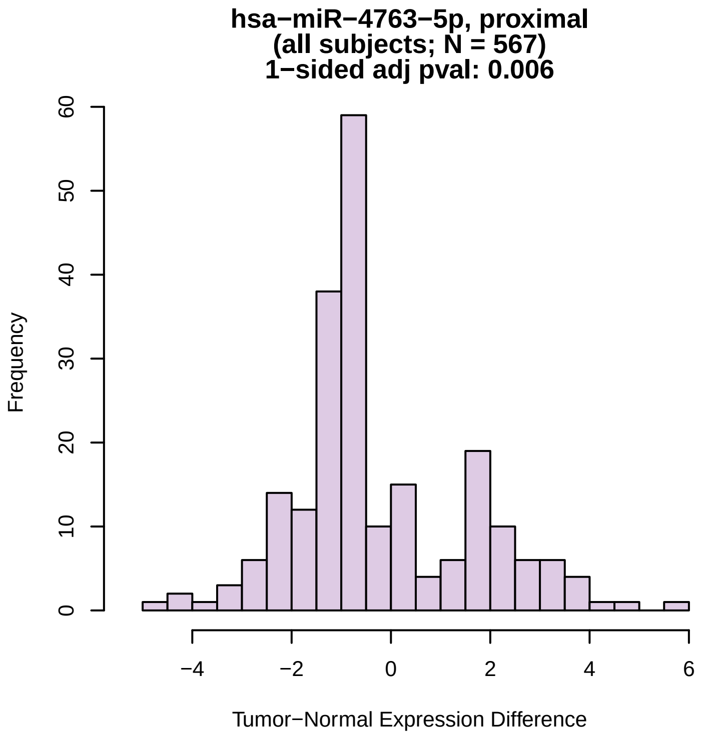
hsa−miR−4763−5p, proximal
(all subjects; N = 567)
1−sided adj pval: 0.006
Frequency
Tumor−Normal Expression Difference
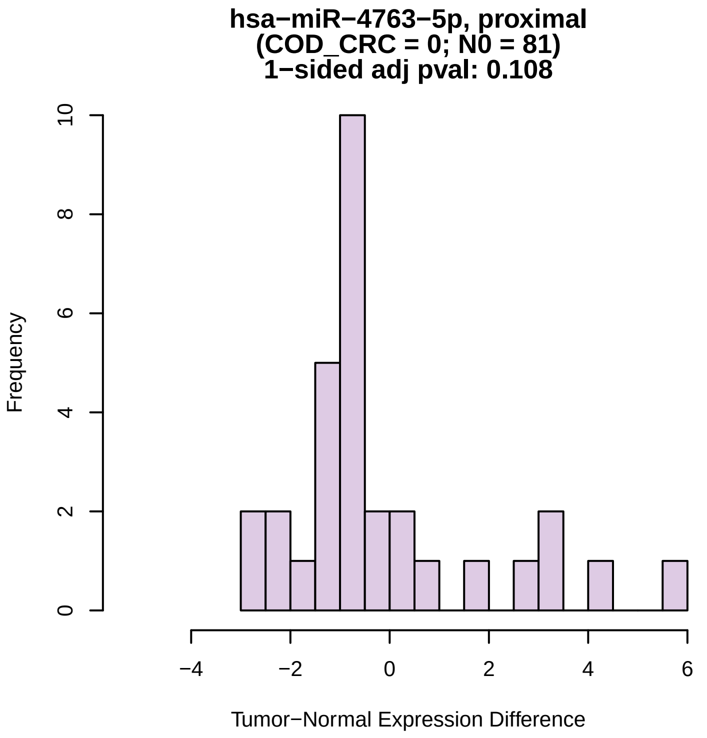
hsa−miR−4763−5p, proximal
(COD_CRC = 0; N0 = 81)
1−sided adj pval: 0.108
Frequency
Tumor−Normal Expression Difference
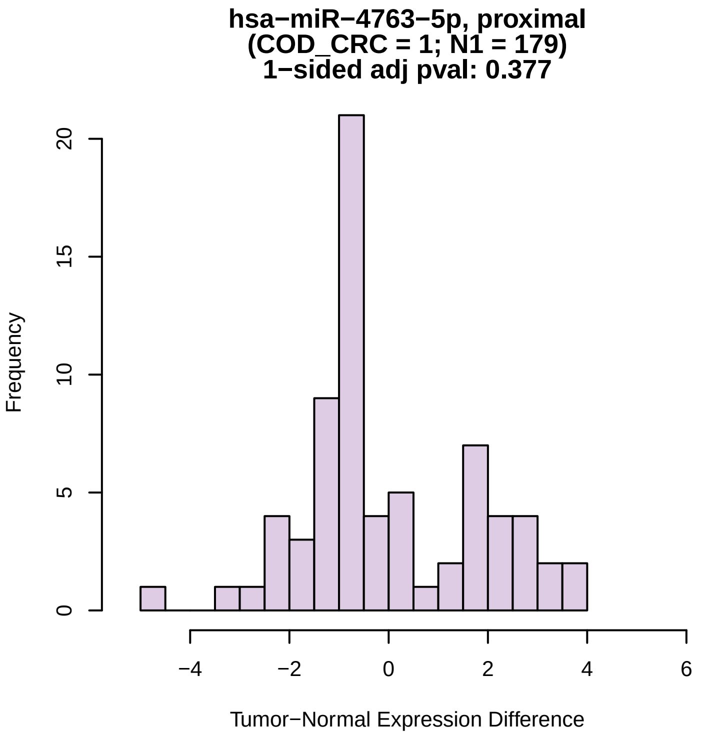
hsa−miR−4763−5p, proximal
(COD_CRC = 1; N1 = 179)
1−sided adj pval: 0.377
Frequency
Tumor−Normal Expression Difference

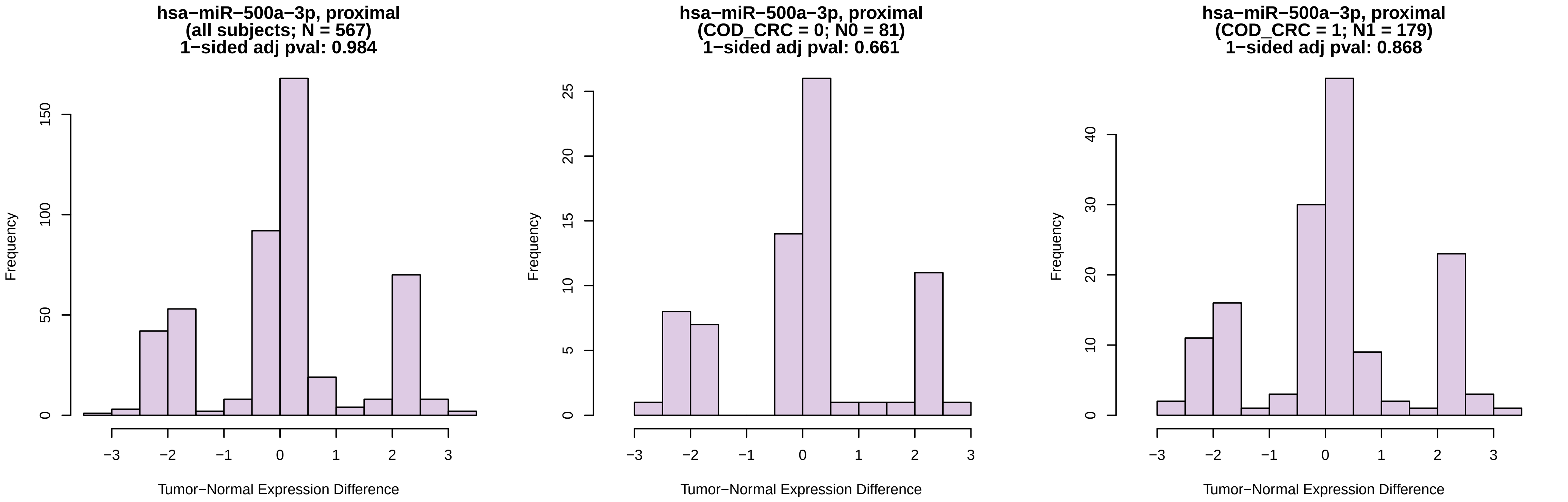

hsa−miR−500a−3p, proximal
(all subjects; N = 567)
1−sided adj pval: 0.984
Frequency
Tumor−Normal Expression Difference
hsa−miR−500a−3p, proximal
(COD_CRC = 0; N0 = 81)
1−sided adj pval: 0.661
Frequency
Tumor−Normal Expression Difference
hsa−miR−500a−3p, proximal
(COD_CRC = 1; N1 = 179)
1−sided adj pval: 0.868
Frequency
Tumor−Normal Expression Difference

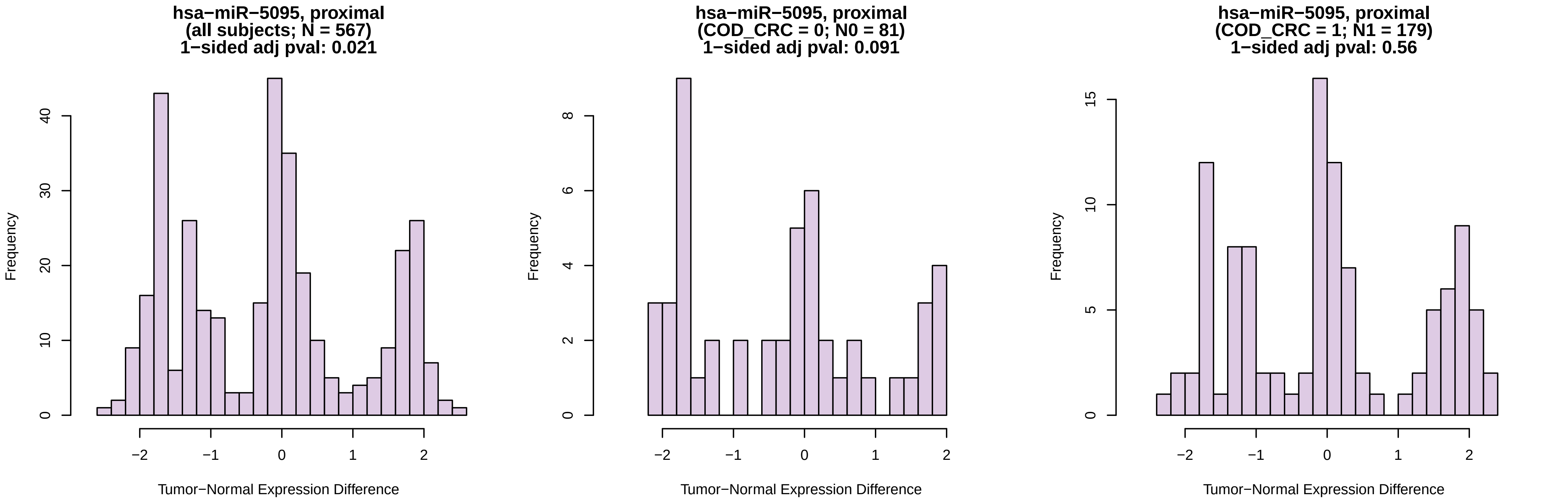
hsa−miR−5095, proximal
(all subjects; N = 567)
1−sided adj pval: 0.021
Frequency
0
10
20
30
40
−2
−1
0
1
2
Tumor−Normal Expression Difference
hsa−miR−5095, proximal
(COD_CRC = 0; N0 = 81)
1−sided adj pval: 0.091
Frequency
0
2
4
6
8
−2
−1
0
1
2
Tumor−Normal Expression Difference
hsa−miR−5095, proximal
(COD_CRC = 1; N1 = 179)
1−sided adj pval: 0.56
Frequency
0
5
10
15
−2
−1
0
1
2
Tumor−Normal Expression Difference

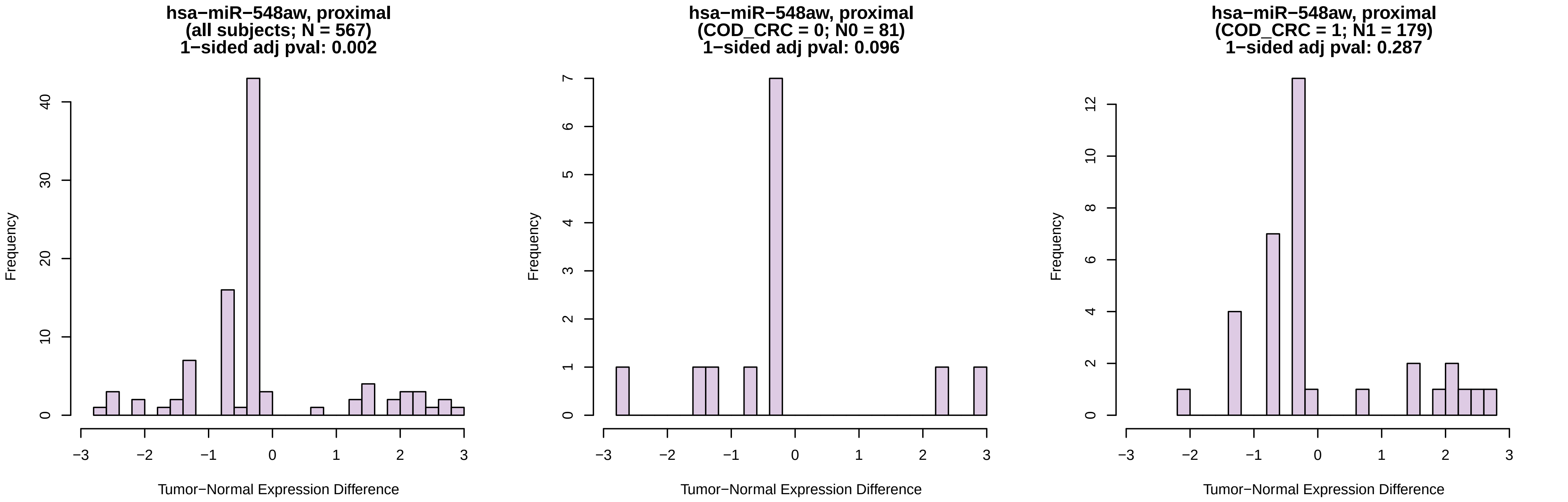
hsa−miR−548aw, proximal
(all subjects; N = 567)
1−sided adj pval: 0.002
Frequency
Tumor−Normal Expression Difference
hsa−miR−548aw, proximal
(COD_CRC = 0; N0 = 81)
1−sided adj pval: 0.096
Frequency
Tumor−Normal Expression Difference
hsa−miR−548aw, proximal
(COD_CRC = 1; N1 = 179)
1−sided adj pval: 0.287
Frequency
Tumor−Normal Expression Difference

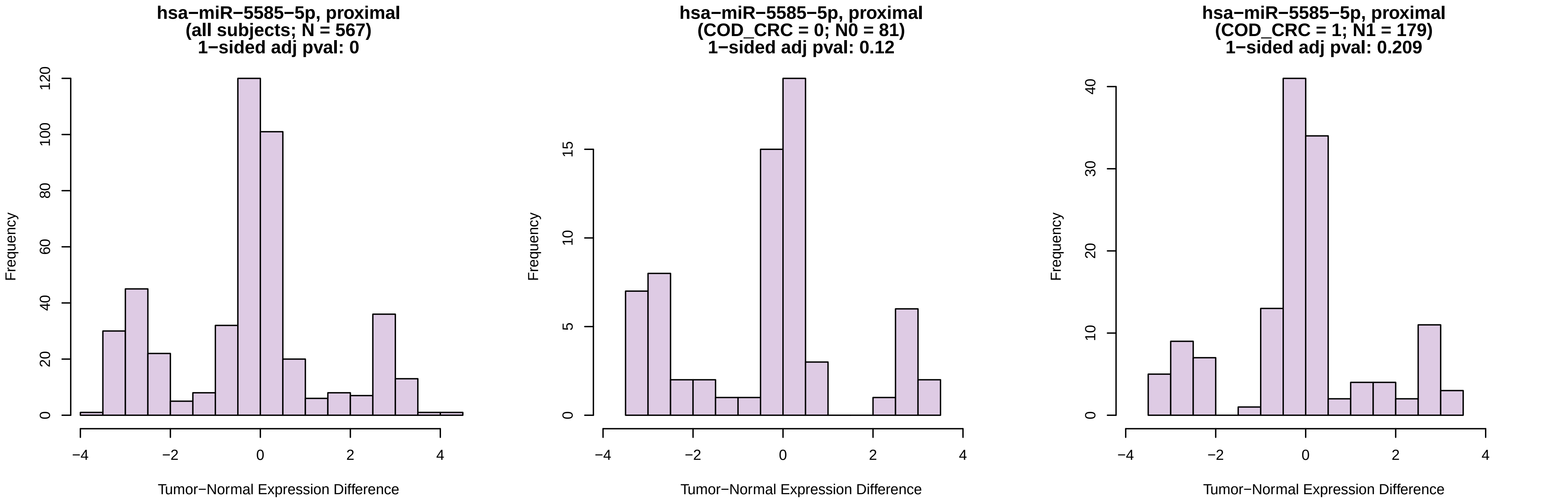

hsa−miR−5585−5p, proximal
(all subjects; N = 567)
1−sided adj pval: 0
Frequency
Tumor−Normal Expression Difference
hsa−miR−5585−5p, proximal
(COD_CRC = 0; N0 = 81)
1−sided adj pval: 0.12
Frequency
Tumor−Normal Expression Difference
hsa−miR−5585−5p, proximal
(COD_CRC = 1; N1 = 179)
1−sided adj pval: 0.209
Frequency
Tumor−Normal Expression Difference

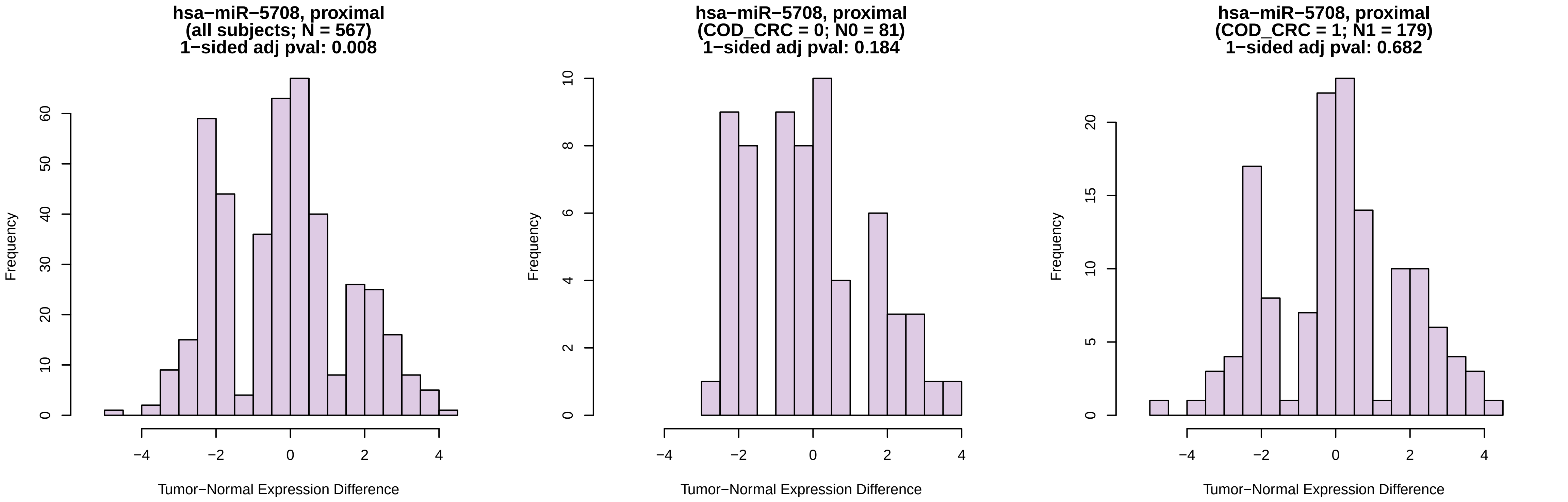
hsa−miR−5708, proximal
(all subjects; N = 567)
1−sided adj pval: 0.008
Frequency
Tumor−Normal Expression Difference
hsa−miR−5708, proximal
(COD_CRC = 0; N0 = 81)
1−sided adj pval: 0.184
Frequency
Tumor−Normal Expression Difference
hsa−miR−5708, proximal
(COD_CRC = 1; N1 = 179)
1−sided adj pval: 0.682
Frequency
Tumor−Normal Expression Difference

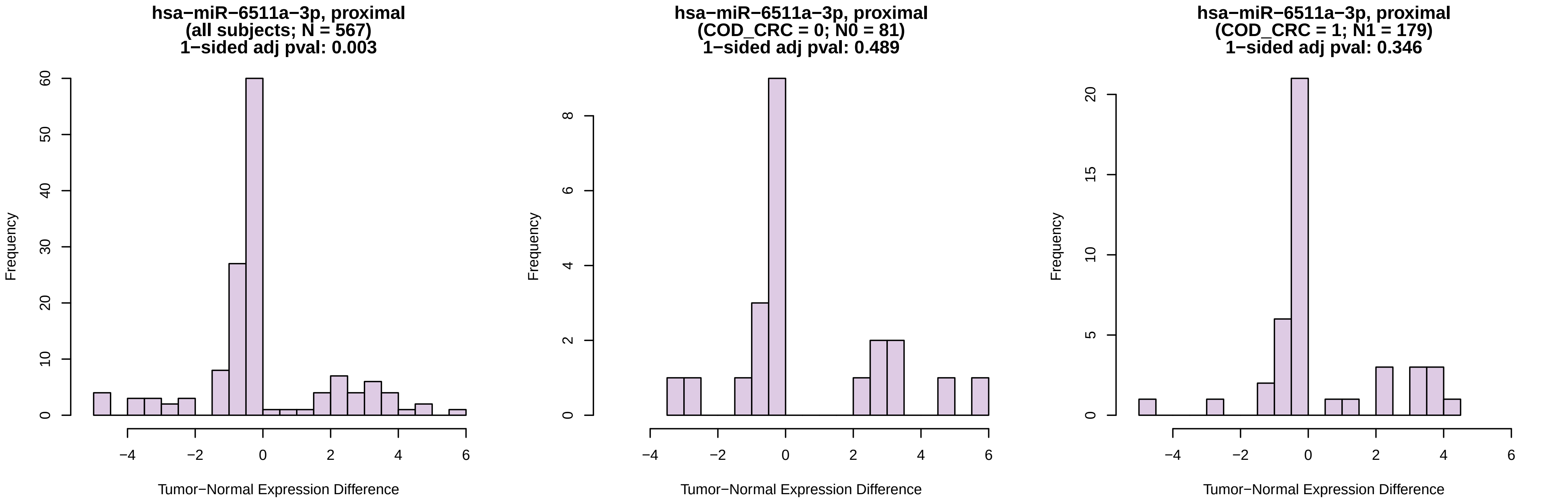

hsa−miR−6511a−3p, proximal
(all subjects; N = 567)
1−sided adj pval: 0.003
Frequency
Tumor−Normal Expression Difference
hsa−miR−6511a−3p, proximal
(COD_CRC = 0; N0 = 81)
1−sided adj pval: 0.489
Frequency
Tumor−Normal Expression Difference
hsa−miR−6511a−3p, proximal
(COD_CRC = 1; N1 = 179)
1−sided adj pval: 0.346
Frequency
Tumor−Normal Expression Difference

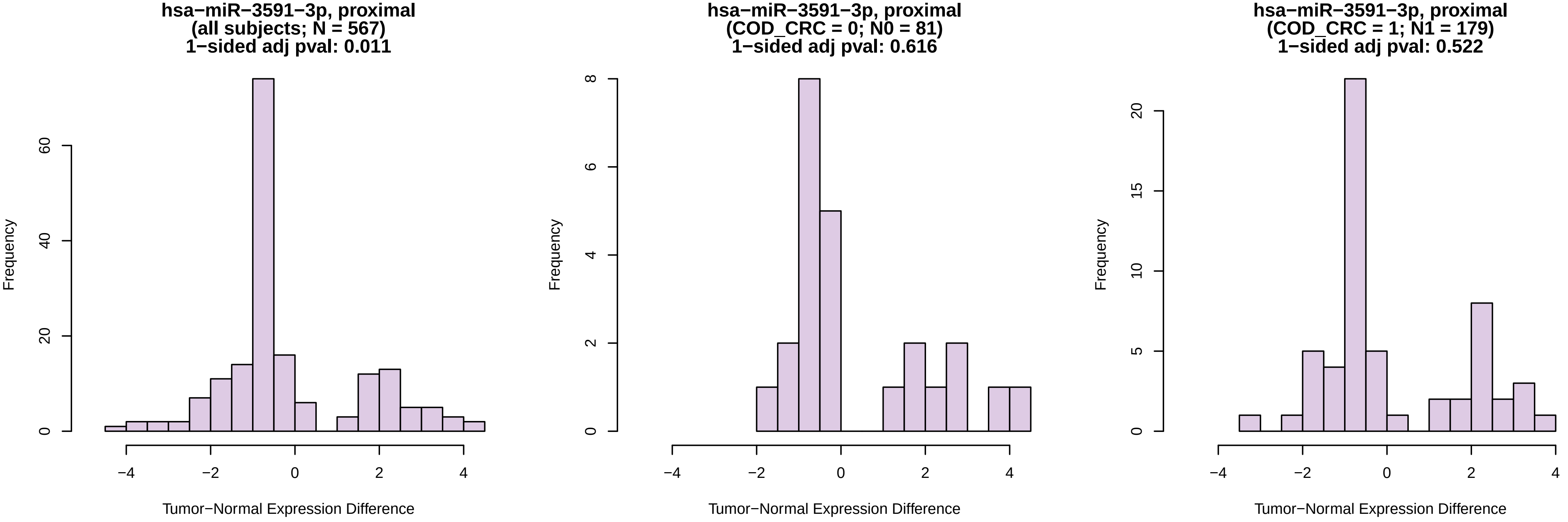
hsa−miR−3591−3p, proximal
(all subjects; N = 567)
1−sided adj pval: 0.011
Frequency
0
20
40
60
−4
−2
0
2
4
Tumor−Normal Expression Difference
hsa−miR−3591−3p, proximal
(COD_CRC = 0; N0 = 81)
1−sided adj pval: 0.616
Frequency
0
2
4
6
8
−4
−2
0
2
4
Tumor−Normal Expression Difference
hsa−miR−3591−3p, proximal
(COD_CRC = 1; N1 = 179)
1−sided adj pval: 0.522
Frequency
0
5
10
15
20
−4
−2
0
2
4
Tumor−Normal Expression Difference

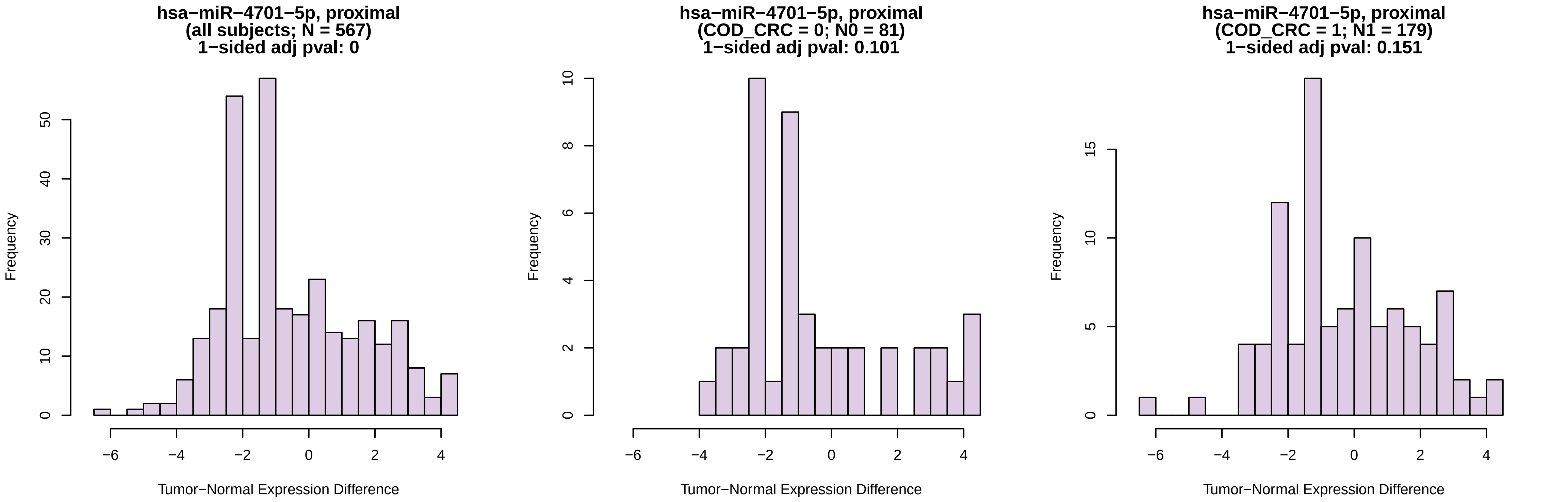
hsa−miR−4701−5p, proximal
(all subjects; N = 567)
1−sided adj pval: 0
Frequency
Tumor−Normal Expression Difference
hsa−miR−4701−5p, proximal
(COD_CRC = 0; N0 = 81)
1−sided adj pval: 0.101
Frequency
Tumor−Normal Expression Difference
hsa−miR−4701−5p, proximal
(COD_CRC = 1; N1 = 179)
1−sided adj pval: 0.151
Frequency
Tumor−Normal Expression Difference

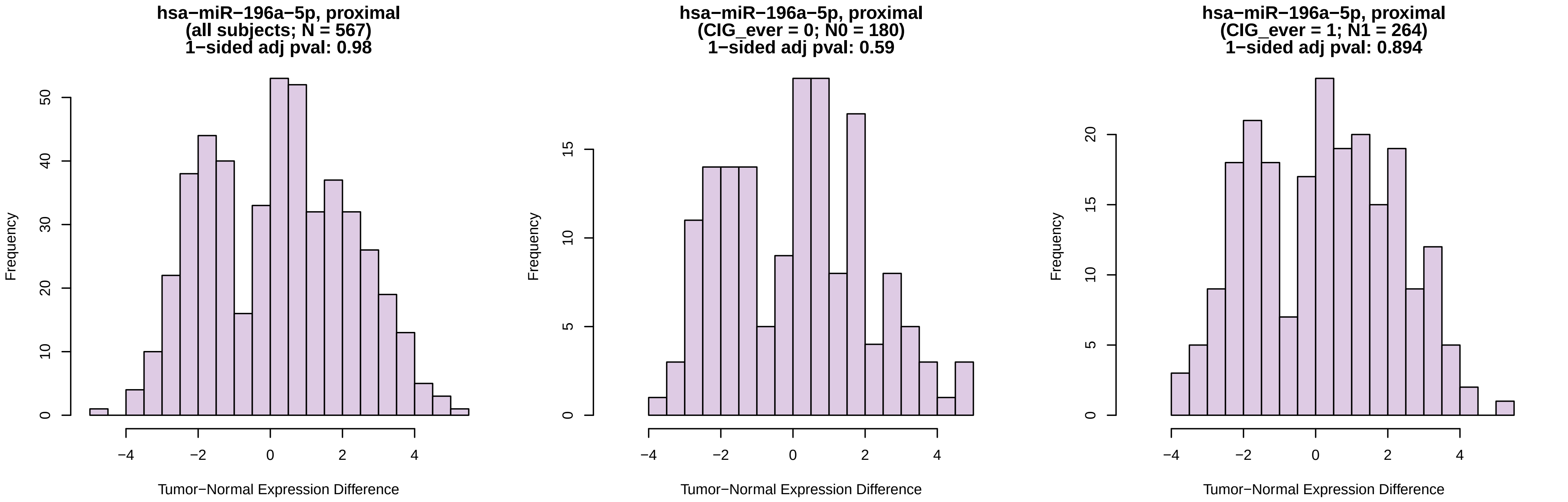
hsa−miR−196a−5p, proximal
(all subjects; N = 567)
1−sided adj pval: 0.98
hsa−miR−196a−5p, proximal
(CIG_ever = 0; N0 = 180)
1−sided adj pval: 0.59
hsa−miR−196a−5p, proximal
(CIG_ever = 1; N1 = 264)
1−sided adj pval: 0.894
Frequency
Tumor−Normal Expression Difference
0
10
20
30
40
50
5
15
−4
−2
2
4

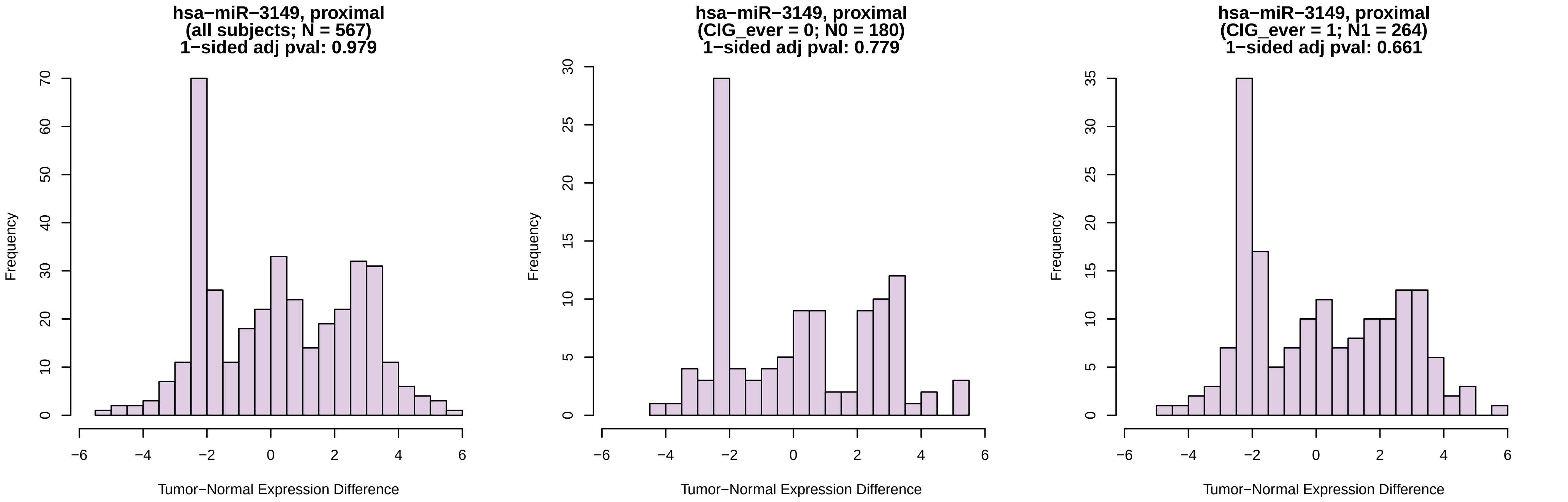
hsa−miR−3149, proximal
(all subjects; N = 567)
1−sided adj pval: 0.979
hsa−miR−3149, proximal
(CIG_ever = 0; N0 = 180)
1−sided adj pval: 0.779
hsa−miR−3149, proximal
(CIG_ever = 1; N1 = 264)
1−sided adj pval: 0.661
Frequency
Tumor−Normal Expression Difference
0
10
20
30
40
50
60
70
0
5
10
15
20
25
30
35
−6
−4
−2
0
2
4
6

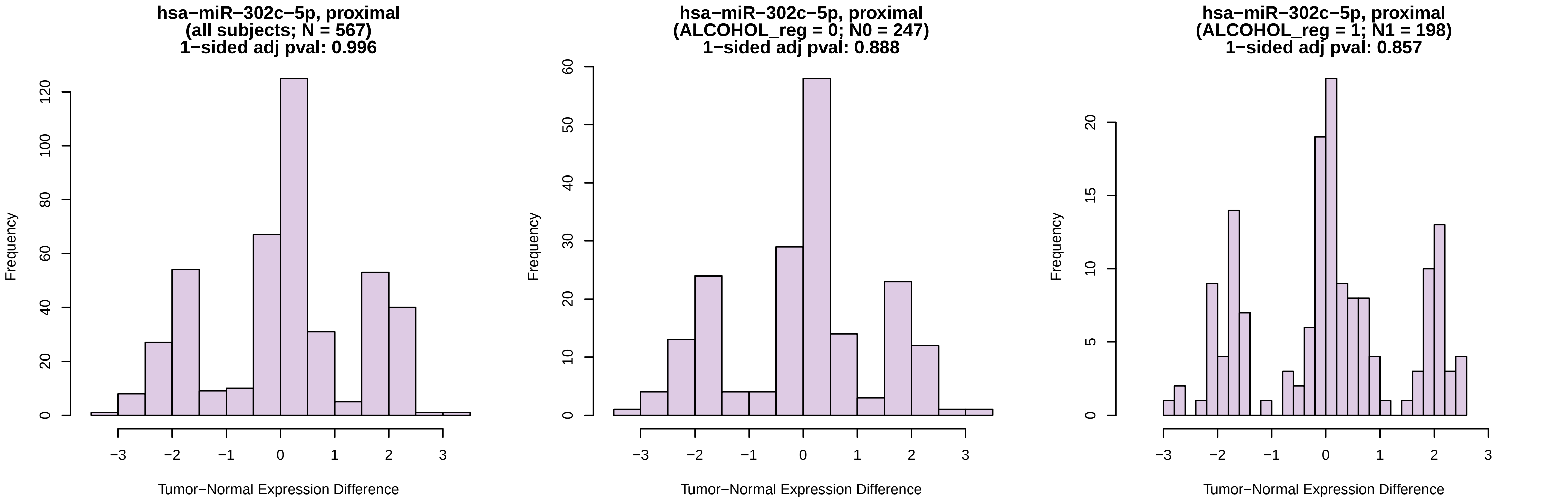
hsa−miR−302c−5p, proximal
(all subjects; N = 567)
1−sided adj pval: 0.996
Frequency
Tumor−Normal Expression Difference
hsa−miR−302c−5p, proximal
(ALCOHOL_reg = 0; N0 = 247)
1−sided adj pval: 0.888
Frequency
Tumor−Normal Expression Difference
hsa−miR−302c−5p, proximal
(ALCOHOL_reg = 1; N1 = 198)
1−sided adj pval: 0.857
Frequency
Tumor−Normal Expression Difference

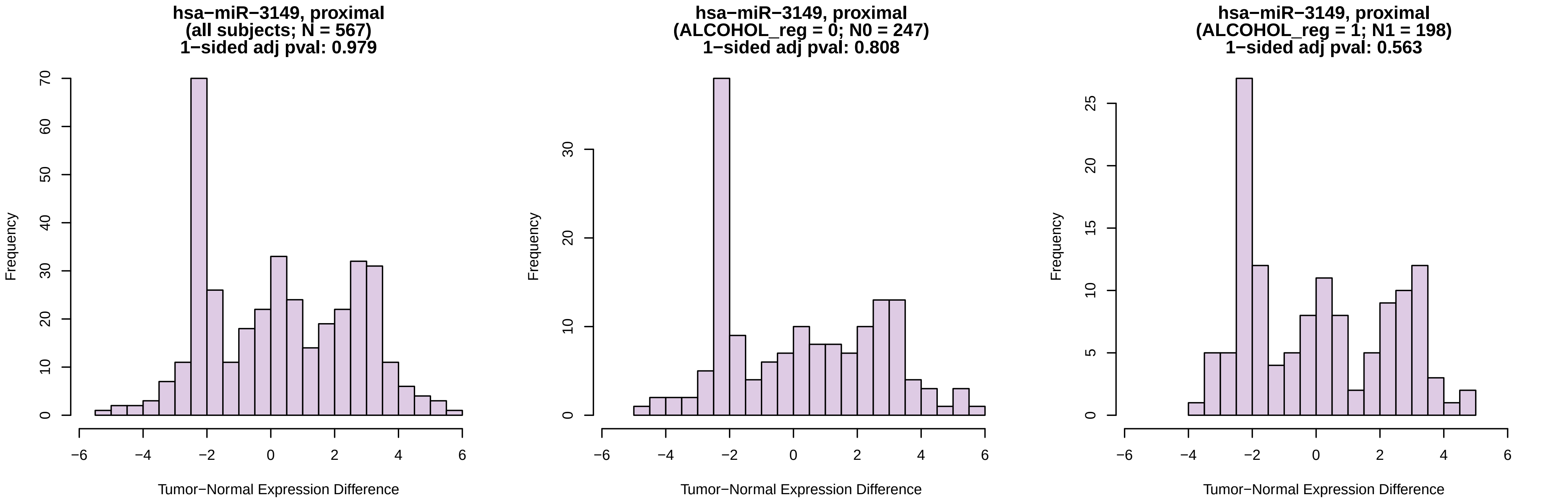
hsa−miR−3149, proximal
(all subjects; N = 567)
1−sided adj pval: 0.979
Frequency
Tumor−Normal Expression Difference
hsa−miR−3149, proximal
(ALCOHOL_reg = 0; N0 = 247)
1−sided adj pval: 0.808
Frequency
Tumor−Normal Expression Difference
hsa−miR−3149, proximal
(ALCOHOL_reg = 1; N1 = 198)
1−sided adj pval: 0.563
Frequency
Tumor−Normal Expression Difference

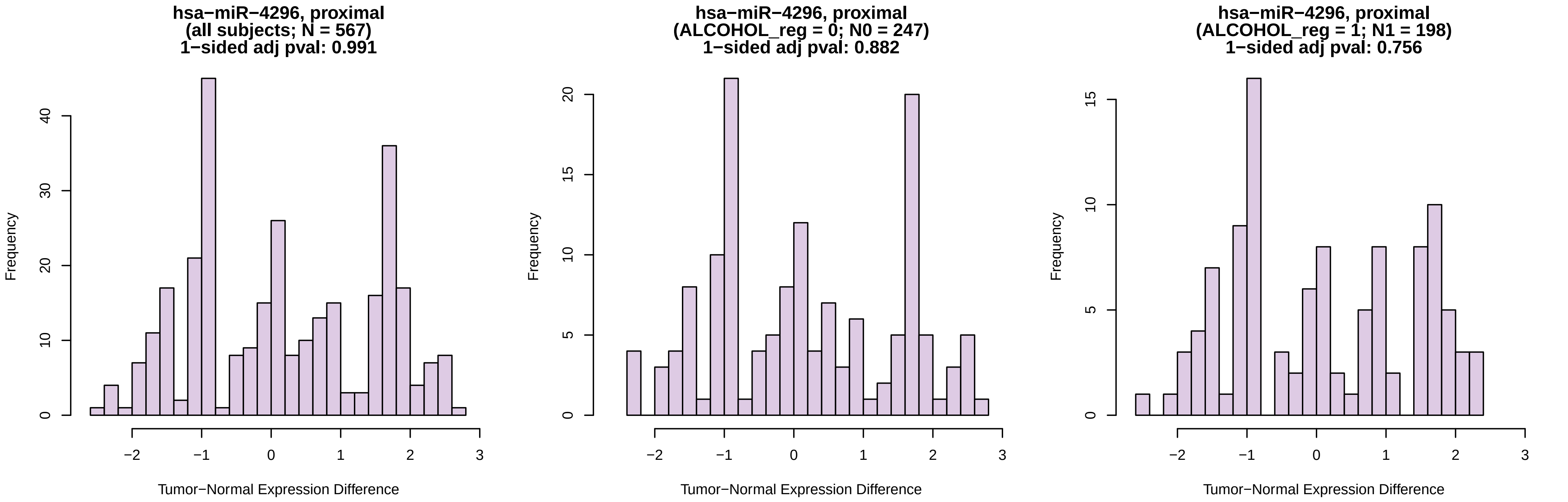
hsa−miR−4296, proximal
(all subjects; N = 567)
1−sided adj pval: 0.991
hsa−miR−4296, proximal
(ALCOHOL_reg = 0; N0 = 247)
1−sided adj pval: 0.882
hsa−miR−4296, proximal
(ALCOHOL_reg = 1; N1 = 198)
1−sided adj pval: 0.756
Frequency
Tumor−Normal Expression Difference
0
10
20
30
40
0
5
10
15
20
0
5
10
15
−2
−1
0
1
2
3

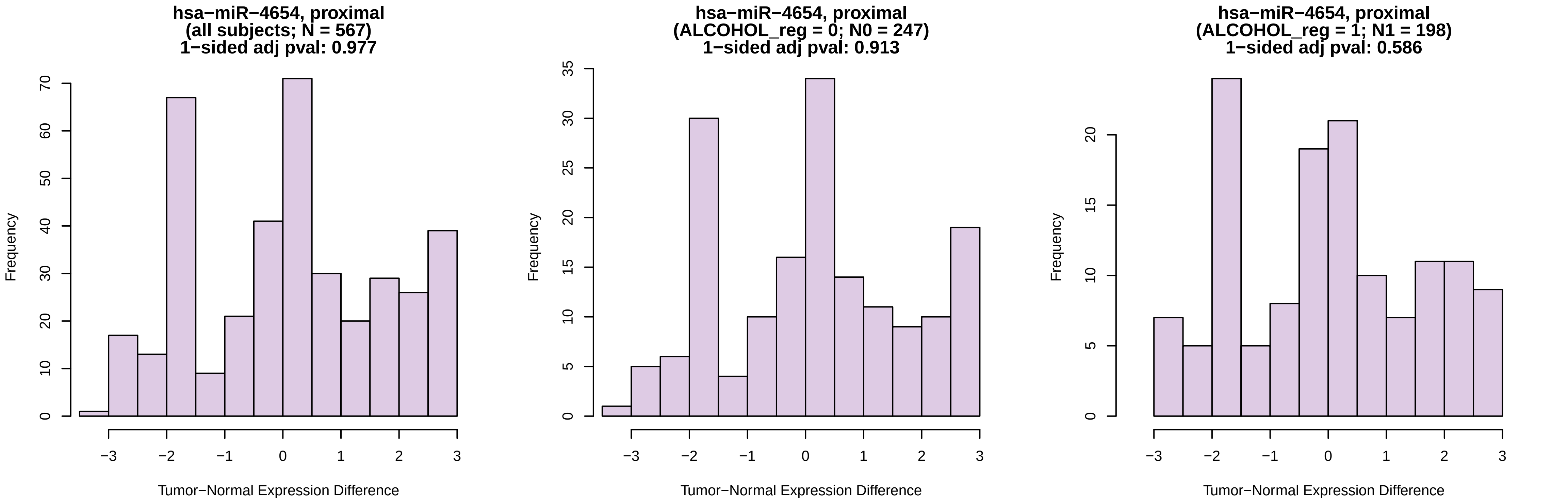
hsa−miR−4654, proximal
(all subjects; N = 567)
1−sided adj pval: 0.977
Frequency
Tumor−Normal Expression Difference
hsa−miR−4654, proximal
(ALCOHOL_reg = 0; N0 = 247)
1−sided adj pval: 0.913
Frequency
Tumor−Normal Expression Difference
hsa−miR−4654, proximal
(ALCOHOL_reg = 1; N1 = 198)
1−sided adj pval: 0.586
Frequency
Tumor−Normal Expression Difference

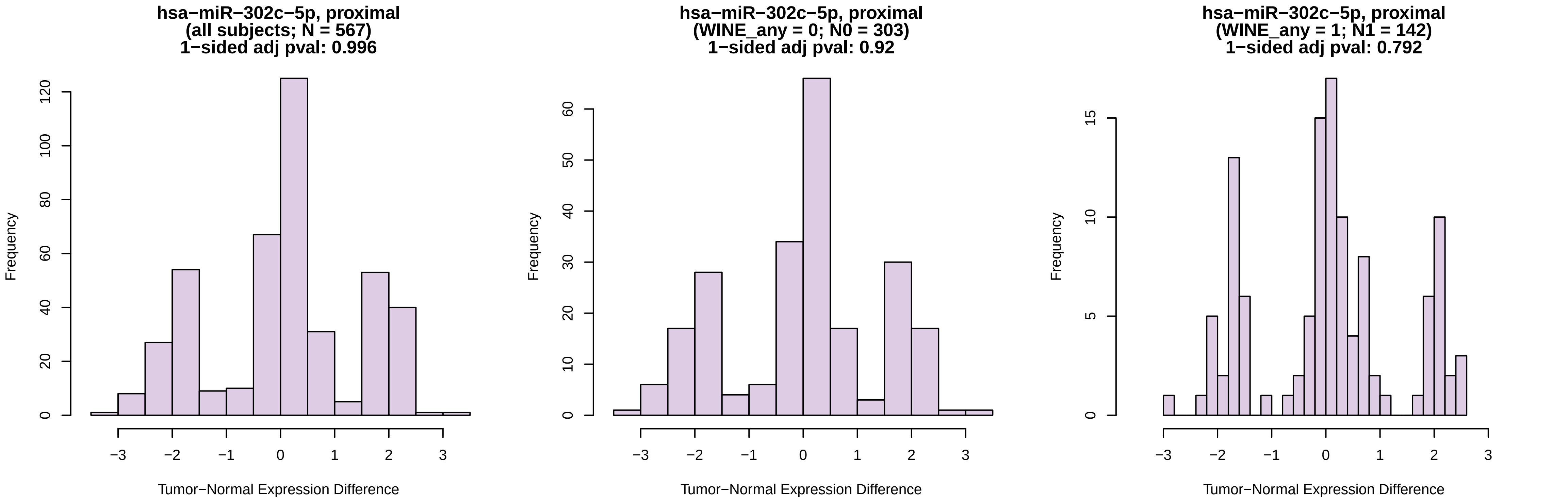

hsa−miR−302c−5p, proximal
(all subjects; N = 567)
1−sided adj pval: 0.996
Frequency
0
20
40
60
80
100
120
−3
−2
−1
0
1
2
3
Tumor−Normal Expression Difference
hsa−miR−302c−5p, proximal
(WINE_any = 0; N0 = 303)
1−sided adj pval: 0.92
Frequency
0
10
20
30
40
50
60
−3
−2
−1
0
1
2
3
Tumor−Normal Expression Difference
hsa−miR−302c−5p, proximal
(WINE_any = 1; N1 = 142)
1−sided adj pval: 0.792
Frequency
0
5
10
15
−3
−2
−1
0
1
2
3
Tumor−Normal Expression Difference

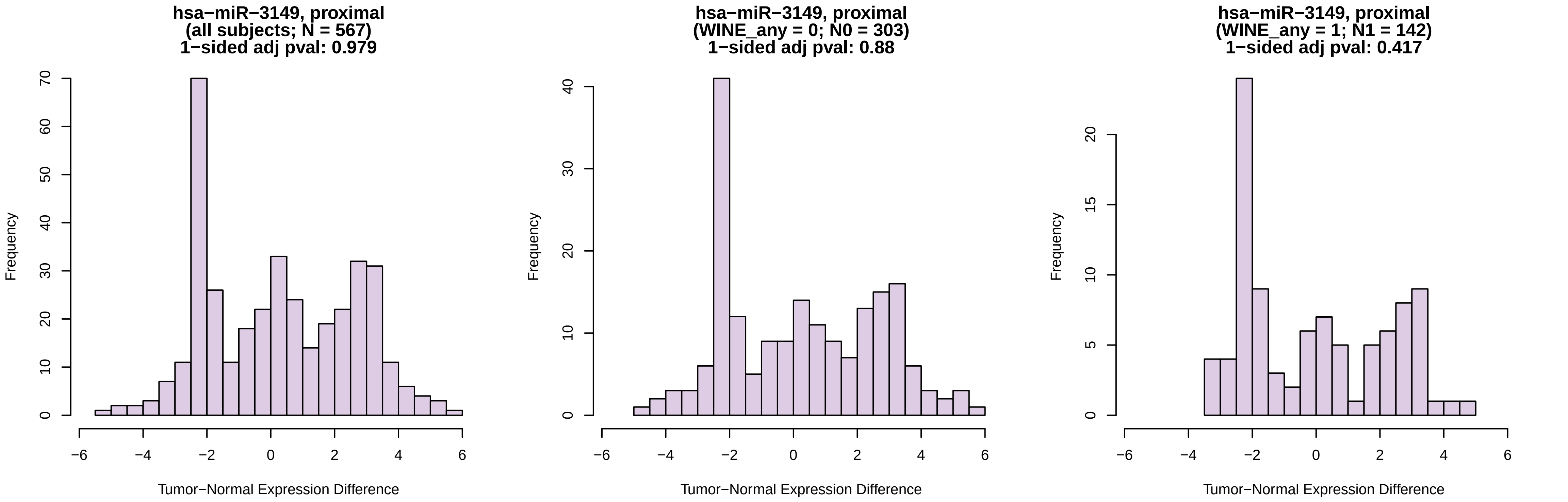
hsa−miR−3149, proximal
(all subjects; N = 567)
1−sided adj pval: 0.979
Frequency
Tumor−Normal Expression Difference
hsa−miR−3149, proximal
(WINE_any = 0; N0 = 303)
1−sided adj pval: 0.88
Frequency
Tumor−Normal Expression Difference
hsa−miR−3149, proximal
(WINE_any = 1; N1 = 142)
1−sided adj pval: 0.417
Frequency
Tumor−Normal Expression Difference

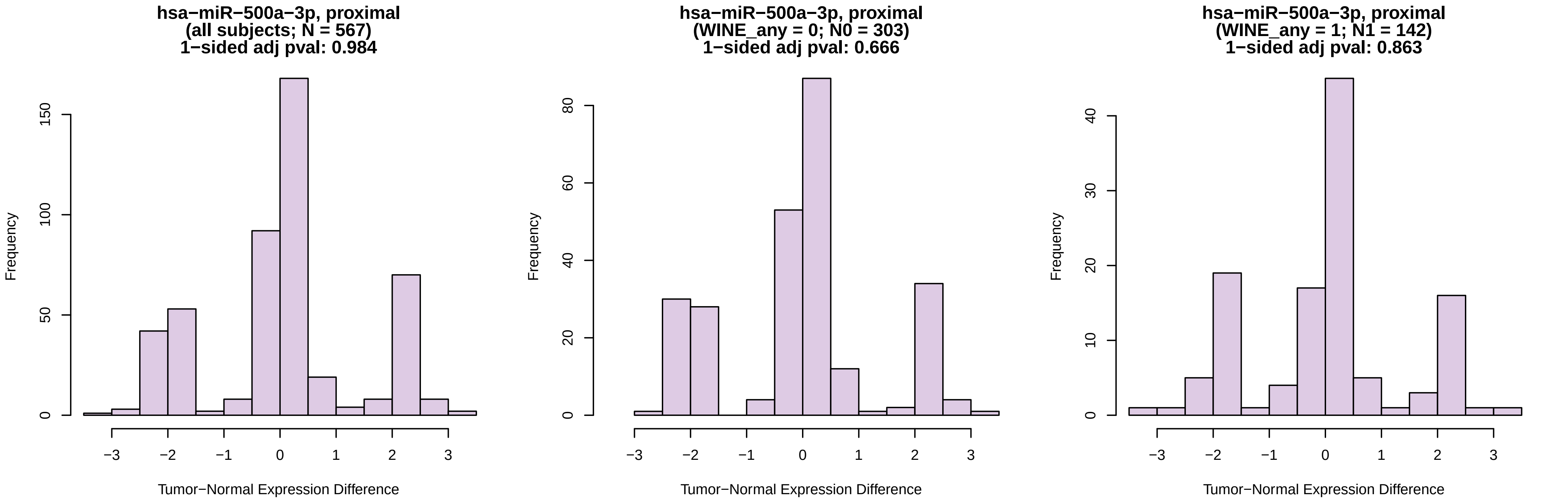
hsa−miR−500a−3p, proximal
(all subjects; N = 567)
1−sided adj pval: 0.984
Frequency
Tumor−Normal Expression Difference
hsa−miR−500a−3p, proximal
(WINE_any = 0; N0 = 303)
1−sided adj pval: 0.666
Frequency
Tumor−Normal Expression Difference
hsa−miR−500a−3p, proximal
(WINE_any = 1; N1 = 142)
1−sided adj pval: 0.863
Frequency
Tumor−Normal Expression Difference

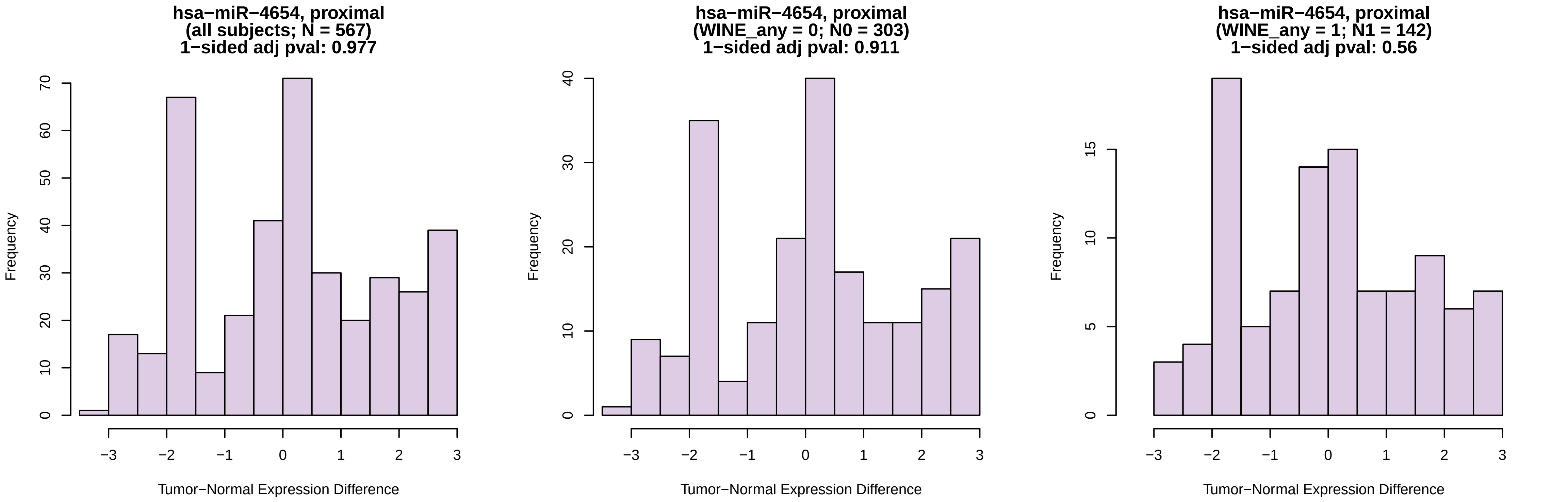

hsa−miR−4654, proximal
(all subjects; N = 567)
1−sided adj pval: 0.977
Frequency
0
10
20
30
40
50
60
70
−3
−2
−1
0
1
2
3
Tumor−Normal Expression Difference
hsa−miR−4654, proximal
(WINE_any = 0; N0 = 303)
1−sided adj pval: 0.911
Frequency
0
10
20
30
40
−3
−2
−1
0
1
2
3
Tumor−Normal Expression Difference
hsa−miR−4654, proximal
(WINE_any = 1; N1 = 142)
1−sided adj pval: 0.56
Frequency
0
5
10
15
−3
−2
−1
0
1
2
3
Tumor−Normal Expression Difference

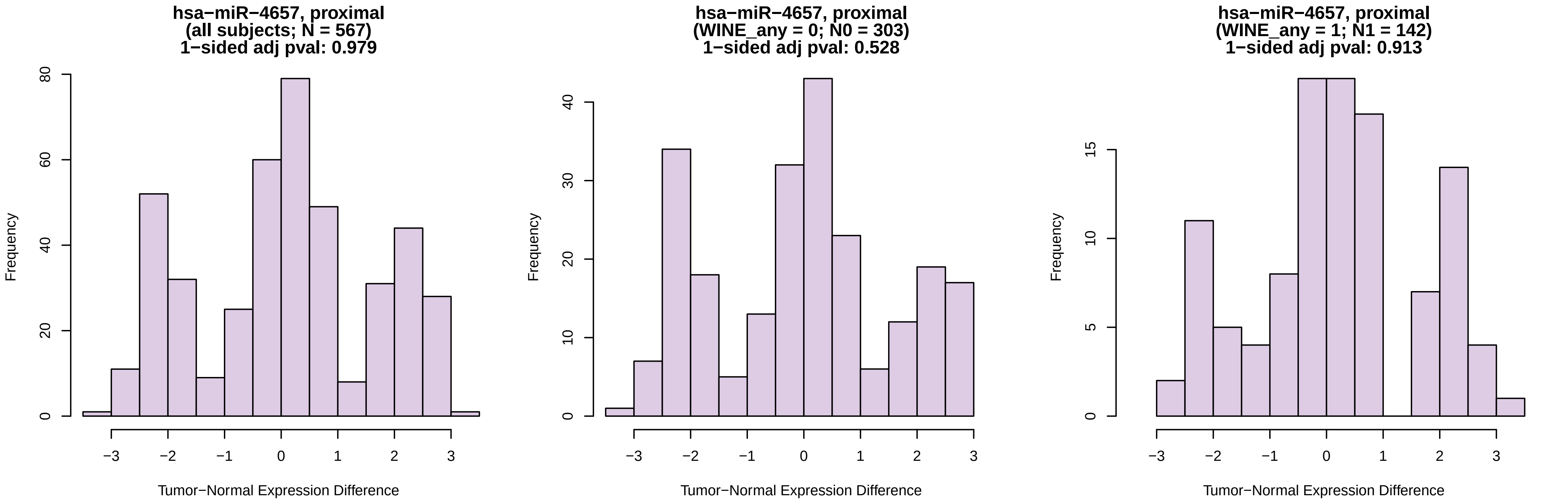
hsa−miR−4657, proximal
(all subjects; N = 567)
1−sided adj pval: 0.979
Frequency
Tumor−Normal Expression Difference
hsa−miR−4657, proximal
(WINE_any = 0; N0 = 303)
1−sided adj pval: 0.528
Frequency
Tumor−Normal Expression Difference
hsa−miR−4657, proximal
(WINE_any = 1; N1 = 142)
1−sided adj pval: 0.913
Frequency
Tumor−Normal Expression Difference

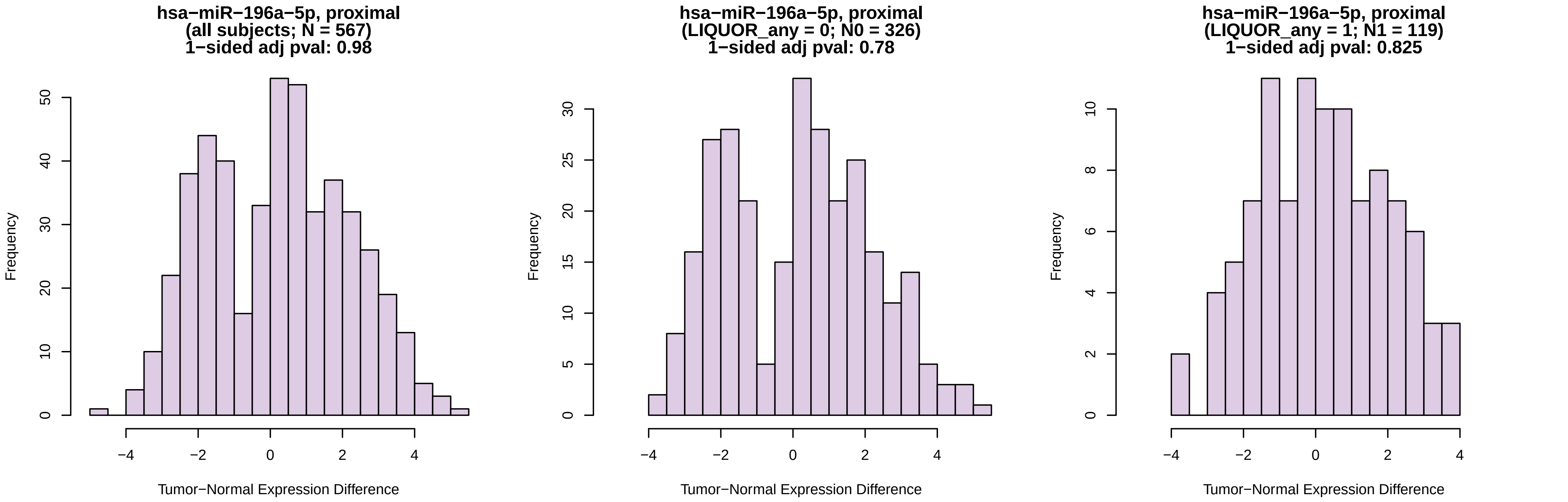

hsa−miR−196a−5p, proximal
(all subjects; N = 567)
1−sided adj pval: 0.98
Frequency
Tumor−Normal Expression Difference
hsa−miR−196a−5p, proximal
(LIQUOR_any = 0; N0 = 326)
1−sided adj pval: 0.78
Frequency
Tumor−Normal Expression Difference
hsa−miR−196a−5p, proximal
(LIQUOR_any = 1; N1 = 119)
1−sided adj pval: 0.825
Frequency
Tumor−Normal Expression Difference

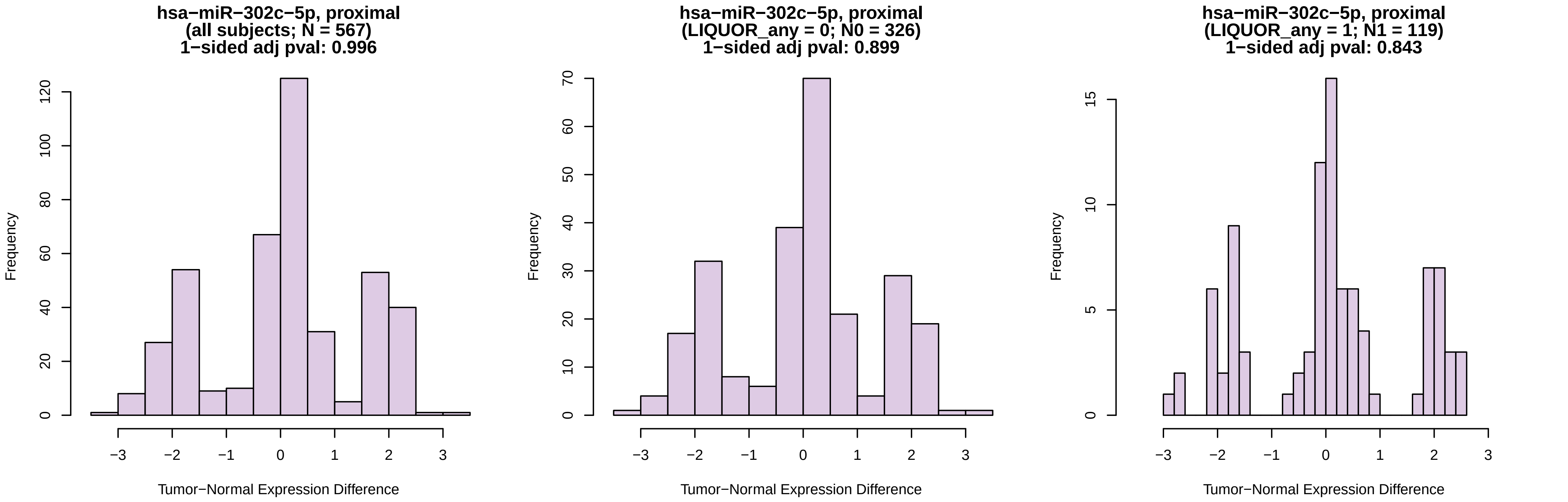
hsa−miR−302c−5p, proximal
(all subjects; N = 567)
1−sided adj pval: 0.996
Frequency
Tumor−Normal Expression Difference
hsa−miR−302c−5p, proximal
(LIQUOR_any = 0; N0 = 326)
1−sided adj pval: 0.899
Frequency
Tumor−Normal Expression Difference
hsa−miR−302c−5p, proximal
(LIQUOR_any = 1; N1 = 119)
1−sided adj pval: 0.843
Frequency
Tumor−Normal Expression Difference

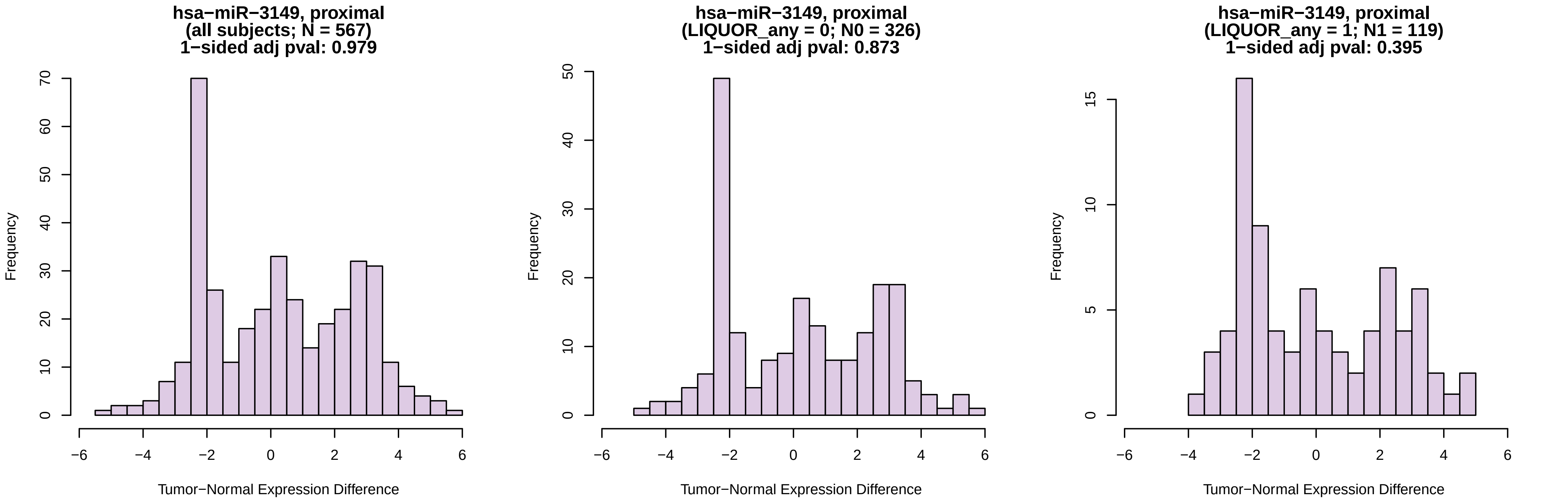

hsa−miR−3149, proximal
(all subjects; N = 567)
1−sided adj pval: 0.979
Frequency
Tumor−Normal Expression Difference
hsa−miR−3149, proximal
(LIQUOR_any = 0; N0 = 326)
1−sided adj pval: 0.873
Frequency
Tumor−Normal Expression Difference
hsa−miR−3149, proximal
(LIQUOR_any = 1; N1 = 119)
1−sided adj pval: 0.395
Frequency
Tumor−Normal Expression Difference

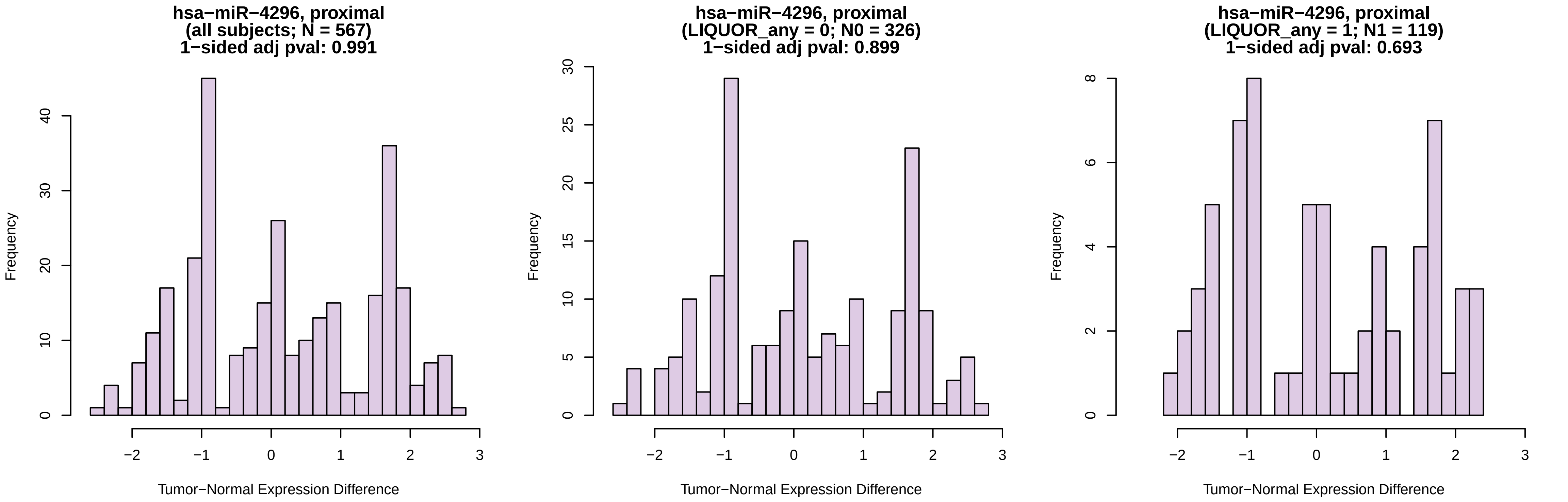

hsa−miR−4296, proximal
(all subjects; N = 567)
1−sided adj pval: 0.991
Frequency
Tumor−Normal Expression Difference
hsa−miR−4296, proximal
(LIQUOR_any = 0; N0 = 326)
1−sided adj pval: 0.899
Frequency
Tumor−Normal Expression Difference
hsa−miR−4296, proximal
(LIQUOR_any = 1; N1 = 119)
1−sided adj pval: 0.693
Frequency
Tumor−Normal Expression Difference

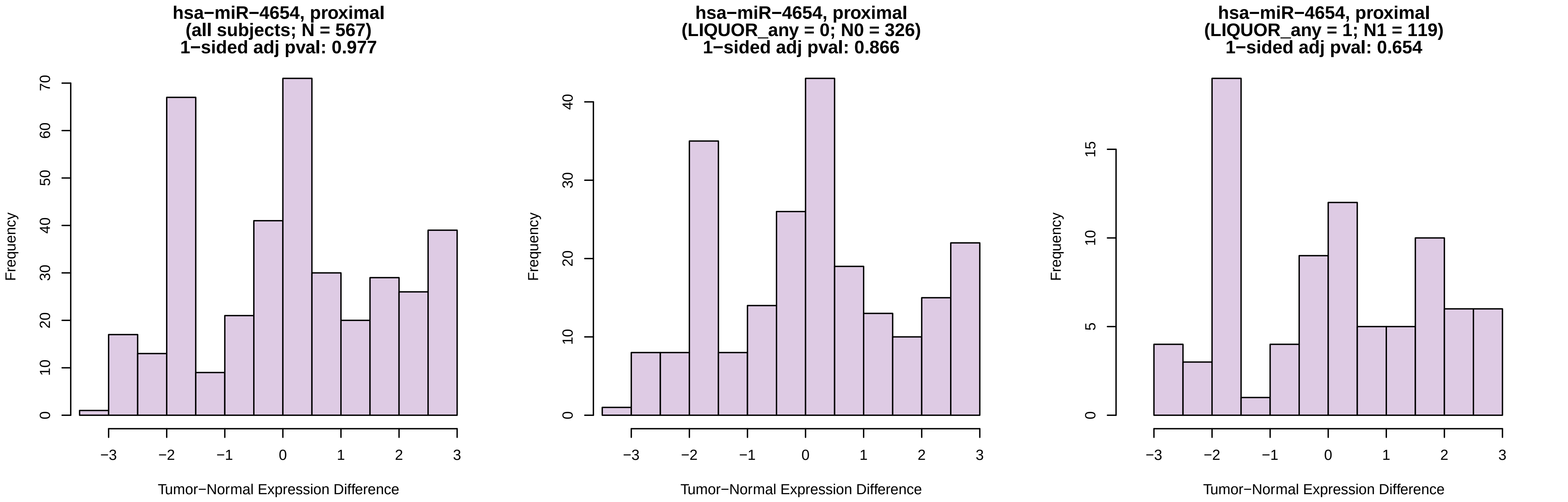

hsa−miR−4654, proximal
(all subjects; N = 567)
1−sided adj pval: 0.977
Frequency
Tumor−Normal Expression Difference
hsa−miR−4654, proximal
(LIQUOR_any = 0; N0 = 326)
1−sided adj pval: 0.866
Frequency
Tumor−Normal Expression Difference
hsa−miR−4654, proximal
(LIQUOR_any = 1; N1 = 119)
1−sided adj pval: 0.654
Frequency
Tumor−Normal Expression Difference

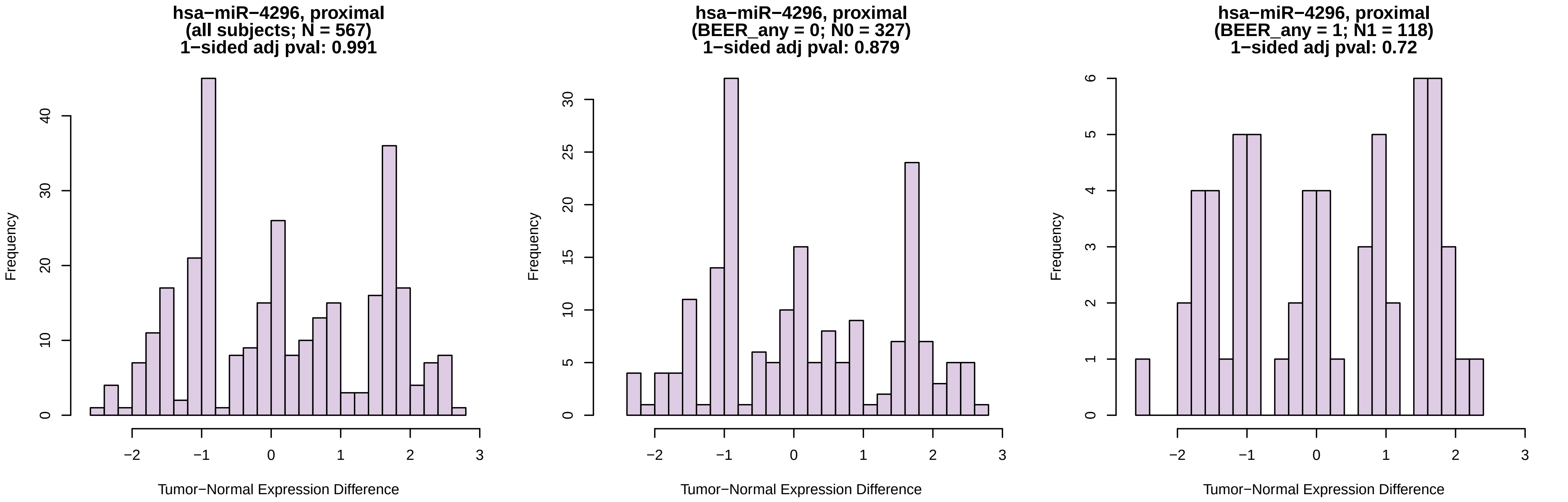
hsa−miR−4296, proximal
(all subjects; N = 567)
1−sided adj pval: 0.991
hsa−miR−4296, proximal
(BEER_any = 0; N0 = 327)
1−sided adj pval: 0.879
hsa−miR−4296, proximal
(BEER_any = 1; N1 = 118)
1−sided adj pval: 0.72
Frequency
Tumor−Normal Expression Difference

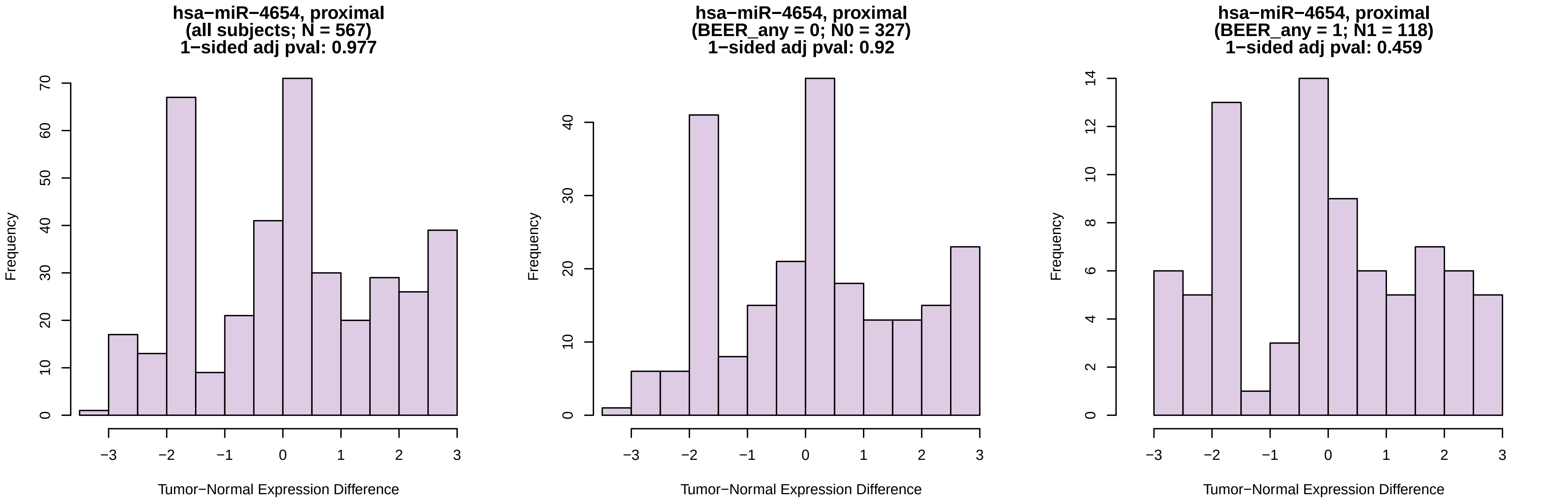
hsa−miR−4654, proximal
(all subjects; N = 567)
1−sided adj pval: 0.977
Frequency
Tumor−Normal Expression Difference
hsa−miR−4654, proximal
(BEER_any = 0; N0 = 327)
1−sided adj pval: 0.92
Frequency
Tumor−Normal Expression Difference
hsa−miR−4654, proximal
(BEER_any = 1; N1 = 118)
1−sided adj pval: 0.459
Frequency
Tumor−Normal Expression Difference

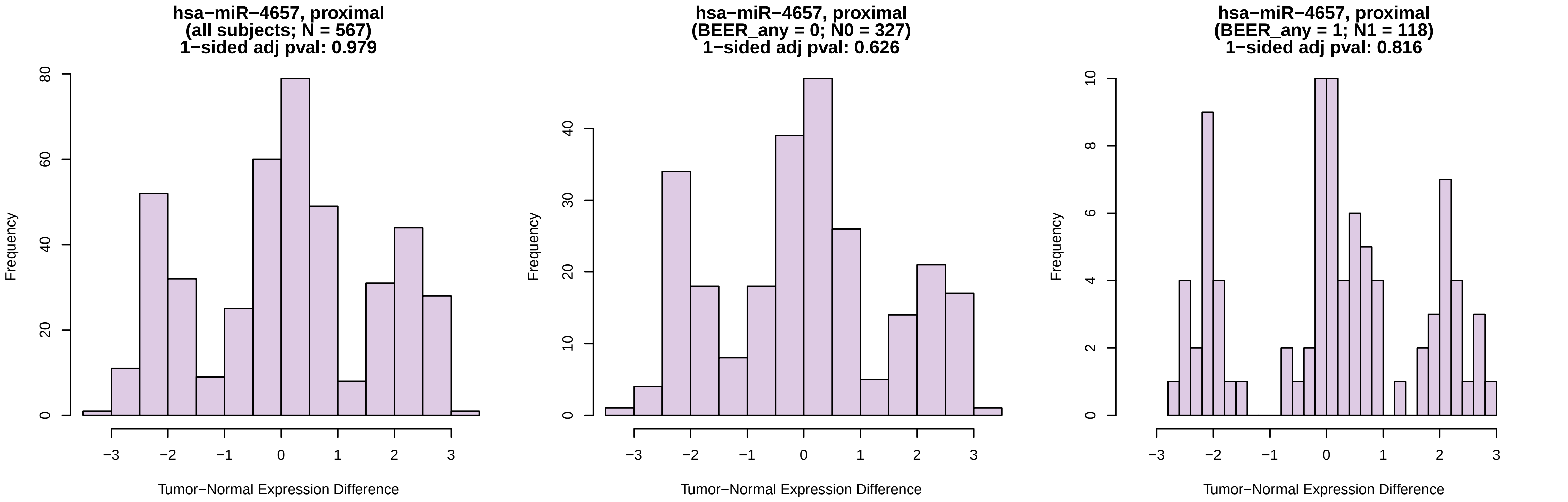

hsa−miR−4657, proximal
(all subjects; N = 567)
1−sided adj pval: 0.979
Frequency
Tumor−Normal Expression Difference
hsa−miR−4657, proximal
(BEER_any = 0; N0 = 327)
1−sided adj pval: 0.626
Frequency
Tumor−Normal Expression Difference
hsa−miR−4657, proximal
(BEER_any = 1; N1 = 118)
1−sided adj pval: 0.816
Frequency
Tumor−Normal Expression Difference

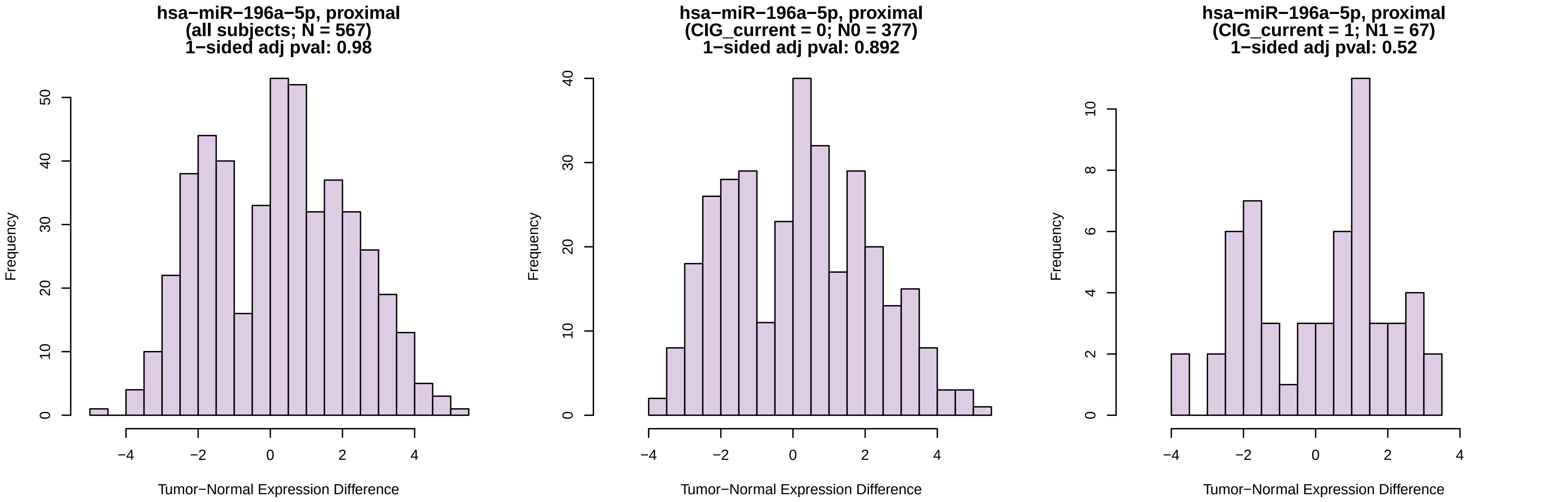

hsa−miR−196a−5p, proximal
(all subjects; N = 567)
1−sided adj pval: 0.98
Frequency
Tumor−Normal Expression Difference
hsa−miR−196a−5p, proximal
(CIG_current = 0; N0 = 377)
1−sided adj pval: 0.892
Frequency
Tumor−Normal Expression Difference
hsa−miR−196a−5p, proximal
(CIG_current = 1; N1 = 67)
1−sided adj pval: 0.52
Frequency
Tumor−Normal Expression Difference

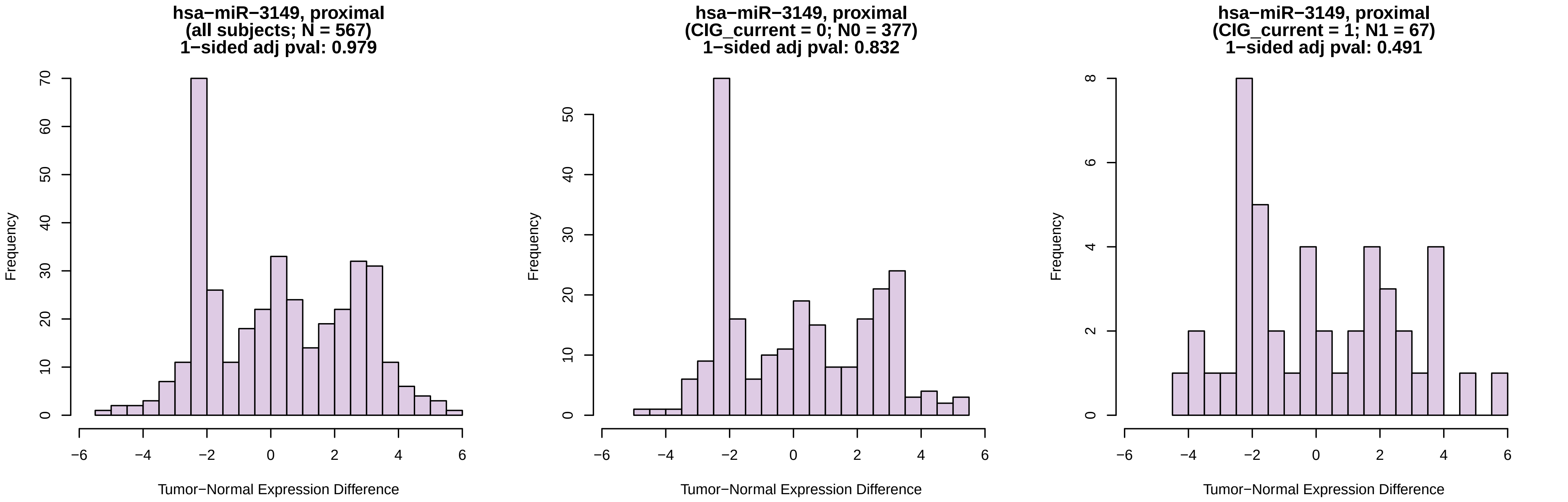

hsa−miR−3149, proximal
(all subjects; N = 567)
1−sided adj pval: 0.979
Frequency
Tumor−Normal Expression Difference
hsa−miR−3149, proximal
(CIG_current = 0; N0 = 377)
1−sided adj pval: 0.832
Frequency
Tumor−Normal Expression Difference
hsa−miR−3149, proximal
(CIG_current = 1; N1 = 67)
1−sided adj pval: 0.491
Frequency
Tumor−Normal Expression Difference

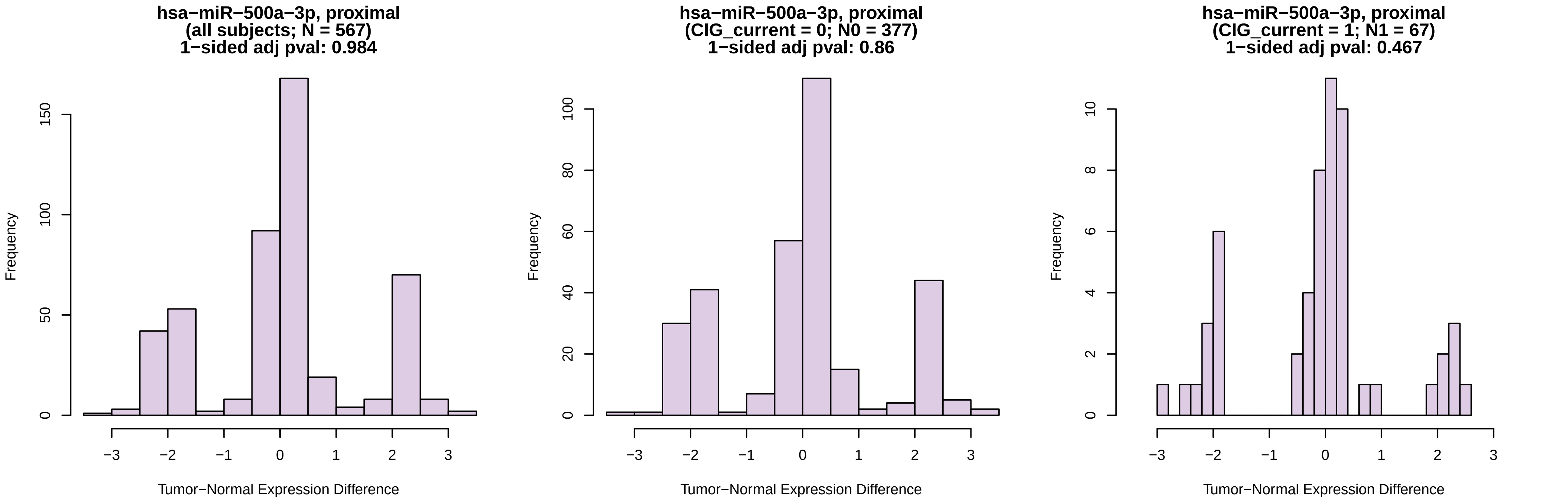

hsa−miR−500a−3p, proximal
(all subjects; N = 567)
1−sided adj pval: 0.984
Frequency
0
50
100
150
−3
−2
−1
0
1
2
3
Tumor−Normal Expression Difference
hsa−miR−500a−3p, proximal
(CIG_current = 0; N0 = 377)
1−sided adj pval: 0.86
Frequency
0
20
40
60
80
100
−3
−2
−1
0
1
2
3
Tumor−Normal Expression Difference
hsa−miR−500a−3p, proximal
(CIG_current = 1; N1 = 67)
1−sided adj pval: 0.467
Frequency
0
2
4
6
8
10
−3
−2
−1
0
1
2
3
Tumor−Normal Expression Difference

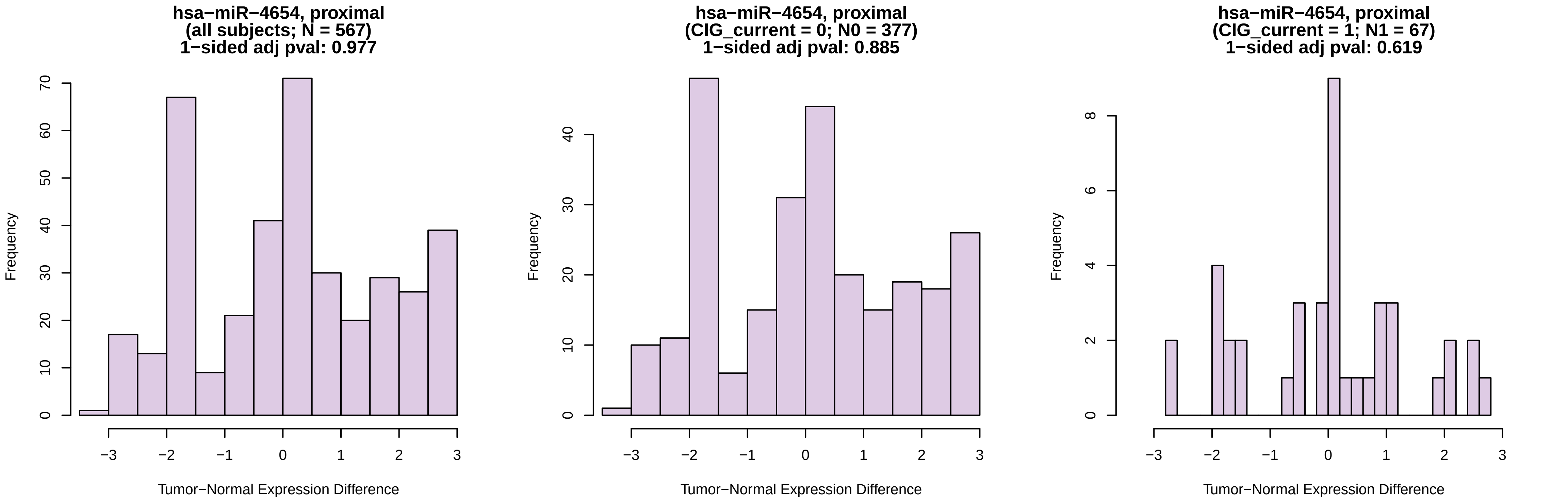
hsa−miR−4654, proximal
(all subjects; N = 567)
1−sided adj pval: 0.977
Frequency
Tumor−Normal Expression Difference
hsa−miR−4654, proximal
(CIG_current = 0; N0 = 377)
1−sided adj pval: 0.885
Frequency
Tumor−Normal Expression Difference
hsa−miR−4654, proximal
(CIG_current = 1; N1 = 67)
1−sided adj pval: 0.619
Frequency
Tumor−Normal Expression Difference

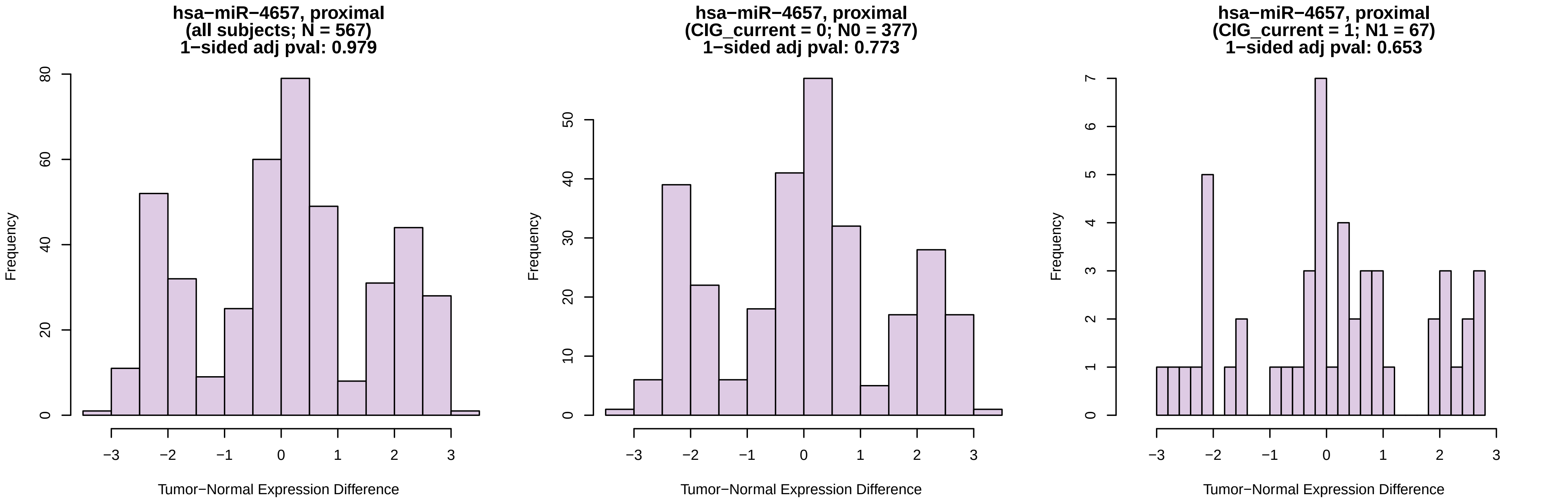
hsa−miR−4657, proximal
(all subjects; N = 567)
1−sided adj pval: 0.979
Frequency
0
20
40
60
80
−3
−2
−1
0
1
2
3
Tumor−Normal Expression Difference
hsa−miR−4657, proximal
(CIG_current = 0; N0 = 377)
1−sided adj pval: 0.773
Frequency
0
10
20
30
40
50
−3
−2
−1
0
1
2
3
Tumor−Normal Expression Difference
hsa−miR−4657, proximal
(CIG_current = 1; N1 = 67)
1−sided adj pval: 0.653
Frequency
0
1
2
3
4
5
6
7
−3
−2
−1
0
1
2
3
Tumor−Normal Expression Difference

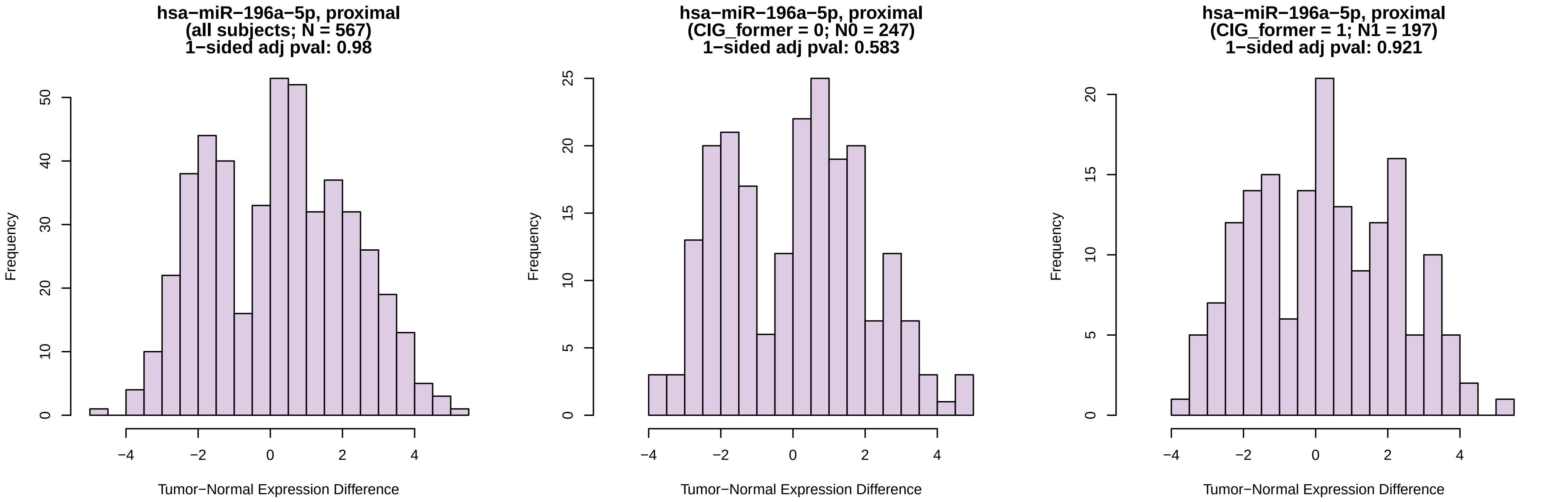
hsa−miR−196a−5p, proximal
(all subjects; N = 567)
1−sided adj pval: 0.98
Frequency
Tumor−Normal Expression Difference
hsa−miR−196a−5p, proximal
(CIG_former = 0; N0 = 247)
1−sided adj pval: 0.583
Frequency
Tumor−Normal Expression Difference
hsa−miR−196a−5p, proximal
(CIG_former = 1; N1 = 197)
1−sided adj pval: 0.921
Frequency
Tumor−Normal Expression Difference

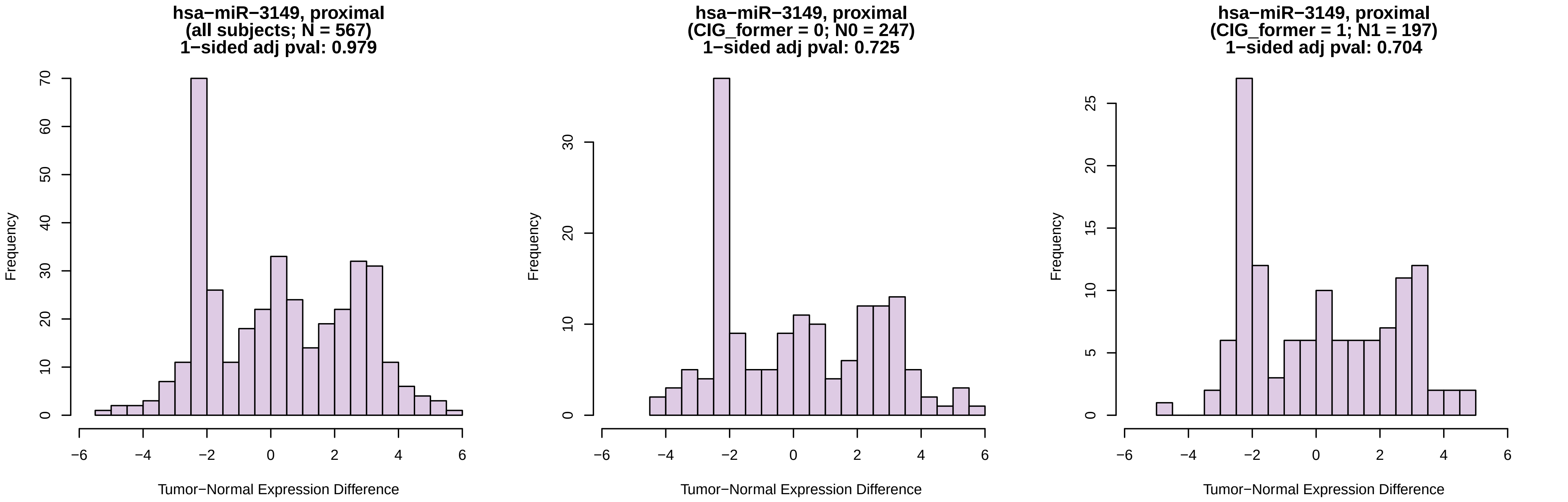

hsa−miR−3149, proximal
(all subjects; N = 567)
1−sided adj pval: 0.979
Frequency
0
10
20
30
40
50
60
70
−6
−4
−2
0
2
4
6
Tumor−Normal Expression Difference
hsa−miR−3149, proximal
(CIG_former = 0; N0 = 247)
1−sided adj pval: 0.725
Frequency
0
10
20
30
−6
−4
−2
0
2
4
6
Tumor−Normal Expression Difference
hsa−miR−3149, proximal
(CIG_former = 1; N1 = 197)
1−sided adj pval: 0.704
Frequency
0
5
10
15
20
25
−6
−4
−2
0
2
4
6
Tumor−Normal Expression Difference

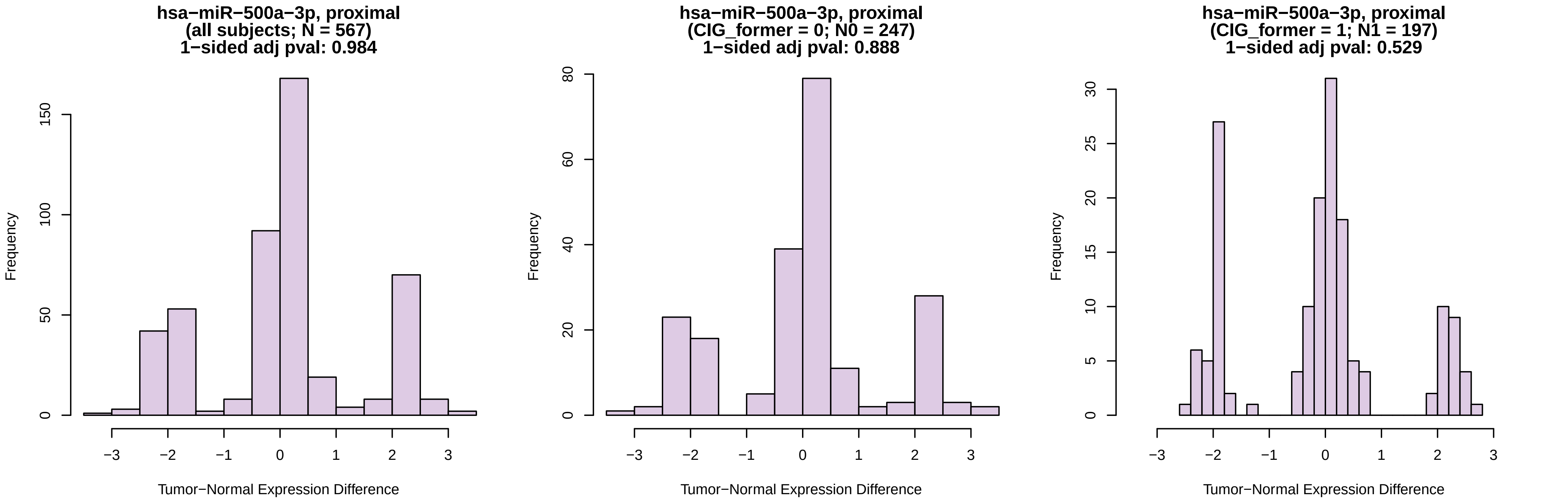
hsa−miR−500a−3p, proximal
(all subjects; N = 567)
1−sided adj pval: 0.984
Frequency
0
50
100
150
−3
−2
−1
0
1
2
3
Tumor−Normal Expression Difference
hsa−miR−500a−3p, proximal
(CIG_former = 0; N0 = 247)
1−sided adj pval: 0.888
Frequency
0
20
40
60
80
−3
−2
−1
0
1
2
3
Tumor−Normal Expression Difference
hsa−miR−500a−3p, proximal
(CIG_former = 1; N1 = 197)
1−sided adj pval: 0.529
Frequency
0
5
10
15
20
25
30
−3
−2
−1
0
1
2
3
Tumor−Normal Expression Difference

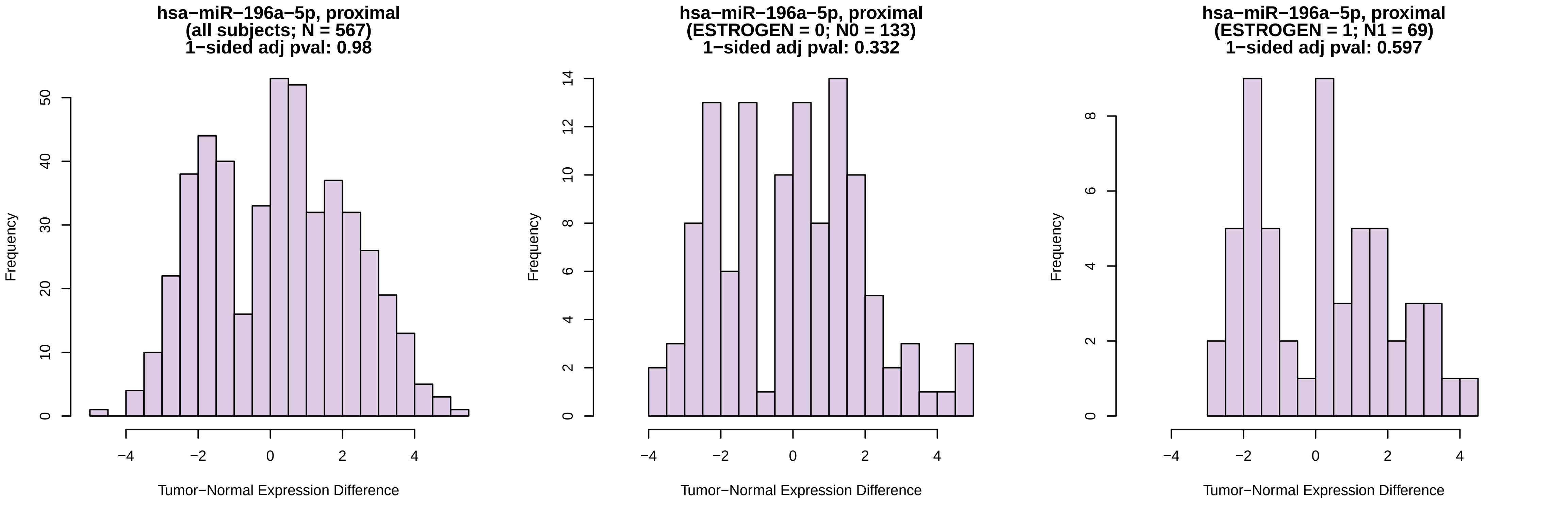
hsa−miR−196a−5p, proximal
(all subjects; N = 567)
1−sided adj pval: 0.98
Frequency
Tumor−Normal Expression Difference
hsa−miR−196a−5p, proximal
(ESTROGEN = 0; N0 = 133)
1−sided adj pval: 0.332
Frequency
Tumor−Normal Expression Difference
hsa−miR−196a−5p, proximal
(ESTROGEN = 1; N1 = 69)
1−sided adj pval: 0.597
Frequency
Tumor−Normal Expression Difference

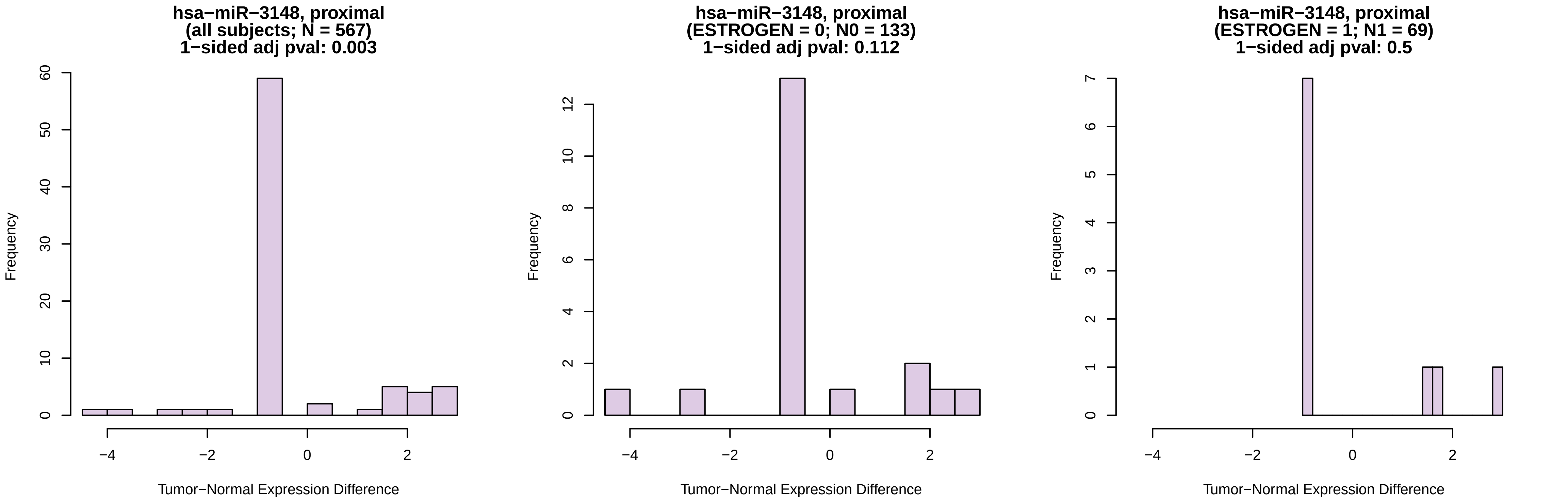

hsa−miR−3148, proximal
(all subjects; N = 567)
1−sided adj pval: 0.003
Frequency
0
10
20
30
40
50
60
−4
−2
0
2
Tumor−Normal Expression Difference
hsa−miR−3148, proximal
(ESTROGEN = 0; N0 = 133)
1−sided adj pval: 0.112
Frequency
0
2
4
6
8
10
12
−4
−2
0
2
Tumor−Normal Expression Difference
hsa−miR−3148, proximal
(ESTROGEN = 1; N1 = 69)
1−sided adj pval: 0.5
Frequency
0
1
2
3
4
5
6
7
−4
−2
0
2
Tumor−Normal Expression Difference

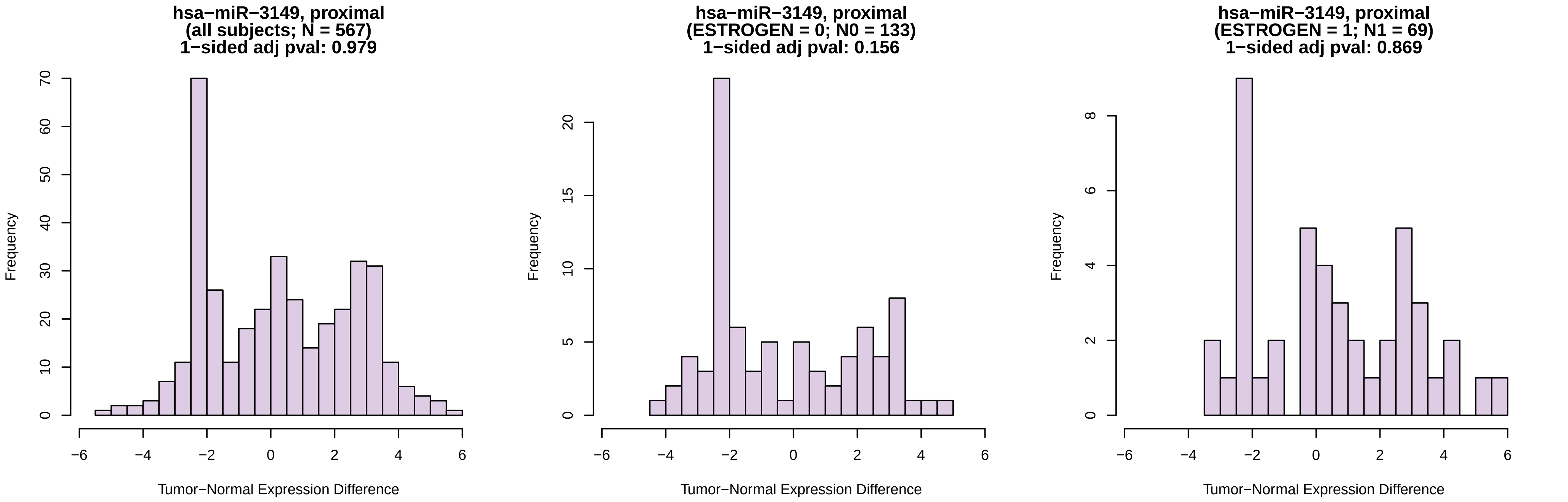

hsa−miR−3149, proximal
(all subjects; N = 567)
1−sided adj pval: 0.979
Frequency
Tumor−Normal Expression Difference
hsa−miR−3149, proximal
(ESTROGEN = 0; N0 = 133)
1−sided adj pval: 0.156
Frequency
Tumor−Normal Expression Difference
hsa−miR−3149, proximal
(ESTROGEN = 1; N1 = 69)
1−sided adj pval: 0.869
Frequency
Tumor−Normal Expression Difference

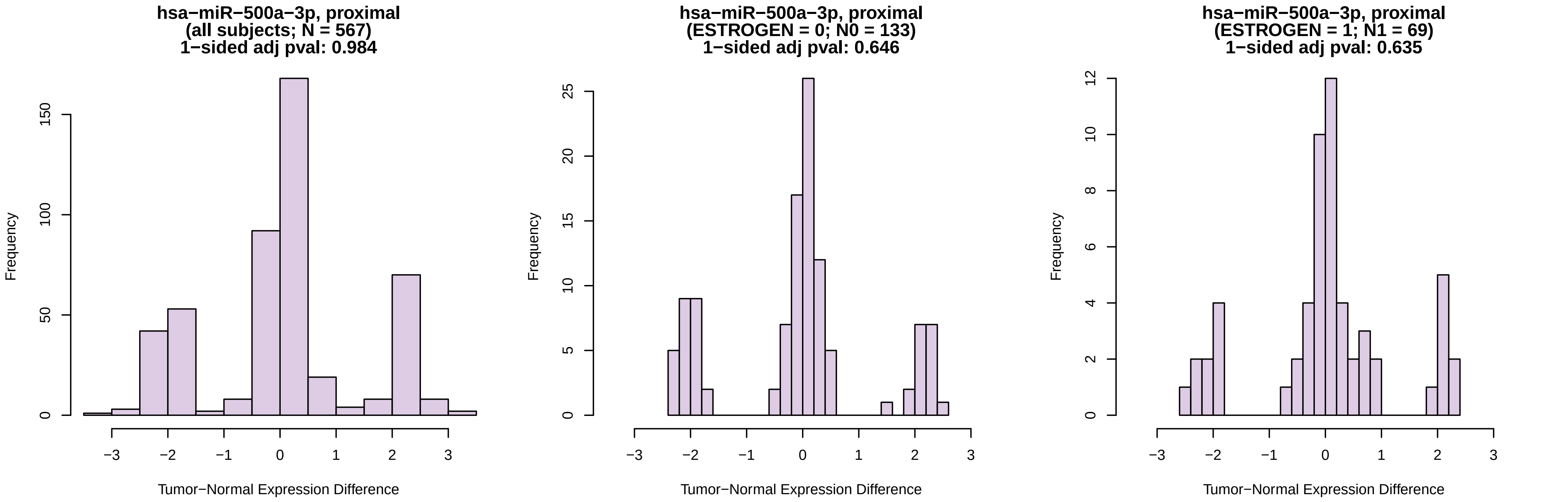
hsa−miR−500a−3p, proximal
(all subjects; N = 567)
1−sided adj pval: 0.984
Frequency
Tumor−Normal Expression Difference
hsa−miR−500a−3p, proximal
(ESTROGEN = 0; N0 = 133)
1−sided adj pval: 0.646
Frequency
Tumor−Normal Expression Difference
hsa−miR−500a−3p, proximal
(ESTROGEN = 1; N1 = 69)
1−sided adj pval: 0.635
Frequency
Tumor−Normal Expression Difference

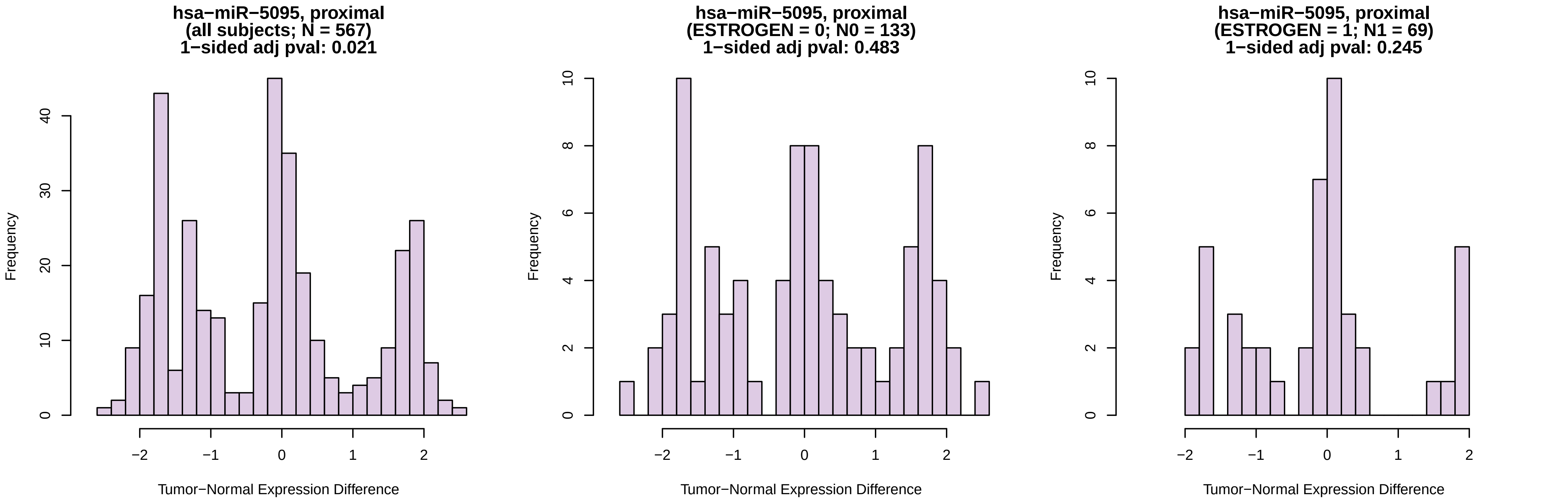
hsa−miR−5095, proximal
(all subjects; N = 567)
1−sided adj pval: 0.021
Frequency
Tumor−Normal Expression Difference
hsa−miR−5095, proximal
(ESTROGEN = 0; N0 = 133)
1−sided adj pval: 0.483
Frequency
Tumor−Normal Expression Difference
hsa−miR−5095, proximal
(ESTROGEN = 1; N1 = 69)
1−sided adj pval: 0.245
Frequency
Tumor−Normal Expression Difference

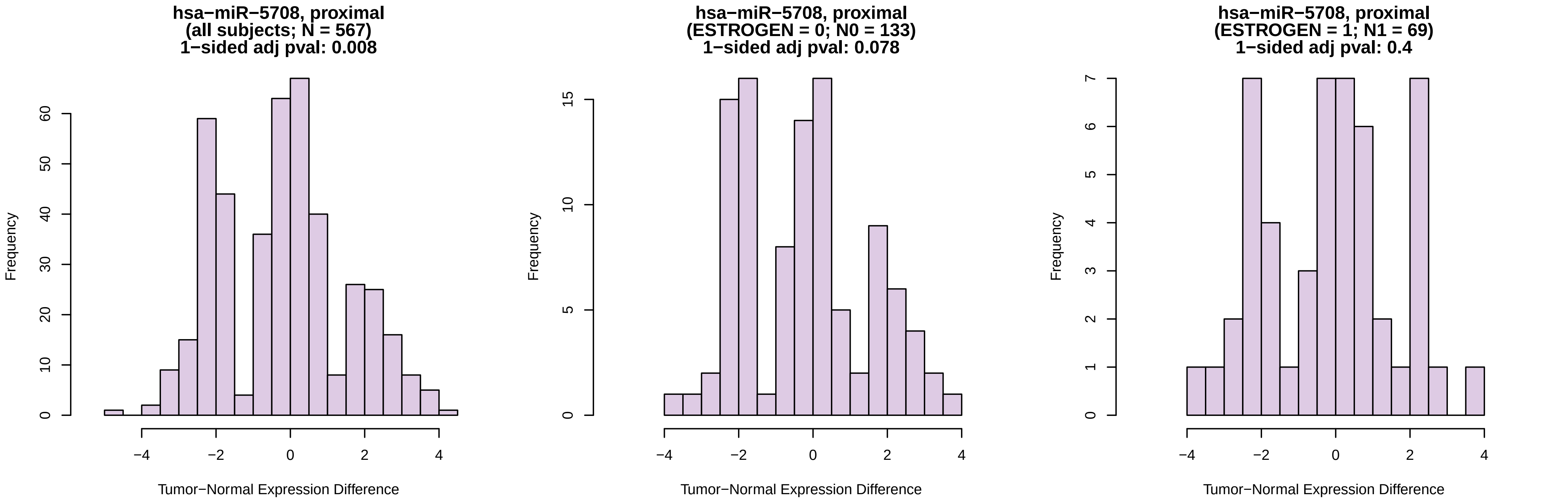
hsa−miR−5708, proximal
(all subjects; N = 567)
1−sided adj pval: 0.008
Frequency
Tumor−Normal Expression Difference
hsa−miR−5708, proximal
(ESTROGEN = 0; N0 = 133)
1−sided adj pval: 0.078
Frequency
Tumor−Normal Expression Difference
hsa−miR−5708, proximal
(ESTROGEN = 1; N1 = 69)
1−sided adj pval: 0.4
Frequency
Tumor−Normal Expression Difference

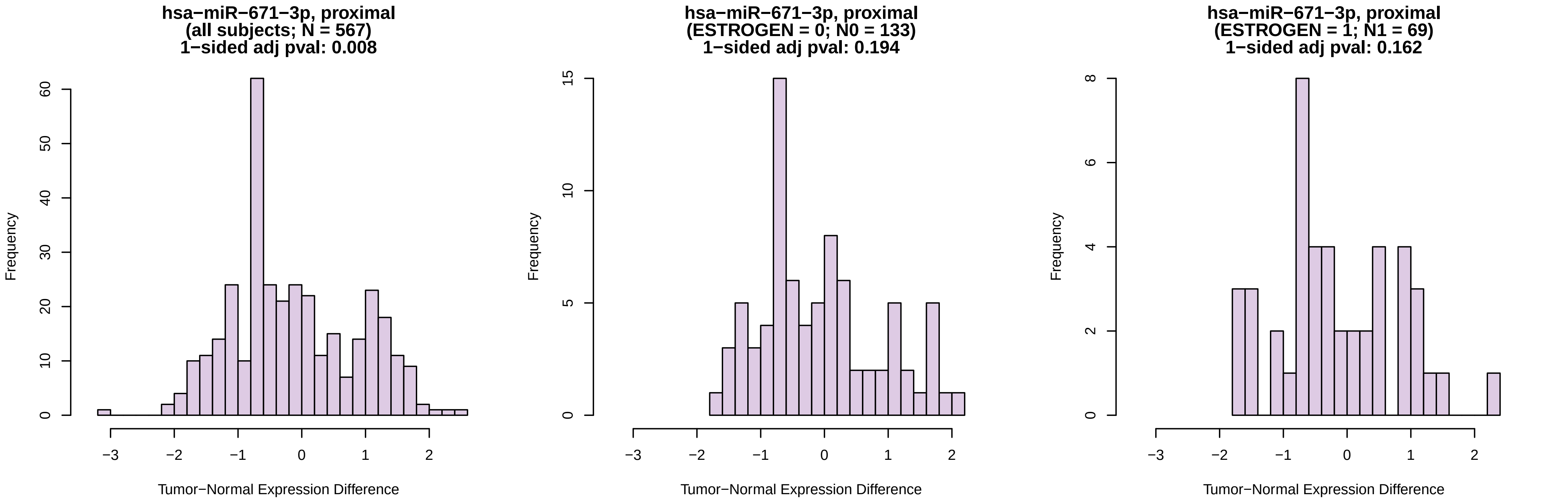
hsa−miR−671−3p, proximal
(all subjects; N = 567)
1−sided adj pval: 0.008
Frequency
Tumor−Normal Expression Difference
hsa−miR−671−3p, proximal
(ESTROGEN = 0; N0 = 133)
1−sided adj pval: 0.194
Frequency
Tumor−Normal Expression Difference
hsa−miR−671−3p, proximal
(ESTROGEN = 1; N1 = 69)
1−sided adj pval: 0.162
Frequency
Tumor−Normal Expression Difference

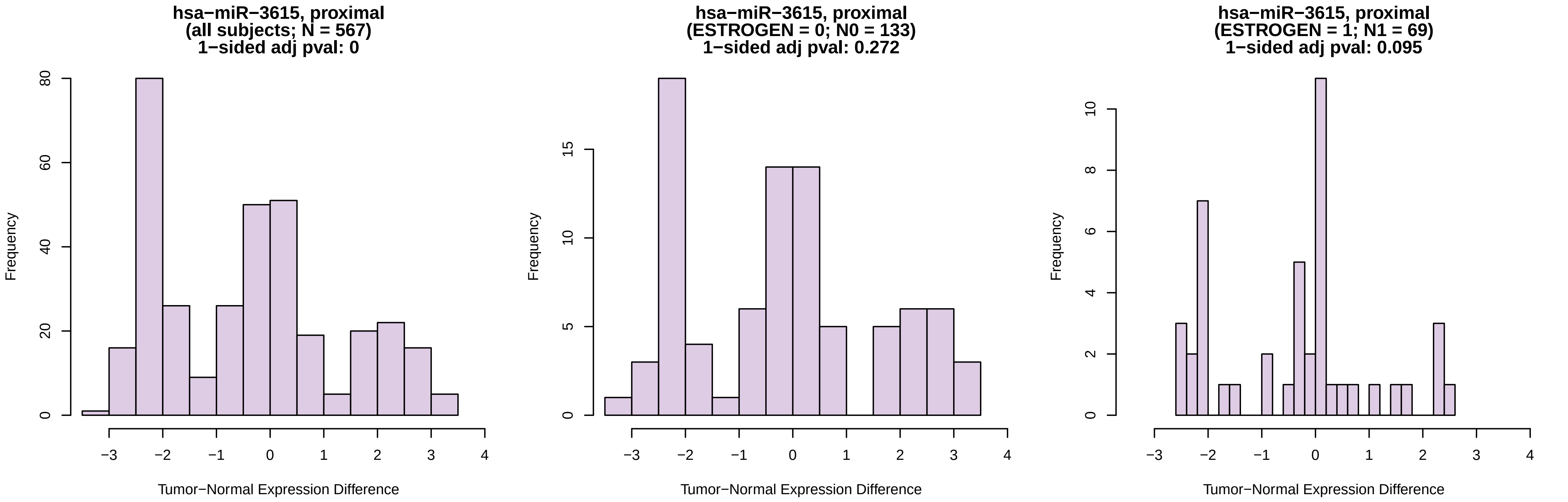
hsa−miR−3615, proximal
(all subjects; N = 567)
1−sided adj pval: 0
Frequency
0
20
40
60
80
−3
−2
−1
0
1
2
3
4
Tumor−Normal Expression Difference
hsa−miR−3615, proximal
(ESTROGEN = 0; N0 = 133)
1−sided adj pval: 0.272
Frequency
0
5
10
15
−3
−2
−1
0
1
2
3
4
Tumor−Normal Expression Difference
hsa−miR−3615, proximal
(ESTROGEN = 1; N1 = 69)
1−sided adj pval: 0.095
Frequency
0
2
4
6
8
10
−3
−2
−1
0
1
2
3
4
Tumor−Normal Expression Difference

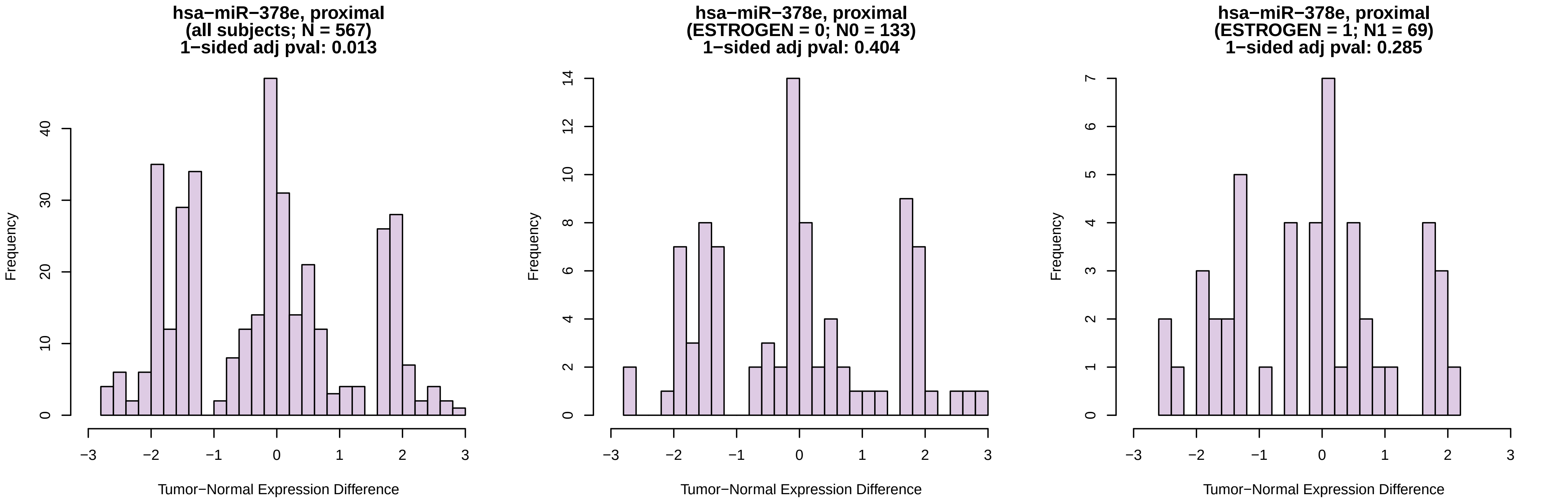

hsa−miR−378e, proximal
(all subjects; N = 567)
1−sided adj pval: 0.013
Frequency
Tumor−Normal Expression Difference
hsa−miR−378e, proximal
(ESTROGEN = 0; N0 = 133)
1−sided adj pval: 0.404
Frequency
Tumor−Normal Expression Difference
hsa−miR−378e, proximal
(ESTROGEN = 1; N1 = 69)
1−sided adj pval: 0.285
Frequency
Tumor−Normal Expression Difference

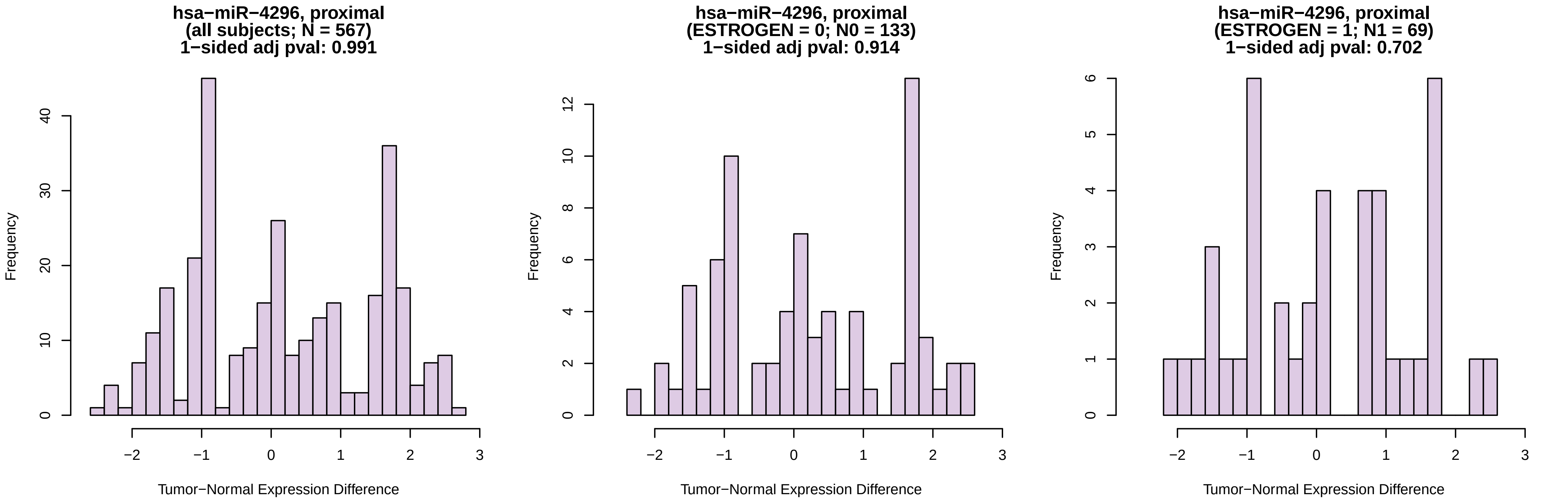
hsa−miR−4296, proximal
(all subjects; N = 567)
1−sided adj pval: 0.991
Frequency
Tumor−Normal Expression Difference
hsa−miR−4296, proximal
(ESTROGEN = 0; N0 = 133)
1−sided adj pval: 0.914
Frequency
Tumor−Normal Expression Difference
hsa−miR−4296, proximal
(ESTROGEN = 1; N1 = 69)
1−sided adj pval: 0.702
Frequency
Tumor−Normal Expression Difference

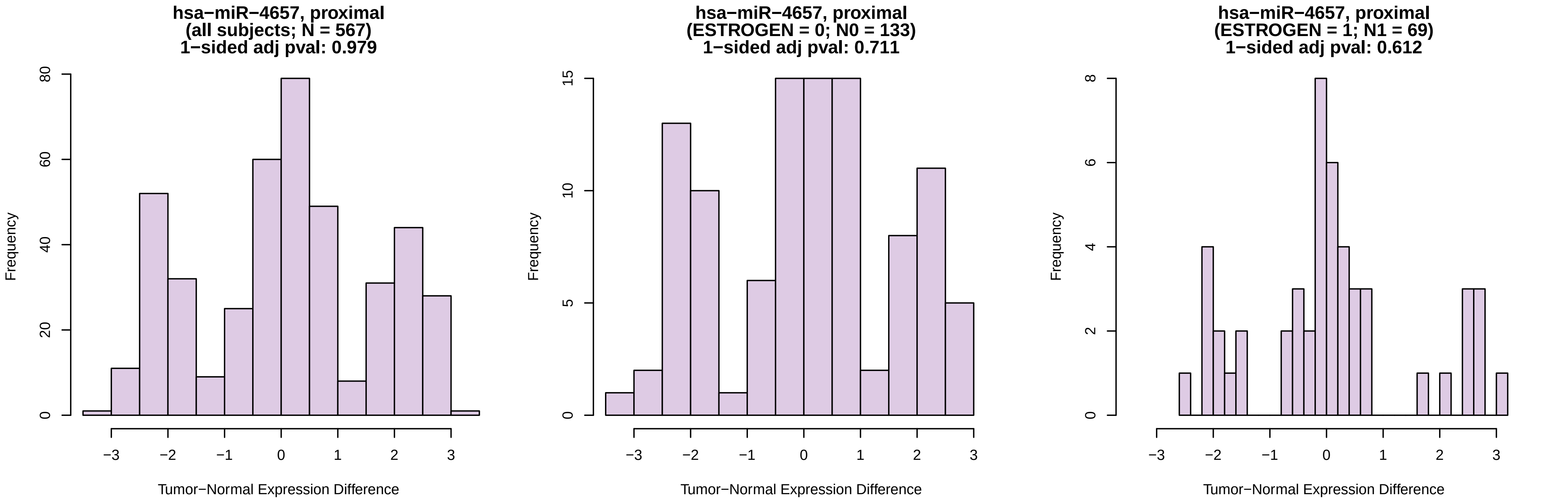

hsa−miR−4657, proximal
(all subjects; N = 567)
1−sided adj pval: 0.979
Frequency
0
20
40
60
80
−3
−2
−1
0
1
2
3
Tumor−Normal Expression Difference
hsa−miR−4657, proximal
(ESTROGEN = 0; N0 = 133)
1−sided adj pval: 0.711
Frequency
0
5
10
15
Tumor−Normal Expression Difference
hsa−miR−4657, proximal
(ESTROGEN = 1; N1 = 69)
1−sided adj pval: 0.612
Frequency
0
2
4
6
8
Tumor−Normal Expression Difference

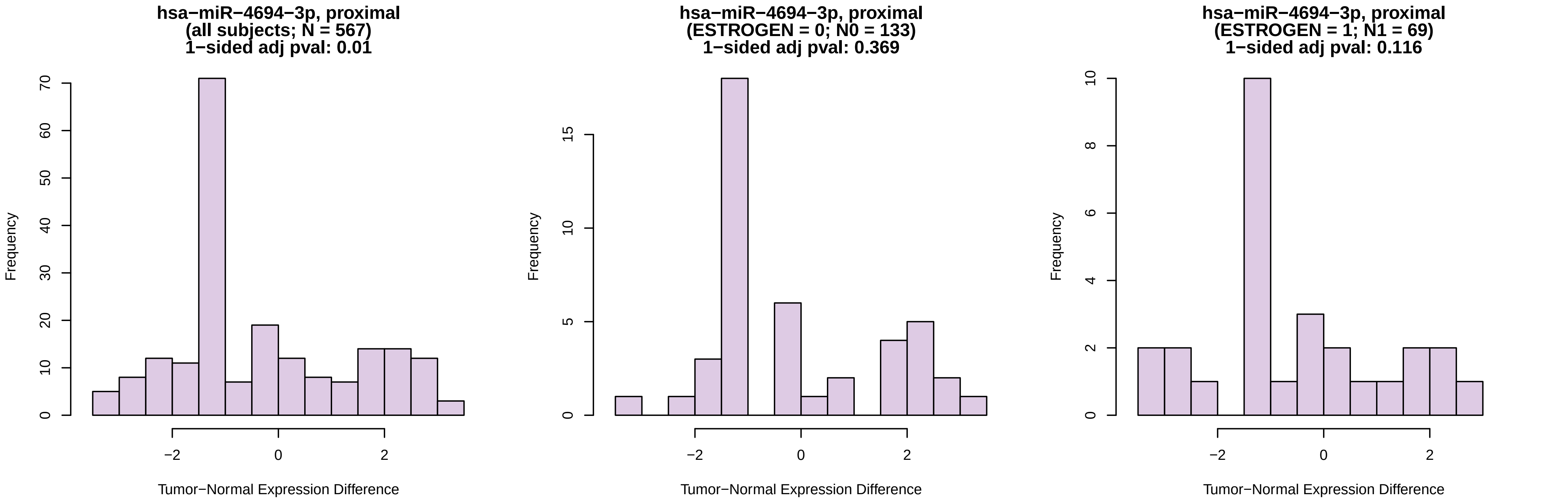
hsa−miR−4694−3p, proximal
(all subjects; N = 567)
1−sided adj pval: 0.01
Frequency
0
10
20
30
40
50
60
70
−2
0
2
Tumor−Normal Expression Difference
hsa−miR−4694−3p, proximal
(ESTROGEN = 0; N0 = 133)
1−sided adj pval: 0.369
Frequency
0
5
10
15
−2
0
2
Tumor−Normal Expression Difference
hsa−miR−4694−3p, proximal
(ESTROGEN = 1; N1 = 69)
1−sided adj pval: 0.116
Frequency
0
2
4
6
8
10
−2
0
2
Tumor−Normal Expression Difference

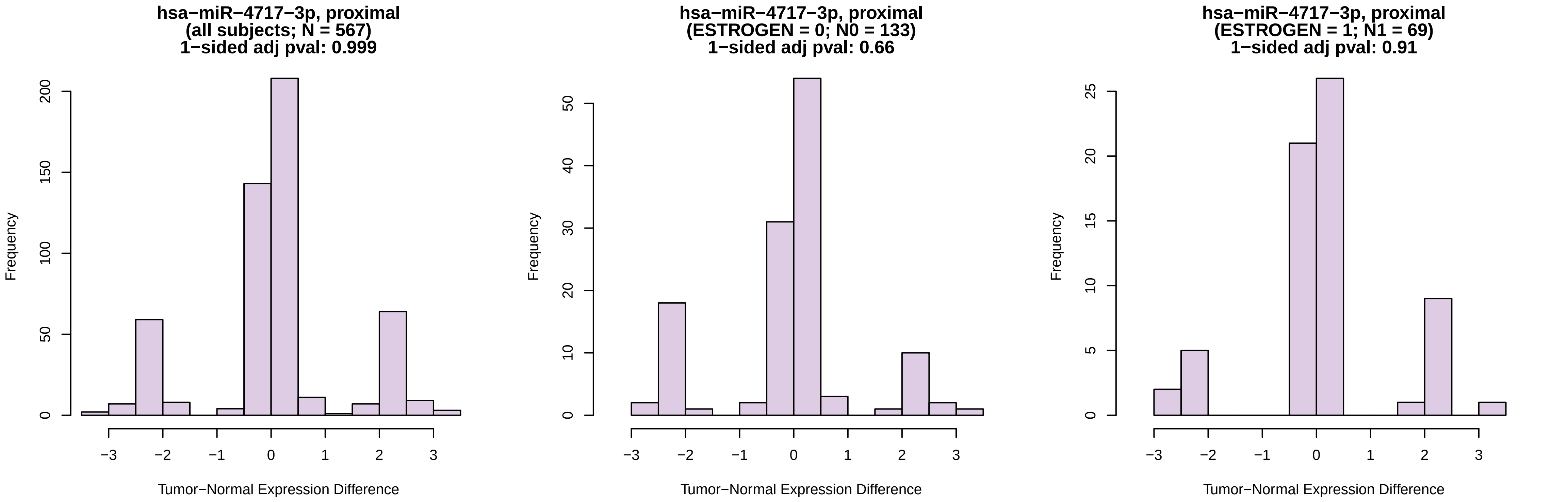
hsa−miR−4717−3p, proximal
(all subjects; N = 567)
1−sided adj pval: 0.999
Frequency
Tumor−Normal Expression Difference
hsa−miR−4717−3p, proximal
(ESTROGEN = 0; N0 = 133)
1−sided adj pval: 0.66
Frequency
Tumor−Normal Expression Difference
hsa−miR−4717−3p, proximal
(ESTROGEN = 1; N1 = 69)
1−sided adj pval: 0.91
Frequency
Tumor−Normal Expression Difference

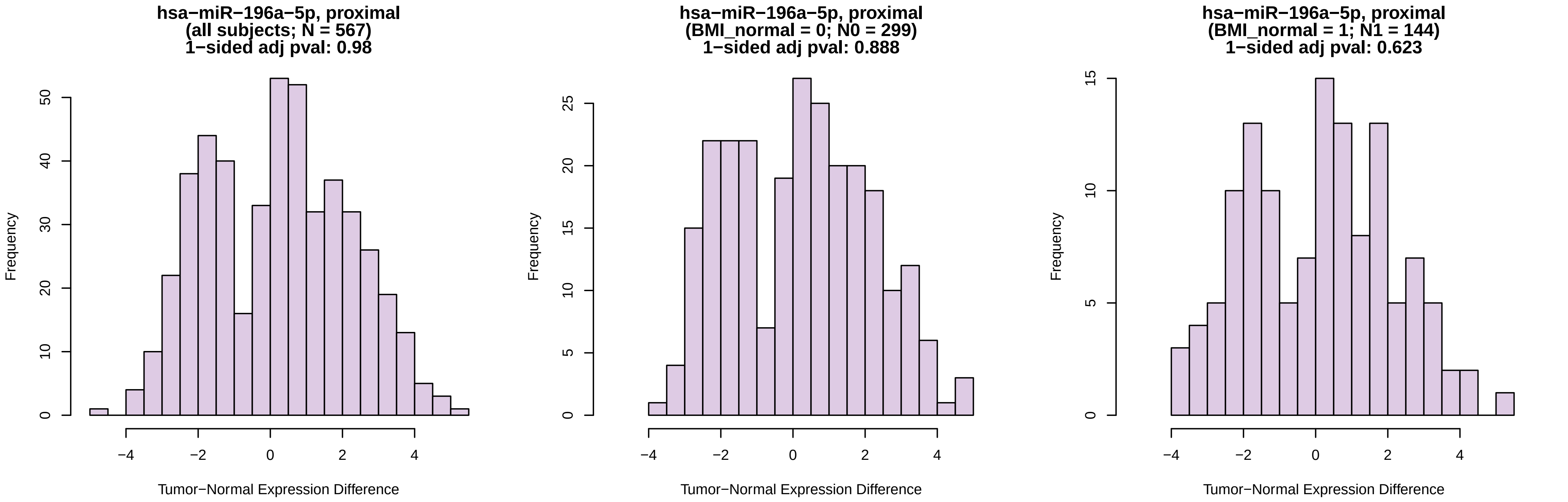

hsa−miR−196a−5p, proximal
(all subjects; N = 567)
1−sided adj pval: 0.98
Frequency
Tumor−Normal Expression Difference
hsa−miR−196a−5p, proximal
(BMI_normal = 0; N0 = 299)
1−sided adj pval: 0.888
Frequency
Tumor−Normal Expression Difference
hsa−miR−196a−5p, proximal
(BMI_normal = 1; N1 = 144)
1−sided adj pval: 0.623
Frequency
Tumor−Normal Expression Difference

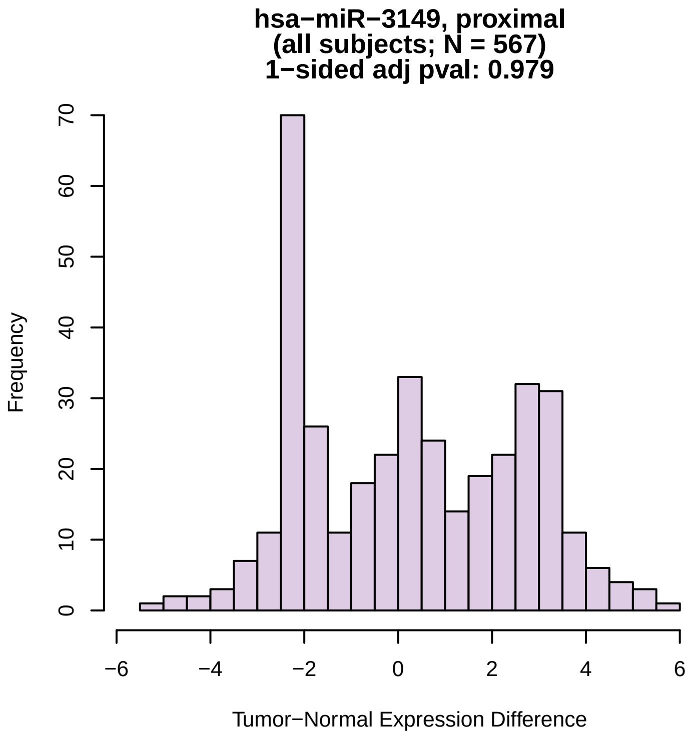
hsa−miR−3149, proximal
(all subjects; N = 567)
1−sided adj pval: 0.979
Frequency
Tumor−Normal Expression Difference

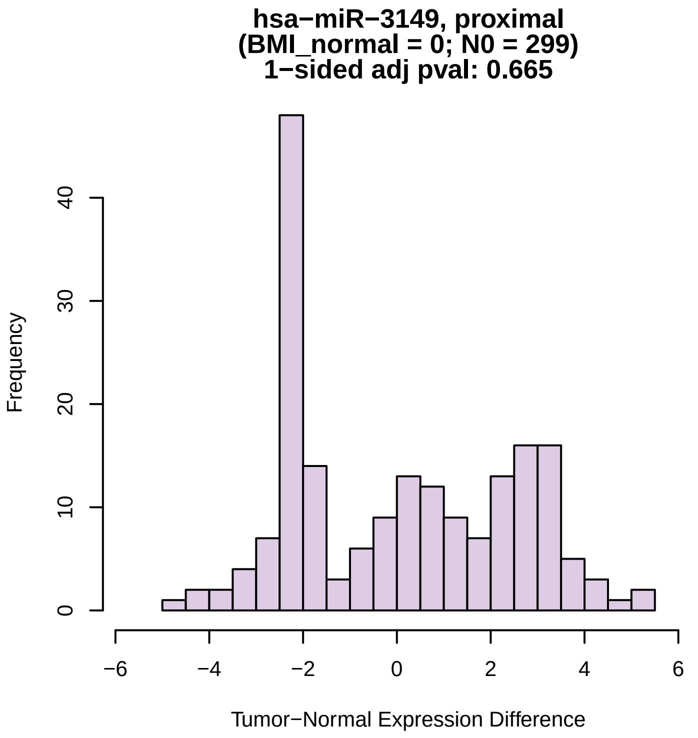
hsa−miR−3149, proximal
(BMI_normal = 0; N0 = 299)
1−sided adj pval: 0.665
Frequency
Tumor−Normal Expression Difference

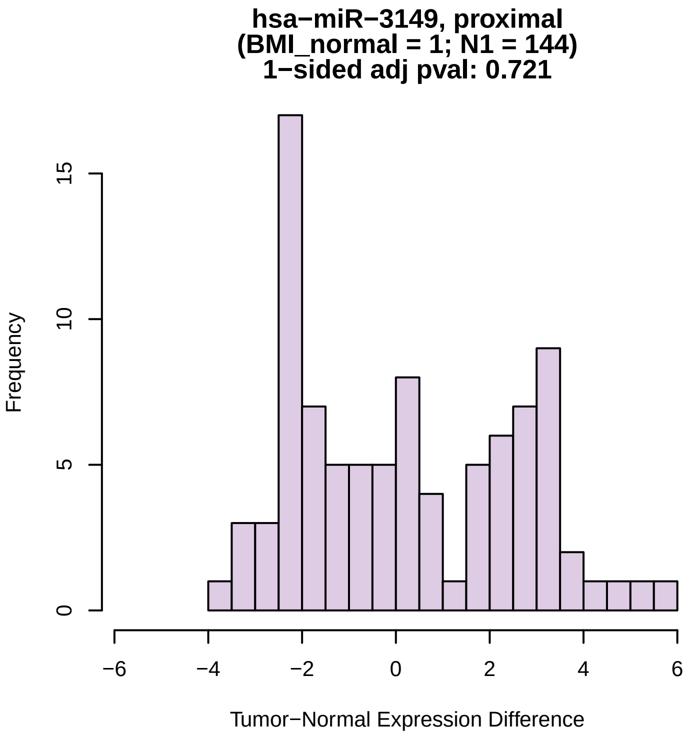
hsa−miR−3149, proximal
(BMI_normal = 1; N1 = 144)
1−sided adj pval: 0.721
Frequency
Tumor−Normal Expression Difference

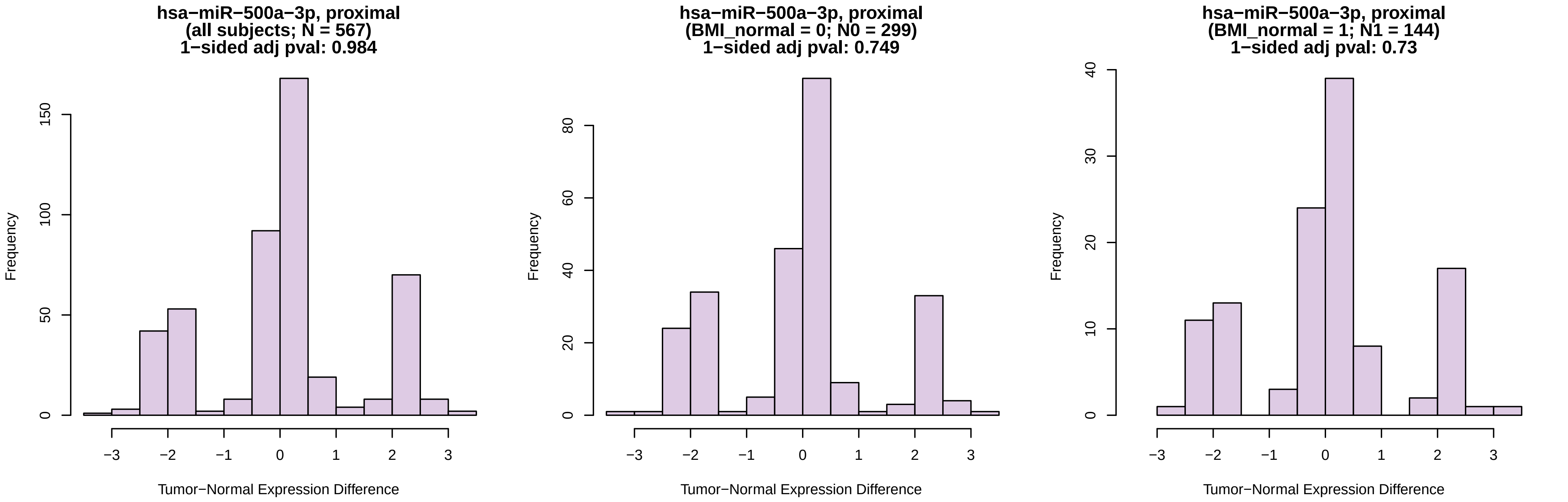
hsa−miR−500a−3p, proximal
(all subjects; N = 567)
1−sided adj pval: 0.984
Frequency
0
50
100
150
−3
−2
−1
0
1
2
3
Tumor−Normal Expression Difference
hsa−miR−500a−3p, proximal
(BMI_normal = 0; N0 = 299)
1−sided adj pval: 0.749
Frequency
0
20
40
60
80
−3
−2
−1
0
1
2
3
Tumor−Normal Expression Difference
hsa−miR−500a−3p, proximal
(BMI_normal = 1; N1 = 144)
1−sided adj pval: 0.73
Frequency
0
10
20
30
40
−3
−2
−1
0
1
2
3
Tumor−Normal Expression Difference

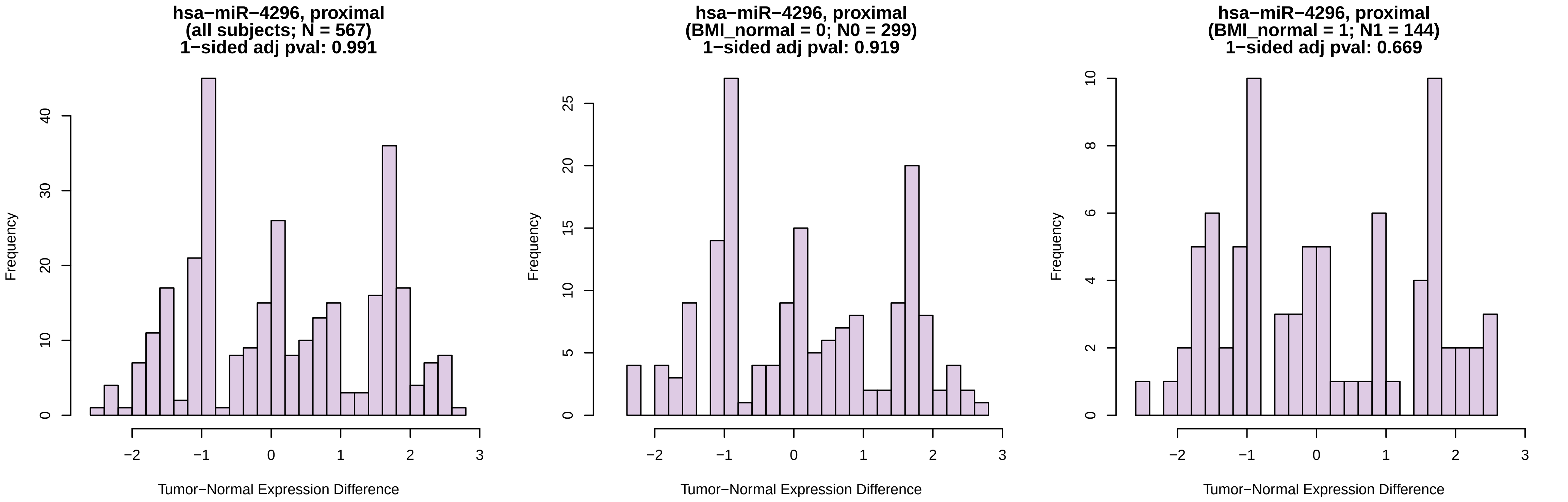
hsa−miR−4296, proximal
(all subjects; N = 567)
1−sided adj pval: 0.991
hsa−miR−4296, proximal
(BMI_normal = 0; N0 = 299)
1−sided adj pval: 0.919
hsa−miR−4296, proximal
(BMI_normal = 1; N1 = 144)
1−sided adj pval: 0.669
Frequency
Tumor−Normal Expression Difference
0
10
20
30
40
5
15
25
2
4
6
8
−2
−1
1
3

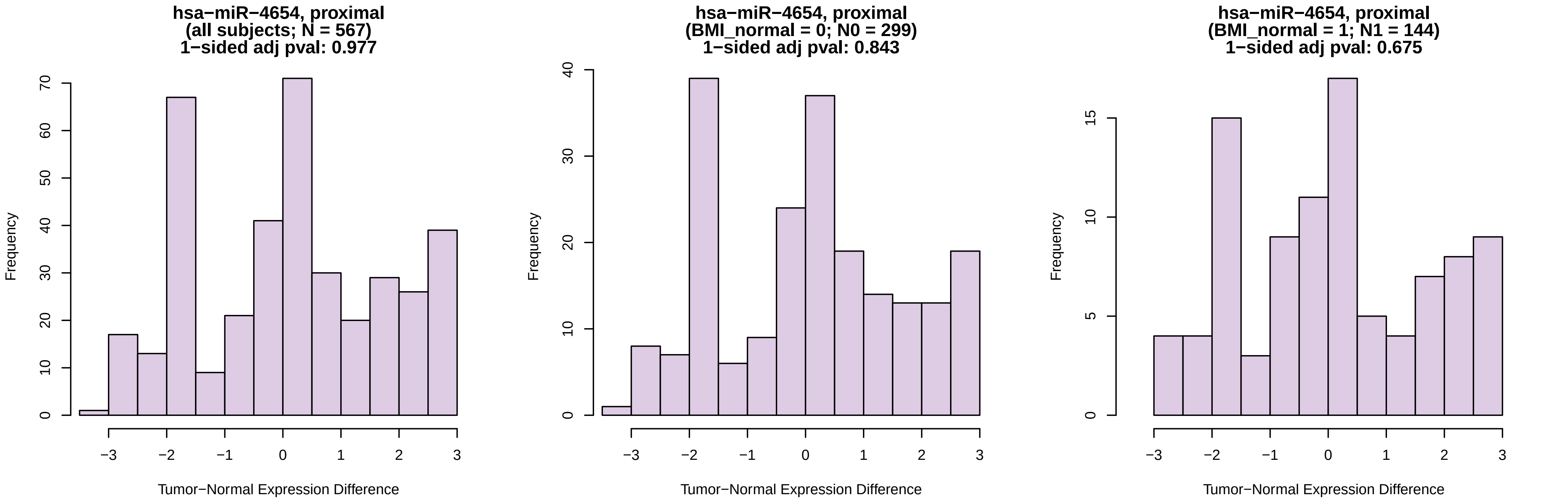
hsa−miR−4654, proximal
(all subjects; N = 567)
1−sided adj pval: 0.977
Frequency
Tumor−Normal Expression Difference
hsa−miR−4654, proximal
(BMI_normal = 0; N0 = 299)
1−sided adj pval: 0.843
Frequency
Tumor−Normal Expression Difference
hsa−miR−4654, proximal
(BMI_normal = 1; N1 = 144)
1−sided adj pval: 0.675
Frequency
Tumor−Normal Expression Difference

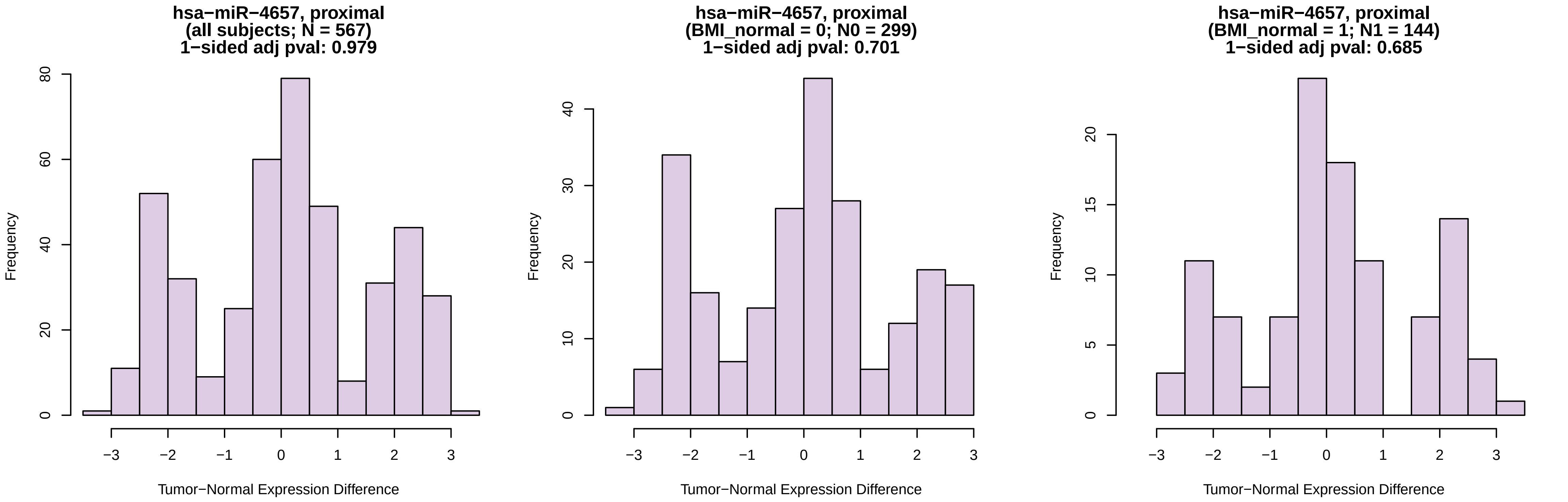
hsa−miR−4657, proximal
(all subjects; N = 567)
1−sided adj pval: 0.979
Frequency
0
20
40
60
80
−3
−2
−1
0
1
2
3
Tumor−Normal Expression Difference
hsa−miR−4657, proximal
(BMI_normal = 0; N0 = 299)
1−sided adj pval: 0.701
Frequency
0
10
20
30
40
−3
−2
−1
0
1
2
3
Tumor−Normal Expression Difference
hsa−miR−4657, proximal
(BMI_normal = 1; N1 = 144)
1−sided adj pval: 0.685
Frequency
0
5
10
15
20
−3
−2
−1
0
1
2
3
Tumor−Normal Expression Difference

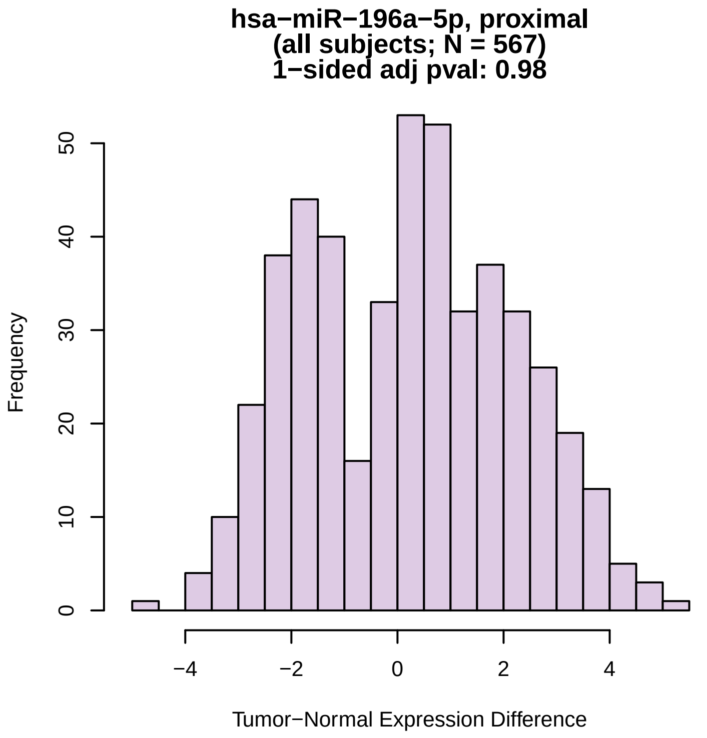

hsa−miR−196a−5p, proximal
(all subjects; N = 567)
1−sided adj pval: 0.98
Frequency
0
10
20
30
40
50
−4
−2
0
2
4
Tumor−Normal Expression Difference

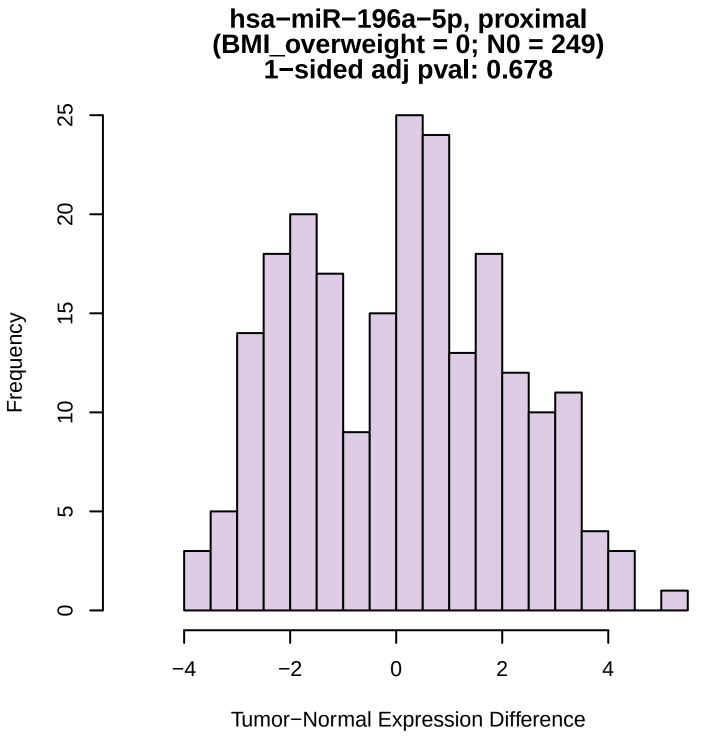

hsa−miR−196a−5p, proximal
(BMI_overweight = 0; N0 = 249)
1−sided adj pval: 0.678
Frequency
0
5
10
15
20
25
−4
−2
0
2
4
Tumor−Normal Expression Difference

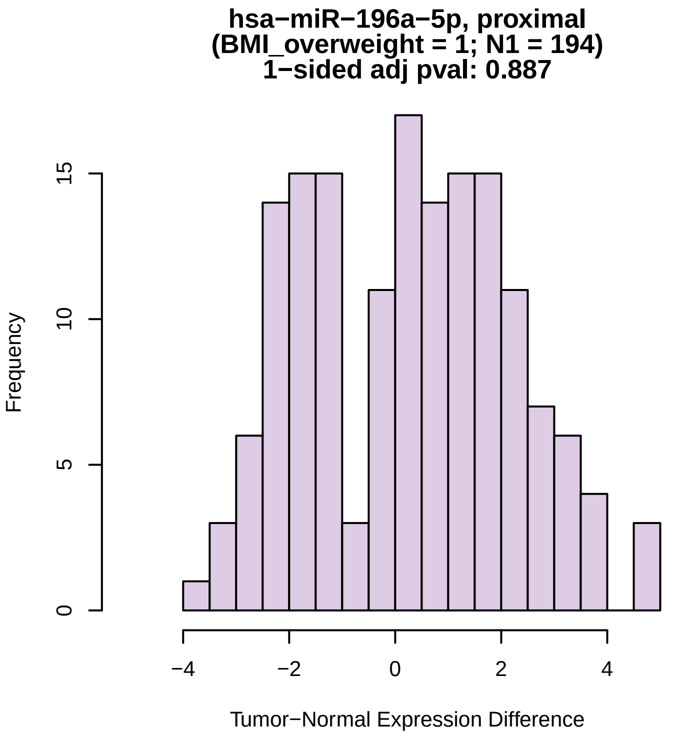

hsa−miR−196a−5p, proximal
(BMI_overweight = 1; N1 = 194)
1−sided adj pval: 0.887
Frequency
0
5
10
15
−4
−2
0
2
4
Tumor−Normal Expression Difference

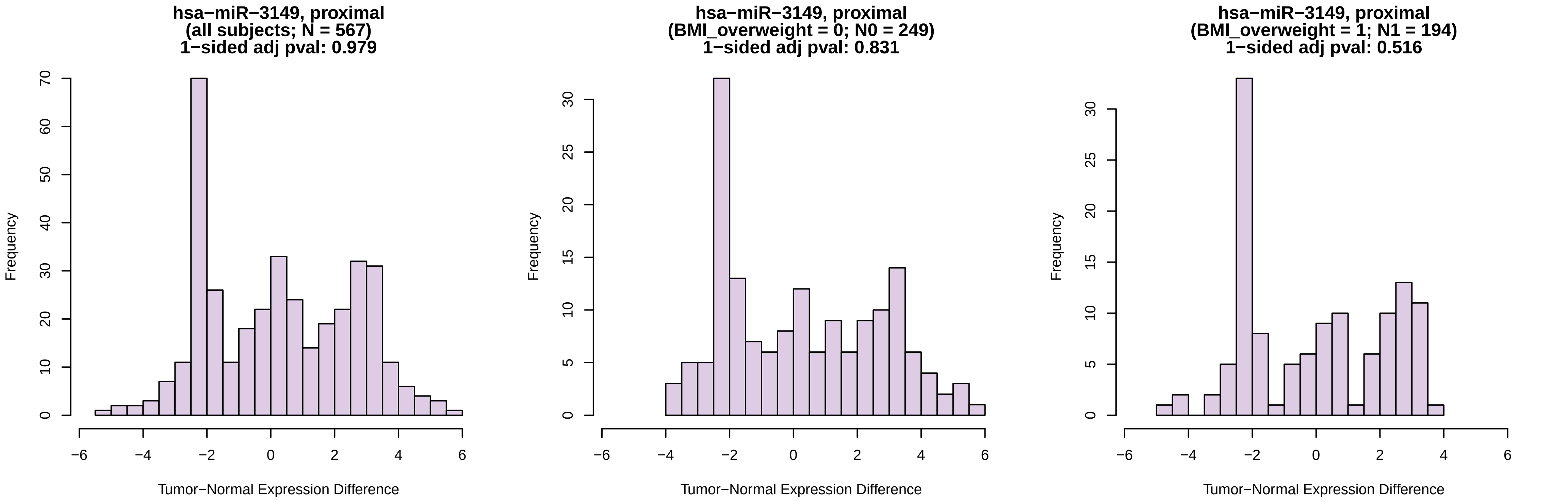
hsa−miR−3149, proximal
(all subjects; N = 567)
1−sided adj pval: 0.979
Frequency
Tumor−Normal Expression Difference
hsa−miR−3149, proximal
(BMI_overweight = 0; N0 = 249)
1−sided adj pval: 0.831
Frequency
Tumor−Normal Expression Difference
hsa−miR−3149, proximal
(BMI_overweight = 1; N1 = 194)
1−sided adj pval: 0.516
Frequency
Tumor−Normal Expression Difference

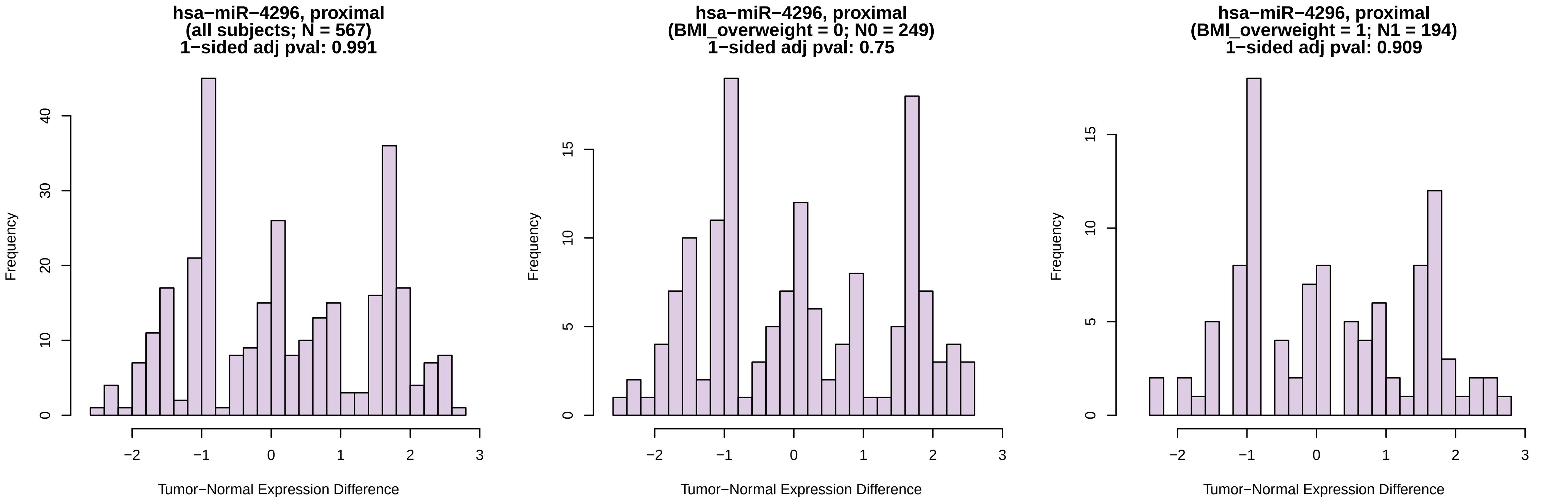

hsa−miR−4296, proximal
(all subjects; N = 567)
1−sided adj pval: 0.991
Frequency
Tumor−Normal Expression Difference
hsa−miR−4296, proximal
(BMI_overweight = 0; N0 = 249)
1−sided adj pval: 0.75
Frequency
Tumor−Normal Expression Difference
hsa−miR−4296, proximal
(BMI_overweight = 1; N1 = 194)
1−sided adj pval: 0.909
Frequency
Tumor−Normal Expression Difference

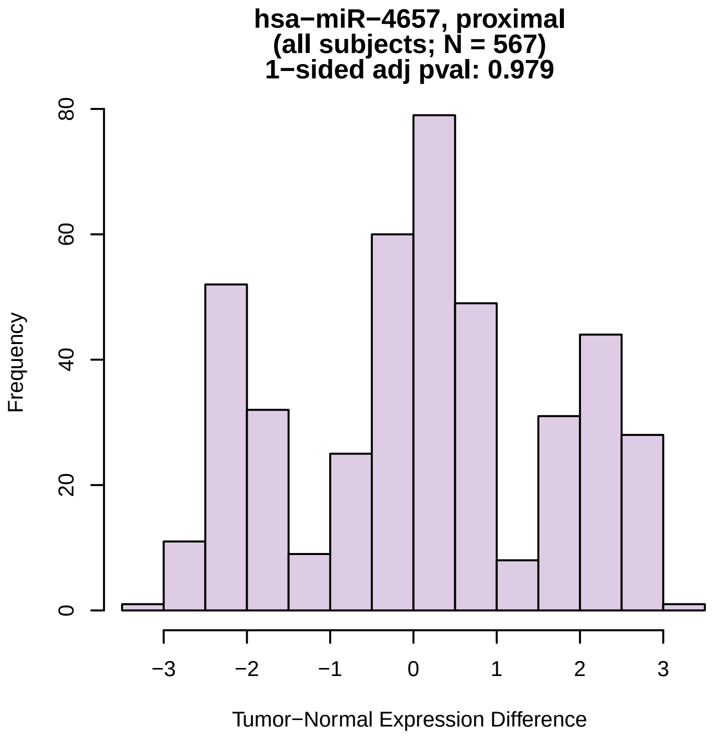
hsa−miR−4657, proximal
(all subjects; N = 567)
1−sided adj pval: 0.979
Frequency
Tumor−Normal Expression Difference
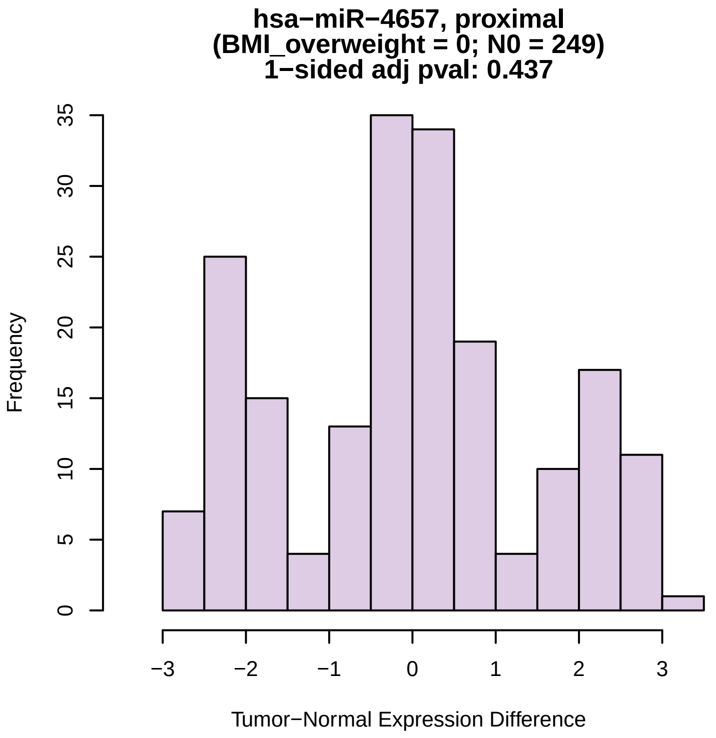
hsa−miR−4657, proximal
(BMI_overweight = 0; N0 = 249)
1−sided adj pval: 0.437
Frequency
Tumor−Normal Expression Difference
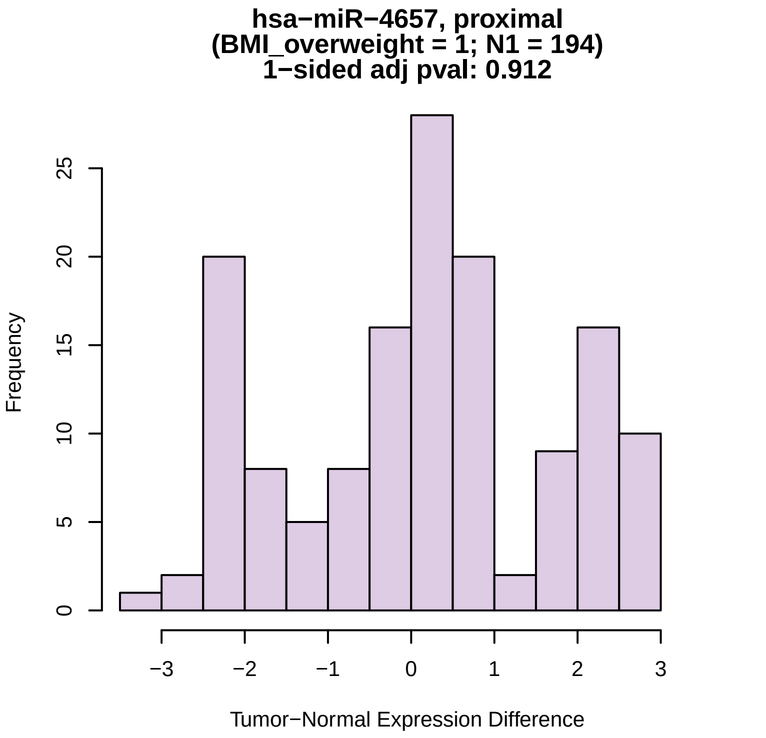
hsa−miR−4657, proximal
(BMI_overweight = 1; N1 = 194)
1−sided adj pval: 0.912
Frequency
Tumor−Normal Expression Difference

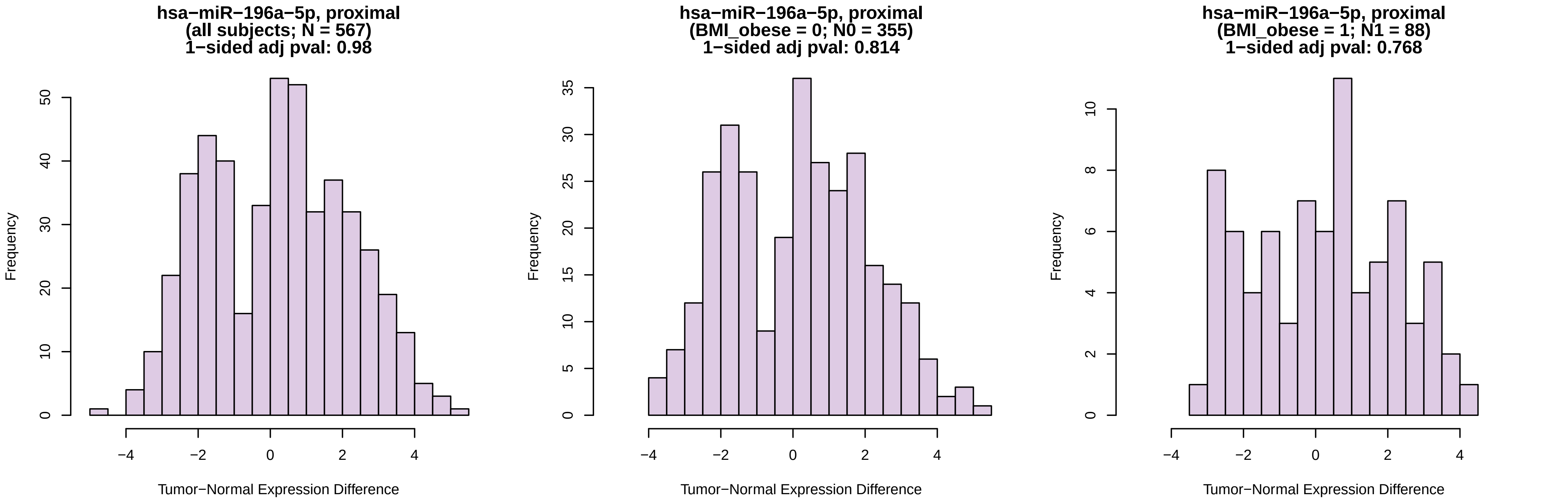
hsa−miR−196a−5p, proximal
(all subjects; N = 567)
1−sided adj pval: 0.98
Frequency
Tumor−Normal Expression Difference
hsa−miR−196a−5p, proximal
(BMI_obese = 0; N0 = 355)
1−sided adj pval: 0.814
Frequency
Tumor−Normal Expression Difference
hsa−miR−196a−5p, proximal
(BMI_obese = 1; N1 = 88)
1−sided adj pval: 0.768
Frequency
Tumor−Normal Expression Difference

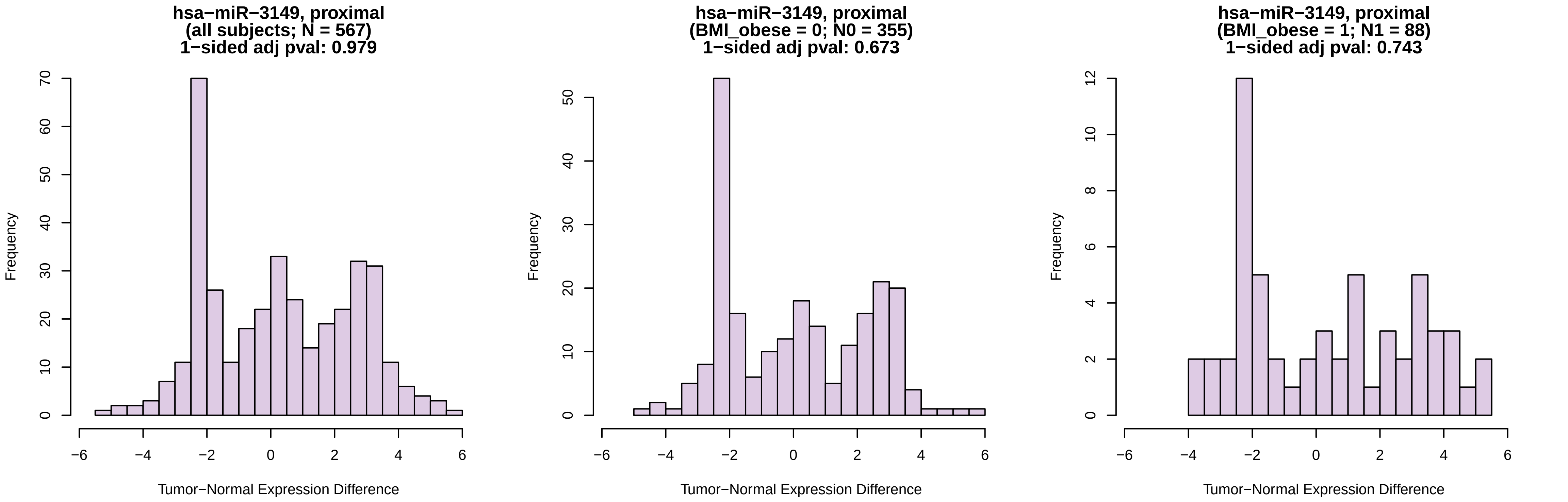
hsa−miR−3149, proximal
(all subjects; N = 567)
1−sided adj pval: 0.979
Frequency
Tumor−Normal Expression Difference
hsa−miR−3149, proximal
(BMI_obese = 0; N0 = 355)
1−sided adj pval: 0.673
Frequency
Tumor−Normal Expression Difference
hsa−miR−3149, proximal
(BMI_obese = 1; N1 = 88)
1−sided adj pval: 0.743
Frequency
Tumor−Normal Expression Difference

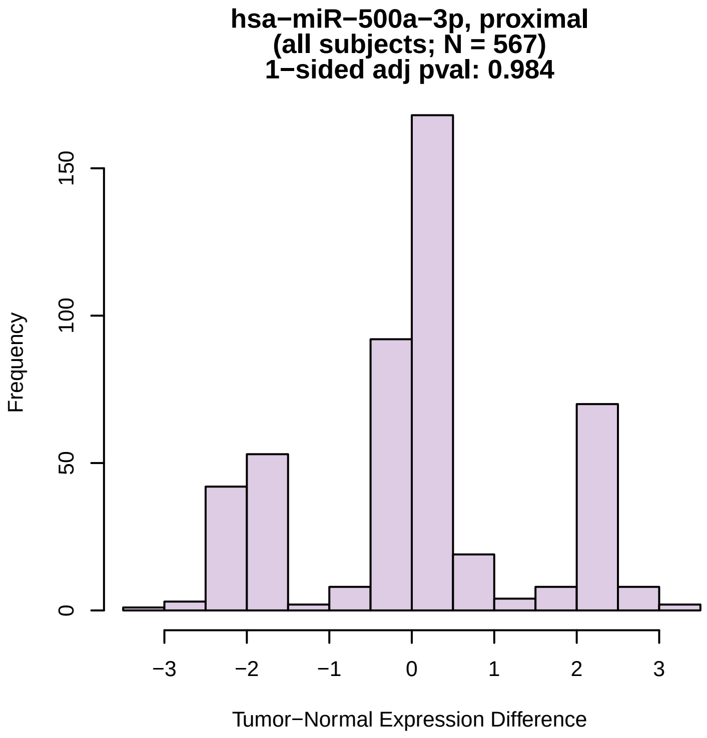
hsa−miR−500a−3p, proximal
(all subjects; N = 567)
1−sided adj pval: 0.984
Frequency
0
50
100
150
−3
−2
−1
0
1
2
3
Tumor−Normal Expression Difference

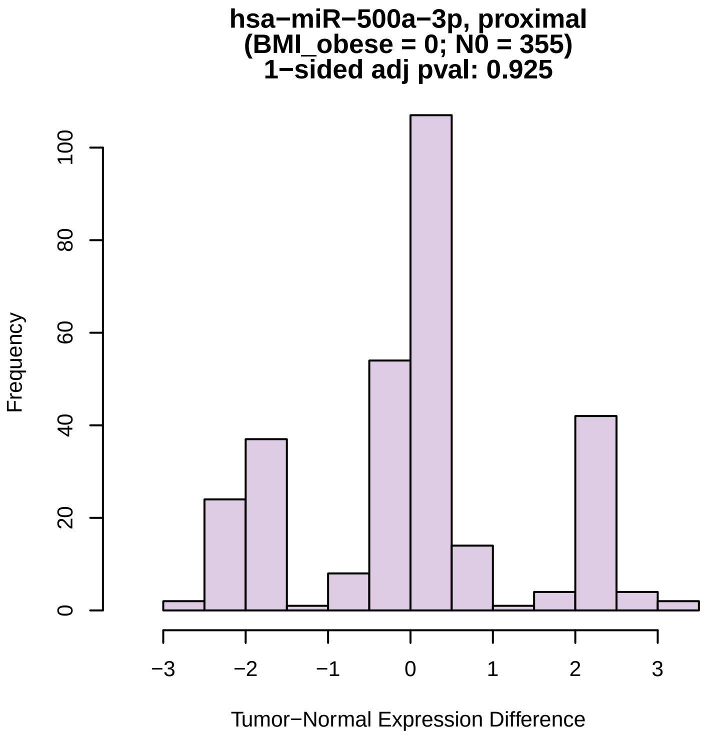
hsa−miR−500a−3p, proximal
(BMI_obese = 0; N0 = 355)
1−sided adj pval: 0.925
Frequency
0
20
40
60
80
100
−3
−2
−1
0
1
2
3
Tumor−Normal Expression Difference

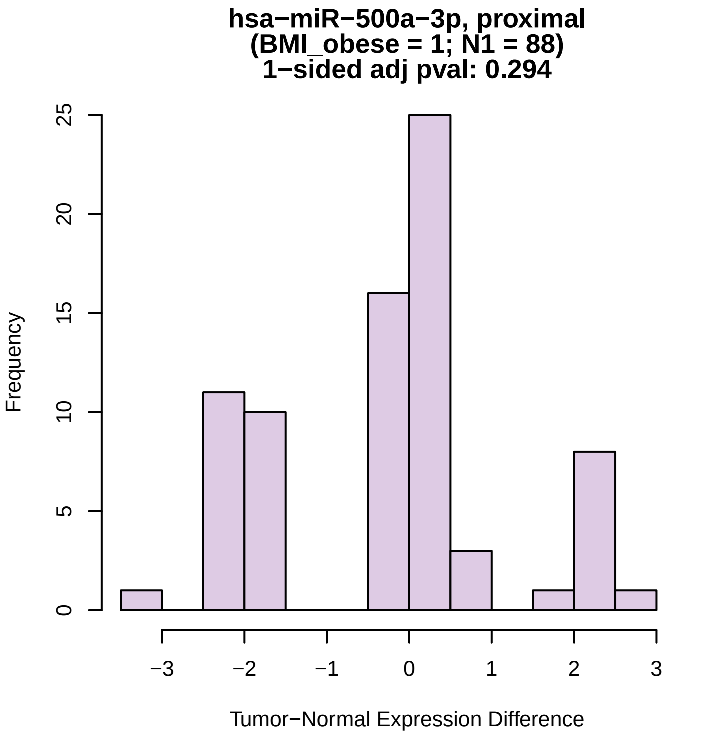
hsa−miR−500a−3p, proximal
(BMI_obese = 1; N1 = 88)
1−sided adj pval: 0.294
Frequency
0
5
10
15
20
25
−3
−2
−1
0
1
2
3
Tumor−Normal Expression Difference

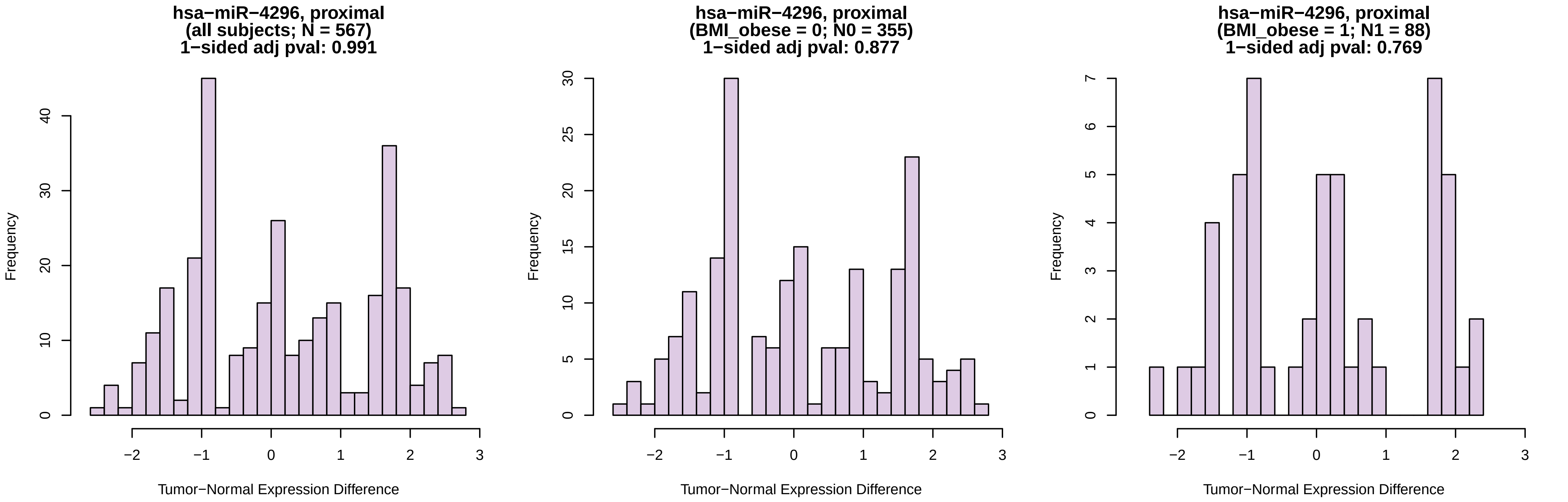
hsa−miR−4296, proximal
(all subjects; N = 567)
1−sided adj pval: 0.991
Frequency
Tumor−Normal Expression Difference
hsa−miR−4296, proximal
(BMI_obese = 0; N0 = 355)
1−sided adj pval: 0.877
Frequency
Tumor−Normal Expression Difference
hsa−miR−4296, proximal
(BMI_obese = 1; N1 = 88)
1−sided adj pval: 0.769
Frequency
Tumor−Normal Expression Difference

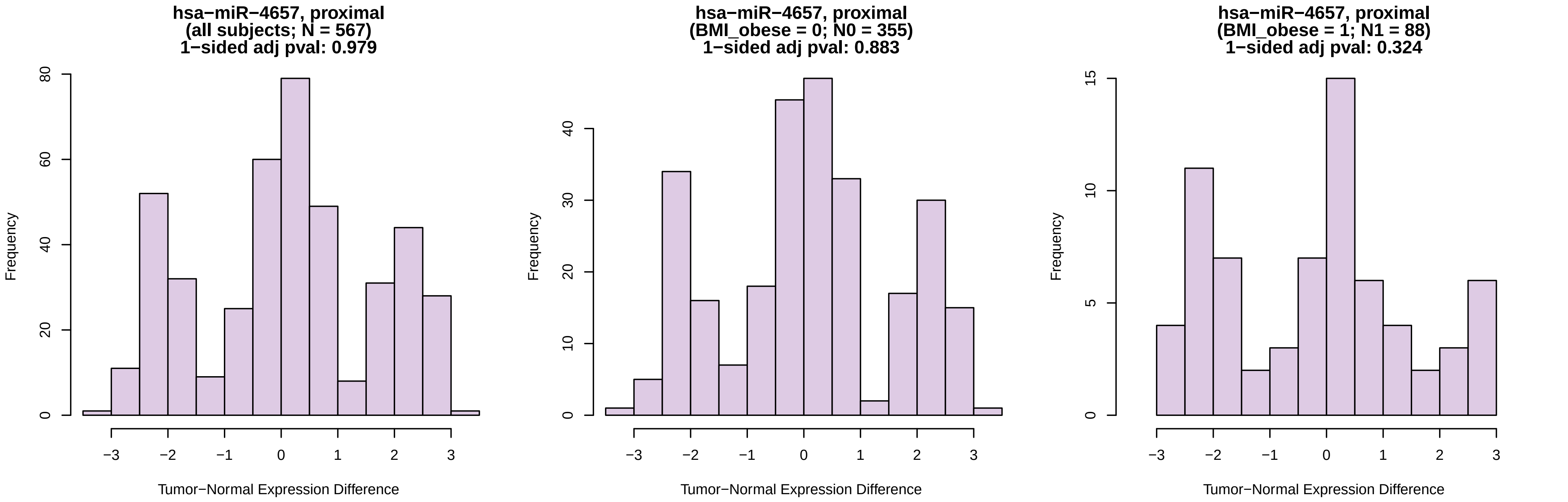

hsa−miR−4657, proximal
(all subjects; N = 567)
1−sided adj pval: 0.979
Frequency
0
20
40
60
80
−3
−2
−1
0
1
2
3
Tumor−Normal Expression Difference
hsa−miR−4657, proximal
(BMI_obese = 0; N0 = 355)
1−sided adj pval: 0.883
Frequency
0
10
20
30
40
−3
−2
−1
0
1
2
3
Tumor−Normal Expression Difference
hsa−miR−4657, proximal
(BMI_obese = 1; N1 = 88)
1−sided adj pval: 0.324
Frequency
0
5
10
15
−3
−2
−1
0
1
2
3
Tumor−Normal Expression Difference

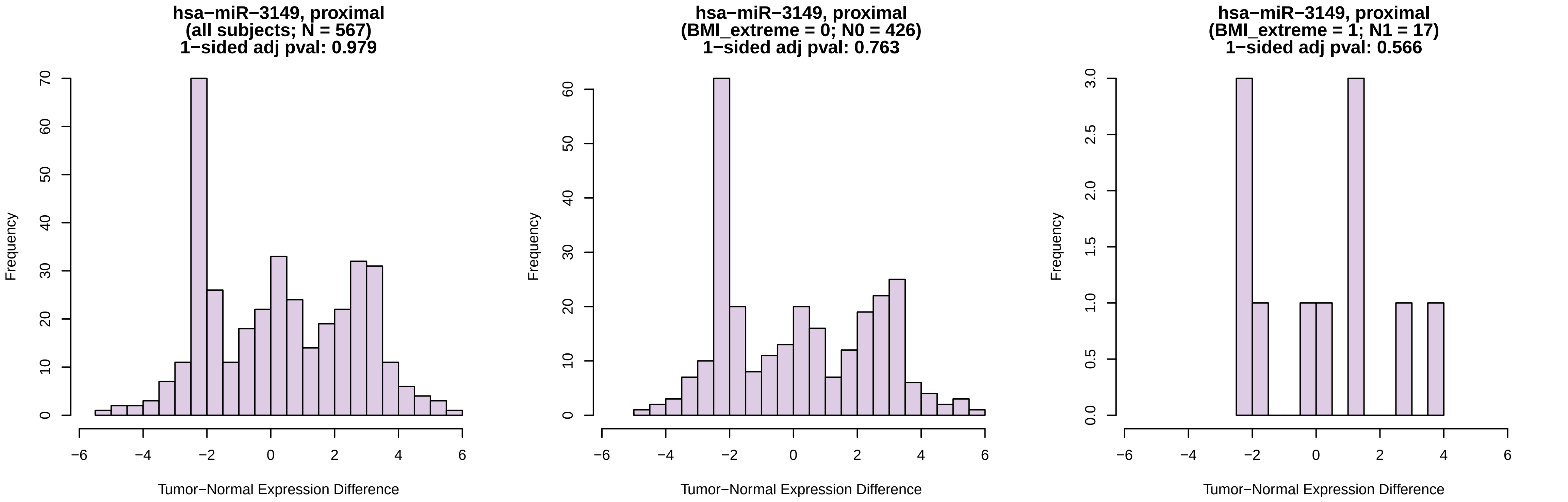

hsa−miR−3149, proximal
(all subjects; N = 567)
1−sided adj pval: 0.979
Frequency
Tumor−Normal Expression Difference
hsa−miR−3149, proximal
(BMI_extreme = 0; N0 = 426)
1−sided adj pval: 0.763
Frequency
Tumor−Normal Expression Difference
hsa−miR−3149, proximal
(BMI_extreme = 1; N1 = 17)
1−sided adj pval: 0.566
Frequency
Tumor−Normal Expression Difference

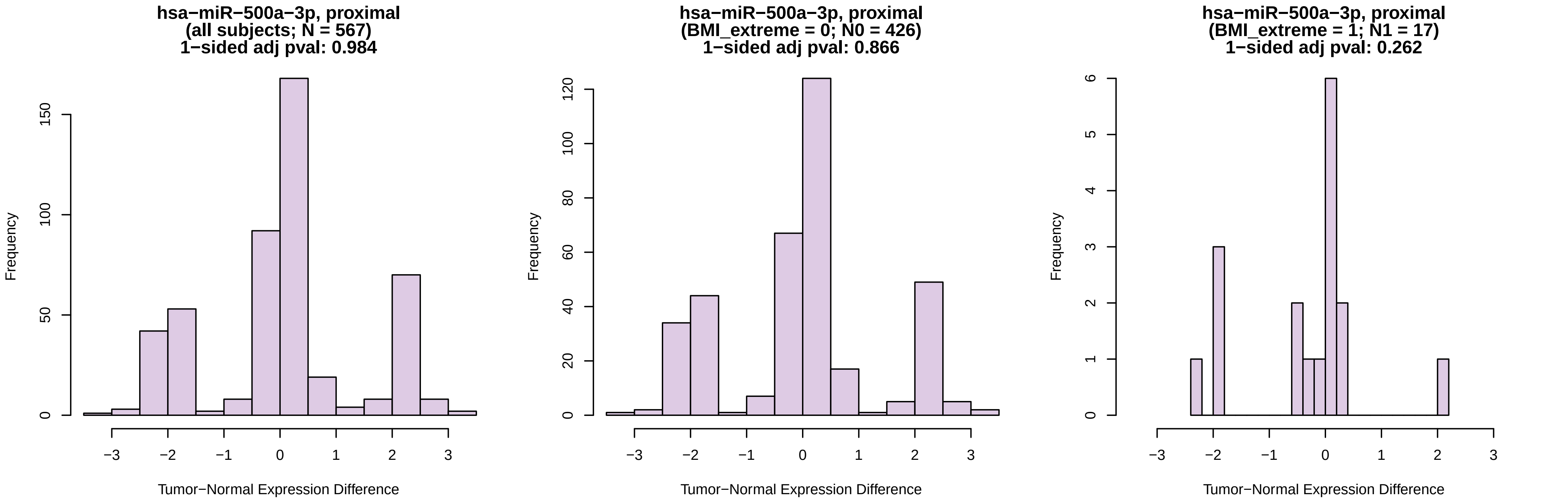
hsa−miR−500a−3p, proximal
(all subjects; N = 567)
1−sided adj pval: 0.984
Frequency
Tumor−Normal Expression Difference
hsa−miR−500a−3p, proximal
(BMI_extreme = 0; N0 = 426)
1−sided adj pval: 0.866
Frequency
Tumor−Normal Expression Difference
hsa−miR−500a−3p, proximal
(BMI_extreme = 1; N1 = 17)
1−sided adj pval: 0.262
Frequency
Tumor−Normal Expression Difference

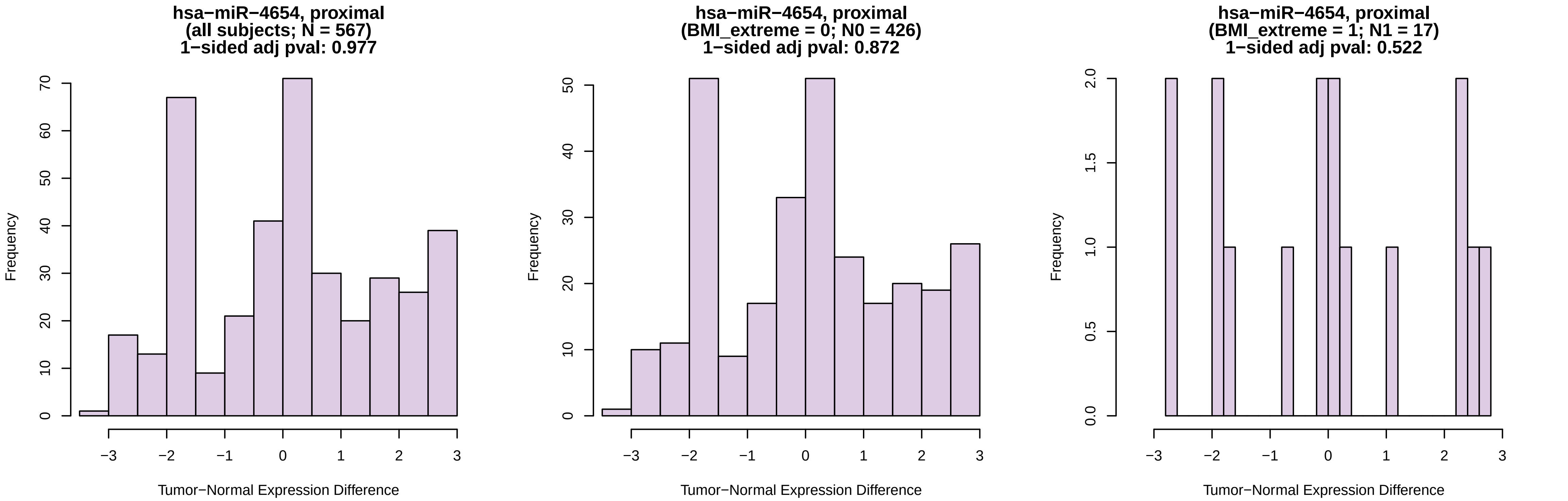
hsa−miR−4654, proximal
(all subjects; N = 567)
1−sided adj pval: 0.977
Frequency
0 10 20 30 40 50 60 70
−3 −2 −1 0 1 2 3
Tumor−Normal Expression Difference
hsa−miR−4654, proximal
(BMI_extreme = 0; N0 = 426)
1−sided adj pval: 0.872
Frequency
0 10 20 30 40 50
−3 −2 −1 0 1 2 3
Tumor−Normal Expression Difference
hsa−miR−4654, proximal
(BMI_extreme = 1; N1 = 17)
1−sided adj pval: 0.522
Frequency
0.0 0.5 1.0 1.5 2.0
−3 −2 −1 0 1 2 3
Tumor−Normal Expression Difference

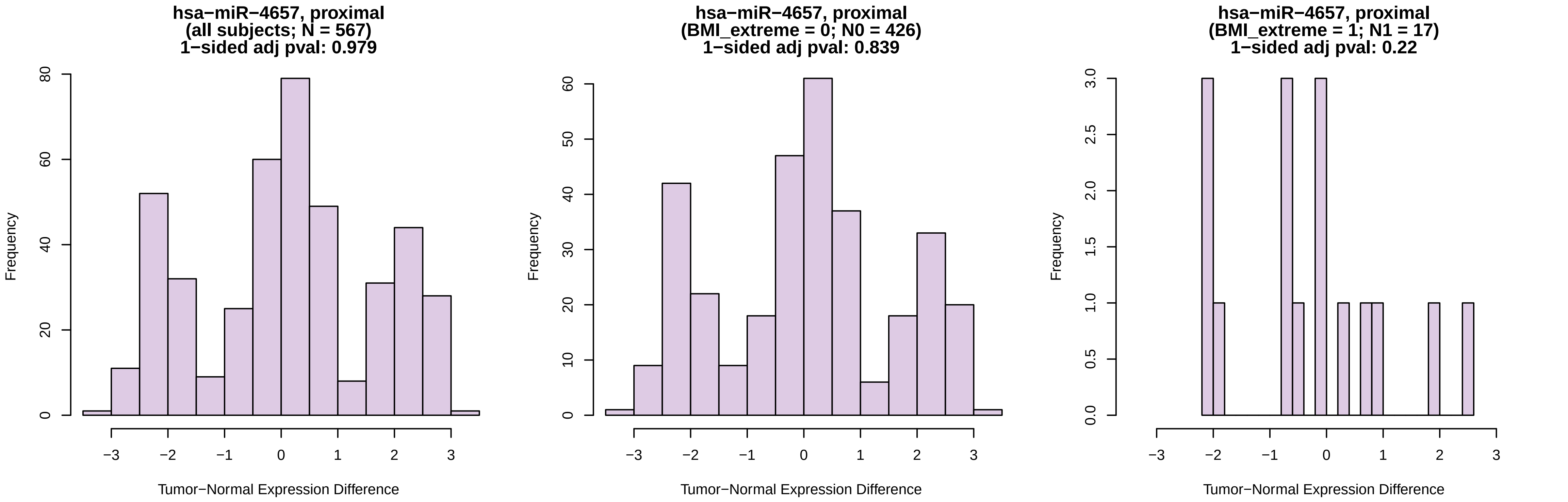

hsa−miR−4657, proximal
(all subjects; N = 567)
1−sided adj pval: 0.979
Frequency
Tumor−Normal Expression Difference
hsa−miR−4657, proximal
(BMI_extreme = 0; N0 = 426)
1−sided adj pval: 0.839
Frequency
Tumor−Normal Expression Difference
hsa−miR−4657, proximal
(BMI_extreme = 1; N1 = 17)
1−sided adj pval: 0.22
Frequency
Tumor−Normal Expression Difference

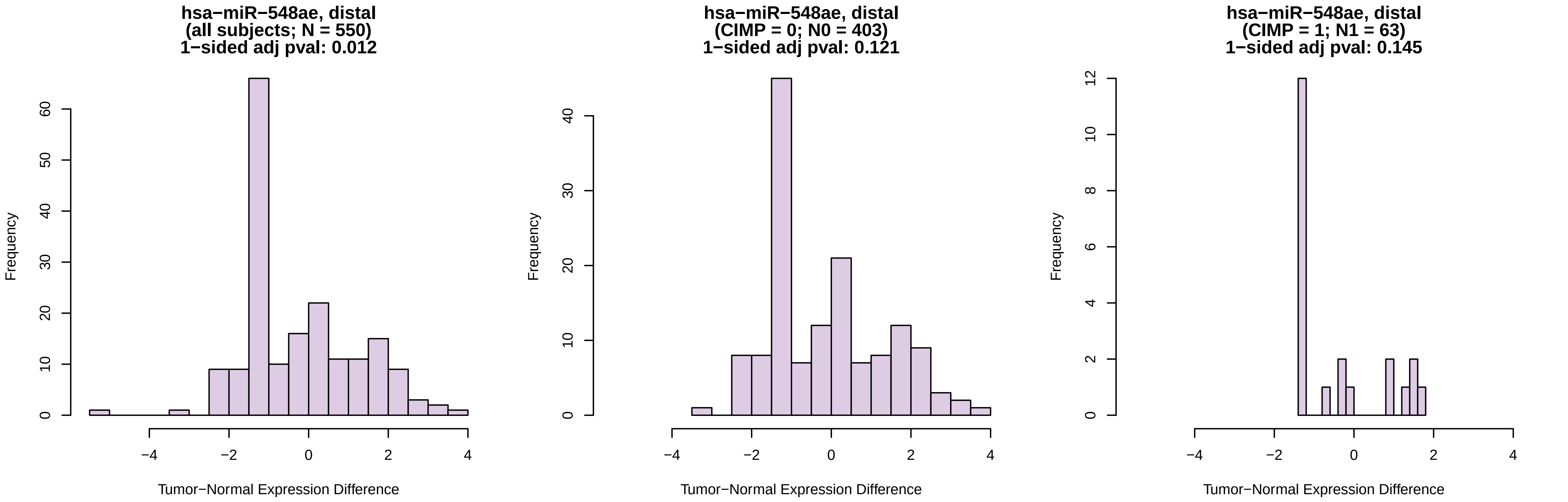
hsa−miR−548ae, distal
(all subjects; N = 550)
1−sided adj pval: 0.012
Frequency
Tumor−Normal Expression Difference
hsa−miR−548ae, distal
(CIMP = 0; N0 = 403)
1−sided adj pval: 0.121
Frequency
Tumor−Normal Expression Difference
hsa−miR−548ae, distal
(CIMP = 1; N1 = 63)
1−sided adj pval: 0.145
Frequency
Tumor−Normal Expression Difference

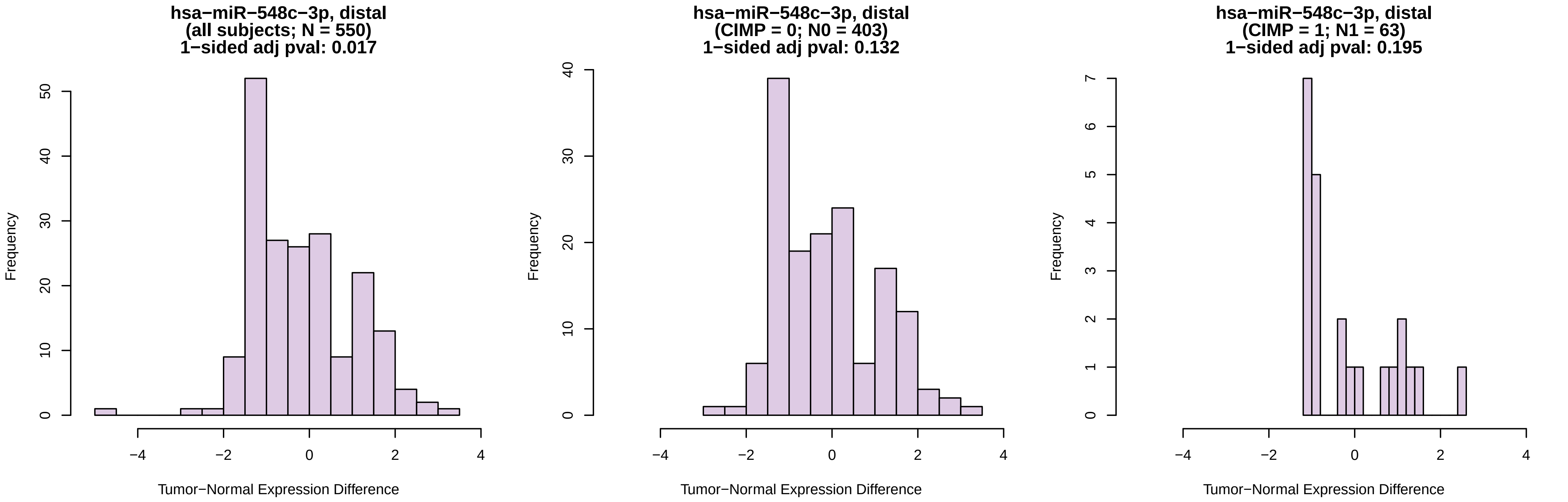
hsa−miR−548c−3p, distal
(all subjects; N = 550)
1−sided adj pval: 0.017
Frequency
Tumor−Normal Expression Difference
hsa−miR−548c−3p, distal
(CIMP = 0; N0 = 403)
1−sided adj pval: 0.132
Frequency
Tumor−Normal Expression Difference
hsa−miR−548c−3p, distal
(CIMP = 1; N1 = 63)
1−sided adj pval: 0.195
Frequency
Tumor−Normal Expression Difference

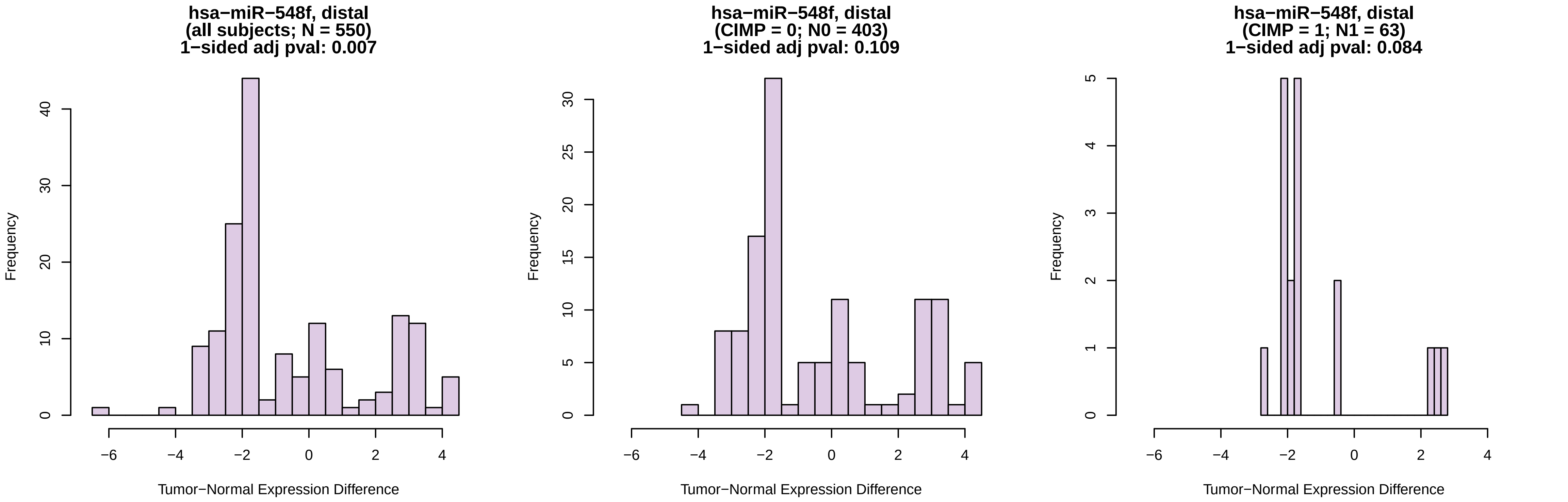
hsa−miR−548f, distal
(all subjects; N = 550)
1−sided adj pval: 0.007
Frequency
Tumor−Normal Expression Difference
hsa−miR−548f, distal
(CIMP = 0; N0 = 403)
1−sided adj pval: 0.109
Frequency
Tumor−Normal Expression Difference
hsa−miR−548f, distal
(CIMP = 1; N1 = 63)
1−sided adj pval: 0.084
Frequency
Tumor−Normal Expression Difference

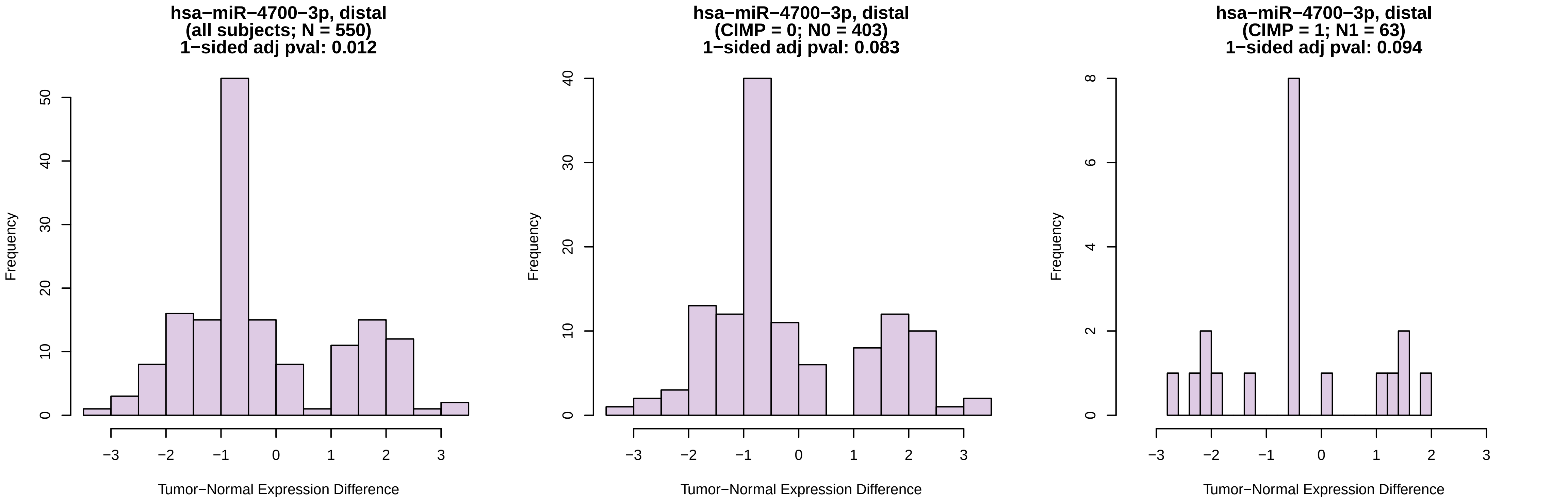
hsa−miR−4700−3p, distal
(all subjects; N = 550)
1−sided adj pval: 0.012
Frequency
0
10
20
30
40
50
−3
−2
−1
0
1
2
3
Tumor−Normal Expression Difference
hsa−miR−4700−3p, distal
(CIMP = 0; N0 = 403)
1−sided adj pval: 0.083
Frequency
0
10
20
30
40
−3
−2
−1
0
1
2
3
Tumor−Normal Expression Difference
hsa−miR−4700−3p, distal
(CIMP = 1; N1 = 63)
1−sided adj pval: 0.094
Frequency
0
2
4
6
8
−3
−2
−1
0
1
2
3
Tumor−Normal Expression Difference

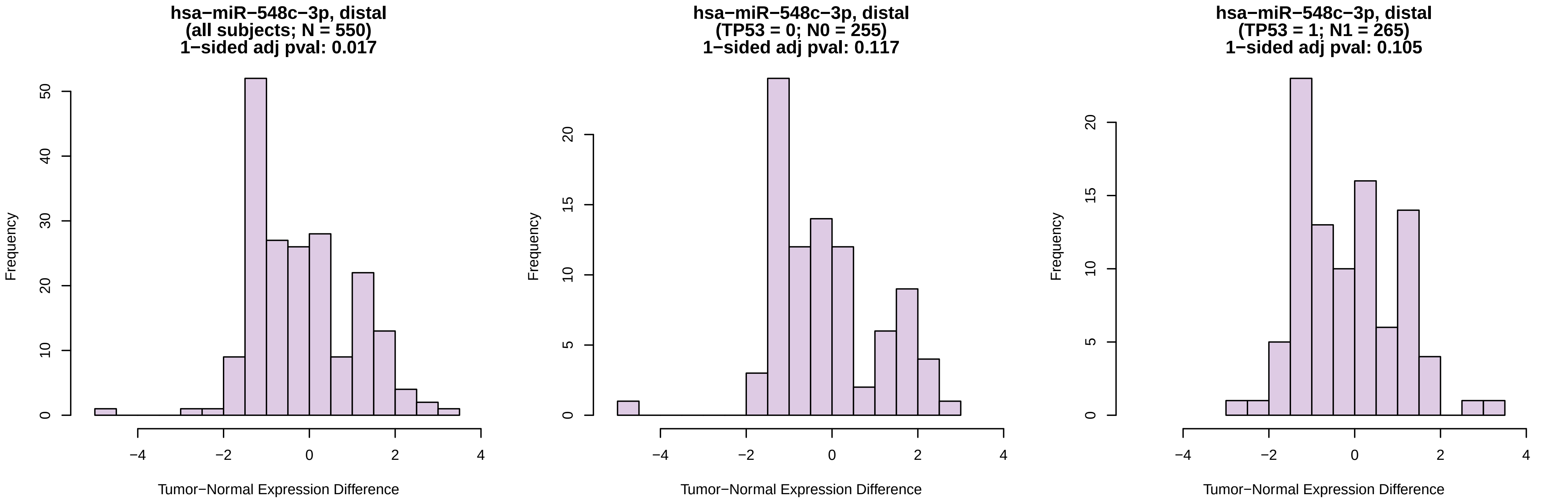

hsa−miR−548c−3p, distal
(all subjects; N = 550)
1−sided adj pval: 0.017
Frequency
Tumor−Normal Expression Difference
hsa−miR−548c−3p, distal
(TP53 = 0; N0 = 255)
1−sided adj pval: 0.117
Frequency
Tumor−Normal Expression Difference
hsa−miR−548c−3p, distal
(TP53 = 1; N1 = 265)
1−sided adj pval: 0.105
Frequency
Tumor−Normal Expression Difference

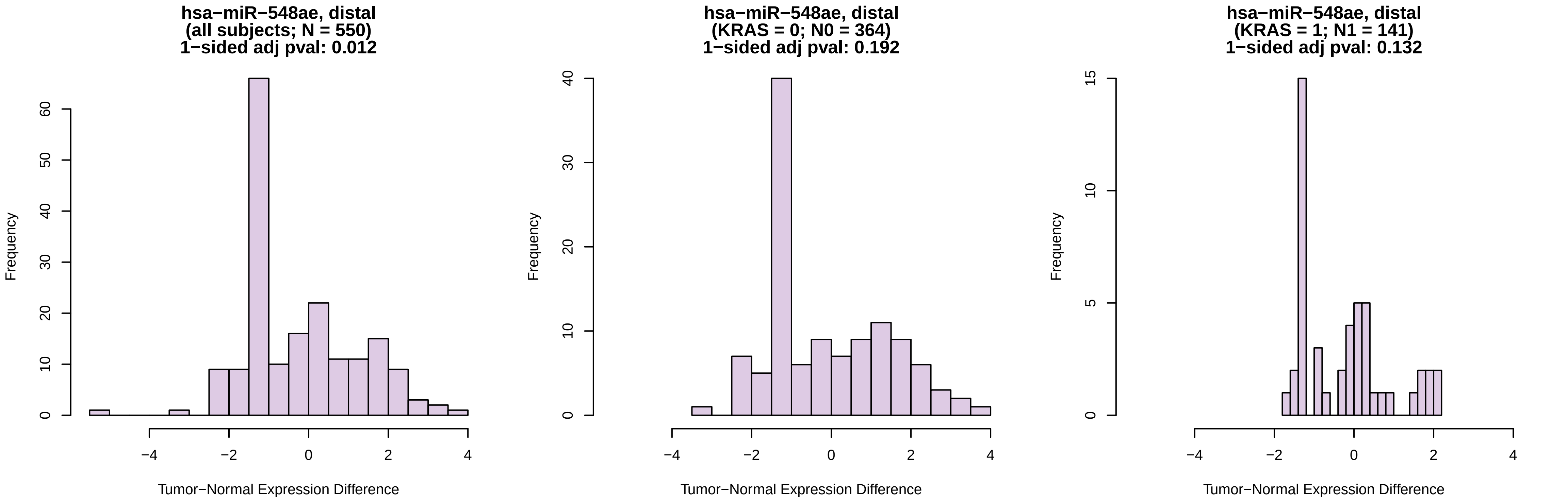
hsa−miR−548ae, distal
(all subjects; N = 550)
1−sided adj pval: 0.012
Frequency
0
10
20
30
40
50
60
−4
−2
0
2
4
Tumor−Normal Expression Difference
hsa−miR−548ae, distal
(KRAS = 0; N0 = 364)
1−sided adj pval: 0.192
Frequency
0
10
20
30
40
−4
−2
0
2
4
Tumor−Normal Expression Difference
hsa−miR−548ae, distal
(KRAS = 1; N1 = 141)
1−sided adj pval: 0.132
Frequency
0
5
10
15
−4
−2
0
2
4
Tumor−Normal Expression Difference

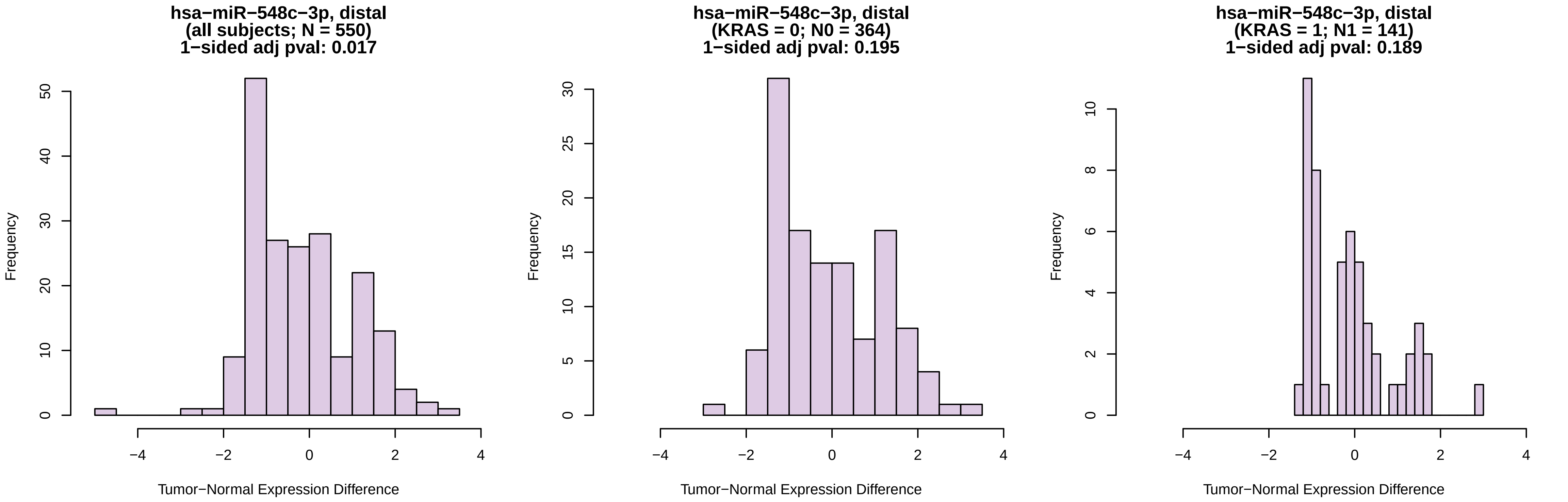
hsa−miR−548c−3p, distal
(all subjects; N = 550)
1−sided adj pval: 0.017
Frequency
Tumor−Normal Expression Difference
hsa−miR−548c−3p, distal
(KRAS = 0; N0 = 364)
1−sided adj pval: 0.195
Frequency
Tumor−Normal Expression Difference
hsa−miR−548c−3p, distal
(KRAS = 1; N1 = 141)
1−sided adj pval: 0.189
Frequency
Tumor−Normal Expression Difference

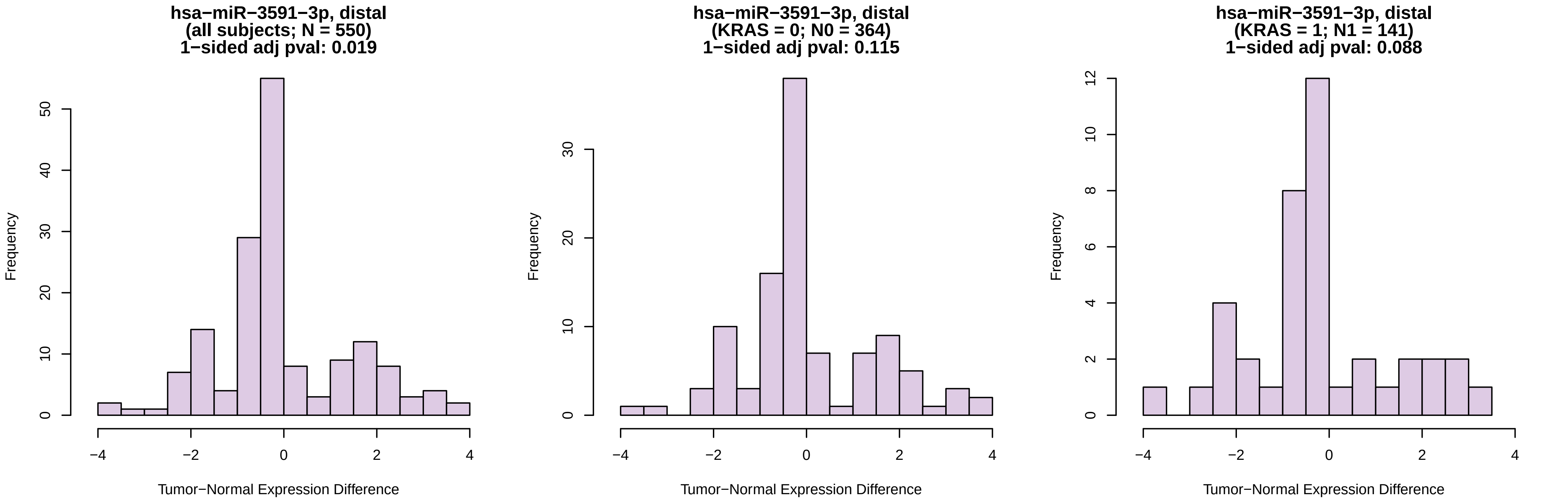

hsa−miR−3591−3p, distal
(all subjects; N = 550)
1−sided adj pval: 0.019
hsa−miR−3591−3p, distal
(KRAS = 0; N0 = 364)
1−sided adj pval: 0.115
hsa−miR−3591−3p, distal
(KRAS = 1; N1 = 141)
1−sided adj pval: 0.088
Frequency
Tumor−Normal Expression Difference

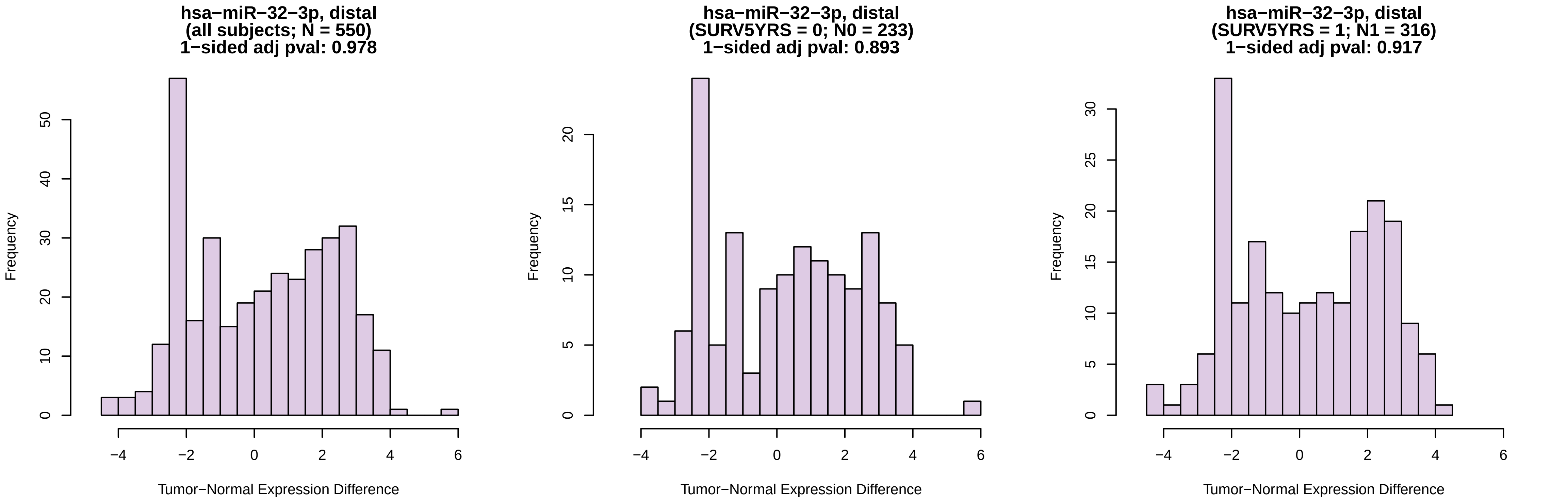

hsa−miR−32−3p, distal
(all subjects; N = 550)
1−sided adj pval: 0.978
Frequency
0
10
20
30
40
50
−4
−2
0
2
4
6
Tumor−Normal Expression Difference
hsa−miR−32−3p, distal
(SURV5YRS = 0; N0 = 233)
1−sided adj pval: 0.893
Frequency
0
5
10
15
20
−4
−2
0
2
4
6
Tumor−Normal Expression Difference
hsa−miR−32−3p, distal
(SURV5YRS = 1; N1 = 316)
1−sided adj pval: 0.917
Frequency
0
5
10
15
20
25
30
−4
−2
0
2
4
6
Tumor−Normal Expression Difference

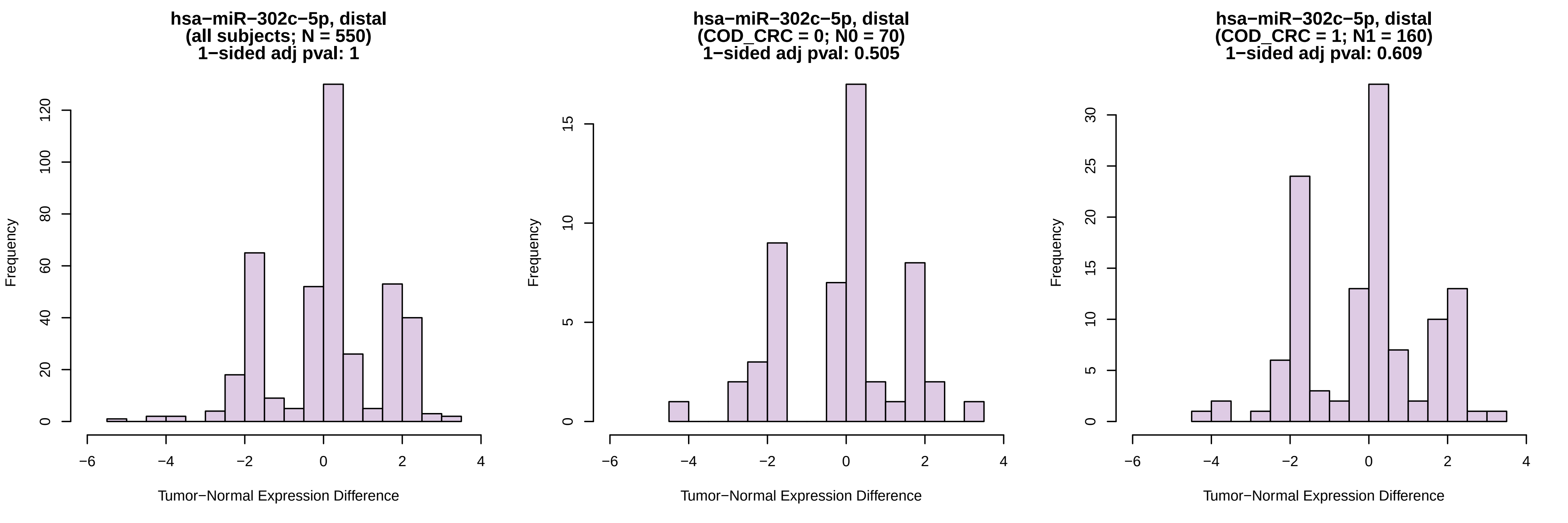
hsa−miR−302c−5p, distal
(all subjects; N = 550)
1−sided adj pval: 1
Frequency
Tumor−Normal Expression Difference
hsa−miR−302c−5p, distal
(COD_CRC = 0; N0 = 70)
1−sided adj pval: 0.505
Frequency
Tumor−Normal Expression Difference
hsa−miR−302c−5p, distal
(COD_CRC = 1; N1 = 160)
1−sided adj pval: 0.609
Frequency
Tumor−Normal Expression Difference

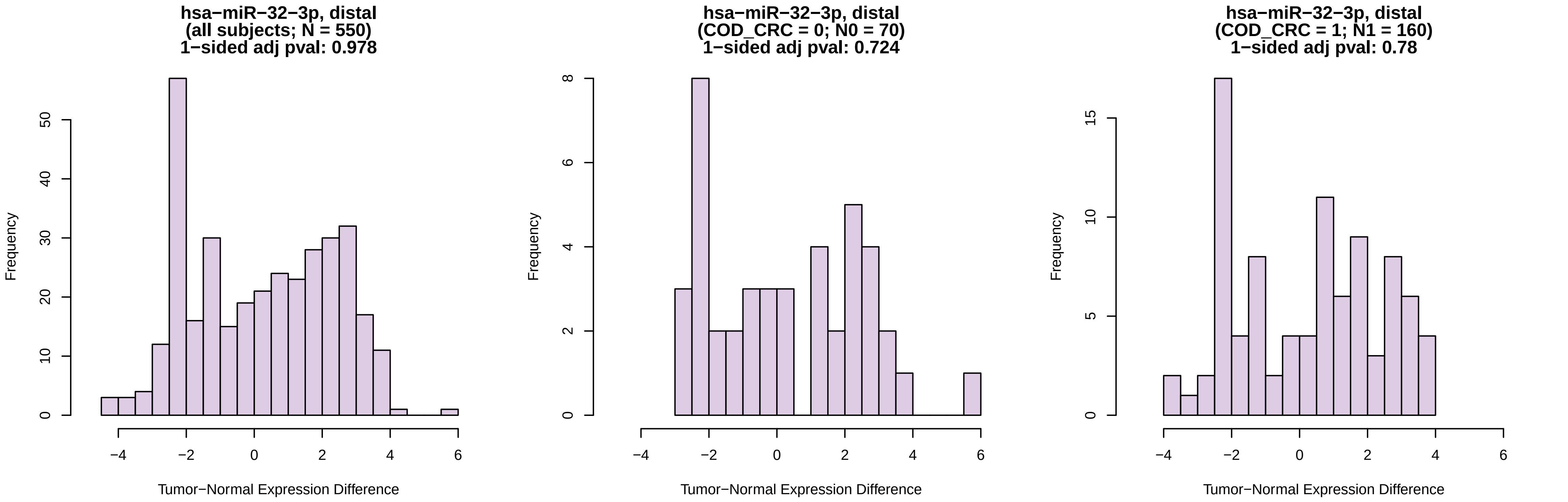
hsa−miR−32−3p, distal
(all subjects; N = 550)
1−sided adj pval: 0.978
Frequency
Tumor−Normal Expression Difference
hsa−miR−32−3p, distal
(COD_CRC = 0; N0 = 70)
1−sided adj pval: 0.724
Frequency
Tumor−Normal Expression Difference
hsa−miR−32−3p, distal
(COD_CRC = 1; N1 = 160)
1−sided adj pval: 0.78
Frequency
Tumor−Normal Expression Difference

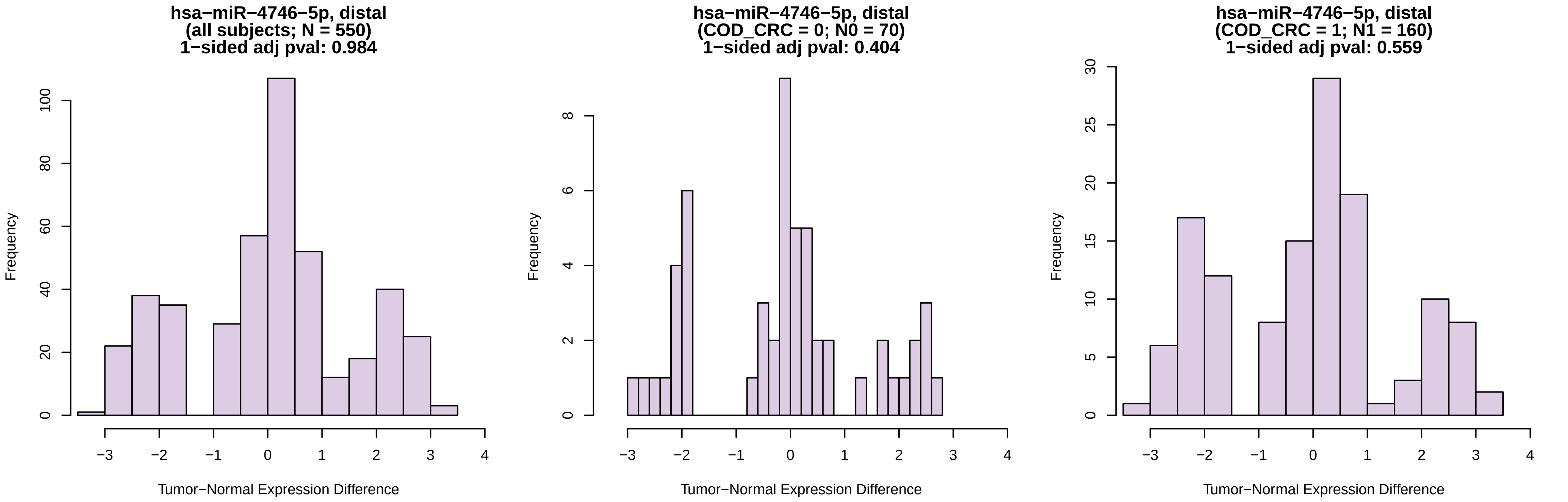
hsa−miR−4746−5p, distal
(all subjects; N = 550)
1−sided adj pval: 0.984
Frequency
Tumor−Normal Expression Difference
hsa−miR−4746−5p, distal
(COD_CRC = 0; N0 = 70)
1−sided adj pval: 0.404
Frequency
Tumor−Normal Expression Difference
hsa−miR−4746−5p, distal
(COD_CRC = 1; N1 = 160)
1−sided adj pval: 0.559
Frequency
Tumor−Normal Expression Difference

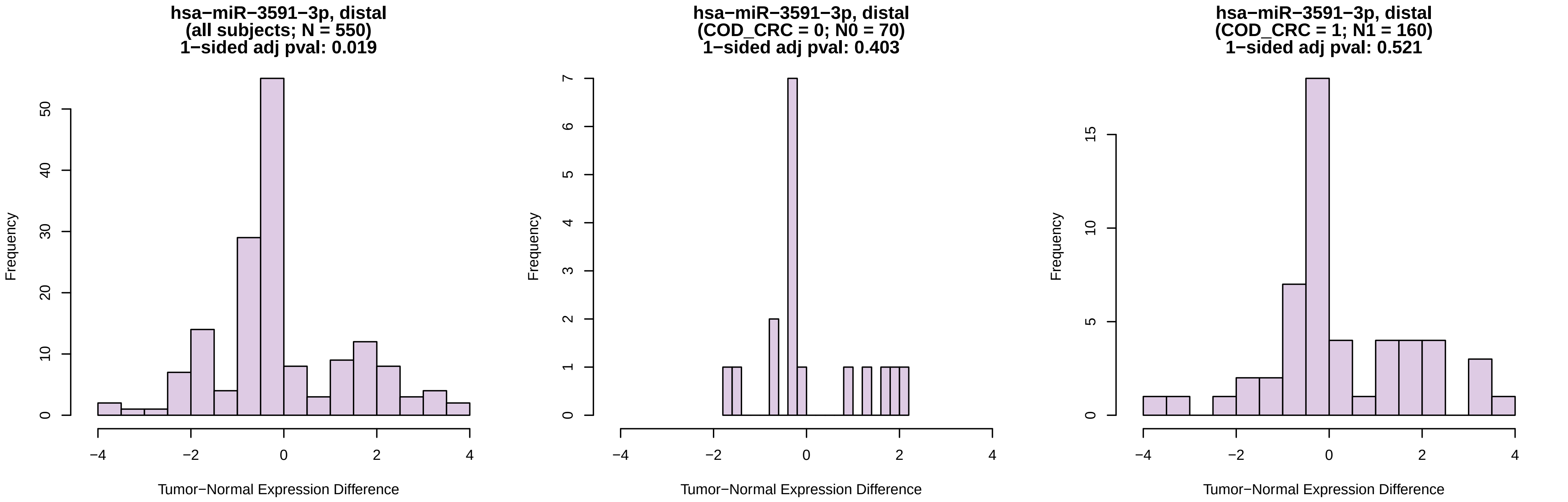

hsa−miR−3591−3p, distal
(all subjects; N = 550)
1−sided adj pval: 0.019
Frequency
Tumor−Normal Expression Difference
hsa−miR−3591−3p, distal
(COD_CRC = 0; N0 = 70)
1−sided adj pval: 0.403
Frequency
Tumor−Normal Expression Difference
hsa−miR−3591−3p, distal
(COD_CRC = 1; N1 = 160)
1−sided adj pval: 0.521
Frequency
Tumor−Normal Expression Difference

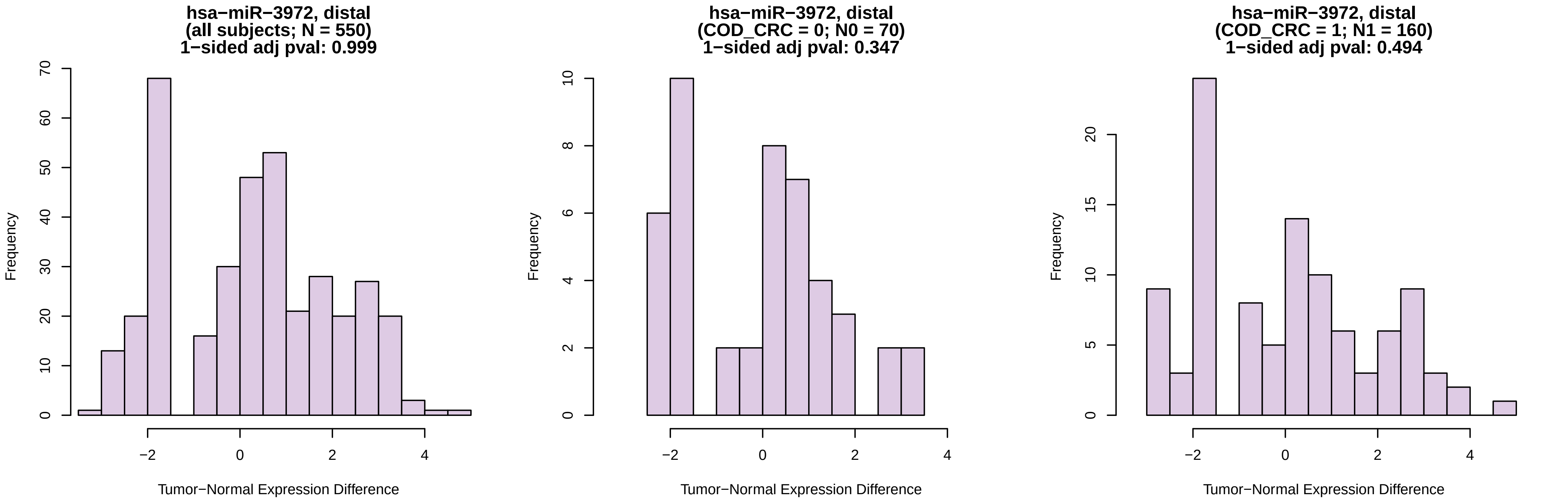

hsa−miR−3972, distal
(all subjects; N = 550)
1−sided adj pval: 0.999
Frequency
0
10
20
30
40
50
60
70
−2
0
2
4
Tumor−Normal Expression Difference
hsa−miR−3972, distal
(COD_CRC = 0; N0 = 70)
1−sided adj pval: 0.347
Frequency
0
2
4
6
8
10
−2
0
2
4
Tumor−Normal Expression Difference
hsa−miR−3972, distal
(COD_CRC = 1; N1 = 160)
1−sided adj pval: 0.494
Frequency
0
5
10
15
20
−2
0
2
4
Tumor−Normal Expression Difference

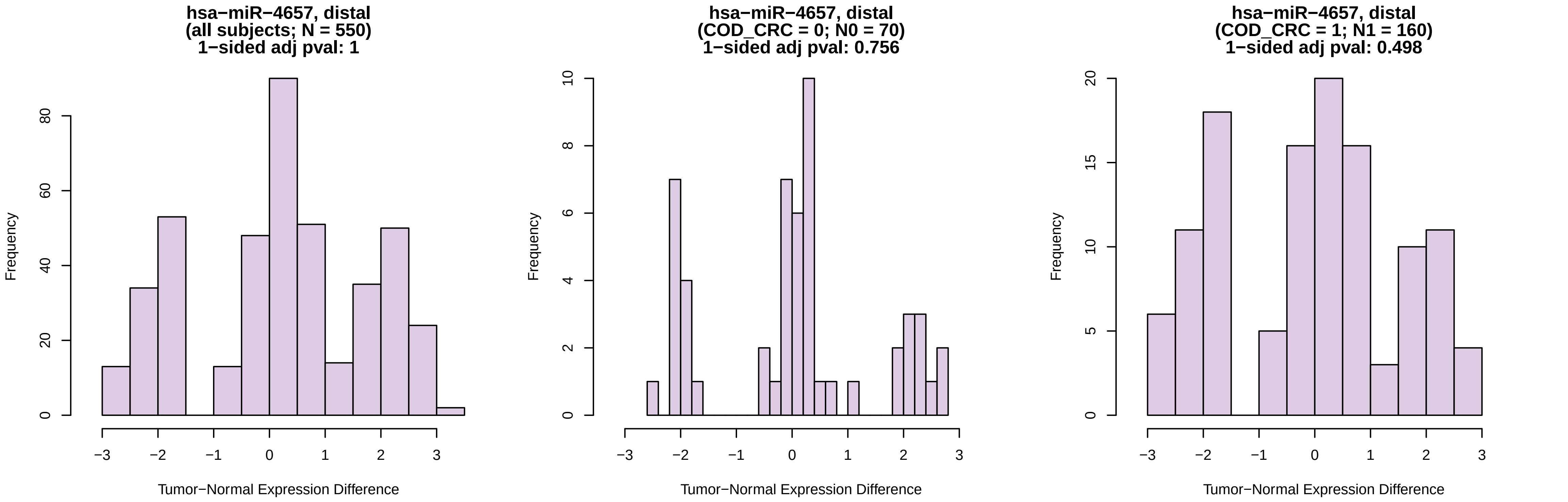
hsa−miR−4657, distal
(all subjects; N = 550)
1−sided adj pval: 1
Frequency
0
20
40
60
80
−3
−2
−1
0
1
2
3
Tumor−Normal Expression Difference
hsa−miR−4657, distal
(COD_CRC = 0; N0 = 70)
1−sided adj pval: 0.756
Frequency
0
2
4
6
8
10
−3
−2
−1
0
1
2
3
Tumor−Normal Expression Difference
hsa−miR−4657, distal
(COD_CRC = 1; N1 = 160)
1−sided adj pval: 0.498
Frequency
0
5
10
15
20
−3
−2
−1
0
1
2
3
Tumor−Normal Expression Difference

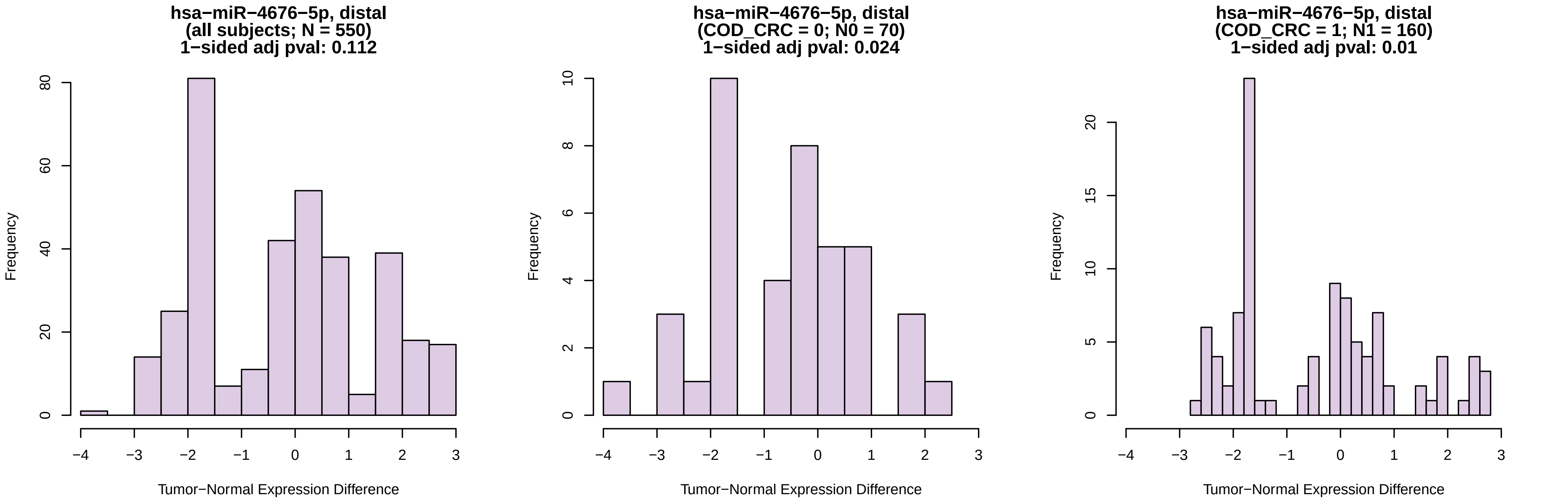

hsa−miR−4676−5p, distal
(all subjects; N = 550)
1−sided adj pval: 0.112
Frequency
Tumor−Normal Expression Difference
hsa−miR−4676−5p, distal
(COD_CRC = 0; N0 = 70)
1−sided adj pval: 0.024
Frequency
Tumor−Normal Expression Difference
hsa−miR−4676−5p, distal
(COD_CRC = 1; N1 = 160)
1−sided adj pval: 0.01
Frequency
Tumor−Normal Expression Difference

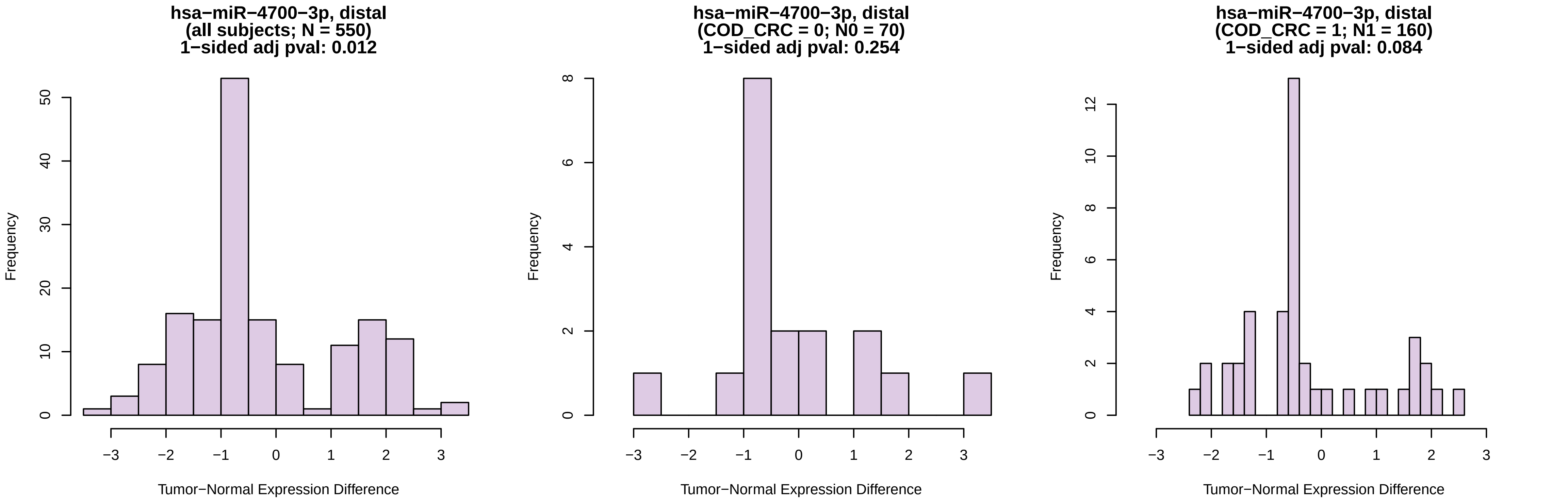

hsa−miR−4700−3p, distal
(all subjects; N = 550)
1−sided adj pval: 0.012
Frequency
0
10
20
30
40
50
−3
−2
−1
0
1
2
3
Tumor−Normal Expression Difference
hsa−miR−4700−3p, distal
(COD_CRC = 0; N0 = 70)
1−sided adj pval: 0.254
Frequency
0
2
4
6
8
−3
−2
−1
0
1
2
3
Tumor−Normal Expression Difference
hsa−miR−4700−3p, distal
(COD_CRC = 1; N1 = 160)
1−sided adj pval: 0.084
Frequency
0
2
4
6
8
10
12
−3
−2
−1
0
1
2
3
Tumor−Normal Expression Difference

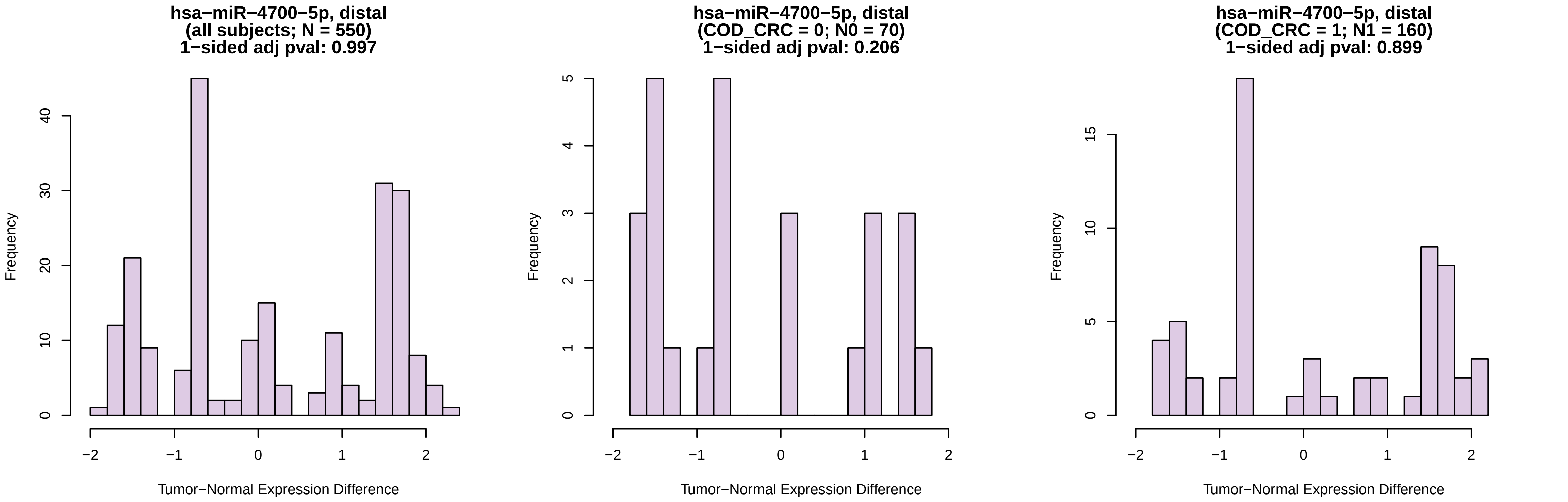

hsa−miR−4700−5p, distal
(all subjects; N = 550)
1−sided adj pval: 0.997
Frequency
0
10
20
30
40
−2
−1
0
1
2
Tumor−Normal Expression Difference
hsa−miR−4700−5p, distal
(COD_CRC = 0; N0 = 70)
1−sided adj pval: 0.206
Frequency
0
1
2
3
4
5
−2
−1
0
1
2
Tumor−Normal Expression Difference
hsa−miR−4700−5p, distal
(COD_CRC = 1; N1 = 160)
1−sided adj pval: 0.899
Frequency
0
5
10
15
−2
−1
0
1
2
Tumor−Normal Expression Difference

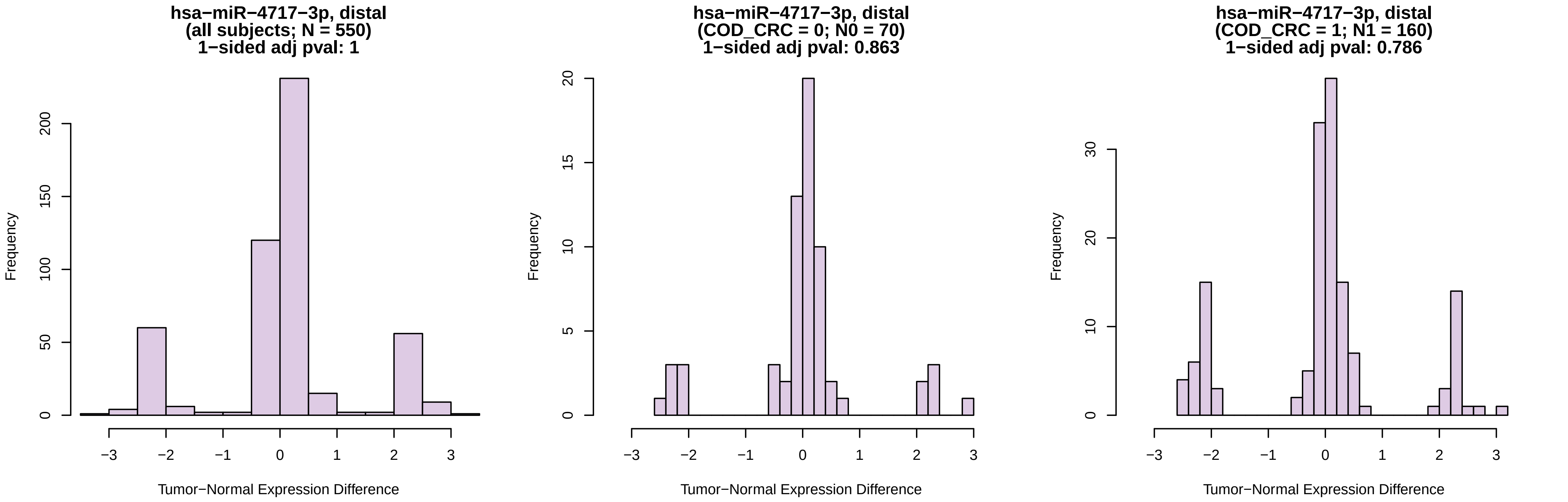

hsa−miR−4717−3p, distal
(all subjects; N = 550)
1−sided adj pval: 1
Frequency
0
50
100
150
200
−3
−2
−1
0
1
2
3
Tumor−Normal Expression Difference
hsa−miR−4717−3p, distal
(COD_CRC = 0; N0 = 70)
1−sided adj pval: 0.863
Frequency
0
5
10
15
20
−3
−2
−1
0
1
2
3
Tumor−Normal Expression Difference
hsa−miR−4717−3p, distal
(COD_CRC = 1; N1 = 160)
1−sided adj pval: 0.786
Frequency
0
10
20
30
−3
−2
−1
0
1
2
3
Tumor−Normal Expression Difference

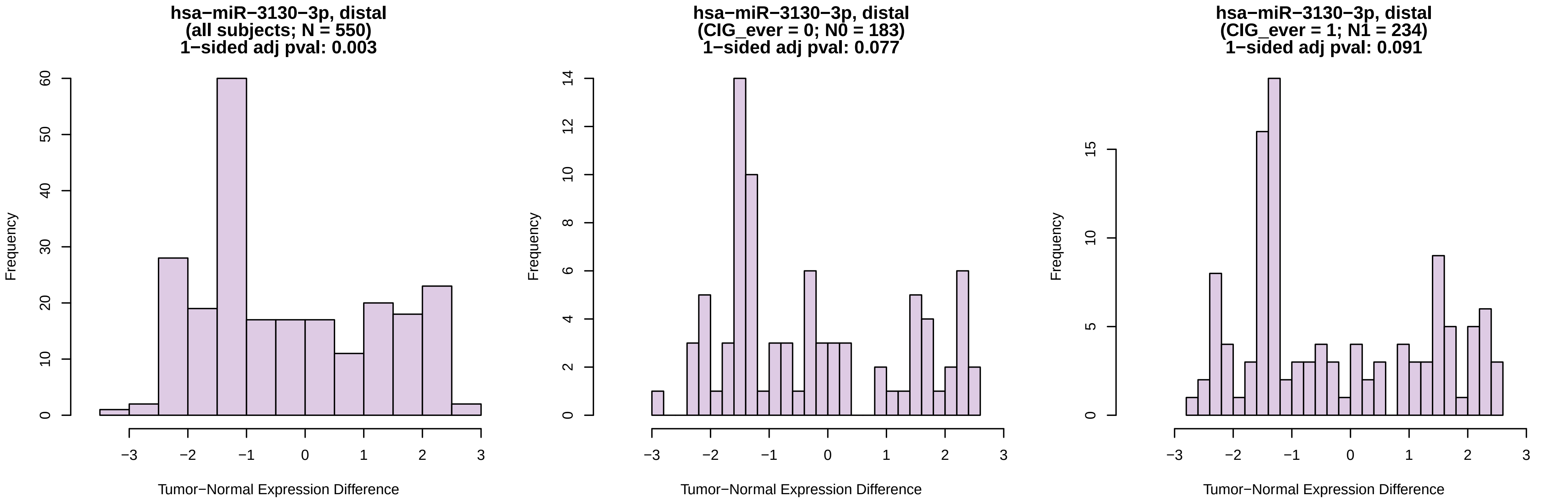

hsa−miR−3130−3p, distal
(all subjects; N = 550)
1−sided adj pval: 0.003
Frequency
Tumor−Normal Expression Difference
hsa−miR−3130−3p, distal
(CIG_ever = 0; N0 = 183)
1−sided adj pval: 0.077
Frequency
Tumor−Normal Expression Difference
hsa−miR−3130−3p, distal
(CIG_ever = 1; N1 = 234)
1−sided adj pval: 0.091
Frequency
Tumor−Normal Expression Difference

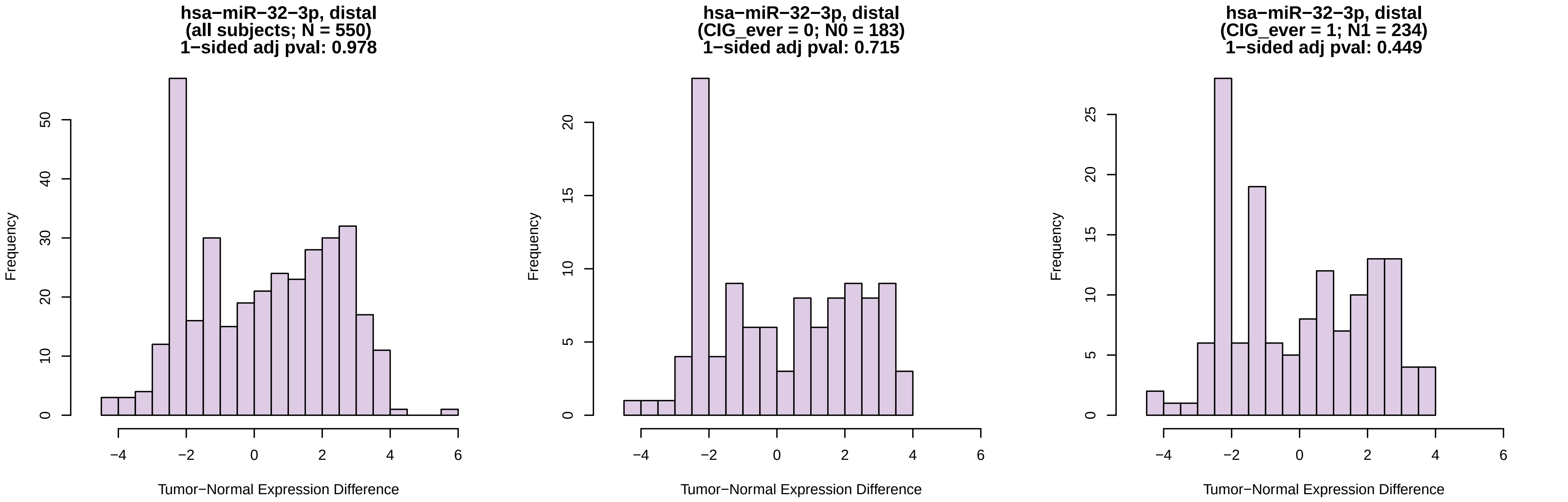
hsa−miR−32−3p, distal
(all subjects; N = 550)
1−sided adj pval: 0.978
Frequency
Tumor−Normal Expression Difference
hsa−miR−32−3p, distal
(CIG_ever = 0; N0 = 183)
1−sided adj pval: 0.715
Frequency
Tumor−Normal Expression Difference
hsa−miR−32−3p, distal
(CIG_ever = 1; N1 = 234)
1−sided adj pval: 0.449
Frequency
Tumor−Normal Expression Difference

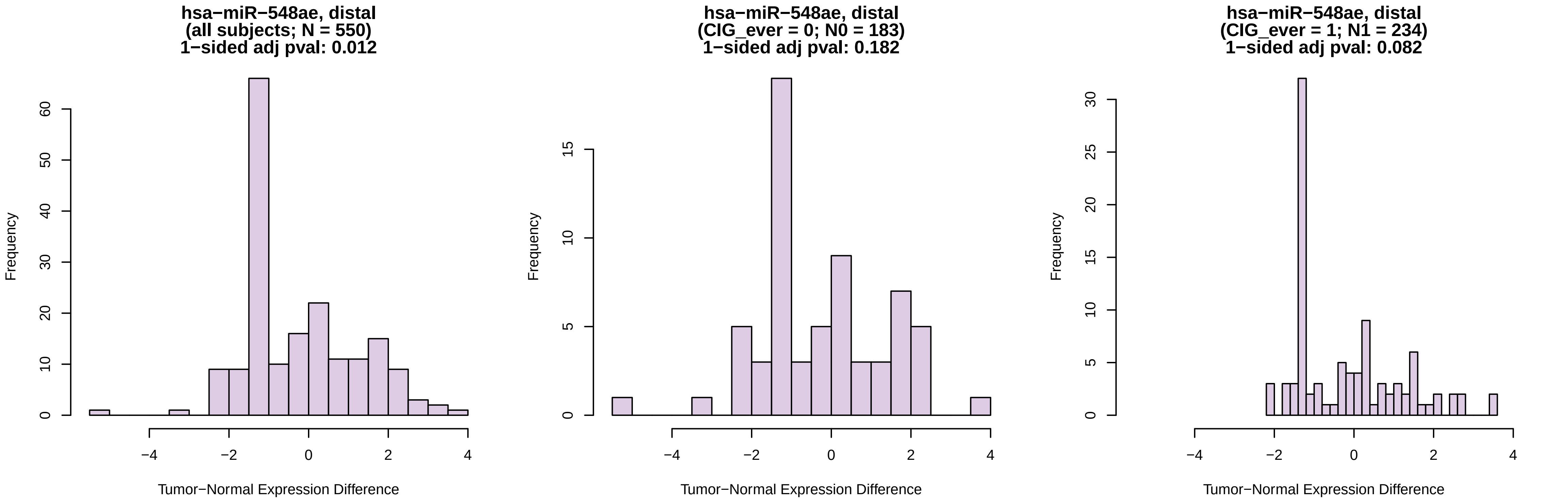

hsa−miR−548ae, distal
(all subjects; N = 550)
1−sided adj pval: 0.012
Frequency
0
10
20
30
40
50
60
−4
−2
0
2
4
Tumor−Normal Expression Difference
hsa−miR−548ae, distal
(CIG_ever = 0; N0 = 183)
1−sided adj pval: 0.182
Frequency
0
5
10
15
−4
−2
0
2
4
Tumor−Normal Expression Difference
hsa−miR−548ae, distal
(CIG_ever = 1; N1 = 234)
1−sided adj pval: 0.082
Frequency
0
5
10
15
20
25
30
−4
−2
0
2
4
Tumor−Normal Expression Difference

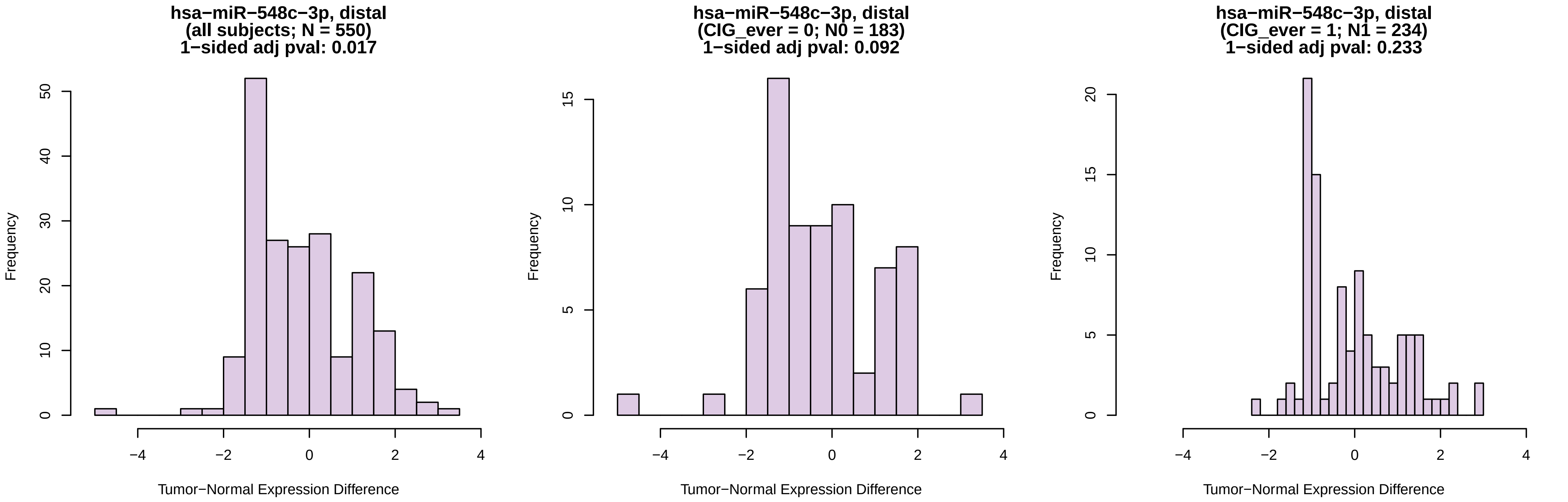
hsa−miR−548c−3p, distal
(all subjects; N = 550)
1−sided adj pval: 0.017
hsa−miR−548c−3p, distal
(CIG_ever = 0; N0 = 183)
1−sided adj pval: 0.092
hsa−miR−548c−3p, distal
(CIG_ever = 1; N1 = 234)
1−sided adj pval: 0.233
Frequency
Tumor−Normal Expression Difference

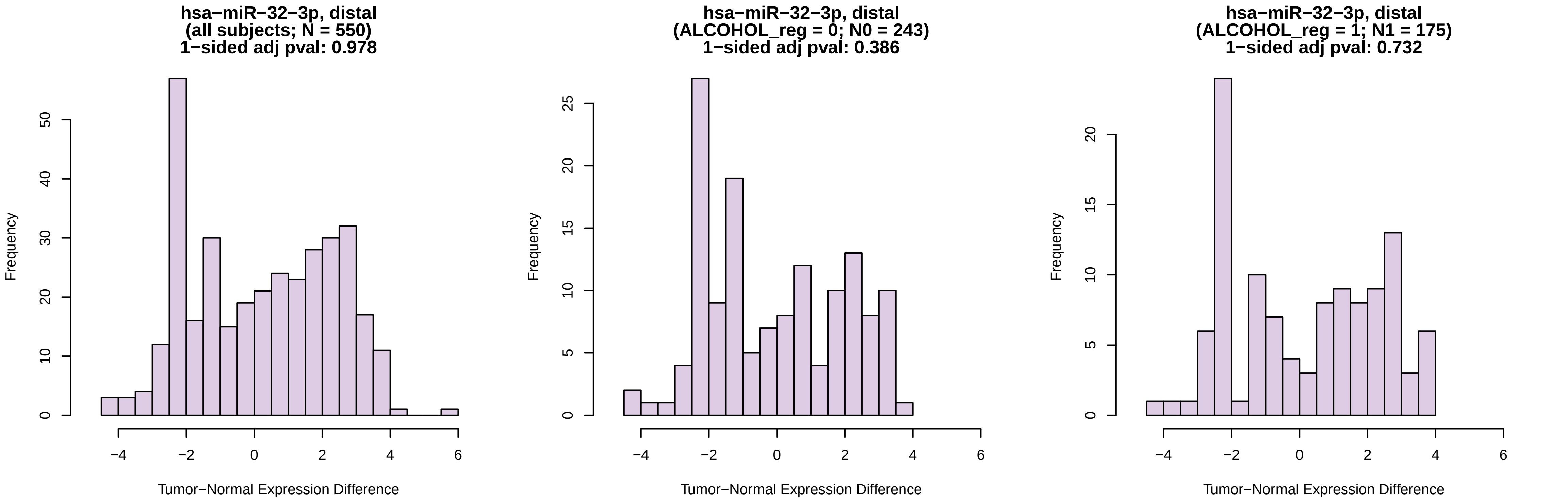

hsa−miR−32−3p, distal
(all subjects; N = 550)
1−sided adj pval: 0.978
Frequency
Tumor−Normal Expression Difference
hsa−miR−32−3p, distal
(ALCOHOL_reg = 0; N0 = 243)
1−sided adj pval: 0.386
Frequency
Tumor−Normal Expression Difference
hsa−miR−32−3p, distal
(ALCOHOL_reg = 1; N1 = 175)
1−sided adj pval: 0.732
Frequency
Tumor−Normal Expression Difference

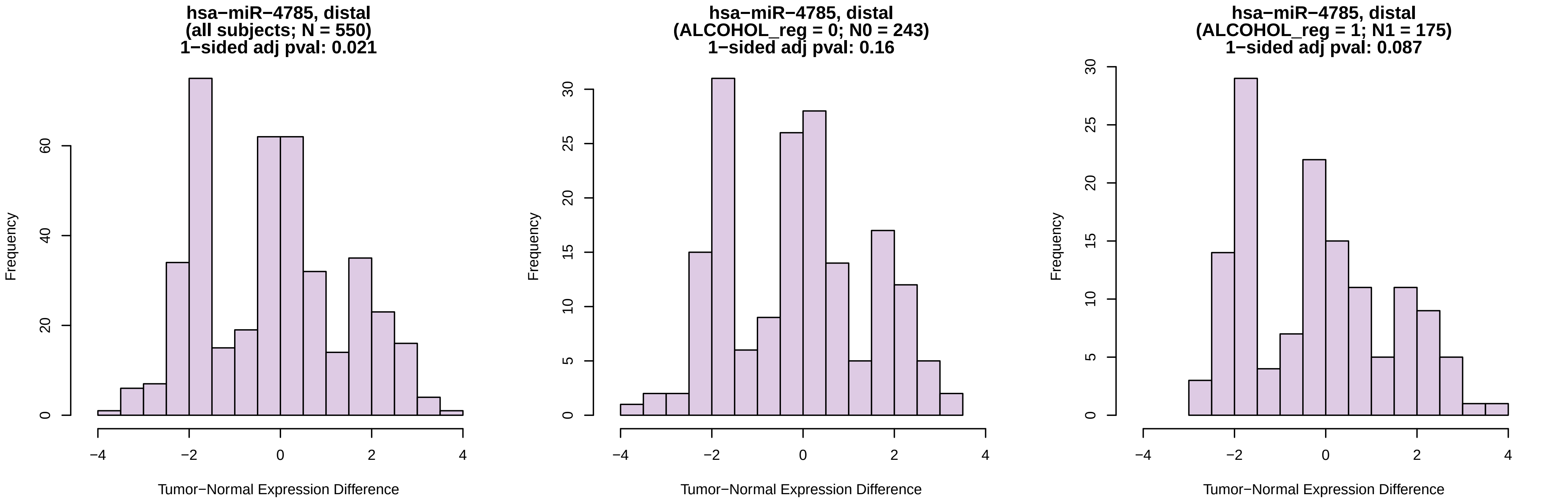
hsa−miR−4785, distal
(all subjects; N = 550)
1−sided adj pval: 0.021
Frequency
0
20
40
60
−4
−2
0
2
4
Tumor−Normal Expression Difference
hsa−miR−4785, distal
(ALCOHOL_reg = 0; N0 = 243)
1−sided adj pval: 0.16
Frequency
0
5
10
15
20
25
30
−4
−2
0
2
4
Tumor−Normal Expression Difference
hsa−miR−4785, distal
(ALCOHOL_reg = 1; N1 = 175)
1−sided adj pval: 0.087
Frequency
0
5
10
15
20
25
30
−4
−2
0
2
4
Tumor−Normal Expression Difference

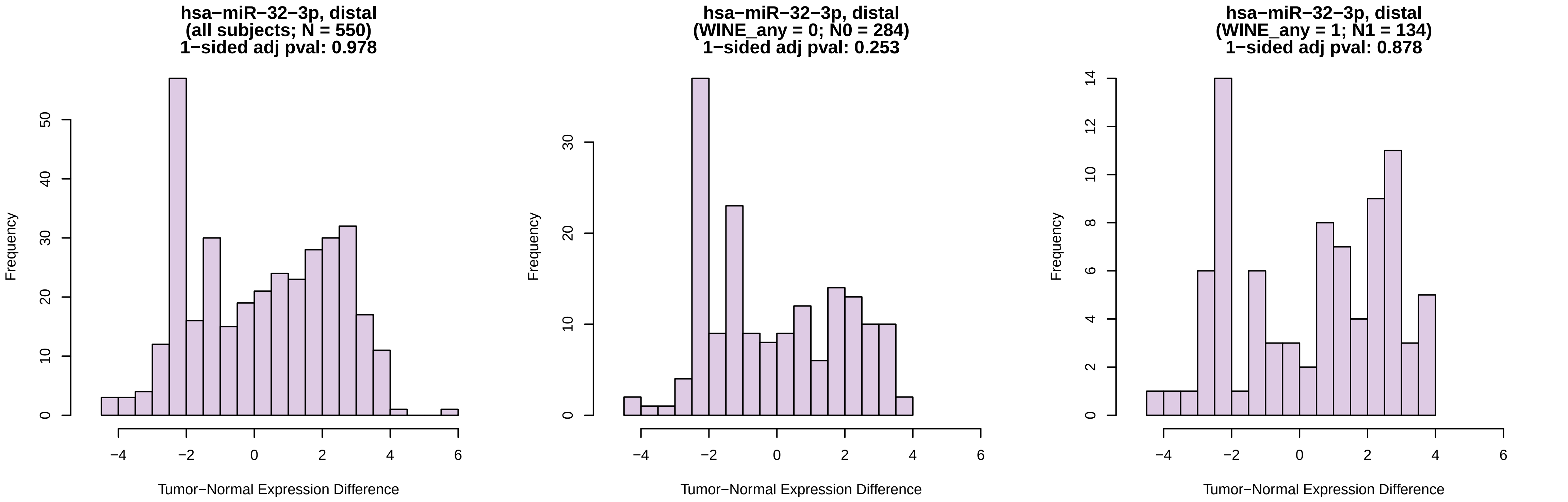

hsa−miR−32−3p, distal
(all subjects; N = 550)
1−sided adj pval: 0.978
Frequency
Tumor−Normal Expression Difference
hsa−miR−32−3p, distal
(WINE_any = 0; N0 = 284)
1−sided adj pval: 0.253
Frequency
Tumor−Normal Expression Difference
hsa−miR−32−3p, distal
(WINE_any = 1; N1 = 134)
1−sided adj pval: 0.878
Frequency
Tumor−Normal Expression Difference

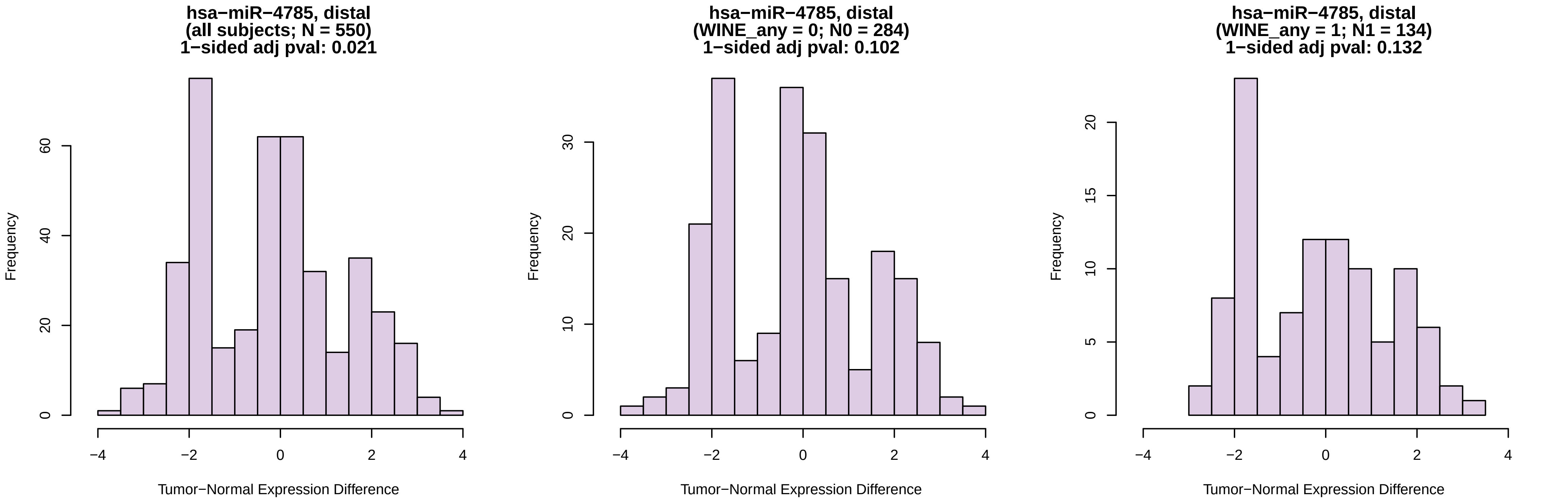
hsa−miR−4785, distal
(all subjects; N = 550)
1−sided adj pval: 0.021
Frequency
0
20
40
60
−4
−2
0
2
4
Tumor−Normal Expression Difference
hsa−miR−4785, distal
(WINE_any = 0; N0 = 284)
1−sided adj pval: 0.102
Frequency
0
10
20
30
−4
−2
0
2
4
Tumor−Normal Expression Difference
hsa−miR−4785, distal
(WINE_any = 1; N1 = 134)
1−sided adj pval: 0.132
Frequency
0
5
10
15
20
−4
−2
0
2
4
Tumor−Normal Expression Difference

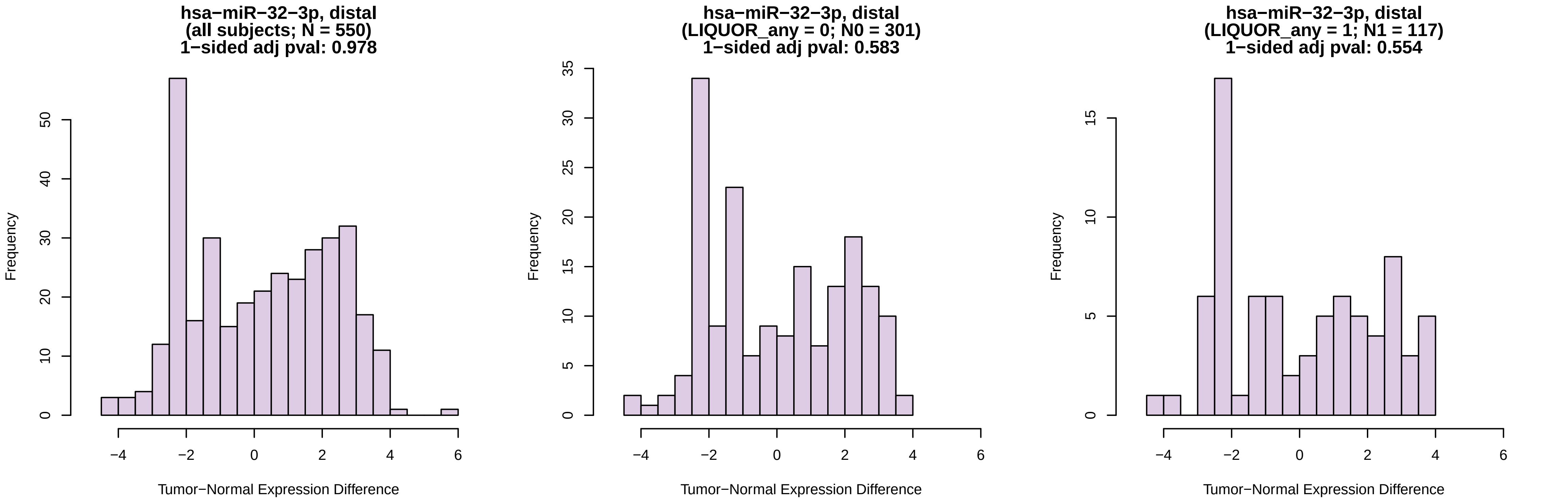

hsa−miR−32−3p, distal
(all subjects; N = 550)
1−sided adj pval: 0.978
Frequency
Tumor−Normal Expression Difference
hsa−miR−32−3p, distal
(LIQUOR_any = 0; N0 = 301)
1−sided adj pval: 0.583
Frequency
Tumor−Normal Expression Difference
hsa−miR−32−3p, distal
(LIQUOR_any = 1; N1 = 117)
1−sided adj pval: 0.554
Frequency
Tumor−Normal Expression Difference

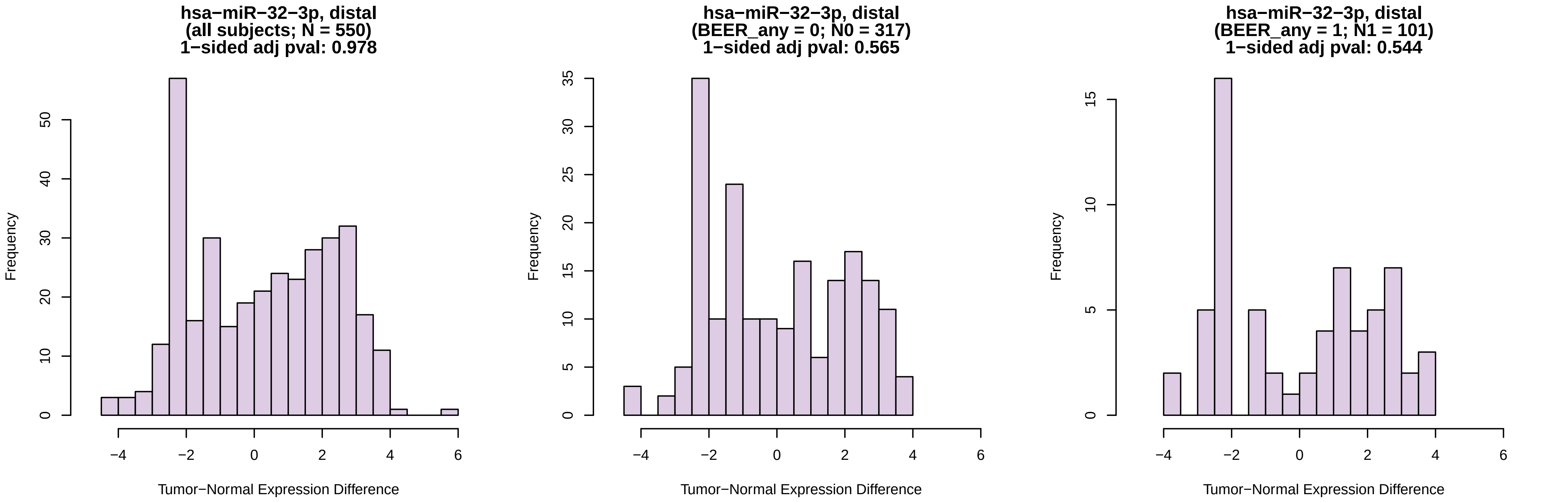

hsa−miR−32−3p, distal
(all subjects; N = 550)
1−sided adj pval: 0.978
Frequency
Tumor−Normal Expression Difference
hsa−miR−32−3p, distal
(BEER_any = 0; N0 = 317)
1−sided adj pval: 0.565
Frequency
Tumor−Normal Expression Difference
hsa−miR−32−3p, distal
(BEER_any = 1; N1 = 101)
1−sided adj pval: 0.544
Frequency
Tumor−Normal Expression Difference

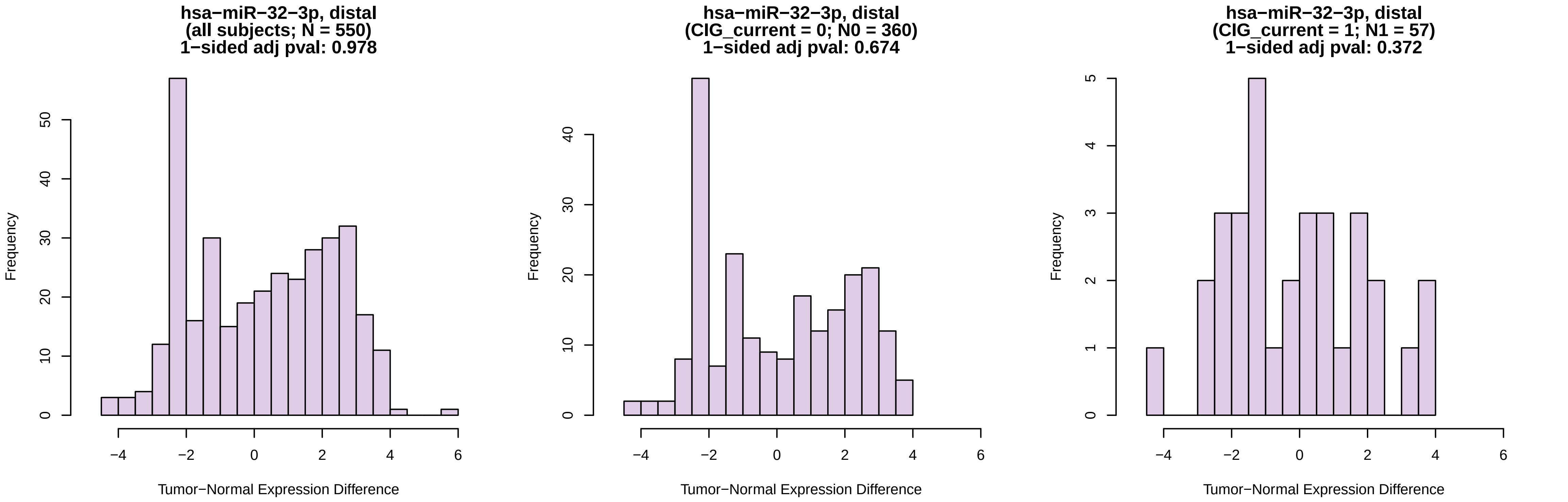
hsa−miR−32−3p, distal
(all subjects; N = 550)
1−sided adj pval: 0.978
Frequency
Tumor−Normal Expression Difference
hsa−miR−32−3p, distal
(CIG_current = 0; N0 = 360)
1−sided adj pval: 0.674
Frequency
Tumor−Normal Expression Difference
hsa−miR−32−3p, distal
(CIG_current = 1; N1 = 57)
1−sided adj pval: 0.372
Frequency
Tumor−Normal Expression Difference

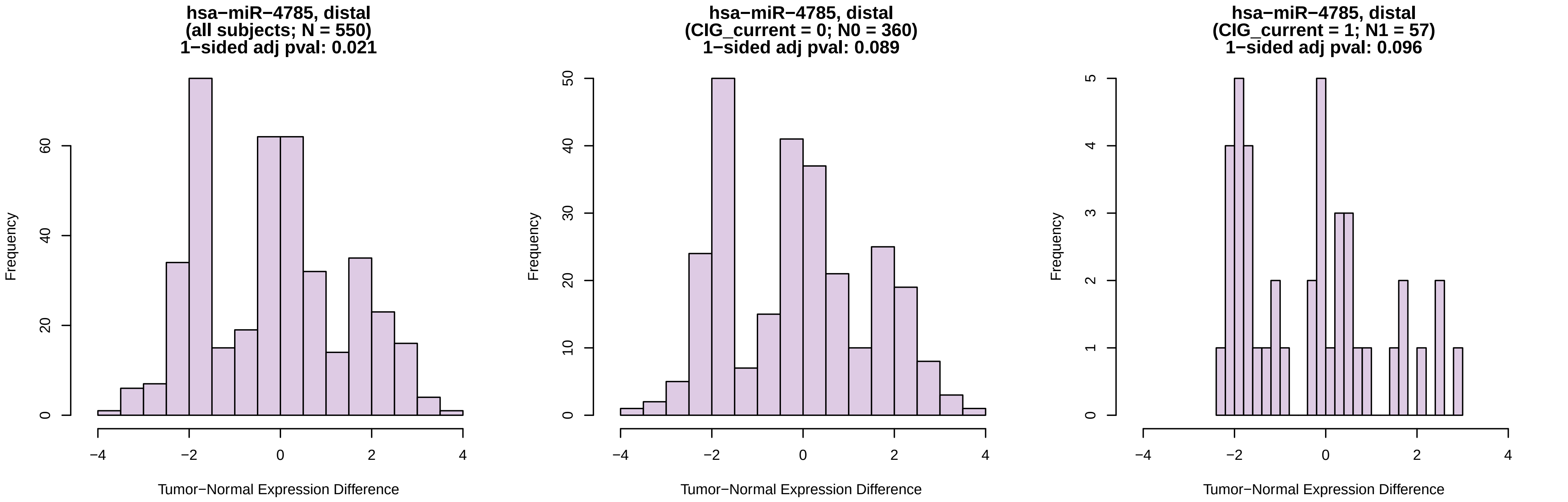
hsa−miR−4785, distal
(all subjects; N = 550)
1−sided adj pval: 0.021
Frequency
0
20
40
60
−4
−2
0
2
4
Tumor−Normal Expression Difference
hsa−miR−4785, distal
(CIG_current = 0; N0 = 360)
1−sided adj pval: 0.089
Frequency
0
10
20
30
40
50
−4
−2
0
2
4
Tumor−Normal Expression Difference
hsa−miR−4785, distal
(CIG_current = 1; N1 = 57)
1−sided adj pval: 0.096
Frequency
0
1
2
3
4
5
−4
−2
0
2
4
Tumor−Normal Expression Difference

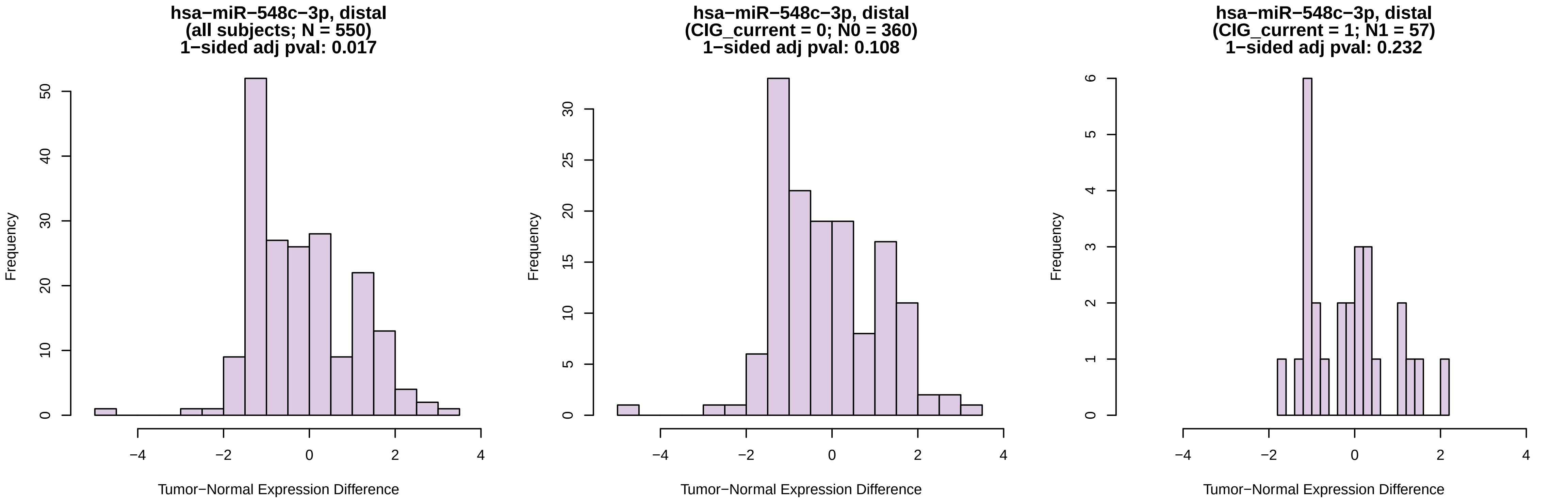
hsa−miR−548c−3p, distal
(all subjects; N = 550)
1−sided adj pval: 0.017
Frequency
Tumor−Normal Expression Difference
hsa−miR−548c−3p, distal
(CIG_current = 0; N0 = 360)
1−sided adj pval: 0.108
Frequency
Tumor−Normal Expression Difference
hsa−miR−548c−3p, distal
(CIG_current = 1; N1 = 57)
1−sided adj pval: 0.232
Frequency
Tumor−Normal Expression Difference

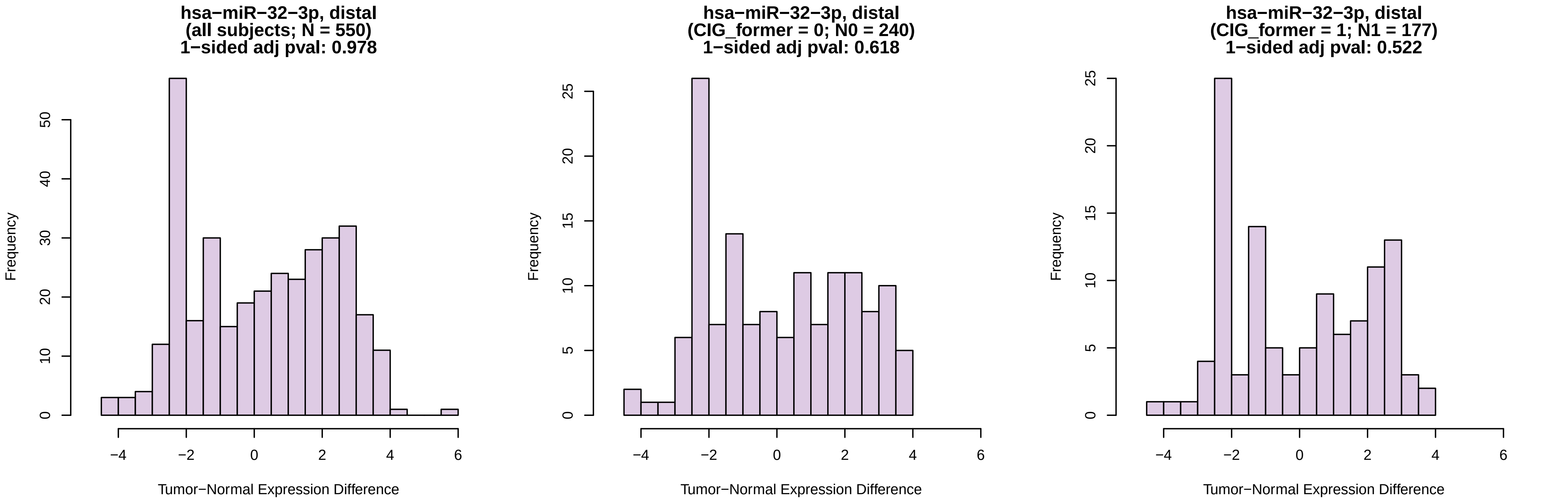

hsa−miR−32−3p, distal
(all subjects; N = 550)
1−sided adj pval: 0.978
Frequency
0
10
20
30
40
50
−4
−2
0
2
4
6
Tumor−Normal Expression Difference
hsa−miR−32−3p, distal
(CIG_former = 0; N0 = 240)
1−sided adj pval: 0.618
Frequency
0
5
10
15
20
25
−4
−2
0
2
4
6
Tumor−Normal Expression Difference
hsa−miR−32−3p, distal
(CIG_former = 1; N1 = 177)
1−sided adj pval: 0.522
Frequency
0
5
10
15
20
25
−4
−2
0
2
4
6
Tumor−Normal Expression Difference

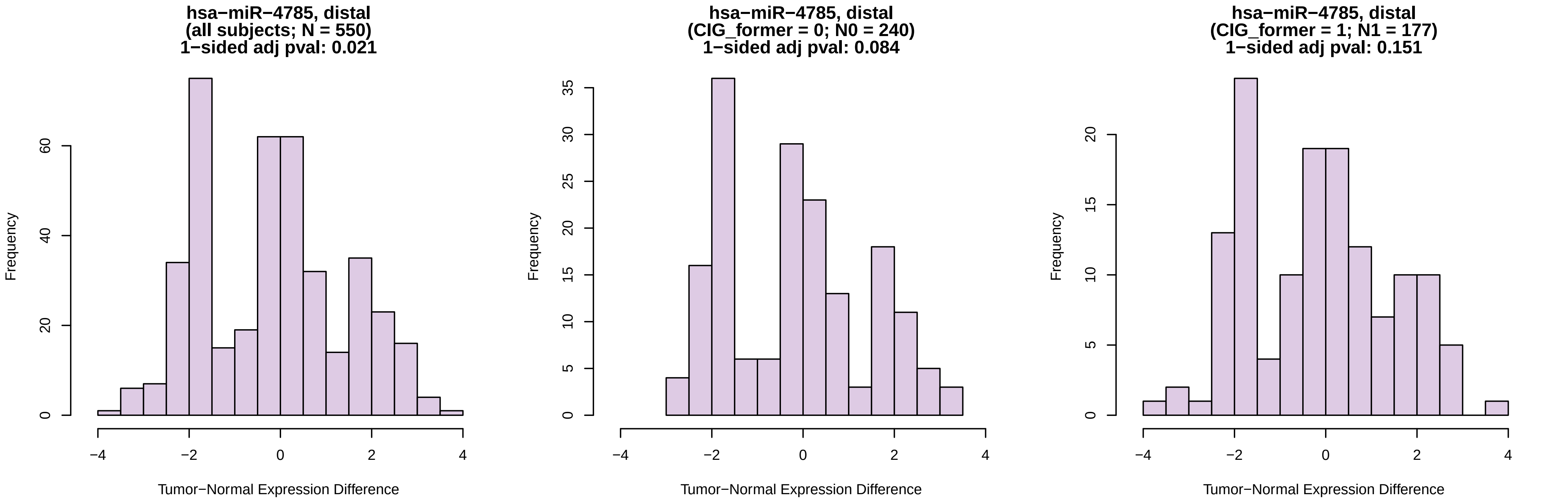

hsa−miR−4785, distal
(all subjects; N = 550)
1−sided adj pval: 0.021
Frequency
0
20
40
60
−4
−2
0
2
4
Tumor−Normal Expression Difference
hsa−miR−4785, distal
(CIG_former = 0; N0 = 240)
1−sided adj pval: 0.084
Frequency
0
5
10
15
20
25
30
35
−4
−2
0
2
4
Tumor−Normal Expression Difference
hsa−miR−4785, distal
(CIG_former = 1; N1 = 177)
1−sided adj pval: 0.151
Frequency
0
5
10
15
20
−4
−2
0
2
4
Tumor−Normal Expression Difference

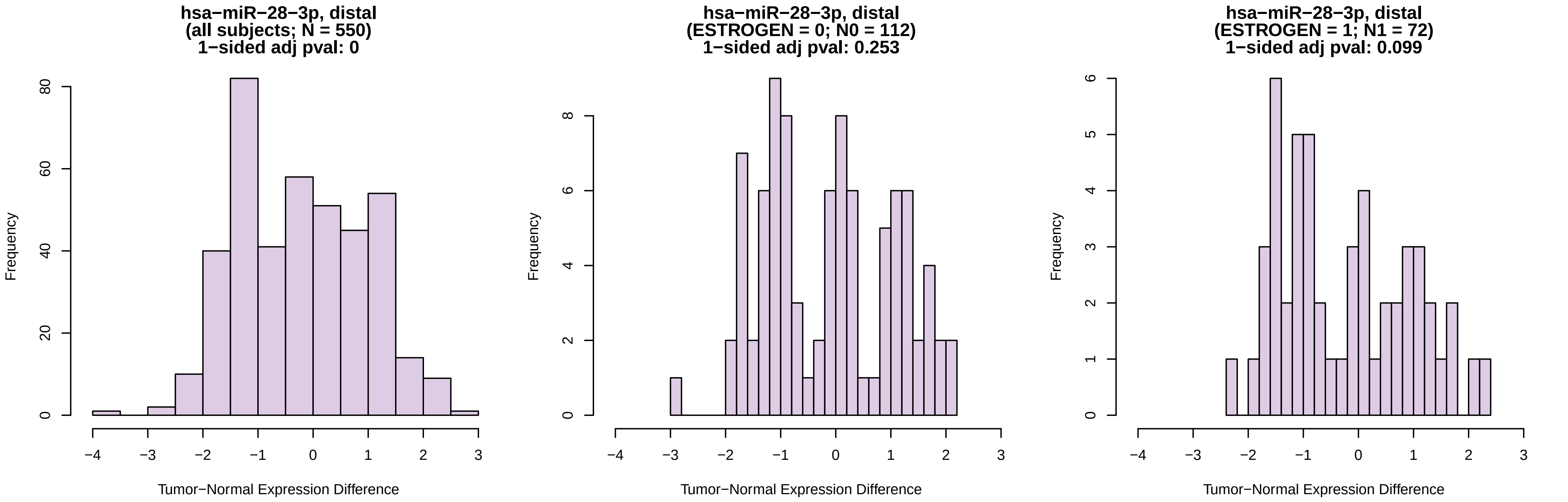

hsa−miR−28−3p, distal
(all subjects; N = 550)
1−sided adj pval: 0
Frequency
Tumor−Normal Expression Difference
hsa−miR−28−3p, distal
(ESTROGEN = 0; N0 = 112)
1−sided adj pval: 0.253
Frequency
Tumor−Normal Expression Difference
hsa−miR−28−3p, distal
(ESTROGEN = 1; N1 = 72)
1−sided adj pval: 0.099
Frequency
Tumor−Normal Expression Difference

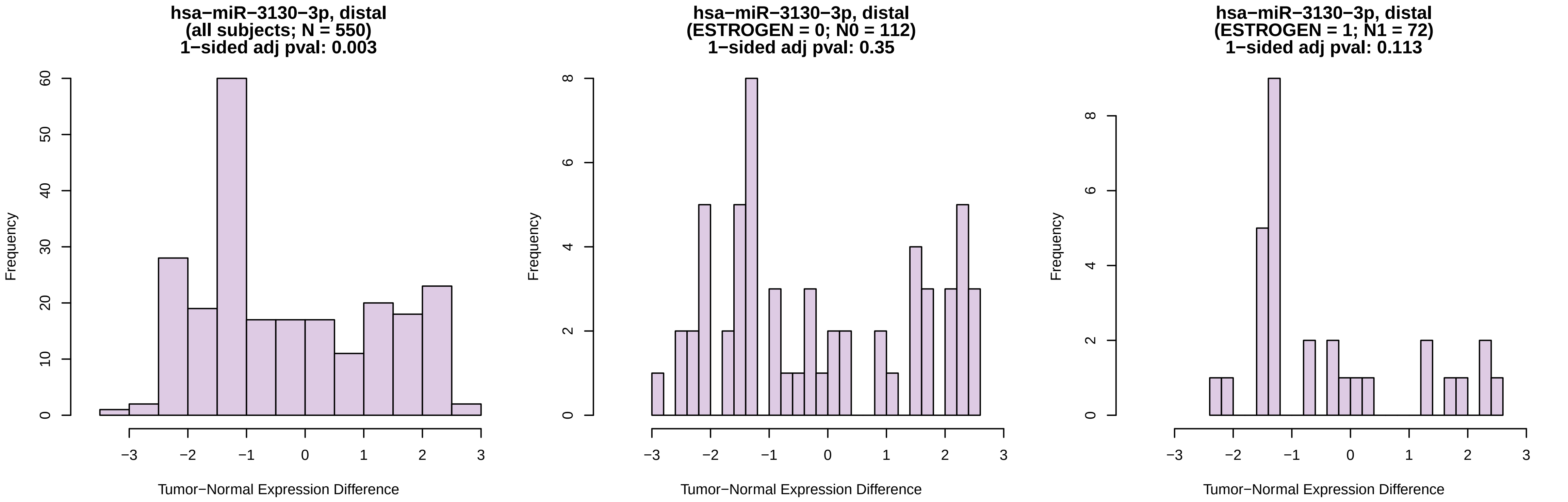

hsa−miR−3130−3p, distal
(all subjects; N = 550)
1−sided adj pval: 0.003
Frequency
Tumor−Normal Expression Difference
hsa−miR−3130−3p, distal
(ESTROGEN = 0; N0 = 112)
1−sided adj pval: 0.35
Frequency
Tumor−Normal Expression Difference
hsa−miR−3130−3p, distal
(ESTROGEN = 1; N1 = 72)
1−sided adj pval: 0.113
Frequency
Tumor−Normal Expression Difference

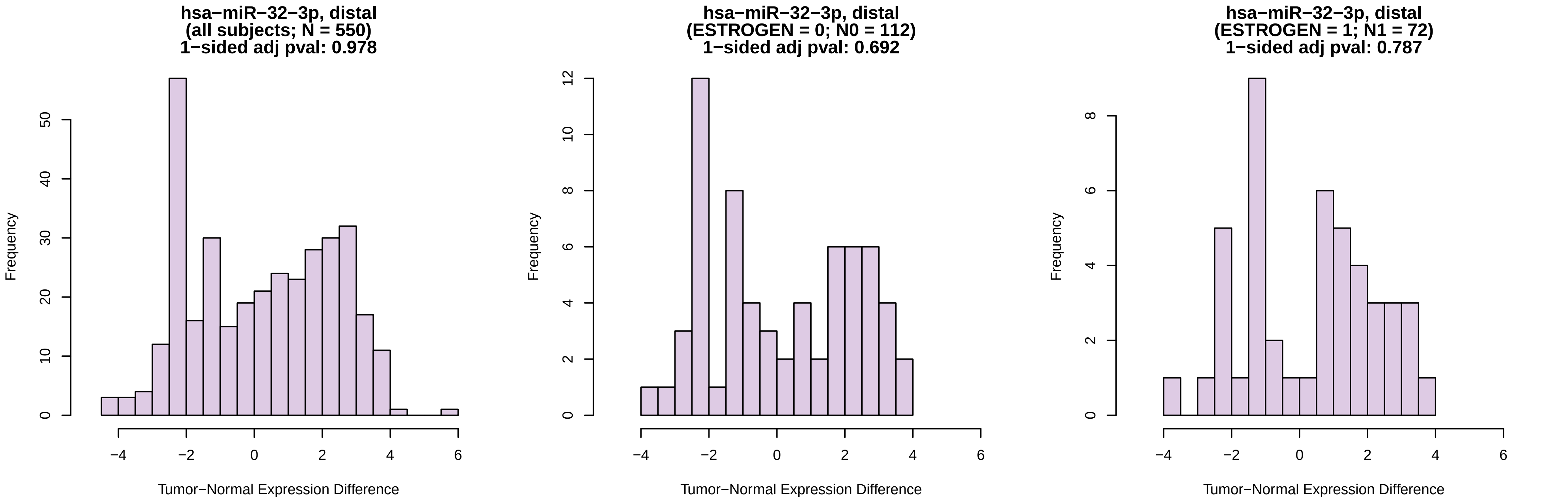
hsa−miR−32−3p, distal
(all subjects; N = 550)
1−sided adj pval: 0.978
Frequency
0
10
20
30
40
50
−4
−2
0
2
4
6
Tumor−Normal Expression Difference
hsa−miR−32−3p, distal
(ESTROGEN = 0; N0 = 112)
1−sided adj pval: 0.692
Frequency
0
2
4
6
8
10
12
−4
−2
0
2
4
6
Tumor−Normal Expression Difference
hsa−miR−32−3p, distal
(ESTROGEN = 1; N1 = 72)
1−sided adj pval: 0.787
Frequency
0
2
4
6
8
−4
−2
0
2
4
6
Tumor−Normal Expression Difference

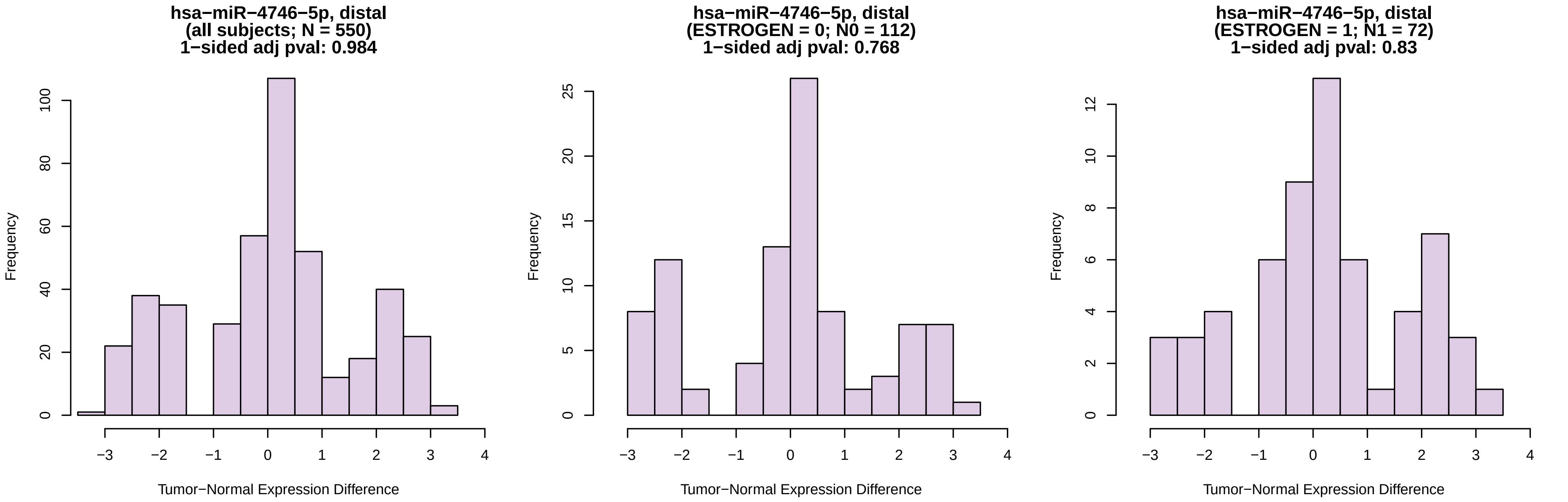
hsa−miR−4746−5p, distal
(all subjects; N = 550)
1−sided adj pval: 0.984
Frequency
Tumor−Normal Expression Difference
hsa−miR−4746−5p, distal
(ESTROGEN = 0; N0 = 112)
1−sided adj pval: 0.768
Frequency
Tumor−Normal Expression Difference
hsa−miR−4746−5p, distal
(ESTROGEN = 1; N1 = 72)
1−sided adj pval: 0.83
Frequency
Tumor−Normal Expression Difference

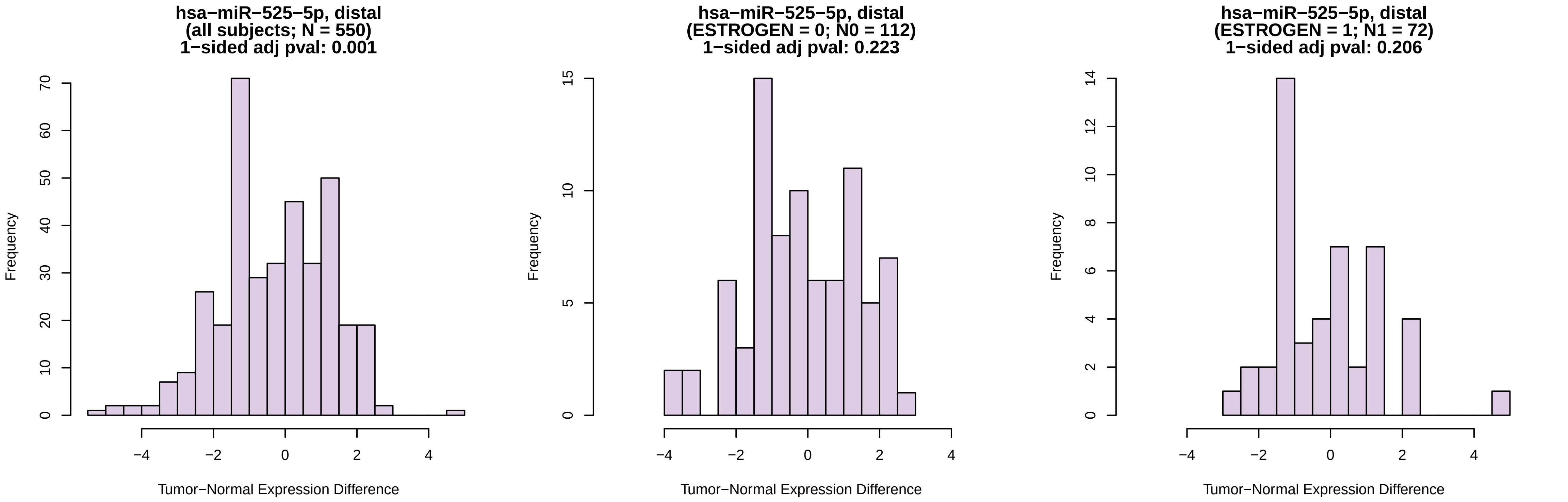
hsa−miR−525−5p, distal
(all subjects; N = 550)
1−sided adj pval: 0.001
Frequency
0
10
20
30
40
50
60
70
−4
−2
0
2
4
Tumor−Normal Expression Difference
hsa−miR−525−5p, distal
(ESTROGEN = 0; N0 = 112)
1−sided adj pval: 0.223
Frequency
0
5
10
15
−4
−2
0
2
4
Tumor−Normal Expression Difference
hsa−miR−525−5p, distal
(ESTROGEN = 1; N1 = 72)
1−sided adj pval: 0.206
Frequency
0
2
4
6
8
10
12
14
−4
−2
0
2
4
Tumor−Normal Expression Difference

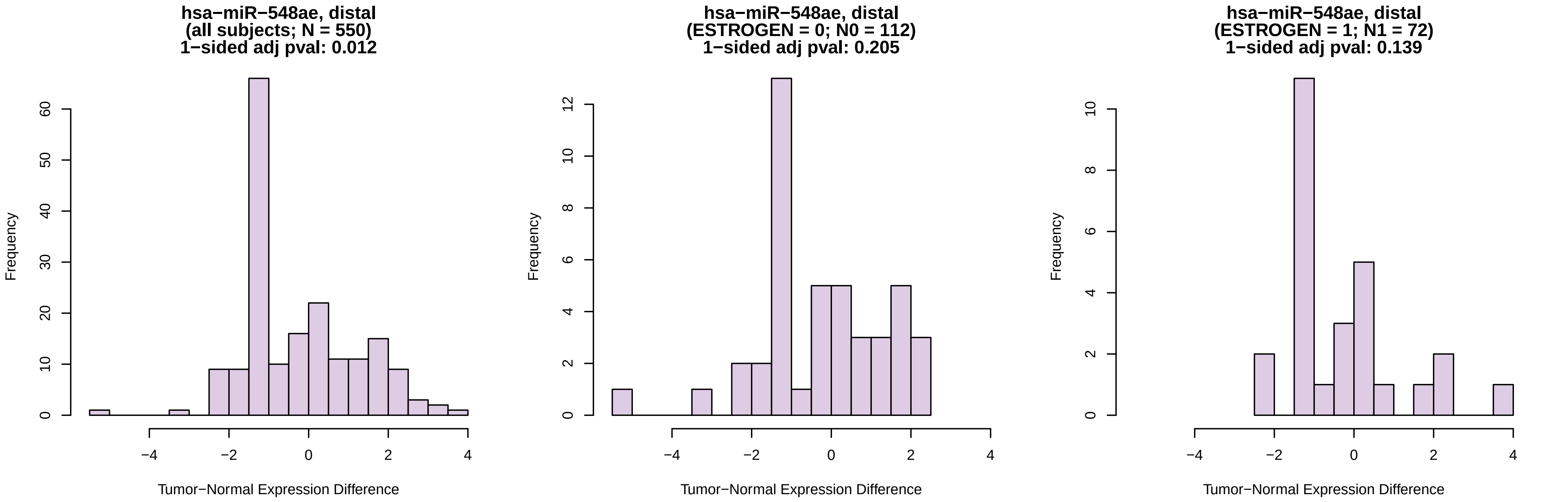
hsa−miR−548ae, distal
(all subjects; N = 550)
1−sided adj pval: 0.012
Frequency
0
10
20
30
40
50
60
−4
−2
0
2
4
Tumor−Normal Expression Difference
hsa−miR−548ae, distal
(ESTROGEN = 0; N0 = 112)
1−sided adj pval: 0.205
Frequency
0
2
4
6
8
10
12
−4
−2
0
2
4
Tumor−Normal Expression Difference
hsa−miR−548ae, distal
(ESTROGEN = 1; N1 = 72)
1−sided adj pval: 0.139
Frequency
0
2
4
6
8
10
−4
−2
0
2
4
Tumor−Normal Expression Difference

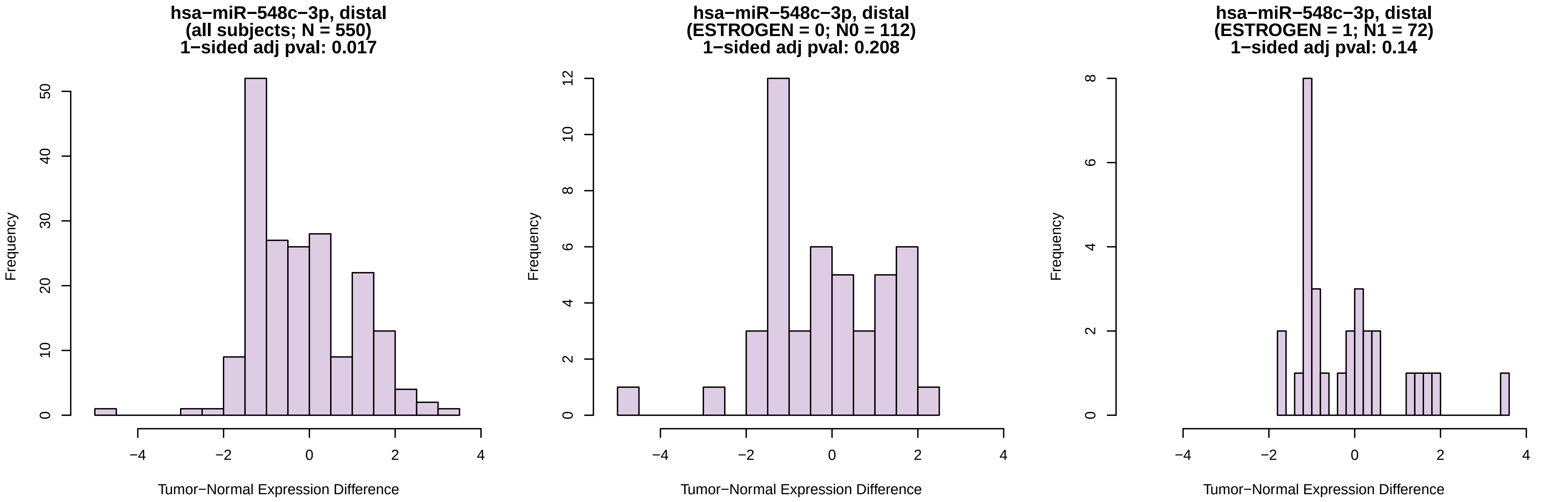

hsa−miR−548c−3p, distal
(all subjects; N = 550)
1−sided adj pval: 0.017
hsa−miR−548c−3p, distal
(ESTROGEN = 0; N0 = 112)
1−sided adj pval: 0.208
hsa−miR−548c−3p, distal
(ESTROGEN = 1; N1 = 72)
1−sided adj pval: 0.14
Frequency
Tumor−Normal Expression Difference

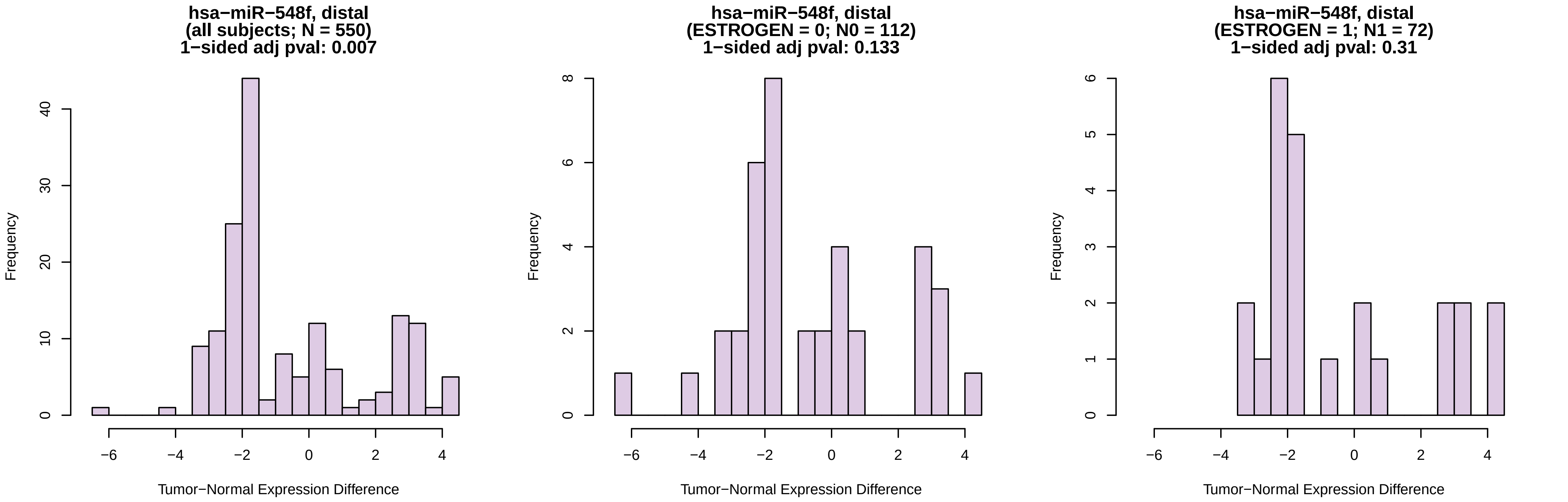
hsa−miR−548f, distal
(all subjects; N = 550)
1−sided adj pval: 0.007
Frequency
0
10
20
30
40
−6
−4
−2
0
2
4
Tumor−Normal Expression Difference
hsa−miR−548f, distal
(ESTROGEN = 0; N0 = 112)
1−sided adj pval: 0.133
Frequency
0
2
4
6
8
−6
−4
−2
0
2
4
Tumor−Normal Expression Difference
hsa−miR−548f, distal
(ESTROGEN = 1; N1 = 72)
1−sided adj pval: 0.31
Frequency
0
1
2
3
4
5
6
−6
−4
−2
0
2
4
Tumor−Normal Expression Difference

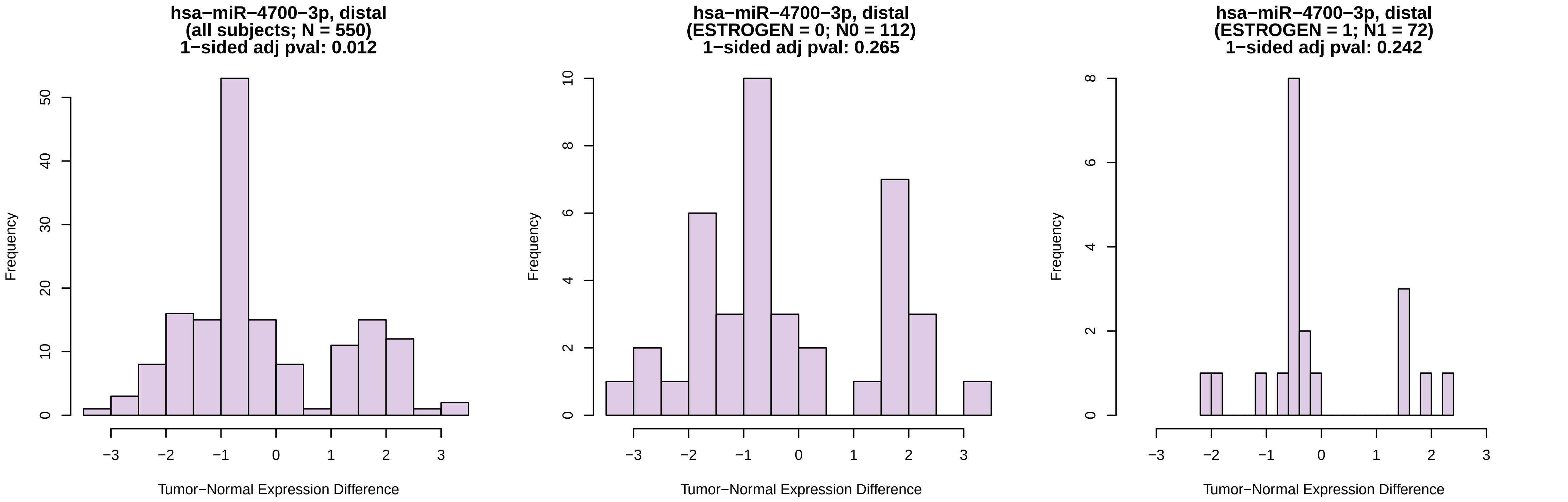
hsa−miR−4700−3p, distal
(all subjects; N = 550)
1−sided adj pval: 0.012
Frequency
0
10
20
30
40
50
−3
−2
−1
0
1
2
3
Tumor−Normal Expression Difference
hsa−miR−4700−3p, distal
(ESTROGEN = 0; N0 = 112)
1−sided adj pval: 0.265
Frequency
0
2
4
6
8
10
−3
−2
−1
0
1
2
3
Tumor−Normal Expression Difference
hsa−miR−4700−3p, distal
(ESTROGEN = 1; N1 = 72)
1−sided adj pval: 0.242
Frequency
0
2
4
6
8
−3
−2
−1
0
1
2
3
Tumor−Normal Expression Difference

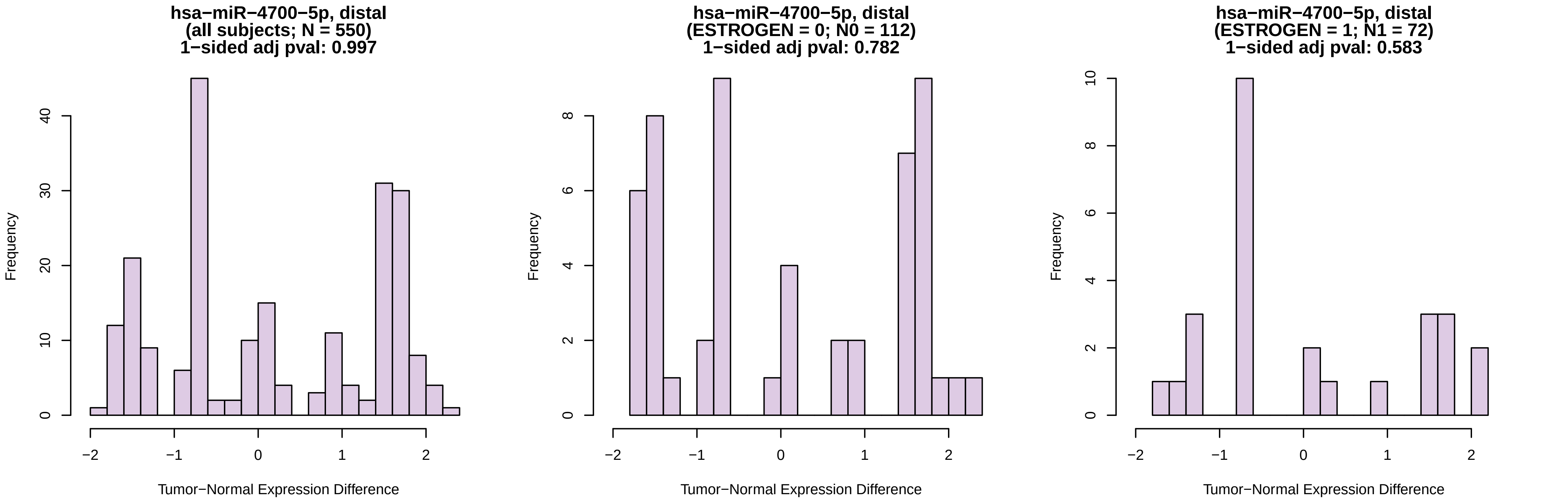

hsa−miR−4700−5p, distal
(all subjects; N = 550)
1−sided adj pval: 0.997
Frequency
Tumor−Normal Expression Difference
hsa−miR−4700−5p, distal
(ESTROGEN = 0; N0 = 112)
1−sided adj pval: 0.782
Frequency
Tumor−Normal Expression Difference
hsa−miR−4700−5p, distal
(ESTROGEN = 1; N1 = 72)
1−sided adj pval: 0.583
Frequency
Tumor−Normal Expression Difference

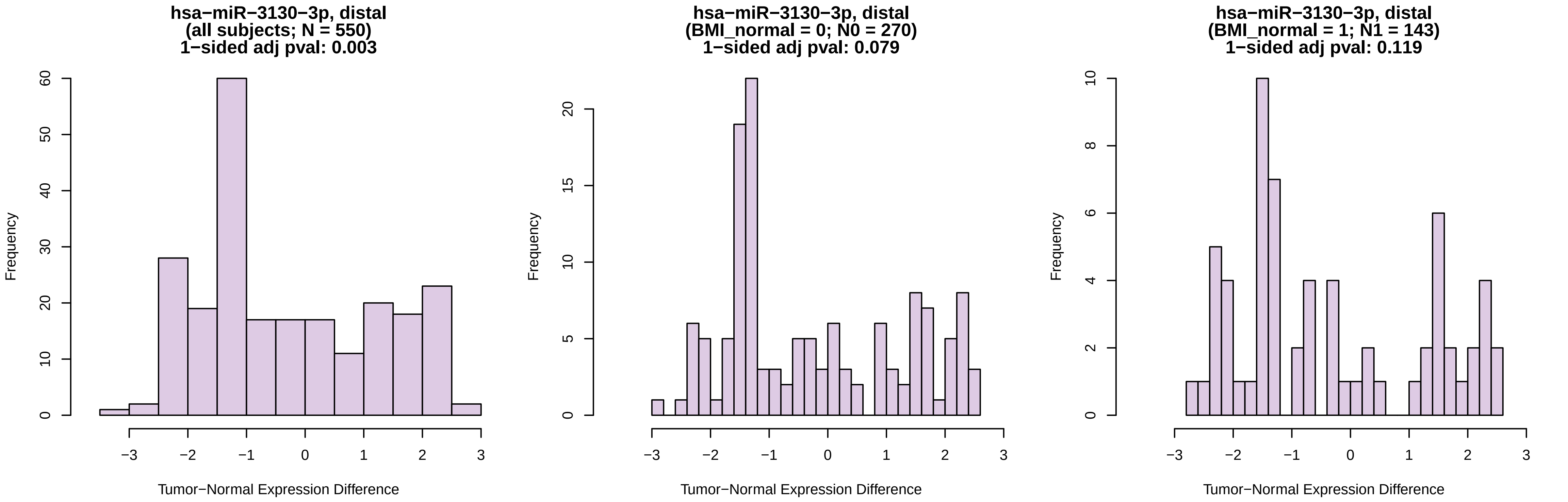

hsa−miR−3130−3p, distal
(all subjects; N = 550)
1−sided adj pval: 0.003
Frequency
Tumor−Normal Expression Difference
hsa−miR−3130−3p, distal
(BMI_normal = 0; N0 = 270)
1−sided adj pval: 0.079
Frequency
Tumor−Normal Expression Difference
hsa−miR−3130−3p, distal
(BMI_normal = 1; N1 = 143)
1−sided adj pval: 0.119
Frequency
Tumor−Normal Expression Difference

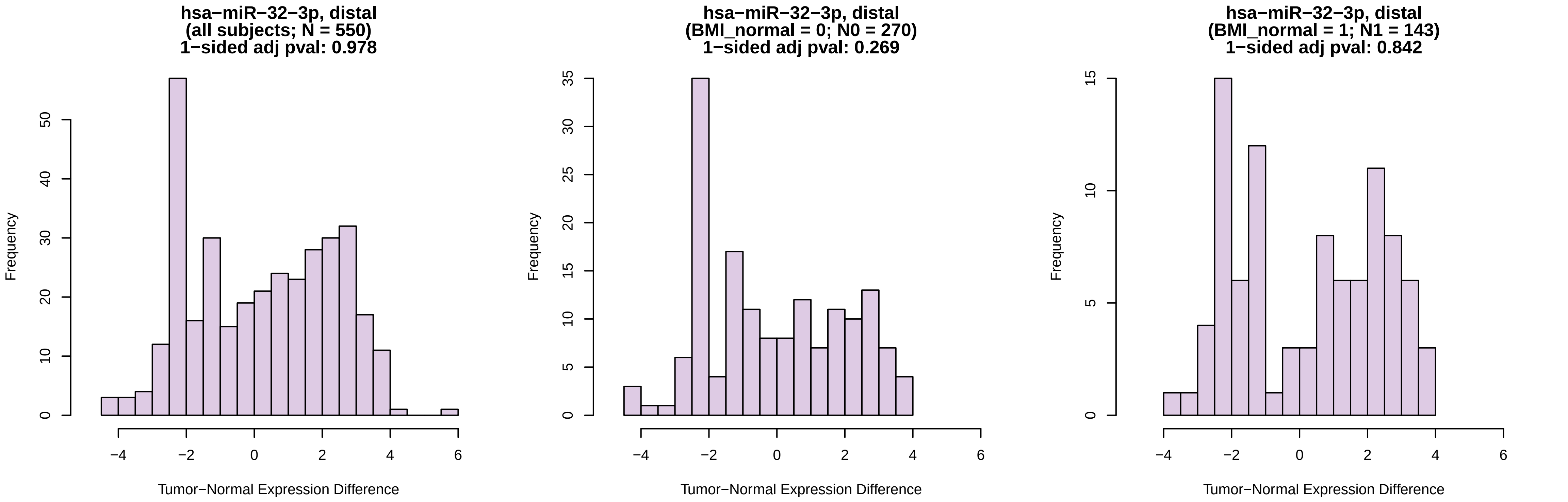
hsa−miR−32−3p, distal
(all subjects; N = 550)
1−sided adj pval: 0.978
hsa−miR−32−3p, distal
(BMI_normal = 0; N0 = 270)
1−sided adj pval: 0.269
hsa−miR−32−3p, distal
(BMI_normal = 1; N1 = 143)
1−sided adj pval: 0.842
Frequency
Tumor−Normal Expression Difference

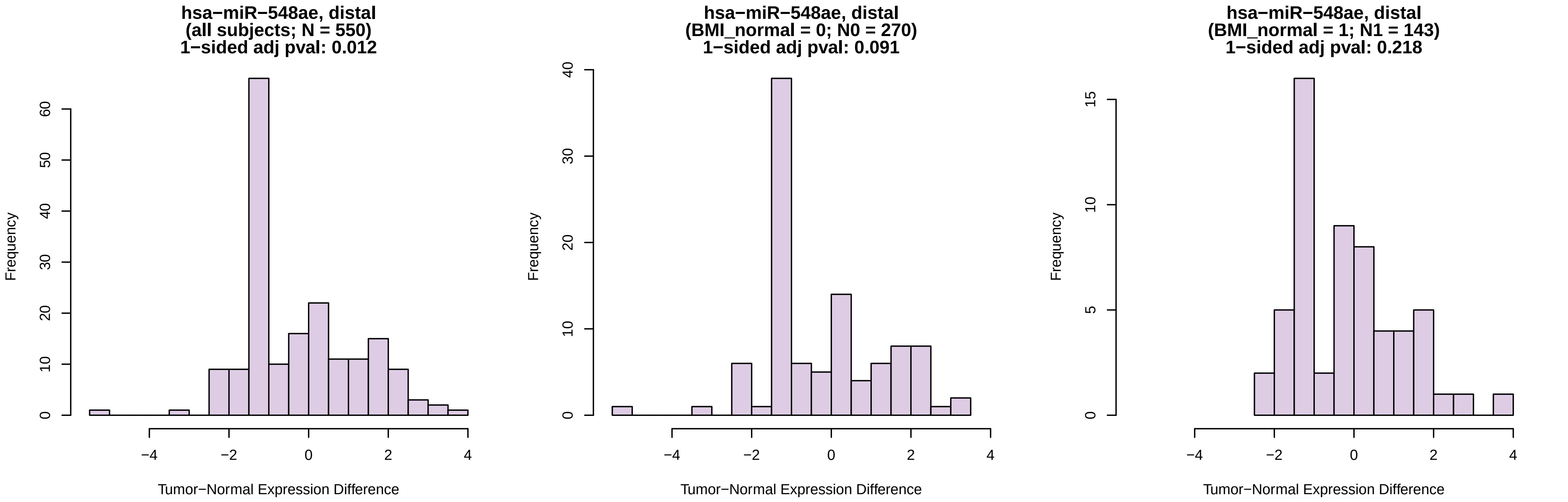

hsa−miR−548ae, distal
(all subjects; N = 550)
1−sided adj pval: 0.012
Frequency
0
10
20
30
40
50
60
−4
−2
0
2
4
Tumor−Normal Expression Difference
hsa−miR−548ae, distal
(BMI_normal = 0; N0 = 270)
1−sided adj pval: 0.091
Frequency
0
10
20
30
40
−4
−2
0
2
4
Tumor−Normal Expression Difference
hsa−miR−548ae, distal
(BMI_normal = 1; N1 = 143)
1−sided adj pval: 0.218
Frequency
0
5
10
15
−4
−2
0
2
4
Tumor−Normal Expression Difference

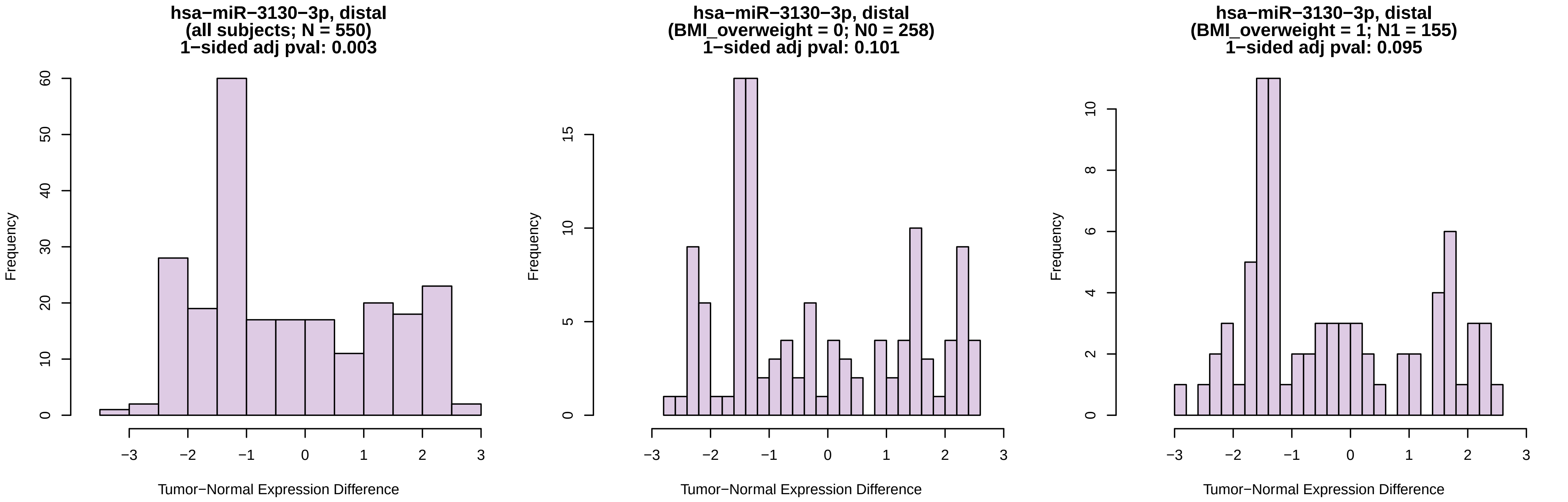
hsa−miR−3130−3p, distal
(all subjects; N = 550)
1−sided adj pval: 0.003
Frequency
Tumor−Normal Expression Difference
hsa−miR−3130−3p, distal
(BMI_overweight = 0; N0 = 258)
1−sided adj pval: 0.101
Frequency
Tumor−Normal Expression Difference
hsa−miR−3130−3p, distal
(BMI_overweight = 1; N1 = 155)
1−sided adj pval: 0.095
Frequency
Tumor−Normal Expression Difference

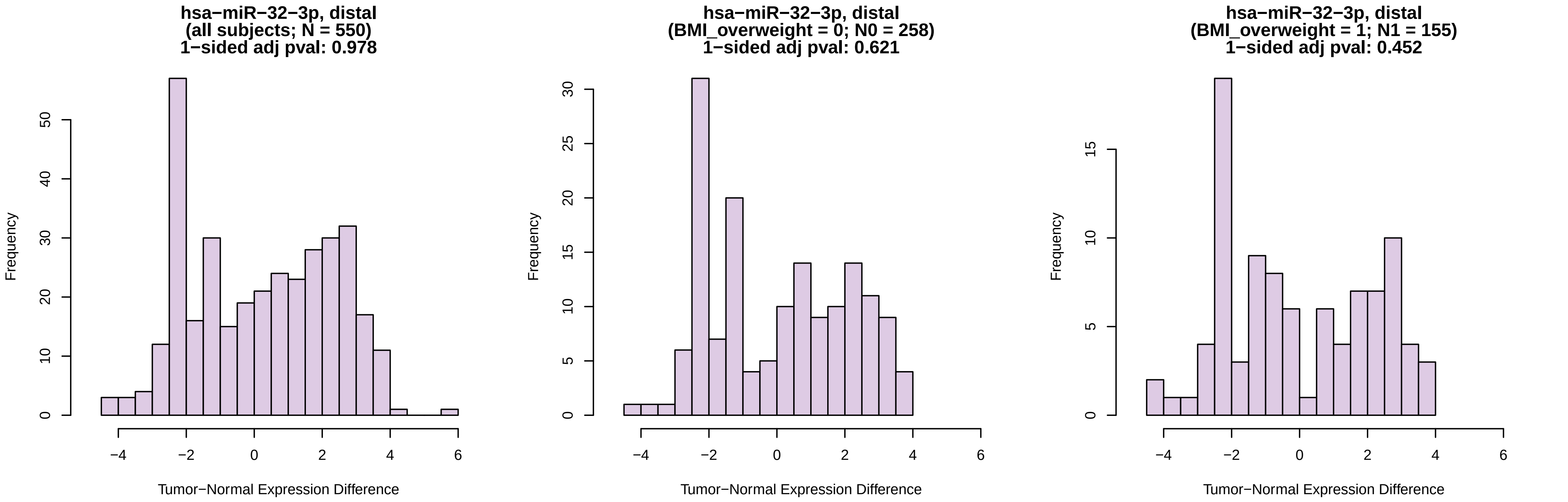

hsa−miR−32−3p, distal
(all subjects; N = 550)
1−sided adj pval: 0.978
Frequency
0
10
20
30
40
50
−4
−2
0
2
4
6
Tumor−Normal Expression Difference
hsa−miR−32−3p, distal
(BMI_overweight = 0; N0 = 258)
1−sided adj pval: 0.621
Frequency
0
5
10
15
20
25
30
−4
−2
0
2
4
6
Tumor−Normal Expression Difference
hsa−miR−32−3p, distal
(BMI_overweight = 1; N1 = 155)
1−sided adj pval: 0.452
Frequency
0
5
10
15
−4
−2
0
2
4
6
Tumor−Normal Expression Difference

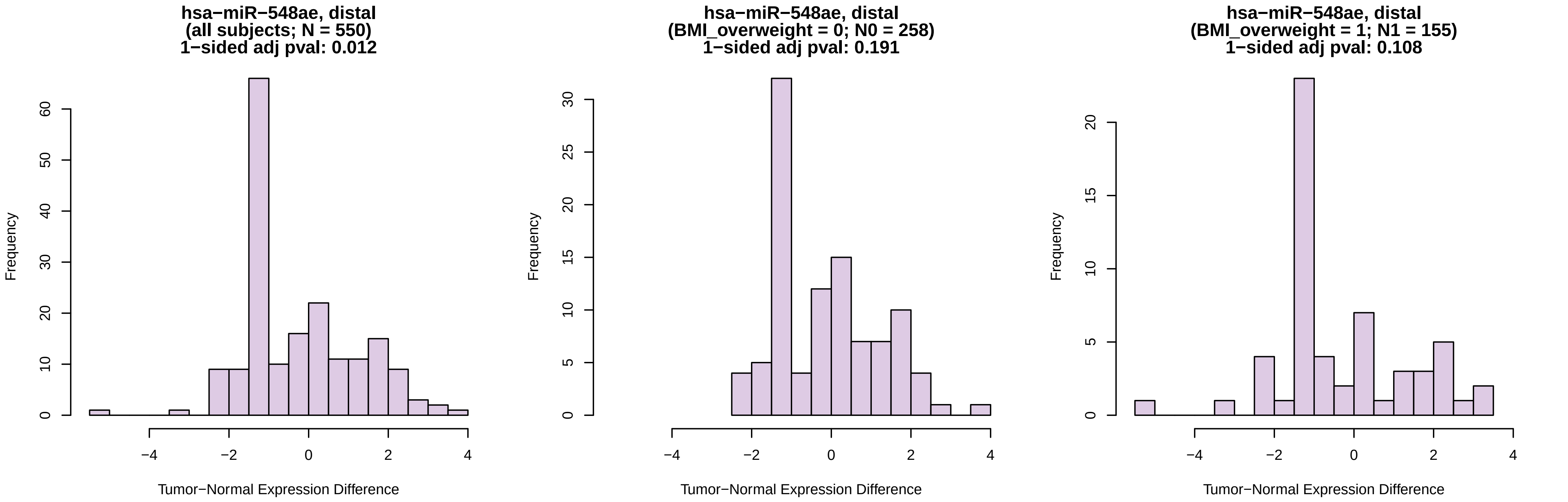
hsa−miR−548ae, distal
(all subjects; N = 550)
1−sided adj pval: 0.012
Frequency
0
10
20
30
40
50
60
−4
−2
0
2
4
Tumor−Normal Expression Difference
hsa−miR−548ae, distal
(BMI_overweight = 0; N0 = 258)
1−sided adj pval: 0.191
Frequency
0
5
10
15
20
25
30
−4
−2
0
2
4
Tumor−Normal Expression Difference
hsa−miR−548ae, distal
(BMI_overweight = 1; N1 = 155)
1−sided adj pval: 0.108
Frequency
0
5
10
15
20
−4
−2
0
2
4
Tumor−Normal Expression Difference

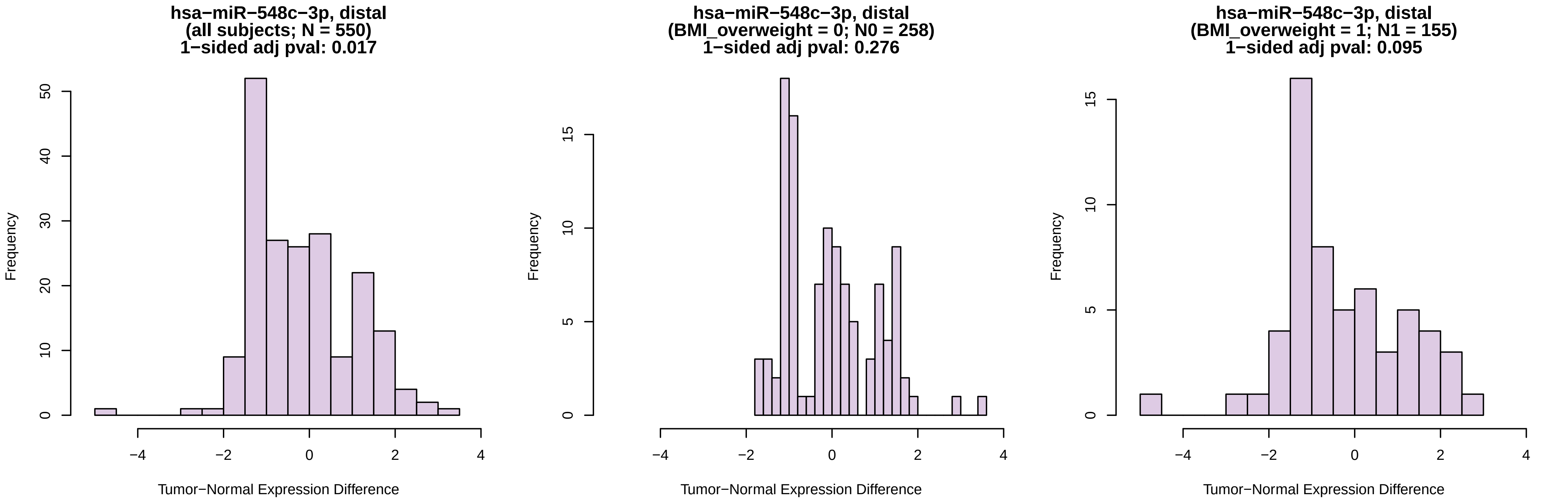
hsa−miR−548c−3p, distal
(all subjects; N = 550)
1−sided adj pval: 0.017
Frequency
Tumor−Normal Expression Difference
hsa−miR−548c−3p, distal
(BMI_overweight = 0; N0 = 258)
1−sided adj pval: 0.276
Frequency
Tumor−Normal Expression Difference
hsa−miR−548c−3p, distal
(BMI_overweight = 1; N1 = 155)
1−sided adj pval: 0.095
Frequency
Tumor−Normal Expression Difference

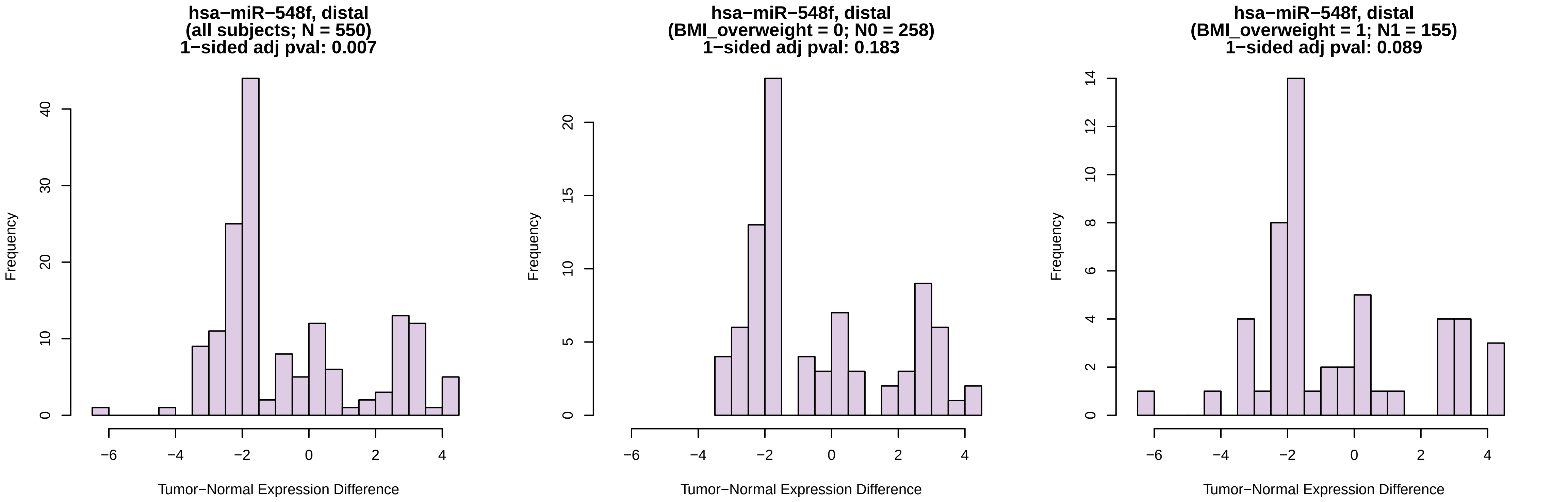

hsa−miR−548f, distal
(all subjects; N = 550)
1−sided adj pval: 0.007
Frequency
Tumor−Normal Expression Difference
hsa−miR−548f, distal
(BMI_overweight = 0; N0 = 258)
1−sided adj pval: 0.183
Frequency
Tumor−Normal Expression Difference
hsa−miR−548f, distal
(BMI_overweight = 1; N1 = 155)
1−sided adj pval: 0.089
Frequency
Tumor−Normal Expression Difference

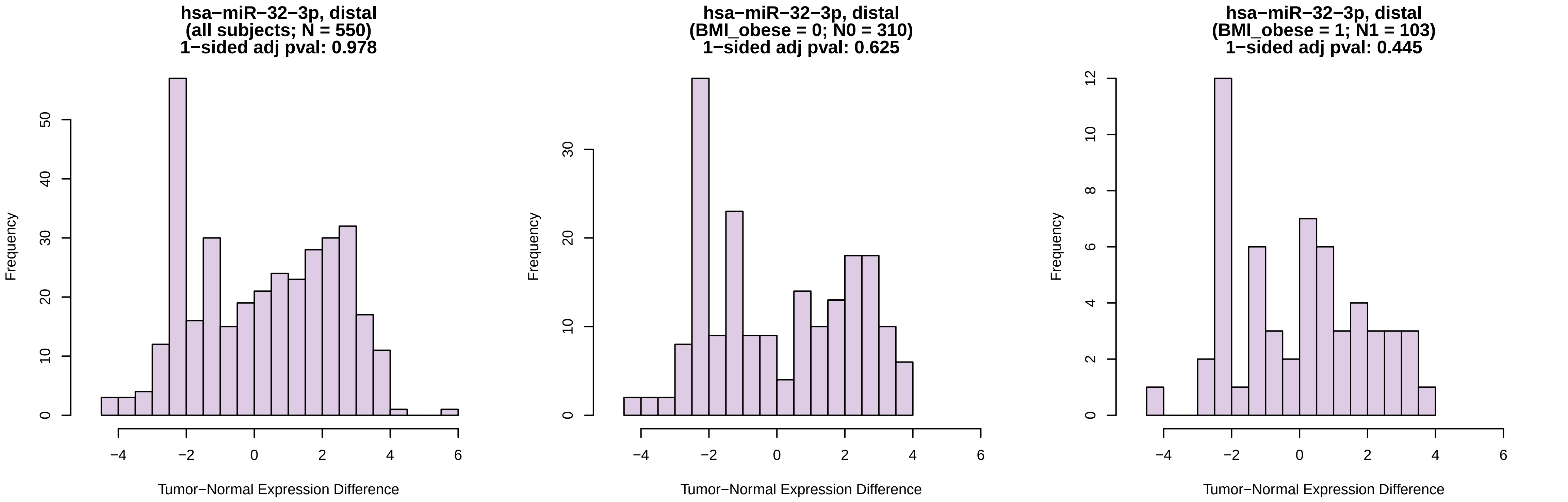
hsa−miR−32−3p, distal
(all subjects; N = 550)
1−sided adj pval: 0.978
Frequency
Tumor−Normal Expression Difference
hsa−miR−32−3p, distal
(BMI_obese = 0; N0 = 310)
1−sided adj pval: 0.625
Frequency
Tumor−Normal Expression Difference
hsa−miR−32−3p, distal
(BMI_obese = 1; N1 = 103)
1−sided adj pval: 0.445
Frequency
Tumor−Normal Expression Difference

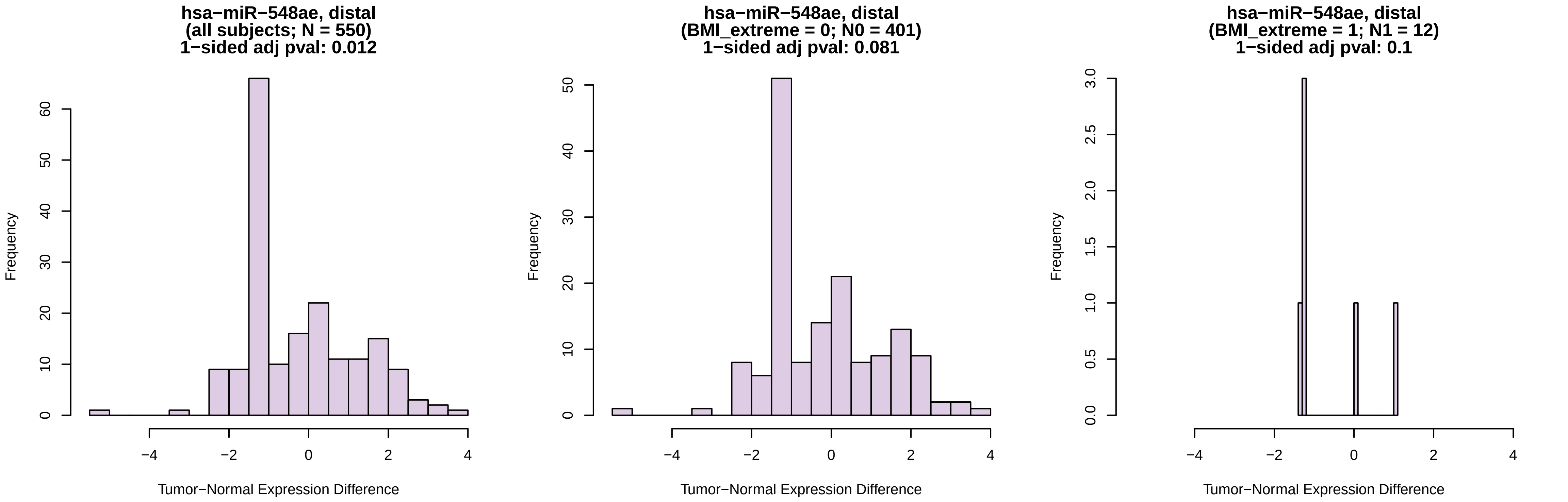
hsa−miR−548ae, distal
(all subjects; N = 550)
1−sided adj pval: 0.012
Frequency
Tumor−Normal Expression Difference
hsa−miR−548ae, distal
(BMI_extreme = 0; N0 = 401)
1−sided adj pval: 0.081
Frequency
Tumor−Normal Expression Difference
hsa−miR−548ae, distal
(BMI_extreme = 1; N1 = 12)
1−sided adj pval: 0.1
Frequency
Tumor−Normal Expression Difference

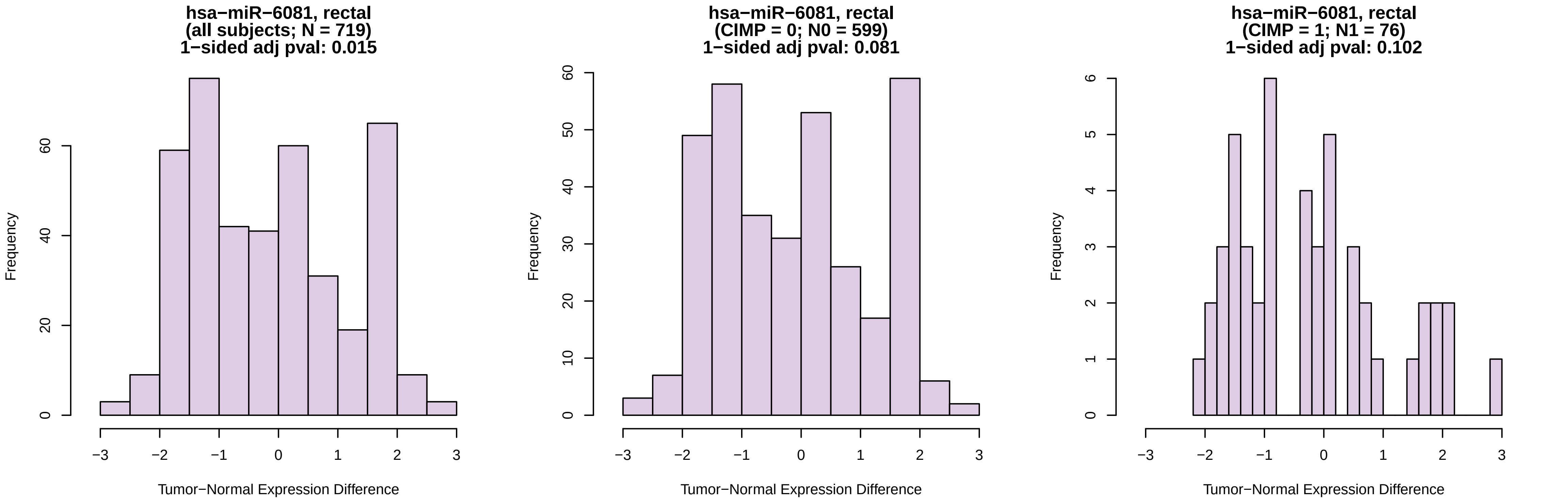

hsa−miR−6081, rectal
(all subjects; N = 719)
1−sided adj pval: 0.015
Frequency
0
20
40
60
−3
−2
−1
0
1
2
3
Tumor−Normal Expression Difference
hsa−miR−6081, rectal
(CIMP = 0; N0 = 599)
1−sided adj pval: 0.081
Frequency
0
10
20
30
40
50
60
−3
−2
−1
0
1
2
3
Tumor−Normal Expression Difference
hsa−miR−6081, rectal
(CIMP = 1; N1 = 76)
1−sided adj pval: 0.102
Frequency
0
1
2
3
4
5
6
−3
−2
−1
0
1
2
3
Tumor−Normal Expression Difference

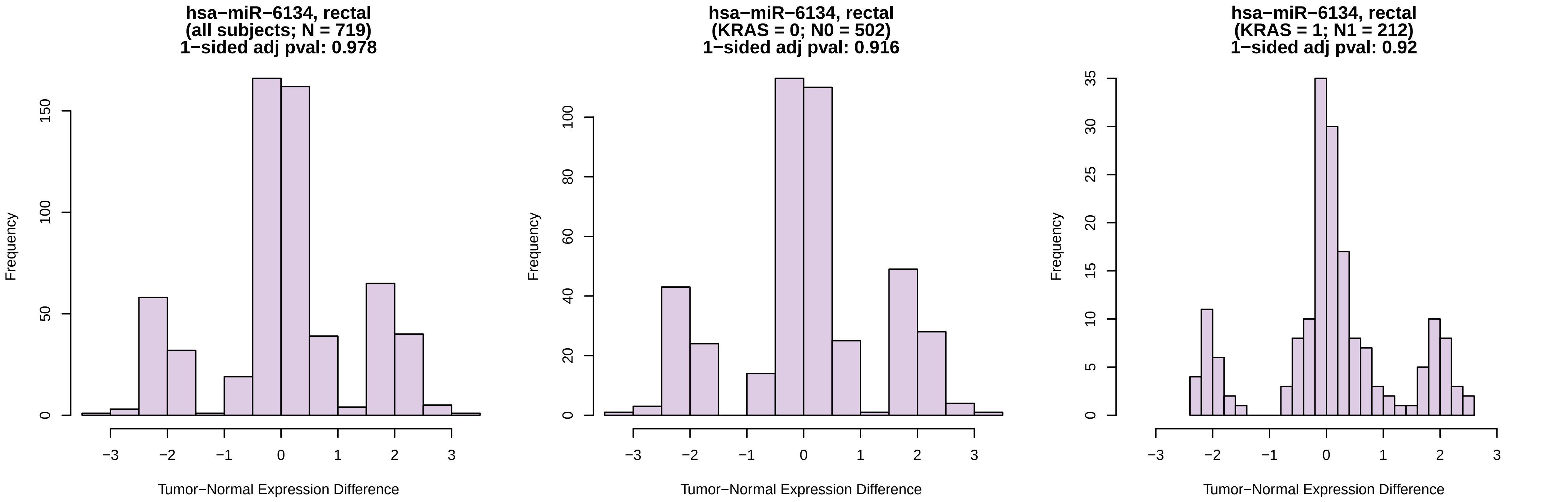
hsa−miR−6134, rectal
(all subjects; N = 719)
1−sided adj pval: 0.978
Frequency
Tumor−Normal Expression Difference
hsa−miR−6134, rectal
(KRAS = 0; N0 = 502)
1−sided adj pval: 0.916
Frequency
Tumor−Normal Expression Difference
hsa−miR−6134, rectal
(KRAS = 1; N1 = 212)
1−sided adj pval: 0.92
Frequency
Tumor−Normal Expression Difference

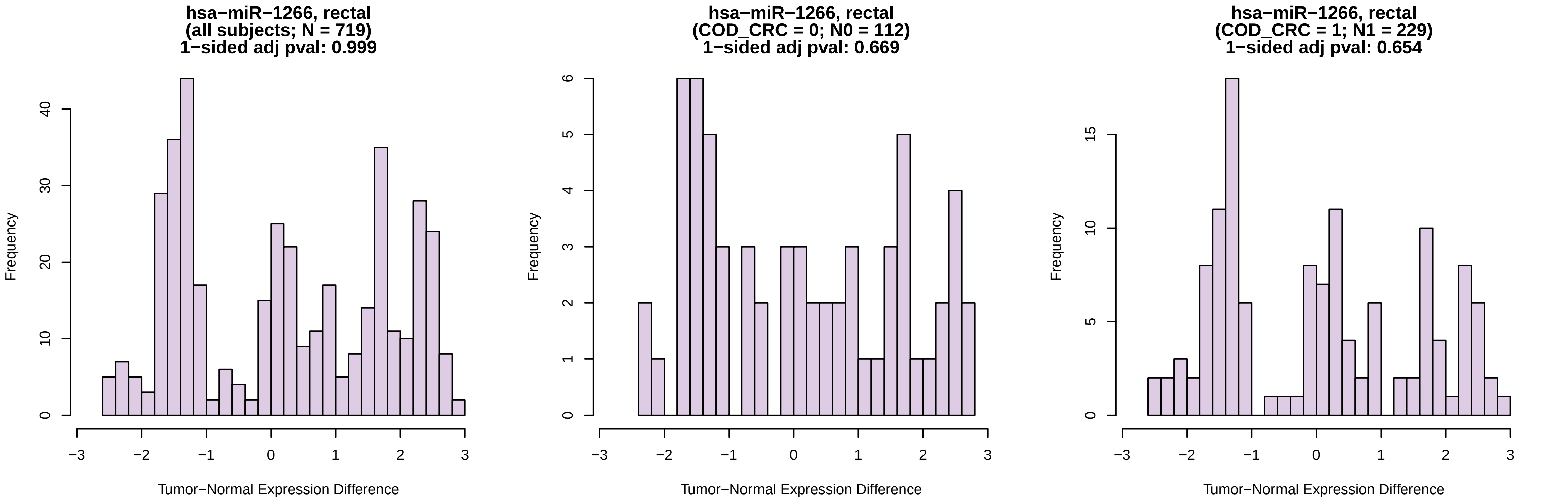

hsa−miR−1266, rectal
(all subjects; N = 719)
1−sided adj pval: 0.999
hsa−miR−1266, rectal
(COD_CRC = 0; N0 = 112)
1−sided adj pval: 0.669
hsa−miR−1266, rectal
(COD_CRC = 1; N1 = 229)
1−sided adj pval: 0.654
Frequency
Tumor−Normal Expression Difference

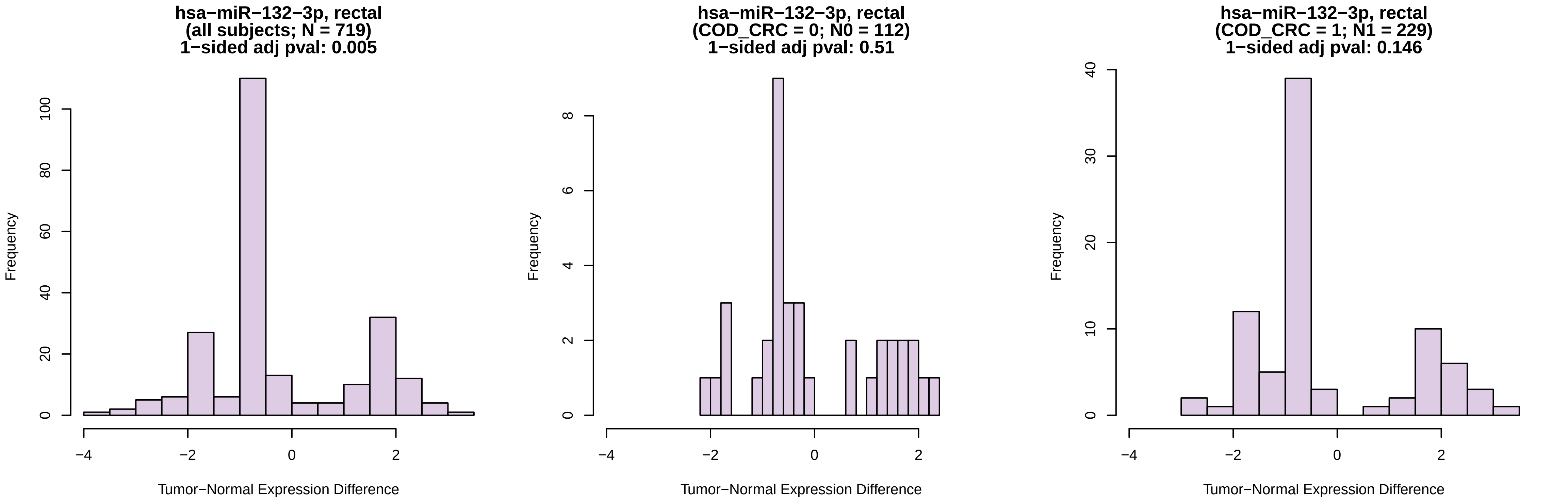
hsa−miR−132−3p, rectal
(all subjects; N = 719)
1−sided adj pval: 0.005
Frequency
Tumor−Normal Expression Difference
hsa−miR−132−3p, rectal
(COD_CRC = 0; N0 = 112)
1−sided adj pval: 0.51
Frequency
Tumor−Normal Expression Difference
hsa−miR−132−3p, rectal
(COD_CRC = 1; N1 = 229)
1−sided adj pval: 0.146
Frequency
Tumor−Normal Expression Difference

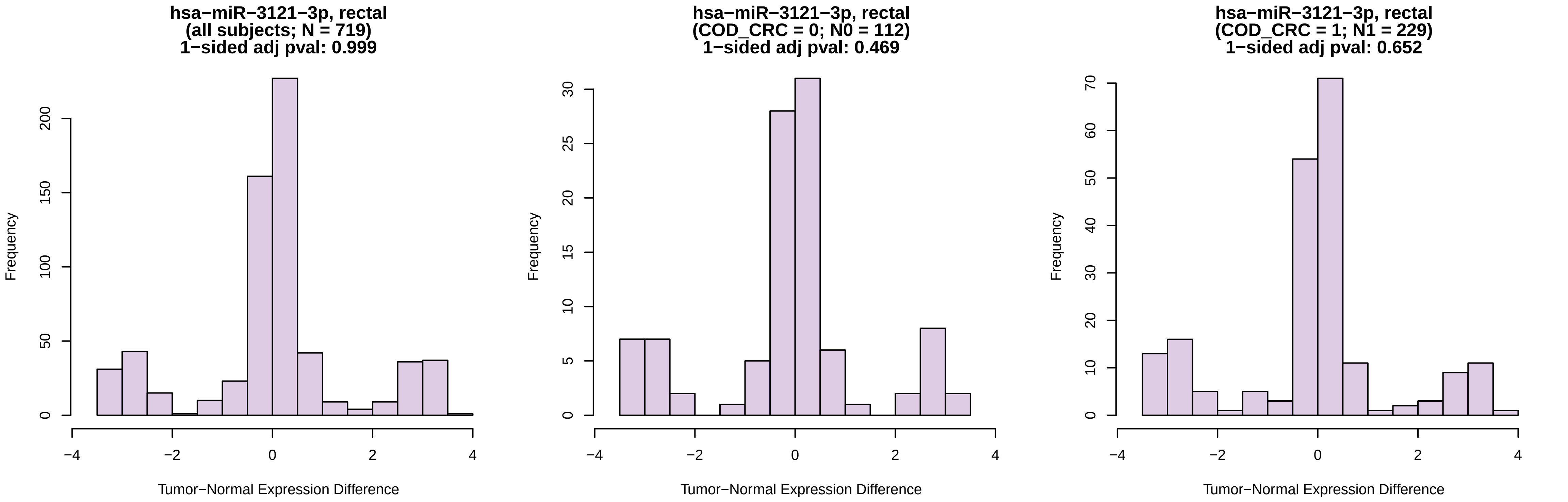
hsa−miR−3121−3p, rectal
(all subjects; N = 719)
1−sided adj pval: 0.999
Frequency
0
50
100
150
200
−4
−2
0
2
4
Tumor−Normal Expression Difference
hsa−miR−3121−3p, rectal
(COD_CRC = 0; N0 = 112)
1−sided adj pval: 0.469
Frequency
0
5
10
15
20
25
30
−4
−2
0
2
4
Tumor−Normal Expression Difference
hsa−miR−3121−3p, rectal
(COD_CRC = 1; N1 = 229)
1−sided adj pval: 0.652
Frequency
0
10
20
30
40
50
60
70
−4
−2
0
2
4
Tumor−Normal Expression Difference

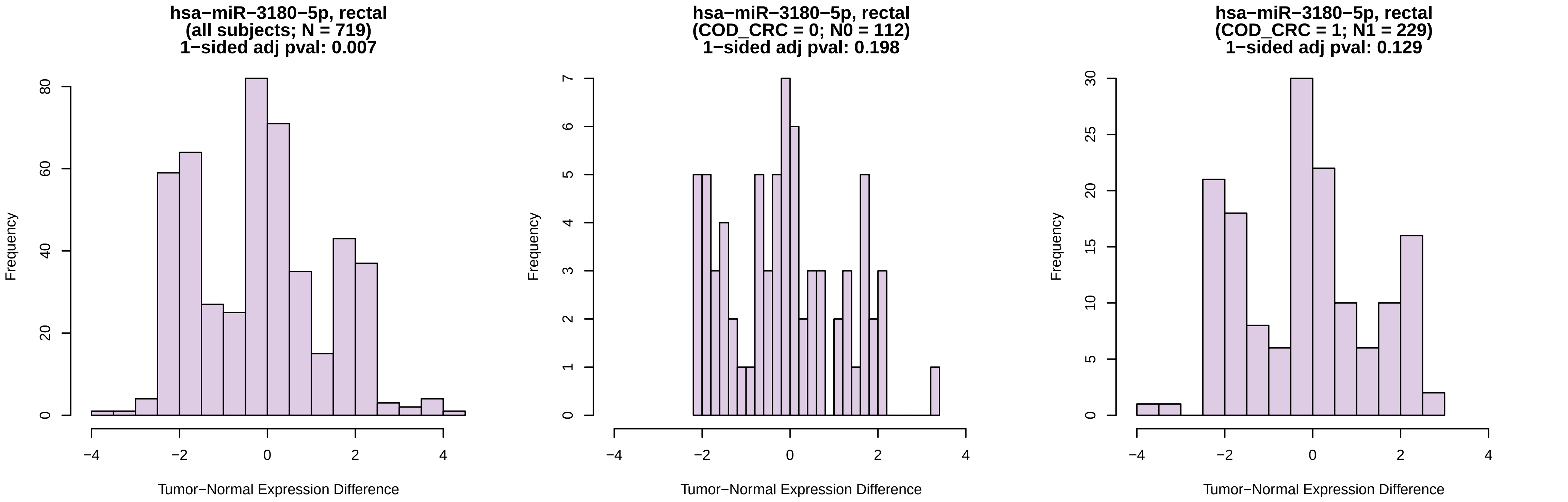

hsa−miR−3180−5p, rectal
(all subjects; N = 719)
1−sided adj pval: 0.007
hsa−miR−3180−5p, rectal
(COD_CRC = 0; N0 = 112)
1−sided adj pval: 0.198
hsa−miR−3180−5p, rectal
(COD_CRC = 1; N1 = 229)
1−sided adj pval: 0.129
Frequency
Tumor−Normal Expression Difference

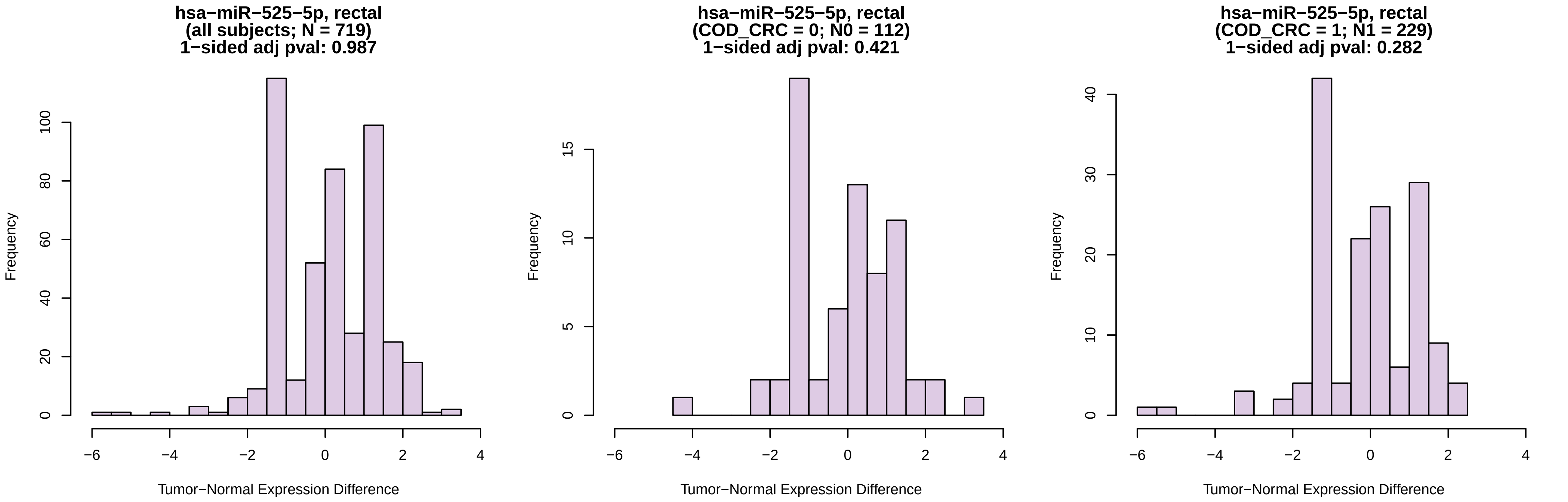

hsa−miR−525−5p, rectal
(all subjects; N = 719)
1−sided adj pval: 0.987
Frequency
Tumor−Normal Expression Difference
hsa−miR−525−5p, rectal
(COD_CRC = 0; N0 = 112)
1−sided adj pval: 0.421
Frequency
Tumor−Normal Expression Difference
hsa−miR−525−5p, rectal
(COD_CRC = 1; N1 = 229)
1−sided adj pval: 0.282
Frequency
Tumor−Normal Expression Difference

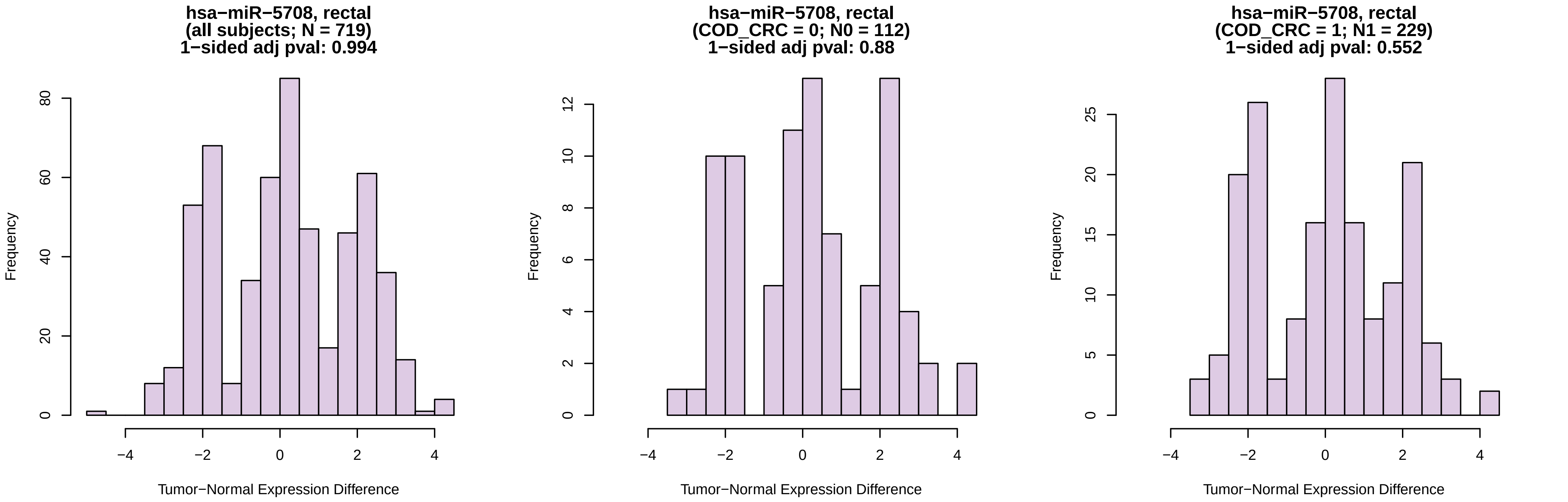
hsa−miR−5708, rectal
(all subjects; N = 719)
1−sided adj pval: 0.994
Frequency
0
20
40
60
80
−4
−2
0
2
4
Tumor−Normal Expression Difference
hsa−miR−5708, rectal
(COD_CRC = 0; N0 = 112)
1−sided adj pval: 0.88
Frequency
0
2
4
6
8
10
12
−4
−2
0
2
4
Tumor−Normal Expression Difference
hsa−miR−5708, rectal
(COD_CRC = 1; N1 = 229)
1−sided adj pval: 0.552
Frequency
0
5
10
15
20
25
−4
−2
0
2
4
Tumor−Normal Expression Difference

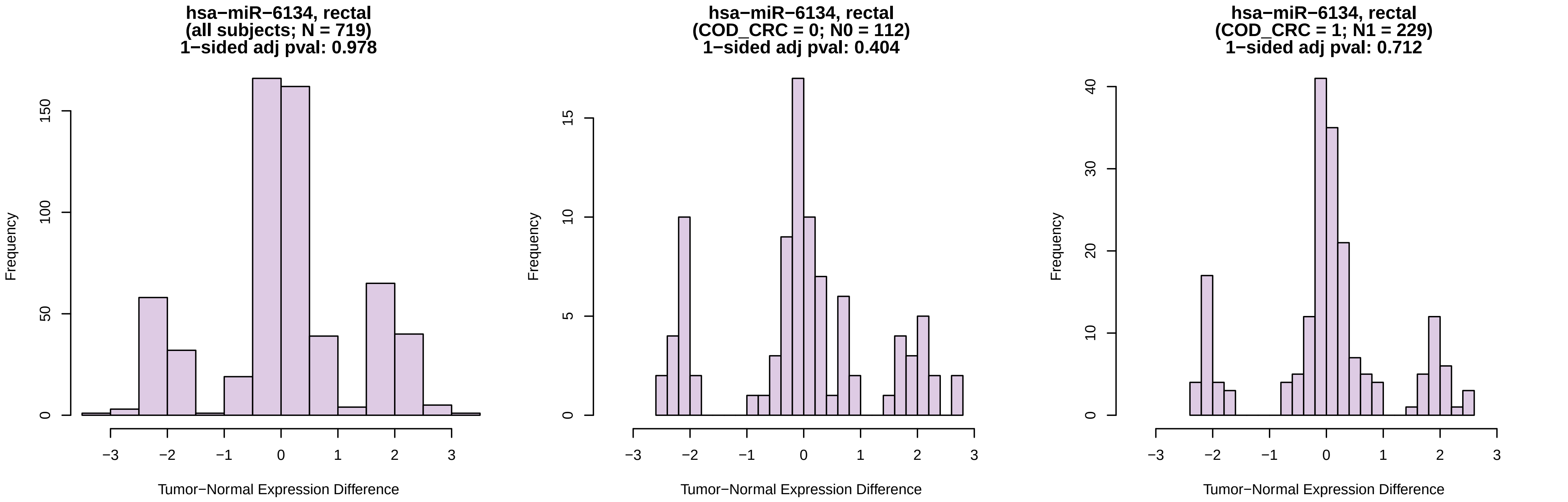
hsa−miR−6134, rectal
(all subjects; N = 719)
1−sided adj pval: 0.978
Frequency
Tumor−Normal Expression Difference
hsa−miR−6134, rectal
(COD_CRC = 0; N0 = 112)
1−sided adj pval: 0.404
Frequency
Tumor−Normal Expression Difference
hsa−miR−6134, rectal
(COD_CRC = 1; N1 = 229)
1−sided adj pval: 0.712
Frequency
Tumor−Normal Expression Difference

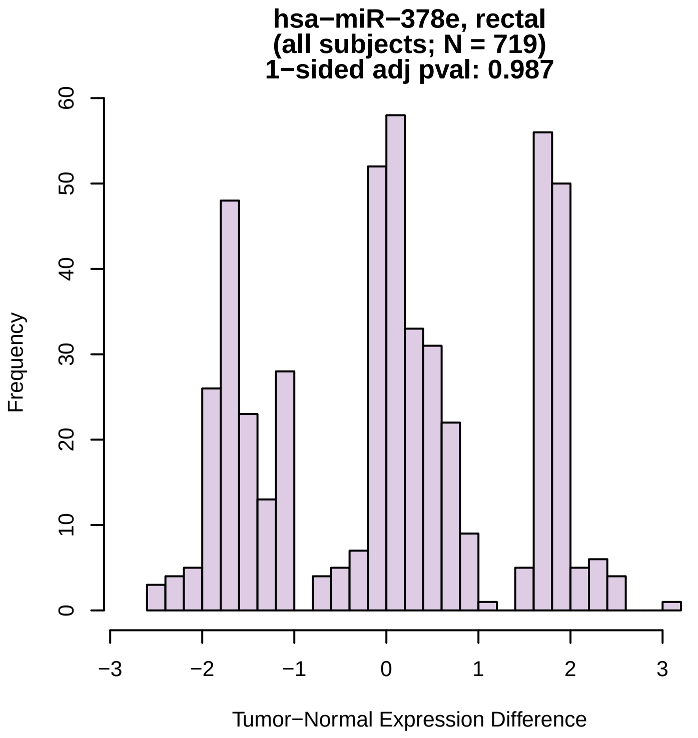

hsa−miR−378e, rectal
(all subjects; N = 719)
1−sided adj pval: 0.987
Frequency
Tumor−Normal Expression Difference

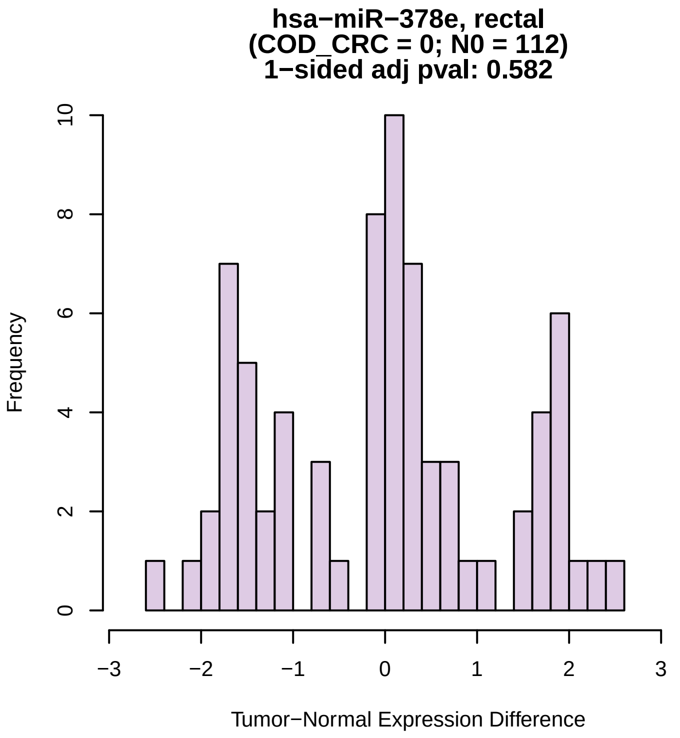

hsa−miR−378e, rectal
(COD_CRC = 0; N0 = 112)
1−sided adj pval: 0.582
Frequency
Tumor−Normal Expression Difference

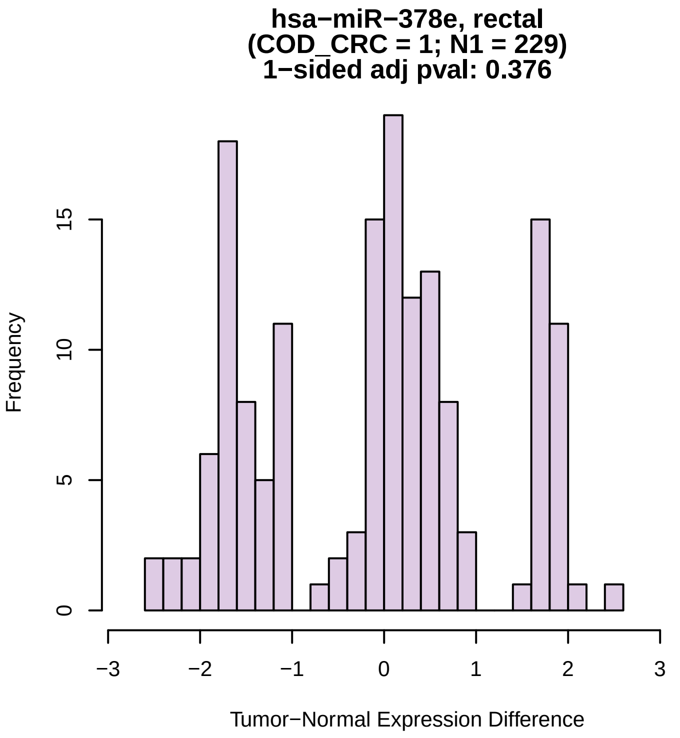

hsa−miR−378e, rectal
(COD_CRC = 1; N1 = 229)
1−sided adj pval: 0.376
Frequency
Tumor−Normal Expression Difference

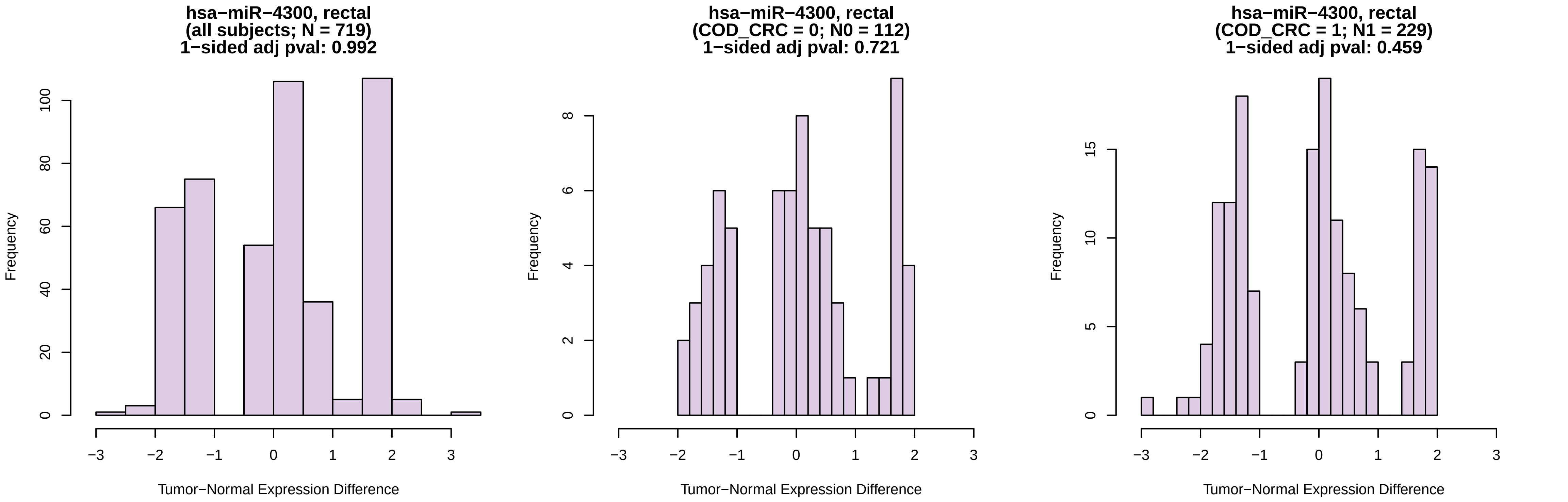
hsa−miR−4300, rectal
(all subjects; N = 719)
1−sided adj pval: 0.992
Frequency
Tumor−Normal Expression Difference
hsa−miR−4300, rectal
(COD_CRC = 0; N0 = 112)
1−sided adj pval: 0.721
Frequency
Tumor−Normal Expression Difference
hsa−miR−4300, rectal
(COD_CRC = 1; N1 = 229)
1−sided adj pval: 0.459
Frequency
Tumor−Normal Expression Difference

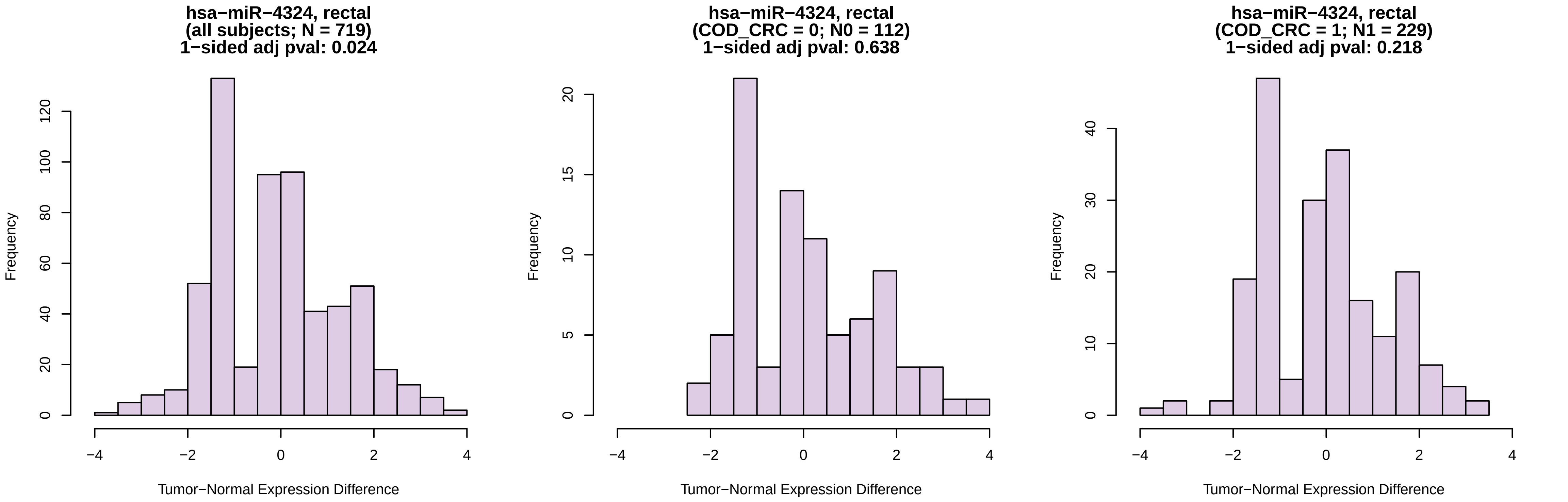

hsa−miR−4324, rectal
(all subjects; N = 719)
1−sided adj pval: 0.024
Frequency
Tumor−Normal Expression Difference
hsa−miR−4324, rectal
(COD_CRC = 0; N0 = 112)
1−sided adj pval: 0.638
Frequency
Tumor−Normal Expression Difference
hsa−miR−4324, rectal
(COD_CRC = 1; N1 = 229)
1−sided adj pval: 0.218
Frequency
Tumor−Normal Expression Difference

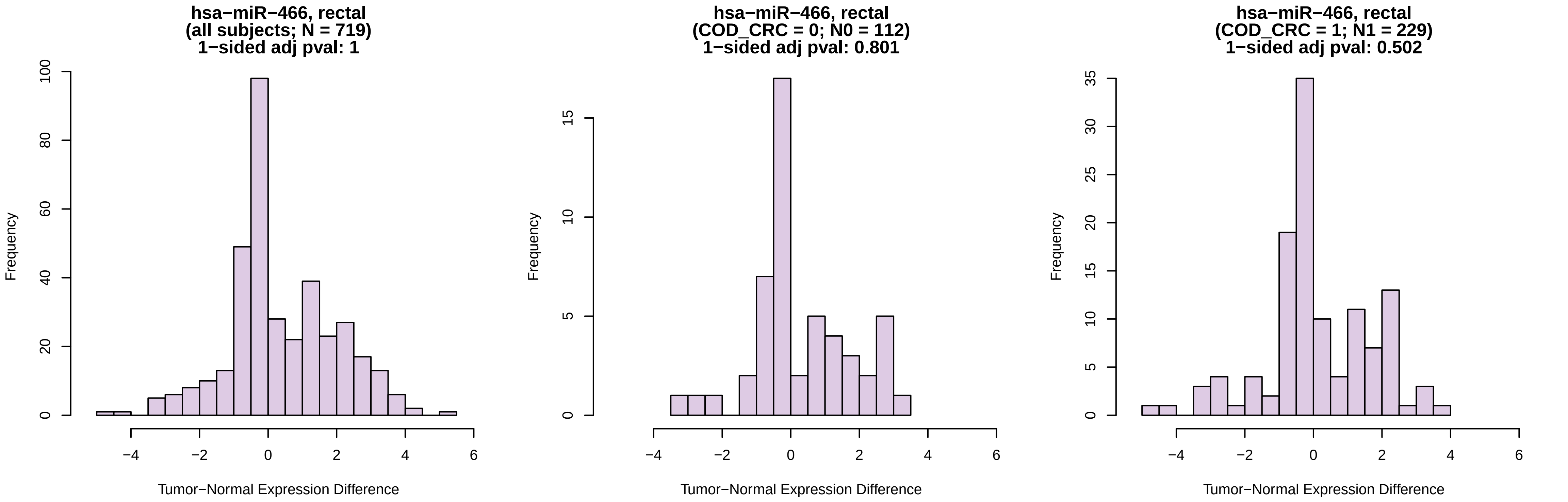

hsa−miR−466, rectal
(all subjects; N = 719)
1−sided adj pval: 1
Frequency
Tumor−Normal Expression Difference
hsa−miR−466, rectal
(COD_CRC = 0; N0 = 112)
1−sided adj pval: 0.801
Frequency
Tumor−Normal Expression Difference
hsa−miR−466, rectal
(COD_CRC = 1; N1 = 229)
1−sided adj pval: 0.502
Frequency
Tumor−Normal Expression Difference

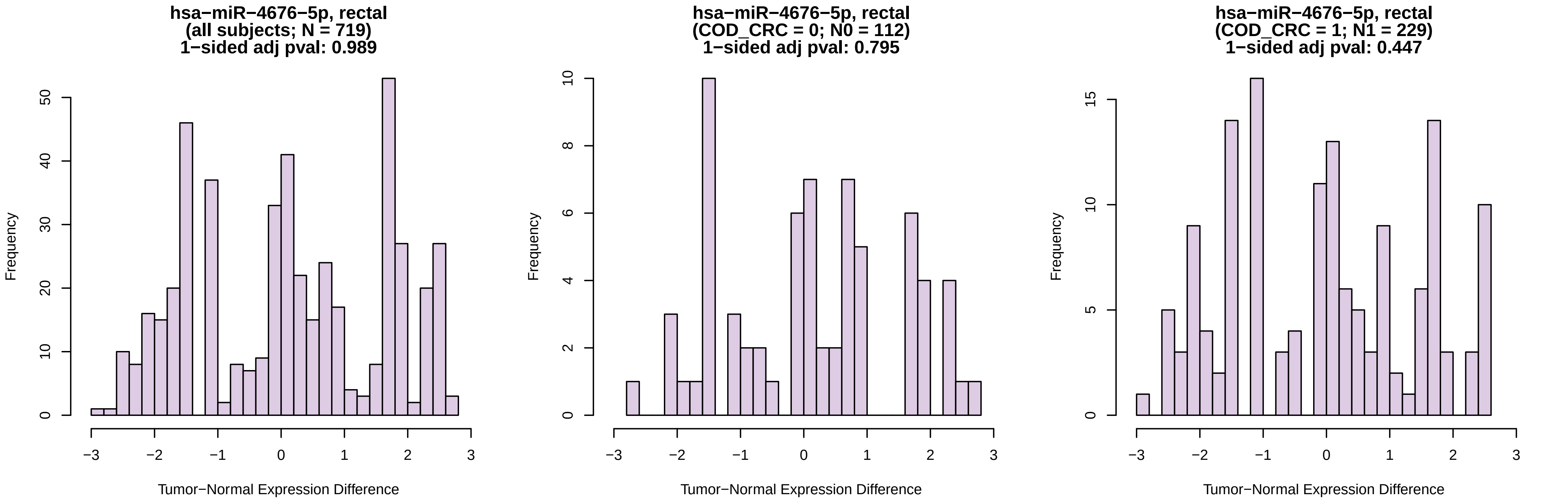
hsa−miR−4676−5p, rectal
(all subjects; N = 719)
1−sided adj pval: 0.989
hsa−miR−4676−5p, rectal
(COD_CRC = 0; N0 = 112)
1−sided adj pval: 0.795
hsa−miR−4676−5p, rectal
(COD_CRC = 1; N1 = 229)
1−sided adj pval: 0.447
Frequency
Tumor−Normal Expression Difference

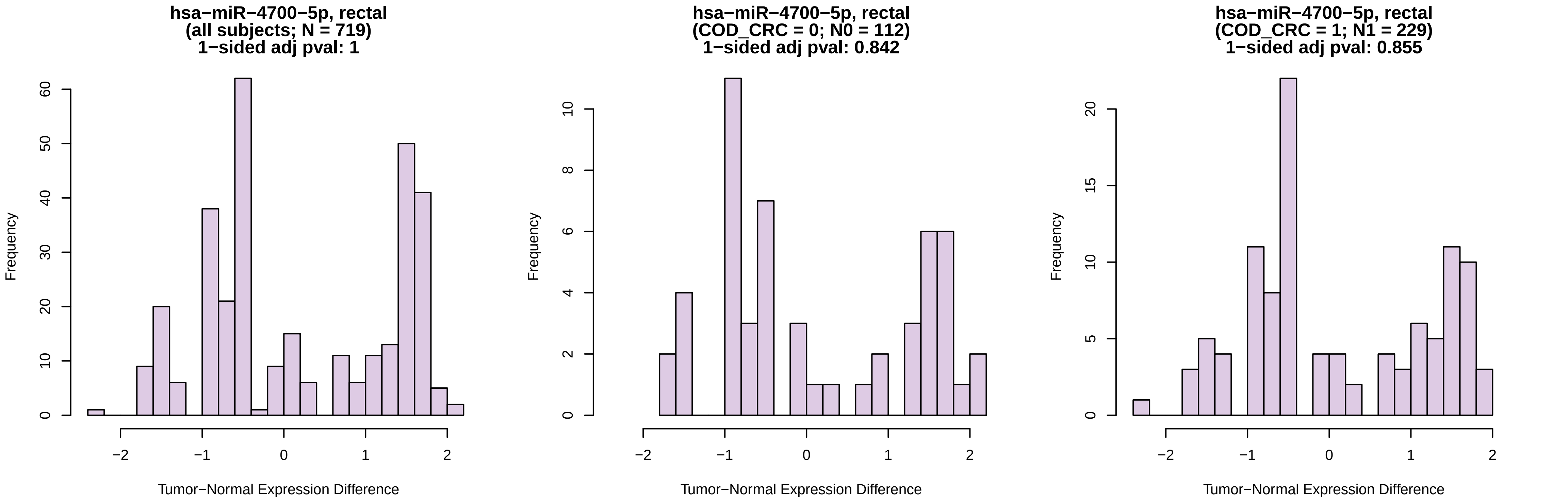
hsa−miR−4700−5p, rectal
(all subjects; N = 719)
1−sided adj pval: 1
Frequency
Tumor−Normal Expression Difference
hsa−miR−4700−5p, rectal
(COD_CRC = 0; N0 = 112)
1−sided adj pval: 0.842
Frequency
Tumor−Normal Expression Difference
hsa−miR−4700−5p, rectal
(COD_CRC = 1; N1 = 229)
1−sided adj pval: 0.855
Frequency
Tumor−Normal Expression Difference

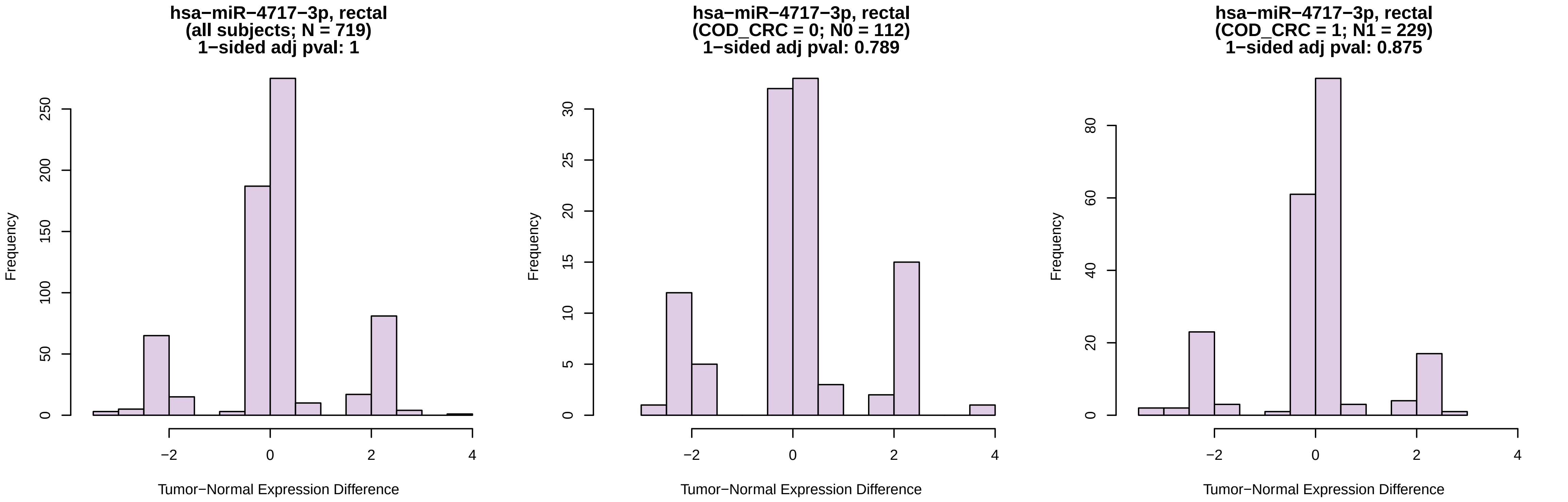
hsa−miR−4717−3p, rectal
(all subjects; N = 719)
1−sided adj pval: 1
Frequency
Tumor−Normal Expression Difference
hsa−miR−4717−3p, rectal
(COD_CRC = 0; N0 = 112)
1−sided adj pval: 0.789
Frequency
Tumor−Normal Expression Difference
hsa−miR−4717−3p, rectal
(COD_CRC = 1; N1 = 229)
1−sided adj pval: 0.875
Frequency
Tumor−Normal Expression Difference

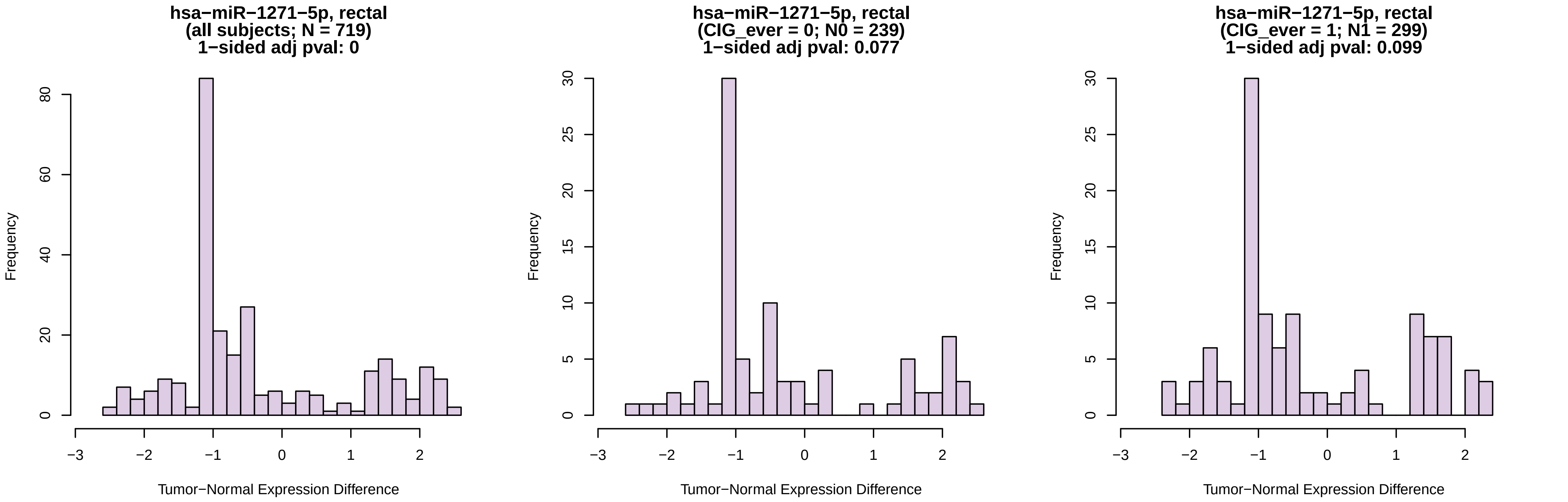

hsa−miR−1271−5p, rectal
(all subjects; N = 719)
1−sided adj pval: 0
Frequency
0
20
40
60
80
−3
−2
−1
0
1
2
Tumor−Normal Expression Difference
hsa−miR−1271−5p, rectal
(CIG_ever = 0; N0 = 239)
1−sided adj pval: 0.077
Frequency
0
5
10
15
20
25
30
−3
−2
−1
0
1
2
Tumor−Normal Expression Difference
hsa−miR−1271−5p, rectal
(CIG_ever = 1; N1 = 299)
1−sided adj pval: 0.099
Frequency
0
5
10
15
20
25
30
−3
−2
−1
0
1
2
Tumor−Normal Expression Difference

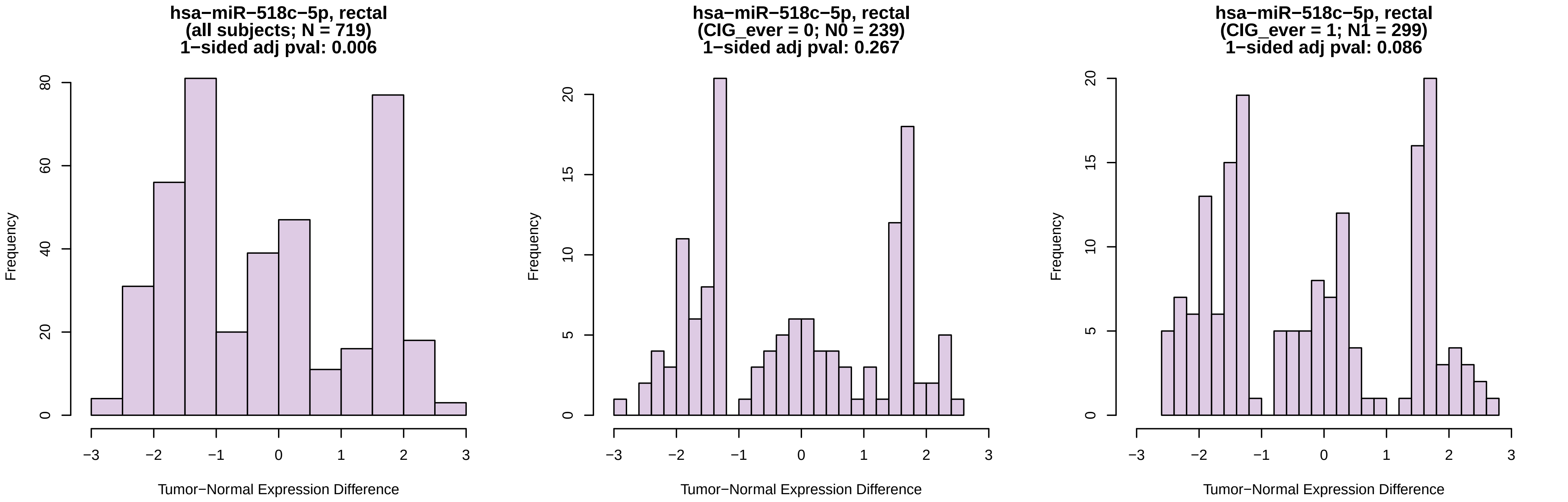
hsa−miR−518c−5p, rectal
(all subjects; N = 719)
1−sided adj pval: 0.006
hsa−miR−518c−5p, rectal
(CIG_ever = 0; N0 = 239)
1−sided adj pval: 0.267
hsa−miR−518c−5p, rectal
(CIG_ever = 1; N1 = 299)
1−sided adj pval: 0.086
Frequency
Tumor−Normal Expression Difference

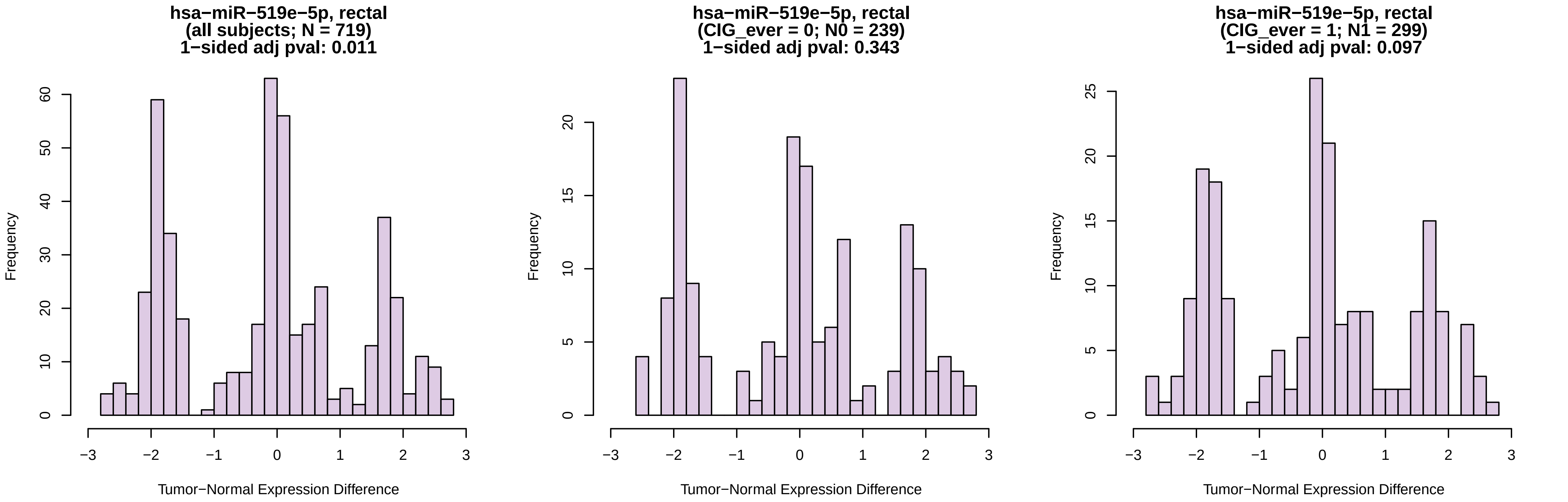
hsa−miR−519e−5p, rectal
(all subjects; N = 719)
1−sided adj pval: 0.011
Frequency
Tumor−Normal Expression Difference
hsa−miR−519e−5p, rectal
(CIG_ever = 0; N0 = 239)
1−sided adj pval: 0.343
Frequency
Tumor−Normal Expression Difference
hsa−miR−519e−5p, rectal
(CIG_ever = 1; N1 = 299)
1−sided adj pval: 0.097
Frequency
Tumor−Normal Expression Difference

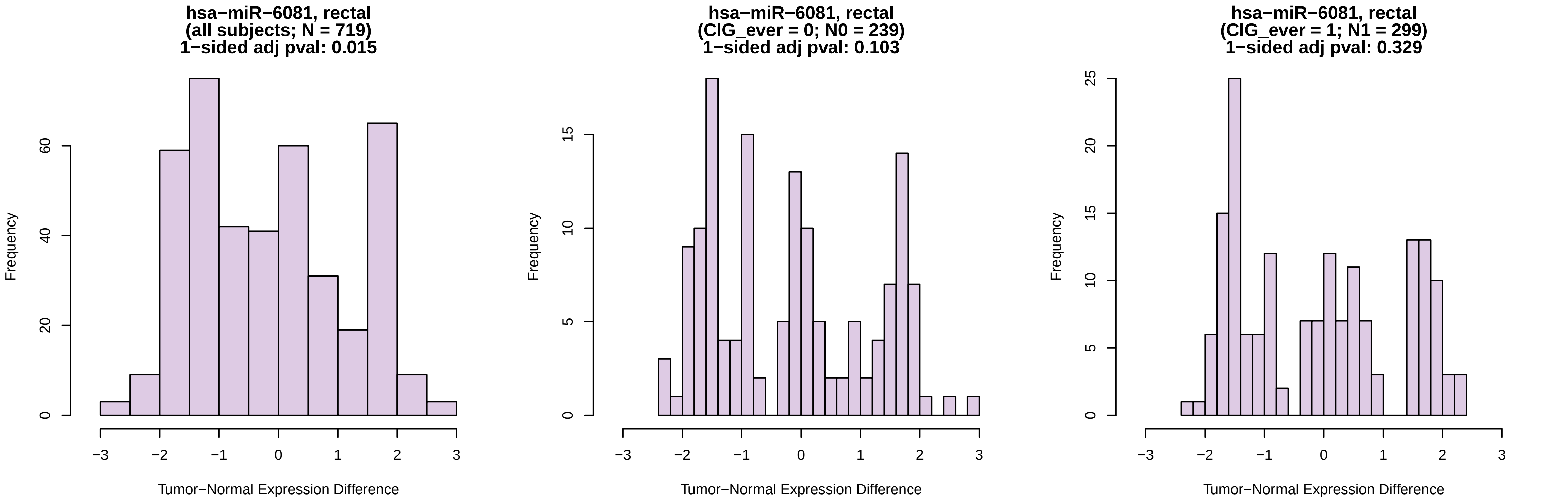
hsa−miR−6081, rectal
(all subjects; N = 719)
1−sided adj pval: 0.015
Frequency
Tumor−Normal Expression Difference
hsa−miR−6081, rectal
(CIG_ever = 0; N0 = 239)
1−sided adj pval: 0.103
Frequency
Tumor−Normal Expression Difference
hsa−miR−6081, rectal
(CIG_ever = 1; N1 = 299)
1−sided adj pval: 0.329
Frequency
Tumor−Normal Expression Difference

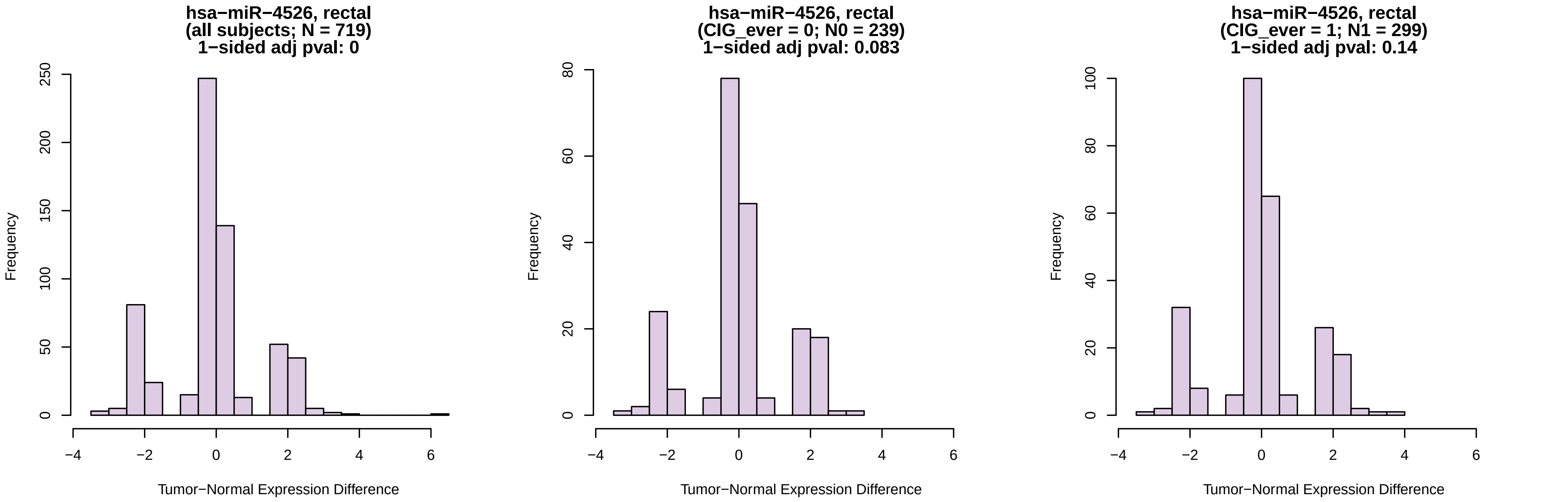
hsa−miR−4526, rectal
(all subjects; N = 719)
1−sided adj pval: 0
Frequency
Tumor−Normal Expression Difference
hsa−miR−4526, rectal
(CIG_ever = 0; N0 = 239)
1−sided adj pval: 0.083
Frequency
Tumor−Normal Expression Difference
hsa−miR−4526, rectal
(CIG_ever = 1; N1 = 299)
1−sided adj pval: 0.14
Frequency
Tumor−Normal Expression Difference

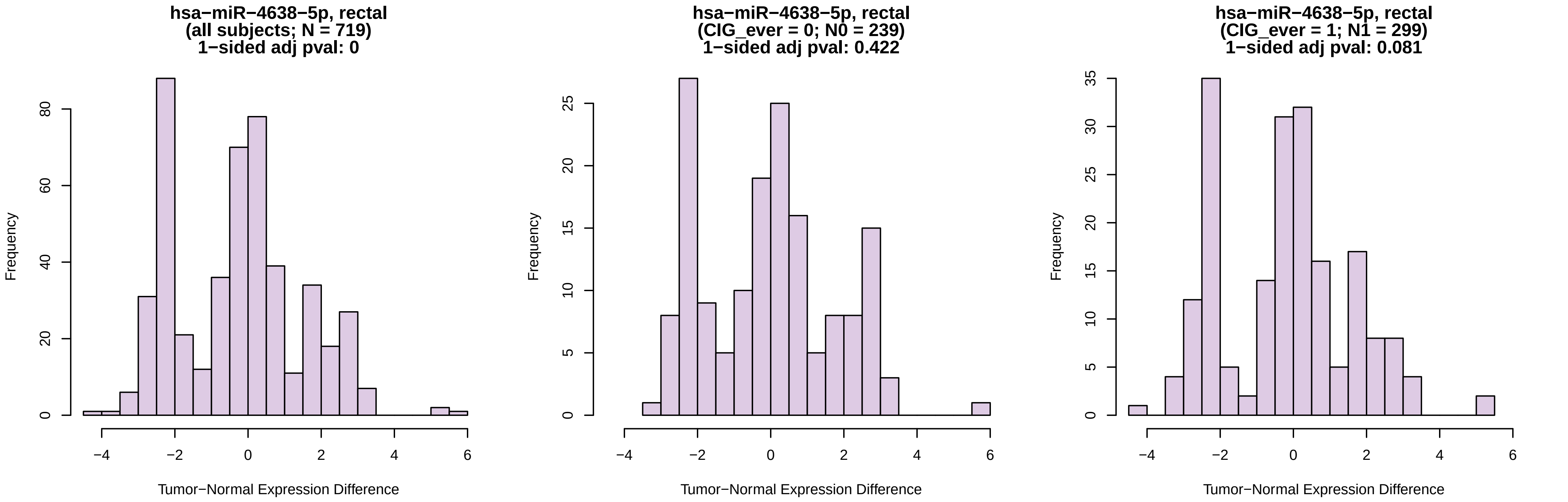
hsa−miR−4638−5p, rectal
(all subjects; N = 719)
1−sided adj pval: 0
Frequency
Tumor−Normal Expression Difference
hsa−miR−4638−5p, rectal
(CIG_ever = 0; N0 = 239)
1−sided adj pval: 0.422
Frequency
Tumor−Normal Expression Difference
hsa−miR−4638−5p, rectal
(CIG_ever = 1; N1 = 299)
1−sided adj pval: 0.081
Frequency
Tumor−Normal Expression Difference

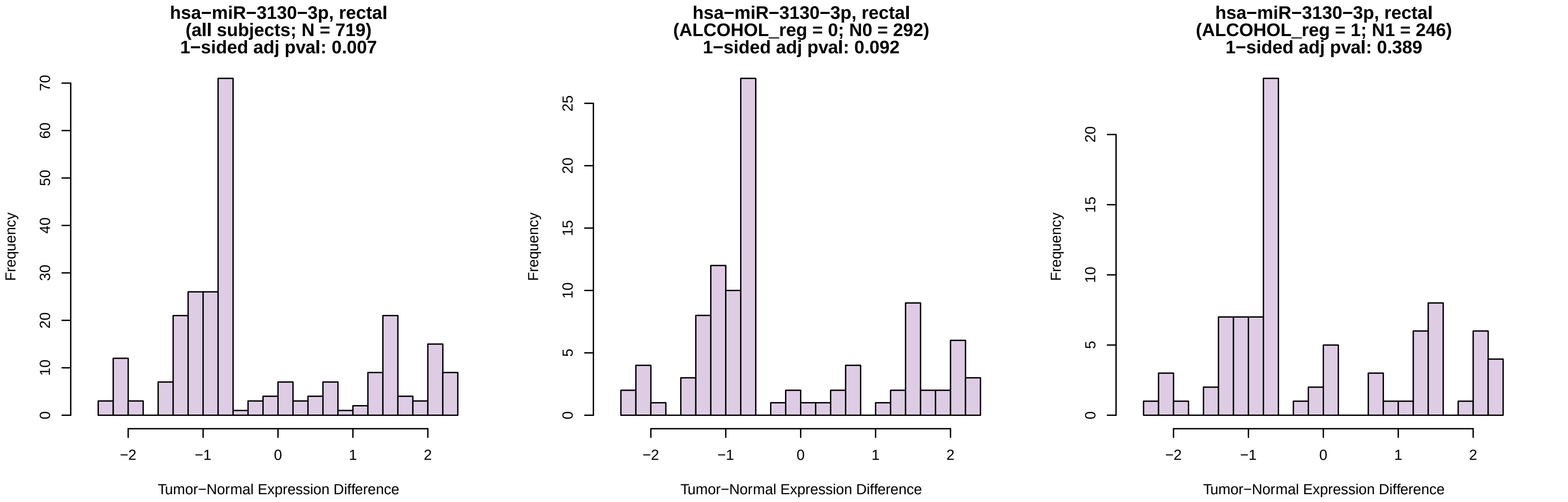
hsa−miR−3130−3p, rectal
(all subjects; N = 719)
1−sided adj pval: 0.007
Frequency
Tumor−Normal Expression Difference
hsa−miR−3130−3p, rectal
(ALCOHOL_reg = 0; N0 = 292)
1−sided adj pval: 0.092
Frequency
Tumor−Normal Expression Difference
hsa−miR−3130−3p, rectal
(ALCOHOL_reg = 1; N1 = 246)
1−sided adj pval: 0.389
Frequency
Tumor−Normal Expression Difference

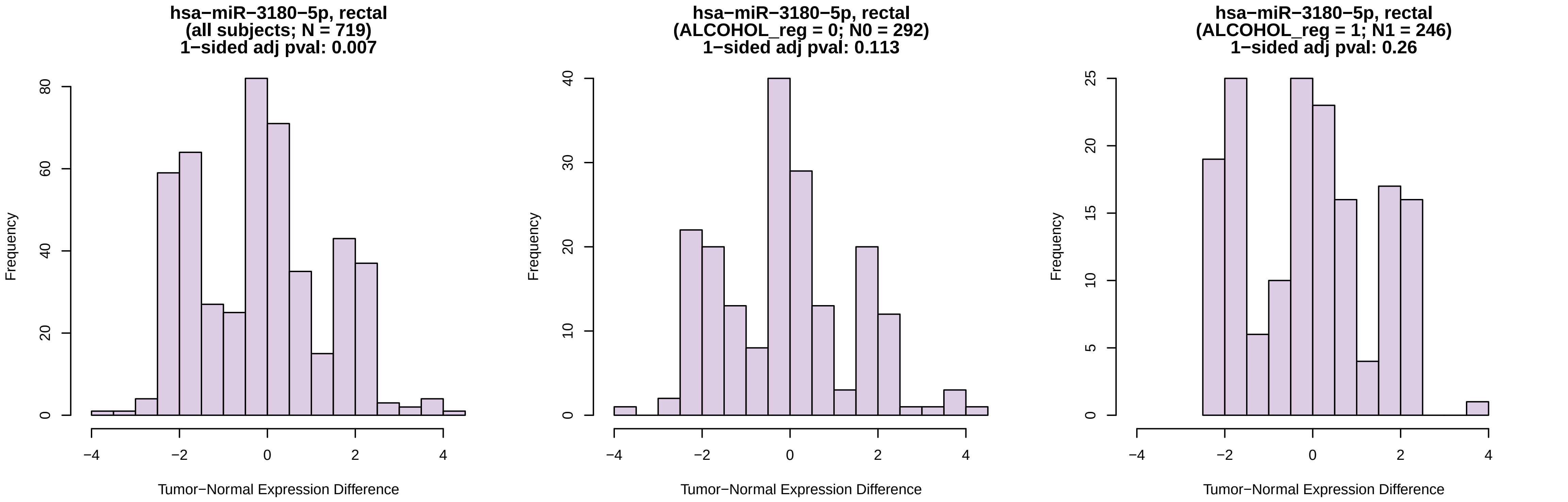

hsa−miR−3180−5p, rectal
(all subjects; N = 719)
1−sided adj pval: 0.007
Frequency
0
20
40
60
80
−4
−2
0
2
4
Tumor−Normal Expression Difference
hsa−miR−3180−5p, rectal
(ALCOHOL_reg = 0; N0 = 292)
1−sided adj pval: 0.113
Frequency
0
10
20
30
40
−4
−2
0
2
4
Tumor−Normal Expression Difference
hsa−miR−3180−5p, rectal
(ALCOHOL_reg = 1; N1 = 246)
1−sided adj pval: 0.26
Frequency
0
5
10
15
20
25
−4
−2
0
2
4
Tumor−Normal Expression Difference

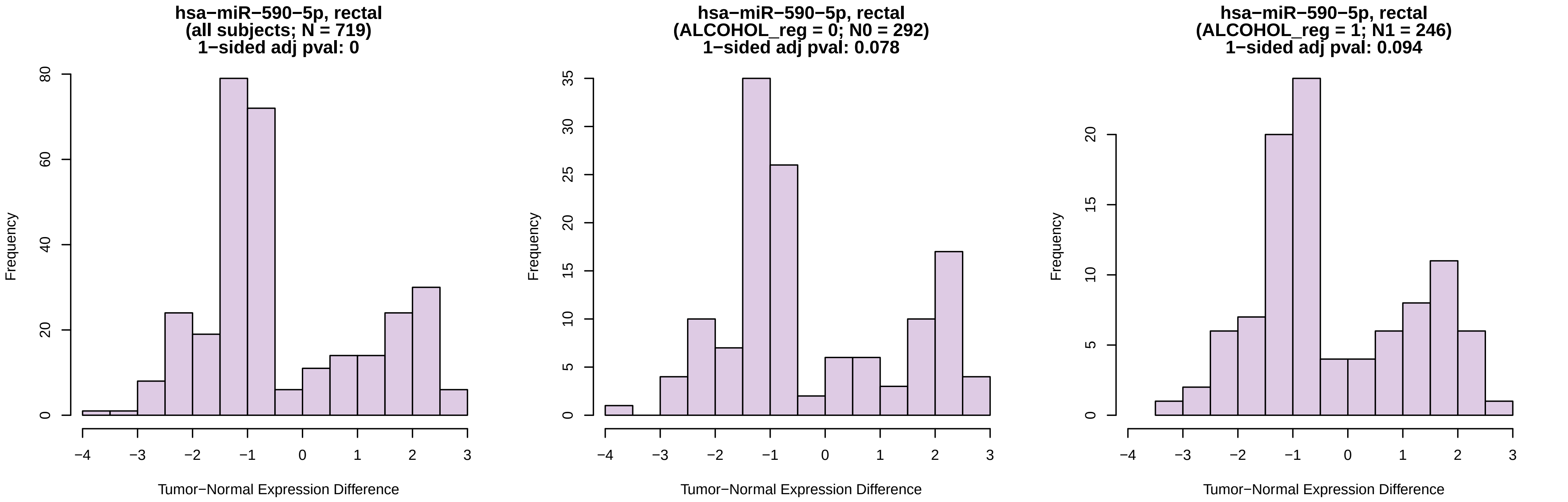

hsa−miR−590−5p, rectal
(all subjects; N = 719)
1−sided adj pval: 0
hsa−miR−590−5p, rectal
(ALCOHOL_reg = 0; N0 = 292)
1−sided adj pval: 0.078
hsa−miR−590−5p, rectal
(ALCOHOL_reg = 1; N1 = 246)
1−sided adj pval: 0.094
Frequency
Tumor−Normal Expression Difference

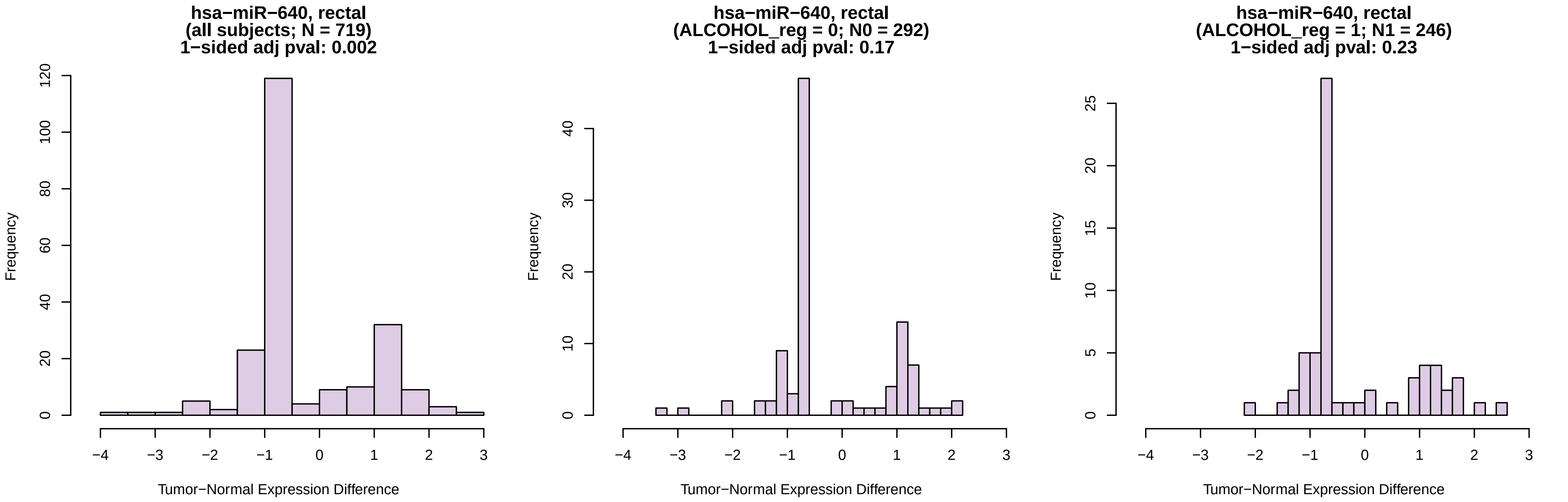
hsa−miR−640, rectal
(all subjects; N = 719)
1−sided adj pval: 0.002
hsa−miR−640, rectal
(ALCOHOL_reg = 0; N0 = 292)
1−sided adj pval: 0.17
hsa−miR−640, rectal
(ALCOHOL_reg = 1; N1 = 246)
1−sided adj pval: 0.23
Frequency
Tumor−Normal Expression Difference

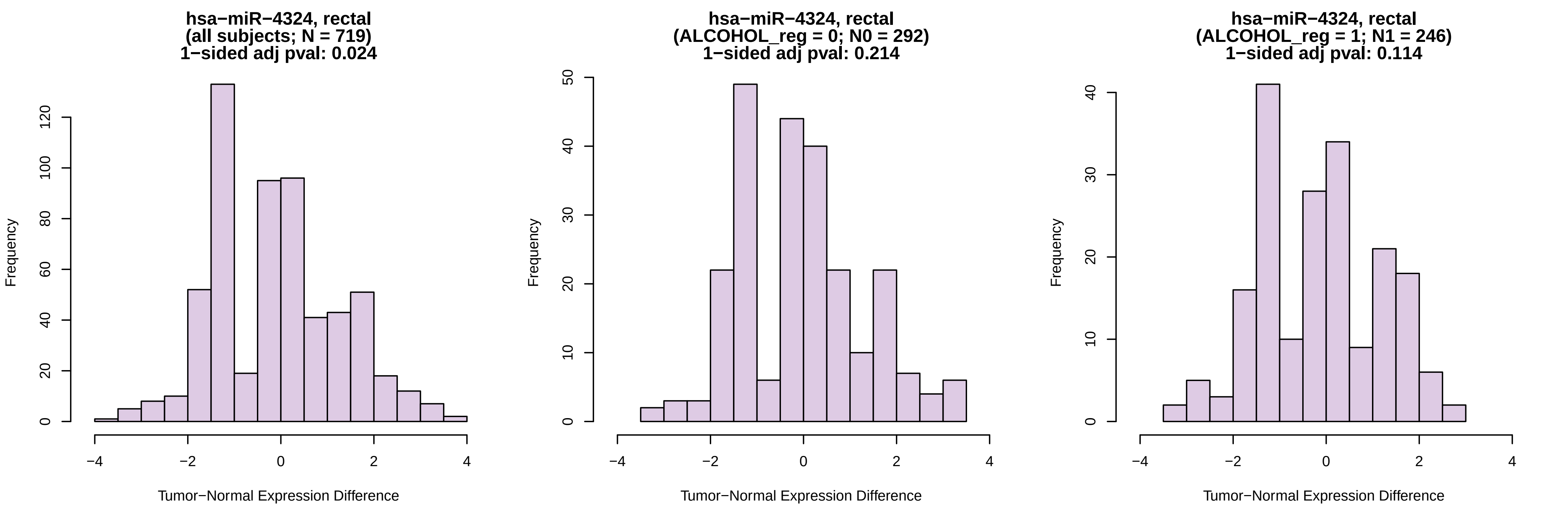
hsa−miR−4324, rectal
(all subjects; N = 719)
1−sided adj pval: 0.024
Frequency
Tumor−Normal Expression Difference
hsa−miR−4324, rectal
(ALCOHOL_reg = 0; N0 = 292)
1−sided adj pval: 0.214
Frequency
Tumor−Normal Expression Difference
hsa−miR−4324, rectal
(ALCOHOL_reg = 1; N1 = 246)
1−sided adj pval: 0.114
Frequency
Tumor−Normal Expression Difference

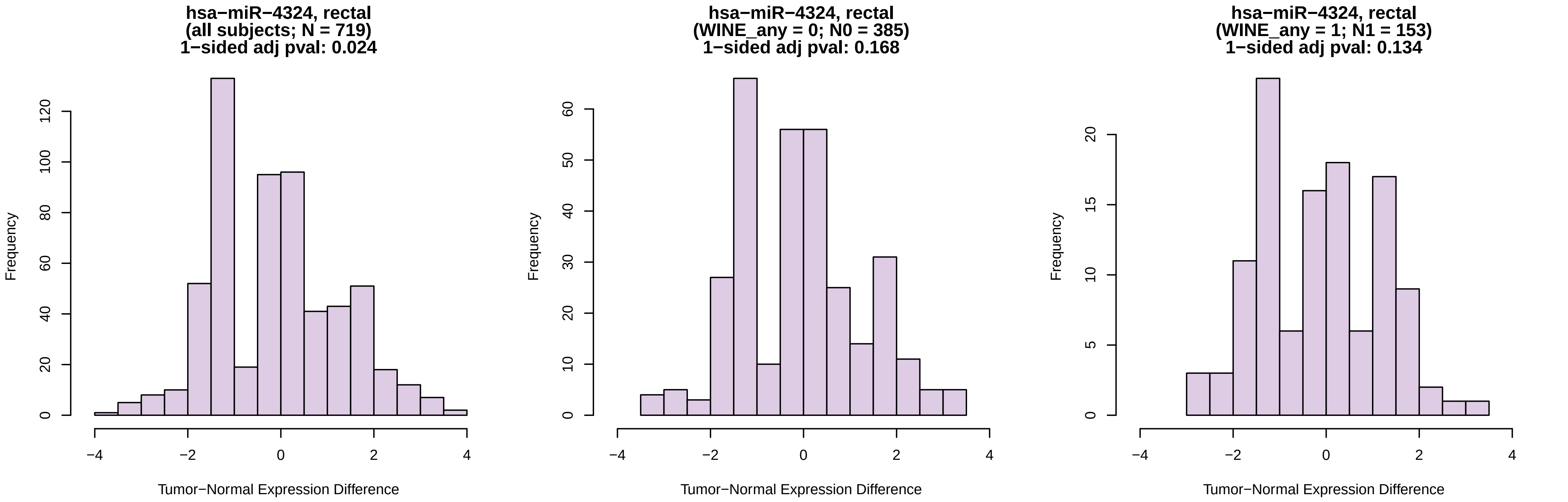
hsa−miR−4324, rectal
(all subjects; N = 719)
1−sided adj pval: 0.024
Frequency
Tumor−Normal Expression Difference
hsa−miR−4324, rectal
(WINE_any = 0; N0 = 385)
1−sided adj pval: 0.168
Frequency
Tumor−Normal Expression Difference
hsa−miR−4324, rectal
(WINE_any = 1; N1 = 153)
1−sided adj pval: 0.134
Frequency
Tumor−Normal Expression Difference

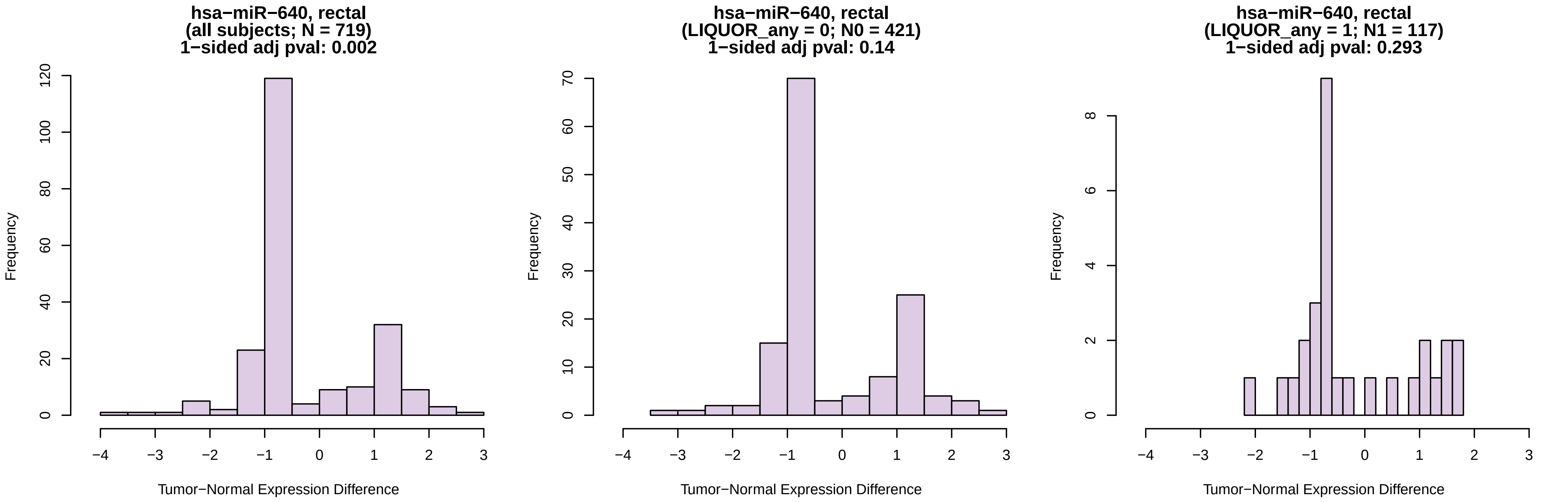

hsa−miR−640, rectal
(all subjects; N = 719)
1−sided adj pval: 0.002
Frequency
Tumor−Normal Expression Difference
hsa−miR−640, rectal
(LIQUOR_any = 0; N0 = 421)
1−sided adj pval: 0.14
Frequency
Tumor−Normal Expression Difference
hsa−miR−640, rectal
(LIQUOR_any = 1; N1 = 117)
1−sided adj pval: 0.293
Frequency
Tumor−Normal Expression Difference

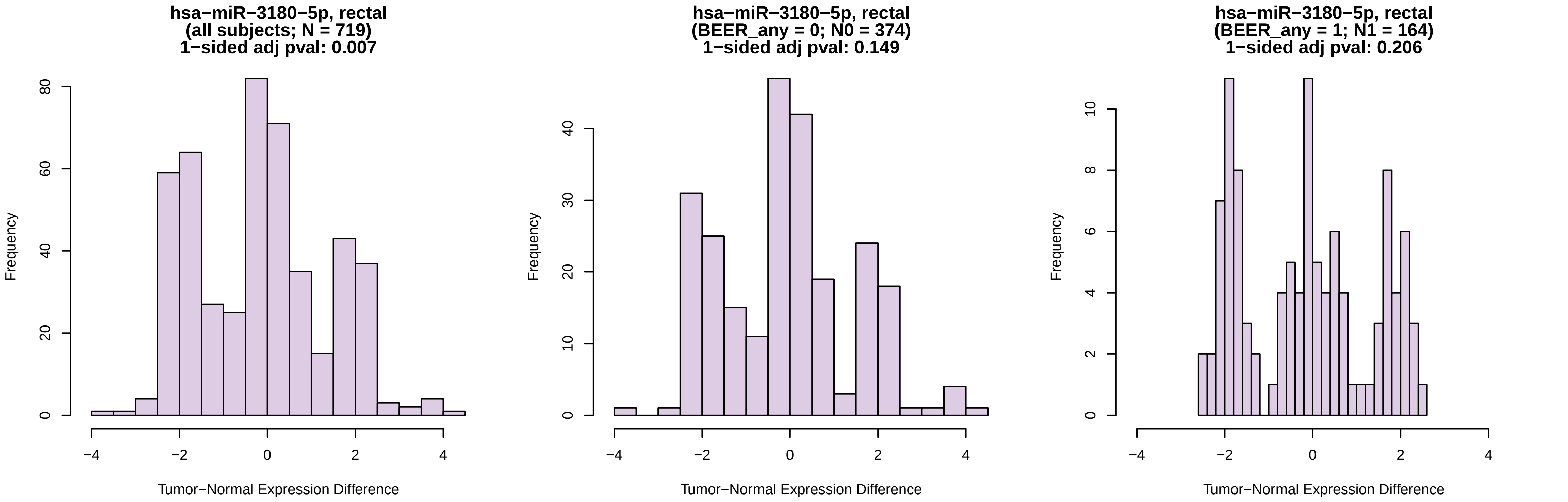

hsa−miR−3180−5p, rectal
(all subjects; N = 719)
1−sided adj pval: 0.007
Frequency
Tumor−Normal Expression Difference
hsa−miR−3180−5p, rectal
(BEER_any = 0; N0 = 374)
1−sided adj pval: 0.149
Frequency
Tumor−Normal Expression Difference
hsa−miR−3180−5p, rectal
(BEER_any = 1; N1 = 164)
1−sided adj pval: 0.206
Frequency
Tumor−Normal Expression Difference

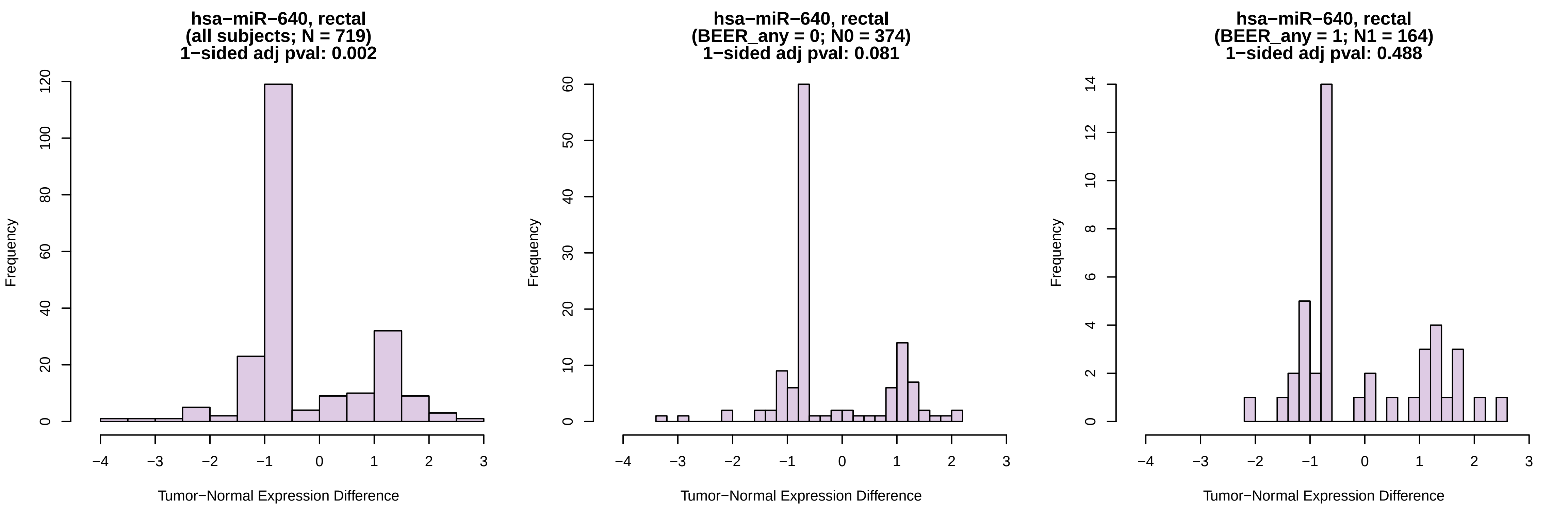
hsa−miR−640, rectal
(all subjects; N = 719)
1−sided adj pval: 0.002
Frequency
Tumor−Normal Expression Difference
hsa−miR−640, rectal
(BEER_any = 0; N0 = 374)
1−sided adj pval: 0.081
Frequency
Tumor−Normal Expression Difference
hsa−miR−640, rectal
(BEER_any = 1; N1 = 164)
1−sided adj pval: 0.488
Frequency
Tumor−Normal Expression Difference

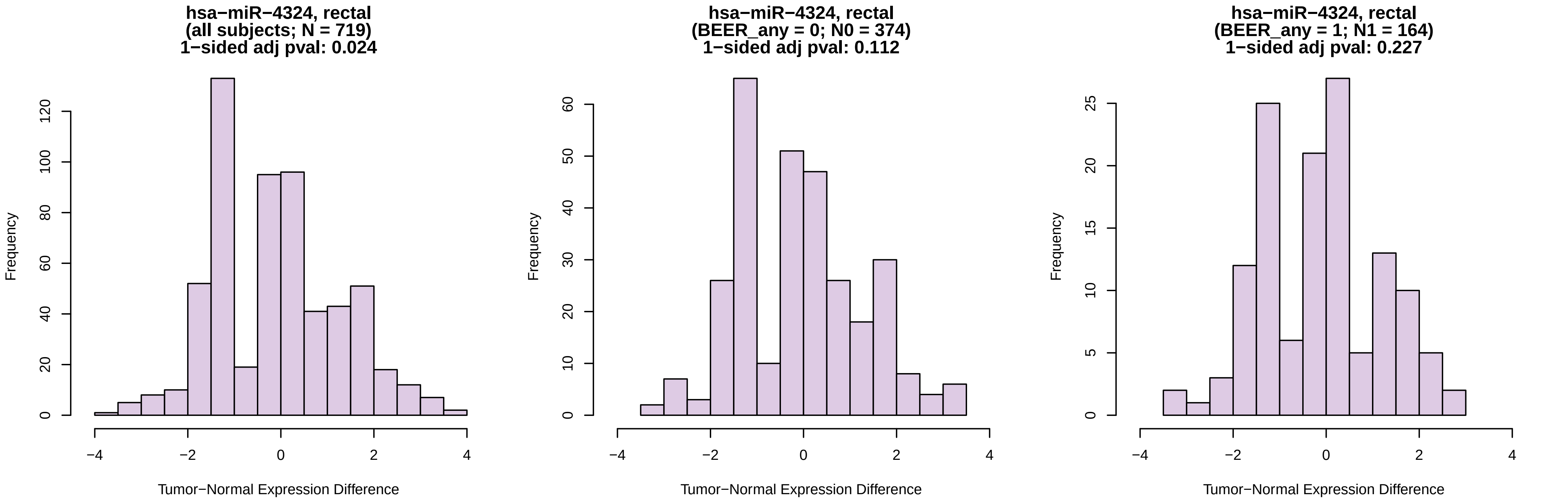

hsa−miR−4324, rectal
(all subjects; N = 719)
1−sided adj pval: 0.024
Frequency
Tumor−Normal Expression Difference
hsa−miR−4324, rectal
(BEER_any = 0; N0 = 374)
1−sided adj pval: 0.112
Frequency
Tumor−Normal Expression Difference
hsa−miR−4324, rectal
(BEER_any = 1; N1 = 164)
1−sided adj pval: 0.227
Frequency
Tumor−Normal Expression Difference

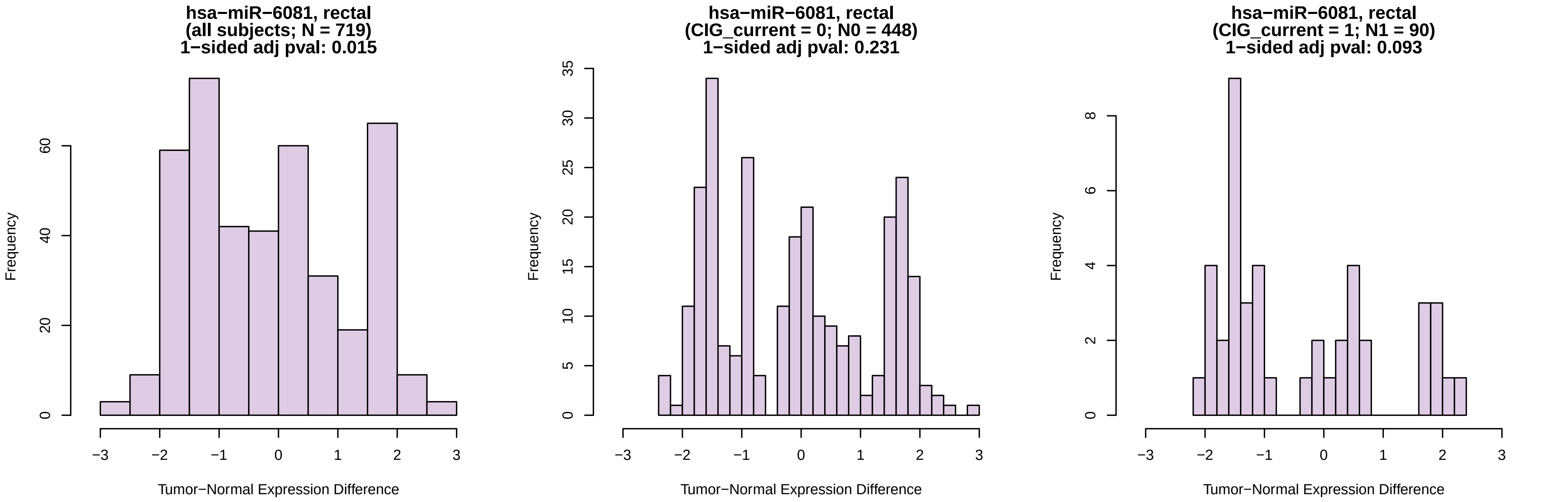
hsa−miR−6081, rectal
(all subjects; N = 719)
1−sided adj pval: 0.015
Frequency
Tumor−Normal Expression Difference
hsa−miR−6081, rectal
(CIG_current = 0; N0 = 448)
1−sided adj pval: 0.231
Frequency
Tumor−Normal Expression Difference
hsa−miR−6081, rectal
(CIG_current = 1; N1 = 90)
1−sided adj pval: 0.093
Frequency
Tumor−Normal Expression Difference

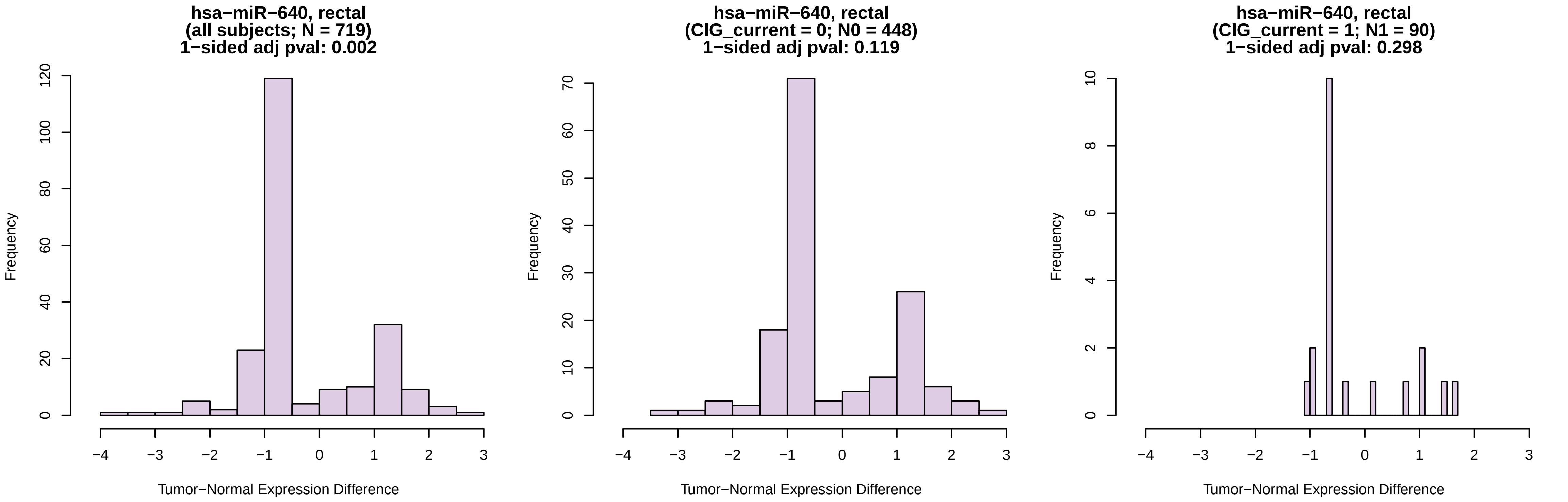

hsa−miR−640, rectal
(all subjects; N = 719)
1−sided adj pval: 0.002
Frequency
Tumor−Normal Expression Difference
hsa−miR−640, rectal
(CIG_current = 0; N0 = 448)
1−sided adj pval: 0.119
Frequency
Tumor−Normal Expression Difference
hsa−miR−640, rectal
(CIG_current = 1; N1 = 90)
1−sided adj pval: 0.298
Frequency
Tumor−Normal Expression Difference

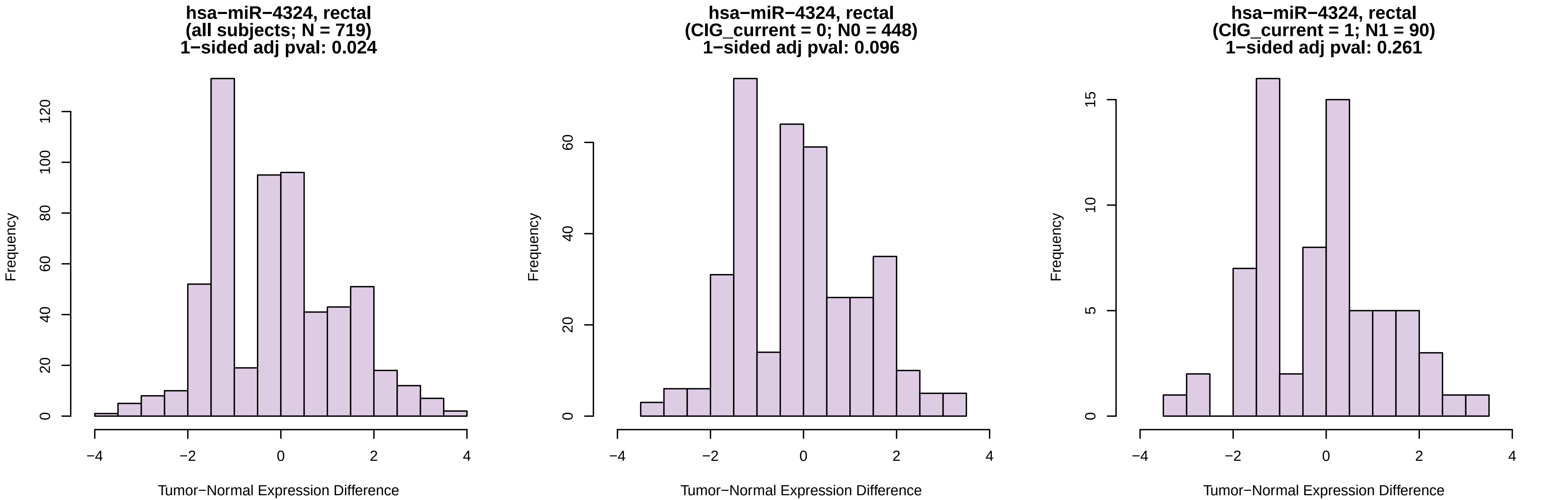
hsa−miR−4324, rectal
(all subjects; N = 719)
1−sided adj pval: 0.024
hsa−miR−4324, rectal
(CIG_current = 0; N0 = 448)
1−sided adj pval: 0.096
hsa−miR−4324, rectal
(CIG_current = 1; N1 = 90)
1−sided adj pval: 0.261
Frequency
Tumor−Normal Expression Difference

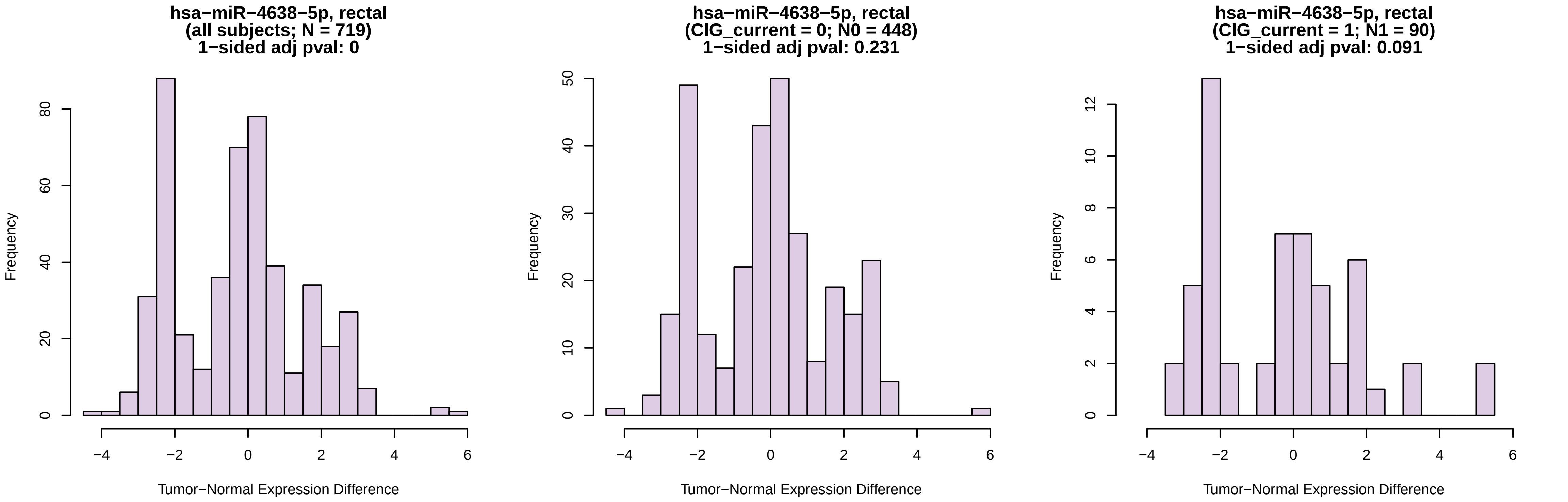

hsa−miR−4638−5p, rectal
(all subjects; N = 719)
1−sided adj pval: 0
hsa−miR−4638−5p, rectal
(CIG_current = 0; N0 = 448)
1−sided adj pval: 0.231
hsa−miR−4638−5p, rectal
(CIG_current = 1; N1 = 90)
1−sided adj pval: 0.091
Frequency
Tumor−Normal Expression Difference

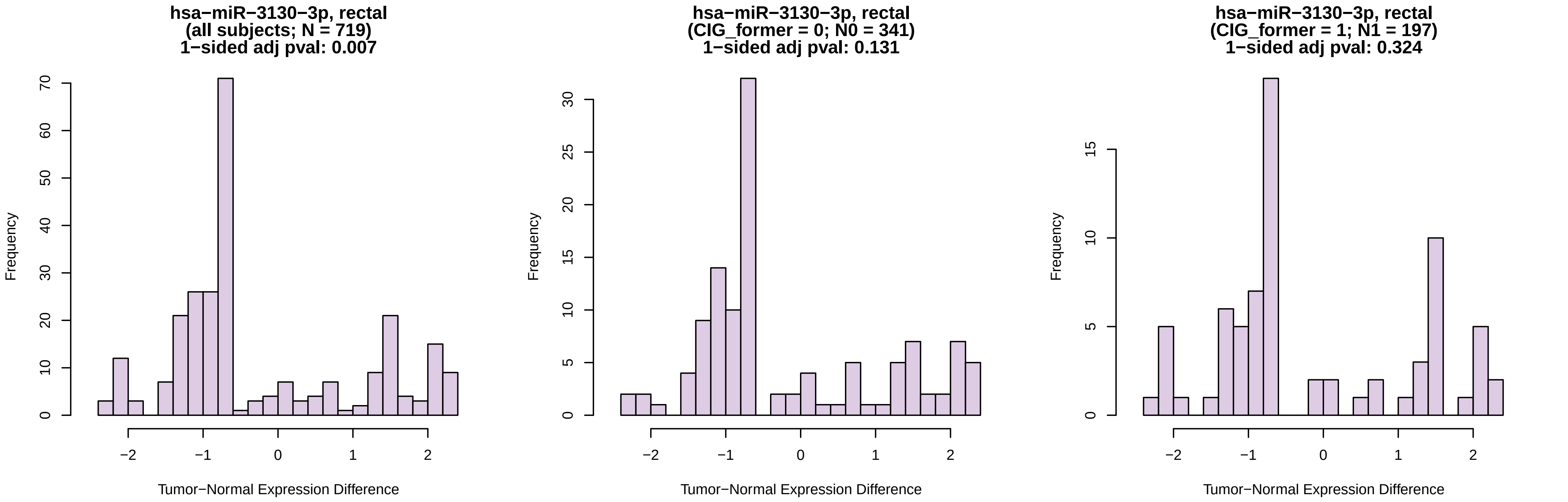
hsa−miR−3130−3p, rectal
(all subjects; N = 719)
1−sided adj pval: 0.007
Frequency
0
10
20
30
40
50
60
70
−2
−1
0
1
2
Tumor−Normal Expression Difference
hsa−miR−3130−3p, rectal
(CIG_former = 0; N0 = 341)
1−sided adj pval: 0.131
Frequency
0
5
10
15
20
25
30
−2
−1
0
1
2
Tumor−Normal Expression Difference
hsa−miR−3130−3p, rectal
(CIG_former = 1; N1 = 197)
1−sided adj pval: 0.324
Frequency
0
5
10
15
−2
−1
0
1
2
Tumor−Normal Expression Difference

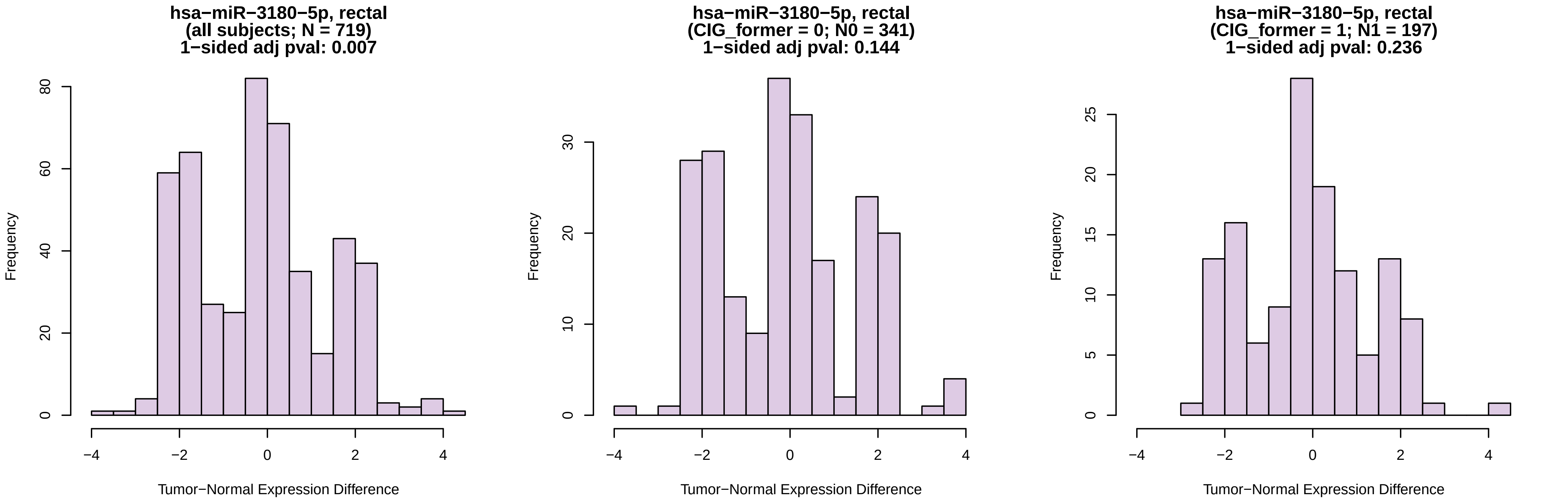
hsa−miR−3180−5p, rectal
(all subjects; N = 719)
1−sided adj pval: 0.007
Frequency
Tumor−Normal Expression Difference
hsa−miR−3180−5p, rectal
(CIG_former = 0; N0 = 341)
1−sided adj pval: 0.144
Frequency
Tumor−Normal Expression Difference
hsa−miR−3180−5p, rectal
(CIG_former = 1; N1 = 197)
1−sided adj pval: 0.236
Frequency
Tumor−Normal Expression Difference

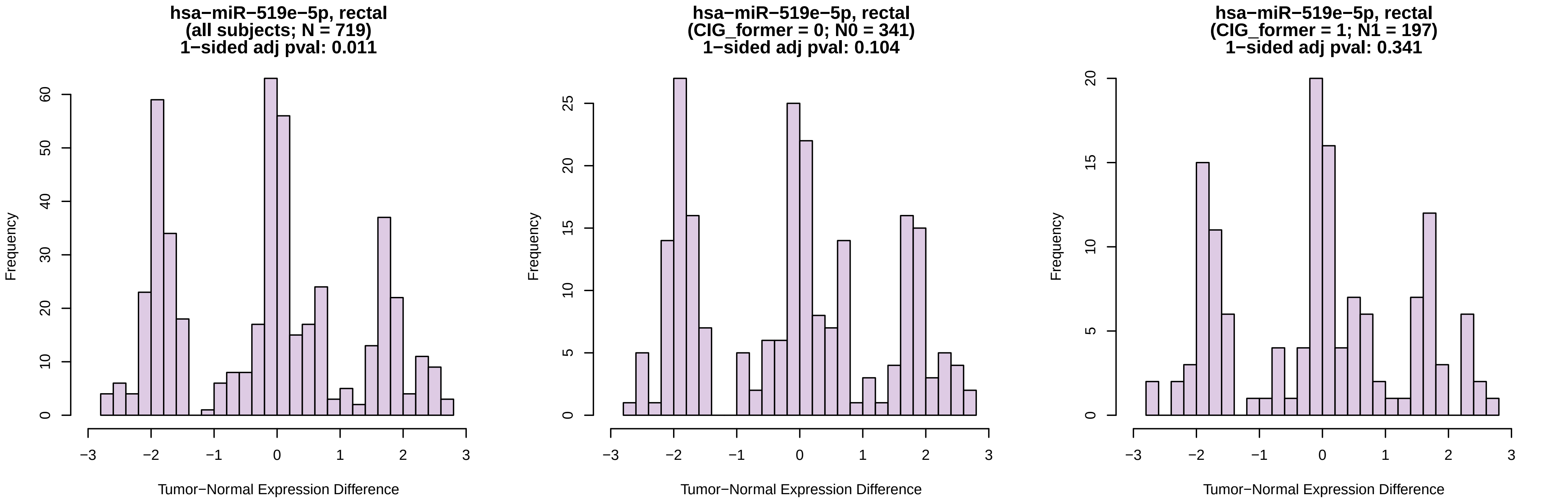

hsa−miR−519e−5p, rectal
(all subjects; N = 719)
1−sided adj pval: 0.011
Frequency
Tumor−Normal Expression Difference
hsa−miR−519e−5p, rectal
(CIG_former = 0; N0 = 341)
1−sided adj pval: 0.104
Frequency
Tumor−Normal Expression Difference
hsa−miR−519e−5p, rectal
(CIG_former = 1; N1 = 197)
1−sided adj pval: 0.341
Frequency
Tumor−Normal Expression Difference

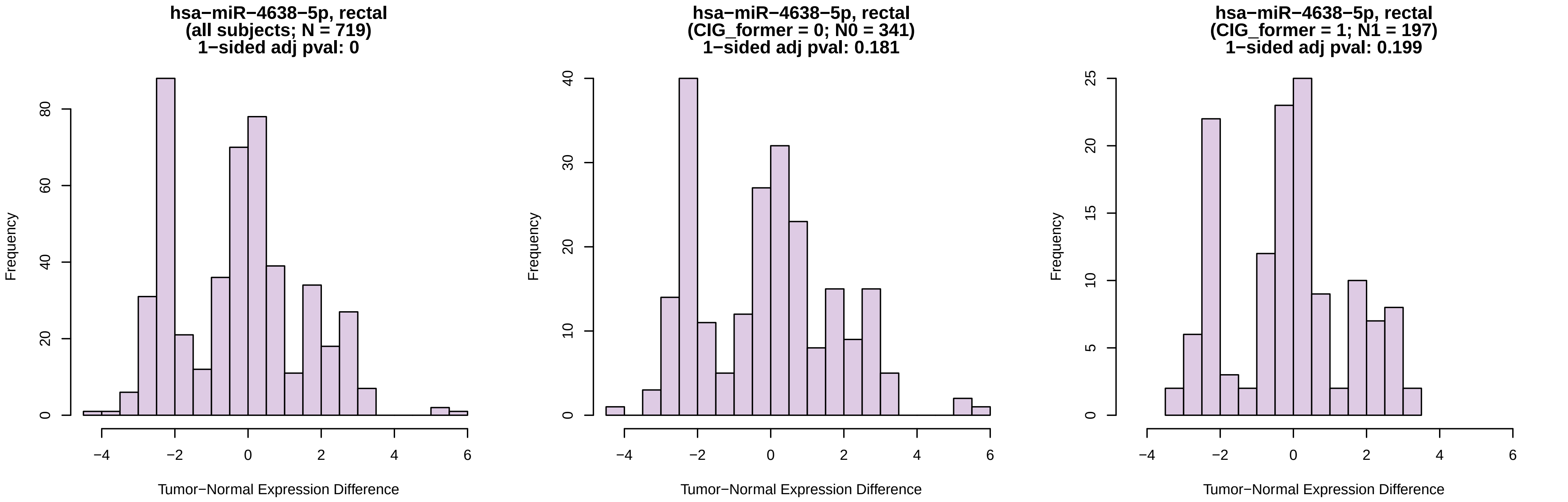
hsa−miR−4638−5p, rectal
(all subjects; N = 719)
1−sided adj pval: 0
Frequency
Tumor−Normal Expression Difference
hsa−miR−4638−5p, rectal
(CIG_former = 0; N0 = 341)
1−sided adj pval: 0.181
Frequency
Tumor−Normal Expression Difference
hsa−miR−4638−5p, rectal
(CIG_former = 1; N1 = 197)
1−sided adj pval: 0.199
Frequency
Tumor−Normal Expression Difference

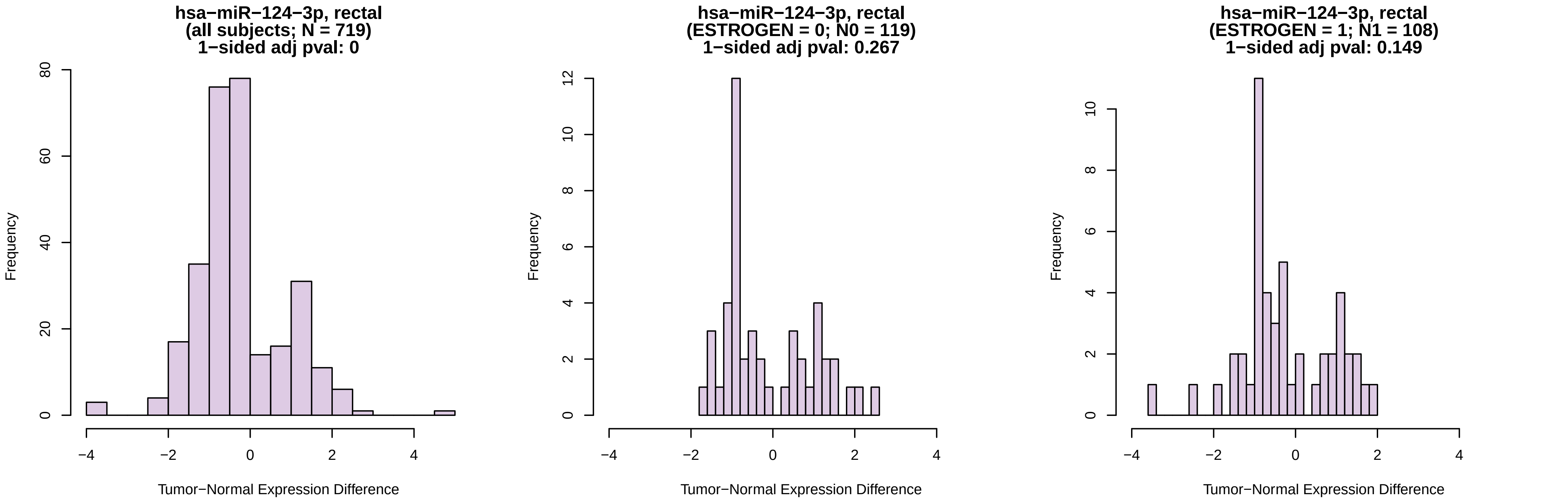
hsa−miR−124−3p, rectal
(all subjects; N = 719)
1−sided adj pval: 0
Frequency
Tumor−Normal Expression Difference
hsa−miR−124−3p, rectal
(ESTROGEN = 0; N0 = 119)
1−sided adj pval: 0.267
Frequency
Tumor−Normal Expression Difference
hsa−miR−124−3p, rectal
(ESTROGEN = 1; N1 = 108)
1−sided adj pval: 0.149
Frequency
Tumor−Normal Expression Difference

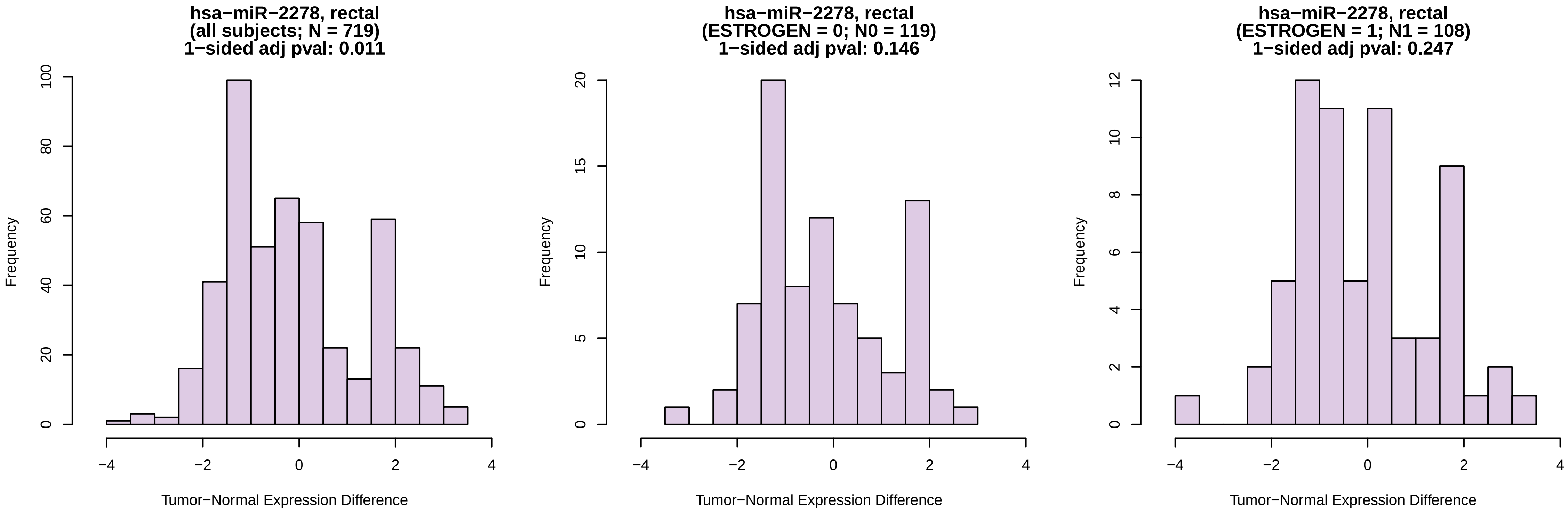
hsa−miR−2278, rectal
(all subjects; N = 719)
1−sided adj pval: 0.011
Frequency
Tumor−Normal Expression Difference
hsa−miR−2278, rectal
(ESTROGEN = 0; N0 = 119)
1−sided adj pval: 0.146
Frequency
Tumor−Normal Expression Difference
hsa−miR−2278, rectal
(ESTROGEN = 1; N1 = 108)
1−sided adj pval: 0.247
Frequency
Tumor−Normal Expression Difference

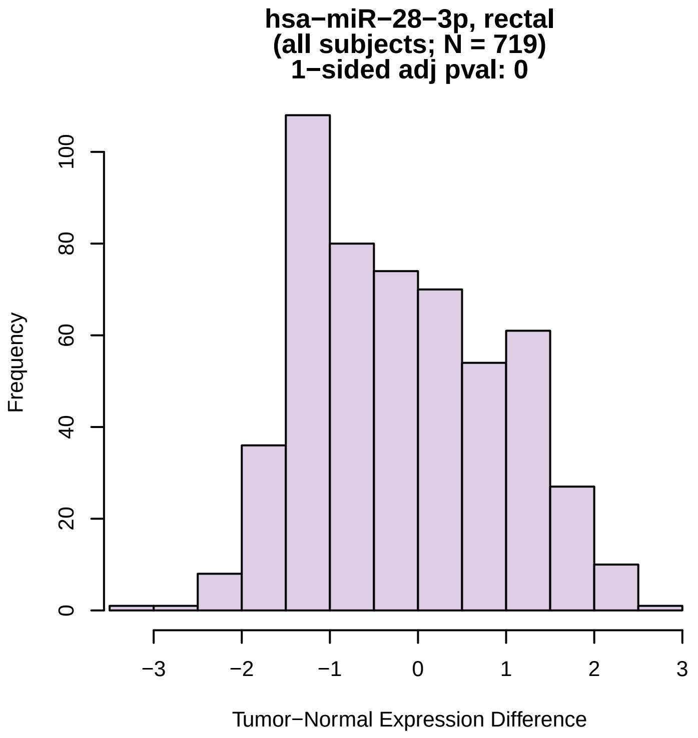
hsa−miR−28−3p, rectal
(all subjects; N = 719)
1−sided adj pval: 0
Frequency
Tumor−Normal Expression Difference
0
20
40
60
80
100
−3
−2
−1
0
1
2
3

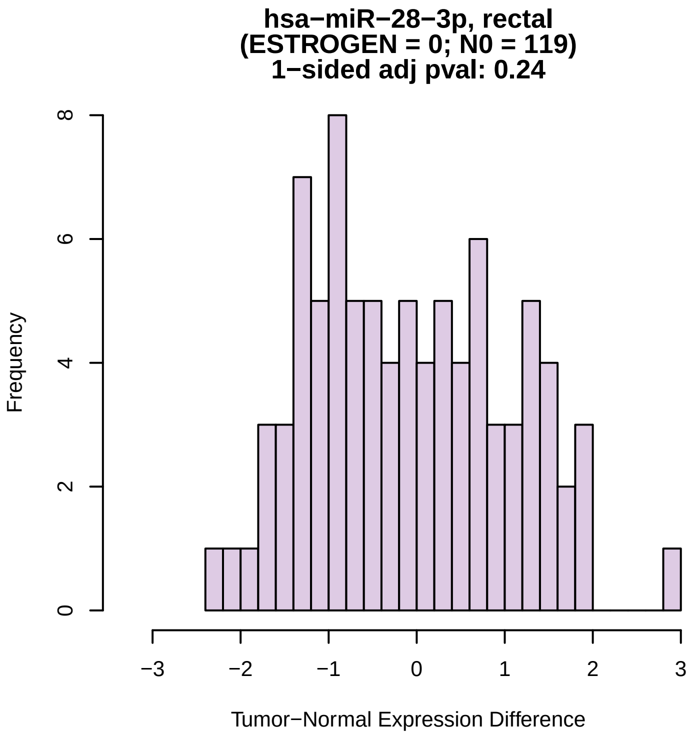
hsa−miR−28−3p, rectal
(ESTROGEN = 0; N0 = 119)
1−sided adj pval: 0.24
Frequency
Tumor−Normal Expression Difference
0
2
4
6
8
−3
−2
−1
0
1
2
3

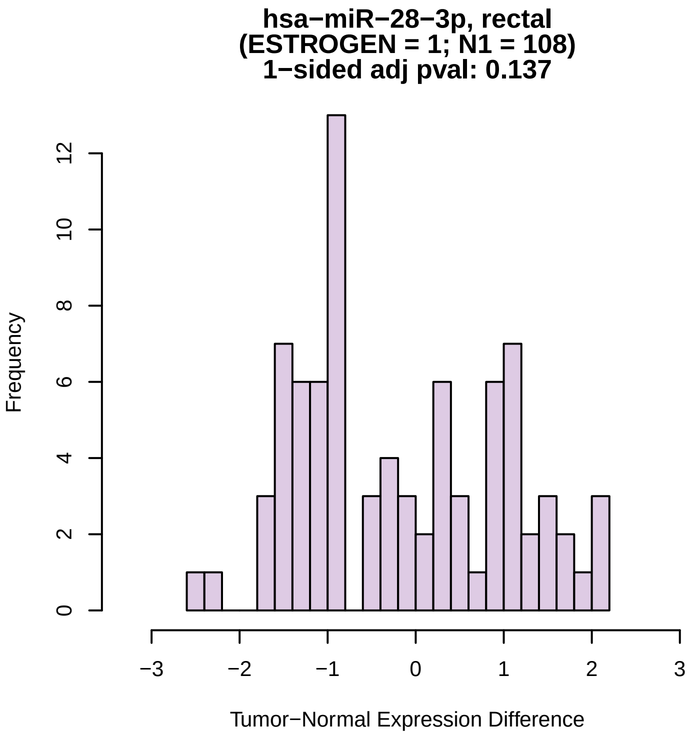
hsa−miR−28−3p, rectal
(ESTROGEN = 1; N1 = 108)
1−sided adj pval: 0.137
Frequency
Tumor−Normal Expression Difference
0
2
4
6
8
10
12
−3
−2
−1
0
1
2
3

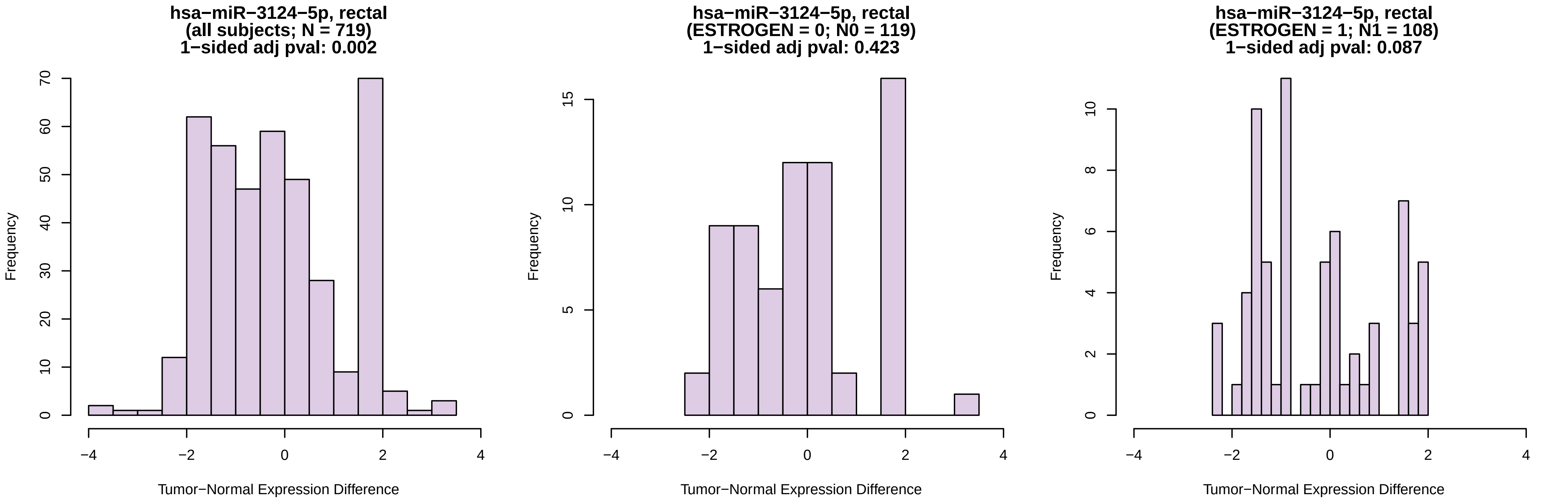
hsa−miR−3124−5p, rectal
(all subjects; N = 719)
1−sided adj pval: 0.002
Frequency
0
10
20
30
40
50
60
70
−4
−2
0
2
4
Tumor−Normal Expression Difference
hsa−miR−3124−5p, rectal
(ESTROGEN = 0; N0 = 119)
1−sided adj pval: 0.423
Frequency
0
5
10
15
−4
−2
0
2
4
Tumor−Normal Expression Difference
hsa−miR−3124−5p, rectal
(ESTROGEN = 1; N1 = 108)
1−sided adj pval: 0.087
Frequency
0
2
4
6
8
10
−4
−2
0
2
4
Tumor−Normal Expression Difference

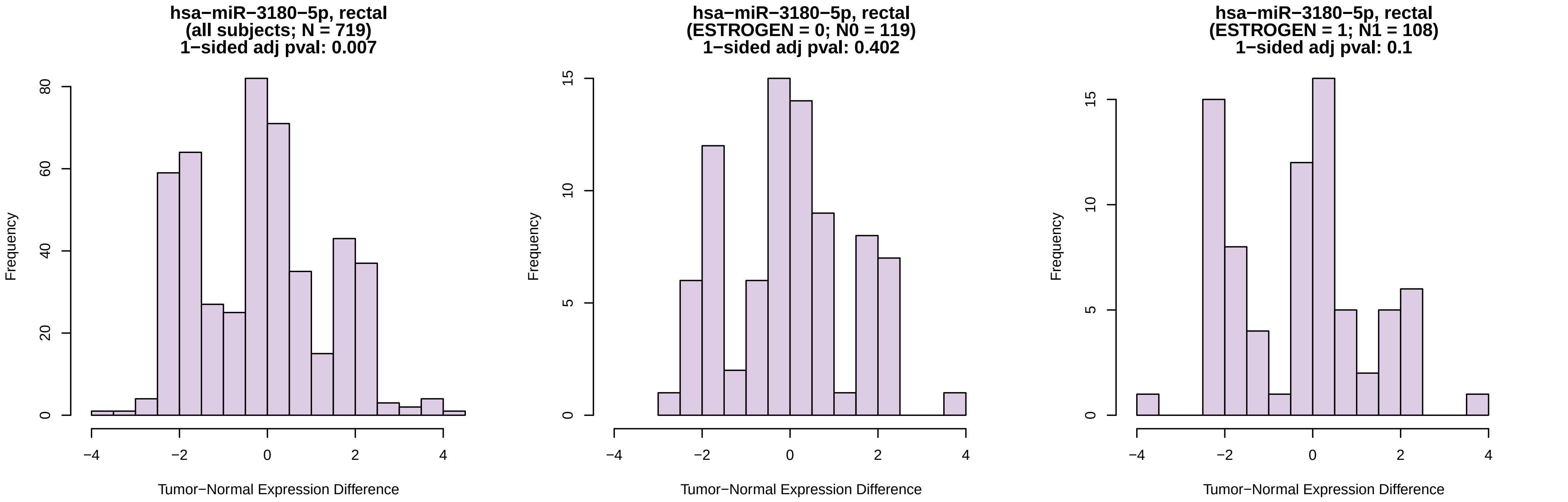
hsa−miR−3180−5p, rectal
(all subjects; N = 719)
1−sided adj pval: 0.007
hsa−miR−3180−5p, rectal
(ESTROGEN = 0; N0 = 119)
1−sided adj pval: 0.402
hsa−miR−3180−5p, rectal
(ESTROGEN = 1; N1 = 108)
1−sided adj pval: 0.1
Frequency
Tumor−Normal Expression Difference

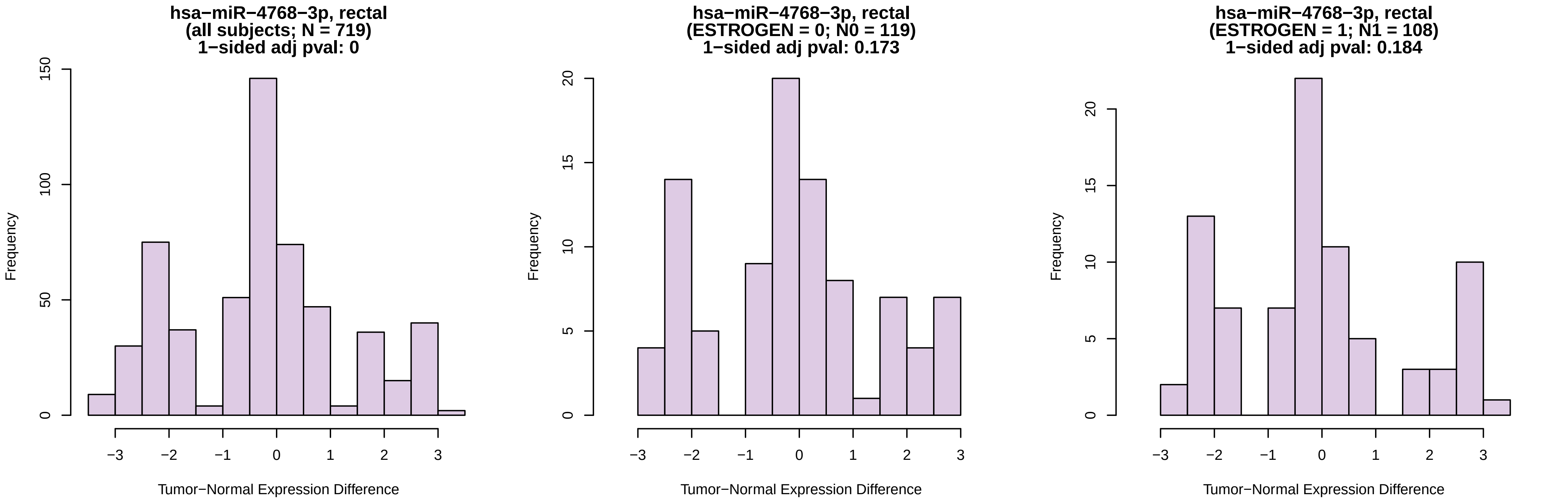
hsa−miR−4768−3p, rectal
(all subjects; N = 719)
1−sided adj pval: 0
Frequency
Tumor−Normal Expression Difference
hsa−miR−4768−3p, rectal
(ESTROGEN = 0; N0 = 119)
1−sided adj pval: 0.173
Frequency
Tumor−Normal Expression Difference
hsa−miR−4768−3p, rectal
(ESTROGEN = 1; N1 = 108)
1−sided adj pval: 0.184
Frequency
Tumor−Normal Expression Difference

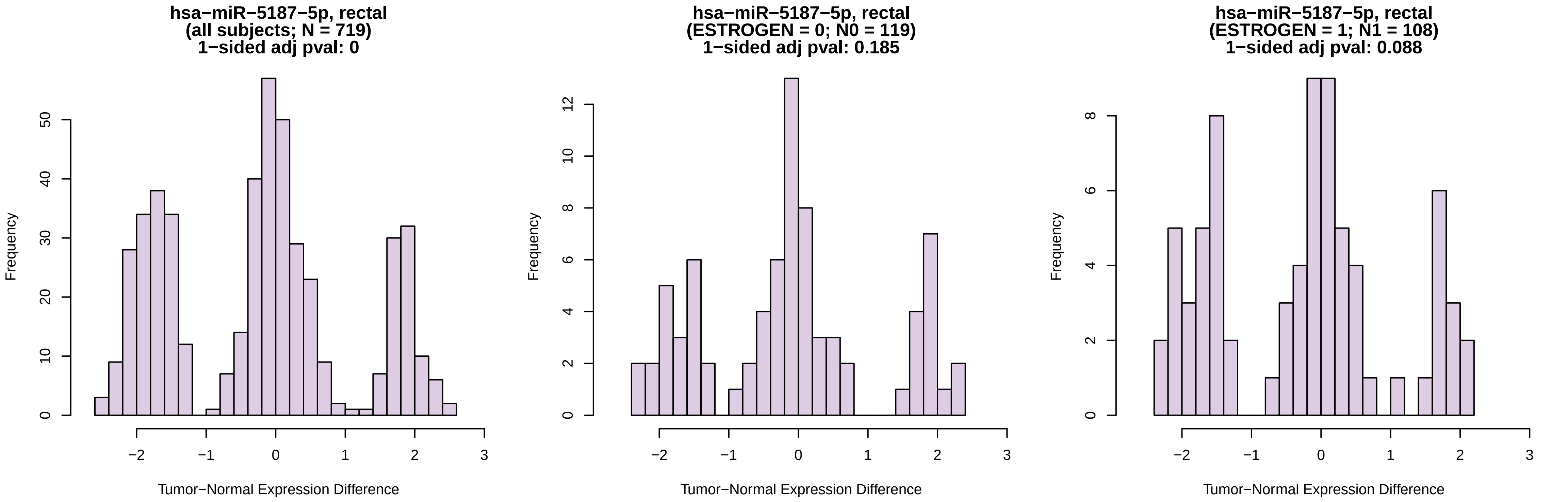
hsa−miR−5187−5p, rectal
(all subjects; N = 719)
1−sided adj pval: 0
Frequency
0
10
20
30
40
50
−2
−1
0
1
2
3
Tumor−Normal Expression Difference
hsa−miR−5187−5p, rectal
(ESTROGEN = 0; N0 = 119)
1−sided adj pval: 0.185
Frequency
0
2
4
6
8
10
12
−2
−1
0
1
2
3
Tumor−Normal Expression Difference
hsa−miR−5187−5p, rectal
(ESTROGEN = 1; N1 = 108)
1−sided adj pval: 0.088
Frequency
0
2
4
6
8
−2
−1
0
1
2
3
Tumor−Normal Expression Difference

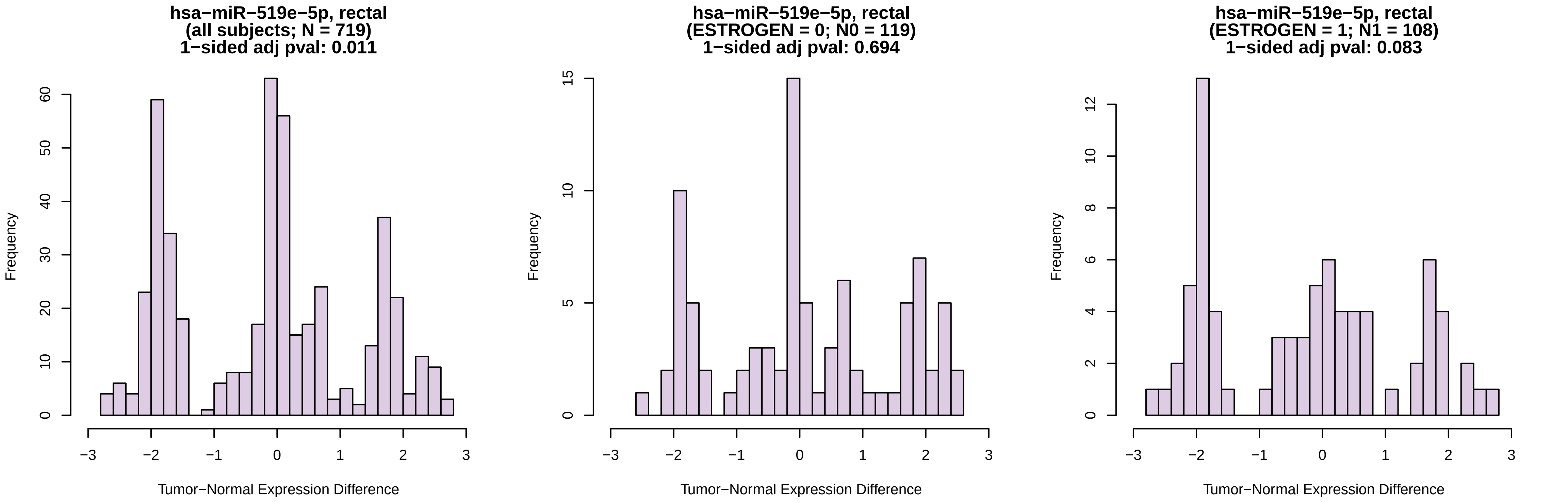
hsa−miR−519e−5p, rectal
(all subjects; N = 719)
1−sided adj pval: 0.011
Frequency
Tumor−Normal Expression Difference
hsa−miR−519e−5p, rectal
(ESTROGEN = 0; N0 = 119)
1−sided adj pval: 0.694
Frequency
Tumor−Normal Expression Difference
hsa−miR−519e−5p, rectal
(ESTROGEN = 1; N1 = 108)
1−sided adj pval: 0.083
Frequency
Tumor−Normal Expression Difference

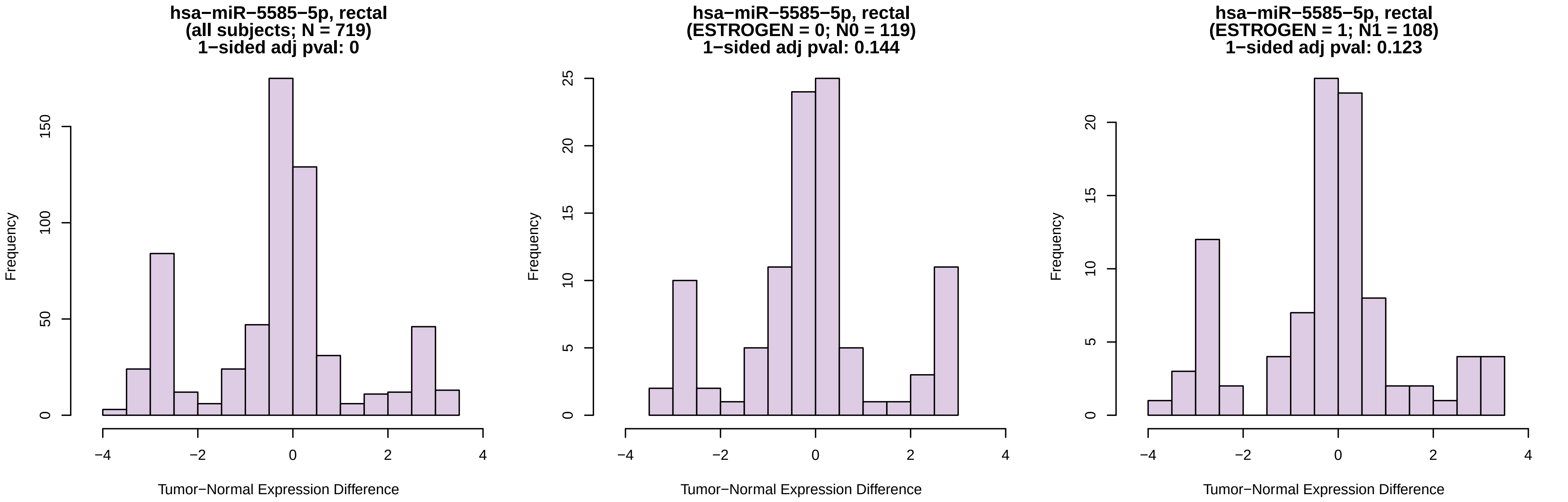
hsa−miR−5585−5p, rectal
(all subjects; N = 719)
1−sided adj pval: 0
hsa−miR−5585−5p, rectal
(ESTROGEN = 0; N0 = 119)
1−sided adj pval: 0.144
hsa−miR−5585−5p, rectal
(ESTROGEN = 1; N1 = 108)
1−sided adj pval: 0.123
Frequency
Tumor−Normal Expression Difference

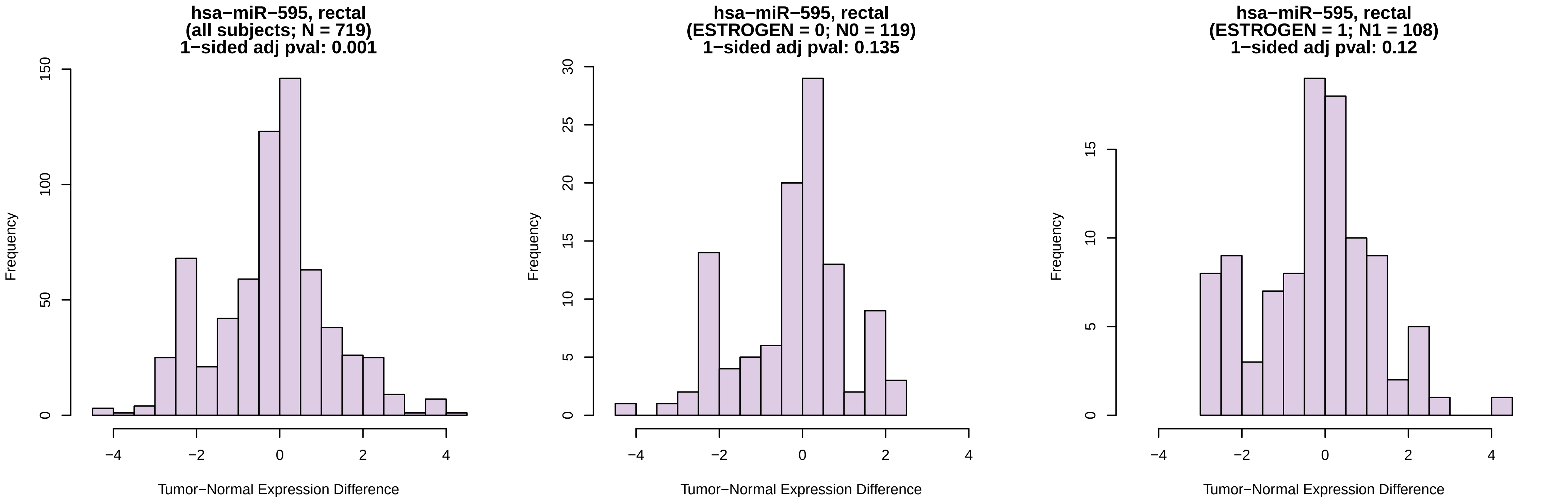

hsa−miR−595, rectal
(all subjects; N = 719)
1−sided adj pval: 0.001
Frequency
0
50
100
150
−4
−2
0
2
4
Tumor−Normal Expression Difference
hsa−miR−595, rectal
(ESTROGEN = 0; N0 = 119)
1−sided adj pval: 0.135
Frequency
0
5
10
15
20
25
30
−4
−2
0
2
4
Tumor−Normal Expression Difference
hsa−miR−595, rectal
(ESTROGEN = 1; N1 = 108)
1−sided adj pval: 0.12
Frequency
0
5
10
15
−4
−2
0
2
4
Tumor−Normal Expression Difference

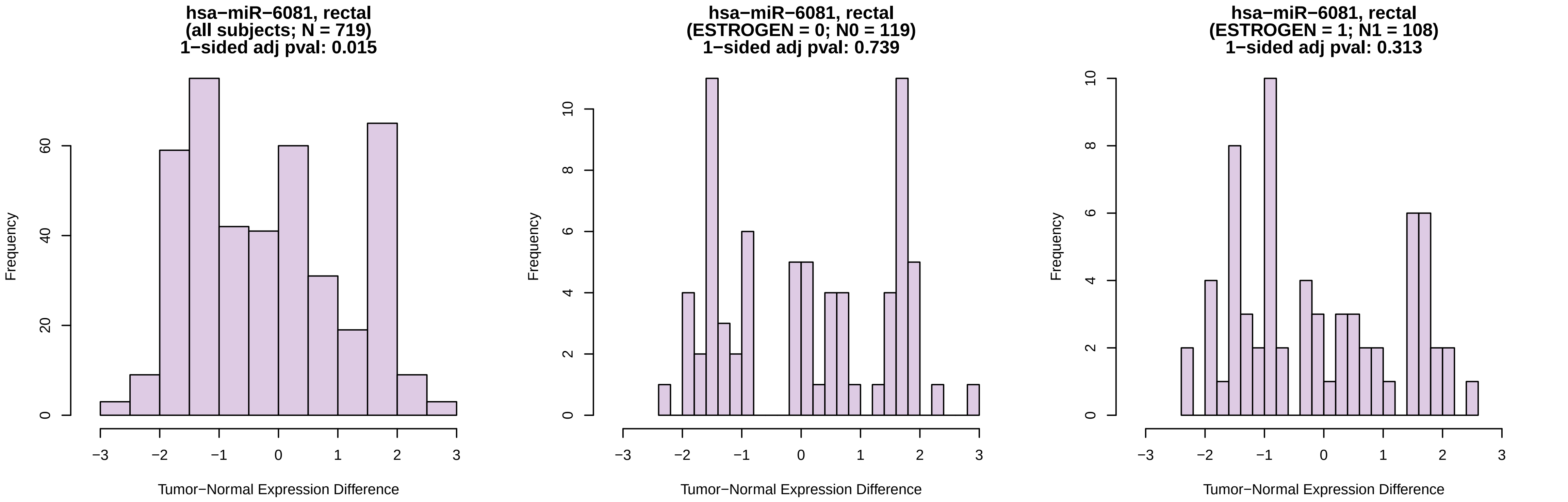
hsa−miR−6081, rectal
(all subjects; N = 719)
1−sided adj pval: 0.015
hsa−miR−6081, rectal
(ESTROGEN = 0; N0 = 119)
1−sided adj pval: 0.739
hsa−miR−6081, rectal
(ESTROGEN = 1; N1 = 108)
1−sided adj pval: 0.313
Frequency
Tumor−Normal Expression Difference

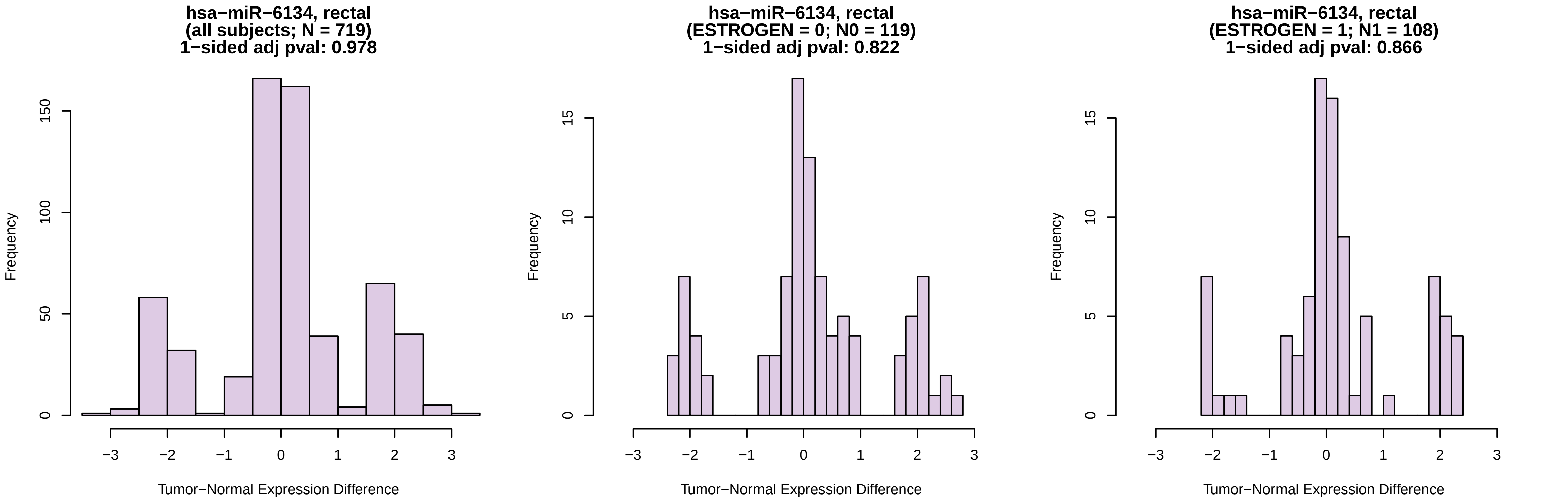
hsa−miR−6134, rectal
(all subjects; N = 719)
1−sided adj pval: 0.978
hsa−miR−6134, rectal
(ESTROGEN = 0; N0 = 119)
1−sided adj pval: 0.822
hsa−miR−6134, rectal
(ESTROGEN = 1; N1 = 108)
1−sided adj pval: 0.866
Frequency
Tumor−Normal Expression Difference
0
50
100
150
5
10
15
−3
−2
−1
0
1
2
3

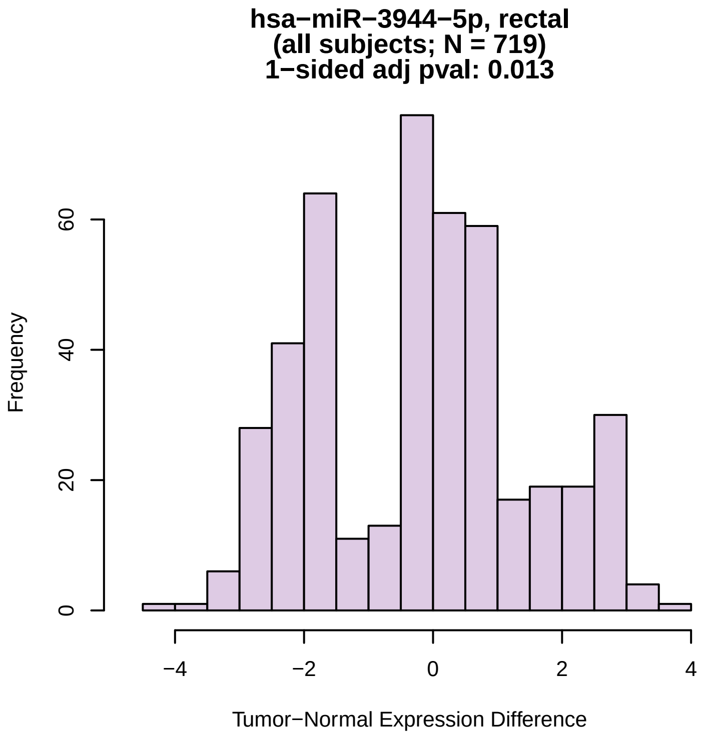
hsa−miR−3944−5p, rectal
(all subjects; N = 719)
1−sided adj pval: 0.013
Frequency
Tumor−Normal Expression Difference

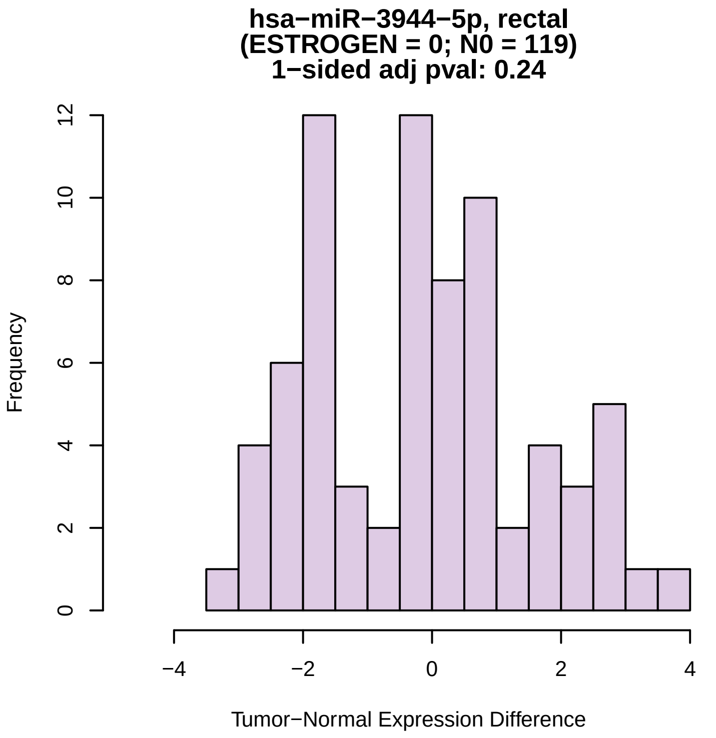
hsa−miR−3944−5p, rectal
(ESTROGEN = 0; N0 = 119)
1−sided adj pval: 0.24
Frequency
Tumor−Normal Expression Difference

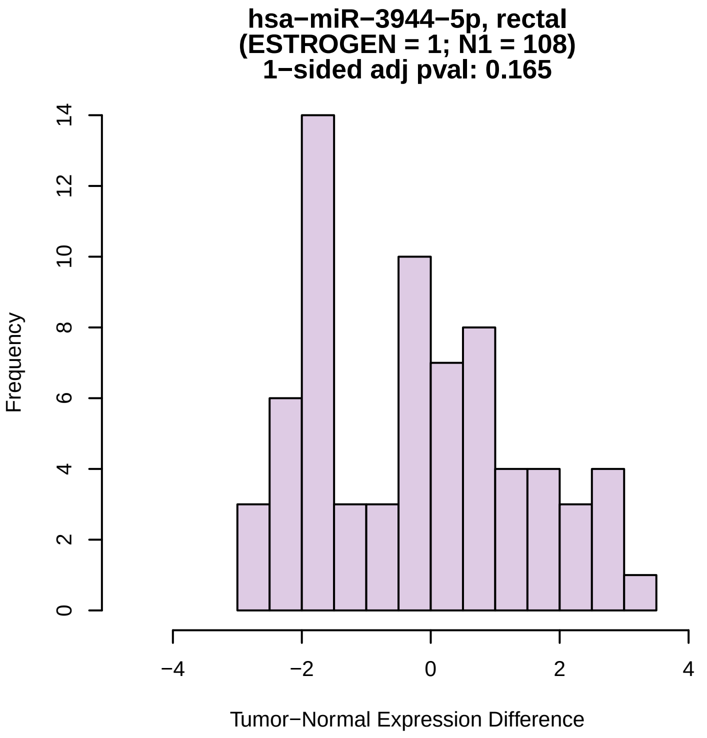
hsa−miR−3944−5p, rectal
(ESTROGEN = 1; N1 = 108)
1−sided adj pval: 0.165
Frequency
Tumor−Normal Expression Difference

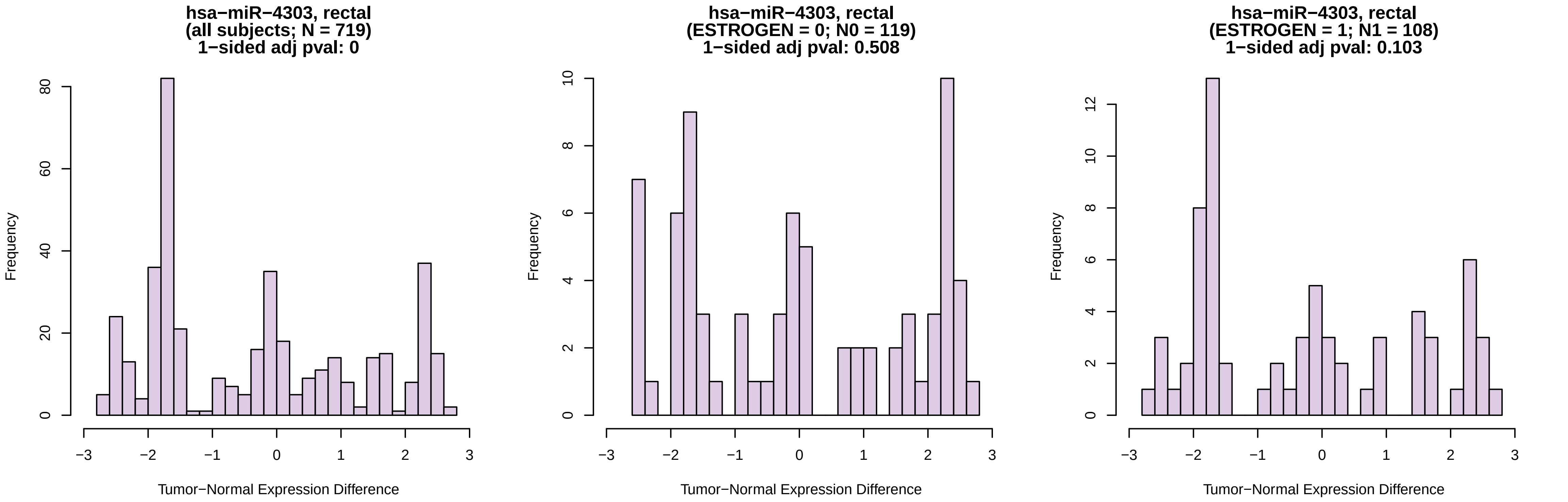

hsa−miR−4303, rectal
(all subjects; N = 719)
1−sided adj pval: 0
hsa−miR−4303, rectal
(ESTROGEN = 0; N0 = 119)
1−sided adj pval: 0.508
hsa−miR−4303, rectal
(ESTROGEN = 1; N1 = 108)
1−sided adj pval: 0.103
Frequency
Tumor−Normal Expression Difference

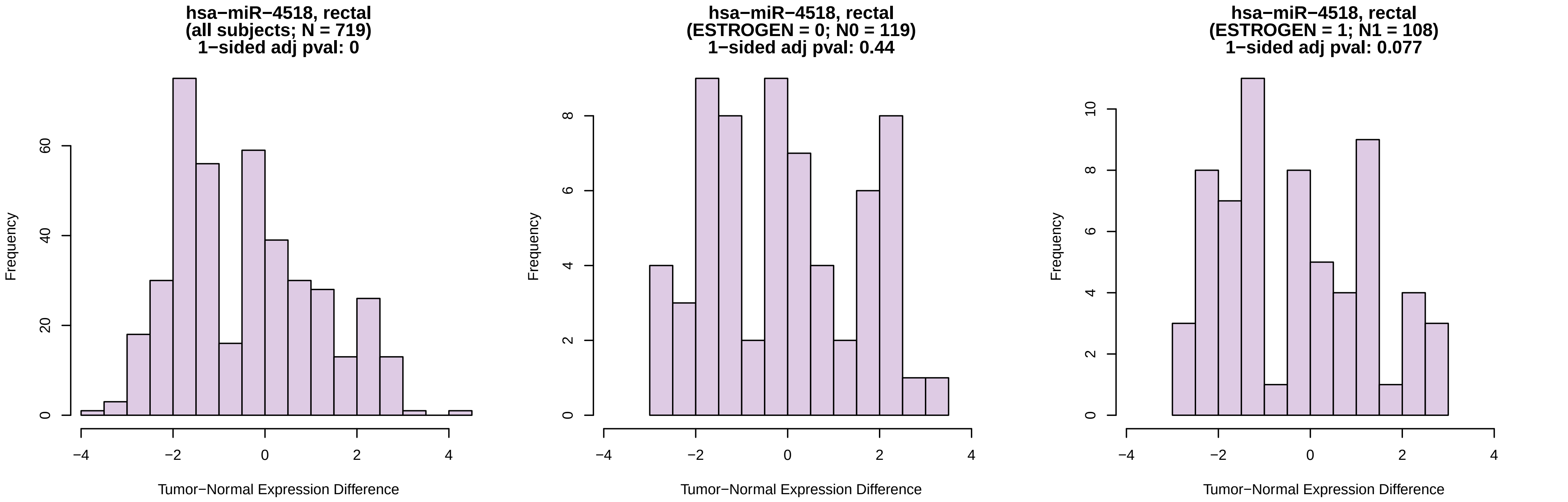
hsa−miR−4518, rectal
(all subjects; N = 719)
1−sided adj pval: 0
Frequency
0
20
40
60
−4
−2
0
2
4
Tumor−Normal Expression Difference
hsa−miR−4518, rectal
(ESTROGEN = 0; N0 = 119)
1−sided adj pval: 0.44
Frequency
0
2
4
6
8
−4
−2
0
2
4
Tumor−Normal Expression Difference
hsa−miR−4518, rectal
(ESTROGEN = 1; N1 = 108)
1−sided adj pval: 0.077
Frequency
0
2
4
6
8
10
−4
−2
0
2
4
Tumor−Normal Expression Difference

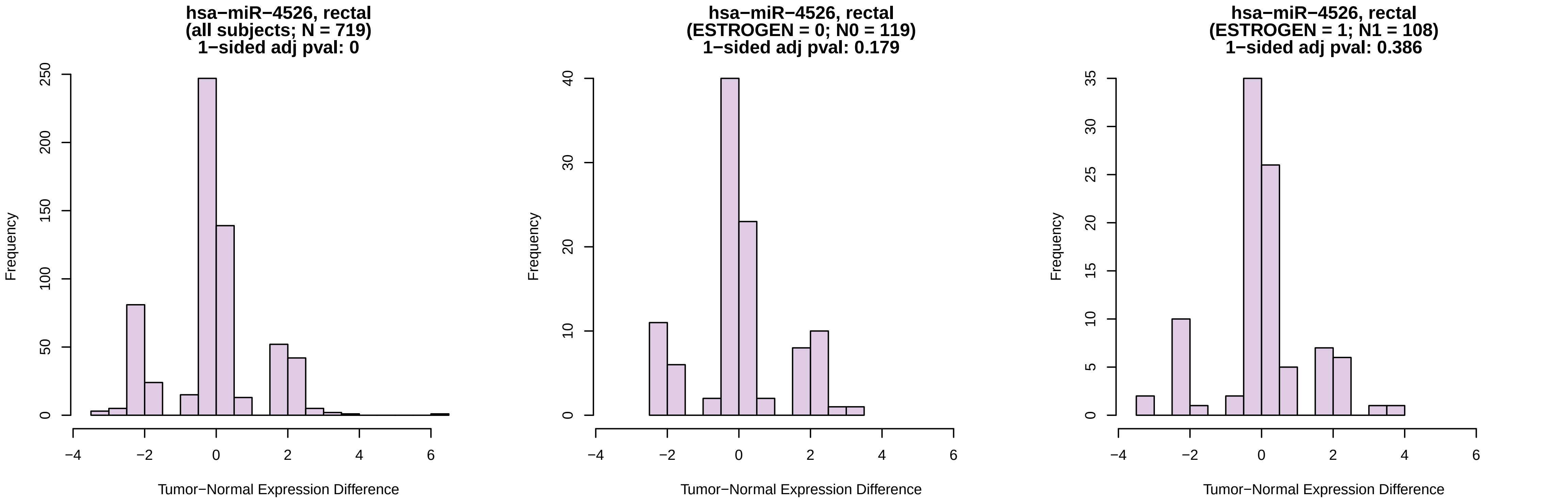

hsa−miR−4526, rectal
(all subjects; N = 719)
1−sided adj pval: 0
Frequency
Tumor−Normal Expression Difference
hsa−miR−4526, rectal
(ESTROGEN = 0; N0 = 119)
1−sided adj pval: 0.179
Frequency
Tumor−Normal Expression Difference
hsa−miR−4526, rectal
(ESTROGEN = 1; N1 = 108)
1−sided adj pval: 0.386
Frequency
Tumor−Normal Expression Difference

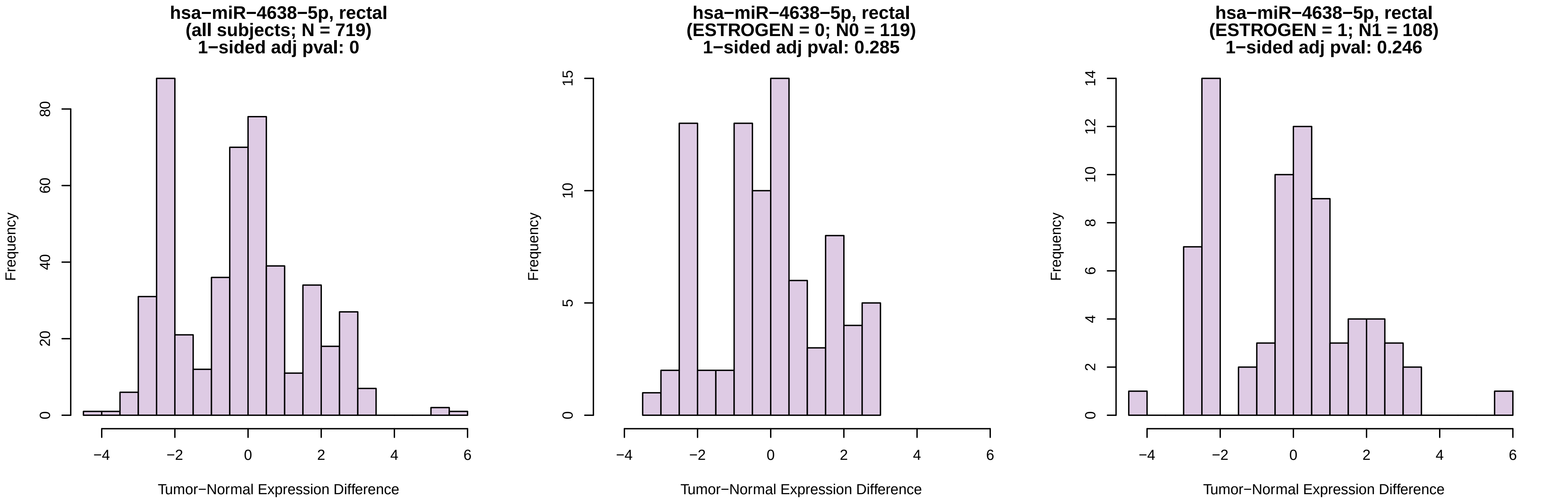
hsa−miR−4638−5p, rectal
(all subjects; N = 719)
1−sided adj pval: 0
Frequency
Tumor−Normal Expression Difference
hsa−miR−4638−5p, rectal
(ESTROGEN = 0; N0 = 119)
1−sided adj pval: 0.285
Frequency
Tumor−Normal Expression Difference
hsa−miR−4638−5p, rectal
(ESTROGEN = 1; N1 = 108)
1−sided adj pval: 0.246
Frequency
Tumor−Normal Expression Difference

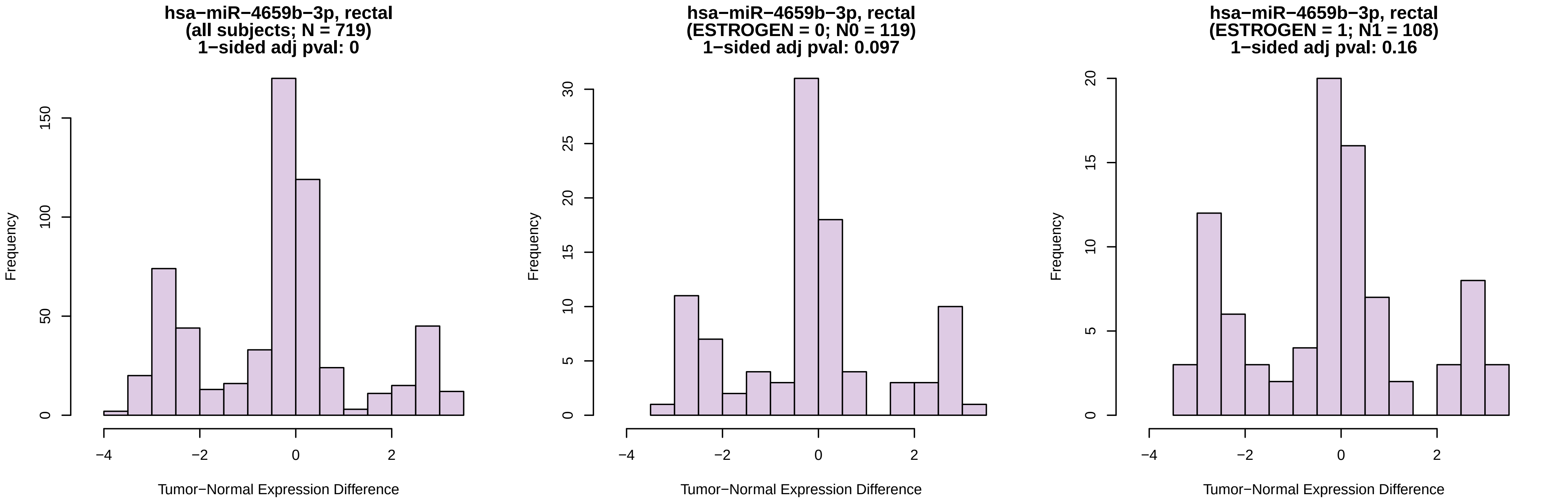

hsa−miR−4659b−3p, rectal
(all subjects; N = 719)
1−sided adj pval: 0
hsa−miR−4659b−3p, rectal
(ESTROGEN = 0; N0 = 119)
1−sided adj pval: 0.097
hsa−miR−4659b−3p, rectal
(ESTROGEN = 1; N1 = 108)
1−sided adj pval: 0.16
Frequency
Tumor−Normal Expression Difference

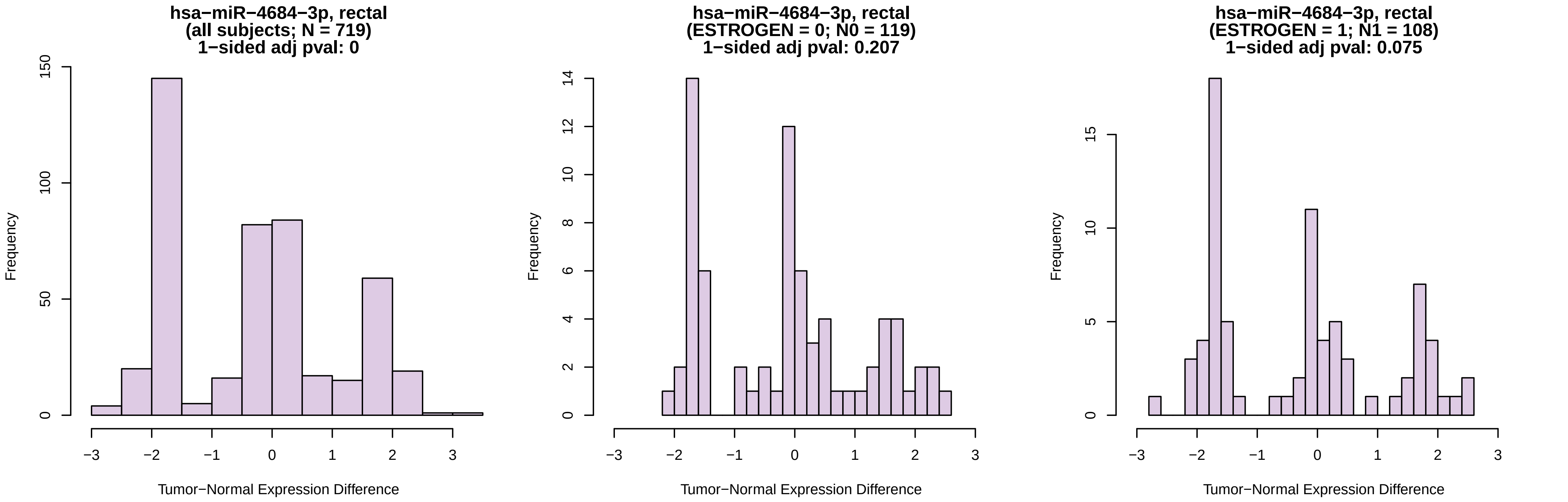
hsa−miR−4684−3p, rectal
(all subjects; N = 719)
1−sided adj pval: 0
Frequency
Tumor−Normal Expression Difference
hsa−miR−4684−3p, rectal
(ESTROGEN = 0; N0 = 119)
1−sided adj pval: 0.207
Frequency
Tumor−Normal Expression Difference
hsa−miR−4684−3p, rectal
(ESTROGEN = 1; N1 = 108)
1−sided adj pval: 0.075
Frequency
Tumor−Normal Expression Difference

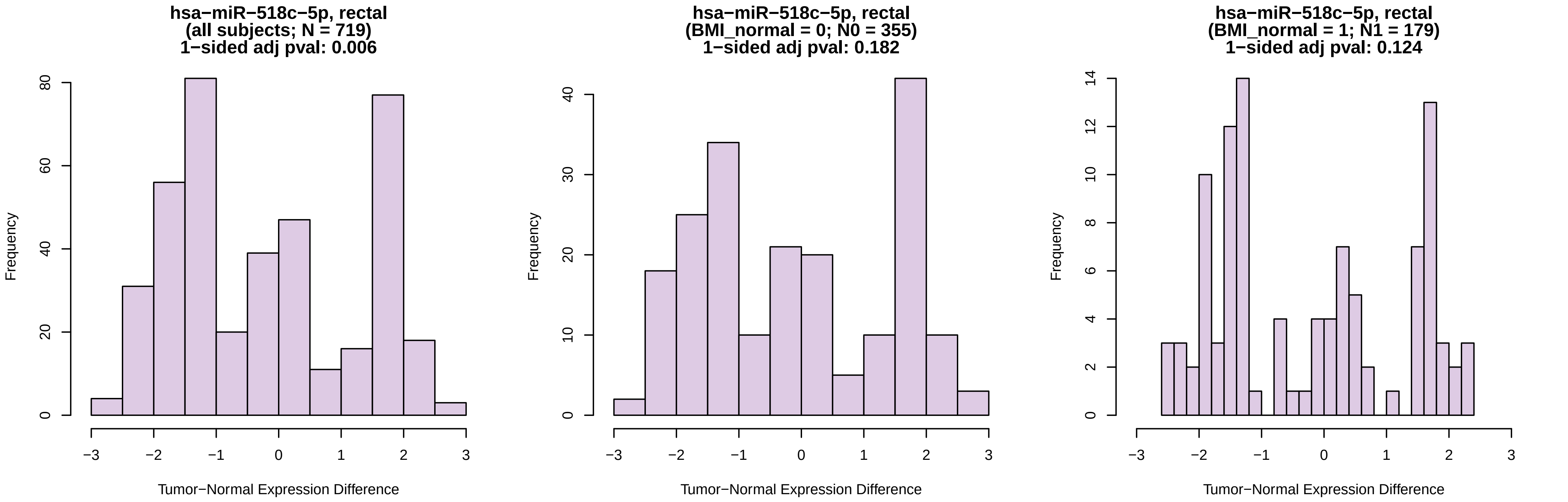

hsa−miR−518c−5p, rectal
(all subjects; N = 719)
1−sided adj pval: 0.006
hsa−miR−518c−5p, rectal
(BMI_normal = 0; N0 = 355)
1−sided adj pval: 0.182
hsa−miR−518c−5p, rectal
(BMI_normal = 1; N1 = 179)
1−sided adj pval: 0.124
Frequency
Tumor−Normal Expression Difference

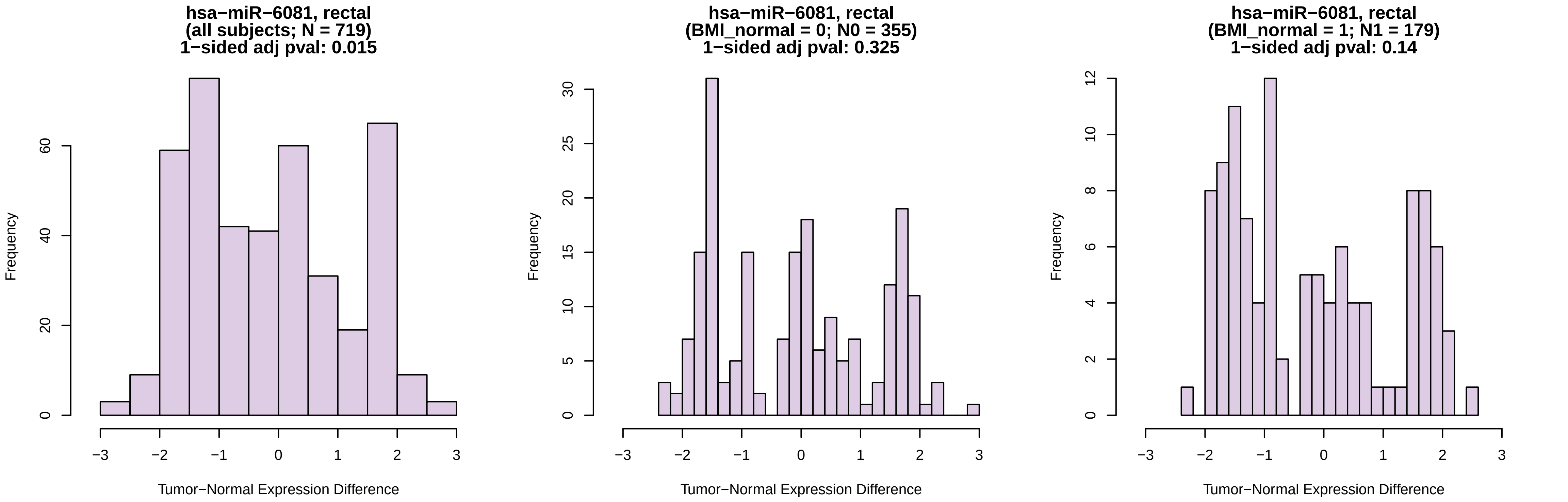

hsa−miR−6081, rectal
(all subjects; N = 719)
1−sided adj pval: 0.015
Frequency
0
20
40
60
−3
−2
−1
0
1
2
3
Tumor−Normal Expression Difference
hsa−miR−6081, rectal
(BMI_normal = 0; N0 = 355)
1−sided adj pval: 0.325
Frequency
0
5
10
15
20
25
30
−3
−2
−1
0
1
2
3
Tumor−Normal Expression Difference
hsa−miR−6081, rectal
(BMI_normal = 1; N1 = 179)
1−sided adj pval: 0.14
Frequency
0
2
4
6
8
10
12
−3
−2
−1
0
1
2
3
Tumor−Normal Expression Difference

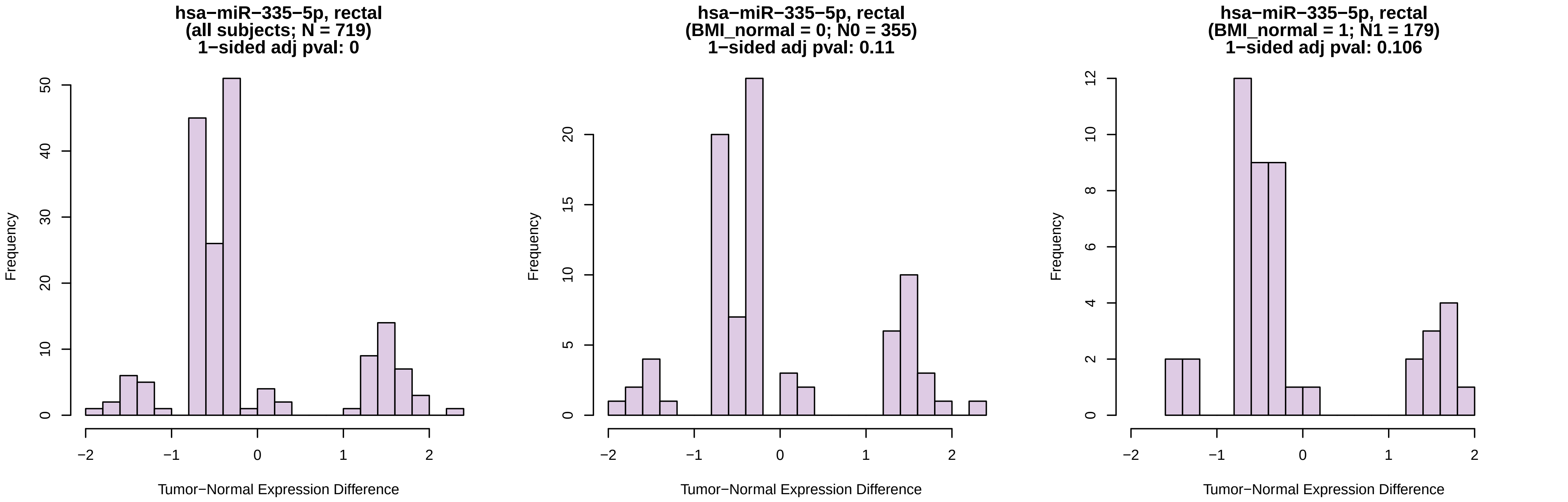

hsa−miR−335−5p, rectal
(all subjects; N = 719)
1−sided adj pval: 0
Frequency
Tumor−Normal Expression Difference
hsa−miR−335−5p, rectal
(BMI_normal = 0; N0 = 355)
1−sided adj pval: 0.11
Frequency
Tumor−Normal Expression Difference
hsa−miR−335−5p, rectal
(BMI_normal = 1; N1 = 179)
1−sided adj pval: 0.106
Frequency
Tumor−Normal Expression Difference

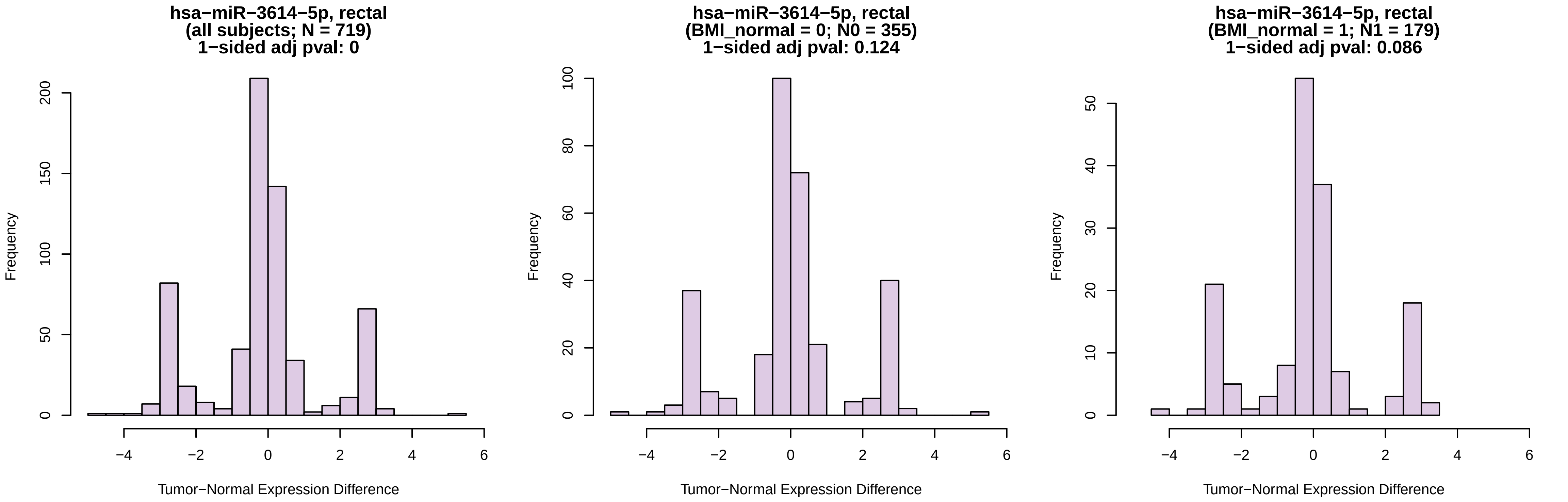
hsa−miR−3614−5p, rectal
(all subjects; N = 719)
1−sided adj pval: 0
Frequency
Tumor−Normal Expression Difference
hsa−miR−3614−5p, rectal
(BMI_normal = 0; N0 = 355)
1−sided adj pval: 0.124
Frequency
Tumor−Normal Expression Difference
hsa−miR−3614−5p, rectal
(BMI_normal = 1; N1 = 179)
1−sided adj pval: 0.086
Frequency
Tumor−Normal Expression Difference

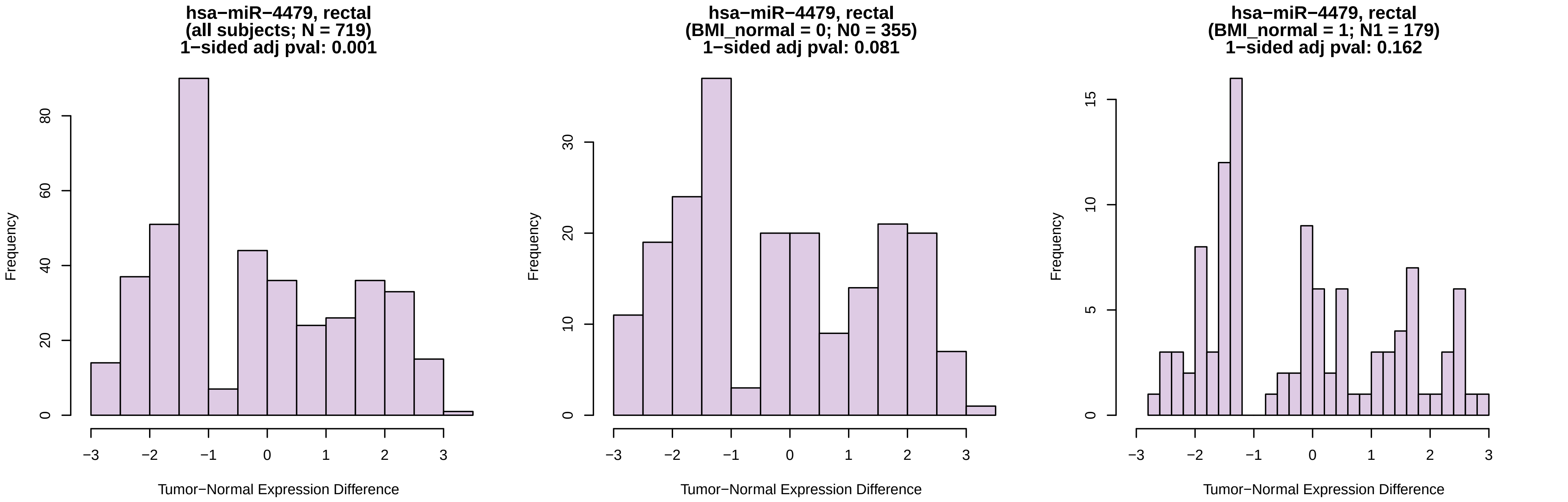
hsa−miR−4479, rectal
(all subjects; N = 719)
1−sided adj pval: 0.001
Frequency
Tumor−Normal Expression Difference
hsa−miR−4479, rectal
(BMI_normal = 0; N0 = 355)
1−sided adj pval: 0.081
Frequency
Tumor−Normal Expression Difference
hsa−miR−4479, rectal
(BMI_normal = 1; N1 = 179)
1−sided adj pval: 0.162
Frequency
Tumor−Normal Expression Difference

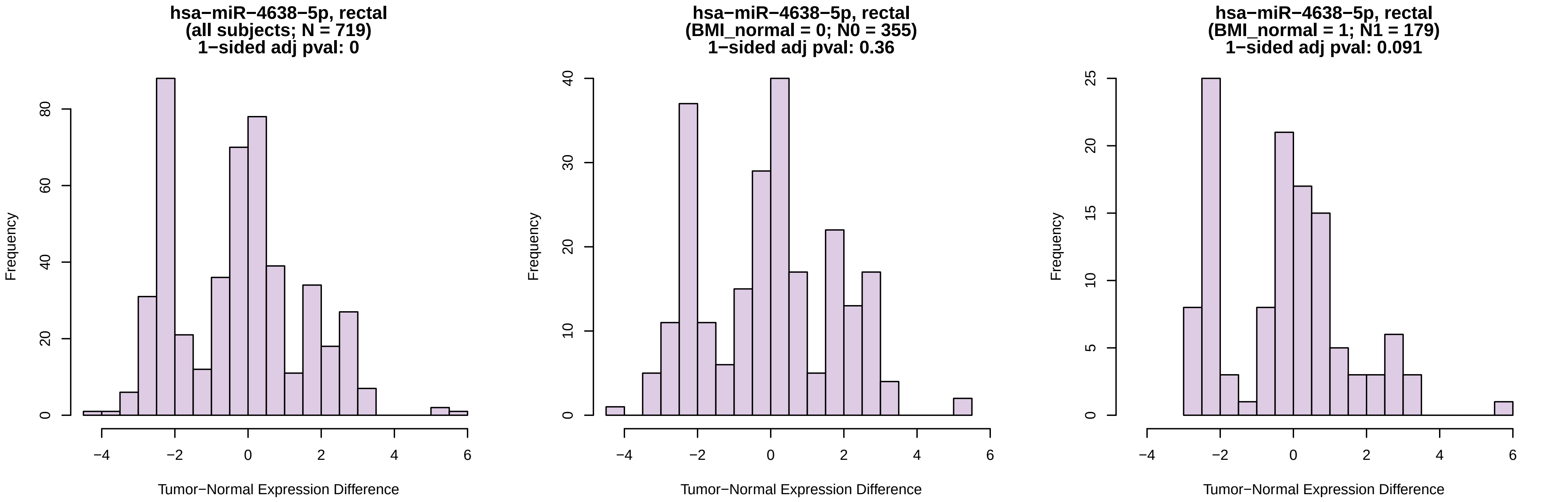
hsa−miR−4638−5p, rectal
(all subjects; N = 719)
1−sided adj pval: 0
Frequency
Tumor−Normal Expression Difference
hsa−miR−4638−5p, rectal
(BMI_normal = 0; N0 = 355)
1−sided adj pval: 0.36
Frequency
Tumor−Normal Expression Difference
hsa−miR−4638−5p, rectal
(BMI_normal = 1; N1 = 179)
1−sided adj pval: 0.091
Frequency
Tumor−Normal Expression Difference

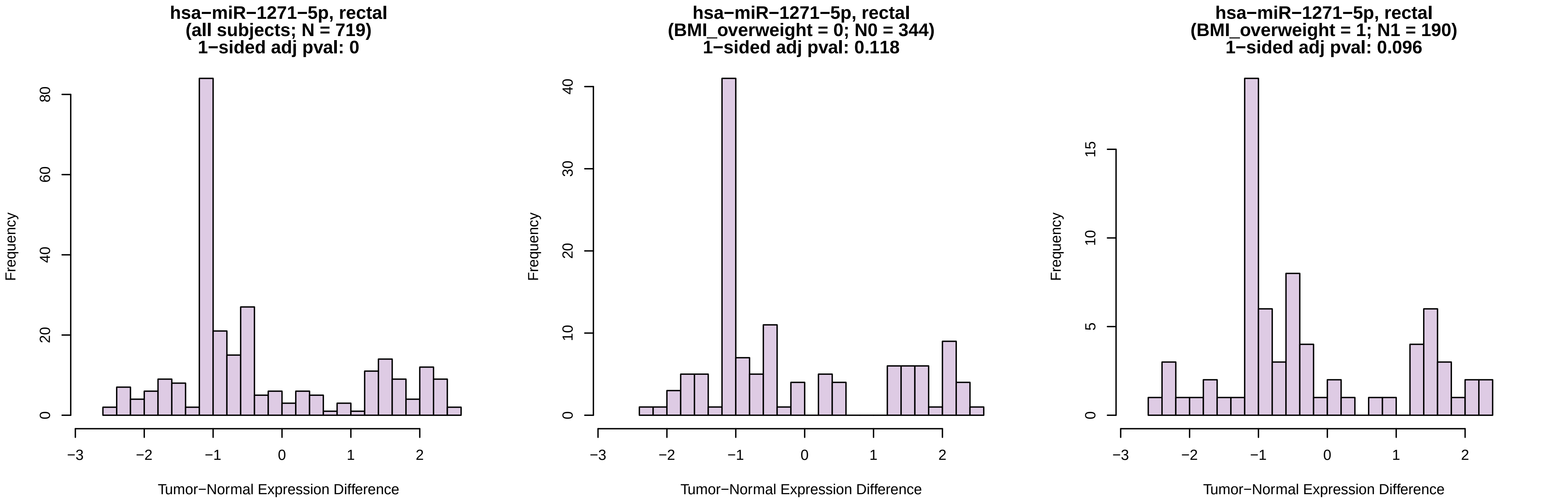

hsa−miR−1271−5p, rectal
(all subjects; N = 719)
1−sided adj pval: 0
Frequency
Tumor−Normal Expression Difference
hsa−miR−1271−5p, rectal
(BMI_overweight = 0; N0 = 344)
1−sided adj pval: 0.118
Frequency
Tumor−Normal Expression Difference
hsa−miR−1271−5p, rectal
(BMI_overweight = 1; N1 = 190)
1−sided adj pval: 0.096
Frequency
Tumor−Normal Expression Difference

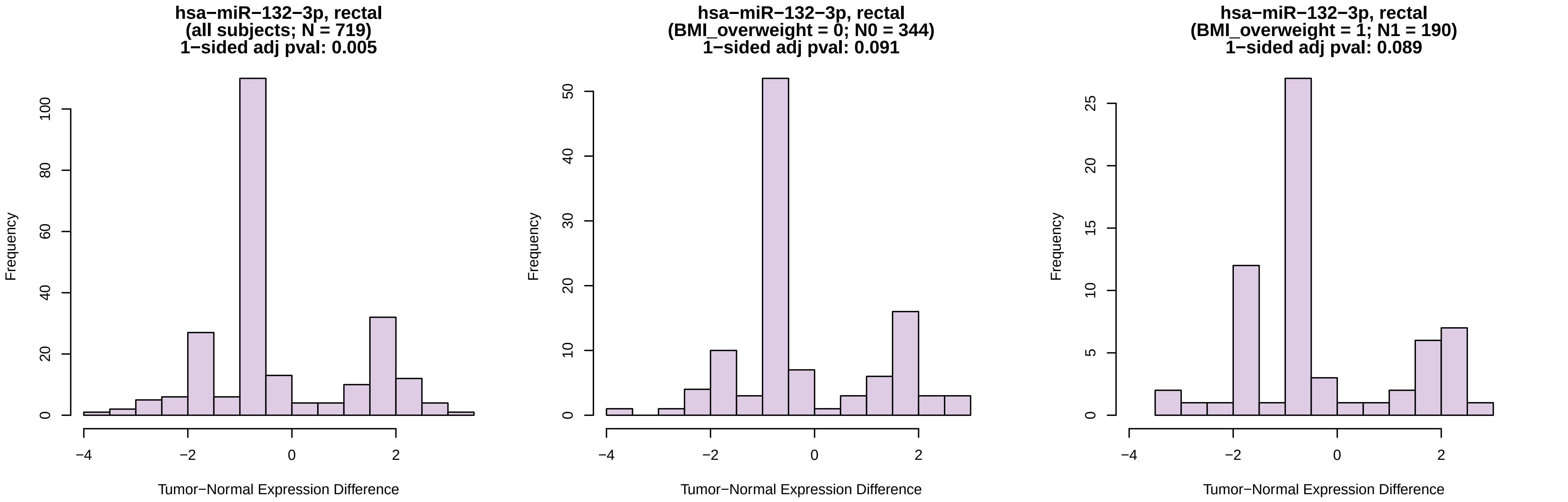
hsa−miR−132−3p, rectal
(all subjects; N = 719)
1−sided adj pval: 0.005
Frequency
Tumor−Normal Expression Difference
hsa−miR−132−3p, rectal
(BMI_overweight = 0; N0 = 344)
1−sided adj pval: 0.091
Frequency
Tumor−Normal Expression Difference
hsa−miR−132−3p, rectal
(BMI_overweight = 1; N1 = 190)
1−sided adj pval: 0.089
Frequency
Tumor−Normal Expression Difference

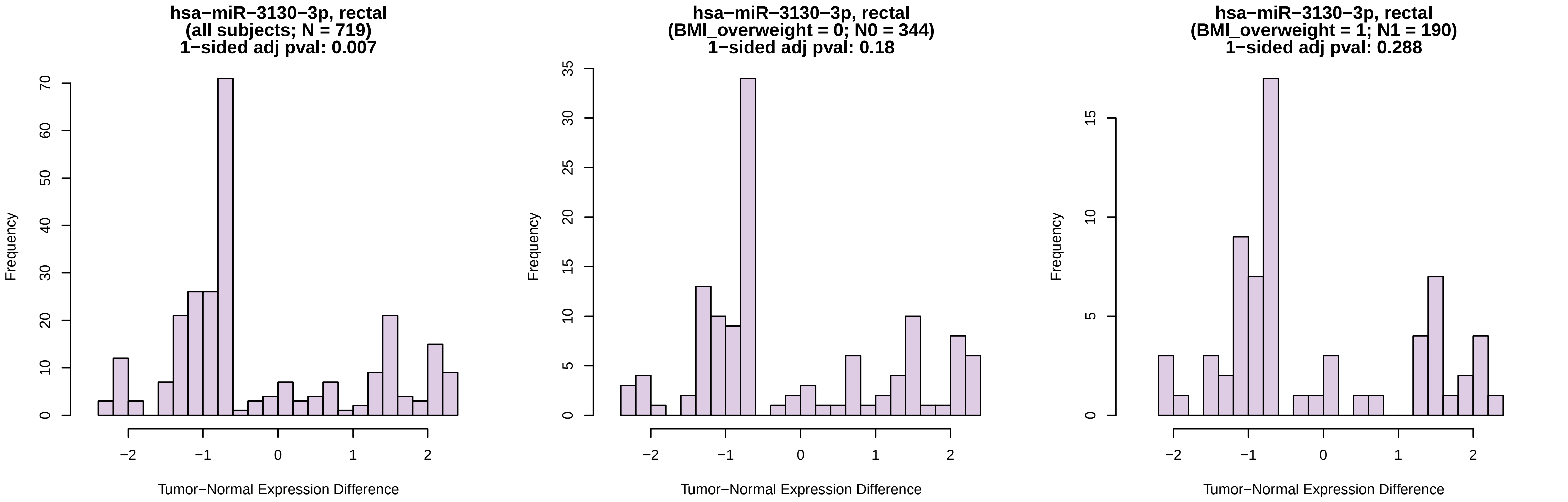

hsa−miR−3130−3p, rectal
(all subjects; N = 719)
1−sided adj pval: 0.007
hsa−miR−3130−3p, rectal
(BMI_overweight = 0; N0 = 344)
1−sided adj pval: 0.18
hsa−miR−3130−3p, rectal
(BMI_overweight = 1; N1 = 190)
1−sided adj pval: 0.288
Frequency
Tumor−Normal Expression Difference

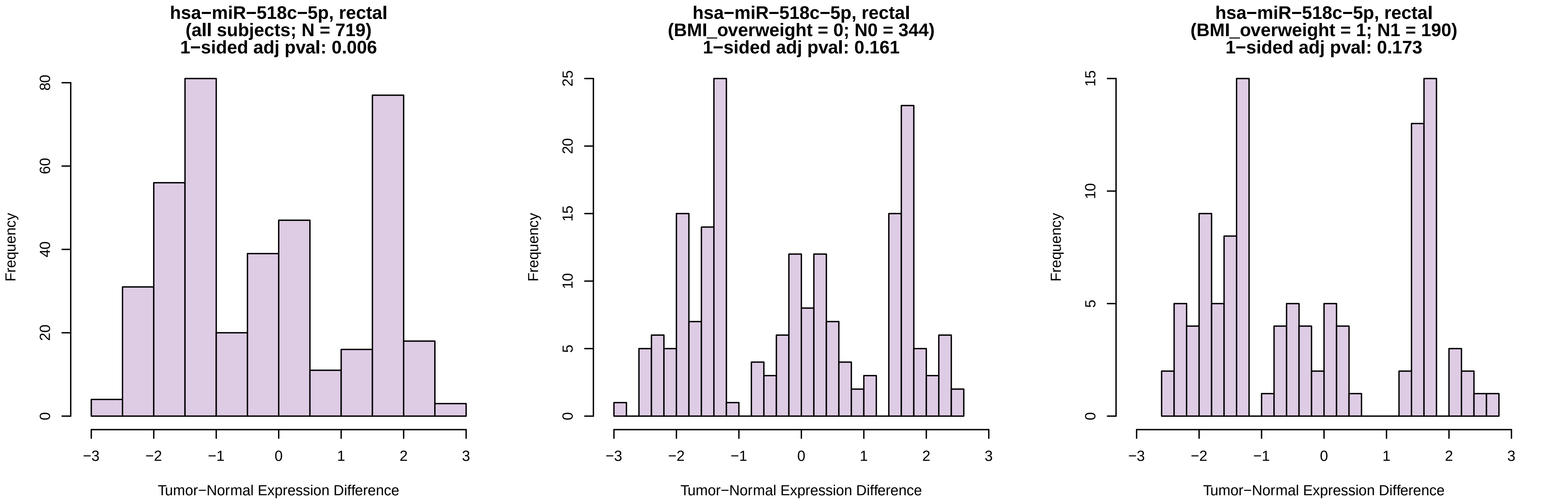

hsa−miR−518c−5p, rectal
(all subjects; N = 719)
1−sided adj pval: 0.006
Frequency
Tumor−Normal Expression Difference
hsa−miR−518c−5p, rectal
(BMI_overweight = 0; N0 = 344)
1−sided adj pval: 0.161
Frequency
Tumor−Normal Expression Difference
hsa−miR−518c−5p, rectal
(BMI_overweight = 1; N1 = 190)
1−sided adj pval: 0.173
Frequency
Tumor−Normal Expression Difference

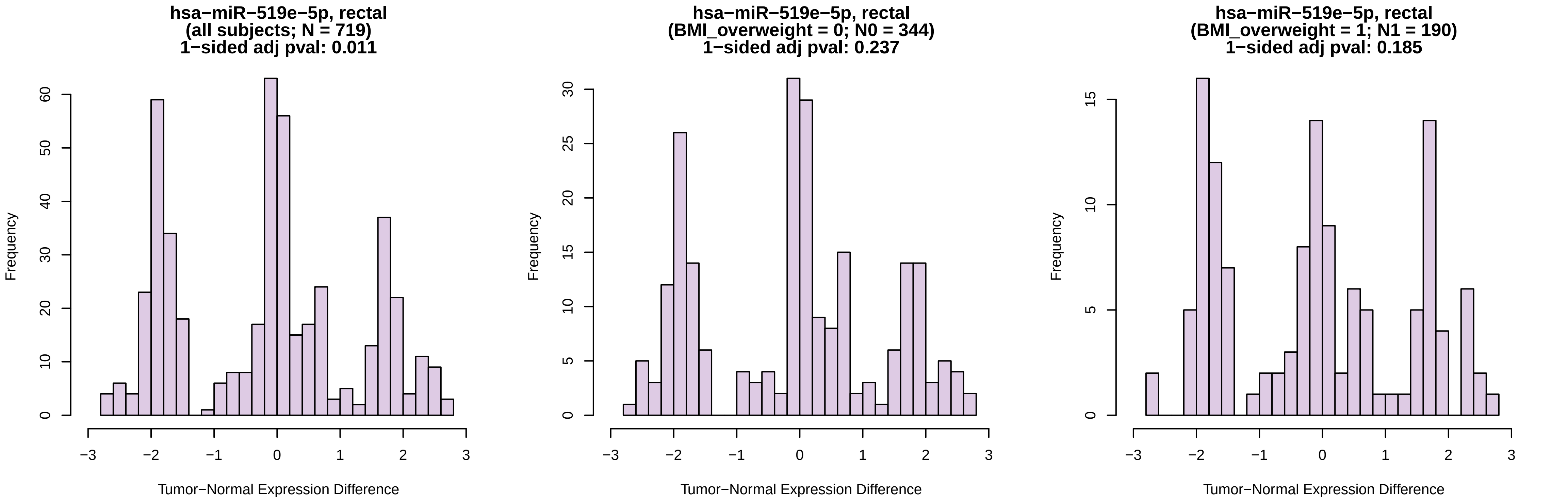
hsa−miR−519e−5p, rectal
(all subjects; N = 719)
1−sided adj pval: 0.011
Frequency
0
10
20
30
40
50
60
−3
−2
−1
0
1
2
3
Tumor−Normal Expression Difference
hsa−miR−519e−5p, rectal
(BMI_overweight = 0; N0 = 344)
1−sided adj pval: 0.237
Frequency
0
5
10
15
20
25
30
−3
−2
−1
0
1
2
3
Tumor−Normal Expression Difference
hsa−miR−519e−5p, rectal
(BMI_overweight = 1; N1 = 190)
1−sided adj pval: 0.185
Frequency
0
5
10
15
−3
−2
−1
0
1
2
3
Tumor−Normal Expression Difference

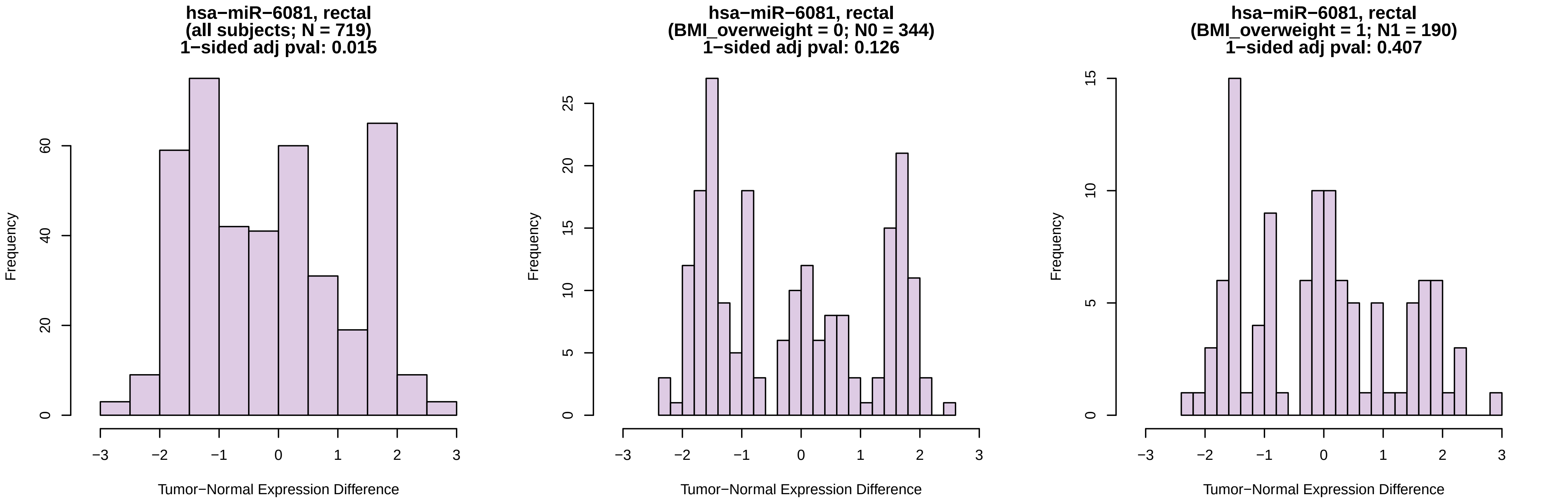

hsa−miR−6081, rectal
(all subjects; N = 719)
1−sided adj pval: 0.015
hsa−miR−6081, rectal
(BMI_overweight = 0; N0 = 344)
1−sided adj pval: 0.126
hsa−miR−6081, rectal
(BMI_overweight = 1; N1 = 190)
1−sided adj pval: 0.407
Frequency
Tumor−Normal Expression Difference

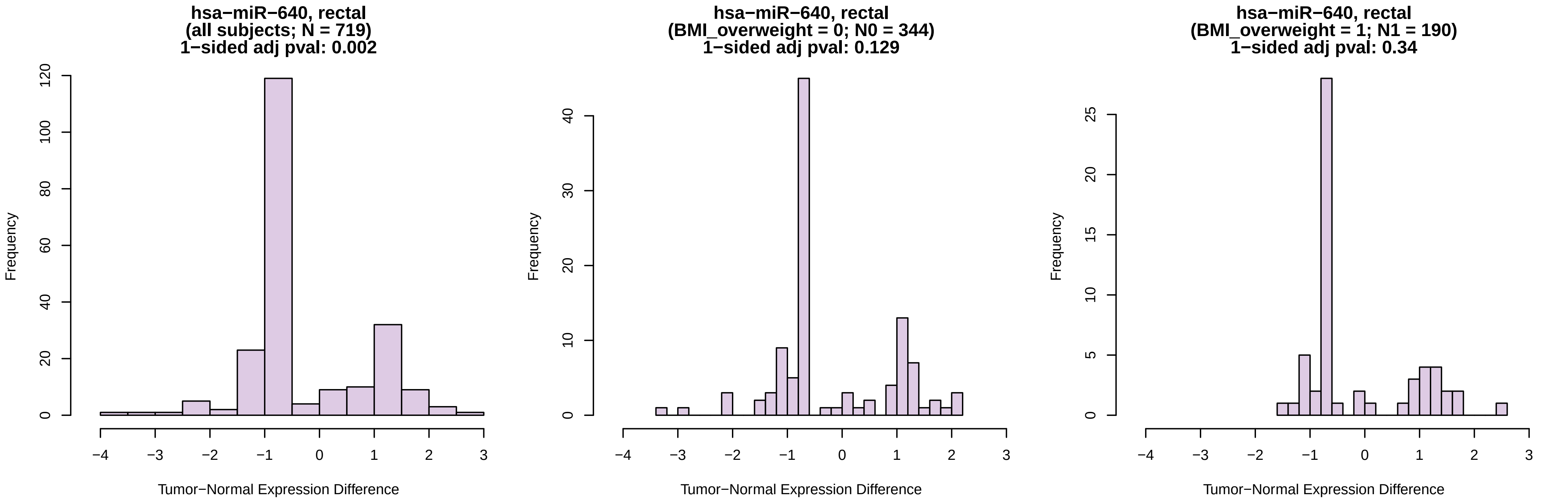
hsa−miR−640, rectal
(all subjects; N = 719)
1−sided adj pval: 0.002
Frequency
Tumor−Normal Expression Difference
hsa−miR−640, rectal
(BMI_overweight = 0; N0 = 344)
1−sided adj pval: 0.129
Frequency
Tumor−Normal Expression Difference
hsa−miR−640, rectal
(BMI_overweight = 1; N1 = 190)
1−sided adj pval: 0.34
Frequency
Tumor−Normal Expression Difference

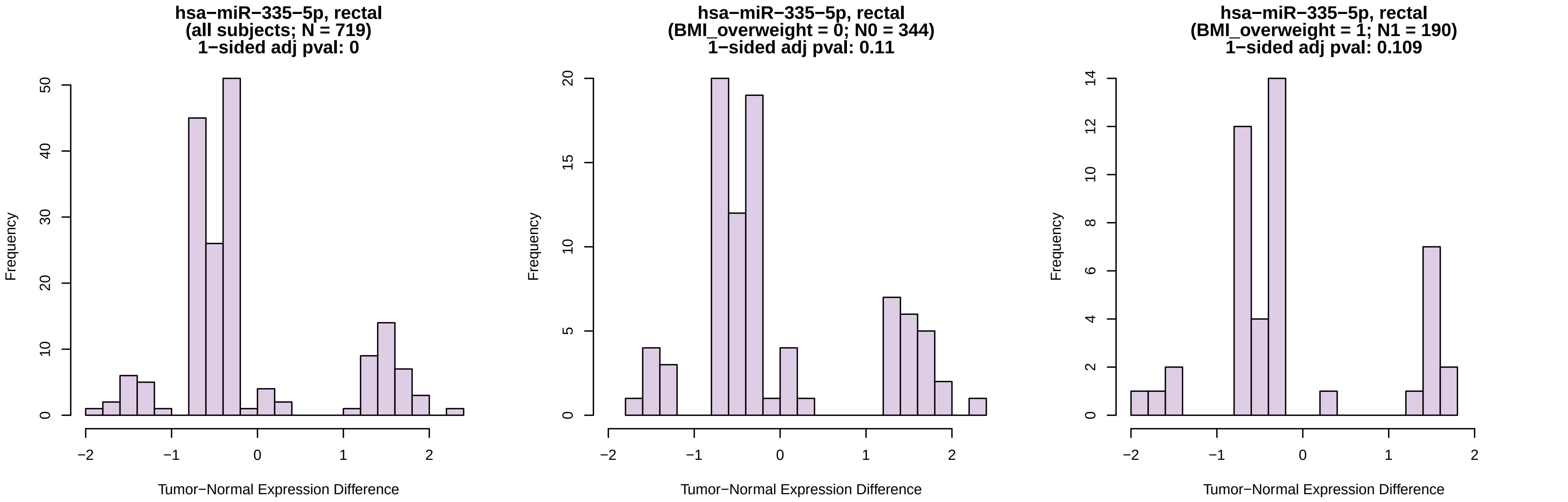

hsa−miR−335−5p, rectal
(all subjects; N = 719)
1−sided adj pval: 0
Frequency
Tumor−Normal Expression Difference
hsa−miR−335−5p, rectal
(BMI_overweight = 0; N0 = 344)
1−sided adj pval: 0.11
Frequency
Tumor−Normal Expression Difference
hsa−miR−335−5p, rectal
(BMI_overweight = 1; N1 = 190)
1−sided adj pval: 0.109
Frequency
Tumor−Normal Expression Difference

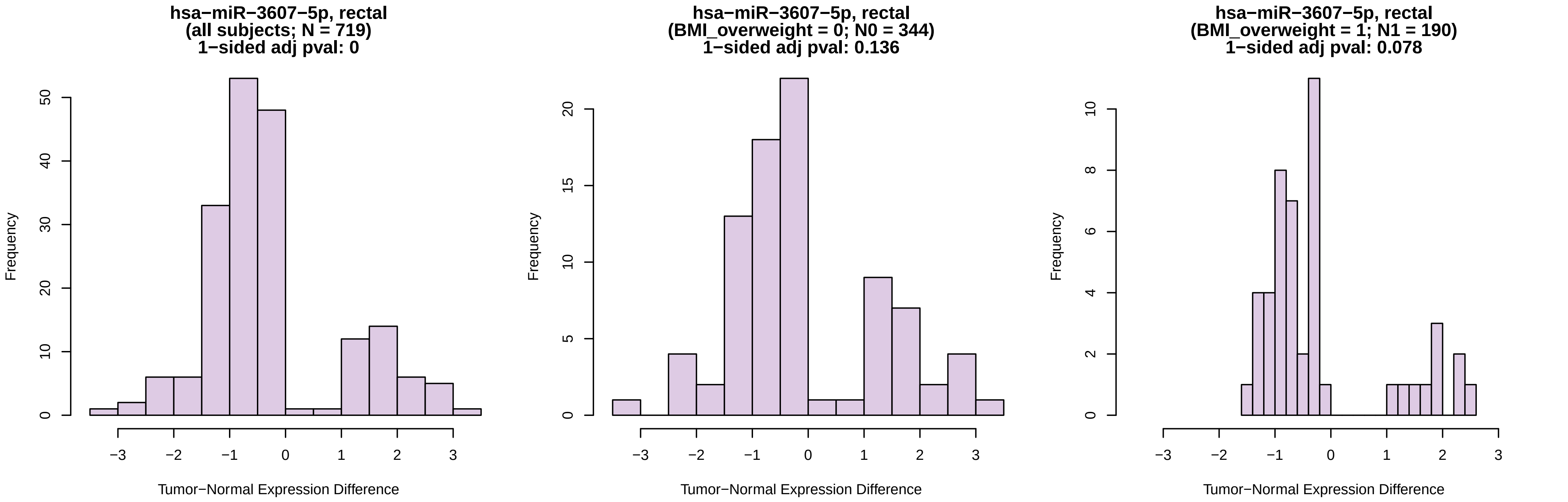

hsa−miR−3607−5p, rectal
(all subjects; N = 719)
1−sided adj pval: 0
hsa−miR−3607−5p, rectal
(BMI_overweight = 0; N0 = 344)
1−sided adj pval: 0.136
hsa−miR−3607−5p, rectal
(BMI_overweight = 1; N1 = 190)
1−sided adj pval: 0.078
Frequency
Tumor−Normal Expression Difference

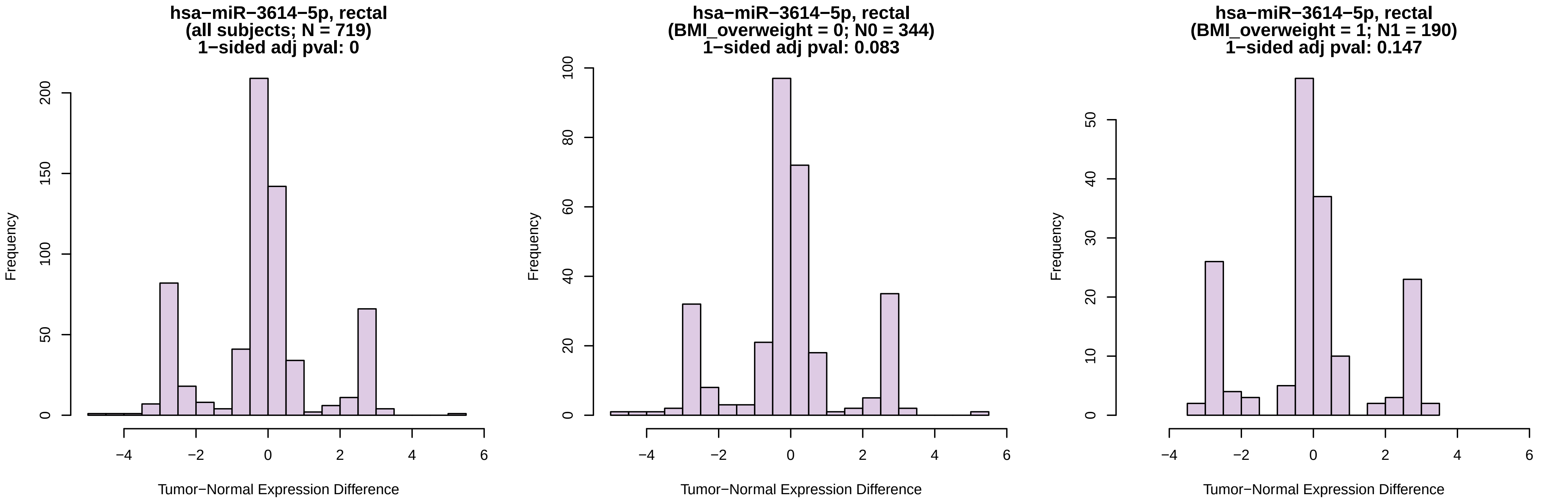
hsa−miR−3614−5p, rectal
(all subjects; N = 719)
1−sided adj pval: 0
Frequency
Tumor−Normal Expression Difference
hsa−miR−3614−5p, rectal
(BMI_overweight = 0; N0 = 344)
1−sided adj pval: 0.083
Frequency
Tumor−Normal Expression Difference
hsa−miR−3614−5p, rectal
(BMI_overweight = 1; N1 = 190)
1−sided adj pval: 0.147
Frequency
Tumor−Normal Expression Difference

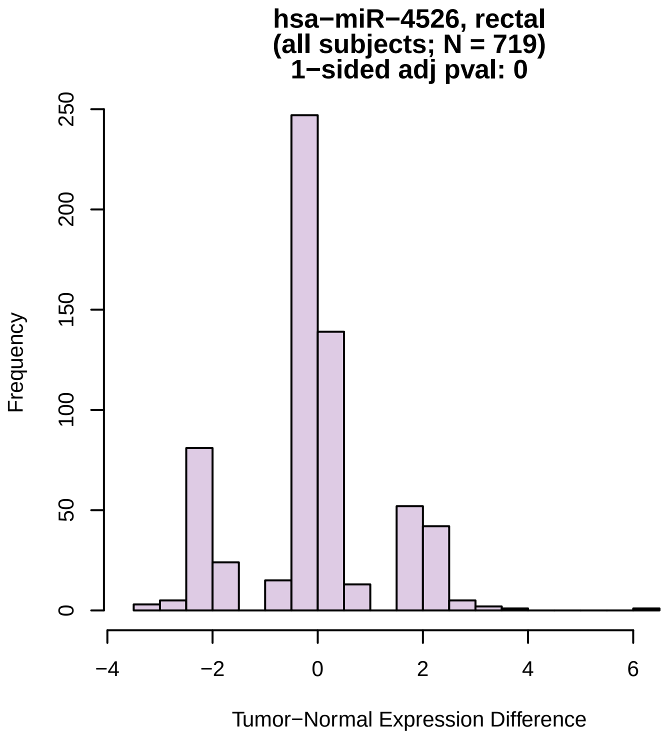
hsa−miR−4526, rectal
(all subjects; N = 719)
1−sided adj pval: 0
Frequency
0
50
100
150
200
250
−4
−2
0
2
4
6
Tumor−Normal Expression Difference

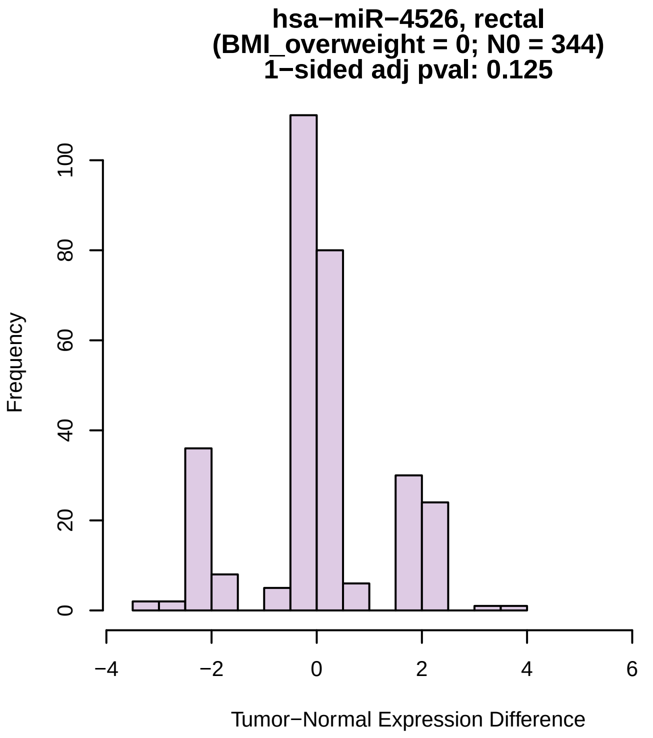
hsa−miR−4526, rectal
(BMI_overweight = 0; N0 = 344)
1−sided adj pval: 0.125
Frequency
0
20
40
60
80
100
−4
−2
0
2
4
6
Tumor−Normal Expression Difference

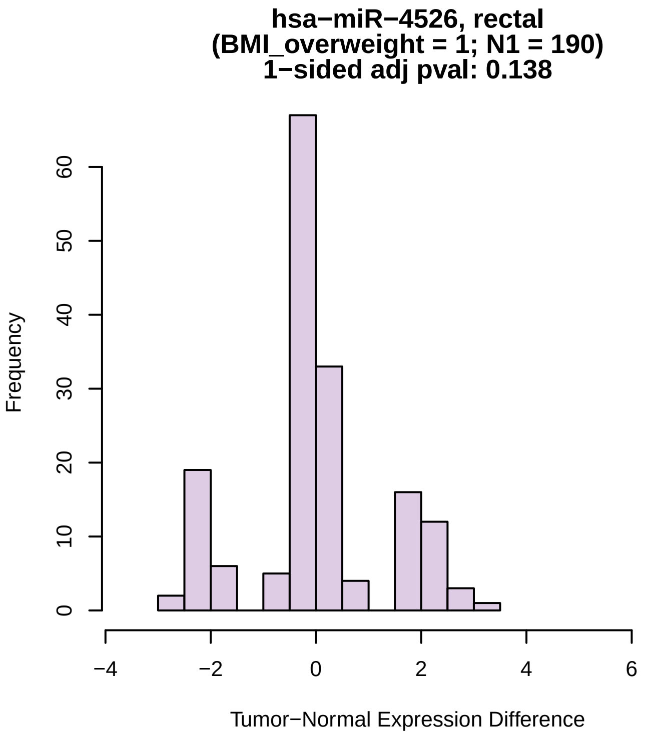
hsa−miR−4526, rectal
(BMI_overweight = 1; N1 = 190)
1−sided adj pval: 0.138
Frequency
0
10
20
30
40
50
60
−4
−2
0
2
4
6
Tumor−Normal Expression Difference

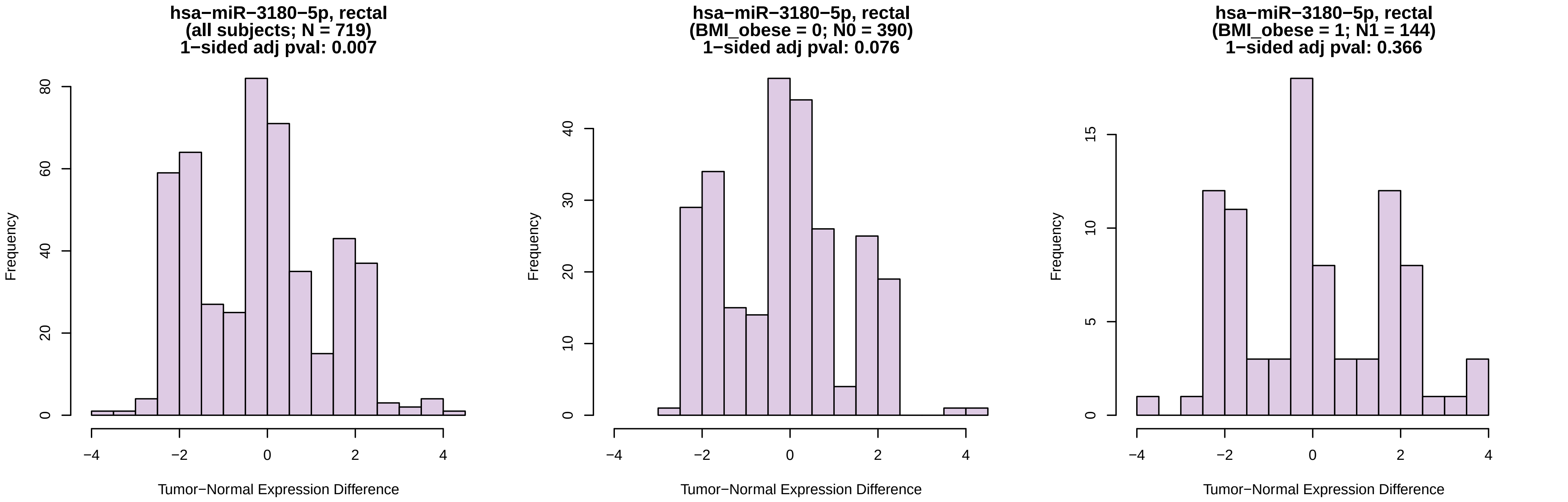
hsa−miR−3180−5p, rectal
(all subjects; N = 719)
1−sided adj pval: 0.007
hsa−miR−3180−5p, rectal
(BMI_obese = 0; N0 = 390)
1−sided adj pval: 0.076
hsa−miR−3180−5p, rectal
(BMI_obese = 1; N1 = 144)
1−sided adj pval: 0.366
Frequency
Tumor−Normal Expression Difference

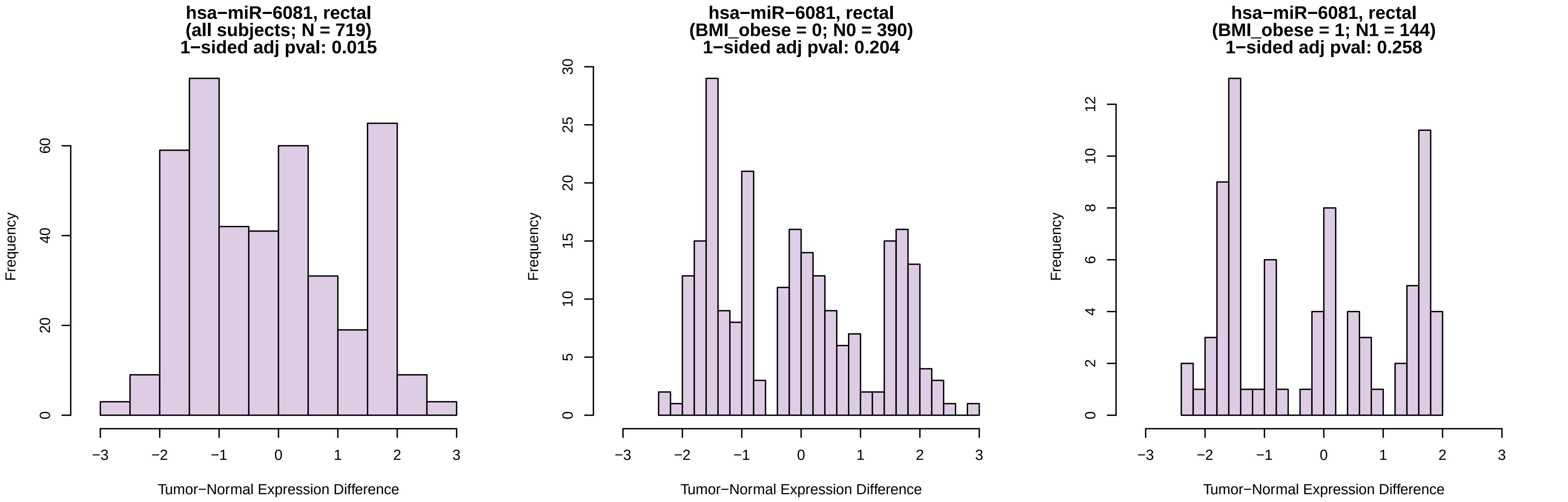
hsa−miR−6081, rectal
(all subjects; N = 719)
1−sided adj pval: 0.015
Frequency
0
20
40
60
−3
−2
−1
0
1
2
3
Tumor−Normal Expression Difference
hsa−miR−6081, rectal
(BMI_obese = 0; N0 = 390)
1−sided adj pval: 0.204
Frequency
0
5
10
15
20
25
30
−3
−2
−1
0
1
2
3
Tumor−Normal Expression Difference
hsa−miR−6081, rectal
(BMI_obese = 1; N1 = 144)
1−sided adj pval: 0.258
Frequency
0
2
4
6
8
10
12
−3
−2
−1
0
1
2
3
Tumor−Normal Expression Difference

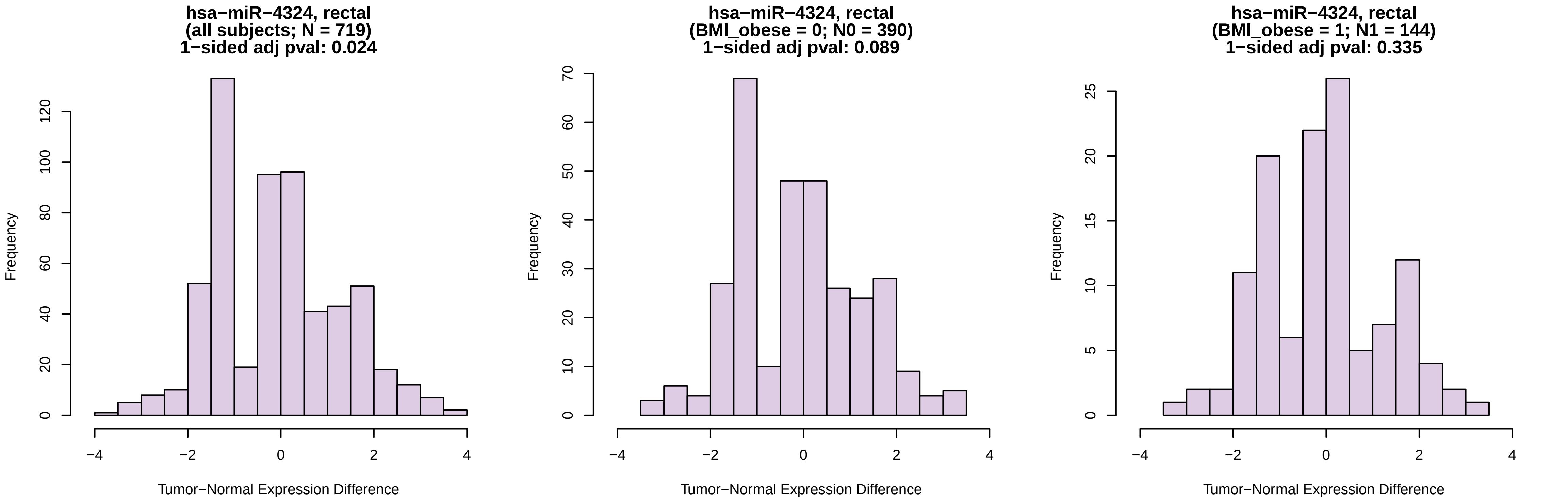
hsa−miR−4324, rectal
(all subjects; N = 719)
1−sided adj pval: 0.024
Frequency
0
20
40
60
80
100
120
−4
−2
0
2
4
Tumor−Normal Expression Difference
hsa−miR−4324, rectal
(BMI_obese = 0; N0 = 390)
1−sided adj pval: 0.089
Frequency
0
10
20
30
40
50
60
70
−4
−2
0
2
4
Tumor−Normal Expression Difference
hsa−miR−4324, rectal
(BMI_obese = 1; N1 = 144)
1−sided adj pval: 0.335
Frequency
0
5
10
15
20
25
−4
−2
0
2
4
Tumor−Normal Expression Difference

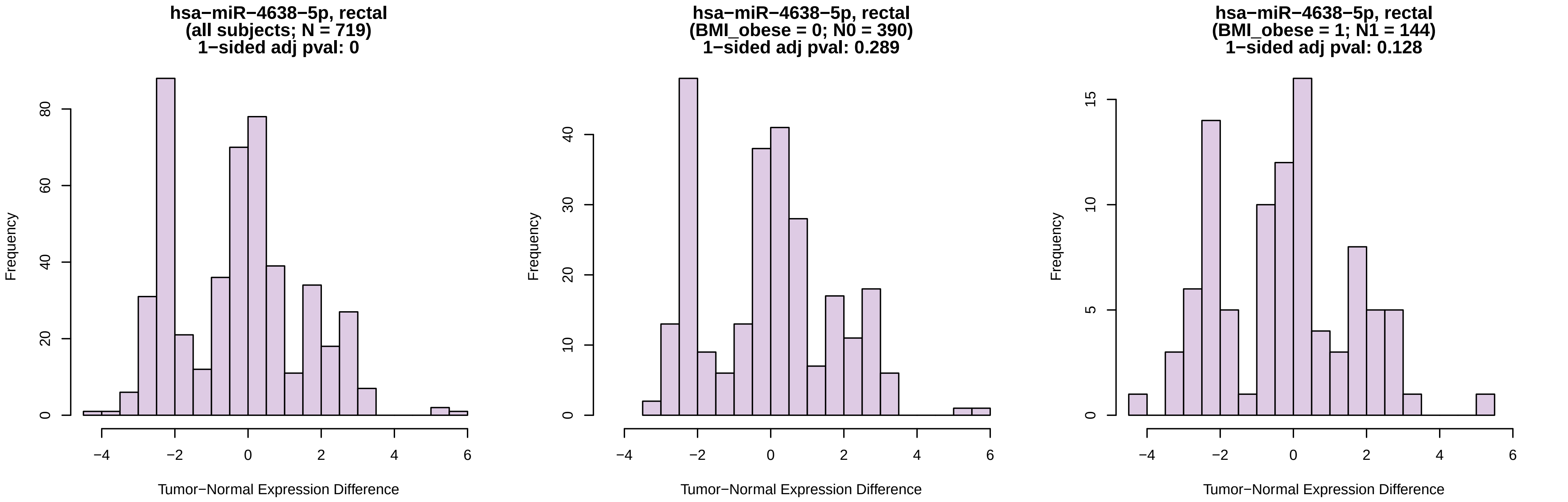
hsa−miR−4638−5p, rectal
(all subjects; N = 719)
1−sided adj pval: 0
Frequency
Tumor−Normal Expression Difference
hsa−miR−4638−5p, rectal
(BMI_obese = 0; N0 = 390)
1−sided adj pval: 0.289
Frequency
Tumor−Normal Expression Difference
hsa−miR−4638−5p, rectal
(BMI_obese = 1; N1 = 144)
1−sided adj pval: 0.128
Frequency
Tumor−Normal Expression Difference

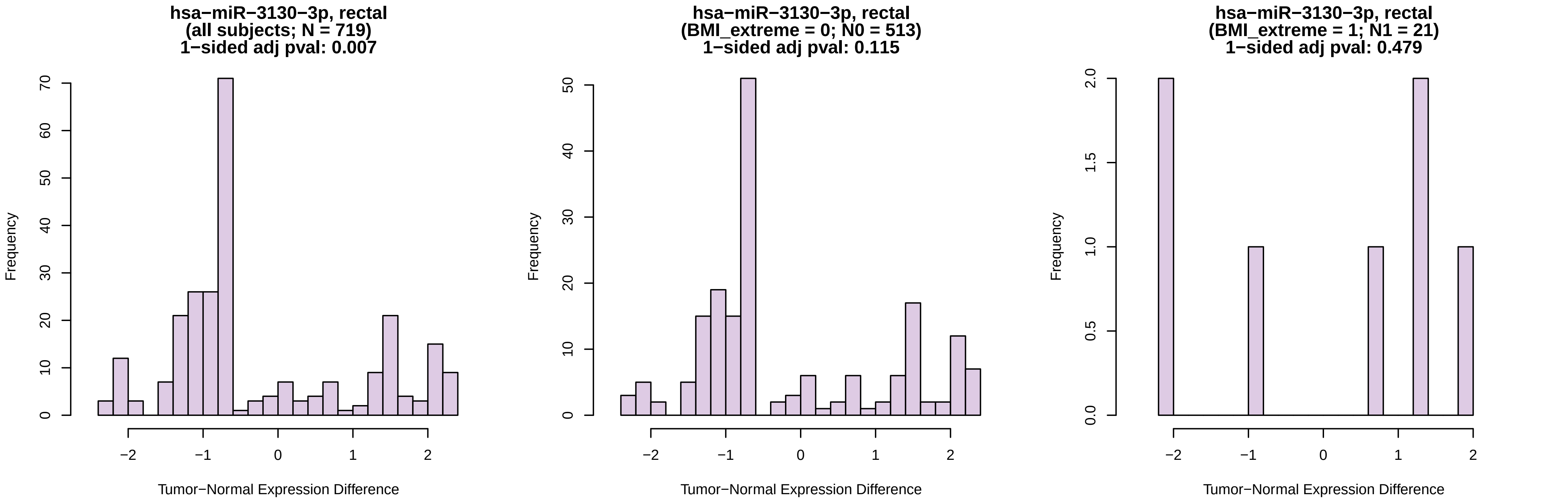

hsa−miR−3130−3p, rectal
(all subjects; N = 719)
1−sided adj pval: 0.007
Frequency
Tumor−Normal Expression Difference
hsa−miR−3130−3p, rectal
(BMI_extreme = 0; N0 = 513)
1−sided adj pval: 0.115
Frequency
Tumor−Normal Expression Difference
hsa−miR−3130−3p, rectal
(BMI_extreme = 1; N1 = 21)
1−sided adj pval: 0.479
Frequency
Tumor−Normal Expression Difference

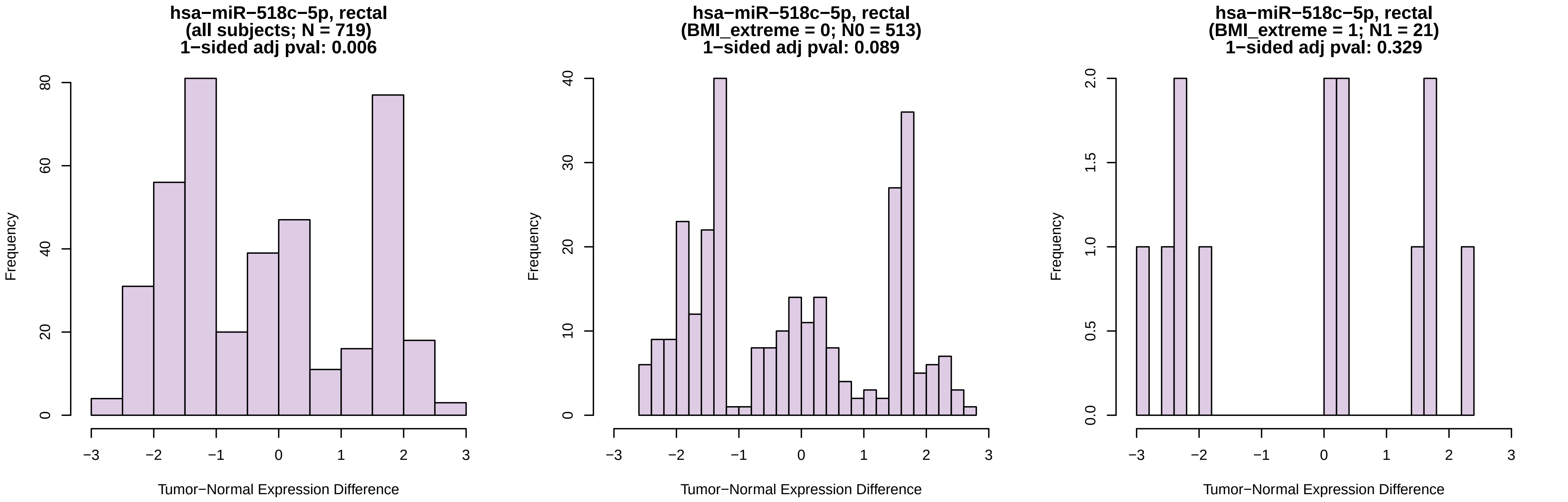
hsa−miR−518c−5p, rectal
(all subjects; N = 719)
1−sided adj pval: 0.006
Frequency
Tumor−Normal Expression Difference
hsa−miR−518c−5p, rectal
(BMI_extreme = 0; N0 = 513)
1−sided adj pval: 0.089
Frequency
Tumor−Normal Expression Difference
hsa−miR−518c−5p, rectal
(BMI_extreme = 1; N1 = 21)
1−sided adj pval: 0.329
Frequency
Tumor−Normal Expression Difference

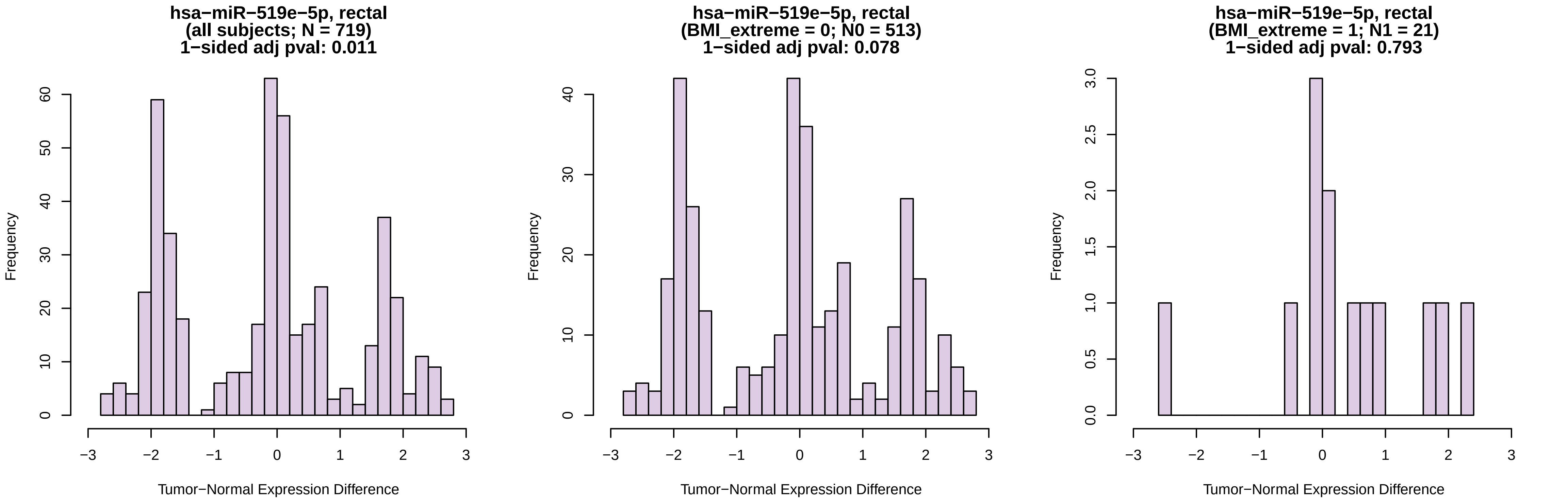
hsa−miR−519e−5p, rectal
(all subjects; N = 719)
1−sided adj pval: 0.011
Frequency
Tumor−Normal Expression Difference
hsa−miR−519e−5p, rectal
(BMI_extreme = 0; N0 = 513)
1−sided adj pval: 0.078
Frequency
Tumor−Normal Expression Difference
hsa−miR−519e−5p, rectal
(BMI_extreme = 1; N1 = 21)
1−sided adj pval: 0.793
Frequency
Tumor−Normal Expression Difference

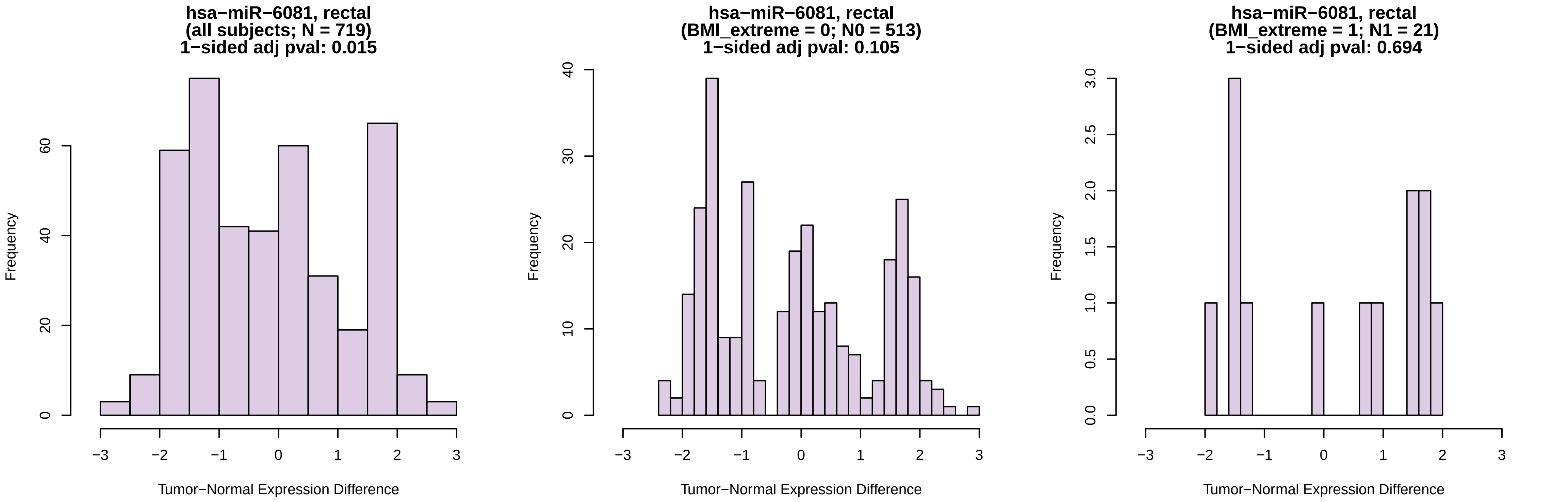

hsa−miR−6081, rectal
(all subjects; N = 719)
1−sided adj pval: 0.015
hsa−miR−6081, rectal
(BMI_extreme = 0; N0 = 513)
1−sided adj pval: 0.105
hsa−miR−6081, rectal
(BMI_extreme = 1; N1 = 21)
1−sided adj pval: 0.694
Frequency
Tumor−Normal Expression Difference

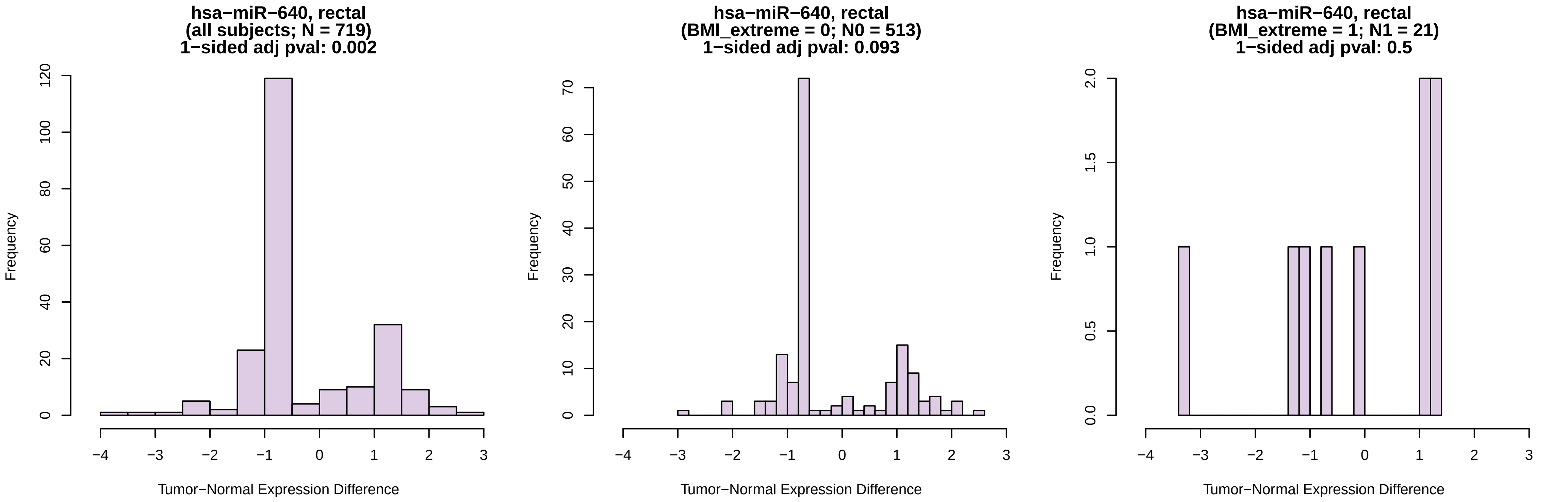
hsa−miR−640, rectal
(all subjects; N = 719)
1−sided adj pval: 0.002
Frequency
Tumor−Normal Expression Difference
hsa−miR−640, rectal
(BMI_extreme = 0; N0 = 513)
1−sided adj pval: 0.093
Frequency
Tumor−Normal Expression Difference
hsa−miR−640, rectal
(BMI_extreme = 1; N1 = 21)
1−sided adj pval: 0.5
Frequency
Tumor−Normal Expression Difference

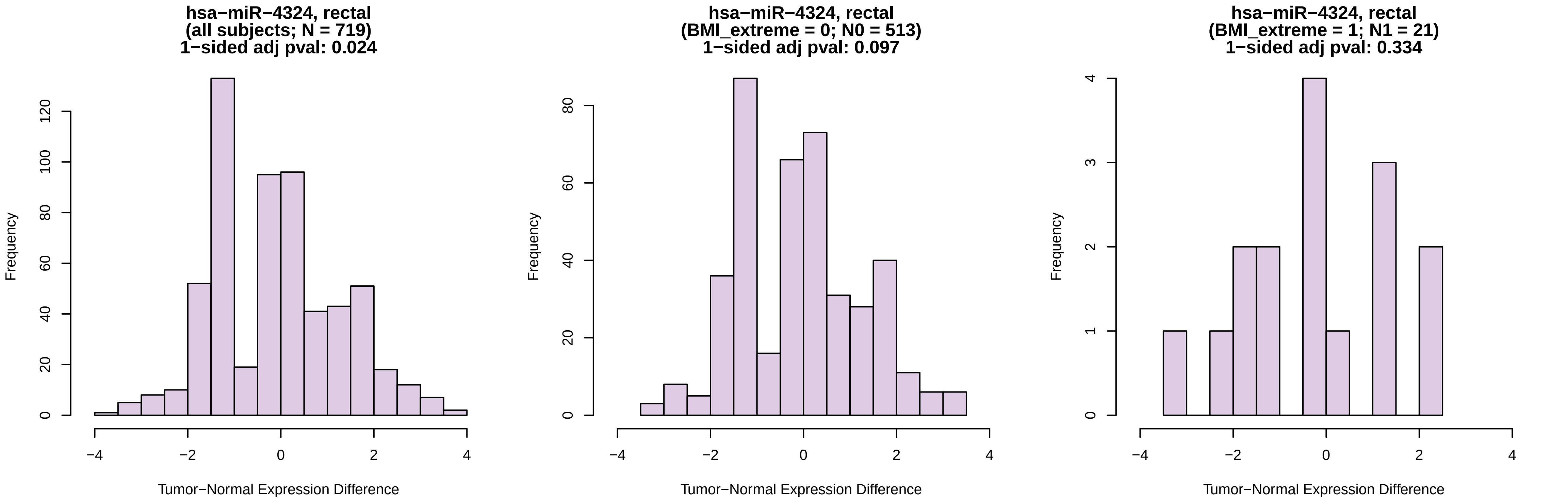
hsa−miR−4324, rectal
(all subjects; N = 719)
1−sided adj pval: 0.024
Frequency
Tumor−Normal Expression Difference
hsa−miR−4324, rectal
(BMI_extreme = 0; N0 = 513)
1−sided adj pval: 0.097
Frequency
Tumor−Normal Expression Difference
hsa−miR−4324, rectal
(BMI_extreme = 1; N1 = 21)
1−sided adj pval: 0.334
Frequency
Tumor−Normal Expression Difference
