# Supplementary material for: Identifying factors associated with the direction and significance of microRNA tumor-normal expression differences in colorectal cancer
Source: BMC Cancer. 2017 Oct 30;17:707. doi: 10.1186/s12885-017-3690-x (PMC5663119; doi:10.1186/s12885-017-3690-x)
Supplement: Supplementary file 7 — (AF7_results.csv) Spreadsheet file for all outcomes of interest, including the FDR-adjusted p-values and sample sizes for all site / factor / microRNA combinations that were classified to one of the five categories (colors) in Table 2. (CSV 199 kb) [file 12885_2017_3690_MOESM6_ESM.pdf]

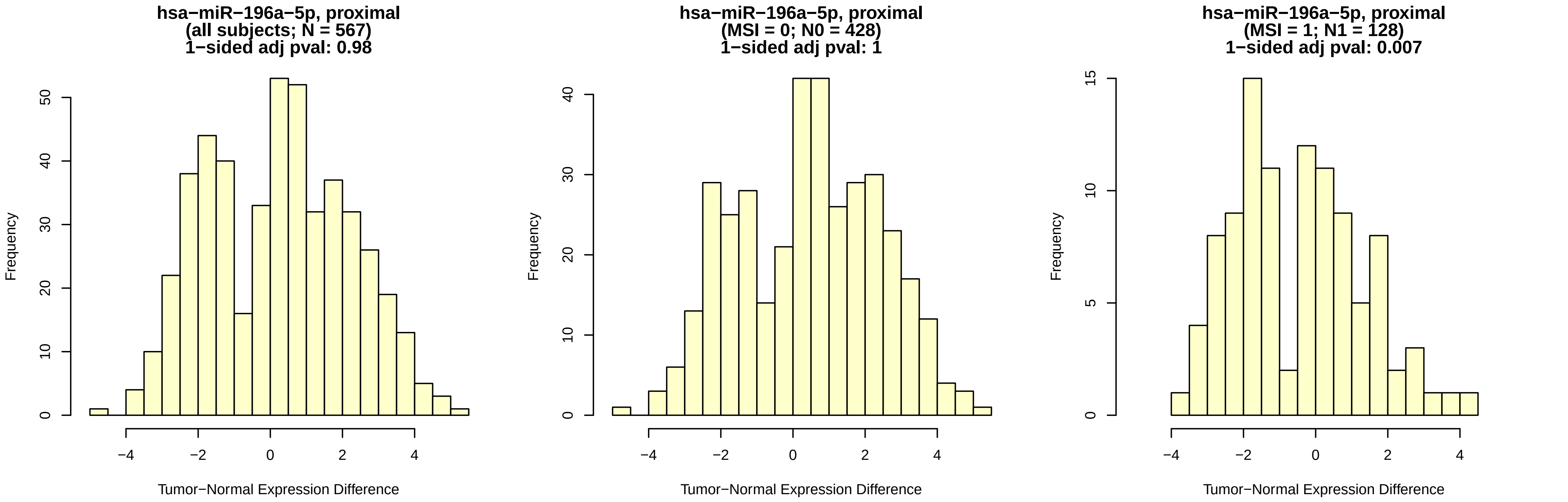
hsa−miR−196a−5p, proximal
(all subjects; N = 567)
1−sided adj pval: 0.98
Frequency
Tumor−Normal Expression Difference
hsa−miR−196a−5p, proximal
(MSI = 0; N0 = 428)
1−sided adj pval: 1
Frequency
Tumor−Normal Expression Difference
hsa−miR−196a−5p, proximal
(MSI = 1; N1 = 128)
1−sided adj pval: 0.007
Frequency
Tumor−Normal Expression Difference

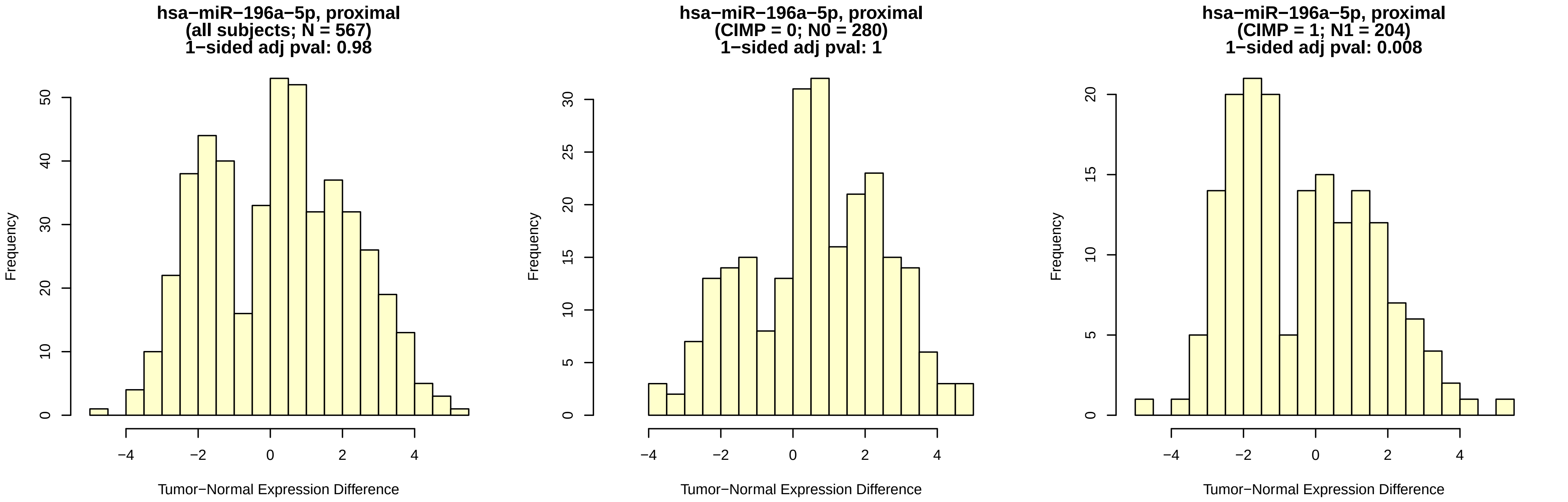
hsa−miR−196a−5p, proximal
(all subjects; N = 567)
1−sided adj pval: 0.98
Frequency
Tumor−Normal Expression Difference
hsa−miR−196a−5p, proximal
(CIMP = 0; N0 = 280)
1−sided adj pval: 1
Frequency
Tumor−Normal Expression Difference
hsa−miR−196a−5p, proximal
(CIMP = 1; N1 = 204)
1−sided adj pval: 0.008
Frequency
Tumor−Normal Expression Difference

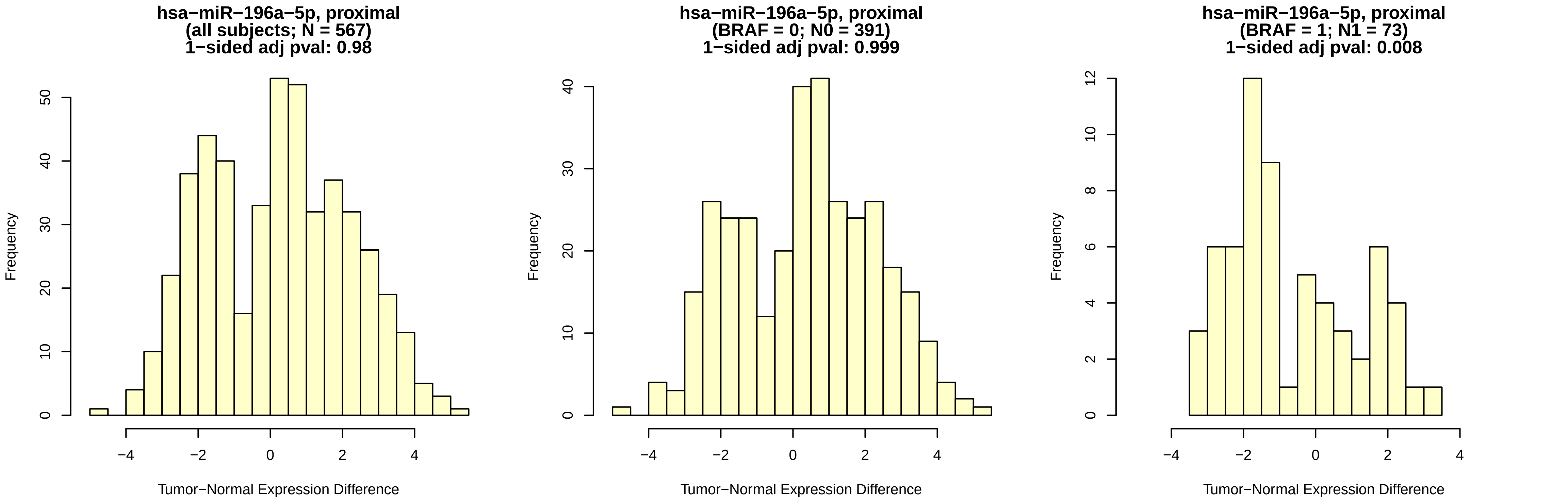
hsa−miR−196a−5p, proximal
(all subjects; N = 567)
1−sided adj pval: 0.98
Frequency
Tumor−Normal Expression Difference
hsa−miR−196a−5p, proximal
(BRAF = 0; N0 = 391)
1−sided adj pval: 0.999
Frequency
Tumor−Normal Expression Difference
hsa−miR−196a−5p, proximal
(BRAF = 1; N1 = 73)
1−sided adj pval: 0.008
Frequency
Tumor−Normal Expression Difference

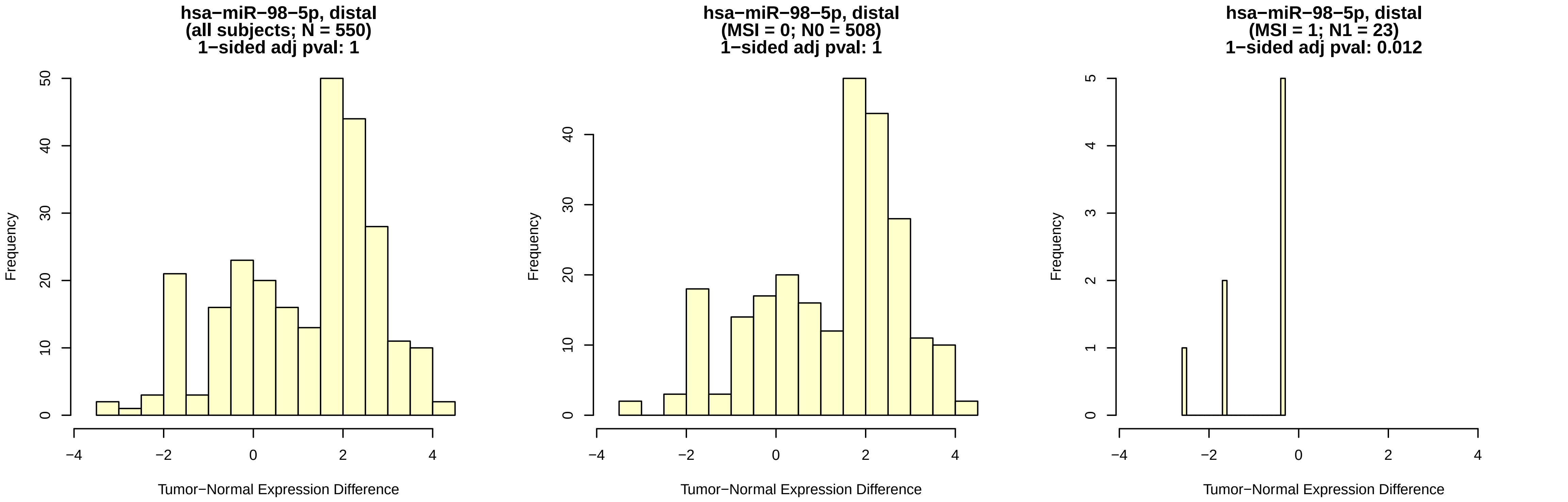

hsa−miR−98−5p, distal
(all subjects; N = 550)
1−sided adj pval: 1
Frequency
Tumor−Normal Expression Difference
hsa−miR−98−5p, distal
(MSI = 0; N0 = 508)
1−sided adj pval: 1
Frequency
Tumor−Normal Expression Difference
hsa−miR−98−5p, distal
(MSI = 1; N1 = 23)
1−sided adj pval: 0.012
Frequency
Tumor−Normal Expression Difference

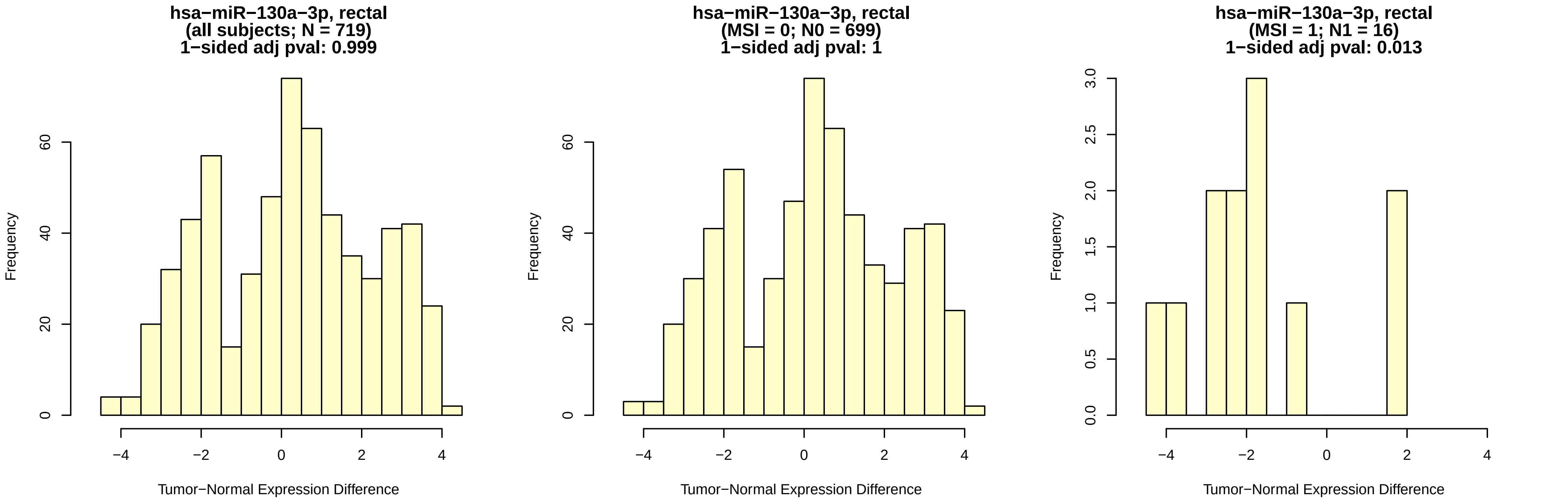
hsa−miR−130a−3p, rectal
(all subjects; N = 719)
1−sided adj pval: 0.999
Frequency
0
20
40
60
−4
−2
0
2
4
Tumor−Normal Expression Difference
hsa−miR−130a−3p, rectal
(MSI = 0; N0 = 699)
1−sided adj pval: 1
Frequency
0
20
40
60
−4
−2
0
2
4
Tumor−Normal Expression Difference
hsa−miR−130a−3p, rectal
(MSI = 1; N1 = 16)
1−sided adj pval: 0.013
Frequency
0.0
0.5
1.0
1.5
2.0
2.5
3.0
−4
−2
0
2
4
Tumor−Normal Expression Difference

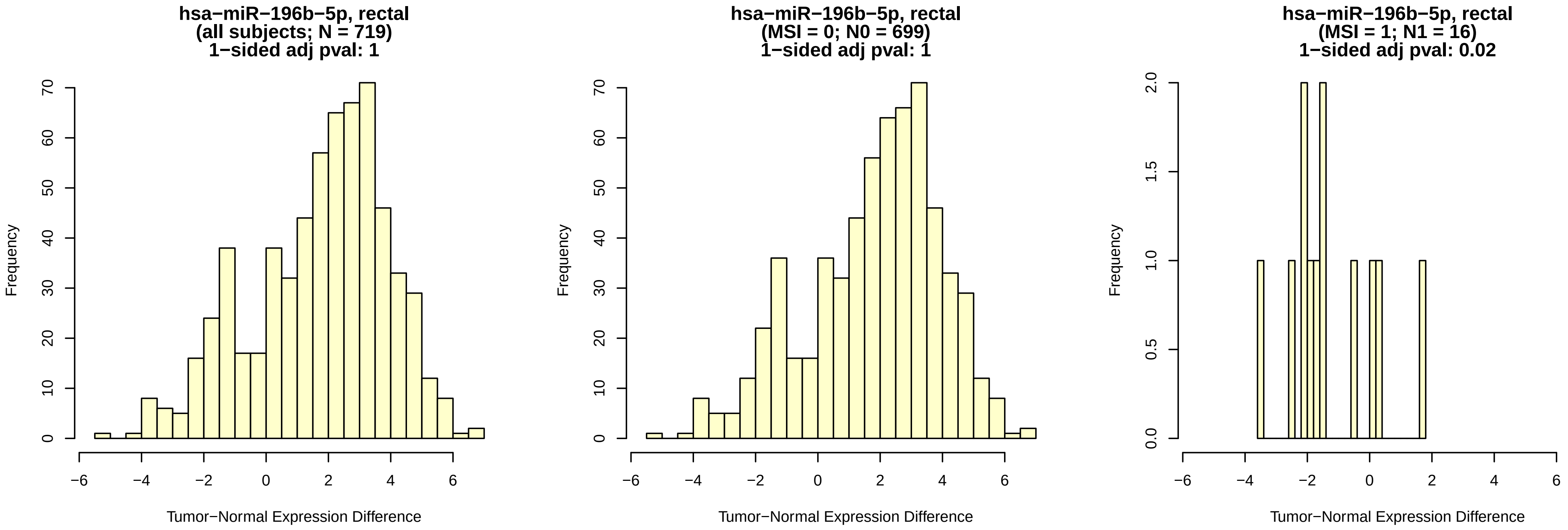

hsa−miR−196b−5p, rectal
(all subjects; N = 719)
1−sided adj pval: 1
Frequency
Tumor−Normal Expression Difference
hsa−miR−196b−5p, rectal
(MSI = 0; N0 = 699)
1−sided adj pval: 1
Frequency
Tumor−Normal Expression Difference
hsa−miR−196b−5p, rectal
(MSI = 1; N1 = 16)
1−sided adj pval: 0.02
Frequency
Tumor−Normal Expression Difference

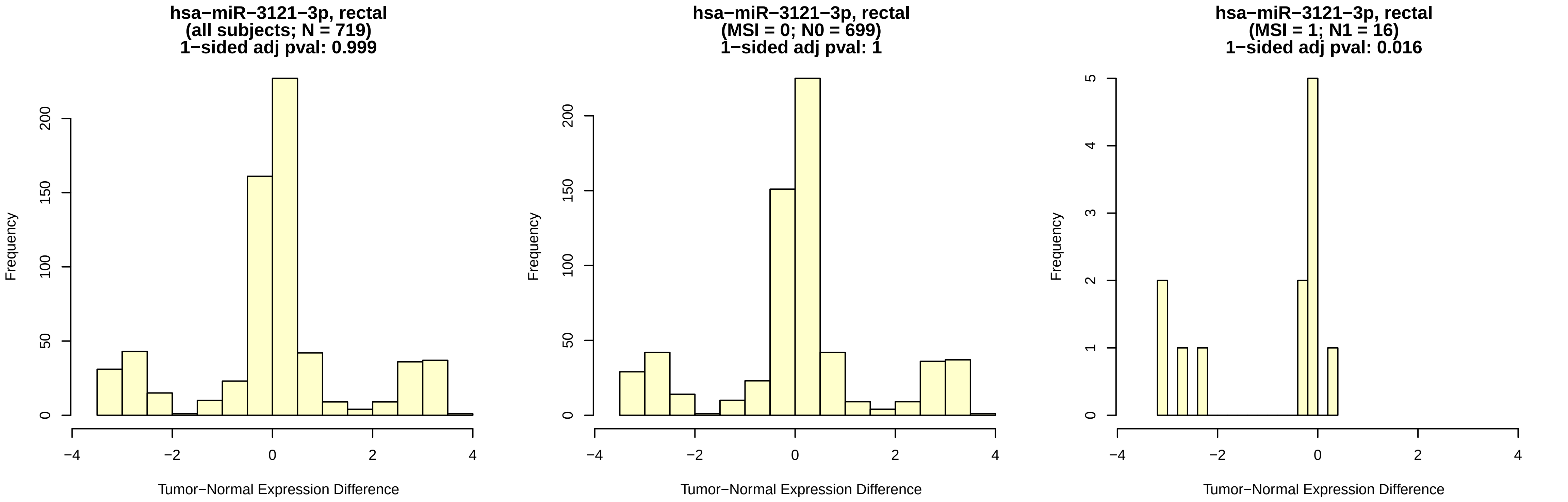
hsa−miR−3121−3p, rectal
(all subjects; N = 719)
1−sided adj pval: 0.999
Frequency
0
50
100
150
200
−4
−2
0
2
4
Tumor−Normal Expression Difference
hsa−miR−3121−3p, rectal
(MSI = 0; N0 = 699)
1−sided adj pval: 1
Frequency
0
50
100
150
200
−4
−2
0
2
4
Tumor−Normal Expression Difference
hsa−miR−3121−3p, rectal
(MSI = 1; N1 = 16)
1−sided adj pval: 0.016
Frequency
0
1
2
3
4
5
−4
−2
0
2
4
Tumor−Normal Expression Difference

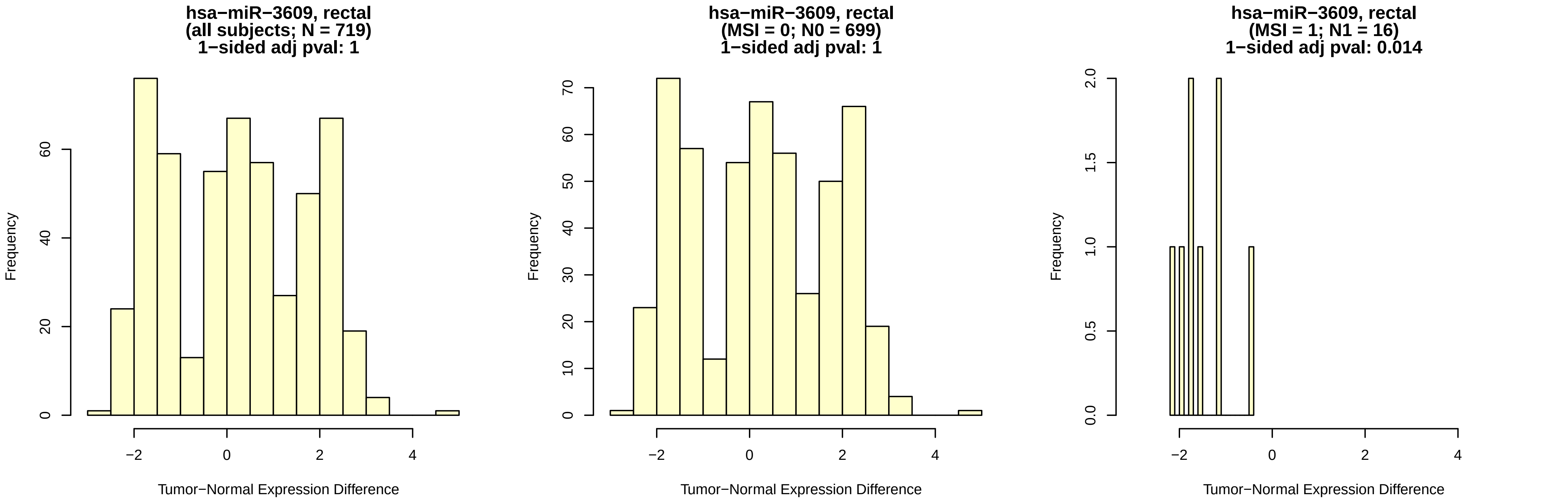
hsa−miR−3609, rectal
(all subjects; N = 719)
1−sided adj pval: 1
Frequency
Tumor−Normal Expression Difference
hsa−miR−3609, rectal
(MSI = 0; N0 = 699)
1−sided adj pval: 1
Frequency
Tumor−Normal Expression Difference
hsa−miR−3609, rectal
(MSI = 1; N1 = 16)
1−sided adj pval: 0.014
Frequency
Tumor−Normal Expression Difference

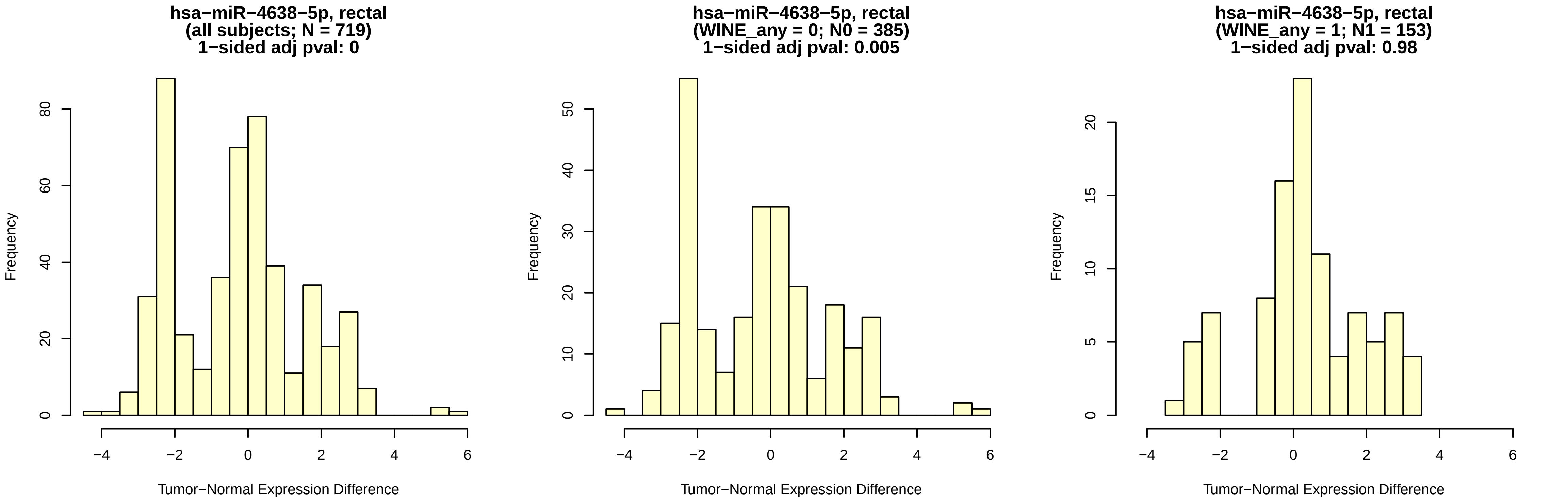

hsa−miR−4638−5p, rectal
(all subjects; N = 719)
1−sided adj pval: 0
Frequency
0
20
40
60
80
−4
−2
0
2
4
6
Tumor−Normal Expression Difference
hsa−miR−4638−5p, rectal
(WINE_any = 0; N0 = 385)
1−sided adj pval: 0.005
Frequency
0
10
20
30
40
50
−4
−2
0
2
4
6
Tumor−Normal Expression Difference
hsa−miR−4638−5p, rectal
(WINE_any = 1; N1 = 153)
1−sided adj pval: 0.98
Frequency
0
5
10
15
20
−4
−2
0
2
4
6
Tumor−Normal Expression Difference

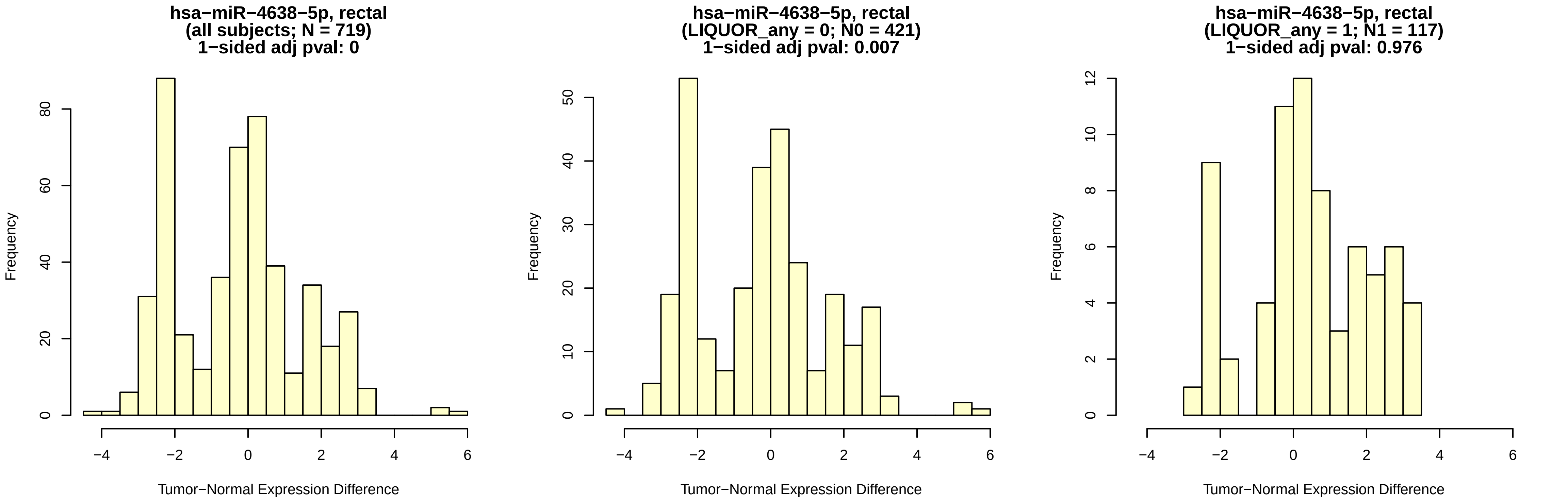

hsa−miR−4638−5p, rectal
(all subjects; N = 719)
1−sided adj pval: 0
Frequency
Tumor−Normal Expression Difference
hsa−miR−4638−5p, rectal
(LIQUOR_any = 0; N0 = 421)
1−sided adj pval: 0.007
Frequency
Tumor−Normal Expression Difference
hsa−miR−4638−5p, rectal
(LIQUOR_any = 1; N1 = 117)
1−sided adj pval: 0.976
Frequency
Tumor−Normal Expression Difference
