# Supplementary material for: Identifying factors associated with the direction and significance of microRNA tumor-normal expression differences in colorectal cancer
Source: BMC Cancer. 2017 Oct 30;17:707. doi: 10.1186/s12885-017-3690-x (PMC5663119; doi:10.1186/s12885-017-3690-x)
Supplement: Supplementary file 3 — (AF3_green.pdf) Visualizations of “green” outcomes of interest – microRNAs that are overall not significant in the tumor-normal test of differential expression, but significant in only one factor level. Each page of this file is in the same format as explained for each row in Fig. 2. (PDF 243 kb) [file 12885_2017_3690_MOESM2_ESM.pdf]

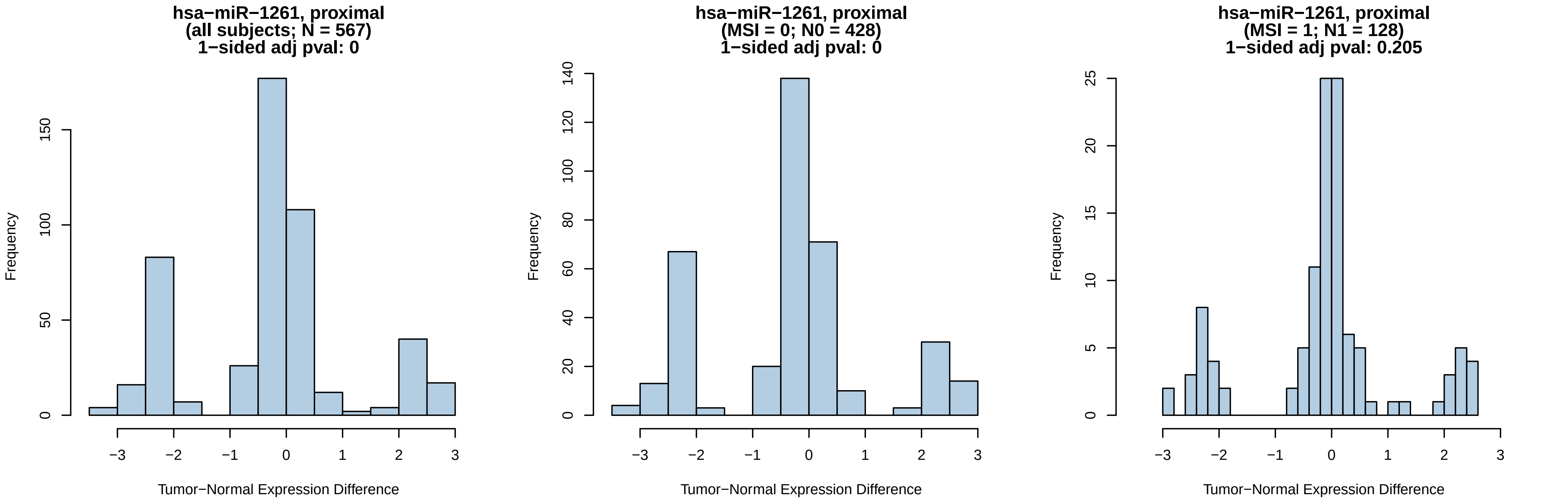
hsa−miR−1261, proximal
(all subjects; N = 567)
1−sided adj pval: 0
Frequency
Tumor−Normal Expression Difference
hsa−miR−1261, proximal
(MSI = 0; N0 = 428)
1−sided adj pval: 0
Frequency
Tumor−Normal Expression Difference
hsa−miR−1261, proximal
(MSI = 1; N1 = 128)
1−sided adj pval: 0.205
Frequency
Tumor−Normal Expression Difference

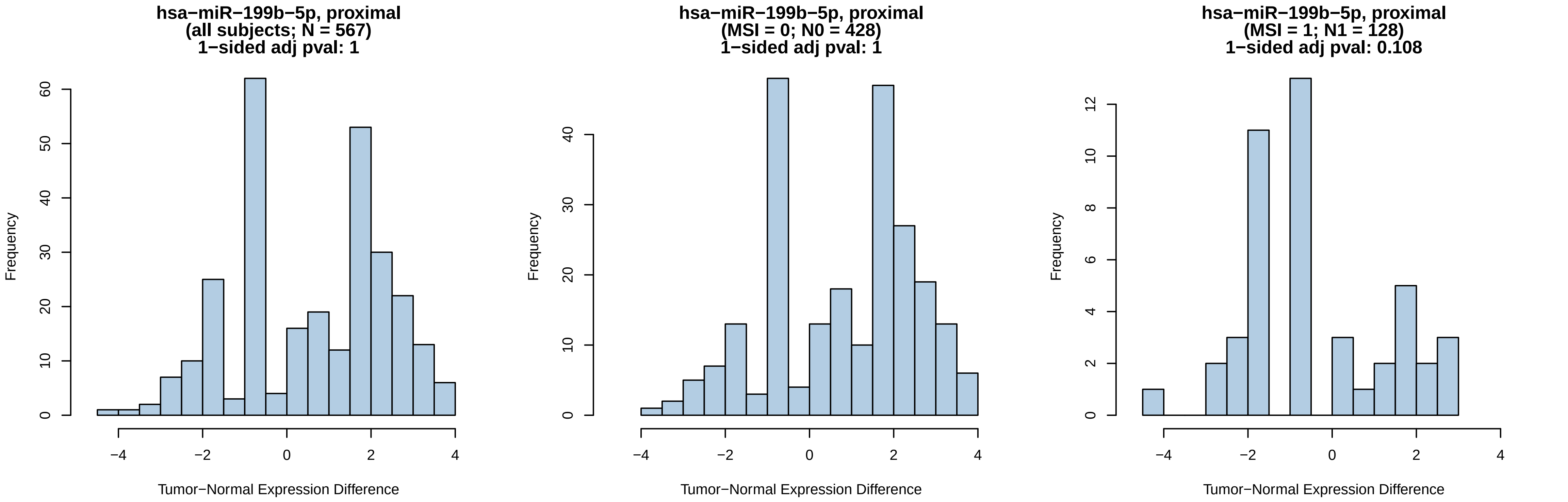
hsa−miR−199b−5p, proximal
(all subjects; N = 567)
1−sided adj pval: 1
Frequency
Tumor−Normal Expression Difference
hsa−miR−199b−5p, proximal
(MSI = 0; N0 = 428)
1−sided adj pval: 1
Frequency
Tumor−Normal Expression Difference
hsa−miR−199b−5p, proximal
(MSI = 1; N1 = 128)
1−sided adj pval: 0.108
Frequency
Tumor−Normal Expression Difference

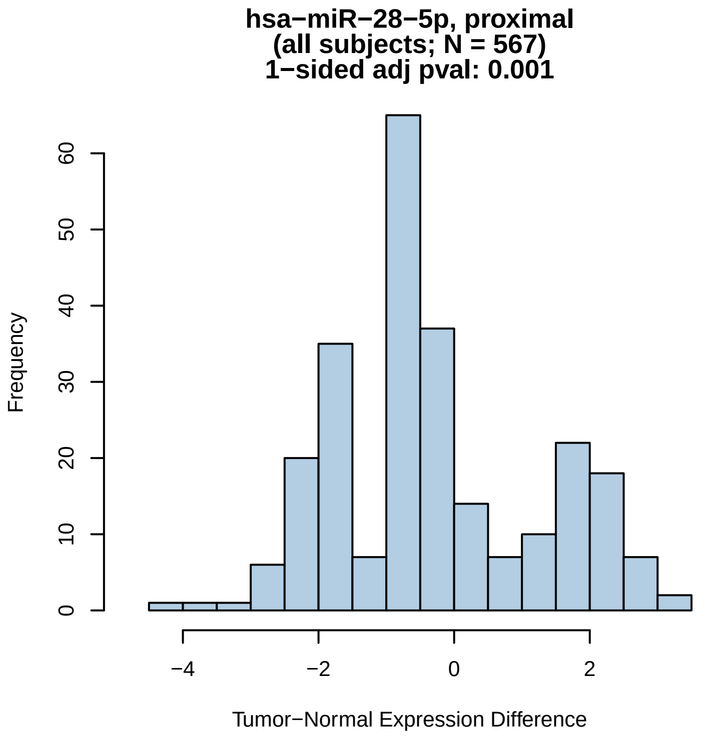
hsa−miR−28−5p, proximal
(all subjects; N = 567)
1−sided adj pval: 0.001
Frequency
0
10
20
30
40
50
60
−4
−2
0
2
Tumor−Normal Expression Difference

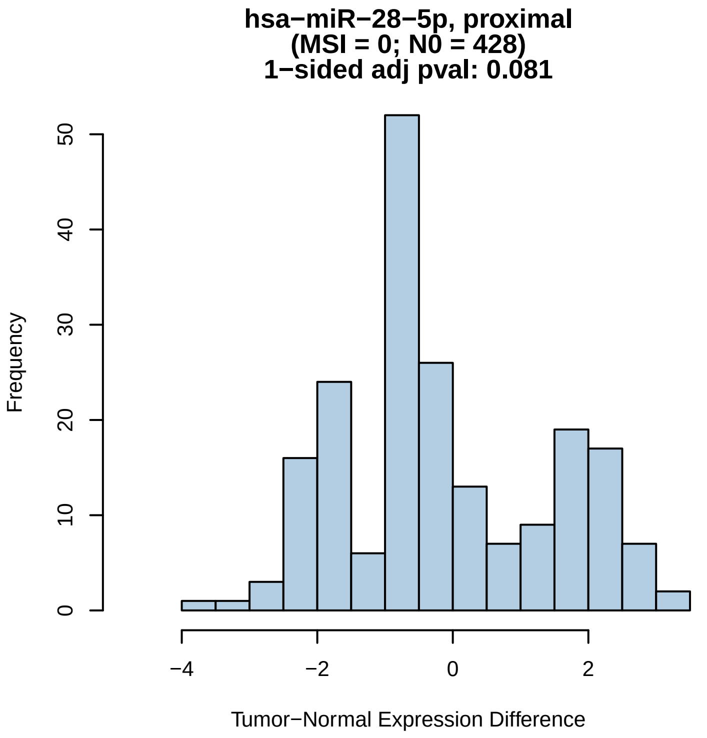
hsa−miR−28−5p, proximal
(MSI = 0; N0 = 428)
1−sided adj pval: 0.081
Frequency
0
10
20
30
40
50
−4
−2
0
2
Tumor−Normal Expression Difference

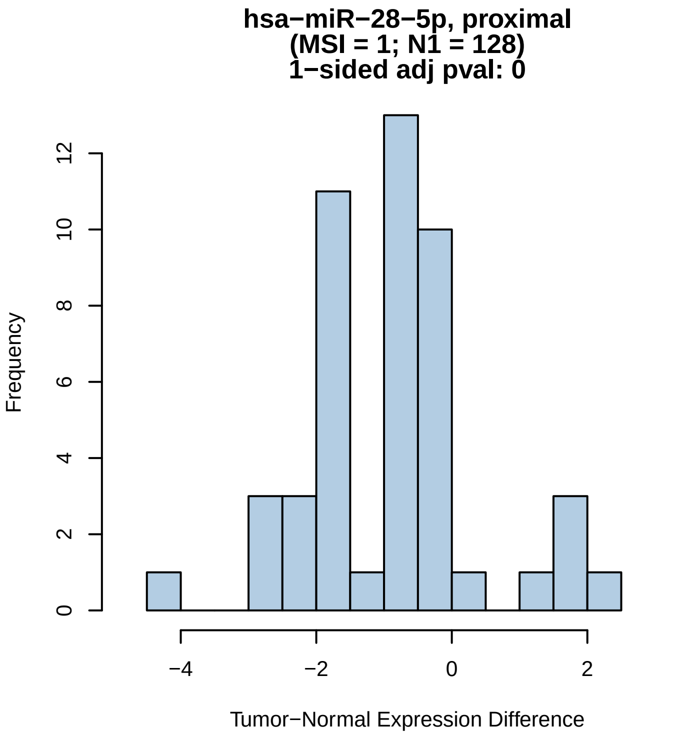
hsa−miR−28−5p, proximal
(MSI = 1; N1 = 128)
1−sided adj pval: 0
Frequency
0
2
4
6
8
10
12
−4
−2
0
2
Tumor−Normal Expression Difference

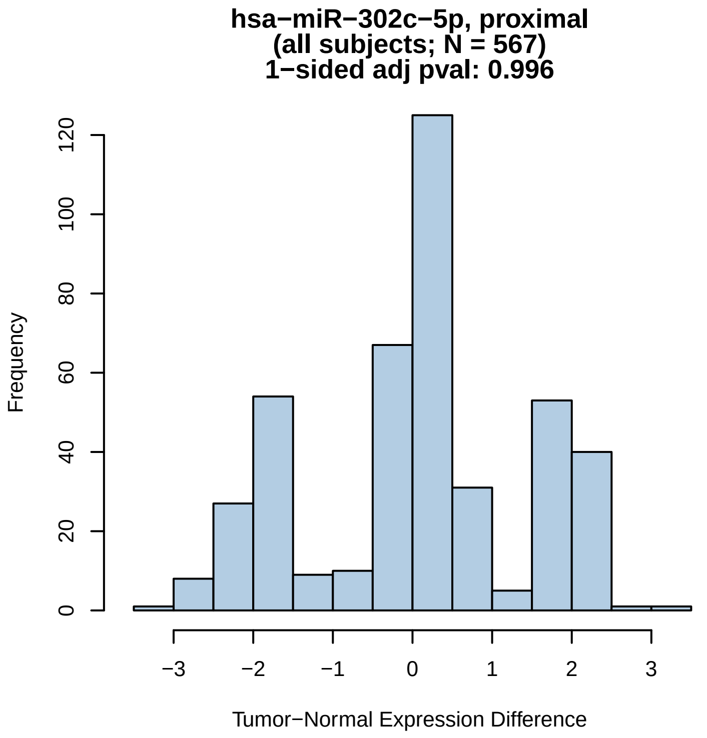
hsa−miR−302c−5p, proximal
(all subjects; N = 567)
1−sided adj pval: 0.996
Frequency
Tumor−Normal Expression Difference

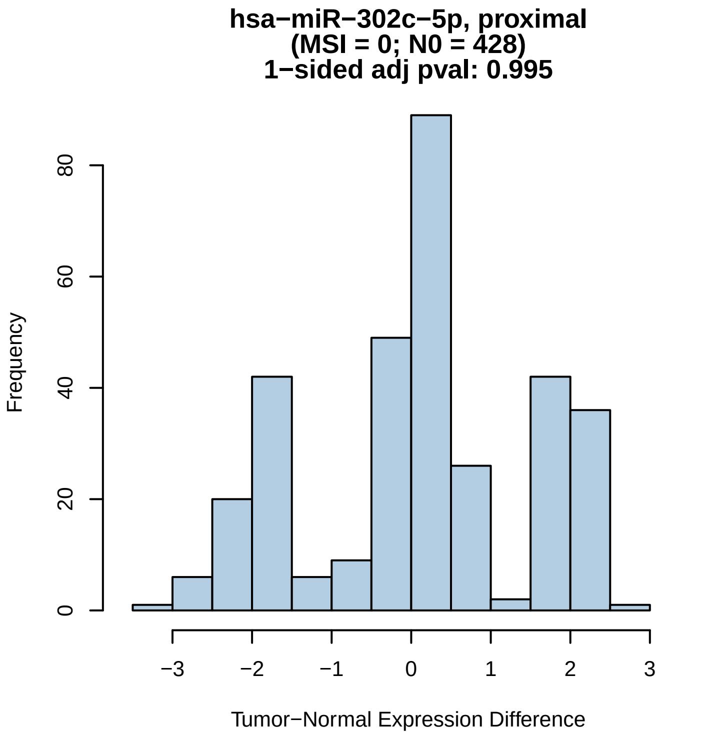
hsa−miR−302c−5p, proximal
(MSI = 0; N0 = 428)
1−sided adj pval: 0.995
Frequency
Tumor−Normal Expression Difference

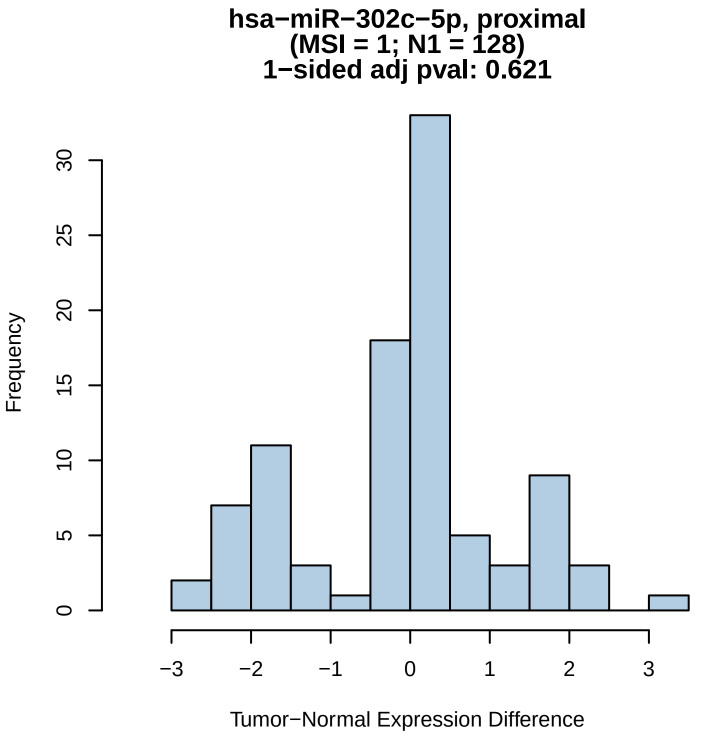
hsa−miR−302c−5p, proximal
(MSI = 1; N1 = 128)
1−sided adj pval: 0.621
Frequency
Tumor−Normal Expression Difference

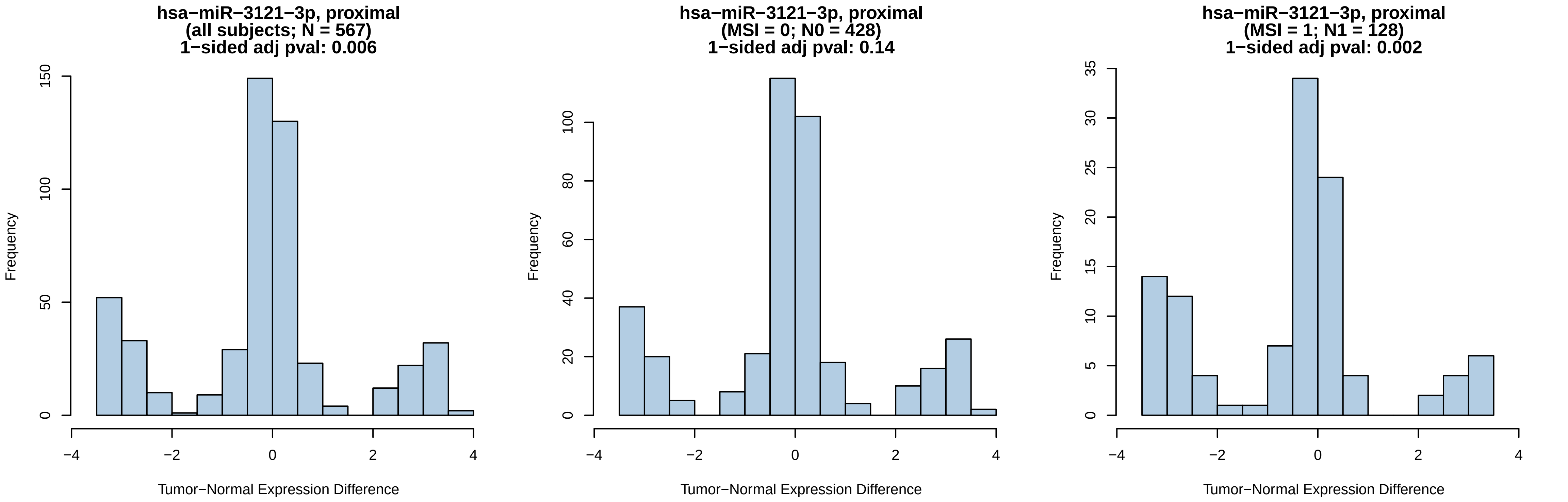
hsa−miR−3121−3p, proximal
(all subjects; N = 567)
1−sided adj pval: 0.006
Frequency
Tumor−Normal Expression Difference
hsa−miR−3121−3p, proximal
(MSI = 0; N0 = 428)
1−sided adj pval: 0.14
Frequency
Tumor−Normal Expression Difference
hsa−miR−3121−3p, proximal
(MSI = 1; N1 = 128)
1−sided adj pval: 0.002
Frequency
Tumor−Normal Expression Difference

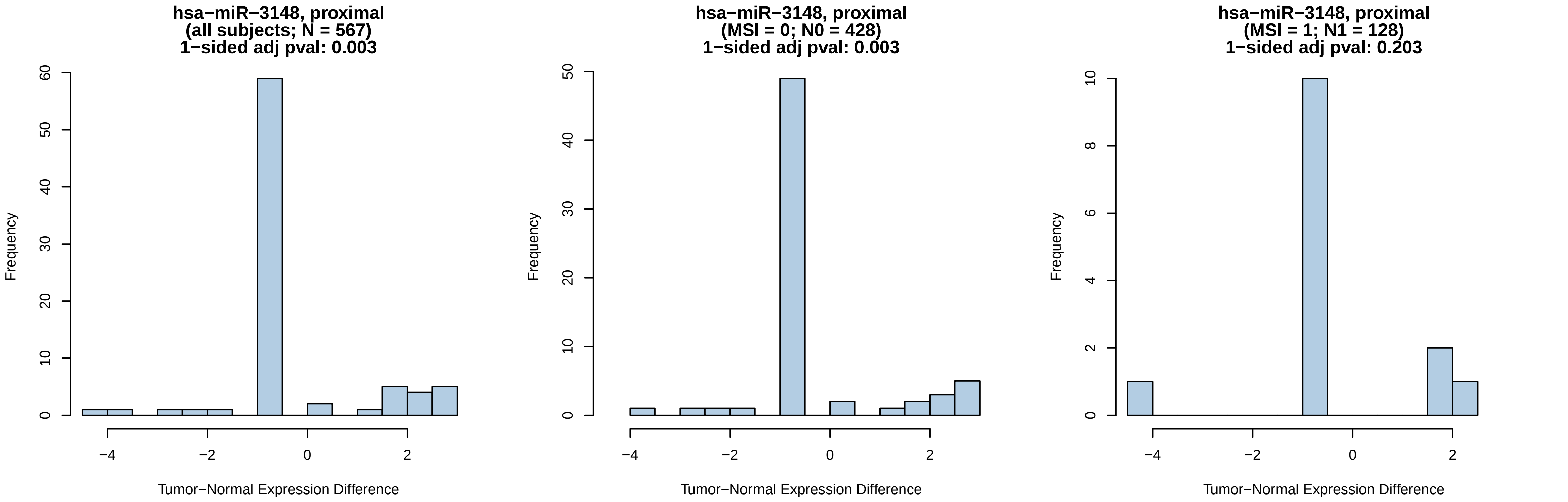

hsa−miR−3148, proximal
(all subjects; N = 567)
1−sided adj pval: 0.003
Frequency
0
10
20
30
40
50
60
−4
−2
0
2
Tumor−Normal Expression Difference
hsa−miR−3148, proximal
(MSI = 0; N0 = 428)
1−sided adj pval: 0.003
Frequency
0
10
20
30
40
50
−4
−2
0
2
Tumor−Normal Expression Difference
hsa−miR−3148, proximal
(MSI = 1; N1 = 128)
1−sided adj pval: 0.203
Frequency
0
2
4
6
8
10
−4
−2
0
2
Tumor−Normal Expression Difference

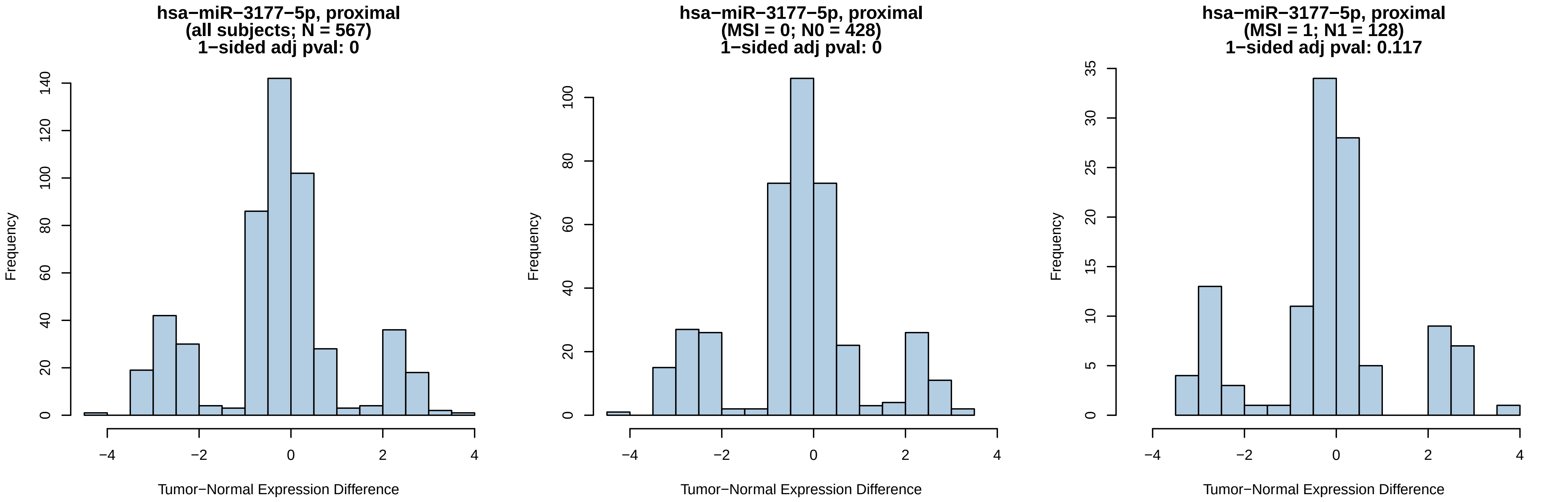
hsa−miR−3177−5p, proximal
(all subjects; N = 567)
1−sided adj pval: 0
Frequency
Tumor−Normal Expression Difference
hsa−miR−3177−5p, proximal
(MSI = 0; N0 = 428)
1−sided adj pval: 0
Frequency
Tumor−Normal Expression Difference
hsa−miR−3177−5p, proximal
(MSI = 1; N1 = 128)
1−sided adj pval: 0.117
Frequency
Tumor−Normal Expression Difference

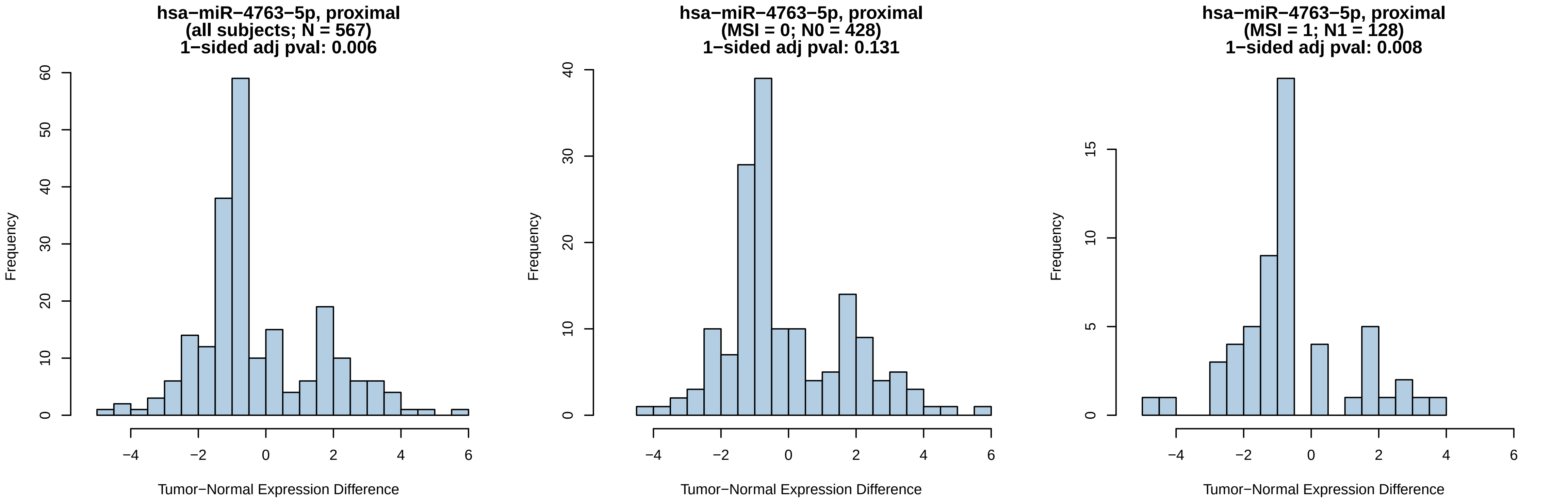
hsa−miR−4763−5p, proximal
(all subjects; N = 567)
1−sided adj pval: 0.006
Frequency
Tumor−Normal Expression Difference
hsa−miR−4763−5p, proximal
(MSI = 0; N0 = 428)
1−sided adj pval: 0.131
Frequency
Tumor−Normal Expression Difference
hsa−miR−4763−5p, proximal
(MSI = 1; N1 = 128)
1−sided adj pval: 0.008
Frequency
Tumor−Normal Expression Difference

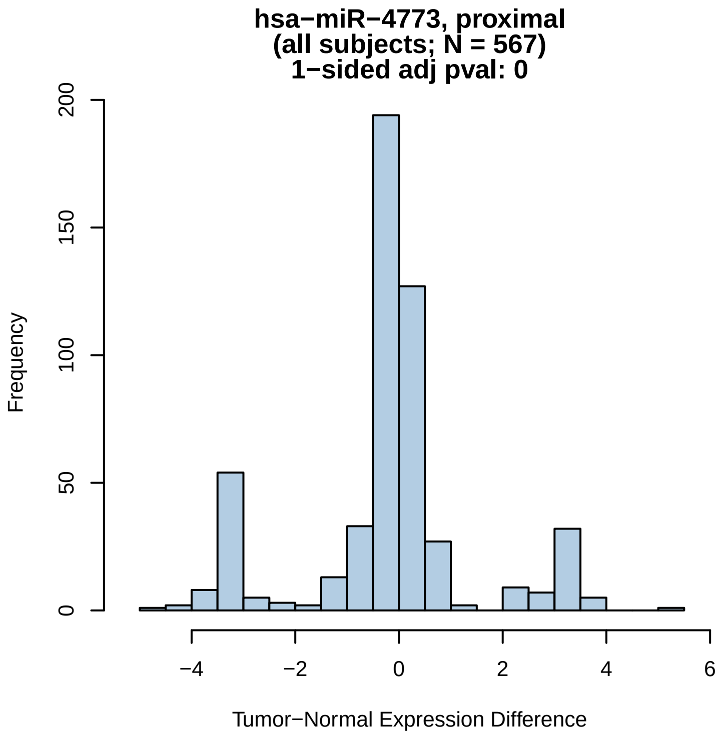
hsa−miR−4773, proximal
(all subjects; N = 567)
1−sided adj pval: 0
Frequency
Tumor−Normal Expression Difference

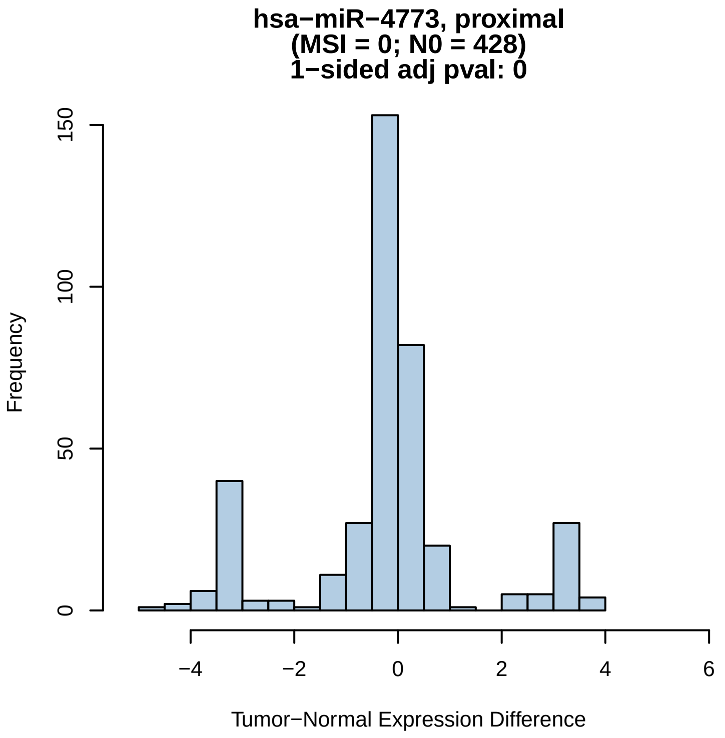
hsa−miR−4773, proximal
(MSI = 0; N0 = 428)
1−sided adj pval: 0
Frequency
Tumor−Normal Expression Difference

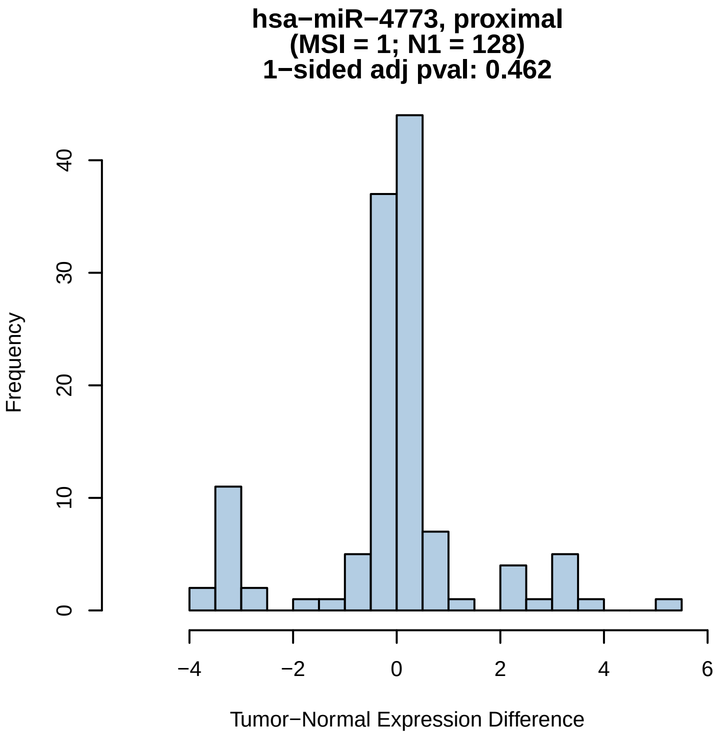
hsa−miR−4773, proximal
(MSI = 1; N1 = 128)
1−sided adj pval: 0.462
Frequency
Tumor−Normal Expression Difference

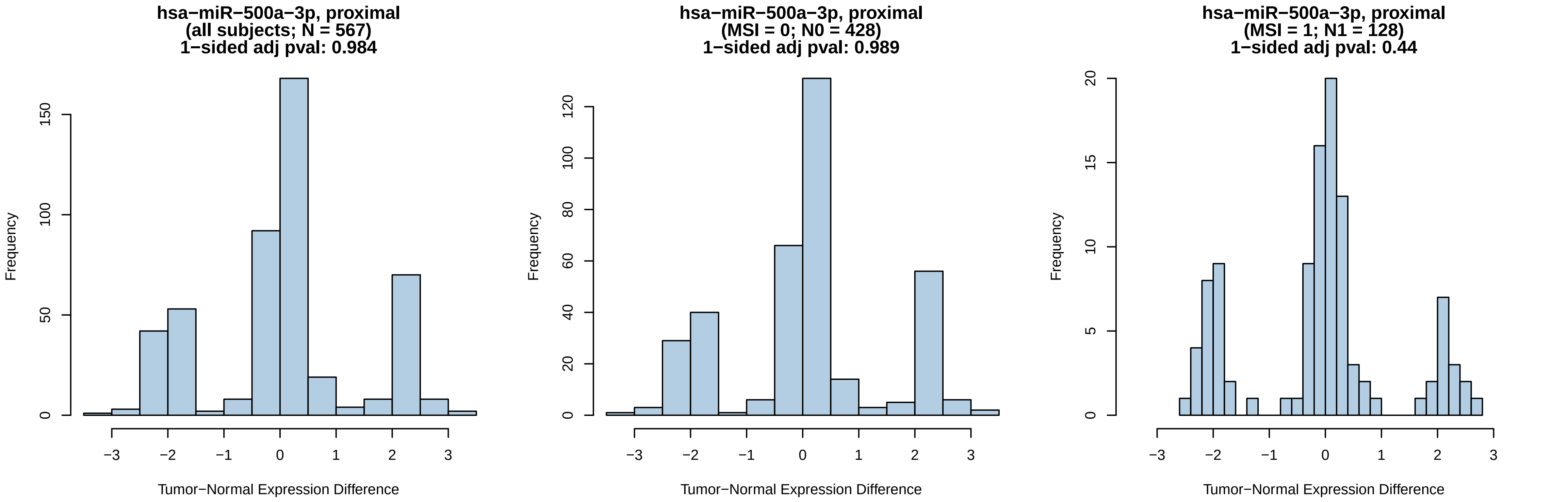
hsa−miR−500a−3p, proximal
(all subjects; N = 567)
1−sided adj pval: 0.984
Frequency
Tumor−Normal Expression Difference
hsa−miR−500a−3p, proximal
(MSI = 0; N0 = 428)
1−sided adj pval: 0.989
Frequency
Tumor−Normal Expression Difference
hsa−miR−500a−3p, proximal
(MSI = 1; N1 = 128)
1−sided adj pval: 0.44
Frequency
Tumor−Normal Expression Difference

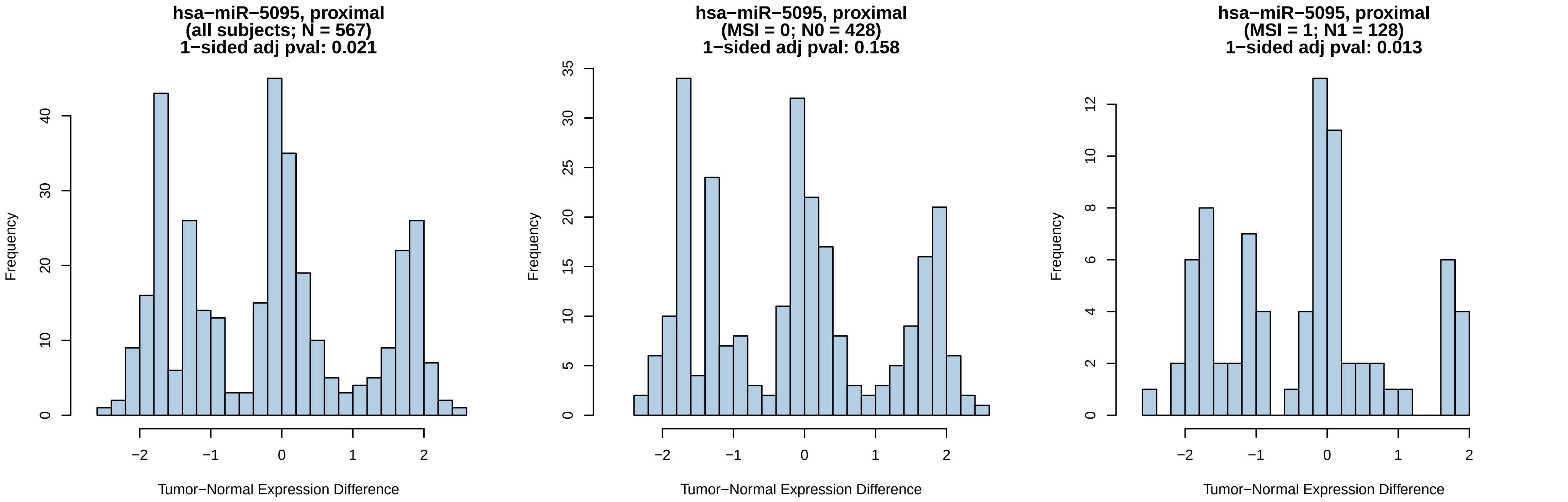
hsa−miR−5095, proximal
(all subjects; N = 567)
1−sided adj pval: 0.021
Frequency
Tumor−Normal Expression Difference
hsa−miR−5095, proximal
(MSI = 0; N0 = 428)
1−sided adj pval: 0.158
Frequency
Tumor−Normal Expression Difference
hsa−miR−5095, proximal
(MSI = 1; N1 = 128)
1−sided adj pval: 0.013
Frequency
Tumor−Normal Expression Difference

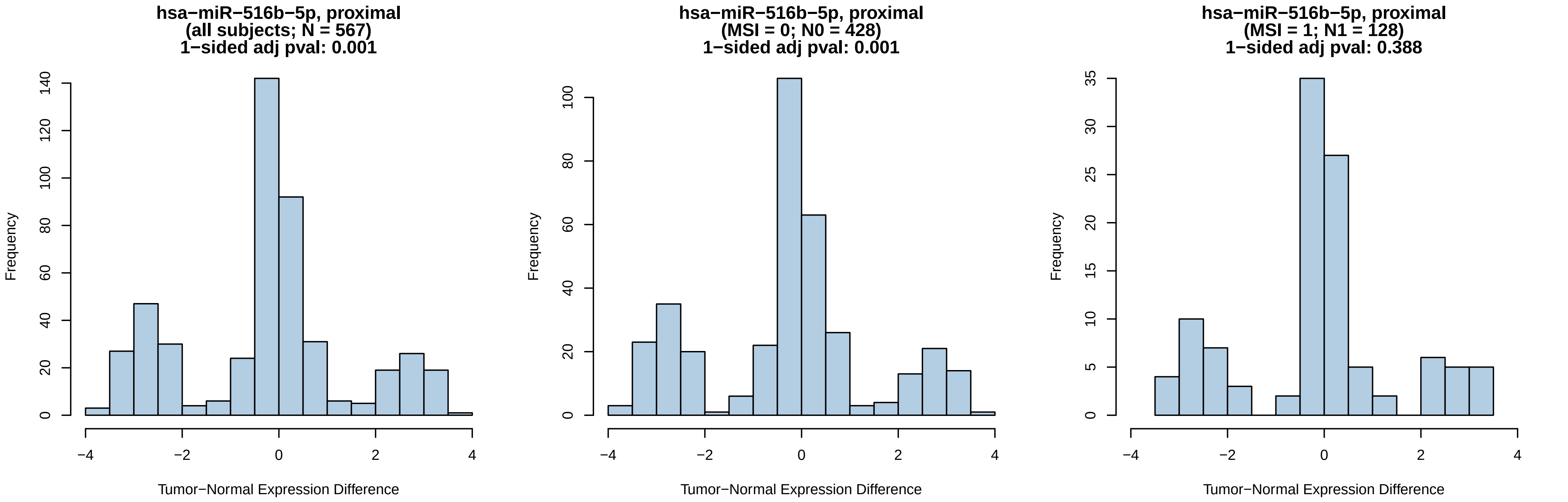
hsa−miR−516b−5p, proximal
(all subjects; N = 567)
1−sided adj pval: 0.001
Frequency
0
20
40
60
80
100
120
140
−4
−2
0
2
4
Tumor−Normal Expression Difference
hsa−miR−516b−5p, proximal
(MSI = 0; N0 = 428)
1−sided adj pval: 0.001
Frequency
0
20
40
60
80
100
−4
−2
0
2
4
Tumor−Normal Expression Difference
hsa−miR−516b−5p, proximal
(MSI = 1; N1 = 128)
1−sided adj pval: 0.388
Frequency
0
5
10
15
20
25
30
35
−4
−2
0
2
4
Tumor−Normal Expression Difference

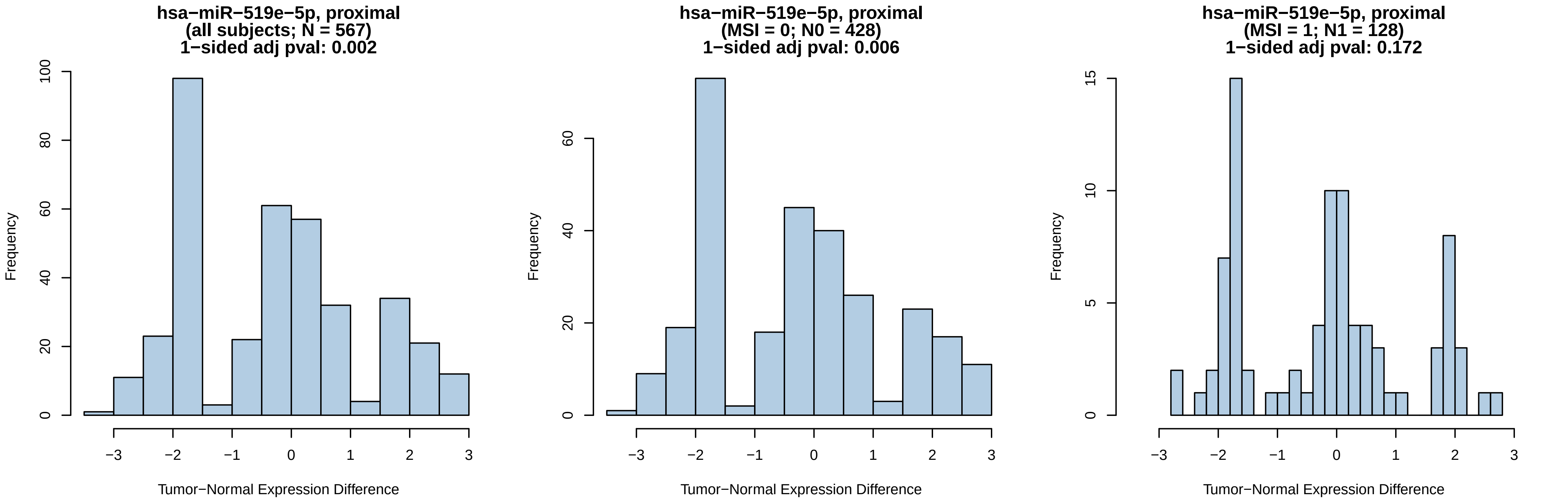

hsa−miR−519e−5p, proximal
(all subjects; N = 567)
1−sided adj pval: 0.002
Frequency
Tumor−Normal Expression Difference
hsa−miR−519e−5p, proximal
(MSI = 0; N0 = 428)
1−sided adj pval: 0.006
Frequency
Tumor−Normal Expression Difference
hsa−miR−519e−5p, proximal
(MSI = 1; N1 = 128)
1−sided adj pval: 0.172
Frequency
Tumor−Normal Expression Difference

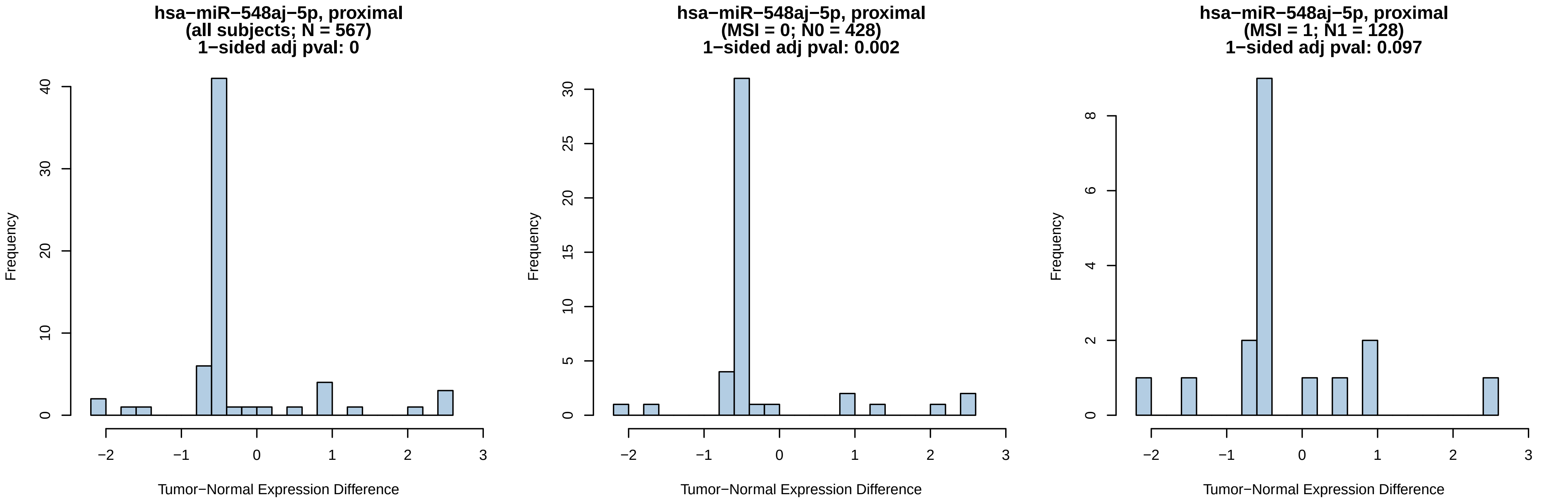
hsa−miR−548aj−5p, proximal
(all subjects; N = 567)
1−sided adj pval: 0
Frequency
0
10
20
30
40
−2
−1
0
1
2
3
Tumor−Normal Expression Difference
hsa−miR−548aj−5p, proximal
(MSI = 0; N0 = 428)
1−sided adj pval: 0.002
Frequency
0
5
10
15
20
25
30
−2
−1
0
1
2
3
Tumor−Normal Expression Difference
hsa−miR−548aj−5p, proximal
(MSI = 1; N1 = 128)
1−sided adj pval: 0.097
Frequency
0
2
4
6
8
−2
−1
0
1
2
3
Tumor−Normal Expression Difference

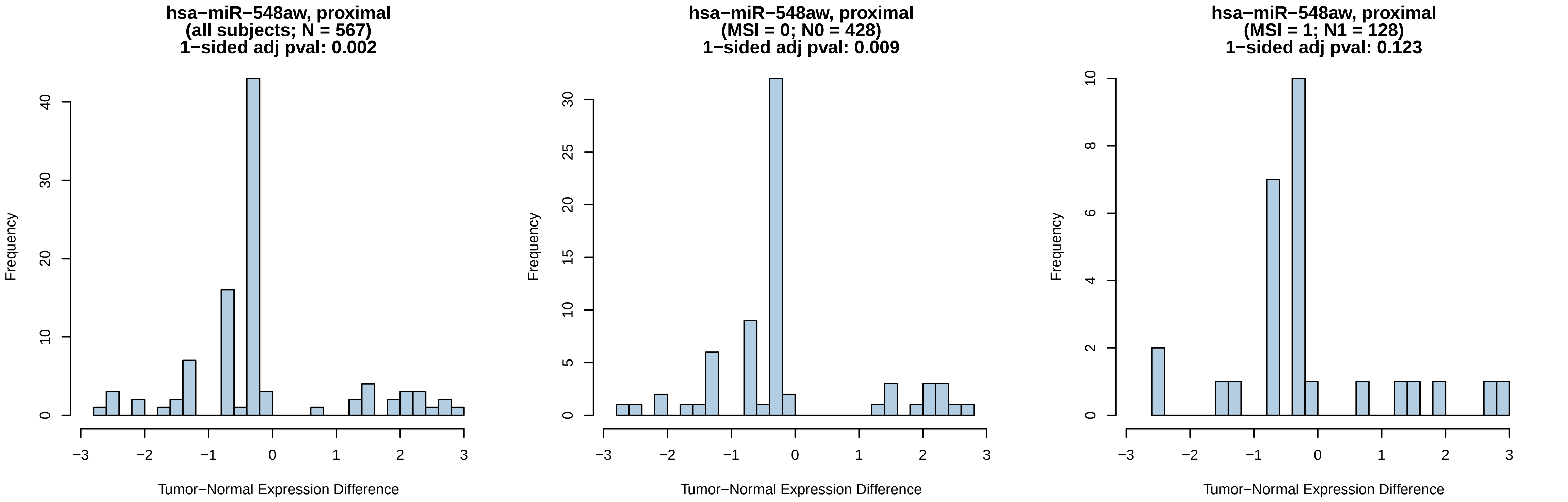
hsa−miR−548aw, proximal
(all subjects; N = 567)
1−sided adj pval: 0.002
hsa−miR−548aw, proximal
(MSI = 0; N0 = 428)
1−sided adj pval: 0.009
hsa−miR−548aw, proximal
(MSI = 1; N1 = 128)
1−sided adj pval: 0.123
Frequency
Tumor−Normal Expression Difference

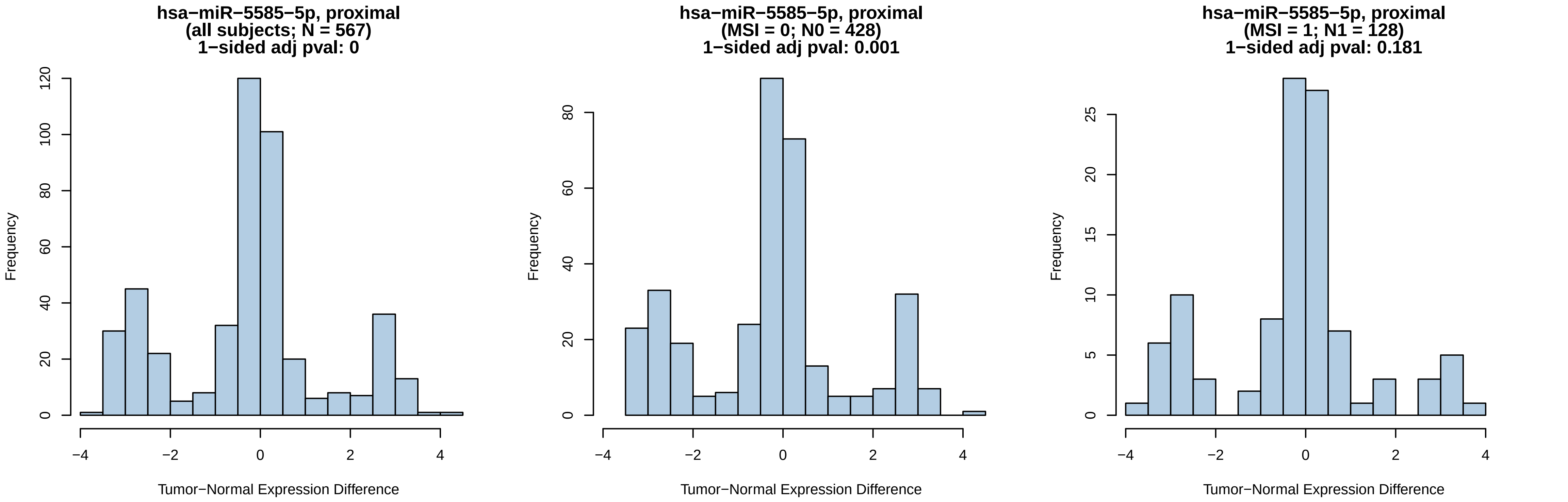
hsa−miR−5585−5p, proximal
(all subjects; N = 567)
1−sided adj pval: 0
Frequency
Tumor−Normal Expression Difference
hsa−miR−5585−5p, proximal
(MSI = 0; N0 = 428)
1−sided adj pval: 0.001
Frequency
Tumor−Normal Expression Difference
hsa−miR−5585−5p, proximal
(MSI = 1; N1 = 128)
1−sided adj pval: 0.181
Frequency
Tumor−Normal Expression Difference

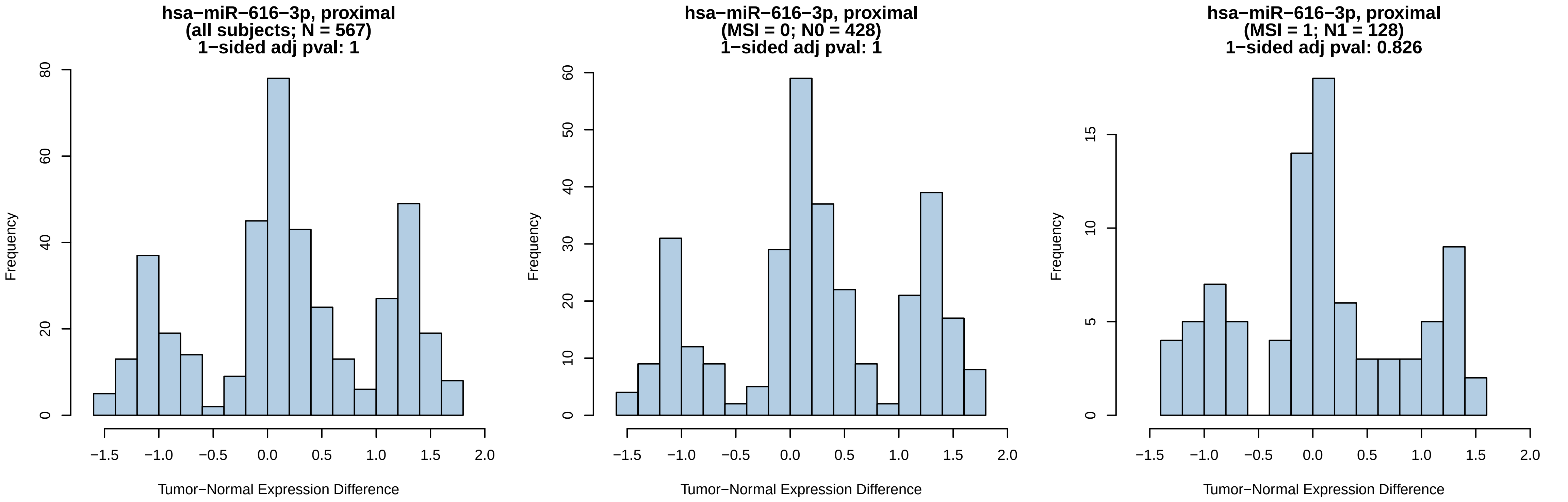
hsa−miR−616−3p, proximal
(all subjects; N = 567)
1−sided adj pval: 1
hsa−miR−616−3p, proximal
(MSI = 0; N0 = 428)
1−sided adj pval: 1
hsa−miR−616−3p, proximal
(MSI = 1; N1 = 128)
1−sided adj pval: 0.826
Frequency
Tumor−Normal Expression Difference

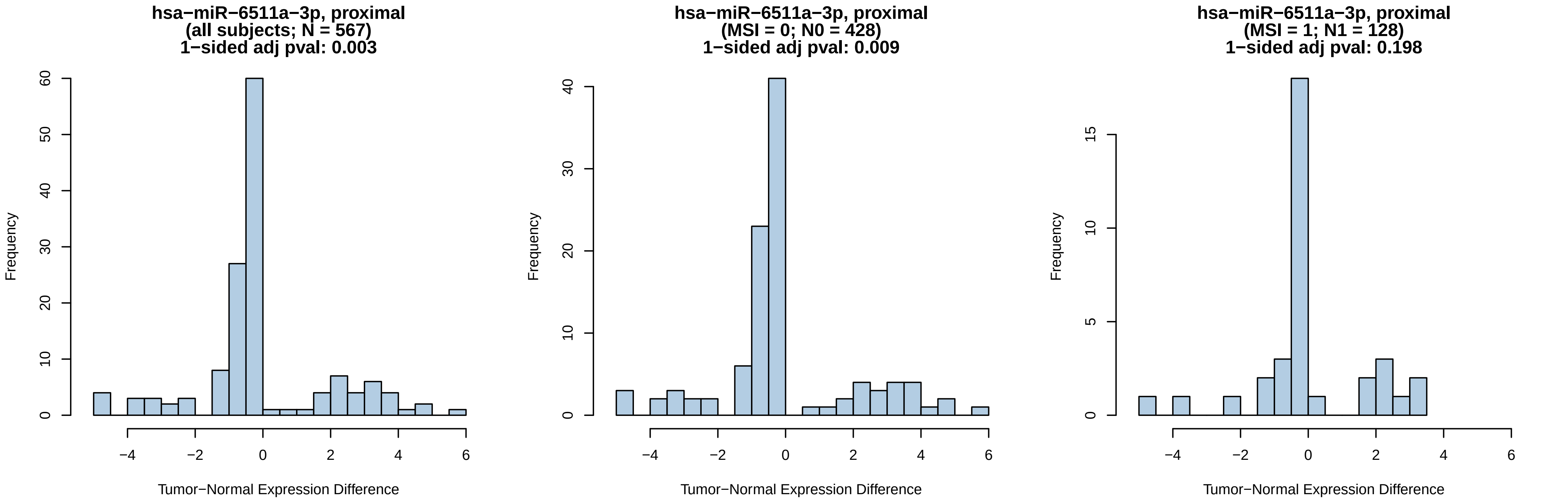
hsa−miR−6511a−3p, proximal
(all subjects; N = 567)
1−sided adj pval: 0.003
Frequency
Tumor−Normal Expression Difference
hsa−miR−6511a−3p, proximal
(MSI = 0; N0 = 428)
1−sided adj pval: 0.009
Frequency
Tumor−Normal Expression Difference
hsa−miR−6511a−3p, proximal
(MSI = 1; N1 = 128)
1−sided adj pval: 0.198
Frequency
Tumor−Normal Expression Difference

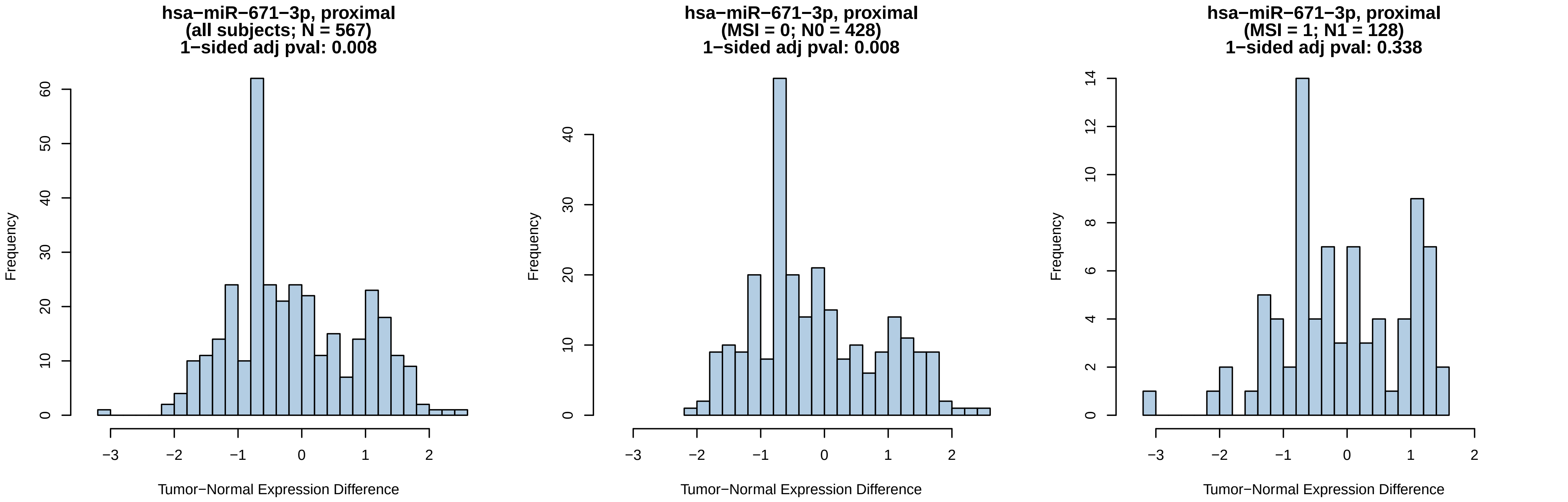
hsa−miR−671−3p, proximal
(all subjects; N = 567)
1−sided adj pval: 0.008
Frequency
Tumor−Normal Expression Difference
hsa−miR−671−3p, proximal
(MSI = 0; N0 = 428)
1−sided adj pval: 0.008
Frequency
Tumor−Normal Expression Difference
hsa−miR−671−3p, proximal
(MSI = 1; N1 = 128)
1−sided adj pval: 0.338
Frequency
Tumor−Normal Expression Difference

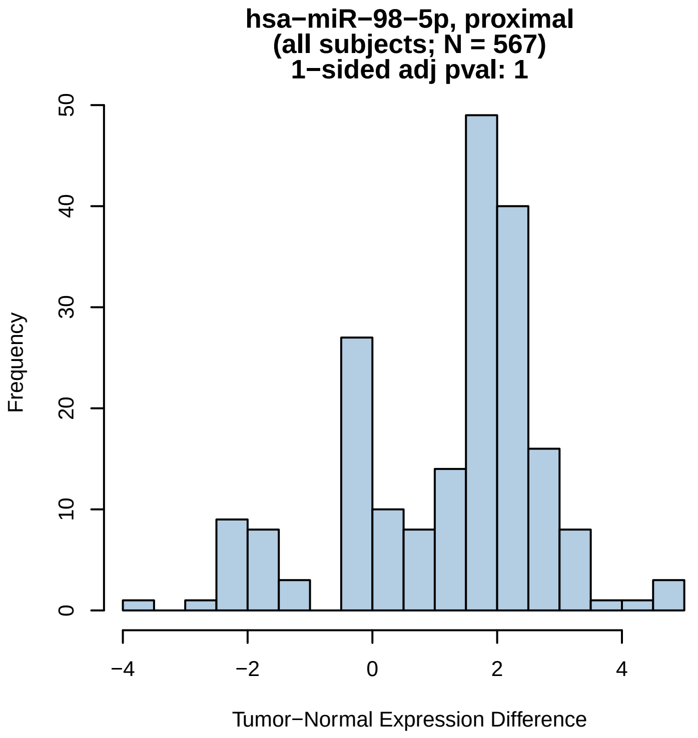
hsa−miR−98−5p, proximal
(all subjects; N = 567)
1−sided adj pval: 1
Frequency
Tumor−Normal Expression Difference
0
10
20
30
40
50
−4
−2
0
2
4

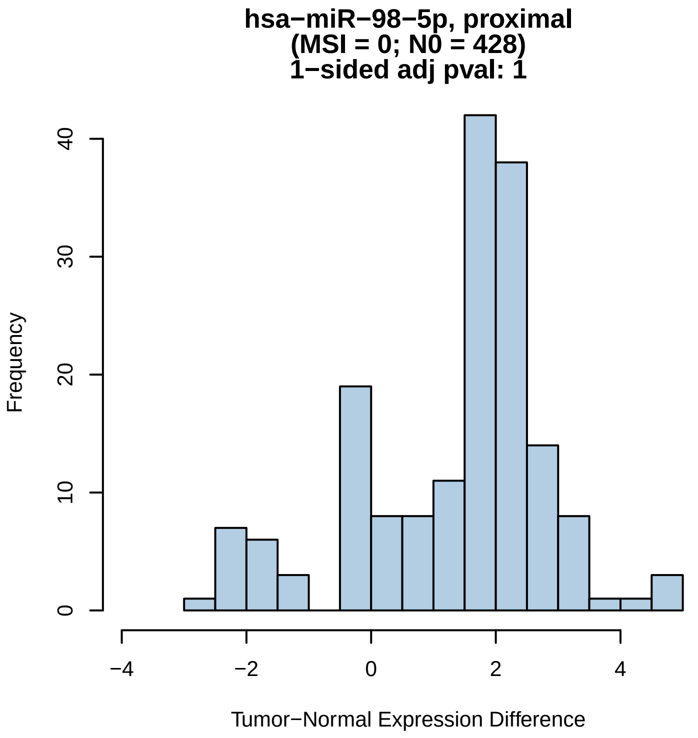
hsa−miR−98−5p, proximal
(MSI = 0; N0 = 428)
1−sided adj pval: 1
Frequency
Tumor−Normal Expression Difference
0
10
20
30
40
−4
−2
0
2
4

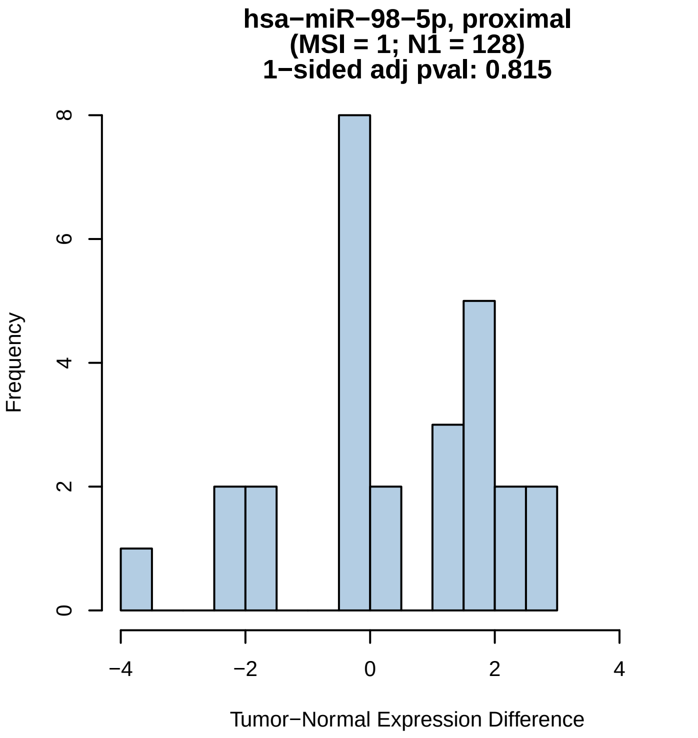
hsa−miR−98−5p, proximal
(MSI = 1; N1 = 128)
1−sided adj pval: 0.815
Frequency
Tumor−Normal Expression Difference
0
2
4
6
8
−4
−2
0
2
4

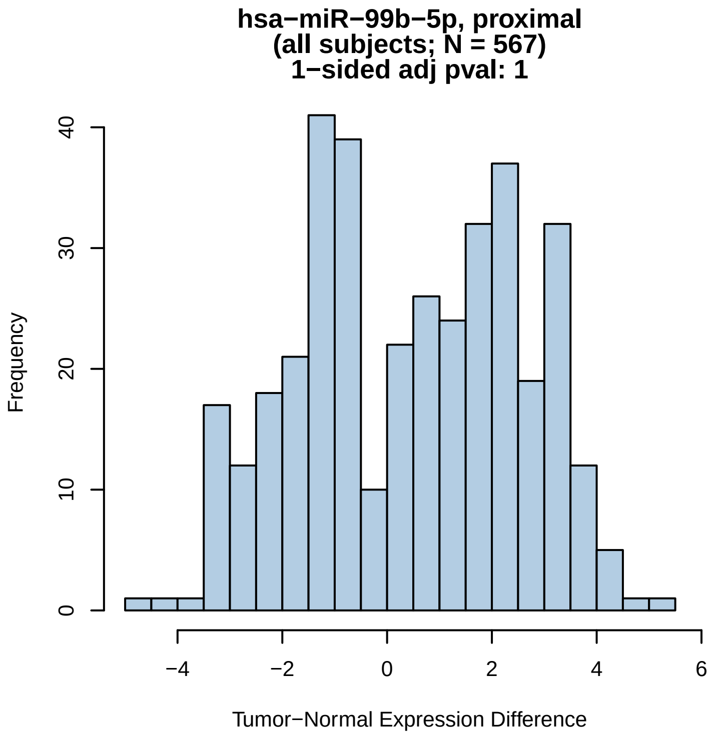
hsa−miR−99b−5p, proximal
(all subjects; N = 567)
1−sided adj pval: 1
Frequency
Tumor−Normal Expression Difference

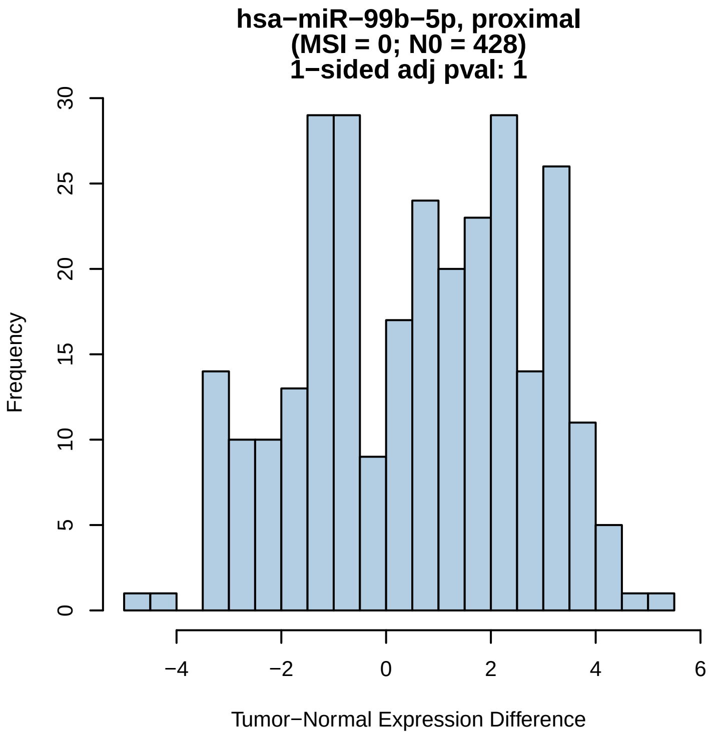
hsa−miR−99b−5p, proximal
(MSI = 0; N0 = 428)
1−sided adj pval: 1
Frequency
Tumor−Normal Expression Difference

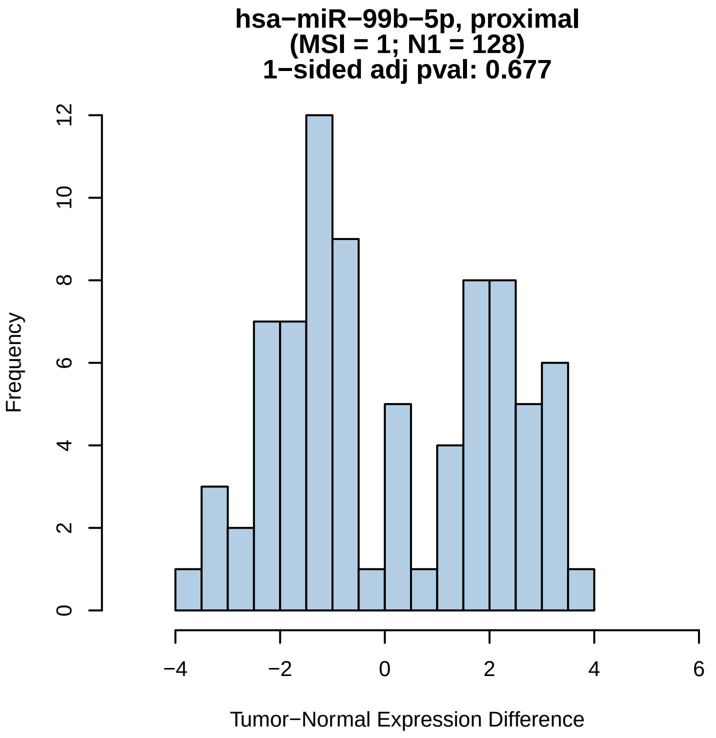
hsa−miR−99b−5p, proximal
(MSI = 1; N1 = 128)
1−sided adj pval: 0.677
Frequency
Tumor−Normal Expression Difference

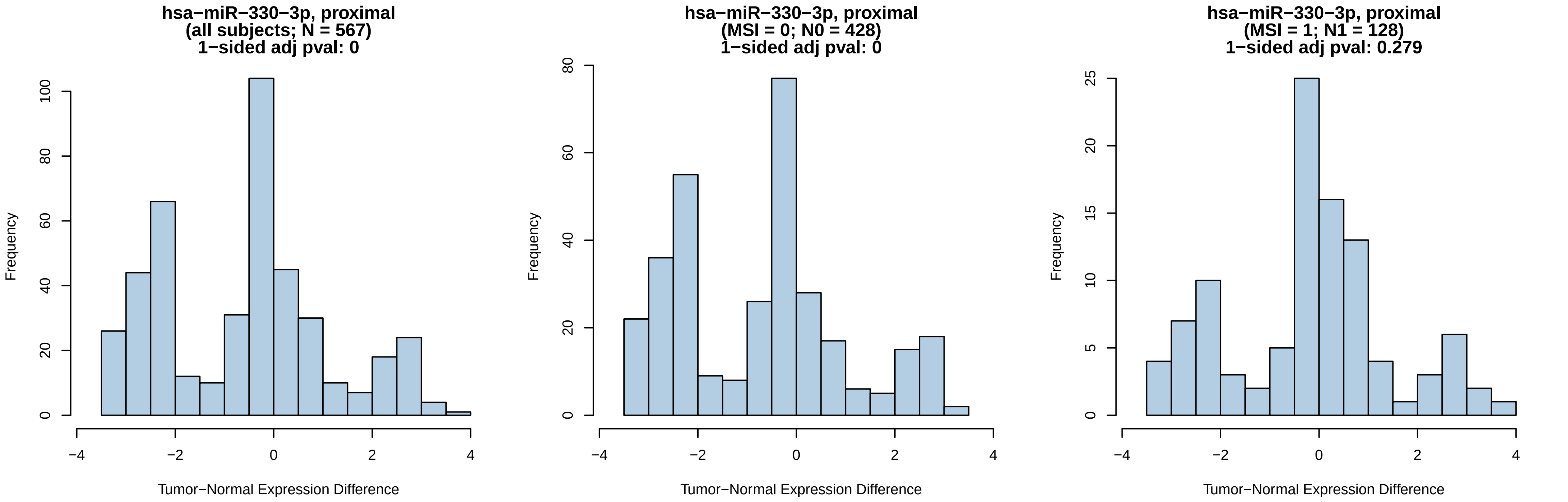
hsa−miR−330−3p, proximal
(all subjects; N = 567)
1−sided adj pval: 0
Frequency
0
20
40
60
80
100
−4
−2
0
2
4
Tumor−Normal Expression Difference
hsa−miR−330−3p, proximal
(MSI = 0; N0 = 428)
1−sided adj pval: 0
Frequency
0
20
40
60
80
−4
−2
0
2
4
Tumor−Normal Expression Difference
hsa−miR−330−3p, proximal
(MSI = 1; N1 = 128)
1−sided adj pval: 0.279
Frequency
0
5
10
15
20
25
−4
−2
0
2
4
Tumor−Normal Expression Difference

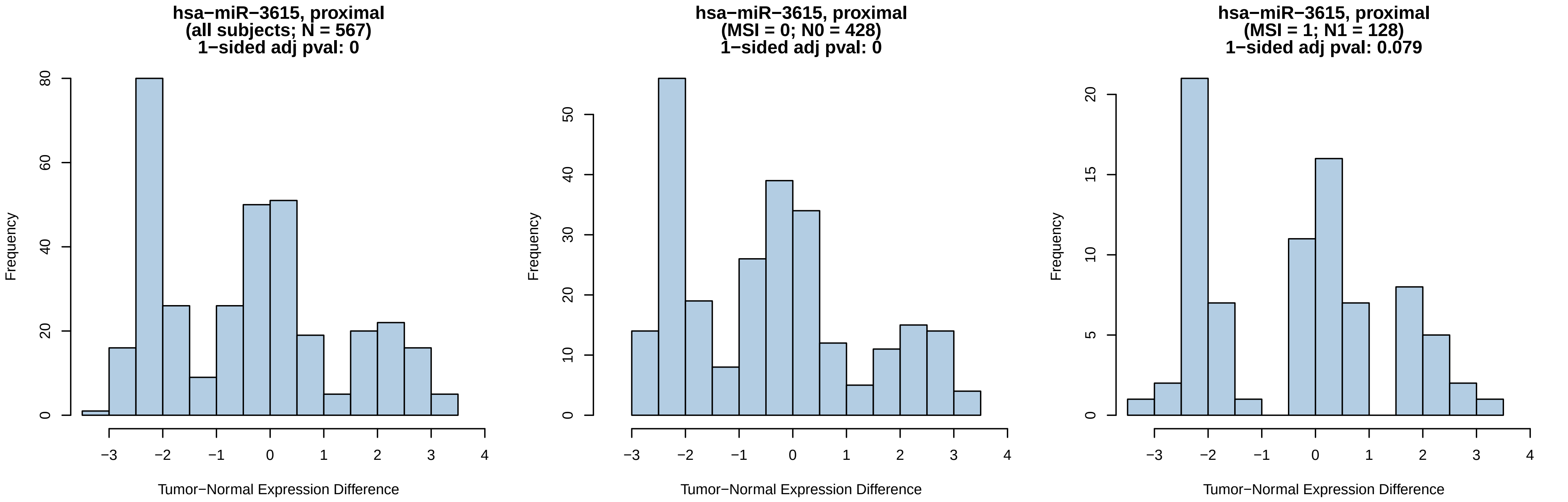
hsa−miR−3615, proximal
(all subjects; N = 567)
1−sided adj pval: 0
hsa−miR−3615, proximal
(MSI = 0; N0 = 428)
1−sided adj pval: 0
hsa−miR−3615, proximal
(MSI = 1; N1 = 128)
1−sided adj pval: 0.079
Frequency
Tumor−Normal Expression Difference

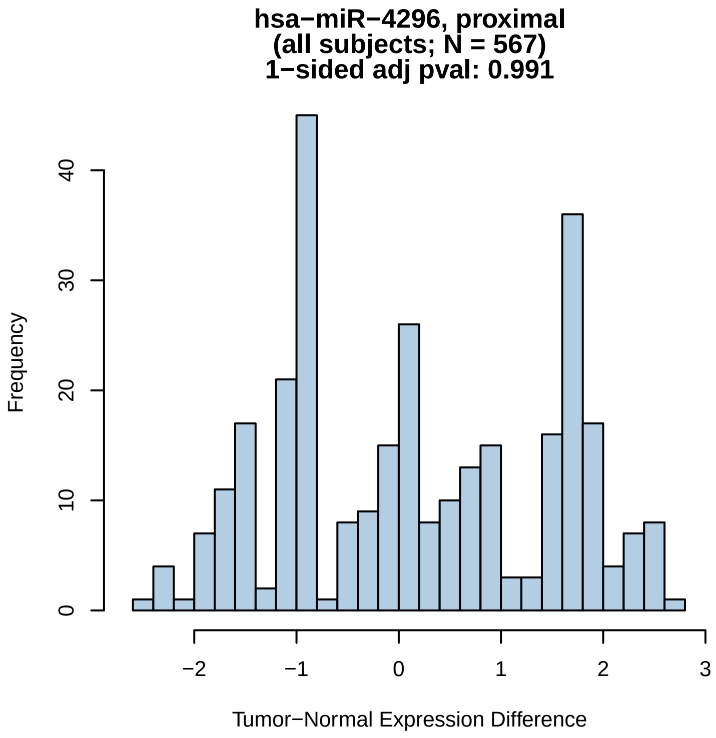
hsa−miR−4296, proximal
(all subjects; N = 567)
1−sided adj pval: 0.991
Frequency
Tumor−Normal Expression Difference

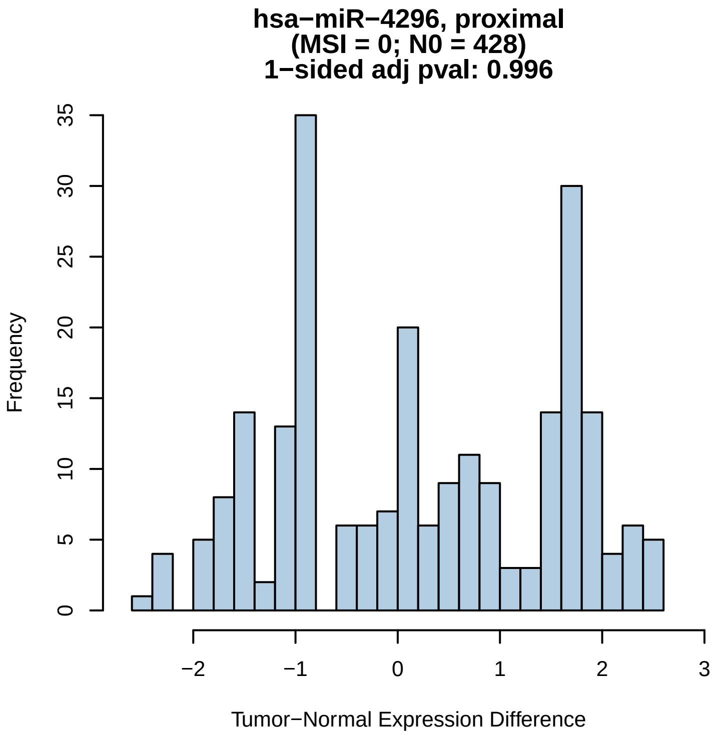
hsa−miR−4296, proximal
(MSI = 0; N0 = 428)
1−sided adj pval: 0.996
Frequency
Tumor−Normal Expression Difference

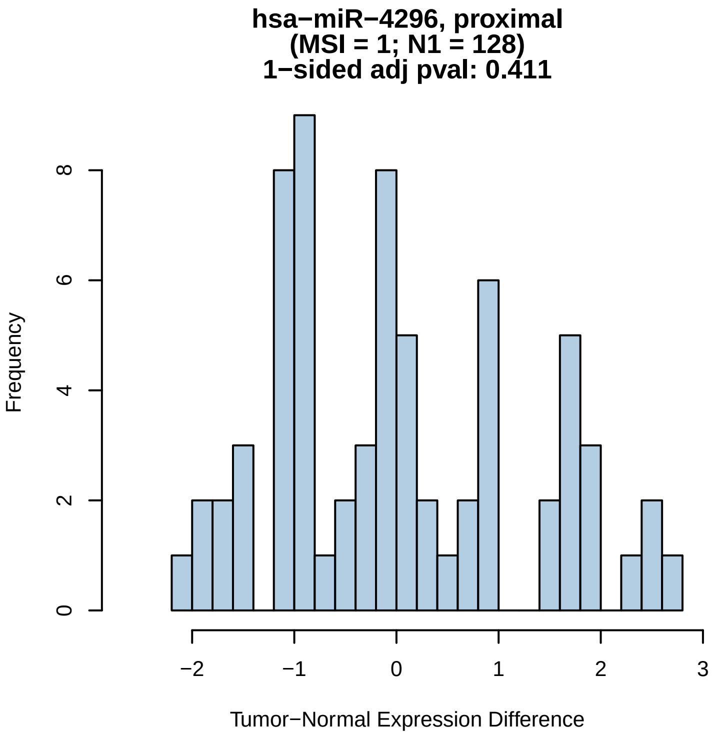
hsa−miR−4296, proximal
(MSI = 1; N1 = 128)
1−sided adj pval: 0.411
Frequency
Tumor−Normal Expression Difference

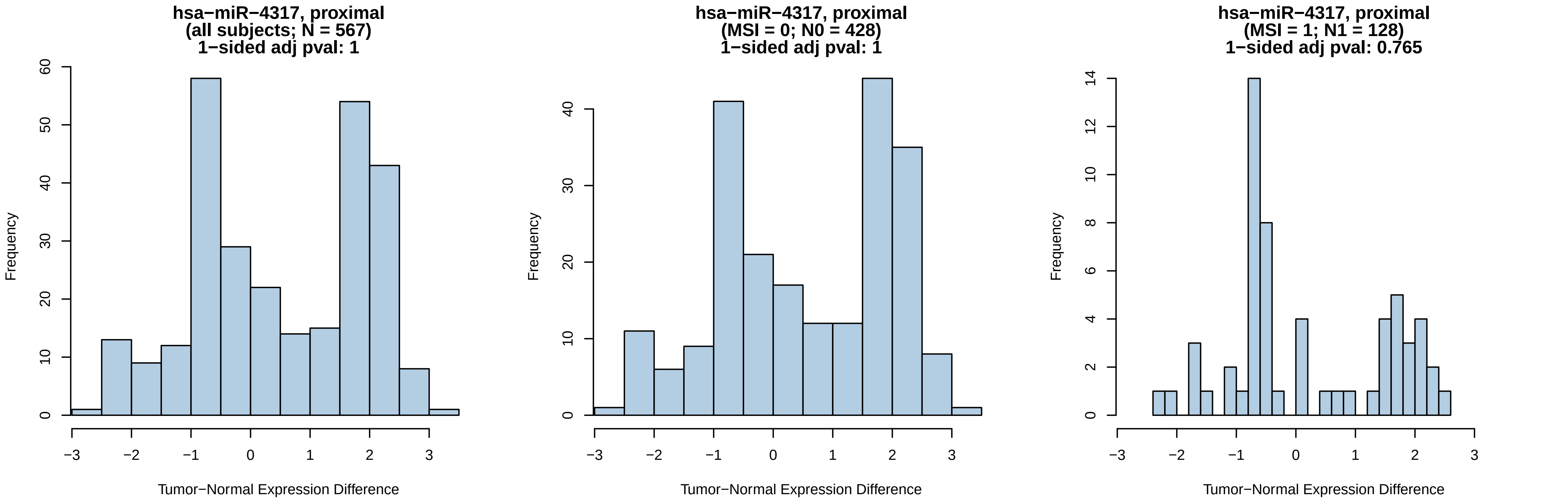
hsa−miR−4317, proximal
(all subjects; N = 567)
1−sided adj pval: 1
Frequency
Tumor−Normal Expression Difference
hsa−miR−4317, proximal
(MSI = 0; N0 = 428)
1−sided adj pval: 1
Frequency
Tumor−Normal Expression Difference
hsa−miR−4317, proximal
(MSI = 1; N1 = 128)
1−sided adj pval: 0.765
Frequency
Tumor−Normal Expression Difference

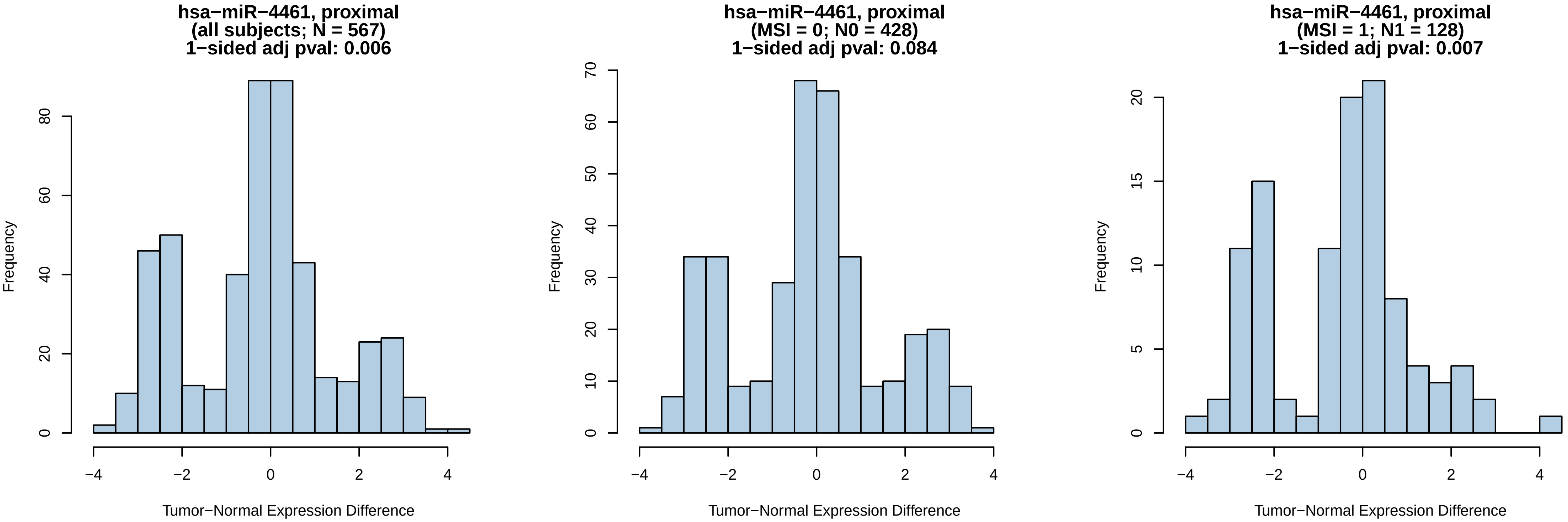
hsa−miR−4461, proximal
(all subjects; N = 567)
1−sided adj pval: 0.006
Frequency
0
20
40
60
80
−4
−2
0
2
4
Tumor−Normal Expression Difference
hsa−miR−4461, proximal
(MSI = 0; N0 = 428)
1−sided adj pval: 0.084
Frequency
0
10
20
30
40
50
60
70
−4
−2
0
2
4
Tumor−Normal Expression Difference
hsa−miR−4461, proximal
(MSI = 1; N1 = 128)
1−sided adj pval: 0.007
Frequency
0
5
10
15
20
−4
−2
0
2
4
Tumor−Normal Expression Difference

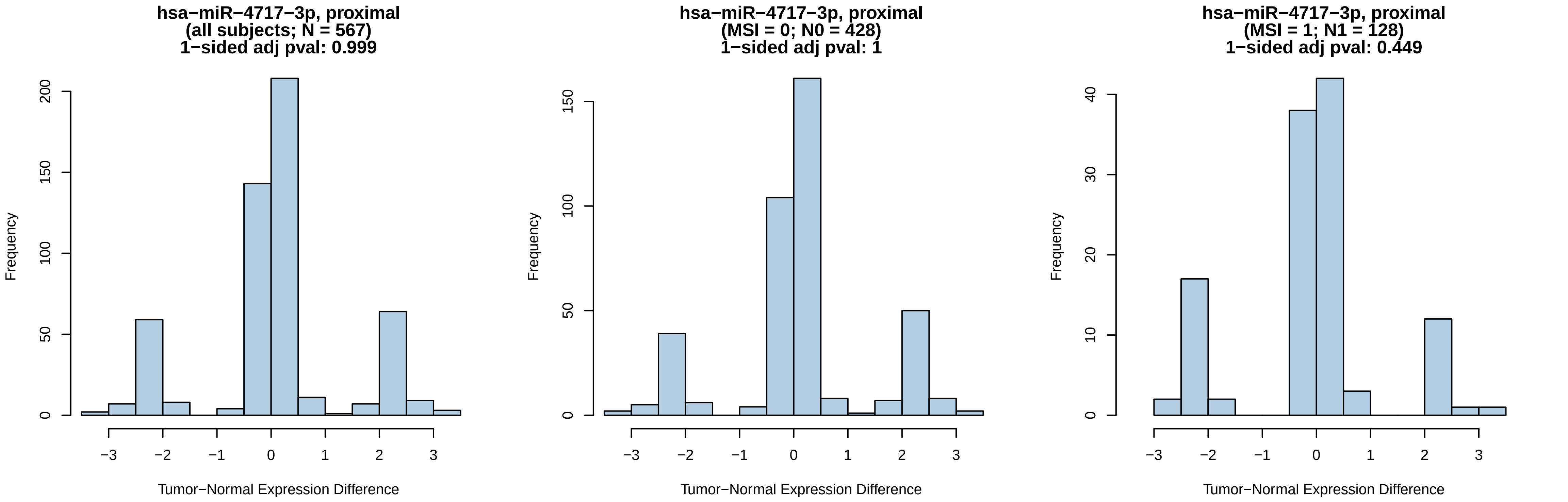

hsa−miR−4717−3p, proximal
(all subjects; N = 567)
1−sided adj pval: 0.999
Frequency
Tumor−Normal Expression Difference
hsa−miR−4717−3p, proximal
(MSI = 0; N0 = 428)
1−sided adj pval: 1
Frequency
Tumor−Normal Expression Difference
hsa−miR−4717−3p, proximal
(MSI = 1; N1 = 128)
1−sided adj pval: 0.449
Frequency
Tumor−Normal Expression Difference

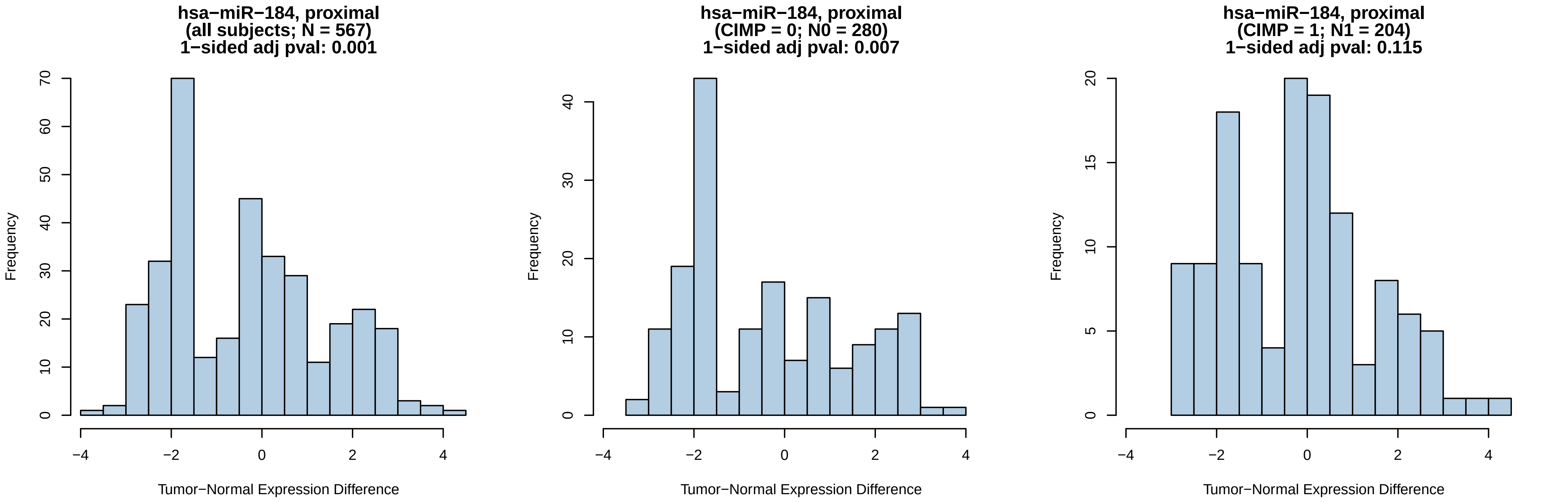
hsa−miR−184, proximal
(all subjects; N = 567)
1−sided adj pval: 0.001
hsa−miR−184, proximal
(CIMP = 0; N0 = 280)
1−sided adj pval: 0.007
hsa−miR−184, proximal
(CIMP = 1; N1 = 204)
1−sided adj pval: 0.115
Frequency
Tumor−Normal Expression Difference

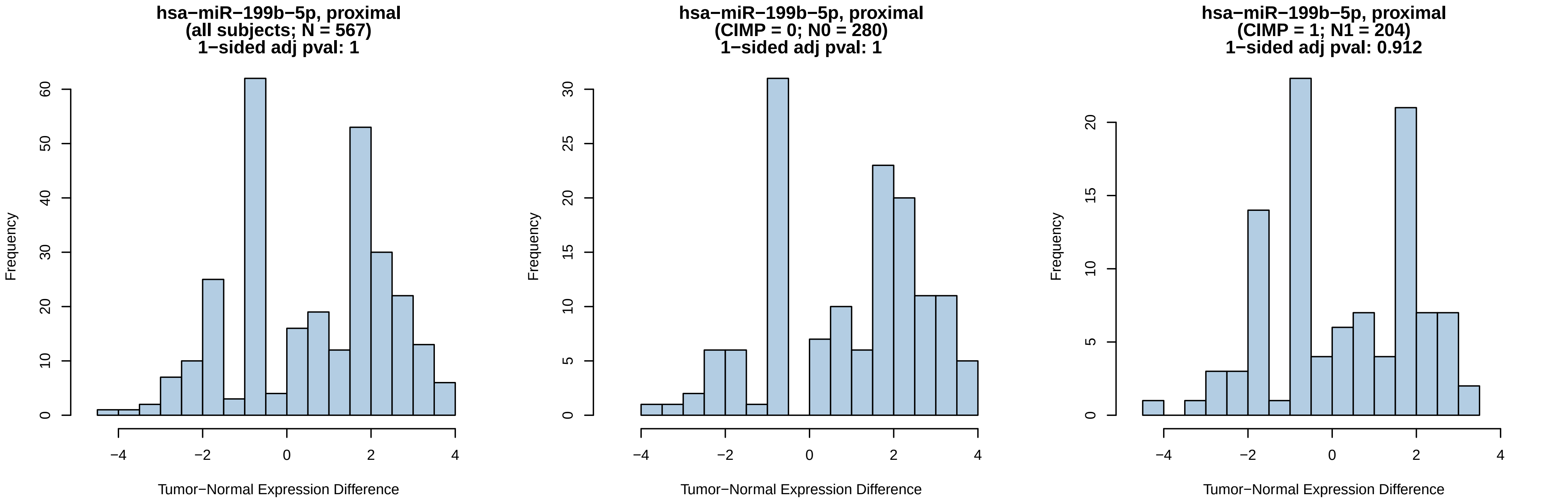
hsa−miR−199b−5p, proximal
(all subjects; N = 567)
1−sided adj pval: 1
Frequency
Tumor−Normal Expression Difference
hsa−miR−199b−5p, proximal
(CIMP = 0; N0 = 280)
1−sided adj pval: 1
Frequency
Tumor−Normal Expression Difference
hsa−miR−199b−5p, proximal
(CIMP = 1; N1 = 204)
1−sided adj pval: 0.912
Frequency
Tumor−Normal Expression Difference

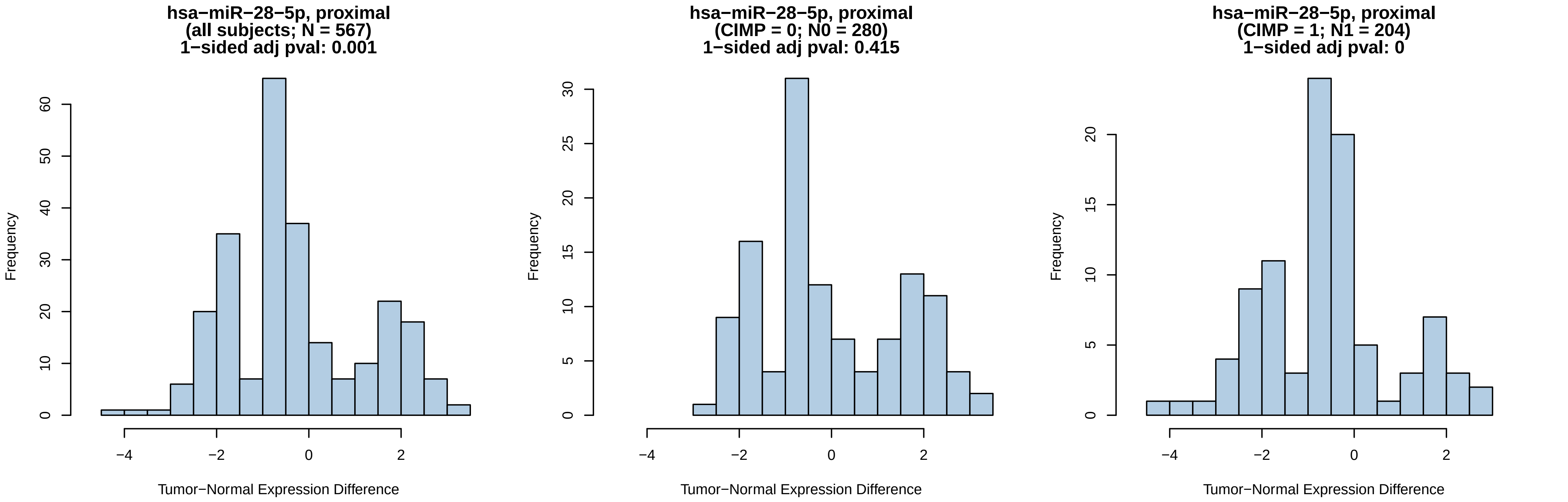
hsa−miR−28−5p, proximal
(all subjects; N = 567)
1−sided adj pval: 0.001
Frequency
Tumor−Normal Expression Difference
hsa−miR−28−5p, proximal
(CIMP = 0; N0 = 280)
1−sided adj pval: 0.415
Frequency
Tumor−Normal Expression Difference
hsa−miR−28−5p, proximal
(CIMP = 1; N1 = 204)
1−sided adj pval: 0
Frequency
Tumor−Normal Expression Difference

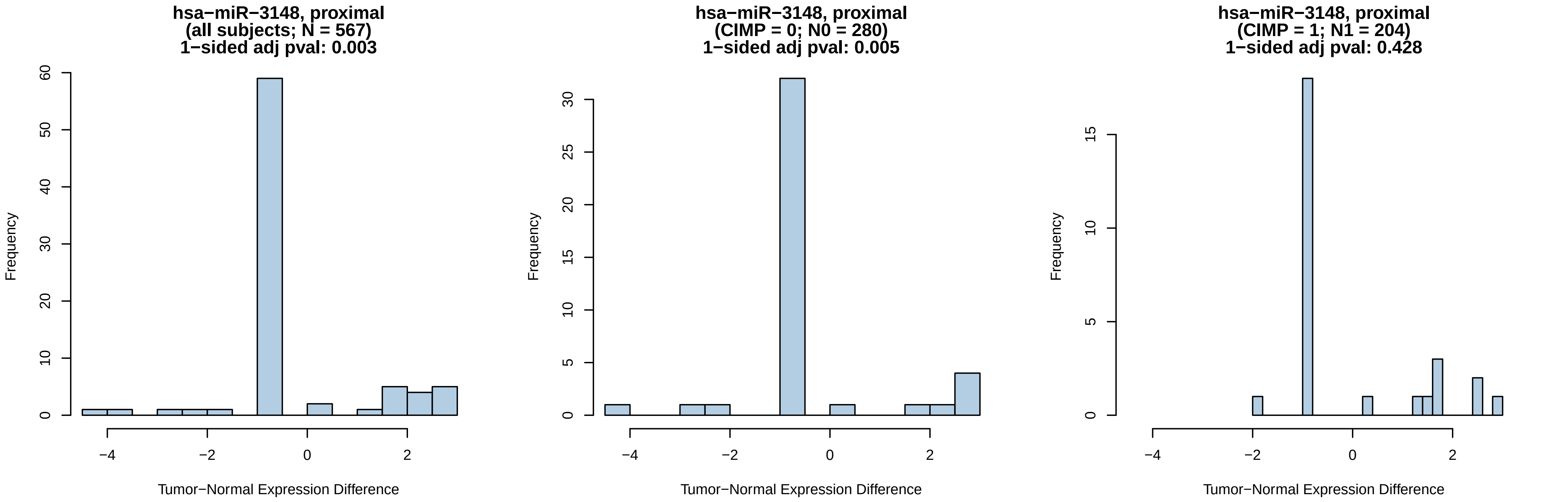
hsa−miR−3148, proximal
(all subjects; N = 567)
1−sided adj pval: 0.003
Frequency
Tumor−Normal Expression Difference
hsa−miR−3148, proximal
(CIMP = 0; N0 = 280)
1−sided adj pval: 0.005
Frequency
Tumor−Normal Expression Difference
hsa−miR−3148, proximal
(CIMP = 1; N1 = 204)
1−sided adj pval: 0.428
Frequency
Tumor−Normal Expression Difference

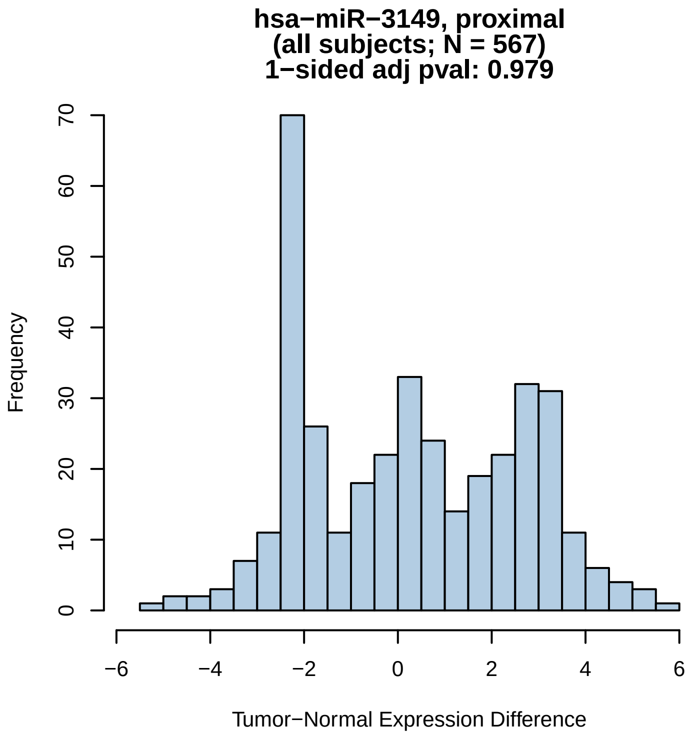
hsa−miR−3149, proximal
(all subjects; N = 567)
1−sided adj pval: 0.979
Frequency
Tumor−Normal Expression Difference

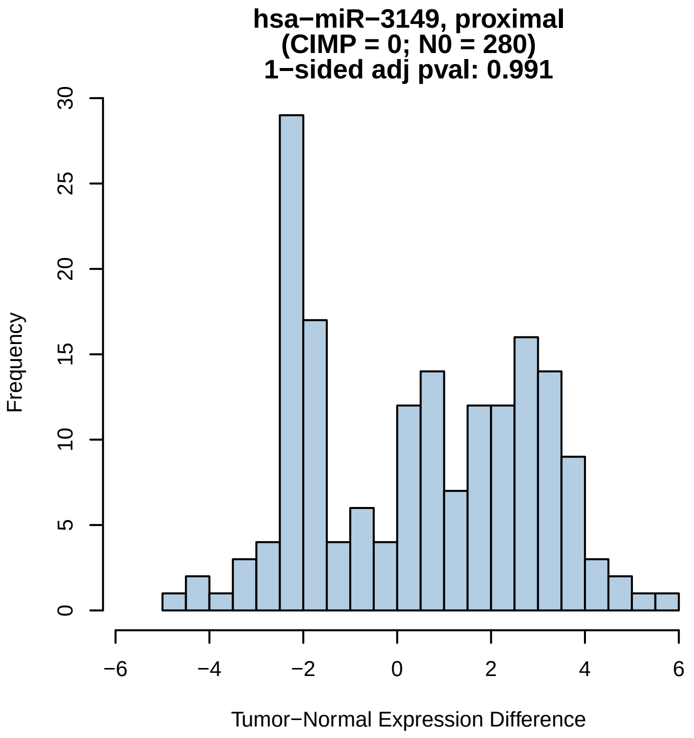
hsa−miR−3149, proximal
(CIMP = 0; N0 = 280)
1−sided adj pval: 0.991
Frequency
Tumor−Normal Expression Difference

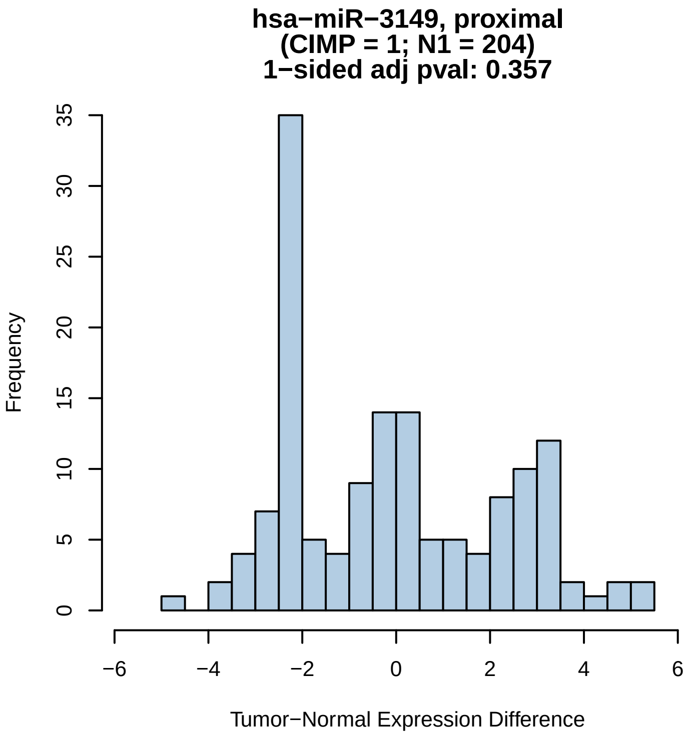
hsa−miR−3149, proximal
(CIMP = 1; N1 = 204)
1−sided adj pval: 0.357
Frequency
Tumor−Normal Expression Difference

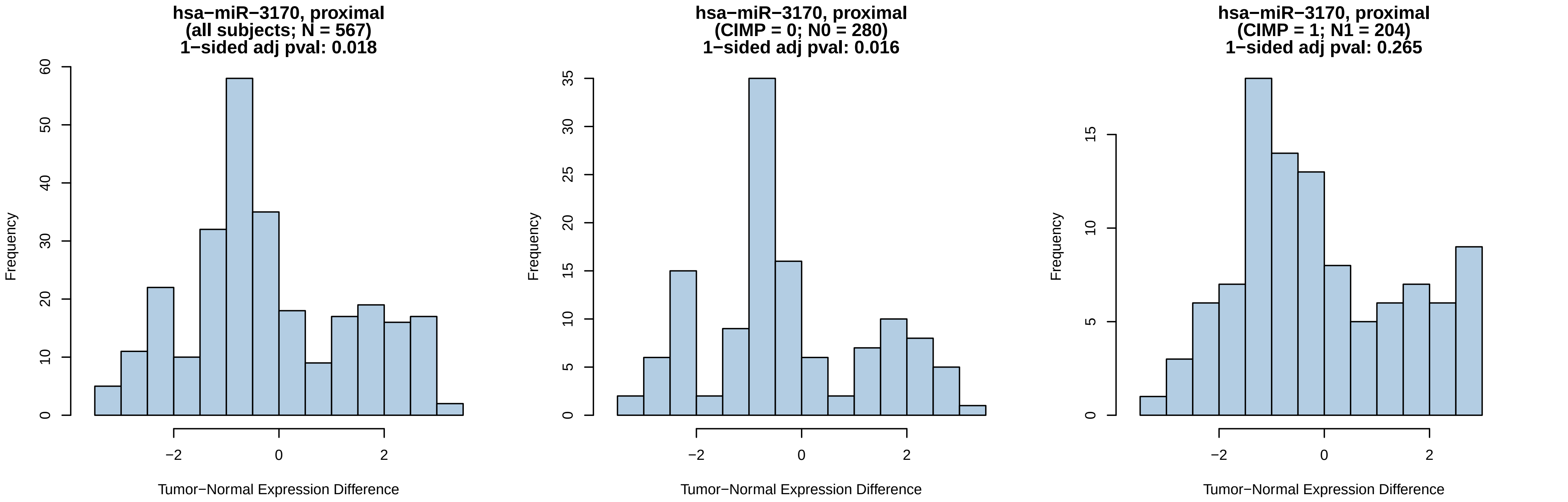

hsa−miR−3170, proximal
(all subjects; N = 567)
1−sided adj pval: 0.018
Frequency
Tumor−Normal Expression Difference
hsa−miR−3170, proximal
(CIMP = 0; N0 = 280)
1−sided adj pval: 0.016
Frequency
Tumor−Normal Expression Difference
hsa−miR−3170, proximal
(CIMP = 1; N1 = 204)
1−sided adj pval: 0.265
Frequency
Tumor−Normal Expression Difference

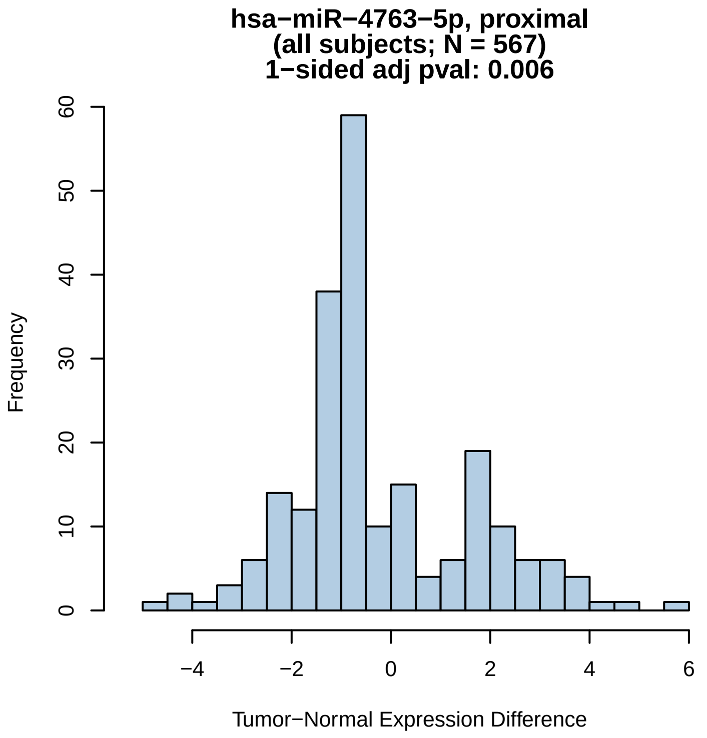
hsa−miR−4763−5p, proximal
(all subjects; N = 567)
1−sided adj pval: 0.006
Frequency
Tumor−Normal Expression Difference

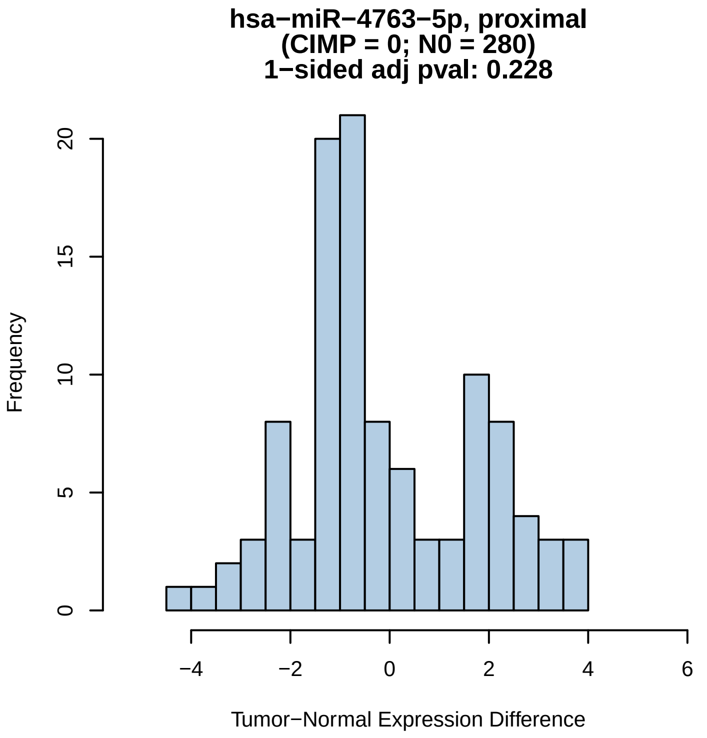
hsa−miR−4763−5p, proximal
(CIMP = 0; N0 = 280)
1−sided adj pval: 0.228
Frequency
Tumor−Normal Expression Difference

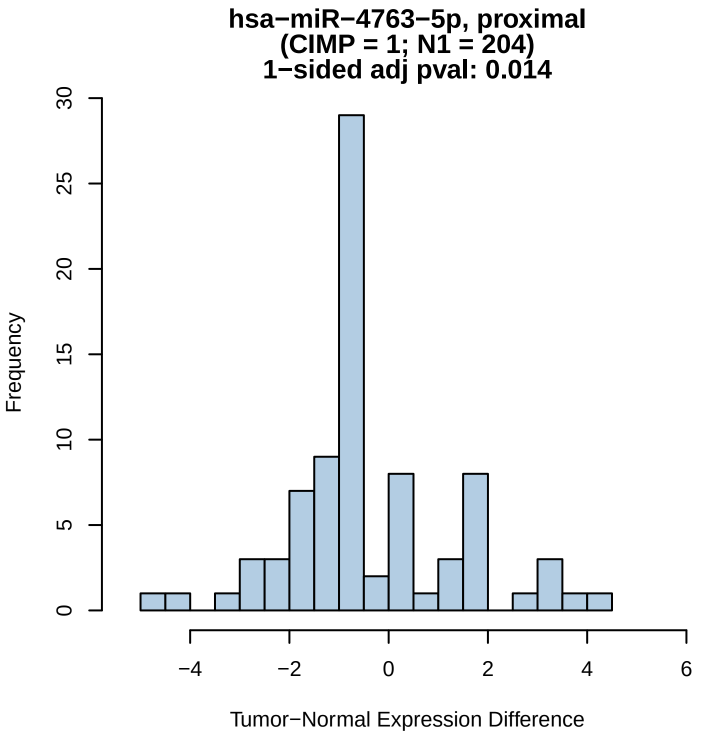
hsa−miR−4763−5p, proximal
(CIMP = 1; N1 = 204)
1−sided adj pval: 0.014
Frequency
Tumor−Normal Expression Difference

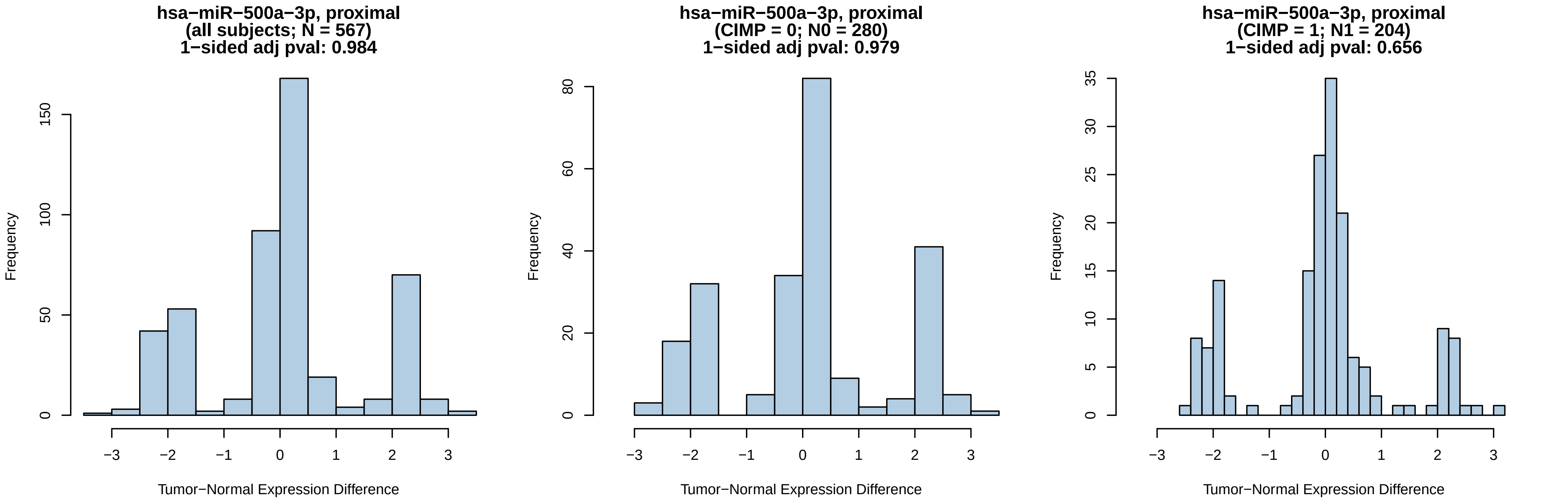
hsa−miR−500a−3p, proximal
(all subjects; N = 567)
1−sided adj pval: 0.984
Frequency
0
50
100
150
−3
−2
−1
0
1
2
3
Tumor−Normal Expression Difference
hsa−miR−500a−3p, proximal
(CIMP = 0; N0 = 280)
1−sided adj pval: 0.979
Frequency
0
20
40
60
80
−3
−2
−1
0
1
2
3
Tumor−Normal Expression Difference
hsa−miR−500a−3p, proximal
(CIMP = 1; N1 = 204)
1−sided adj pval: 0.656
Frequency
0
5
10
15
20
25
30
35
−3
−2
−1
0
1
2
3
Tumor−Normal Expression Difference

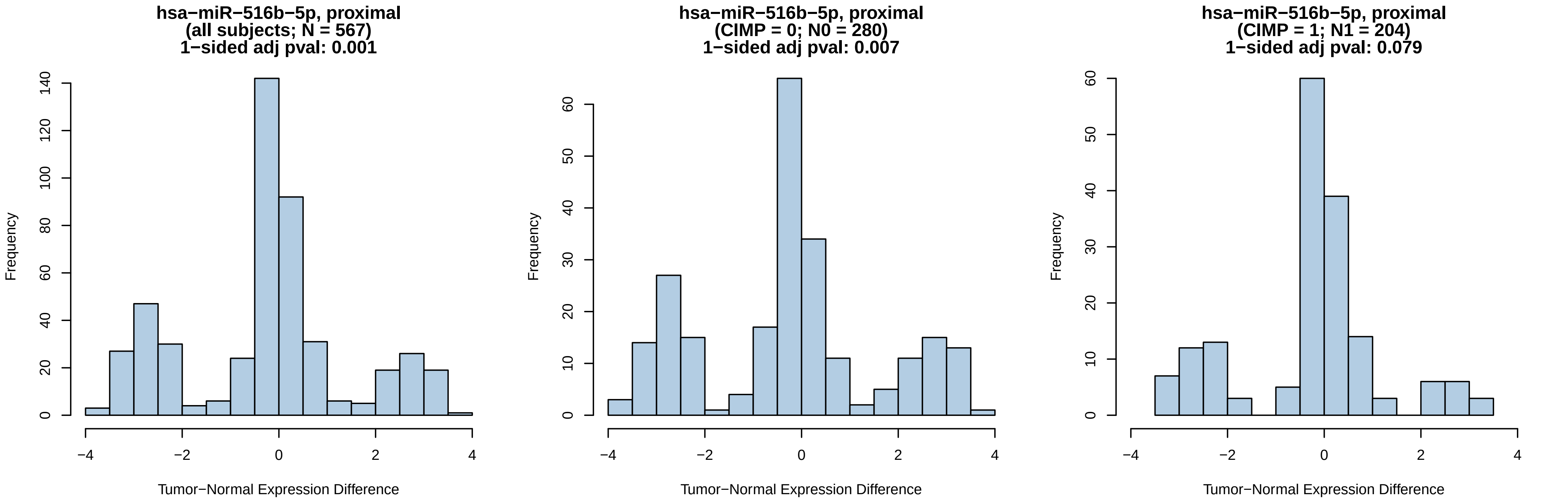
hsa−miR−516b−5p, proximal
(all subjects; N = 567)
1−sided adj pval: 0.001
Frequency
0
20
40
60
80
100
120
140
−4
−2
0
2
4
Tumor−Normal Expression Difference
hsa−miR−516b−5p, proximal
(CIMP = 0; N0 = 280)
1−sided adj pval: 0.007
Frequency
0
10
20
30
40
50
60
−4
−2
0
2
4
Tumor−Normal Expression Difference
hsa−miR−516b−5p, proximal
(CIMP = 1; N1 = 204)
1−sided adj pval: 0.079
Frequency
0
10
20
30
40
50
60
−4
−2
0
2
4
Tumor−Normal Expression Difference

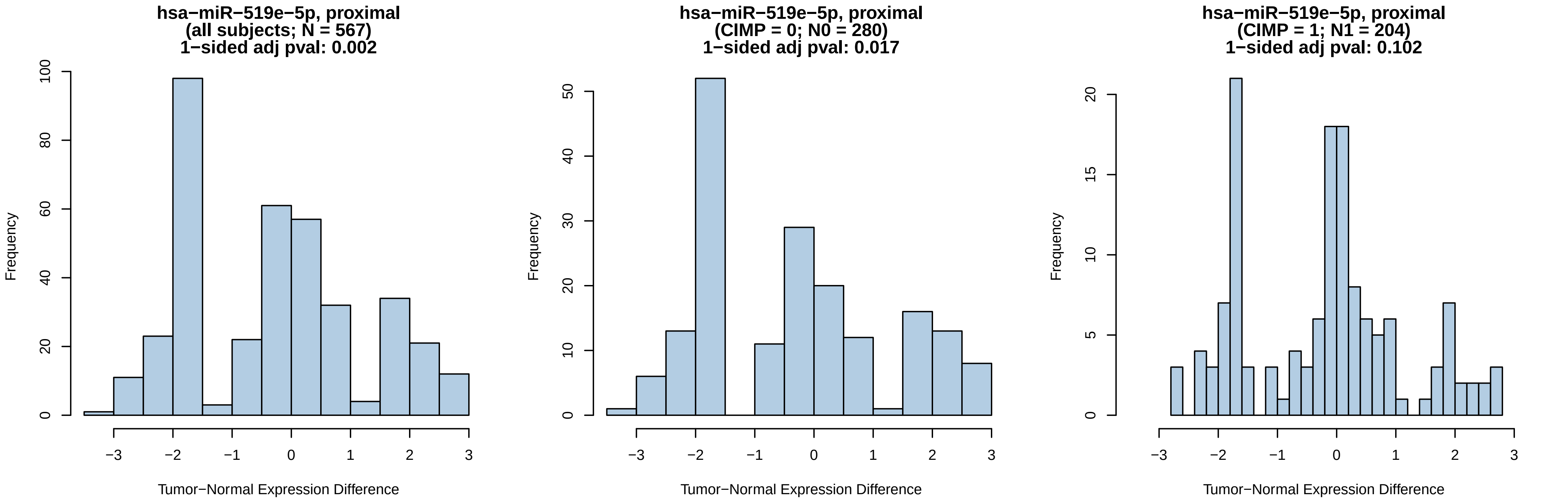
hsa−miR−519e−5p, proximal
(all subjects; N = 567)
1−sided adj pval: 0.002
Frequency
Tumor−Normal Expression Difference
hsa−miR−519e−5p, proximal
(CIMP = 0; N0 = 280)
1−sided adj pval: 0.017
Frequency
Tumor−Normal Expression Difference
hsa−miR−519e−5p, proximal
(CIMP = 1; N1 = 204)
1−sided adj pval: 0.102
Frequency
Tumor−Normal Expression Difference

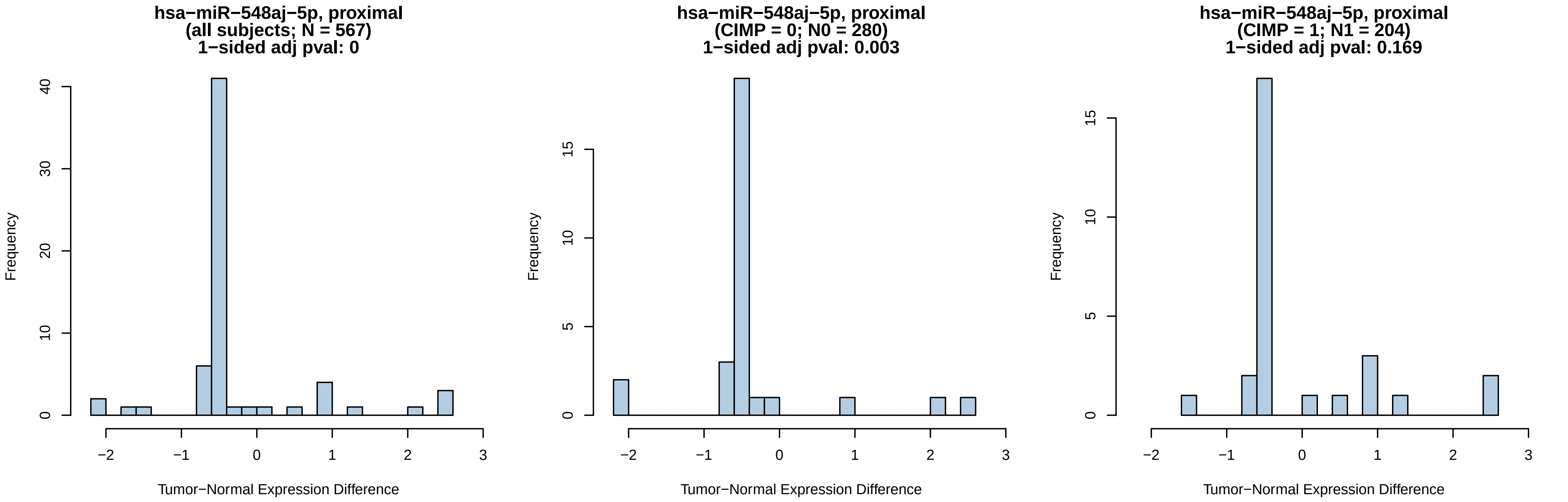

hsa−miR−548aj−5p, proximal
(all subjects; N = 567)
1−sided adj pval: 0
Frequency
Tumor−Normal Expression Difference
hsa−miR−548aj−5p, proximal
(CIMP = 0; N0 = 280)
1−sided adj pval: 0.003
Frequency
Tumor−Normal Expression Difference
hsa−miR−548aj−5p, proximal
(CIMP = 1; N1 = 204)
1−sided adj pval: 0.169
Frequency
Tumor−Normal Expression Difference

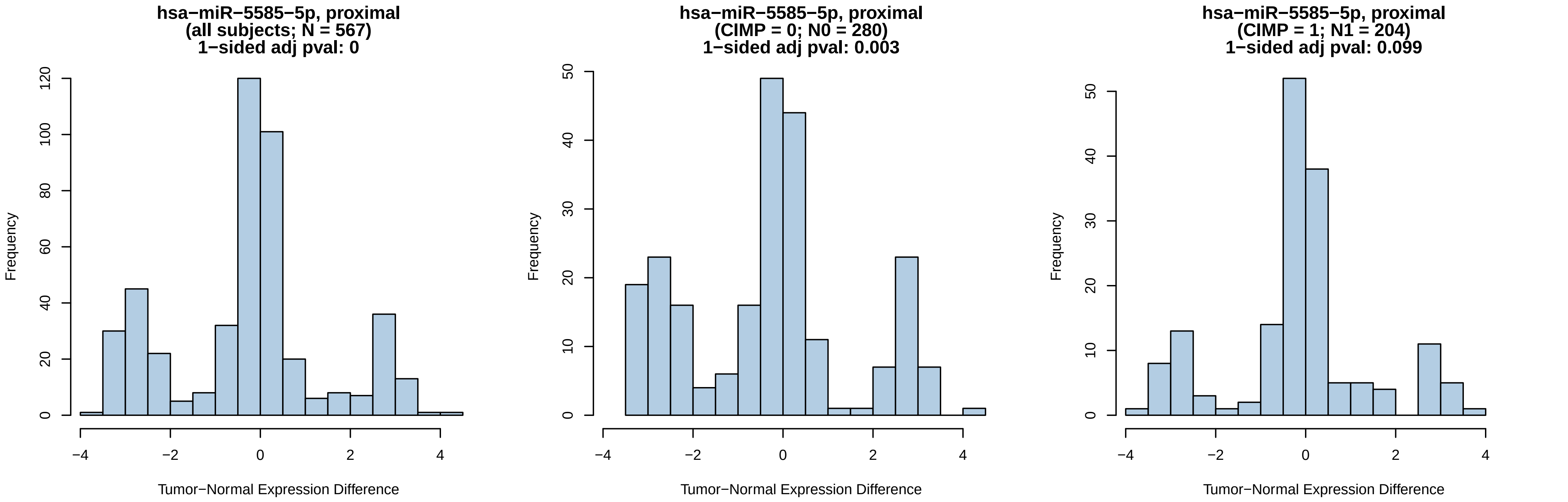
hsa−miR−5585−5p, proximal
(all subjects; N = 567)
1−sided adj pval: 0
hsa−miR−5585−5p, proximal
(CIMP = 0; N0 = 280)
1−sided adj pval: 0.003
hsa−miR−5585−5p, proximal
(CIMP = 1; N1 = 204)
1−sided adj pval: 0.099
Frequency
Tumor−Normal Expression Difference

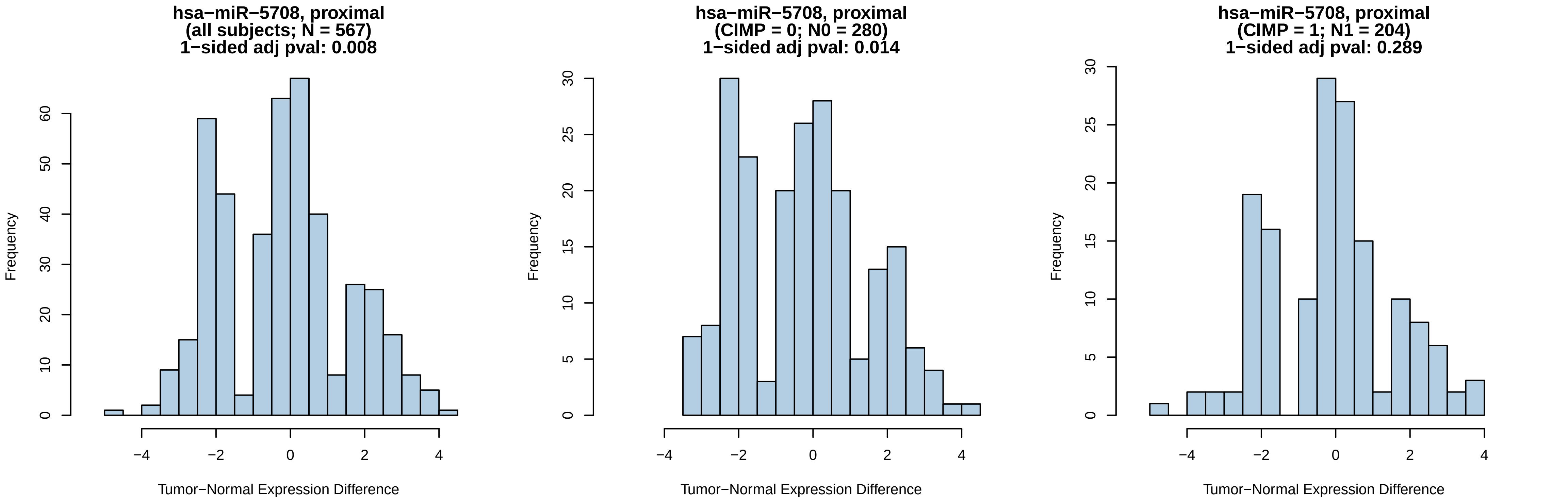
hsa−miR−5708, proximal
(all subjects; N = 567)
1−sided adj pval: 0.008
Frequency
Tumor−Normal Expression Difference
hsa−miR−5708, proximal
(CIMP = 0; N0 = 280)
1−sided adj pval: 0.014
Frequency
Tumor−Normal Expression Difference
hsa−miR−5708, proximal
(CIMP = 1; N1 = 204)
1−sided adj pval: 0.289
Frequency
Tumor−Normal Expression Difference

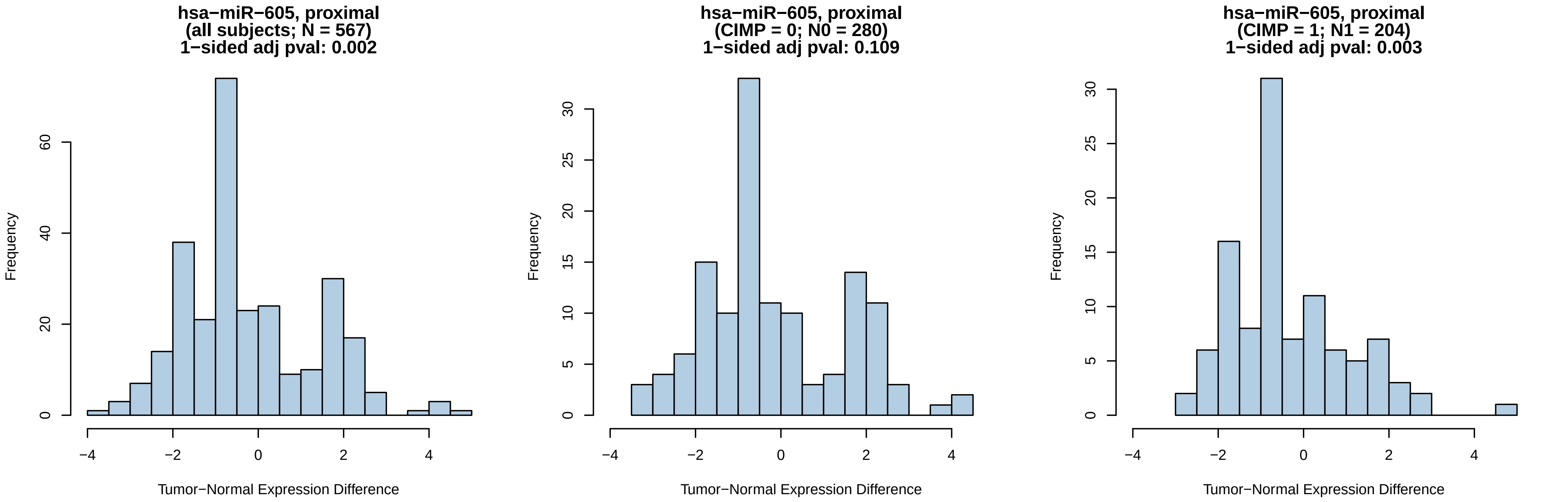
hsa−miR−605, proximal
(all subjects; N = 567)
1−sided adj pval: 0.002
Frequency
Tumor−Normal Expression Difference
hsa−miR−605, proximal
(CIMP = 0; N0 = 280)
1−sided adj pval: 0.109
Frequency
Tumor−Normal Expression Difference
hsa−miR−605, proximal
(CIMP = 1; N1 = 204)
1−sided adj pval: 0.003
Frequency
Tumor−Normal Expression Difference

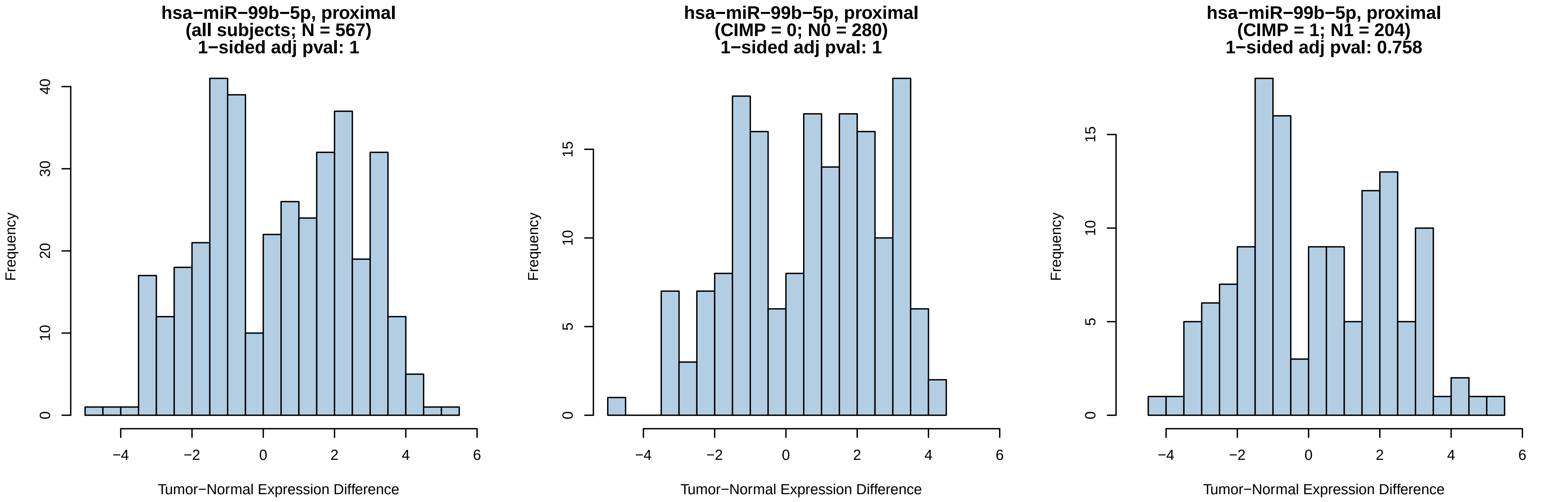
hsa−miR−99b−5p, proximal
(all subjects; N = 567)
1−sided adj pval: 1
hsa−miR−99b−5p, proximal
(CIMP = 0; N0 = 280)
1−sided adj pval: 1
hsa−miR−99b−5p, proximal
(CIMP = 1; N1 = 204)
1−sided adj pval: 0.758
Frequency
Tumor−Normal Expression Difference

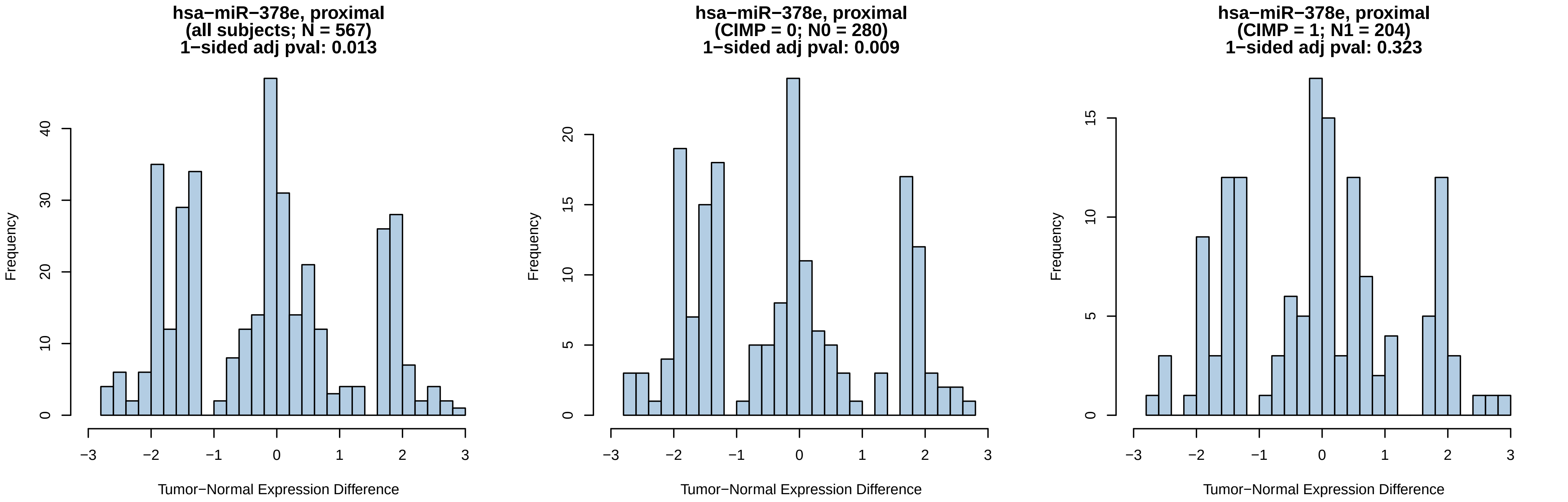
hsa−miR−378e, proximal
(all subjects; N = 567)
1−sided adj pval: 0.013
hsa−miR−378e, proximal
(CIMP = 0; N0 = 280)
1−sided adj pval: 0.009
hsa−miR−378e, proximal
(CIMP = 1; N1 = 204)
1−sided adj pval: 0.323
Frequency
Tumor−Normal Expression Difference
0
10
20
30
40
0
5
10
15
20
0
5
10
15
−3
−2
−1
0
1
2
3

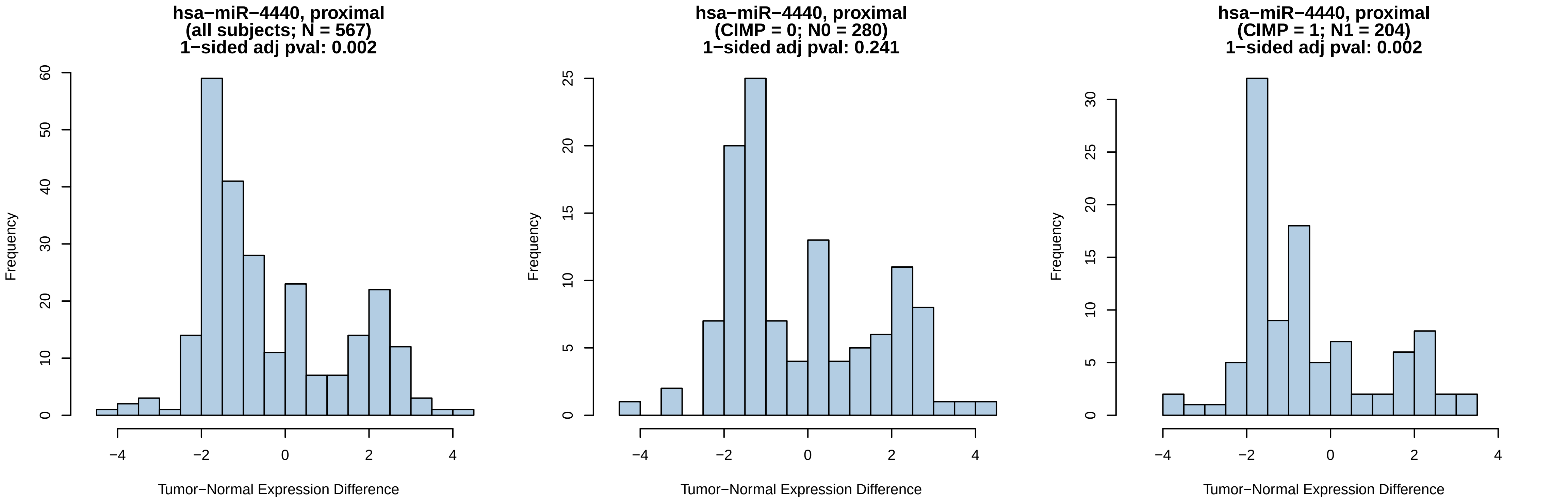
hsa−miR−4440, proximal
(all subjects; N = 567)
1−sided adj pval: 0.002
Frequency
Tumor−Normal Expression Difference
hsa−miR−4440, proximal
(CIMP = 0; N0 = 280)
1−sided adj pval: 0.241
Frequency
Tumor−Normal Expression Difference
hsa−miR−4440, proximal
(CIMP = 1; N1 = 204)
1−sided adj pval: 0.002
Frequency
Tumor−Normal Expression Difference

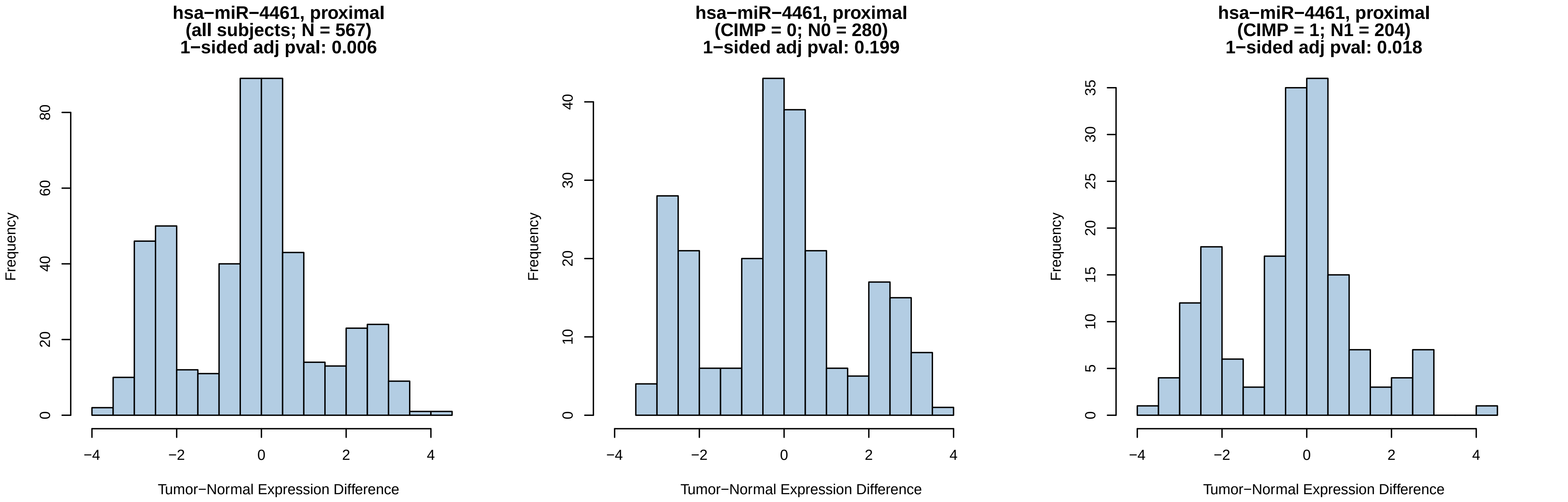
hsa−miR−4461, proximal
(all subjects; N = 567)
1−sided adj pval: 0.006
hsa−miR−4461, proximal
(CIMP = 0; N0 = 280)
1−sided adj pval: 0.199
hsa−miR−4461, proximal
(CIMP = 1; N1 = 204)
1−sided adj pval: 0.018
Frequency
Tumor−Normal Expression Difference

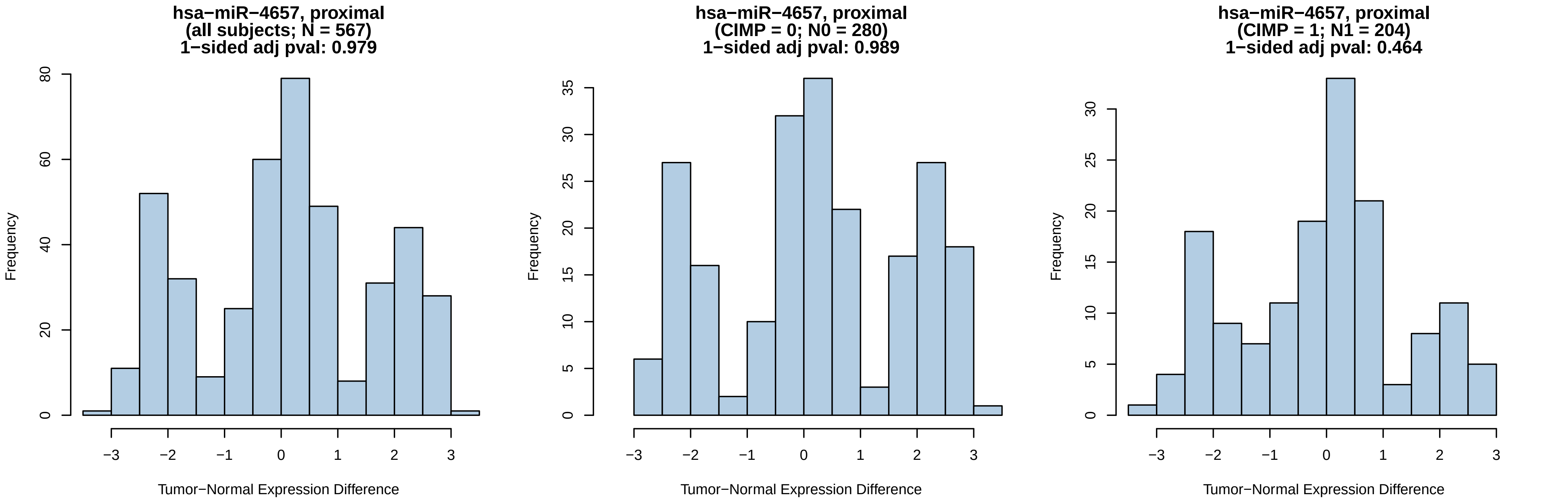
hsa−miR−4657, proximal
(all subjects; N = 567)
1−sided adj pval: 0.979
Frequency
Tumor−Normal Expression Difference
hsa−miR−4657, proximal
(CIMP = 0; N0 = 280)
1−sided adj pval: 0.989
Frequency
Tumor−Normal Expression Difference
hsa−miR−4657, proximal
(CIMP = 1; N1 = 204)
1−sided adj pval: 0.464
Frequency
Tumor−Normal Expression Difference

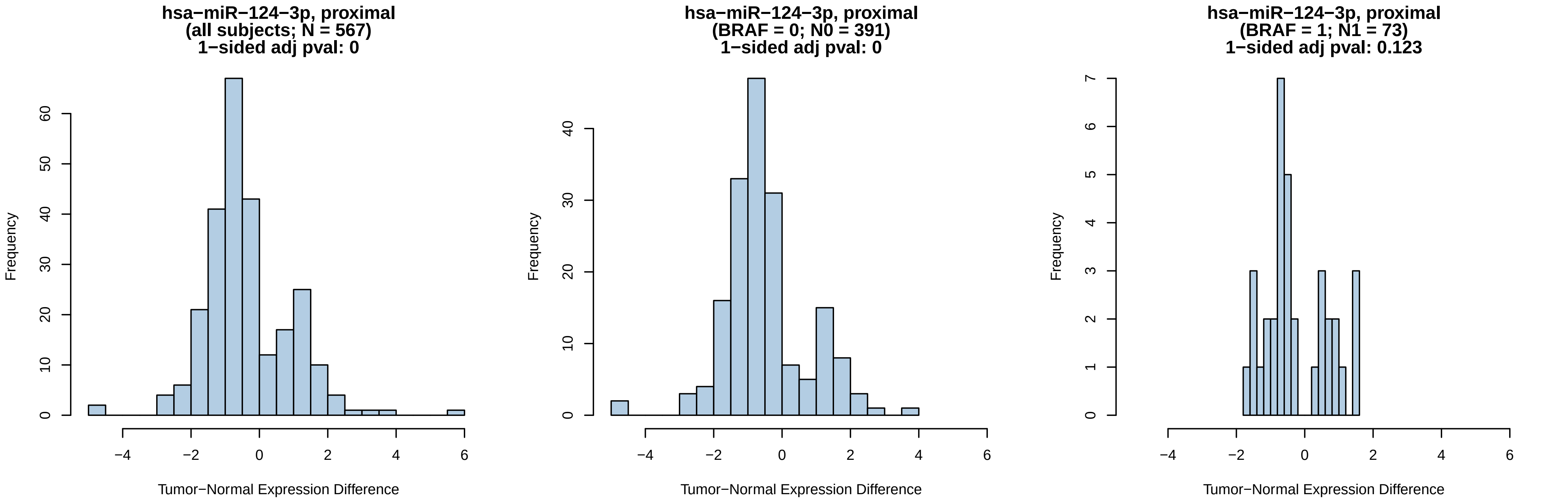
hsa−miR−124−3p, proximal
(all subjects; N = 567)
1−sided adj pval: 0
Frequency
Tumor−Normal Expression Difference
hsa−miR−124−3p, proximal
(BRAF = 0; N0 = 391)
1−sided adj pval: 0
Frequency
Tumor−Normal Expression Difference
hsa−miR−124−3p, proximal
(BRAF = 1; N1 = 73)
1−sided adj pval: 0.123
Frequency
Tumor−Normal Expression Difference

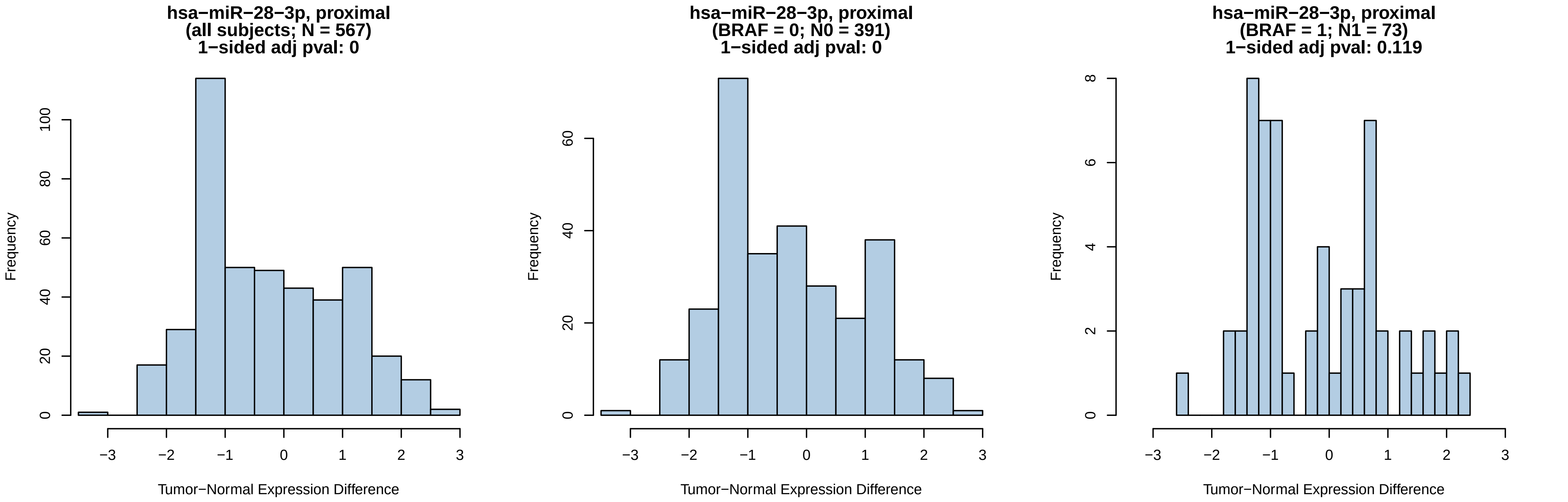
hsa−miR−28−3p, proximal
(all subjects; N = 567)
1−sided adj pval: 0
Frequency
Tumor−Normal Expression Difference
hsa−miR−28−3p, proximal
(BRAF = 0; N0 = 391)
1−sided adj pval: 0
Frequency
Tumor−Normal Expression Difference
hsa−miR−28−3p, proximal
(BRAF = 1; N1 = 73)
1−sided adj pval: 0.119
Frequency
Tumor−Normal Expression Difference

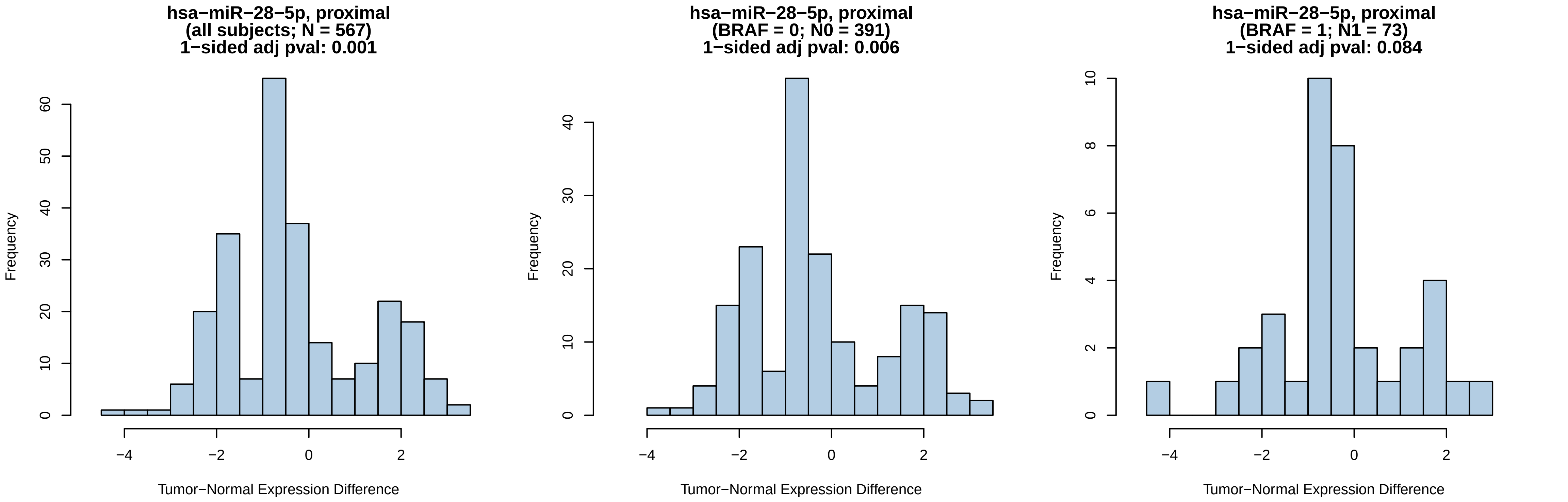
hsa−miR−28−5p, proximal
(all subjects; N = 567)
1−sided adj pval: 0.001
Frequency
Tumor−Normal Expression Difference
hsa−miR−28−5p, proximal
(BRAF = 0; N0 = 391)
1−sided adj pval: 0.006
Frequency
Tumor−Normal Expression Difference
hsa−miR−28−5p, proximal
(BRAF = 1; N1 = 73)
1−sided adj pval: 0.084
Frequency
Tumor−Normal Expression Difference

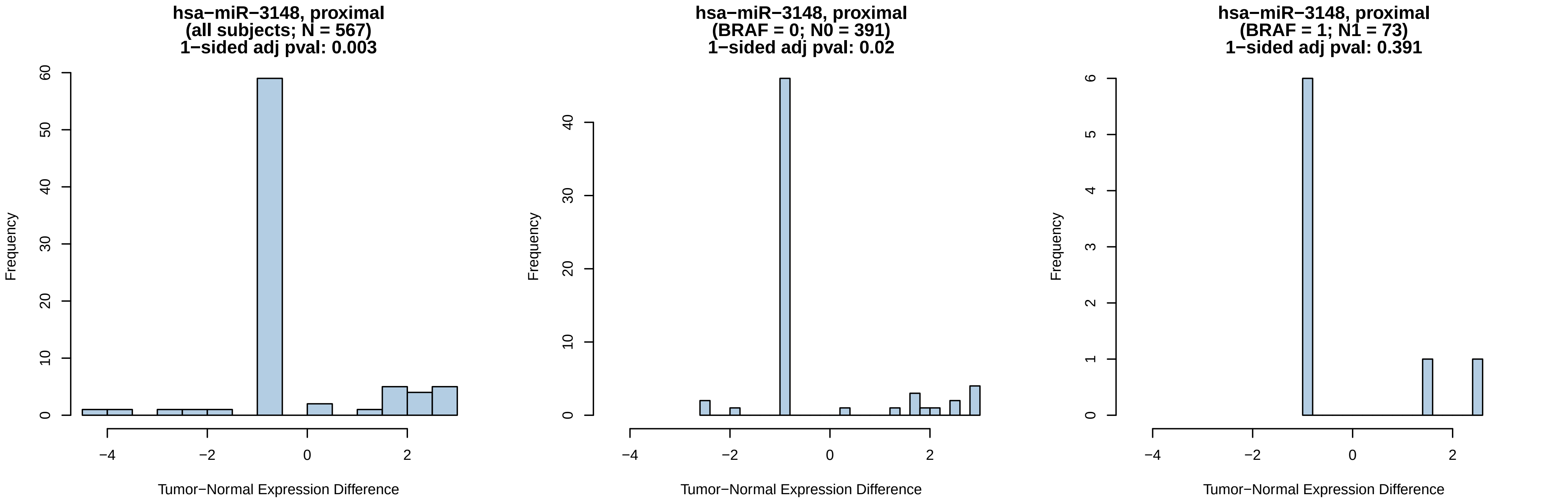
hsa−miR−3148, proximal
(all subjects; N = 567)
1−sided adj pval: 0.003
hsa−miR−3148, proximal
(BRAF = 0; N0 = 391)
1−sided adj pval: 0.02
hsa−miR−3148, proximal
(BRAF = 1; N1 = 73)
1−sided adj pval: 0.391
Frequency
Tumor−Normal Expression Difference

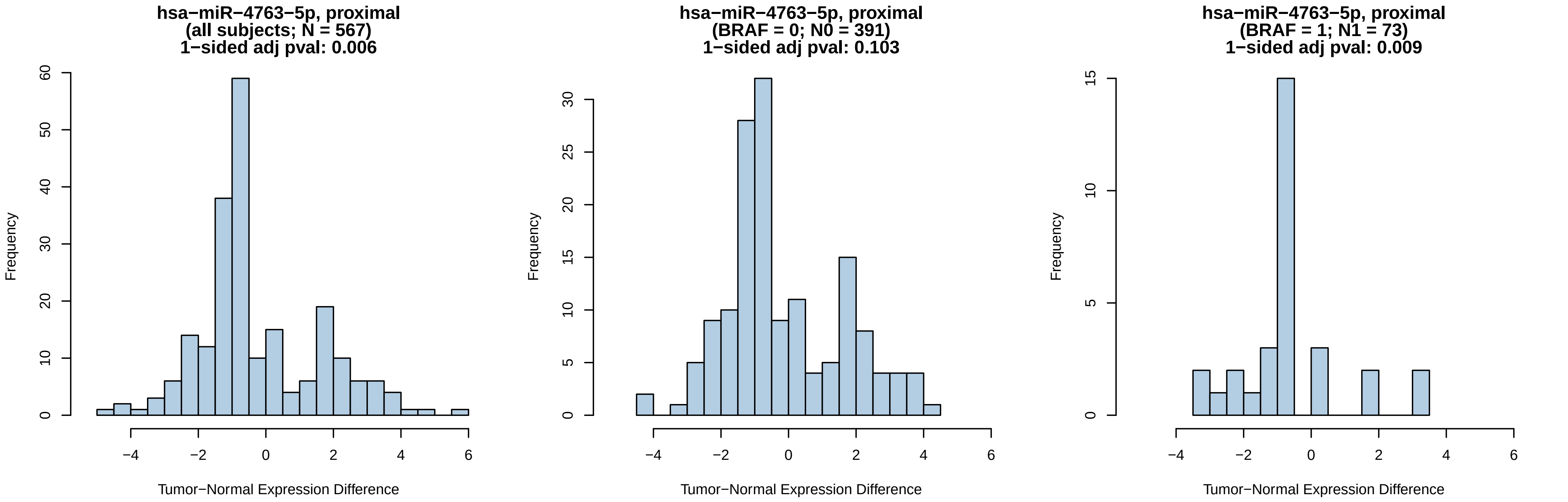
hsa−miR−4763−5p, proximal
(all subjects; N = 567)
1−sided adj pval: 0.006
Frequency
Tumor−Normal Expression Difference
hsa−miR−4763−5p, proximal
(BRAF = 0; N0 = 391)
1−sided adj pval: 0.103
Frequency
Tumor−Normal Expression Difference
hsa−miR−4763−5p, proximal
(BRAF = 1; N1 = 73)
1−sided adj pval: 0.009
Frequency
Tumor−Normal Expression Difference

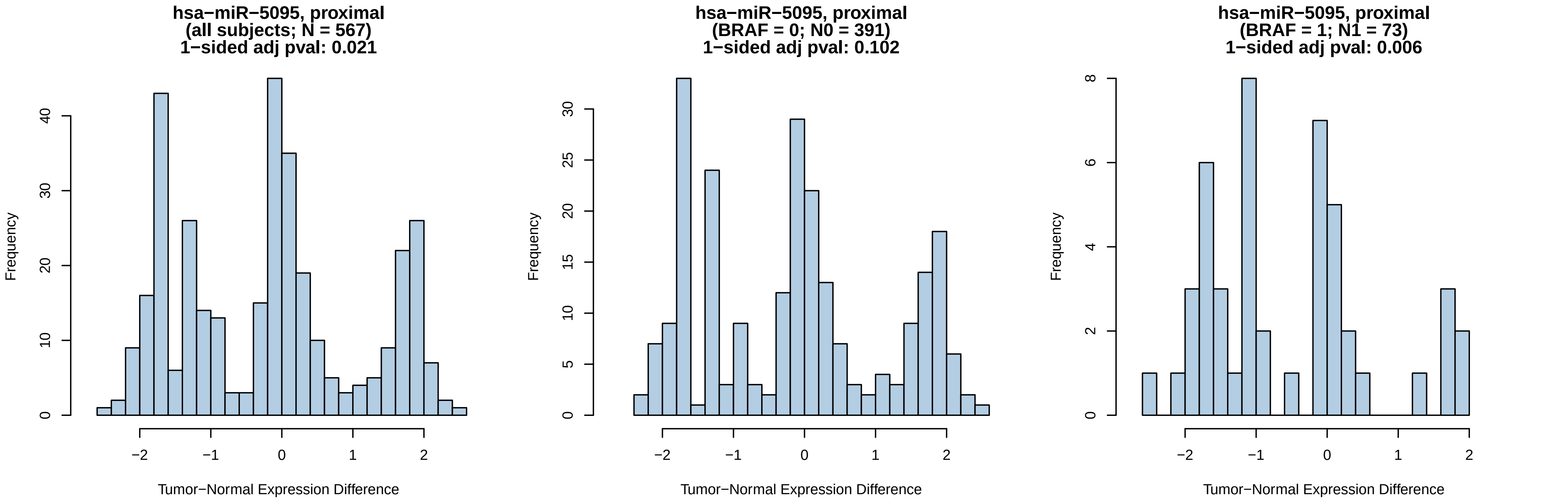
hsa−miR−5095, proximal
(all subjects; N = 567)
1−sided adj pval: 0.021
hsa−miR−5095, proximal
(BRAF = 0; N0 = 391)
1−sided adj pval: 0.102
hsa−miR−5095, proximal
(BRAF = 1; N1 = 73)
1−sided adj pval: 0.006
Frequency
Tumor−Normal Expression Difference

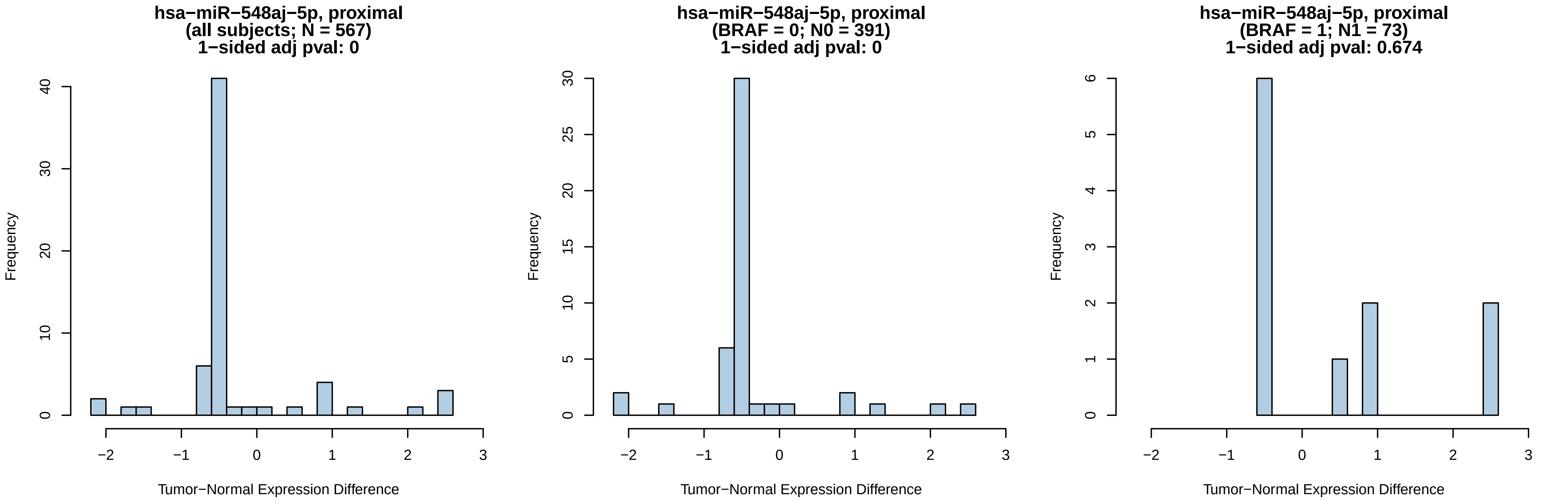
hsa−miR−548aj−5p, proximal
(all subjects; N = 567)
1−sided adj pval: 0
Frequency
Tumor−Normal Expression Difference
hsa−miR−548aj−5p, proximal
(BRAF = 0; N0 = 391)
1−sided adj pval: 0
Frequency
Tumor−Normal Expression Difference
hsa−miR−548aj−5p, proximal
(BRAF = 1; N1 = 73)
1−sided adj pval: 0.674
Frequency
Tumor−Normal Expression Difference

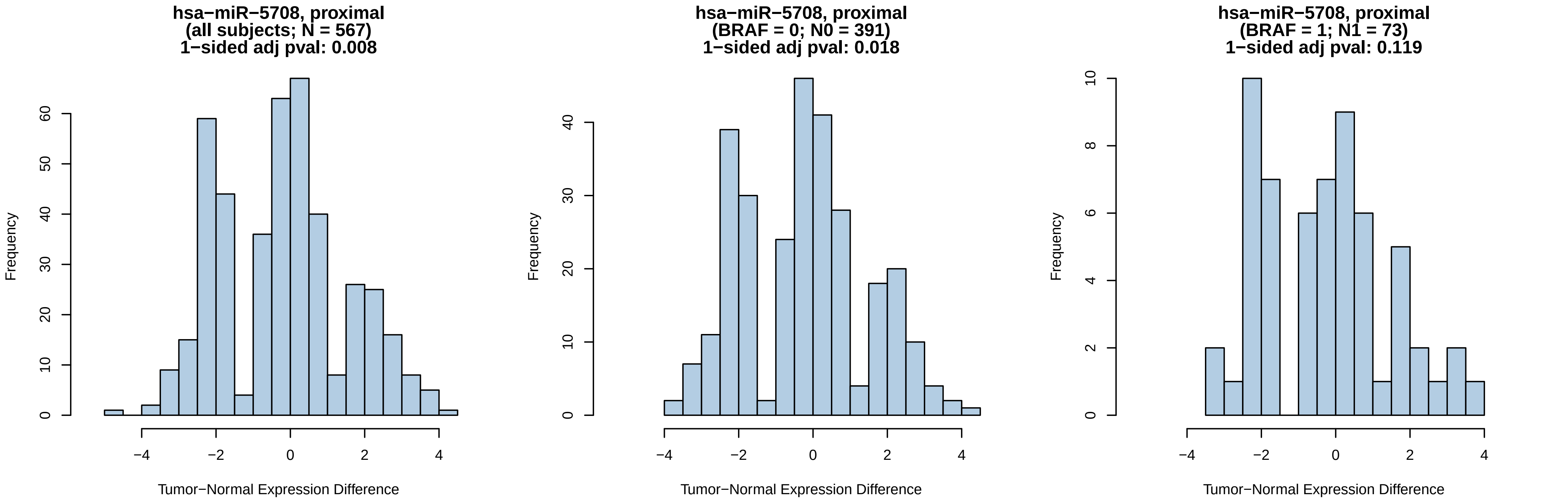
hsa−miR−5708, proximal
(all subjects; N = 567)
1−sided adj pval: 0.008
Frequency
Tumor−Normal Expression Difference
hsa−miR−5708, proximal
(BRAF = 0; N0 = 391)
1−sided adj pval: 0.018
Frequency
Tumor−Normal Expression Difference
hsa−miR−5708, proximal
(BRAF = 1; N1 = 73)
1−sided adj pval: 0.119
Frequency
Tumor−Normal Expression Difference

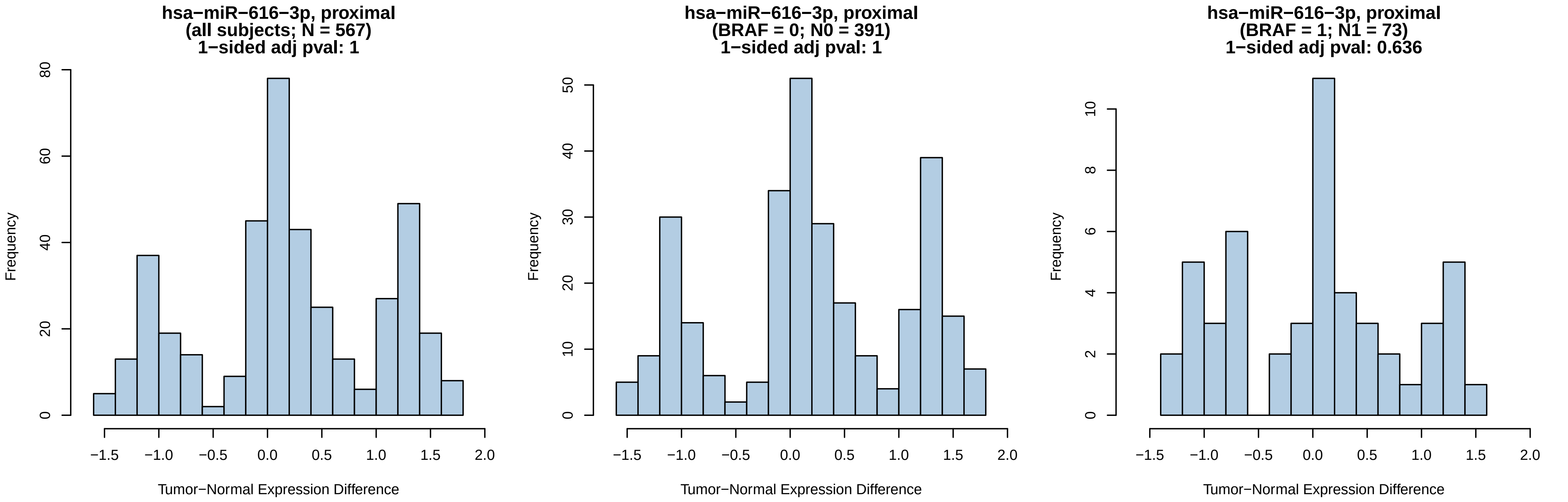
hsa−miR−616−3p, proximal
(all subjects; N = 567)
1−sided adj pval: 1
hsa−miR−616−3p, proximal
(BRAF = 0; N0 = 391)
1−sided adj pval: 1
hsa−miR−616−3p, proximal
(BRAF = 1; N1 = 73)
1−sided adj pval: 0.636
Frequency
Tumor−Normal Expression Difference

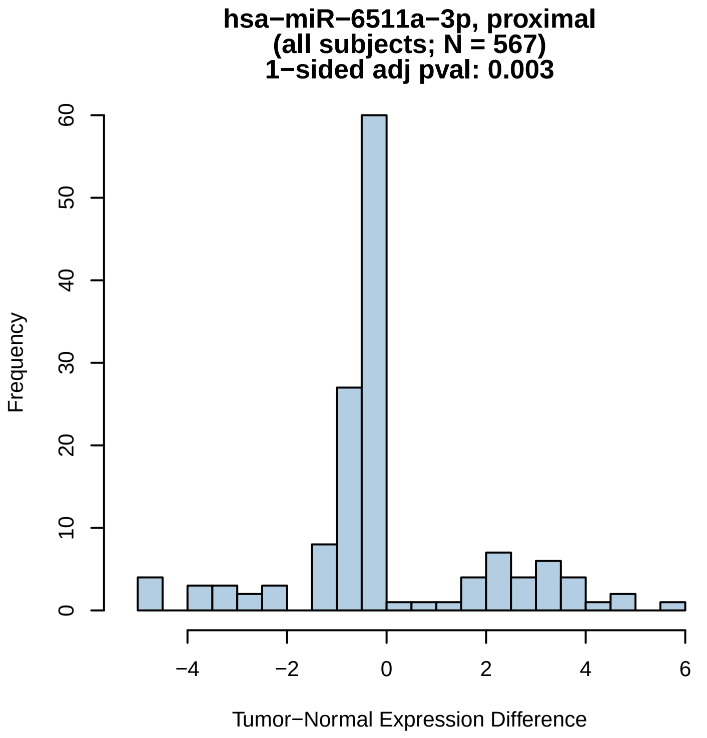
hsa−miR−6511a−3p, proximal
(all subjects; N = 567)
1−sided adj pval: 0.003
Frequency
Tumor−Normal Expression Difference

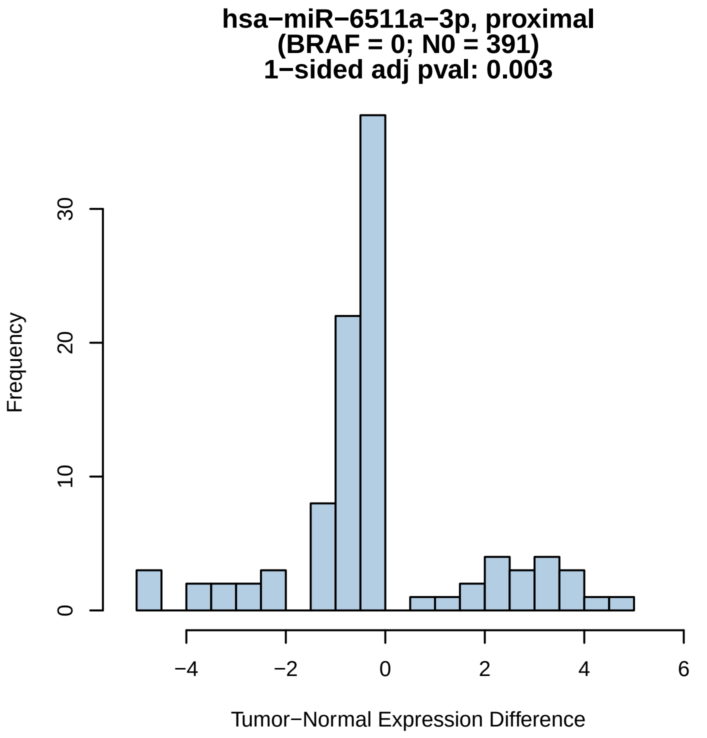
hsa−miR−6511a−3p, proximal
(BRAF = 0; N0 = 391)
1−sided adj pval: 0.003
Frequency
Tumor−Normal Expression Difference

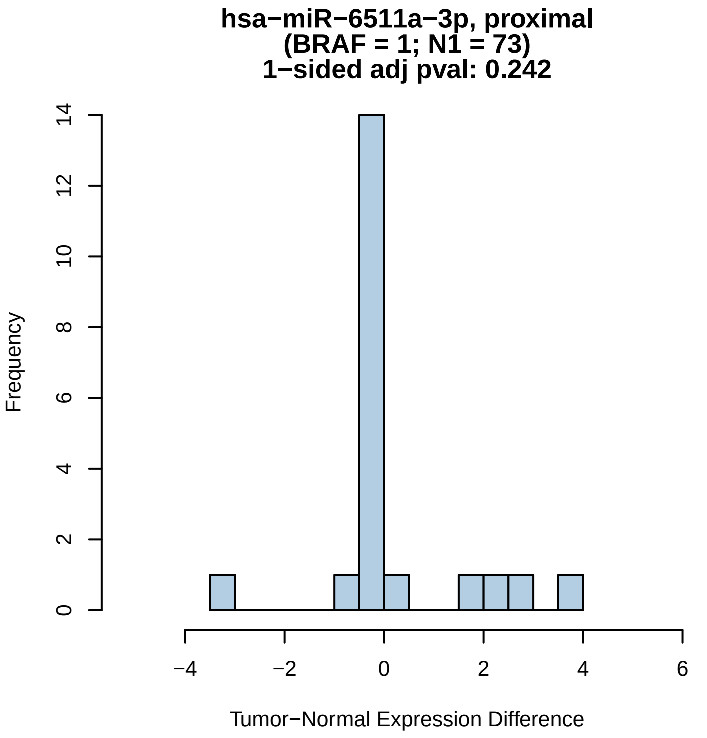
hsa−miR−6511a−3p, proximal
(BRAF = 1; N1 = 73)
1−sided adj pval: 0.242
Frequency
Tumor−Normal Expression Difference

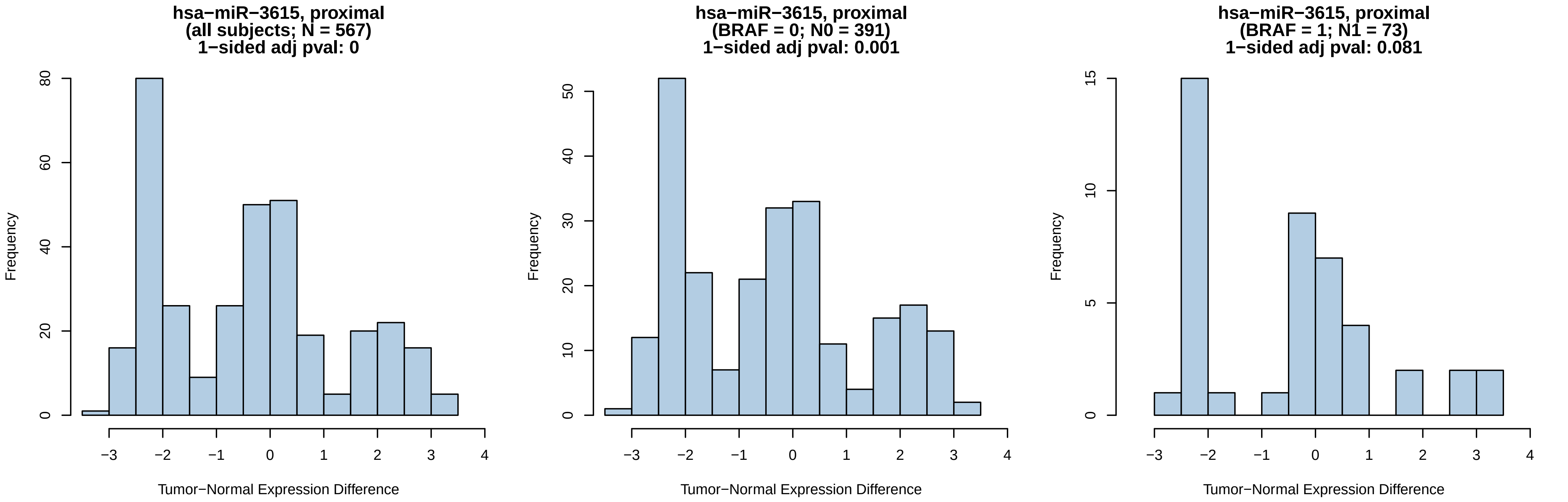
hsa−miR−3615, proximal
(all subjects; N = 567)
1−sided adj pval: 0
Frequency
Tumor−Normal Expression Difference
hsa−miR−3615, proximal
(BRAF = 0; N0 = 391)
1−sided adj pval: 0.001
Frequency
Tumor−Normal Expression Difference
hsa−miR−3615, proximal
(BRAF = 1; N1 = 73)
1−sided adj pval: 0.081
Frequency
Tumor−Normal Expression Difference

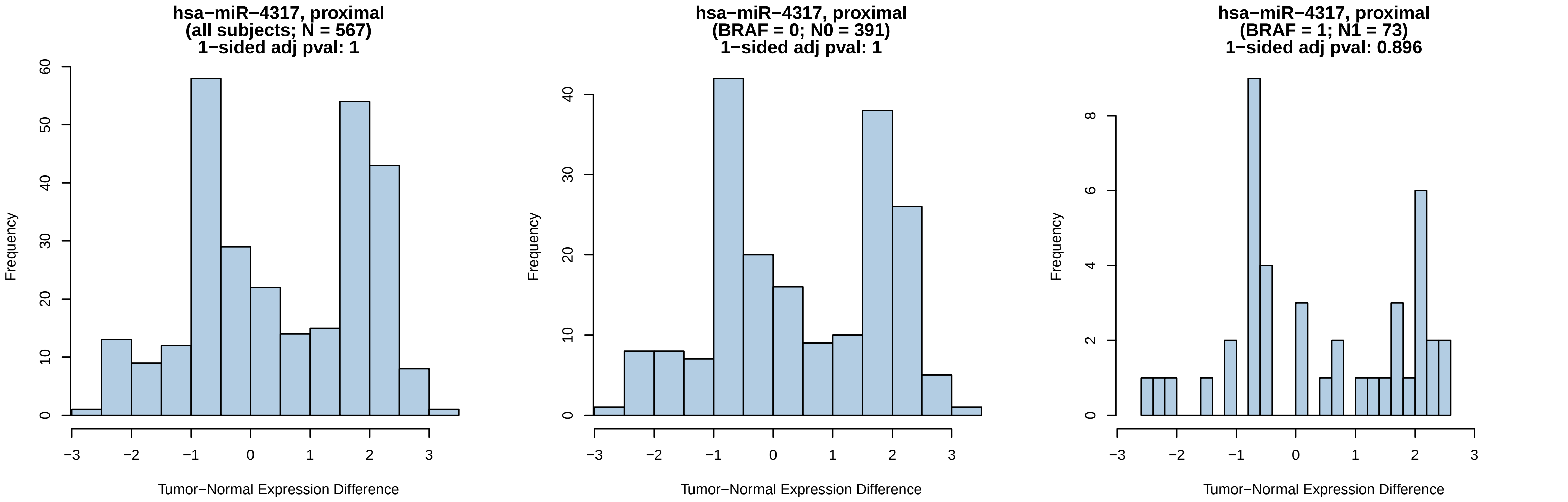
hsa−miR−4317, proximal
(all subjects; N = 567)
1−sided adj pval: 1
Frequency
Tumor−Normal Expression Difference
hsa−miR−4317, proximal
(BRAF = 0; N0 = 391)
1−sided adj pval: 1
Frequency
Tumor−Normal Expression Difference
hsa−miR−4317, proximal
(BRAF = 1; N1 = 73)
1−sided adj pval: 0.896
Frequency
Tumor−Normal Expression Difference

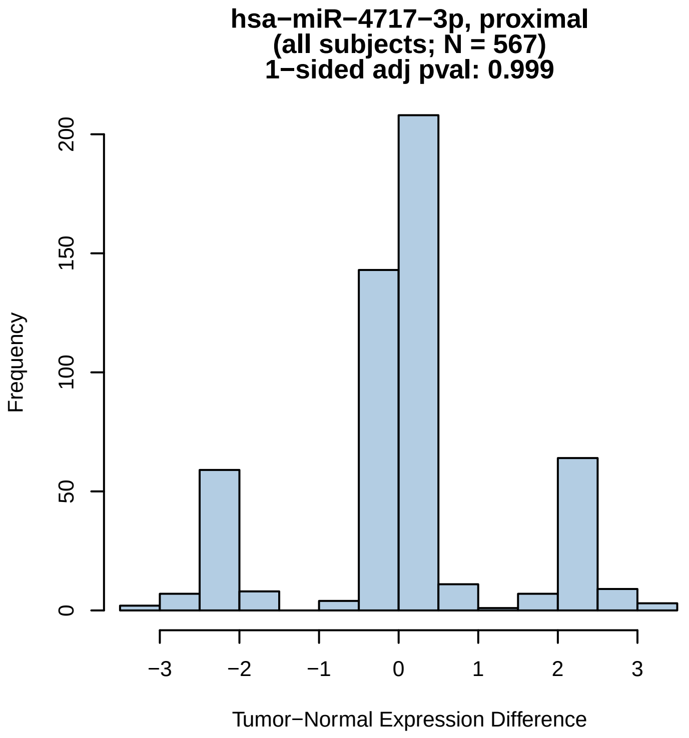

hsa−miR−4717−3p, proximal
(all subjects; N = 567)
1−sided adj pval: 0.999
Frequency
0
50
100
150
200
−3
−2
−1
0
1
2
3
Tumor−Normal Expression Difference


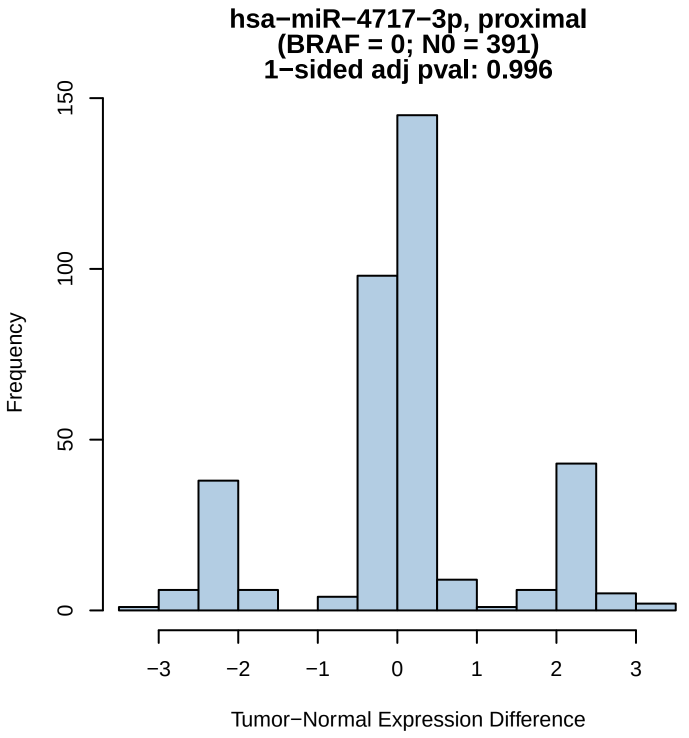

hsa−miR−4717−3p, proximal
(BRAF = 0; N0 = 391)
1−sided adj pval: 0.996
Frequency
0
50
100
150
−3
−2
−1
0
1
2
3
Tumor−Normal Expression Difference


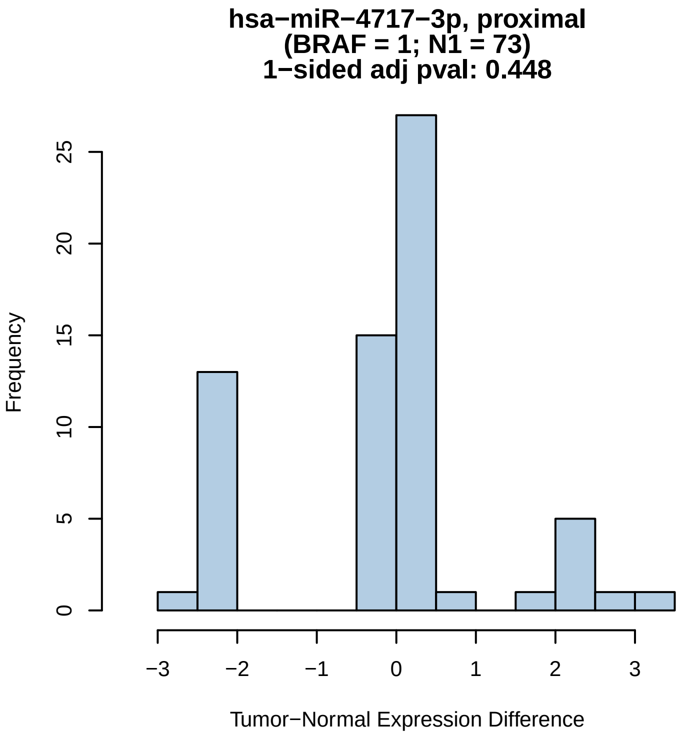

hsa−miR−4717−3p, proximal
(BRAF = 1; N1 = 73)
1−sided adj pval: 0.448
Frequency
0
5
10
15
20
25
−3
−2
−1
0
1
2
3
Tumor−Normal Expression Difference

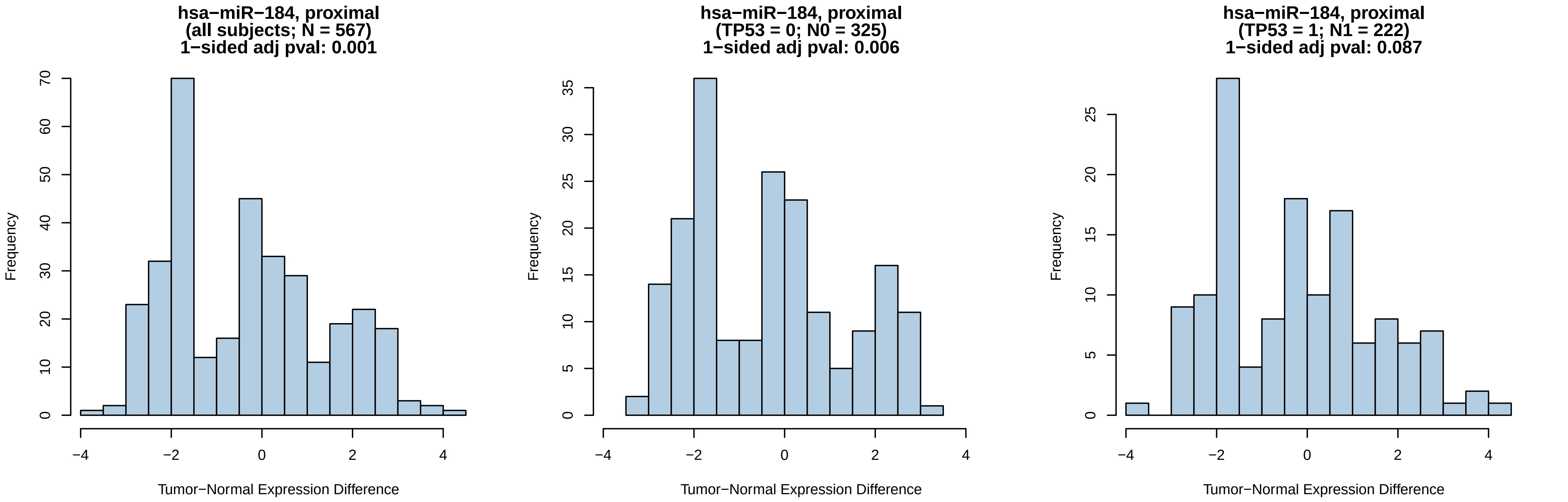
hsa−miR−184, proximal
(all subjects; N = 567)
1−sided adj pval: 0.001
hsa−miR−184, proximal
(TP53 = 0; N0 = 325)
1−sided adj pval: 0.006
hsa−miR−184, proximal
(TP53 = 1; N1 = 222)
1−sided adj pval: 0.087
Frequency
Tumor−Normal Expression Difference

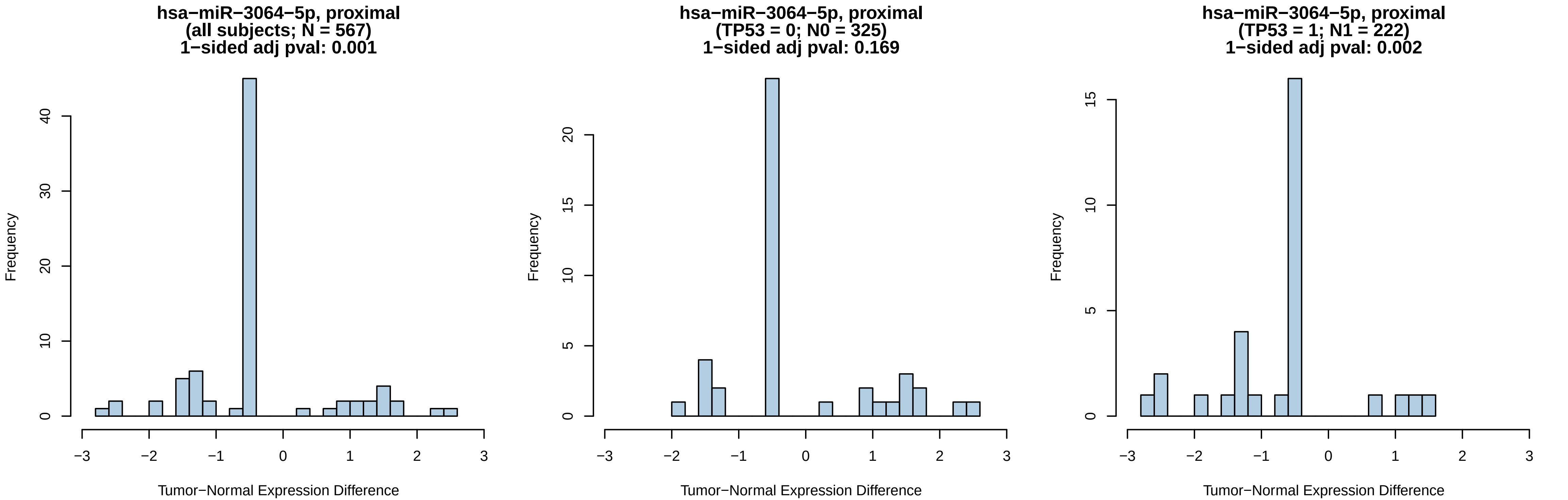

hsa−miR−3064−5p, proximal
(all subjects; N = 567)
1−sided adj pval: 0.001
Frequency
Tumor−Normal Expression Difference
hsa−miR−3064−5p, proximal
(TP53 = 0; N0 = 325)
1−sided adj pval: 0.169
Frequency
Tumor−Normal Expression Difference
hsa−miR−3064−5p, proximal
(TP53 = 1; N1 = 222)
1−sided adj pval: 0.002
Frequency
Tumor−Normal Expression Difference

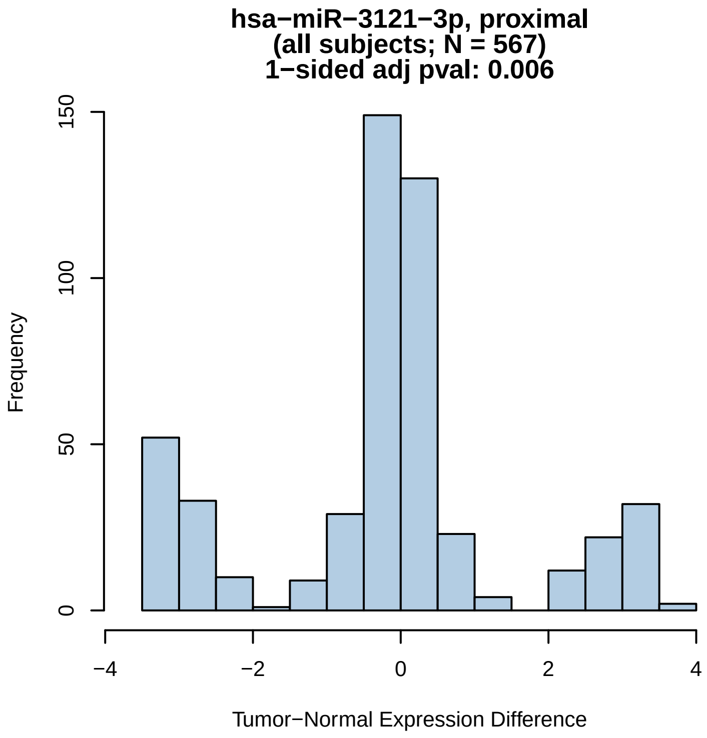

hsa−miR−3121−3p, proximal
(all subjects; N = 567)
1−sided adj pval: 0.006
Frequency
Tumor−Normal Expression Difference


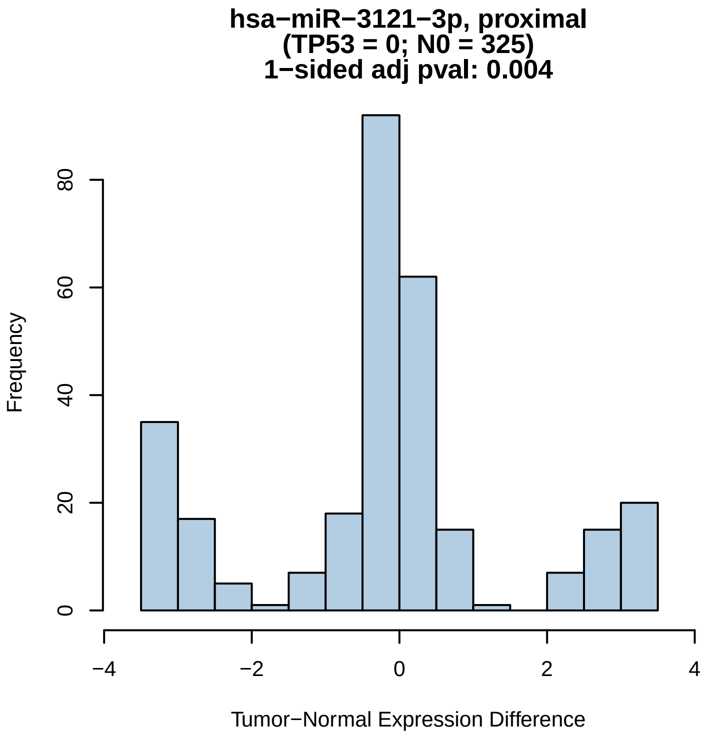

hsa−miR−3121−3p, proximal
(TP53 = 0; N0 = 325)
1−sided adj pval: 0.004
Frequency
Tumor−Normal Expression Difference


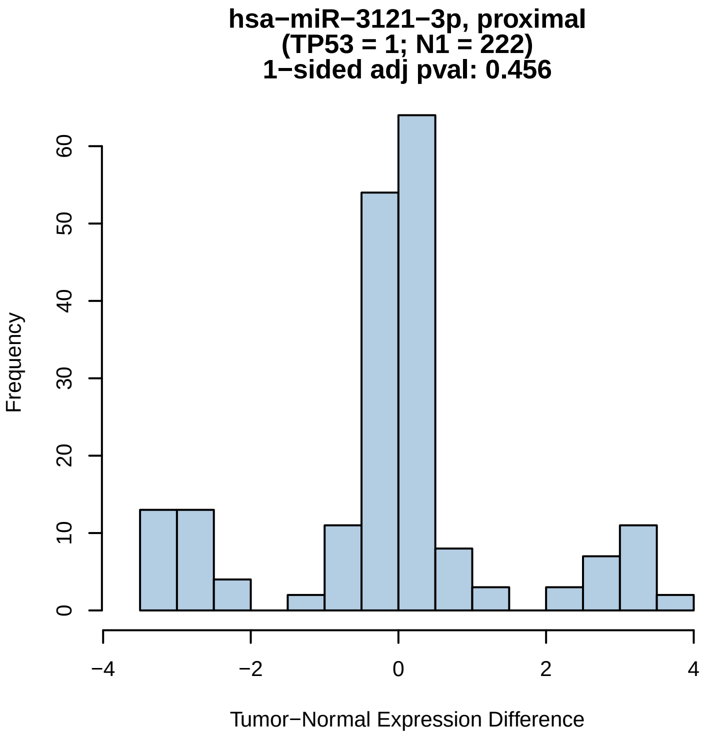

hsa−miR−3121−3p, proximal
(TP53 = 1; N1 = 222)
1−sided adj pval: 0.456
Frequency
Tumor−Normal Expression Difference

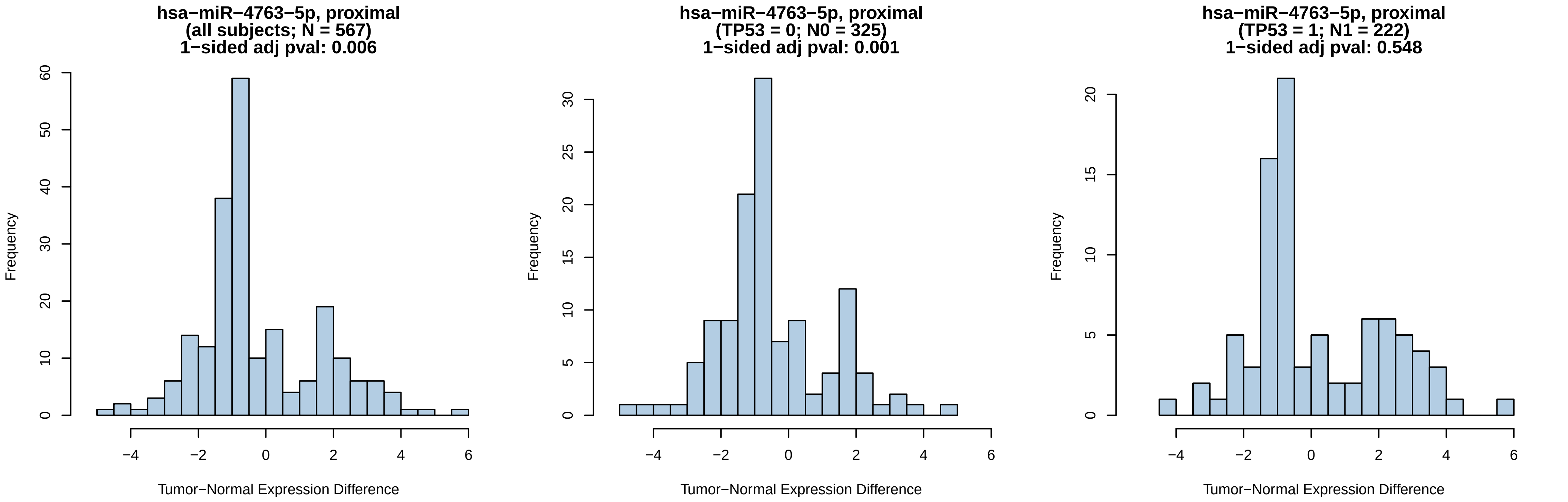
hsa−miR−4763−5p, proximal
(all subjects; N = 567)
1−sided adj pval: 0.006
Frequency
Tumor−Normal Expression Difference
hsa−miR−4763−5p, proximal
(TP53 = 0; N0 = 325)
1−sided adj pval: 0.001
Frequency
Tumor−Normal Expression Difference
hsa−miR−4763−5p, proximal
(TP53 = 1; N1 = 222)
1−sided adj pval: 0.548
Frequency
Tumor−Normal Expression Difference

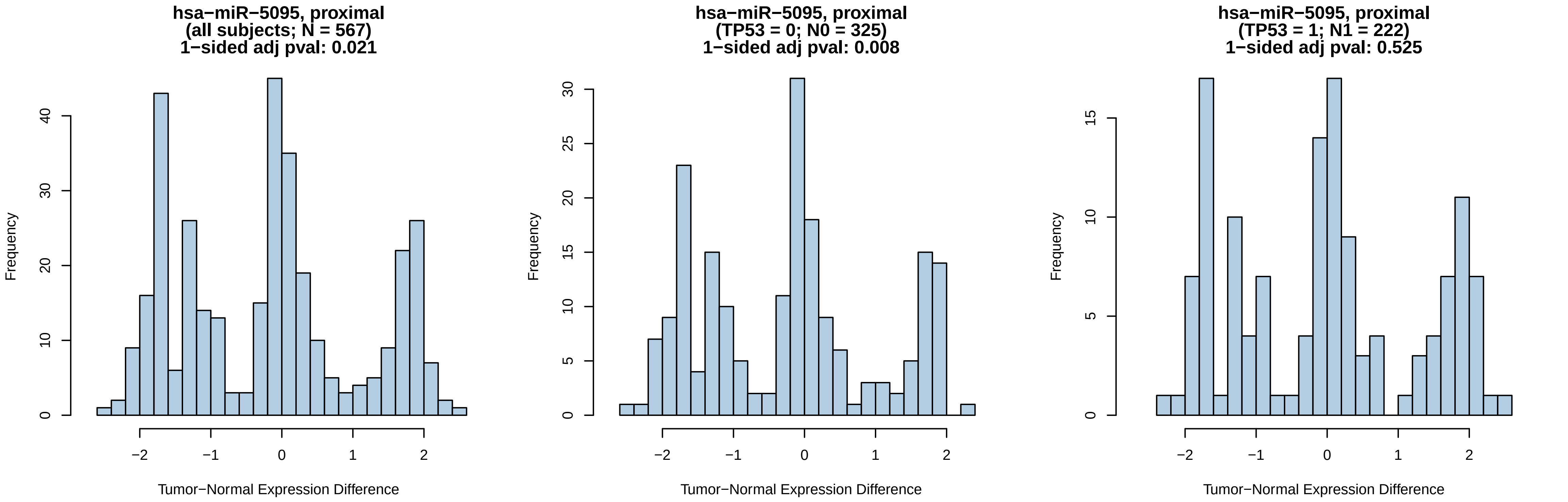
hsa−miR−5095, proximal
(all subjects; N = 567)
1−sided adj pval: 0.021
Frequency
Tumor−Normal Expression Difference
hsa−miR−5095, proximal
(TP53 = 0; N0 = 325)
1−sided adj pval: 0.008
Frequency
Tumor−Normal Expression Difference
hsa−miR−5095, proximal
(TP53 = 1; N1 = 222)
1−sided adj pval: 0.525
Frequency
Tumor−Normal Expression Difference

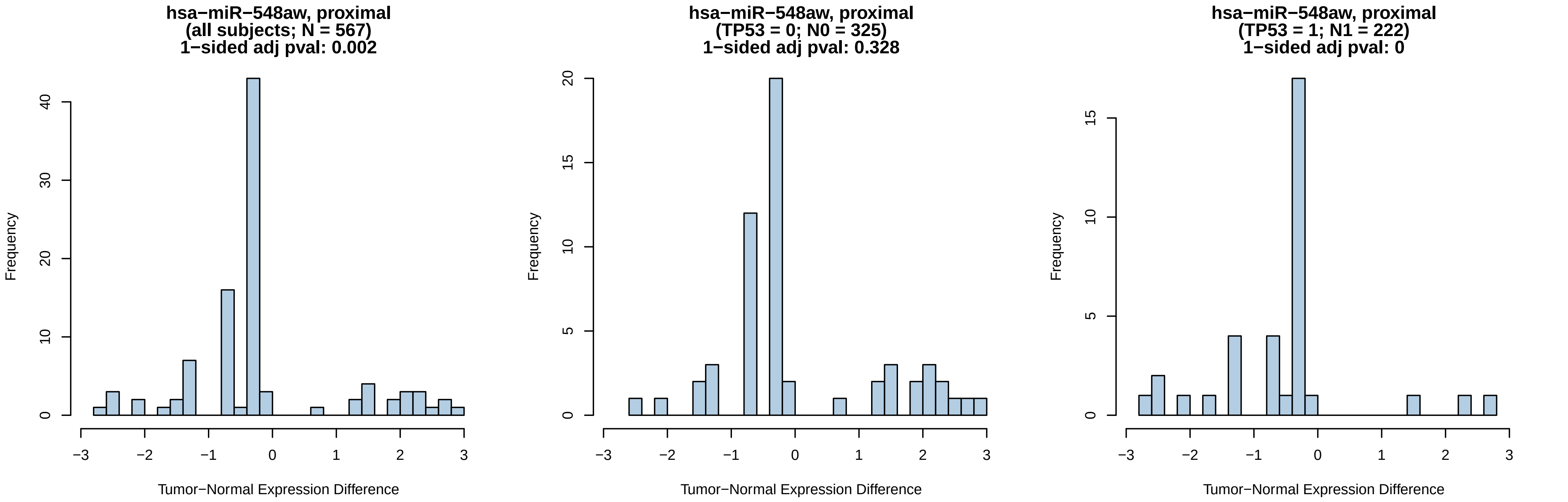
hsa−miR−548aw, proximal
(all subjects; N = 567)
1−sided adj pval: 0.002
Frequency
Tumor−Normal Expression Difference
hsa−miR−548aw, proximal
(TP53 = 0; N0 = 325)
1−sided adj pval: 0.328
Frequency
Tumor−Normal Expression Difference
hsa−miR−548aw, proximal
(TP53 = 1; N1 = 222)
1−sided adj pval: 0
Frequency
Tumor−Normal Expression Difference

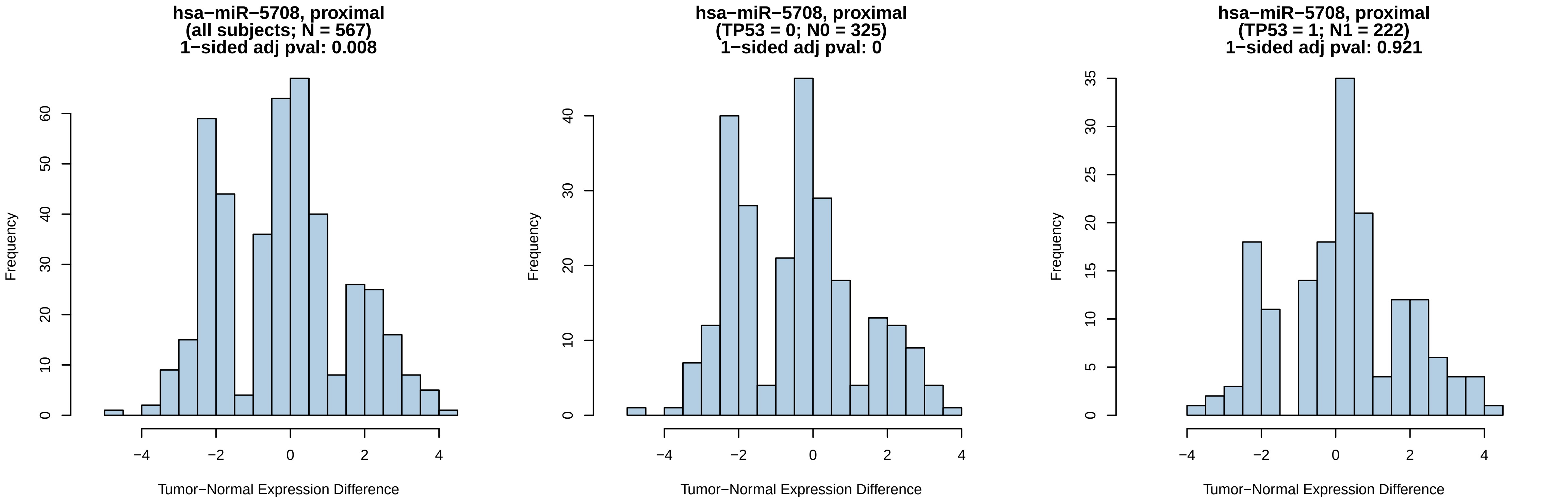
hsa−miR−5708, proximal
(all subjects; N = 567)
1−sided adj pval: 0.008
Frequency
Tumor−Normal Expression Difference
hsa−miR−5708, proximal
(TP53 = 0; N0 = 325)
1−sided adj pval: 0
Frequency
Tumor−Normal Expression Difference
hsa−miR−5708, proximal
(TP53 = 1; N1 = 222)
1−sided adj pval: 0.921
Frequency
Tumor−Normal Expression Difference

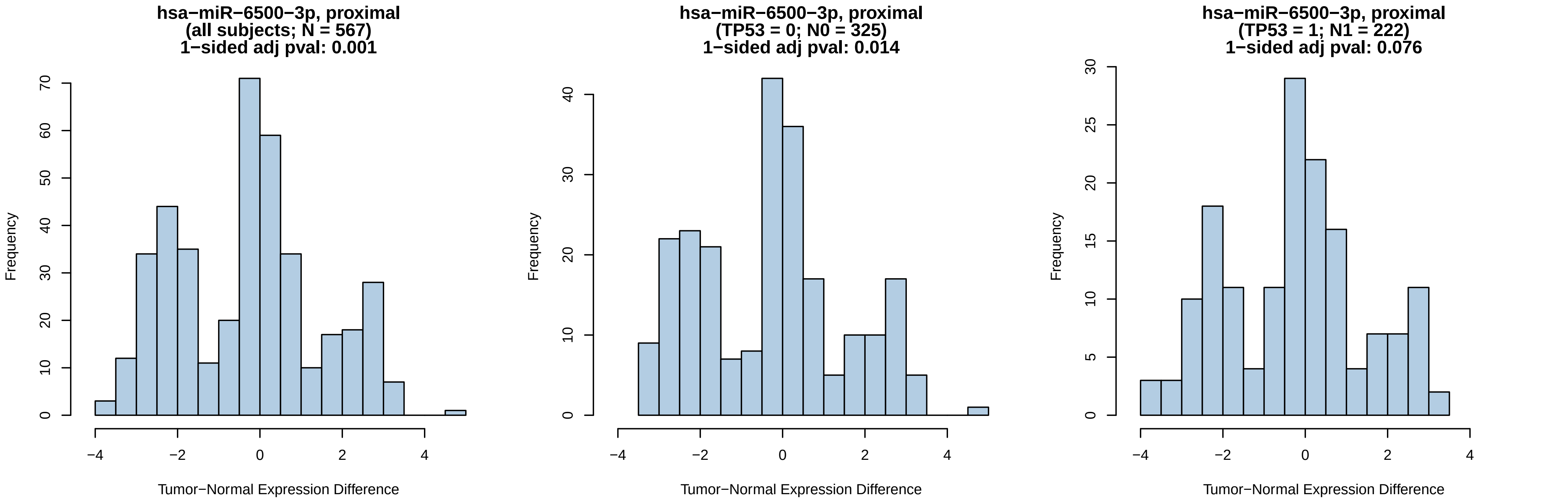

hsa−miR−6500−3p, proximal
(all subjects; N = 567)
1−sided adj pval: 0.001
Frequency
Tumor−Normal Expression Difference
hsa−miR−6500−3p, proximal
(TP53 = 0; N0 = 325)
1−sided adj pval: 0.014
Frequency
Tumor−Normal Expression Difference
hsa−miR−6500−3p, proximal
(TP53 = 1; N1 = 222)
1−sided adj pval: 0.076
Frequency
Tumor−Normal Expression Difference

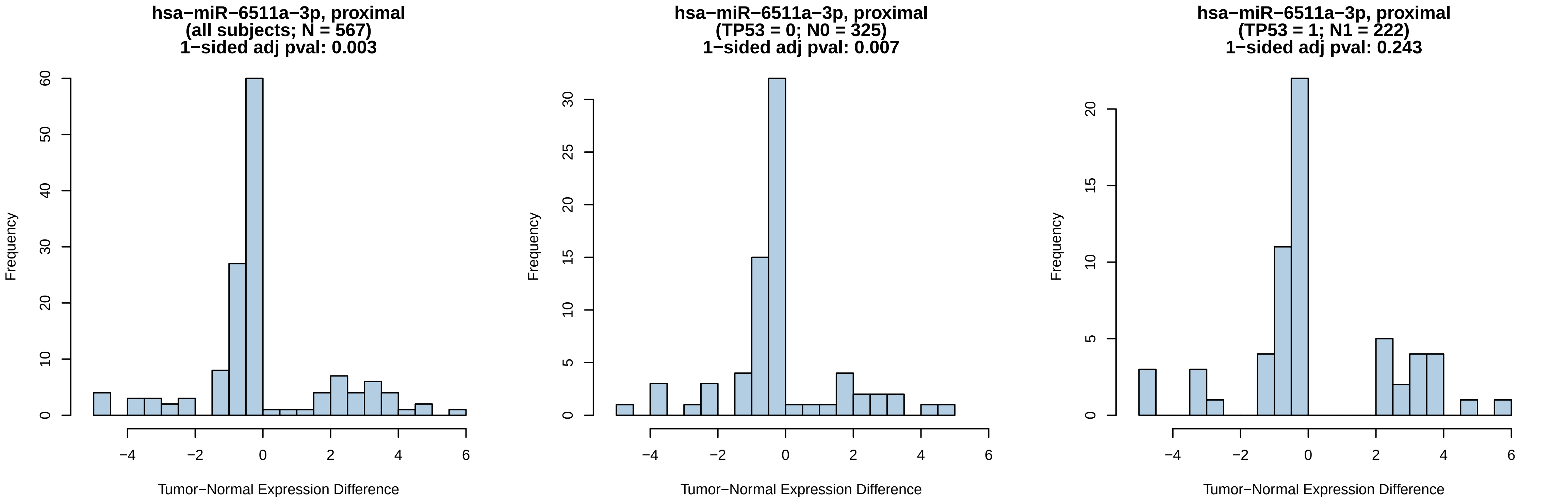
hsa−miR−6511a−3p, proximal
(all subjects; N = 567)
1−sided adj pval: 0.003
hsa−miR−6511a−3p, proximal
(TP53 = 0; N0 = 325)
1−sided adj pval: 0.007
hsa−miR−6511a−3p, proximal
(TP53 = 1; N1 = 222)
1−sided adj pval: 0.243
Frequency
Tumor−Normal Expression Difference

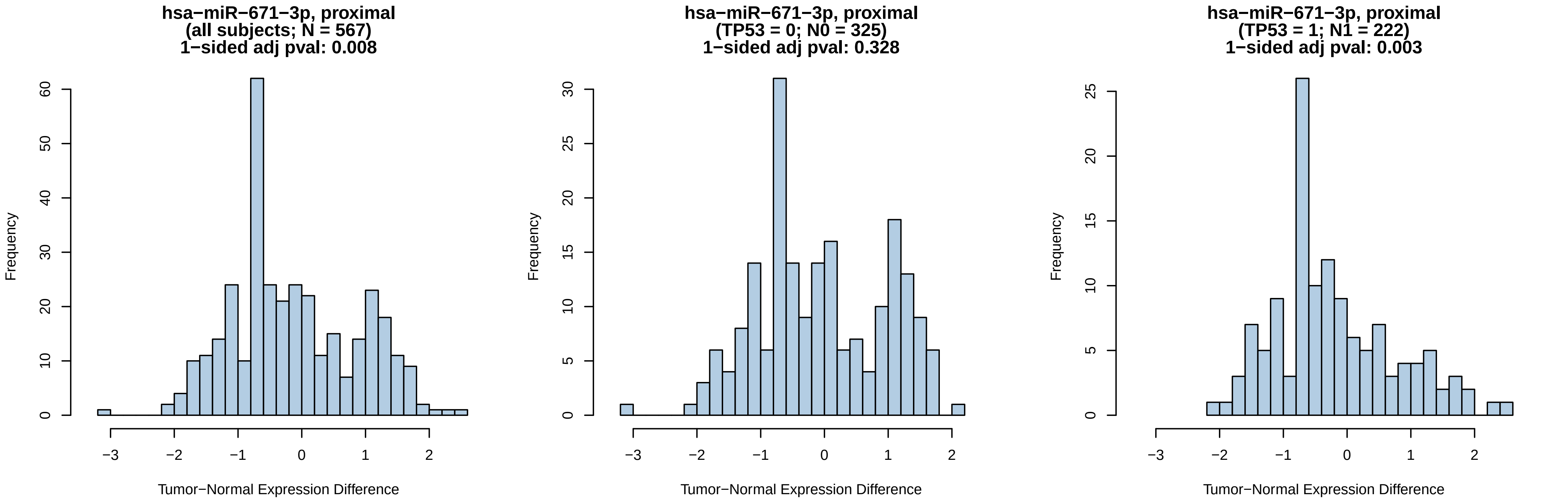
hsa−miR−671−3p, proximal
(all subjects; N = 567)
1−sided adj pval: 0.008
Frequency
Tumor−Normal Expression Difference
hsa−miR−671−3p, proximal
(TP53 = 0; N0 = 325)
1−sided adj pval: 0.328
Frequency
Tumor−Normal Expression Difference
hsa−miR−671−3p, proximal
(TP53 = 1; N1 = 222)
1−sided adj pval: 0.003
Frequency
Tumor−Normal Expression Difference

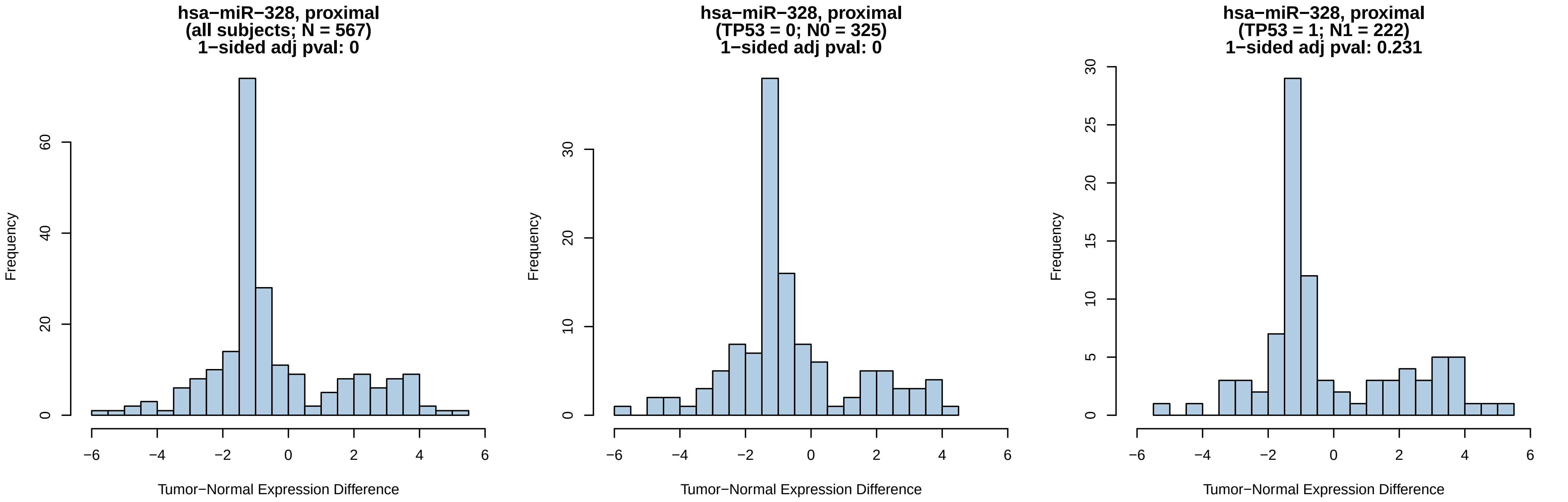

hsa−miR−328, proximal
(all subjects; N = 567)
1−sided adj pval: 0
hsa−miR−328, proximal
(TP53 = 0; N0 = 325)
1−sided adj pval: 0
hsa−miR−328, proximal
(TP53 = 1; N1 = 222)
1−sided adj pval: 0.231
Frequency
Tumor−Normal Expression Difference

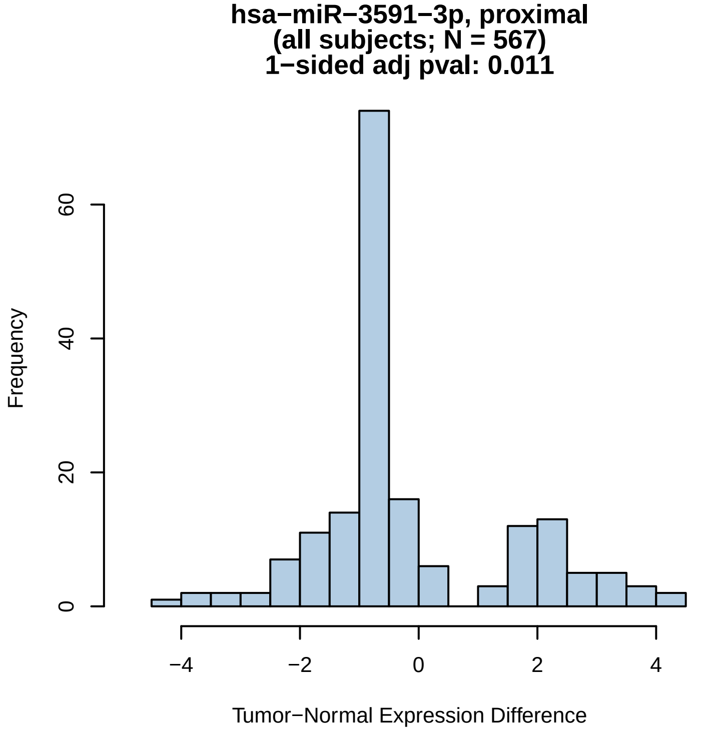

hsa−miR−3591−3p, proximal
(all subjects; N = 567)
1−sided adj pval: 0.011
Frequency
Tumor−Normal Expression Difference


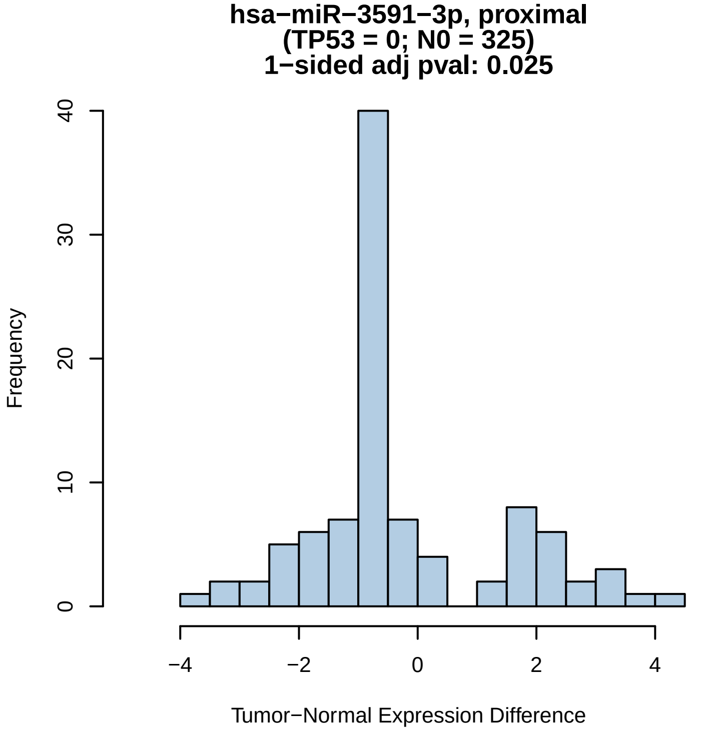

hsa−miR−3591−3p, proximal
(TP53 = 0; N0 = 325)
1−sided adj pval: 0.025
Frequency
Tumor−Normal Expression Difference


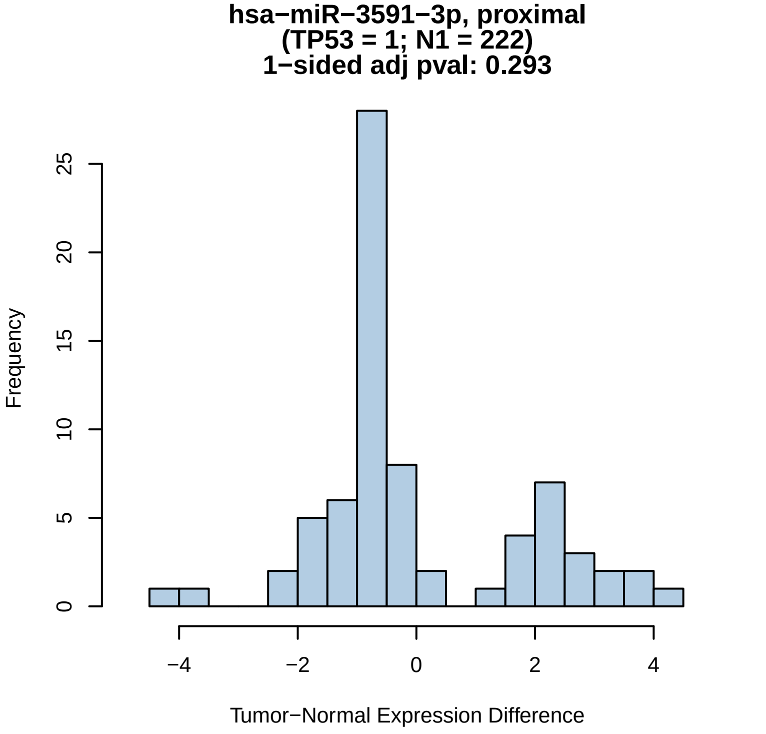

hsa−miR−3591−3p, proximal
(TP53 = 1; N1 = 222)
1−sided adj pval: 0.293
Frequency
Tumor−Normal Expression Difference

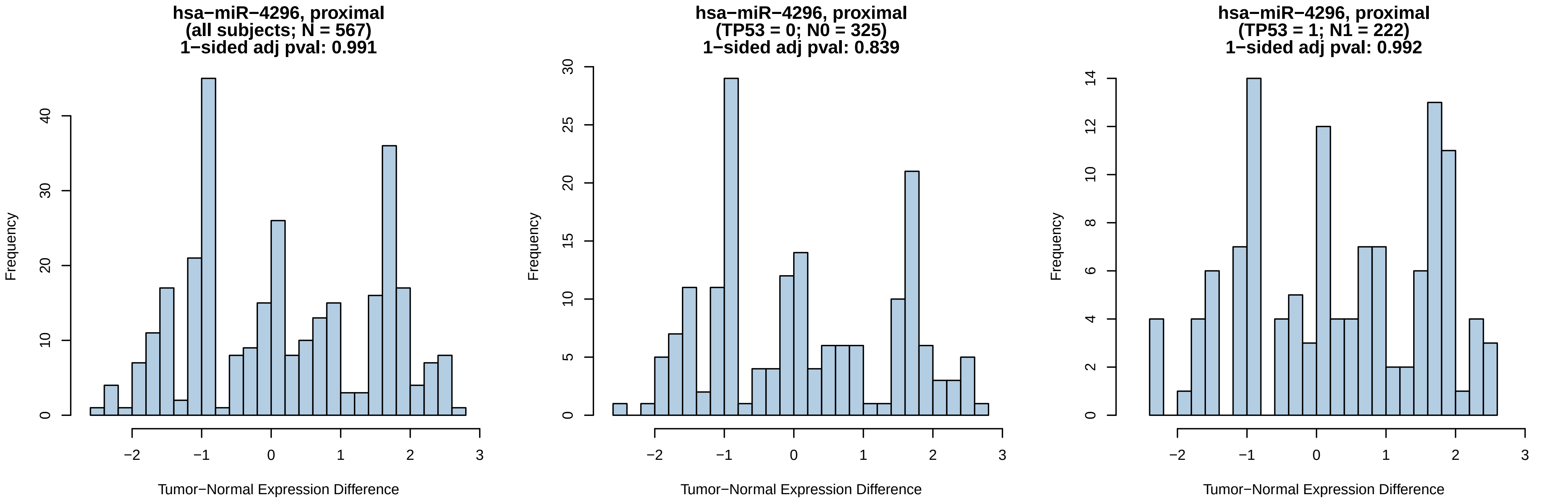
hsa−miR−4296, proximal
(all subjects; N = 567)
1−sided adj pval: 0.991
Frequency
Tumor−Normal Expression Difference
hsa−miR−4296, proximal
(TP53 = 0; N0 = 325)
1−sided adj pval: 0.839
Frequency
Tumor−Normal Expression Difference
hsa−miR−4296, proximal
(TP53 = 1; N1 = 222)
1−sided adj pval: 0.992
Frequency
Tumor−Normal Expression Difference

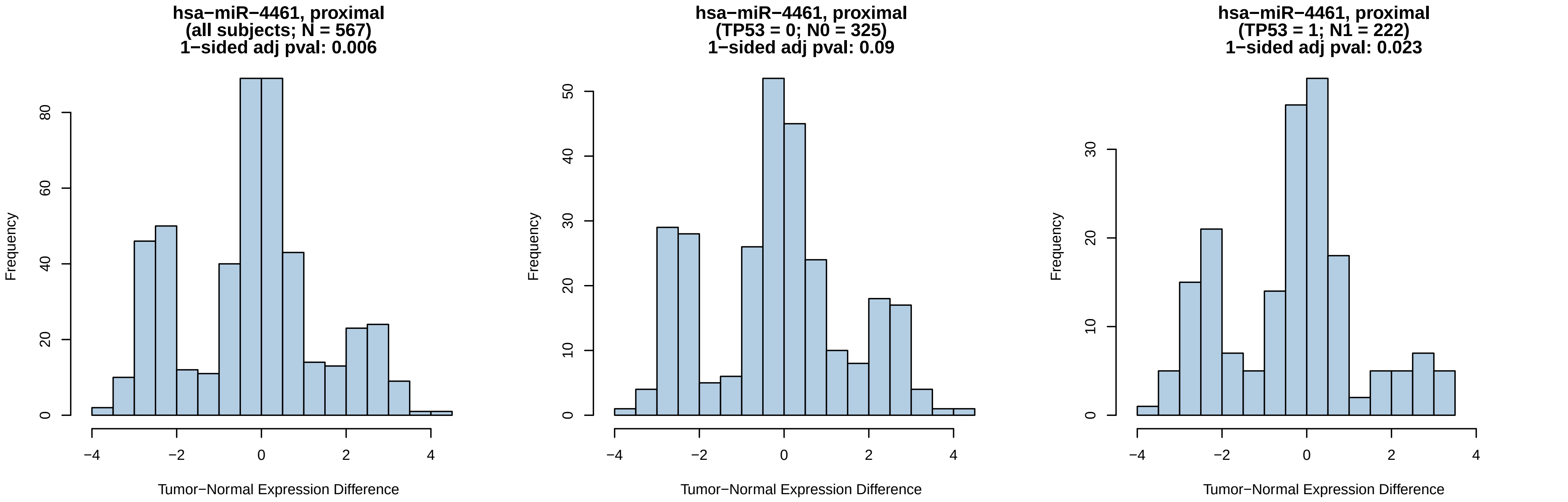

hsa−miR−4461, proximal
(all subjects; N = 567)
1−sided adj pval: 0.006
Frequency
Tumor−Normal Expression Difference
hsa−miR−4461, proximal
(TP53 = 0; N0 = 325)
1−sided adj pval: 0.09
Frequency
Tumor−Normal Expression Difference
hsa−miR−4461, proximal
(TP53 = 1; N1 = 222)
1−sided adj pval: 0.023
Frequency
Tumor−Normal Expression Difference

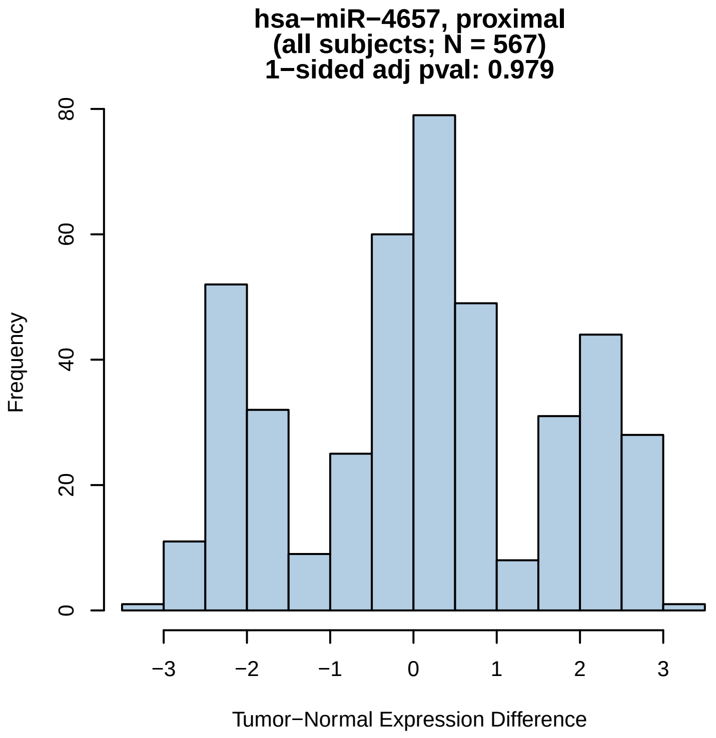
hsa−miR−4657, proximal
(all subjects; N = 567)
1−sided adj pval: 0.979
Frequency
Tumor−Normal Expression Difference

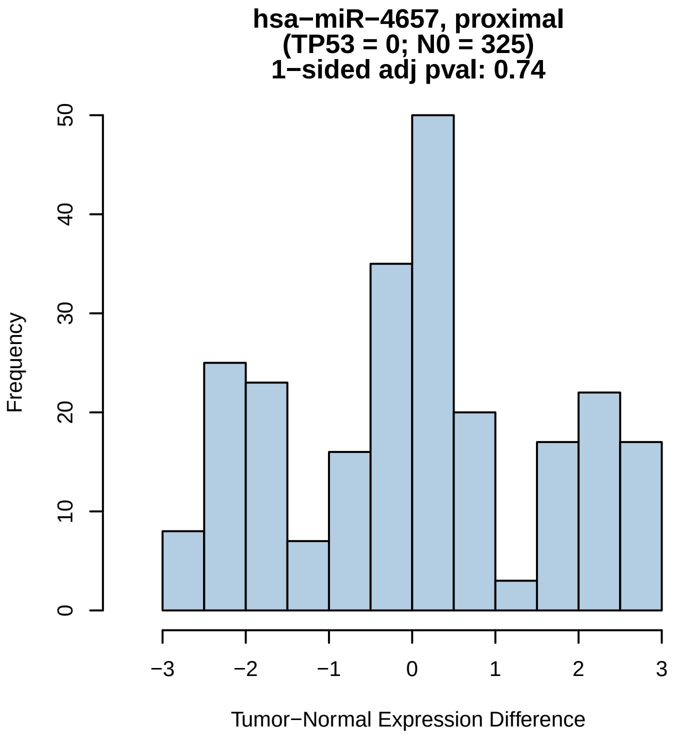
hsa−miR−4657, proximal
(TP53 = 0; N0 = 325)
1−sided adj pval: 0.74
Frequency
Tumor−Normal Expression Difference

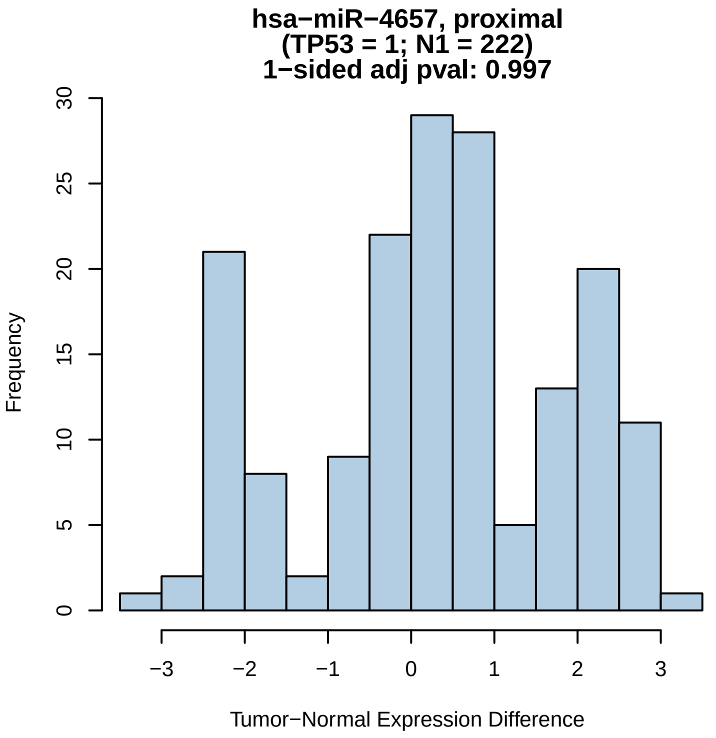
hsa−miR−4657, proximal
(TP53 = 1; N1 = 222)
1−sided adj pval: 0.997
Frequency
Tumor−Normal Expression Difference

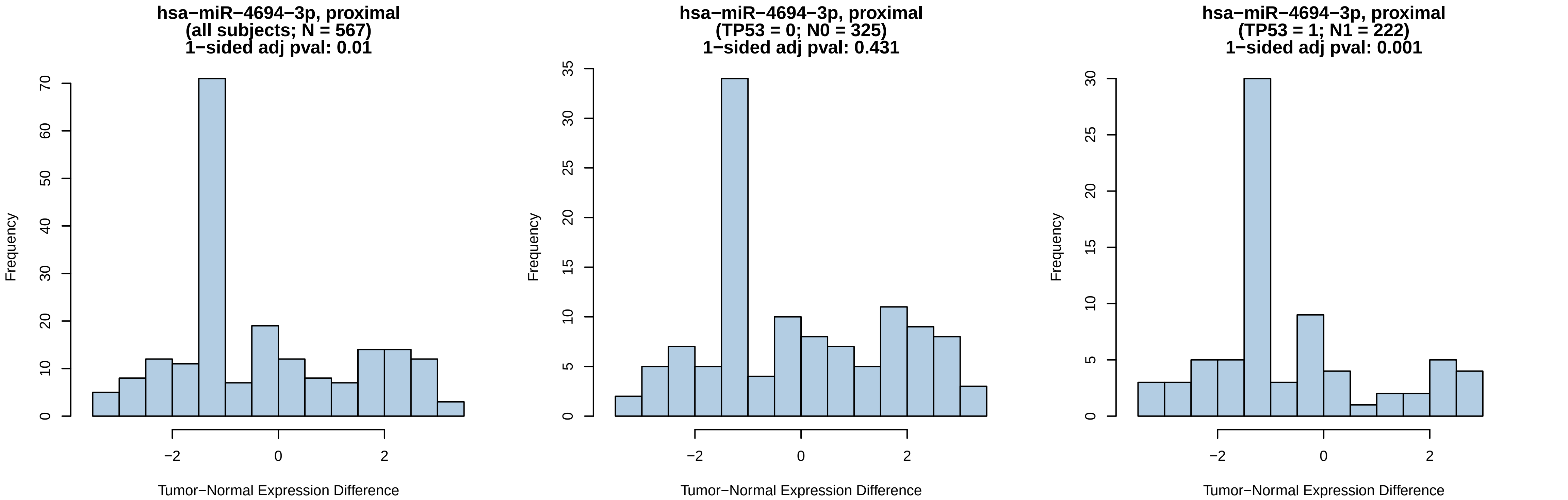

hsa−miR−4694−3p, proximal
(all subjects; N = 567)
1−sided adj pval: 0.01
Frequency
Tumor−Normal Expression Difference
hsa−miR−4694−3p, proximal
(TP53 = 0; N0 = 325)
1−sided adj pval: 0.431
Frequency
Tumor−Normal Expression Difference
hsa−miR−4694−3p, proximal
(TP53 = 1; N1 = 222)
1−sided adj pval: 0.001
Frequency
Tumor−Normal Expression Difference

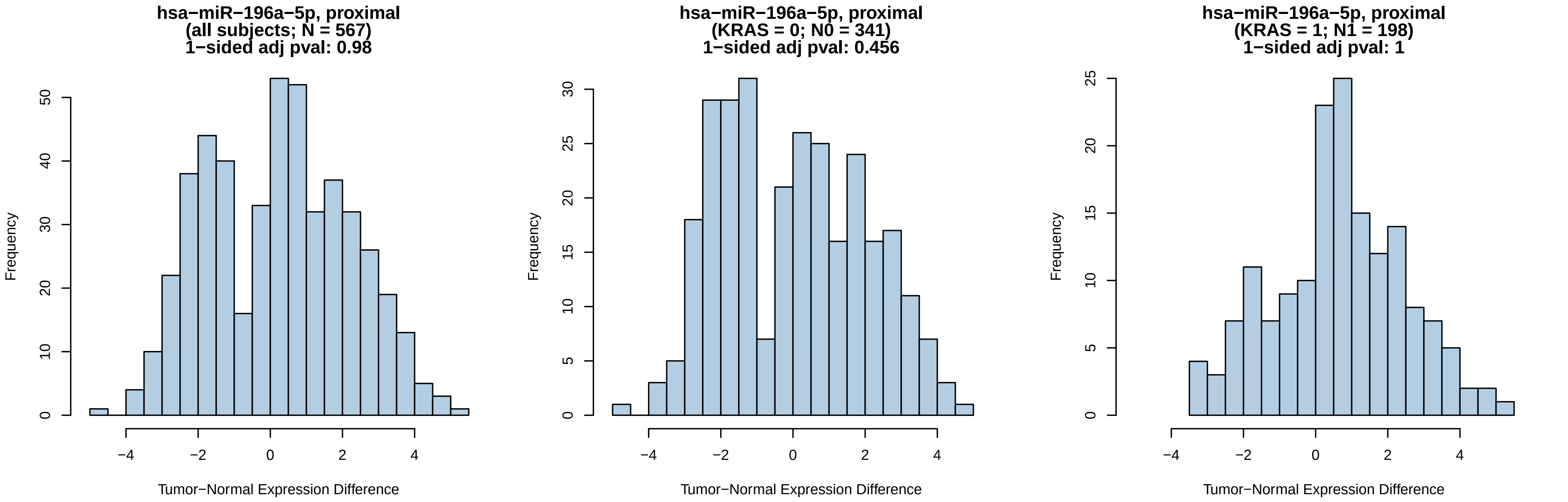
hsa−miR−196a−5p, proximal
(all subjects; N = 567)
1−sided adj pval: 0.98
hsa−miR−196a−5p, proximal
(KRAS = 0; N0 = 341)
1−sided adj pval: 0.456
hsa−miR−196a−5p, proximal
(KRAS = 1; N1 = 198)
1−sided adj pval: 1
Frequency
Tumor−Normal Expression Difference

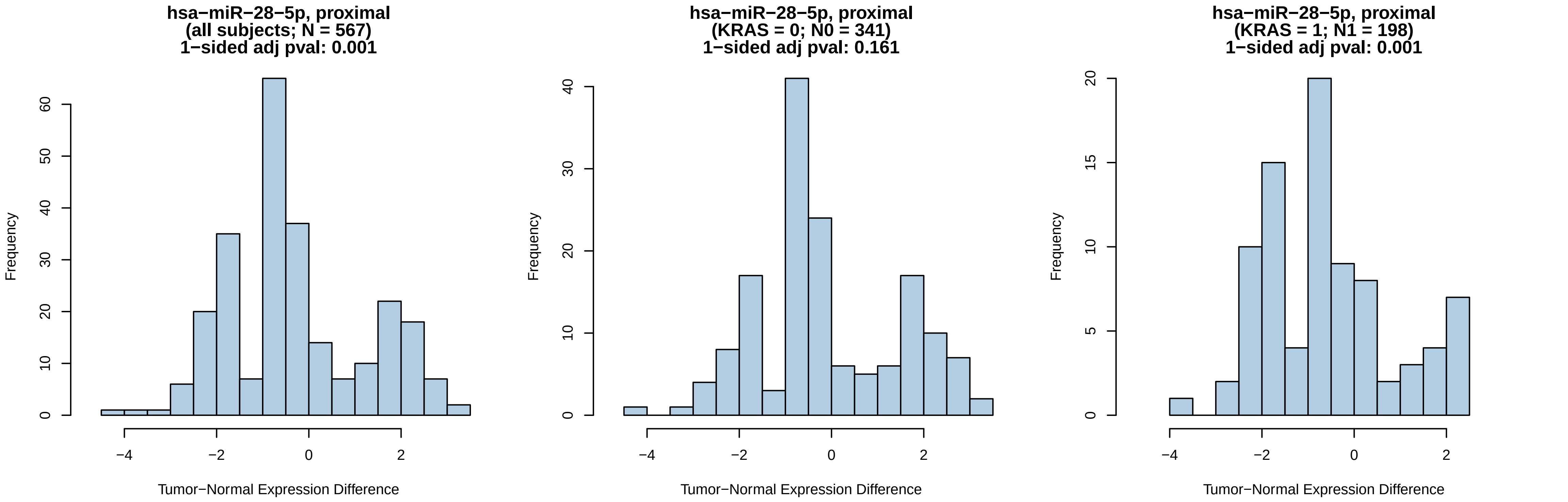
hsa−miR−28−5p, proximal
(all subjects; N = 567)
1−sided adj pval: 0.001
hsa−miR−28−5p, proximal
(KRAS = 0; N0 = 341)
1−sided adj pval: 0.161
hsa−miR−28−5p, proximal
(KRAS = 1; N1 = 198)
1−sided adj pval: 0.001
Frequency
Tumor−Normal Expression Difference
0
10
20
30
40
50
60
5
15
−4
−2
2

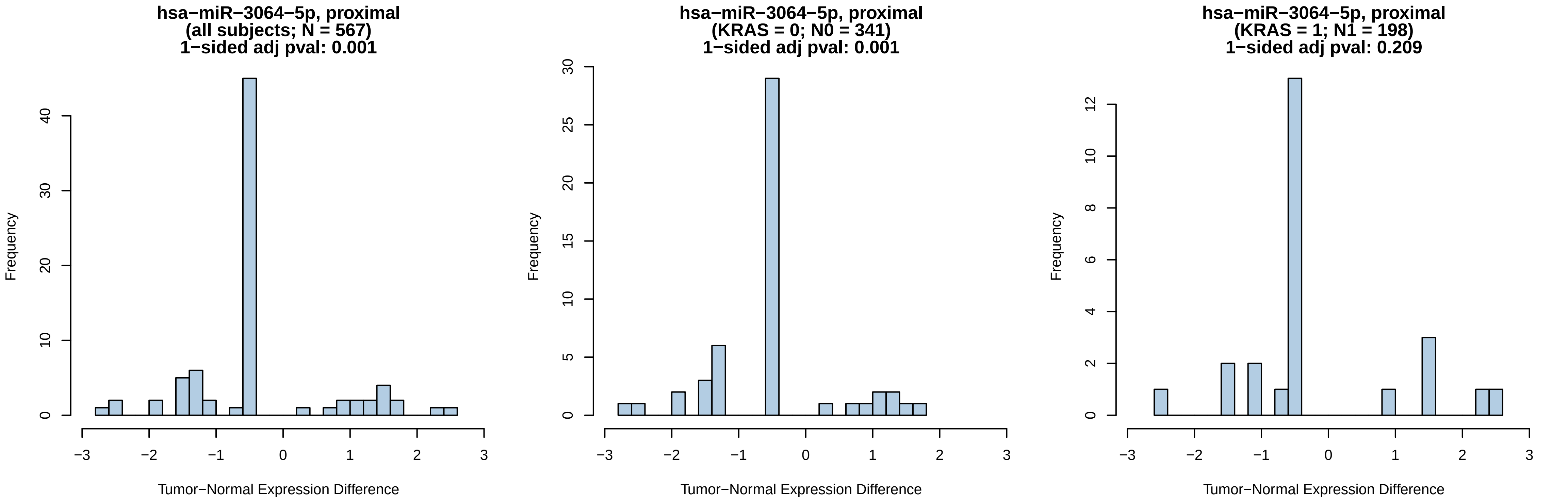
hsa−miR−3064−5p, proximal
(all subjects; N = 567)
1−sided adj pval: 0.001
hsa−miR−3064−5p, proximal
(KRAS = 0; N0 = 341)
1−sided adj pval: 0.001
hsa−miR−3064−5p, proximal
(KRAS = 1; N1 = 198)
1−sided adj pval: 0.209
Frequency
Tumor−Normal Expression Difference

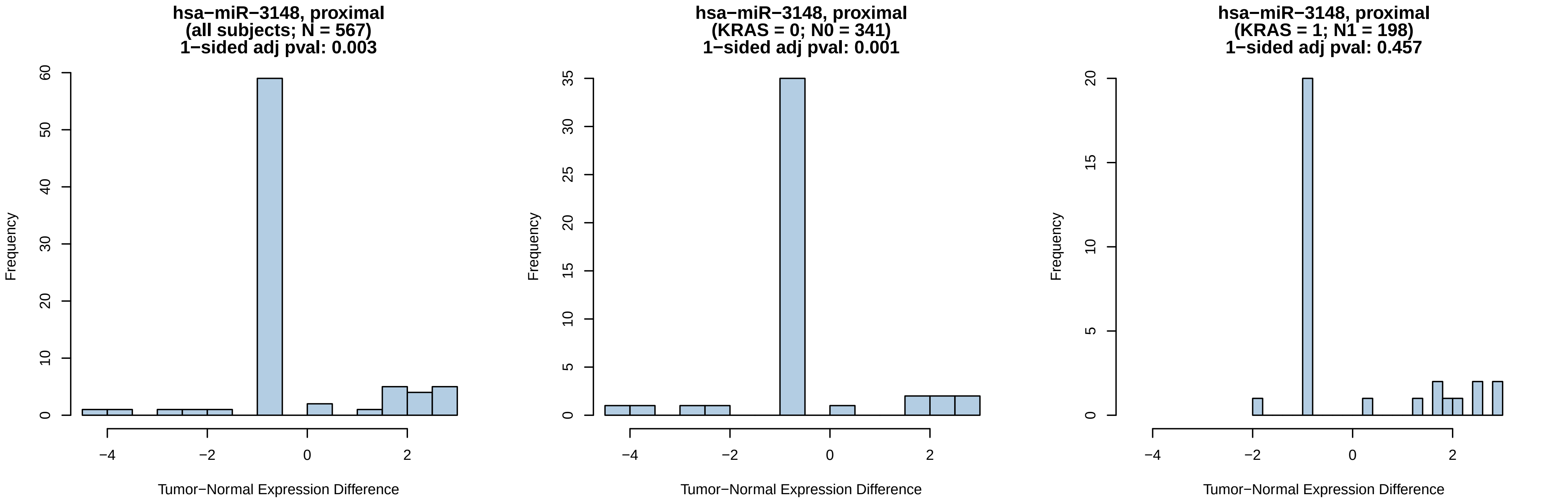

hsa−miR−3148, proximal
(all subjects; N = 567)
1−sided adj pval: 0.003
Frequency
Tumor−Normal Expression Difference
hsa−miR−3148, proximal
(KRAS = 0; N0 = 341)
1−sided adj pval: 0.001
Frequency
Tumor−Normal Expression Difference
hsa−miR−3148, proximal
(KRAS = 1; N1 = 198)
1−sided adj pval: 0.457
Frequency
Tumor−Normal Expression Difference

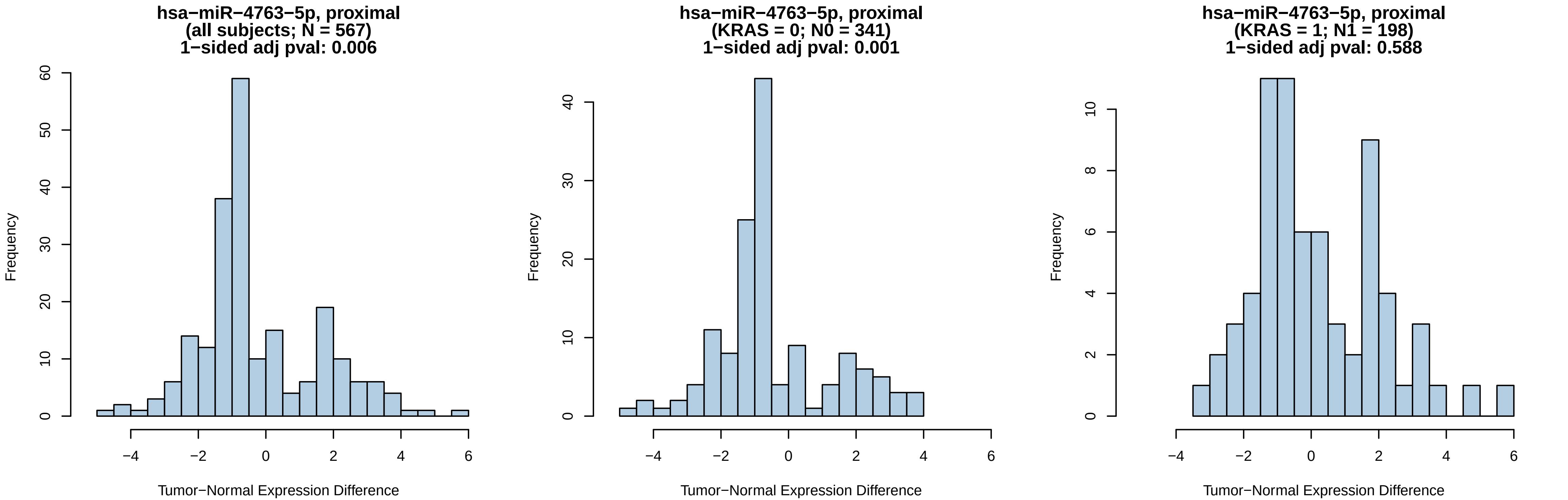
hsa−miR−4763−5p, proximal
(all subjects; N = 567)
1−sided adj pval: 0.006
hsa−miR−4763−5p, proximal
(KRAS = 0; N0 = 341)
1−sided adj pval: 0.001
hsa−miR−4763−5p, proximal
(KRAS = 1; N1 = 198)
1−sided adj pval: 0.588
Frequency
Tumor−Normal Expression Difference

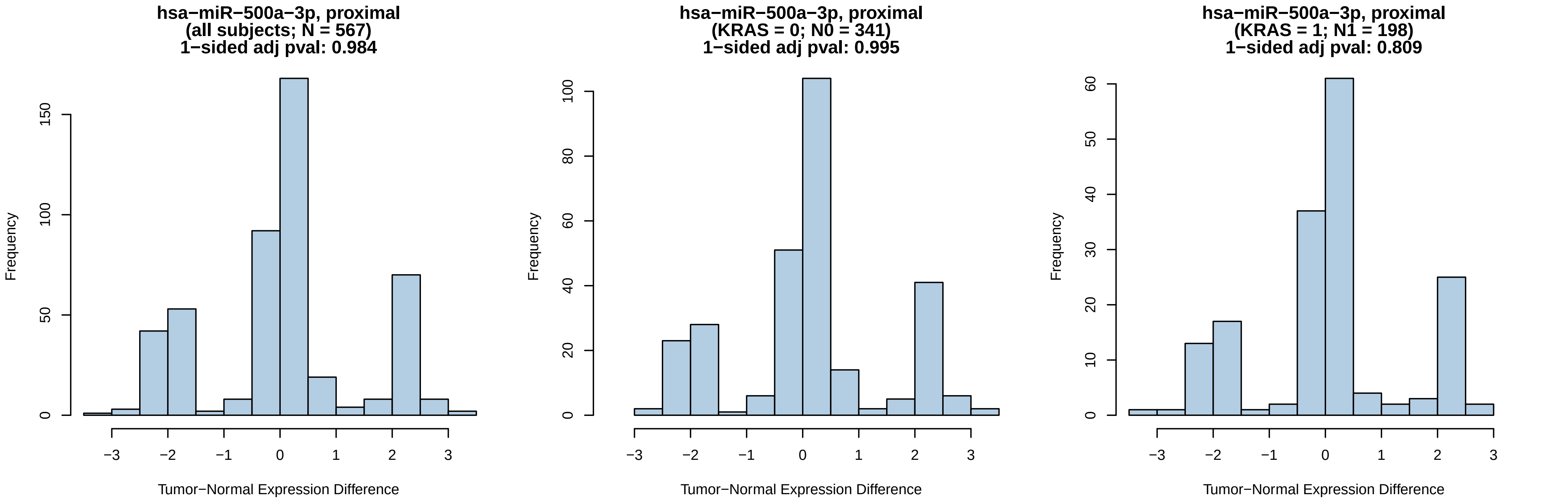

hsa−miR−500a−3p, proximal
(all subjects; N = 567)
1−sided adj pval: 0.984
Frequency
0
50
100
150
−3
−2
−1
0
1
2
3
Tumor−Normal Expression Difference
hsa−miR−500a−3p, proximal
(KRAS = 0; N0 = 341)
1−sided adj pval: 0.995
Frequency
0
20
40
60
80
100
−3
−2
−1
0
1
2
3
Tumor−Normal Expression Difference
hsa−miR−500a−3p, proximal
(KRAS = 1; N1 = 198)
1−sided adj pval: 0.809
Frequency
0
10
20
30
40
50
60
−3
−2
−1
0
1
2
3
Tumor−Normal Expression Difference

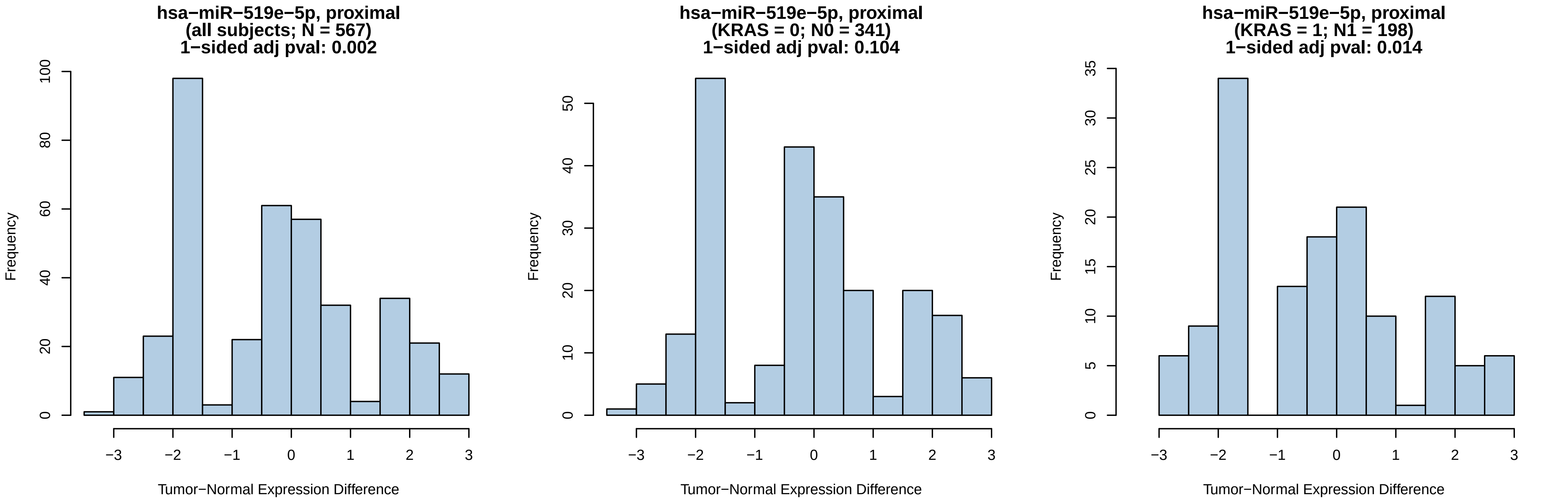

hsa−miR−519e−5p, proximal
(all subjects; N = 567)
1−sided adj pval: 0.002
hsa−miR−519e−5p, proximal
(KRAS = 0; N0 = 341)
1−sided adj pval: 0.104
hsa−miR−519e−5p, proximal
(KRAS = 1; N1 = 198)
1−sided adj pval: 0.014
Frequency
Tumor−Normal Expression Difference

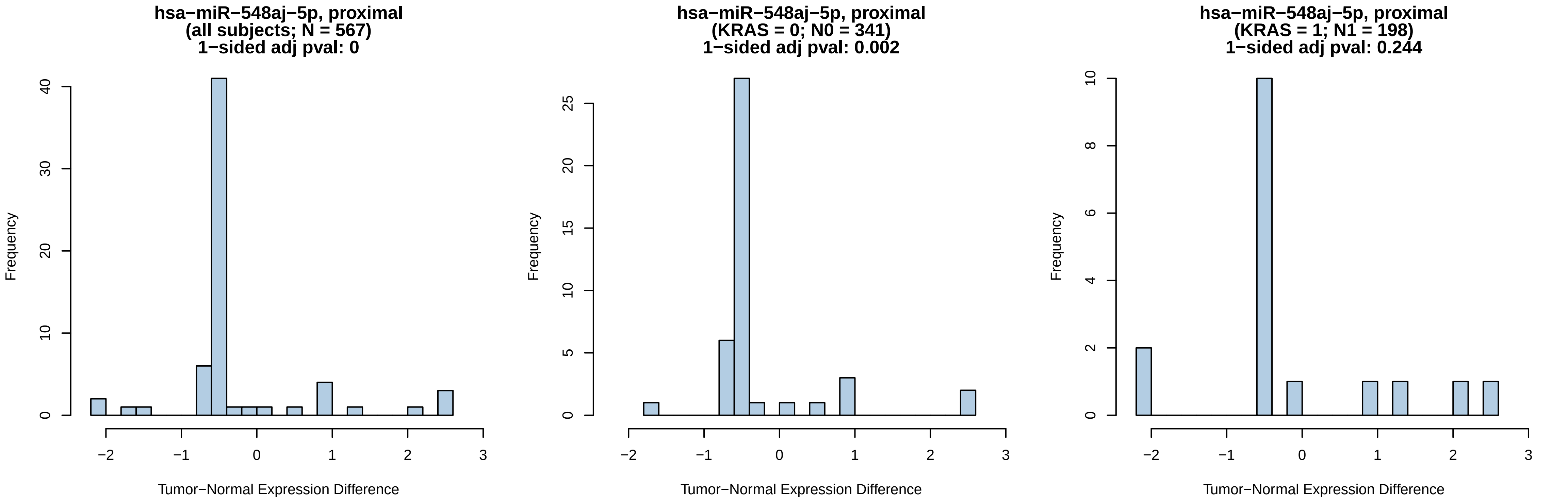

hsa−miR−548aj−5p, proximal
(all subjects; N = 567)
1−sided adj pval: 0
hsa−miR−548aj−5p, proximal
(KRAS = 0; N0 = 341)
1−sided adj pval: 0.002
hsa−miR−548aj−5p, proximal
(KRAS = 1; N1 = 198)
1−sided adj pval: 0.244
Frequency
Tumor−Normal Expression Difference

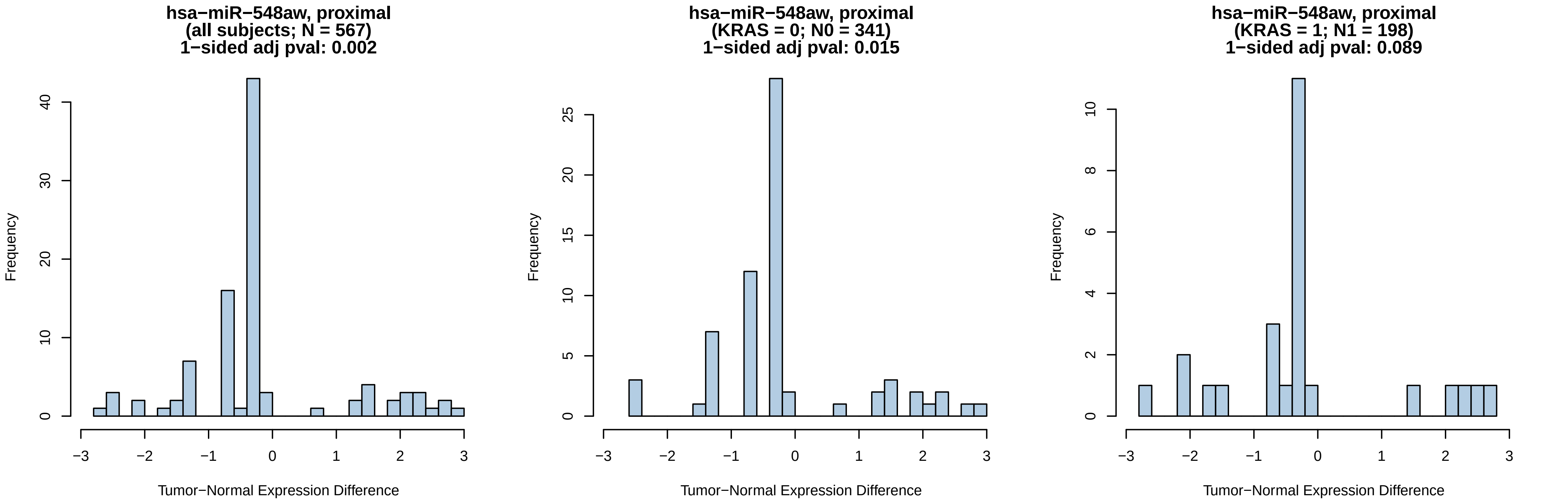
hsa−miR−548aw, proximal
(all subjects; N = 567)
1−sided adj pval: 0.002
Frequency
Tumor−Normal Expression Difference
hsa−miR−548aw, proximal
(KRAS = 0; N0 = 341)
1−sided adj pval: 0.015
Frequency
Tumor−Normal Expression Difference
hsa−miR−548aw, proximal
(KRAS = 1; N1 = 198)
1−sided adj pval: 0.089
Frequency
Tumor−Normal Expression Difference

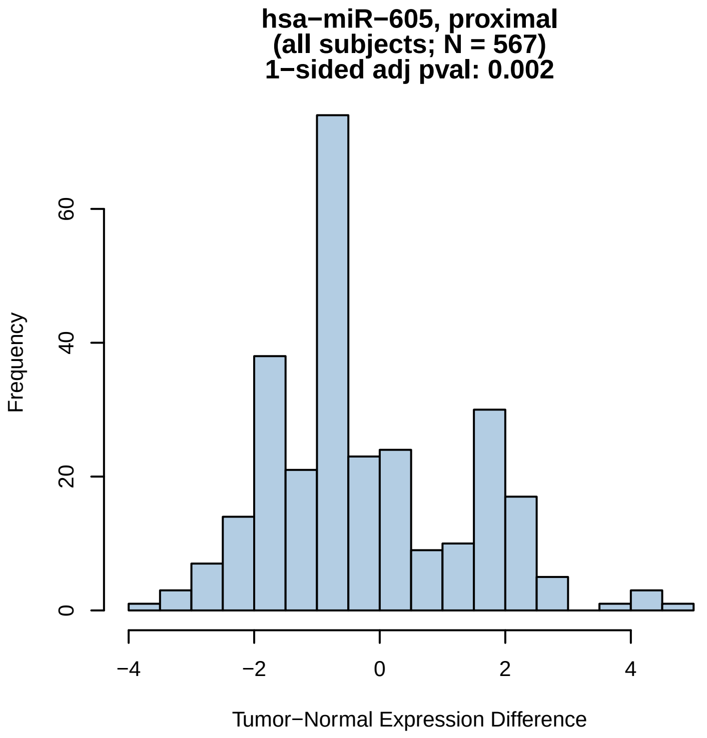
hsa−miR−605, proximal
(all subjects; N = 567)
1−sided adj pval: 0.002
Frequency
0
20
40
60
−4
−2
0
2
4
Tumor−Normal Expression Difference

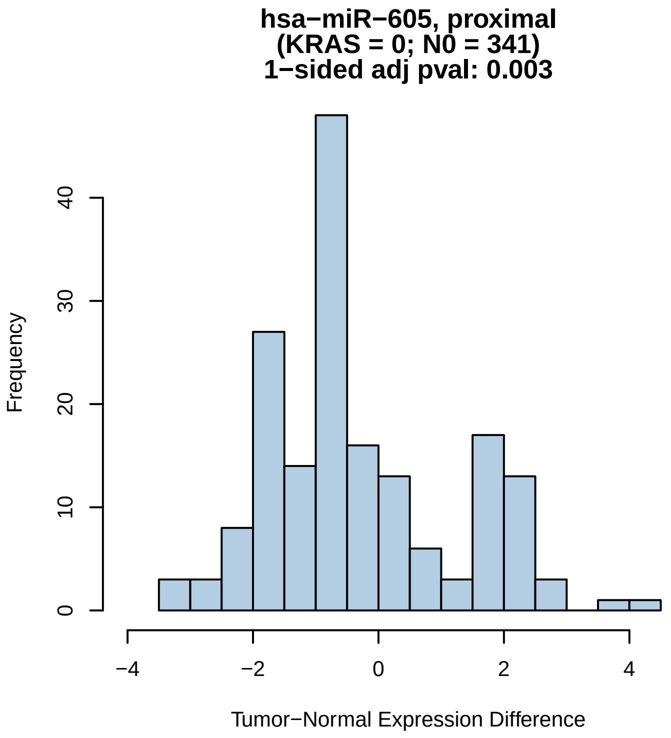
hsa−miR−605, proximal
(KRAS = 0; N0 = 341)
1−sided adj pval: 0.003
Frequency
0
10
20
30
40
−4
−2
0
2
4
Tumor−Normal Expression Difference

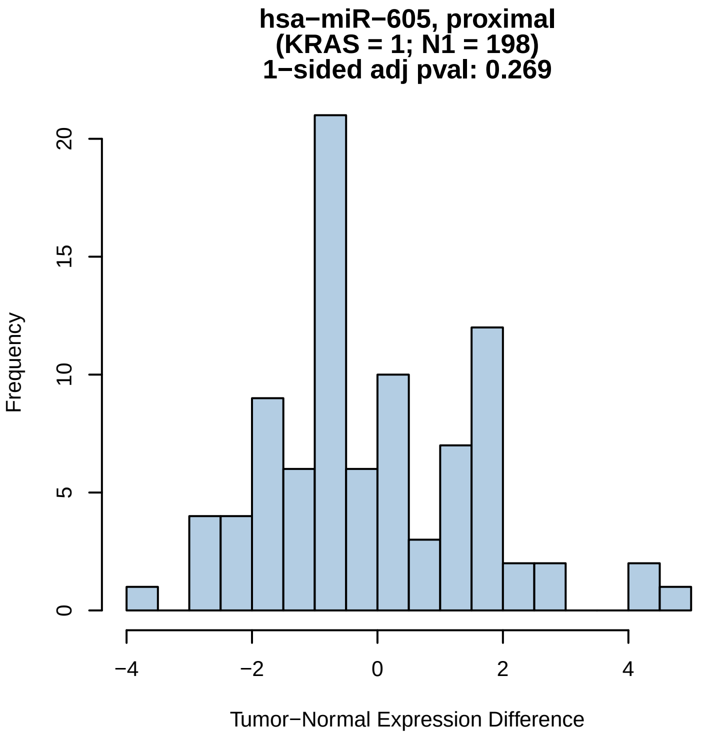
hsa−miR−605, proximal
(KRAS = 1; N1 = 198)
1−sided adj pval: 0.269
Frequency
0
5
10
15
20
−4
−2
0
2
4
Tumor−Normal Expression Difference

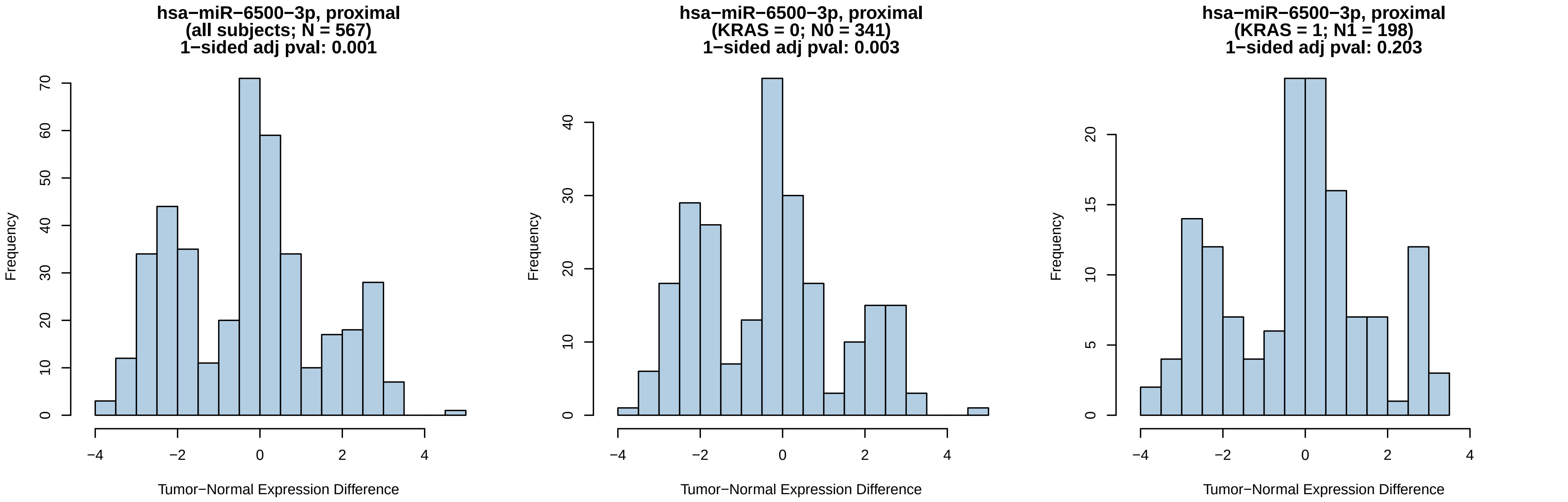
hsa−miR−6500−3p, proximal
(all subjects; N = 567)
1−sided adj pval: 0.001
Frequency
Tumor−Normal Expression Difference
hsa−miR−6500−3p, proximal
(KRAS = 0; N0 = 341)
1−sided adj pval: 0.003
Frequency
Tumor−Normal Expression Difference
hsa−miR−6500−3p, proximal
(KRAS = 1; N1 = 198)
1−sided adj pval: 0.203
Frequency
Tumor−Normal Expression Difference

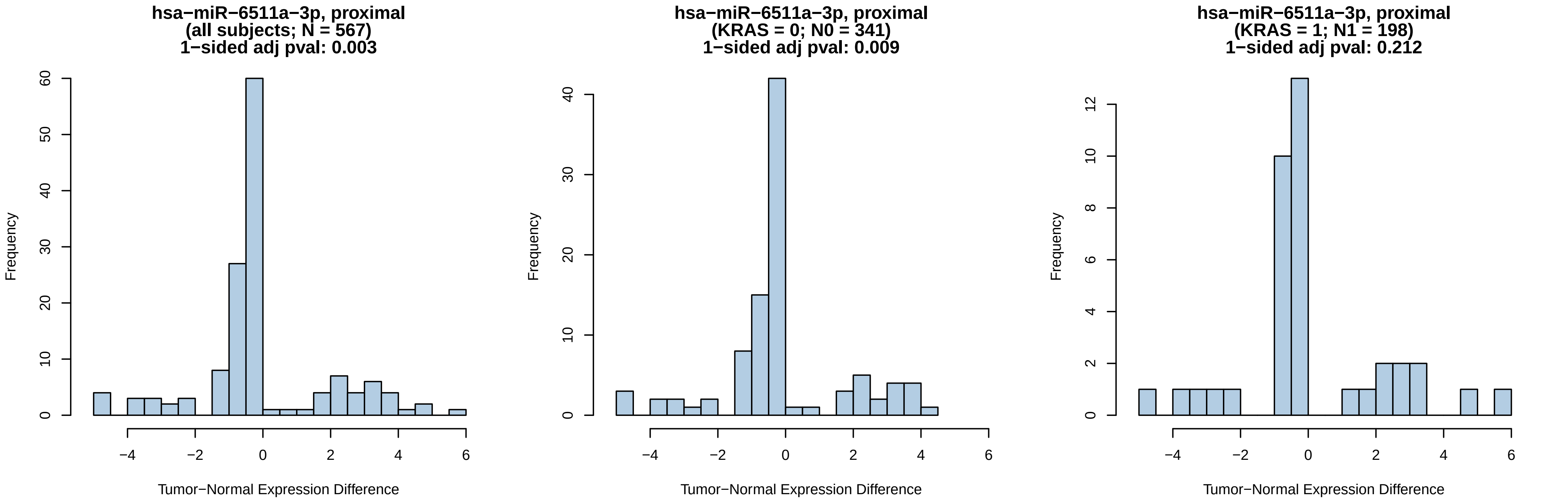
hsa−miR−6511a−3p, proximal
(all subjects; N = 567)
1−sided adj pval: 0.003
hsa−miR−6511a−3p, proximal
(KRAS = 0; N0 = 341)
1−sided adj pval: 0.009
hsa−miR−6511a−3p, proximal
(KRAS = 1; N1 = 198)
1−sided adj pval: 0.212
Frequency
Tumor−Normal Expression Difference

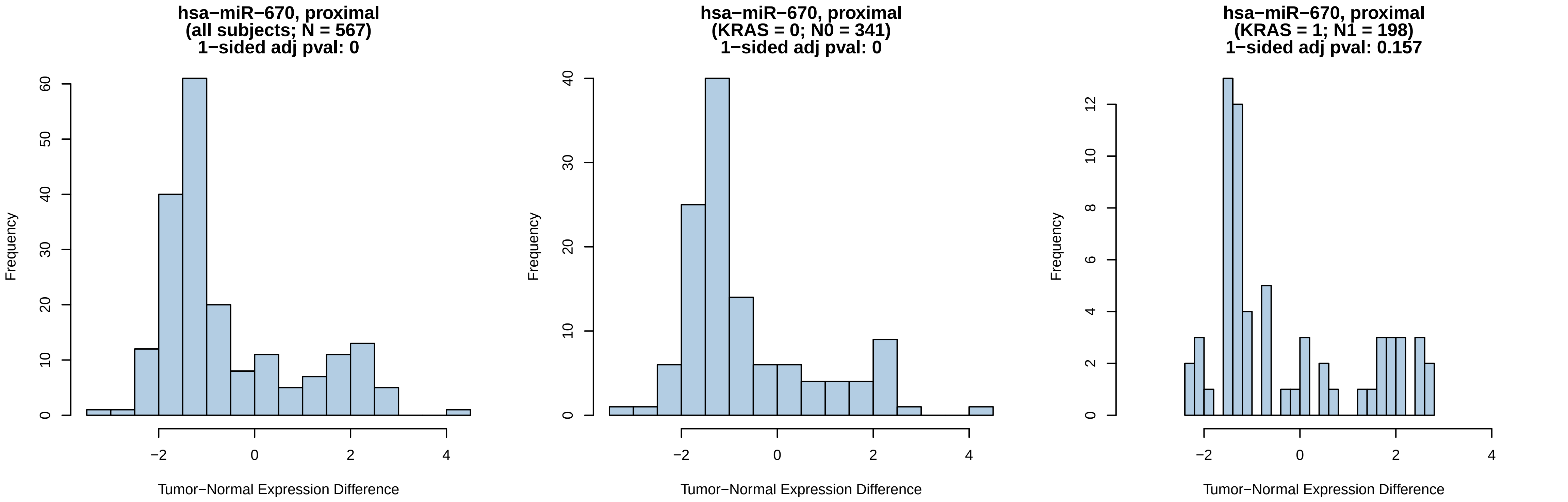

hsa−miR−670, proximal
(all subjects; N = 567)
1−sided adj pval: 0
Frequency
Tumor−Normal Expression Difference
hsa−miR−670, proximal
(KRAS = 0; N0 = 341)
1−sided adj pval: 0
Frequency
Tumor−Normal Expression Difference
hsa−miR−670, proximal
(KRAS = 1; N1 = 198)
1−sided adj pval: 0.157
Frequency
Tumor−Normal Expression Difference

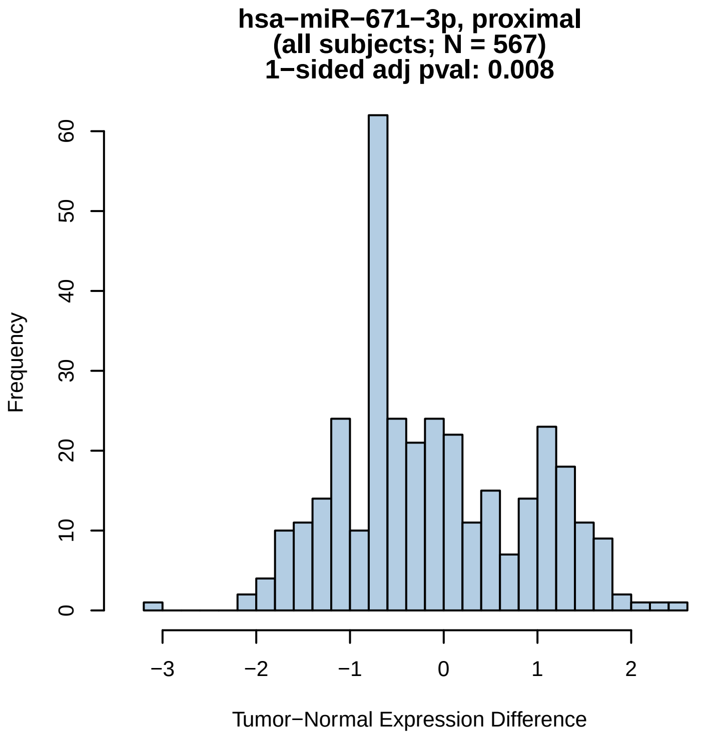
hsa−miR−671−3p, proximal
(all subjects; N = 567)
1−sided adj pval: 0.008
Frequency
0
10
20
30
40
50
60
−3
−2
−1
0
1
2
Tumor−Normal Expression Difference

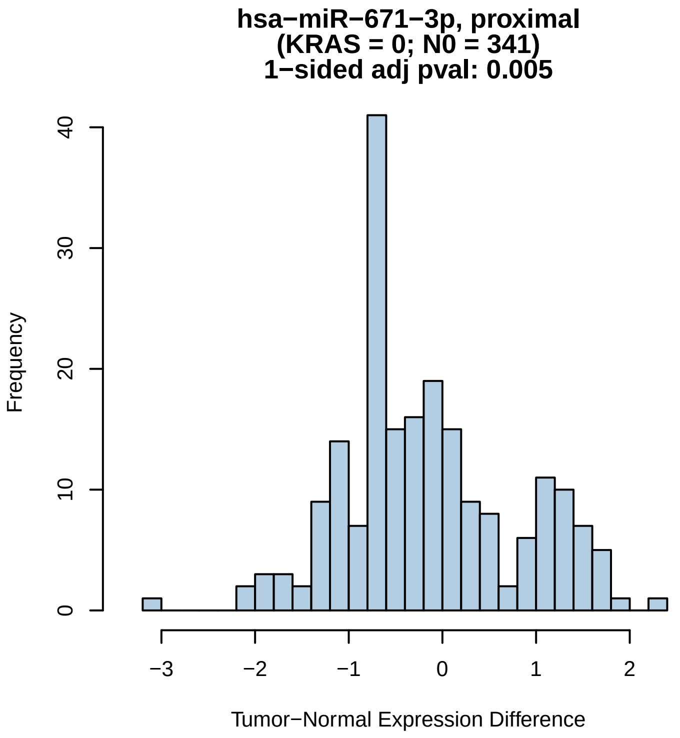
hsa−miR−671−3p, proximal
(KRAS = 0; N0 = 341)
1−sided adj pval: 0.005
Frequency
0
10
20
30
40
−3
−2
−1
0
1
2
Tumor−Normal Expression Difference

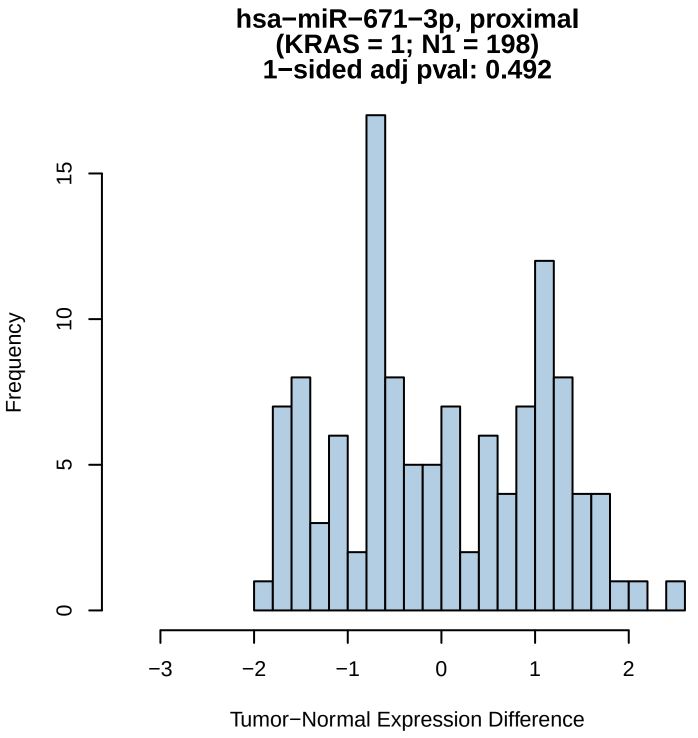
hsa−miR−671−3p, proximal
(KRAS = 1; N1 = 198)
1−sided adj pval: 0.492
Frequency
0
5
10
15
−3
−2
−1
0
1
2
Tumor−Normal Expression Difference

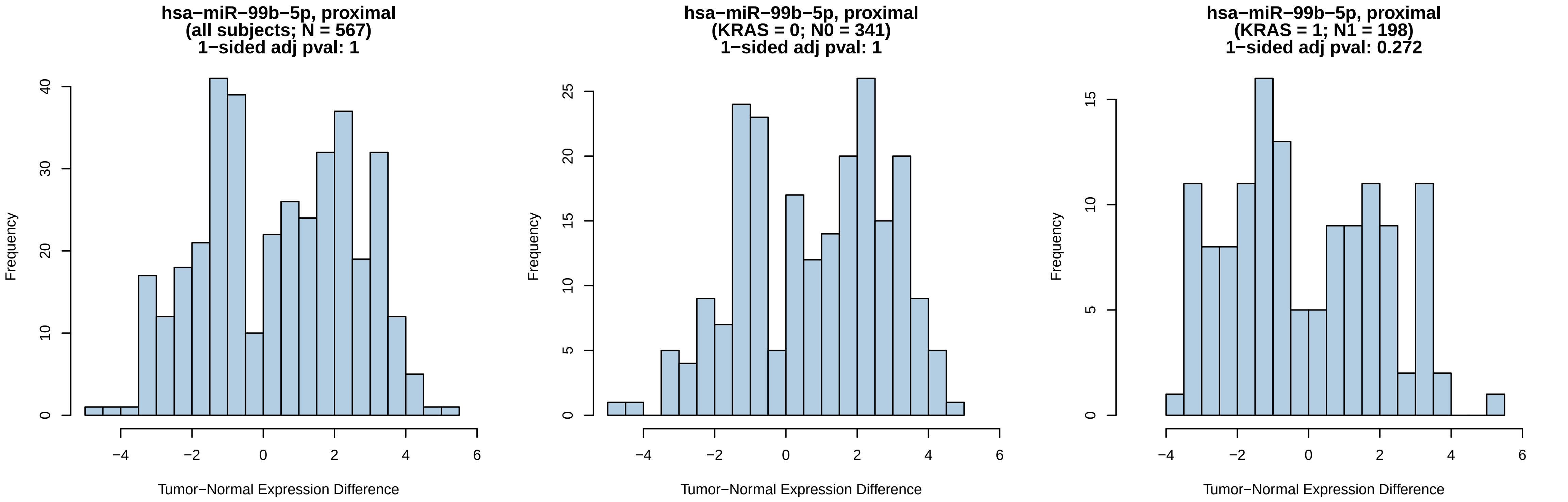
hsa−miR−99b−5p, proximal
(all subjects; N = 567)
1−sided adj pval: 1
Frequency
Tumor−Normal Expression Difference
hsa−miR−99b−5p, proximal
(KRAS = 0; N0 = 341)
1−sided adj pval: 1
Frequency
Tumor−Normal Expression Difference
hsa−miR−99b−5p, proximal
(KRAS = 1; N1 = 198)
1−sided adj pval: 0.272
Frequency
Tumor−Normal Expression Difference

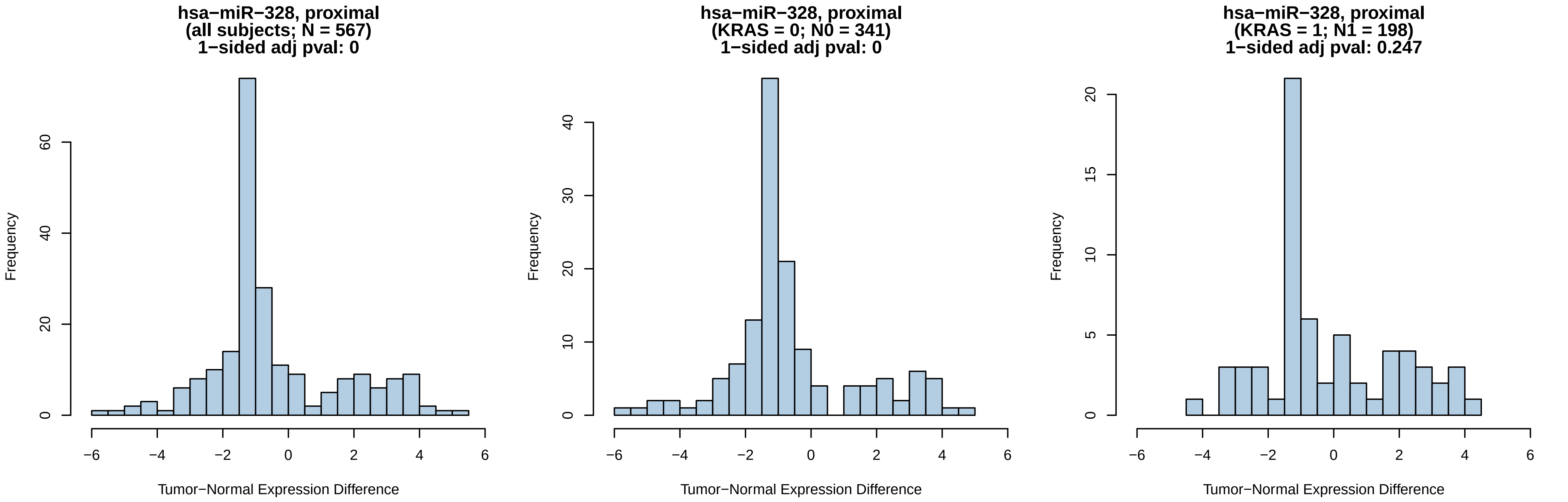

hsa−miR−328, proximal
(all subjects; N = 567)
1−sided adj pval: 0
Frequency
0
20
40
60
−6
−4
−2
0
2
4
6
Tumor−Normal Expression Difference
hsa−miR−328, proximal
(KRAS = 0; N0 = 341)
1−sided adj pval: 0
Frequency
0
10
20
30
40
−6
−4
−2
0
2
4
6
Tumor−Normal Expression Difference
hsa−miR−328, proximal
(KRAS = 1; N1 = 198)
1−sided adj pval: 0.247
Frequency
0
5
10
15
20
−6
−4
−2
0
2
4
6
Tumor−Normal Expression Difference

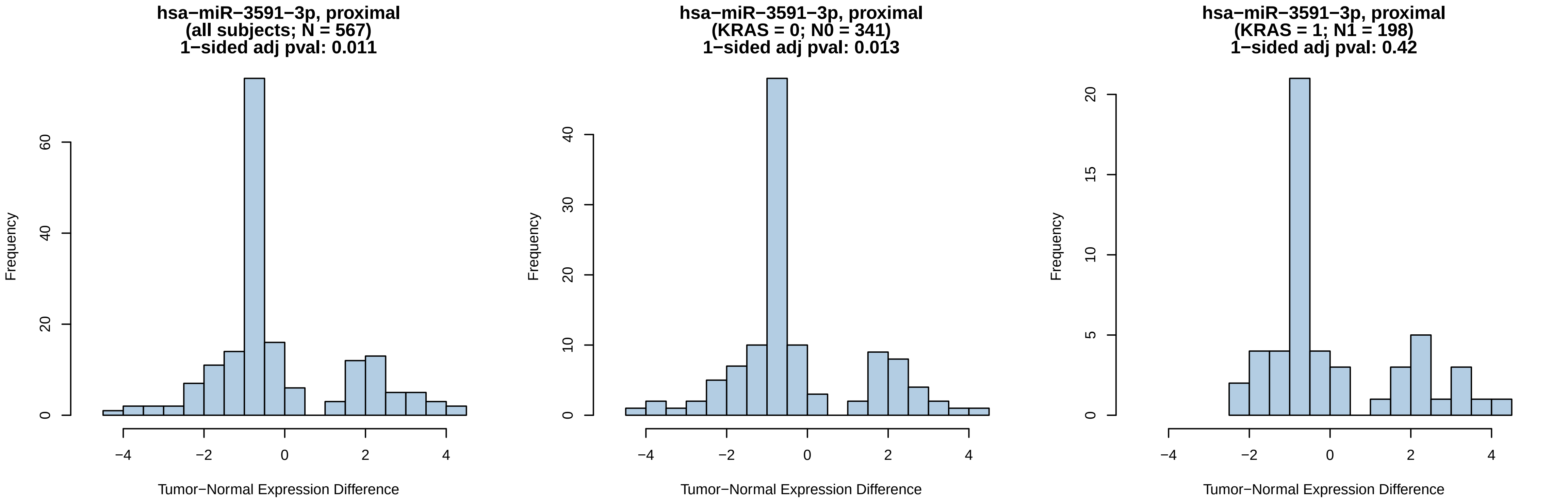
hsa−miR−3591−3p, proximal
(all subjects; N = 567)
1−sided adj pval: 0.011
Frequency
0
20
40
60
−4
−2
0
2
4
Tumor−Normal Expression Difference
hsa−miR−3591−3p, proximal
(KRAS = 0; N0 = 341)
1−sided adj pval: 0.013
Frequency
0
10
20
30
40
−4
−2
0
2
4
Tumor−Normal Expression Difference
hsa−miR−3591−3p, proximal
(KRAS = 1; N1 = 198)
1−sided adj pval: 0.42
Frequency
0
5
10
15
20
−4
−2
0
2
4
Tumor−Normal Expression Difference

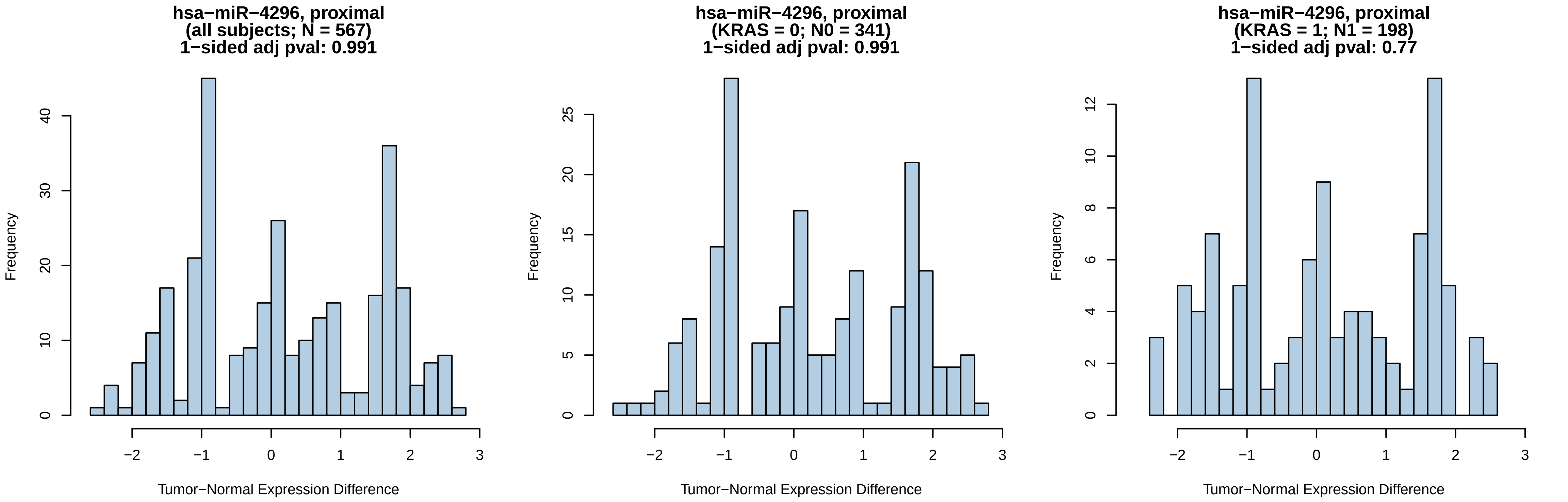

hsa−miR−4296, proximal
(all subjects; N = 567)
1−sided adj pval: 0.991
hsa−miR−4296, proximal
(KRAS = 0; N0 = 341)
1−sided adj pval: 0.991
hsa−miR−4296, proximal
(KRAS = 1; N1 = 198)
1−sided adj pval: 0.77
Frequency
Tumor−Normal Expression Difference

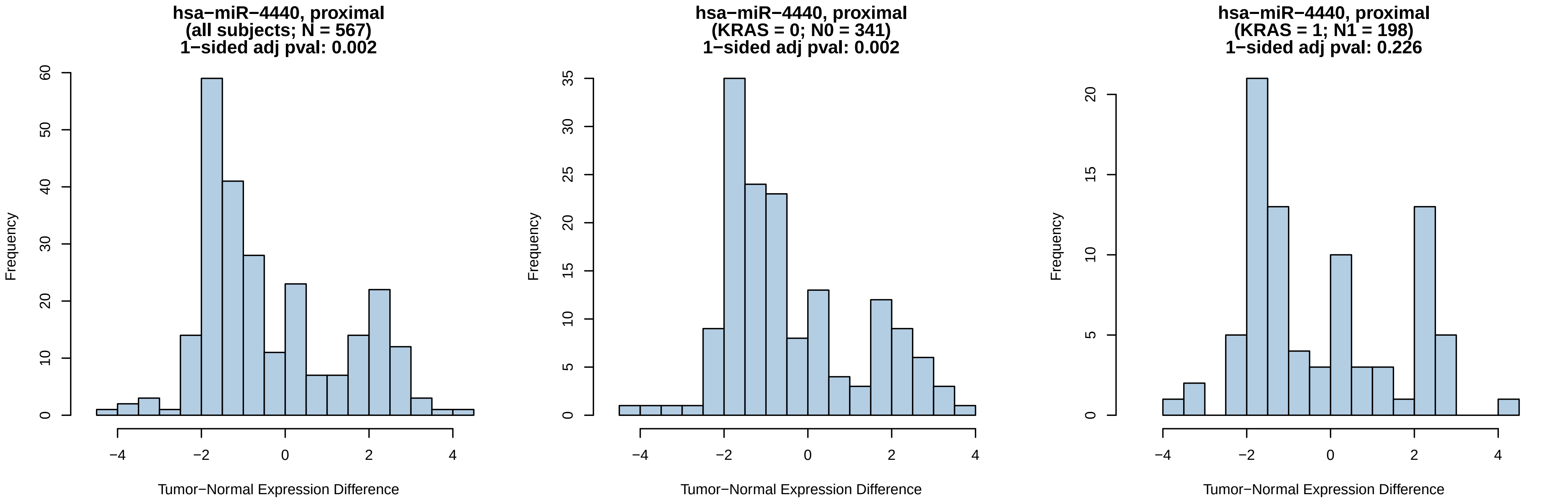
hsa−miR−4440, proximal
(all subjects; N = 567)
1−sided adj pval: 0.002
Frequency
0
10
20
30
40
50
60
−4
−2
0
2
4
Tumor−Normal Expression Difference
hsa−miR−4440, proximal
(KRAS = 0; N0 = 341)
1−sided adj pval: 0.002
Frequency
0
5
10
15
20
25
30
35
−4
−2
0
2
4
Tumor−Normal Expression Difference
hsa−miR−4440, proximal
(KRAS = 1; N1 = 198)
1−sided adj pval: 0.226
Frequency
0
5
10
15
20
−4
−2
0
2
4
Tumor−Normal Expression Difference

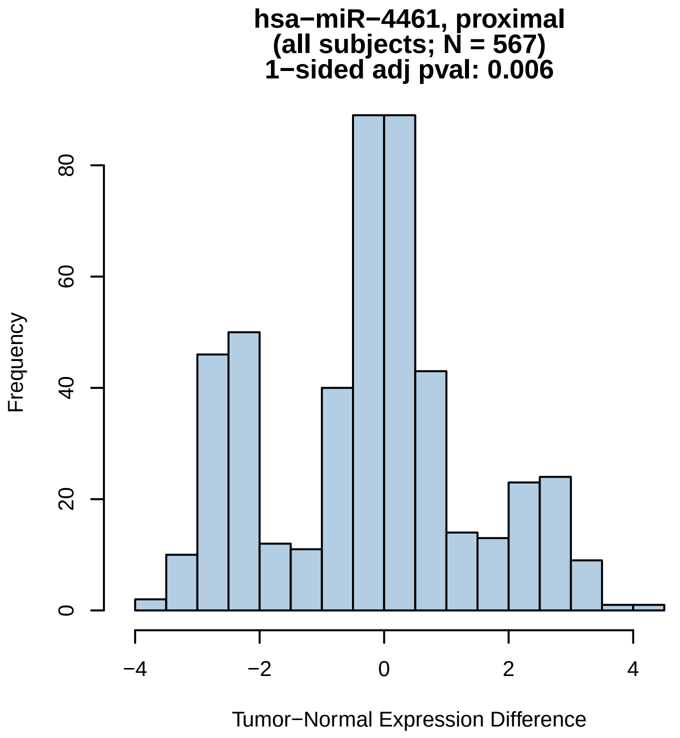
hsa−miR−4461, proximal
(all subjects; N = 567)
1−sided adj pval: 0.006
Frequency
0
20
40
60
80
−4
−2
0
2
4
Tumor−Normal Expression Difference

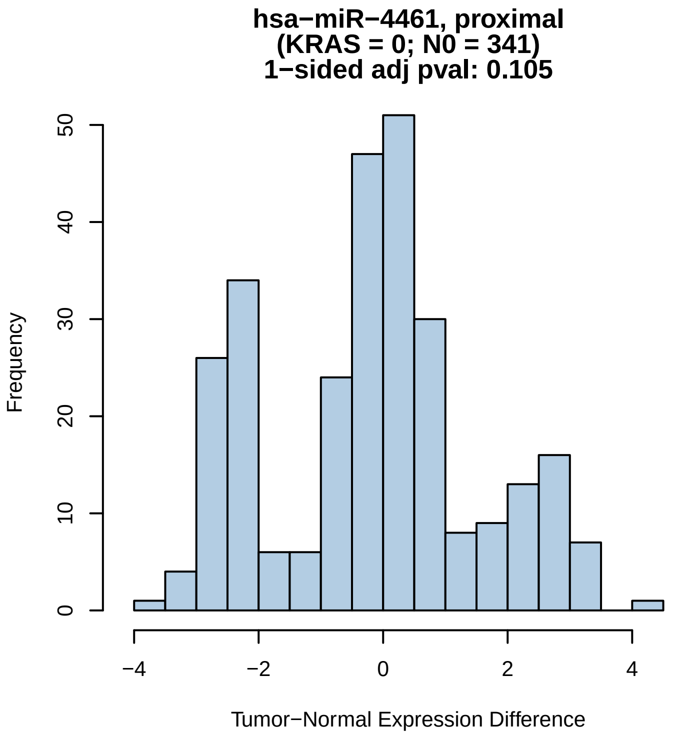
hsa−miR−4461, proximal
(KRAS = 0; N0 = 341)
1−sided adj pval: 0.105
Frequency
0
10
20
30
40
50
−4
−2
0
2
4
Tumor−Normal Expression Difference

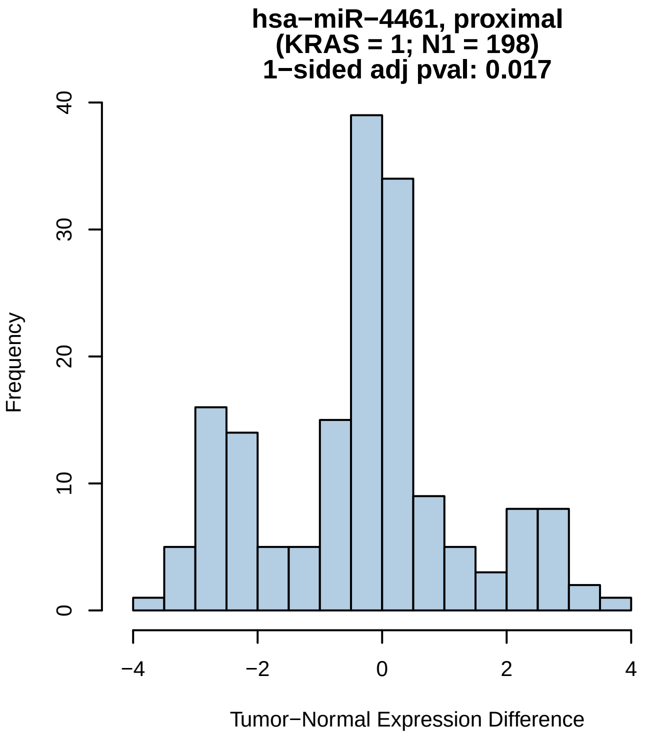
hsa−miR−4461, proximal
(KRAS = 1; N1 = 198)
1−sided adj pval: 0.017
Frequency
0
10
20
30
40
−4
−2
0
2
4
Tumor−Normal Expression Difference

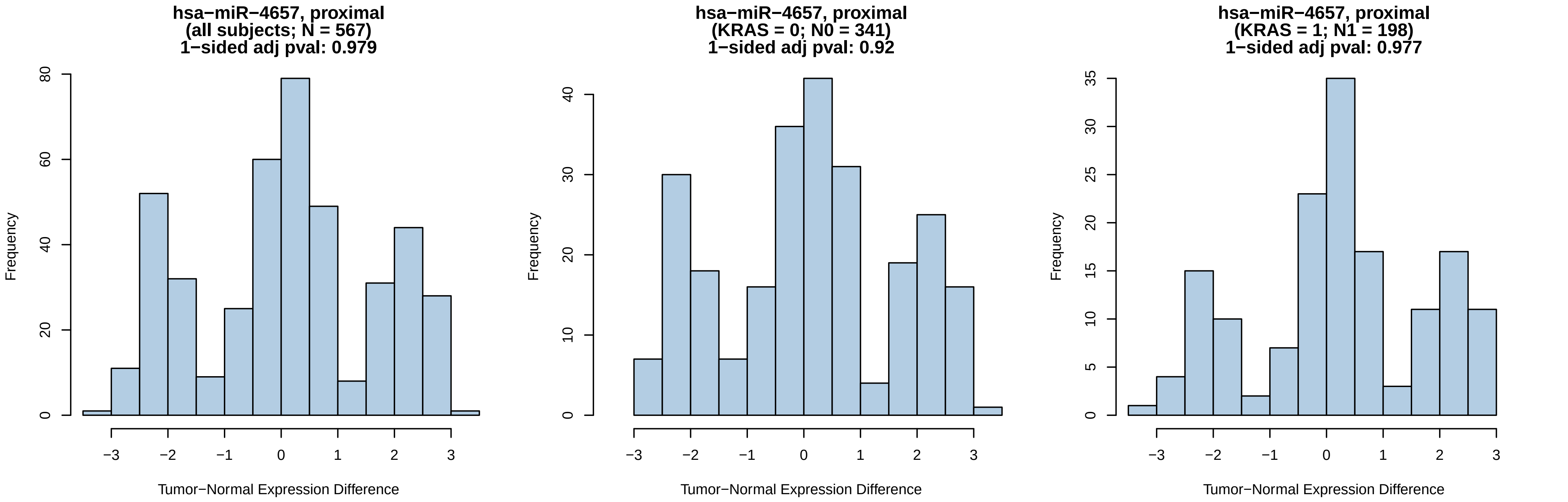

hsa−miR−4657, proximal
(all subjects; N = 567)
1−sided adj pval: 0.979
Frequency
Tumor−Normal Expression Difference
hsa−miR−4657, proximal
(KRAS = 0; N0 = 341)
1−sided adj pval: 0.92
Frequency
Tumor−Normal Expression Difference
hsa−miR−4657, proximal
(KRAS = 1; N1 = 198)
1−sided adj pval: 0.977
Frequency
Tumor−Normal Expression Difference

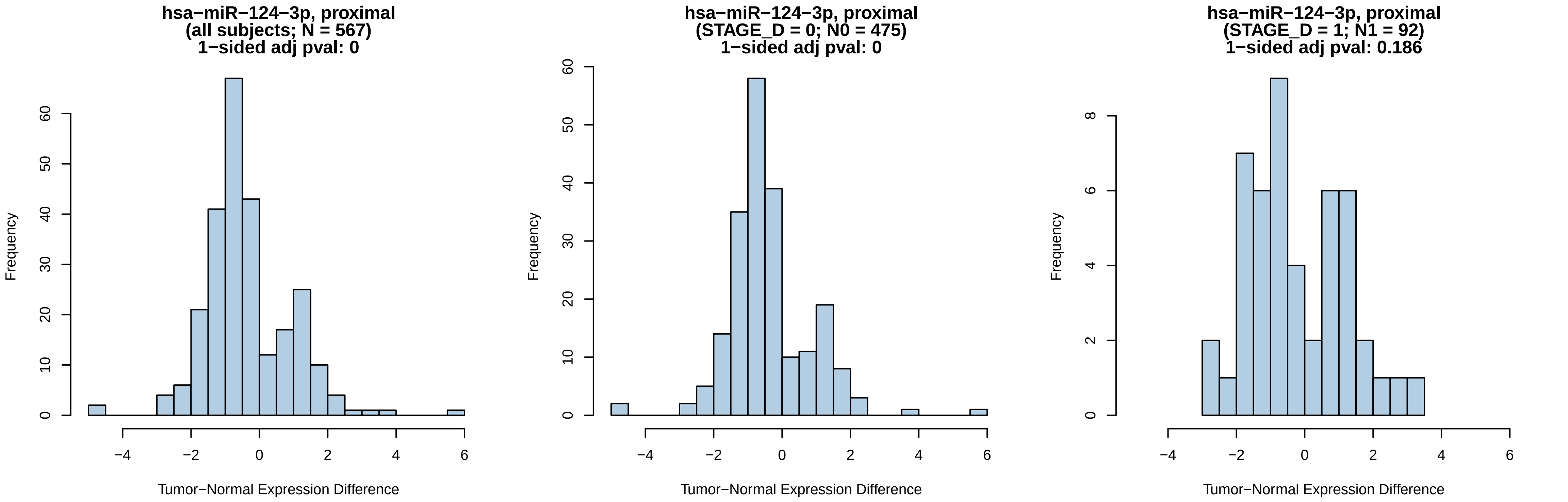

hsa−miR−124−3p, proximal
(all subjects; N = 567)
1−sided adj pval: 0
Frequency
0
10
20
30
40
50
60
−4
−2
0
2
4
6
Tumor−Normal Expression Difference
hsa−miR−124−3p, proximal
(STAGE_D = 0; N0 = 475)
1−sided adj pval: 0
Frequency
0
10
20
30
40
50
60
−4
−2
0
2
4
6
Tumor−Normal Expression Difference
hsa−miR−124−3p, proximal
(STAGE_D = 1; N1 = 92)
1−sided adj pval: 0.186
Frequency
0
2
4
6
8
−4
−2
0
2
4
6
Tumor−Normal Expression Difference

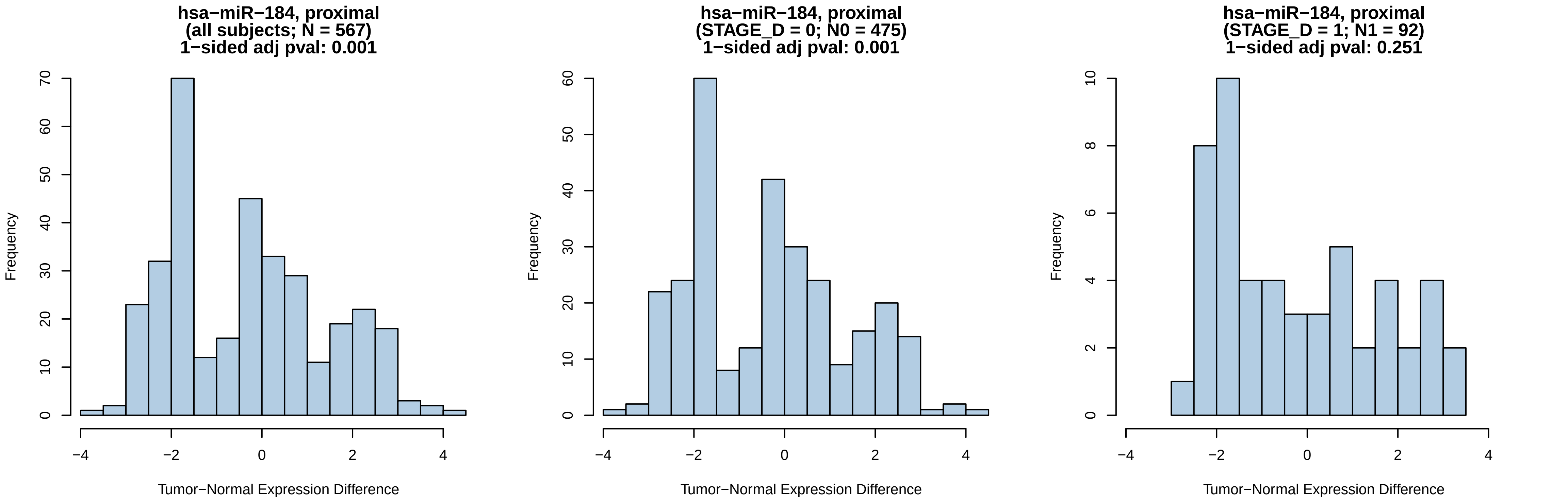

hsa−miR−184, proximal
(all subjects; N = 567)
1−sided adj pval: 0.001
Frequency
Tumor−Normal Expression Difference
hsa−miR−184, proximal
(STAGE_D = 0; N0 = 475)
1−sided adj pval: 0.001
Frequency
Tumor−Normal Expression Difference
hsa−miR−184, proximal
(STAGE_D = 1; N1 = 92)
1−sided adj pval: 0.251
Frequency
Tumor−Normal Expression Difference

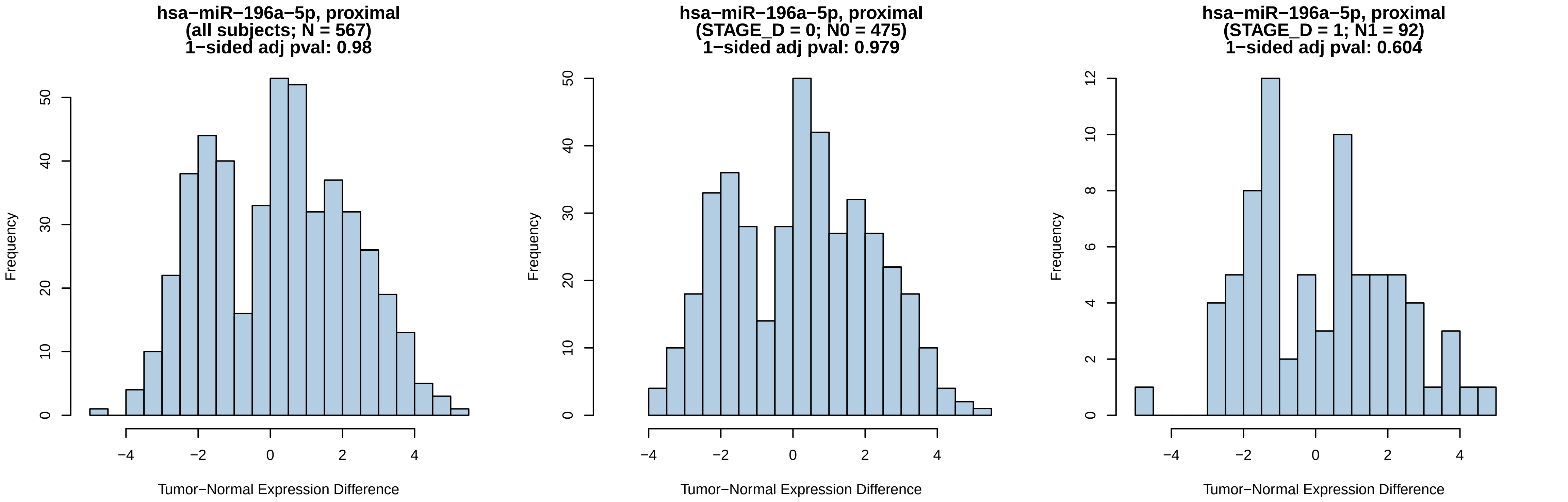

hsa−miR−196a−5p, proximal
(all subjects; N = 567)
1−sided adj pval: 0.98
Frequency
Tumor−Normal Expression Difference
hsa−miR−196a−5p, proximal
(STAGE_D = 0; N0 = 475)
1−sided adj pval: 0.979
Frequency
Tumor−Normal Expression Difference
hsa−miR−196a−5p, proximal
(STAGE_D = 1; N1 = 92)
1−sided adj pval: 0.604
Frequency
Tumor−Normal Expression Difference

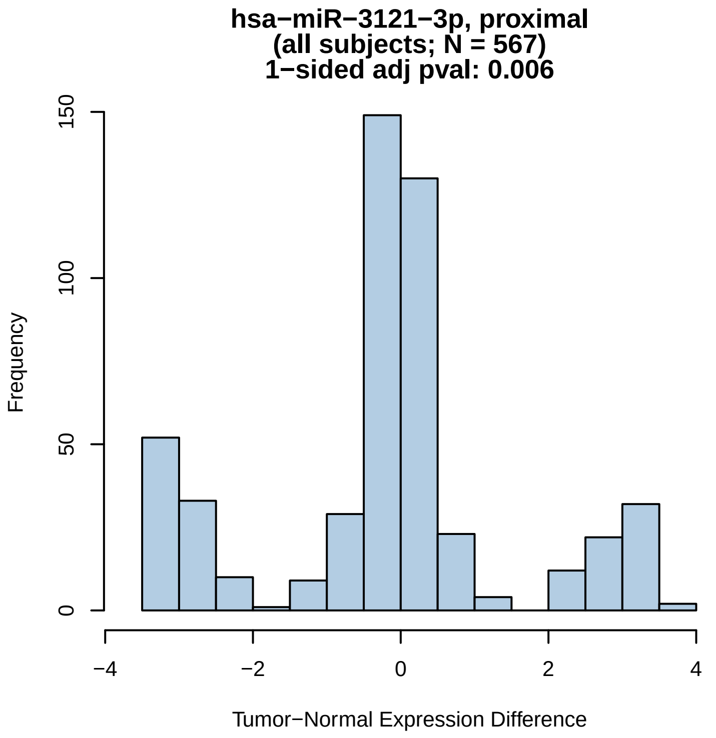
hsa−miR−3121−3p, proximal
(all subjects; N = 567)
1−sided adj pval: 0.006
Frequency
0
50
100
150
−4
−2
0
2
4
Tumor−Normal Expression Difference

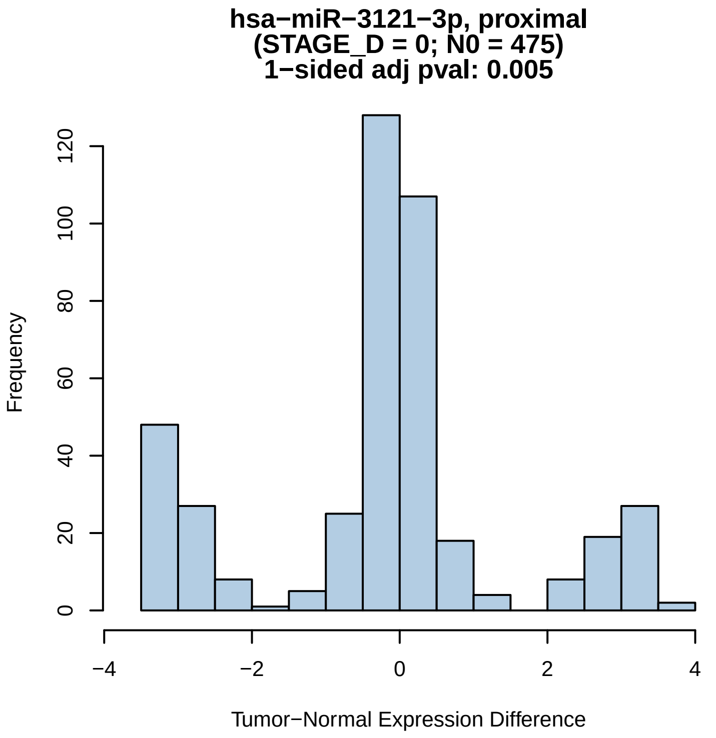
hsa−miR−3121−3p, proximal
(STAGE_D = 0; N0 = 475)
1−sided adj pval: 0.005
Frequency
0
20
40
60
80
100
120
−4
−2
0
2
4
Tumor−Normal Expression Difference

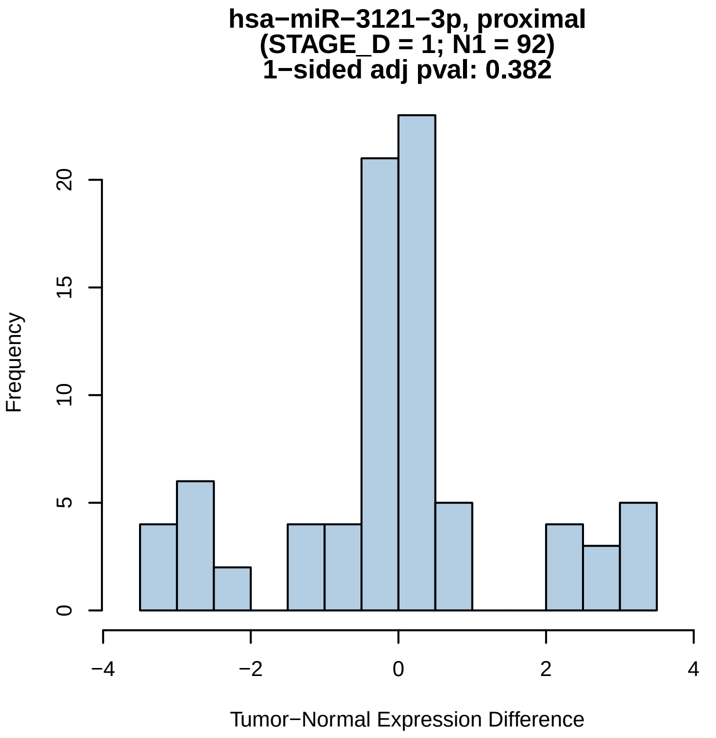
hsa−miR−3121−3p, proximal
(STAGE_D = 1; N1 = 92)
1−sided adj pval: 0.382
Frequency
0
5
10
15
20
−4
−2
0
2
4
Tumor−Normal Expression Difference

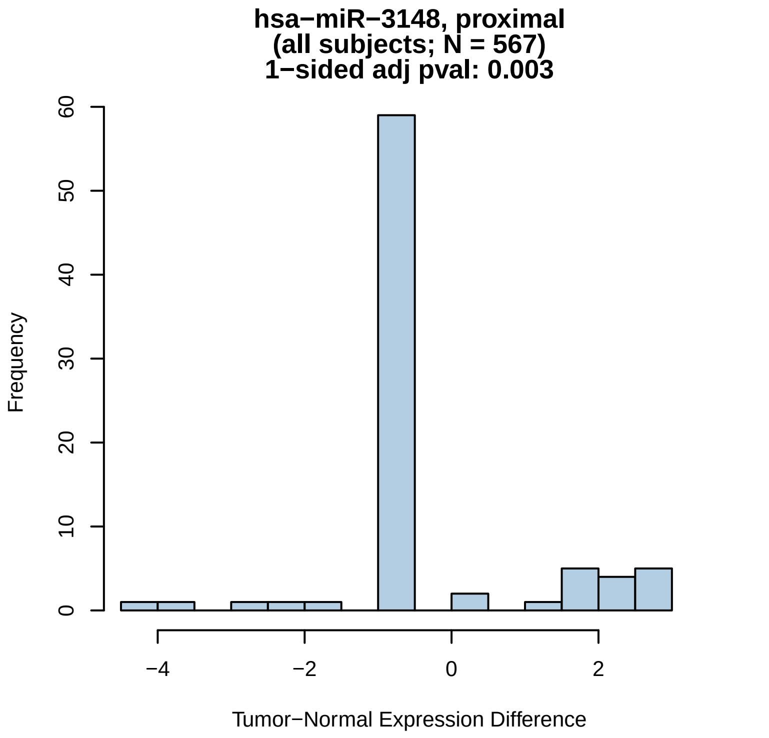
hsa−miR−3148, proximal
(all subjects; N = 567)
1−sided adj pval: 0.003
Frequency
Tumor−Normal Expression Difference

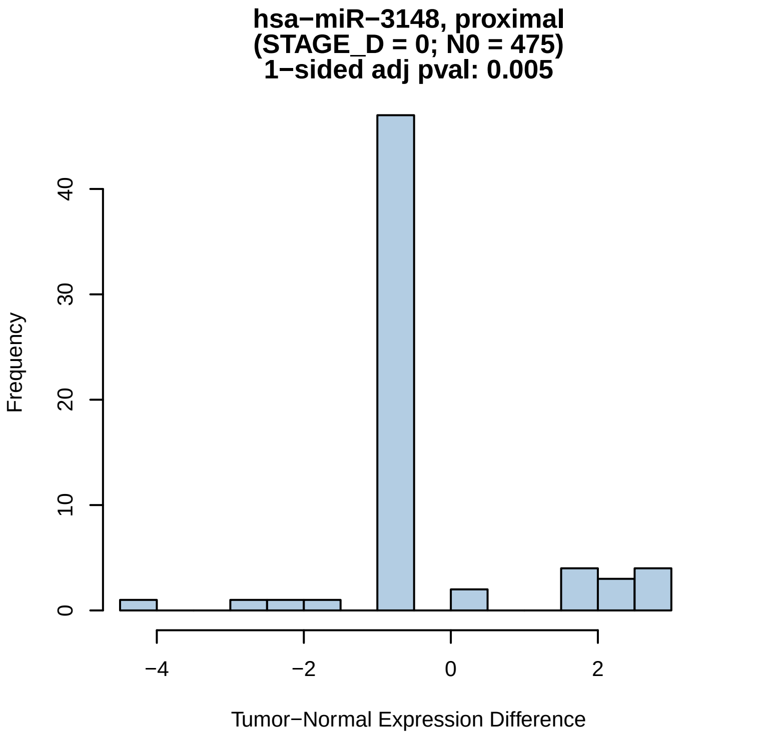
hsa−miR−3148, proximal
(STAGE_D = 0; N0 = 475)
1−sided adj pval: 0.005
Frequency
Tumor−Normal Expression Difference

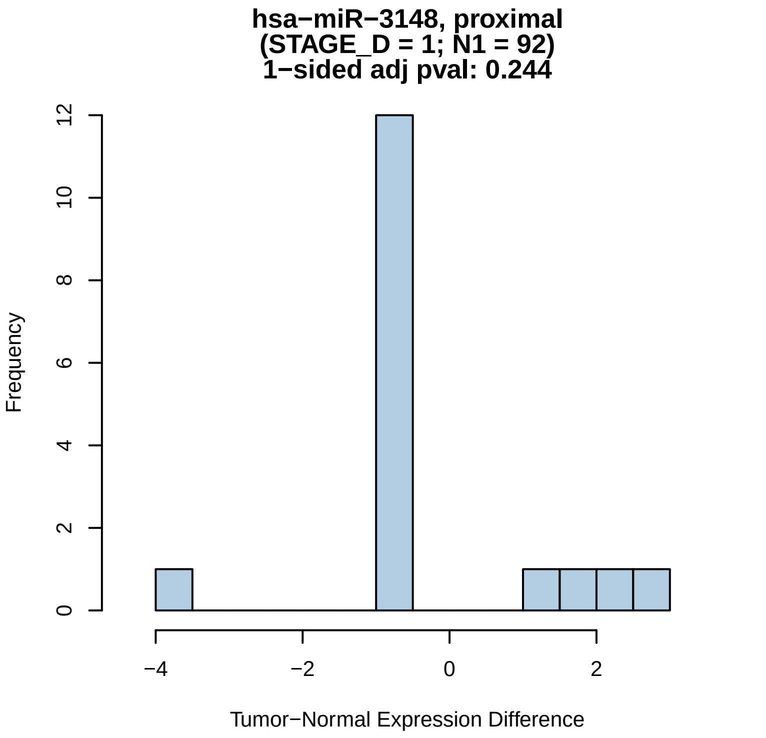
hsa−miR−3148, proximal
(STAGE_D = 1; N1 = 92)
1−sided adj pval: 0.244
Frequency
Tumor−Normal Expression Difference

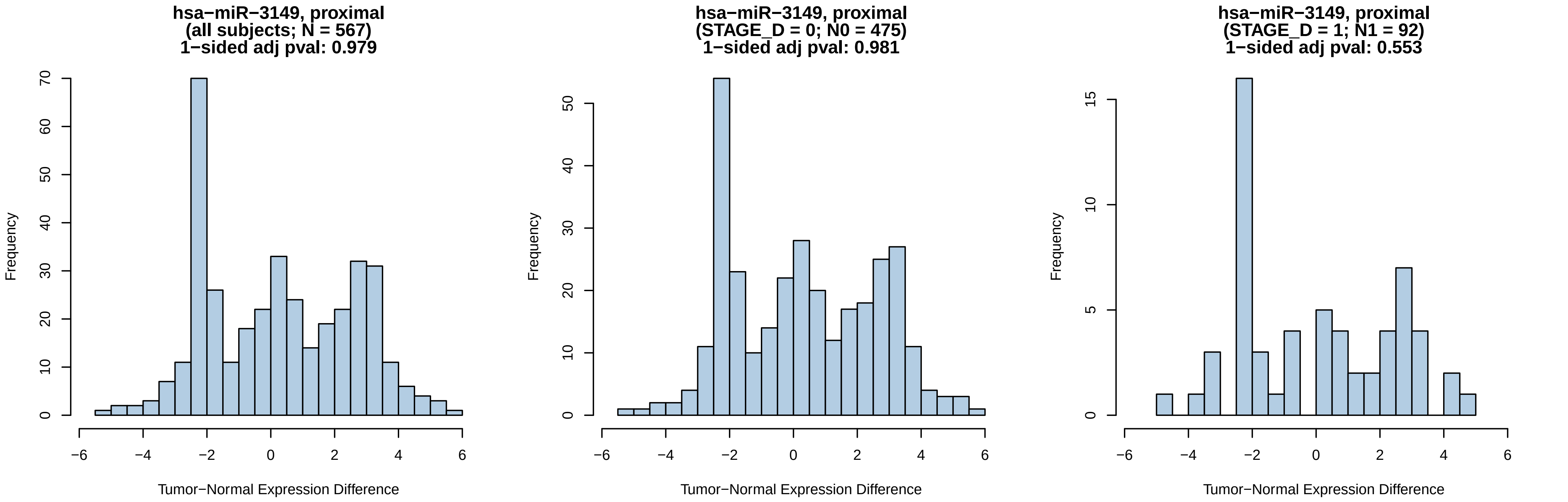
hsa−miR−3149, proximal
(all subjects; N = 567)
1−sided adj pval: 0.979
Frequency
Tumor−Normal Expression Difference
hsa−miR−3149, proximal
(STAGE_D = 0; N0 = 475)
1−sided adj pval: 0.981
Frequency
Tumor−Normal Expression Difference
hsa−miR−3149, proximal
(STAGE_D = 1; N1 = 92)
1−sided adj pval: 0.553
Frequency
Tumor−Normal Expression Difference

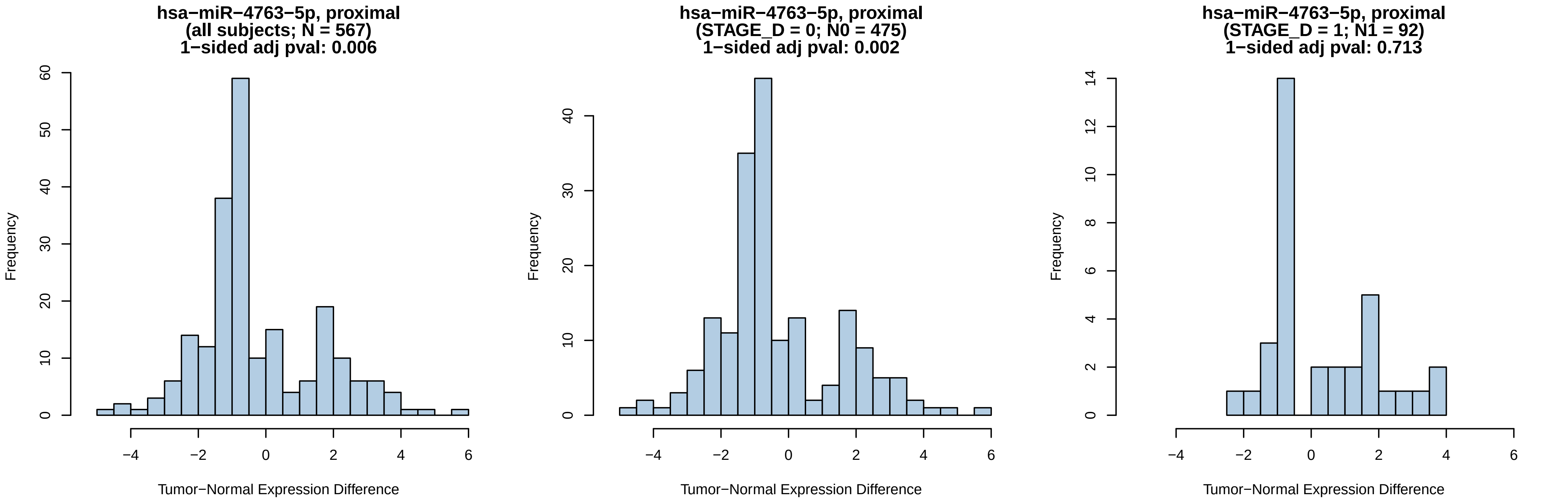
hsa−miR−4763−5p, proximal
(all subjects; N = 567)
1−sided adj pval: 0.006
Frequency
Tumor−Normal Expression Difference
hsa−miR−4763−5p, proximal
(STAGE_D = 0; N0 = 475)
1−sided adj pval: 0.002
Frequency
Tumor−Normal Expression Difference
hsa−miR−4763−5p, proximal
(STAGE_D = 1; N1 = 92)
1−sided adj pval: 0.713
Frequency
Tumor−Normal Expression Difference

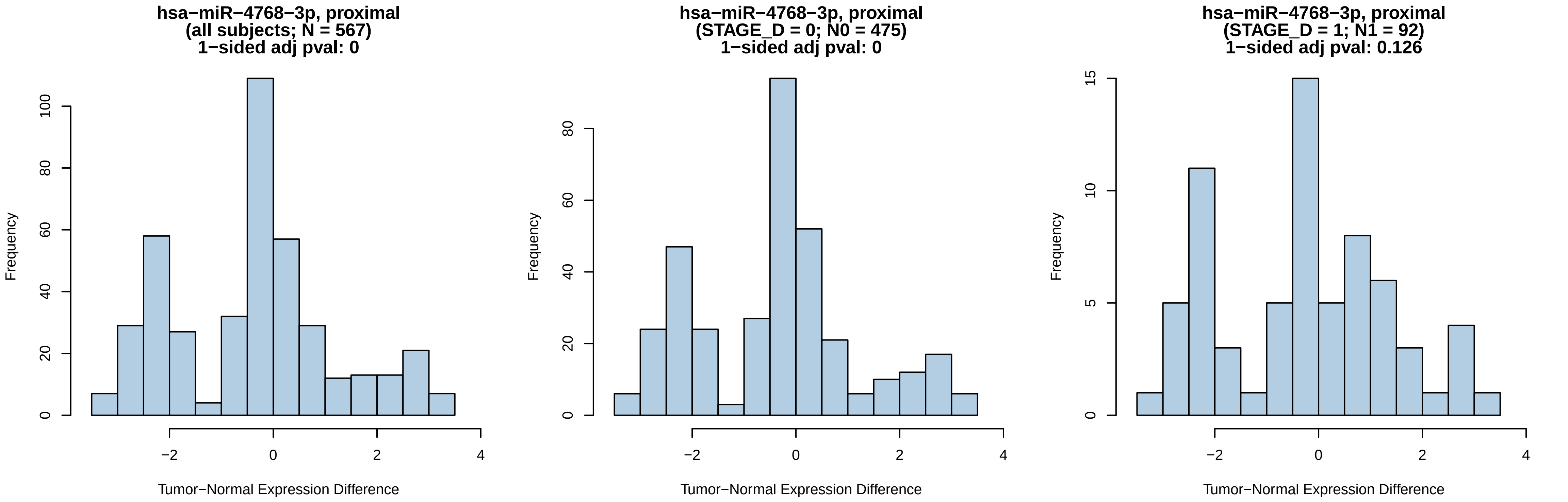
hsa−miR−4768−3p, proximal
(all subjects; N = 567)
1−sided adj pval: 0
Frequency
Tumor−Normal Expression Difference
hsa−miR−4768−3p, proximal
(STAGE_D = 0; N0 = 475)
1−sided adj pval: 0
Frequency
Tumor−Normal Expression Difference
hsa−miR−4768−3p, proximal
(STAGE_D = 1; N1 = 92)
1−sided adj pval: 0.126
Frequency
Tumor−Normal Expression Difference

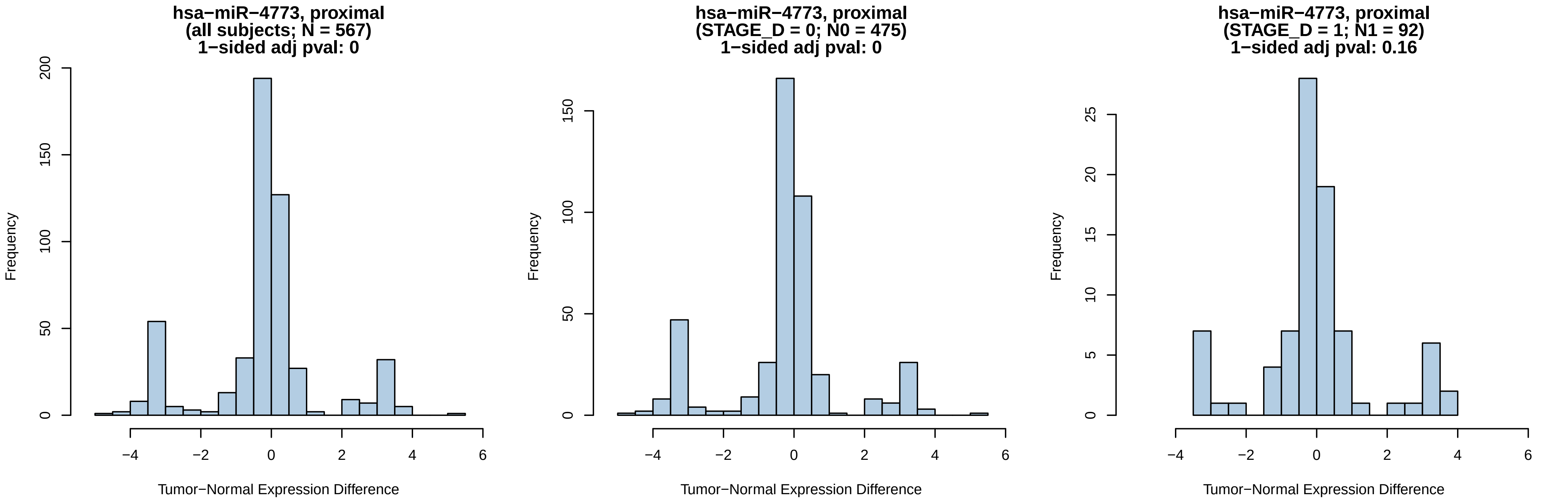

hsa−miR−4773, proximal
(all subjects; N = 567)
1−sided adj pval: 0
Frequency
Tumor−Normal Expression Difference
hsa−miR−4773, proximal
(STAGE_D = 0; N0 = 475)
1−sided adj pval: 0
Frequency
Tumor−Normal Expression Difference
hsa−miR−4773, proximal
(STAGE_D = 1; N1 = 92)
1−sided adj pval: 0.16
Frequency
Tumor−Normal Expression Difference

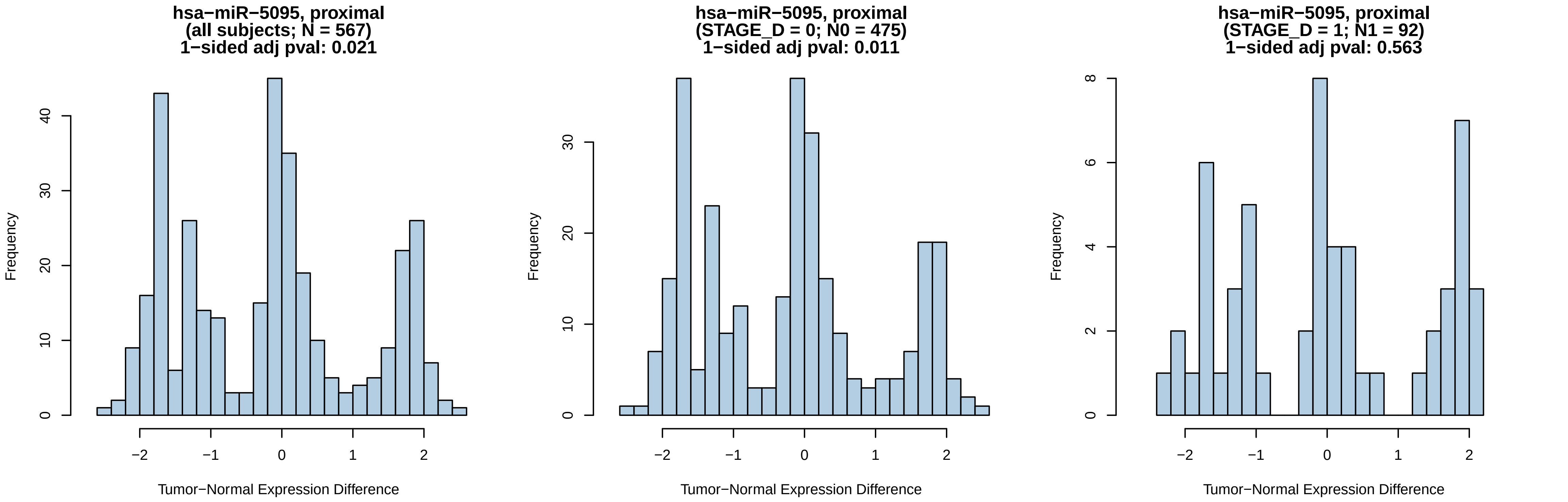

hsa−miR−5095, proximal
(all subjects; N = 567)
1−sided adj pval: 0.021
Frequency
Tumor−Normal Expression Difference
hsa−miR−5095, proximal
(STAGE_D = 0; N0 = 475)
1−sided adj pval: 0.011
Frequency
Tumor−Normal Expression Difference
hsa−miR−5095, proximal
(STAGE_D = 1; N1 = 92)
1−sided adj pval: 0.563
Frequency
Tumor−Normal Expression Difference

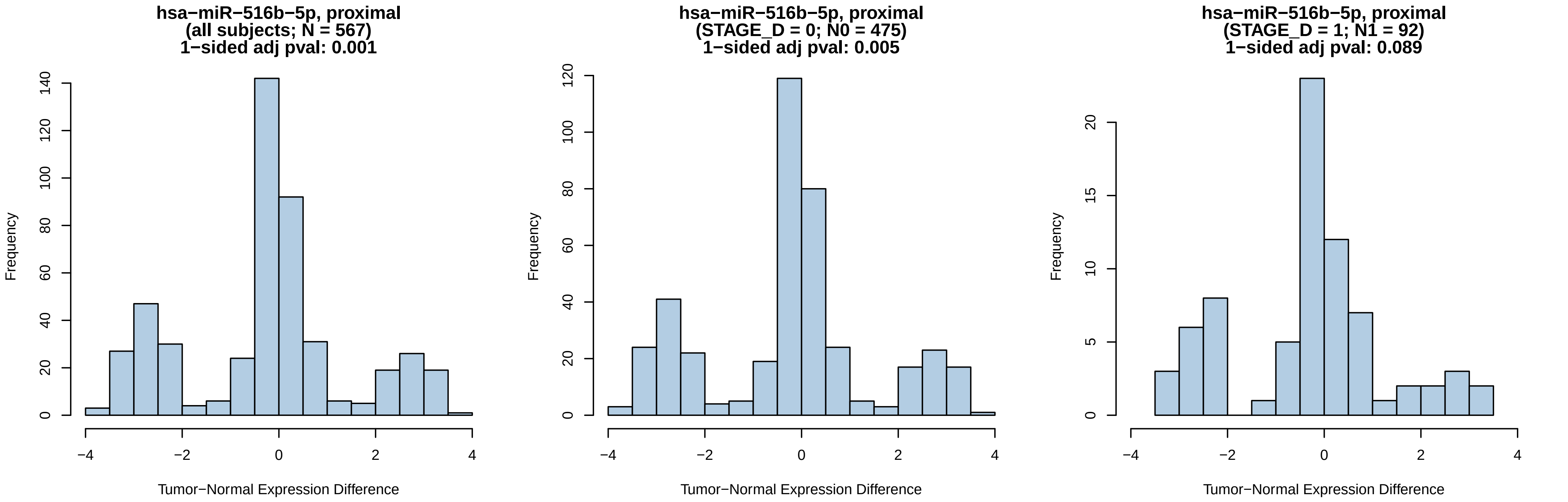

hsa−miR−516b−5p, proximal
(all subjects; N = 567)
1−sided adj pval: 0.001
Frequency
Tumor−Normal Expression Difference
hsa−miR−516b−5p, proximal
(STAGE_D = 0; N0 = 475)
1−sided adj pval: 0.005
Frequency
Tumor−Normal Expression Difference
hsa−miR−516b−5p, proximal
(STAGE_D = 1; N1 = 92)
1−sided adj pval: 0.089
Frequency
Tumor−Normal Expression Difference

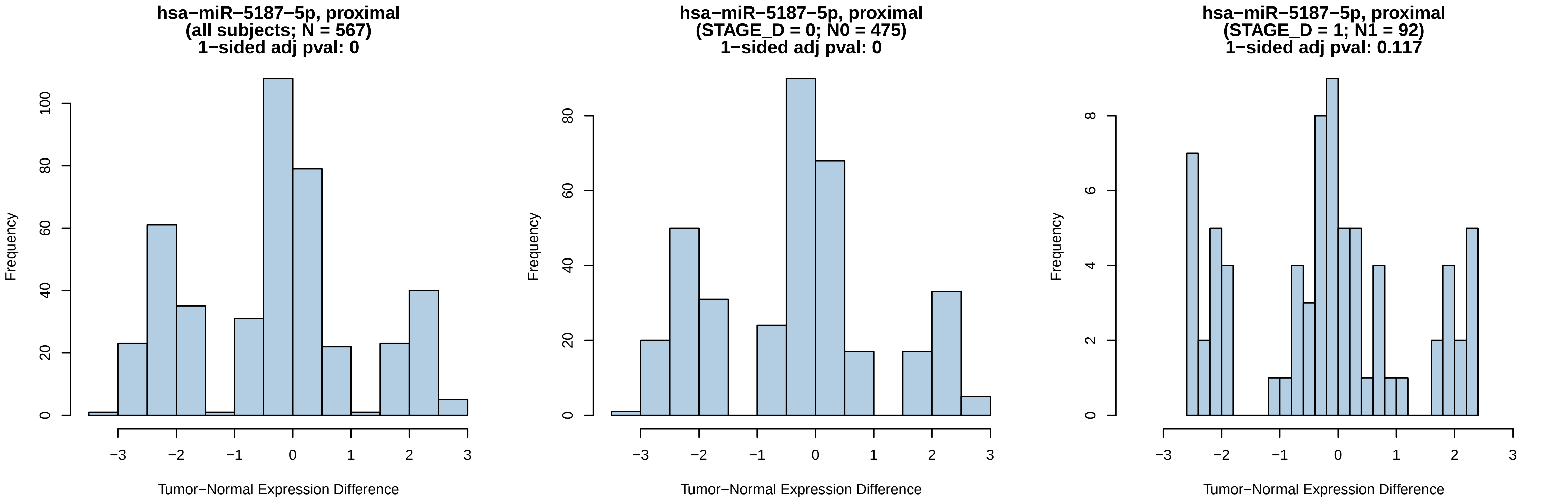
hsa−miR−5187−5p, proximal
(all subjects; N = 567)
1−sided adj pval: 0
Frequency
0
20
40
60
80
100
−3
−2
−1
0
1
2
3
Tumor−Normal Expression Difference
hsa−miR−5187−5p, proximal
(STAGE_D = 0; N0 = 475)
1−sided adj pval: 0
Frequency
0
20
40
60
80
−3
−2
−1
0
1
2
3
Tumor−Normal Expression Difference
hsa−miR−5187−5p, proximal
(STAGE_D = 1; N1 = 92)
1−sided adj pval: 0.117
Frequency
0
2
4
6
8
−3
−2
−1
0
1
2
3
Tumor−Normal Expression Difference

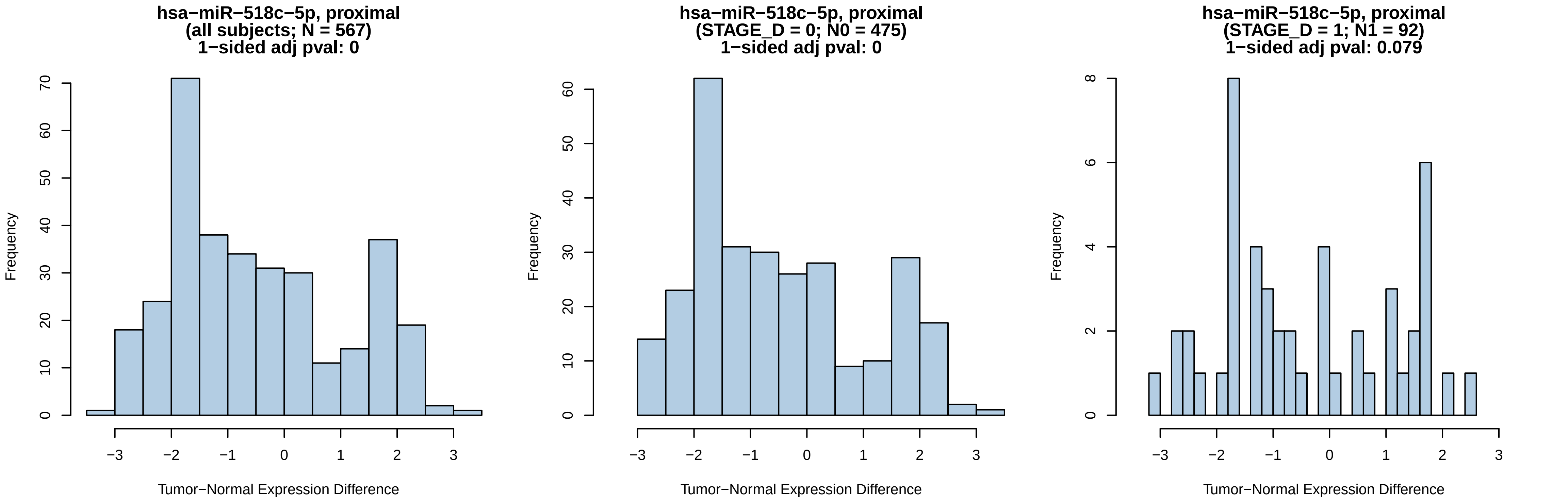
hsa−miR−518c−5p, proximal
(all subjects; N = 567)
1−sided adj pval: 0
Frequency
Tumor−Normal Expression Difference
hsa−miR−518c−5p, proximal
(STAGE_D = 0; N0 = 475)
1−sided adj pval: 0
Frequency
Tumor−Normal Expression Difference
hsa−miR−518c−5p, proximal
(STAGE_D = 1; N1 = 92)
1−sided adj pval: 0.079
Frequency
Tumor−Normal Expression Difference

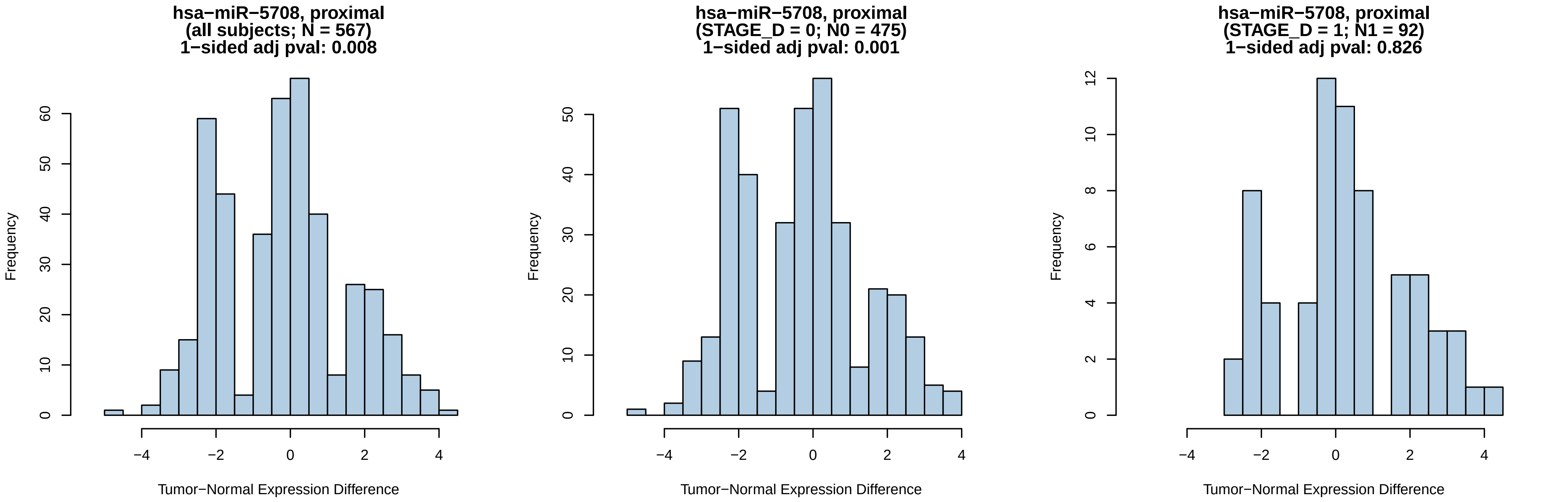
hsa−miR−5708, proximal
(all subjects; N = 567)
1−sided adj pval: 0.008
Frequency
Tumor−Normal Expression Difference
hsa−miR−5708, proximal
(STAGE_D = 0; N0 = 475)
1−sided adj pval: 0.001
Frequency
Tumor−Normal Expression Difference
hsa−miR−5708, proximal
(STAGE_D = 1; N1 = 92)
1−sided adj pval: 0.826
Frequency
Tumor−Normal Expression Difference

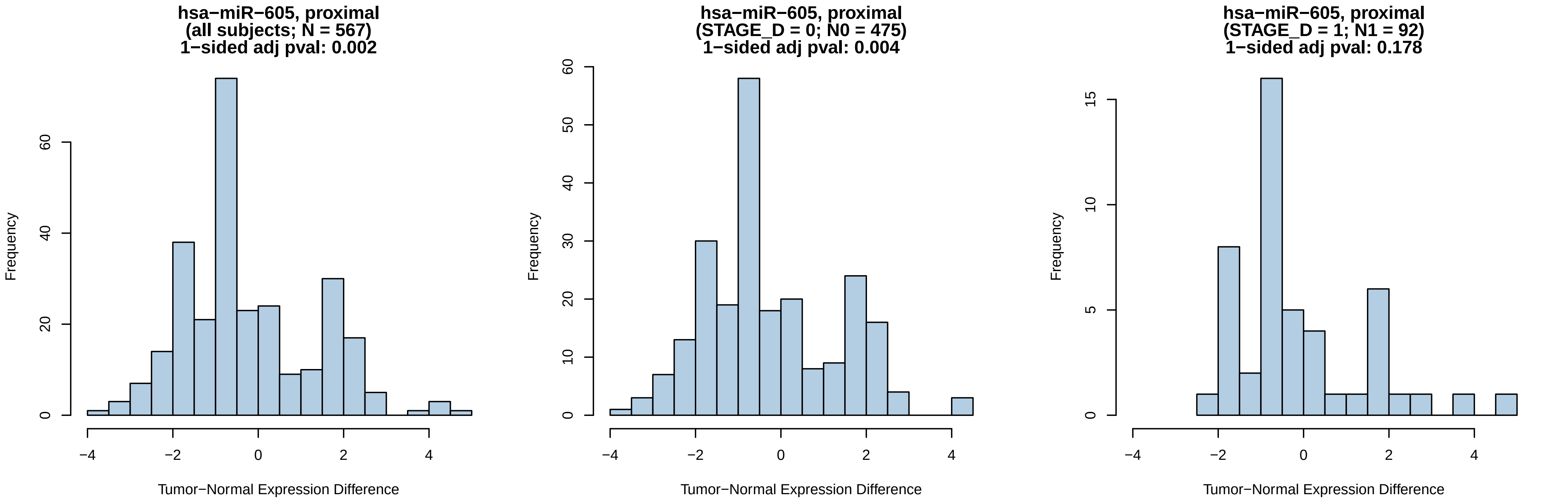

hsa−miR−605, proximal
(all subjects; N = 567)
1−sided adj pval: 0.002
Frequency
Tumor−Normal Expression Difference
hsa−miR−605, proximal
(STAGE_D = 0; N0 = 475)
1−sided adj pval: 0.004
Frequency
Tumor−Normal Expression Difference
hsa−miR−605, proximal
(STAGE_D = 1; N1 = 92)
1−sided adj pval: 0.178
Frequency
Tumor−Normal Expression Difference

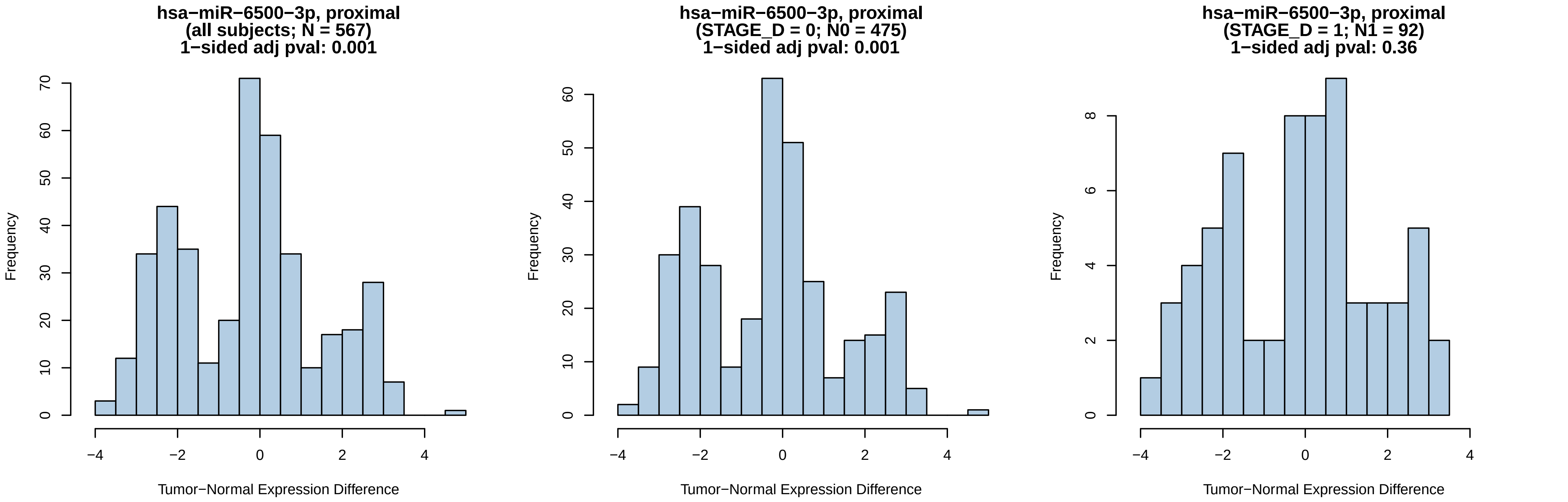
hsa−miR−6500−3p, proximal
(all subjects; N = 567)
1−sided adj pval: 0.001
Frequency
0
10
20
30
40
50
60
70
−4
−2
0
2
4
Tumor−Normal Expression Difference
hsa−miR−6500−3p, proximal
(STAGE_D = 0; N0 = 475)
1−sided adj pval: 0.001
Frequency
0
10
20
30
40
50
60
−4
−2
0
2
4
Tumor−Normal Expression Difference
hsa−miR−6500−3p, proximal
(STAGE_D = 1; N1 = 92)
1−sided adj pval: 0.36
Frequency
0
2
4
6
8
−4
−2
0
2
4
Tumor−Normal Expression Difference

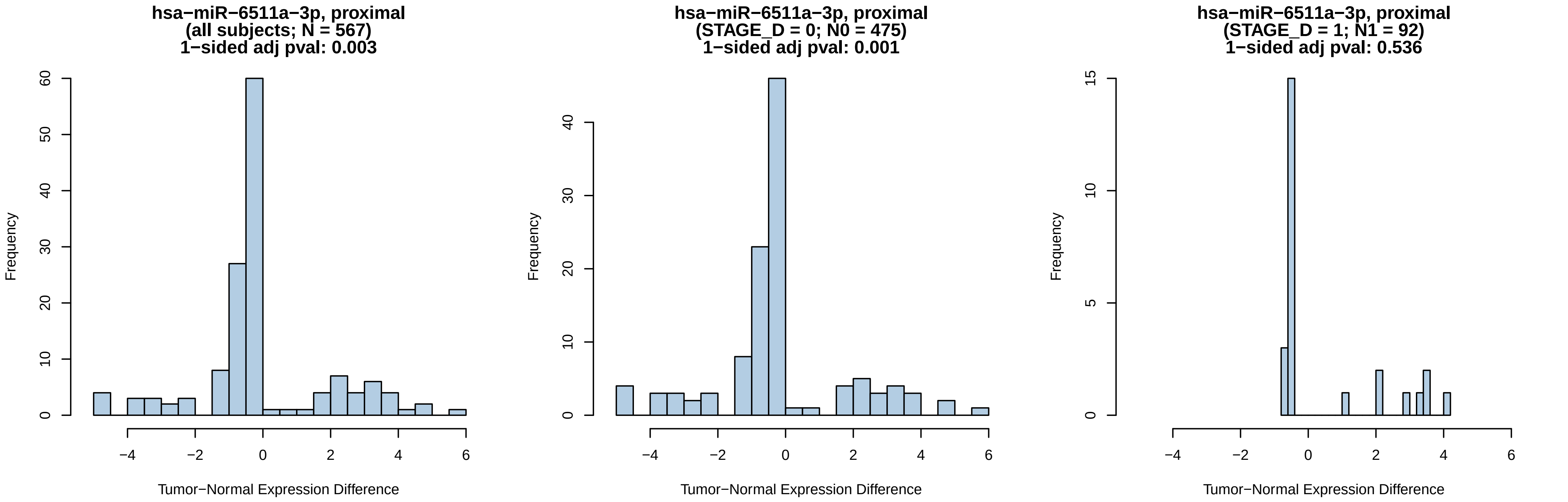
hsa−miR−6511a−3p, proximal
(all subjects; N = 567)
1−sided adj pval: 0.003
hsa−miR−6511a−3p, proximal
(STAGE_D = 0; N0 = 475)
1−sided adj pval: 0.001
hsa−miR−6511a−3p, proximal
(STAGE_D = 1; N1 = 92)
1−sided adj pval: 0.536
Frequency
Tumor−Normal Expression Difference

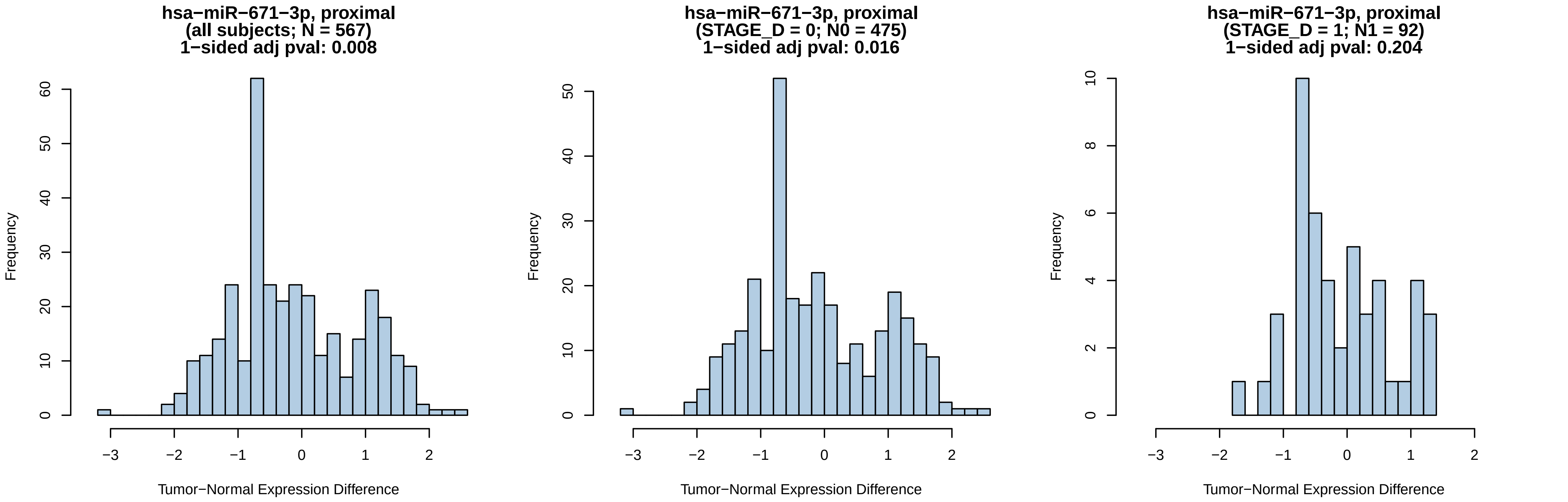
hsa−miR−671−3p, proximal
(all subjects; N = 567)
1−sided adj pval: 0.008
Frequency
Tumor−Normal Expression Difference
hsa−miR−671−3p, proximal
(STAGE_D = 0; N0 = 475)
1−sided adj pval: 0.016
Frequency
Tumor−Normal Expression Difference
hsa−miR−671−3p, proximal
(STAGE_D = 1; N1 = 92)
1−sided adj pval: 0.204
Frequency
Tumor−Normal Expression Difference

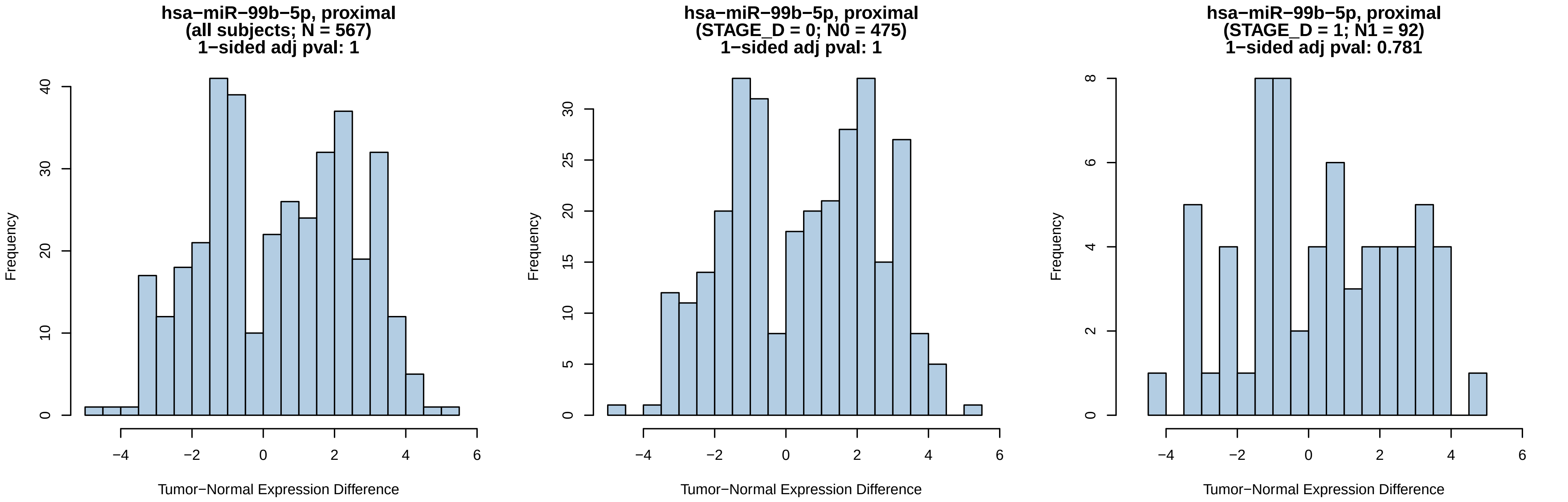
hsa−miR−99b−5p, proximal
(all subjects; N = 567)
1−sided adj pval: 1
hsa−miR−99b−5p, proximal
(STAGE_D = 0; N0 = 475)
1−sided adj pval: 1
hsa−miR−99b−5p, proximal
(STAGE_D = 1; N1 = 92)
1−sided adj pval: 0.781
Frequency
Tumor−Normal Expression Difference

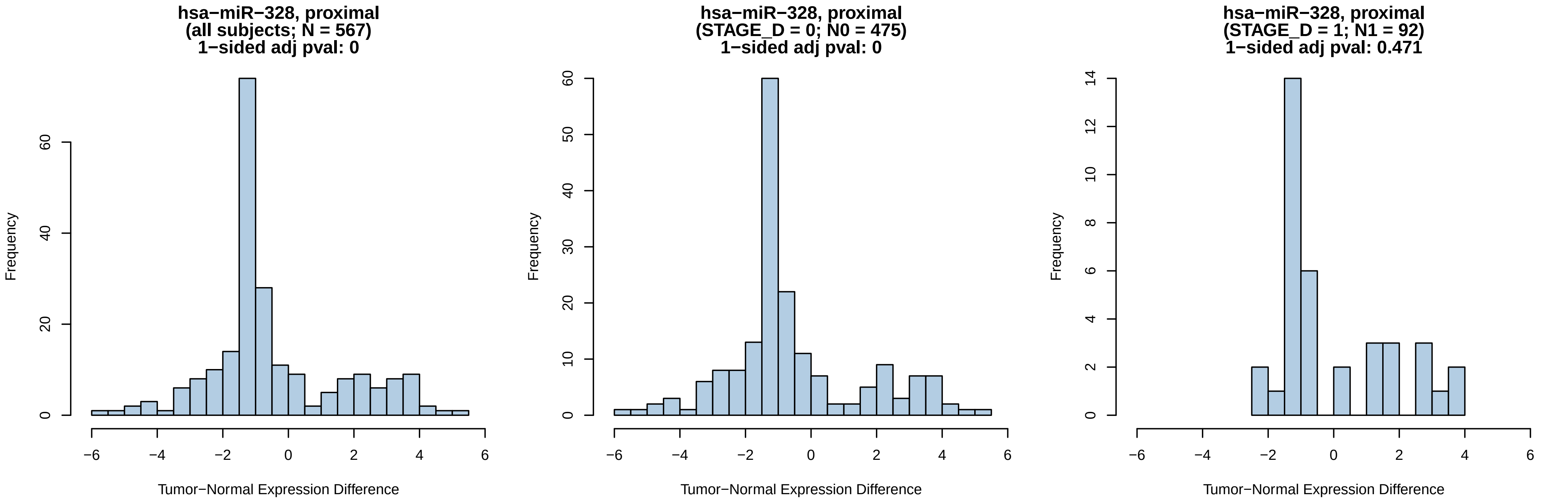

hsa−miR−328, proximal
(all subjects; N = 567)
1−sided adj pval: 0
Frequency
Tumor−Normal Expression Difference
hsa−miR−328, proximal
(STAGE_D = 0; N0 = 475)
1−sided adj pval: 0
Frequency
Tumor−Normal Expression Difference
hsa−miR−328, proximal
(STAGE_D = 1; N1 = 92)
1−sided adj pval: 0.471
Frequency
Tumor−Normal Expression Difference

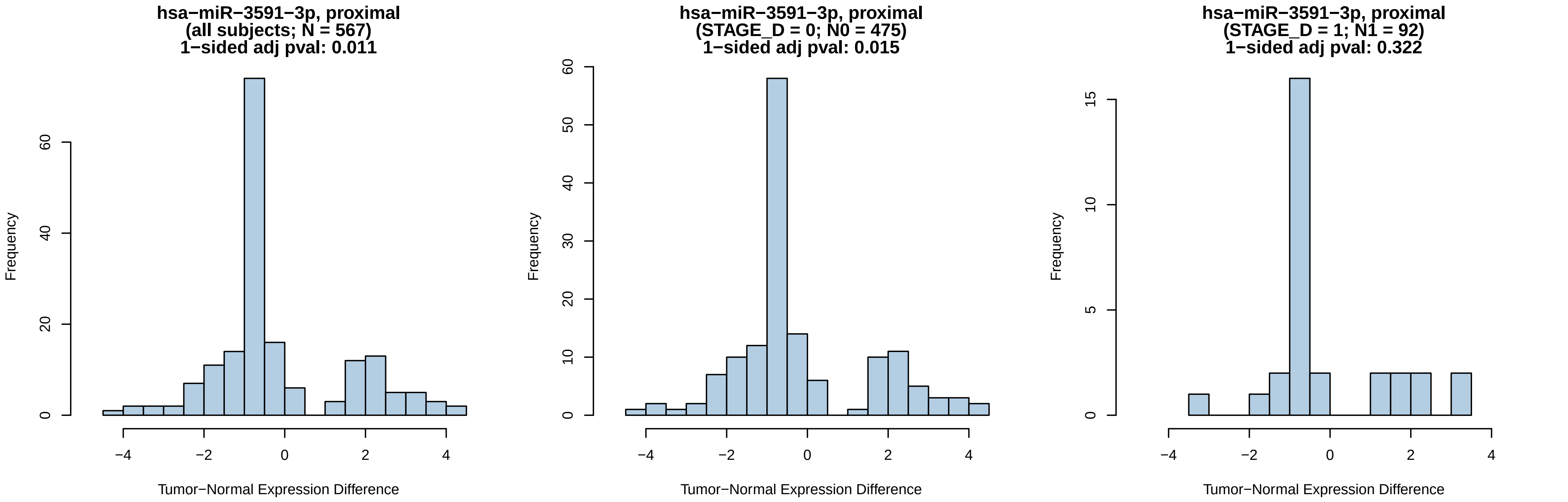

hsa−miR−3591−3p, proximal
(all subjects; N = 567)
1−sided adj pval: 0.011
Frequency
0
20
40
60
−4
−2
0
2
4
Tumor−Normal Expression Difference
hsa−miR−3591−3p, proximal
(STAGE_D = 0; N0 = 475)
1−sided adj pval: 0.015
Frequency
0
10
20
30
40
50
60
−4
−2
0
2
4
Tumor−Normal Expression Difference
hsa−miR−3591−3p, proximal
(STAGE_D = 1; N1 = 92)
1−sided adj pval: 0.322
Frequency
0
5
10
15
−4
−2
0
2
4
Tumor−Normal Expression Difference

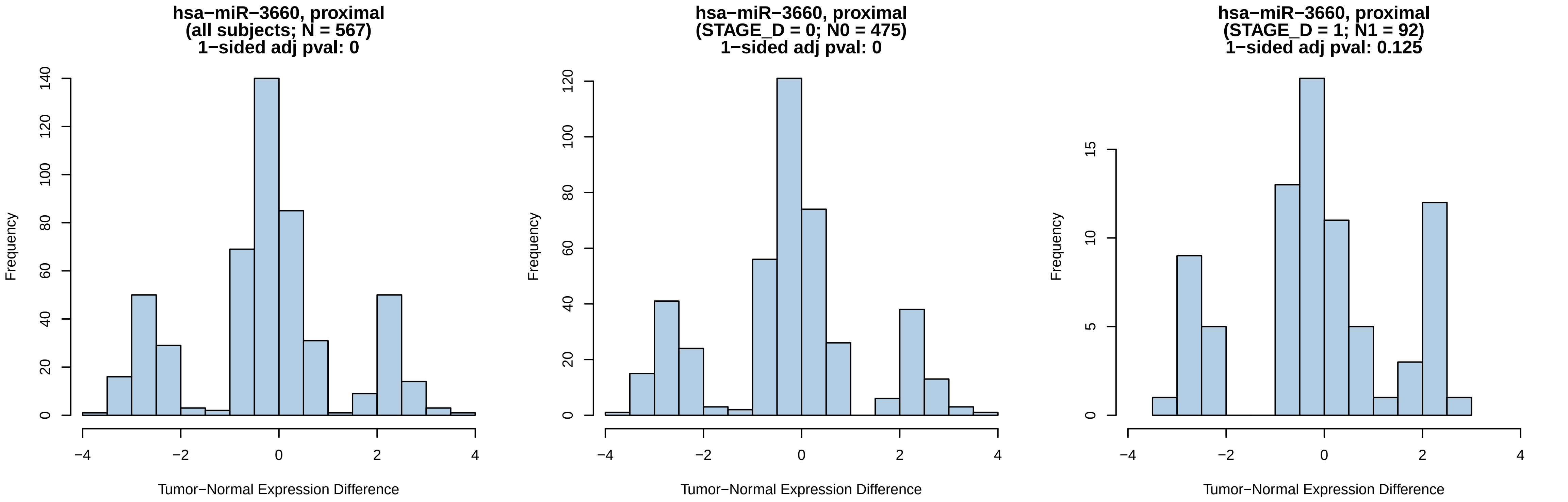

hsa−miR−3660, proximal
(all subjects; N = 567)
1−sided adj pval: 0
Frequency
Tumor−Normal Expression Difference
hsa−miR−3660, proximal
(STAGE_D = 0; N0 = 475)
1−sided adj pval: 0
Frequency
Tumor−Normal Expression Difference
hsa−miR−3660, proximal
(STAGE_D = 1; N1 = 92)
1−sided adj pval: 0.125
Frequency
Tumor−Normal Expression Difference

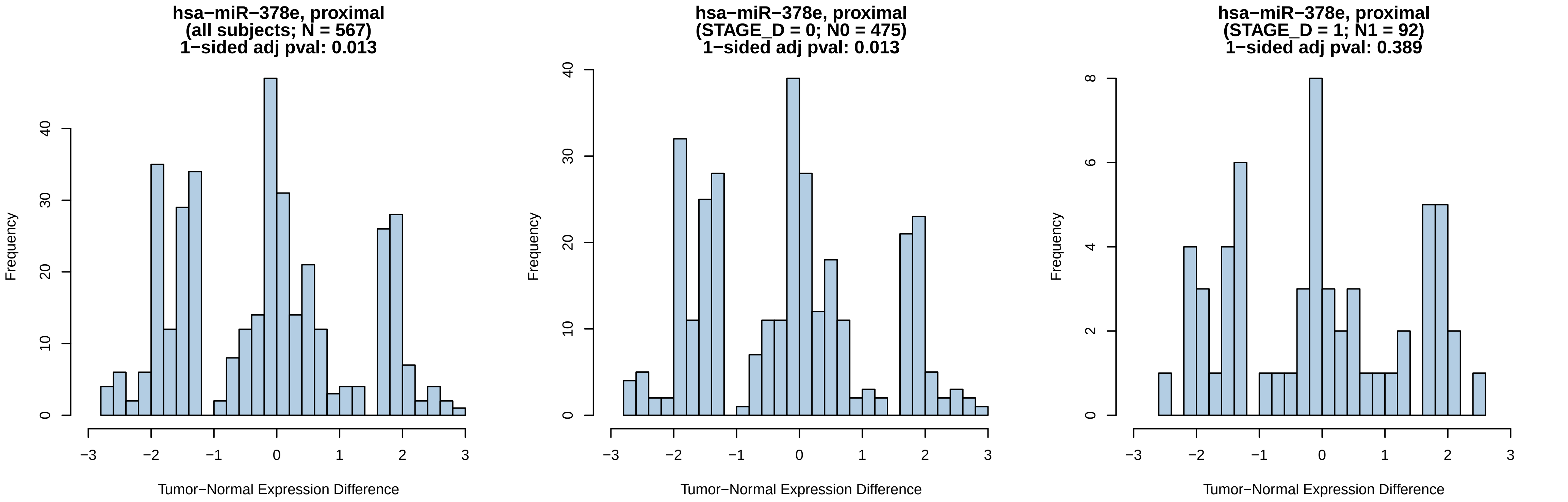

hsa−miR−378e, proximal
(all subjects; N = 567)
1−sided adj pval: 0.013
hsa−miR−378e, proximal
(STAGE_D = 0; N0 = 475)
1−sided adj pval: 0.013
hsa−miR−378e, proximal
(STAGE_D = 1; N1 = 92)
1−sided adj pval: 0.389
Frequency
Tumor−Normal Expression Difference

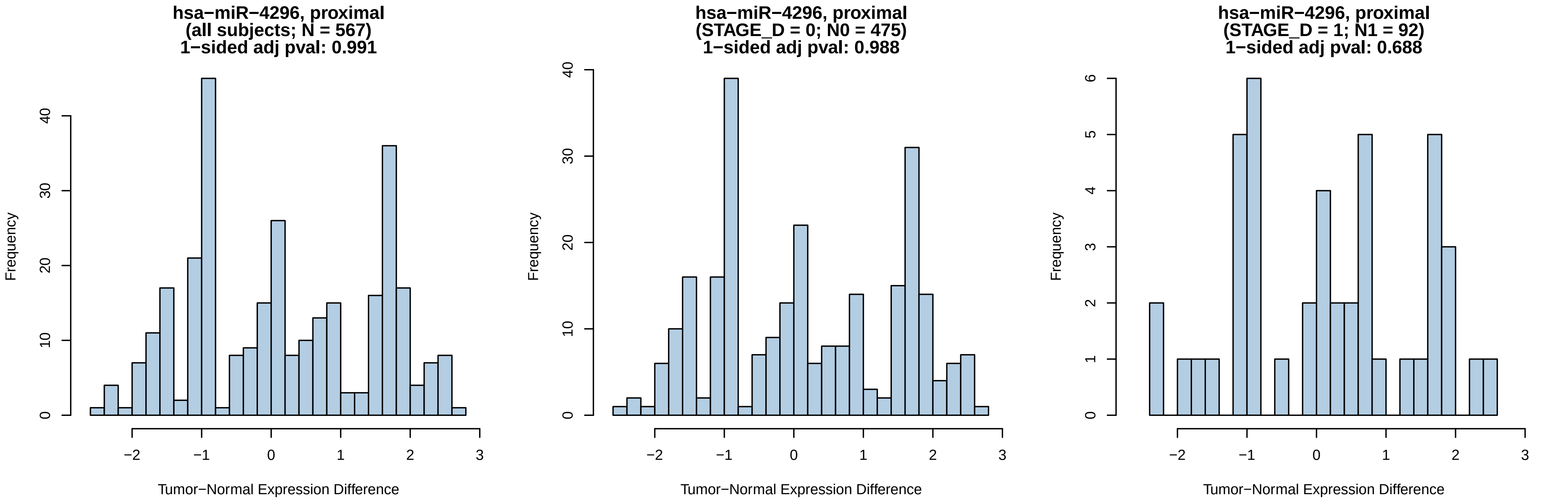

hsa−miR−4296, proximal
(all subjects; N = 567)
1−sided adj pval: 0.991
Frequency
Tumor−Normal Expression Difference
hsa−miR−4296, proximal
(STAGE_D = 0; N0 = 475)
1−sided adj pval: 0.988
Frequency
Tumor−Normal Expression Difference
hsa−miR−4296, proximal
(STAGE_D = 1; N1 = 92)
1−sided adj pval: 0.688
Frequency
Tumor−Normal Expression Difference

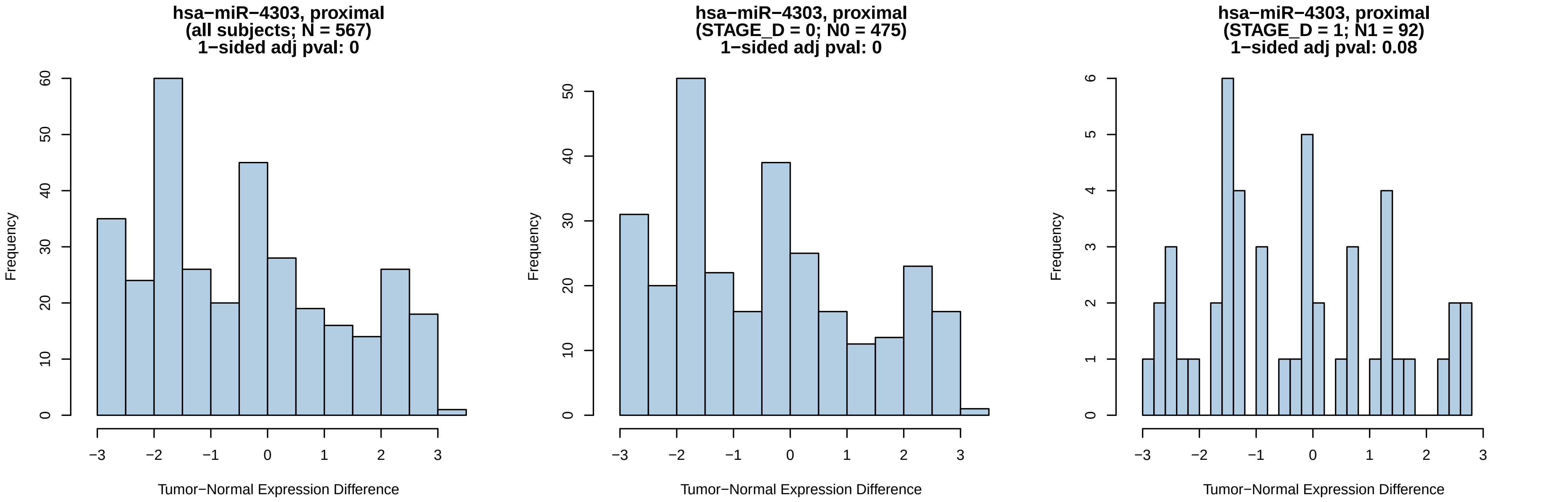

hsa−miR−4303, proximal
(all subjects; N = 567)
1−sided adj pval: 0
hsa−miR−4303, proximal
(STAGE_D = 0; N0 = 475)
1−sided adj pval: 0
hsa−miR−4303, proximal
(STAGE_D = 1; N1 = 92)
1−sided adj pval: 0.08
Frequency
Tumor−Normal Expression Difference

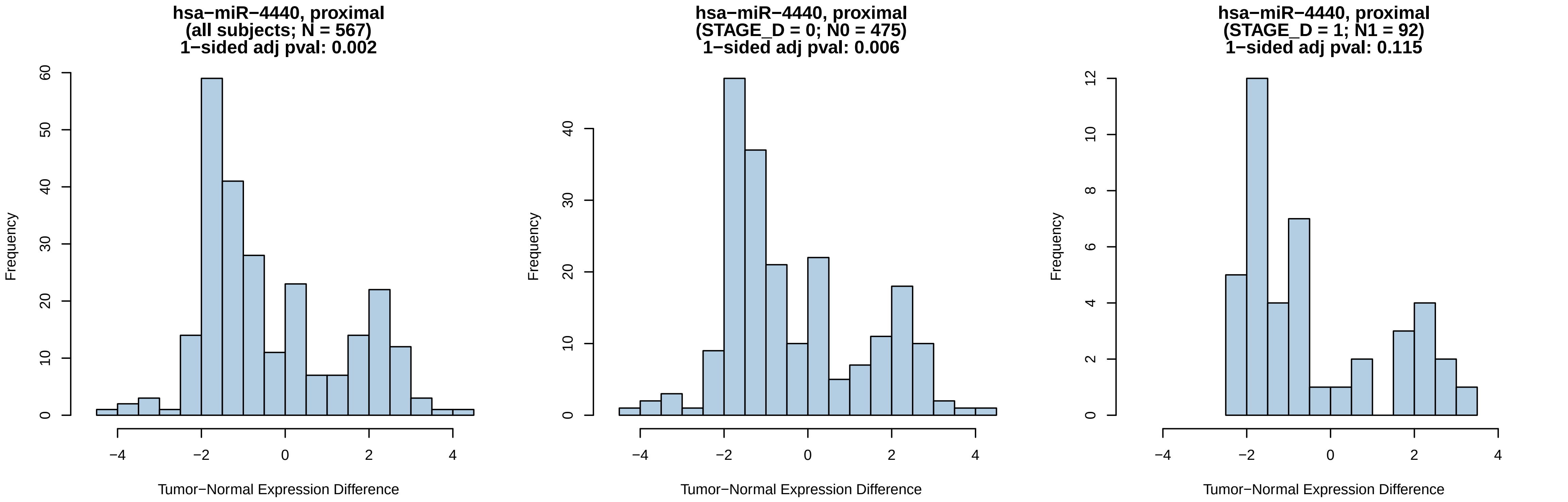

hsa−miR−4440, proximal
(all subjects; N = 567)
1−sided adj pval: 0.002
Frequency
Tumor−Normal Expression Difference
hsa−miR−4440, proximal
(STAGE_D = 0; N0 = 475)
1−sided adj pval: 0.006
Frequency
Tumor−Normal Expression Difference
hsa−miR−4440, proximal
(STAGE_D = 1; N1 = 92)
1−sided adj pval: 0.115
Frequency
Tumor−Normal Expression Difference

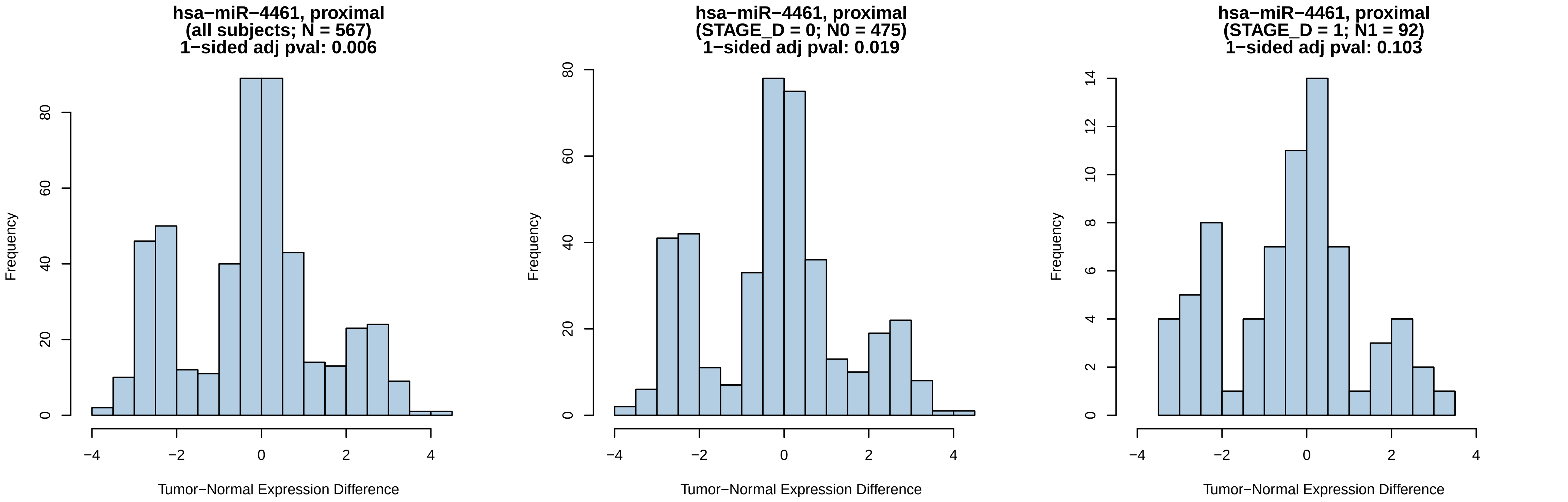
hsa−miR−4461, proximal
(all subjects; N = 567)
1−sided adj pval: 0.006
Frequency
Tumor−Normal Expression Difference
hsa−miR−4461, proximal
(STAGE_D = 0; N0 = 475)
1−sided adj pval: 0.019
Frequency
Tumor−Normal Expression Difference
hsa−miR−4461, proximal
(STAGE_D = 1; N1 = 92)
1−sided adj pval: 0.103
Frequency
Tumor−Normal Expression Difference

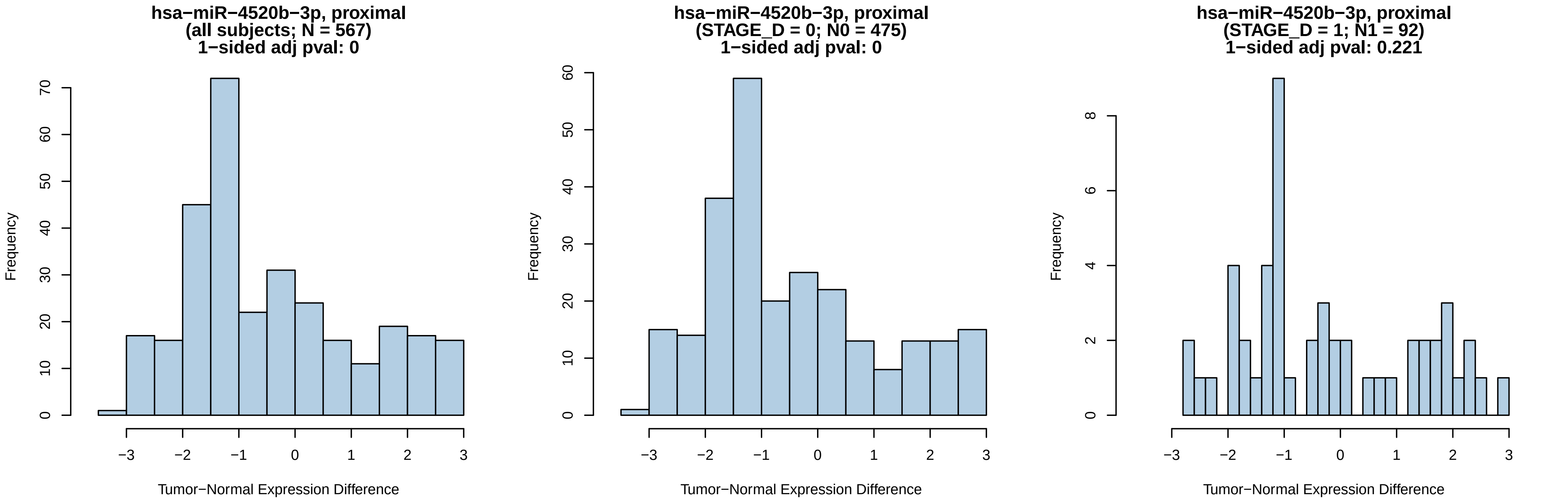
hsa−miR−4520b−3p, proximal
(all subjects; N = 567)
1−sided adj pval: 0
Frequency
Tumor−Normal Expression Difference
hsa−miR−4520b−3p, proximal
(STAGE_D = 0; N0 = 475)
1−sided adj pval: 0
Frequency
Tumor−Normal Expression Difference
hsa−miR−4520b−3p, proximal
(STAGE_D = 1; N1 = 92)
1−sided adj pval: 0.221
Frequency
Tumor−Normal Expression Difference

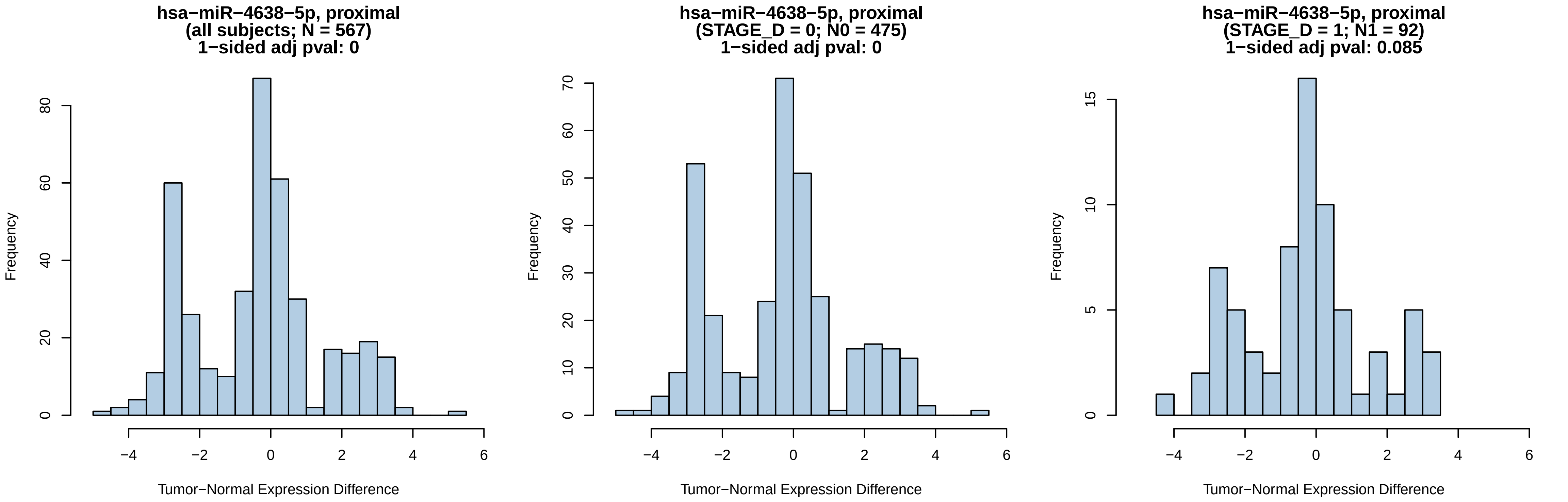
hsa−miR−4638−5p, proximal
(all subjects; N = 567)
1−sided adj pval: 0
Frequency
Tumor−Normal Expression Difference
hsa−miR−4638−5p, proximal
(STAGE_D = 0; N0 = 475)
1−sided adj pval: 0
Frequency
Tumor−Normal Expression Difference
hsa−miR−4638−5p, proximal
(STAGE_D = 1; N1 = 92)
1−sided adj pval: 0.085
Frequency
Tumor−Normal Expression Difference

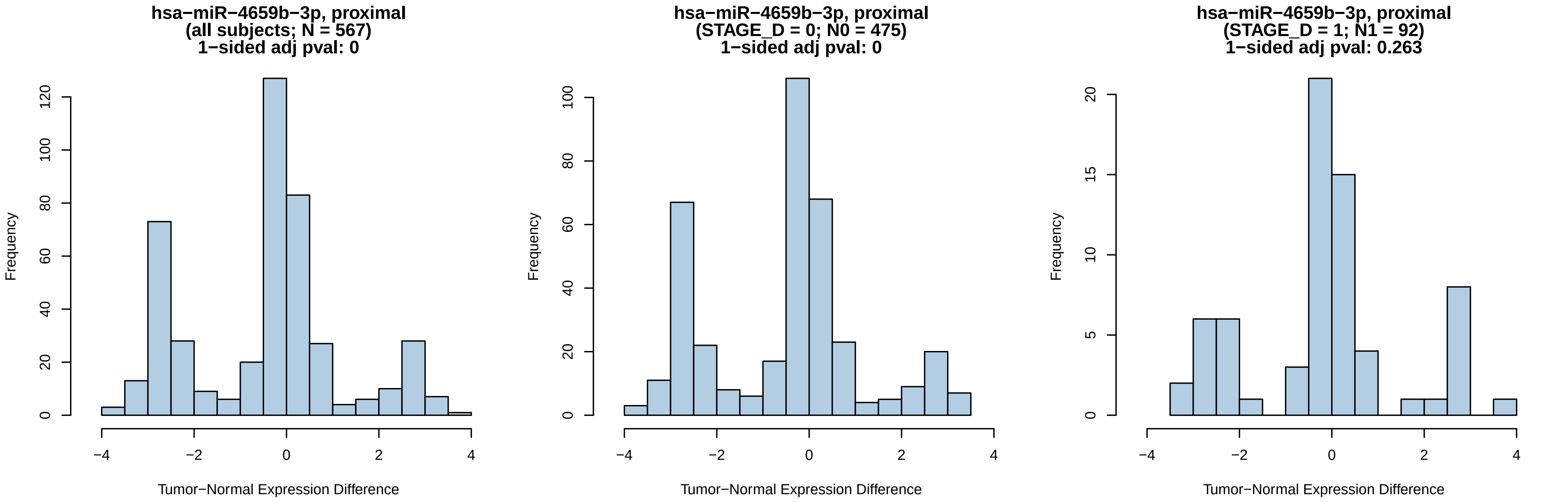
hsa−miR−4659b−3p, proximal
(all subjects; N = 567)
1−sided adj pval: 0
Frequency
Tumor−Normal Expression Difference
hsa−miR−4659b−3p, proximal
(STAGE_D = 0; N0 = 475)
1−sided adj pval: 0
Frequency
Tumor−Normal Expression Difference
hsa−miR−4659b−3p, proximal
(STAGE_D = 1; N1 = 92)
1−sided adj pval: 0.263
Frequency
Tumor−Normal Expression Difference

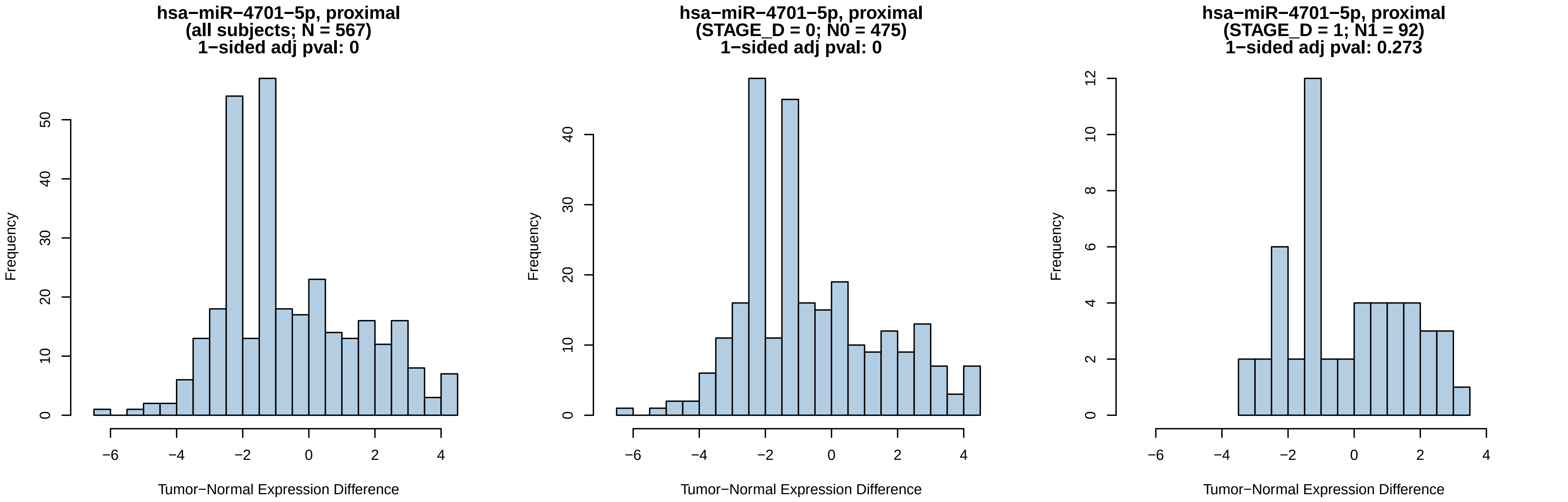
hsa−miR−4701−5p, proximal
(all subjects; N = 567)
1−sided adj pval: 0
Frequency
Tumor−Normal Expression Difference
hsa−miR−4701−5p, proximal
(STAGE_D = 0; N0 = 475)
1−sided adj pval: 0
Frequency
Tumor−Normal Expression Difference
hsa−miR−4701−5p, proximal
(STAGE_D = 1; N1 = 92)
1−sided adj pval: 0.273
Frequency
Tumor−Normal Expression Difference

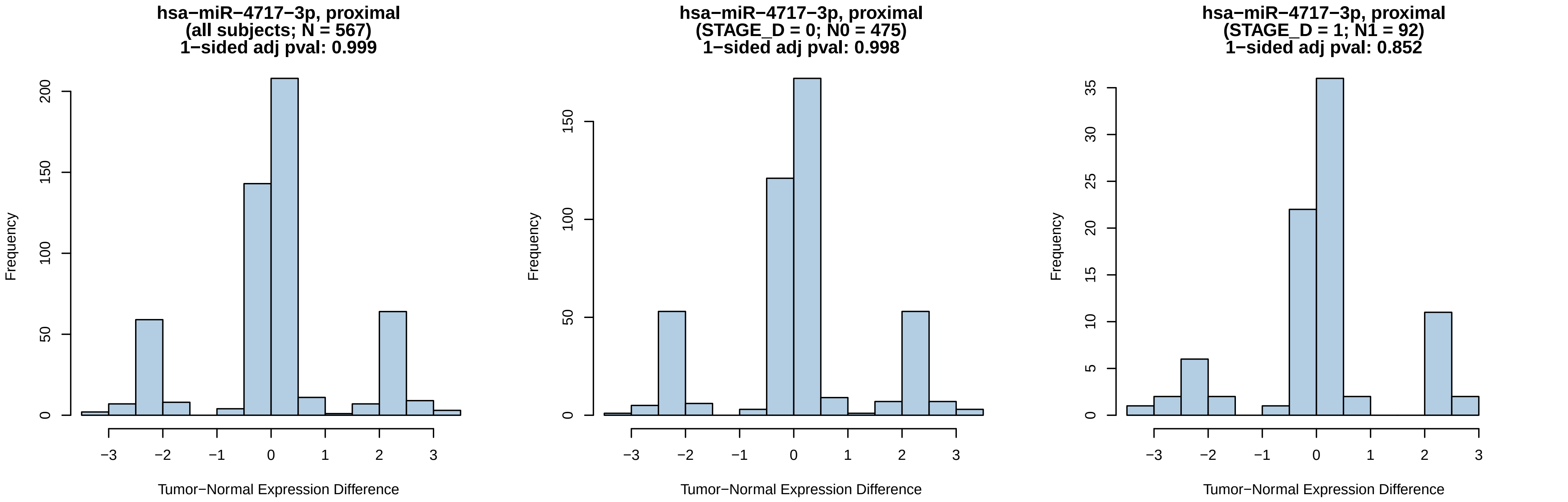
hsa−miR−4717−3p, proximal
(all subjects; N = 567)
1−sided adj pval: 0.999
Frequency
Tumor−Normal Expression Difference
hsa−miR−4717−3p, proximal
(STAGE_D = 0; N0 = 475)
1−sided adj pval: 0.998
Frequency
Tumor−Normal Expression Difference
hsa−miR−4717−3p, proximal
(STAGE_D = 1; N1 = 92)
1−sided adj pval: 0.852
Frequency
Tumor−Normal Expression Difference

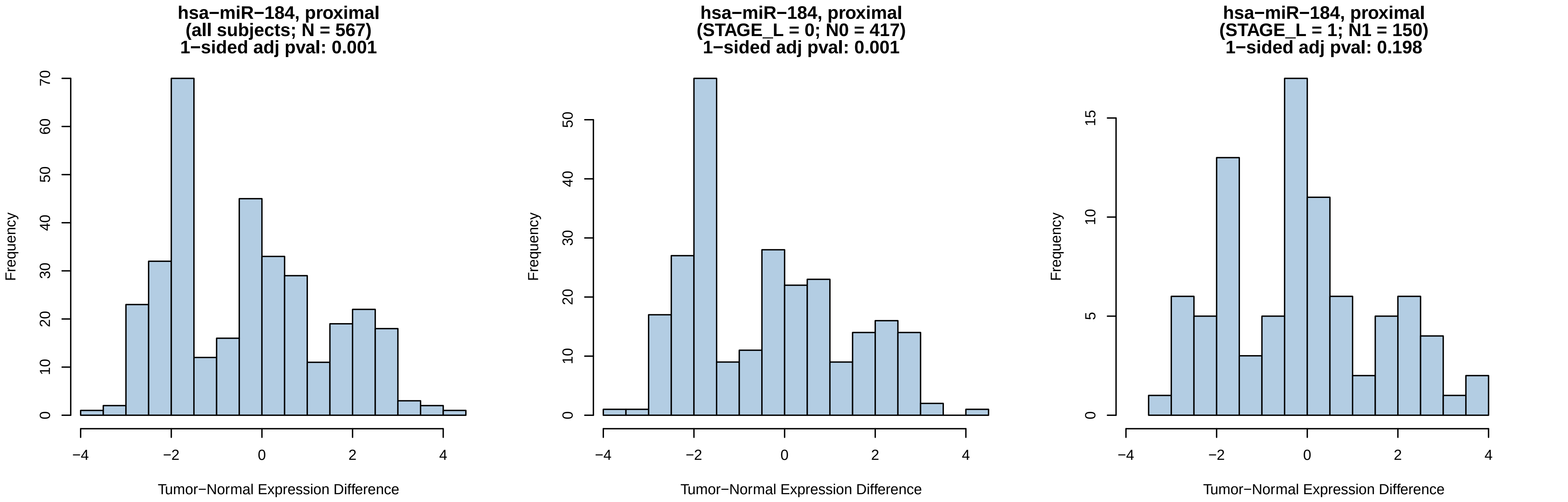

hsa−miR−184, proximal
(all subjects; N = 567)
1−sided adj pval: 0.001
Frequency
Tumor−Normal Expression Difference
hsa−miR−184, proximal
(STAGE_L = 0; N0 = 417)
1−sided adj pval: 0.001
Frequency
Tumor−Normal Expression Difference
hsa−miR−184, proximal
(STAGE_L = 1; N1 = 150)
1−sided adj pval: 0.198
Frequency
Tumor−Normal Expression Difference

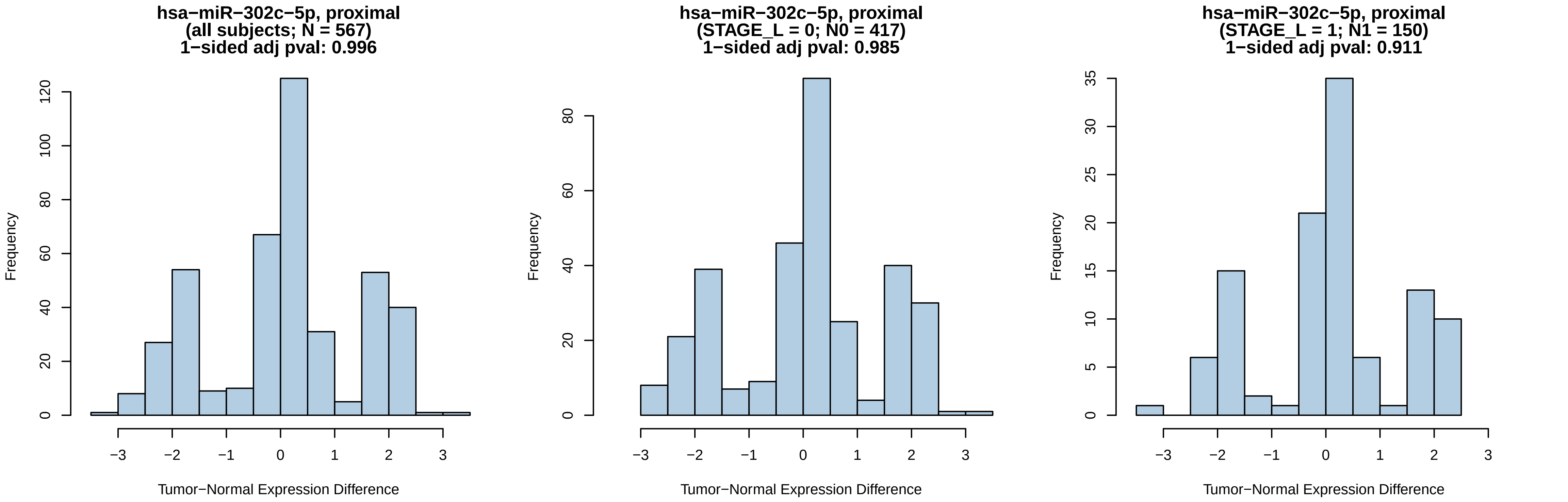

hsa−miR−302c−5p, proximal
(all subjects; N = 567)
1−sided adj pval: 0.996
Frequency
Tumor−Normal Expression Difference
hsa−miR−302c−5p, proximal
(STAGE_L = 0; N0 = 417)
1−sided adj pval: 0.985
Frequency
Tumor−Normal Expression Difference
hsa−miR−302c−5p, proximal
(STAGE_L = 1; N1 = 150)
1−sided adj pval: 0.911
Frequency
Tumor−Normal Expression Difference

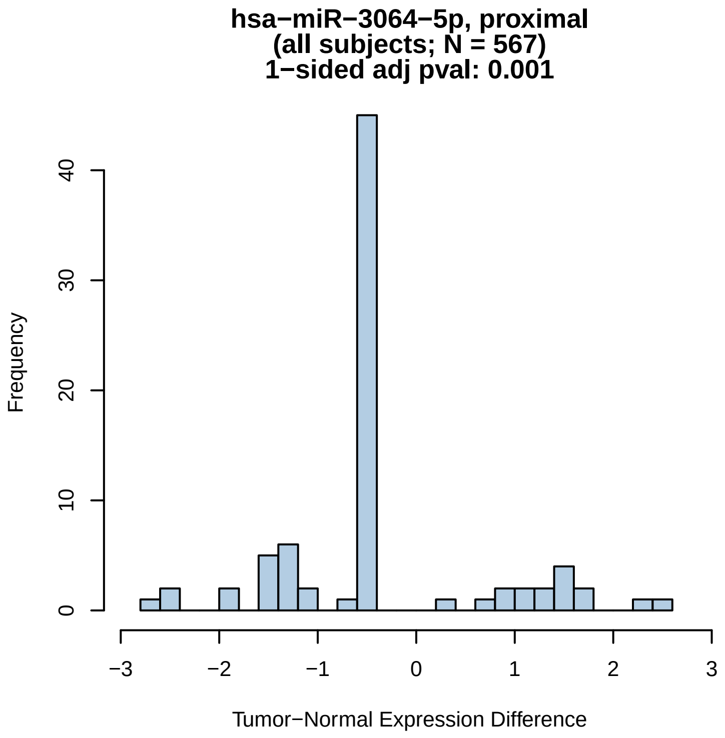
hsa−miR−3064−5p, proximal
(all subjects; N = 567)
1−sided adj pval: 0.001
Frequency
0
10
20
30
40
−3
−2
−1
0
1
2
3
Tumor−Normal Expression Difference

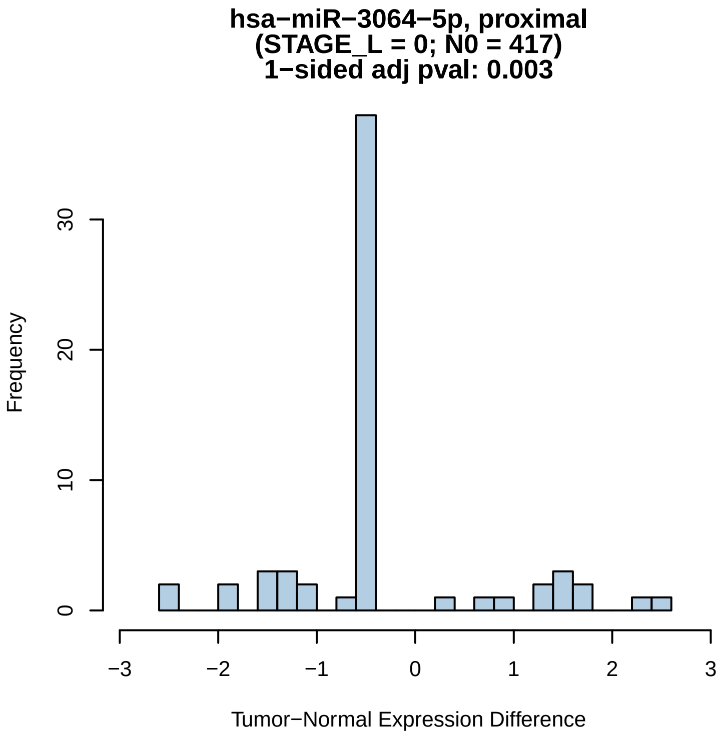
hsa−miR−3064−5p, proximal
(STAGE_L = 0; N0 = 417)
1−sided adj pval: 0.003
Frequency
0
10
20
30
−3
−2
−1
0
1
2
3
Tumor−Normal Expression Difference

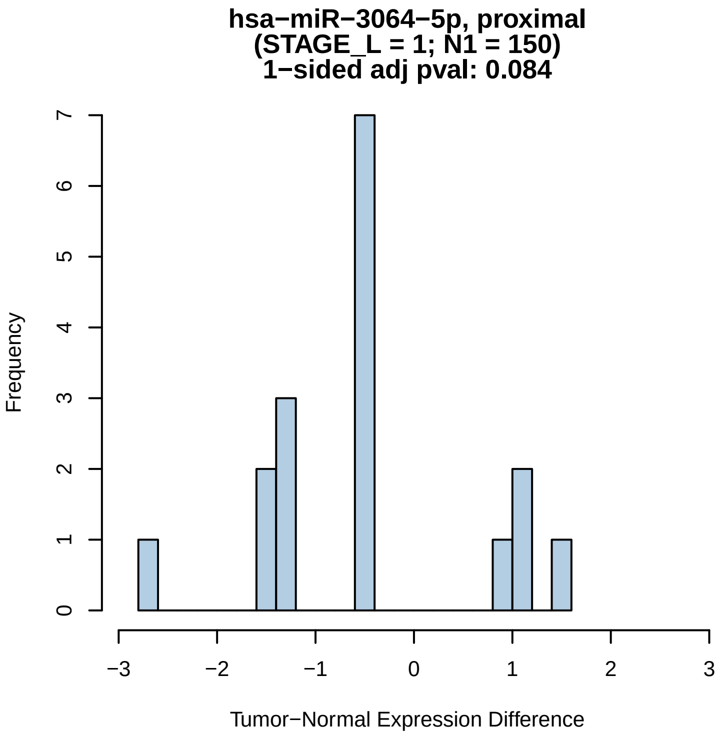
hsa−miR−3064−5p, proximal
(STAGE_L = 1; N1 = 150)
1−sided adj pval: 0.084
Frequency
0
1
2
3
4
5
6
7
−3
−2
−1
0
1
2
3
Tumor−Normal Expression Difference

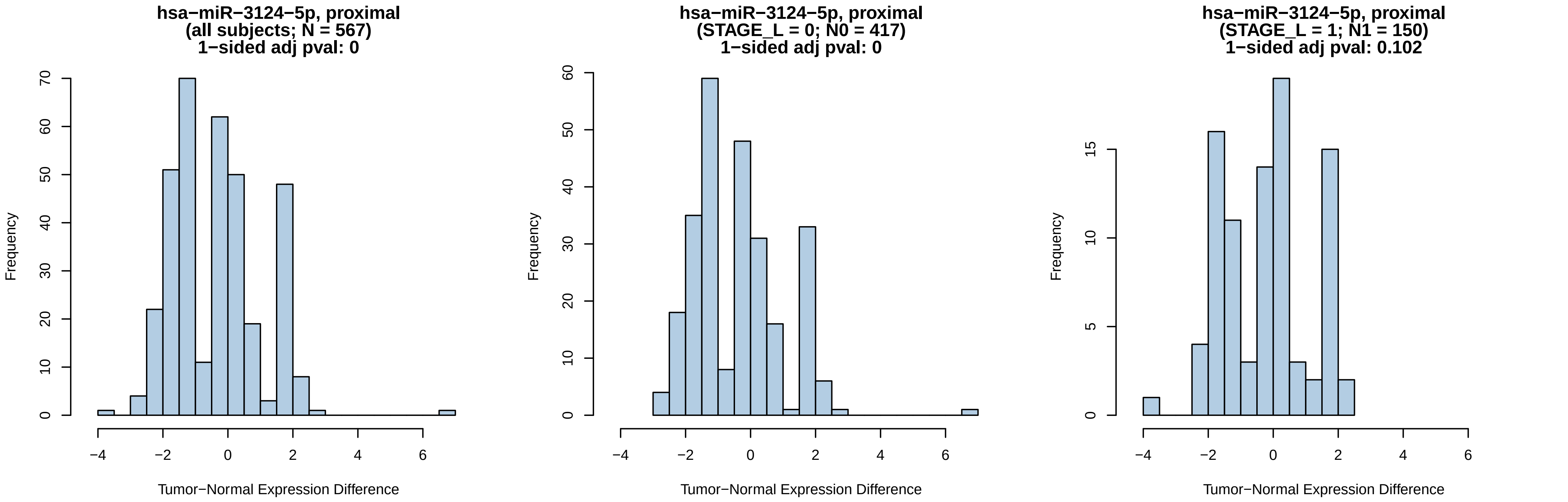
hsa−miR−3124−5p, proximal
(all subjects; N = 567)
1−sided adj pval: 0
Frequency
Tumor−Normal Expression Difference
hsa−miR−3124−5p, proximal
(STAGE_L = 0; N0 = 417)
1−sided adj pval: 0
Frequency
Tumor−Normal Expression Difference
hsa−miR−3124−5p, proximal
(STAGE_L = 1; N1 = 150)
1−sided adj pval: 0.102
Frequency
Tumor−Normal Expression Difference

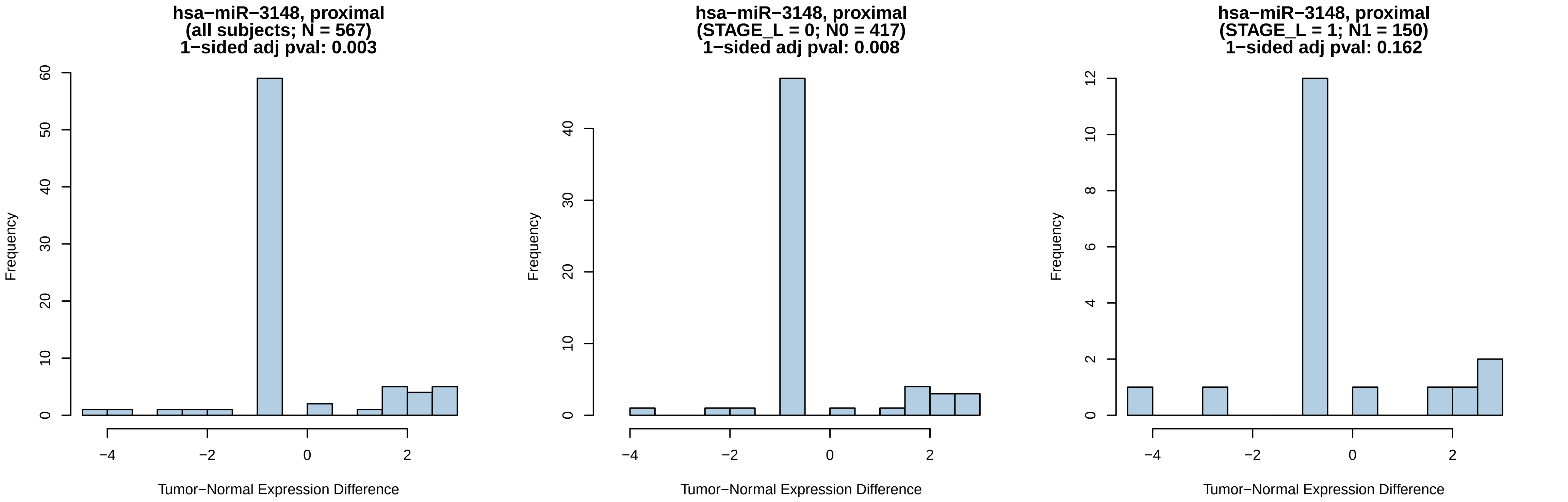
hsa−miR−3148, proximal
(all subjects; N = 567)
1−sided adj pval: 0.003
Frequency
0
10
20
30
40
50
60
−4
−2
0
2
Tumor−Normal Expression Difference
hsa−miR−3148, proximal
(STAGE_L = 0; N0 = 417)
1−sided adj pval: 0.008
Frequency
0
10
20
30
40
−4
−2
0
2
Tumor−Normal Expression Difference
hsa−miR−3148, proximal
(STAGE_L = 1; N1 = 150)
1−sided adj pval: 0.162
Frequency
0
2
4
6
8
10
12
−4
−2
0
2
Tumor−Normal Expression Difference

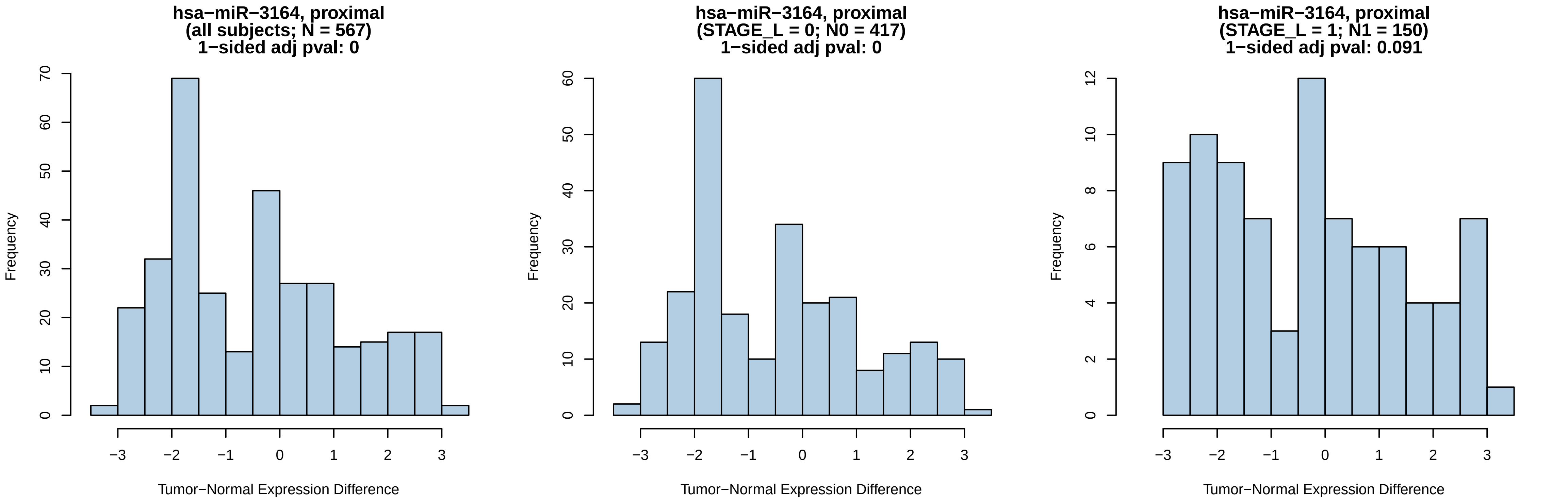
hsa−miR−3164, proximal
(all subjects; N = 567)
1−sided adj pval: 0
hsa−miR−3164, proximal
(STAGE_L = 0; N0 = 417)
1−sided adj pval: 0
hsa−miR−3164, proximal
(STAGE_L = 1; N1 = 150)
1−sided adj pval: 0.091
Frequency
Tumor−Normal Expression Difference

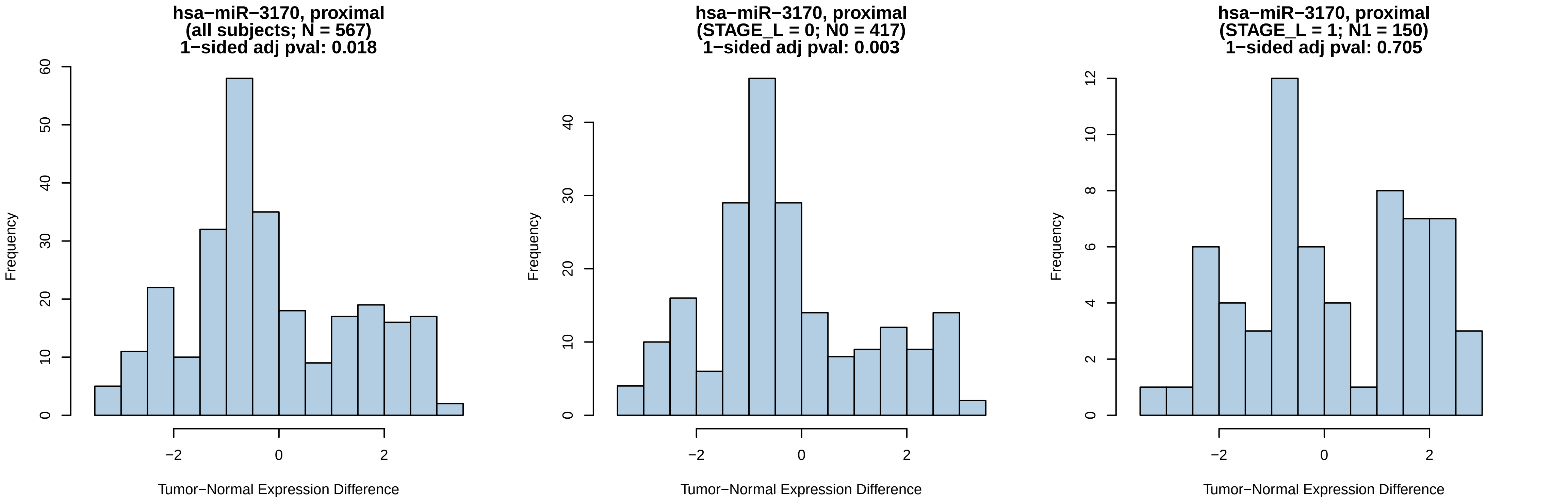
hsa−miR−3170, proximal
(all subjects; N = 567)
1−sided adj pval: 0.018
hsa−miR−3170, proximal
(STAGE_L = 0; N0 = 417)
1−sided adj pval: 0.003
hsa−miR−3170, proximal
(STAGE_L = 1; N1 = 150)
1−sided adj pval: 0.705
Frequency
Tumor−Normal Expression Difference

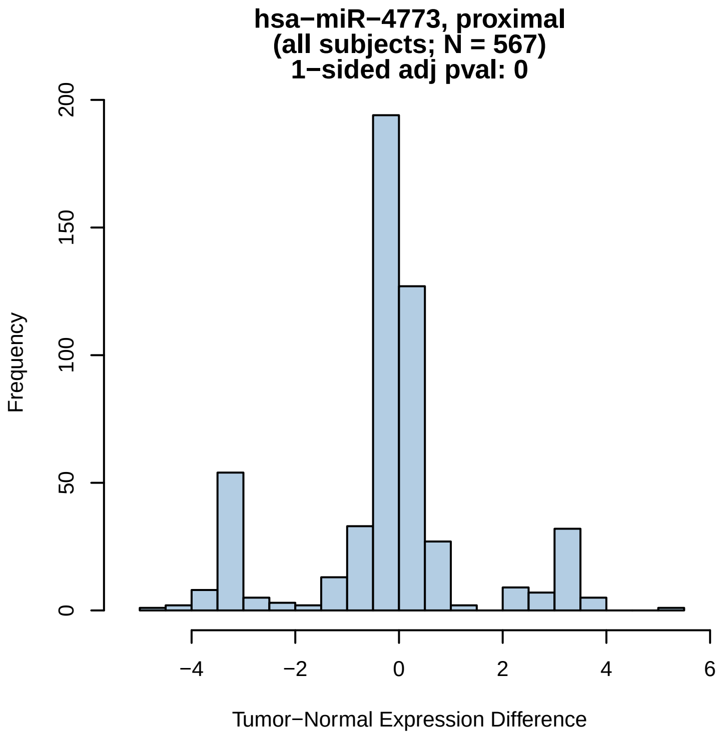
hsa−miR−4773, proximal
(all subjects; N = 567)
1−sided adj pval: 0
Frequency
0
50
100
150
200
−4
−2
0
2
4
6
Tumor−Normal Expression Difference
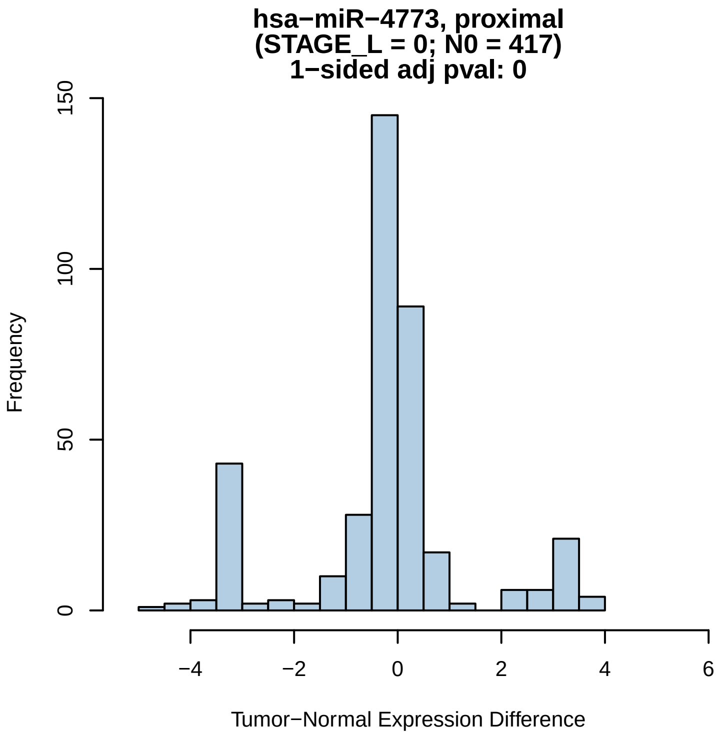
hsa−miR−4773, proximal
(STAGE_L = 0; N0 = 417)
1−sided adj pval: 0
Frequency
0
50
100
150
−4
−2
0
2
4
6
Tumor−Normal Expression Difference
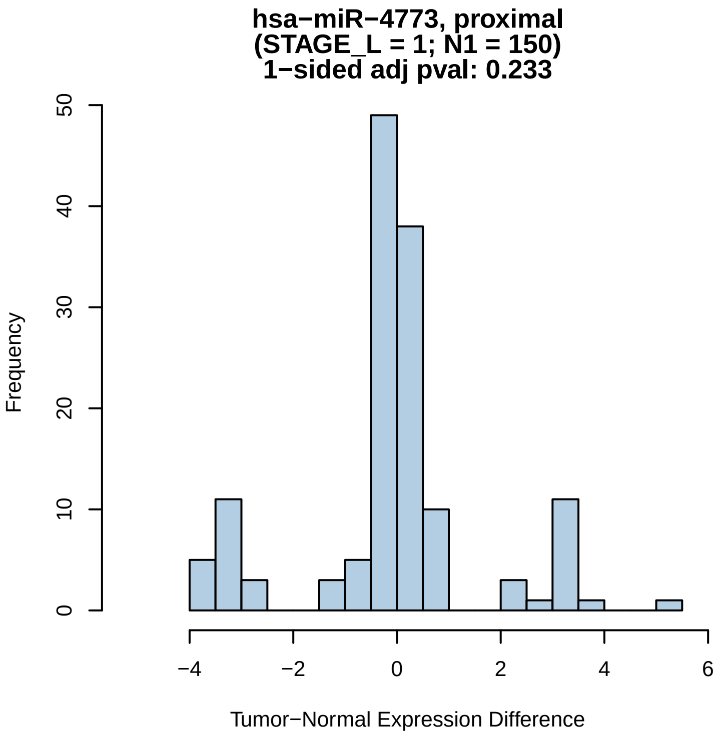
hsa−miR−4773, proximal
(STAGE_L = 1; N1 = 150)
1−sided adj pval: 0.233
Frequency
0
10
20
30
40
50
−4
−2
0
2
4
6
Tumor−Normal Expression Difference

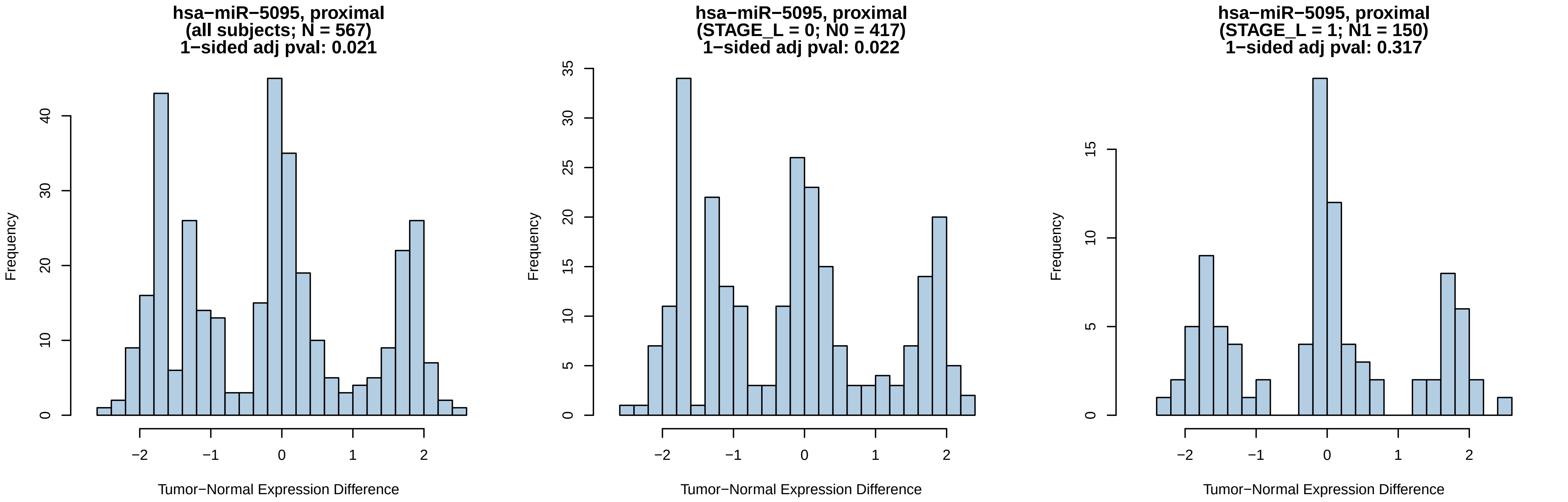
hsa−miR−5095, proximal
(all subjects; N = 567)
1−sided adj pval: 0.021
hsa−miR−5095, proximal
(STAGE_L = 0; N0 = 417)
1−sided adj pval: 0.022
hsa−miR−5095, proximal
(STAGE_L = 1; N1 = 150)
1−sided adj pval: 0.317
Frequency
Tumor−Normal Expression Difference

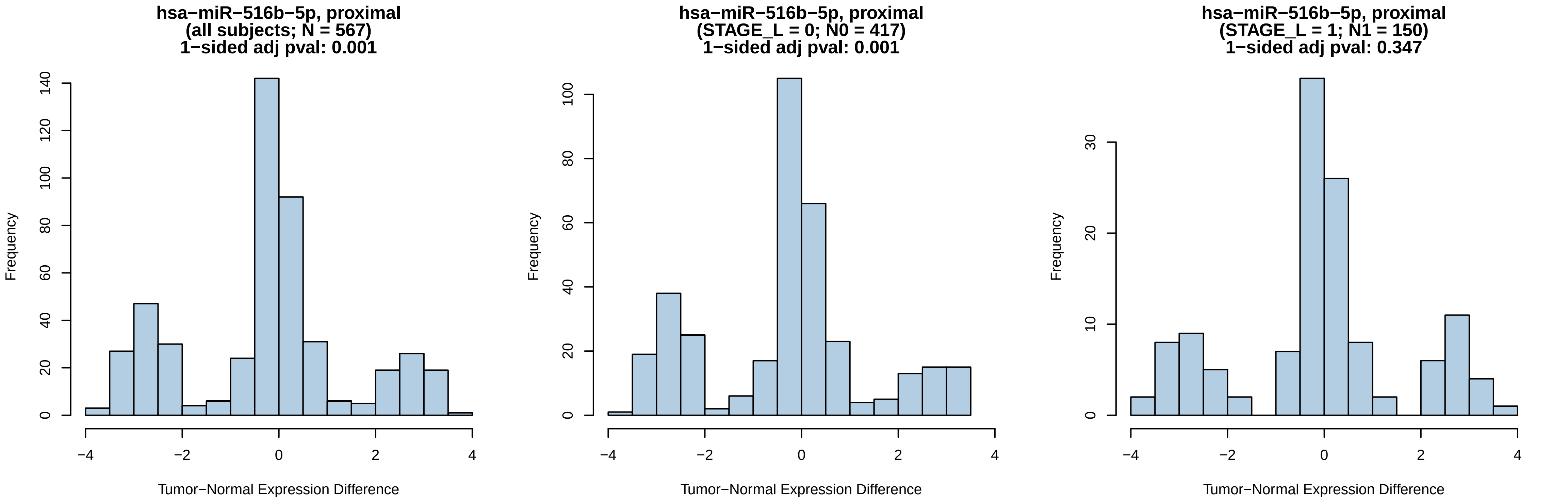
hsa−miR−516b−5p, proximal
(all subjects; N = 567)
1−sided adj pval: 0.001
Frequency
0
20
40
60
80
100
120
140
−4
−2
0
2
4
Tumor−Normal Expression Difference
hsa−miR−516b−5p, proximal
(STAGE_L = 0; N0 = 417)
1−sided adj pval: 0.001
Frequency
0
20
40
60
80
100
−4
−2
0
2
4
Tumor−Normal Expression Difference
hsa−miR−516b−5p, proximal
(STAGE_L = 1; N1 = 150)
1−sided adj pval: 0.347
Frequency
0
10
20
30
−4
−2
0
2
4
Tumor−Normal Expression Difference

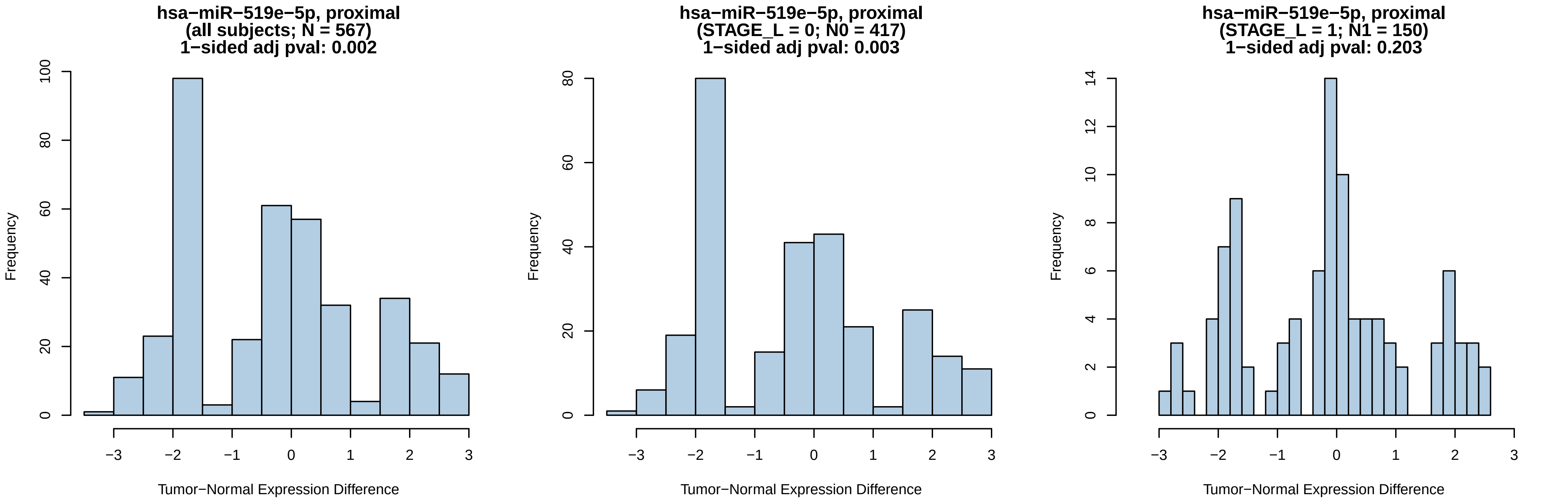
hsa−miR−519e−5p, proximal
(all subjects; N = 567)
1−sided adj pval: 0.002
Frequency
Tumor−Normal Expression Difference
hsa−miR−519e−5p, proximal
(STAGE_L = 0; N0 = 417)
1−sided adj pval: 0.003
Frequency
Tumor−Normal Expression Difference
hsa−miR−519e−5p, proximal
(STAGE_L = 1; N1 = 150)
1−sided adj pval: 0.203
Frequency
Tumor−Normal Expression Difference

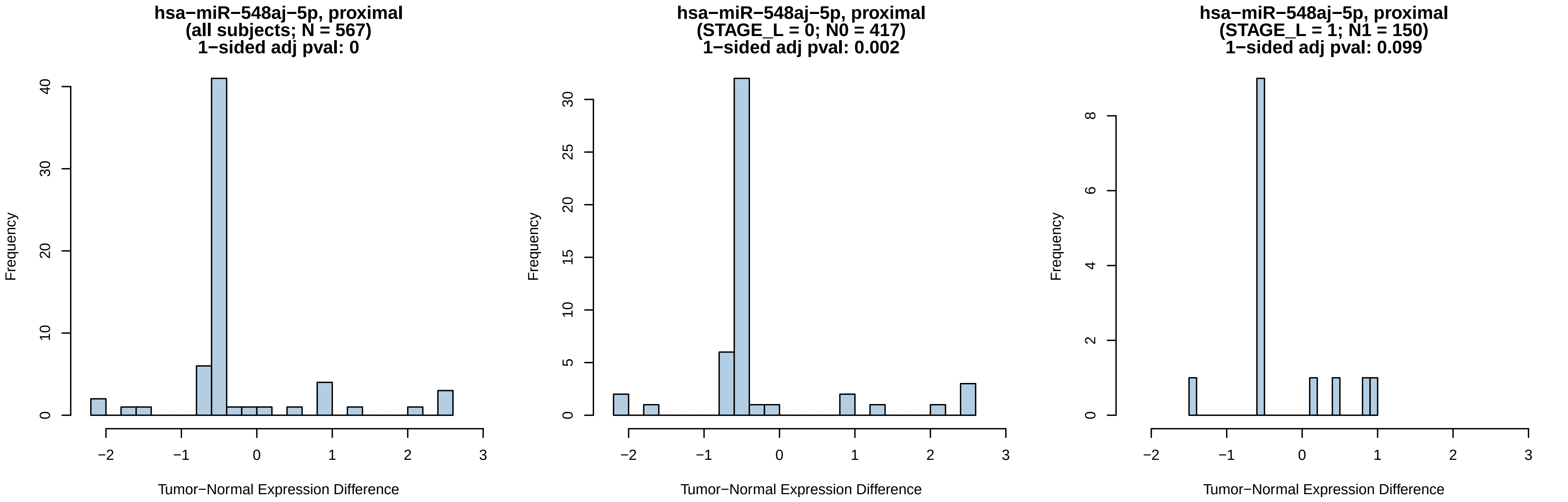

hsa−miR−548aj−5p, proximal
(all subjects; N = 567)
1−sided adj pval: 0
Frequency
Tumor−Normal Expression Difference
hsa−miR−548aj−5p, proximal
(STAGE_L = 0; N0 = 417)
1−sided adj pval: 0.002
Frequency
Tumor−Normal Expression Difference
hsa−miR−548aj−5p, proximal
(STAGE_L = 1; N1 = 150)
1−sided adj pval: 0.099
Frequency
Tumor−Normal Expression Difference

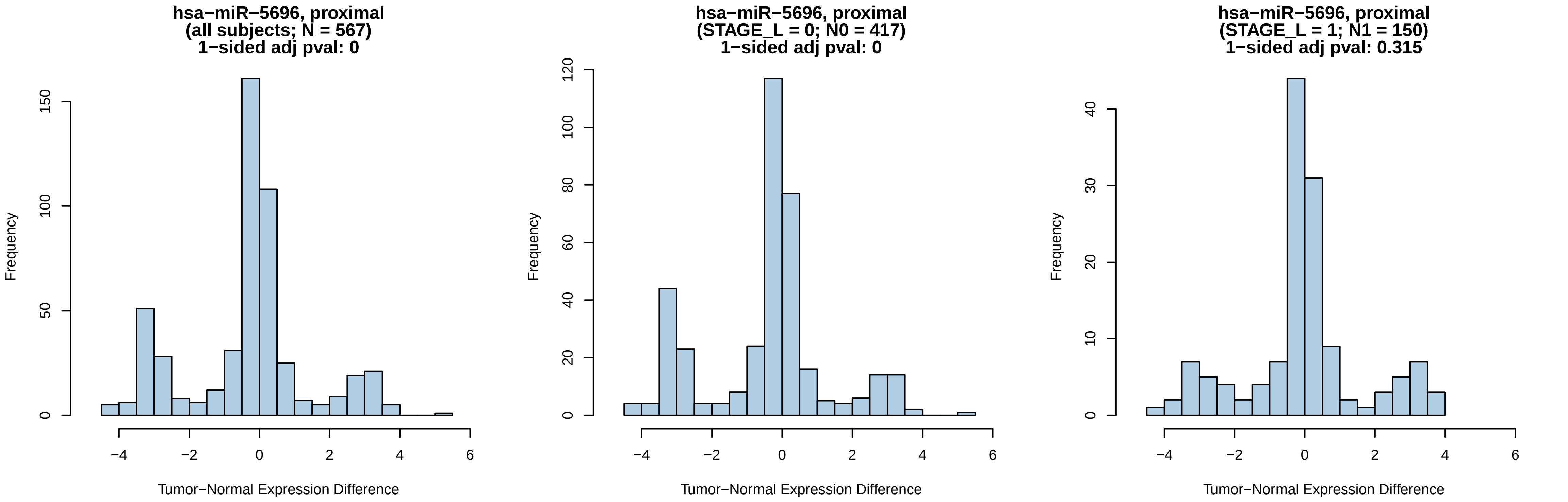
hsa−miR−5696, proximal
(all subjects; N = 567)
1−sided adj pval: 0
hsa−miR−5696, proximal
(STAGE_L = 0; N0 = 417)
1−sided adj pval: 0
hsa−miR−5696, proximal
(STAGE_L = 1; N1 = 150)
1−sided adj pval: 0.315
Frequency
Tumor−Normal Expression Difference

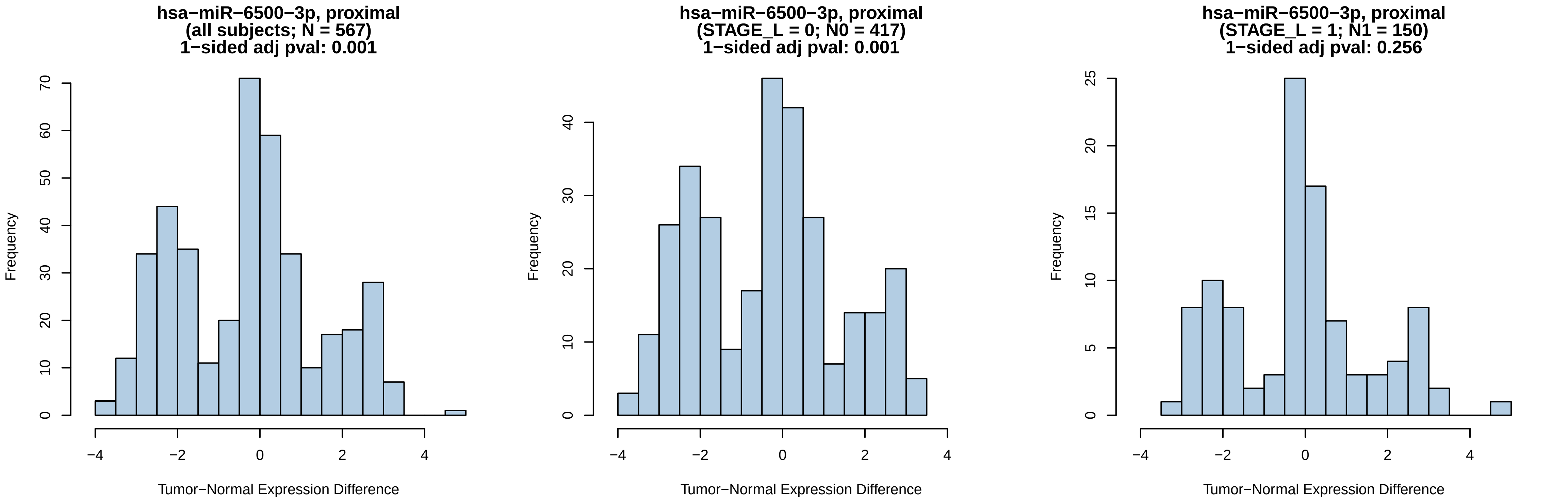
hsa−miR−6500−3p, proximal
(all subjects; N = 567)
1−sided adj pval: 0.001
Frequency
0
10
20
30
40
50
60
70
−4
−2
0
2
4
Tumor−Normal Expression Difference
hsa−miR−6500−3p, proximal
(STAGE_L = 0; N0 = 417)
1−sided adj pval: 0.001
Frequency
0
10
20
30
40
−4
−2
0
2
4
Tumor−Normal Expression Difference
hsa−miR−6500−3p, proximal
(STAGE_L = 1; N1 = 150)
1−sided adj pval: 0.256
Frequency
0
5
10
15
20
25
−4
−2
0
2
4
Tumor−Normal Expression Difference

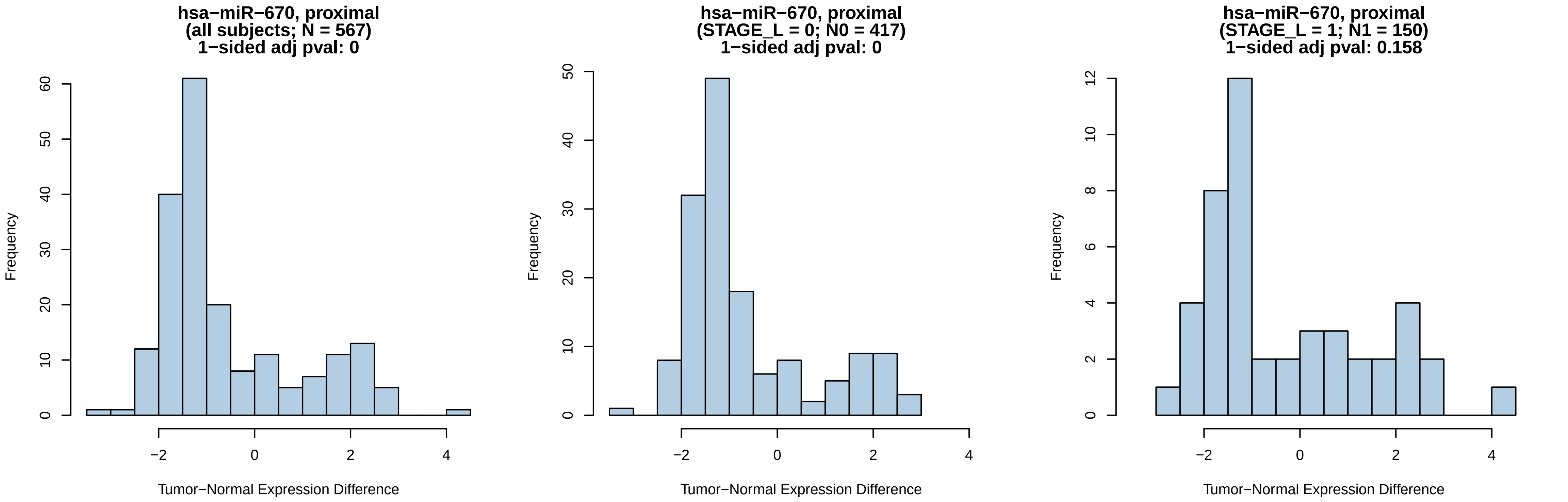

hsa−miR−670, proximal
(all subjects; N = 567)
1−sided adj pval: 0
Frequency
Tumor−Normal Expression Difference
hsa−miR−670, proximal
(STAGE_L = 0; N0 = 417)
1−sided adj pval: 0
Frequency
Tumor−Normal Expression Difference
hsa−miR−670, proximal
(STAGE_L = 1; N1 = 150)
1−sided adj pval: 0.158
Frequency
Tumor−Normal Expression Difference

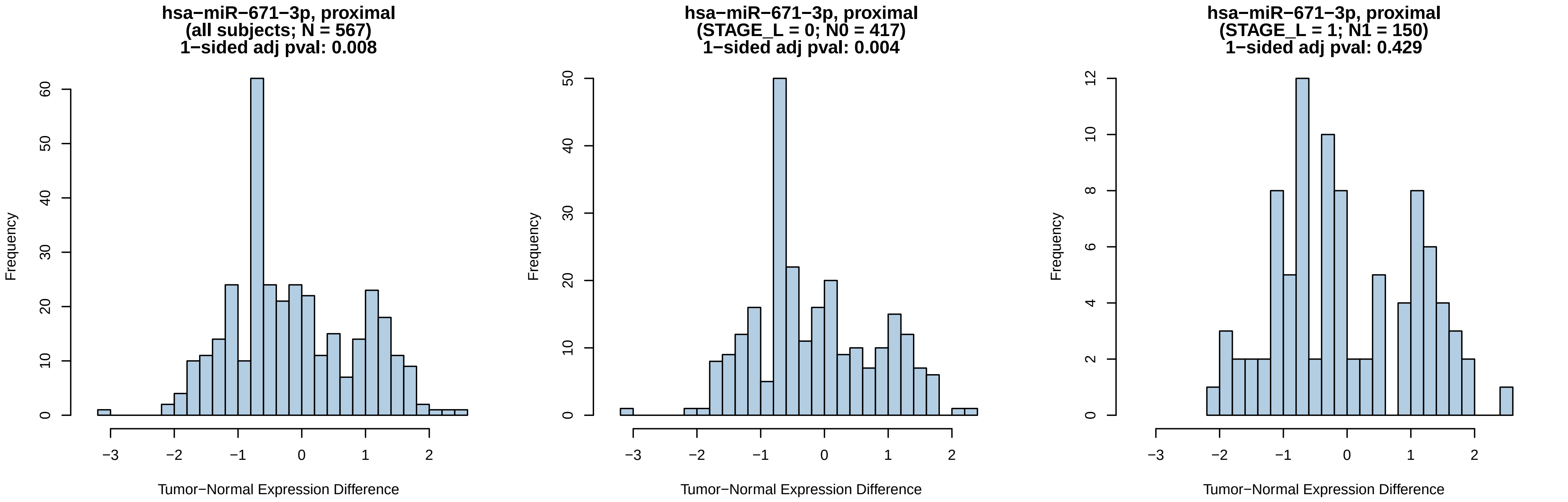
hsa−miR−671−3p, proximal
(all subjects; N = 567)
1−sided adj pval: 0.008
Frequency
Tumor−Normal Expression Difference
hsa−miR−671−3p, proximal
(STAGE_L = 0; N0 = 417)
1−sided adj pval: 0.004
Frequency
Tumor−Normal Expression Difference
hsa−miR−671−3p, proximal
(STAGE_L = 1; N1 = 150)
1−sided adj pval: 0.429
Frequency
Tumor−Normal Expression Difference

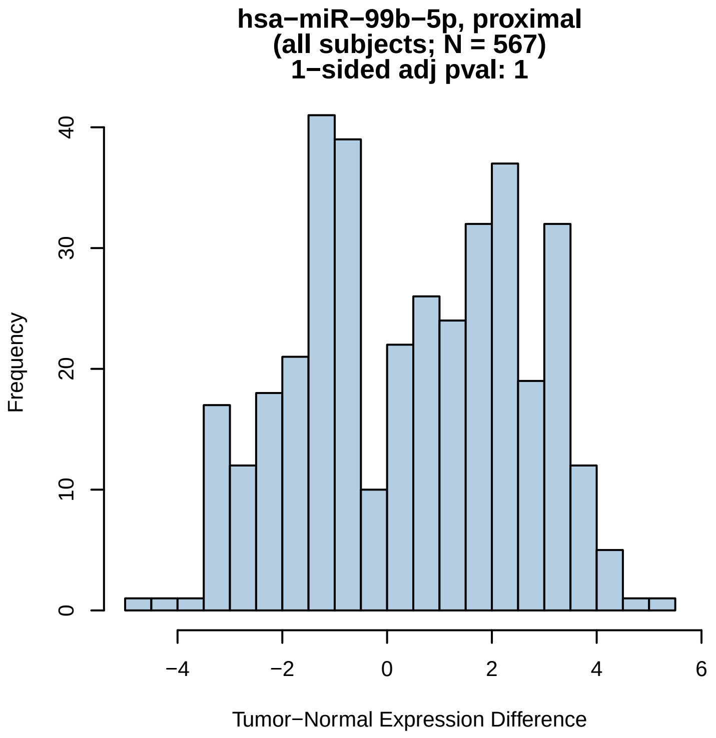
hsa−miR−99b−5p, proximal
(all subjects; N = 567)
1−sided adj pval: 1
Frequency
Tumor−Normal Expression Difference

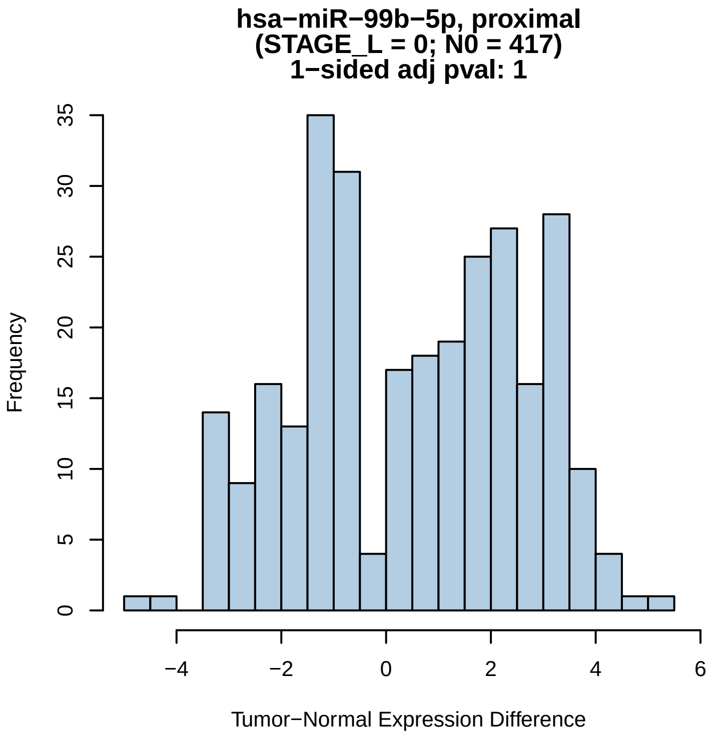
hsa−miR−99b−5p, proximal
(STAGE_L = 0; N0 = 417)
1−sided adj pval: 1
Frequency
Tumor−Normal Expression Difference

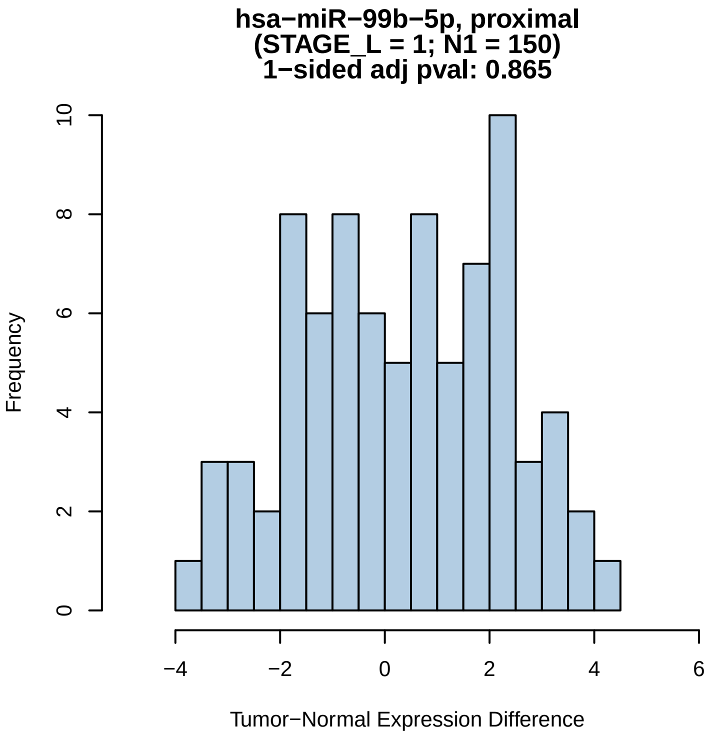
hsa−miR−99b−5p, proximal
(STAGE_L = 1; N1 = 150)
1−sided adj pval: 0.865
Frequency
Tumor−Normal Expression Difference

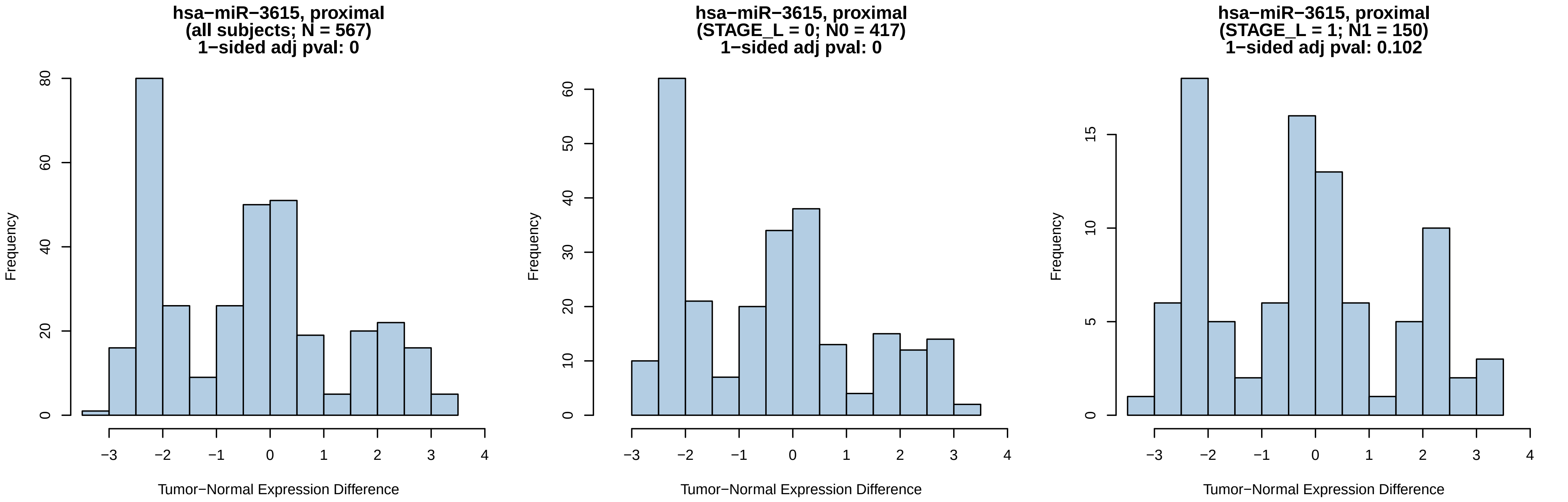
hsa−miR−3615, proximal
(all subjects; N = 567)
1−sided adj pval: 0
hsa−miR−3615, proximal
(STAGE_L = 0; N0 = 417)
1−sided adj pval: 0
hsa−miR−3615, proximal
(STAGE_L = 1; N1 = 150)
1−sided adj pval: 0.102
Frequency
Tumor−Normal Expression Difference

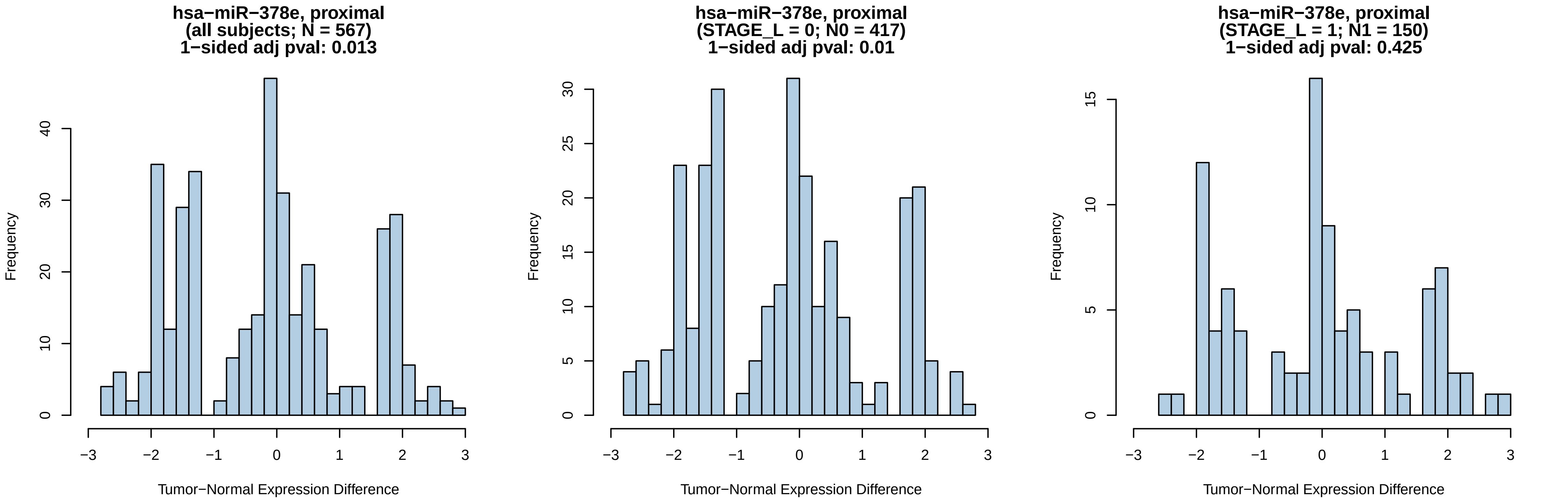

hsa−miR−378e, proximal
(all subjects; N = 567)
1−sided adj pval: 0.013
hsa−miR−378e, proximal
(STAGE_L = 0; N0 = 417)
1−sided adj pval: 0.01
hsa−miR−378e, proximal
(STAGE_L = 1; N1 = 150)
1−sided adj pval: 0.425
Frequency
Tumor−Normal Expression Difference

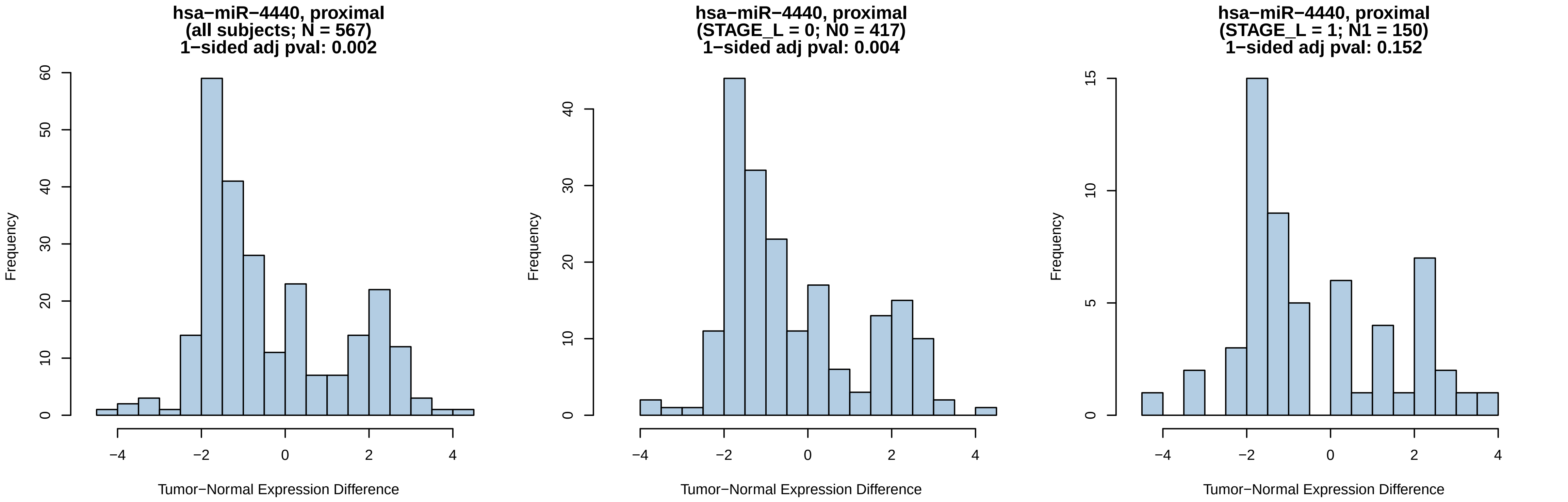

hsa−miR−4440, proximal
(all subjects; N = 567)
1−sided adj pval: 0.002
Frequency
Tumor−Normal Expression Difference
hsa−miR−4440, proximal
(STAGE_L = 0; N0 = 417)
1−sided adj pval: 0.004
Frequency
Tumor−Normal Expression Difference
hsa−miR−4440, proximal
(STAGE_L = 1; N1 = 150)
1−sided adj pval: 0.152
Frequency
Tumor−Normal Expression Difference

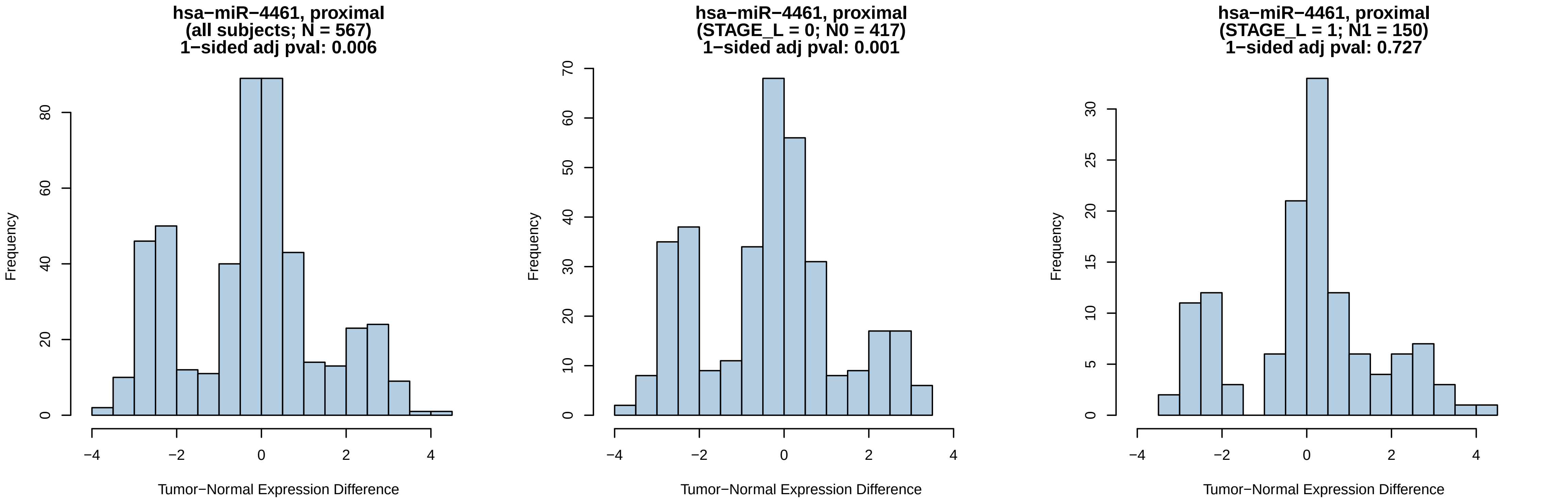

hsa−miR−4461, proximal
(all subjects; N = 567)
1−sided adj pval: 0.006
Frequency
Tumor−Normal Expression Difference
hsa−miR−4461, proximal
(STAGE_L = 0; N0 = 417)
1−sided adj pval: 0.001
Frequency
Tumor−Normal Expression Difference
hsa−miR−4461, proximal
(STAGE_L = 1; N1 = 150)
1−sided adj pval: 0.727
Frequency
Tumor−Normal Expression Difference

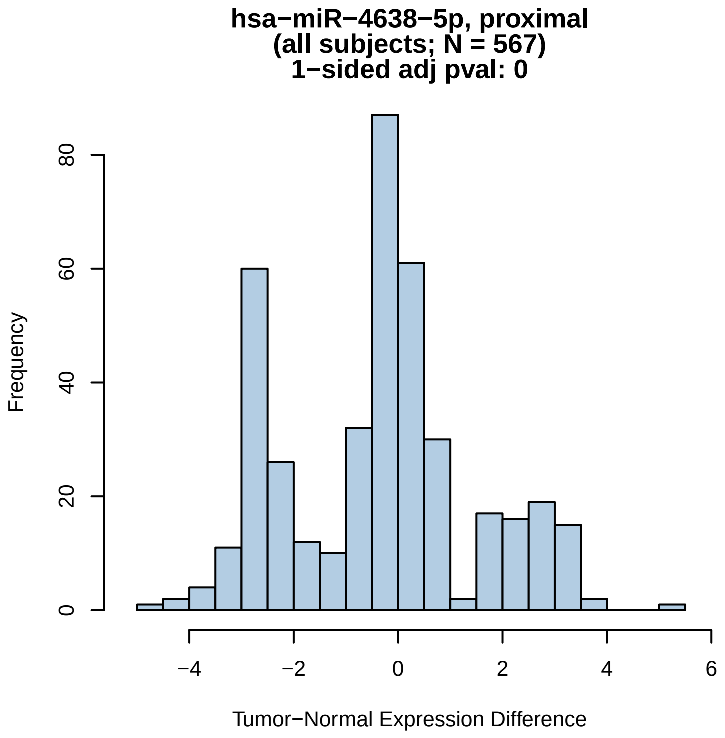
hsa−miR−4638−5p, proximal
(all subjects; N = 567)
1−sided adj pval: 0
Frequency
Tumor−Normal Expression Difference
0
20
40
60
80
−4
−2
0
2
4
6

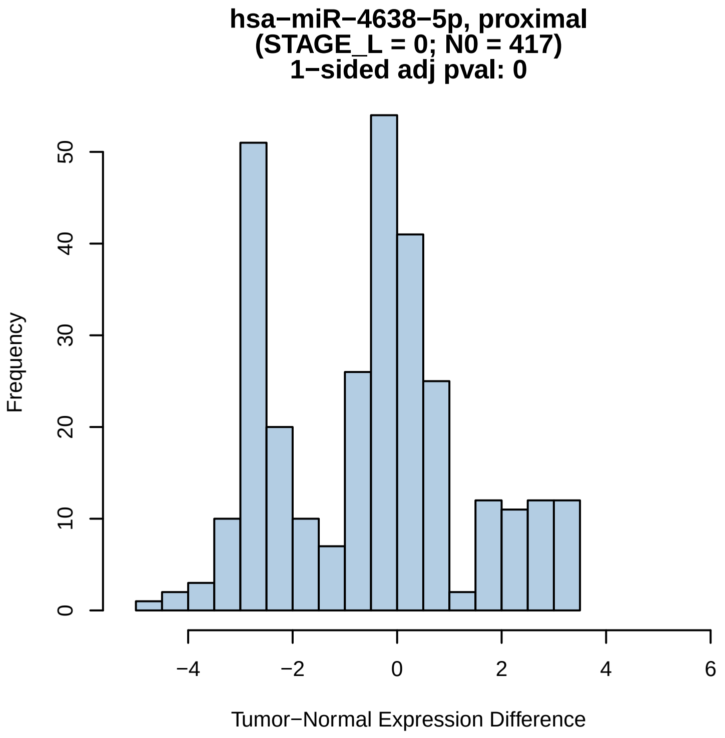
hsa−miR−4638−5p, proximal
(STAGE_L = 0; N0 = 417)
1−sided adj pval: 0
Frequency
Tumor−Normal Expression Difference
0
10
20
30
40
50
−4
−2
0
2
4
6

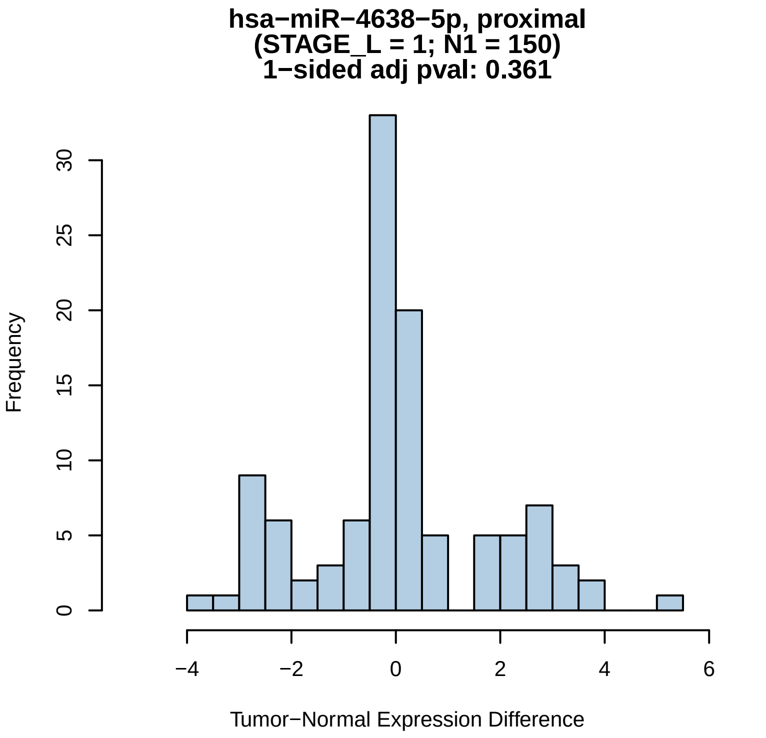
hsa−miR−4638−5p, proximal
(STAGE_L = 1; N1 = 150)
1−sided adj pval: 0.361
Frequency
Tumor−Normal Expression Difference
0
5
10
15
20
25
30
−4
−2
0
2
4
6

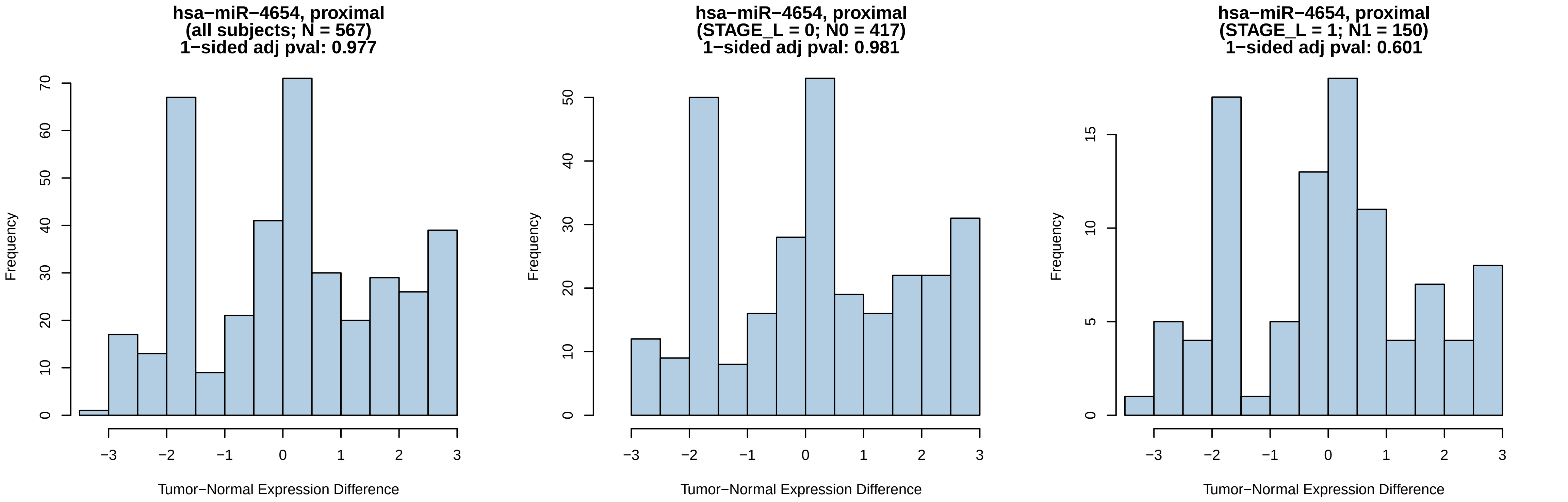

hsa−miR−4654, proximal
(all subjects; N = 567)
1−sided adj pval: 0.977
Frequency
Tumor−Normal Expression Difference
hsa−miR−4654, proximal
(STAGE_L = 0; N0 = 417)
1−sided adj pval: 0.981
Frequency
Tumor−Normal Expression Difference
hsa−miR−4654, proximal
(STAGE_L = 1; N1 = 150)
1−sided adj pval: 0.601
Frequency
Tumor−Normal Expression Difference

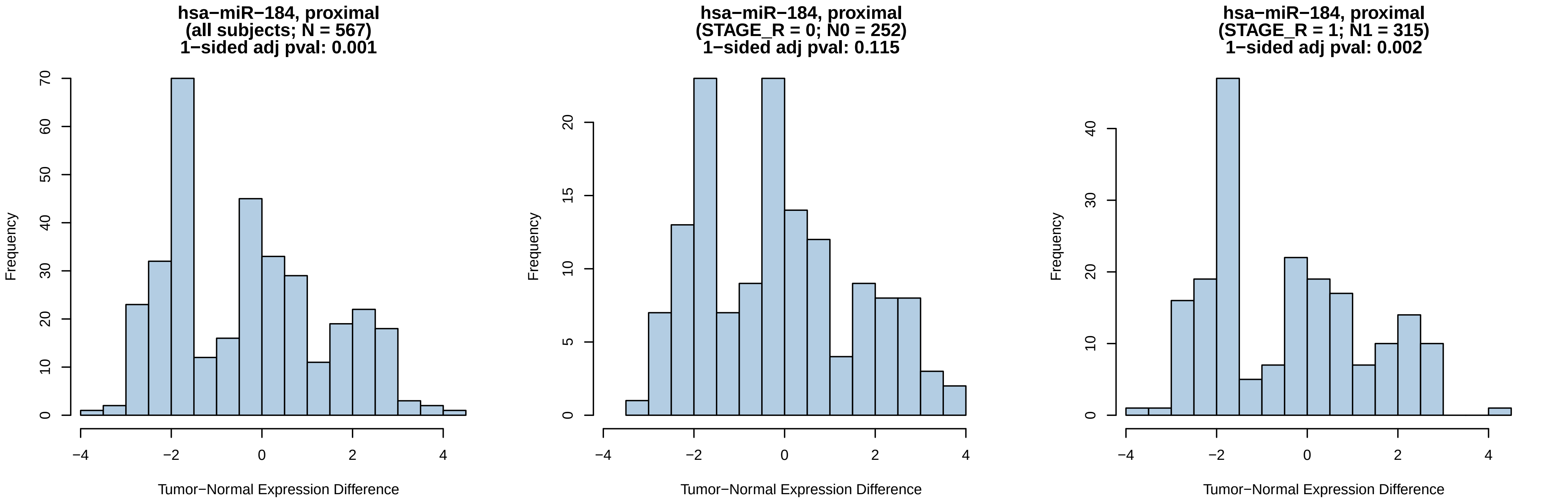

hsa−miR−184, proximal
(all subjects; N = 567)
1−sided adj pval: 0.001
hsa−miR−184, proximal
(STAGE_R = 0; N0 = 252)
1−sided adj pval: 0.115
hsa−miR−184, proximal
(STAGE_R = 1; N1 = 315)
1−sided adj pval: 0.002
Frequency
Tumor−Normal Expression Difference

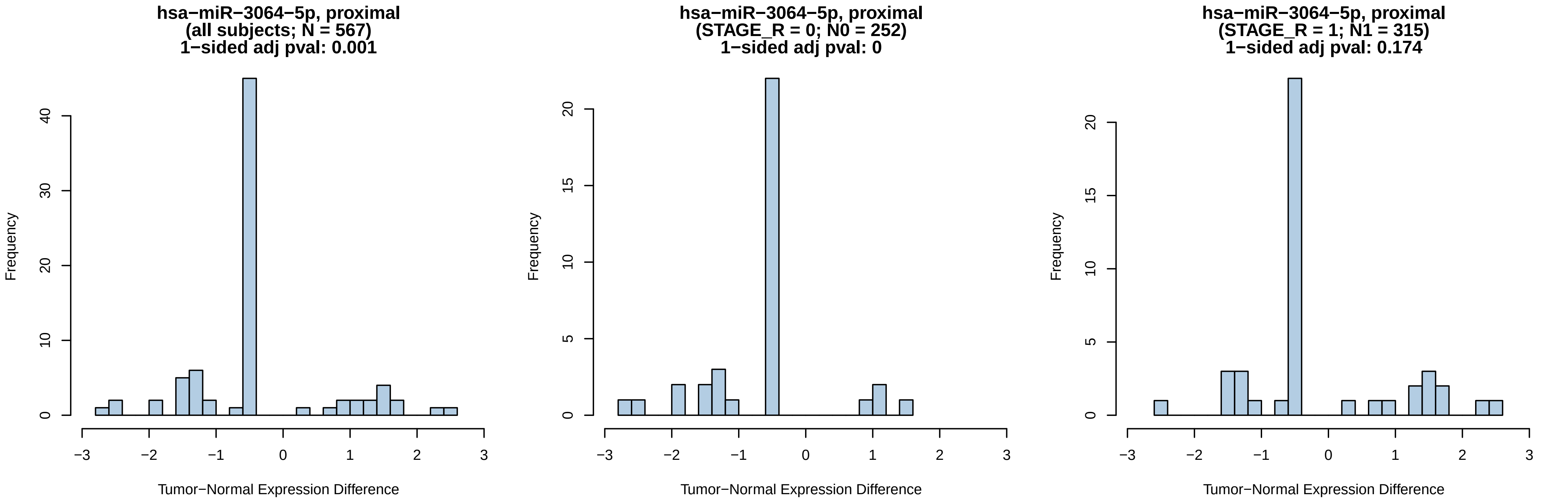
hsa−miR−3064−5p, proximal
(all subjects; N = 567)
1−sided adj pval: 0.001
hsa−miR−3064−5p, proximal
(STAGE_R = 0; N0 = 252)
1−sided adj pval: 0
hsa−miR−3064−5p, proximal
(STAGE_R = 1; N1 = 315)
1−sided adj pval: 0.174
Frequency
Tumor−Normal Expression Difference

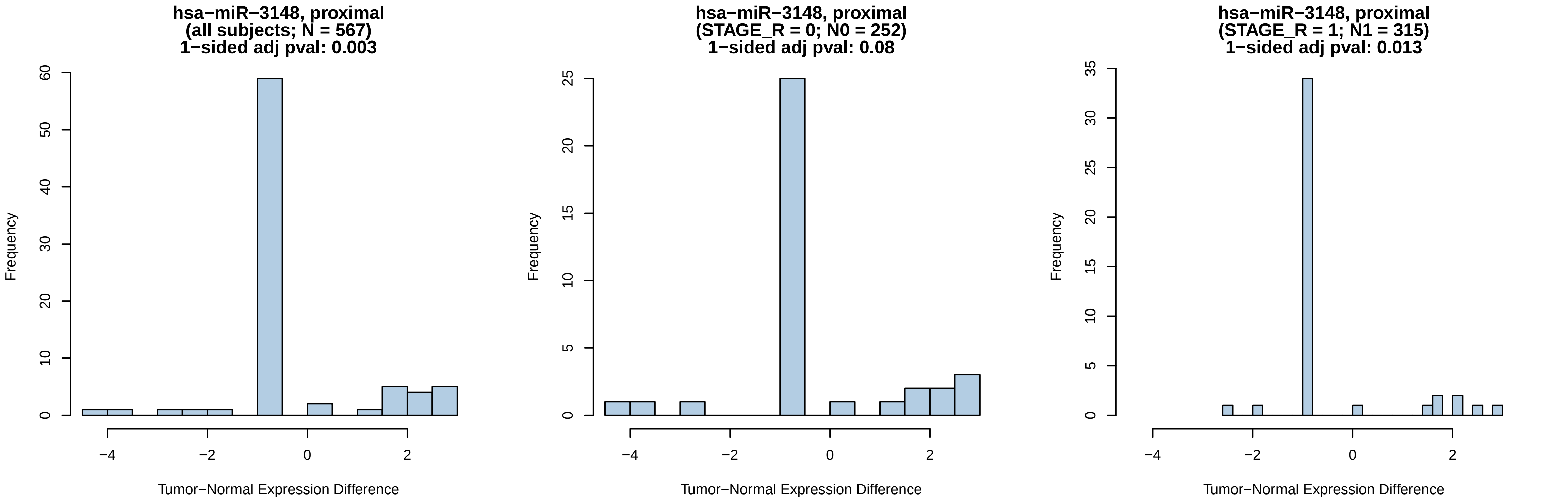

hsa−miR−3148, proximal
(all subjects; N = 567)
1−sided adj pval: 0.003
hsa−miR−3148, proximal
(STAGE_R = 0; N0 = 252)
1−sided adj pval: 0.08
hsa−miR−3148, proximal
(STAGE_R = 1; N1 = 315)
1−sided adj pval: 0.013
Frequency
Tumor−Normal Expression Difference

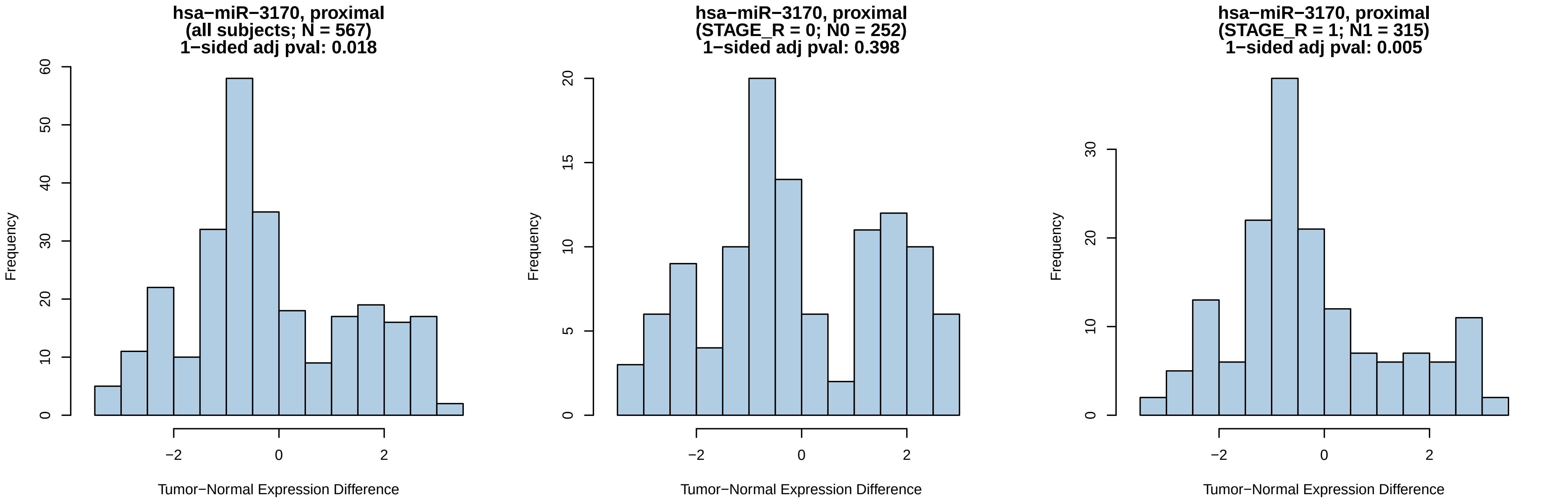
hsa−miR−3170, proximal
(all subjects; N = 567)
1−sided adj pval: 0.018
Frequency
Tumor−Normal Expression Difference
hsa−miR−3170, proximal
(STAGE_R = 0; N0 = 252)
1−sided adj pval: 0.398
Frequency
Tumor−Normal Expression Difference
hsa−miR−3170, proximal
(STAGE_R = 1; N1 = 315)
1−sided adj pval: 0.005
Frequency
Tumor−Normal Expression Difference

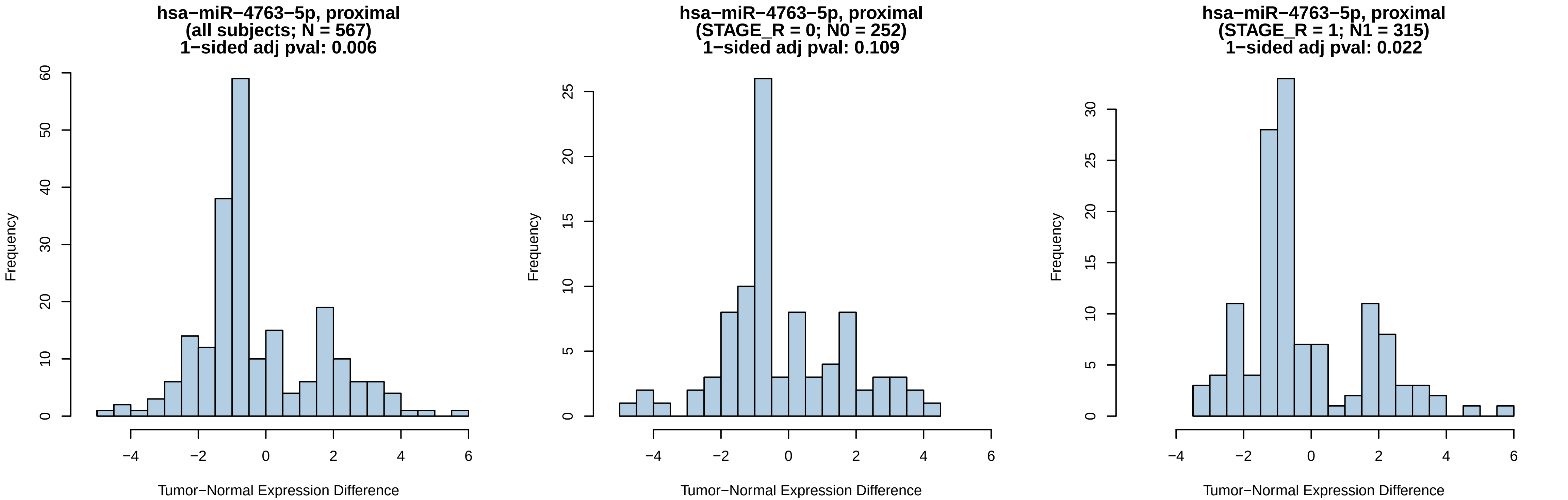
hsa−miR−4763−5p, proximal
(all subjects; N = 567)
1−sided adj pval: 0.006
Frequency
Tumor−Normal Expression Difference
hsa−miR−4763−5p, proximal
(STAGE_R = 0; N0 = 252)
1−sided adj pval: 0.109
Frequency
Tumor−Normal Expression Difference
hsa−miR−4763−5p, proximal
(STAGE_R = 1; N1 = 315)
1−sided adj pval: 0.022
Frequency
Tumor−Normal Expression Difference

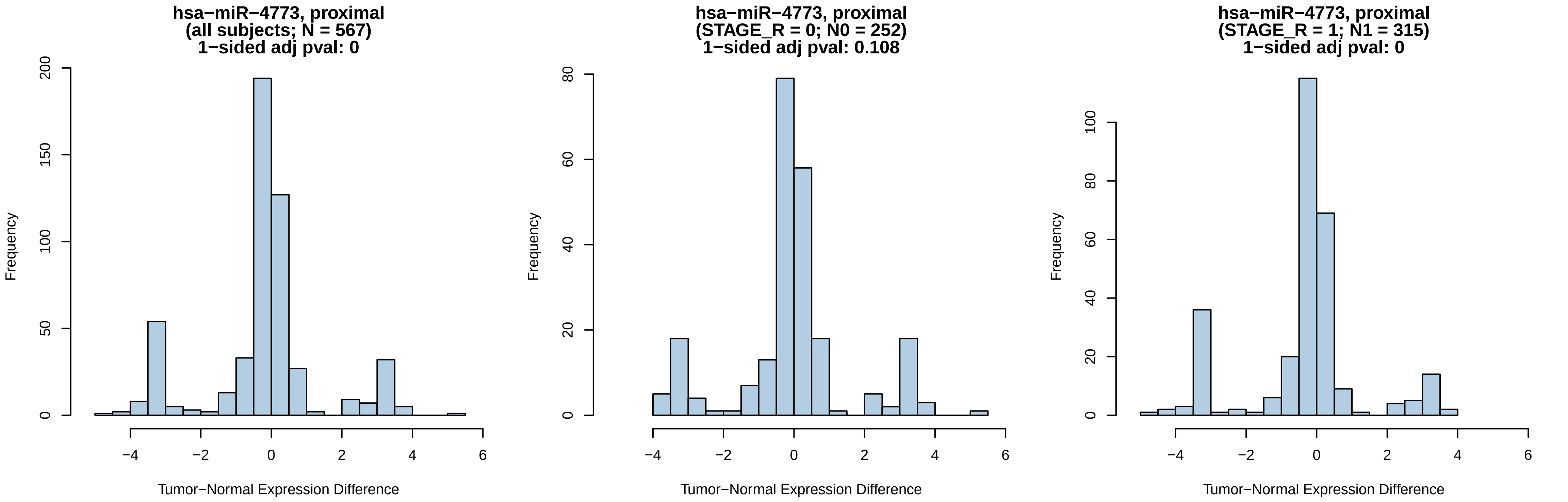
hsa−miR−4773, proximal
(all subjects; N = 567)
1−sided adj pval: 0
hsa−miR−4773, proximal
(STAGE_R = 0; N0 = 252)
1−sided adj pval: 0.108
hsa−miR−4773, proximal
(STAGE_R = 1; N1 = 315)
1−sided adj pval: 0
Frequency
Tumor−Normal Expression Difference

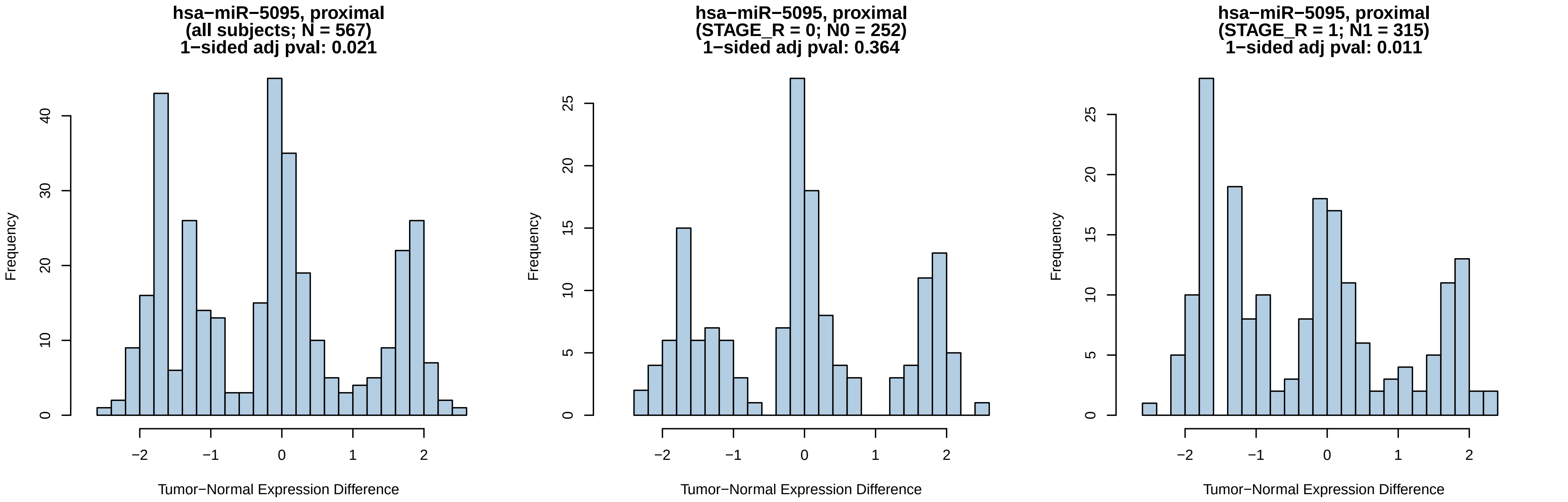
hsa−miR−5095, proximal
(all subjects; N = 567)
1−sided adj pval: 0.021
Frequency
Tumor−Normal Expression Difference
hsa−miR−5095, proximal
(STAGE_R = 0; N0 = 252)
1−sided adj pval: 0.364
Frequency
Tumor−Normal Expression Difference
hsa−miR−5095, proximal
(STAGE_R = 1; N1 = 315)
1−sided adj pval: 0.011
Frequency
Tumor−Normal Expression Difference

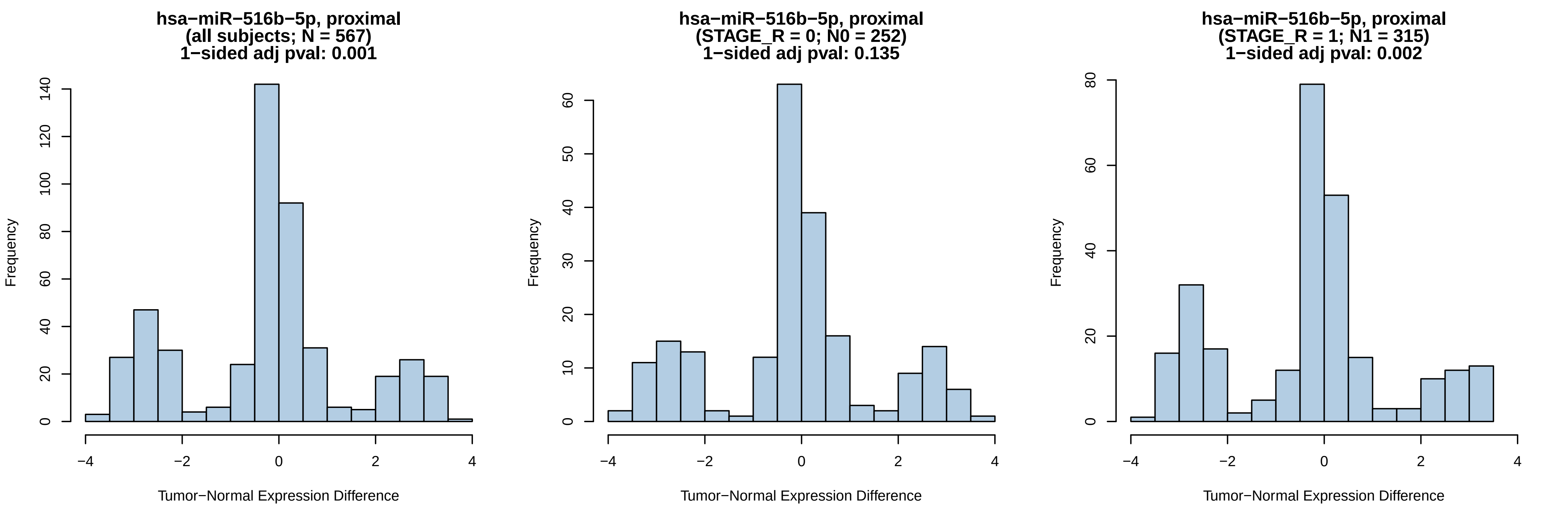
hsa−miR−516b−5p, proximal
(all subjects; N = 567)
1−sided adj pval: 0.001
Frequency
Tumor−Normal Expression Difference
hsa−miR−516b−5p, proximal
(STAGE_R = 0; N0 = 252)
1−sided adj pval: 0.135
Frequency
Tumor−Normal Expression Difference
hsa−miR−516b−5p, proximal
(STAGE_R = 1; N1 = 315)
1−sided adj pval: 0.002
Frequency
Tumor−Normal Expression Difference

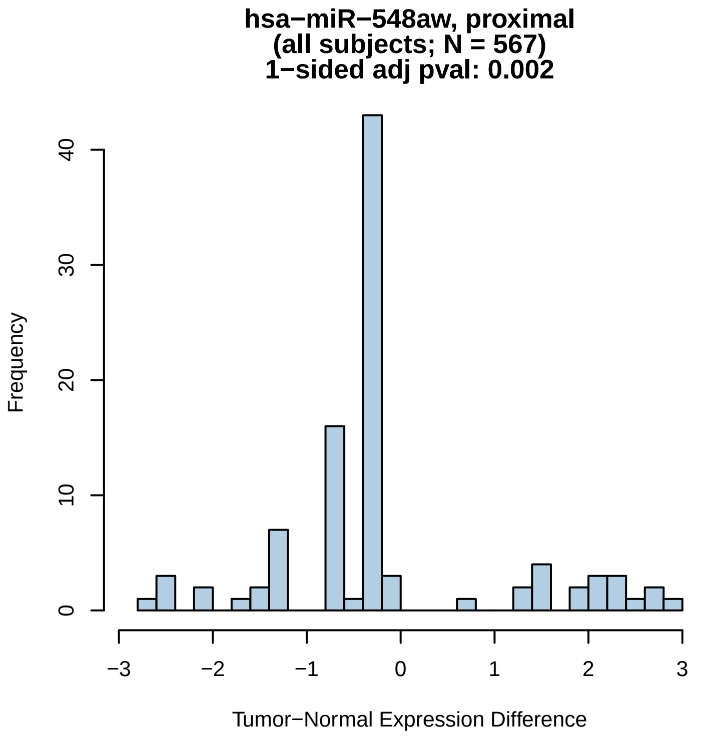
hsa−miR−548aw, proximal
(all subjects; N = 567)
1−sided adj pval: 0.002
Frequency
0
10
20
30
40
−3
−2
−1
0
1
2
3
Tumor−Normal Expression Difference

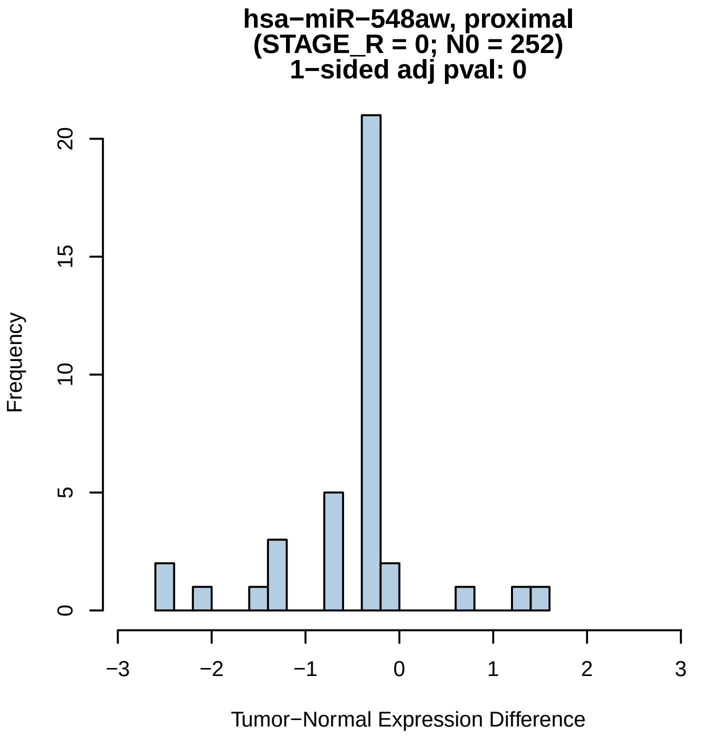
hsa−miR−548aw, proximal
(STAGE_R = 0; N0 = 252)
1−sided adj pval: 0
Frequency
0
5
10
15
20
−3
−2
−1
0
1
2
3
Tumor−Normal Expression Difference

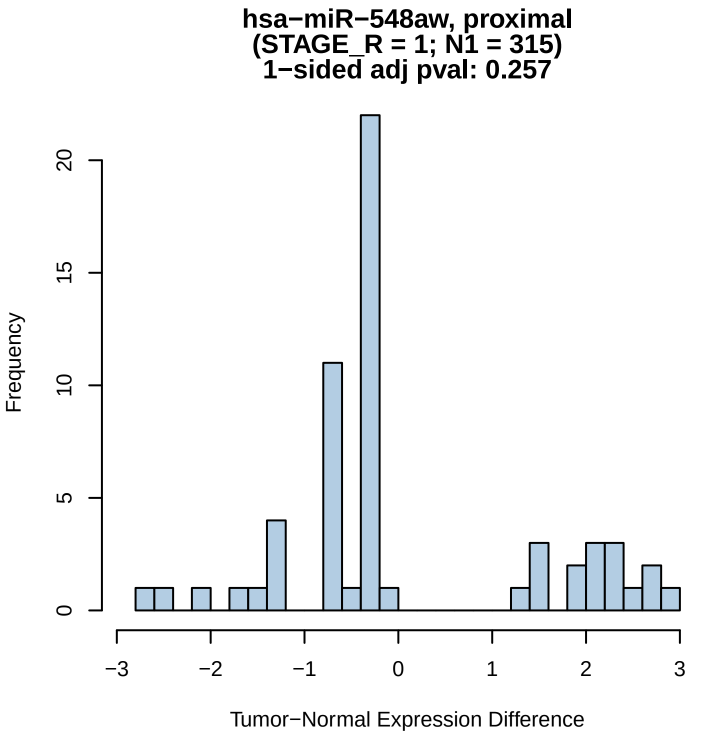
hsa−miR−548aw, proximal
(STAGE_R = 1; N1 = 315)
1−sided adj pval: 0.257
Frequency
0
5
10
15
20
−3
−2
−1
0
1
2
3
Tumor−Normal Expression Difference

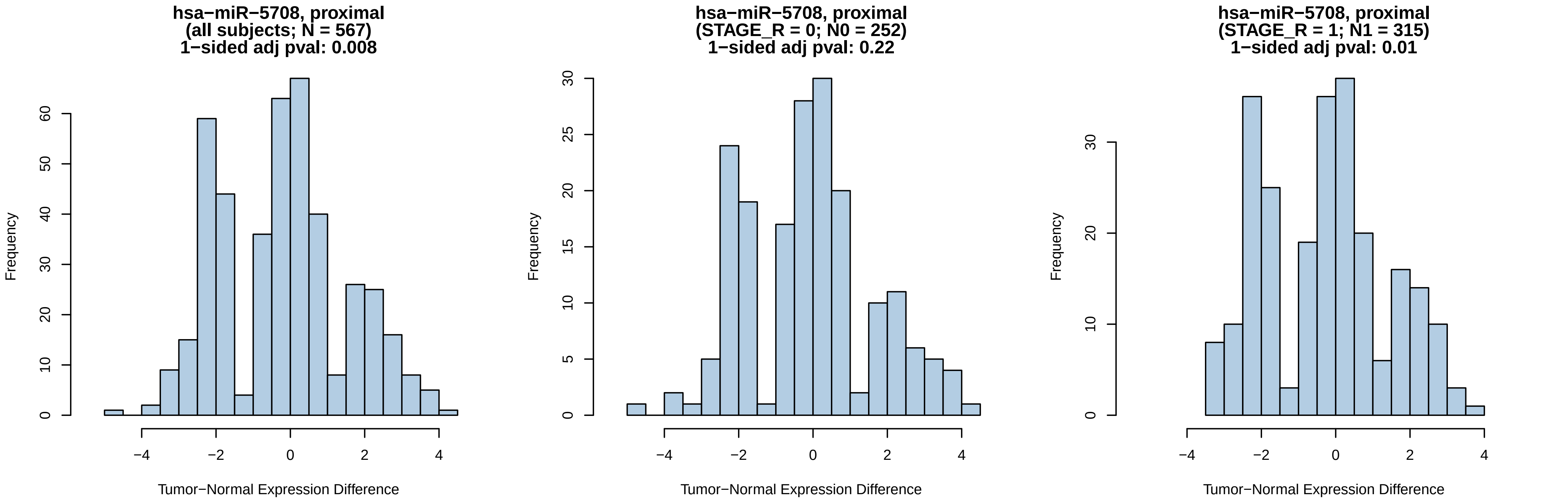
hsa−miR−5708, proximal
(all subjects; N = 567)
1−sided adj pval: 0.008
Frequency
Tumor−Normal Expression Difference
hsa−miR−5708, proximal
(STAGE_R = 0; N0 = 252)
1−sided adj pval: 0.22
Frequency
Tumor−Normal Expression Difference
hsa−miR−5708, proximal
(STAGE_R = 1; N1 = 315)
1−sided adj pval: 0.01
Frequency
Tumor−Normal Expression Difference

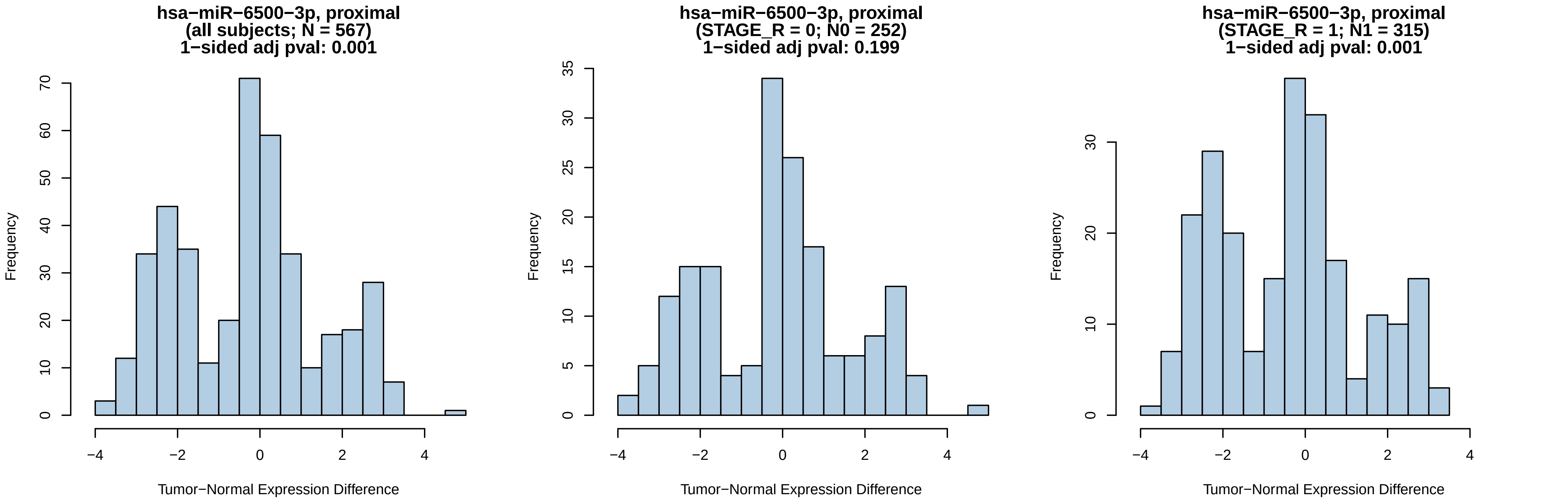

hsa−miR−6500−3p, proximal
(all subjects; N = 567)
1−sided adj pval: 0.001
Frequency
0
10
20
30
40
50
60
70
−4
−2
0
2
4
Tumor−Normal Expression Difference
hsa−miR−6500−3p, proximal
(STAGE_R = 0; N0 = 252)
1−sided adj pval: 0.199
Frequency
0
5
10
15
20
25
30
35
−4
−2
0
2
4
Tumor−Normal Expression Difference
hsa−miR−6500−3p, proximal
(STAGE_R = 1; N1 = 315)
1−sided adj pval: 0.001
Frequency
0
10
20
30
−4
−2
0
2
4
Tumor−Normal Expression Difference

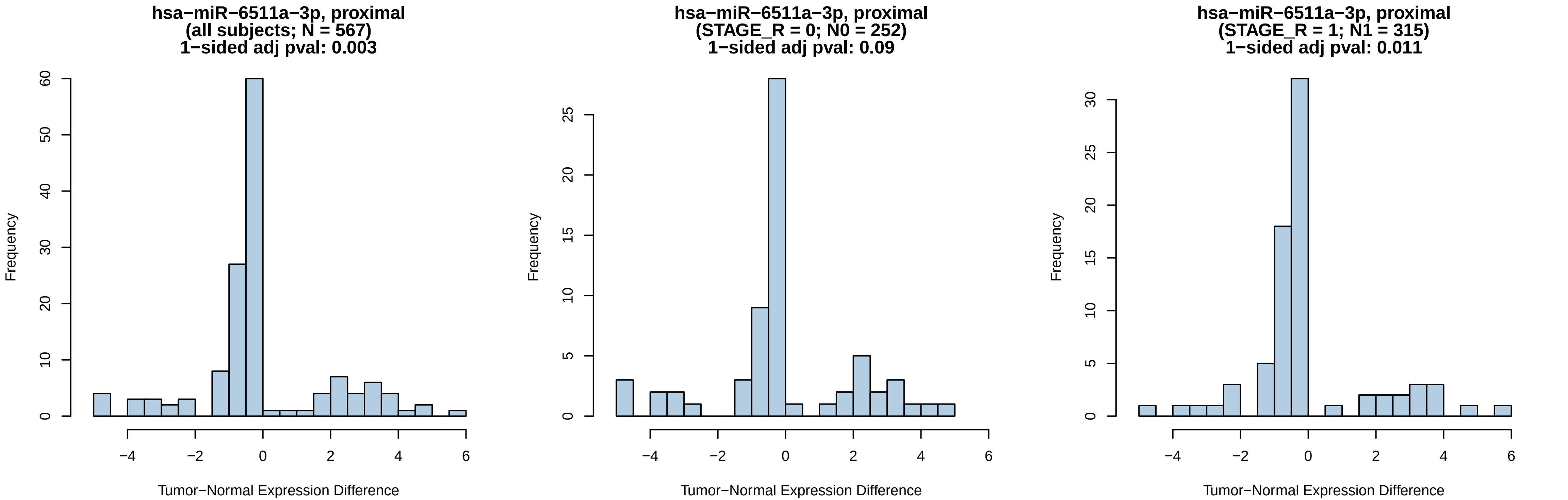
hsa−miR−6511a−3p, proximal
(all subjects; N = 567)
1−sided adj pval: 0.003
hsa−miR−6511a−3p, proximal
(STAGE_R = 0; N0 = 252)
1−sided adj pval: 0.09
hsa−miR−6511a−3p, proximal
(STAGE_R = 1; N1 = 315)
1−sided adj pval: 0.011
Frequency
Tumor−Normal Expression Difference

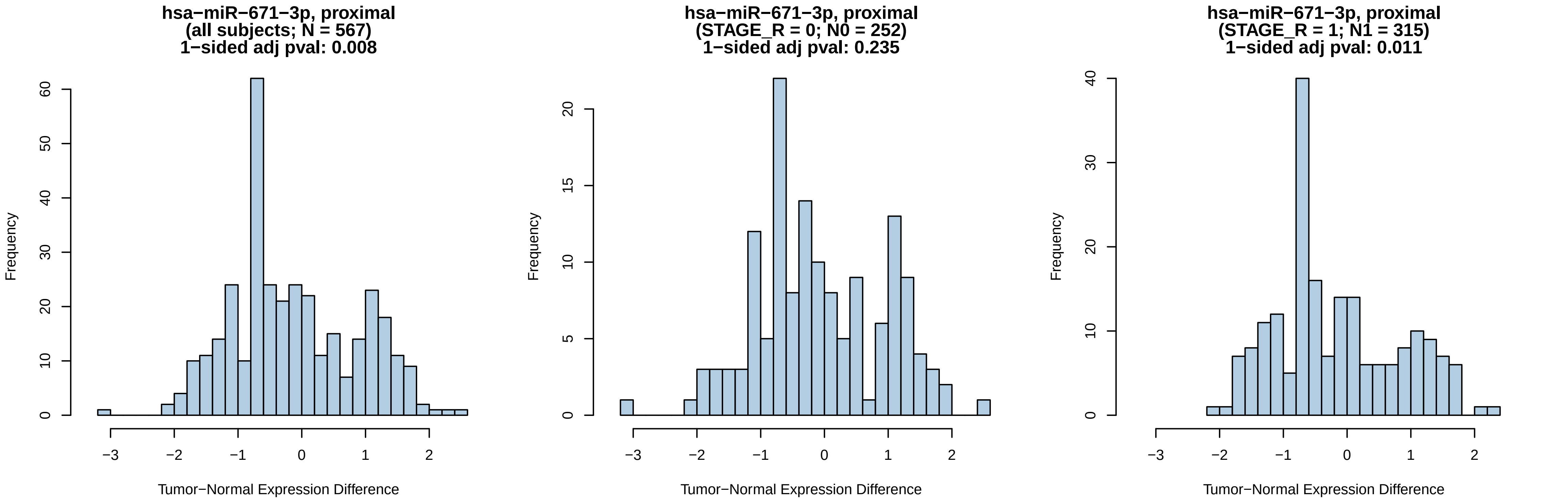
hsa−miR−671−3p, proximal
(all subjects; N = 567)
1−sided adj pval: 0.008
Frequency
Tumor−Normal Expression Difference
hsa−miR−671−3p, proximal
(STAGE_R = 0; N0 = 252)
1−sided adj pval: 0.235
Frequency
Tumor−Normal Expression Difference
hsa−miR−671−3p, proximal
(STAGE_R = 1; N1 = 315)
1−sided adj pval: 0.011
Frequency
Tumor−Normal Expression Difference

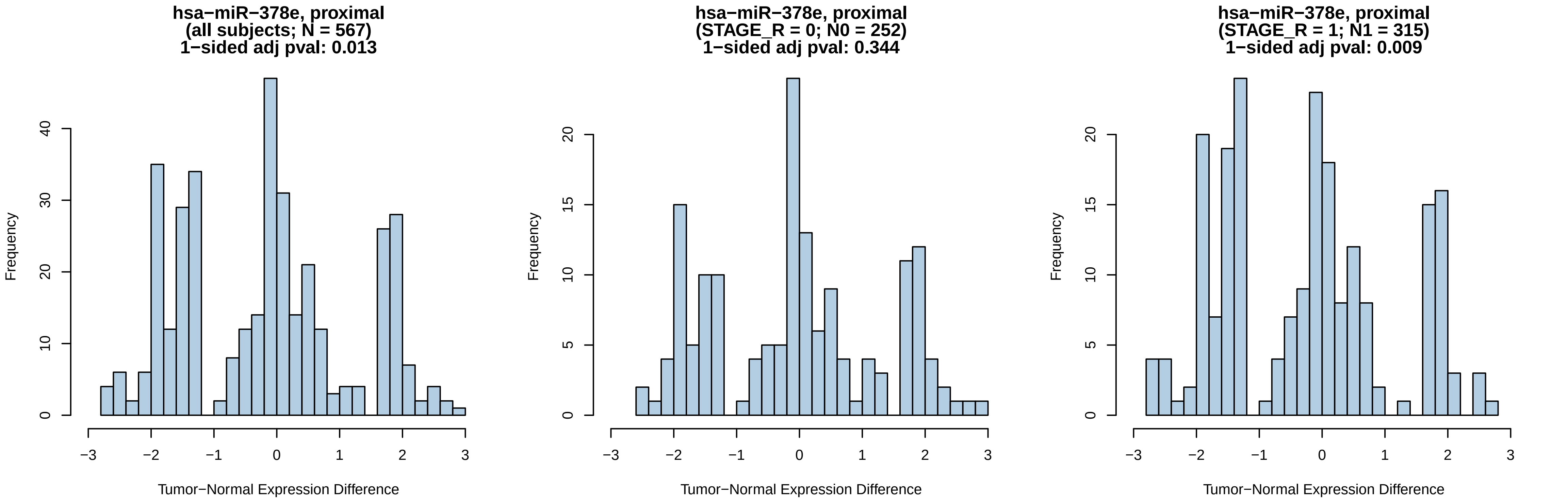
hsa−miR−378e, proximal
(all subjects; N = 567)
1−sided adj pval: 0.013
hsa−miR−378e, proximal
(STAGE_R = 0; N0 = 252)
1−sided adj pval: 0.344
hsa−miR−378e, proximal
(STAGE_R = 1; N1 = 315)
1−sided adj pval: 0.009
Frequency
Tumor−Normal Expression Difference

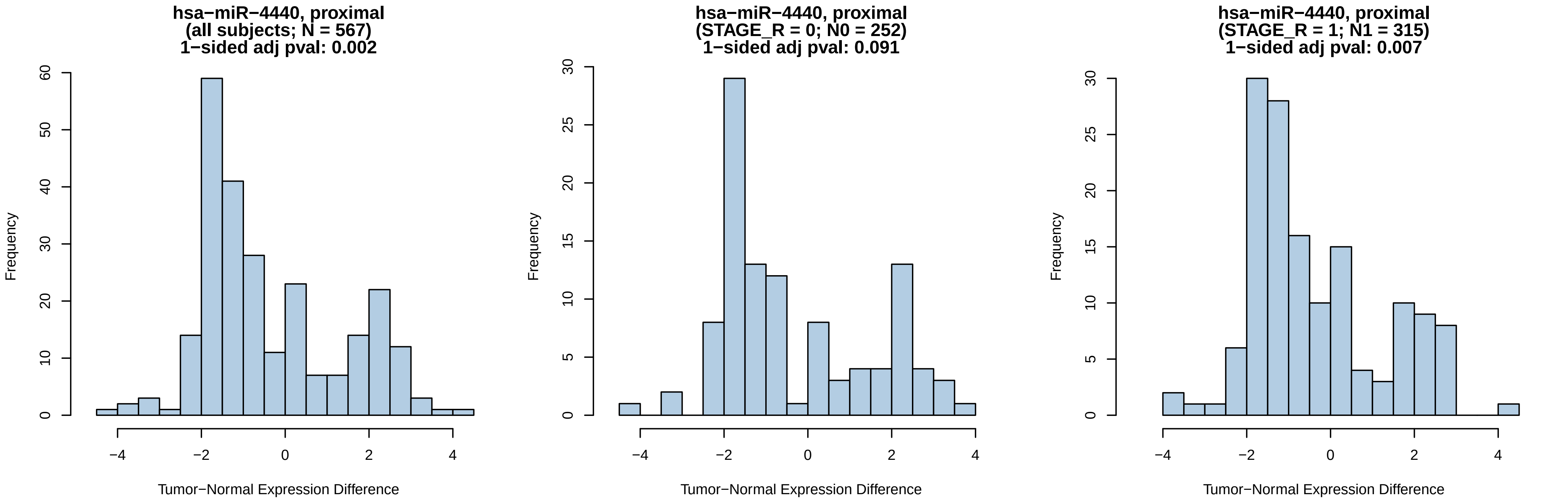
hsa−miR−4440, proximal
(all subjects; N = 567)
1−sided adj pval: 0.002
Frequency
Tumor−Normal Expression Difference
hsa−miR−4440, proximal
(STAGE_R = 0; N0 = 252)
1−sided adj pval: 0.091
Frequency
Tumor−Normal Expression Difference
hsa−miR−4440, proximal
(STAGE_R = 1; N1 = 315)
1−sided adj pval: 0.007
Frequency
Tumor−Normal Expression Difference

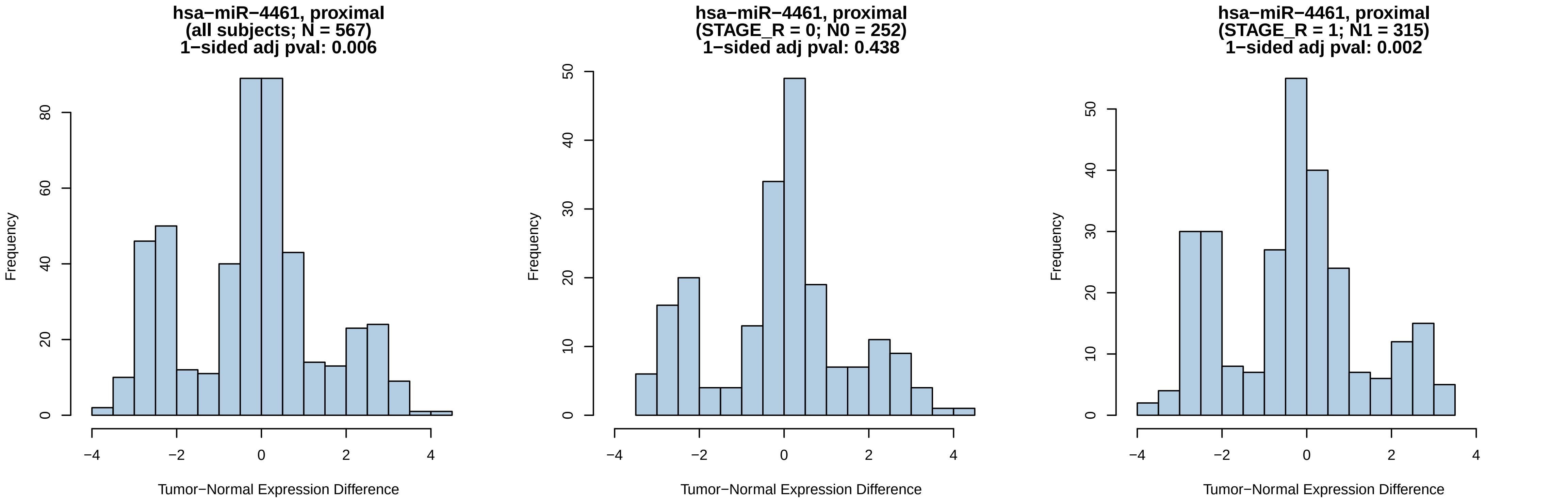

hsa−miR−4461, proximal
(all subjects; N = 567)
1−sided adj pval: 0.006
Frequency
Tumor−Normal Expression Difference
hsa−miR−4461, proximal
(STAGE_R = 0; N0 = 252)
1−sided adj pval: 0.438
Frequency
Tumor−Normal Expression Difference
hsa−miR−4461, proximal
(STAGE_R = 1; N1 = 315)
1−sided adj pval: 0.002
Frequency
Tumor−Normal Expression Difference

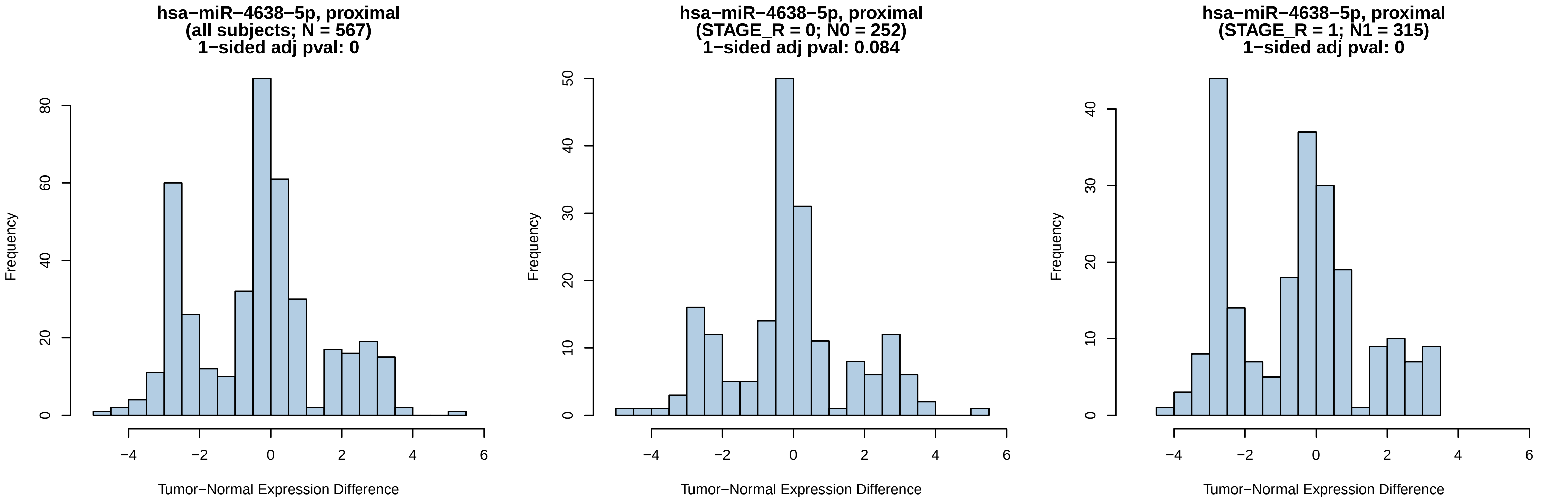
hsa−miR−4638−5p, proximal
(all subjects; N = 567)
1−sided adj pval: 0
Frequency
Tumor−Normal Expression Difference
hsa−miR−4638−5p, proximal
(STAGE_R = 0; N0 = 252)
1−sided adj pval: 0.084
Frequency
Tumor−Normal Expression Difference
hsa−miR−4638−5p, proximal
(STAGE_R = 1; N1 = 315)
1−sided adj pval: 0
Frequency
Tumor−Normal Expression Difference

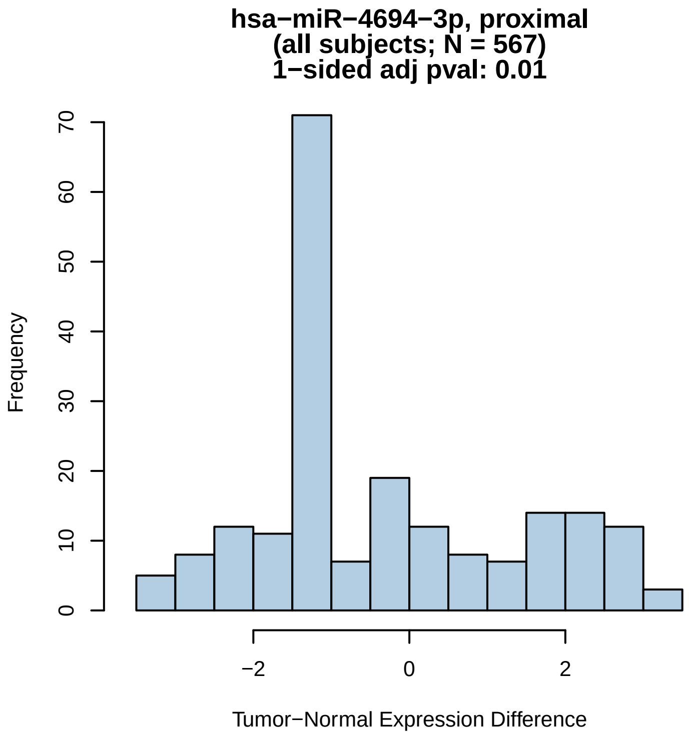
hsa−miR−4694−3p, proximal
(all subjects; N = 567)
1−sided adj pval: 0.01
Frequency
Tumor−Normal Expression Difference

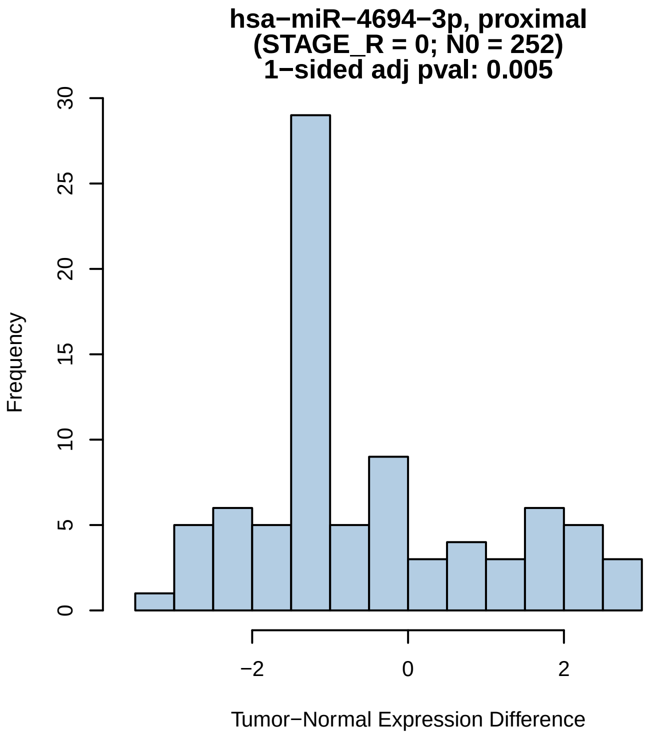
hsa−miR−4694−3p, proximal
(STAGE_R = 0; N0 = 252)
1−sided adj pval: 0.005
Frequency
Tumor−Normal Expression Difference

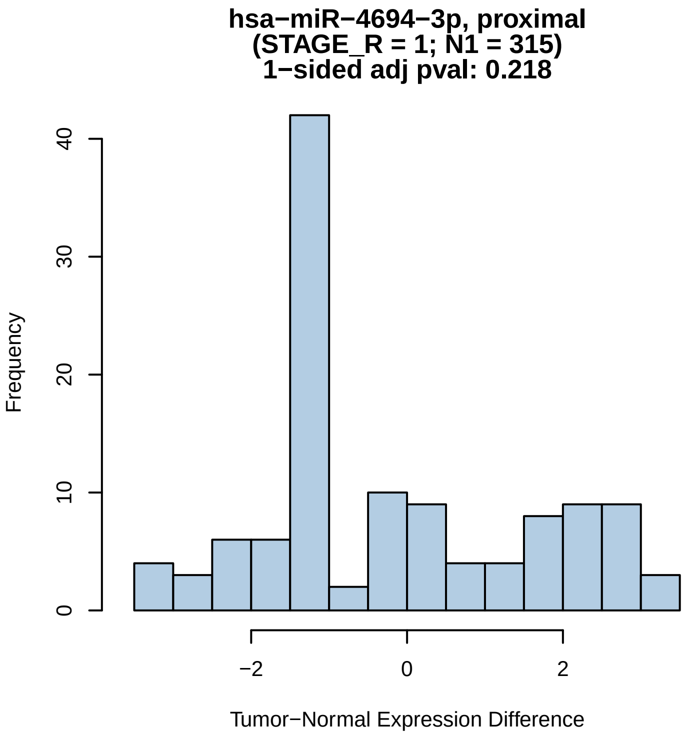
hsa−miR−4694−3p, proximal
(STAGE_R = 1; N1 = 315)
1−sided adj pval: 0.218
Frequency
Tumor−Normal Expression Difference

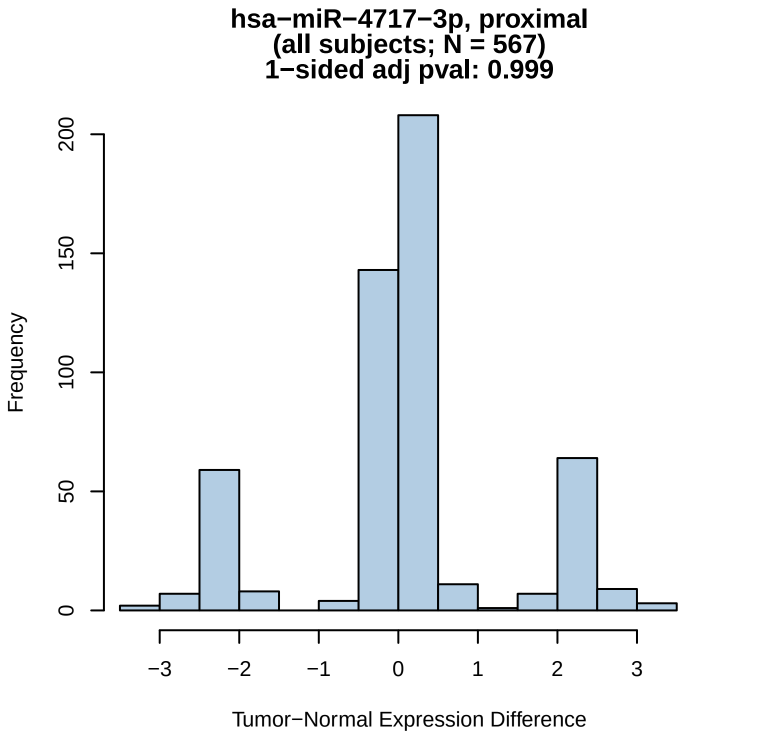
hsa−miR−4717−3p, proximal
(all subjects; N = 567)
1−sided adj pval: 0.999
Frequency
Tumor−Normal Expression Difference

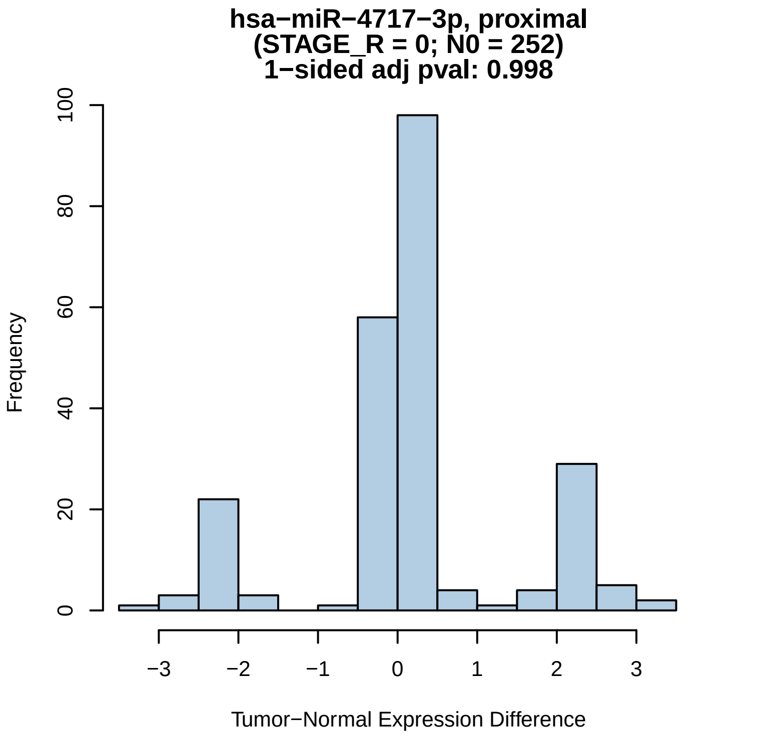
hsa−miR−4717−3p, proximal
(STAGE_R = 0; N0 = 252)
1−sided adj pval: 0.998
Frequency
Tumor−Normal Expression Difference

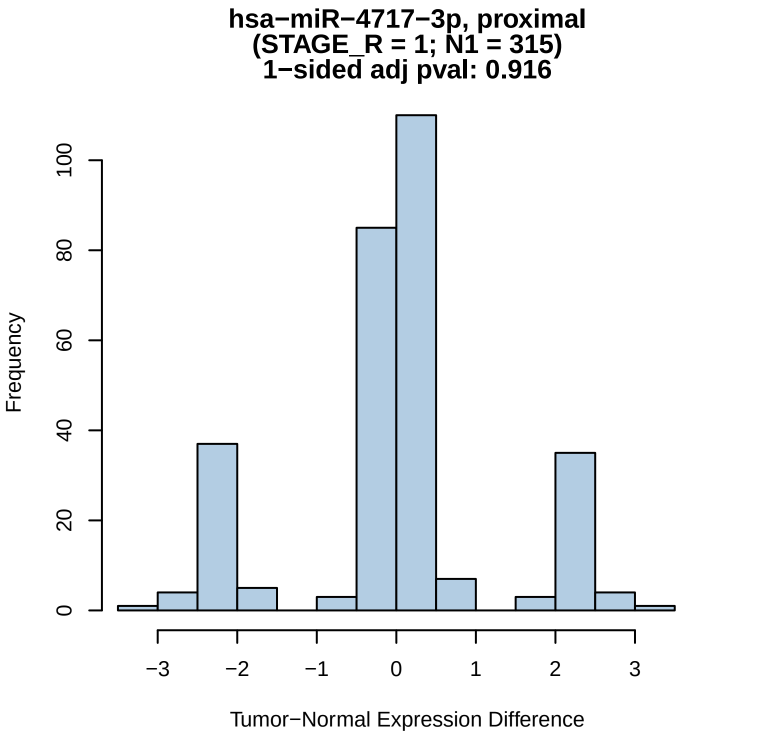
hsa−miR−4717−3p, proximal
(STAGE_R = 1; N1 = 315)
1−sided adj pval: 0.916
Frequency
Tumor−Normal Expression Difference

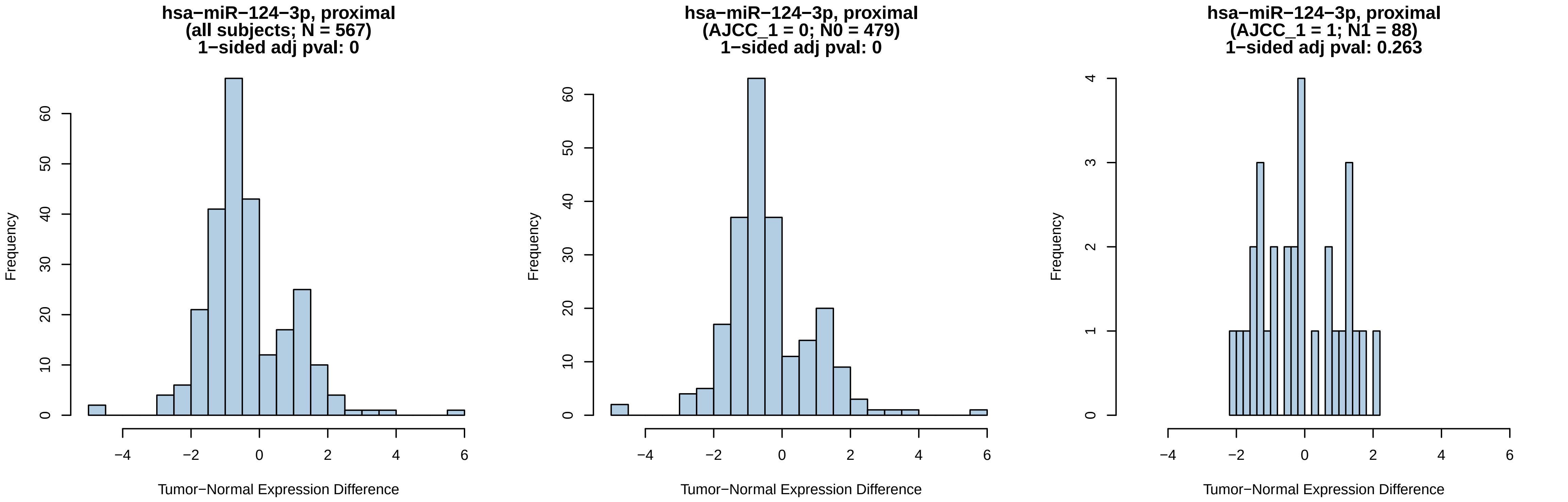

hsa−miR−124−3p, proximal
(all subjects; N = 567)
1−sided adj pval: 0
hsa−miR−124−3p, proximal
(AJCC_1 = 0; N0 = 479)
1−sided adj pval: 0
hsa−miR−124−3p, proximal
(AJCC_1 = 1; N1 = 88)
1−sided adj pval: 0.263
Frequency
Tumor−Normal Expression Difference

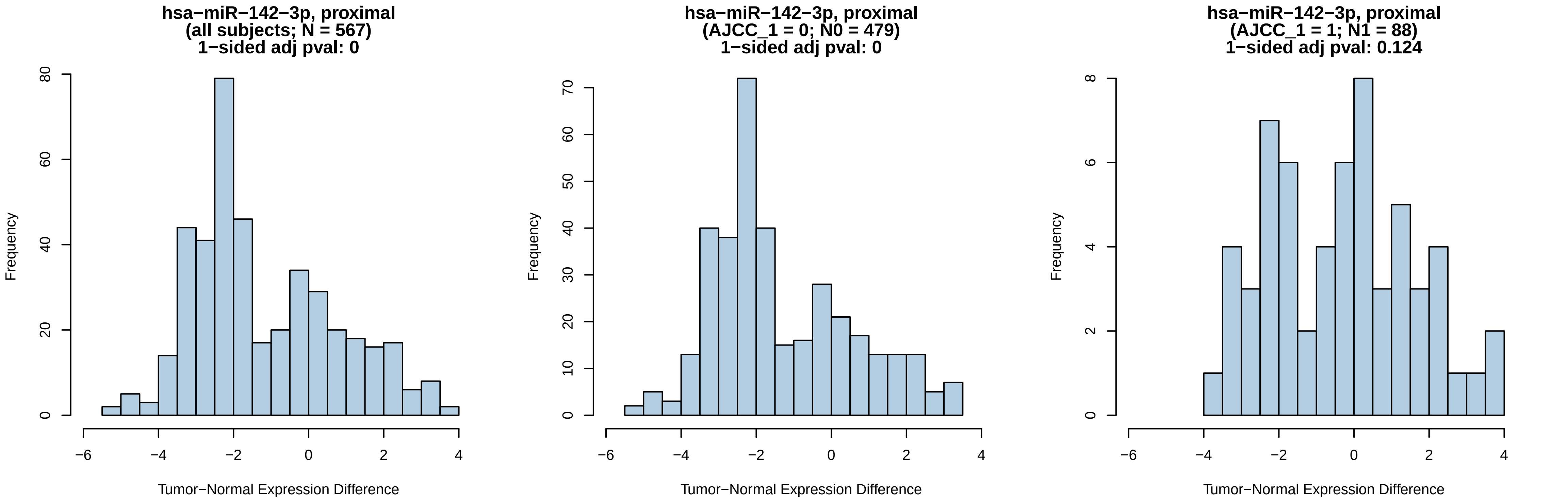
hsa−miR−142−3p, proximal
(all subjects; N = 567)
1−sided adj pval: 0
Frequency
Tumor−Normal Expression Difference
hsa−miR−142−3p, proximal
(AJCC_1 = 0; N0 = 479)
1−sided adj pval: 0
Frequency
Tumor−Normal Expression Difference
hsa−miR−142−3p, proximal
(AJCC_1 = 1; N1 = 88)
1−sided adj pval: 0.124
Frequency
Tumor−Normal Expression Difference

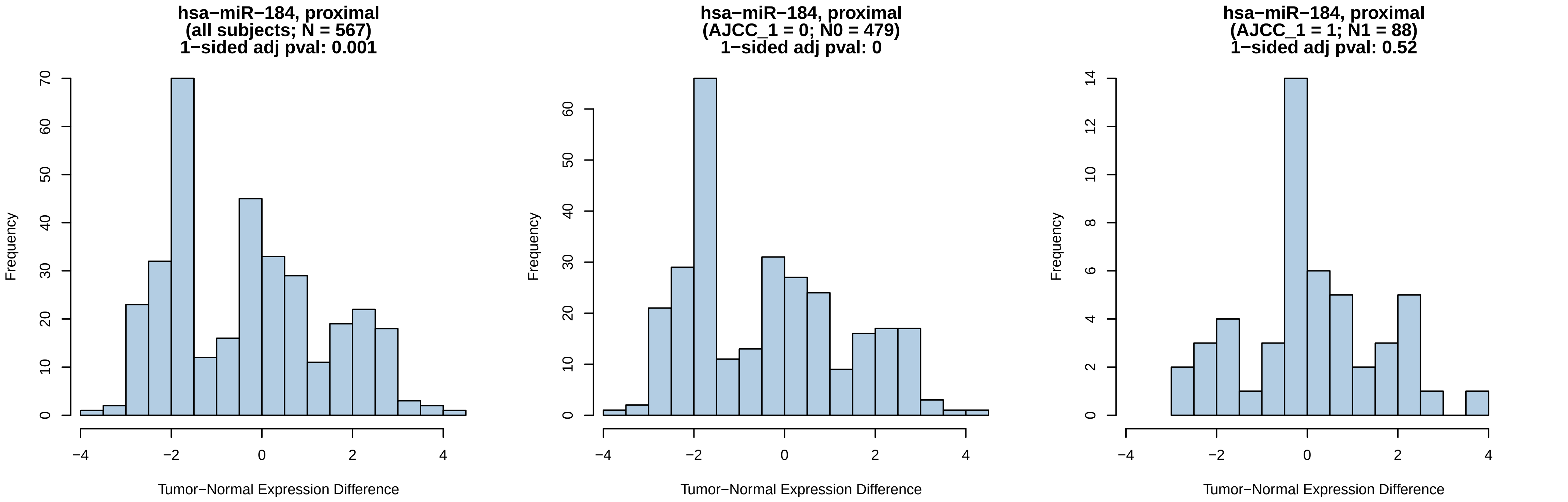
hsa−miR−184, proximal
(all subjects; N = 567)
1−sided adj pval: 0.001
Frequency
Tumor−Normal Expression Difference
hsa−miR−184, proximal
(AJCC_1 = 0; N0 = 479)
1−sided adj pval: 0
Frequency
Tumor−Normal Expression Difference
hsa−miR−184, proximal
(AJCC_1 = 1; N1 = 88)
1−sided adj pval: 0.52
Frequency
Tumor−Normal Expression Difference

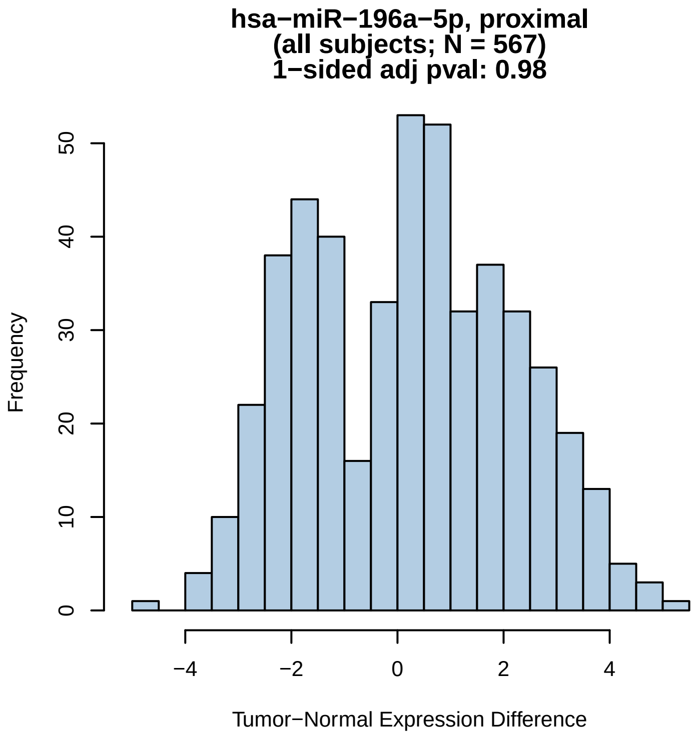
hsa−miR−196a−5p, proximal
(all subjects; N = 567)
1−sided adj pval: 0.98
Frequency
0
10
20
30
40
50
−4
−2
0
2
4
Tumor−Normal Expression Difference

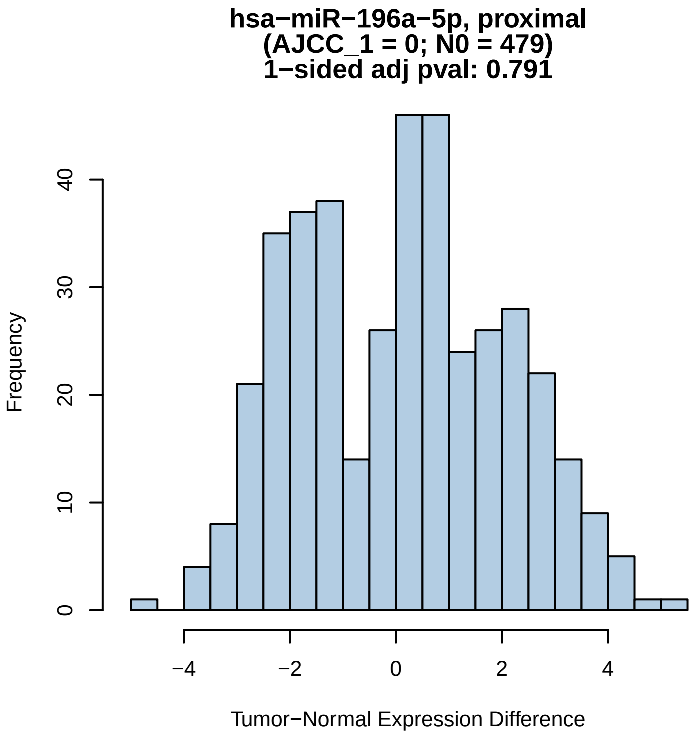
hsa−miR−196a−5p, proximal
(AJCC_1 = 0; N0 = 479)
1−sided adj pval: 0.791
Frequency
0
10
20
30
40
−4
−2
0
2
4
Tumor−Normal Expression Difference

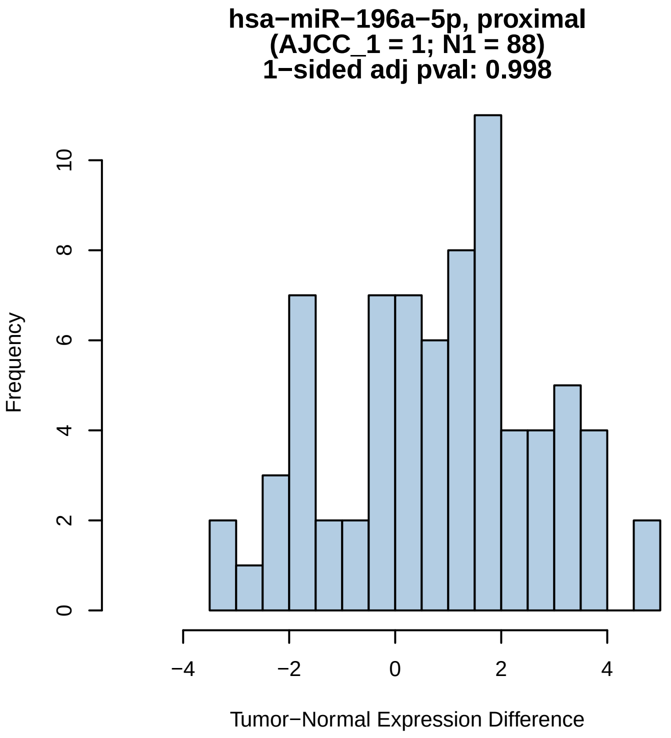
hsa−miR−196a−5p, proximal
(AJCC_1 = 1; N1 = 88)
1−sided adj pval: 0.998
Frequency
0
2
4
6
8
10
−4
−2
0
2
4
Tumor−Normal Expression Difference

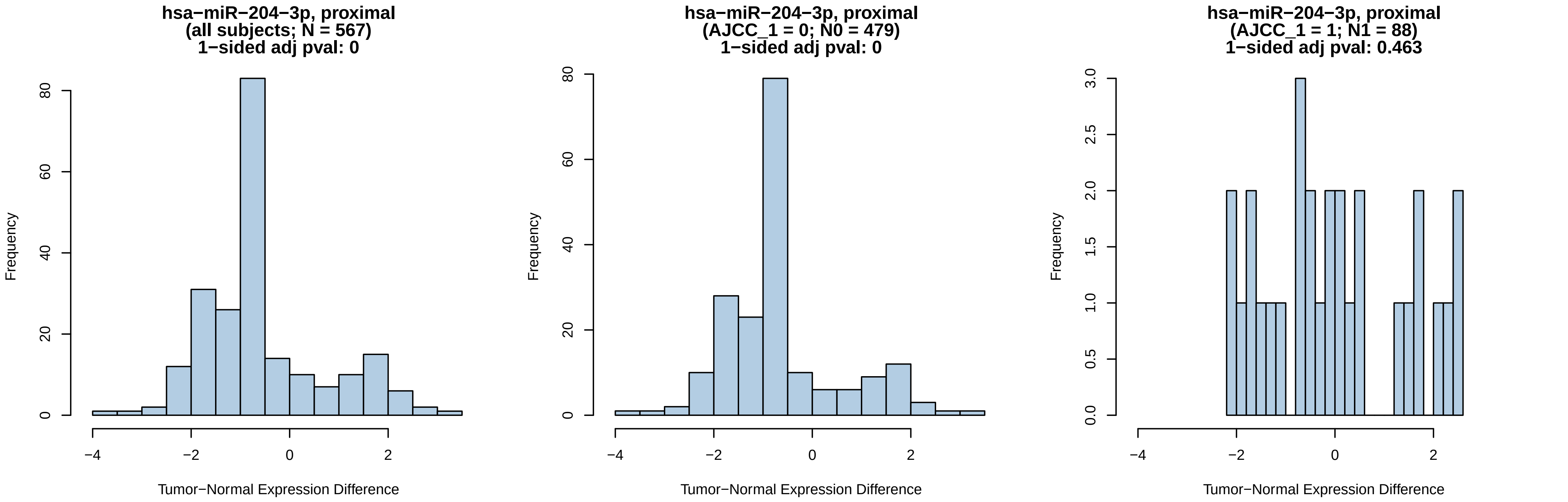
hsa−miR−204−3p, proximal
(all subjects; N = 567)
1−sided adj pval: 0
hsa−miR−204−3p, proximal
(AJCC_1 = 0; N0 = 479)
1−sided adj pval: 0
hsa−miR−204−3p, proximal
(AJCC_1 = 1; N1 = 88)
1−sided adj pval: 0.463
Frequency
Tumor−Normal Expression Difference

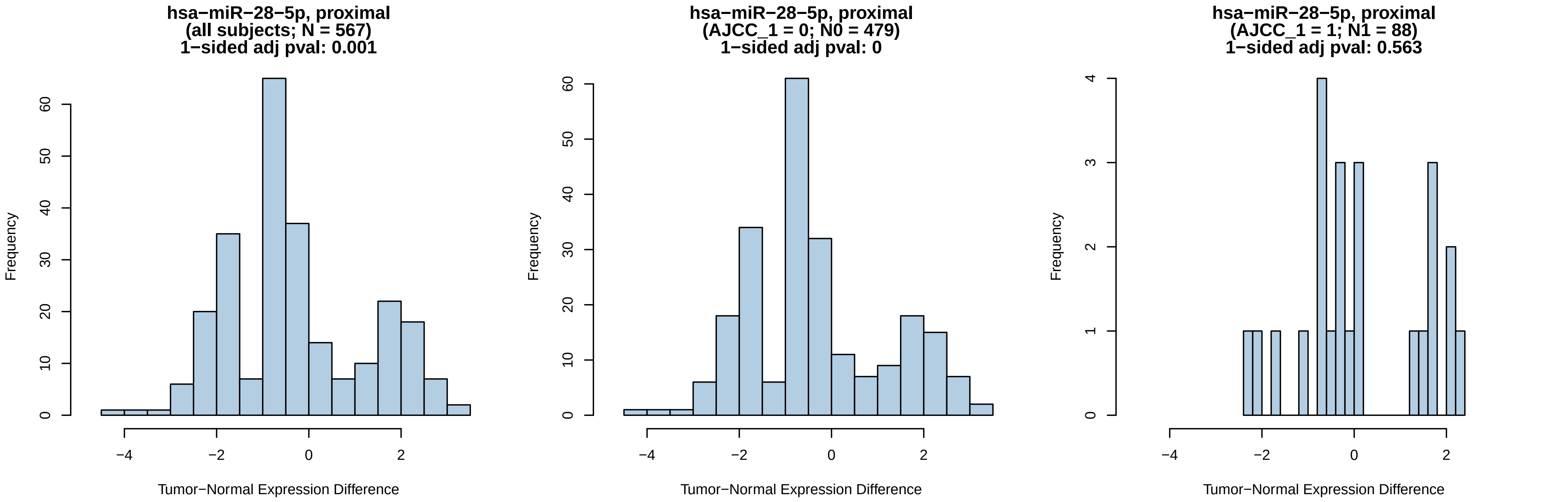
hsa−miR−28−5p, proximal
(all subjects; N = 567)
1−sided adj pval: 0.001
Frequency
Tumor−Normal Expression Difference
hsa−miR−28−5p, proximal
(AJCC_1 = 0; N0 = 479)
1−sided adj pval: 0
Frequency
Tumor−Normal Expression Difference
hsa−miR−28−5p, proximal
(AJCC_1 = 1; N1 = 88)
1−sided adj pval: 0.563
Frequency
Tumor−Normal Expression Difference

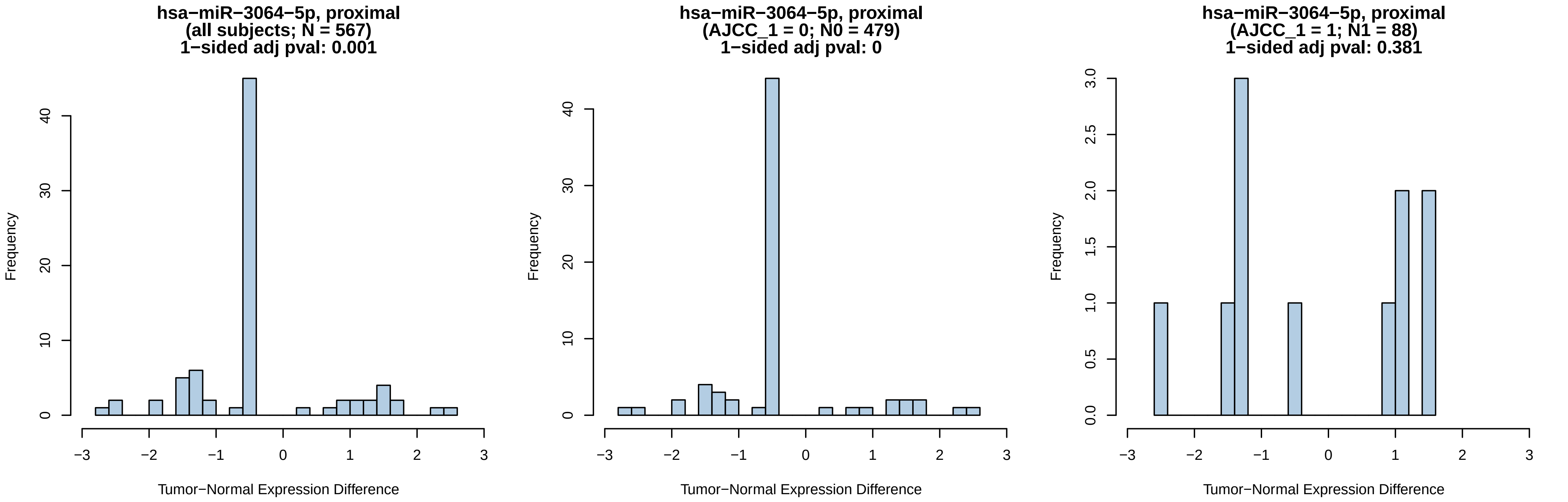
hsa−miR−3064−5p, proximal
(all subjects; N = 567)
1−sided adj pval: 0.001
hsa−miR−3064−5p, proximal
(AJCC_1 = 0; N0 = 479)
1−sided adj pval: 0
hsa−miR−3064−5p, proximal
(AJCC_1 = 1; N1 = 88)
1−sided adj pval: 0.381
Frequency
Tumor−Normal Expression Difference

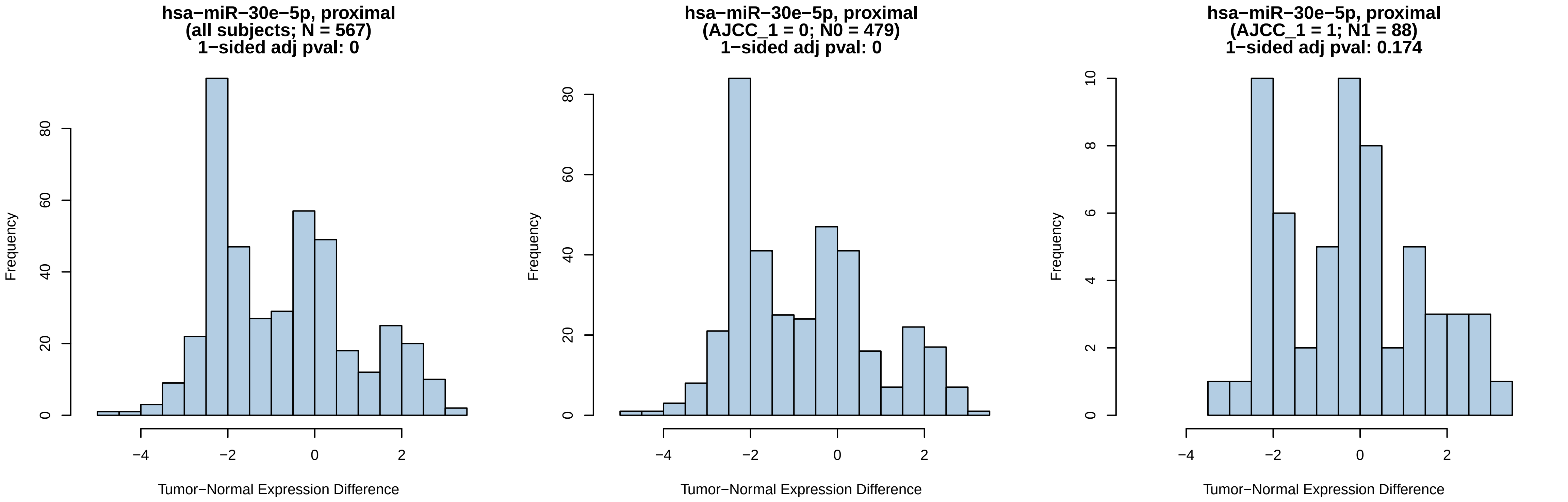

hsa−miR−30e−5p, proximal
(all subjects; N = 567)
1−sided adj pval: 0
hsa−miR−30e−5p, proximal
(AJCC_1 = 0; N0 = 479)
1−sided adj pval: 0
hsa−miR−30e−5p, proximal
(AJCC_1 = 1; N1 = 88)
1−sided adj pval: 0.174
Frequency
Tumor−Normal Expression Difference

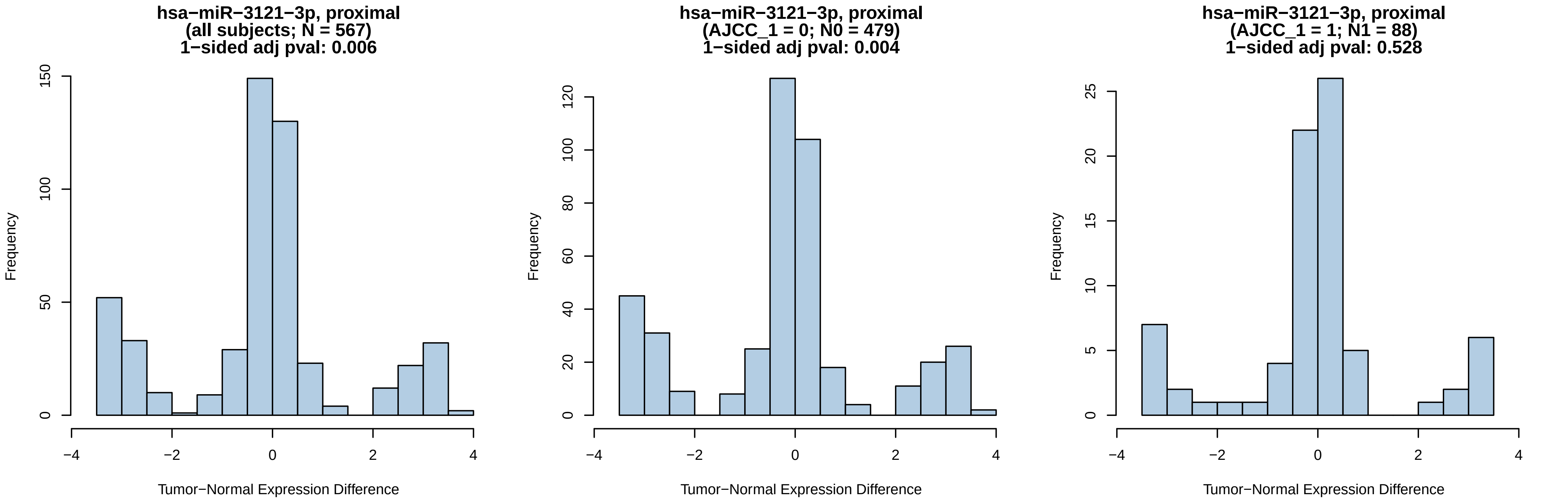
hsa−miR−3121−3p, proximal
(all subjects; N = 567)
1−sided adj pval: 0.006
Frequency
Tumor−Normal Expression Difference
hsa−miR−3121−3p, proximal
(AJCC_1 = 0; N0 = 479)
1−sided adj pval: 0.004
Frequency
Tumor−Normal Expression Difference
hsa−miR−3121−3p, proximal
(AJCC_1 = 1; N1 = 88)
1−sided adj pval: 0.528
Frequency
Tumor−Normal Expression Difference

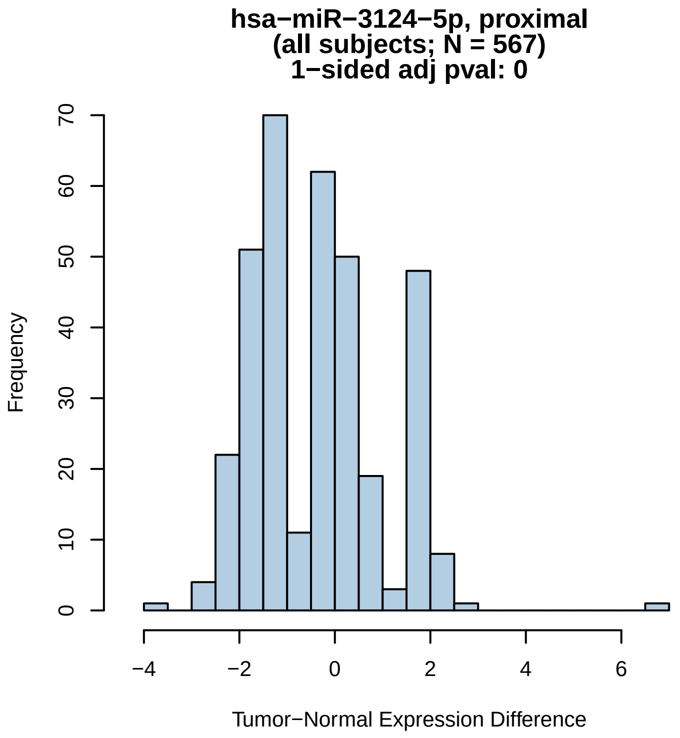
hsa−miR−3124−5p, proximal
(all subjects; N = 567)
1−sided adj pval: 0
Frequency
Tumor−Normal Expression Difference

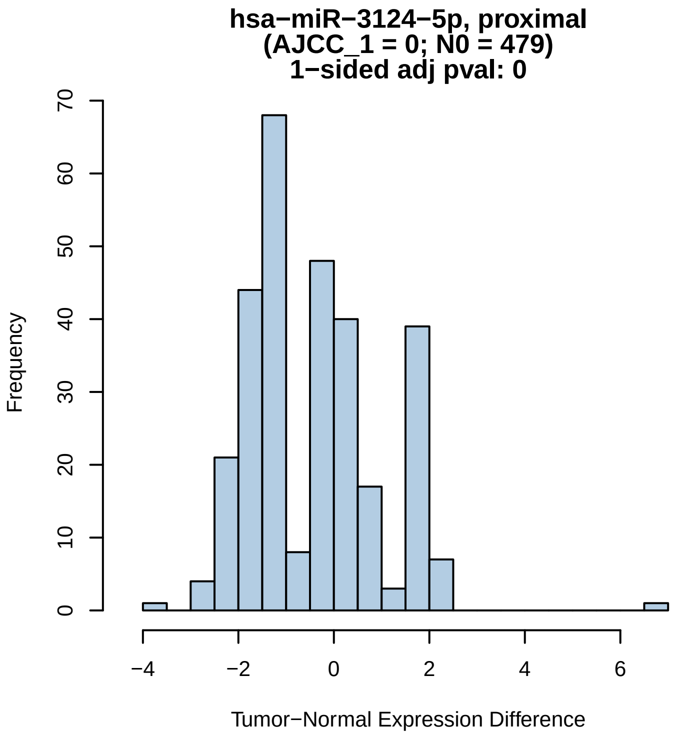
hsa−miR−3124−5p, proximal
(AJCC_1 = 0; N0 = 479)
1−sided adj pval: 0
Frequency
Tumor−Normal Expression Difference

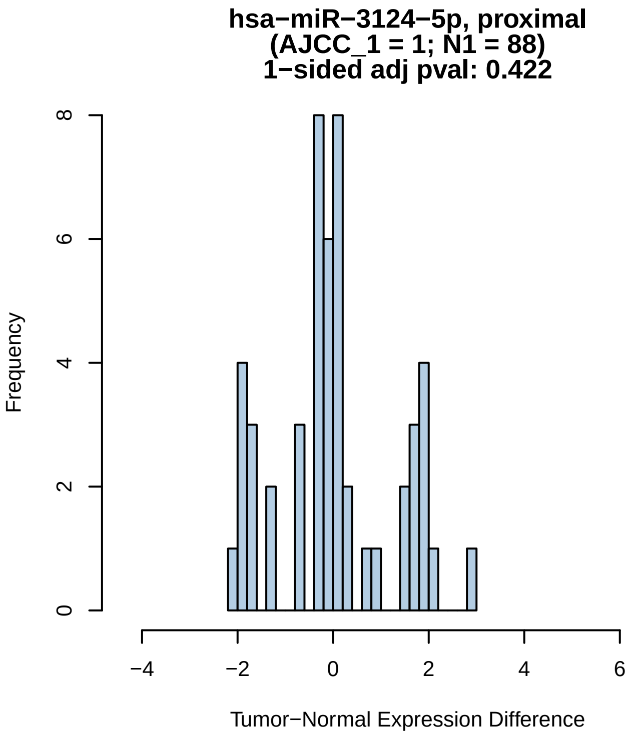
hsa−miR−3124−5p, proximal
(AJCC_1 = 1; N1 = 88)
1−sided adj pval: 0.422
Frequency
Tumor−Normal Expression Difference

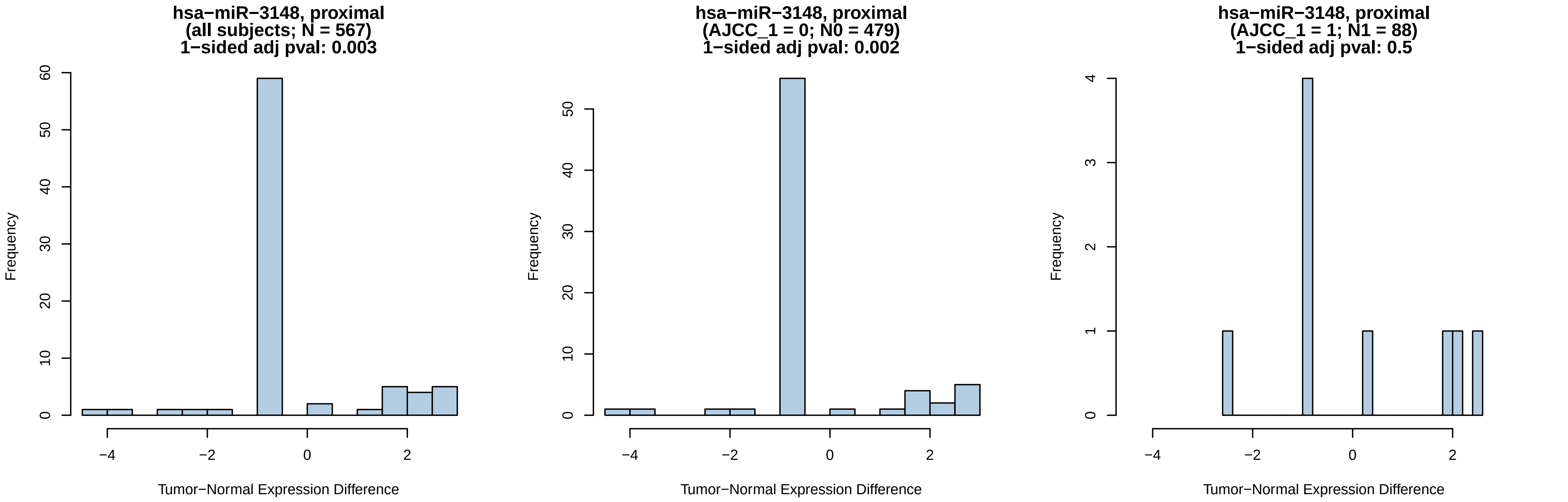
hsa−miR−3148, proximal
(all subjects; N = 567)
1−sided adj pval: 0.003
hsa−miR−3148, proximal
(AJCC_1 = 0; N0 = 479)
1−sided adj pval: 0.002
hsa−miR−3148, proximal
(AJCC_1 = 1; N1 = 88)
1−sided adj pval: 0.5
Frequency
Tumor−Normal Expression Difference

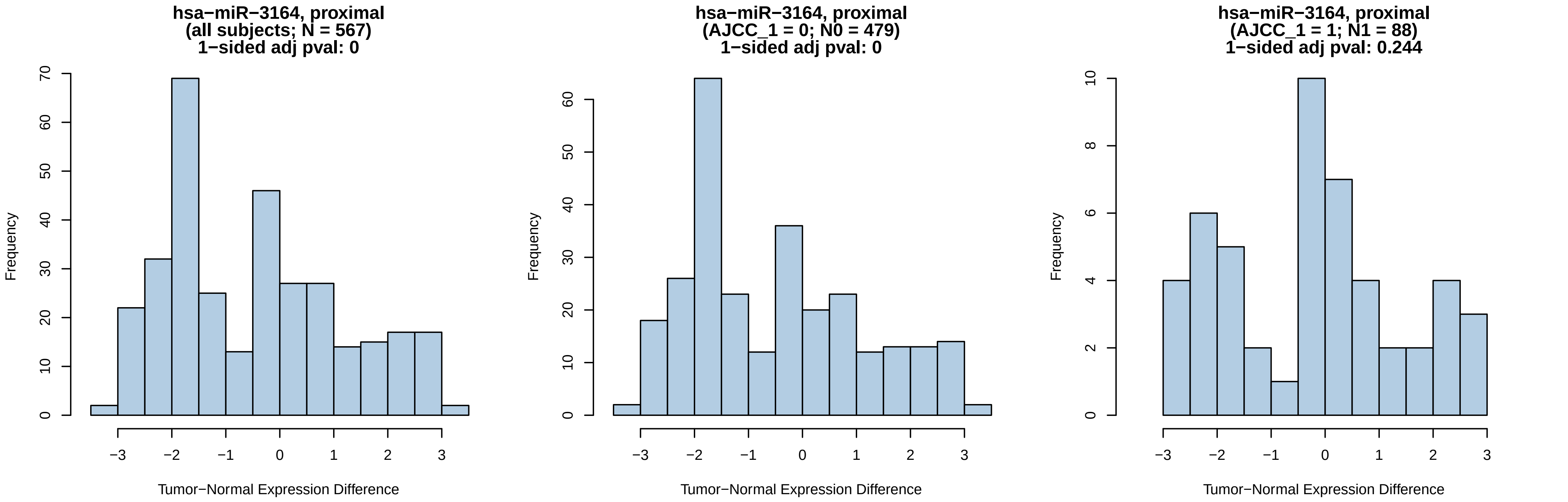
hsa−miR−3164, proximal
(all subjects; N = 567)
1−sided adj pval: 0
Frequency
Tumor−Normal Expression Difference
hsa−miR−3164, proximal
(AJCC_1 = 0; N0 = 479)
1−sided adj pval: 0
Frequency
Tumor−Normal Expression Difference
hsa−miR−3164, proximal
(AJCC_1 = 1; N1 = 88)
1−sided adj pval: 0.244
Frequency
Tumor−Normal Expression Difference

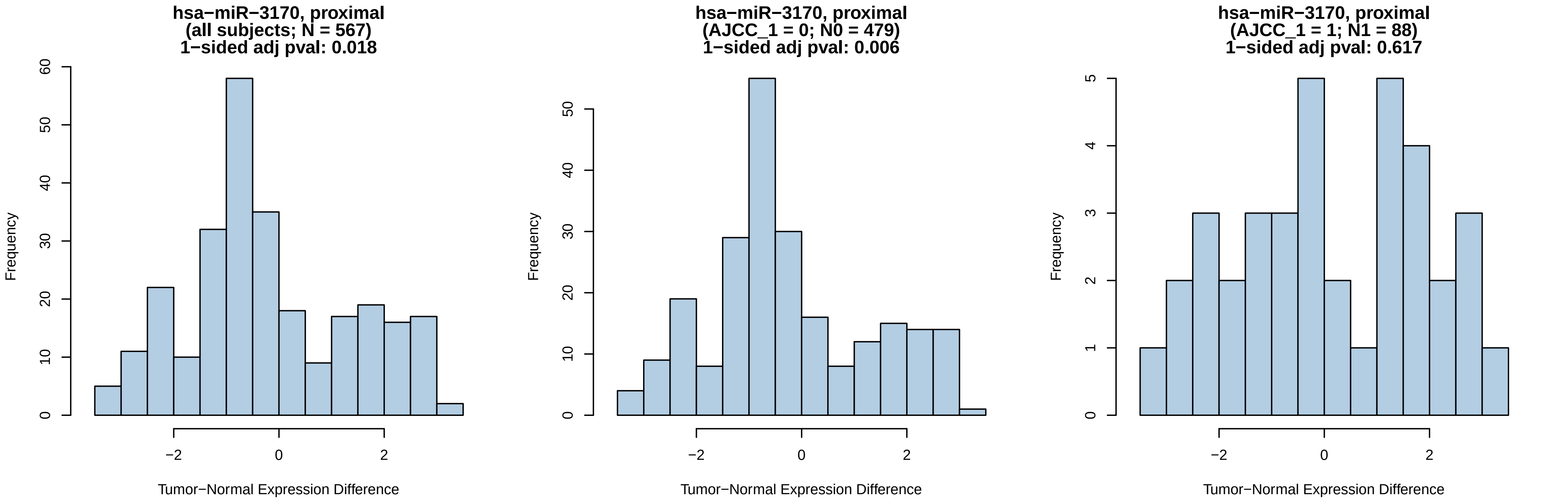
hsa−miR−3170, proximal
(all subjects; N = 567)
1−sided adj pval: 0.018
Frequency
Tumor−Normal Expression Difference
hsa−miR−3170, proximal
(AJCC_1 = 0; N0 = 479)
1−sided adj pval: 0.006
Frequency
Tumor−Normal Expression Difference
hsa−miR−3170, proximal
(AJCC_1 = 1; N1 = 88)
1−sided adj pval: 0.617
Frequency
Tumor−Normal Expression Difference

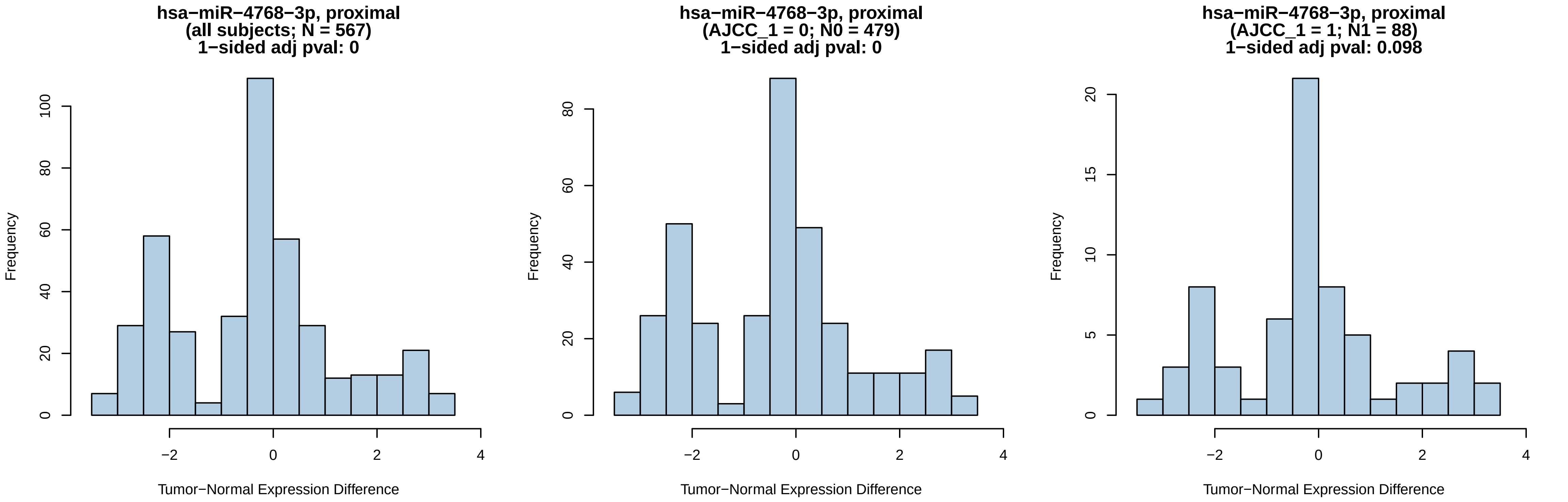
hsa−miR−4768−3p, proximal
(all subjects; N = 567)
1−sided adj pval: 0
Frequency
Tumor−Normal Expression Difference
hsa−miR−4768−3p, proximal
(AJCC_1 = 0; N0 = 479)
1−sided adj pval: 0
Frequency
Tumor−Normal Expression Difference
hsa−miR−4768−3p, proximal
(AJCC_1 = 1; N1 = 88)
1−sided adj pval: 0.098
Frequency
Tumor−Normal Expression Difference

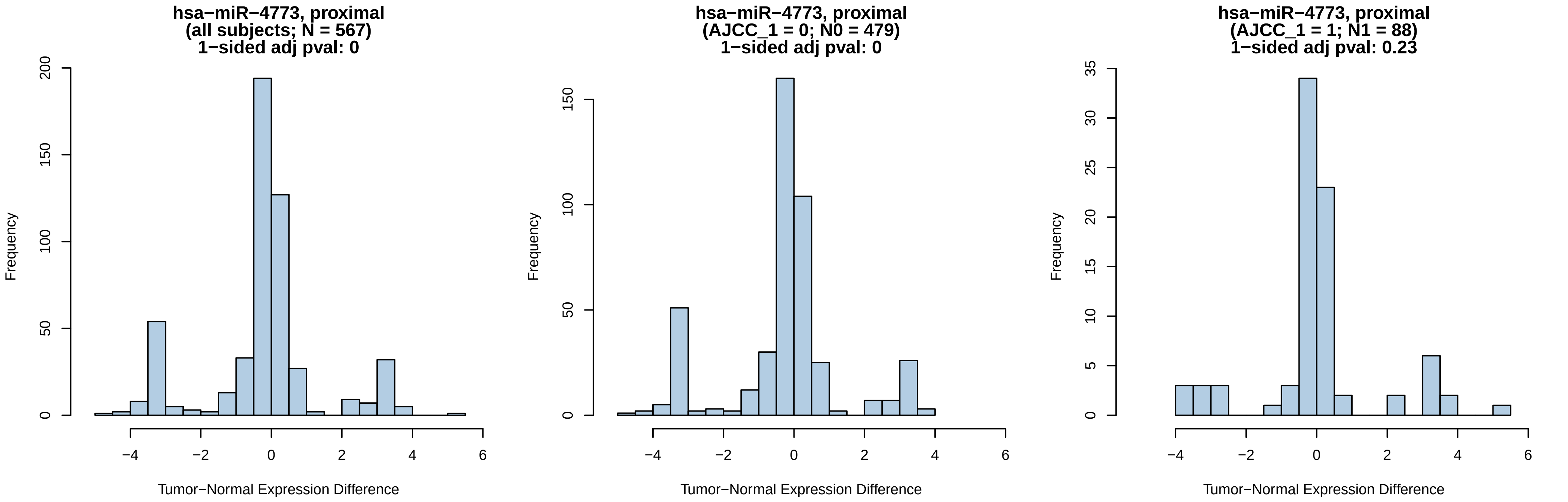
hsa−miR−4773, proximal
(all subjects; N = 567)
1−sided adj pval: 0
Frequency
Tumor−Normal Expression Difference
hsa−miR−4773, proximal
(AJCC_1 = 0; N0 = 479)
1−sided adj pval: 0
Frequency
Tumor−Normal Expression Difference
hsa−miR−4773, proximal
(AJCC_1 = 1; N1 = 88)
1−sided adj pval: 0.23
Frequency
Tumor−Normal Expression Difference

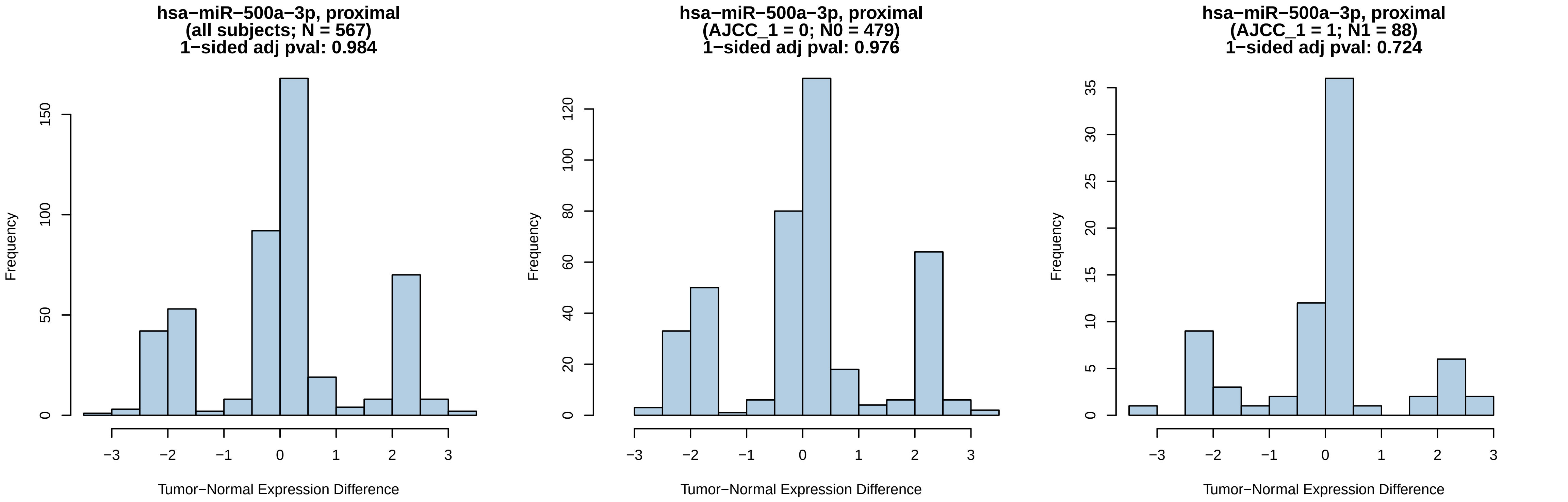

hsa−miR−500a−3p, proximal
(all subjects; N = 567)
1−sided adj pval: 0.984
Frequency
Tumor−Normal Expression Difference
hsa−miR−500a−3p, proximal
(AJCC_1 = 0; N0 = 479)
1−sided adj pval: 0.976
Frequency
Tumor−Normal Expression Difference
hsa−miR−500a−3p, proximal
(AJCC_1 = 1; N1 = 88)
1−sided adj pval: 0.724
Frequency
Tumor−Normal Expression Difference

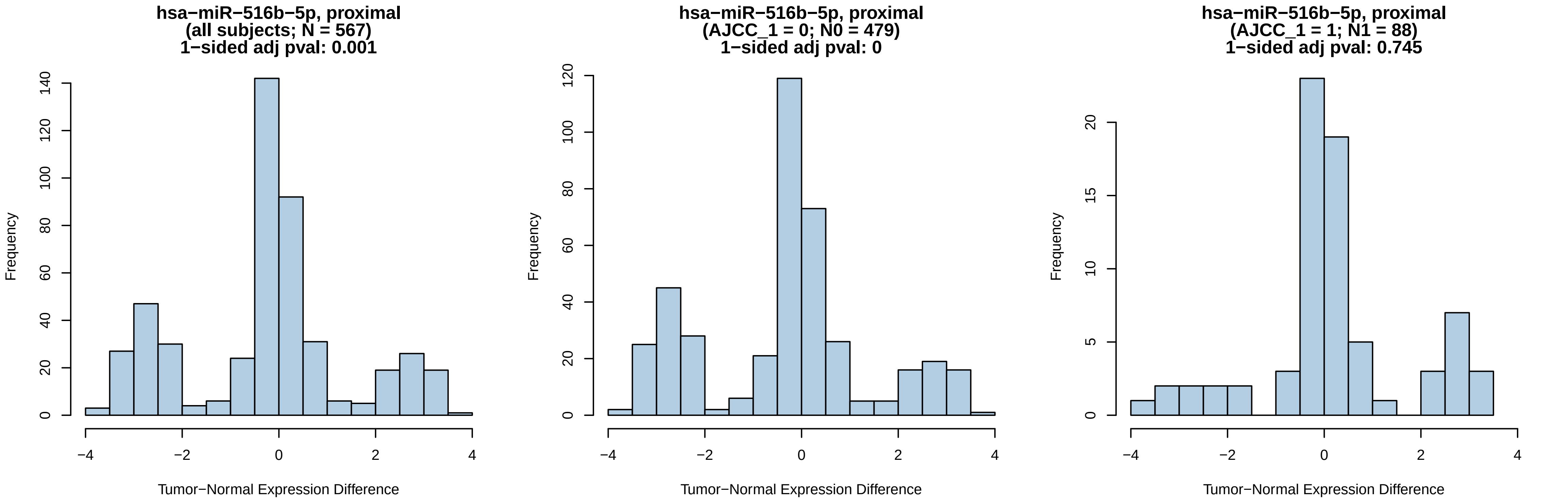
hsa−miR−516b−5p, proximal
(all subjects; N = 567)
1−sided adj pval: 0.001
hsa−miR−516b−5p, proximal
(AJCC_1 = 0; N0 = 479)
1−sided adj pval: 0
hsa−miR−516b−5p, proximal
(AJCC_1 = 1; N1 = 88)
1−sided adj pval: 0.745
Frequency
Tumor−Normal Expression Difference

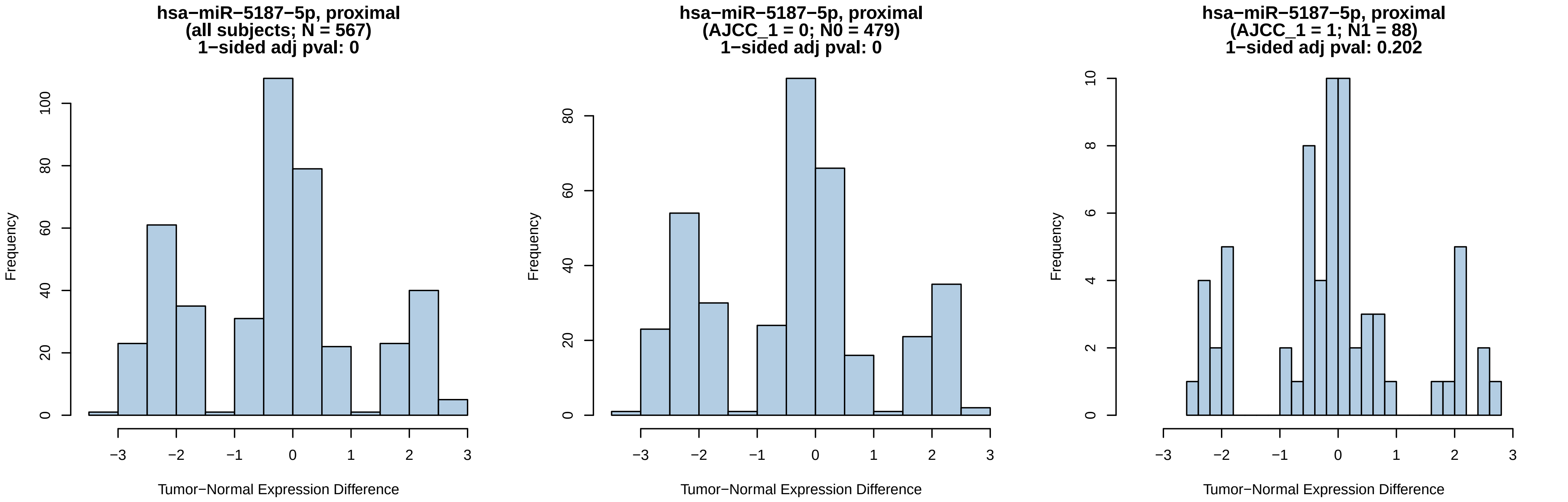
hsa−miR−5187−5p, proximal
(all subjects; N = 567)
1−sided adj pval: 0
hsa−miR−5187−5p, proximal
(AJCC_1 = 0; N0 = 479)
1−sided adj pval: 0
hsa−miR−5187−5p, proximal
(AJCC_1 = 1; N1 = 88)
1−sided adj pval: 0.202
Frequency
Tumor−Normal Expression Difference
0
20
40
60
80
100
0
2
4
6
8
10
−3
−2
−1
0
1
2
3

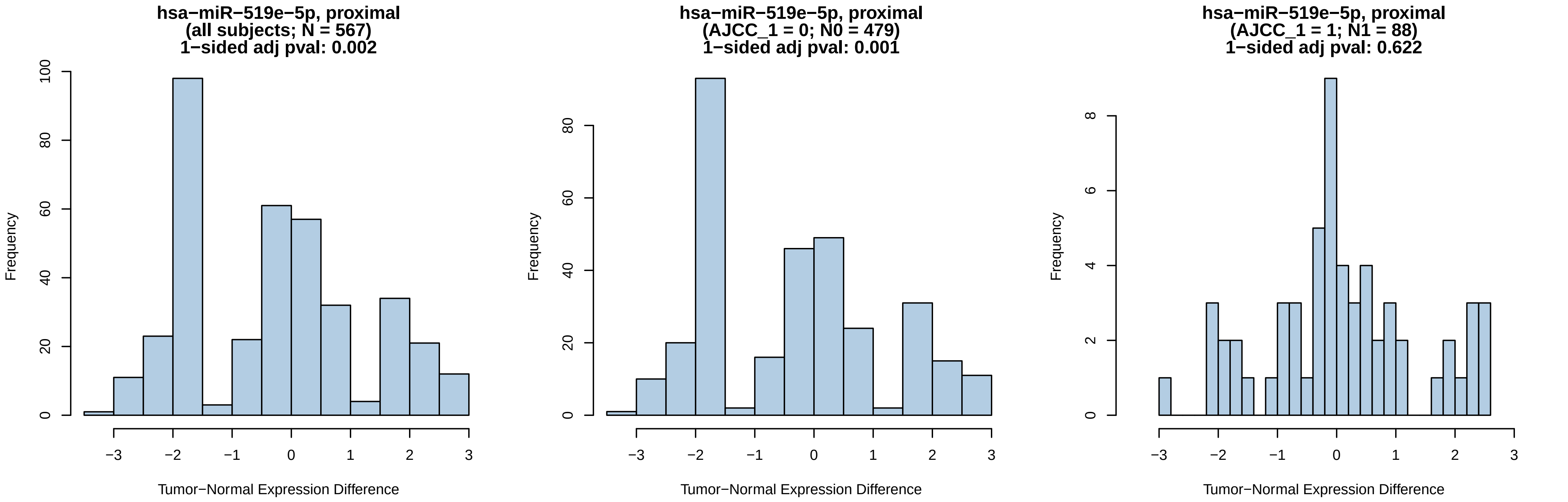
hsa−miR−519e−5p, proximal
(all subjects; N = 567)
1−sided adj pval: 0.002
Frequency
Tumor−Normal Expression Difference
0
20
40
60
80
100
−3
−2
−1
0
1
2
3
hsa−miR−519e−5p, proximal
(AJCC_1 = 0; N0 = 479)
1−sided adj pval: 0.001
Frequency
Tumor−Normal Expression Difference
0
20
40
60
80
hsa−miR−519e−5p, proximal
(AJCC_1 = 1; N1 = 88)
1−sided adj pval: 0.622
Frequency
Tumor−Normal Expression Difference
0
2
4
6
8

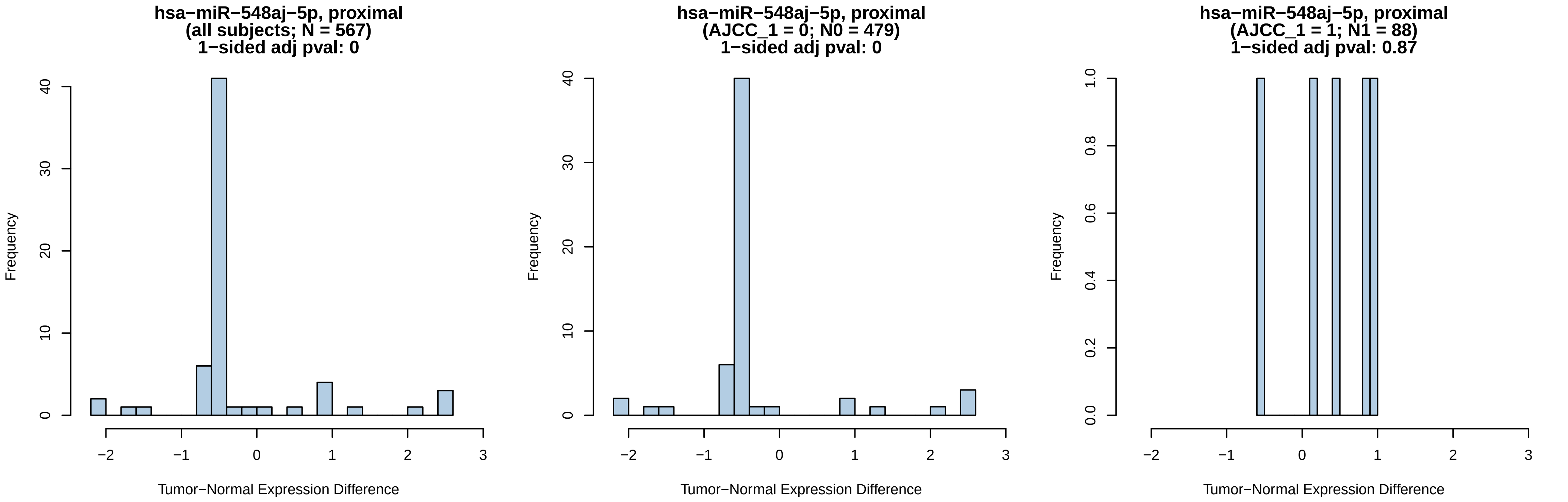
hsa−miR−548aj−5p, proximal
(all subjects; N = 567)
1−sided adj pval: 0
Frequency
Tumor−Normal Expression Difference
hsa−miR−548aj−5p, proximal
(AJCC_1 = 0; N0 = 479)
1−sided adj pval: 0
Frequency
Tumor−Normal Expression Difference
hsa−miR−548aj−5p, proximal
(AJCC_1 = 1; N1 = 88)
1−sided adj pval: 0.87
Frequency
Tumor−Normal Expression Difference

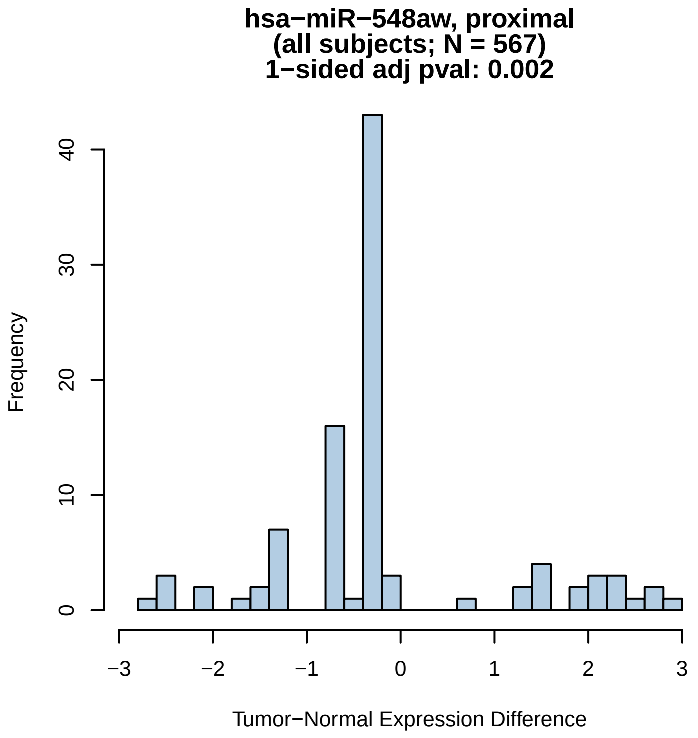

hsa−miR−548aw, proximal
(all subjects; N = 567)
1−sided adj pval: 0.002
Frequency
Tumor−Normal Expression Difference


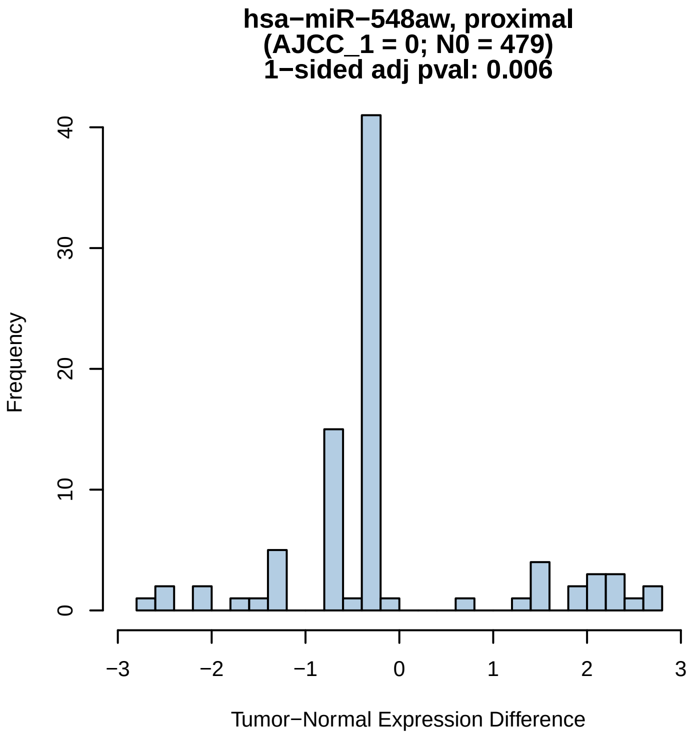

hsa−miR−548aw, proximal
(AJCC_1 = 0; N0 = 479)
1−sided adj pval: 0.006
Frequency
Tumor−Normal Expression Difference


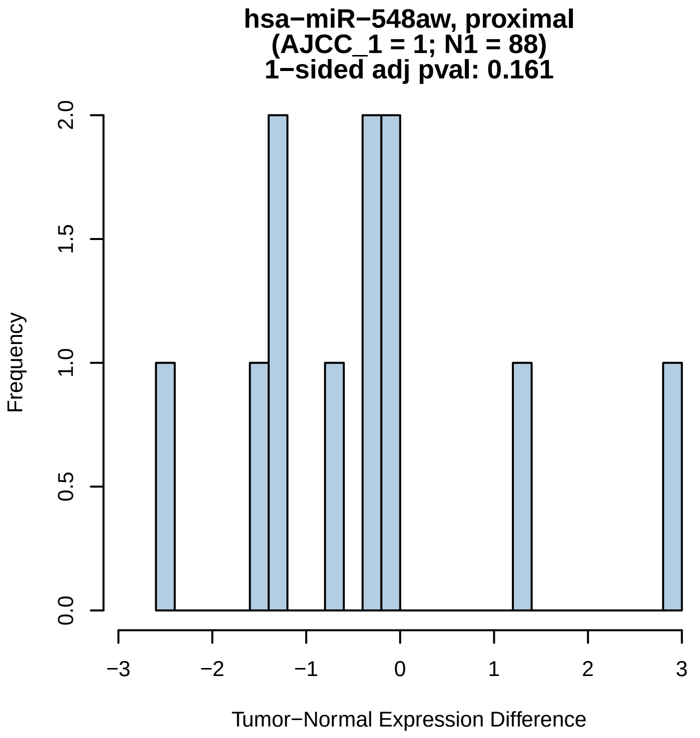

hsa−miR−548aw, proximal
(AJCC_1 = 1; N1 = 88)
1−sided adj pval: 0.161
Frequency
Tumor−Normal Expression Difference

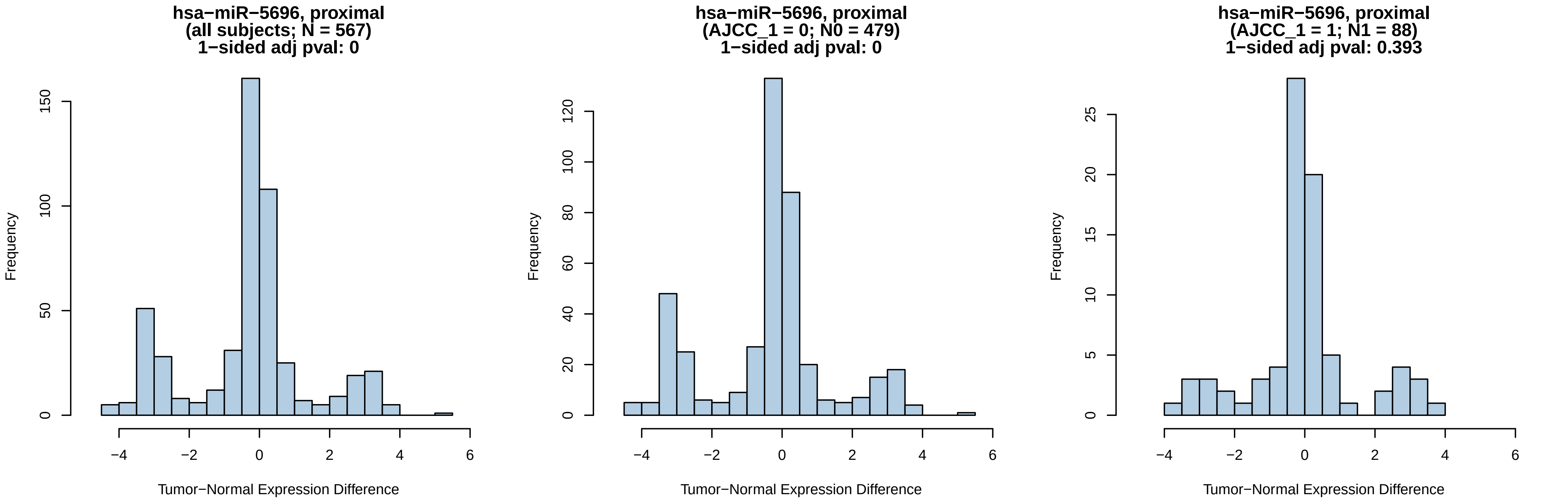
hsa−miR−5696, proximal
(all subjects; N = 567)
1−sided adj pval: 0
Frequency
Tumor−Normal Expression Difference
hsa−miR−5696, proximal
(AJCC_1 = 0; N0 = 479)
1−sided adj pval: 0
Frequency
Tumor−Normal Expression Difference
hsa−miR−5696, proximal
(AJCC_1 = 1; N1 = 88)
1−sided adj pval: 0.393
Frequency
Tumor−Normal Expression Difference

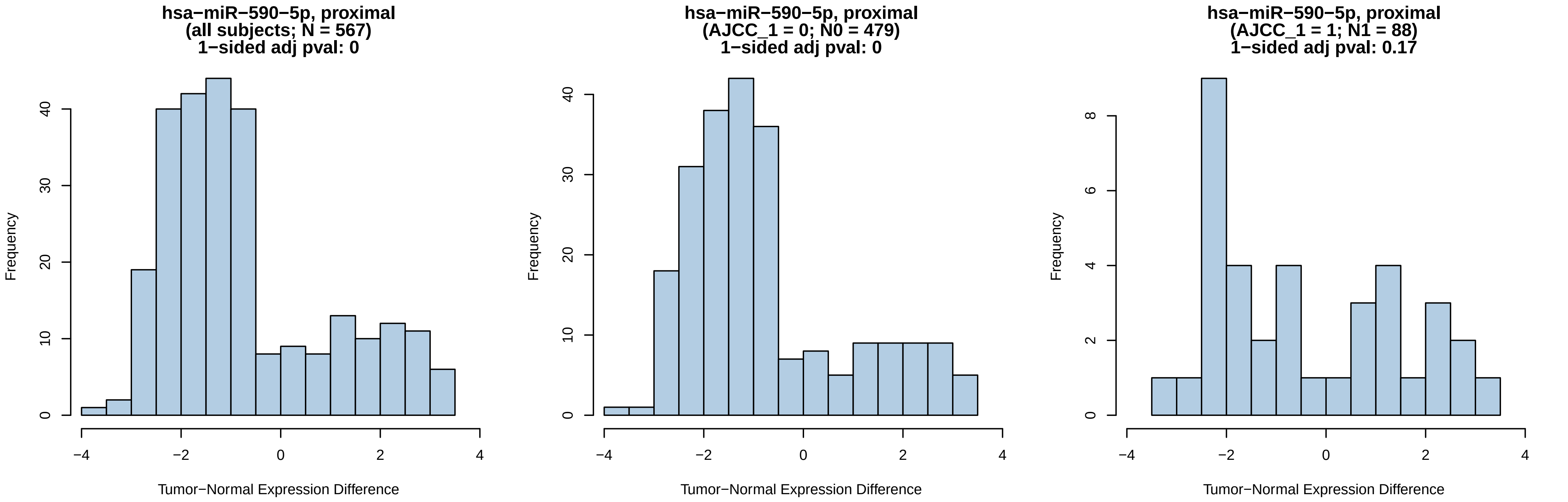
hsa−miR−590−5p, proximal
(all subjects; N = 567)
1−sided adj pval: 0
hsa−miR−590−5p, proximal
(AJCC_1 = 0; N0 = 479)
1−sided adj pval: 0
hsa−miR−590−5p, proximal
(AJCC_1 = 1; N1 = 88)
1−sided adj pval: 0.17
Frequency
Tumor−Normal Expression Difference
−4
−2
0
2
4
0
10
20
30
40
0
2
4
6
8

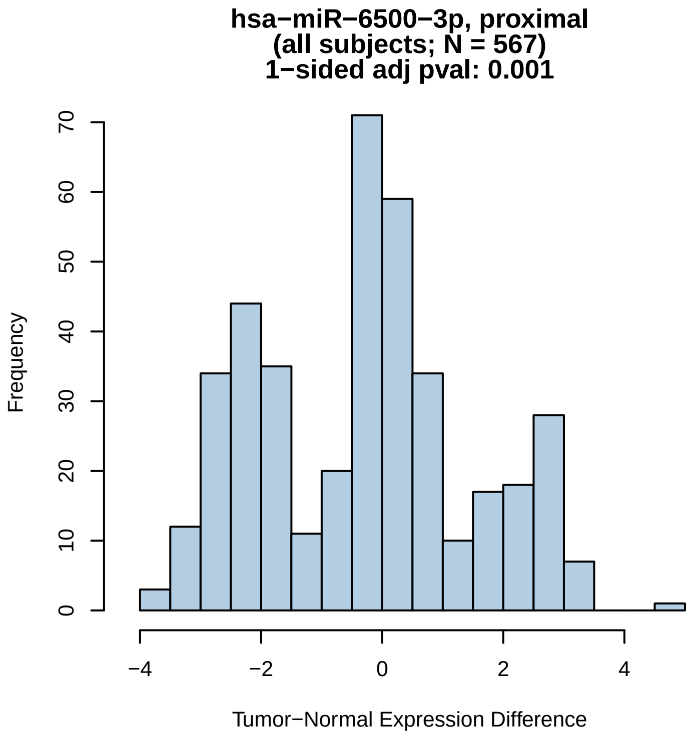
hsa−miR−6500−3p, proximal
(all subjects; N = 567)
1−sided adj pval: 0.001
Frequency
0
10
20
30
40
50
60
70
−4
−2
0
2
4
Tumor−Normal Expression Difference

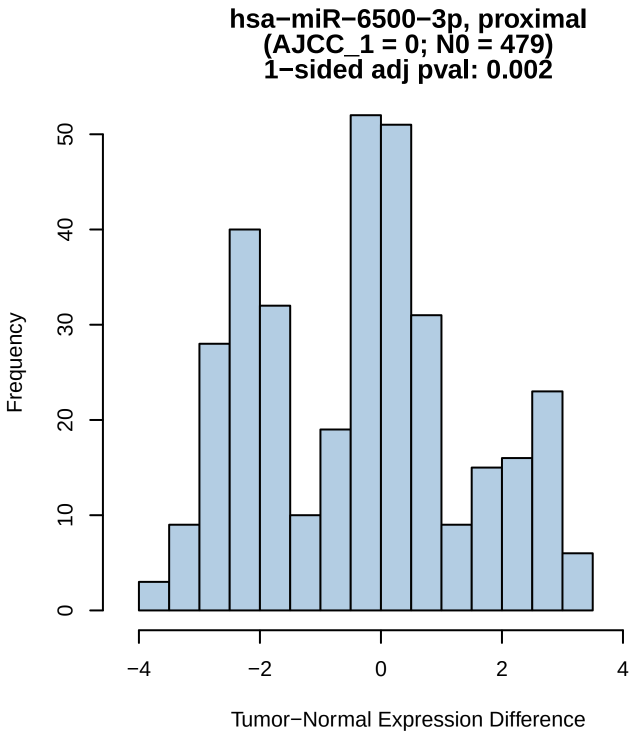
hsa−miR−6500−3p, proximal
(AJCC_1 = 0; N0 = 479)
1−sided adj pval: 0.002
Frequency
0
10
20
30
40
50
−4
−2
0
2
4
Tumor−Normal Expression Difference

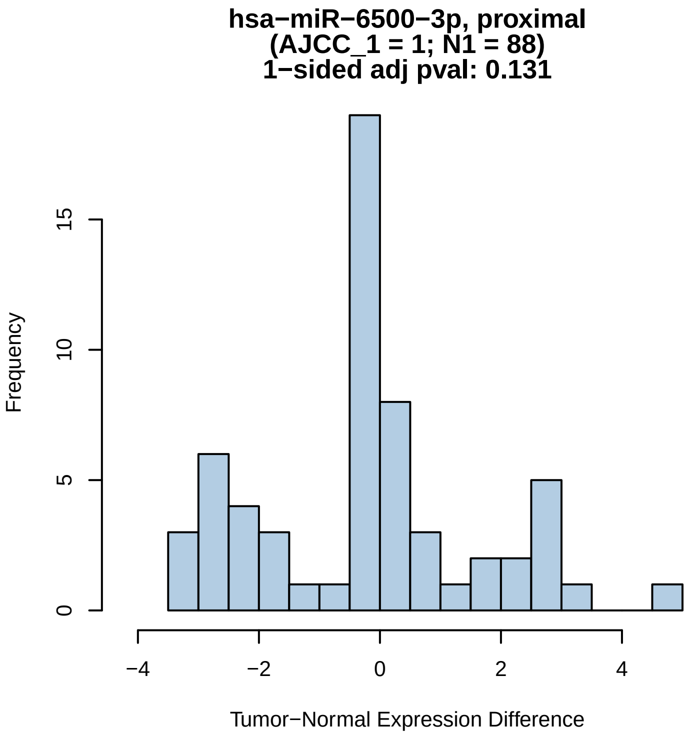
hsa−miR−6500−3p, proximal
(AJCC_1 = 1; N1 = 88)
1−sided adj pval: 0.131
Frequency
0
5
10
15
−4
−2
0
2
4
Tumor−Normal Expression Difference

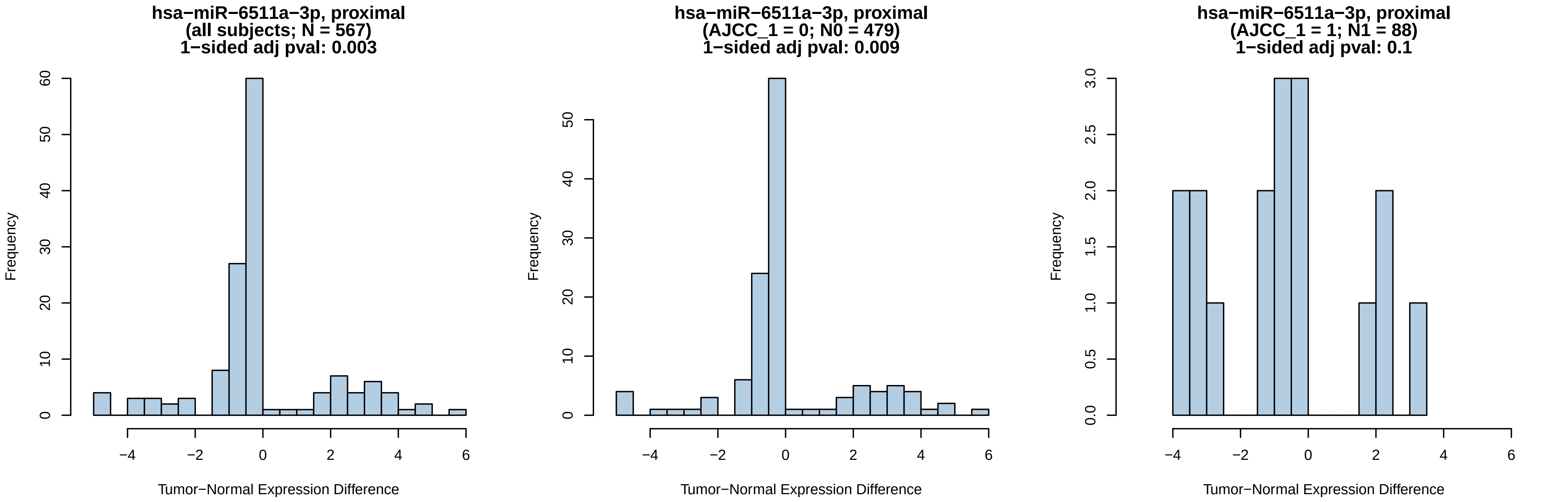
hsa−miR−6511a−3p, proximal
(all subjects; N = 567)
1−sided adj pval: 0.003
hsa−miR−6511a−3p, proximal
(AJCC_1 = 0; N0 = 479)
1−sided adj pval: 0.009
hsa−miR−6511a−3p, proximal
(AJCC_1 = 1; N1 = 88)
1−sided adj pval: 0.1
Frequency
Tumor−Normal Expression Difference

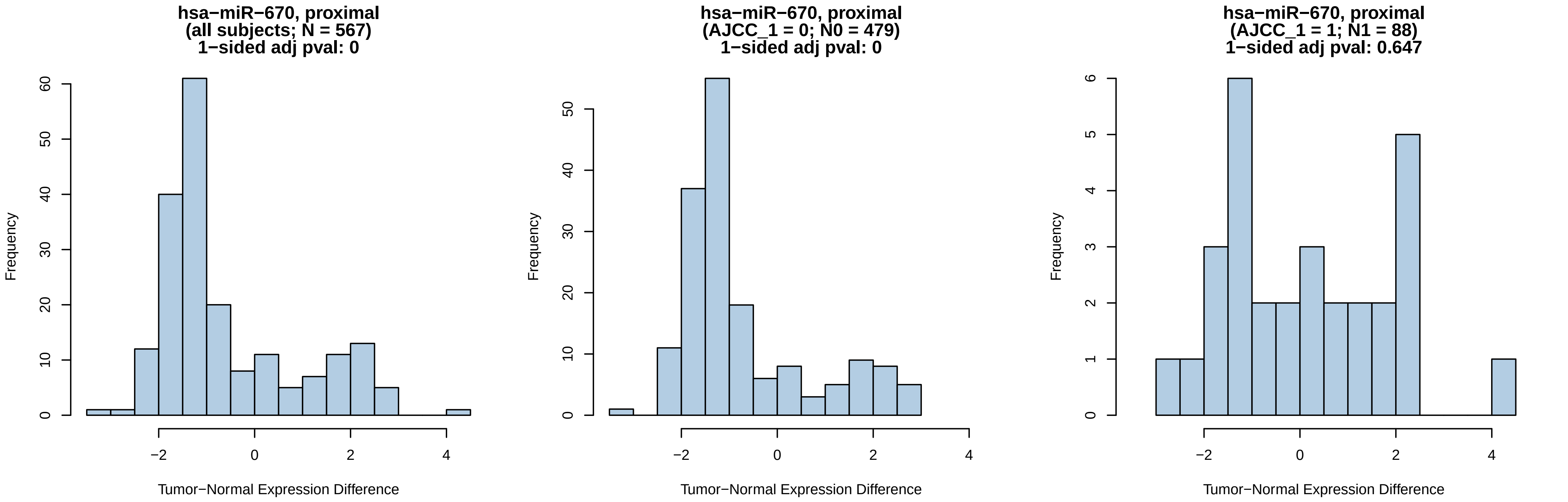

hsa−miR−670, proximal
(all subjects; N = 567)
1−sided adj pval: 0
Frequency
0
10
20
30
40
50
60
−2
0
2
4
Tumor−Normal Expression Difference
hsa−miR−670, proximal
(AJCC_1 = 0; N0 = 479)
1−sided adj pval: 0
Frequency
0
10
20
30
40
50
−2
0
2
4
Tumor−Normal Expression Difference
hsa−miR−670, proximal
(AJCC_1 = 1; N1 = 88)
1−sided adj pval: 0.647
Frequency
0
1
2
3
4
5
6
−2
0
2
4
Tumor−Normal Expression Difference

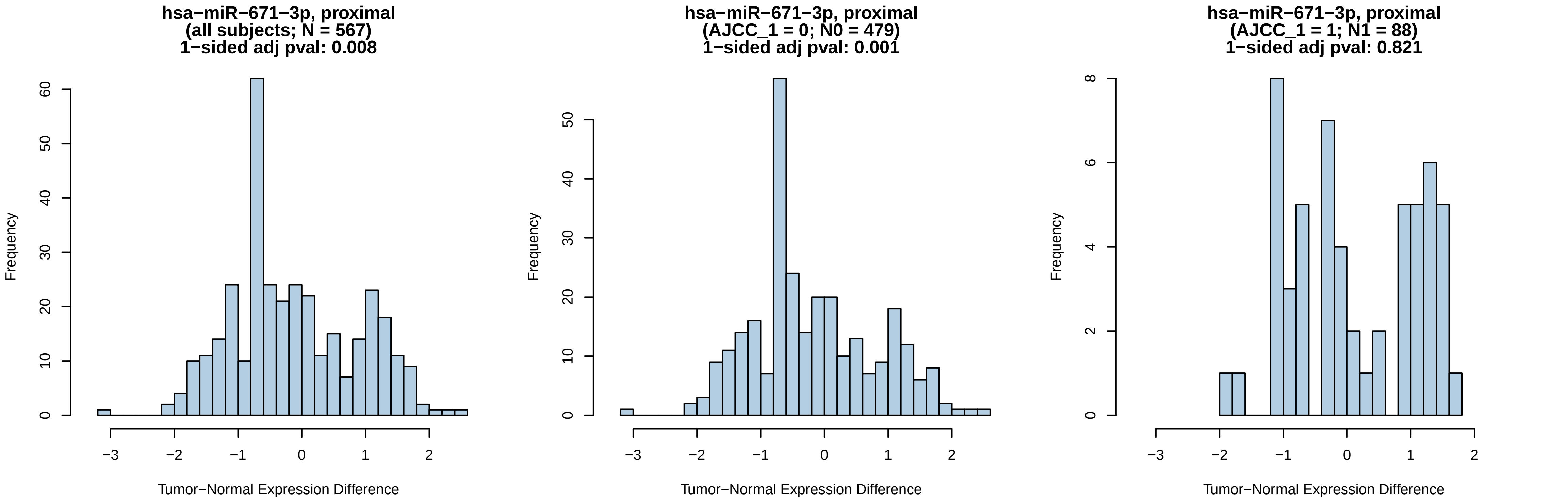
hsa−miR−671−3p, proximal
(all subjects; N = 567)
1−sided adj pval: 0.008
Frequency
0
10
20
30
40
50
60
−3
−2
−1
0
1
2
Tumor−Normal Expression Difference
hsa−miR−671−3p, proximal
(AJCC_1 = 0; N0 = 479)
1−sided adj pval: 0.001
Frequency
0
10
20
30
40
50
−3
−2
−1
0
1
2
Tumor−Normal Expression Difference
hsa−miR−671−3p, proximal
(AJCC_1 = 1; N1 = 88)
1−sided adj pval: 0.821
Frequency
0
2
4
6
8
−3
−2
−1
0
1
2
Tumor−Normal Expression Difference

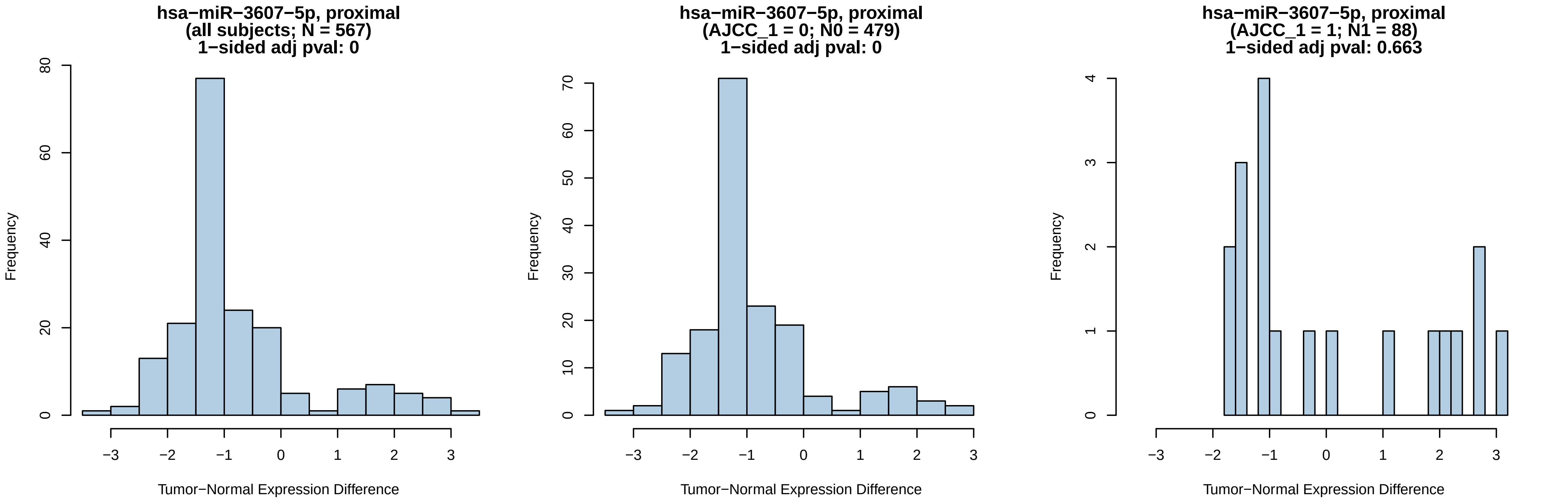
hsa−miR−3607−5p, proximal
(all subjects; N = 567)
1−sided adj pval: 0
hsa−miR−3607−5p, proximal
(AJCC_1 = 0; N0 = 479)
1−sided adj pval: 0
hsa−miR−3607−5p, proximal
(AJCC_1 = 1; N1 = 88)
1−sided adj pval: 0.663
Frequency
Tumor−Normal Expression Difference

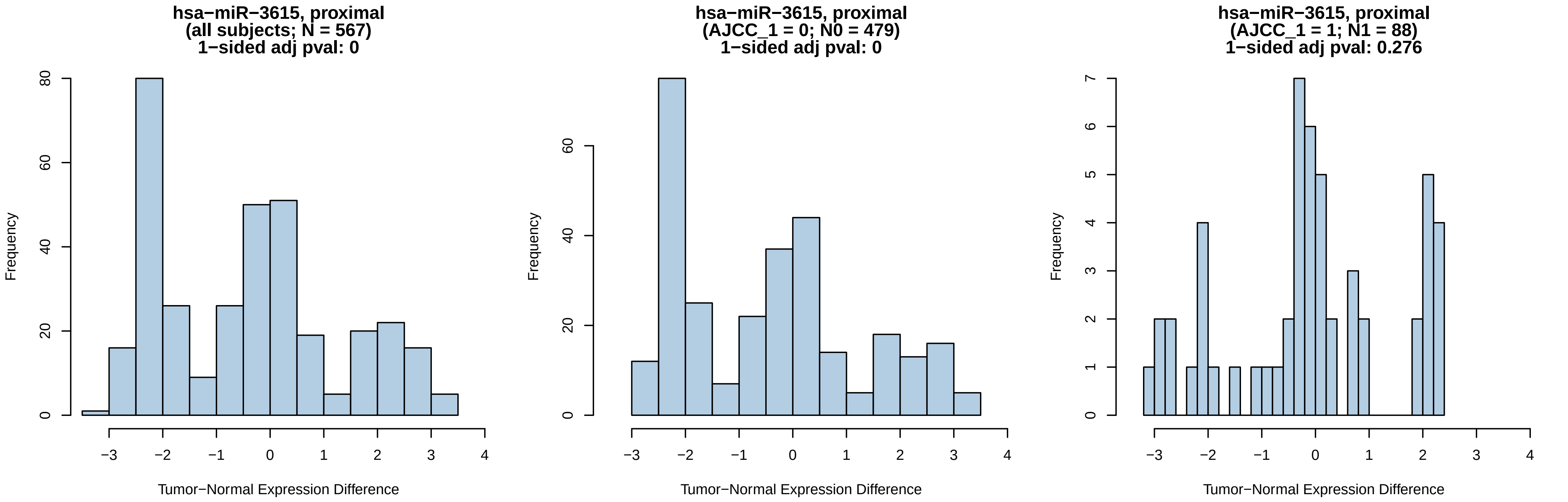

hsa−miR−3615, proximal
(all subjects; N = 567)
1−sided adj pval: 0
Frequency
Tumor−Normal Expression Difference
hsa−miR−3615, proximal
(AJCC_1 = 0; N0 = 479)
1−sided adj pval: 0
Frequency
Tumor−Normal Expression Difference
hsa−miR−3615, proximal
(AJCC_1 = 1; N1 = 88)
1−sided adj pval: 0.276
Frequency
Tumor−Normal Expression Difference

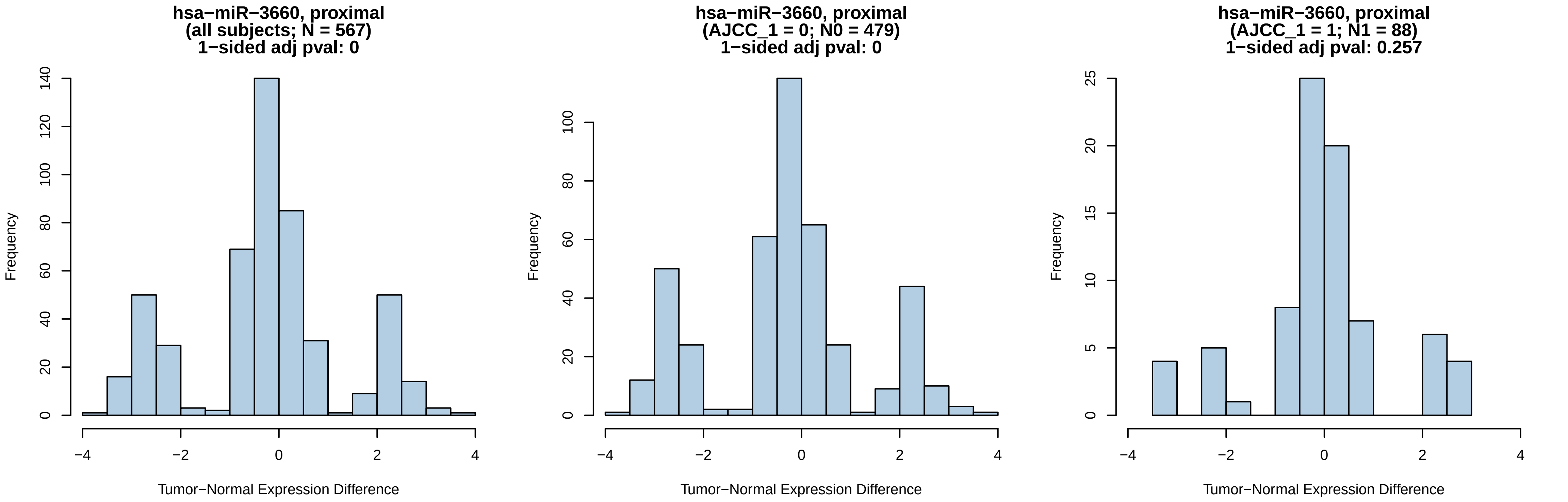

hsa−miR−3660, proximal
(all subjects; N = 567)
1−sided adj pval: 0
Frequency
Tumor−Normal Expression Difference
hsa−miR−3660, proximal
(AJCC_1 = 0; N0 = 479)
1−sided adj pval: 0
Frequency
Tumor−Normal Expression Difference
hsa−miR−3660, proximal
(AJCC_1 = 1; N1 = 88)
1−sided adj pval: 0.257
Frequency
Tumor−Normal Expression Difference

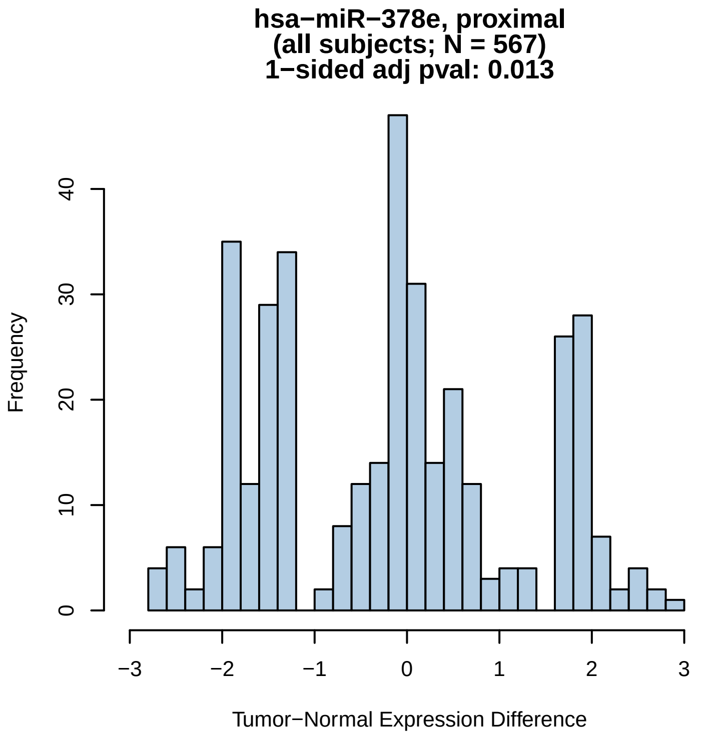
hsa−miR−378e, proximal
(all subjects; N = 567)
1−sided adj pval: 0.013
Frequency
0
10
20
30
40
−3
−2
−1
0
1
2
3
Tumor−Normal Expression Difference

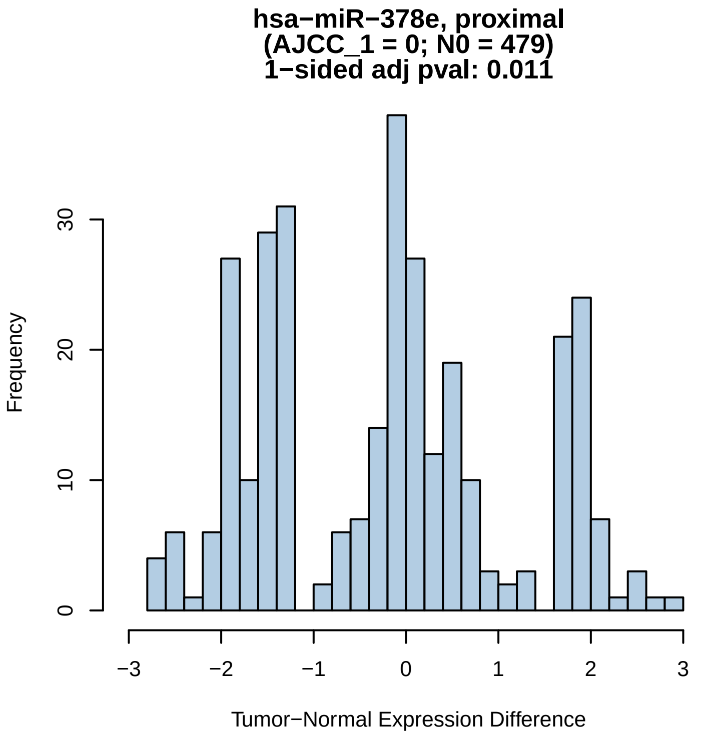
hsa−miR−378e, proximal
(AJCC_1 = 0; N0 = 479)
1−sided adj pval: 0.011
Frequency
0
10
20
30
−3
−2
−1
0
1
2
3
Tumor−Normal Expression Difference

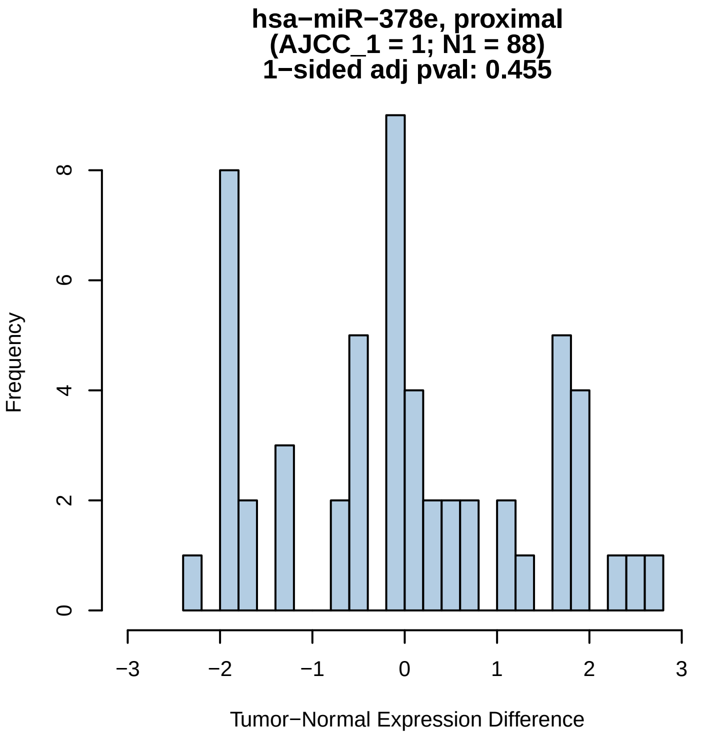
hsa−miR−378e, proximal
(AJCC_1 = 1; N1 = 88)
1−sided adj pval: 0.455
Frequency
0
2
4
6
8
−3
−2
−1
0
1
2
3
Tumor−Normal Expression Difference

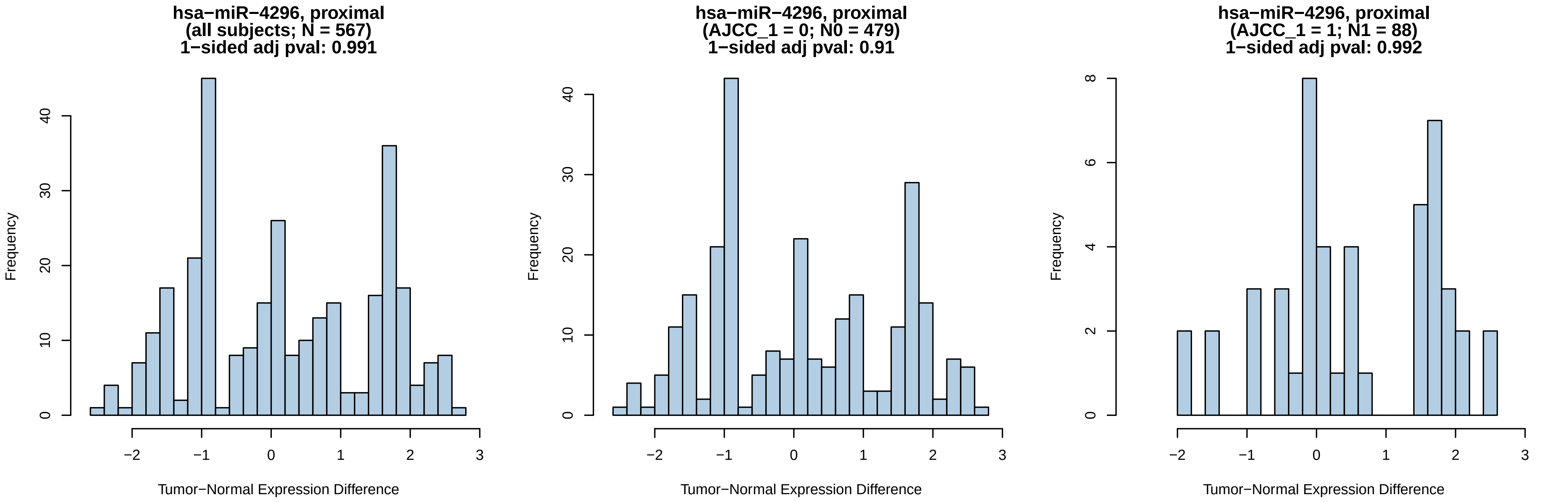
hsa−miR−4296, proximal
(all subjects; N = 567)
1−sided adj pval: 0.991
Frequency
Tumor−Normal Expression Difference
hsa−miR−4296, proximal
(AJCC_1 = 0; N0 = 479)
1−sided adj pval: 0.91
Frequency
Tumor−Normal Expression Difference
hsa−miR−4296, proximal
(AJCC_1 = 1; N1 = 88)
1−sided adj pval: 0.992
Frequency
Tumor−Normal Expression Difference

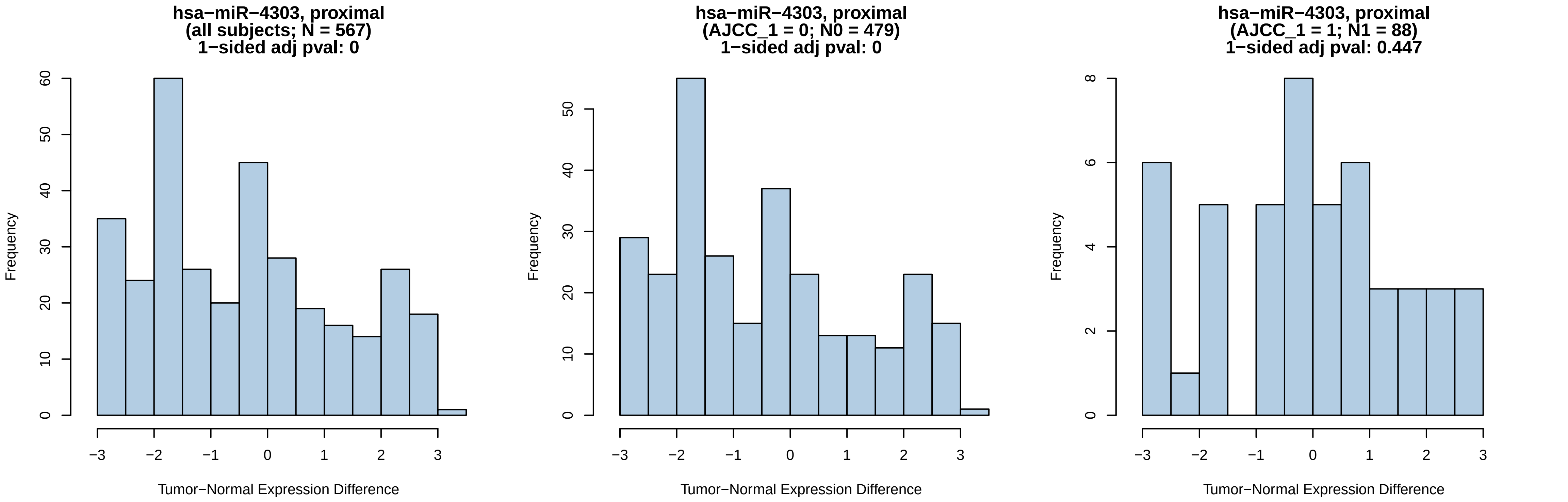
hsa−miR−4303, proximal
(all subjects; N = 567)
1−sided adj pval: 0
Frequency
Tumor−Normal Expression Difference
hsa−miR−4303, proximal
(AJCC_1 = 0; N0 = 479)
1−sided adj pval: 0
Frequency
Tumor−Normal Expression Difference
hsa−miR−4303, proximal
(AJCC_1 = 1; N1 = 88)
1−sided adj pval: 0.447
Frequency
Tumor−Normal Expression Difference

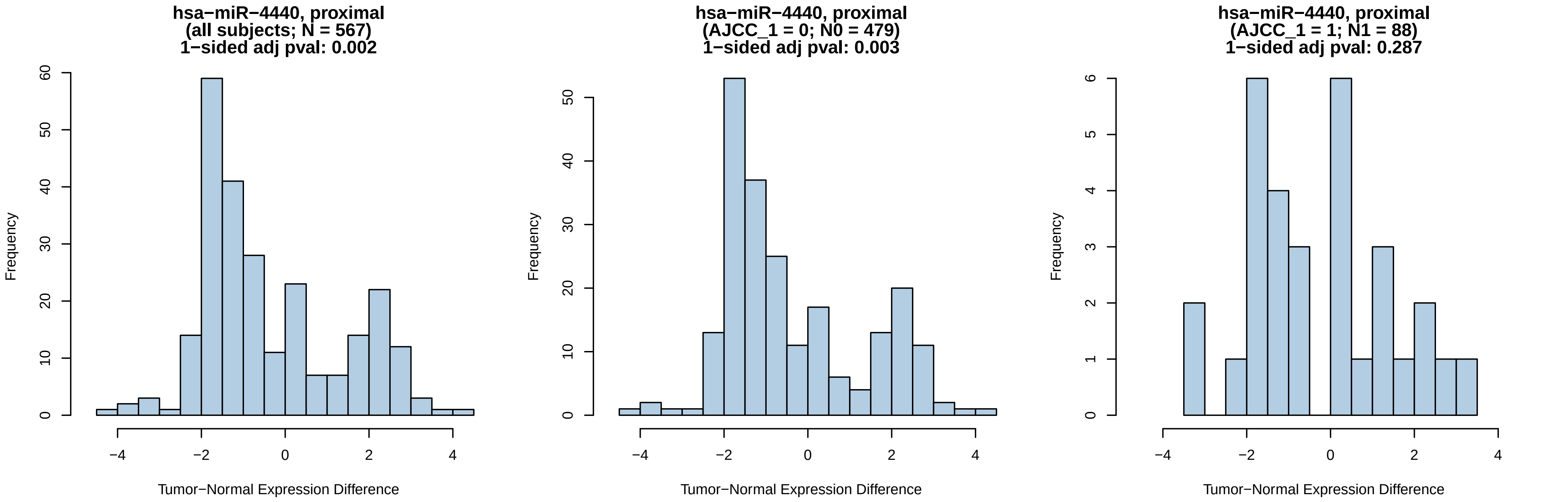
hsa−miR−4440, proximal
(all subjects; N = 567)
1−sided adj pval: 0.002
Frequency
Tumor−Normal Expression Difference
hsa−miR−4440, proximal
(AJCC_1 = 0; N0 = 479)
1−sided adj pval: 0.003
Frequency
Tumor−Normal Expression Difference
hsa−miR−4440, proximal
(AJCC_1 = 1; N1 = 88)
1−sided adj pval: 0.287
Frequency
Tumor−Normal Expression Difference

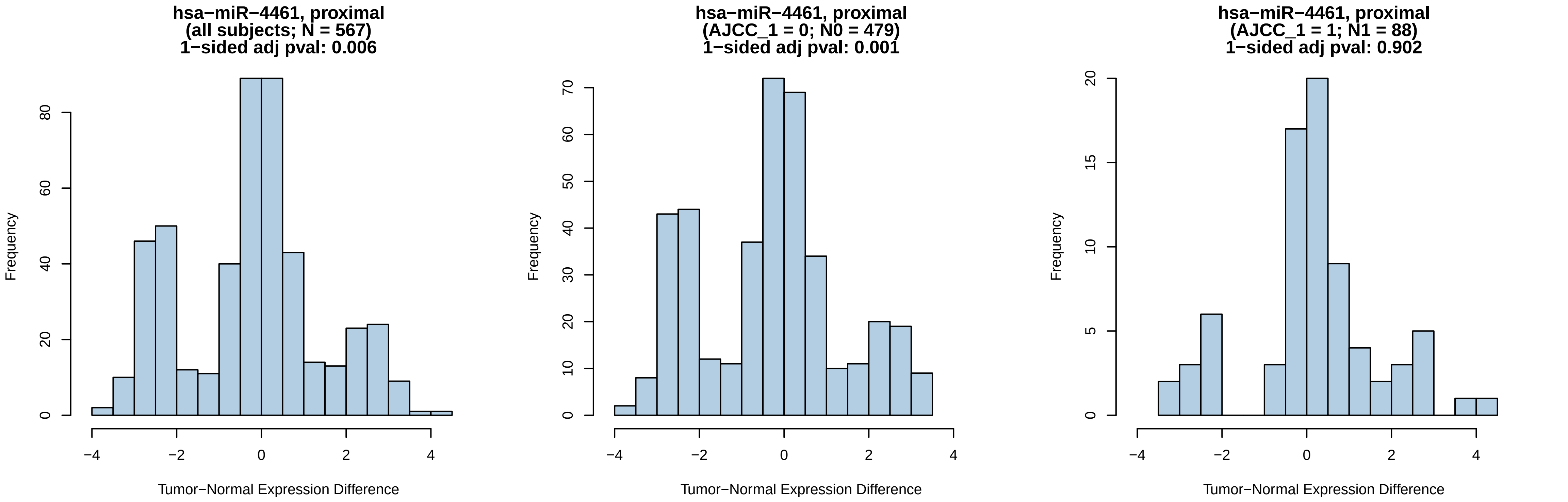
hsa−miR−4461, proximal
(all subjects; N = 567)
1−sided adj pval: 0.006
hsa−miR−4461, proximal
(AJCC_1 = 0; N0 = 479)
1−sided adj pval: 0.001
hsa−miR−4461, proximal
(AJCC_1 = 1; N1 = 88)
1−sided adj pval: 0.902
Frequency
Tumor−Normal Expression Difference

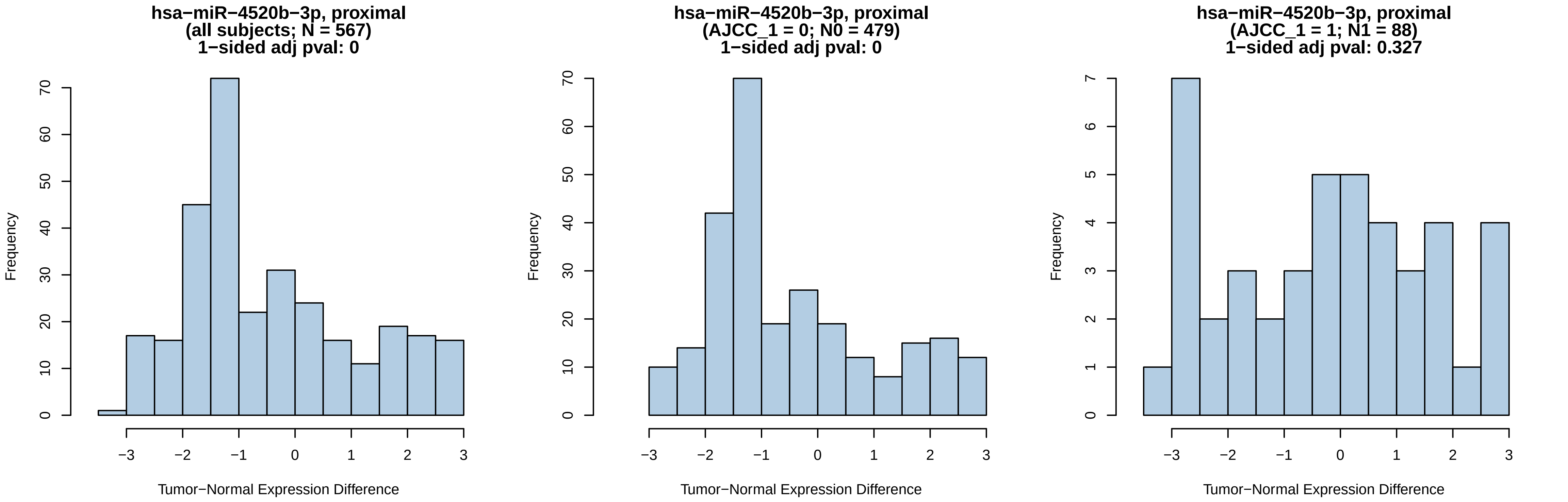
hsa−miR−4520b−3p, proximal
(all subjects; N = 567)
1−sided adj pval: 0
hsa−miR−4520b−3p, proximal
(AJCC_1 = 0; N0 = 479)
1−sided adj pval: 0
hsa−miR−4520b−3p, proximal
(AJCC_1 = 1; N1 = 88)
1−sided adj pval: 0.327
Frequency
Tumor−Normal Expression Difference

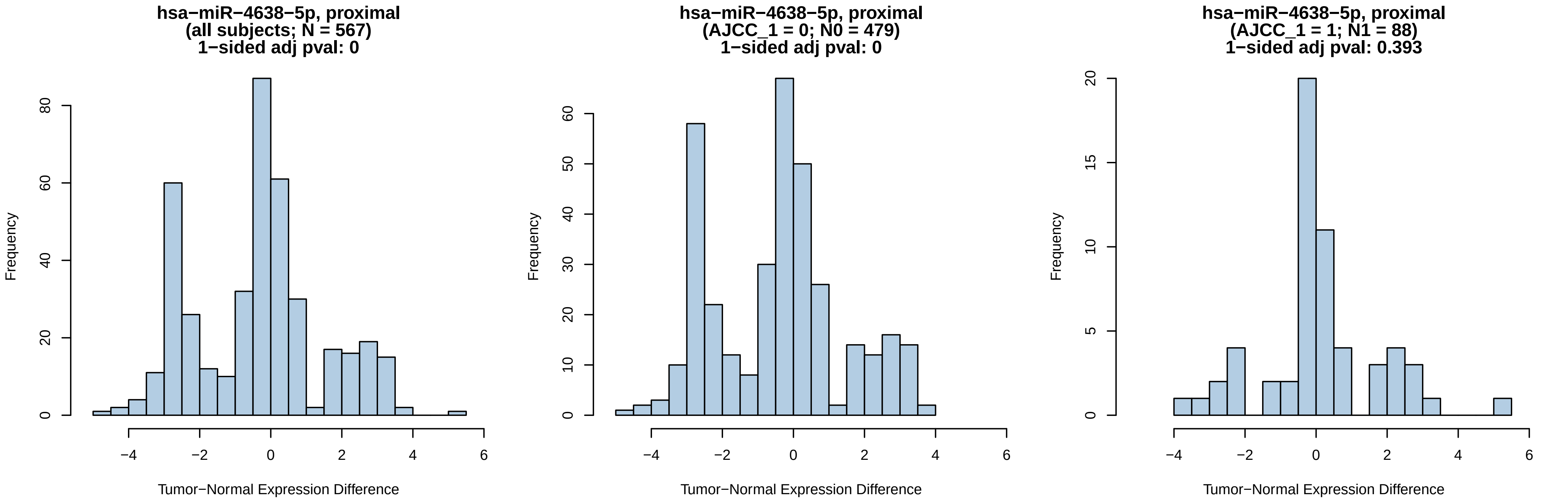
hsa−miR−4638−5p, proximal
(all subjects; N = 567)
1−sided adj pval: 0
hsa−miR−4638−5p, proximal
(AJCC_1 = 0; N0 = 479)
1−sided adj pval: 0
hsa−miR−4638−5p, proximal
(AJCC_1 = 1; N1 = 88)
1−sided adj pval: 0.393
Frequency
Tumor−Normal Expression Difference
Frequency
Tumor−Normal Expression Difference
Frequency
Tumor−Normal Expression Difference

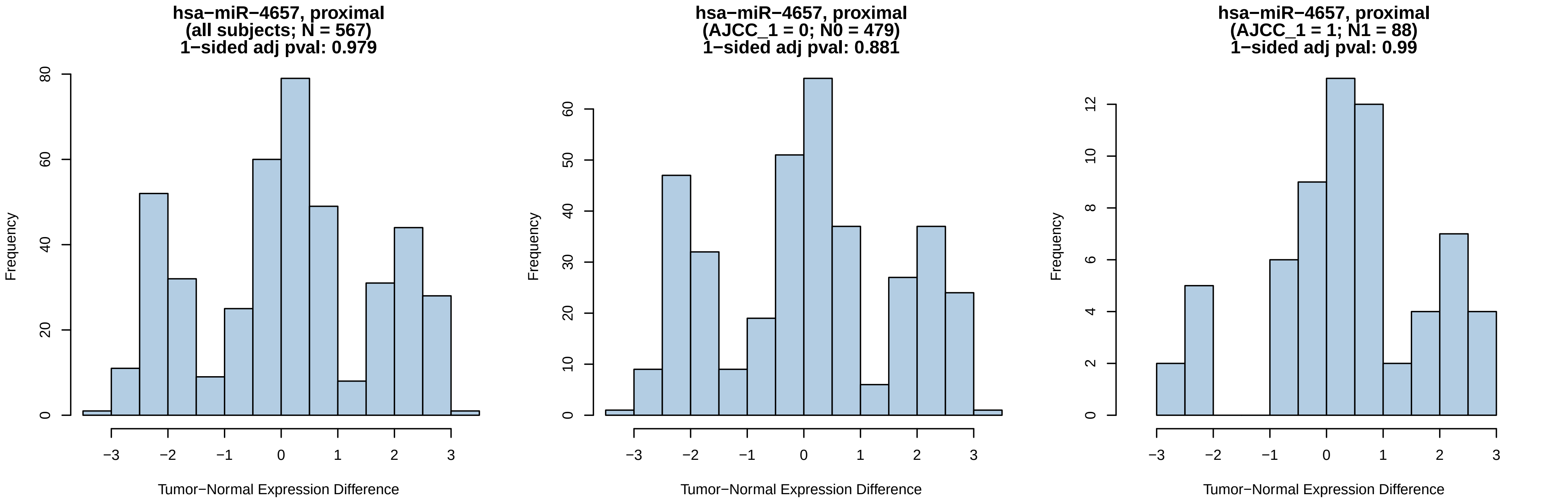
hsa−miR−4657, proximal
(all subjects; N = 567)
1−sided adj pval: 0.979
Frequency
Tumor−Normal Expression Difference
hsa−miR−4657, proximal
(AJCC_1 = 0; N0 = 479)
1−sided adj pval: 0.881
Frequency
Tumor−Normal Expression Difference
hsa−miR−4657, proximal
(AJCC_1 = 1; N1 = 88)
1−sided adj pval: 0.99
Frequency
Tumor−Normal Expression Difference

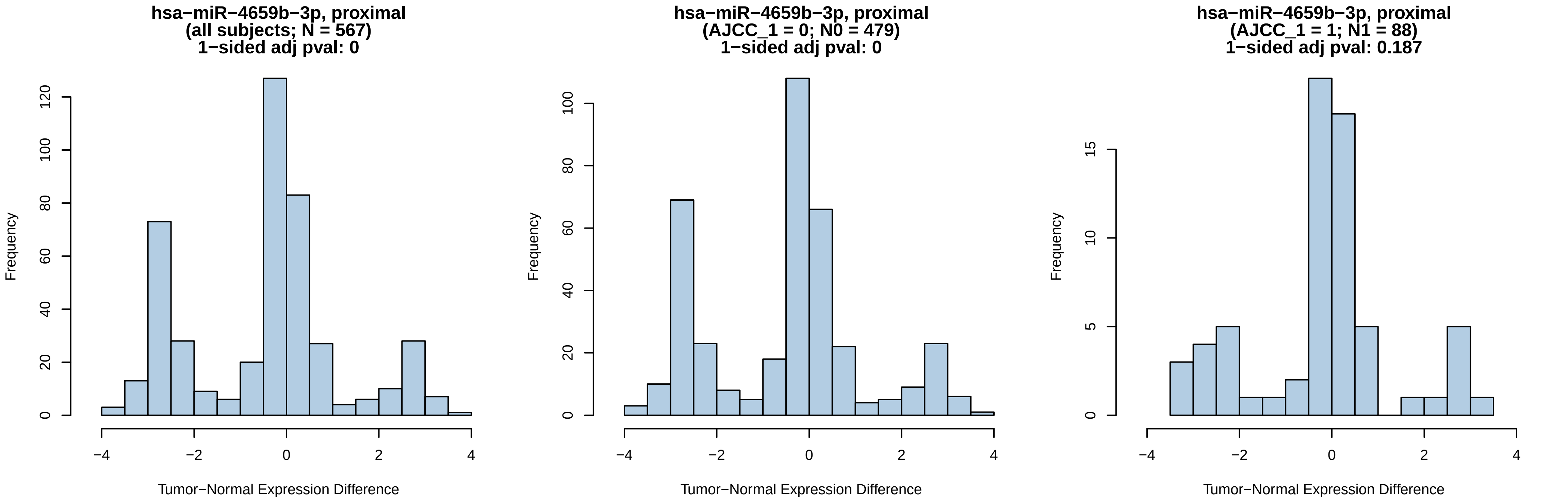

hsa−miR−4659b−3p, proximal
(all subjects; N = 567)
1−sided adj pval: 0
Frequency
Tumor−Normal Expression Difference
hsa−miR−4659b−3p, proximal
(AJCC_1 = 0; N0 = 479)
1−sided adj pval: 0
Frequency
Tumor−Normal Expression Difference
hsa−miR−4659b−3p, proximal
(AJCC_1 = 1; N1 = 88)
1−sided adj pval: 0.187
Frequency
Tumor−Normal Expression Difference

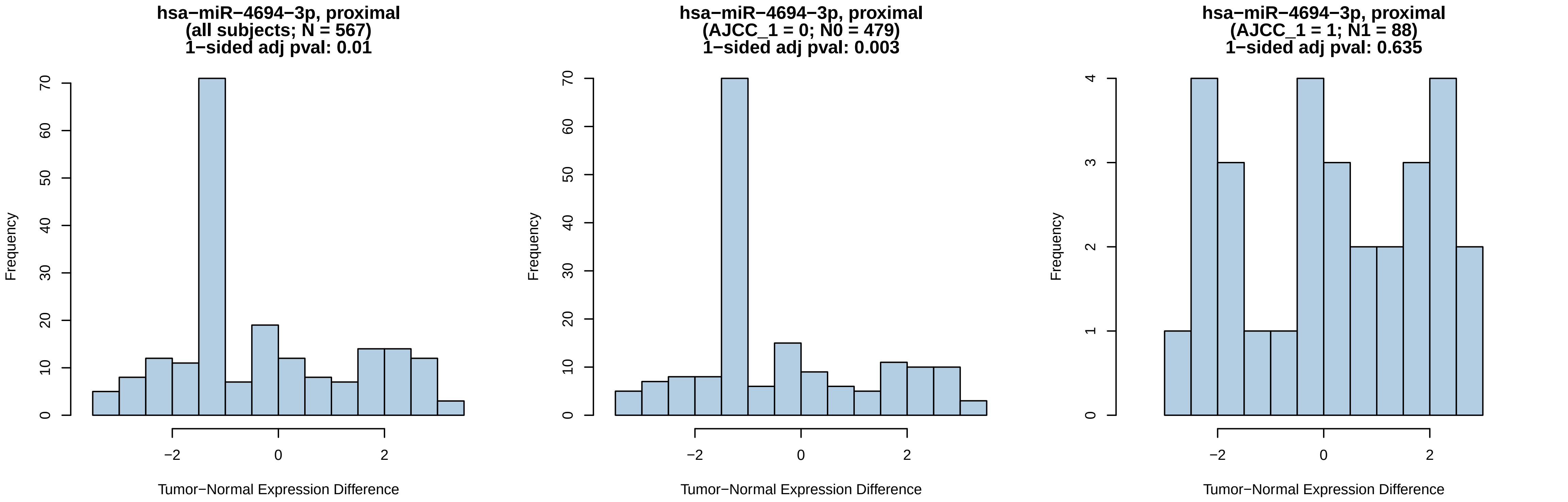

hsa−miR−4694−3p, proximal
(all subjects; N = 567)
1−sided adj pval: 0.01
Frequency
Tumor−Normal Expression Difference
hsa−miR−4694−3p, proximal
(AJCC_1 = 0; N0 = 479)
1−sided adj pval: 0.003
Frequency
Tumor−Normal Expression Difference
hsa−miR−4694−3p, proximal
(AJCC_1 = 1; N1 = 88)
1−sided adj pval: 0.635
Frequency
Tumor−Normal Expression Difference

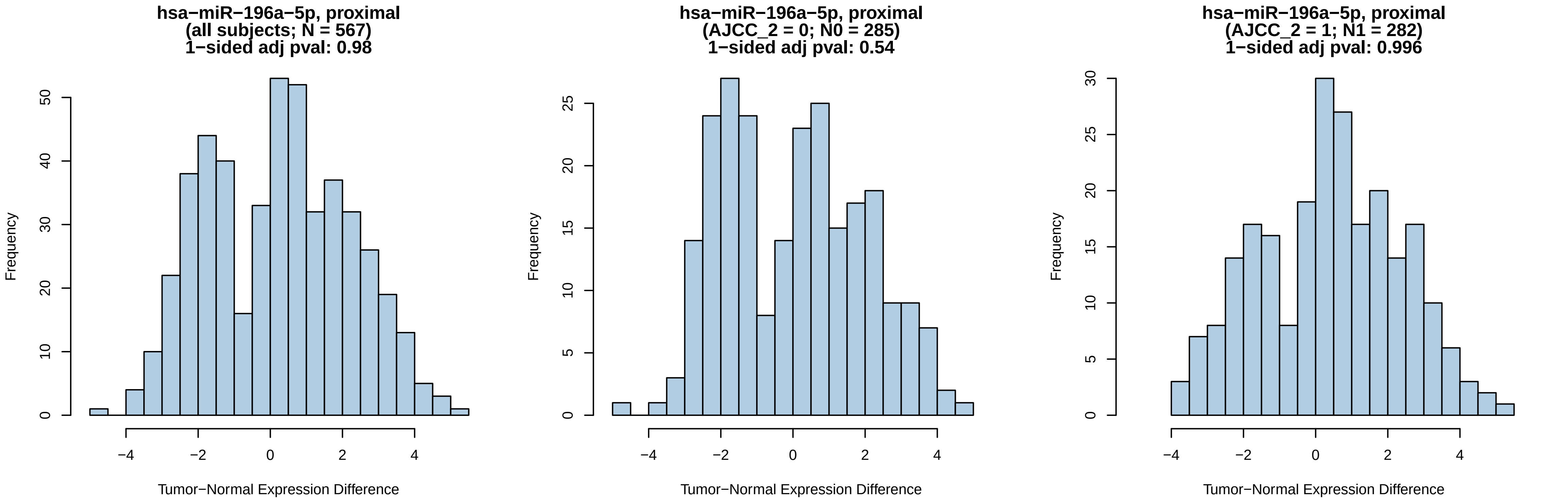

hsa−miR−196a−5p, proximal
(all subjects; N = 567)
1−sided adj pval: 0.98
Frequency
Tumor−Normal Expression Difference
hsa−miR−196a−5p, proximal
(AJCC_2 = 0; N0 = 285)
1−sided adj pval: 0.54
Frequency
Tumor−Normal Expression Difference
hsa−miR−196a−5p, proximal
(AJCC_2 = 1; N1 = 282)
1−sided adj pval: 0.996
Frequency
Tumor−Normal Expression Difference

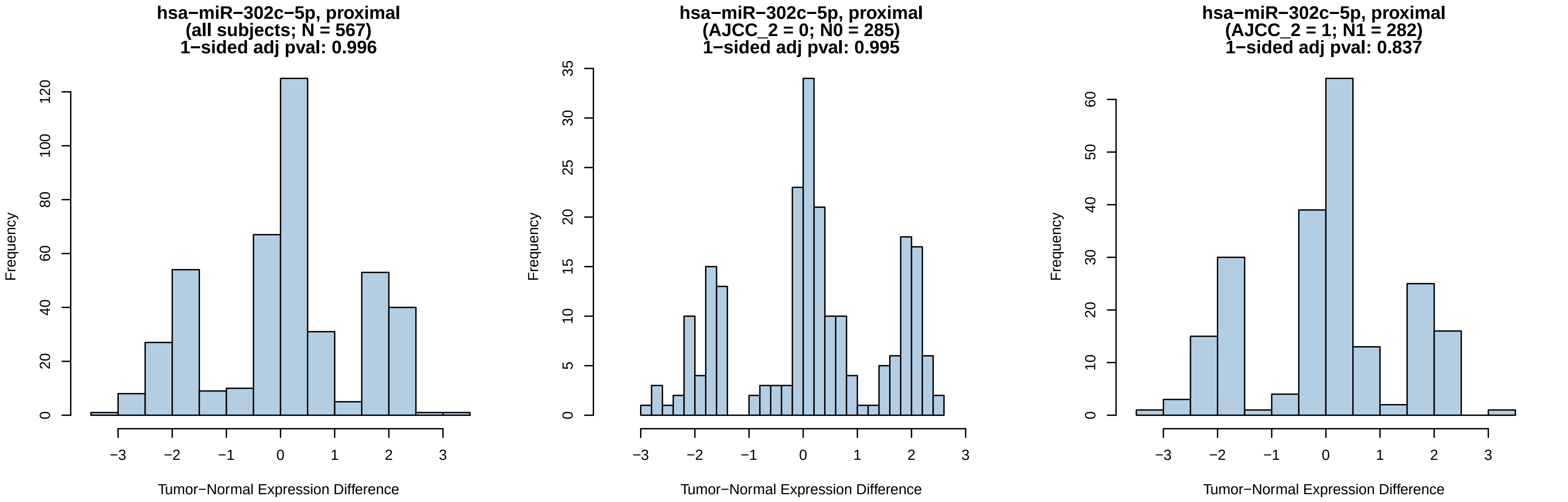

hsa−miR−302c−5p, proximal
(all subjects; N = 567)
1−sided adj pval: 0.996
hsa−miR−302c−5p, proximal
(AJCC_2 = 0; N0 = 285)
1−sided adj pval: 0.995
hsa−miR−302c−5p, proximal
(AJCC_2 = 1; N1 = 282)
1−sided adj pval: 0.837
Frequency
Tumor−Normal Expression Difference

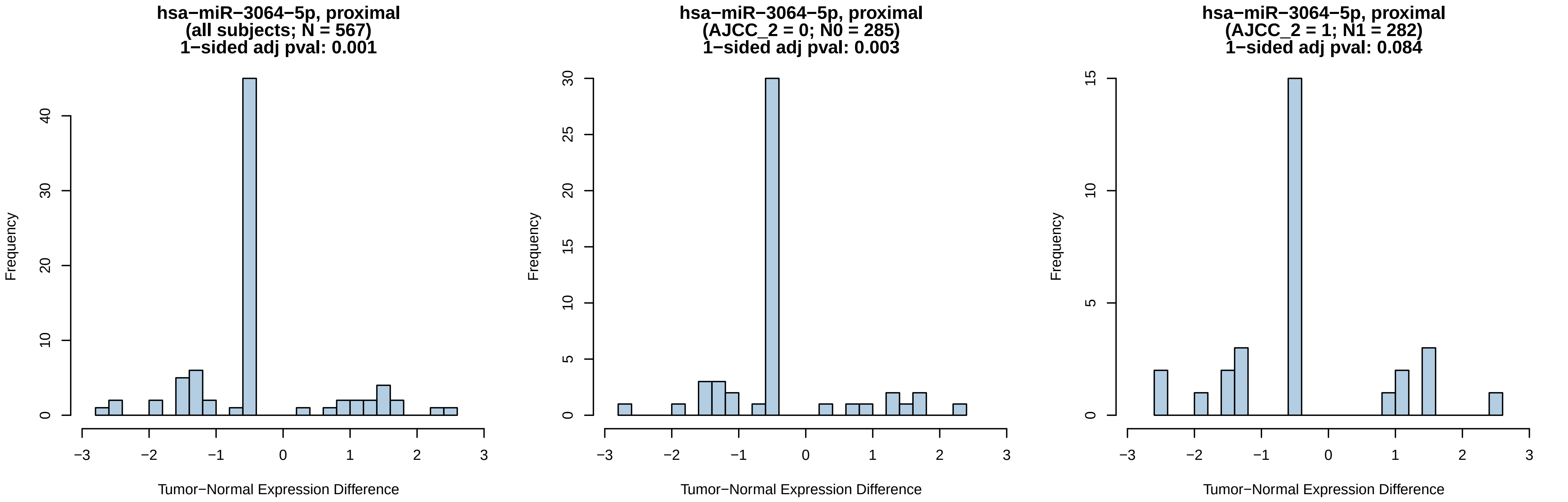

hsa−miR−3064−5p, proximal
(all subjects; N = 567)
1−sided adj pval: 0.001
Frequency
0
10
20
30
40
−3
−2
−1
0
1
2
3
Tumor−Normal Expression Difference
hsa−miR−3064−5p, proximal
(AJCC_2 = 0; N0 = 285)
1−sided adj pval: 0.003
Frequency
0
5
10
15
20
25
30
Tumor−Normal Expression Difference
hsa−miR−3064−5p, proximal
(AJCC_2 = 1; N1 = 282)
1−sided adj pval: 0.084
Frequency
0
5
10
15
Tumor−Normal Expression Difference

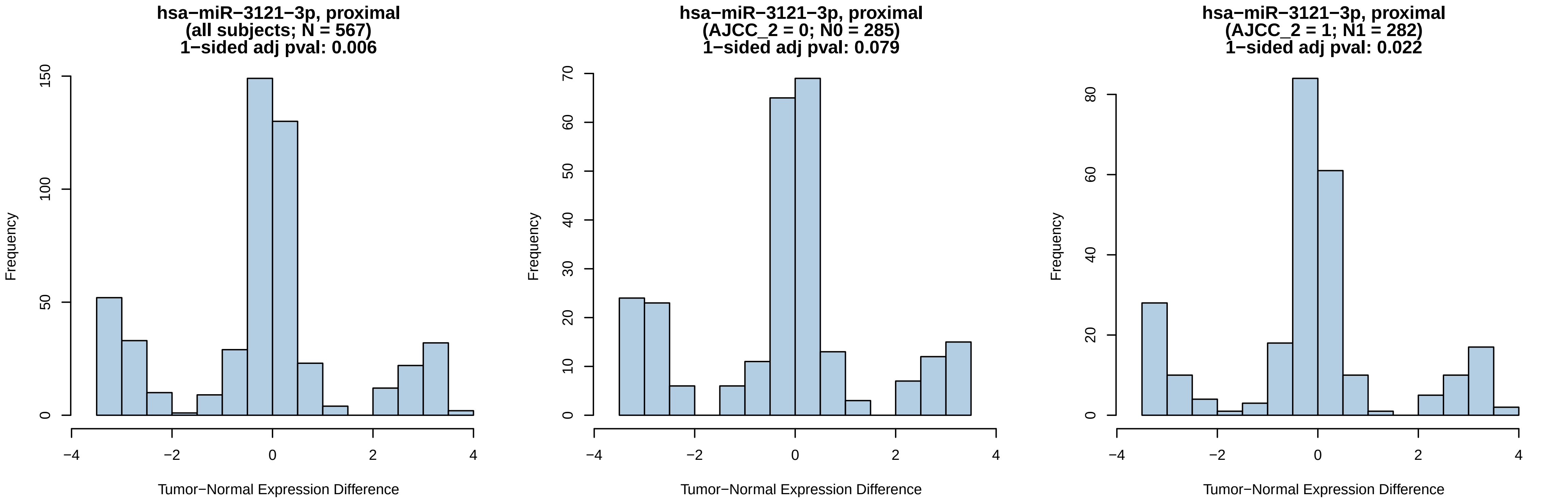

hsa−miR−3121−3p, proximal
(all subjects; N = 567)
1−sided adj pval: 0.006
Frequency
0
50
100
150
−4
−2
0
2
4
Tumor−Normal Expression Difference
hsa−miR−3121−3p, proximal
(AJCC_2 = 0; N0 = 285)
1−sided adj pval: 0.079
Frequency
0
10
20
30
40
50
60
70
−4
−2
0
2
4
Tumor−Normal Expression Difference
hsa−miR−3121−3p, proximal
(AJCC_2 = 1; N1 = 282)
1−sided adj pval: 0.022
Frequency
0
20
40
60
80
−4
−2
0
2
4
Tumor−Normal Expression Difference

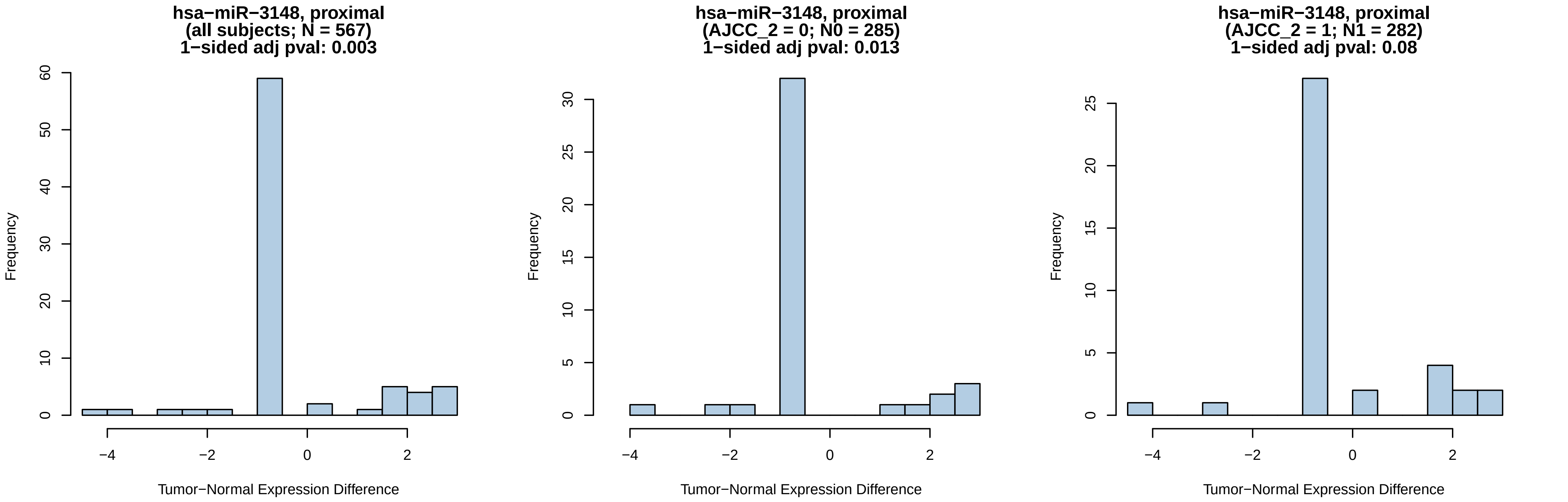

hsa−miR−3148, proximal
(all subjects; N = 567)
1−sided adj pval: 0.003
Frequency
0
10
20
30
40
50
60
−4
−2
0
2
Tumor−Normal Expression Difference
hsa−miR−3148, proximal
(AJCC_2 = 0; N0 = 285)
1−sided adj pval: 0.013
Frequency
0
5
10
15
20
25
30
−4
−2
0
2
Tumor−Normal Expression Difference
hsa−miR−3148, proximal
(AJCC_2 = 1; N1 = 282)
1−sided adj pval: 0.08
Frequency
0
5
10
15
20
25
−4
−2
0
2
Tumor−Normal Expression Difference

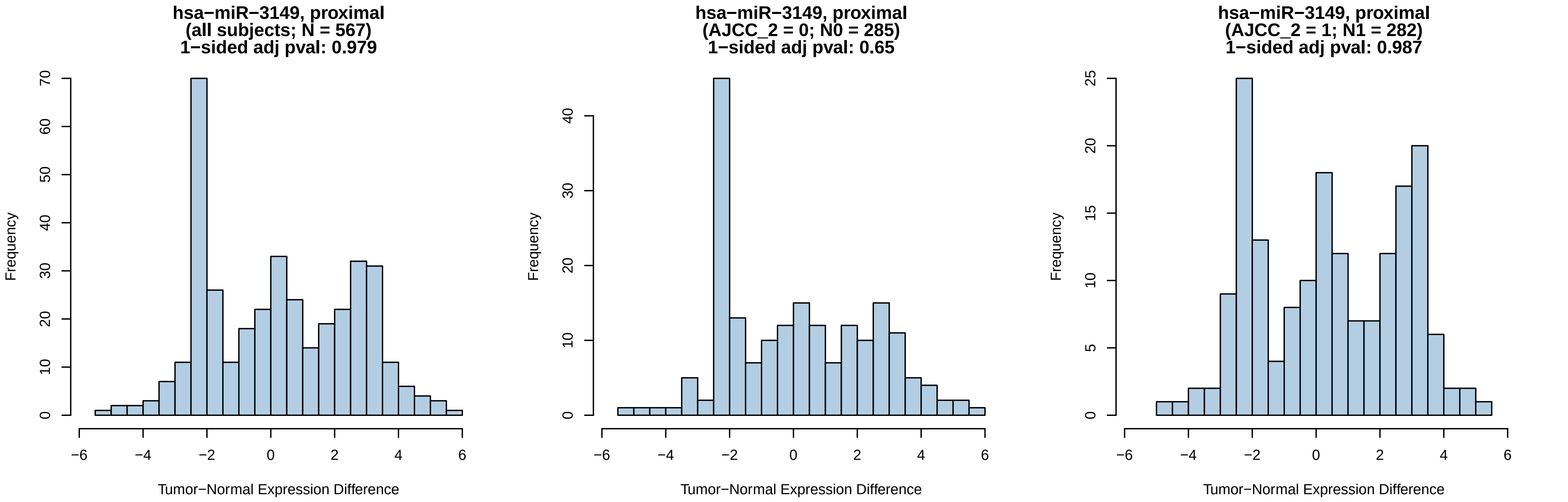
hsa−miR−3149, proximal
(all subjects; N = 567)
1−sided adj pval: 0.979
Frequency
Tumor−Normal Expression Difference
hsa−miR−3149, proximal
(AJCC_2 = 0; N0 = 285)
1−sided adj pval: 0.65
Frequency
Tumor−Normal Expression Difference
hsa−miR−3149, proximal
(AJCC_2 = 1; N1 = 282)
1−sided adj pval: 0.987
Frequency
Tumor−Normal Expression Difference

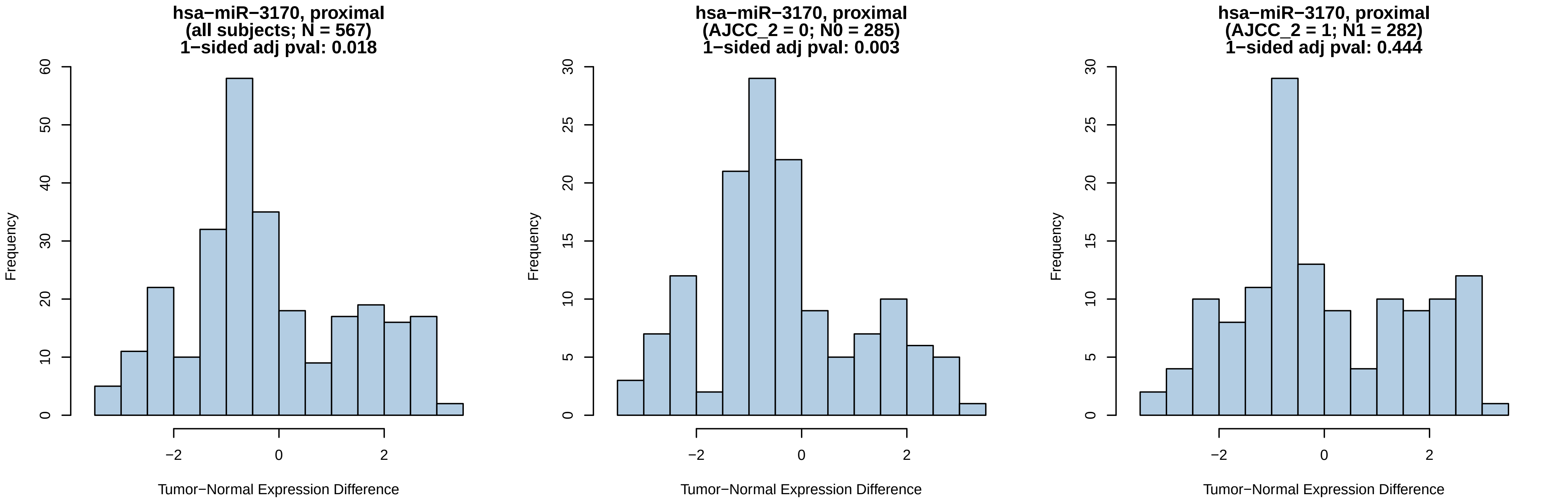
hsa−miR−3170, proximal
(all subjects; N = 567)
1−sided adj pval: 0.018
hsa−miR−3170, proximal
(AJCC_2 = 0; N0 = 285)
1−sided adj pval: 0.003
hsa−miR−3170, proximal
(AJCC_2 = 1; N1 = 282)
1−sided adj pval: 0.444
Frequency
Tumor−Normal Expression Difference

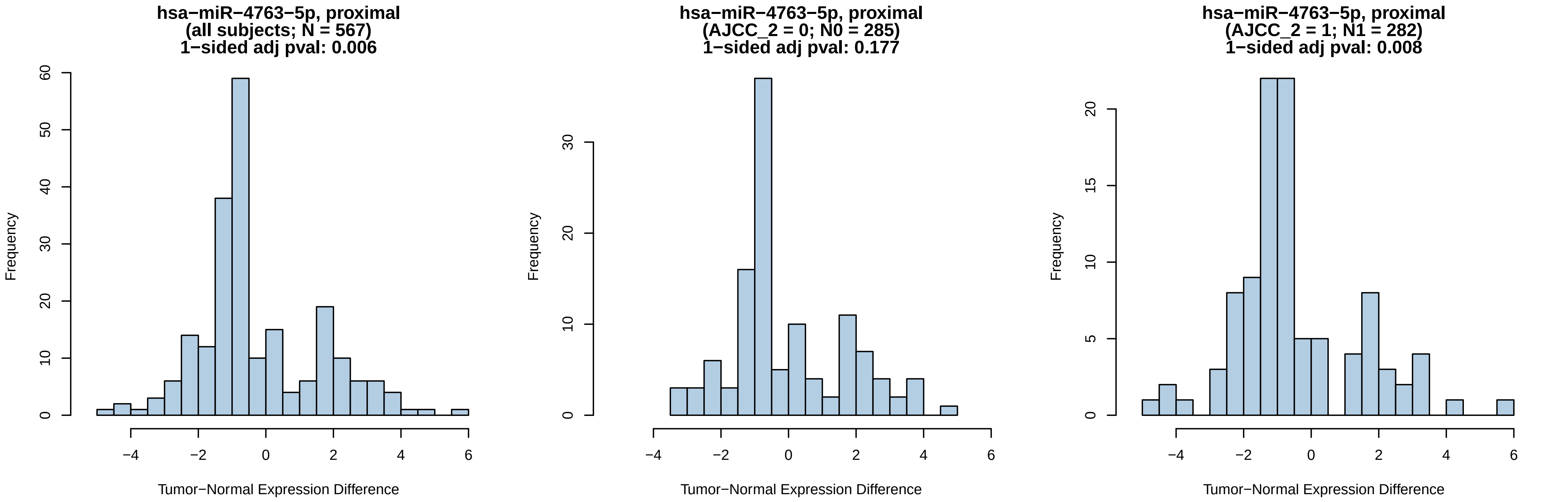
hsa−miR−4763−5p, proximal
(all subjects; N = 567)
1−sided adj pval: 0.006
Frequency
Tumor−Normal Expression Difference
hsa−miR−4763−5p, proximal
(AJCC_2 = 0; N0 = 285)
1−sided adj pval: 0.177
Frequency
Tumor−Normal Expression Difference
hsa−miR−4763−5p, proximal
(AJCC_2 = 1; N1 = 282)
1−sided adj pval: 0.008
Frequency
Tumor−Normal Expression Difference

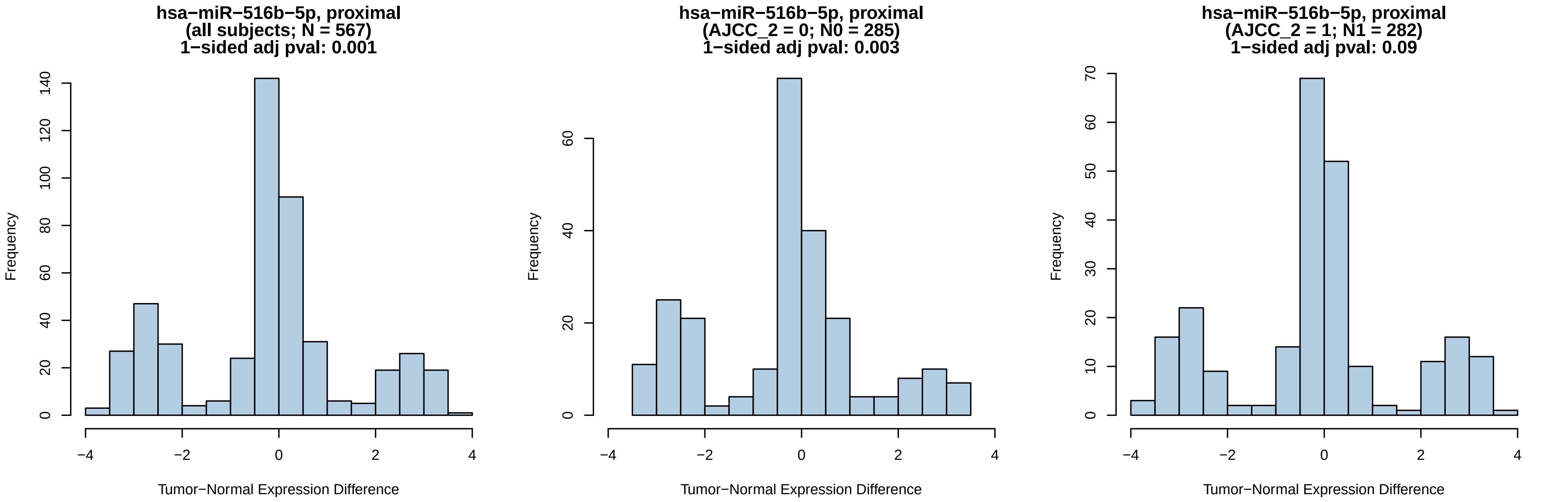

hsa−miR−516b−5p, proximal
(all subjects; N = 567)
1−sided adj pval: 0.001
hsa−miR−516b−5p, proximal
(AJCC_2 = 0; N0 = 285)
1−sided adj pval: 0.003
hsa−miR−516b−5p, proximal
(AJCC_2 = 1; N1 = 282)
1−sided adj pval: 0.09
Frequency
Tumor−Normal Expression Difference

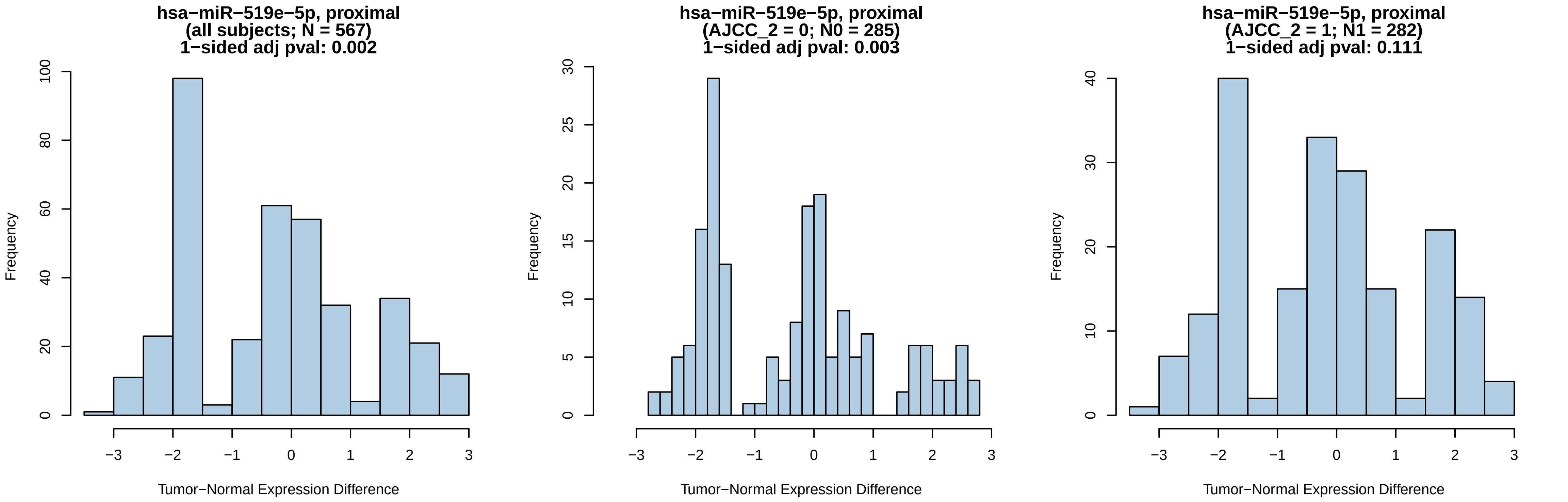

hsa−miR−519e−5p, proximal
(all subjects; N = 567)
1−sided adj pval: 0.002
Frequency
Tumor−Normal Expression Difference
hsa−miR−519e−5p, proximal
(AJCC_2 = 0; N0 = 285)
1−sided adj pval: 0.003
Frequency
Tumor−Normal Expression Difference
hsa−miR−519e−5p, proximal
(AJCC_2 = 1; N1 = 282)
1−sided adj pval: 0.111
Frequency
Tumor−Normal Expression Difference

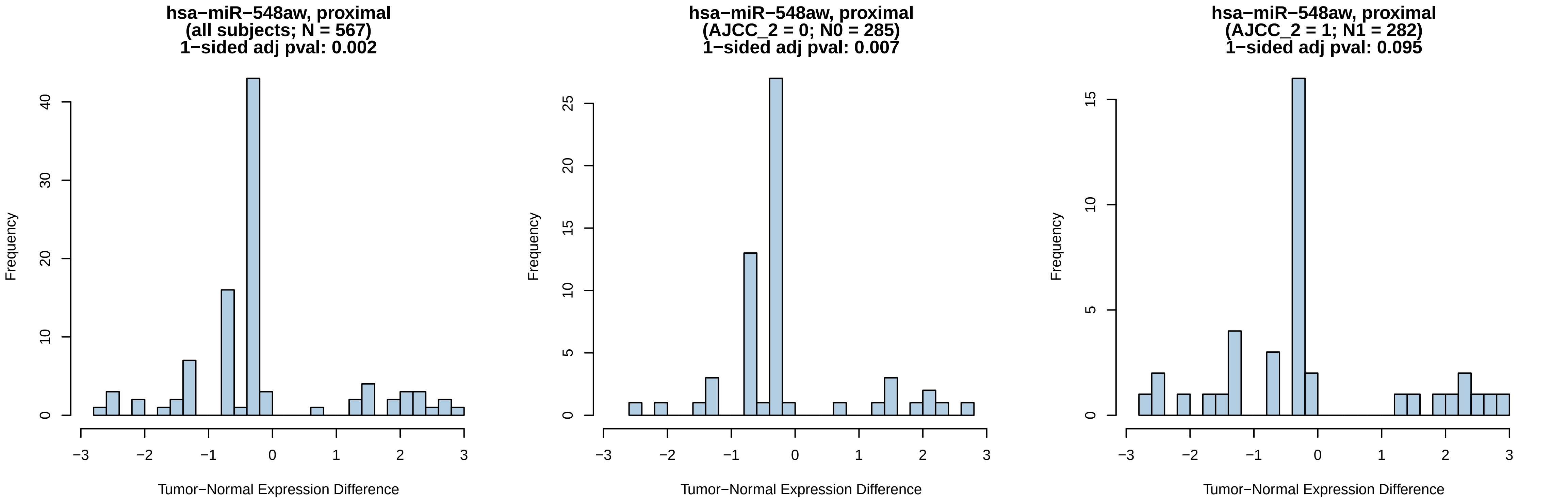

hsa−miR−548aw, proximal
(all subjects; N = 567)
1−sided adj pval: 0.002
Frequency
Tumor−Normal Expression Difference
hsa−miR−548aw, proximal
(AJCC_2 = 0; N0 = 285)
1−sided adj pval: 0.007
Frequency
Tumor−Normal Expression Difference
hsa−miR−548aw, proximal
(AJCC_2 = 1; N1 = 282)
1−sided adj pval: 0.095
Frequency
Tumor−Normal Expression Difference

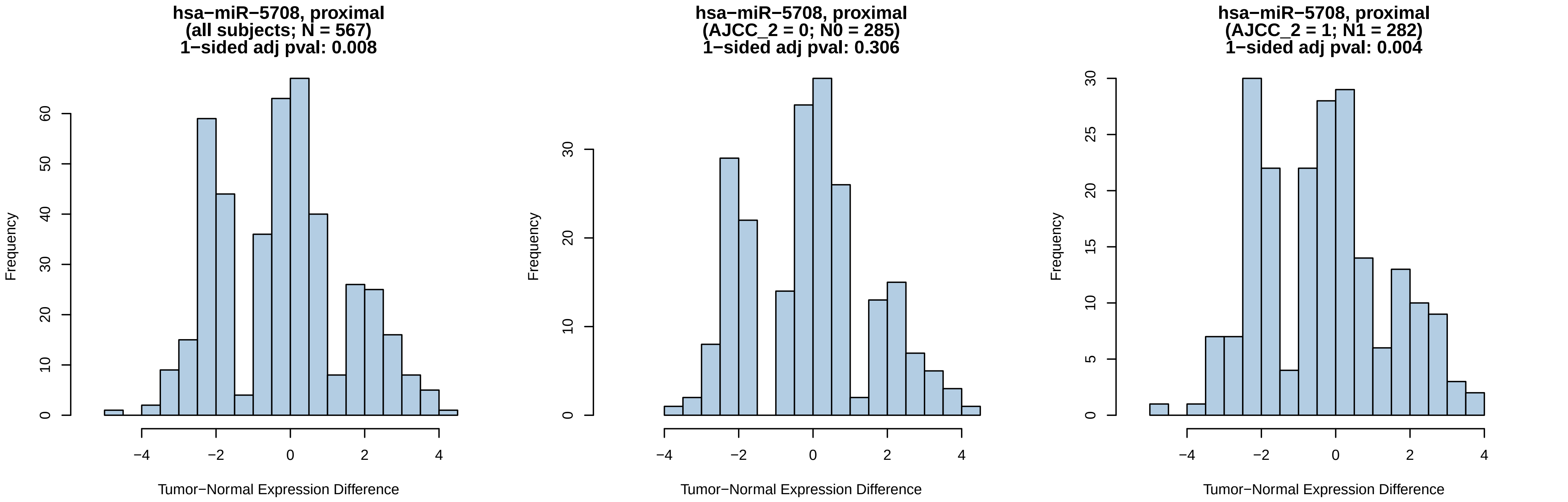
hsa−miR−5708, proximal
(all subjects; N = 567)
1−sided adj pval: 0.008
Frequency
Tumor−Normal Expression Difference
hsa−miR−5708, proximal
(AJCC_2 = 0; N0 = 285)
1−sided adj pval: 0.306
Frequency
Tumor−Normal Expression Difference
hsa−miR−5708, proximal
(AJCC_2 = 1; N1 = 282)
1−sided adj pval: 0.004
Frequency
Tumor−Normal Expression Difference

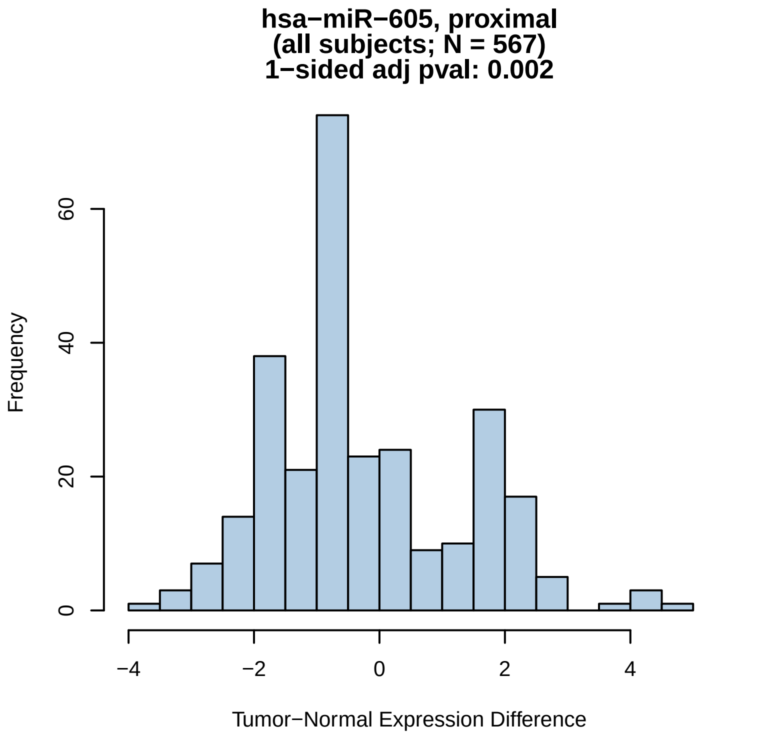
hsa−miR−605, proximal
(all subjects; N = 567)
1−sided adj pval: 0.002
Frequency
Tumor−Normal Expression Difference

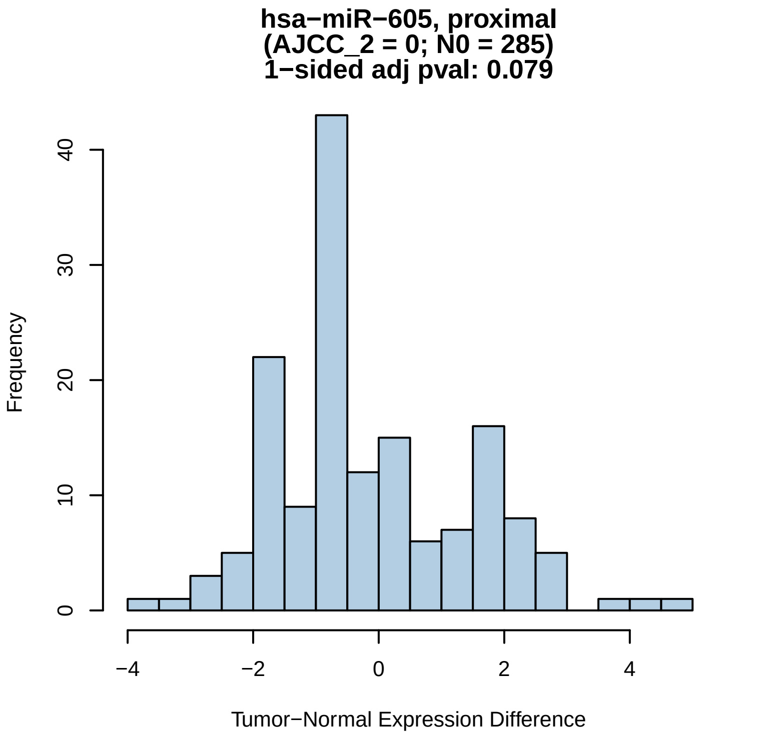
hsa−miR−605, proximal
(AJCC_2 = 0; N0 = 285)
1−sided adj pval: 0.079
Frequency
Tumor−Normal Expression Difference

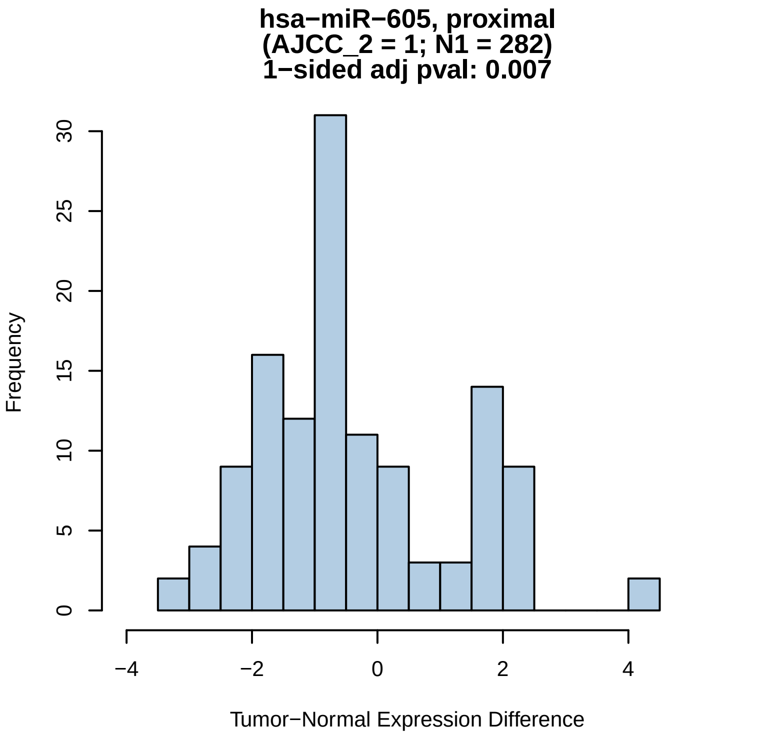
hsa−miR−605, proximal
(AJCC_2 = 1; N1 = 282)
1−sided adj pval: 0.007
Frequency
Tumor−Normal Expression Difference

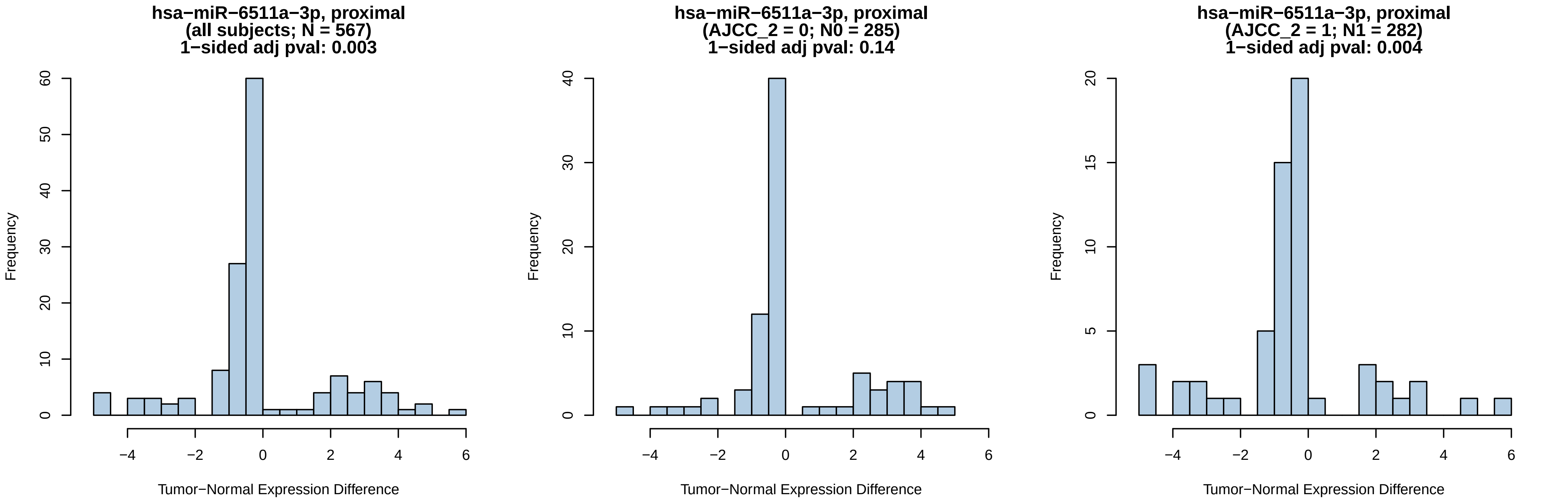

hsa−miR−6511a−3p, proximal
(all subjects; N = 567)
1−sided adj pval: 0.003
hsa−miR−6511a−3p, proximal
(AJCC_2 = 0; N0 = 285)
1−sided adj pval: 0.14
hsa−miR−6511a−3p, proximal
(AJCC_2 = 1; N1 = 282)
1−sided adj pval: 0.004
Frequency
Tumor−Normal Expression Difference

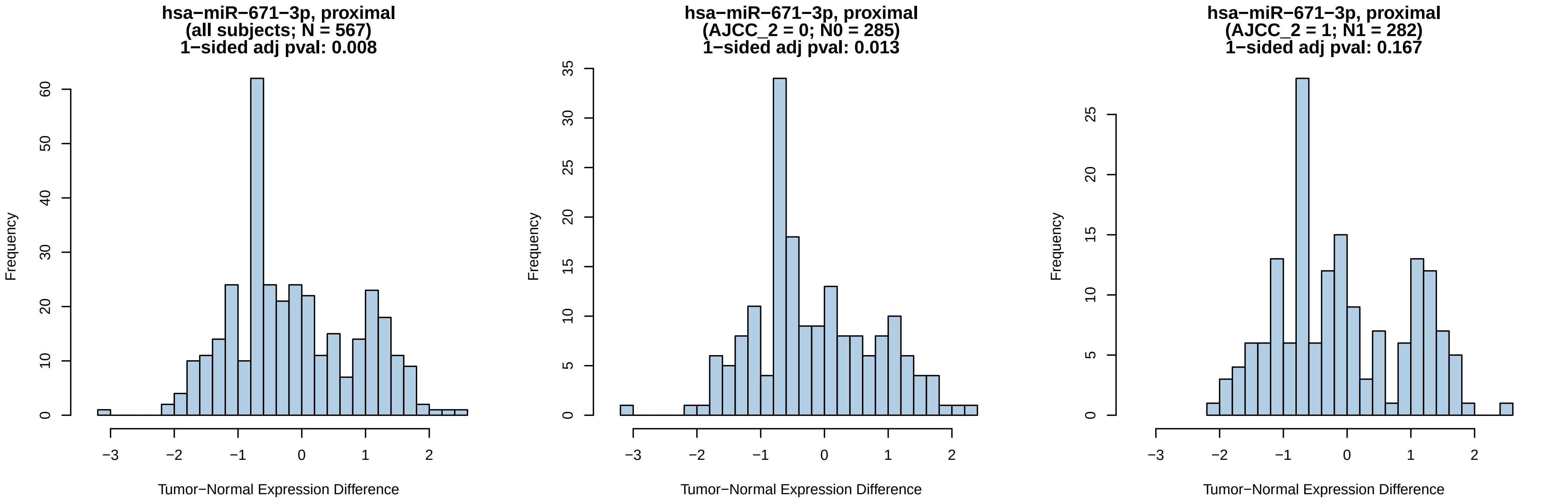

hsa−miR−671−3p, proximal
(all subjects; N = 567)
1−sided adj pval: 0.008
Frequency
Tumor−Normal Expression Difference
hsa−miR−671−3p, proximal
(AJCC_2 = 0; N0 = 285)
1−sided adj pval: 0.013
Frequency
Tumor−Normal Expression Difference
hsa−miR−671−3p, proximal
(AJCC_2 = 1; N1 = 282)
1−sided adj pval: 0.167
Frequency
Tumor−Normal Expression Difference

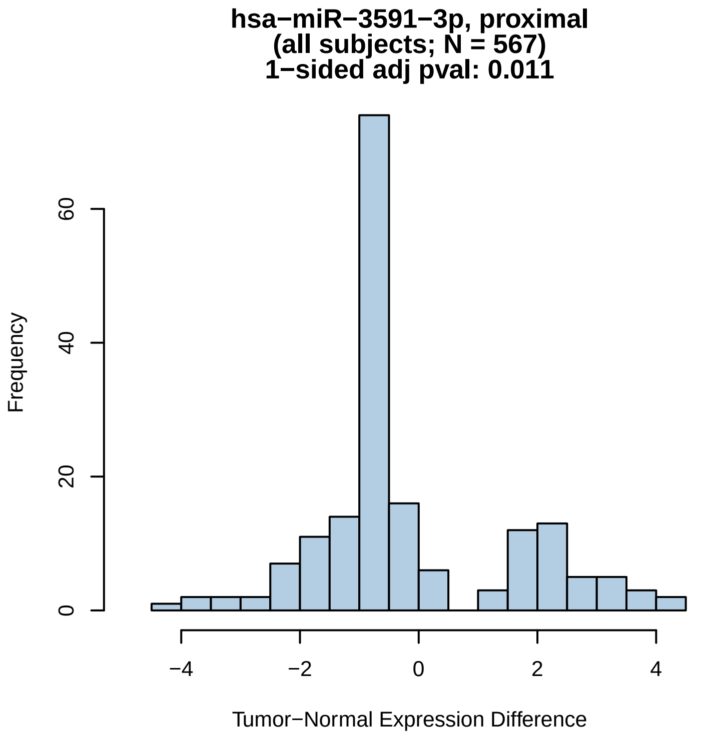
hsa−miR−3591−3p, proximal
(all subjects; N = 567)
1−sided adj pval: 0.011
Frequency
0
20
40
60
−4
−2
0
2
4
Tumor−Normal Expression Difference

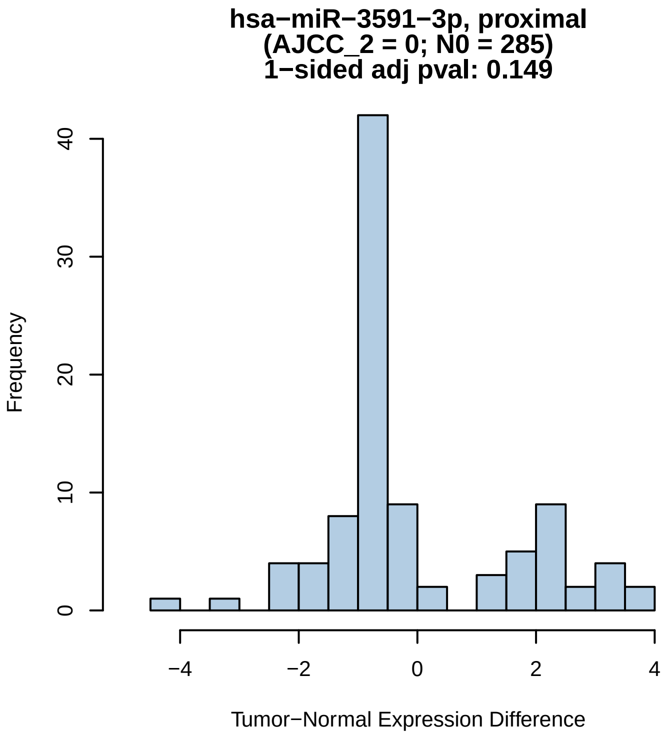
hsa−miR−3591−3p, proximal
(AJCC_2 = 0; N0 = 285)
1−sided adj pval: 0.149
Frequency
0
10
20
30
40
−4
−2
0
2
4
Tumor−Normal Expression Difference

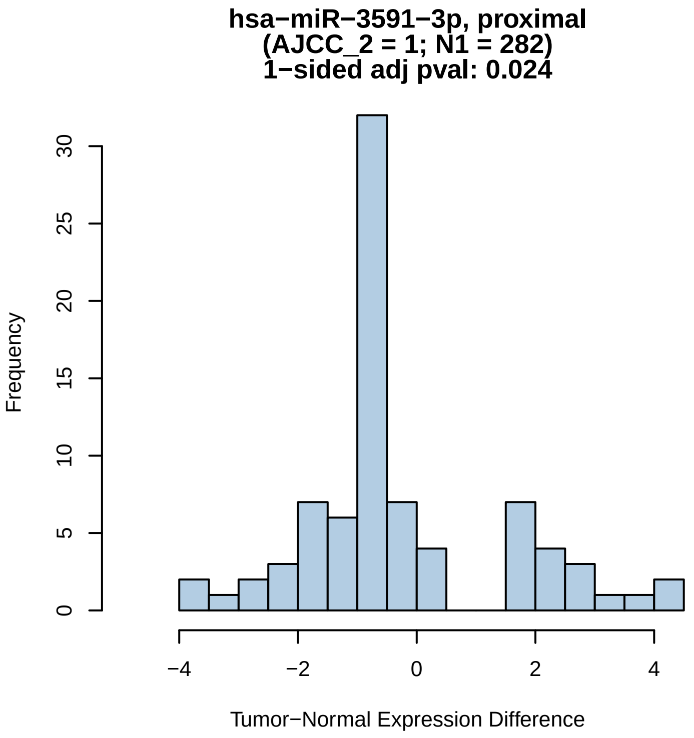
hsa−miR−3591−3p, proximal
(AJCC_2 = 1; N1 = 282)
1−sided adj pval: 0.024
Frequency
0
5
10
15
20
25
30
−4
−2
0
2
4
Tumor−Normal Expression Difference

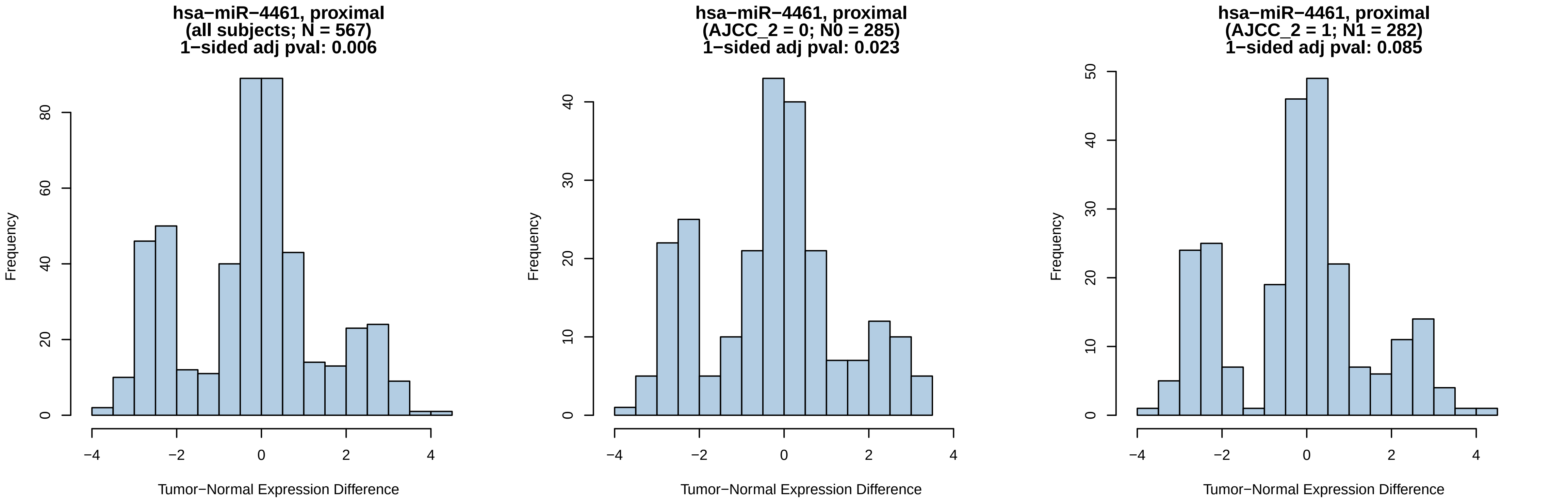
hsa−miR−4461, proximal
(all subjects; N = 567)
1−sided adj pval: 0.006
hsa−miR−4461, proximal
(AJCC_2 = 0; N0 = 285)
1−sided adj pval: 0.023
hsa−miR−4461, proximal
(AJCC_2 = 1; N1 = 282)
1−sided adj pval: 0.085
Frequency
Tumor−Normal Expression Difference

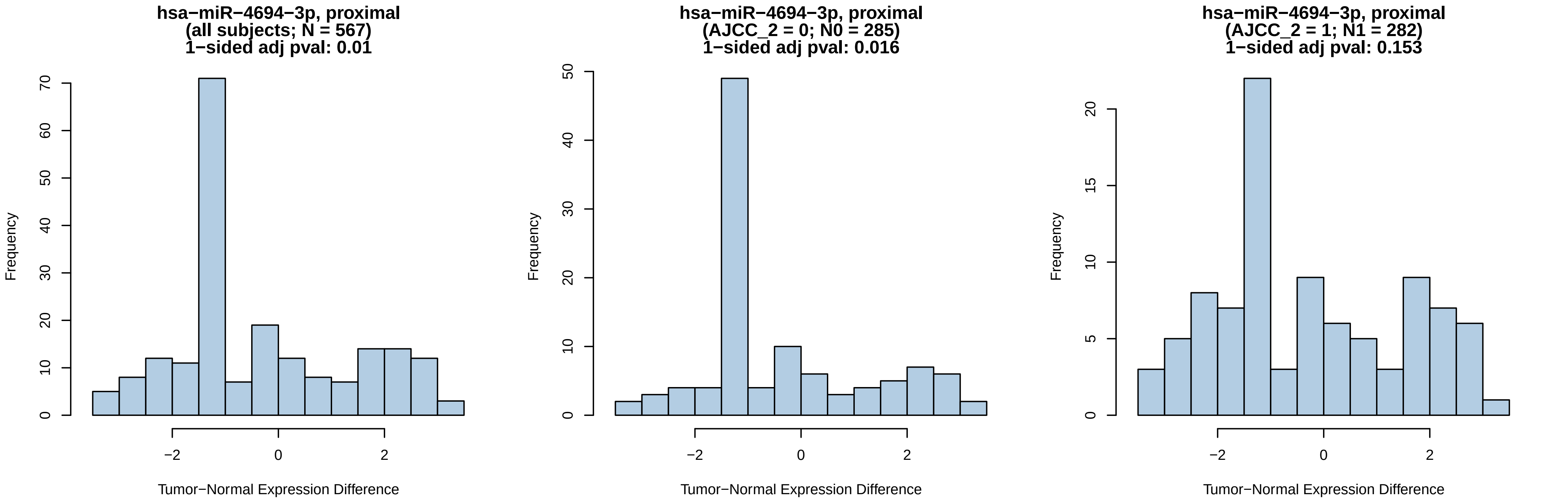

hsa−miR−4694−3p, proximal
(all subjects; N = 567)
1−sided adj pval: 0.01
Frequency
Tumor−Normal Expression Difference
hsa−miR−4694−3p, proximal
(AJCC_2 = 0; N0 = 285)
1−sided adj pval: 0.016
Frequency
Tumor−Normal Expression Difference
hsa−miR−4694−3p, proximal
(AJCC_2 = 1; N1 = 282)
1−sided adj pval: 0.153
Frequency
Tumor−Normal Expression Difference

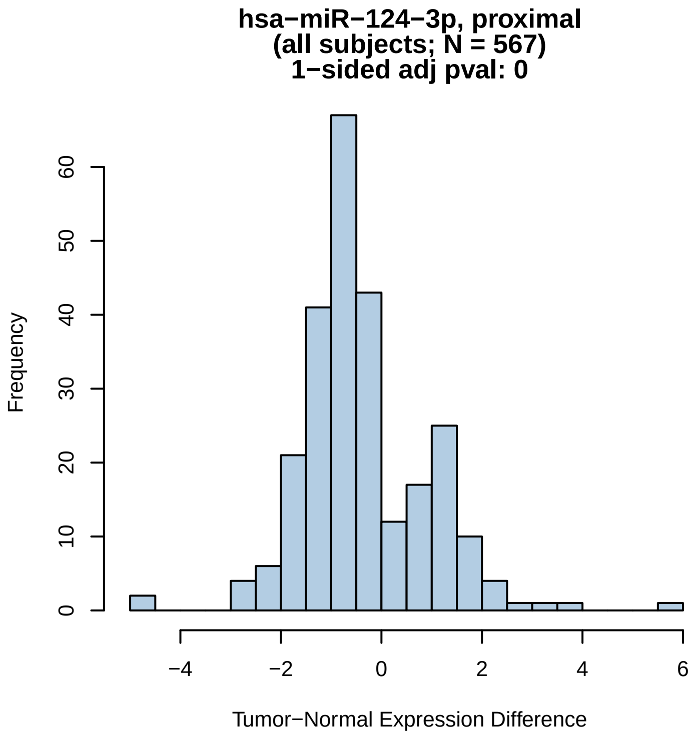
hsa−miR−124−3p, proximal
(all subjects; N = 567)
1−sided adj pval: 0
Frequency
0
10
20
30
40
50
60
−4
−2
0
2
4
6
Tumor−Normal Expression Difference

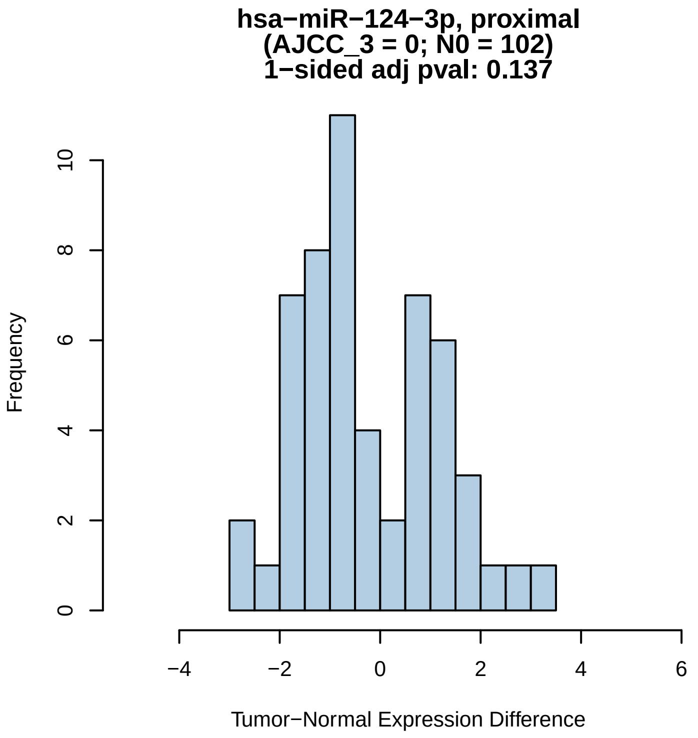
hsa−miR−124−3p, proximal
(AJCC_3 = 0; N0 = 102)
1−sided adj pval: 0.137
Frequency
0
2
4
6
8
10
−4
−2
0
2
4
6
Tumor−Normal Expression Difference

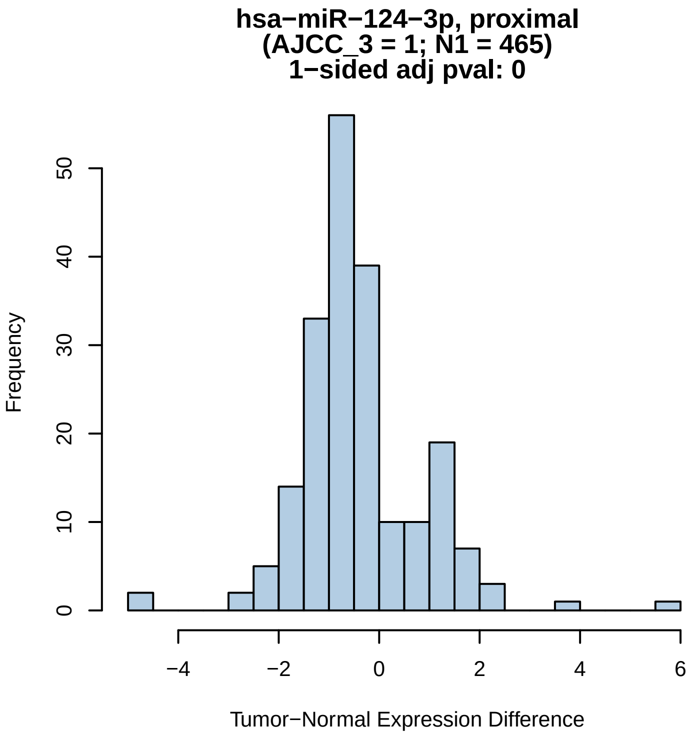
hsa−miR−124−3p, proximal
(AJCC_3 = 1; N1 = 465)
1−sided adj pval: 0
Frequency
0
10
20
30
40
50
−4
−2
0
2
4
6
Tumor−Normal Expression Difference

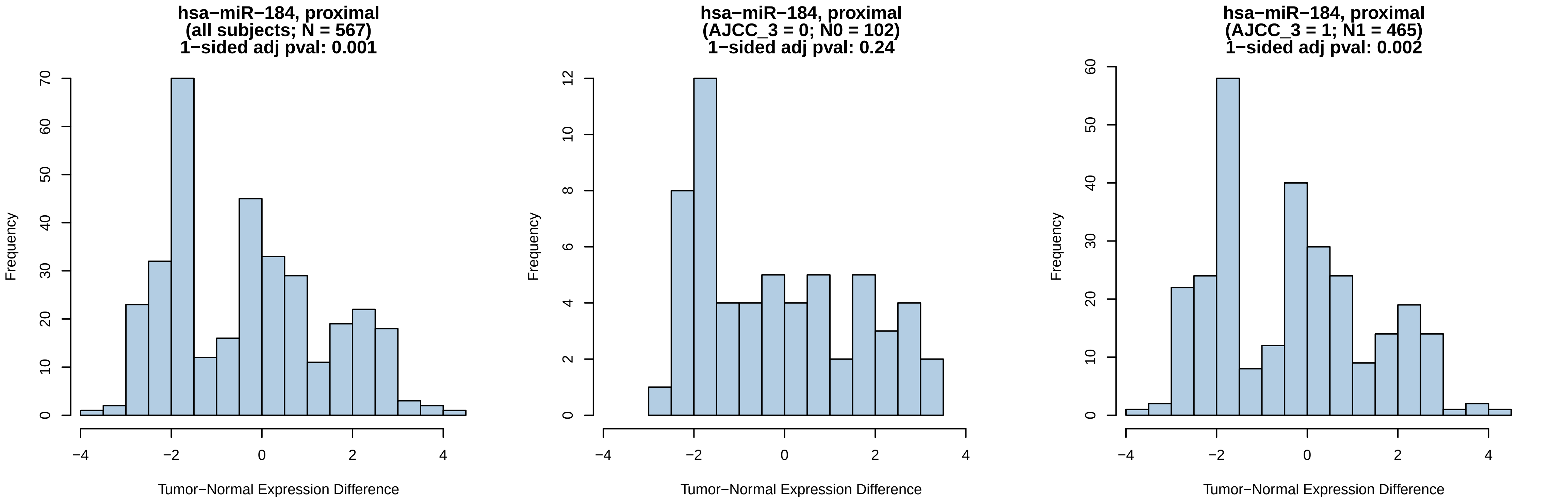
hsa−miR−184, proximal
(all subjects; N = 567)
1−sided adj pval: 0.001
Frequency
0
10
20
30
40
50
60
70
−4
−2
0
2
4
Tumor−Normal Expression Difference
hsa−miR−184, proximal
(AJCC_3 = 0; N0 = 102)
1−sided adj pval: 0.24
Frequency
0
2
4
6
8
10
12
−4
−2
0
2
4
Tumor−Normal Expression Difference
hsa−miR−184, proximal
(AJCC_3 = 1; N1 = 465)
1−sided adj pval: 0.002
Frequency
0
10
20
30
40
50
60
−4
−2
0
2
4
Tumor−Normal Expression Difference

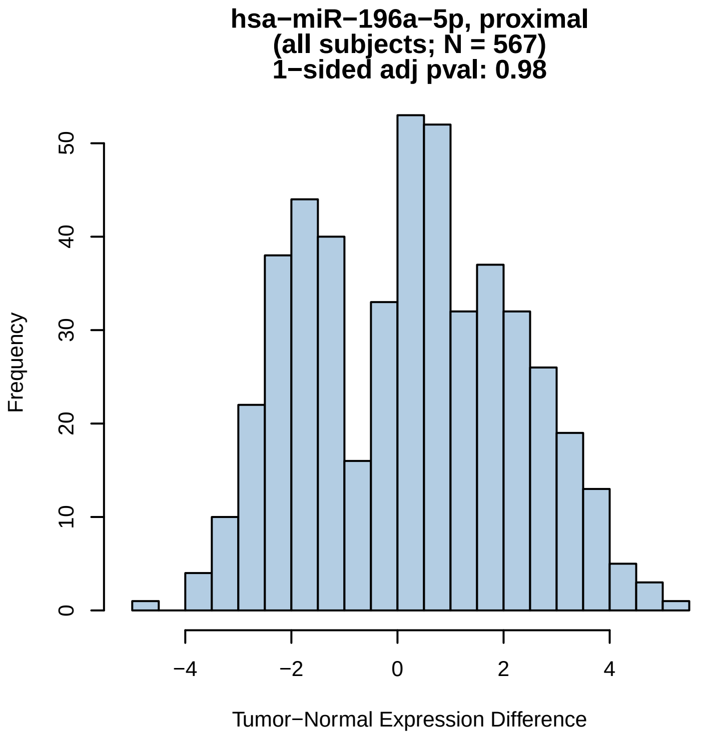
hsa−miR−196a−5p, proximal
(all subjects; N = 567)
1−sided adj pval: 0.98
Frequency
Tumor−Normal Expression Difference

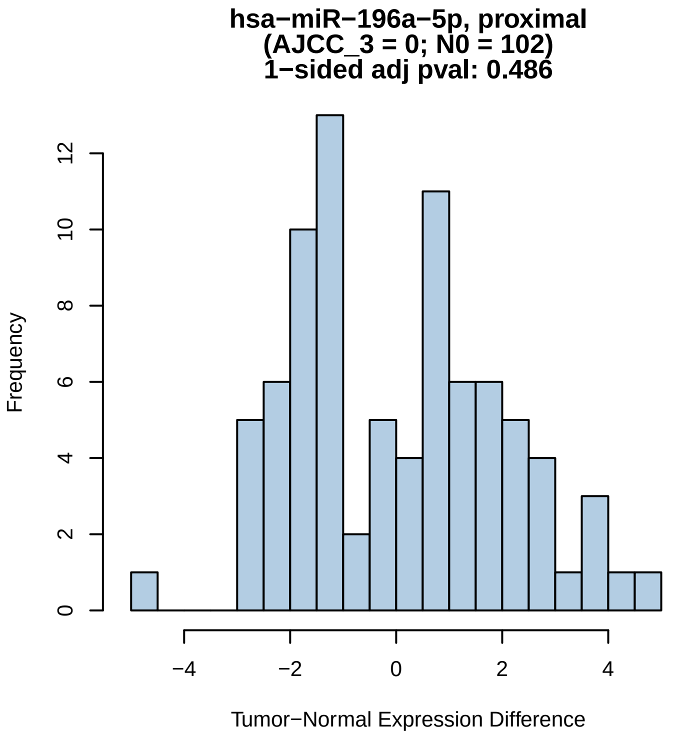
hsa−miR−196a−5p, proximal
(AJCC_3 = 0; N0 = 102)
1−sided adj pval: 0.486
Frequency
Tumor−Normal Expression Difference

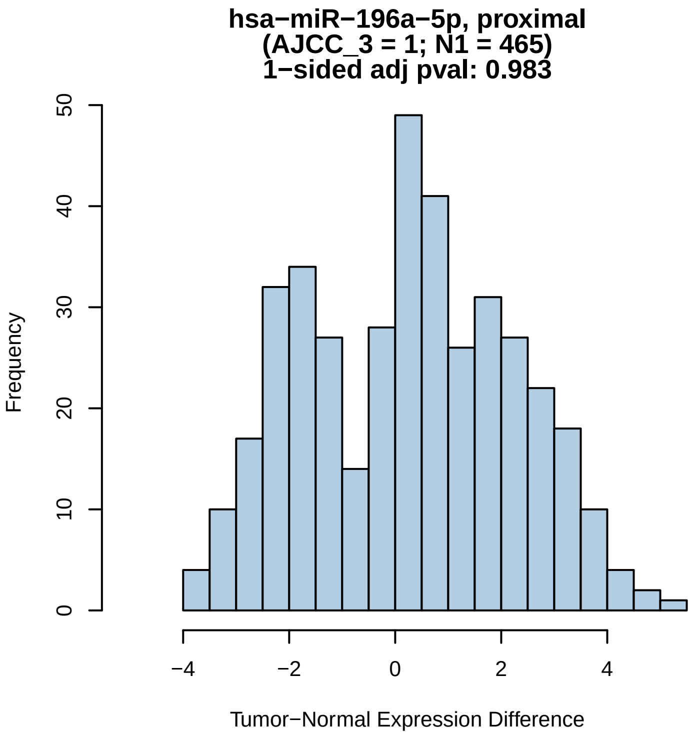
hsa−miR−196a−5p, proximal
(AJCC_3 = 1; N1 = 465)
1−sided adj pval: 0.983
Frequency
Tumor−Normal Expression Difference

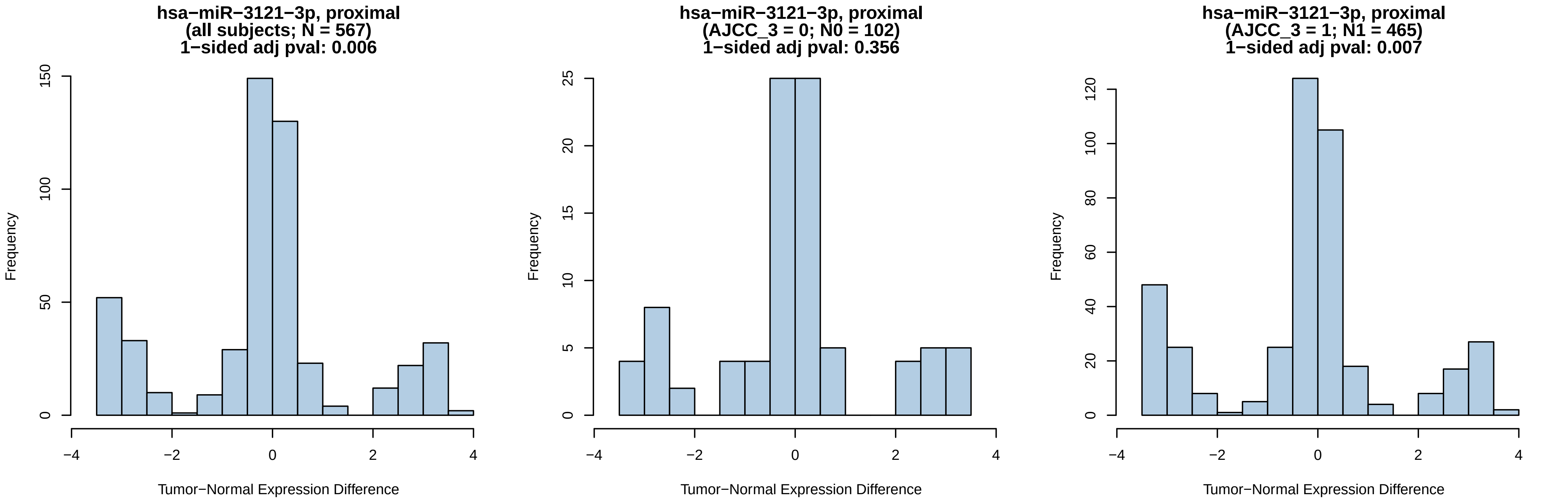

hsa−miR−3121−3p, proximal
(all subjects; N = 567)
1−sided adj pval: 0.006
Frequency
Tumor−Normal Expression Difference
hsa−miR−3121−3p, proximal
(AJCC_3 = 0; N0 = 102)
1−sided adj pval: 0.356
Frequency
Tumor−Normal Expression Difference
hsa−miR−3121−3p, proximal
(AJCC_3 = 1; N1 = 465)
1−sided adj pval: 0.007
Frequency
Tumor−Normal Expression Difference

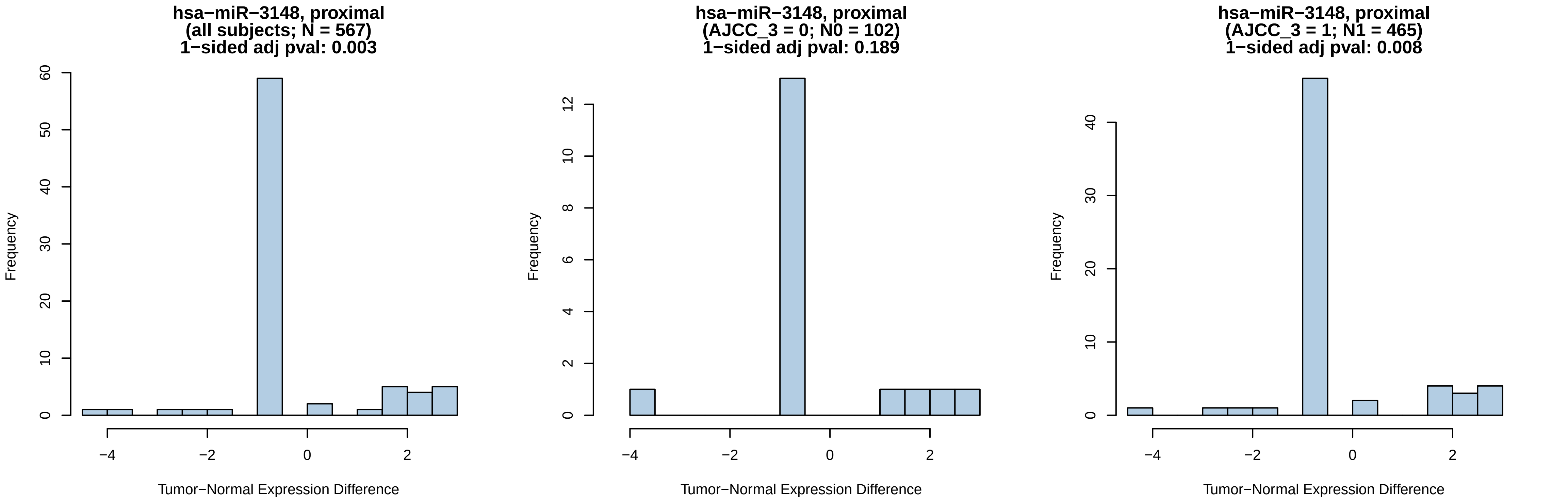

hsa−miR−3148, proximal
(all subjects; N = 567)
1−sided adj pval: 0.003
Frequency
0
10
20
30
40
50
60
−4
−2
0
2
Tumor−Normal Expression Difference
hsa−miR−3148, proximal
(AJCC_3 = 0; N0 = 102)
1−sided adj pval: 0.189
Frequency
0
2
4
6
8
10
12
−4
−2
0
2
Tumor−Normal Expression Difference
hsa−miR−3148, proximal
(AJCC_3 = 1; N1 = 465)
1−sided adj pval: 0.008
Frequency
0
10
20
30
40
−4
−2
0
2
Tumor−Normal Expression Difference

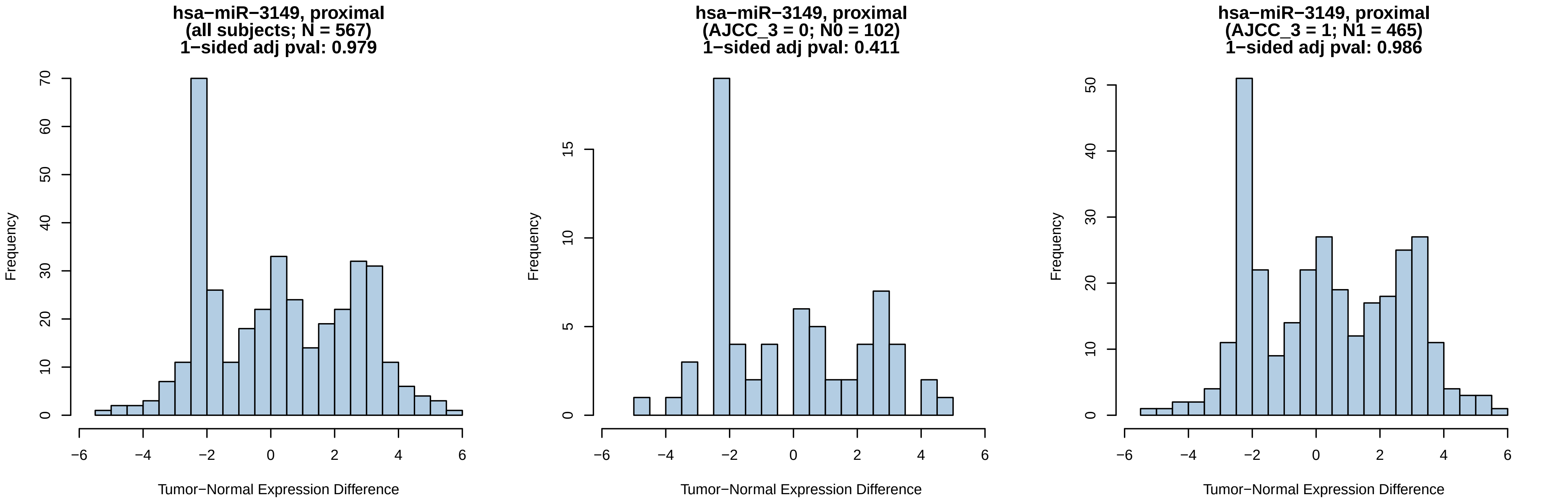
hsa−miR−3149, proximal
(all subjects; N = 567)
1−sided adj pval: 0.979
hsa−miR−3149, proximal
(AJCC_3 = 0; N0 = 102)
1−sided adj pval: 0.411
hsa−miR−3149, proximal
(AJCC_3 = 1; N1 = 465)
1−sided adj pval: 0.986
Frequency
Tumor−Normal Expression Difference

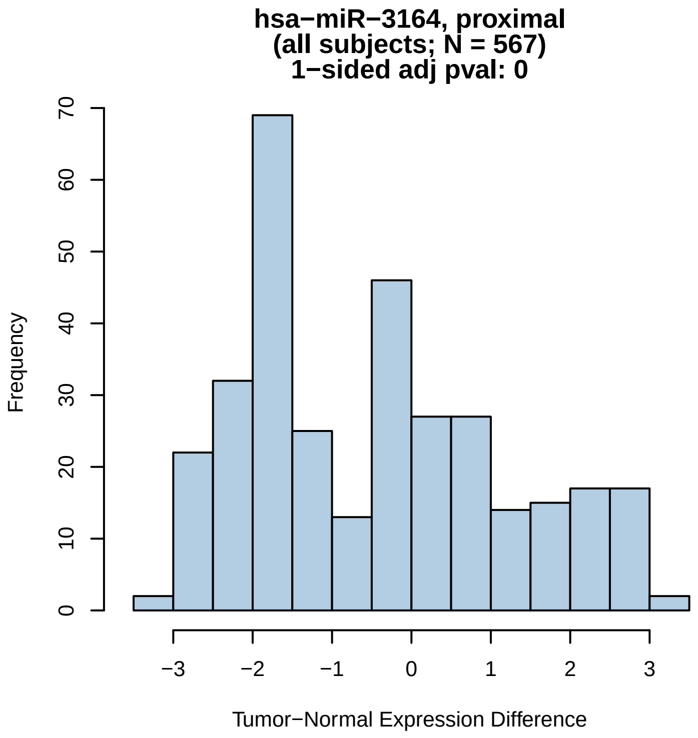
hsa−miR−3164, proximal
(all subjects; N = 567)
1−sided adj pval: 0
Frequency
Tumor−Normal Expression Difference
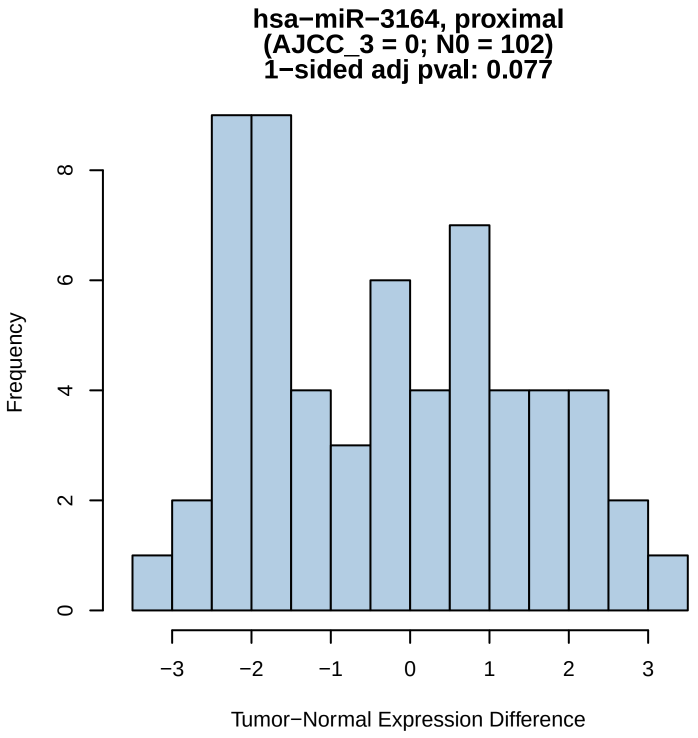
hsa−miR−3164, proximal
(AJCC_3 = 0; N0 = 102)
1−sided adj pval: 0.077
Frequency
Tumor−Normal Expression Difference
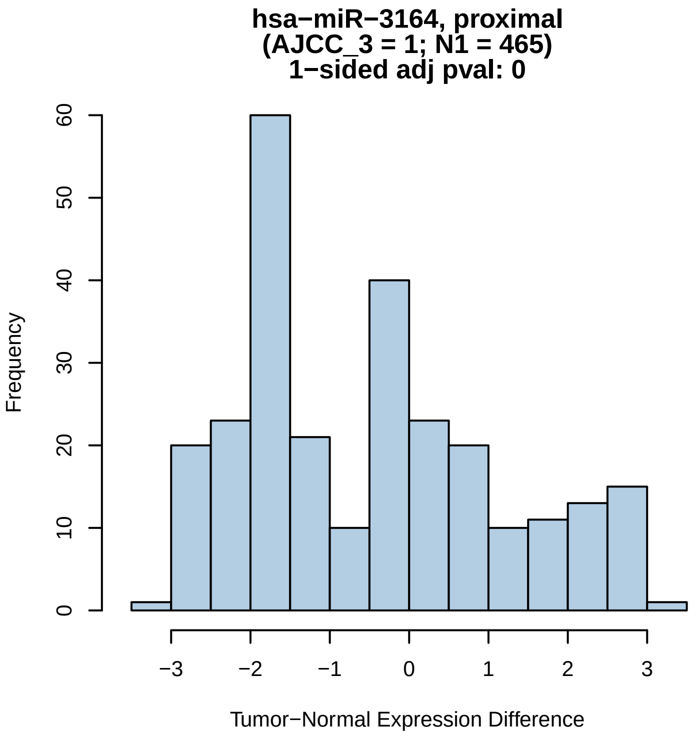
hsa−miR−3164, proximal
(AJCC_3 = 1; N1 = 465)
1−sided adj pval: 0
Frequency
Tumor−Normal Expression Difference

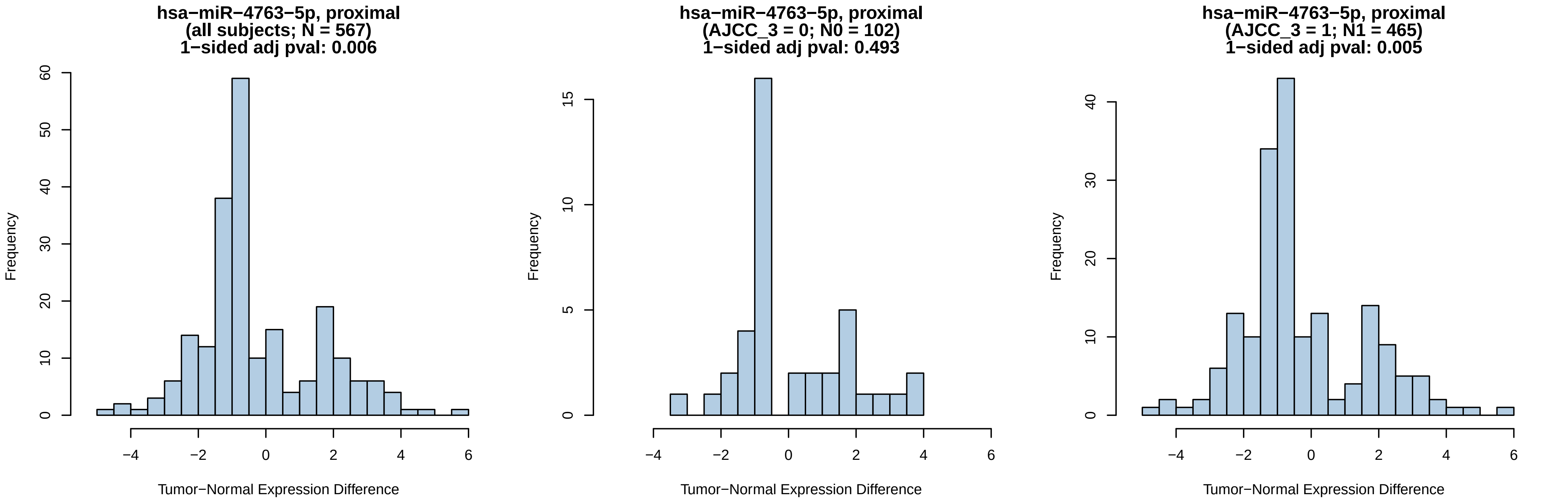

hsa−miR−4763−5p, proximal
(all subjects; N = 567)
1−sided adj pval: 0.006
Frequency
Tumor−Normal Expression Difference
hsa−miR−4763−5p, proximal
(AJCC_3 = 0; N0 = 102)
1−sided adj pval: 0.493
Frequency
Tumor−Normal Expression Difference
hsa−miR−4763−5p, proximal
(AJCC_3 = 1; N1 = 465)
1−sided adj pval: 0.005
Frequency
Tumor−Normal Expression Difference

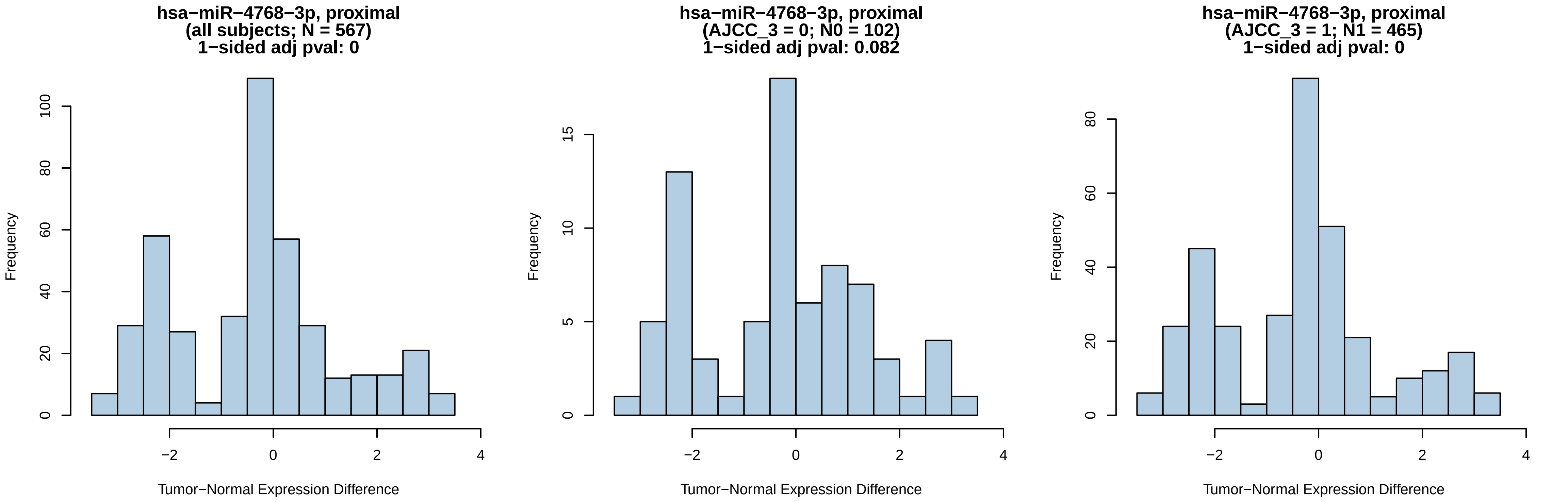
hsa−miR−4768−3p, proximal
(all subjects; N = 567)
1−sided adj pval: 0
Frequency
0
20
40
60
80
100
−2
0
2
4
Tumor−Normal Expression Difference
hsa−miR−4768−3p, proximal
(AJCC_3 = 0; N0 = 102)
1−sided adj pval: 0.082
Frequency
0
5
10
15
−2
0
2
4
Tumor−Normal Expression Difference
hsa−miR−4768−3p, proximal
(AJCC_3 = 1; N1 = 465)
1−sided adj pval: 0
Frequency
0
20
40
60
80
−2
0
2
4
Tumor−Normal Expression Difference

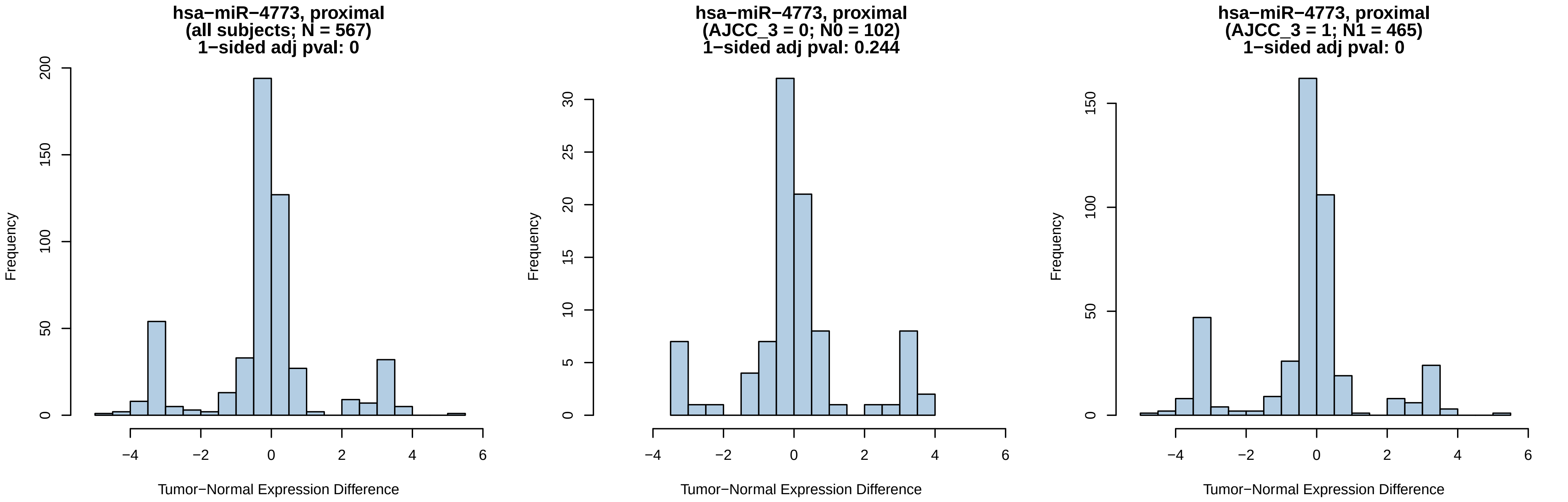
hsa−miR−4773, proximal
(all subjects; N = 567)
1−sided adj pval: 0
hsa−miR−4773, proximal
(AJCC_3 = 0; N0 = 102)
1−sided adj pval: 0.244
hsa−miR−4773, proximal
(AJCC_3 = 1; N1 = 465)
1−sided adj pval: 0
Frequency
Tumor−Normal Expression Difference

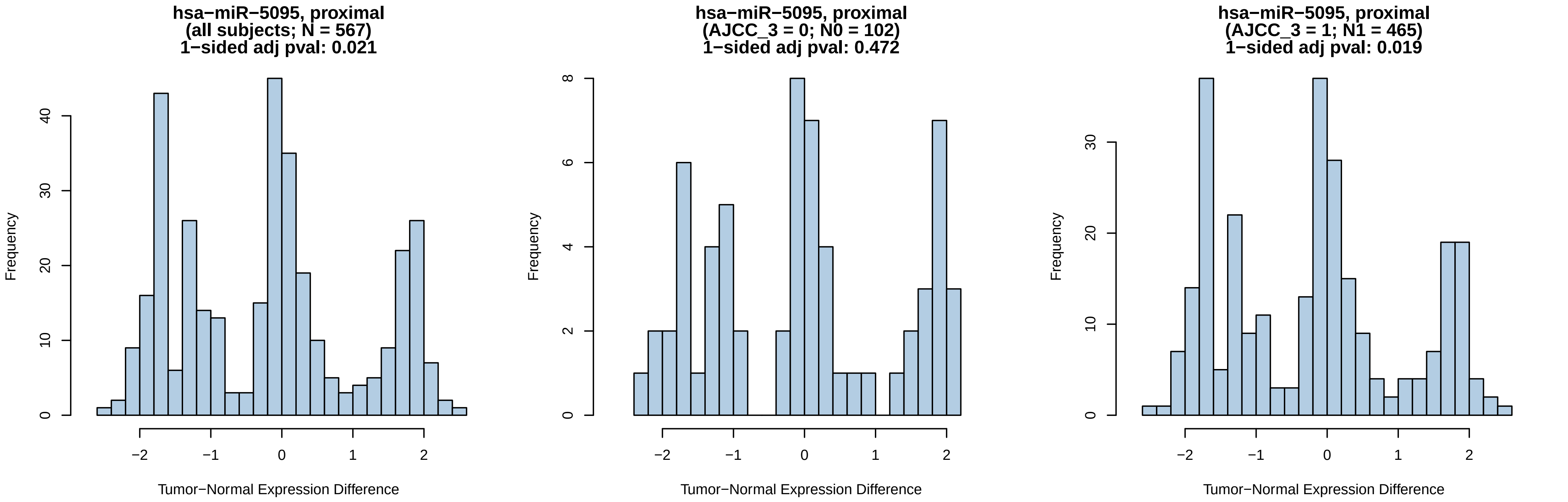
hsa−miR−5095, proximal
(all subjects; N = 567)
1−sided adj pval: 0.021
Frequency
0
10
20
30
40
−2
−1
0
1
2
Tumor−Normal Expression Difference
hsa−miR−5095, proximal
(AJCC_3 = 0; N0 = 102)
1−sided adj pval: 0.472
Frequency
0
2
4
6
8
−2
−1
0
1
2
Tumor−Normal Expression Difference
hsa−miR−5095, proximal
(AJCC_3 = 1; N1 = 465)
1−sided adj pval: 0.019
Frequency
0
10
20
30
−2
−1
0
1
2
Tumor−Normal Expression Difference

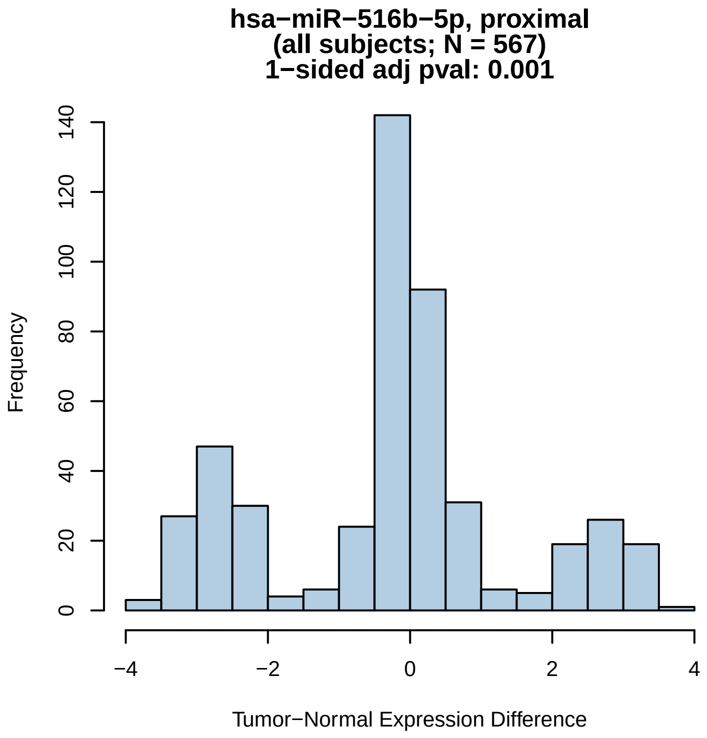
hsa−miR−516b−5p, proximal
(all subjects; N = 567)
1−sided adj pval: 0.001
Frequency
0
20
40
60
80
100
120
140
−4
−2
0
2
4
Tumor−Normal Expression Difference

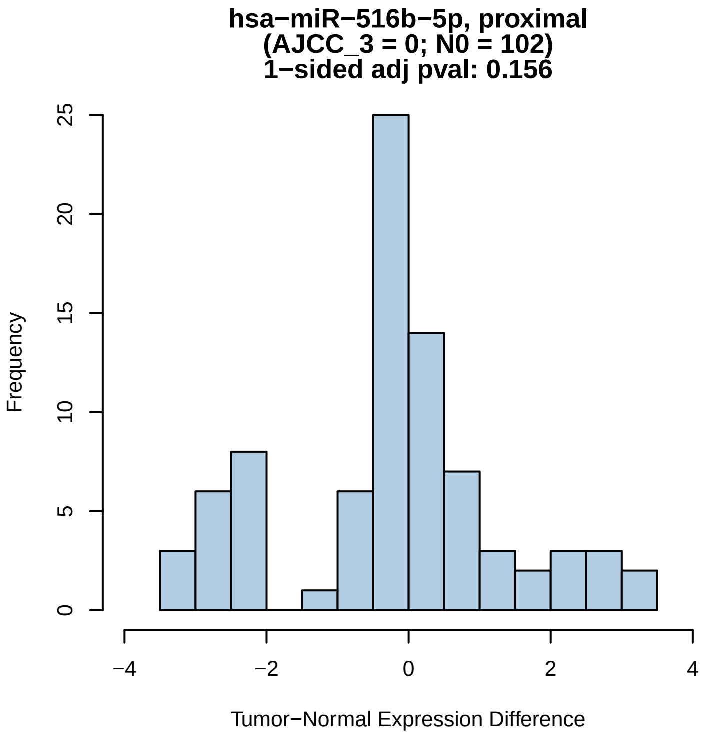
hsa−miR−516b−5p, proximal
(AJCC_3 = 0; N0 = 102)
1−sided adj pval: 0.156
Frequency
0
5
10
15
20
25
−4
−2
0
2
4
Tumor−Normal Expression Difference

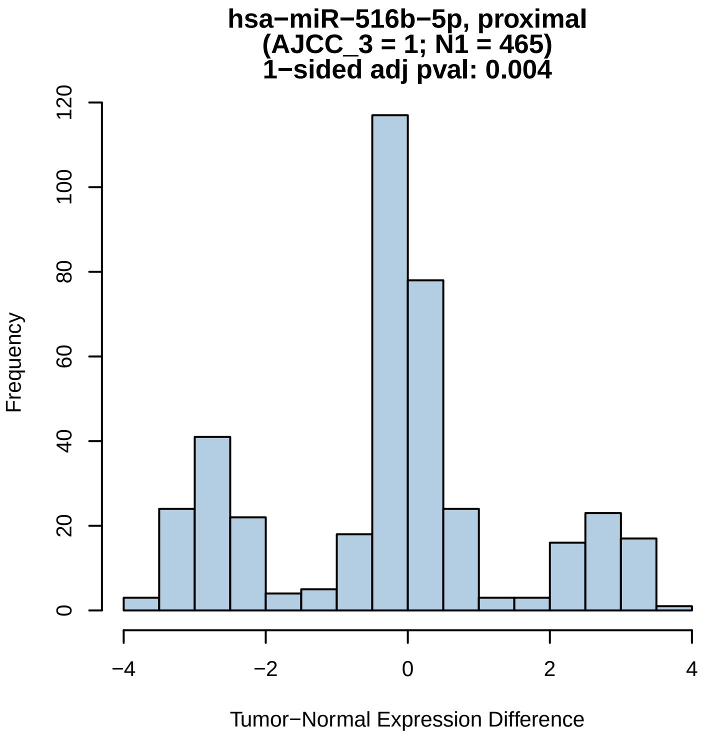
hsa−miR−516b−5p, proximal
(AJCC_3 = 1; N1 = 465)
1−sided adj pval: 0.004
Frequency
0
20
40
60
80
100
120
−4
−2
0
2
4
Tumor−Normal Expression Difference

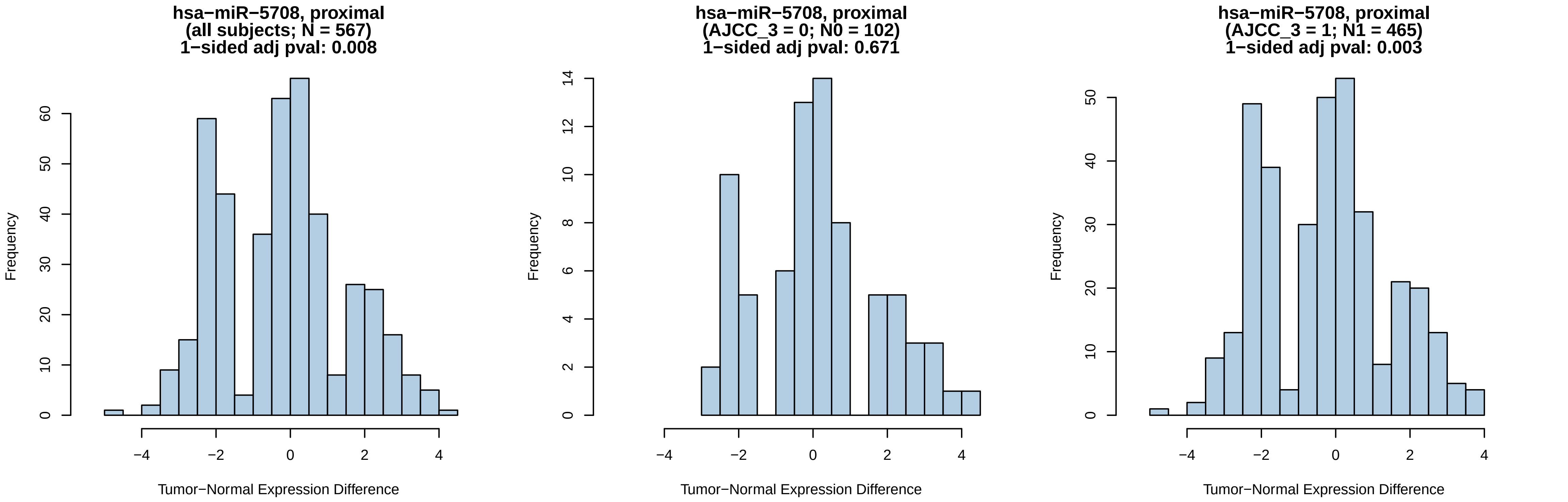

hsa−miR−5708, proximal
(all subjects; N = 567)
1−sided adj pval: 0.008
Frequency
Tumor−Normal Expression Difference
hsa−miR−5708, proximal
(AJCC_3 = 0; N0 = 102)
1−sided adj pval: 0.671
Frequency
Tumor−Normal Expression Difference
hsa−miR−5708, proximal
(AJCC_3 = 1; N1 = 465)
1−sided adj pval: 0.003
Frequency
Tumor−Normal Expression Difference

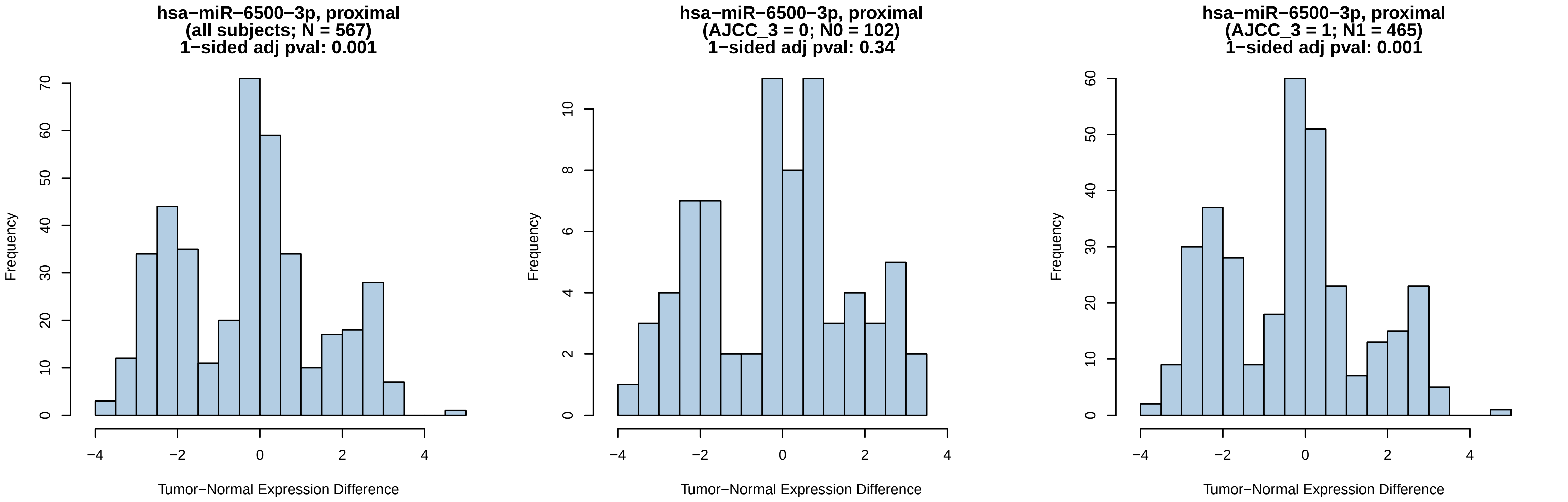

hsa−miR−6500−3p, proximal
(all subjects; N = 567)
1−sided adj pval: 0.001
Frequency
Tumor−Normal Expression Difference
hsa−miR−6500−3p, proximal
(AJCC_3 = 0; N0 = 102)
1−sided adj pval: 0.34
Frequency
Tumor−Normal Expression Difference
hsa−miR−6500−3p, proximal
(AJCC_3 = 1; N1 = 465)
1−sided adj pval: 0.001
Frequency
Tumor−Normal Expression Difference

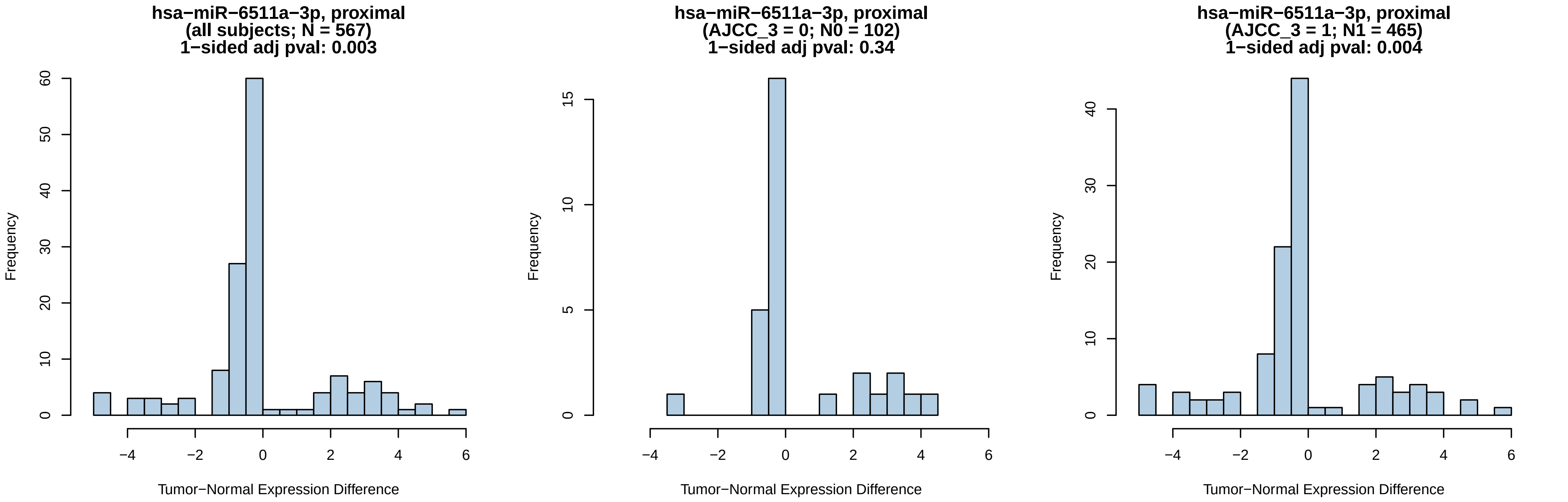

hsa−miR−6511a−3p, proximal
(all subjects; N = 567)
1−sided adj pval: 0.003
hsa−miR−6511a−3p, proximal
(AJCC_3 = 0; N0 = 102)
1−sided adj pval: 0.34
hsa−miR−6511a−3p, proximal
(AJCC_3 = 1; N1 = 465)
1−sided adj pval: 0.004
Frequency
Tumor−Normal Expression Difference

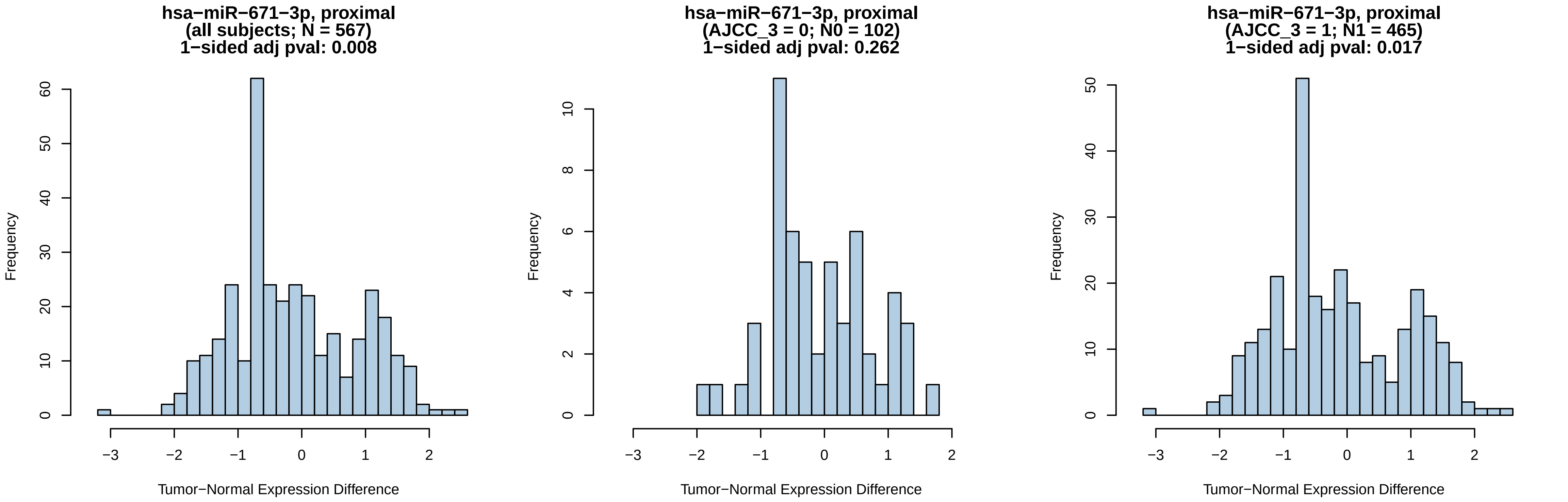

hsa−miR−671−3p, proximal
(all subjects; N = 567)
1−sided adj pval: 0.008
Frequency
Tumor−Normal Expression Difference
hsa−miR−671−3p, proximal
(AJCC_3 = 0; N0 = 102)
1−sided adj pval: 0.262
Frequency
Tumor−Normal Expression Difference
hsa−miR−671−3p, proximal
(AJCC_3 = 1; N1 = 465)
1−sided adj pval: 0.017
Frequency
Tumor−Normal Expression Difference

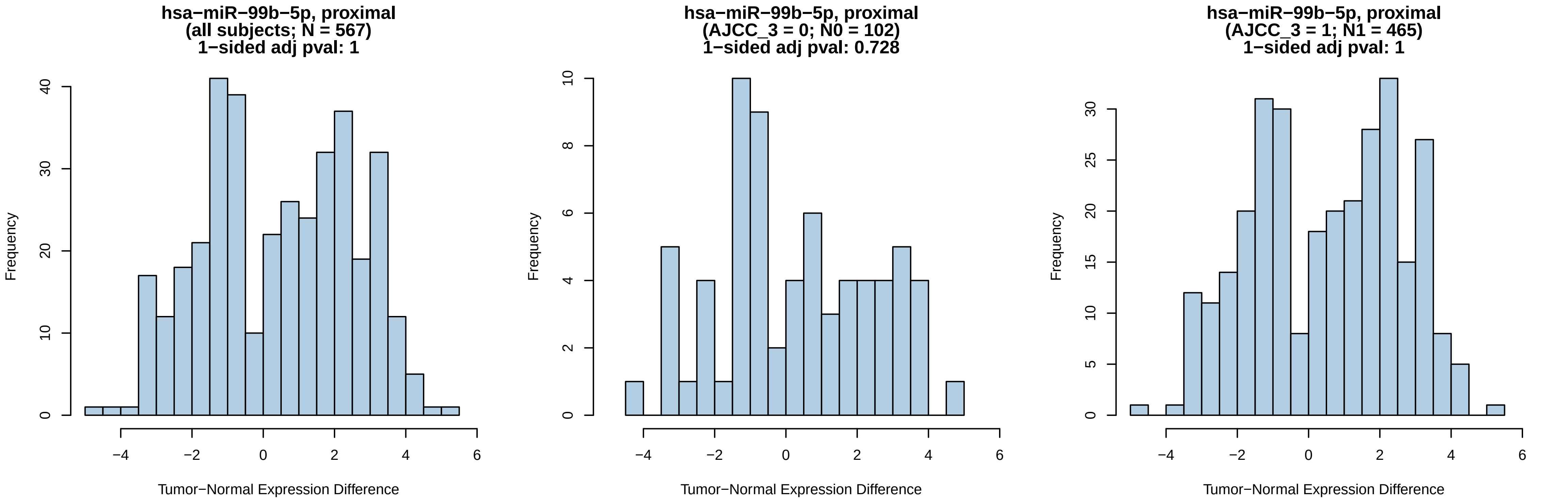
hsa−miR−99b−5p, proximal
(all subjects; N = 567)
1−sided adj pval: 1
Frequency
Tumor−Normal Expression Difference
hsa−miR−99b−5p, proximal
(AJCC_3 = 0; N0 = 102)
1−sided adj pval: 0.728
Frequency
Tumor−Normal Expression Difference
hsa−miR−99b−5p, proximal
(AJCC_3 = 1; N1 = 465)
1−sided adj pval: 1
Frequency
Tumor−Normal Expression Difference

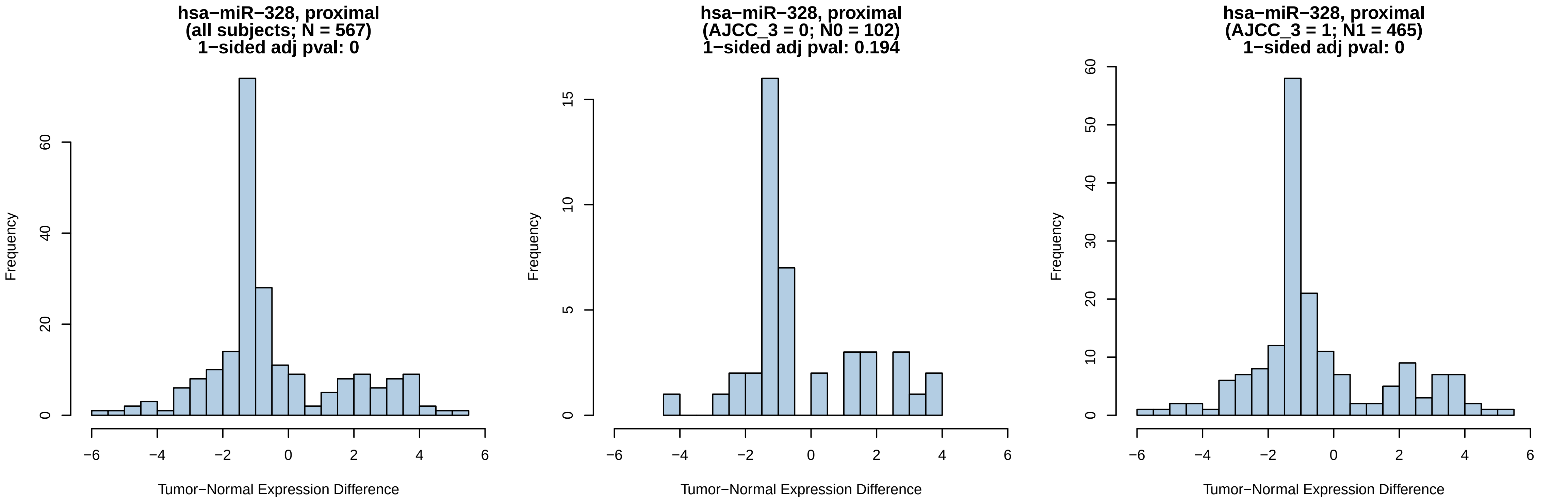
hsa−miR−328, proximal
(all subjects; N = 567)
1−sided adj pval: 0
Frequency
Tumor−Normal Expression Difference
hsa−miR−328, proximal
(AJCC_3 = 0; N0 = 102)
1−sided adj pval: 0.194
Frequency
Tumor−Normal Expression Difference
hsa−miR−328, proximal
(AJCC_3 = 1; N1 = 465)
1−sided adj pval: 0
Frequency
Tumor−Normal Expression Difference

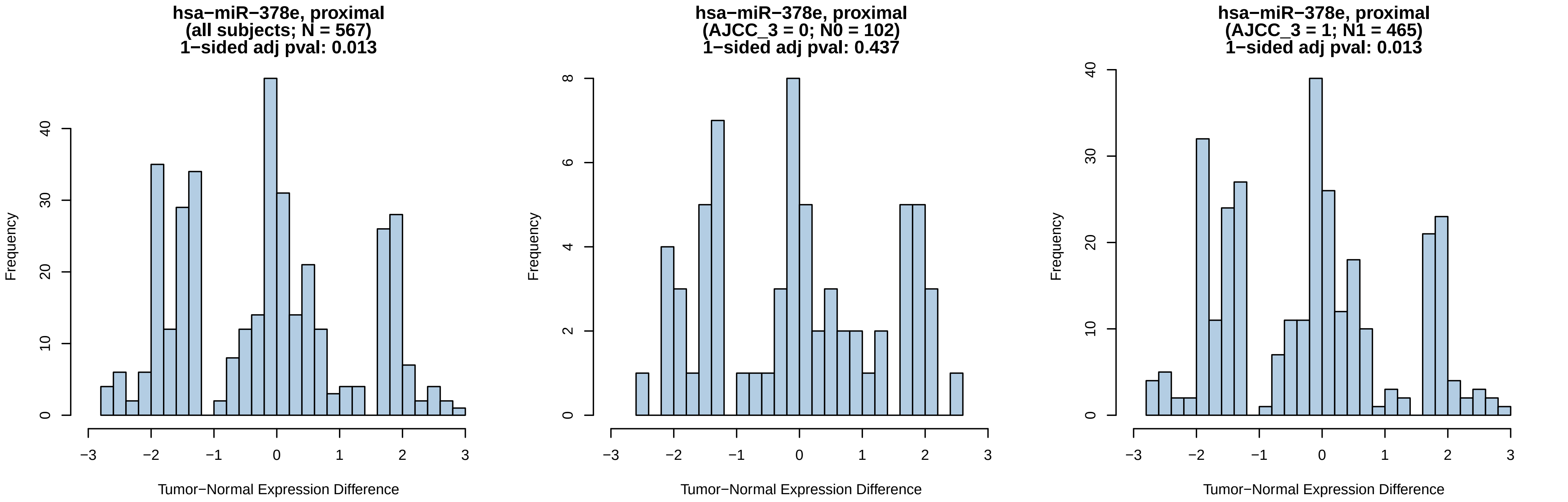
hsa−miR−378e, proximal
(all subjects; N = 567)
1−sided adj pval: 0.013
hsa−miR−378e, proximal
(AJCC_3 = 0; N0 = 102)
1−sided adj pval: 0.437
hsa−miR−378e, proximal
(AJCC_3 = 1; N1 = 465)
1−sided adj pval: 0.013
Frequency
Tumor−Normal Expression Difference

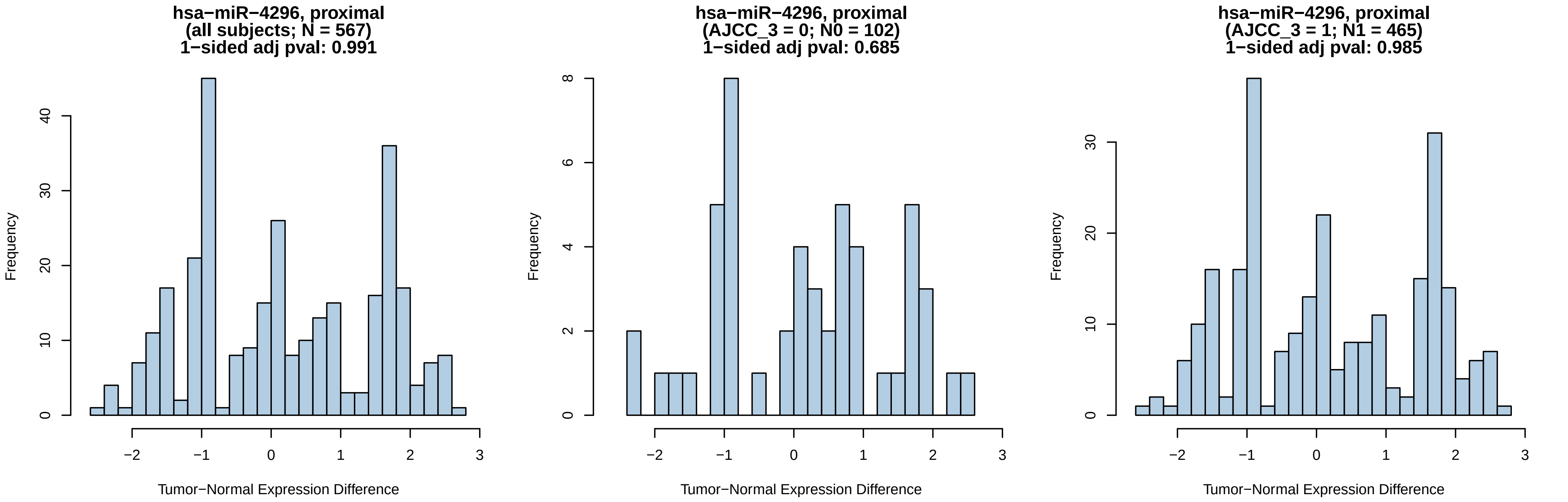

hsa−miR−4296, proximal
(all subjects; N = 567)
1−sided adj pval: 0.991
hsa−miR−4296, proximal
(AJCC_3 = 0; N0 = 102)
1−sided adj pval: 0.685
hsa−miR−4296, proximal
(AJCC_3 = 1; N1 = 465)
1−sided adj pval: 0.985
Frequency
Tumor−Normal Expression Difference

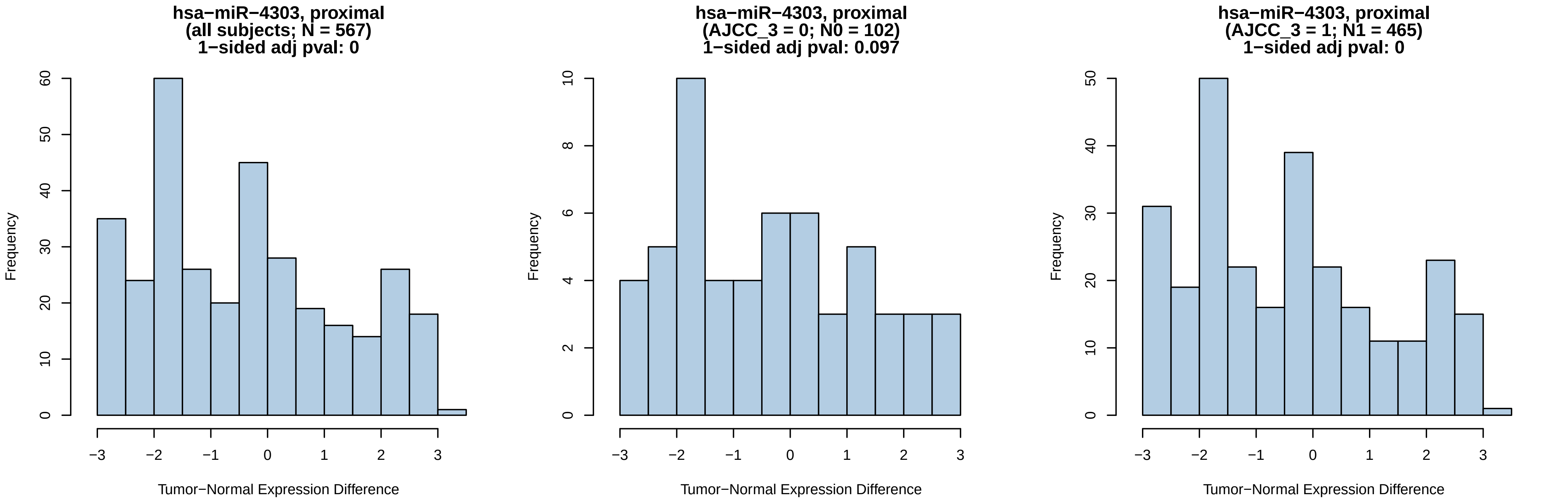

hsa−miR−4303, proximal
(all subjects; N = 567)
1−sided adj pval: 0
Frequency
Tumor−Normal Expression Difference
hsa−miR−4303, proximal
(AJCC_3 = 0; N0 = 102)
1−sided adj pval: 0.097
Frequency
Tumor−Normal Expression Difference
hsa−miR−4303, proximal
(AJCC_3 = 1; N1 = 465)
1−sided adj pval: 0
Frequency
Tumor−Normal Expression Difference

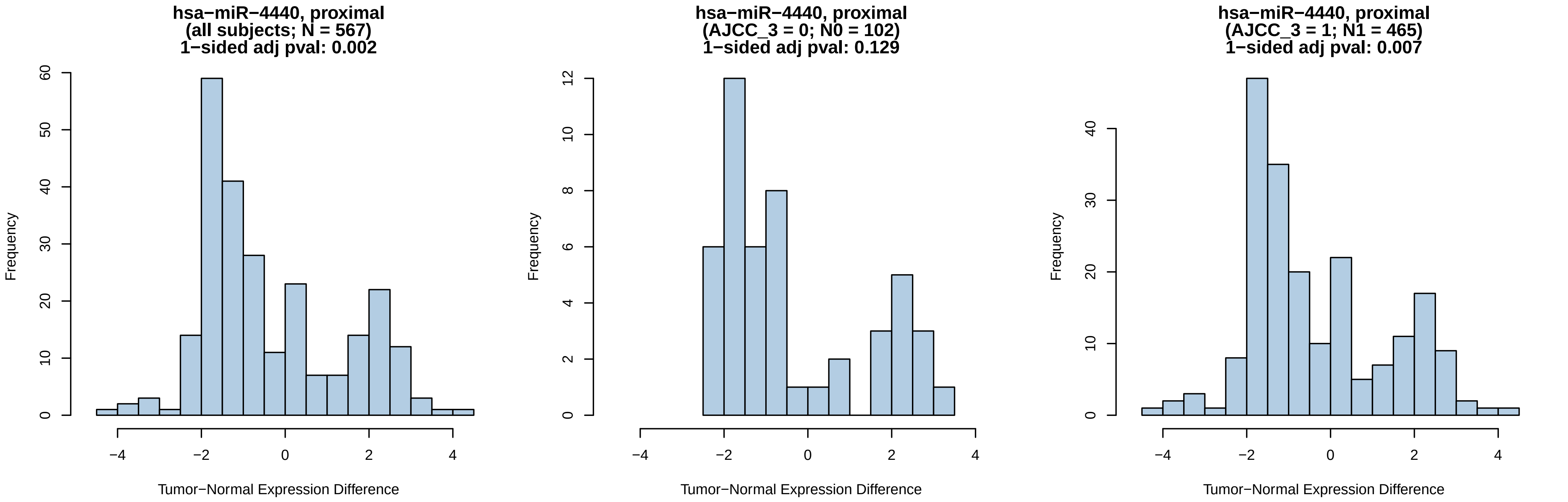
hsa−miR−4440, proximal
(all subjects; N = 567)
1−sided adj pval: 0.002
Frequency
Tumor−Normal Expression Difference
hsa−miR−4440, proximal
(AJCC_3 = 0; N0 = 102)
1−sided adj pval: 0.129
Frequency
Tumor−Normal Expression Difference
hsa−miR−4440, proximal
(AJCC_3 = 1; N1 = 465)
1−sided adj pval: 0.007
Frequency
Tumor−Normal Expression Difference

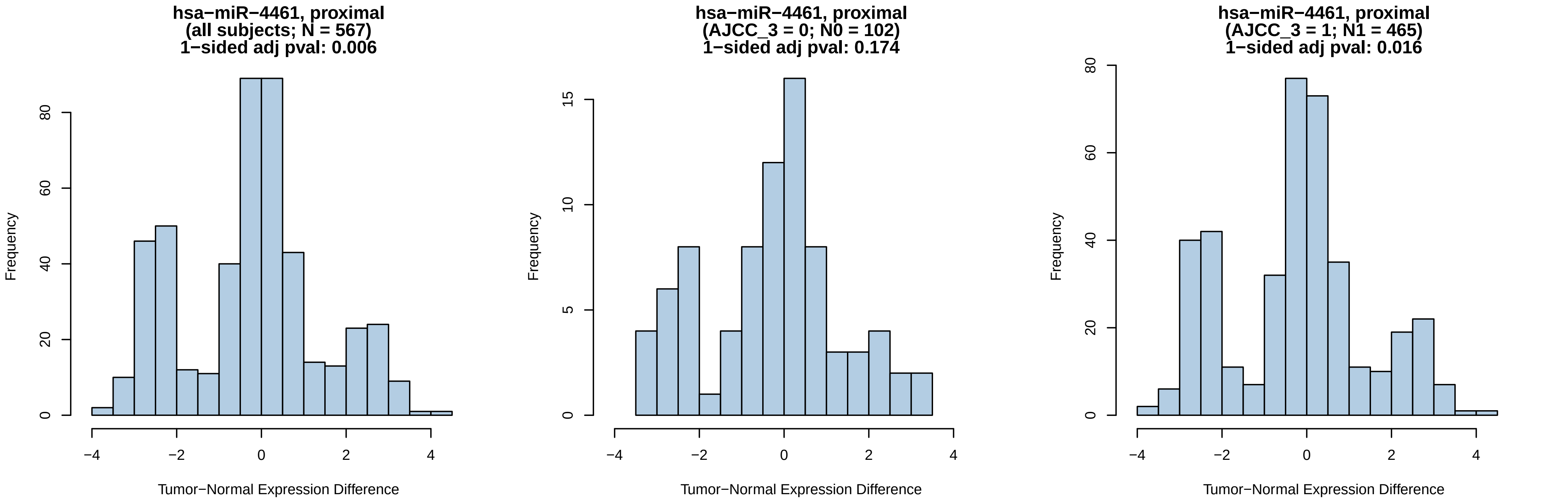
hsa−miR−4461, proximal
(all subjects; N = 567)
1−sided adj pval: 0.006
Frequency
Tumor−Normal Expression Difference
hsa−miR−4461, proximal
(AJCC_3 = 0; N0 = 102)
1−sided adj pval: 0.174
Frequency
Tumor−Normal Expression Difference
hsa−miR−4461, proximal
(AJCC_3 = 1; N1 = 465)
1−sided adj pval: 0.016
Frequency
Tumor−Normal Expression Difference

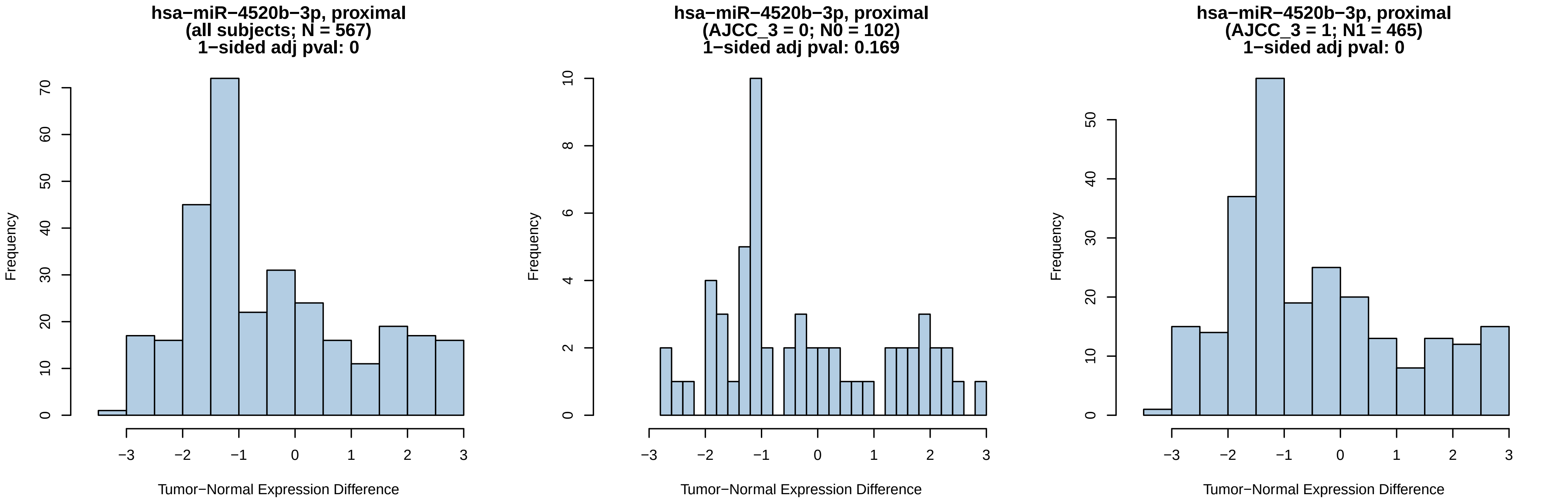
hsa−miR−4520b−3p, proximal
(all subjects; N = 567)
1−sided adj pval: 0
Frequency
Tumor−Normal Expression Difference
hsa−miR−4520b−3p, proximal
(AJCC_3 = 0; N0 = 102)
1−sided adj pval: 0.169
Frequency
Tumor−Normal Expression Difference
hsa−miR−4520b−3p, proximal
(AJCC_3 = 1; N1 = 465)
1−sided adj pval: 0
Frequency
Tumor−Normal Expression Difference

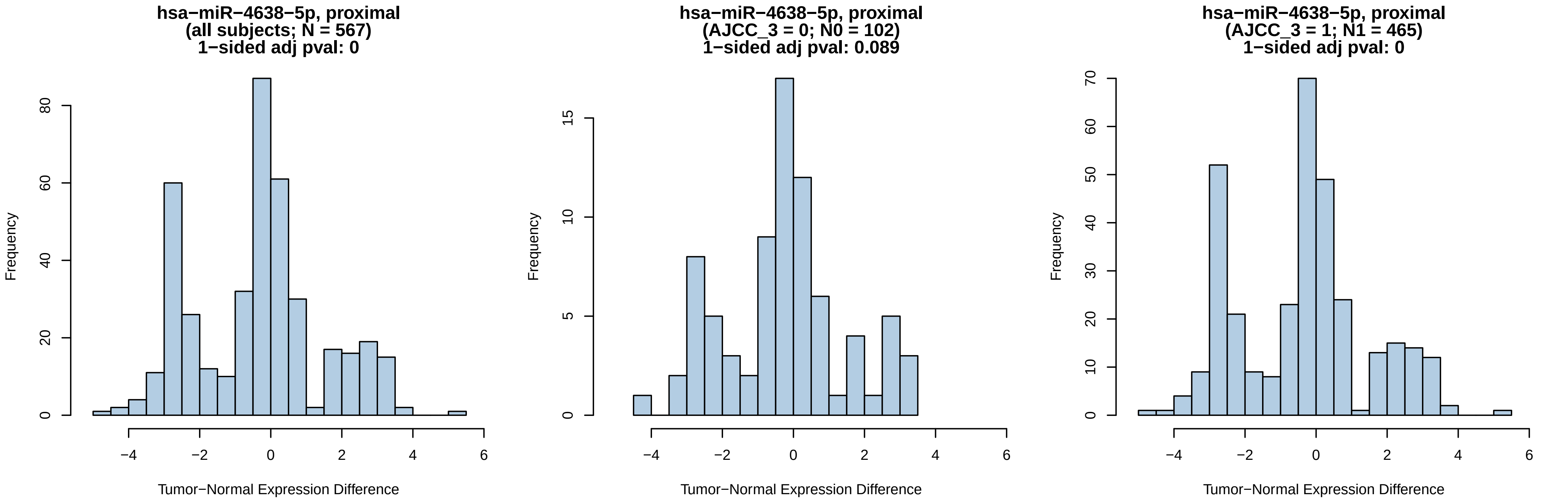
hsa−miR−4638−5p, proximal
(all subjects; N = 567)
1−sided adj pval: 0
hsa−miR−4638−5p, proximal
(AJCC_3 = 0; N0 = 102)
1−sided adj pval: 0.089
hsa−miR−4638−5p, proximal
(AJCC_3 = 1; N1 = 465)
1−sided adj pval: 0
Frequency
Tumor−Normal Expression Difference

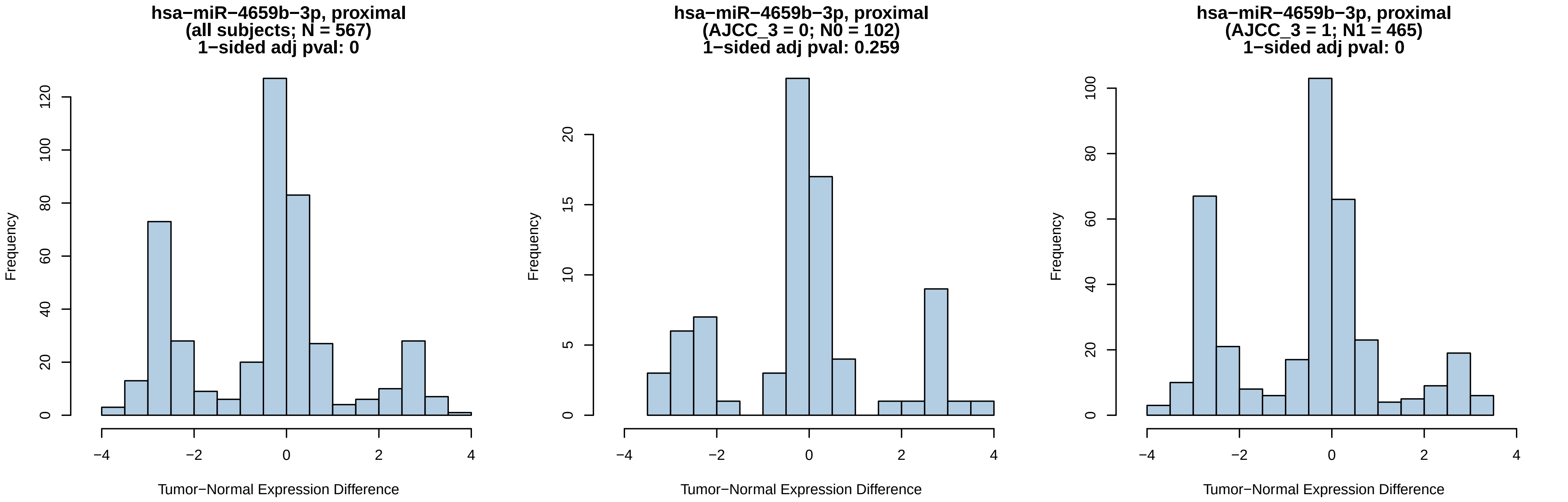
hsa−miR−4659b−3p, proximal
(all subjects; N = 567)
1−sided adj pval: 0
hsa−miR−4659b−3p, proximal
(AJCC_3 = 0; N0 = 102)
1−sided adj pval: 0.259
hsa−miR−4659b−3p, proximal
(AJCC_3 = 1; N1 = 465)
1−sided adj pval: 0
Frequency
Tumor−Normal Expression Difference

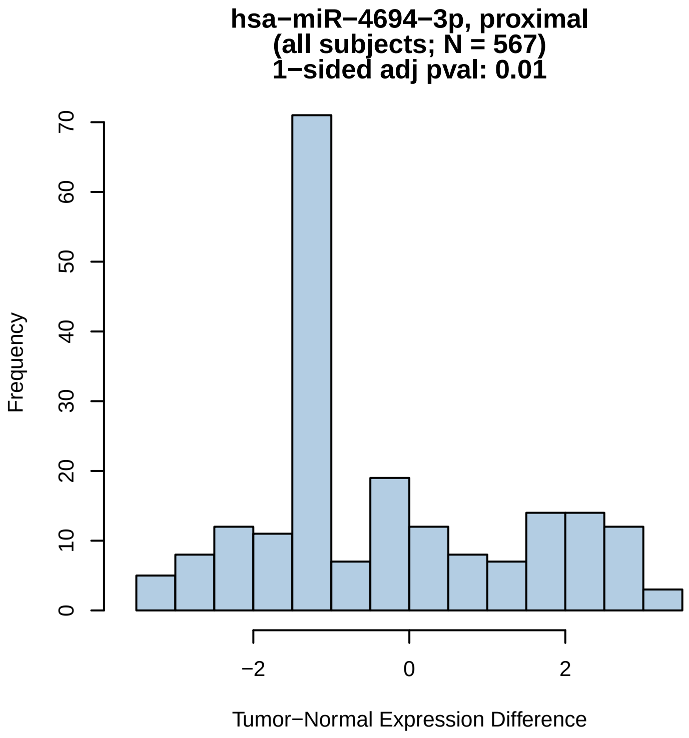

hsa−miR−4694−3p, proximal
(all subjects; N = 567)
1−sided adj pval: 0.01
Frequency
Tumor−Normal Expression Difference


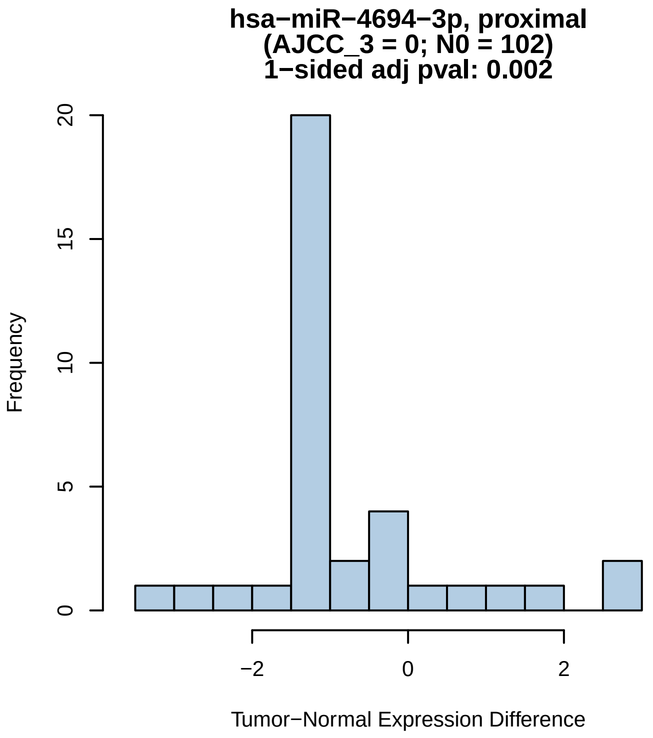

hsa−miR−4694−3p, proximal
(AJCC_3 = 0; N0 = 102)
1−sided adj pval: 0.002
Frequency
Tumor−Normal Expression Difference


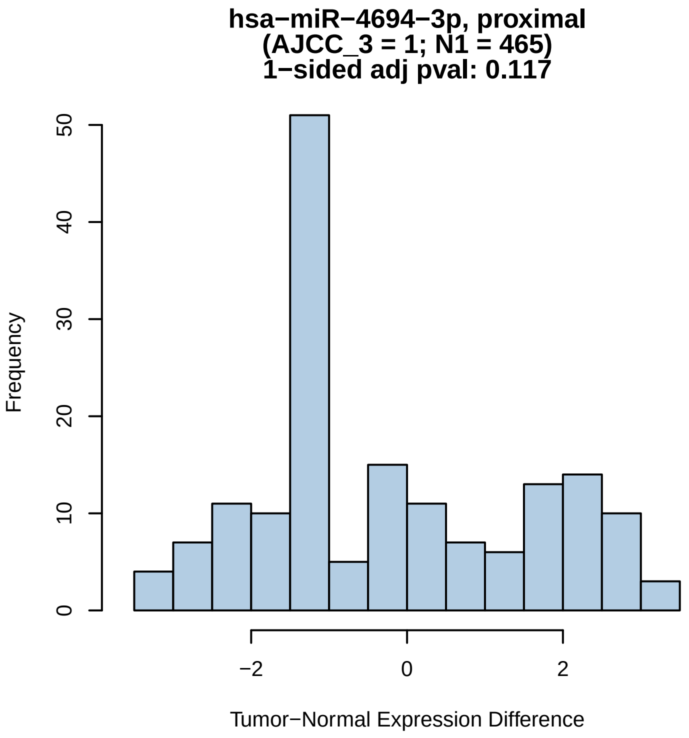

hsa−miR−4694−3p, proximal
(AJCC_3 = 1; N1 = 465)
1−sided adj pval: 0.117
Frequency
Tumor−Normal Expression Difference

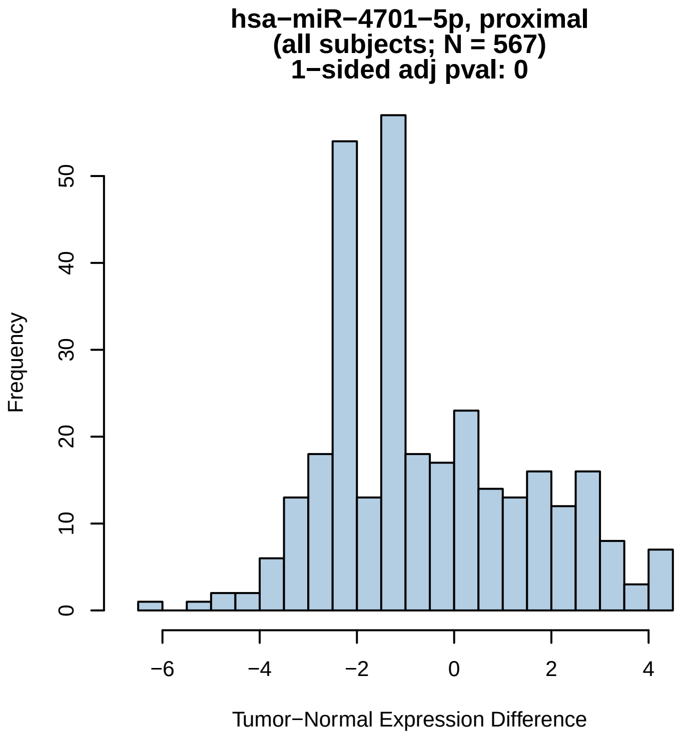

hsa−miR−4701−5p, proximal
(all subjects; N = 567)
1−sided adj pval: 0
Frequency
Tumor−Normal Expression Difference


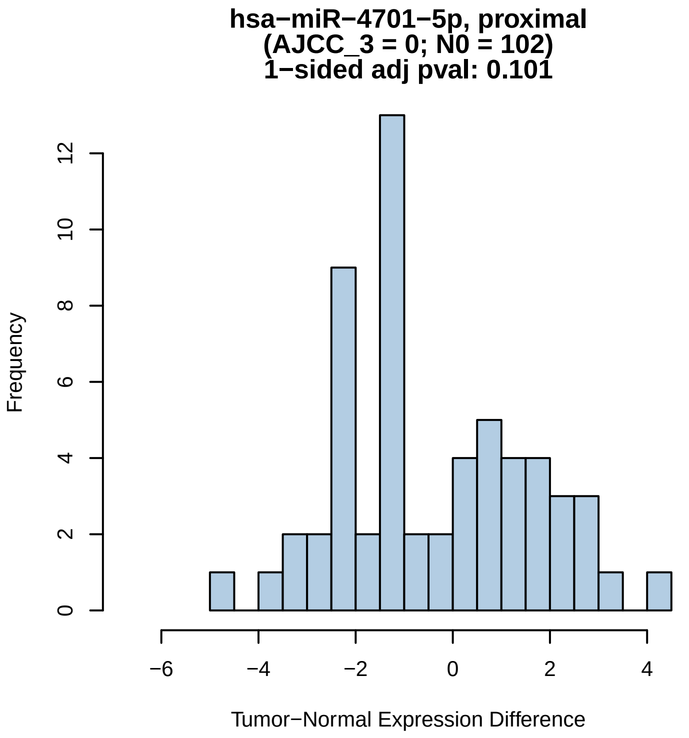

hsa−miR−4701−5p, proximal
(AJCC_3 = 0; N0 = 102)
1−sided adj pval: 0.101
Frequency
Tumor−Normal Expression Difference


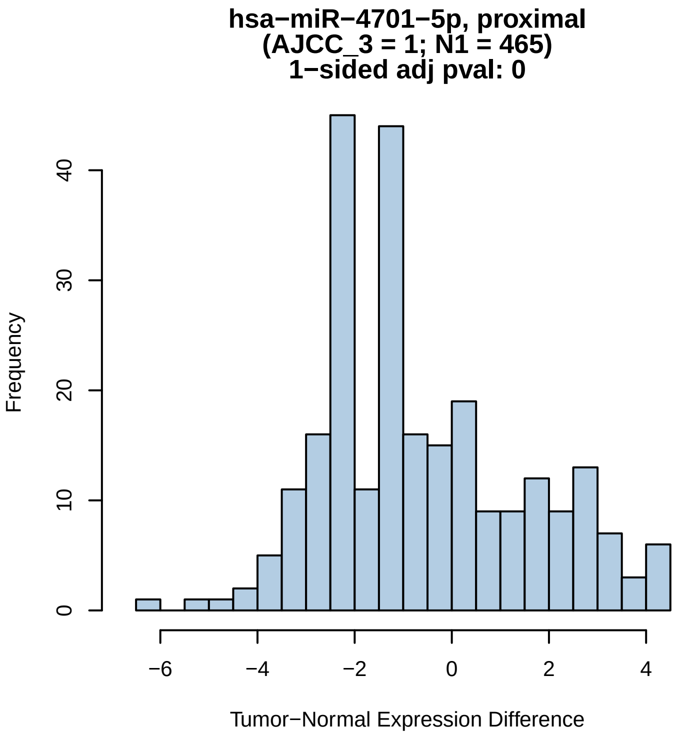

hsa−miR−4701−5p, proximal
(AJCC_3 = 1; N1 = 465)
1−sided adj pval: 0
Frequency
Tumor−Normal Expression Difference

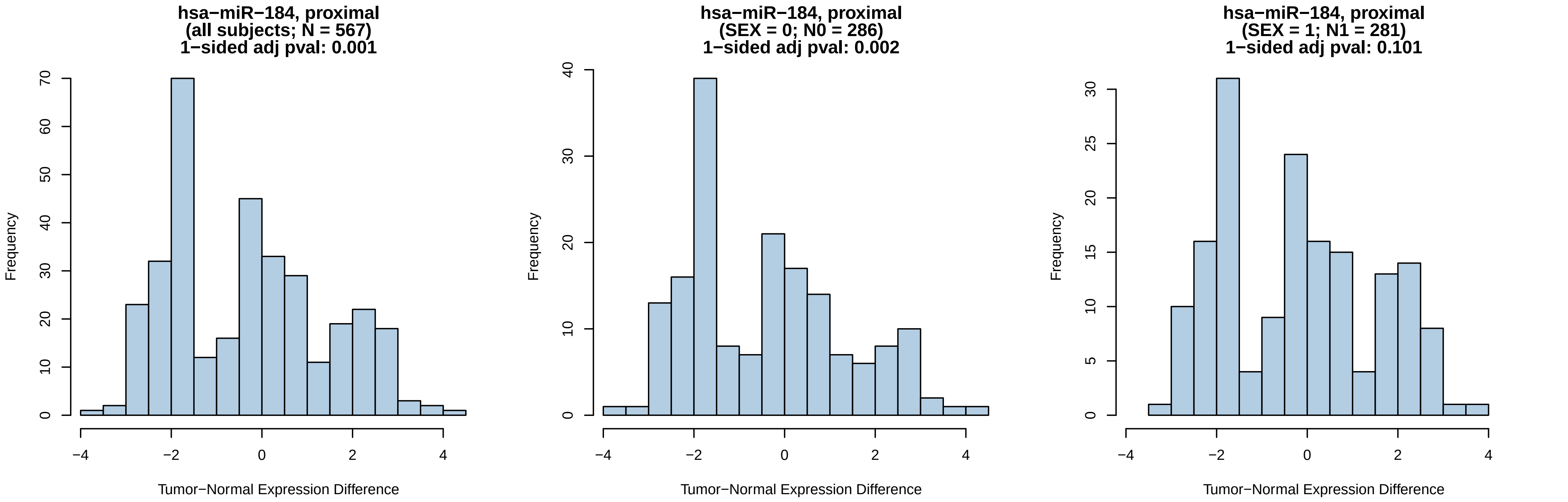
hsa−miR−184, proximal
(all subjects; N = 567)
1−sided adj pval: 0.001
Frequency
Tumor−Normal Expression Difference
hsa−miR−184, proximal
(SEX = 0; N0 = 286)
1−sided adj pval: 0.002
Frequency
Tumor−Normal Expression Difference
hsa−miR−184, proximal
(SEX = 1; N1 = 281)
1−sided adj pval: 0.101
Frequency
Tumor−Normal Expression Difference

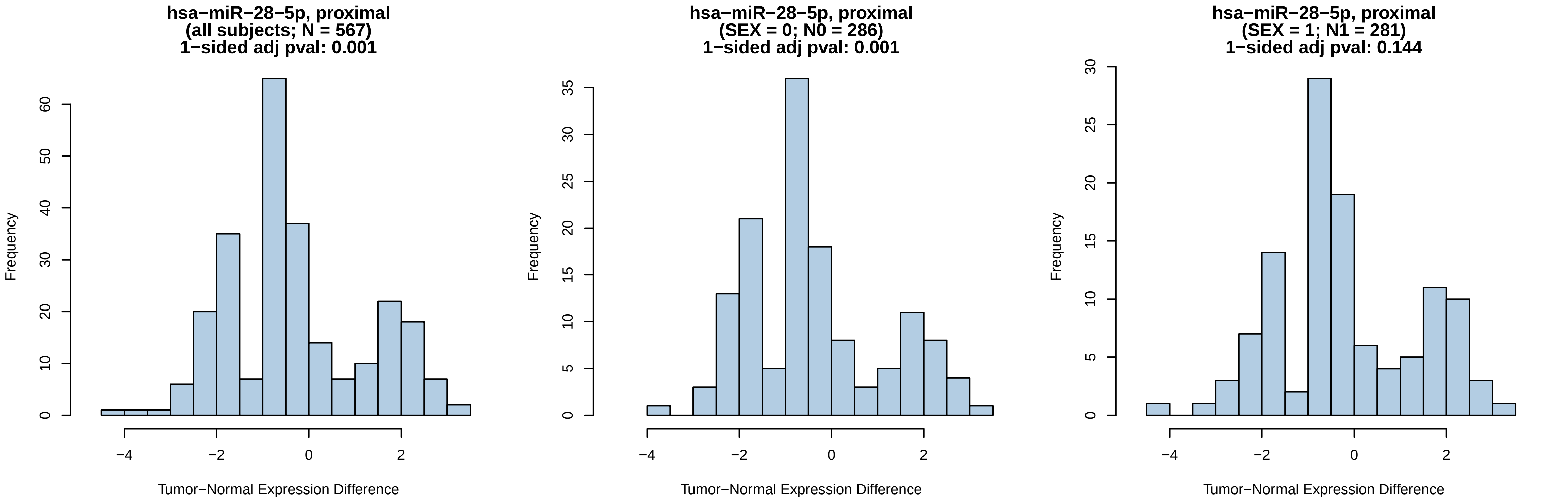
hsa−miR−28−5p, proximal
(all subjects; N = 567)
1−sided adj pval: 0.001
Frequency
Tumor−Normal Expression Difference
hsa−miR−28−5p, proximal
(SEX = 0; N0 = 286)
1−sided adj pval: 0.001
Frequency
Tumor−Normal Expression Difference
hsa−miR−28−5p, proximal
(SEX = 1; N1 = 281)
1−sided adj pval: 0.144
Frequency
Tumor−Normal Expression Difference

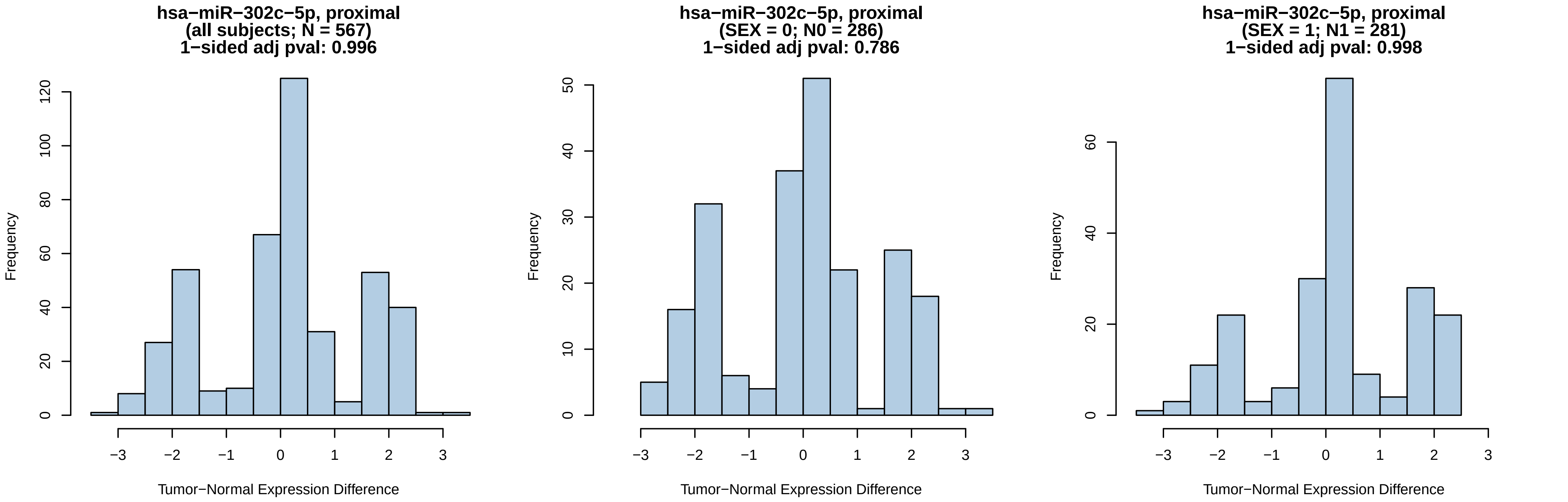
hsa−miR−302c−5p, proximal
(all subjects; N = 567)
1−sided adj pval: 0.996
Frequency
Tumor−Normal Expression Difference
hsa−miR−302c−5p, proximal
(SEX = 0; N0 = 286)
1−sided adj pval: 0.786
Frequency
Tumor−Normal Expression Difference
hsa−miR−302c−5p, proximal
(SEX = 1; N1 = 281)
1−sided adj pval: 0.998
Frequency
Tumor−Normal Expression Difference

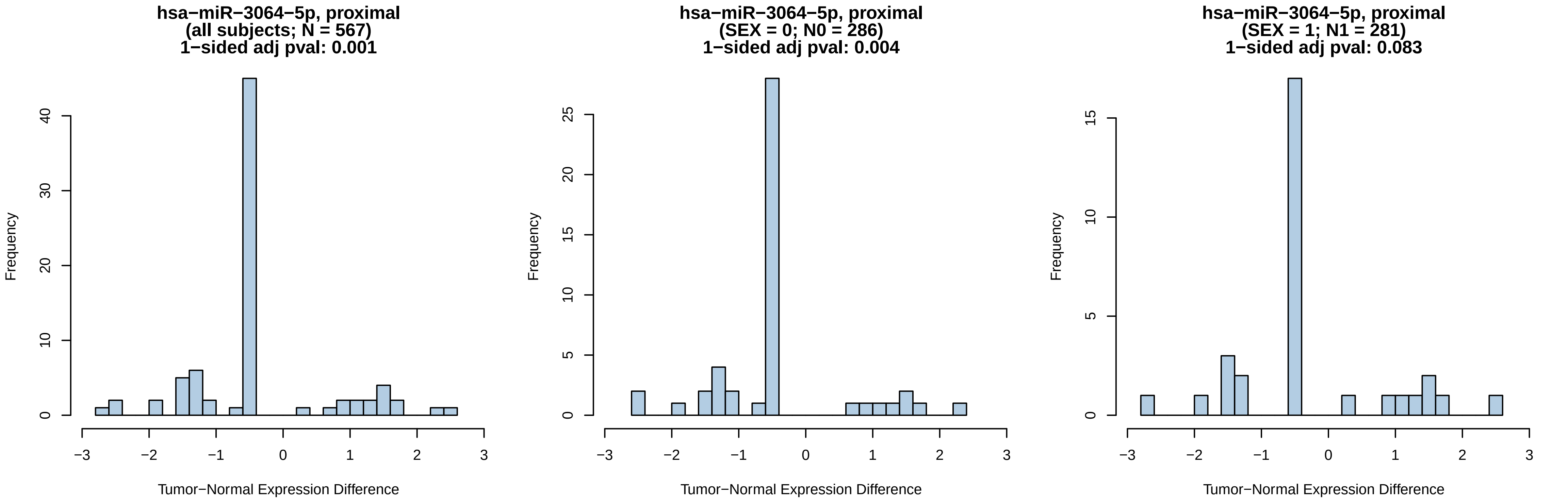
hsa−miR−3064−5p, proximal
(all subjects; N = 567)
1−sided adj pval: 0.001
Frequency
Tumor−Normal Expression Difference
hsa−miR−3064−5p, proximal
(SEX = 0; N0 = 286)
1−sided adj pval: 0.004
Frequency
Tumor−Normal Expression Difference
hsa−miR−3064−5p, proximal
(SEX = 1; N1 = 281)
1−sided adj pval: 0.083
Frequency
Tumor−Normal Expression Difference

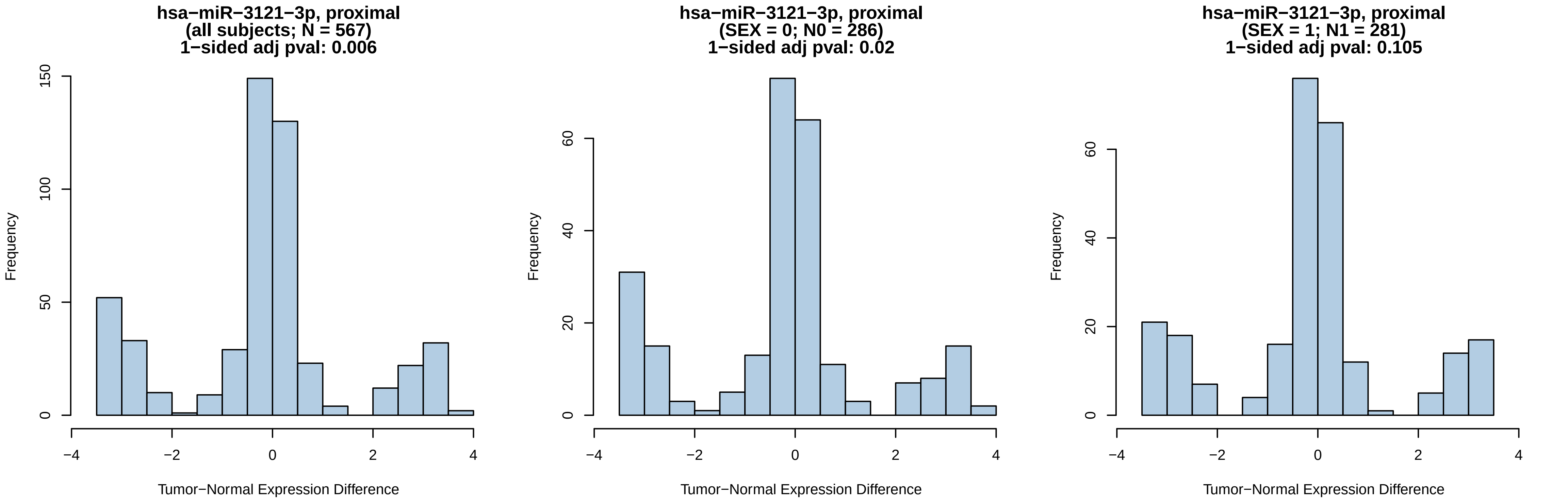
hsa−miR−3121−3p, proximal
(all subjects; N = 567)
1−sided adj pval: 0.006
hsa−miR−3121−3p, proximal
(SEX = 0; N0 = 286)
1−sided adj pval: 0.02
hsa−miR−3121−3p, proximal
(SEX = 1; N1 = 281)
1−sided adj pval: 0.105
Frequency
Tumor−Normal Expression Difference
0
50
100
150
20
40
60
−4
−2
0
2
4

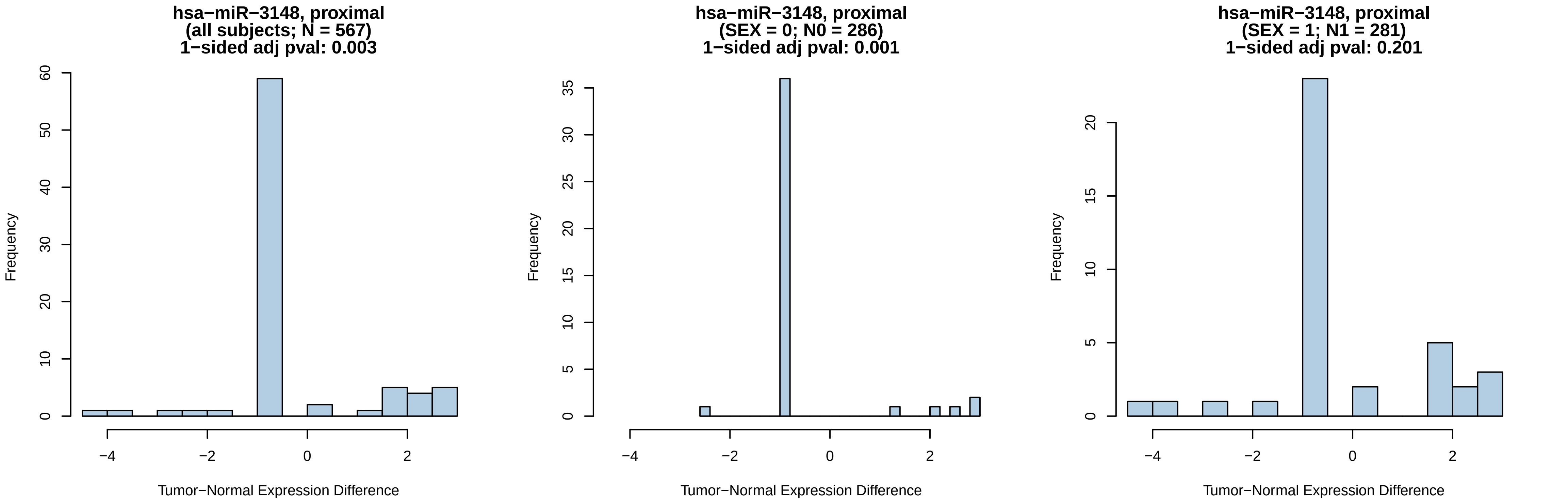

hsa−miR−3148, proximal
(all subjects; N = 567)
1−sided adj pval: 0.003
Frequency
Tumor−Normal Expression Difference
hsa−miR−3148, proximal
(SEX = 0; N0 = 286)
1−sided adj pval: 0.001
Frequency
Tumor−Normal Expression Difference
hsa−miR−3148, proximal
(SEX = 1; N1 = 281)
1−sided adj pval: 0.201
Frequency
Tumor−Normal Expression Difference

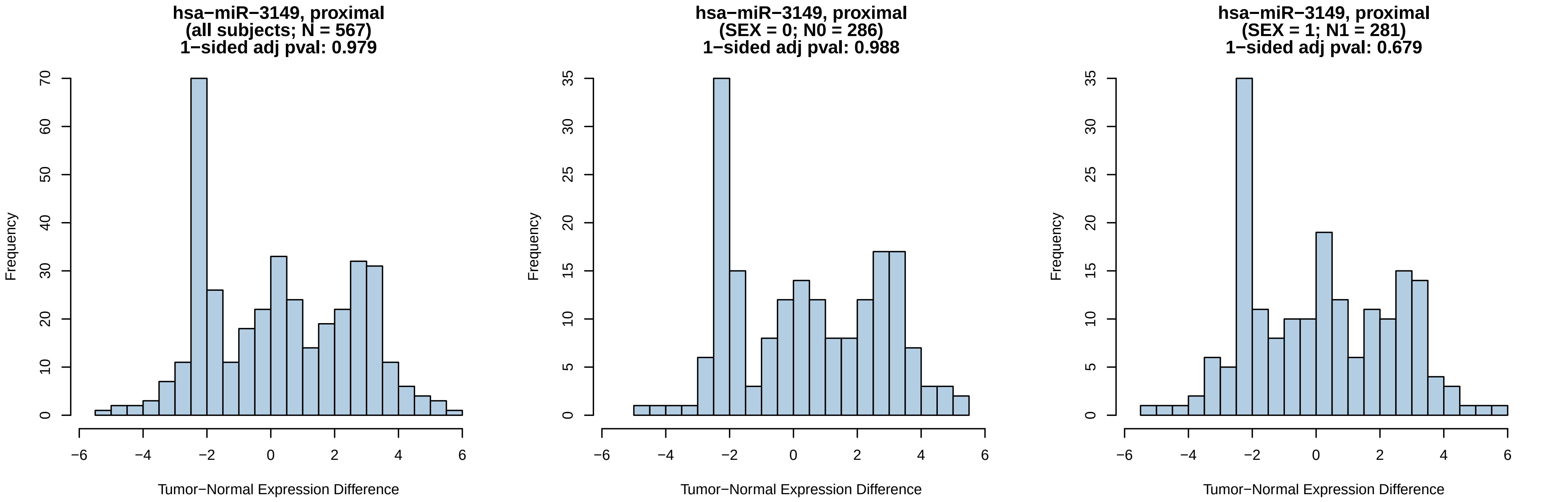
hsa−miR−3149, proximal
(all subjects; N = 567)
1−sided adj pval: 0.979
hsa−miR−3149, proximal
(SEX = 0; N0 = 286)
1−sided adj pval: 0.988
hsa−miR−3149, proximal
(SEX = 1; N1 = 281)
1−sided adj pval: 0.679
Frequency
Tumor−Normal Expression Difference

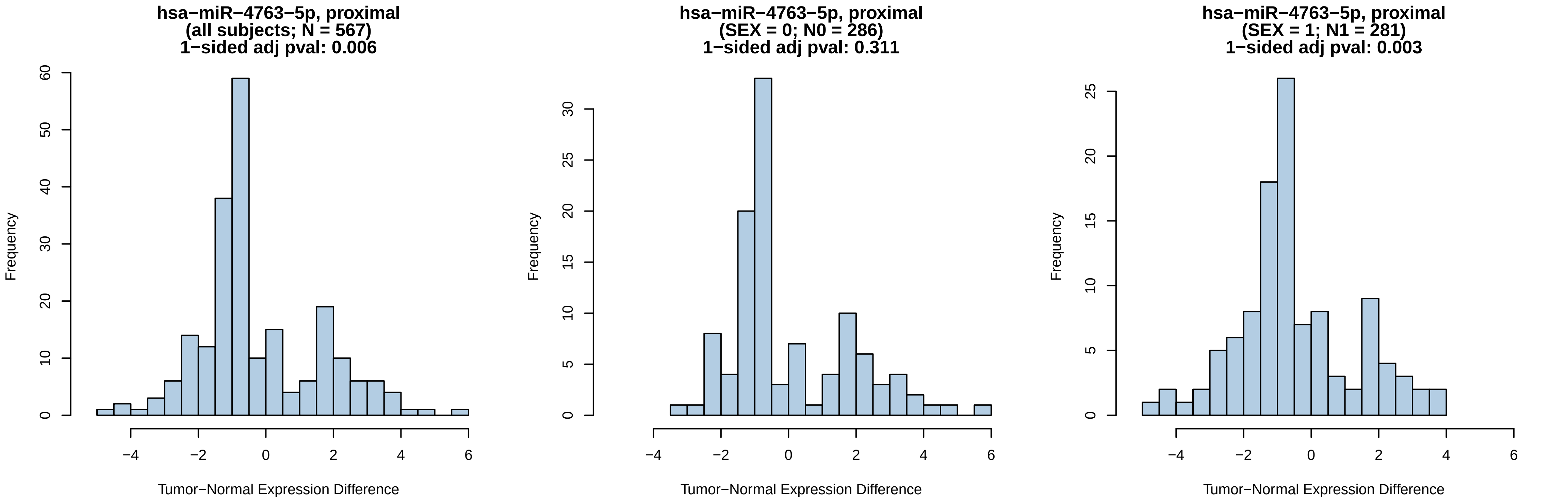
hsa−miR−4763−5p, proximal
(all subjects; N = 567)
1−sided adj pval: 0.006
Frequency
0
10
20
30
40
50
60
−4
−2
0
2
4
6
Tumor−Normal Expression Difference
hsa−miR−4763−5p, proximal
(SEX = 0; N0 = 286)
1−sided adj pval: 0.311
Frequency
0
5
10
15
20
25
30
−4
−2
0
2
4
6
Tumor−Normal Expression Difference
hsa−miR−4763−5p, proximal
(SEX = 1; N1 = 281)
1−sided adj pval: 0.003
Frequency
0
5
10
15
20
25
−4
−2
0
2
4
6
Tumor−Normal Expression Difference

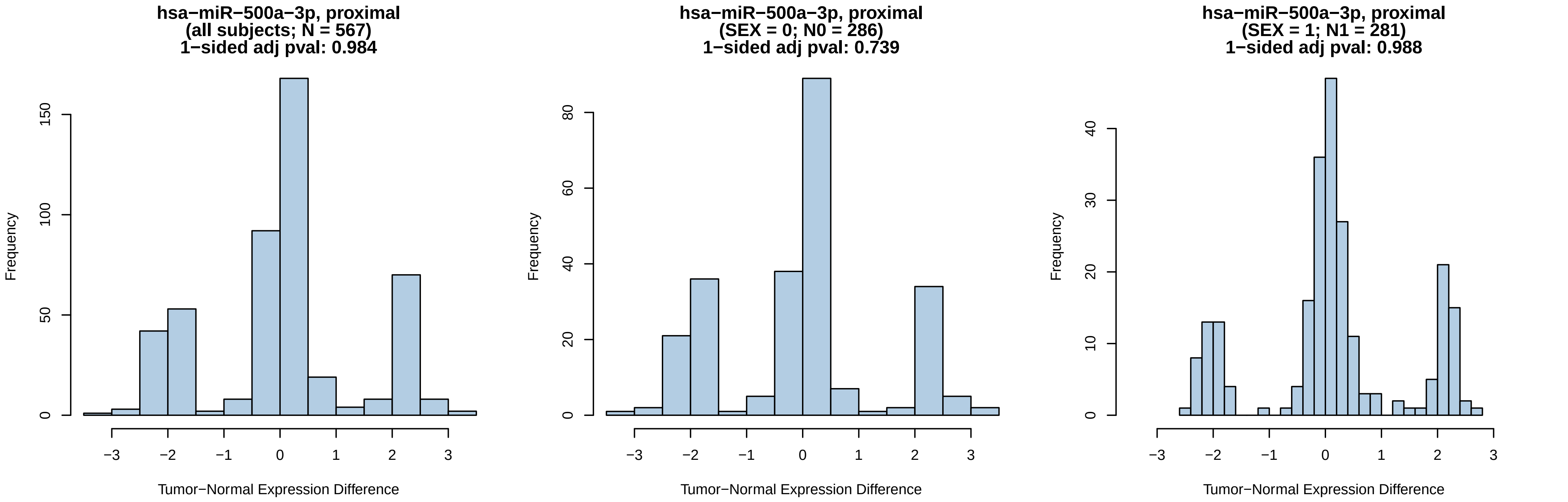
hsa−miR−500a−3p, proximal
(all subjects; N = 567)
1−sided adj pval: 0.984
Frequency
Tumor−Normal Expression Difference
hsa−miR−500a−3p, proximal
(SEX = 0; N0 = 286)
1−sided adj pval: 0.739
Frequency
Tumor−Normal Expression Difference
hsa−miR−500a−3p, proximal
(SEX = 1; N1 = 281)
1−sided adj pval: 0.988
Frequency
Tumor−Normal Expression Difference

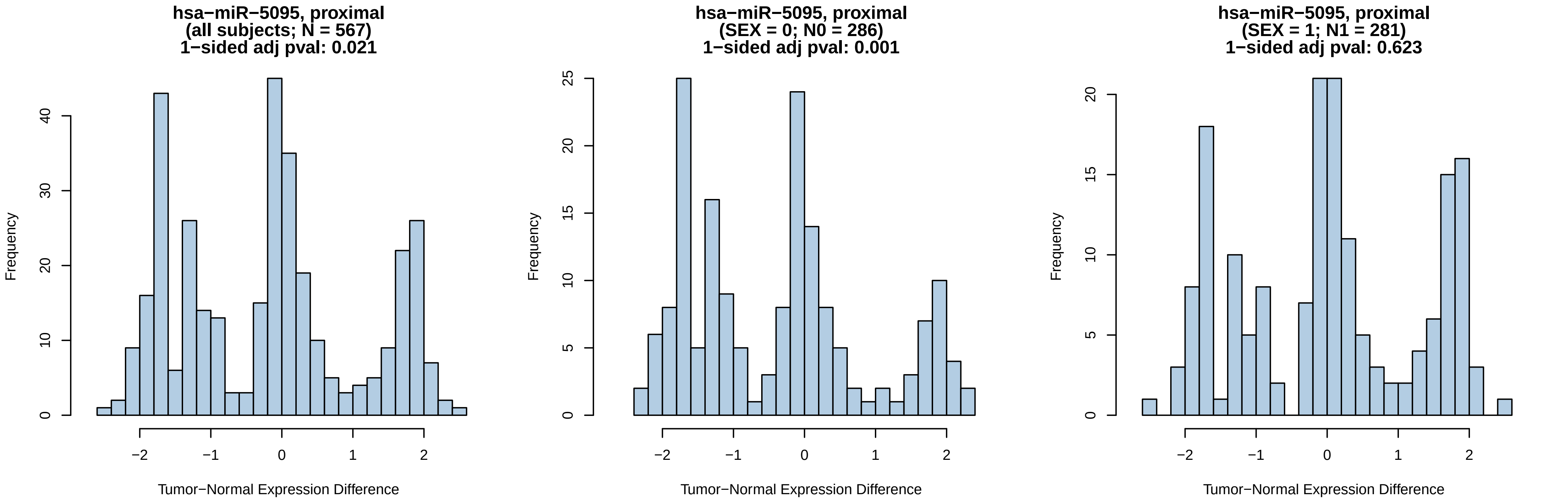

hsa−miR−5095, proximal
(all subjects; N = 567)
1−sided adj pval: 0.021
Frequency
Tumor−Normal Expression Difference
hsa−miR−5095, proximal
(SEX = 0; N0 = 286)
1−sided adj pval: 0.001
Frequency
Tumor−Normal Expression Difference
hsa−miR−5095, proximal
(SEX = 1; N1 = 281)
1−sided adj pval: 0.623
Frequency
Tumor−Normal Expression Difference

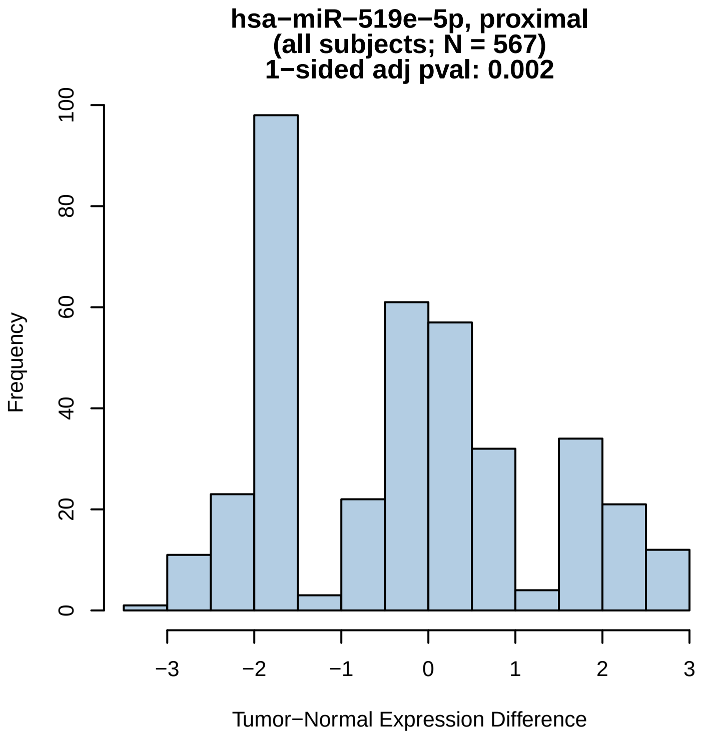
hsa−miR−519e−5p, proximal
(all subjects; N = 567)
1−sided adj pval: 0.002
Frequency
Tumor−Normal Expression Difference

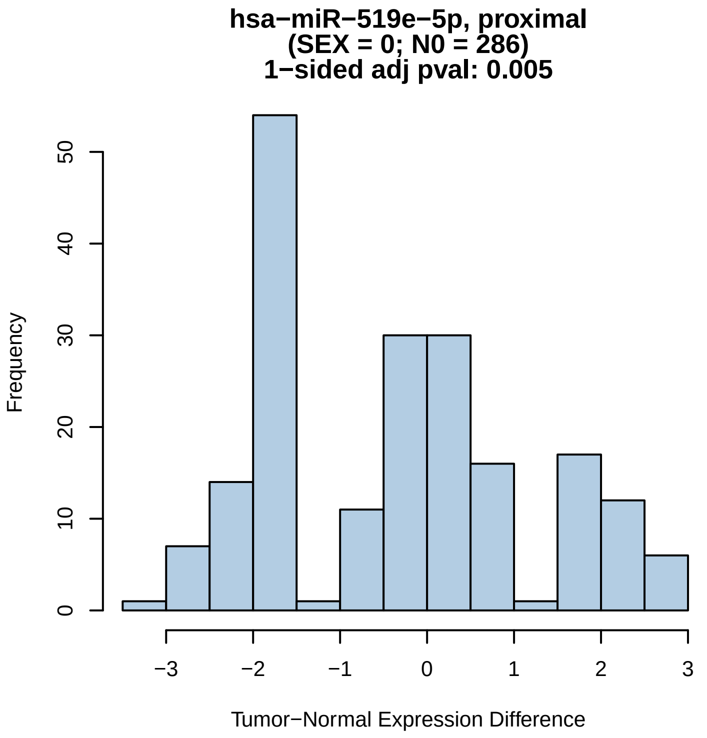
hsa−miR−519e−5p, proximal
(SEX = 0; N0 = 286)
1−sided adj pval: 0.005
Frequency
Tumor−Normal Expression Difference

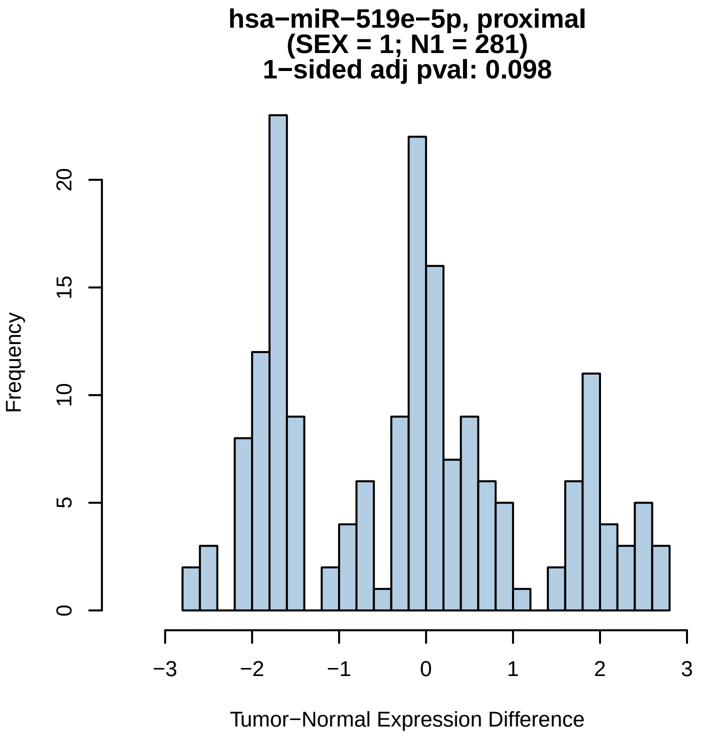
hsa−miR−519e−5p, proximal
(SEX = 1; N1 = 281)
1−sided adj pval: 0.098
Frequency
Tumor−Normal Expression Difference

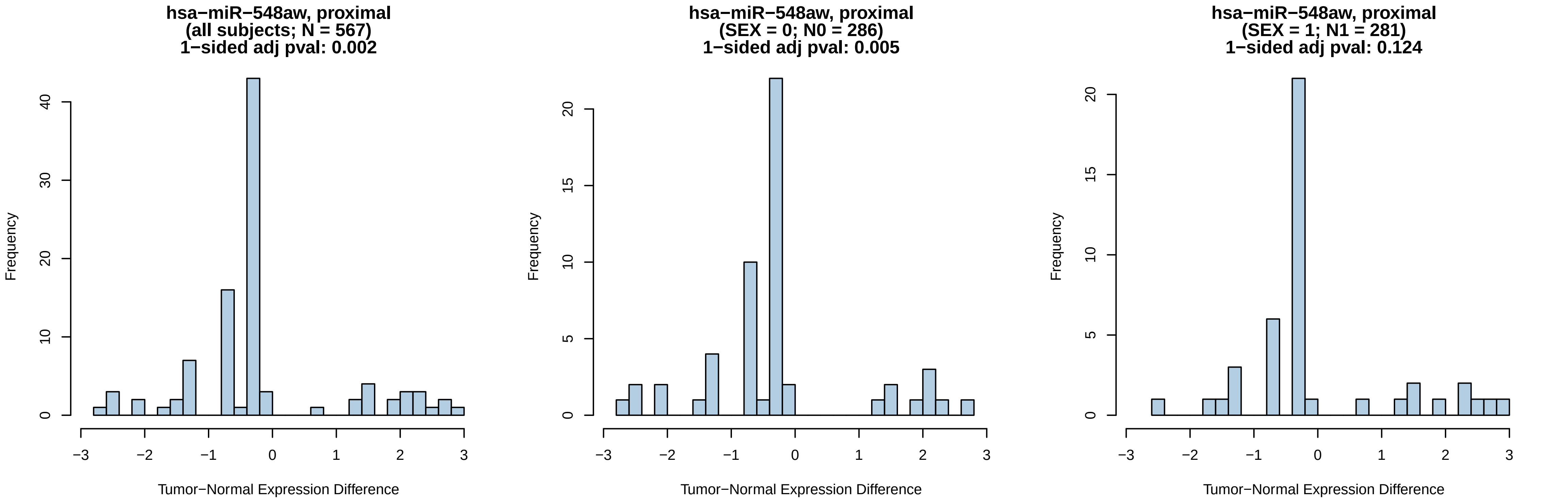
hsa−miR−548aw, proximal
(all subjects; N = 567)
1−sided adj pval: 0.002
Frequency
Tumor−Normal Expression Difference
hsa−miR−548aw, proximal
(SEX = 0; N0 = 286)
1−sided adj pval: 0.005
Frequency
Tumor−Normal Expression Difference
hsa−miR−548aw, proximal
(SEX = 1; N1 = 281)
1−sided adj pval: 0.124
Frequency
Tumor−Normal Expression Difference

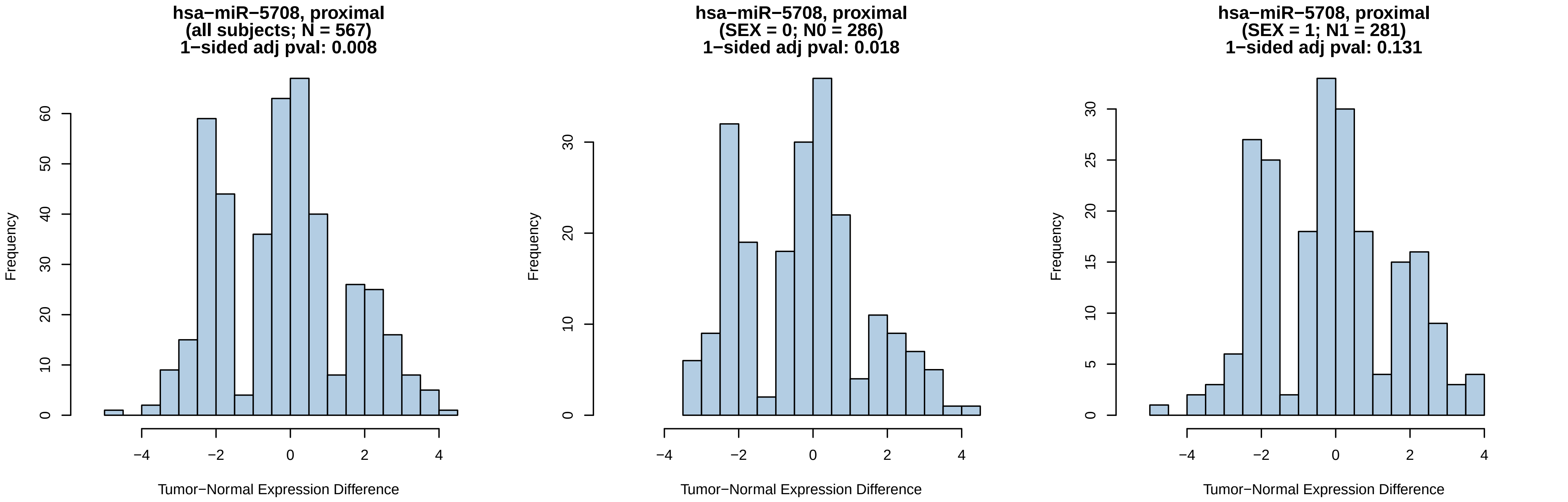
hsa−miR−5708, proximal
(all subjects; N = 567)
1−sided adj pval: 0.008
Frequency
Tumor−Normal Expression Difference
hsa−miR−5708, proximal
(SEX = 0; N0 = 286)
1−sided adj pval: 0.018
Frequency
Tumor−Normal Expression Difference
hsa−miR−5708, proximal
(SEX = 1; N1 = 281)
1−sided adj pval: 0.131
Frequency
Tumor−Normal Expression Difference

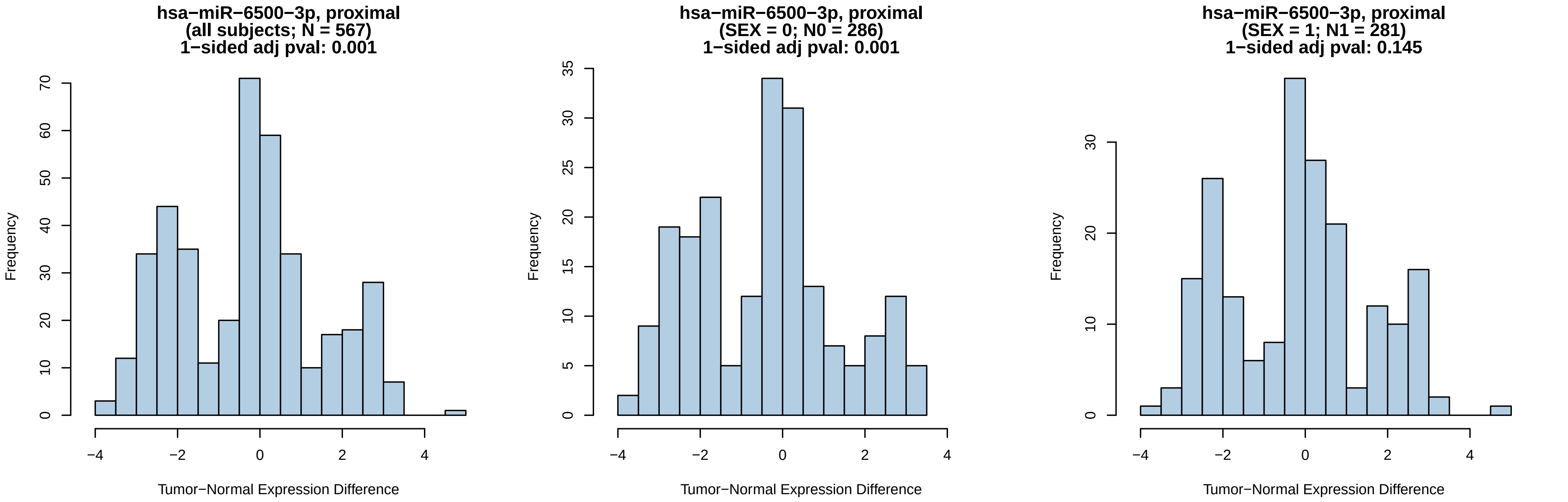

hsa−miR−6500−3p, proximal
(all subjects; N = 567)
1−sided adj pval: 0.001
Frequency
Tumor−Normal Expression Difference
hsa−miR−6500−3p, proximal
(SEX = 0; N0 = 286)
1−sided adj pval: 0.001
Frequency
Tumor−Normal Expression Difference
hsa−miR−6500−3p, proximal
(SEX = 1; N1 = 281)
1−sided adj pval: 0.145
Frequency
Tumor−Normal Expression Difference

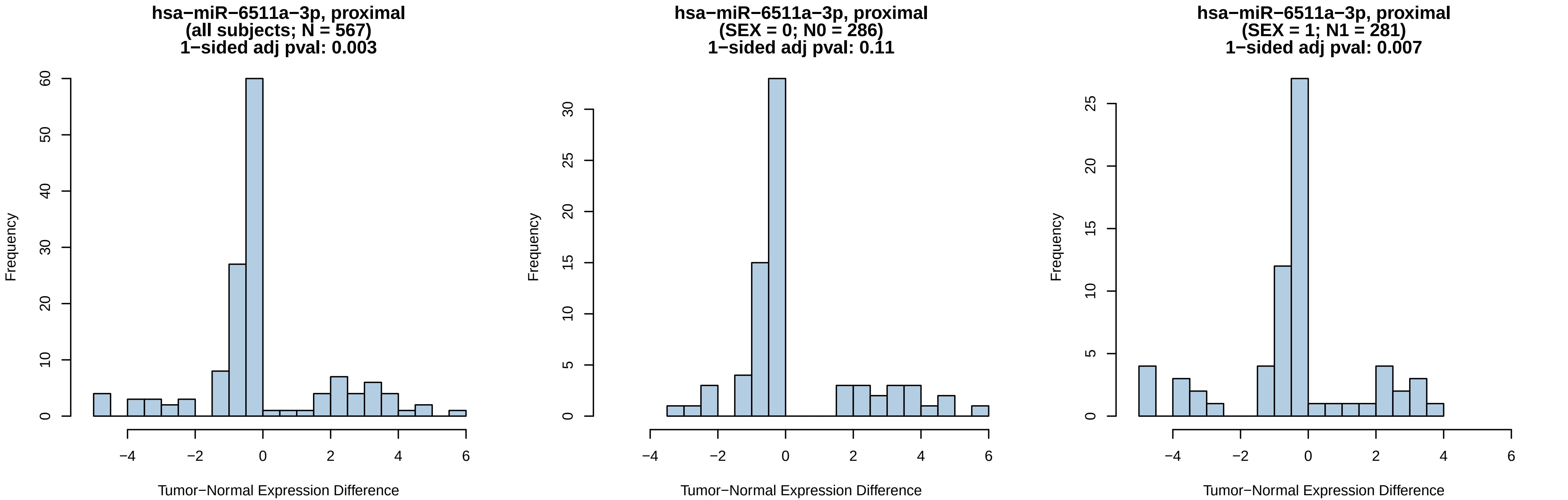

hsa−miR−6511a−3p, proximal
(all subjects; N = 567)
1−sided adj pval: 0.003
Frequency
Tumor−Normal Expression Difference
hsa−miR−6511a−3p, proximal
(SEX = 0; N0 = 286)
1−sided adj pval: 0.11
Frequency
Tumor−Normal Expression Difference
hsa−miR−6511a−3p, proximal
(SEX = 1; N1 = 281)
1−sided adj pval: 0.007
Frequency
Tumor−Normal Expression Difference

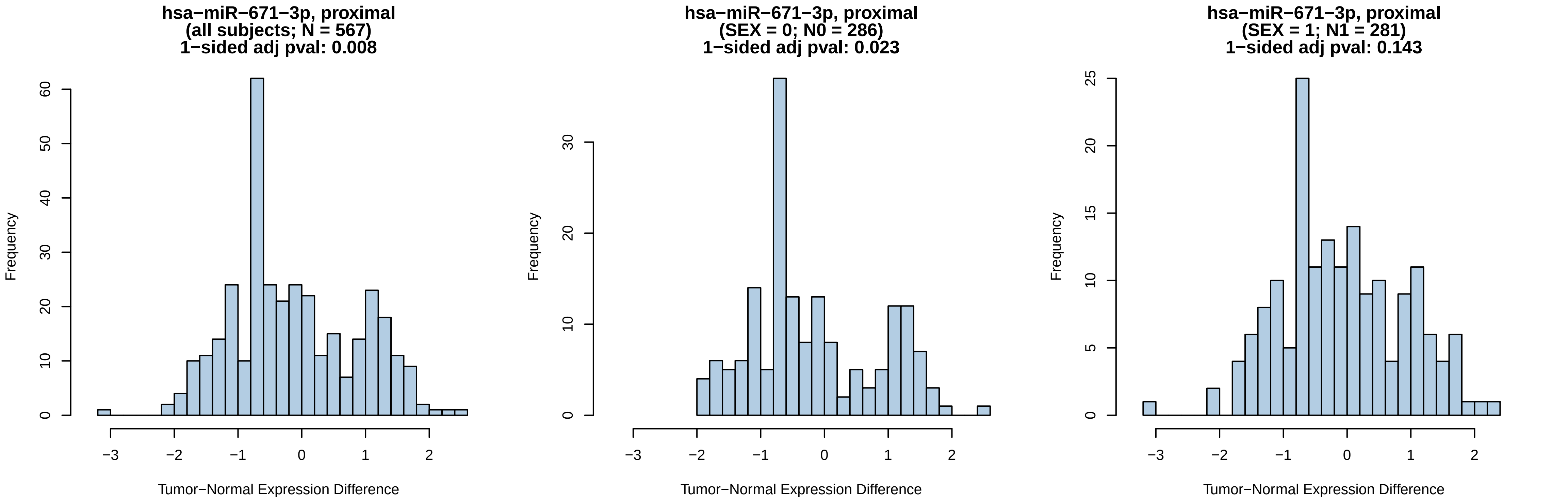

hsa−miR−671−3p, proximal
(all subjects; N = 567)
1−sided adj pval: 0.008
Frequency
Tumor−Normal Expression Difference
hsa−miR−671−3p, proximal
(SEX = 0; N0 = 286)
1−sided adj pval: 0.023
Frequency
Tumor−Normal Expression Difference
hsa−miR−671−3p, proximal
(SEX = 1; N1 = 281)
1−sided adj pval: 0.143
Frequency
Tumor−Normal Expression Difference

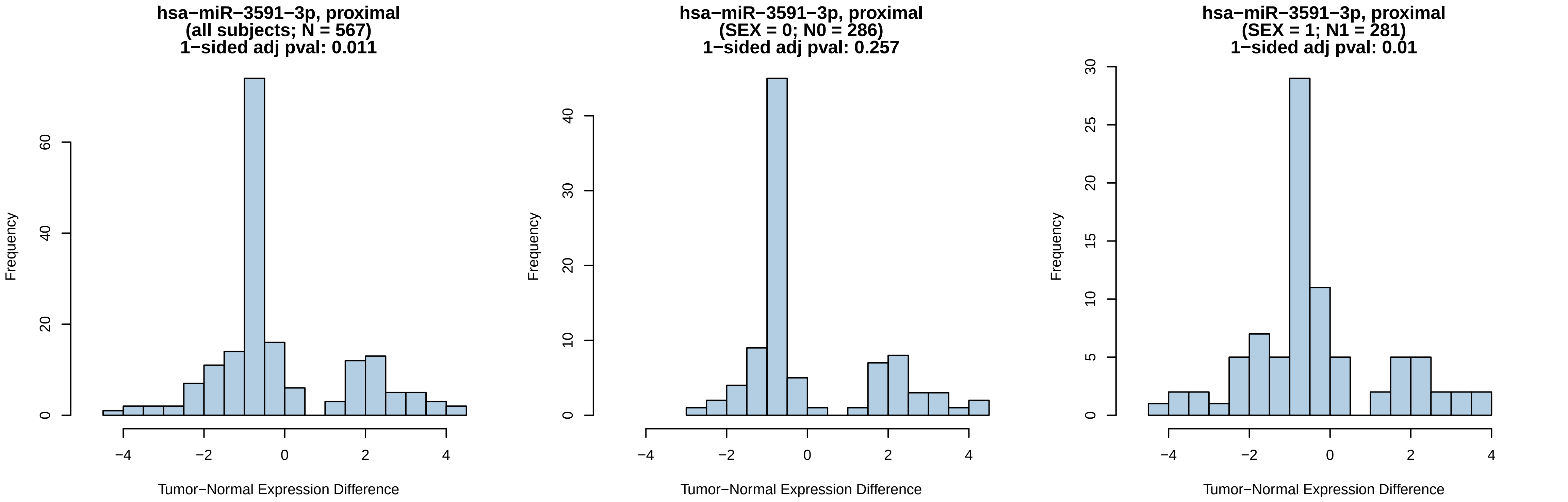
hsa−miR−3591−3p, proximal
(all subjects; N = 567)
1−sided adj pval: 0.011
Frequency
Tumor−Normal Expression Difference
hsa−miR−3591−3p, proximal
(SEX = 0; N0 = 286)
1−sided adj pval: 0.257
Frequency
Tumor−Normal Expression Difference
hsa−miR−3591−3p, proximal
(SEX = 1; N1 = 281)
1−sided adj pval: 0.01
Frequency
Tumor−Normal Expression Difference

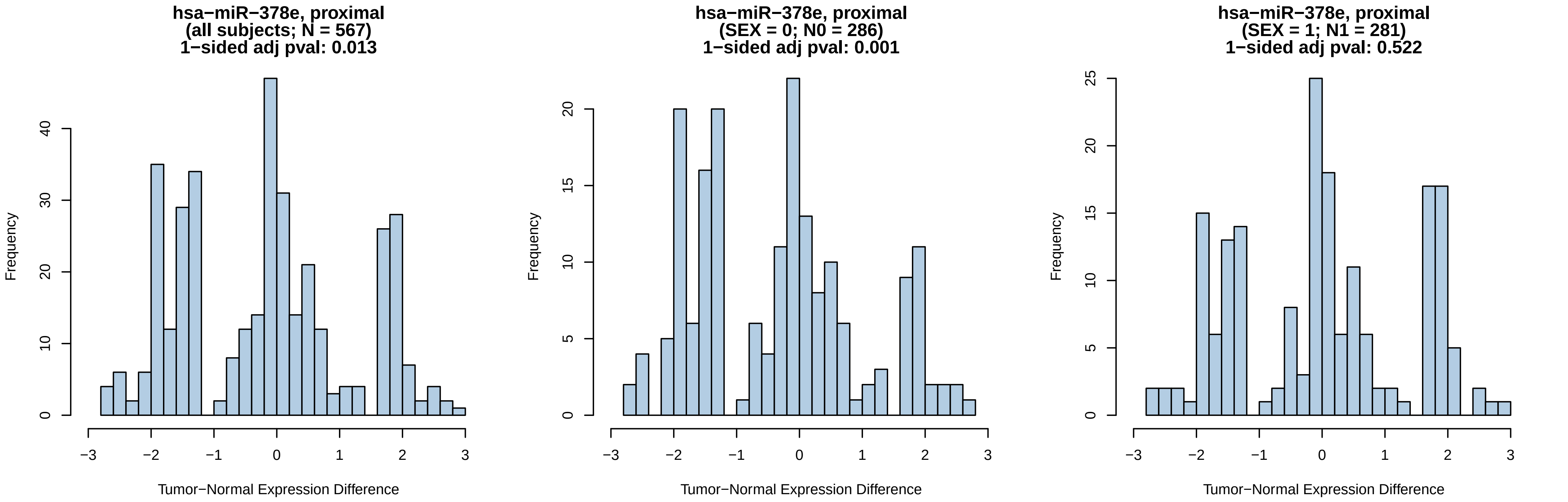
hsa−miR−378e, proximal
(all subjects; N = 567)
1−sided adj pval: 0.013
hsa−miR−378e, proximal
(SEX = 0; N0 = 286)
1−sided adj pval: 0.001
hsa−miR−378e, proximal
(SEX = 1; N1 = 281)
1−sided adj pval: 0.522
Frequency
Tumor−Normal Expression Difference

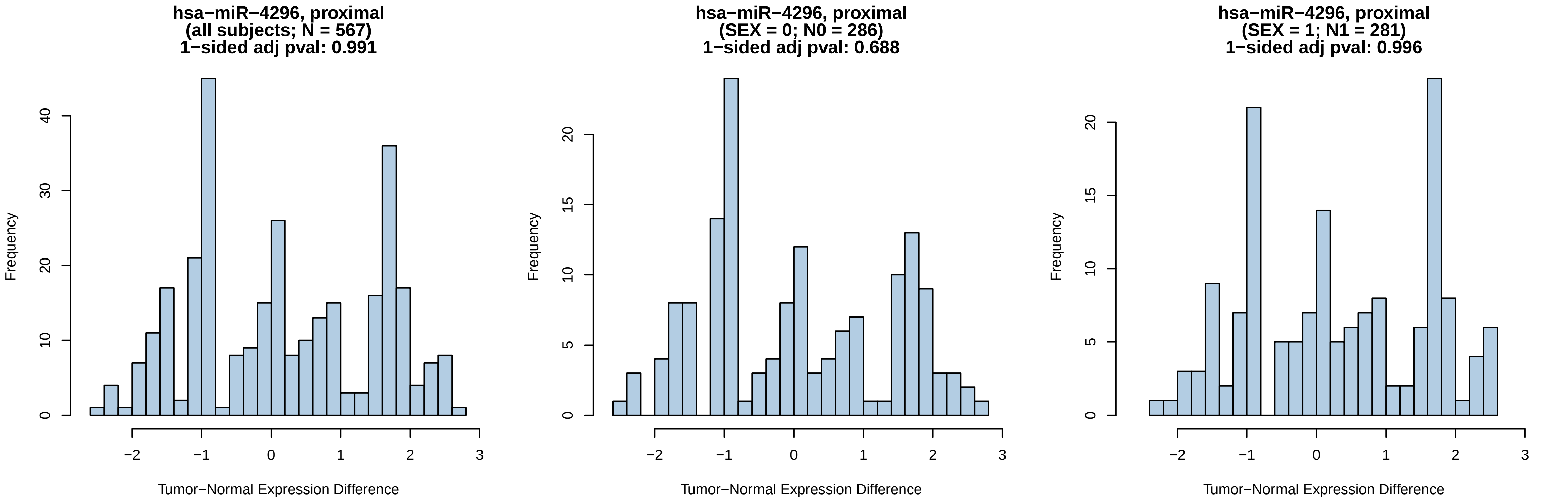

hsa−miR−4296, proximal
(all subjects; N = 567)
1−sided adj pval: 0.991
Frequency
Tumor−Normal Expression Difference
hsa−miR−4296, proximal
(SEX = 0; N0 = 286)
1−sided adj pval: 0.688
Frequency
Tumor−Normal Expression Difference
hsa−miR−4296, proximal
(SEX = 1; N1 = 281)
1−sided adj pval: 0.996
Frequency
Tumor−Normal Expression Difference

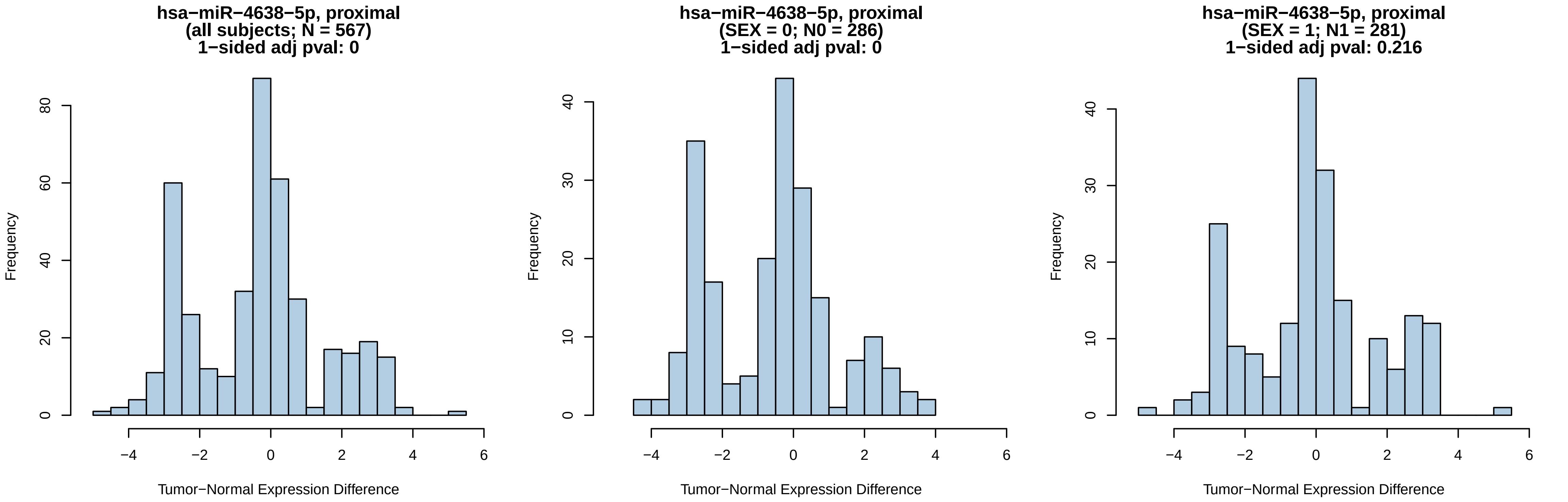
hsa−miR−4638−5p, proximal
(all subjects; N = 567)
1−sided adj pval: 0
Frequency
Tumor−Normal Expression Difference
hsa−miR−4638−5p, proximal
(SEX = 0; N0 = 286)
1−sided adj pval: 0
Frequency
Tumor−Normal Expression Difference
hsa−miR−4638−5p, proximal
(SEX = 1; N1 = 281)
1−sided adj pval: 0.216
Frequency
Tumor−Normal Expression Difference

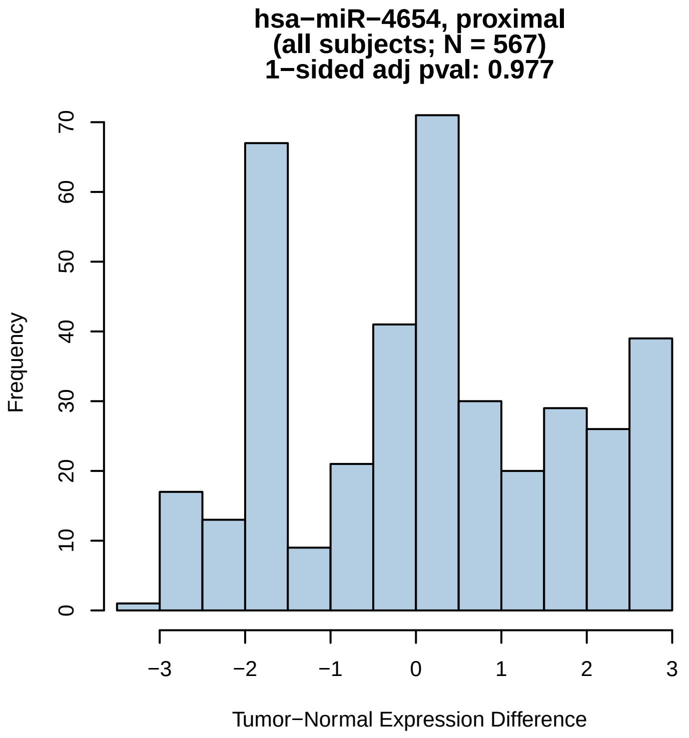
hsa−miR−4654, proximal
(all subjects; N = 567)
1−sided adj pval: 0.977
Frequency
Tumor−Normal Expression Difference
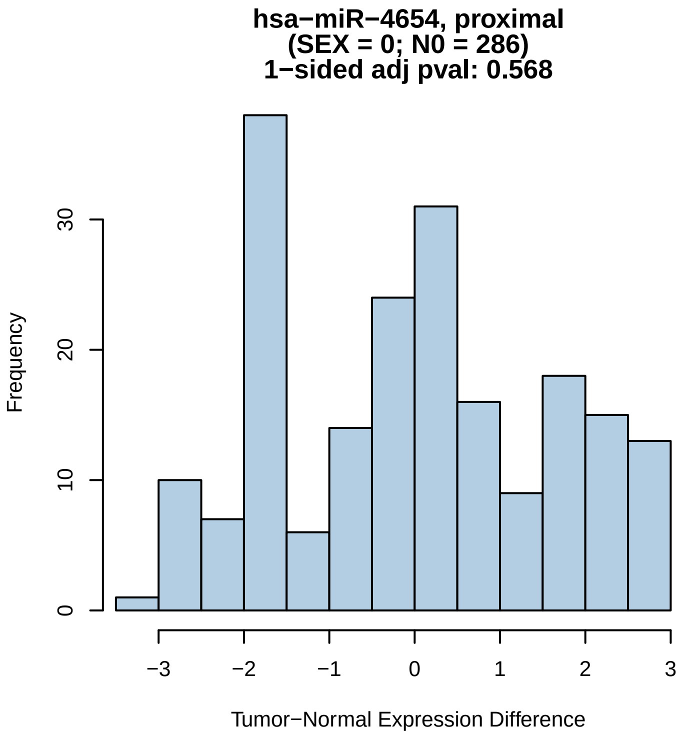
hsa−miR−4654, proximal
(SEX = 0; N0 = 286)
1−sided adj pval: 0.568
Frequency
Tumor−Normal Expression Difference
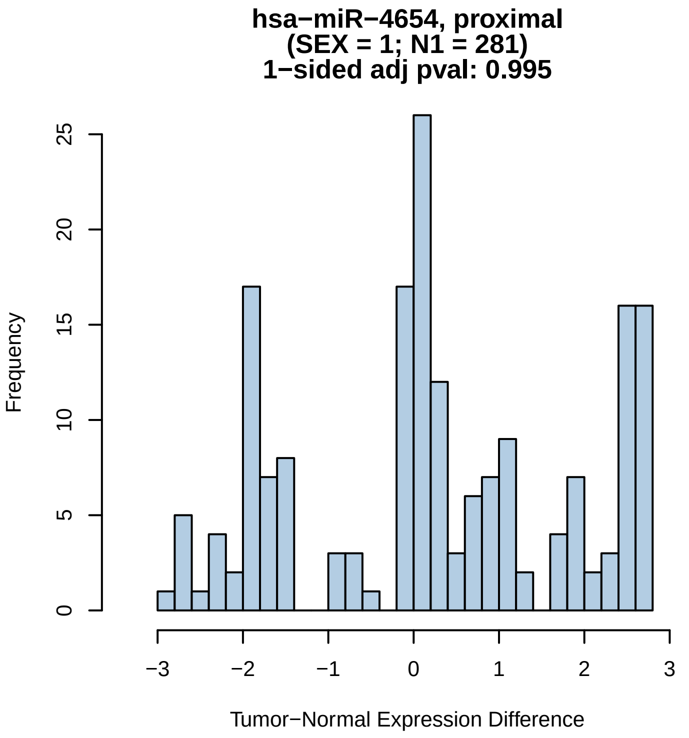
hsa−miR−4654, proximal
(SEX = 1; N1 = 281)
1−sided adj pval: 0.995
Frequency
Tumor−Normal Expression Difference

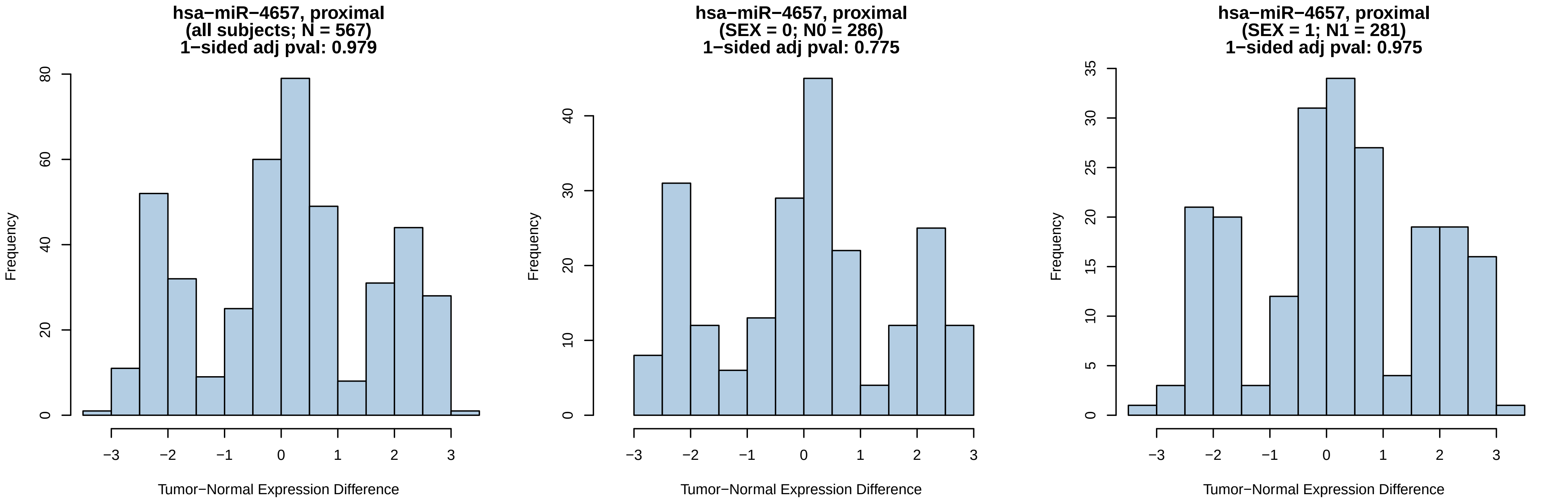

hsa−miR−4657, proximal
(all subjects; N = 567)
1−sided adj pval: 0.979
Frequency
Tumor−Normal Expression Difference
hsa−miR−4657, proximal
(SEX = 0; N0 = 286)
1−sided adj pval: 0.775
Frequency
Tumor−Normal Expression Difference
hsa−miR−4657, proximal
(SEX = 1; N1 = 281)
1−sided adj pval: 0.975
Frequency
Tumor−Normal Expression Difference

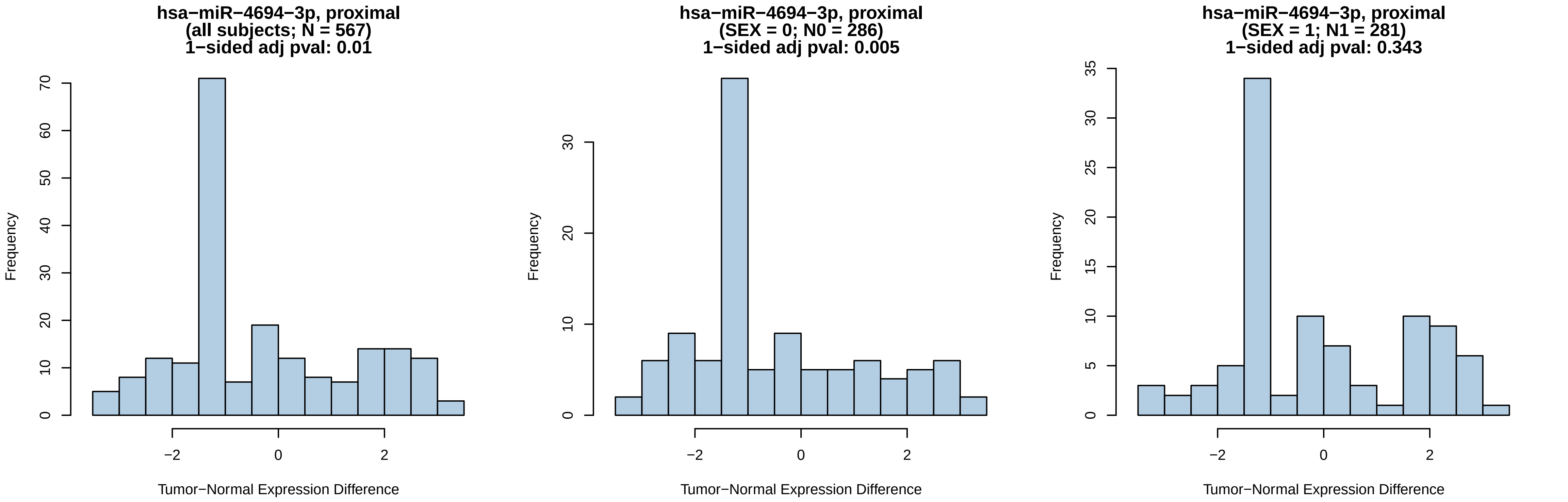
hsa−miR−4694−3p, proximal
(all subjects; N = 567)
1−sided adj pval: 0.01
Frequency
0
10
20
30
40
50
60
70
−2
0
2
Tumor−Normal Expression Difference
hsa−miR−4694−3p, proximal
(SEX = 0; N0 = 286)
1−sided adj pval: 0.005
Frequency
0
10
20
30
−2
0
2
Tumor−Normal Expression Difference
hsa−miR−4694−3p, proximal
(SEX = 1; N1 = 281)
1−sided adj pval: 0.343
Frequency
0
5
10
15
20
25
30
35
−2
0
2
Tumor−Normal Expression Difference

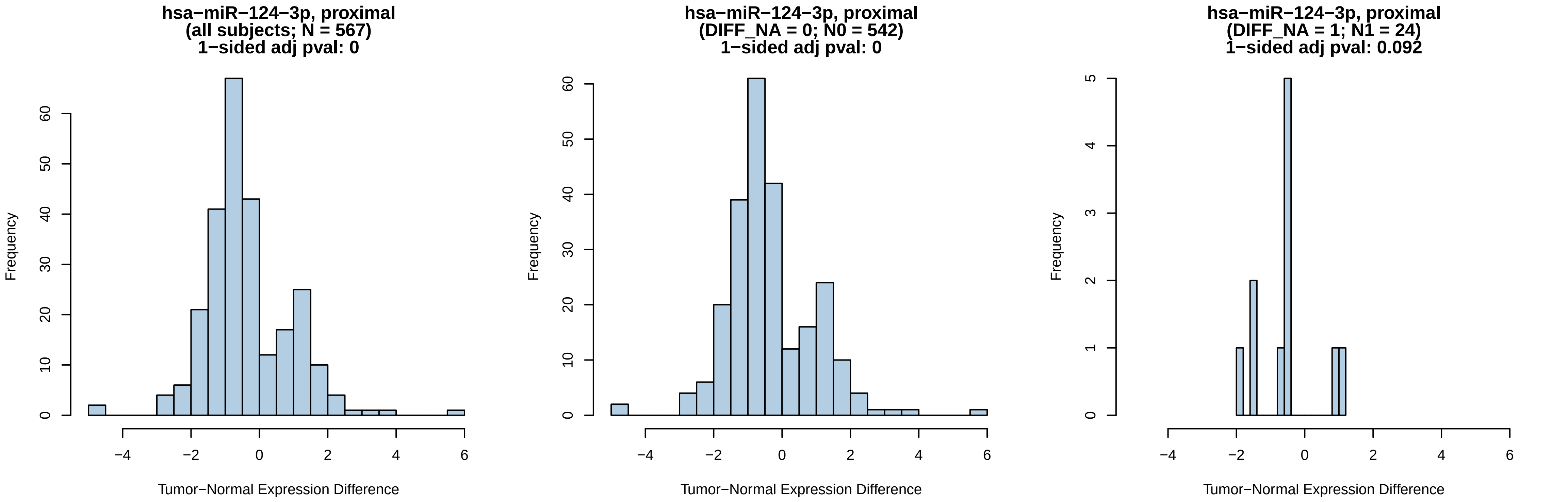
hsa−miR−124−3p, proximal
(all subjects; N = 567)
1−sided adj pval: 0
hsa−miR−124−3p, proximal
(DIFF_NA = 0; N0 = 542)
1−sided adj pval: 0
hsa−miR−124−3p, proximal
(DIFF_NA = 1; N1 = 24)
1−sided adj pval: 0.092
Frequency
Tumor−Normal Expression Difference

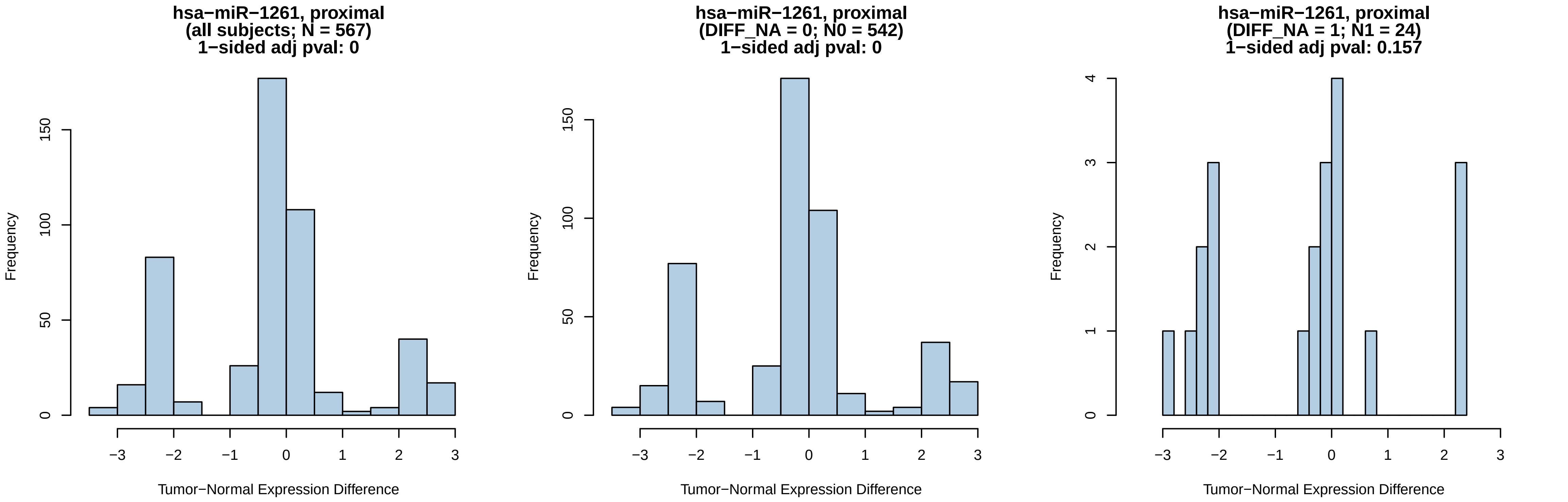
hsa−miR−1261, proximal
(all subjects; N = 567)
1−sided adj pval: 0
Frequency
Tumor−Normal Expression Difference
hsa−miR−1261, proximal
(DIFF_NA = 0; N0 = 542)
1−sided adj pval: 0
Frequency
Tumor−Normal Expression Difference
hsa−miR−1261, proximal
(DIFF_NA = 1; N1 = 24)
1−sided adj pval: 0.157
Frequency
Tumor−Normal Expression Difference

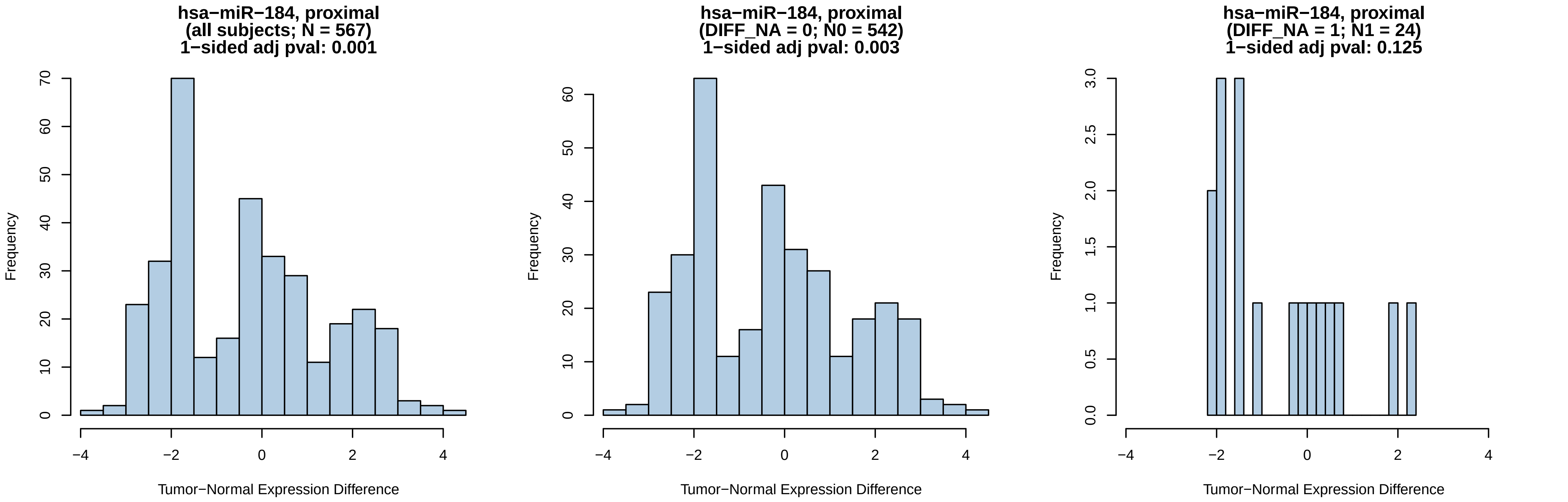

hsa−miR−184, proximal
(all subjects; N = 567)
1−sided adj pval: 0.001
hsa−miR−184, proximal
(DIFF_NA = 0; N0 = 542)
1−sided adj pval: 0.003
hsa−miR−184, proximal
(DIFF_NA = 1; N1 = 24)
1−sided adj pval: 0.125
Frequency
Tumor−Normal Expression Difference

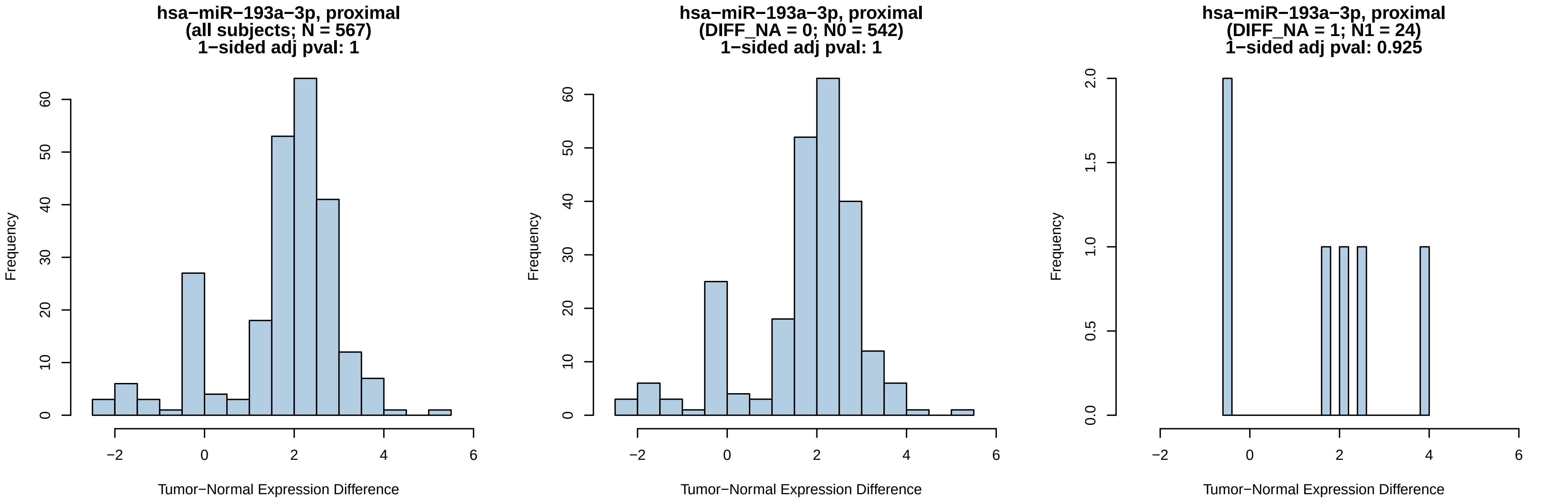
hsa−miR−193a−3p, proximal
(all subjects; N = 567)
1−sided adj pval: 1
Frequency
Tumor−Normal Expression Difference
hsa−miR−193a−3p, proximal
(DIFF_NA = 0; N0 = 542)
1−sided adj pval: 1
Frequency
Tumor−Normal Expression Difference
hsa−miR−193a−3p, proximal
(DIFF_NA = 1; N1 = 24)
1−sided adj pval: 0.925
Frequency
Tumor−Normal Expression Difference

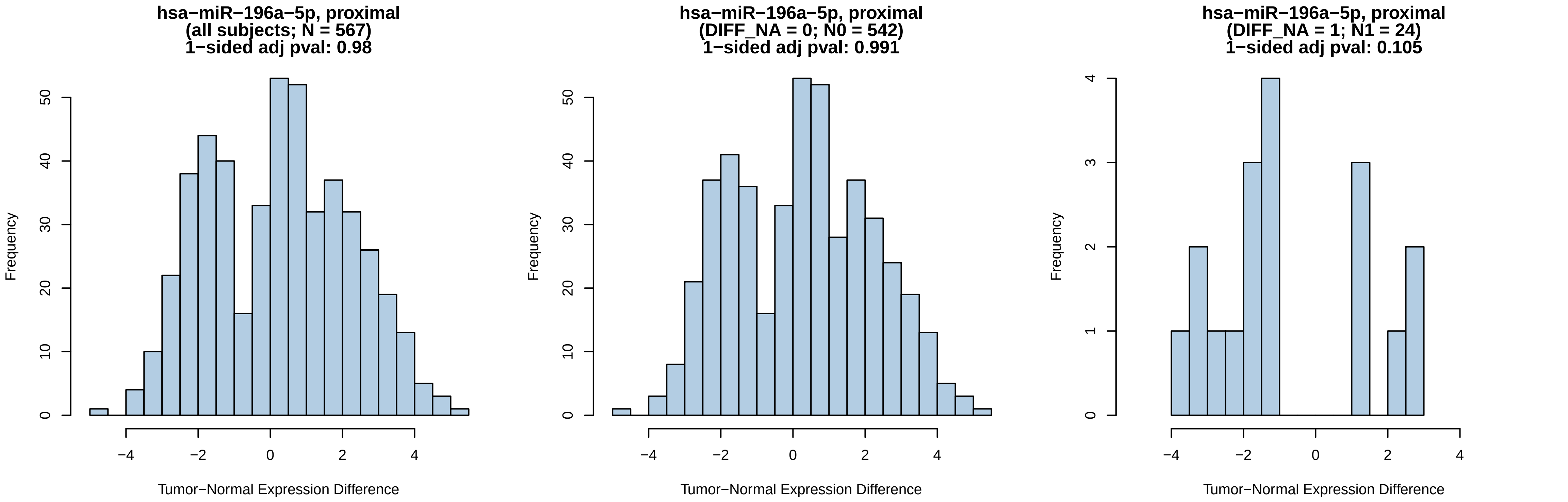
hsa−miR−196a−5p, proximal
(all subjects; N = 567)
1−sided adj pval: 0.98
Frequency
Tumor−Normal Expression Difference
hsa−miR−196a−5p, proximal
(DIFF_NA = 0; N0 = 542)
1−sided adj pval: 0.991
Frequency
Tumor−Normal Expression Difference
hsa−miR−196a−5p, proximal
(DIFF_NA = 1; N1 = 24)
1−sided adj pval: 0.105
Frequency
Tumor−Normal Expression Difference

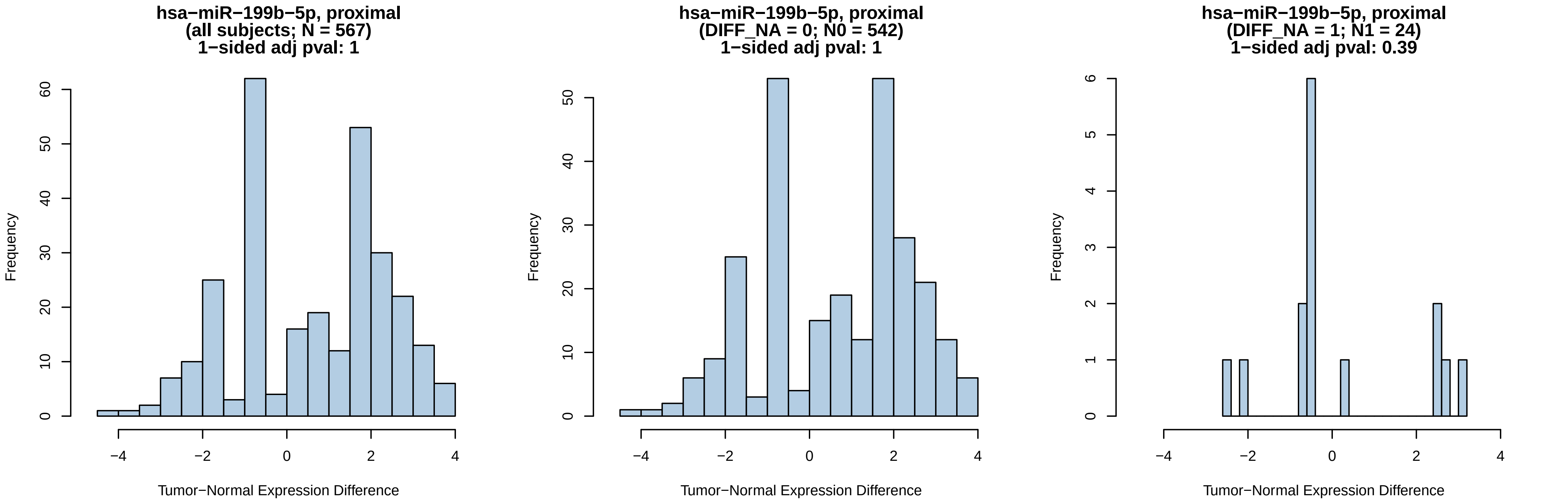
hsa−miR−199b−5p, proximal
(all subjects; N = 567)
1−sided adj pval: 1
hsa−miR−199b−5p, proximal
(DIFF_NA = 0; N0 = 542)
1−sided adj pval: 1
hsa−miR−199b−5p, proximal
(DIFF_NA = 1; N1 = 24)
1−sided adj pval: 0.39
Frequency
Tumor−Normal Expression Difference

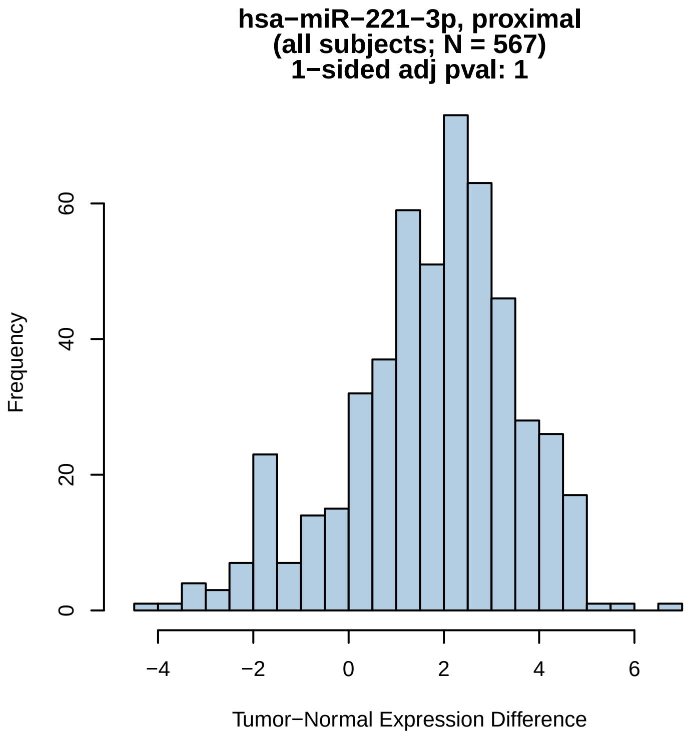
hsa−miR−221−3p, proximal
(all subjects; N = 567)
1−sided adj pval: 1
Frequency
Tumor−Normal Expression Difference
0
20
40
60
−4
−2
0
2
4
6

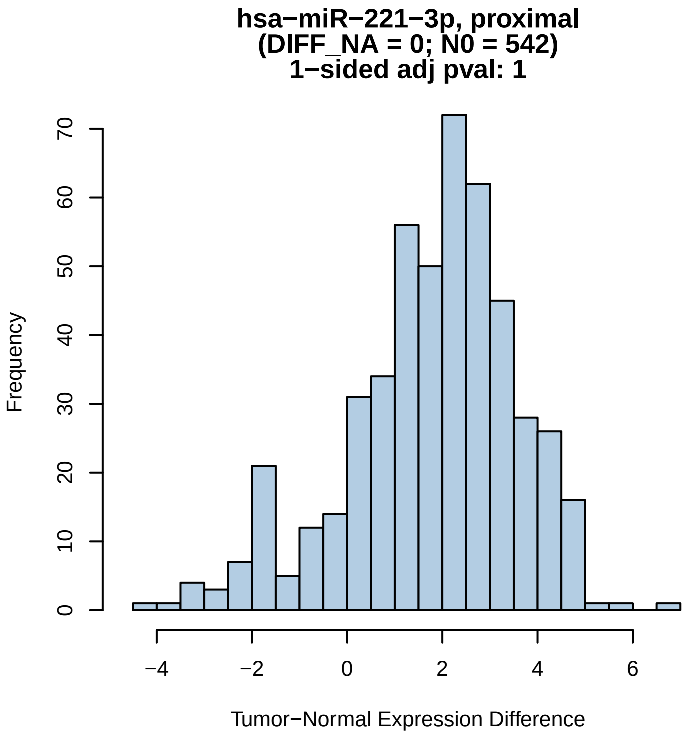
hsa−miR−221−3p, proximal
(DIFF_NA = 0; N0 = 542)
1−sided adj pval: 1
Frequency
Tumor−Normal Expression Difference
0
10
20
30
40
50
60
70
−4
−2
0
2
4
6

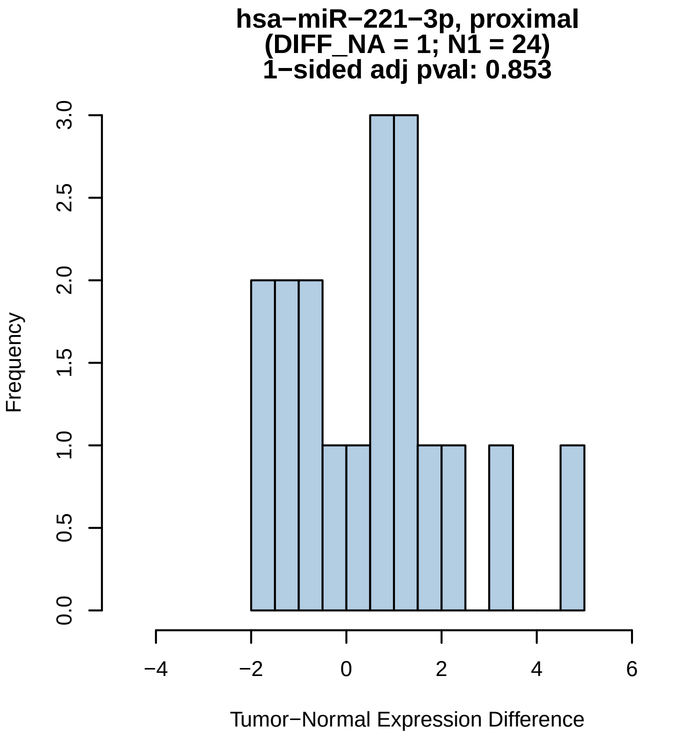
hsa−miR−221−3p, proximal
(DIFF_NA = 1; N1 = 24)
1−sided adj pval: 0.853
Frequency
Tumor−Normal Expression Difference
0.0
0.5
1.0
1.5
2.0
2.5
3.0
−4
−2
0
2
4
6

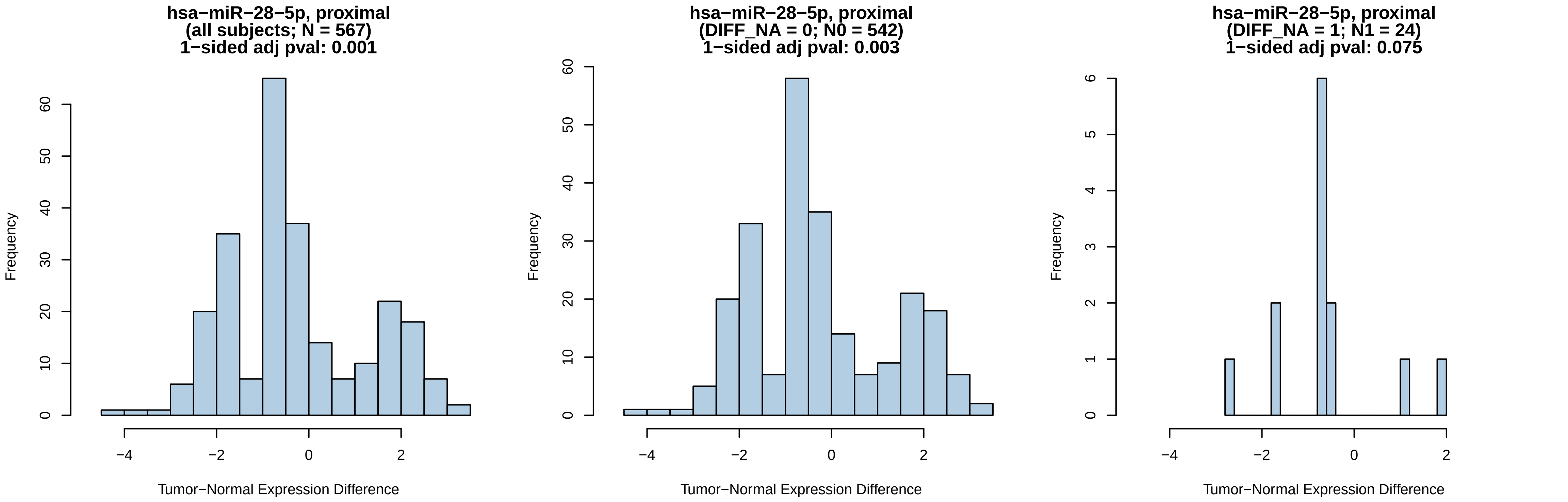

hsa−miR−28−5p, proximal
(all subjects; N = 567)
1−sided adj pval: 0.001
Frequency
0
10
20
30
40
50
60
−4
−2
0
2
Tumor−Normal Expression Difference
hsa−miR−28−5p, proximal
(DIFF_NA = 0; N0 = 542)
1−sided adj pval: 0.003
Frequency
0
10
20
30
40
50
60
−4
−2
0
2
Tumor−Normal Expression Difference
hsa−miR−28−5p, proximal
(DIFF_NA = 1; N1 = 24)
1−sided adj pval: 0.075
Frequency
0
1
2
3
4
5
6
−4
−2
0
2
Tumor−Normal Expression Difference

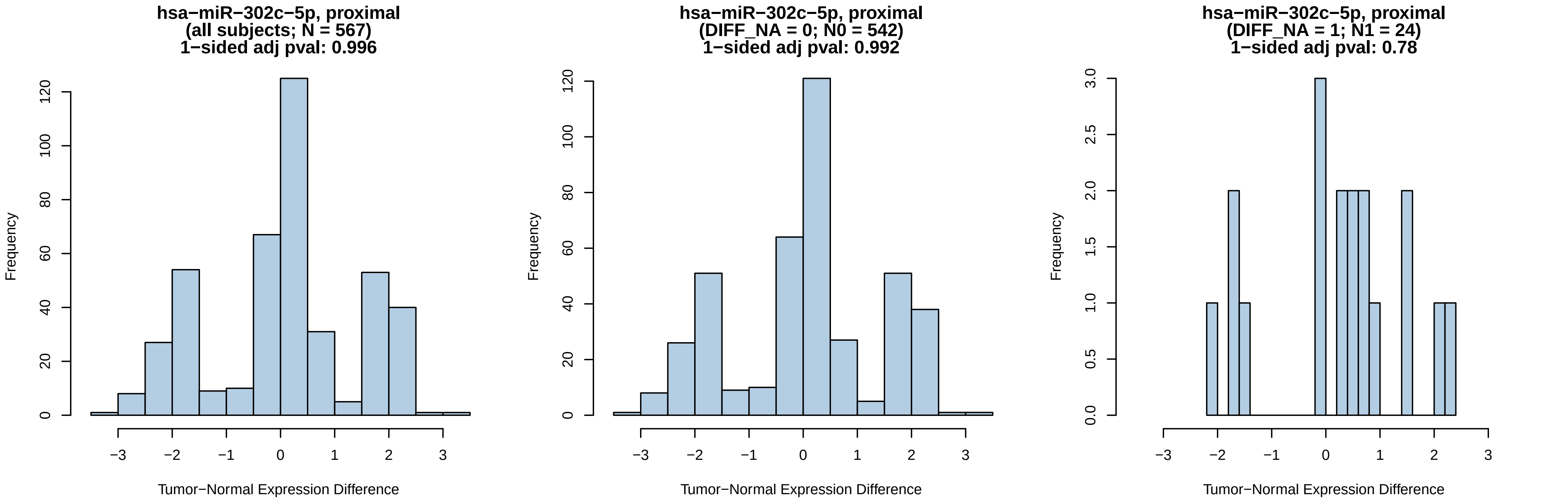

hsa−miR−302c−5p, proximal
(all subjects; N = 567)
1−sided adj pval: 0.996
Frequency
Tumor−Normal Expression Difference
hsa−miR−302c−5p, proximal
(DIFF_NA = 0; N0 = 542)
1−sided adj pval: 0.992
Frequency
Tumor−Normal Expression Difference
hsa−miR−302c−5p, proximal
(DIFF_NA = 1; N1 = 24)
1−sided adj pval: 0.78
Frequency
Tumor−Normal Expression Difference

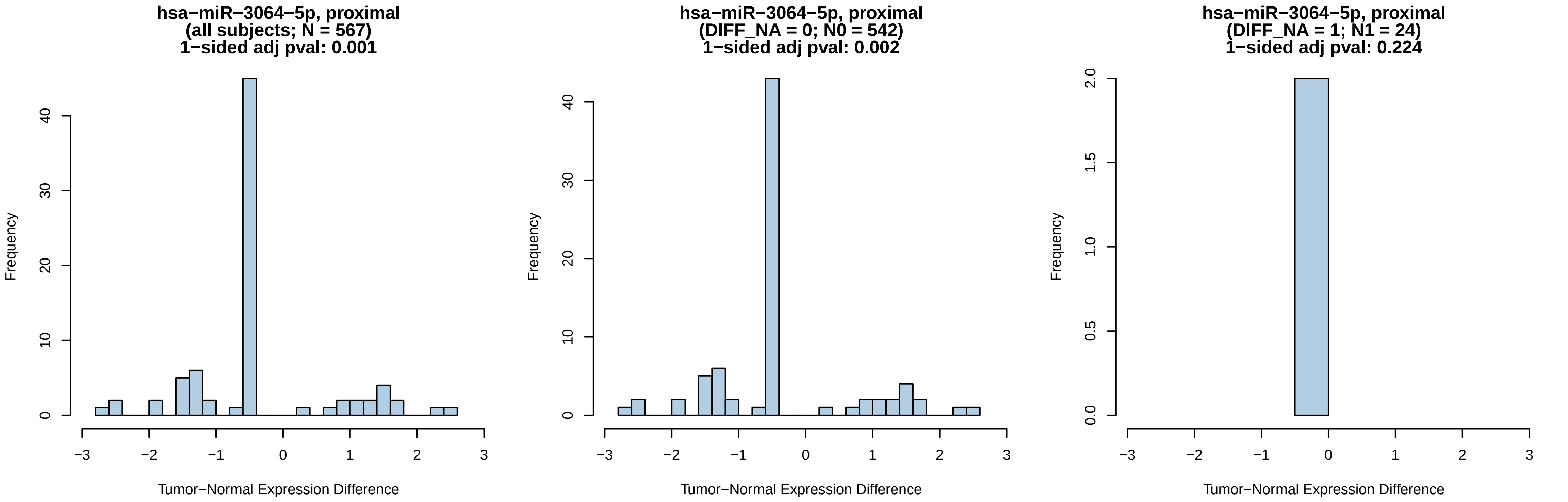
hsa−miR−3064−5p, proximal
(all subjects; N = 567)
1−sided adj pval: 0.001
hsa−miR−3064−5p, proximal
(DIFF_NA = 0; N0 = 542)
1−sided adj pval: 0.002
hsa−miR−3064−5p, proximal
(DIFF_NA = 1; N1 = 24)
1−sided adj pval: 0.224
Frequency
Tumor−Normal Expression Difference

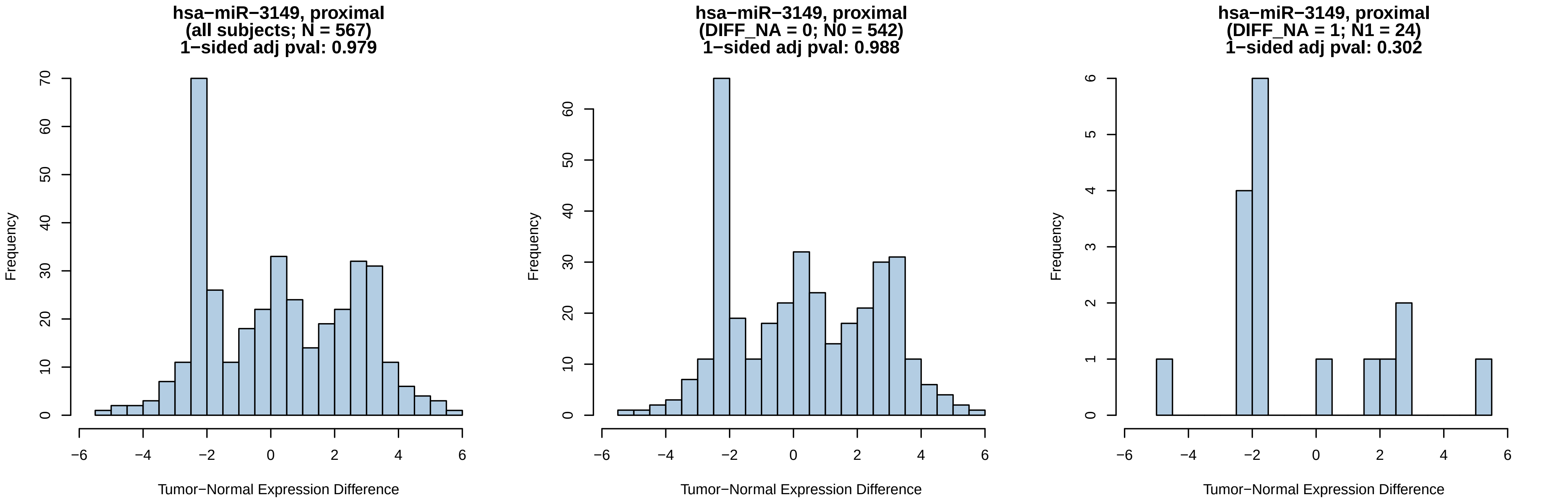

hsa−miR−3149, proximal
(all subjects; N = 567)
1−sided adj pval: 0.979
Frequency
Tumor−Normal Expression Difference
hsa−miR−3149, proximal
(DIFF_NA = 0; N0 = 542)
1−sided adj pval: 0.988
Frequency
Tumor−Normal Expression Difference
hsa−miR−3149, proximal
(DIFF_NA = 1; N1 = 24)
1−sided adj pval: 0.302
Frequency
Tumor−Normal Expression Difference

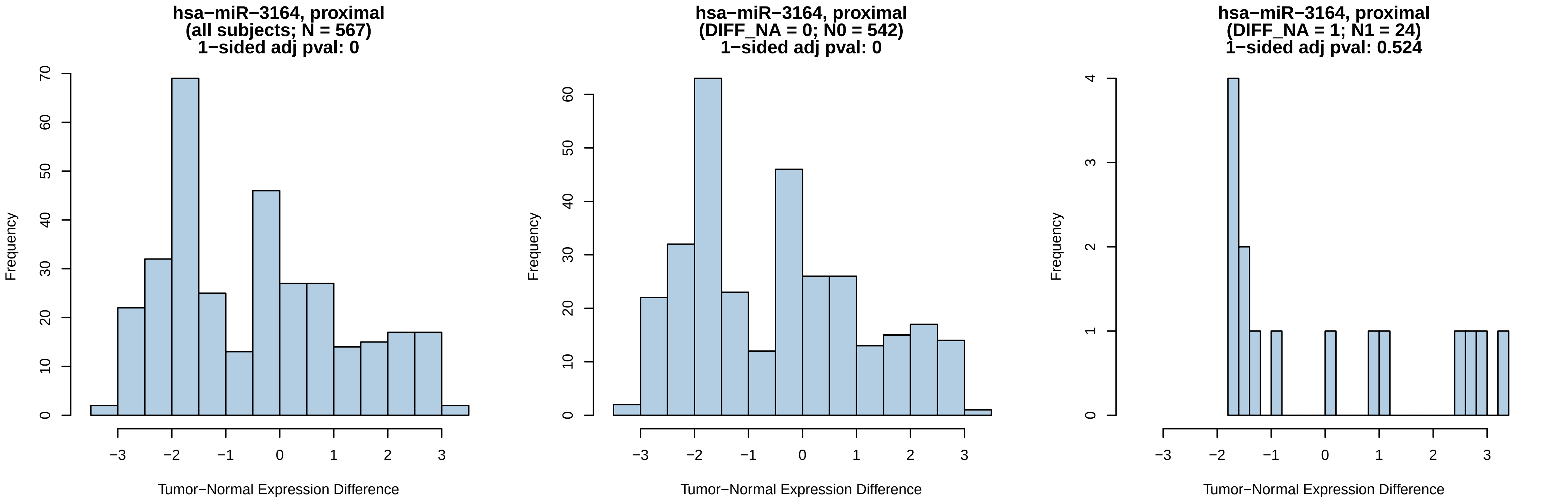
hsa−miR−3164, proximal
(all subjects; N = 567)
1−sided adj pval: 0
Frequency
Tumor−Normal Expression Difference
hsa−miR−3164, proximal
(DIFF_NA = 0; N0 = 542)
1−sided adj pval: 0
Frequency
Tumor−Normal Expression Difference
hsa−miR−3164, proximal
(DIFF_NA = 1; N1 = 24)
1−sided adj pval: 0.524
Frequency
Tumor−Normal Expression Difference

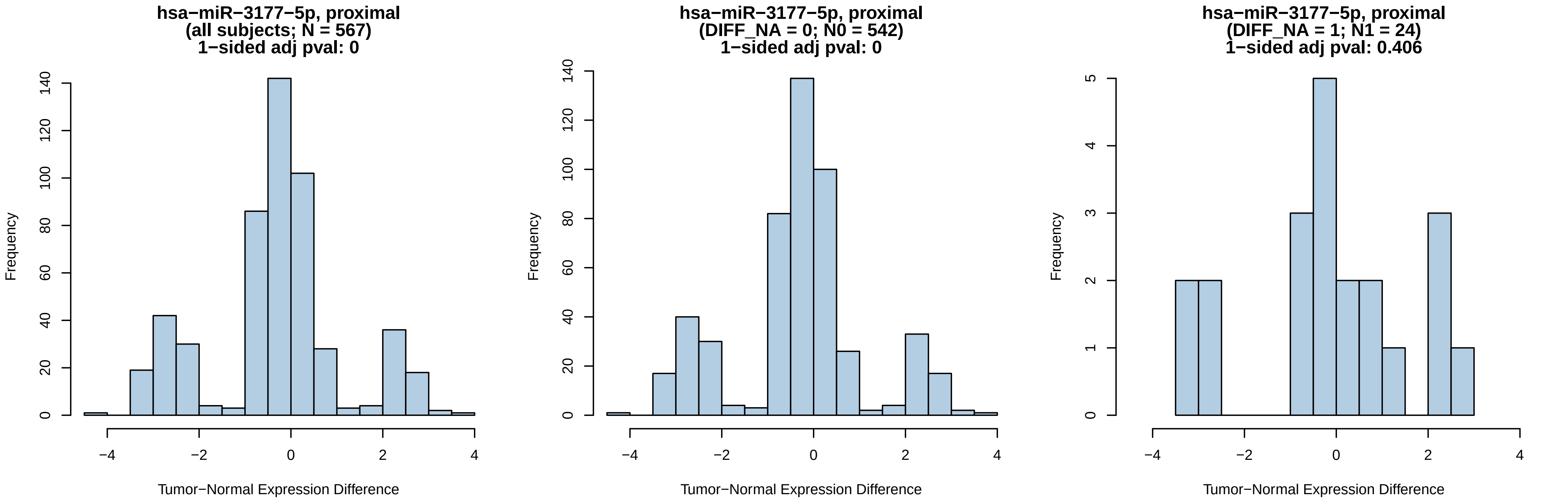

hsa−miR−3177−5p, proximal
(all subjects; N = 567)
1−sided adj pval: 0
Frequency
Tumor−Normal Expression Difference
hsa−miR−3177−5p, proximal
(DIFF_NA = 0; N0 = 542)
1−sided adj pval: 0
Frequency
Tumor−Normal Expression Difference
hsa−miR−3177−5p, proximal
(DIFF_NA = 1; N1 = 24)
1−sided adj pval: 0.406
Frequency
Tumor−Normal Expression Difference

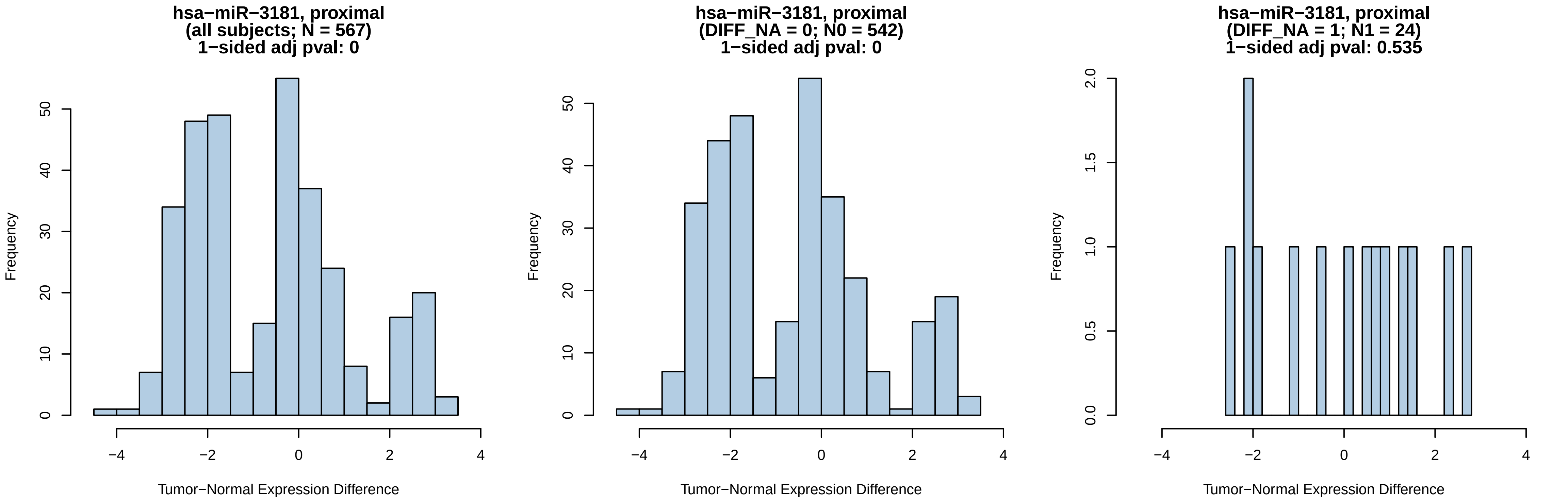
hsa−miR−3181, proximal
(all subjects; N = 567)
1−sided adj pval: 0
hsa−miR−3181, proximal
(DIFF_NA = 0; N0 = 542)
1−sided adj pval: 0
hsa−miR−3181, proximal
(DIFF_NA = 1; N1 = 24)
1−sided adj pval: 0.535
Frequency
Tumor−Normal Expression Difference

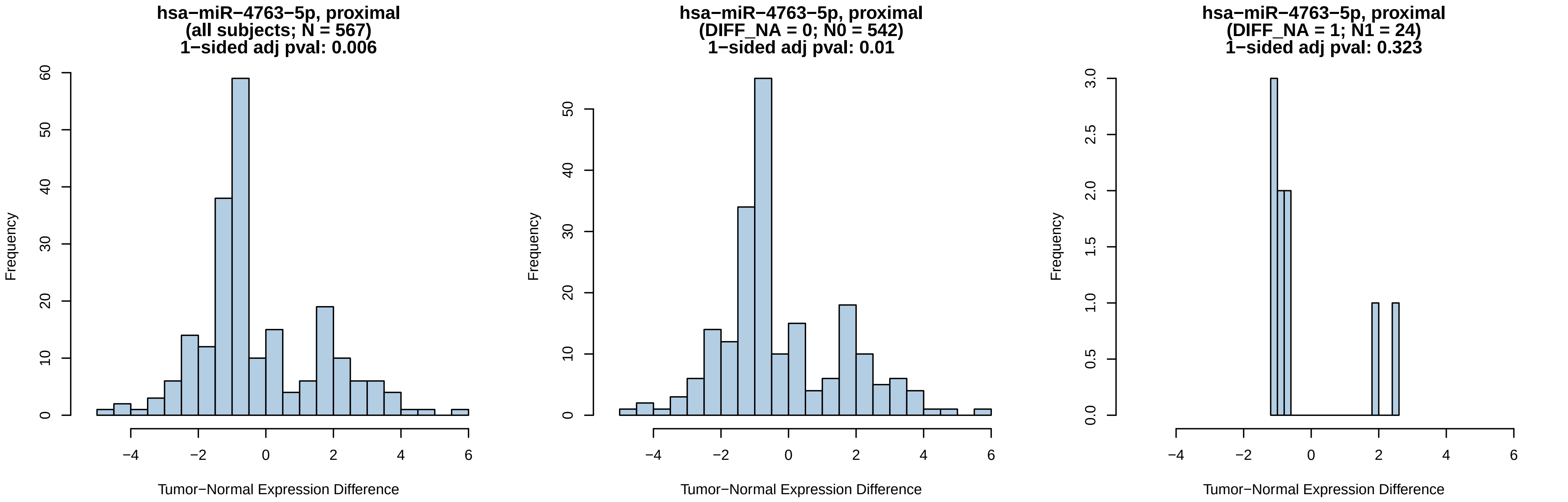
hsa−miR−4763−5p, proximal
(all subjects; N = 567)
1−sided adj pval: 0.006
Frequency
Tumor−Normal Expression Difference
hsa−miR−4763−5p, proximal
(DIFF_NA = 0; N0 = 542)
1−sided adj pval: 0.01
Frequency
Tumor−Normal Expression Difference
hsa−miR−4763−5p, proximal
(DIFF_NA = 1; N1 = 24)
1−sided adj pval: 0.323
Frequency
Tumor−Normal Expression Difference

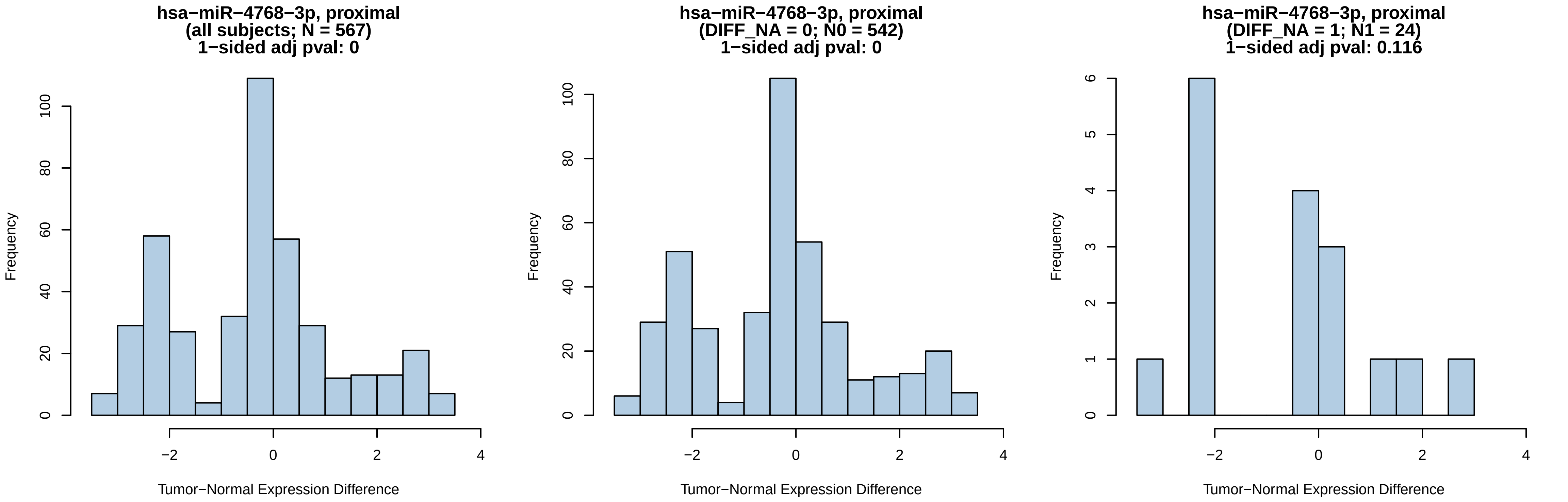

hsa−miR−4768−3p, proximal
(all subjects; N = 567)
1−sided adj pval: 0
Frequency
0
20
40
60
80
100
−2
0
2
4
Tumor−Normal Expression Difference
hsa−miR−4768−3p, proximal
(DIFF_NA = 0; N0 = 542)
1−sided adj pval: 0
Frequency
0
20
40
60
80
100
−2
0
2
4
Tumor−Normal Expression Difference
hsa−miR−4768−3p, proximal
(DIFF_NA = 1; N1 = 24)
1−sided adj pval: 0.116
Frequency
0
1
2
3
4
5
6
−2
0
2
4
Tumor−Normal Expression Difference

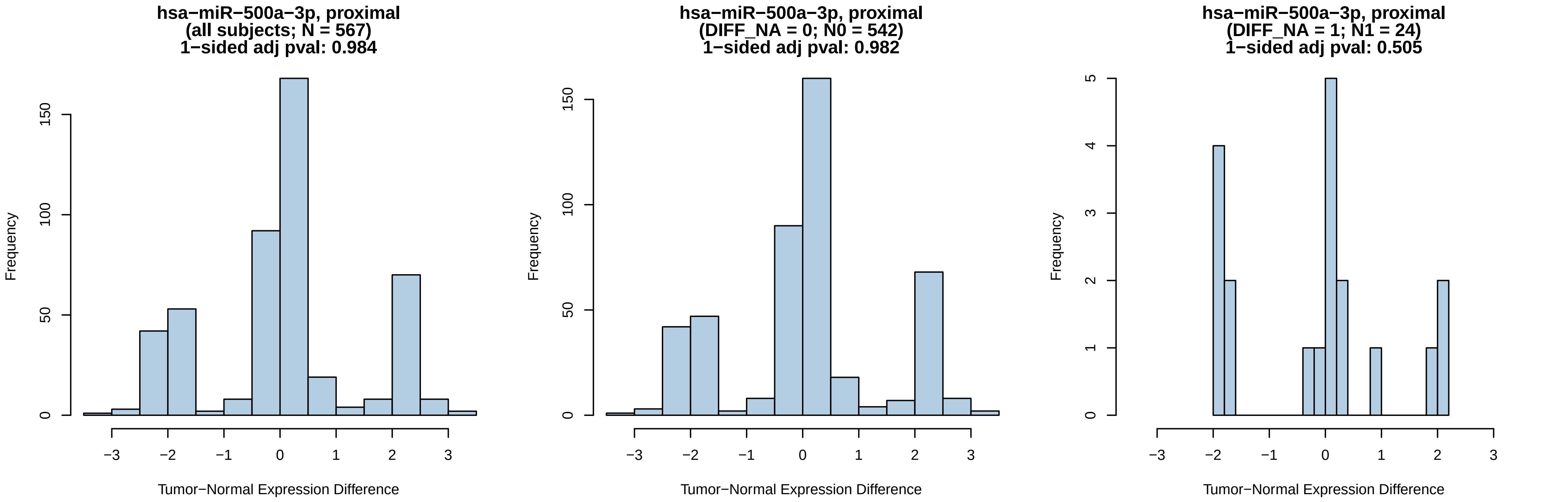

hsa−miR−500a−3p, proximal
(all subjects; N = 567)
1−sided adj pval: 0.984
hsa−miR−500a−3p, proximal
(DIFF_NA = 0; N0 = 542)
1−sided adj pval: 0.982
hsa−miR−500a−3p, proximal
(DIFF_NA = 1; N1 = 24)
1−sided adj pval: 0.505
Frequency
Tumor−Normal Expression Difference

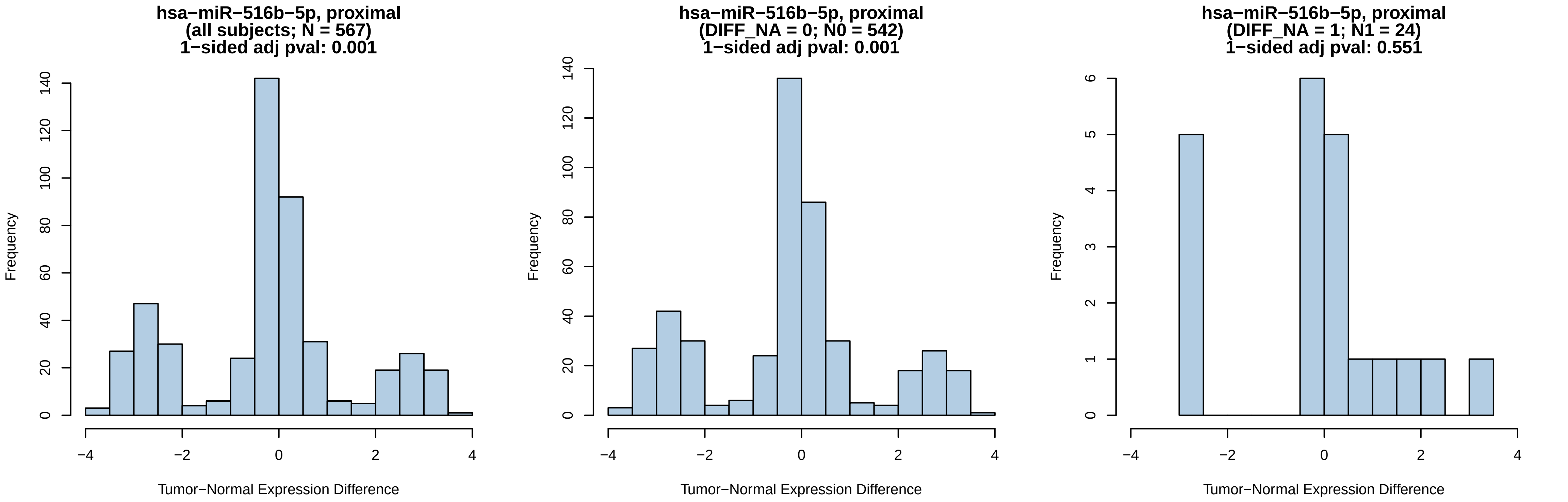
hsa−miR−516b−5p, proximal
(all subjects; N = 567)
1−sided adj pval: 0.001
Frequency
Tumor−Normal Expression Difference
hsa−miR−516b−5p, proximal
(DIFF_NA = 0; N0 = 542)
1−sided adj pval: 0.001
Frequency
Tumor−Normal Expression Difference
hsa−miR−516b−5p, proximal
(DIFF_NA = 1; N1 = 24)
1−sided adj pval: 0.551
Frequency
Tumor−Normal Expression Difference

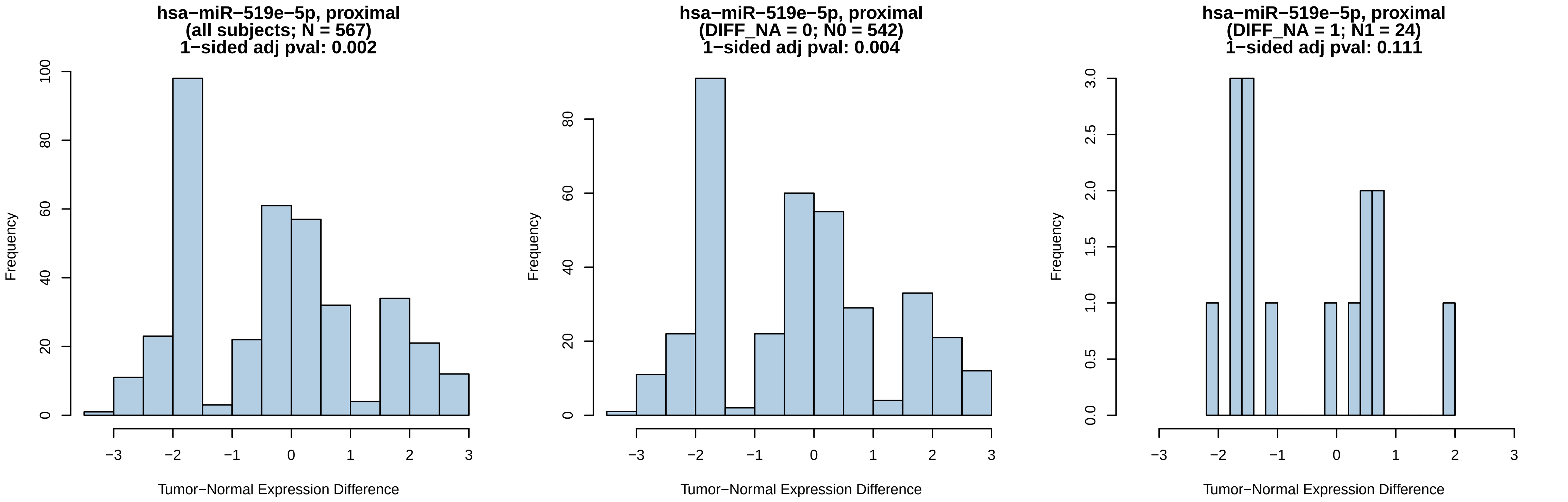
hsa−miR−519e−5p, proximal
(all subjects; N = 567)
1−sided adj pval: 0.002
Frequency
Tumor−Normal Expression Difference
hsa−miR−519e−5p, proximal
(DIFF_NA = 0; N0 = 542)
1−sided adj pval: 0.004
Frequency
Tumor−Normal Expression Difference
hsa−miR−519e−5p, proximal
(DIFF_NA = 1; N1 = 24)
1−sided adj pval: 0.111
Frequency
Tumor−Normal Expression Difference

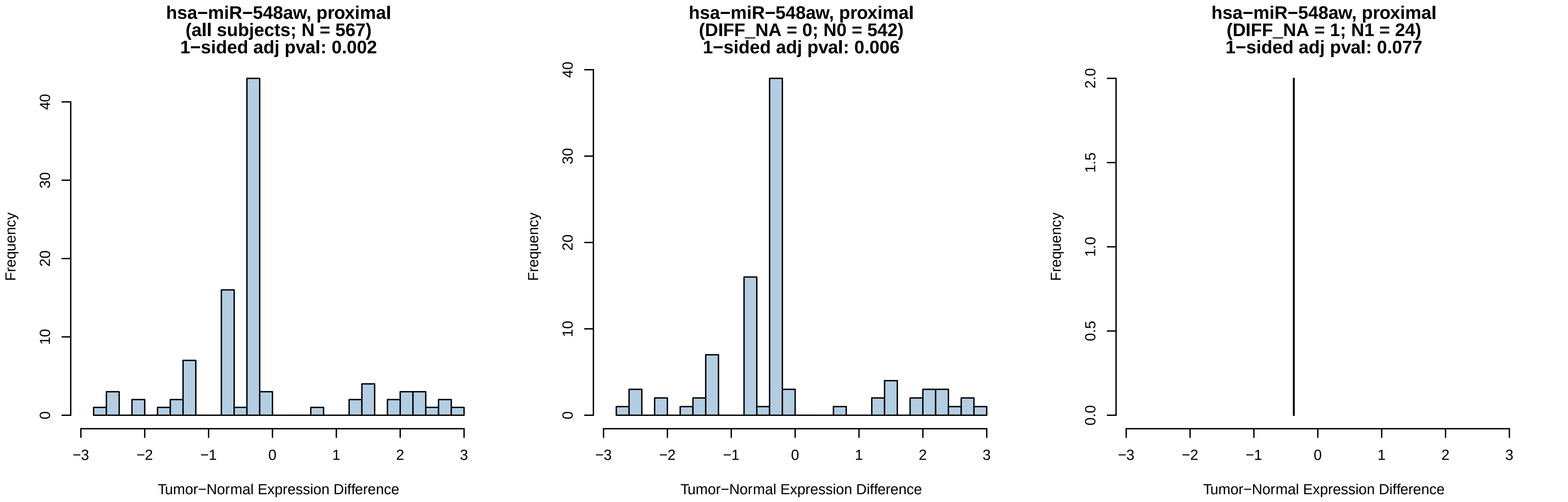
hsa−miR−548aw, proximal
(all subjects; N = 567)
1−sided adj pval: 0.002
Frequency
Tumor−Normal Expression Difference
hsa−miR−548aw, proximal
(DIFF_NA = 0; N0 = 542)
1−sided adj pval: 0.006
Frequency
Tumor−Normal Expression Difference
hsa−miR−548aw, proximal
(DIFF_NA = 1; N1 = 24)
1−sided adj pval: 0.077
Frequency
Tumor−Normal Expression Difference

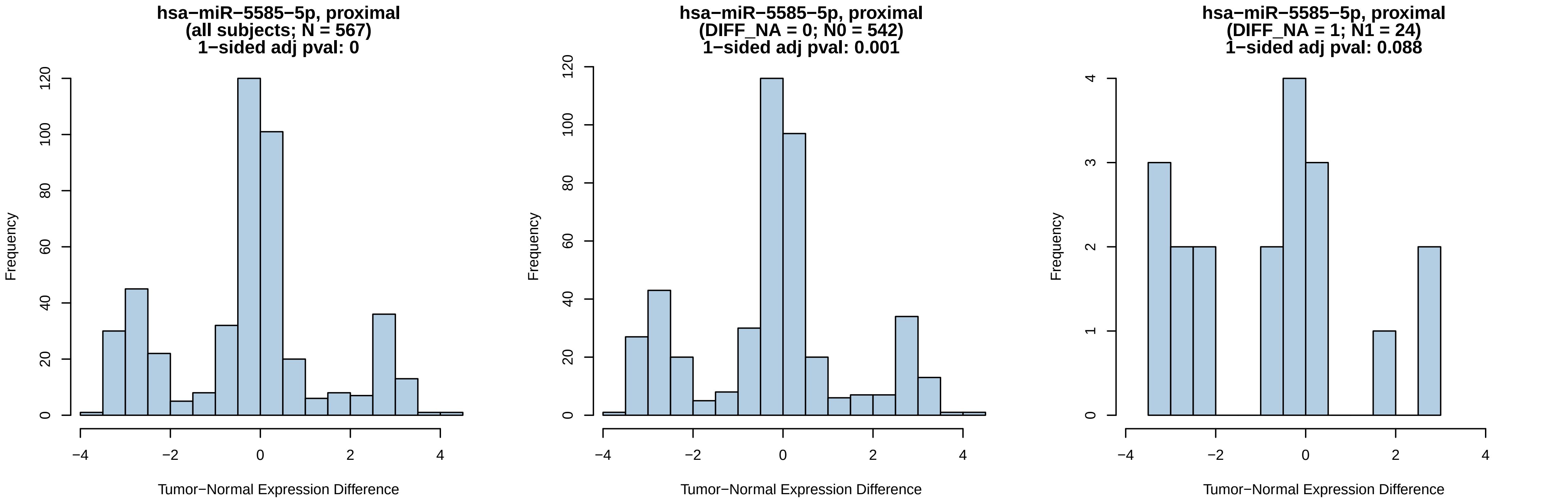
hsa−miR−5585−5p, proximal
(all subjects; N = 567)
1−sided adj pval: 0
hsa−miR−5585−5p, proximal
(DIFF_NA = 0; N0 = 542)
1−sided adj pval: 0.001
hsa−miR−5585−5p, proximal
(DIFF_NA = 1; N1 = 24)
1−sided adj pval: 0.088
Frequency
Tumor−Normal Expression Difference

hsa−miR−5696, proximal
(all subjects; N = 567)
1−sided adj pval: 0

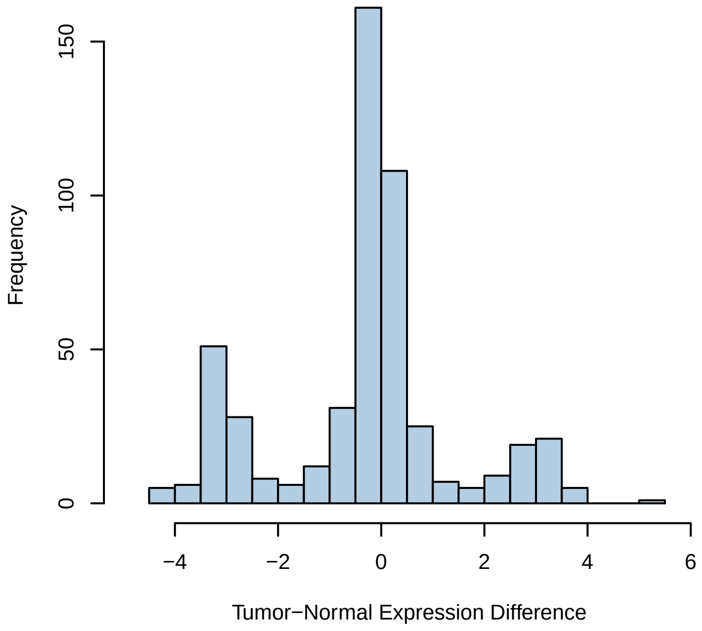


hsa−miR−5696, proximal
(DIFF_NA = 0; N0 = 542)
1−sided adj pval: 0

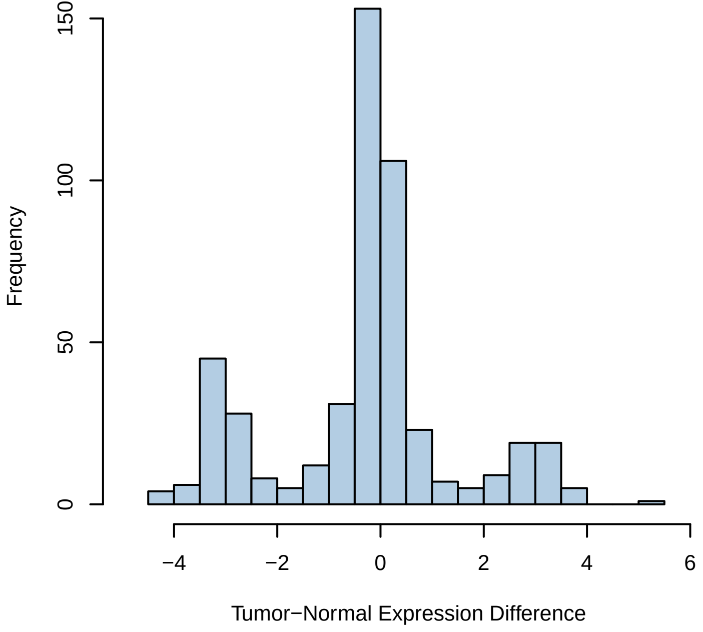


hsa−miR−5696, proximal
(DIFF_NA = 1; N1 = 24)
1−sided adj pval: 0.098

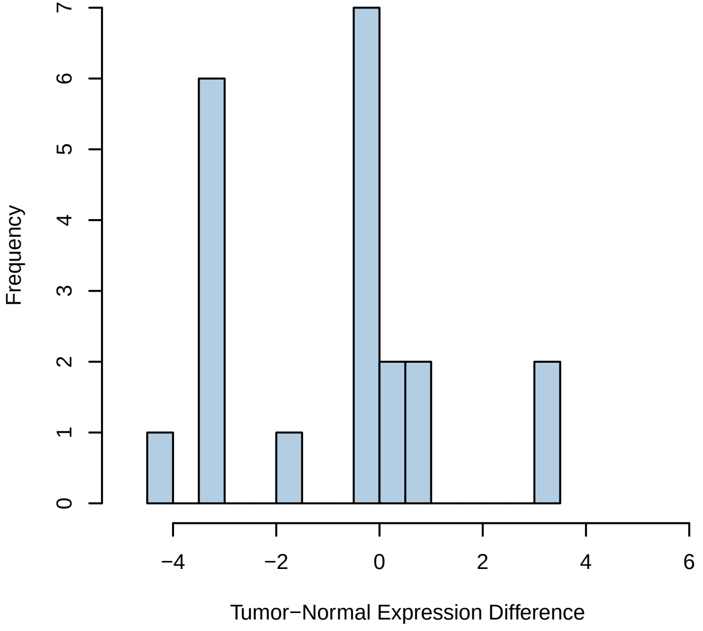

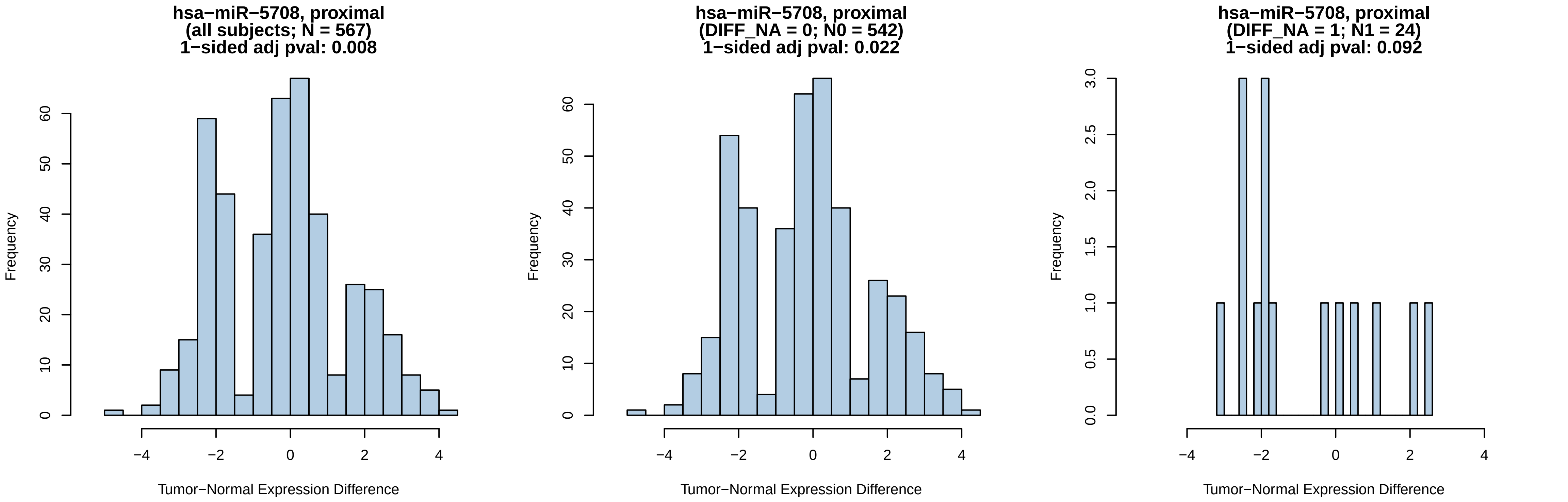

hsa−miR−5708, proximal
(all subjects; N = 567)
1−sided adj pval: 0.008
Frequency
0
10
20
30
40
50
60
−4
−2
0
2
4
Tumor−Normal Expression Difference
hsa−miR−5708, proximal
(DIFF_NA = 0; N0 = 542)
1−sided adj pval: 0.022
Frequency
0
10
20
30
40
50
60
−4
−2
0
2
4
Tumor−Normal Expression Difference
hsa−miR−5708, proximal
(DIFF_NA = 1; N1 = 24)
1−sided adj pval: 0.092
Frequency
0.0
0.5
1.0
1.5
2.0
2.5
3.0
−4
−2
0
2
4
Tumor−Normal Expression Difference

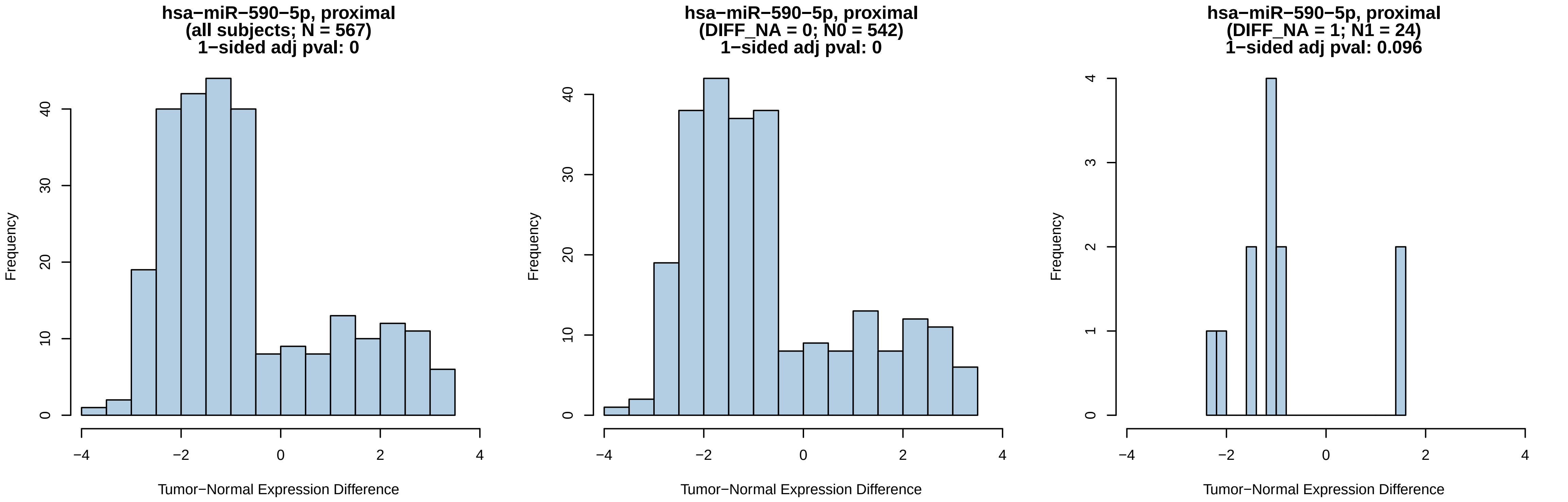

hsa−miR−590−5p, proximal
(all subjects; N = 567)
1−sided adj pval: 0
Frequency
0
10
20
30
40
−4
−2
0
2
4
Tumor−Normal Expression Difference
hsa−miR−590−5p, proximal
(DIFF_NA = 0; N0 = 542)
1−sided adj pval: 0
Frequency
0
10
20
30
40
−4
−2
0
2
4
Tumor−Normal Expression Difference
hsa−miR−590−5p, proximal
(DIFF_NA = 1; N1 = 24)
1−sided adj pval: 0.096
Frequency
0
1
2
3
4
−4
−2
0
2
4
Tumor−Normal Expression Difference

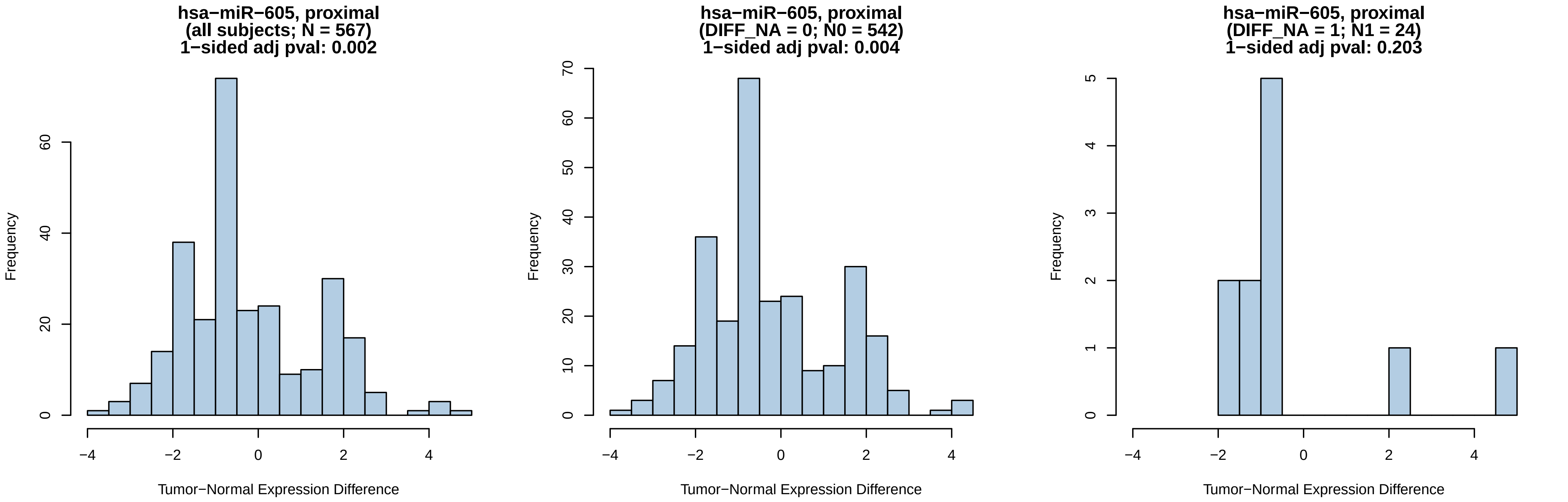
hsa−miR−605, proximal
(all subjects; N = 567)
1−sided adj pval: 0.002
Frequency
Tumor−Normal Expression Difference
hsa−miR−605, proximal
(DIFF_NA = 0; N0 = 542)
1−sided adj pval: 0.004
Frequency
Tumor−Normal Expression Difference
hsa−miR−605, proximal
(DIFF_NA = 1; N1 = 24)
1−sided adj pval: 0.203
Frequency
Tumor−Normal Expression Difference

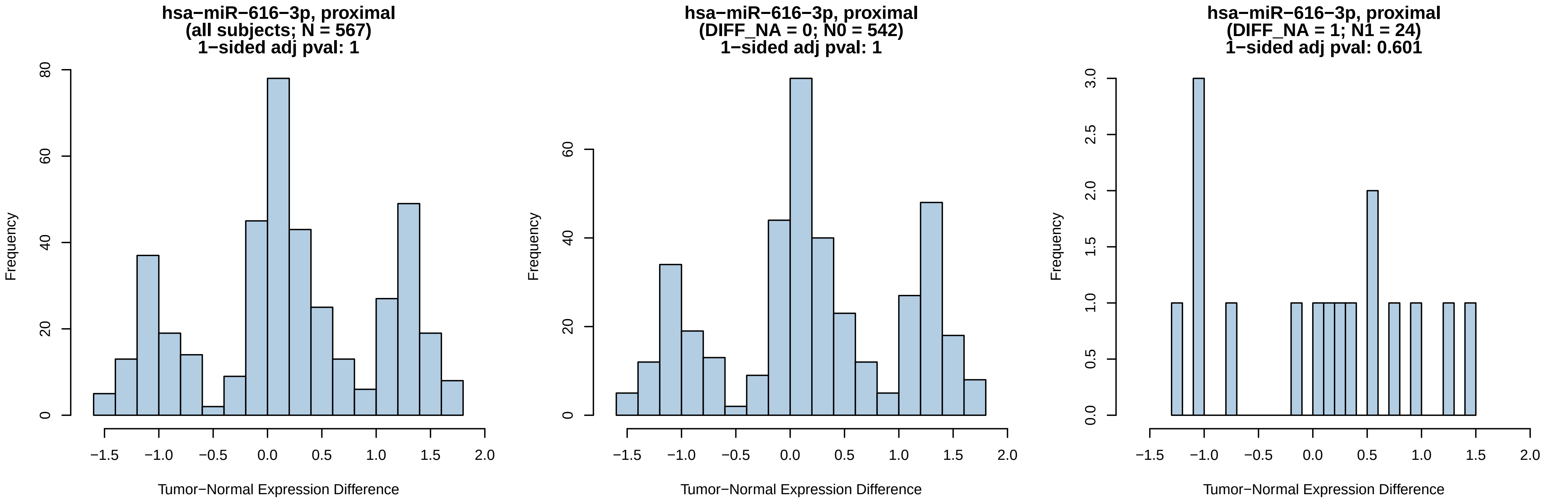

hsa−miR−616−3p, proximal
(all subjects; N = 567)
1−sided adj pval: 1
Frequency
Tumor−Normal Expression Difference
hsa−miR−616−3p, proximal
(DIFF_NA = 0; N0 = 542)
1−sided adj pval: 1
Frequency
Tumor−Normal Expression Difference
hsa−miR−616−3p, proximal
(DIFF_NA = 1; N1 = 24)
1−sided adj pval: 0.601
Frequency
Tumor−Normal Expression Difference

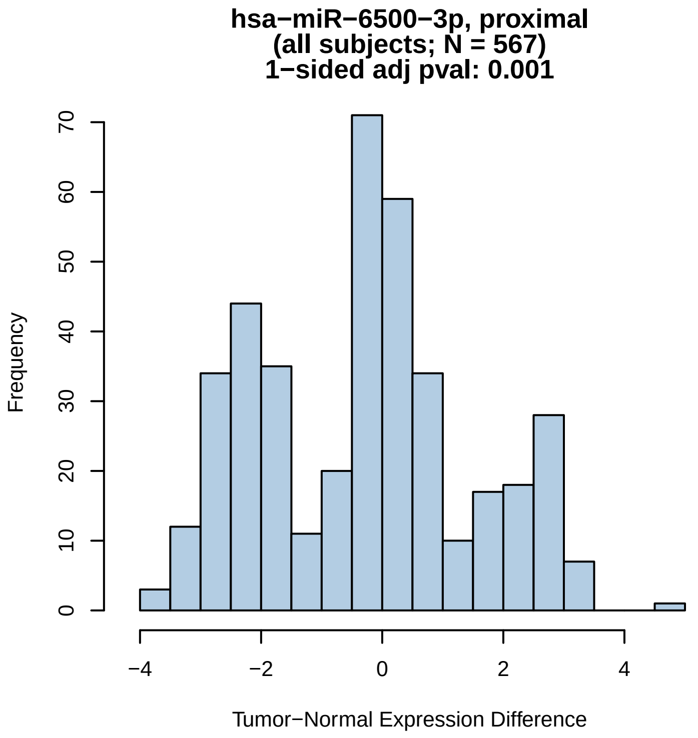
hsa−miR−6500−3p, proximal
(all subjects; N = 567)
1−sided adj pval: 0.001
Frequency
Tumor−Normal Expression Difference

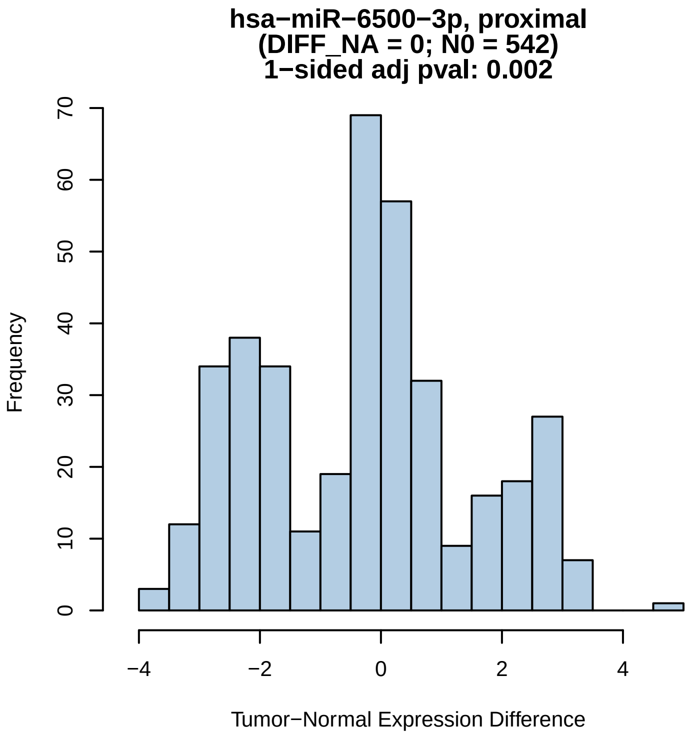
hsa−miR−6500−3p, proximal
(DIFF_NA = 0; N0 = 542)
1−sided adj pval: 0.002
Frequency
Tumor−Normal Expression Difference

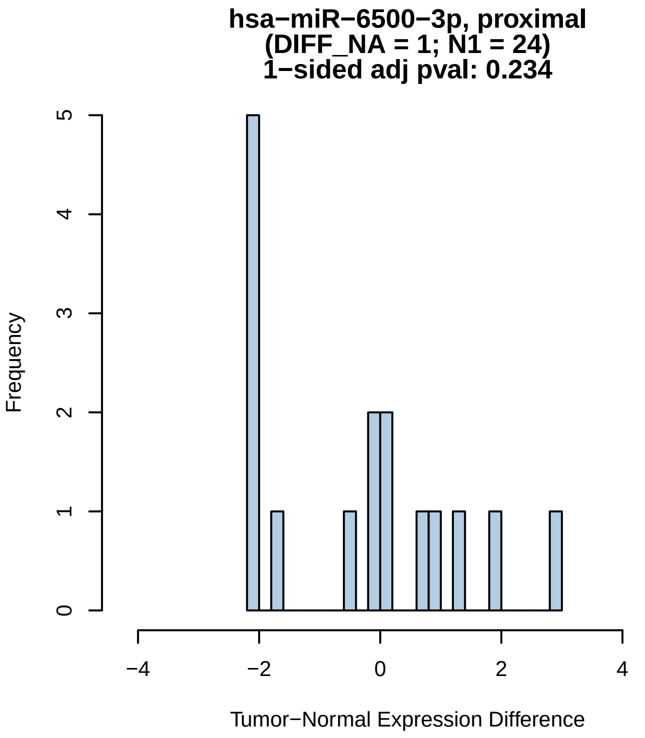
hsa−miR−6500−3p, proximal
(DIFF_NA = 1; N1 = 24)
1−sided adj pval: 0.234
Frequency
Tumor−Normal Expression Difference

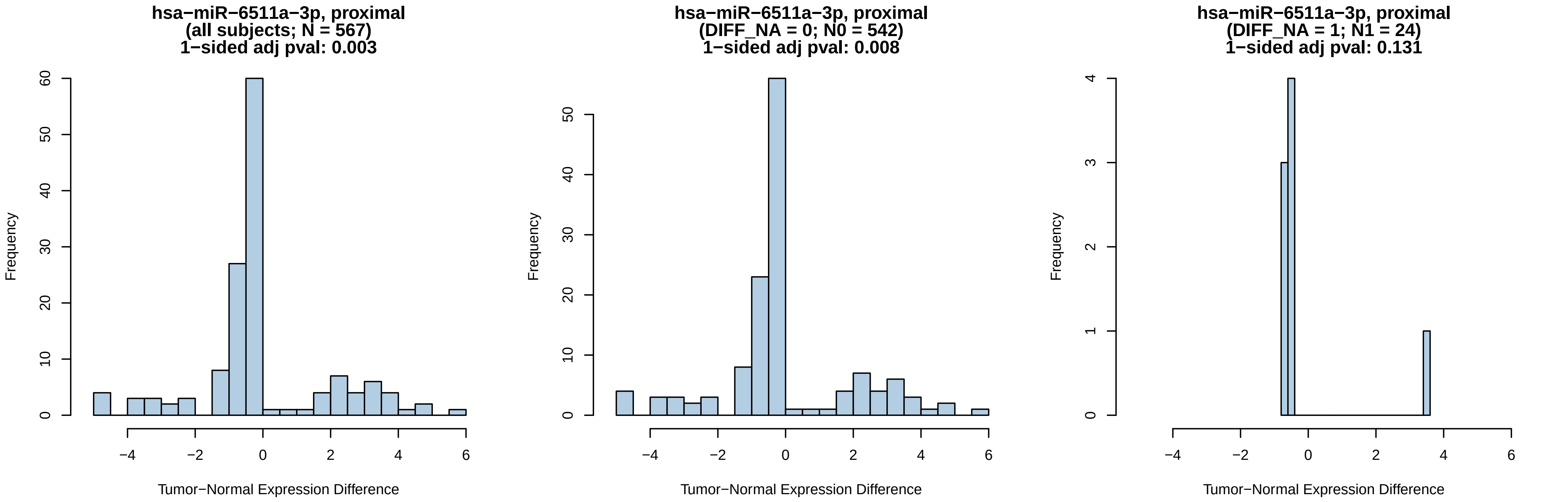
hsa−miR−6511a−3p, proximal
(all subjects; N = 567)
1−sided adj pval: 0.003
hsa−miR−6511a−3p, proximal
(DIFF_NA = 0; N0 = 542)
1−sided adj pval: 0.008
hsa−miR−6511a−3p, proximal
(DIFF_NA = 1; N1 = 24)
1−sided adj pval: 0.131
Frequency
Tumor−Normal Expression Difference

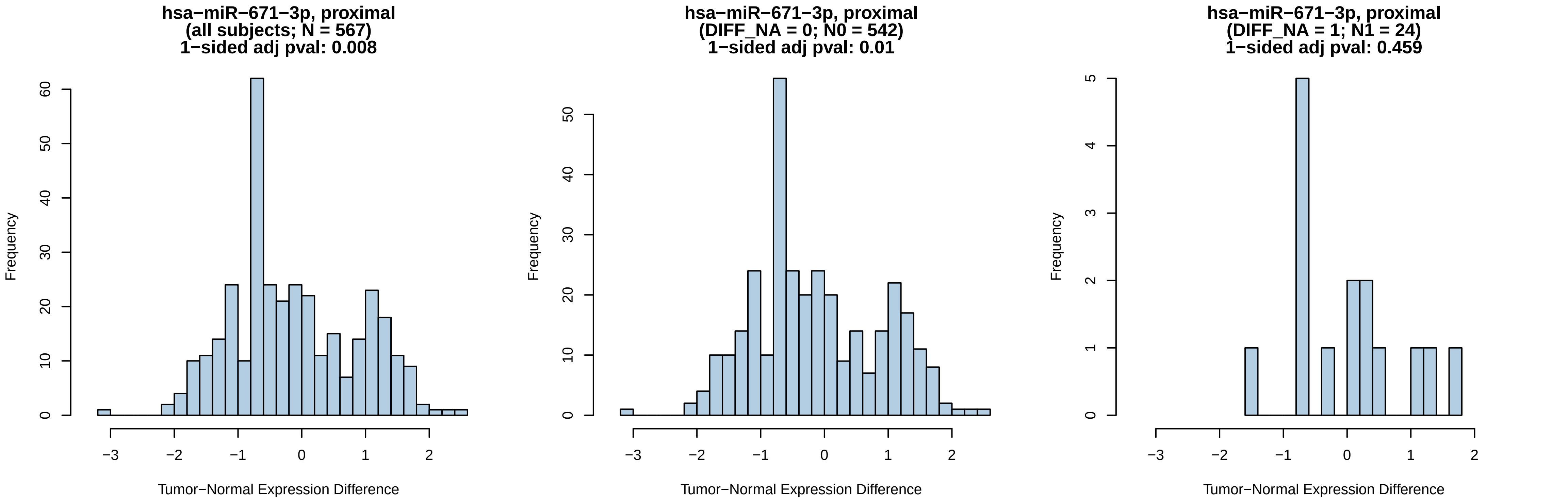

hsa−miR−671−3p, proximal
(all subjects; N = 567)
1−sided adj pval: 0.008
Frequency
Tumor−Normal Expression Difference
hsa−miR−671−3p, proximal
(DIFF_NA = 0; N0 = 542)
1−sided adj pval: 0.01
Frequency
Tumor−Normal Expression Difference
hsa−miR−671−3p, proximal
(DIFF_NA = 1; N1 = 24)
1−sided adj pval: 0.459
Frequency
Tumor−Normal Expression Difference

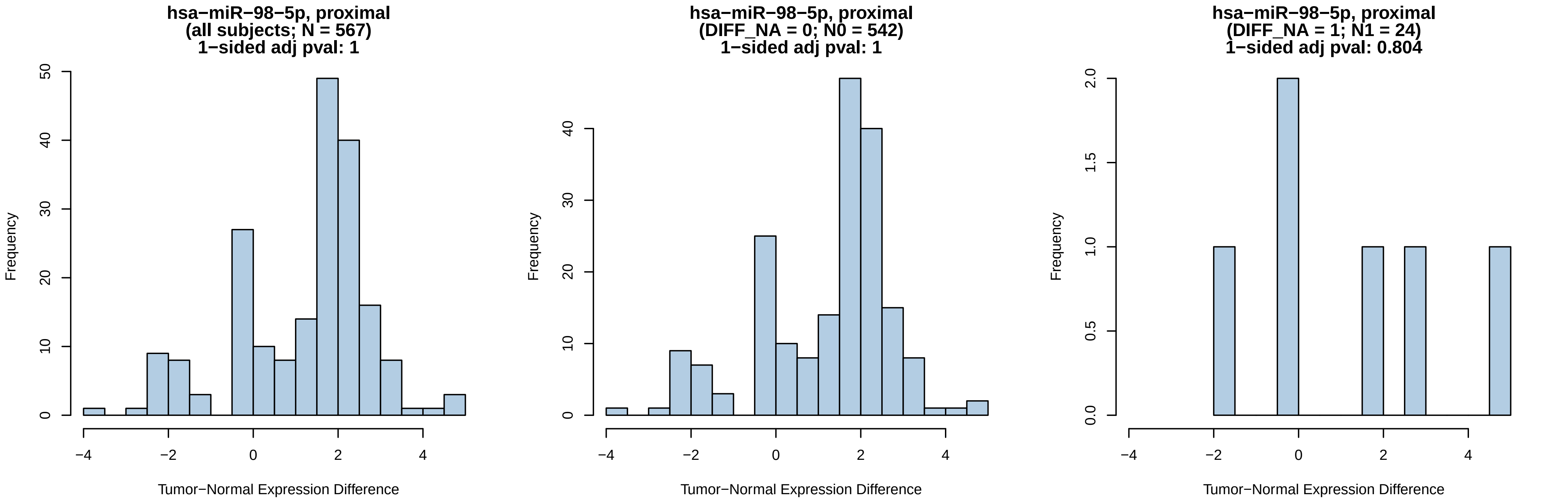
hsa−miR−98−5p, proximal
(all subjects; N = 567)
1−sided adj pval: 1
hsa−miR−98−5p, proximal
(DIFF_NA = 0; N0 = 542)
1−sided adj pval: 1
hsa−miR−98−5p, proximal
(DIFF_NA = 1; N1 = 24)
1−sided adj pval: 0.804
Frequency
Tumor−Normal Expression Difference

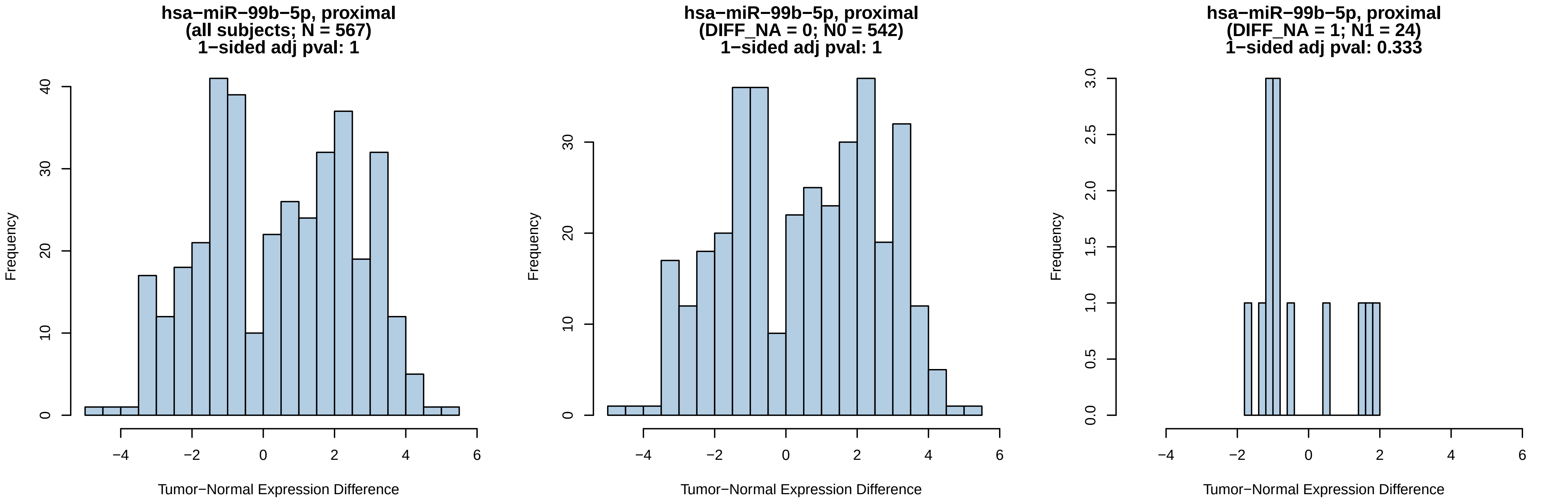
hsa−miR−99b−5p, proximal
(all subjects; N = 567)
1−sided adj pval: 1
Frequency
Tumor−Normal Expression Difference
hsa−miR−99b−5p, proximal
(DIFF_NA = 0; N0 = 542)
1−sided adj pval: 1
Frequency
Tumor−Normal Expression Difference
hsa−miR−99b−5p, proximal
(DIFF_NA = 1; N1 = 24)
1−sided adj pval: 0.333
Frequency
Tumor−Normal Expression Difference

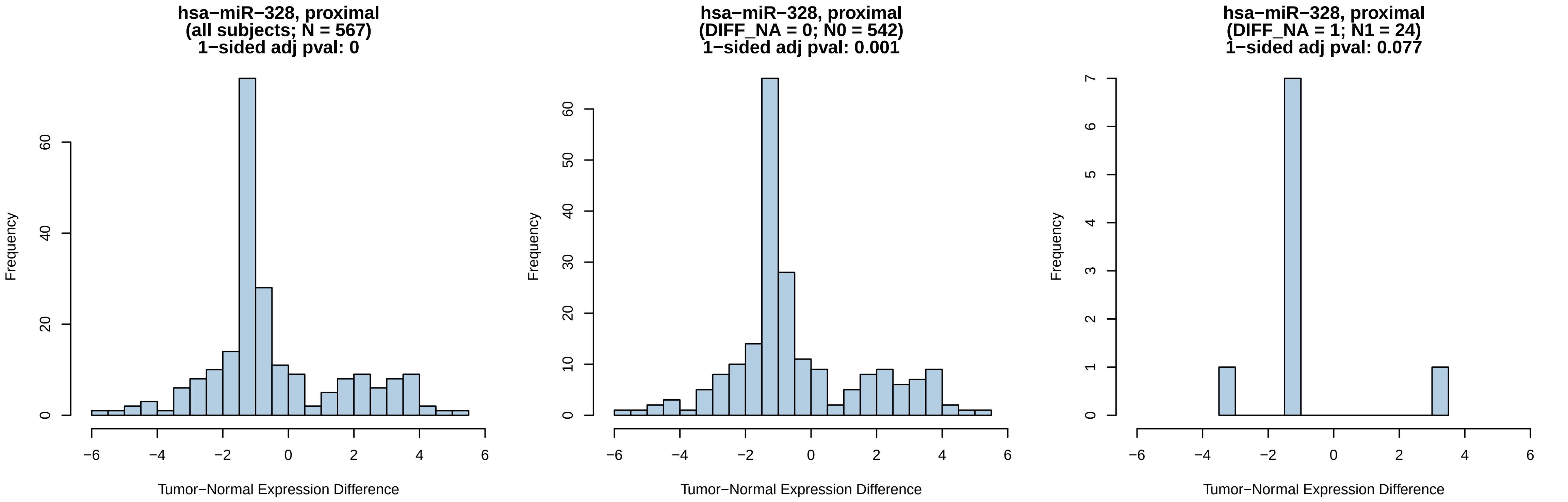

hsa−miR−328, proximal
(all subjects; N = 567)
1−sided adj pval: 0
hsa−miR−328, proximal
(DIFF_NA = 0; N0 = 542)
1−sided adj pval: 0.001
hsa−miR−328, proximal
(DIFF_NA = 1; N1 = 24)
1−sided adj pval: 0.077
Frequency
Tumor−Normal Expression Difference

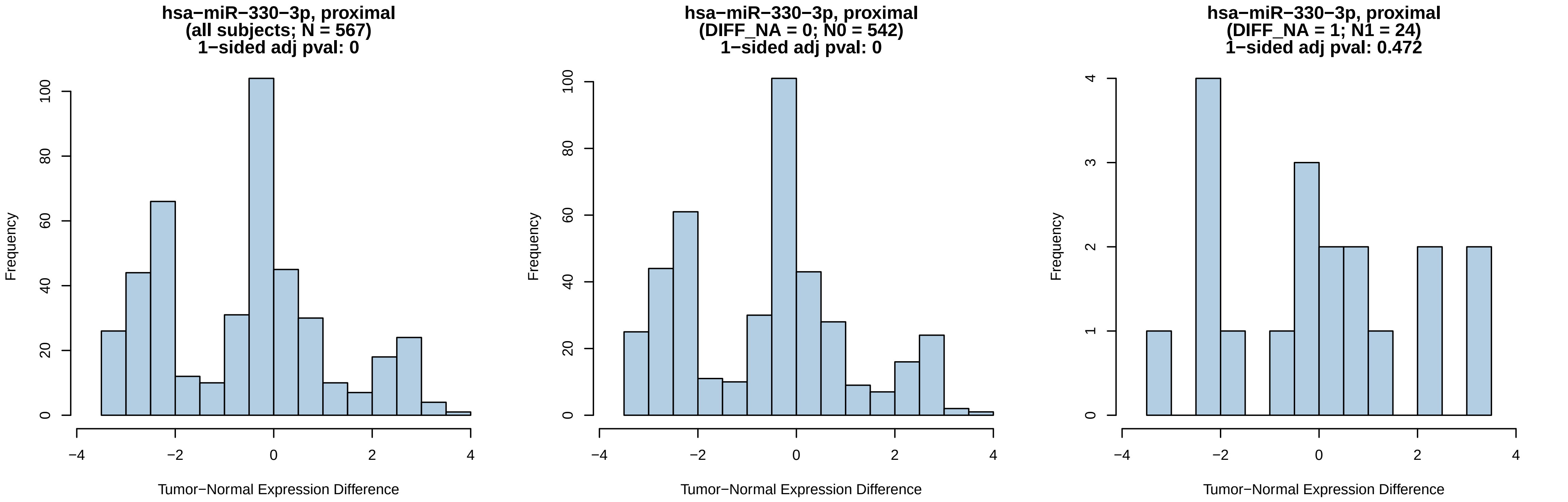
hsa−miR−330−3p, proximal
(all subjects; N = 567)
1−sided adj pval: 0
Frequency
Tumor−Normal Expression Difference
hsa−miR−330−3p, proximal
(DIFF_NA = 0; N0 = 542)
1−sided adj pval: 0
Frequency
Tumor−Normal Expression Difference
hsa−miR−330−3p, proximal
(DIFF_NA = 1; N1 = 24)
1−sided adj pval: 0.472
Frequency
Tumor−Normal Expression Difference

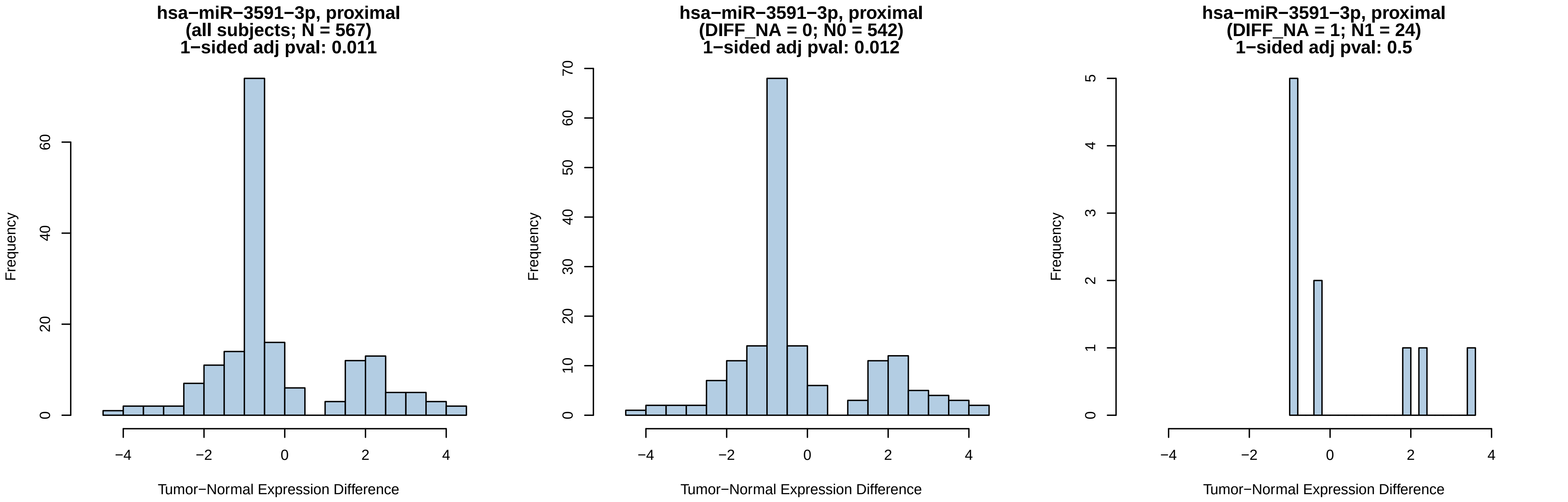

hsa−miR−3591−3p, proximal
(all subjects; N = 567)
1−sided adj pval: 0.011
hsa−miR−3591−3p, proximal
(DIFF_NA = 0; N0 = 542)
1−sided adj pval: 0.012
hsa−miR−3591−3p, proximal
(DIFF_NA = 1; N1 = 24)
1−sided adj pval: 0.5
Frequency
Tumor−Normal Expression Difference

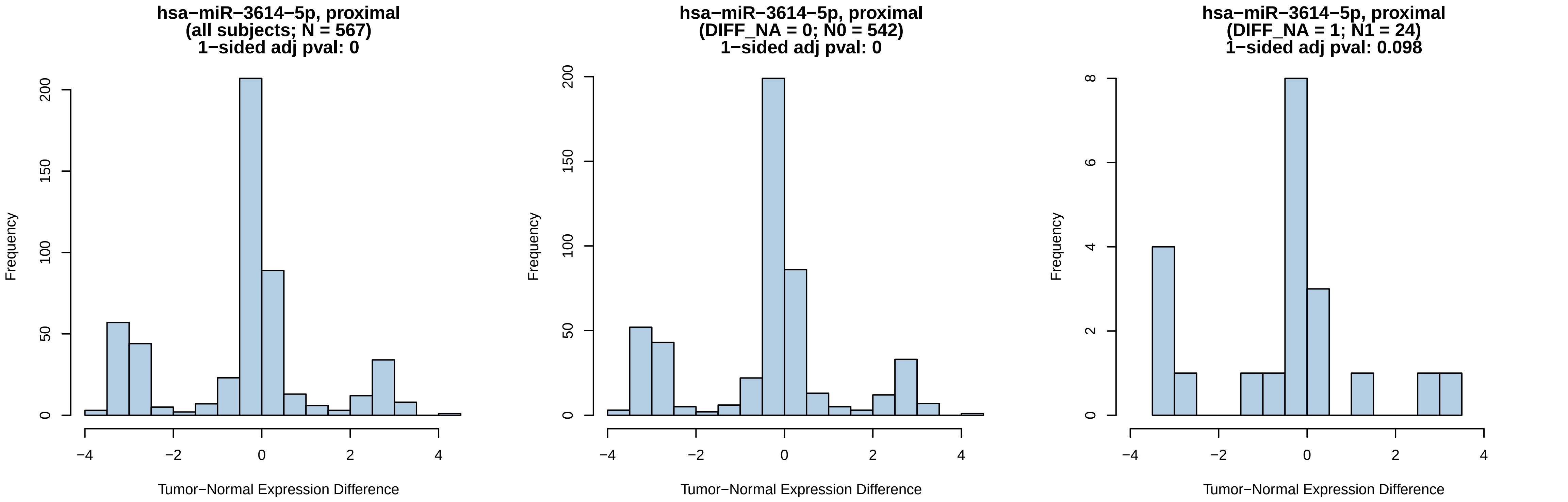

hsa−miR−3614−5p, proximal
(all subjects; N = 567)
1−sided adj pval: 0
Frequency
Tumor−Normal Expression Difference
hsa−miR−3614−5p, proximal
(DIFF_NA = 0; N0 = 542)
1−sided adj pval: 0
Frequency
Tumor−Normal Expression Difference
hsa−miR−3614−5p, proximal
(DIFF_NA = 1; N1 = 24)
1−sided adj pval: 0.098
Frequency
Tumor−Normal Expression Difference

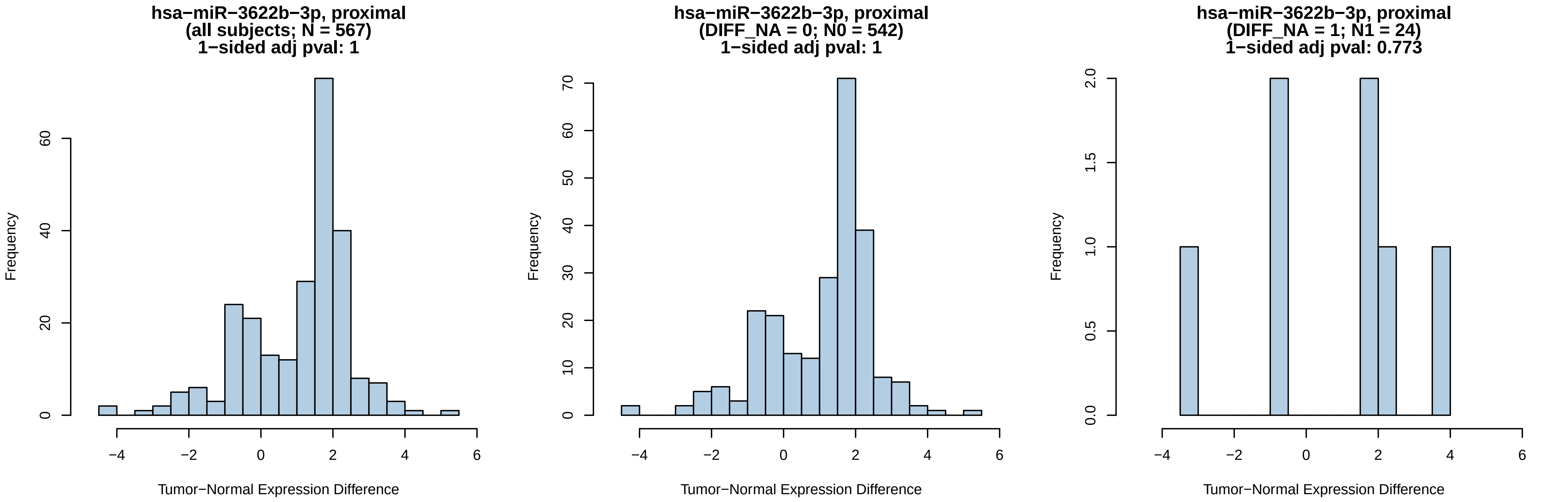
hsa−miR−3622b−3p, proximal
(all subjects; N = 567)
1−sided adj pval: 1
hsa−miR−3622b−3p, proximal
(DIFF_NA = 0; N0 = 542)
1−sided adj pval: 1
hsa−miR−3622b−3p, proximal
(DIFF_NA = 1; N1 = 24)
1−sided adj pval: 0.773
Frequency
Tumor−Normal Expression Difference

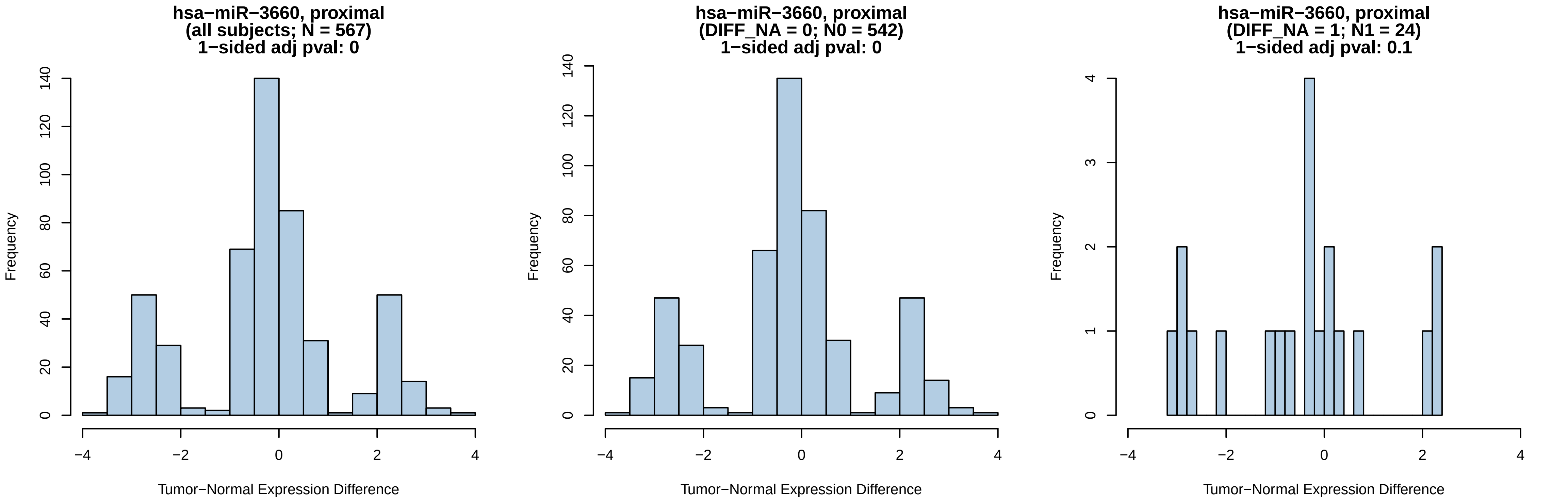

hsa−miR−3660, proximal
(all subjects; N = 567)
1−sided adj pval: 0
Frequency
Tumor−Normal Expression Difference
hsa−miR−3660, proximal
(DIFF_NA = 0; N0 = 542)
1−sided adj pval: 0
Frequency
Tumor−Normal Expression Difference
hsa−miR−3660, proximal
(DIFF_NA = 1; N1 = 24)
1−sided adj pval: 0.1
Frequency
Tumor−Normal Expression Difference

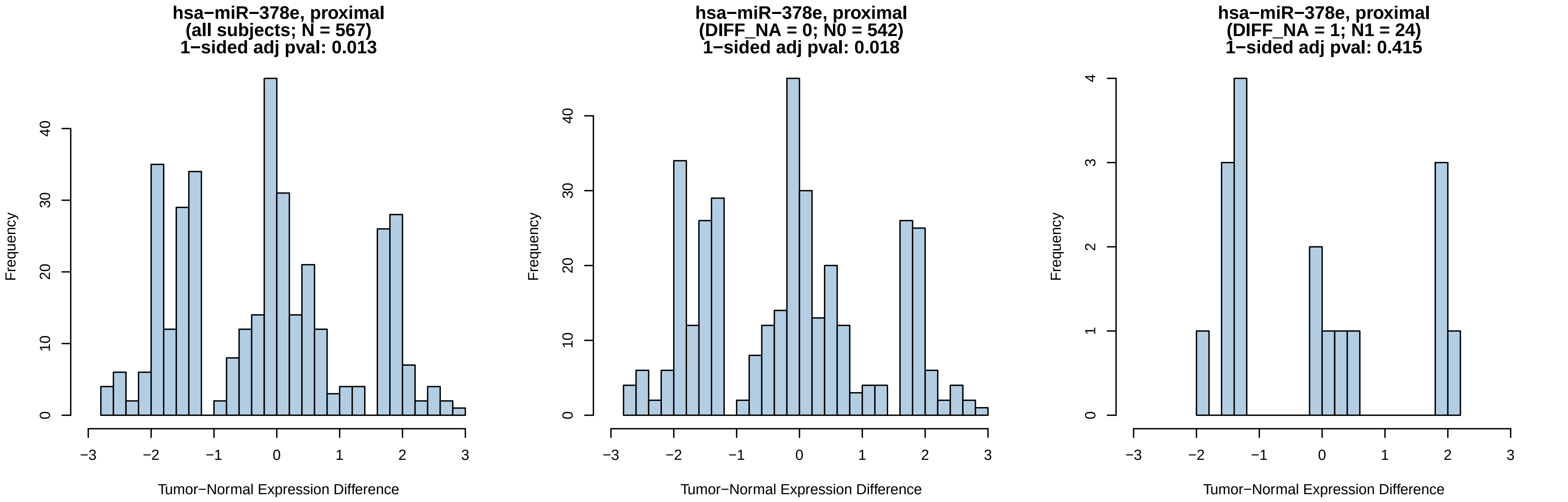
hsa−miR−378e, proximal
(all subjects; N = 567)
1−sided adj pval: 0.013
hsa−miR−378e, proximal
(DIFF_NA = 0; N0 = 542)
1−sided adj pval: 0.018
hsa−miR−378e, proximal
(DIFF_NA = 1; N1 = 24)
1−sided adj pval: 0.415
Frequency
Tumor−Normal Expression Difference

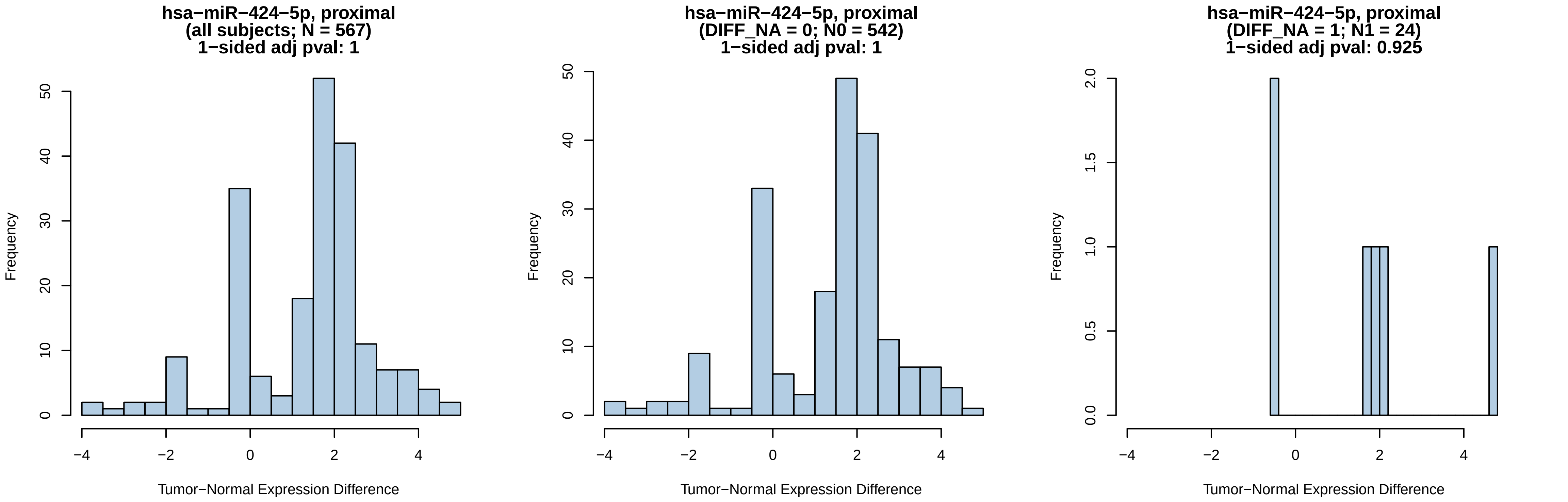
hsa−miR−424−5p, proximal
(all subjects; N = 567)
1−sided adj pval: 1
hsa−miR−424−5p, proximal
(DIFF_NA = 0; N0 = 542)
1−sided adj pval: 1
hsa−miR−424−5p, proximal
(DIFF_NA = 1; N1 = 24)
1−sided adj pval: 0.925
Frequency
Tumor−Normal Expression Difference

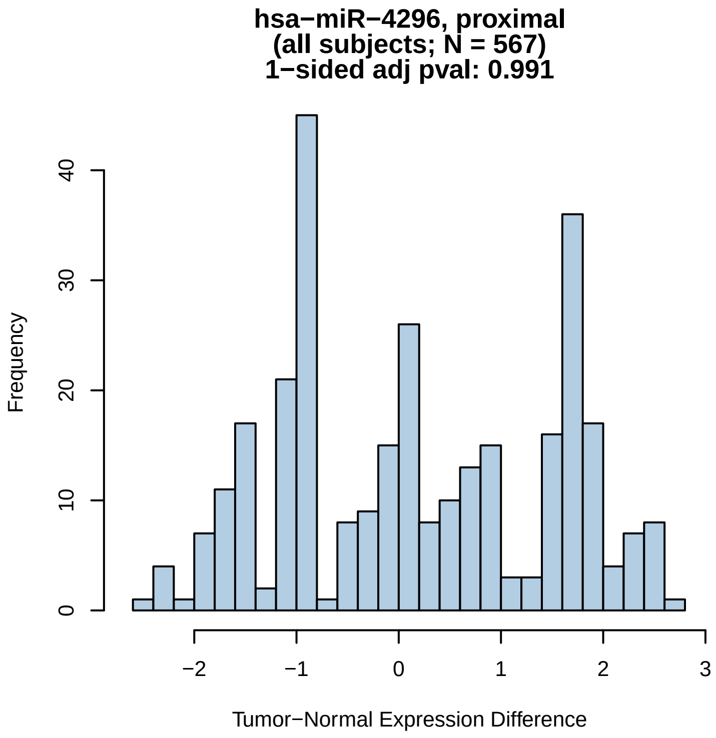
hsa−miR−4296, proximal
(all subjects; N = 567)
1−sided adj pval: 0.991
Frequency
Tumor−Normal Expression Difference

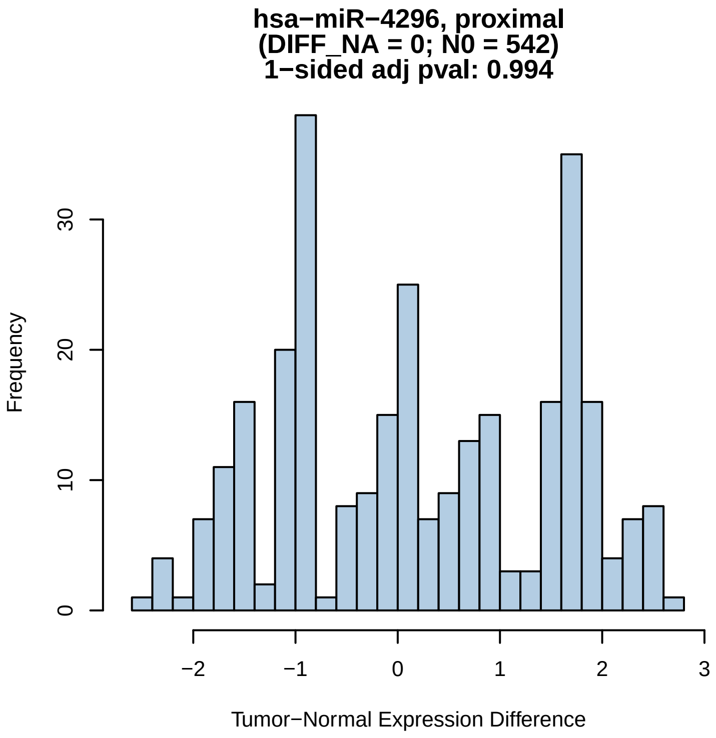
hsa−miR−4296, proximal
(DIFF_NA = 0; N0 = 542)
1−sided adj pval: 0.994
Frequency
Tumor−Normal Expression Difference

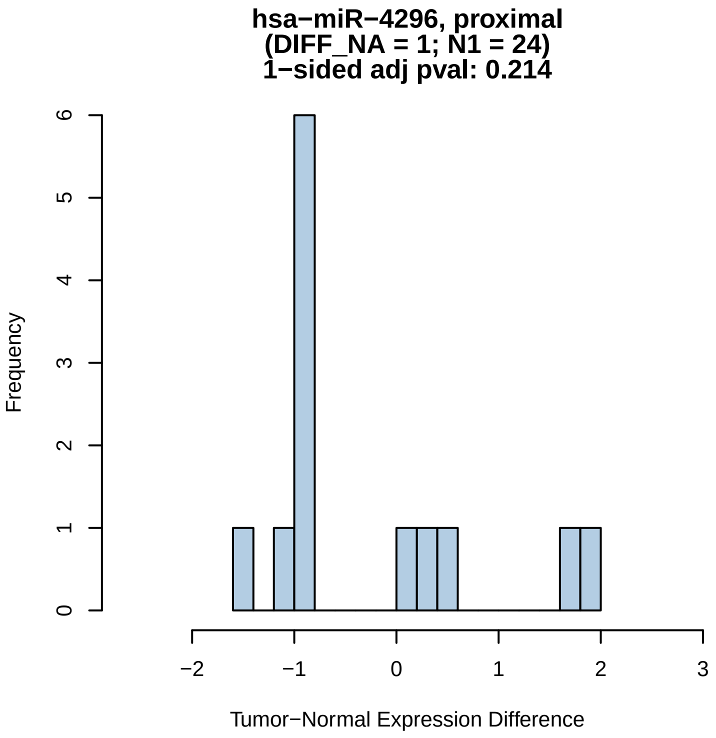
hsa−miR−4296, proximal
(DIFF_NA = 1; N1 = 24)
1−sided adj pval: 0.214
Frequency
Tumor−Normal Expression Difference

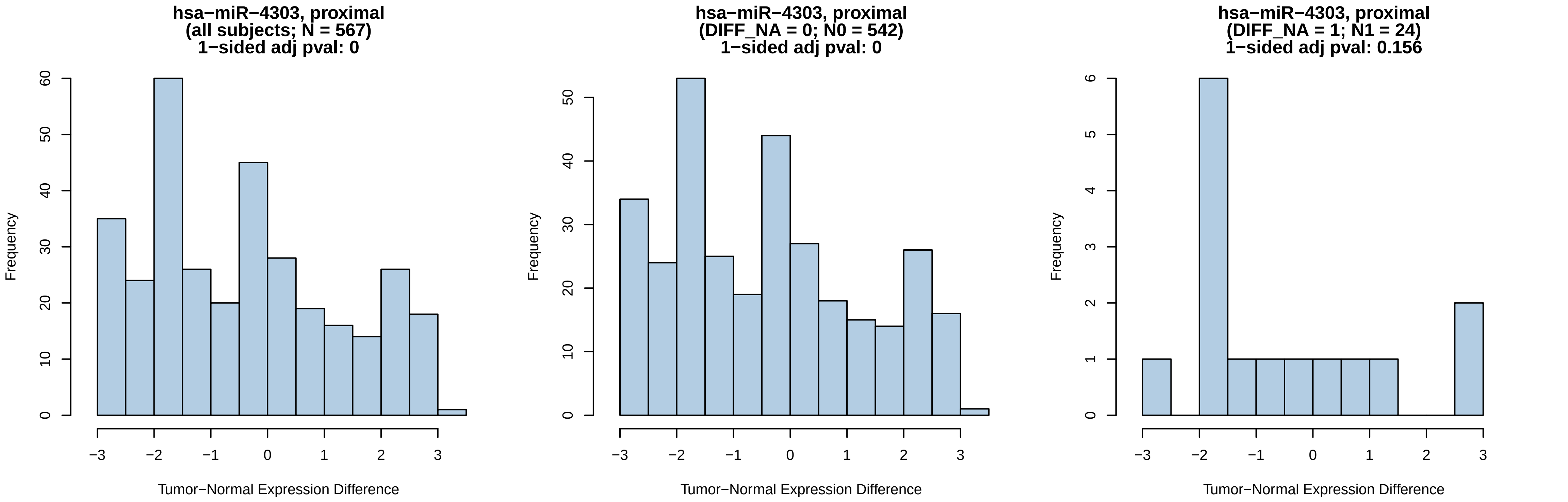
hsa−miR−4303, proximal
(all subjects; N = 567)
1−sided adj pval: 0
hsa−miR−4303, proximal
(DIFF_NA = 0; N0 = 542)
1−sided adj pval: 0
hsa−miR−4303, proximal
(DIFF_NA = 1; N1 = 24)
1−sided adj pval: 0.156
Frequency
Tumor−Normal Expression Difference

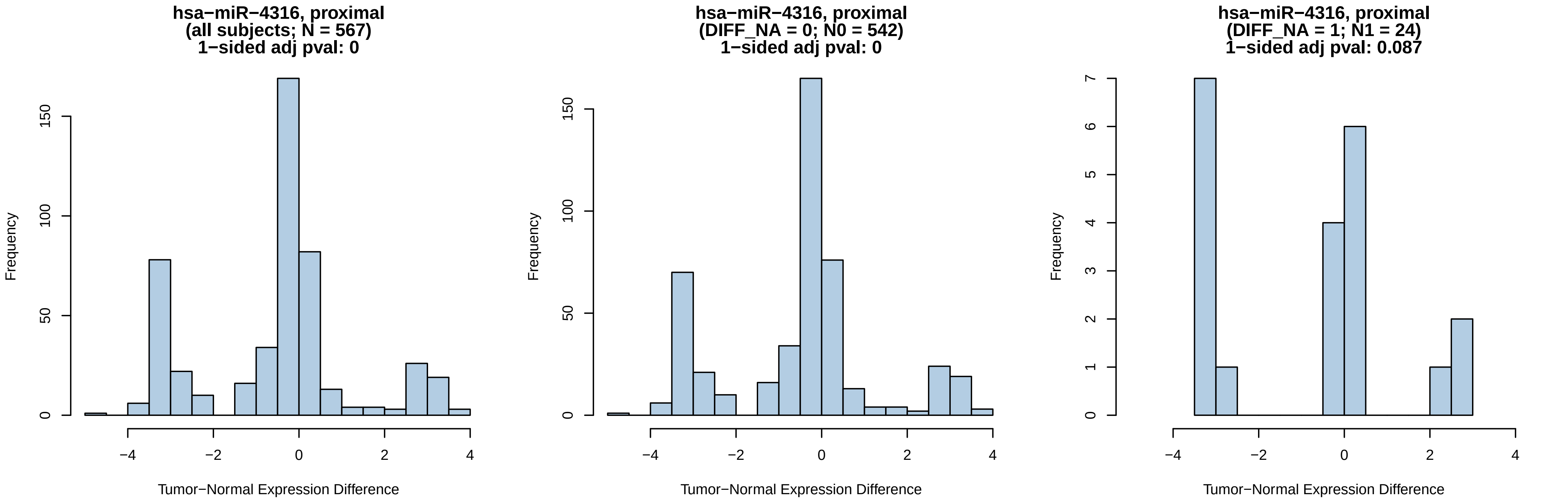

hsa−miR−4316, proximal
(all subjects; N = 567)
1−sided adj pval: 0
Frequency
Tumor−Normal Expression Difference
hsa−miR−4316, proximal
(DIFF_NA = 0; N0 = 542)
1−sided adj pval: 0
Frequency
Tumor−Normal Expression Difference
hsa−miR−4316, proximal
(DIFF_NA = 1; N1 = 24)
1−sided adj pval: 0.087
Frequency
Tumor−Normal Expression Difference

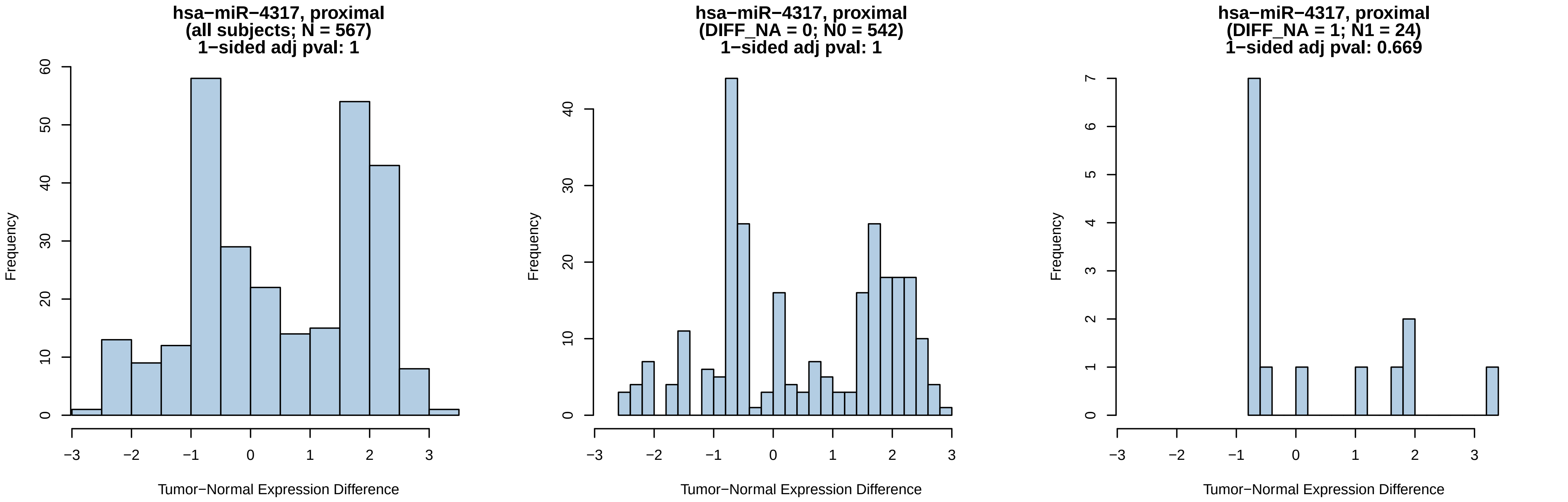
hsa−miR−4317, proximal
(all subjects; N = 567)
1−sided adj pval: 1
Frequency
Tumor−Normal Expression Difference
hsa−miR−4317, proximal
(DIFF_NA = 0; N0 = 542)
1−sided adj pval: 1
Frequency
Tumor−Normal Expression Difference
hsa−miR−4317, proximal
(DIFF_NA = 1; N1 = 24)
1−sided adj pval: 0.669
Frequency
Tumor−Normal Expression Difference

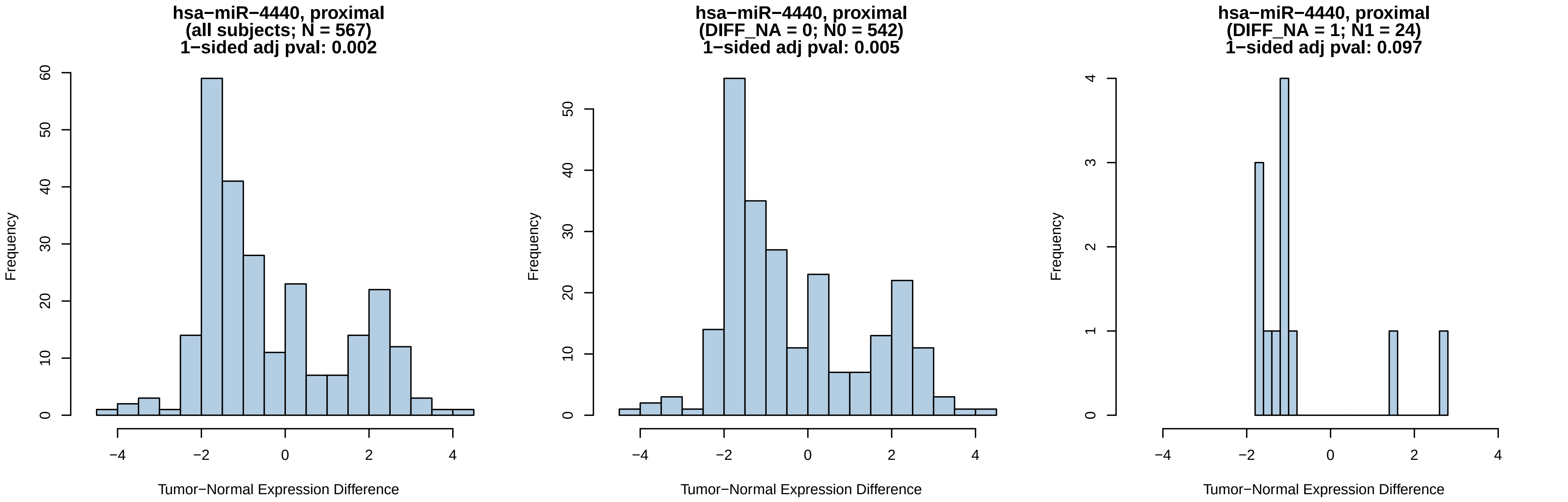

hsa−miR−4440, proximal
(all subjects; N = 567)
1−sided adj pval: 0.002
Frequency
Tumor−Normal Expression Difference
hsa−miR−4440, proximal
(DIFF_NA = 0; N0 = 542)
1−sided adj pval: 0.005
Frequency
Tumor−Normal Expression Difference
hsa−miR−4440, proximal
(DIFF_NA = 1; N1 = 24)
1−sided adj pval: 0.097
Frequency
Tumor−Normal Expression Difference

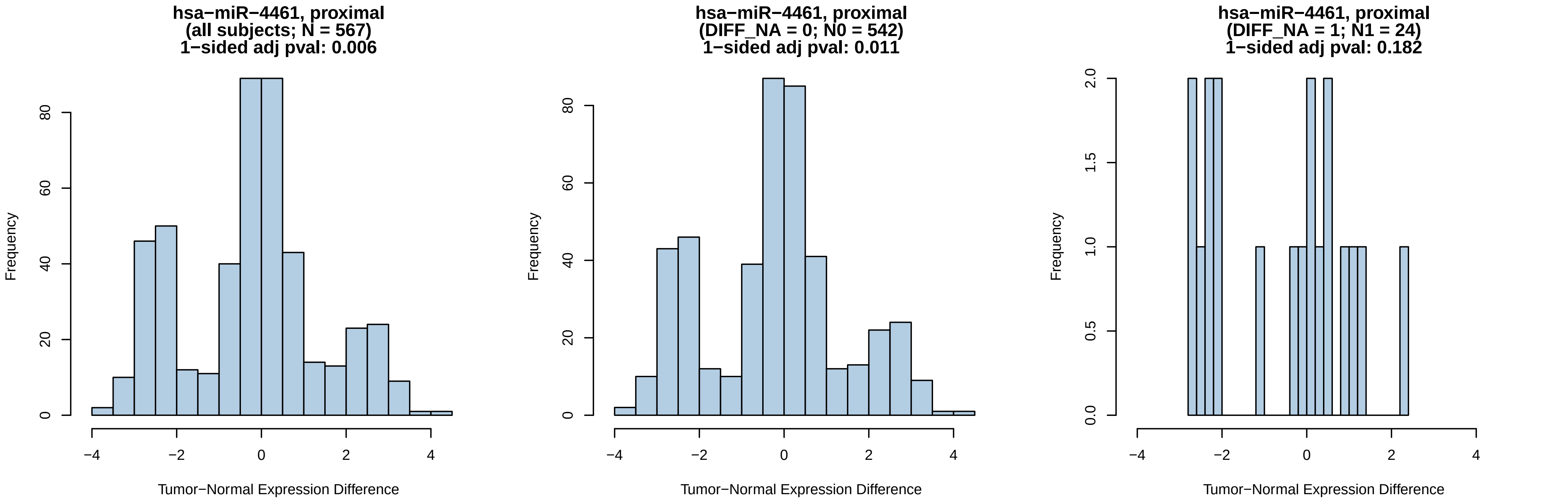
hsa−miR−4461, proximal
(all subjects; N = 567)
1−sided adj pval: 0.006
Frequency
Tumor−Normal Expression Difference
hsa−miR−4461, proximal
(DIFF_NA = 0; N0 = 542)
1−sided adj pval: 0.011
Frequency
Tumor−Normal Expression Difference
hsa−miR−4461, proximal
(DIFF_NA = 1; N1 = 24)
1−sided adj pval: 0.182
Frequency
Tumor−Normal Expression Difference

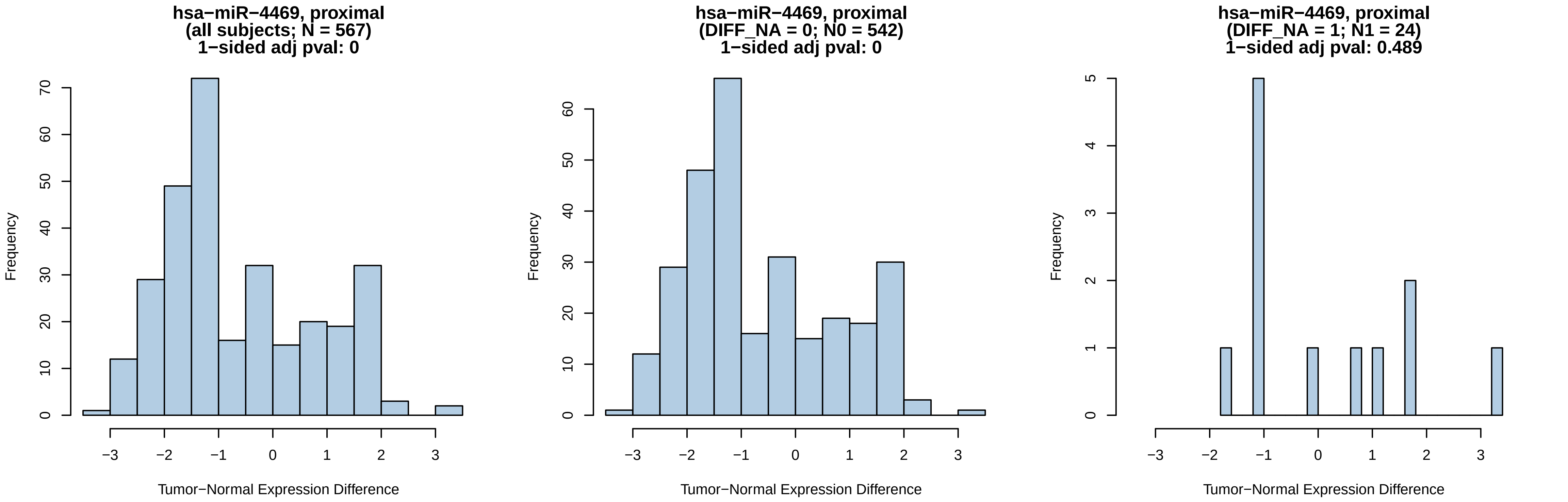
hsa−miR−4469, proximal
(all subjects; N = 567)
1−sided adj pval: 0
Frequency
Tumor−Normal Expression Difference
hsa−miR−4469, proximal
(DIFF_NA = 0; N0 = 542)
1−sided adj pval: 0
Frequency
Tumor−Normal Expression Difference
hsa−miR−4469, proximal
(DIFF_NA = 1; N1 = 24)
1−sided adj pval: 0.489
Frequency
Tumor−Normal Expression Difference

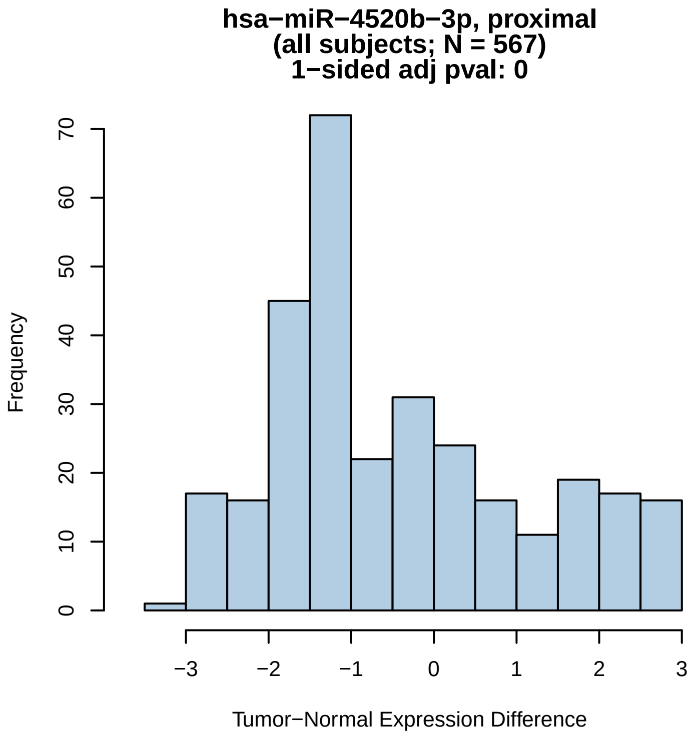
hsa−miR−4520b−3p, proximal
(all subjects; N = 567)
1−sided adj pval: 0
Frequency
0
10
20
30
40
50
60
70
−3
−2
−1
0
1
2
3
Tumor−Normal Expression Difference

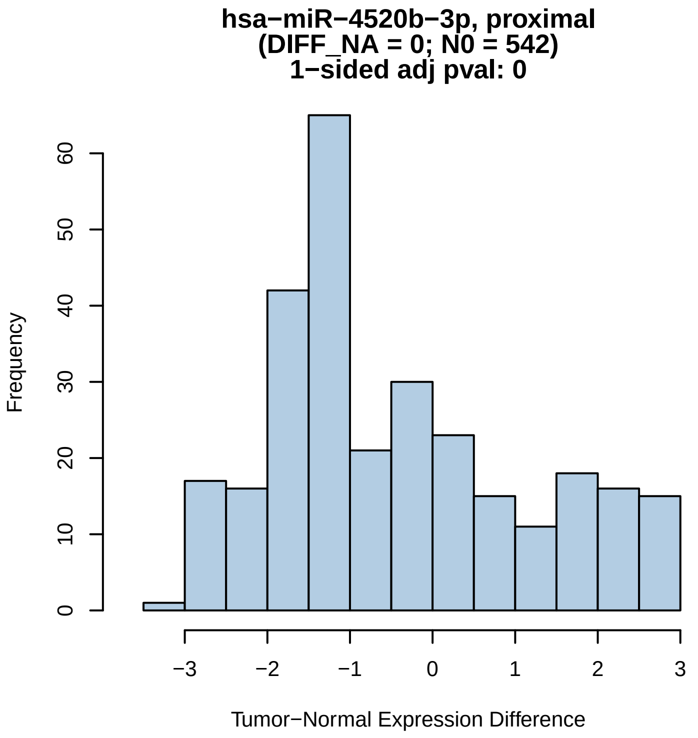
hsa−miR−4520b−3p, proximal
(DIFF_NA = 0; N0 = 542)
1−sided adj pval: 0
Frequency
0
10
20
30
40
50
60
−3
−2
−1
0
1
2
3
Tumor−Normal Expression Difference

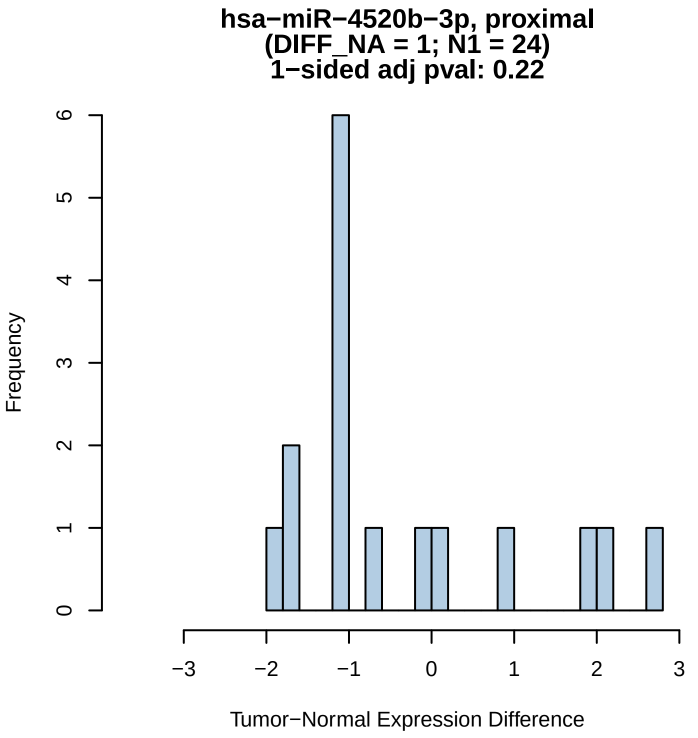
hsa−miR−4520b−3p, proximal
(DIFF_NA = 1; N1 = 24)
1−sided adj pval: 0.22
Frequency
0
1
2
3
4
5
6
−3
−2
−1
0
1
2
3
Tumor−Normal Expression Difference

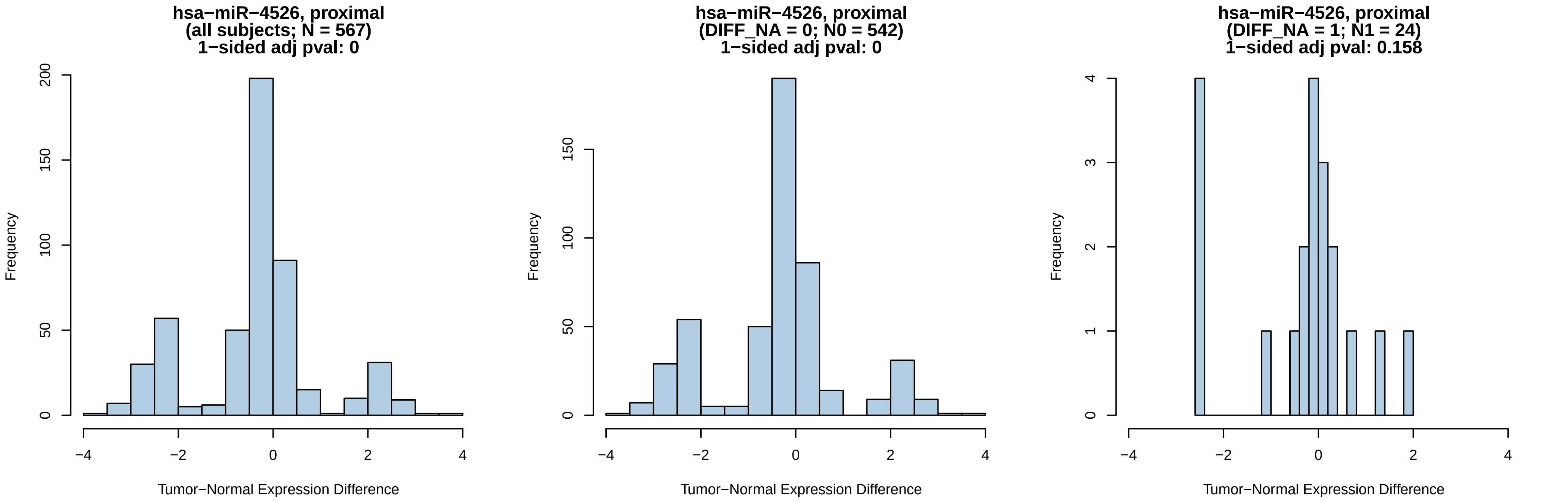

hsa−miR−4526, proximal
(all subjects; N = 567)
1−sided adj pval: 0
Frequency
Tumor−Normal Expression Difference
hsa−miR−4526, proximal
(DIFF_NA = 0; N0 = 542)
1−sided adj pval: 0
Frequency
Tumor−Normal Expression Difference
hsa−miR−4526, proximal
(DIFF_NA = 1; N1 = 24)
1−sided adj pval: 0.158
Frequency
Tumor−Normal Expression Difference

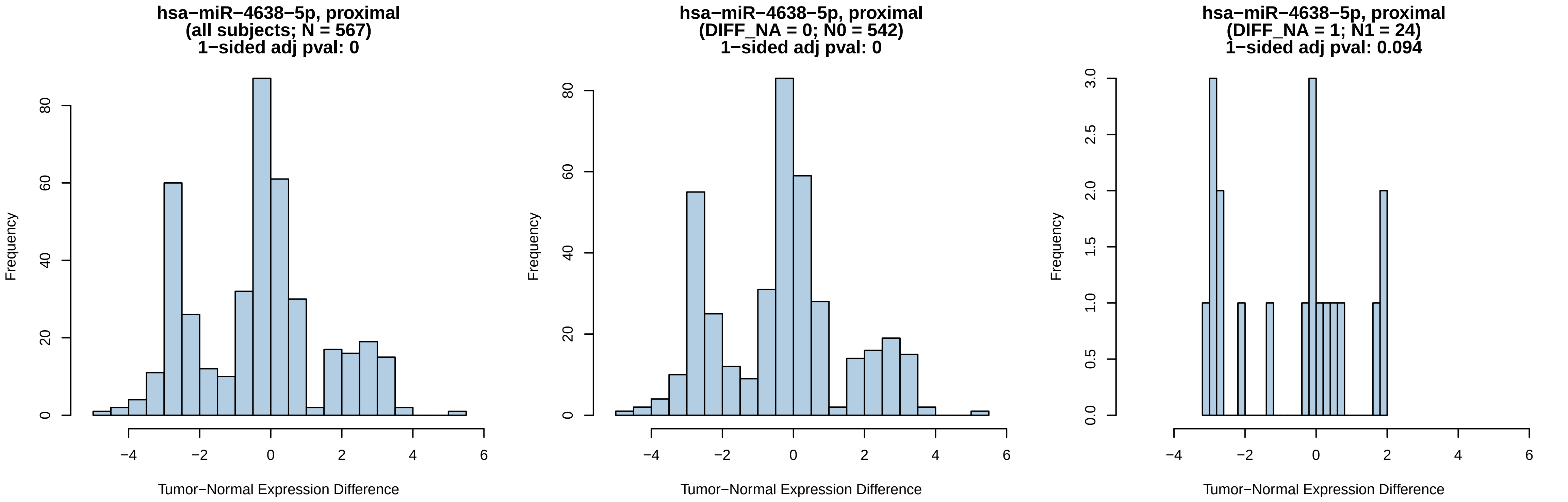

hsa−miR−4638−5p, proximal
(all subjects; N = 567)
1−sided adj pval: 0
Frequency
Tumor−Normal Expression Difference
hsa−miR−4638−5p, proximal
(DIFF_NA = 0; N0 = 542)
1−sided adj pval: 0
Frequency
Tumor−Normal Expression Difference
hsa−miR−4638−5p, proximal
(DIFF_NA = 1; N1 = 24)
1−sided adj pval: 0.094
Frequency
Tumor−Normal Expression Difference

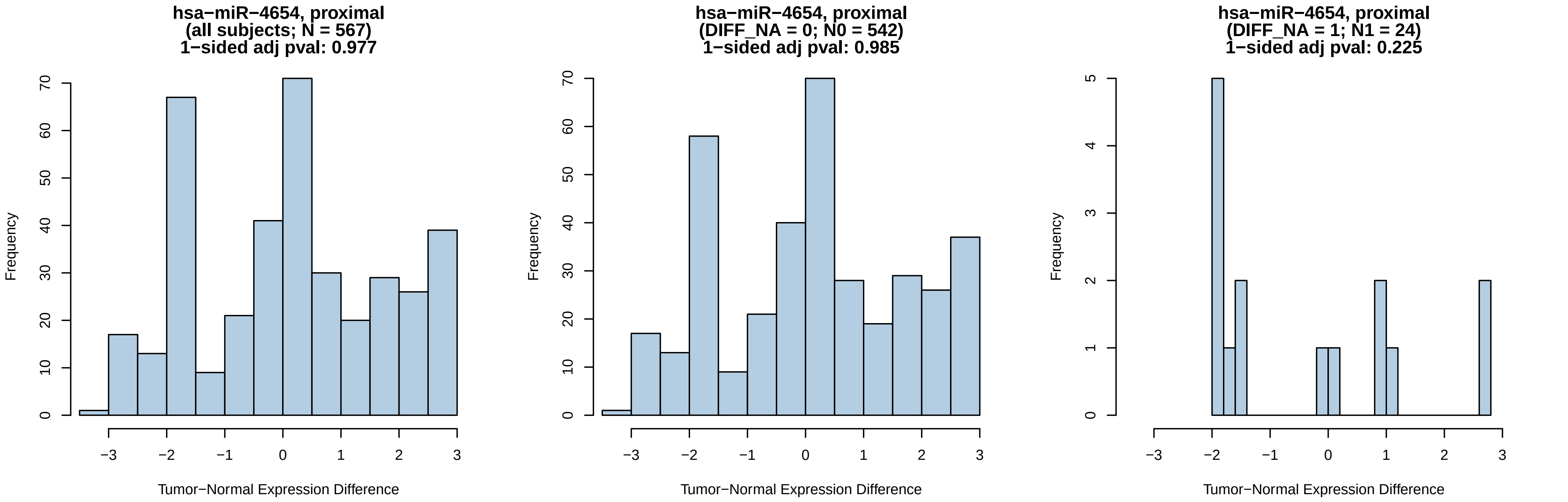
hsa−miR−4654, proximal
(all subjects; N = 567)
1−sided adj pval: 0.977
hsa−miR−4654, proximal
(DIFF_NA = 0; N0 = 542)
1−sided adj pval: 0.985
hsa−miR−4654, proximal
(DIFF_NA = 1; N1 = 24)
1−sided adj pval: 0.225
Frequency
Tumor−Normal Expression Difference

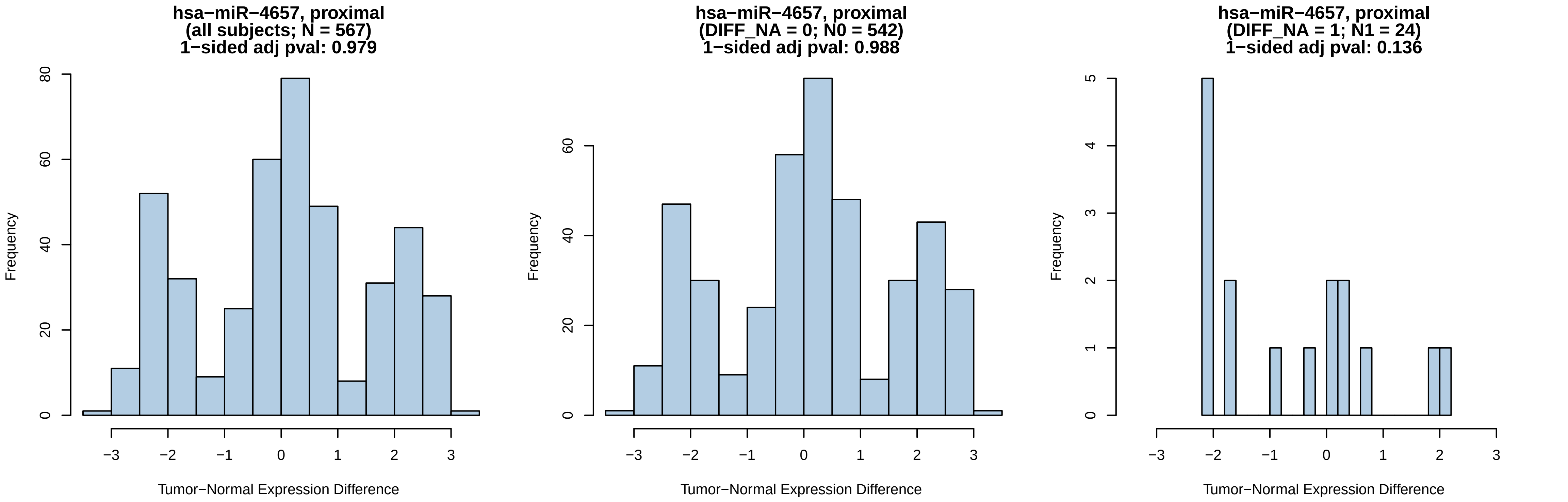
hsa−miR−4657, proximal
(all subjects; N = 567)
1−sided adj pval: 0.979
hsa−miR−4657, proximal
(DIFF_NA = 0; N0 = 542)
1−sided adj pval: 0.988
hsa−miR−4657, proximal
(DIFF_NA = 1; N1 = 24)
1−sided adj pval: 0.136
Frequency
Tumor−Normal Expression Difference

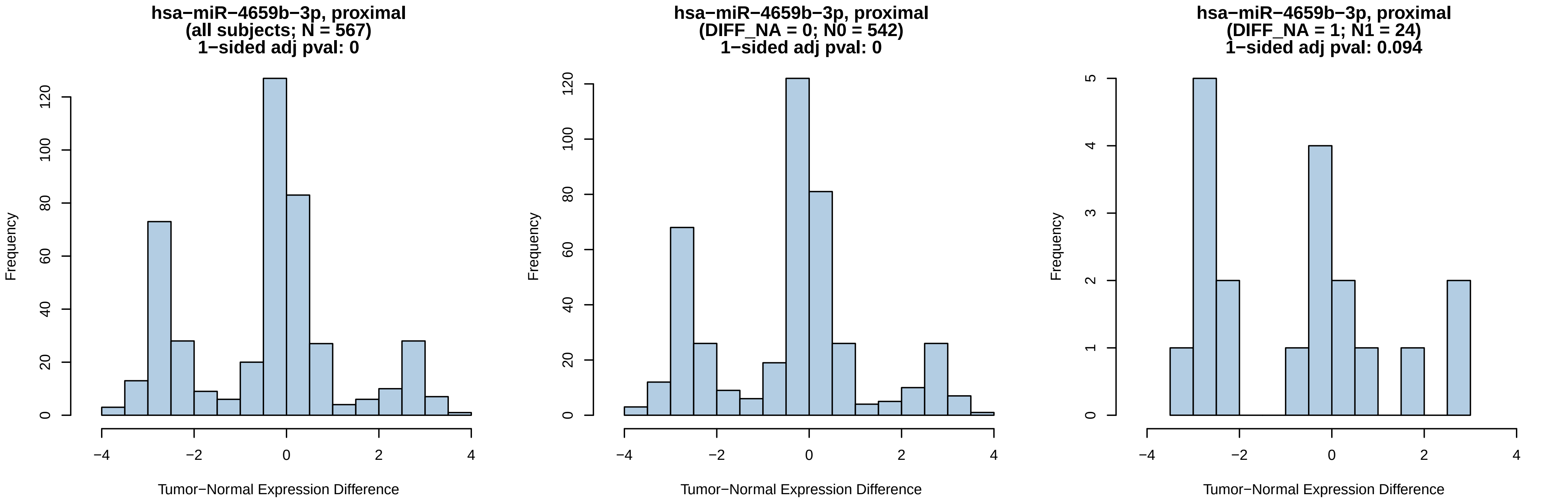
hsa−miR−4659b−3p, proximal
(all subjects; N = 567)
1−sided adj pval: 0
hsa−miR−4659b−3p, proximal
(DIFF_NA = 0; N0 = 542)
1−sided adj pval: 0
hsa−miR−4659b−3p, proximal
(DIFF_NA = 1; N1 = 24)
1−sided adj pval: 0.094
Frequency
Tumor−Normal Expression Difference

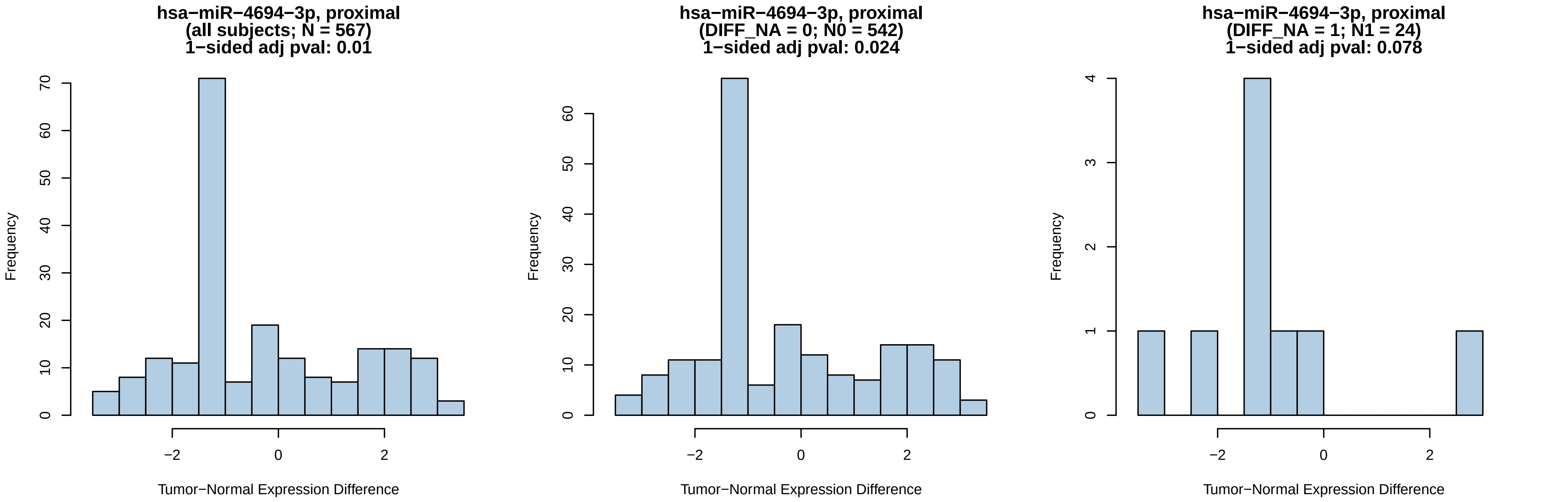
hsa−miR−4694−3p, proximal
(all subjects; N = 567)
1−sided adj pval: 0.01
Frequency
Tumor−Normal Expression Difference
hsa−miR−4694−3p, proximal
(DIFF_NA = 0; N0 = 542)
1−sided adj pval: 0.024
Frequency
Tumor−Normal Expression Difference
hsa−miR−4694−3p, proximal
(DIFF_NA = 1; N1 = 24)
1−sided adj pval: 0.078
Frequency
Tumor−Normal Expression Difference

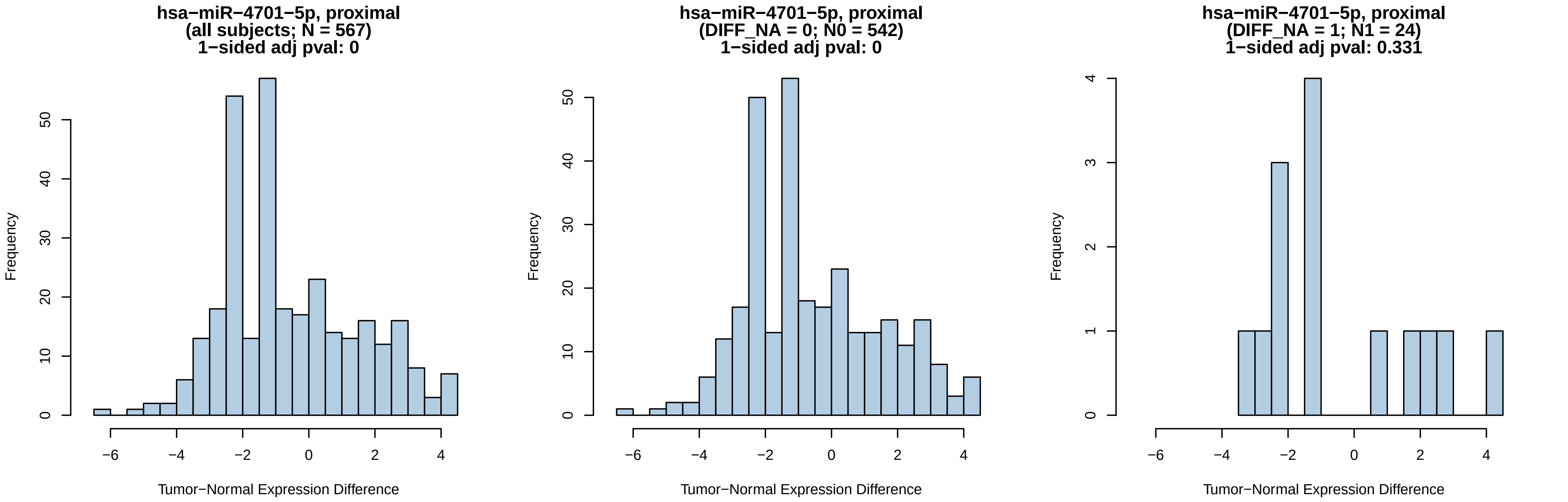

hsa−miR−4701−5p, proximal
(all subjects; N = 567)
1−sided adj pval: 0
Frequency
Tumor−Normal Expression Difference
hsa−miR−4701−5p, proximal
(DIFF_NA = 0; N0 = 542)
1−sided adj pval: 0
Frequency
Tumor−Normal Expression Difference
hsa−miR−4701−5p, proximal
(DIFF_NA = 1; N1 = 24)
1−sided adj pval: 0.331
Frequency
Tumor−Normal Expression Difference

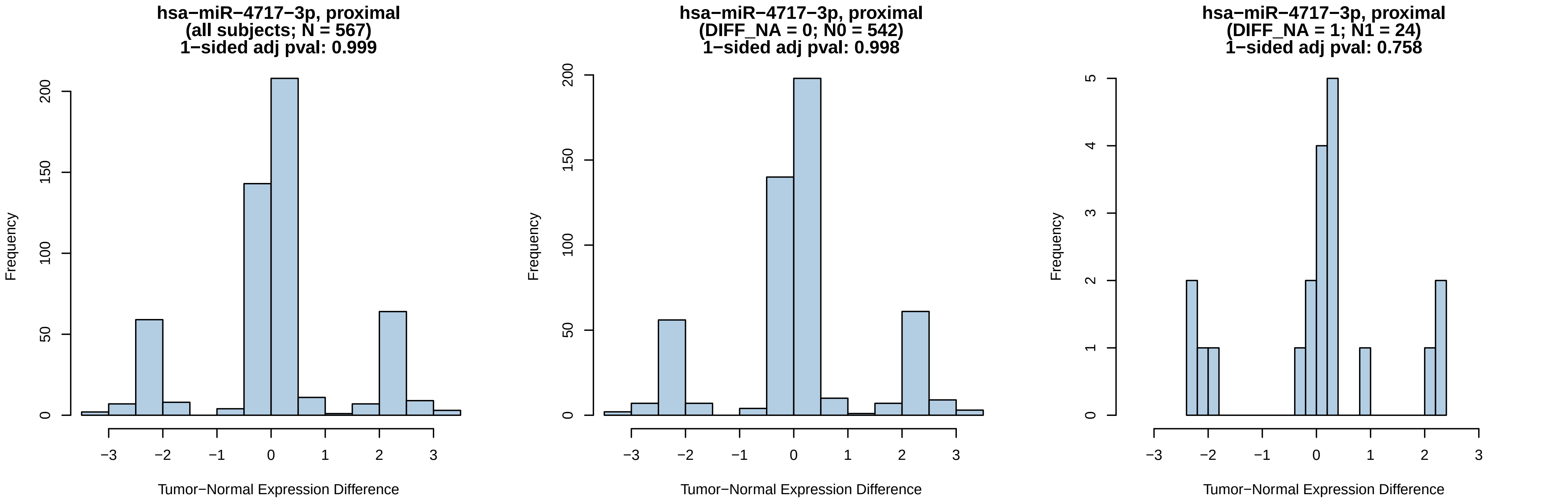
hsa−miR−4717−3p, proximal
(all subjects; N = 567)
1−sided adj pval: 0.999
hsa−miR−4717−3p, proximal
(DIFF_NA = 0; N0 = 542)
1−sided adj pval: 0.998
hsa−miR−4717−3p, proximal
(DIFF_NA = 1; N1 = 24)
1−sided adj pval: 0.758
Frequency
Tumor−Normal Expression Difference

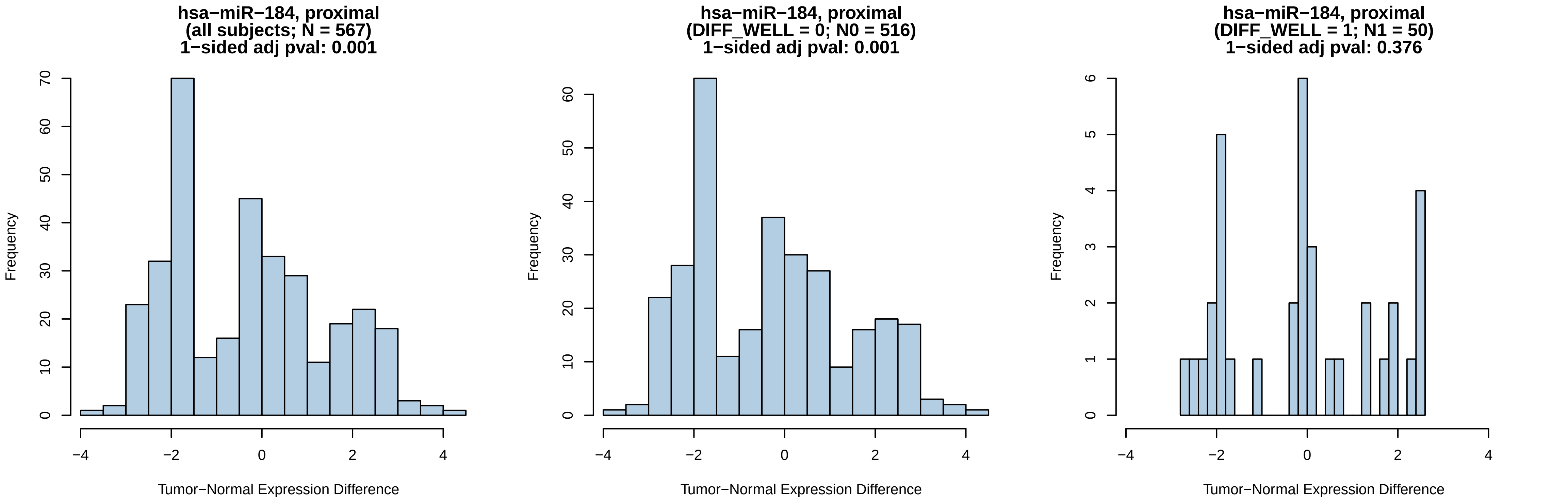
hsa−miR−184, proximal
(all subjects; N = 567)
1−sided adj pval: 0.001
Frequency
Tumor−Normal Expression Difference
hsa−miR−184, proximal
(DIFF_WELL = 0; N0 = 516)
1−sided adj pval: 0.001
Frequency
Tumor−Normal Expression Difference
hsa−miR−184, proximal
(DIFF_WELL = 1; N1 = 50)
1−sided adj pval: 0.376
Frequency
Tumor−Normal Expression Difference

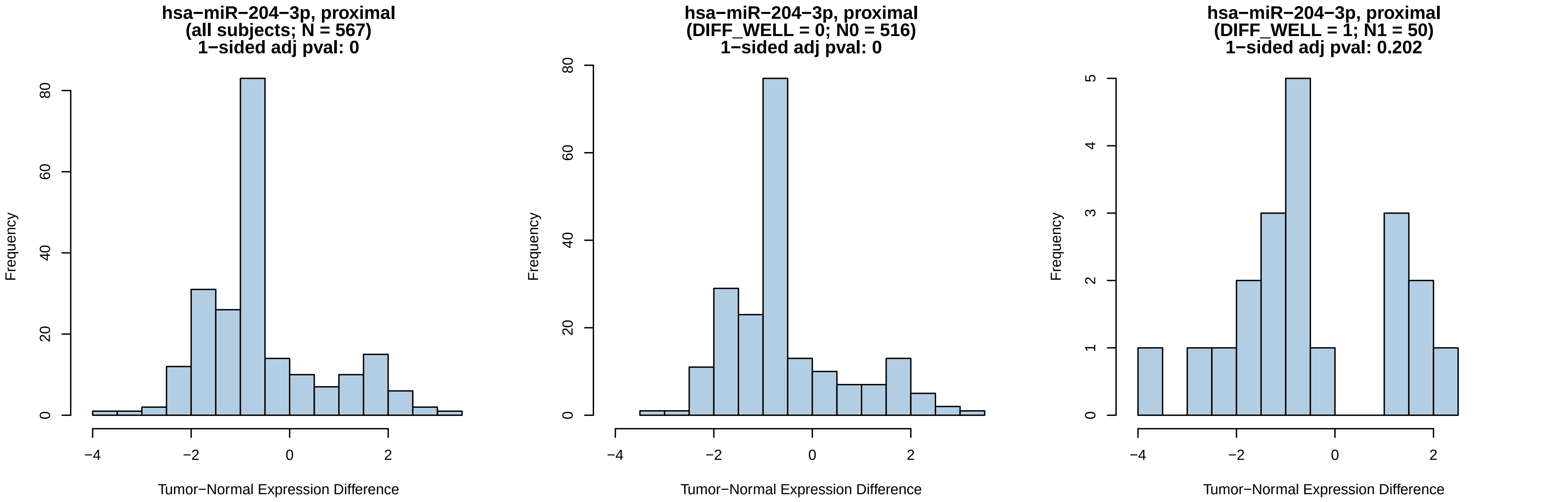

hsa−miR−204−3p, proximal
(all subjects; N = 567)
1−sided adj pval: 0
Frequency
Tumor−Normal Expression Difference
hsa−miR−204−3p, proximal
(DIFF_WELL = 0; N0 = 516)
1−sided adj pval: 0
Frequency
Tumor−Normal Expression Difference
hsa−miR−204−3p, proximal
(DIFF_WELL = 1; N1 = 50)
1−sided adj pval: 0.202
Frequency
Tumor−Normal Expression Difference

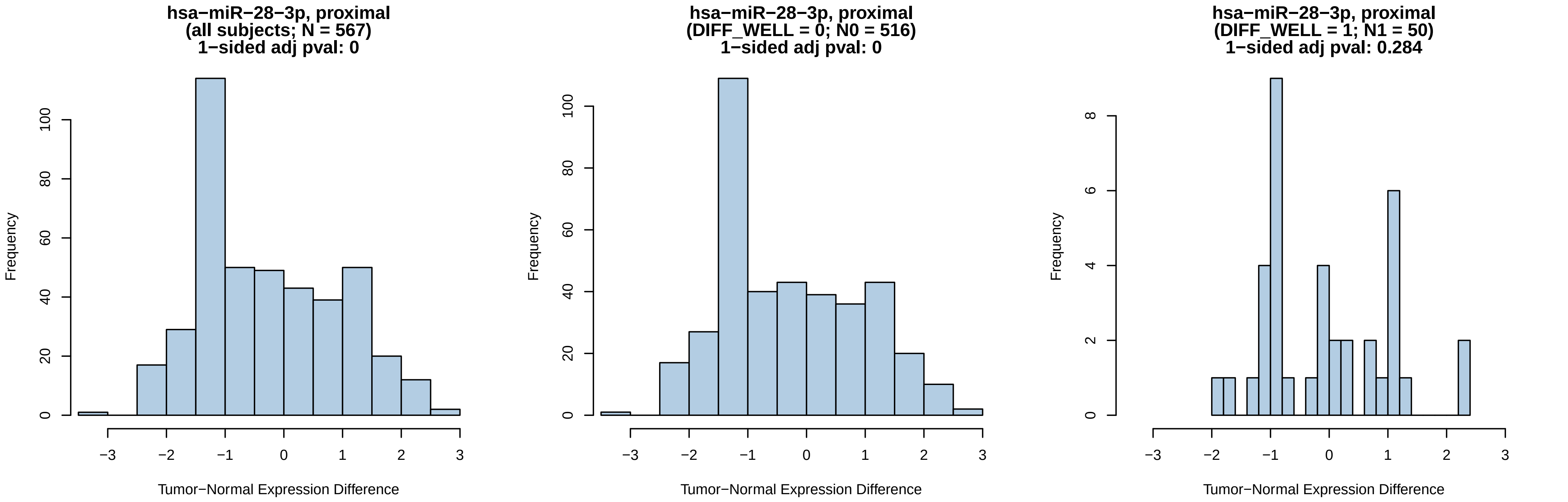
hsa−miR−28−3p, proximal
(all subjects; N = 567)
1−sided adj pval: 0
Frequency
Tumor−Normal Expression Difference
hsa−miR−28−3p, proximal
(DIFF_WELL = 0; N0 = 516)
1−sided adj pval: 0
Frequency
Tumor−Normal Expression Difference
hsa−miR−28−3p, proximal
(DIFF_WELL = 1; N1 = 50)
1−sided adj pval: 0.284
Frequency
Tumor−Normal Expression Difference

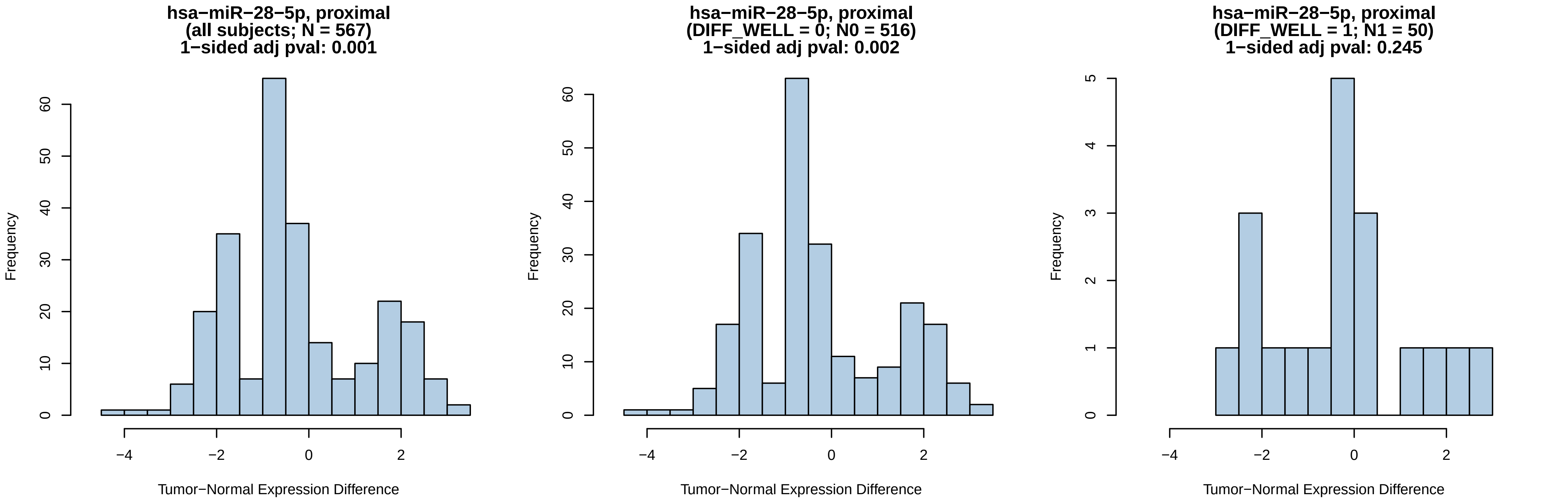
hsa−miR−28−5p, proximal
(all subjects; N = 567)
1−sided adj pval: 0.001
hsa−miR−28−5p, proximal
(DIFF_WELL = 0; N0 = 516)
1−sided adj pval: 0.002
hsa−miR−28−5p, proximal
(DIFF_WELL = 1; N1 = 50)
1−sided adj pval: 0.245
Frequency
Tumor−Normal Expression Difference

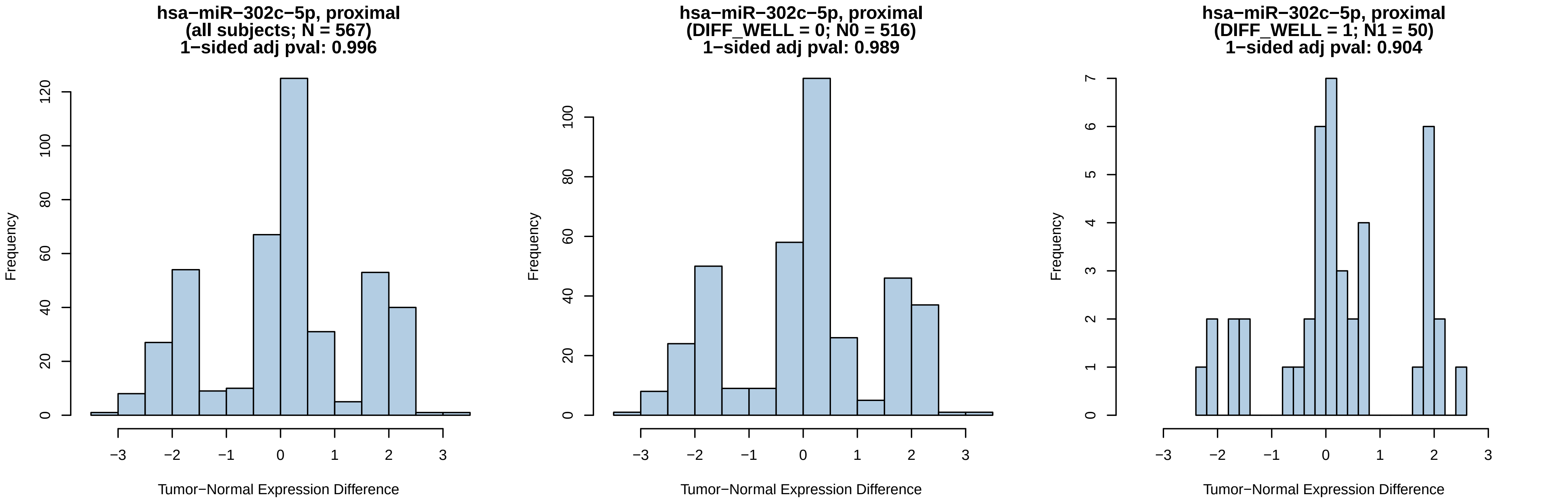

hsa−miR−302c−5p, proximal
(all subjects; N = 567)
1−sided adj pval: 0.996
hsa−miR−302c−5p, proximal
(DIFF_WELL = 0; N0 = 516)
1−sided adj pval: 0.989
hsa−miR−302c−5p, proximal
(DIFF_WELL = 1; N1 = 50)
1−sided adj pval: 0.904
Frequency
Tumor−Normal Expression Difference

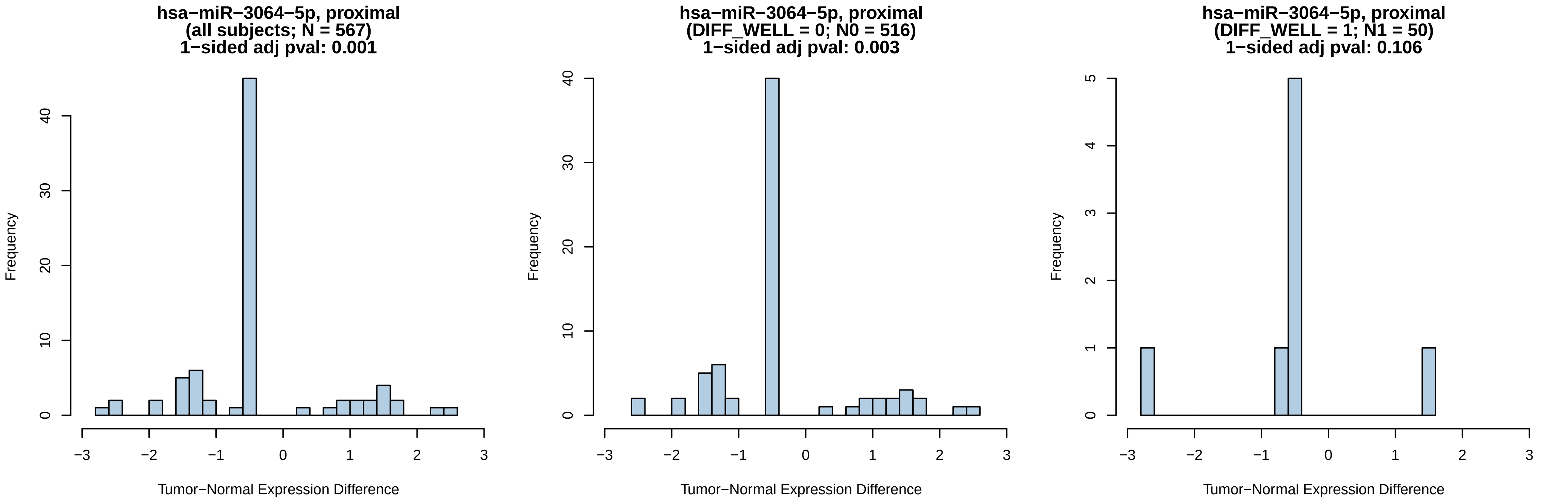
hsa−miR−3064−5p, proximal
(all subjects; N = 567)
1−sided adj pval: 0.001
hsa−miR−3064−5p, proximal
(DIFF_WELL = 0; N0 = 516)
1−sided adj pval: 0.003
hsa−miR−3064−5p, proximal
(DIFF_WELL = 1; N1 = 50)
1−sided adj pval: 0.106
Frequency
Tumor−Normal Expression Difference

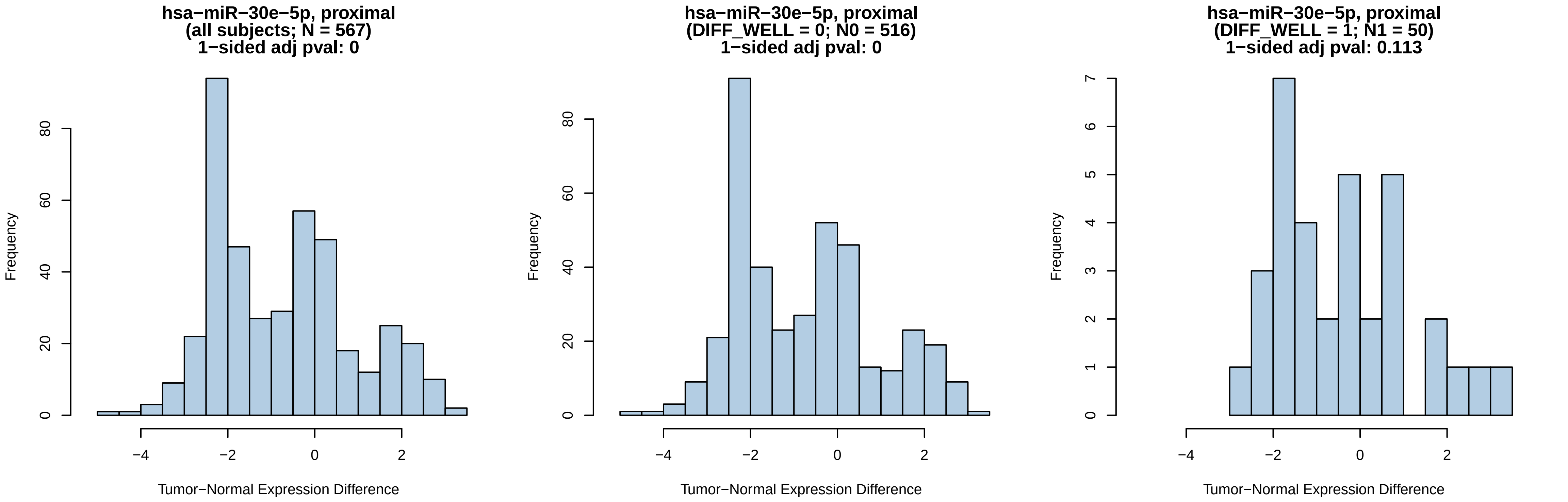

hsa−miR−30e−5p, proximal
(all subjects; N = 567)
1−sided adj pval: 0
hsa−miR−30e−5p, proximal
(DIFF_WELL = 0; N0 = 516)
1−sided adj pval: 0
hsa−miR−30e−5p, proximal
(DIFF_WELL = 1; N1 = 50)
1−sided adj pval: 0.113
Frequency
Tumor−Normal Expression Difference

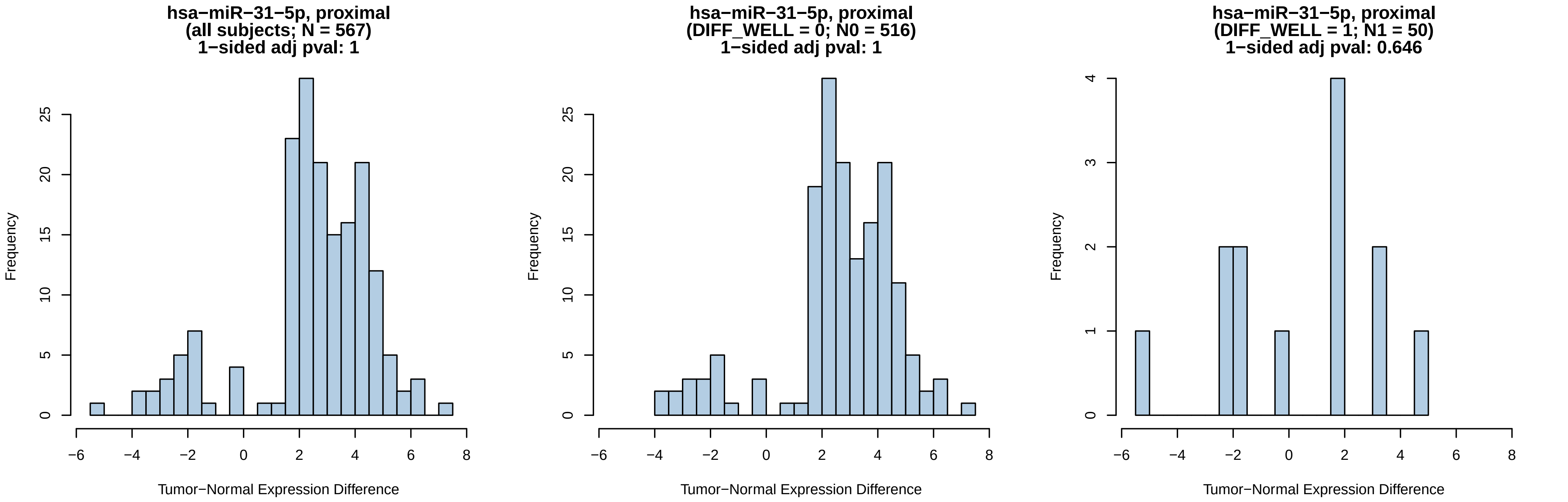
hsa−miR−31−5p, proximal
(all subjects; N = 567)
1−sided adj pval: 1
hsa−miR−31−5p, proximal
(DIFF_WELL = 0; N0 = 516)
1−sided adj pval: 1
hsa−miR−31−5p, proximal
(DIFF_WELL = 1; N1 = 50)
1−sided adj pval: 0.646
Frequency
Tumor−Normal Expression Difference

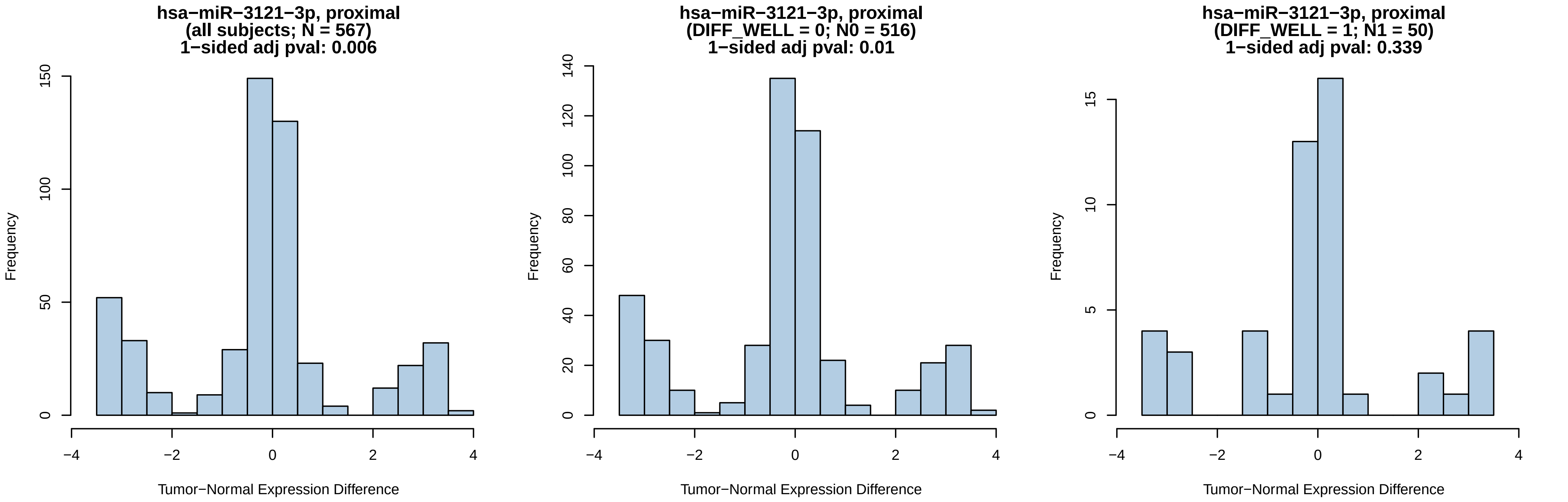

hsa−miR−3121−3p, proximal
(all subjects; N = 567)
1−sided adj pval: 0.006
Frequency
0
50
100
150
−4
−2
0
2
4
Tumor−Normal Expression Difference
hsa−miR−3121−3p, proximal
(DIFF_WELL = 0; N0 = 516)
1−sided adj pval: 0.01
Frequency
0
20
40
60
80
100
120
140
−4
−2
0
2
4
Tumor−Normal Expression Difference
hsa−miR−3121−3p, proximal
(DIFF_WELL = 1; N1 = 50)
1−sided adj pval: 0.339
Frequency
0
5
10
15
−4
−2
0
2
4
Tumor−Normal Expression Difference

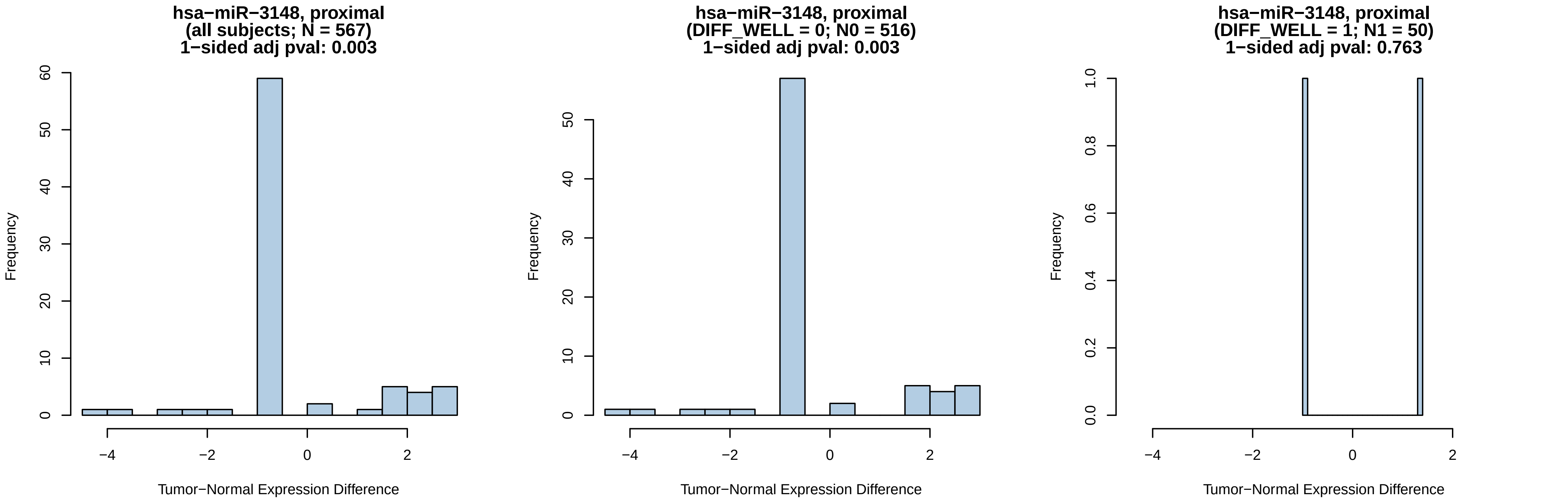
hsa−miR−3148, proximal
(all subjects; N = 567)
1−sided adj pval: 0.003
Frequency
0
10
20
30
40
50
60
−4
−2
0
2
Tumor−Normal Expression Difference
hsa−miR−3148, proximal
(DIFF_WELL = 0; N0 = 516)
1−sided adj pval: 0.003
Frequency
0
10
20
30
40
50
−4
−2
0
2
Tumor−Normal Expression Difference
hsa−miR−3148, proximal
(DIFF_WELL = 1; N1 = 50)
1−sided adj pval: 0.763
Frequency
0.0
0.2
0.4
0.6
0.8
1.0
−4
−2
0
2
Tumor−Normal Expression Difference

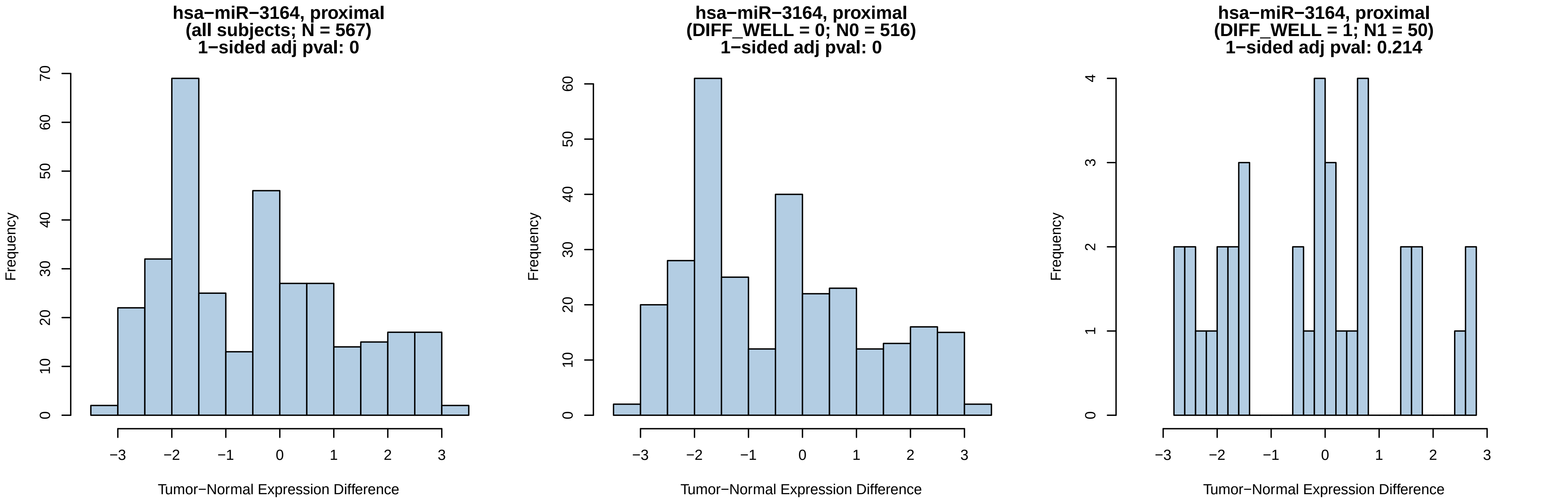
hsa−miR−3164, proximal
(all subjects; N = 567)
1−sided adj pval: 0
Frequency
Tumor−Normal Expression Difference
hsa−miR−3164, proximal
(DIFF_WELL = 0; N0 = 516)
1−sided adj pval: 0
Frequency
Tumor−Normal Expression Difference
hsa−miR−3164, proximal
(DIFF_WELL = 1; N1 = 50)
1−sided adj pval: 0.214
Frequency
Tumor−Normal Expression Difference

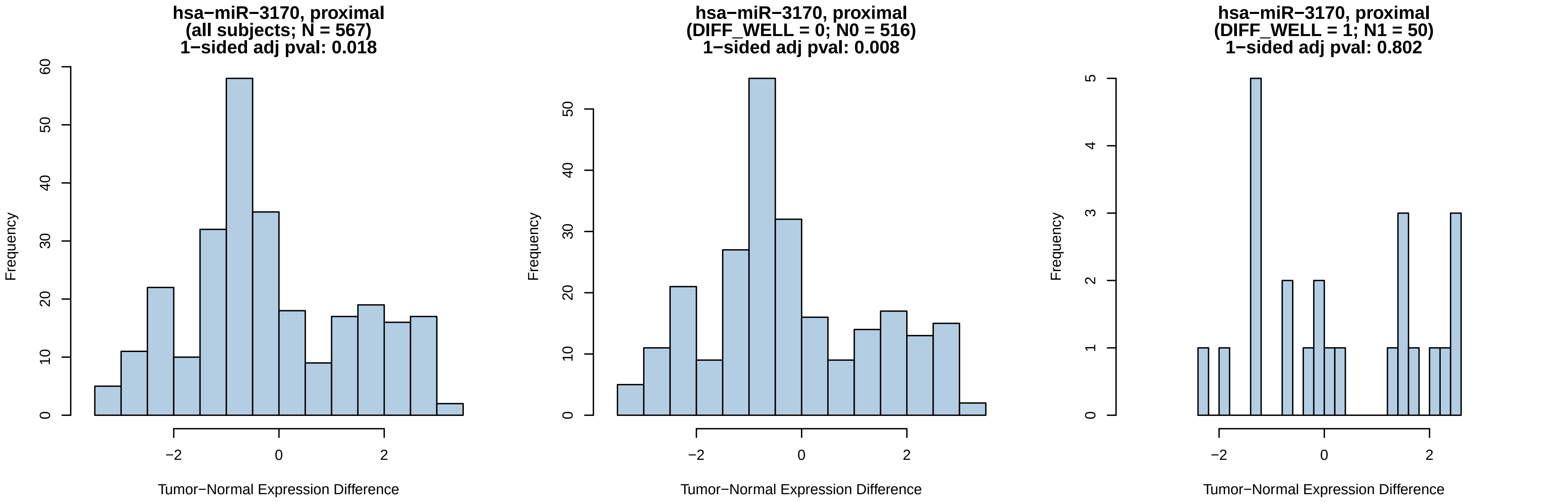
hsa−miR−3170, proximal
(all subjects; N = 567)
1−sided adj pval: 0.018
hsa−miR−3170, proximal
(DIFF_WELL = 0; N0 = 516)
1−sided adj pval: 0.008
hsa−miR−3170, proximal
(DIFF_WELL = 1; N1 = 50)
1−sided adj pval: 0.802
Frequency
Tumor−Normal Expression Difference

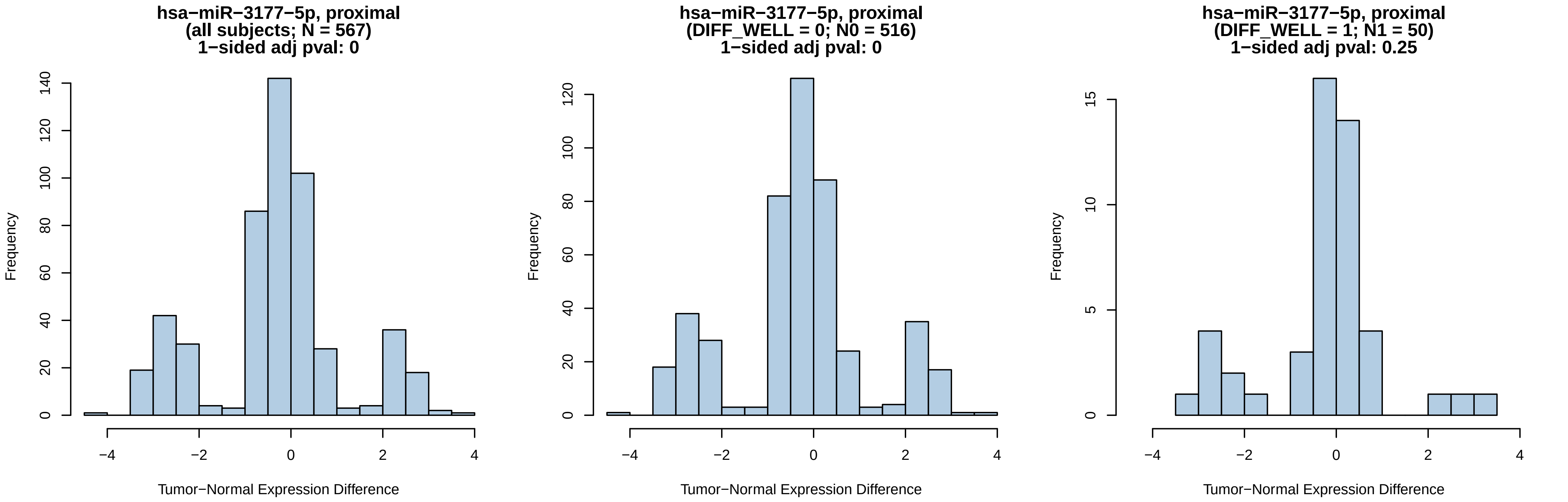
hsa−miR−3177−5p, proximal
(all subjects; N = 567)
1−sided adj pval: 0
hsa−miR−3177−5p, proximal
(DIFF_WELL = 0; N0 = 516)
1−sided adj pval: 0
hsa−miR−3177−5p, proximal
(DIFF_WELL = 1; N1 = 50)
1−sided adj pval: 0.25
Frequency
Tumor−Normal Expression Difference

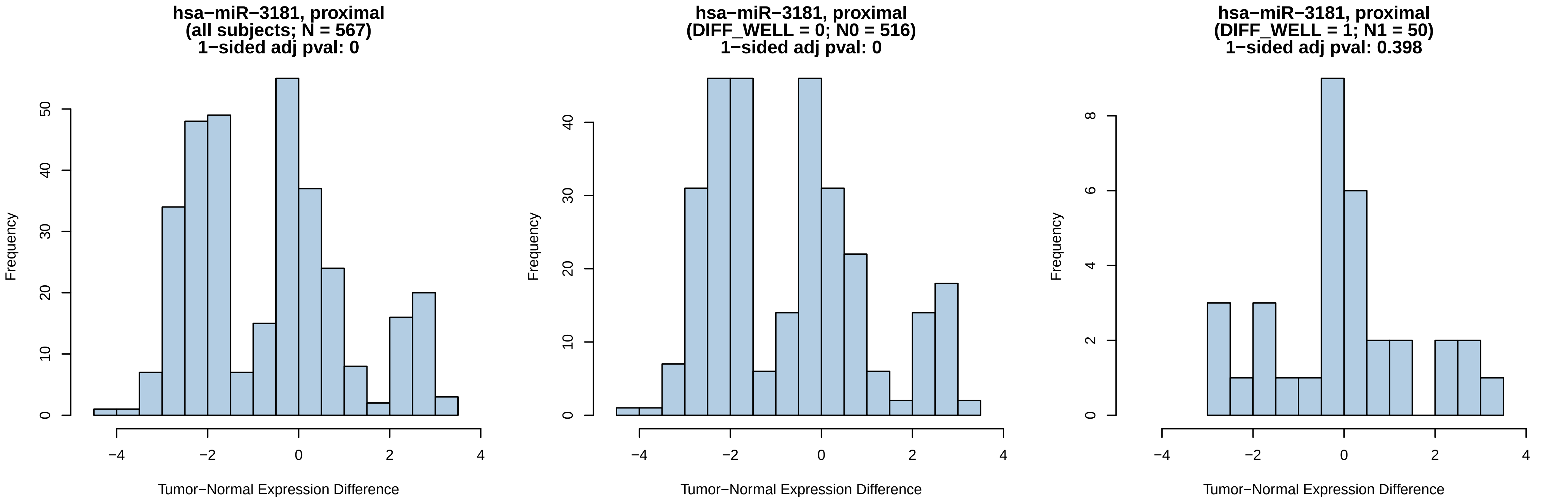
hsa−miR−3181, proximal
(all subjects; N = 567)
1−sided adj pval: 0
hsa−miR−3181, proximal
(DIFF_WELL = 0; N0 = 516)
1−sided adj pval: 0
hsa−miR−3181, proximal
(DIFF_WELL = 1; N1 = 50)
1−sided adj pval: 0.398
Frequency
Tumor−Normal Expression Difference

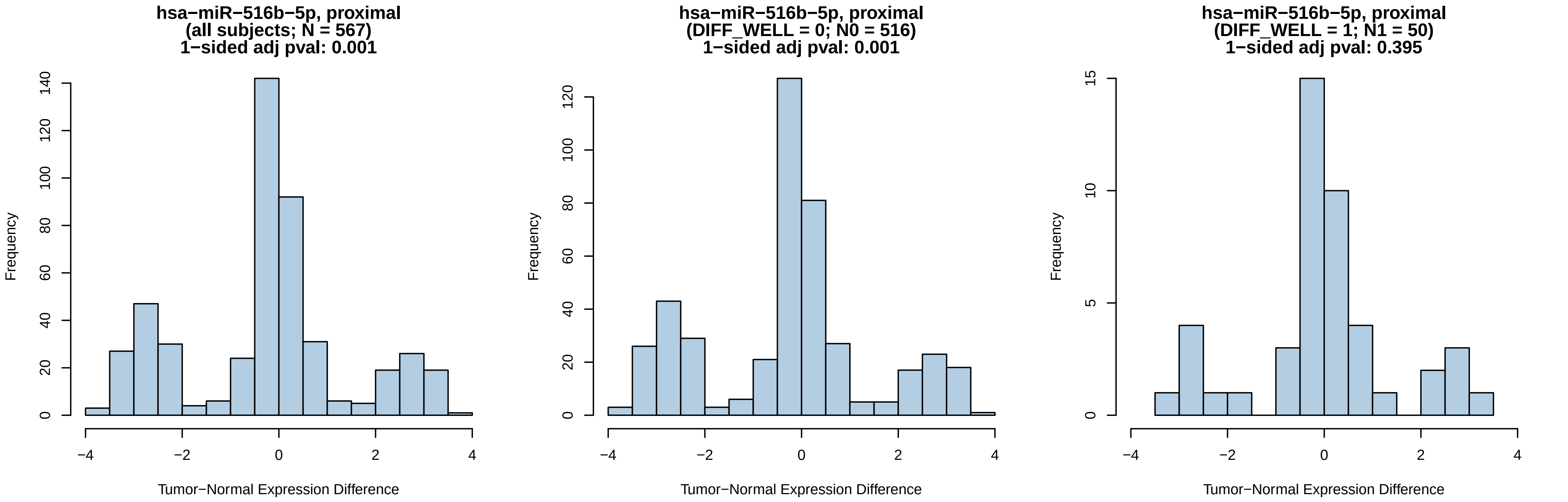
hsa−miR−516b−5p, proximal
(all subjects; N = 567)
1−sided adj pval: 0.001
Frequency
Tumor−Normal Expression Difference
hsa−miR−516b−5p, proximal
(DIFF_WELL = 0; N0 = 516)
1−sided adj pval: 0.001
Frequency
Tumor−Normal Expression Difference
hsa−miR−516b−5p, proximal
(DIFF_WELL = 1; N1 = 50)
1−sided adj pval: 0.395
Frequency
Tumor−Normal Expression Difference

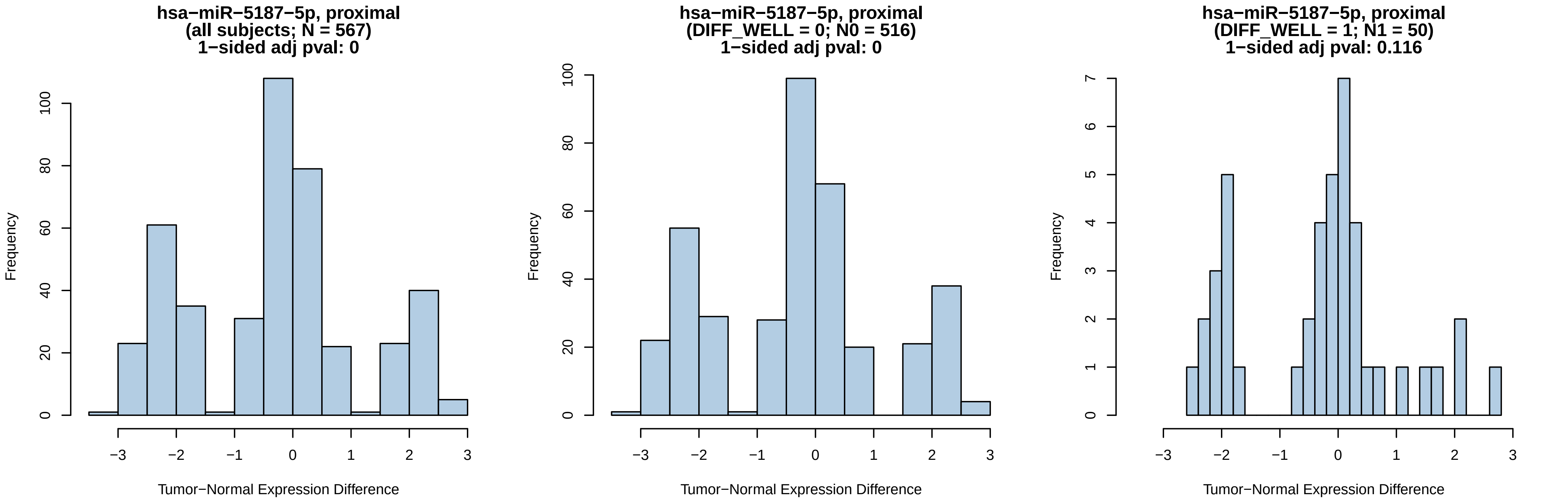

hsa−miR−5187−5p, proximal
(all subjects; N = 567)
1−sided adj pval: 0
Frequency
Tumor−Normal Expression Difference
hsa−miR−5187−5p, proximal
(DIFF_WELL = 0; N0 = 516)
1−sided adj pval: 0
Frequency
Tumor−Normal Expression Difference
hsa−miR−5187−5p, proximal
(DIFF_WELL = 1; N1 = 50)
1−sided adj pval: 0.116
Frequency
Tumor−Normal Expression Difference

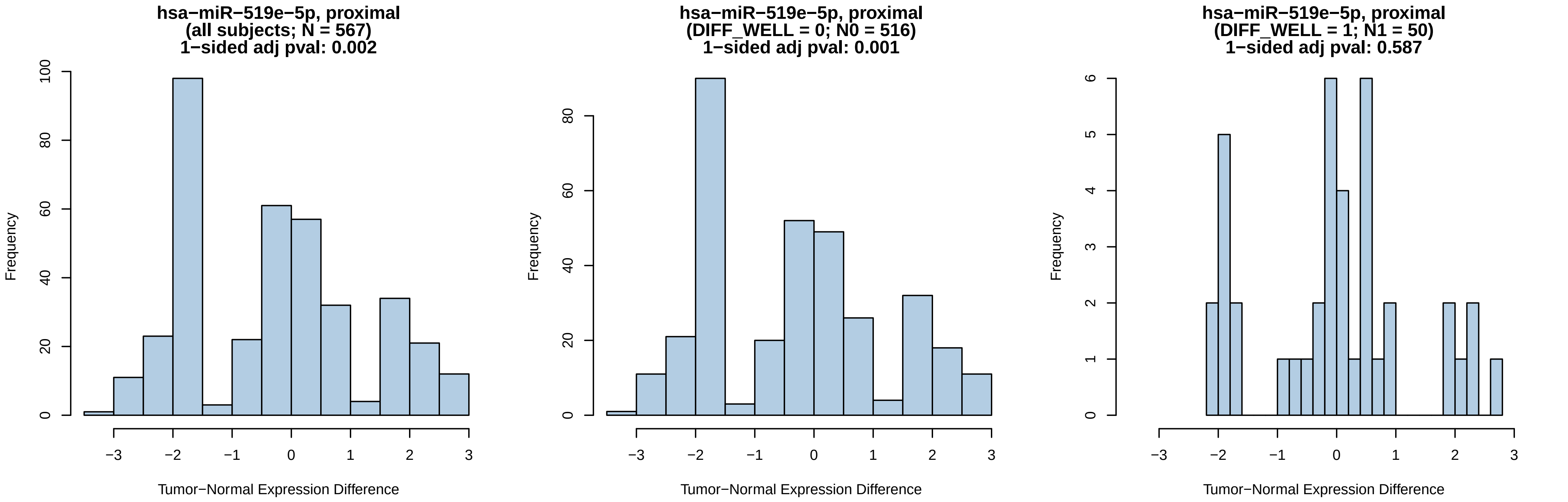
hsa−miR−519e−5p, proximal
(all subjects; N = 567)
1−sided adj pval: 0.002
Frequency
Tumor−Normal Expression Difference
hsa−miR−519e−5p, proximal
(DIFF_WELL = 0; N0 = 516)
1−sided adj pval: 0.001
Frequency
Tumor−Normal Expression Difference
hsa−miR−519e−5p, proximal
(DIFF_WELL = 1; N1 = 50)
1−sided adj pval: 0.587
Frequency
Tumor−Normal Expression Difference

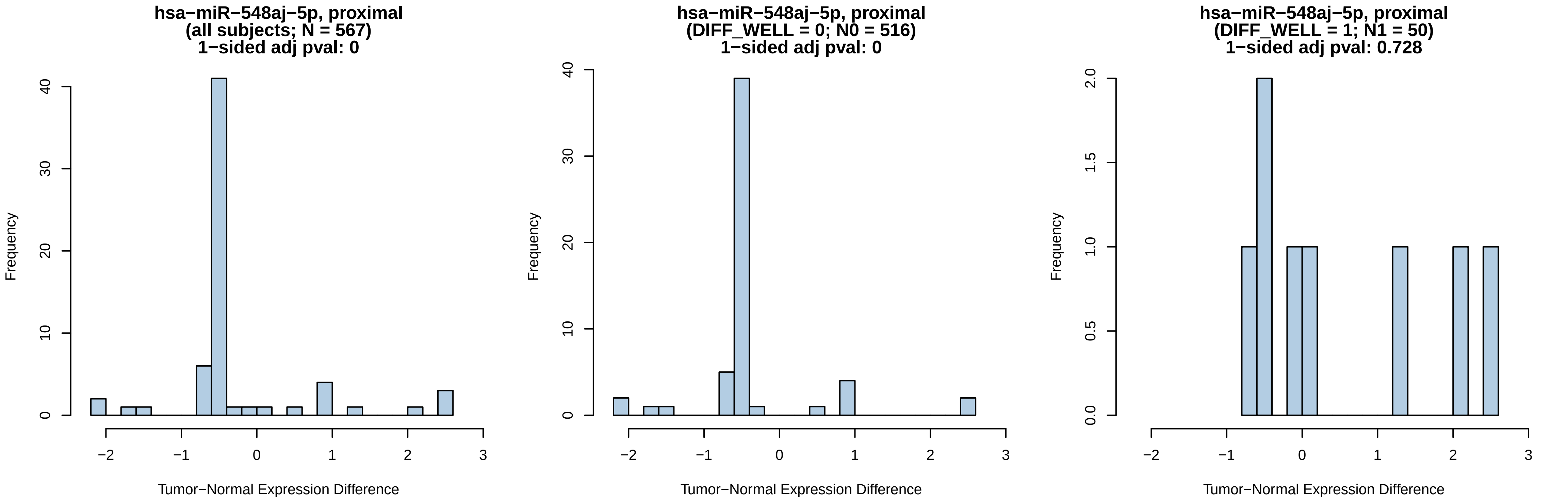

hsa−miR−548aj−5p, proximal
(all subjects; N = 567)
1−sided adj pval: 0
Frequency
Tumor−Normal Expression Difference
hsa−miR−548aj−5p, proximal
(DIFF_WELL = 0; N0 = 516)
1−sided adj pval: 0
Frequency
Tumor−Normal Expression Difference
hsa−miR−548aj−5p, proximal
(DIFF_WELL = 1; N1 = 50)
1−sided adj pval: 0.728
Frequency
Tumor−Normal Expression Difference

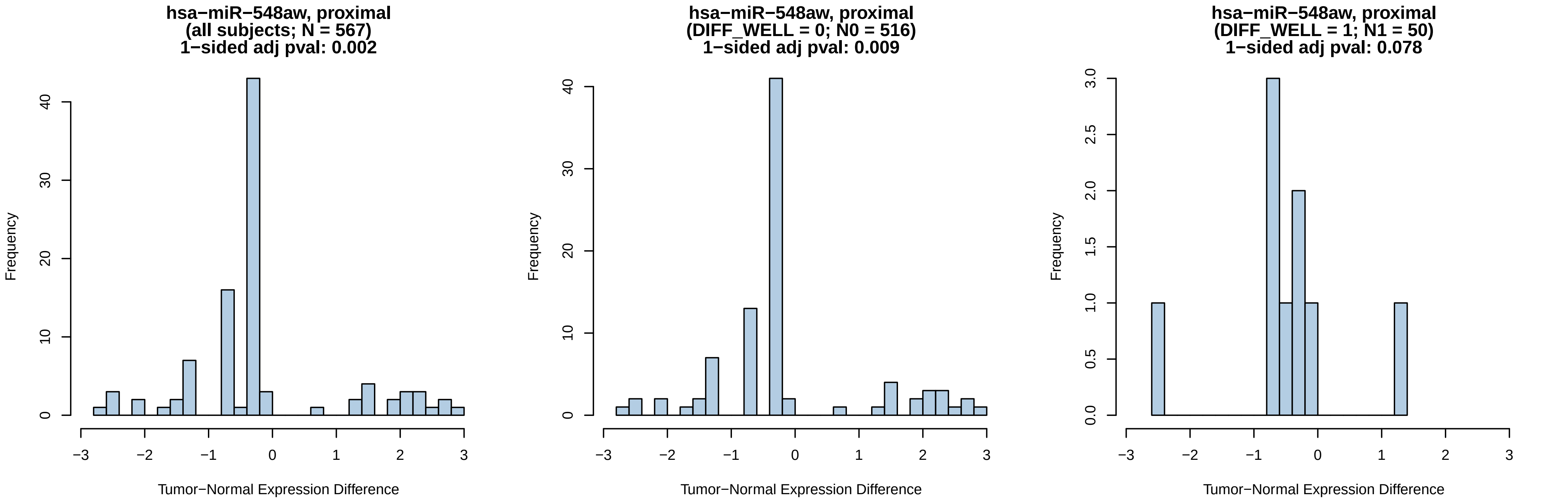

hsa−miR−548aw, proximal
(all subjects; N = 567)
1−sided adj pval: 0.002
hsa−miR−548aw, proximal
(DIFF_WELL = 0; N0 = 516)
1−sided adj pval: 0.009
hsa−miR−548aw, proximal
(DIFF_WELL = 1; N1 = 50)
1−sided adj pval: 0.078
Frequency
Tumor−Normal Expression Difference

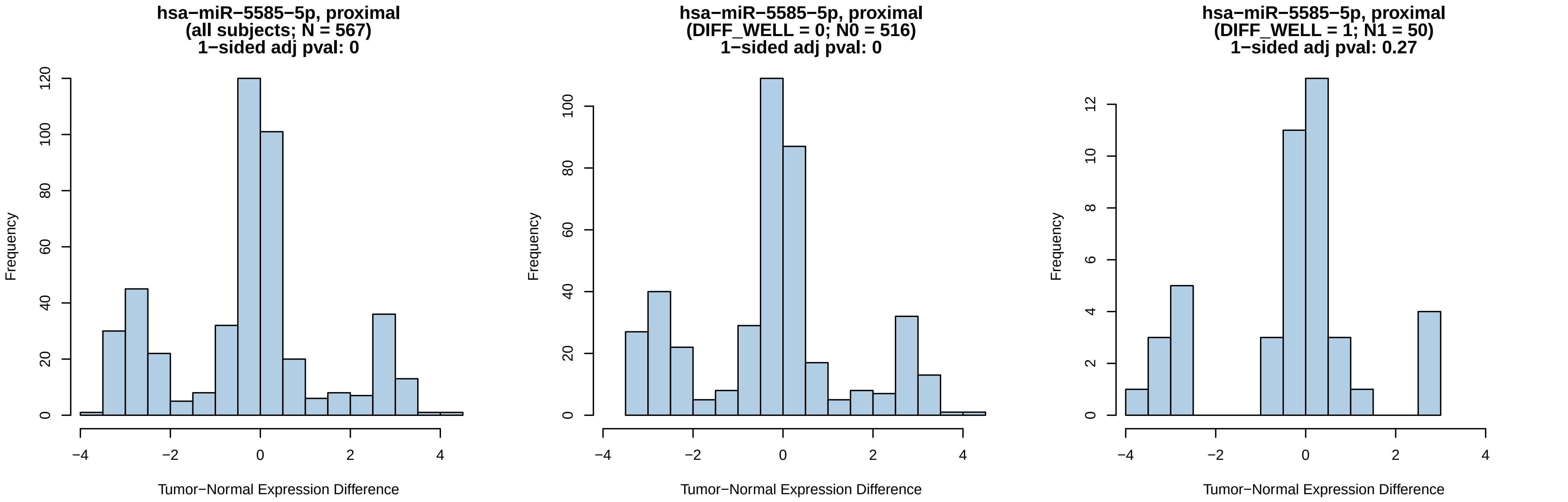

hsa−miR−5585−5p, proximal
(all subjects; N = 567)
1−sided adj pval: 0
Frequency
Tumor−Normal Expression Difference
hsa−miR−5585−5p, proximal
(DIFF_WELL = 0; N0 = 516)
1−sided adj pval: 0
Frequency
Tumor−Normal Expression Difference
hsa−miR−5585−5p, proximal
(DIFF_WELL = 1; N1 = 50)
1−sided adj pval: 0.27
Frequency
Tumor−Normal Expression Difference

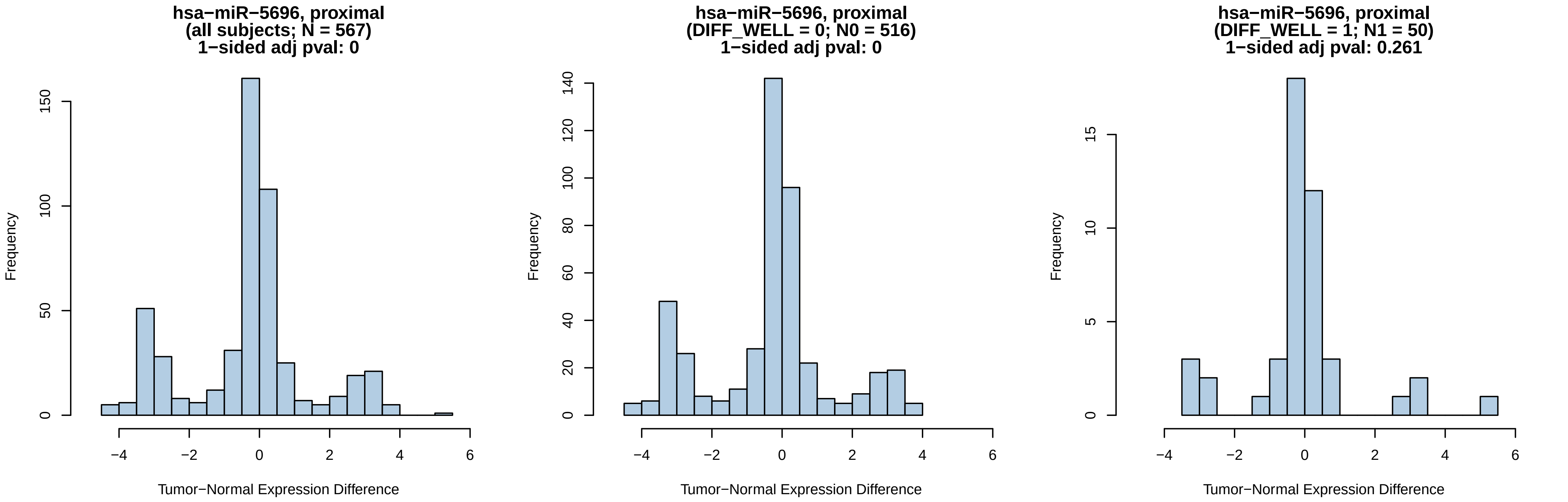
hsa−miR−5696, proximal
(all subjects; N = 567)
1−sided adj pval: 0
Frequency
Tumor−Normal Expression Difference
0
50
100
150
−4
−2
0
2
4
6
hsa−miR−5696, proximal
(DIFF_WELL = 0; N0 = 516)
1−sided adj pval: 0
Frequency
Tumor−Normal Expression Difference
0
20
40
60
80
100
120
140
hsa−miR−5696, proximal
(DIFF_WELL = 1; N1 = 50)
1−sided adj pval: 0.261
Frequency
Tumor−Normal Expression Difference
0
5
10
15

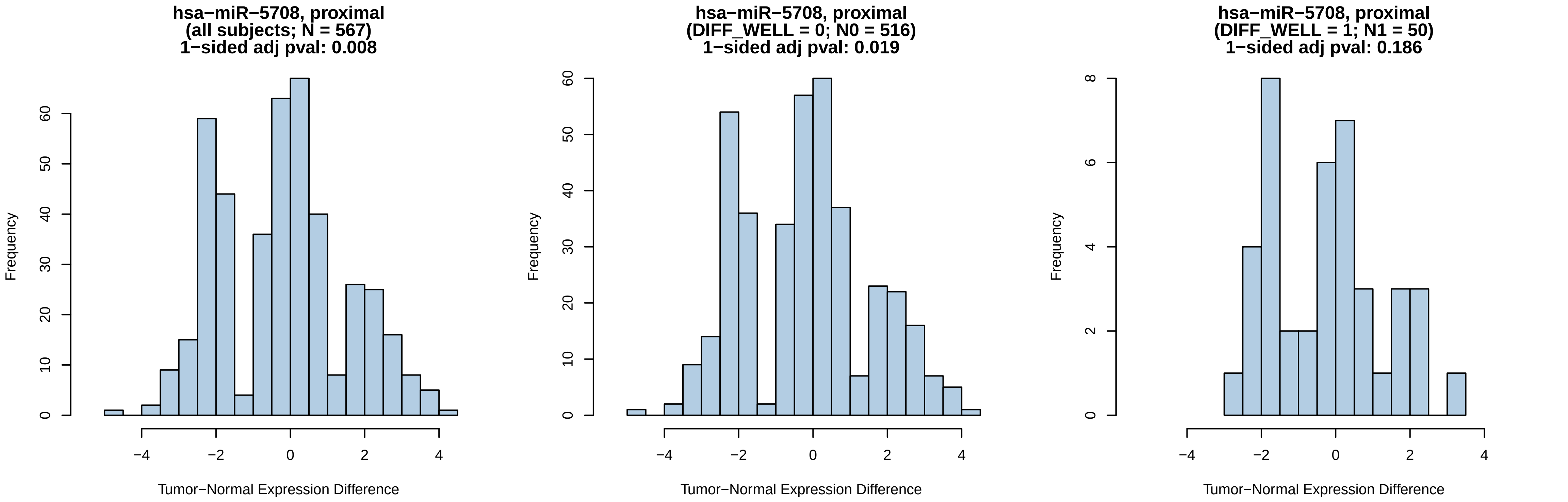
hsa−miR−5708, proximal
(all subjects; N = 567)
1−sided adj pval: 0.008
Frequency
Tumor−Normal Expression Difference
hsa−miR−5708, proximal
(DIFF_WELL = 0; N0 = 516)
1−sided adj pval: 0.019
Frequency
Tumor−Normal Expression Difference
hsa−miR−5708, proximal
(DIFF_WELL = 1; N1 = 50)
1−sided adj pval: 0.186
Frequency
Tumor−Normal Expression Difference

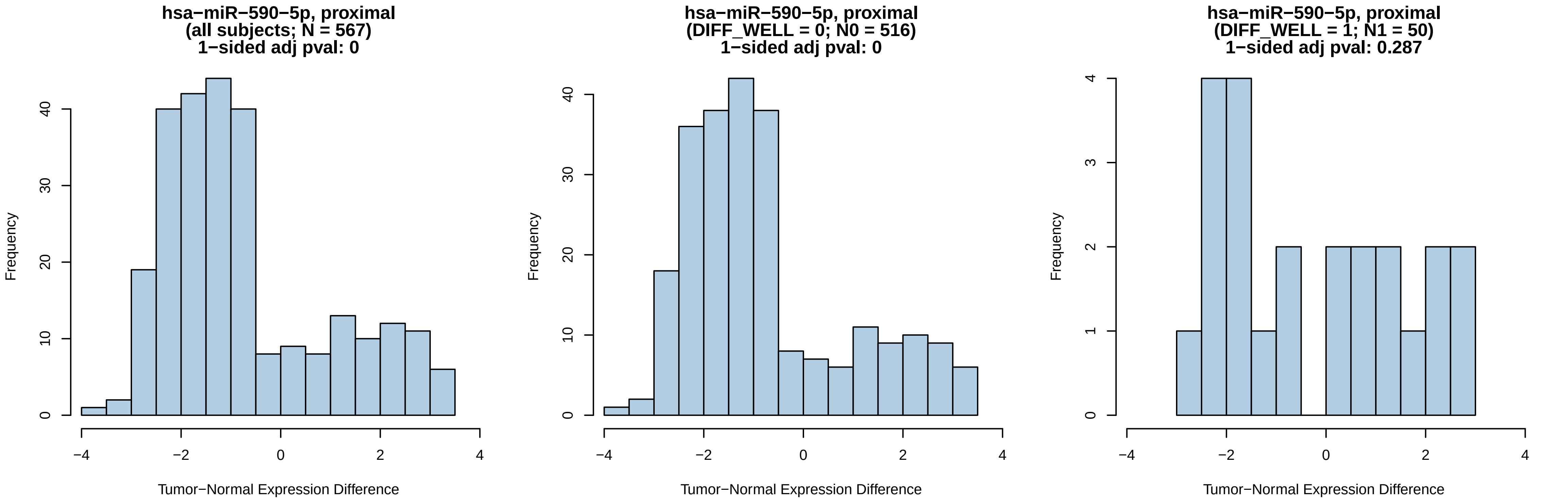

hsa−miR−590−5p, proximal
(all subjects; N = 567)
1−sided adj pval: 0
Frequency
0
10
20
30
40
−4
−2
0
2
4
Tumor−Normal Expression Difference
hsa−miR−590−5p, proximal
(DIFF_WELL = 0; N0 = 516)
1−sided adj pval: 0
Frequency
0
10
20
30
40
−4
−2
0
2
4
Tumor−Normal Expression Difference
hsa−miR−590−5p, proximal
(DIFF_WELL = 1; N1 = 50)
1−sided adj pval: 0.287
Frequency
0
1
2
3
4
−4
−2
0
2
4
Tumor−Normal Expression Difference

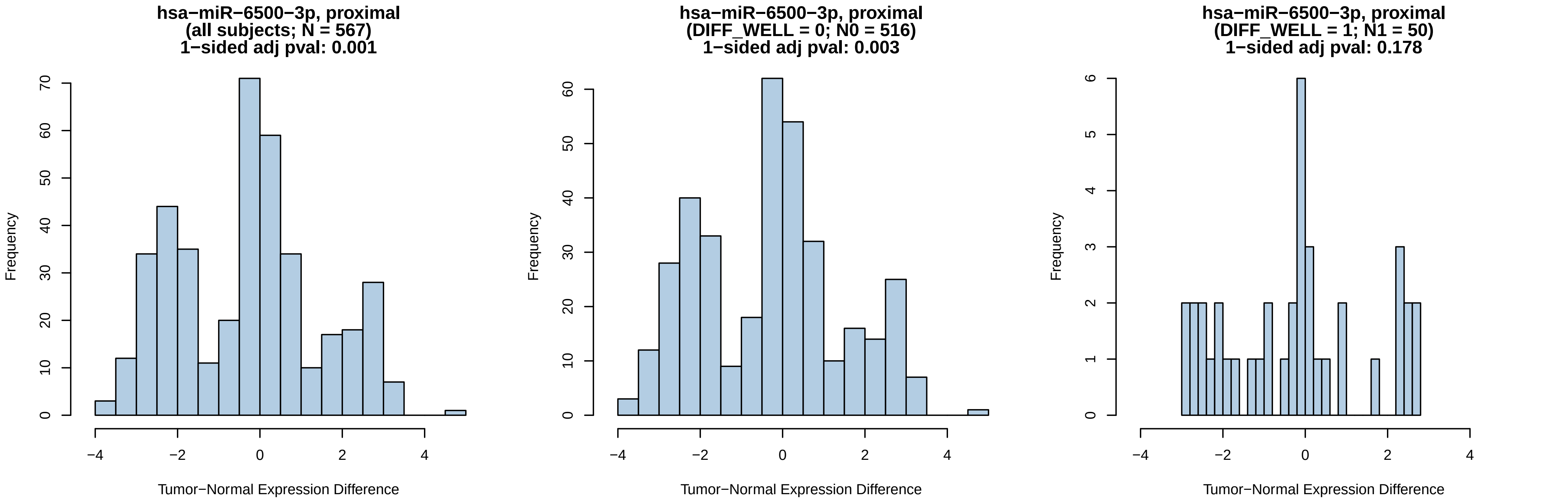
hsa−miR−6500−3p, proximal
(all subjects; N = 567)
1−sided adj pval: 0.001
Frequency
Tumor−Normal Expression Difference
hsa−miR−6500−3p, proximal
(DIFF_WELL = 0; N0 = 516)
1−sided adj pval: 0.003
Frequency
Tumor−Normal Expression Difference
hsa−miR−6500−3p, proximal
(DIFF_WELL = 1; N1 = 50)
1−sided adj pval: 0.178
Frequency
Tumor−Normal Expression Difference

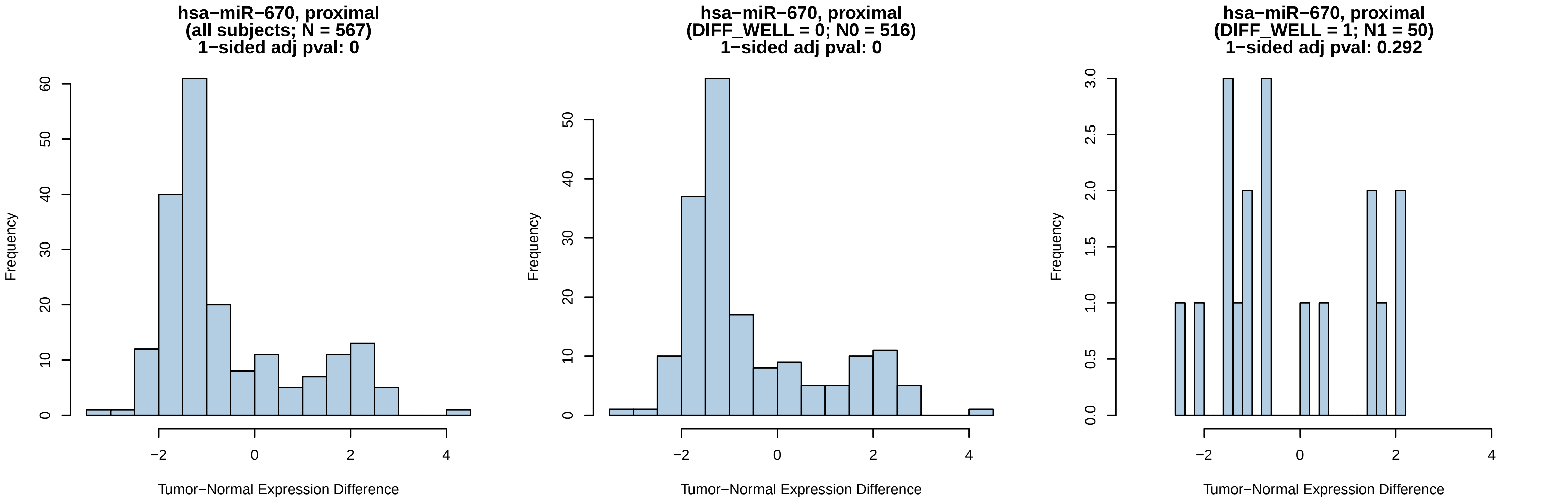
hsa−miR−670, proximal
(all subjects; N = 567)
1−sided adj pval: 0
Frequency
Tumor−Normal Expression Difference
hsa−miR−670, proximal
(DIFF_WELL = 0; N0 = 516)
1−sided adj pval: 0
Frequency
Tumor−Normal Expression Difference
hsa−miR−670, proximal
(DIFF_WELL = 1; N1 = 50)
1−sided adj pval: 0.292
Frequency
Tumor−Normal Expression Difference

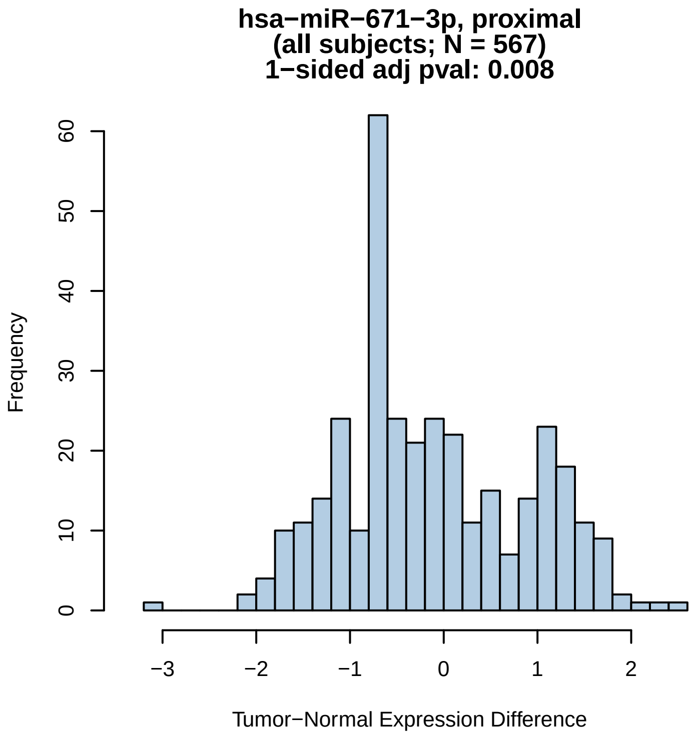
hsa−miR−671−3p, proximal
(all subjects; N = 567)
1−sided adj pval: 0.008
Frequency
0
10
20
30
40
50
60
−3
−2
−1
0
1
2
Tumor−Normal Expression Difference

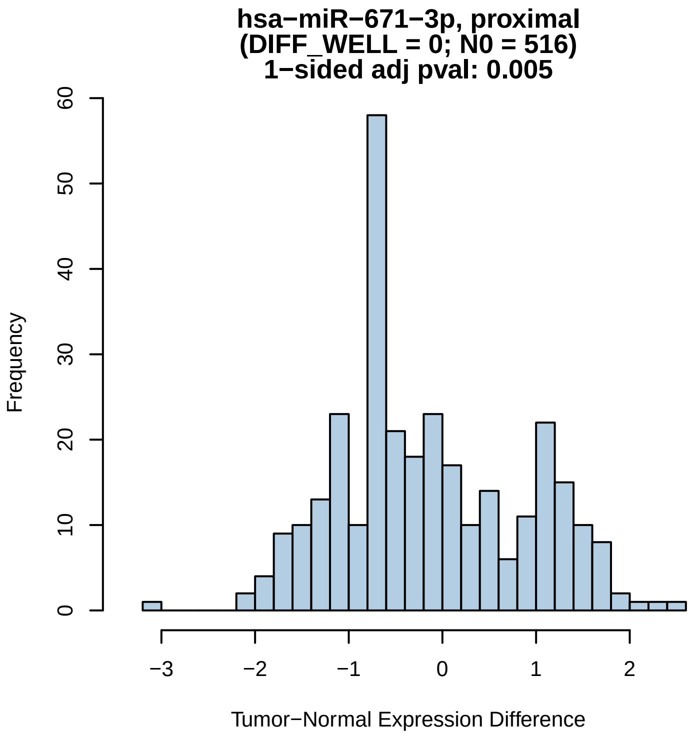
hsa−miR−671−3p, proximal
(DIFF_WELL = 0; N0 = 516)
1−sided adj pval: 0.005
Frequency
0
10
20
30
40
50
60
−3
−2
−1
0
1
2
Tumor−Normal Expression Difference

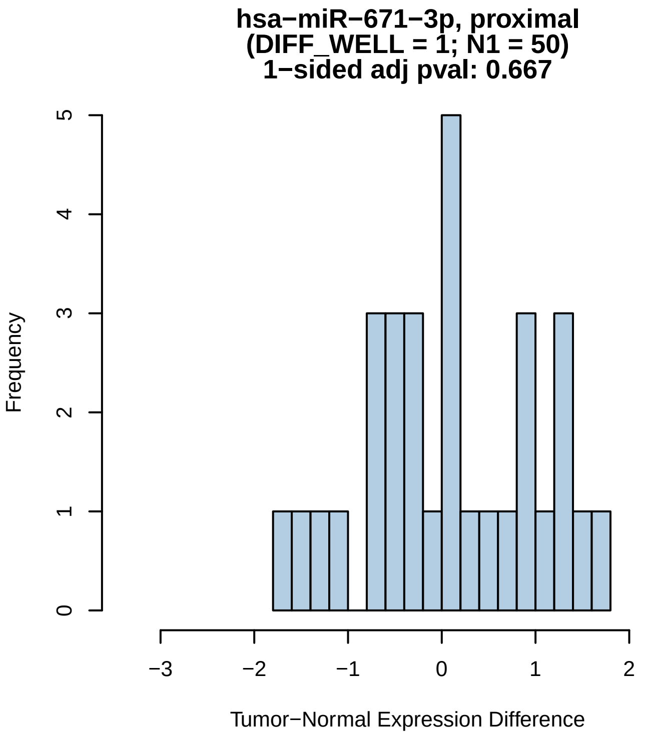
hsa−miR−671−3p, proximal
(DIFF_WELL = 1; N1 = 50)
1−sided adj pval: 0.667
Frequency
0
1
2
3
4
5
−3
−2
−1
0
1
2
Tumor−Normal Expression Difference

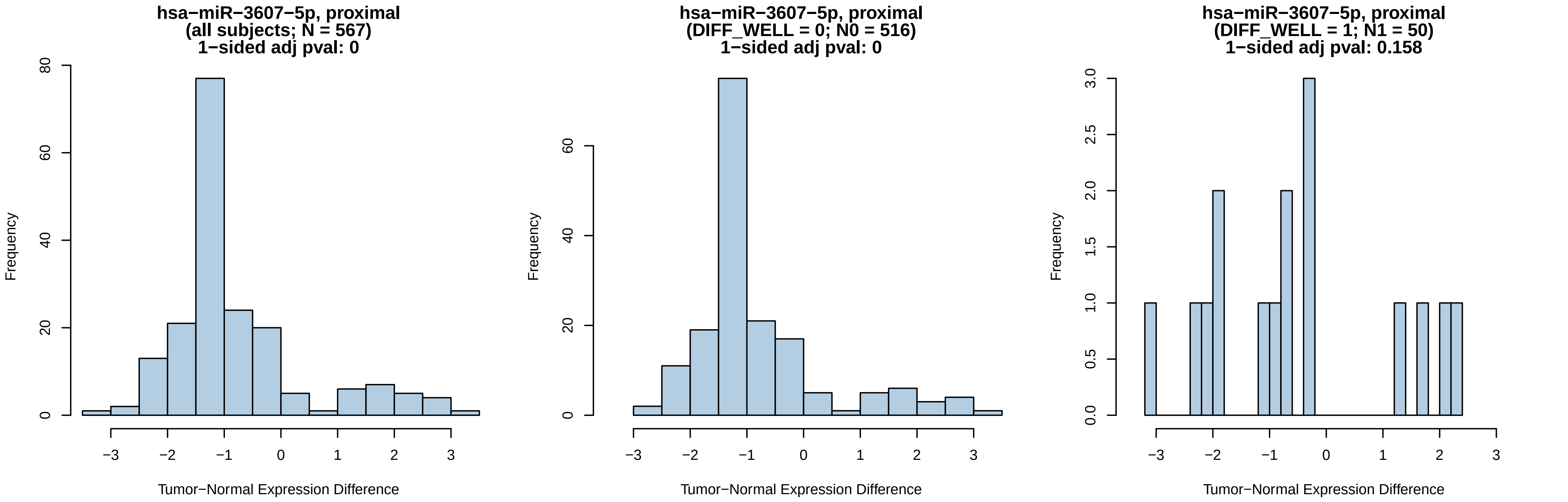
hsa−miR−3607−5p, proximal
(all subjects; N = 567)
1−sided adj pval: 0
hsa−miR−3607−5p, proximal
(DIFF_WELL = 0; N0 = 516)
1−sided adj pval: 0
hsa−miR−3607−5p, proximal
(DIFF_WELL = 1; N1 = 50)
1−sided adj pval: 0.158
Frequency
Tumor−Normal Expression Difference

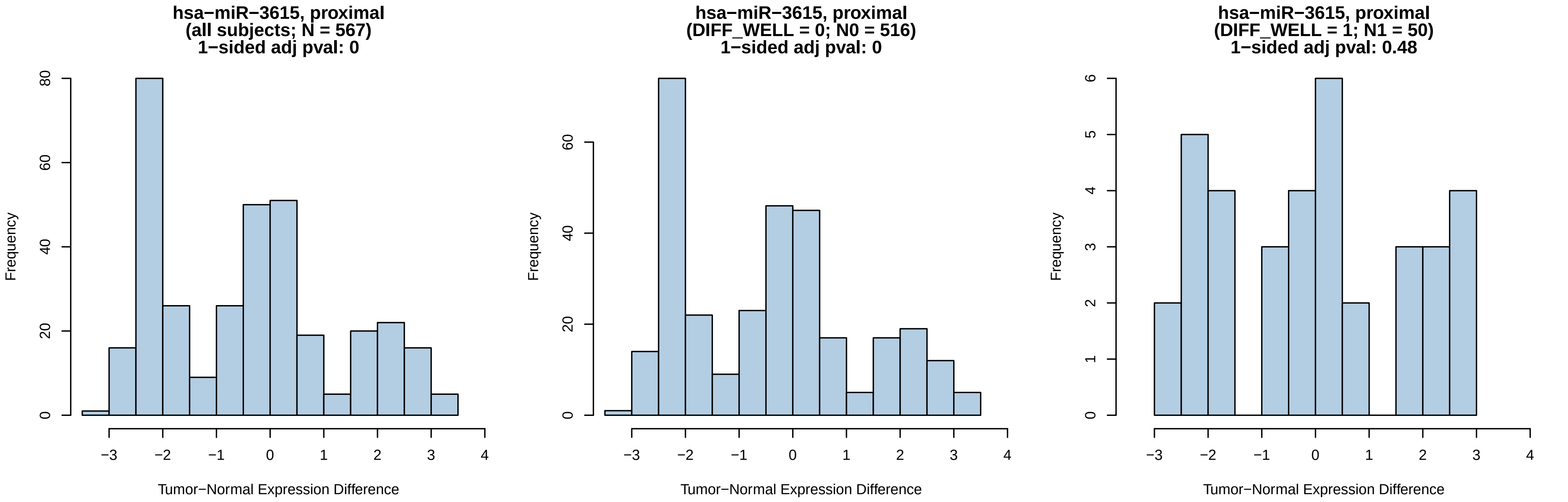
hsa−miR−3615, proximal
(all subjects; N = 567)
1−sided adj pval: 0
Frequency
Tumor−Normal Expression Difference
hsa−miR−3615, proximal
(DIFF_WELL = 0; N0 = 516)
1−sided adj pval: 0
Frequency
Tumor−Normal Expression Difference
hsa−miR−3615, proximal
(DIFF_WELL = 1; N1 = 50)
1−sided adj pval: 0.48
Frequency
Tumor−Normal Expression Difference

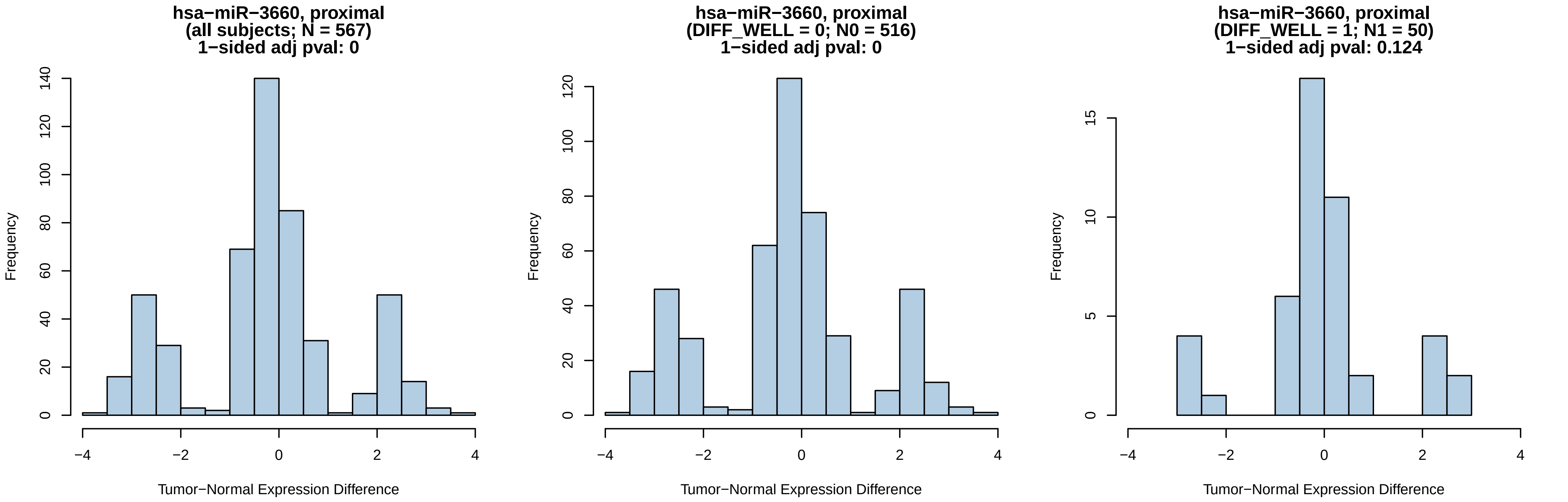

hsa−miR−3660, proximal
(all subjects; N = 567)
1−sided adj pval: 0
Frequency
Tumor−Normal Expression Difference
hsa−miR−3660, proximal
(DIFF_WELL = 0; N0 = 516)
1−sided adj pval: 0
Frequency
Tumor−Normal Expression Difference
hsa−miR−3660, proximal
(DIFF_WELL = 1; N1 = 50)
1−sided adj pval: 0.124
Frequency
Tumor−Normal Expression Difference

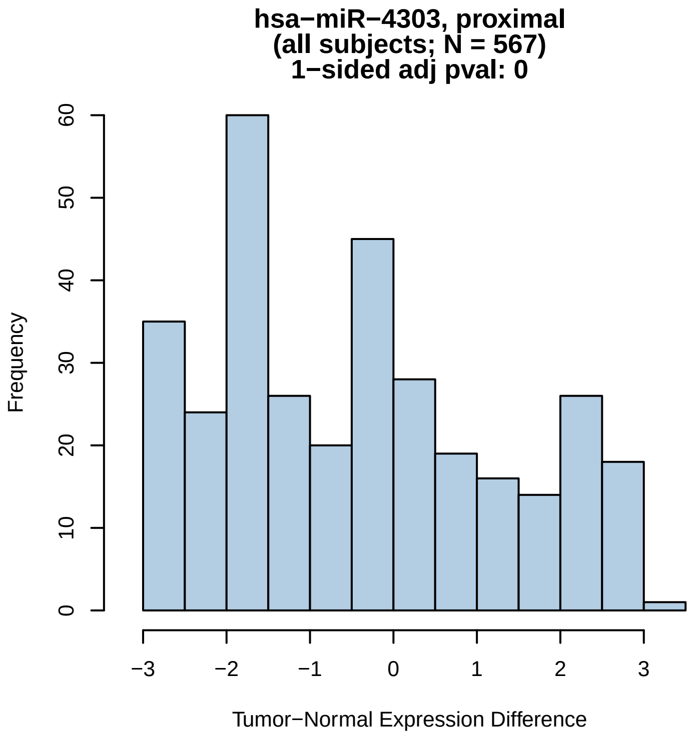
hsa−miR−4303, proximal
(all subjects; N = 567)
1−sided adj pval: 0
Frequency
Tumor−Normal Expression Difference
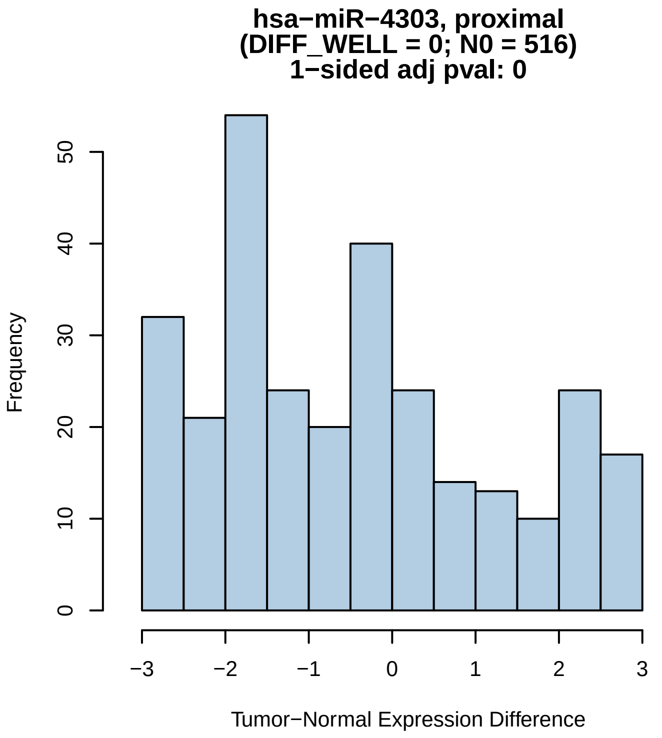
hsa−miR−4303, proximal
(DIFF_WELL = 0; N0 = 516)
1−sided adj pval: 0
Frequency
Tumor−Normal Expression Difference
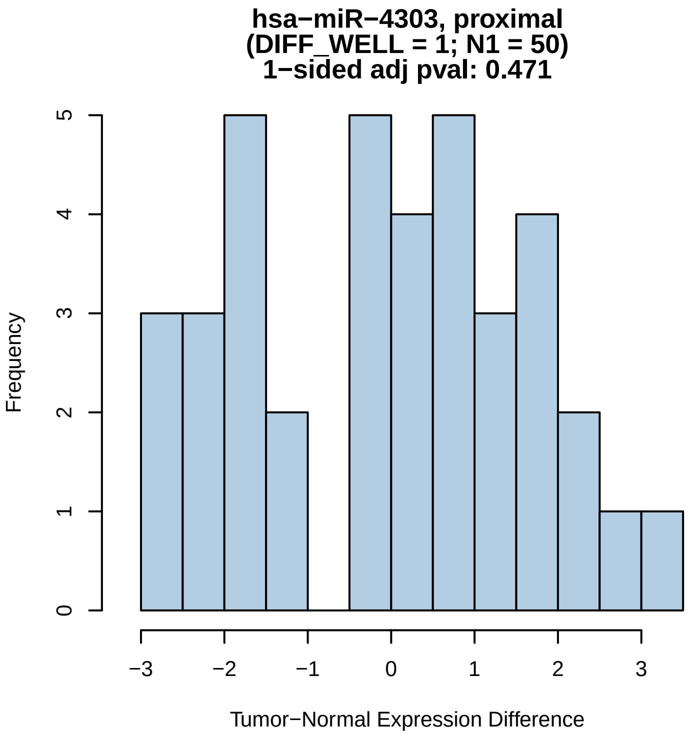
hsa−miR−4303, proximal
(DIFF_WELL = 1; N1 = 50)
1−sided adj pval: 0.471
Frequency
Tumor−Normal Expression Difference

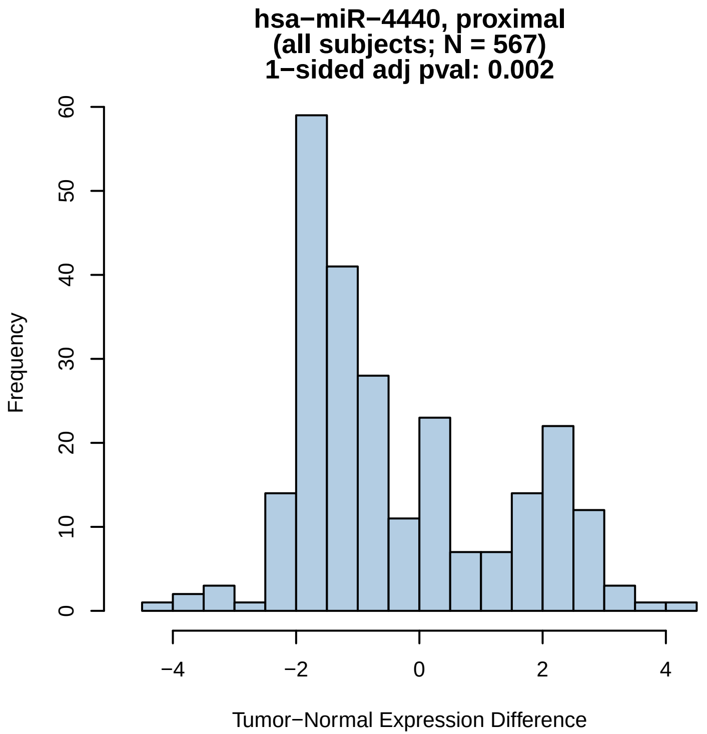

hsa−miR−4440, proximal
(all subjects; N = 567)
1−sided adj pval: 0.002
Frequency
Tumor−Normal Expression Difference
0
10
20
30
40
50
60
−4
−2
0
2
4


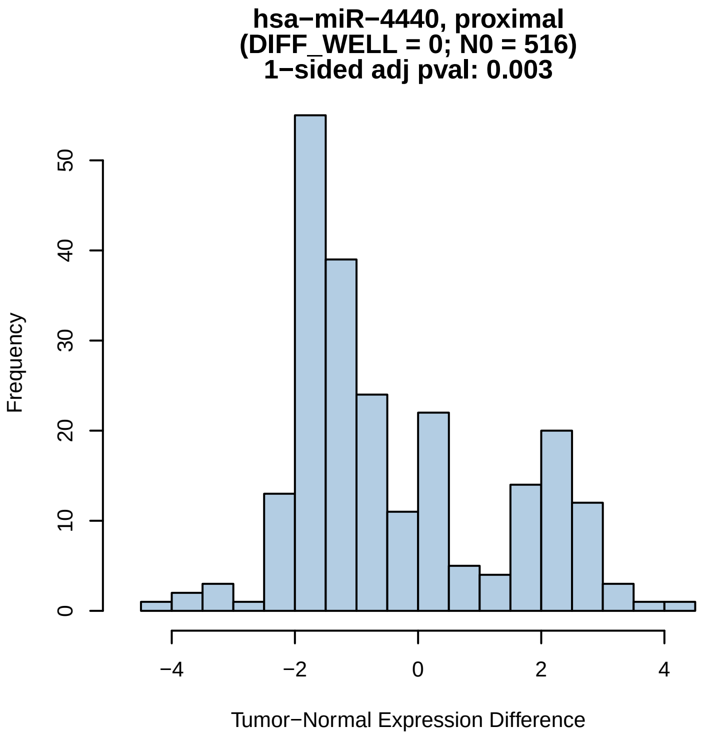

hsa−miR−4440, proximal
(DIFF_WELL = 0; N0 = 516)
1−sided adj pval: 0.003
Frequency
Tumor−Normal Expression Difference
0
10
20
30
40
50
−4
−2
0
2
4


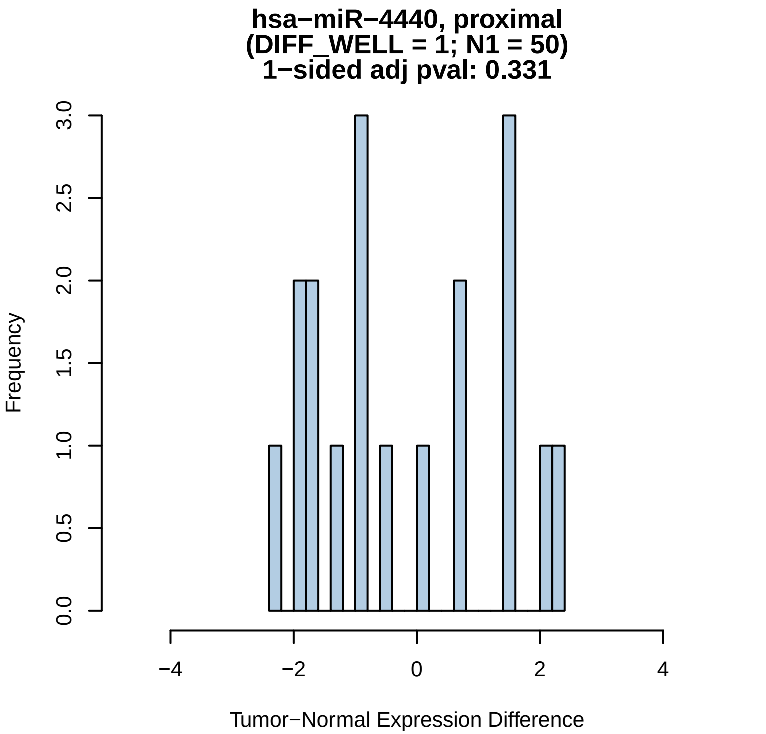

hsa−miR−4440, proximal
(DIFF_WELL = 1; N1 = 50)
1−sided adj pval: 0.331
Frequency
Tumor−Normal Expression Difference
0.0
0.5
1.0
1.5
2.0
2.5
3.0
−4
−2
0
2
4

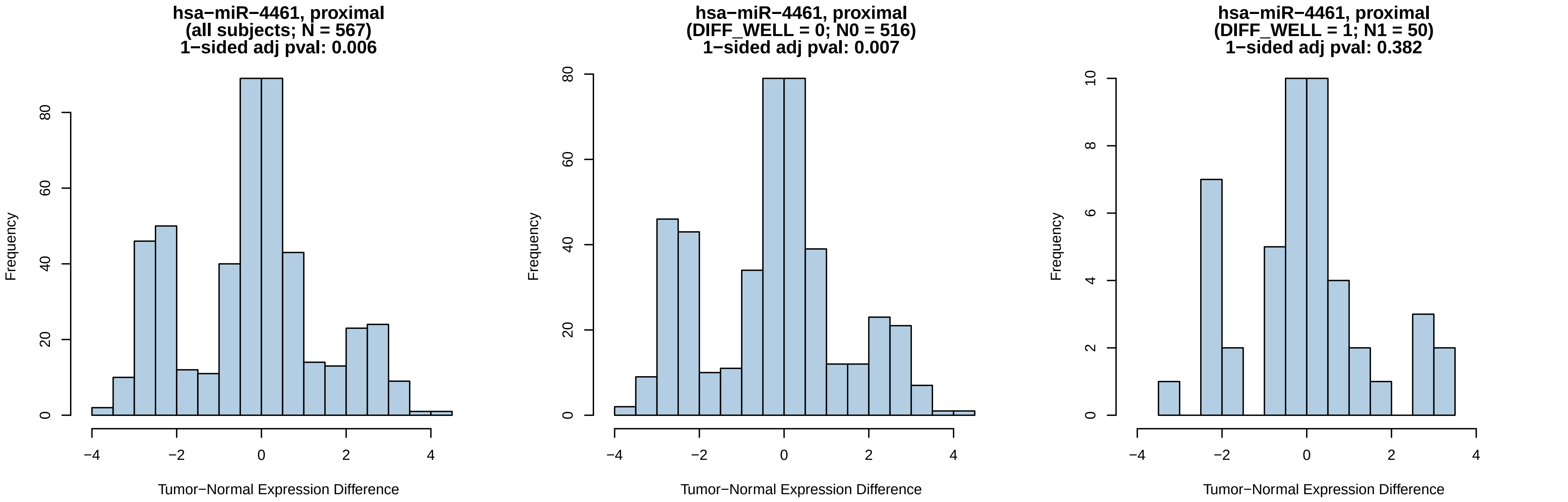
hsa−miR−4461, proximal
(all subjects; N = 567)
1−sided adj pval: 0.006
Frequency
Tumor−Normal Expression Difference
hsa−miR−4461, proximal
(DIFF_WELL = 0; N0 = 516)
1−sided adj pval: 0.007
Frequency
Tumor−Normal Expression Difference
hsa−miR−4461, proximal
(DIFF_WELL = 1; N1 = 50)
1−sided adj pval: 0.382
Frequency
Tumor−Normal Expression Difference

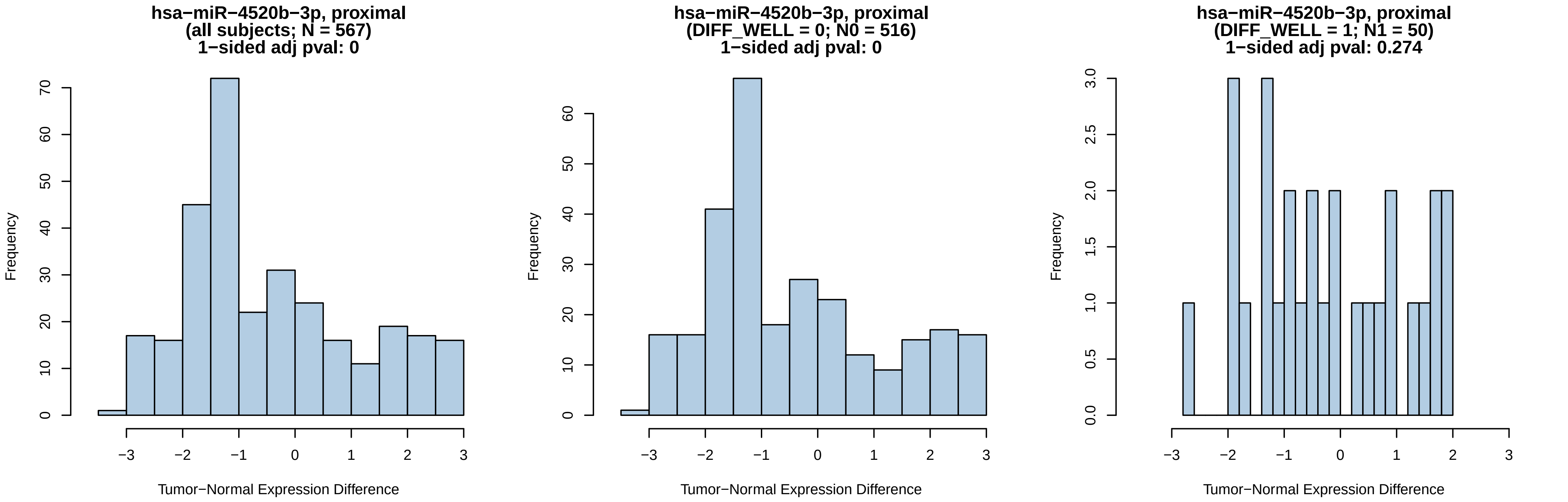

hsa−miR−4520b−3p, proximal
(all subjects; N = 567)
1−sided adj pval: 0
Frequency
0
10
20
30
40
50
60
70
−3
−2
−1
0
1
2
3
Tumor−Normal Expression Difference
hsa−miR−4520b−3p, proximal
(DIFF_WELL = 0; N0 = 516)
1−sided adj pval: 0
Frequency
0
10
20
30
40
50
60
−3
−2
−1
0
1
2
3
Tumor−Normal Expression Difference
hsa−miR−4520b−3p, proximal
(DIFF_WELL = 1; N1 = 50)
1−sided adj pval: 0.274
Frequency
0.0
0.5
1.0
1.5
2.0
2.5
3.0
−3
−2
−1
0
1
2
3
Tumor−Normal Expression Difference

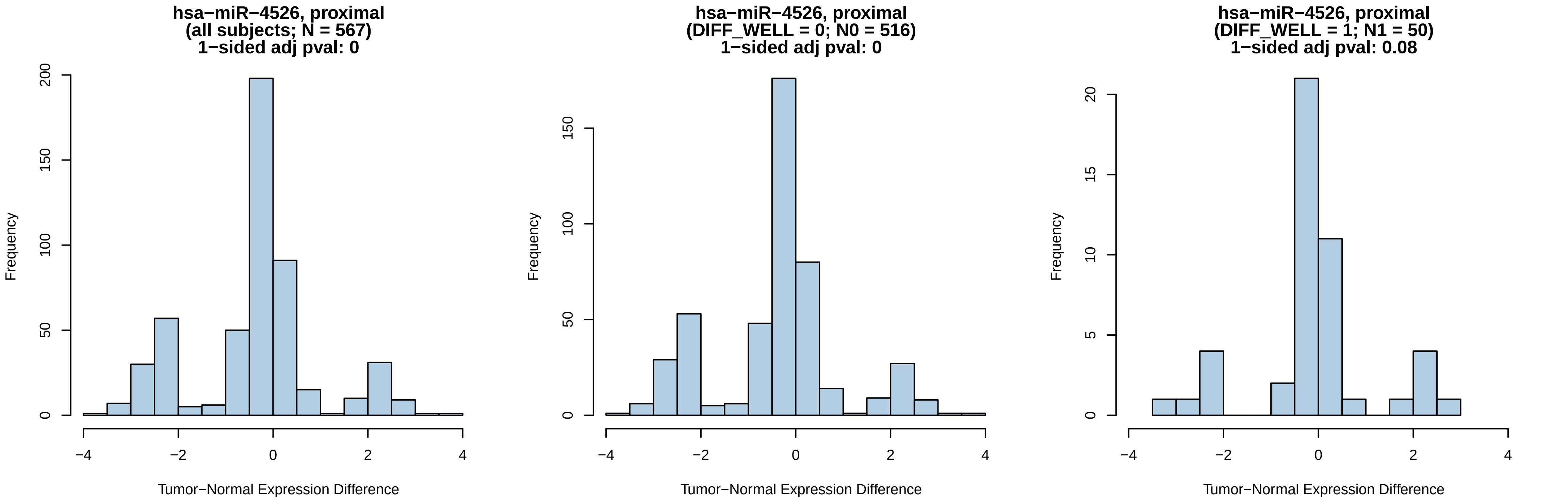
hsa−miR−4526, proximal
(all subjects; N = 567)
1−sided adj pval: 0
hsa−miR−4526, proximal
(DIFF_WELL = 0; N0 = 516)
1−sided adj pval: 0
hsa−miR−4526, proximal
(DIFF_WELL = 1; N1 = 50)
1−sided adj pval: 0.08
Frequency
Tumor−Normal Expression Difference

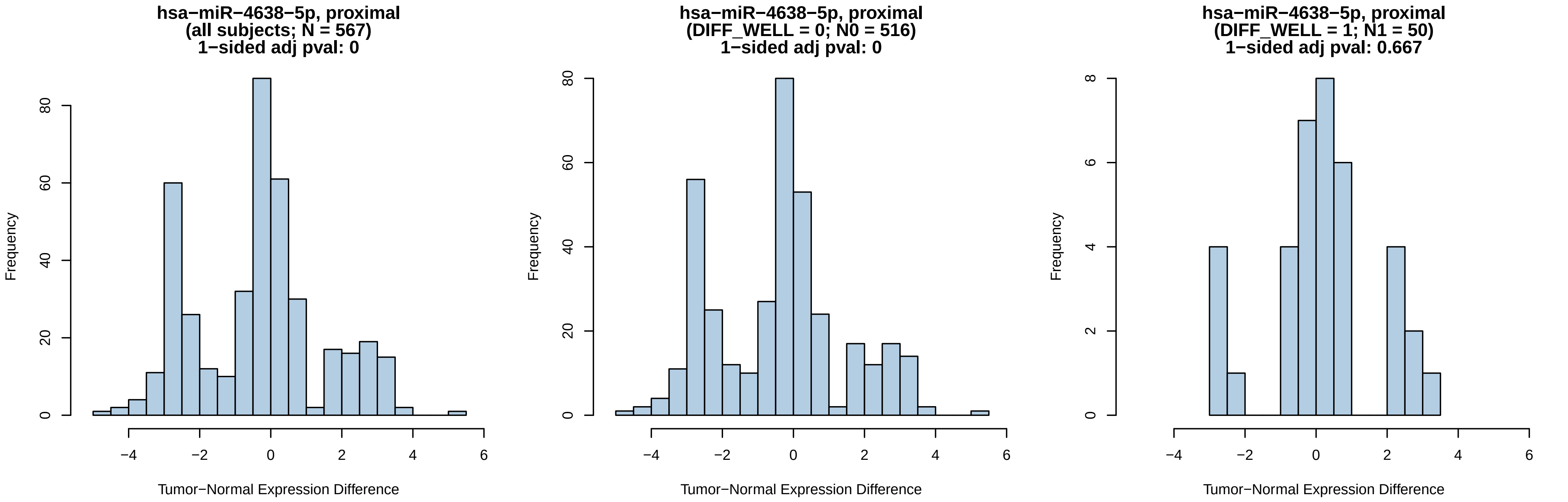

hsa−miR−4638−5p, proximal
(all subjects; N = 567)
1−sided adj pval: 0
Frequency
Tumor−Normal Expression Difference
hsa−miR−4638−5p, proximal
(DIFF_WELL = 0; N0 = 516)
1−sided adj pval: 0
Frequency
Tumor−Normal Expression Difference
hsa−miR−4638−5p, proximal
(DIFF_WELL = 1; N1 = 50)
1−sided adj pval: 0.667
Frequency
Tumor−Normal Expression Difference

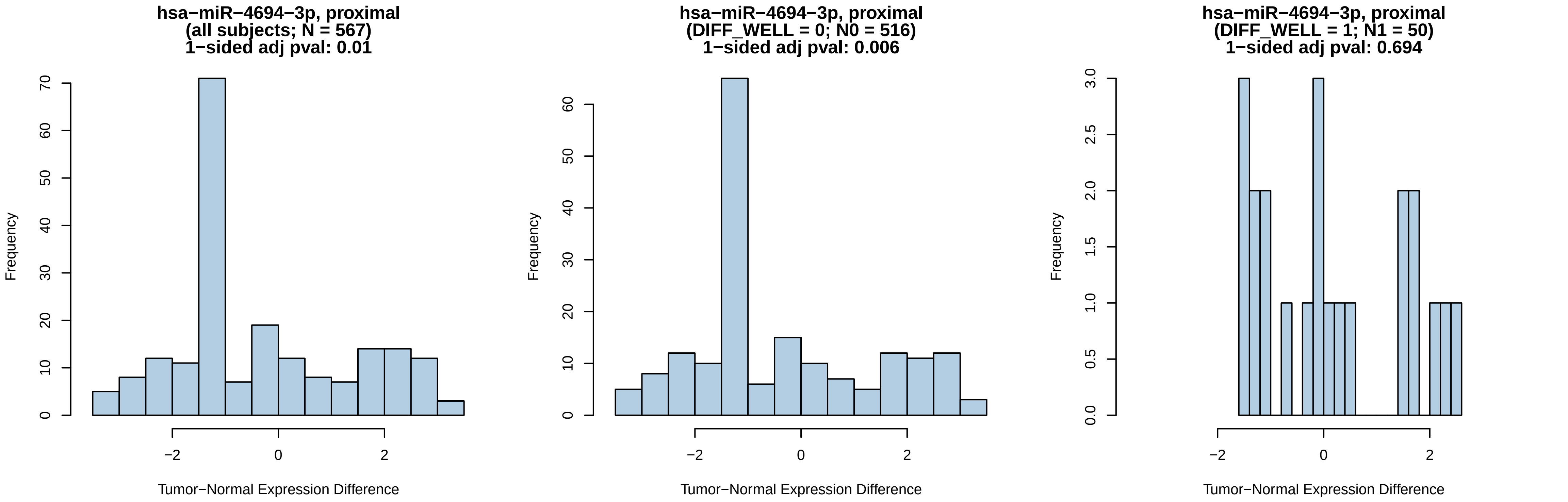
hsa−miR−4694−3p, proximal
(all subjects; N = 567)
1−sided adj pval: 0.01
Frequency
Tumor−Normal Expression Difference
hsa−miR−4694−3p, proximal
(DIFF_WELL = 0; N0 = 516)
1−sided adj pval: 0.006
Frequency
Tumor−Normal Expression Difference
hsa−miR−4694−3p, proximal
(DIFF_WELL = 1; N1 = 50)
1−sided adj pval: 0.694
Frequency
Tumor−Normal Expression Difference

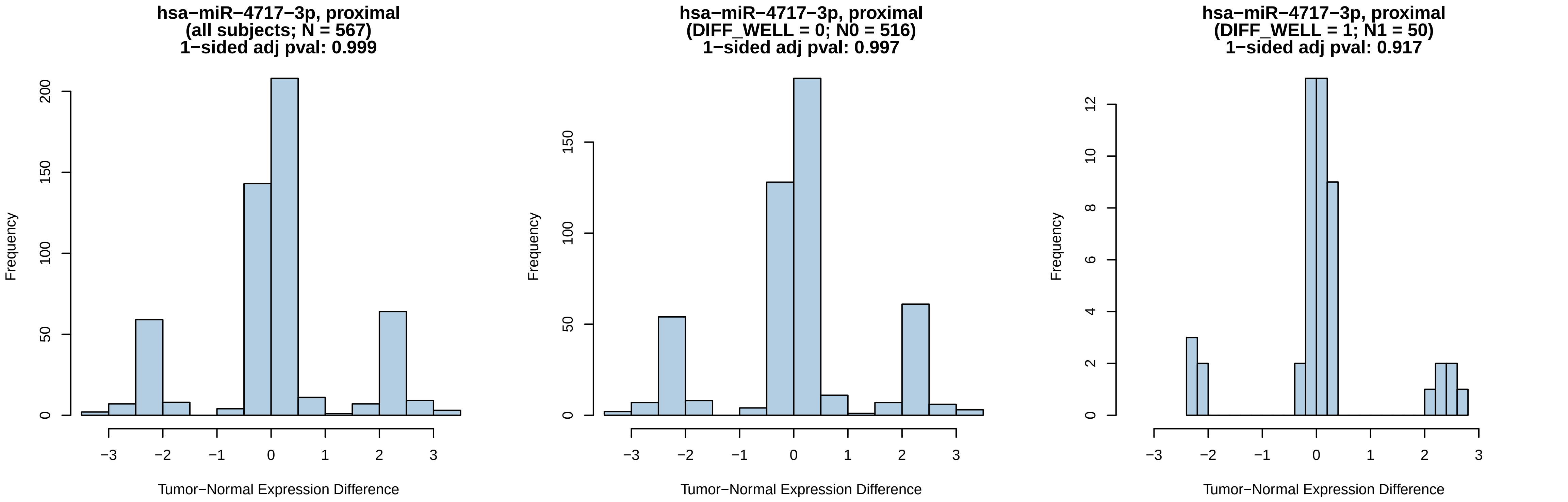

hsa−miR−4717−3p, proximal
(all subjects; N = 567)
1−sided adj pval: 0.999
hsa−miR−4717−3p, proximal
(DIFF_WELL = 0; N0 = 516)
1−sided adj pval: 0.997
hsa−miR−4717−3p, proximal
(DIFF_WELL = 1; N1 = 50)
1−sided adj pval: 0.917
Frequency
Tumor−Normal Expression Difference

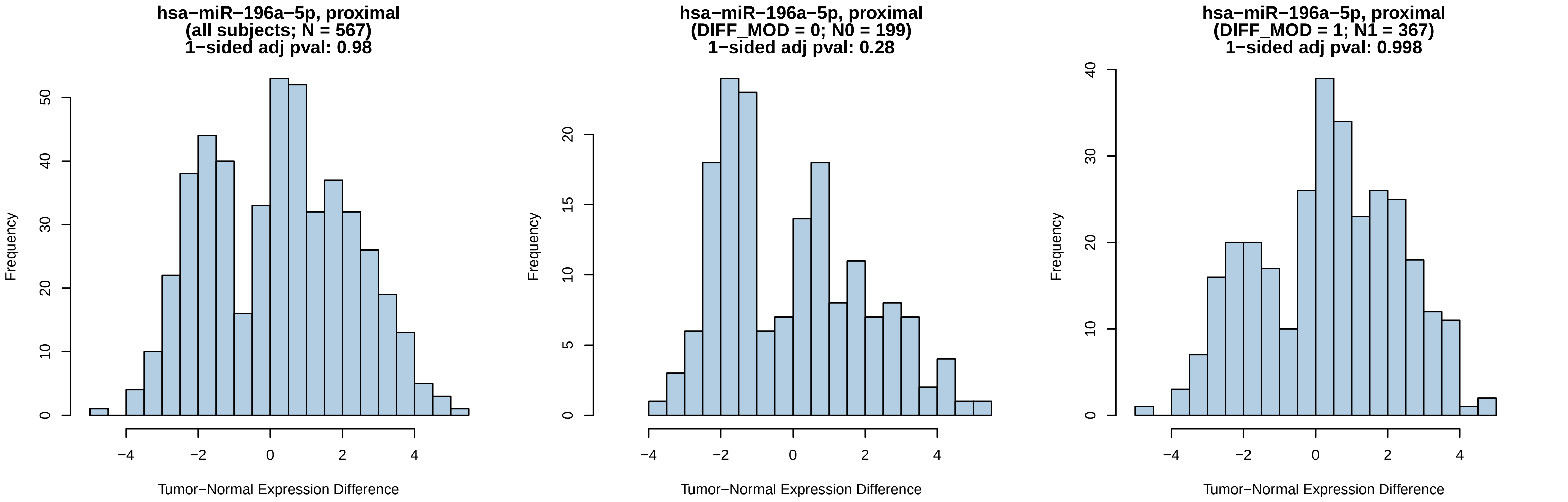

hsa−miR−196a−5p, proximal
(all subjects; N = 567)
1−sided adj pval: 0.98
Frequency
Tumor−Normal Expression Difference
hsa−miR−196a−5p, proximal
(DIFF_MOD = 0; N0 = 199)
1−sided adj pval: 0.28
Frequency
Tumor−Normal Expression Difference
hsa−miR−196a−5p, proximal
(DIFF_MOD = 1; N1 = 367)
1−sided adj pval: 0.998
Frequency
Tumor−Normal Expression Difference

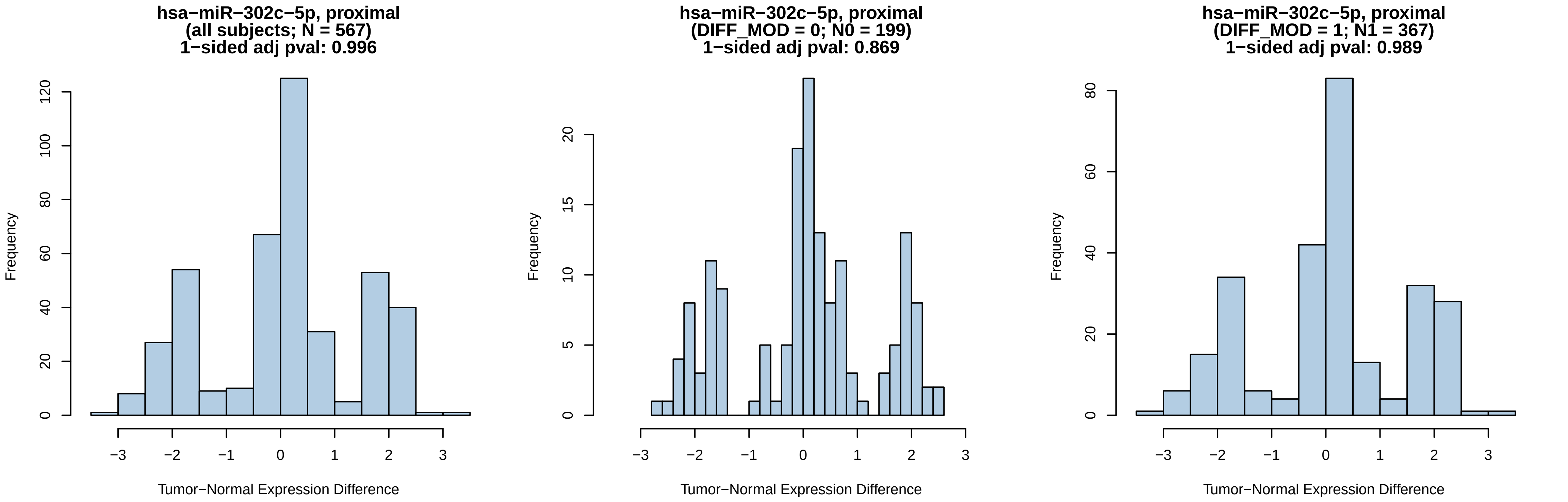

hsa−miR−302c−5p, proximal
(all subjects; N = 567)
1−sided adj pval: 0.996
hsa−miR−302c−5p, proximal
(DIFF_MOD = 0; N0 = 199)
1−sided adj pval: 0.869
hsa−miR−302c−5p, proximal
(DIFF_MOD = 1; N1 = 367)
1−sided adj pval: 0.989
Frequency
Tumor−Normal Expression Difference

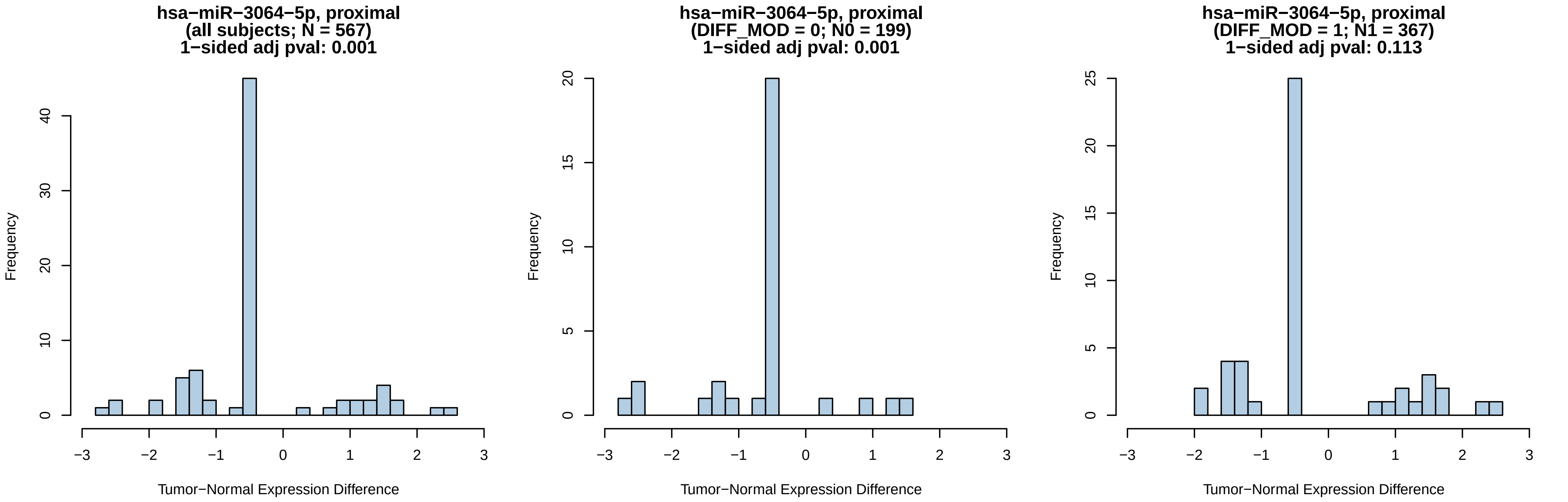
hsa−miR−3064−5p, proximal
(all subjects; N = 567)
1−sided adj pval: 0.001
hsa−miR−3064−5p, proximal
(DIFF_MOD = 0; N0 = 199)
1−sided adj pval: 0.001
hsa−miR−3064−5p, proximal
(DIFF_MOD = 1; N1 = 367)
1−sided adj pval: 0.113
Frequency
Tumor−Normal Expression Difference

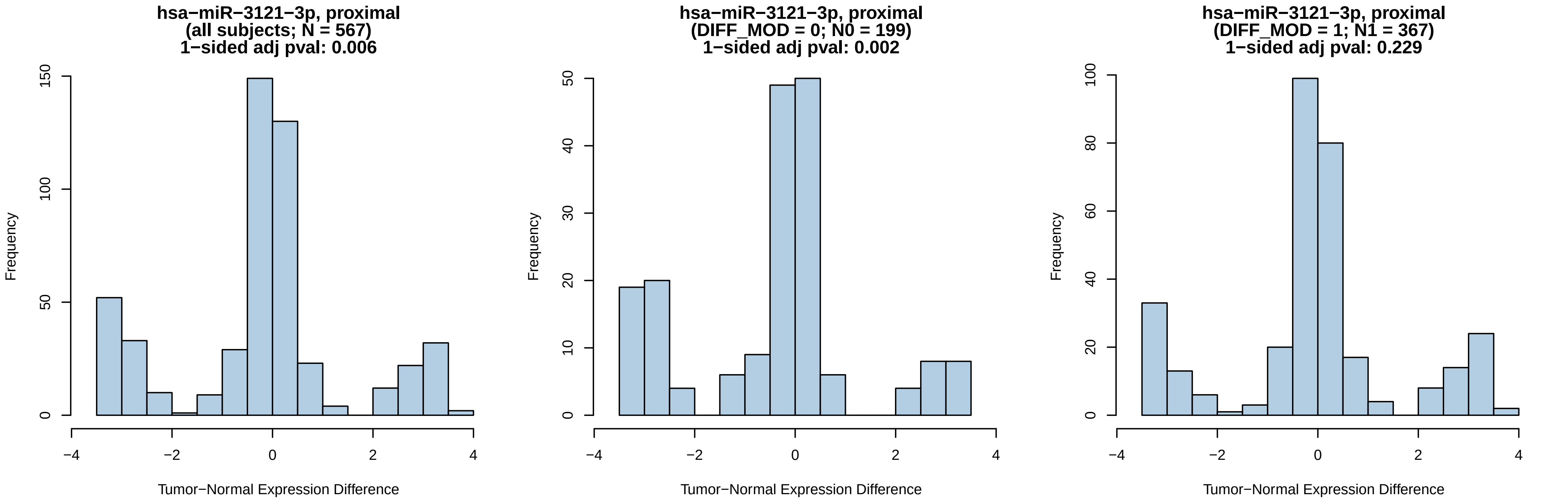

hsa−miR−3121−3p, proximal
(all subjects; N = 567)
1−sided adj pval: 0.006
Frequency
0
50
100
150
−4
−2
0
2
4
Tumor−Normal Expression Difference
hsa−miR−3121−3p, proximal
(DIFF_MOD = 0; N0 = 199)
1−sided adj pval: 0.002
Frequency
0
10
20
30
40
50
−4
−2
0
2
4
Tumor−Normal Expression Difference
hsa−miR−3121−3p, proximal
(DIFF_MOD = 1; N1 = 367)
1−sided adj pval: 0.229
Frequency
0
20
40
60
80
100
−4
−2
0
2
4
Tumor−Normal Expression Difference

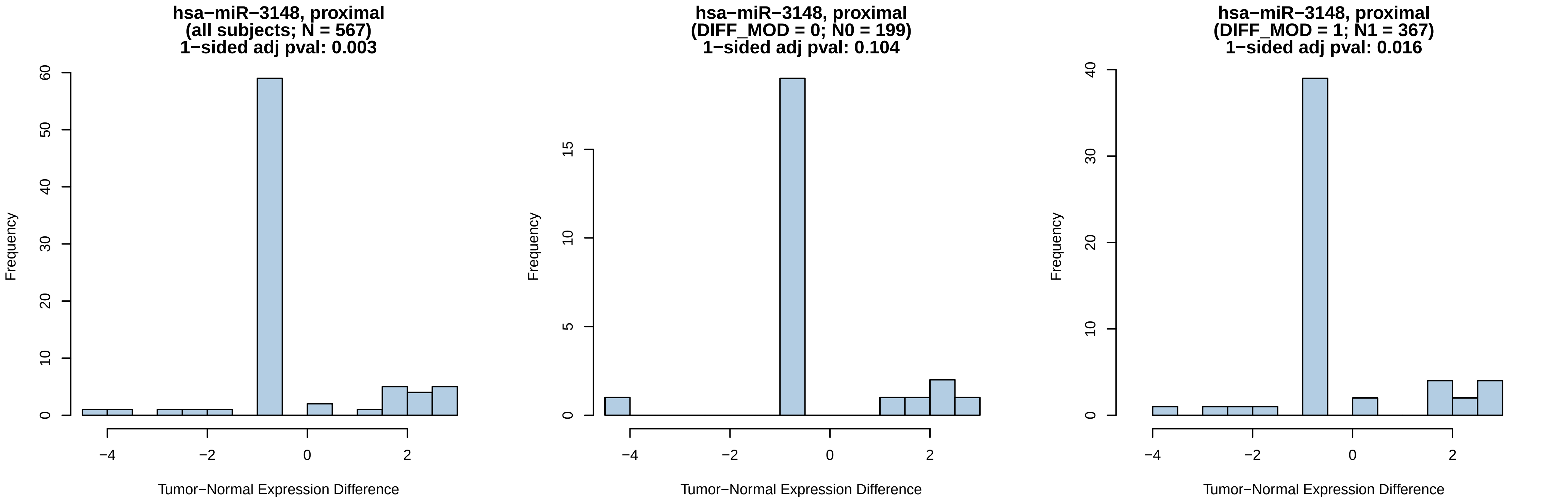

hsa−miR−3148, proximal
(all subjects; N = 567)
1−sided adj pval: 0.003
Frequency
0
10
20
30
40
50
60
−4
−2
0
2
Tumor−Normal Expression Difference
hsa−miR−3148, proximal
(DIFF_MOD = 0; N0 = 199)
1−sided adj pval: 0.104
Frequency
0
5
10
15
−4
−2
0
2
Tumor−Normal Expression Difference
hsa−miR−3148, proximal
(DIFF_MOD = 1; N1 = 367)
1−sided adj pval: 0.016
Frequency
0
10
20
30
40
−4
−2
0
2
Tumor−Normal Expression Difference

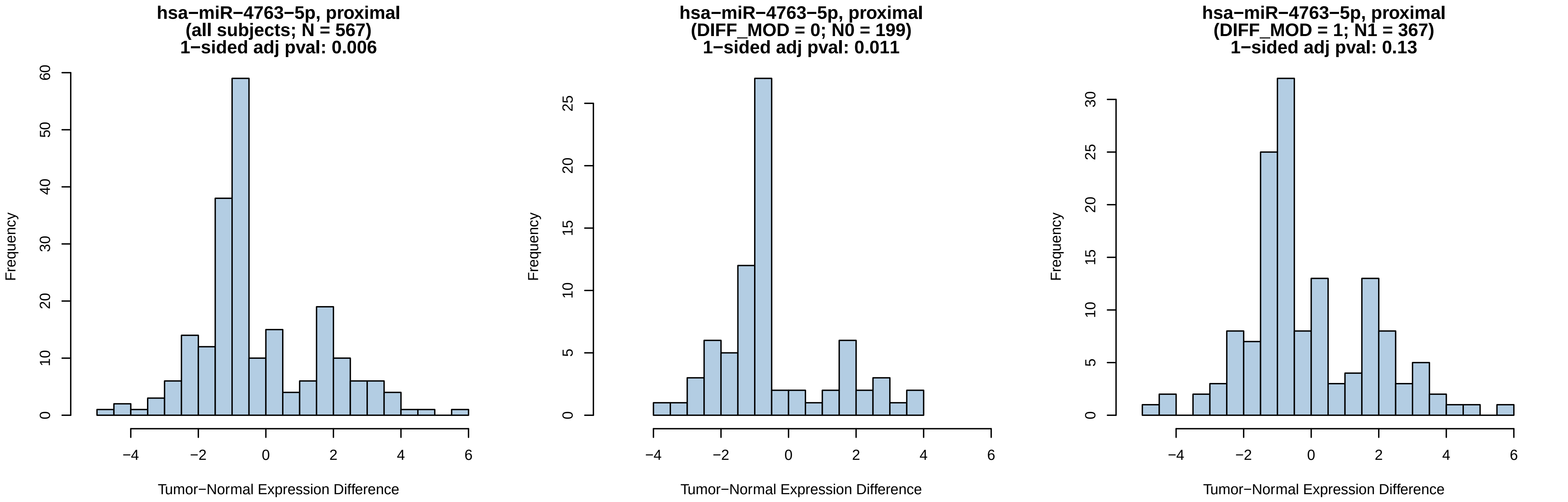
hsa−miR−4763−5p, proximal
(all subjects; N = 567)
1−sided adj pval: 0.006
Frequency
Tumor−Normal Expression Difference
hsa−miR−4763−5p, proximal
(DIFF_MOD = 0; N0 = 199)
1−sided adj pval: 0.011
Frequency
Tumor−Normal Expression Difference
hsa−miR−4763−5p, proximal
(DIFF_MOD = 1; N1 = 367)
1−sided adj pval: 0.13
Frequency
Tumor−Normal Expression Difference

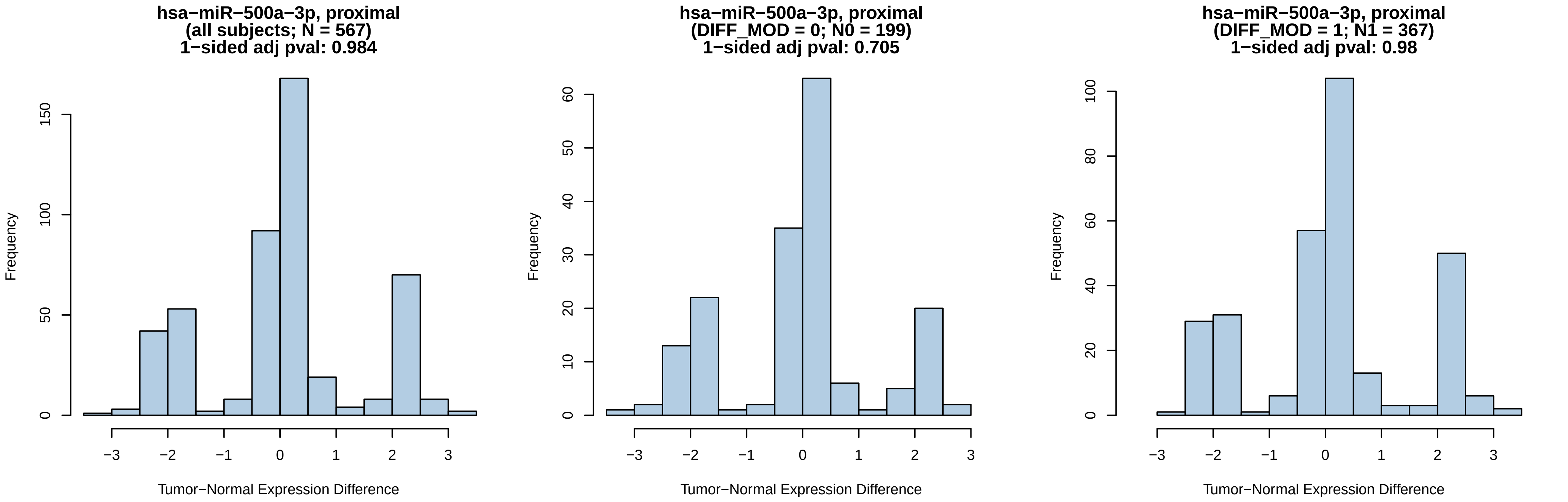
hsa−miR−500a−3p, proximal
(all subjects; N = 567)
1−sided adj pval: 0.984
hsa−miR−500a−3p, proximal
(DIFF_MOD = 0; N0 = 199)
1−sided adj pval: 0.705
hsa−miR−500a−3p, proximal
(DIFF_MOD = 1; N1 = 367)
1−sided adj pval: 0.98
Frequency
Tumor−Normal Expression Difference

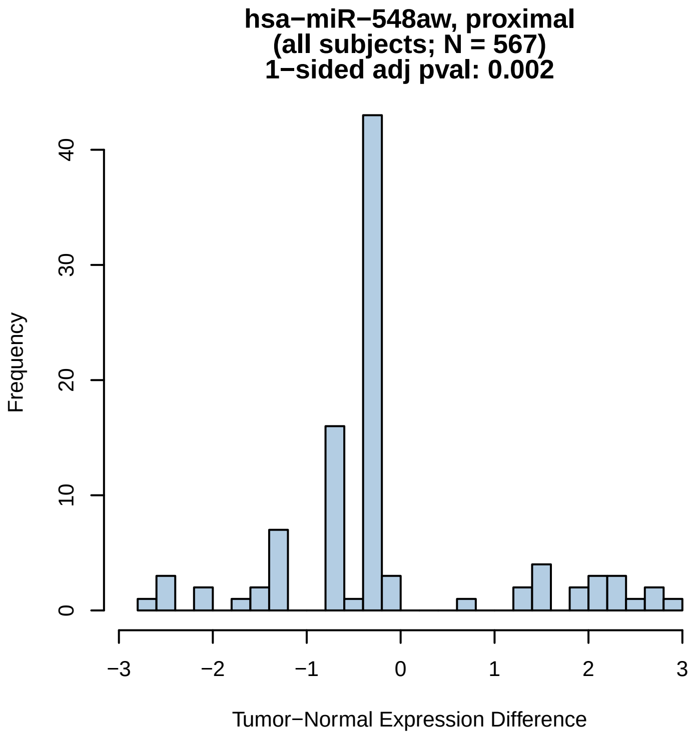

hsa−miR−548aw, proximal
(all subjects; N = 567)
1−sided adj pval: 0.002
Frequency
Tumor−Normal Expression Difference


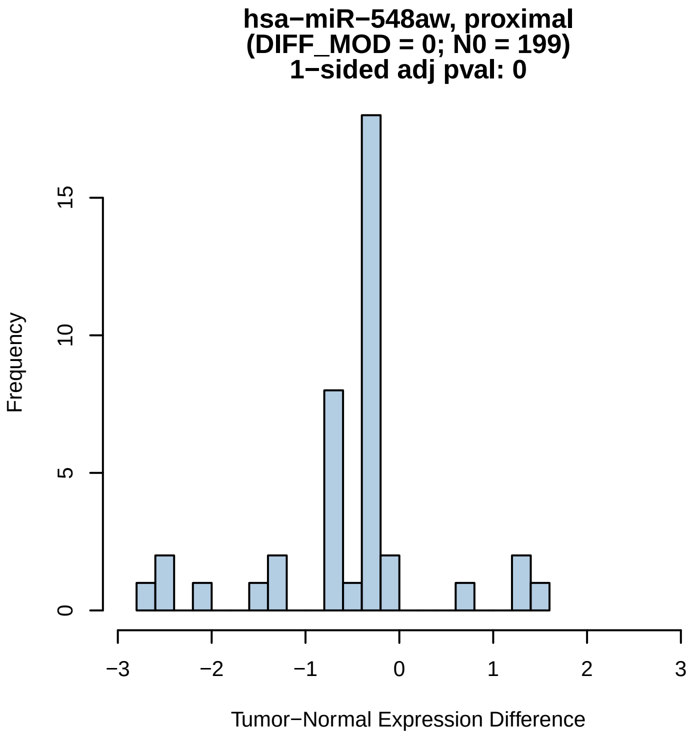

hsa−miR−548aw, proximal
(DIFF_MOD = 0; N0 = 199)
1−sided adj pval: 0
Frequency
Tumor−Normal Expression Difference


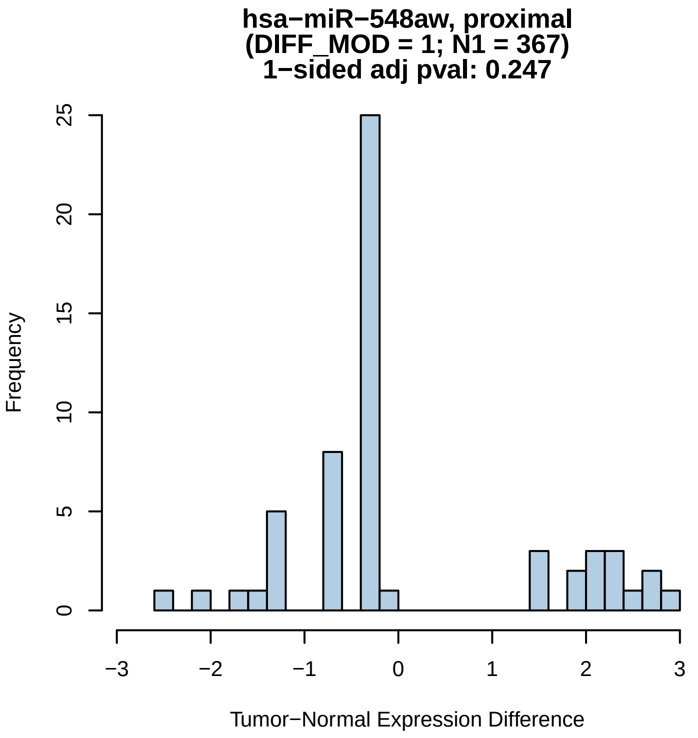

hsa−miR−548aw, proximal
(DIFF_MOD = 1; N1 = 367)
1−sided adj pval: 0.247
Frequency
Tumor−Normal Expression Difference

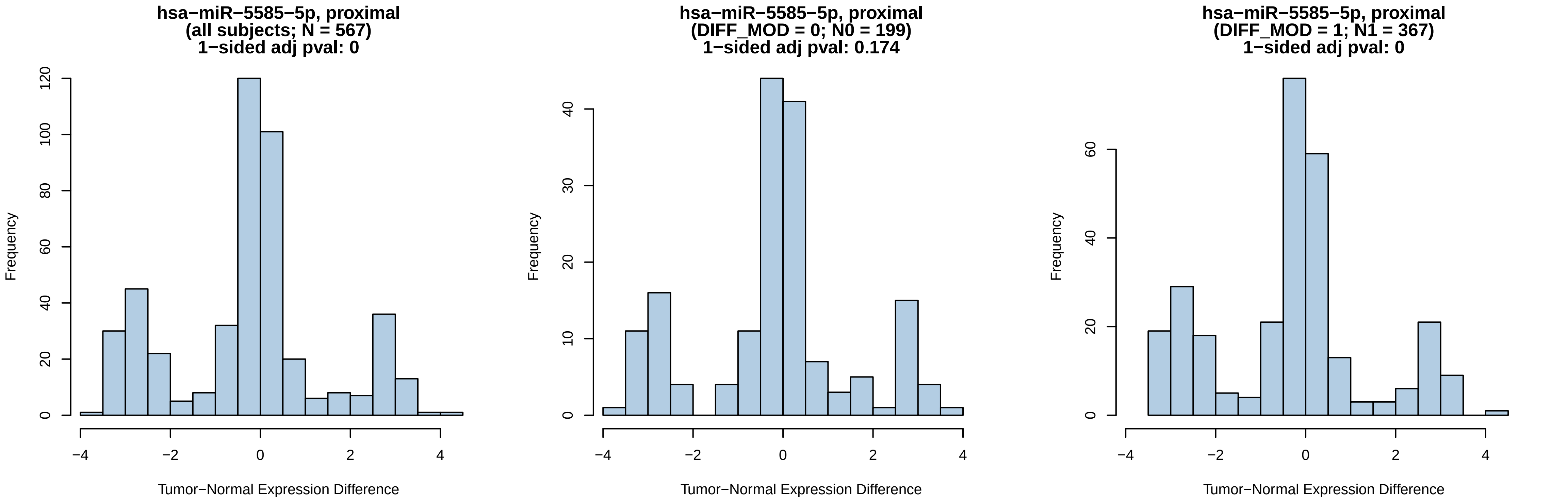
hsa−miR−5585−5p, proximal
(all subjects; N = 567)
1−sided adj pval: 0
Frequency
0
20
40
60
80
100
120
−4
−2
0
2
4
Tumor−Normal Expression Difference
hsa−miR−5585−5p, proximal
(DIFF_MOD = 0; N0 = 199)
1−sided adj pval: 0.174
Frequency
0
10
20
30
40
−4
−2
0
2
4
Tumor−Normal Expression Difference
hsa−miR−5585−5p, proximal
(DIFF_MOD = 1; N1 = 367)
1−sided adj pval: 0
Frequency
0
20
40
60
−4
−2
0
2
4
Tumor−Normal Expression Difference

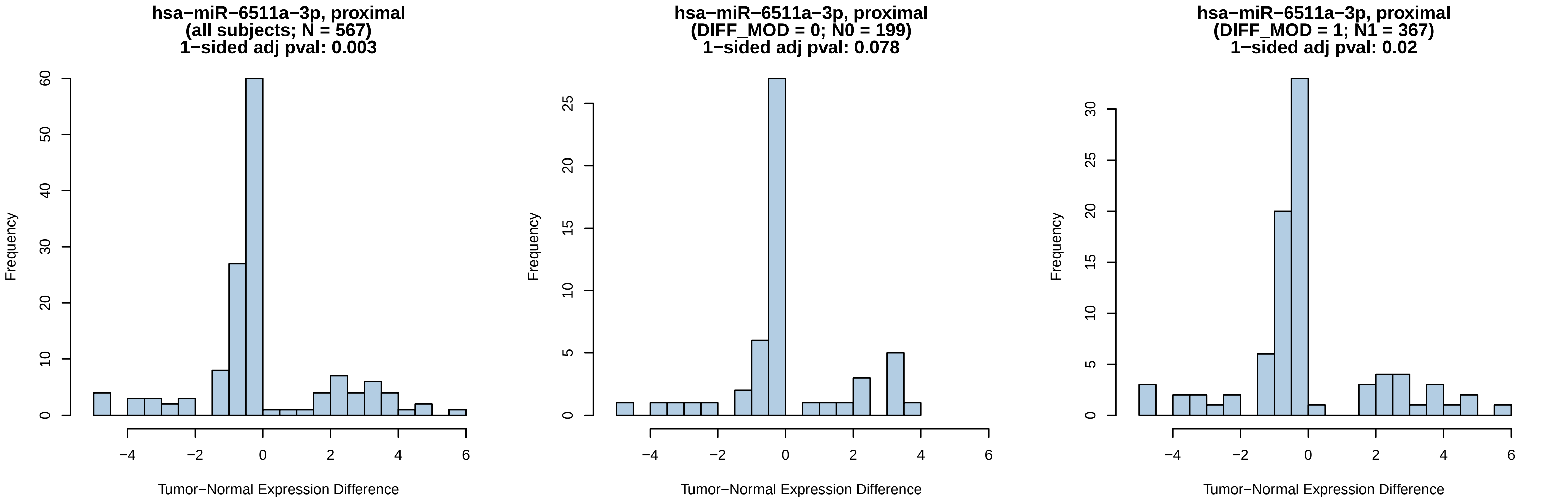
hsa−miR−6511a−3p, proximal
(all subjects; N = 567)
1−sided adj pval: 0.003
Frequency
Tumor−Normal Expression Difference
hsa−miR−6511a−3p, proximal
(DIFF_MOD = 0; N0 = 199)
1−sided adj pval: 0.078
Frequency
Tumor−Normal Expression Difference
hsa−miR−6511a−3p, proximal
(DIFF_MOD = 1; N1 = 367)
1−sided adj pval: 0.02
Frequency
Tumor−Normal Expression Difference

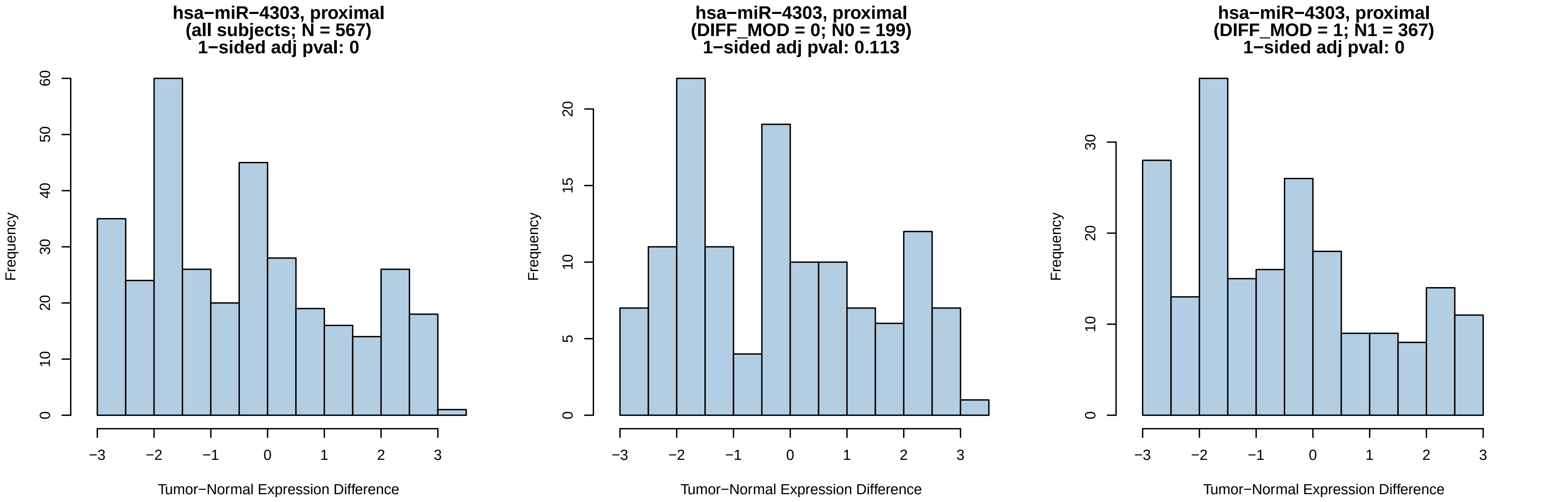

hsa−miR−4303, proximal
(all subjects; N = 567)
1−sided adj pval: 0
hsa−miR−4303, proximal
(DIFF_MOD = 0; N0 = 199)
1−sided adj pval: 0.113
hsa−miR−4303, proximal
(DIFF_MOD = 1; N1 = 367)
1−sided adj pval: 0
Frequency
Tumor−Normal Expression Difference

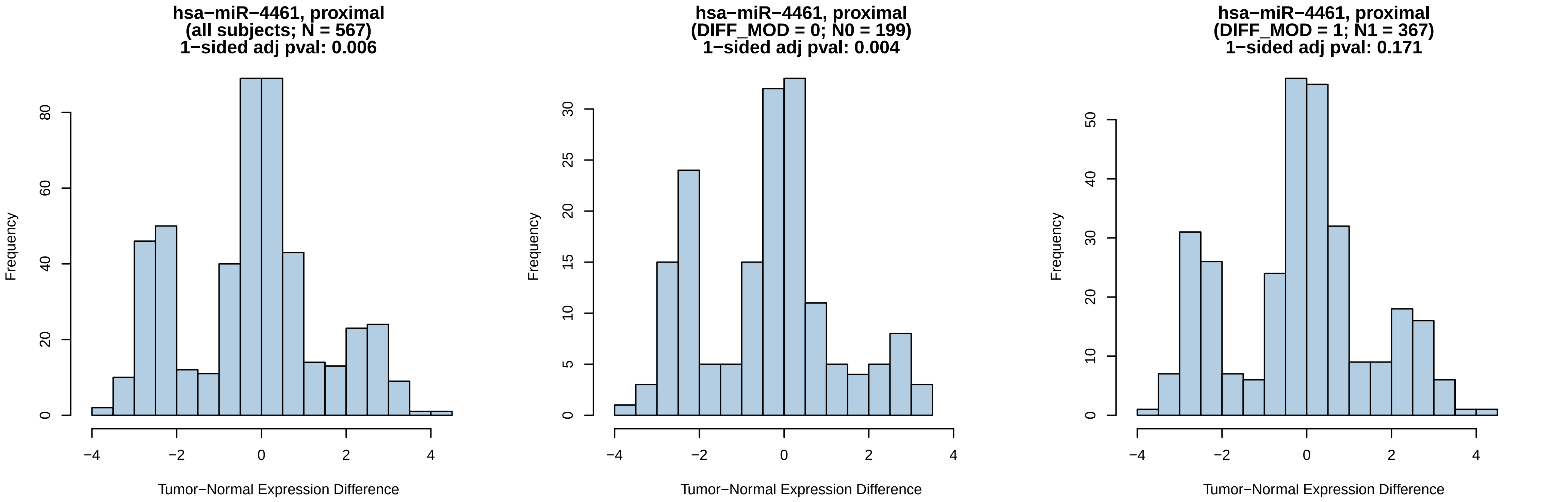

hsa−miR−4461, proximal
(all subjects; N = 567)
1−sided adj pval: 0.006
Frequency
Tumor−Normal Expression Difference
hsa−miR−4461, proximal
(DIFF_MOD = 0; N0 = 199)
1−sided adj pval: 0.004
Frequency
Tumor−Normal Expression Difference
hsa−miR−4461, proximal
(DIFF_MOD = 1; N1 = 367)
1−sided adj pval: 0.171
Frequency
Tumor−Normal Expression Difference

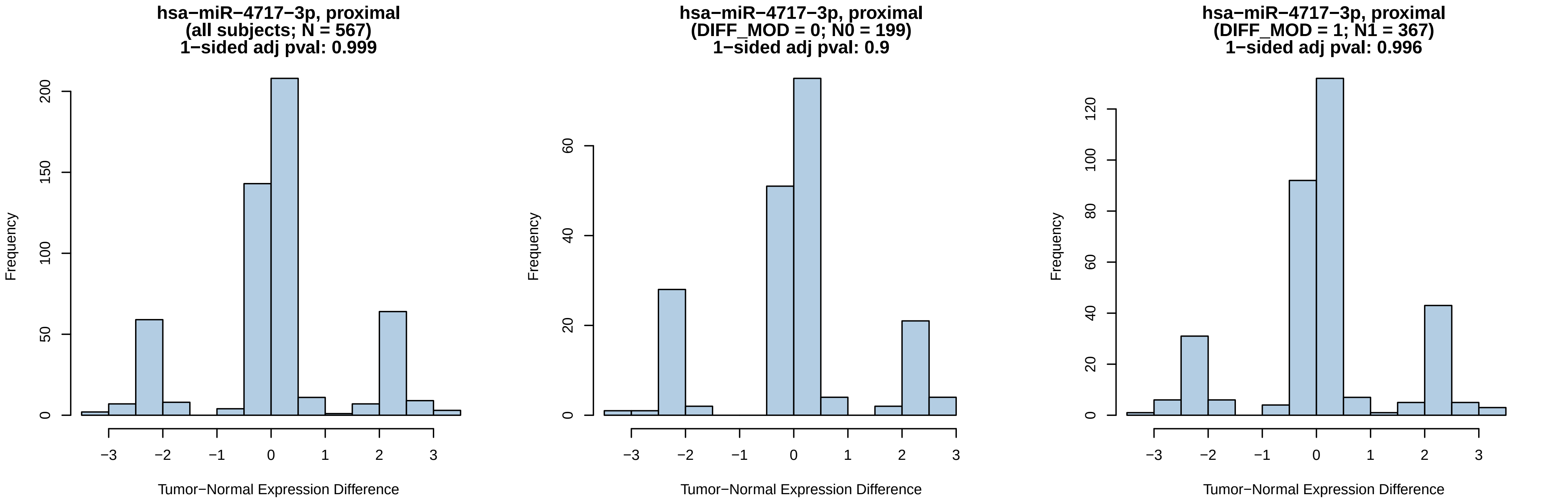
hsa−miR−4717−3p, proximal
(all subjects; N = 567)
1−sided adj pval: 0.999
Frequency
Tumor−Normal Expression Difference
hsa−miR−4717−3p, proximal
(DIFF_MOD = 0; N0 = 199)
1−sided adj pval: 0.9
Frequency
Tumor−Normal Expression Difference
hsa−miR−4717−3p, proximal
(DIFF_MOD = 1; N1 = 367)
1−sided adj pval: 0.996
Frequency
Tumor−Normal Expression Difference

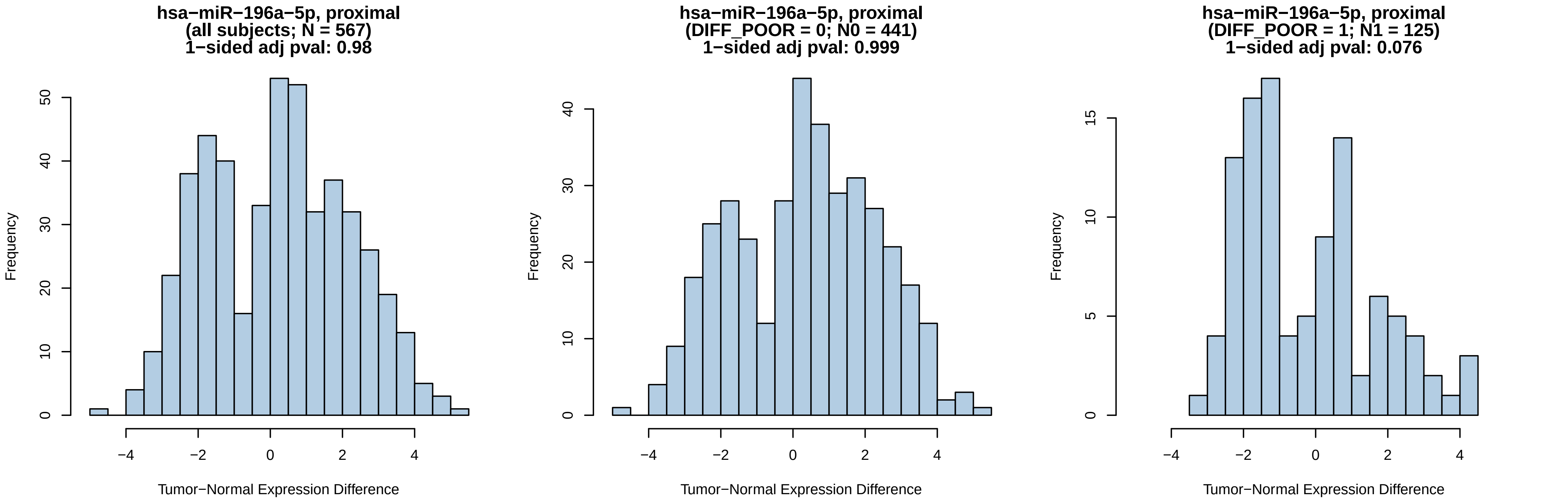

hsa−miR−196a−5p, proximal
(all subjects; N = 567)
1−sided adj pval: 0.98
Frequency
Tumor−Normal Expression Difference
hsa−miR−196a−5p, proximal
(DIFF_POOR = 0; N0 = 441)
1−sided adj pval: 0.999
Frequency
Tumor−Normal Expression Difference
hsa−miR−196a−5p, proximal
(DIFF_POOR = 1; N1 = 125)
1−sided adj pval: 0.076
Frequency
Tumor−Normal Expression Difference

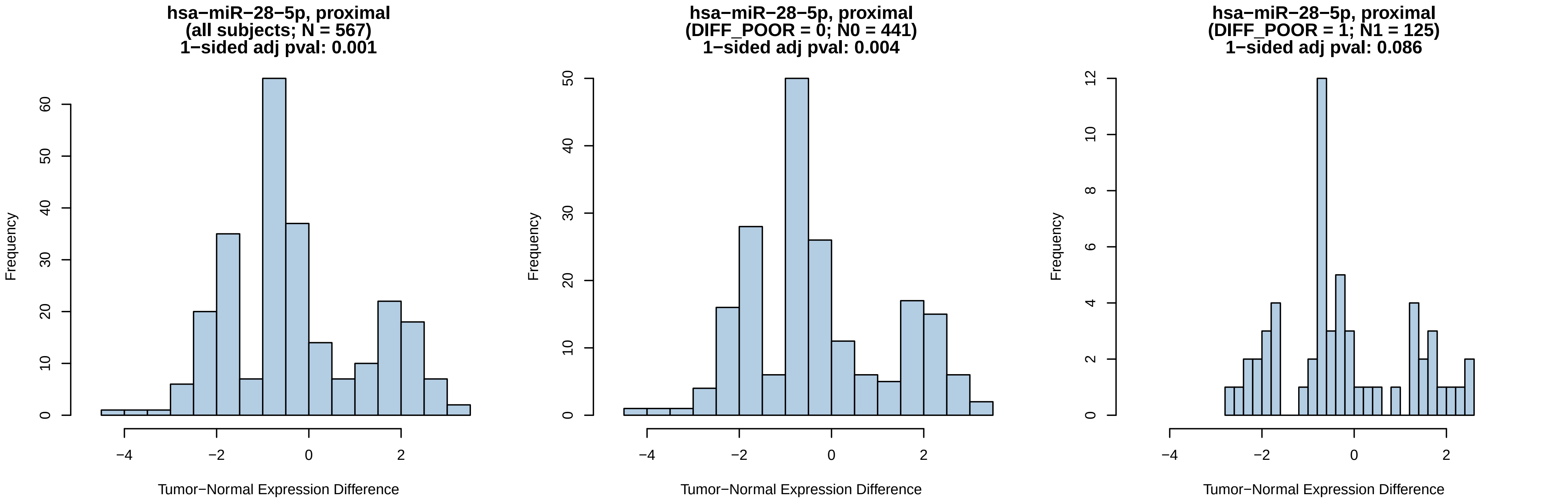
hsa−miR−28−5p, proximal
(all subjects; N = 567)
1−sided adj pval: 0.001
Frequency
Tumor−Normal Expression Difference
hsa−miR−28−5p, proximal
(DIFF_POOR = 0; N0 = 441)
1−sided adj pval: 0.004
Frequency
Tumor−Normal Expression Difference
hsa−miR−28−5p, proximal
(DIFF_POOR = 1; N1 = 125)
1−sided adj pval: 0.086
Frequency
Tumor−Normal Expression Difference

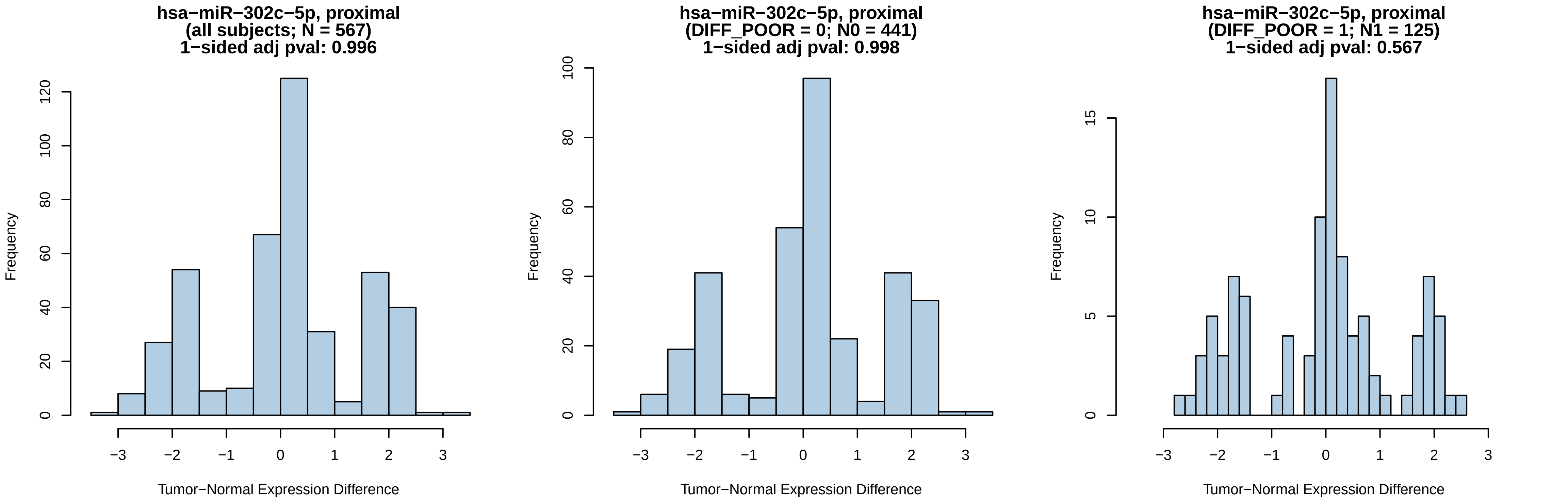
hsa−miR−302c−5p, proximal
(all subjects; N = 567)
1−sided adj pval: 0.996
hsa−miR−302c−5p, proximal
(DIFF_POOR = 0; N0 = 441)
1−sided adj pval: 0.998
hsa−miR−302c−5p, proximal
(DIFF_POOR = 1; N1 = 125)
1−sided adj pval: 0.567
Frequency
Tumor−Normal Expression Difference

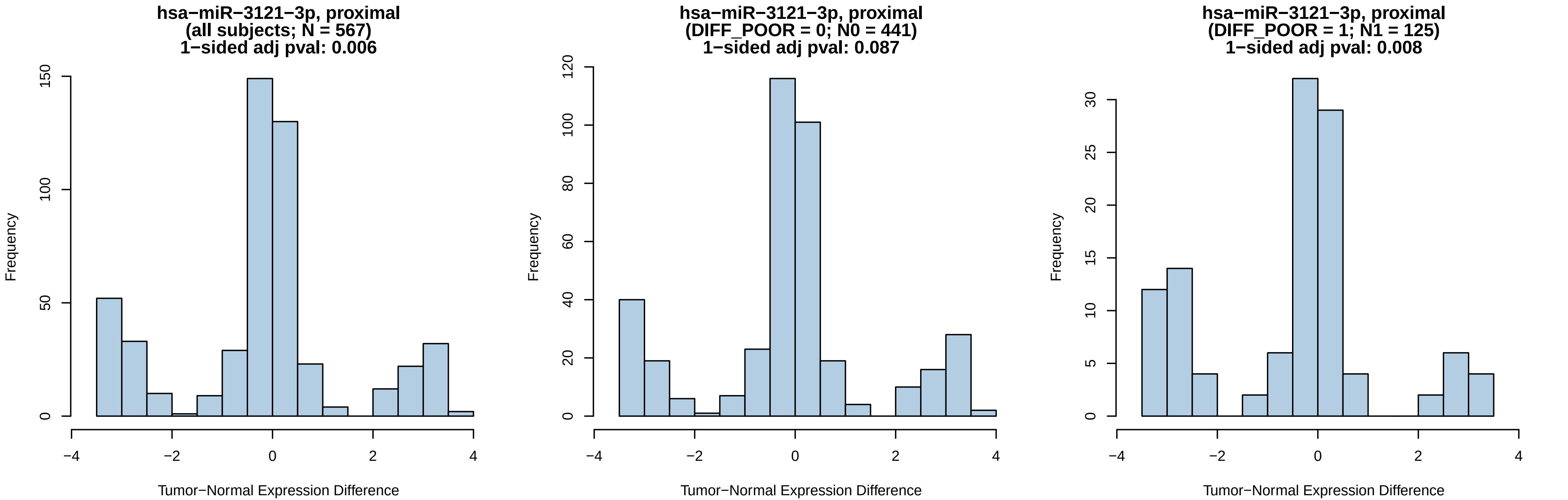
hsa−miR−3121−3p, proximal
(all subjects; N = 567)
1−sided adj pval: 0.006
Frequency
Tumor−Normal Expression Difference
hsa−miR−3121−3p, proximal
(DIFF_POOR = 0; N0 = 441)
1−sided adj pval: 0.087
Frequency
Tumor−Normal Expression Difference
hsa−miR−3121−3p, proximal
(DIFF_POOR = 1; N1 = 125)
1−sided adj pval: 0.008
Frequency
Tumor−Normal Expression Difference

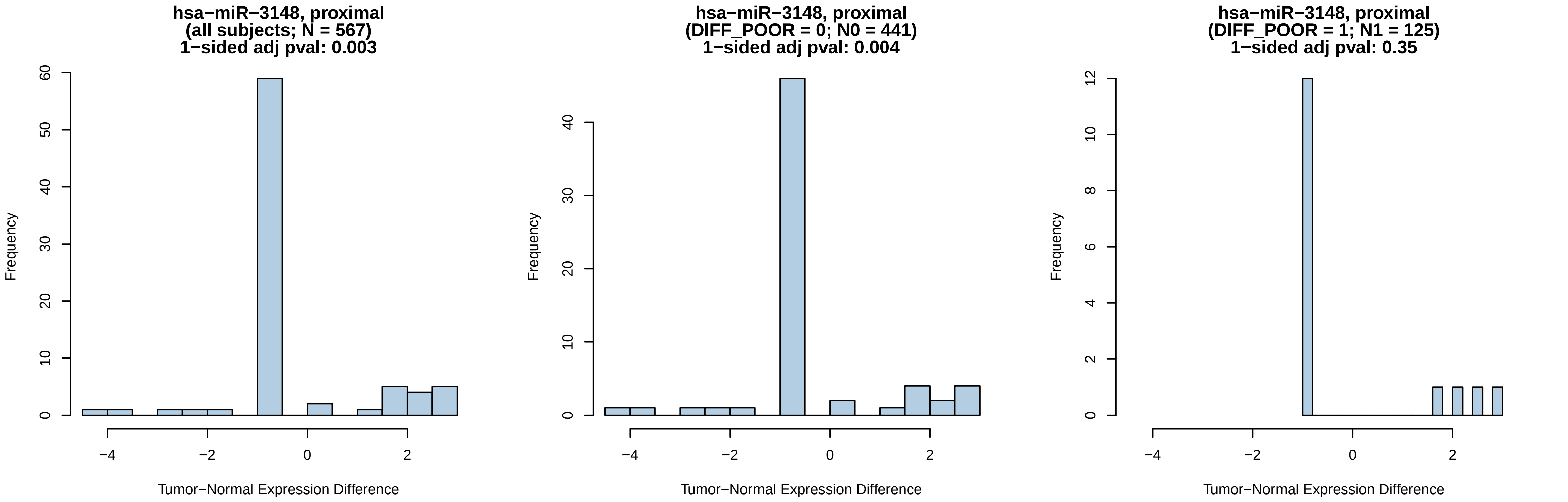
hsa−miR−3148, proximal
(all subjects; N = 567)
1−sided adj pval: 0.003
hsa−miR−3148, proximal
(DIFF_POOR = 0; N0 = 441)
1−sided adj pval: 0.004
hsa−miR−3148, proximal
(DIFF_POOR = 1; N1 = 125)
1−sided adj pval: 0.35
Frequency
Tumor−Normal Expression Difference

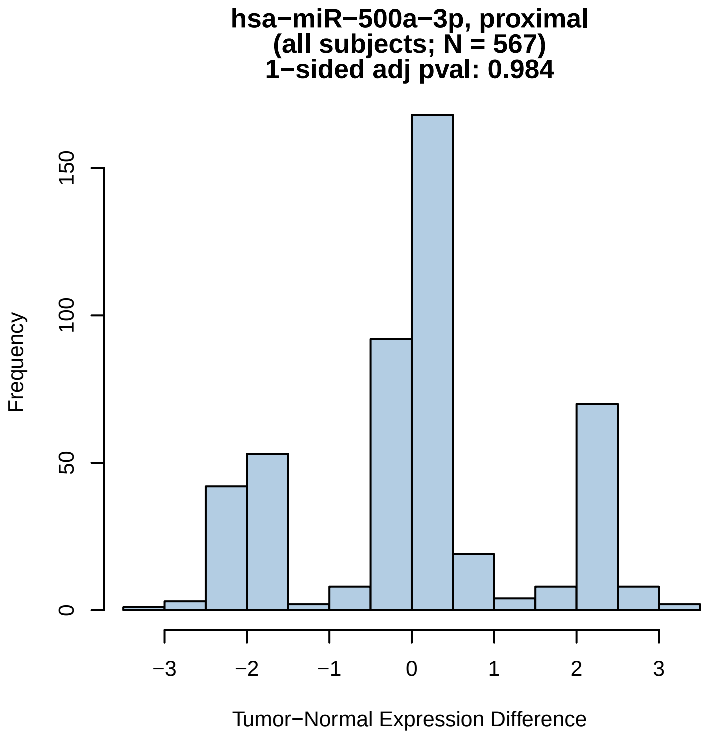
hsa−miR−500a−3p, proximal
(all subjects; N = 567)
1−sided adj pval: 0.984
Frequency
0
50
100
150
−3
−2
−1
0
1
2
3
Tumor−Normal Expression Difference
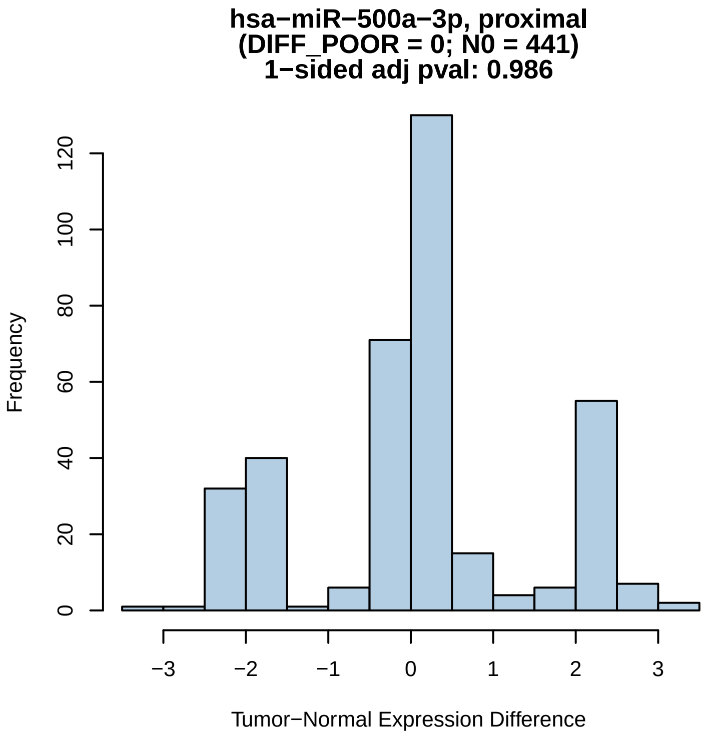
hsa−miR−500a−3p, proximal
(DIFF_POOR = 0; N0 = 441)
1−sided adj pval: 0.986
Frequency
0
20
40
60
80
100
120
−3
−2
−1
0
1
2
3
Tumor−Normal Expression Difference
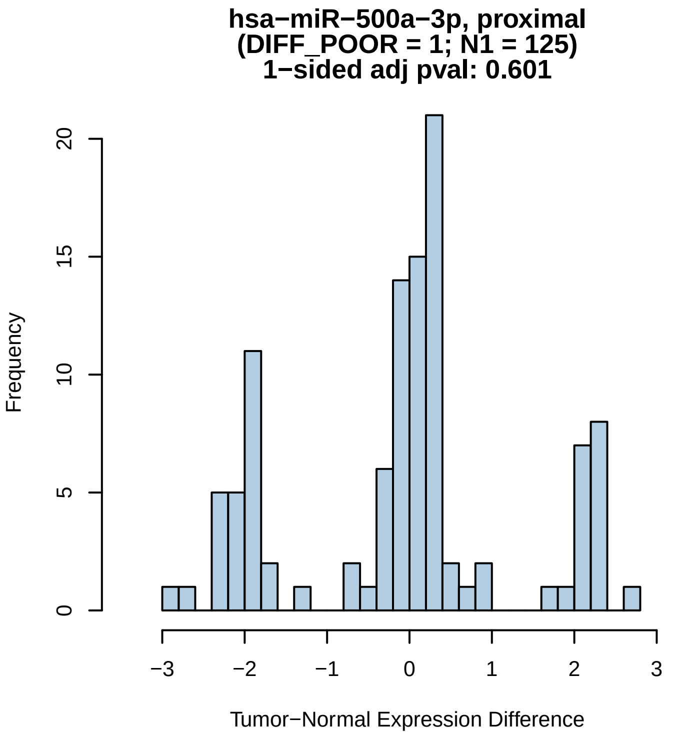
hsa−miR−500a−3p, proximal
(DIFF_POOR = 1; N1 = 125)
1−sided adj pval: 0.601
Frequency
0
5
10
15
20
−3
−2
−1
0
1
2
3
Tumor−Normal Expression Difference

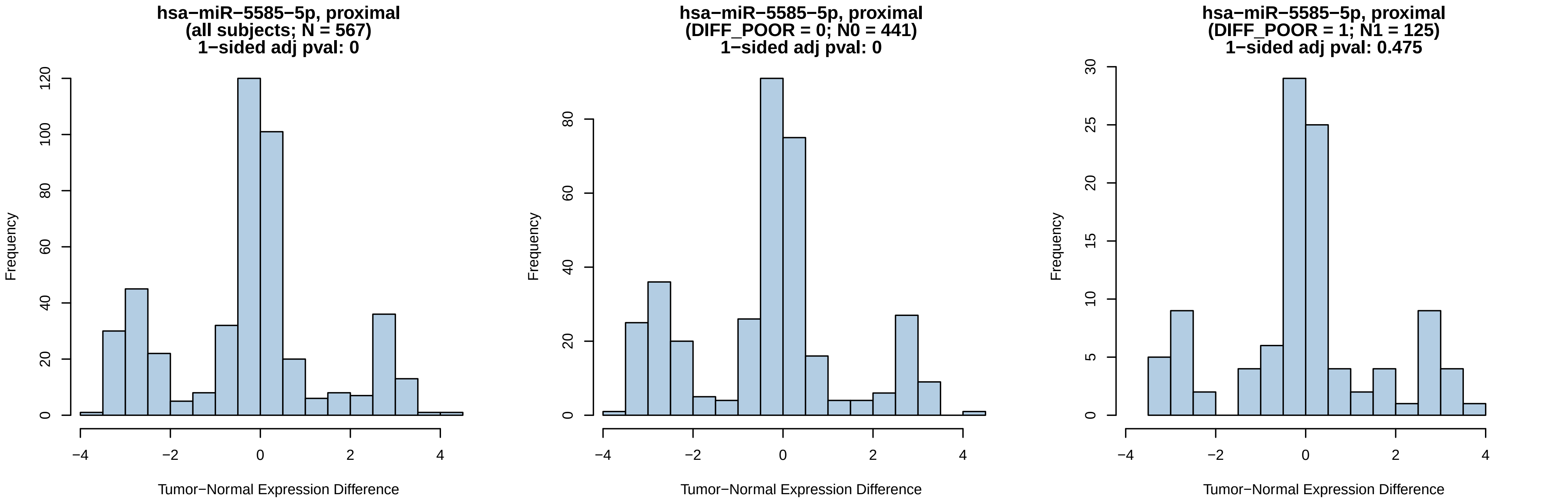
hsa−miR−5585−5p, proximal
(all subjects; N = 567)
1−sided adj pval: 0
Frequency
Tumor−Normal Expression Difference
hsa−miR−5585−5p, proximal
(DIFF_POOR = 0; N0 = 441)
1−sided adj pval: 0
Frequency
Tumor−Normal Expression Difference
hsa−miR−5585−5p, proximal
(DIFF_POOR = 1; N1 = 125)
1−sided adj pval: 0.475
Frequency
Tumor−Normal Expression Difference

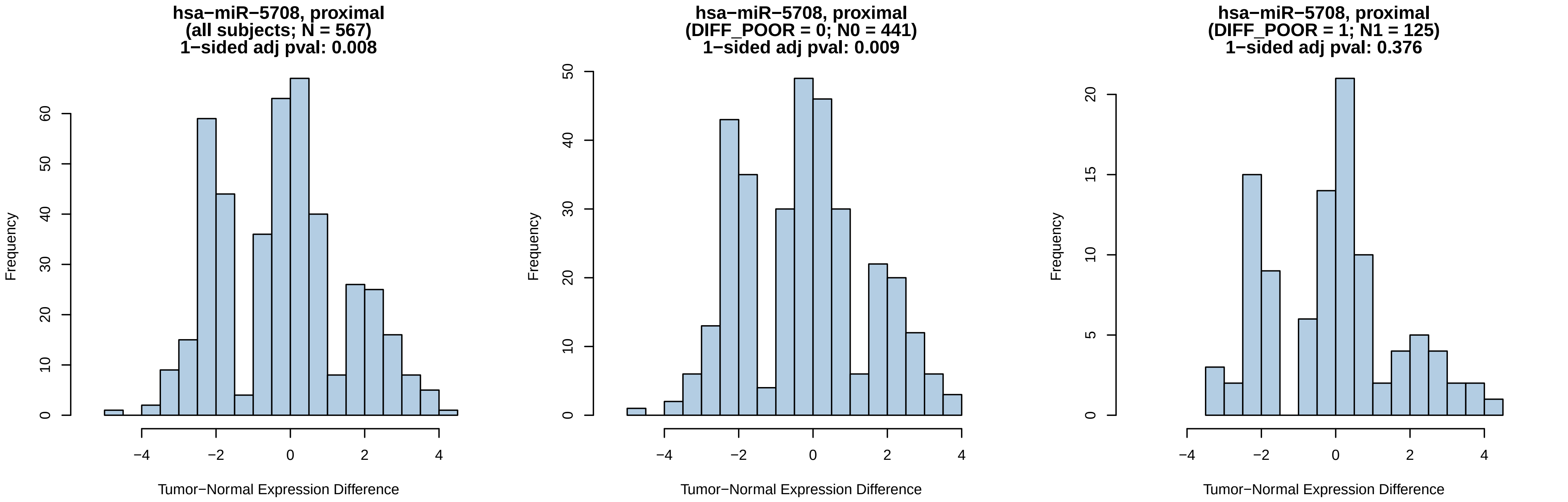

hsa−miR−5708, proximal
(all subjects; N = 567)
1−sided adj pval: 0.008
hsa−miR−5708, proximal
(DIFF_POOR = 0; N0 = 441)
1−sided adj pval: 0.009
hsa−miR−5708, proximal
(DIFF_POOR = 1; N1 = 125)
1−sided adj pval: 0.376
Frequency
Tumor−Normal Expression Difference

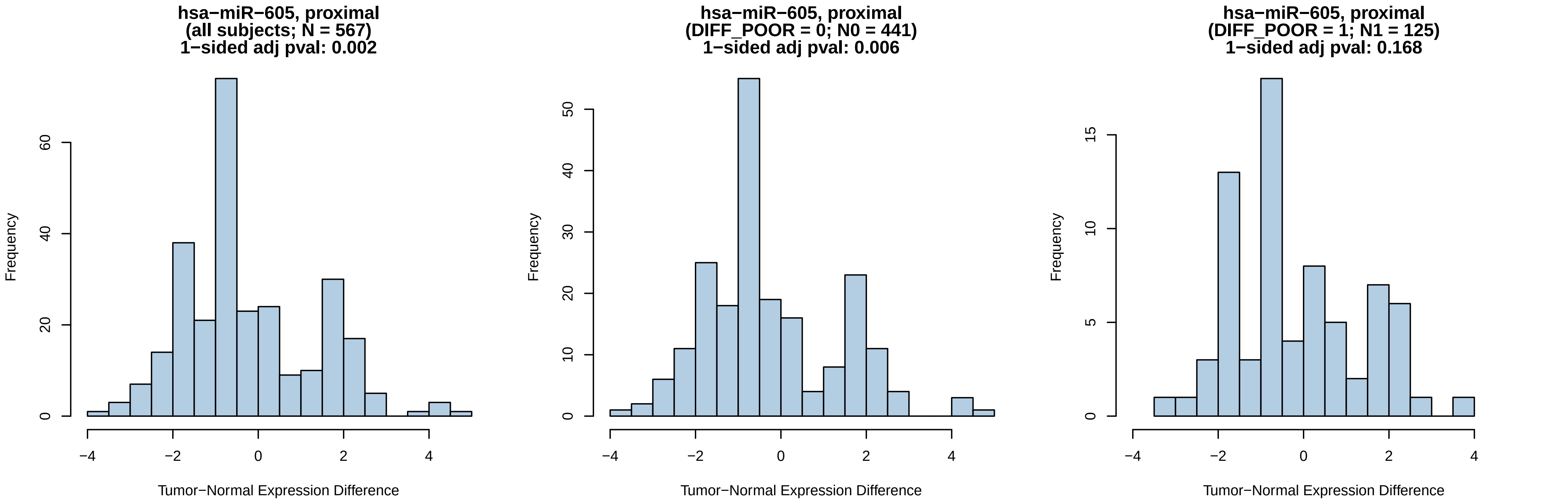

hsa−miR−605, proximal
(all subjects; N = 567)
1−sided adj pval: 0.002
Frequency
Tumor−Normal Expression Difference
hsa−miR−605, proximal
(DIFF_POOR = 0; N0 = 441)
1−sided adj pval: 0.006
Frequency
Tumor−Normal Expression Difference
hsa−miR−605, proximal
(DIFF_POOR = 1; N1 = 125)
1−sided adj pval: 0.168
Frequency
Tumor−Normal Expression Difference

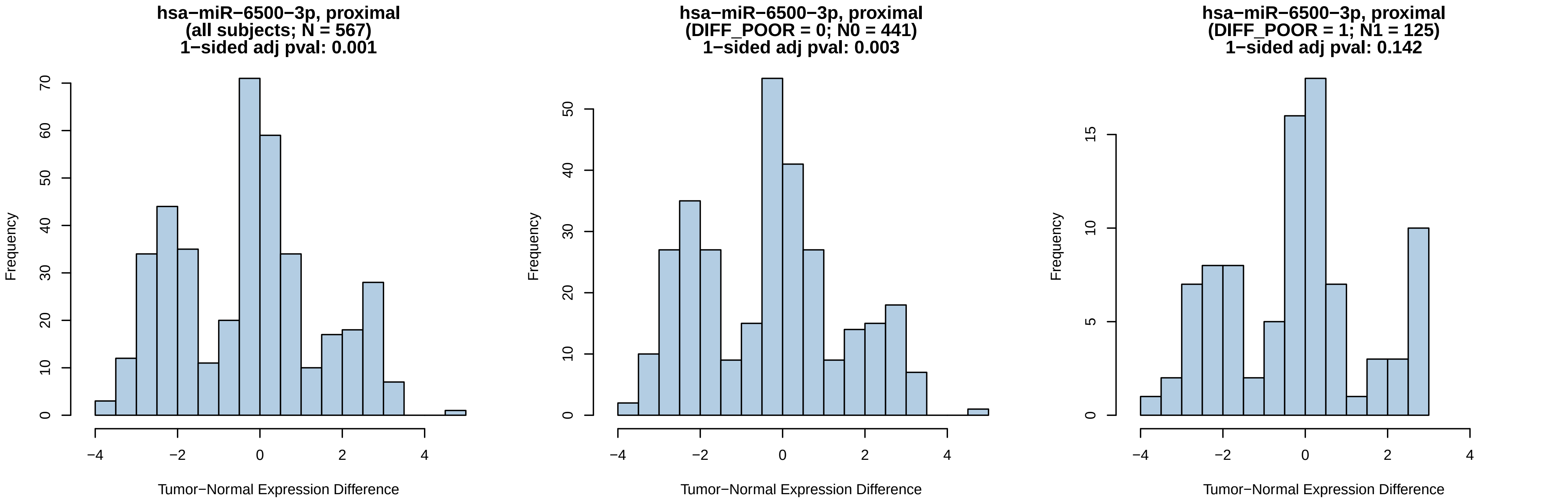
hsa−miR−6500−3p, proximal
(all subjects; N = 567)
1−sided adj pval: 0.001
Frequency
Tumor−Normal Expression Difference
hsa−miR−6500−3p, proximal
(DIFF_POOR = 0; N0 = 441)
1−sided adj pval: 0.003
Frequency
Tumor−Normal Expression Difference
hsa−miR−6500−3p, proximal
(DIFF_POOR = 1; N1 = 125)
1−sided adj pval: 0.142
Frequency
Tumor−Normal Expression Difference

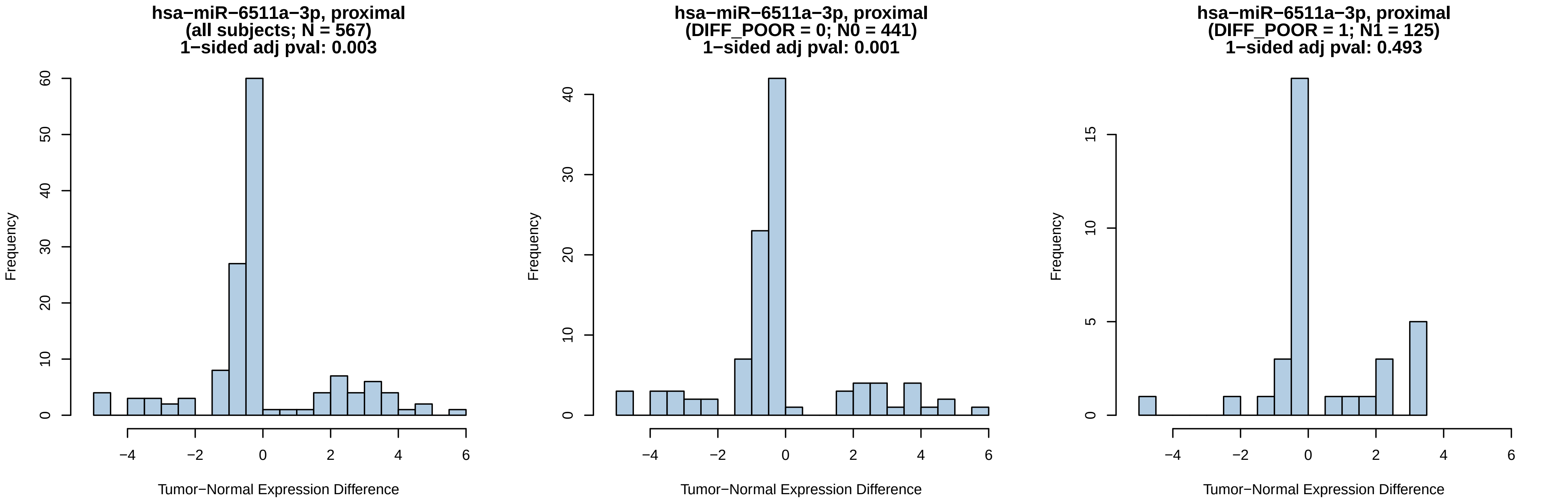

hsa−miR−6511a−3p, proximal
(all subjects; N = 567)
1−sided adj pval: 0.003
Frequency
Tumor−Normal Expression Difference
hsa−miR−6511a−3p, proximal
(DIFF_POOR = 0; N0 = 441)
1−sided adj pval: 0.001
Frequency
Tumor−Normal Expression Difference
hsa−miR−6511a−3p, proximal
(DIFF_POOR = 1; N1 = 125)
1−sided adj pval: 0.493
Frequency
Tumor−Normal Expression Difference

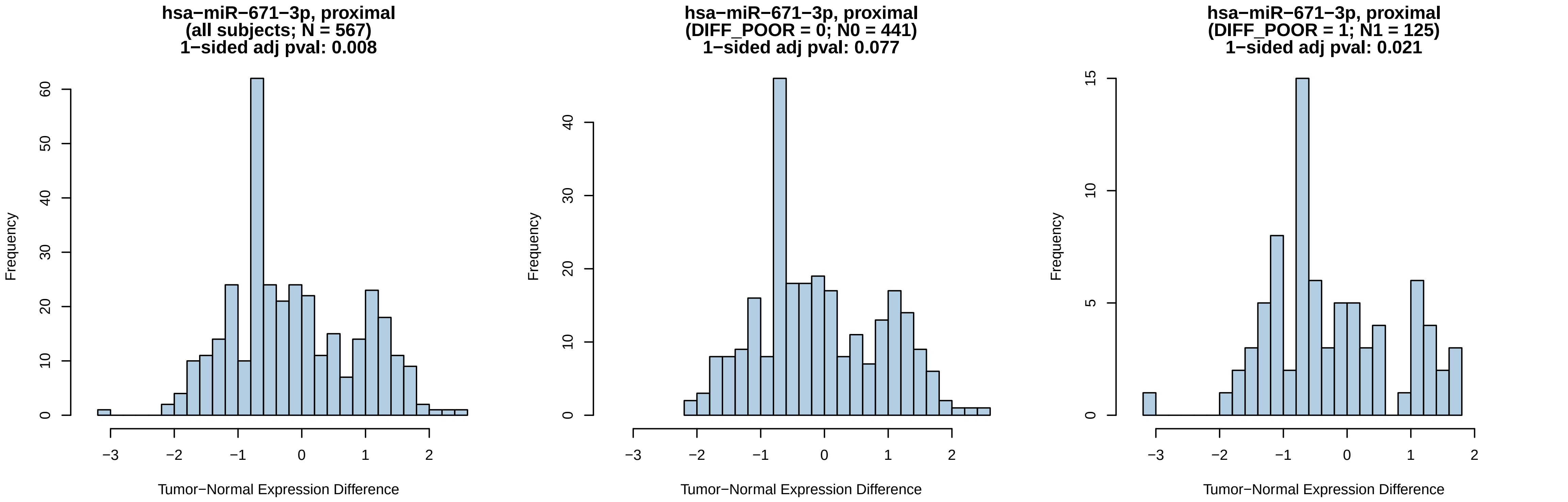
hsa−miR−671−3p, proximal
(all subjects; N = 567)
1−sided adj pval: 0.008
hsa−miR−671−3p, proximal
(DIFF_POOR = 0; N0 = 441)
1−sided adj pval: 0.077
hsa−miR−671−3p, proximal
(DIFF_POOR = 1; N1 = 125)
1−sided adj pval: 0.021
Frequency
Tumor−Normal Expression Difference

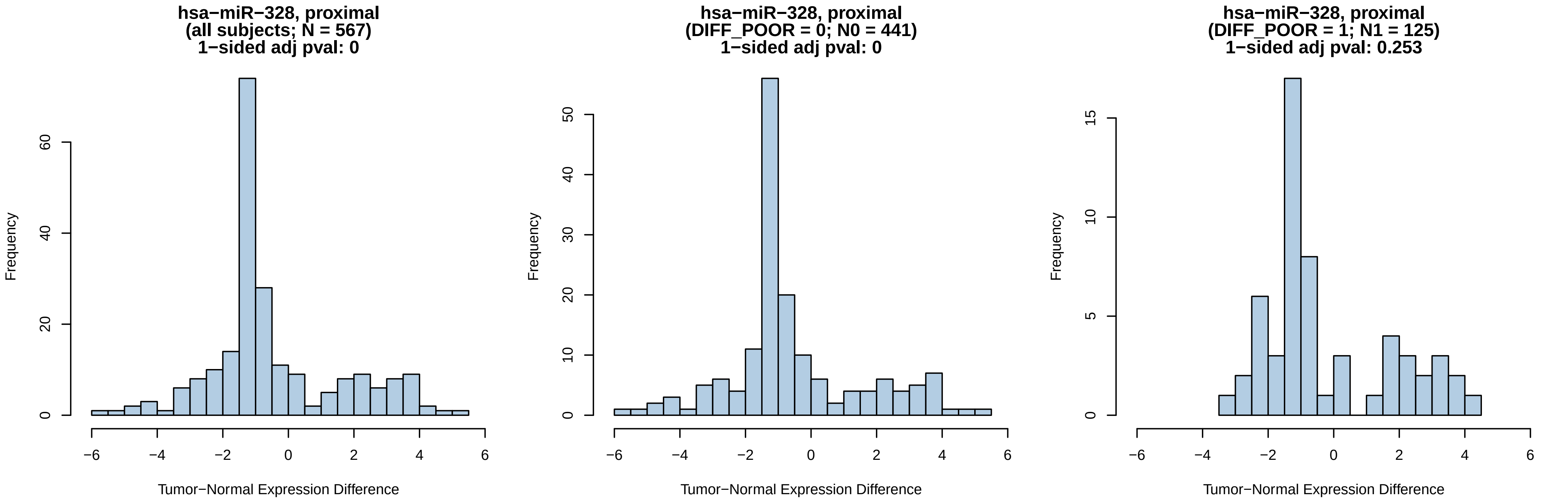
hsa−miR−328, proximal
(all subjects; N = 567)
1−sided adj pval: 0
Frequency
Tumor−Normal Expression Difference
hsa−miR−328, proximal
(DIFF_POOR = 0; N0 = 441)
1−sided adj pval: 0
Frequency
Tumor−Normal Expression Difference
hsa−miR−328, proximal
(DIFF_POOR = 1; N1 = 125)
1−sided adj pval: 0.253
Frequency
Tumor−Normal Expression Difference

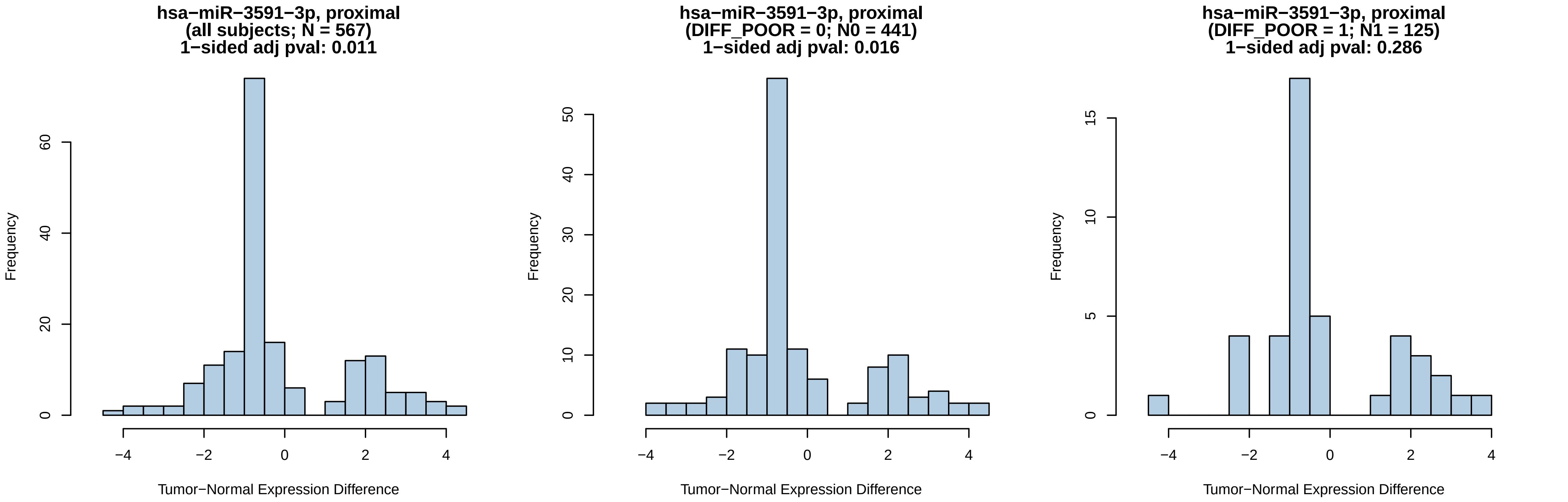
hsa−miR−3591−3p, proximal
(all subjects; N = 567)
1−sided adj pval: 0.011
hsa−miR−3591−3p, proximal
(DIFF_POOR = 0; N0 = 441)
1−sided adj pval: 0.016
hsa−miR−3591−3p, proximal
(DIFF_POOR = 1; N1 = 125)
1−sided adj pval: 0.286
Frequency
Tumor−Normal Expression Difference

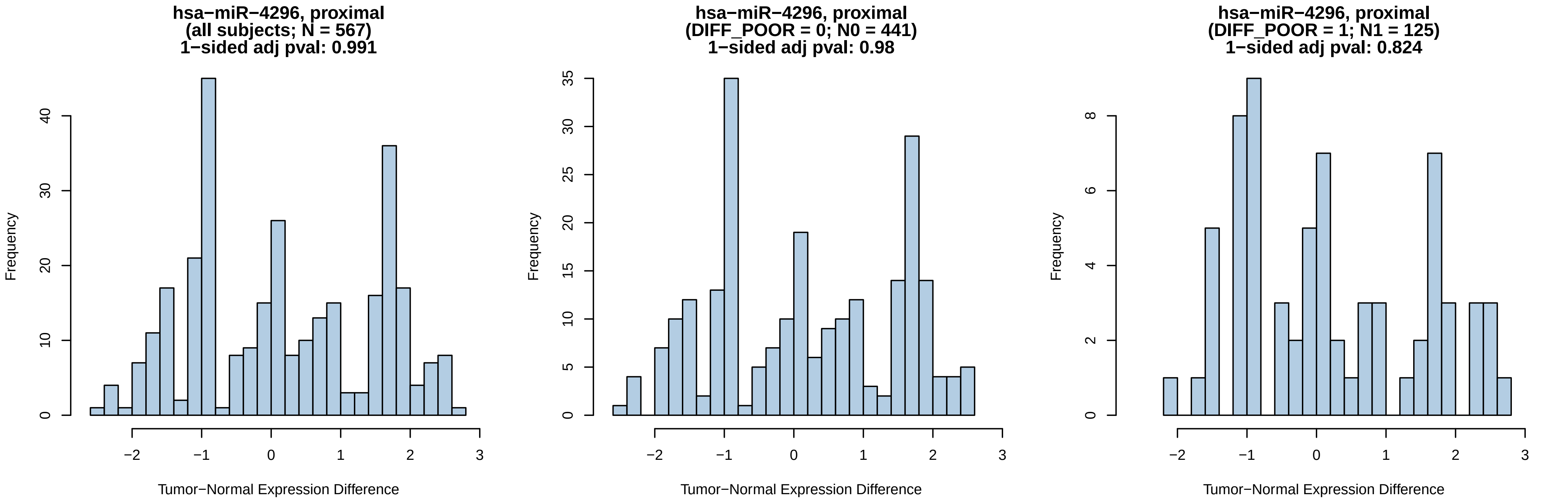
hsa−miR−4296, proximal
(all subjects; N = 567)
1−sided adj pval: 0.991
Frequency
Tumor−Normal Expression Difference
hsa−miR−4296, proximal
(DIFF_POOR = 0; N0 = 441)
1−sided adj pval: 0.98
Frequency
Tumor−Normal Expression Difference
hsa−miR−4296, proximal
(DIFF_POOR = 1; N1 = 125)
1−sided adj pval: 0.824
Frequency
Tumor−Normal Expression Difference

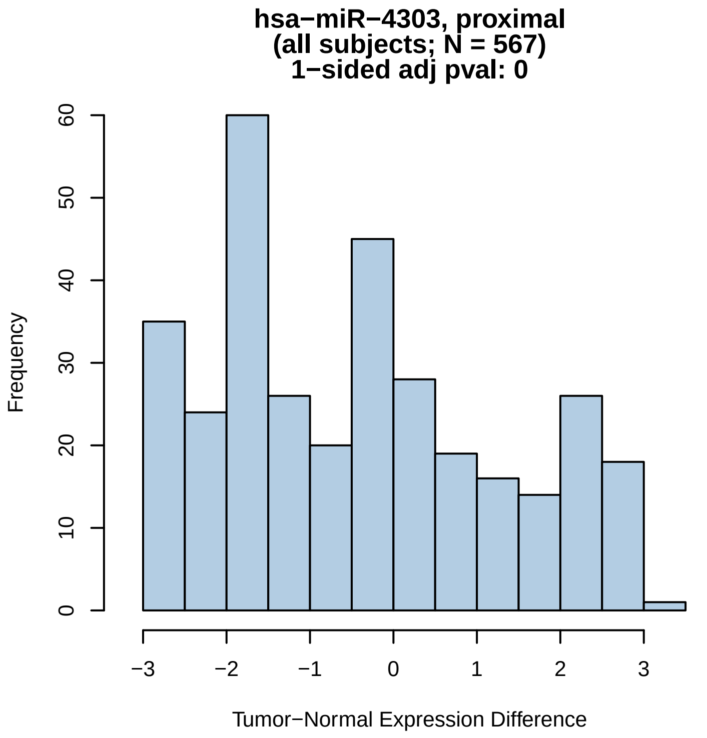
hsa−miR−4303, proximal
(all subjects; N = 567)
1−sided adj pval: 0
Frequency
0
10
20
30
40
50
60
−3
−2
−1
0
1
2
3
Tumor−Normal Expression Difference

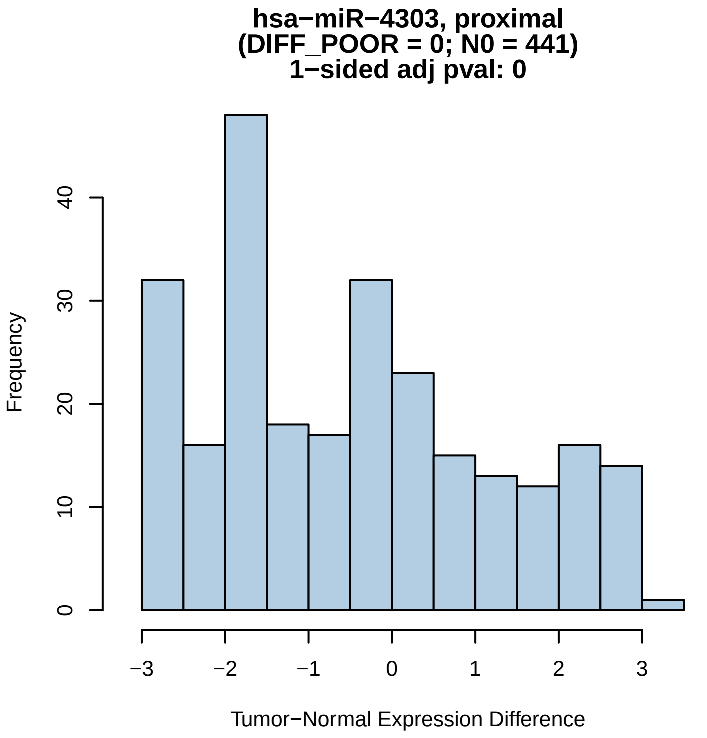
hsa−miR−4303, proximal
(DIFF_POOR = 0; N0 = 441)
1−sided adj pval: 0
Frequency
0
10
20
30
40
−3
−2
−1
0
1
2
3
Tumor−Normal Expression Difference

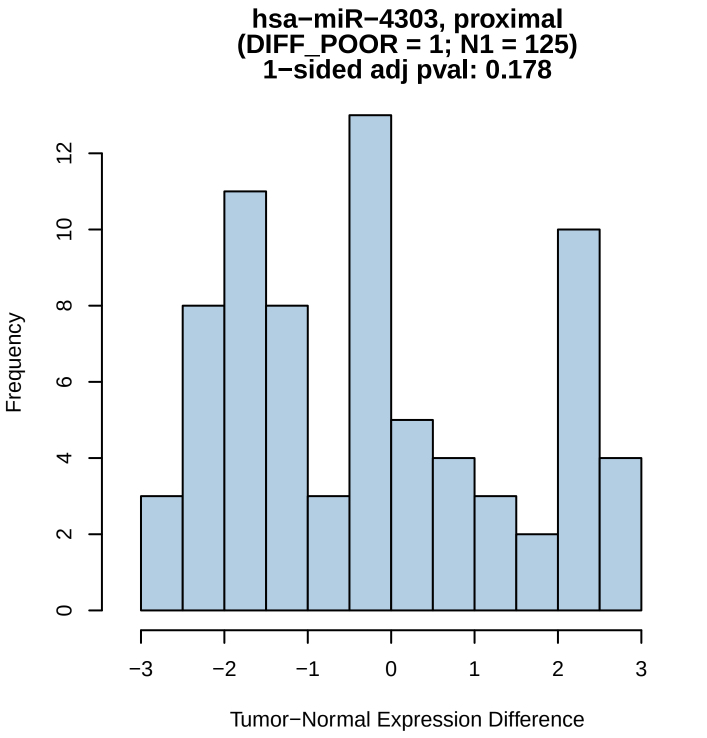
hsa−miR−4303, proximal
(DIFF_POOR = 1; N1 = 125)
1−sided adj pval: 0.178
Frequency
0
2
4
6
8
10
12
−3
−2
−1
0
1
2
3
Tumor−Normal Expression Difference

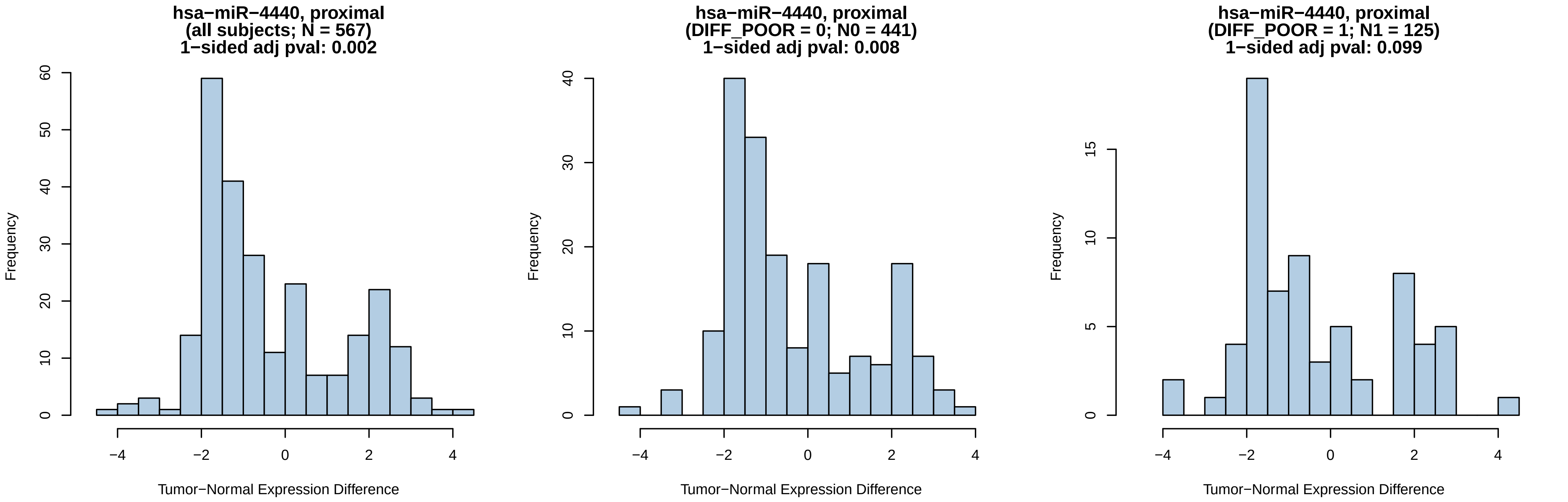
hsa−miR−4440, proximal
(all subjects; N = 567)
1−sided adj pval: 0.002
Frequency
0
10
20
30
40
50
60
−4
−2
0
2
4
Tumor−Normal Expression Difference
hsa−miR−4440, proximal
(DIFF_POOR = 0; N0 = 441)
1−sided adj pval: 0.008
Frequency
0
10
20
30
40
−4
−2
0
2
4
Tumor−Normal Expression Difference
hsa−miR−4440, proximal
(DIFF_POOR = 1; N1 = 125)
1−sided adj pval: 0.099
Frequency
0
5
10
15
−4
−2
0
2
4
Tumor−Normal Expression Difference

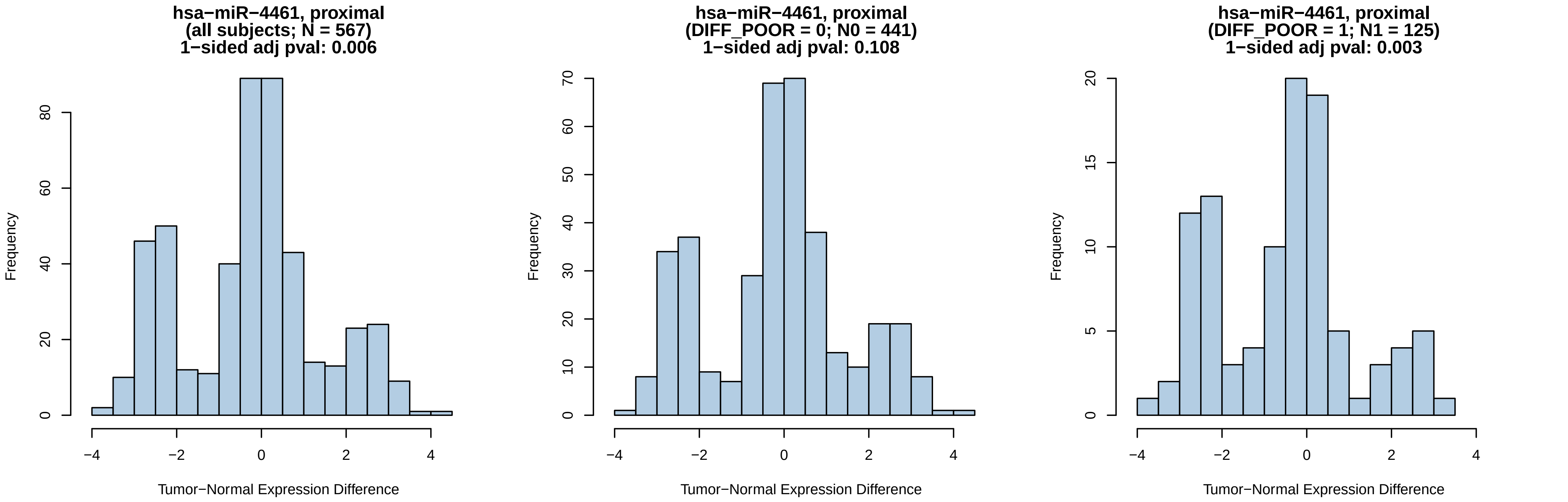
hsa−miR−4461, proximal
(all subjects; N = 567)
1−sided adj pval: 0.006
Frequency
Tumor−Normal Expression Difference
hsa−miR−4461, proximal
(DIFF_POOR = 0; N0 = 441)
1−sided adj pval: 0.108
Frequency
Tumor−Normal Expression Difference
hsa−miR−4461, proximal
(DIFF_POOR = 1; N1 = 125)
1−sided adj pval: 0.003
Frequency
Tumor−Normal Expression Difference

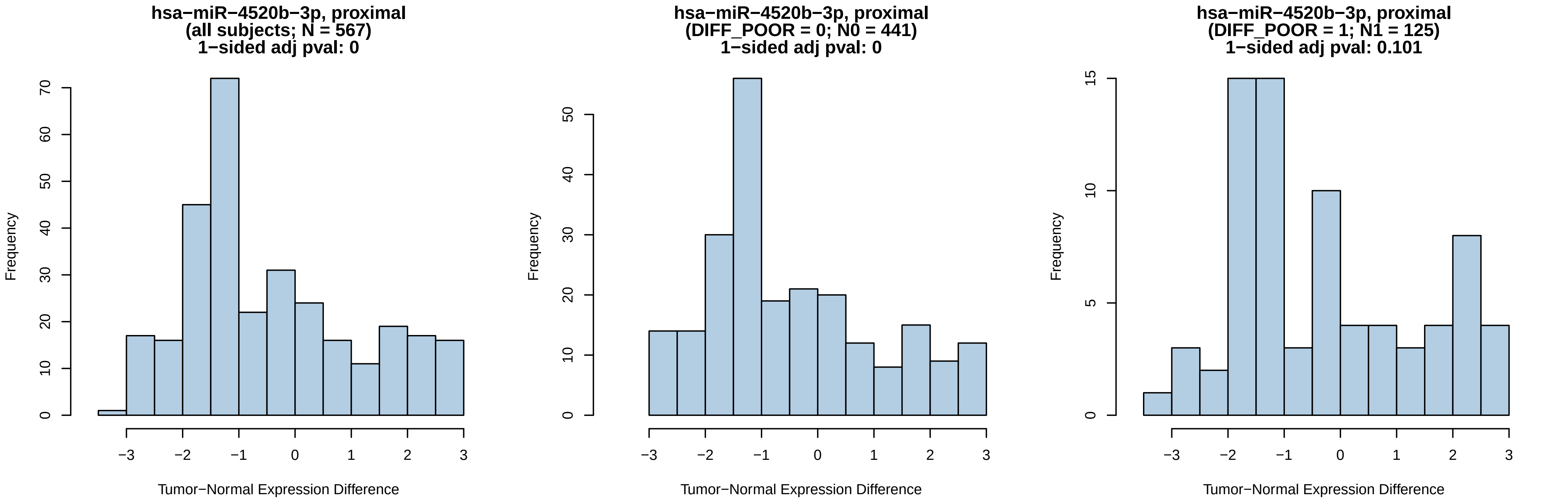
hsa−miR−4520b−3p, proximal
(all subjects; N = 567)
1−sided adj pval: 0
hsa−miR−4520b−3p, proximal
(DIFF_POOR = 0; N0 = 441)
1−sided adj pval: 0
hsa−miR−4520b−3p, proximal
(DIFF_POOR = 1; N1 = 125)
1−sided adj pval: 0.101
Frequency
Tumor−Normal Expression Difference
0
10
20
30
40
50
60
70
5
15
−3
−2
−1
1
2
3

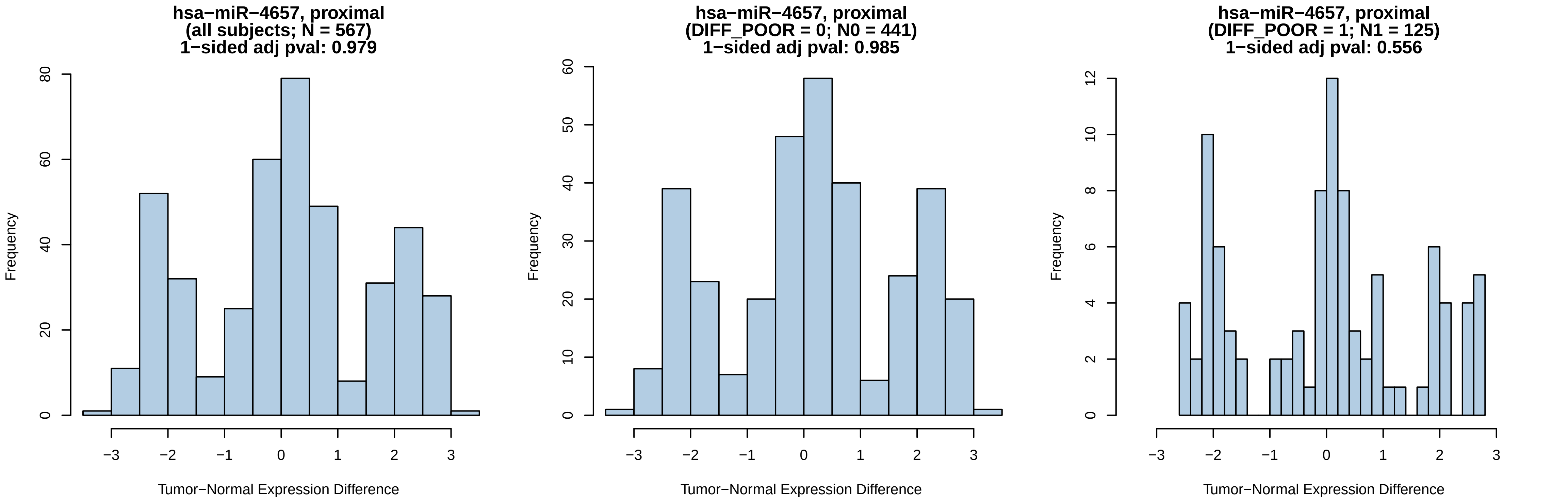
hsa−miR−4657, proximal
(all subjects; N = 567)
1−sided adj pval: 0.979
Frequency
Tumor−Normal Expression Difference
hsa−miR−4657, proximal
(DIFF_POOR = 0; N0 = 441)
1−sided adj pval: 0.985
Frequency
Tumor−Normal Expression Difference
hsa−miR−4657, proximal
(DIFF_POOR = 1; N1 = 125)
1−sided adj pval: 0.556
Frequency
Tumor−Normal Expression Difference

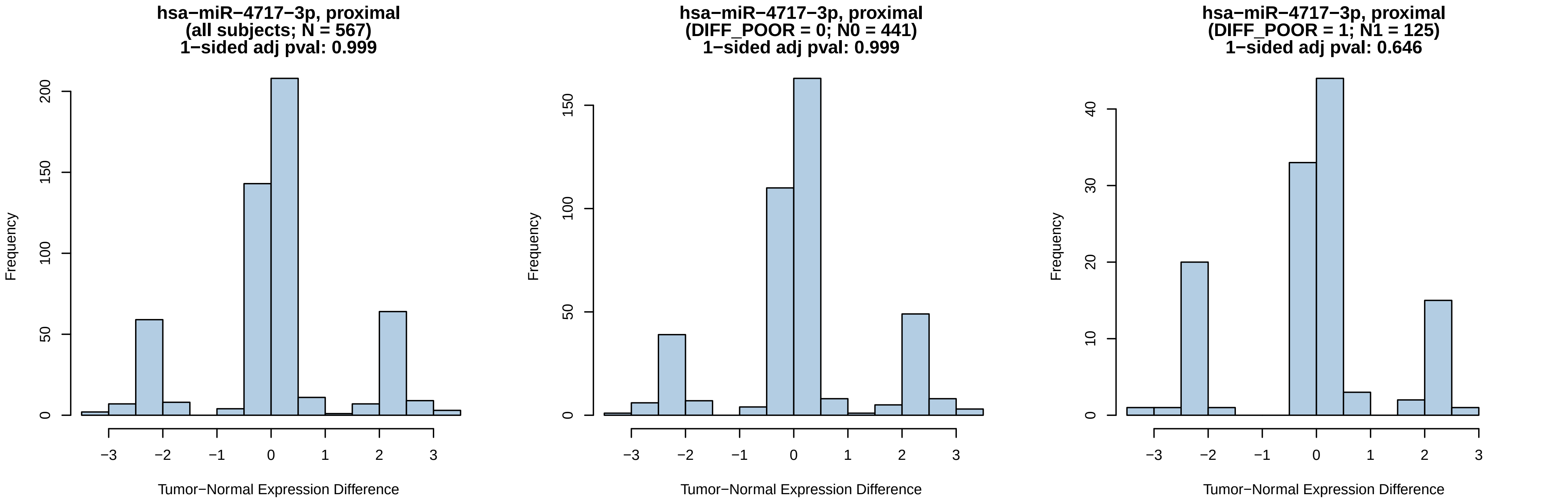
hsa−miR−4717−3p, proximal
(all subjects; N = 567)
1−sided adj pval: 0.999
Frequency
0
50
100
150
200
−3
−2
−1
0
1
2
3
Tumor−Normal Expression Difference
hsa−miR−4717−3p, proximal
(DIFF_POOR = 0; N0 = 441)
1−sided adj pval: 0.999
Frequency
0
50
100
150
−3
−2
−1
0
1
2
3
Tumor−Normal Expression Difference
hsa−miR−4717−3p, proximal
(DIFF_POOR = 1; N1 = 125)
1−sided adj pval: 0.646
Frequency
0
10
20
30
40
−3
−2
−1
0
1
2
3
Tumor−Normal Expression Difference

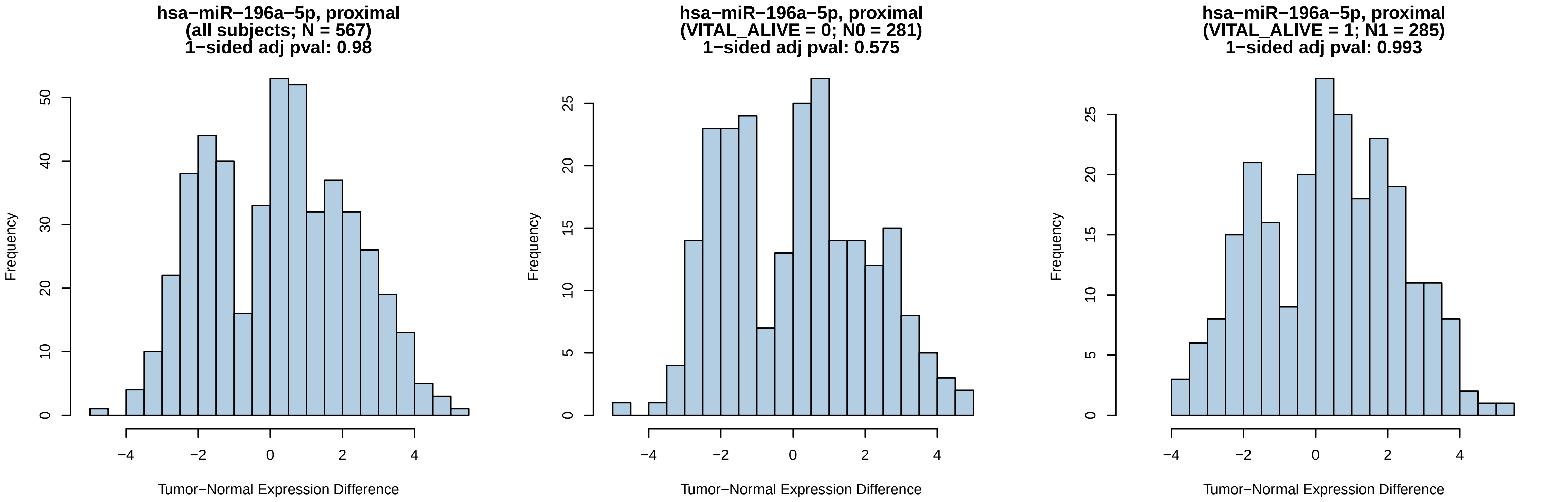
hsa−miR−196a−5p, proximal
(all subjects; N = 567)
1−sided adj pval: 0.98
Frequency
Tumor−Normal Expression Difference
hsa−miR−196a−5p, proximal
(VITAL_ALIVE = 0; N0 = 281)
1−sided adj pval: 0.575
Frequency
Tumor−Normal Expression Difference
hsa−miR−196a−5p, proximal
(VITAL_ALIVE = 1; N1 = 285)
1−sided adj pval: 0.993
Frequency
Tumor−Normal Expression Difference

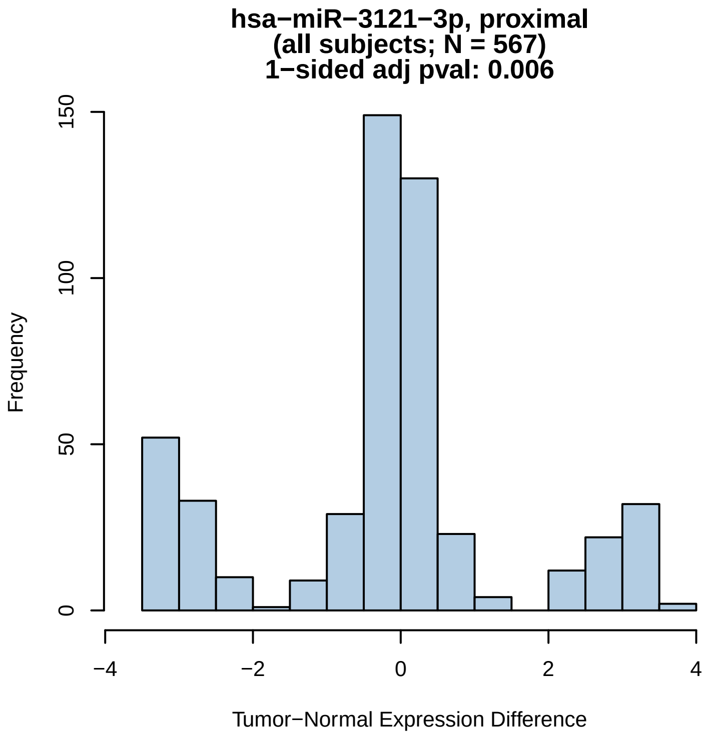
hsa−miR−3121−3p, proximal
(all subjects; N = 567)
1−sided adj pval: 0.006
Frequency
0
50
100
150
−4
−2
0
2
4
Tumor−Normal Expression Difference

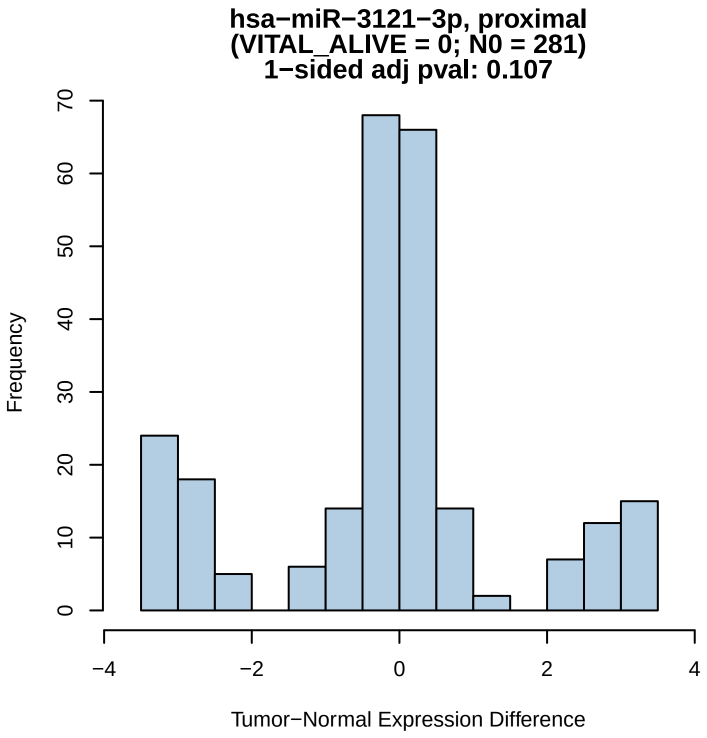
hsa−miR−3121−3p, proximal
(VITAL_ALIVE = 0; N0 = 281)
1−sided adj pval: 0.107
Frequency
0
10
20
30
40
50
60
70
−4
−2
0
2
4
Tumor−Normal Expression Difference

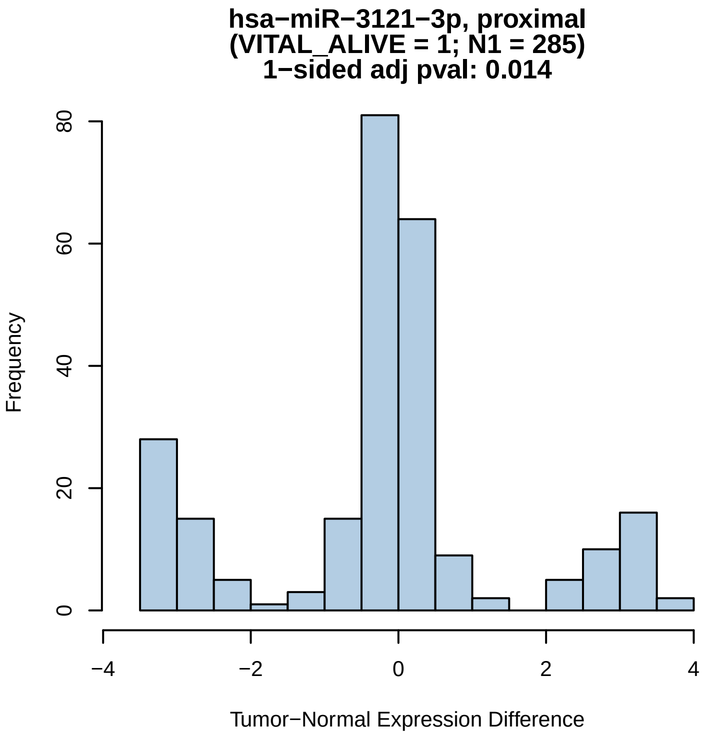
hsa−miR−3121−3p, proximal
(VITAL_ALIVE = 1; N1 = 285)
1−sided adj pval: 0.014
Frequency
0
20
40
60
80
−4
−2
0
2
4
Tumor−Normal Expression Difference

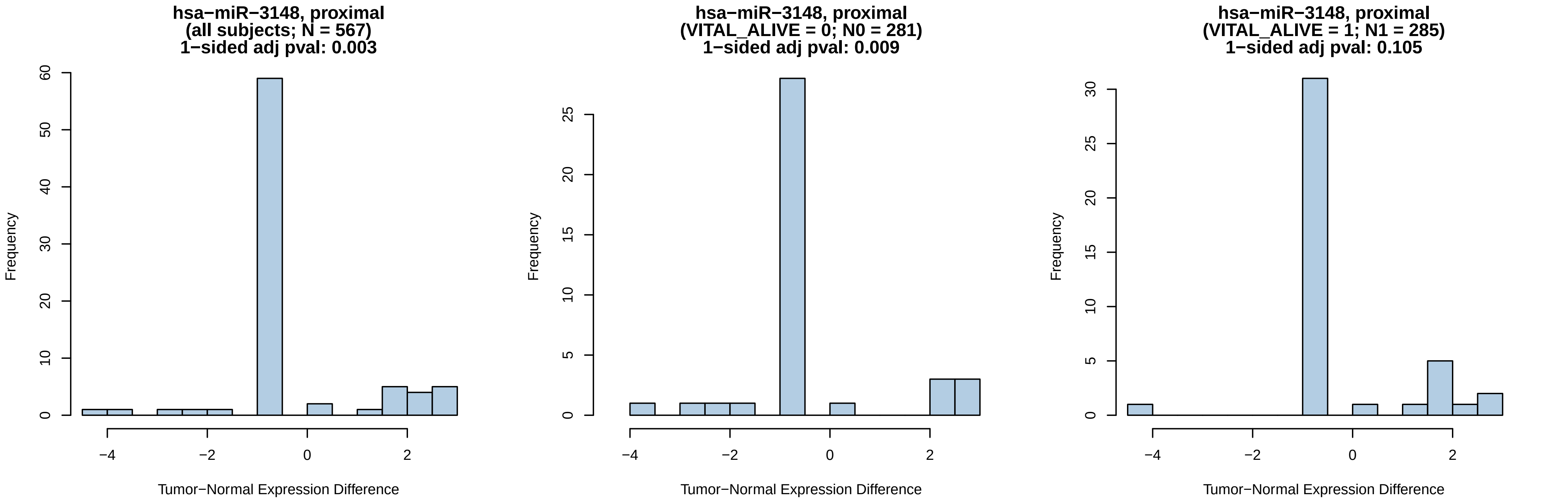
hsa−miR−3148, proximal
(all subjects; N = 567)
1−sided adj pval: 0.003
hsa−miR−3148, proximal
(VITAL_ALIVE = 0; N0 = 281)
1−sided adj pval: 0.009
hsa−miR−3148, proximal
(VITAL_ALIVE = 1; N1 = 285)
1−sided adj pval: 0.105
Frequency
Tumor−Normal Expression Difference

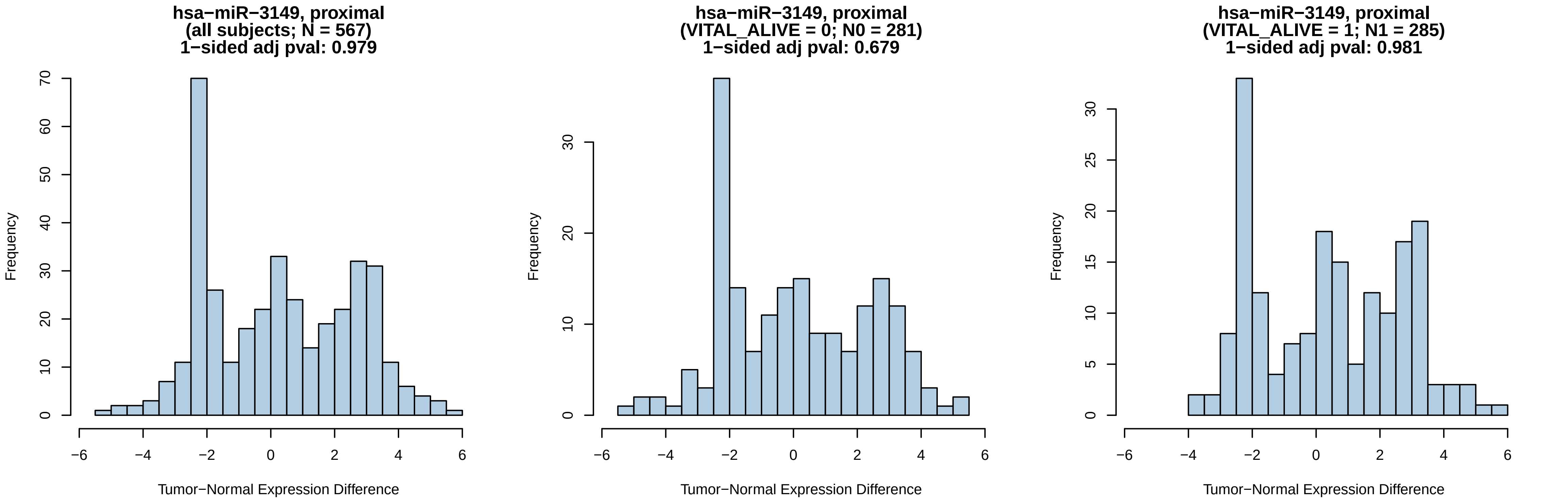
hsa−miR−3149, proximal
(all subjects; N = 567)
1−sided adj pval: 0.979
hsa−miR−3149, proximal
(VITAL_ALIVE = 0; N0 = 281)
1−sided adj pval: 0.679
hsa−miR−3149, proximal
(VITAL_ALIVE = 1; N1 = 285)
1−sided adj pval: 0.981
Frequency
Tumor−Normal Expression Difference

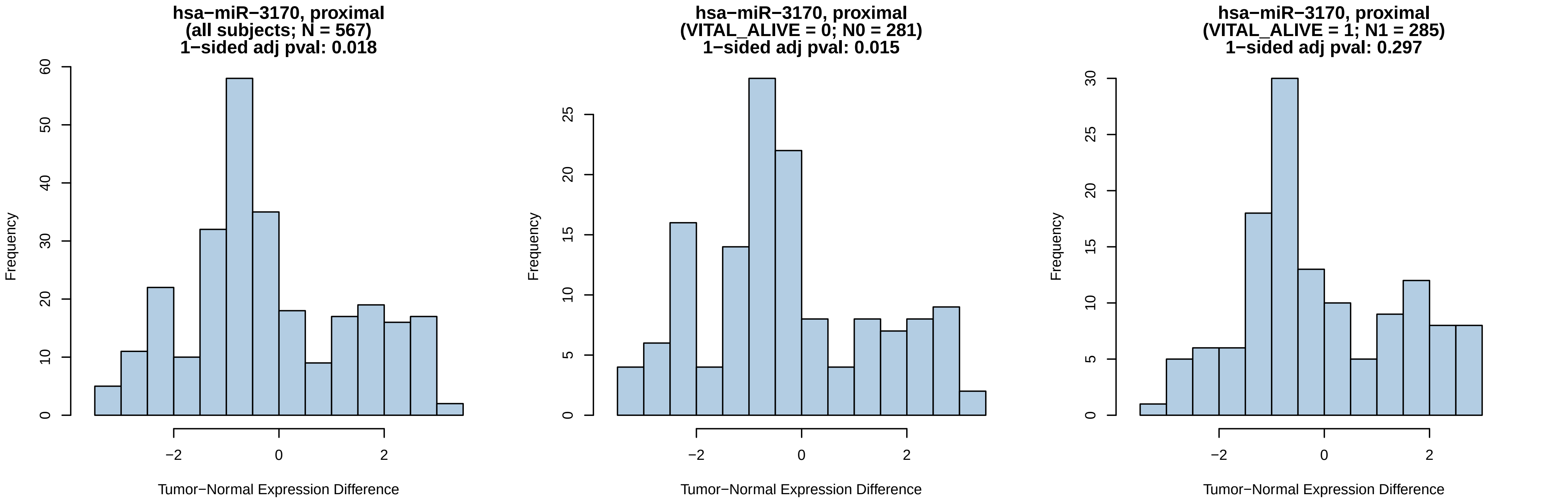

hsa−miR−3170, proximal
(all subjects; N = 567)
1−sided adj pval: 0.018
Frequency
0
10
20
30
40
50
60
−2
0
2
Tumor−Normal Expression Difference
hsa−miR−3170, proximal
(VITAL_ALIVE = 0; N0 = 281)
1−sided adj pval: 0.015
Frequency
0
5
10
15
20
25
−2
0
2
Tumor−Normal Expression Difference
hsa−miR−3170, proximal
(VITAL_ALIVE = 1; N1 = 285)
1−sided adj pval: 0.297
Frequency
0
5
10
15
20
25
30
−2
0
2
Tumor−Normal Expression Difference

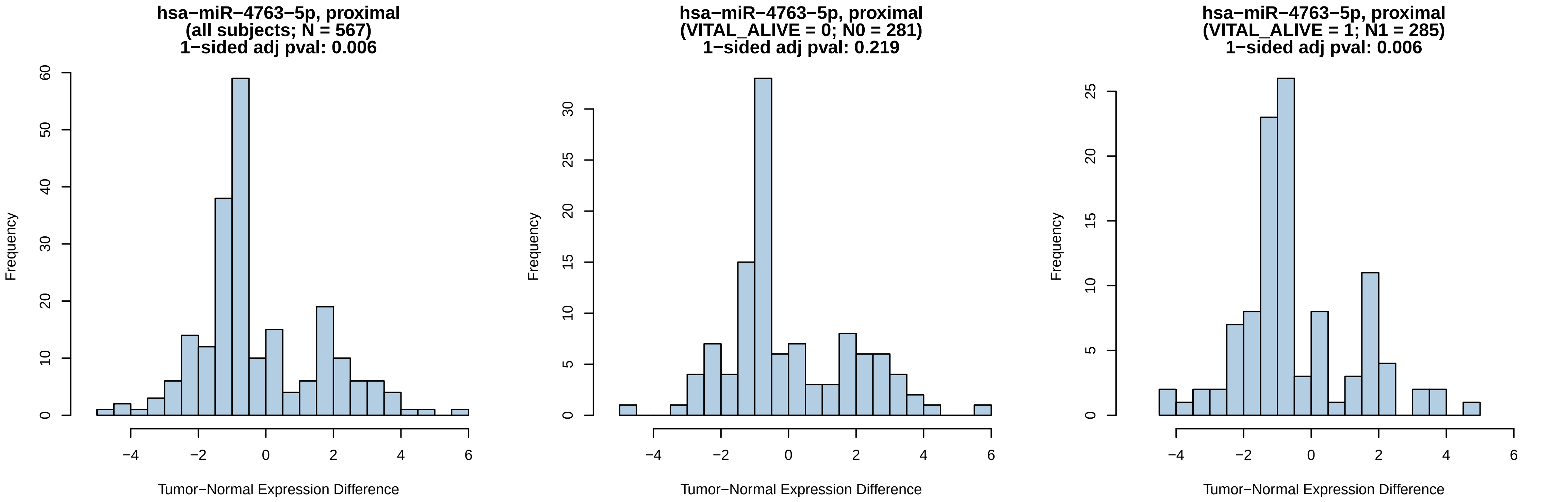
hsa−miR−4763−5p, proximal
(all subjects; N = 567)
1−sided adj pval: 0.006
hsa−miR−4763−5p, proximal
(VITAL_ALIVE = 0; N0 = 281)
1−sided adj pval: 0.219
hsa−miR−4763−5p, proximal
(VITAL_ALIVE = 1; N1 = 285)
1−sided adj pval: 0.006
Frequency
Tumor−Normal Expression Difference

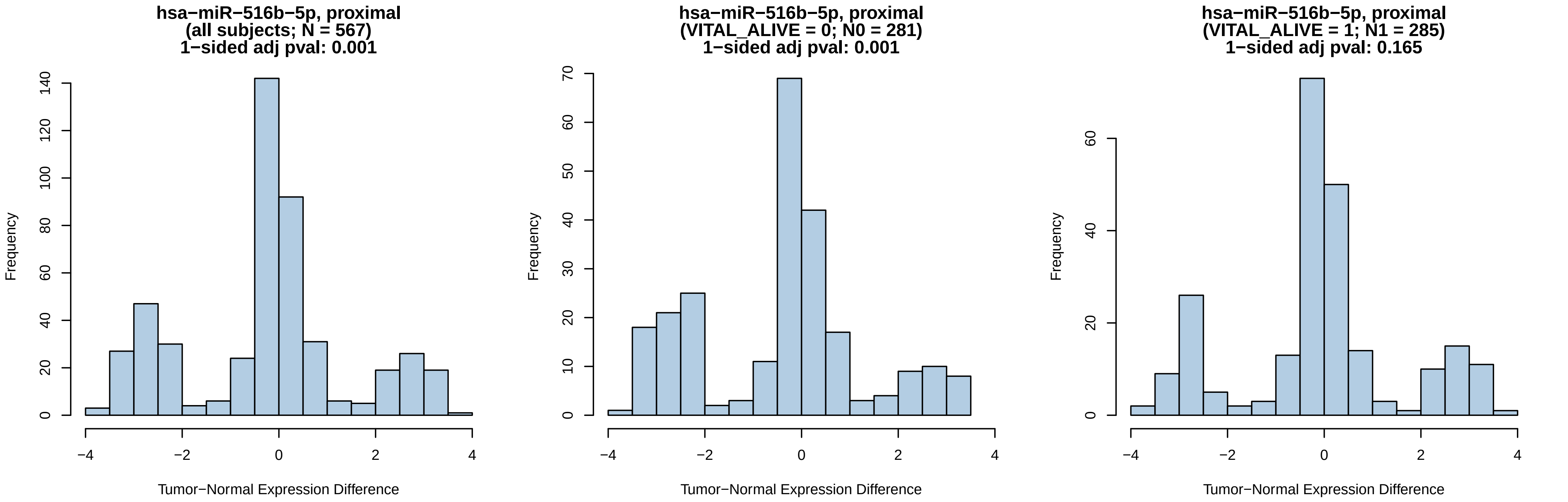
hsa−miR−516b−5p, proximal
(all subjects; N = 567)
1−sided adj pval: 0.001
hsa−miR−516b−5p, proximal
(VITAL_ALIVE = 0; N0 = 281)
1−sided adj pval: 0.001
hsa−miR−516b−5p, proximal
(VITAL_ALIVE = 1; N1 = 285)
1−sided adj pval: 0.165
Frequency
Tumor−Normal Expression Difference

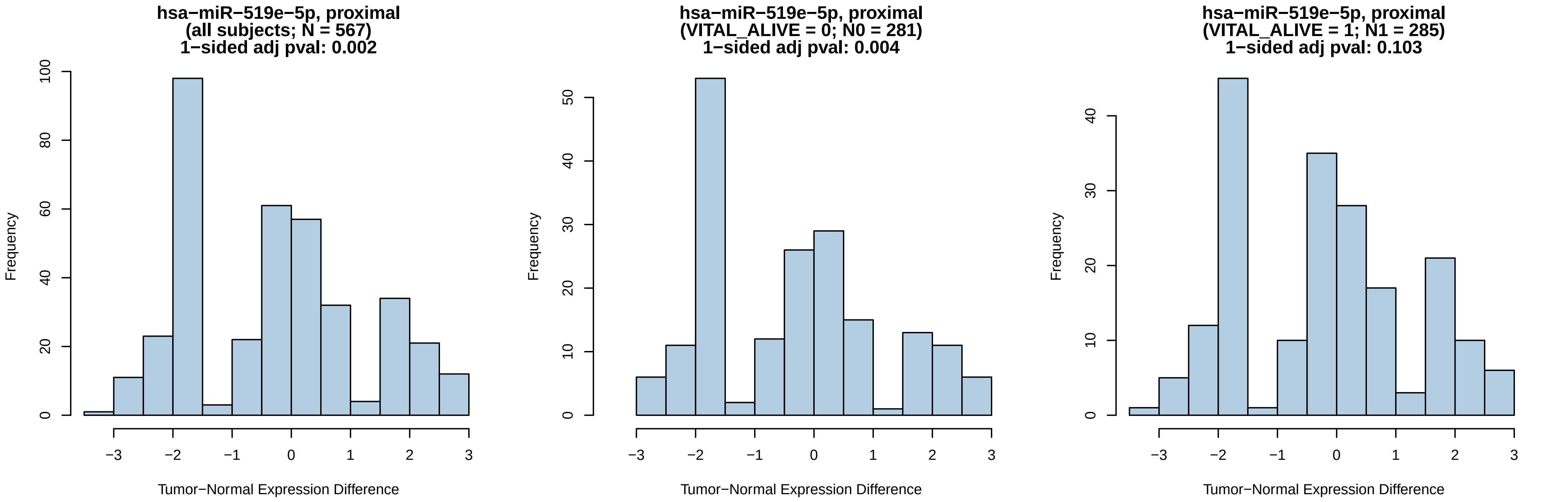
hsa−miR−519e−5p, proximal
(all subjects; N = 567)
1−sided adj pval: 0.002
Frequency
0
20
40
60
80
100
−3
−2
−1
0
1
2
3
Tumor−Normal Expression Difference
hsa−miR−519e−5p, proximal
(VITAL_ALIVE = 0; N0 = 281)
1−sided adj pval: 0.004
Frequency
0
10
20
30
40
50
−3
−2
−1
0
1
2
3
Tumor−Normal Expression Difference
hsa−miR−519e−5p, proximal
(VITAL_ALIVE = 1; N1 = 285)
1−sided adj pval: 0.103
Frequency
0
10
20
30
40
−3
−2
−1
0
1
2
3
Tumor−Normal Expression Difference

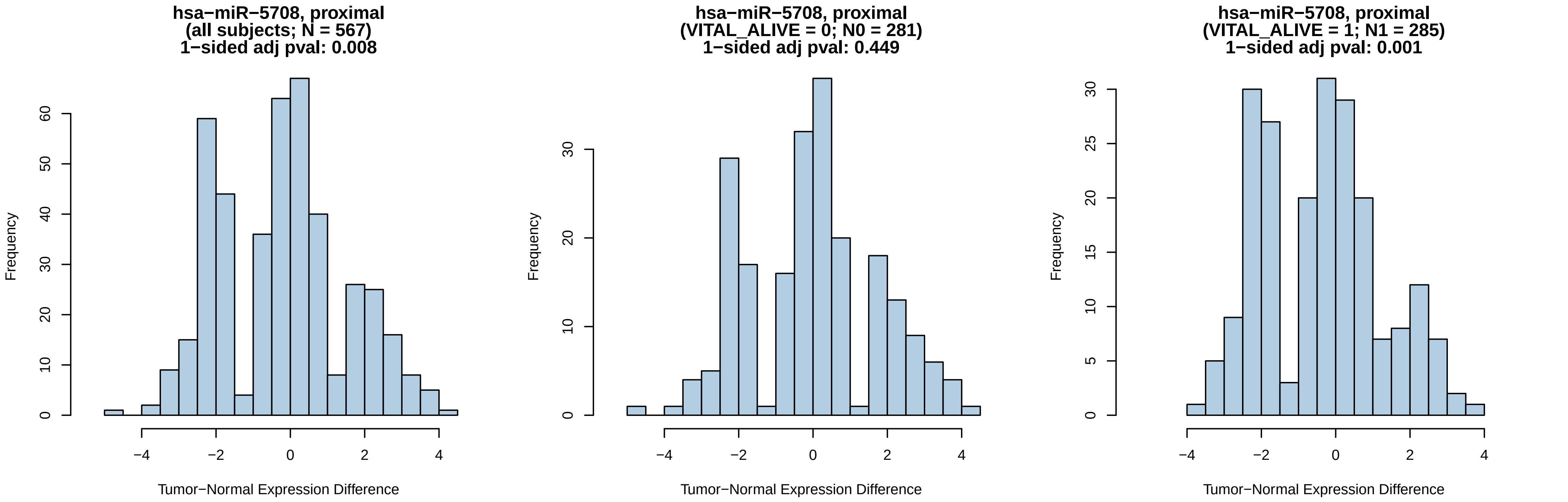
hsa−miR−5708, proximal
(all subjects; N = 567)
1−sided adj pval: 0.008
hsa−miR−5708, proximal
(VITAL_ALIVE = 0; N0 = 281)
1−sided adj pval: 0.449
hsa−miR−5708, proximal
(VITAL_ALIVE = 1; N1 = 285)
1−sided adj pval: 0.001
Frequency
Tumor−Normal Expression Difference

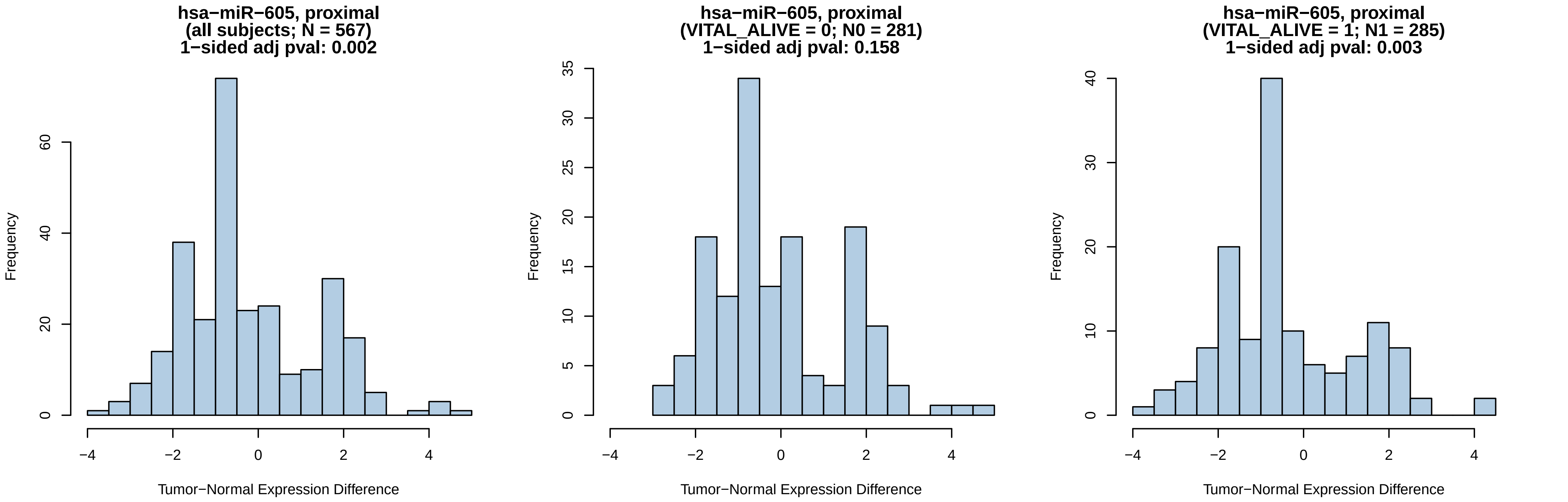

hsa−miR−605, proximal
(all subjects; N = 567)
1−sided adj pval: 0.002
Frequency
Tumor−Normal Expression Difference
hsa−miR−605, proximal
(VITAL_ALIVE = 0; N0 = 281)
1−sided adj pval: 0.158
Frequency
Tumor−Normal Expression Difference
hsa−miR−605, proximal
(VITAL_ALIVE = 1; N1 = 285)
1−sided adj pval: 0.003
Frequency
Tumor−Normal Expression Difference

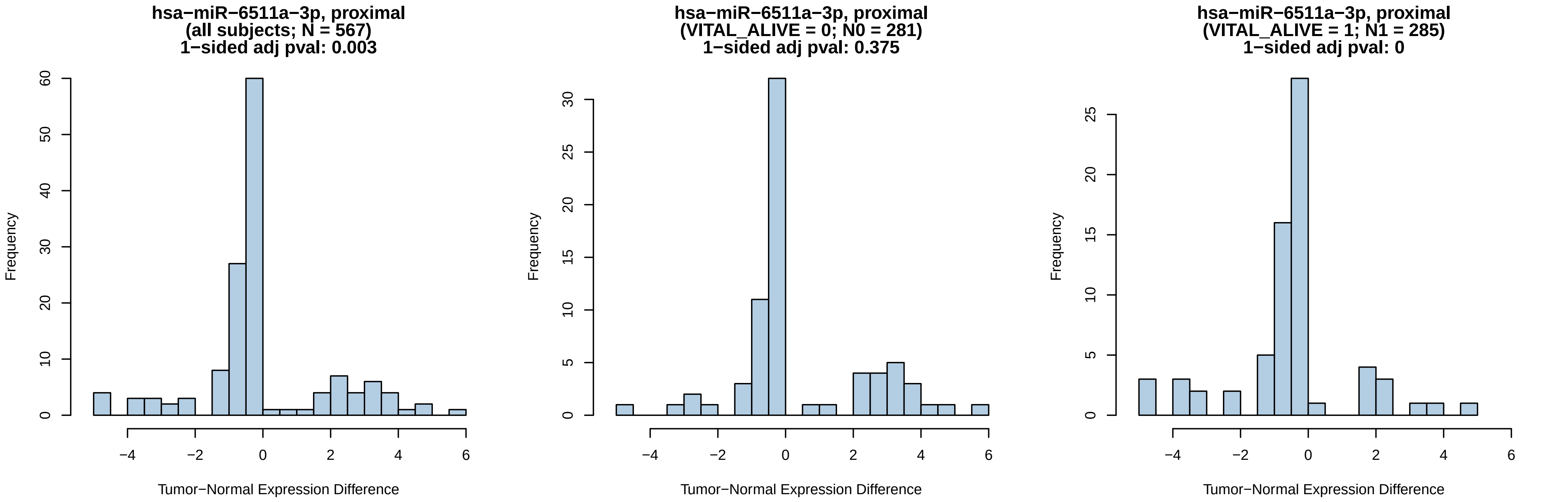
hsa−miR−6511a−3p, proximal
(all subjects; N = 567)
1−sided adj pval: 0.003
Frequency
Tumor−Normal Expression Difference
hsa−miR−6511a−3p, proximal
(VITAL_ALIVE = 0; N0 = 281)
1−sided adj pval: 0.375
Frequency
Tumor−Normal Expression Difference
hsa−miR−6511a−3p, proximal
(VITAL_ALIVE = 1; N1 = 285)
1−sided adj pval: 0
Frequency
Tumor−Normal Expression Difference

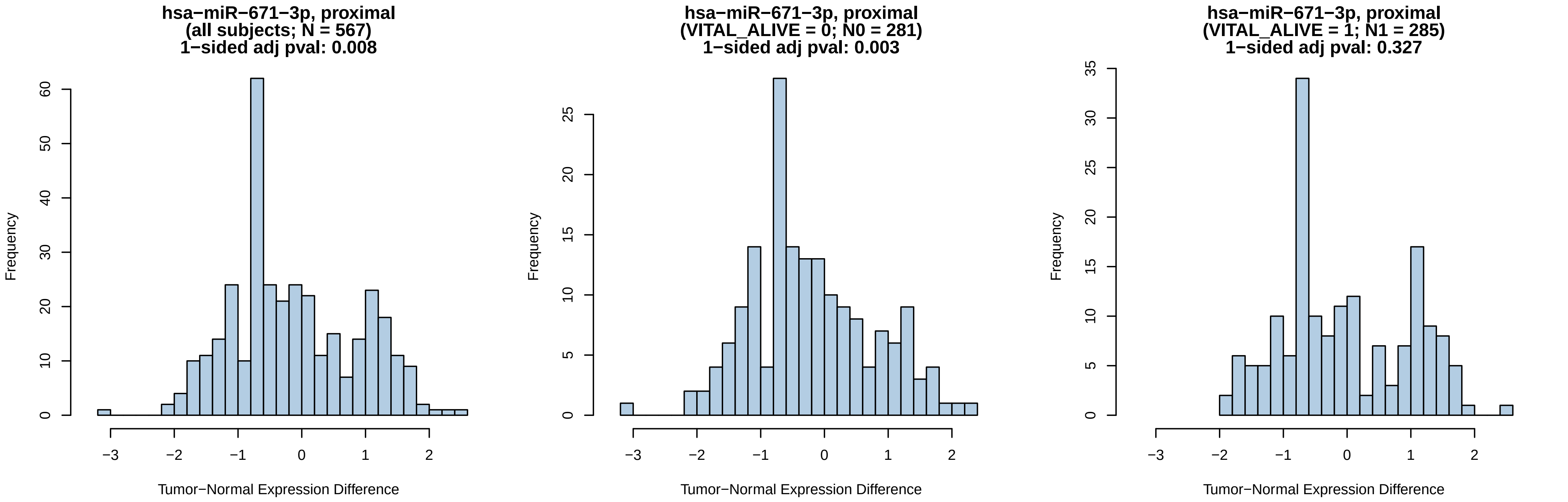
hsa−miR−671−3p, proximal
(all subjects; N = 567)
1−sided adj pval: 0.008
Frequency
Tumor−Normal Expression Difference
hsa−miR−671−3p, proximal
(VITAL_ALIVE = 0; N0 = 281)
1−sided adj pval: 0.003
Frequency
Tumor−Normal Expression Difference
hsa−miR−671−3p, proximal
(VITAL_ALIVE = 1; N1 = 285)
1−sided adj pval: 0.327
Frequency
Tumor−Normal Expression Difference

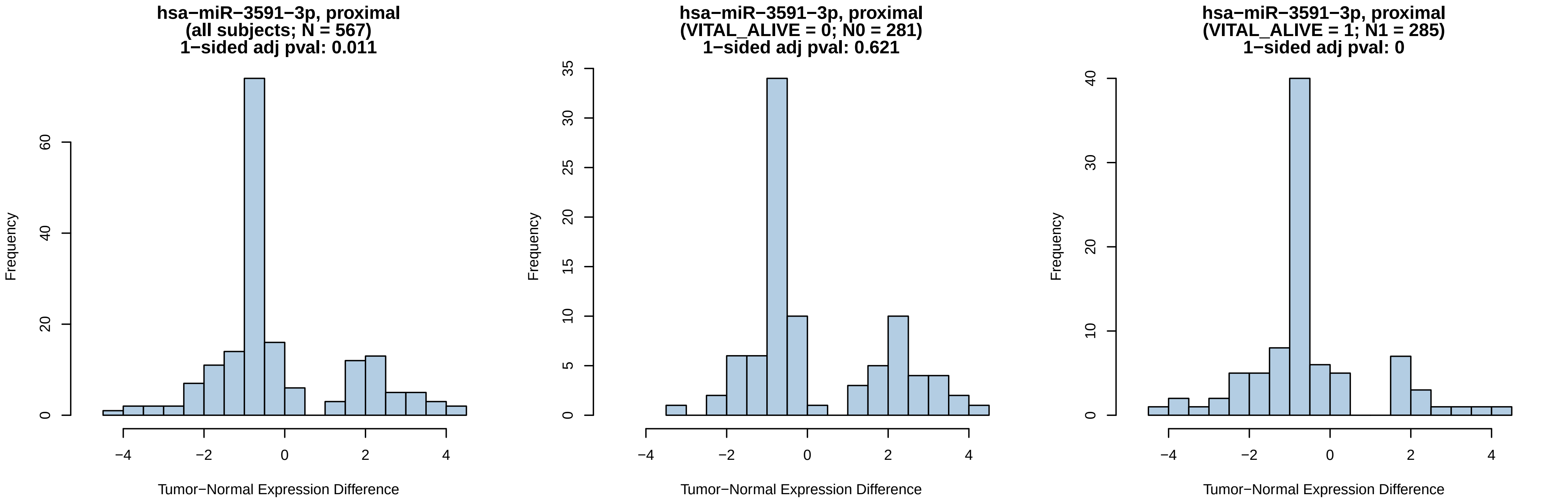

hsa−miR−3591−3p, proximal
(all subjects; N = 567)
1−sided adj pval: 0.011
Frequency
Tumor−Normal Expression Difference
hsa−miR−3591−3p, proximal
(VITAL_ALIVE = 0; N0 = 281)
1−sided adj pval: 0.621
Frequency
Tumor−Normal Expression Difference
hsa−miR−3591−3p, proximal
(VITAL_ALIVE = 1; N1 = 285)
1−sided adj pval: 0
Frequency
Tumor−Normal Expression Difference

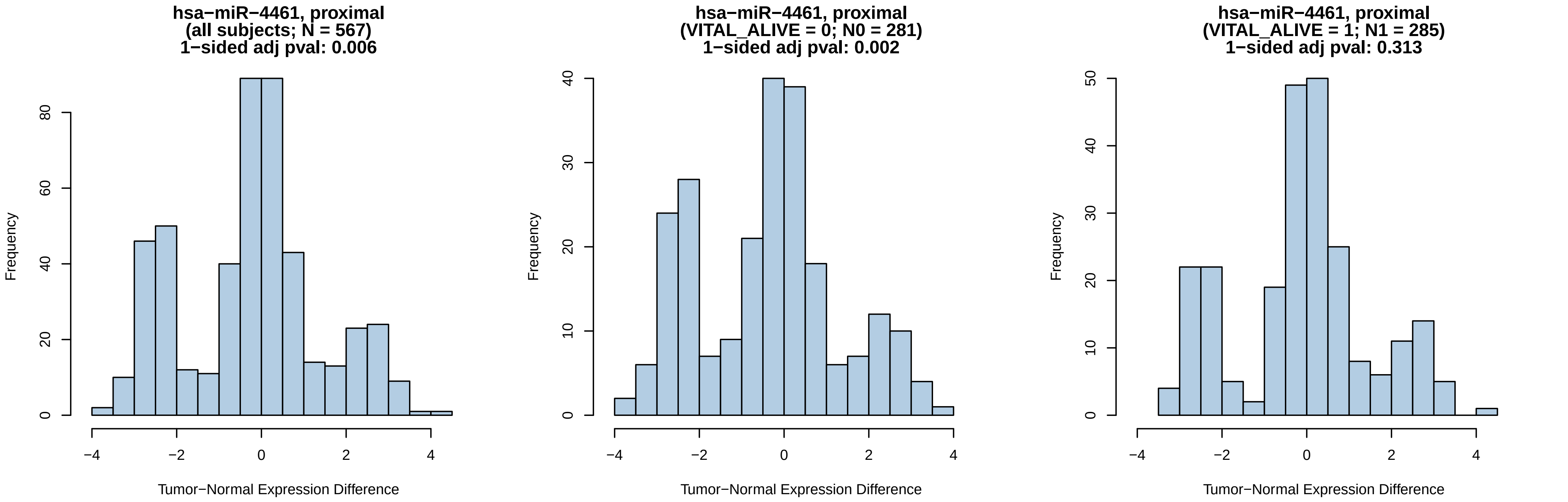

hsa−miR−4461, proximal
(all subjects; N = 567)
1−sided adj pval: 0.006
Frequency
Tumor−Normal Expression Difference
hsa−miR−4461, proximal
(VITAL_ALIVE = 0; N0 = 281)
1−sided adj pval: 0.002
Frequency
Tumor−Normal Expression Difference
hsa−miR−4461, proximal
(VITAL_ALIVE = 1; N1 = 285)
1−sided adj pval: 0.313
Frequency
Tumor−Normal Expression Difference

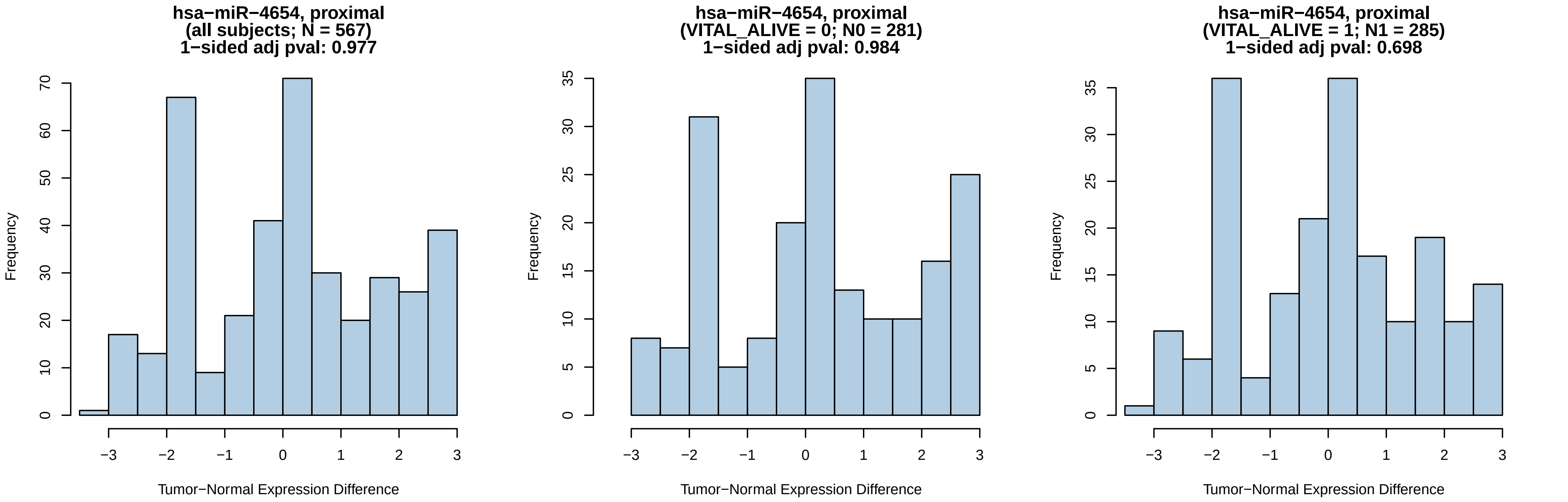
hsa−miR−4654, proximal
(all subjects; N = 567)
1−sided adj pval: 0.977
hsa−miR−4654, proximal
(VITAL_ALIVE = 0; N0 = 281)
1−sided adj pval: 0.984
hsa−miR−4654, proximal
(VITAL_ALIVE = 1; N1 = 285)
1−sided adj pval: 0.698
Frequency
Tumor−Normal Expression Difference
0
10
20
30
40
50
60
70
0
5
10
15
20
25
30
35
−3
−2
−1
0
1
2
3

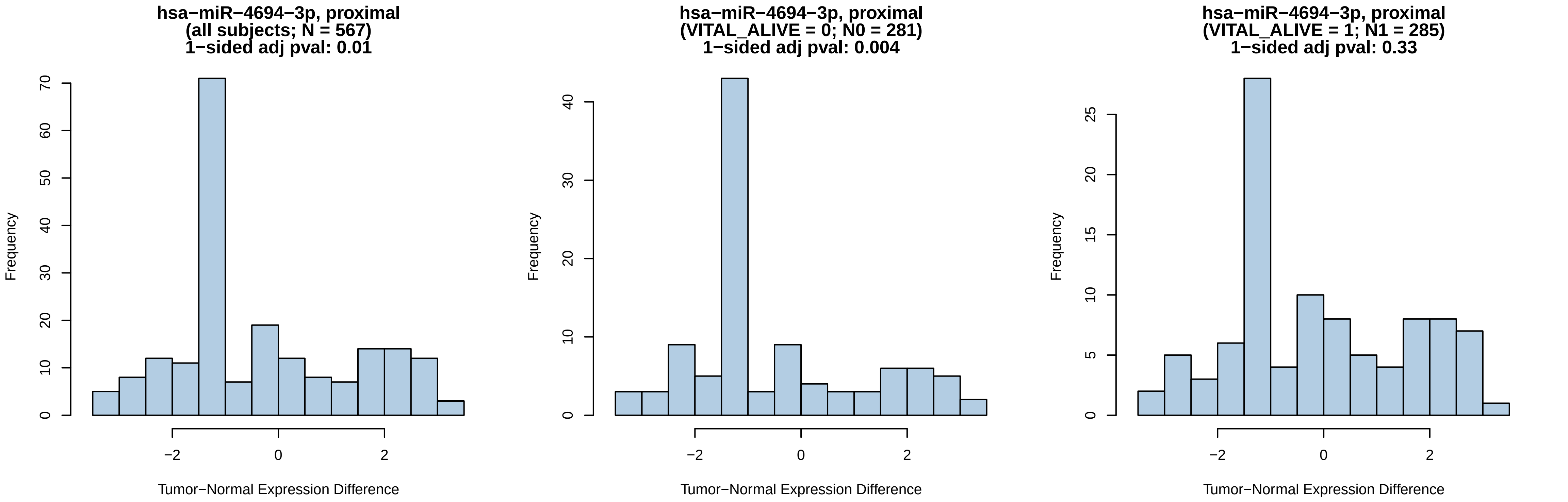
hsa−miR−4694−3p, proximal
(all subjects; N = 567)
1−sided adj pval: 0.01
hsa−miR−4694−3p, proximal
(VITAL_ALIVE = 0; N0 = 281)
1−sided adj pval: 0.004
hsa−miR−4694−3p, proximal
(VITAL_ALIVE = 1; N1 = 285)
1−sided adj pval: 0.33
Frequency
Tumor−Normal Expression Difference

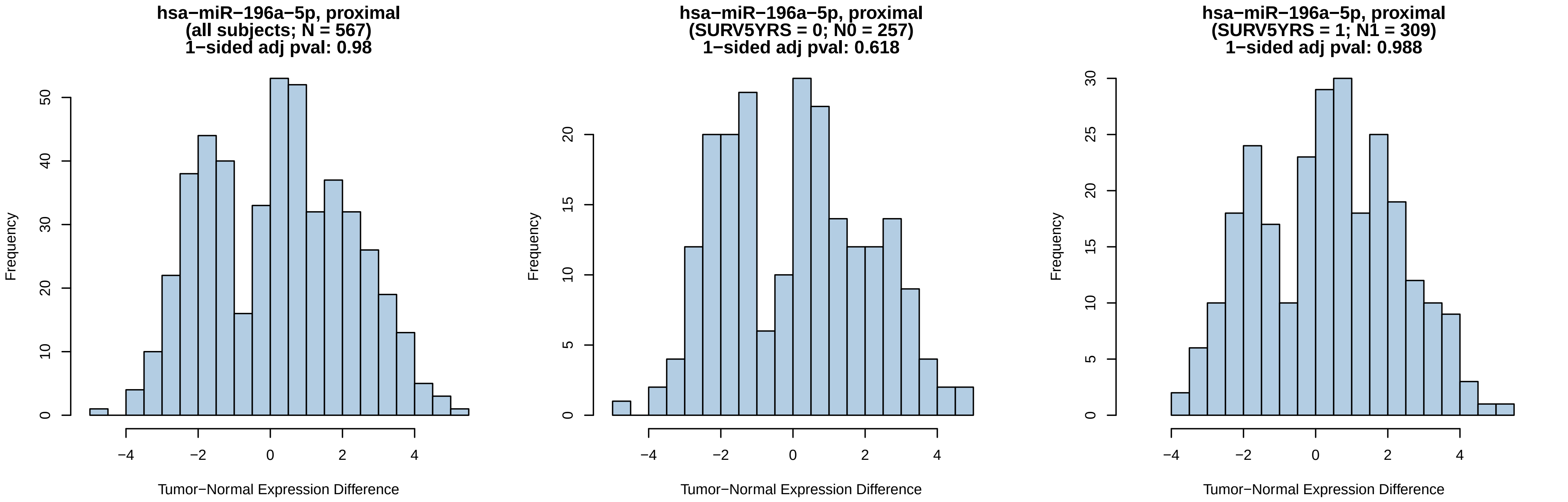

hsa−miR−196a−5p, proximal
(all subjects; N = 567)
1−sided adj pval: 0.98
Frequency
Tumor−Normal Expression Difference
hsa−miR−196a−5p, proximal
(SURV5YRS = 0; N0 = 257)
1−sided adj pval: 0.618
Frequency
Tumor−Normal Expression Difference
hsa−miR−196a−5p, proximal
(SURV5YRS = 1; N1 = 309)
1−sided adj pval: 0.988
Frequency
Tumor−Normal Expression Difference

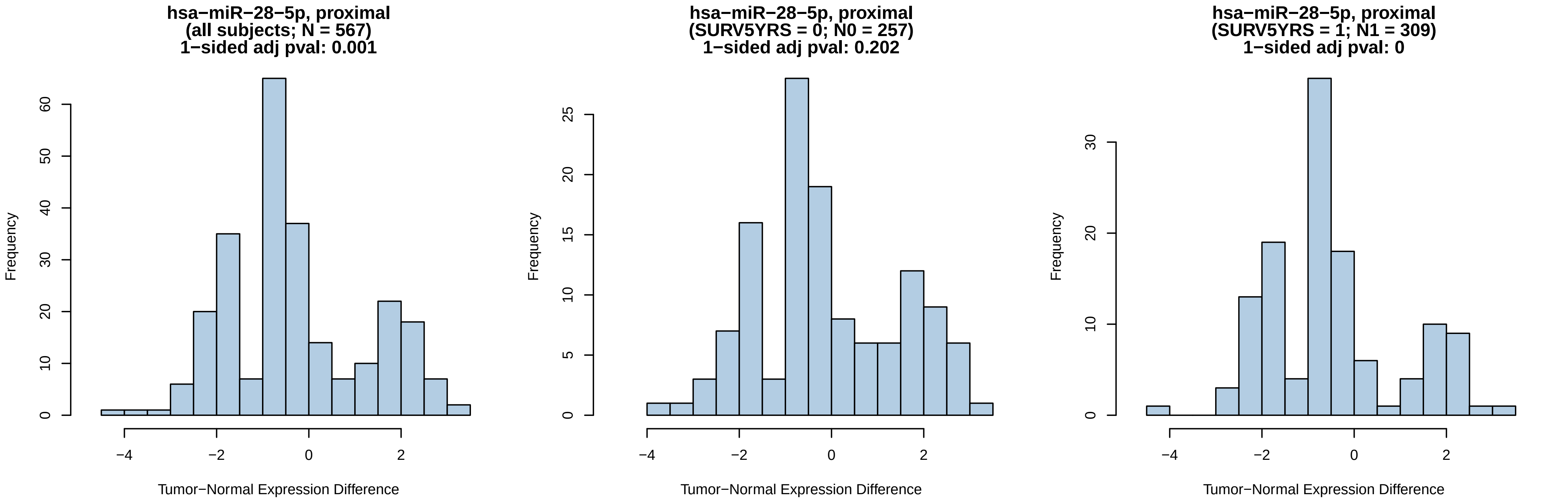

hsa−miR−28−5p, proximal
(all subjects; N = 567)
1−sided adj pval: 0.001
Frequency
Tumor−Normal Expression Difference
hsa−miR−28−5p, proximal
(SURV5YRS = 0; N0 = 257)
1−sided adj pval: 0.202
Frequency
Tumor−Normal Expression Difference
hsa−miR−28−5p, proximal
(SURV5YRS = 1; N1 = 309)
1−sided adj pval: 0
Frequency
Tumor−Normal Expression Difference

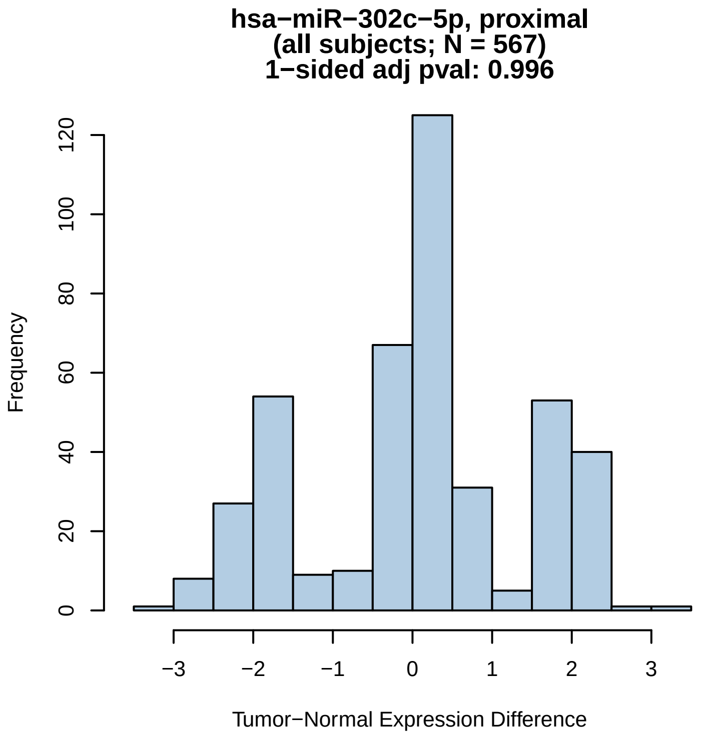

hsa−miR−302c−5p, proximal
(all subjects; N = 567)
1−sided adj pval: 0.996
Frequency
Tumor−Normal Expression Difference


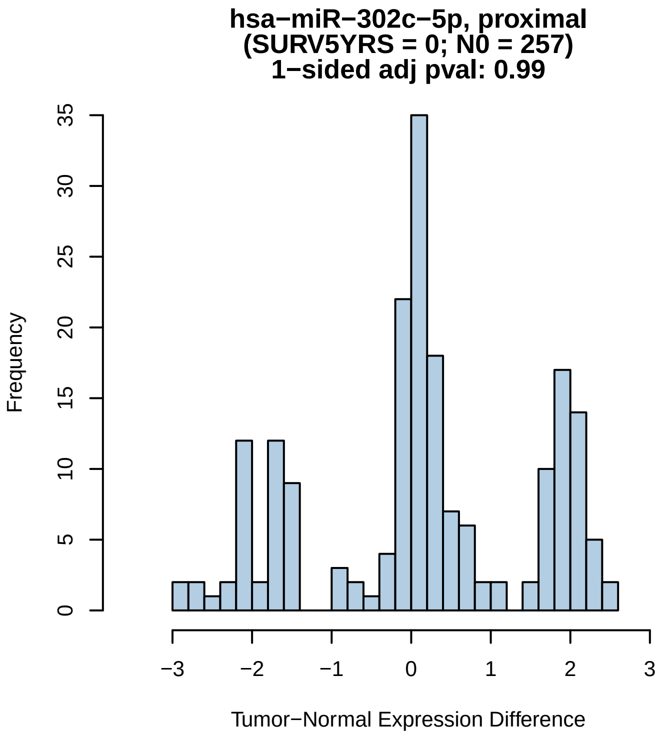

hsa−miR−302c−5p, proximal
(SURV5YRS = 0; N0 = 257)
1−sided adj pval: 0.99
Frequency
Tumor−Normal Expression Difference


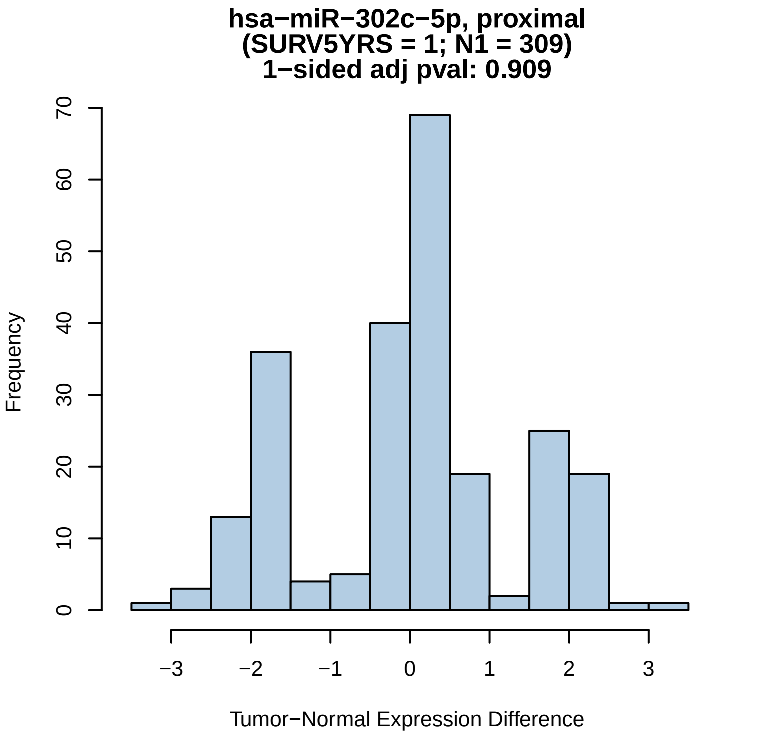

hsa−miR−302c−5p, proximal
(SURV5YRS = 1; N1 = 309)
1−sided adj pval: 0.909
Frequency
Tumor−Normal Expression Difference

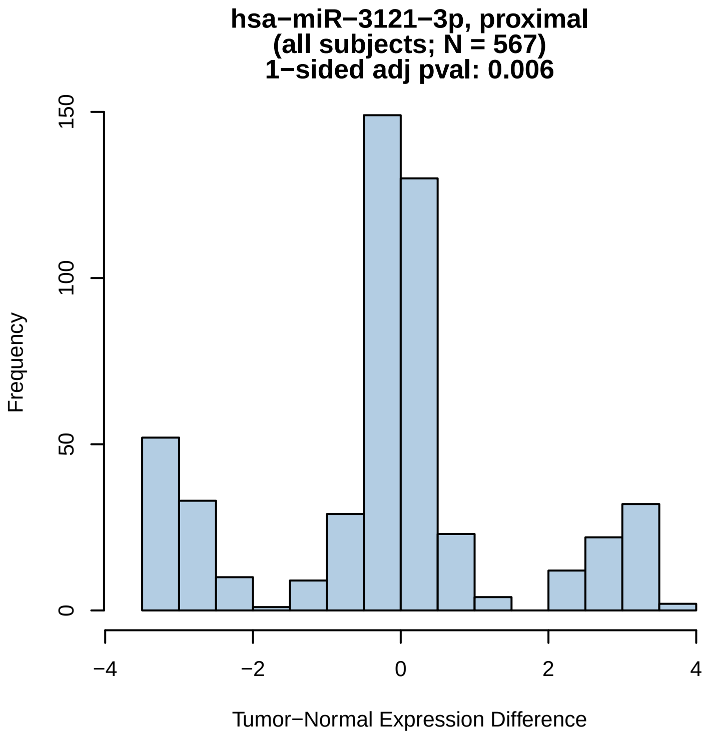

hsa−miR−3121−3p, proximal
(all subjects; N = 567)
1−sided adj pval: 0.006
Frequency
Tumor−Normal Expression Difference


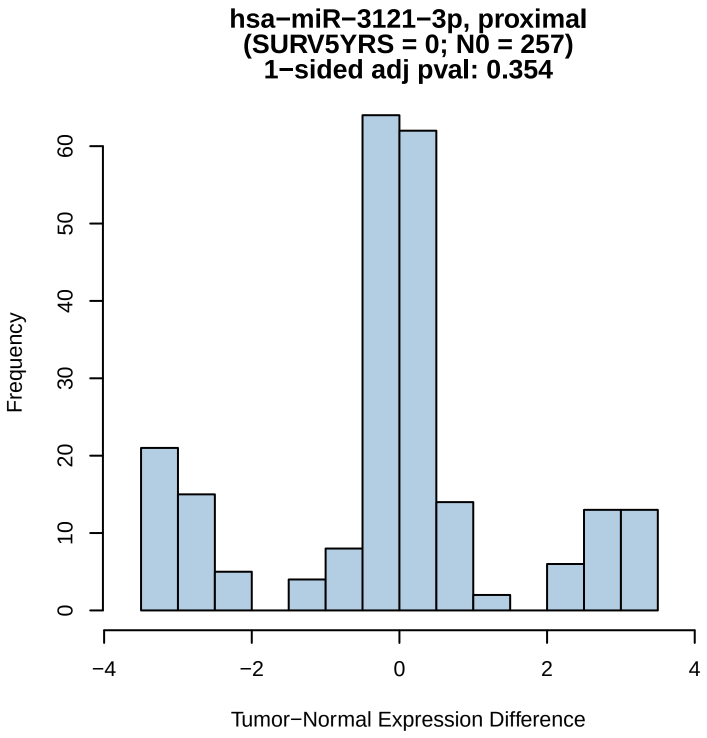

hsa−miR−3121−3p, proximal
(SURV5YRS = 0; N0 = 257)
1−sided adj pval: 0.354
Frequency
Tumor−Normal Expression Difference


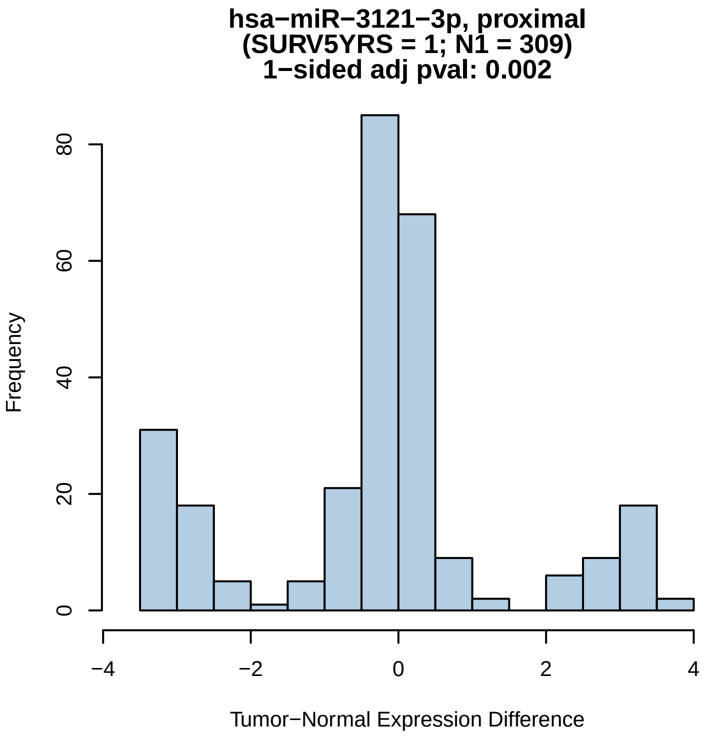

hsa−miR−3121−3p, proximal
(SURV5YRS = 1; N1 = 309)
1−sided adj pval: 0.002
Frequency
Tumor−Normal Expression Difference

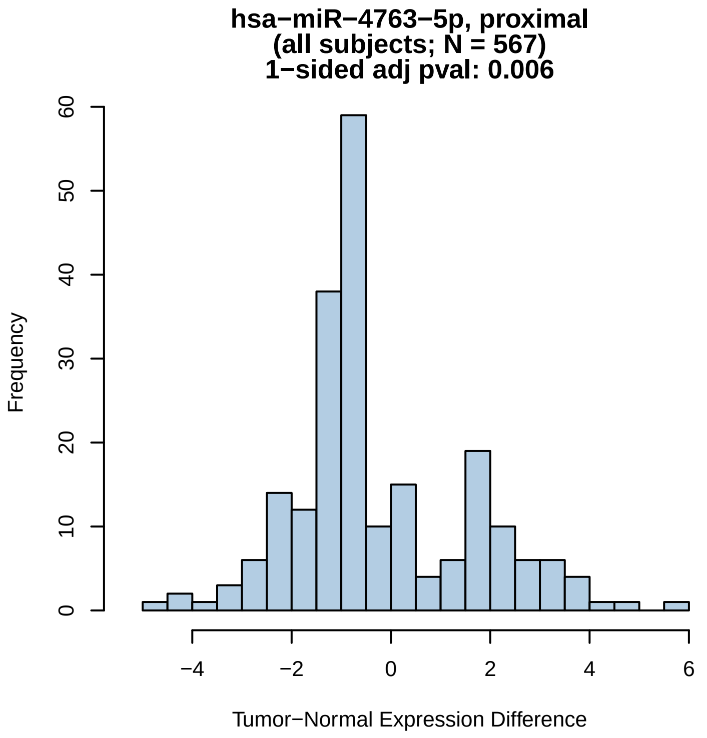
hsa−miR−4763−5p, proximal
(all subjects; N = 567)
1−sided adj pval: 0.006
Frequency
Tumor−Normal Expression Difference
0
10
20
30
40
50
60
−4
−2
0
2
4
6

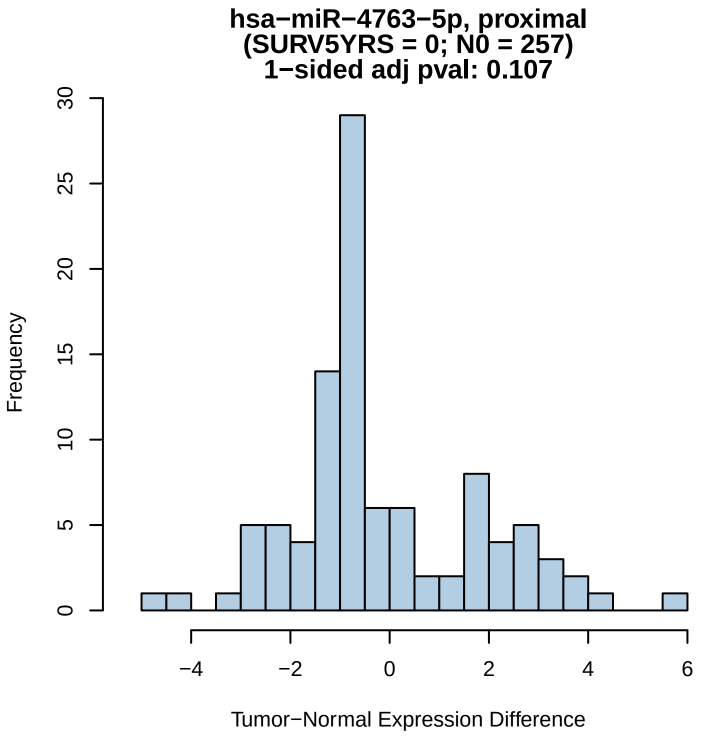
hsa−miR−4763−5p, proximal
(SURV5YRS = 0; N0 = 257)
1−sided adj pval: 0.107
Frequency
Tumor−Normal Expression Difference
0
5
10
15
20
25
30
−4
−2
0
2
4
6

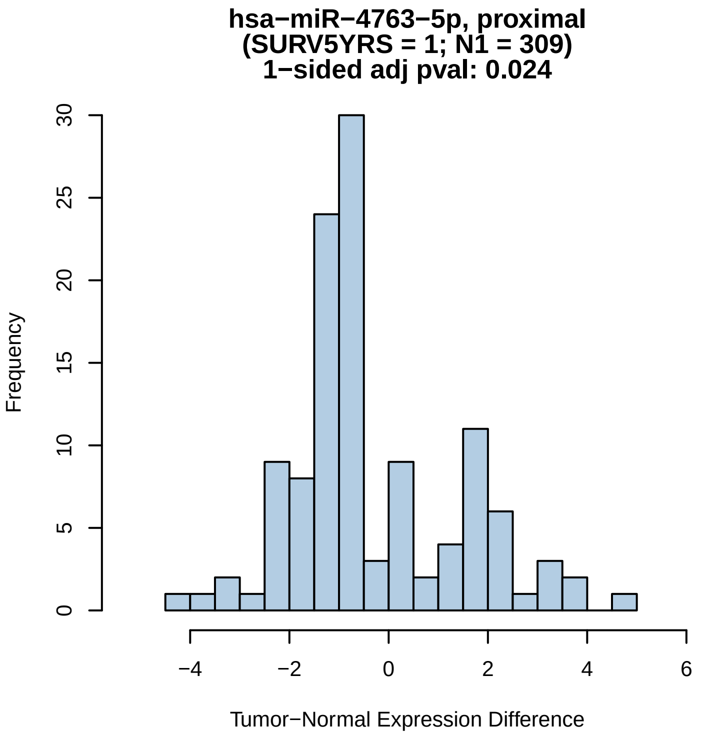
hsa−miR−4763−5p, proximal
(SURV5YRS = 1; N1 = 309)
1−sided adj pval: 0.024
Frequency
Tumor−Normal Expression Difference
0
5
10
15
20
25
30
−4
−2
0
2
4
6

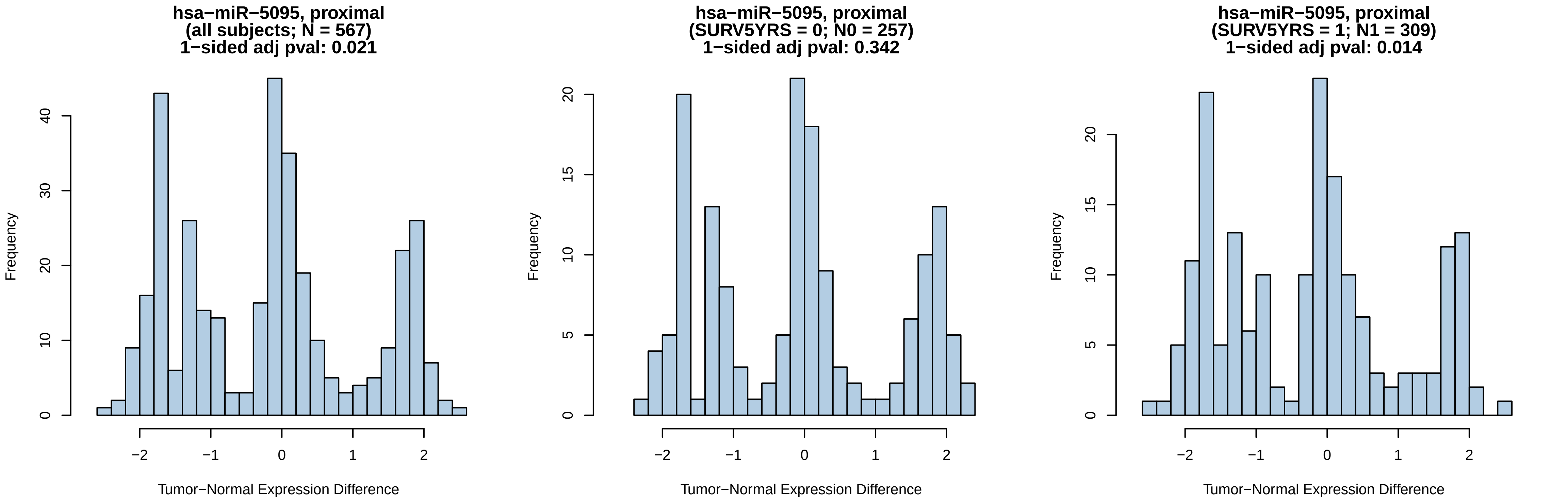
hsa−miR−5095, proximal
(all subjects; N = 567)
1−sided adj pval: 0.021
Frequency
Tumor−Normal Expression Difference
hsa−miR−5095, proximal
(SURV5YRS = 0; N0 = 257)
1−sided adj pval: 0.342
Frequency
Tumor−Normal Expression Difference
hsa−miR−5095, proximal
(SURV5YRS = 1; N1 = 309)
1−sided adj pval: 0.014
Frequency
Tumor−Normal Expression Difference

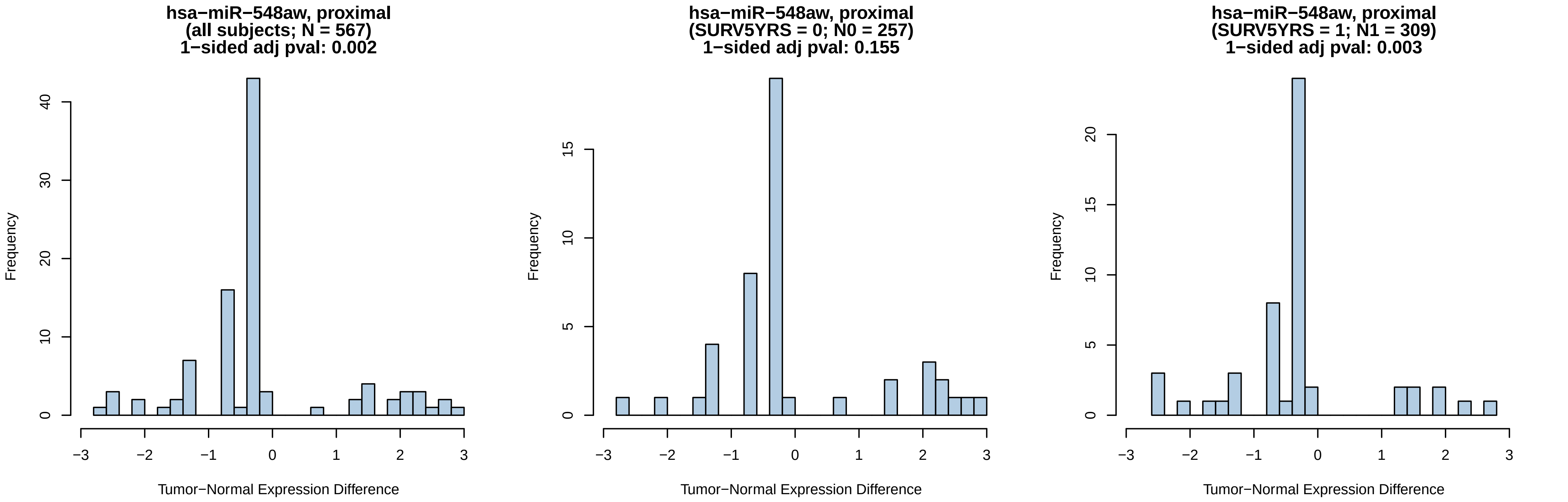
hsa−miR−548aw, proximal
(all subjects; N = 567)
1−sided adj pval: 0.002
Frequency
Tumor−Normal Expression Difference
hsa−miR−548aw, proximal
(SURV5YRS = 0; N0 = 257)
1−sided adj pval: 0.155
Frequency
Tumor−Normal Expression Difference
hsa−miR−548aw, proximal
(SURV5YRS = 1; N1 = 309)
1−sided adj pval: 0.003
Frequency
Tumor−Normal Expression Difference

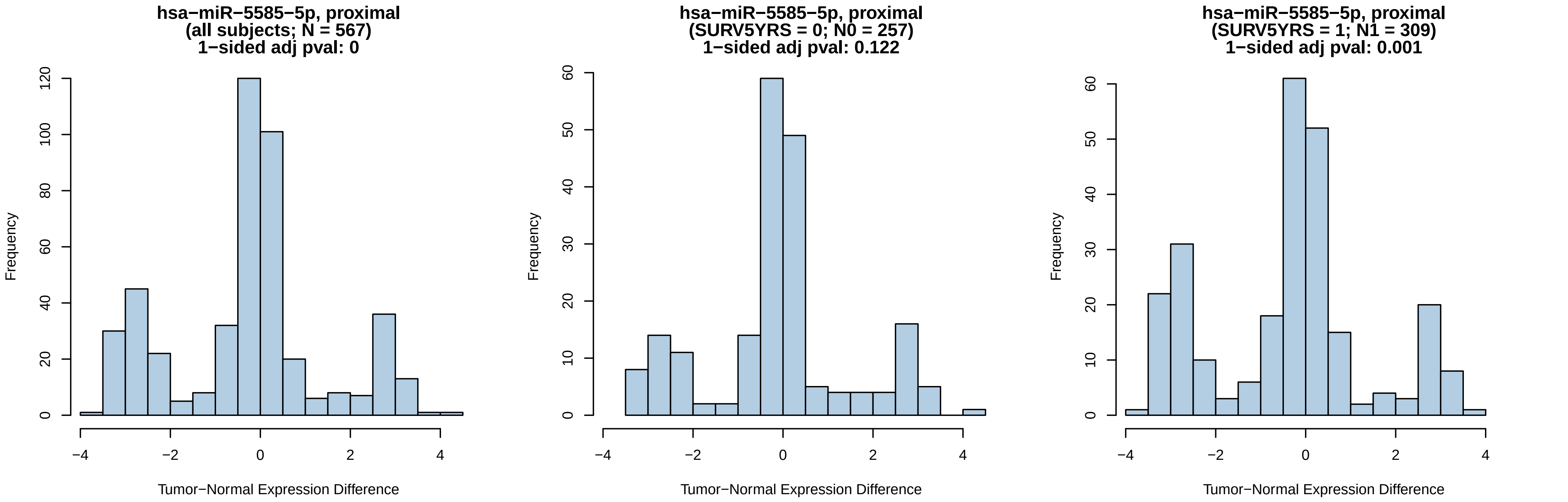
hsa−miR−5585−5p, proximal
(all subjects; N = 567)
1−sided adj pval: 0
Frequency
Tumor−Normal Expression Difference
hsa−miR−5585−5p, proximal
(SURV5YRS = 0; N0 = 257)
1−sided adj pval: 0.122
Frequency
Tumor−Normal Expression Difference
hsa−miR−5585−5p, proximal
(SURV5YRS = 1; N1 = 309)
1−sided adj pval: 0.001
Frequency
Tumor−Normal Expression Difference

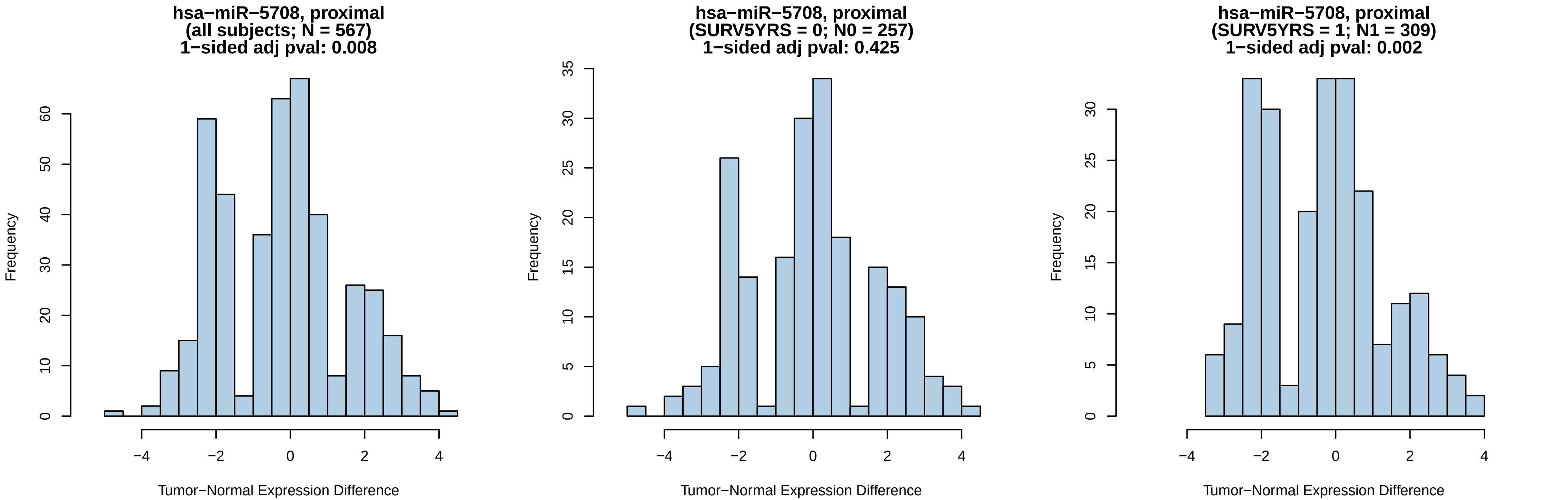
hsa−miR−5708, proximal
(all subjects; N = 567)
1−sided adj pval: 0.008
Frequency
Tumor−Normal Expression Difference
hsa−miR−5708, proximal
(SURV5YRS = 0; N0 = 257)
1−sided adj pval: 0.425
Frequency
Tumor−Normal Expression Difference
hsa−miR−5708, proximal
(SURV5YRS = 1; N1 = 309)
1−sided adj pval: 0.002
Frequency
Tumor−Normal Expression Difference

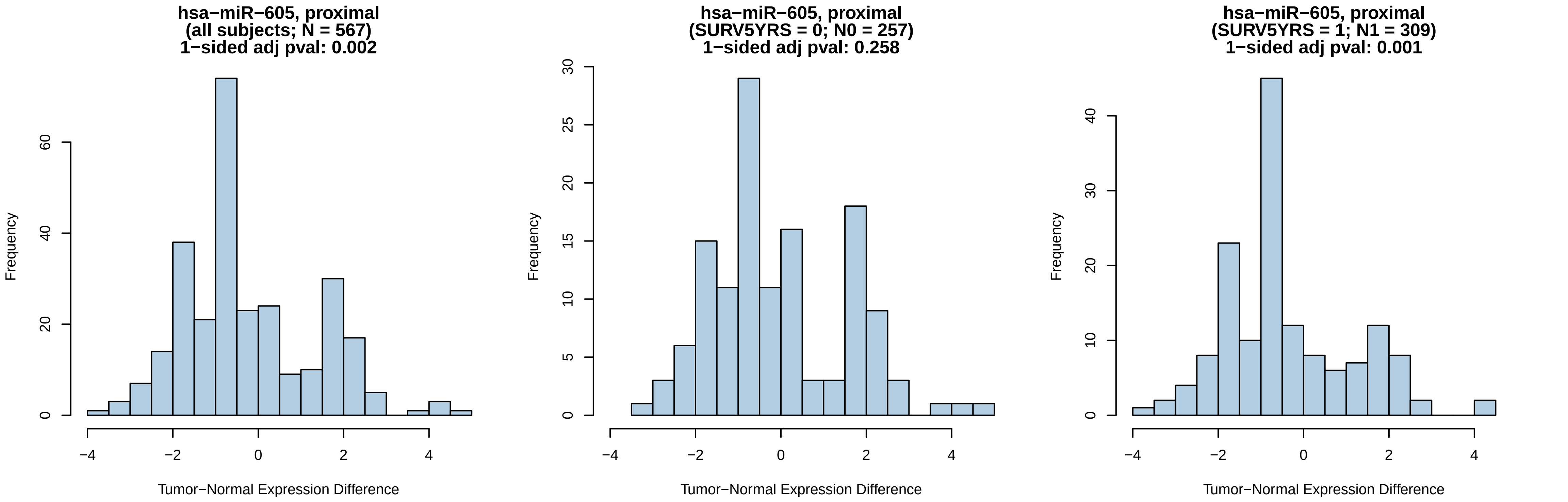

hsa−miR−605, proximal
(all subjects; N = 567)
1−sided adj pval: 0.002
Frequency
Tumor−Normal Expression Difference
hsa−miR−605, proximal
(SURV5YRS = 0; N0 = 257)
1−sided adj pval: 0.258
Frequency
Tumor−Normal Expression Difference
hsa−miR−605, proximal
(SURV5YRS = 1; N1 = 309)
1−sided adj pval: 0.001
Frequency
Tumor−Normal Expression Difference

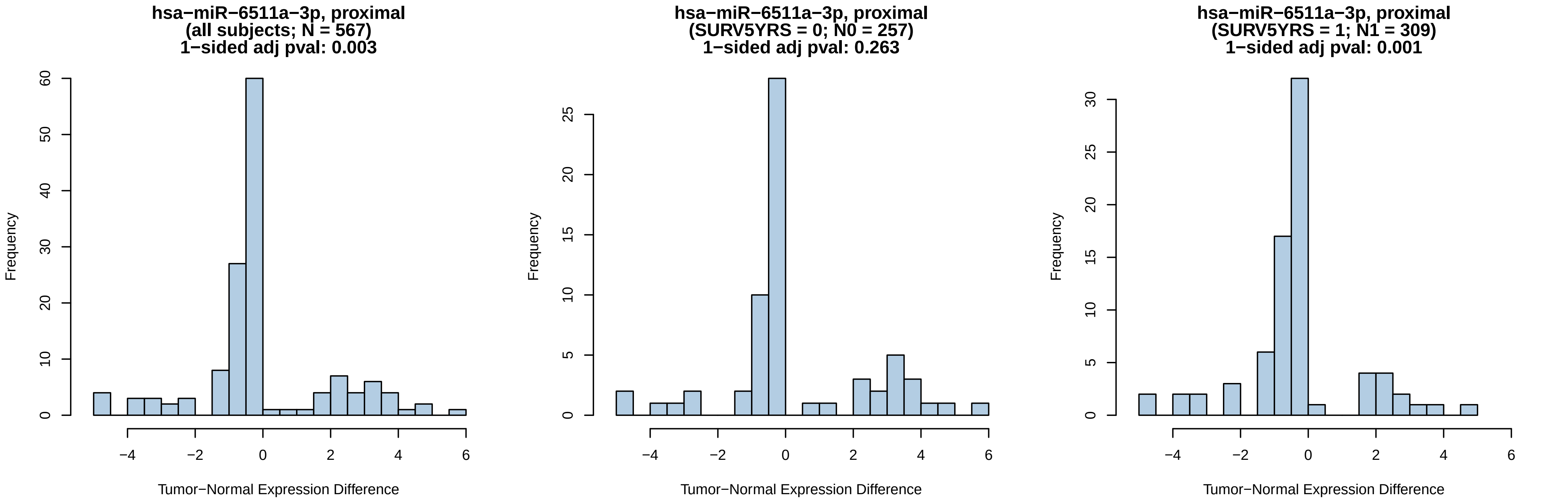
hsa−miR−6511a−3p, proximal
(all subjects; N = 567)
1−sided adj pval: 0.003
Frequency
Tumor−Normal Expression Difference
0
10
20
30
40
50
60
−4
−2
0
2
4
6
hsa−miR−6511a−3p, proximal
(SURV5YRS = 0; N0 = 257)
1−sided adj pval: 0.263
Frequency
Tumor−Normal Expression Difference
0
5
10
15
20
25
−4
−2
0
2
4
6
hsa−miR−6511a−3p, proximal
(SURV5YRS = 1; N1 = 309)
1−sided adj pval: 0.001
Frequency
Tumor−Normal Expression Difference
0
5
10
15
20
25
30
−4
−2
0
2
4
6

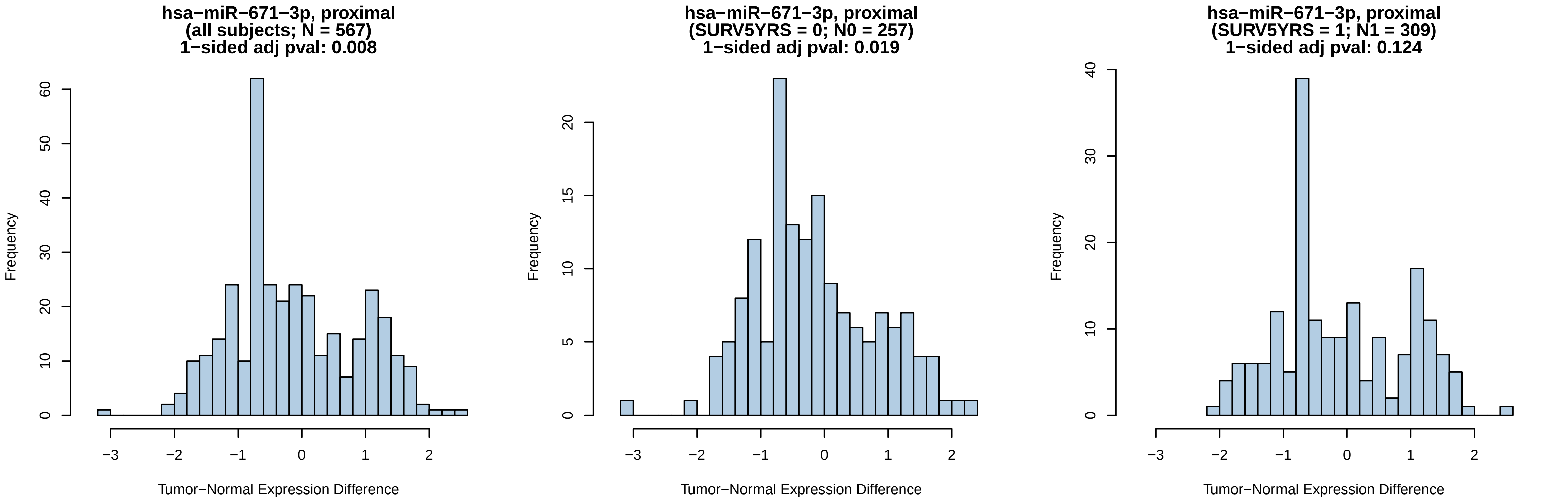

hsa−miR−671−3p, proximal
(all subjects; N = 567)
1−sided adj pval: 0.008
hsa−miR−671−3p, proximal
(SURV5YRS = 0; N0 = 257)
1−sided adj pval: 0.019
hsa−miR−671−3p, proximal
(SURV5YRS = 1; N1 = 309)
1−sided adj pval: 0.124
Frequency
Tumor−Normal Expression Difference

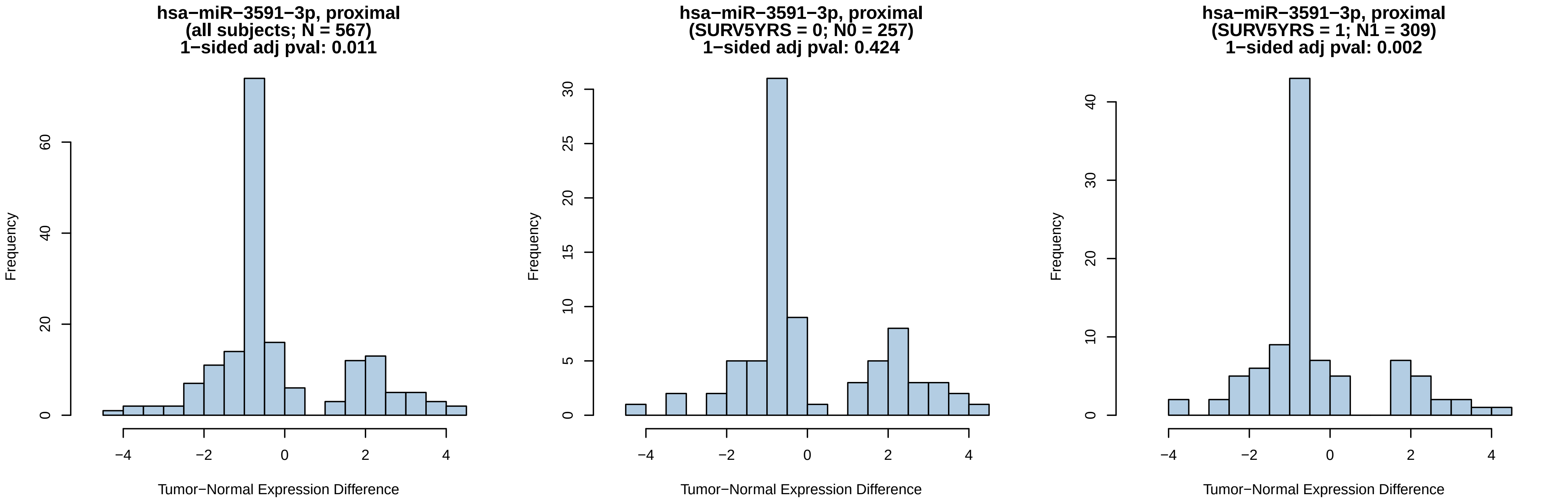
hsa−miR−3591−3p, proximal
(all subjects; N = 567)
1−sided adj pval: 0.011
Frequency
Tumor−Normal Expression Difference
hsa−miR−3591−3p, proximal
(SURV5YRS = 0; N0 = 257)
1−sided adj pval: 0.424
Frequency
Tumor−Normal Expression Difference
hsa−miR−3591−3p, proximal
(SURV5YRS = 1; N1 = 309)
1−sided adj pval: 0.002
Frequency
Tumor−Normal Expression Difference

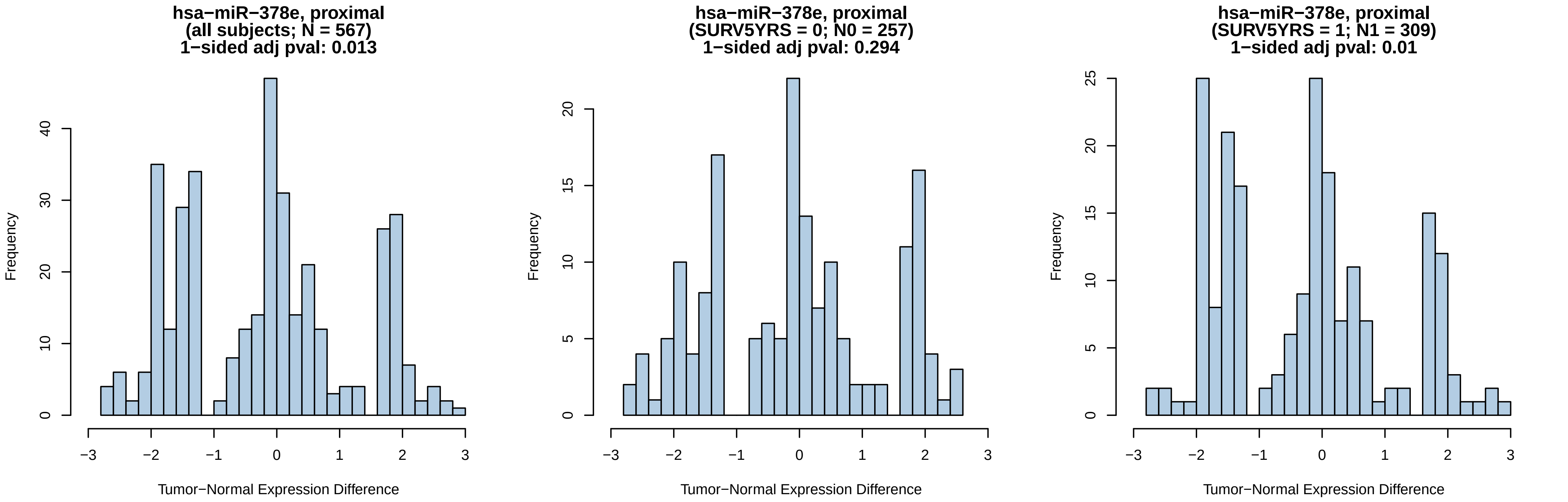
hsa−miR−378e, proximal
(all subjects; N = 567)
1−sided adj pval: 0.013
hsa−miR−378e, proximal
(SURV5YRS = 0; N0 = 257)
1−sided adj pval: 0.294
hsa−miR−378e, proximal
(SURV5YRS = 1; N1 = 309)
1−sided adj pval: 0.01
Frequency
Tumor−Normal Expression Difference

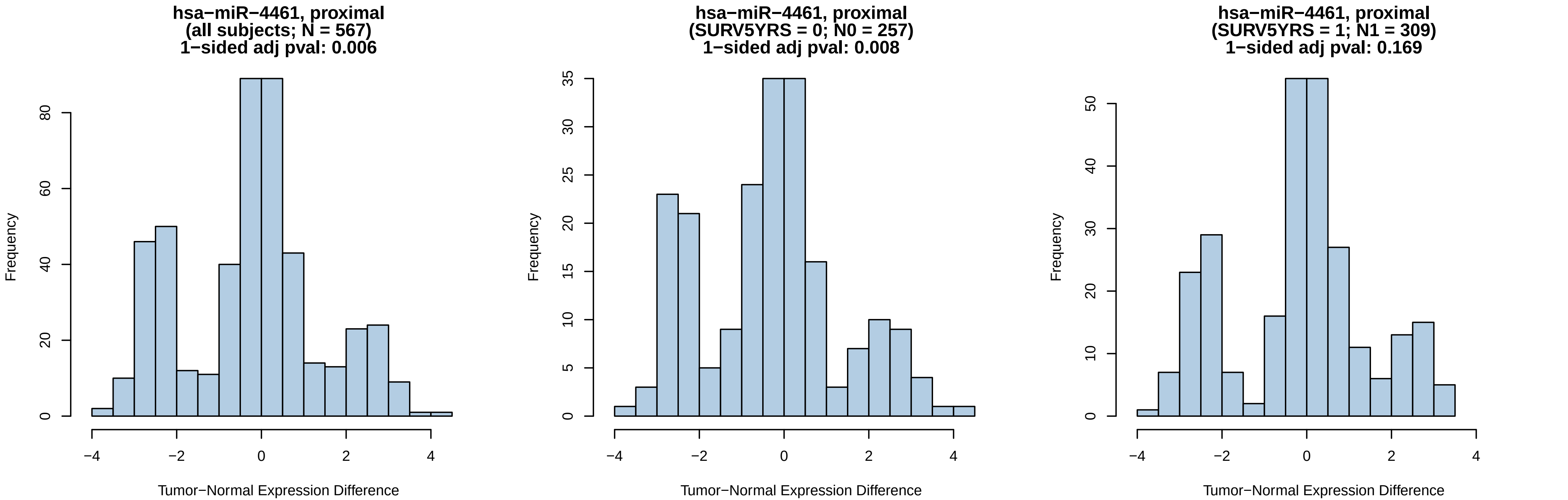
hsa−miR−4461, proximal
(all subjects; N = 567)
1−sided adj pval: 0.006
hsa−miR−4461, proximal
(SURV5YRS = 0; N0 = 257)
1−sided adj pval: 0.008
hsa−miR−4461, proximal
(SURV5YRS = 1; N1 = 309)
1−sided adj pval: 0.169
Frequency
Tumor−Normal Expression Difference
0
20
40
60
80
0
5
10
15
20
25
30
35
0
10
20
30
40
50
−4
−2
0
2
4

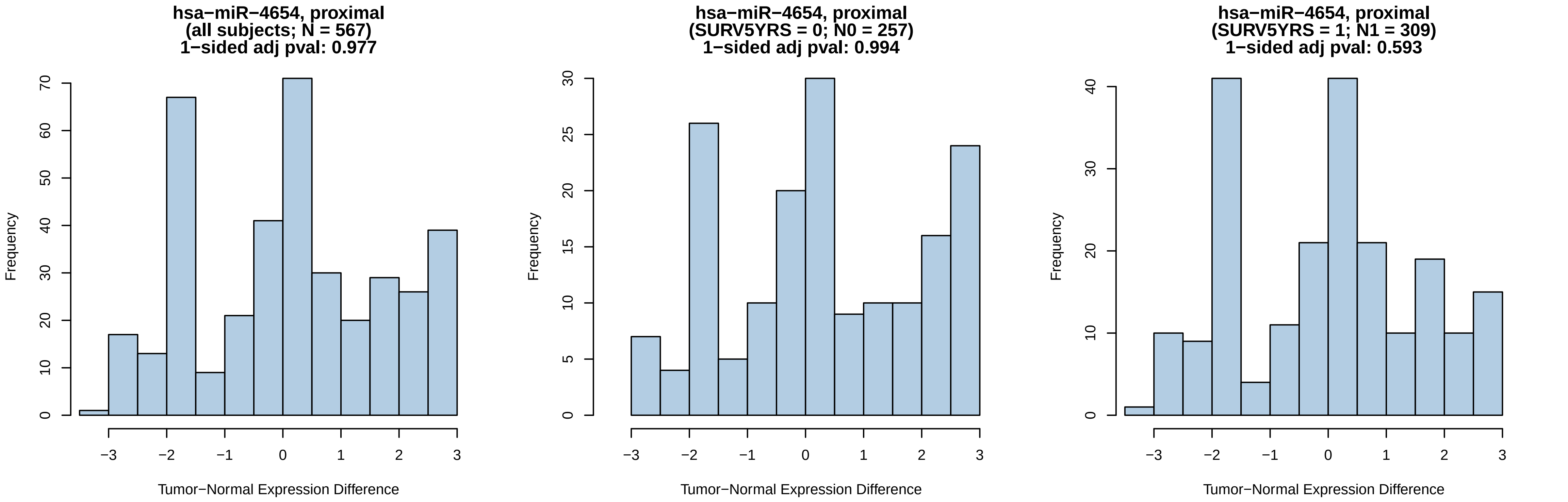
hsa−miR−4654, proximal
(all subjects; N = 567)
1−sided adj pval: 0.977
Frequency
Tumor−Normal Expression Difference
hsa−miR−4654, proximal
(SURV5YRS = 0; N0 = 257)
1−sided adj pval: 0.994
Frequency
Tumor−Normal Expression Difference
hsa−miR−4654, proximal
(SURV5YRS = 1; N1 = 309)
1−sided adj pval: 0.593
Frequency
Tumor−Normal Expression Difference

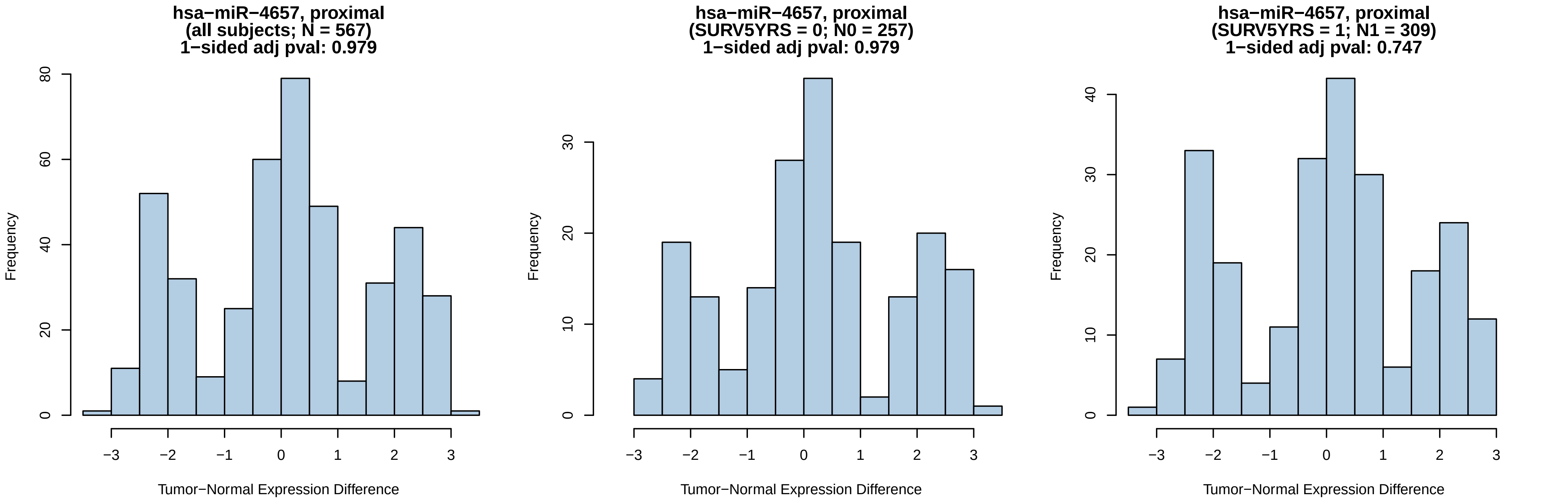
hsa−miR−4657, proximal
(all subjects; N = 567)
1−sided adj pval: 0.979
Frequency
Tumor−Normal Expression Difference
hsa−miR−4657, proximal
(SURV5YRS = 0; N0 = 257)
1−sided adj pval: 0.979
Frequency
Tumor−Normal Expression Difference
hsa−miR−4657, proximal
(SURV5YRS = 1; N1 = 309)
1−sided adj pval: 0.747
Frequency
Tumor−Normal Expression Difference

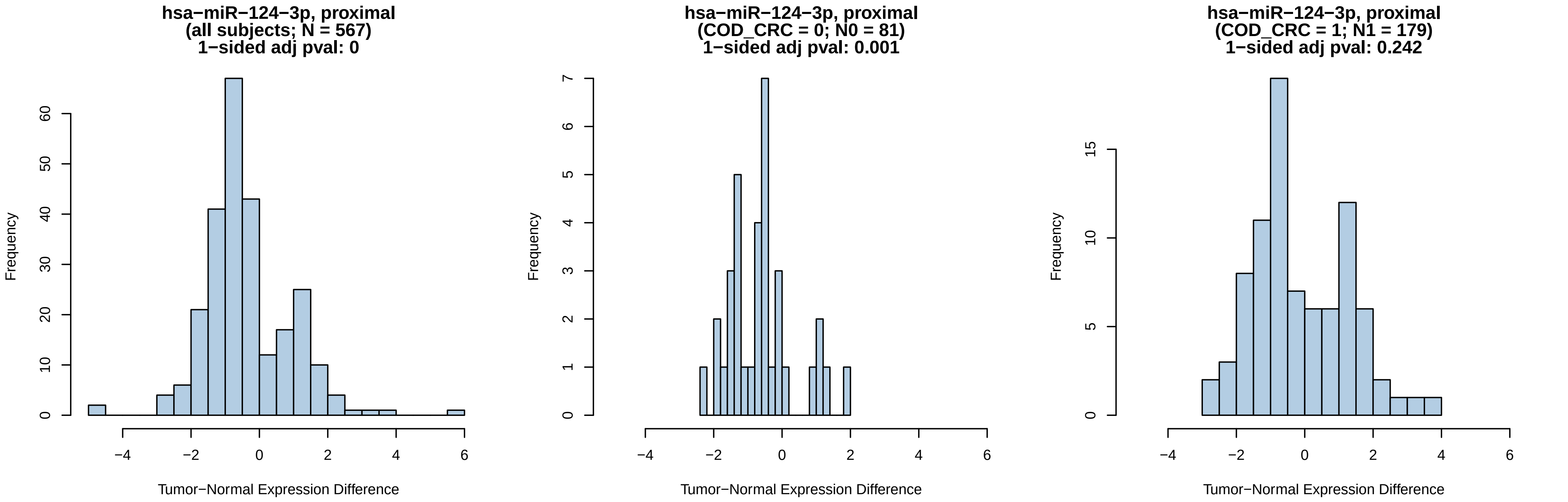
hsa−miR−124−3p, proximal
(all subjects; N = 567)
1−sided adj pval: 0
Frequency
Tumor−Normal Expression Difference
hsa−miR−124−3p, proximal
(COD_CRC = 0; N0 = 81)
1−sided adj pval: 0.001
Frequency
Tumor−Normal Expression Difference
hsa−miR−124−3p, proximal
(COD_CRC = 1; N1 = 179)
1−sided adj pval: 0.242
Frequency
Tumor−Normal Expression Difference

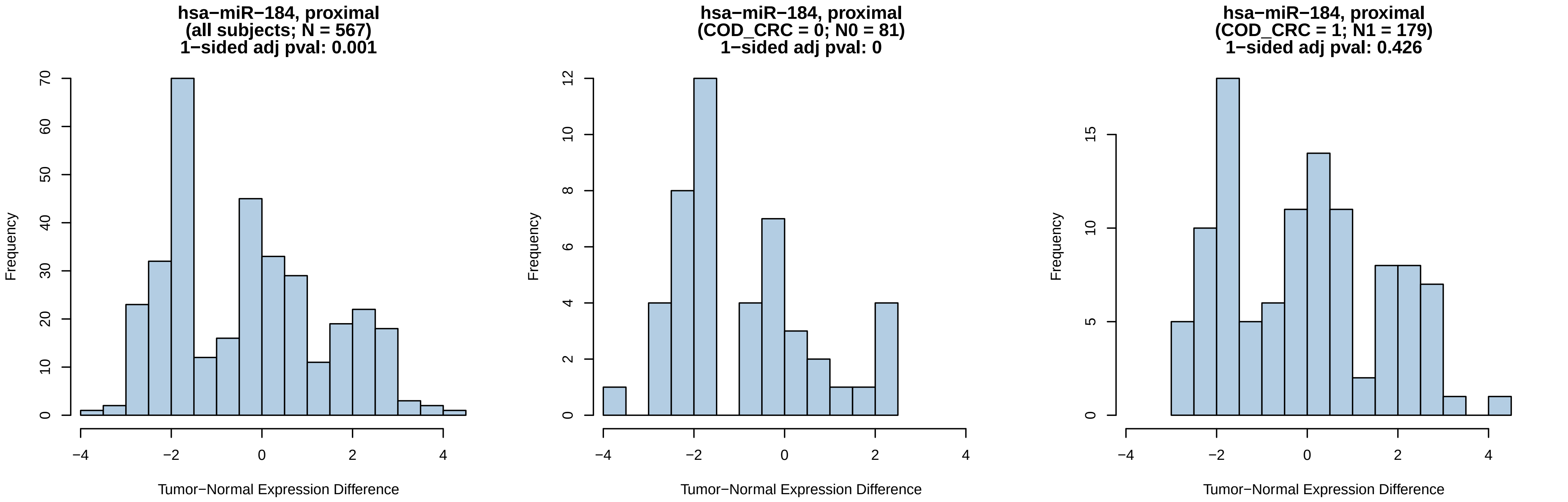
hsa−miR−184, proximal
(all subjects; N = 567)
1−sided adj pval: 0.001
Frequency
Tumor−Normal Expression Difference
hsa−miR−184, proximal
(COD_CRC = 0; N0 = 81)
1−sided adj pval: 0
Frequency
Tumor−Normal Expression Difference
hsa−miR−184, proximal
(COD_CRC = 1; N1 = 179)
1−sided adj pval: 0.426
Frequency
Tumor−Normal Expression Difference

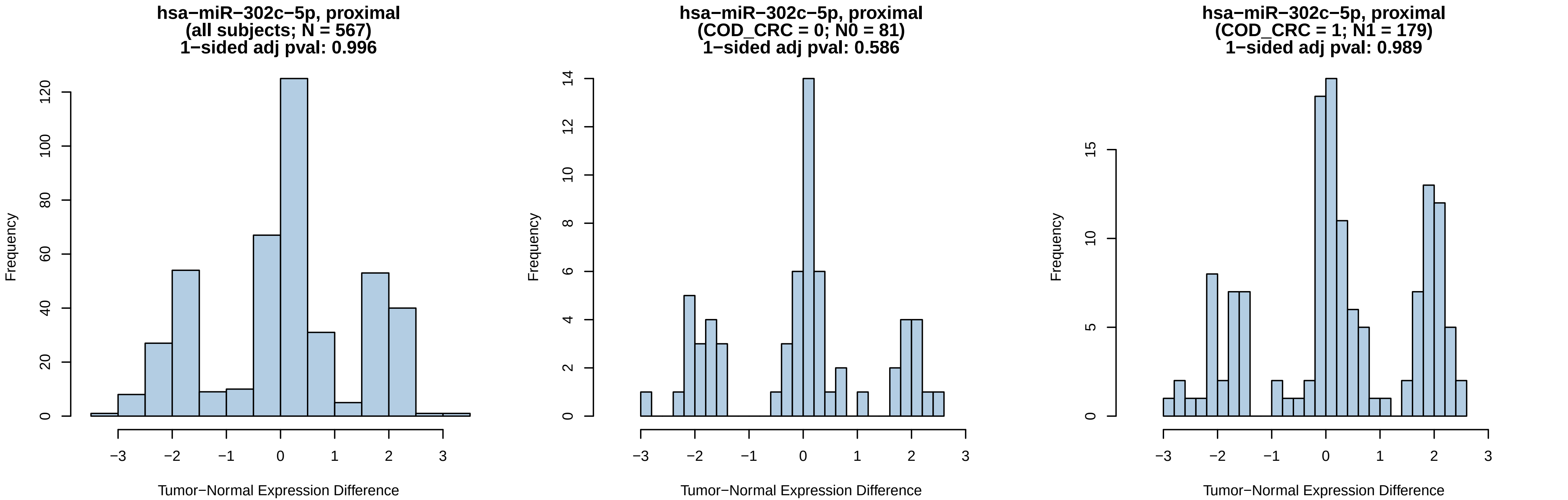

hsa−miR−302c−5p, proximal
(all subjects; N = 567)
1−sided adj pval: 0.996
Frequency
Tumor−Normal Expression Difference
hsa−miR−302c−5p, proximal
(COD_CRC = 0; N0 = 81)
1−sided adj pval: 0.586
Frequency
Tumor−Normal Expression Difference
hsa−miR−302c−5p, proximal
(COD_CRC = 1; N1 = 179)
1−sided adj pval: 0.989
Frequency
Tumor−Normal Expression Difference

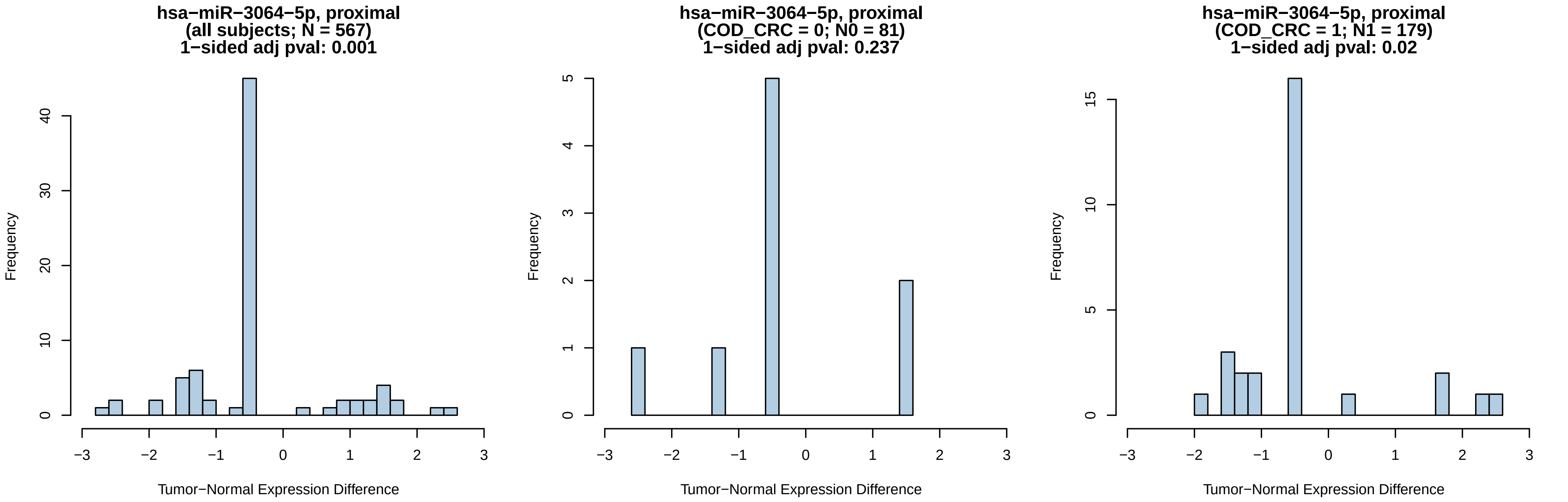
hsa−miR−3064−5p, proximal
(all subjects; N = 567)
1−sided adj pval: 0.001
hsa−miR−3064−5p, proximal
(COD_CRC = 0; N0 = 81)
1−sided adj pval: 0.237
hsa−miR−3064−5p, proximal
(COD_CRC = 1; N1 = 179)
1−sided adj pval: 0.02
Frequency
Tumor−Normal Expression Difference

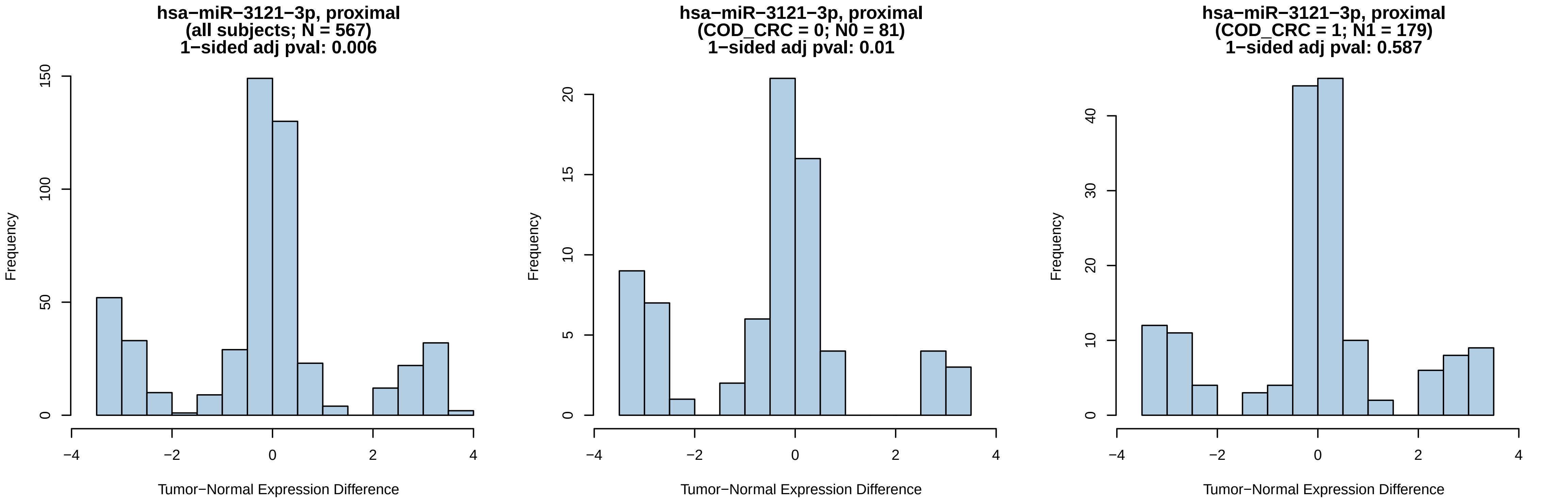
hsa−miR−3121−3p, proximal
(all subjects; N = 567)
1−sided adj pval: 0.006
hsa−miR−3121−3p, proximal
(COD_CRC = 0; N0 = 81)
1−sided adj pval: 0.01
hsa−miR−3121−3p, proximal
(COD_CRC = 1; N1 = 179)
1−sided adj pval: 0.587
Frequency
Tumor−Normal Expression Difference

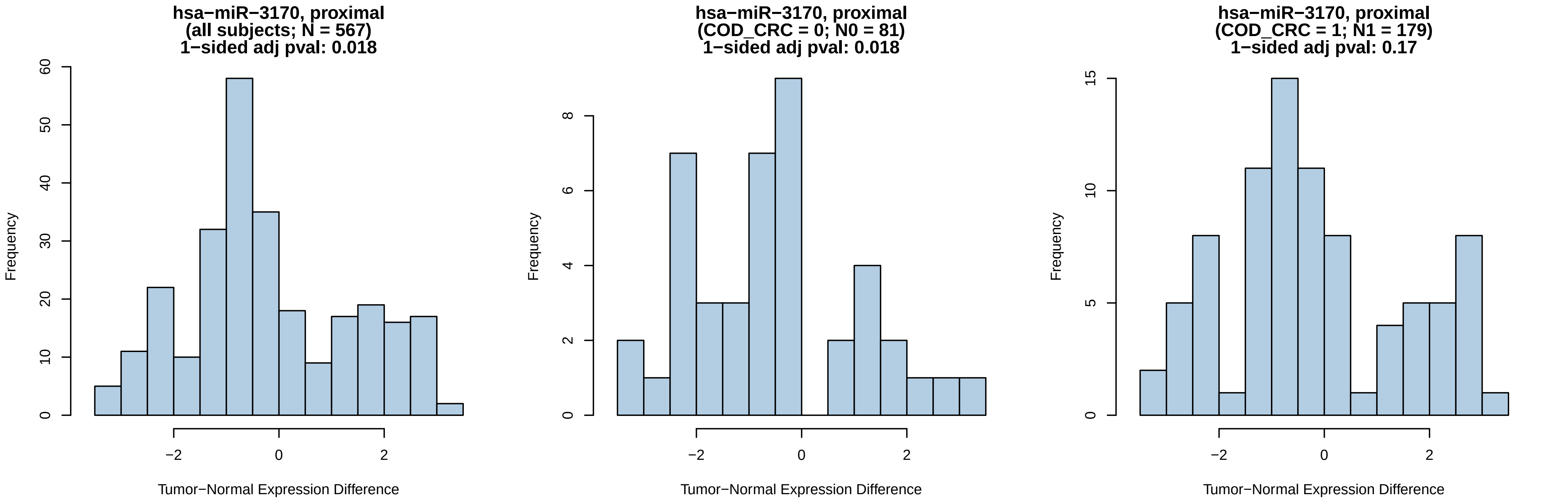
hsa−miR−3170, proximal
(all subjects; N = 567)
1−sided adj pval: 0.018
hsa−miR−3170, proximal
(COD_CRC = 0; N0 = 81)
1−sided adj pval: 0.018
hsa−miR−3170, proximal
(COD_CRC = 1; N1 = 179)
1−sided adj pval: 0.17
Frequency
Tumor−Normal Expression Difference

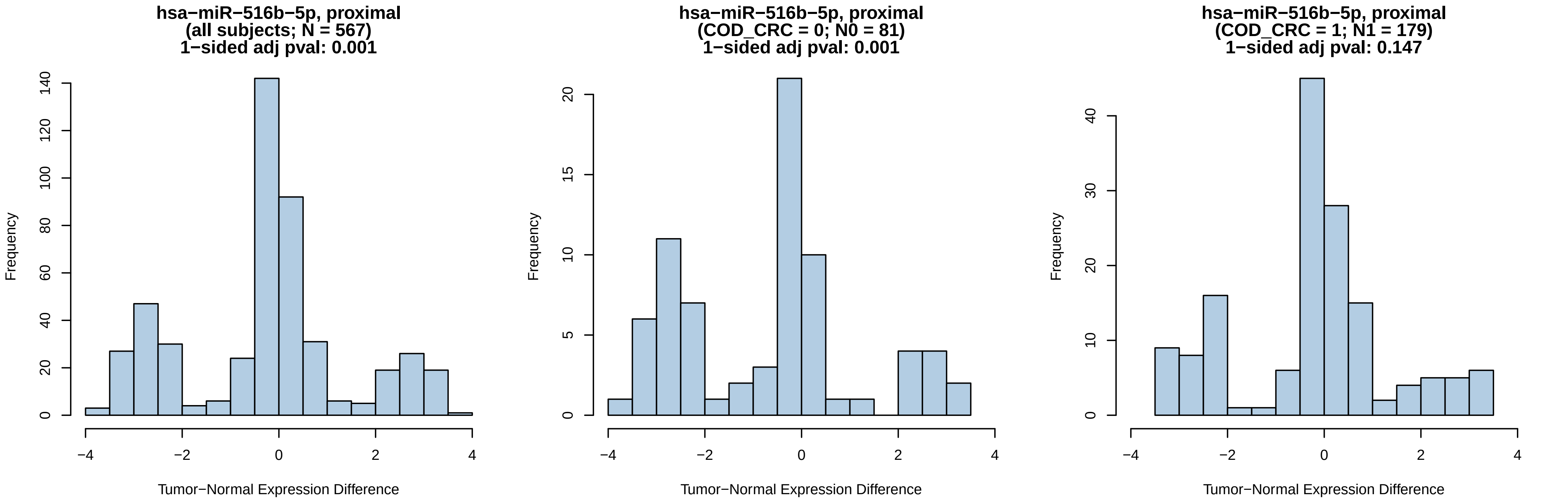

hsa−miR−516b−5p, proximal
(all subjects; N = 567)
1−sided adj pval: 0.001
Frequency
Tumor−Normal Expression Difference
hsa−miR−516b−5p, proximal
(COD_CRC = 0; N0 = 81)
1−sided adj pval: 0.001
Frequency
Tumor−Normal Expression Difference
hsa−miR−516b−5p, proximal
(COD_CRC = 1; N1 = 179)
1−sided adj pval: 0.147
Frequency
Tumor−Normal Expression Difference

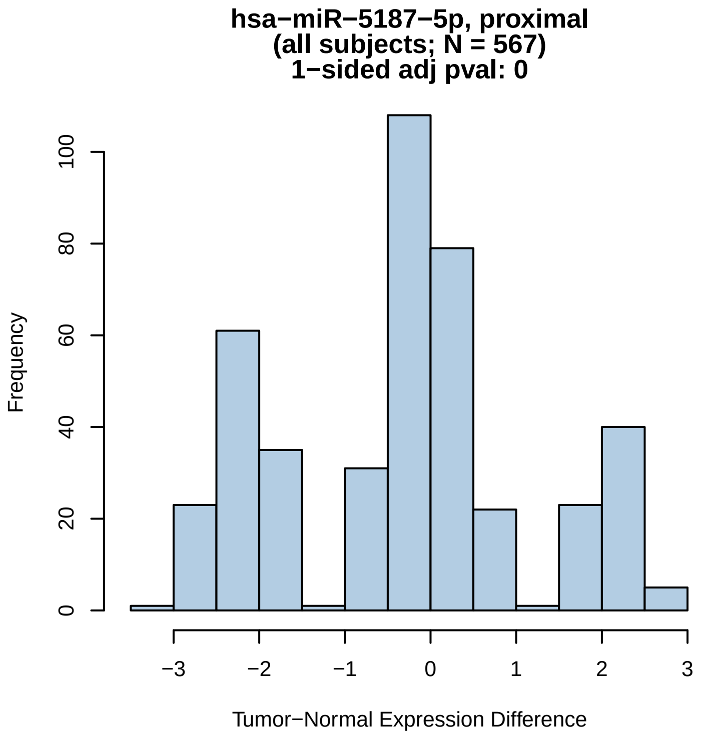
hsa−miR−5187−5p, proximal
(all subjects; N = 567)
1−sided adj pval: 0
Frequency
0
20
40
60
80
100
−3
−2
−1
0
1
2
3
Tumor−Normal Expression Difference

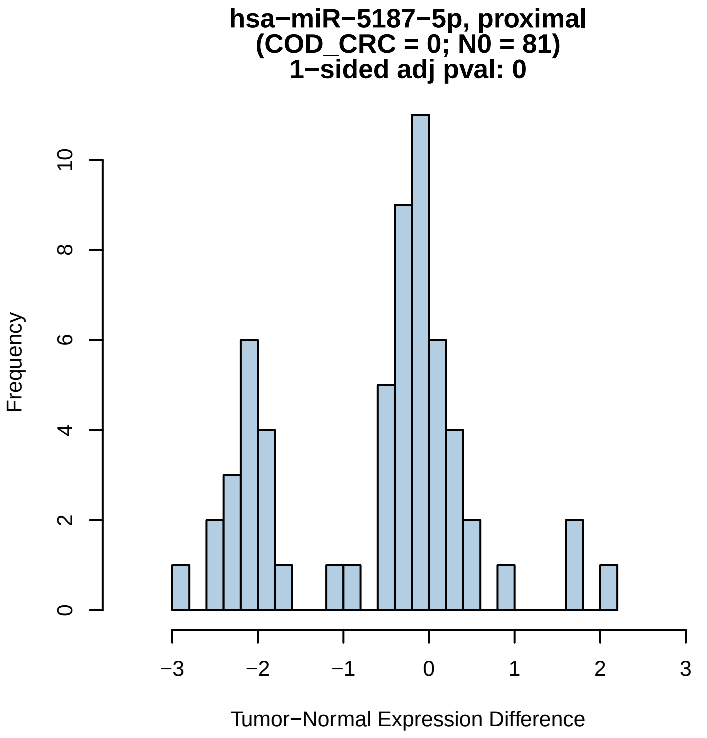
hsa−miR−5187−5p, proximal
(COD_CRC = 0; N0 = 81)
1−sided adj pval: 0
Frequency
0
2
4
6
8
10
−3
−2
−1
0
1
2
3
Tumor−Normal Expression Difference

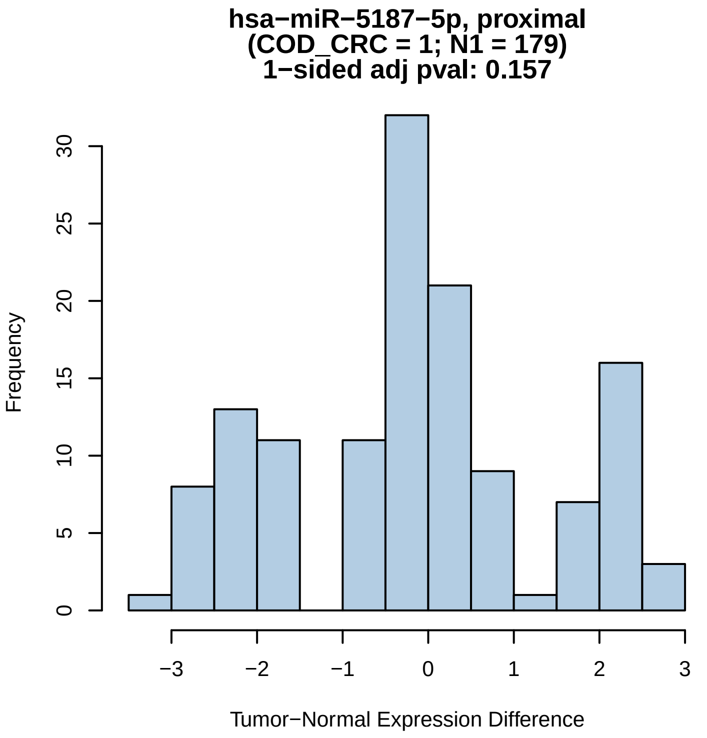
hsa−miR−5187−5p, proximal
(COD_CRC = 1; N1 = 179)
1−sided adj pval: 0.157
Frequency
0
5
10
15
20
25
30
−3
−2
−1
0
1
2
3
Tumor−Normal Expression Difference

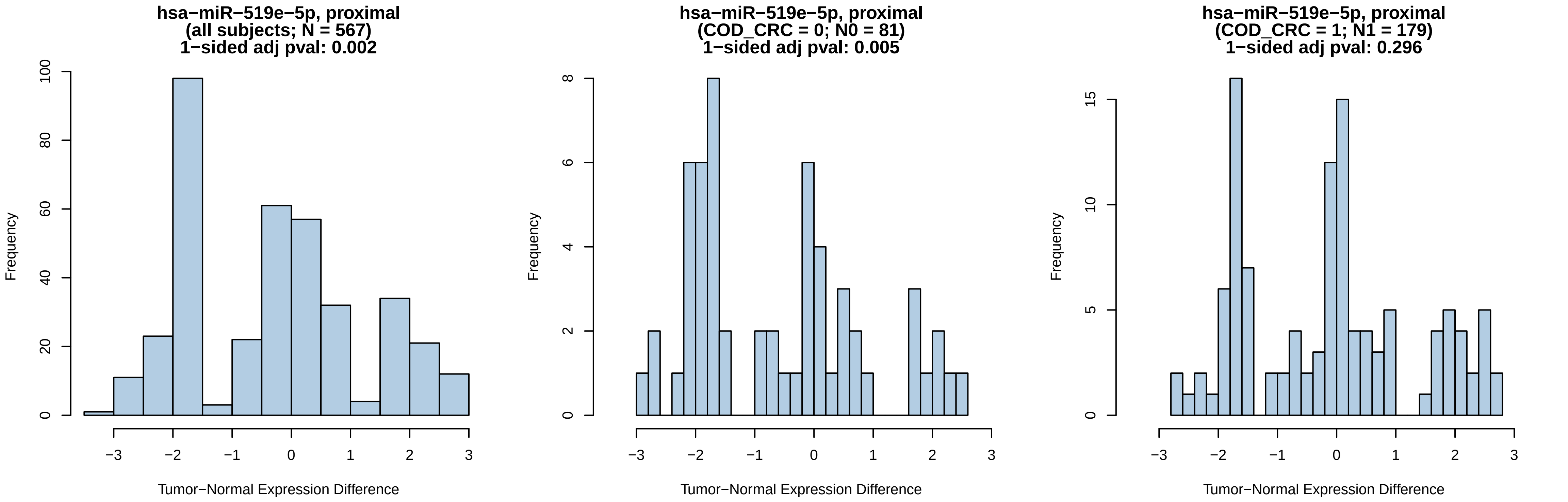
hsa−miR−519e−5p, proximal
(all subjects; N = 567)
1−sided adj pval: 0.002
Frequency
0
20
40
60
80
100
−3
−2
−1
0
1
2
3
Tumor−Normal Expression Difference
hsa−miR−519e−5p, proximal
(COD_CRC = 0; N0 = 81)
1−sided adj pval: 0.005
Frequency
0
2
4
6
8
−3
−2
−1
0
1
2
3
Tumor−Normal Expression Difference
hsa−miR−519e−5p, proximal
(COD_CRC = 1; N1 = 179)
1−sided adj pval: 0.296
Frequency
0
5
10
15
−3
−2
−1
0
1
2
3
Tumor−Normal Expression Difference

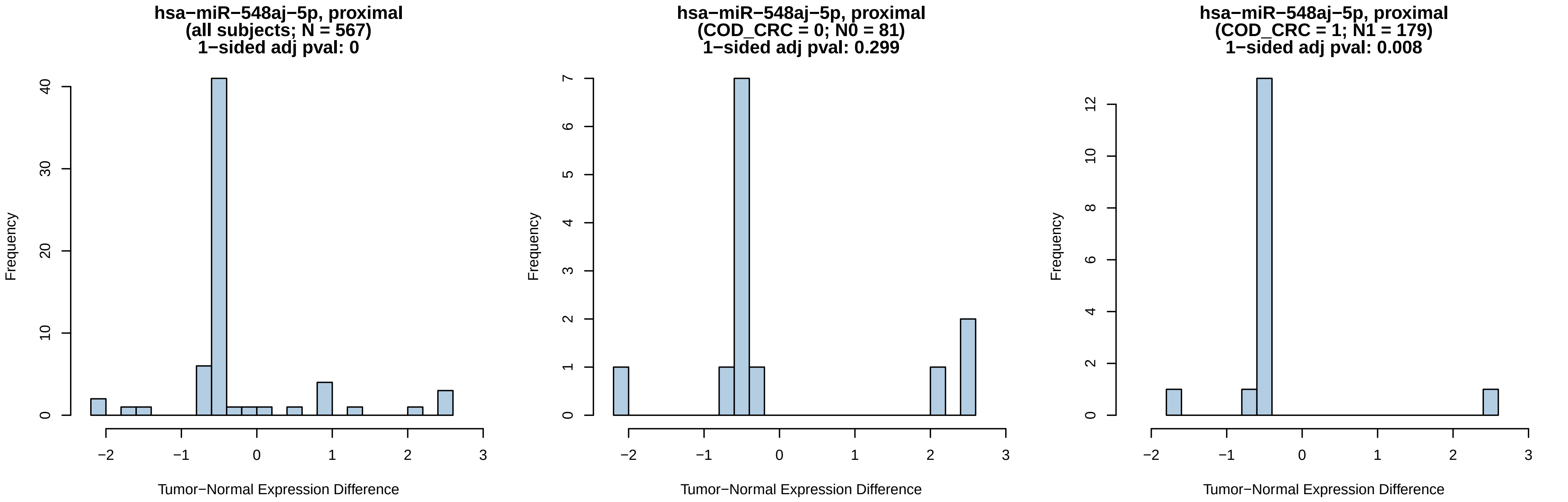
hsa−miR−548aj−5p, proximal
(all subjects; N = 567)
1−sided adj pval: 0
Frequency
Tumor−Normal Expression Difference
hsa−miR−548aj−5p, proximal
(COD_CRC = 0; N0 = 81)
1−sided adj pval: 0.299
Frequency
Tumor−Normal Expression Difference
hsa−miR−548aj−5p, proximal
(COD_CRC = 1; N1 = 179)
1−sided adj pval: 0.008
Frequency
Tumor−Normal Expression Difference

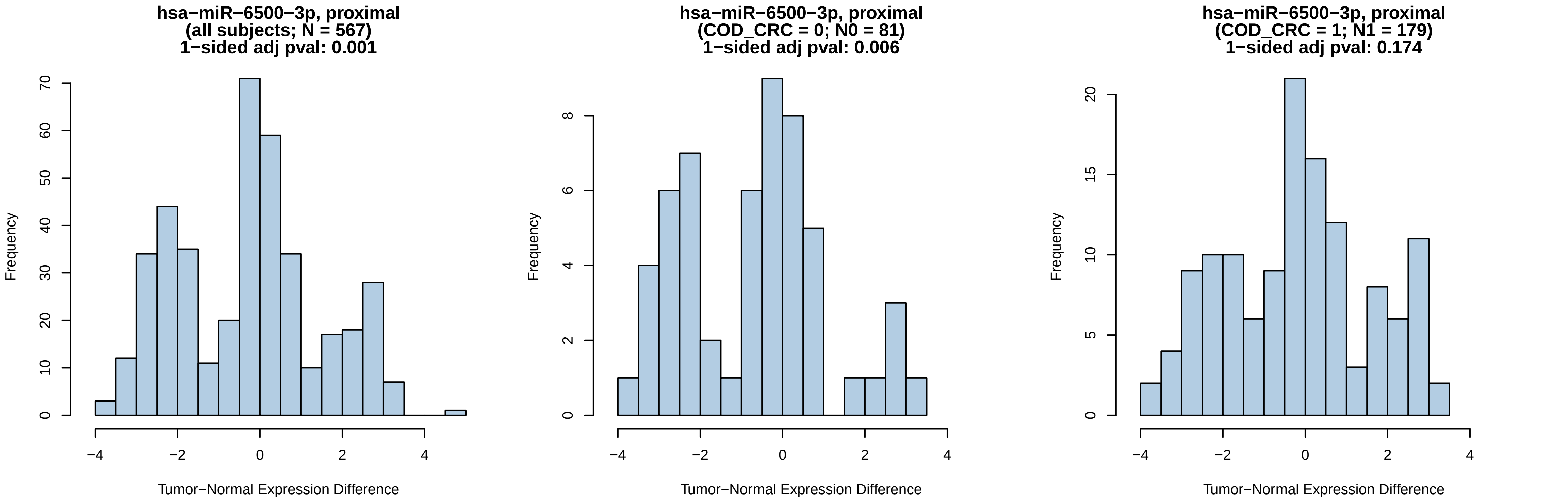

hsa−miR−6500−3p, proximal
(all subjects; N = 567)
1−sided adj pval: 0.001
Frequency
Tumor−Normal Expression Difference
hsa−miR−6500−3p, proximal
(COD_CRC = 0; N0 = 81)
1−sided adj pval: 0.006
Frequency
Tumor−Normal Expression Difference
hsa−miR−6500−3p, proximal
(COD_CRC = 1; N1 = 179)
1−sided adj pval: 0.174
Frequency
Tumor−Normal Expression Difference

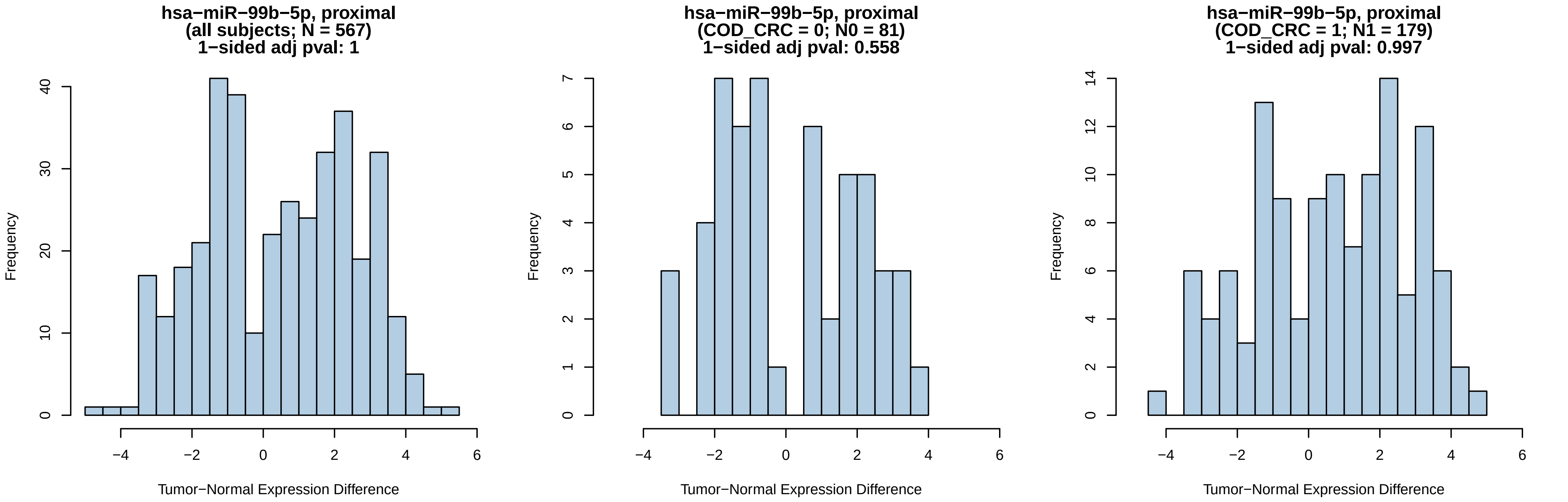

hsa−miR−99b−5p, proximal
(all subjects; N = 567)
1−sided adj pval: 1
Frequency
Tumor−Normal Expression Difference
hsa−miR−99b−5p, proximal
(COD_CRC = 0; N0 = 81)
1−sided adj pval: 0.558
Frequency
Tumor−Normal Expression Difference
hsa−miR−99b−5p, proximal
(COD_CRC = 1; N1 = 179)
1−sided adj pval: 0.997
Frequency
Tumor−Normal Expression Difference

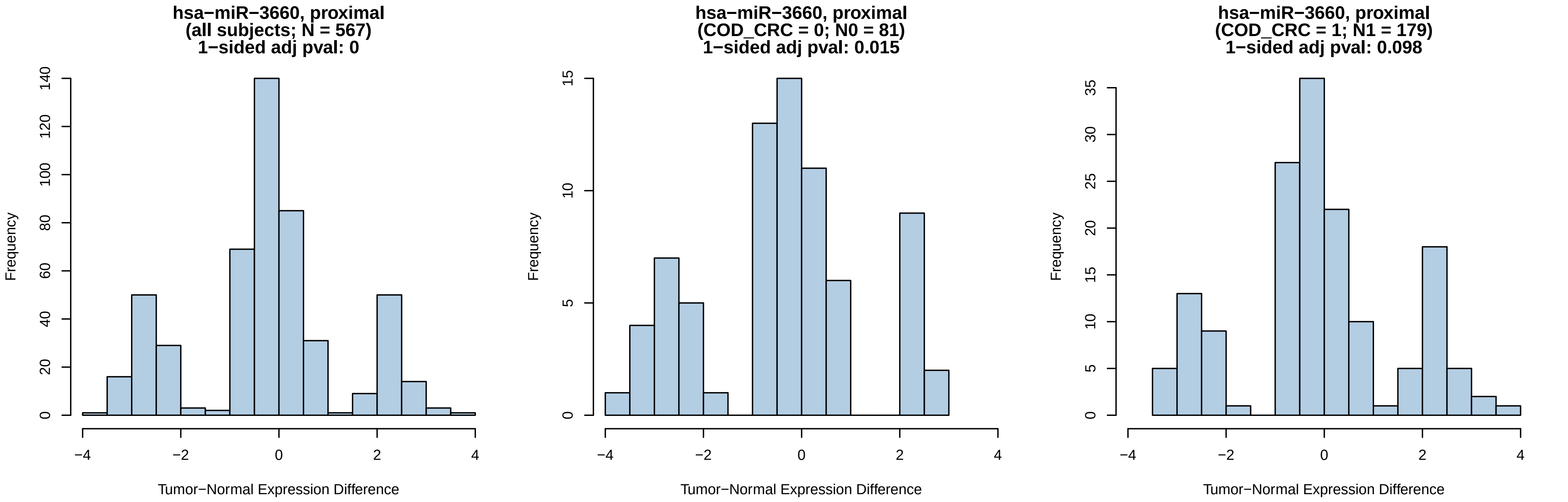

hsa−miR−3660, proximal
(all subjects; N = 567)
1−sided adj pval: 0
Frequency
Tumor−Normal Expression Difference
hsa−miR−3660, proximal
(COD_CRC = 0; N0 = 81)
1−sided adj pval: 0.015
Frequency
Tumor−Normal Expression Difference
hsa−miR−3660, proximal
(COD_CRC = 1; N1 = 179)
1−sided adj pval: 0.098
Frequency
Tumor−Normal Expression Difference

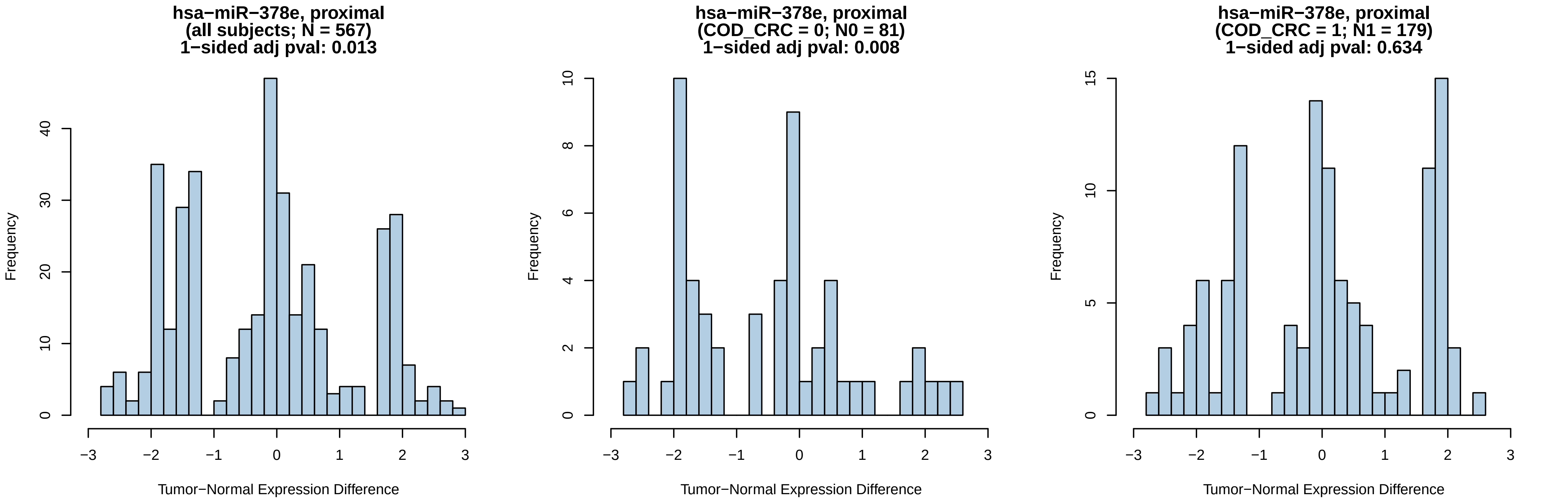
hsa−miR−378e, proximal
(all subjects; N = 567)
1−sided adj pval: 0.013
Frequency
Tumor−Normal Expression Difference
0
10
20
30
40
−3
−2
−1
0
1
2
3
hsa−miR−378e, proximal
(COD_CRC = 0; N0 = 81)
1−sided adj pval: 0.008
Frequency
Tumor−Normal Expression Difference
0
2
4
6
8
10
hsa−miR−378e, proximal
(COD_CRC = 1; N1 = 179)
1−sided adj pval: 0.634
Frequency
Tumor−Normal Expression Difference
0
5
10
15

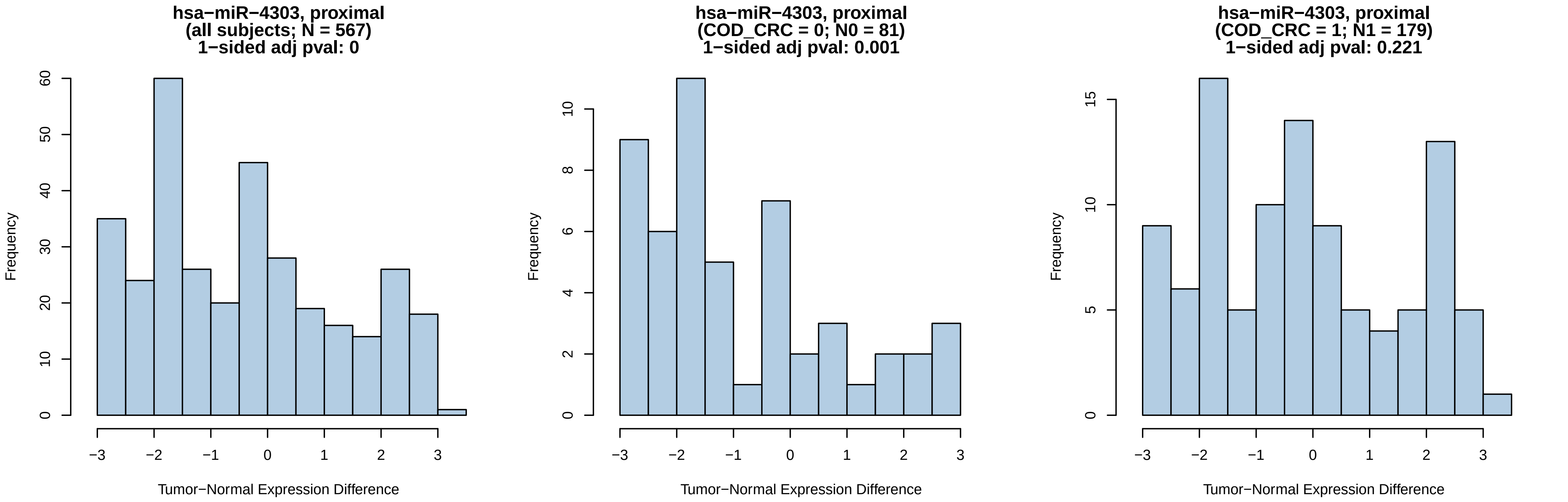

hsa−miR−4303, proximal
(all subjects; N = 567)
1−sided adj pval: 0
hsa−miR−4303, proximal
(COD_CRC = 0; N0 = 81)
1−sided adj pval: 0.001
hsa−miR−4303, proximal
(COD_CRC = 1; N1 = 179)
1−sided adj pval: 0.221
Frequency
Tumor−Normal Expression Difference

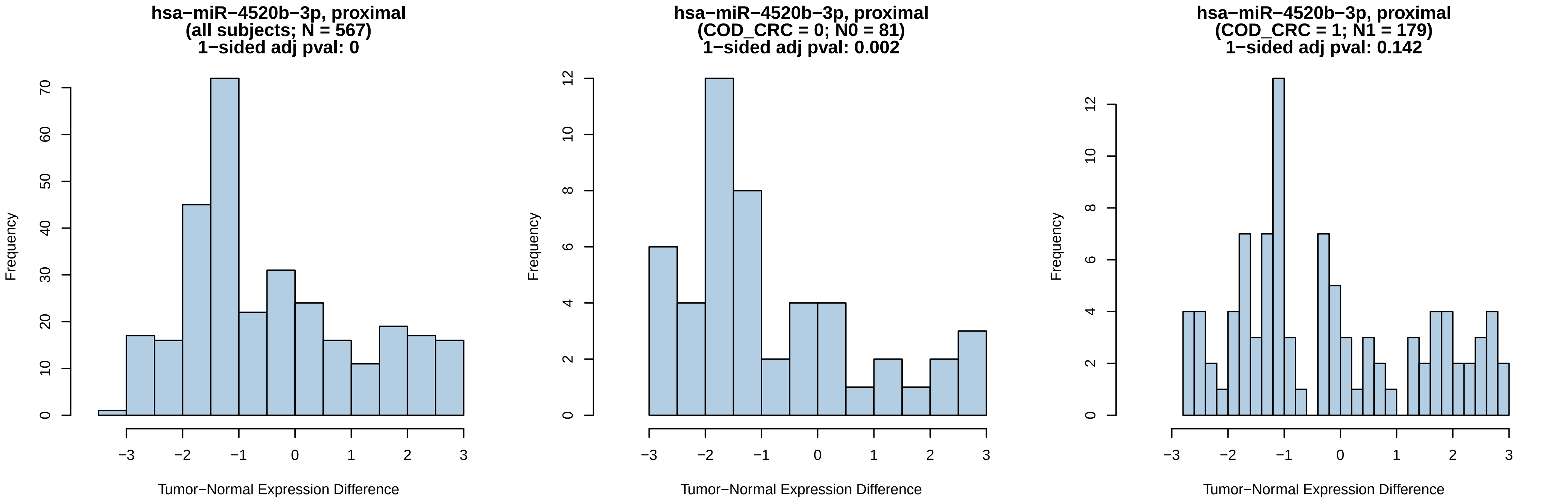

hsa−miR−4520b−3p, proximal
(all subjects; N = 567)
1−sided adj pval: 0
hsa−miR−4520b−3p, proximal
(COD_CRC = 0; N0 = 81)
1−sided adj pval: 0.002
hsa−miR−4520b−3p, proximal
(COD_CRC = 1; N1 = 179)
1−sided adj pval: 0.142
Frequency
Tumor−Normal Expression Difference

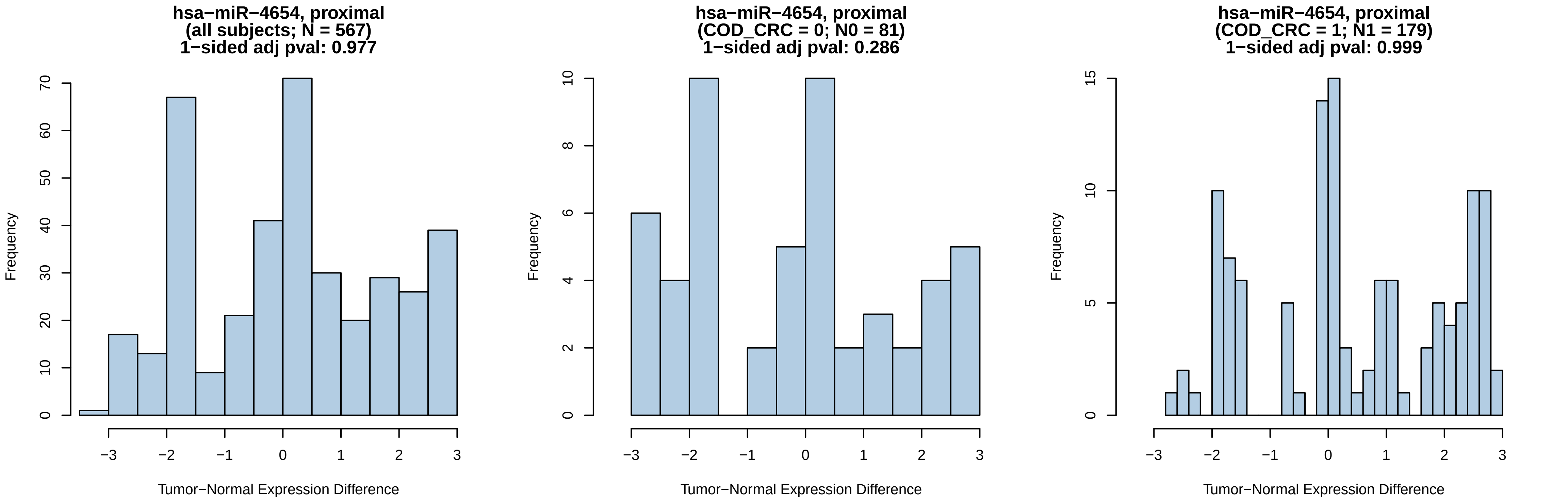

hsa−miR−4654, proximal
(all subjects; N = 567)
1−sided adj pval: 0.977
Frequency
0
10
20
30
40
50
60
70
−3
−2
−1
0
1
2
3
Tumor−Normal Expression Difference
hsa−miR−4654, proximal
(COD_CRC = 0; N0 = 81)
1−sided adj pval: 0.286
Frequency
0
2
4
6
8
10
−3
−2
−1
0
1
2
3
Tumor−Normal Expression Difference
hsa−miR−4654, proximal
(COD_CRC = 1; N1 = 179)
1−sided adj pval: 0.999
Frequency
0
5
10
15
−3
−2
−1
0
1
2
3
Tumor−Normal Expression Difference

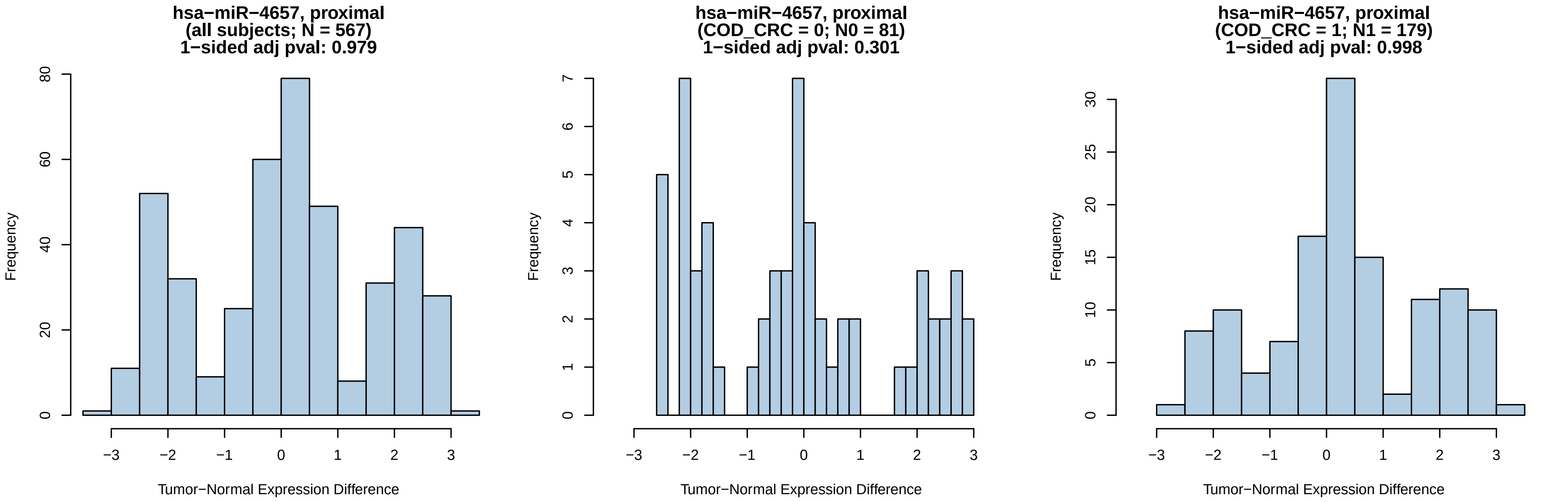
hsa−miR−4657, proximal
(all subjects; N = 567)
1−sided adj pval: 0.979
hsa−miR−4657, proximal
(COD_CRC = 0; N0 = 81)
1−sided adj pval: 0.301
hsa−miR−4657, proximal
(COD_CRC = 1; N1 = 179)
1−sided adj pval: 0.998
Frequency
Tumor−Normal Expression Difference

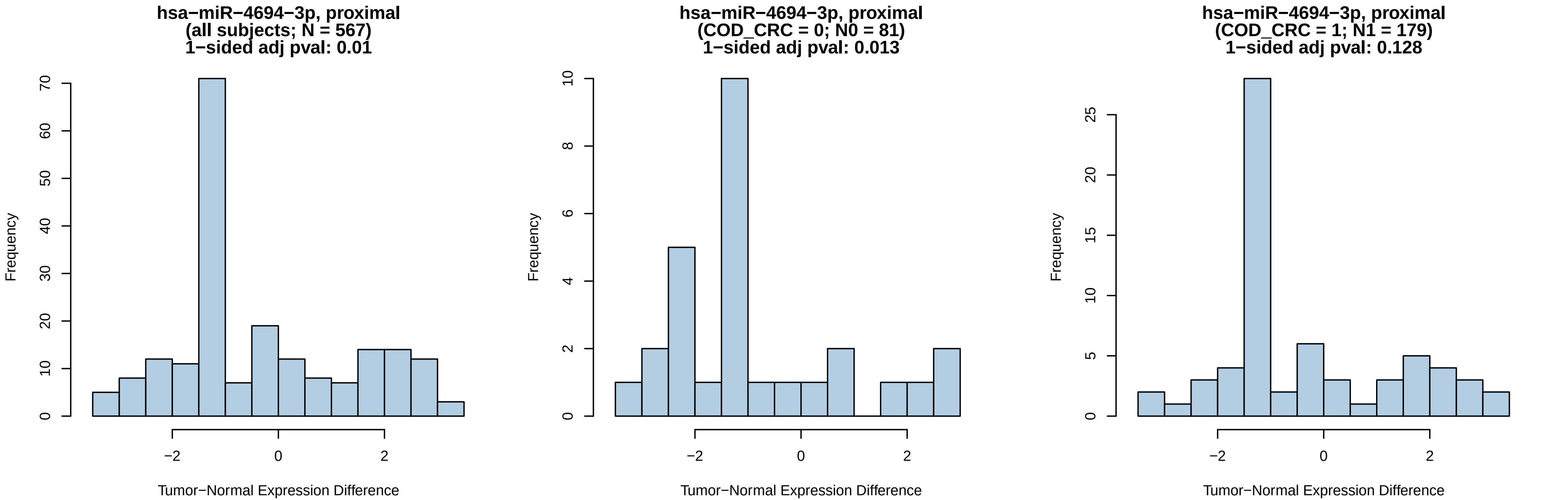
hsa−miR−4694−3p, proximal
(all subjects; N = 567)
1−sided adj pval: 0.01
Frequency
Tumor−Normal Expression Difference
hsa−miR−4694−3p, proximal
(COD_CRC = 0; N0 = 81)
1−sided adj pval: 0.013
Frequency
Tumor−Normal Expression Difference
hsa−miR−4694−3p, proximal
(COD_CRC = 1; N1 = 179)
1−sided adj pval: 0.128
Frequency
Tumor−Normal Expression Difference

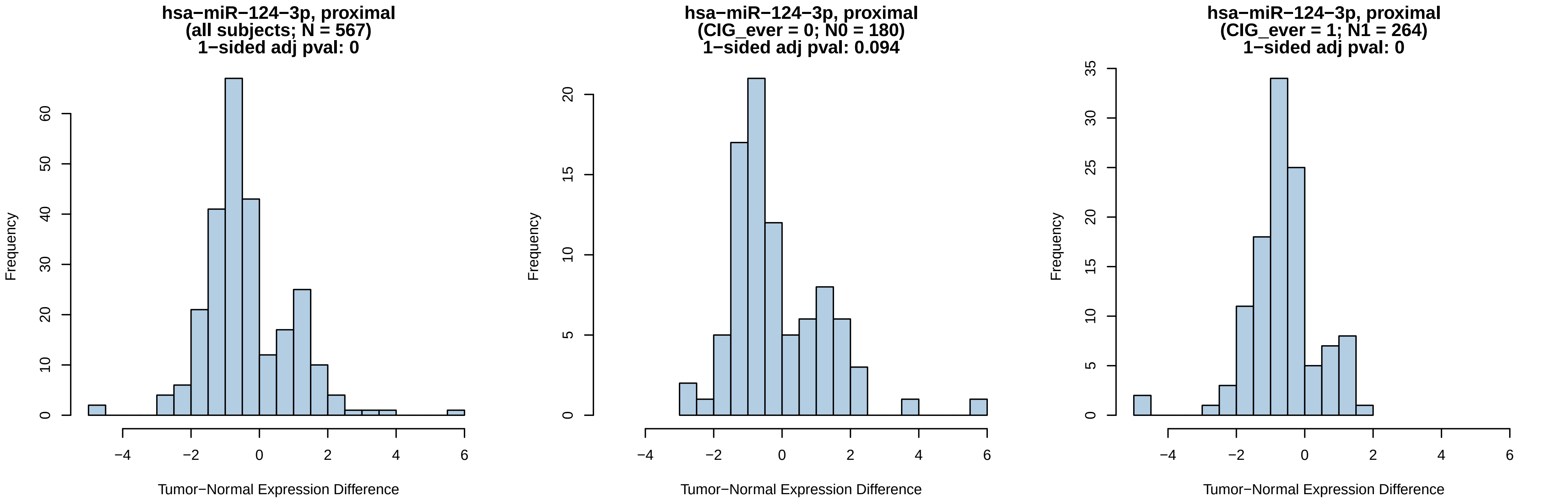
hsa−miR−124−3p, proximal
(all subjects; N = 567)
1−sided adj pval: 0
hsa−miR−124−3p, proximal
(CIG_ever = 0; N0 = 180)
1−sided adj pval: 0.094
hsa−miR−124−3p, proximal
(CIG_ever = 1; N1 = 264)
1−sided adj pval: 0
Frequency
Tumor−Normal Expression Difference

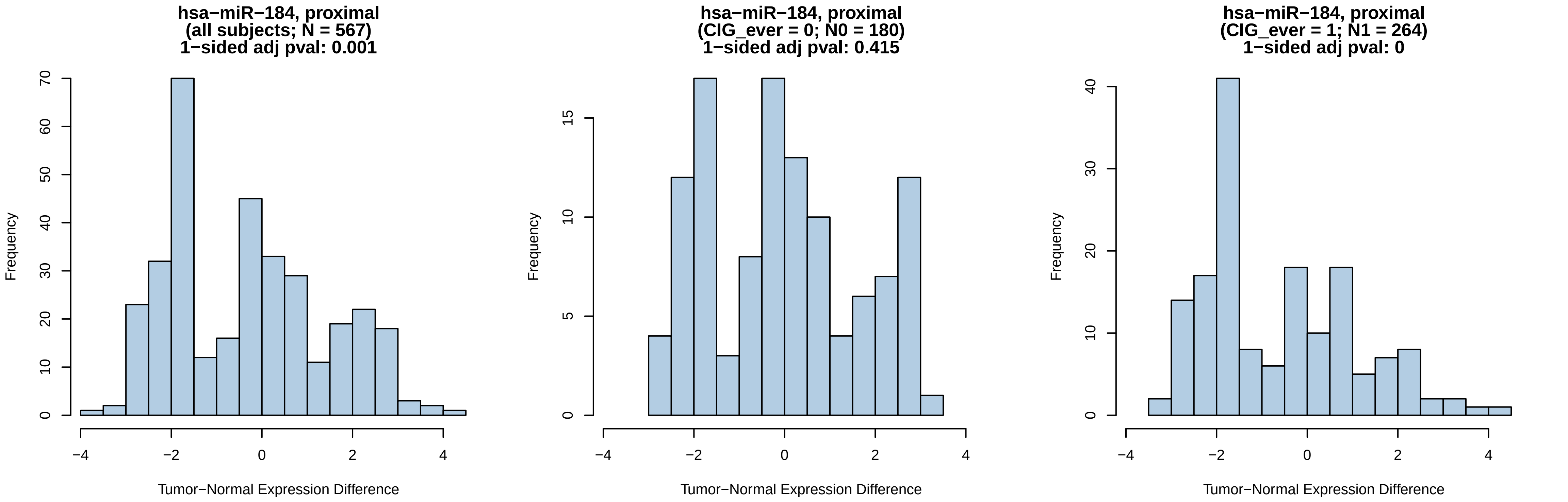
hsa−miR−184, proximal
(all subjects; N = 567)
1−sided adj pval: 0.001
hsa−miR−184, proximal
(CIG_ever = 0; N0 = 180)
1−sided adj pval: 0.415
hsa−miR−184, proximal
(CIG_ever = 1; N1 = 264)
1−sided adj pval: 0
Frequency
Tumor−Normal Expression Difference

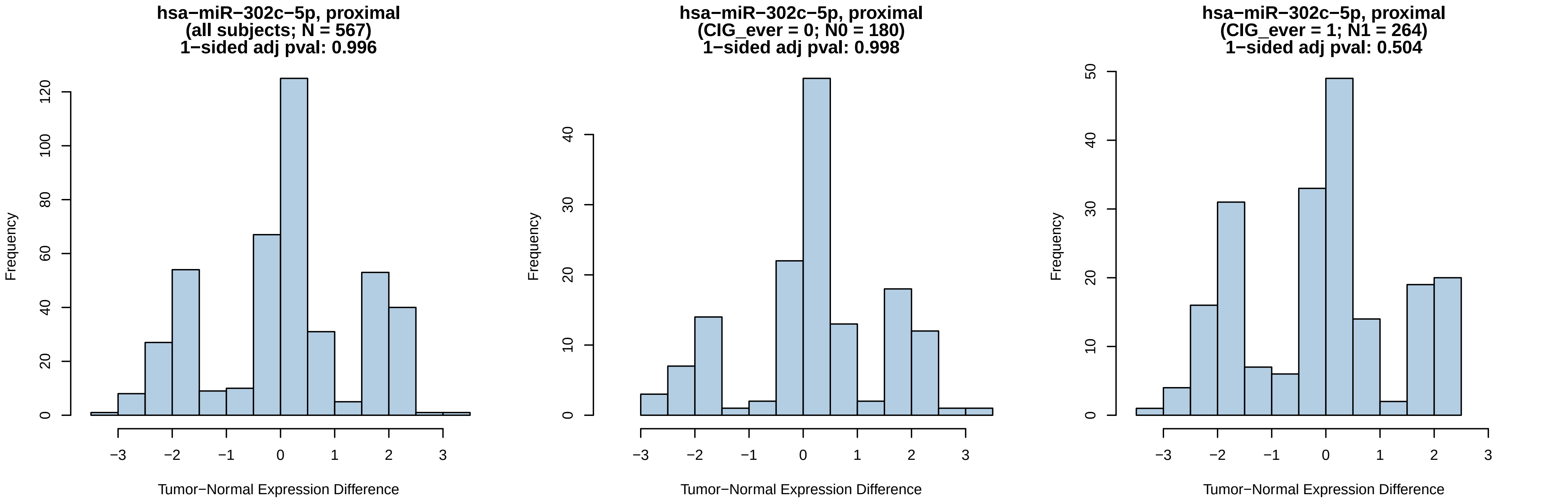

hsa−miR−302c−5p, proximal
(all subjects; N = 567)
1−sided adj pval: 0.996
Frequency
Tumor−Normal Expression Difference
hsa−miR−302c−5p, proximal
(CIG_ever = 0; N0 = 180)
1−sided adj pval: 0.998
Frequency
Tumor−Normal Expression Difference
hsa−miR−302c−5p, proximal
(CIG_ever = 1; N1 = 264)
1−sided adj pval: 0.504
Frequency
Tumor−Normal Expression Difference

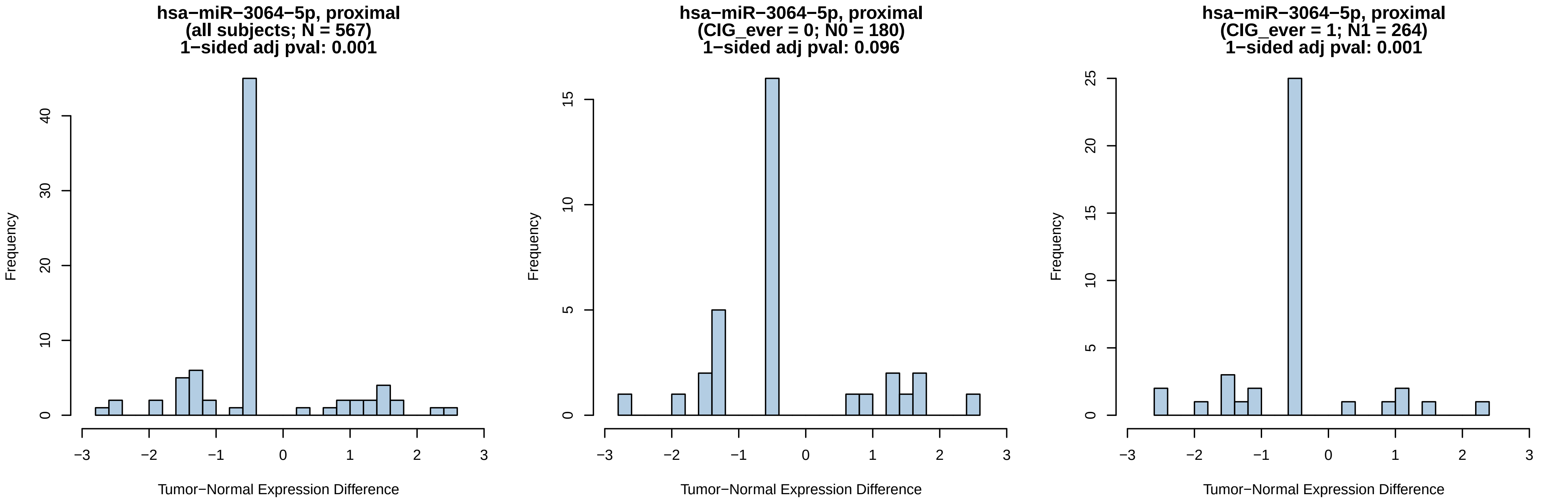
hsa−miR−3064−5p, proximal
(all subjects; N = 567)
1−sided adj pval: 0.001
Frequency
Tumor−Normal Expression Difference
hsa−miR−3064−5p, proximal
(CIG_ever = 0; N0 = 180)
1−sided adj pval: 0.096
Frequency
Tumor−Normal Expression Difference
hsa−miR−3064−5p, proximal
(CIG_ever = 1; N1 = 264)
1−sided adj pval: 0.001
Frequency
Tumor−Normal Expression Difference

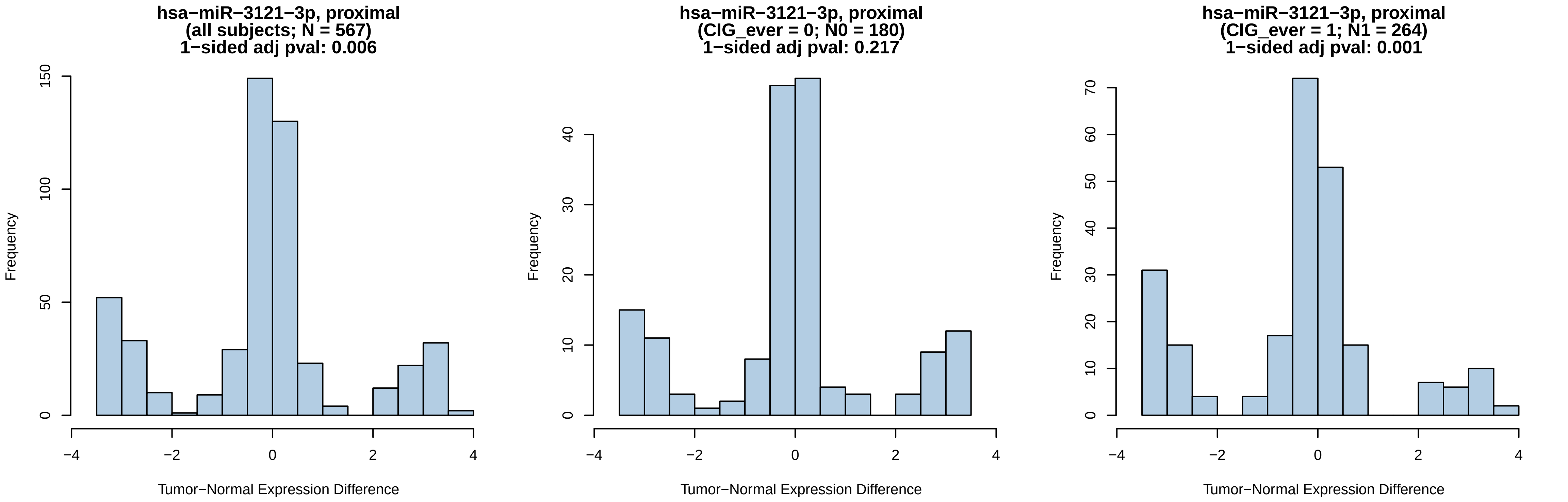
hsa−miR−3121−3p, proximal
(all subjects; N = 567)
1−sided adj pval: 0.006
Frequency
Tumor−Normal Expression Difference
hsa−miR−3121−3p, proximal
(CIG_ever = 0; N0 = 180)
1−sided adj pval: 0.217
Frequency
Tumor−Normal Expression Difference
hsa−miR−3121−3p, proximal
(CIG_ever = 1; N1 = 264)
1−sided adj pval: 0.001
Frequency
Tumor−Normal Expression Difference

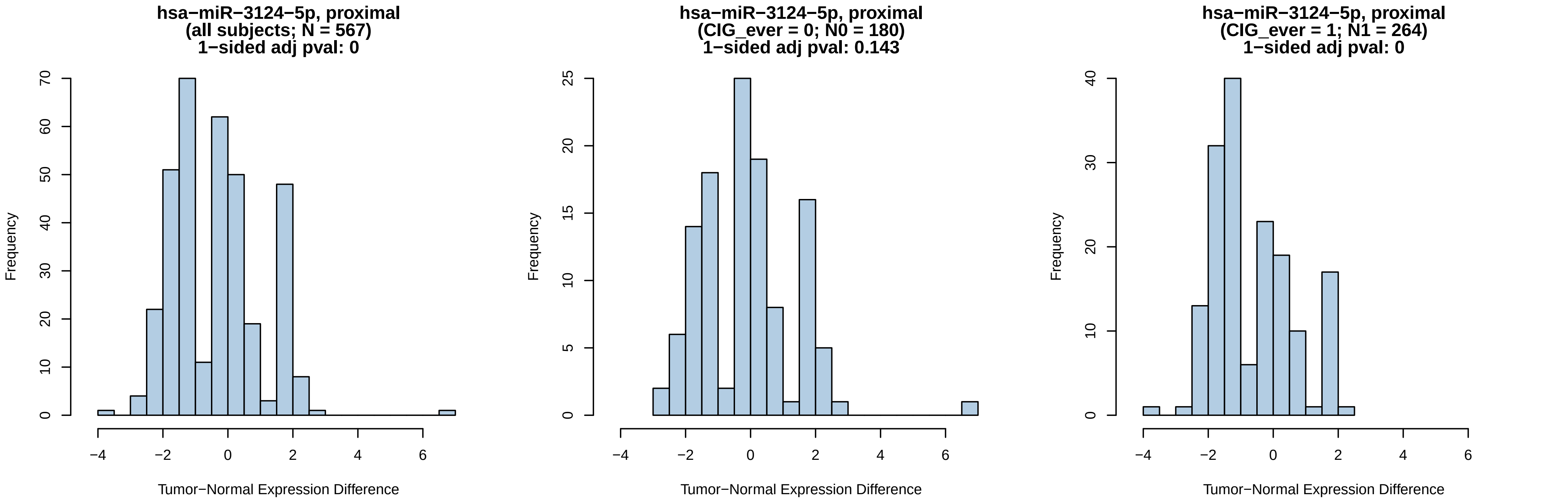
hsa−miR−3124−5p, proximal
(all subjects; N = 567)
1−sided adj pval: 0
hsa−miR−3124−5p, proximal
(CIG_ever = 0; N0 = 180)
1−sided adj pval: 0.143
hsa−miR−3124−5p, proximal
(CIG_ever = 1; N1 = 264)
1−sided adj pval: 0
Frequency
Tumor−Normal Expression Difference

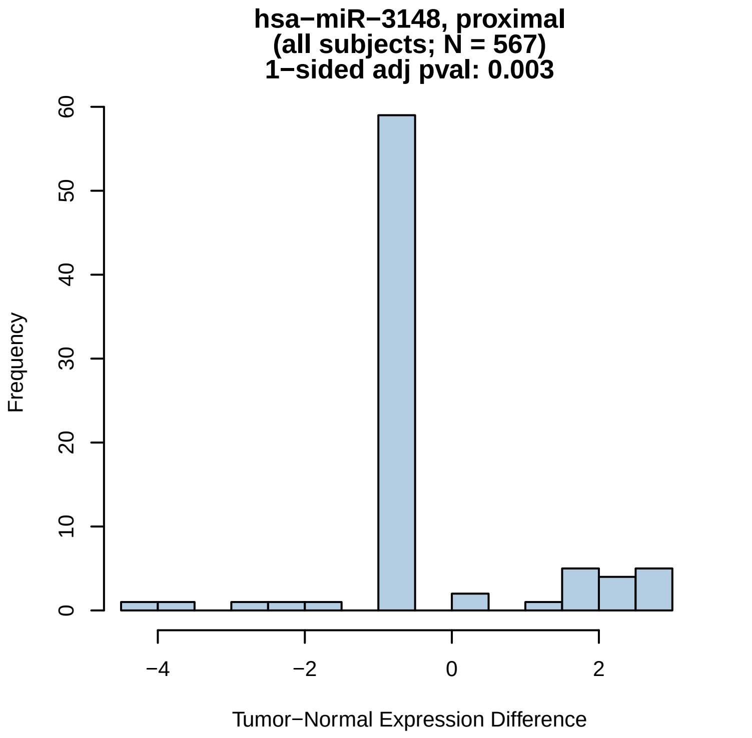
hsa−miR−3148, proximal
(all subjects; N = 567)
1−sided adj pval: 0.003
Frequency
Tumor−Normal Expression Difference

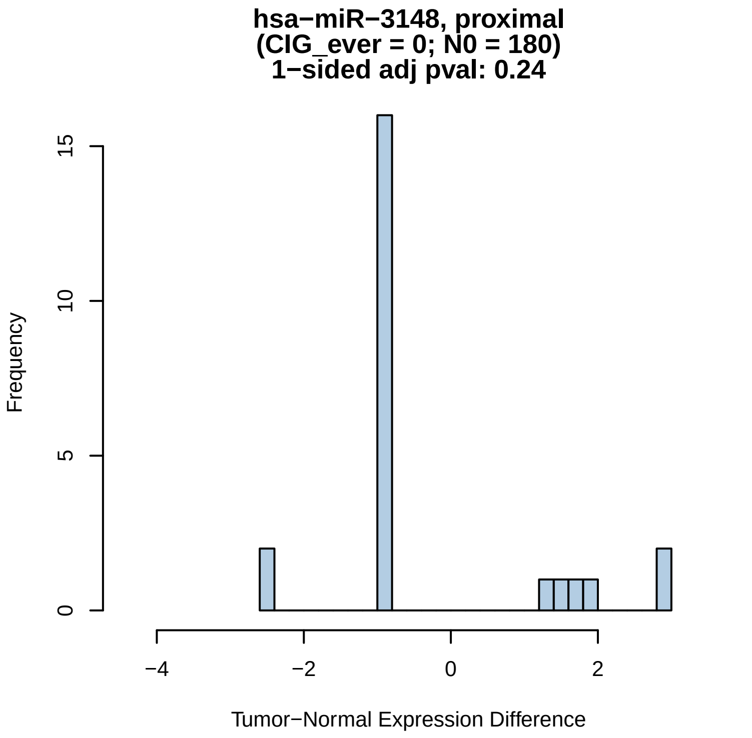
hsa−miR−3148, proximal
(CIG_ever = 0; N0 = 180)
1−sided adj pval: 0.24
Frequency
Tumor−Normal Expression Difference

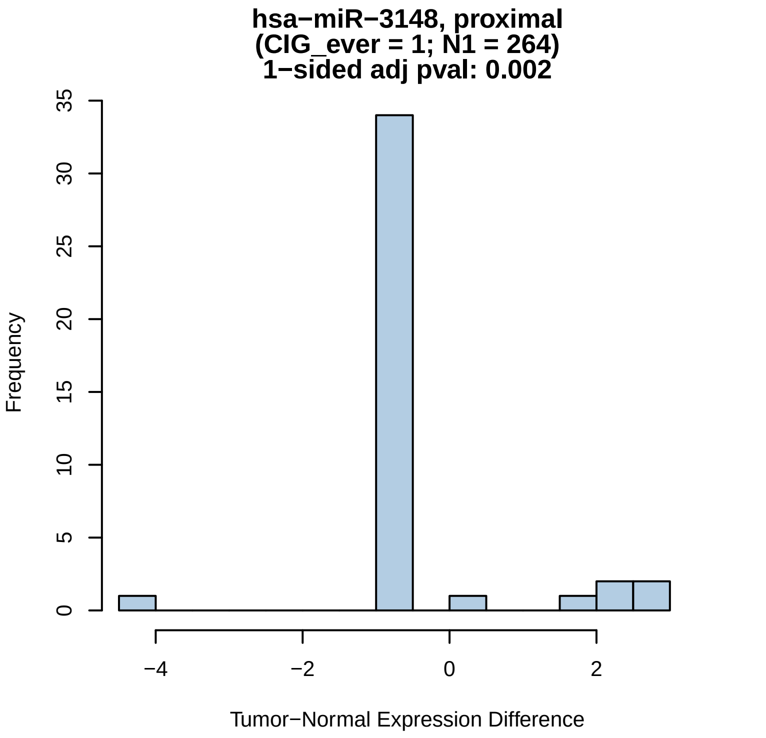
hsa−miR−3148, proximal
(CIG_ever = 1; N1 = 264)
1−sided adj pval: 0.002
Frequency
Tumor−Normal Expression Difference

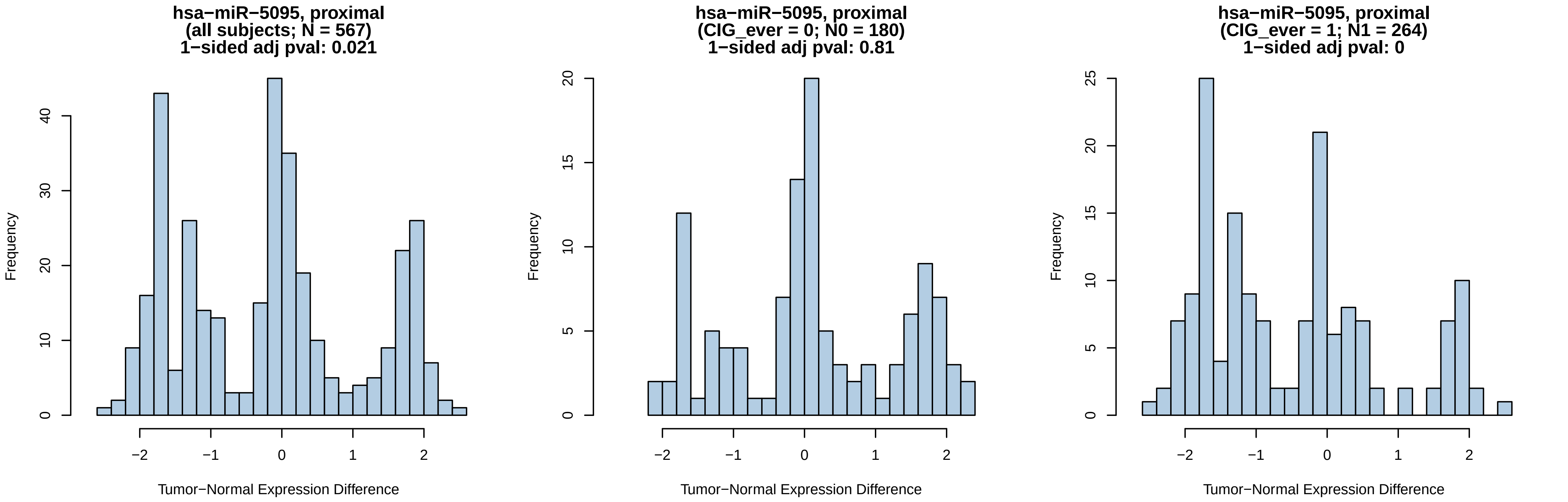
hsa−miR−5095, proximal
(all subjects; N = 567)
1−sided adj pval: 0.021
hsa−miR−5095, proximal
(CIG_ever = 0; N0 = 180)
1−sided adj pval: 0.81
hsa−miR−5095, proximal
(CIG_ever = 1; N1 = 264)
1−sided adj pval: 0
Frequency
Tumor−Normal Expression Difference

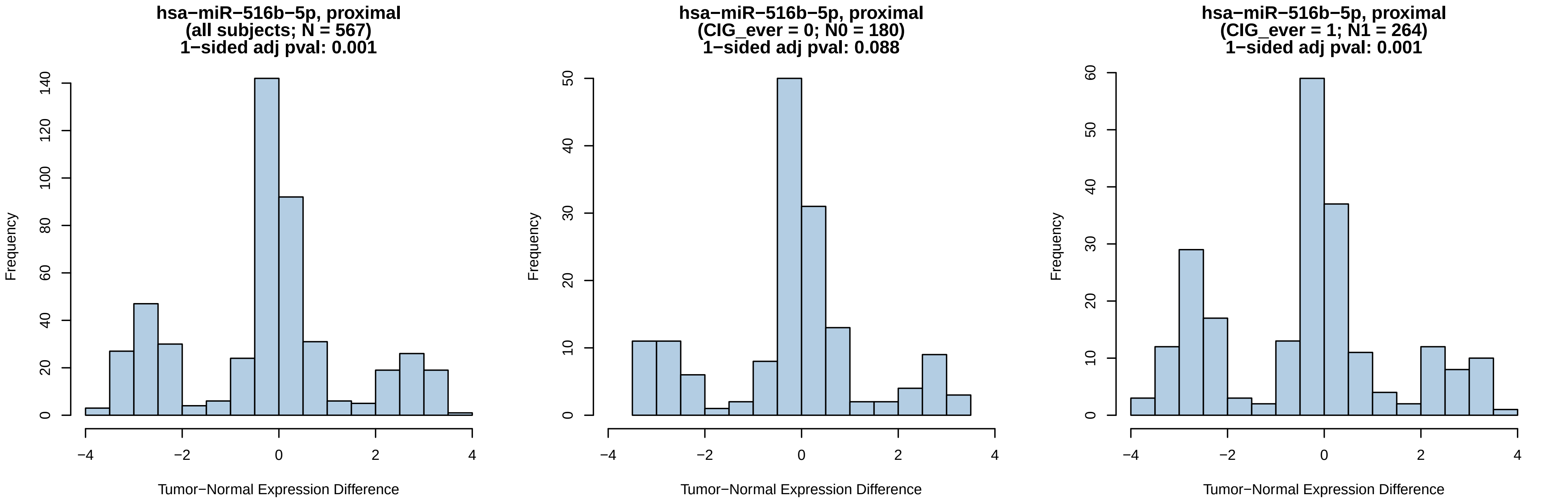
hsa−miR−516b−5p, proximal
(all subjects; N = 567)
1−sided adj pval: 0.001
hsa−miR−516b−5p, proximal
(CIG_ever = 0; N0 = 180)
1−sided adj pval: 0.088
hsa−miR−516b−5p, proximal
(CIG_ever = 1; N1 = 264)
1−sided adj pval: 0.001
Frequency
Tumor−Normal Expression Difference
0
20
40
60
80
100
120
140
0
10
20
30
40
50
60
−4
−2
0
2
4

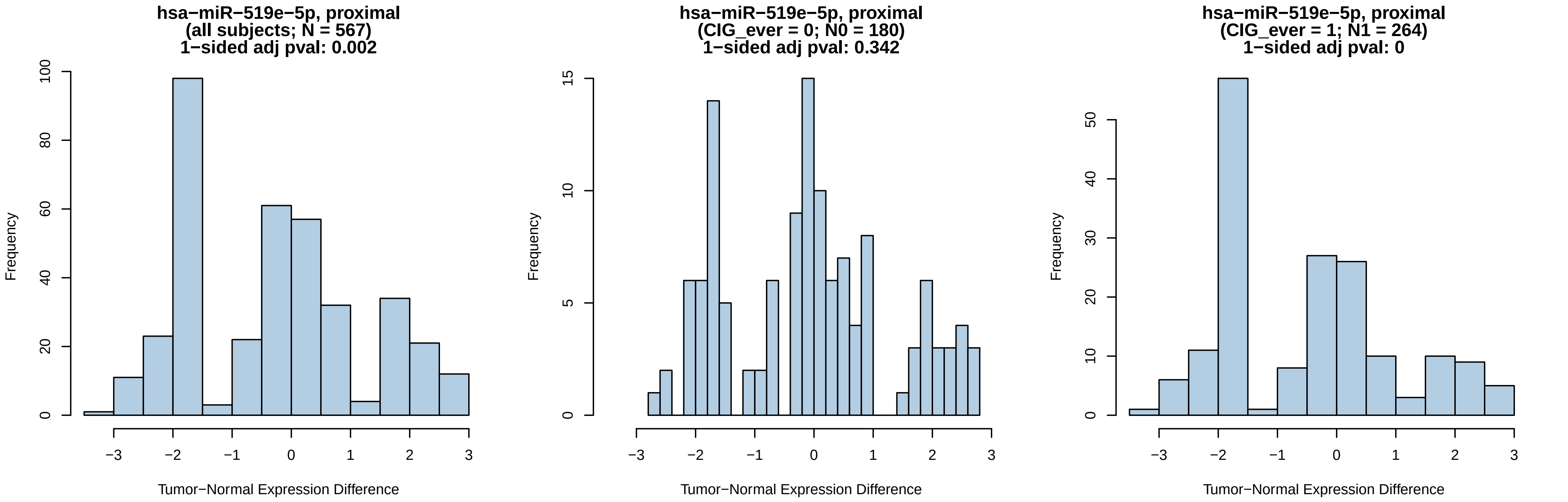
hsa−miR−519e−5p, proximal
(all subjects; N = 567)
1−sided adj pval: 0.002
Frequency
Tumor−Normal Expression Difference
hsa−miR−519e−5p, proximal
(CIG_ever = 0; N0 = 180)
1−sided adj pval: 0.342
Frequency
Tumor−Normal Expression Difference
hsa−miR−519e−5p, proximal
(CIG_ever = 1; N1 = 264)
1−sided adj pval: 0
Frequency
Tumor−Normal Expression Difference

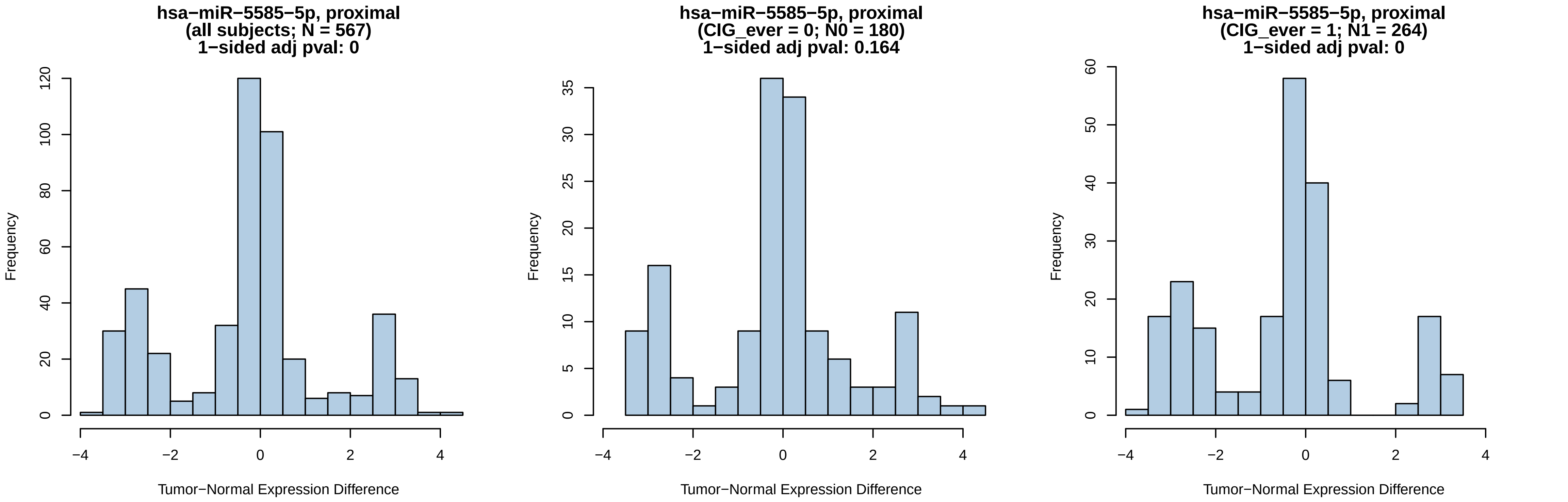
hsa−miR−5585−5p, proximal
(all subjects; N = 567)
1−sided adj pval: 0
hsa−miR−5585−5p, proximal
(CIG_ever = 0; N0 = 180)
1−sided adj pval: 0.164
hsa−miR−5585−5p, proximal
(CIG_ever = 1; N1 = 264)
1−sided adj pval: 0
Frequency
Tumor−Normal Expression Difference

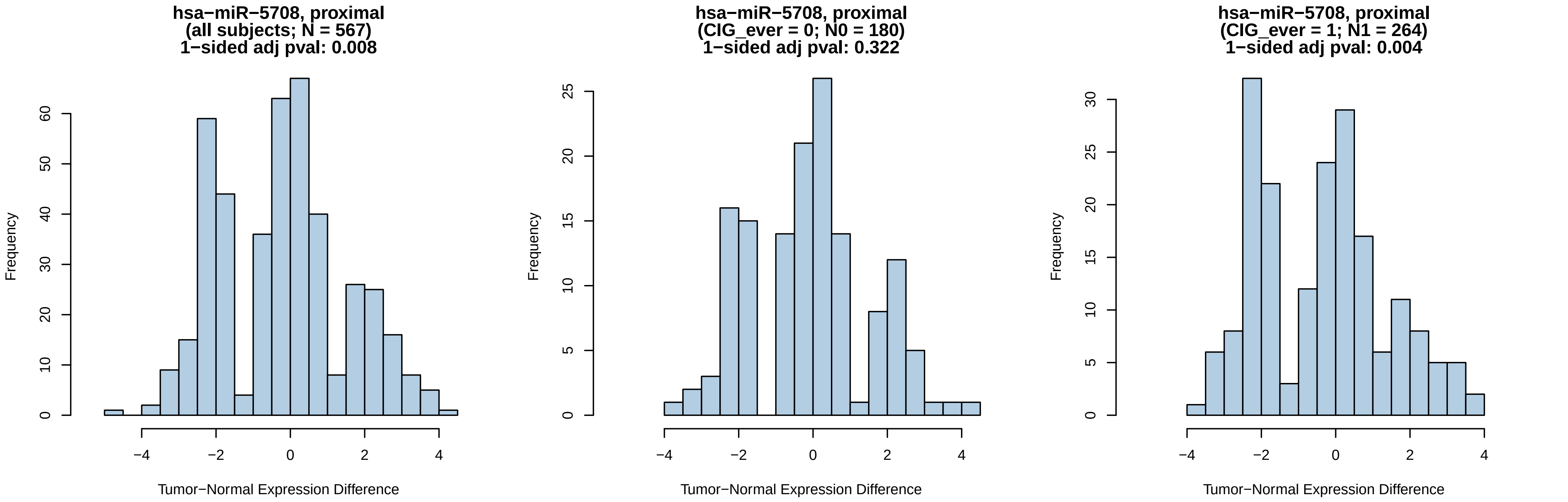
hsa−miR−5708, proximal
(all subjects; N = 567)
1−sided adj pval: 0.008
Frequency
Tumor−Normal Expression Difference
hsa−miR−5708, proximal
(CIG_ever = 0; N0 = 180)
1−sided adj pval: 0.322
Frequency
Tumor−Normal Expression Difference
hsa−miR−5708, proximal
(CIG_ever = 1; N1 = 264)
1−sided adj pval: 0.004
Frequency
Tumor−Normal Expression Difference

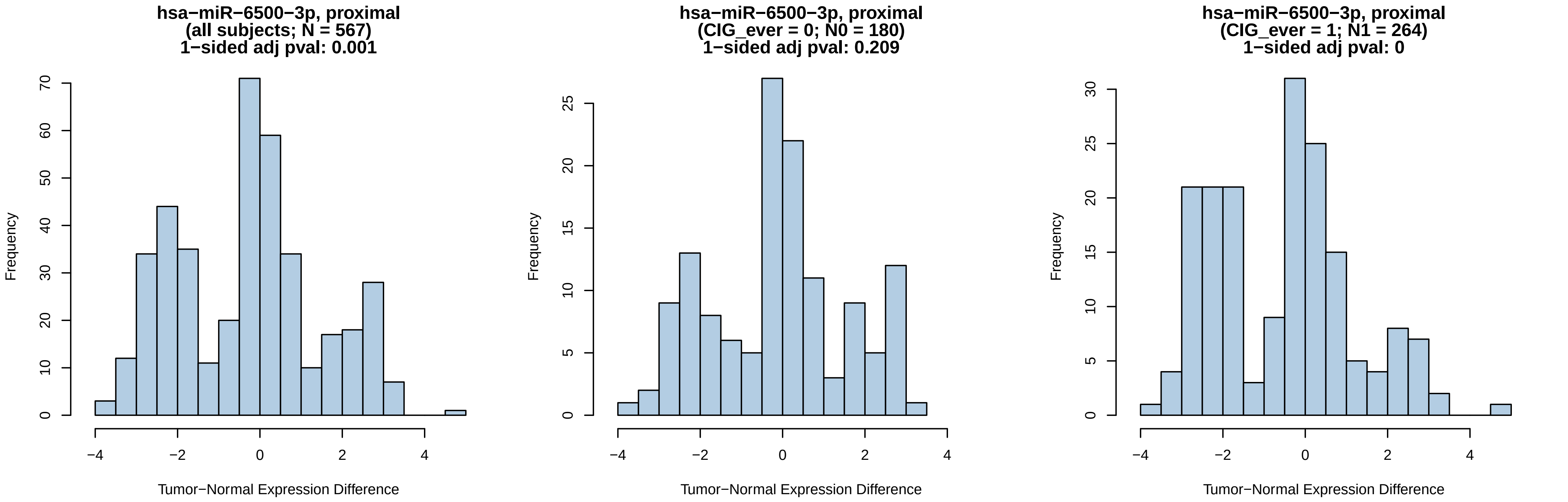
hsa−miR−6500−3p, proximal
(all subjects; N = 567)
1−sided adj pval: 0.001
Frequency
Tumor−Normal Expression Difference
hsa−miR−6500−3p, proximal
(CIG_ever = 0; N0 = 180)
1−sided adj pval: 0.209
Frequency
Tumor−Normal Expression Difference
hsa−miR−6500−3p, proximal
(CIG_ever = 1; N1 = 264)
1−sided adj pval: 0
Frequency
Tumor−Normal Expression Difference

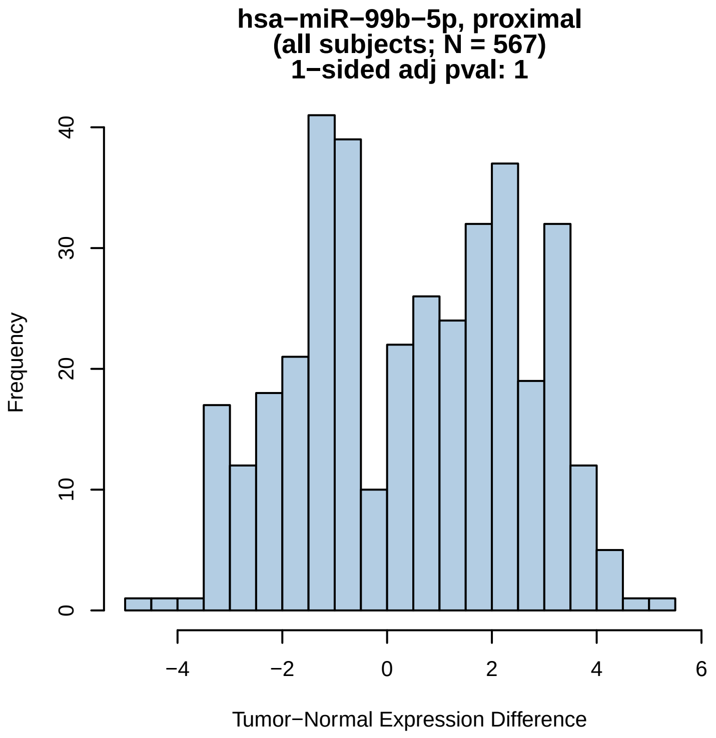

hsa−miR−99b−5p, proximal
(all subjects; N = 567)
1−sided adj pval: 1
Frequency
Tumor−Normal Expression Difference

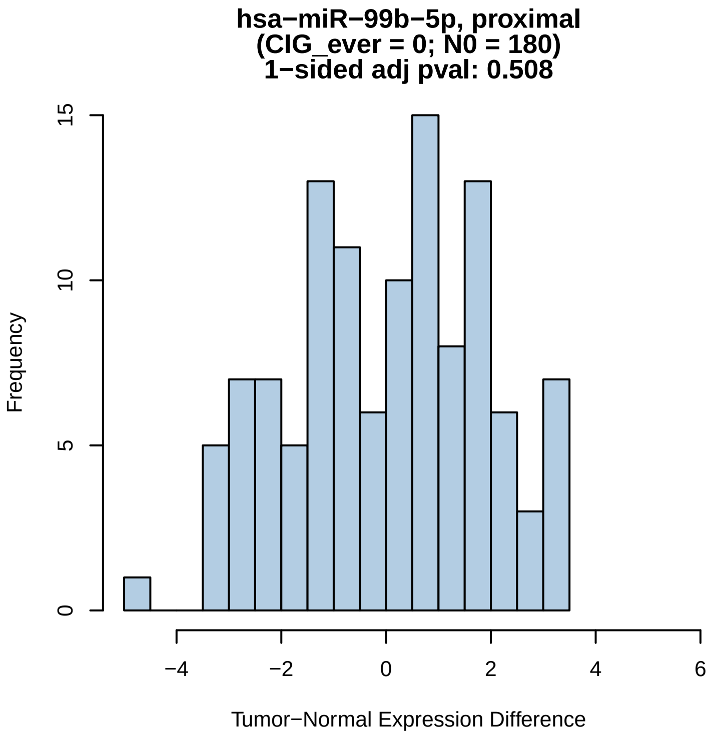

hsa−miR−99b−5p, proximal
(CIG_ever = 0; N0 = 180)
1−sided adj pval: 0.508
Frequency
Tumor−Normal Expression Difference

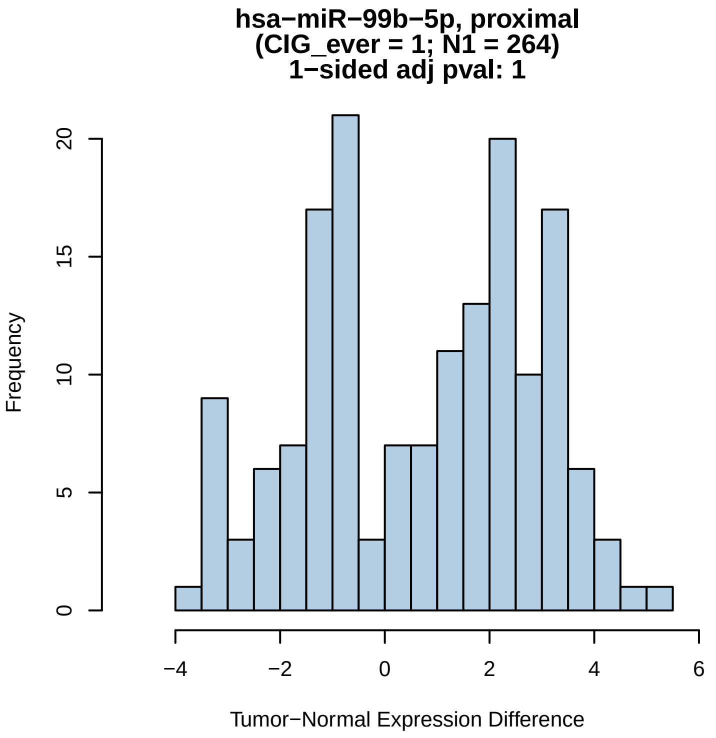

hsa−miR−99b−5p, proximal
(CIG_ever = 1; N1 = 264)
1−sided adj pval: 1
Frequency
Tumor−Normal Expression Difference

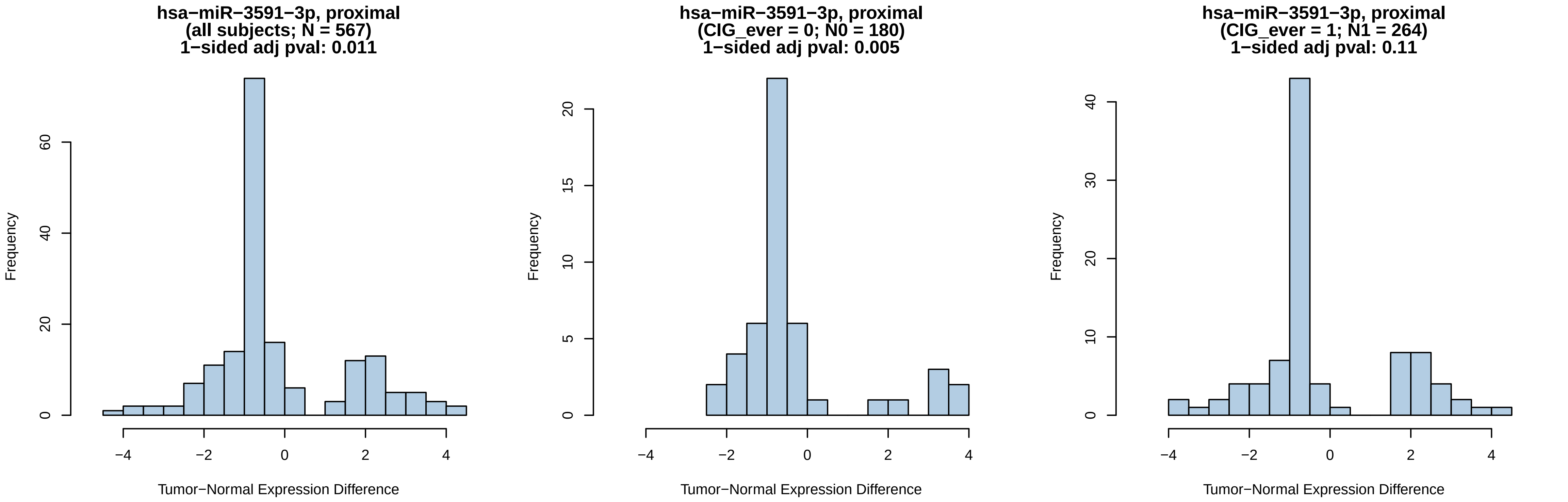

hsa−miR−3591−3p, proximal
(all subjects; N = 567)
1−sided adj pval: 0.011
Frequency
Tumor−Normal Expression Difference
hsa−miR−3591−3p, proximal
(CIG_ever = 0; N0 = 180)
1−sided adj pval: 0.005
Frequency
Tumor−Normal Expression Difference
hsa−miR−3591−3p, proximal
(CIG_ever = 1; N1 = 264)
1−sided adj pval: 0.11
Frequency
Tumor−Normal Expression Difference

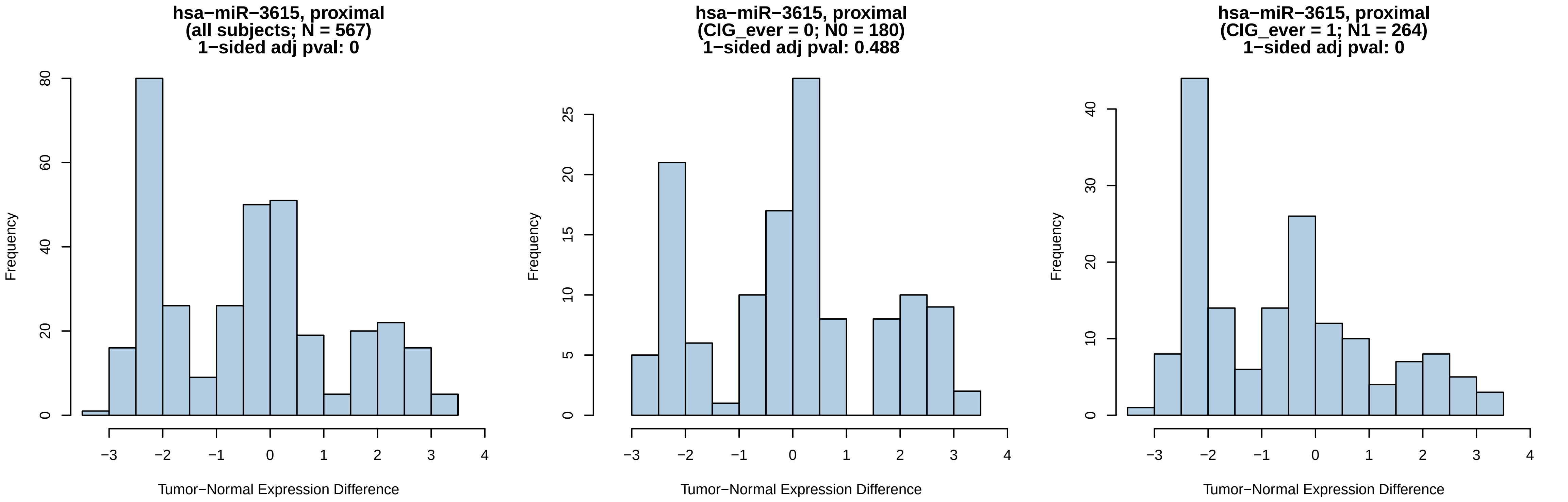
hsa−miR−3615, proximal
(all subjects; N = 567)
1−sided adj pval: 0
hsa−miR−3615, proximal
(CIG_ever = 0; N0 = 180)
1−sided adj pval: 0.488
hsa−miR−3615, proximal
(CIG_ever = 1; N1 = 264)
1−sided adj pval: 0
Frequency
Tumor−Normal Expression Difference

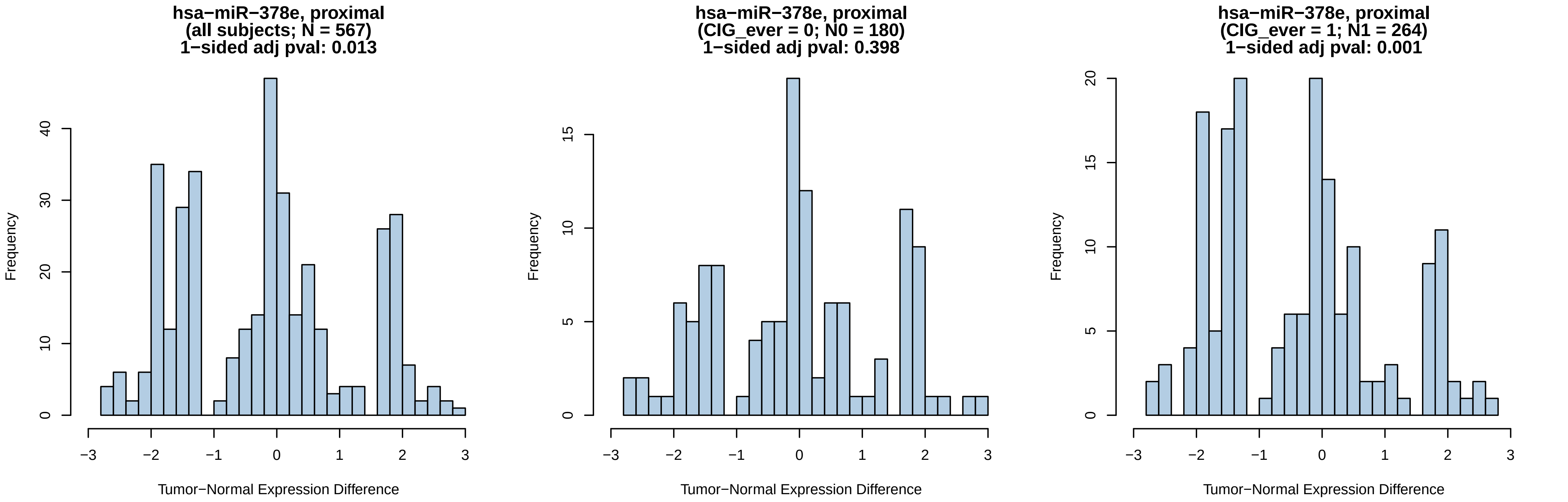

hsa−miR−378e, proximal
(all subjects; N = 567)
1−sided adj pval: 0.013
Frequency
Tumor−Normal Expression Difference
hsa−miR−378e, proximal
(CIG_ever = 0; N0 = 180)
1−sided adj pval: 0.398
Frequency
Tumor−Normal Expression Difference
hsa−miR−378e, proximal
(CIG_ever = 1; N1 = 264)
1−sided adj pval: 0.001
Frequency
Tumor−Normal Expression Difference

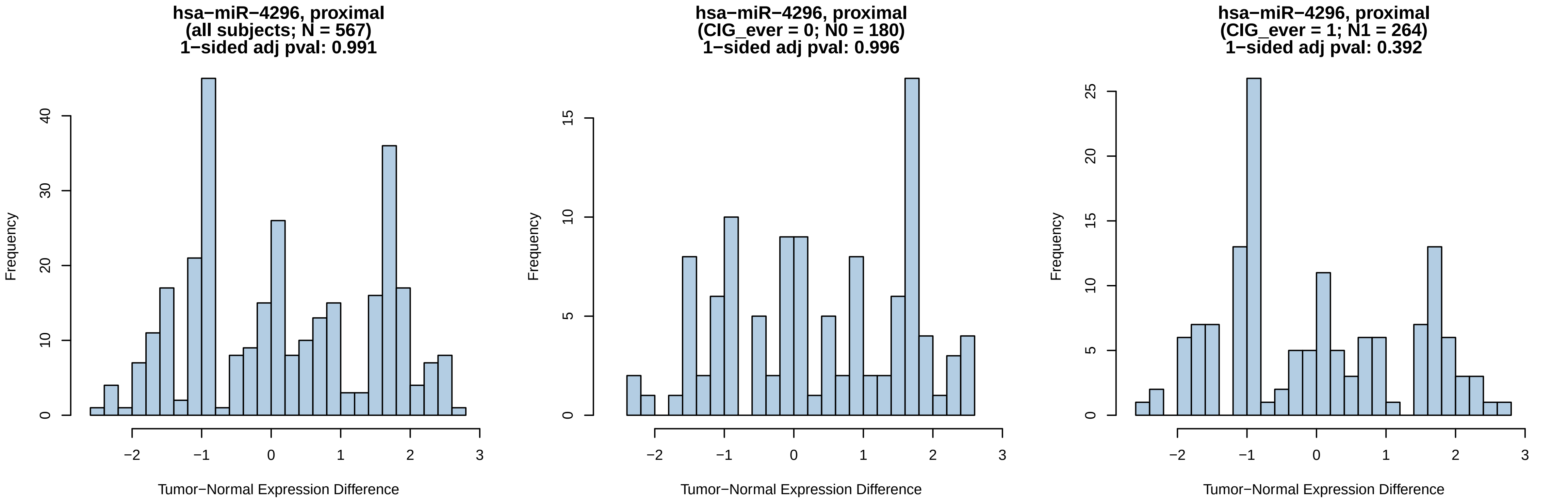
hsa−miR−4296, proximal
(all subjects; N = 567)
1−sided adj pval: 0.991
Frequency
Tumor−Normal Expression Difference
hsa−miR−4296, proximal
(CIG_ever = 0; N0 = 180)
1−sided adj pval: 0.996
Frequency
Tumor−Normal Expression Difference
hsa−miR−4296, proximal
(CIG_ever = 1; N1 = 264)
1−sided adj pval: 0.392
Frequency
Tumor−Normal Expression Difference

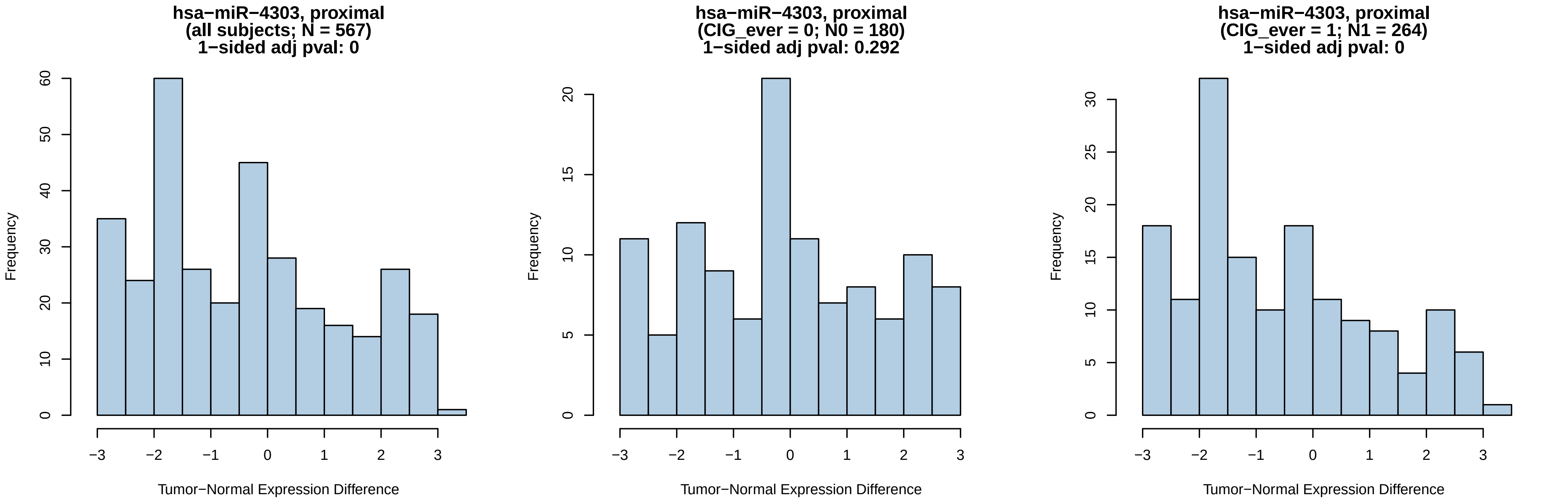
hsa−miR−4303, proximal
(all subjects; N = 567)
1−sided adj pval: 0
Frequency
Tumor−Normal Expression Difference
hsa−miR−4303, proximal
(CIG_ever = 0; N0 = 180)
1−sided adj pval: 0.292
Frequency
Tumor−Normal Expression Difference
hsa−miR−4303, proximal
(CIG_ever = 1; N1 = 264)
1−sided adj pval: 0
Frequency
Tumor−Normal Expression Difference

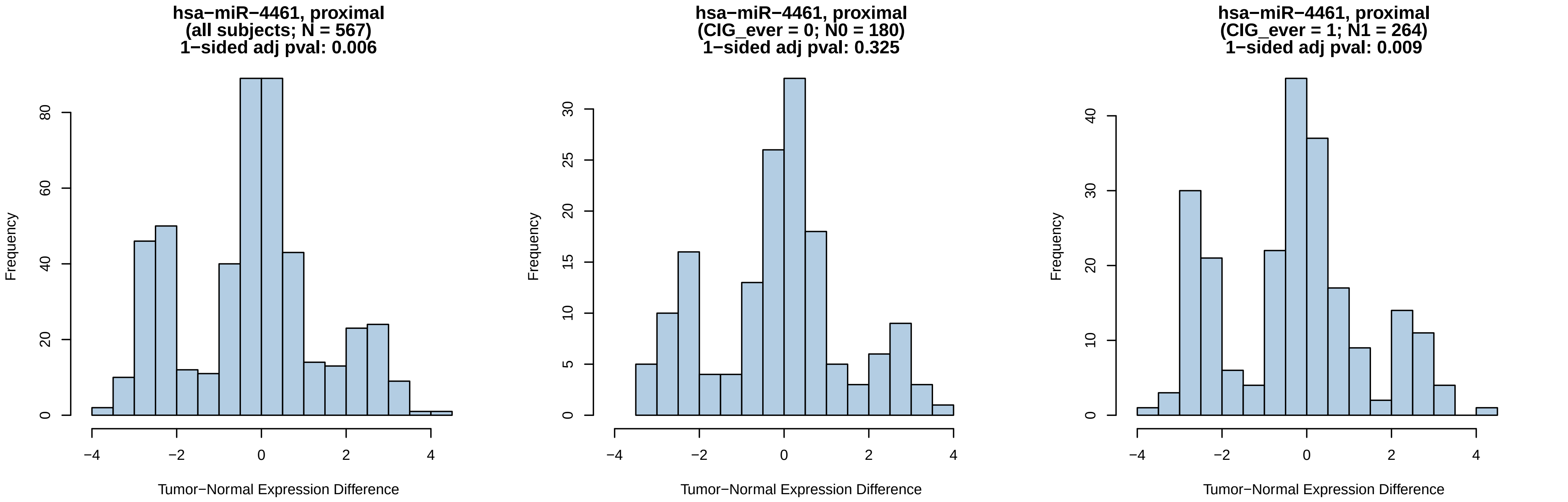
hsa−miR−4461, proximal
(all subjects; N = 567)
1−sided adj pval: 0.006
Frequency
Tumor−Normal Expression Difference
hsa−miR−4461, proximal
(CIG_ever = 0; N0 = 180)
1−sided adj pval: 0.325
Frequency
Tumor−Normal Expression Difference
hsa−miR−4461, proximal
(CIG_ever = 1; N1 = 264)
1−sided adj pval: 0.009
Frequency
Tumor−Normal Expression Difference

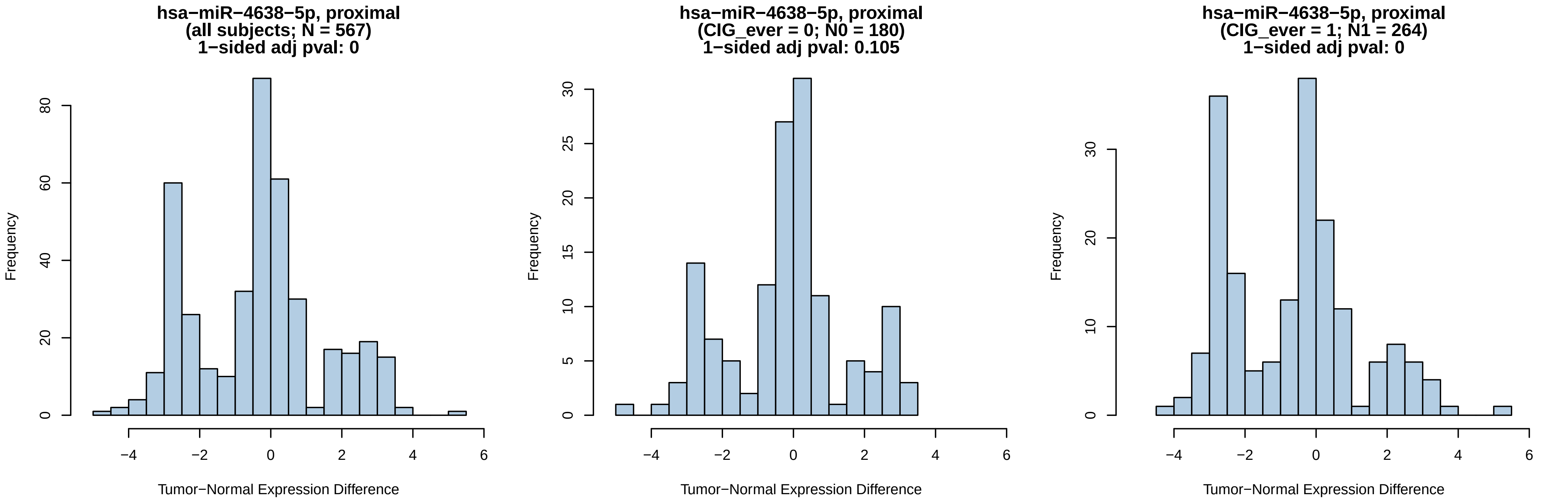

hsa−miR−4638−5p, proximal
(all subjects; N = 567)
1−sided adj pval: 0
Frequency
Tumor−Normal Expression Difference
hsa−miR−4638−5p, proximal
(CIG_ever = 0; N0 = 180)
1−sided adj pval: 0.105
Frequency
Tumor−Normal Expression Difference
hsa−miR−4638−5p, proximal
(CIG_ever = 1; N1 = 264)
1−sided adj pval: 0
Frequency
Tumor−Normal Expression Difference

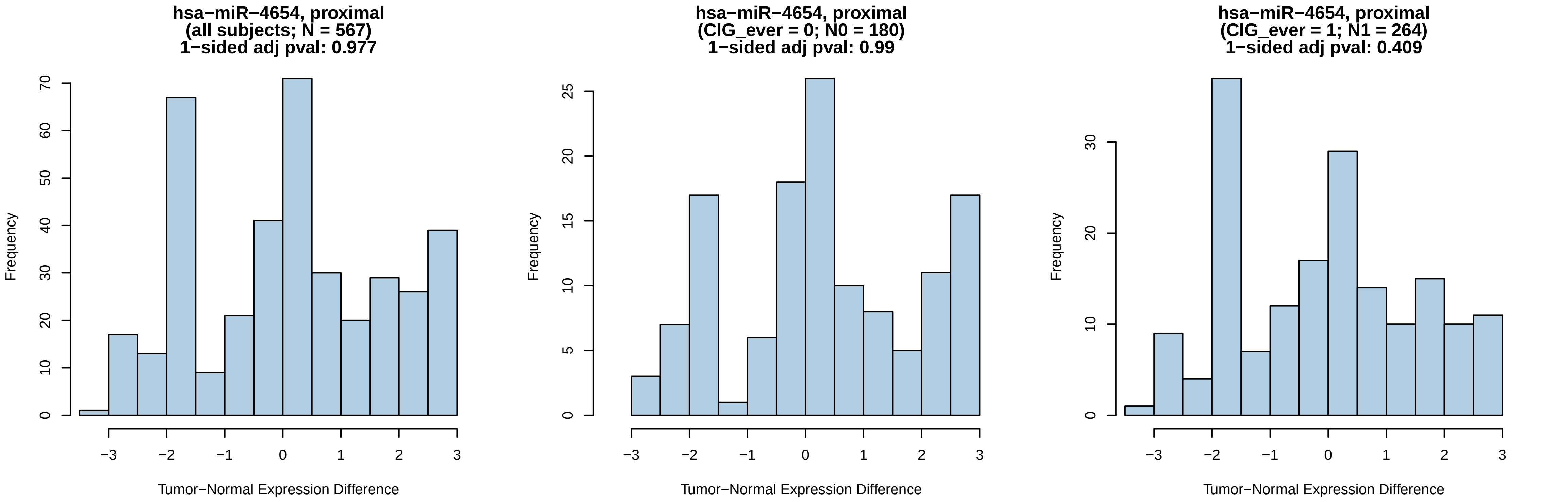
hsa−miR−4654, proximal
(all subjects; N = 567)
1−sided adj pval: 0.977
hsa−miR−4654, proximal
(CIG_ever = 0; N0 = 180)
1−sided adj pval: 0.99
hsa−miR−4654, proximal
(CIG_ever = 1; N1 = 264)
1−sided adj pval: 0.409
Frequency
Tumor−Normal Expression Difference

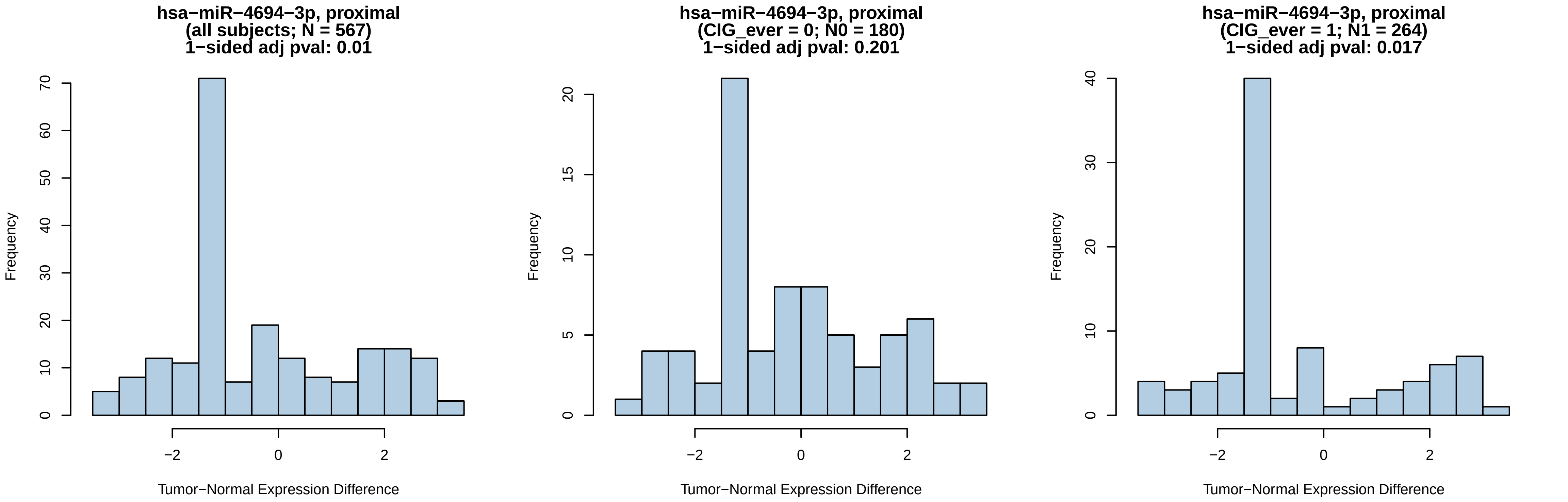
hsa−miR−4694−3p, proximal
(all subjects; N = 567)
1−sided adj pval: 0.01
Frequency
Tumor−Normal Expression Difference
hsa−miR−4694−3p, proximal
(CIG_ever = 0; N0 = 180)
1−sided adj pval: 0.201
Frequency
Tumor−Normal Expression Difference
hsa−miR−4694−3p, proximal
(CIG_ever = 1; N1 = 264)
1−sided adj pval: 0.017
Frequency
Tumor−Normal Expression Difference

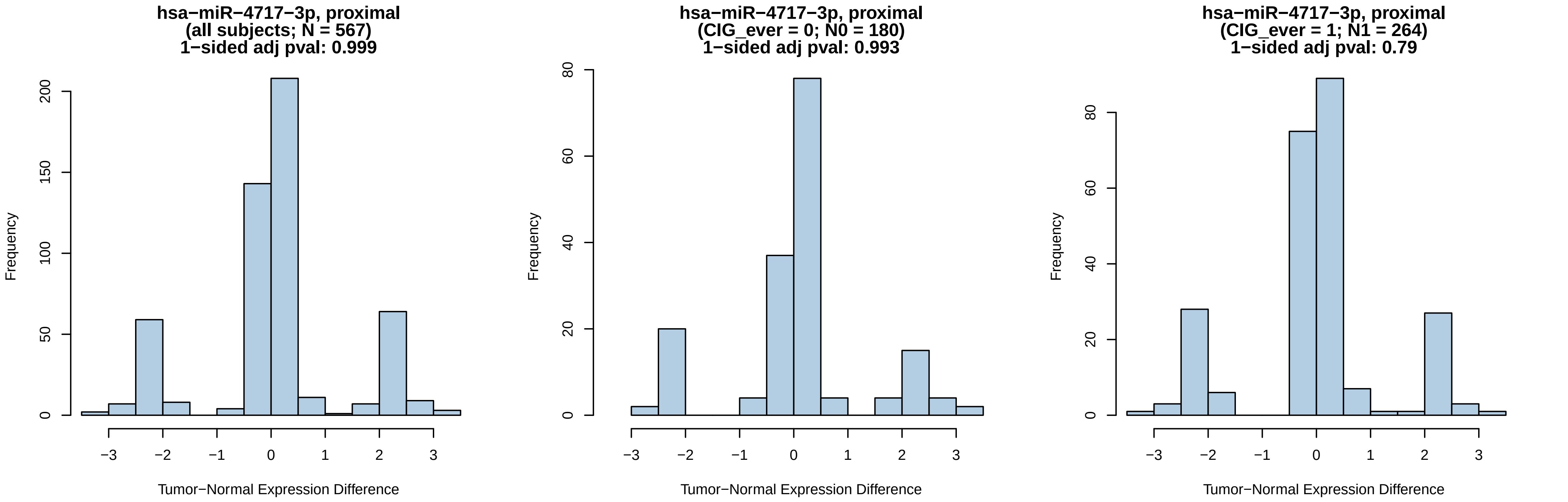
hsa−miR−4717−3p, proximal
(all subjects; N = 567)
1−sided adj pval: 0.999
Frequency
0
50
100
150
200
−3
−2
−1
0
1
2
3
Tumor−Normal Expression Difference
hsa−miR−4717−3p, proximal
(CIG_ever = 0; N0 = 180)
1−sided adj pval: 0.993
Frequency
0
20
40
60
80
−3
−2
−1
0
1
2
3
Tumor−Normal Expression Difference
hsa−miR−4717−3p, proximal
(CIG_ever = 1; N1 = 264)
1−sided adj pval: 0.79
Frequency
0
20
40
60
80
−3
−2
−1
0
1
2
3
Tumor−Normal Expression Difference

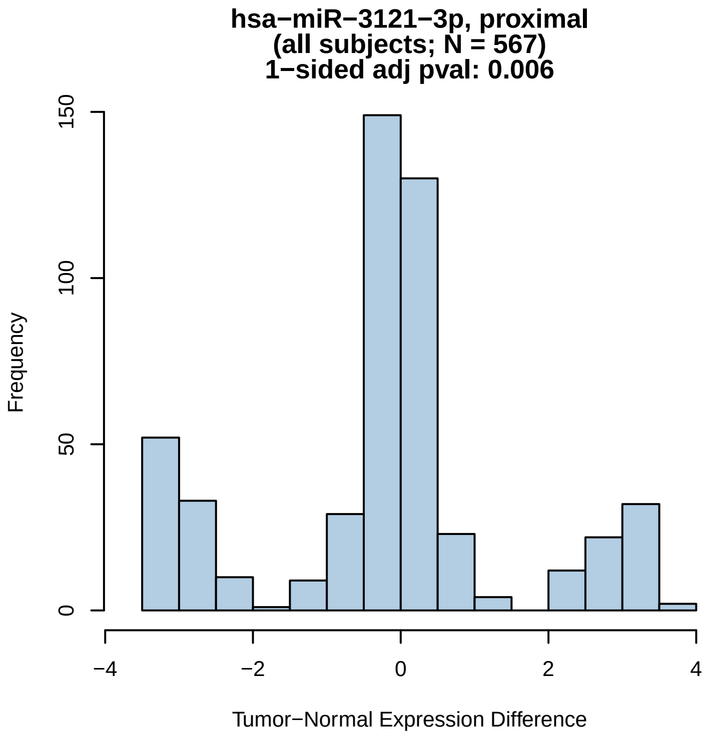
hsa−miR−3121−3p, proximal
(all subjects; N = 567)
1−sided adj pval: 0.006
Frequency
0
50
100
150
−4
−2
0
2
4
Tumor−Normal Expression Difference

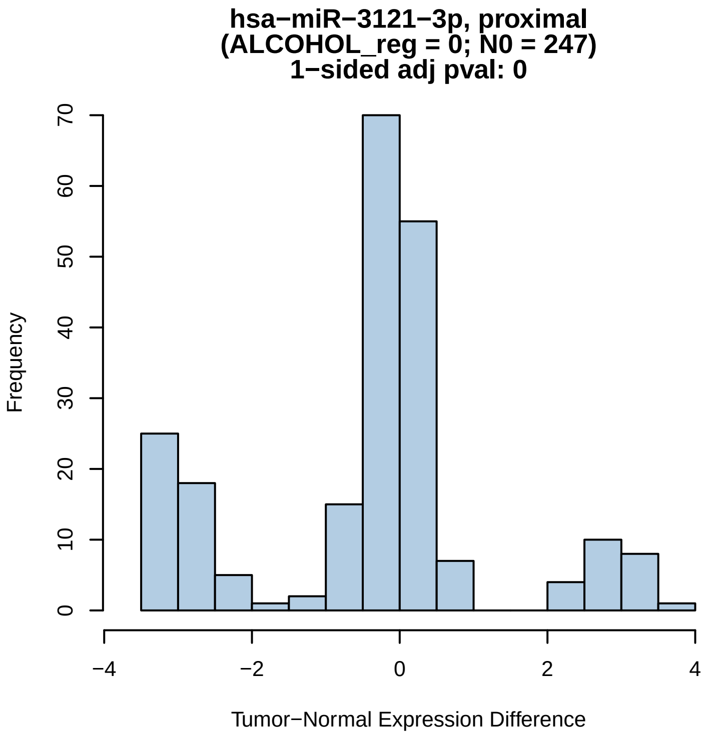
hsa−miR−3121−3p, proximal
(ALCOHOL_reg = 0; N0 = 247)
1−sided adj pval: 0
Frequency
0
10
20
30
40
50
60
70
−4
−2
0
2
4
Tumor−Normal Expression Difference

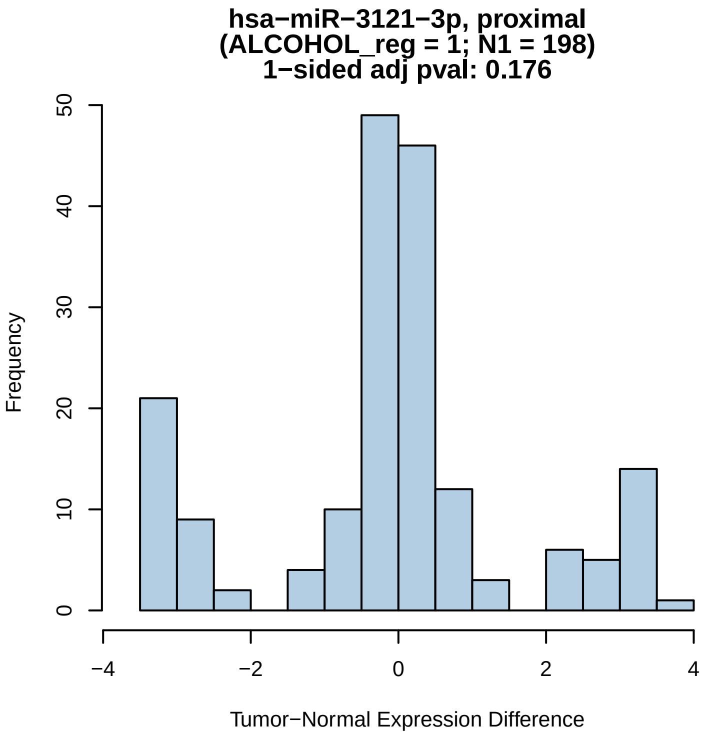
hsa−miR−3121−3p, proximal
(ALCOHOL_reg = 1; N1 = 198)
1−sided adj pval: 0.176
Frequency
0
10
20
30
40
50
−4
−2
0
2
4
Tumor−Normal Expression Difference

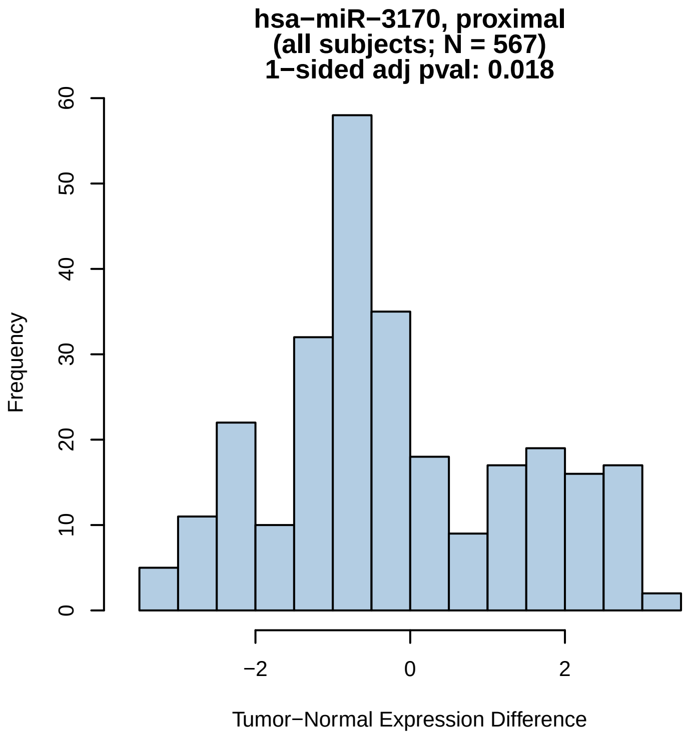

hsa−miR−3170, proximal
(all subjects; N = 567)
1−sided adj pval: 0.018
Frequency
0
10
20
30
40
50
60
−2
0
2
Tumor−Normal Expression Difference


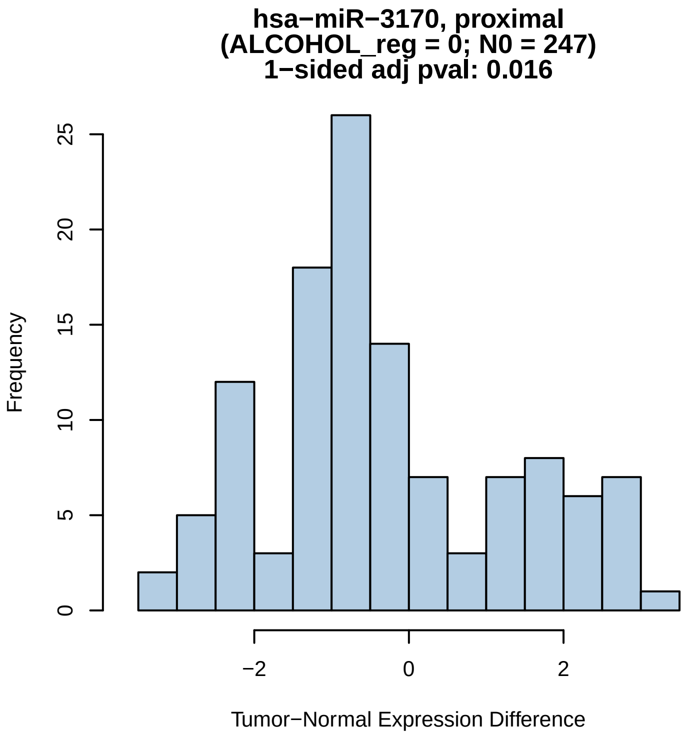

hsa−miR−3170, proximal
(ALCOHOL_reg = 0; N0 = 247)
1−sided adj pval: 0.016
Frequency
0
5
10
15
20
25
−2
0
2
Tumor−Normal Expression Difference


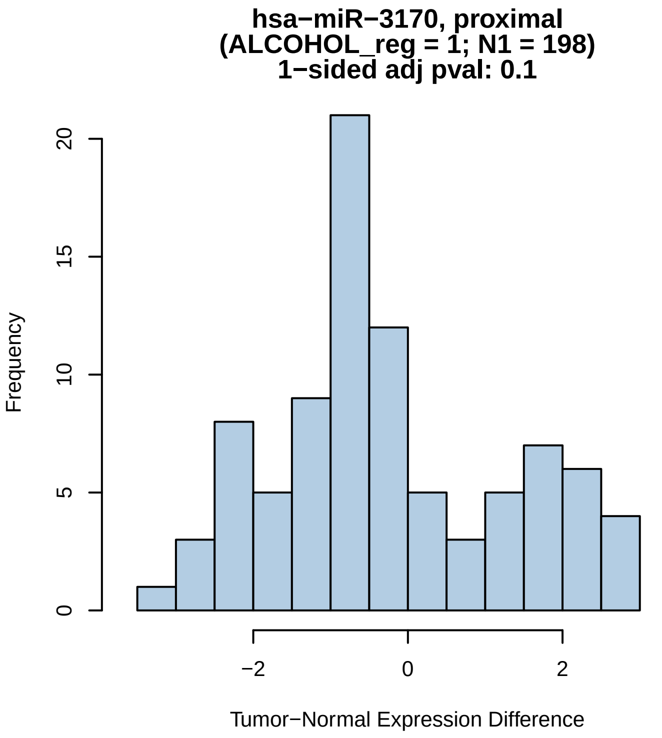

hsa−miR−3170, proximal
(ALCOHOL_reg = 1; N1 = 198)
1−sided adj pval: 0.1
Frequency
0
5
10
15
20
−2
0
2
Tumor−Normal Expression Difference

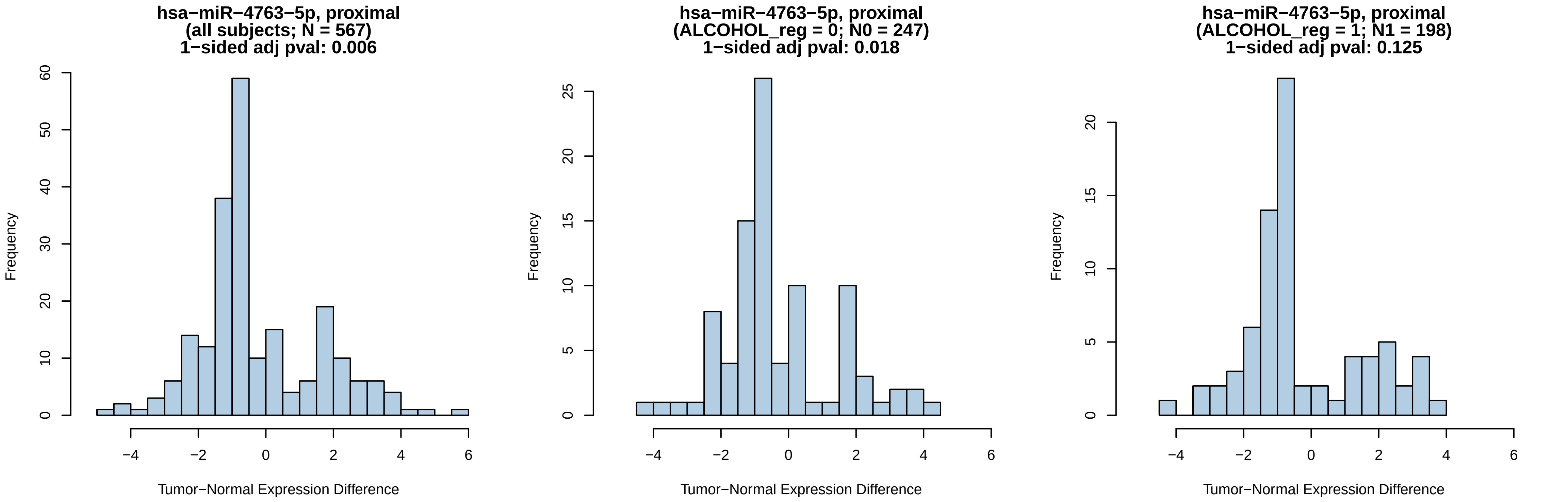
hsa−miR−4763−5p, proximal
(all subjects; N = 567)
1−sided adj pval: 0.006
Frequency
Tumor−Normal Expression Difference
hsa−miR−4763−5p, proximal
(ALCOHOL_reg = 0; N0 = 247)
1−sided adj pval: 0.018
Frequency
Tumor−Normal Expression Difference
hsa−miR−4763−5p, proximal
(ALCOHOL_reg = 1; N1 = 198)
1−sided adj pval: 0.125
Frequency
Tumor−Normal Expression Difference

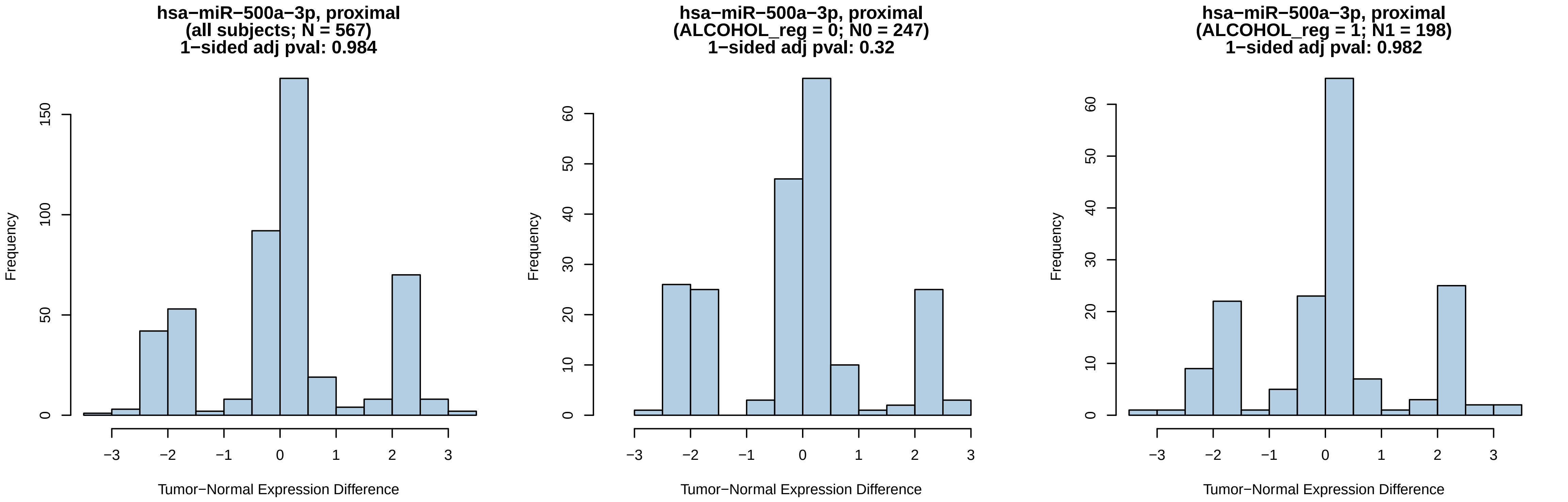

hsa−miR−500a−3p, proximal
(all subjects; N = 567)
1−sided adj pval: 0.984
hsa−miR−500a−3p, proximal
(ALCOHOL_reg = 0; N0 = 247)
1−sided adj pval: 0.32
hsa−miR−500a−3p, proximal
(ALCOHOL_reg = 1; N1 = 198)
1−sided adj pval: 0.982
Frequency
Tumor−Normal Expression Difference

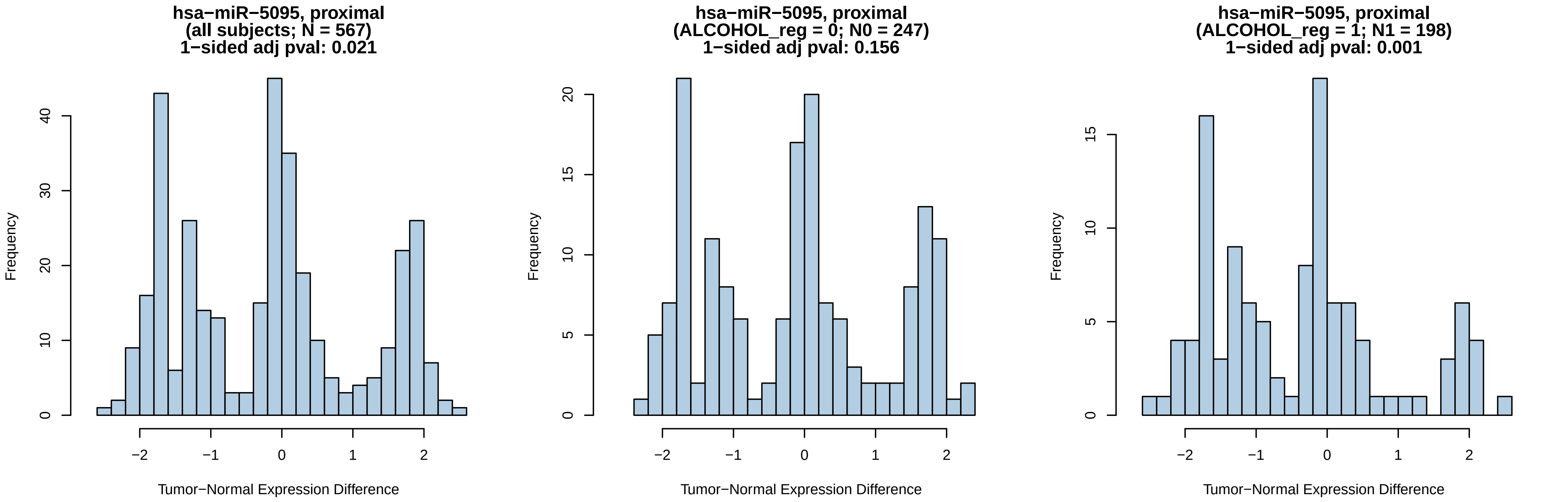
hsa−miR−5095, proximal
(all subjects; N = 567)
1−sided adj pval: 0.021
hsa−miR−5095, proximal
(ALCOHOL_reg = 0; N0 = 247)
1−sided adj pval: 0.156
hsa−miR−5095, proximal
(ALCOHOL_reg = 1; N1 = 198)
1−sided adj pval: 0.001
Frequency
Tumor−Normal Expression Difference

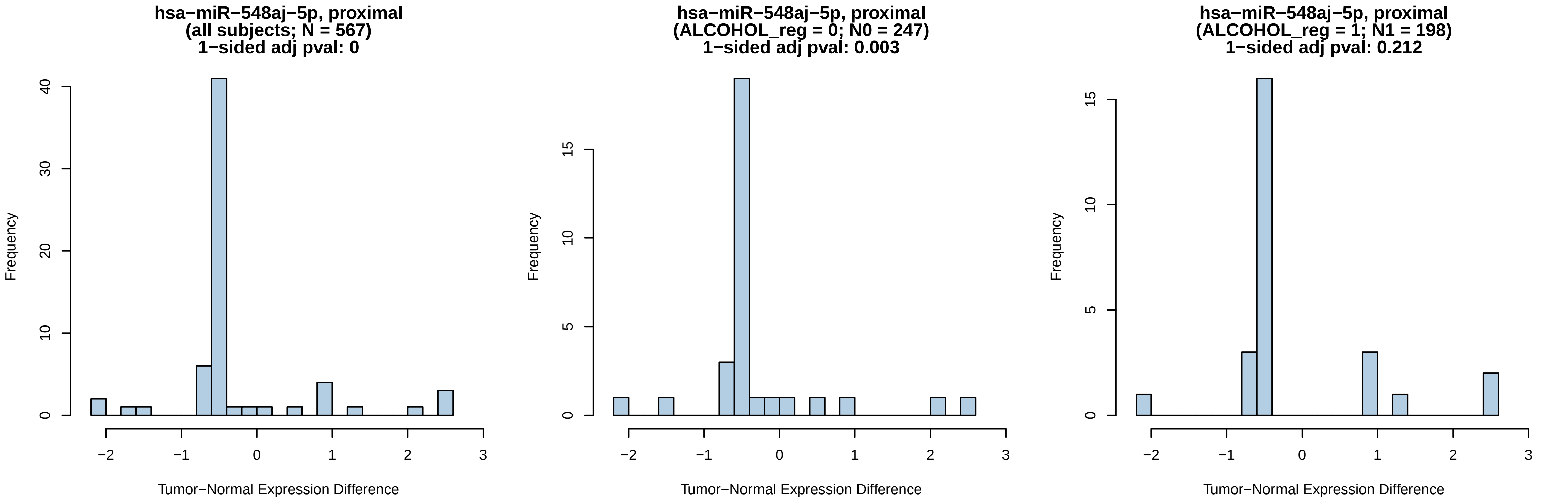
hsa−miR−548aj−5p, proximal
(all subjects; N = 567)
1−sided adj pval: 0
Frequency
0
10
20
30
40
−2
−1
0
1
2
3
Tumor−Normal Expression Difference
hsa−miR−548aj−5p, proximal
(ALCOHOL_reg = 0; N0 = 247)
1−sided adj pval: 0.003
Frequency
0
5
10
15
−2
−1
0
1
2
3
Tumor−Normal Expression Difference
hsa−miR−548aj−5p, proximal
(ALCOHOL_reg = 1; N1 = 198)
1−sided adj pval: 0.212
Frequency
0
5
10
15
−2
−1
0
1
2
3
Tumor−Normal Expression Difference

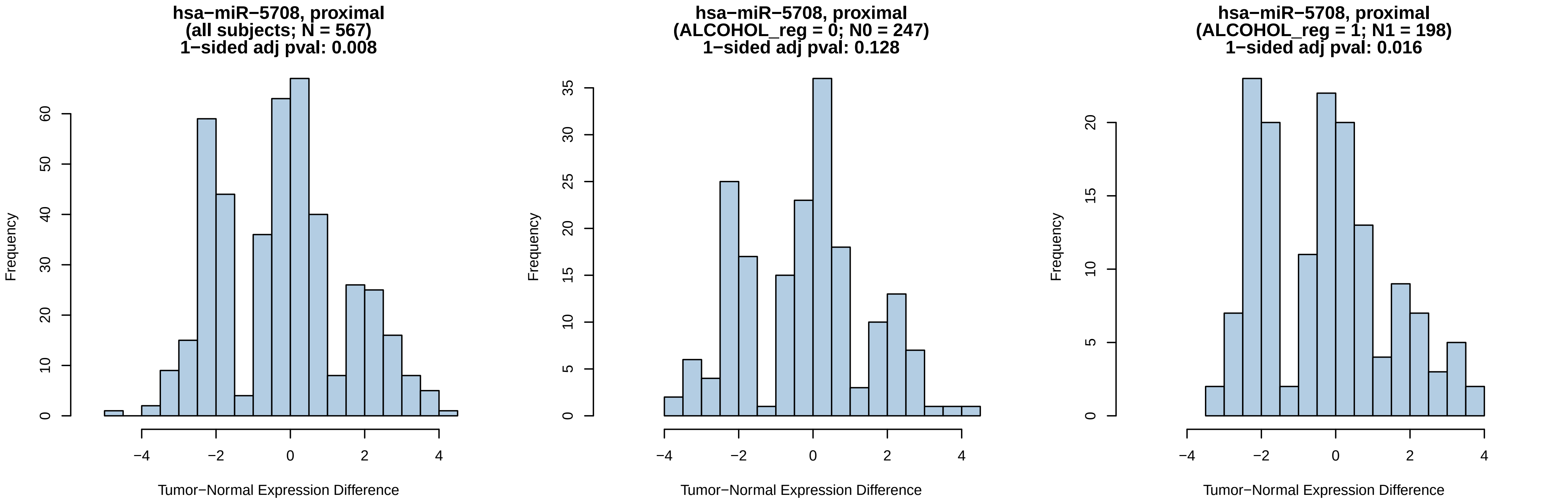
hsa−miR−5708, proximal
(all subjects; N = 567)
1−sided adj pval: 0.008
Frequency
Tumor−Normal Expression Difference
hsa−miR−5708, proximal
(ALCOHOL_reg = 0; N0 = 247)
1−sided adj pval: 0.128
Frequency
Tumor−Normal Expression Difference
hsa−miR−5708, proximal
(ALCOHOL_reg = 1; N1 = 198)
1−sided adj pval: 0.016
Frequency
Tumor−Normal Expression Difference

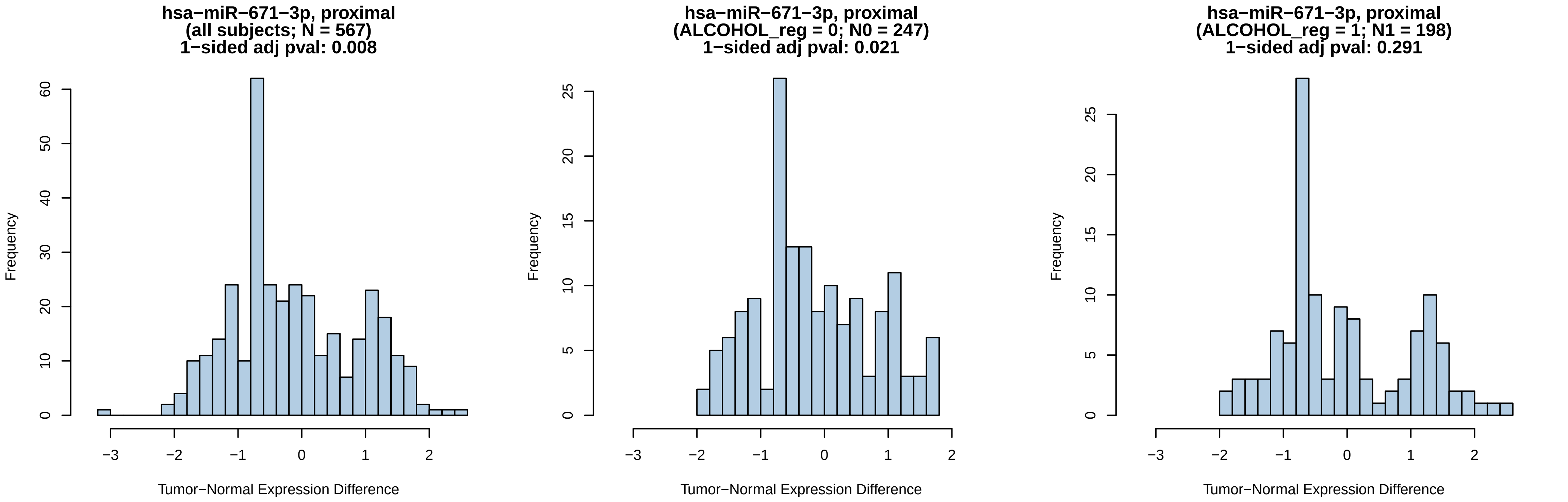

hsa−miR−671−3p, proximal
(all subjects; N = 567)
1−sided adj pval: 0.008
Frequency
Tumor−Normal Expression Difference
hsa−miR−671−3p, proximal
(ALCOHOL_reg = 0; N0 = 247)
1−sided adj pval: 0.021
Frequency
Tumor−Normal Expression Difference
hsa−miR−671−3p, proximal
(ALCOHOL_reg = 1; N1 = 198)
1−sided adj pval: 0.291
Frequency
Tumor−Normal Expression Difference

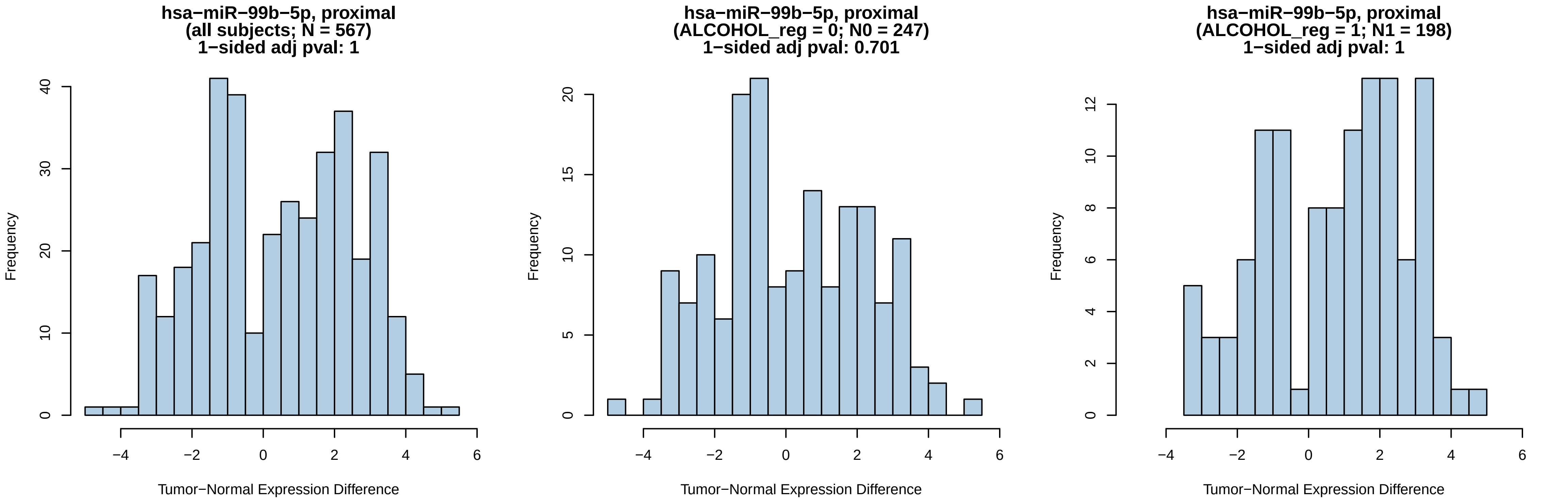
hsa−miR−99b−5p, proximal
(all subjects; N = 567)
1−sided adj pval: 1
hsa−miR−99b−5p, proximal
(ALCOHOL_reg = 0; N0 = 247)
1−sided adj pval: 0.701
hsa−miR−99b−5p, proximal
(ALCOHOL_reg = 1; N1 = 198)
1−sided adj pval: 1
Frequency
Tumor−Normal Expression Difference

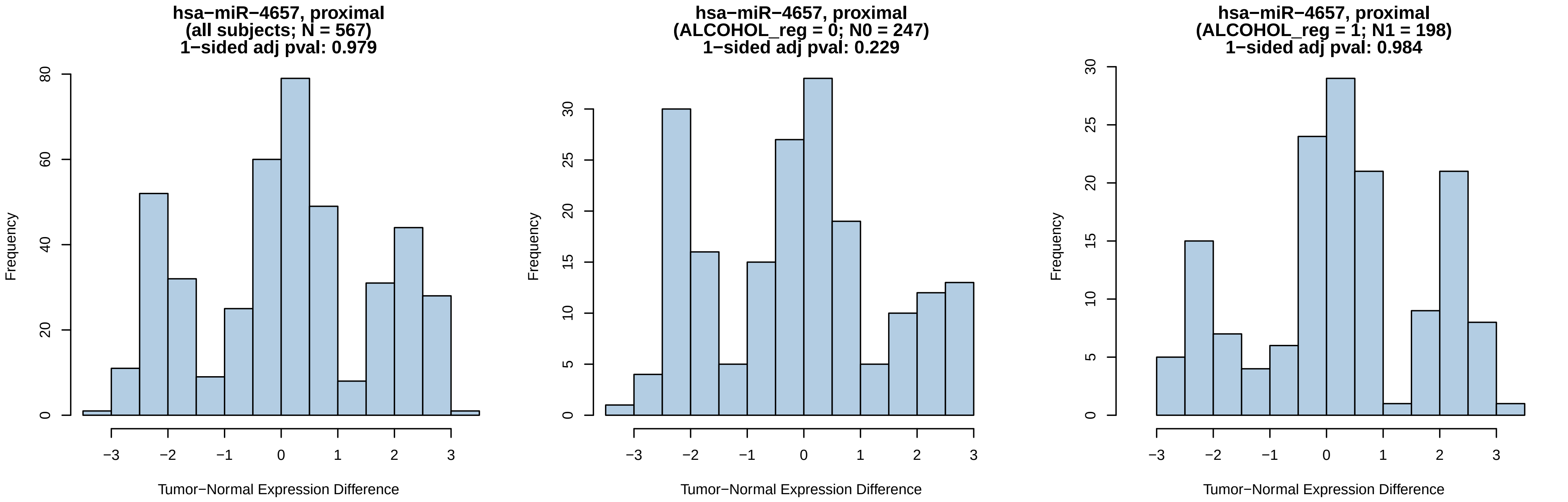
hsa−miR−4657, proximal
(all subjects; N = 567)
1−sided adj pval: 0.979
Frequency
Tumor−Normal Expression Difference
hsa−miR−4657, proximal
(ALCOHOL_reg = 0; N0 = 247)
1−sided adj pval: 0.229
Frequency
Tumor−Normal Expression Difference
hsa−miR−4657, proximal
(ALCOHOL_reg = 1; N1 = 198)
1−sided adj pval: 0.984
Frequency
Tumor−Normal Expression Difference

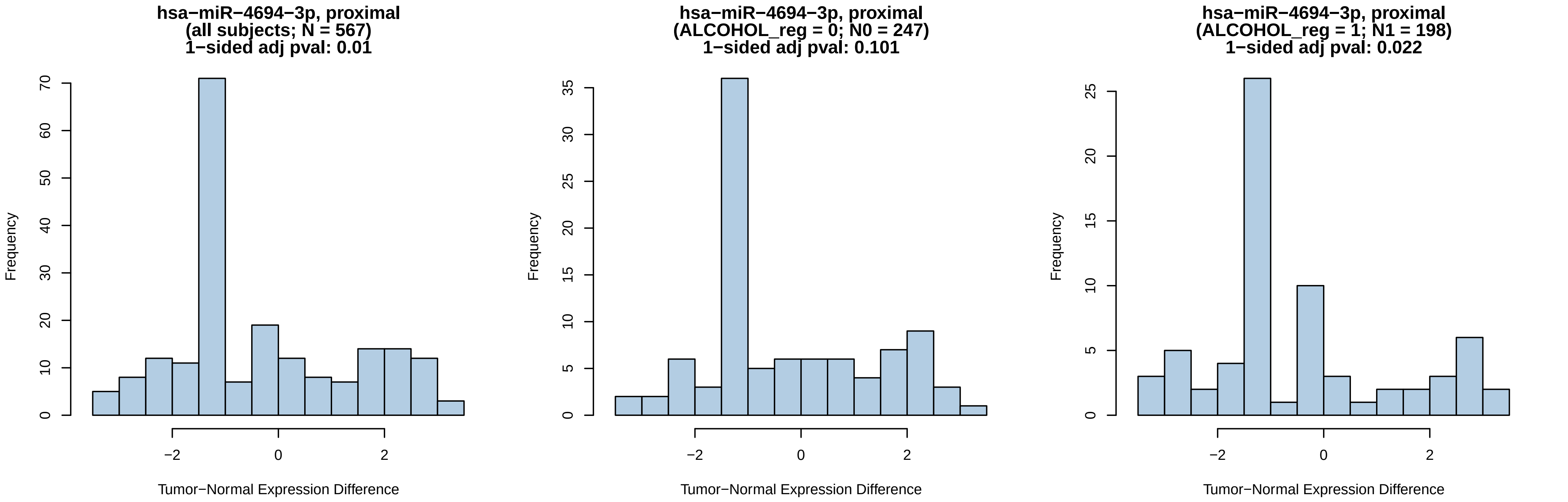

hsa−miR−4694−3p, proximal
(all subjects; N = 567)
1−sided adj pval: 0.01
Frequency
Tumor−Normal Expression Difference
hsa−miR−4694−3p, proximal
(ALCOHOL_reg = 0; N0 = 247)
1−sided adj pval: 0.101
Frequency
Tumor−Normal Expression Difference
hsa−miR−4694−3p, proximal
(ALCOHOL_reg = 1; N1 = 198)
1−sided adj pval: 0.022
Frequency
Tumor−Normal Expression Difference

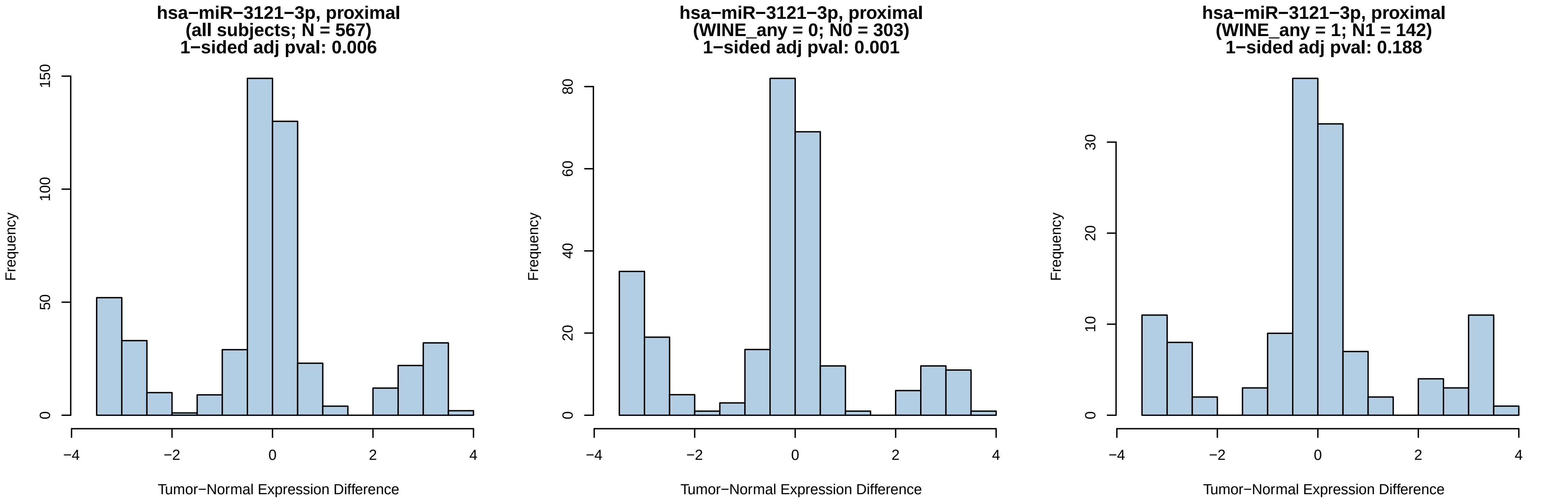
hsa−miR−3121−3p, proximal
(all subjects; N = 567)
1−sided adj pval: 0.006
Frequency
0
50
100
150
−4
−2
0
2
4
Tumor−Normal Expression Difference
hsa−miR−3121−3p, proximal
(WINE_any = 0; N0 = 303)
1−sided adj pval: 0.001
Frequency
0
20
40
60
80
−4
−2
0
2
4
Tumor−Normal Expression Difference
hsa−miR−3121−3p, proximal
(WINE_any = 1; N1 = 142)
1−sided adj pval: 0.188
Frequency
0
10
20
30
−4
−2
0
2
4
Tumor−Normal Expression Difference

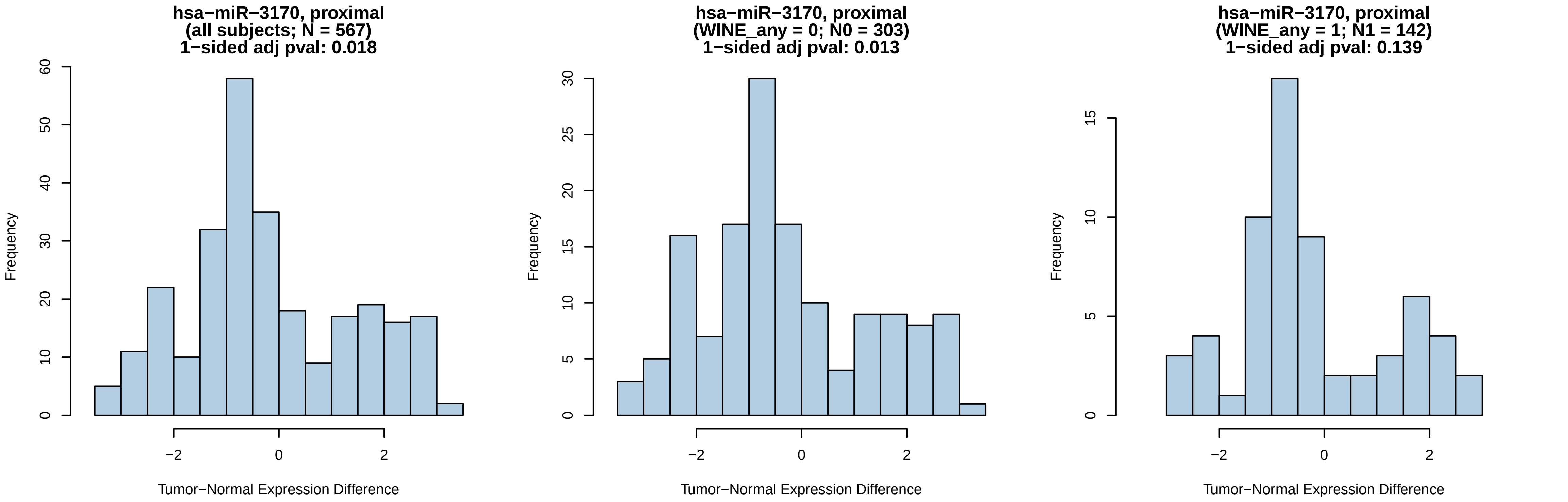
hsa−miR−3170, proximal
(all subjects; N = 567)
1−sided adj pval: 0.018
Frequency
Tumor−Normal Expression Difference
hsa−miR−3170, proximal
(WINE_any = 0; N0 = 303)
1−sided adj pval: 0.013
Frequency
Tumor−Normal Expression Difference
hsa−miR−3170, proximal
(WINE_any = 1; N1 = 142)
1−sided adj pval: 0.139
Frequency
Tumor−Normal Expression Difference

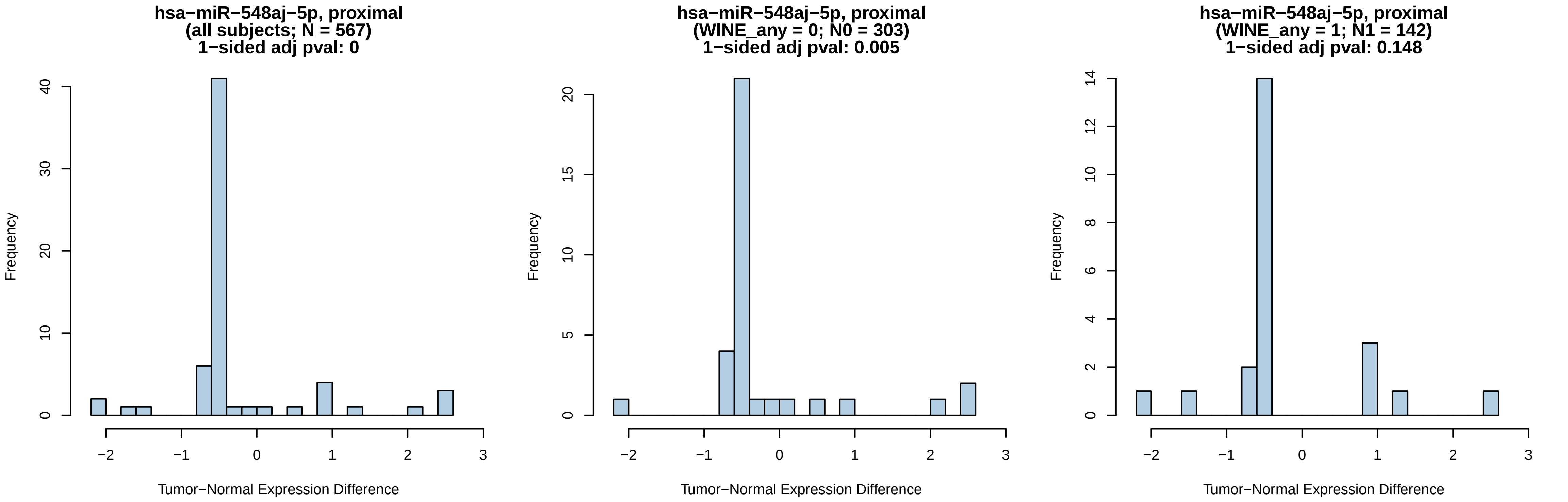

hsa−miR−548aj−5p, proximal
(all subjects; N = 567)
1−sided adj pval: 0
Frequency
Tumor−Normal Expression Difference
hsa−miR−548aj−5p, proximal
(WINE_any = 0; N0 = 303)
1−sided adj pval: 0.005
Frequency
Tumor−Normal Expression Difference
hsa−miR−548aj−5p, proximal
(WINE_any = 1; N1 = 142)
1−sided adj pval: 0.148
Frequency
Tumor−Normal Expression Difference

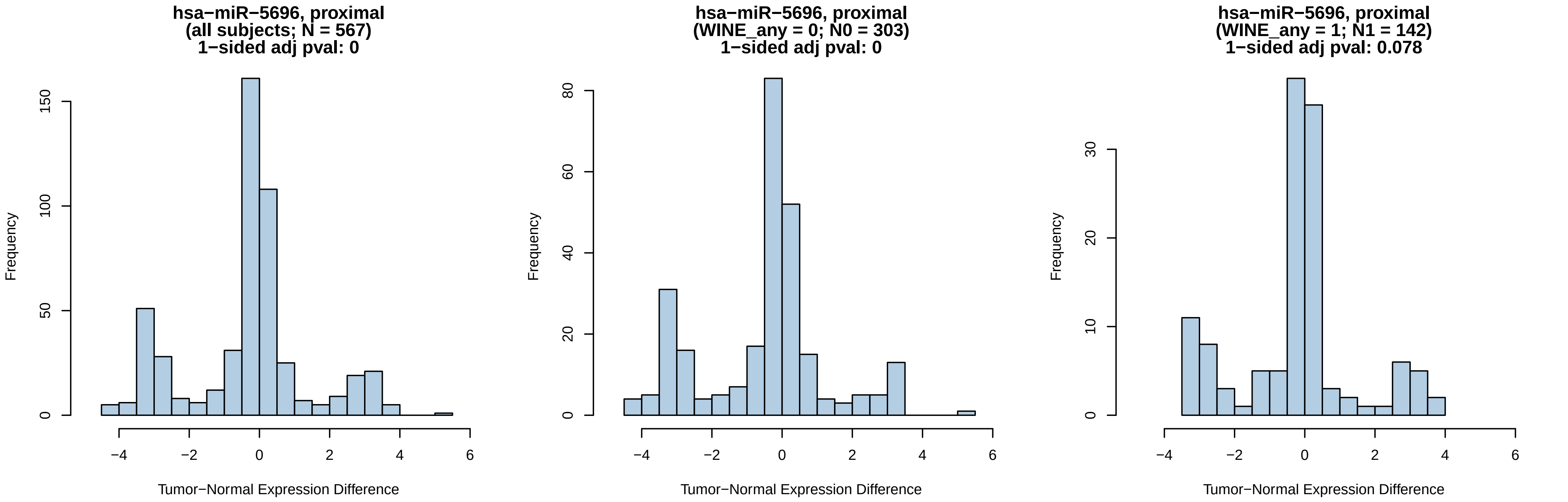

hsa−miR−5696, proximal
(all subjects; N = 567)
1−sided adj pval: 0
Frequency
Tumor−Normal Expression Difference
hsa−miR−5696, proximal
(WINE_any = 0; N0 = 303)
1−sided adj pval: 0
Frequency
Tumor−Normal Expression Difference
hsa−miR−5696, proximal
(WINE_any = 1; N1 = 142)
1−sided adj pval: 0.078
Frequency
Tumor−Normal Expression Difference

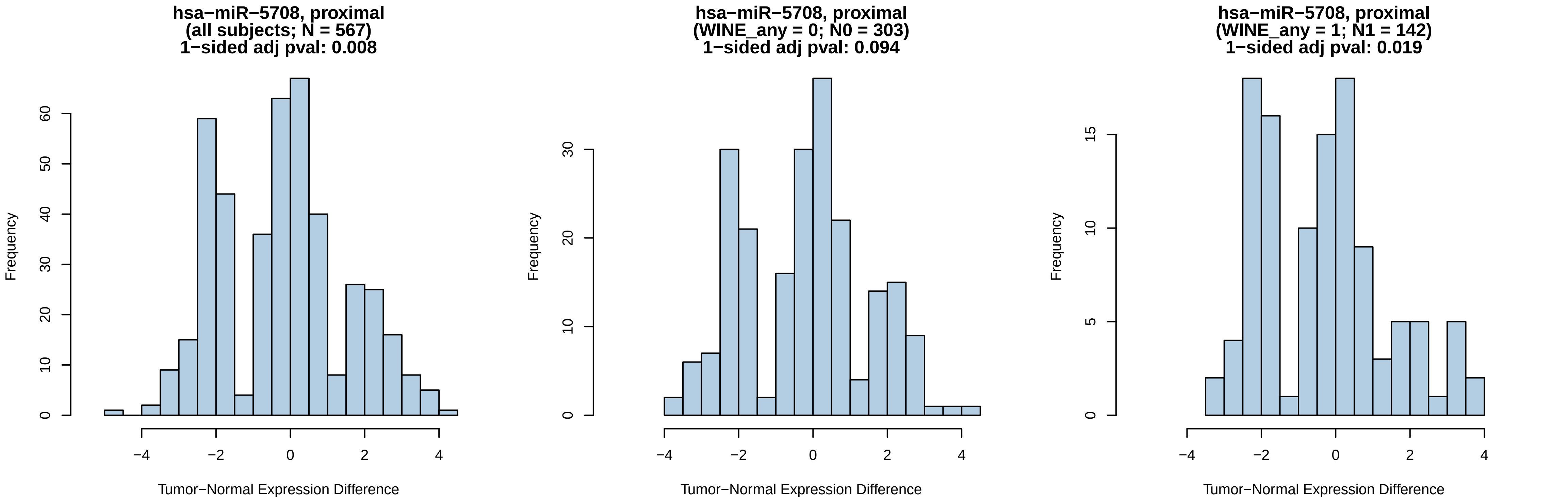

hsa−miR−5708, proximal
(all subjects; N = 567)
1−sided adj pval: 0.008
Frequency
Tumor−Normal Expression Difference
hsa−miR−5708, proximal
(WINE_any = 0; N0 = 303)
1−sided adj pval: 0.094
Frequency
Tumor−Normal Expression Difference
hsa−miR−5708, proximal
(WINE_any = 1; N1 = 142)
1−sided adj pval: 0.019
Frequency
Tumor−Normal Expression Difference

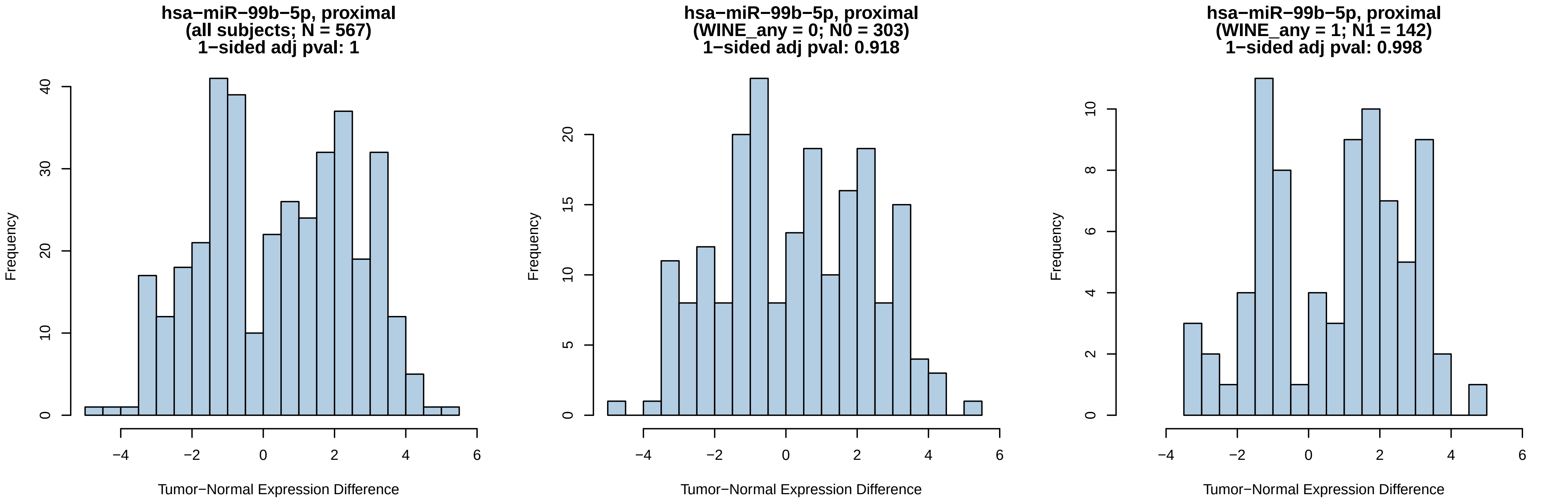
hsa−miR−99b−5p, proximal
(all subjects; N = 567)
1−sided adj pval: 1
hsa−miR−99b−5p, proximal
(WINE_any = 0; N0 = 303)
1−sided adj pval: 0.918
hsa−miR−99b−5p, proximal
(WINE_any = 1; N1 = 142)
1−sided adj pval: 0.998
Frequency
Tumor−Normal Expression Difference
0
10
20
30
40
5
15
2
4
6
8
−4
−2

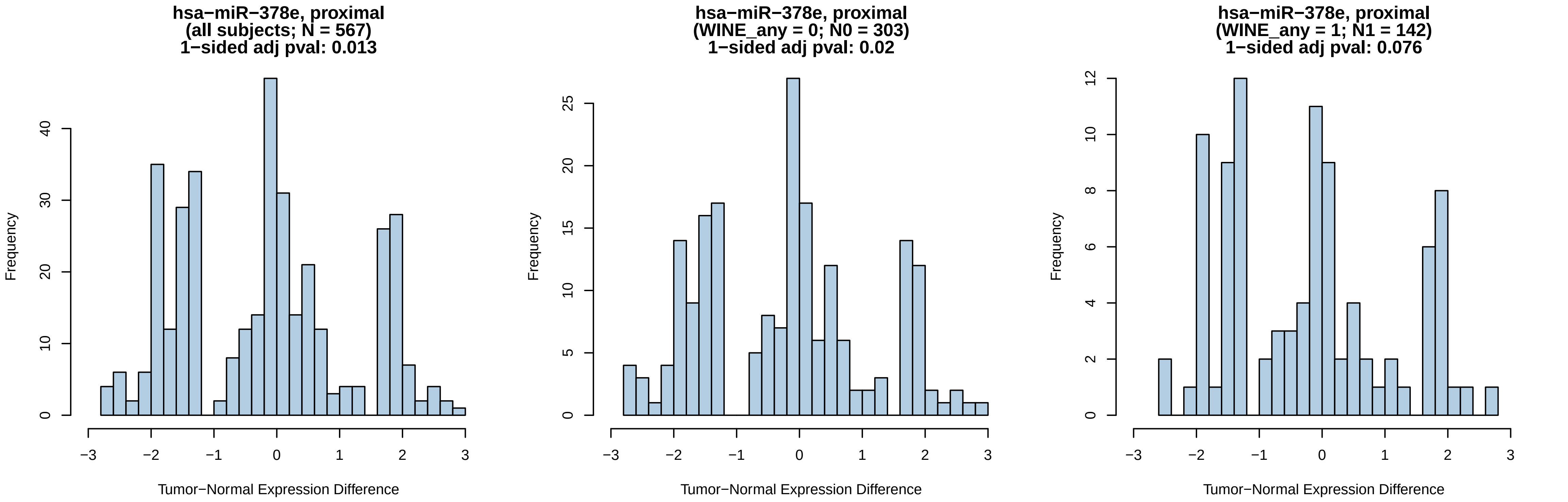
hsa−miR−378e, proximal
(all subjects; N = 567)
1−sided adj pval: 0.013
hsa−miR−378e, proximal
(WINE_any = 0; N0 = 303)
1−sided adj pval: 0.02
hsa−miR−378e, proximal
(WINE_any = 1; N1 = 142)
1−sided adj pval: 0.076
Frequency
Tumor−Normal Expression Difference

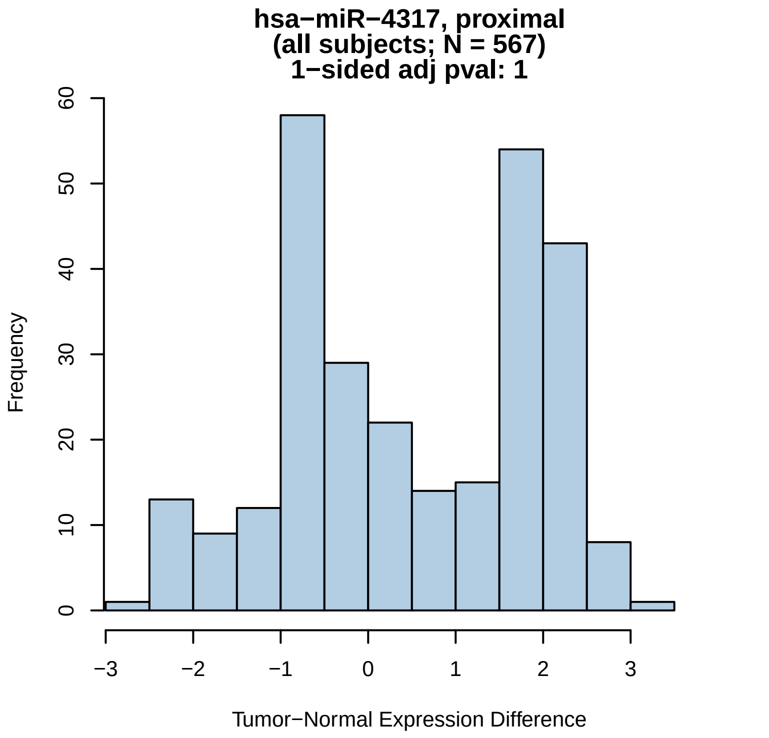
hsa−miR−4317, proximal
(all subjects; N = 567)
1−sided adj pval: 1
Frequency
0
10
20
30
40
50
60
−3
−2
−1
0
1
2
3
Tumor−Normal Expression Difference

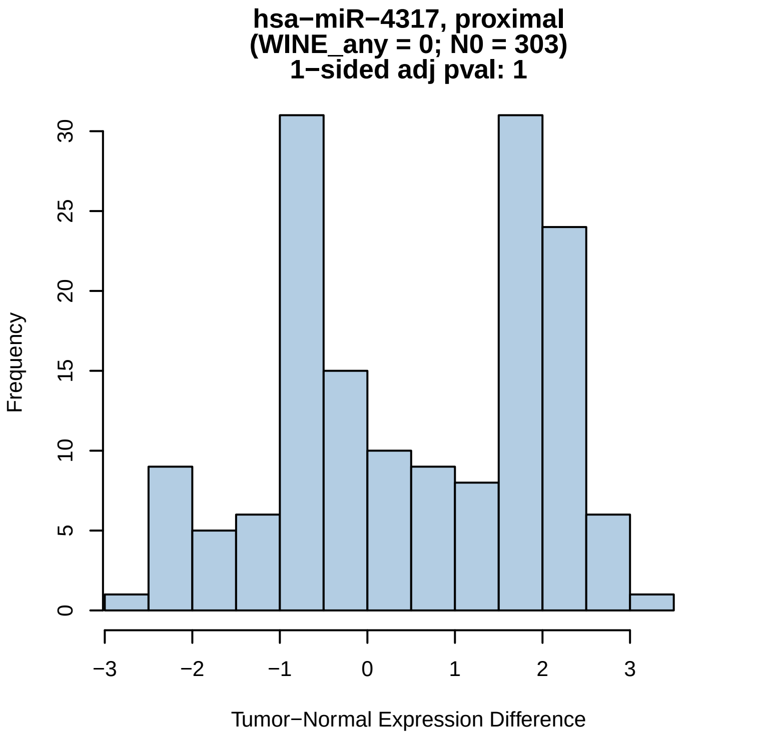
hsa−miR−4317, proximal
(WINE_any = 0; N0 = 303)
1−sided adj pval: 1
Frequency
0
5
10
15
20
25
30
−3
−2
−1
0
1
2
3
Tumor−Normal Expression Difference

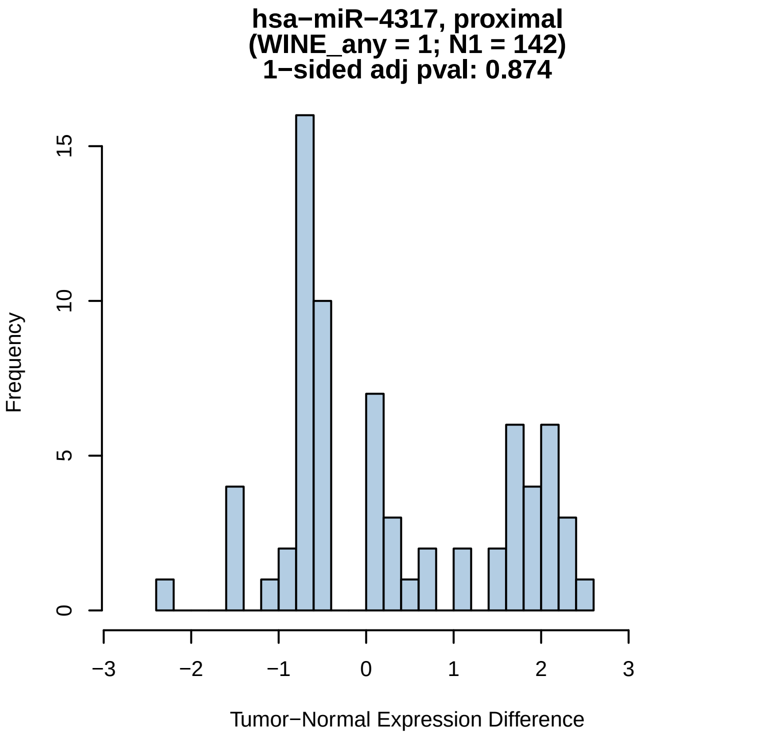
hsa−miR−4317, proximal
(WINE_any = 1; N1 = 142)
1−sided adj pval: 0.874
Frequency
0
5
10
15
−3
−2
−1
0
1
2
3
Tumor−Normal Expression Difference

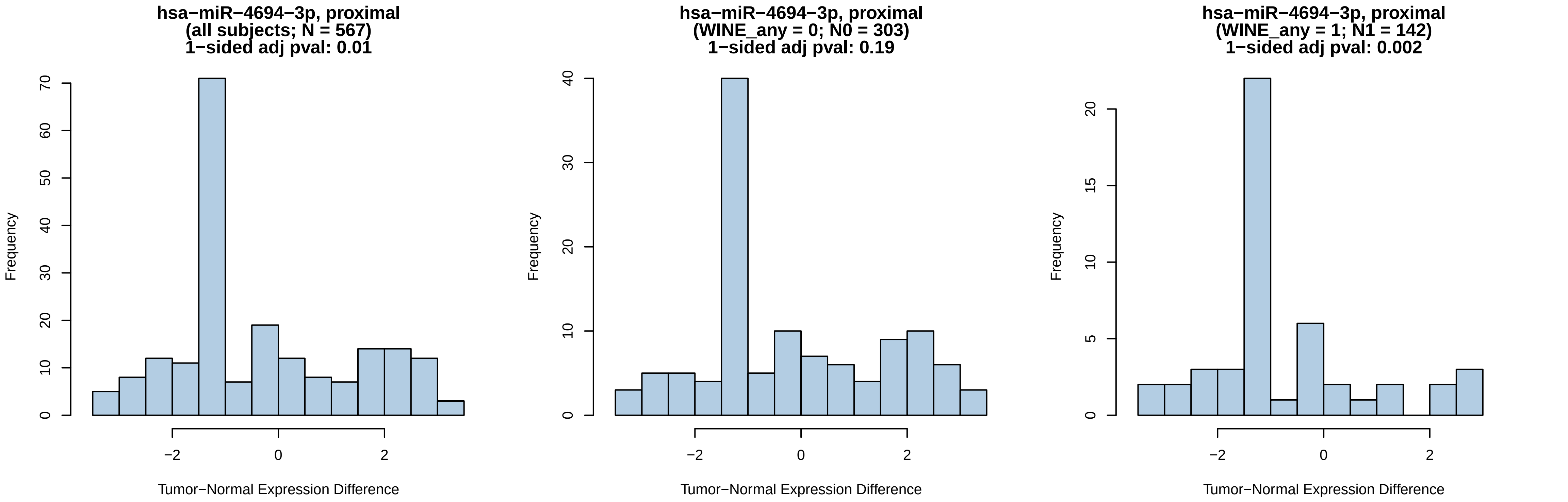
hsa−miR−4694−3p, proximal
(all subjects; N = 567)
1−sided adj pval: 0.01
Frequency
0
10
20
30
40
50
60
70
−2
0
2
Tumor−Normal Expression Difference
hsa−miR−4694−3p, proximal
(WINE_any = 0; N0 = 303)
1−sided adj pval: 0.19
Frequency
0
10
20
30
40
−2
0
2
Tumor−Normal Expression Difference
hsa−miR−4694−3p, proximal
(WINE_any = 1; N1 = 142)
1−sided adj pval: 0.002
Frequency
0
5
10
15
20
−2
0
2
Tumor−Normal Expression Difference

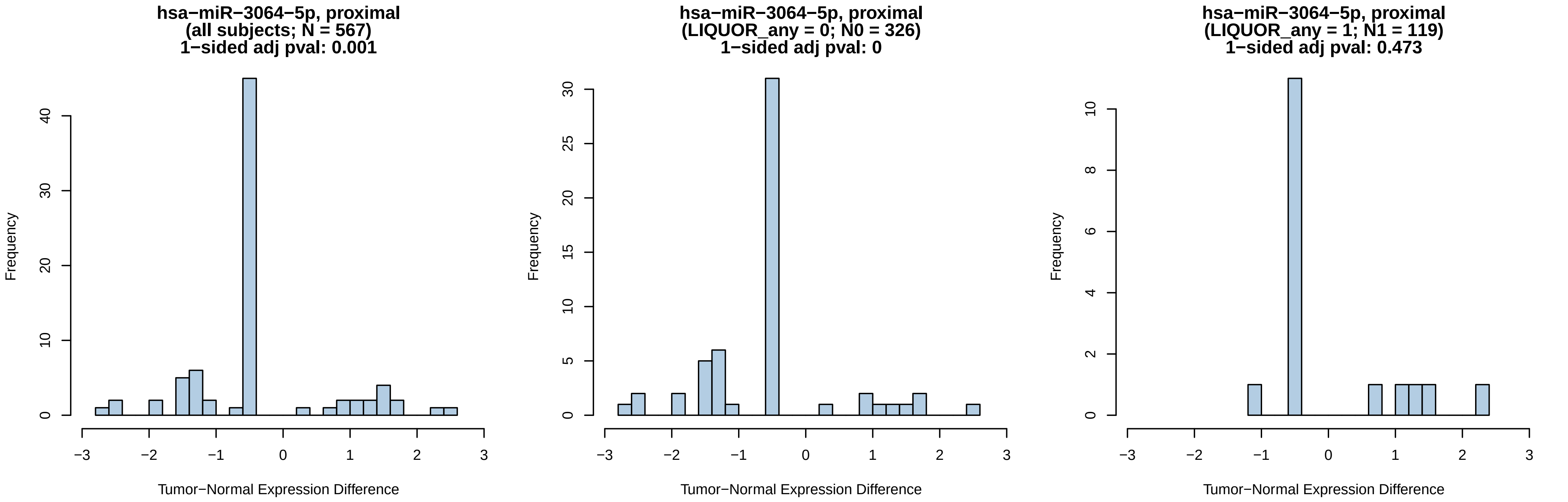
hsa−miR−3064−5p, proximal
(all subjects; N = 567)
1−sided adj pval: 0.001
Frequency
Tumor−Normal Expression Difference
hsa−miR−3064−5p, proximal
(LIQUOR_any = 0; N0 = 326)
1−sided adj pval: 0
Frequency
Tumor−Normal Expression Difference
hsa−miR−3064−5p, proximal
(LIQUOR_any = 1; N1 = 119)
1−sided adj pval: 0.473
Frequency
Tumor−Normal Expression Difference

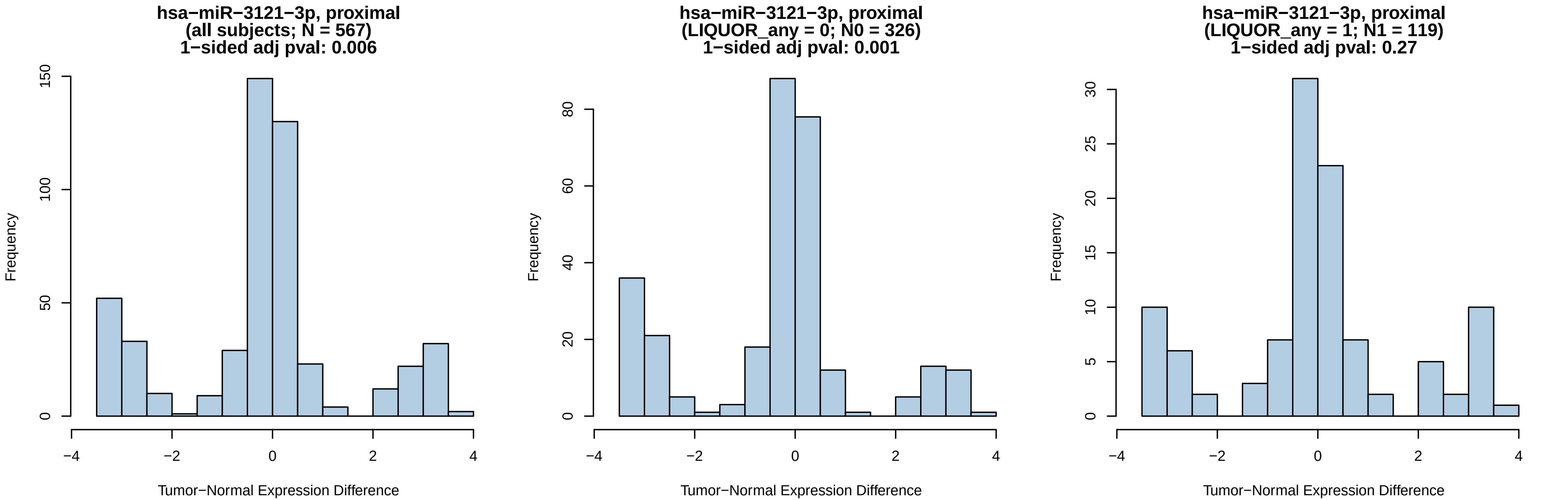

hsa−miR−3121−3p, proximal
(all subjects; N = 567)
1−sided adj pval: 0.006
Frequency
Tumor−Normal Expression Difference
hsa−miR−3121−3p, proximal
(LIQUOR_any = 0; N0 = 326)
1−sided adj pval: 0.001
Frequency
Tumor−Normal Expression Difference
hsa−miR−3121−3p, proximal
(LIQUOR_any = 1; N1 = 119)
1−sided adj pval: 0.27
Frequency
Tumor−Normal Expression Difference

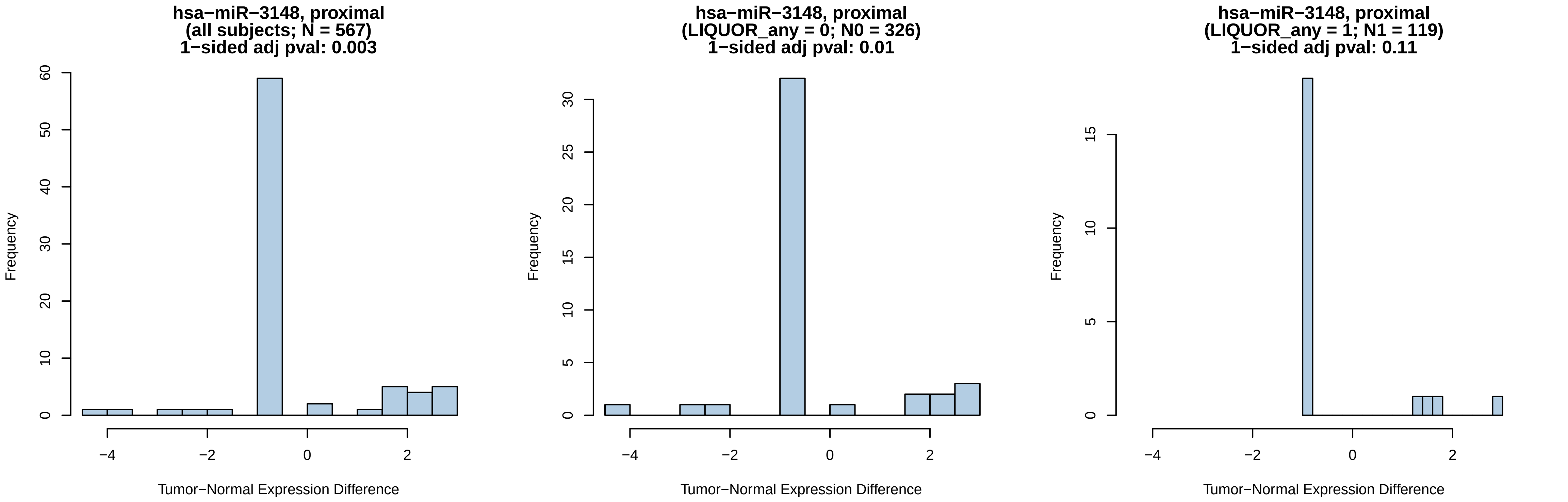
hsa−miR−3148, proximal
(all subjects; N = 567)
1−sided adj pval: 0.003
hsa−miR−3148, proximal
(LIQUOR_any = 0; N0 = 326)
1−sided adj pval: 0.01
hsa−miR−3148, proximal
(LIQUOR_any = 1; N1 = 119)
1−sided adj pval: 0.11
Frequency
Tumor−Normal Expression Difference

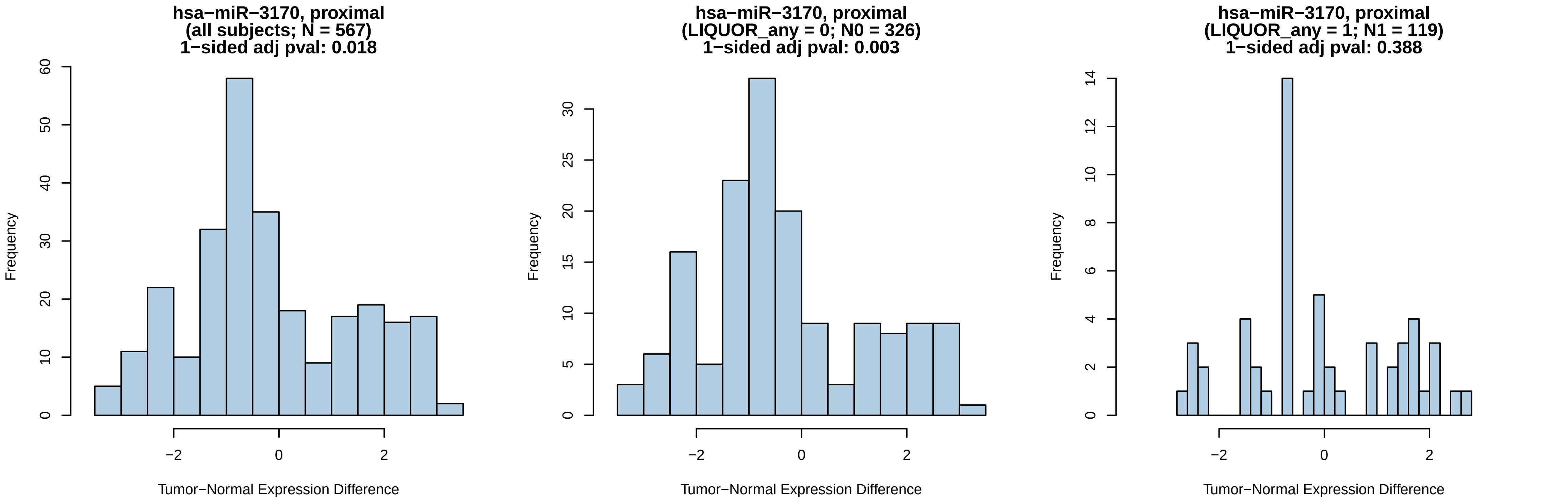
hsa−miR−3170, proximal
(all subjects; N = 567)
1−sided adj pval: 0.018
Frequency
Tumor−Normal Expression Difference
hsa−miR−3170, proximal
(LIQUOR_any = 0; N0 = 326)
1−sided adj pval: 0.003
Frequency
Tumor−Normal Expression Difference
hsa−miR−3170, proximal
(LIQUOR_any = 1; N1 = 119)
1−sided adj pval: 0.388
Frequency
Tumor−Normal Expression Difference

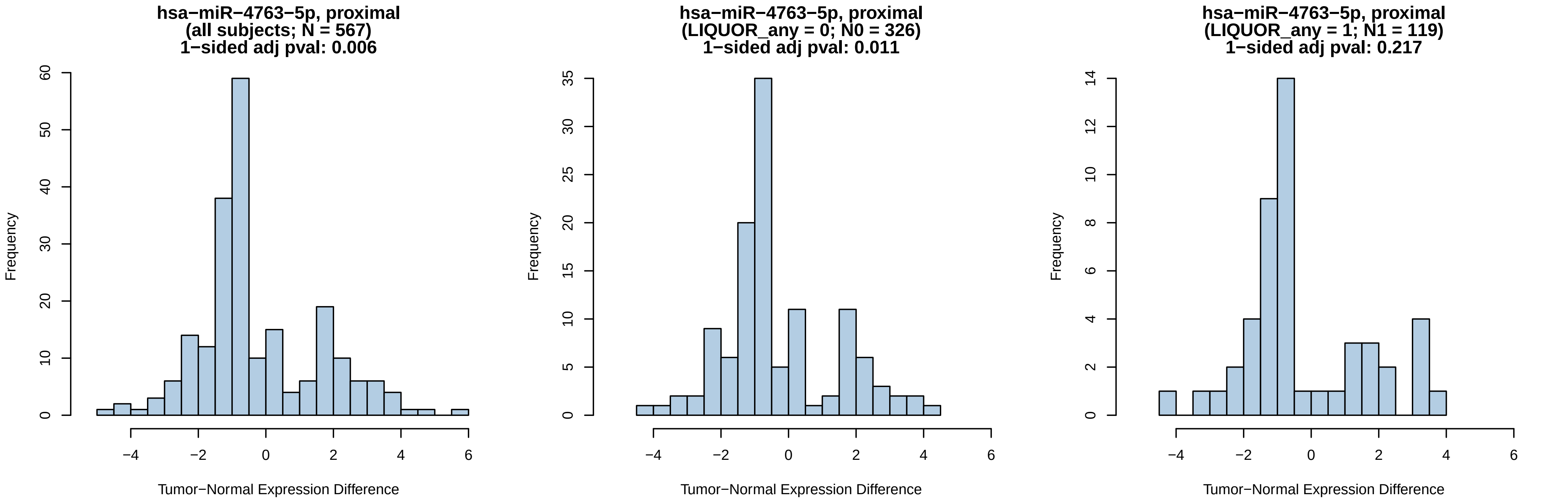
hsa−miR−4763−5p, proximal
(all subjects; N = 567)
1−sided adj pval: 0.006
hsa−miR−4763−5p, proximal
(LIQUOR_any = 0; N0 = 326)
1−sided adj pval: 0.011
hsa−miR−4763−5p, proximal
(LIQUOR_any = 1; N1 = 119)
1−sided adj pval: 0.217
Frequency
Tumor−Normal Expression Difference

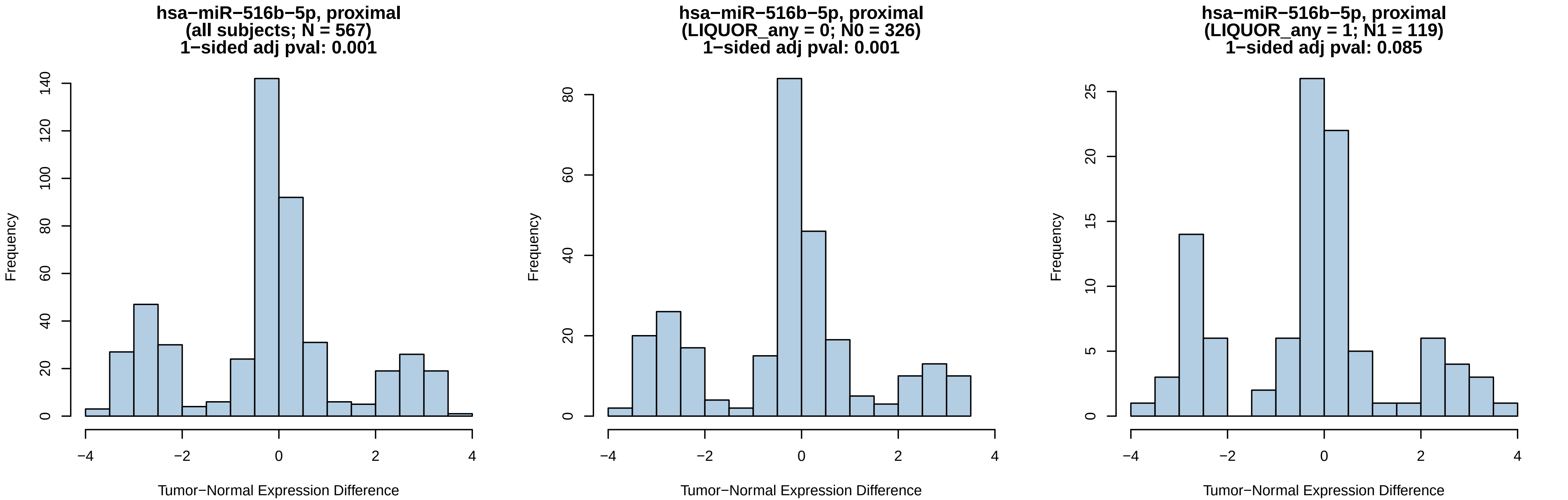
hsa−miR−516b−5p, proximal
(all subjects; N = 567)
1−sided adj pval: 0.001
hsa−miR−516b−5p, proximal
(LIQUOR_any = 0; N0 = 326)
1−sided adj pval: 0.001
hsa−miR−516b−5p, proximal
(LIQUOR_any = 1; N1 = 119)
1−sided adj pval: 0.085
Frequency
Tumor−Normal Expression Difference

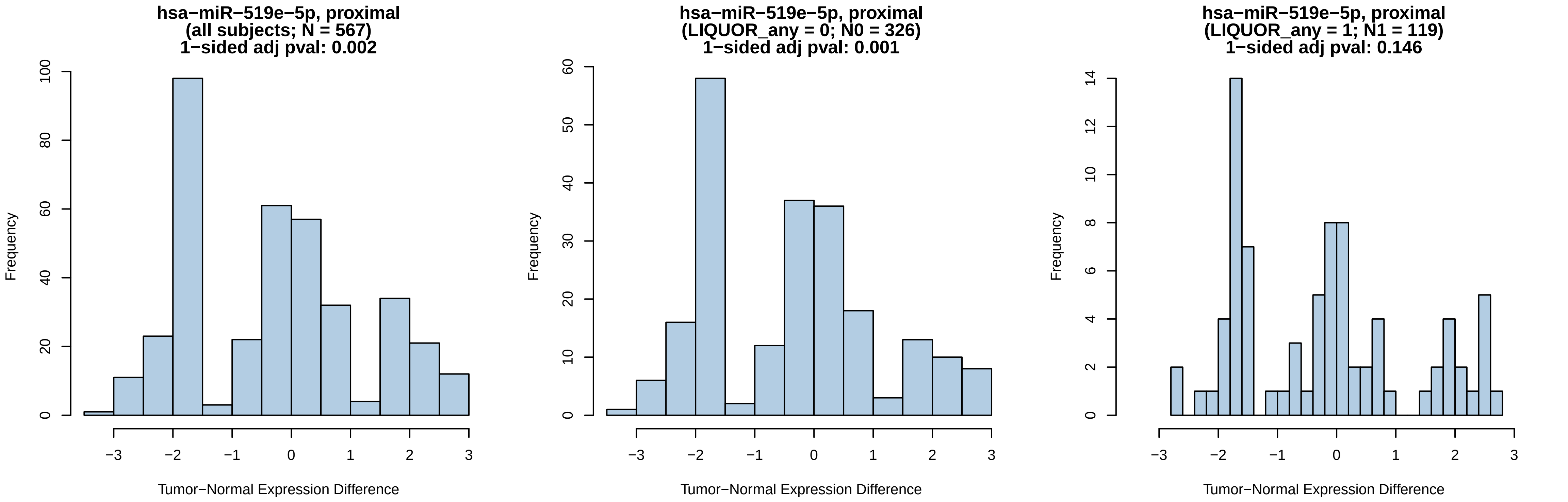
hsa−miR−519e−5p, proximal
(all subjects; N = 567)
1−sided adj pval: 0.002
Frequency
Tumor−Normal Expression Difference
hsa−miR−519e−5p, proximal
(LIQUOR_any = 0; N0 = 326)
1−sided adj pval: 0.001
Frequency
Tumor−Normal Expression Difference
hsa−miR−519e−5p, proximal
(LIQUOR_any = 1; N1 = 119)
1−sided adj pval: 0.146
Frequency
Tumor−Normal Expression Difference

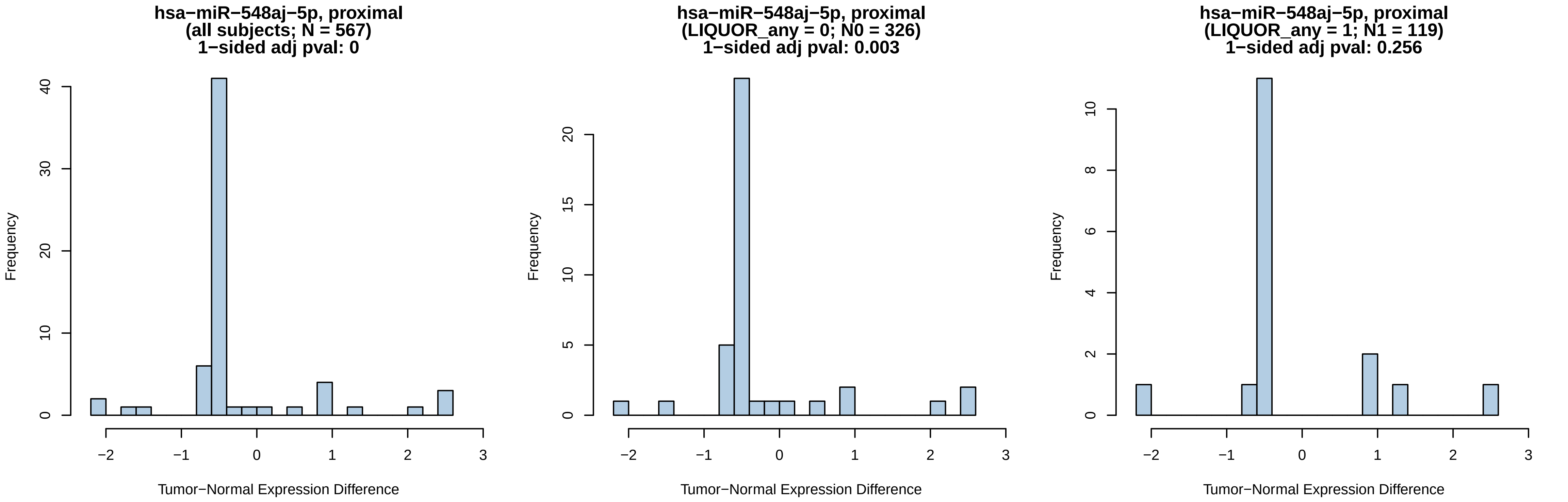
hsa−miR−548aj−5p, proximal
(all subjects; N = 567)
1−sided adj pval: 0
Frequency
Tumor−Normal Expression Difference
hsa−miR−548aj−5p, proximal
(LIQUOR_any = 0; N0 = 326)
1−sided adj pval: 0.003
Frequency
Tumor−Normal Expression Difference
hsa−miR−548aj−5p, proximal
(LIQUOR_any = 1; N1 = 119)
1−sided adj pval: 0.256
Frequency
Tumor−Normal Expression Difference

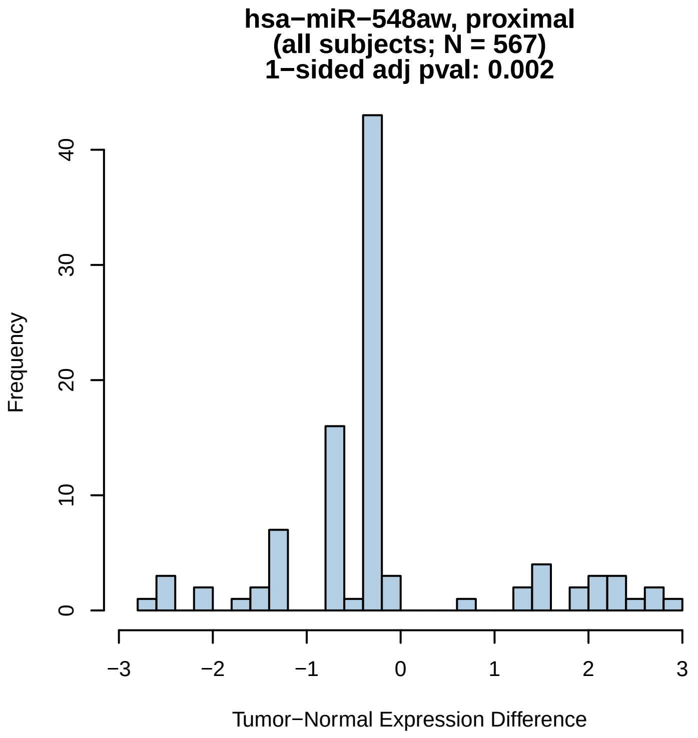
hsa−miR−548aw, proximal
(all subjects; N = 567)
1−sided adj pval: 0.002
Frequency
0
10
20
30
40
−3
−2
−1
0
1
2
3
Tumor−Normal Expression Difference

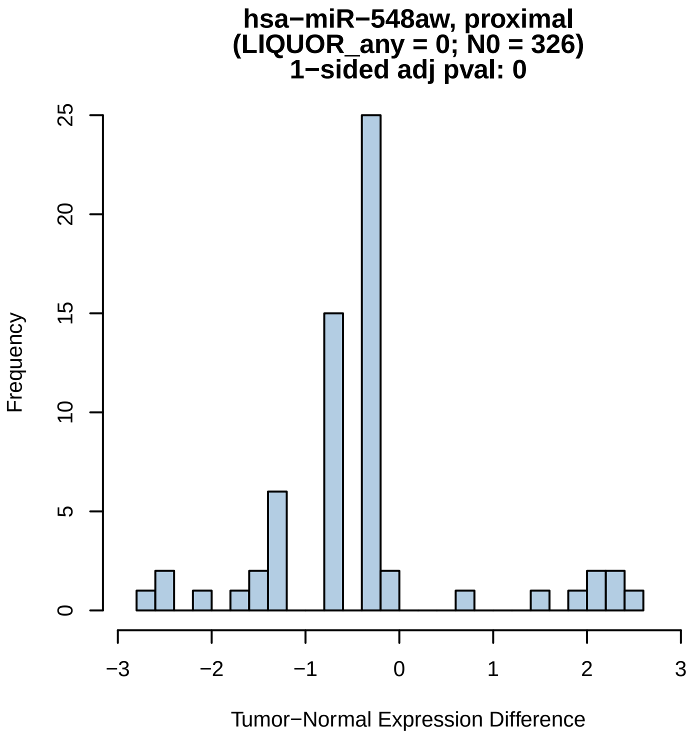
hsa−miR−548aw, proximal
(LIQUOR_any = 0; N0 = 326)
1−sided adj pval: 0
Frequency
0
5
10
15
20
25
−3
−2
−1
0
1
2
3
Tumor−Normal Expression Difference

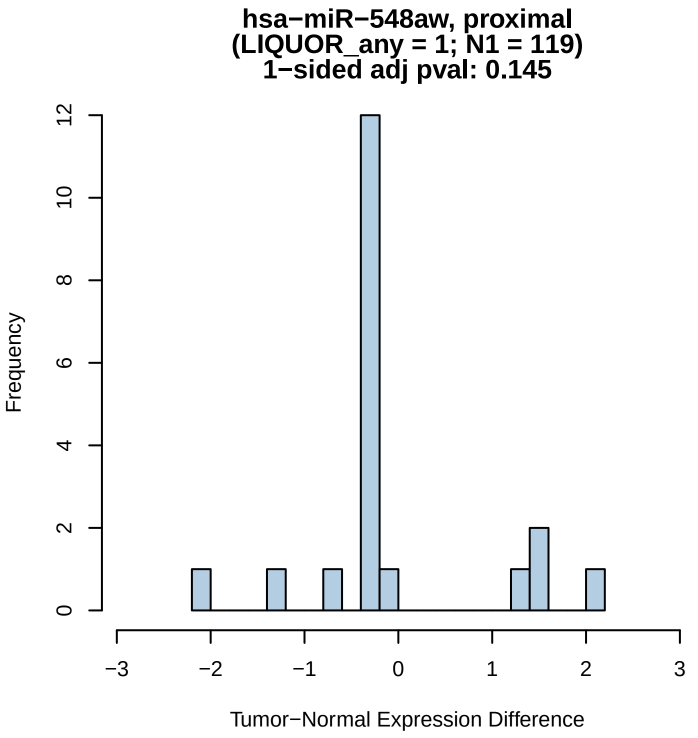
hsa−miR−548aw, proximal
(LIQUOR_any = 1; N1 = 119)
1−sided adj pval: 0.145
Frequency
0
2
4
6
8
10
12
−3
−2
−1
0
1
2
3
Tumor−Normal Expression Difference

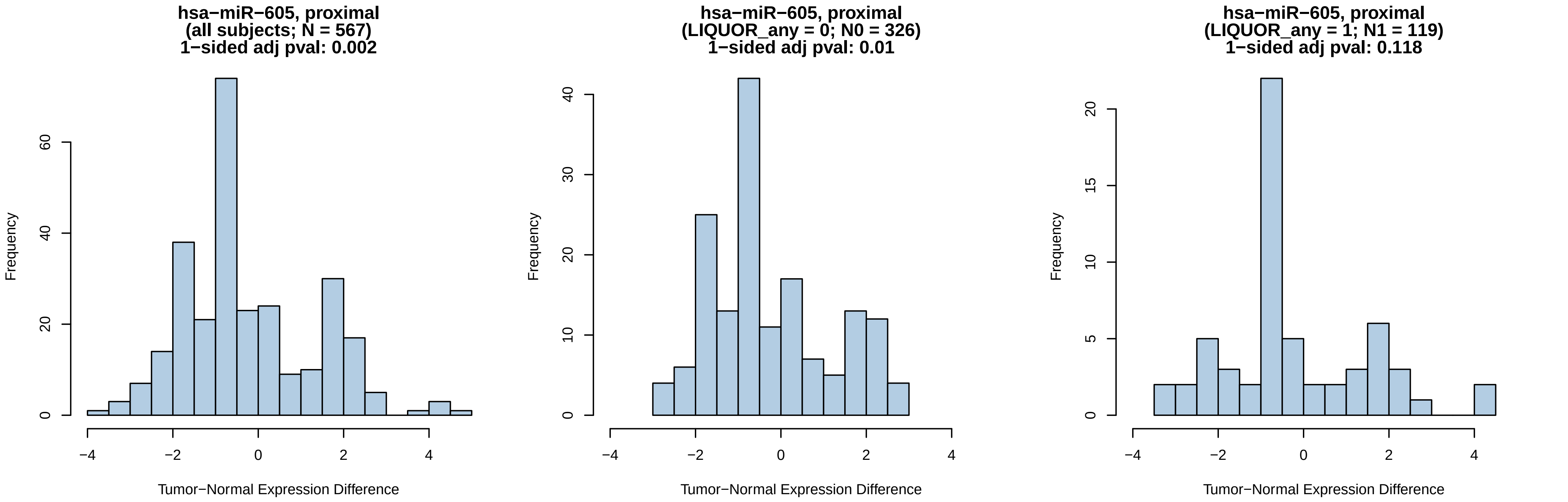

hsa−miR−605, proximal
(all subjects; N = 567)
1−sided adj pval: 0.002
Frequency
0
20
40
60
−4
−2
0
2
4
Tumor−Normal Expression Difference
hsa−miR−605, proximal
(LIQUOR_any = 0; N0 = 326)
1−sided adj pval: 0.01
Frequency
0
10
20
30
40
−4
−2
0
2
4
Tumor−Normal Expression Difference
hsa−miR−605, proximal
(LIQUOR_any = 1; N1 = 119)
1−sided adj pval: 0.118
Frequency
0
5
10
15
20
−4
−2
0
2
4
Tumor−Normal Expression Difference

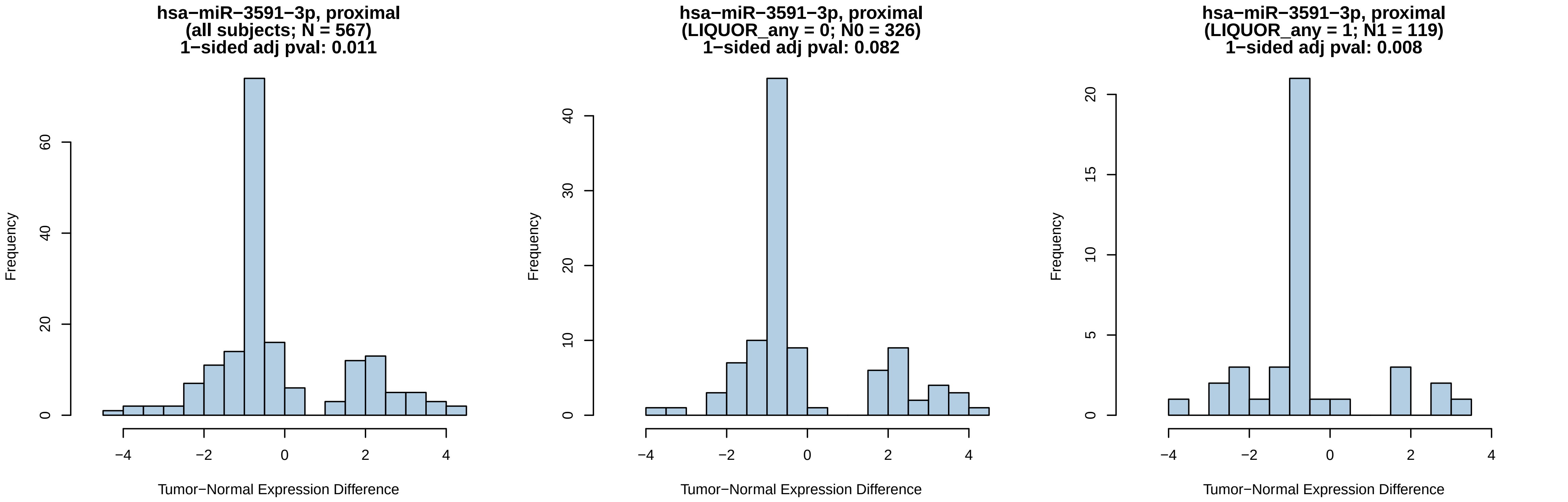

hsa−miR−3591−3p, proximal
(all subjects; N = 567)
1−sided adj pval: 0.011
Frequency
0
20
40
60
−4
−2
0
2
4
Tumor−Normal Expression Difference
hsa−miR−3591−3p, proximal
(LIQUOR_any = 0; N0 = 326)
1−sided adj pval: 0.082
Frequency
0
10
20
30
40
−4
−2
0
2
4
Tumor−Normal Expression Difference
hsa−miR−3591−3p, proximal
(LIQUOR_any = 1; N1 = 119)
1−sided adj pval: 0.008
Frequency
0
5
10
15
20
−4
−2
0
2
4
Tumor−Normal Expression Difference

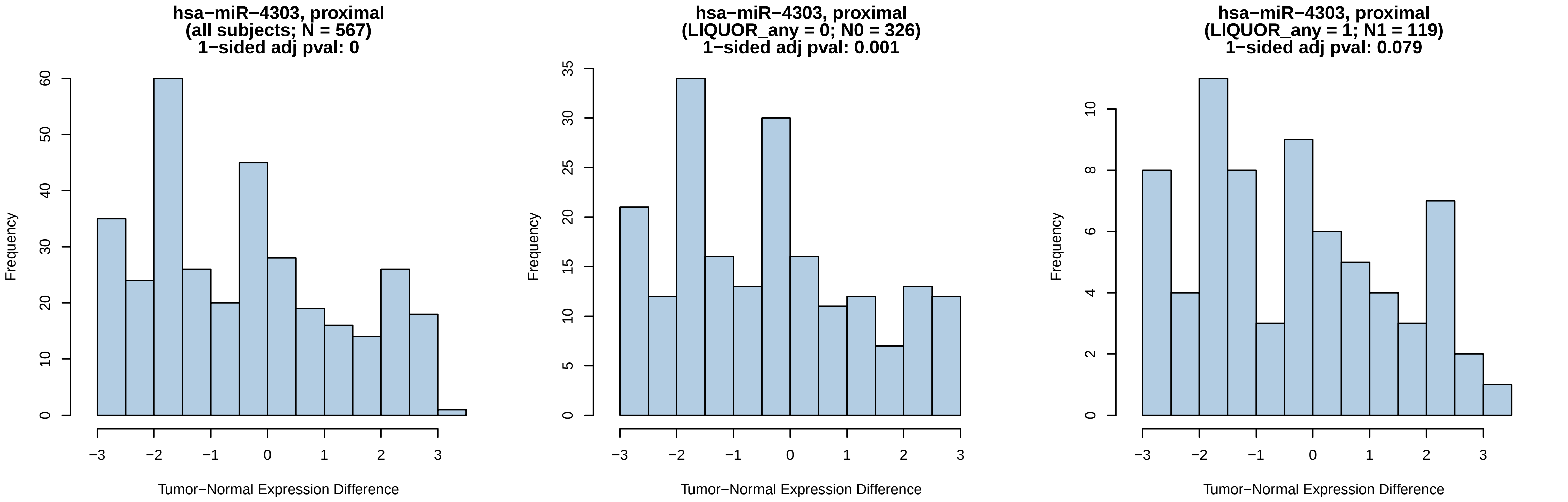
hsa−miR−4303, proximal
(all subjects; N = 567)
1−sided adj pval: 0
Frequency
0
10
20
30
40
50
60
−3
−2
−1
0
1
2
3
Tumor−Normal Expression Difference
hsa−miR−4303, proximal
(LIQUOR_any = 0; N0 = 326)
1−sided adj pval: 0.001
Frequency
0
5
10
15
20
25
30
35
−3
−2
−1
0
1
2
3
Tumor−Normal Expression Difference
hsa−miR−4303, proximal
(LIQUOR_any = 1; N1 = 119)
1−sided adj pval: 0.079
Frequency
0
2
4
6
8
10
−3
−2
−1
0
1
2
3
Tumor−Normal Expression Difference

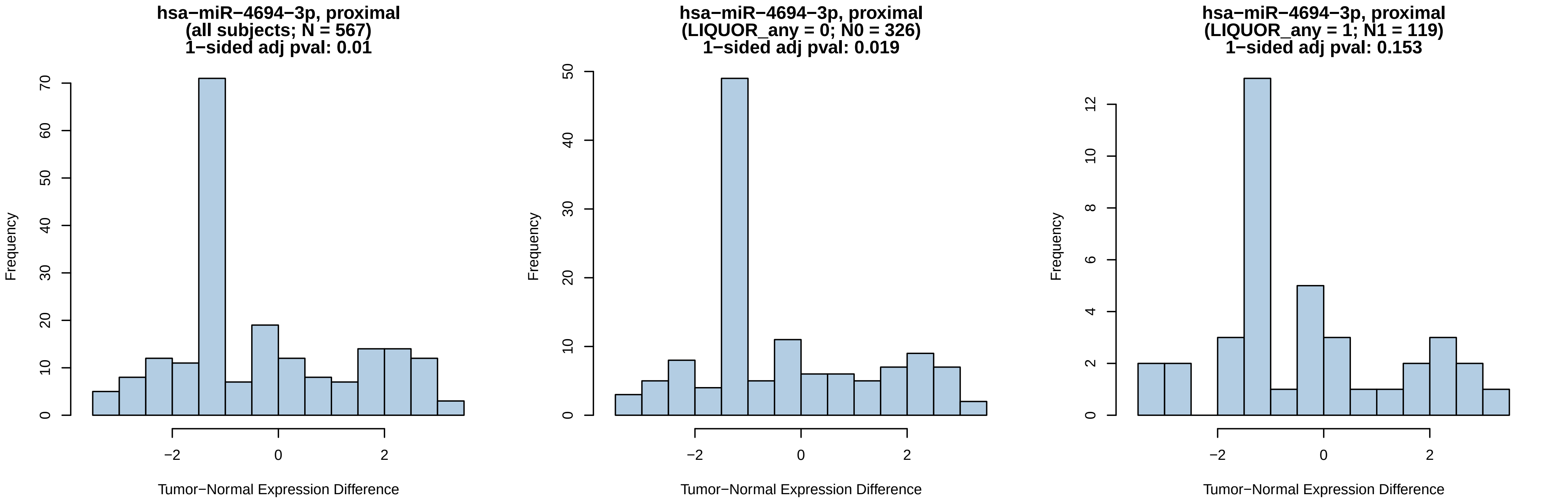
hsa−miR−4694−3p, proximal
(all subjects; N = 567)
1−sided adj pval: 0.01
hsa−miR−4694−3p, proximal
(LIQUOR_any = 0; N0 = 326)
1−sided adj pval: 0.019
hsa−miR−4694−3p, proximal
(LIQUOR_any = 1; N1 = 119)
1−sided adj pval: 0.153
Frequency
Tumor−Normal Expression Difference

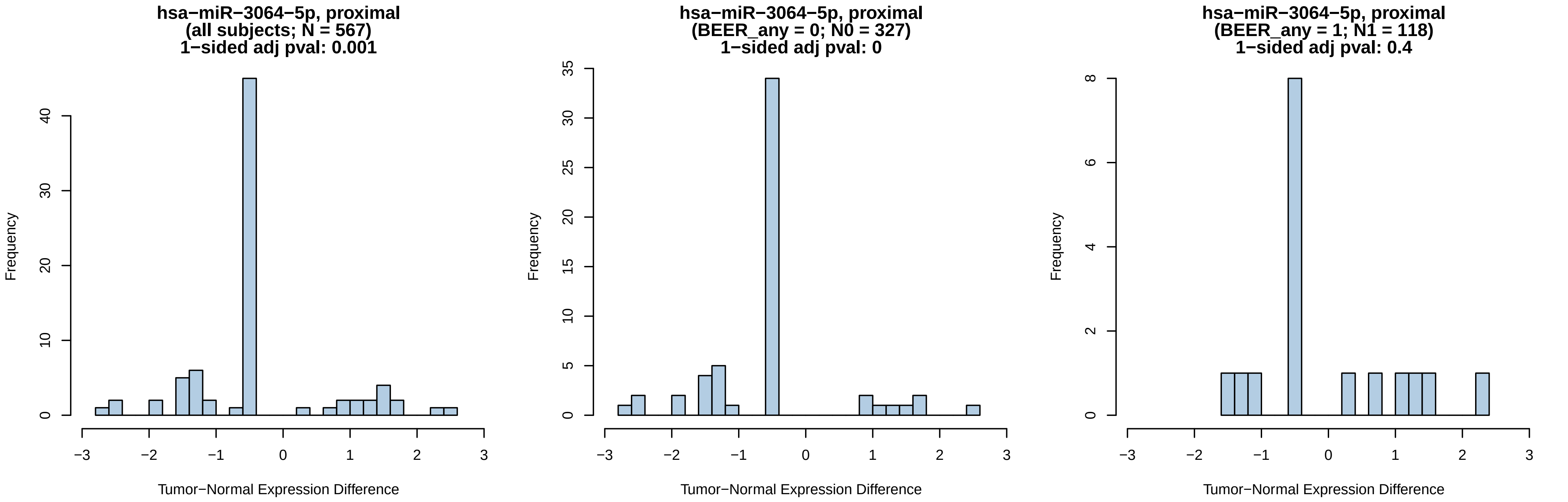
hsa−miR−3064−5p, proximal
(all subjects; N = 567)
1−sided adj pval: 0.001
Frequency
Tumor−Normal Expression Difference
hsa−miR−3064−5p, proximal
(BEER_any = 0; N0 = 327)
1−sided adj pval: 0
Frequency
Tumor−Normal Expression Difference
hsa−miR−3064−5p, proximal
(BEER_any = 1; N1 = 118)
1−sided adj pval: 0.4
Frequency
Tumor−Normal Expression Difference

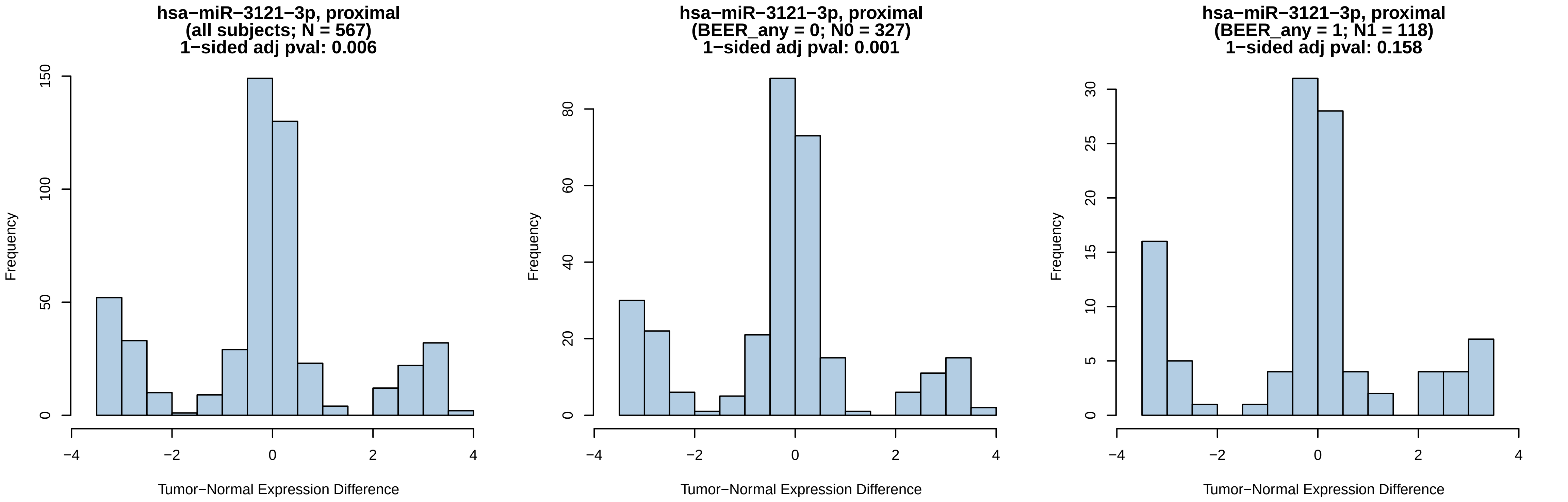

hsa−miR−3121−3p, proximal
(all subjects; N = 567)
1−sided adj pval: 0.006
Frequency
0
50
100
150
−4
−2
0
2
4
Tumor−Normal Expression Difference
hsa−miR−3121−3p, proximal
(BEER_any = 0; N0 = 327)
1−sided adj pval: 0.001
Frequency
0
20
40
60
80
−4
−2
0
2
4
Tumor−Normal Expression Difference
hsa−miR−3121−3p, proximal
(BEER_any = 1; N1 = 118)
1−sided adj pval: 0.158
Frequency
0
5
10
15
20
25
30
−4
−2
0
2
4
Tumor−Normal Expression Difference

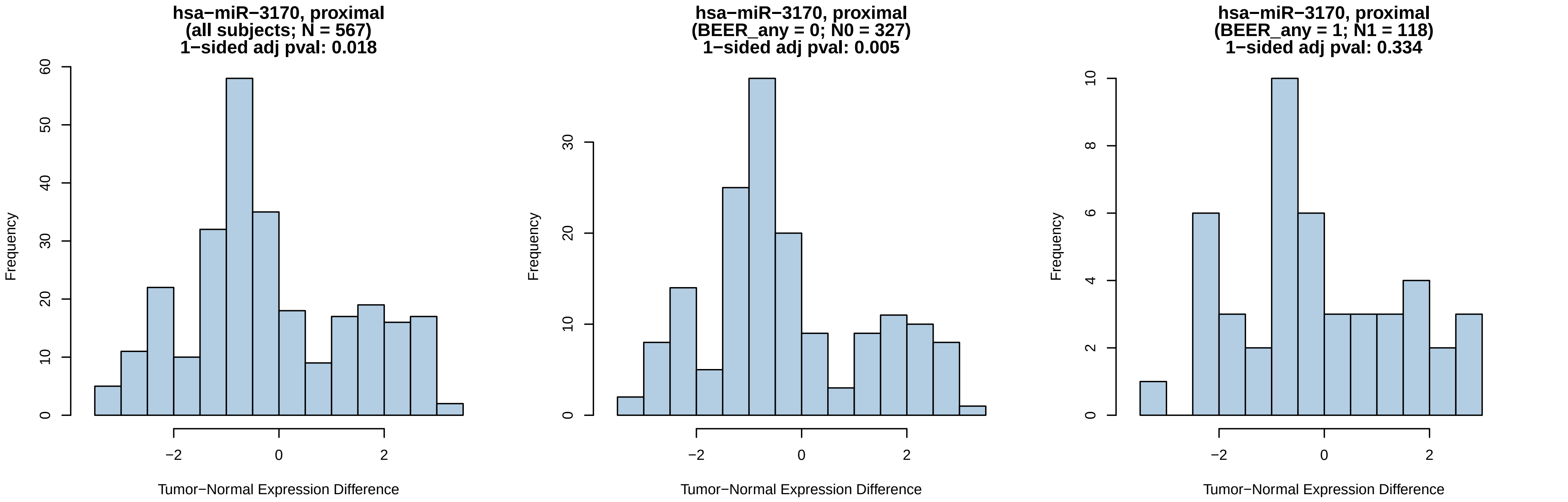

hsa−miR−3170, proximal
(all subjects; N = 567)
1−sided adj pval: 0.018
Frequency
Tumor−Normal Expression Difference
hsa−miR−3170, proximal
(BEER_any = 0; N0 = 327)
1−sided adj pval: 0.005
Frequency
Tumor−Normal Expression Difference
hsa−miR−3170, proximal
(BEER_any = 1; N1 = 118)
1−sided adj pval: 0.334
Frequency
Tumor−Normal Expression Difference

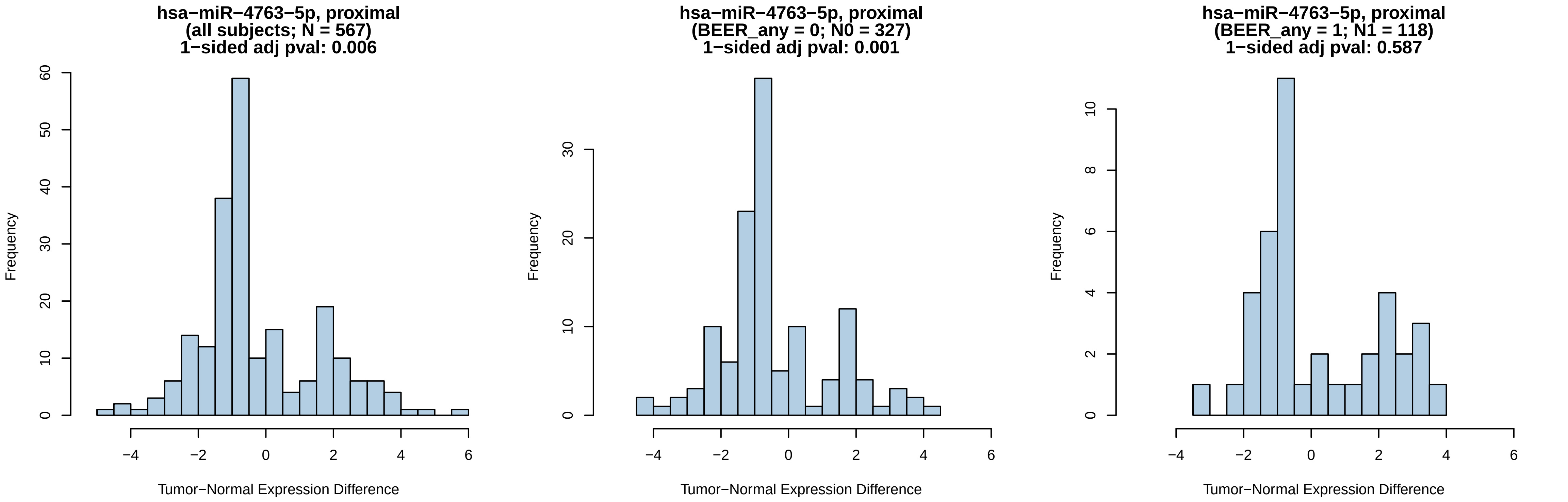

hsa−miR−4763−5p, proximal
(all subjects; N = 567)
1−sided adj pval: 0.006
Frequency
Tumor−Normal Expression Difference
hsa−miR−4763−5p, proximal
(BEER_any = 0; N0 = 327)
1−sided adj pval: 0.001
Frequency
Tumor−Normal Expression Difference
hsa−miR−4763−5p, proximal
(BEER_any = 1; N1 = 118)
1−sided adj pval: 0.587
Frequency
Tumor−Normal Expression Difference

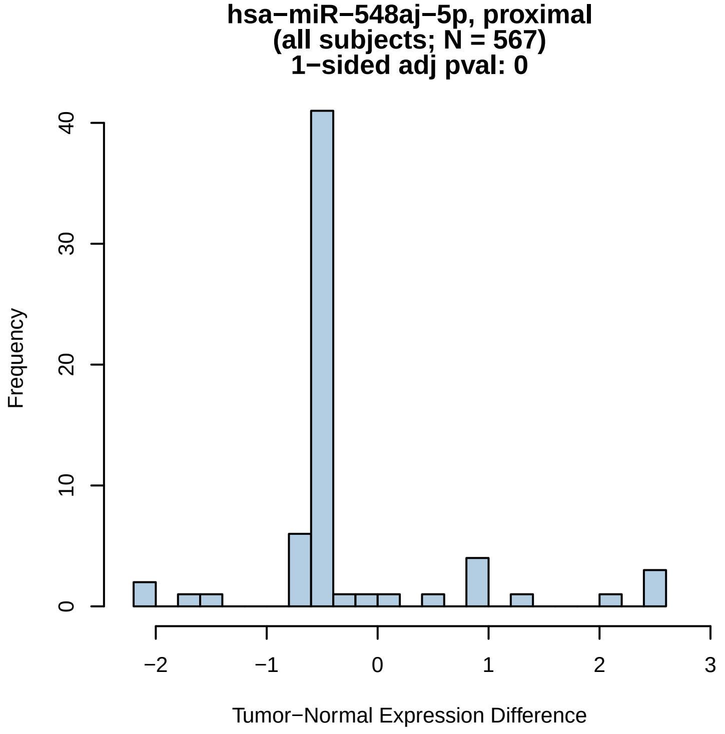
hsa−miR−548aj−5p, proximal
(all subjects; N = 567)
1−sided adj pval: 0
Frequency
0
10
20
30
40
−2
−1
0
1
2
3
Tumor−Normal Expression Difference

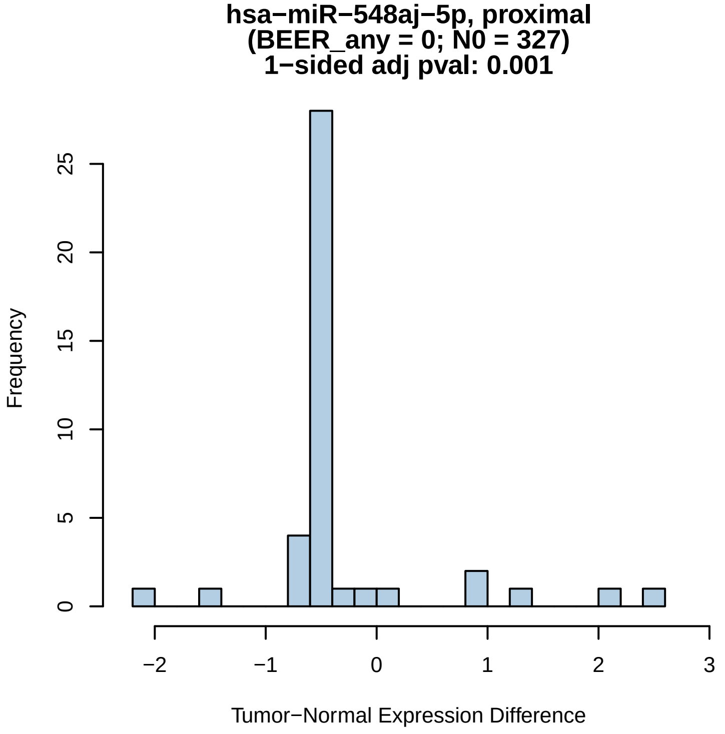
hsa−miR−548aj−5p, proximal
(BEER_any = 0; N0 = 327)
1−sided adj pval: 0.001
Frequency
0
5
10
15
20
25
−2
−1
0
1
2
3
Tumor−Normal Expression Difference

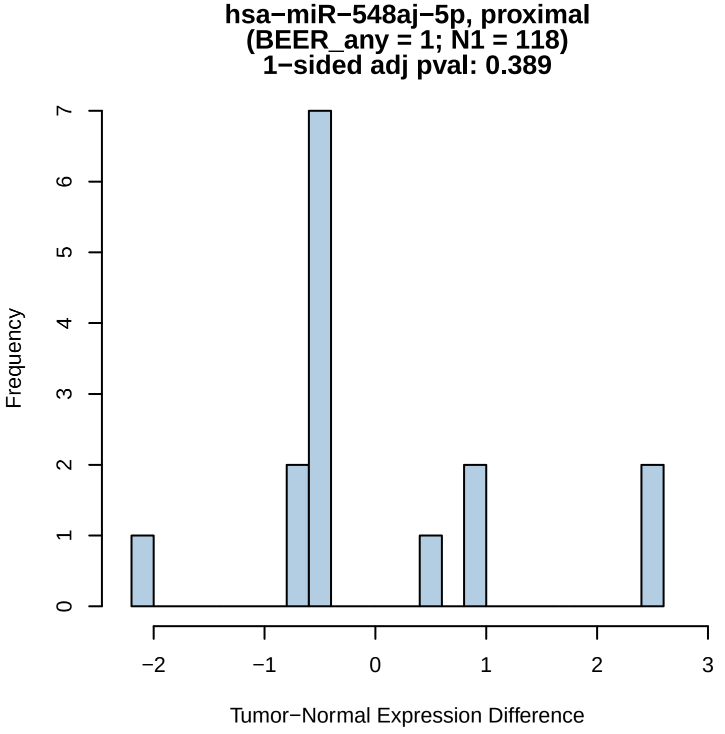
hsa−miR−548aj−5p, proximal
(BEER_any = 1; N1 = 118)
1−sided adj pval: 0.389
Frequency
0
1
2
3
4
5
6
7
−2
−1
0
1
2
3
Tumor−Normal Expression Difference

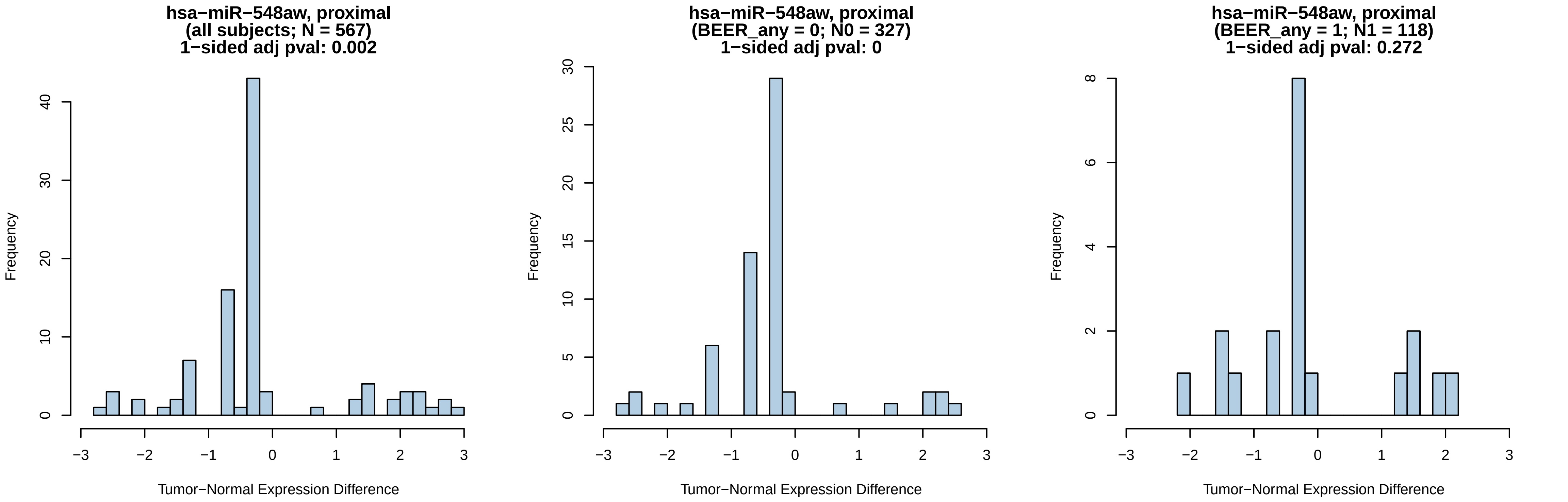
hsa−miR−548aw, proximal
(all subjects; N = 567)
1−sided adj pval: 0.002
Frequency
Tumor−Normal Expression Difference
hsa−miR−548aw, proximal
(BEER_any = 0; N0 = 327)
1−sided adj pval: 0
Frequency
Tumor−Normal Expression Difference
hsa−miR−548aw, proximal
(BEER_any = 1; N1 = 118)
1−sided adj pval: 0.272
Frequency
Tumor−Normal Expression Difference

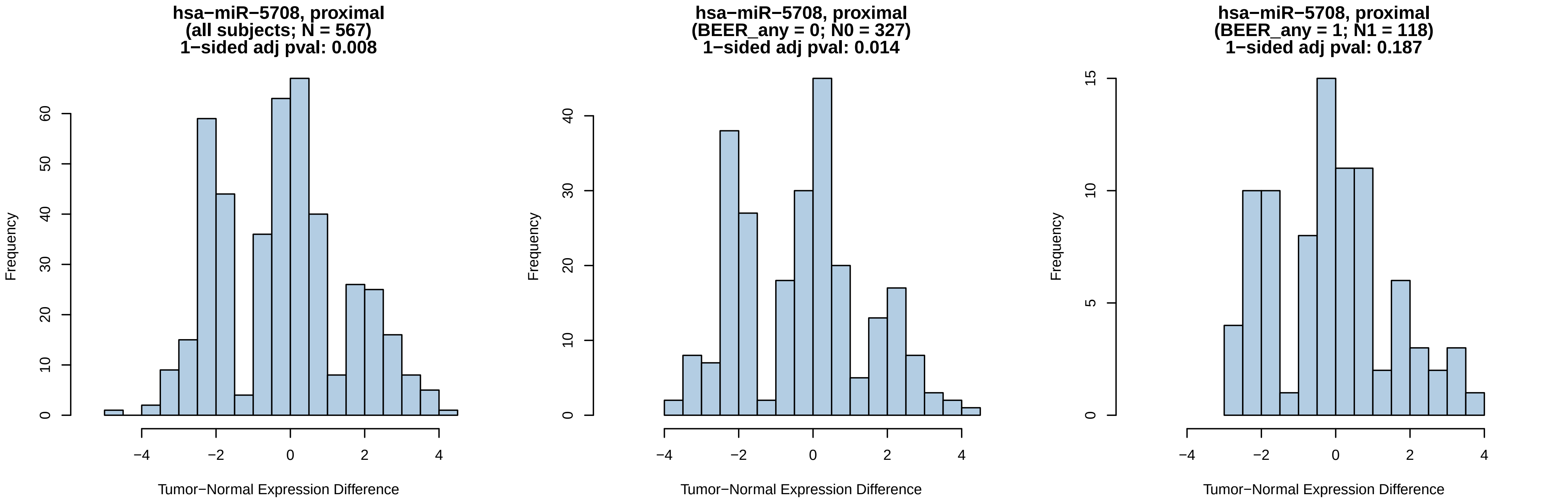

hsa−miR−5708, proximal
(all subjects; N = 567)
1−sided adj pval: 0.008
Frequency
0
10
20
30
40
50
60
−4
−2
0
2
4
Tumor−Normal Expression Difference
hsa−miR−5708, proximal
(BEER_any = 0; N0 = 327)
1−sided adj pval: 0.014
Frequency
0
10
20
30
40
−4
−2
0
2
4
Tumor−Normal Expression Difference
hsa−miR−5708, proximal
(BEER_any = 1; N1 = 118)
1−sided adj pval: 0.187
Frequency
0
5
10
15
−4
−2
0
2
4
Tumor−Normal Expression Difference

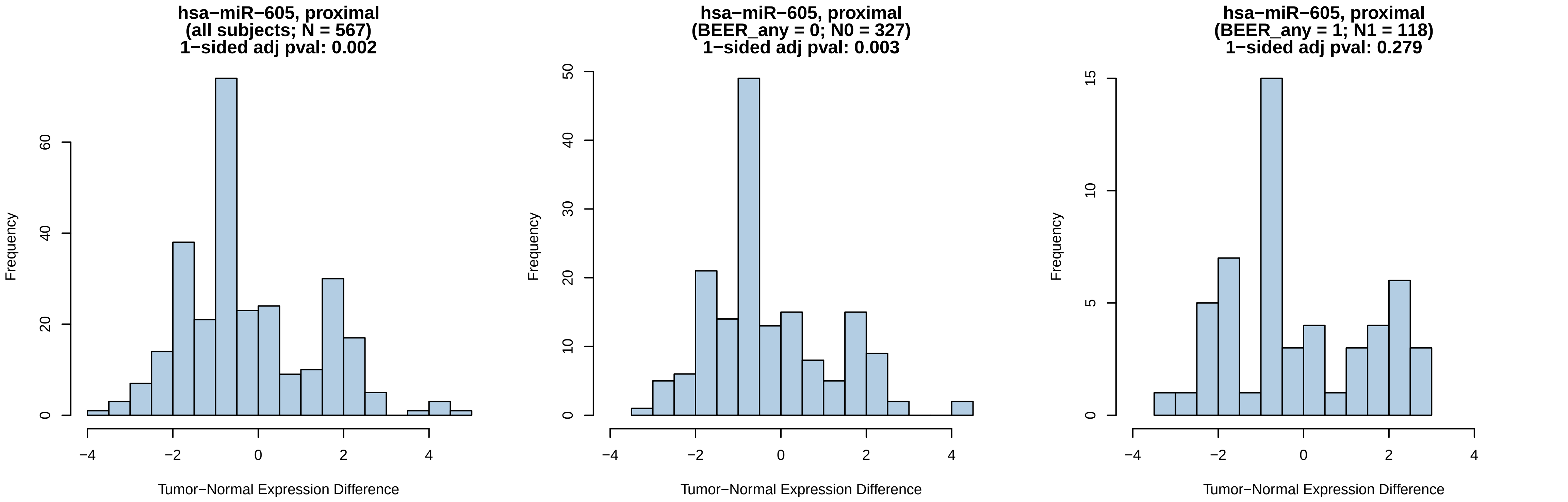
hsa−miR−605, proximal
(all subjects; N = 567)
1−sided adj pval: 0.002
Frequency
Tumor−Normal Expression Difference
hsa−miR−605, proximal
(BEER_any = 0; N0 = 327)
1−sided adj pval: 0.003
Frequency
Tumor−Normal Expression Difference
hsa−miR−605, proximal
(BEER_any = 1; N1 = 118)
1−sided adj pval: 0.279
Frequency
Tumor−Normal Expression Difference

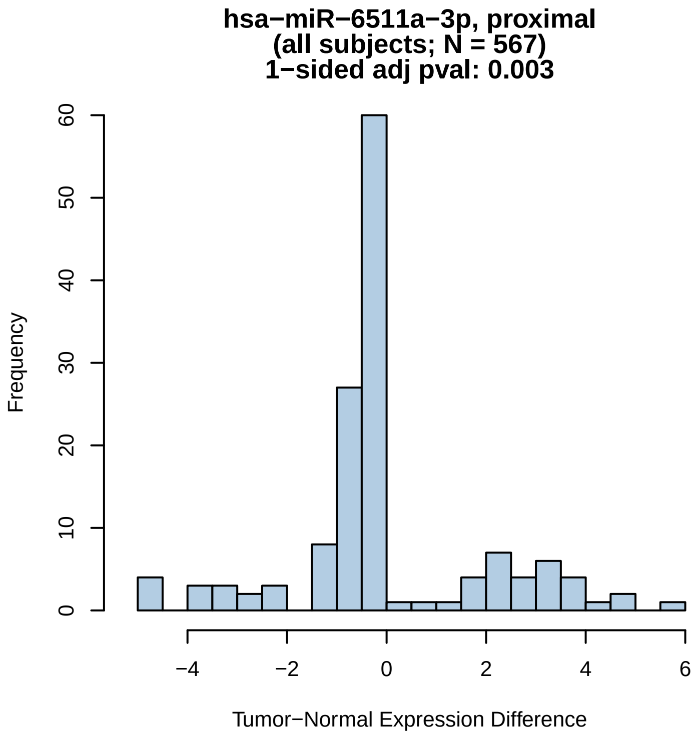
hsa−miR−6511a−3p, proximal
(all subjects; N = 567)
1−sided adj pval: 0.003
Frequency
Tumor−Normal Expression Difference

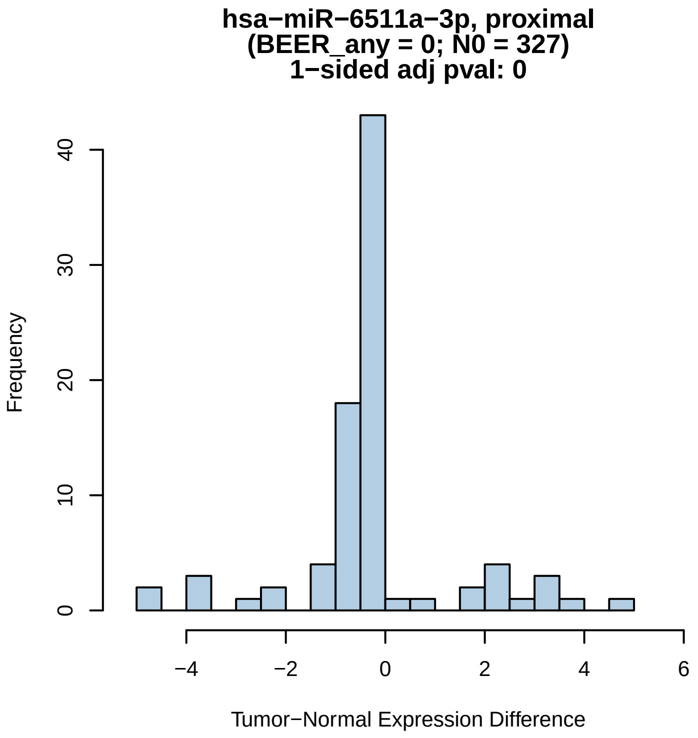
hsa−miR−6511a−3p, proximal
(BEER_any = 0; N0 = 327)
1−sided adj pval: 0
Frequency
Tumor−Normal Expression Difference

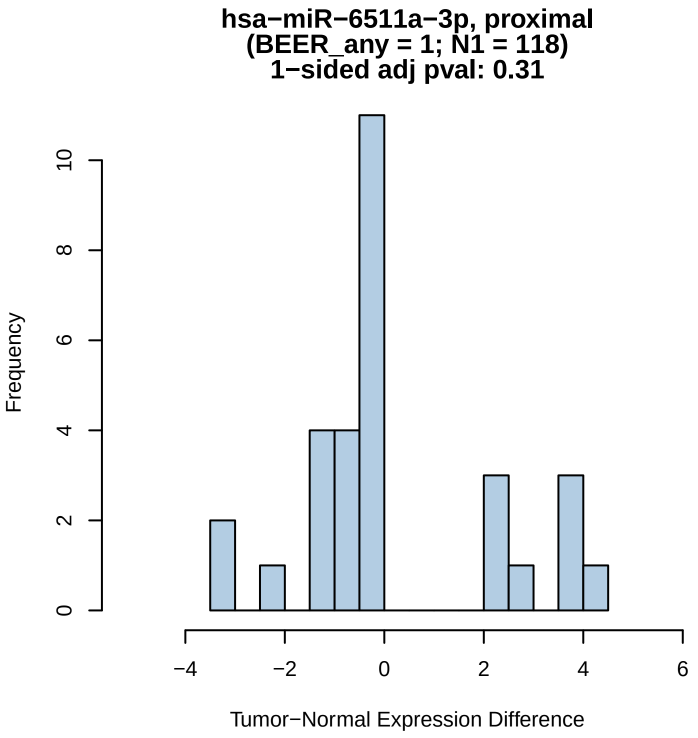
hsa−miR−6511a−3p, proximal
(BEER_any = 1; N1 = 118)
1−sided adj pval: 0.31
Frequency
Tumor−Normal Expression Difference

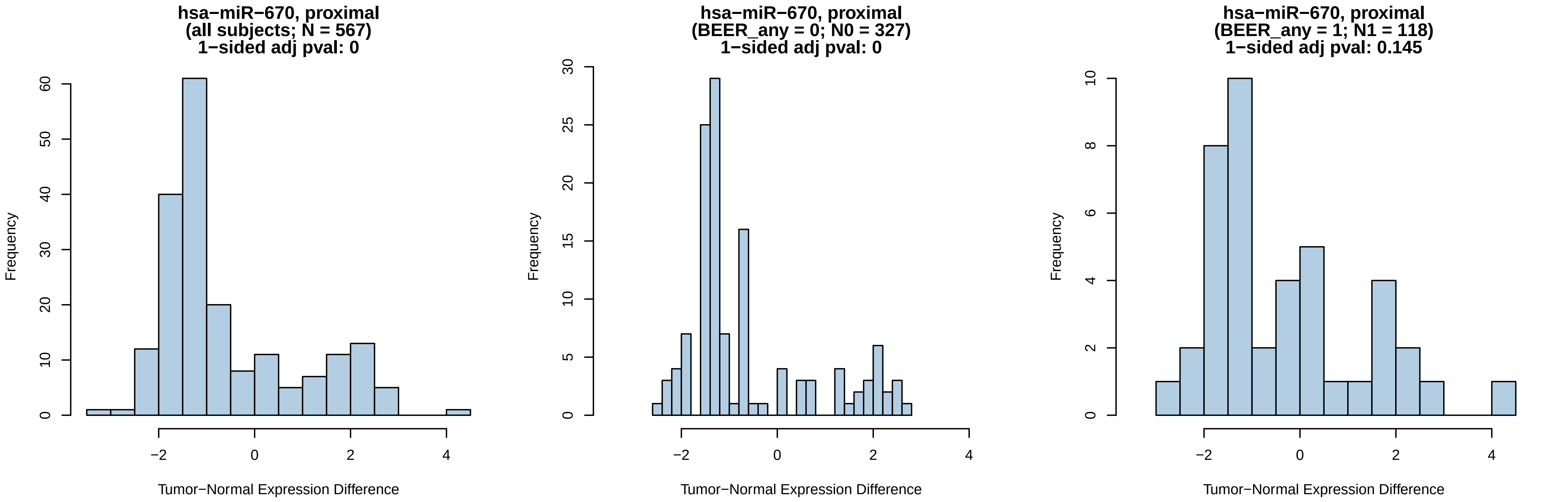

hsa−miR−670, proximal
(all subjects; N = 567)
1−sided adj pval: 0
Frequency
Tumor−Normal Expression Difference
hsa−miR−670, proximal
(BEER_any = 0; N0 = 327)
1−sided adj pval: 0
Frequency
Tumor−Normal Expression Difference
hsa−miR−670, proximal
(BEER_any = 1; N1 = 118)
1−sided adj pval: 0.145
Frequency
Tumor−Normal Expression Difference

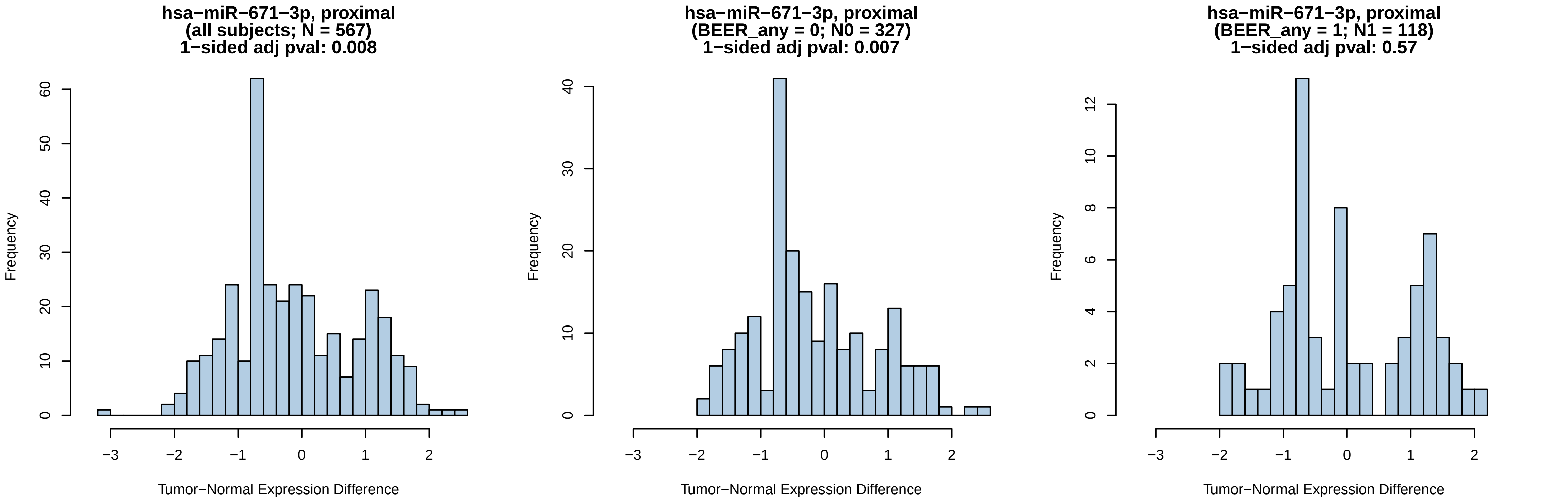
hsa−miR−671−3p, proximal
(all subjects; N = 567)
1−sided adj pval: 0.008
Frequency
Tumor−Normal Expression Difference
hsa−miR−671−3p, proximal
(BEER_any = 0; N0 = 327)
1−sided adj pval: 0.007
Frequency
Tumor−Normal Expression Difference
hsa−miR−671−3p, proximal
(BEER_any = 1; N1 = 118)
1−sided adj pval: 0.57
Frequency
Tumor−Normal Expression Difference

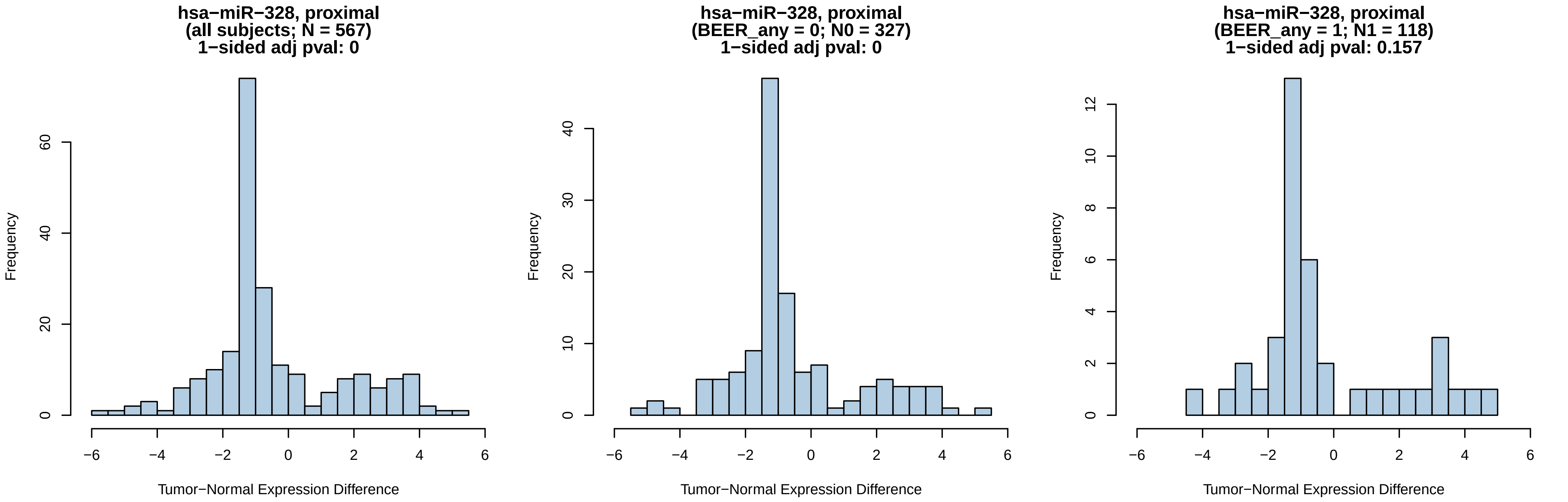

hsa−miR−328, proximal
(all subjects; N = 567)
1−sided adj pval: 0
hsa−miR−328, proximal
(BEER_any = 0; N0 = 327)
1−sided adj pval: 0
hsa−miR−328, proximal
(BEER_any = 1; N1 = 118)
1−sided adj pval: 0.157
Frequency
Tumor−Normal Expression Difference

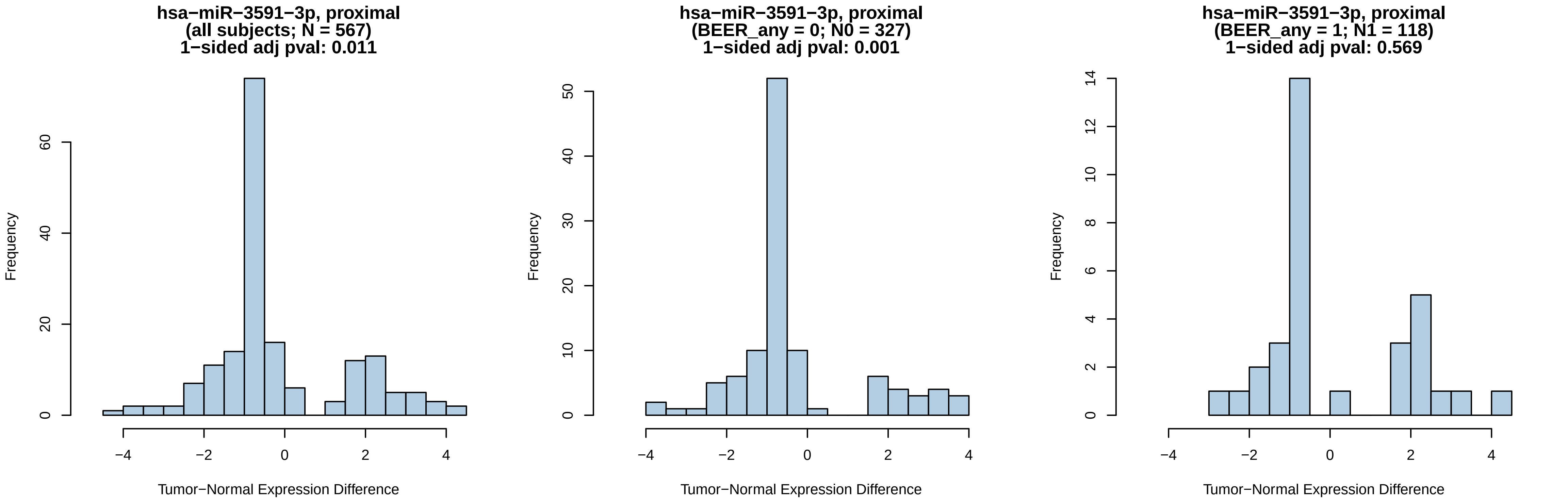

hsa−miR−3591−3p, proximal
(all subjects; N = 567)
1−sided adj pval: 0.011
Frequency
Tumor−Normal Expression Difference
hsa−miR−3591−3p, proximal
(BEER_any = 0; N0 = 327)
1−sided adj pval: 0.001
Frequency
Tumor−Normal Expression Difference
hsa−miR−3591−3p, proximal
(BEER_any = 1; N1 = 118)
1−sided adj pval: 0.569
Frequency
Tumor−Normal Expression Difference

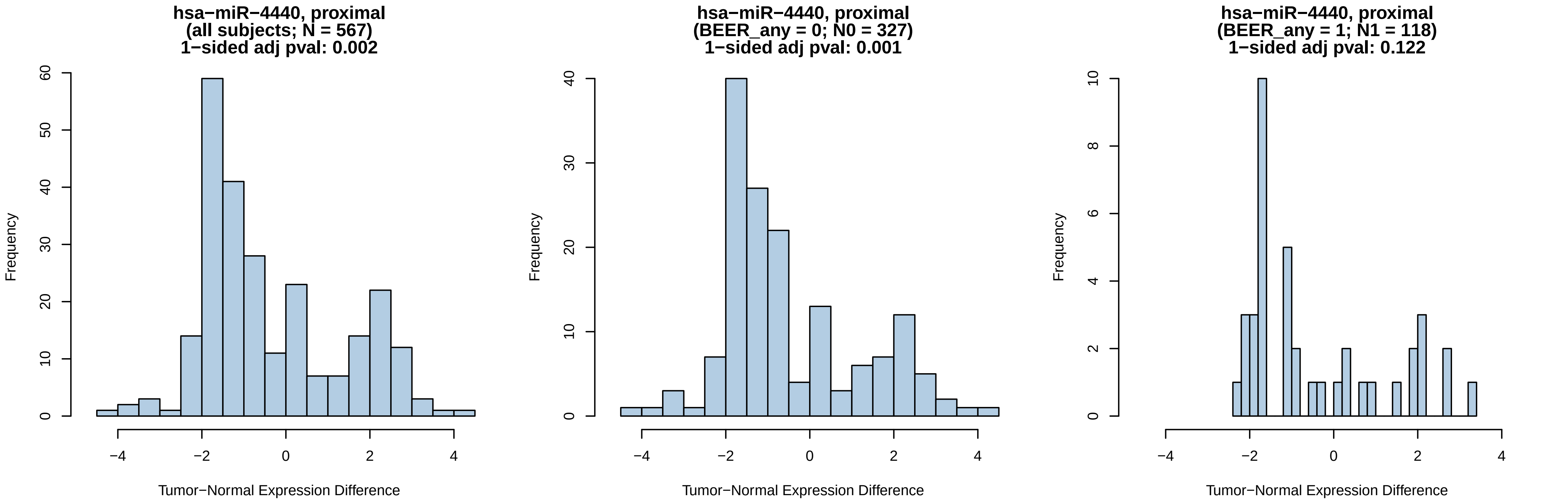

hsa−miR−4440, proximal
(all subjects; N = 567)
1−sided adj pval: 0.002
Frequency
Tumor−Normal Expression Difference
hsa−miR−4440, proximal
(BEER_any = 0; N0 = 327)
1−sided adj pval: 0.001
Frequency
Tumor−Normal Expression Difference
hsa−miR−4440, proximal
(BEER_any = 1; N1 = 118)
1−sided adj pval: 0.122
Frequency
Tumor−Normal Expression Difference

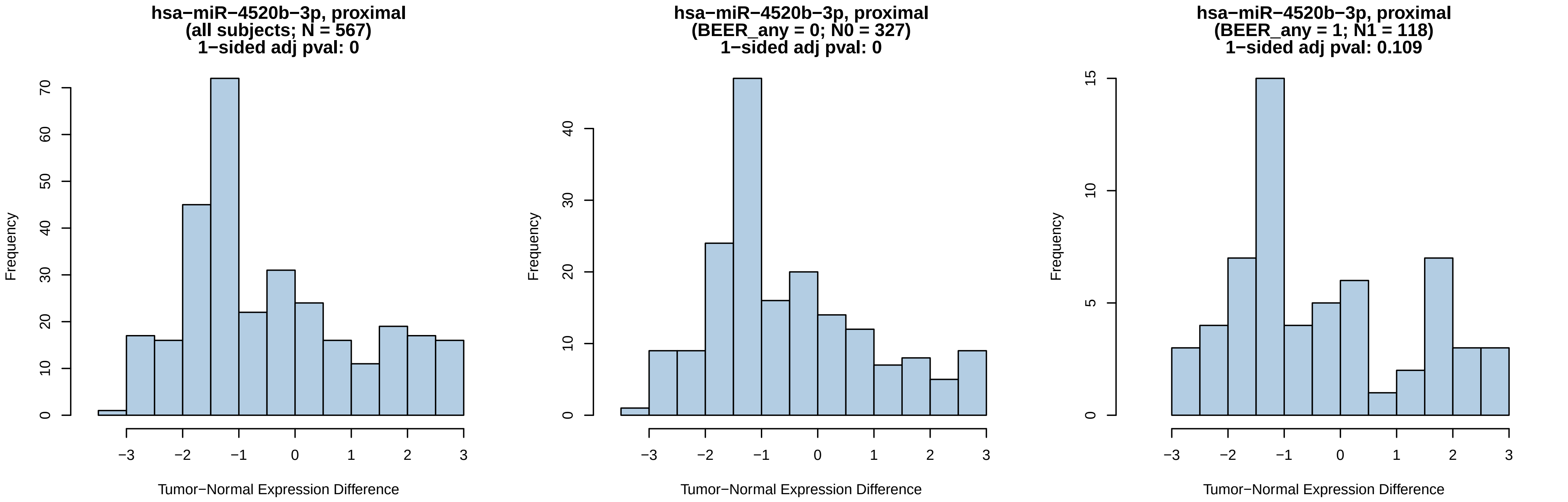

hsa−miR−4520b−3p, proximal
(all subjects; N = 567)
1−sided adj pval: 0
Frequency
Tumor−Normal Expression Difference
hsa−miR−4520b−3p, proximal
(BEER_any = 0; N0 = 327)
1−sided adj pval: 0
Frequency
Tumor−Normal Expression Difference
hsa−miR−4520b−3p, proximal
(BEER_any = 1; N1 = 118)
1−sided adj pval: 0.109
Frequency
Tumor−Normal Expression Difference

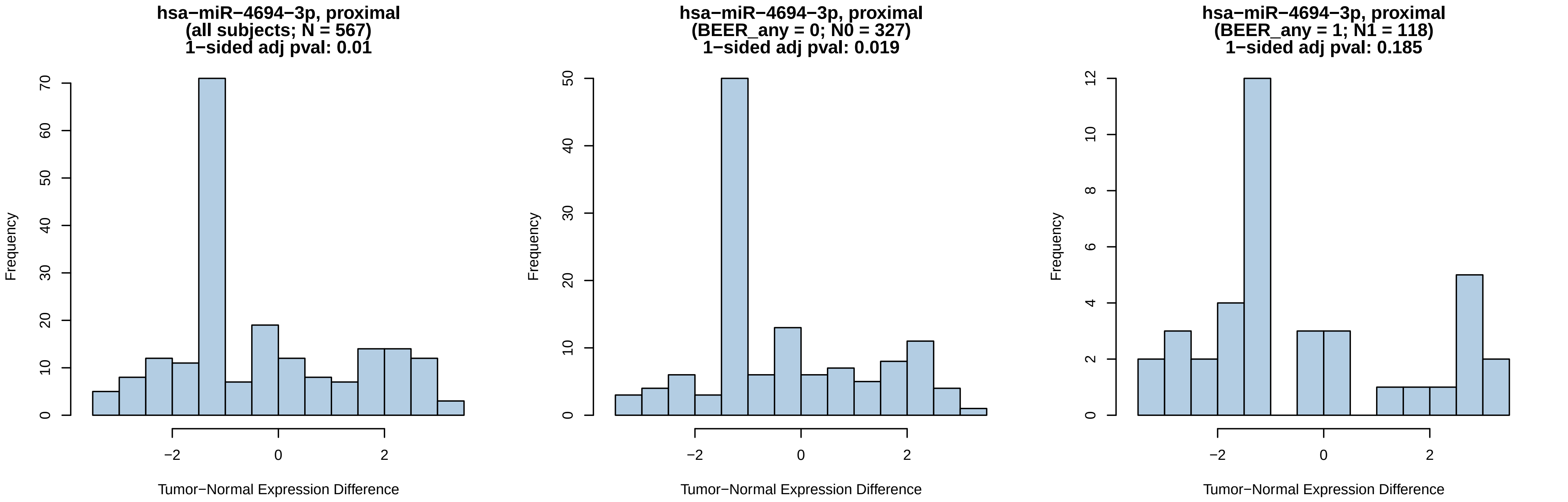
hsa−miR−4694−3p, proximal
(all subjects; N = 567)
1−sided adj pval: 0.01
Frequency
Tumor−Normal Expression Difference
hsa−miR−4694−3p, proximal
(BEER_any = 0; N0 = 327)
1−sided adj pval: 0.019
Frequency
Tumor−Normal Expression Difference
hsa−miR−4694−3p, proximal
(BEER_any = 1; N1 = 118)
1−sided adj pval: 0.185
Frequency
Tumor−Normal Expression Difference

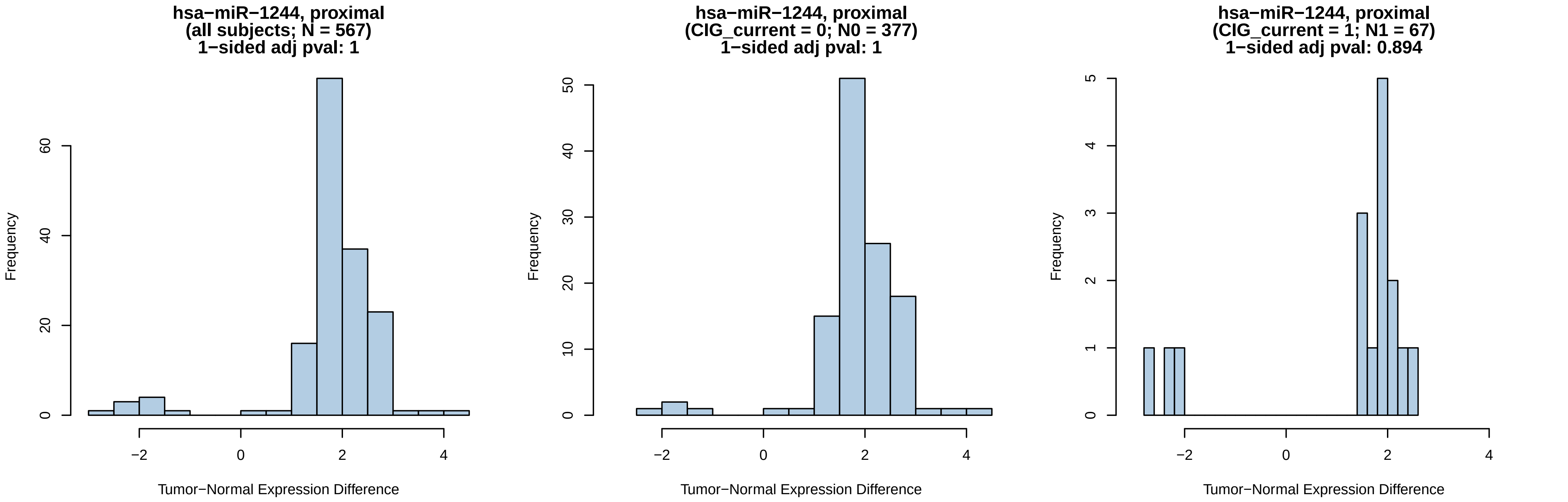

hsa−miR−1244, proximal
(all subjects; N = 567)
1−sided adj pval: 1
Frequency
0
20
40
60
−2
0
2
4
Tumor−Normal Expression Difference
hsa−miR−1244, proximal
(CIG_current = 0; N0 = 377)
1−sided adj pval: 1
Frequency
0
10
20
30
40
50
−2
0
2
4
Tumor−Normal Expression Difference
hsa−miR−1244, proximal
(CIG_current = 1; N1 = 67)
1−sided adj pval: 0.894
Frequency
0
1
2
3
4
5
−2
0
2
4
Tumor−Normal Expression Difference

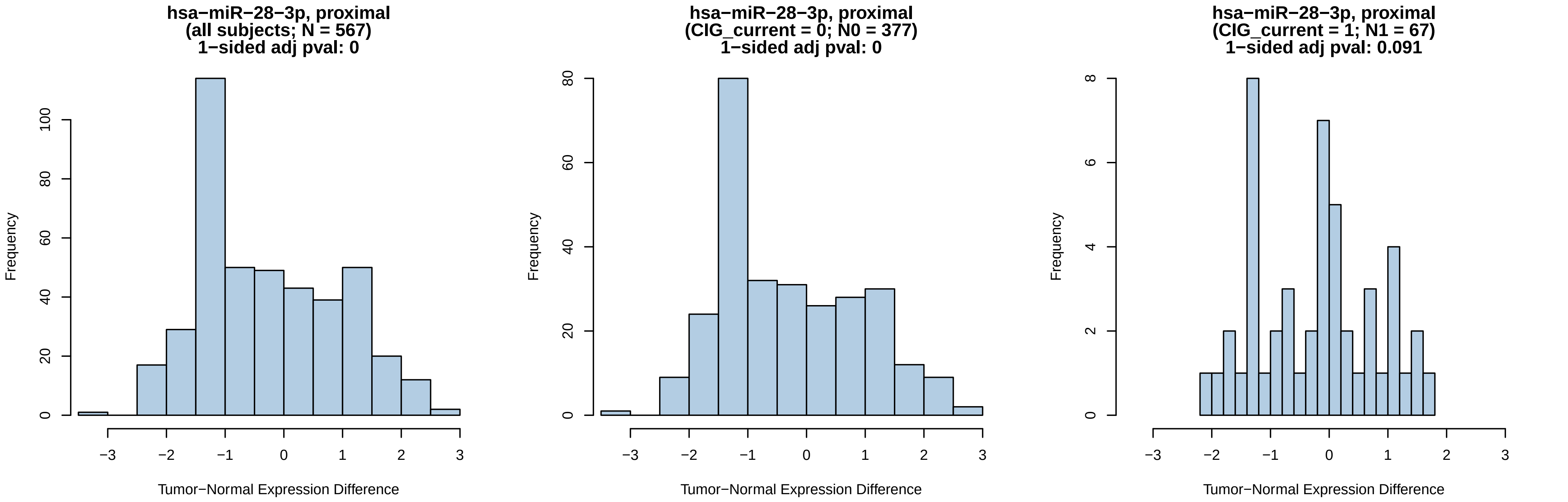
hsa−miR−28−3p, proximal
(all subjects; N = 567)
1−sided adj pval: 0
Frequency
0
20
40
60
80
100
−3
−2
−1
0
1
2
3
Tumor−Normal Expression Difference
hsa−miR−28−3p, proximal
(CIG_current = 0; N0 = 377)
1−sided adj pval: 0
Frequency
0
20
40
60
80
−3
−2
−1
0
1
2
3
Tumor−Normal Expression Difference
hsa−miR−28−3p, proximal
(CIG_current = 1; N1 = 67)
1−sided adj pval: 0.091
Frequency
0
2
4
6
8
−3
−2
−1
0
1
2
3
Tumor−Normal Expression Difference

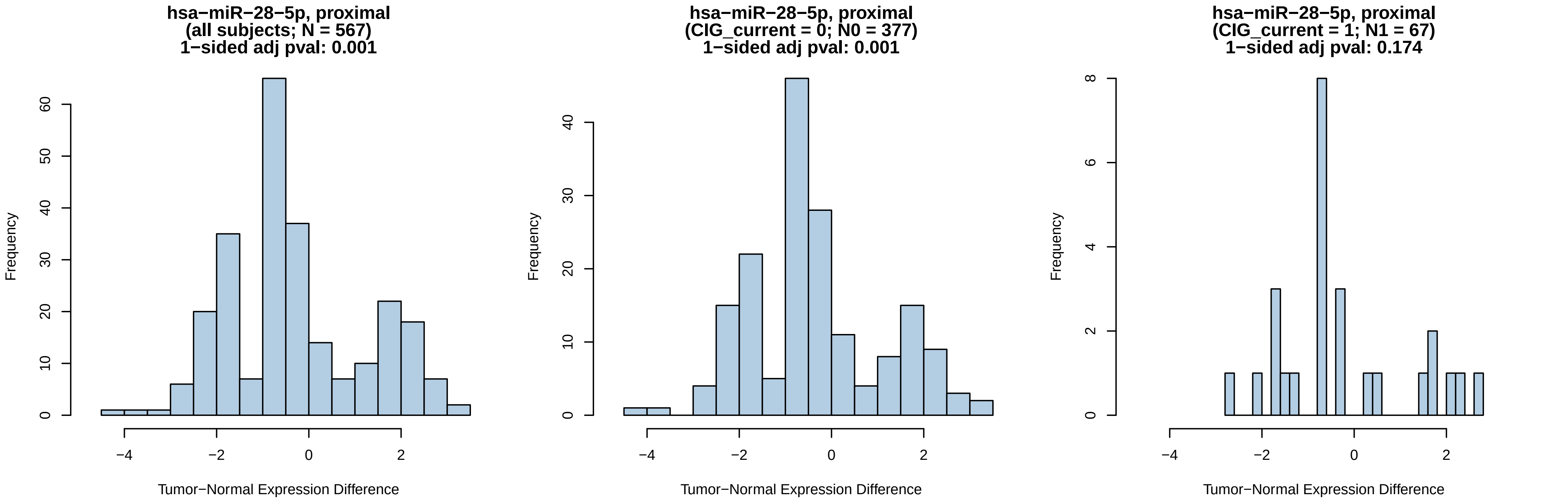

hsa−miR−28−5p, proximal
(all subjects; N = 567)
1−sided adj pval: 0.001
Frequency
Tumor−Normal Expression Difference
hsa−miR−28−5p, proximal
(CIG_current = 0; N0 = 377)
1−sided adj pval: 0.001
Frequency
Tumor−Normal Expression Difference
hsa−miR−28−5p, proximal
(CIG_current = 1; N1 = 67)
1−sided adj pval: 0.174
Frequency
Tumor−Normal Expression Difference

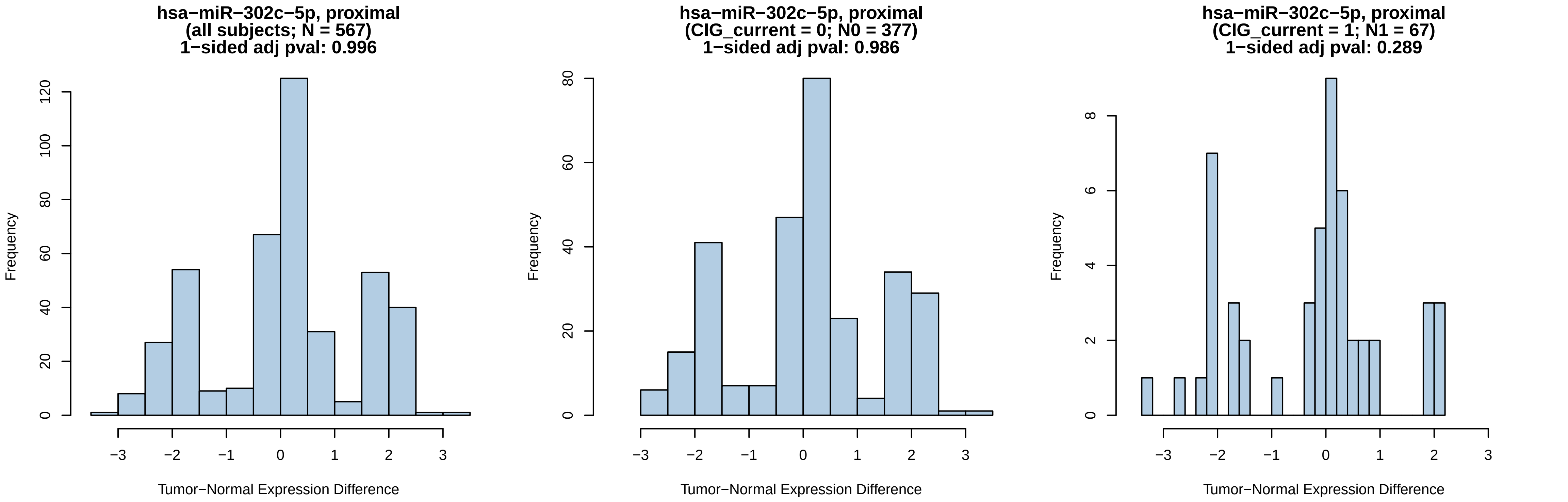
hsa−miR−302c−5p, proximal
(all subjects; N = 567)
1−sided adj pval: 0.996
Frequency
0
20
40
60
80
100
120
−3
−2
−1
0
1
2
3
Tumor−Normal Expression Difference
hsa−miR−302c−5p, proximal
(CIG_current = 0; N0 = 377)
1−sided adj pval: 0.986
Frequency
0
20
40
60
80
−3
−2
−1
0
1
2
3
Tumor−Normal Expression Difference
hsa−miR−302c−5p, proximal
(CIG_current = 1; N1 = 67)
1−sided adj pval: 0.289
Frequency
0
2
4
6
8
−3
−2
−1
0
1
2
3
Tumor−Normal Expression Difference

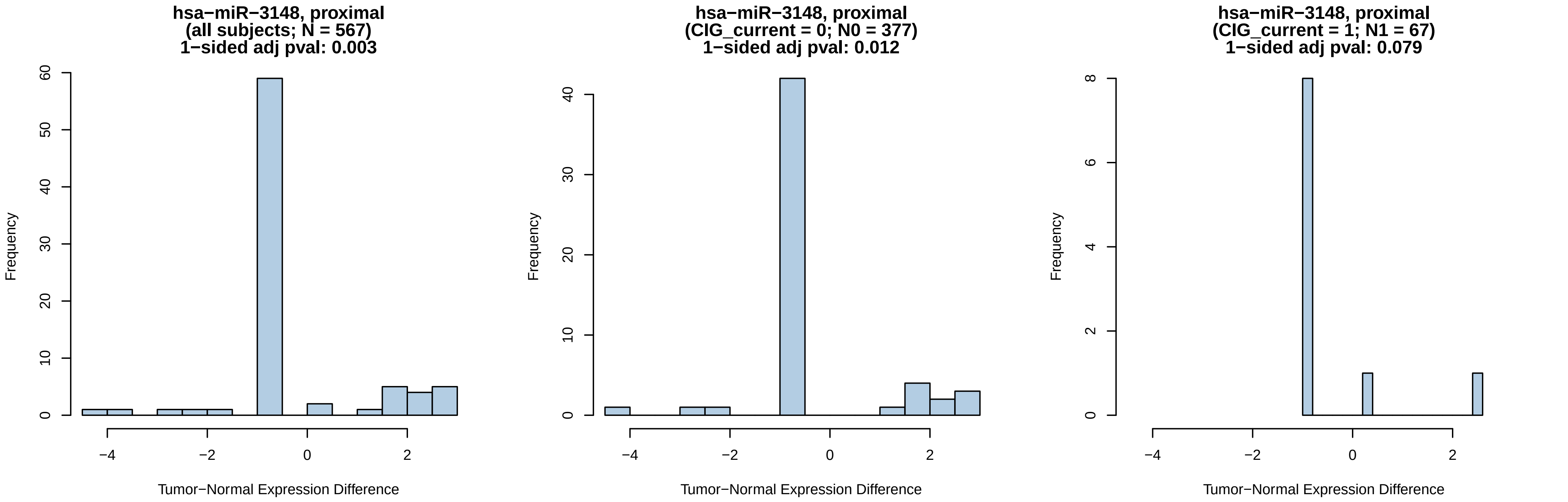

hsa−miR−3148, proximal
(all subjects; N = 567)
1−sided adj pval: 0.003
Frequency
Tumor−Normal Expression Difference
hsa−miR−3148, proximal
(CIG_current = 0; N0 = 377)
1−sided adj pval: 0.012
Frequency
Tumor−Normal Expression Difference
hsa−miR−3148, proximal
(CIG_current = 1; N1 = 67)
1−sided adj pval: 0.079
Frequency
Tumor−Normal Expression Difference

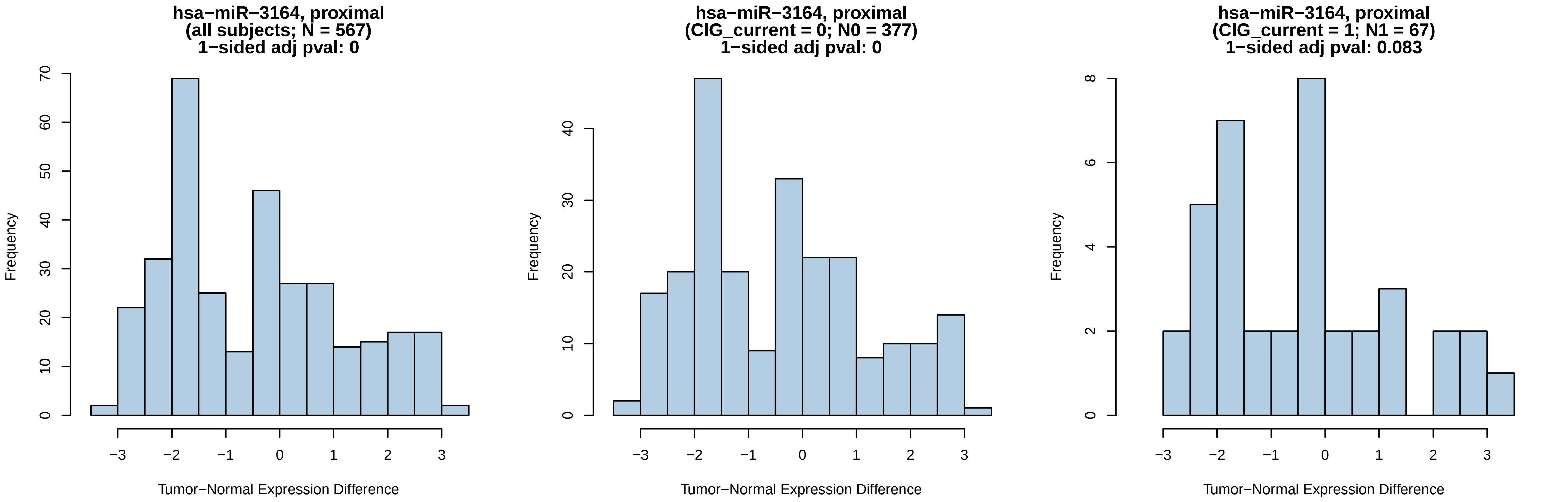

hsa−miR−3164, proximal
(all subjects; N = 567)
1−sided adj pval: 0
Frequency
Tumor−Normal Expression Difference
hsa−miR−3164, proximal
(CIG_current = 0; N0 = 377)
1−sided adj pval: 0
Frequency
Tumor−Normal Expression Difference
hsa−miR−3164, proximal
(CIG_current = 1; N1 = 67)
1−sided adj pval: 0.083
Frequency
Tumor−Normal Expression Difference

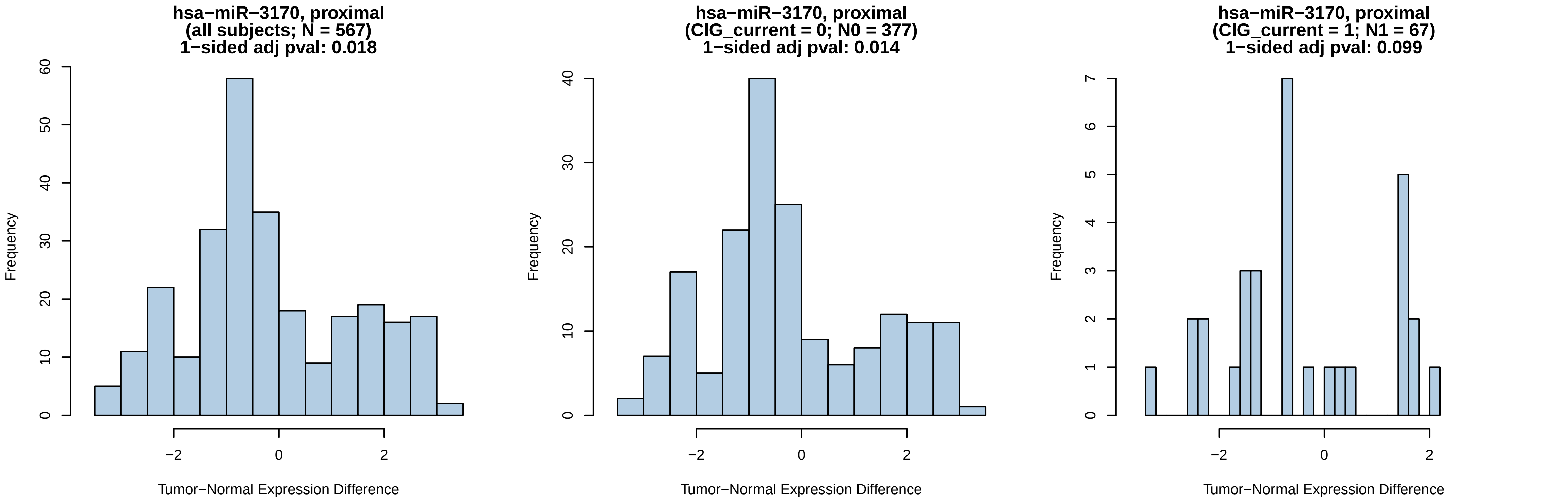
hsa−miR−3170, proximal
(all subjects; N = 567)
1−sided adj pval: 0.018
Frequency
Tumor−Normal Expression Difference
hsa−miR−3170, proximal
(CIG_current = 0; N0 = 377)
1−sided adj pval: 0.014
Frequency
Tumor−Normal Expression Difference
hsa−miR−3170, proximal
(CIG_current = 1; N1 = 67)
1−sided adj pval: 0.099
Frequency
Tumor−Normal Expression Difference

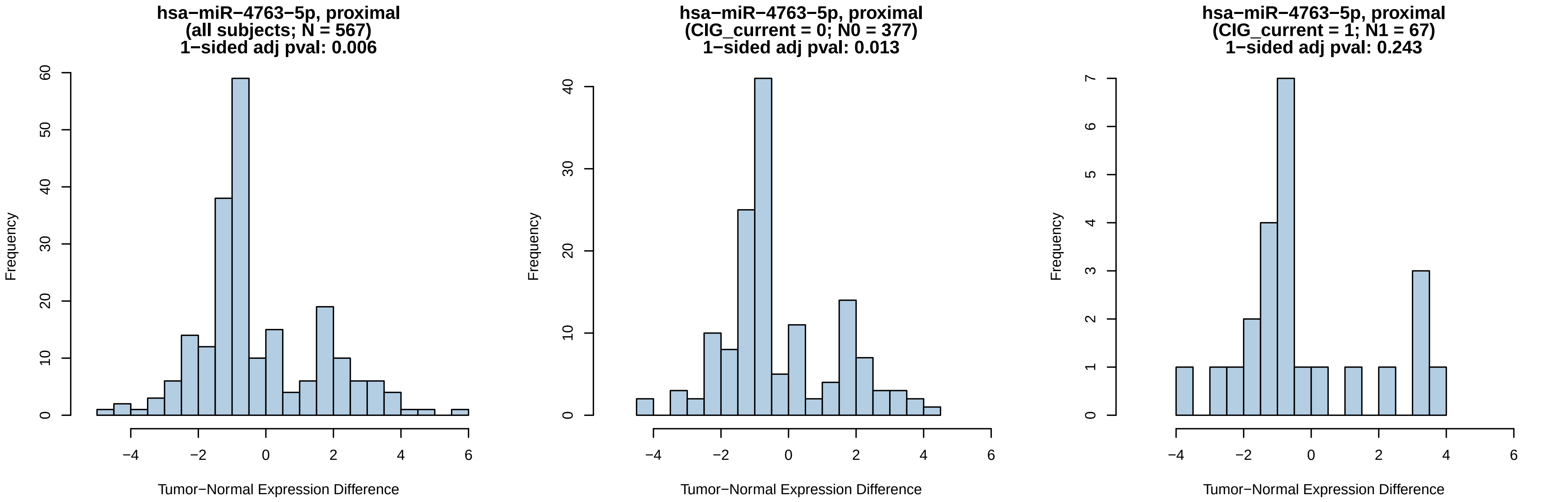
hsa−miR−4763−5p, proximal
(all subjects; N = 567)
1−sided adj pval: 0.006
hsa−miR−4763−5p, proximal
(CIG_current = 0; N0 = 377)
1−sided adj pval: 0.013
hsa−miR−4763−5p, proximal
(CIG_current = 1; N1 = 67)
1−sided adj pval: 0.243
Frequency
Tumor−Normal Expression Difference

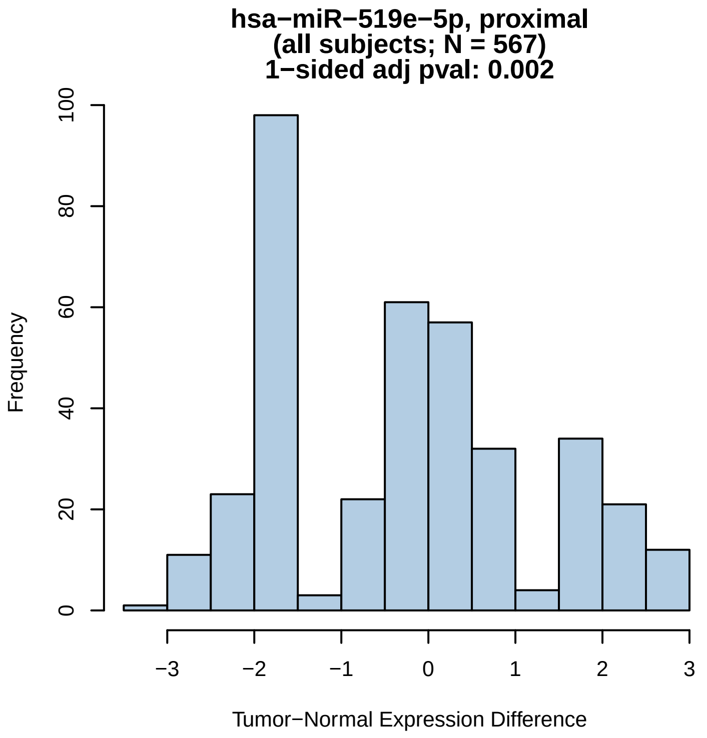
hsa−miR−519e−5p, proximal
(all subjects; N = 567)
1−sided adj pval: 0.002
Frequency
Tumor−Normal Expression Difference

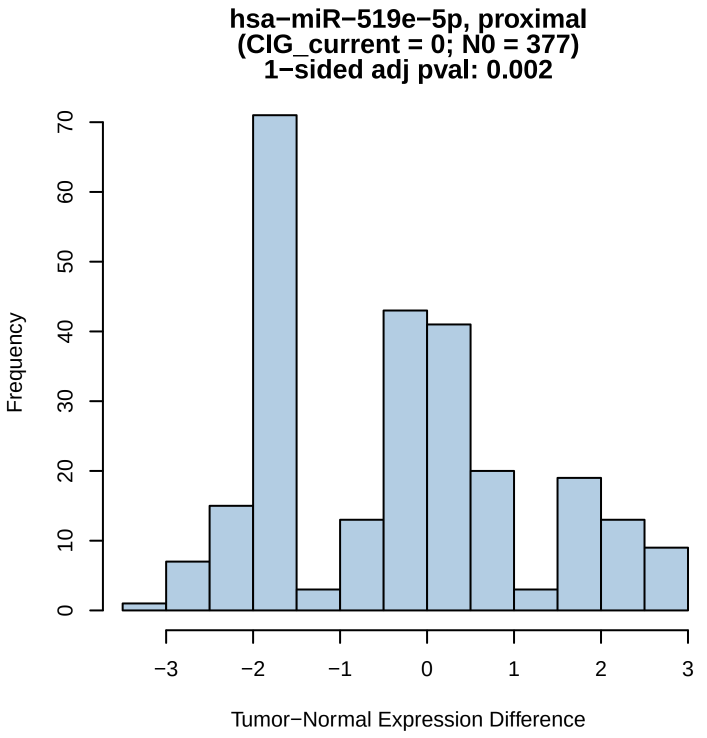
hsa−miR−519e−5p, proximal
(CIG_current = 0; N0 = 377)
1−sided adj pval: 0.002
Frequency
Tumor−Normal Expression Difference

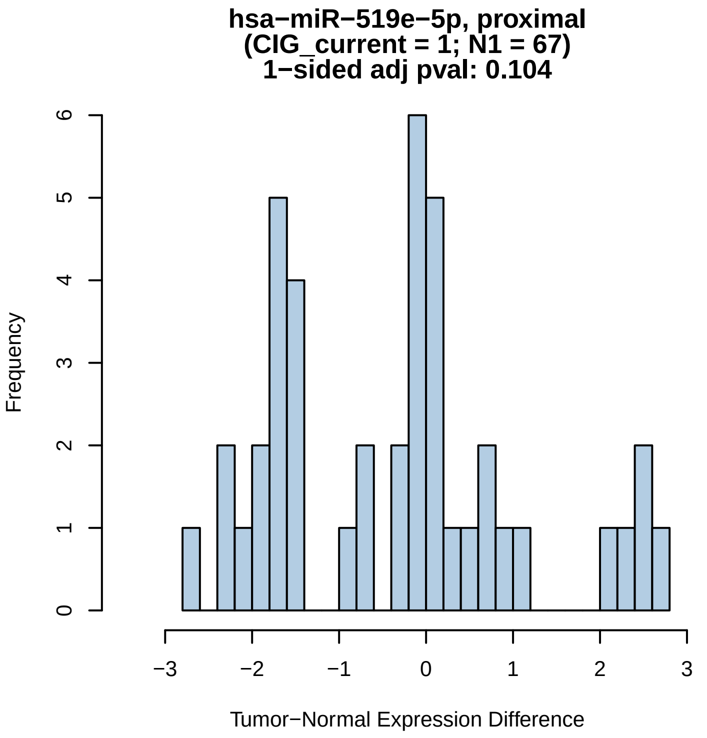
hsa−miR−519e−5p, proximal
(CIG_current = 1; N1 = 67)
1−sided adj pval: 0.104
Frequency
Tumor−Normal Expression Difference

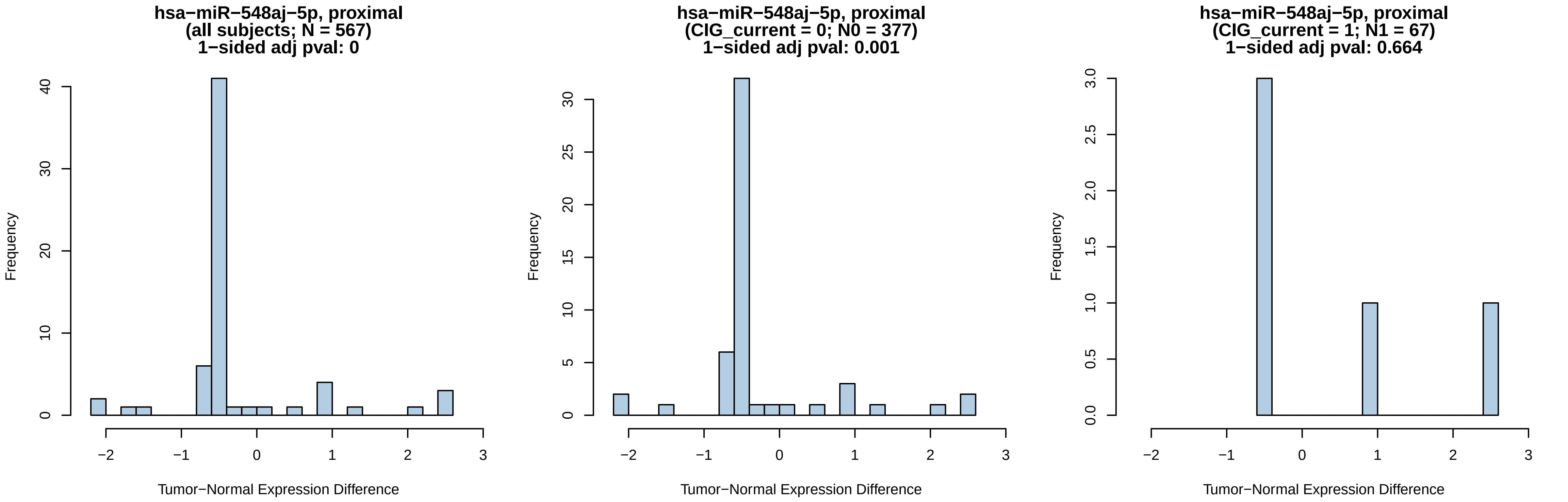
hsa−miR−548aj−5p, proximal
(all subjects; N = 567)
1−sided adj pval: 0
hsa−miR−548aj−5p, proximal
(CIG_current = 0; N0 = 377)
1−sided adj pval: 0.001
hsa−miR−548aj−5p, proximal
(CIG_current = 1; N1 = 67)
1−sided adj pval: 0.664
Frequency
Tumor−Normal Expression Difference

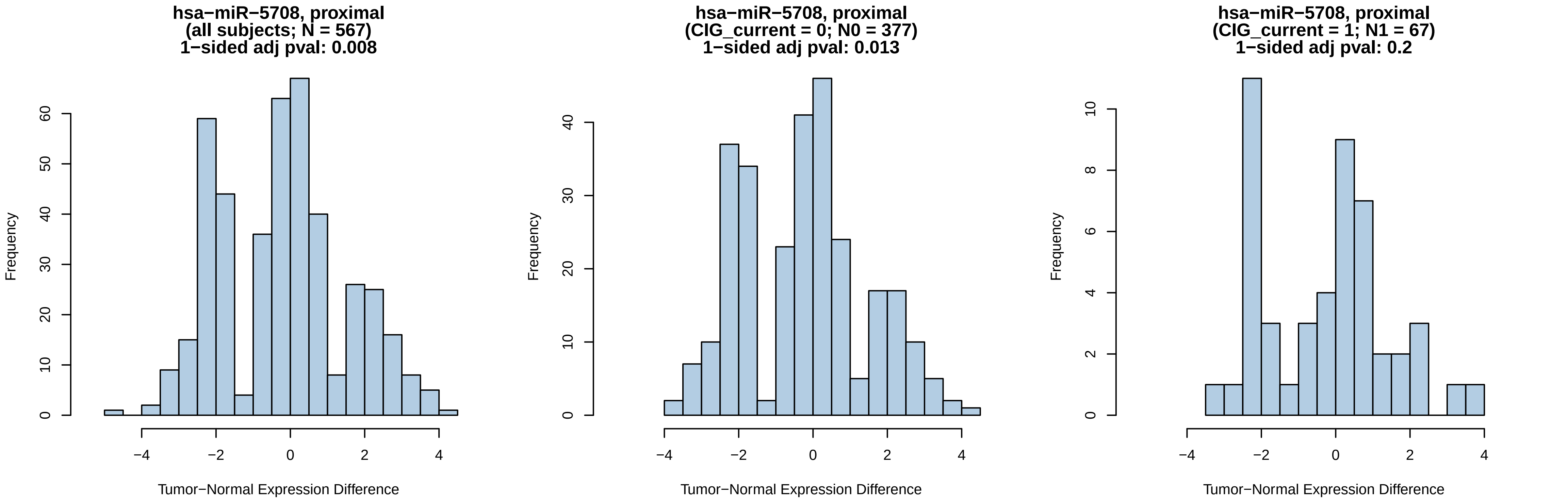
hsa−miR−5708, proximal
(all subjects; N = 567)
1−sided adj pval: 0.008
Frequency
0
10
20
30
40
50
60
−4
−2
0
2
4
Tumor−Normal Expression Difference
hsa−miR−5708, proximal
(CIG_current = 0; N0 = 377)
1−sided adj pval: 0.013
Frequency
0
10
20
30
40
−4
−2
0
2
4
Tumor−Normal Expression Difference
hsa−miR−5708, proximal
(CIG_current = 1; N1 = 67)
1−sided adj pval: 0.2
Frequency
0
2
4
6
8
10
−4
−2
0
2
4
Tumor−Normal Expression Difference

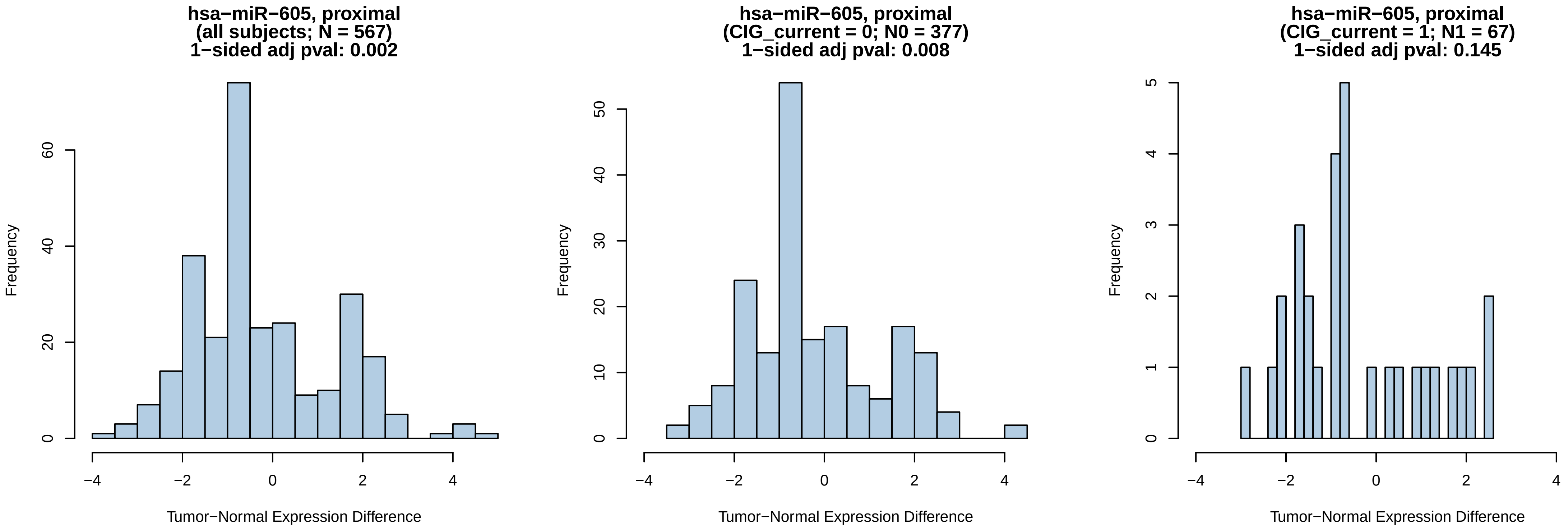

hsa−miR−605, proximal
(all subjects; N = 567)
1−sided adj pval: 0.002
Frequency
0
20
40
60
−4
−2
0
2
4
Tumor−Normal Expression Difference
hsa−miR−605, proximal
(CIG_current = 0; N0 = 377)
1−sided adj pval: 0.008
Frequency
0
10
20
30
40
50
−4
−2
0
2
4
Tumor−Normal Expression Difference
hsa−miR−605, proximal
(CIG_current = 1; N1 = 67)
1−sided adj pval: 0.145
Frequency
0
1
2
3
4
5
−4
−2
0
2
4
Tumor−Normal Expression Difference

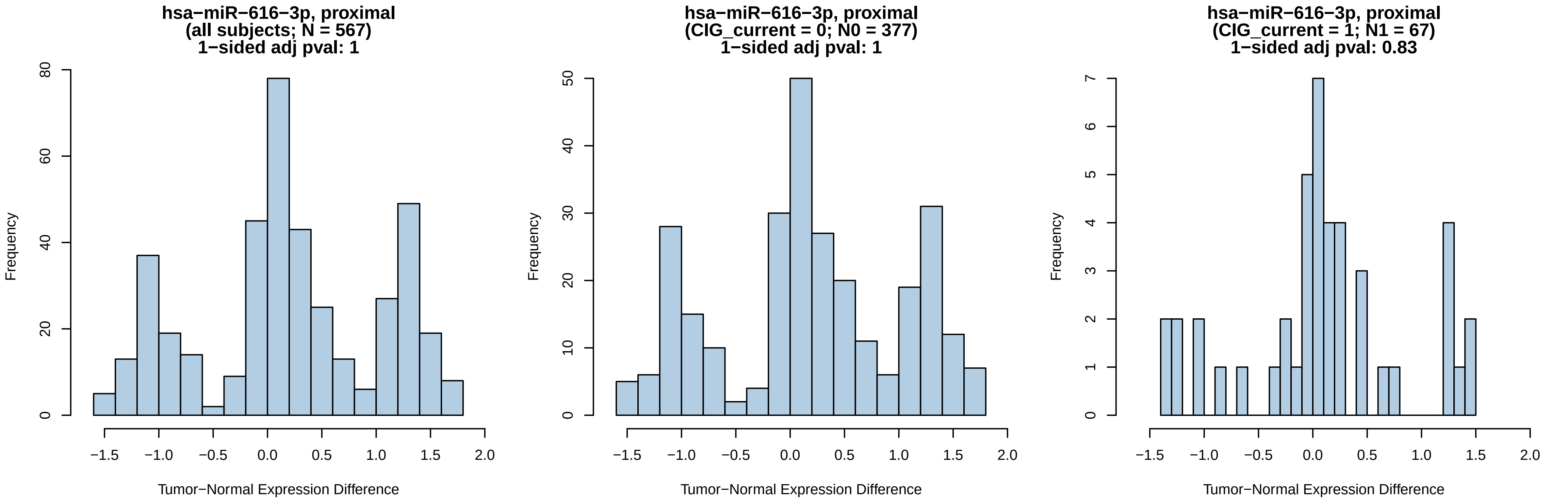

hsa−miR−616−3p, proximal
(all subjects; N = 567)
1−sided adj pval: 1
Frequency
Tumor−Normal Expression Difference
hsa−miR−616−3p, proximal
(CIG_current = 0; N0 = 377)
1−sided adj pval: 1
Frequency
Tumor−Normal Expression Difference
hsa−miR−616−3p, proximal
(CIG_current = 1; N1 = 67)
1−sided adj pval: 0.83
Frequency
Tumor−Normal Expression Difference

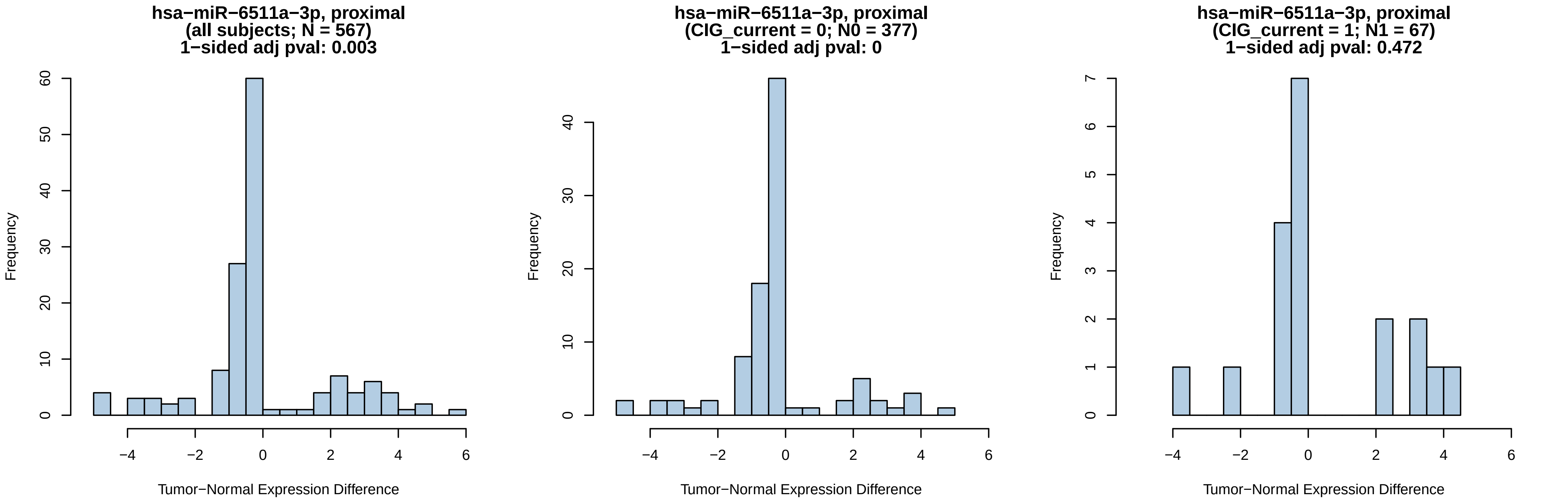

hsa−miR−6511a−3p, proximal
(all subjects; N = 567)
1−sided adj pval: 0.003
Frequency
Tumor−Normal Expression Difference
hsa−miR−6511a−3p, proximal
(CIG_current = 0; N0 = 377)
1−sided adj pval: 0
Frequency
Tumor−Normal Expression Difference
hsa−miR−6511a−3p, proximal
(CIG_current = 1; N1 = 67)
1−sided adj pval: 0.472
Frequency
Tumor−Normal Expression Difference

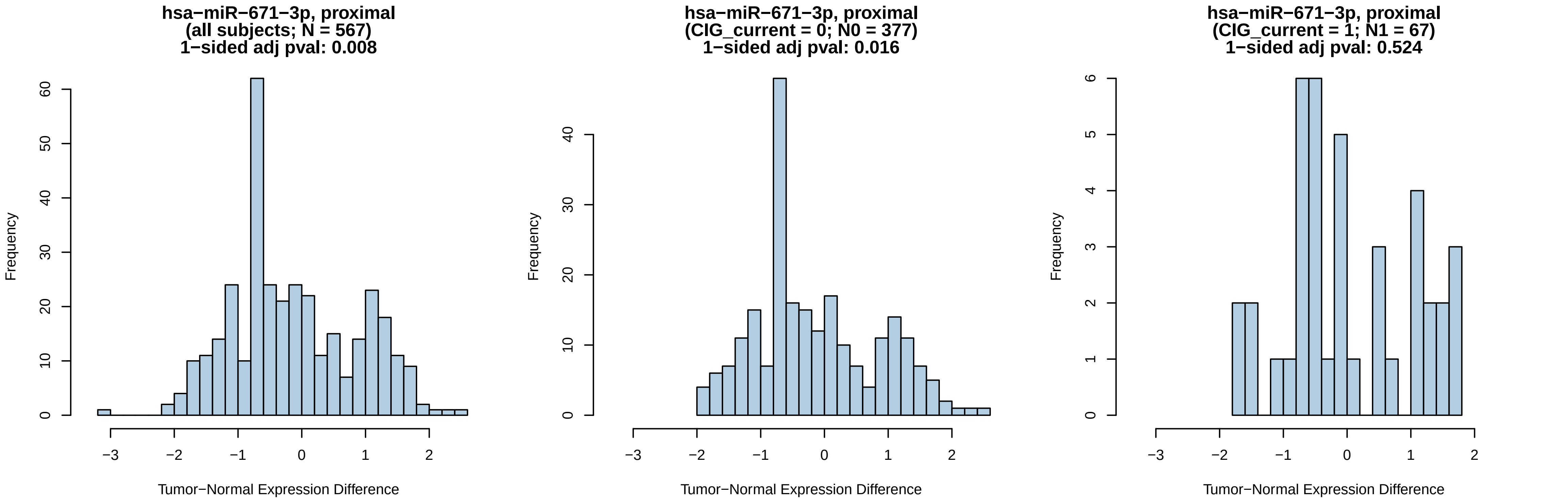

hsa−miR−671−3p, proximal
(all subjects; N = 567)
1−sided adj pval: 0.008
Frequency
Tumor−Normal Expression Difference
hsa−miR−671−3p, proximal
(CIG_current = 0; N0 = 377)
1−sided adj pval: 0.016
Frequency
Tumor−Normal Expression Difference
hsa−miR−671−3p, proximal
(CIG_current = 1; N1 = 67)
1−sided adj pval: 0.524
Frequency
Tumor−Normal Expression Difference

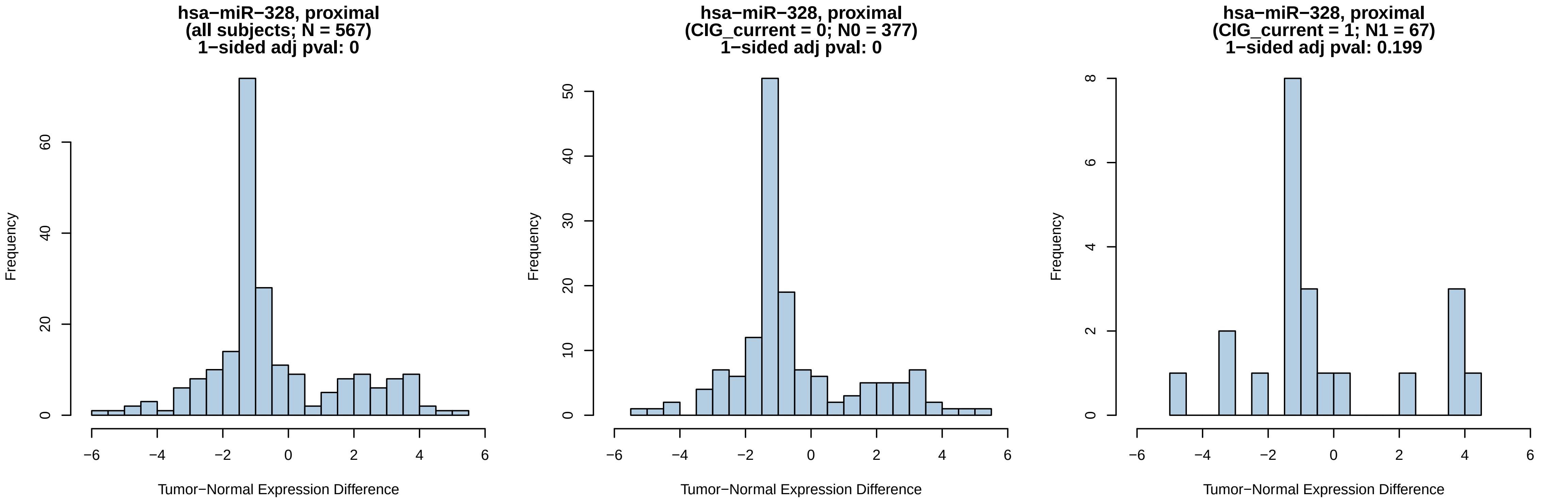
hsa−miR−328, proximal
(all subjects; N = 567)
1−sided adj pval: 0
Frequency
Tumor−Normal Expression Difference
hsa−miR−328, proximal
(CIG_current = 0; N0 = 377)
1−sided adj pval: 0
Frequency
Tumor−Normal Expression Difference
hsa−miR−328, proximal
(CIG_current = 1; N1 = 67)
1−sided adj pval: 0.199
Frequency
Tumor−Normal Expression Difference

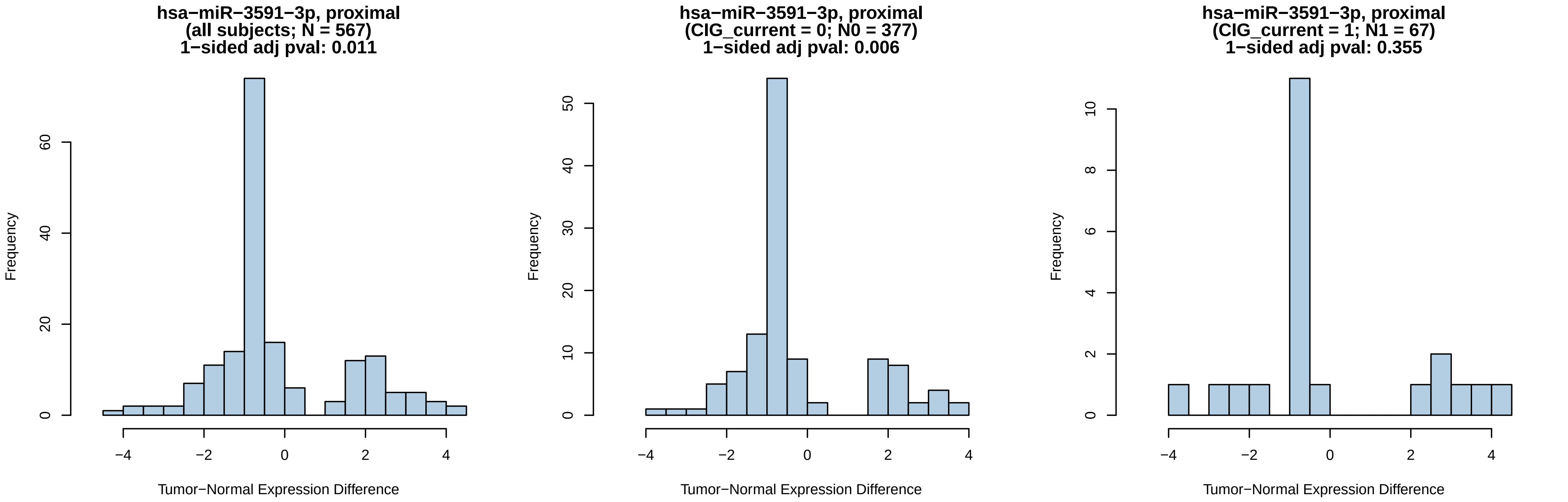

hsa−miR−3591−3p, proximal
(all subjects; N = 567)
1−sided adj pval: 0.011
Frequency
0
20
40
60
−4
−2
0
2
4
Tumor−Normal Expression Difference
hsa−miR−3591−3p, proximal
(CIG_current = 0; N0 = 377)
1−sided adj pval: 0.006
Frequency
0
10
20
30
40
50
−4
−2
0
2
4
Tumor−Normal Expression Difference
hsa−miR−3591−3p, proximal
(CIG_current = 1; N1 = 67)
1−sided adj pval: 0.355
Frequency
0
2
4
6
8
10
−4
−2
0
2
4
Tumor−Normal Expression Difference

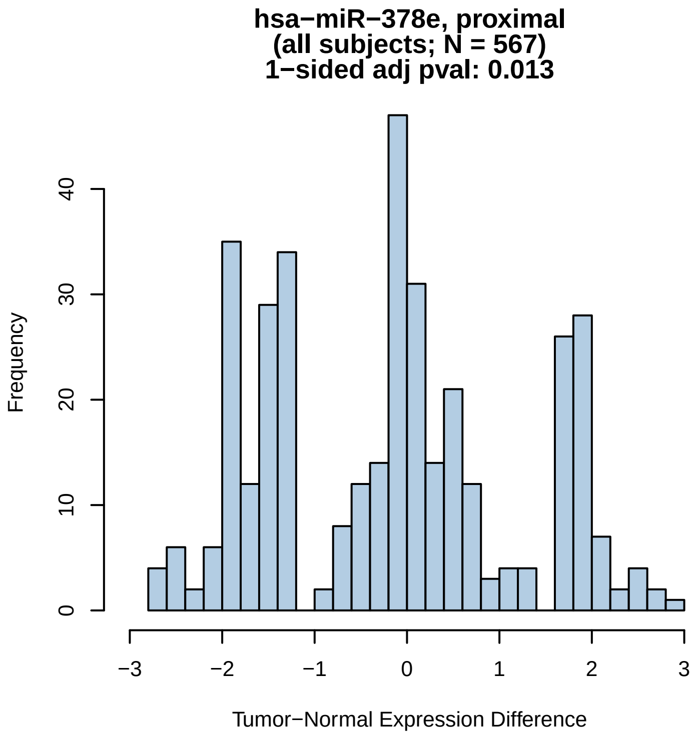
hsa−miR−378e, proximal
(all subjects; N = 567)
1−sided adj pval: 0.013
Frequency
Tumor−Normal Expression Difference

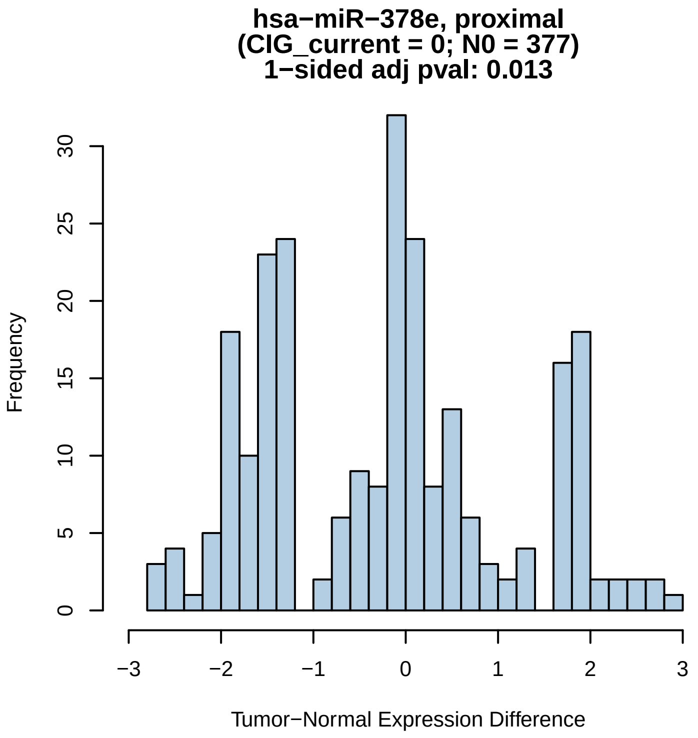
hsa−miR−378e, proximal
(CIG_current = 0; N0 = 377)
1−sided adj pval: 0.013
Frequency
Tumor−Normal Expression Difference

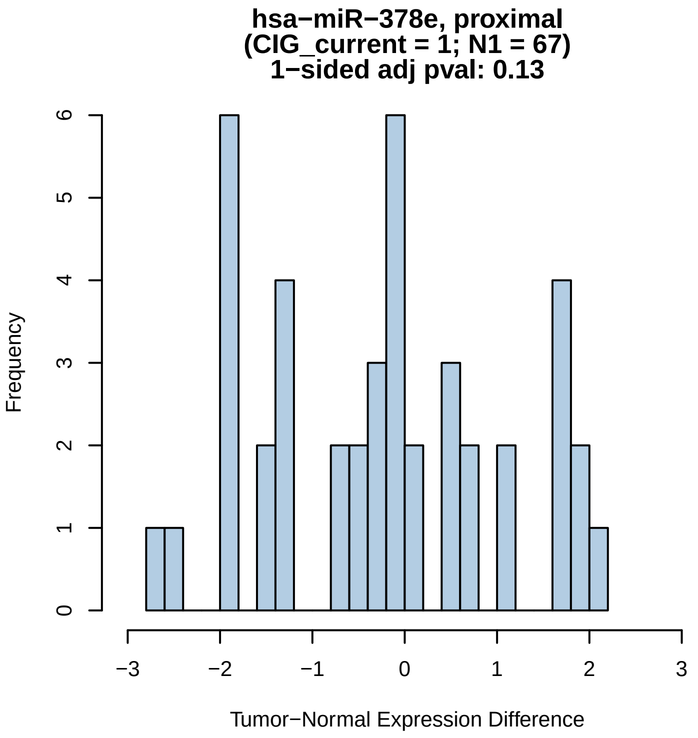
hsa−miR−378e, proximal
(CIG_current = 1; N1 = 67)
1−sided adj pval: 0.13
Frequency
Tumor−Normal Expression Difference

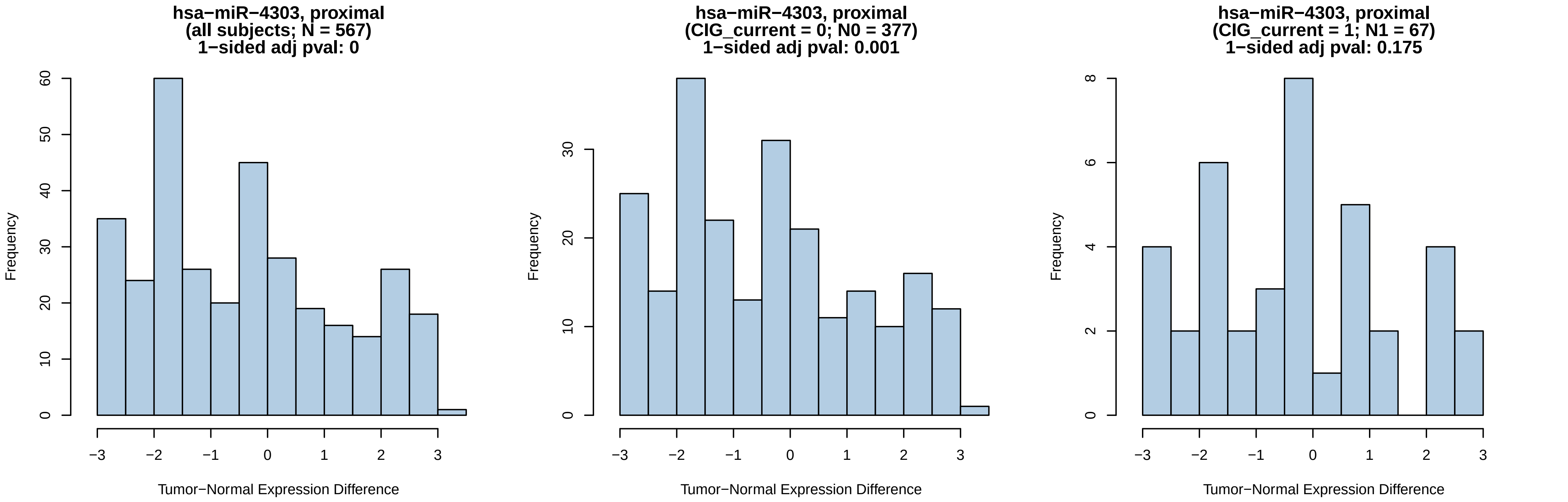

hsa−miR−4303, proximal
(all subjects; N = 567)
1−sided adj pval: 0
Frequency
0
10
20
30
40
50
60
−3
−2
−1
0
1
2
3
Tumor−Normal Expression Difference
hsa−miR−4303, proximal
(CIG_current = 0; N0 = 377)
1−sided adj pval: 0.001
Frequency
0
10
20
30
Tumor−Normal Expression Difference
hsa−miR−4303, proximal
(CIG_current = 1; N1 = 67)
1−sided adj pval: 0.175
Frequency
0
2
4
6
8
Tumor−Normal Expression Difference

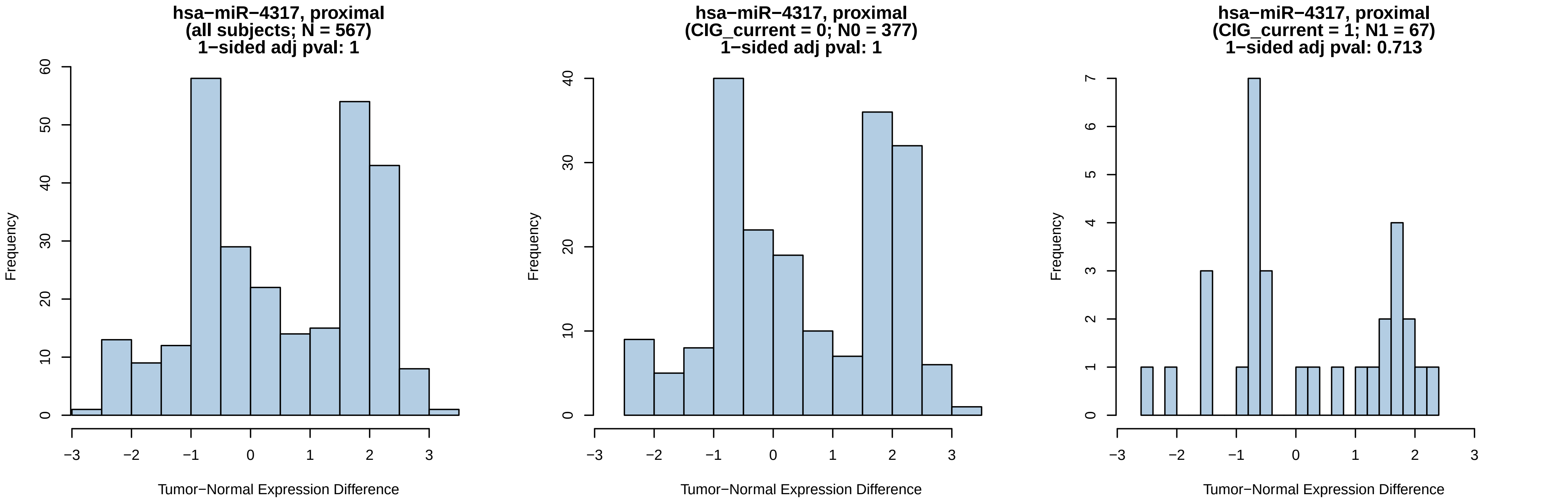
hsa−miR−4317, proximal
(all subjects; N = 567)
1−sided adj pval: 1
Frequency
Tumor−Normal Expression Difference
hsa−miR−4317, proximal
(CIG_current = 0; N0 = 377)
1−sided adj pval: 1
Frequency
Tumor−Normal Expression Difference
hsa−miR−4317, proximal
(CIG_current = 1; N1 = 67)
1−sided adj pval: 0.713
Frequency
Tumor−Normal Expression Difference

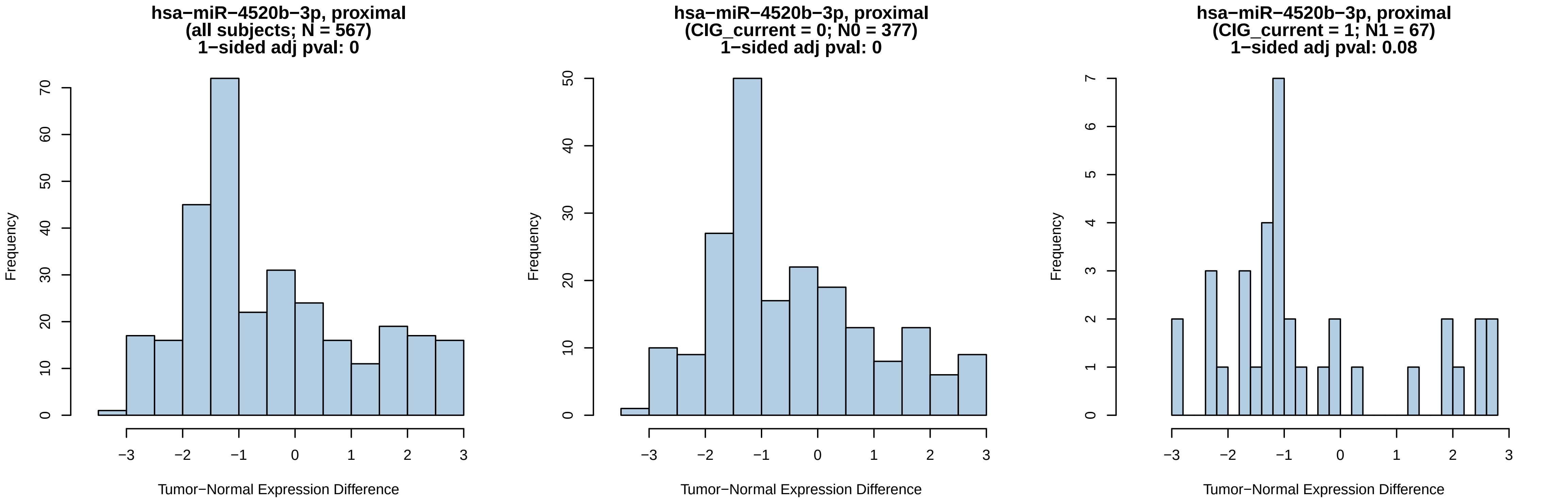
hsa−miR−4520b−3p, proximal
(all subjects; N = 567)
1−sided adj pval: 0
Frequency
Tumor−Normal Expression Difference
hsa−miR−4520b−3p, proximal
(CIG_current = 0; N0 = 377)
1−sided adj pval: 0
Frequency
Tumor−Normal Expression Difference
hsa−miR−4520b−3p, proximal
(CIG_current = 1; N1 = 67)
1−sided adj pval: 0.08
Frequency
Tumor−Normal Expression Difference

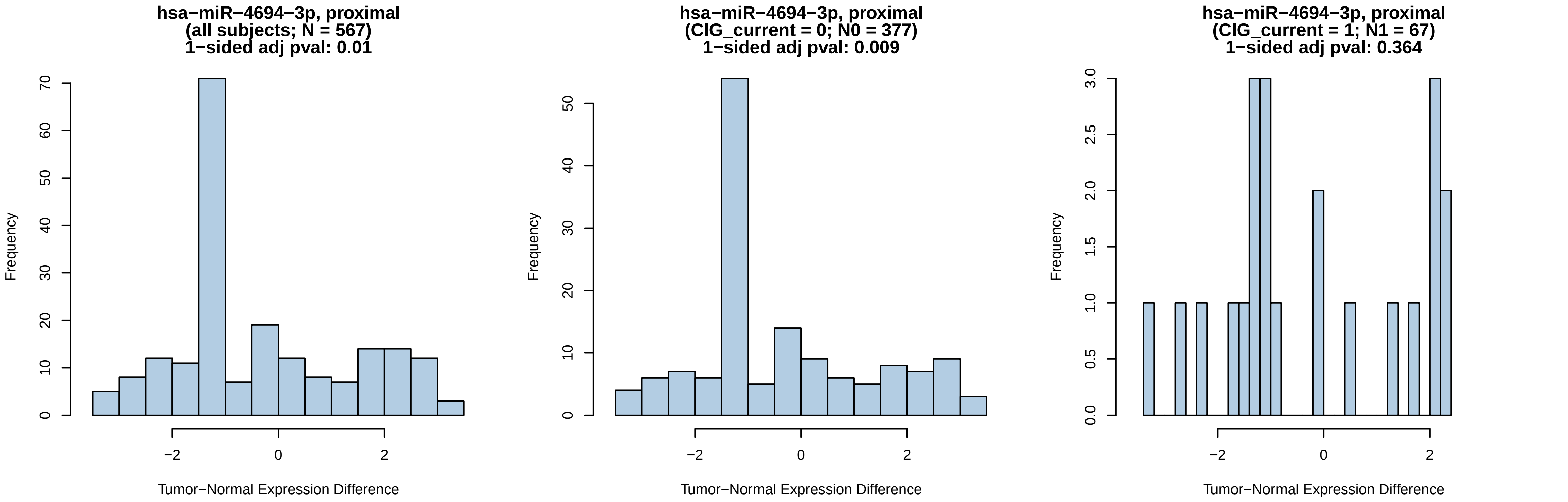

hsa−miR−4694−3p, proximal
(all subjects; N = 567)
1−sided adj pval: 0.01
hsa−miR−4694−3p, proximal
(CIG_current = 0; N0 = 377)
1−sided adj pval: 0.009
hsa−miR−4694−3p, proximal
(CIG_current = 1; N1 = 67)
1−sided adj pval: 0.364
Frequency
Tumor−Normal Expression Difference
0
10
20
30
40
50
60
70
0.0
0.5
1.0
1.5
2.0
2.5
3.0
−2
0
2

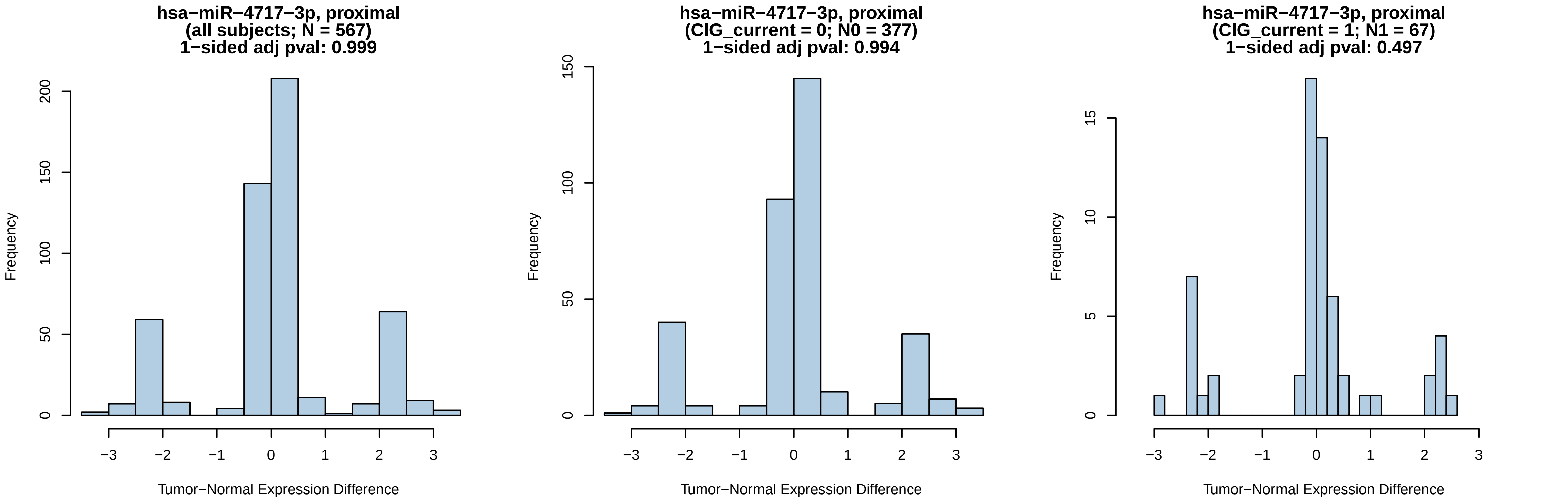
hsa−miR−4717−3p, proximal
(all subjects; N = 567)
1−sided adj pval: 0.999
Frequency
Tumor−Normal Expression Difference
hsa−miR−4717−3p, proximal
(CIG_current = 0; N0 = 377)
1−sided adj pval: 0.994
Frequency
Tumor−Normal Expression Difference
hsa−miR−4717−3p, proximal
(CIG_current = 1; N1 = 67)
1−sided adj pval: 0.497
Frequency
Tumor−Normal Expression Difference

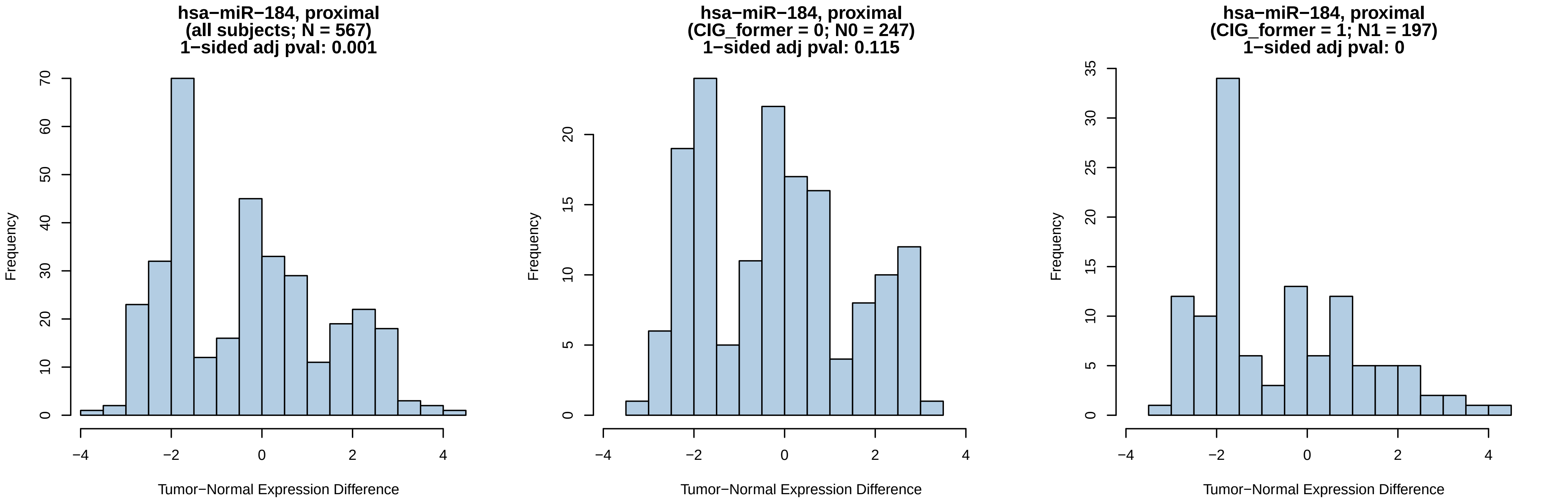
hsa−miR−184, proximal
(all subjects; N = 567)
1−sided adj pval: 0.001
Frequency
Tumor−Normal Expression Difference
hsa−miR−184, proximal
(CIG_former = 0; N0 = 247)
1−sided adj pval: 0.115
Frequency
Tumor−Normal Expression Difference
hsa−miR−184, proximal
(CIG_former = 1; N1 = 197)
1−sided adj pval: 0
Frequency
Tumor−Normal Expression Difference

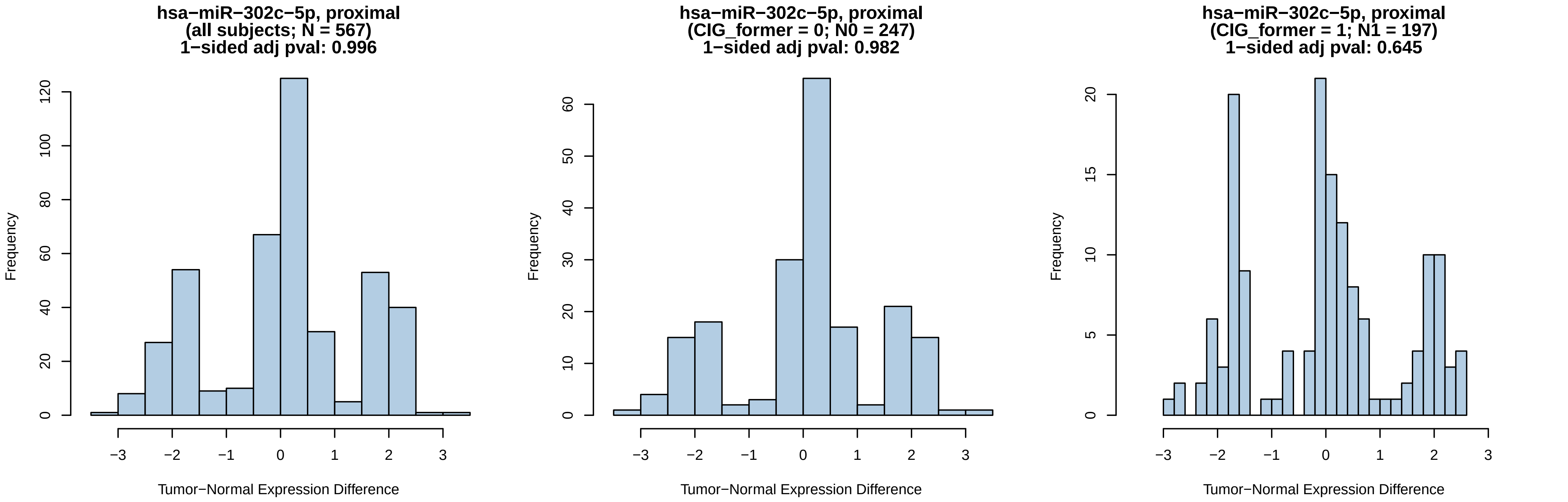

hsa−miR−302c−5p, proximal
(all subjects; N = 567)
1−sided adj pval: 0.996
Frequency
Tumor−Normal Expression Difference
hsa−miR−302c−5p, proximal
(CIG_former = 0; N0 = 247)
1−sided adj pval: 0.982
Frequency
Tumor−Normal Expression Difference
hsa−miR−302c−5p, proximal
(CIG_former = 1; N1 = 197)
1−sided adj pval: 0.645
Frequency
Tumor−Normal Expression Difference

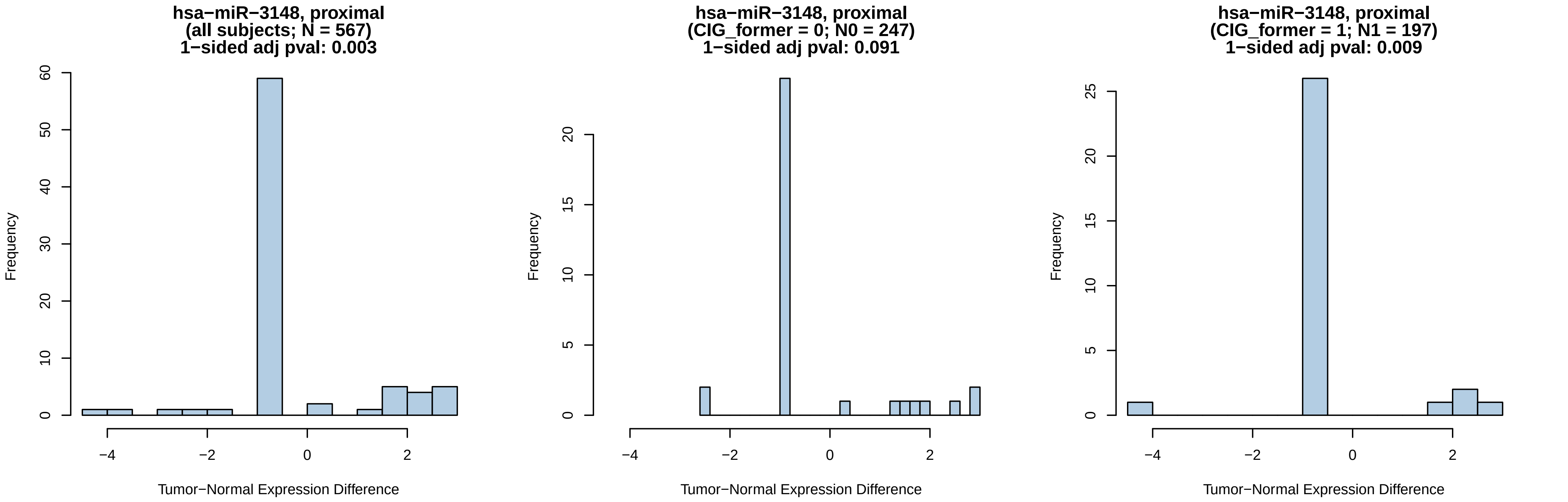

hsa−miR−3148, proximal
(all subjects; N = 567)
1−sided adj pval: 0.003
Frequency
Tumor−Normal Expression Difference
hsa−miR−3148, proximal
(CIG_former = 0; N0 = 247)
1−sided adj pval: 0.091
Frequency
Tumor−Normal Expression Difference
hsa−miR−3148, proximal
(CIG_former = 1; N1 = 197)
1−sided adj pval: 0.009
Frequency
Tumor−Normal Expression Difference

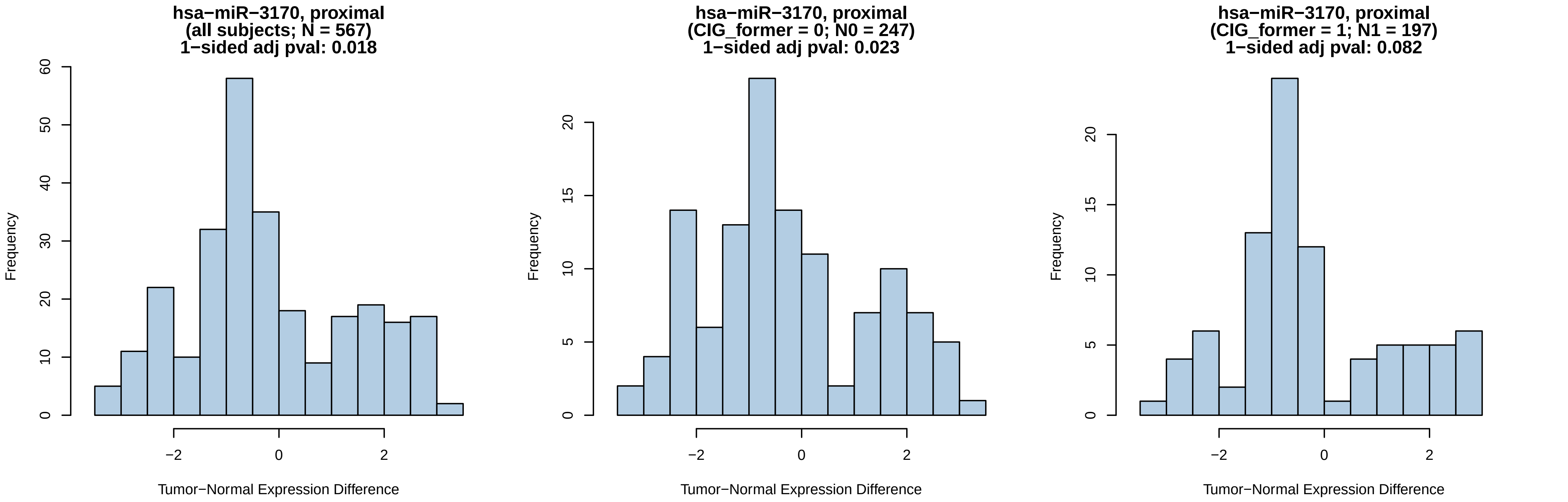

hsa−miR−3170, proximal
(all subjects; N = 567)
1−sided adj pval: 0.018
Frequency
0
10
20
30
40
50
60
−2
0
2
Tumor−Normal Expression Difference
hsa−miR−3170, proximal
(CIG_former = 0; N0 = 247)
1−sided adj pval: 0.023
Frequency
0
5
10
15
20
−2
0
2
Tumor−Normal Expression Difference
hsa−miR−3170, proximal
(CIG_former = 1; N1 = 197)
1−sided adj pval: 0.082
Frequency
0
5
10
15
20
−2
0
2
Tumor−Normal Expression Difference

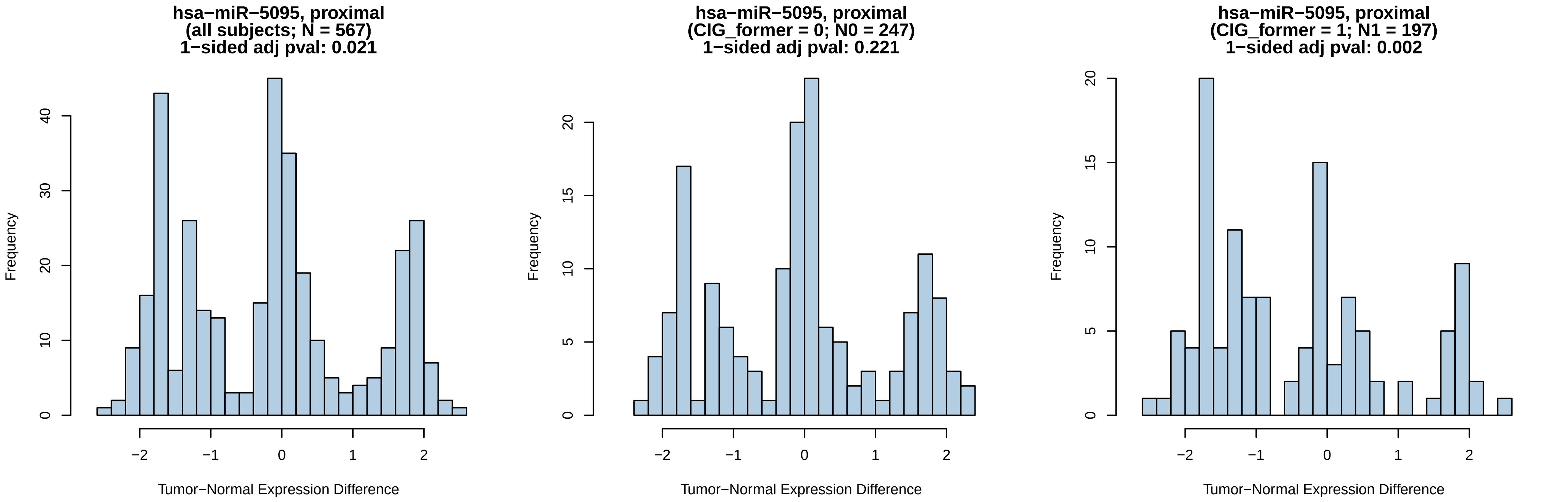
hsa−miR−5095, proximal
(all subjects; N = 567)
1−sided adj pval: 0.021
Frequency
Tumor−Normal Expression Difference
hsa−miR−5095, proximal
(CIG_former = 0; N0 = 247)
1−sided adj pval: 0.221
Frequency
Tumor−Normal Expression Difference
hsa−miR−5095, proximal
(CIG_former = 1; N1 = 197)
1−sided adj pval: 0.002
Frequency
Tumor−Normal Expression Difference

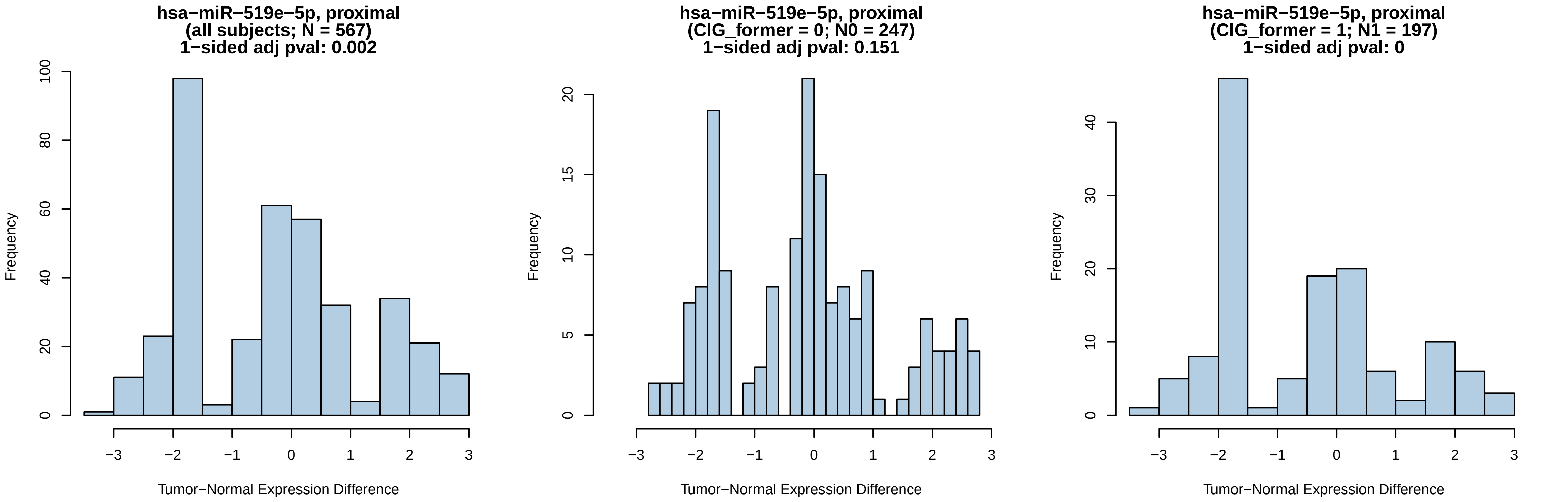
hsa−miR−519e−5p, proximal
(all subjects; N = 567)
1−sided adj pval: 0.002
Frequency
Tumor−Normal Expression Difference
hsa−miR−519e−5p, proximal
(CIG_former = 0; N0 = 247)
1−sided adj pval: 0.151
Frequency
Tumor−Normal Expression Difference
hsa−miR−519e−5p, proximal
(CIG_former = 1; N1 = 197)
1−sided adj pval: 0
Frequency
Tumor−Normal Expression Difference

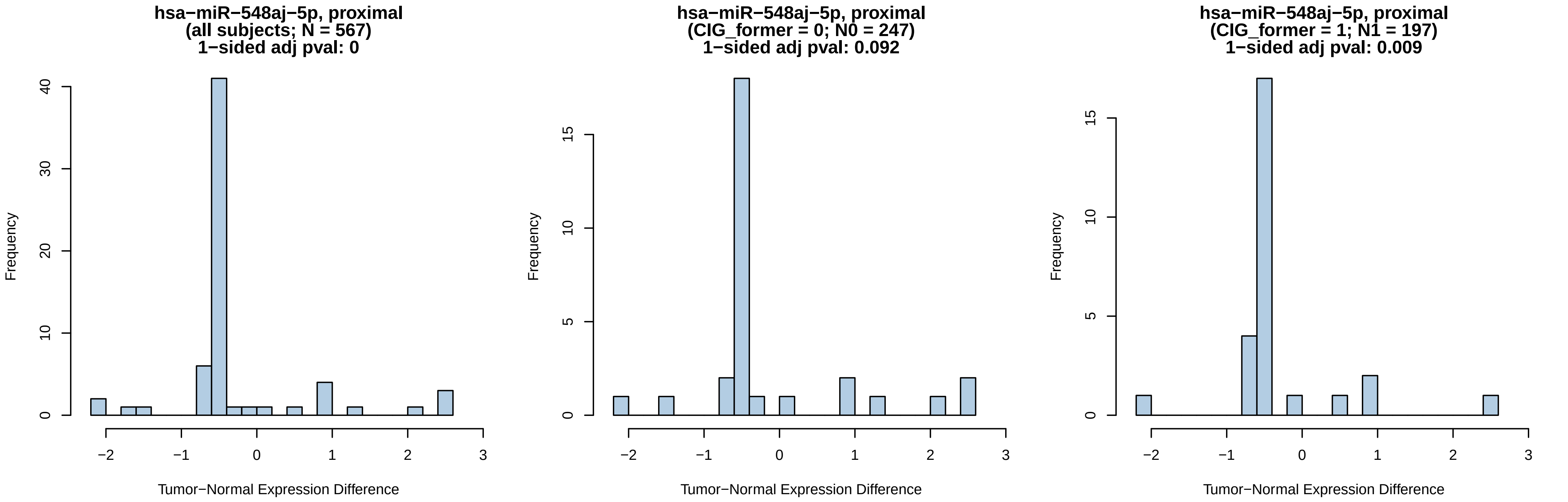

hsa−miR−548aj−5p, proximal
(all subjects; N = 567)
1−sided adj pval: 0
Frequency
Tumor−Normal Expression Difference
hsa−miR−548aj−5p, proximal
(CIG_former = 0; N0 = 247)
1−sided adj pval: 0.092
Frequency
Tumor−Normal Expression Difference
hsa−miR−548aj−5p, proximal
(CIG_former = 1; N1 = 197)
1−sided adj pval: 0.009
Frequency
Tumor−Normal Expression Difference

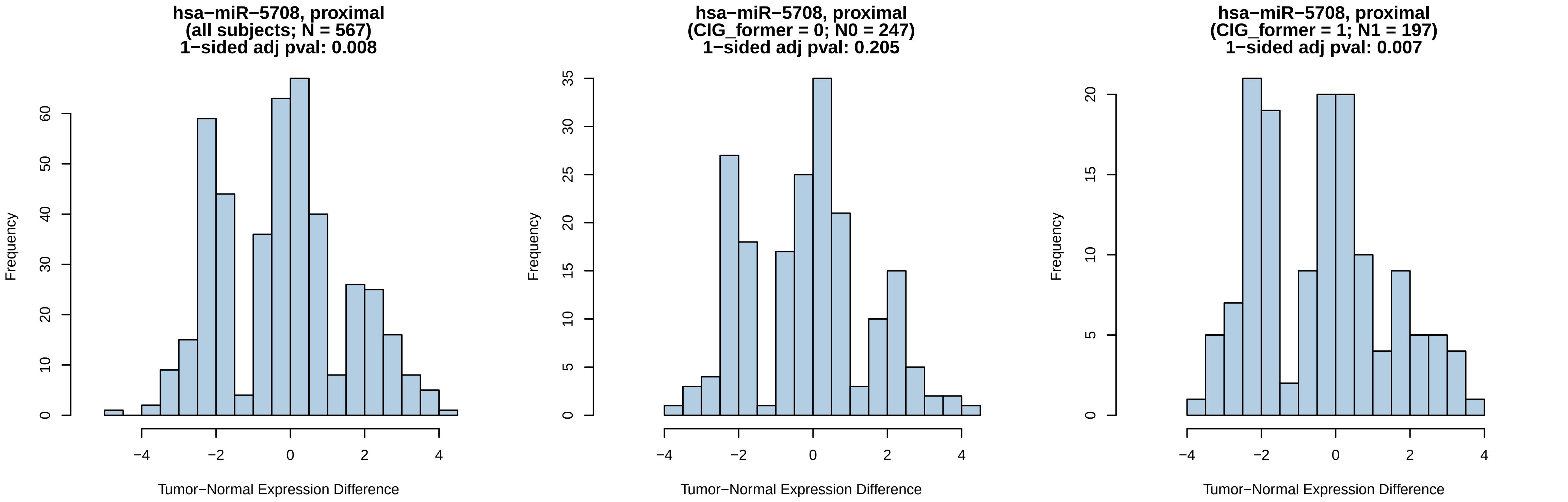
hsa−miR−5708, proximal
(all subjects; N = 567)
1−sided adj pval: 0.008
Frequency
Tumor−Normal Expression Difference
hsa−miR−5708, proximal
(CIG_former = 0; N0 = 247)
1−sided adj pval: 0.205
Frequency
Tumor−Normal Expression Difference
hsa−miR−5708, proximal
(CIG_former = 1; N1 = 197)
1−sided adj pval: 0.007
Frequency
Tumor−Normal Expression Difference

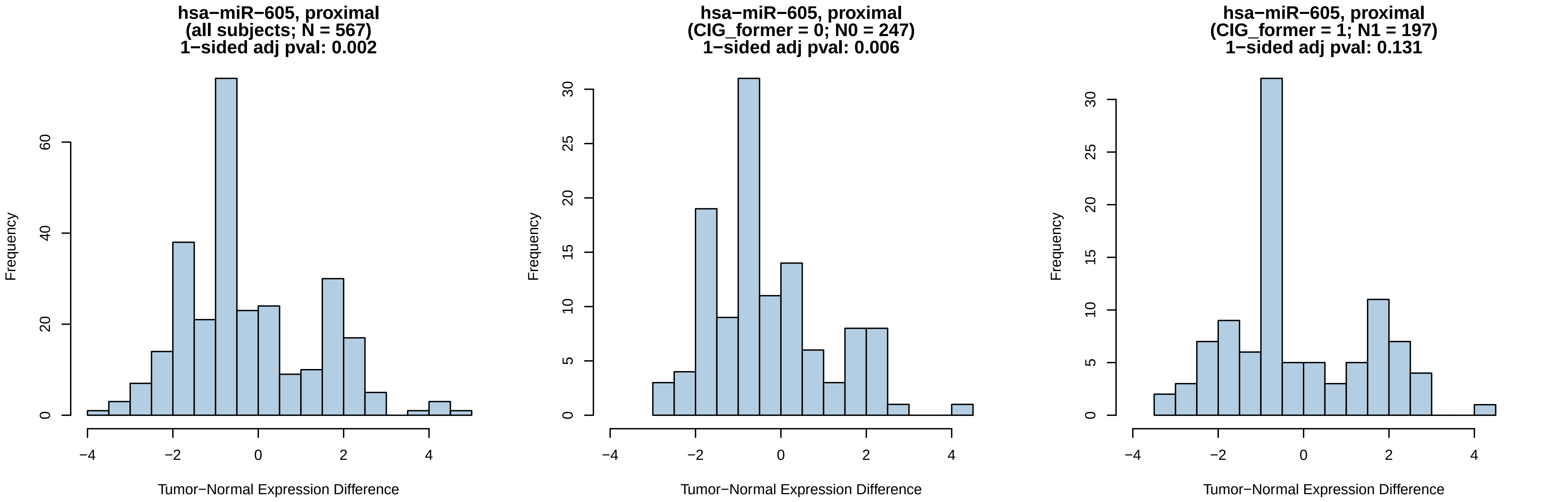

hsa−miR−605, proximal
(all subjects; N = 567)
1−sided adj pval: 0.002
Frequency
Tumor−Normal Expression Difference
hsa−miR−605, proximal
(CIG_former = 0; N0 = 247)
1−sided adj pval: 0.006
Frequency
Tumor−Normal Expression Difference
hsa−miR−605, proximal
(CIG_former = 1; N1 = 197)
1−sided adj pval: 0.131
Frequency
Tumor−Normal Expression Difference

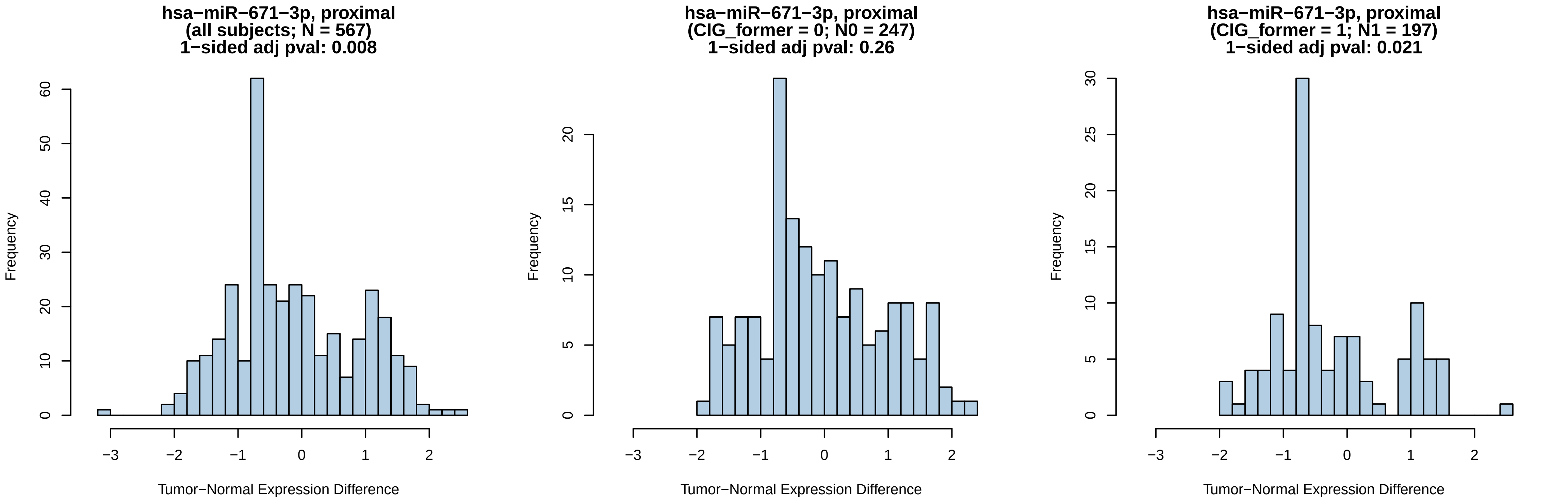

hsa−miR−671−3p, proximal
(all subjects; N = 567)
1−sided adj pval: 0.008
Frequency
Tumor−Normal Expression Difference
hsa−miR−671−3p, proximal
(CIG_former = 0; N0 = 247)
1−sided adj pval: 0.26
Frequency
Tumor−Normal Expression Difference
hsa−miR−671−3p, proximal
(CIG_former = 1; N1 = 197)
1−sided adj pval: 0.021
Frequency
Tumor−Normal Expression Difference

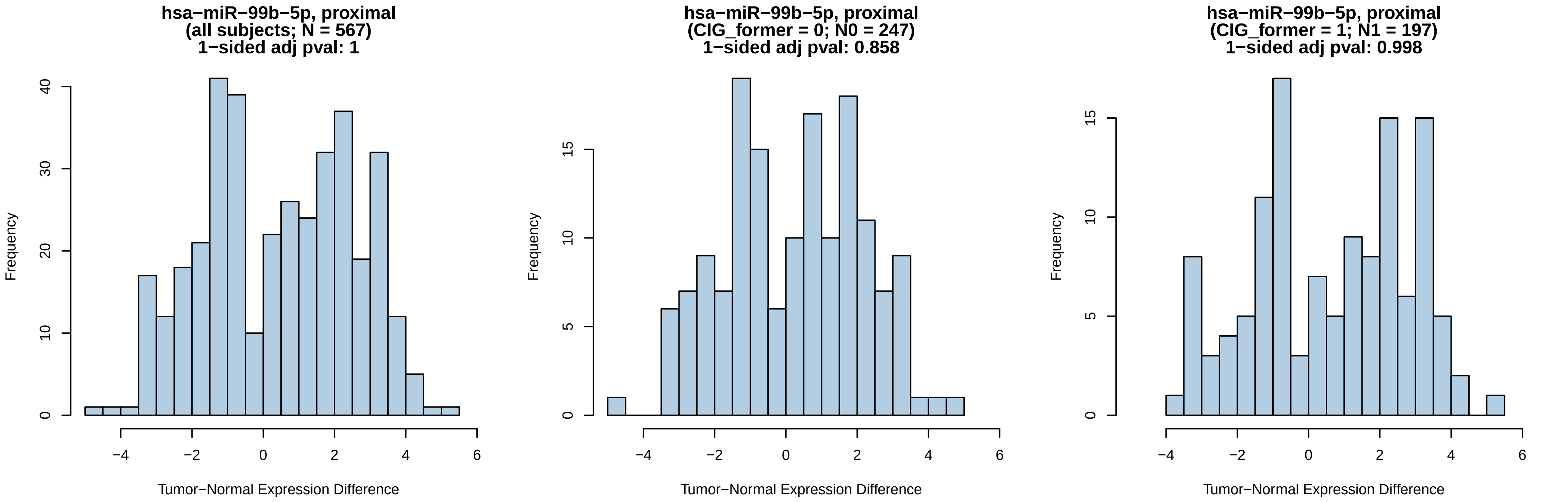
hsa−miR−99b−5p, proximal
(all subjects; N = 567)
1−sided adj pval: 1
Frequency
Tumor−Normal Expression Difference
hsa−miR−99b−5p, proximal
(CIG_former = 0; N0 = 247)
1−sided adj pval: 0.858
Frequency
Tumor−Normal Expression Difference
hsa−miR−99b−5p, proximal
(CIG_former = 1; N1 = 197)
1−sided adj pval: 0.998
Frequency
Tumor−Normal Expression Difference

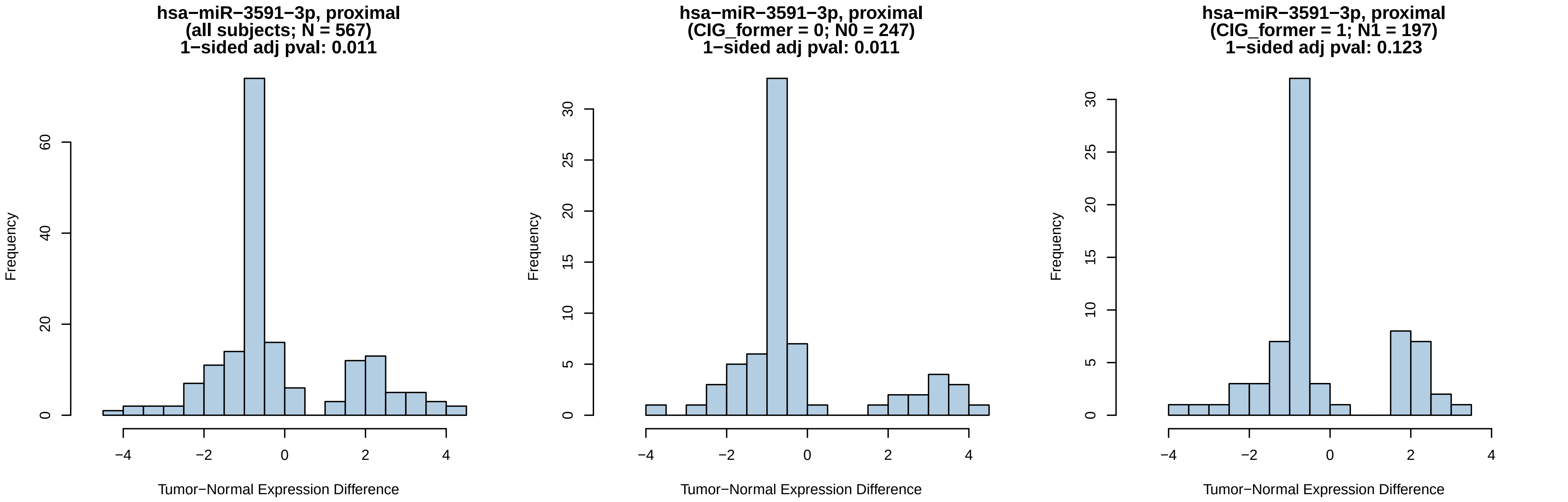
hsa−miR−3591−3p, proximal
(all subjects; N = 567)
1−sided adj pval: 0.011
Frequency
0
20
40
60
−4
−2
0
2
4
Tumor−Normal Expression Difference
hsa−miR−3591−3p, proximal
(CIG_former = 0; N0 = 247)
1−sided adj pval: 0.011
Frequency
0
5
10
15
20
25
30
−4
−2
0
2
4
Tumor−Normal Expression Difference
hsa−miR−3591−3p, proximal
(CIG_former = 1; N1 = 197)
1−sided adj pval: 0.123
Frequency
0
5
10
15
20
25
30
−4
−2
0
2
4
Tumor−Normal Expression Difference

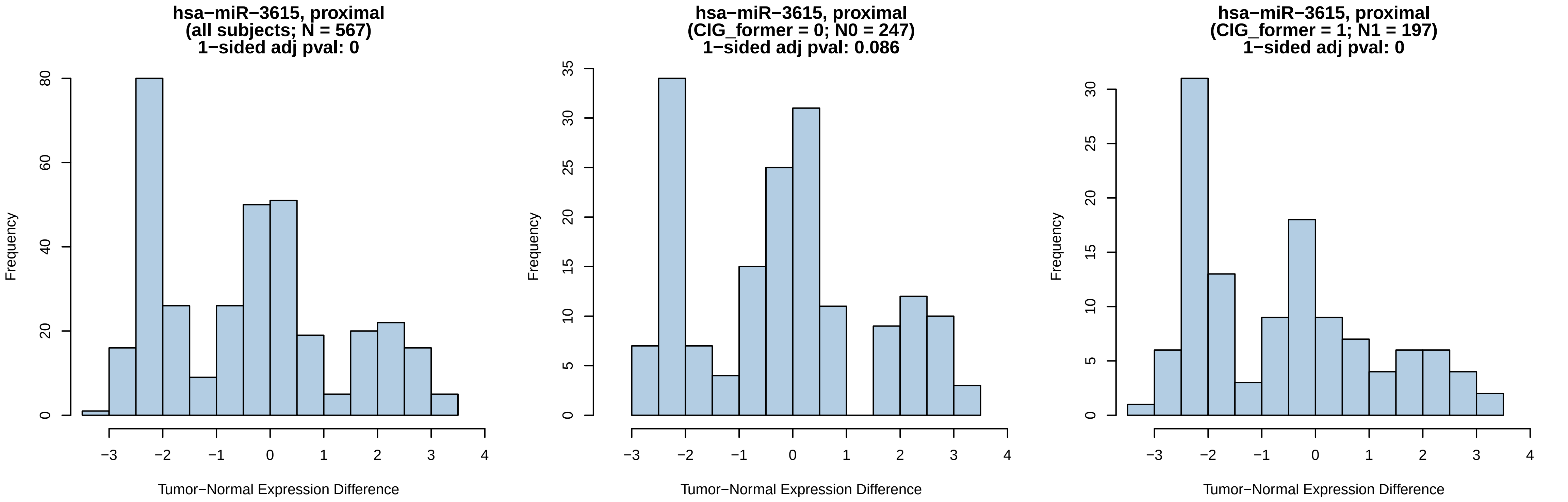

hsa−miR−3615, proximal
(all subjects; N = 567)
1−sided adj pval: 0
Frequency
Tumor−Normal Expression Difference
hsa−miR−3615, proximal
(CIG_former = 0; N0 = 247)
1−sided adj pval: 0.086
Frequency
Tumor−Normal Expression Difference
hsa−miR−3615, proximal
(CIG_former = 1; N1 = 197)
1−sided adj pval: 0
Frequency
Tumor−Normal Expression Difference

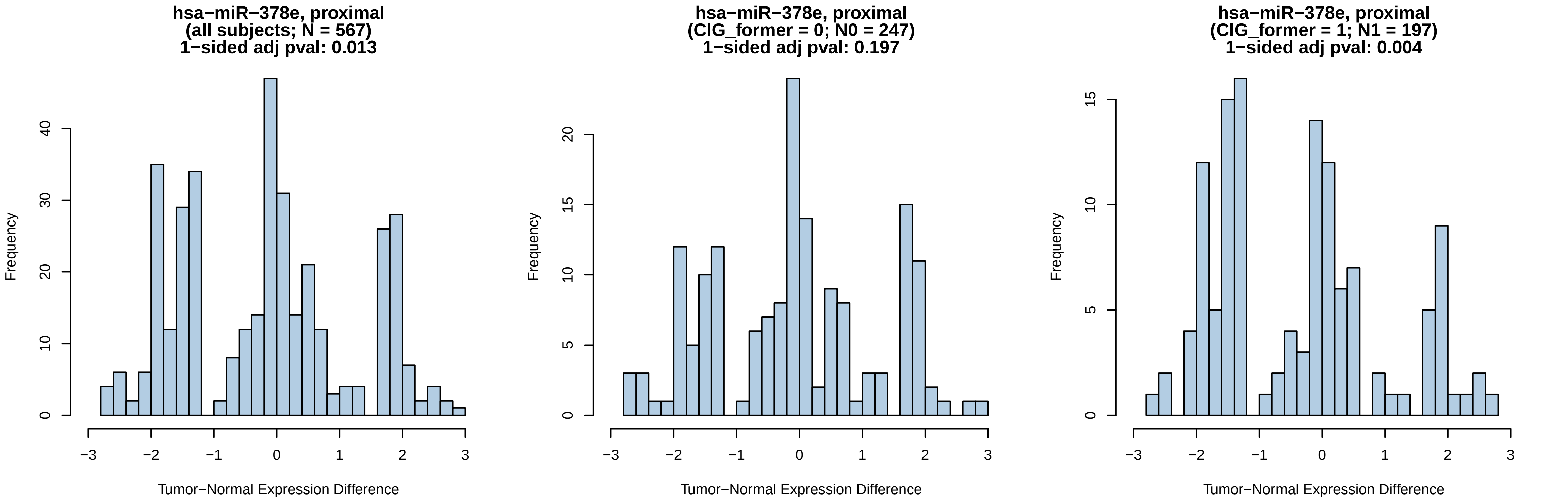
hsa−miR−378e, proximal
(all subjects; N = 567)
1−sided adj pval: 0.013
Frequency
Tumor−Normal Expression Difference
hsa−miR−378e, proximal
(CIG_former = 0; N0 = 247)
1−sided adj pval: 0.197
Frequency
Tumor−Normal Expression Difference
hsa−miR−378e, proximal
(CIG_former = 1; N1 = 197)
1−sided adj pval: 0.004
Frequency
Tumor−Normal Expression Difference

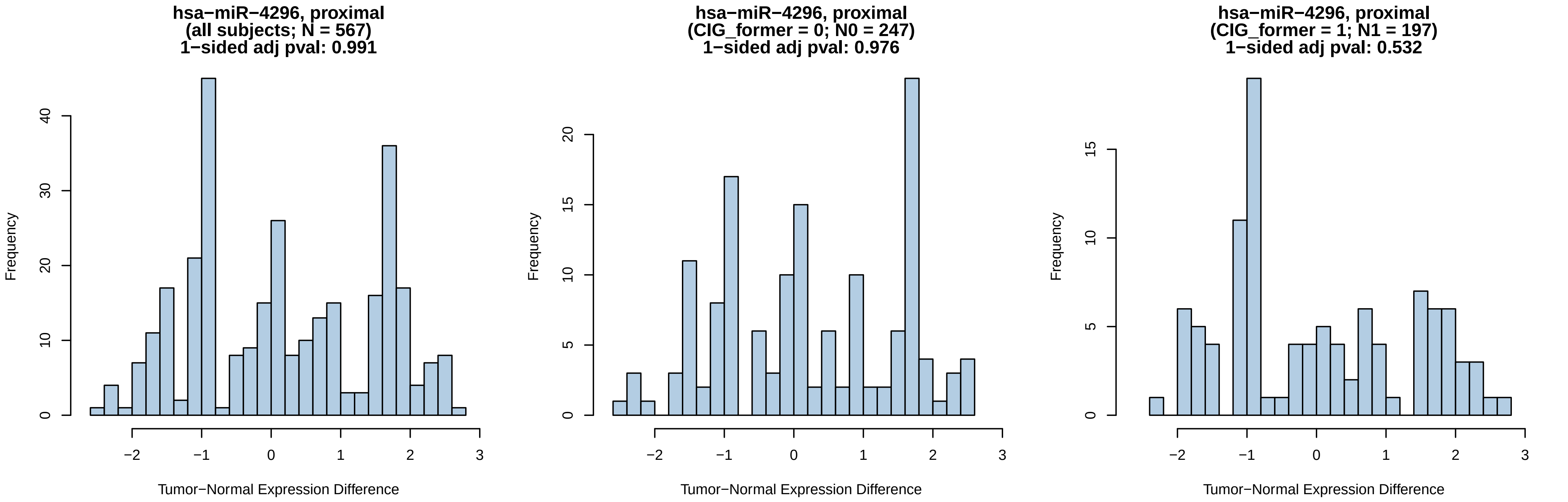
hsa−miR−4296, proximal
(all subjects; N = 567)
1−sided adj pval: 0.991
Frequency
Tumor−Normal Expression Difference
hsa−miR−4296, proximal
(CIG_former = 0; N0 = 247)
1−sided adj pval: 0.976
Frequency
Tumor−Normal Expression Difference
hsa−miR−4296, proximal
(CIG_former = 1; N1 = 197)
1−sided adj pval: 0.532
Frequency
Tumor−Normal Expression Difference

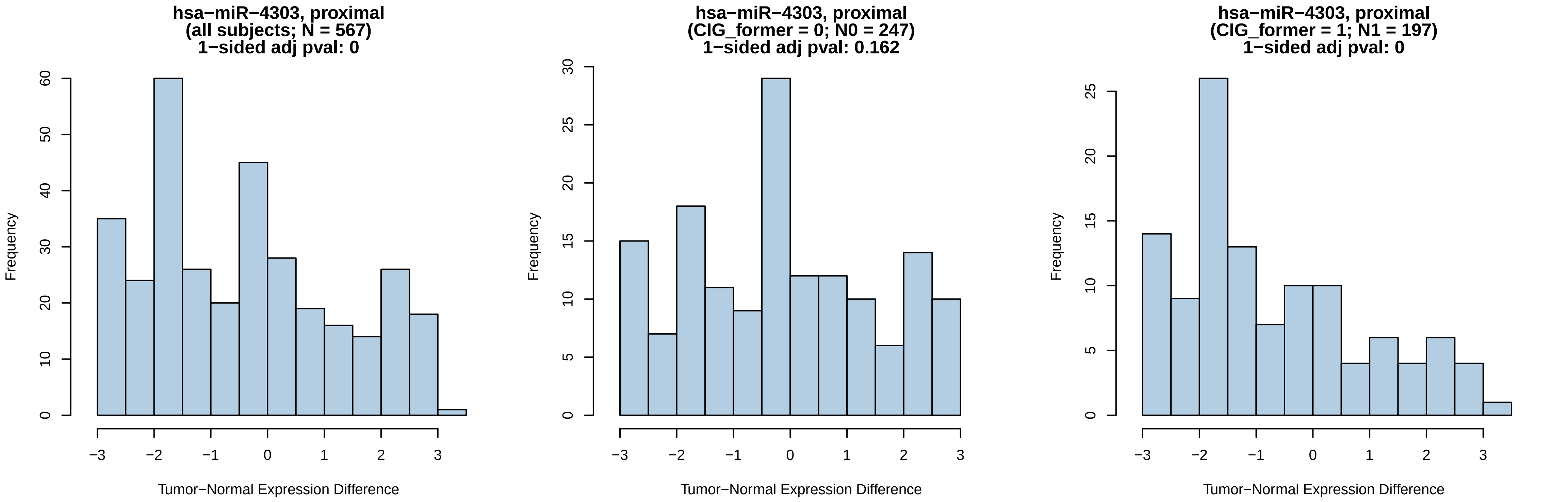
hsa−miR−4303, proximal
(all subjects; N = 567)
1−sided adj pval: 0
hsa−miR−4303, proximal
(CIG_former = 0; N0 = 247)
1−sided adj pval: 0.162
hsa−miR−4303, proximal
(CIG_former = 1; N1 = 197)
1−sided adj pval: 0
Frequency
Tumor−Normal Expression Difference

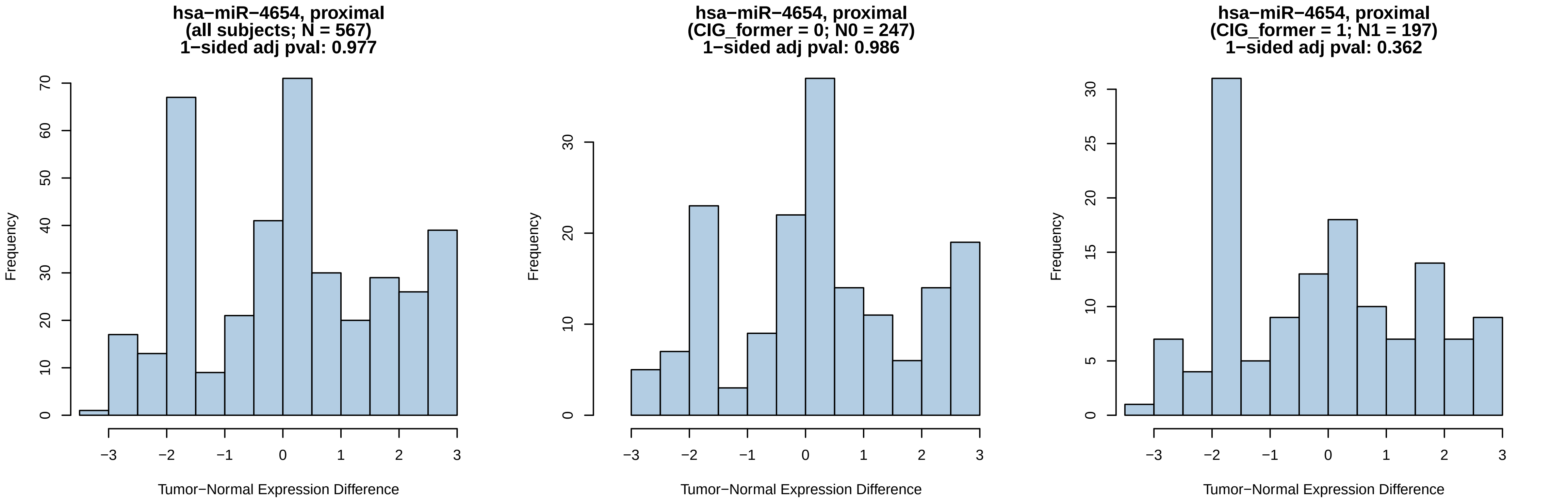
hsa−miR−4654, proximal
(all subjects; N = 567)
1−sided adj pval: 0.977
Frequency
Tumor−Normal Expression Difference
hsa−miR−4654, proximal
(CIG_former = 0; N0 = 247)
1−sided adj pval: 0.986
Frequency
Tumor−Normal Expression Difference
hsa−miR−4654, proximal
(CIG_former = 1; N1 = 197)
1−sided adj pval: 0.362
Frequency
Tumor−Normal Expression Difference

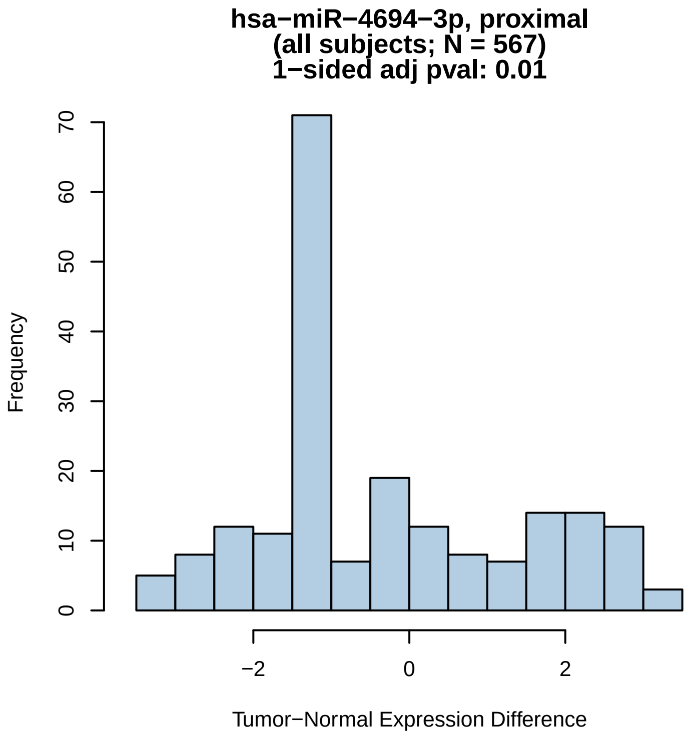

hsa−miR−4694−3p, proximal
(all subjects; N = 567)
1−sided adj pval: 0.01
Frequency
0
10
20
30
40
50
60
70
−2
0
2
Tumor−Normal Expression Difference


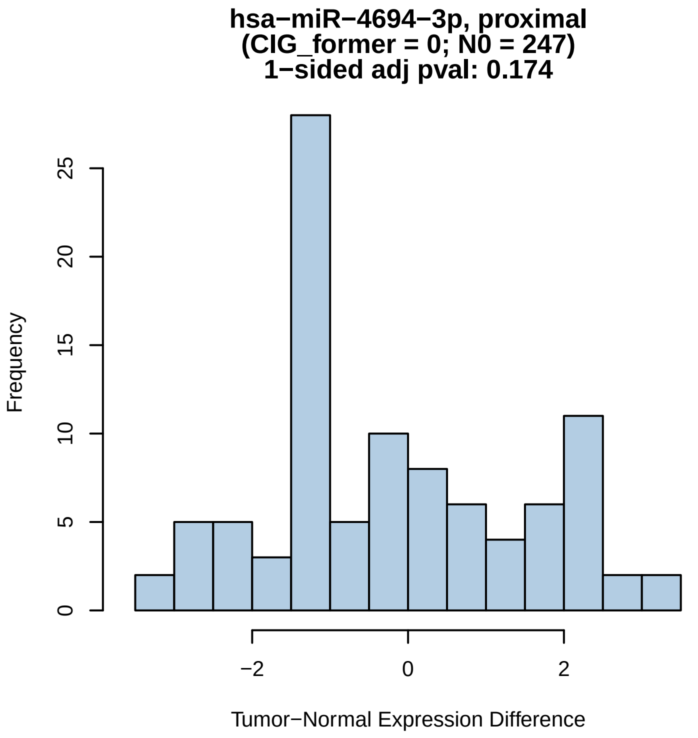

hsa−miR−4694−3p, proximal
(CIG_former = 0; N0 = 247)
1−sided adj pval: 0.174
Frequency
0
5
10
15
20
25
−2
0
2
Tumor−Normal Expression Difference


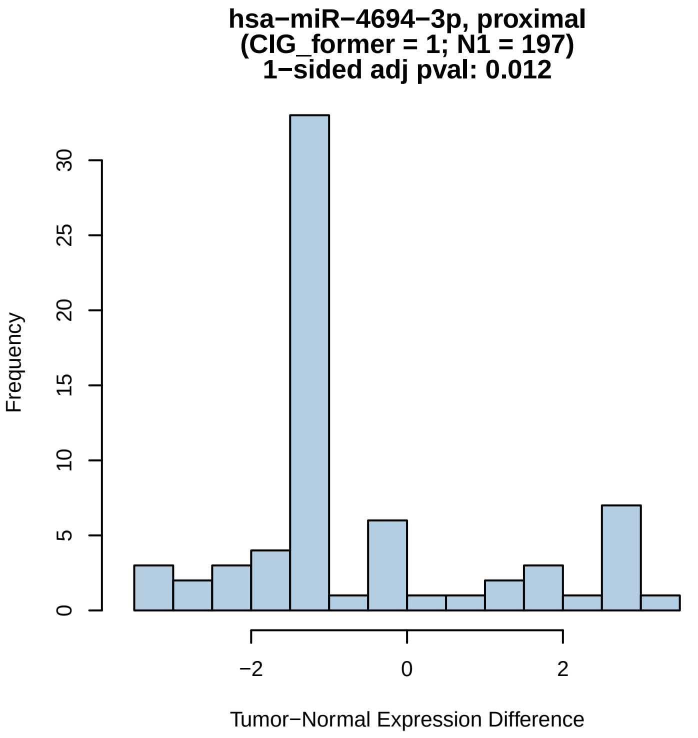

hsa−miR−4694−3p, proximal
(CIG_former = 1; N1 = 197)
1−sided adj pval: 0.012
Frequency
0
5
10
15
20
25
30
−2
0
2
Tumor−Normal Expression Difference

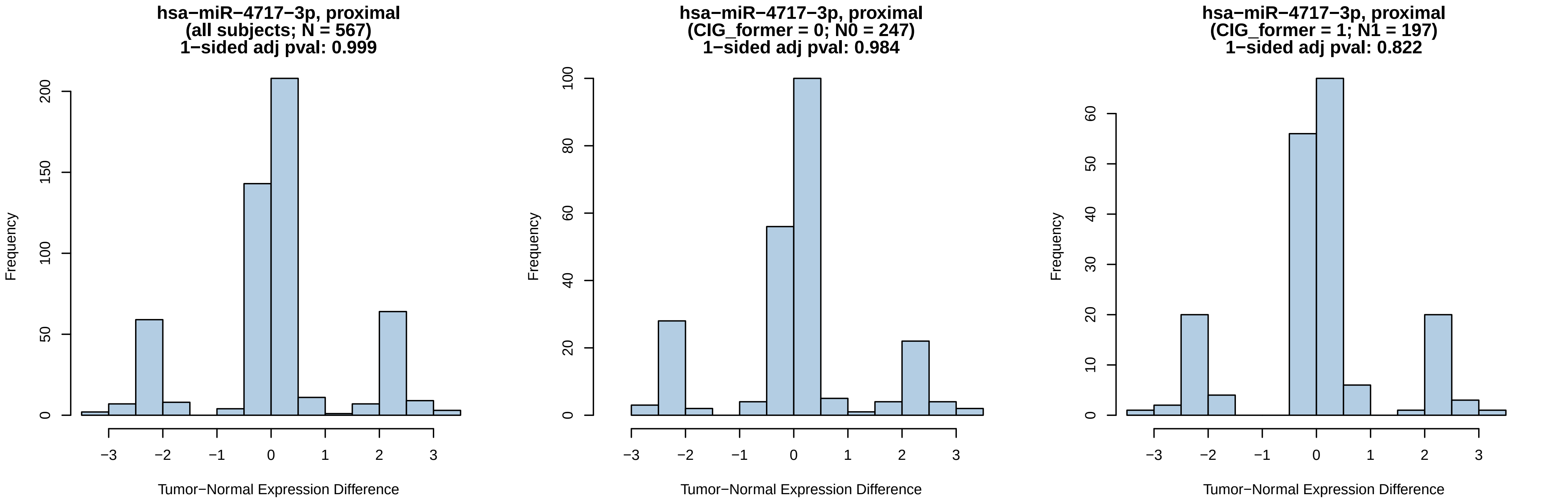
hsa−miR−4717−3p, proximal
(all subjects; N = 567)
1−sided adj pval: 0.999
Frequency
0
50
100
150
200
−3
−2
−1
0
1
2
3
Tumor−Normal Expression Difference
hsa−miR−4717−3p, proximal
(CIG_former = 0; N0 = 247)
1−sided adj pval: 0.984
Frequency
0
20
40
60
80
100
−3
−2
−1
0
1
2
3
Tumor−Normal Expression Difference
hsa−miR−4717−3p, proximal
(CIG_former = 1; N1 = 197)
1−sided adj pval: 0.822
Frequency
0
10
20
30
40
50
60
−3
−2
−1
0
1
2
3
Tumor−Normal Expression Difference

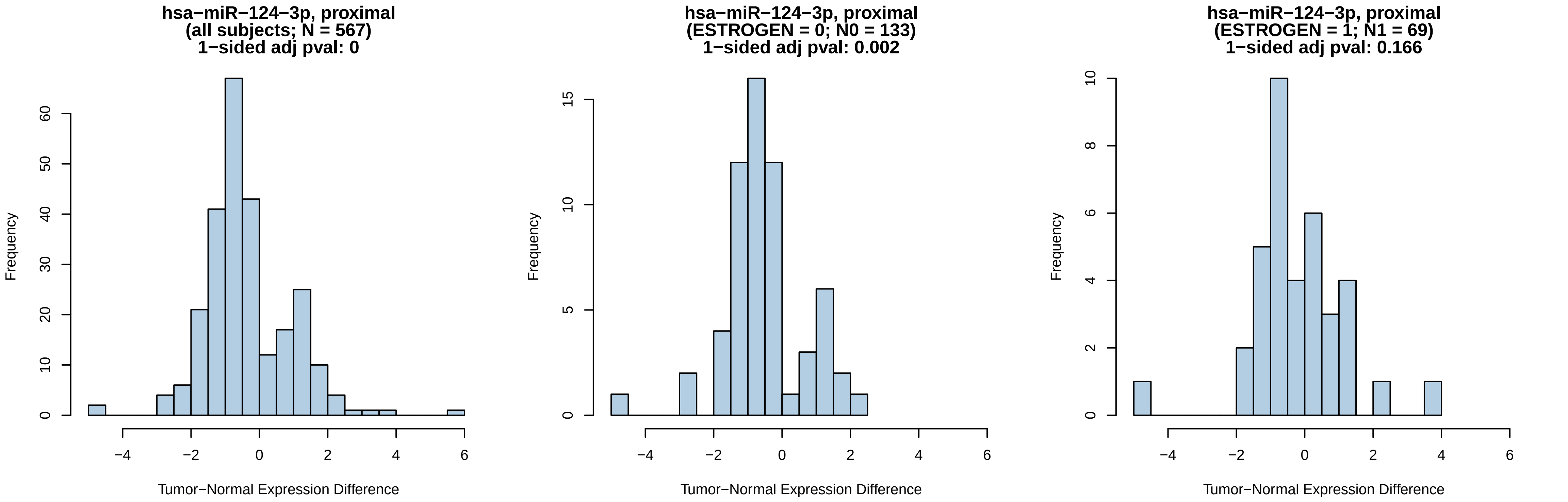

hsa−miR−124−3p, proximal
(all subjects; N = 567)
1−sided adj pval: 0
hsa−miR−124−3p, proximal
(ESTROGEN = 0; N0 = 133)
1−sided adj pval: 0.002
hsa−miR−124−3p, proximal
(ESTROGEN = 1; N1 = 69)
1−sided adj pval: 0.166
Frequency
Tumor−Normal Expression Difference

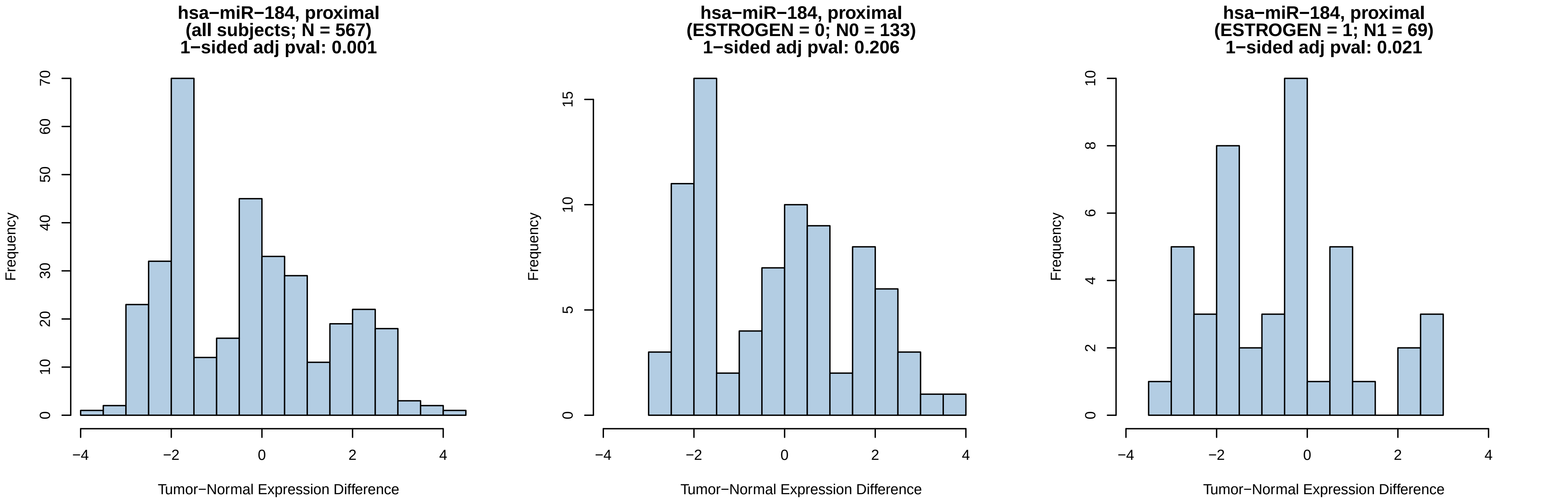
hsa−miR−184, proximal
(all subjects; N = 567)
1−sided adj pval: 0.001
Frequency
Tumor−Normal Expression Difference
hsa−miR−184, proximal
(ESTROGEN = 0; N0 = 133)
1−sided adj pval: 0.206
Frequency
Tumor−Normal Expression Difference
hsa−miR−184, proximal
(ESTROGEN = 1; N1 = 69)
1−sided adj pval: 0.021
Frequency
Tumor−Normal Expression Difference

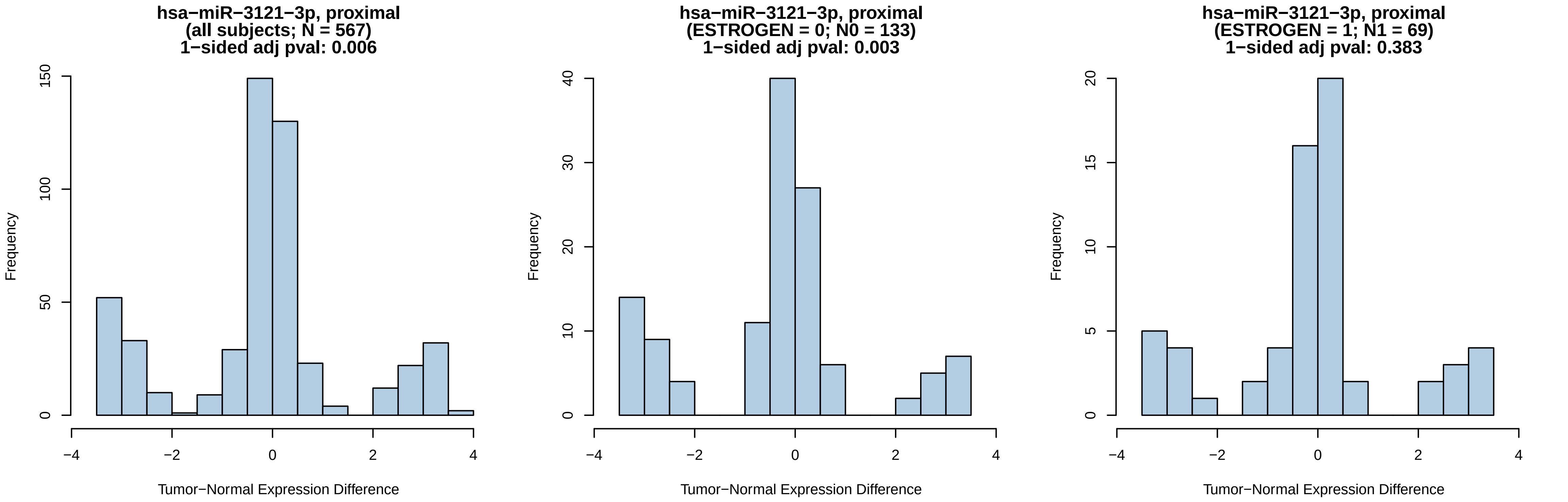

hsa−miR−3121−3p, proximal
(all subjects; N = 567)
1−sided adj pval: 0.006
hsa−miR−3121−3p, proximal
(ESTROGEN = 0; N0 = 133)
1−sided adj pval: 0.003
hsa−miR−3121−3p, proximal
(ESTROGEN = 1; N1 = 69)
1−sided adj pval: 0.383
Frequency
Tumor−Normal Expression Difference

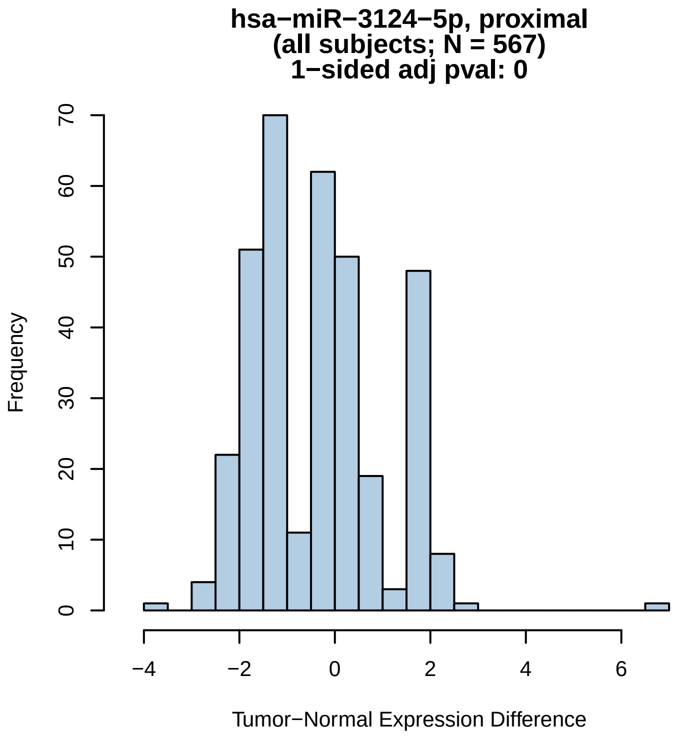

hsa−miR−3124−5p, proximal
(all subjects; N = 567)
1−sided adj pval: 0
Frequency
Tumor−Normal Expression Difference


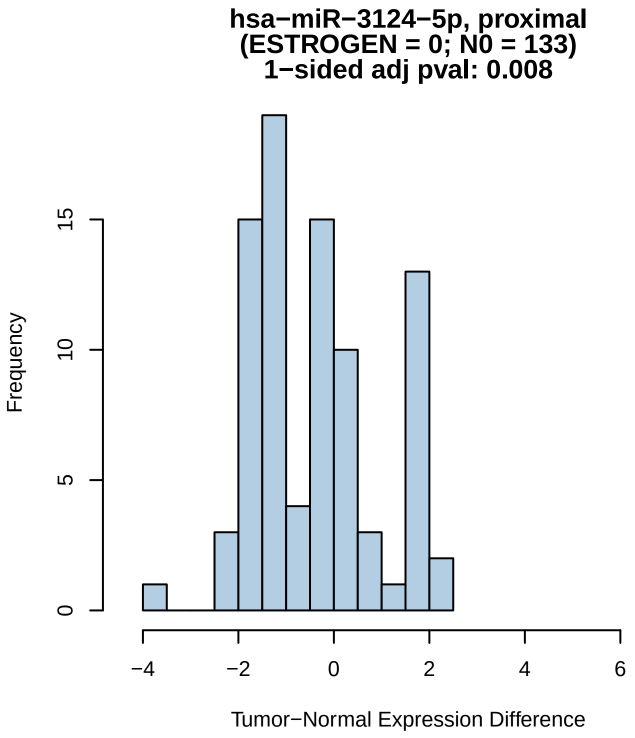

hsa−miR−3124−5p, proximal
(ESTROGEN = 0; N0 = 133)
1−sided adj pval: 0.008
Frequency
Tumor−Normal Expression Difference


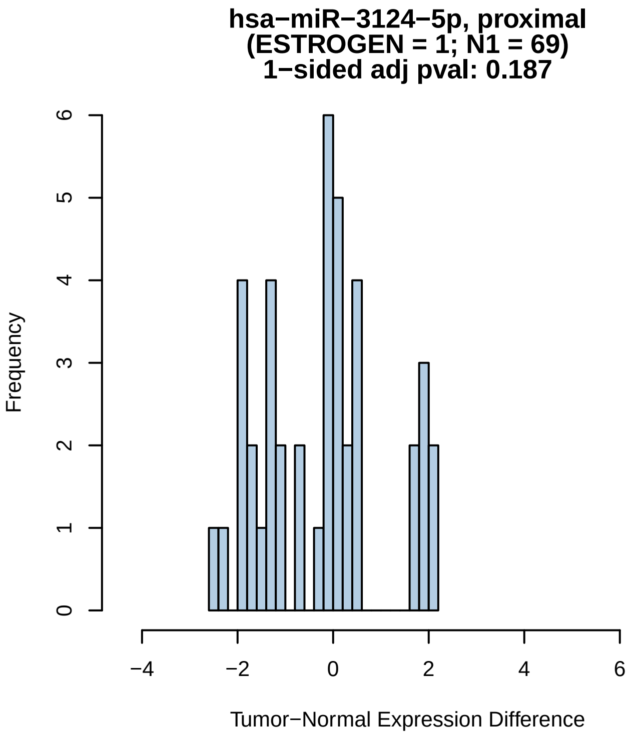

hsa−miR−3124−5p, proximal
(ESTROGEN = 1; N1 = 69)
1−sided adj pval: 0.187
Frequency
Tumor−Normal Expression Difference

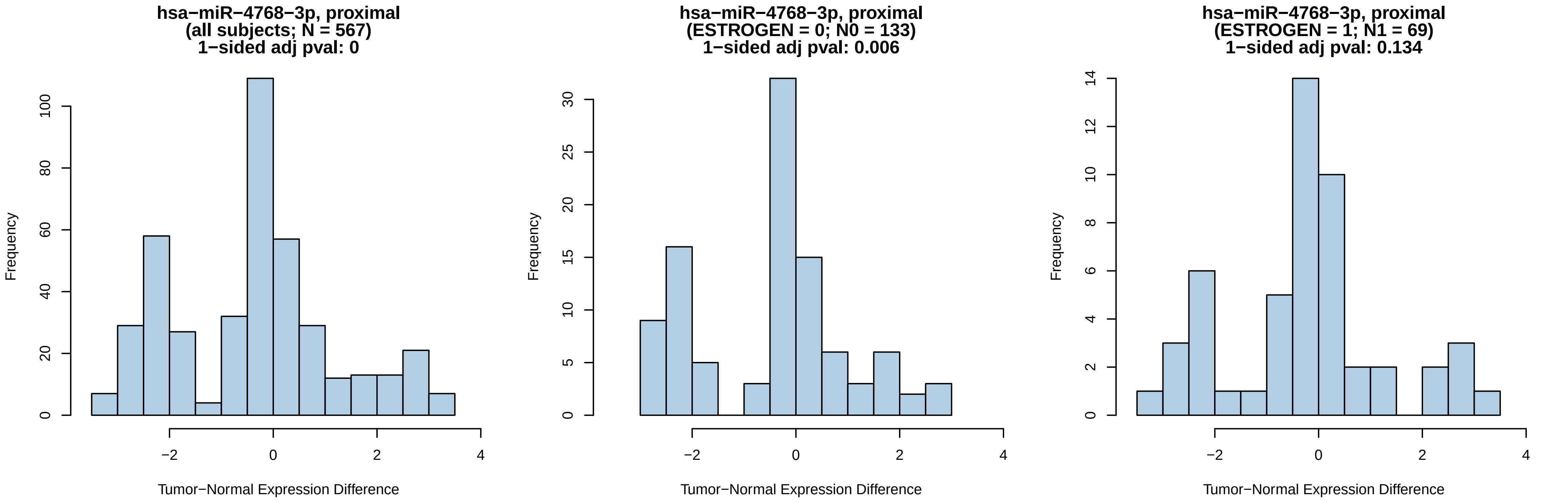
hsa−miR−4768−3p, proximal
(all subjects; N = 567)
1−sided adj pval: 0
Frequency
Tumor−Normal Expression Difference
hsa−miR−4768−3p, proximal
(ESTROGEN = 0; N0 = 133)
1−sided adj pval: 0.006
Frequency
Tumor−Normal Expression Difference
hsa−miR−4768−3p, proximal
(ESTROGEN = 1; N1 = 69)
1−sided adj pval: 0.134
Frequency
Tumor−Normal Expression Difference

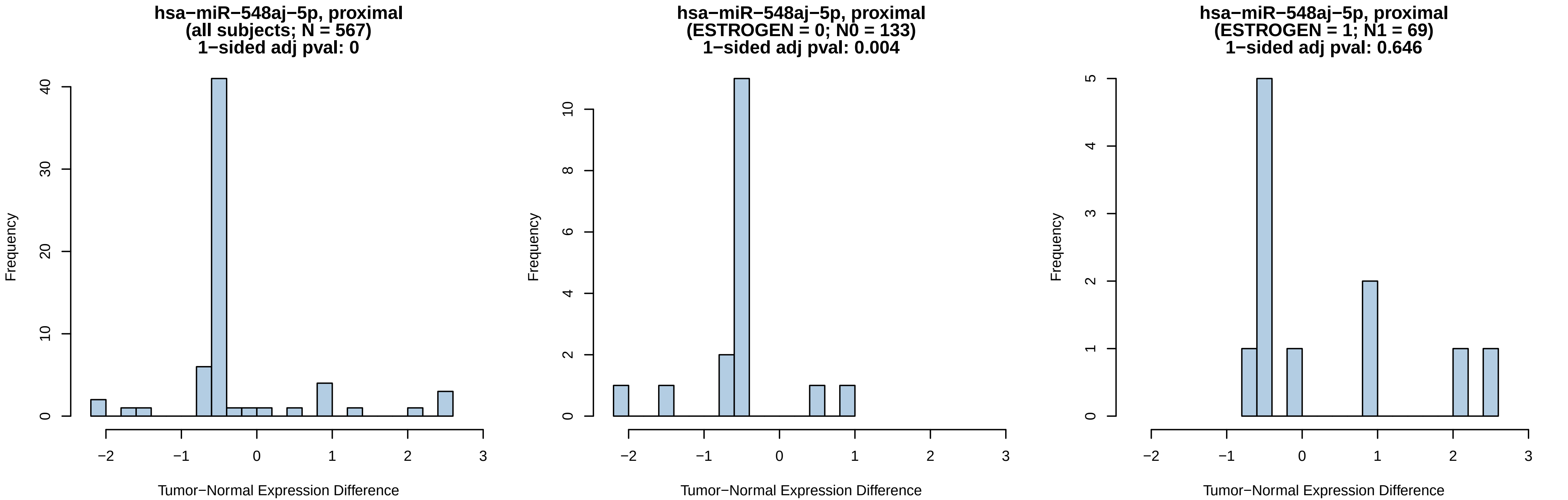
hsa−miR−548aj−5p, proximal
(all subjects; N = 567)
1−sided adj pval: 0
Frequency
Tumor−Normal Expression Difference
hsa−miR−548aj−5p, proximal
(ESTROGEN = 0; N0 = 133)
1−sided adj pval: 0.004
Frequency
Tumor−Normal Expression Difference
hsa−miR−548aj−5p, proximal
(ESTROGEN = 1; N1 = 69)
1−sided adj pval: 0.646
Frequency
Tumor−Normal Expression Difference

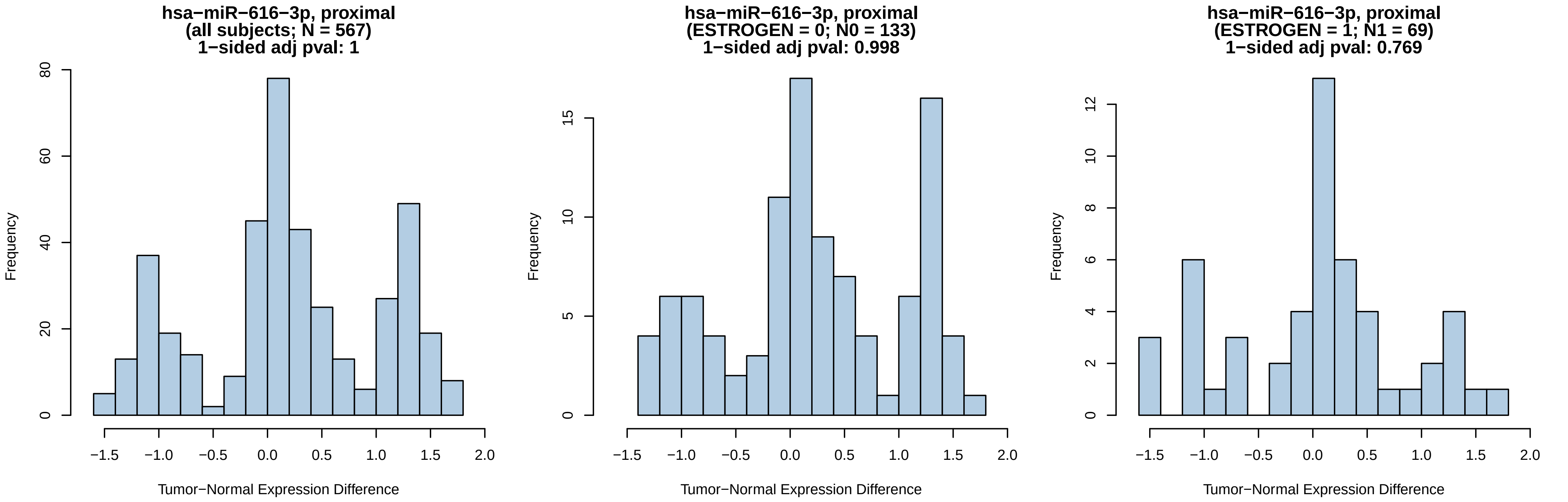
hsa−miR−616−3p, proximal
(all subjects; N = 567)
1−sided adj pval: 1
Frequency
Tumor−Normal Expression Difference
hsa−miR−616−3p, proximal
(ESTROGEN = 0; N0 = 133)
1−sided adj pval: 0.998
Frequency
Tumor−Normal Expression Difference
hsa−miR−616−3p, proximal
(ESTROGEN = 1; N1 = 69)
1−sided adj pval: 0.769
Frequency
Tumor−Normal Expression Difference

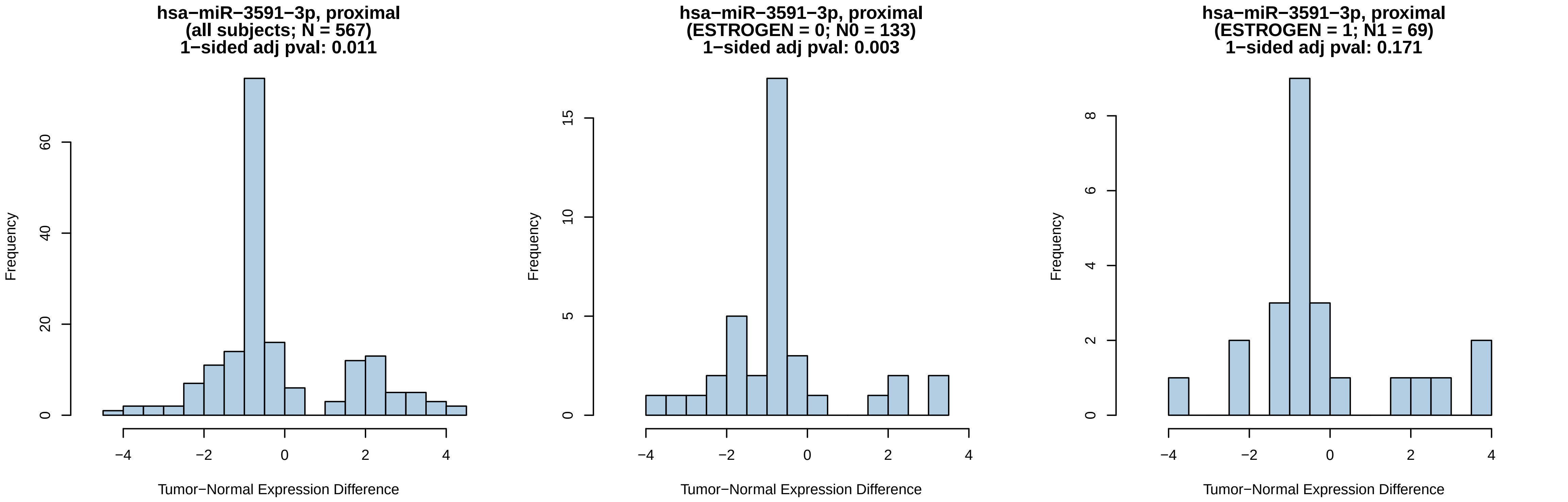
hsa−miR−3591−3p, proximal
(all subjects; N = 567)
1−sided adj pval: 0.011
Frequency
0
20
40
60
−4
−2
0
2
4
Tumor−Normal Expression Difference
hsa−miR−3591−3p, proximal
(ESTROGEN = 0; N0 = 133)
1−sided adj pval: 0.003
Frequency
0
5
10
15
−4
−2
0
2
4
Tumor−Normal Expression Difference
hsa−miR−3591−3p, proximal
(ESTROGEN = 1; N1 = 69)
1−sided adj pval: 0.171
Frequency
0
2
4
6
8
−4
−2
0
2
4
Tumor−Normal Expression Difference

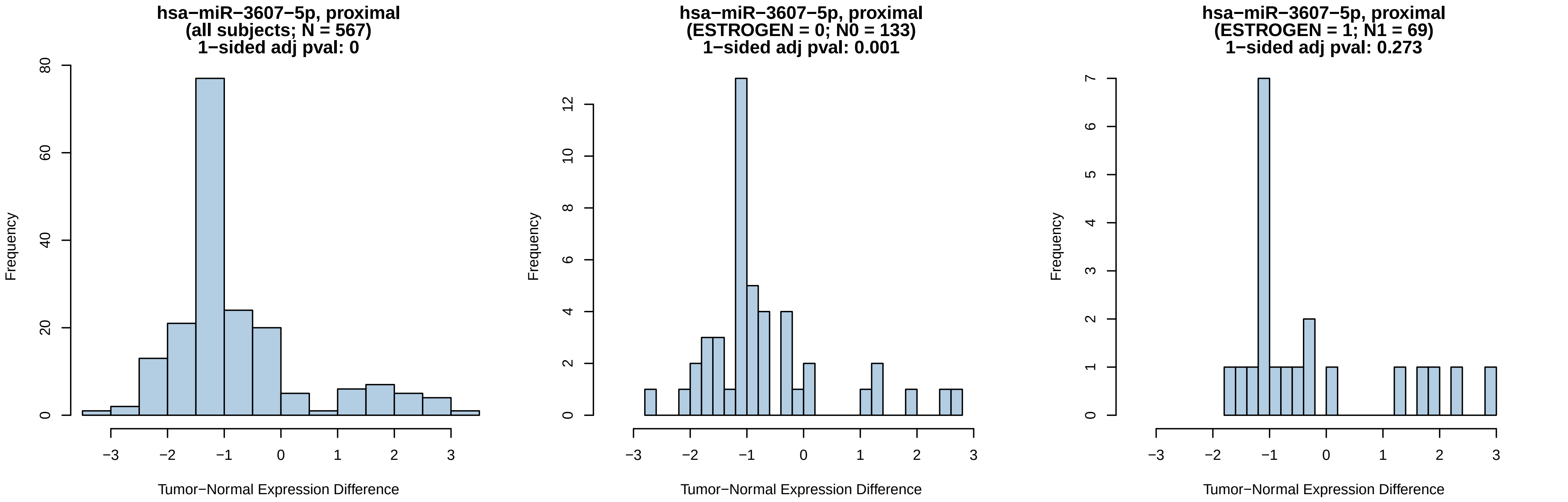
hsa−miR−3607−5p, proximal
(all subjects; N = 567)
1−sided adj pval: 0
Frequency
Tumor−Normal Expression Difference
hsa−miR−3607−5p, proximal
(ESTROGEN = 0; N0 = 133)
1−sided adj pval: 0.001
Frequency
Tumor−Normal Expression Difference
hsa−miR−3607−5p, proximal
(ESTROGEN = 1; N1 = 69)
1−sided adj pval: 0.273
Frequency
Tumor−Normal Expression Difference

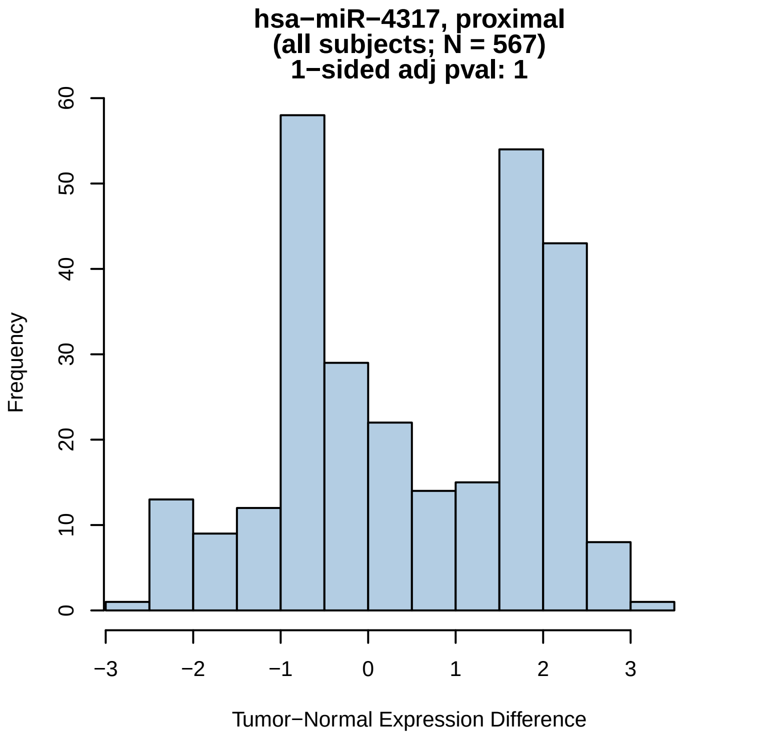
hsa−miR−4317, proximal
(all subjects; N = 567)
1−sided adj pval: 1
Frequency
Tumor−Normal Expression Difference

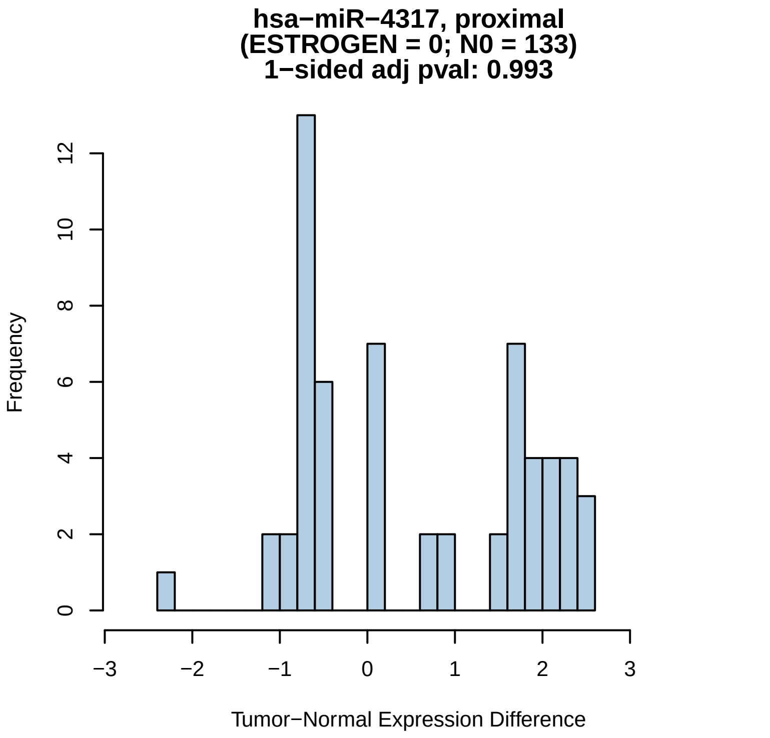
hsa−miR−4317, proximal
(ESTROGEN = 0; N0 = 133)
1−sided adj pval: 0.993
Frequency
Tumor−Normal Expression Difference

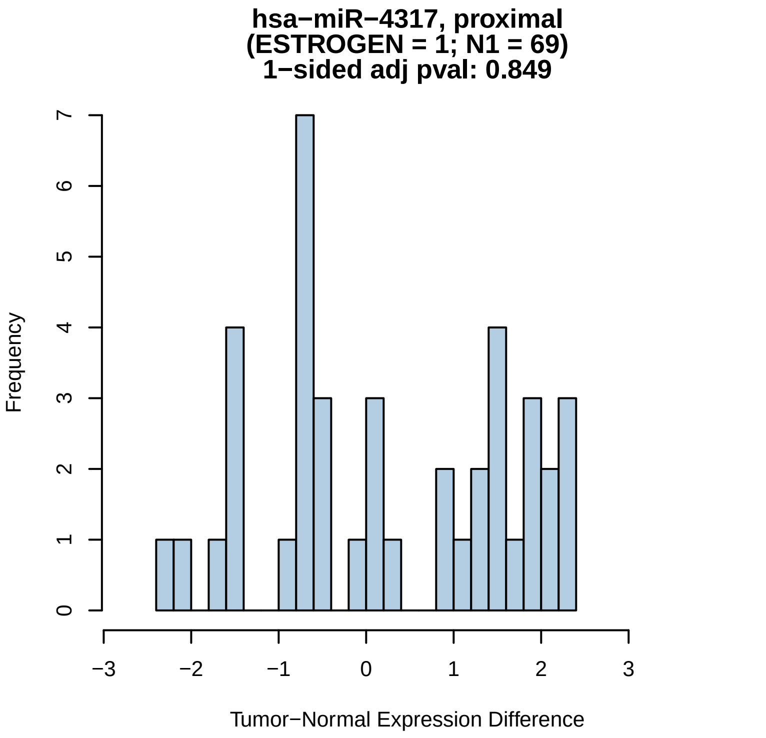
hsa−miR−4317, proximal
(ESTROGEN = 1; N1 = 69)
1−sided adj pval: 0.849
Frequency
Tumor−Normal Expression Difference

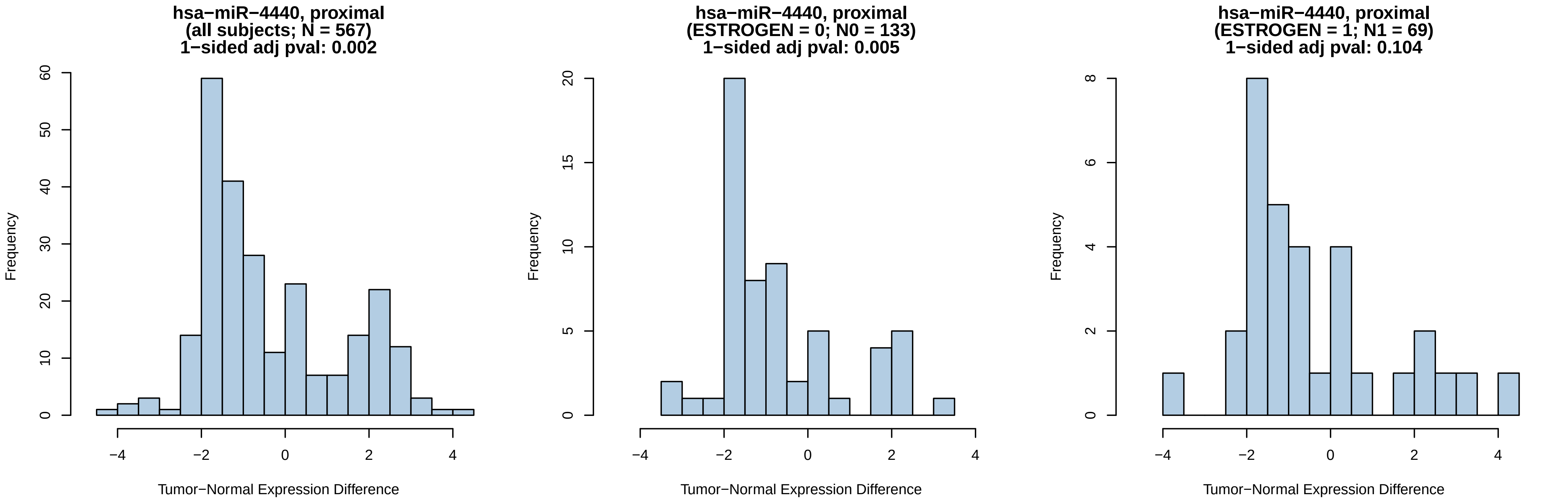

hsa−miR−4440, proximal
(all subjects; N = 567)
1−sided adj pval: 0.002
Frequency
Tumor−Normal Expression Difference
hsa−miR−4440, proximal
(ESTROGEN = 0; N0 = 133)
1−sided adj pval: 0.005
Frequency
Tumor−Normal Expression Difference
hsa−miR−4440, proximal
(ESTROGEN = 1; N1 = 69)
1−sided adj pval: 0.104
Frequency
Tumor−Normal Expression Difference

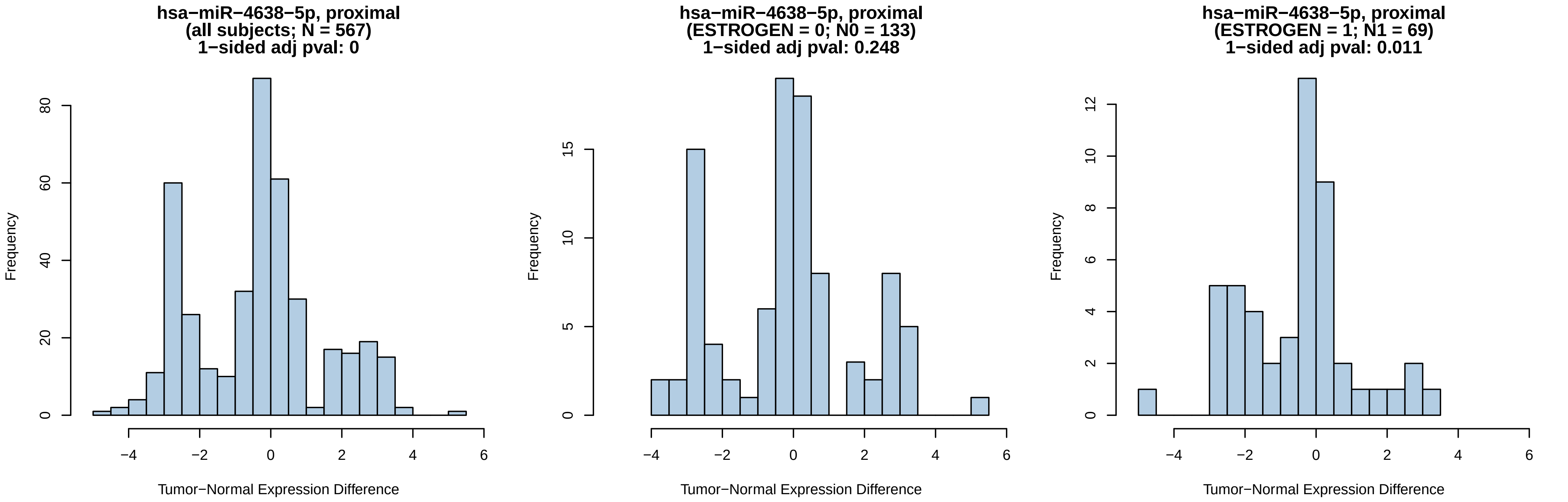

hsa−miR−4638−5p, proximal
(all subjects; N = 567)
1−sided adj pval: 0
hsa−miR−4638−5p, proximal
(ESTROGEN = 0; N0 = 133)
1−sided adj pval: 0.248
hsa−miR−4638−5p, proximal
(ESTROGEN = 1; N1 = 69)
1−sided adj pval: 0.011
Frequency
Tumor−Normal Expression Difference

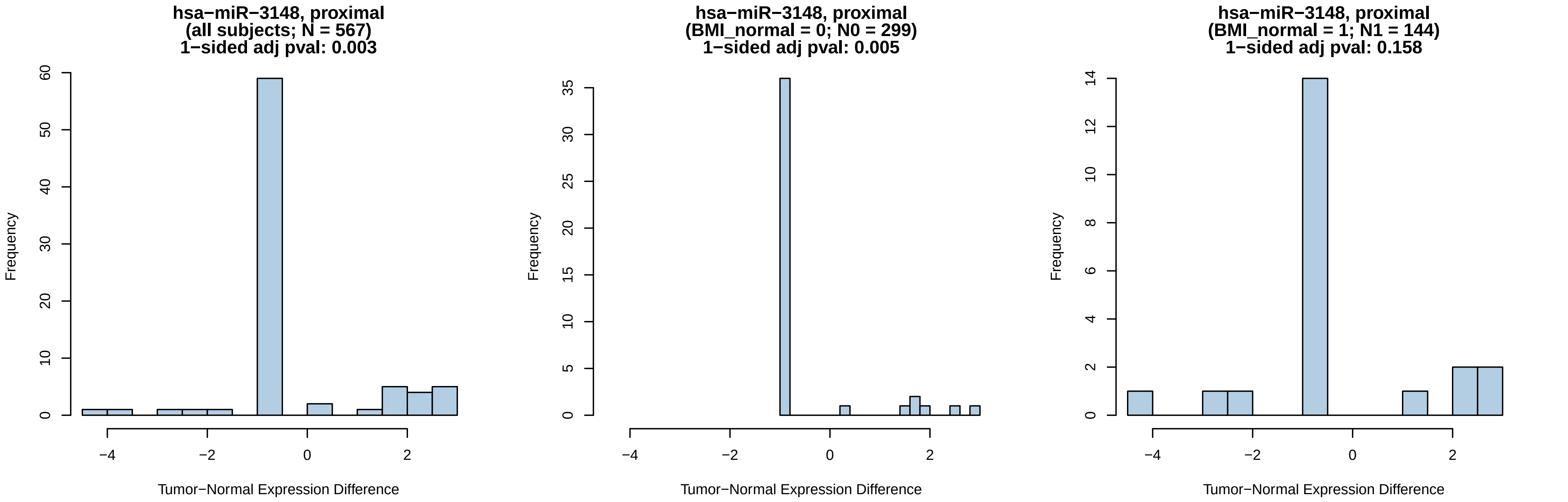

hsa−miR−3148, proximal
(all subjects; N = 567)
1−sided adj pval: 0.003
Frequency
Tumor−Normal Expression Difference
hsa−miR−3148, proximal
(BMI_normal = 0; N0 = 299)
1−sided adj pval: 0.005
Frequency
Tumor−Normal Expression Difference
hsa−miR−3148, proximal
(BMI_normal = 1; N1 = 144)
1−sided adj pval: 0.158
Frequency
Tumor−Normal Expression Difference

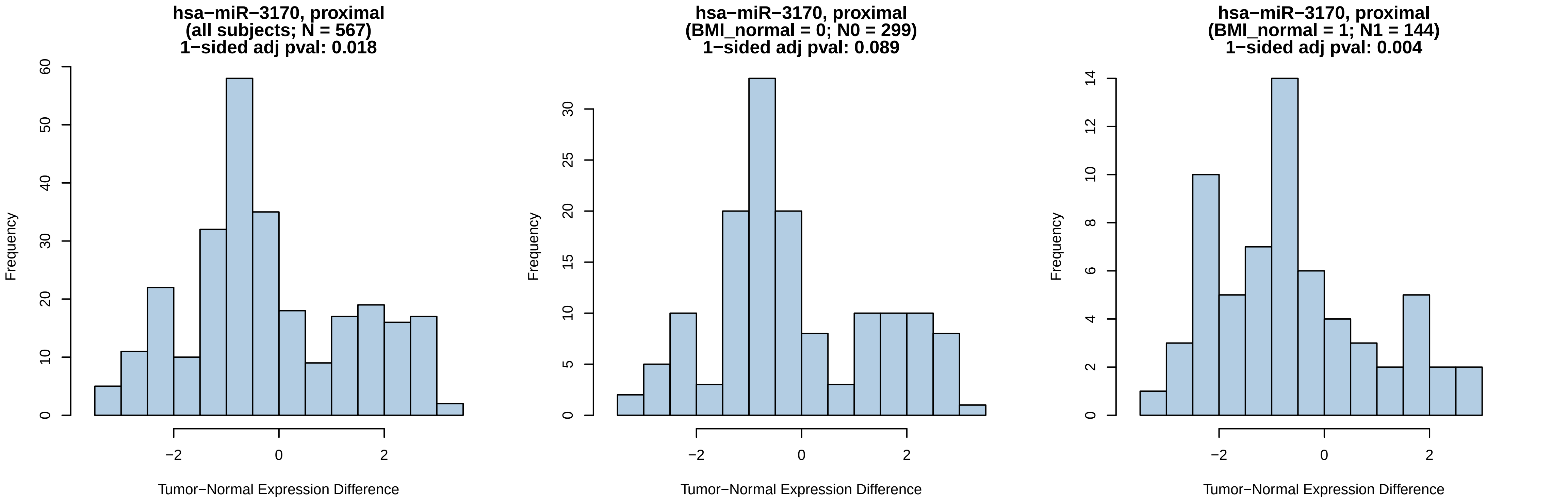

hsa−miR−3170, proximal
(all subjects; N = 567)
1−sided adj pval: 0.018
Frequency
0
10
20
30
40
50
60
−2
0
2
Tumor−Normal Expression Difference
hsa−miR−3170, proximal
(BMI_normal = 0; N0 = 299)
1−sided adj pval: 0.089
Frequency
0
5
10
15
20
25
30
−2
0
2
Tumor−Normal Expression Difference
hsa−miR−3170, proximal
(BMI_normal = 1; N1 = 144)
1−sided adj pval: 0.004
Frequency
0
2
4
6
8
10
12
14
−2
0
2
Tumor−Normal Expression Difference

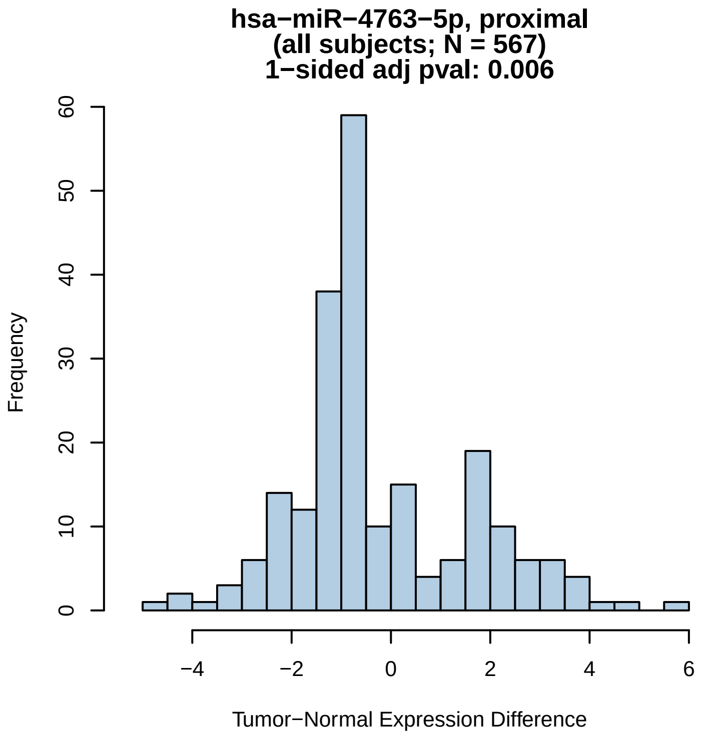
hsa−miR−4763−5p, proximal
(all subjects; N = 567)
1−sided adj pval: 0.006
Frequency
Tumor−Normal Expression Difference

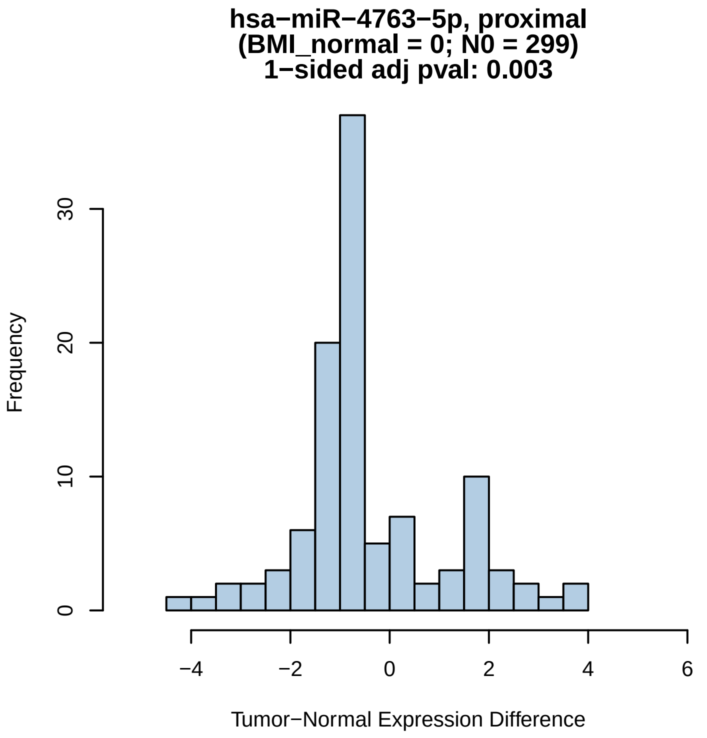
hsa−miR−4763−5p, proximal
(BMI_normal = 0; N0 = 299)
1−sided adj pval: 0.003
Frequency
Tumor−Normal Expression Difference

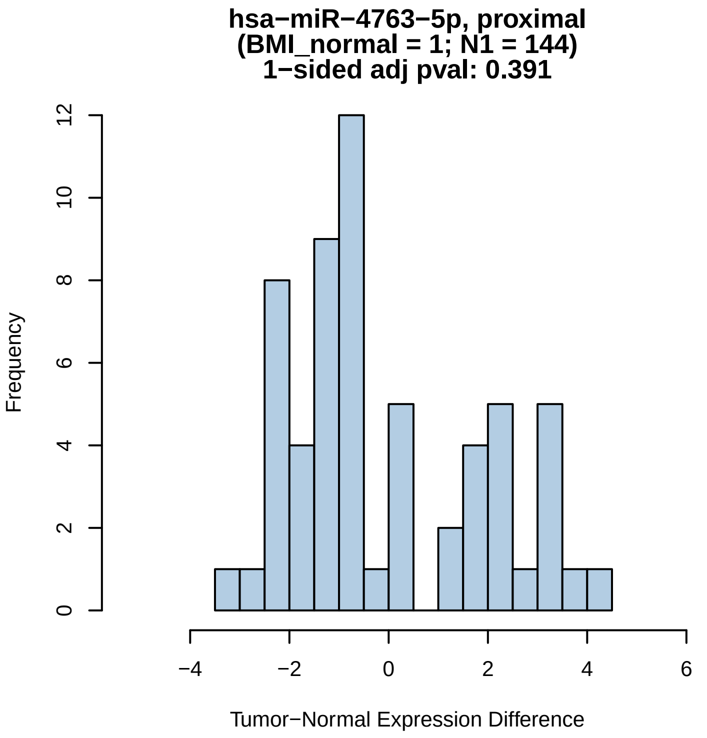
hsa−miR−4763−5p, proximal
(BMI_normal = 1; N1 = 144)
1−sided adj pval: 0.391
Frequency
Tumor−Normal Expression Difference

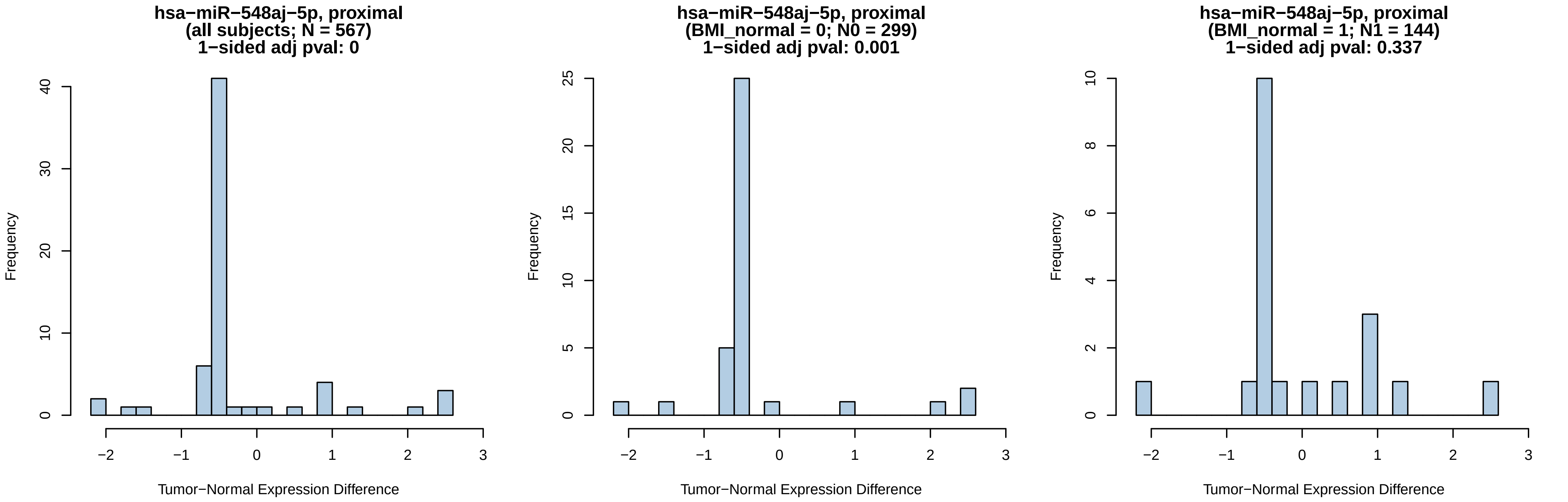
hsa−miR−548aj−5p, proximal
(all subjects; N = 567)
1−sided adj pval: 0
Frequency
Tumor−Normal Expression Difference
hsa−miR−548aj−5p, proximal
(BMI_normal = 0; N0 = 299)
1−sided adj pval: 0.001
Frequency
Tumor−Normal Expression Difference
hsa−miR−548aj−5p, proximal
(BMI_normal = 1; N1 = 144)
1−sided adj pval: 0.337
Frequency
Tumor−Normal Expression Difference

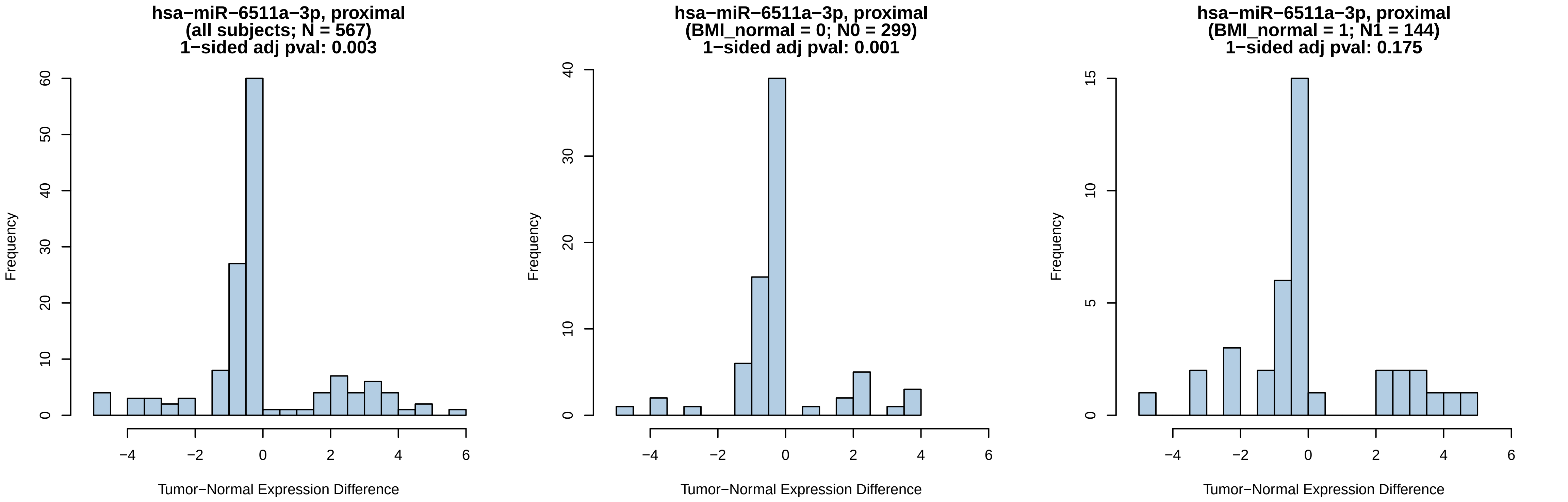
hsa−miR−6511a−3p, proximal
(all subjects; N = 567)
1−sided adj pval: 0.003
hsa−miR−6511a−3p, proximal
(BMI_normal = 0; N0 = 299)
1−sided adj pval: 0.001
hsa−miR−6511a−3p, proximal
(BMI_normal = 1; N1 = 144)
1−sided adj pval: 0.175
Frequency
Tumor−Normal Expression Difference

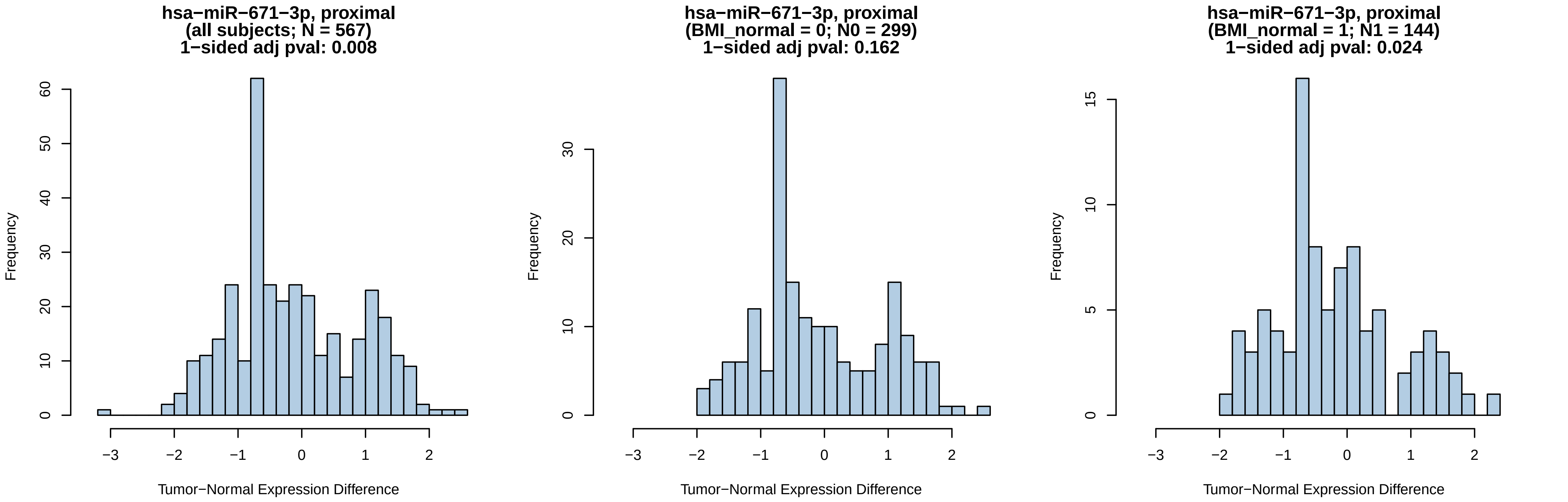
hsa−miR−671−3p, proximal
(all subjects; N = 567)
1−sided adj pval: 0.008
Frequency
Tumor−Normal Expression Difference
hsa−miR−671−3p, proximal
(BMI_normal = 0; N0 = 299)
1−sided adj pval: 0.162
Frequency
Tumor−Normal Expression Difference
hsa−miR−671−3p, proximal
(BMI_normal = 1; N1 = 144)
1−sided adj pval: 0.024
Frequency
Tumor−Normal Expression Difference

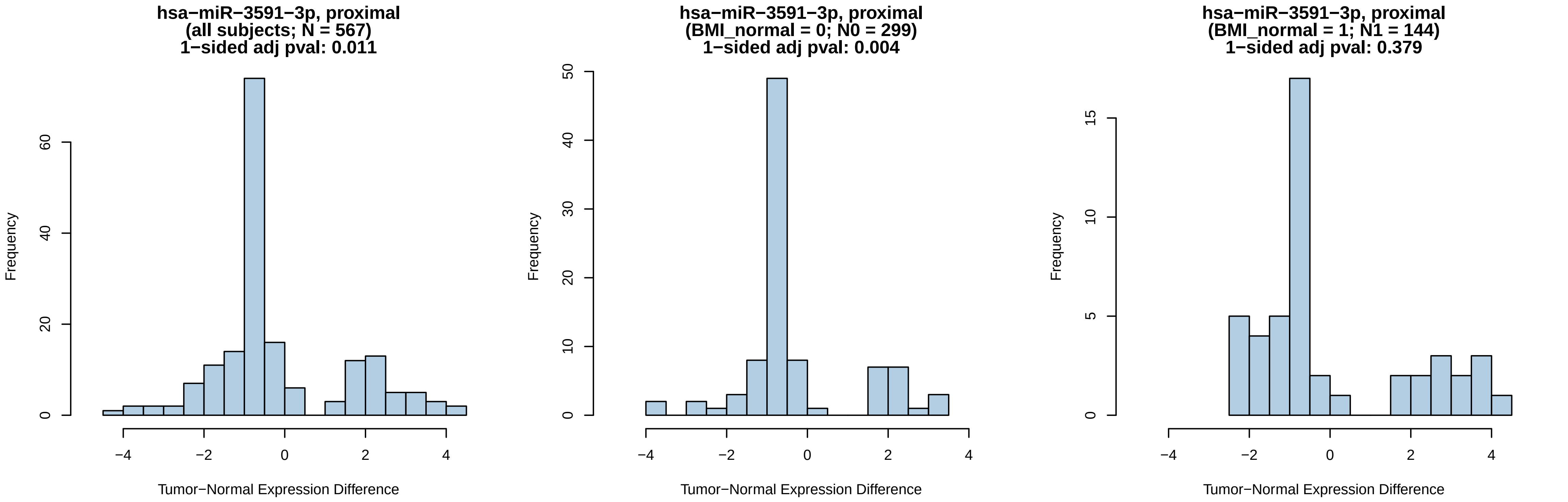

hsa−miR−3591−3p, proximal
(all subjects; N = 567)
1−sided adj pval: 0.011
Frequency
0
20
40
60
−4
−2
0
2
4
Tumor−Normal Expression Difference
hsa−miR−3591−3p, proximal
(BMI_normal = 0; N0 = 299)
1−sided adj pval: 0.004
Frequency
0
10
20
30
40
50
−4
−2
0
2
4
Tumor−Normal Expression Difference
hsa−miR−3591−3p, proximal
(BMI_normal = 1; N1 = 144)
1−sided adj pval: 0.379
Frequency
0
5
10
15
−4
−2
0
2
4
Tumor−Normal Expression Difference

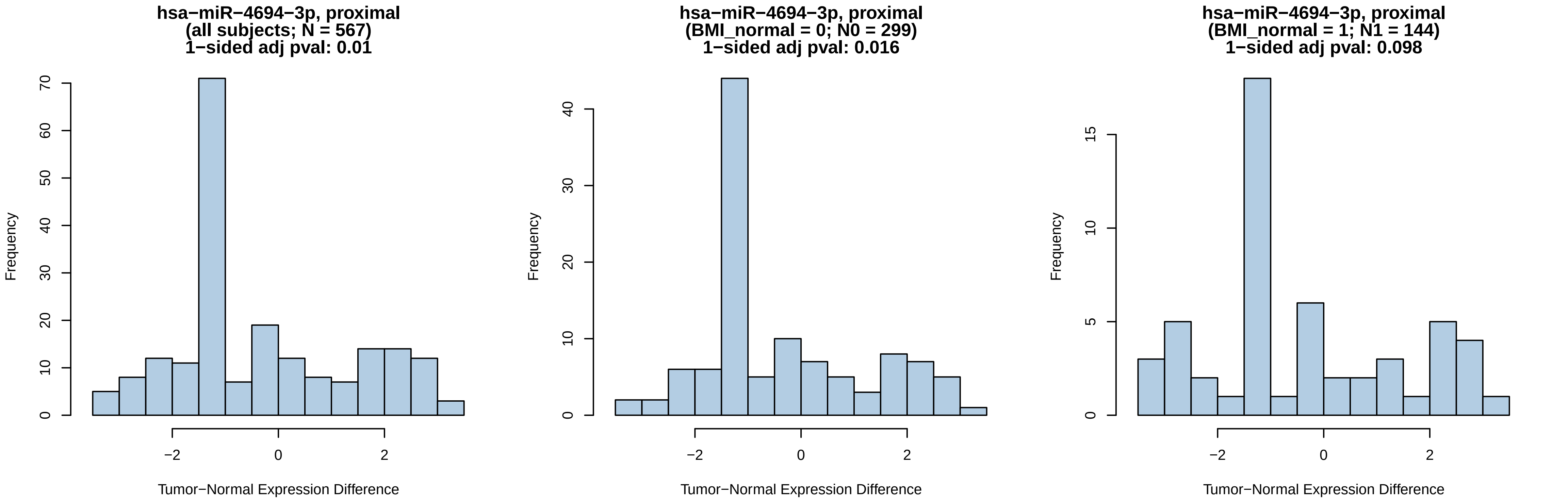

hsa−miR−4694−3p, proximal
(all subjects; N = 567)
1−sided adj pval: 0.01
hsa−miR−4694−3p, proximal
(BMI_normal = 0; N0 = 299)
1−sided adj pval: 0.016
hsa−miR−4694−3p, proximal
(BMI_normal = 1; N1 = 144)
1−sided adj pval: 0.098
Frequency
Tumor−Normal Expression Difference

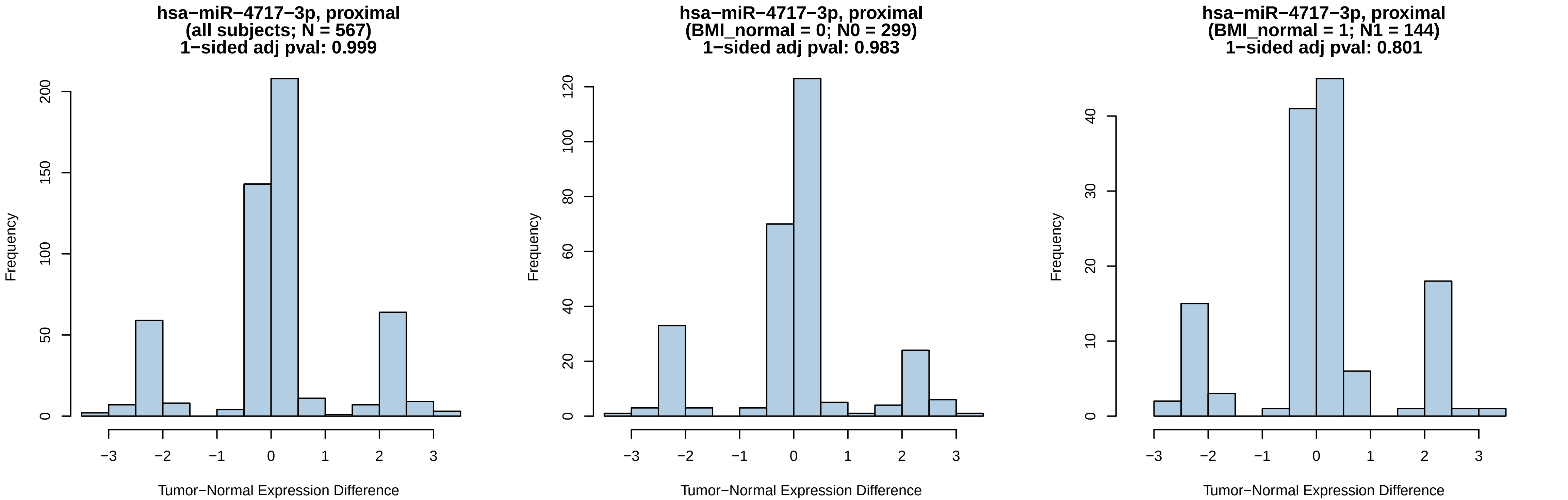
hsa−miR−4717−3p, proximal
(all subjects; N = 567)
1−sided adj pval: 0.999
Frequency
Tumor−Normal Expression Difference
hsa−miR−4717−3p, proximal
(BMI_normal = 0; N0 = 299)
1−sided adj pval: 0.983
Frequency
Tumor−Normal Expression Difference
hsa−miR−4717−3p, proximal
(BMI_normal = 1; N1 = 144)
1−sided adj pval: 0.801
Frequency
Tumor−Normal Expression Difference

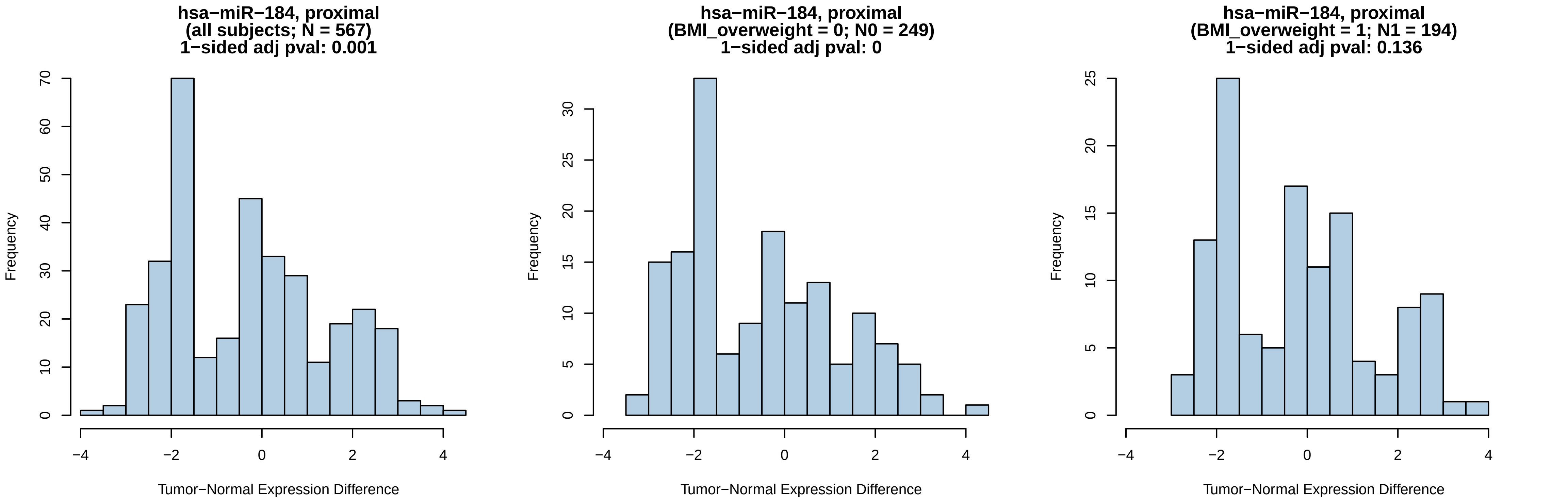
hsa−miR−184, proximal
(all subjects; N = 567)
1−sided adj pval: 0.001
Frequency
Tumor−Normal Expression Difference
hsa−miR−184, proximal
(BMI_overweight = 0; N0 = 249)
1−sided adj pval: 0
Frequency
Tumor−Normal Expression Difference
hsa−miR−184, proximal
(BMI_overweight = 1; N1 = 194)
1−sided adj pval: 0.136
Frequency
Tumor−Normal Expression Difference

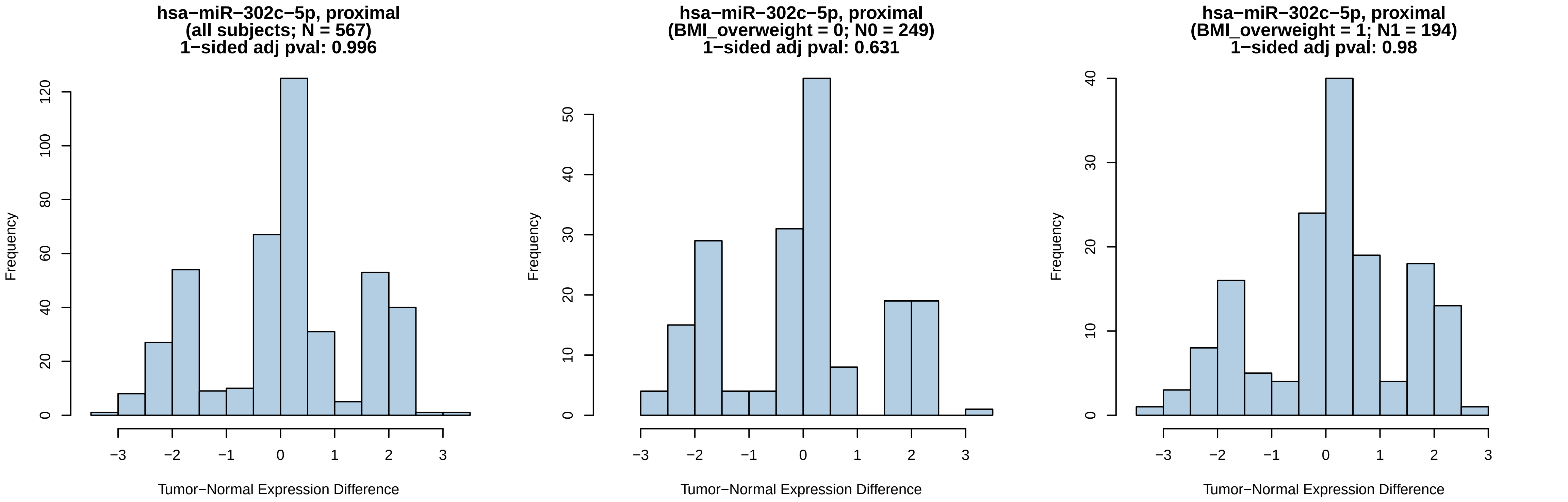

hsa−miR−302c−5p, proximal
(all subjects; N = 567)
1−sided adj pval: 0.996
hsa−miR−302c−5p, proximal
(BMI_overweight = 0; N0 = 249)
1−sided adj pval: 0.631
hsa−miR−302c−5p, proximal
(BMI_overweight = 1; N1 = 194)
1−sided adj pval: 0.98
Frequency
Tumor−Normal Expression Difference

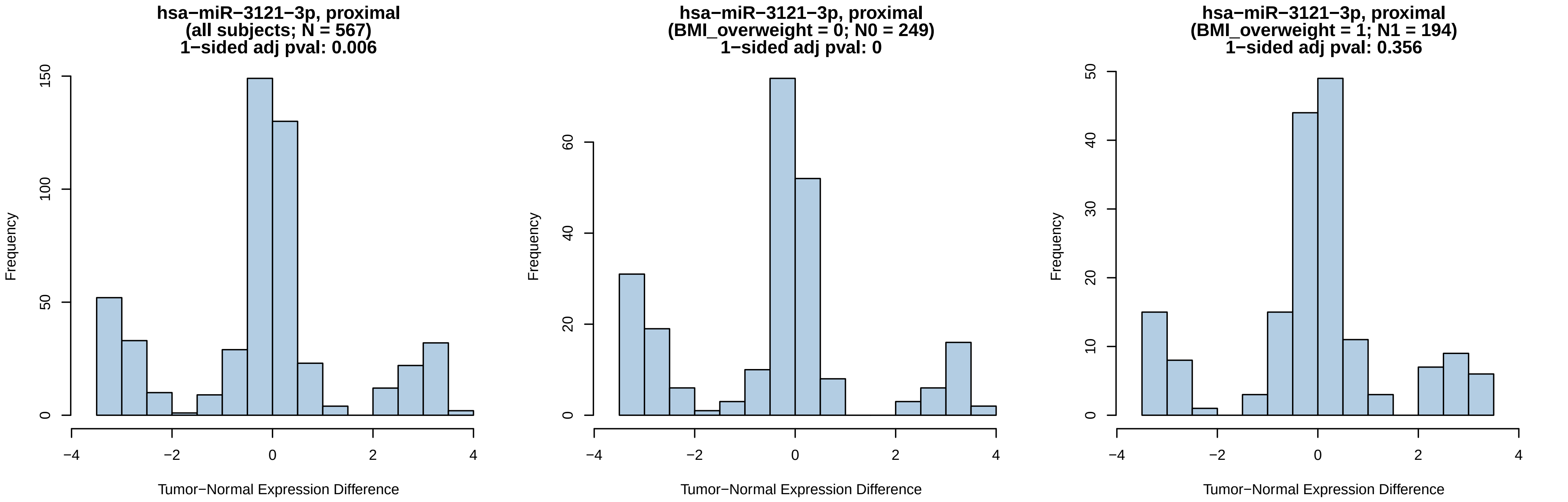

hsa−miR−3121−3p, proximal
(all subjects; N = 567)
1−sided adj pval: 0.006
Frequency
0
50
100
150
−4
−2
0
2
4
Tumor−Normal Expression Difference
hsa−miR−3121−3p, proximal
(BMI_overweight = 0; N0 = 249)
1−sided adj pval: 0
Frequency
0
20
40
60
−4
−2
0
2
4
Tumor−Normal Expression Difference
hsa−miR−3121−3p, proximal
(BMI_overweight = 1; N1 = 194)
1−sided adj pval: 0.356
Frequency
0
10
20
30
40
50
−4
−2
0
2
4
Tumor−Normal Expression Difference

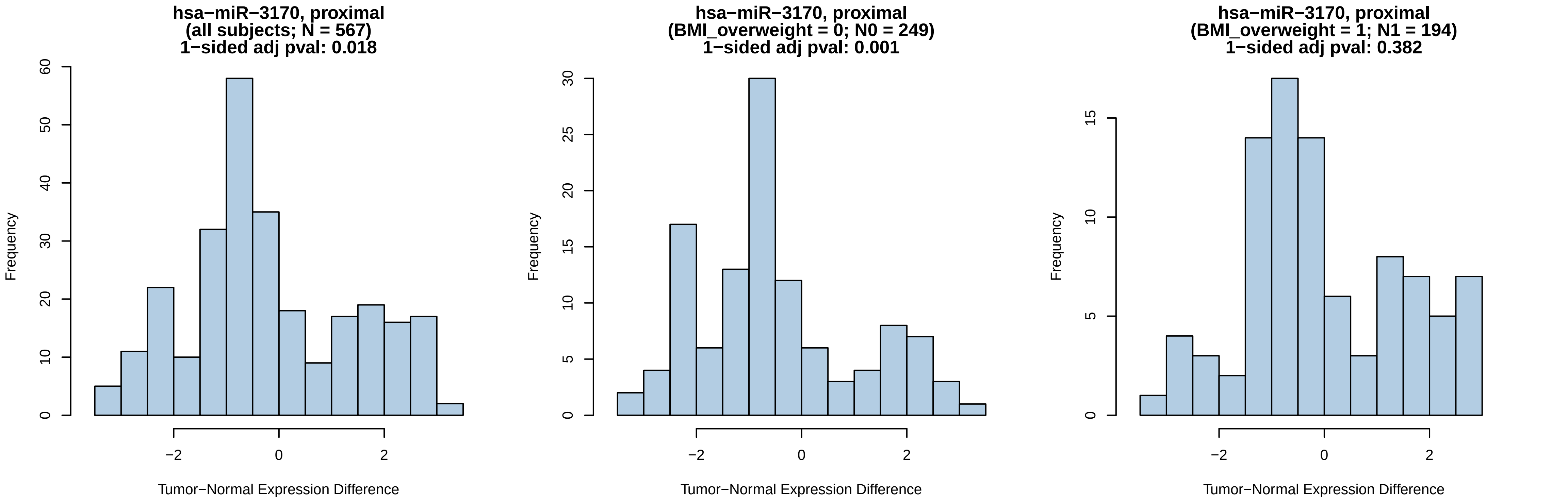

hsa−miR−3170, proximal
(all subjects; N = 567)
1−sided adj pval: 0.018
Frequency
0
10
20
30
40
50
60
−2
0
2
Tumor−Normal Expression Difference
hsa−miR−3170, proximal
(BMI_overweight = 0; N0 = 249)
1−sided adj pval: 0.001
Frequency
0
5
10
15
20
25
30
−2
0
2
Tumor−Normal Expression Difference
hsa−miR−3170, proximal
(BMI_overweight = 1; N1 = 194)
1−sided adj pval: 0.382
Frequency
0
5
10
15
−2
0
2
Tumor−Normal Expression Difference

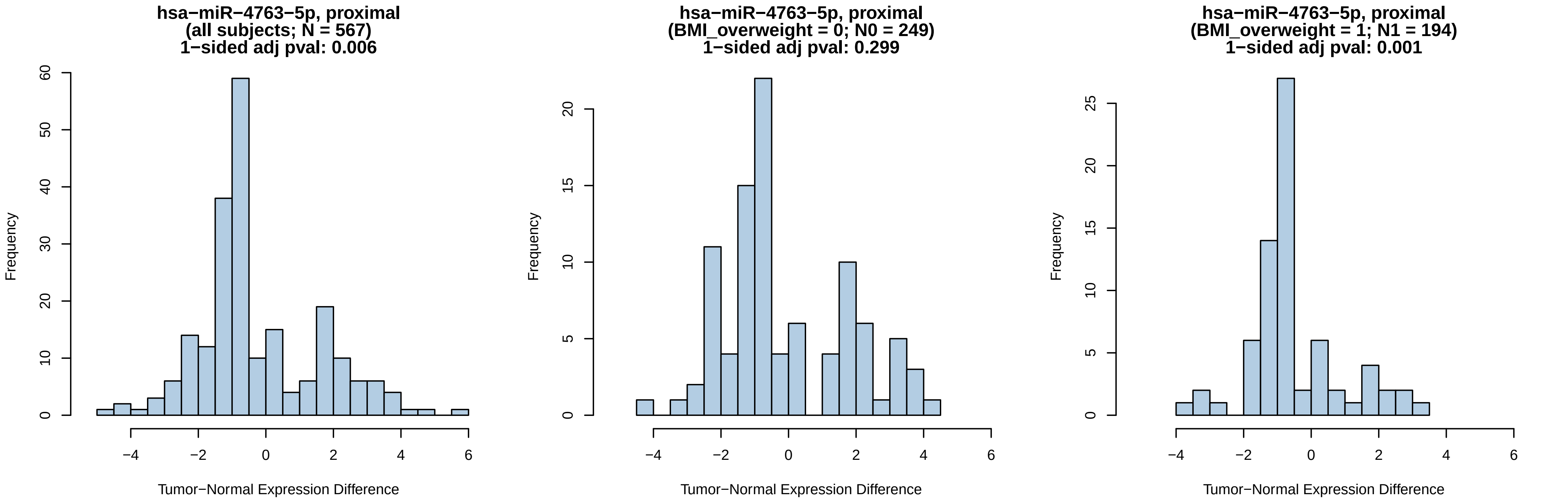
hsa−miR−4763−5p, proximal
(all subjects; N = 567)
1−sided adj pval: 0.006
hsa−miR−4763−5p, proximal
(BMI_overweight = 0; N0 = 249)
1−sided adj pval: 0.299
hsa−miR−4763−5p, proximal
(BMI_overweight = 1; N1 = 194)
1−sided adj pval: 0.001
Frequency
Tumor−Normal Expression Difference

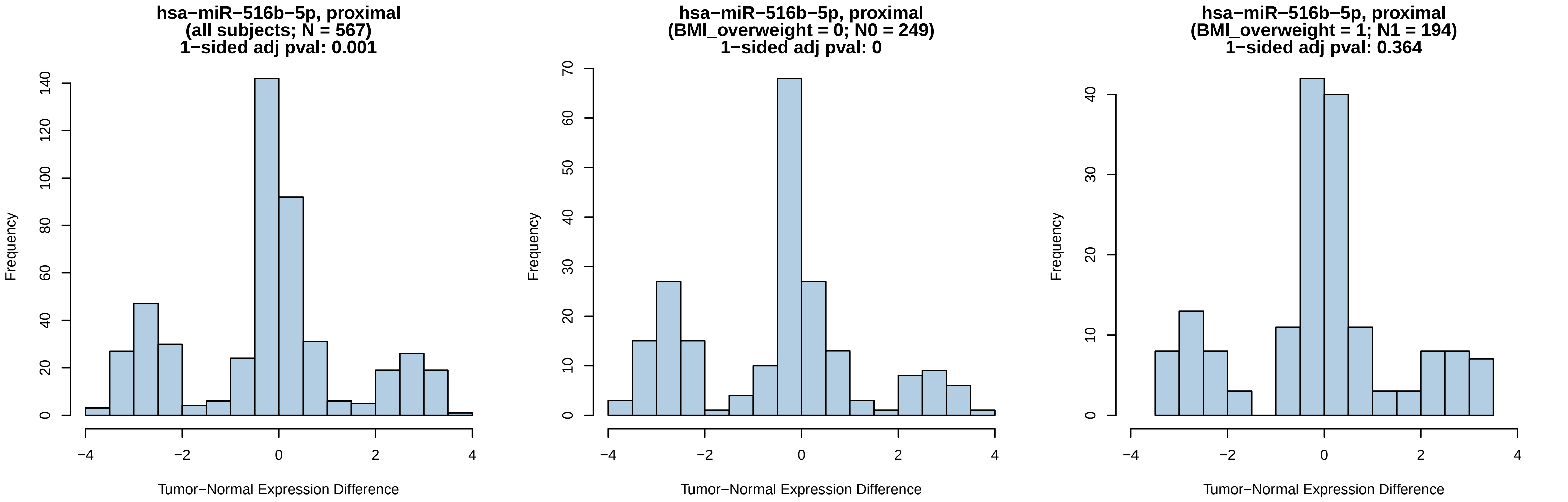
hsa−miR−516b−5p, proximal
(all subjects; N = 567)
1−sided adj pval: 0.001
hsa−miR−516b−5p, proximal
(BMI_overweight = 0; N0 = 249)
1−sided adj pval: 0
hsa−miR−516b−5p, proximal
(BMI_overweight = 1; N1 = 194)
1−sided adj pval: 0.364
Frequency
Tumor−Normal Expression Difference

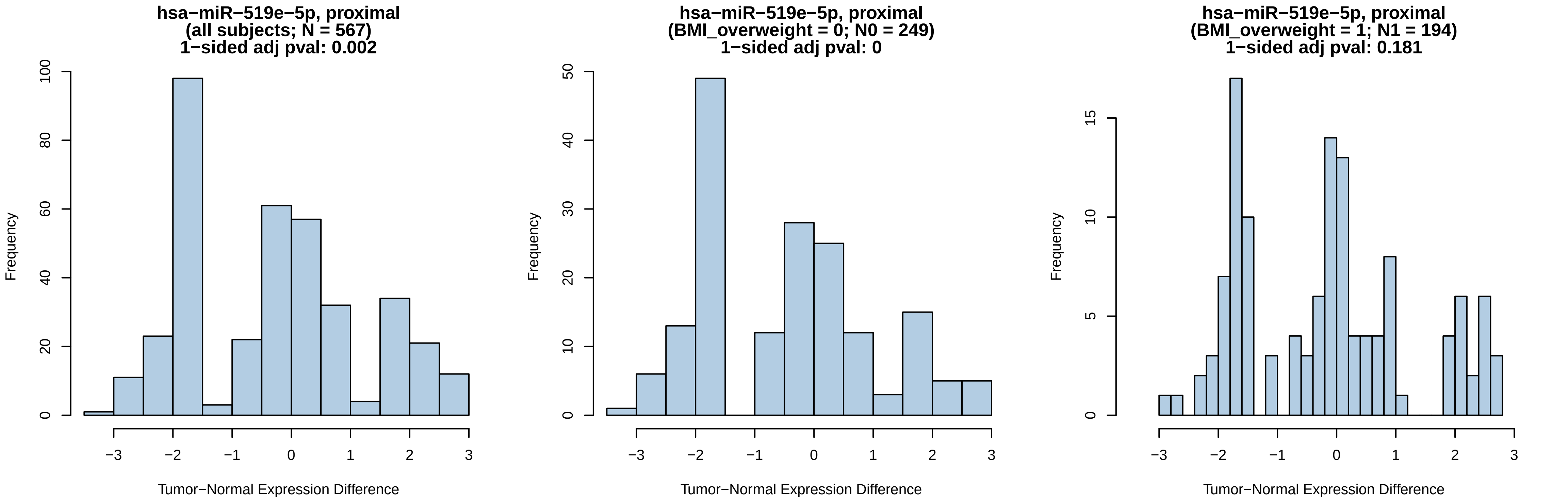

hsa−miR−519e−5p, proximal
(all subjects; N = 567)
1−sided adj pval: 0.002
hsa−miR−519e−5p, proximal
(BMI_overweight = 0; N0 = 249)
1−sided adj pval: 0
hsa−miR−519e−5p, proximal
(BMI_overweight = 1; N1 = 194)
1−sided adj pval: 0.181
Frequency
Tumor−Normal Expression Difference

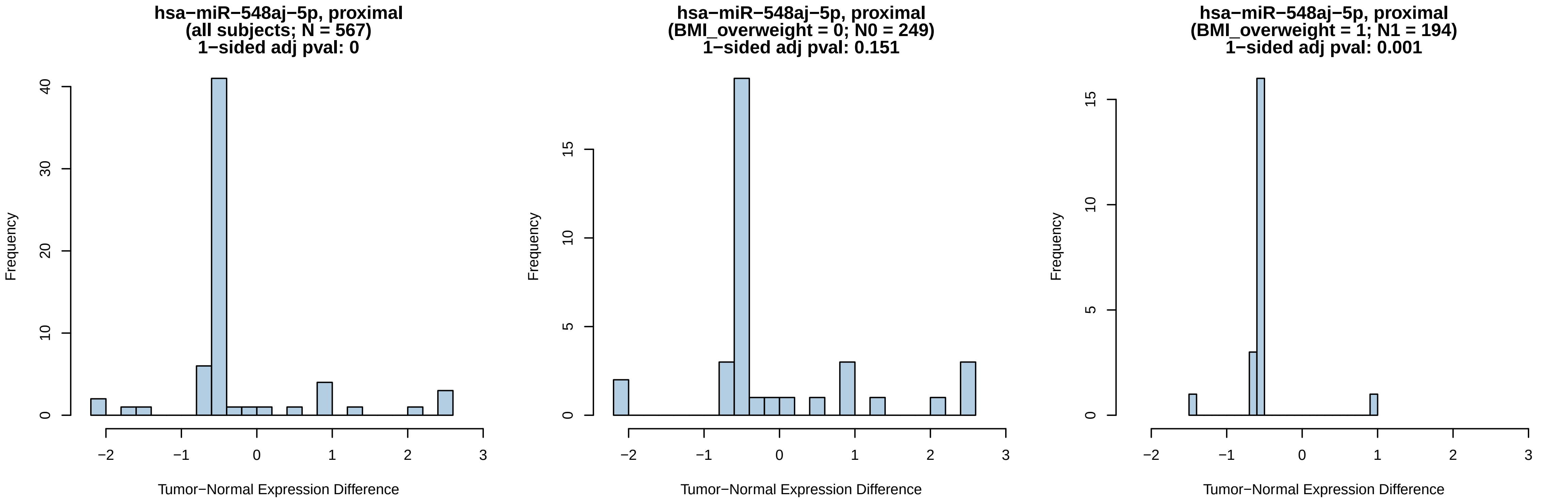
hsa−miR−548aj−5p, proximal
(all subjects; N = 567)
1−sided adj pval: 0
Frequency
Tumor−Normal Expression Difference
hsa−miR−548aj−5p, proximal
(BMI_overweight = 0; N0 = 249)
1−sided adj pval: 0.151
Frequency
Tumor−Normal Expression Difference
hsa−miR−548aj−5p, proximal
(BMI_overweight = 1; N1 = 194)
1−sided adj pval: 0.001
Frequency
Tumor−Normal Expression Difference

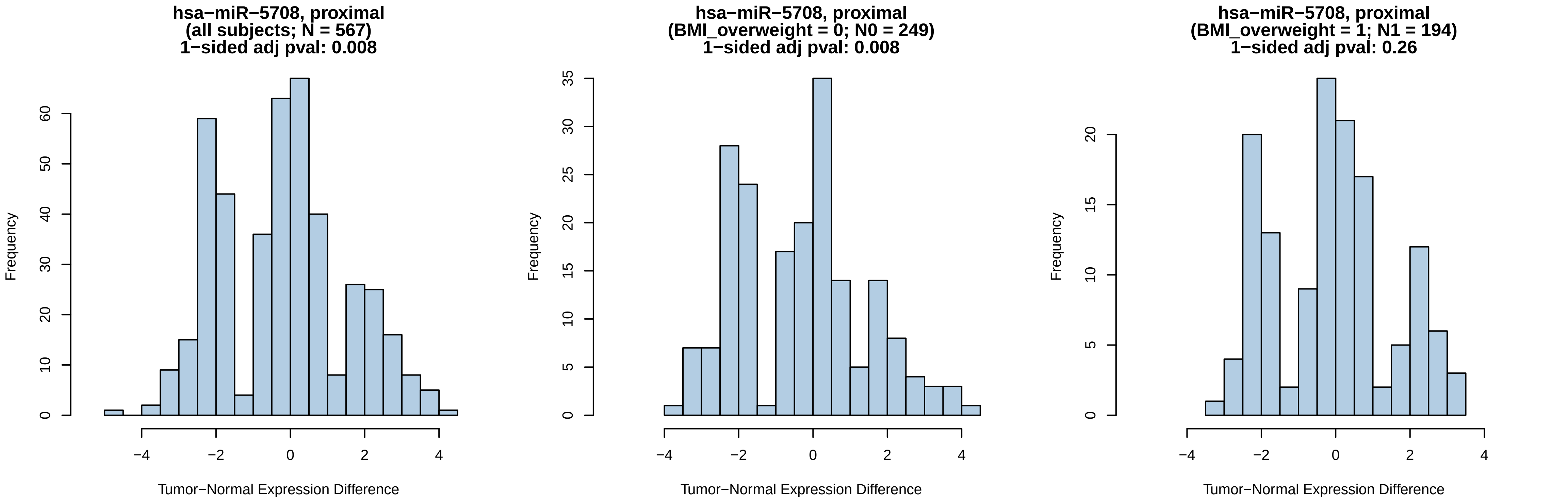
hsa−miR−5708, proximal
(all subjects; N = 567)
1−sided adj pval: 0.008
Frequency
Tumor−Normal Expression Difference
hsa−miR−5708, proximal
(BMI_overweight = 0; N0 = 249)
1−sided adj pval: 0.008
Frequency
Tumor−Normal Expression Difference
hsa−miR−5708, proximal
(BMI_overweight = 1; N1 = 194)
1−sided adj pval: 0.26
Frequency
Tumor−Normal Expression Difference

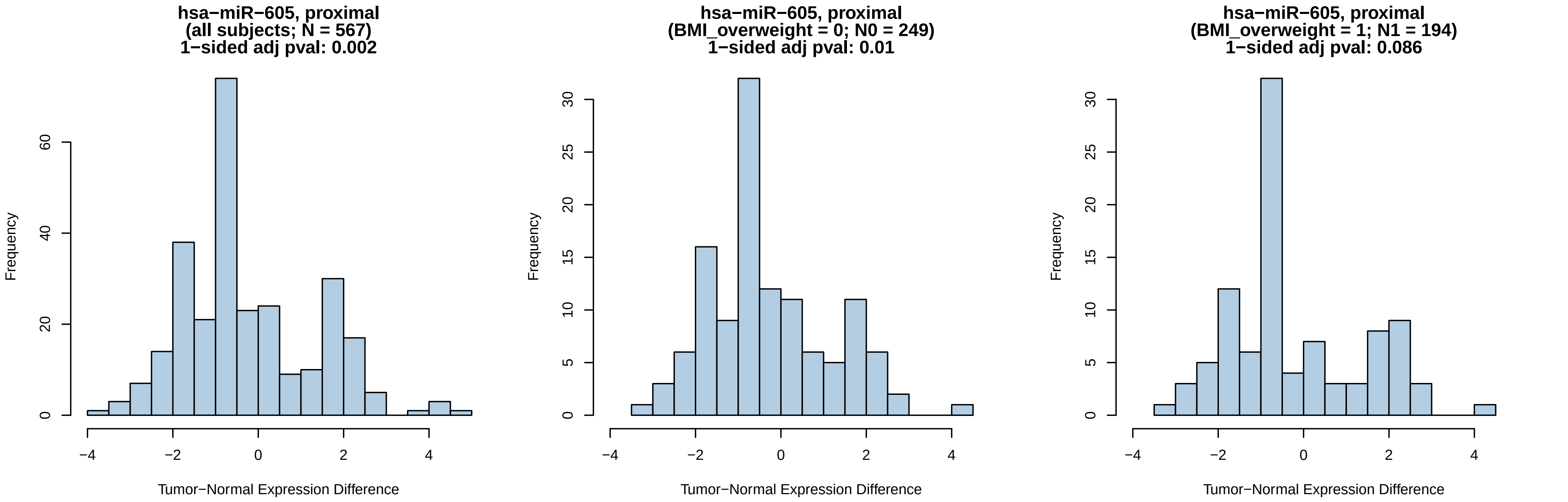
hsa−miR−605, proximal
(all subjects; N = 567)
1−sided adj pval: 0.002
Frequency
Tumor−Normal Expression Difference
hsa−miR−605, proximal
(BMI_overweight = 0; N0 = 249)
1−sided adj pval: 0.01
Frequency
Tumor−Normal Expression Difference
hsa−miR−605, proximal
(BMI_overweight = 1; N1 = 194)
1−sided adj pval: 0.086
Frequency
Tumor−Normal Expression Difference

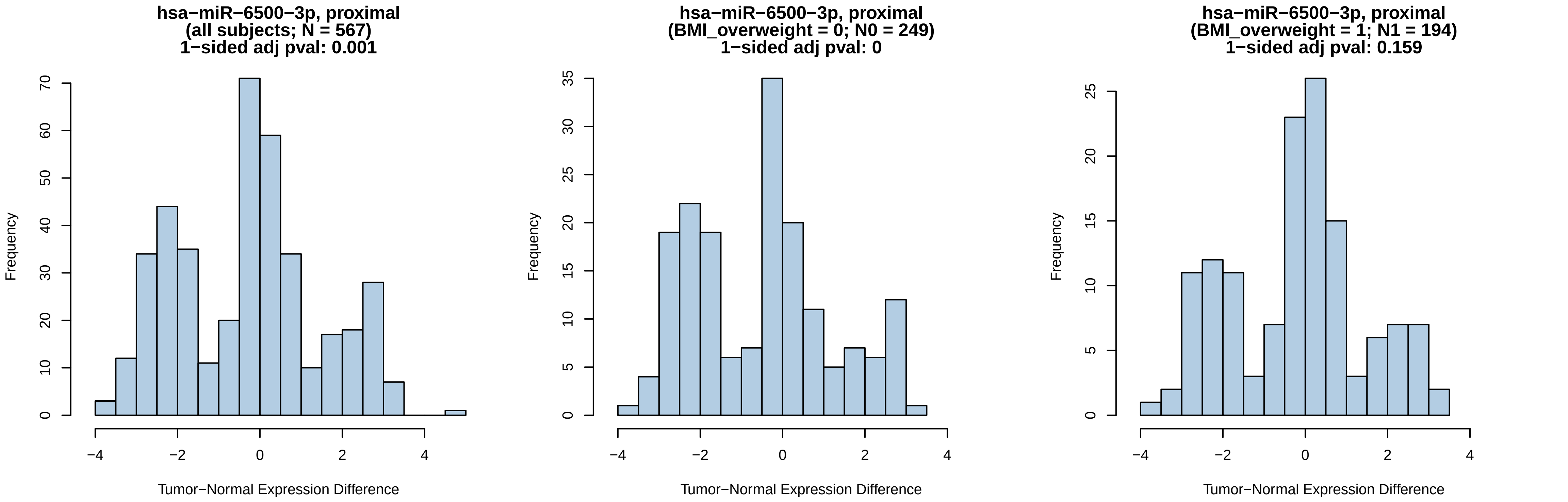
hsa−miR−6500−3p, proximal
(all subjects; N = 567)
1−sided adj pval: 0.001
Frequency
Tumor−Normal Expression Difference
hsa−miR−6500−3p, proximal
(BMI_overweight = 0; N0 = 249)
1−sided adj pval: 0
Frequency
Tumor−Normal Expression Difference
hsa−miR−6500−3p, proximal
(BMI_overweight = 1; N1 = 194)
1−sided adj pval: 0.159
Frequency
Tumor−Normal Expression Difference

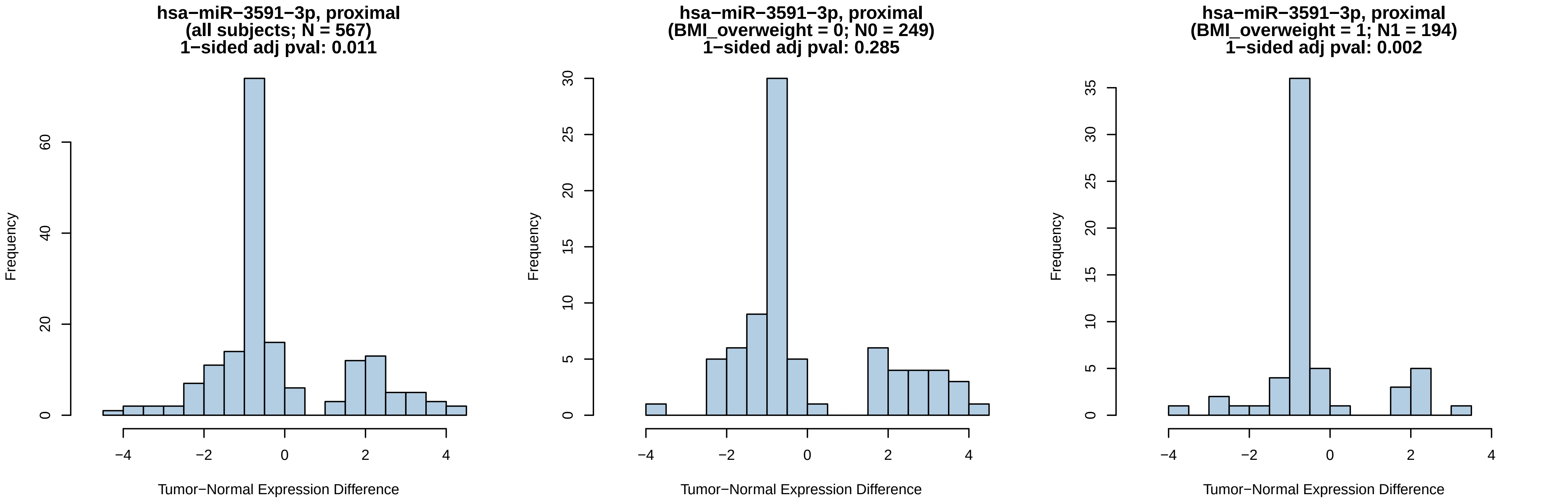
hsa−miR−3591−3p, proximal
(all subjects; N = 567)
1−sided adj pval: 0.011
Frequency
Tumor−Normal Expression Difference
hsa−miR−3591−3p, proximal
(BMI_overweight = 0; N0 = 249)
1−sided adj pval: 0.285
Frequency
Tumor−Normal Expression Difference
hsa−miR−3591−3p, proximal
(BMI_overweight = 1; N1 = 194)
1−sided adj pval: 0.002
Frequency
Tumor−Normal Expression Difference

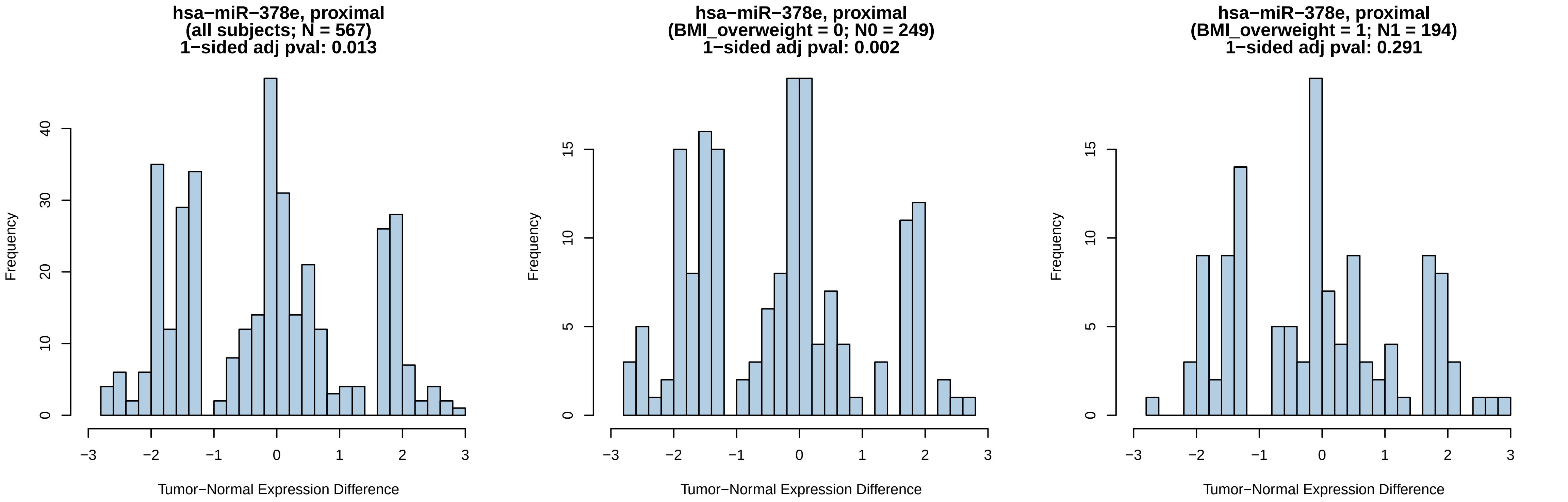
hsa−miR−378e, proximal
(all subjects; N = 567)
1−sided adj pval: 0.013
hsa−miR−378e, proximal
(BMI_overweight = 0; N0 = 249)
1−sided adj pval: 0.002
hsa−miR−378e, proximal
(BMI_overweight = 1; N1 = 194)
1−sided adj pval: 0.291
Frequency
Tumor−Normal Expression Difference

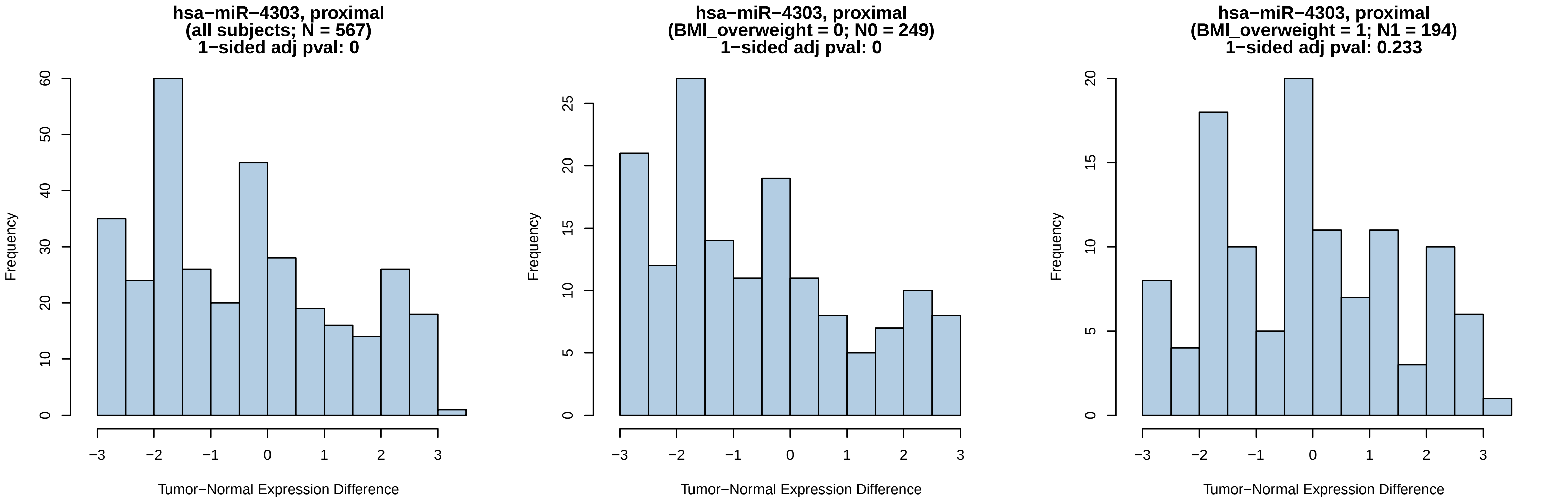
hsa−miR−4303, proximal
(all subjects; N = 567)
1−sided adj pval: 0
hsa−miR−4303, proximal
(BMI_overweight = 0; N0 = 249)
1−sided adj pval: 0
hsa−miR−4303, proximal
(BMI_overweight = 1; N1 = 194)
1−sided adj pval: 0.233
Frequency
Tumor−Normal Expression Difference

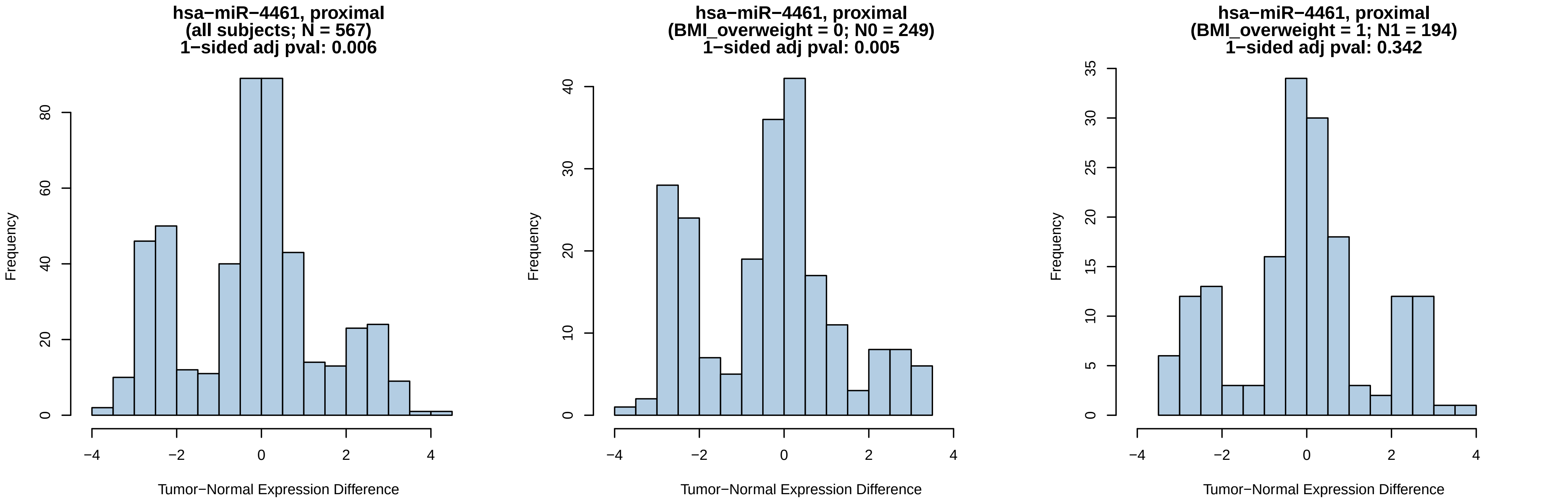
hsa−miR−4461, proximal
(all subjects; N = 567)
1−sided adj pval: 0.006
Frequency
Tumor−Normal Expression Difference
hsa−miR−4461, proximal
(BMI_overweight = 0; N0 = 249)
1−sided adj pval: 0.005
Frequency
Tumor−Normal Expression Difference
hsa−miR−4461, proximal
(BMI_overweight = 1; N1 = 194)
1−sided adj pval: 0.342
Frequency
Tumor−Normal Expression Difference

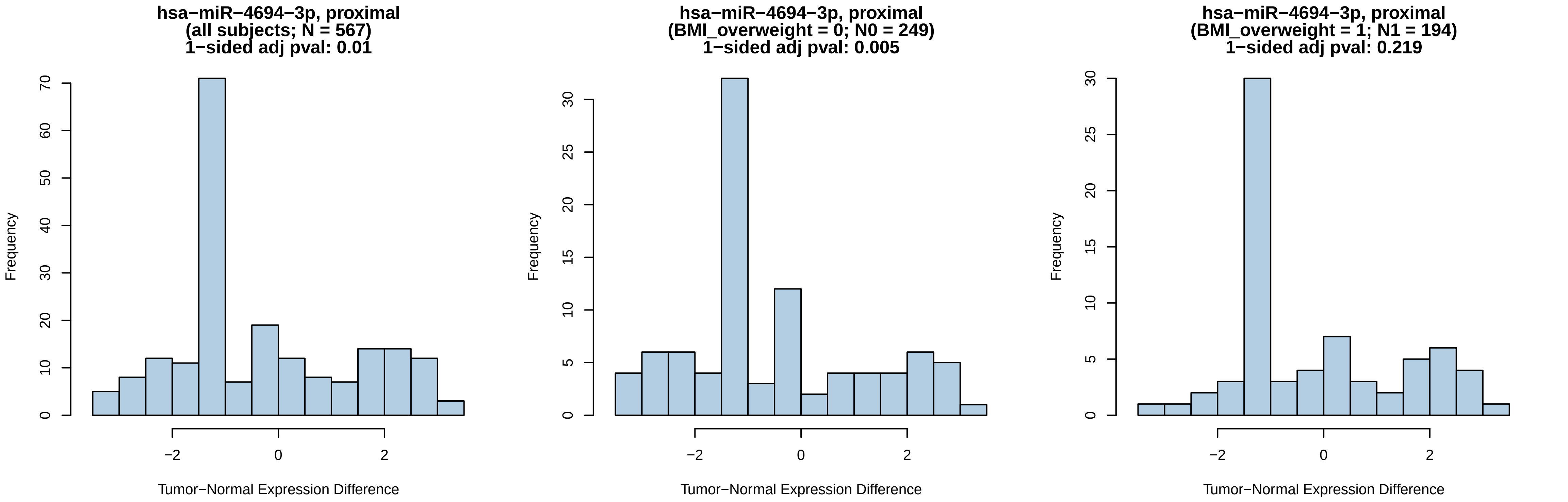

hsa−miR−4694−3p, proximal
(all subjects; N = 567)
1−sided adj pval: 0.01
Frequency
Tumor−Normal Expression Difference
hsa−miR−4694−3p, proximal
(BMI_overweight = 0; N0 = 249)
1−sided adj pval: 0.005
Frequency
Tumor−Normal Expression Difference
hsa−miR−4694−3p, proximal
(BMI_overweight = 1; N1 = 194)
1−sided adj pval: 0.219
Frequency
Tumor−Normal Expression Difference

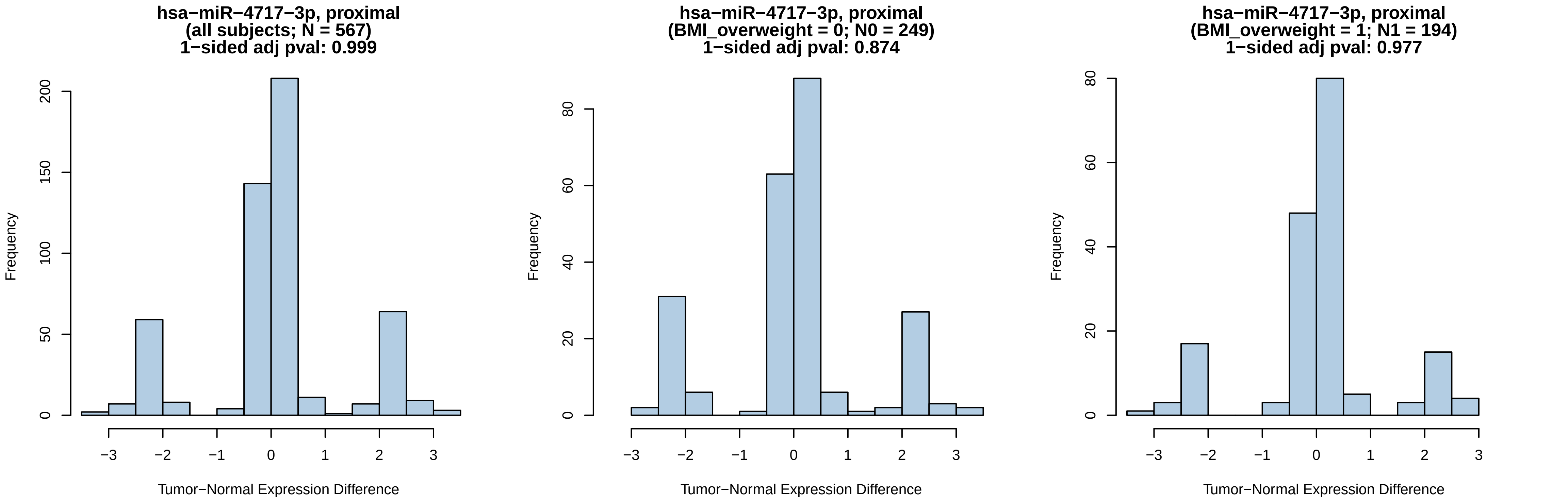
hsa−miR−4717−3p, proximal
(all subjects; N = 567)
1−sided adj pval: 0.999
Frequency
0
50
100
150
200
−3
−2
−1
0
1
2
3
Tumor−Normal Expression Difference
hsa−miR−4717−3p, proximal
(BMI_overweight = 0; N0 = 249)
1−sided adj pval: 0.874
Frequency
0
20
40
60
80
−3
−2
−1
0
1
2
3
Tumor−Normal Expression Difference
hsa−miR−4717−3p, proximal
(BMI_overweight = 1; N1 = 194)
1−sided adj pval: 0.977
Frequency
0
20
40
60
80
−3
−2
−1
0
1
2
3
Tumor−Normal Expression Difference

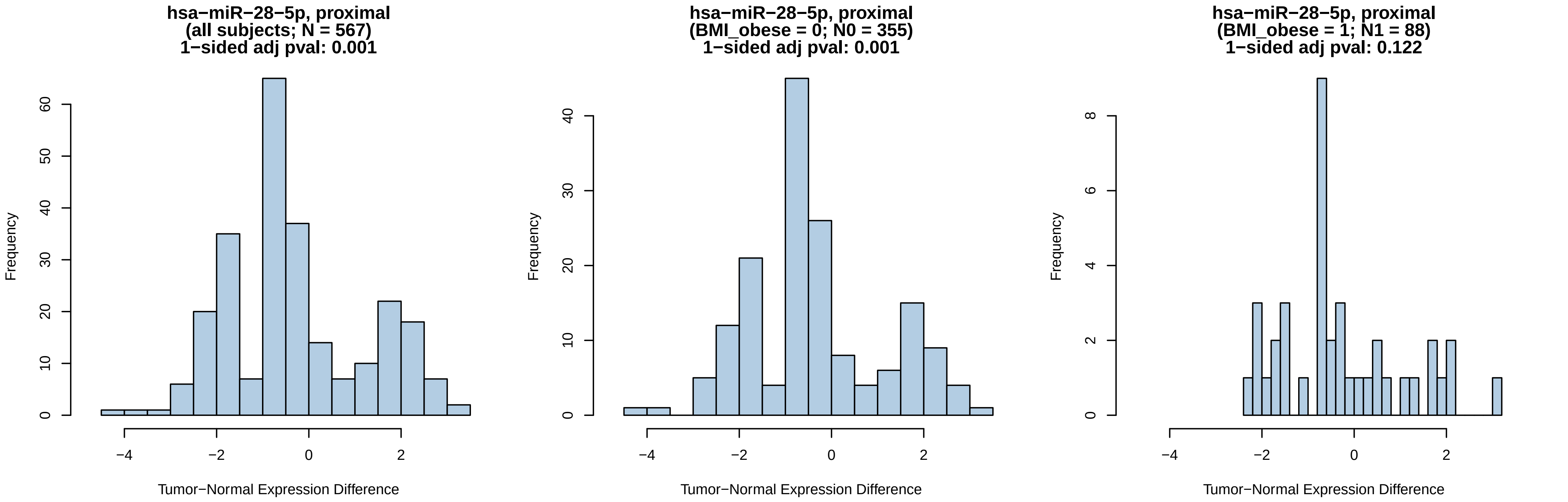

hsa−miR−28−5p, proximal
(all subjects; N = 567)
1−sided adj pval: 0.001
hsa−miR−28−5p, proximal
(BMI_obese = 0; N0 = 355)
1−sided adj pval: 0.001
hsa−miR−28−5p, proximal
(BMI_obese = 1; N1 = 88)
1−sided adj pval: 0.122
Frequency
Tumor−Normal Expression Difference

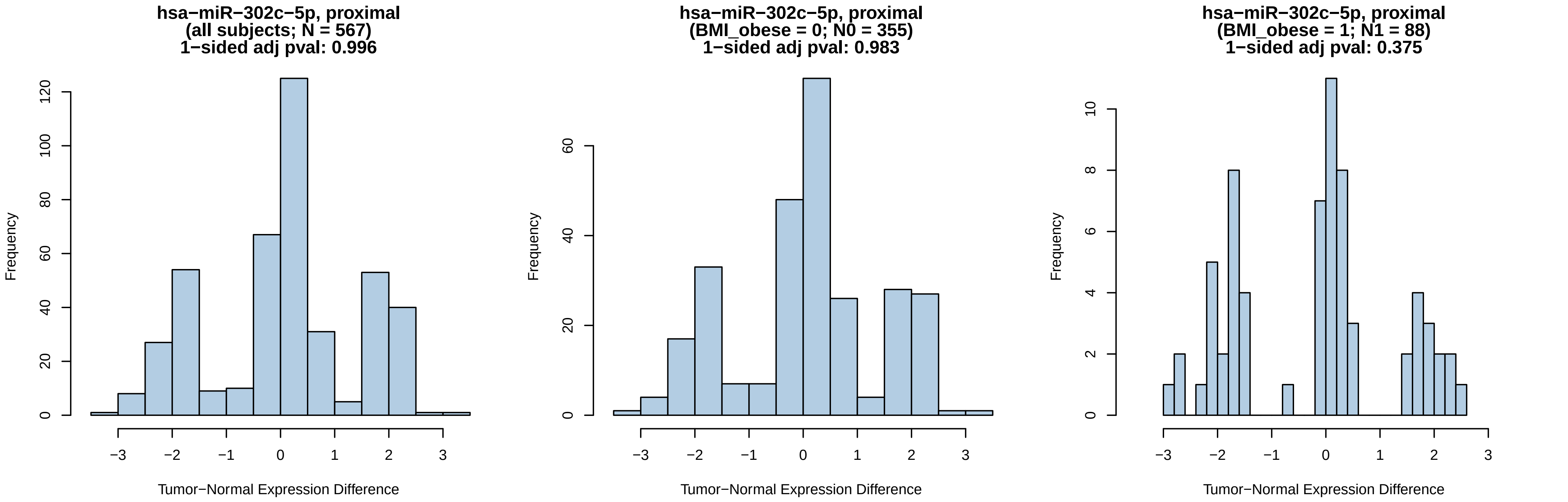
hsa−miR−302c−5p, proximal
(all subjects; N = 567)
1−sided adj pval: 0.996
Frequency
Tumor−Normal Expression Difference
hsa−miR−302c−5p, proximal
(BMI_obese = 0; N0 = 355)
1−sided adj pval: 0.983
Frequency
Tumor−Normal Expression Difference
hsa−miR−302c−5p, proximal
(BMI_obese = 1; N1 = 88)
1−sided adj pval: 0.375
Frequency
Tumor−Normal Expression Difference

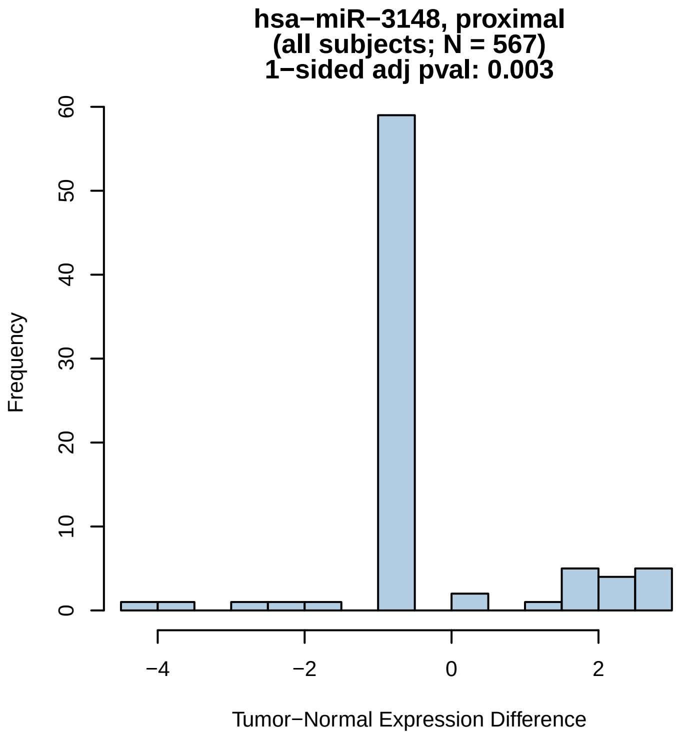

hsa−miR−3148, proximal
(all subjects; N = 567)
1−sided adj pval: 0.003
Frequency
0
10
20
30
40
50
60
−4
−2
0
2
Tumor−Normal Expression Difference


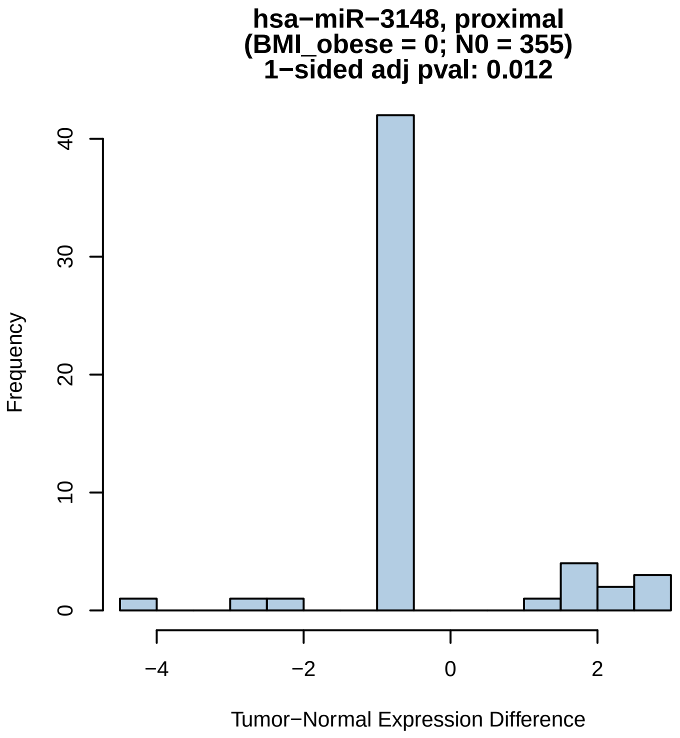

hsa−miR−3148, proximal
(BMI_obese = 0; N0 = 355)
1−sided adj pval: 0.012
Frequency
0
10
20
30
40
−4
−2
0
2
Tumor−Normal Expression Difference


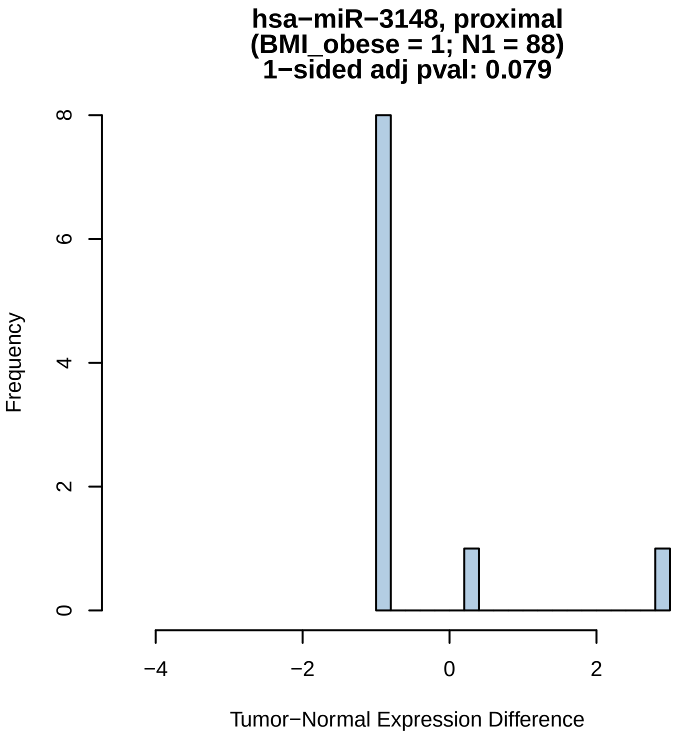

hsa−miR−3148, proximal
(BMI_obese = 1; N1 = 88)
1−sided adj pval: 0.079
Frequency
0
2
4
6
8
−4
−2
0
2
Tumor−Normal Expression Difference

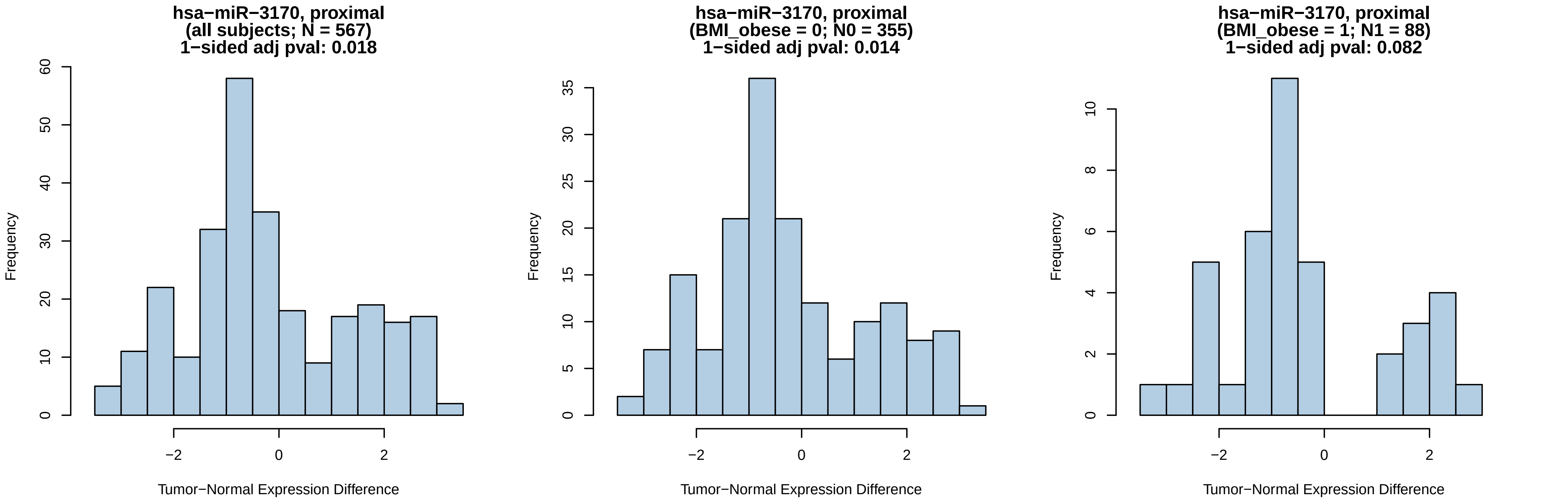

hsa−miR−3170, proximal
(all subjects; N = 567)
1−sided adj pval: 0.018
Frequency
0
10
20
30
40
50
60
−2
0
2
Tumor−Normal Expression Difference
hsa−miR−3170, proximal
(BMI_obese = 0; N0 = 355)
1−sided adj pval: 0.014
Frequency
0
5
10
15
20
25
30
35
−2
0
2
Tumor−Normal Expression Difference
hsa−miR−3170, proximal
(BMI_obese = 1; N1 = 88)
1−sided adj pval: 0.082
Frequency
0
2
4
6
8
10
−2
0
2
Tumor−Normal Expression Difference

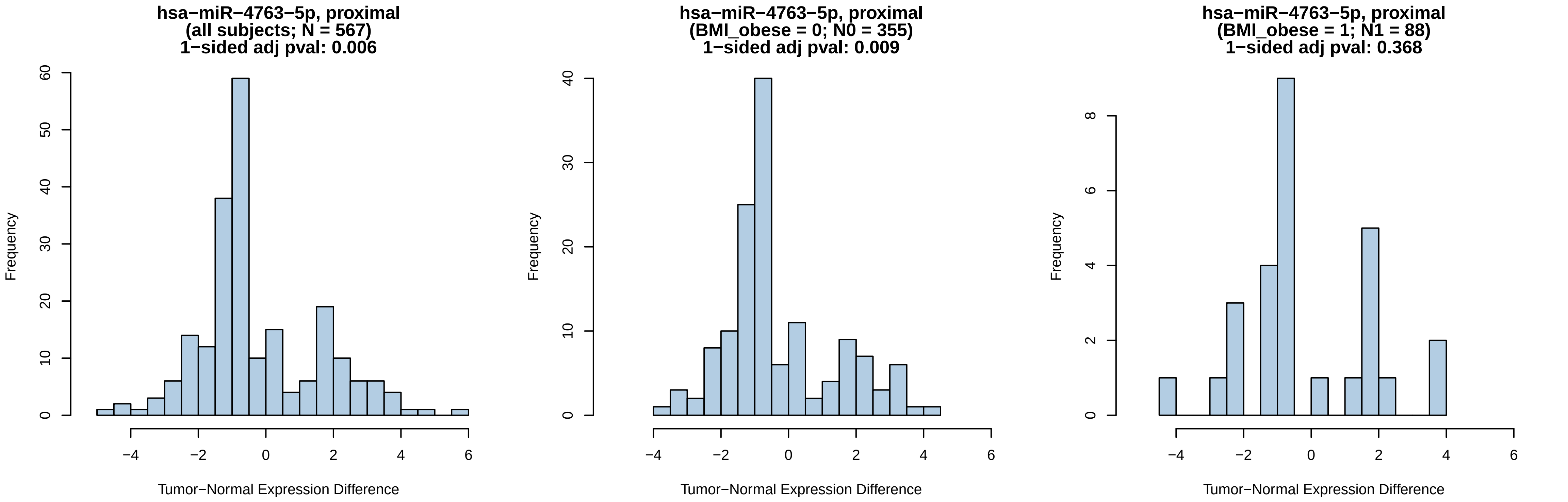
hsa−miR−4763−5p, proximal
(all subjects; N = 567)
1−sided adj pval: 0.006
Frequency
Tumor−Normal Expression Difference
hsa−miR−4763−5p, proximal
(BMI_obese = 0; N0 = 355)
1−sided adj pval: 0.009
Frequency
Tumor−Normal Expression Difference
hsa−miR−4763−5p, proximal
(BMI_obese = 1; N1 = 88)
1−sided adj pval: 0.368
Frequency
Tumor−Normal Expression Difference

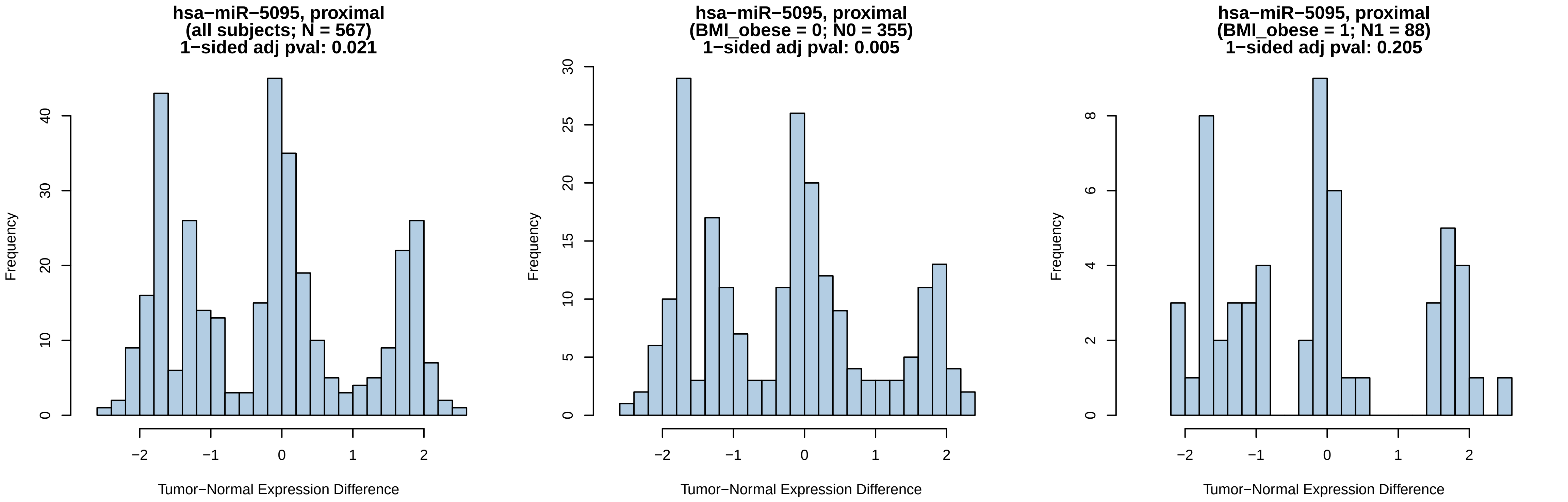
hsa−miR−5095, proximal
(all subjects; N = 567)
1−sided adj pval: 0.021
Frequency
0
10
20
30
40
−2
−1
0
1
2
Tumor−Normal Expression Difference
hsa−miR−5095, proximal
(BMI_obese = 0; N0 = 355)
1−sided adj pval: 0.005
Frequency
0
5
10
15
20
25
30
−2
−1
0
1
2
Tumor−Normal Expression Difference
hsa−miR−5095, proximal
(BMI_obese = 1; N1 = 88)
1−sided adj pval: 0.205
Frequency
0
2
4
6
8
−2
−1
0
1
2
Tumor−Normal Expression Difference

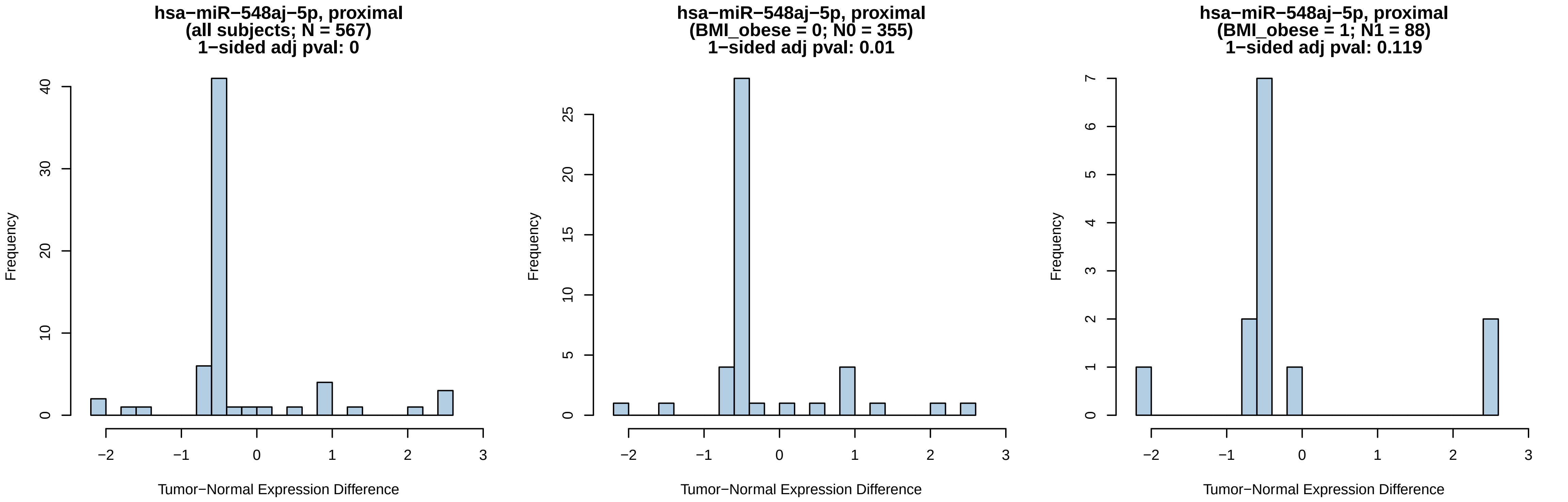
hsa−miR−548aj−5p, proximal
(all subjects; N = 567)
1−sided adj pval: 0
Frequency
Tumor−Normal Expression Difference
hsa−miR−548aj−5p, proximal
(BMI_obese = 0; N0 = 355)
1−sided adj pval: 0.01
Frequency
Tumor−Normal Expression Difference
hsa−miR−548aj−5p, proximal
(BMI_obese = 1; N1 = 88)
1−sided adj pval: 0.119
Frequency
Tumor−Normal Expression Difference

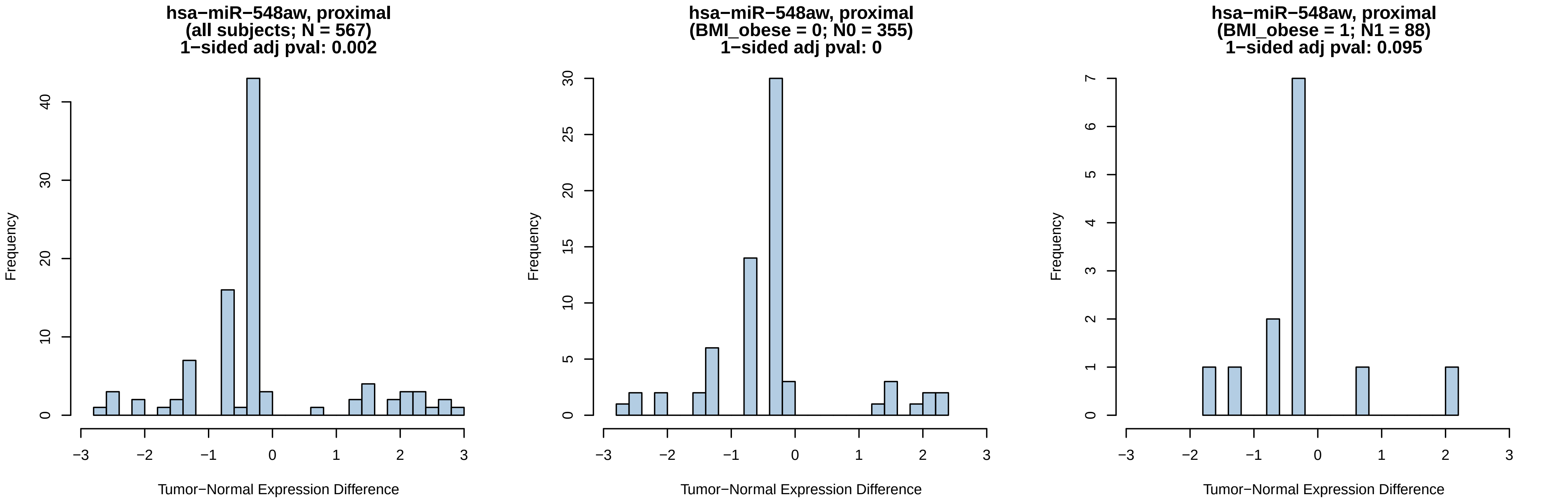
hsa−miR−548aw, proximal
(all subjects; N = 567)
1−sided adj pval: 0.002
Frequency
Tumor−Normal Expression Difference
hsa−miR−548aw, proximal
(BMI_obese = 0; N0 = 355)
1−sided adj pval: 0
Frequency
Tumor−Normal Expression Difference
hsa−miR−548aw, proximal
(BMI_obese = 1; N1 = 88)
1−sided adj pval: 0.095
Frequency
Tumor−Normal Expression Difference

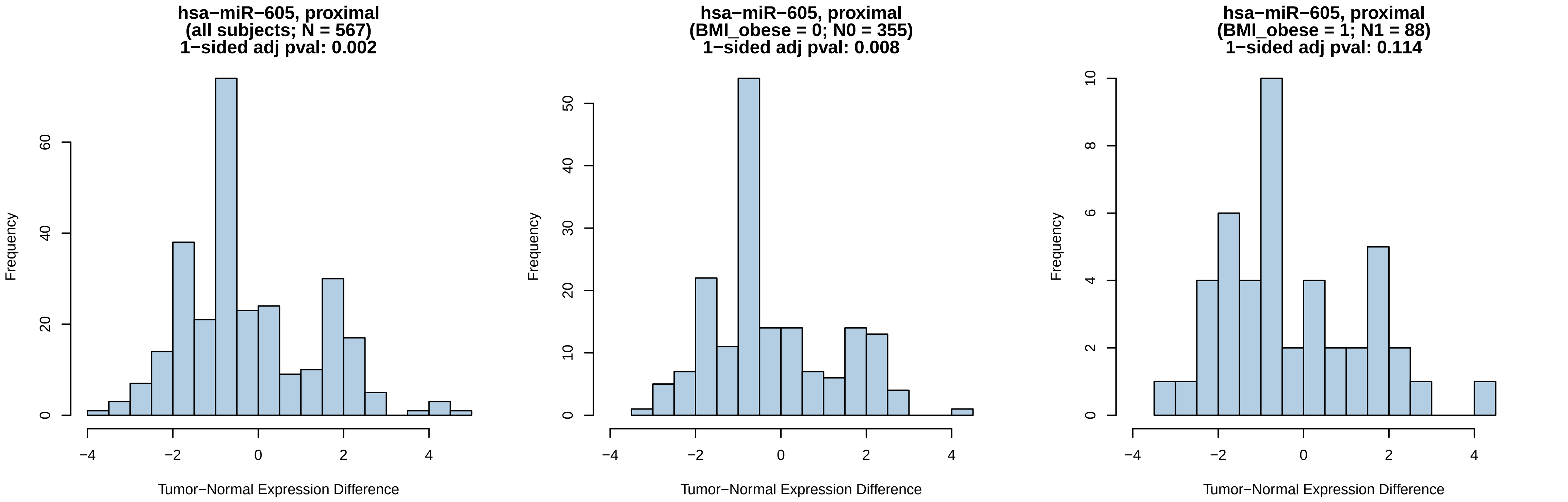
hsa−miR−605, proximal
(all subjects; N = 567)
1−sided adj pval: 0.002
hsa−miR−605, proximal
(BMI_obese = 0; N0 = 355)
1−sided adj pval: 0.008
hsa−miR−605, proximal
(BMI_obese = 1; N1 = 88)
1−sided adj pval: 0.114
Frequency
Tumor−Normal Expression Difference

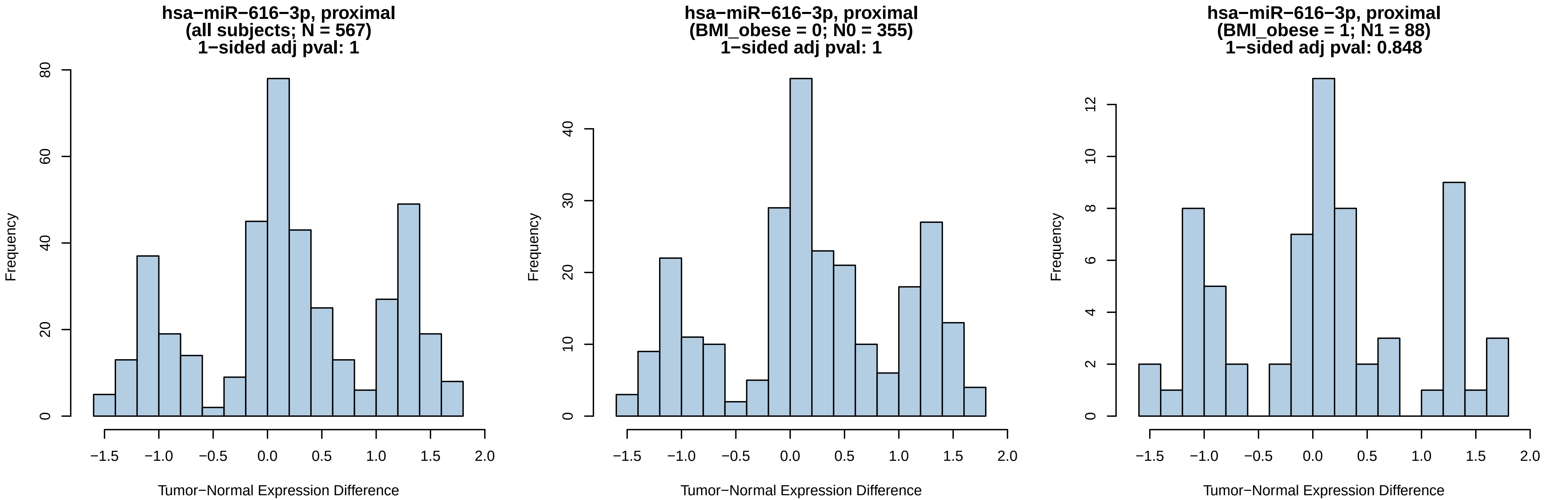

hsa−miR−616−3p, proximal
(all subjects; N = 567)
1−sided adj pval: 1
Frequency
Tumor−Normal Expression Difference
hsa−miR−616−3p, proximal
(BMI_obese = 0; N0 = 355)
1−sided adj pval: 1
Frequency
Tumor−Normal Expression Difference
hsa−miR−616−3p, proximal
(BMI_obese = 1; N1 = 88)
1−sided adj pval: 0.848
Frequency
Tumor−Normal Expression Difference

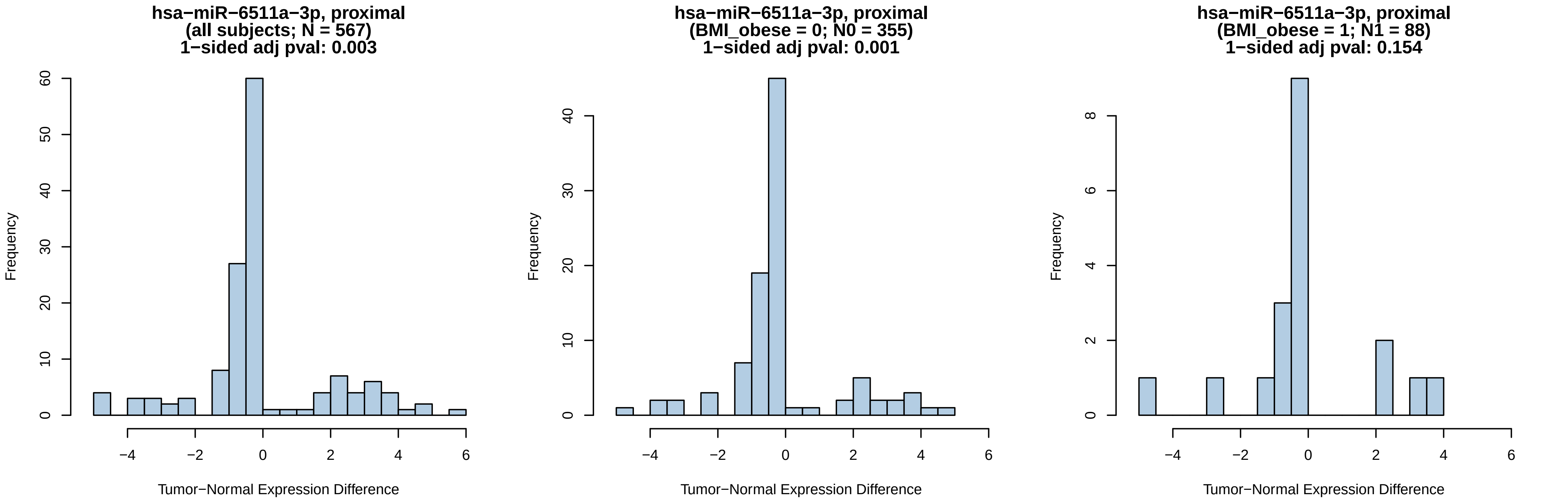
hsa−miR−6511a−3p, proximal
(all subjects; N = 567)
1−sided adj pval: 0.003
Frequency
Tumor−Normal Expression Difference
hsa−miR−6511a−3p, proximal
(BMI_obese = 0; N0 = 355)
1−sided adj pval: 0.001
Frequency
Tumor−Normal Expression Difference
hsa−miR−6511a−3p, proximal
(BMI_obese = 1; N1 = 88)
1−sided adj pval: 0.154
Frequency
Tumor−Normal Expression Difference

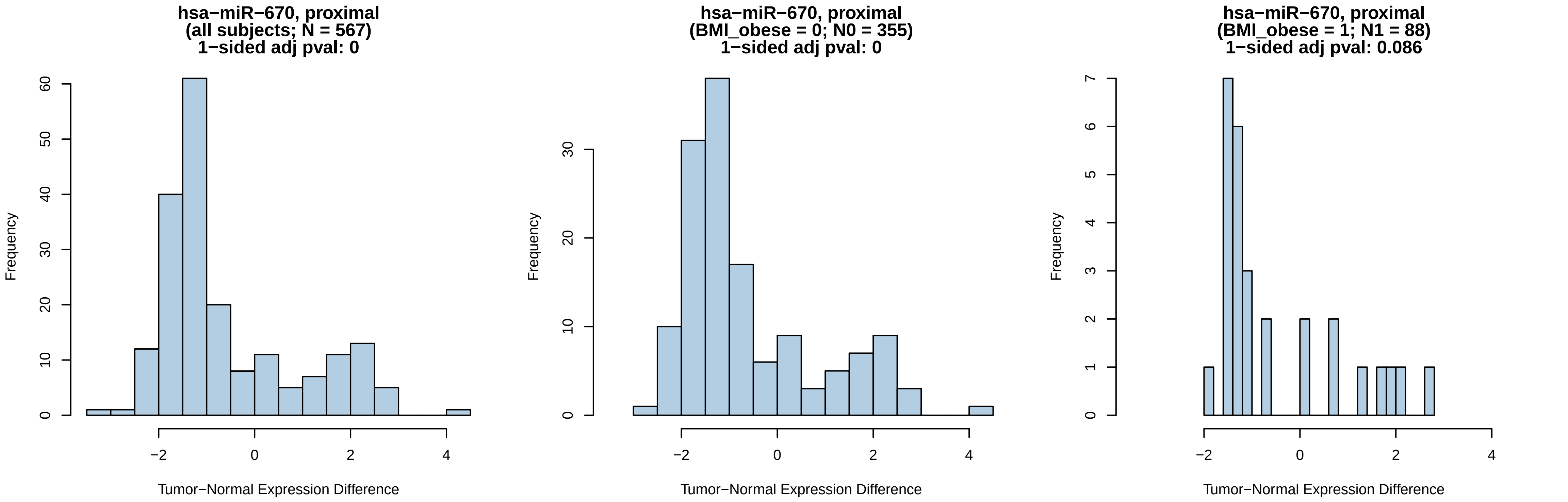
hsa−miR−670, proximal
(all subjects; N = 567)
1−sided adj pval: 0
Frequency
Tumor−Normal Expression Difference
hsa−miR−670, proximal
(BMI_obese = 0; N0 = 355)
1−sided adj pval: 0
Frequency
Tumor−Normal Expression Difference
hsa−miR−670, proximal
(BMI_obese = 1; N1 = 88)
1−sided adj pval: 0.086
Frequency
Tumor−Normal Expression Difference

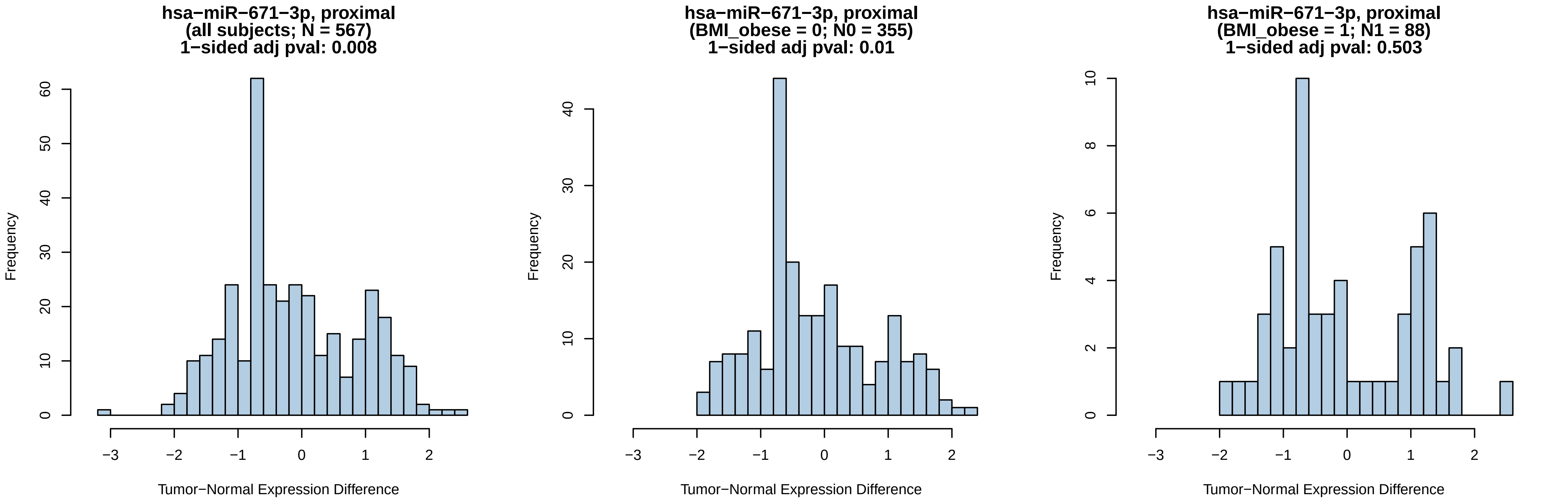

hsa−miR−671−3p, proximal
(all subjects; N = 567)
1−sided adj pval: 0.008
Frequency
Tumor−Normal Expression Difference
hsa−miR−671−3p, proximal
(BMI_obese = 0; N0 = 355)
1−sided adj pval: 0.01
Frequency
Tumor−Normal Expression Difference
hsa−miR−671−3p, proximal
(BMI_obese = 1; N1 = 88)
1−sided adj pval: 0.503
Frequency
Tumor−Normal Expression Difference

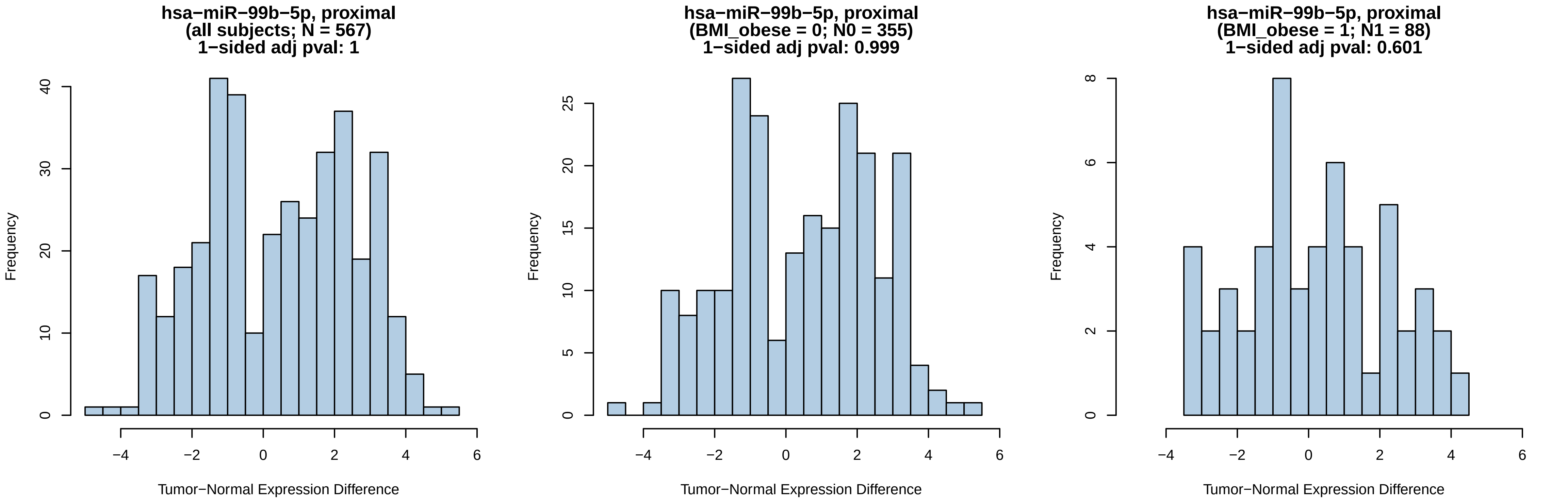
hsa−miR−99b−5p, proximal
(all subjects; N = 567)
1−sided adj pval: 1
Frequency
Tumor−Normal Expression Difference
hsa−miR−99b−5p, proximal
(BMI_obese = 0; N0 = 355)
1−sided adj pval: 0.999
Frequency
Tumor−Normal Expression Difference
hsa−miR−99b−5p, proximal
(BMI_obese = 1; N1 = 88)
1−sided adj pval: 0.601
Frequency
Tumor−Normal Expression Difference

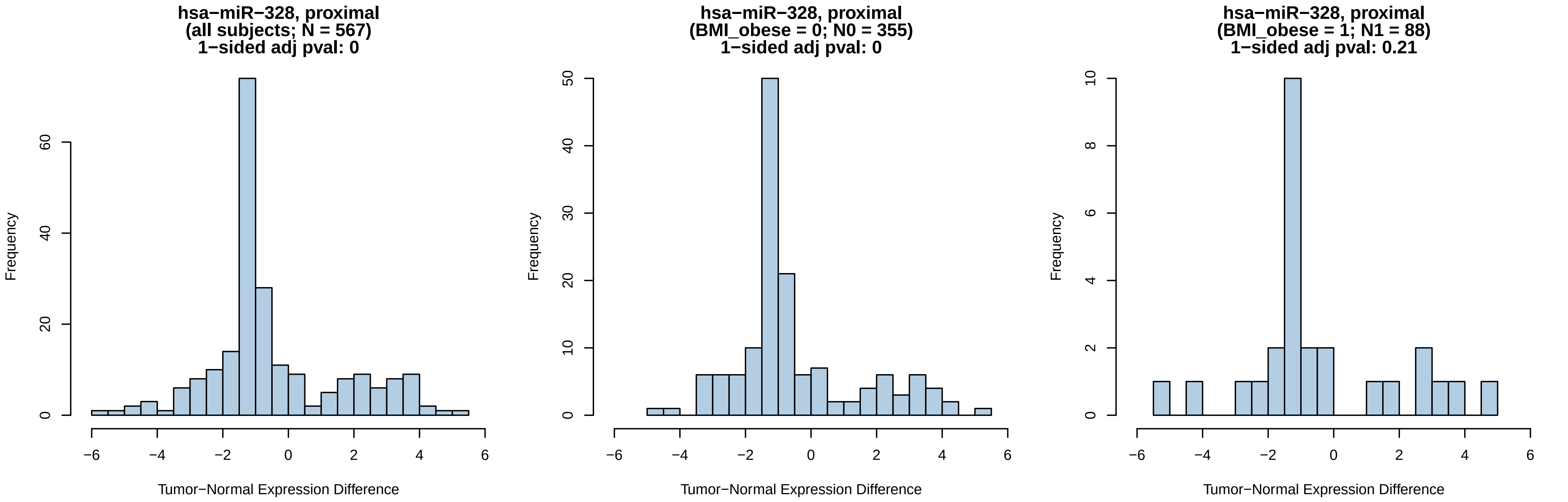
hsa−miR−328, proximal
(all subjects; N = 567)
1−sided adj pval: 0
hsa−miR−328, proximal
(BMI_obese = 0; N0 = 355)
1−sided adj pval: 0
hsa−miR−328, proximal
(BMI_obese = 1; N1 = 88)
1−sided adj pval: 0.21
Frequency
Tumor−Normal Expression Difference
0
20
40
60
10
30
50
2
4
6
8
−6
−4
−2
0
2
4
6

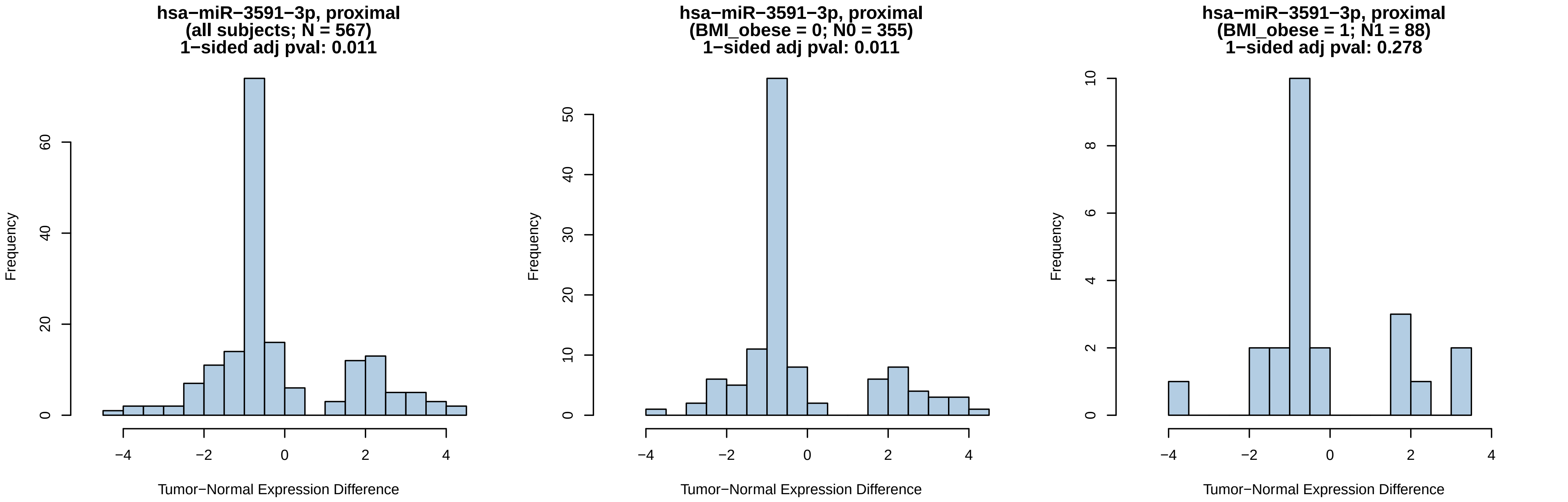
hsa−miR−3591−3p, proximal
(all subjects; N = 567)
1−sided adj pval: 0.011
Frequency
Tumor−Normal Expression Difference
hsa−miR−3591−3p, proximal
(BMI_obese = 0; N0 = 355)
1−sided adj pval: 0.011
Frequency
Tumor−Normal Expression Difference
hsa−miR−3591−3p, proximal
(BMI_obese = 1; N1 = 88)
1−sided adj pval: 0.278
Frequency
Tumor−Normal Expression Difference

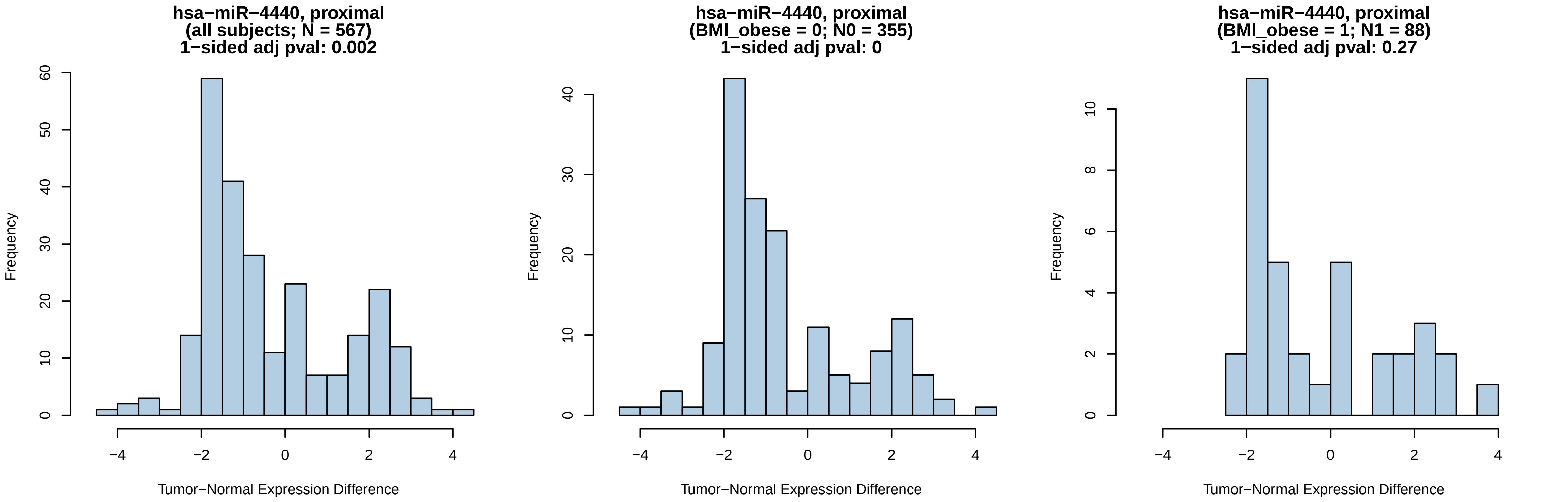
hsa−miR−4440, proximal
(all subjects; N = 567)
1−sided adj pval: 0.002
Frequency
Tumor−Normal Expression Difference
hsa−miR−4440, proximal
(BMI_obese = 0; N0 = 355)
1−sided adj pval: 0
Frequency
Tumor−Normal Expression Difference
hsa−miR−4440, proximal
(BMI_obese = 1; N1 = 88)
1−sided adj pval: 0.27
Frequency
Tumor−Normal Expression Difference

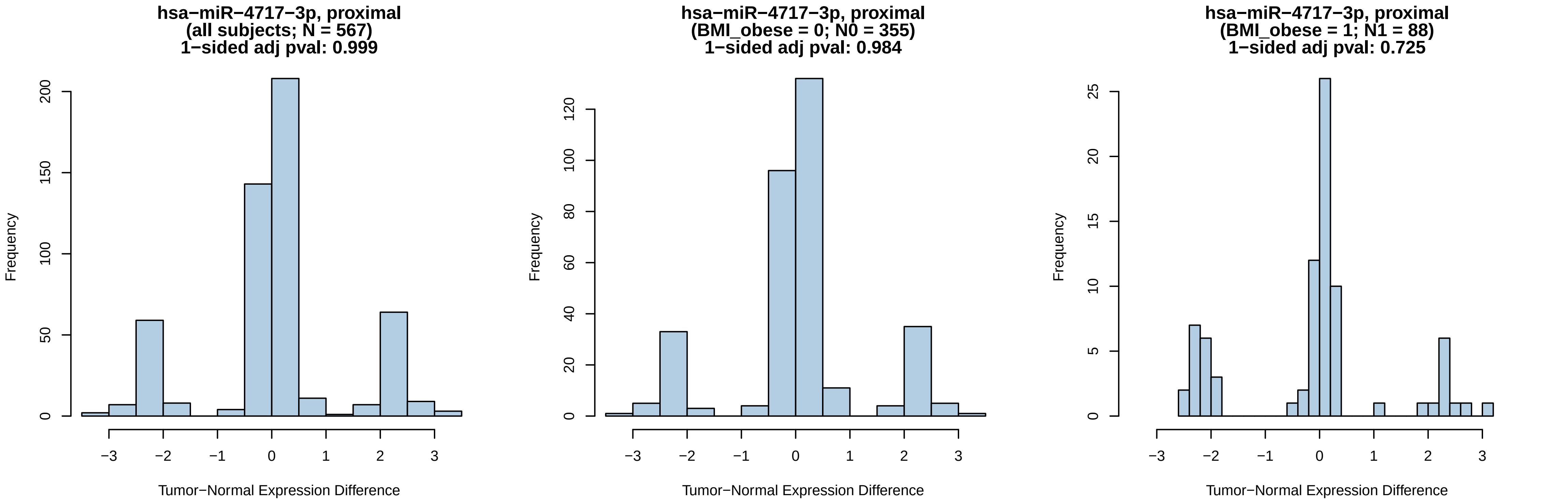
hsa−miR−4717−3p, proximal
(all subjects; N = 567)
1−sided adj pval: 0.999
Frequency
Tumor−Normal Expression Difference
hsa−miR−4717−3p, proximal
(BMI_obese = 0; N0 = 355)
1−sided adj pval: 0.984
Frequency
Tumor−Normal Expression Difference
hsa−miR−4717−3p, proximal
(BMI_obese = 1; N1 = 88)
1−sided adj pval: 0.725
Frequency
Tumor−Normal Expression Difference

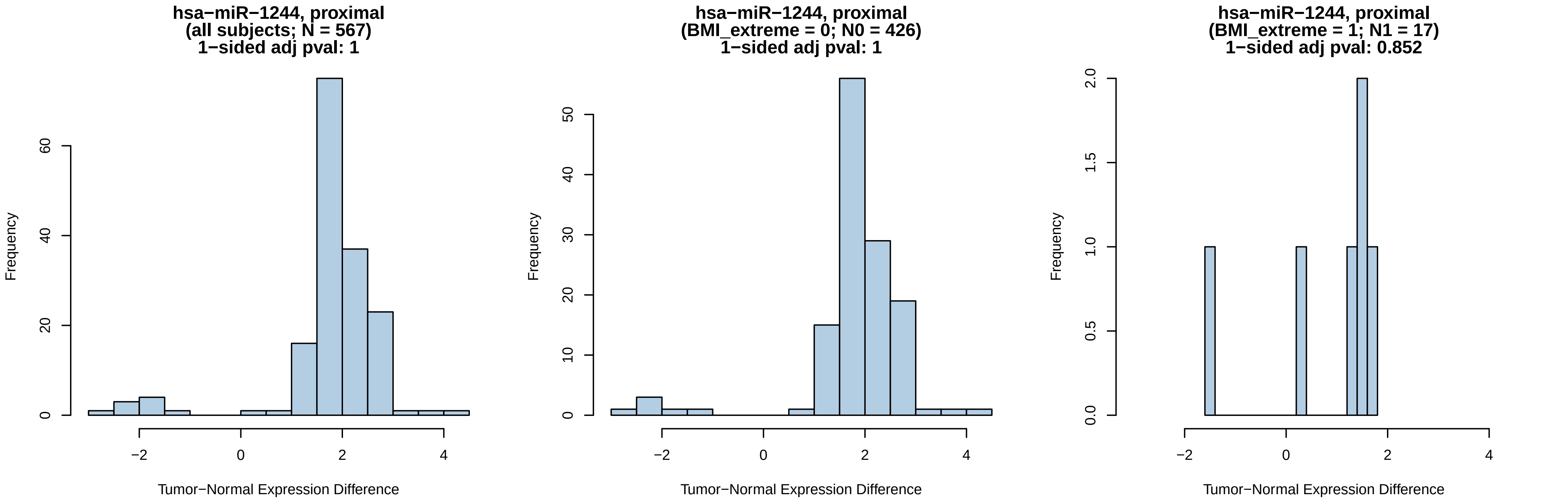
hsa−miR−1244, proximal
(all subjects; N = 567)
1−sided adj pval: 1
Frequency
0
20
40
60
−2
0
2
4
Tumor−Normal Expression Difference
hsa−miR−1244, proximal
(BMI_extreme = 0; N0 = 426)
1−sided adj pval: 1
Frequency
0
10
20
30
40
50
−2
0
2
4
Tumor−Normal Expression Difference
hsa−miR−1244, proximal
(BMI_extreme = 1; N1 = 17)
1−sided adj pval: 0.852
Frequency
0.0
0.5
1.0
1.5
2.0
−2
0
2
4
Tumor−Normal Expression Difference

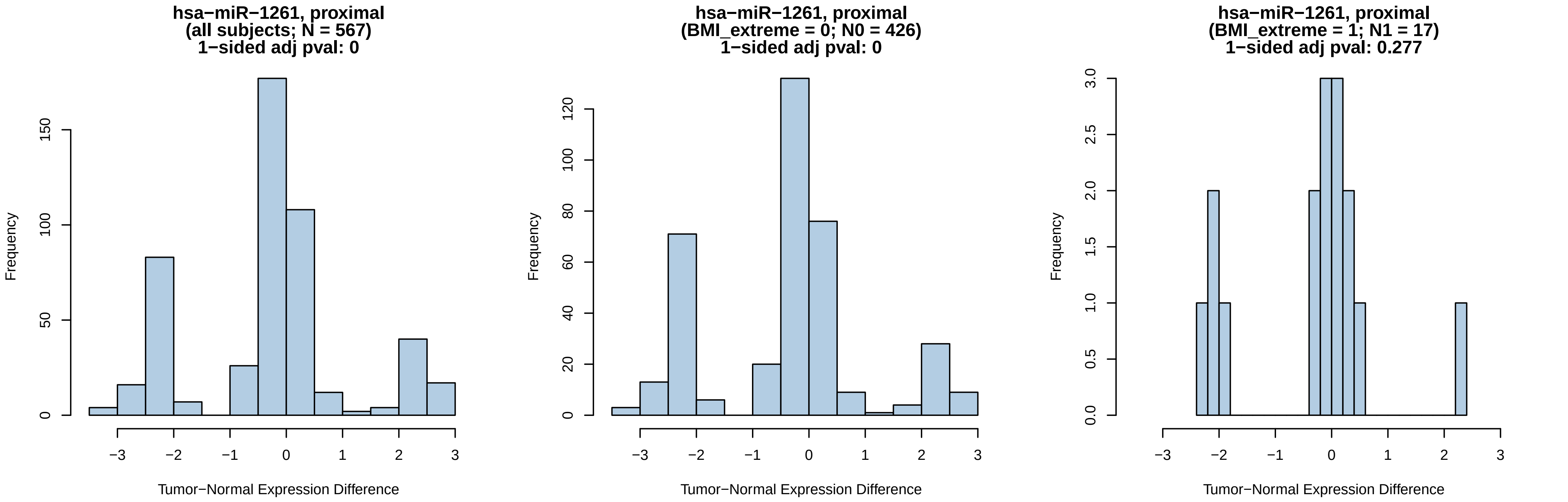
hsa−miR−1261, proximal
(all subjects; N = 567)
1−sided adj pval: 0
Frequency
Tumor−Normal Expression Difference
hsa−miR−1261, proximal
(BMI_extreme = 0; N0 = 426)
1−sided adj pval: 0
Frequency
Tumor−Normal Expression Difference
hsa−miR−1261, proximal
(BMI_extreme = 1; N1 = 17)
1−sided adj pval: 0.277
Frequency
Tumor−Normal Expression Difference

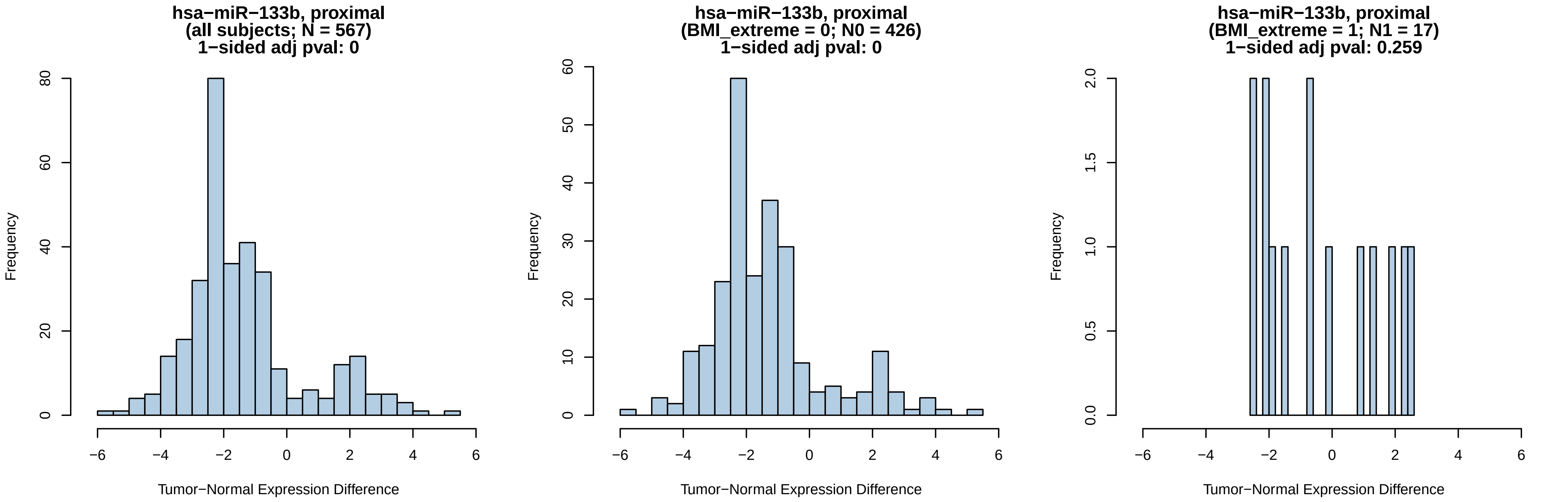
hsa−miR−133b, proximal
(all subjects; N = 567)
1−sided adj pval: 0
Frequency
Tumor−Normal Expression Difference
hsa−miR−133b, proximal
(BMI_extreme = 0; N0 = 426)
1−sided adj pval: 0
Frequency
Tumor−Normal Expression Difference
hsa−miR−133b, proximal
(BMI_extreme = 1; N1 = 17)
1−sided adj pval: 0.259
Frequency
Tumor−Normal Expression Difference

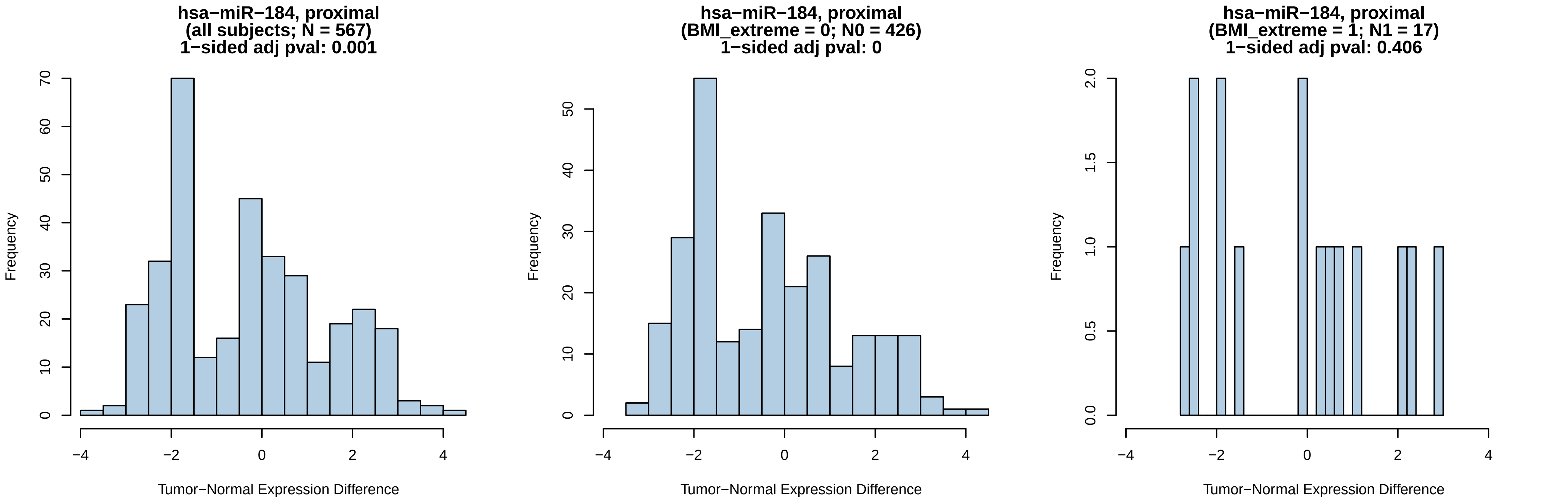

hsa−miR−184, proximal
(all subjects; N = 567)
1−sided adj pval: 0.001
Frequency
Tumor−Normal Expression Difference
hsa−miR−184, proximal
(BMI_extreme = 0; N0 = 426)
1−sided adj pval: 0
Frequency
Tumor−Normal Expression Difference
hsa−miR−184, proximal
(BMI_extreme = 1; N1 = 17)
1−sided adj pval: 0.406
Frequency
Tumor−Normal Expression Difference

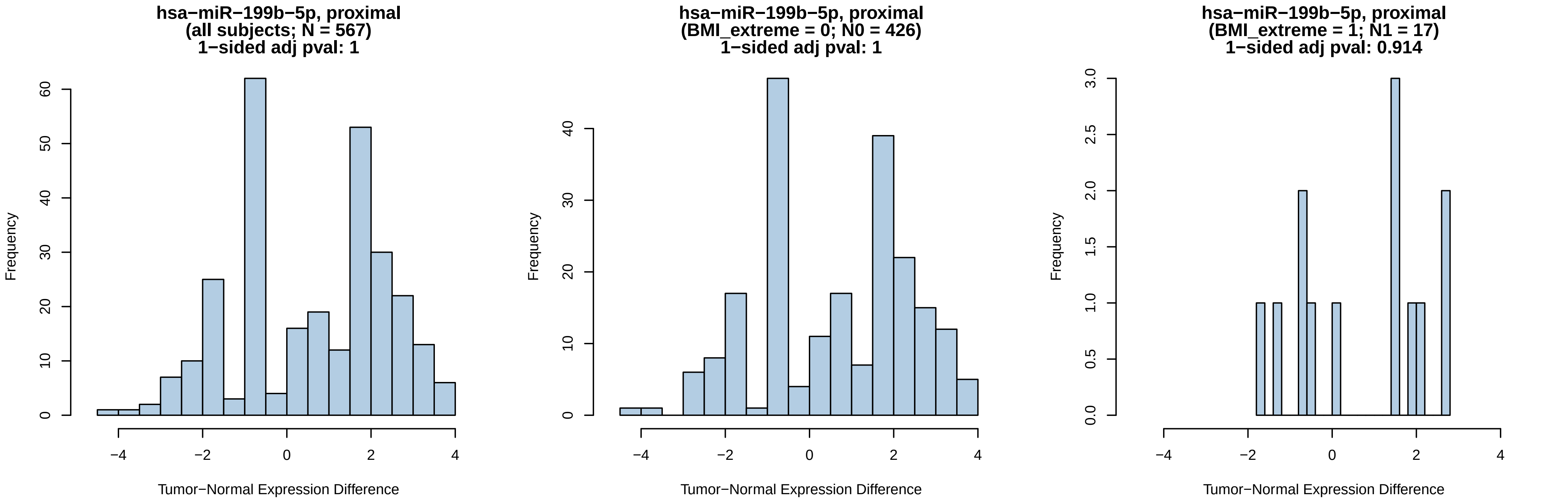

hsa−miR−199b−5p, proximal
(all subjects; N = 567)
1−sided adj pval: 1
Frequency
0
10
20
30
40
50
60
−4
−2
0
2
4
Tumor−Normal Expression Difference
hsa−miR−199b−5p, proximal
(BMI_extreme = 0; N0 = 426)
1−sided adj pval: 1
Frequency
0
10
20
30
40
−4
−2
0
2
4
Tumor−Normal Expression Difference
hsa−miR−199b−5p, proximal
(BMI_extreme = 1; N1 = 17)
1−sided adj pval: 0.914
Frequency
0.0
0.5
1.0
1.5
2.0
2.5
3.0
−4
−2
0
2
4
Tumor−Normal Expression Difference

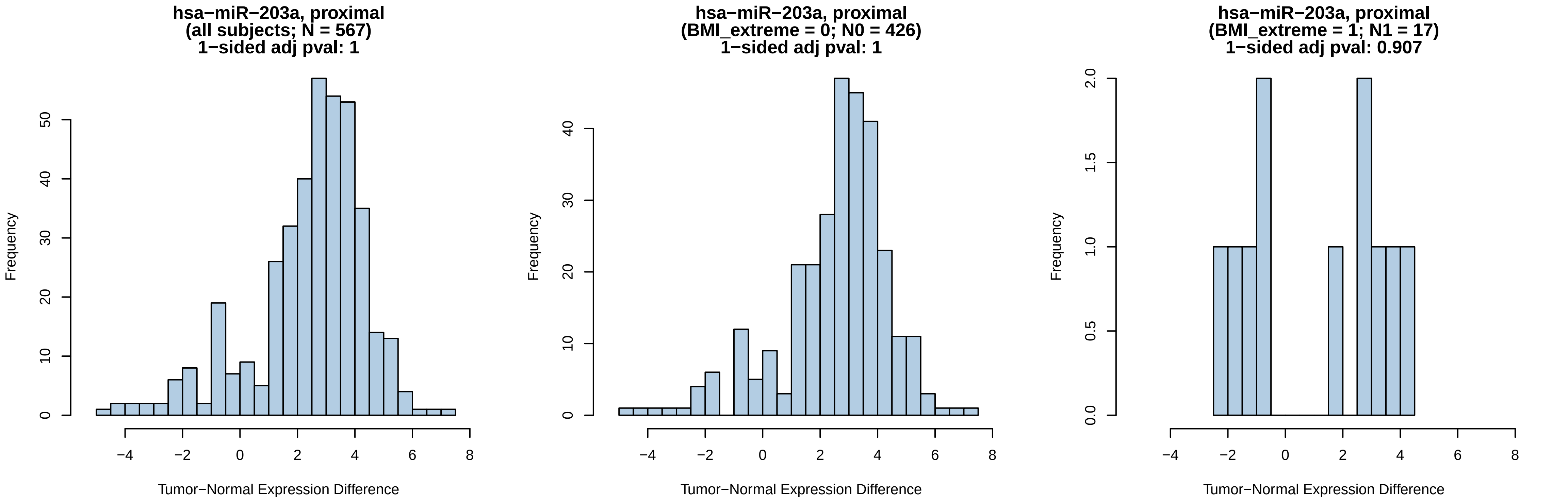

hsa−miR−203a, proximal
(all subjects; N = 567)
1−sided adj pval: 1
hsa−miR−203a, proximal
(BMI_extreme = 0; N0 = 426)
1−sided adj pval: 1
hsa−miR−203a, proximal
(BMI_extreme = 1; N1 = 17)
1−sided adj pval: 0.907
Frequency
Tumor−Normal Expression Difference

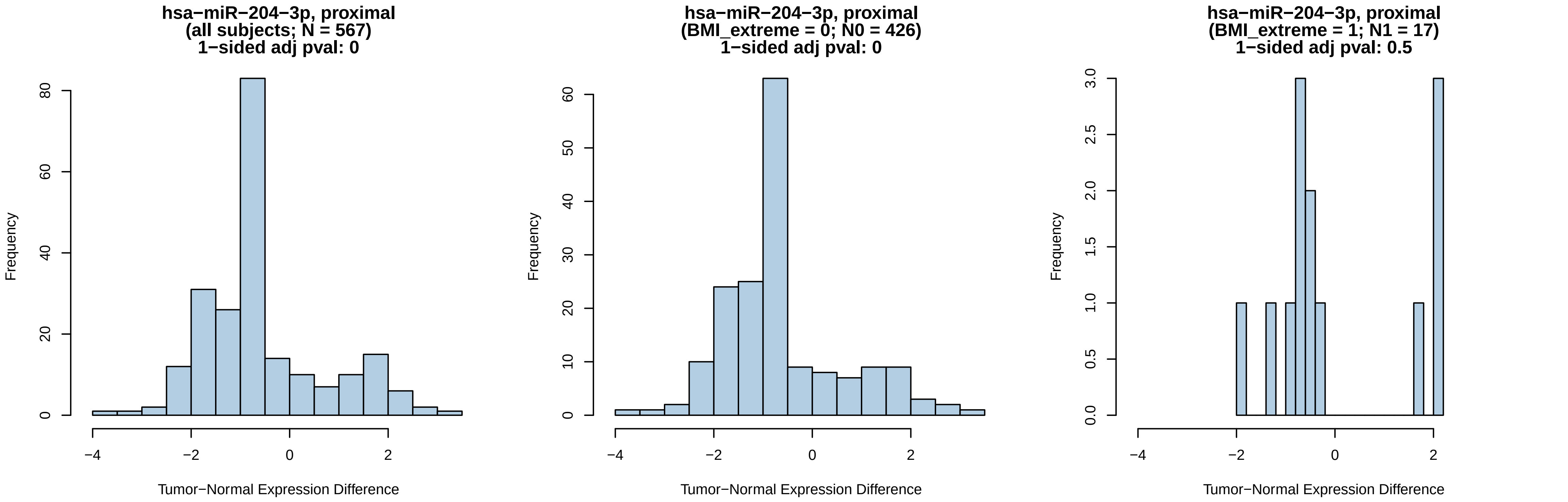
hsa−miR−204−3p, proximal
(all subjects; N = 567)
1−sided adj pval: 0
Frequency
Tumor−Normal Expression Difference
hsa−miR−204−3p, proximal
(BMI_extreme = 0; N0 = 426)
1−sided adj pval: 0
Frequency
Tumor−Normal Expression Difference
hsa−miR−204−3p, proximal
(BMI_extreme = 1; N1 = 17)
1−sided adj pval: 0.5
Frequency
Tumor−Normal Expression Difference

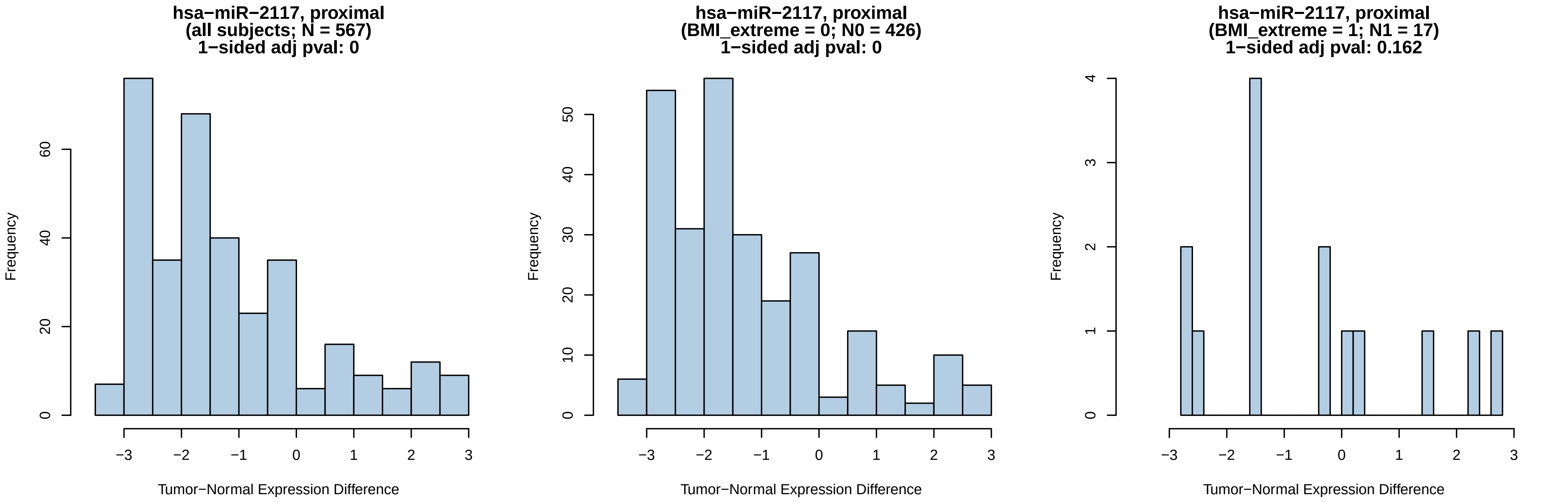
hsa−miR−2117, proximal
(all subjects; N = 567)
1−sided adj pval: 0
Frequency
Tumor−Normal Expression Difference
hsa−miR−2117, proximal
(BMI_extreme = 0; N0 = 426)
1−sided adj pval: 0
Frequency
Tumor−Normal Expression Difference
hsa−miR−2117, proximal
(BMI_extreme = 1; N1 = 17)
1−sided adj pval: 0.162
Frequency
Tumor−Normal Expression Difference

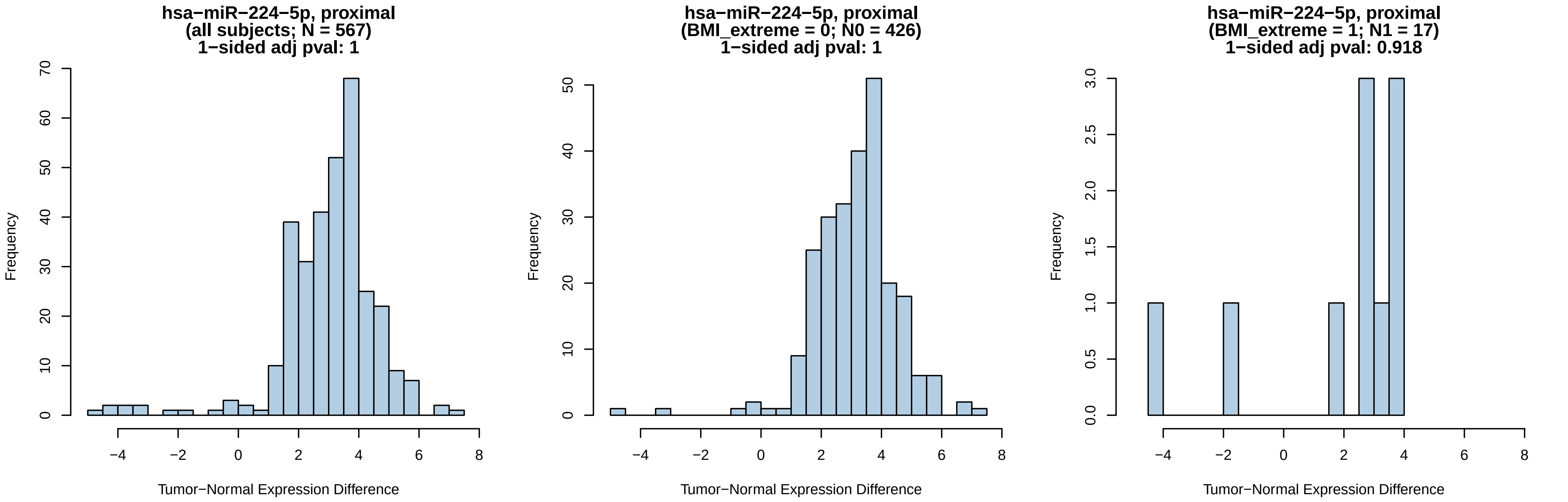
hsa−miR−224−5p, proximal
(all subjects; N = 567)
1−sided adj pval: 1
Frequency
Tumor−Normal Expression Difference
hsa−miR−224−5p, proximal
(BMI_extreme = 0; N0 = 426)
1−sided adj pval: 1
Frequency
Tumor−Normal Expression Difference
hsa−miR−224−5p, proximal
(BMI_extreme = 1; N1 = 17)
1−sided adj pval: 0.918
Frequency
Tumor−Normal Expression Difference

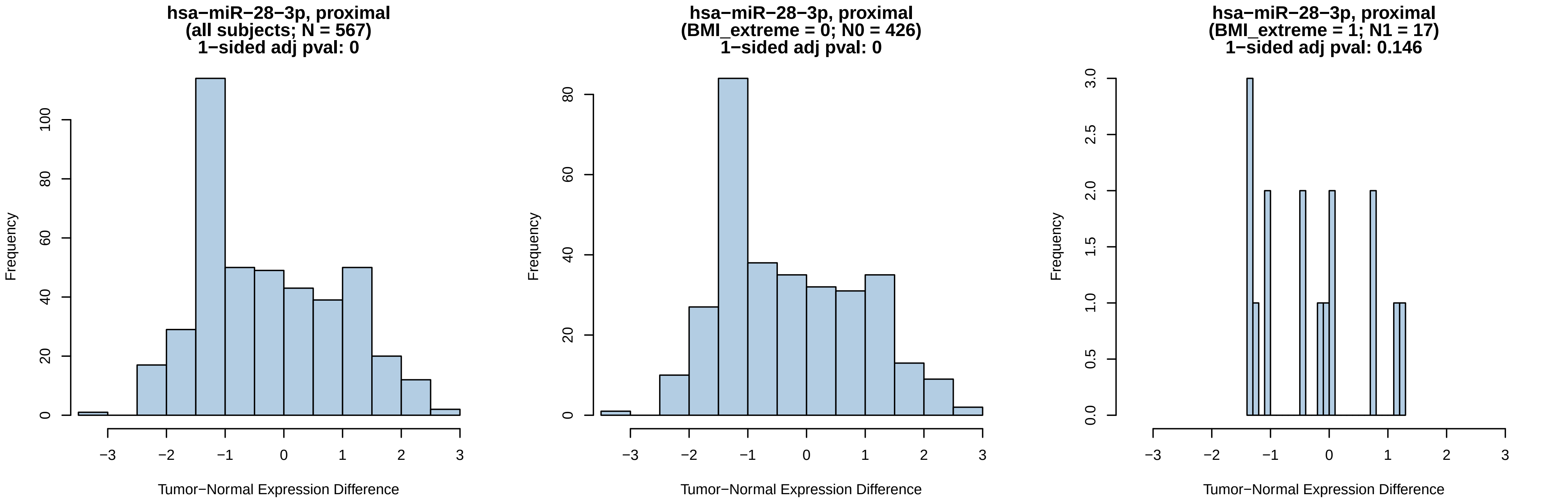
hsa−miR−28−3p, proximal
(all subjects; N = 567)
1−sided adj pval: 0
Frequency
0
20
40
60
80
100
−3
−2
−1
0
1
2
3
Tumor−Normal Expression Difference
hsa−miR−28−3p, proximal
(BMI_extreme = 0; N0 = 426)
1−sided adj pval: 0
Frequency
0
20
40
60
80
−3
−2
−1
0
1
2
3
Tumor−Normal Expression Difference
hsa−miR−28−3p, proximal
(BMI_extreme = 1; N1 = 17)
1−sided adj pval: 0.146
Frequency
0.0
0.5
1.0
1.5
2.0
2.5
3.0
−3
−2
−1
0
1
2
3
Tumor−Normal Expression Difference

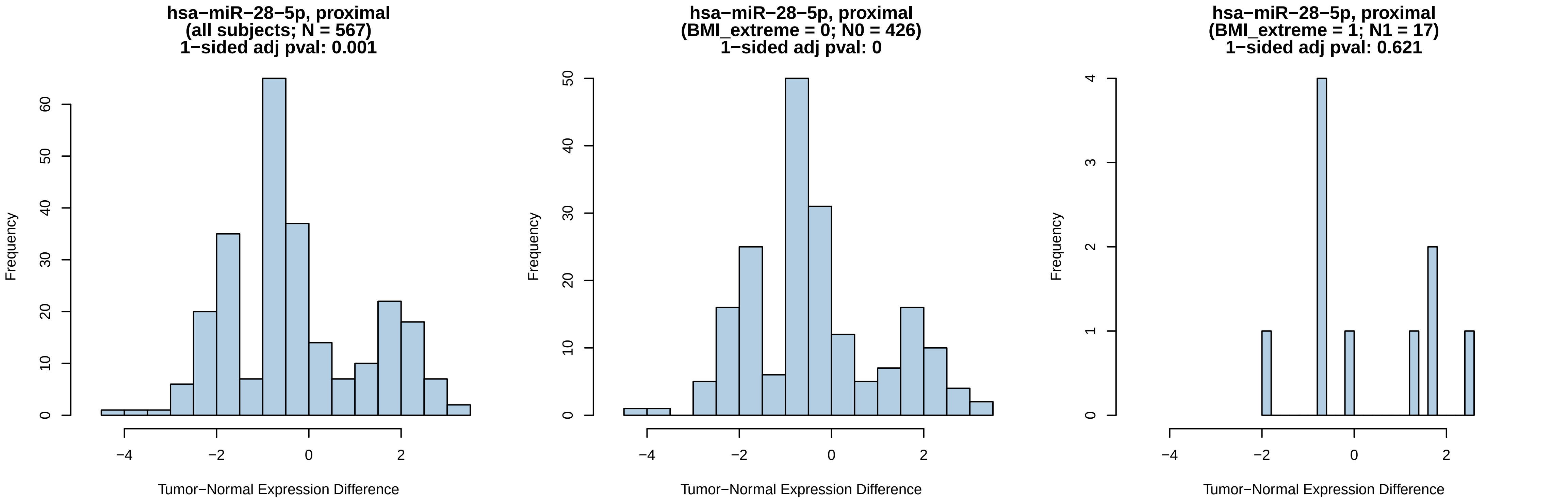
hsa−miR−28−5p, proximal
(all subjects; N = 567)
1−sided adj pval: 0.001
hsa−miR−28−5p, proximal
(BMI_extreme = 0; N0 = 426)
1−sided adj pval: 0
hsa−miR−28−5p, proximal
(BMI_extreme = 1; N1 = 17)
1−sided adj pval: 0.621
Frequency
Tumor−Normal Expression Difference

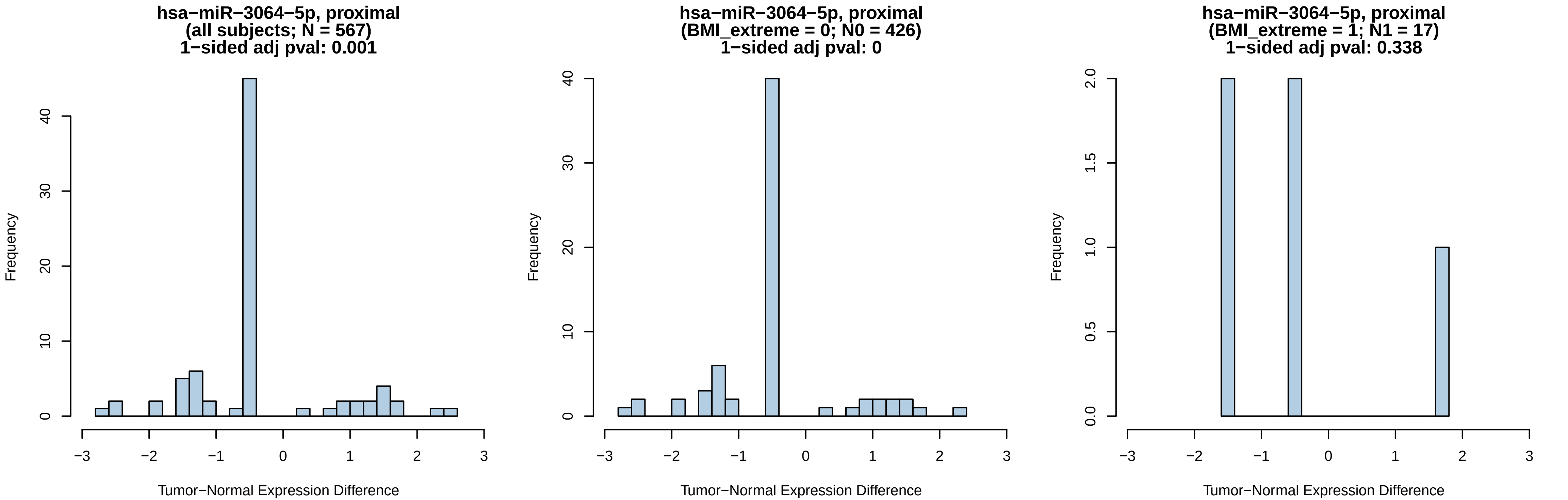

hsa−miR−3064−5p, proximal
(all subjects; N = 567)
1−sided adj pval: 0.001
Frequency
Tumor−Normal Expression Difference
hsa−miR−3064−5p, proximal
(BMI_extreme = 0; N0 = 426)
1−sided adj pval: 0
Frequency
Tumor−Normal Expression Difference
hsa−miR−3064−5p, proximal
(BMI_extreme = 1; N1 = 17)
1−sided adj pval: 0.338
Frequency
Tumor−Normal Expression Difference

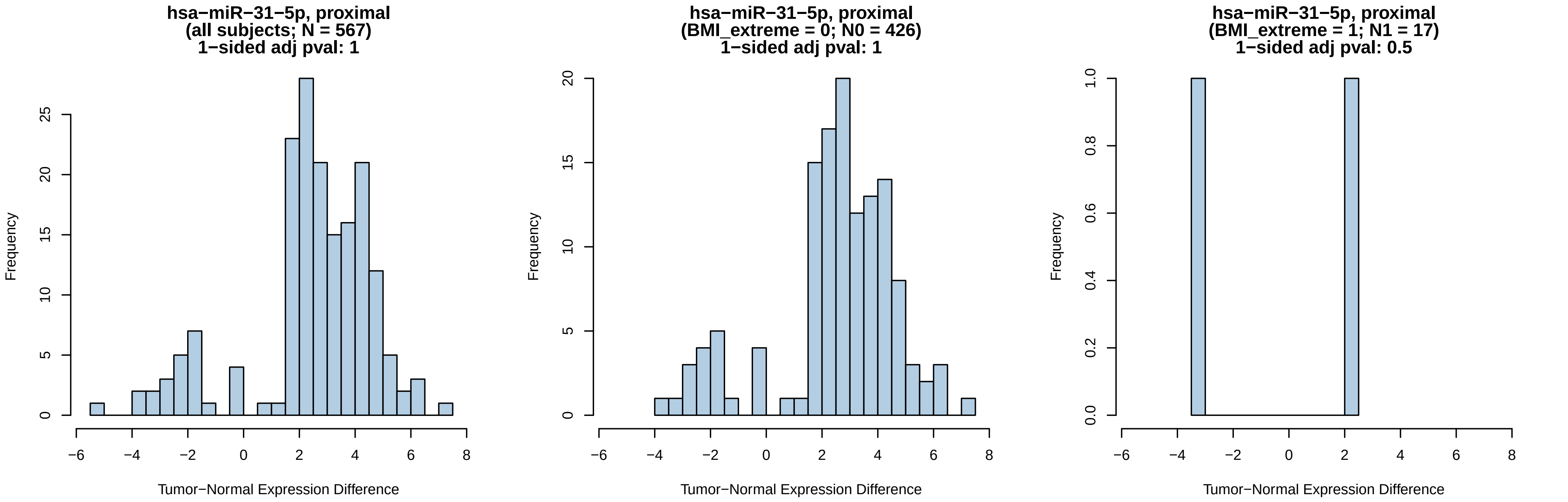
hsa−miR−31−5p, proximal
(all subjects; N = 567)
1−sided adj pval: 1
Frequency
0
5
10
15
20
25
−6
−4
−2
0
2
4
6
8
Tumor−Normal Expression Difference
hsa−miR−31−5p, proximal
(BMI_extreme = 0; N0 = 426)
1−sided adj pval: 1
Frequency
0
5
10
15
20
−6
−4
−2
0
2
4
6
8
Tumor−Normal Expression Difference
hsa−miR−31−5p, proximal
(BMI_extreme = 1; N1 = 17)
1−sided adj pval: 0.5
Frequency
0.0
0.2
0.4
0.6
0.8
1.0
−6
−4
−2
0
2
4
6
8
Tumor−Normal Expression Difference

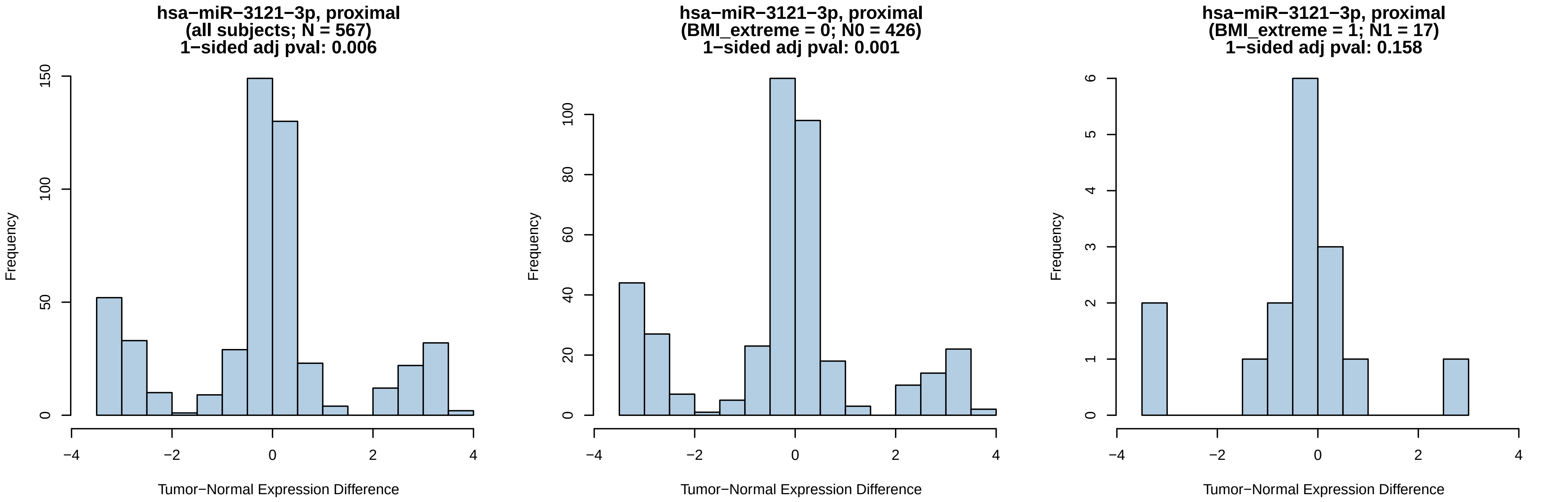
hsa−miR−3121−3p, proximal
(all subjects; N = 567)
1−sided adj pval: 0.006
hsa−miR−3121−3p, proximal
(BMI_extreme = 0; N0 = 426)
1−sided adj pval: 0.001
hsa−miR−3121−3p, proximal
(BMI_extreme = 1; N1 = 17)
1−sided adj pval: 0.158
Frequency
Tumor−Normal Expression Difference

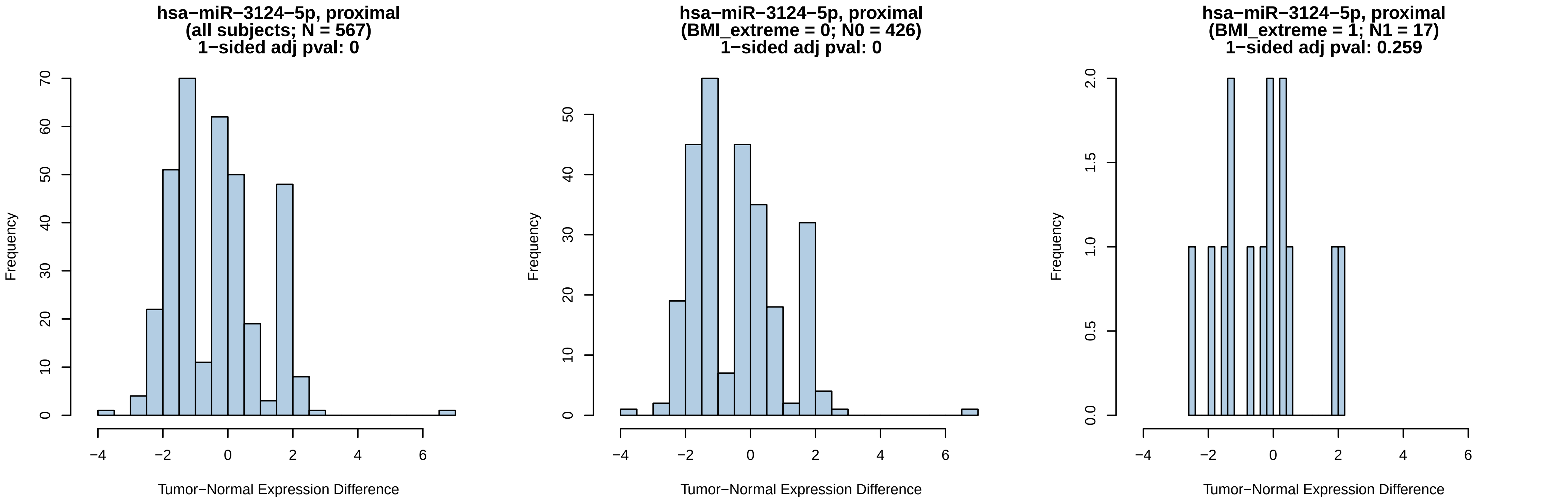

hsa−miR−3124−5p, proximal
(all subjects; N = 567)
1−sided adj pval: 0
Frequency
Tumor−Normal Expression Difference
hsa−miR−3124−5p, proximal
(BMI_extreme = 0; N0 = 426)
1−sided adj pval: 0
Frequency
Tumor−Normal Expression Difference
hsa−miR−3124−5p, proximal
(BMI_extreme = 1; N1 = 17)
1−sided adj pval: 0.259
Frequency
Tumor−Normal Expression Difference

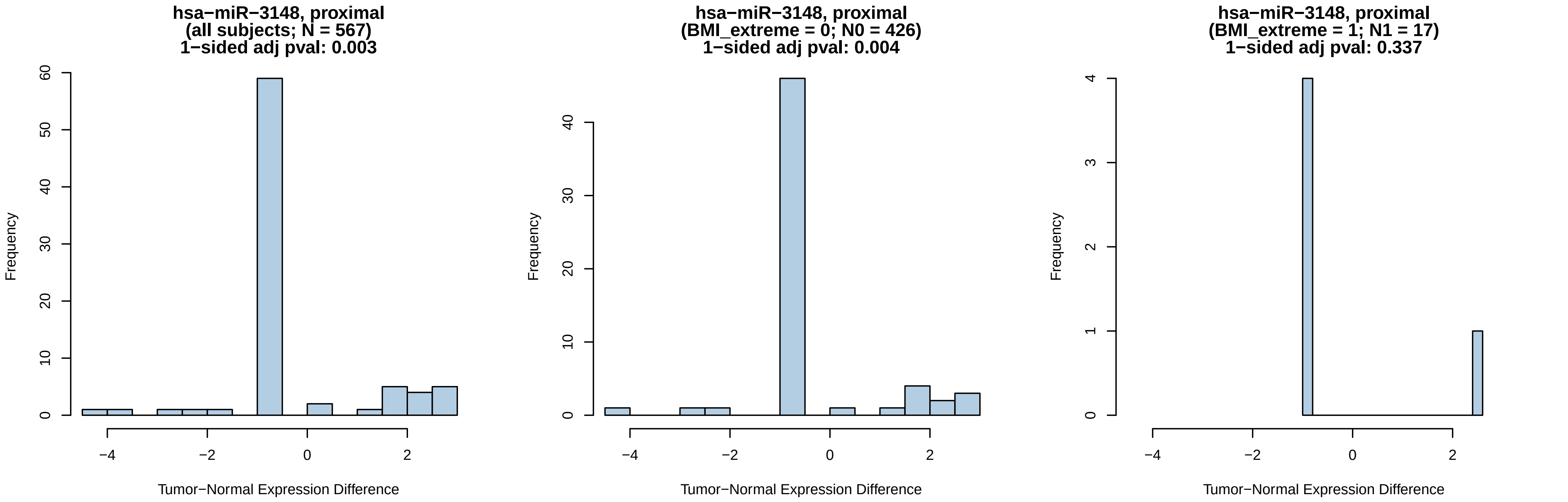

hsa−miR−3148, proximal
(all subjects; N = 567)
1−sided adj pval: 0.003
Frequency
Tumor−Normal Expression Difference
hsa−miR−3148, proximal
(BMI_extreme = 0; N0 = 426)
1−sided adj pval: 0.004
Frequency
Tumor−Normal Expression Difference
hsa−miR−3148, proximal
(BMI_extreme = 1; N1 = 17)
1−sided adj pval: 0.337
Frequency
Tumor−Normal Expression Difference

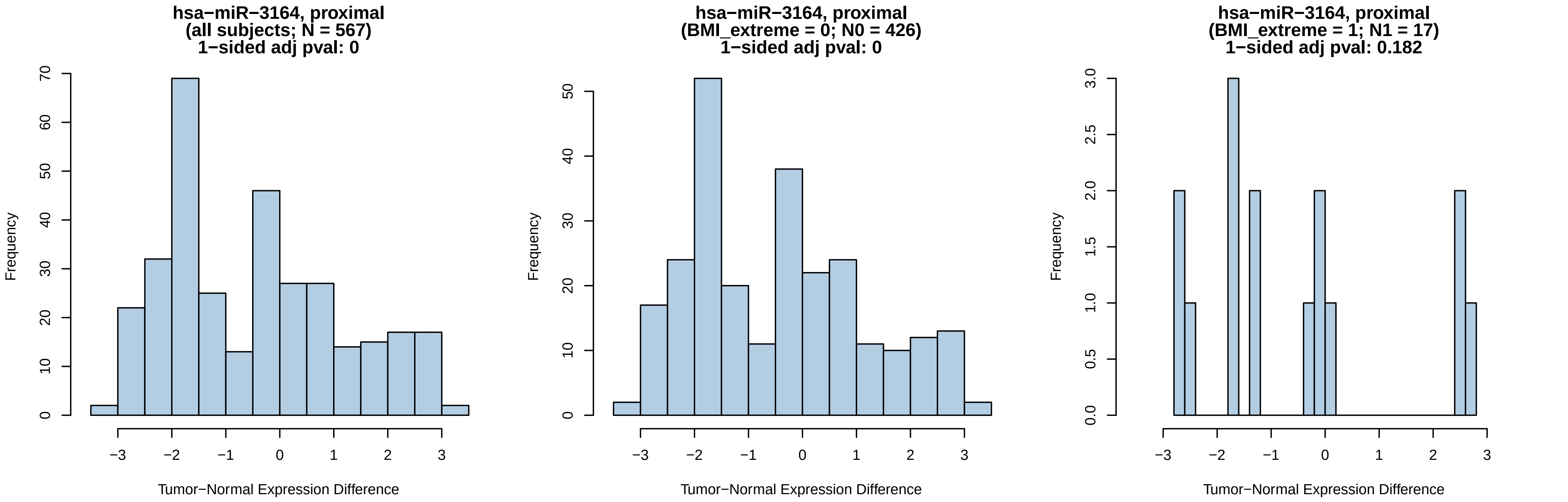
hsa−miR−3164, proximal
(all subjects; N = 567)
1−sided adj pval: 0
hsa−miR−3164, proximal
(BMI_extreme = 0; N0 = 426)
1−sided adj pval: 0
hsa−miR−3164, proximal
(BMI_extreme = 1; N1 = 17)
1−sided adj pval: 0.182
Frequency
Tumor−Normal Expression Difference

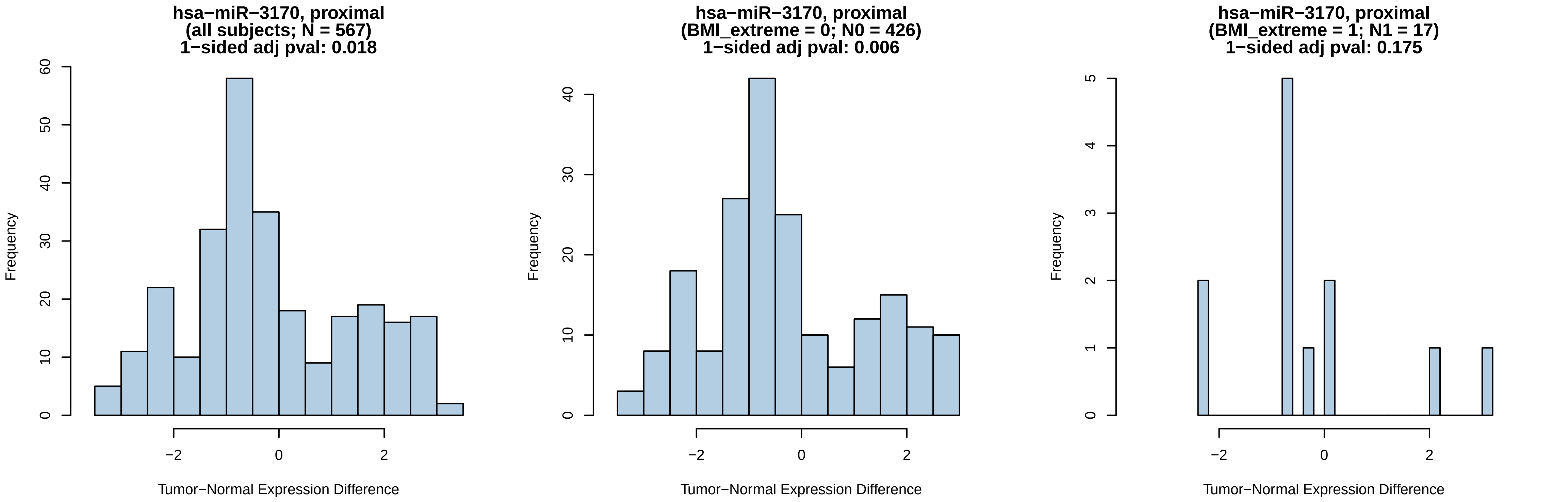
hsa−miR−3170, proximal
(all subjects; N = 567)
1−sided adj pval: 0.018
Frequency
0
10
20
30
40
50
60
−2
0
2
Tumor−Normal Expression Difference
hsa−miR−3170, proximal
(BMI_extreme = 0; N0 = 426)
1−sided adj pval: 0.006
Frequency
0
10
20
30
40
−2
0
2
Tumor−Normal Expression Difference
hsa−miR−3170, proximal
(BMI_extreme = 1; N1 = 17)
1−sided adj pval: 0.175
Frequency
0
1
2
3
4
5
−2
0
2
Tumor−Normal Expression Difference

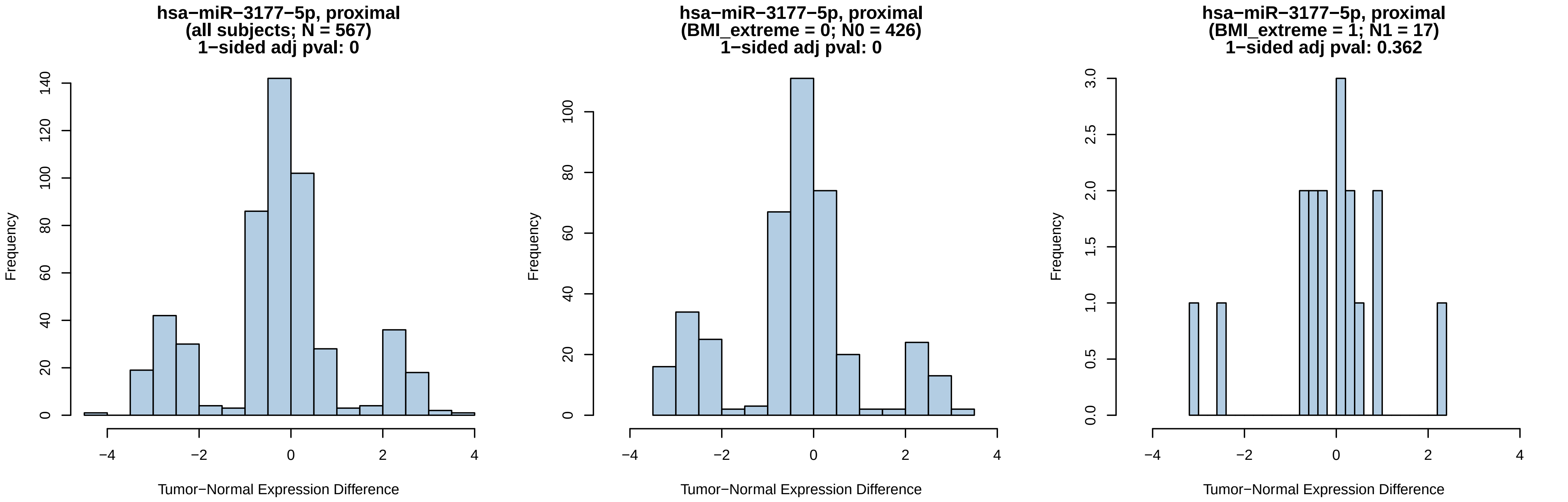
hsa−miR−3177−5p, proximal
(all subjects; N = 567)
1−sided adj pval: 0
Frequency
Tumor−Normal Expression Difference
hsa−miR−3177−5p, proximal
(BMI_extreme = 0; N0 = 426)
1−sided adj pval: 0
Frequency
Tumor−Normal Expression Difference
hsa−miR−3177−5p, proximal
(BMI_extreme = 1; N1 = 17)
1−sided adj pval: 0.362
Frequency
Tumor−Normal Expression Difference

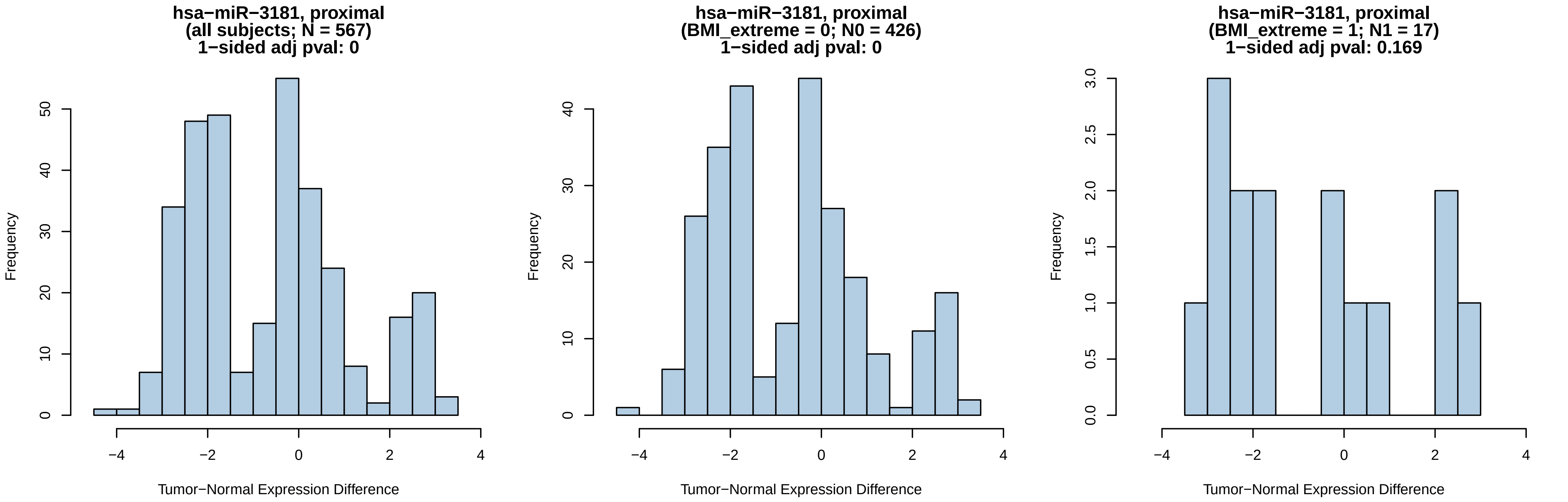

hsa−miR−3181, proximal
(all subjects; N = 567)
1−sided adj pval: 0
Frequency
Tumor−Normal Expression Difference
hsa−miR−3181, proximal
(BMI_extreme = 0; N0 = 426)
1−sided adj pval: 0
Frequency
Tumor−Normal Expression Difference
hsa−miR−3181, proximal
(BMI_extreme = 1; N1 = 17)
1−sided adj pval: 0.169
Frequency
Tumor−Normal Expression Difference

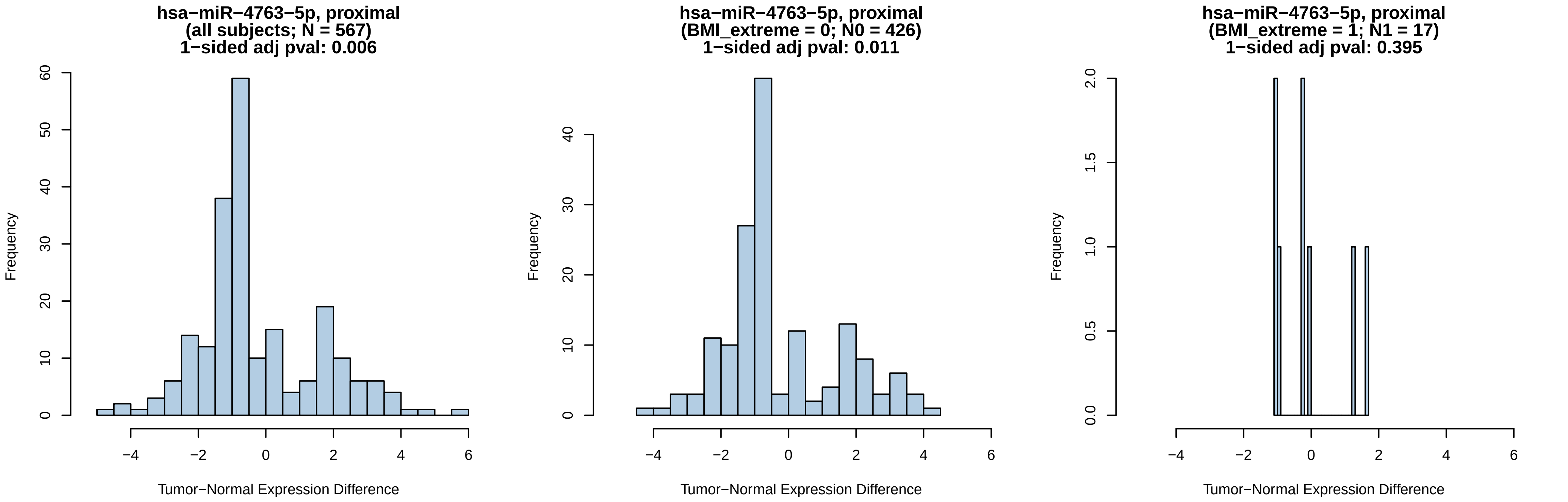
hsa−miR−4763−5p, proximal
(all subjects; N = 567)
1−sided adj pval: 0.006
hsa−miR−4763−5p, proximal
(BMI_extreme = 0; N0 = 426)
1−sided adj pval: 0.011
hsa−miR−4763−5p, proximal
(BMI_extreme = 1; N1 = 17)
1−sided adj pval: 0.395
Frequency
Tumor−Normal Expression Difference

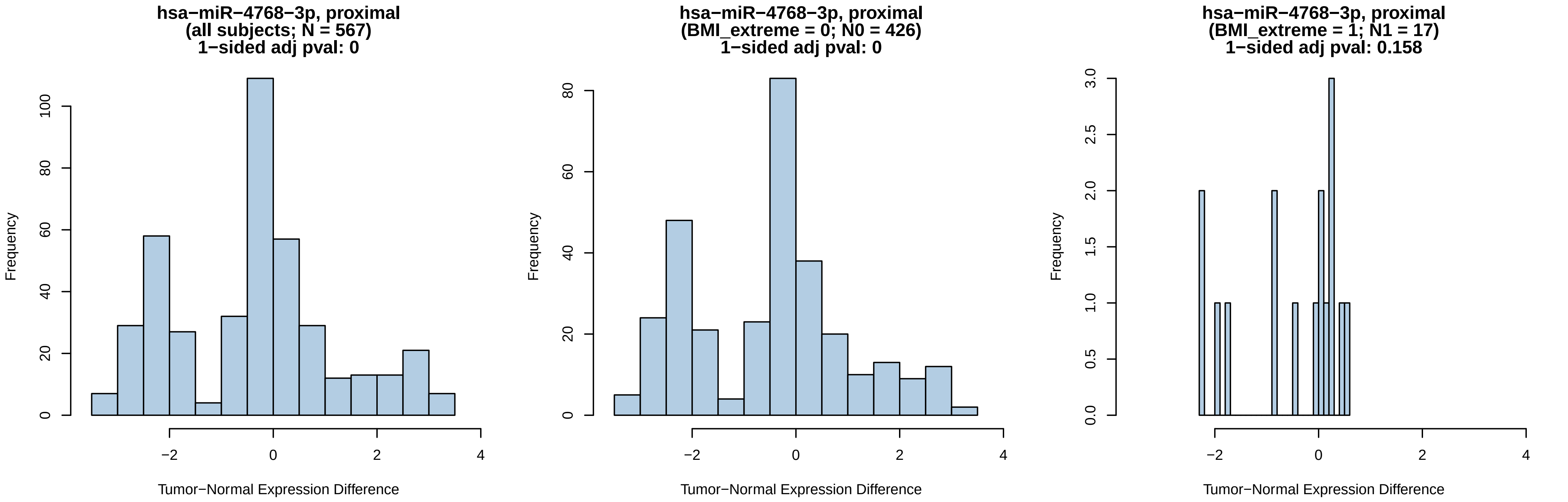
hsa−miR−4768−3p, proximal
(all subjects; N = 567)
1−sided adj pval: 0
Frequency
Tumor−Normal Expression Difference
hsa−miR−4768−3p, proximal
(BMI_extreme = 0; N0 = 426)
1−sided adj pval: 0
Frequency
Tumor−Normal Expression Difference
hsa−miR−4768−3p, proximal
(BMI_extreme = 1; N1 = 17)
1−sided adj pval: 0.158
Frequency
Tumor−Normal Expression Difference

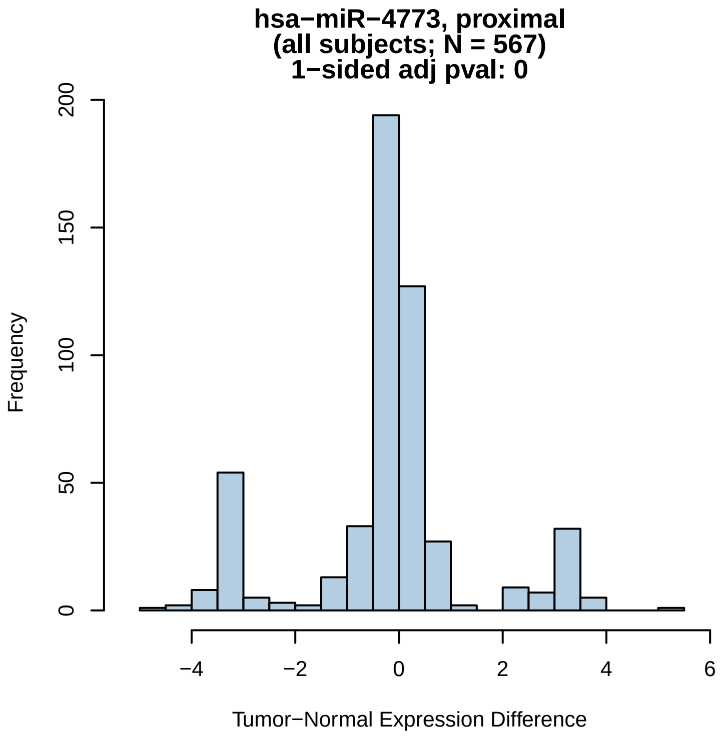
hsa−miR−4773, proximal
(all subjects; N = 567)
1−sided adj pval: 0
Frequency
0
50
100
150
200
−4
−2
0
2
4
6
Tumor−Normal Expression Difference

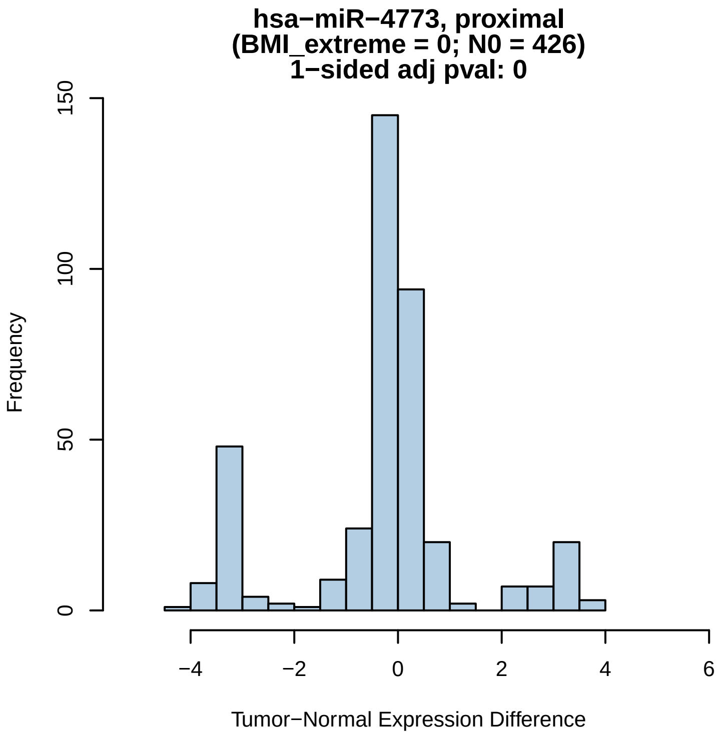
hsa−miR−4773, proximal
(BMI_extreme = 0; N0 = 426)
1−sided adj pval: 0
Frequency
0
50
100
150
−4
−2
0
2
4
6
Tumor−Normal Expression Difference

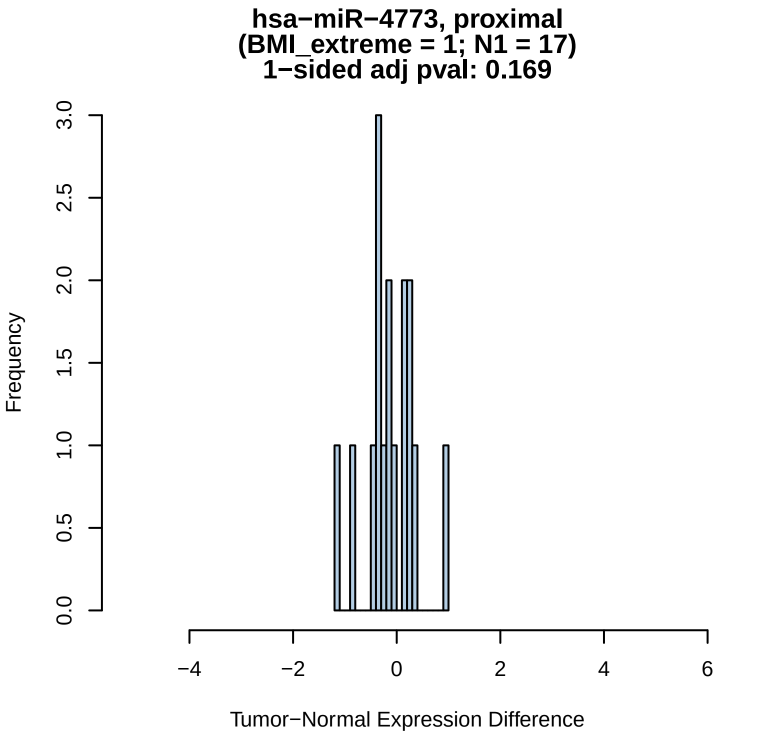
hsa−miR−4773, proximal
(BMI_extreme = 1; N1 = 17)
1−sided adj pval: 0.169
Frequency
0.0
0.5
1.0
1.5
2.0
2.5
3.0
−4
−2
0
2
4
6
Tumor−Normal Expression Difference

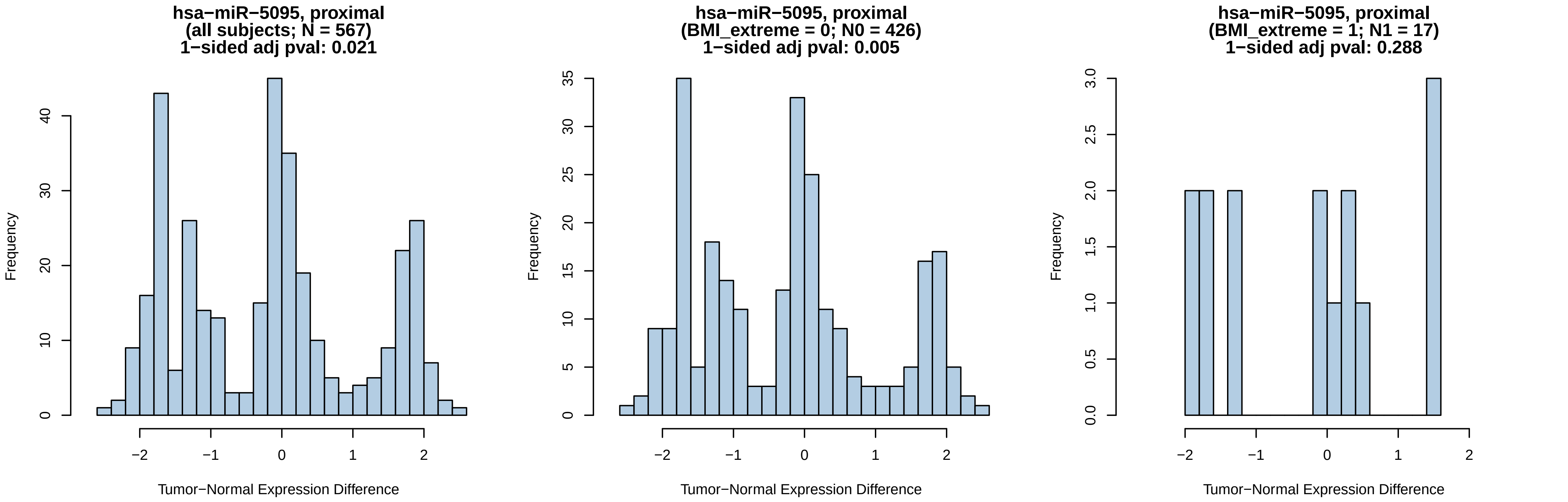

hsa−miR−5095, proximal
(all subjects; N = 567)
1−sided adj pval: 0.021
Frequency
Tumor−Normal Expression Difference
hsa−miR−5095, proximal
(BMI_extreme = 0; N0 = 426)
1−sided adj pval: 0.005
Frequency
Tumor−Normal Expression Difference
hsa−miR−5095, proximal
(BMI_extreme = 1; N1 = 17)
1−sided adj pval: 0.288
Frequency
Tumor−Normal Expression Difference

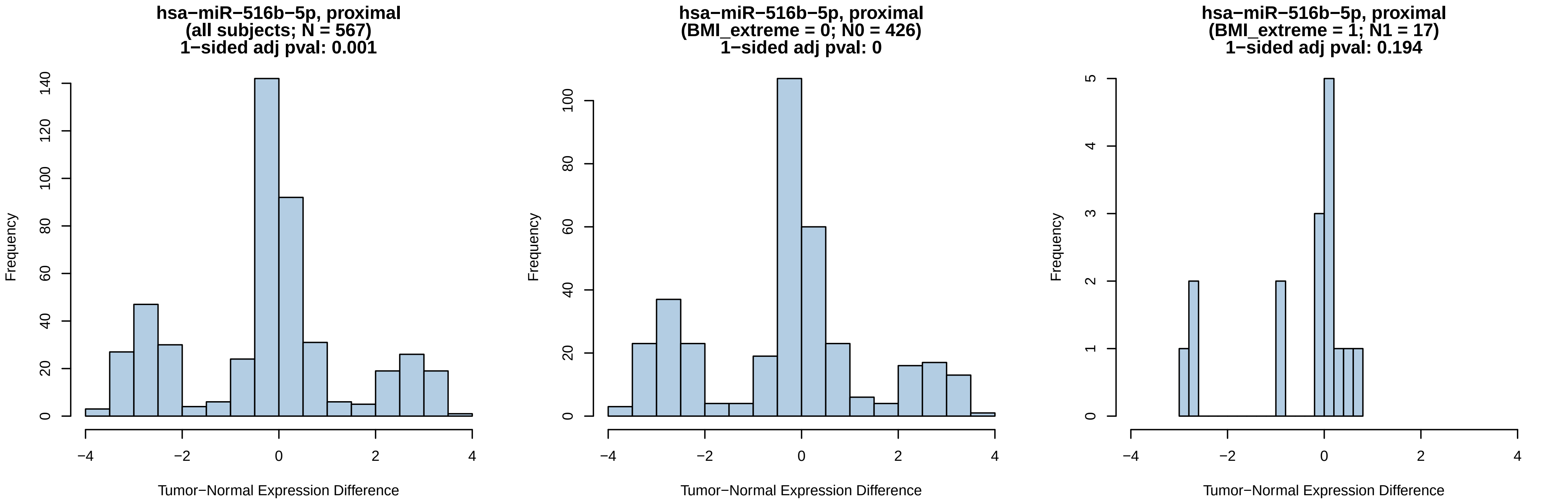

hsa−miR−516b−5p, proximal
(all subjects; N = 567)
1−sided adj pval: 0.001
Frequency
Tumor−Normal Expression Difference
hsa−miR−516b−5p, proximal
(BMI_extreme = 0; N0 = 426)
1−sided adj pval: 0
Frequency
Tumor−Normal Expression Difference
hsa−miR−516b−5p, proximal
(BMI_extreme = 1; N1 = 17)
1−sided adj pval: 0.194
Frequency
Tumor−Normal Expression Difference

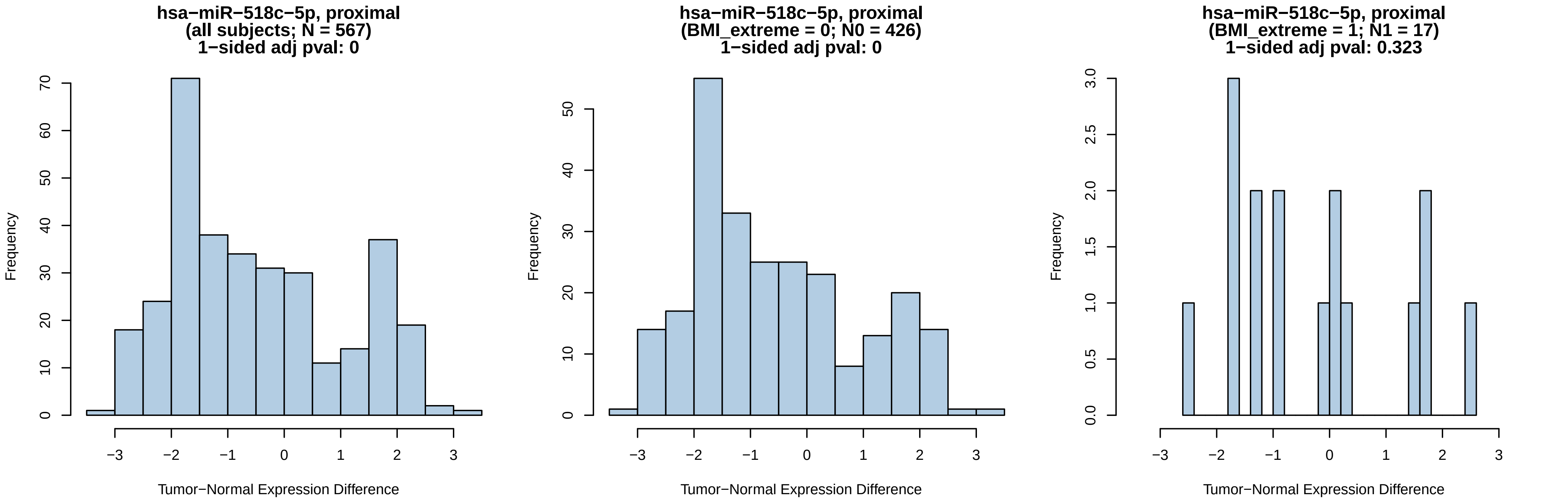

hsa−miR−518c−5p, proximal
(all subjects; N = 567)
1−sided adj pval: 0
Frequency
Tumor−Normal Expression Difference
hsa−miR−518c−5p, proximal
(BMI_extreme = 0; N0 = 426)
1−sided adj pval: 0
Frequency
Tumor−Normal Expression Difference
hsa−miR−518c−5p, proximal
(BMI_extreme = 1; N1 = 17)
1−sided adj pval: 0.323
Frequency
Tumor−Normal Expression Difference

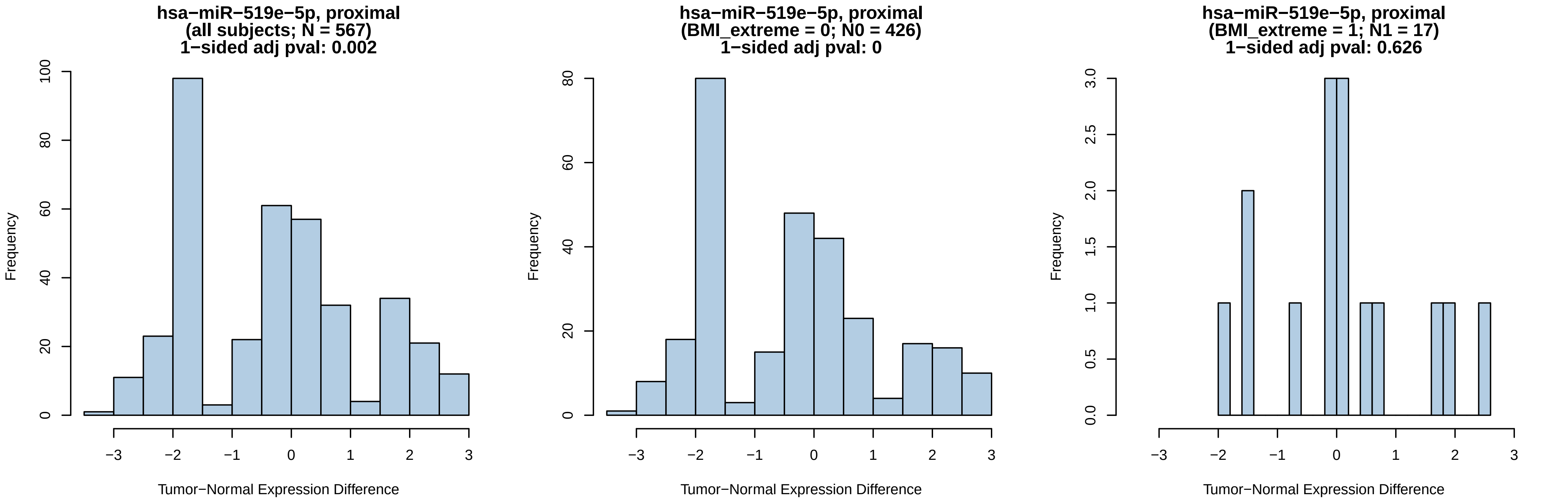

hsa−miR−519e−5p, proximal
(all subjects; N = 567)
1−sided adj pval: 0.002
Frequency
0
20
40
60
80
100
−3
−2
−1
0
1
2
3
Tumor−Normal Expression Difference
hsa−miR−519e−5p, proximal
(BMI_extreme = 0; N0 = 426)
1−sided adj pval: 0
Frequency
0
20
40
60
80
−3
−2
−1
0
1
2
3
Tumor−Normal Expression Difference
hsa−miR−519e−5p, proximal
(BMI_extreme = 1; N1 = 17)
1−sided adj pval: 0.626
Frequency
0.0
0.5
1.0
1.5
2.0
2.5
3.0
−3
−2
−1
0
1
2
3
Tumor−Normal Expression Difference

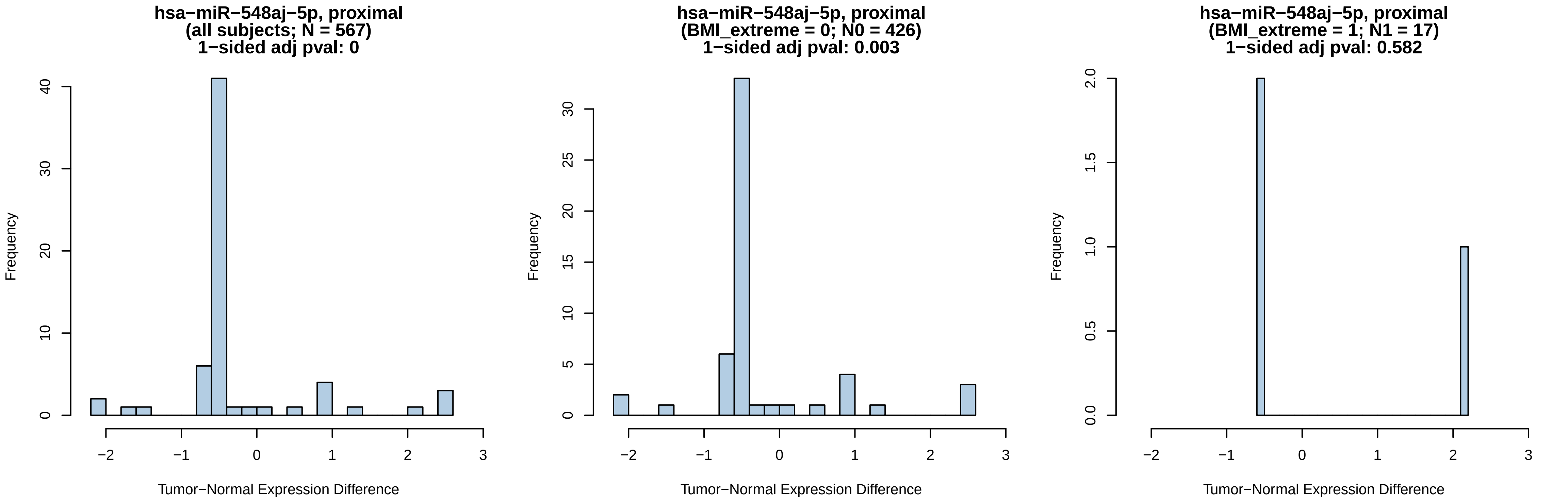

hsa−miR−548aj−5p, proximal
(all subjects; N = 567)
1−sided adj pval: 0
Frequency
Tumor−Normal Expression Difference
hsa−miR−548aj−5p, proximal
(BMI_extreme = 0; N0 = 426)
1−sided adj pval: 0.003
Frequency
Tumor−Normal Expression Difference
hsa−miR−548aj−5p, proximal
(BMI_extreme = 1; N1 = 17)
1−sided adj pval: 0.582
Frequency
Tumor−Normal Expression Difference

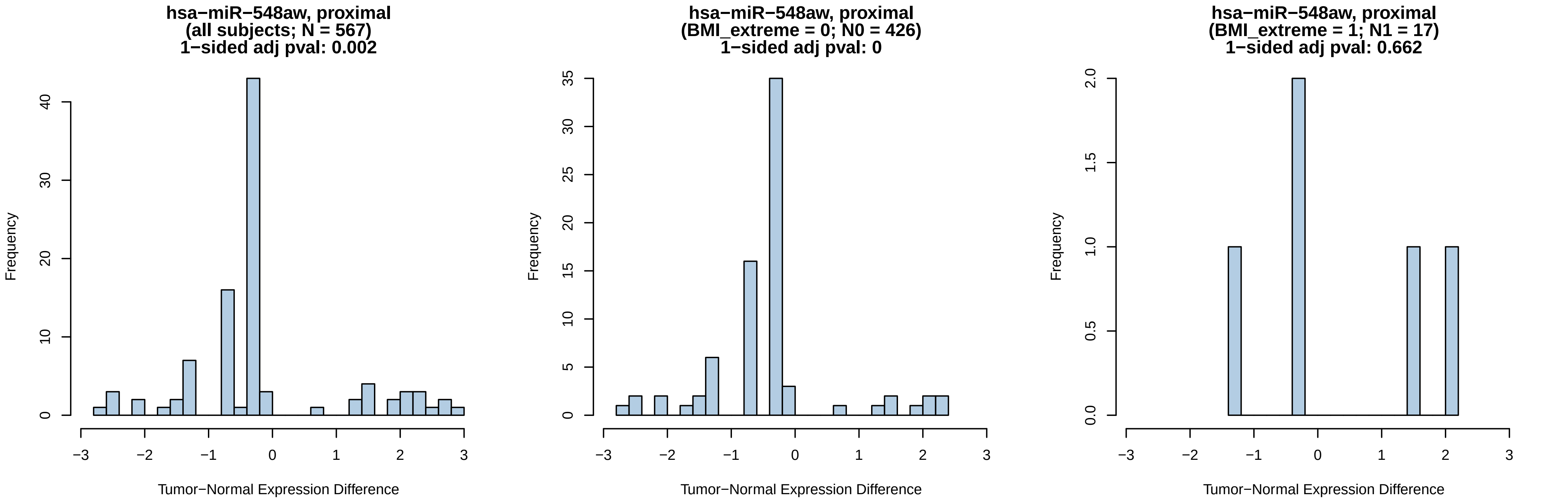
hsa−miR−548aw, proximal
(all subjects; N = 567)
1−sided adj pval: 0.002
Frequency
Tumor−Normal Expression Difference
hsa−miR−548aw, proximal
(BMI_extreme = 0; N0 = 426)
1−sided adj pval: 0
Frequency
Tumor−Normal Expression Difference
hsa−miR−548aw, proximal
(BMI_extreme = 1; N1 = 17)
1−sided adj pval: 0.662
Frequency
Tumor−Normal Expression Difference

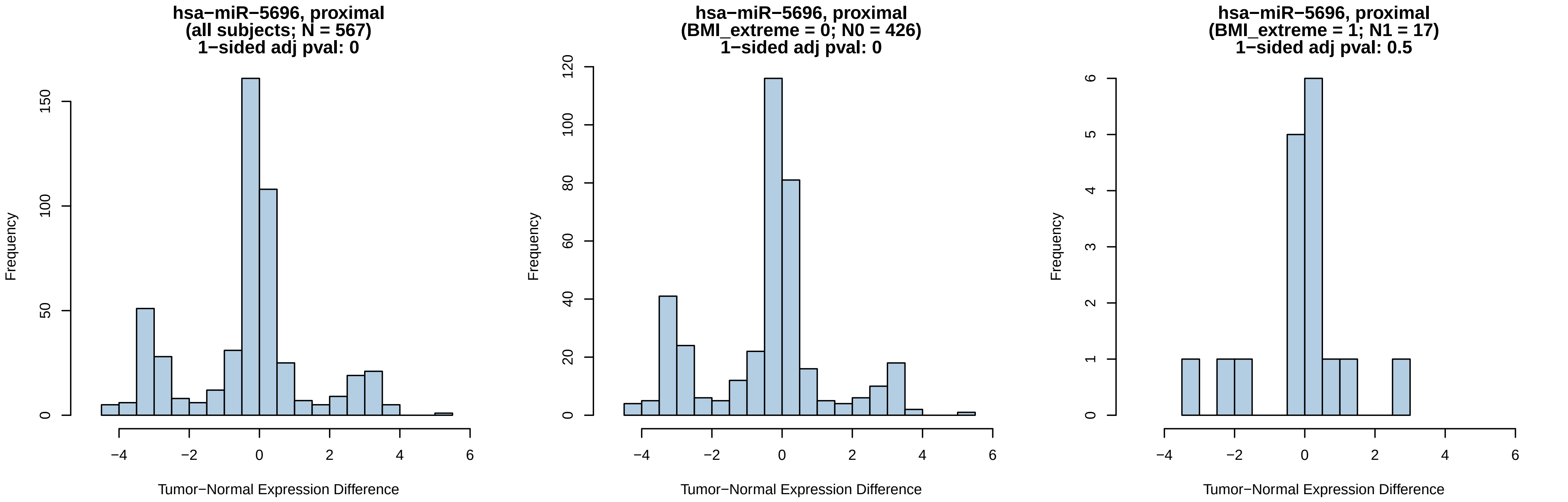
hsa−miR−5696, proximal
(all subjects; N = 567)
1−sided adj pval: 0
Frequency
0
50
100
150
−4
−2
0
2
4
6
Tumor−Normal Expression Difference
hsa−miR−5696, proximal
(BMI_extreme = 0; N0 = 426)
1−sided adj pval: 0
Frequency
0
20
40
60
80
100
120
−4
−2
0
2
4
6
Tumor−Normal Expression Difference
hsa−miR−5696, proximal
(BMI_extreme = 1; N1 = 17)
1−sided adj pval: 0.5
Frequency
0
1
2
3
4
5
6
−4
−2
0
2
4
6
Tumor−Normal Expression Difference

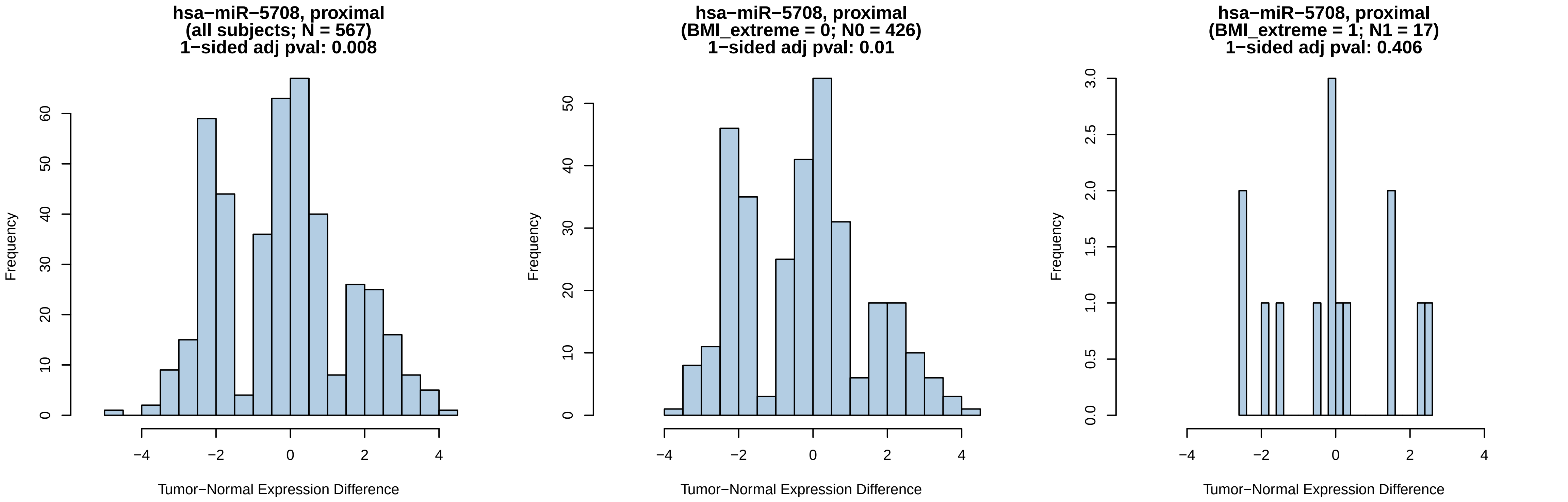
hsa−miR−5708, proximal
(all subjects; N = 567)
1−sided adj pval: 0.008
hsa−miR−5708, proximal
(BMI_extreme = 0; N0 = 426)
1−sided adj pval: 0.01
hsa−miR−5708, proximal
(BMI_extreme = 1; N1 = 17)
1−sided adj pval: 0.406
Frequency
Tumor−Normal Expression Difference

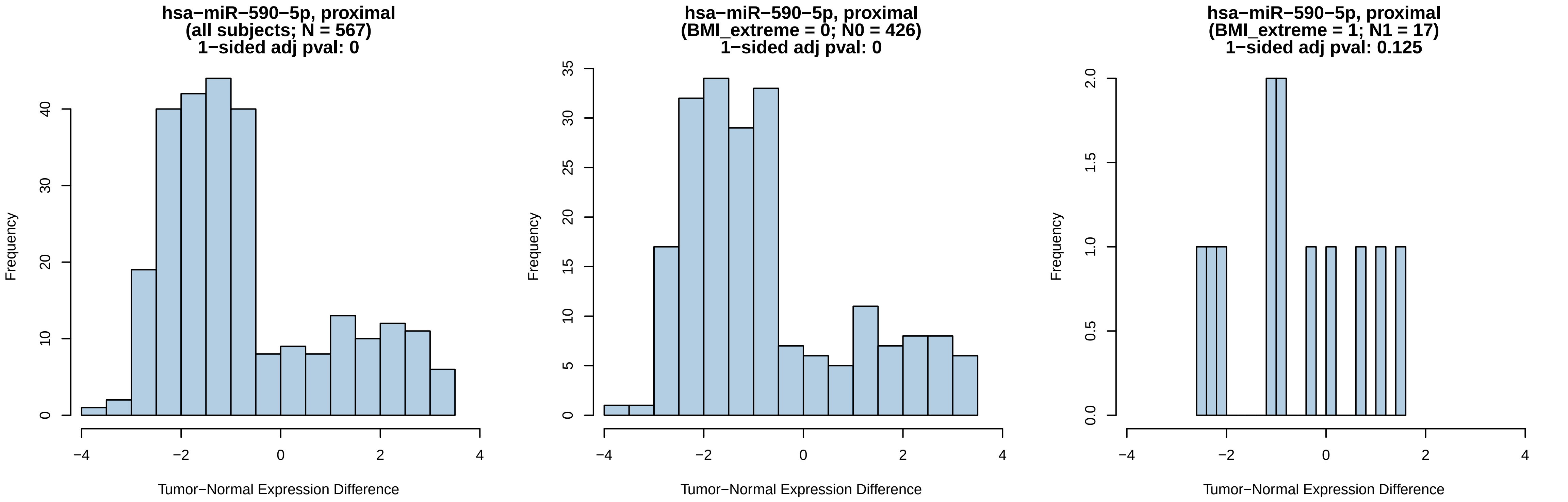
hsa−miR−590−5p, proximal
(all subjects; N = 567)
1−sided adj pval: 0
Frequency
Tumor−Normal Expression Difference
hsa−miR−590−5p, proximal
(BMI_extreme = 0; N0 = 426)
1−sided adj pval: 0
Frequency
Tumor−Normal Expression Difference
hsa−miR−590−5p, proximal
(BMI_extreme = 1; N1 = 17)
1−sided adj pval: 0.125
Frequency
Tumor−Normal Expression Difference

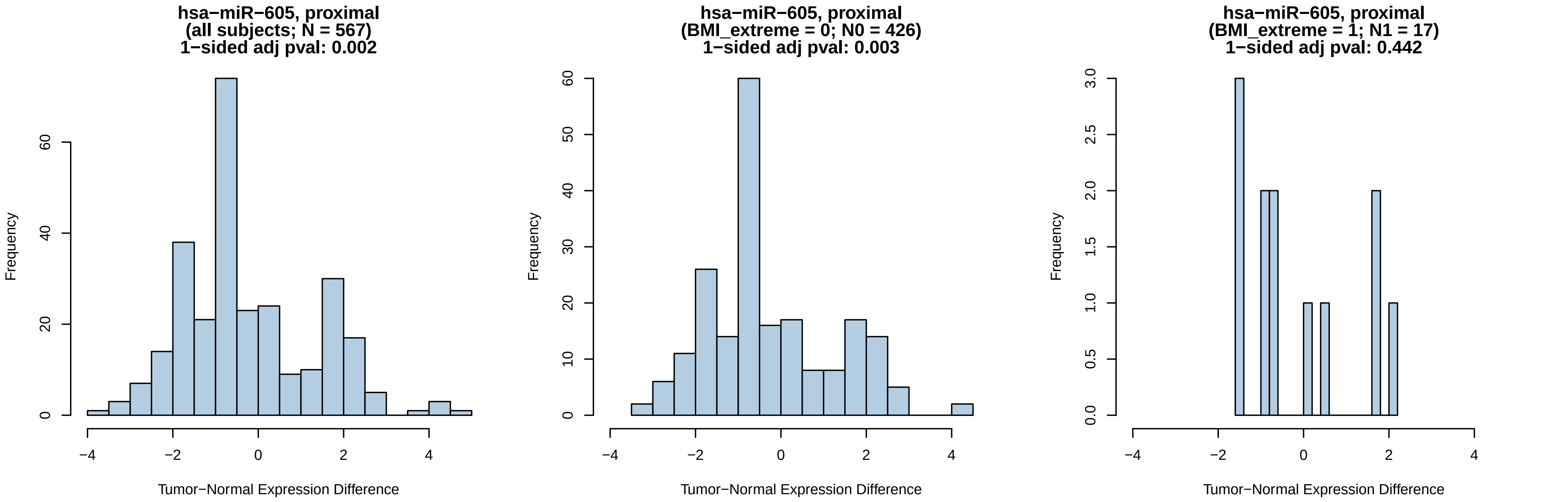
hsa−miR−605, proximal
(all subjects; N = 567)
1−sided adj pval: 0.002
Frequency
0
20
40
60
−4
−2
0
2
4
Tumor−Normal Expression Difference
hsa−miR−605, proximal
(BMI_extreme = 0; N0 = 426)
1−sided adj pval: 0.003
Frequency
0
10
20
30
40
50
60
−4
−2
0
2
4
Tumor−Normal Expression Difference
hsa−miR−605, proximal
(BMI_extreme = 1; N1 = 17)
1−sided adj pval: 0.442
Frequency
0.0
0.5
1.0
1.5
2.0
2.5
3.0
−4
−2
0
2
4
Tumor−Normal Expression Difference

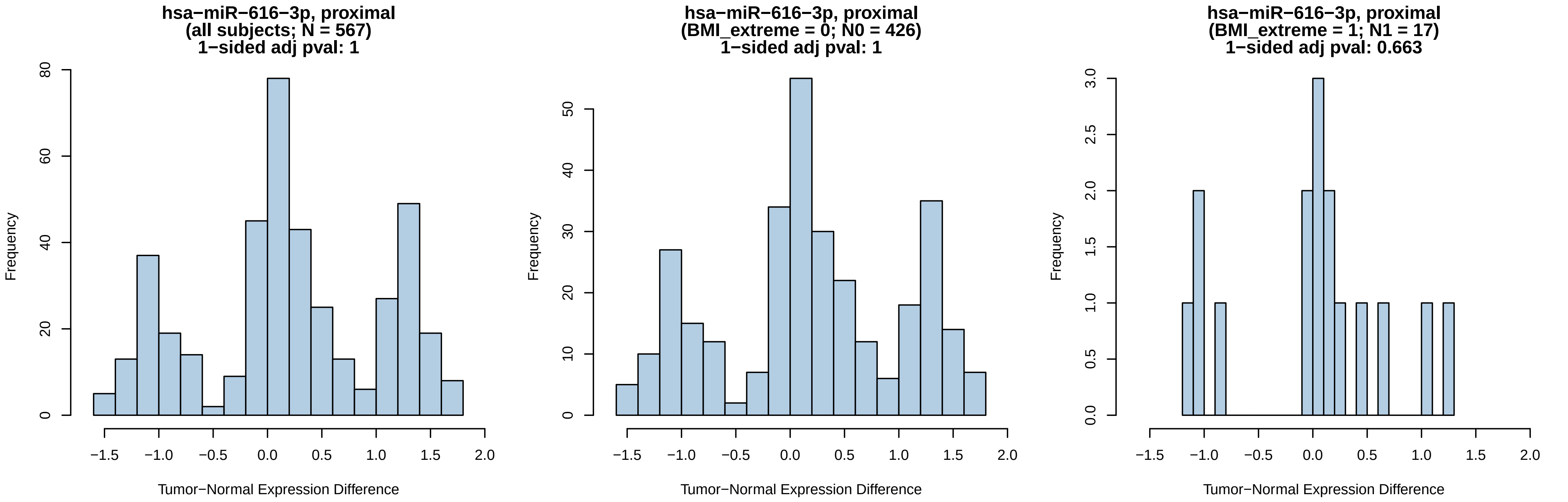

hsa−miR−616−3p, proximal
(all subjects; N = 567)
1−sided adj pval: 1
Frequency
0
20
40
60
80
−1.5
−1.0
−0.5
0.0
0.5
1.0
1.5
2.0
Tumor−Normal Expression Difference
hsa−miR−616−3p, proximal
(BMI_extreme = 0; N0 = 426)
1−sided adj pval: 1
Frequency
0
10
20
30
40
50
−1.5
−1.0
−0.5
0.0
0.5
1.0
1.5
2.0
Tumor−Normal Expression Difference
hsa−miR−616−3p, proximal
(BMI_extreme = 1; N1 = 17)
1−sided adj pval: 0.663
Frequency
0.0
0.5
1.0
1.5
2.0
2.5
3.0
−1.5
−1.0
−0.5
0.0
0.5
1.0
1.5
2.0
Tumor−Normal Expression Difference

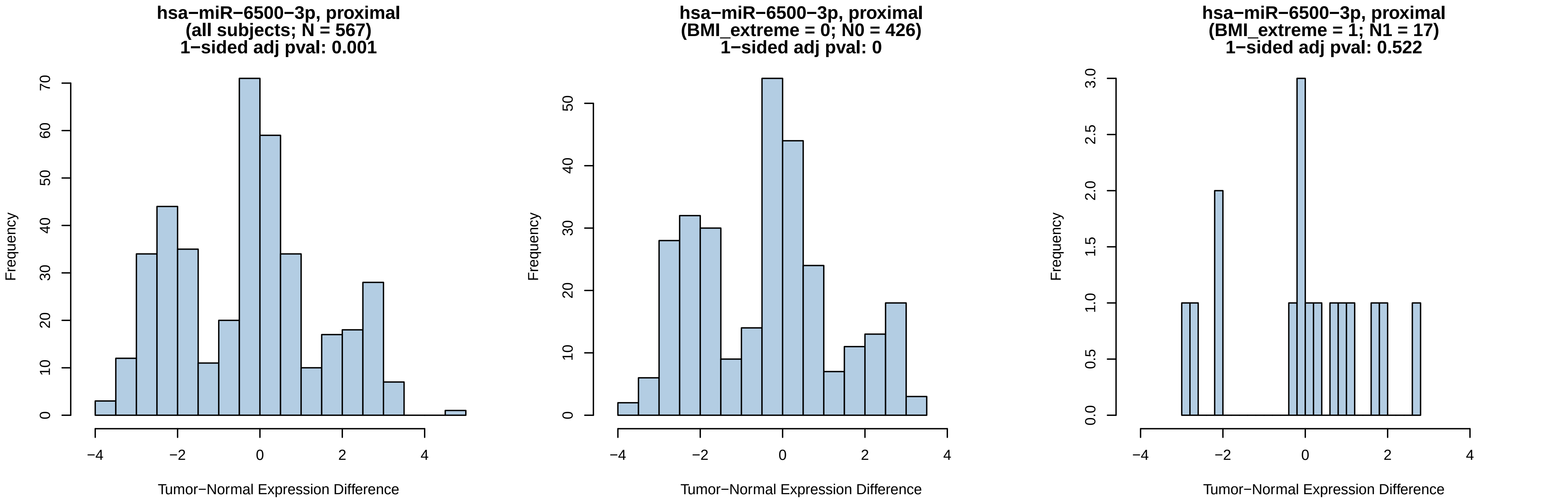
hsa−miR−6500−3p, proximal
(all subjects; N = 567)
1−sided adj pval: 0.001
hsa−miR−6500−3p, proximal
(BMI_extreme = 0; N0 = 426)
1−sided adj pval: 0
hsa−miR−6500−3p, proximal
(BMI_extreme = 1; N1 = 17)
1−sided adj pval: 0.522
Frequency
Tumor−Normal Expression Difference

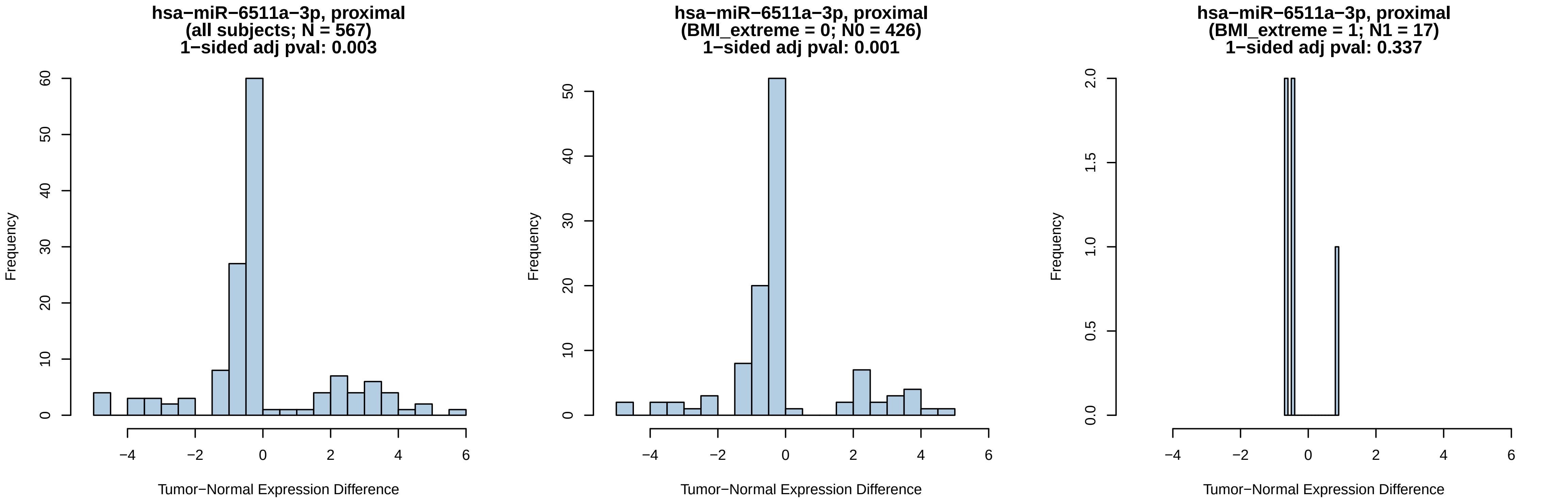

hsa−miR−6511a−3p, proximal
(all subjects; N = 567)
1−sided adj pval: 0.003
Frequency
Tumor−Normal Expression Difference
hsa−miR−6511a−3p, proximal
(BMI_extreme = 0; N0 = 426)
1−sided adj pval: 0.001
Frequency
Tumor−Normal Expression Difference
hsa−miR−6511a−3p, proximal
(BMI_extreme = 1; N1 = 17)
1−sided adj pval: 0.337
Frequency
Tumor−Normal Expression Difference

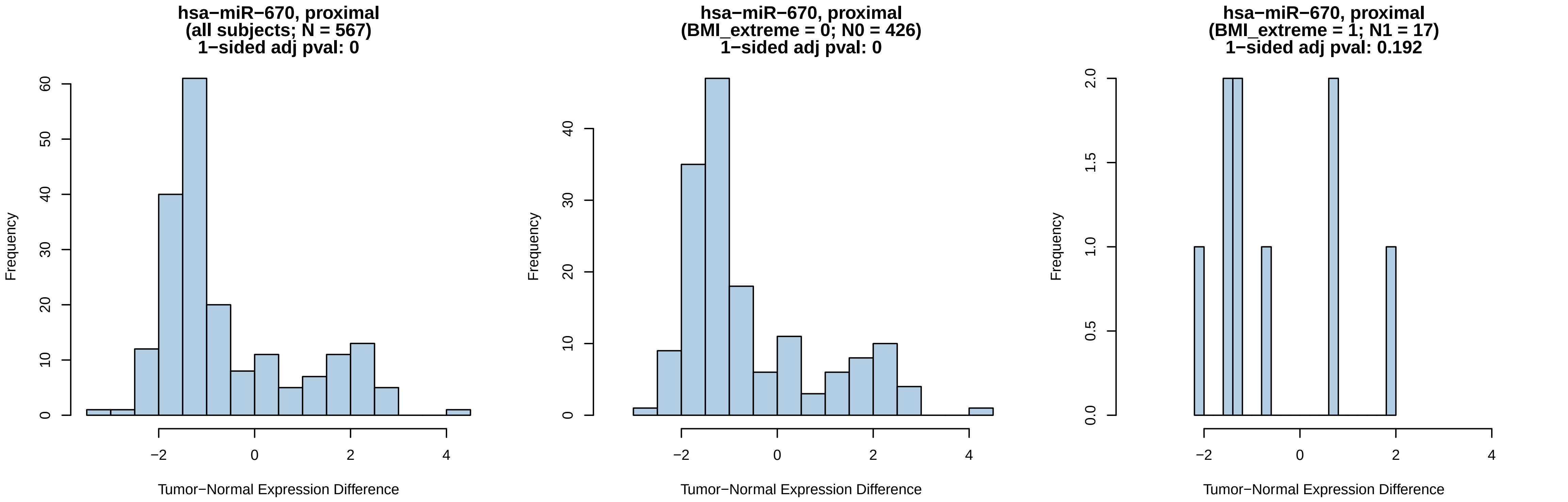

hsa−miR−670, proximal
(all subjects; N = 567)
1−sided adj pval: 0
Frequency
Tumor−Normal Expression Difference
hsa−miR−670, proximal
(BMI_extreme = 0; N0 = 426)
1−sided adj pval: 0
Frequency
Tumor−Normal Expression Difference
hsa−miR−670, proximal
(BMI_extreme = 1; N1 = 17)
1−sided adj pval: 0.192
Frequency
Tumor−Normal Expression Difference

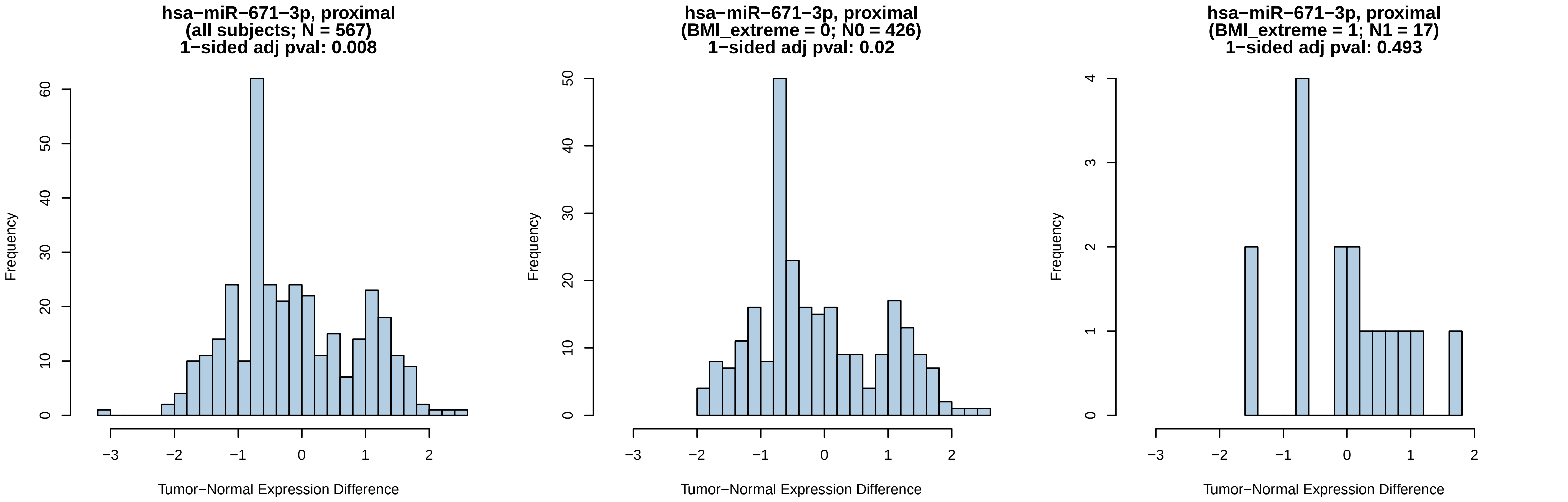

hsa−miR−671−3p, proximal
(all subjects; N = 567)
1−sided adj pval: 0.008
Frequency
Tumor−Normal Expression Difference
hsa−miR−671−3p, proximal
(BMI_extreme = 0; N0 = 426)
1−sided adj pval: 0.02
Frequency
Tumor−Normal Expression Difference
hsa−miR−671−3p, proximal
(BMI_extreme = 1; N1 = 17)
1−sided adj pval: 0.493
Frequency
Tumor−Normal Expression Difference

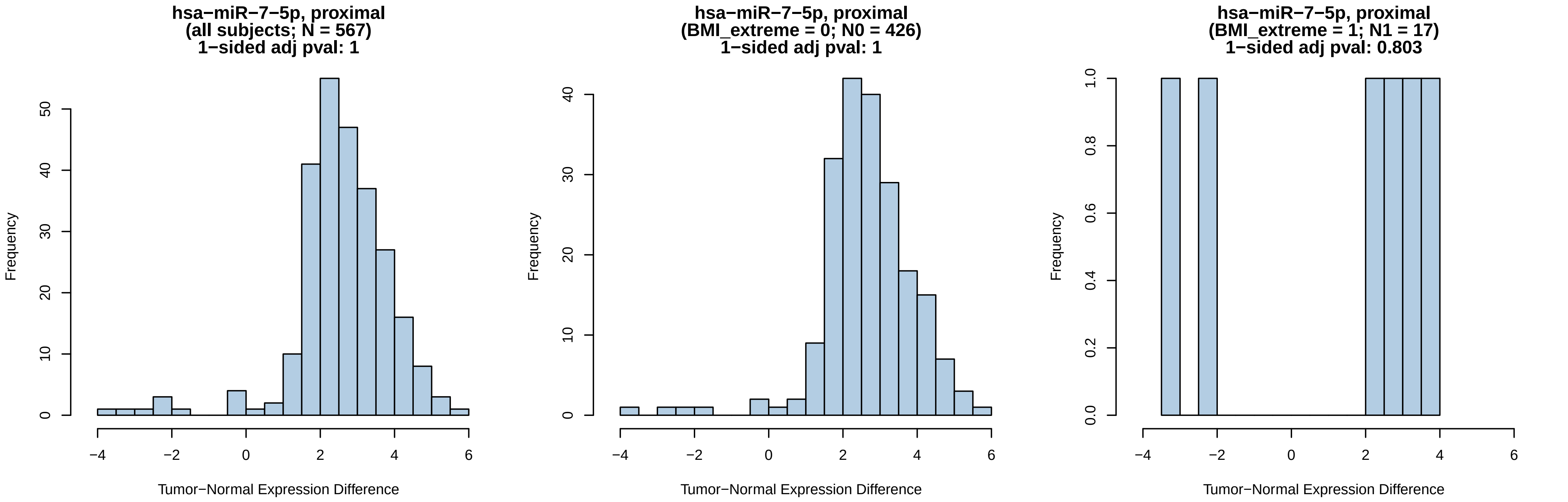
hsa−miR−7−5p, proximal
(all subjects; N = 567)
1−sided adj pval: 1
Frequency
Tumor−Normal Expression Difference
hsa−miR−7−5p, proximal
(BMI_extreme = 0; N0 = 426)
1−sided adj pval: 1
Frequency
Tumor−Normal Expression Difference
hsa−miR−7−5p, proximal
(BMI_extreme = 1; N1 = 17)
1−sided adj pval: 0.803
Frequency
Tumor−Normal Expression Difference

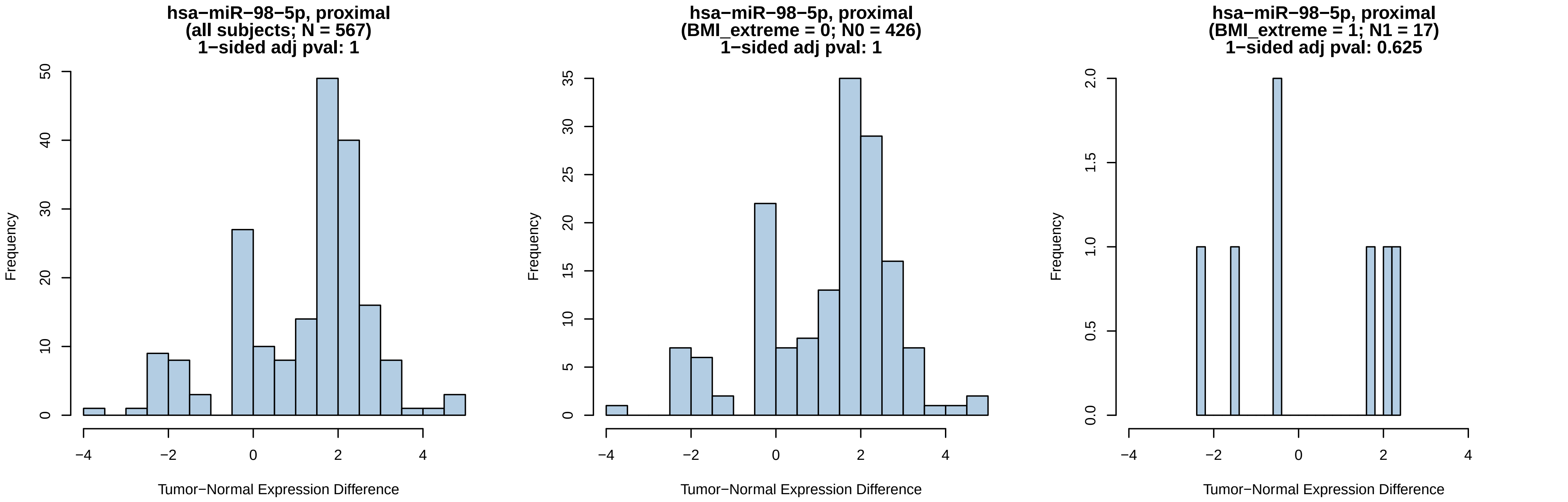

hsa−miR−98−5p, proximal
(all subjects; N = 567)
1−sided adj pval: 1
Frequency
Tumor−Normal Expression Difference
hsa−miR−98−5p, proximal
(BMI_extreme = 0; N0 = 426)
1−sided adj pval: 1
Frequency
Tumor−Normal Expression Difference
hsa−miR−98−5p, proximal
(BMI_extreme = 1; N1 = 17)
1−sided adj pval: 0.625
Frequency
Tumor−Normal Expression Difference

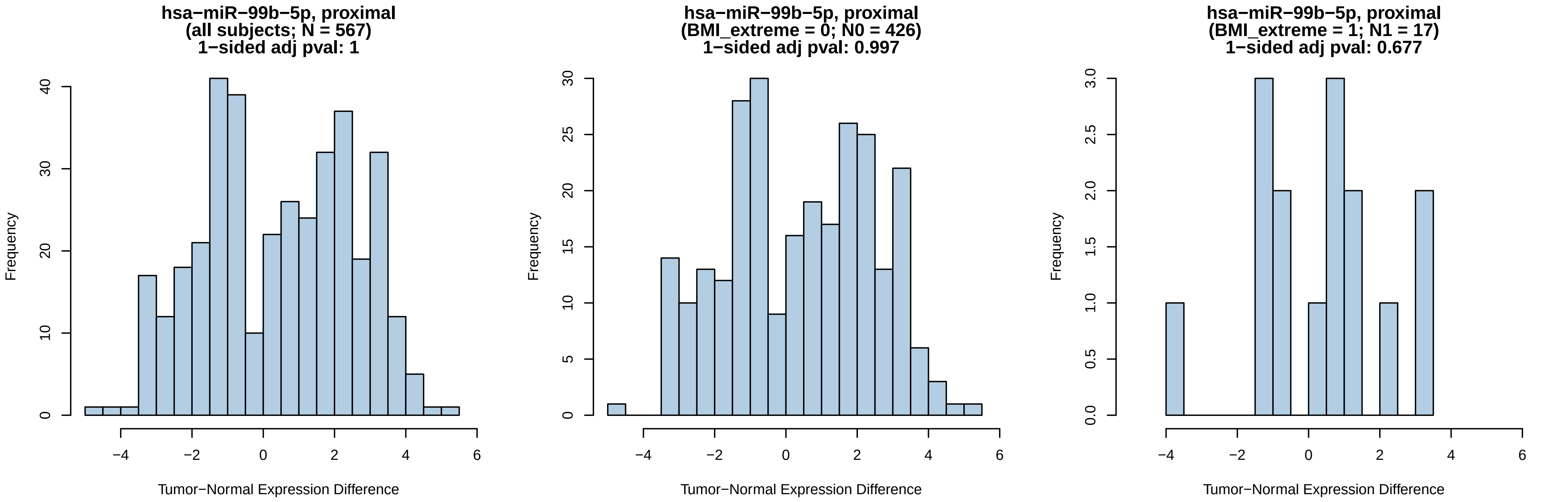

hsa−miR−99b−5p, proximal
(all subjects; N = 567)
1−sided adj pval: 1
Frequency
Tumor−Normal Expression Difference
hsa−miR−99b−5p, proximal
(BMI_extreme = 0; N0 = 426)
1−sided adj pval: 0.997
Frequency
Tumor−Normal Expression Difference
hsa−miR−99b−5p, proximal
(BMI_extreme = 1; N1 = 17)
1−sided adj pval: 0.677
Frequency
Tumor−Normal Expression Difference

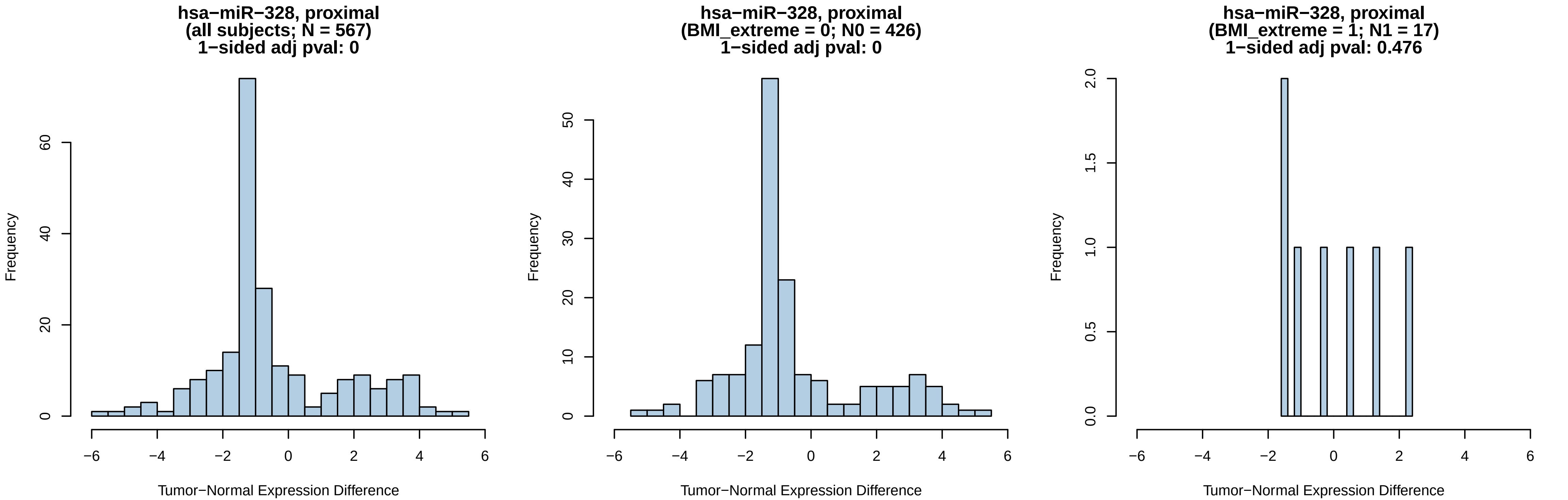
hsa−miR−328, proximal
(all subjects; N = 567)
1−sided adj pval: 0
hsa−miR−328, proximal
(BMI_extreme = 0; N0 = 426)
1−sided adj pval: 0
hsa−miR−328, proximal
(BMI_extreme = 1; N1 = 17)
1−sided adj pval: 0.476
Frequency
Tumor−Normal Expression Difference

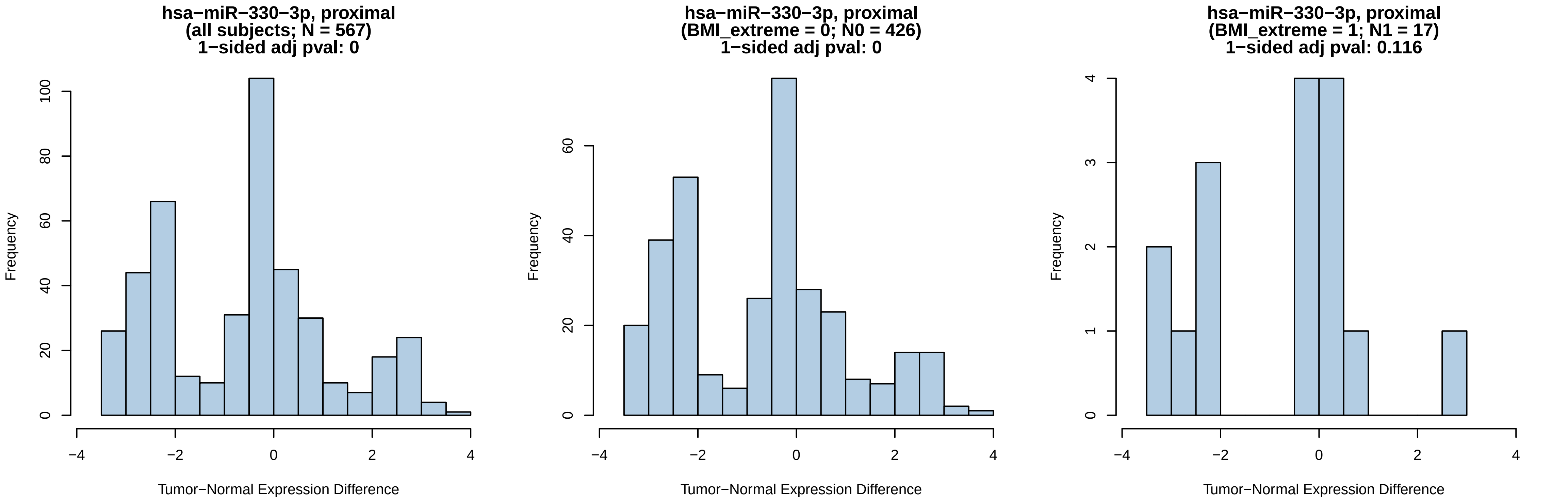
hsa−miR−330−3p, proximal
(all subjects; N = 567)
1−sided adj pval: 0
Frequency
Tumor−Normal Expression Difference
hsa−miR−330−3p, proximal
(BMI_extreme = 0; N0 = 426)
1−sided adj pval: 0
Frequency
Tumor−Normal Expression Difference
hsa−miR−330−3p, proximal
(BMI_extreme = 1; N1 = 17)
1−sided adj pval: 0.116
Frequency
Tumor−Normal Expression Difference

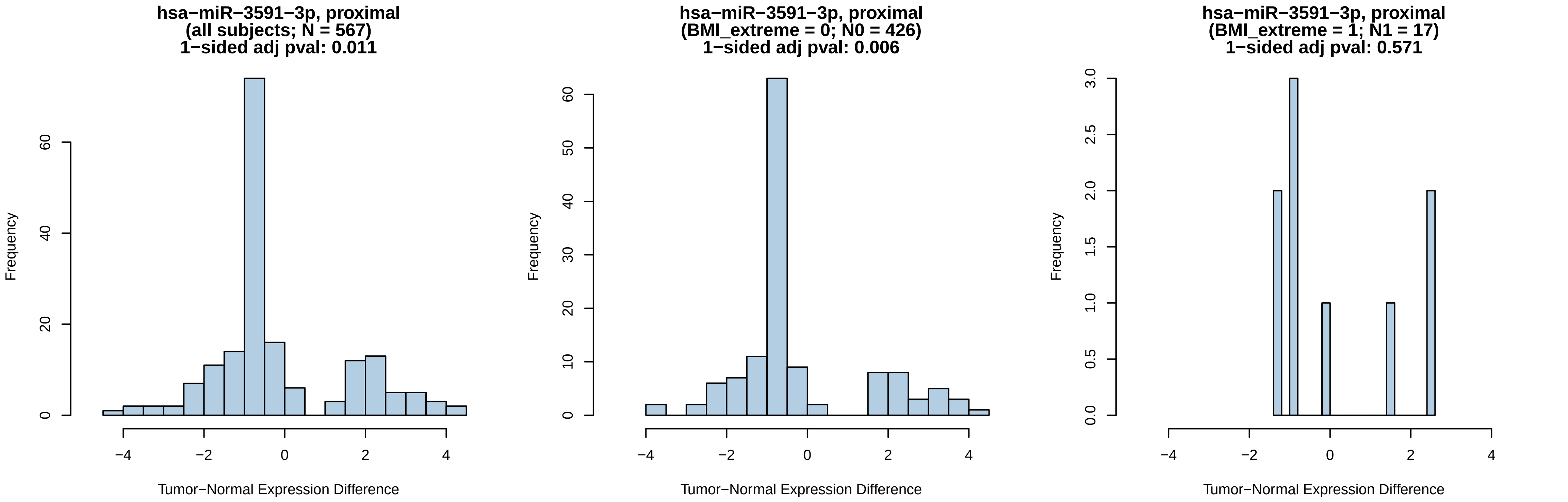
hsa−miR−3591−3p, proximal
(all subjects; N = 567)
1−sided adj pval: 0.011
Frequency
Tumor−Normal Expression Difference
hsa−miR−3591−3p, proximal
(BMI_extreme = 0; N0 = 426)
1−sided adj pval: 0.006
Frequency
Tumor−Normal Expression Difference
hsa−miR−3591−3p, proximal
(BMI_extreme = 1; N1 = 17)
1−sided adj pval: 0.571
Frequency
Tumor−Normal Expression Difference

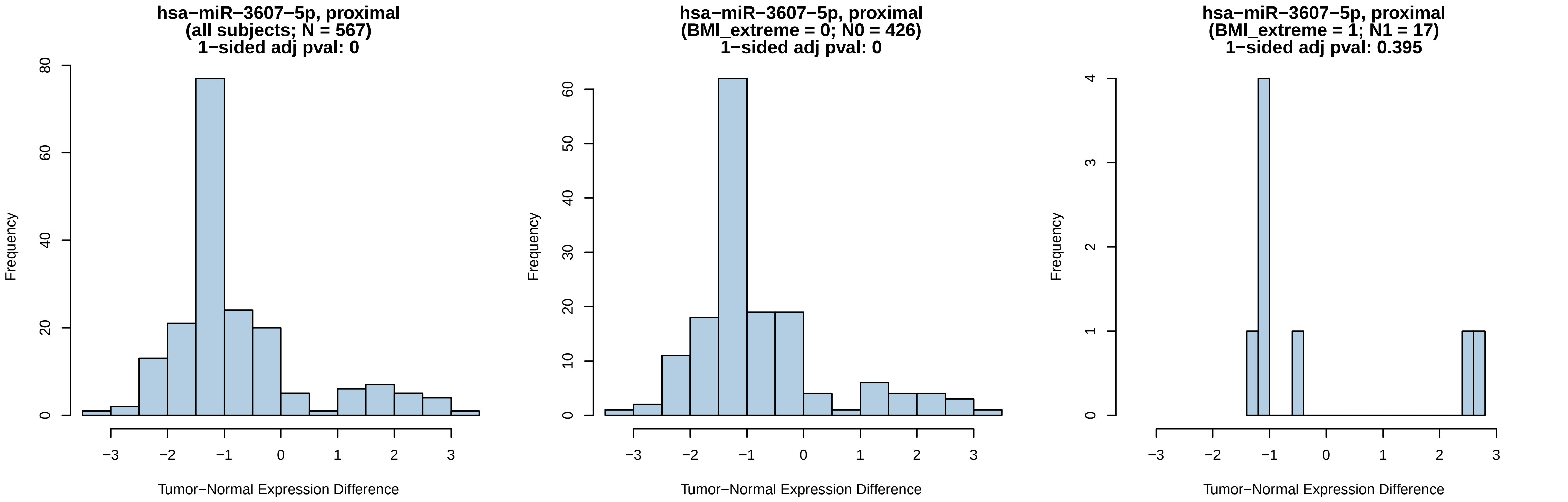
hsa−miR−3607−5p, proximal
(all subjects; N = 567)
1−sided adj pval: 0
Frequency
Tumor−Normal Expression Difference
hsa−miR−3607−5p, proximal
(BMI_extreme = 0; N0 = 426)
1−sided adj pval: 0
Frequency
Tumor−Normal Expression Difference
hsa−miR−3607−5p, proximal
(BMI_extreme = 1; N1 = 17)
1−sided adj pval: 0.395
Frequency
Tumor−Normal Expression Difference

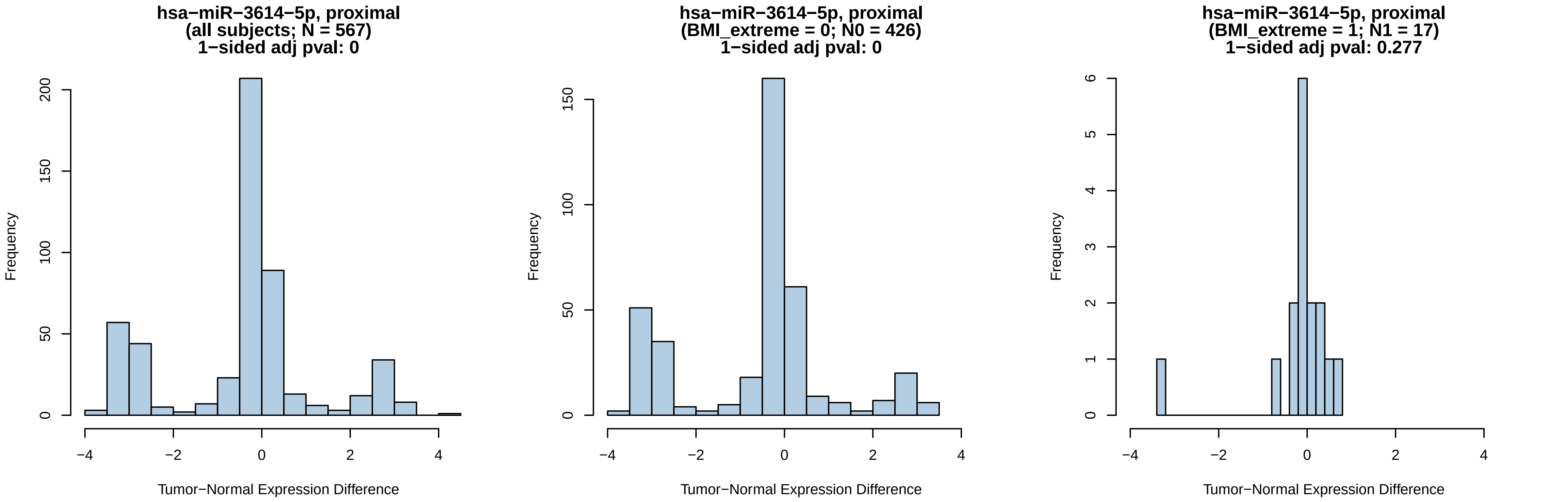

hsa−miR−3614−5p, proximal
(all subjects; N = 567)
1−sided adj pval: 0
Frequency
0
50
100
150
200
−4
−2
0
2
4
Tumor−Normal Expression Difference
hsa−miR−3614−5p, proximal
(BMI_extreme = 0; N0 = 426)
1−sided adj pval: 0
Frequency
0
50
100
150
−4
−2
0
2
4
Tumor−Normal Expression Difference
hsa−miR−3614−5p, proximal
(BMI_extreme = 1; N1 = 17)
1−sided adj pval: 0.277
Frequency
0
1
2
3
4
5
6
−4
−2
0
2
4
Tumor−Normal Expression Difference

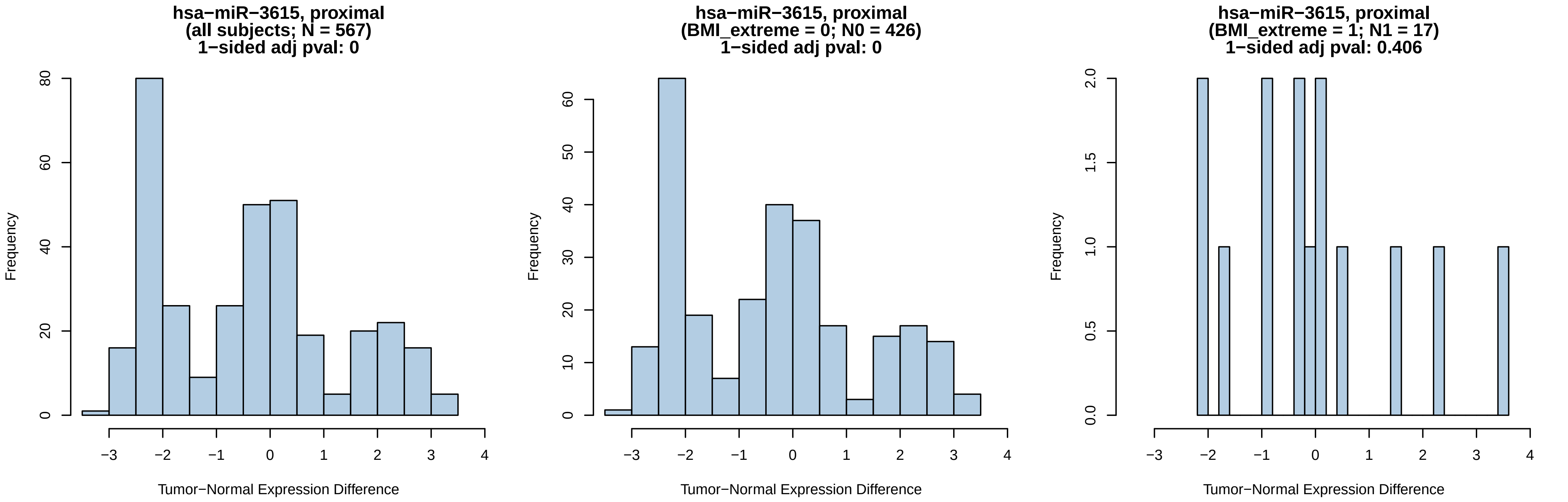

hsa−miR−3615, proximal
(all subjects; N = 567)
1−sided adj pval: 0
Frequency
0
20
40
60
80
−3
−2
−1
0
1
2
3
4
Tumor−Normal Expression Difference
hsa−miR−3615, proximal
(BMI_extreme = 0; N0 = 426)
1−sided adj pval: 0
Frequency
0
10
20
30
40
50
60
−3
−2
−1
0
1
2
3
4
Tumor−Normal Expression Difference
hsa−miR−3615, proximal
(BMI_extreme = 1; N1 = 17)
1−sided adj pval: 0.406
Frequency
0.0
0.5
1.0
1.5
2.0
−3
−2
−1
0
1
2
3
4
Tumor−Normal Expression Difference

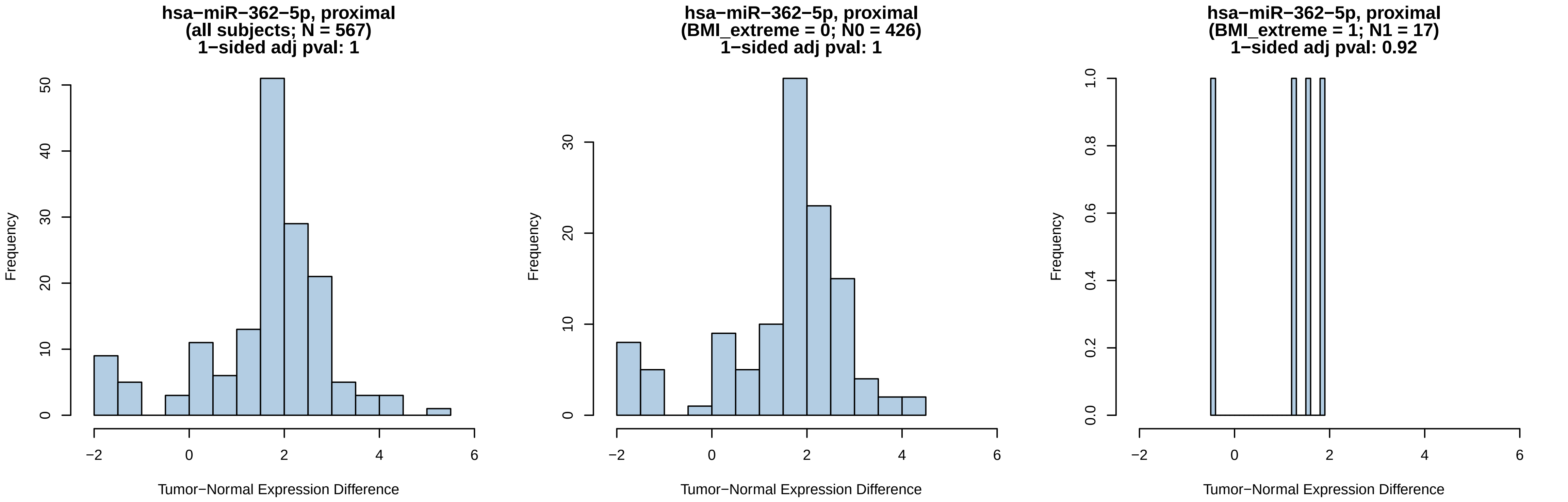
hsa−miR−362−5p, proximal
(all subjects; N = 567)
1−sided adj pval: 1
hsa−miR−362−5p, proximal
(BMI_extreme = 0; N0 = 426)
1−sided adj pval: 1
hsa−miR−362−5p, proximal
(BMI_extreme = 1; N1 = 17)
1−sided adj pval: 0.92
Frequency
Tumor−Normal Expression Difference

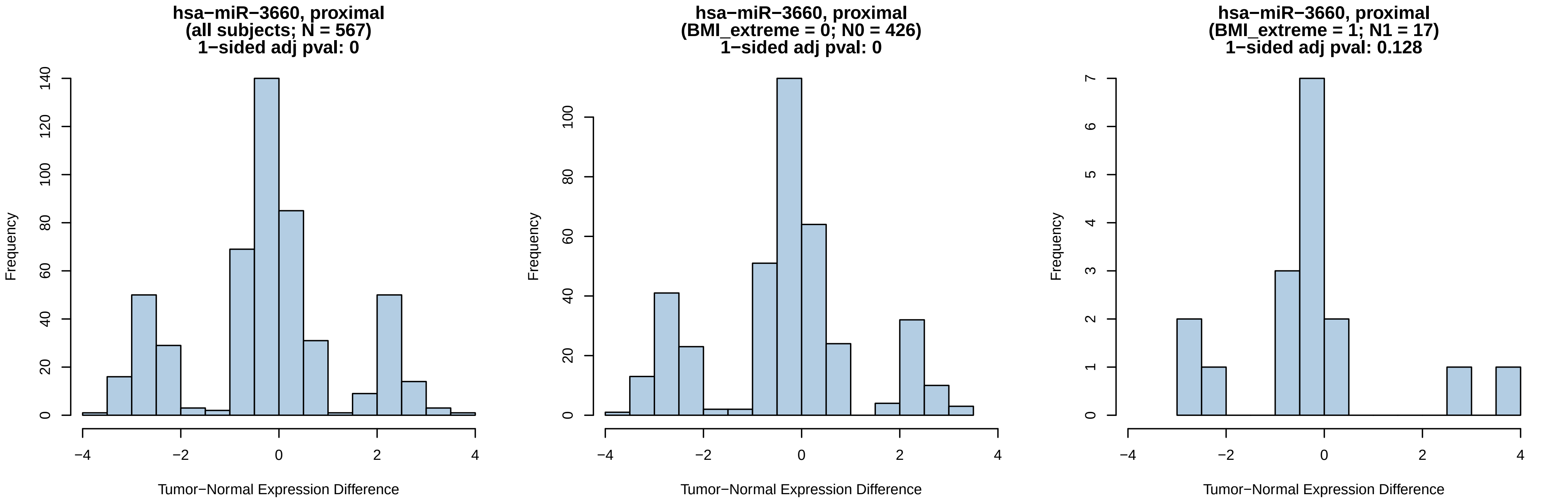
hsa−miR−3660, proximal
(all subjects; N = 567)
1−sided adj pval: 0
hsa−miR−3660, proximal
(BMI_extreme = 0; N0 = 426)
1−sided adj pval: 0
hsa−miR−3660, proximal
(BMI_extreme = 1; N1 = 17)
1−sided adj pval: 0.128
Frequency
Tumor−Normal Expression Difference

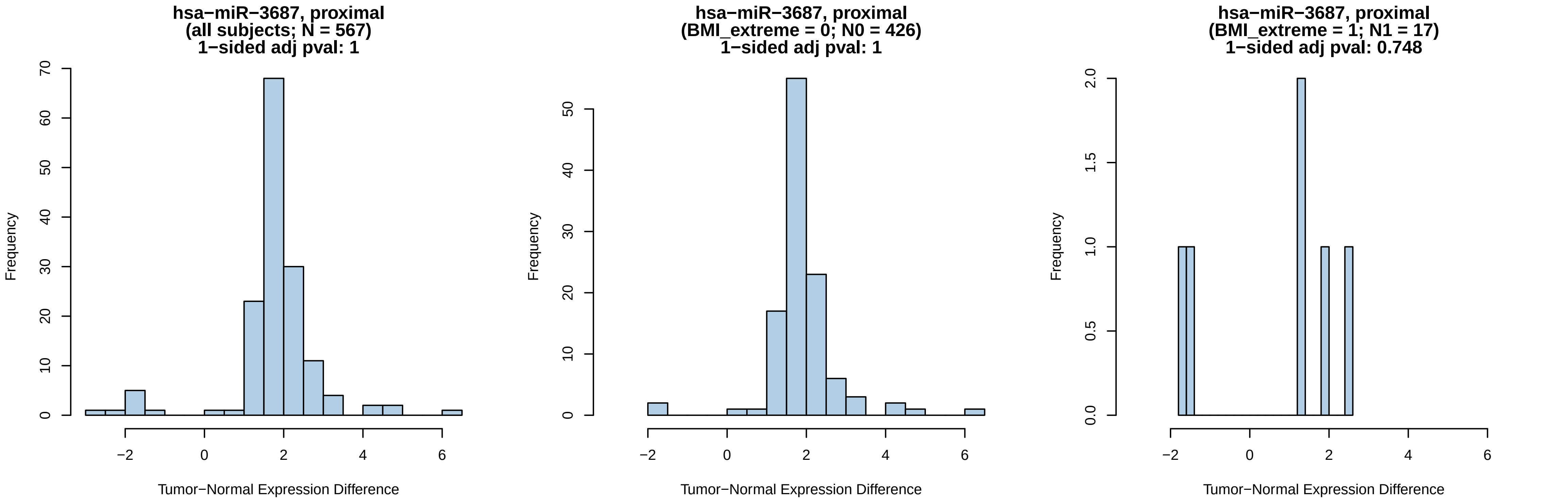
hsa−miR−3687, proximal
(all subjects; N = 567)
1−sided adj pval: 1
Frequency
Tumor−Normal Expression Difference
hsa−miR−3687, proximal
(BMI_extreme = 0; N0 = 426)
1−sided adj pval: 1
Frequency
Tumor−Normal Expression Difference
hsa−miR−3687, proximal
(BMI_extreme = 1; N1 = 17)
1−sided adj pval: 0.748
Frequency
Tumor−Normal Expression Difference

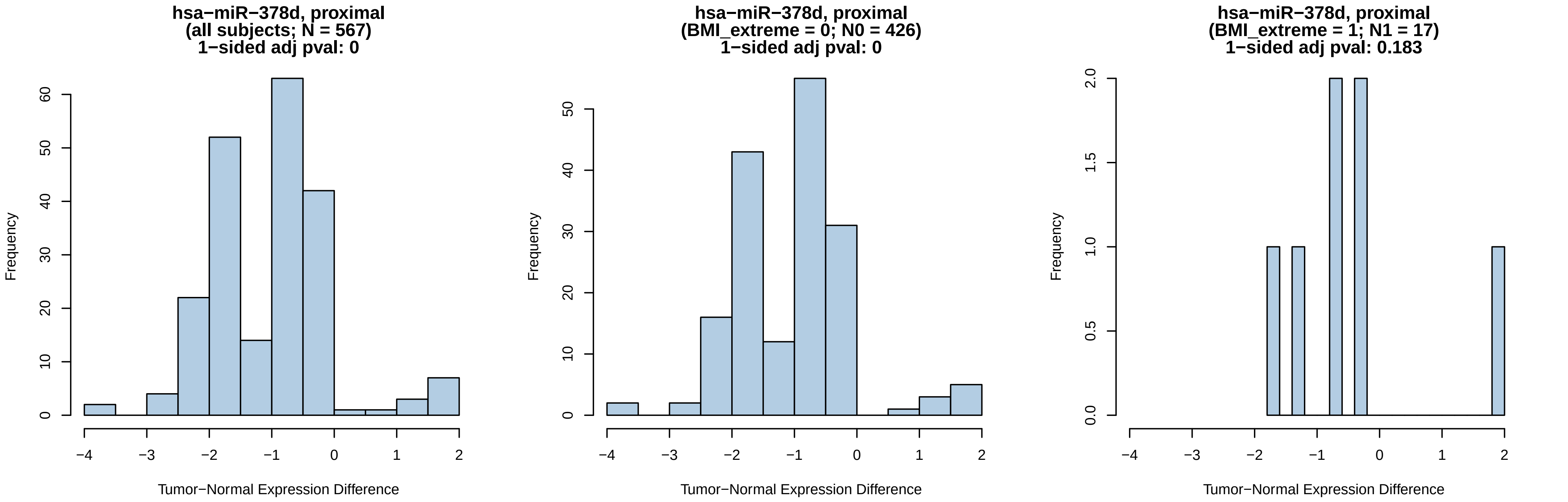

hsa−miR−378d, proximal
(all subjects; N = 567)
1−sided adj pval: 0
hsa−miR−378d, proximal
(BMI_extreme = 0; N0 = 426)
1−sided adj pval: 0
hsa−miR−378d, proximal
(BMI_extreme = 1; N1 = 17)
1−sided adj pval: 0.183
Frequency
Tumor−Normal Expression Difference

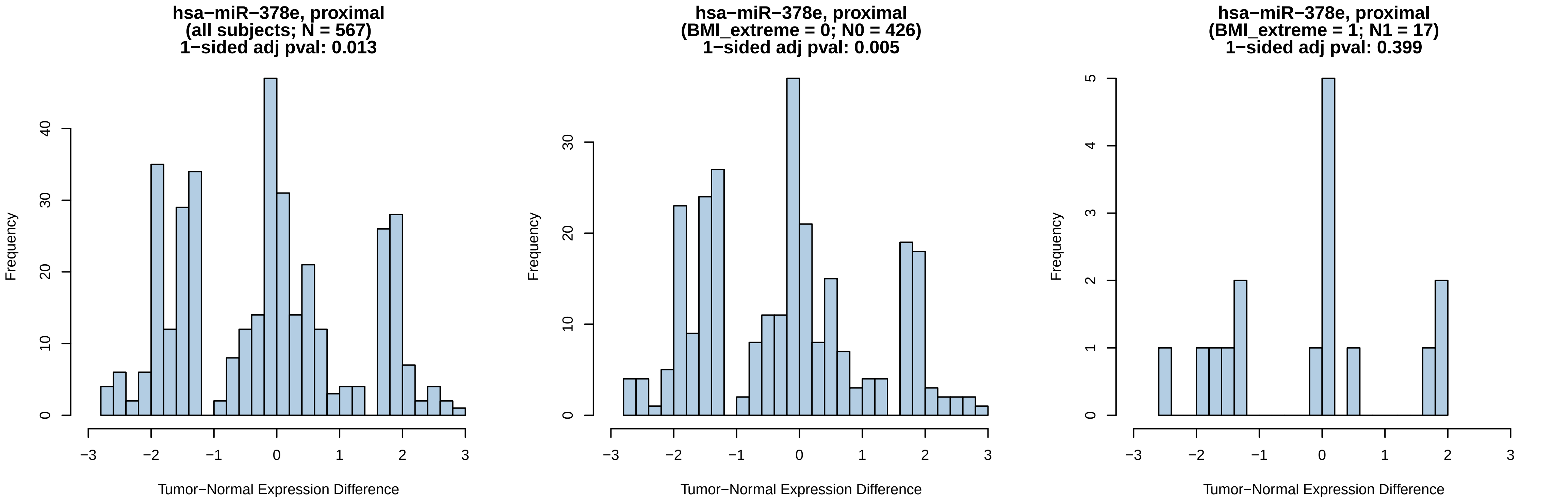
hsa−miR−378e, proximal
(all subjects; N = 567)
1−sided adj pval: 0.013
hsa−miR−378e, proximal
(BMI_extreme = 0; N0 = 426)
1−sided adj pval: 0.005
hsa−miR−378e, proximal
(BMI_extreme = 1; N1 = 17)
1−sided adj pval: 0.399
Frequency
Tumor−Normal Expression Difference

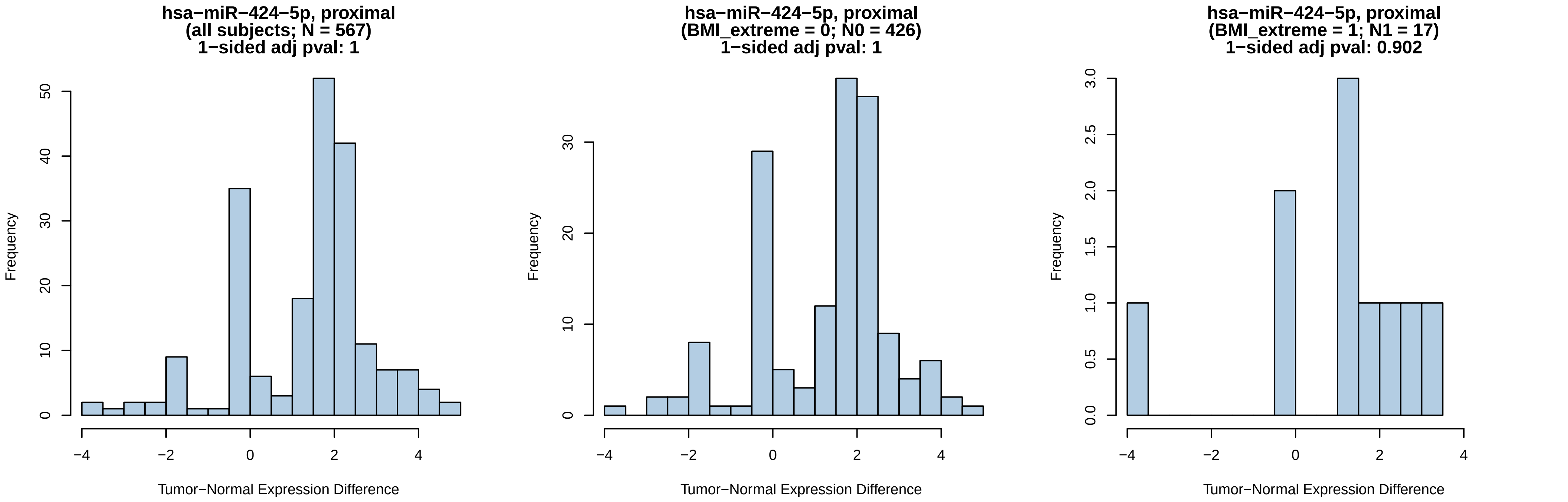
hsa−miR−424−5p, proximal
(all subjects; N = 567)
1−sided adj pval: 1
Frequency
Tumor−Normal Expression Difference
hsa−miR−424−5p, proximal
(BMI_extreme = 0; N0 = 426)
1−sided adj pval: 1
Frequency
Tumor−Normal Expression Difference
hsa−miR−424−5p, proximal
(BMI_extreme = 1; N1 = 17)
1−sided adj pval: 0.902
Frequency
Tumor−Normal Expression Difference

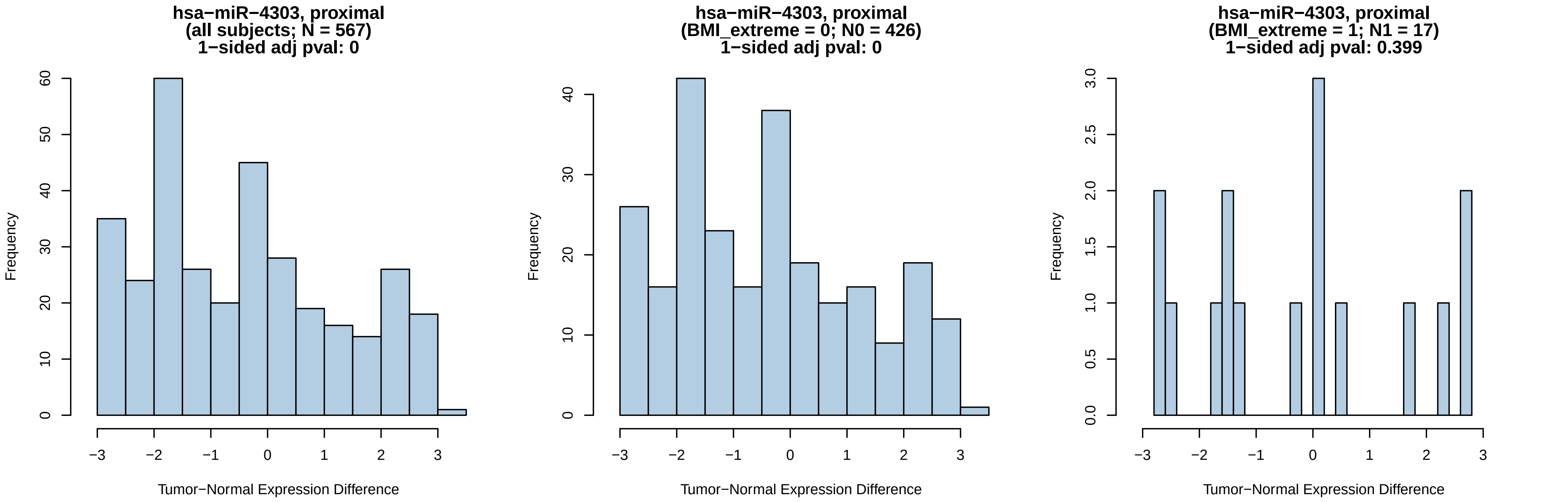

hsa−miR−4303, proximal
(all subjects; N = 567)
1−sided adj pval: 0
Frequency
0
10
20
30
40
50
60
−3
−2
−1
0
1
2
3
Tumor−Normal Expression Difference
hsa−miR−4303, proximal
(BMI_extreme = 0; N0 = 426)
1−sided adj pval: 0
Frequency
0
10
20
30
40
−3
−2
−1
0
1
2
3
Tumor−Normal Expression Difference
hsa−miR−4303, proximal
(BMI_extreme = 1; N1 = 17)
1−sided adj pval: 0.399
Frequency
0.0
0.5
1.0
1.5
2.0
2.5
3.0
−3
−2
−1
0
1
2
3
Tumor−Normal Expression Difference

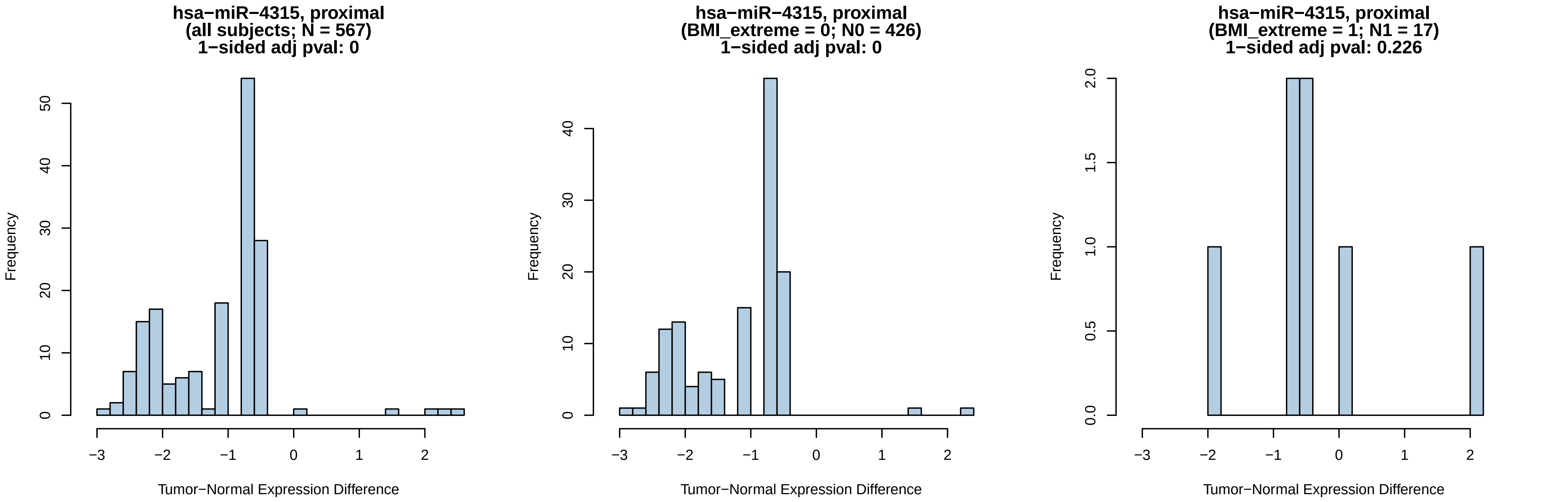

hsa−miR−4315, proximal
(all subjects; N = 567)
1−sided adj pval: 0
Frequency
Tumor−Normal Expression Difference
hsa−miR−4315, proximal
(BMI_extreme = 0; N0 = 426)
1−sided adj pval: 0
Frequency
Tumor−Normal Expression Difference
hsa−miR−4315, proximal
(BMI_extreme = 1; N1 = 17)
1−sided adj pval: 0.226
Frequency
Tumor−Normal Expression Difference

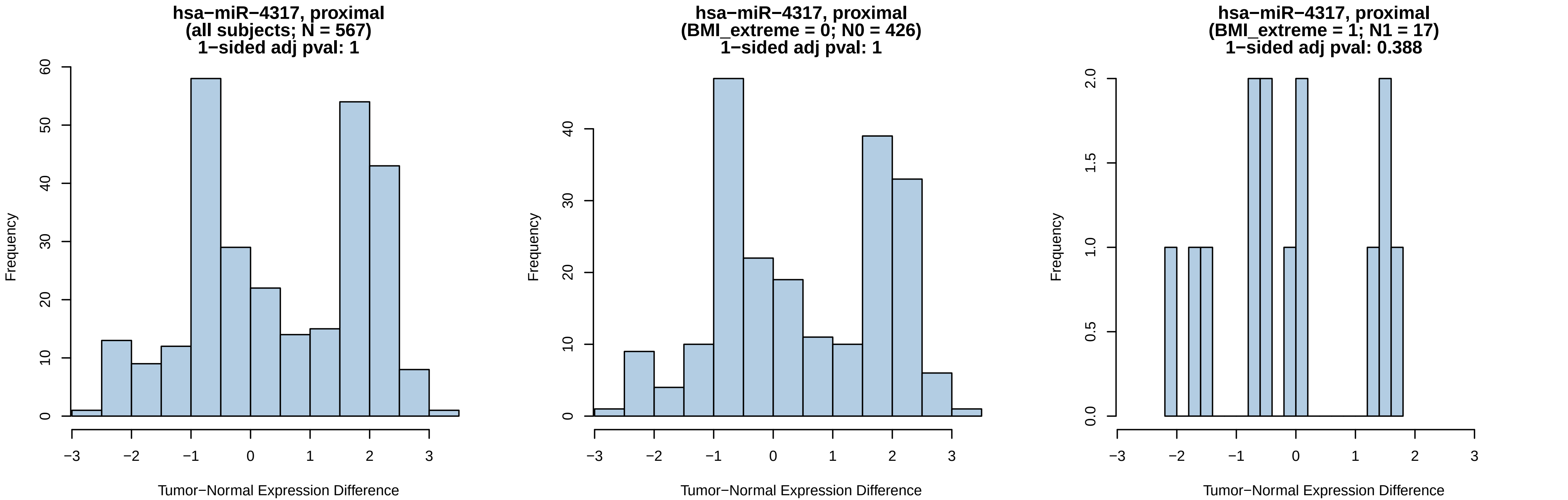
hsa−miR−4317, proximal
(all subjects; N = 567)
1−sided adj pval: 1
hsa−miR−4317, proximal
(BMI_extreme = 0; N0 = 426)
1−sided adj pval: 1
hsa−miR−4317, proximal
(BMI_extreme = 1; N1 = 17)
1−sided adj pval: 0.388
Frequency
Tumor−Normal Expression Difference

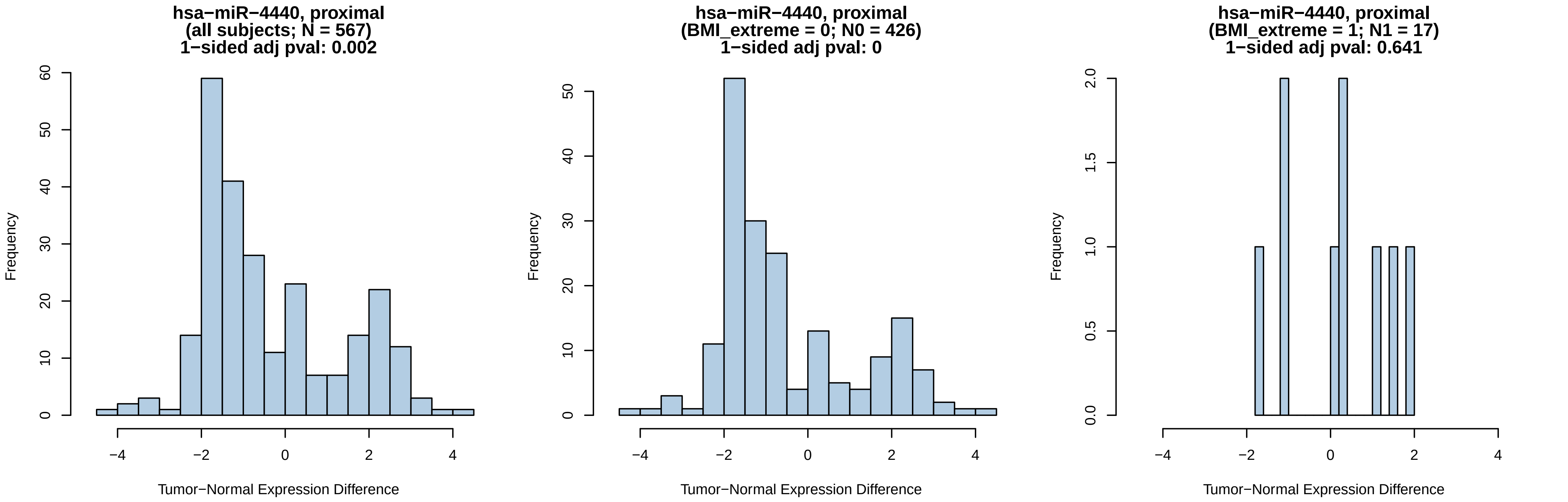

hsa−miR−4440, proximal
(all subjects; N = 567)
1−sided adj pval: 0.002
Frequency
Tumor−Normal Expression Difference
hsa−miR−4440, proximal
(BMI_extreme = 0; N0 = 426)
1−sided adj pval: 0
Frequency
Tumor−Normal Expression Difference
hsa−miR−4440, proximal
(BMI_extreme = 1; N1 = 17)
1−sided adj pval: 0.641
Frequency
Tumor−Normal Expression Difference

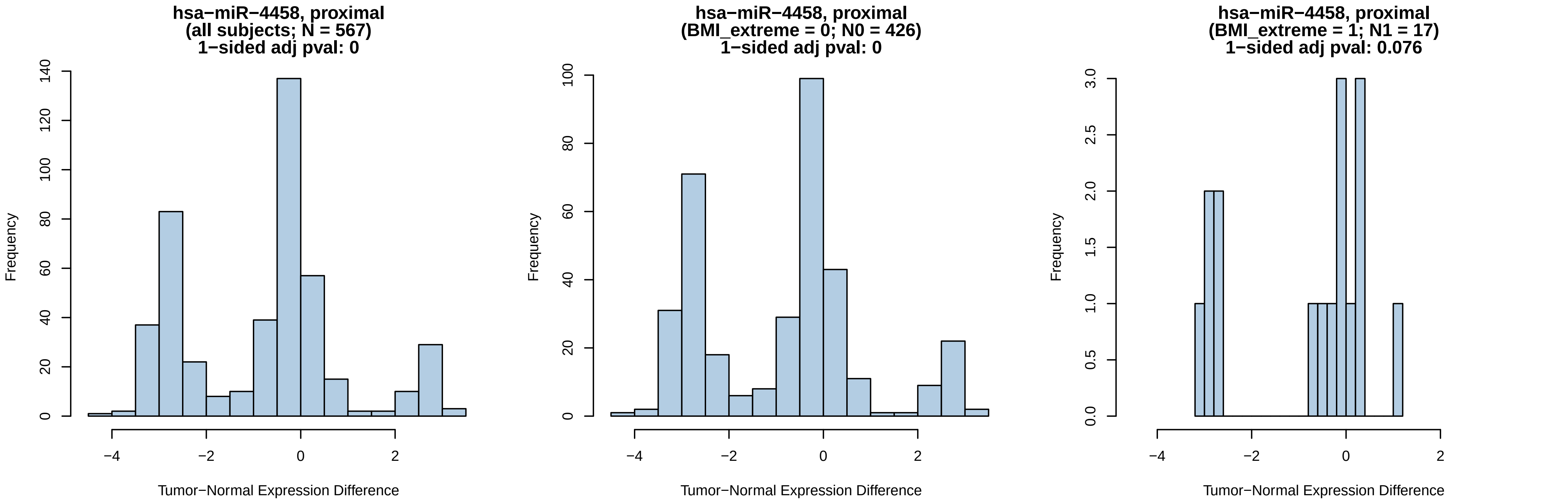

hsa−miR−4458, proximal
(all subjects; N = 567)
1−sided adj pval: 0
Frequency
Tumor−Normal Expression Difference
hsa−miR−4458, proximal
(BMI_extreme = 0; N0 = 426)
1−sided adj pval: 0
Frequency
Tumor−Normal Expression Difference
hsa−miR−4458, proximal
(BMI_extreme = 1; N1 = 17)
1−sided adj pval: 0.076
Frequency
Tumor−Normal Expression Difference

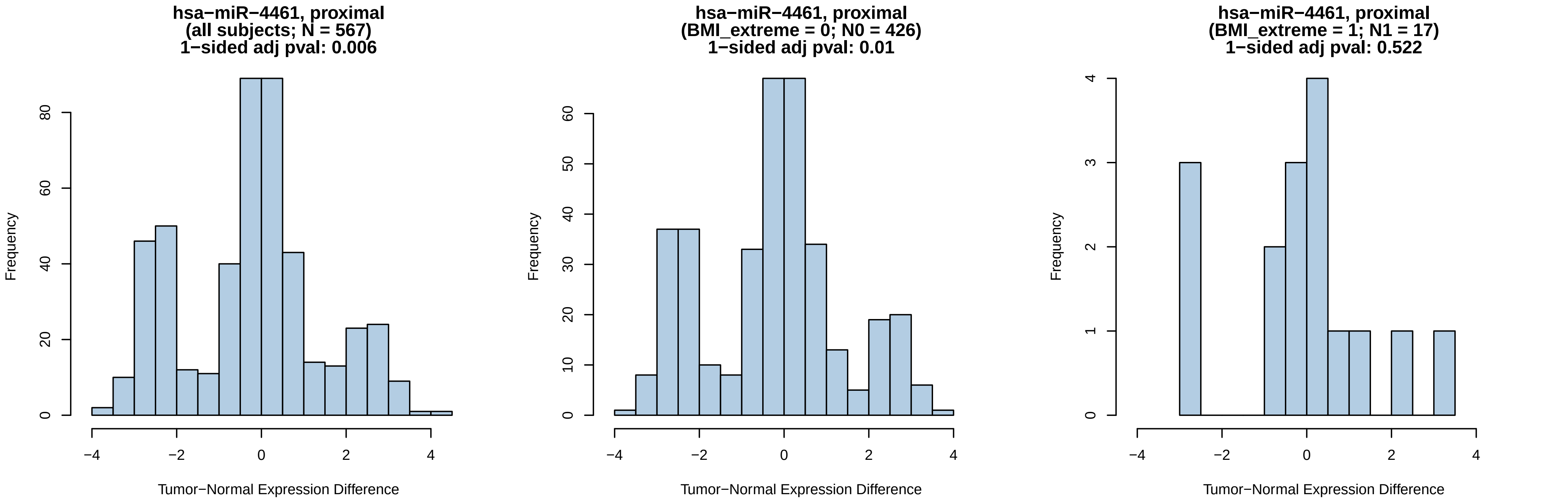
hsa−miR−4461, proximal
(all subjects; N = 567)
1−sided adj pval: 0.006
Frequency
Tumor−Normal Expression Difference
hsa−miR−4461, proximal
(BMI_extreme = 0; N0 = 426)
1−sided adj pval: 0.01
Frequency
Tumor−Normal Expression Difference
hsa−miR−4461, proximal
(BMI_extreme = 1; N1 = 17)
1−sided adj pval: 0.522
Frequency
Tumor−Normal Expression Difference

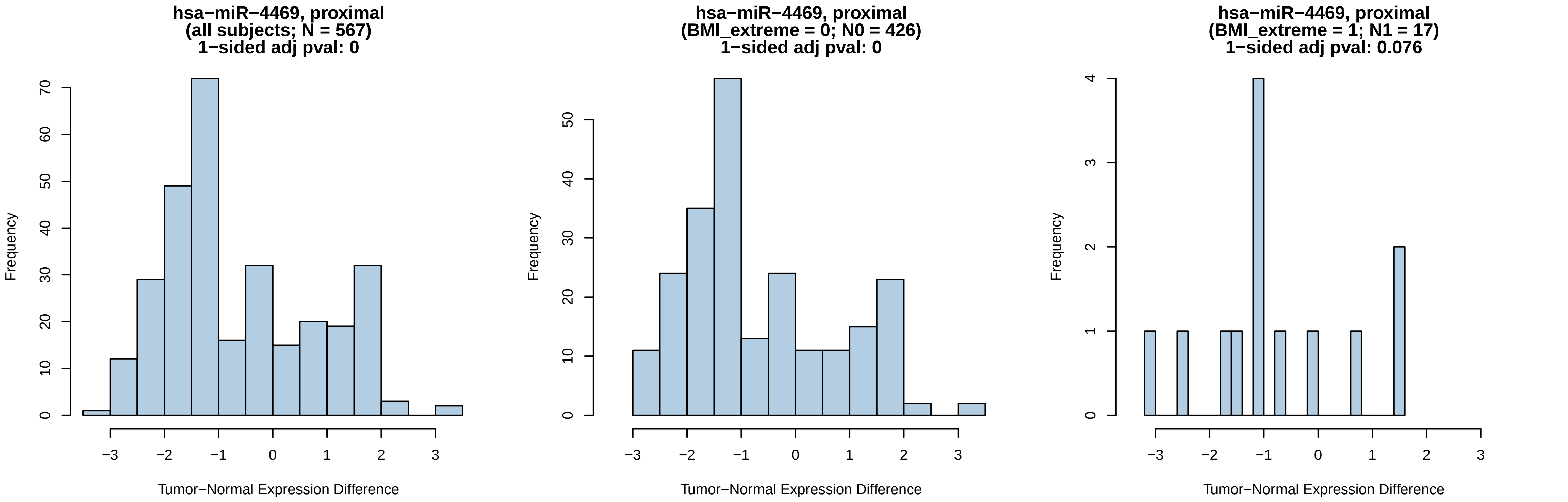
hsa−miR−4469, proximal
(all subjects; N = 567)
1−sided adj pval: 0
Frequency
Tumor−Normal Expression Difference
hsa−miR−4469, proximal
(BMI_extreme = 0; N0 = 426)
1−sided adj pval: 0
Frequency
Tumor−Normal Expression Difference
hsa−miR−4469, proximal
(BMI_extreme = 1; N1 = 17)
1−sided adj pval: 0.076
Frequency
Tumor−Normal Expression Difference

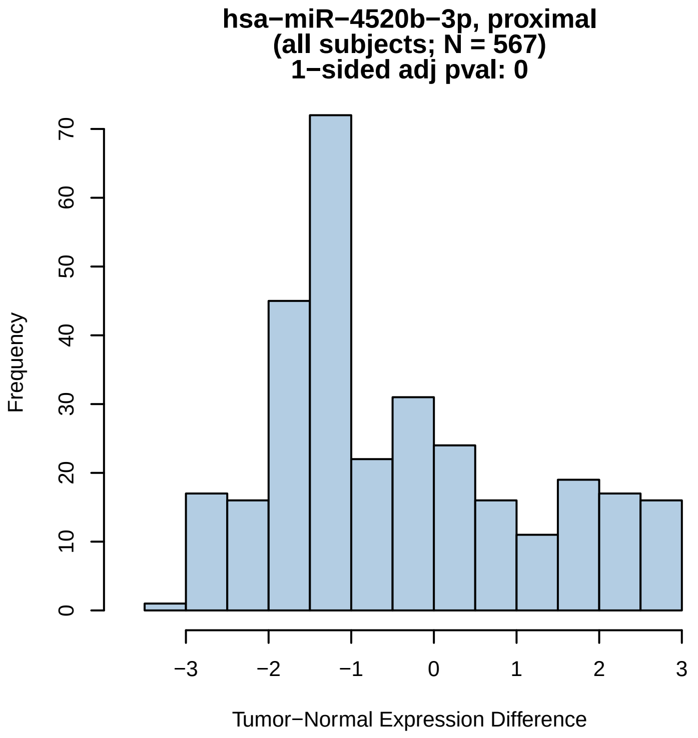
hsa−miR−4520b−3p, proximal
(all subjects; N = 567)
1−sided adj pval: 0
Frequency
0
10
20
30
40
50
60
70
−3
−2
−1
0
1
2
3
Tumor−Normal Expression Difference

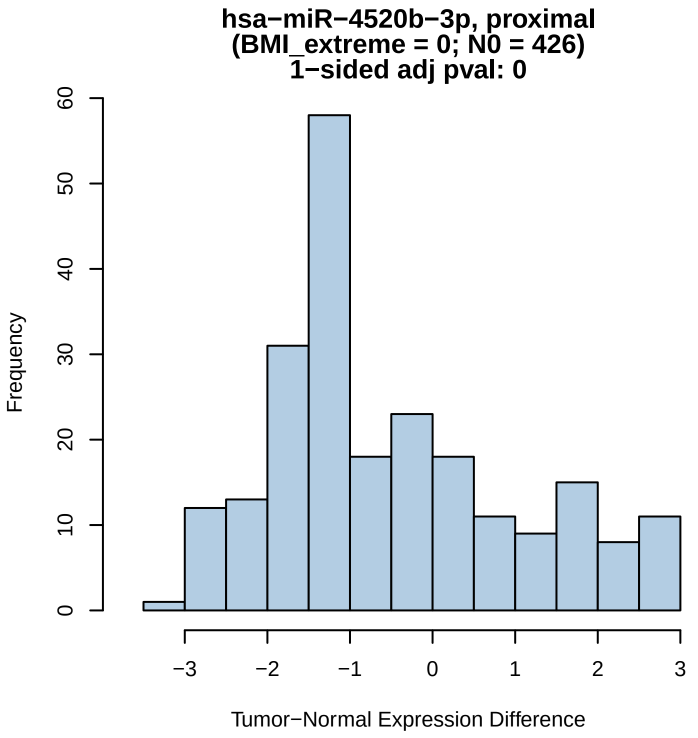
hsa−miR−4520b−3p, proximal
(BMI_extreme = 0; N0 = 426)
1−sided adj pval: 0
Frequency
0
10
20
30
40
50
60
−3
−2
−1
0
1
2
3
Tumor−Normal Expression Difference

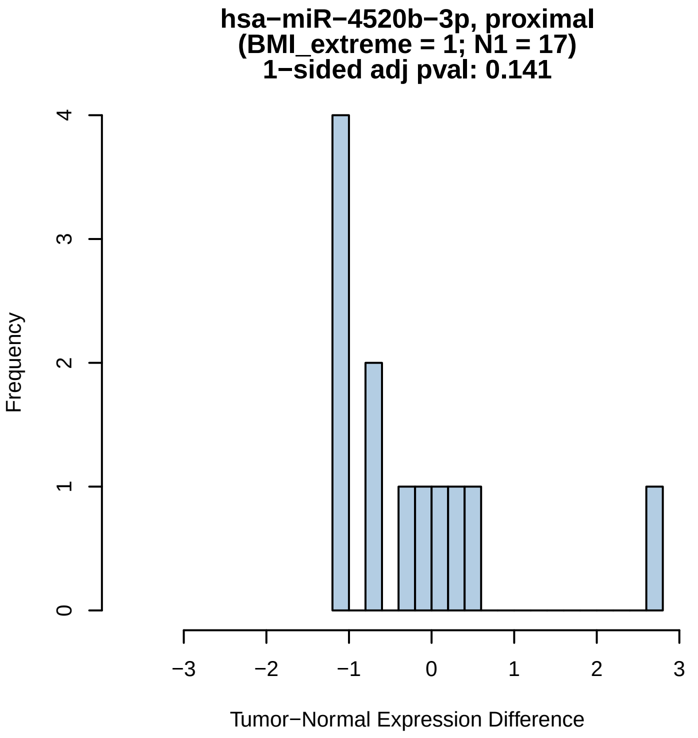
hsa−miR−4520b−3p, proximal
(BMI_extreme = 1; N1 = 17)
1−sided adj pval: 0.141
Frequency
0
1
2
3
4
−3
−2
−1
0
1
2
3
Tumor−Normal Expression Difference

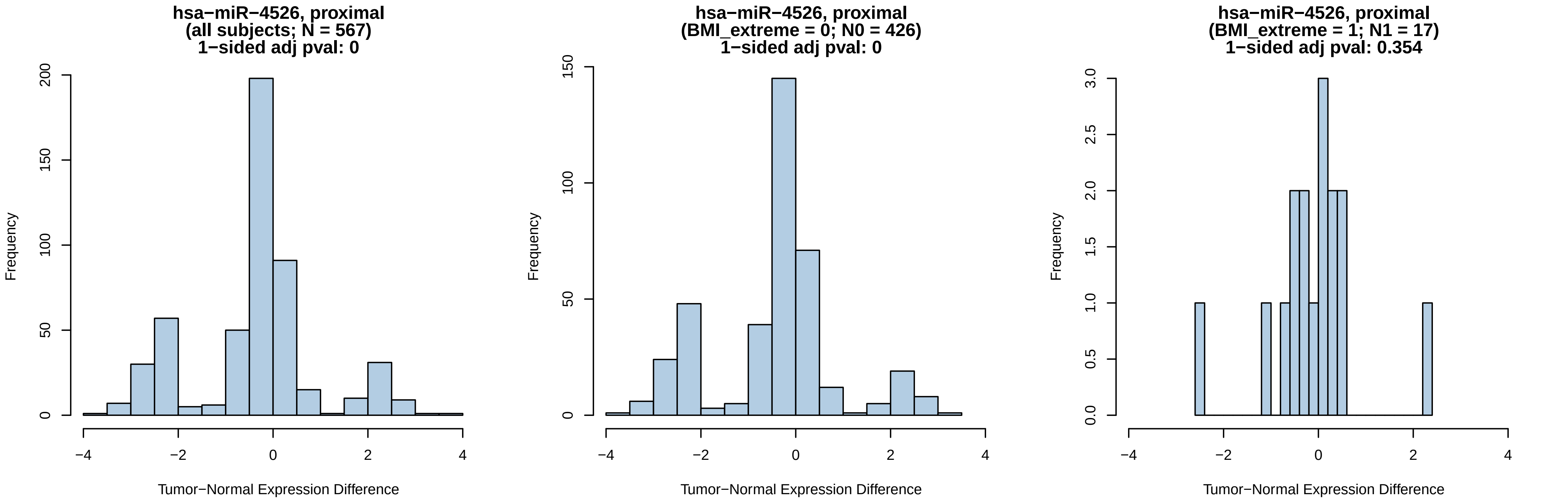
hsa−miR−4526, proximal
(all subjects; N = 567)
1−sided adj pval: 0
Frequency
Tumor−Normal Expression Difference
hsa−miR−4526, proximal
(BMI_extreme = 0; N0 = 426)
1−sided adj pval: 0
Frequency
Tumor−Normal Expression Difference
hsa−miR−4526, proximal
(BMI_extreme = 1; N1 = 17)
1−sided adj pval: 0.354
Frequency
Tumor−Normal Expression Difference

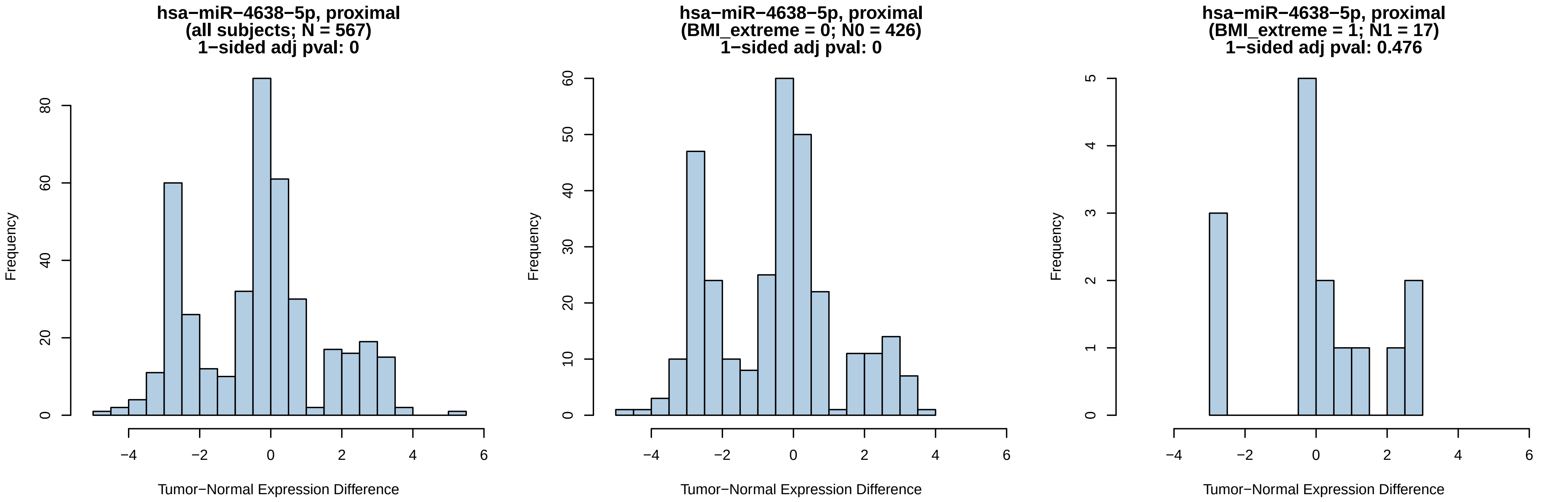

hsa−miR−4638−5p, proximal
(all subjects; N = 567)
1−sided adj pval: 0
Frequency
Tumor−Normal Expression Difference
hsa−miR−4638−5p, proximal
(BMI_extreme = 0; N0 = 426)
1−sided adj pval: 0
Frequency
Tumor−Normal Expression Difference
hsa−miR−4638−5p, proximal
(BMI_extreme = 1; N1 = 17)
1−sided adj pval: 0.476
Frequency
Tumor−Normal Expression Difference

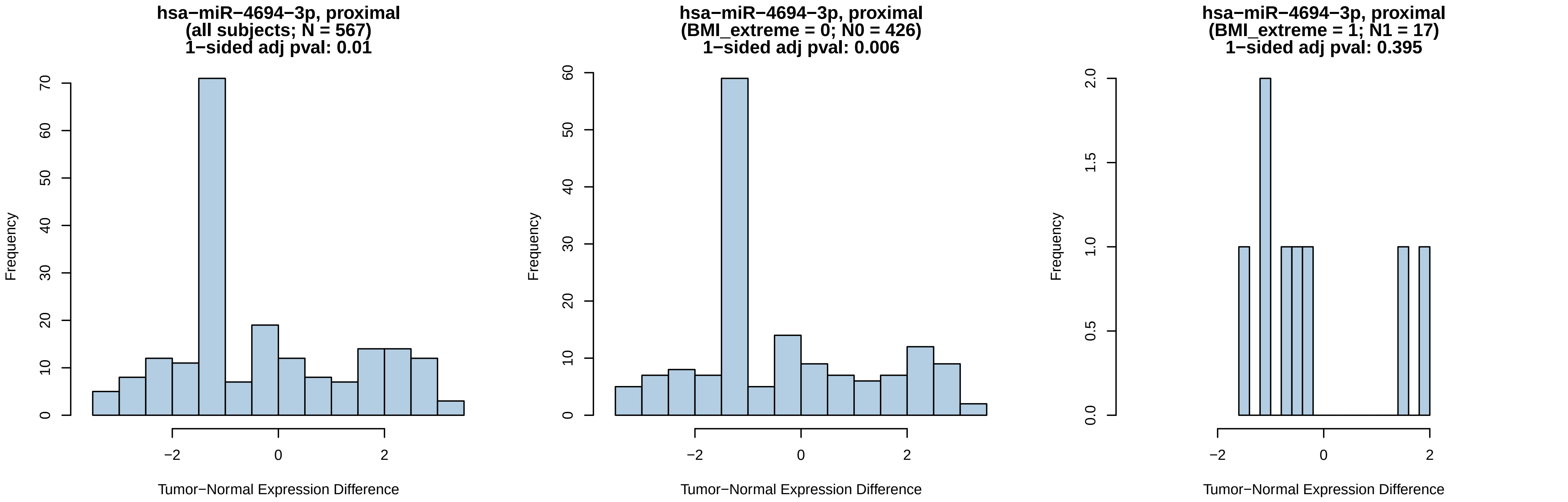
hsa−miR−4694−3p, proximal
(all subjects; N = 567)
1−sided adj pval: 0.01
Frequency
0
10
20
30
40
50
60
70
−2
0
2
Tumor−Normal Expression Difference
hsa−miR−4694−3p, proximal
(BMI_extreme = 0; N0 = 426)
1−sided adj pval: 0.006
Frequency
0
10
20
30
40
50
60
−2
0
2
Tumor−Normal Expression Difference
hsa−miR−4694−3p, proximal
(BMI_extreme = 1; N1 = 17)
1−sided adj pval: 0.395
Frequency
0.0
0.5
1.0
1.5
2.0
−2
0
2
Tumor−Normal Expression Difference

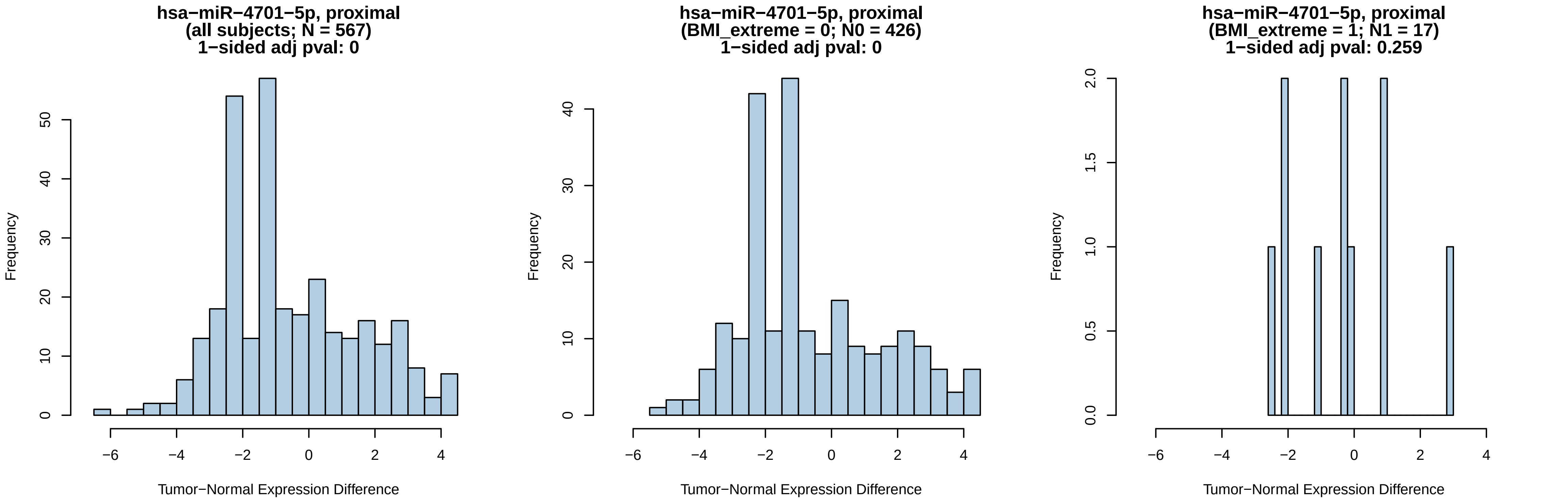
hsa−miR−4701−5p, proximal
(all subjects; N = 567)
1−sided adj pval: 0
Frequency
Tumor−Normal Expression Difference
hsa−miR−4701−5p, proximal
(BMI_extreme = 0; N0 = 426)
1−sided adj pval: 0
Frequency
Tumor−Normal Expression Difference
hsa−miR−4701−5p, proximal
(BMI_extreme = 1; N1 = 17)
1−sided adj pval: 0.259
Frequency
Tumor−Normal Expression Difference

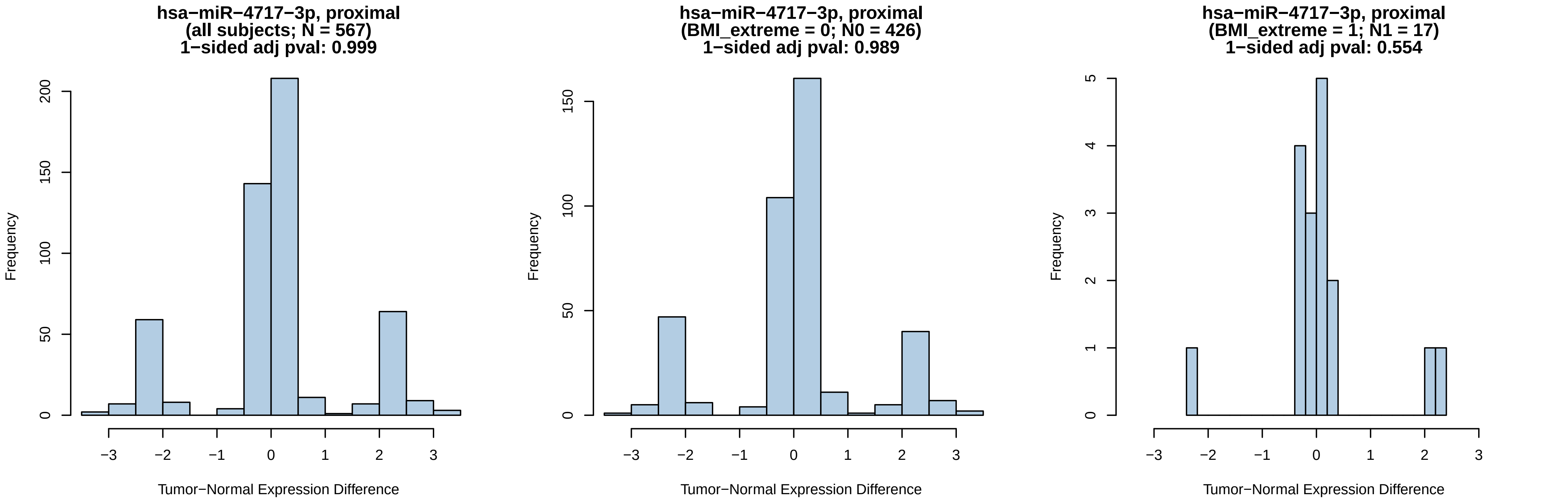
hsa−miR−4717−3p, proximal
(all subjects; N = 567)
1−sided adj pval: 0.999
Frequency
Tumor−Normal Expression Difference
hsa−miR−4717−3p, proximal
(BMI_extreme = 0; N0 = 426)
1−sided adj pval: 0.989
Frequency
Tumor−Normal Expression Difference
hsa−miR−4717−3p, proximal
(BMI_extreme = 1; N1 = 17)
1−sided adj pval: 0.554
Frequency
Tumor−Normal Expression Difference

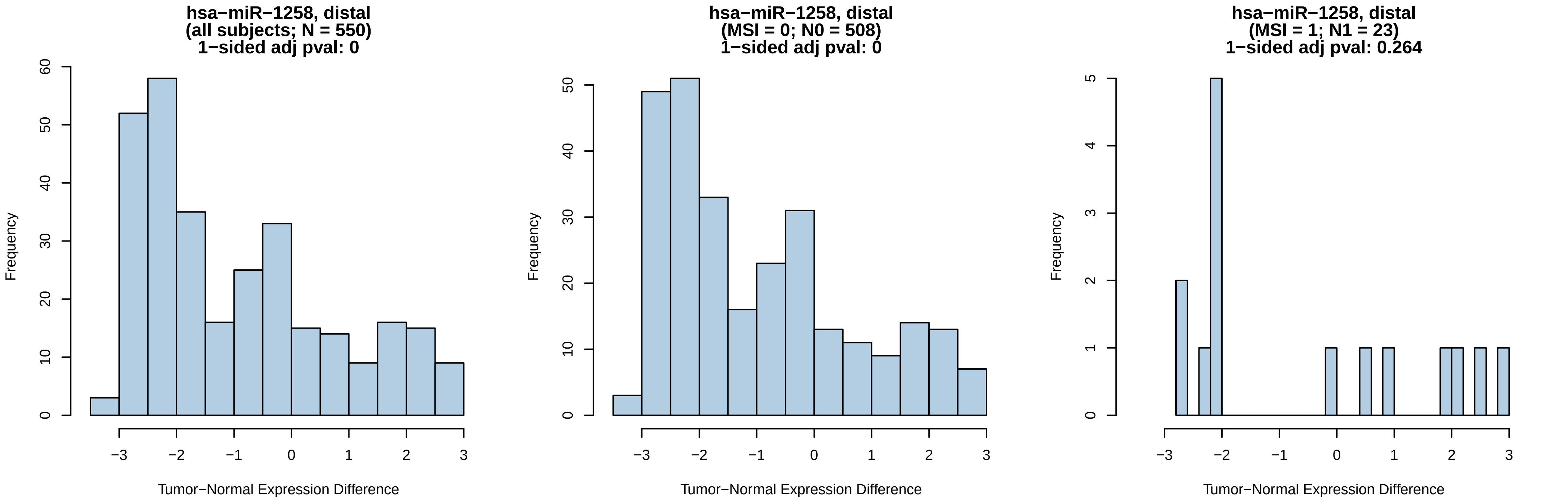

hsa−miR−1258, distal
(all subjects; N = 550)
1−sided adj pval: 0
Frequency
Tumor−Normal Expression Difference
hsa−miR−1258, distal
(MSI = 0; N0 = 508)
1−sided adj pval: 0
Frequency
Tumor−Normal Expression Difference
hsa−miR−1258, distal
(MSI = 1; N1 = 23)
1−sided adj pval: 0.264
Frequency
Tumor−Normal Expression Difference

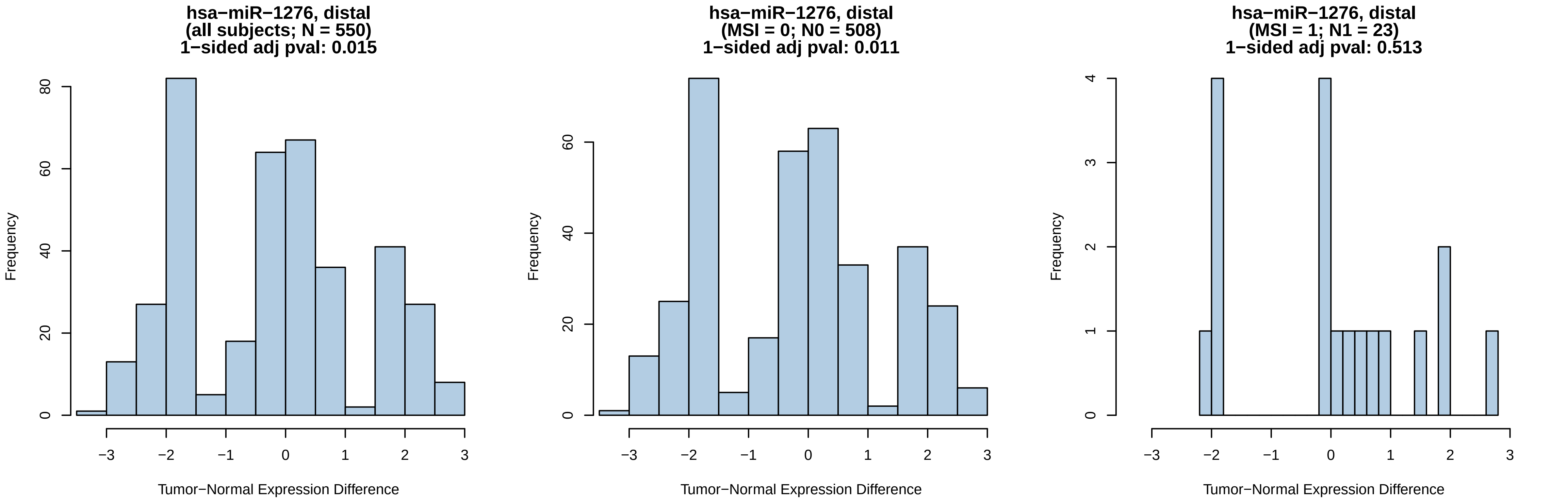
hsa−miR−1276, distal
(all subjects; N = 550)
1−sided adj pval: 0.015
hsa−miR−1276, distal
(MSI = 0; N0 = 508)
1−sided adj pval: 0.011
hsa−miR−1276, distal
(MSI = 1; N1 = 23)
1−sided adj pval: 0.513
Frequency
Tumor−Normal Expression Difference
−3
−2
−1
0
1
2
3
0
20
40
60
80
1
2
4

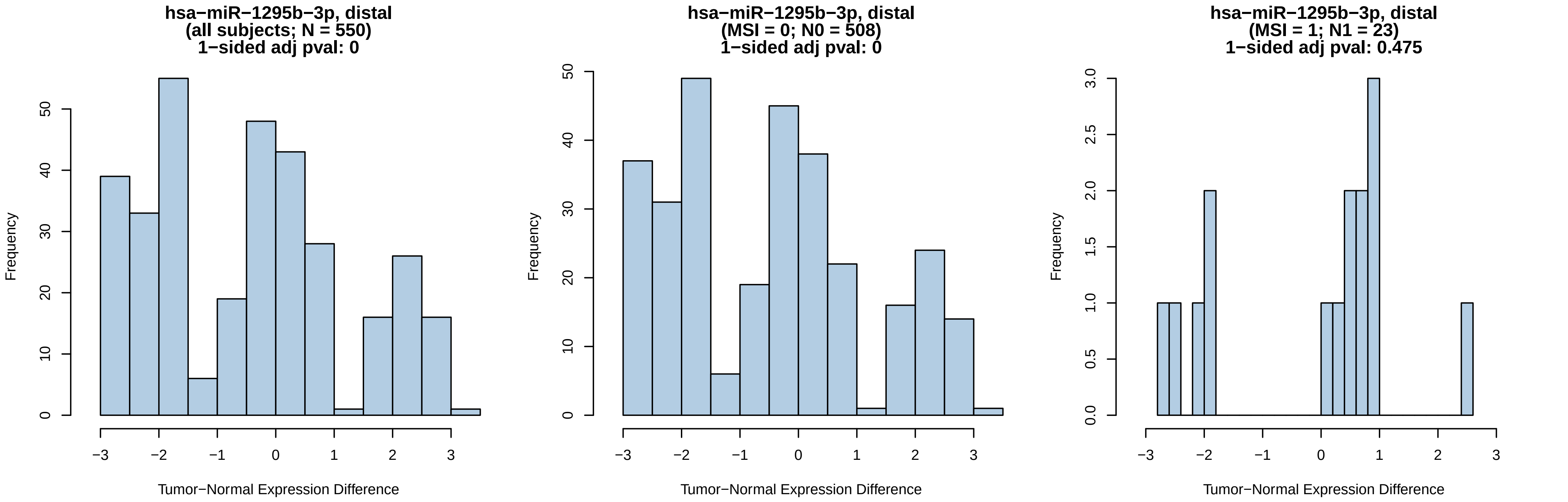

hsa−miR−1295b−3p, distal
(all subjects; N = 550)
1−sided adj pval: 0
Frequency
Tumor−Normal Expression Difference
hsa−miR−1295b−3p, distal
(MSI = 0; N0 = 508)
1−sided adj pval: 0
Frequency
Tumor−Normal Expression Difference
hsa−miR−1295b−3p, distal
(MSI = 1; N1 = 23)
1−sided adj pval: 0.475
Frequency
Tumor−Normal Expression Difference

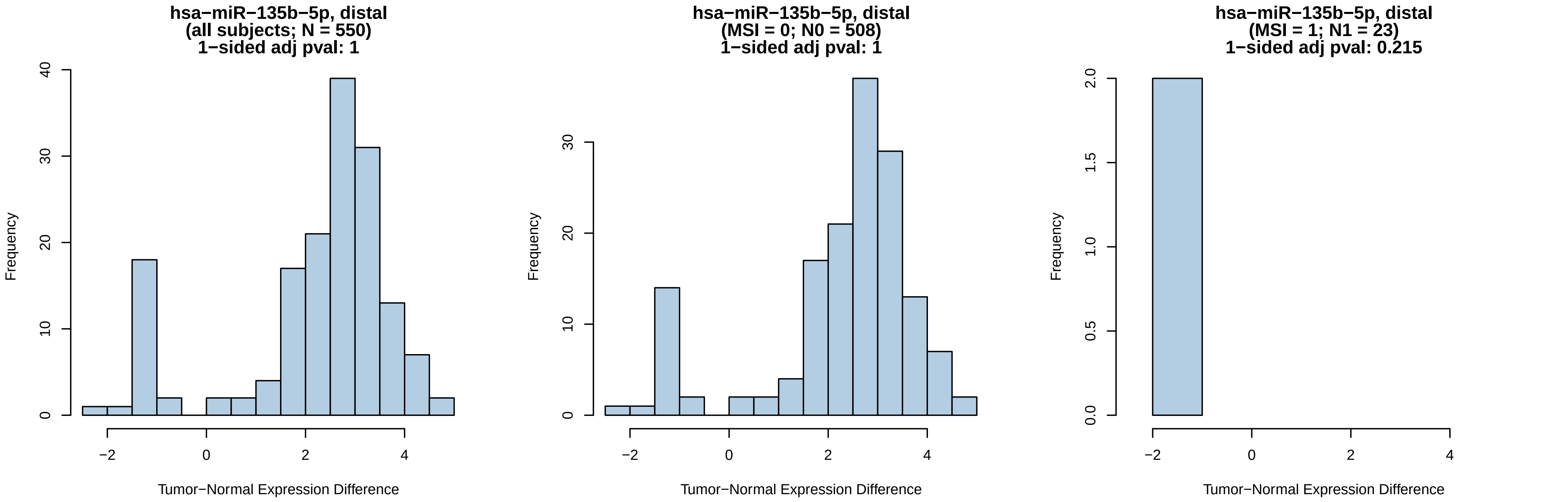
hsa−miR−135b−5p, distal
(all subjects; N = 550)
1−sided adj pval: 1
hsa−miR−135b−5p, distal
(MSI = 0; N0 = 508)
1−sided adj pval: 1
hsa−miR−135b−5p, distal
(MSI = 1; N1 = 23)
1−sided adj pval: 0.215
Frequency
Tumor−Normal Expression Difference
0
10
20
30
40
0.0
0.5
1.0
1.5
2.0
−2
0
2
4

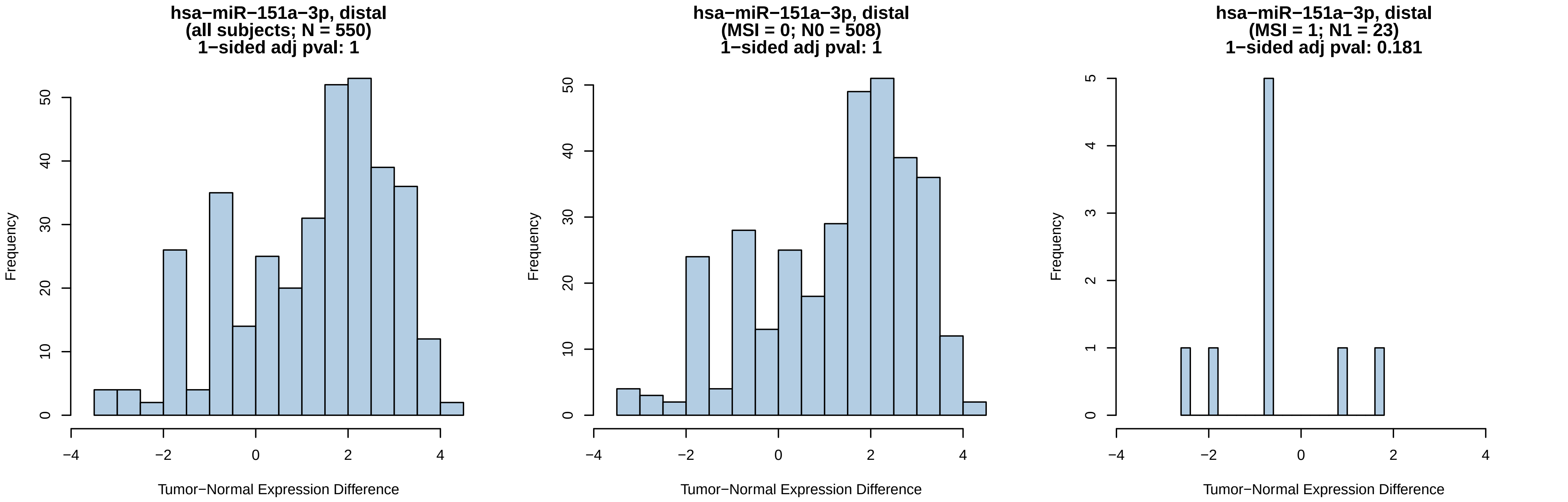
hsa−miR−151a−3p, distal
(all subjects; N = 550)
1−sided adj pval: 1
Frequency
Tumor−Normal Expression Difference
hsa−miR−151a−3p, distal
(MSI = 0; N0 = 508)
1−sided adj pval: 1
Frequency
Tumor−Normal Expression Difference
hsa−miR−151a−3p, distal
(MSI = 1; N1 = 23)
1−sided adj pval: 0.181
Frequency
Tumor−Normal Expression Difference

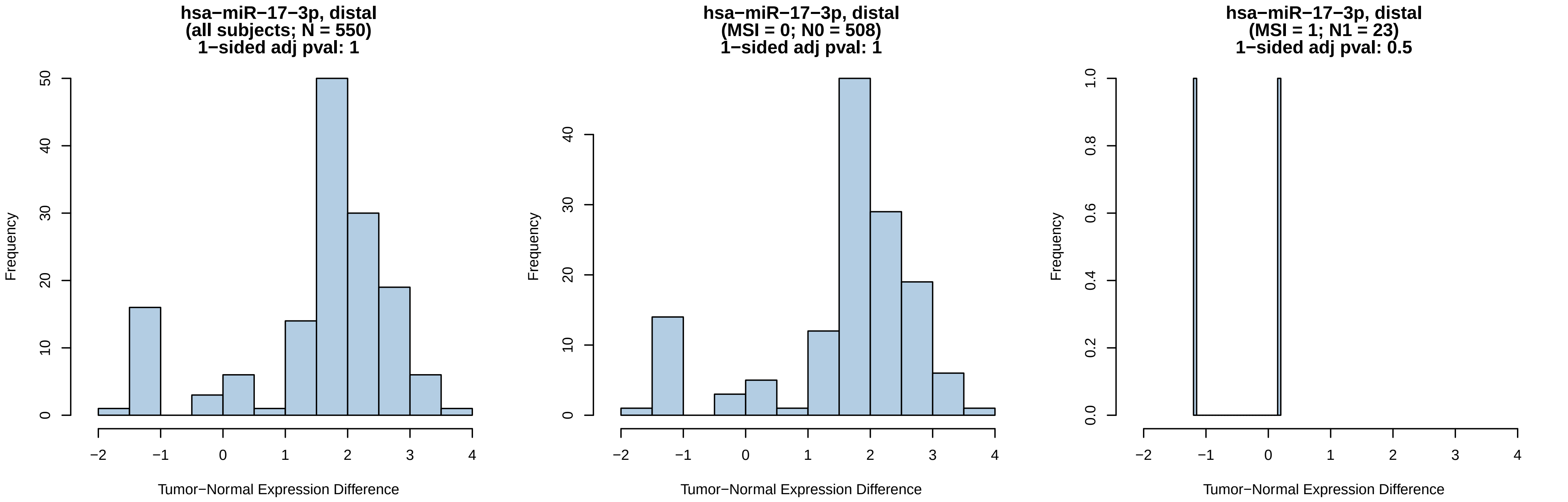
hsa−miR−17−3p, distal
(all subjects; N = 550)
1−sided adj pval: 1
Frequency
Tumor−Normal Expression Difference
hsa−miR−17−3p, distal
(MSI = 0; N0 = 508)
1−sided adj pval: 1
Frequency
Tumor−Normal Expression Difference
hsa−miR−17−3p, distal
(MSI = 1; N1 = 23)
1−sided adj pval: 0.5
Frequency
Tumor−Normal Expression Difference

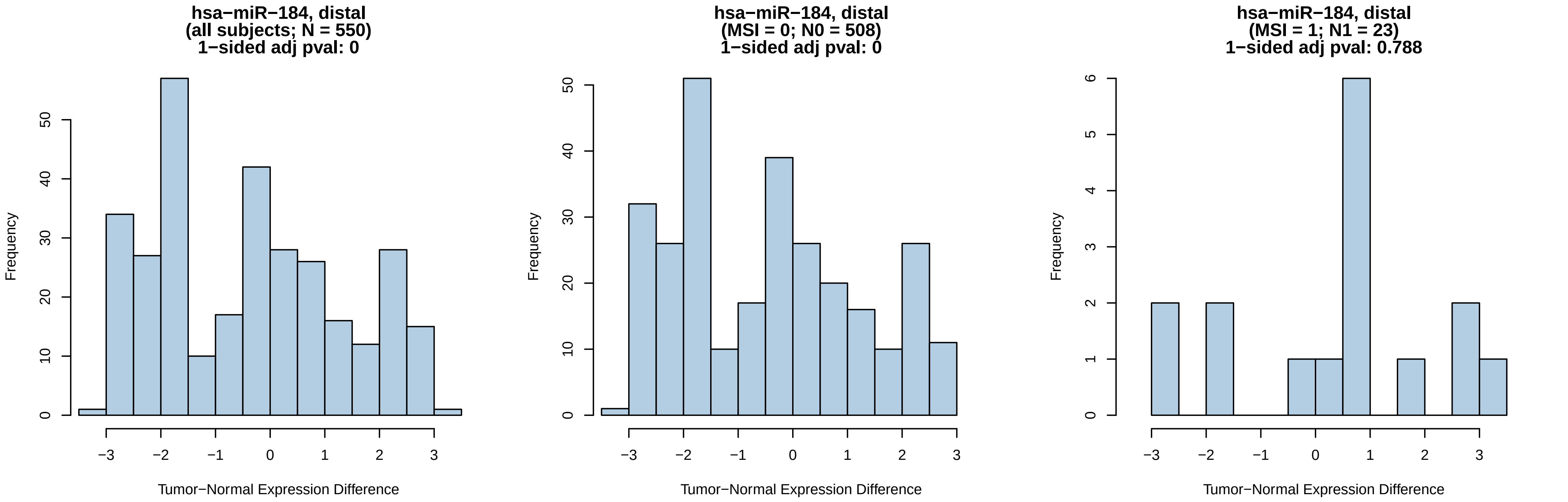

hsa−miR−184, distal
(all subjects; N = 550)
1−sided adj pval: 0
Frequency
Tumor−Normal Expression Difference
hsa−miR−184, distal
(MSI = 0; N0 = 508)
1−sided adj pval: 0
Frequency
Tumor−Normal Expression Difference
hsa−miR−184, distal
(MSI = 1; N1 = 23)
1−sided adj pval: 0.788
Frequency
Tumor−Normal Expression Difference

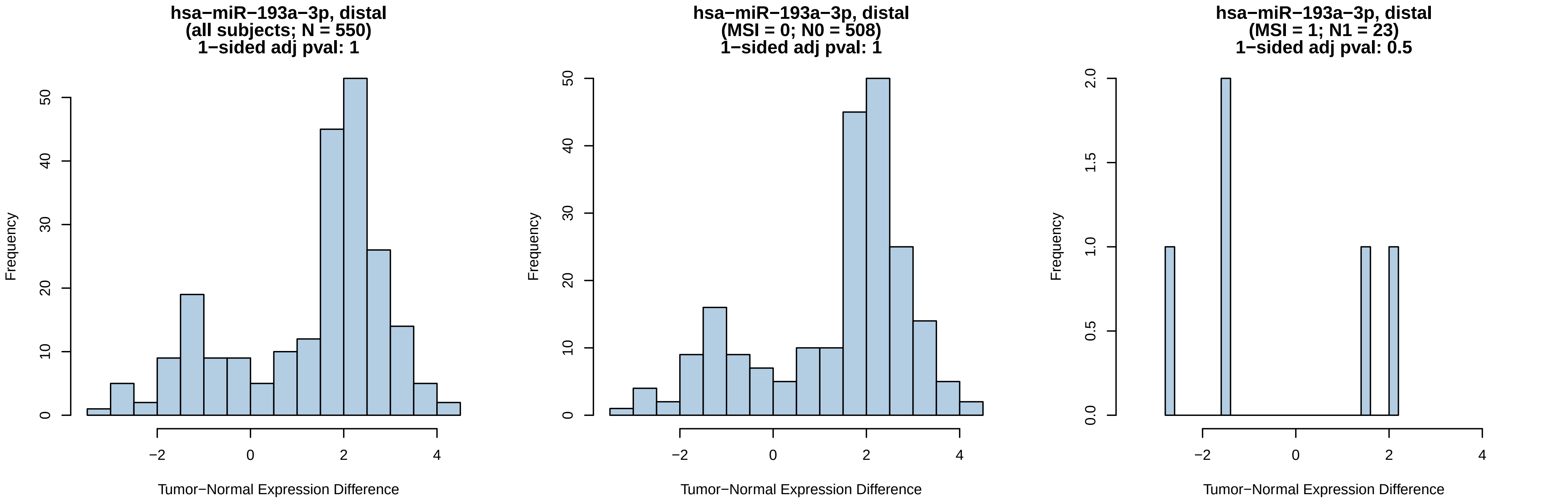

hsa−miR−193a−3p, distal
(all subjects; N = 550)
1−sided adj pval: 1
Frequency
0
10
20
30
40
50
−2
0
2
4
Tumor−Normal Expression Difference
hsa−miR−193a−3p, distal
(MSI = 0; N0 = 508)
1−sided adj pval: 1
Frequency
0
10
20
30
40
50
−2
0
2
4
Tumor−Normal Expression Difference
hsa−miR−193a−3p, distal
(MSI = 1; N1 = 23)
1−sided adj pval: 0.5
Frequency
0.0
0.5
1.0
1.5
2.0
−2
0
2
4
Tumor−Normal Expression Difference

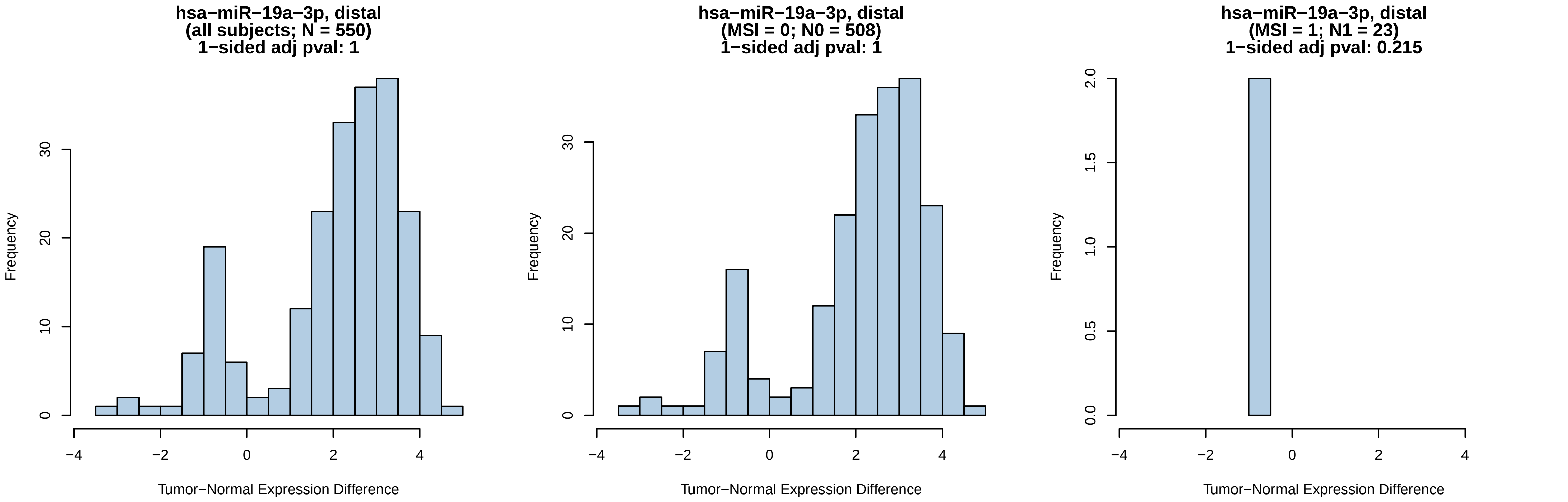

hsa−miR−19a−3p, distal
(all subjects; N = 550)
1−sided adj pval: 1
Frequency
Tumor−Normal Expression Difference
hsa−miR−19a−3p, distal
(MSI = 0; N0 = 508)
1−sided adj pval: 1
Frequency
Tumor−Normal Expression Difference
hsa−miR−19a−3p, distal
(MSI = 1; N1 = 23)
1−sided adj pval: 0.215
Frequency
Tumor−Normal Expression Difference

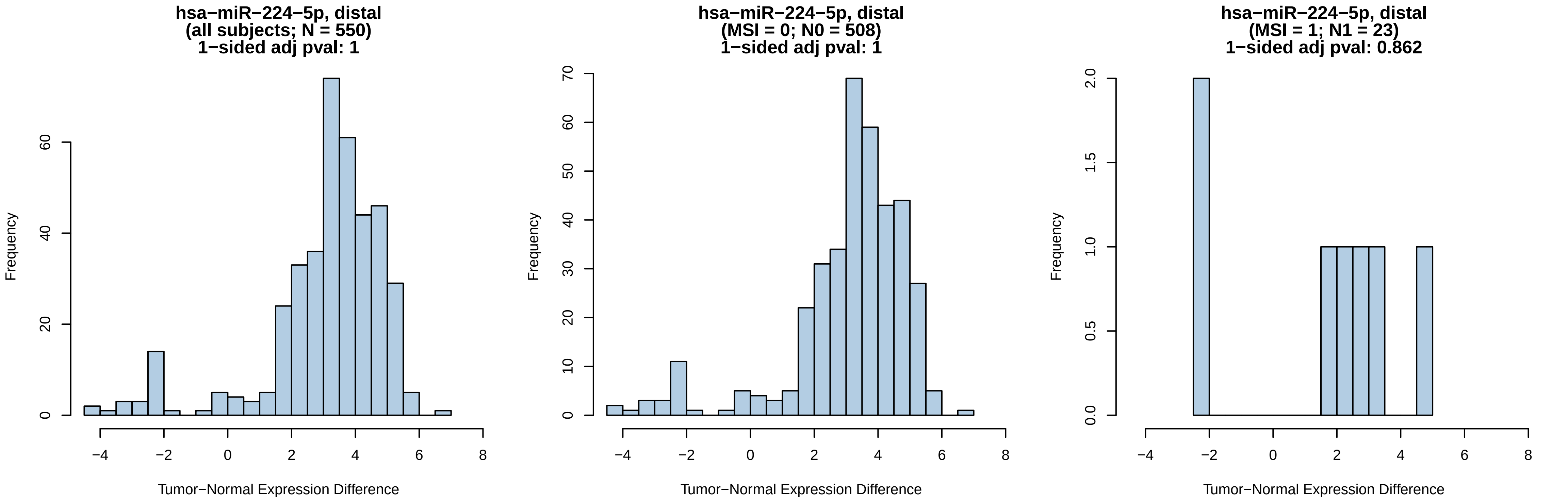

hsa−miR−224−5p, distal
(all subjects; N = 550)
1−sided adj pval: 1
hsa−miR−224−5p, distal
(MSI = 0; N0 = 508)
1−sided adj pval: 1
hsa−miR−224−5p, distal
(MSI = 1; N1 = 23)
1−sided adj pval: 0.862
Frequency
Tumor−Normal Expression Difference

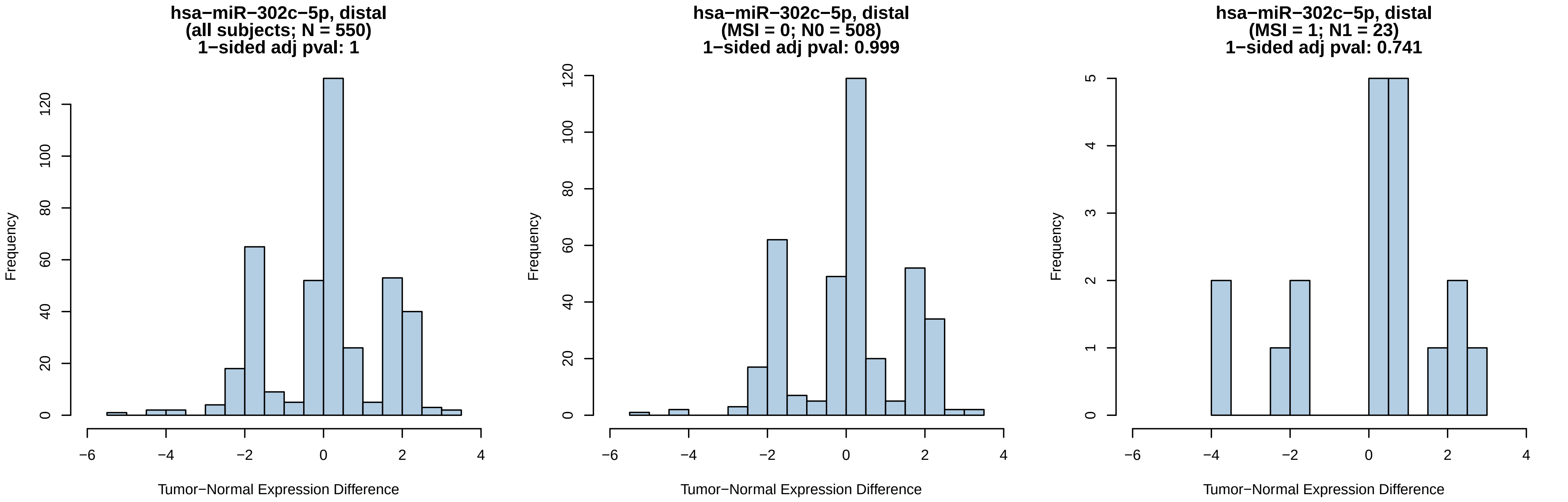
hsa−miR−302c−5p, distal
(all subjects; N = 550)
1−sided adj pval: 1
hsa−miR−302c−5p, distal
(MSI = 0; N0 = 508)
1−sided adj pval: 0.999
hsa−miR−302c−5p, distal
(MSI = 1; N1 = 23)
1−sided adj pval: 0.741
Frequency
Tumor−Normal Expression Difference

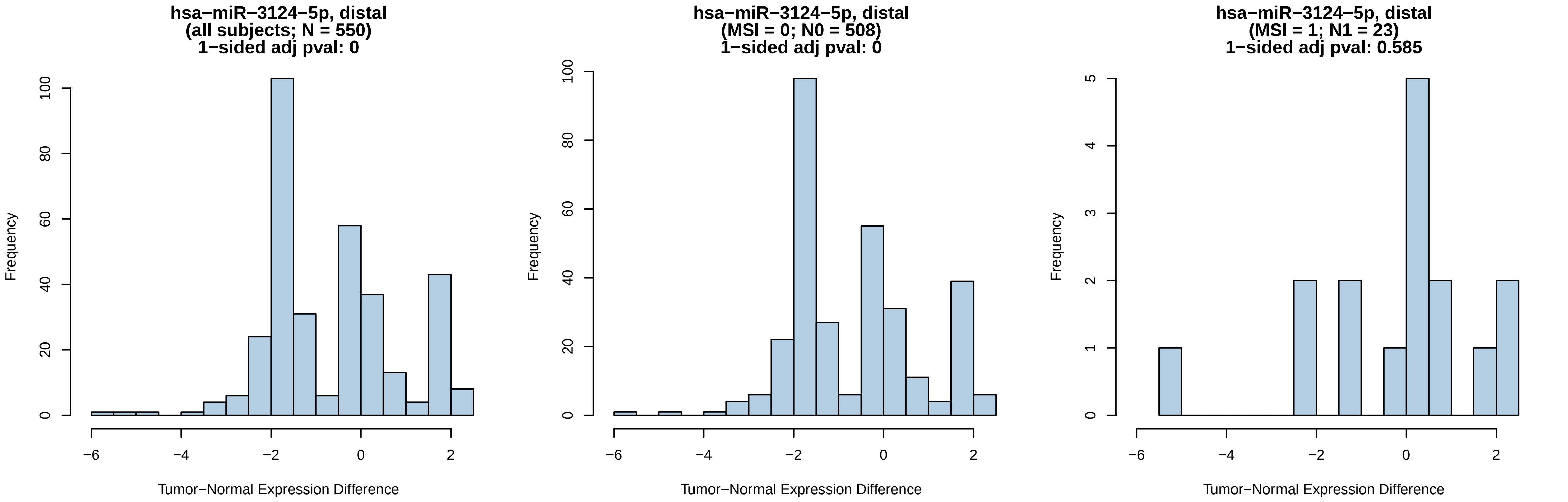
hsa−miR−3124−5p, distal
(all subjects; N = 550)
1−sided adj pval: 0
hsa−miR−3124−5p, distal
(MSI = 0; N0 = 508)
1−sided adj pval: 0
hsa−miR−3124−5p, distal
(MSI = 1; N1 = 23)
1−sided adj pval: 0.585
Frequency
Tumor−Normal Expression Difference

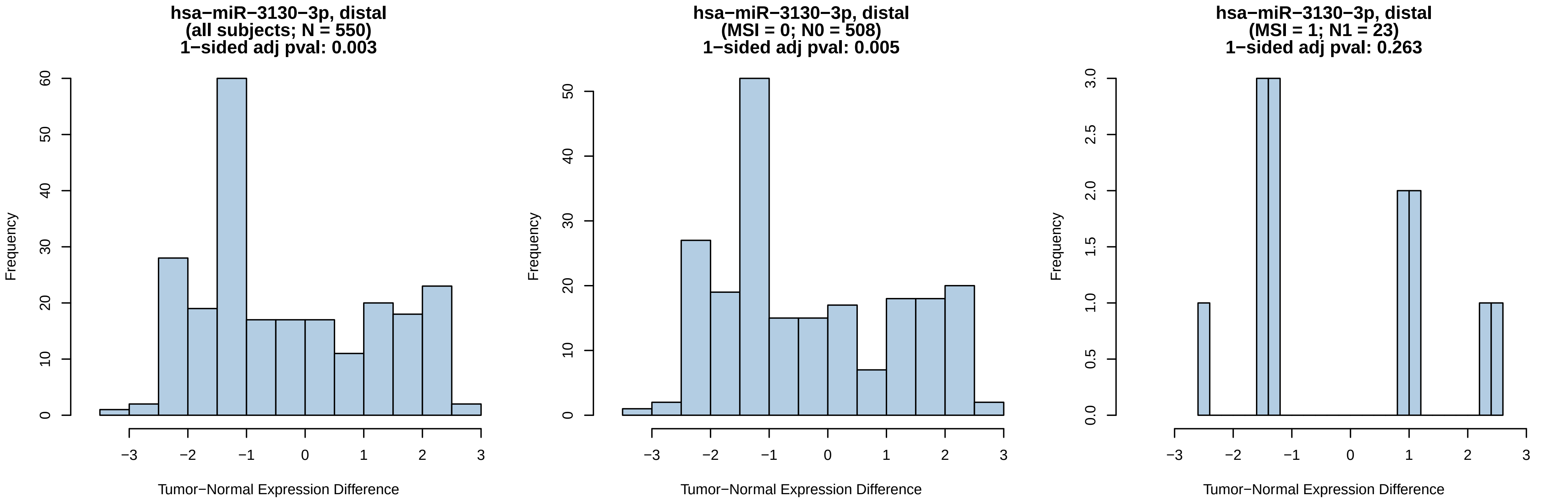
hsa−miR−3130−3p, distal
(all subjects; N = 550)
1−sided adj pval: 0.003
Frequency
Tumor−Normal Expression Difference
hsa−miR−3130−3p, distal
(MSI = 0; N0 = 508)
1−sided adj pval: 0.005
Frequency
Tumor−Normal Expression Difference
hsa−miR−3130−3p, distal
(MSI = 1; N1 = 23)
1−sided adj pval: 0.263
Frequency
Tumor−Normal Expression Difference

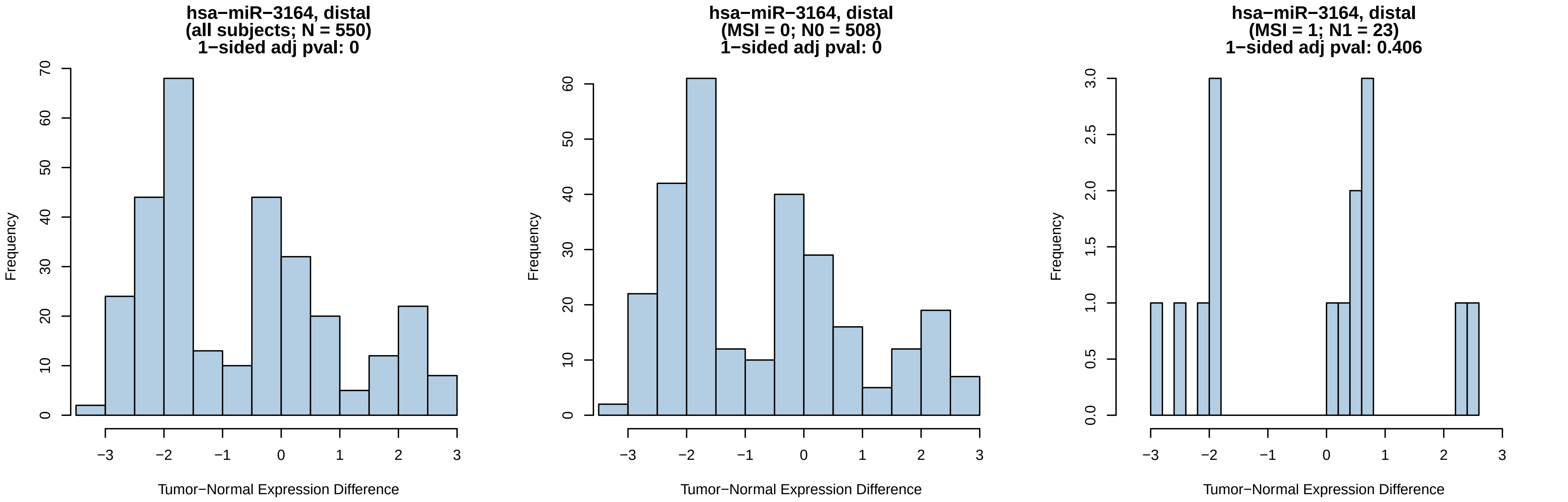
hsa−miR−3164, distal
(all subjects; N = 550)
1−sided adj pval: 0
Frequency
Tumor−Normal Expression Difference
hsa−miR−3164, distal
(MSI = 0; N0 = 508)
1−sided adj pval: 0
Frequency
Tumor−Normal Expression Difference
hsa−miR−3164, distal
(MSI = 1; N1 = 23)
1−sided adj pval: 0.406
Frequency
Tumor−Normal Expression Difference

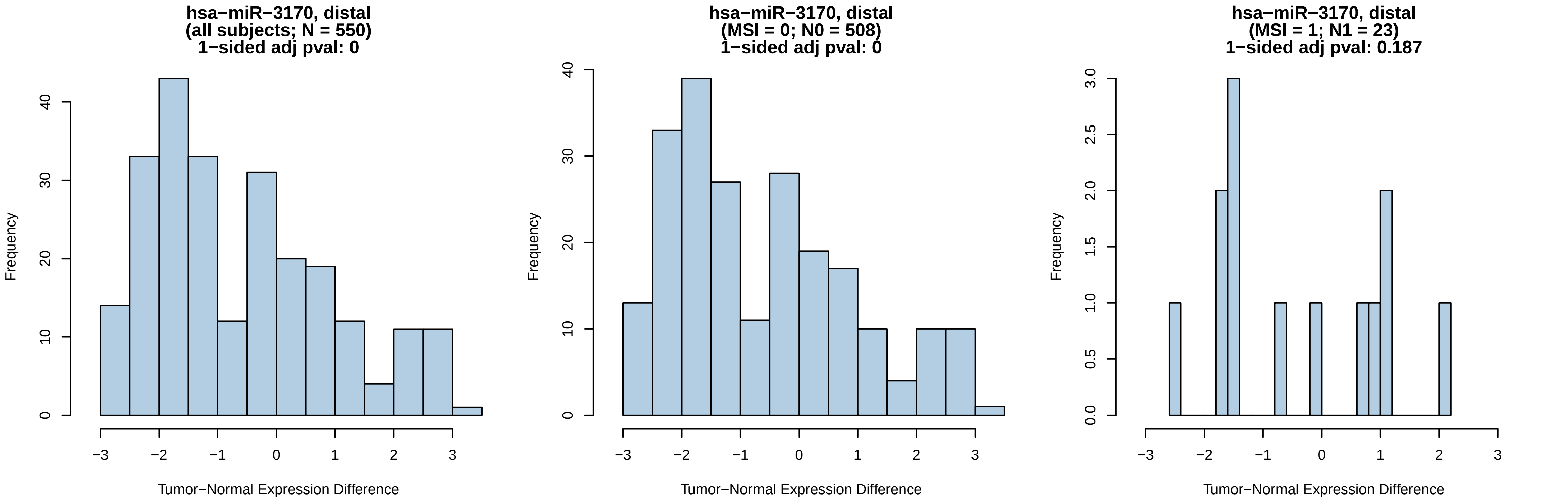

hsa−miR−3170, distal
(all subjects; N = 550)
1−sided adj pval: 0
Frequency
Tumor−Normal Expression Difference
hsa−miR−3170, distal
(MSI = 0; N0 = 508)
1−sided adj pval: 0
Frequency
Tumor−Normal Expression Difference
hsa−miR−3170, distal
(MSI = 1; N1 = 23)
1−sided adj pval: 0.187
Frequency
Tumor−Normal Expression Difference

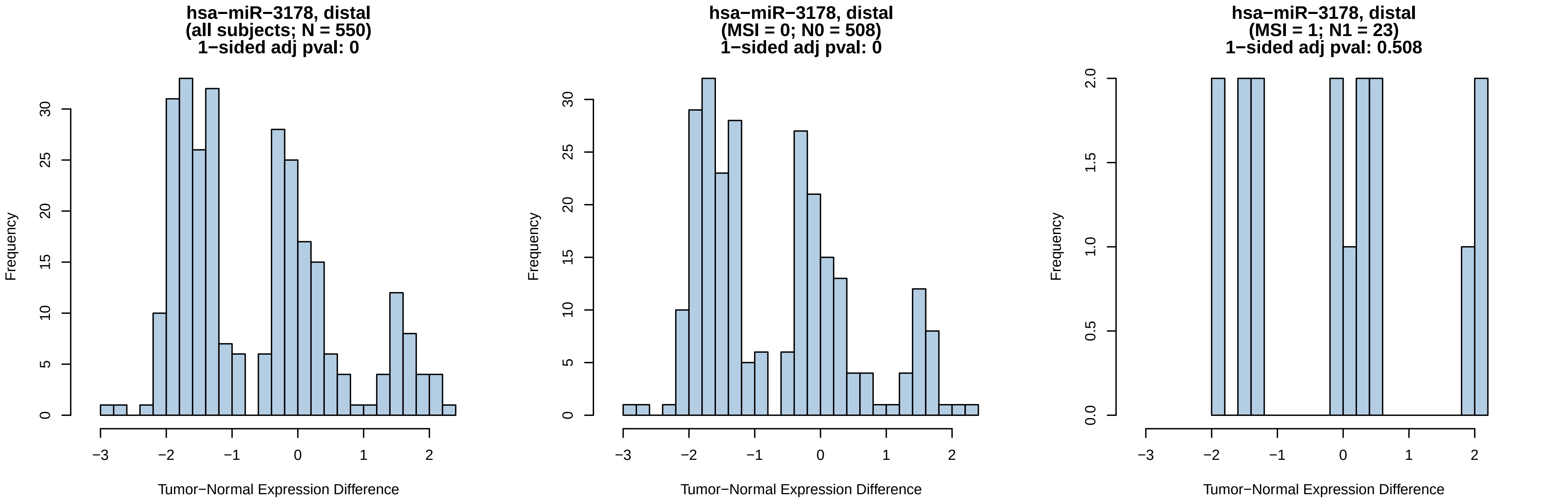
hsa−miR−3178, distal
(all subjects; N = 550)
1−sided adj pval: 0
Frequency
Tumor−Normal Expression Difference
hsa−miR−3178, distal
(MSI = 0; N0 = 508)
1−sided adj pval: 0
Frequency
Tumor−Normal Expression Difference
hsa−miR−3178, distal
(MSI = 1; N1 = 23)
1−sided adj pval: 0.508
Frequency
Tumor−Normal Expression Difference

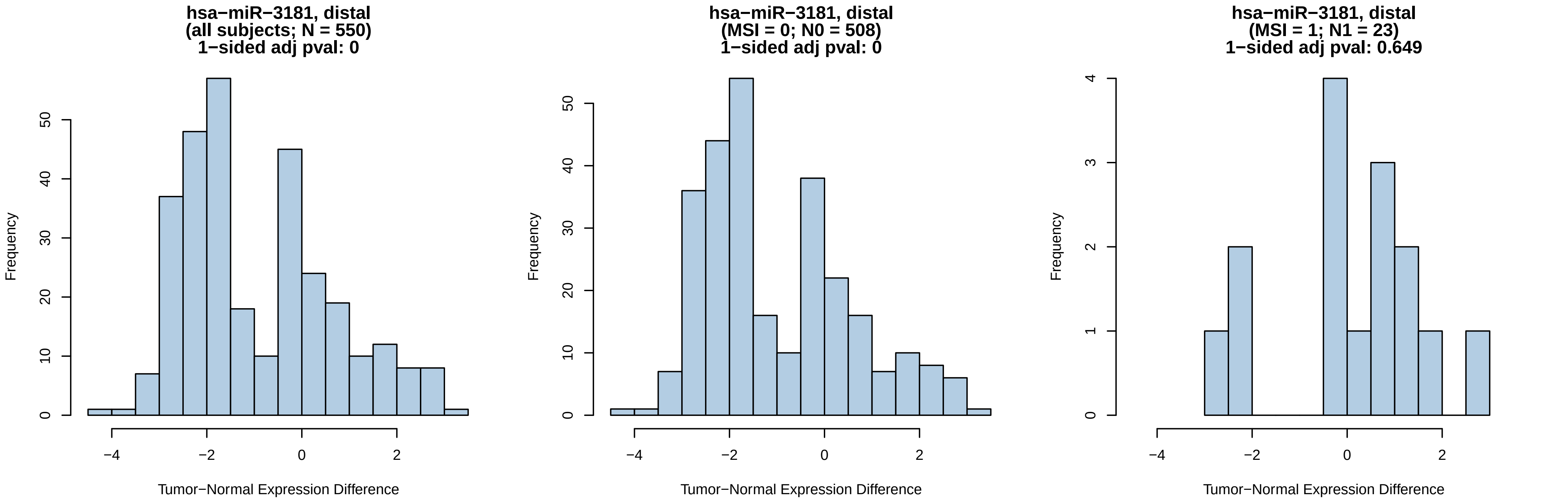
hsa−miR−3181, distal
(all subjects; N = 550)
1−sided adj pval: 0
hsa−miR−3181, distal
(MSI = 0; N0 = 508)
1−sided adj pval: 0
hsa−miR−3181, distal
(MSI = 1; N1 = 23)
1−sided adj pval: 0.649
Frequency
Tumor−Normal Expression Difference
0
10
20
30
40
50
1
2
3
4
−4
−2
0
2

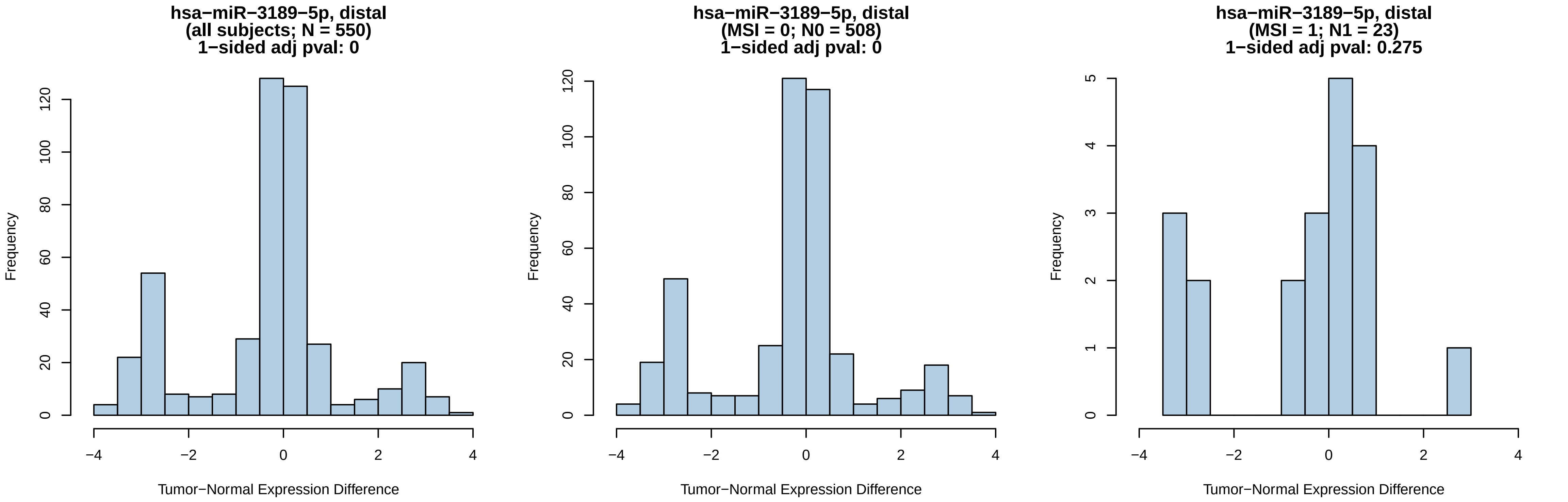

hsa−miR−3189−5p, distal
(all subjects; N = 550)
1−sided adj pval: 0
Frequency
Tumor−Normal Expression Difference
hsa−miR−3189−5p, distal
(MSI = 0; N0 = 508)
1−sided adj pval: 0
Frequency
Tumor−Normal Expression Difference
hsa−miR−3189−5p, distal
(MSI = 1; N1 = 23)
1−sided adj pval: 0.275
Frequency
Tumor−Normal Expression Difference

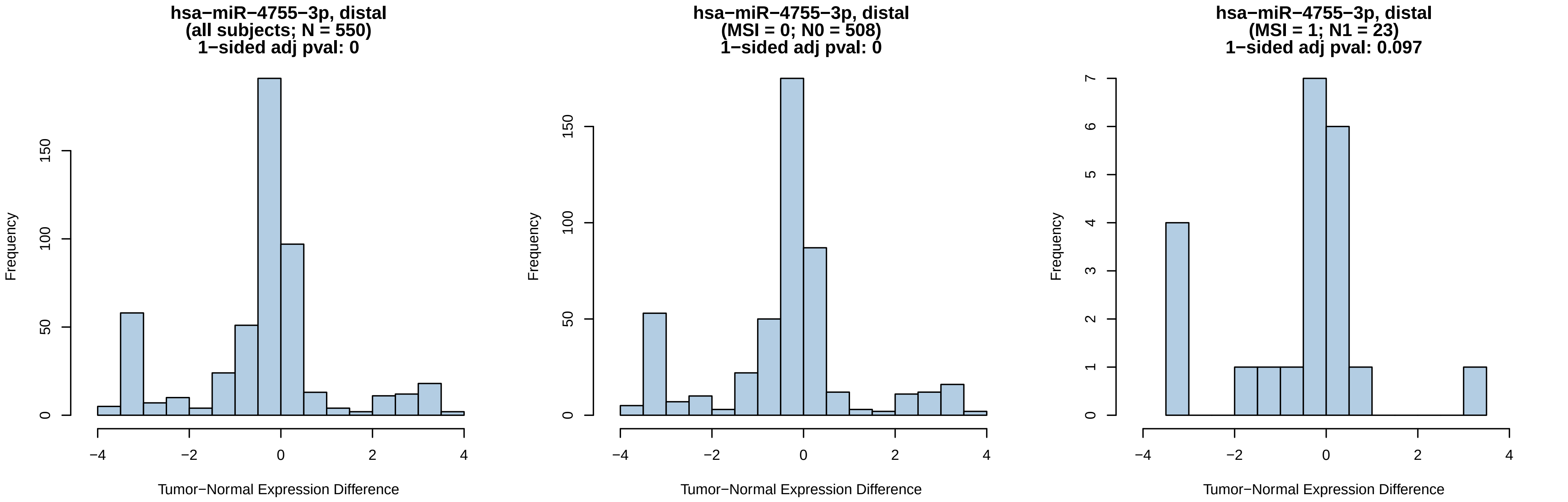
hsa−miR−4755−3p, distal
(all subjects; N = 550)
1−sided adj pval: 0
Frequency
Tumor−Normal Expression Difference
hsa−miR−4755−3p, distal
(MSI = 0; N0 = 508)
1−sided adj pval: 0
Frequency
Tumor−Normal Expression Difference
hsa−miR−4755−3p, distal
(MSI = 1; N1 = 23)
1−sided adj pval: 0.097
Frequency
Tumor−Normal Expression Difference

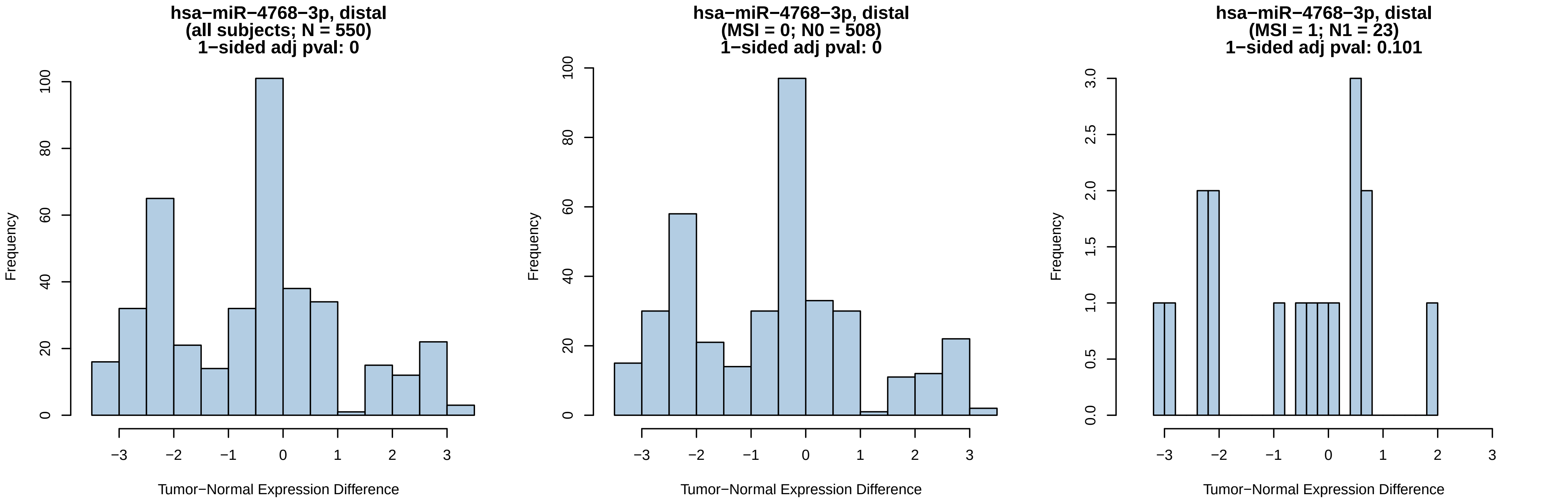
hsa−miR−4768−3p, distal
(all subjects; N = 550)
1−sided adj pval: 0
Frequency
Tumor−Normal Expression Difference
hsa−miR−4768−3p, distal
(MSI = 0; N0 = 508)
1−sided adj pval: 0
Frequency
Tumor−Normal Expression Difference
hsa−miR−4768−3p, distal
(MSI = 1; N1 = 23)
1−sided adj pval: 0.101
Frequency
Tumor−Normal Expression Difference

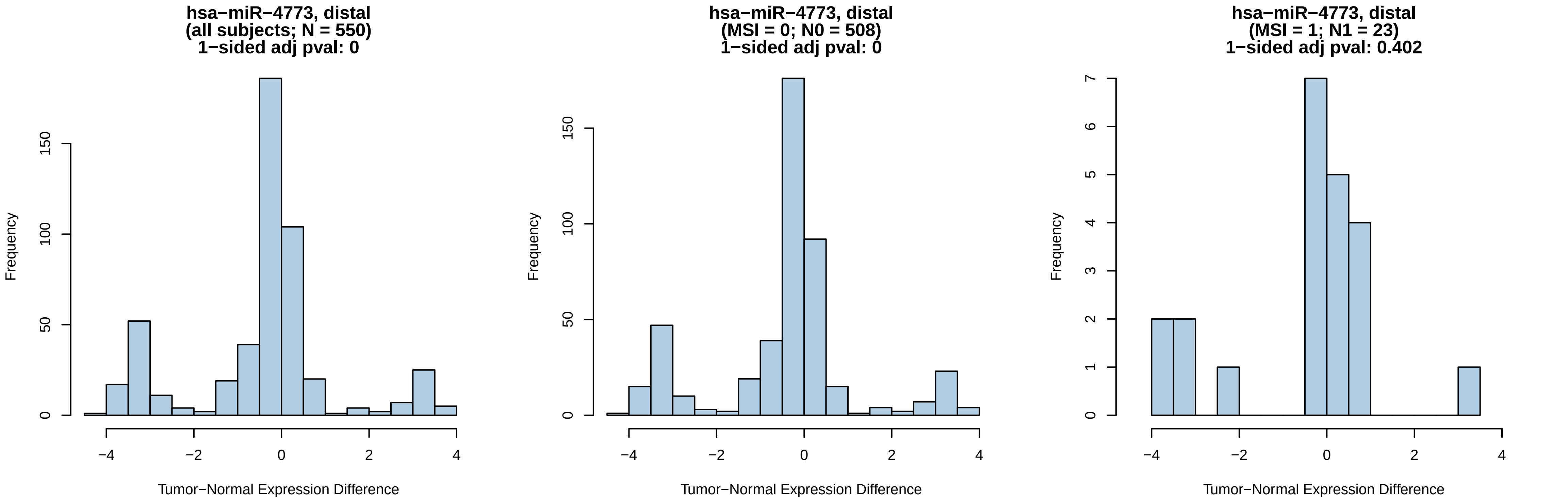
hsa−miR−4773, distal
(all subjects; N = 550)
1−sided adj pval: 0
Frequency
Tumor−Normal Expression Difference
hsa−miR−4773, distal
(MSI = 0; N0 = 508)
1−sided adj pval: 0
Frequency
Tumor−Normal Expression Difference
hsa−miR−4773, distal
(MSI = 1; N1 = 23)
1−sided adj pval: 0.402
Frequency
Tumor−Normal Expression Difference

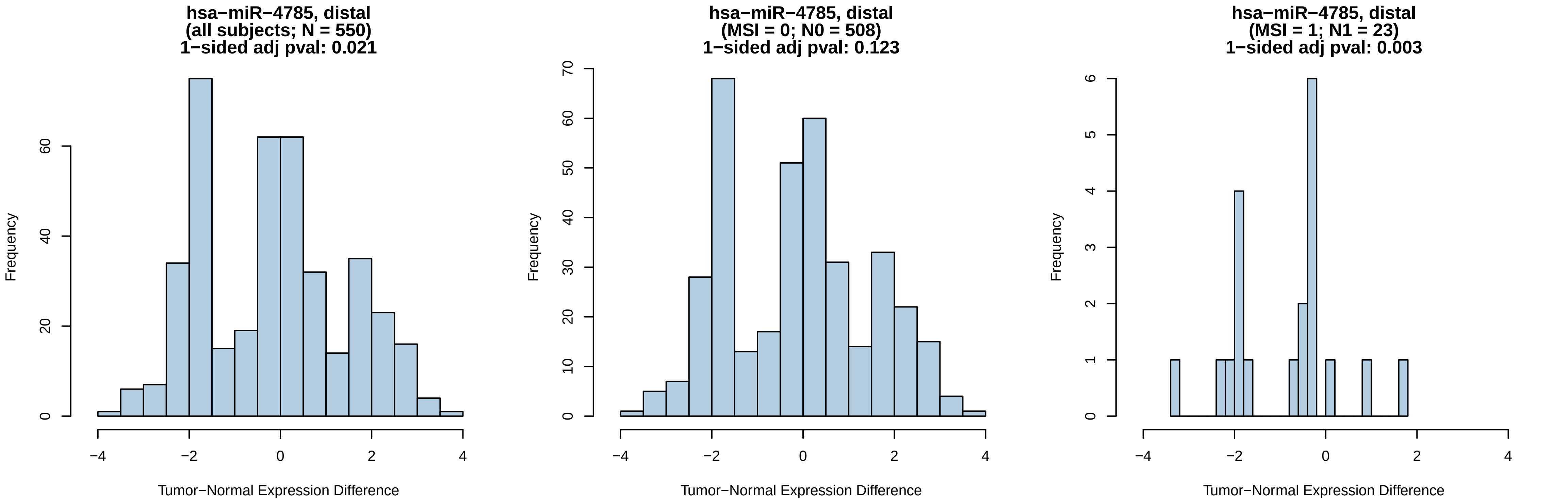
hsa−miR−4785, distal
(all subjects; N = 550)
1−sided adj pval: 0.021
Frequency
Tumor−Normal Expression Difference
hsa−miR−4785, distal
(MSI = 0; N0 = 508)
1−sided adj pval: 0.123
Frequency
Tumor−Normal Expression Difference
hsa−miR−4785, distal
(MSI = 1; N1 = 23)
1−sided adj pval: 0.003
Frequency
Tumor−Normal Expression Difference

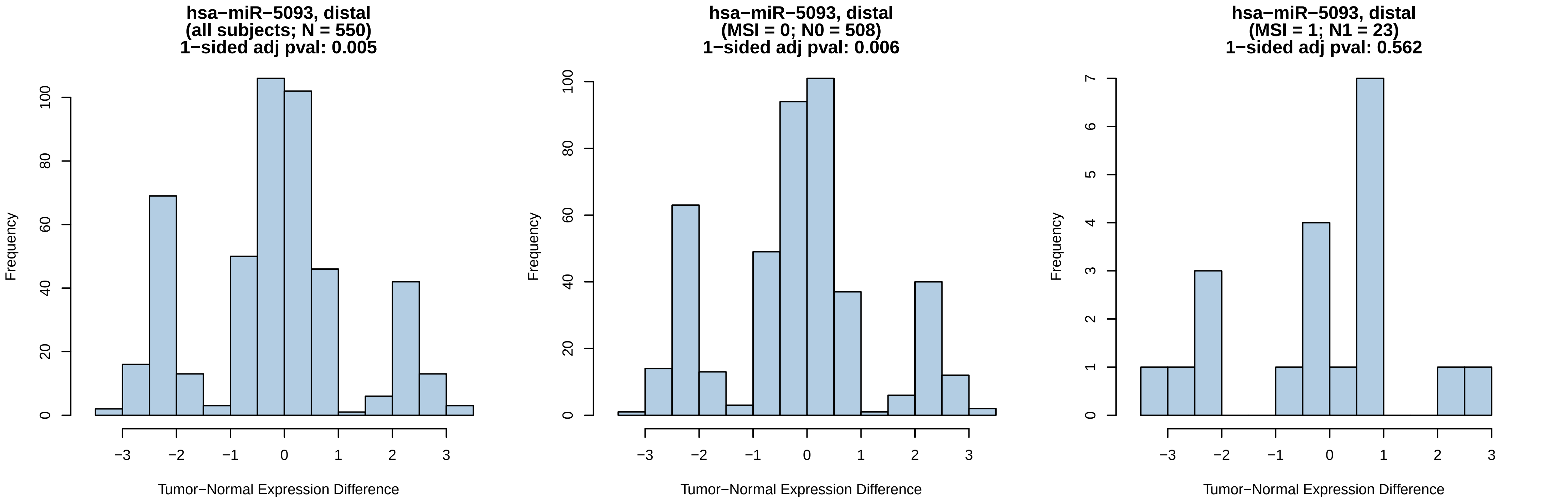

hsa−miR−5093, distal
(all subjects; N = 550)
1−sided adj pval: 0.005
Frequency
Tumor−Normal Expression Difference
hsa−miR−5093, distal
(MSI = 0; N0 = 508)
1−sided adj pval: 0.006
Frequency
Tumor−Normal Expression Difference
hsa−miR−5093, distal
(MSI = 1; N1 = 23)
1−sided adj pval: 0.562
Frequency
Tumor−Normal Expression Difference

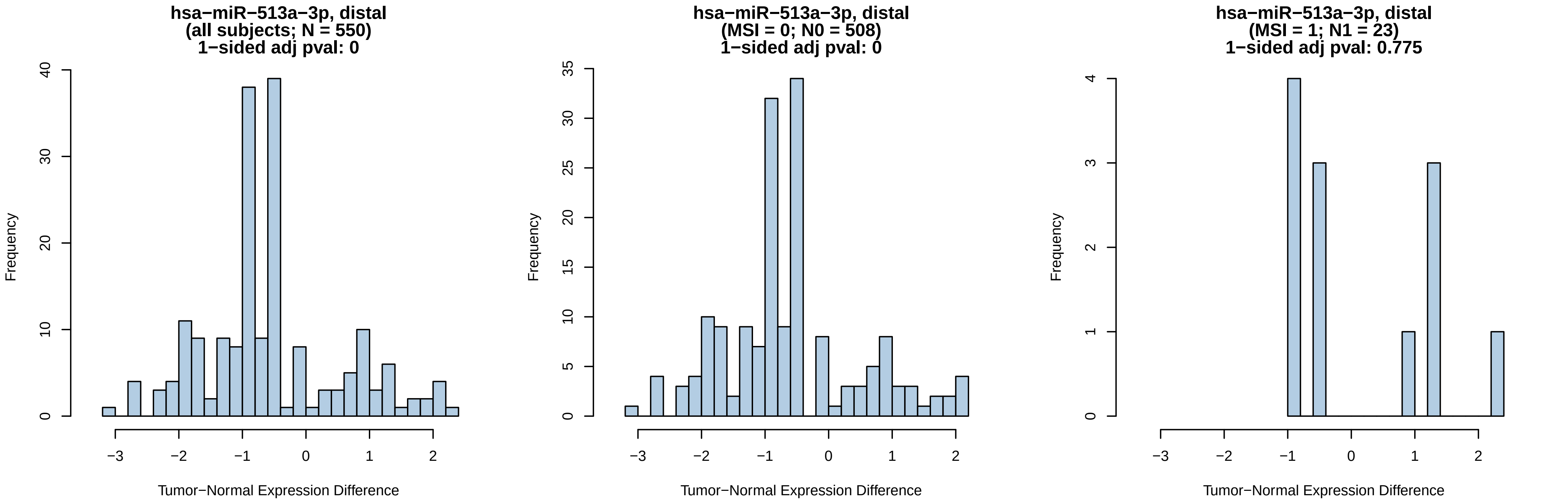

hsa−miR−513a−3p, distal
(all subjects; N = 550)
1−sided adj pval: 0
Frequency
Tumor−Normal Expression Difference
hsa−miR−513a−3p, distal
(MSI = 0; N0 = 508)
1−sided adj pval: 0
Frequency
Tumor−Normal Expression Difference
hsa−miR−513a−3p, distal
(MSI = 1; N1 = 23)
1−sided adj pval: 0.775
Frequency
Tumor−Normal Expression Difference

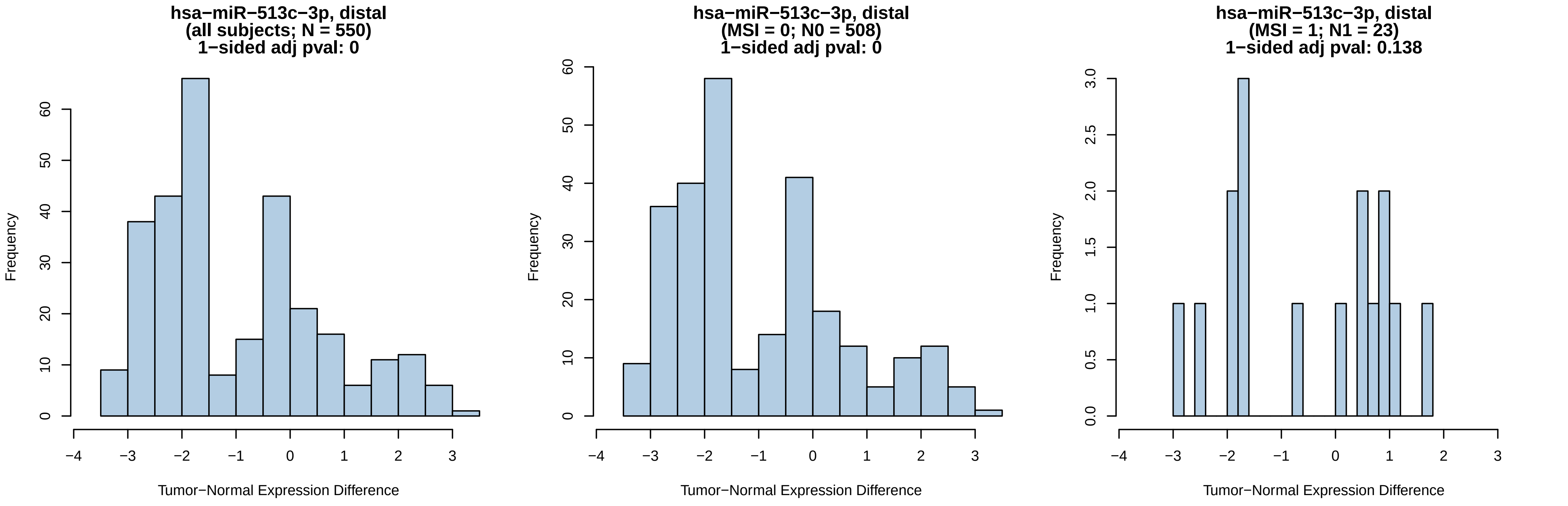
hsa−miR−513c−3p, distal
(all subjects; N = 550)
1−sided adj pval: 0
hsa−miR−513c−3p, distal
(MSI = 0; N0 = 508)
1−sided adj pval: 0
hsa−miR−513c−3p, distal
(MSI = 1; N1 = 23)
1−sided adj pval: 0.138
Frequency
Tumor−Normal Expression Difference

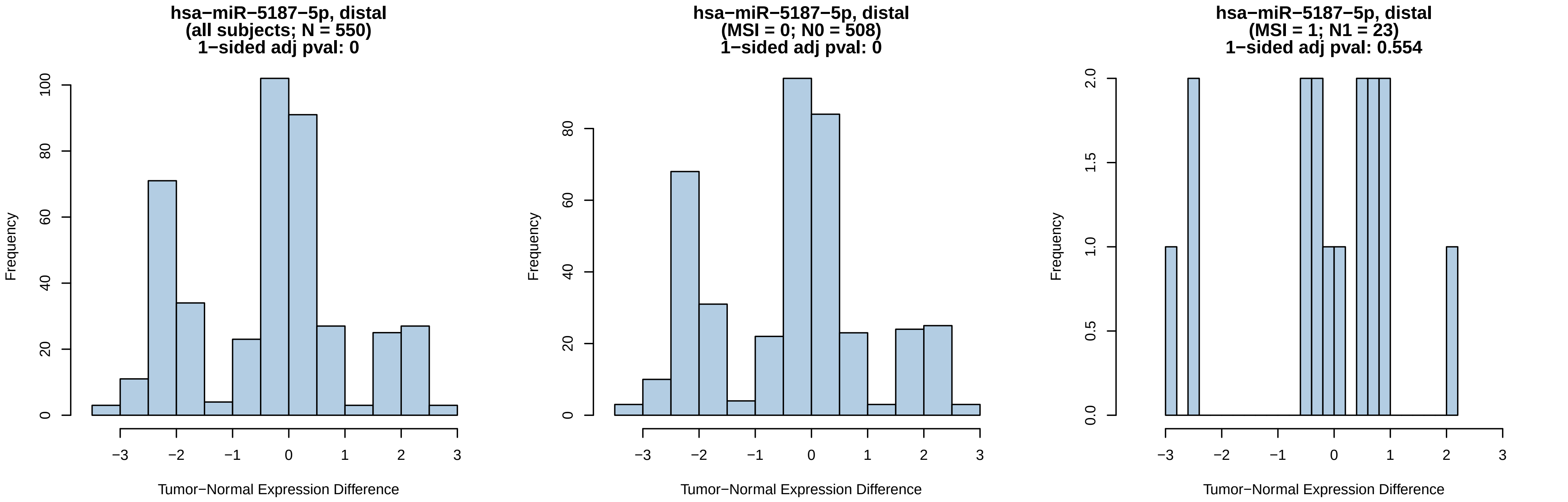
hsa−miR−5187−5p, distal
(all subjects; N = 550)
1−sided adj pval: 0
Frequency
Tumor−Normal Expression Difference
hsa−miR−5187−5p, distal
(MSI = 0; N0 = 508)
1−sided adj pval: 0
Frequency
Tumor−Normal Expression Difference
hsa−miR−5187−5p, distal
(MSI = 1; N1 = 23)
1−sided adj pval: 0.554
Frequency
Tumor−Normal Expression Difference

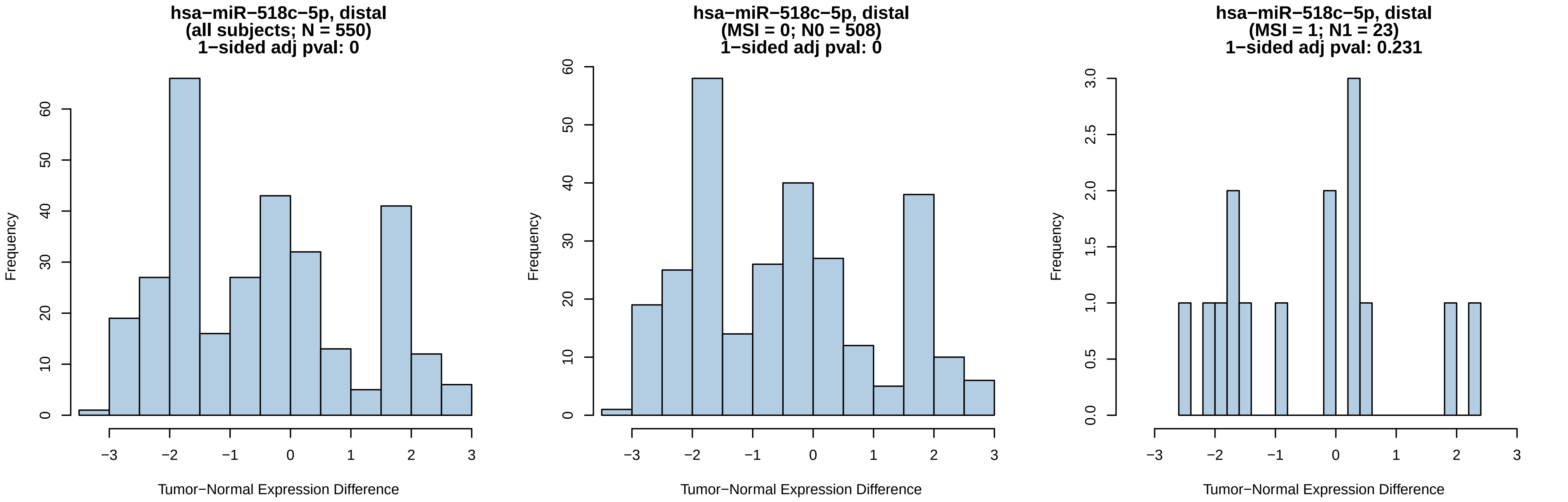

hsa−miR−518c−5p, distal
(all subjects; N = 550)
1−sided adj pval: 0
hsa−miR−518c−5p, distal
(MSI = 0; N0 = 508)
1−sided adj pval: 0
hsa−miR−518c−5p, distal
(MSI = 1; N1 = 23)
1−sided adj pval: 0.231
Frequency
Tumor−Normal Expression Difference

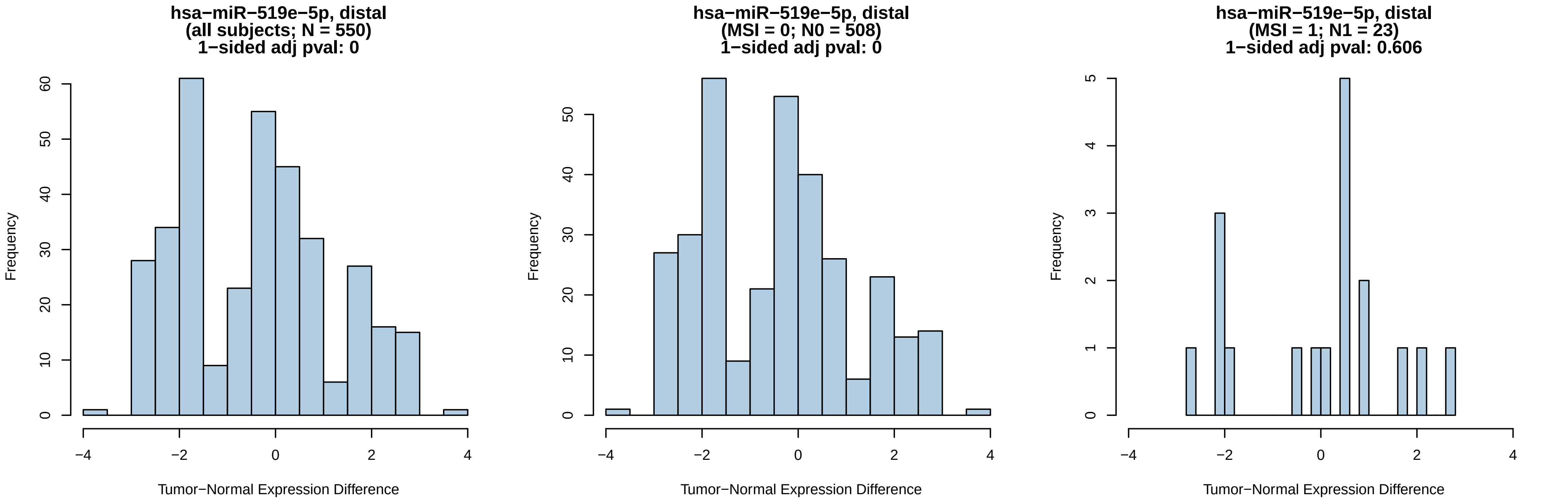
hsa−miR−519e−5p, distal
(all subjects; N = 550)
1−sided adj pval: 0
Frequency
Tumor−Normal Expression Difference
hsa−miR−519e−5p, distal
(MSI = 0; N0 = 508)
1−sided adj pval: 0
Frequency
Tumor−Normal Expression Difference
hsa−miR−519e−5p, distal
(MSI = 1; N1 = 23)
1−sided adj pval: 0.606
Frequency
Tumor−Normal Expression Difference

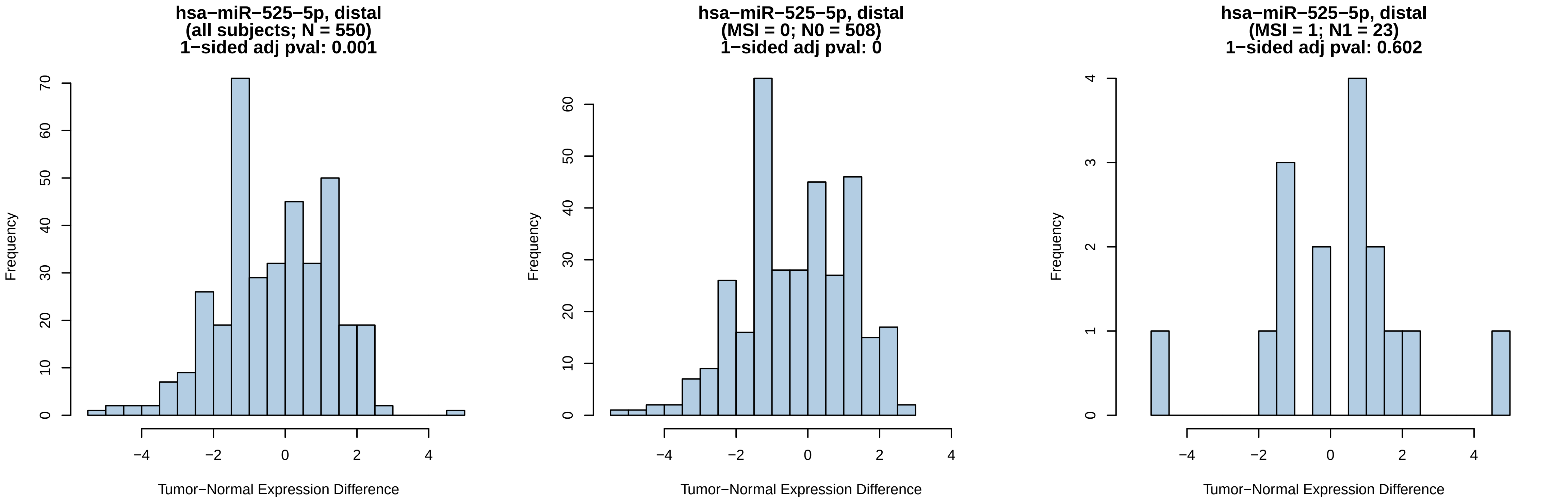

hsa−miR−525−5p, distal
(all subjects; N = 550)
1−sided adj pval: 0.001
Frequency
Tumor−Normal Expression Difference
hsa−miR−525−5p, distal
(MSI = 0; N0 = 508)
1−sided adj pval: 0
Frequency
Tumor−Normal Expression Difference
hsa−miR−525−5p, distal
(MSI = 1; N1 = 23)
1−sided adj pval: 0.602
Frequency
Tumor−Normal Expression Difference

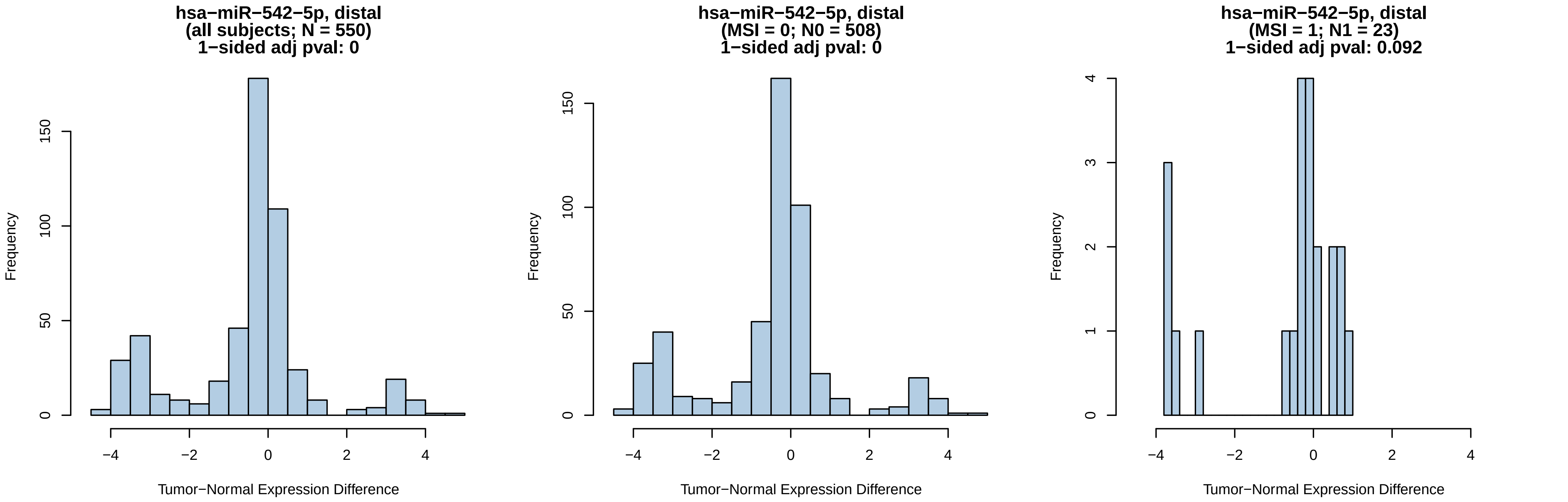
hsa−miR−542−5p, distal
(all subjects; N = 550)
1−sided adj pval: 0
Frequency
Tumor−Normal Expression Difference
hsa−miR−542−5p, distal
(MSI = 0; N0 = 508)
1−sided adj pval: 0
Frequency
Tumor−Normal Expression Difference
hsa−miR−542−5p, distal
(MSI = 1; N1 = 23)
1−sided adj pval: 0.092
Frequency
Tumor−Normal Expression Difference

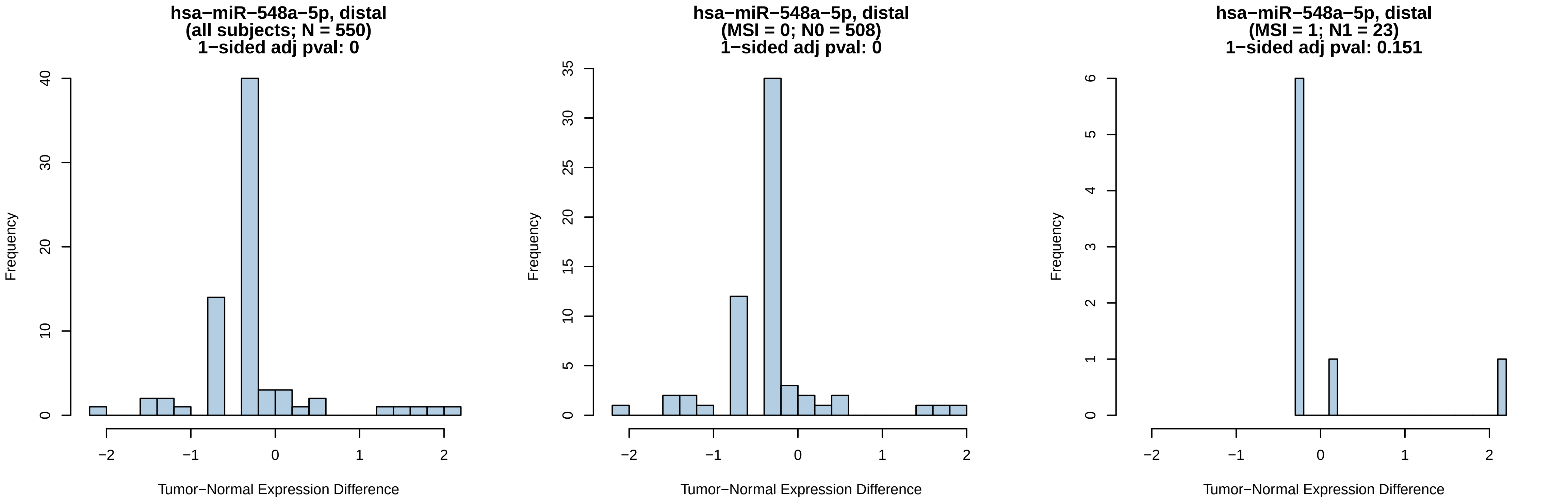
hsa−miR−548a−5p, distal
(all subjects; N = 550)
1−sided adj pval: 0
Frequency
Tumor−Normal Expression Difference
hsa−miR−548a−5p, distal
(MSI = 0; N0 = 508)
1−sided adj pval: 0
Frequency
Tumor−Normal Expression Difference
hsa−miR−548a−5p, distal
(MSI = 1; N1 = 23)
1−sided adj pval: 0.151
Frequency
Tumor−Normal Expression Difference

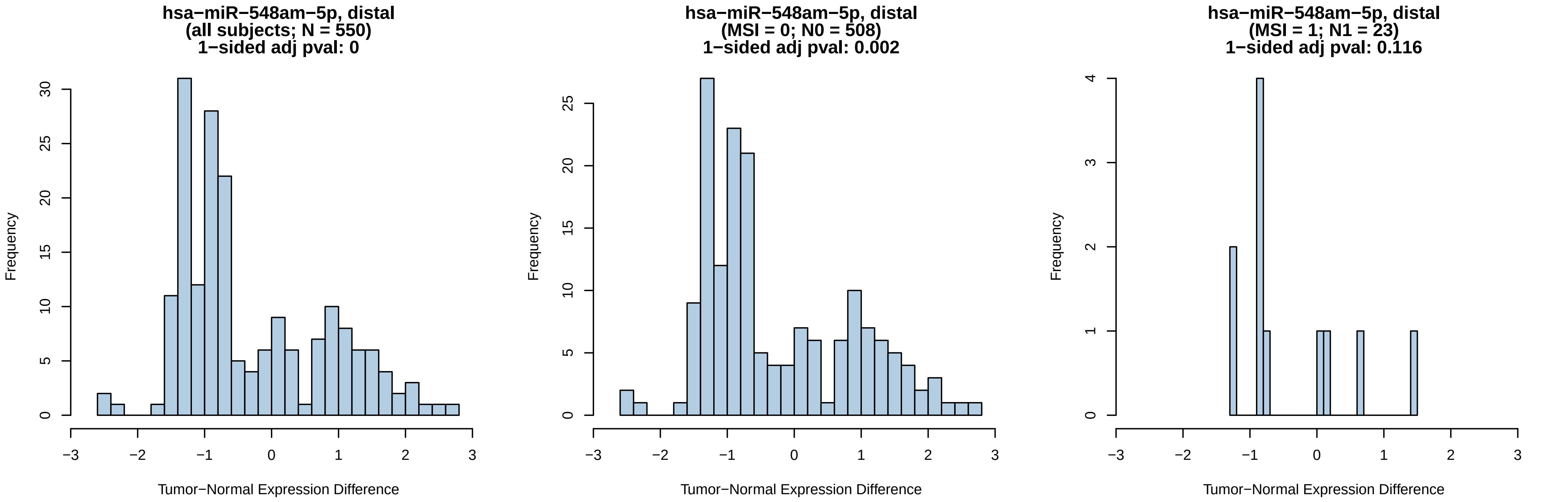
hsa−miR−548am−5p, distal
(all subjects; N = 550)
1−sided adj pval: 0
hsa−miR−548am−5p, distal
(MSI = 0; N0 = 508)
1−sided adj pval: 0.002
hsa−miR−548am−5p, distal
(MSI = 1; N1 = 23)
1−sided adj pval: 0.116
Frequency
Tumor−Normal Expression Difference

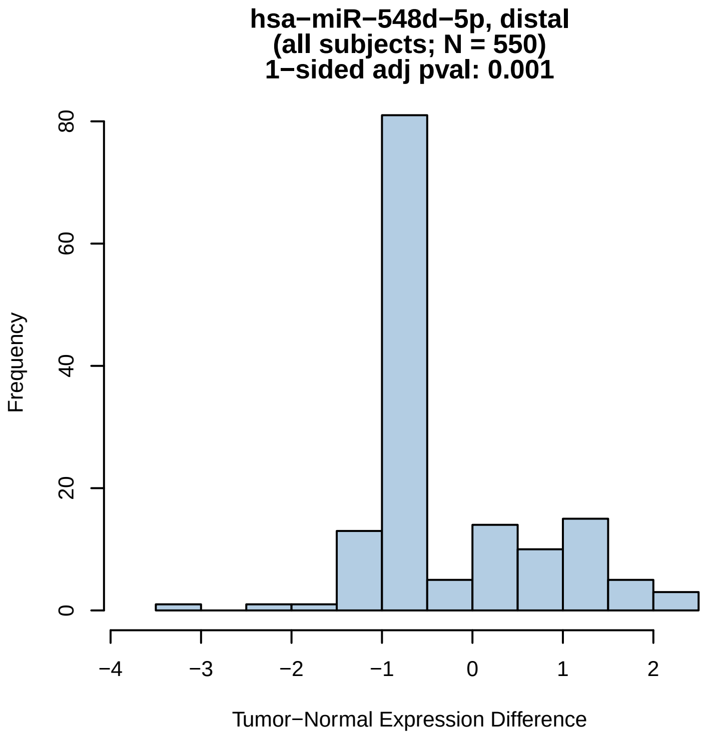
hsa−miR−548d−5p, distal
(all subjects; N = 550)
1−sided adj pval: 0.001
Frequency
Tumor−Normal Expression Difference
0
20
40
60
80
−4
−3
−2
−1
0
1
2

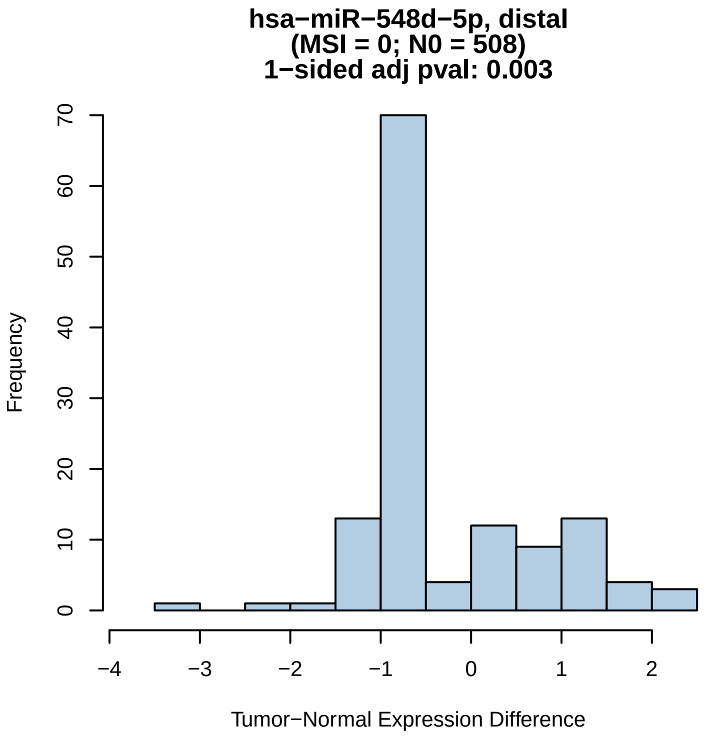
hsa−miR−548d−5p, distal
(MSI = 0; N0 = 508)
1−sided adj pval: 0.003
Frequency
Tumor−Normal Expression Difference
0
10
20
30
40
50
60
70
−4
−3
−2
−1
0
1
2

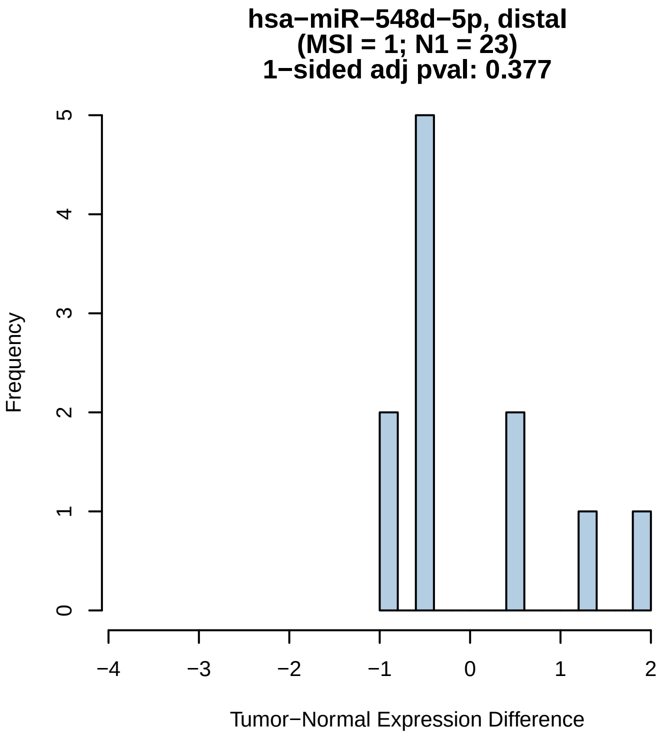
hsa−miR−548d−5p, distal
(MSI = 1; N1 = 23)
1−sided adj pval: 0.377
Frequency
Tumor−Normal Expression Difference
0
1
2
3
4
5
−4
−3
−2
−1
0
1
2

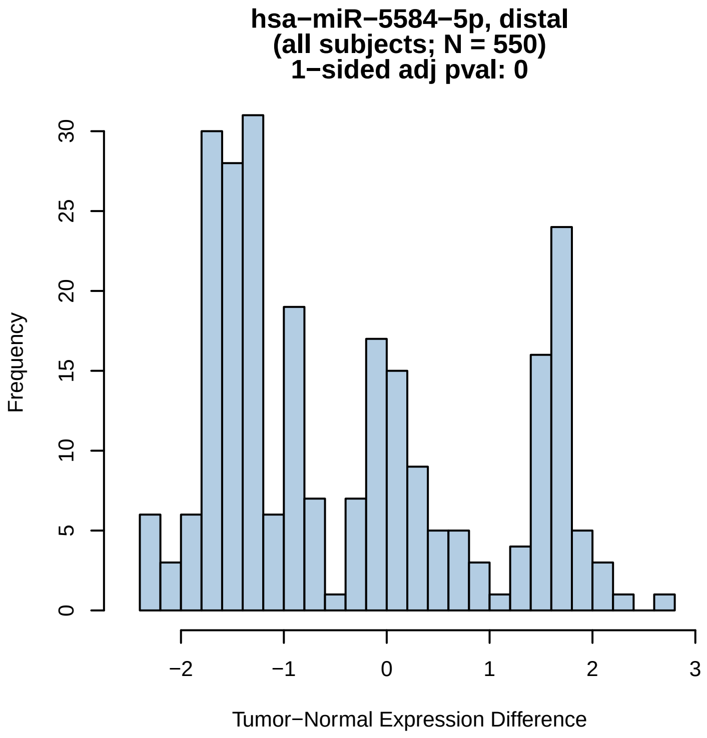
hsa−miR−5584−5p, distal
(all subjects; N = 550)
1−sided adj pval: 0
Frequency
Tumor−Normal Expression Difference

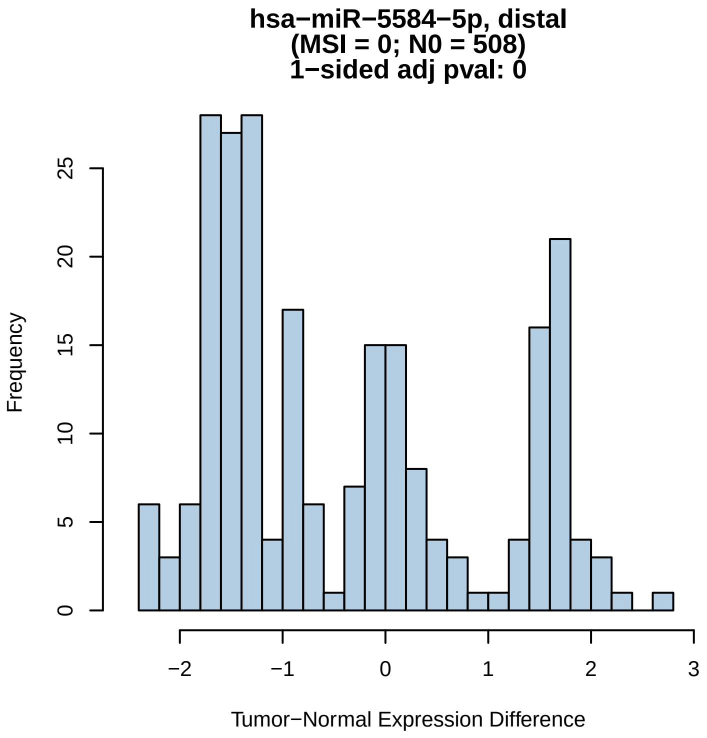
hsa−miR−5584−5p, distal
(MSI = 0; N0 = 508)
1−sided adj pval: 0
Frequency
Tumor−Normal Expression Difference

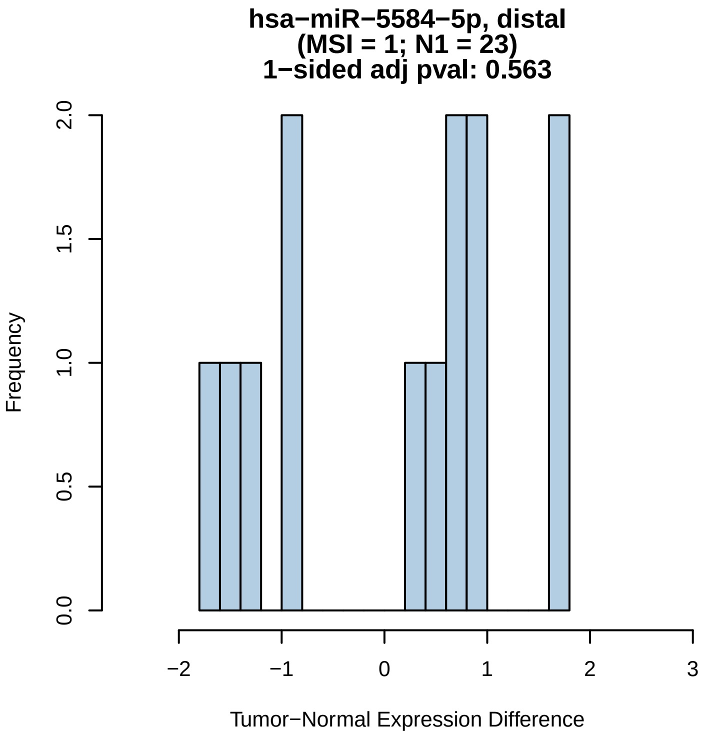
hsa−miR−5584−5p, distal
(MSI = 1; N1 = 23)
1−sided adj pval: 0.563
Frequency
Tumor−Normal Expression Difference

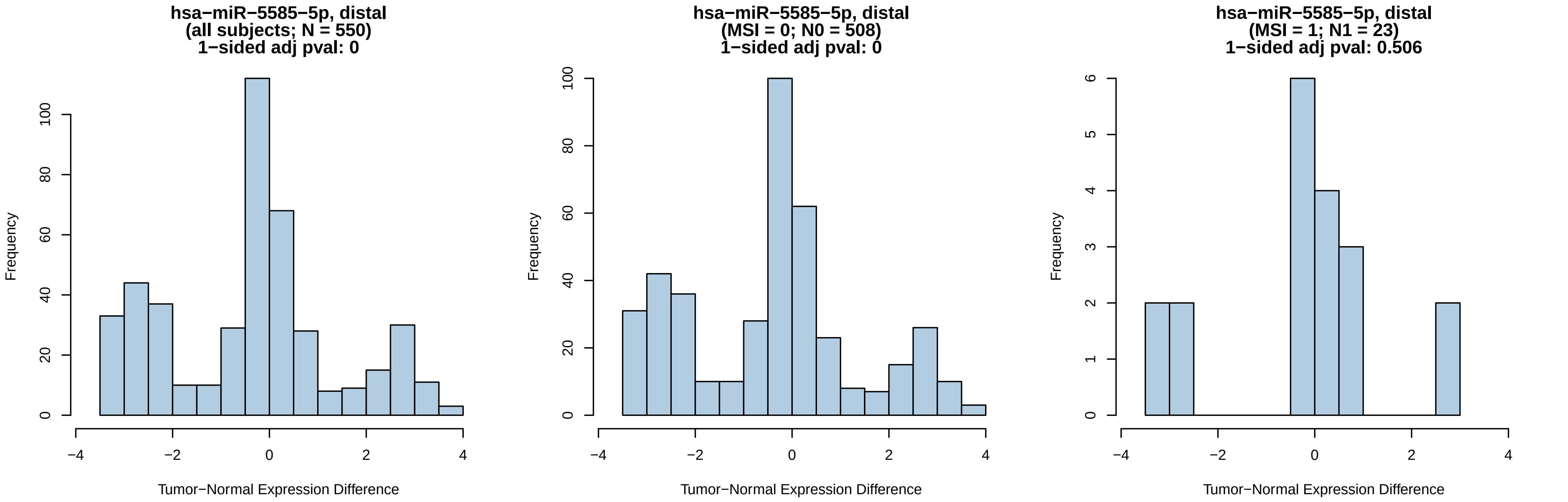
hsa−miR−5585−5p, distal
(all subjects; N = 550)
1−sided adj pval: 0
Frequency
Tumor−Normal Expression Difference
hsa−miR−5585−5p, distal
(MSI = 0; N0 = 508)
1−sided adj pval: 0
Frequency
Tumor−Normal Expression Difference
hsa−miR−5585−5p, distal
(MSI = 1; N1 = 23)
1−sided adj pval: 0.506
Frequency
Tumor−Normal Expression Difference

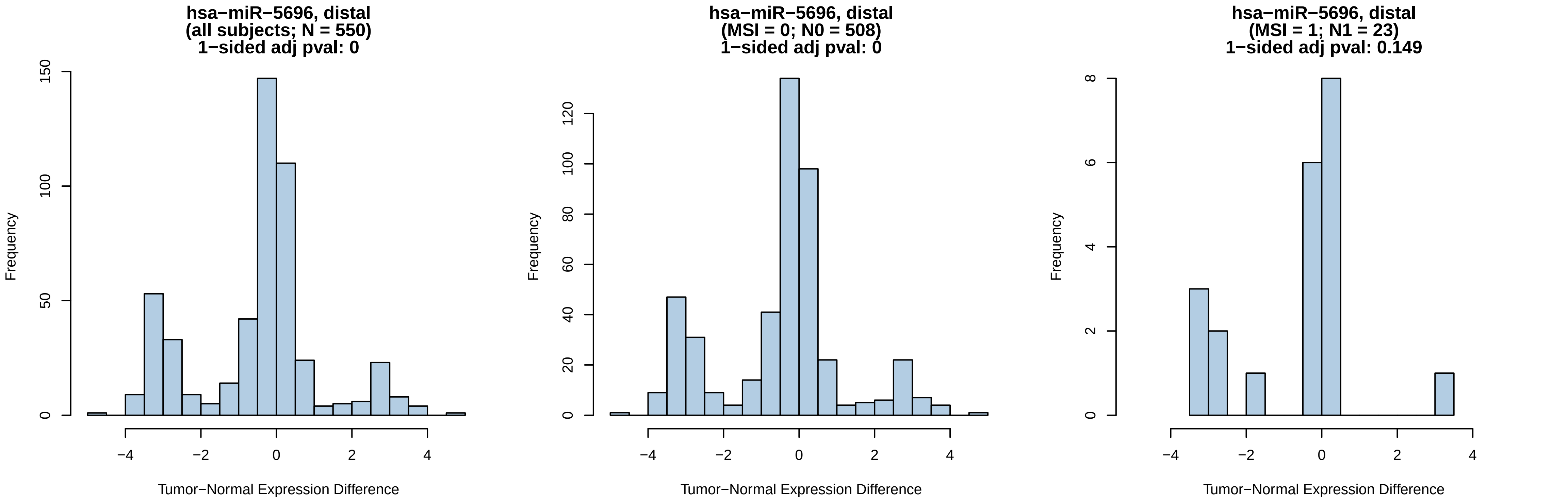

hsa−miR−5696, distal
(all subjects; N = 550)
1−sided adj pval: 0
Frequency
Tumor−Normal Expression Difference
hsa−miR−5696, distal
(MSI = 0; N0 = 508)
1−sided adj pval: 0
Frequency
Tumor−Normal Expression Difference
hsa−miR−5696, distal
(MSI = 1; N1 = 23)
1−sided adj pval: 0.149
Frequency
Tumor−Normal Expression Difference

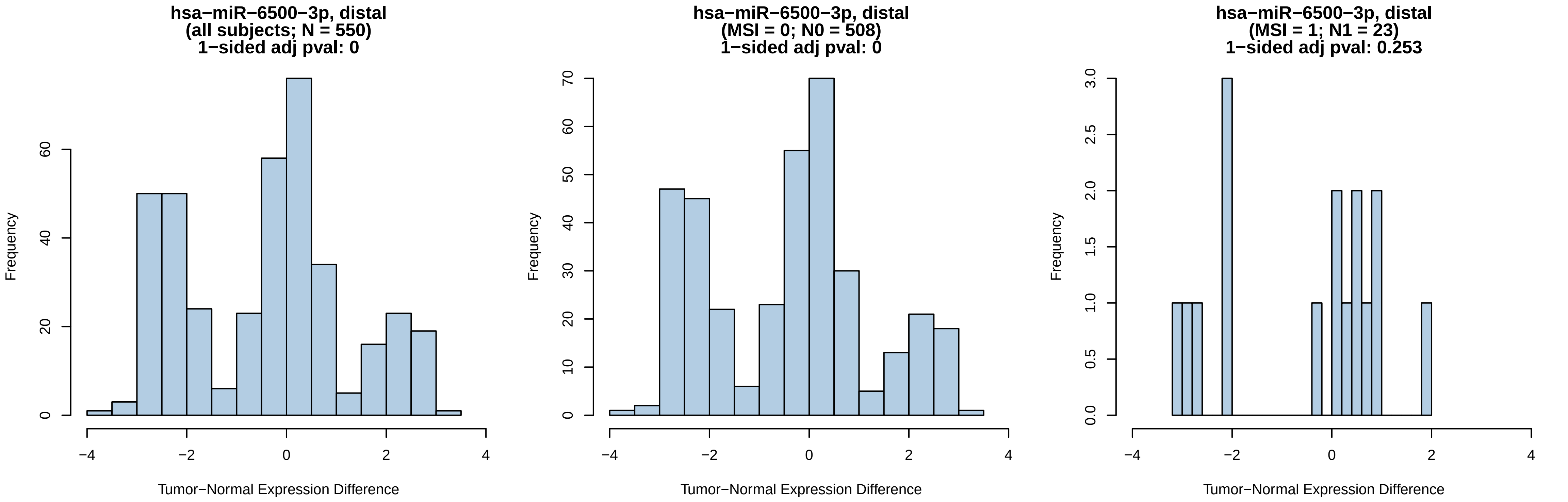
hsa−miR−6500−3p, distal
(all subjects; N = 550)
1−sided adj pval: 0
Frequency
Tumor−Normal Expression Difference
hsa−miR−6500−3p, distal
(MSI = 0; N0 = 508)
1−sided adj pval: 0
Frequency
Tumor−Normal Expression Difference
hsa−miR−6500−3p, distal
(MSI = 1; N1 = 23)
1−sided adj pval: 0.253
Frequency
Tumor−Normal Expression Difference

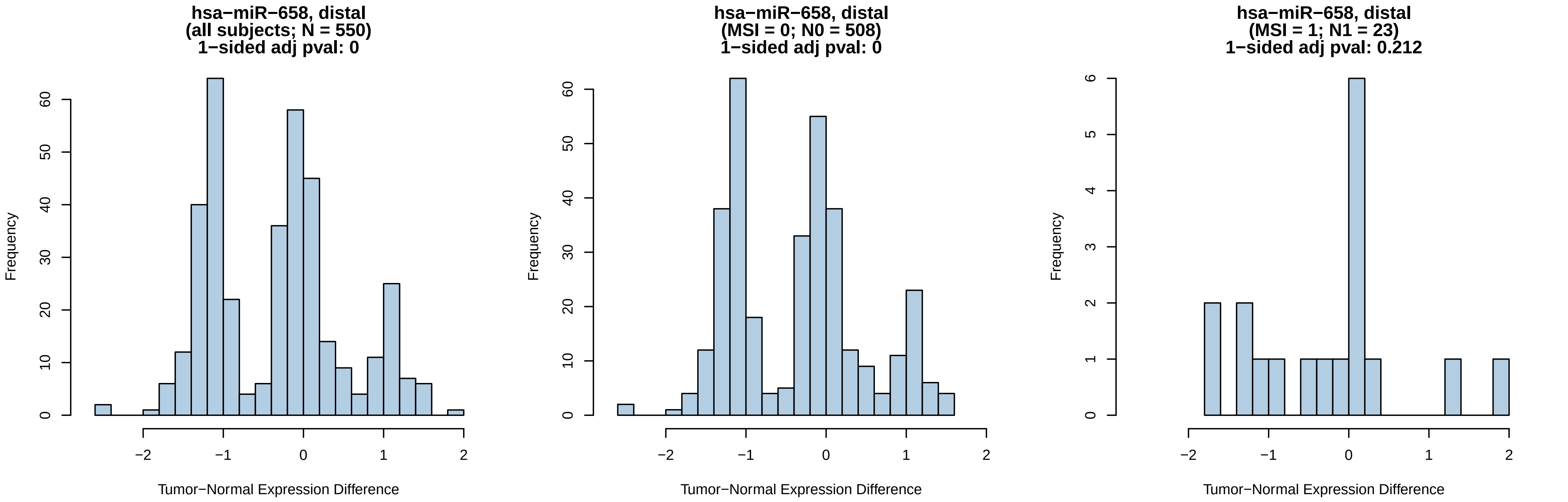

hsa−miR−658, distal
(all subjects; N = 550)
1−sided adj pval: 0
Frequency
Tumor−Normal Expression Difference
hsa−miR−658, distal
(MSI = 0; N0 = 508)
1−sided adj pval: 0
Frequency
Tumor−Normal Expression Difference
hsa−miR−658, distal
(MSI = 1; N1 = 23)
1−sided adj pval: 0.212
Frequency
Tumor−Normal Expression Difference

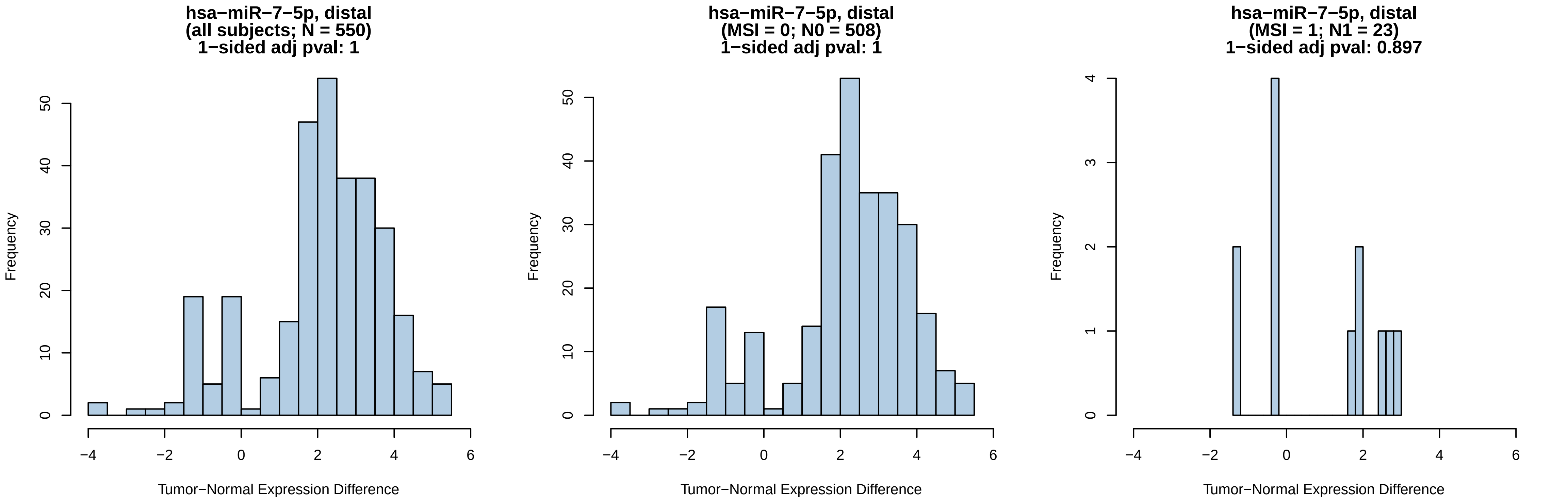

hsa−miR−7−5p, distal
(all subjects; N = 550)
1−sided adj pval: 1
hsa−miR−7−5p, distal
(MSI = 0; N0 = 508)
1−sided adj pval: 1
hsa−miR−7−5p, distal
(MSI = 1; N1 = 23)
1−sided adj pval: 0.897
Frequency
Tumor−Normal Expression Difference

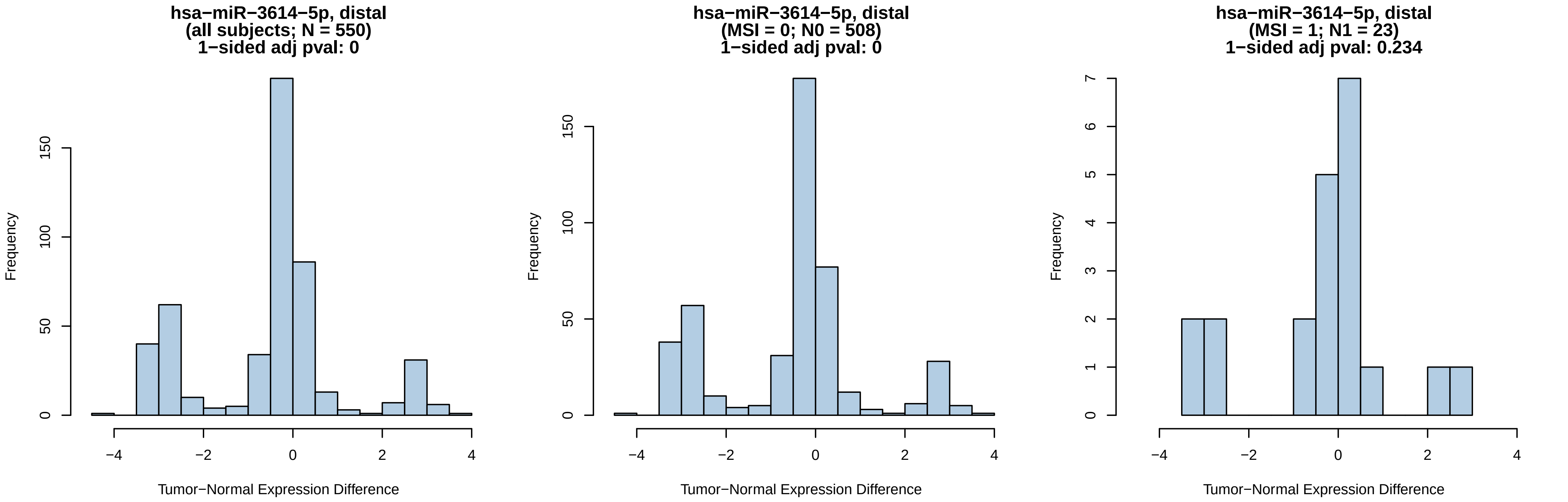

hsa−miR−3614−5p, distal
(all subjects; N = 550)
1−sided adj pval: 0
hsa−miR−3614−5p, distal
(MSI = 0; N0 = 508)
1−sided adj pval: 0
hsa−miR−3614−5p, distal
(MSI = 1; N1 = 23)
1−sided adj pval: 0.234
Frequency
Tumor−Normal Expression Difference

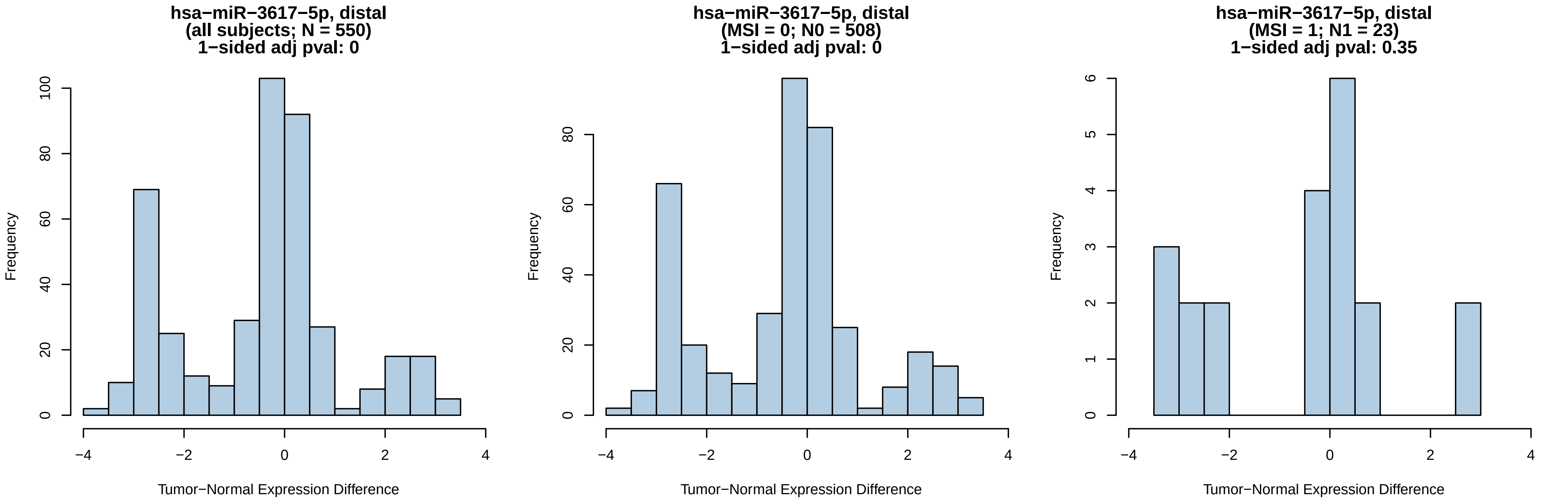
hsa−miR−3617−5p, distal
(all subjects; N = 550)
1−sided adj pval: 0
Frequency
Tumor−Normal Expression Difference
hsa−miR−3617−5p, distal
(MSI = 0; N0 = 508)
1−sided adj pval: 0
Frequency
Tumor−Normal Expression Difference
hsa−miR−3617−5p, distal
(MSI = 1; N1 = 23)
1−sided adj pval: 0.35
Frequency
Tumor−Normal Expression Difference

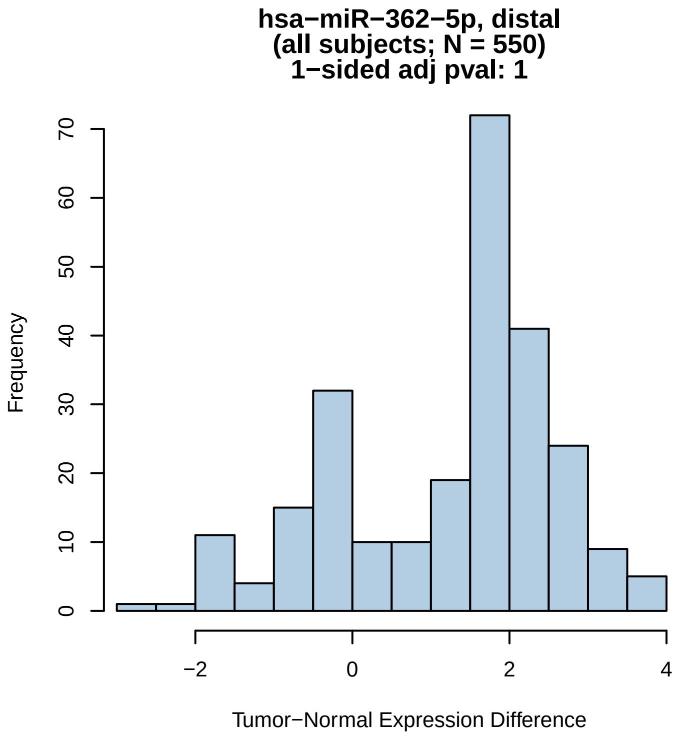
hsa−miR−362−5p, distal
(all subjects; N = 550)
1−sided adj pval: 1
Frequency
0
10
20
30
40
50
60
70
−2
0
2
4
Tumor−Normal Expression Difference

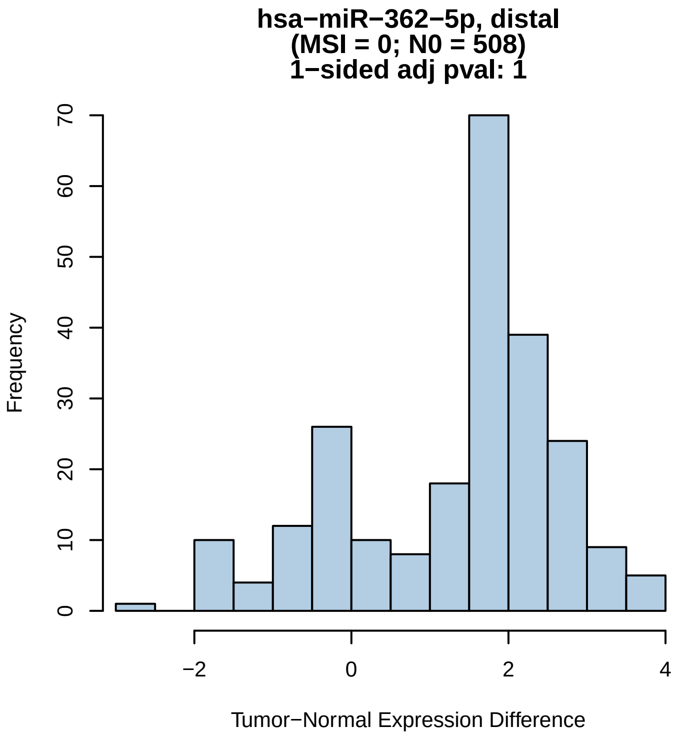
hsa−miR−362−5p, distal
(MSI = 0; N0 = 508)
1−sided adj pval: 1
Frequency
0
10
20
30
40
50
60
70
−2
0
2
4
Tumor−Normal Expression Difference

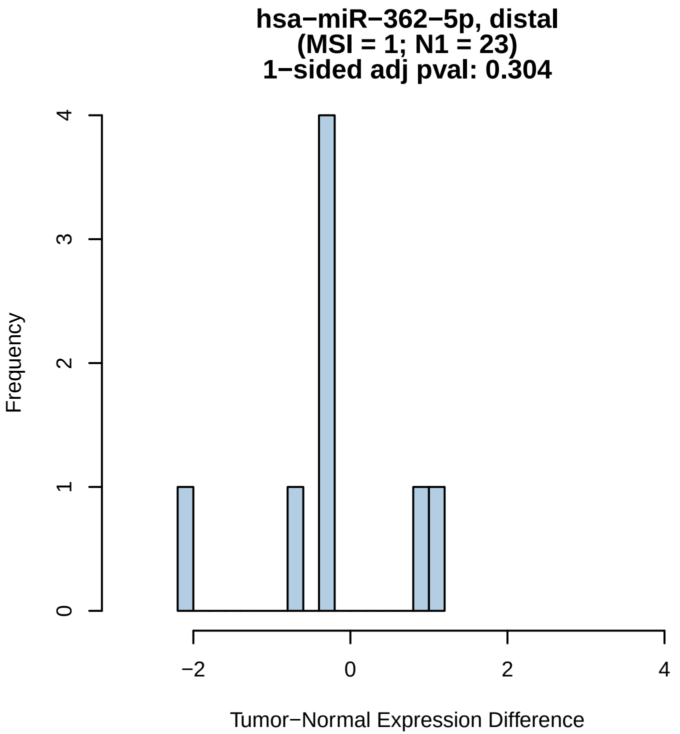
hsa−miR−362−5p, distal
(MSI = 1; N1 = 23)
1−sided adj pval: 0.304
Frequency
0
1
2
3
4
−2
0
2
4
Tumor−Normal Expression Difference

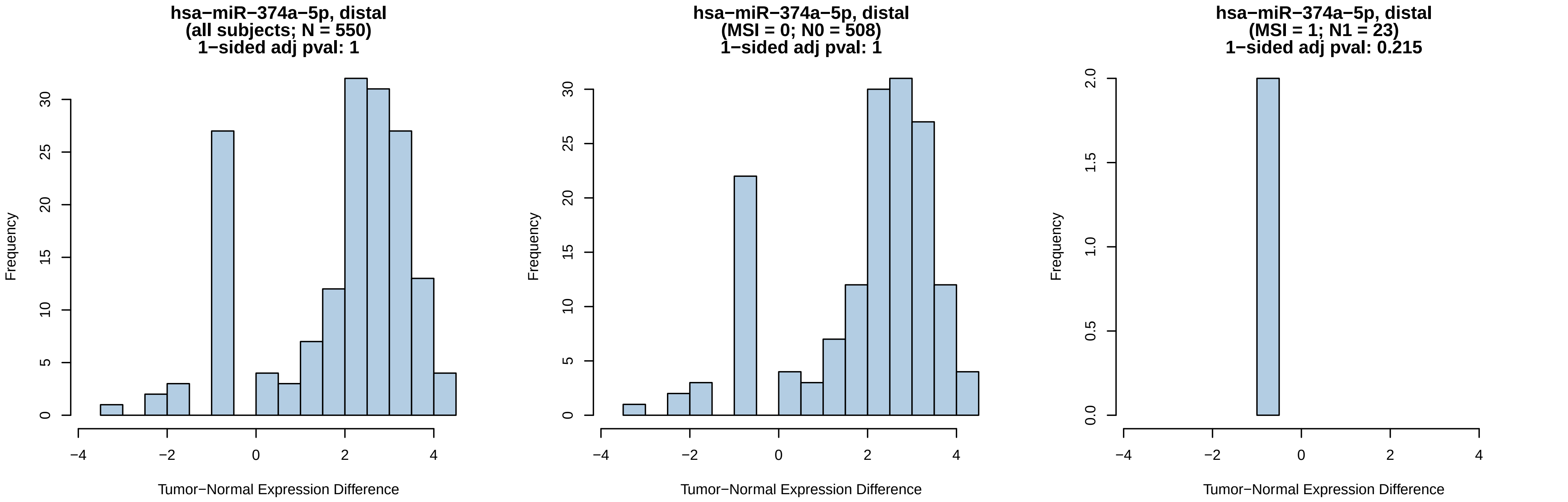

hsa−miR−374a−5p, distal
(all subjects; N = 550)
1−sided adj pval: 1
Frequency
Tumor−Normal Expression Difference
hsa−miR−374a−5p, distal
(MSI = 0; N0 = 508)
1−sided adj pval: 1
Frequency
Tumor−Normal Expression Difference
hsa−miR−374a−5p, distal
(MSI = 1; N1 = 23)
1−sided adj pval: 0.215
Frequency
Tumor−Normal Expression Difference

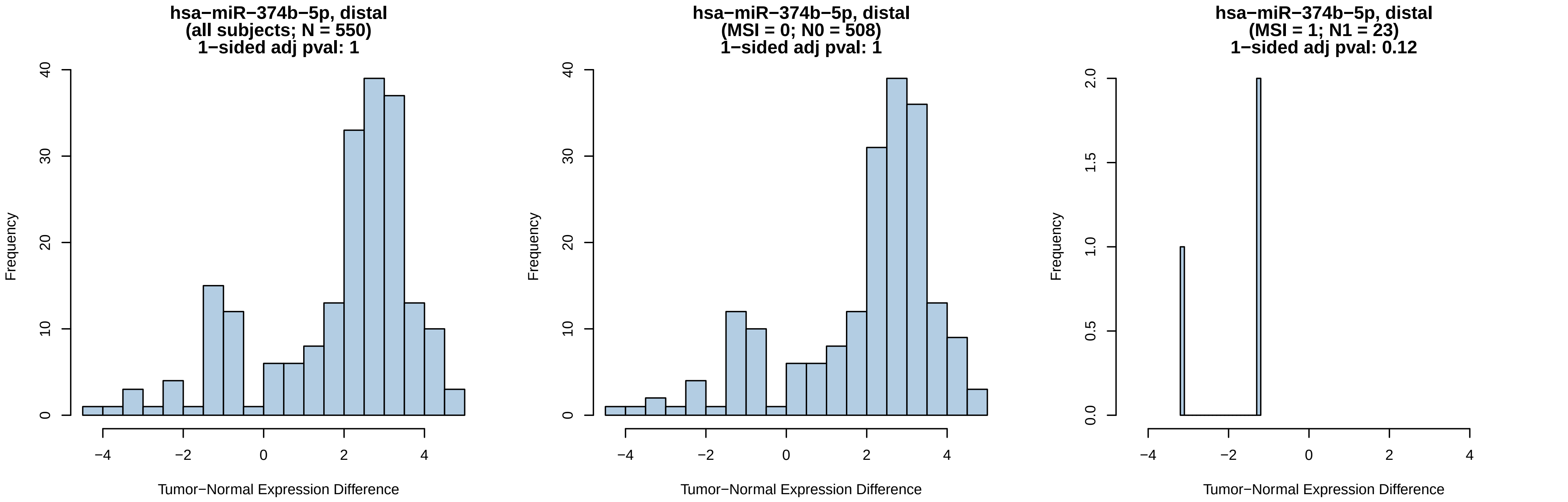
hsa−miR−374b−5p, distal
(all subjects; N = 550)
1−sided adj pval: 1
Frequency
Tumor−Normal Expression Difference
hsa−miR−374b−5p, distal
(MSI = 0; N0 = 508)
1−sided adj pval: 1
Frequency
Tumor−Normal Expression Difference
hsa−miR−374b−5p, distal
(MSI = 1; N1 = 23)
1−sided adj pval: 0.12
Frequency
Tumor−Normal Expression Difference

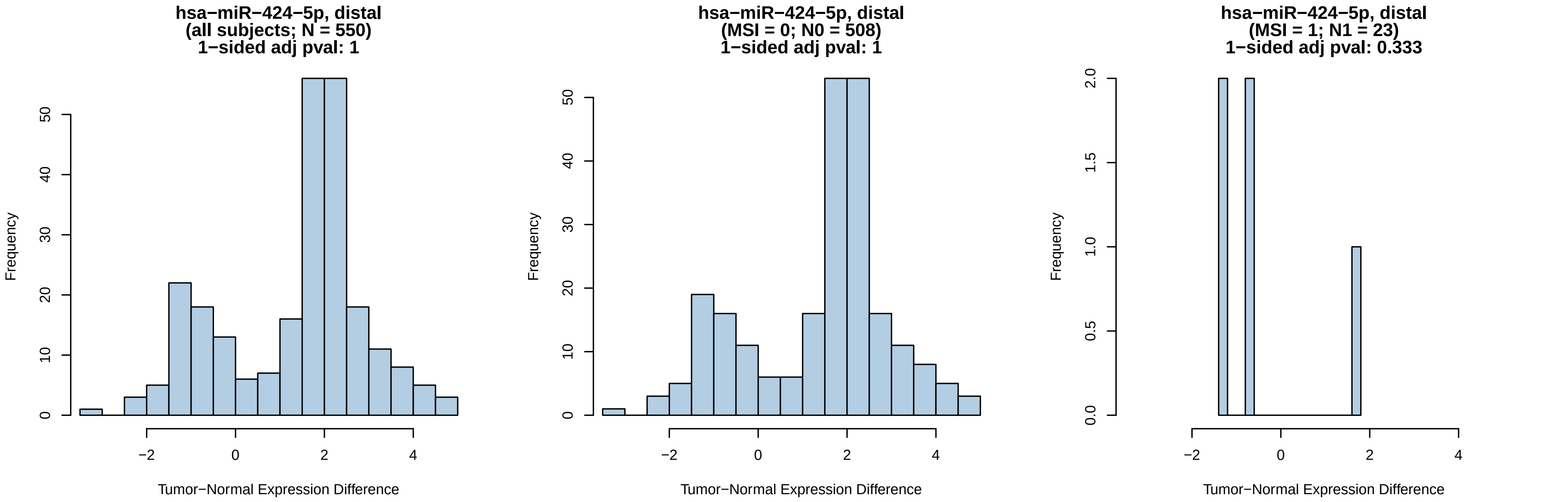

hsa−miR−424−5p, distal
(all subjects; N = 550)
1−sided adj pval: 1
Frequency
Tumor−Normal Expression Difference
hsa−miR−424−5p, distal
(MSI = 0; N0 = 508)
1−sided adj pval: 1
Frequency
Tumor−Normal Expression Difference
hsa−miR−424−5p, distal
(MSI = 1; N1 = 23)
1−sided adj pval: 0.333
Frequency
Tumor−Normal Expression Difference

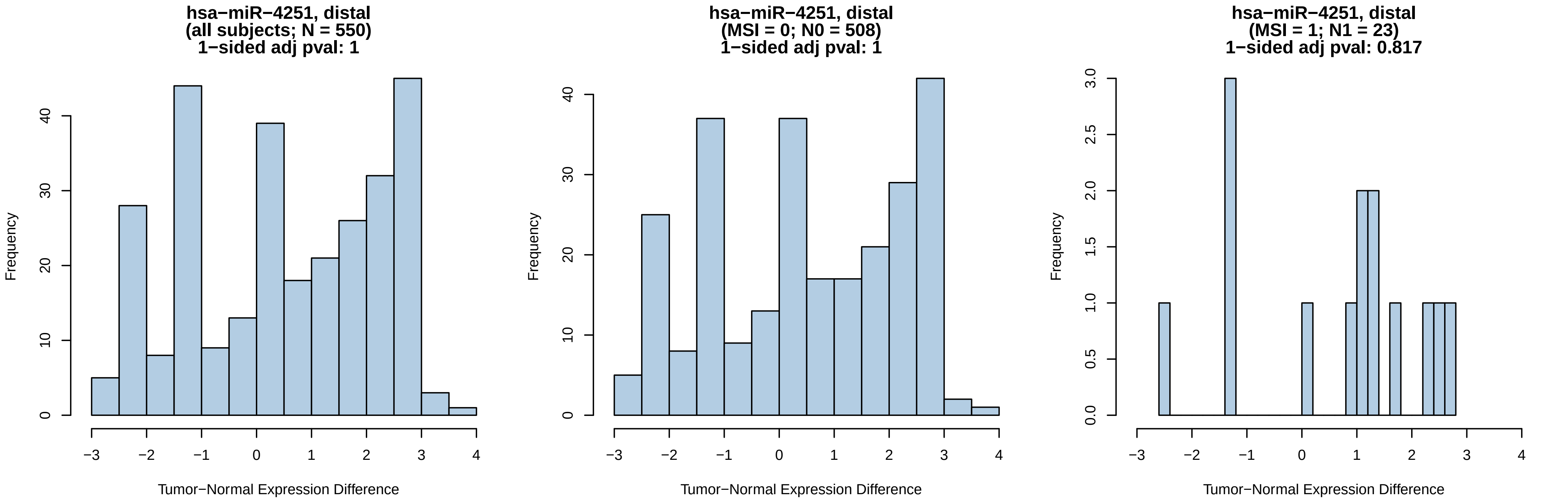
hsa−miR−4251, distal
(all subjects; N = 550)
1−sided adj pval: 1
Frequency
Tumor−Normal Expression Difference
hsa−miR−4251, distal
(MSI = 0; N0 = 508)
1−sided adj pval: 1
Frequency
Tumor−Normal Expression Difference
hsa−miR−4251, distal
(MSI = 1; N1 = 23)
1−sided adj pval: 0.817
Frequency
Tumor−Normal Expression Difference

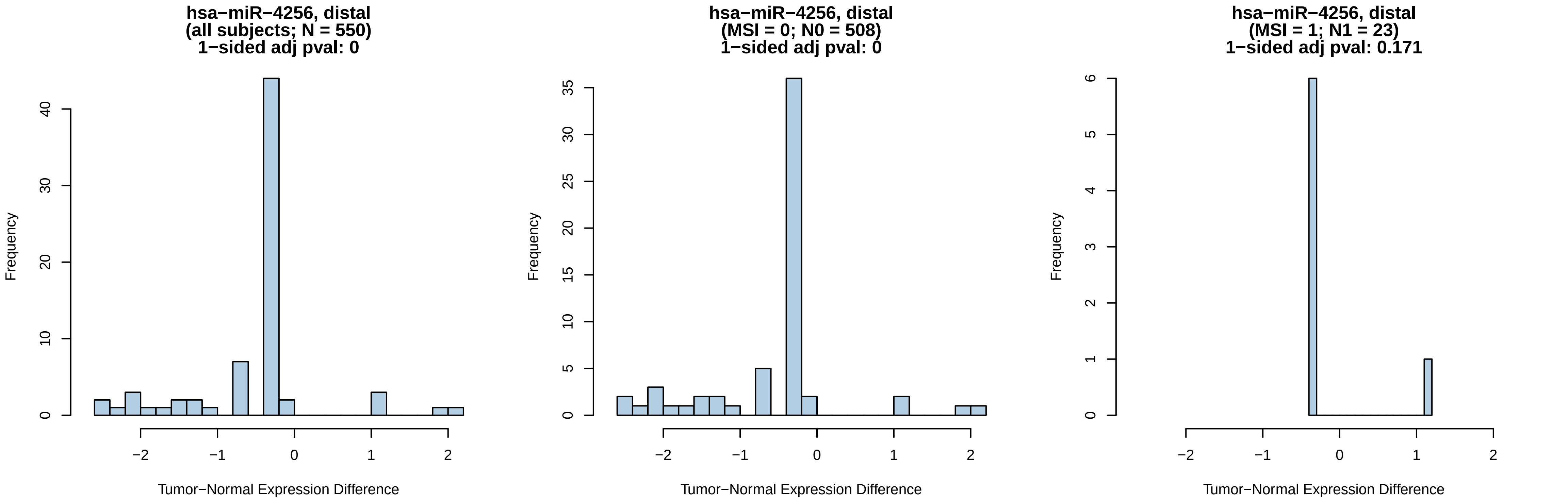
hsa−miR−4256, distal
(all subjects; N = 550)
1−sided adj pval: 0
Frequency
Tumor−Normal Expression Difference
hsa−miR−4256, distal
(MSI = 0; N0 = 508)
1−sided adj pval: 0
Frequency
Tumor−Normal Expression Difference
hsa−miR−4256, distal
(MSI = 1; N1 = 23)
1−sided adj pval: 0.171
Frequency
Tumor−Normal Expression Difference

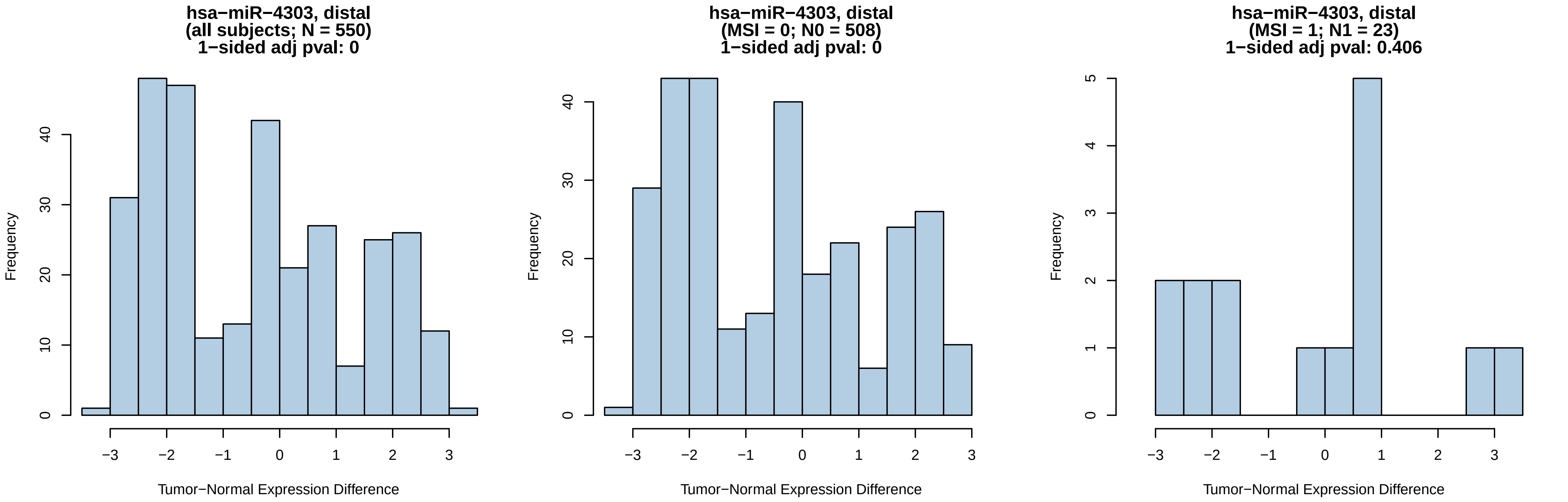
hsa−miR−4303, distal
(all subjects; N = 550)
1−sided adj pval: 0
hsa−miR−4303, distal
(MSI = 0; N0 = 508)
1−sided adj pval: 0
hsa−miR−4303, distal
(MSI = 1; N1 = 23)
1−sided adj pval: 0.406
Frequency
Tumor−Normal Expression Difference

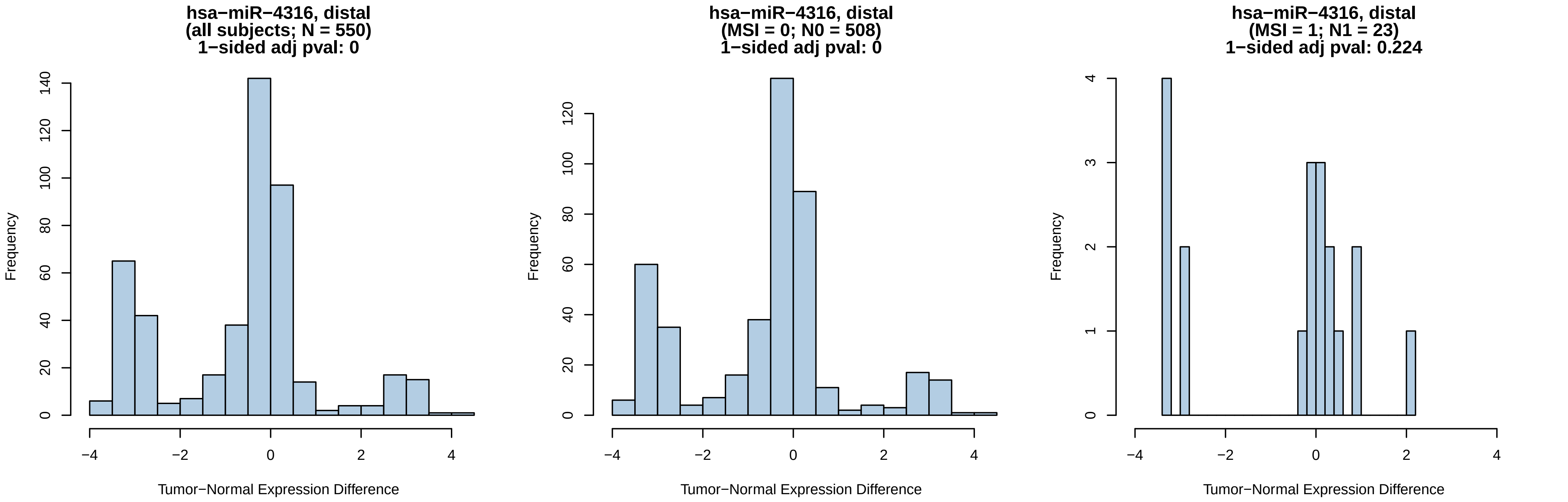

hsa−miR−4316, distal
(all subjects; N = 550)
1−sided adj pval: 0
Frequency
Tumor−Normal Expression Difference
hsa−miR−4316, distal
(MSI = 0; N0 = 508)
1−sided adj pval: 0
Frequency
Tumor−Normal Expression Difference
hsa−miR−4316, distal
(MSI = 1; N1 = 23)
1−sided adj pval: 0.224
Frequency
Tumor−Normal Expression Difference

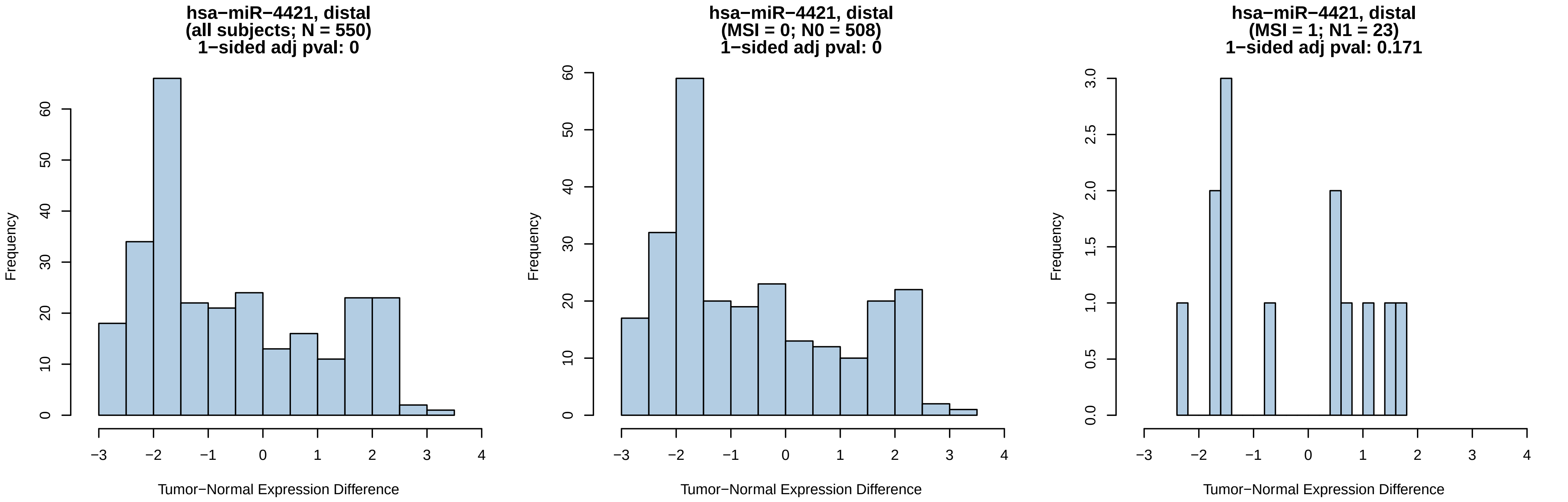
hsa−miR−4421, distal
(all subjects; N = 550)
1−sided adj pval: 0
Frequency
Tumor−Normal Expression Difference
hsa−miR−4421, distal
(MSI = 0; N0 = 508)
1−sided adj pval: 0
Frequency
Tumor−Normal Expression Difference
hsa−miR−4421, distal
(MSI = 1; N1 = 23)
1−sided adj pval: 0.171
Frequency
Tumor−Normal Expression Difference

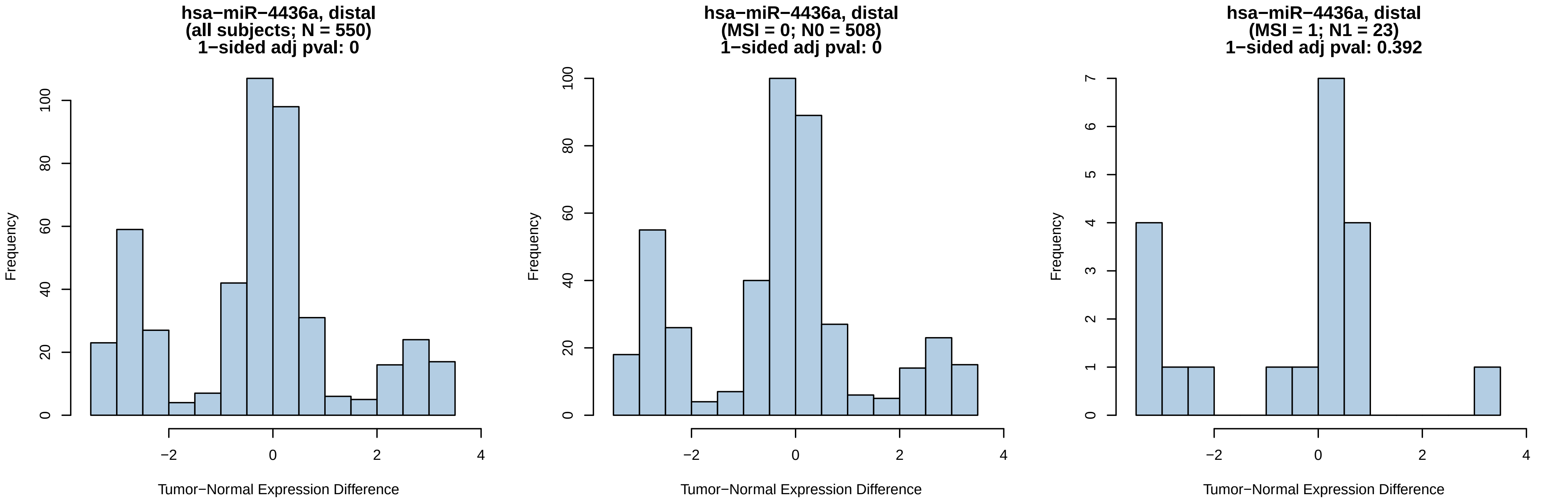

hsa−miR−4436a, distal
(all subjects; N = 550)
1−sided adj pval: 0
Frequency
Tumor−Normal Expression Difference
hsa−miR−4436a, distal
(MSI = 0; N0 = 508)
1−sided adj pval: 0
Frequency
Tumor−Normal Expression Difference
hsa−miR−4436a, distal
(MSI = 1; N1 = 23)
1−sided adj pval: 0.392
Frequency
Tumor−Normal Expression Difference

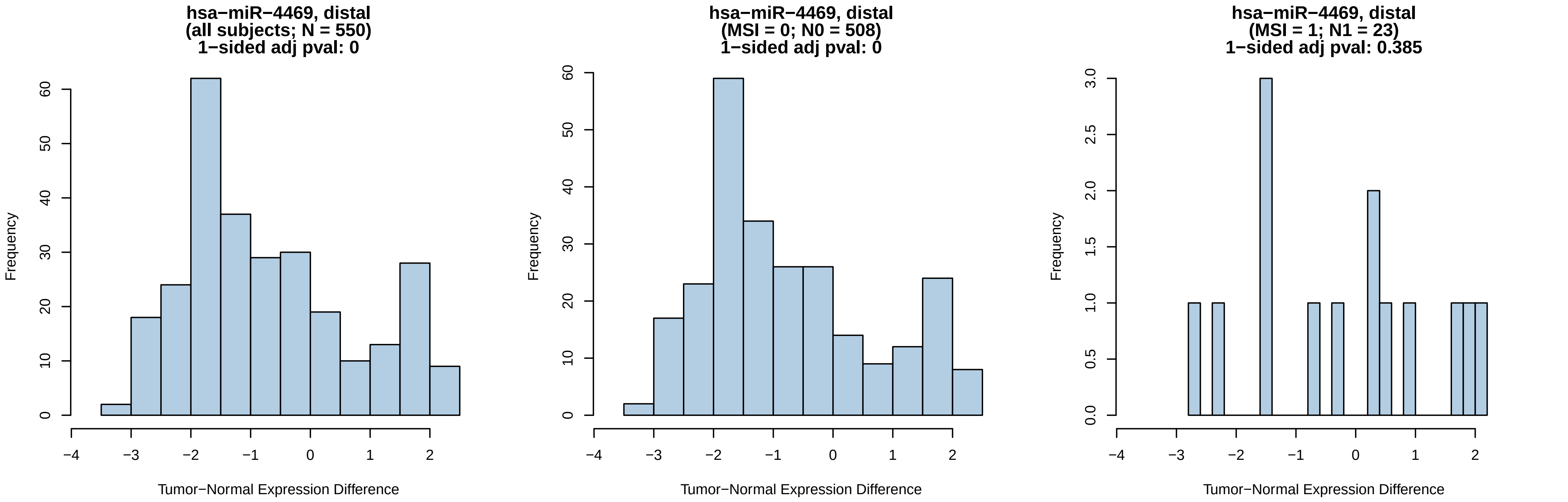
hsa−miR−4469, distal
(all subjects; N = 550)
1−sided adj pval: 0
hsa−miR−4469, distal
(MSI = 0; N0 = 508)
1−sided adj pval: 0
hsa−miR−4469, distal
(MSI = 1; N1 = 23)
1−sided adj pval: 0.385
Frequency
Tumor−Normal Expression Difference

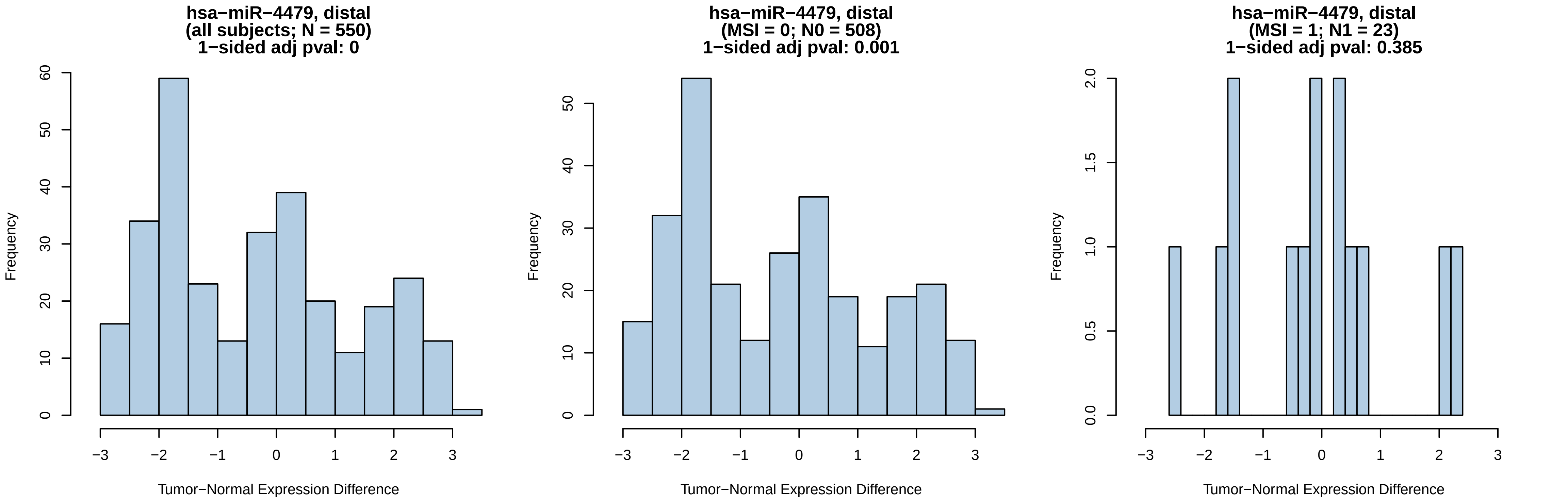

hsa−miR−4479, distal
(all subjects; N = 550)
1−sided adj pval: 0
hsa−miR−4479, distal
(MSI = 0; N0 = 508)
1−sided adj pval: 0.001
hsa−miR−4479, distal
(MSI = 1; N1 = 23)
1−sided adj pval: 0.385
Frequency
Tumor−Normal Expression Difference

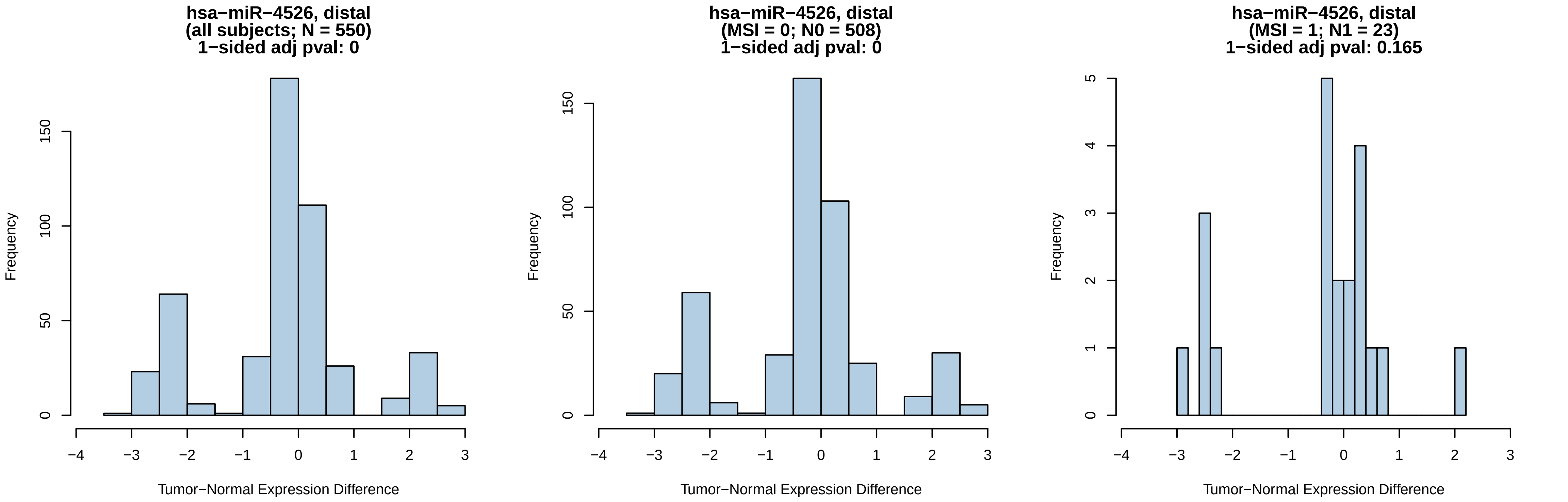

hsa−miR−4526, distal
(all subjects; N = 550)
1−sided adj pval: 0
hsa−miR−4526, distal
(MSI = 0; N0 = 508)
1−sided adj pval: 0
hsa−miR−4526, distal
(MSI = 1; N1 = 23)
1−sided adj pval: 0.165
Frequency
Tumor−Normal Expression Difference

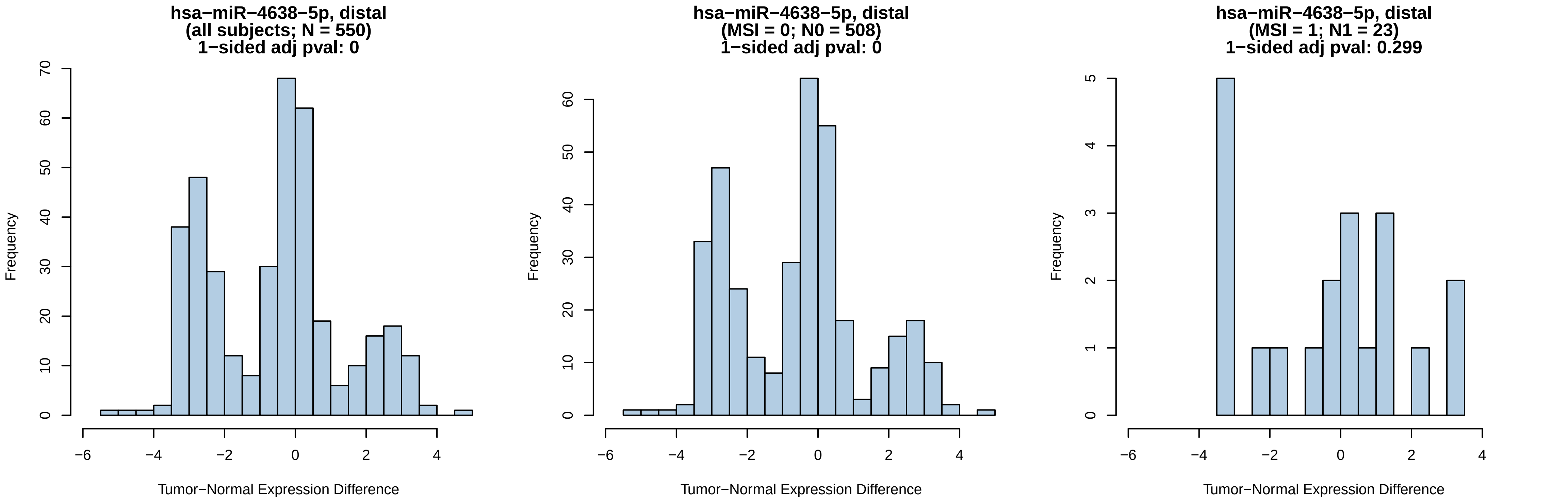

hsa−miR−4638−5p, distal
(all subjects; N = 550)
1−sided adj pval: 0
Frequency
Tumor−Normal Expression Difference
hsa−miR−4638−5p, distal
(MSI = 0; N0 = 508)
1−sided adj pval: 0
Frequency
Tumor−Normal Expression Difference
hsa−miR−4638−5p, distal
(MSI = 1; N1 = 23)
1−sided adj pval: 0.299
Frequency
Tumor−Normal Expression Difference

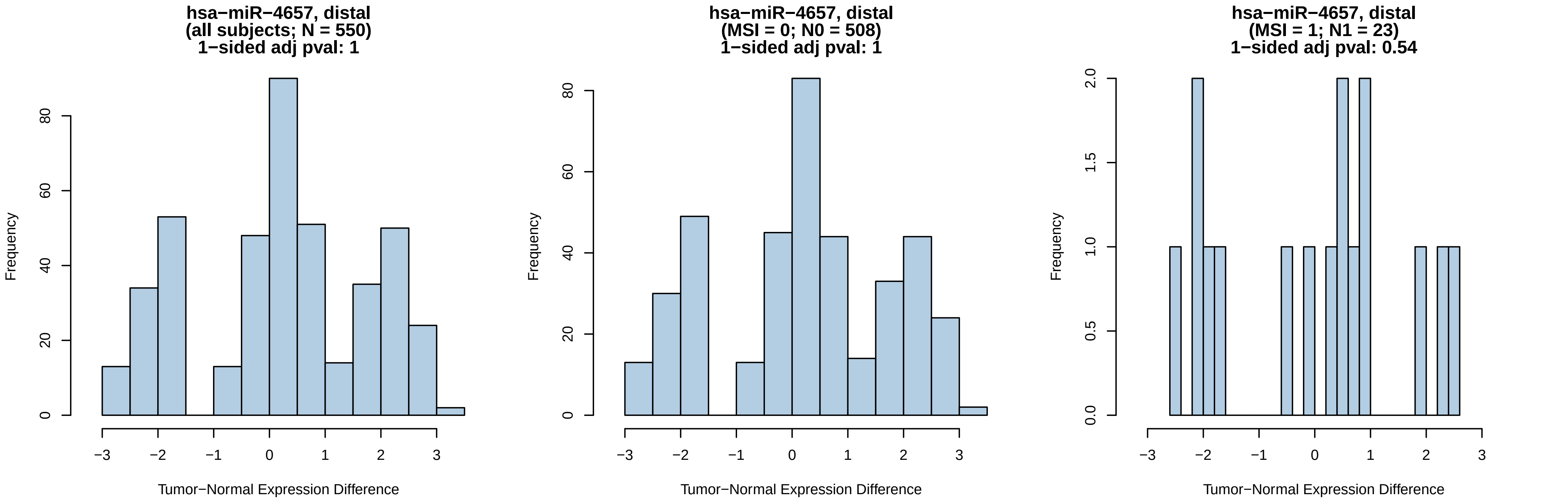

hsa−miR−4657, distal
(all subjects; N = 550)
1−sided adj pval: 1
Frequency
Tumor−Normal Expression Difference
hsa−miR−4657, distal
(MSI = 0; N0 = 508)
1−sided adj pval: 1
Frequency
Tumor−Normal Expression Difference
hsa−miR−4657, distal
(MSI = 1; N1 = 23)
1−sided adj pval: 0.54
Frequency
Tumor−Normal Expression Difference

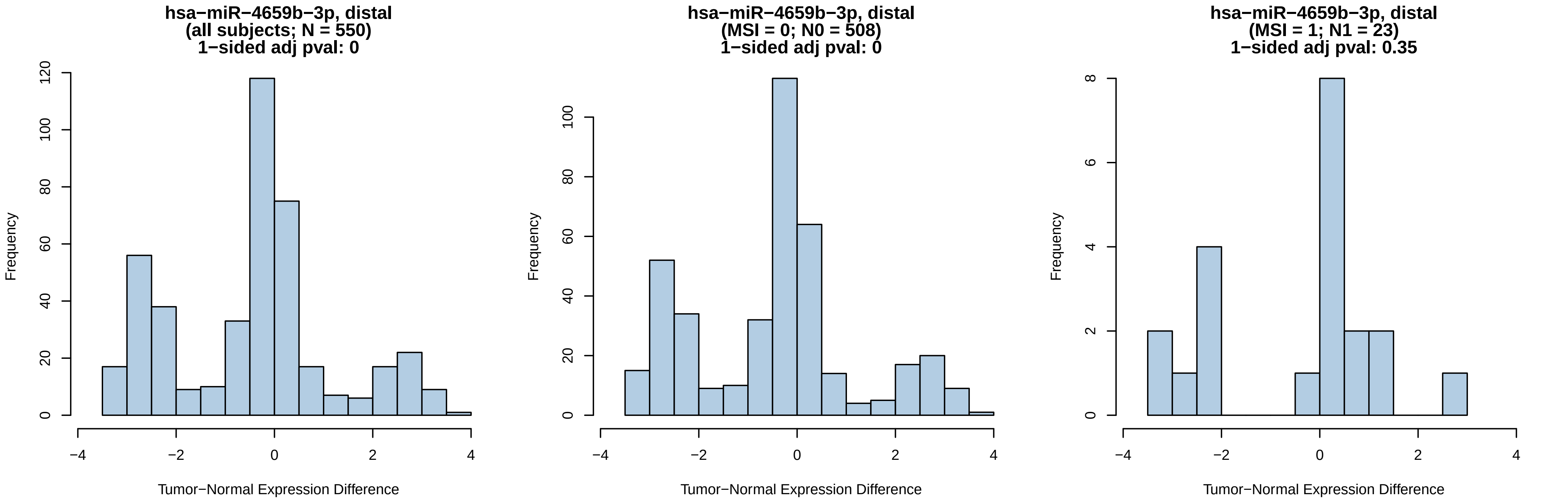
hsa−miR−4659b−3p, distal
(all subjects; N = 550)
1−sided adj pval: 0
Frequency
Tumor−Normal Expression Difference
hsa−miR−4659b−3p, distal
(MSI = 0; N0 = 508)
1−sided adj pval: 0
Frequency
Tumor−Normal Expression Difference
hsa−miR−4659b−3p, distal
(MSI = 1; N1 = 23)
1−sided adj pval: 0.35
Frequency
Tumor−Normal Expression Difference

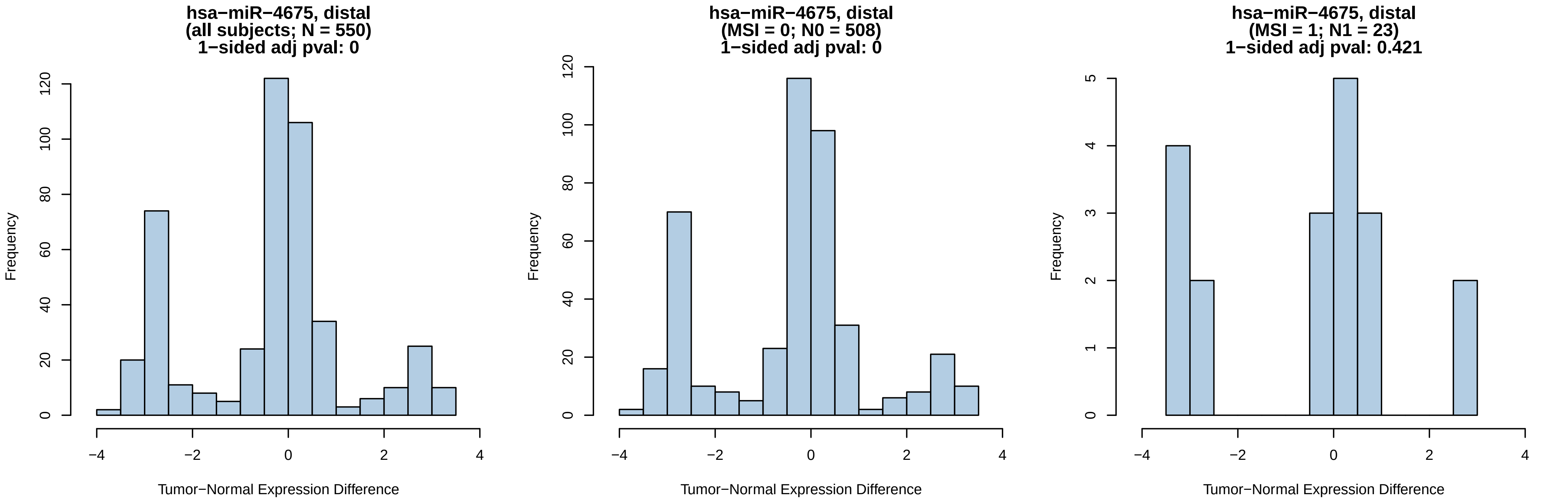
hsa−miR−4675, distal
(all subjects; N = 550)
1−sided adj pval: 0
hsa−miR−4675, distal
(MSI = 0; N0 = 508)
1−sided adj pval: 0
hsa−miR−4675, distal
(MSI = 1; N1 = 23)
1−sided adj pval: 0.421
Frequency
Tumor−Normal Expression Difference

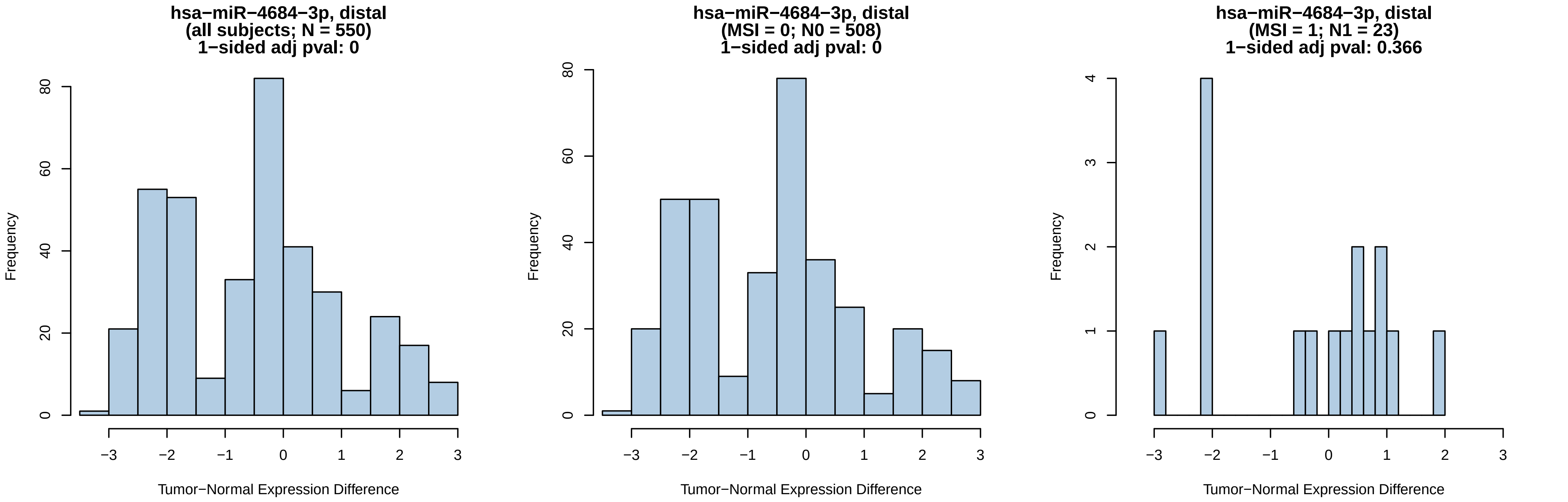
hsa−miR−4684−3p, distal
(all subjects; N = 550)
1−sided adj pval: 0
hsa−miR−4684−3p, distal
(MSI = 0; N0 = 508)
1−sided adj pval: 0
hsa−miR−4684−3p, distal
(MSI = 1; N1 = 23)
1−sided adj pval: 0.366
Frequency
Tumor−Normal Expression Difference

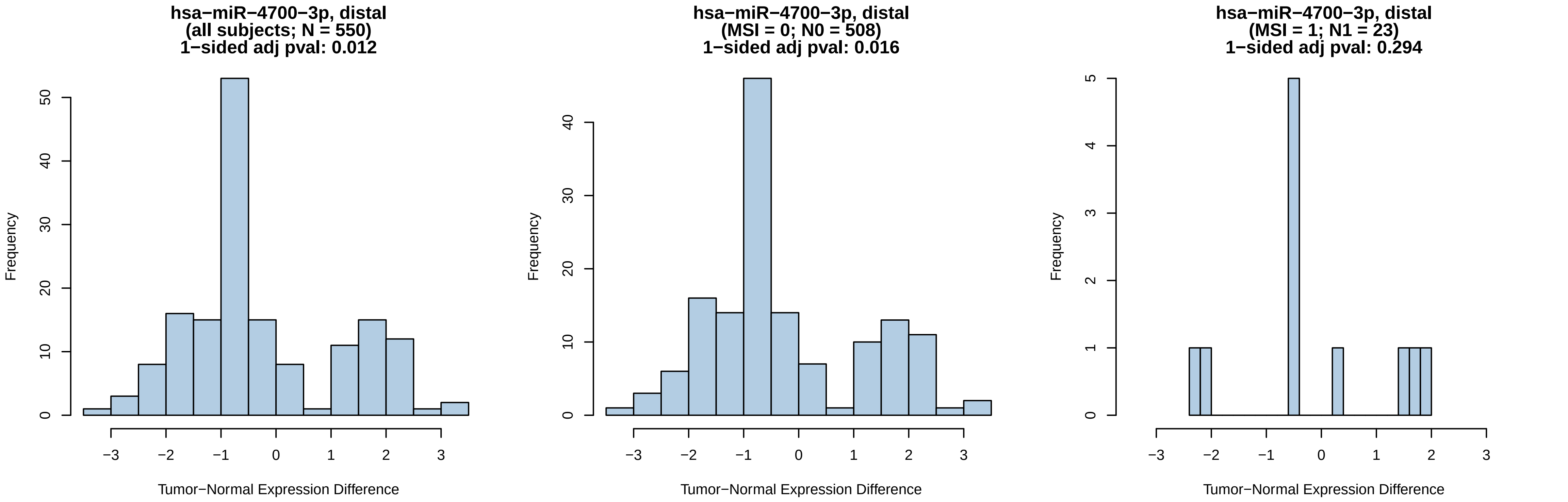

hsa−miR−4700−3p, distal
(all subjects; N = 550)
1−sided adj pval: 0.012
hsa−miR−4700−3p, distal
(MSI = 0; N0 = 508)
1−sided adj pval: 0.016
hsa−miR−4700−3p, distal
(MSI = 1; N1 = 23)
1−sided adj pval: 0.294
Frequency
Tumor−Normal Expression Difference

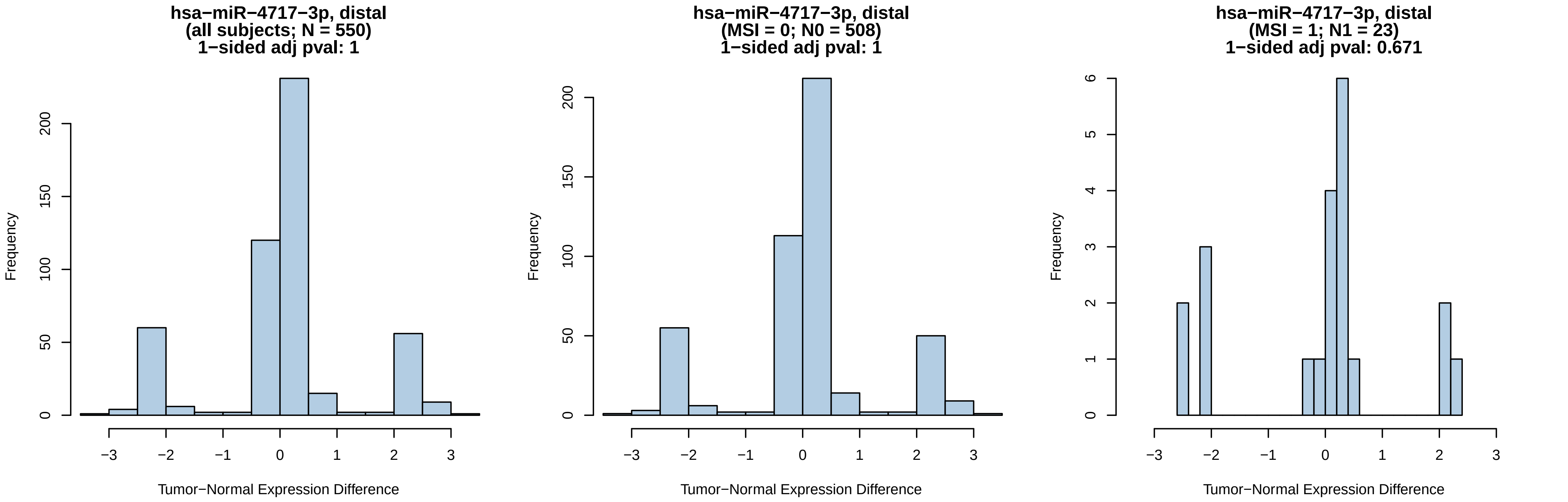

hsa−miR−4717−3p, distal
(all subjects; N = 550)
1−sided adj pval: 1
Frequency
Tumor−Normal Expression Difference
hsa−miR−4717−3p, distal
(MSI = 0; N0 = 508)
1−sided adj pval: 1
Frequency
Tumor−Normal Expression Difference
hsa−miR−4717−3p, distal
(MSI = 1; N1 = 23)
1−sided adj pval: 0.671
Frequency
Tumor−Normal Expression Difference

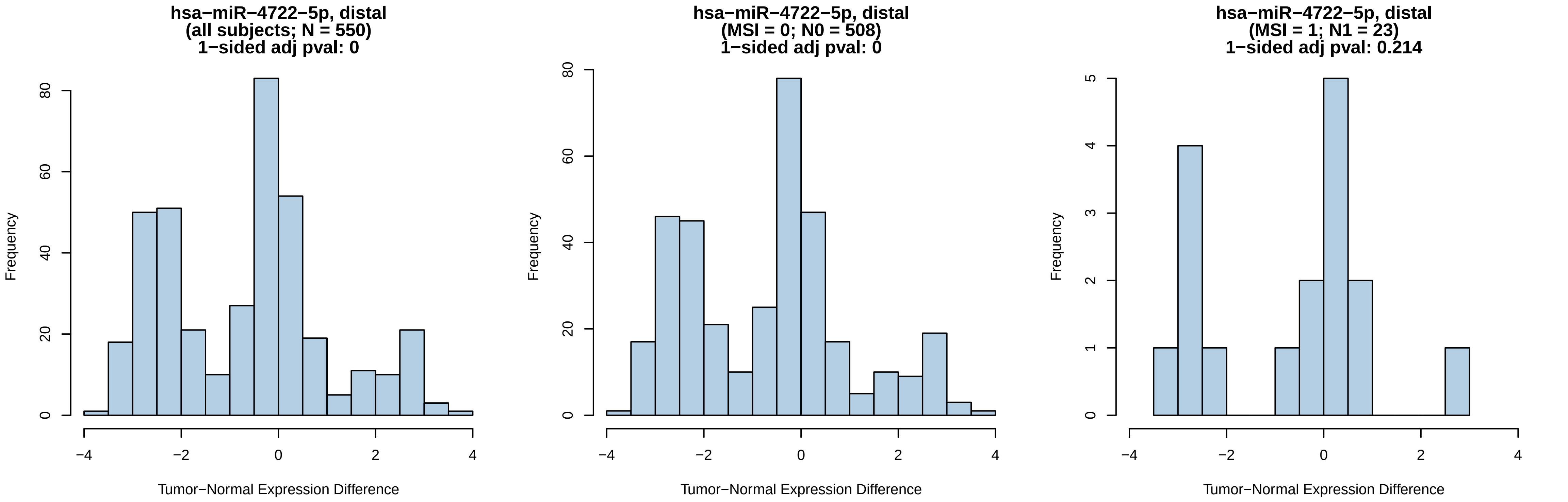

hsa−miR−4722−5p, distal
(all subjects; N = 550)
1−sided adj pval: 0
Frequency
Tumor−Normal Expression Difference
hsa−miR−4722−5p, distal
(MSI = 0; N0 = 508)
1−sided adj pval: 0
Frequency
Tumor−Normal Expression Difference
hsa−miR−4722−5p, distal
(MSI = 1; N1 = 23)
1−sided adj pval: 0.214
Frequency
Tumor−Normal Expression Difference

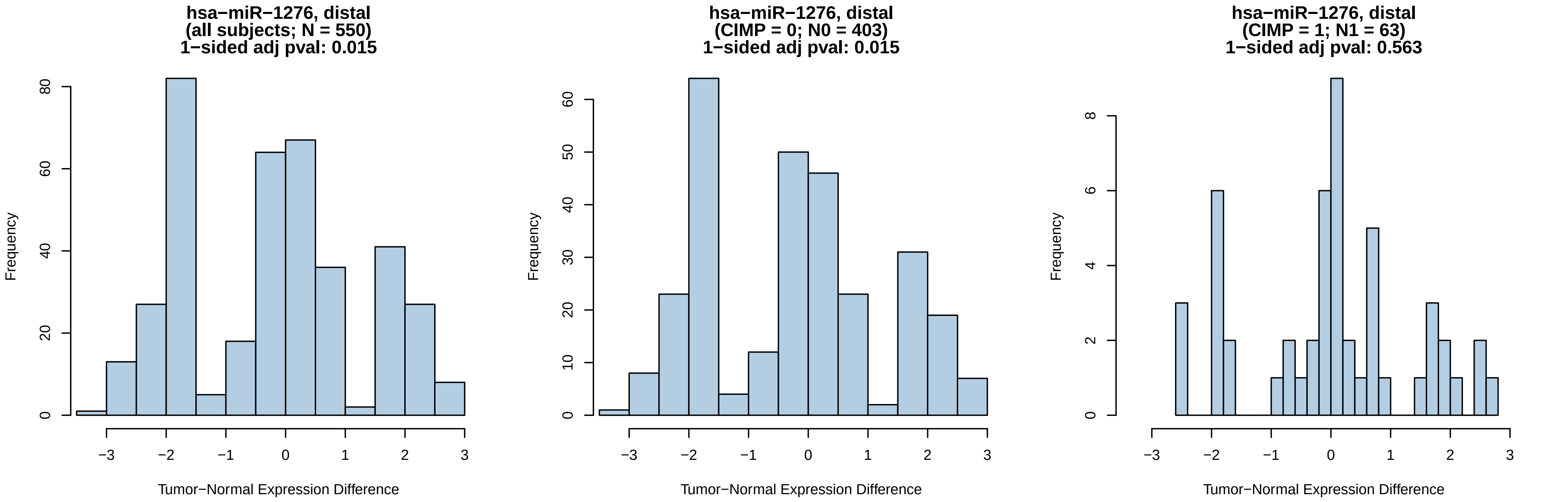
hsa−miR−1276, distal
(all subjects; N = 550)
1−sided adj pval: 0.015
Frequency
Tumor−Normal Expression Difference
hsa−miR−1276, distal
(CIMP = 0; N0 = 403)
1−sided adj pval: 0.015
Frequency
Tumor−Normal Expression Difference
hsa−miR−1276, distal
(CIMP = 1; N1 = 63)
1−sided adj pval: 0.563
Frequency
Tumor−Normal Expression Difference

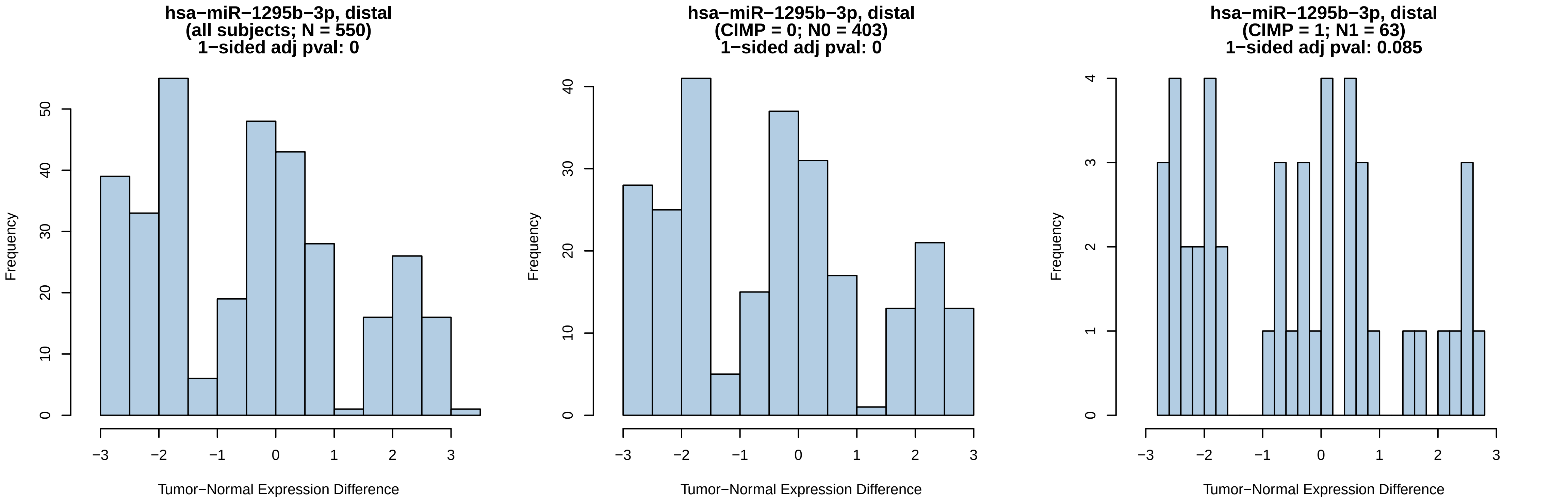

hsa−miR−1295b−3p, distal
(all subjects; N = 550)
1−sided adj pval: 0
Frequency
Tumor−Normal Expression Difference
hsa−miR−1295b−3p, distal
(CIMP = 0; N0 = 403)
1−sided adj pval: 0
Frequency
Tumor−Normal Expression Difference
hsa−miR−1295b−3p, distal
(CIMP = 1; N1 = 63)
1−sided adj pval: 0.085
Frequency
Tumor−Normal Expression Difference

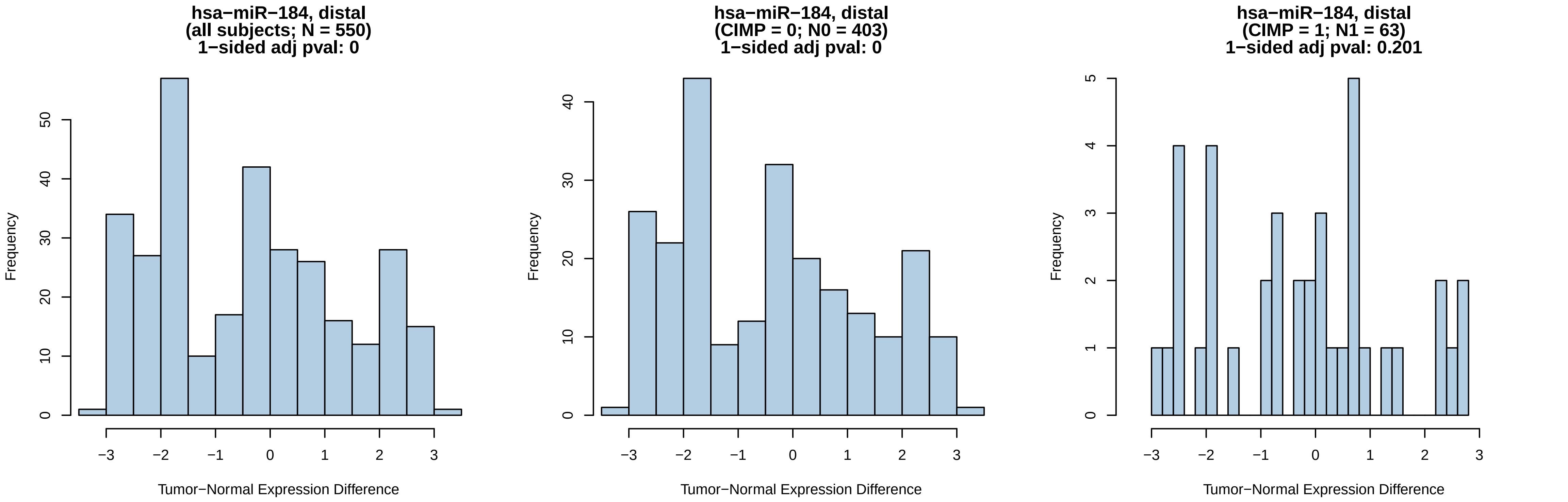

hsa−miR−184, distal
(all subjects; N = 550)
1−sided adj pval: 0
Frequency
Tumor−Normal Expression Difference
hsa−miR−184, distal
(CIMP = 0; N0 = 403)
1−sided adj pval: 0
Frequency
Tumor−Normal Expression Difference
hsa−miR−184, distal
(CIMP = 1; N1 = 63)
1−sided adj pval: 0.201
Frequency
Tumor−Normal Expression Difference

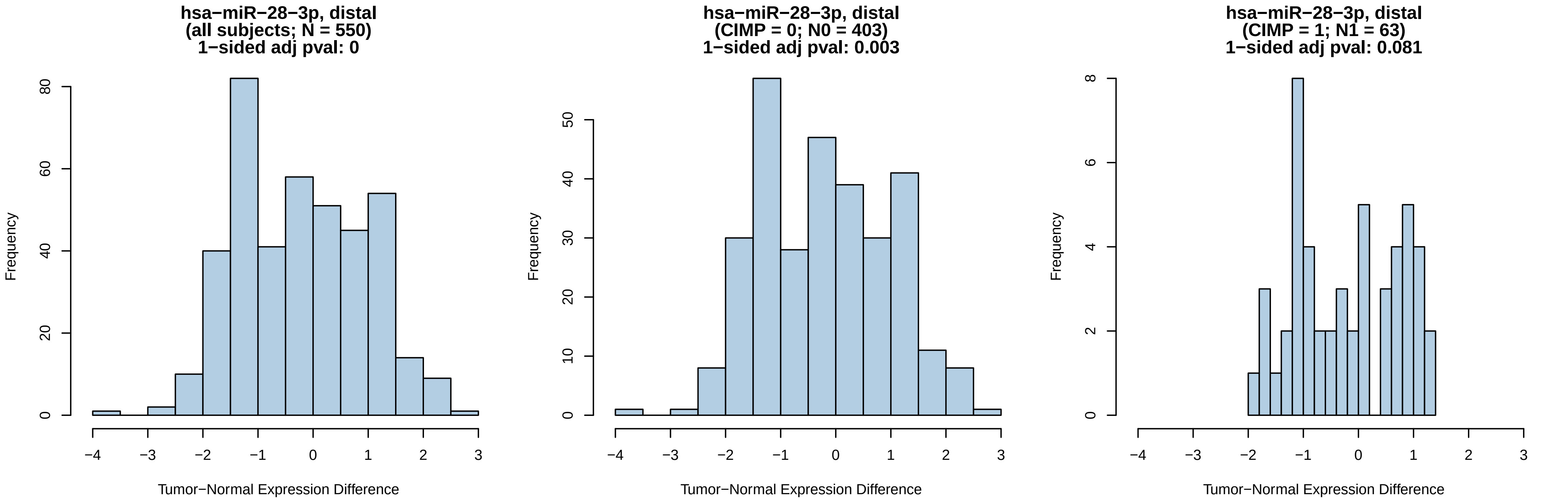
hsa−miR−28−3p, distal
(all subjects; N = 550)
1−sided adj pval: 0
Frequency
Tumor−Normal Expression Difference
hsa−miR−28−3p, distal
(CIMP = 0; N0 = 403)
1−sided adj pval: 0.003
Frequency
Tumor−Normal Expression Difference
hsa−miR−28−3p, distal
(CIMP = 1; N1 = 63)
1−sided adj pval: 0.081
Frequency
Tumor−Normal Expression Difference

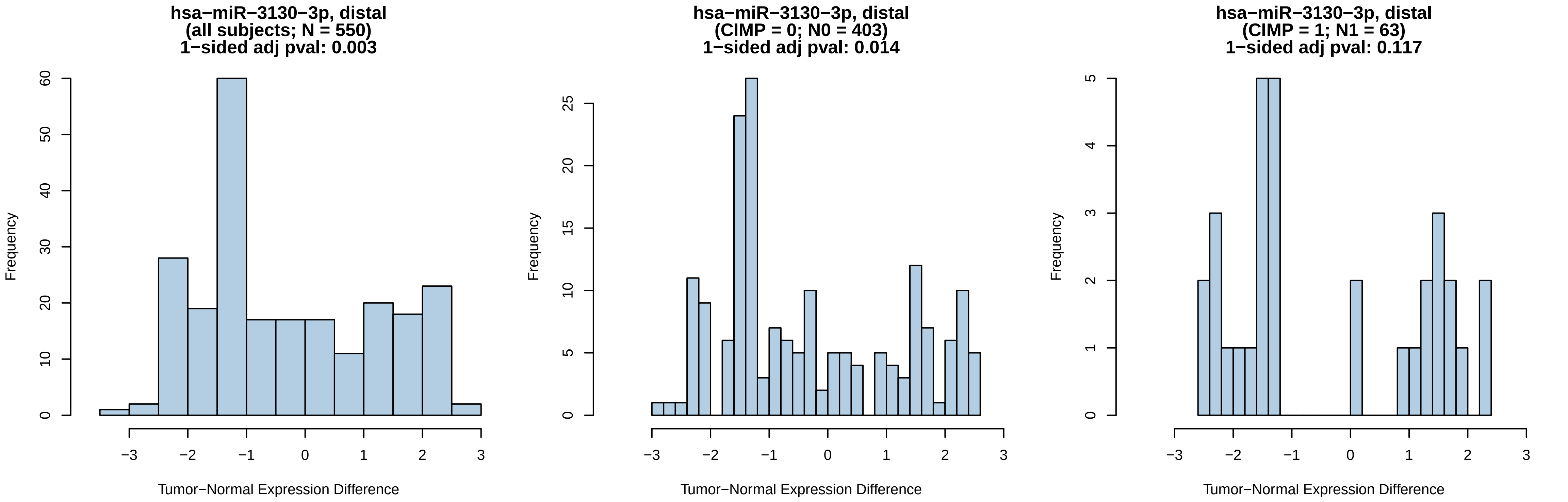
hsa−miR−3130−3p, distal
(all subjects; N = 550)
1−sided adj pval: 0.003
hsa−miR−3130−3p, distal
(CIMP = 0; N0 = 403)
1−sided adj pval: 0.014
hsa−miR−3130−3p, distal
(CIMP = 1; N1 = 63)
1−sided adj pval: 0.117
Frequency
Tumor−Normal Expression Difference

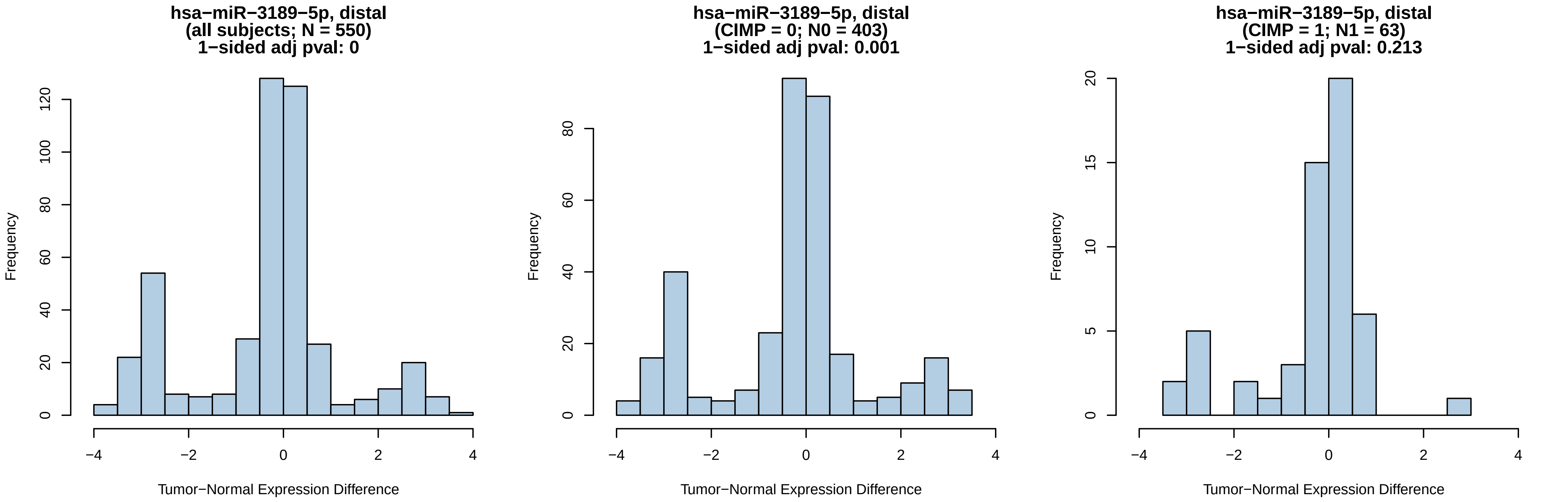
hsa−miR−3189−5p, distal
(all subjects; N = 550)
1−sided adj pval: 0
Frequency
Tumor−Normal Expression Difference
hsa−miR−3189−5p, distal
(CIMP = 0; N0 = 403)
1−sided adj pval: 0.001
Frequency
Tumor−Normal Expression Difference
hsa−miR−3189−5p, distal
(CIMP = 1; N1 = 63)
1−sided adj pval: 0.213
Frequency
Tumor−Normal Expression Difference

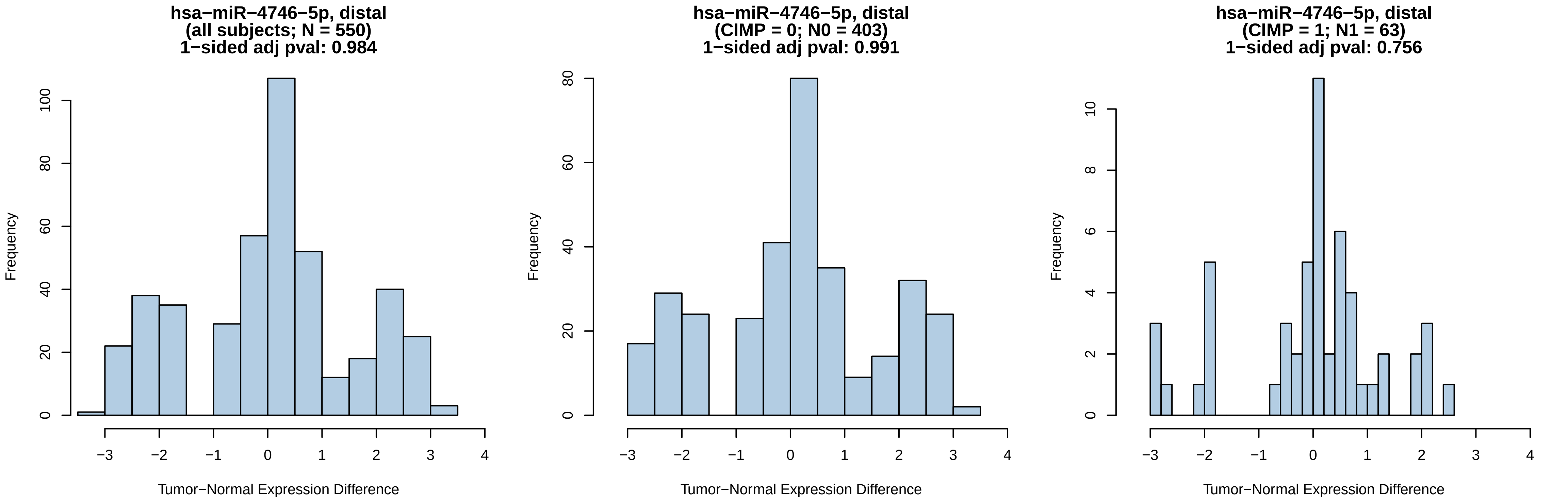
hsa−miR−4746−5p, distal
(all subjects; N = 550)
1−sided adj pval: 0.984
Frequency
Tumor−Normal Expression Difference
hsa−miR−4746−5p, distal
(CIMP = 0; N0 = 403)
1−sided adj pval: 0.991
Frequency
Tumor−Normal Expression Difference
hsa−miR−4746−5p, distal
(CIMP = 1; N1 = 63)
1−sided adj pval: 0.756
Frequency
Tumor−Normal Expression Difference

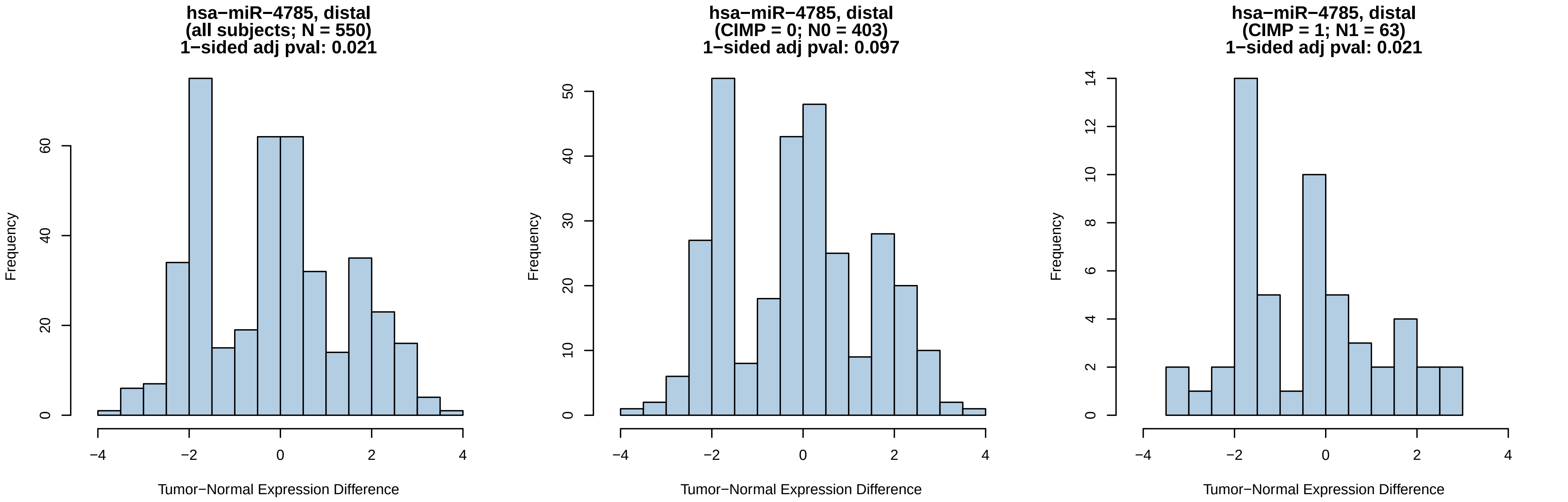
hsa−miR−4785, distal
(all subjects; N = 550)
1−sided adj pval: 0.021
Frequency
0
20
40
60
−4
−2
0
2
4
Tumor−Normal Expression Difference
hsa−miR−4785, distal
(CIMP = 0; N0 = 403)
1−sided adj pval: 0.097
Frequency
0
10
20
30
40
50
−4
−2
0
2
4
Tumor−Normal Expression Difference
hsa−miR−4785, distal
(CIMP = 1; N1 = 63)
1−sided adj pval: 0.021
Frequency
0
2
4
6
8
10
12
14
−4
−2
0
2
4
Tumor−Normal Expression Difference

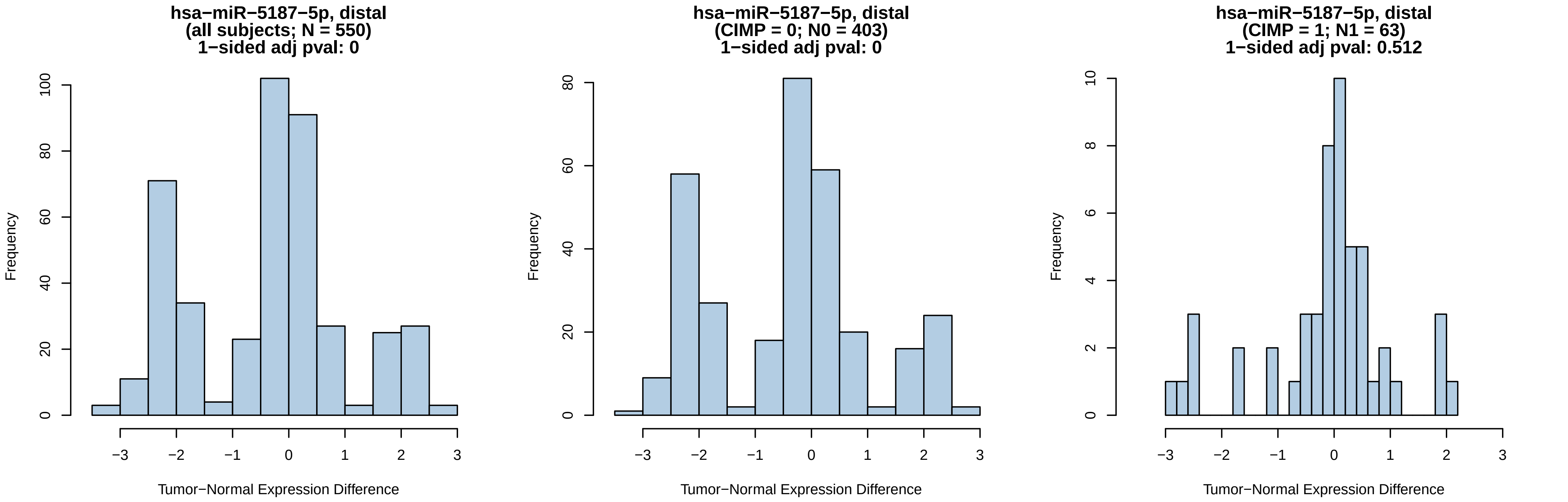
hsa−miR−5187−5p, distal
(all subjects; N = 550)
1−sided adj pval: 0
Frequency
0
20
40
60
80
100
−3
−2
−1
0
1
2
3
Tumor−Normal Expression Difference
hsa−miR−5187−5p, distal
(CIMP = 0; N0 = 403)
1−sided adj pval: 0
Frequency
0
20
40
60
80
−3
−2
−1
0
1
2
3
Tumor−Normal Expression Difference
hsa−miR−5187−5p, distal
(CIMP = 1; N1 = 63)
1−sided adj pval: 0.512
Frequency
0
2
4
6
8
10
−3
−2
−1
0
1
2
3
Tumor−Normal Expression Difference

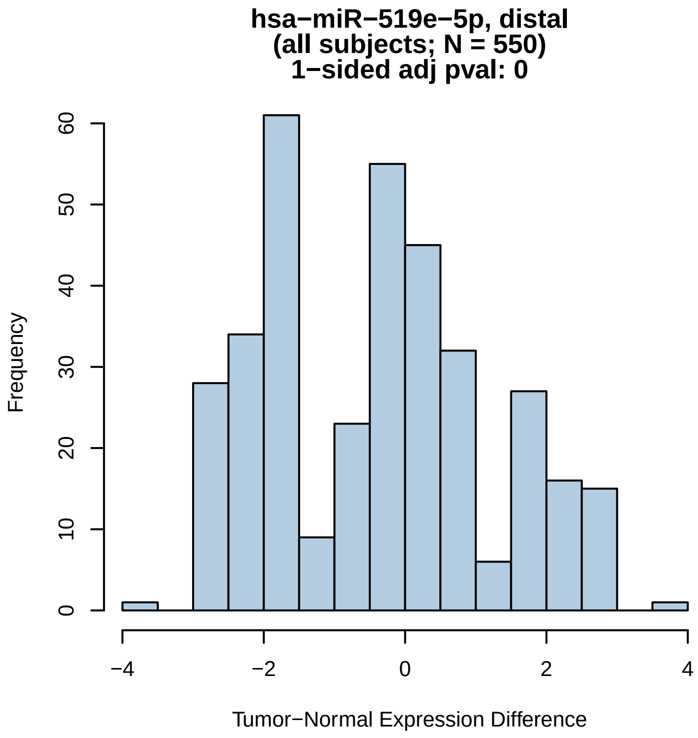

hsa−miR−519e−5p, distal
(all subjects; N = 550)
1−sided adj pval: 0
Frequency
0
10
20
30
40
50
60
−4
−2
0
2
4
Tumor−Normal Expression Difference


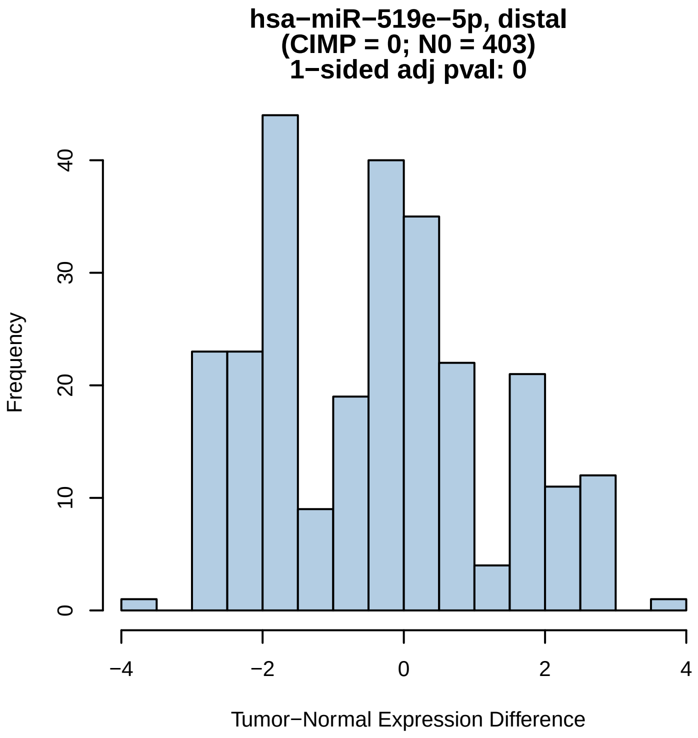

hsa−miR−519e−5p, distal
(CIMP = 0; N0 = 403)
1−sided adj pval: 0
Frequency
0
10
20
30
40
−4
−2
0
2
4
Tumor−Normal Expression Difference


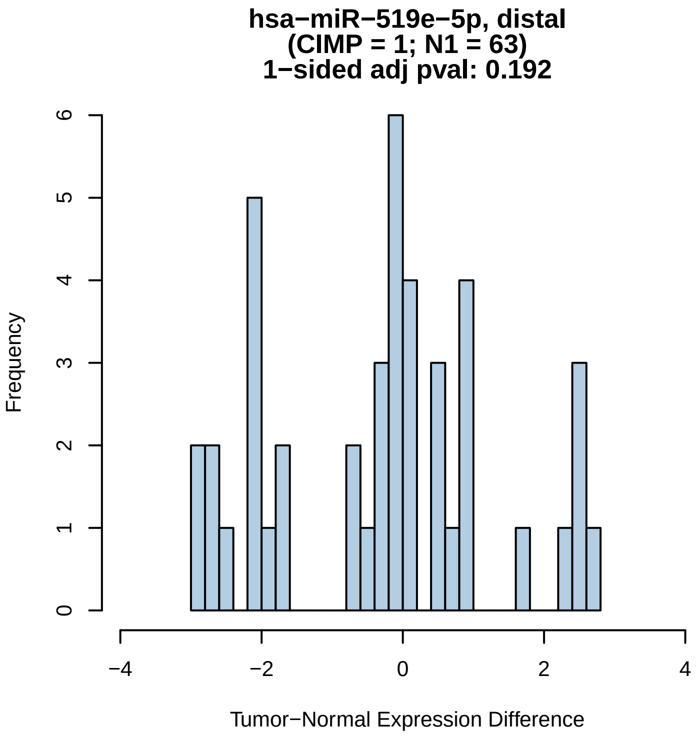

hsa−miR−519e−5p, distal
(CIMP = 1; N1 = 63)
1−sided adj pval: 0.192
Frequency
0
1
2
3
4
5
6
−4
−2
0
2
4
Tumor−Normal Expression Difference

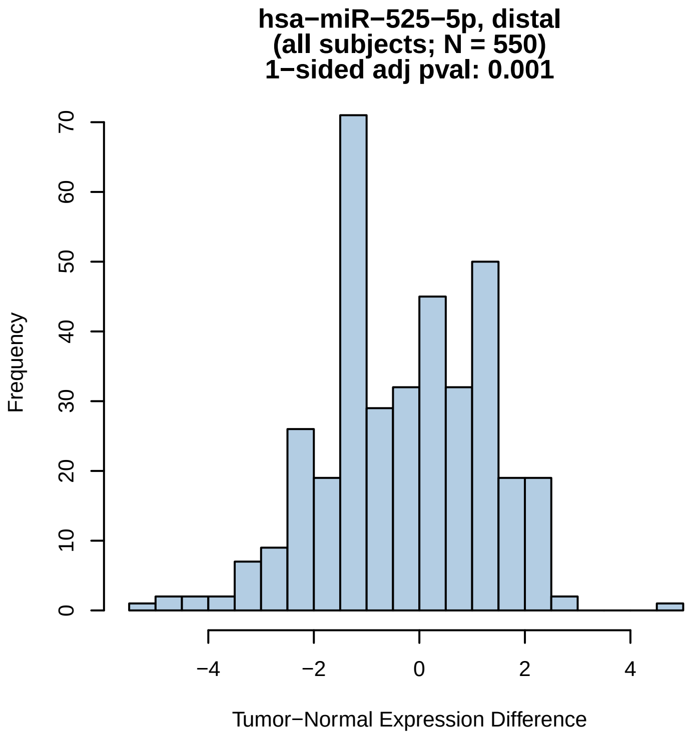
hsa−miR−525−5p, distal
(all subjects; N = 550)
1−sided adj pval: 0.001
Frequency
Tumor−Normal Expression Difference

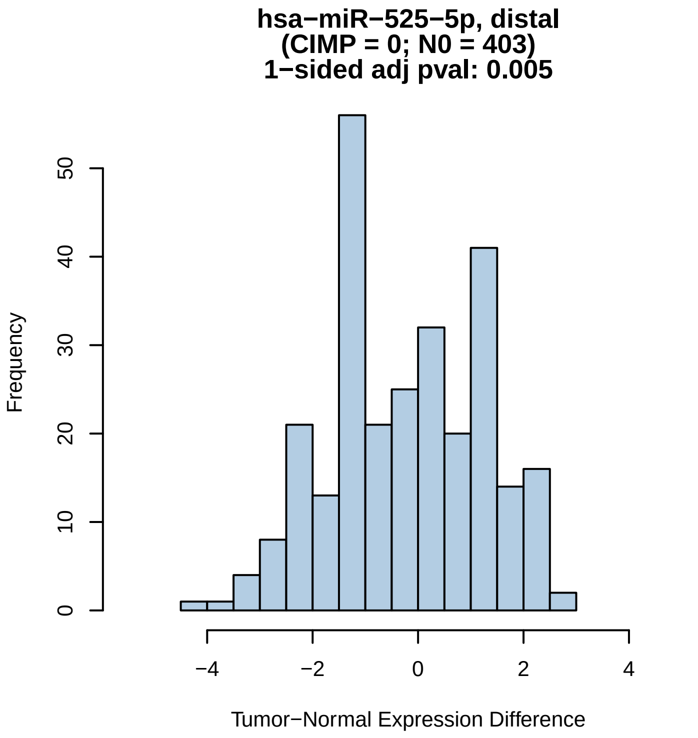
hsa−miR−525−5p, distal
(CIMP = 0; N0 = 403)
1−sided adj pval: 0.005
Frequency
Tumor−Normal Expression Difference

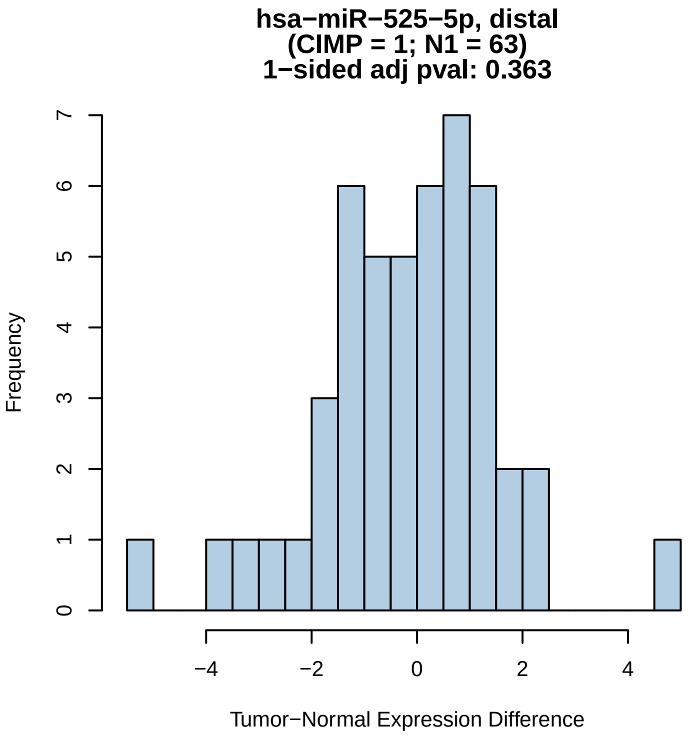
hsa−miR−525−5p, distal
(CIMP = 1; N1 = 63)
1−sided adj pval: 0.363
Frequency
Tumor−Normal Expression Difference

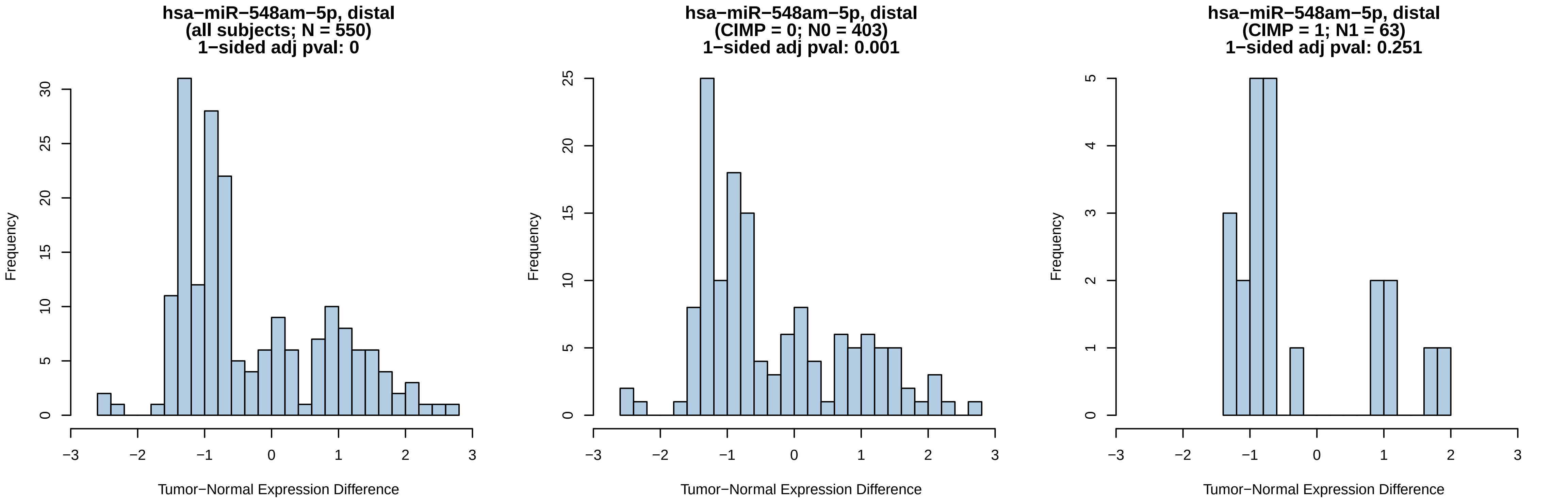

hsa−miR−548am−5p, distal
(all subjects; N = 550)
1−sided adj pval: 0
hsa−miR−548am−5p, distal
(CIMP = 0; N0 = 403)
1−sided adj pval: 0.001
hsa−miR−548am−5p, distal
(CIMP = 1; N1 = 63)
1−sided adj pval: 0.251
Frequency
Tumor−Normal Expression Difference

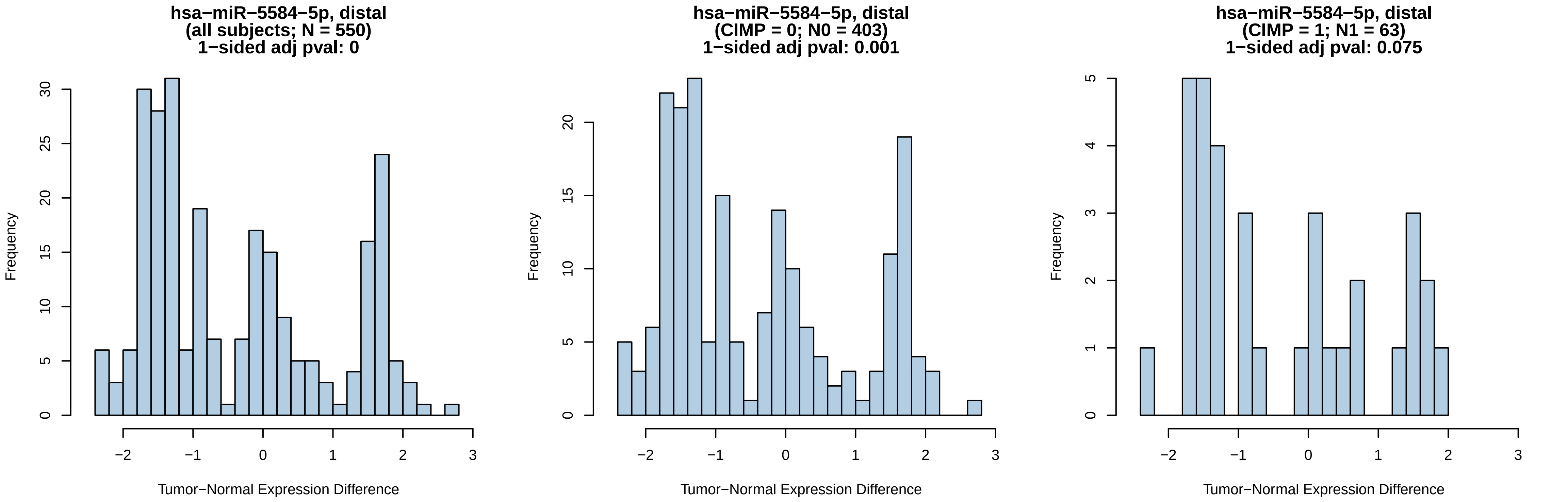
hsa−miR−5584−5p, distal
(all subjects; N = 550)
1−sided adj pval: 0
Frequency
Tumor−Normal Expression Difference
hsa−miR−5584−5p, distal
(CIMP = 0; N0 = 403)
1−sided adj pval: 0.001
Frequency
Tumor−Normal Expression Difference
hsa−miR−5584−5p, distal
(CIMP = 1; N1 = 63)
1−sided adj pval: 0.075
Frequency
Tumor−Normal Expression Difference

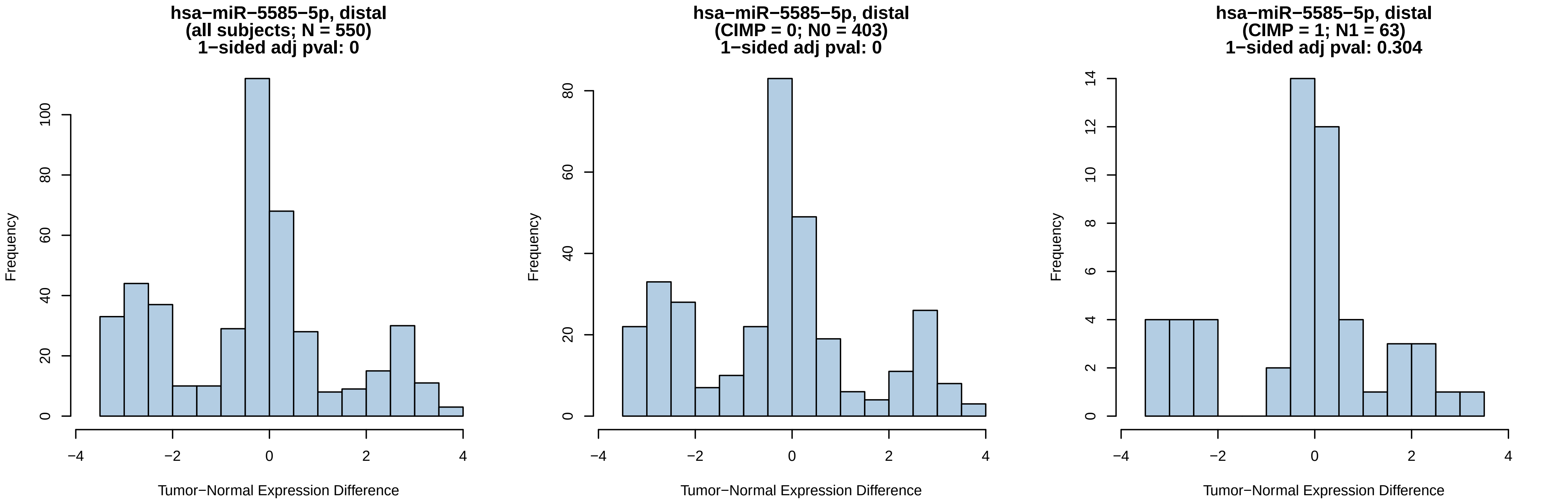
hsa−miR−5585−5p, distal
(all subjects; N = 550)
1−sided adj pval: 0
Frequency
Tumor−Normal Expression Difference
hsa−miR−5585−5p, distal
(CIMP = 0; N0 = 403)
1−sided adj pval: 0
Frequency
Tumor−Normal Expression Difference
hsa−miR−5585−5p, distal
(CIMP = 1; N1 = 63)
1−sided adj pval: 0.304
Frequency
Tumor−Normal Expression Difference

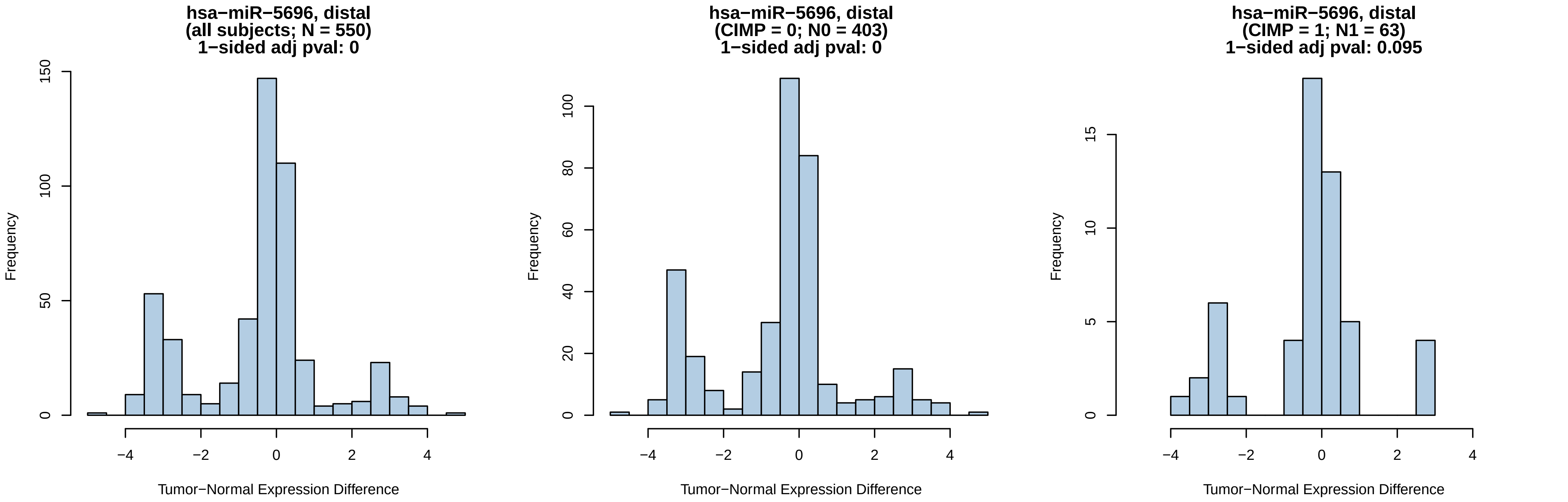
hsa−miR−5696, distal
(all subjects; N = 550)
1−sided adj pval: 0
hsa−miR−5696, distal
(CIMP = 0; N0 = 403)
1−sided adj pval: 0
hsa−miR−5696, distal
(CIMP = 1; N1 = 63)
1−sided adj pval: 0.095
Frequency
Tumor−Normal Expression Difference

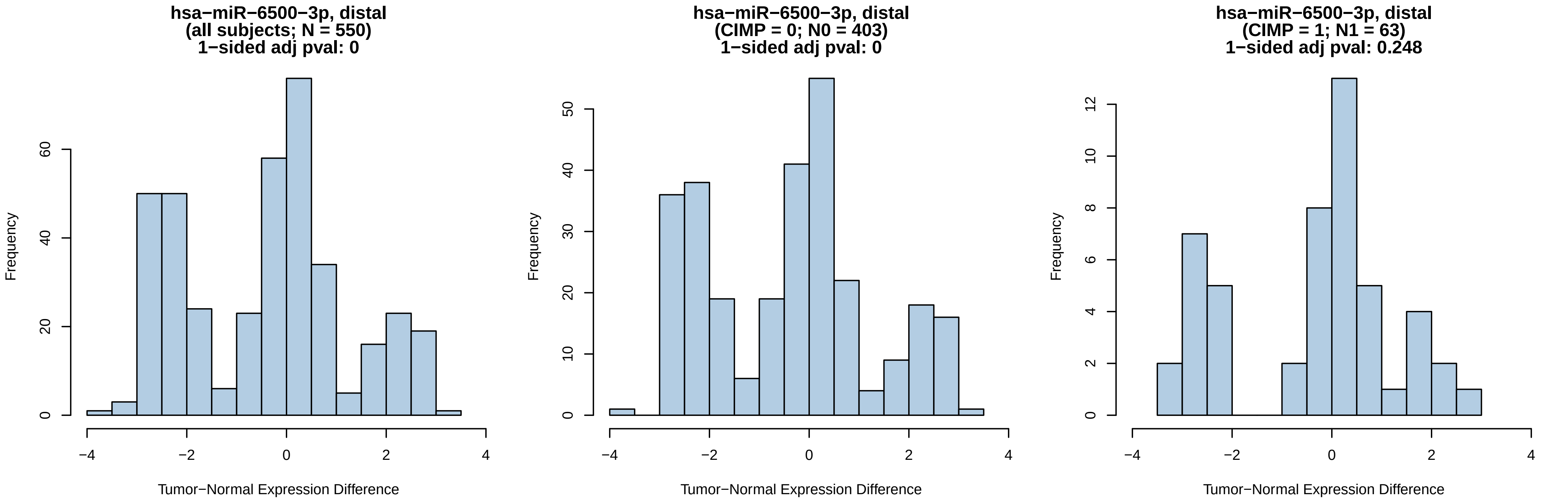
hsa−miR−6500−3p, distal
(all subjects; N = 550)
1−sided adj pval: 0
Frequency
0
20
40
60
−4
−2
0
2
4
Tumor−Normal Expression Difference
hsa−miR−6500−3p, distal
(CIMP = 0; N0 = 403)
1−sided adj pval: 0
Frequency
0
10
20
30
40
50
−4
−2
0
2
4
Tumor−Normal Expression Difference
hsa−miR−6500−3p, distal
(CIMP = 1; N1 = 63)
1−sided adj pval: 0.248
Frequency
0
2
4
6
8
10
12
−4
−2
0
2
4
Tumor−Normal Expression Difference

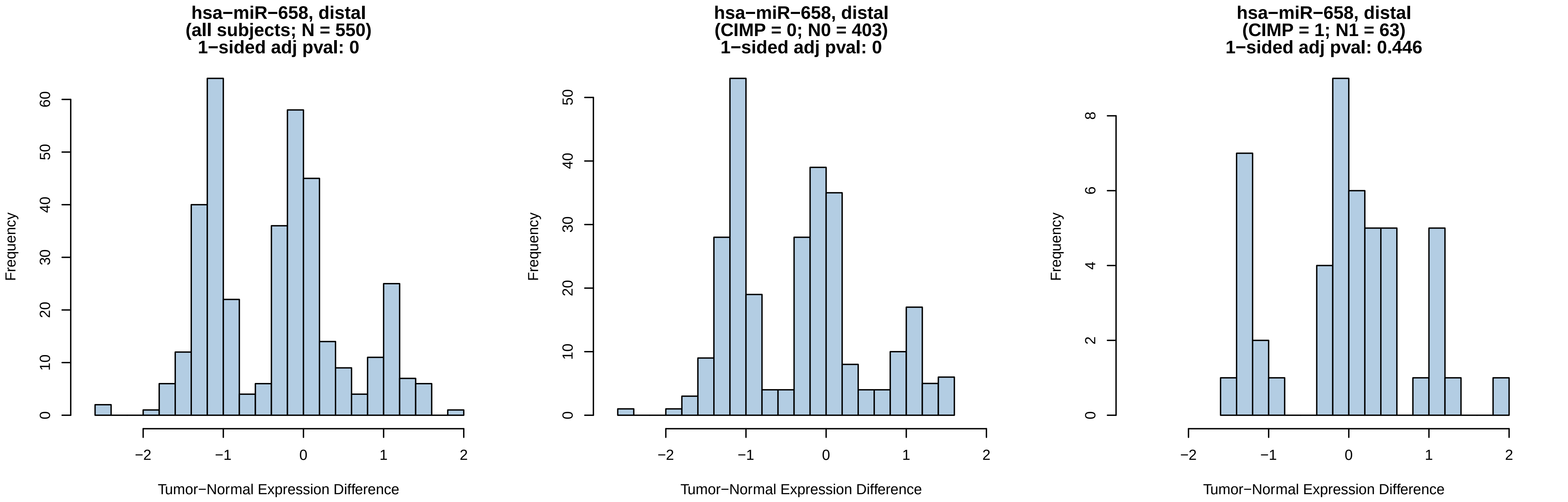
hsa−miR−658, distal
(all subjects; N = 550)
1−sided adj pval: 0
Frequency
Tumor−Normal Expression Difference
hsa−miR−658, distal
(CIMP = 0; N0 = 403)
1−sided adj pval: 0
Frequency
Tumor−Normal Expression Difference
hsa−miR−658, distal
(CIMP = 1; N1 = 63)
1−sided adj pval: 0.446
Frequency
Tumor−Normal Expression Difference

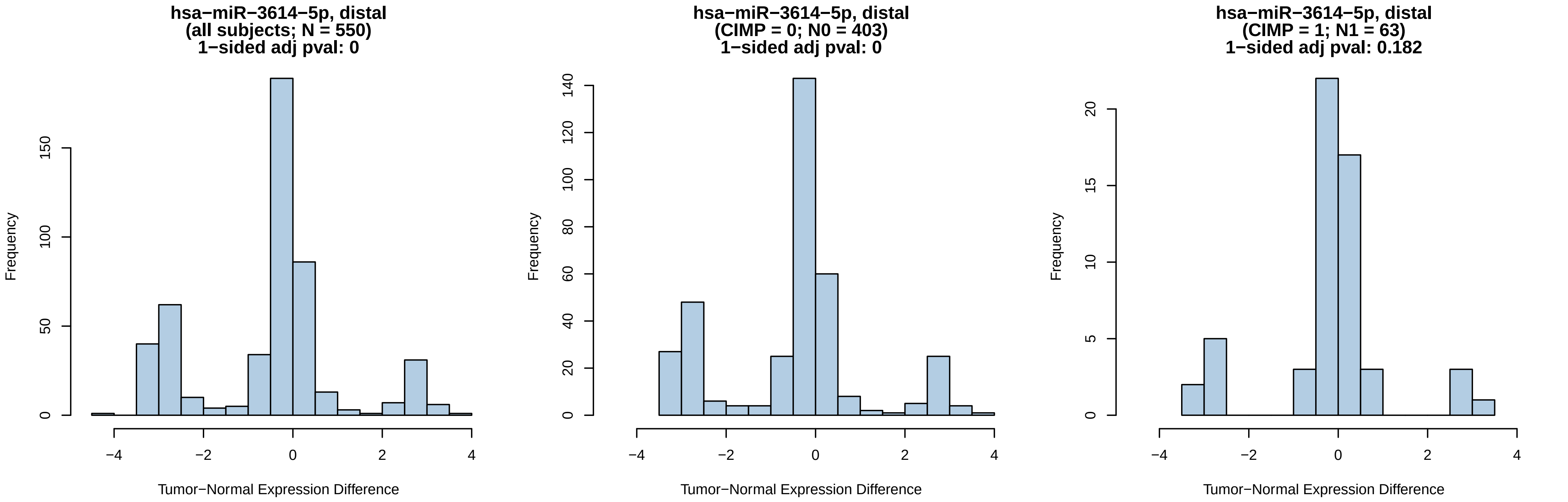
hsa−miR−3614−5p, distal
(all subjects; N = 550)
1−sided adj pval: 0
Frequency
Tumor−Normal Expression Difference
hsa−miR−3614−5p, distal
(CIMP = 0; N0 = 403)
1−sided adj pval: 0
Frequency
Tumor−Normal Expression Difference
hsa−miR−3614−5p, distal
(CIMP = 1; N1 = 63)
1−sided adj pval: 0.182
Frequency
Tumor−Normal Expression Difference

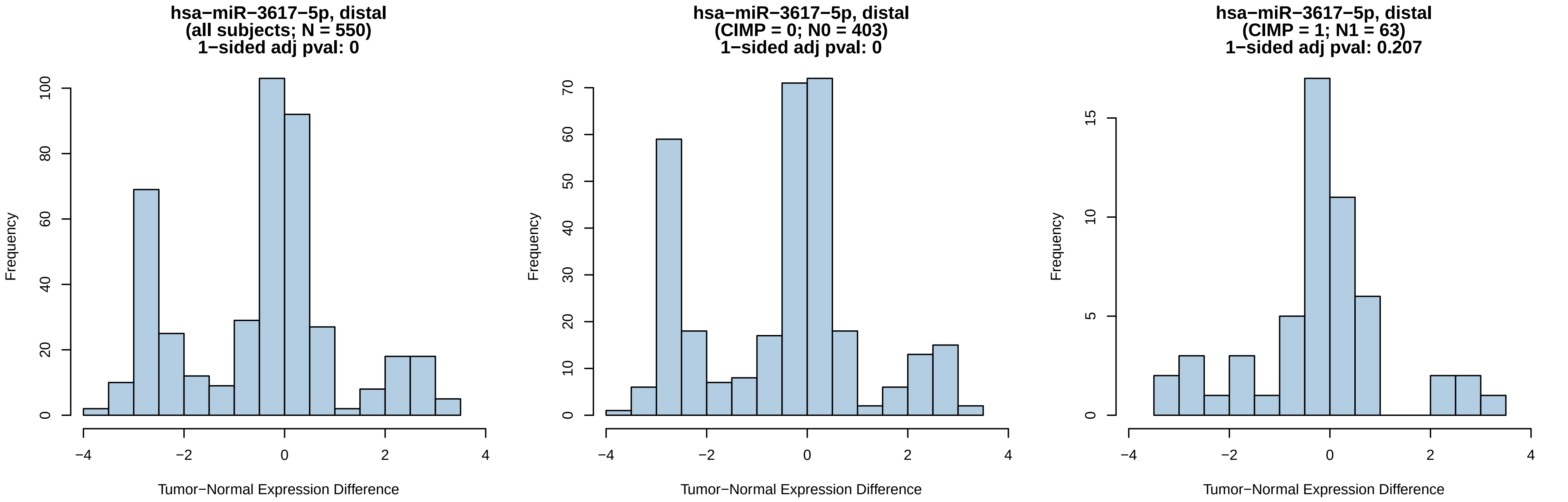

hsa−miR−3617−5p, distal
(all subjects; N = 550)
1−sided adj pval: 0
Frequency
Tumor−Normal Expression Difference
hsa−miR−3617−5p, distal
(CIMP = 0; N0 = 403)
1−sided adj pval: 0
Frequency
Tumor−Normal Expression Difference
hsa−miR−3617−5p, distal
(CIMP = 1; N1 = 63)
1−sided adj pval: 0.207
Frequency
Tumor−Normal Expression Difference

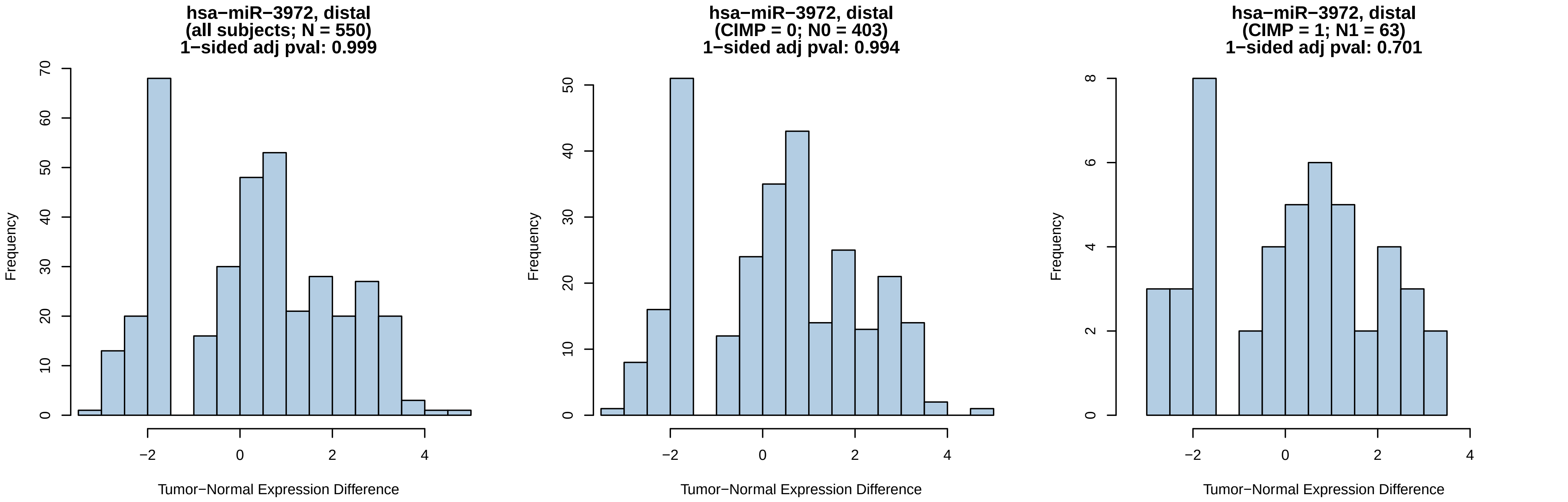
hsa−miR−3972, distal
(all subjects; N = 550)
1−sided adj pval: 0.999
hsa−miR−3972, distal
(CIMP = 0; N0 = 403)
1−sided adj pval: 0.994
hsa−miR−3972, distal
(CIMP = 1; N1 = 63)
1−sided adj pval: 0.701
Frequency
Tumor−Normal Expression Difference

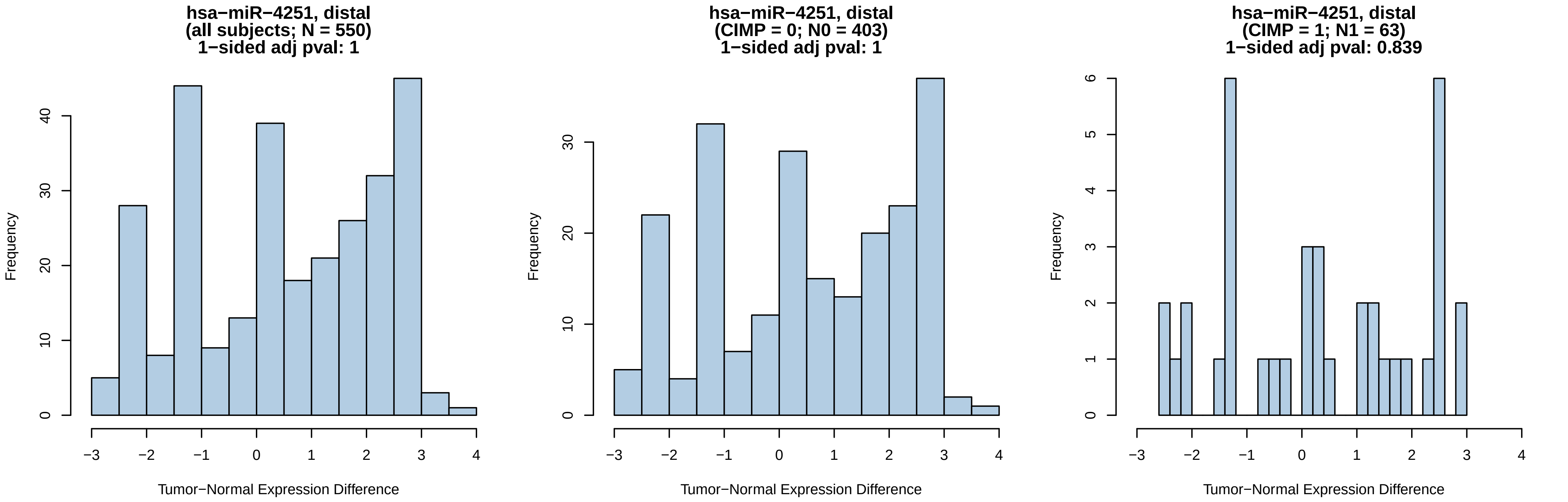
hsa−miR−4251, distal
(all subjects; N = 550)
1−sided adj pval: 1
hsa−miR−4251, distal
(CIMP = 0; N0 = 403)
1−sided adj pval: 1
hsa−miR−4251, distal
(CIMP = 1; N1 = 63)
1−sided adj pval: 0.839
Frequency
Tumor−Normal Expression Difference

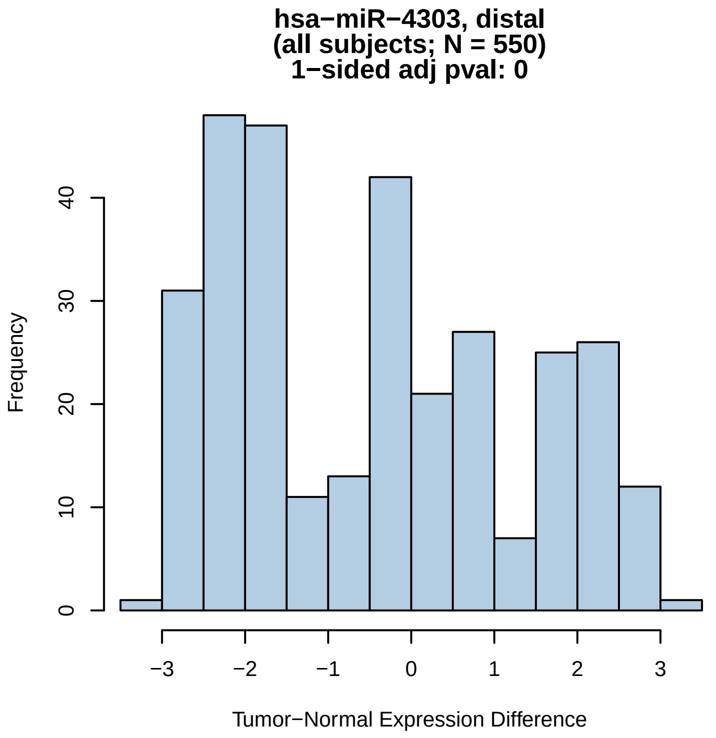
hsa−miR−4303, distal
(all subjects; N = 550)
1−sided adj pval: 0
Frequency
0
10
20
30
40
−3
−2
−1
0
1
2
3
Tumor−Normal Expression Difference

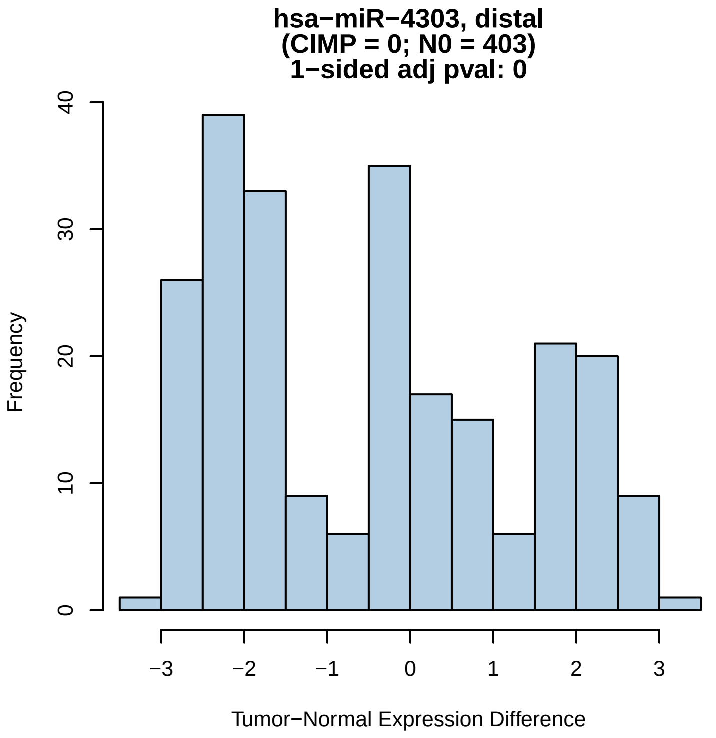
hsa−miR−4303, distal
(CIMP = 0; N0 = 403)
1−sided adj pval: 0
Frequency
0
10
20
30
40
−3
−2
−1
0
1
2
3
Tumor−Normal Expression Difference

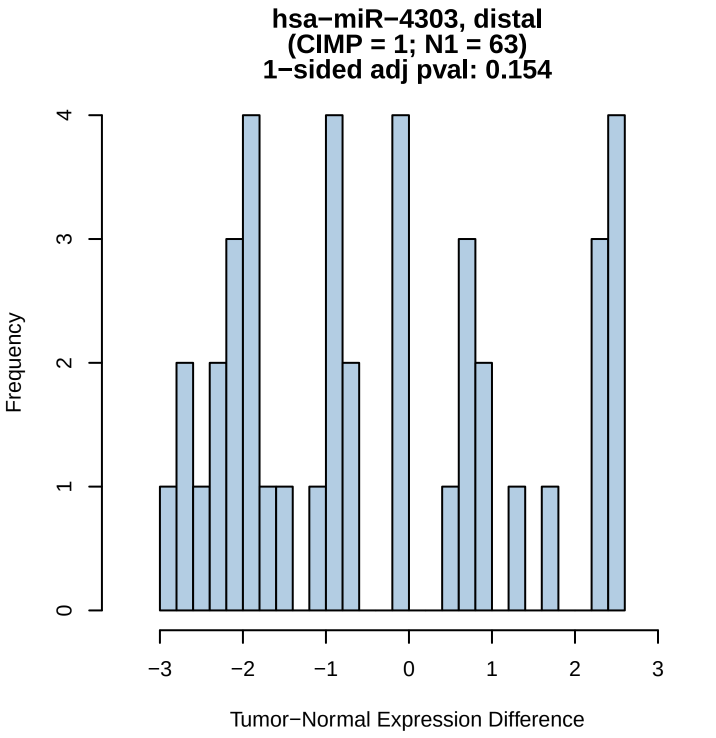
hsa−miR−4303, distal
(CIMP = 1; N1 = 63)
1−sided adj pval: 0.154
Frequency
0
1
2
3
4
−3
−2
−1
0
1
2
3
Tumor−Normal Expression Difference

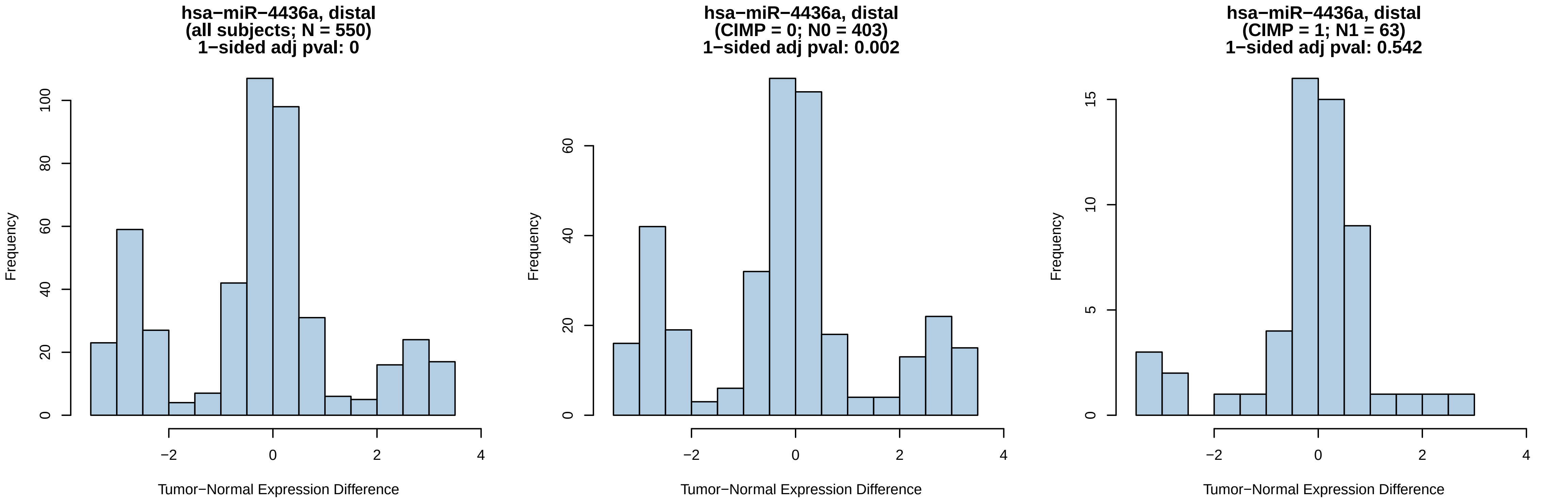
hsa−miR−4436a, distal
(all subjects; N = 550)
1−sided adj pval: 0
hsa−miR−4436a, distal
(CIMP = 0; N0 = 403)
1−sided adj pval: 0.002
hsa−miR−4436a, distal
(CIMP = 1; N1 = 63)
1−sided adj pval: 0.542
Frequency
Tumor−Normal Expression Difference

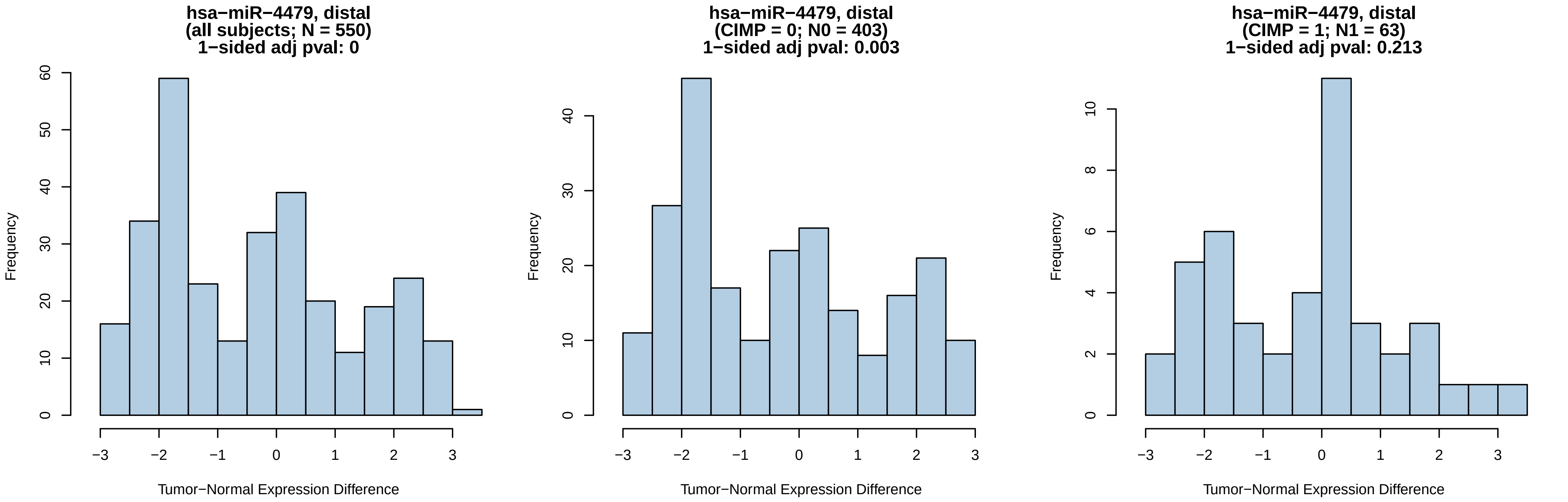

hsa−miR−4479, distal
(all subjects; N = 550)
1−sided adj pval: 0
hsa−miR−4479, distal
(CIMP = 0; N0 = 403)
1−sided adj pval: 0.003
hsa−miR−4479, distal
(CIMP = 1; N1 = 63)
1−sided adj pval: 0.213
Frequency
Tumor−Normal Expression Difference

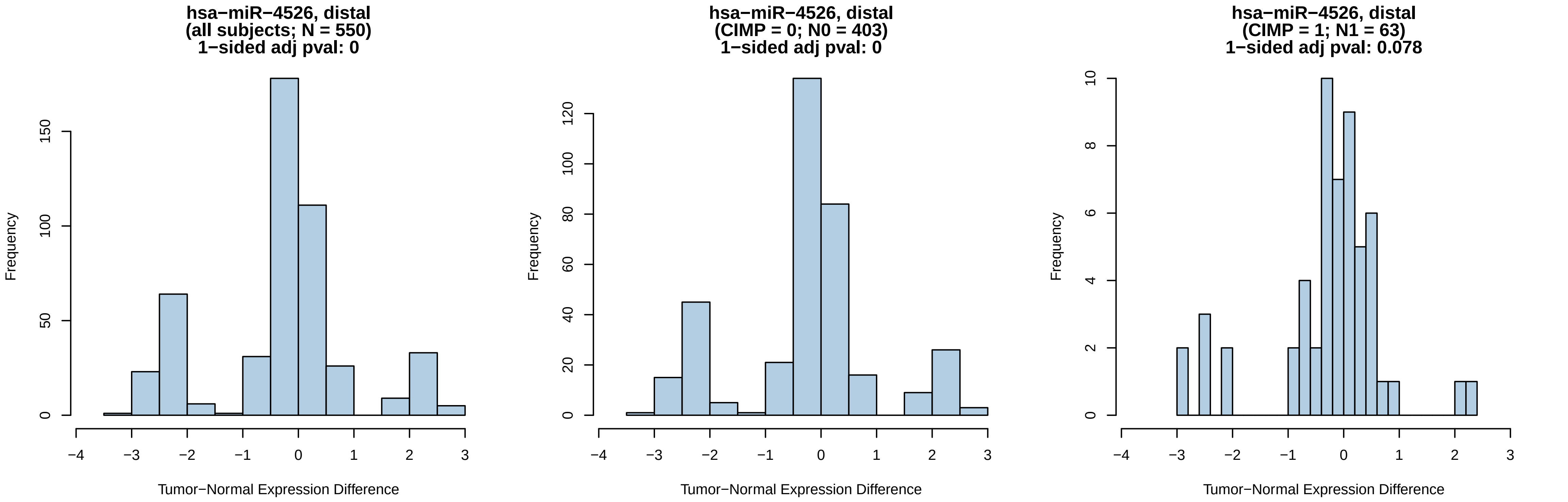
hsa−miR−4526, distal
(all subjects; N = 550)
1−sided adj pval: 0
hsa−miR−4526, distal
(CIMP = 0; N0 = 403)
1−sided adj pval: 0
hsa−miR−4526, distal
(CIMP = 1; N1 = 63)
1−sided adj pval: 0.078
Frequency
Tumor−Normal Expression Difference

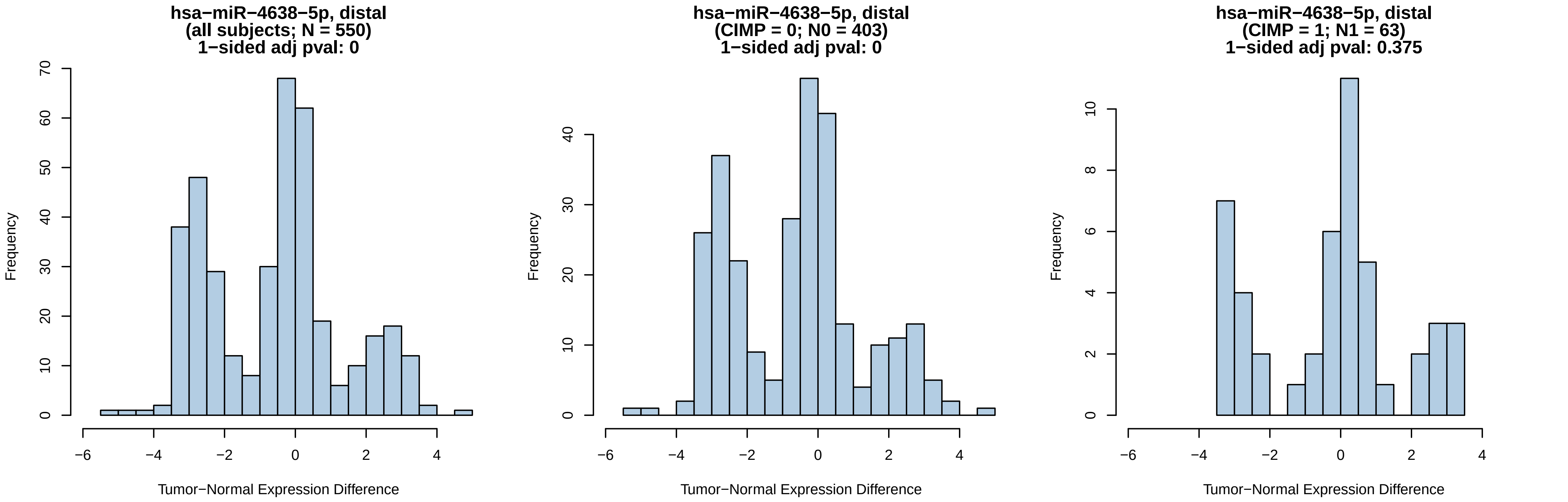

hsa−miR−4638−5p, distal
(all subjects; N = 550)
1−sided adj pval: 0
Frequency
Tumor−Normal Expression Difference
hsa−miR−4638−5p, distal
(CIMP = 0; N0 = 403)
1−sided adj pval: 0
Frequency
Tumor−Normal Expression Difference
hsa−miR−4638−5p, distal
(CIMP = 1; N1 = 63)
1−sided adj pval: 0.375
Frequency
Tumor−Normal Expression Difference

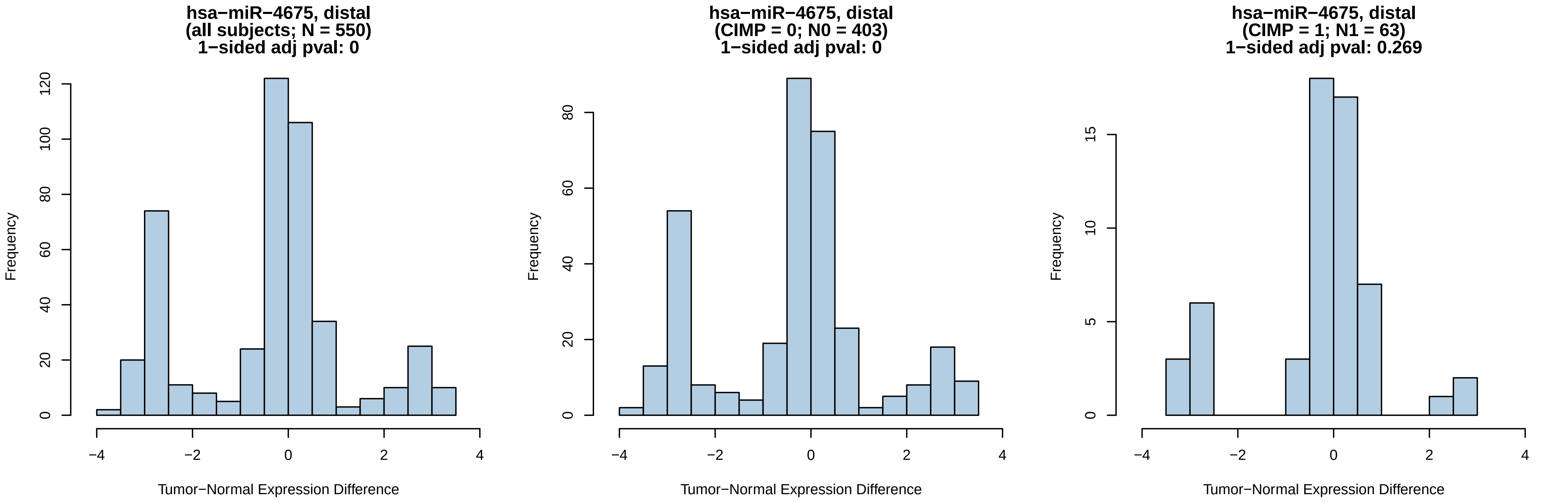
hsa−miR−4675, distal
(all subjects; N = 550)
1−sided adj pval: 0
hsa−miR−4675, distal
(CIMP = 0; N0 = 403)
1−sided adj pval: 0
hsa−miR−4675, distal
(CIMP = 1; N1 = 63)
1−sided adj pval: 0.269
Frequency
Tumor−Normal Expression Difference
0
20
40
60
80
100
120
5
10
15
−4
−2
0
2
4

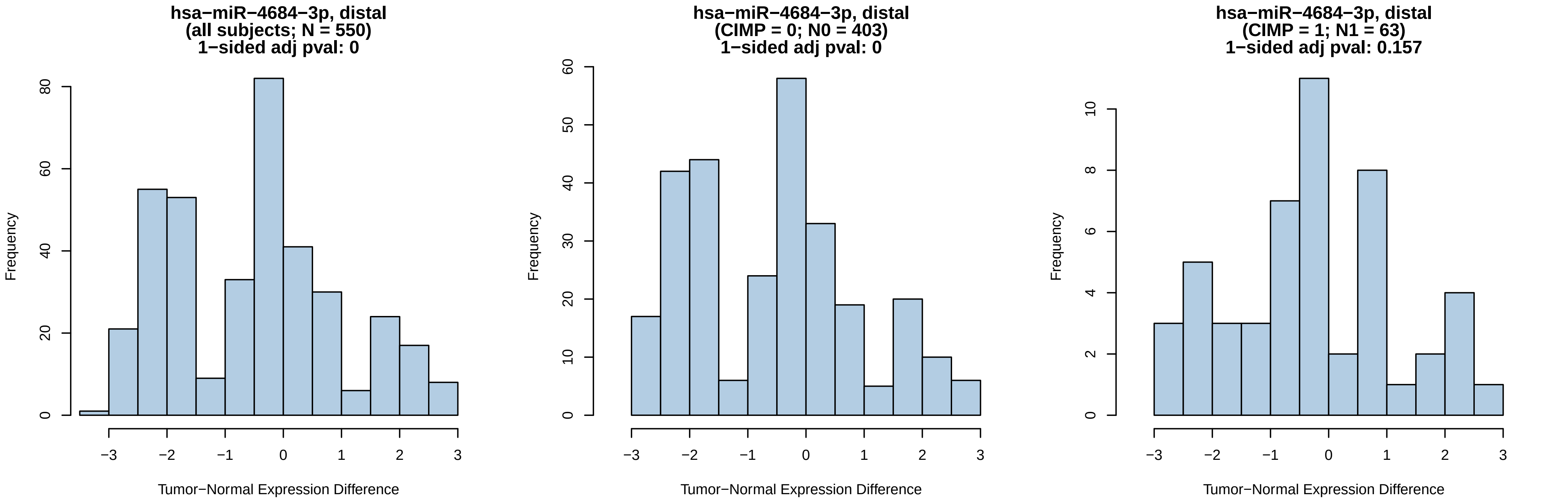
hsa−miR−4684−3p, distal
(all subjects; N = 550)
1−sided adj pval: 0
hsa−miR−4684−3p, distal
(CIMP = 0; N0 = 403)
1−sided adj pval: 0
hsa−miR−4684−3p, distal
(CIMP = 1; N1 = 63)
1−sided adj pval: 0.157
Frequency
Tumor−Normal Expression Difference

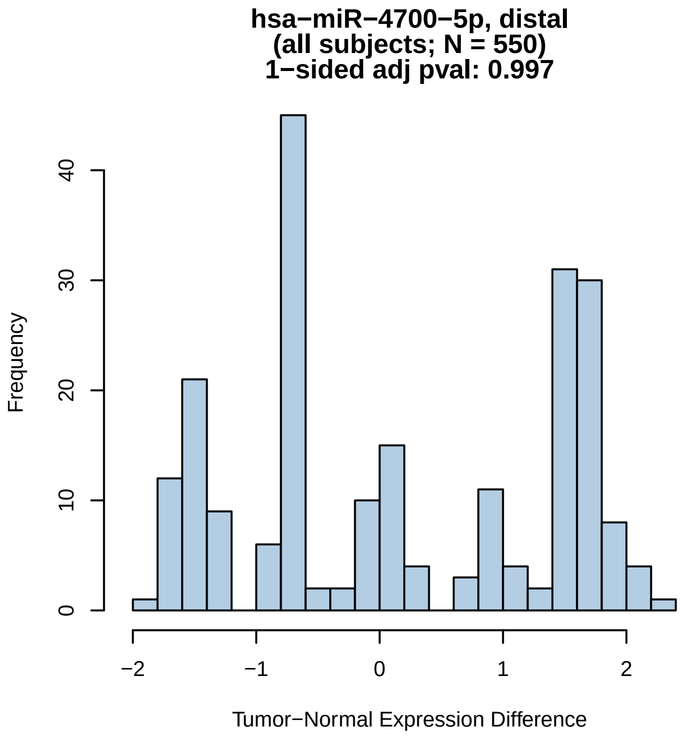
hsa−miR−4700−5p, distal
(all subjects; N = 550)
1−sided adj pval: 0.997
Frequency
Tumor−Normal Expression Difference
0
10
20
30
40
−2
−1
0
1
2

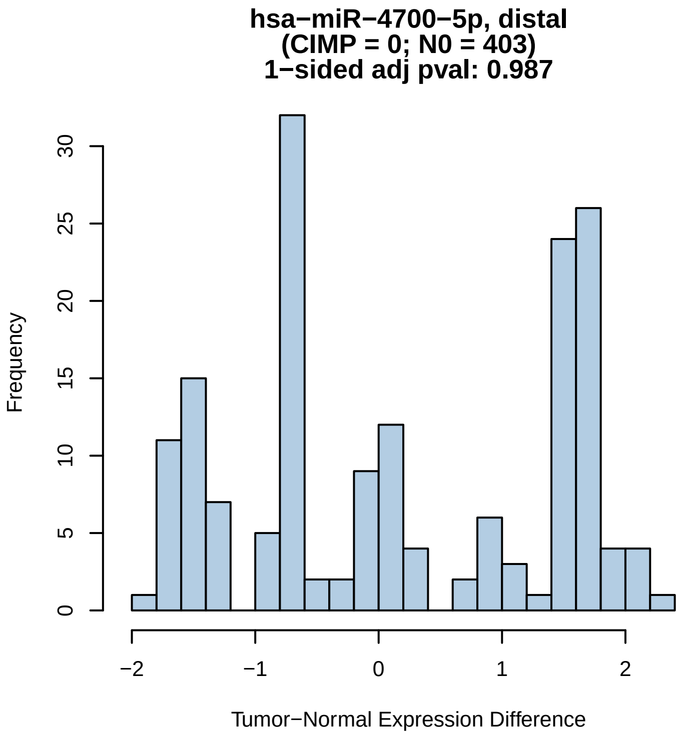
hsa−miR−4700−5p, distal
(CIMP = 0; N0 = 403)
1−sided adj pval: 0.987
Frequency
Tumor−Normal Expression Difference
0
5
10
15
20
25
30
−2
−1
0
1
2

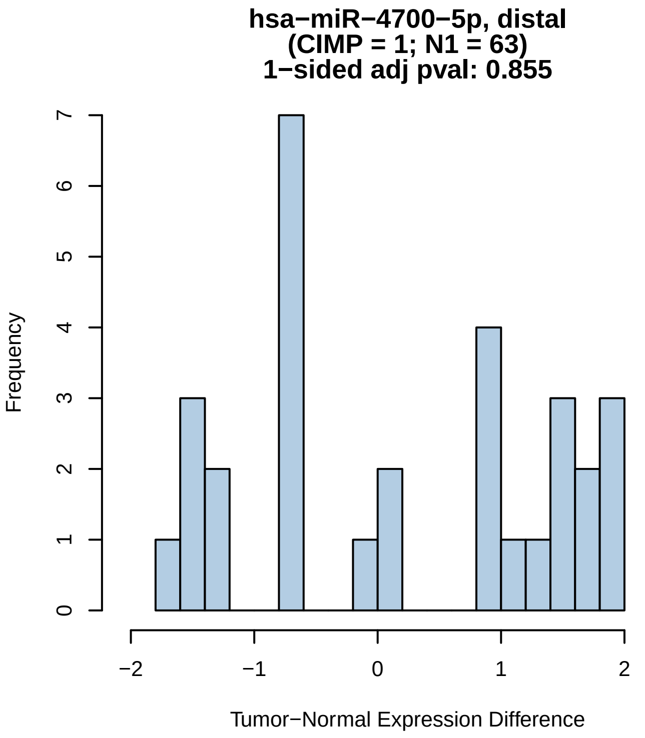
hsa−miR−4700−5p, distal
(CIMP = 1; N1 = 63)
1−sided adj pval: 0.855
Frequency
Tumor−Normal Expression Difference
0
1
2
3
4
5
6
7
−2
−1
0
1
2

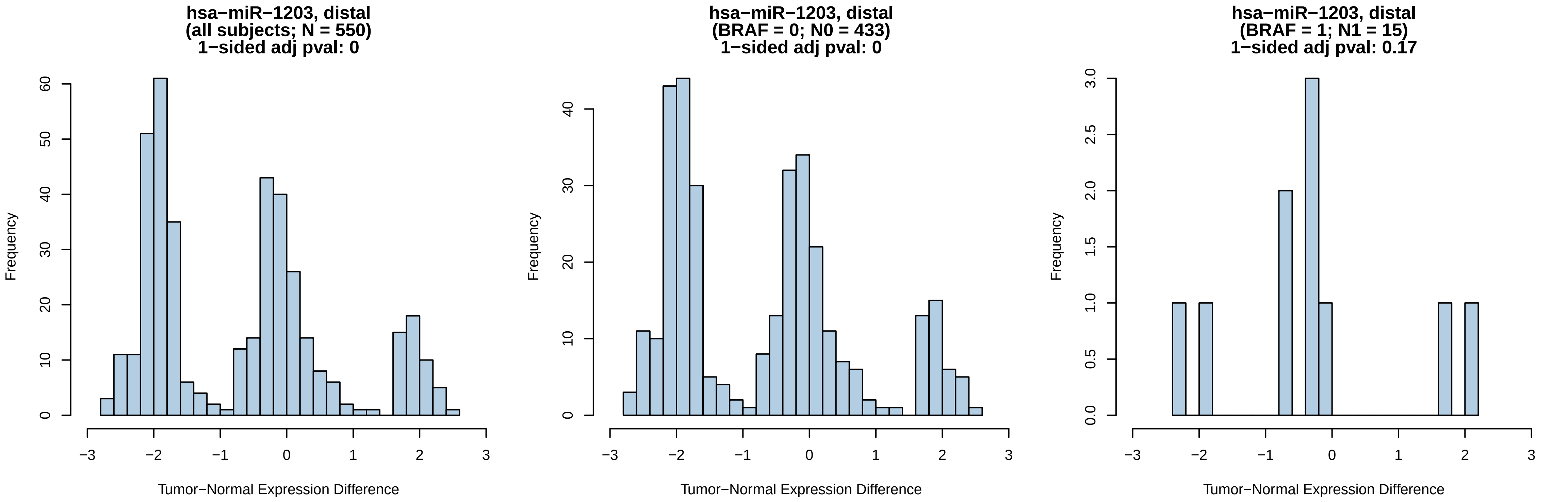
hsa−miR−1203, distal
(all subjects; N = 550)
1−sided adj pval: 0
hsa−miR−1203, distal
(BRAF = 0; N0 = 433)
1−sided adj pval: 0
hsa−miR−1203, distal
(BRAF = 1; N1 = 15)
1−sided adj pval: 0.17
Frequency
Tumor−Normal Expression Difference

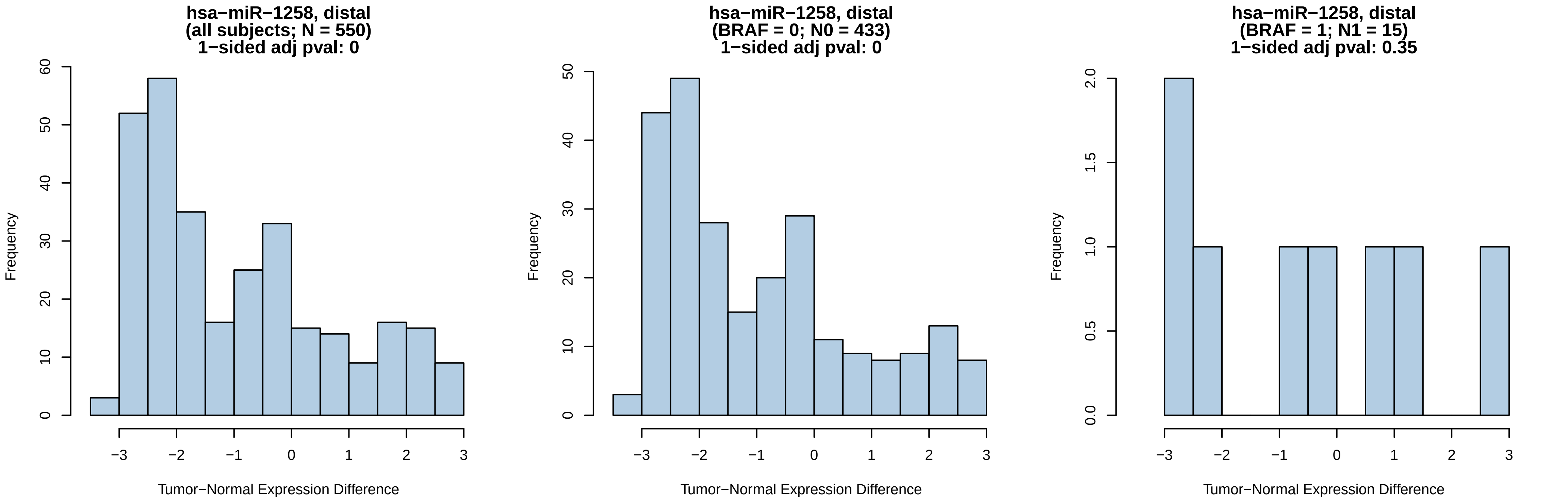

hsa−miR−1258, distal
(all subjects; N = 550)
1−sided adj pval: 0
hsa−miR−1258, distal
(BRAF = 0; N0 = 433)
1−sided adj pval: 0
hsa−miR−1258, distal
(BRAF = 1; N1 = 15)
1−sided adj pval: 0.35
Frequency
Tumor−Normal Expression Difference

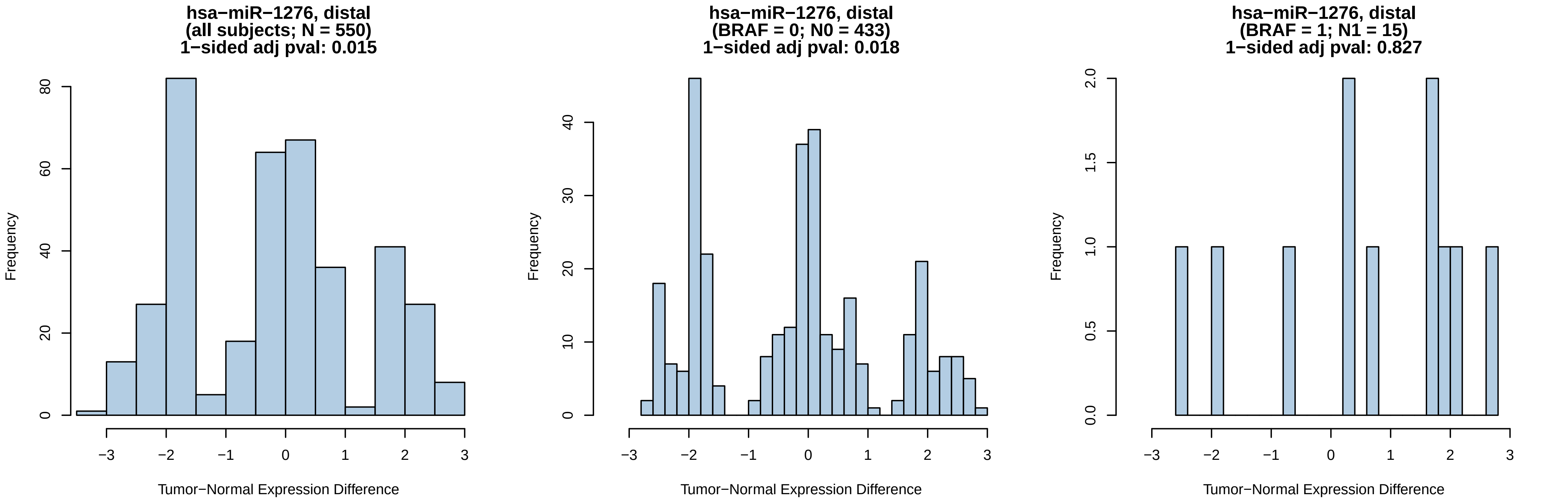

hsa−miR−1276, distal
(all subjects; N = 550)
1−sided adj pval: 0.015
hsa−miR−1276, distal
(BRAF = 0; N0 = 433)
1−sided adj pval: 0.018
hsa−miR−1276, distal
(BRAF = 1; N1 = 15)
1−sided adj pval: 0.827
Frequency
Tumor−Normal Expression Difference

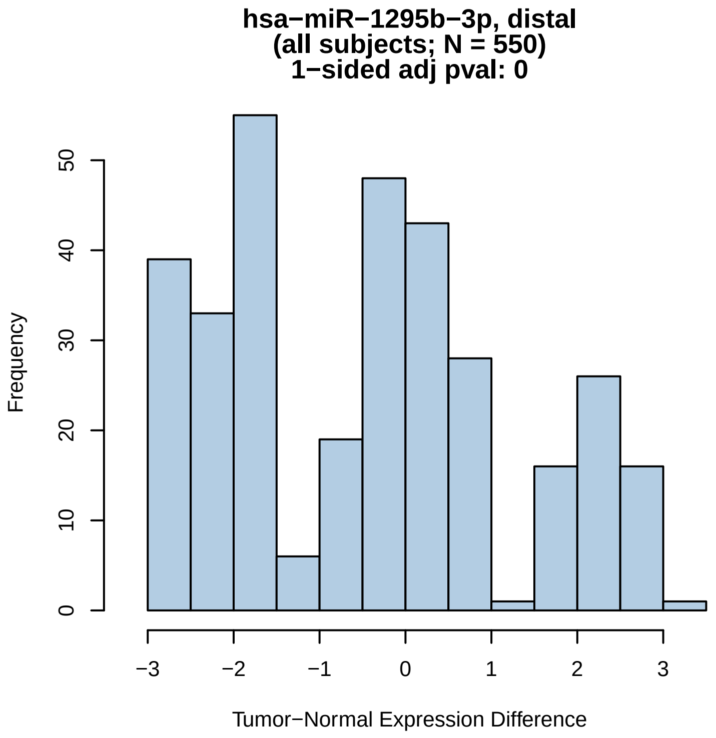
hsa−miR−1295b−3p, distal
(all subjects; N = 550)
1−sided adj pval: 0
Frequency
0
10
20
30
40
50
−3
−2
−1
0
1
2
3
Tumor−Normal Expression Difference

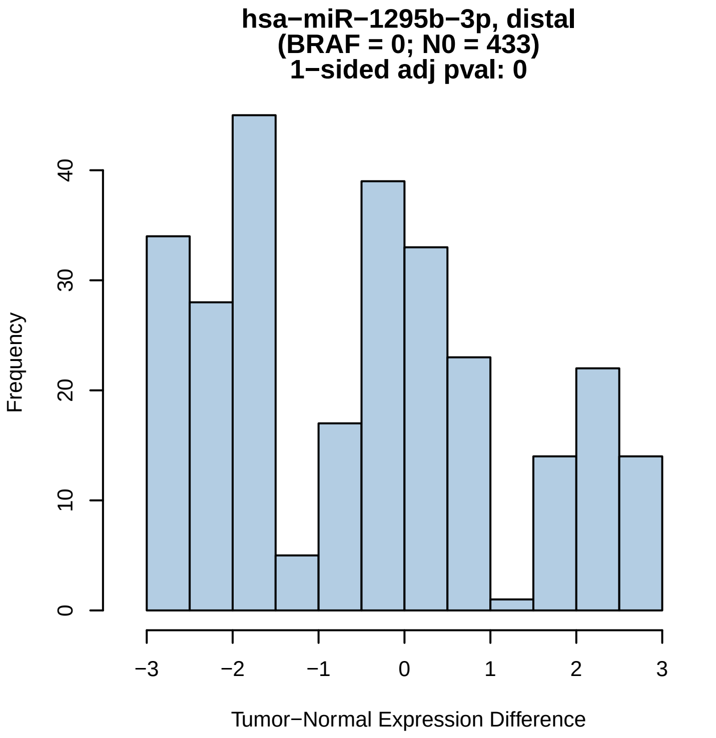
hsa−miR−1295b−3p, distal
(BRAF = 0; N0 = 433)
1−sided adj pval: 0
Frequency
0
10
20
30
40
−3
−2
−1
0
1
2
3
Tumor−Normal Expression Difference

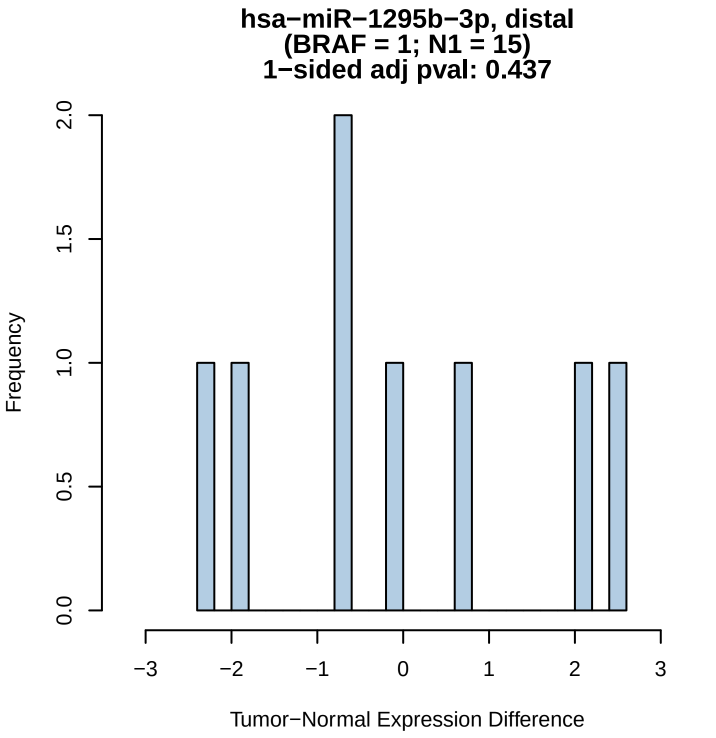
hsa−miR−1295b−3p, distal
(BRAF = 1; N1 = 15)
1−sided adj pval: 0.437
Frequency
0.0
0.5
1.0
1.5
2.0
−3
−2
−1
0
1
2
3
Tumor−Normal Expression Difference

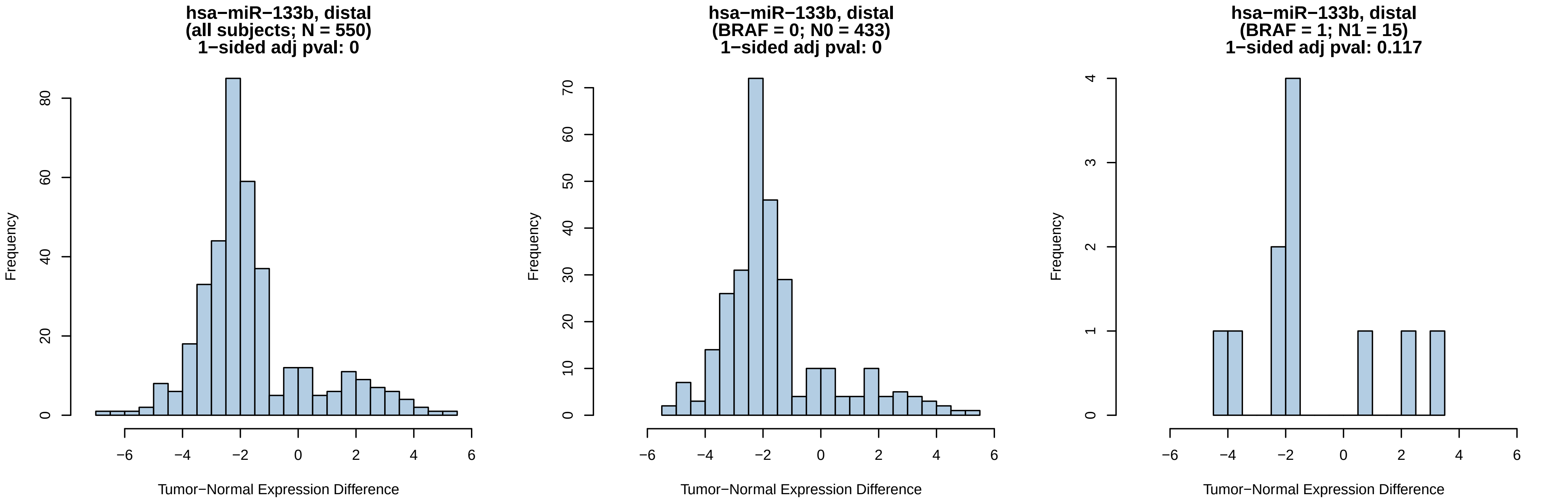

hsa−miR−133b, distal
(all subjects; N = 550)
1−sided adj pval: 0
hsa−miR−133b, distal
(BRAF = 0; N0 = 433)
1−sided adj pval: 0
hsa−miR−133b, distal
(BRAF = 1; N1 = 15)
1−sided adj pval: 0.117
Frequency
Tumor−Normal Expression Difference

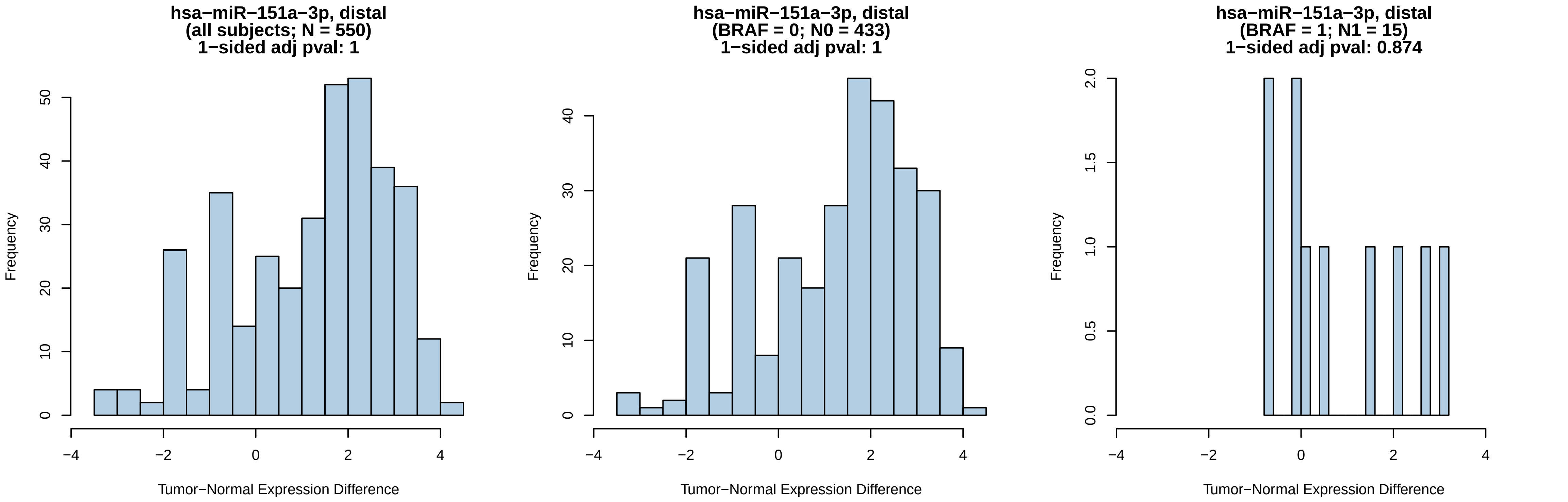
hsa−miR−151a−3p, distal
(all subjects; N = 550)
1−sided adj pval: 1
hsa−miR−151a−3p, distal
(BRAF = 0; N0 = 433)
1−sided adj pval: 1
hsa−miR−151a−3p, distal
(BRAF = 1; N1 = 15)
1−sided adj pval: 0.874
Frequency
Tumor−Normal Expression Difference
0
10
20
30
40
50
0.0
0.5
1.0
1.5
2.0
−4
−2
0
2
4

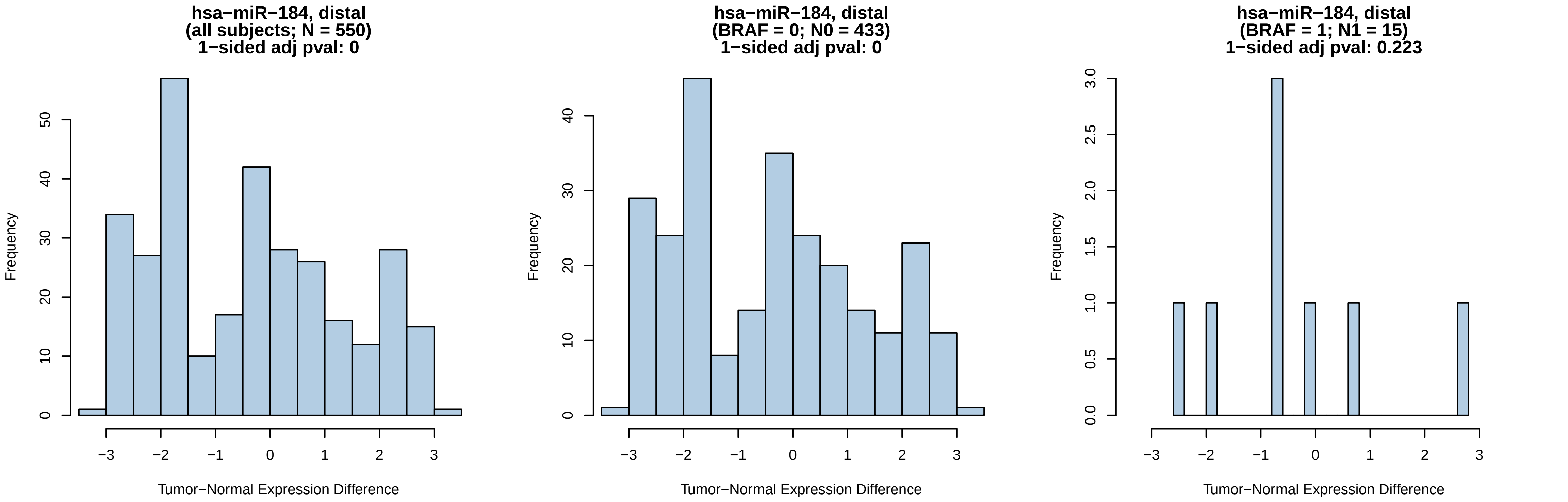
hsa−miR−184, distal
(all subjects; N = 550)
1−sided adj pval: 0
Frequency
Tumor−Normal Expression Difference
hsa−miR−184, distal
(BRAF = 0; N0 = 433)
1−sided adj pval: 0
Frequency
Tumor−Normal Expression Difference
hsa−miR−184, distal
(BRAF = 1; N1 = 15)
1−sided adj pval: 0.223
Frequency
Tumor−Normal Expression Difference

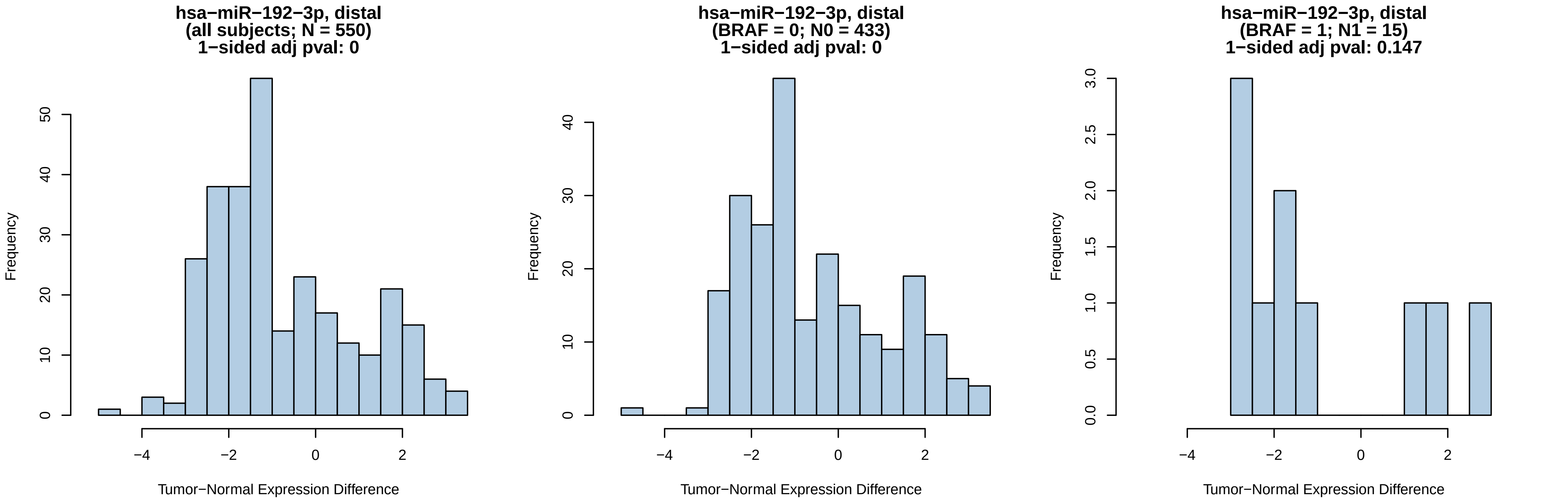
hsa−miR−192−3p, distal
(all subjects; N = 550)
1−sided adj pval: 0
Frequency
Tumor−Normal Expression Difference
hsa−miR−192−3p, distal
(BRAF = 0; N0 = 433)
1−sided adj pval: 0
Frequency
Tumor−Normal Expression Difference
hsa−miR−192−3p, distal
(BRAF = 1; N1 = 15)
1−sided adj pval: 0.147
Frequency
Tumor−Normal Expression Difference

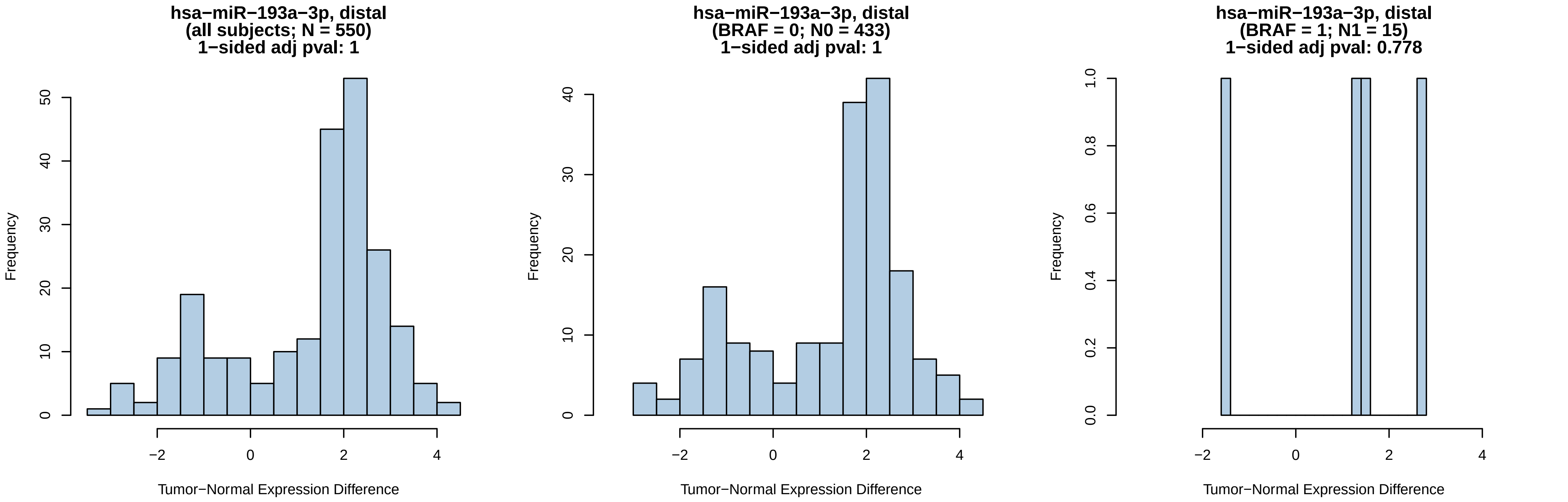
hsa−miR−193a−3p, distal
(all subjects; N = 550)
1−sided adj pval: 1
Frequency
0
10
20
30
40
50
−2
0
2
4
Tumor−Normal Expression Difference
hsa−miR−193a−3p, distal
(BRAF = 0; N0 = 433)
1−sided adj pval: 1
Frequency
0
10
20
30
40
−2
0
2
4
Tumor−Normal Expression Difference
hsa−miR−193a−3p, distal
(BRAF = 1; N1 = 15)
1−sided adj pval: 0.778
Frequency
0.0
0.2
0.4
0.6
0.8
1.0
−2
0
2
4
Tumor−Normal Expression Difference

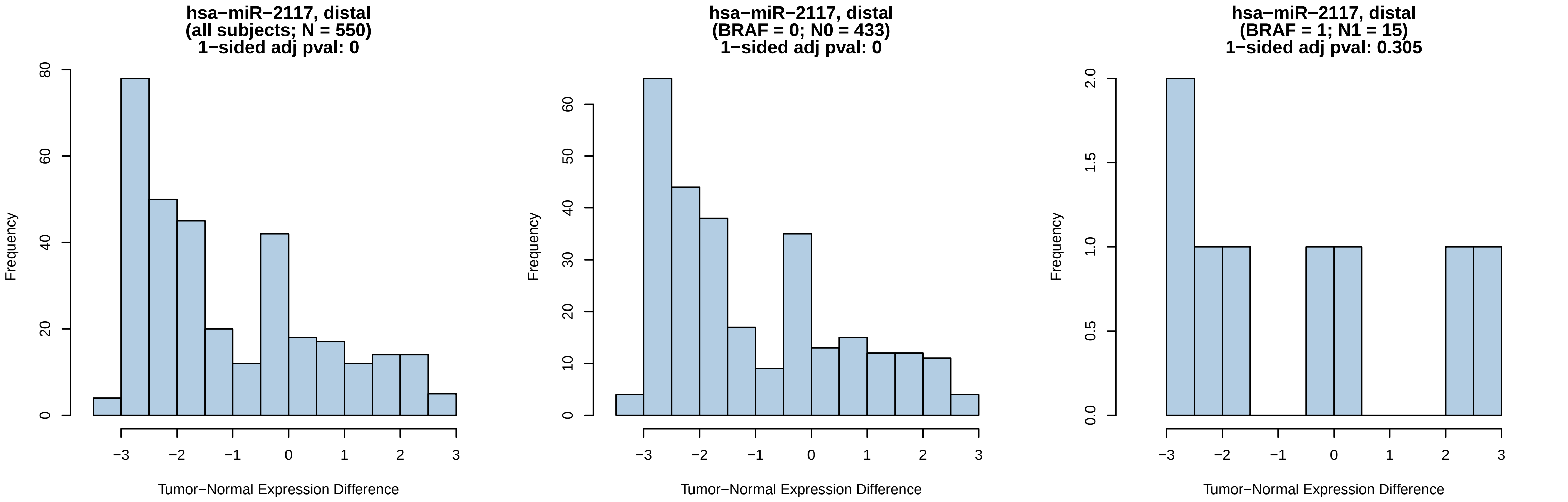
hsa−miR−2117, distal
(all subjects; N = 550)
1−sided adj pval: 0
Frequency
0
20
40
60
80
−3
−2
−1
0
1
2
3
Tumor−Normal Expression Difference
hsa−miR−2117, distal
(BRAF = 0; N0 = 433)
1−sided adj pval: 0
Frequency
0
10
20
30
40
50
60
−3
−2
−1
0
1
2
3
Tumor−Normal Expression Difference
hsa−miR−2117, distal
(BRAF = 1; N1 = 15)
1−sided adj pval: 0.305
Frequency
0.0
0.5
1.0
1.5
2.0
−3
−2
−1
0
1
2
3
Tumor−Normal Expression Difference

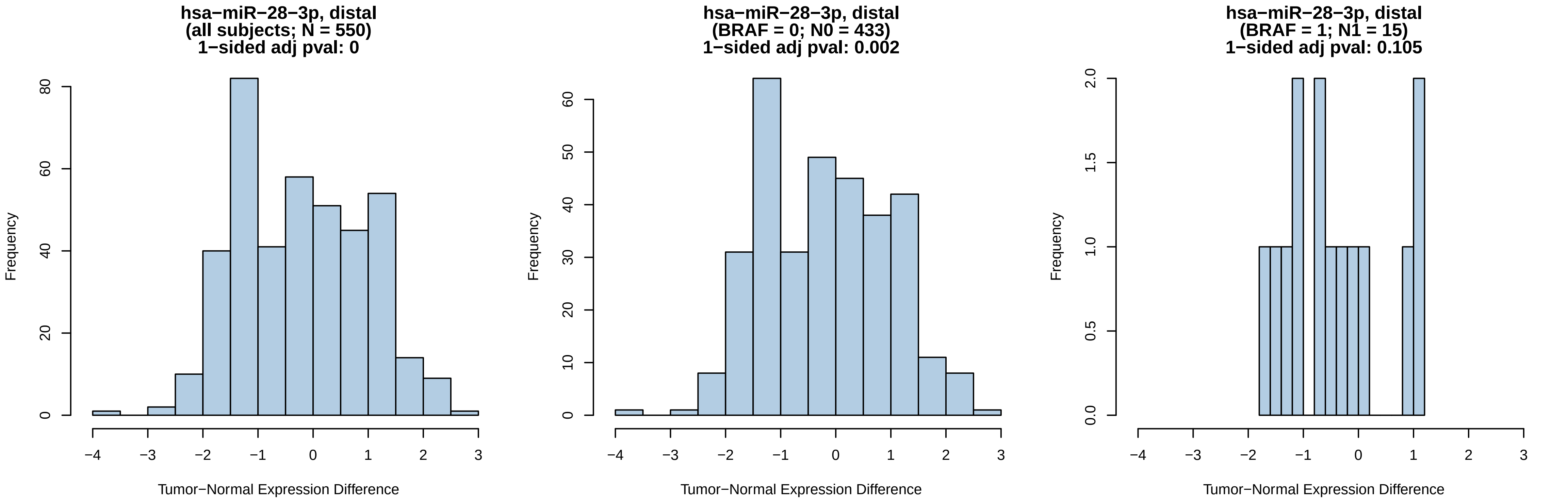

hsa−miR−28−3p, distal
(all subjects; N = 550)
1−sided adj pval: 0
Frequency
Tumor−Normal Expression Difference
hsa−miR−28−3p, distal
(BRAF = 0; N0 = 433)
1−sided adj pval: 0.002
Frequency
Tumor−Normal Expression Difference
hsa−miR−28−3p, distal
(BRAF = 1; N1 = 15)
1−sided adj pval: 0.105
Frequency
Tumor−Normal Expression Difference

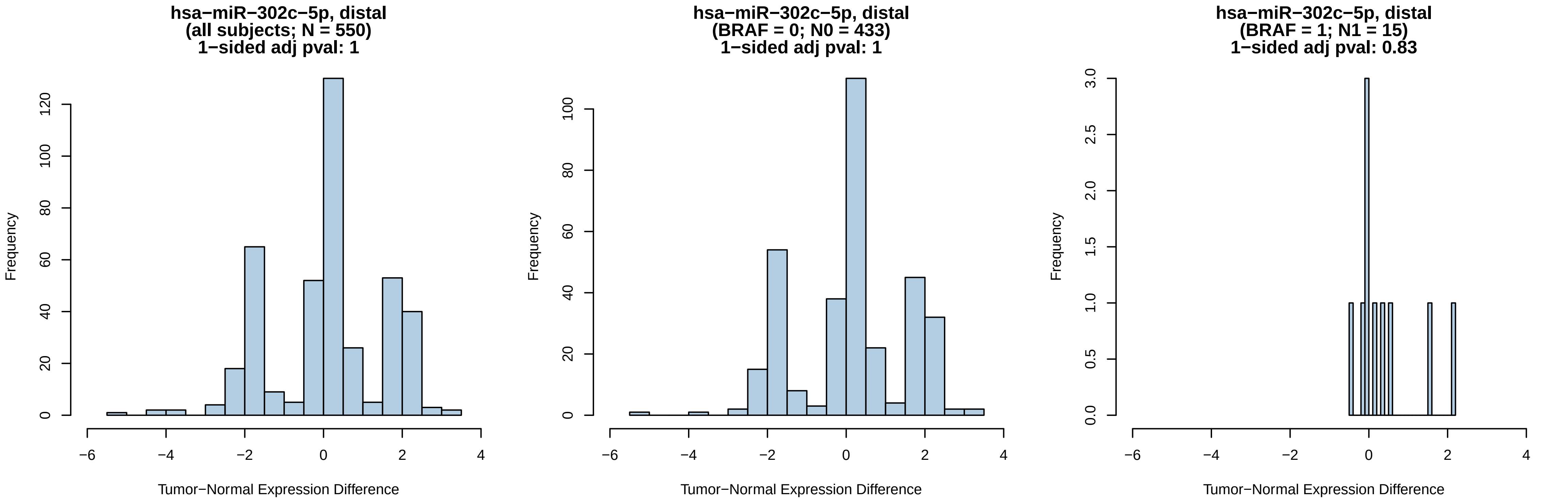

hsa−miR−302c−5p, distal
(all subjects; N = 550)
1−sided adj pval: 1
hsa−miR−302c−5p, distal
(BRAF = 0; N0 = 433)
1−sided adj pval: 1
hsa−miR−302c−5p, distal
(BRAF = 1; N1 = 15)
1−sided adj pval: 0.83
Frequency
Tumor−Normal Expression Difference

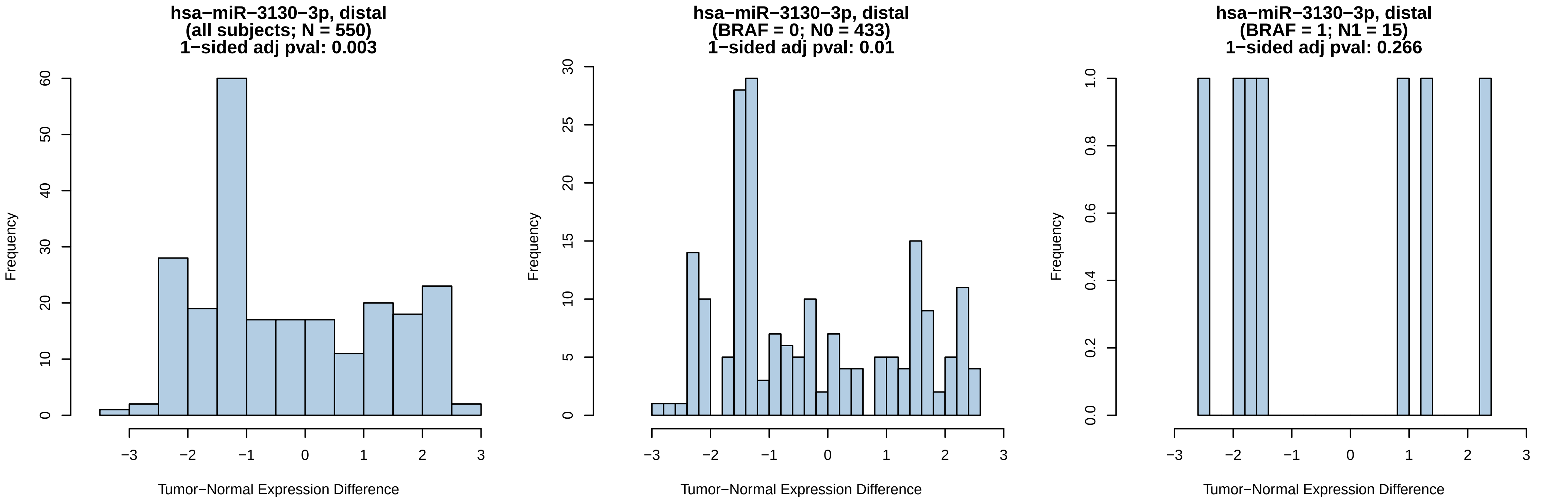

hsa−miR−3130−3p, distal
(all subjects; N = 550)
1−sided adj pval: 0.003
Frequency
Tumor−Normal Expression Difference
hsa−miR−3130−3p, distal
(BRAF = 0; N0 = 433)
1−sided adj pval: 0.01
Frequency
Tumor−Normal Expression Difference
hsa−miR−3130−3p, distal
(BRAF = 1; N1 = 15)
1−sided adj pval: 0.266
Frequency
Tumor−Normal Expression Difference

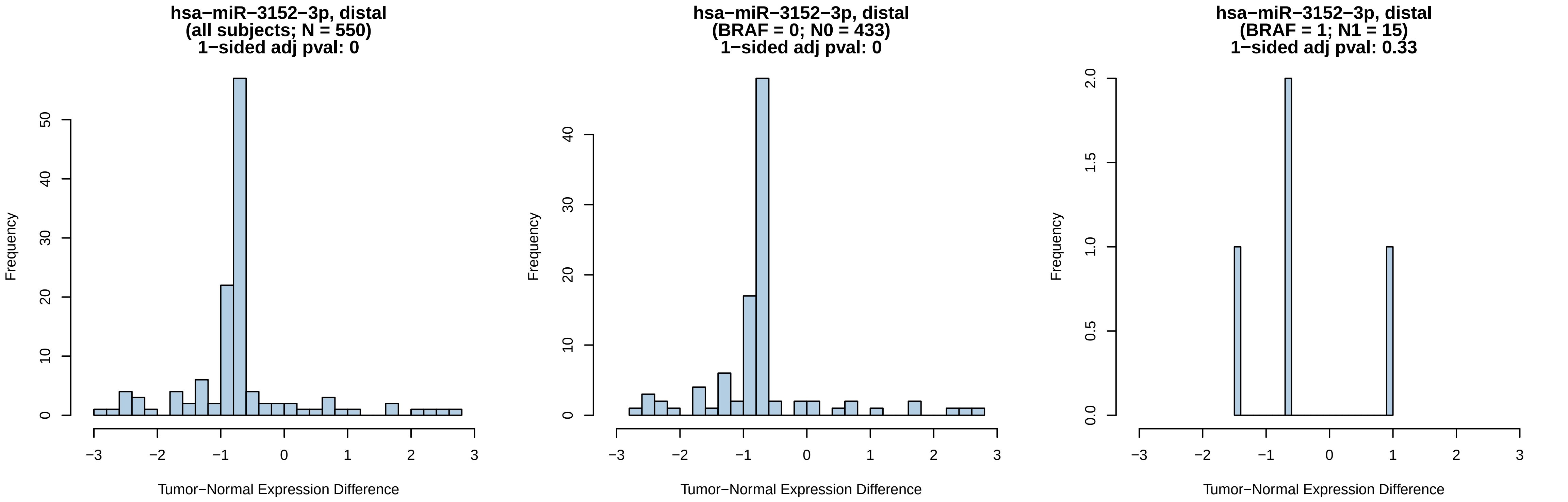
hsa−miR−3152−3p, distal
(all subjects; N = 550)
1−sided adj pval: 0
hsa−miR−3152−3p, distal
(BRAF = 0; N0 = 433)
1−sided adj pval: 0
hsa−miR−3152−3p, distal
(BRAF = 1; N1 = 15)
1−sided adj pval: 0.33
Frequency
Tumor−Normal Expression Difference
0
10
20
30
40
50
0.0
0.5
1.0
1.5
2.0
−3
−2
−1
0
1
2
3

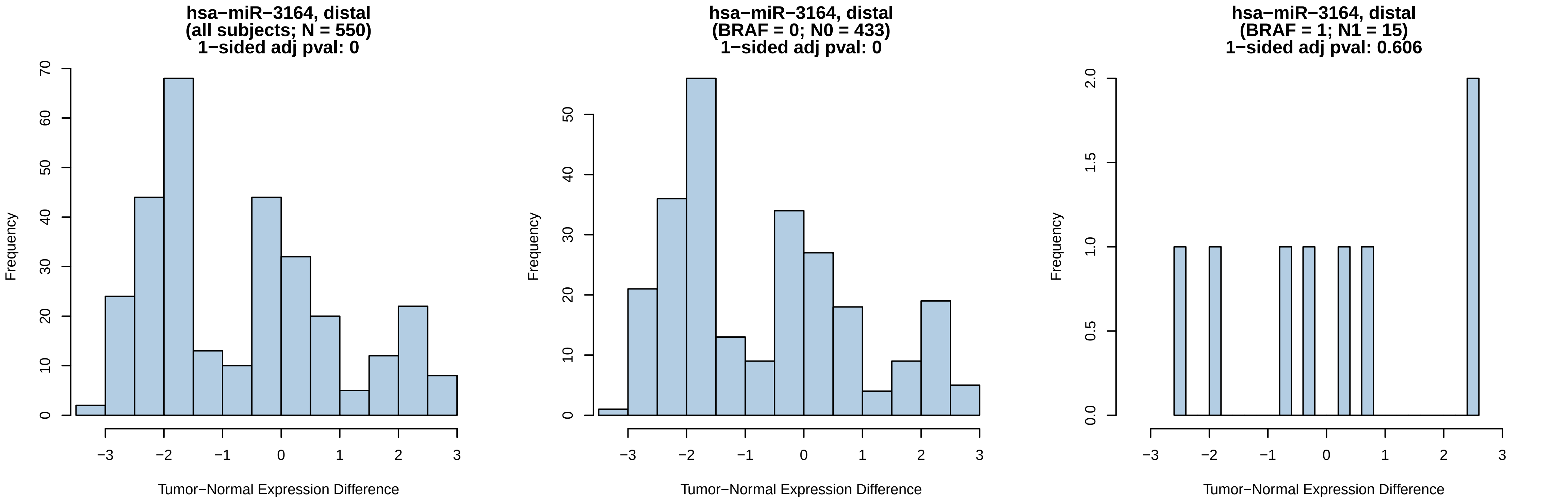
hsa−miR−3164, distal
(all subjects; N = 550)
1−sided adj pval: 0
Frequency
0
10
20
30
40
50
60
70
−3
−2
−1
0
1
2
3
Tumor−Normal Expression Difference
hsa−miR−3164, distal
(BRAF = 0; N0 = 433)
1−sided adj pval: 0
Frequency
0
10
20
30
40
50
−3
−2
−1
0
1
2
3
Tumor−Normal Expression Difference
hsa−miR−3164, distal
(BRAF = 1; N1 = 15)
1−sided adj pval: 0.606
Frequency
0.0
0.5
1.0
1.5
2.0
−3
−2
−1
0
1
2
3
Tumor−Normal Expression Difference

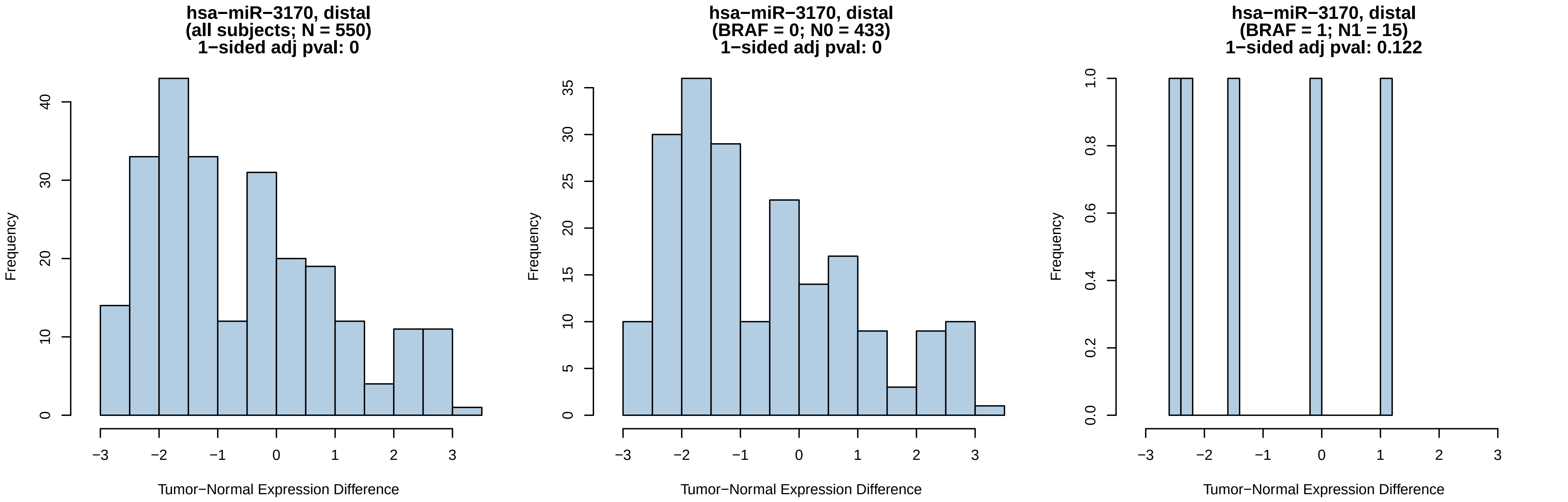
hsa−miR−3170, distal
(all subjects; N = 550)
1−sided adj pval: 0
hsa−miR−3170, distal
(BRAF = 0; N0 = 433)
1−sided adj pval: 0
hsa−miR−3170, distal
(BRAF = 1; N1 = 15)
1−sided adj pval: 0.122
Frequency
Tumor−Normal Expression Difference
Frequency
Tumor−Normal Expression Difference
Frequency
Tumor−Normal Expression Difference

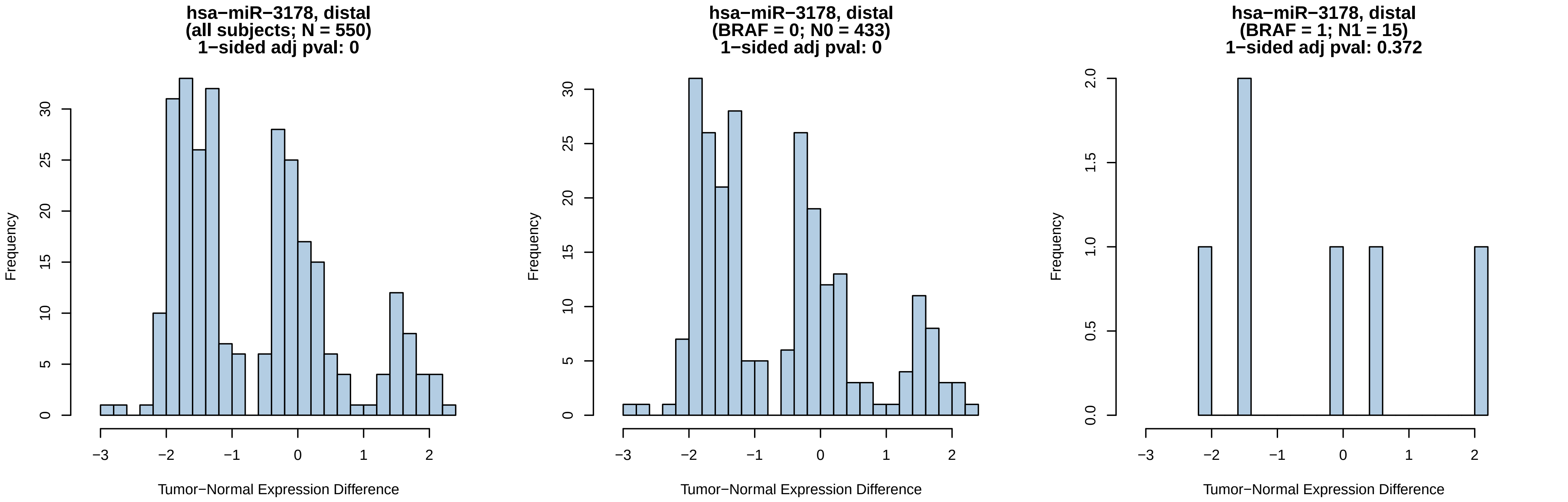

hsa−miR−3178, distal
(all subjects; N = 550)
1−sided adj pval: 0
Frequency
Tumor−Normal Expression Difference
hsa−miR−3178, distal
(BRAF = 0; N0 = 433)
1−sided adj pval: 0
Frequency
Tumor−Normal Expression Difference
hsa−miR−3178, distal
(BRAF = 1; N1 = 15)
1−sided adj pval: 0.372
Frequency
Tumor−Normal Expression Difference

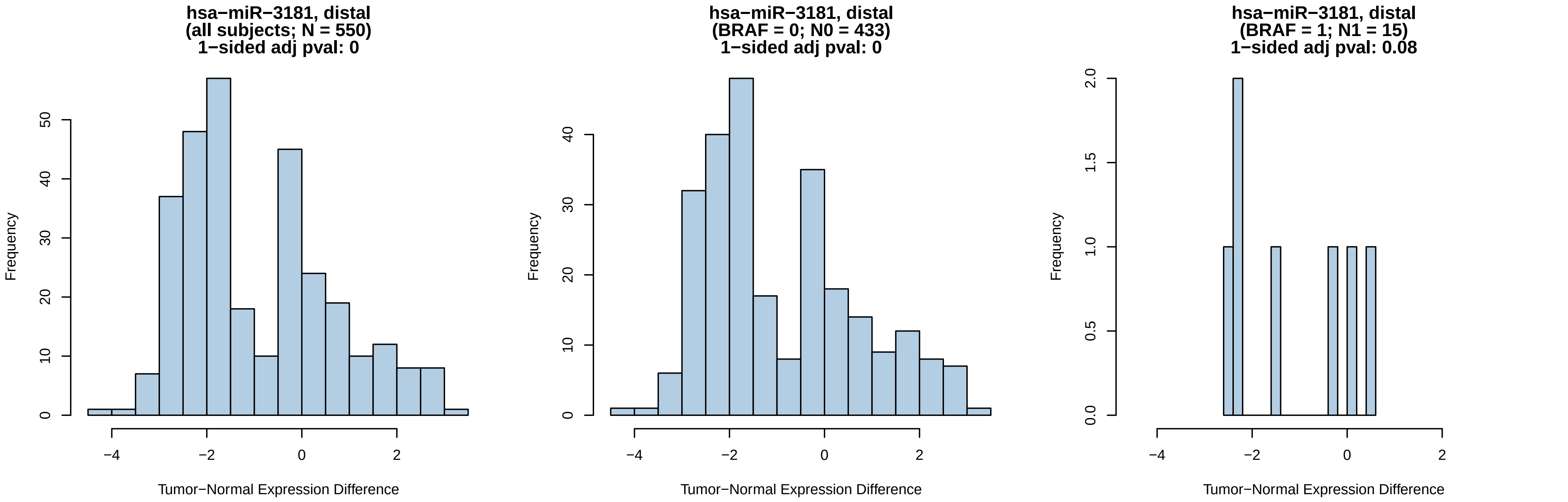
hsa−miR−3181, distal
(all subjects; N = 550)
1−sided adj pval: 0
Frequency
Tumor−Normal Expression Difference
hsa−miR−3181, distal
(BRAF = 0; N0 = 433)
1−sided adj pval: 0
Frequency
Tumor−Normal Expression Difference
hsa−miR−3181, distal
(BRAF = 1; N1 = 15)
1−sided adj pval: 0.08
Frequency
Tumor−Normal Expression Difference

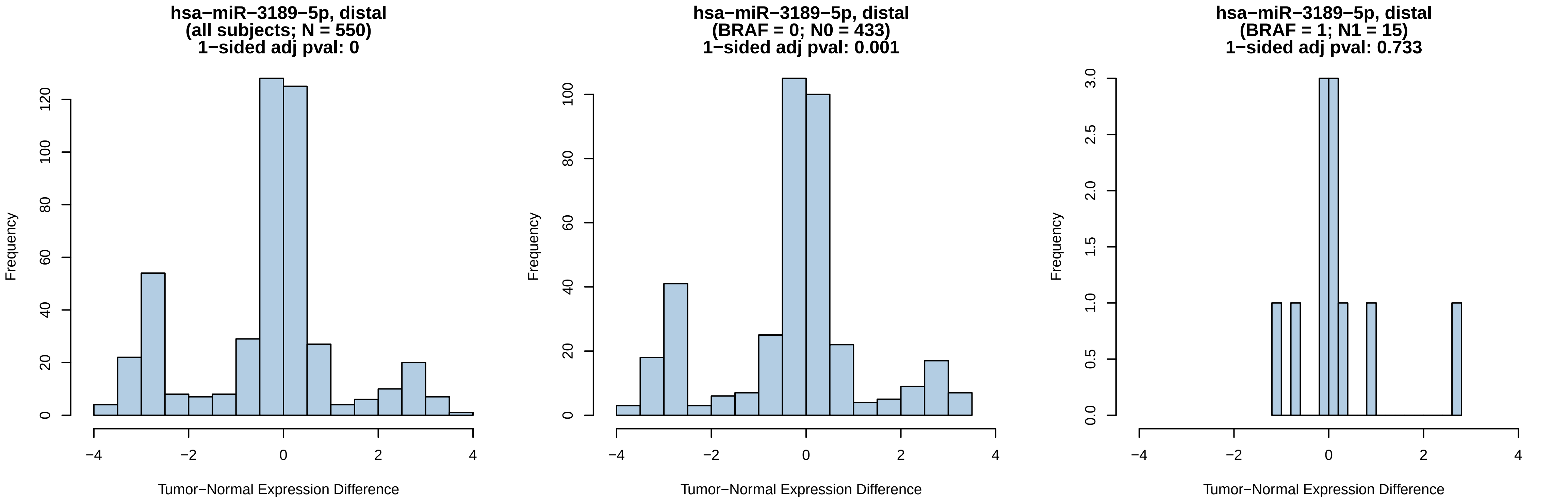
hsa−miR−3189−5p, distal
(all subjects; N = 550)
1−sided adj pval: 0
hsa−miR−3189−5p, distal
(BRAF = 0; N0 = 433)
1−sided adj pval: 0.001
hsa−miR−3189−5p, distal
(BRAF = 1; N1 = 15)
1−sided adj pval: 0.733
Frequency
Tumor−Normal Expression Difference

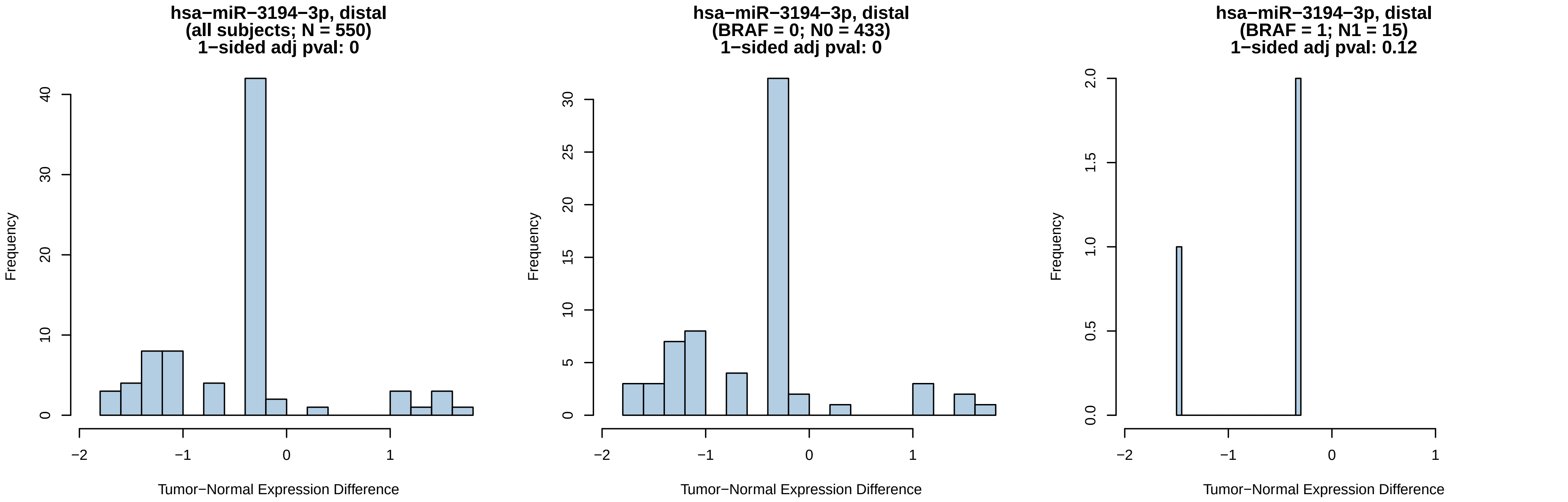
hsa−miR−3194−3p, distal
(all subjects; N = 550)
1−sided adj pval: 0
hsa−miR−3194−3p, distal
(BRAF = 0; N0 = 433)
1−sided adj pval: 0
hsa−miR−3194−3p, distal
(BRAF = 1; N1 = 15)
1−sided adj pval: 0.12
Frequency
Tumor−Normal Expression Difference

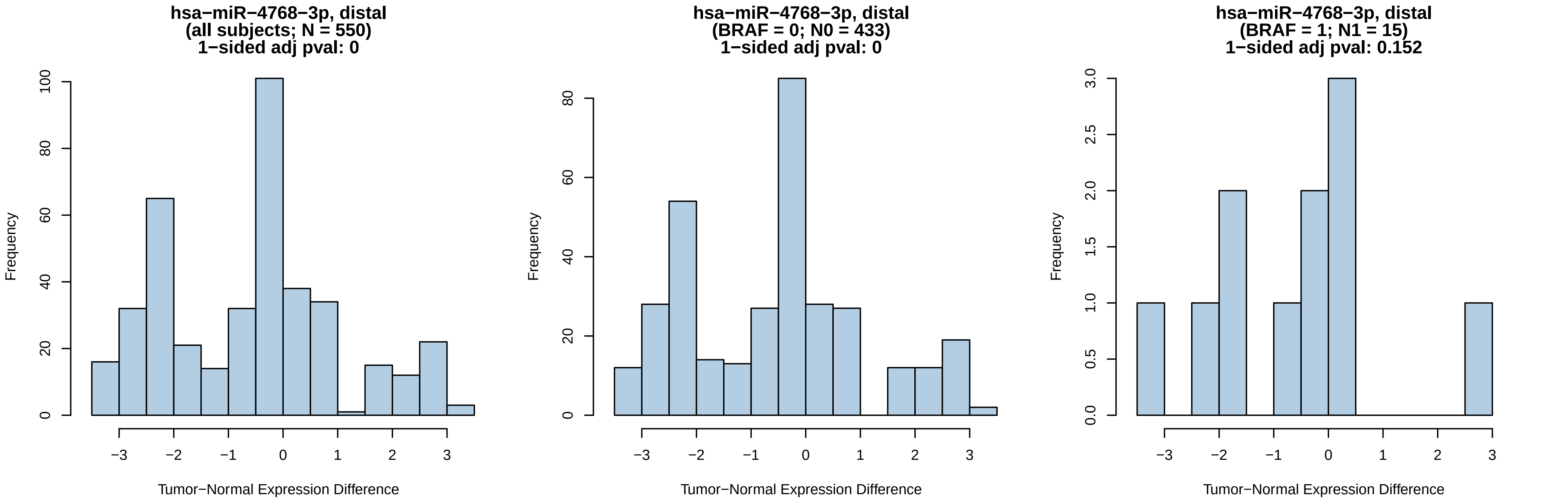
hsa−miR−4768−3p, distal
(all subjects; N = 550)
1−sided adj pval: 0
Frequency
Tumor−Normal Expression Difference
hsa−miR−4768−3p, distal
(BRAF = 0; N0 = 433)
1−sided adj pval: 0
Frequency
Tumor−Normal Expression Difference
hsa−miR−4768−3p, distal
(BRAF = 1; N1 = 15)
1−sided adj pval: 0.152
Frequency
Tumor−Normal Expression Difference

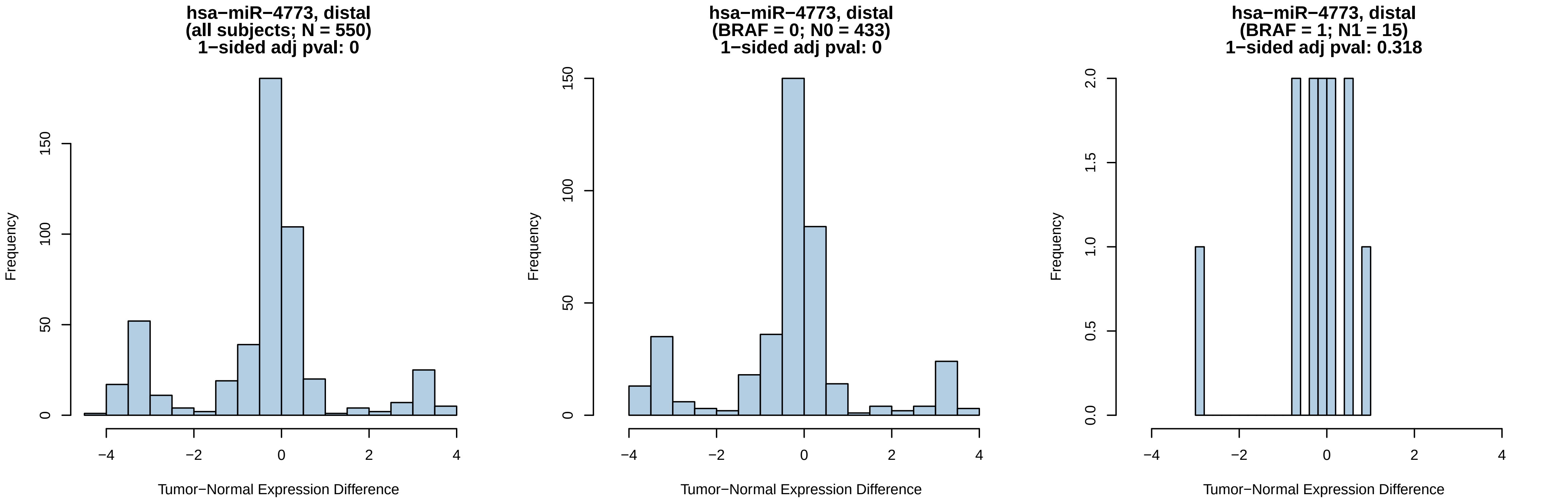

hsa−miR−4773, distal
(all subjects; N = 550)
1−sided adj pval: 0
Frequency
Tumor−Normal Expression Difference
hsa−miR−4773, distal
(BRAF = 0; N0 = 433)
1−sided adj pval: 0
Frequency
Tumor−Normal Expression Difference
hsa−miR−4773, distal
(BRAF = 1; N1 = 15)
1−sided adj pval: 0.318
Frequency
Tumor−Normal Expression Difference

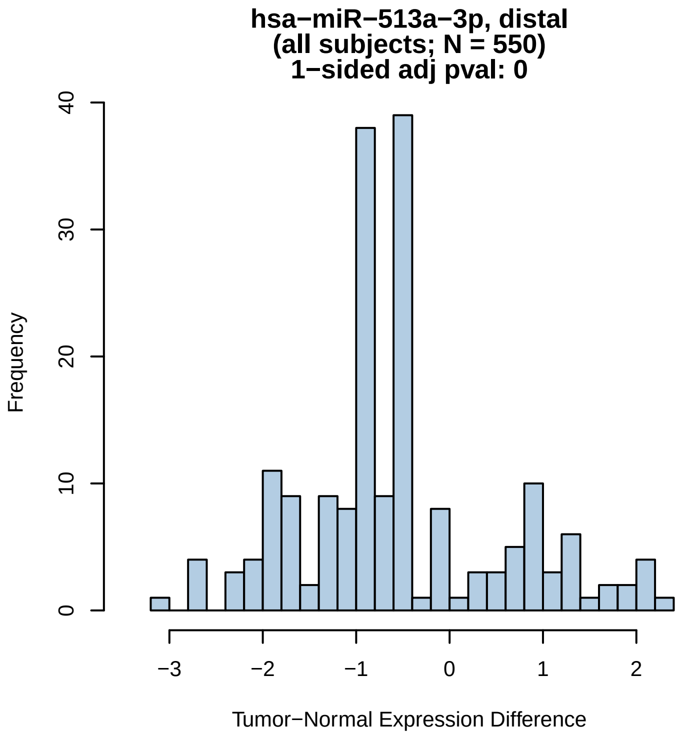

hsa−miR−513a−3p, distal
(all subjects; N = 550)
1−sided adj pval: 0
Frequency
Tumor−Normal Expression Difference


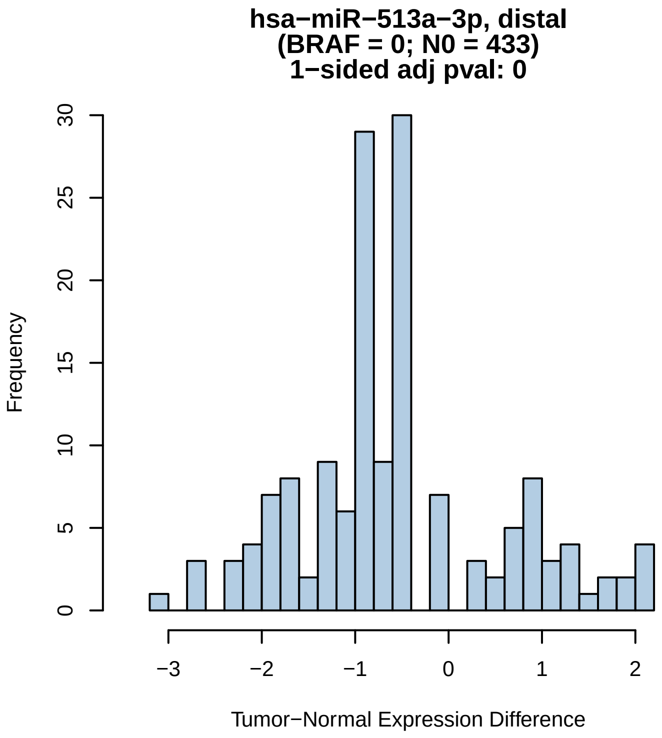

hsa−miR−513a−3p, distal
(BRAF = 0; N0 = 433)
1−sided adj pval: 0
Frequency
Tumor−Normal Expression Difference


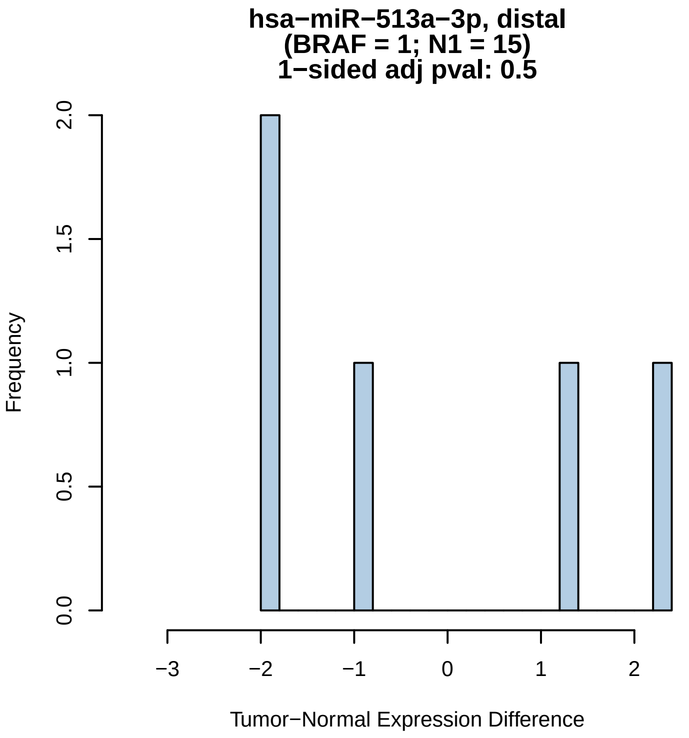

hsa−miR−513a−3p, distal
(BRAF = 1; N1 = 15)
1−sided adj pval: 0.5
Frequency
Tumor−Normal Expression Difference

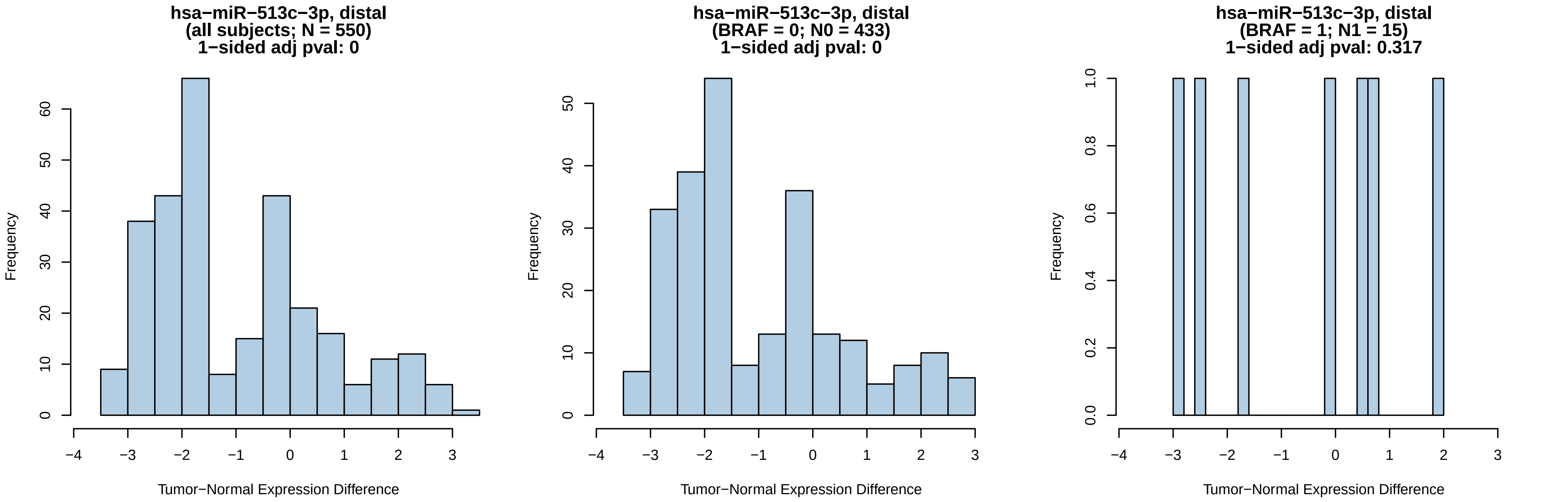
hsa−miR−513c−3p, distal
(all subjects; N = 550)
1−sided adj pval: 0
Frequency
Tumor−Normal Expression Difference
hsa−miR−513c−3p, distal
(BRAF = 0; N0 = 433)
1−sided adj pval: 0
Frequency
Tumor−Normal Expression Difference
hsa−miR−513c−3p, distal
(BRAF = 1; N1 = 15)
1−sided adj pval: 0.317
Frequency
Tumor−Normal Expression Difference

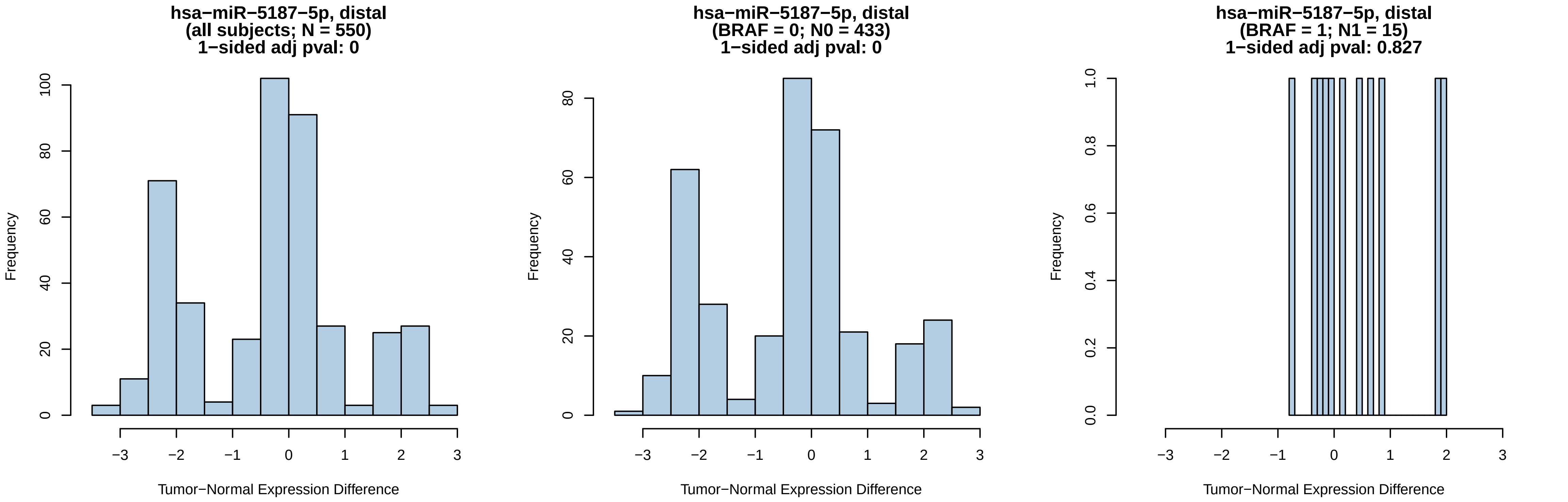
hsa−miR−5187−5p, distal
(all subjects; N = 550)
1−sided adj pval: 0
Frequency
Tumor−Normal Expression Difference
hsa−miR−5187−5p, distal
(BRAF = 0; N0 = 433)
1−sided adj pval: 0
Frequency
Tumor−Normal Expression Difference
hsa−miR−5187−5p, distal
(BRAF = 1; N1 = 15)
1−sided adj pval: 0.827
Frequency
Tumor−Normal Expression Difference

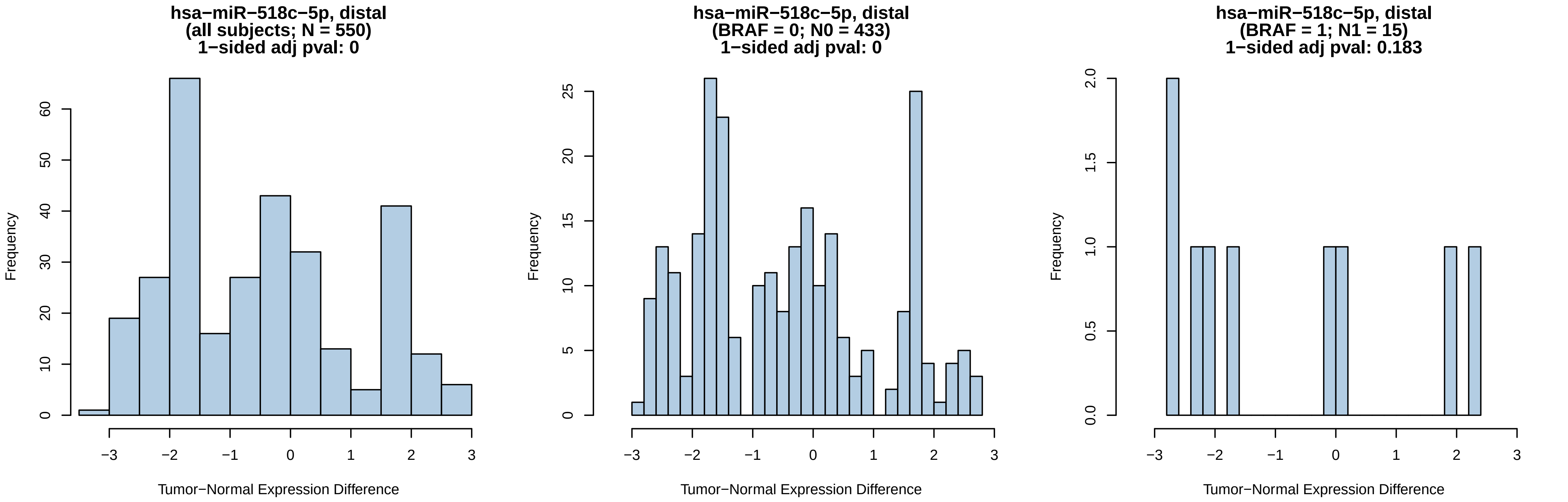
hsa−miR−518c−5p, distal
(all subjects; N = 550)
1−sided adj pval: 0
Frequency
Tumor−Normal Expression Difference
hsa−miR−518c−5p, distal
(BRAF = 0; N0 = 433)
1−sided adj pval: 0
Frequency
Tumor−Normal Expression Difference
hsa−miR−518c−5p, distal
(BRAF = 1; N1 = 15)
1−sided adj pval: 0.183
Frequency
Tumor−Normal Expression Difference

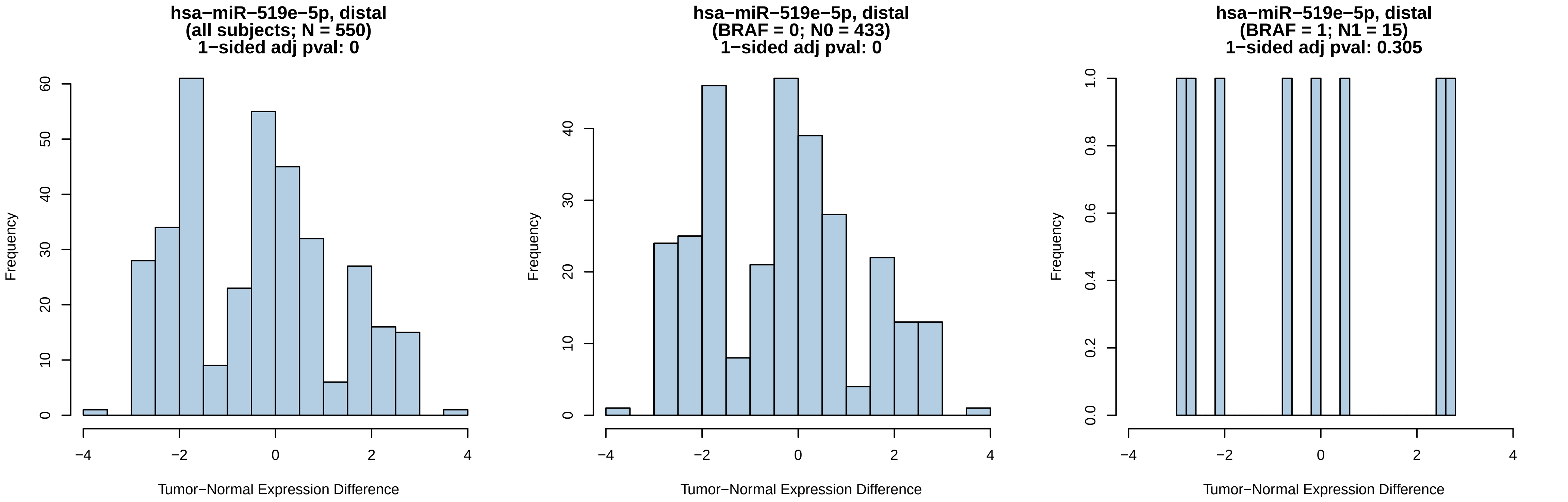

hsa−miR−519e−5p, distal
(all subjects; N = 550)
1−sided adj pval: 0
Frequency
Tumor−Normal Expression Difference
hsa−miR−519e−5p, distal
(BRAF = 0; N0 = 433)
1−sided adj pval: 0
Frequency
Tumor−Normal Expression Difference
hsa−miR−519e−5p, distal
(BRAF = 1; N1 = 15)
1−sided adj pval: 0.305
Frequency
Tumor−Normal Expression Difference

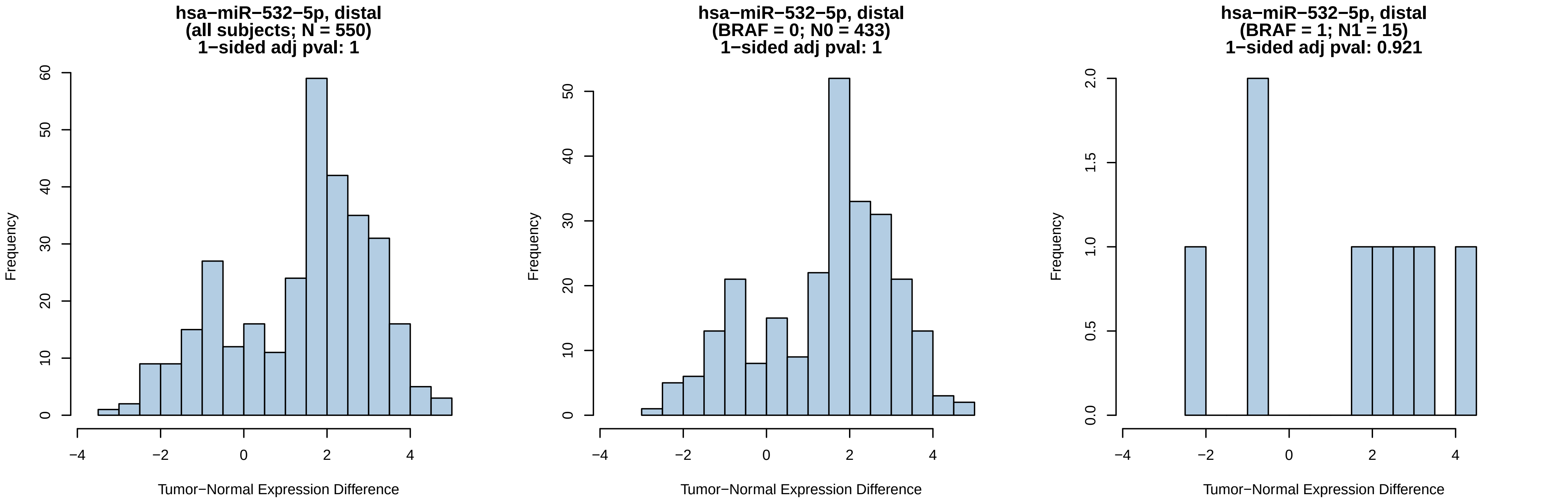
hsa−miR−532−5p, distal
(all subjects; N = 550)
1−sided adj pval: 1
Frequency
Tumor−Normal Expression Difference
hsa−miR−532−5p, distal
(BRAF = 0; N0 = 433)
1−sided adj pval: 1
Frequency
Tumor−Normal Expression Difference
hsa−miR−532−5p, distal
(BRAF = 1; N1 = 15)
1−sided adj pval: 0.921
Frequency
Tumor−Normal Expression Difference

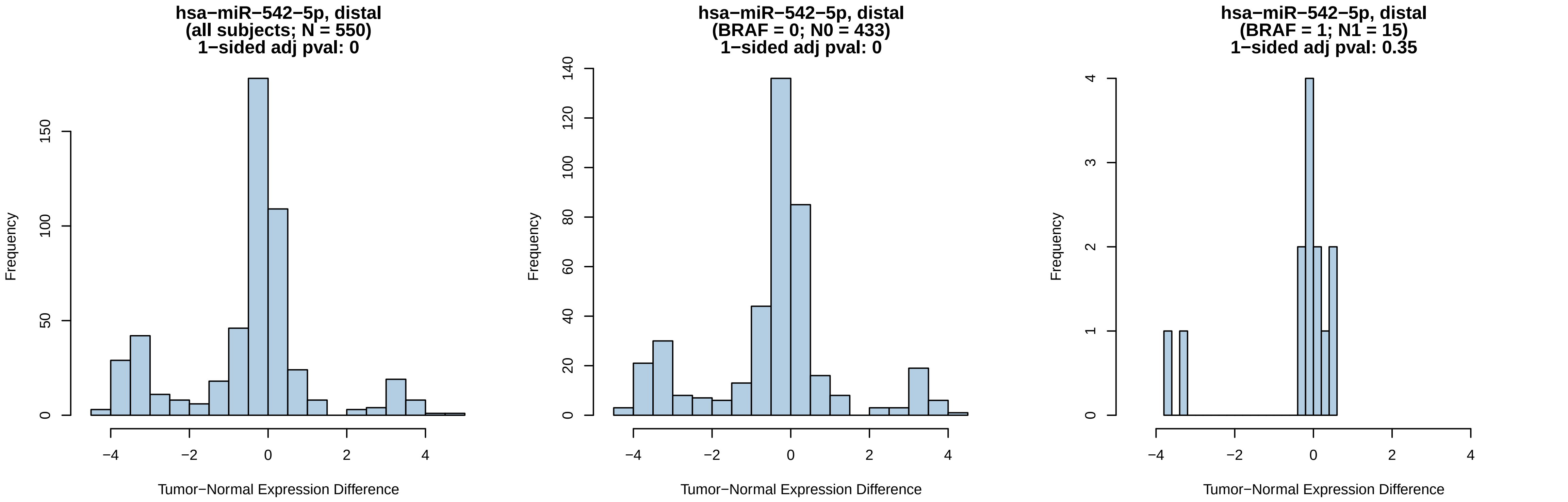

hsa−miR−542−5p, distal
(all subjects; N = 550)
1−sided adj pval: 0
hsa−miR−542−5p, distal
(BRAF = 0; N0 = 433)
1−sided adj pval: 0
hsa−miR−542−5p, distal
(BRAF = 1; N1 = 15)
1−sided adj pval: 0.35
Frequency
Tumor−Normal Expression Difference

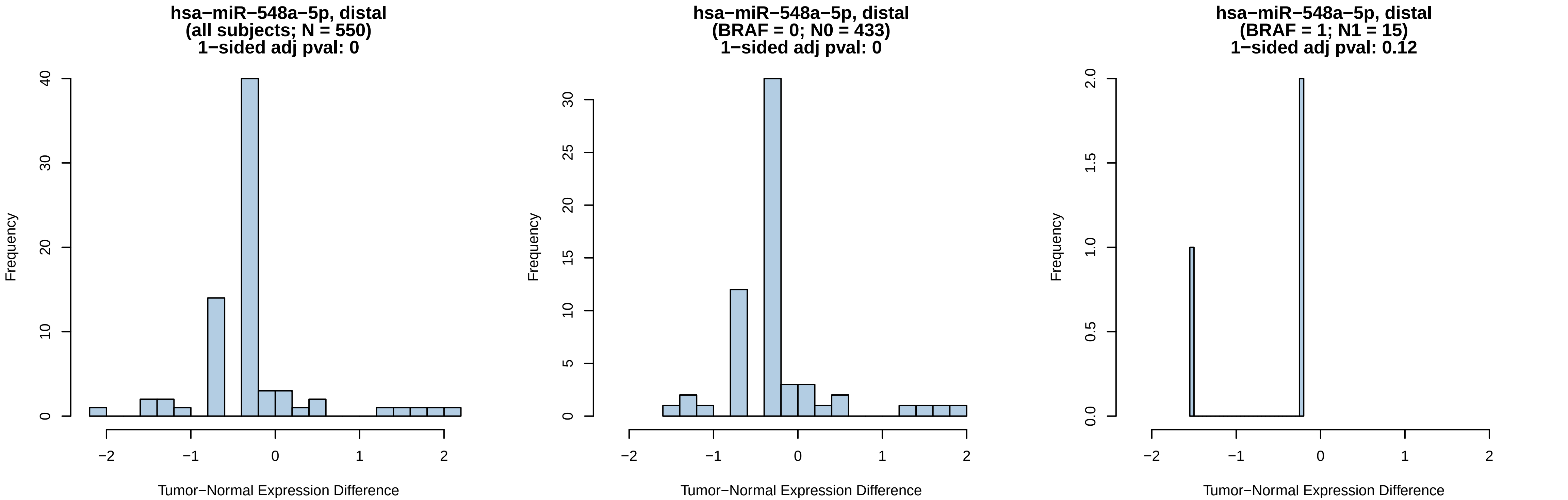

hsa−miR−548a−5p, distal
(all subjects; N = 550)
1−sided adj pval: 0
Frequency
Tumor−Normal Expression Difference
hsa−miR−548a−5p, distal
(BRAF = 0; N0 = 433)
1−sided adj pval: 0
Frequency
Tumor−Normal Expression Difference
hsa−miR−548a−5p, distal
(BRAF = 1; N1 = 15)
1−sided adj pval: 0.12
Frequency
Tumor−Normal Expression Difference

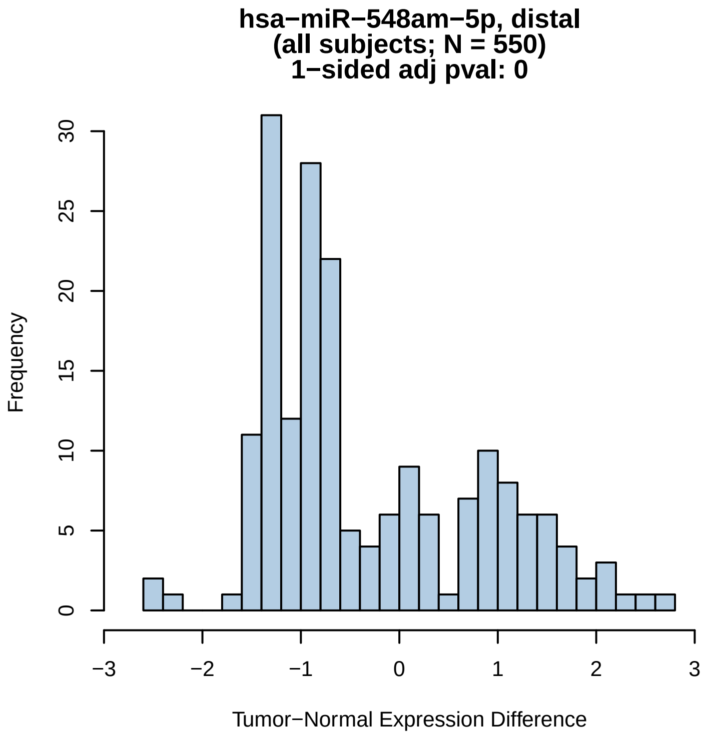

hsa−miR−548am−5p, distal
(all subjects; N = 550)
1−sided adj pval: 0
Frequency
Tumor−Normal Expression Difference


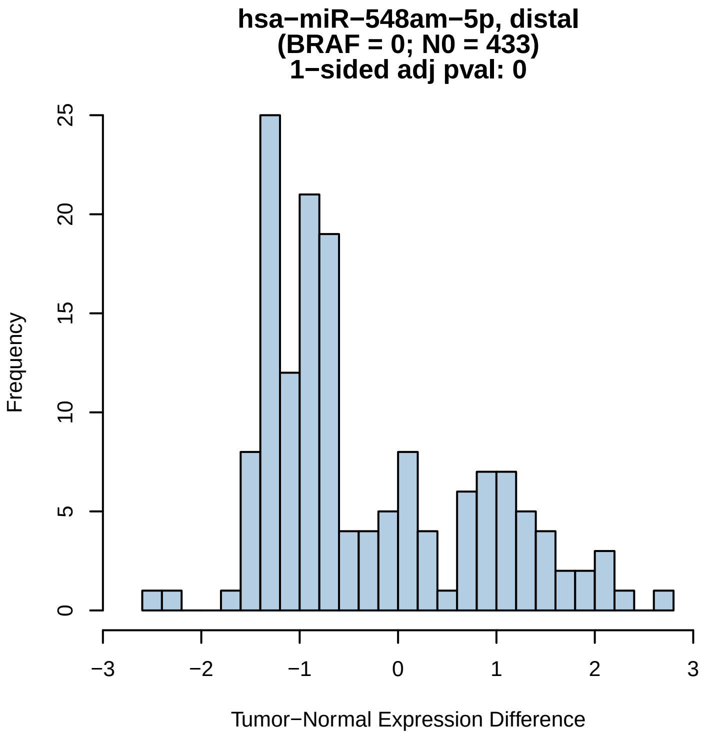

hsa−miR−548am−5p, distal
(BRAF = 0; N0 = 433)
1−sided adj pval: 0
Frequency
Tumor−Normal Expression Difference


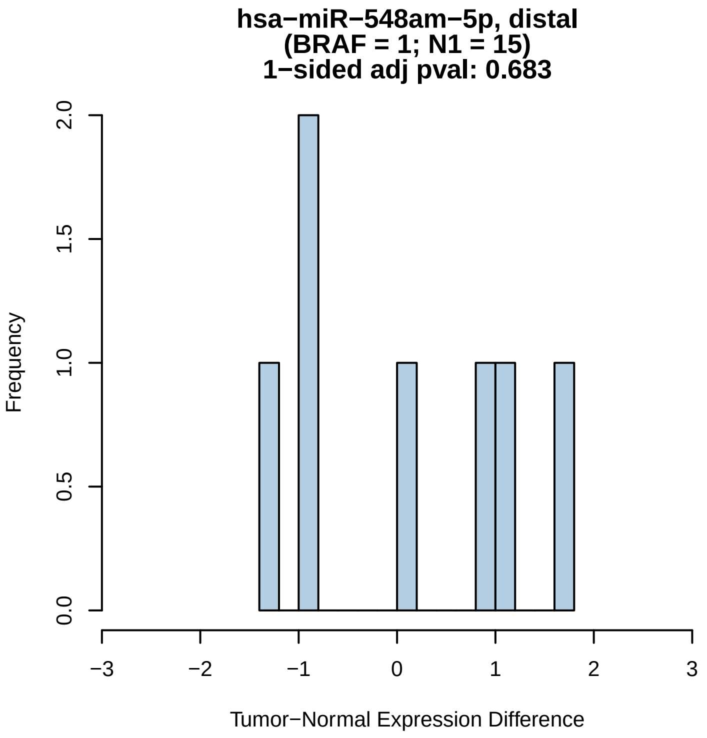

hsa−miR−548am−5p, distal
(BRAF = 1; N1 = 15)
1−sided adj pval: 0.683
Frequency
Tumor−Normal Expression Difference

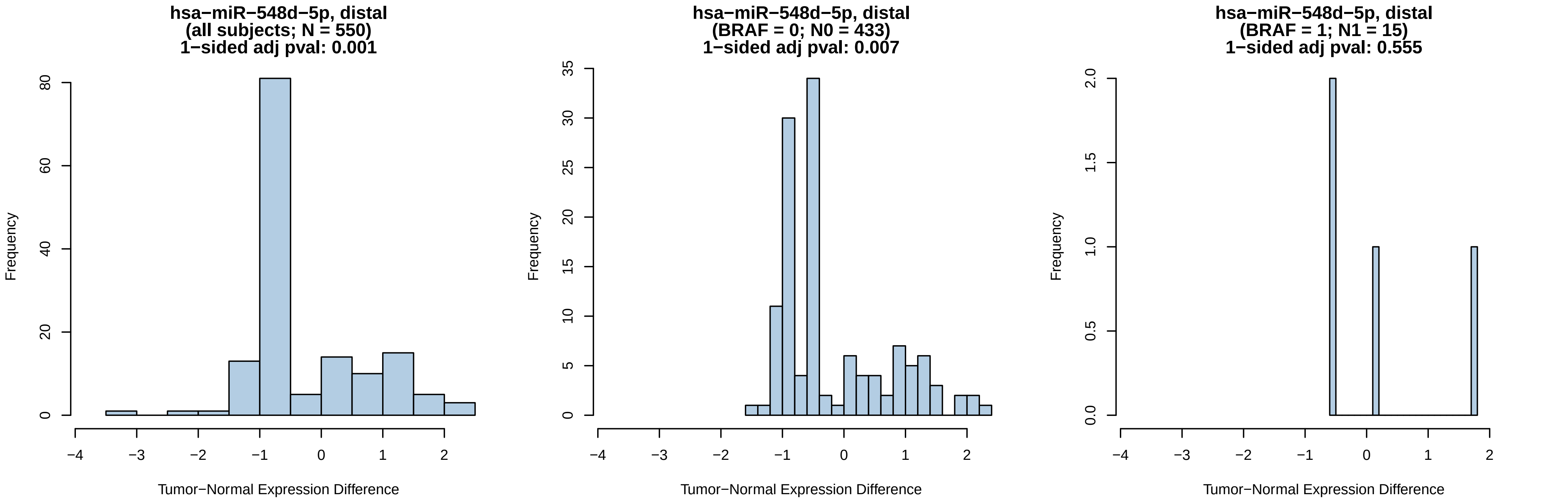
hsa−miR−548d−5p, distal
(all subjects; N = 550)
1−sided adj pval: 0.001
Frequency
Tumor−Normal Expression Difference
hsa−miR−548d−5p, distal
(BRAF = 0; N0 = 433)
1−sided adj pval: 0.007
Frequency
Tumor−Normal Expression Difference
hsa−miR−548d−5p, distal
(BRAF = 1; N1 = 15)
1−sided adj pval: 0.555
Frequency
Tumor−Normal Expression Difference

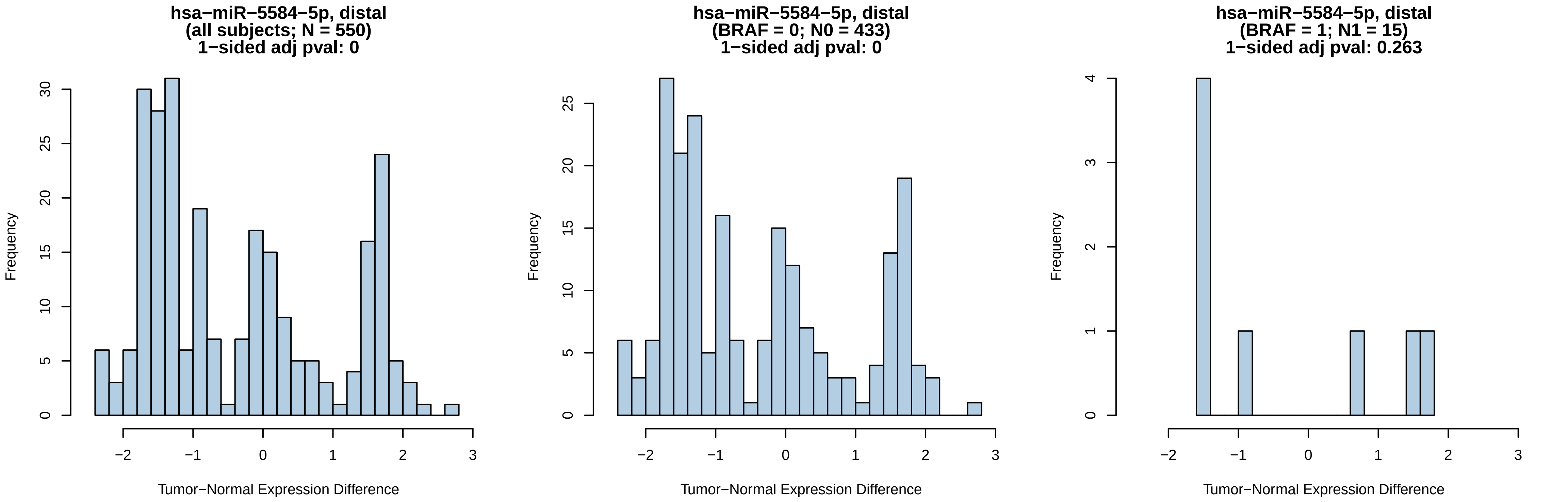
hsa−miR−5584−5p, distal
(all subjects; N = 550)
1−sided adj pval: 0
Frequency
Tumor−Normal Expression Difference
hsa−miR−5584−5p, distal
(BRAF = 0; N0 = 433)
1−sided adj pval: 0
Frequency
Tumor−Normal Expression Difference
hsa−miR−5584−5p, distal
(BRAF = 1; N1 = 15)
1−sided adj pval: 0.263
Frequency
Tumor−Normal Expression Difference

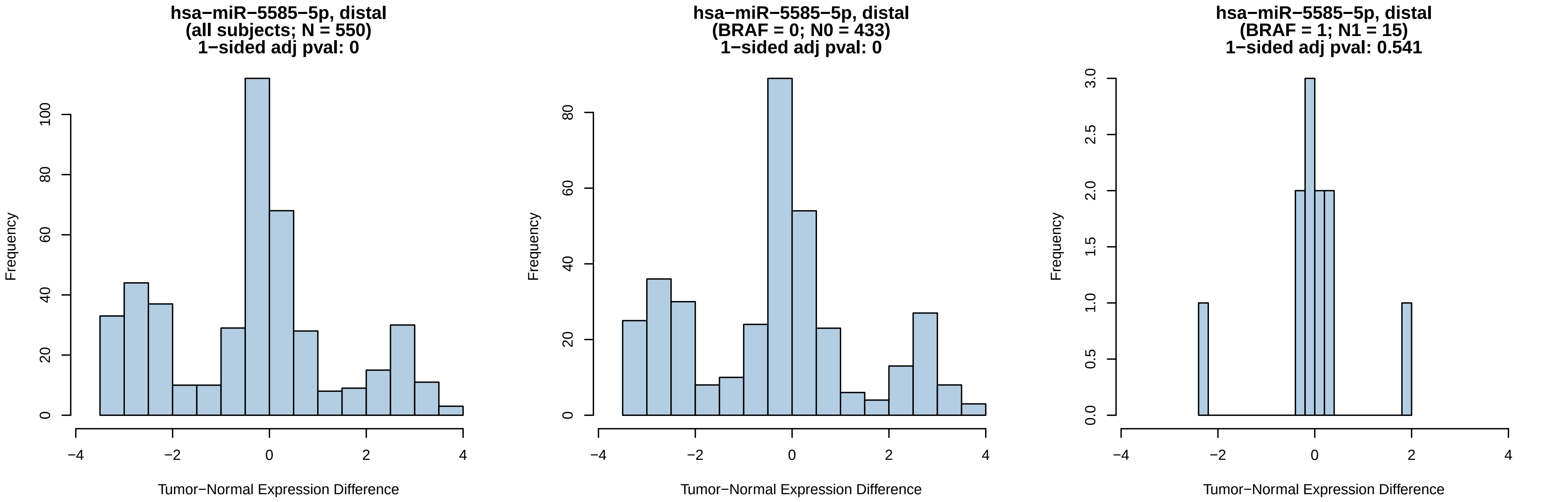
hsa−miR−5585−5p, distal
(all subjects; N = 550)
1−sided adj pval: 0
hsa−miR−5585−5p, distal
(BRAF = 0; N0 = 433)
1−sided adj pval: 0
hsa−miR−5585−5p, distal
(BRAF = 1; N1 = 15)
1−sided adj pval: 0.541
Frequency
Tumor−Normal Expression Difference

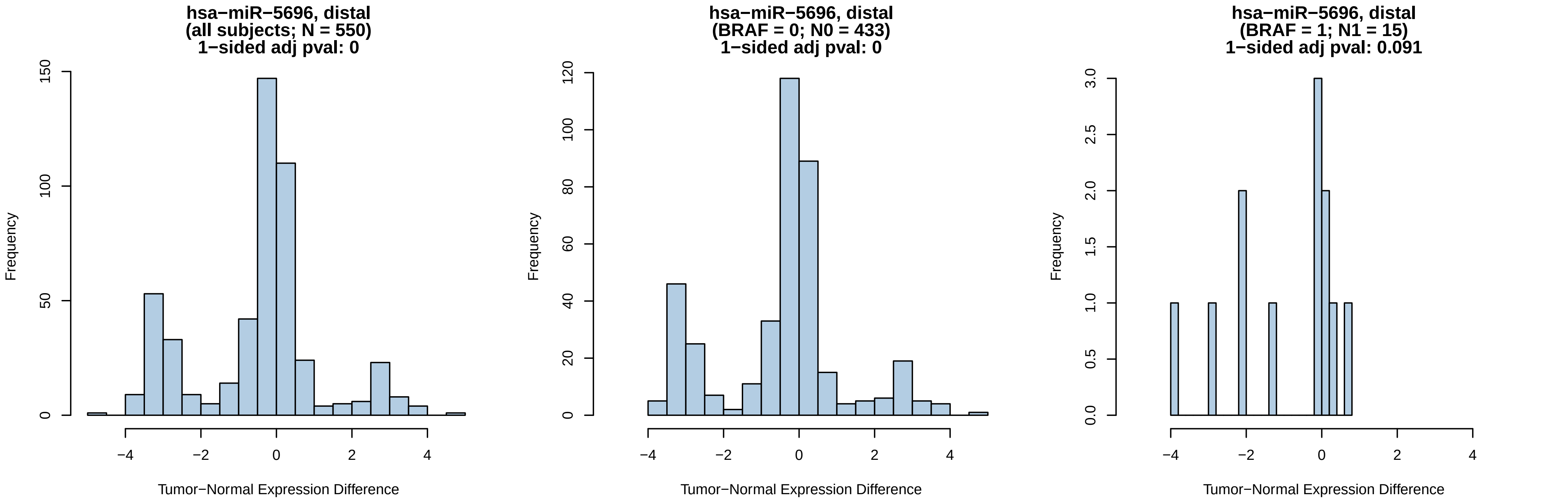
hsa−miR−5696, distal
(all subjects; N = 550)
1−sided adj pval: 0
Frequency
Tumor−Normal Expression Difference
hsa−miR−5696, distal
(BRAF = 0; N0 = 433)
1−sided adj pval: 0
Frequency
Tumor−Normal Expression Difference
hsa−miR−5696, distal
(BRAF = 1; N1 = 15)
1−sided adj pval: 0.091
Frequency
Tumor−Normal Expression Difference

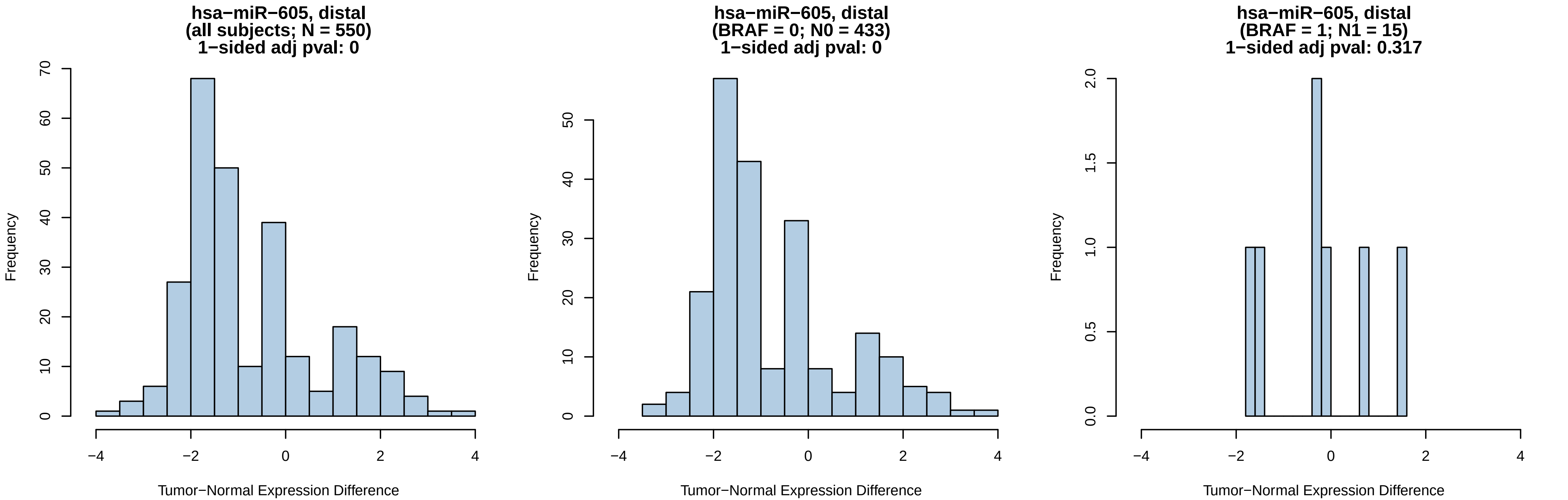

hsa−miR−605, distal
(all subjects; N = 550)
1−sided adj pval: 0
hsa−miR−605, distal
(BRAF = 0; N0 = 433)
1−sided adj pval: 0
hsa−miR−605, distal
(BRAF = 1; N1 = 15)
1−sided adj pval: 0.317
Frequency
Tumor−Normal Expression Difference

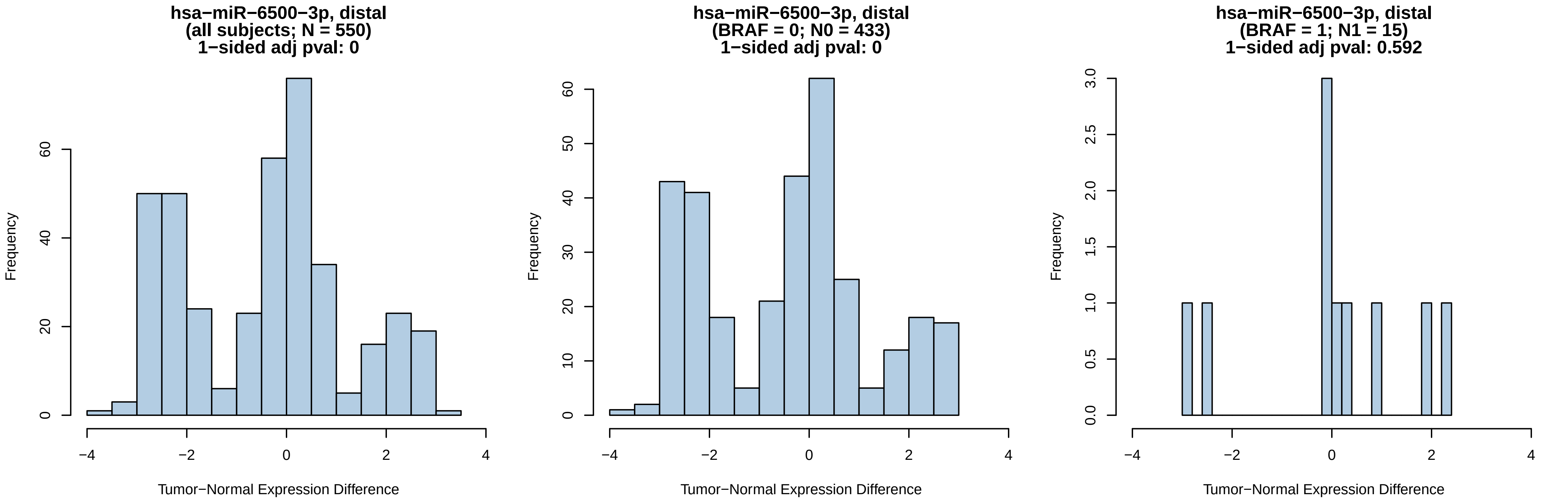

hsa−miR−6500−3p, distal
(all subjects; N = 550)
1−sided adj pval: 0
Frequency
Tumor−Normal Expression Difference
hsa−miR−6500−3p, distal
(BRAF = 0; N0 = 433)
1−sided adj pval: 0
Frequency
Tumor−Normal Expression Difference
hsa−miR−6500−3p, distal
(BRAF = 1; N1 = 15)
1−sided adj pval: 0.592
Frequency
Tumor−Normal Expression Difference

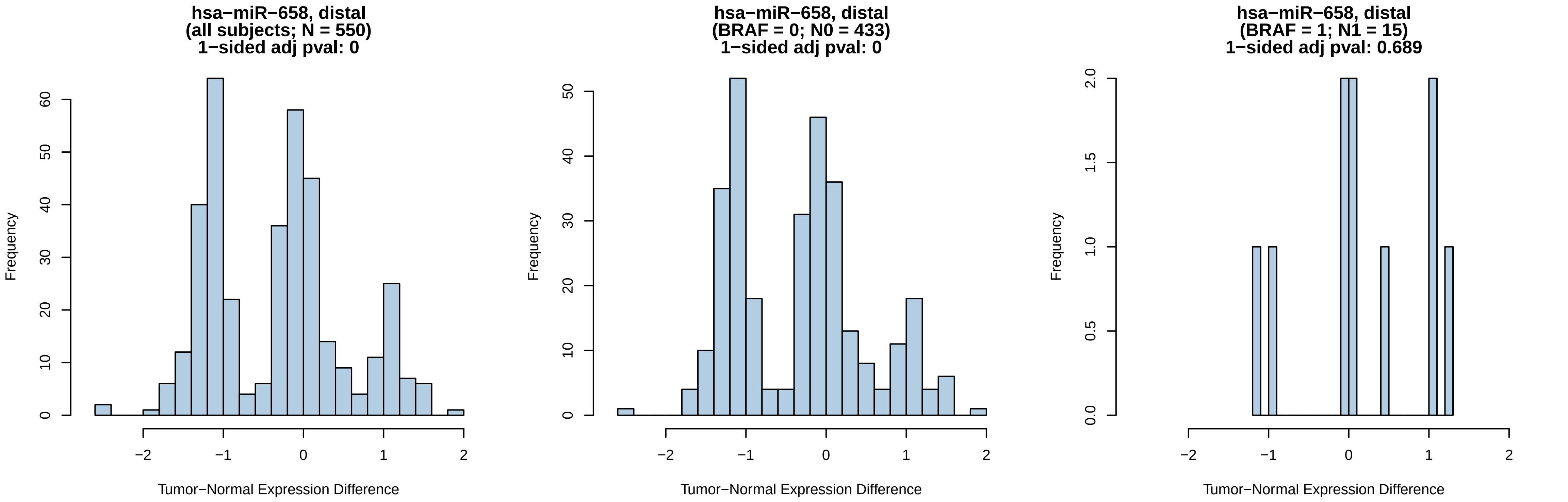
hsa−miR−658, distal
(all subjects; N = 550)
1−sided adj pval: 0
Frequency
0
10
20
30
40
50
60
−2
−1
0
1
2
Tumor−Normal Expression Difference
hsa−miR−658, distal
(BRAF = 0; N0 = 433)
1−sided adj pval: 0
Frequency
0
10
20
30
40
50
−2
−1
0
1
2
Tumor−Normal Expression Difference
hsa−miR−658, distal
(BRAF = 1; N1 = 15)
1−sided adj pval: 0.689
Frequency
0.0
0.5
1.0
1.5
2.0
−2
−1
0
1
2
Tumor−Normal Expression Difference

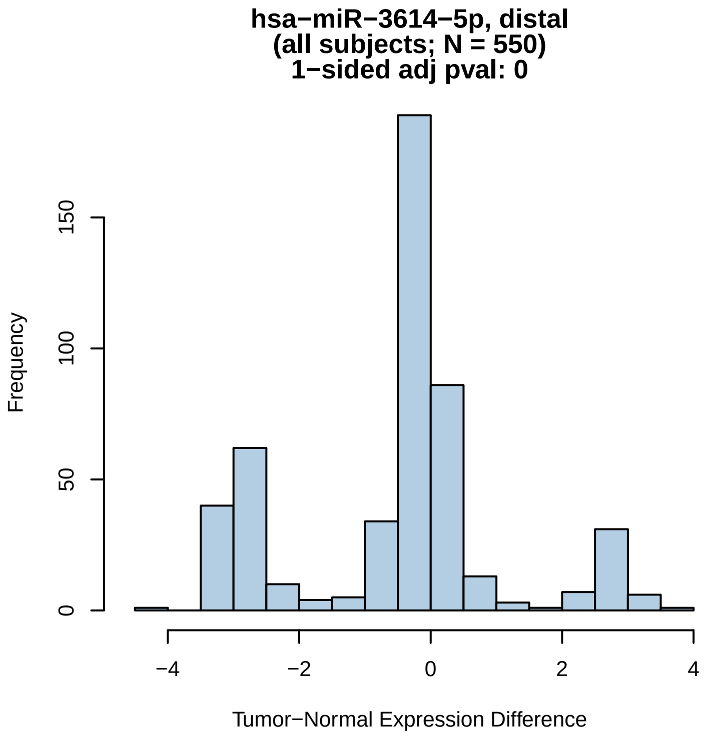

hsa−miR−3614−5p, distal
(all subjects; N = 550)
1−sided adj pval: 0
Frequency
Tumor−Normal Expression Difference


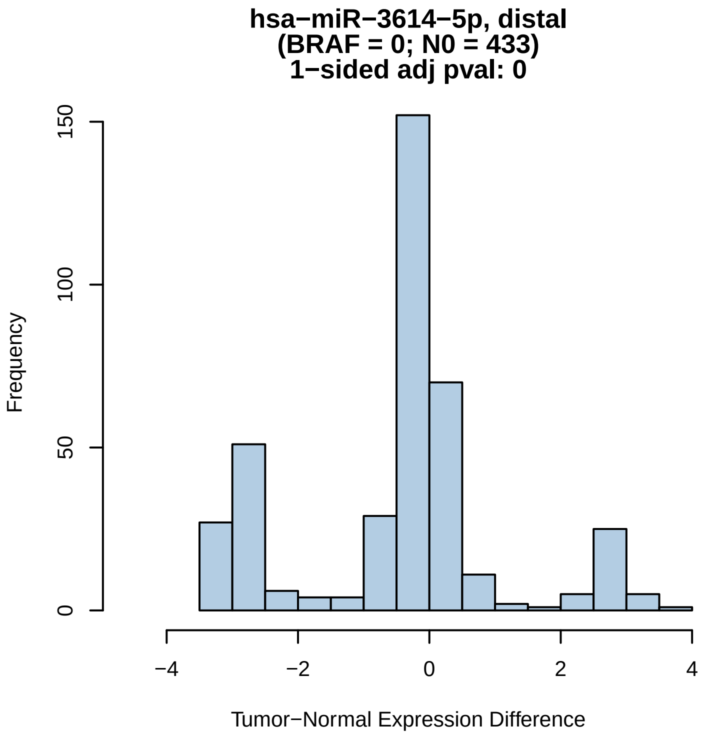

hsa−miR−3614−5p, distal
(BRAF = 0; N0 = 433)
1−sided adj pval: 0
Frequency
Tumor−Normal Expression Difference


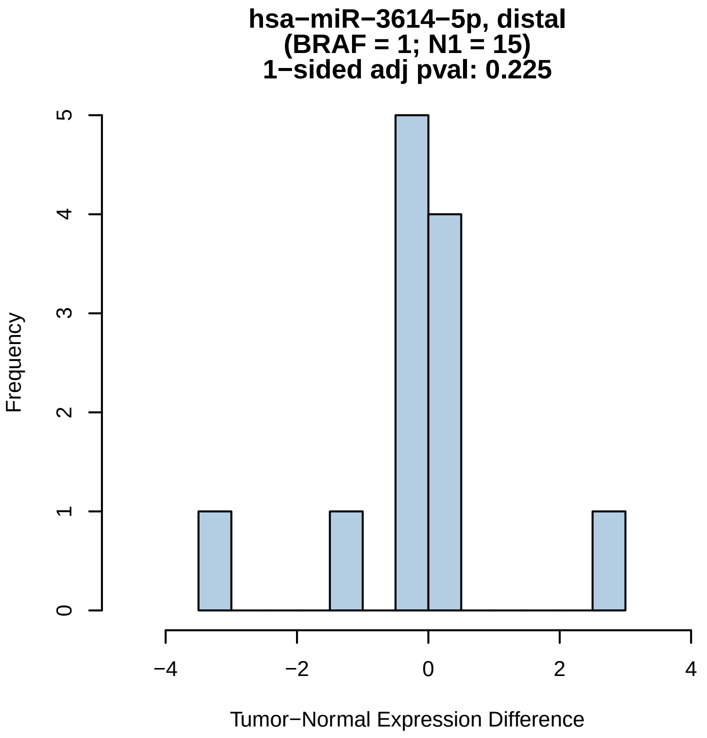

hsa−miR−3614−5p, distal
(BRAF = 1; N1 = 15)
1−sided adj pval: 0.225
Frequency
Tumor−Normal Expression Difference

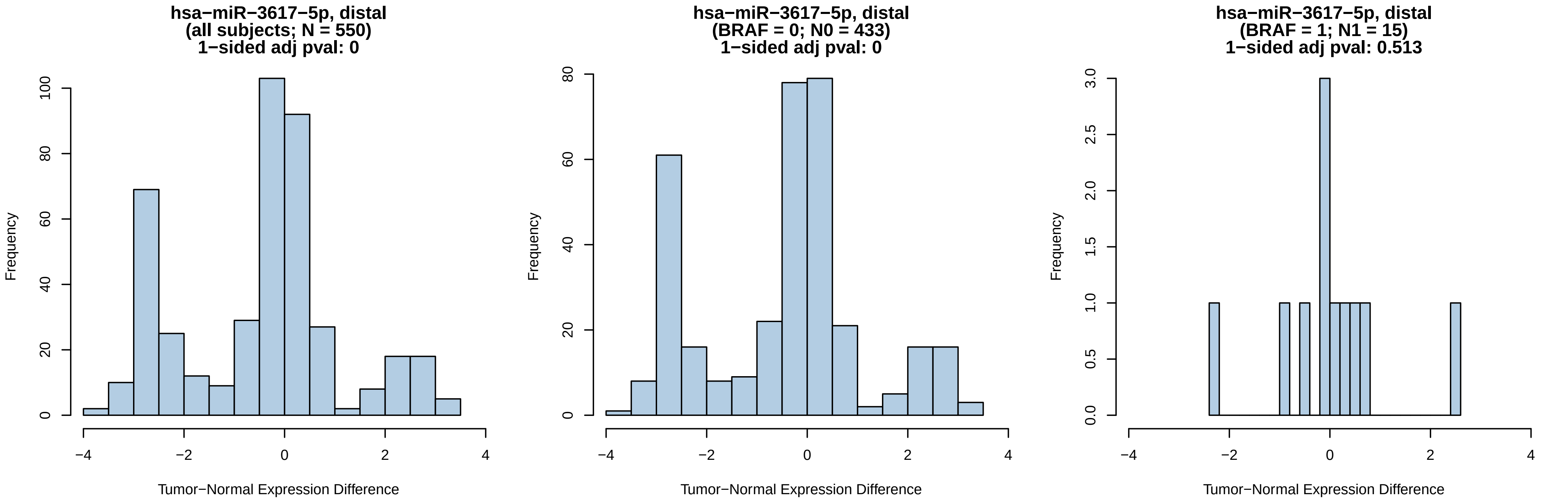

hsa−miR−3617−5p, distal
(all subjects; N = 550)
1−sided adj pval: 0
Frequency
Tumor−Normal Expression Difference
hsa−miR−3617−5p, distal
(BRAF = 0; N0 = 433)
1−sided adj pval: 0
Frequency
Tumor−Normal Expression Difference
hsa−miR−3617−5p, distal
(BRAF = 1; N1 = 15)
1−sided adj pval: 0.513
Frequency
Tumor−Normal Expression Difference

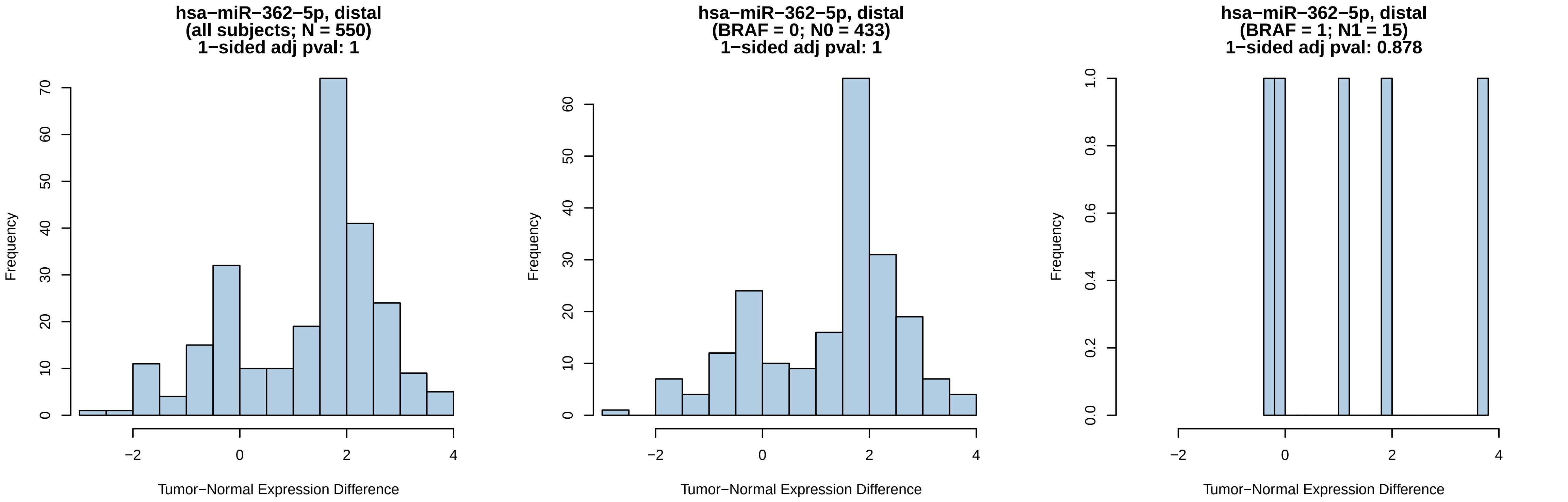
hsa−miR−362−5p, distal
(all subjects; N = 550)
1−sided adj pval: 1
Frequency
Tumor−Normal Expression Difference
hsa−miR−362−5p, distal
(BRAF = 0; N0 = 433)
1−sided adj pval: 1
Frequency
Tumor−Normal Expression Difference
hsa−miR−362−5p, distal
(BRAF = 1; N1 = 15)
1−sided adj pval: 0.878
Frequency
Tumor−Normal Expression Difference

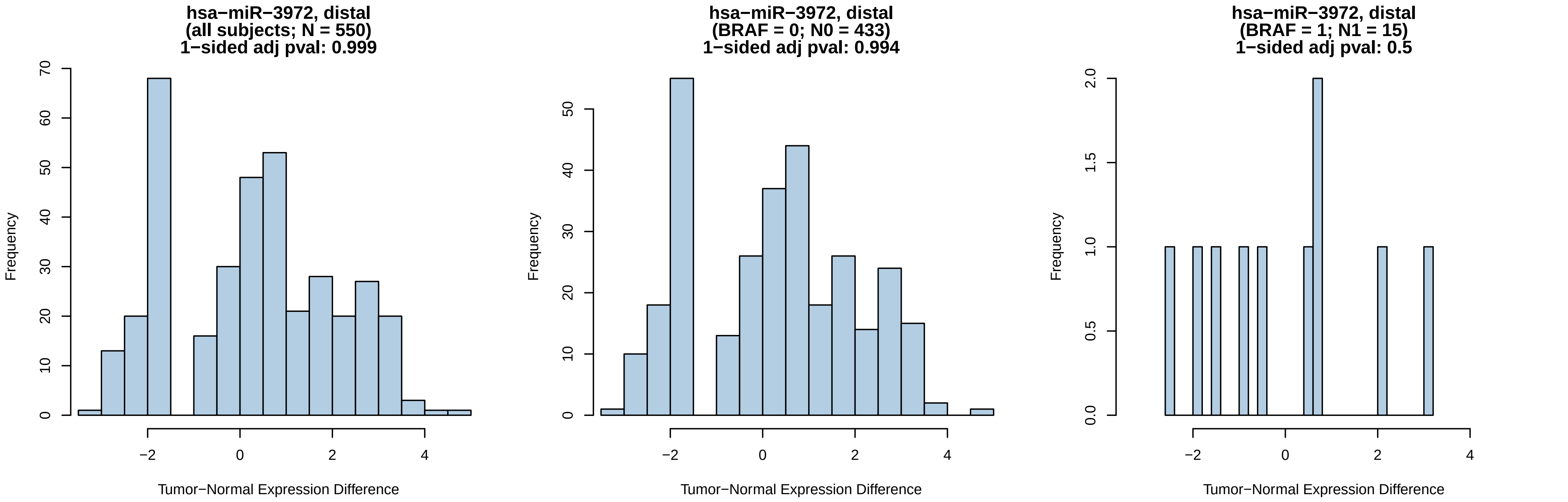
hsa−miR−3972, distal
(all subjects; N = 550)
1−sided adj pval: 0.999
Frequency
Tumor−Normal Expression Difference
hsa−miR−3972, distal
(BRAF = 0; N0 = 433)
1−sided adj pval: 0.994
Frequency
Tumor−Normal Expression Difference
hsa−miR−3972, distal
(BRAF = 1; N1 = 15)
1−sided adj pval: 0.5
Frequency
Tumor−Normal Expression Difference

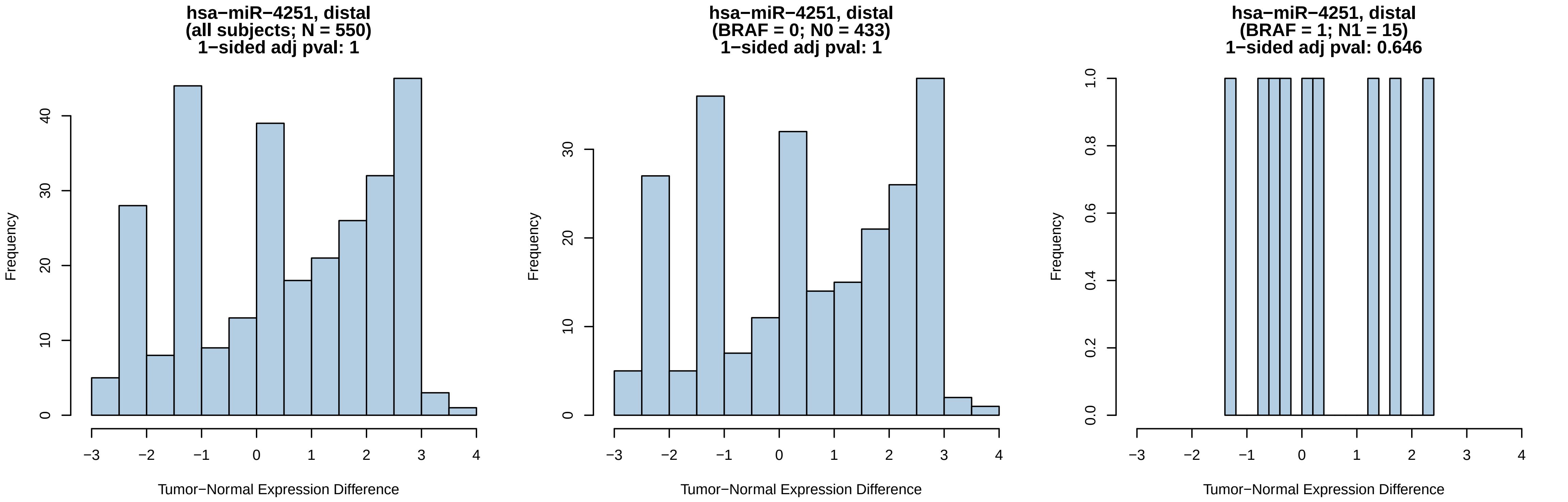
hsa−miR−4251, distal
(all subjects; N = 550)
1−sided adj pval: 1
Frequency
0
10
20
30
40
−3
−2
−1
0
1
2
3
4
Tumor−Normal Expression Difference
hsa−miR−4251, distal
(BRAF = 0; N0 = 433)
1−sided adj pval: 1
Frequency
0
10
20
30
−3
−2
−1
0
1
2
3
4
Tumor−Normal Expression Difference
hsa−miR−4251, distal
(BRAF = 1; N1 = 15)
1−sided adj pval: 0.646
Frequency
0.0
0.2
0.4
0.6
0.8
1.0
−3
−2
−1
0
1
2
3
4
Tumor−Normal Expression Difference

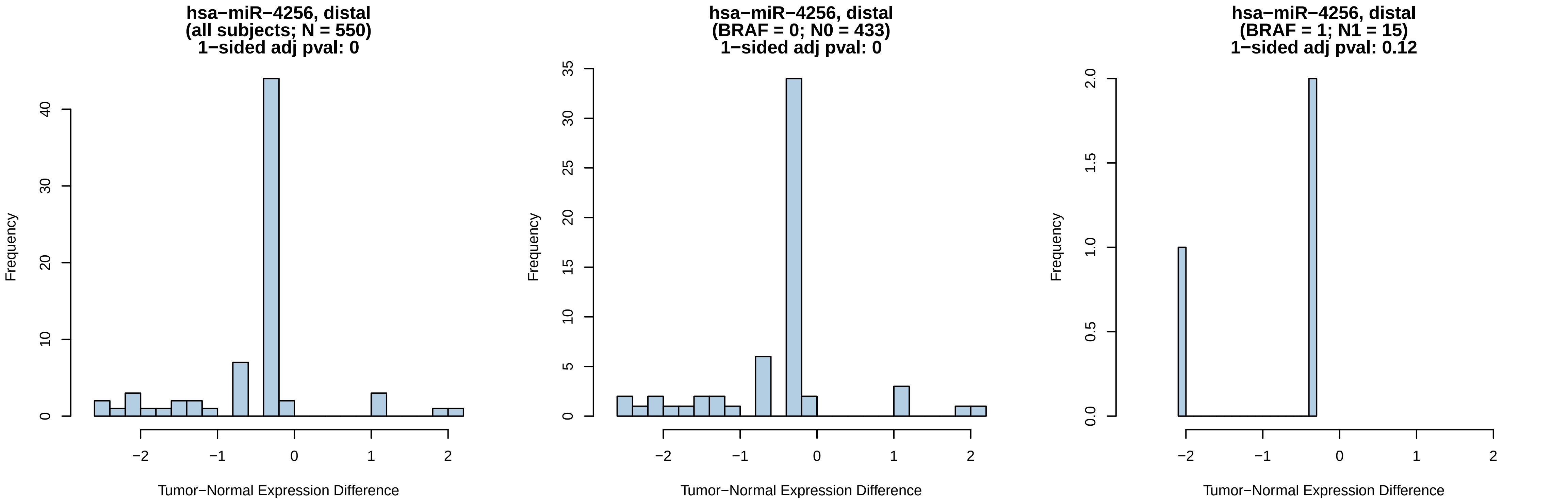
hsa−miR−4256, distal
(all subjects; N = 550)
1−sided adj pval: 0
Frequency
Tumor−Normal Expression Difference
hsa−miR−4256, distal
(BRAF = 0; N0 = 433)
1−sided adj pval: 0
Frequency
Tumor−Normal Expression Difference
hsa−miR−4256, distal
(BRAF = 1; N1 = 15)
1−sided adj pval: 0.12
Frequency
Tumor−Normal Expression Difference

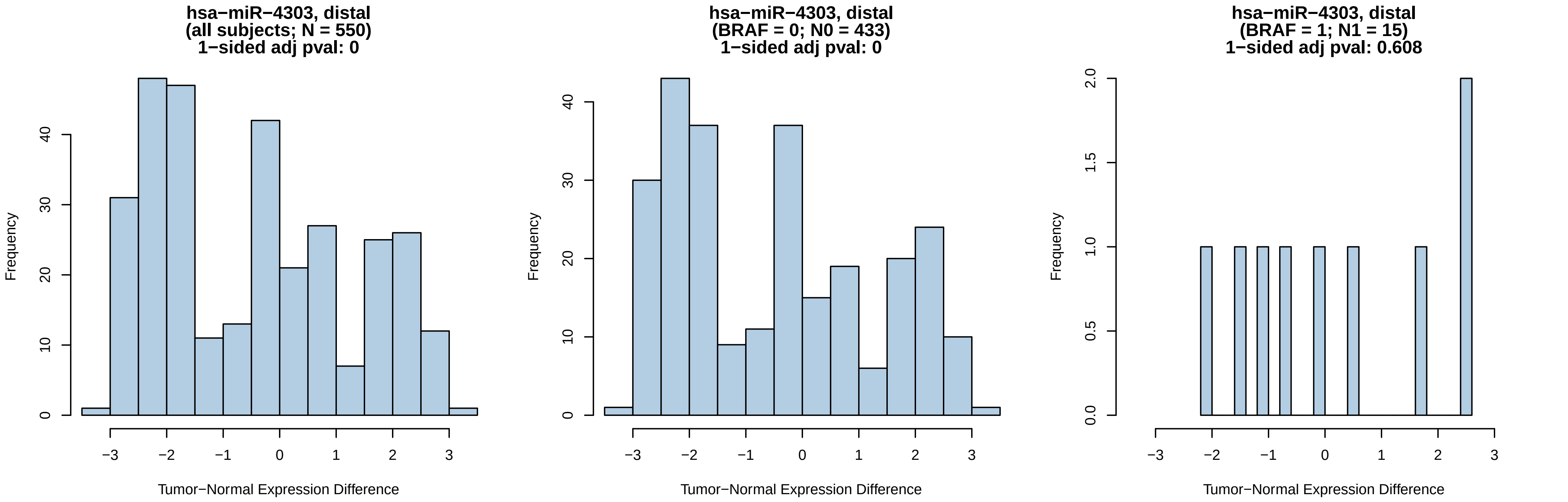
hsa−miR−4303, distal
(all subjects; N = 550)
1−sided adj pval: 0
Frequency
Tumor−Normal Expression Difference
hsa−miR−4303, distal
(BRAF = 0; N0 = 433)
1−sided adj pval: 0
Frequency
Tumor−Normal Expression Difference
hsa−miR−4303, distal
(BRAF = 1; N1 = 15)
1−sided adj pval: 0.608
Frequency
Tumor−Normal Expression Difference

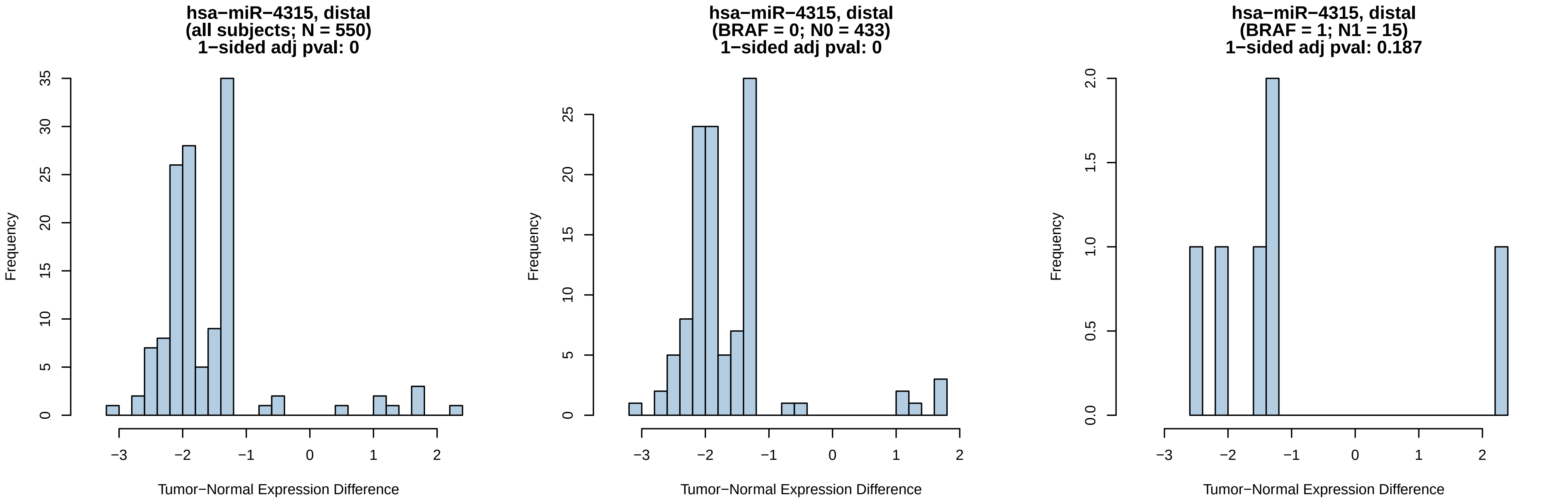
hsa−miR−4315, distal
(all subjects; N = 550)
1−sided adj pval: 0
hsa−miR−4315, distal
(BRAF = 0; N0 = 433)
1−sided adj pval: 0
hsa−miR−4315, distal
(BRAF = 1; N1 = 15)
1−sided adj pval: 0.187
Frequency
Tumor−Normal Expression Difference

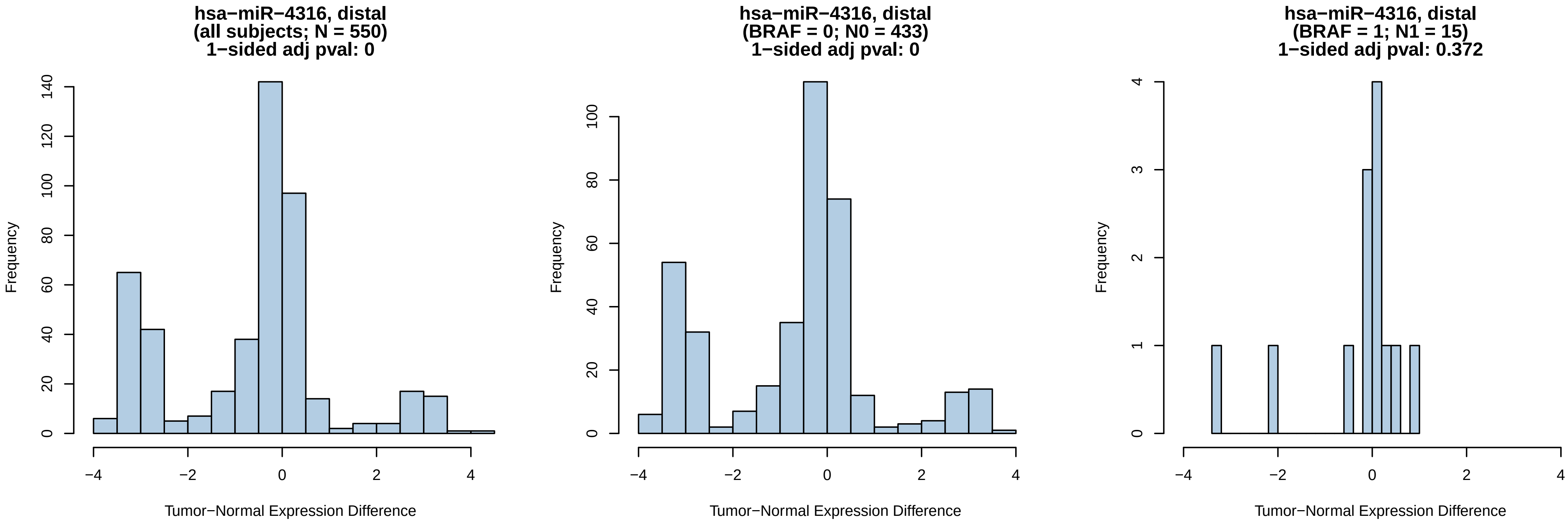
hsa−miR−4316, distal
(all subjects; N = 550)
1−sided adj pval: 0
Frequency
0
20
40
60
80
100
120
140
−4
−2
0
2
4
Tumor−Normal Expression Difference
hsa−miR−4316, distal
(BRAF = 0; N0 = 433)
1−sided adj pval: 0
Frequency
0
20
40
60
80
100
−4
−2
0
2
4
Tumor−Normal Expression Difference
hsa−miR−4316, distal
(BRAF = 1; N1 = 15)
1−sided adj pval: 0.372
Frequency
0
1
2
3
4
−4
−2
0
2
4
Tumor−Normal Expression Difference

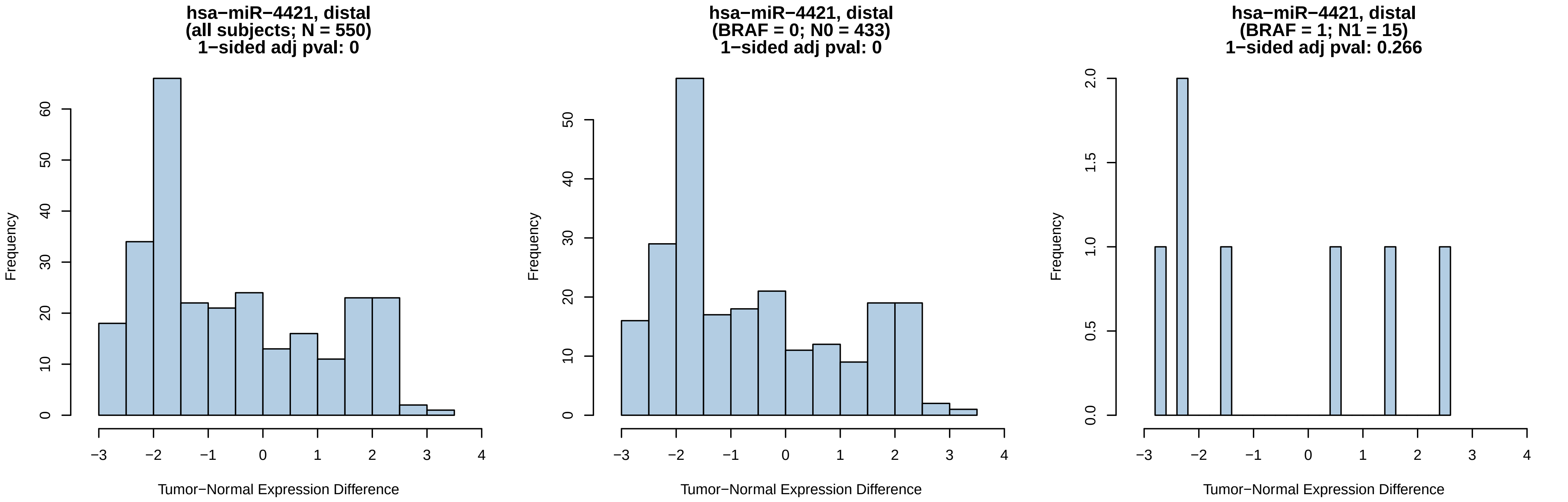
hsa−miR−4421, distal
(all subjects; N = 550)
1−sided adj pval: 0
hsa−miR−4421, distal
(BRAF = 0; N0 = 433)
1−sided adj pval: 0
hsa−miR−4421, distal
(BRAF = 1; N1 = 15)
1−sided adj pval: 0.266
Frequency
Tumor−Normal Expression Difference

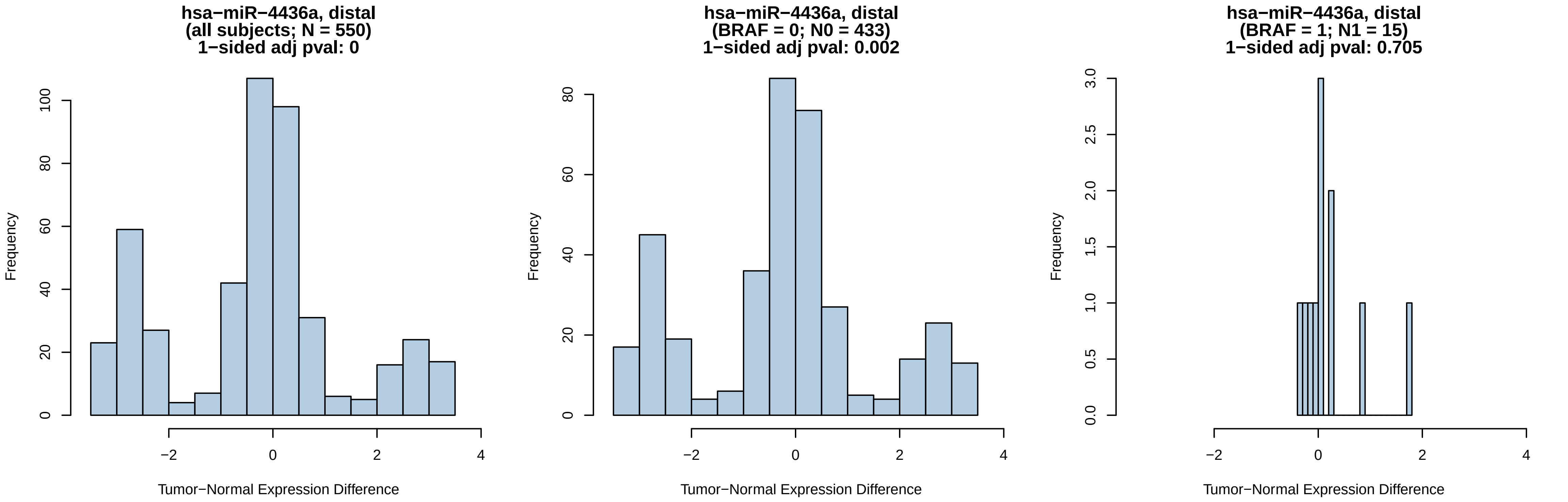

hsa−miR−4436a, distal
(all subjects; N = 550)
1−sided adj pval: 0
Frequency
Tumor−Normal Expression Difference
hsa−miR−4436a, distal
(BRAF = 0; N0 = 433)
1−sided adj pval: 0.002
Frequency
Tumor−Normal Expression Difference
hsa−miR−4436a, distal
(BRAF = 1; N1 = 15)
1−sided adj pval: 0.705
Frequency
Tumor−Normal Expression Difference

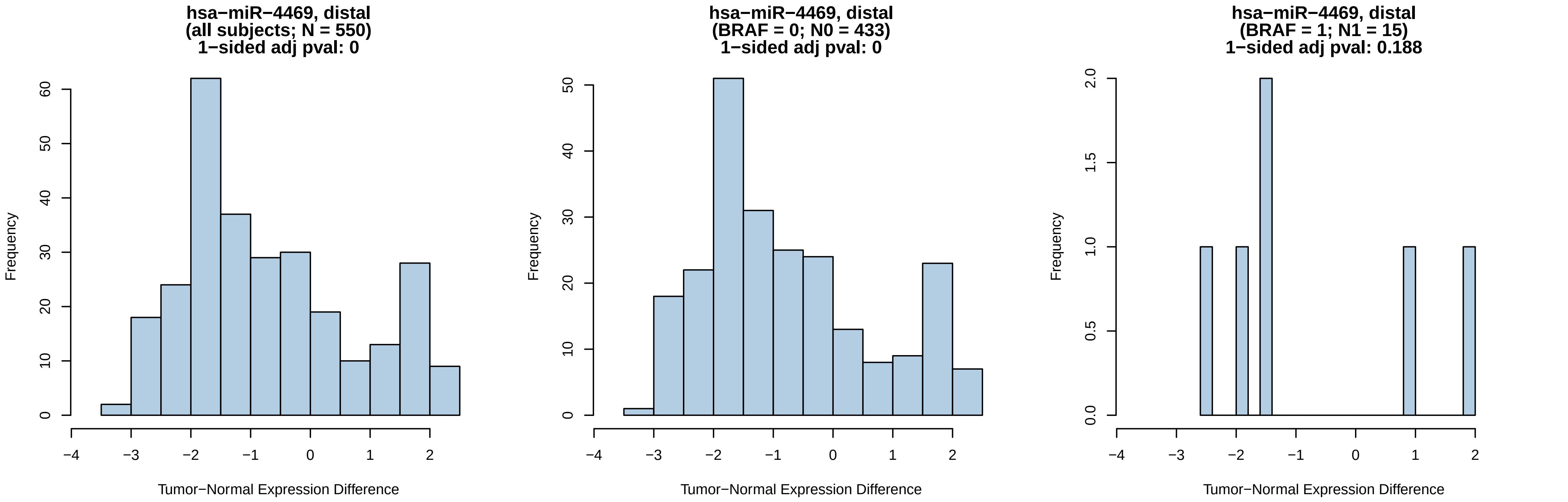

hsa−miR−4469, distal
(all subjects; N = 550)
1−sided adj pval: 0
hsa−miR−4469, distal
(BRAF = 0; N0 = 433)
1−sided adj pval: 0
hsa−miR−4469, distal
(BRAF = 1; N1 = 15)
1−sided adj pval: 0.188
Frequency
Tumor−Normal Expression Difference

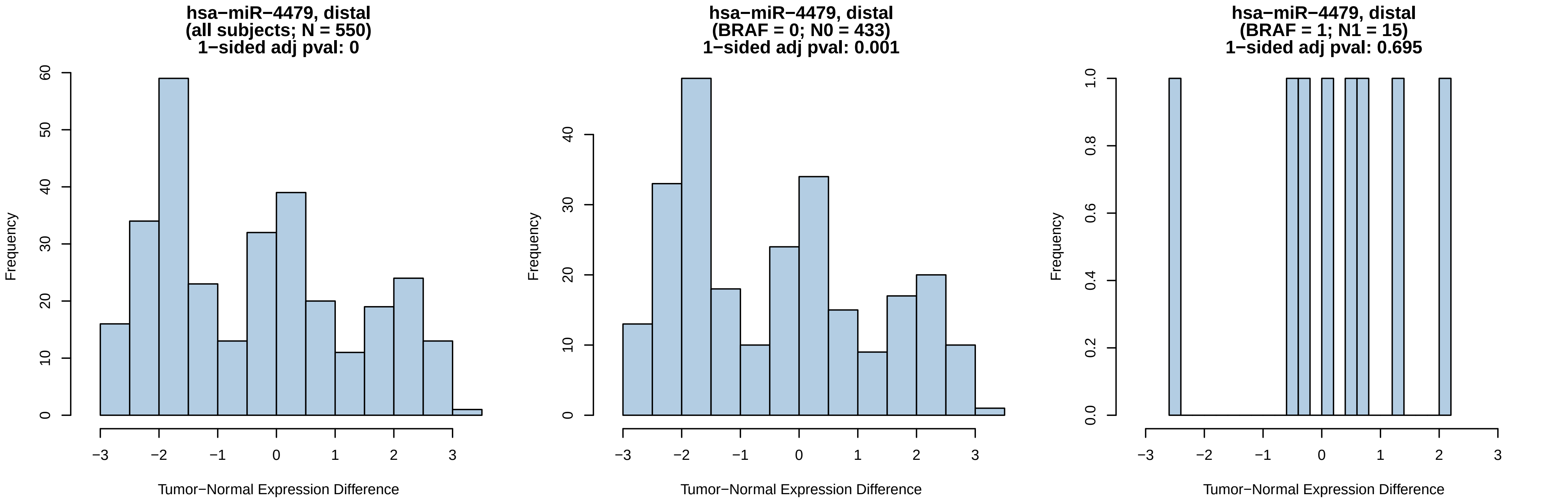
hsa−miR−4479, distal
(all subjects; N = 550)
1−sided adj pval: 0
hsa−miR−4479, distal
(BRAF = 0; N0 = 433)
1−sided adj pval: 0.001
hsa−miR−4479, distal
(BRAF = 1; N1 = 15)
1−sided adj pval: 0.695
Frequency
Tumor−Normal Expression Difference

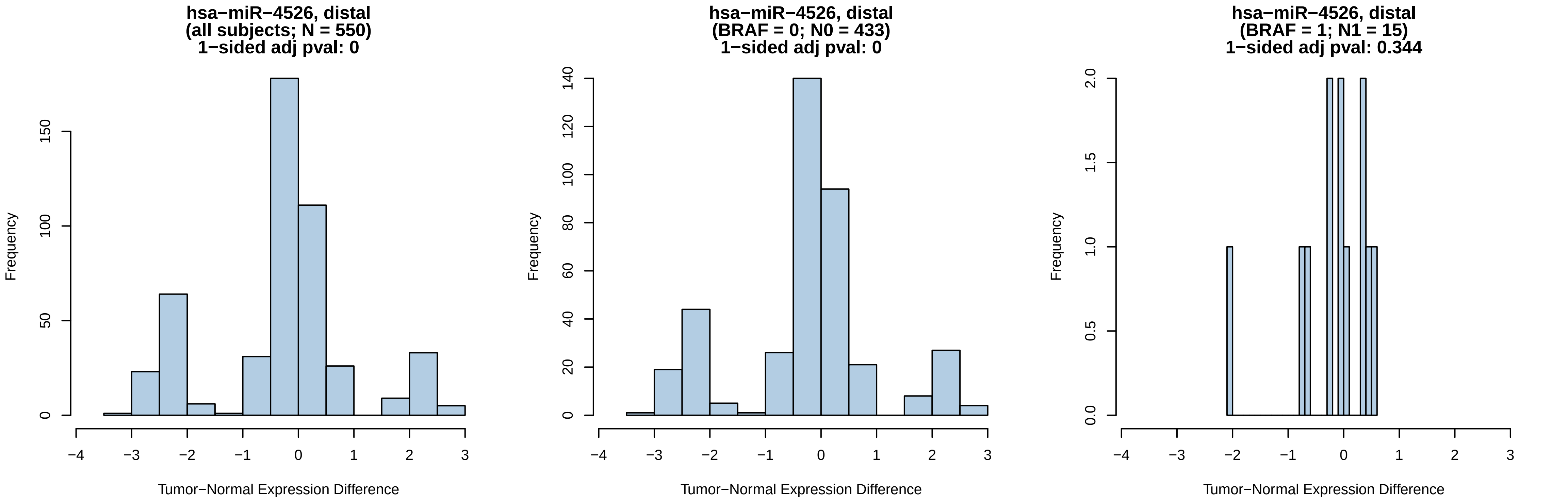
hsa−miR−4526, distal
(all subjects; N = 550)
1−sided adj pval: 0
Frequency
0
50
100
150
−4
−3
−2
−1
0
1
2
3
Tumor−Normal Expression Difference
hsa−miR−4526, distal
(BRAF = 0; N0 = 433)
1−sided adj pval: 0
Frequency
0
20
40
60
80
100
120
140
−4
−3
−2
−1
0
1
2
3
Tumor−Normal Expression Difference
hsa−miR−4526, distal
(BRAF = 1; N1 = 15)
1−sided adj pval: 0.344
Frequency
0.0
0.5
1.0
1.5
2.0
−4
−3
−2
−1
0
1
2
3
Tumor−Normal Expression Difference

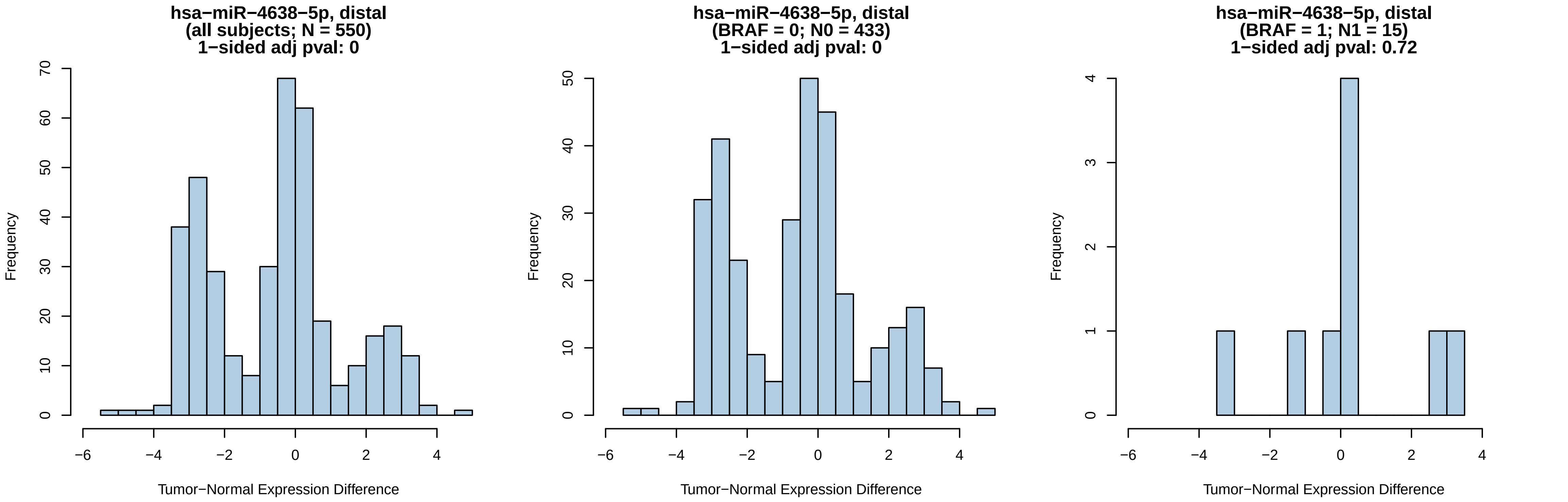
hsa−miR−4638−5p, distal
(all subjects; N = 550)
1−sided adj pval: 0
Frequency
Tumor−Normal Expression Difference
hsa−miR−4638−5p, distal
(BRAF = 0; N0 = 433)
1−sided adj pval: 0
Frequency
Tumor−Normal Expression Difference
hsa−miR−4638−5p, distal
(BRAF = 1; N1 = 15)
1−sided adj pval: 0.72
Frequency
Tumor−Normal Expression Difference

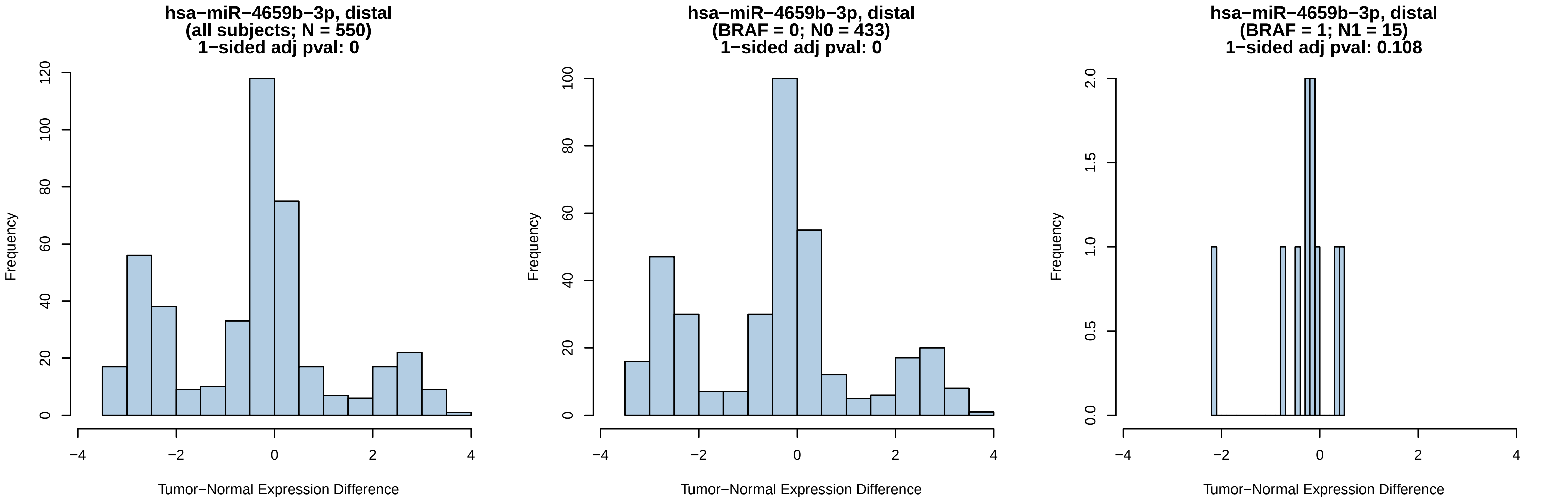
hsa−miR−4659b−3p, distal
(all subjects; N = 550)
1−sided adj pval: 0
Frequency
0
20
40
60
80
100
120
−4
−2
0
2
4
Tumor−Normal Expression Difference
hsa−miR−4659b−3p, distal
(BRAF = 0; N0 = 433)
1−sided adj pval: 0
Frequency
0
20
40
60
80
100
−4
−2
0
2
4
Tumor−Normal Expression Difference
hsa−miR−4659b−3p, distal
(BRAF = 1; N1 = 15)
1−sided adj pval: 0.108
Frequency
0.0
0.5
1.0
1.5
2.0
−4
−2
0
2
4
Tumor−Normal Expression Difference

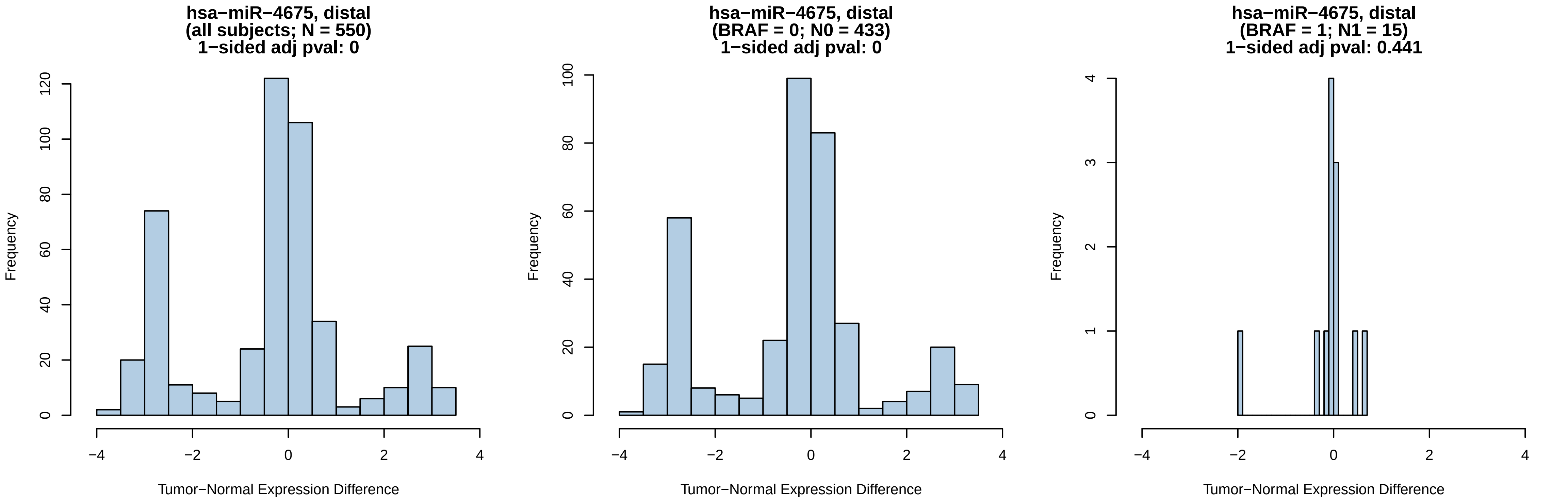

hsa−miR−4675, distal
(all subjects; N = 550)
1−sided adj pval: 0
Frequency
Tumor−Normal Expression Difference
hsa−miR−4675, distal
(BRAF = 0; N0 = 433)
1−sided adj pval: 0
Frequency
Tumor−Normal Expression Difference
hsa−miR−4675, distal
(BRAF = 1; N1 = 15)
1−sided adj pval: 0.441
Frequency
Tumor−Normal Expression Difference

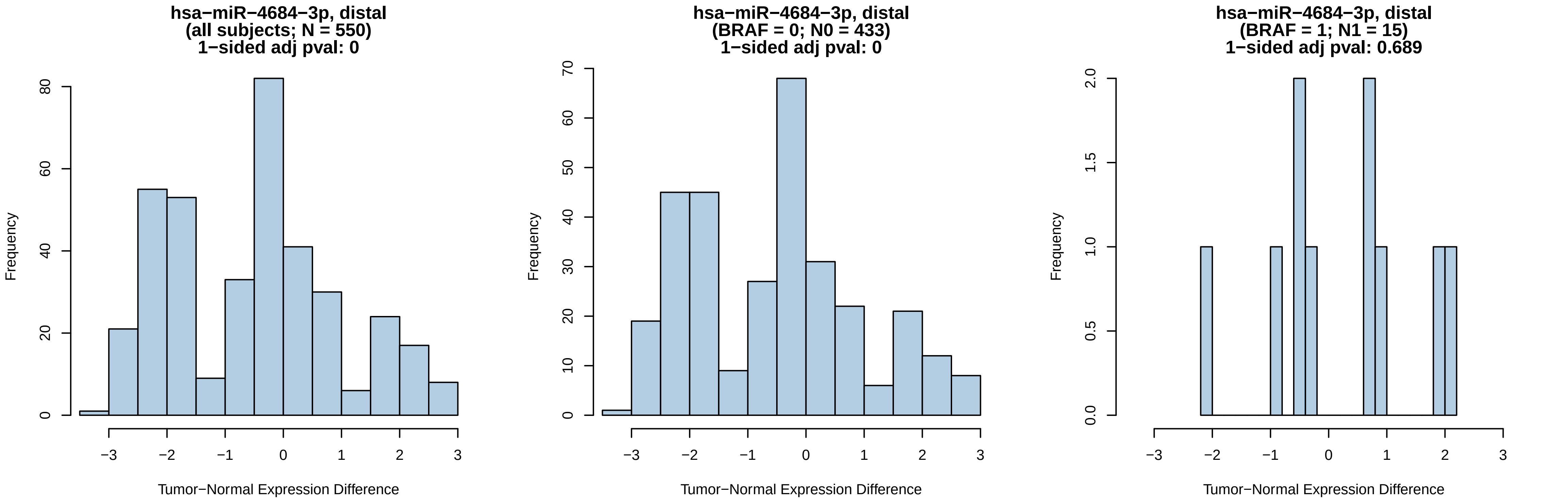
hsa−miR−4684−3p, distal
(all subjects; N = 550)
1−sided adj pval: 0
Frequency
Tumor−Normal Expression Difference
hsa−miR−4684−3p, distal
(BRAF = 0; N0 = 433)
1−sided adj pval: 0
Frequency
Tumor−Normal Expression Difference
hsa−miR−4684−3p, distal
(BRAF = 1; N1 = 15)
1−sided adj pval: 0.689
Frequency
Tumor−Normal Expression Difference

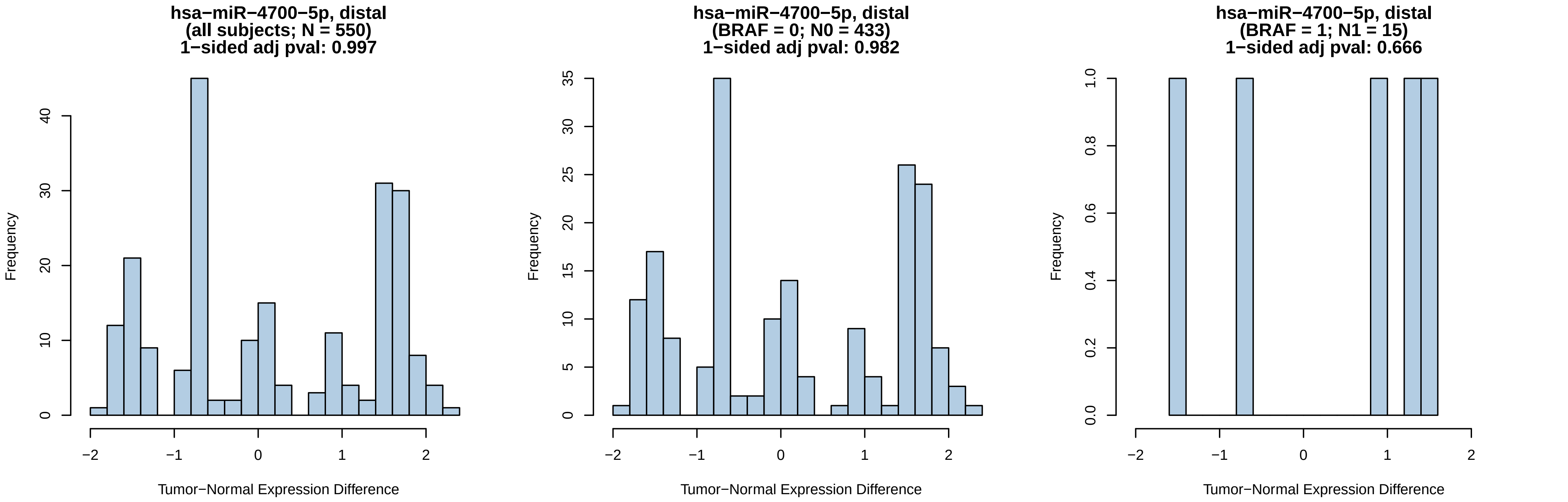

hsa−miR−4700−5p, distal
(all subjects; N = 550)
1−sided adj pval: 0.997
Frequency
Tumor−Normal Expression Difference
hsa−miR−4700−5p, distal
(BRAF = 0; N0 = 433)
1−sided adj pval: 0.982
Frequency
Tumor−Normal Expression Difference
hsa−miR−4700−5p, distal
(BRAF = 1; N1 = 15)
1−sided adj pval: 0.666
Frequency
Tumor−Normal Expression Difference

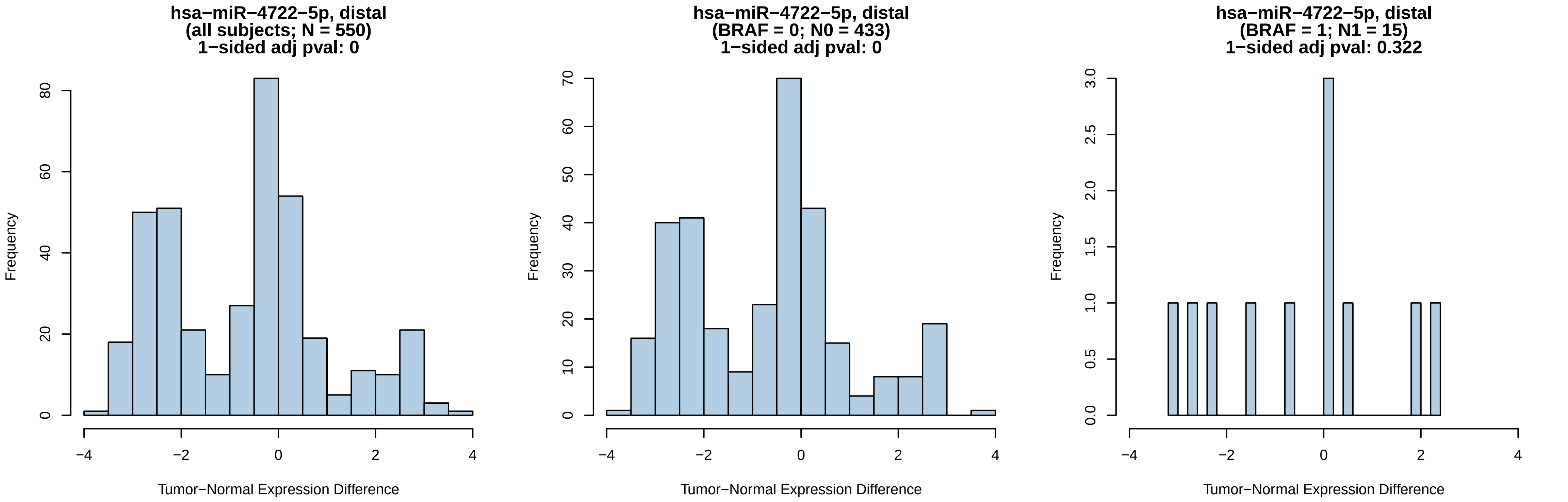
hsa−miR−4722−5p, distal
(all subjects; N = 550)
1−sided adj pval: 0
Frequency
Tumor−Normal Expression Difference
hsa−miR−4722−5p, distal
(BRAF = 0; N0 = 433)
1−sided adj pval: 0
Frequency
Tumor−Normal Expression Difference
hsa−miR−4722−5p, distal
(BRAF = 1; N1 = 15)
1−sided adj pval: 0.322
Frequency
Tumor−Normal Expression Difference

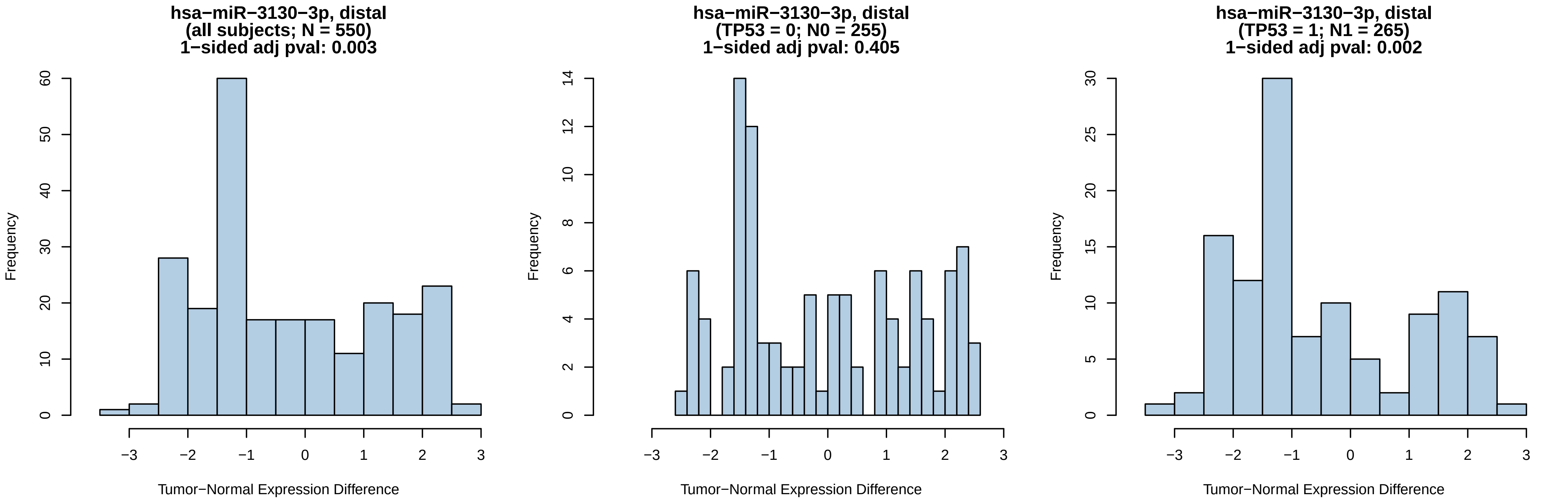
hsa−miR−3130−3p, distal
(all subjects; N = 550)
1−sided adj pval: 0.003
hsa−miR−3130−3p, distal
(TP53 = 0; N0 = 255)
1−sided adj pval: 0.405
hsa−miR−3130−3p, distal
(TP53 = 1; N1 = 265)
1−sided adj pval: 0.002
Frequency
Tumor−Normal Expression Difference

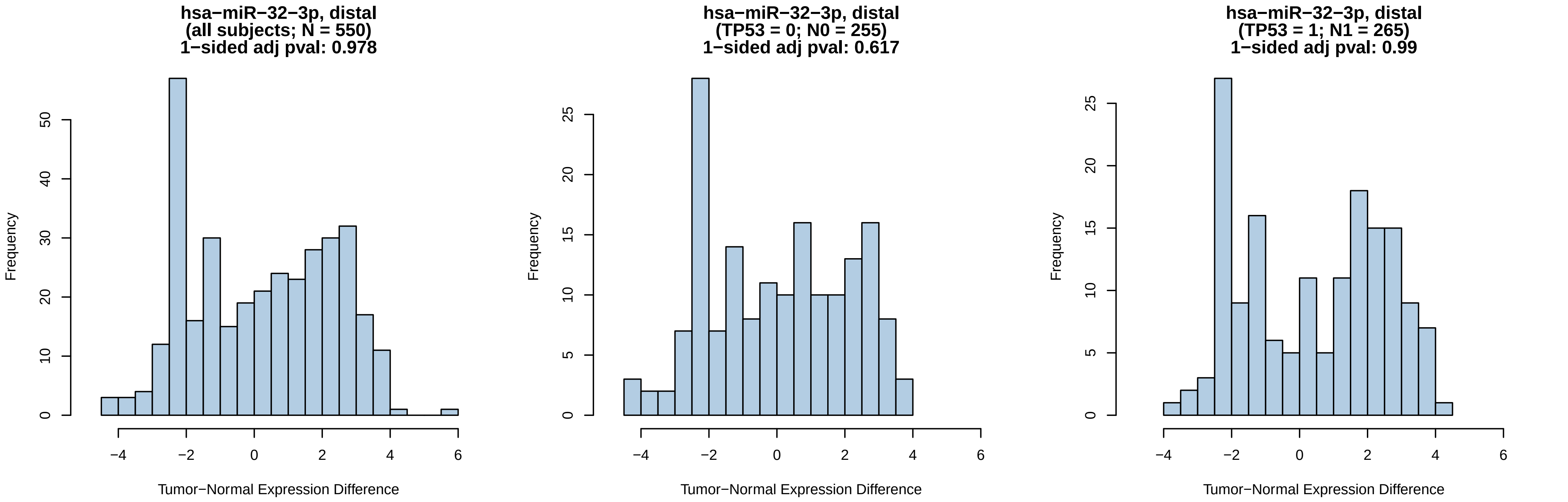

hsa−miR−32−3p, distal
(all subjects; N = 550)
1−sided adj pval: 0.978
Frequency
Tumor−Normal Expression Difference
hsa−miR−32−3p, distal
(TP53 = 0; N0 = 255)
1−sided adj pval: 0.617
Frequency
Tumor−Normal Expression Difference
hsa−miR−32−3p, distal
(TP53 = 1; N1 = 265)
1−sided adj pval: 0.99
Frequency
Tumor−Normal Expression Difference

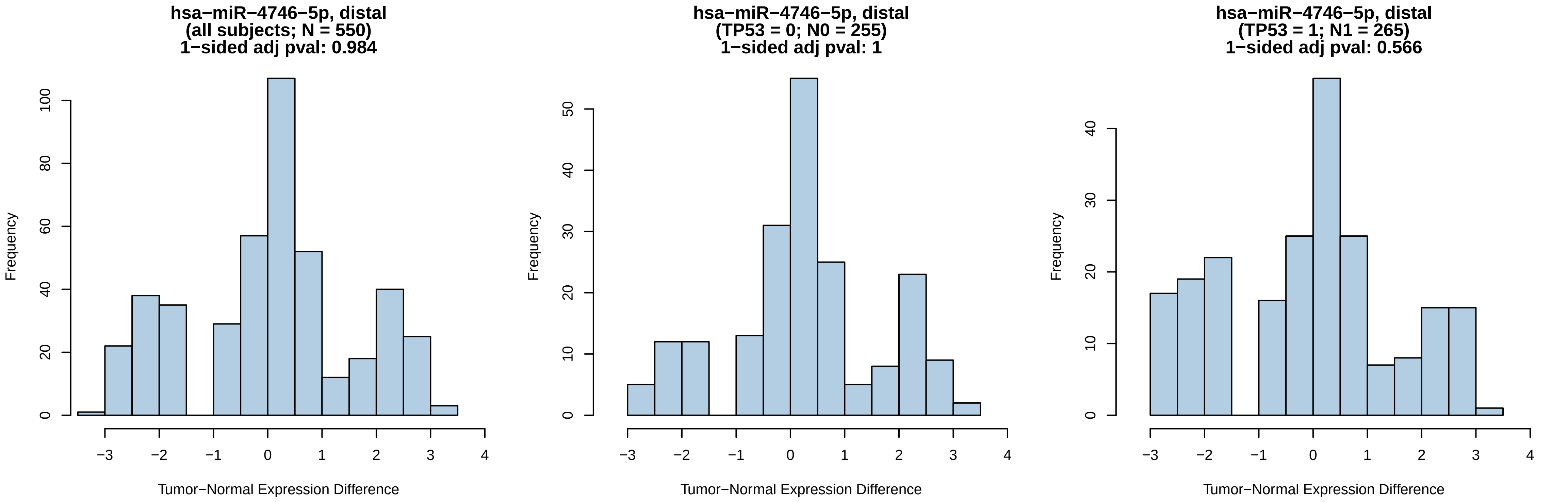

hsa−miR−4746−5p, distal
(all subjects; N = 550)
1−sided adj pval: 0.984
Frequency
Tumor−Normal Expression Difference
hsa−miR−4746−5p, distal
(TP53 = 0; N0 = 255)
1−sided adj pval: 1
Frequency
Tumor−Normal Expression Difference
hsa−miR−4746−5p, distal
(TP53 = 1; N1 = 265)
1−sided adj pval: 0.566
Frequency
Tumor−Normal Expression Difference

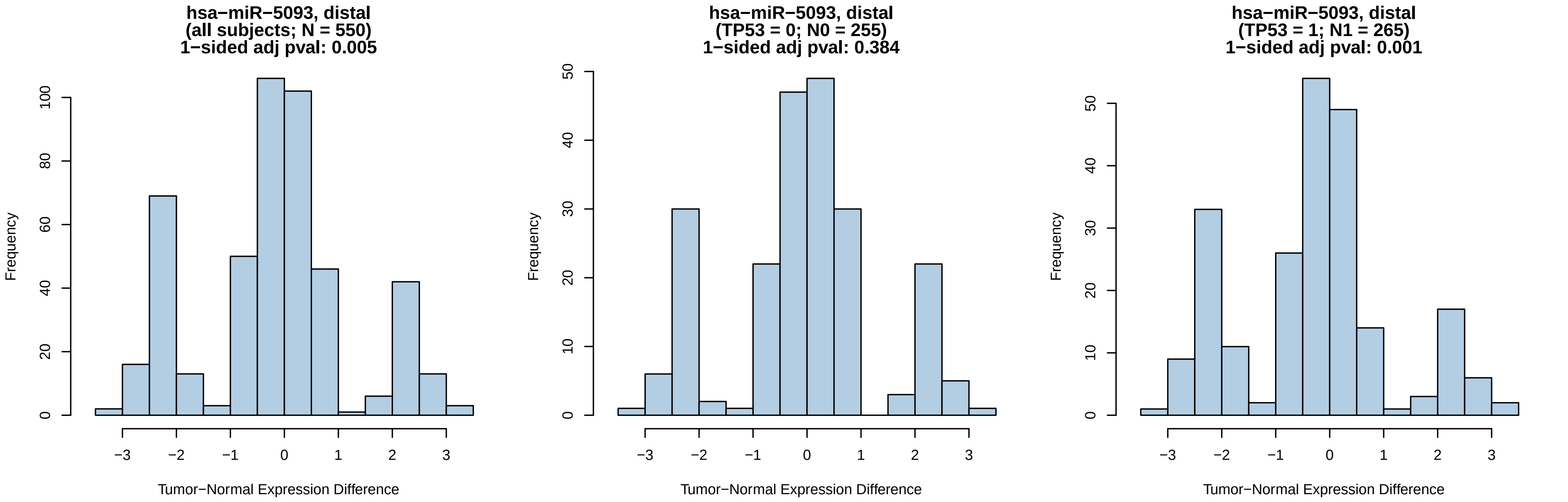
hsa−miR−5093, distal
(all subjects; N = 550)
1−sided adj pval: 0.005
Frequency
Tumor−Normal Expression Difference
hsa−miR−5093, distal
(TP53 = 0; N0 = 255)
1−sided adj pval: 0.384
Frequency
Tumor−Normal Expression Difference
hsa−miR−5093, distal
(TP53 = 1; N1 = 265)
1−sided adj pval: 0.001
Frequency
Tumor−Normal Expression Difference

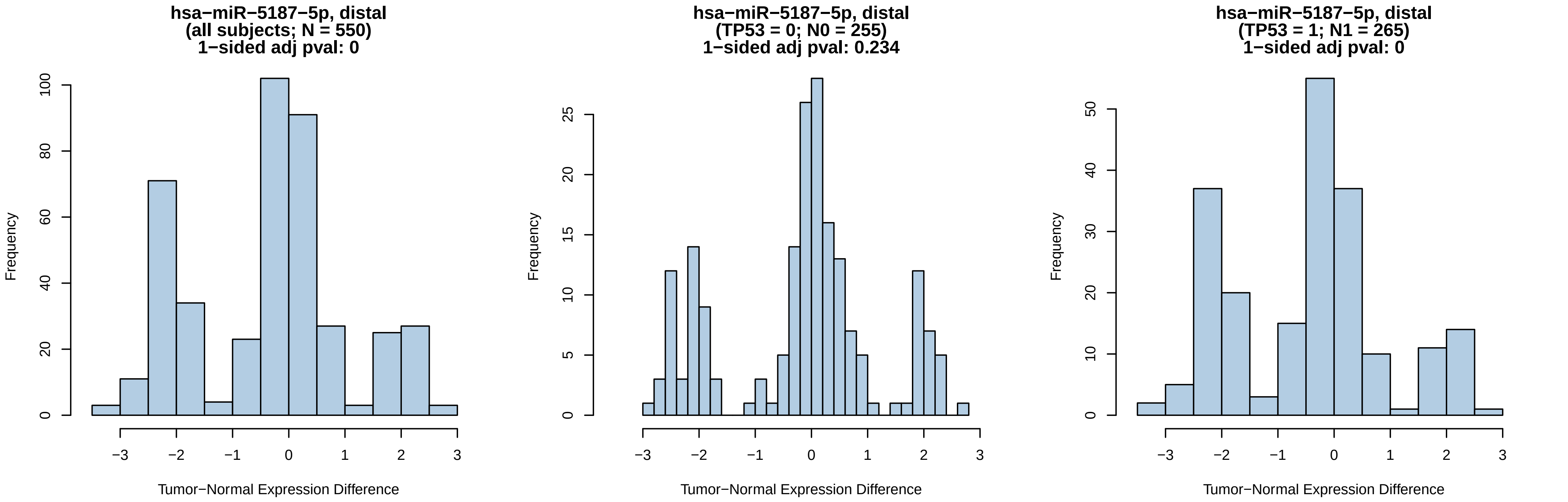
hsa−miR−5187−5p, distal
(all subjects; N = 550)
1−sided adj pval: 0
Frequency
Tumor−Normal Expression Difference
hsa−miR−5187−5p, distal
(TP53 = 0; N0 = 255)
1−sided adj pval: 0.234
Frequency
Tumor−Normal Expression Difference
hsa−miR−5187−5p, distal
(TP53 = 1; N1 = 265)
1−sided adj pval: 0
Frequency
Tumor−Normal Expression Difference

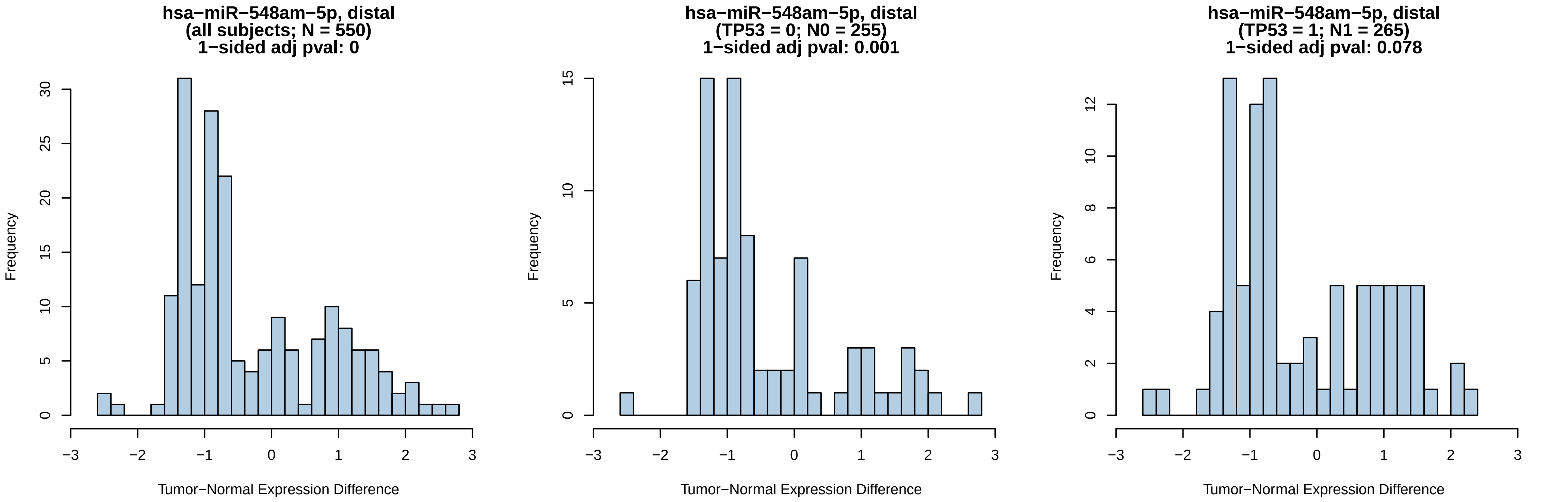

hsa−miR−548am−5p, distal
(all subjects; N = 550)
1−sided adj pval: 0
Frequency
Tumor−Normal Expression Difference
hsa−miR−548am−5p, distal
(TP53 = 0; N0 = 255)
1−sided adj pval: 0.001
Frequency
Tumor−Normal Expression Difference
hsa−miR−548am−5p, distal
(TP53 = 1; N1 = 265)
1−sided adj pval: 0.078
Frequency
Tumor−Normal Expression Difference

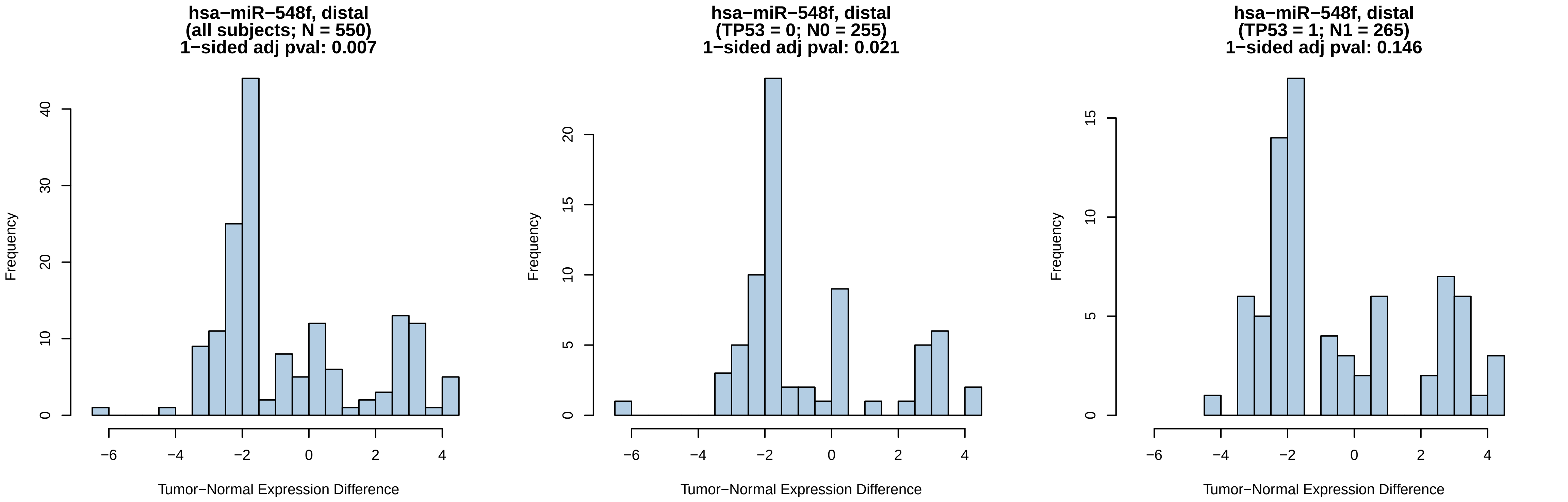
hsa−miR−548f, distal
(all subjects; N = 550)
1−sided adj pval: 0.007
Frequency
Tumor−Normal Expression Difference
hsa−miR−548f, distal
(TP53 = 0; N0 = 255)
1−sided adj pval: 0.021
Frequency
Tumor−Normal Expression Difference
hsa−miR−548f, distal
(TP53 = 1; N1 = 265)
1−sided adj pval: 0.146
Frequency
Tumor−Normal Expression Difference

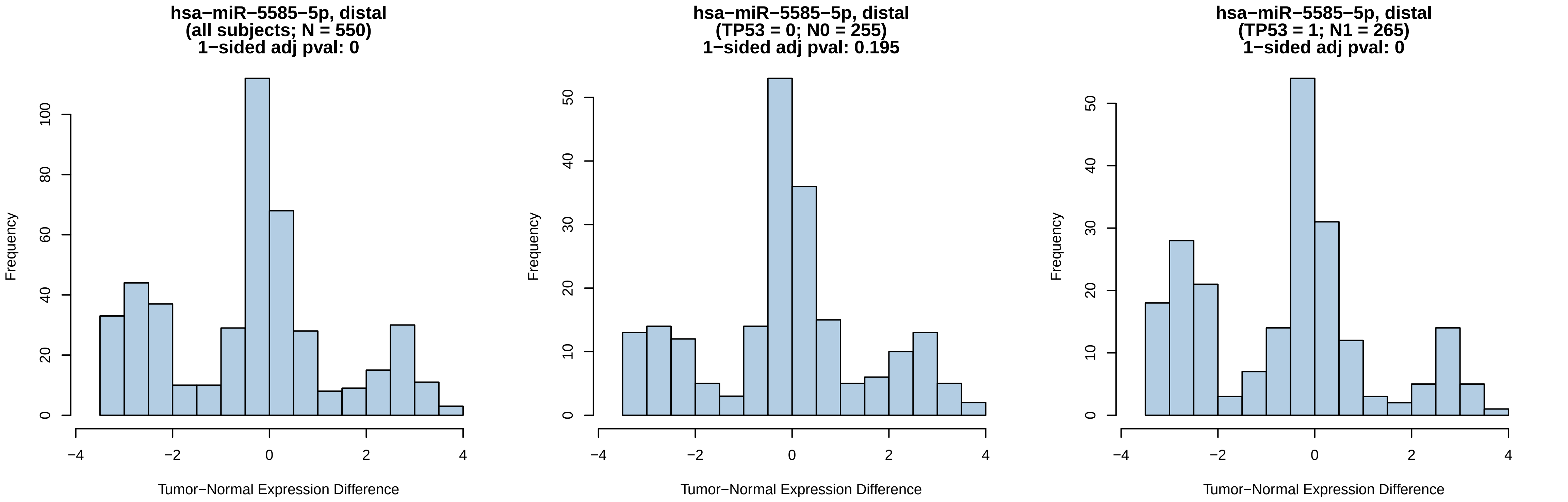
hsa−miR−5585−5p, distal
(all subjects; N = 550)
1−sided adj pval: 0
hsa−miR−5585−5p, distal
(TP53 = 0; N0 = 255)
1−sided adj pval: 0.195
hsa−miR−5585−5p, distal
(TP53 = 1; N1 = 265)
1−sided adj pval: 0
Frequency
Tumor−Normal Expression Difference

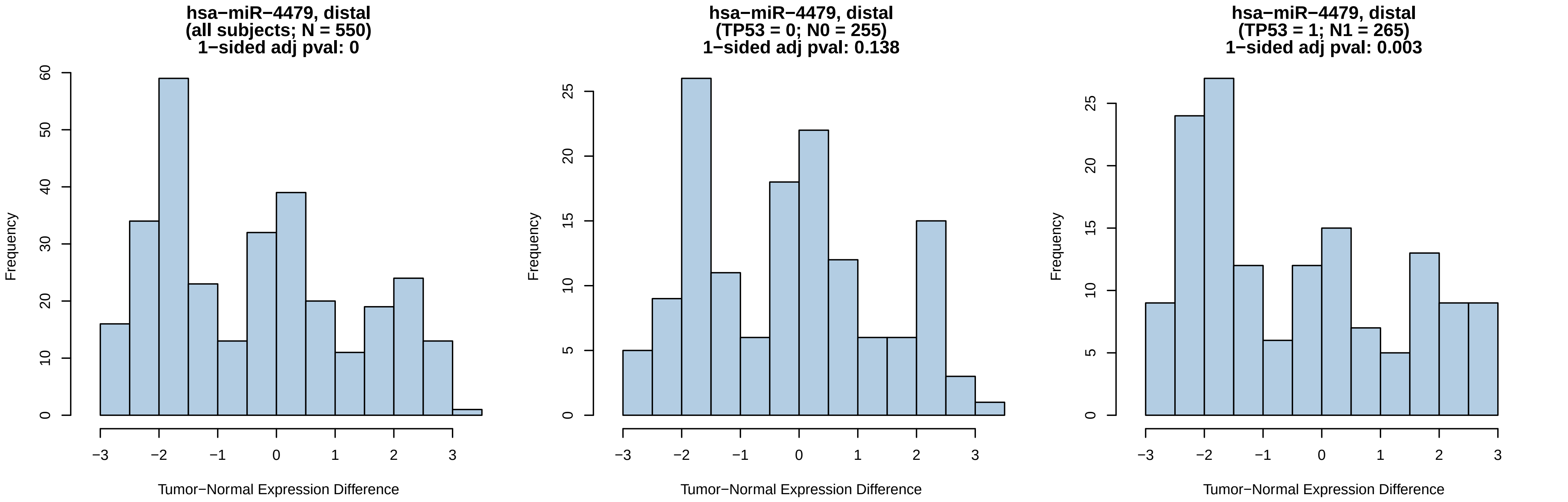
hsa−miR−4479, distal
(all subjects; N = 550)
1−sided adj pval: 0
Frequency
0
10
20
30
40
50
60
−3
−2
−1
0
1
2
3
Tumor−Normal Expression Difference
hsa−miR−4479, distal
(TP53 = 0; N0 = 255)
1−sided adj pval: 0.138
Frequency
0
5
10
15
20
25
−3
−2
−1
0
1
2
3
Tumor−Normal Expression Difference
hsa−miR−4479, distal
(TP53 = 1; N1 = 265)
1−sided adj pval: 0.003
Frequency
0
5
10
15
20
25
−3
−2
−1
0
1
2
3
Tumor−Normal Expression Difference

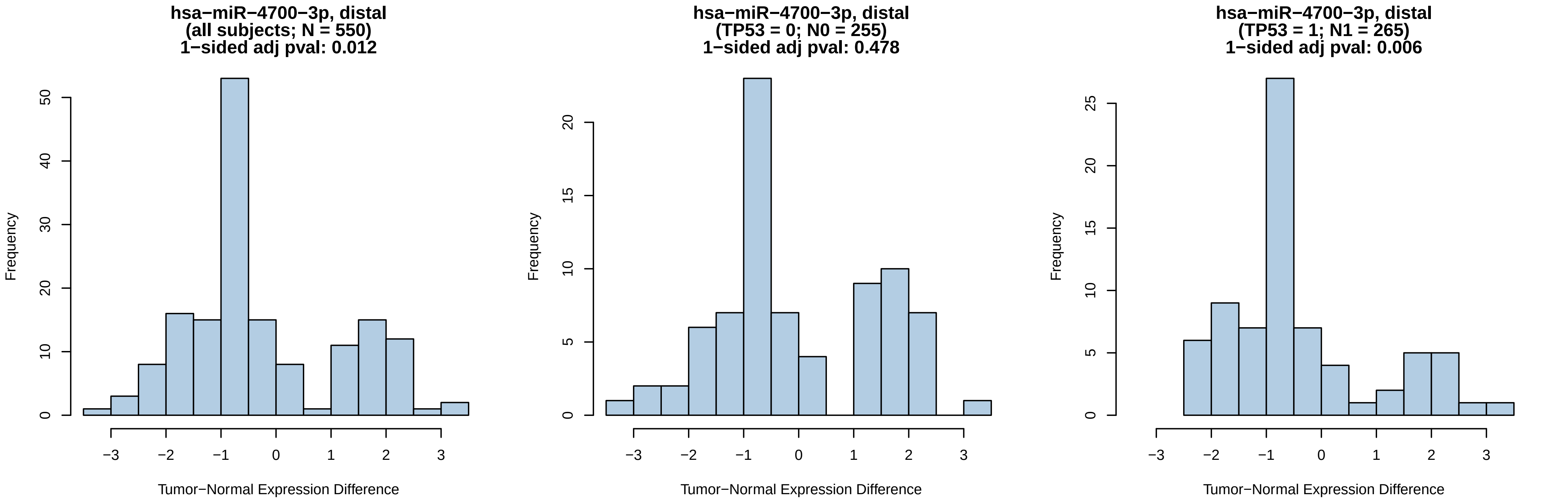

hsa−miR−4700−3p, distal
(all subjects; N = 550)
1−sided adj pval: 0.012
Frequency
Tumor−Normal Expression Difference
hsa−miR−4700−3p, distal
(TP53 = 0; N0 = 255)
1−sided adj pval: 0.478
Frequency
Tumor−Normal Expression Difference
hsa−miR−4700−3p, distal
(TP53 = 1; N1 = 265)
1−sided adj pval: 0.006
Frequency
Tumor−Normal Expression Difference

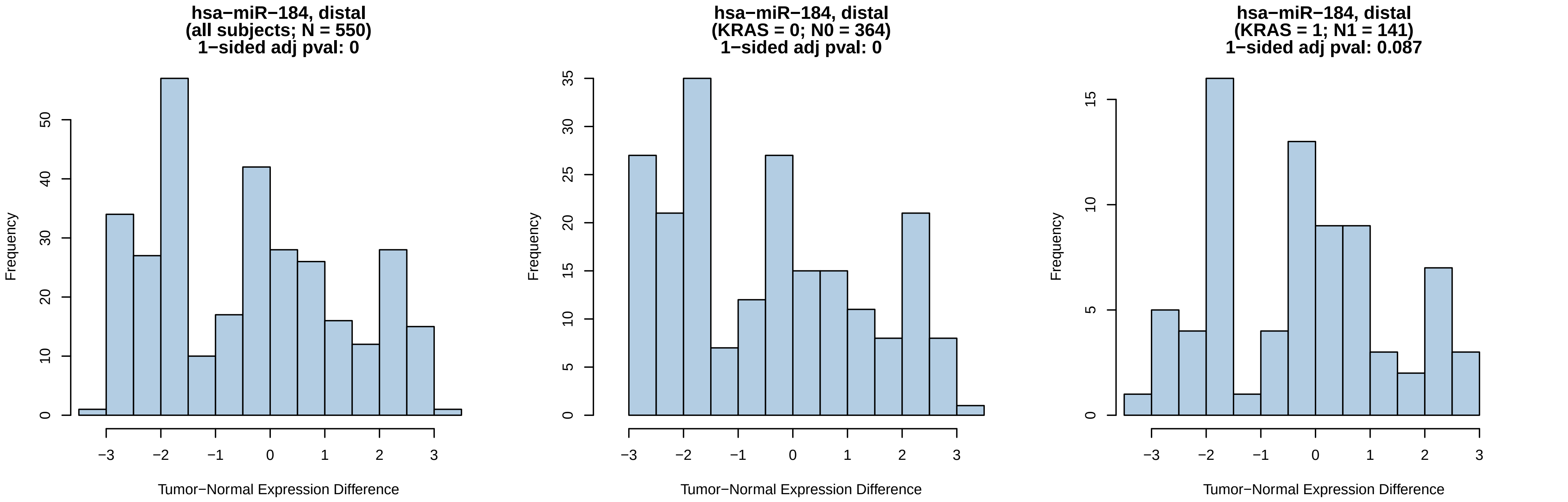
hsa−miR−184, distal
(all subjects; N = 550)
1−sided adj pval: 0
hsa−miR−184, distal
(KRAS = 0; N0 = 364)
1−sided adj pval: 0
hsa−miR−184, distal
(KRAS = 1; N1 = 141)
1−sided adj pval: 0.087
Frequency
Tumor−Normal Expression Difference
0
10
20
30
40
50
5
15
25
35
−3
−2
−1
0
1
2
3

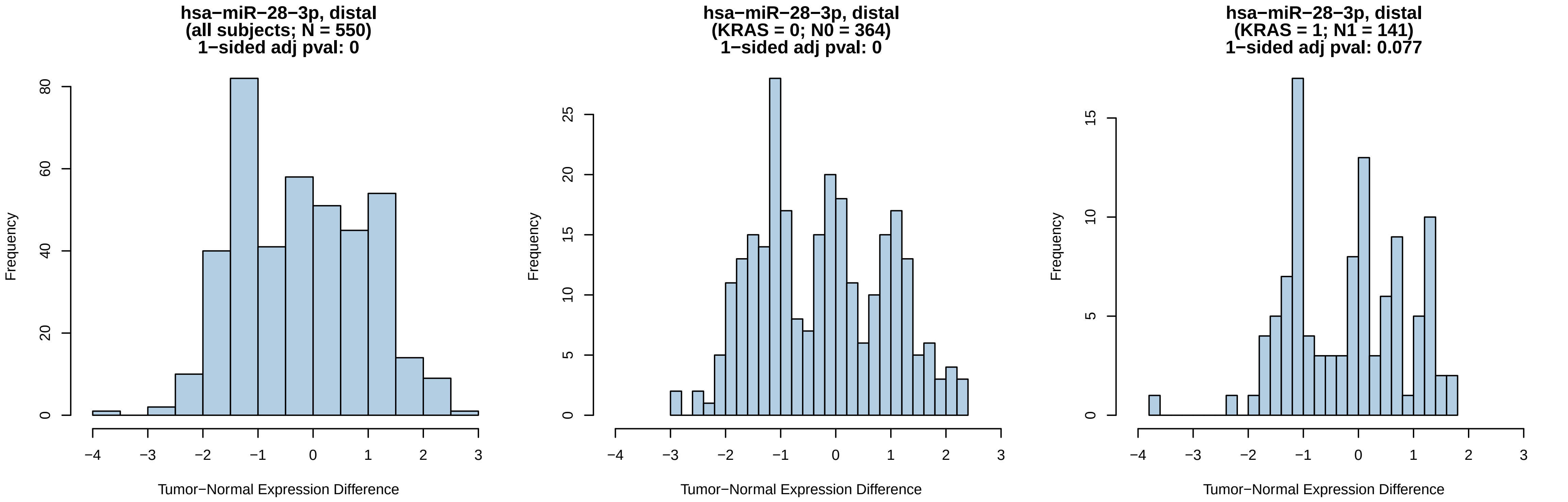
hsa−miR−28−3p, distal
(all subjects; N = 550)
1−sided adj pval: 0
Frequency
Tumor−Normal Expression Difference
hsa−miR−28−3p, distal
(KRAS = 0; N0 = 364)
1−sided adj pval: 0
Frequency
Tumor−Normal Expression Difference
hsa−miR−28−3p, distal
(KRAS = 1; N1 = 141)
1−sided adj pval: 0.077
Frequency
Tumor−Normal Expression Difference

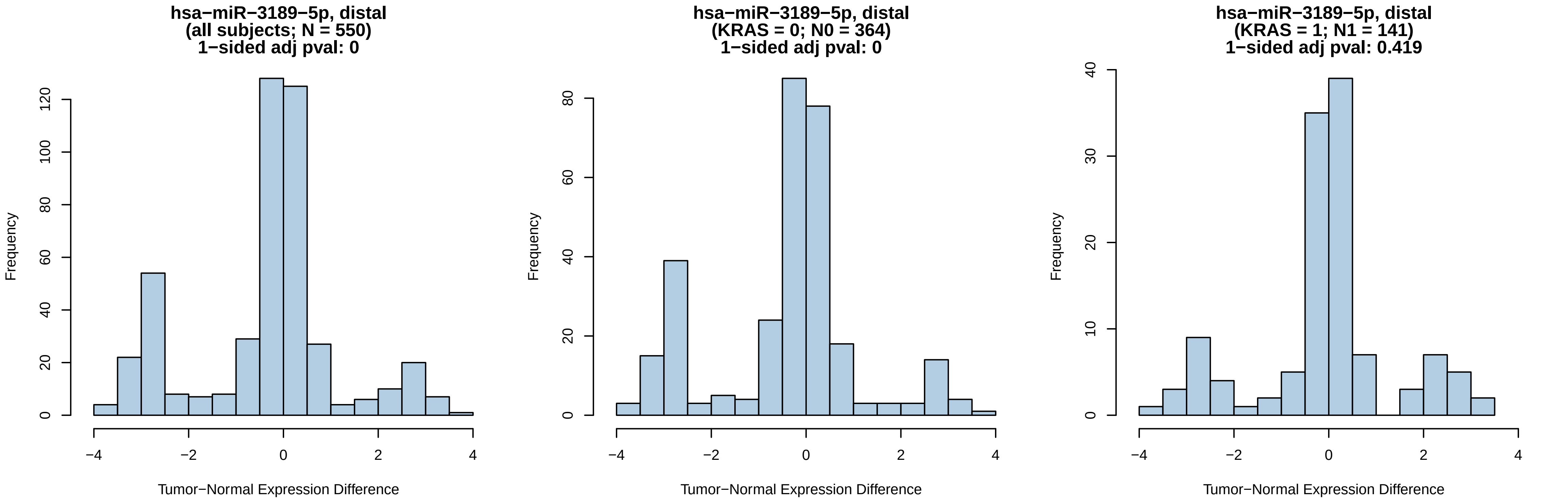
hsa−miR−3189−5p, distal
(all subjects; N = 550)
1−sided adj pval: 0
Frequency
Tumor−Normal Expression Difference
hsa−miR−3189−5p, distal
(KRAS = 0; N0 = 364)
1−sided adj pval: 0
Frequency
Tumor−Normal Expression Difference
hsa−miR−3189−5p, distal
(KRAS = 1; N1 = 141)
1−sided adj pval: 0.419
Frequency
Tumor−Normal Expression Difference

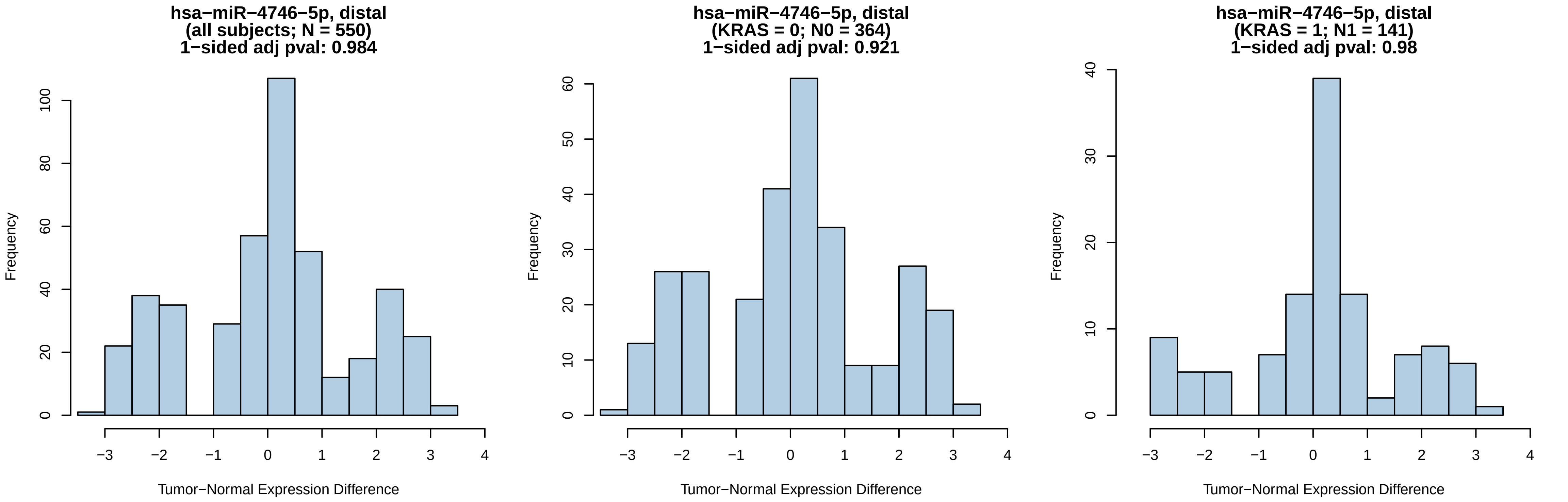

hsa−miR−4746−5p, distal
(all subjects; N = 550)
1−sided adj pval: 0.984
Frequency
Tumor−Normal Expression Difference
hsa−miR−4746−5p, distal
(KRAS = 0; N0 = 364)
1−sided adj pval: 0.921
Frequency
Tumor−Normal Expression Difference
hsa−miR−4746−5p, distal
(KRAS = 1; N1 = 141)
1−sided adj pval: 0.98
Frequency
Tumor−Normal Expression Difference

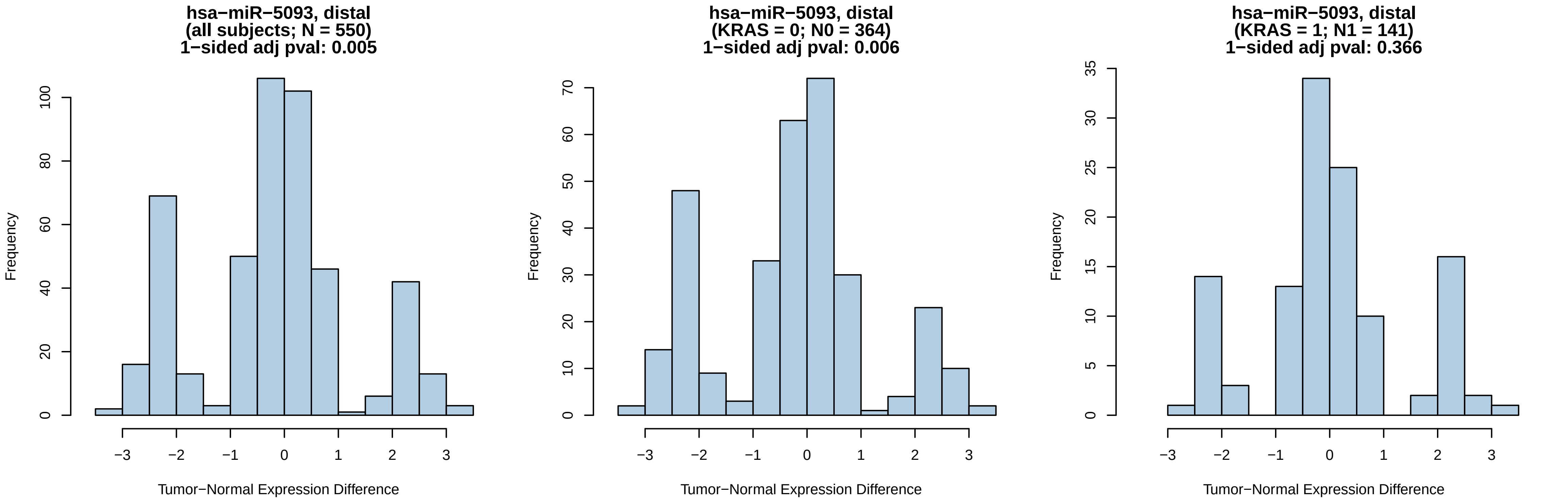
hsa−miR−5093, distal
(all subjects; N = 550)
1−sided adj pval: 0.005
hsa−miR−5093, distal
(KRAS = 0; N0 = 364)
1−sided adj pval: 0.006
hsa−miR−5093, distal
(KRAS = 1; N1 = 141)
1−sided adj pval: 0.366
Frequency
Tumor−Normal Expression Difference

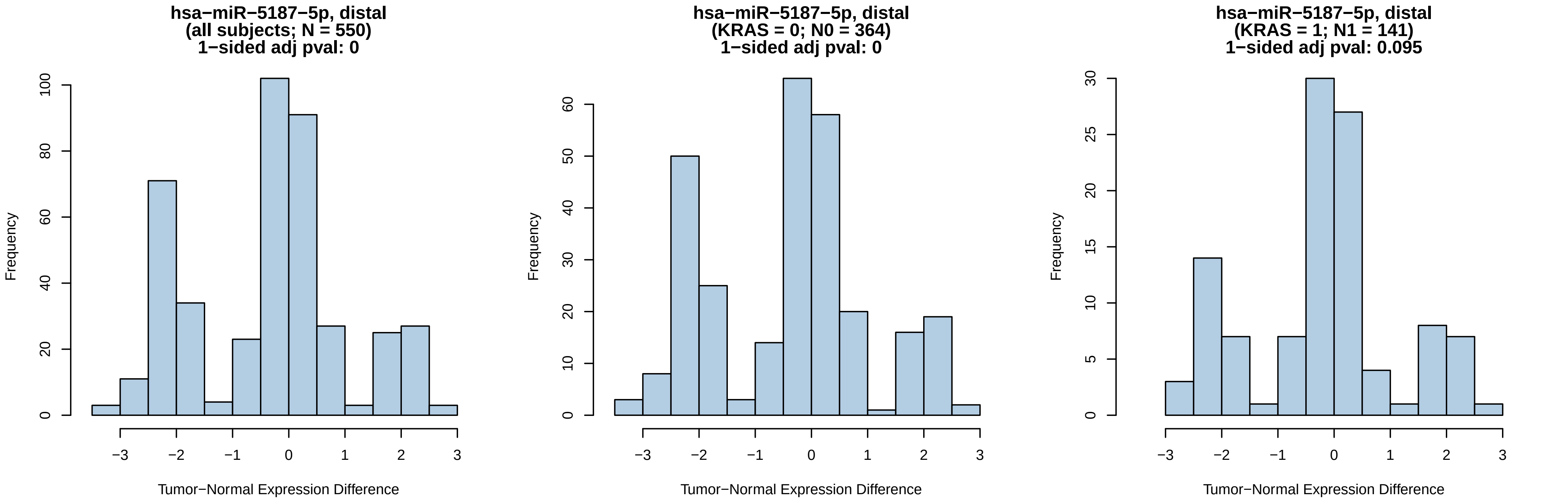

hsa−miR−5187−5p, distal
(all subjects; N = 550)
1−sided adj pval: 0
Frequency
Tumor−Normal Expression Difference
hsa−miR−5187−5p, distal
(KRAS = 0; N0 = 364)
1−sided adj pval: 0
Frequency
Tumor−Normal Expression Difference
hsa−miR−5187−5p, distal
(KRAS = 1; N1 = 141)
1−sided adj pval: 0.095
Frequency
Tumor−Normal Expression Difference

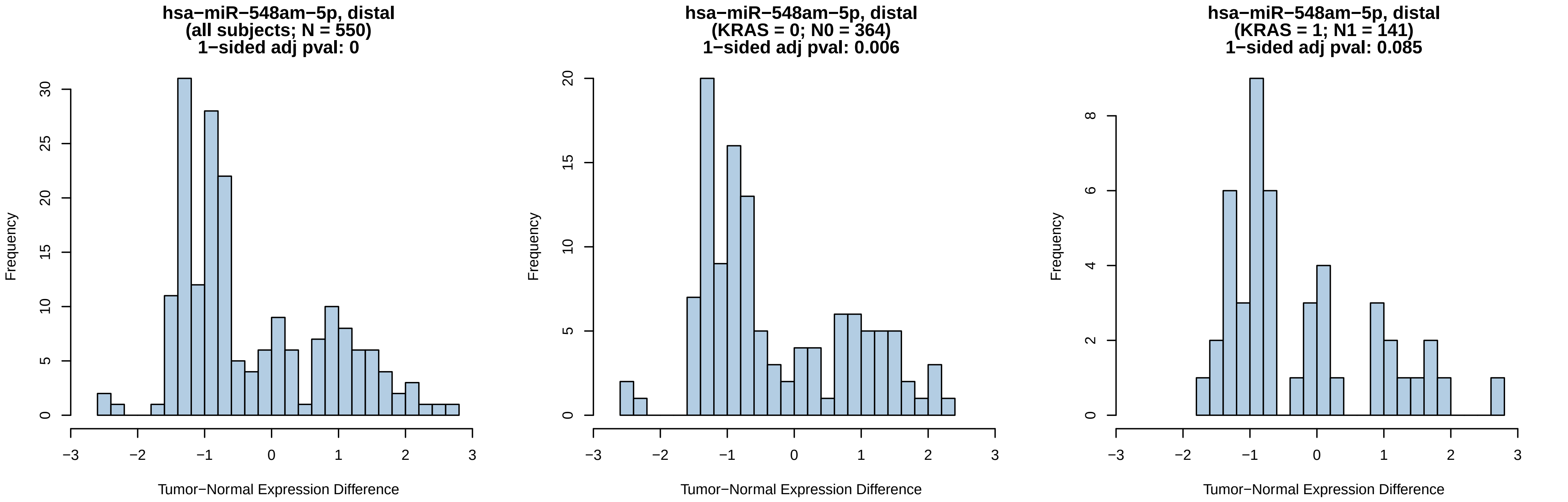
hsa−miR−548am−5p, distal
(all subjects; N = 550)
1−sided adj pval: 0
Frequency
Tumor−Normal Expression Difference
hsa−miR−548am−5p, distal
(KRAS = 0; N0 = 364)
1−sided adj pval: 0.006
Frequency
Tumor−Normal Expression Difference
hsa−miR−548am−5p, distal
(KRAS = 1; N1 = 141)
1−sided adj pval: 0.085
Frequency
Tumor−Normal Expression Difference

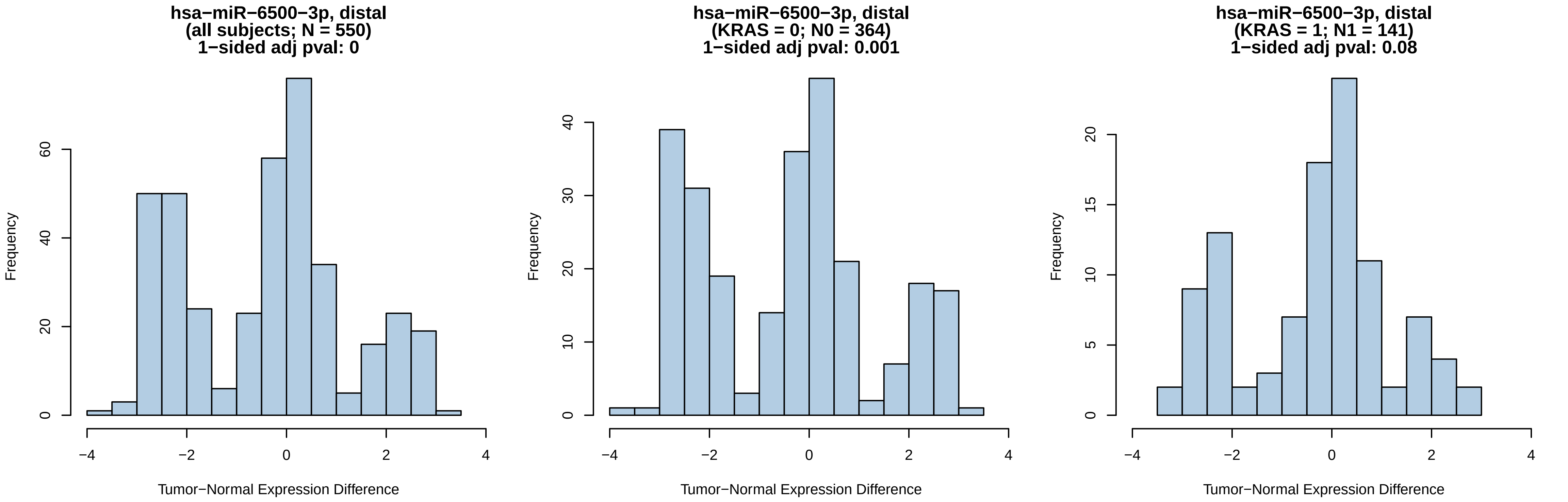
hsa−miR−6500−3p, distal
(all subjects; N = 550)
1−sided adj pval: 0
hsa−miR−6500−3p, distal
(KRAS = 0; N0 = 364)
1−sided adj pval: 0.001
hsa−miR−6500−3p, distal
(KRAS = 1; N1 = 141)
1−sided adj pval: 0.08
Frequency
Tumor−Normal Expression Difference

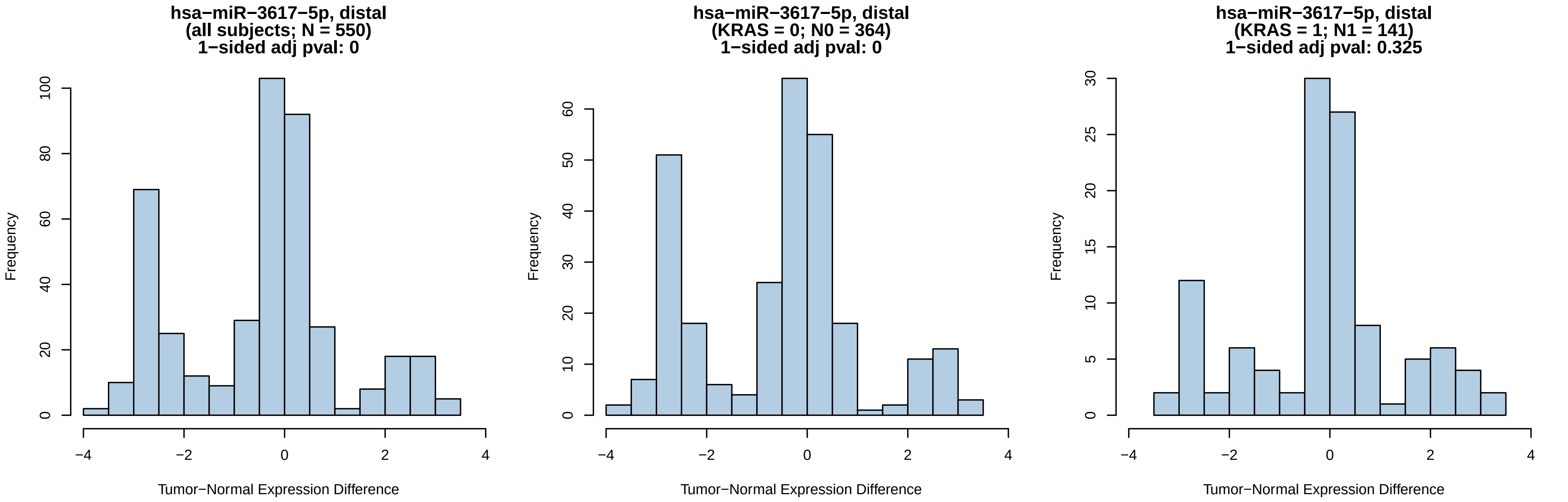
hsa−miR−3617−5p, distal
(all subjects; N = 550)
1−sided adj pval: 0
Frequency
Tumor−Normal Expression Difference
hsa−miR−3617−5p, distal
(KRAS = 0; N0 = 364)
1−sided adj pval: 0
Frequency
Tumor−Normal Expression Difference
hsa−miR−3617−5p, distal
(KRAS = 1; N1 = 141)
1−sided adj pval: 0.325
Frequency
Tumor−Normal Expression Difference

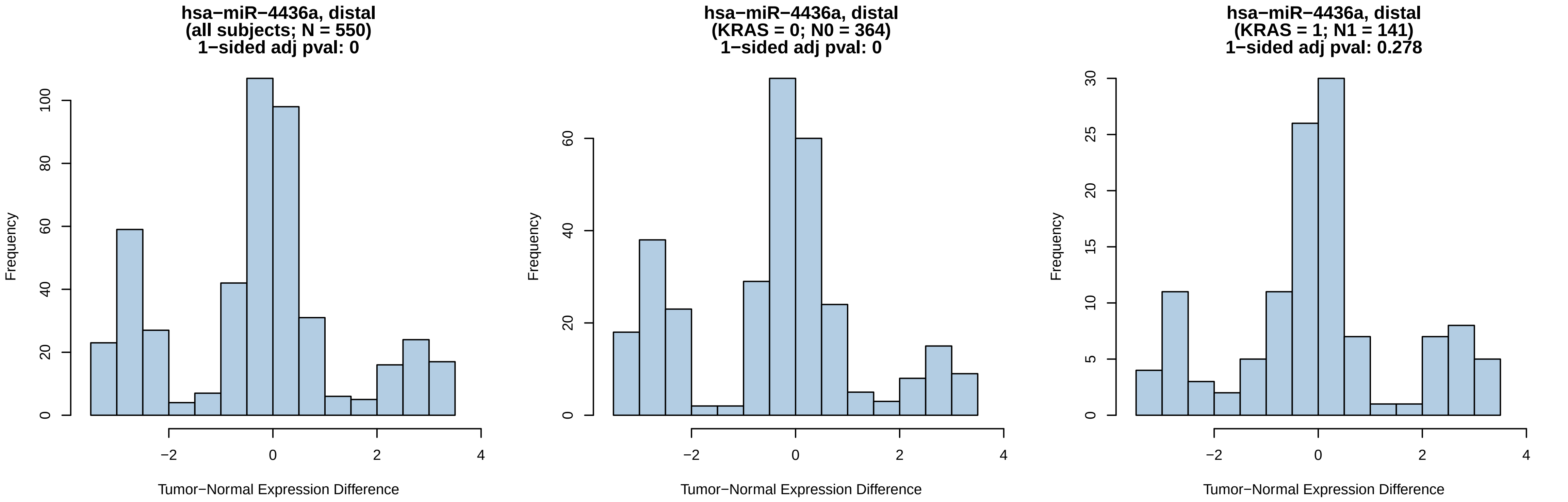
hsa−miR−4436a, distal
(all subjects; N = 550)
1−sided adj pval: 0
hsa−miR−4436a, distal
(KRAS = 0; N0 = 364)
1−sided adj pval: 0
hsa−miR−4436a, distal
(KRAS = 1; N1 = 141)
1−sided adj pval: 0.278
Frequency
Tumor−Normal Expression Difference

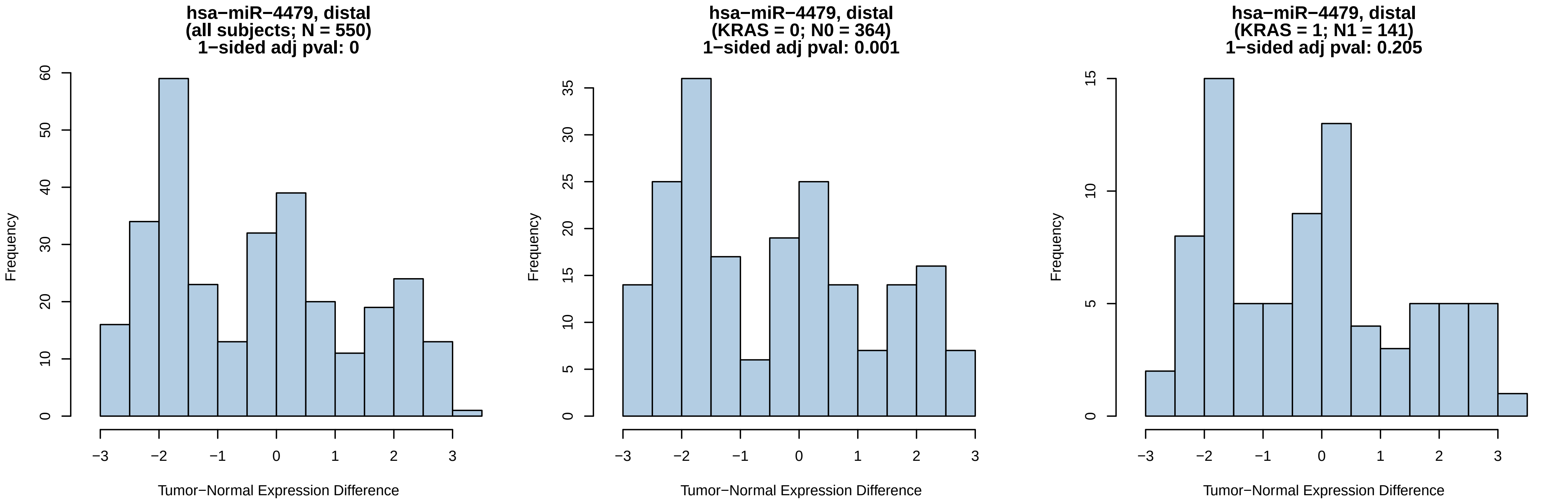
hsa−miR−4479, distal
(all subjects; N = 550)
1−sided adj pval: 0
Frequency
Tumor−Normal Expression Difference
hsa−miR−4479, distal
(KRAS = 0; N0 = 364)
1−sided adj pval: 0.001
Frequency
Tumor−Normal Expression Difference
hsa−miR−4479, distal
(KRAS = 1; N1 = 141)
1−sided adj pval: 0.205
Frequency
Tumor−Normal Expression Difference

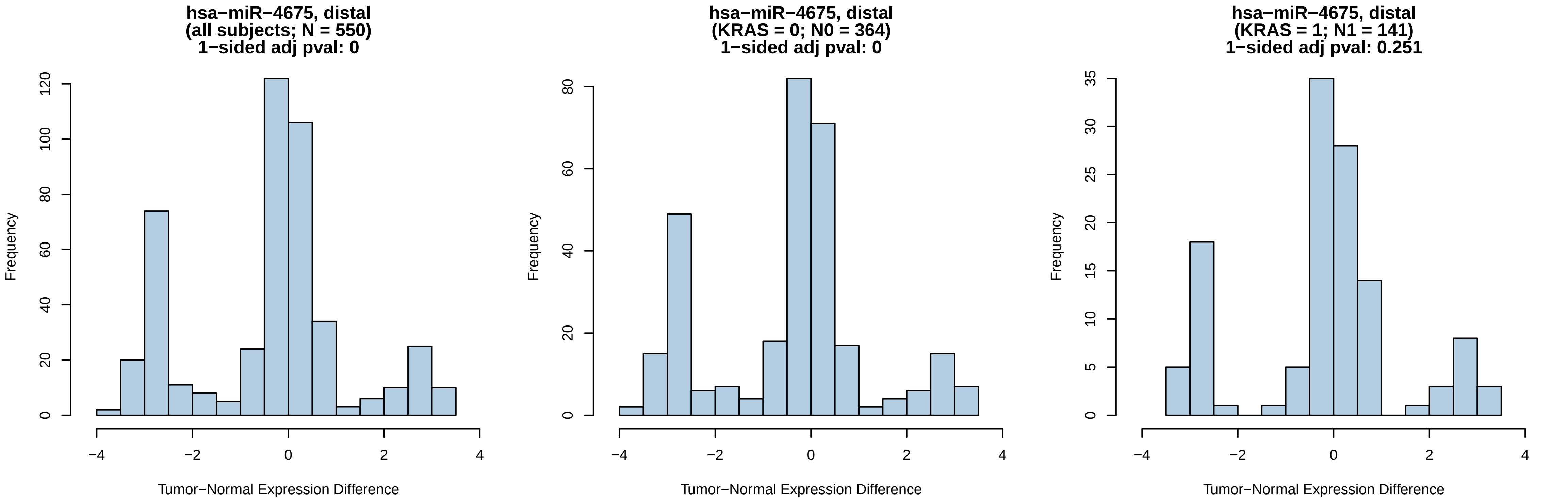

hsa−miR−4675, distal
(all subjects; N = 550)
1−sided adj pval: 0
Frequency
Tumor−Normal Expression Difference
hsa−miR−4675, distal
(KRAS = 0; N0 = 364)
1−sided adj pval: 0
Frequency
Tumor−Normal Expression Difference
hsa−miR−4675, distal
(KRAS = 1; N1 = 141)
1−sided adj pval: 0.251
Frequency
Tumor−Normal Expression Difference

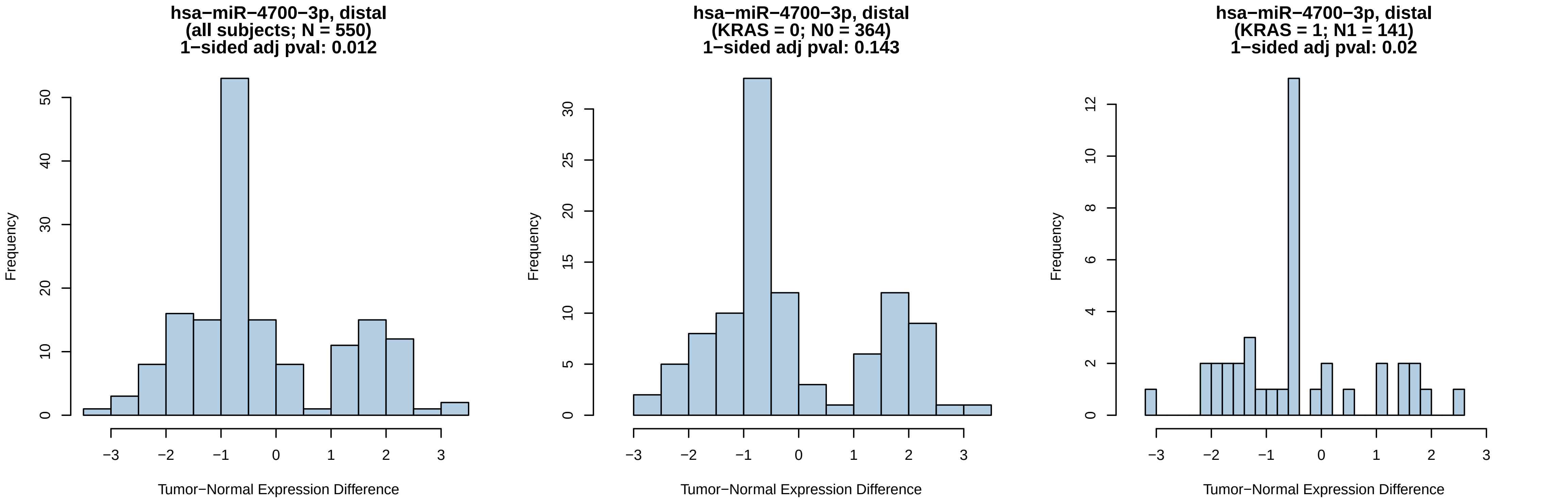
hsa−miR−4700−3p, distal
(all subjects; N = 550)
1−sided adj pval: 0.012
Frequency
Tumor−Normal Expression Difference
hsa−miR−4700−3p, distal
(KRAS = 0; N0 = 364)
1−sided adj pval: 0.143
Frequency
Tumor−Normal Expression Difference
hsa−miR−4700−3p, distal
(KRAS = 1; N1 = 141)
1−sided adj pval: 0.02
Frequency
Tumor−Normal Expression Difference

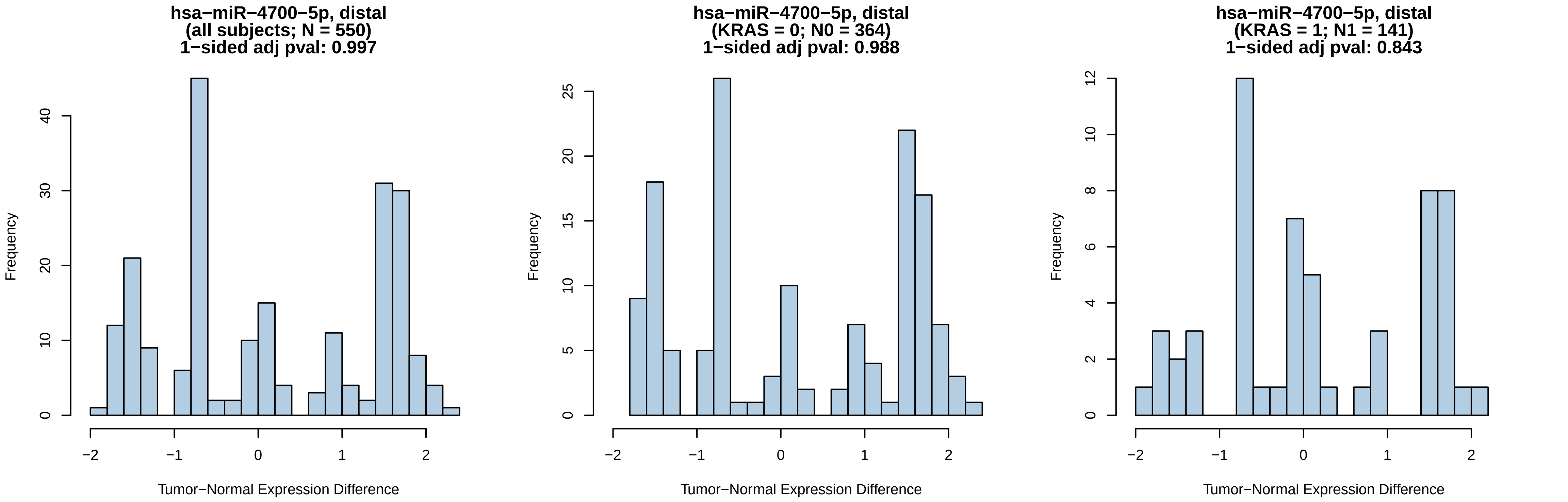

hsa−miR−4700−5p, distal
(all subjects; N = 550)
1−sided adj pval: 0.997
Frequency
0
10
20
30
40
−2
−1
0
1
2
Tumor−Normal Expression Difference
hsa−miR−4700−5p, distal
(KRAS = 0; N0 = 364)
1−sided adj pval: 0.988
Frequency
0
5
10
15
20
25
−2
−1
0
1
2
Tumor−Normal Expression Difference
hsa−miR−4700−5p, distal
(KRAS = 1; N1 = 141)
1−sided adj pval: 0.843
Frequency
0
2
4
6
8
10
12
−2
−1
0
1
2
Tumor−Normal Expression Difference

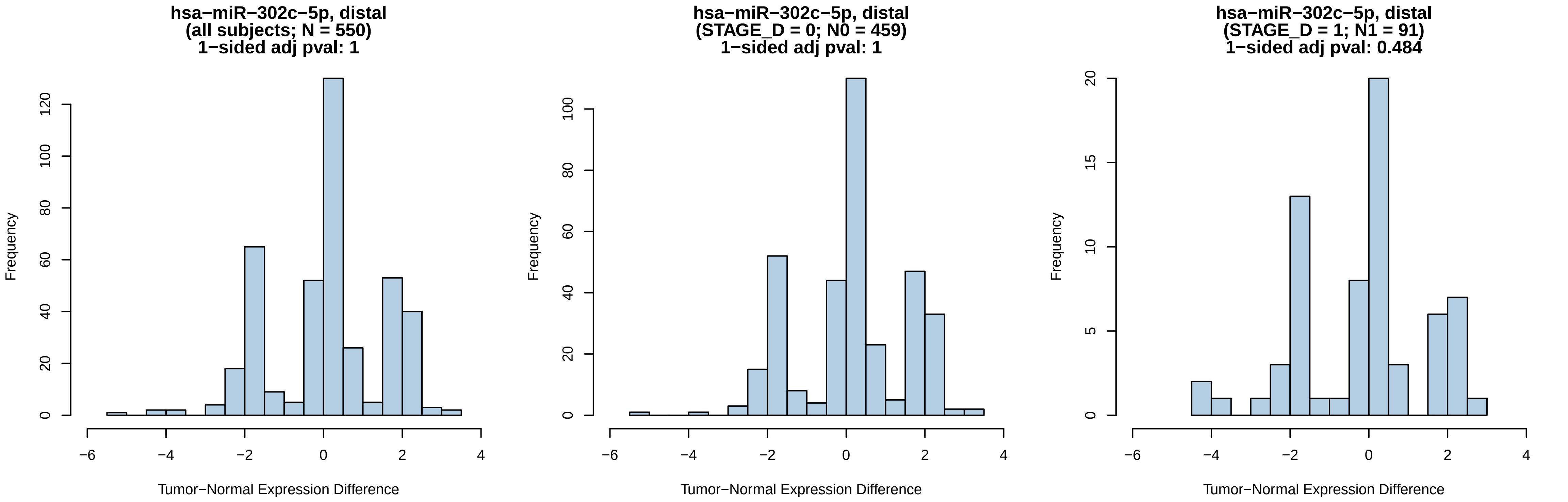

hsa−miR−302c−5p, distal
(all subjects; N = 550)
1−sided adj pval: 1
hsa−miR−302c−5p, distal
(STAGE_D = 0; N0 = 459)
1−sided adj pval: 1
hsa−miR−302c−5p, distal
(STAGE_D = 1; N1 = 91)
1−sided adj pval: 0.484
Frequency
Tumor−Normal Expression Difference

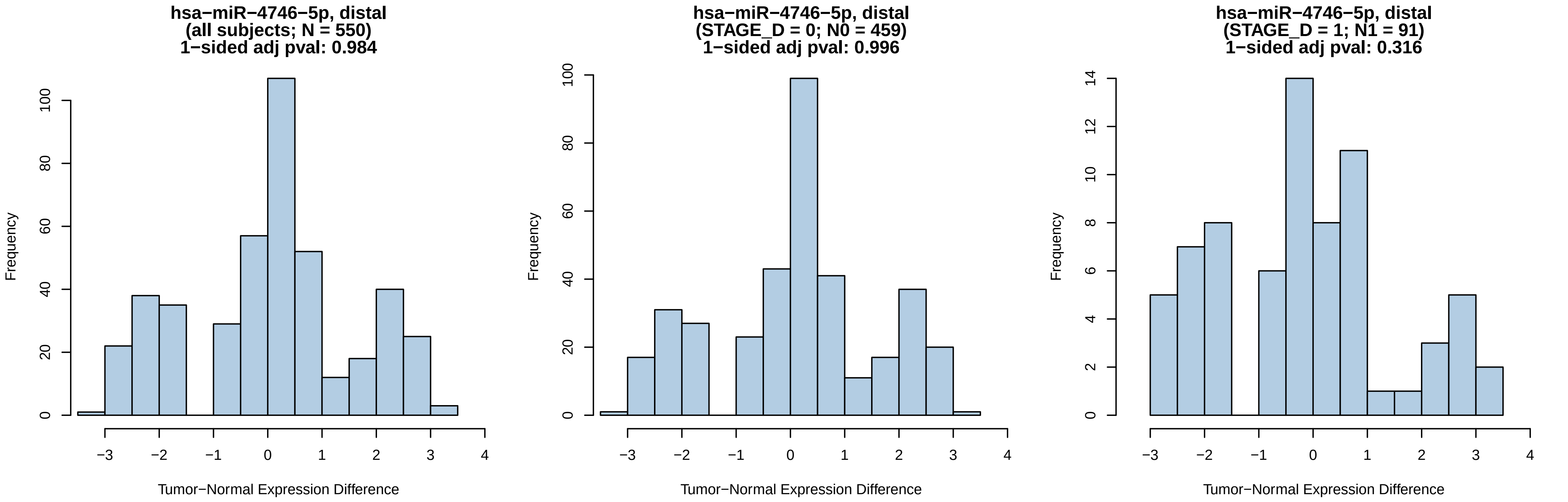

hsa−miR−4746−5p, distal
(all subjects; N = 550)
1−sided adj pval: 0.984
Frequency
Tumor−Normal Expression Difference
hsa−miR−4746−5p, distal
(STAGE_D = 0; N0 = 459)
1−sided adj pval: 0.996
Frequency
Tumor−Normal Expression Difference
hsa−miR−4746−5p, distal
(STAGE_D = 1; N1 = 91)
1−sided adj pval: 0.316
Frequency
Tumor−Normal Expression Difference

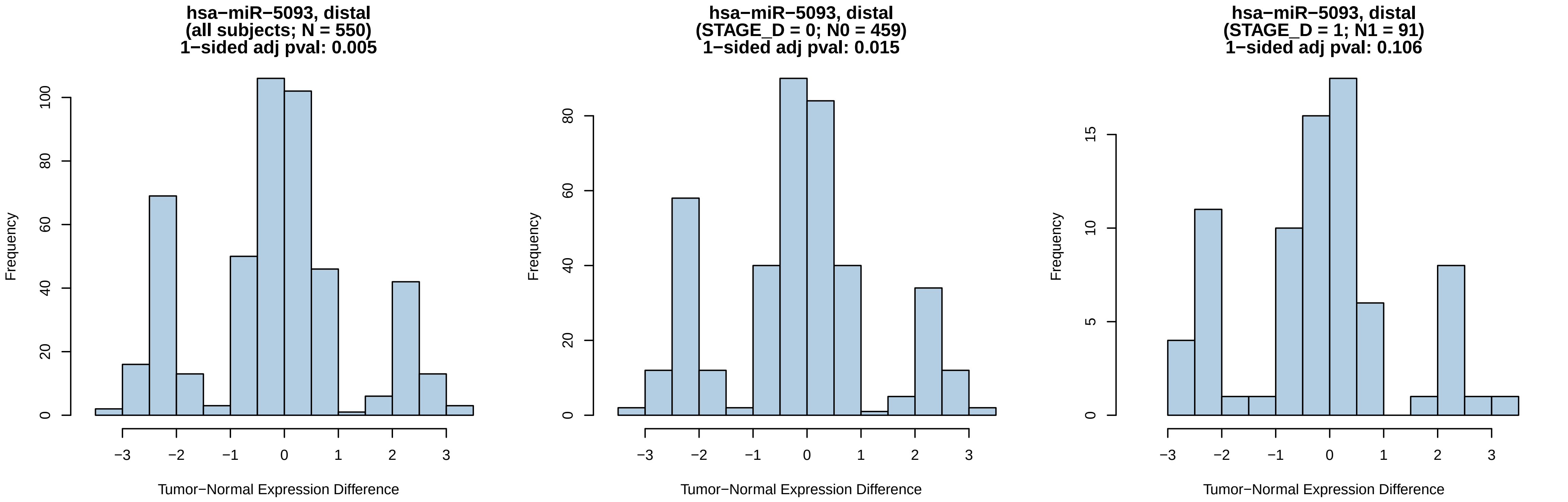

hsa−miR−5093, distal
(all subjects; N = 550)
1−sided adj pval: 0.005
Frequency
0
20
40
60
80
100
−3
−2
−1
0
1
2
3
Tumor−Normal Expression Difference
hsa−miR−5093, distal
(STAGE_D = 0; N0 = 459)
1−sided adj pval: 0.015
Frequency
0
20
40
60
80
−3
−2
−1
0
1
2
3
Tumor−Normal Expression Difference
hsa−miR−5093, distal
(STAGE_D = 1; N1 = 91)
1−sided adj pval: 0.106
Frequency
0
5
10
15
−3
−2
−1
0
1
2
3
Tumor−Normal Expression Difference

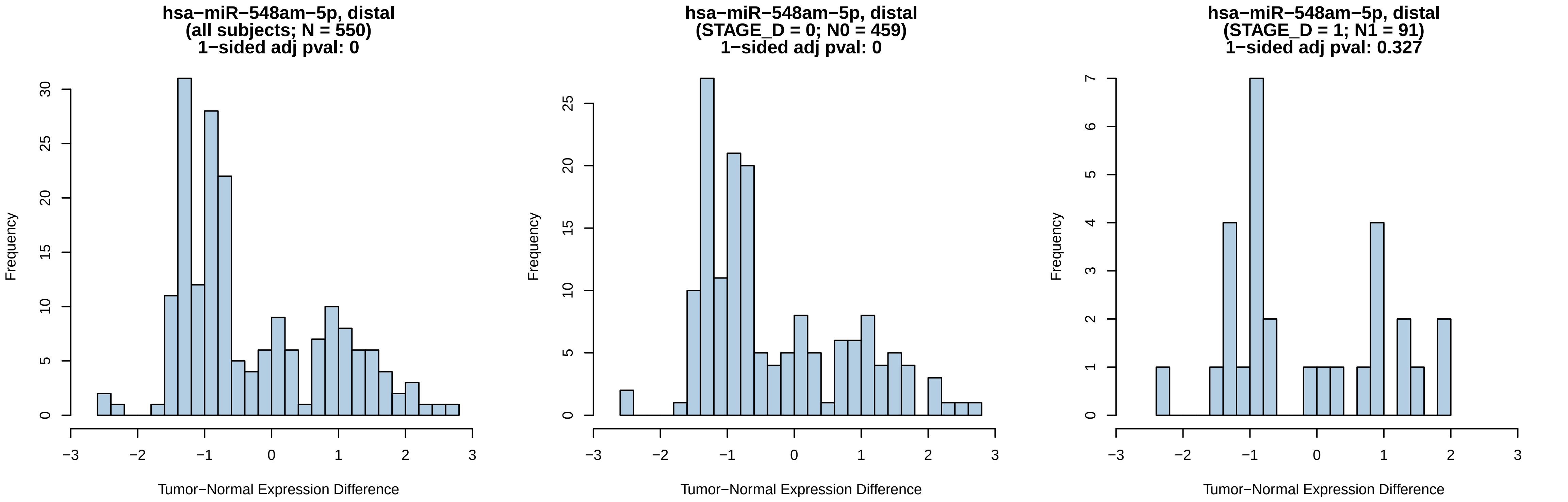

hsa−miR−548am−5p, distal
(all subjects; N = 550)
1−sided adj pval: 0
hsa−miR−548am−5p, distal
(STAGE_D = 0; N0 = 459)
1−sided adj pval: 0
hsa−miR−548am−5p, distal
(STAGE_D = 1; N1 = 91)
1−sided adj pval: 0.327
Frequency
Tumor−Normal Expression Difference

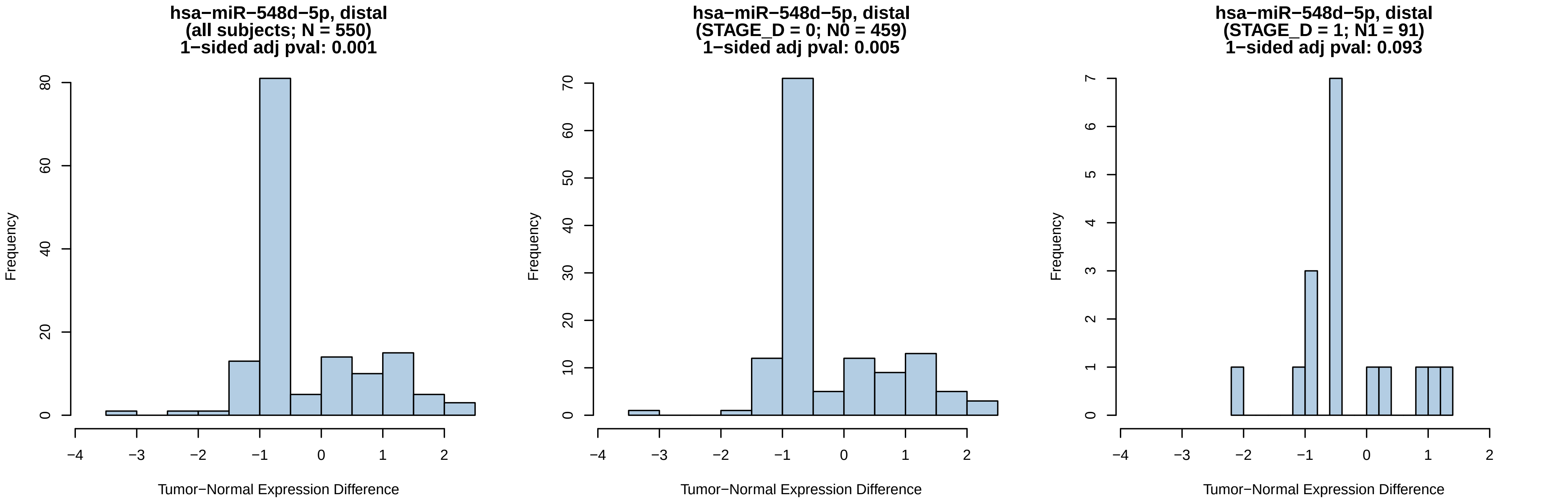
hsa−miR−548d−5p, distal
(all subjects; N = 550)
1−sided adj pval: 0.001
hsa−miR−548d−5p, distal
(STAGE_D = 0; N0 = 459)
1−sided adj pval: 0.005
hsa−miR−548d−5p, distal
(STAGE_D = 1; N1 = 91)
1−sided adj pval: 0.093
Frequency
Tumor−Normal Expression Difference

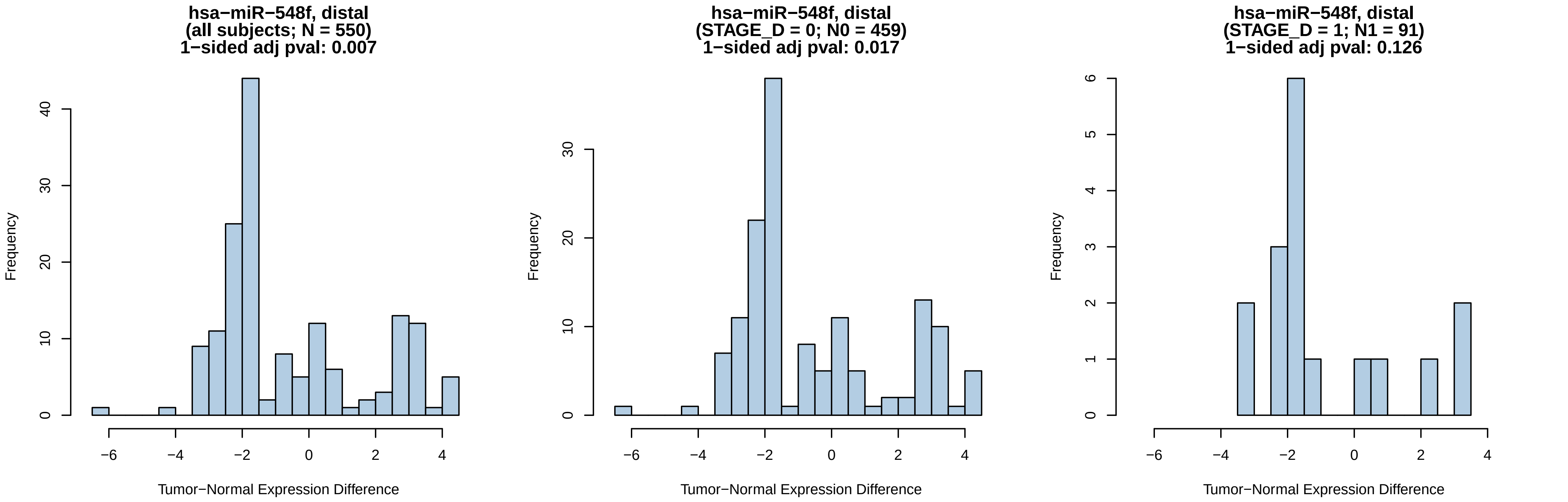
hsa−miR−548f, distal
(all subjects; N = 550)
1−sided adj pval: 0.007
Frequency
Tumor−Normal Expression Difference
hsa−miR−548f, distal
(STAGE_D = 0; N0 = 459)
1−sided adj pval: 0.017
Frequency
Tumor−Normal Expression Difference
hsa−miR−548f, distal
(STAGE_D = 1; N1 = 91)
1−sided adj pval: 0.126
Frequency
Tumor−Normal Expression Difference

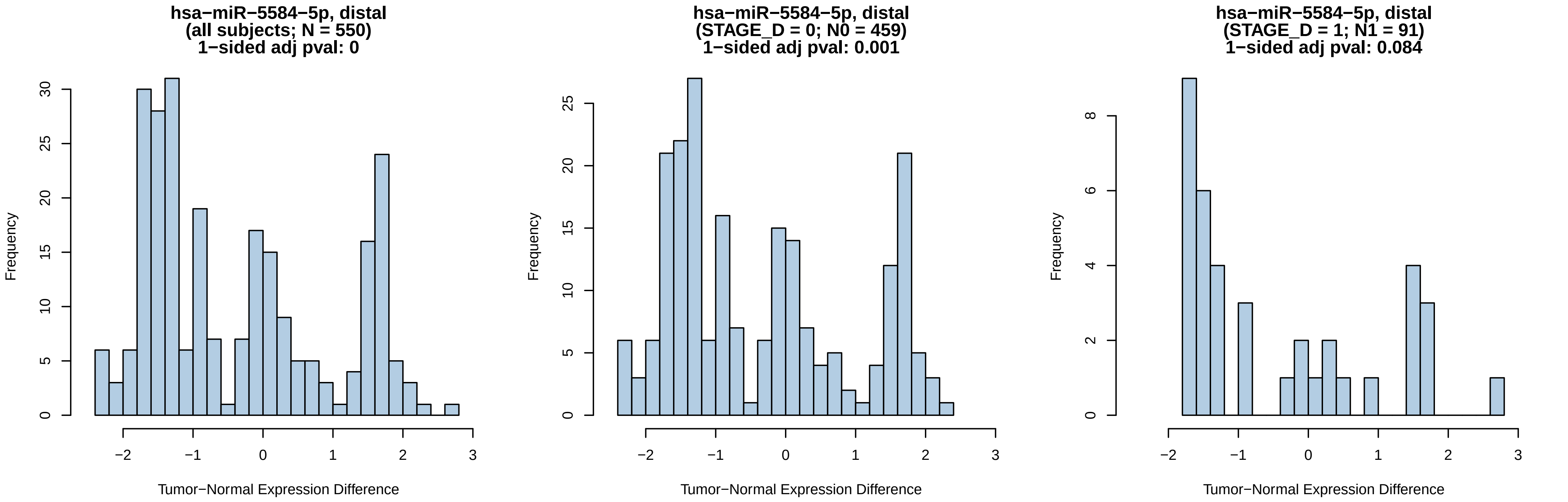
hsa−miR−5584−5p, distal
(all subjects; N = 550)
1−sided adj pval: 0
Frequency
0
5
10
15
20
25
30
−2
−1
0
1
2
3
Tumor−Normal Expression Difference
hsa−miR−5584−5p, distal
(STAGE_D = 0; N0 = 459)
1−sided adj pval: 0.001
Frequency
0
5
10
15
20
25
−2
−1
0
1
2
3
Tumor−Normal Expression Difference
hsa−miR−5584−5p, distal
(STAGE_D = 1; N1 = 91)
1−sided adj pval: 0.084
Frequency
0
2
4
6
8
−2
−1
0
1
2
3
Tumor−Normal Expression Difference

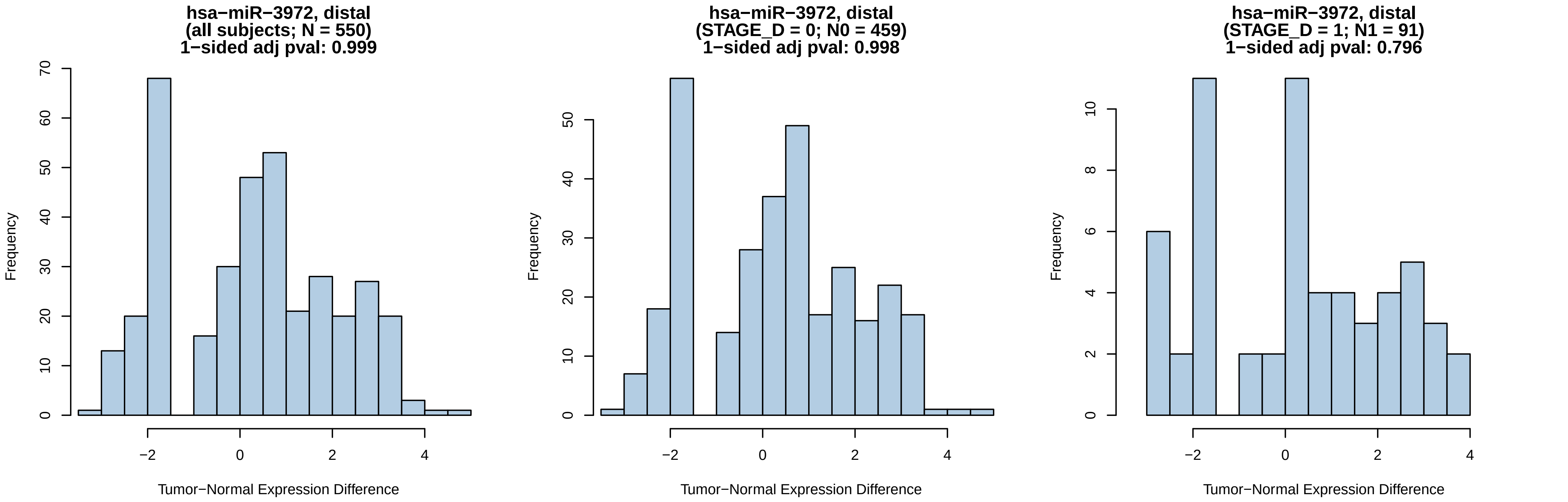
hsa−miR−3972, distal
(all subjects; N = 550)
1−sided adj pval: 0.999
hsa−miR−3972, distal
(STAGE_D = 0; N0 = 459)
1−sided adj pval: 0.998
hsa−miR−3972, distal
(STAGE_D = 1; N1 = 91)
1−sided adj pval: 0.796
Frequency
Tumor−Normal Expression Difference
0
10
20
30
40
50
60
70
−2
0
2
4
8
6

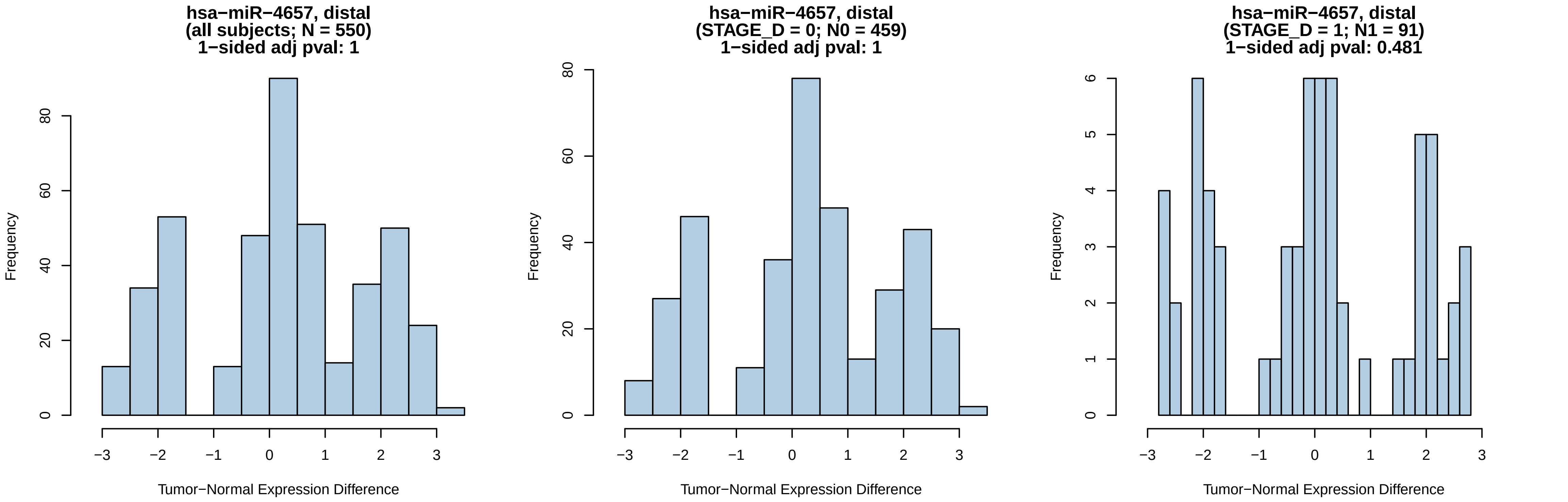
hsa−miR−4657, distal
(all subjects; N = 550)
1−sided adj pval: 1
Frequency
Tumor−Normal Expression Difference
hsa−miR−4657, distal
(STAGE_D = 0; N0 = 459)
1−sided adj pval: 1
Frequency
Tumor−Normal Expression Difference
hsa−miR−4657, distal
(STAGE_D = 1; N1 = 91)
1−sided adj pval: 0.481
Frequency
Tumor−Normal Expression Difference

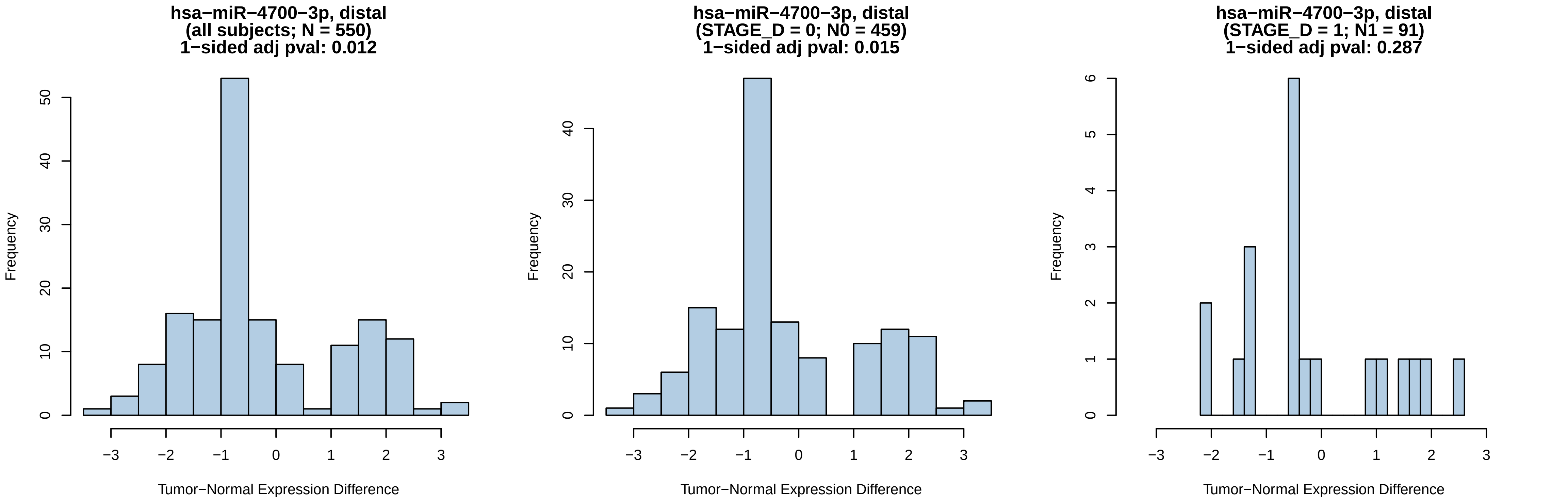

hsa−miR−4700−3p, distal
(all subjects; N = 550)
1−sided adj pval: 0.012
hsa−miR−4700−3p, distal
(STAGE_D = 0; N0 = 459)
1−sided adj pval: 0.015
hsa−miR−4700−3p, distal
(STAGE_D = 1; N1 = 91)
1−sided adj pval: 0.287
Frequency
Tumor−Normal Expression Difference

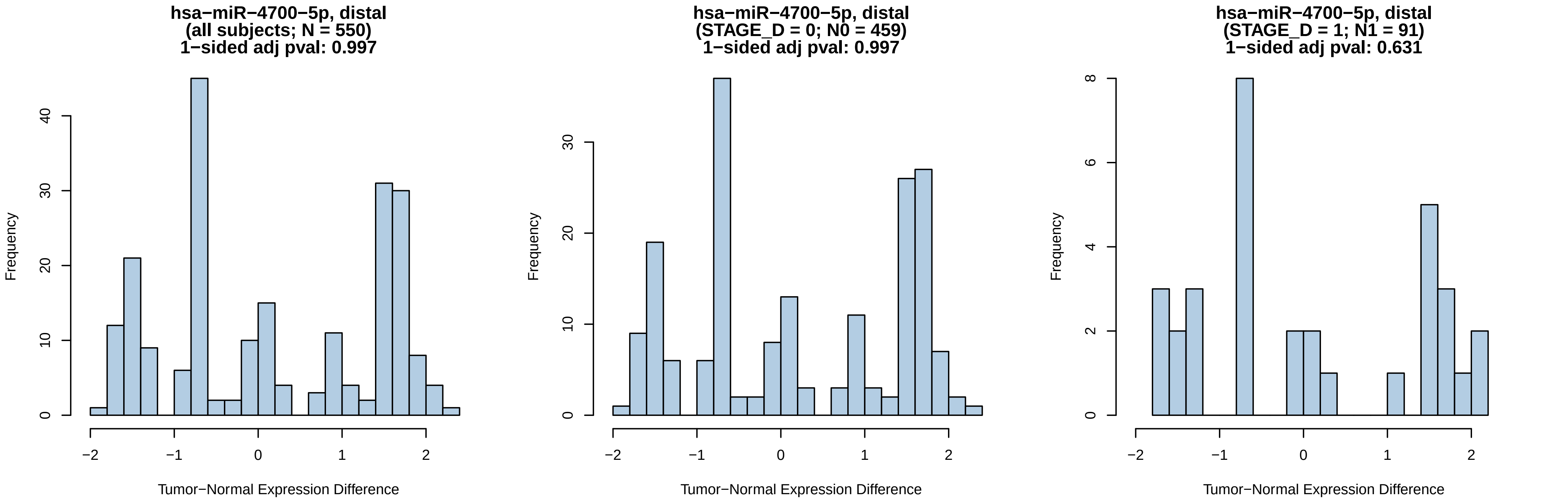

hsa−miR−4700−5p, distal
(all subjects; N = 550)
1−sided adj pval: 0.997
Frequency
0
10
20
30
40
−2
−1
0
1
2
Tumor−Normal Expression Difference
hsa−miR−4700−5p, distal
(STAGE_D = 0; N0 = 459)
1−sided adj pval: 0.997
Frequency
0
10
20
30
−2
−1
0
1
2
Tumor−Normal Expression Difference
hsa−miR−4700−5p, distal
(STAGE_D = 1; N1 = 91)
1−sided adj pval: 0.631
Frequency
0
2
4
6
8
−2
−1
0
1
2
Tumor−Normal Expression Difference

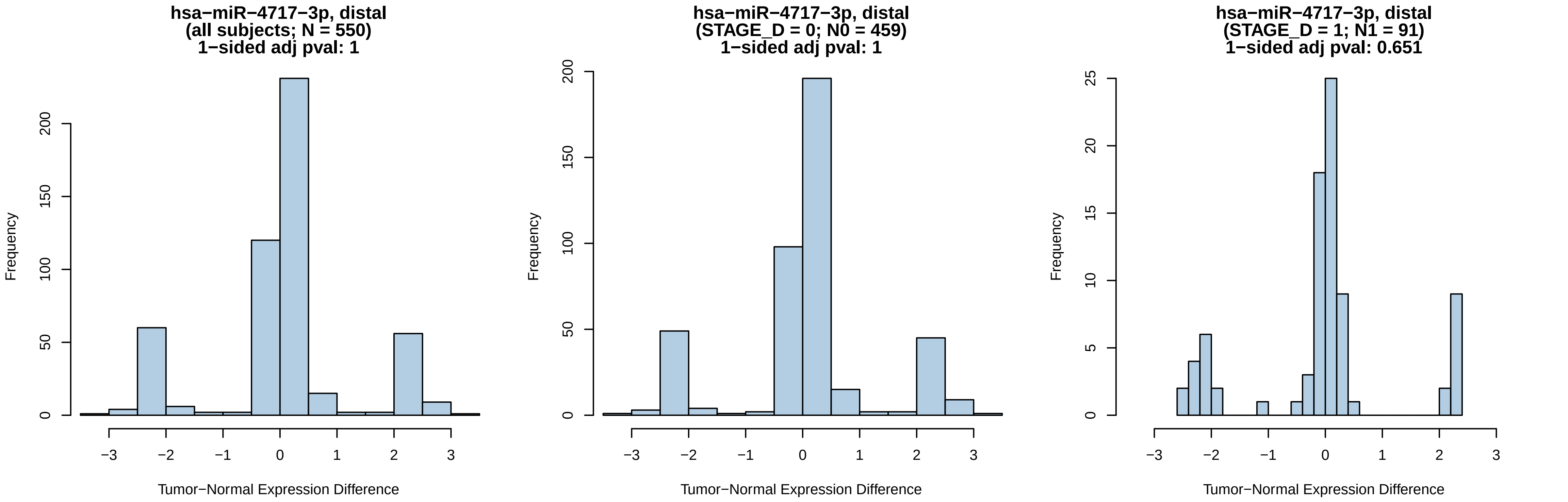
hsa−miR−4717−3p, distal
(all subjects; N = 550)
1−sided adj pval: 1
Frequency
0
50
100
150
200
−3
−2
−1
0
1
2
3
Tumor−Normal Expression Difference
hsa−miR−4717−3p, distal
(STAGE_D = 0; N0 = 459)
1−sided adj pval: 1
Frequency
0
50
100
150
200
−3
−2
−1
0
1
2
3
Tumor−Normal Expression Difference
hsa−miR−4717−3p, distal
(STAGE_D = 1; N1 = 91)
1−sided adj pval: 0.651
Frequency
0
5
10
15
20
25
−3
−2
−1
0
1
2
3
Tumor−Normal Expression Difference

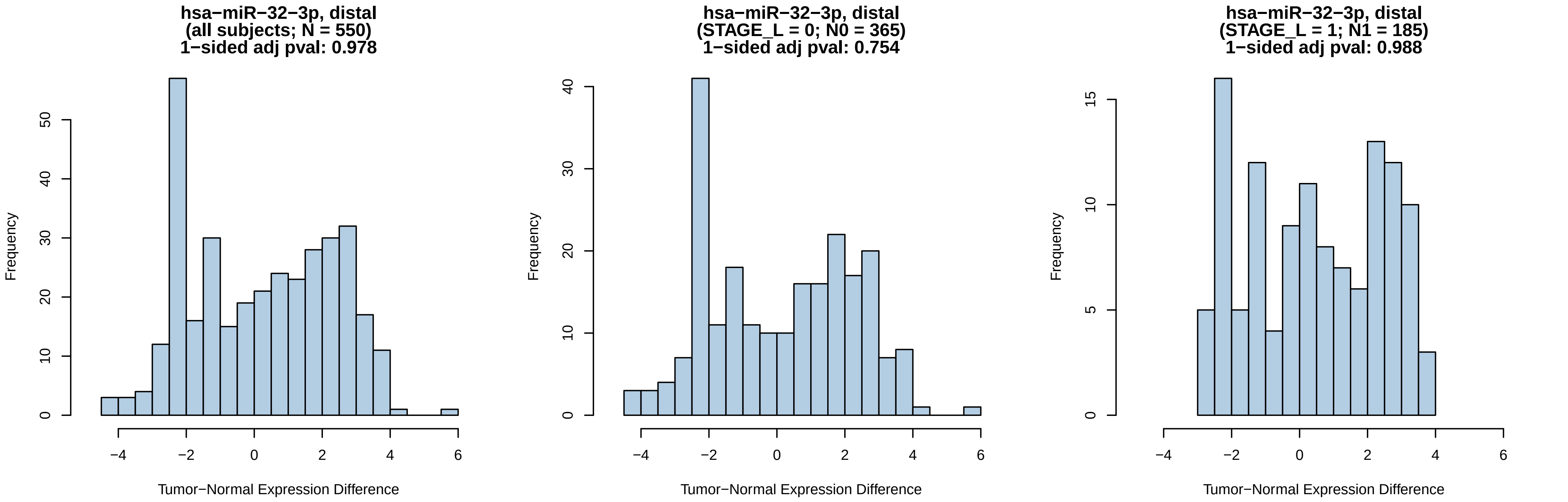

hsa−miR−32−3p, distal
(all subjects; N = 550)
1−sided adj pval: 0.978
Frequency
0
10
20
30
40
50
−4
−2
0
2
4
6
Tumor−Normal Expression Difference
hsa−miR−32−3p, distal
(STAGE_L = 0; N0 = 365)
1−sided adj pval: 0.754
Frequency
0
10
20
30
40
−4
−2
0
2
4
6
Tumor−Normal Expression Difference
hsa−miR−32−3p, distal
(STAGE_L = 1; N1 = 185)
1−sided adj pval: 0.988
Frequency
0
5
10
15
−4
−2
0
2
4
6
Tumor−Normal Expression Difference

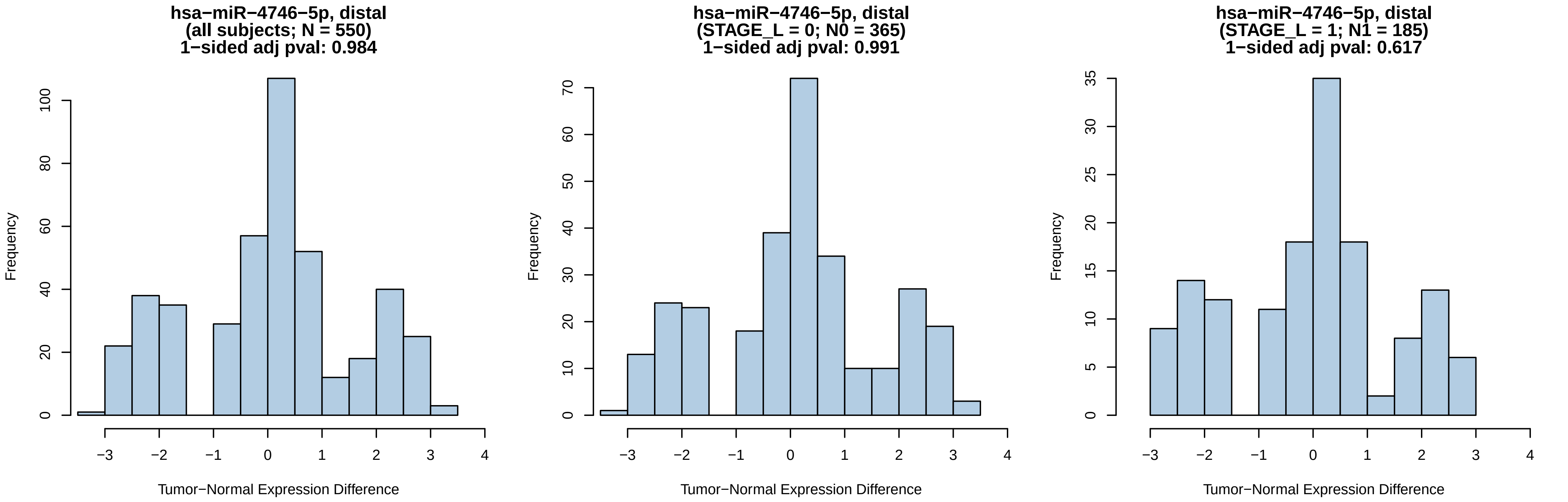

hsa−miR−4746−5p, distal
(all subjects; N = 550)
1−sided adj pval: 0.984
Frequency
Tumor−Normal Expression Difference
hsa−miR−4746−5p, distal
(STAGE_L = 0; N0 = 365)
1−sided adj pval: 0.991
Frequency
Tumor−Normal Expression Difference
hsa−miR−4746−5p, distal
(STAGE_L = 1; N1 = 185)
1−sided adj pval: 0.617
Frequency
Tumor−Normal Expression Difference

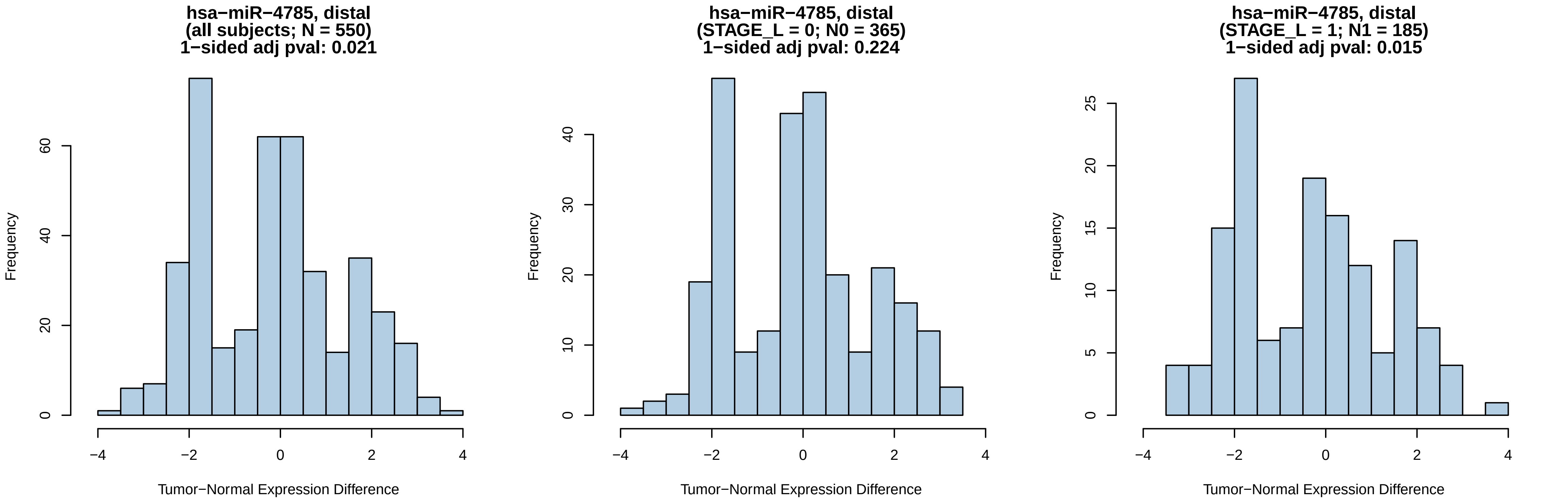
hsa−miR−4785, distal
(all subjects; N = 550)
1−sided adj pval: 0.021
Frequency
Tumor−Normal Expression Difference
hsa−miR−4785, distal
(STAGE_L = 0; N0 = 365)
1−sided adj pval: 0.224
Frequency
Tumor−Normal Expression Difference
hsa−miR−4785, distal
(STAGE_L = 1; N1 = 185)
1−sided adj pval: 0.015
Frequency
Tumor−Normal Expression Difference

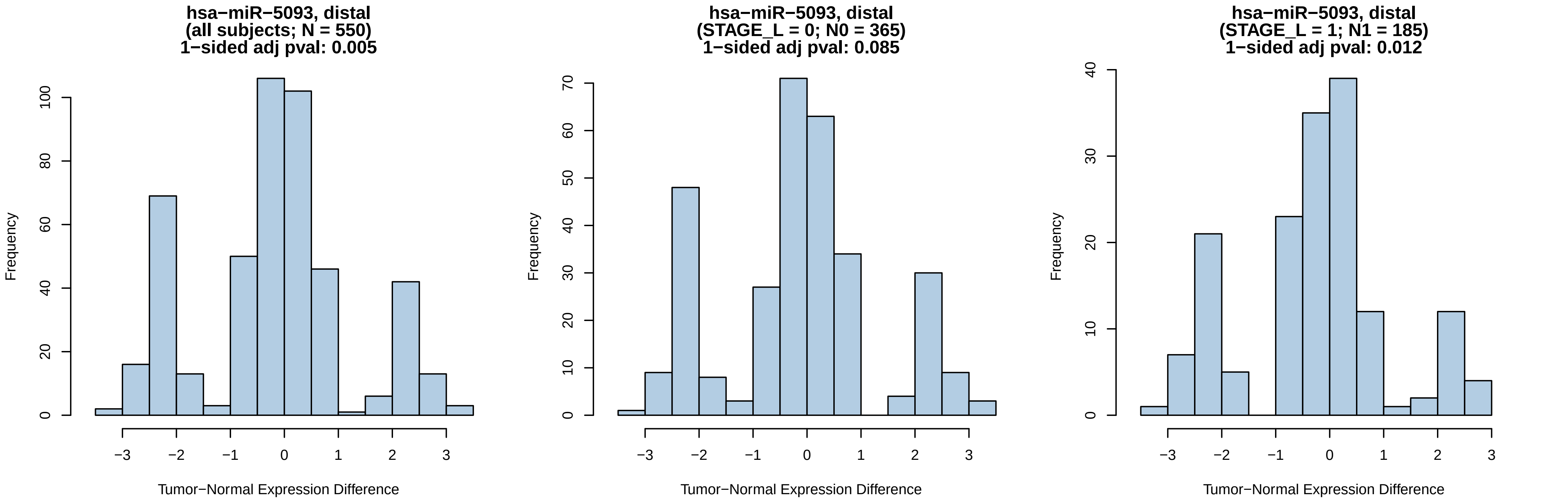
hsa−miR−5093, distal
(all subjects; N = 550)
1−sided adj pval: 0.005
hsa−miR−5093, distal
(STAGE_L = 0; N0 = 365)
1−sided adj pval: 0.085
hsa−miR−5093, distal
(STAGE_L = 1; N1 = 185)
1−sided adj pval: 0.012
Frequency
Tumor−Normal Expression Difference

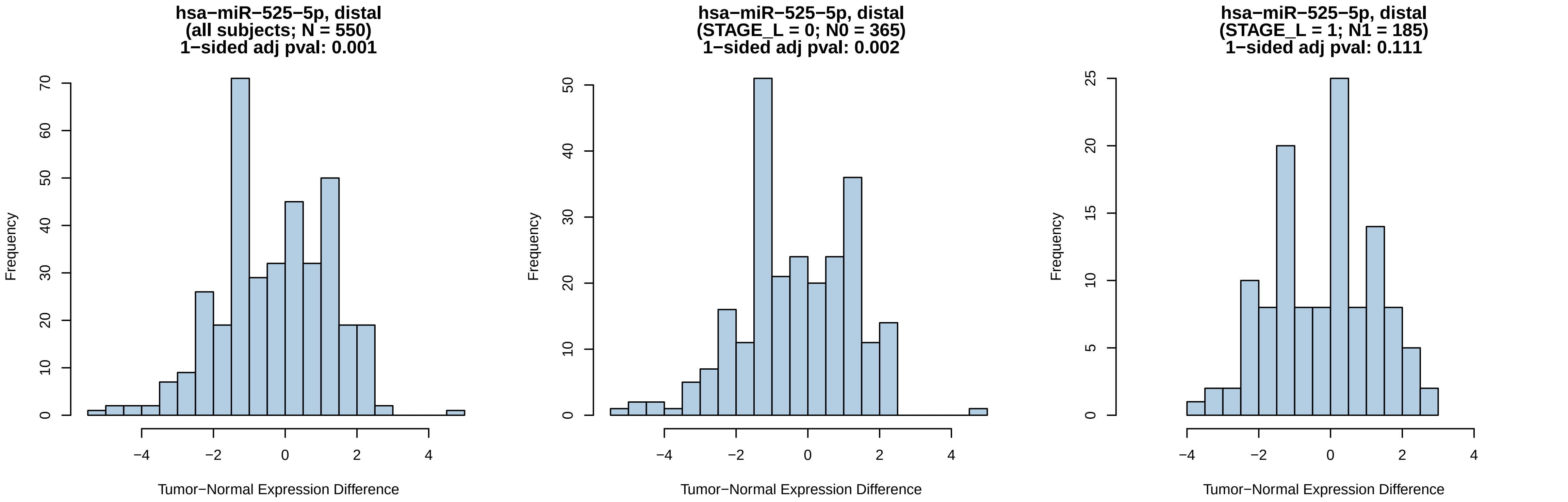

hsa−miR−525−5p, distal
(all subjects; N = 550)
1−sided adj pval: 0.001
Frequency
Tumor−Normal Expression Difference
hsa−miR−525−5p, distal
(STAGE_L = 0; N0 = 365)
1−sided adj pval: 0.002
Frequency
Tumor−Normal Expression Difference
hsa−miR−525−5p, distal
(STAGE_L = 1; N1 = 185)
1−sided adj pval: 0.111
Frequency
Tumor−Normal Expression Difference

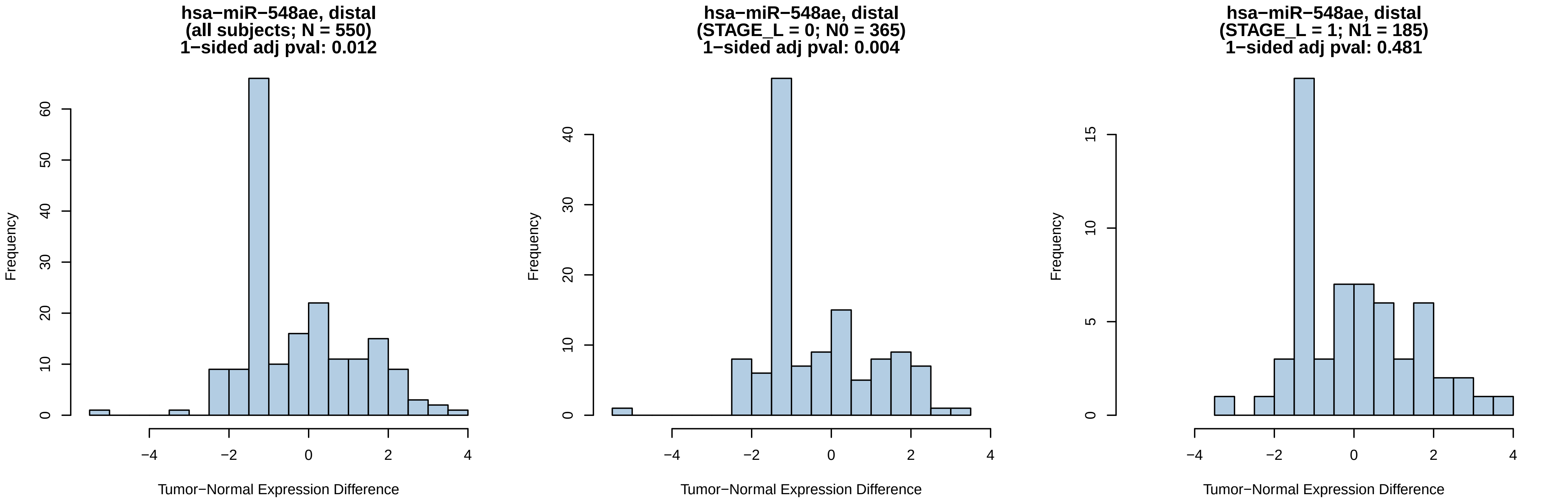
hsa−miR−548ae, distal
(all subjects; N = 550)
1−sided adj pval: 0.012
hsa−miR−548ae, distal
(STAGE_L = 0; N0 = 365)
1−sided adj pval: 0.004
hsa−miR−548ae, distal
(STAGE_L = 1; N1 = 185)
1−sided adj pval: 0.481
Frequency
Tumor−Normal Expression Difference

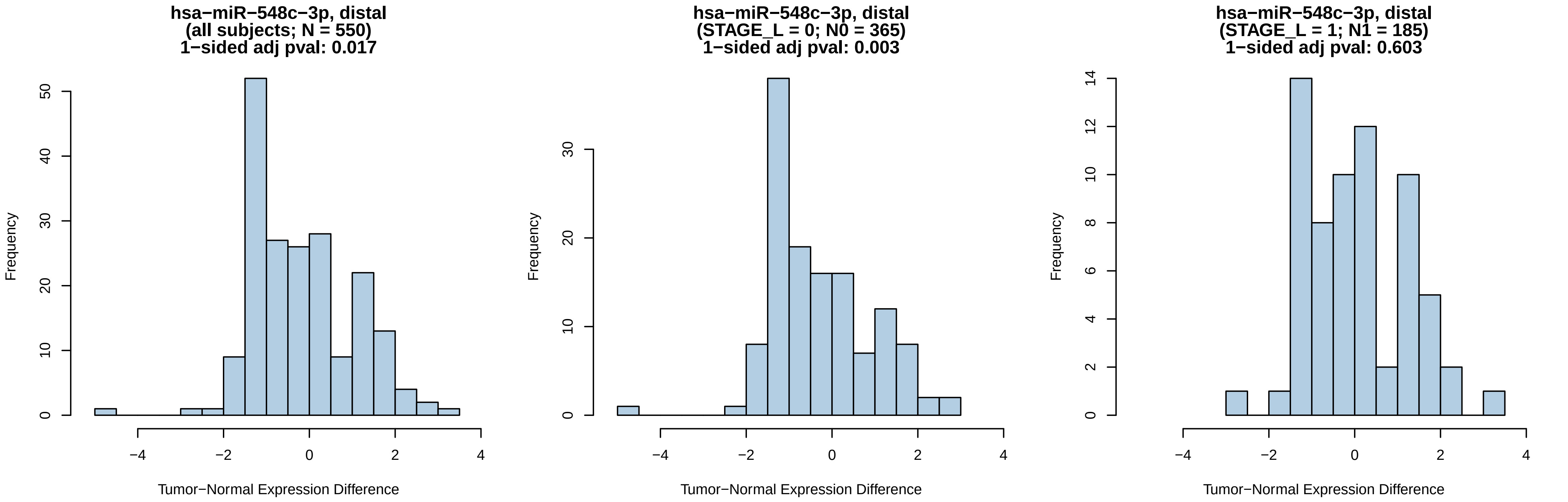

hsa−miR−548c−3p, distal
(all subjects; N = 550)
1−sided adj pval: 0.017
Frequency
Tumor−Normal Expression Difference
hsa−miR−548c−3p, distal
(STAGE_L = 0; N0 = 365)
1−sided adj pval: 0.003
Frequency
Tumor−Normal Expression Difference
hsa−miR−548c−3p, distal
(STAGE_L = 1; N1 = 185)
1−sided adj pval: 0.603
Frequency
Tumor−Normal Expression Difference

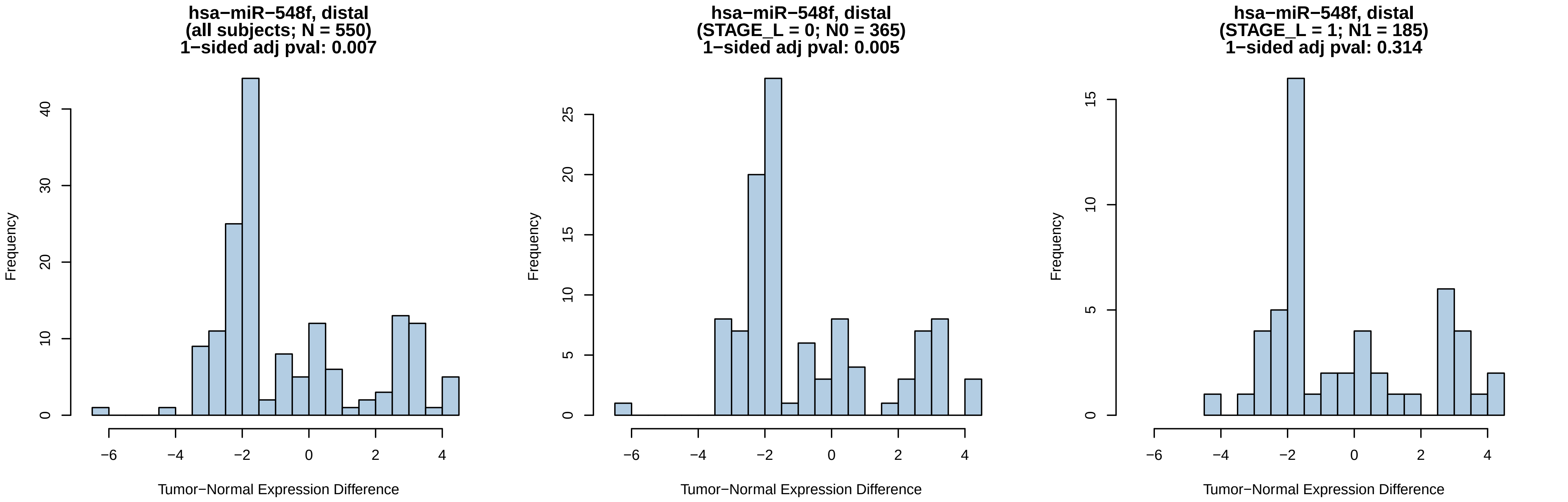
hsa−miR−548f, distal
(all subjects; N = 550)
1−sided adj pval: 0.007
Frequency
0
10
20
30
40
−6
−4
−2
0
2
4
Tumor−Normal Expression Difference
hsa−miR−548f, distal
(STAGE_L = 0; N0 = 365)
1−sided adj pval: 0.005
Frequency
0
5
10
15
20
25
−6
−4
−2
0
2
4
Tumor−Normal Expression Difference
hsa−miR−548f, distal
(STAGE_L = 1; N1 = 185)
1−sided adj pval: 0.314
Frequency
0
5
10
15
−6
−4
−2
0
2
4
Tumor−Normal Expression Difference

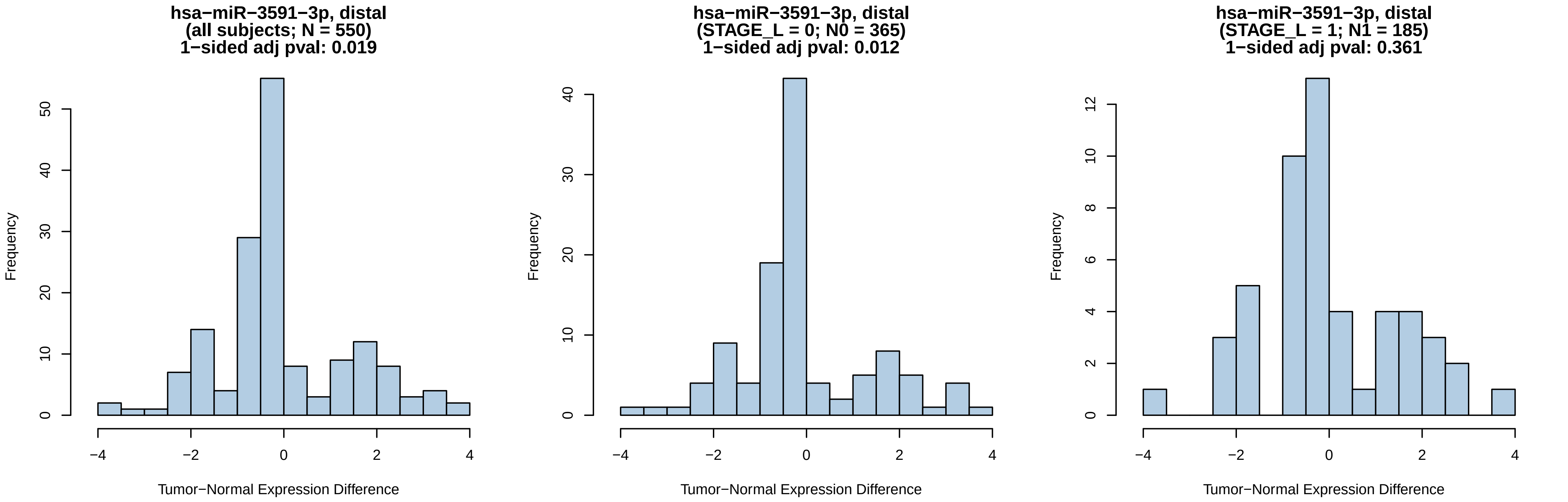
hsa−miR−3591−3p, distal
(all subjects; N = 550)
1−sided adj pval: 0.019
Frequency
Tumor−Normal Expression Difference
hsa−miR−3591−3p, distal
(STAGE_L = 0; N0 = 365)
1−sided adj pval: 0.012
Frequency
Tumor−Normal Expression Difference
hsa−miR−3591−3p, distal
(STAGE_L = 1; N1 = 185)
1−sided adj pval: 0.361
Frequency
Tumor−Normal Expression Difference

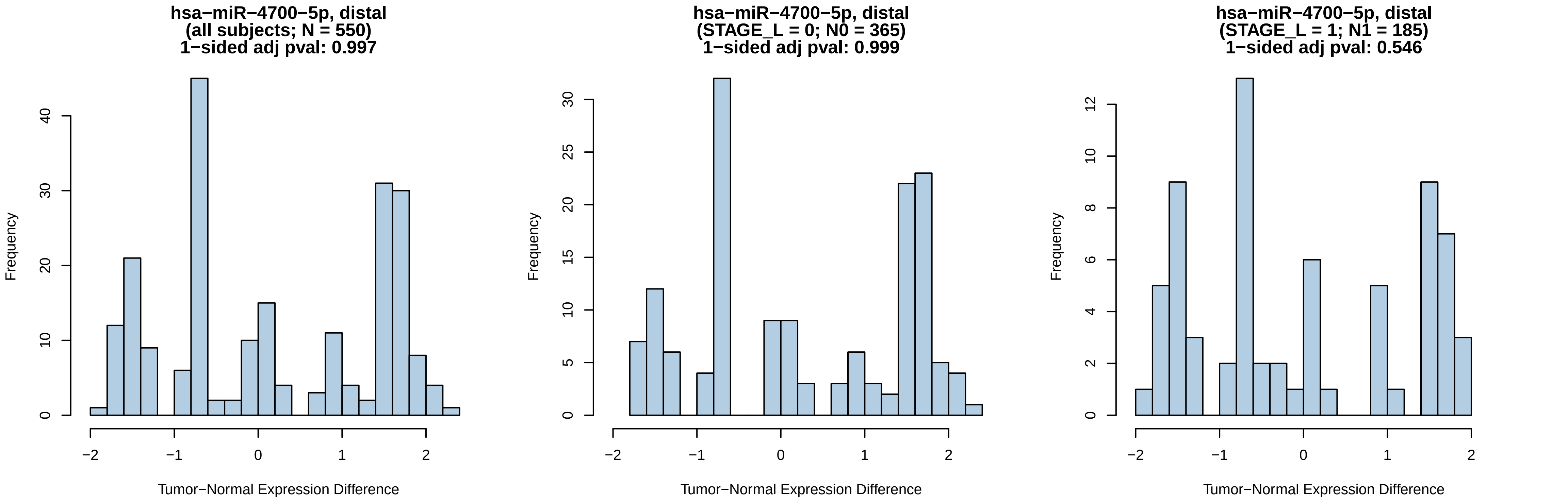
hsa−miR−4700−5p, distal
(all subjects; N = 550)
1−sided adj pval: 0.997
Frequency
Tumor−Normal Expression Difference
hsa−miR−4700−5p, distal
(STAGE_L = 0; N0 = 365)
1−sided adj pval: 0.999
Frequency
Tumor−Normal Expression Difference
hsa−miR−4700−5p, distal
(STAGE_L = 1; N1 = 185)
1−sided adj pval: 0.546
Frequency
Tumor−Normal Expression Difference

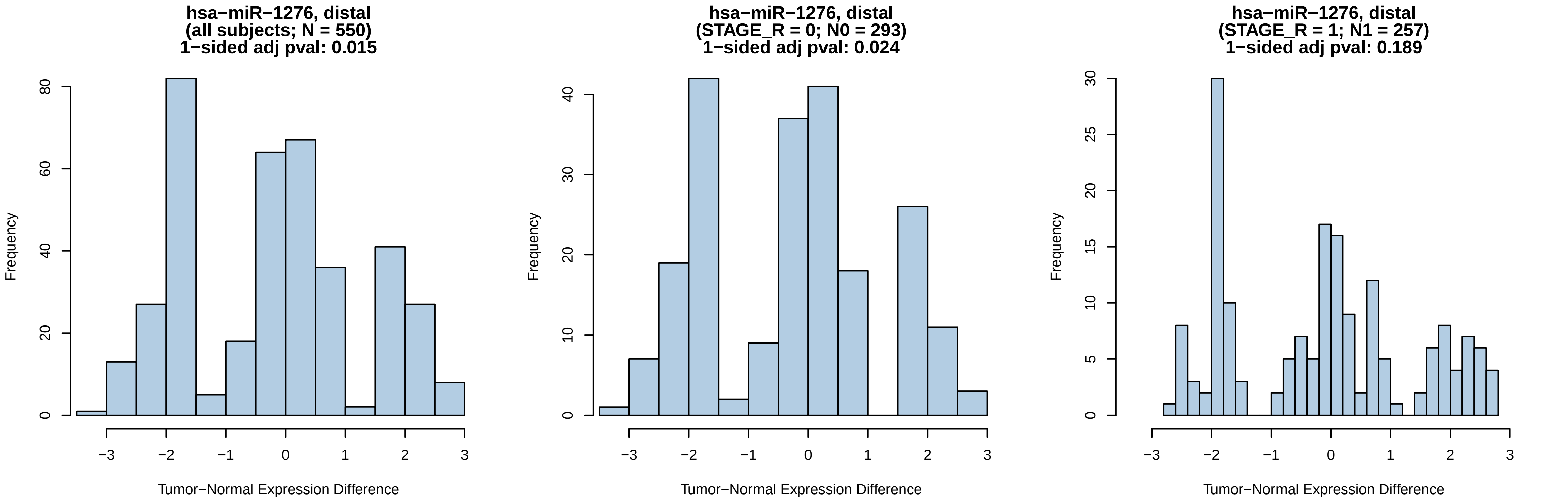

hsa−miR−1276, distal
(all subjects; N = 550)
1−sided adj pval: 0.015
hsa−miR−1276, distal
(STAGE_R = 0; N0 = 293)
1−sided adj pval: 0.024
hsa−miR−1276, distal
(STAGE_R = 1; N1 = 257)
1−sided adj pval: 0.189
Frequency
Tumor−Normal Expression Difference

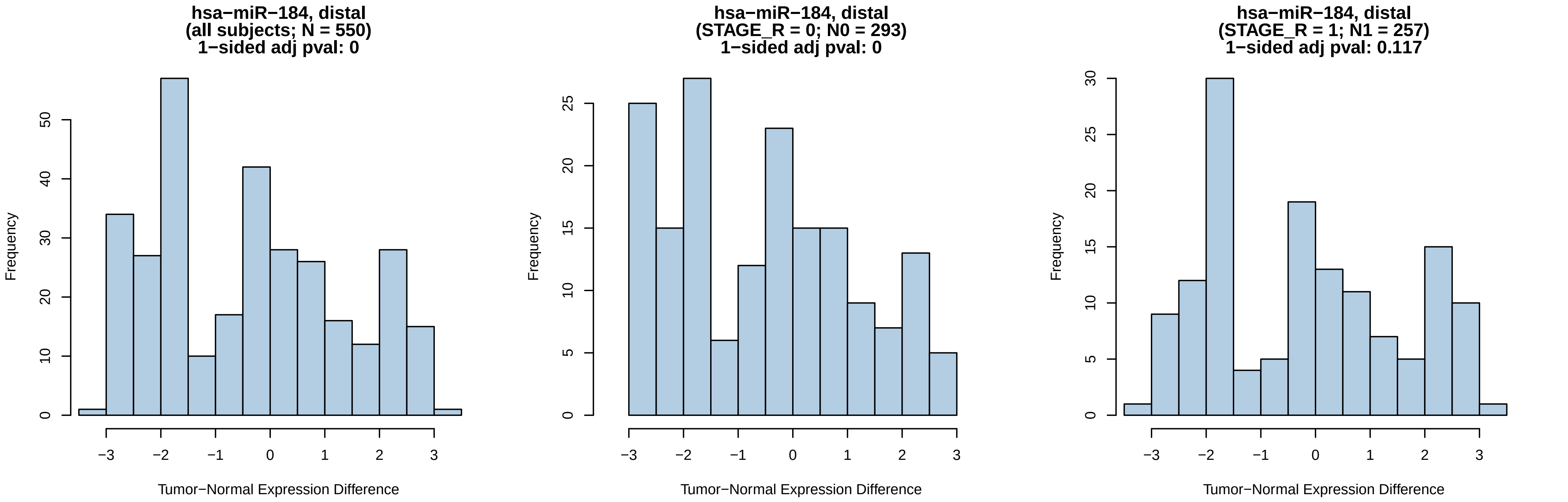
hsa−miR−184, distal
(all subjects; N = 550)
1−sided adj pval: 0
Frequency
Tumor−Normal Expression Difference
hsa−miR−184, distal
(STAGE_R = 0; N0 = 293)
1−sided adj pval: 0
Frequency
Tumor−Normal Expression Difference
hsa−miR−184, distal
(STAGE_R = 1; N1 = 257)
1−sided adj pval: 0.117
Frequency
Tumor−Normal Expression Difference

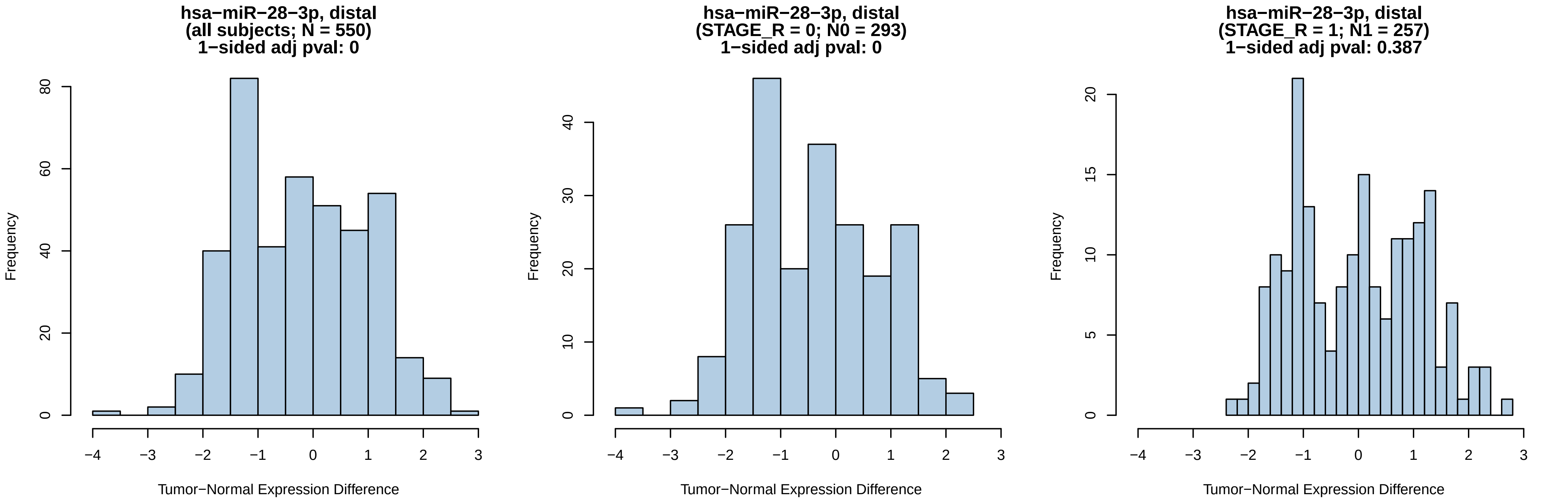
hsa−miR−28−3p, distal
(all subjects; N = 550)
1−sided adj pval: 0
hsa−miR−28−3p, distal
(STAGE_R = 0; N0 = 293)
1−sided adj pval: 0
hsa−miR−28−3p, distal
(STAGE_R = 1; N1 = 257)
1−sided adj pval: 0.387
Frequency
Tumor−Normal Expression Difference

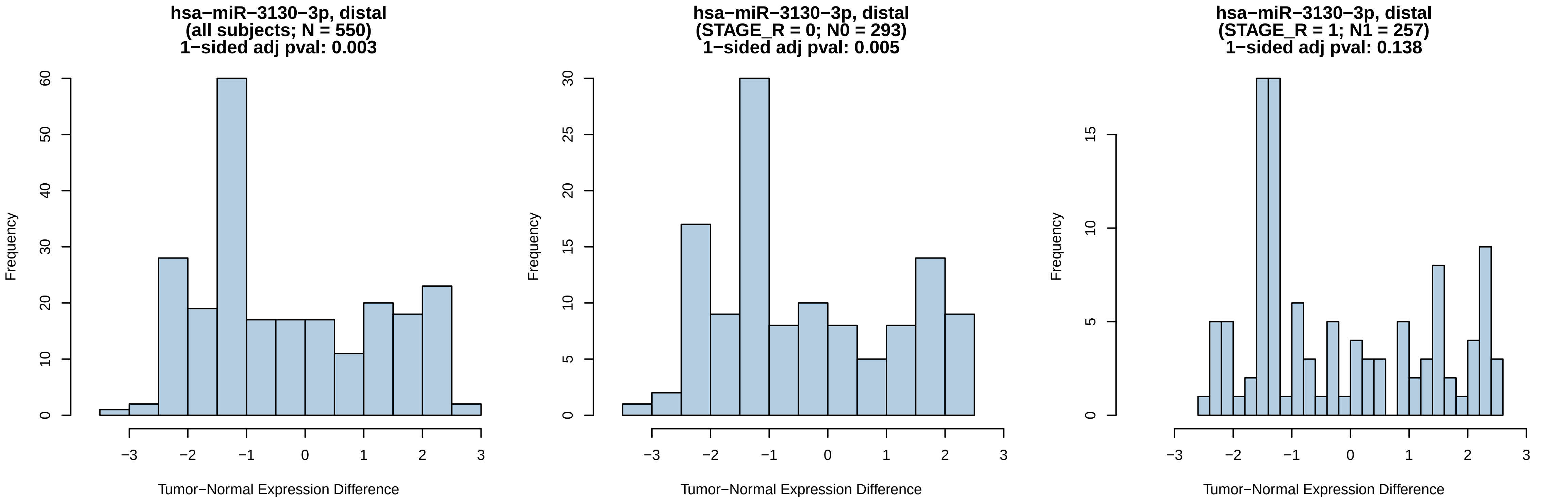

hsa−miR−3130−3p, distal
(all subjects; N = 550)
1−sided adj pval: 0.003
Frequency
Tumor−Normal Expression Difference
hsa−miR−3130−3p, distal
(STAGE_R = 0; N0 = 293)
1−sided adj pval: 0.005
Frequency
Tumor−Normal Expression Difference
hsa−miR−3130−3p, distal
(STAGE_R = 1; N1 = 257)
1−sided adj pval: 0.138
Frequency
Tumor−Normal Expression Difference

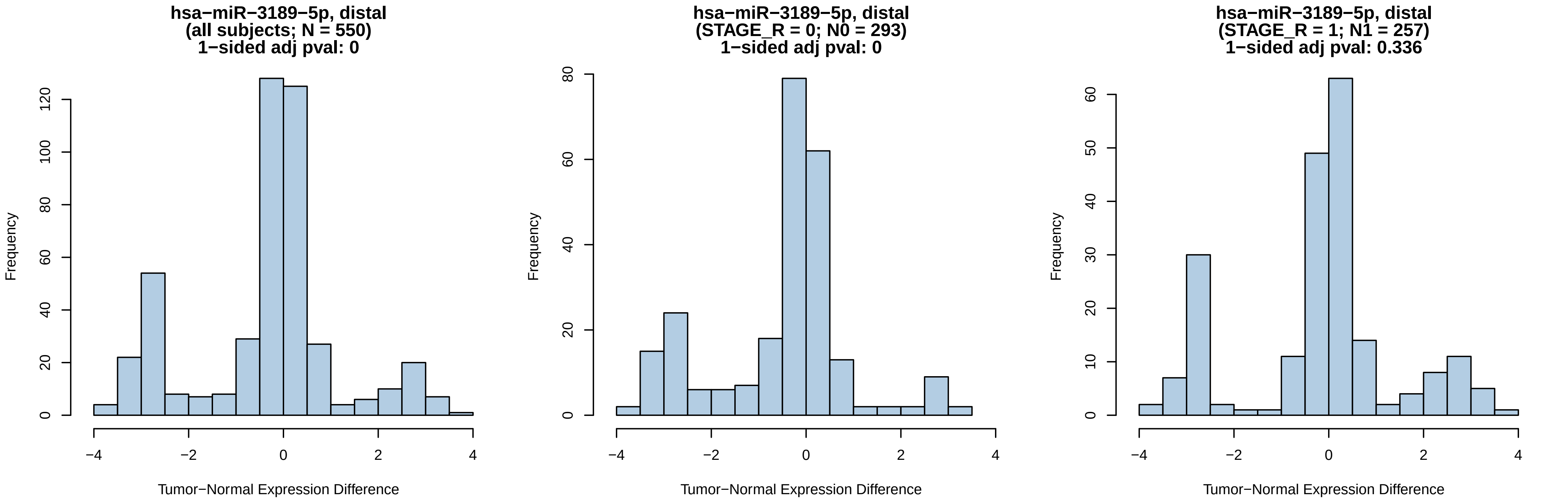
hsa−miR−3189−5p, distal
(all subjects; N = 550)
1−sided adj pval: 0
Frequency
Tumor−Normal Expression Difference
hsa−miR−3189−5p, distal
(STAGE_R = 0; N0 = 293)
1−sided adj pval: 0
Frequency
Tumor−Normal Expression Difference
hsa−miR−3189−5p, distal
(STAGE_R = 1; N1 = 257)
1−sided adj pval: 0.336
Frequency
Tumor−Normal Expression Difference

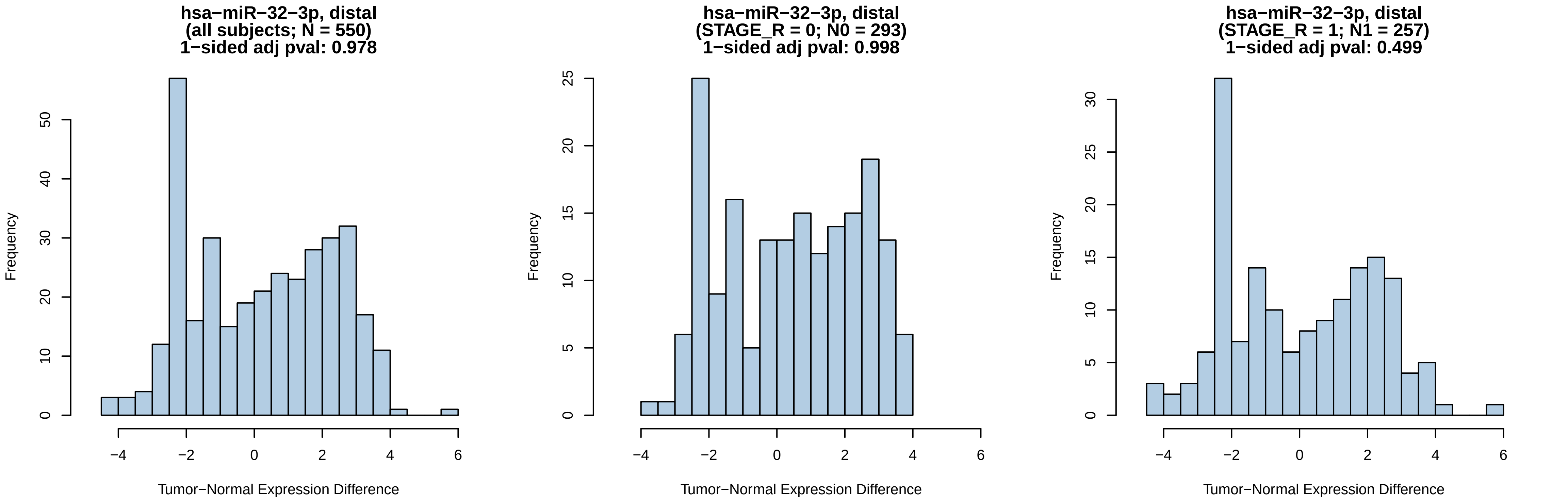
hsa−miR−32−3p, distal
(all subjects; N = 550)
1−sided adj pval: 0.978
hsa−miR−32−3p, distal
(STAGE_R = 0; N0 = 293)
1−sided adj pval: 0.998
hsa−miR−32−3p, distal
(STAGE_R = 1; N1 = 257)
1−sided adj pval: 0.499
Frequency
Tumor−Normal Expression Difference

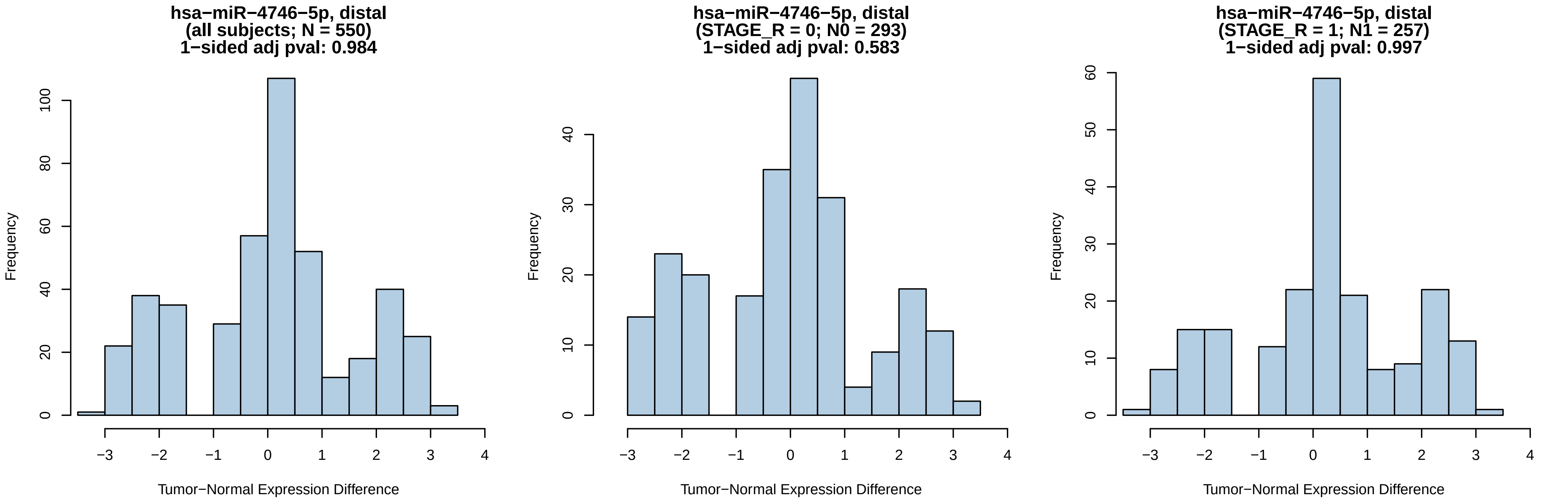
hsa−miR−4746−5p, distal
(all subjects; N = 550)
1−sided adj pval: 0.984
Frequency
Tumor−Normal Expression Difference
hsa−miR−4746−5p, distal
(STAGE_R = 0; N0 = 293)
1−sided adj pval: 0.583
Frequency
Tumor−Normal Expression Difference
hsa−miR−4746−5p, distal
(STAGE_R = 1; N1 = 257)
1−sided adj pval: 0.997
Frequency
Tumor−Normal Expression Difference

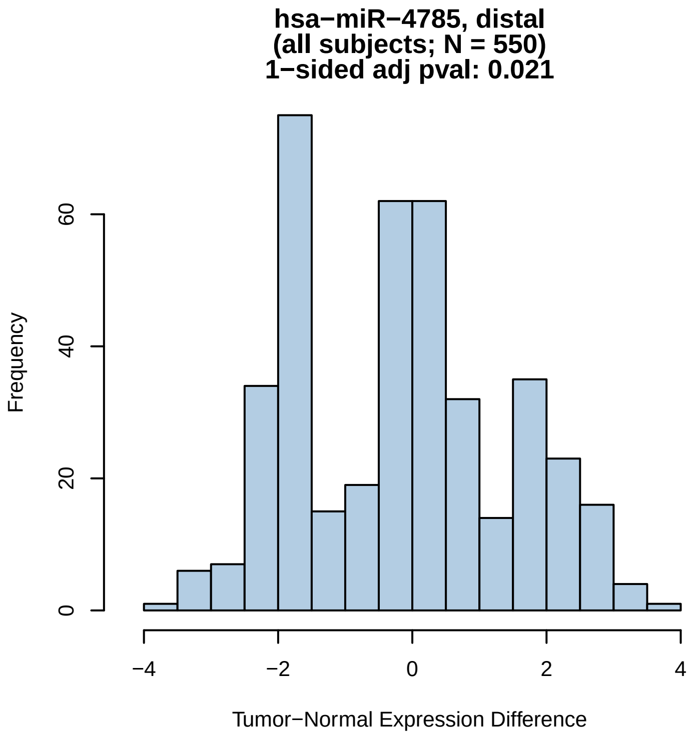
hsa−miR−4785, distal
(all subjects; N = 550)
1−sided adj pval: 0.021
Frequency
0
20
40
60
−4
−2
0
2
4
Tumor−Normal Expression Difference

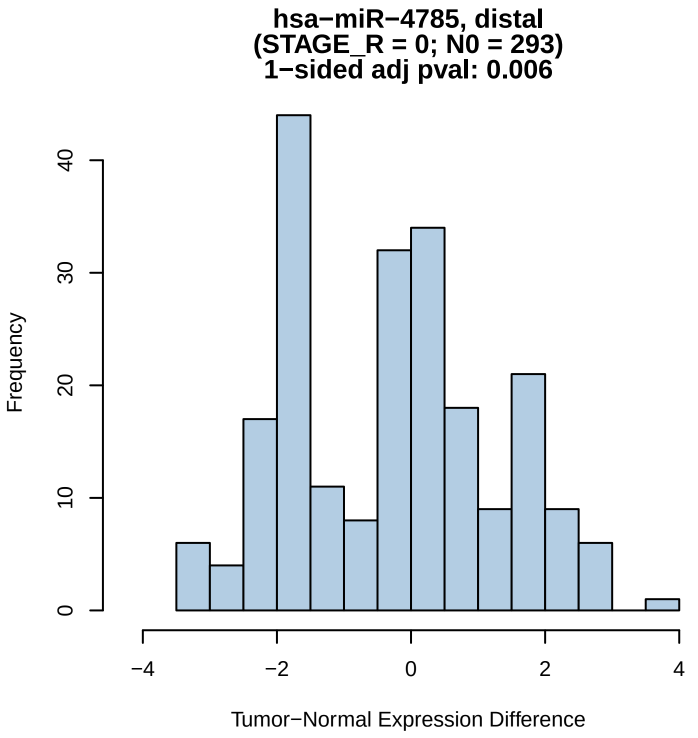
hsa−miR−4785, distal
(STAGE_R = 0; N0 = 293)
1−sided adj pval: 0.006
Frequency
0
10
20
30
40
−4
−2
0
2
4
Tumor−Normal Expression Difference

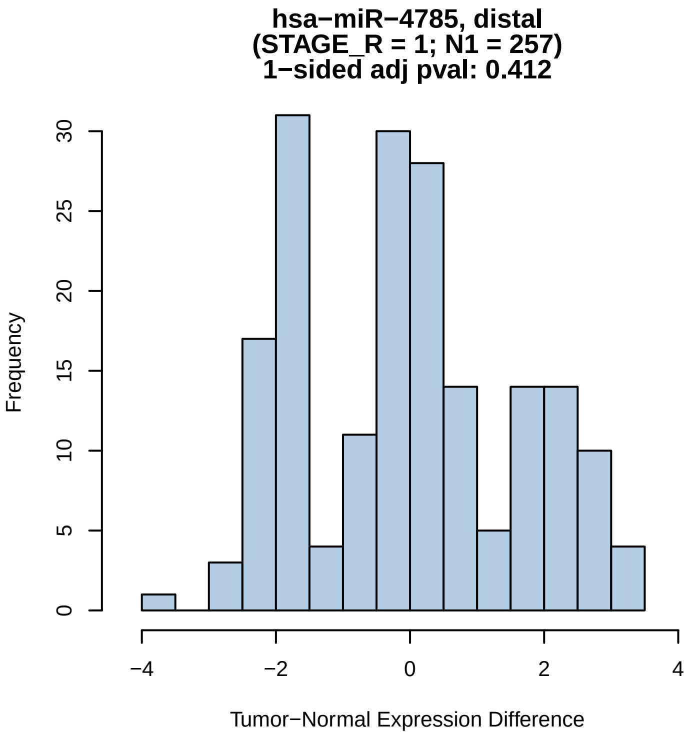
hsa−miR−4785, distal
(STAGE_R = 1; N1 = 257)
1−sided adj pval: 0.412
Frequency
0
5
10
15
20
25
30
−4
−2
0
2
4
Tumor−Normal Expression Difference

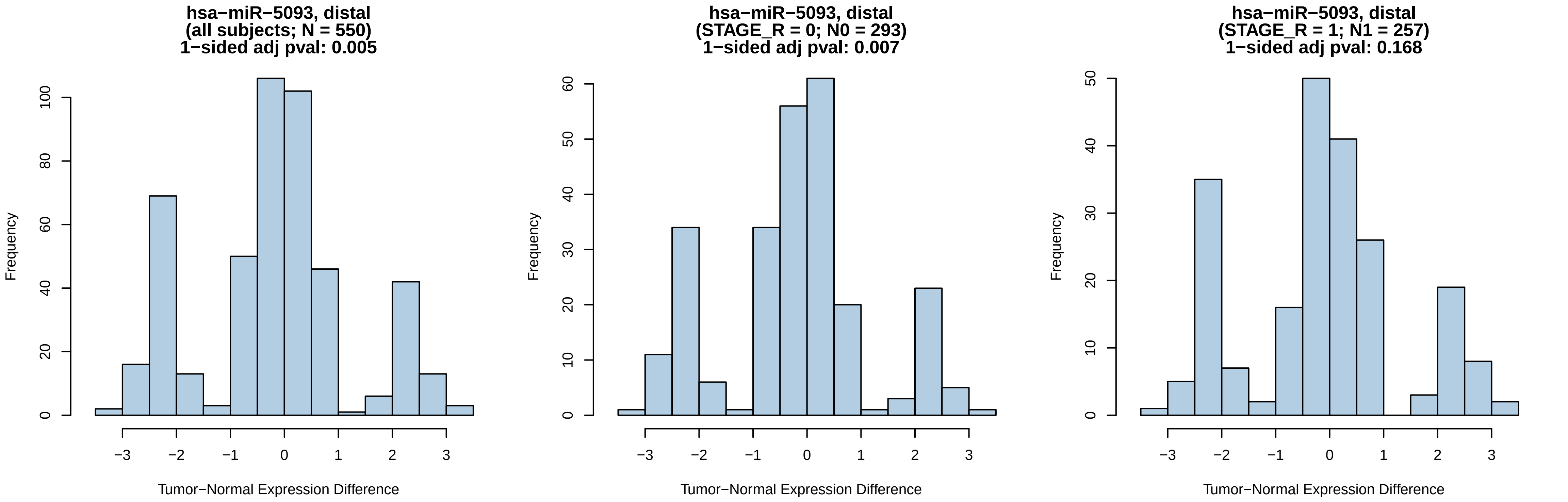

hsa−miR−5093, distal
(all subjects; N = 550)
1−sided adj pval: 0.005
hsa−miR−5093, distal
(STAGE_R = 0; N0 = 293)
1−sided adj pval: 0.007
hsa−miR−5093, distal
(STAGE_R = 1; N1 = 257)
1−sided adj pval: 0.168
Frequency
Tumor−Normal Expression Difference

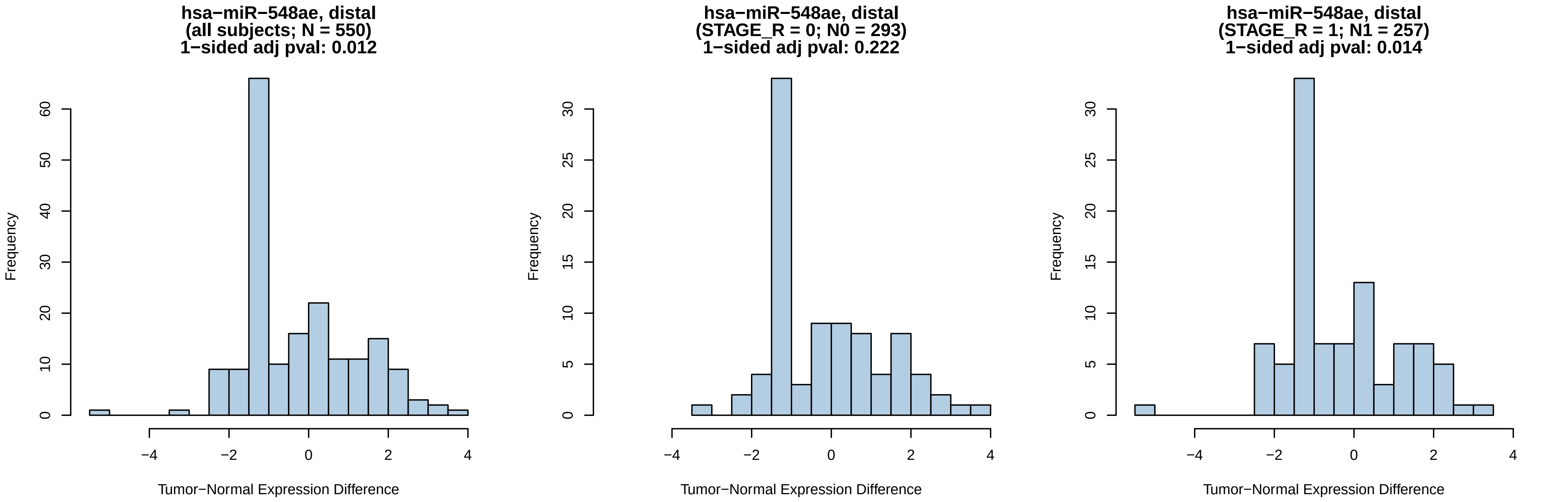

hsa−miR−548ae, distal
(all subjects; N = 550)
1−sided adj pval: 0.012
Frequency
0
10
20
30
40
50
60
−4
−2
0
2
4
Tumor−Normal Expression Difference
hsa−miR−548ae, distal
(STAGE_R = 0; N0 = 293)
1−sided adj pval: 0.222
Frequency
0
5
10
15
20
25
30
−4
−2
0
2
4
Tumor−Normal Expression Difference
hsa−miR−548ae, distal
(STAGE_R = 1; N1 = 257)
1−sided adj pval: 0.014
Frequency
0
5
10
15
20
25
30
−4
−2
0
2
4
Tumor−Normal Expression Difference

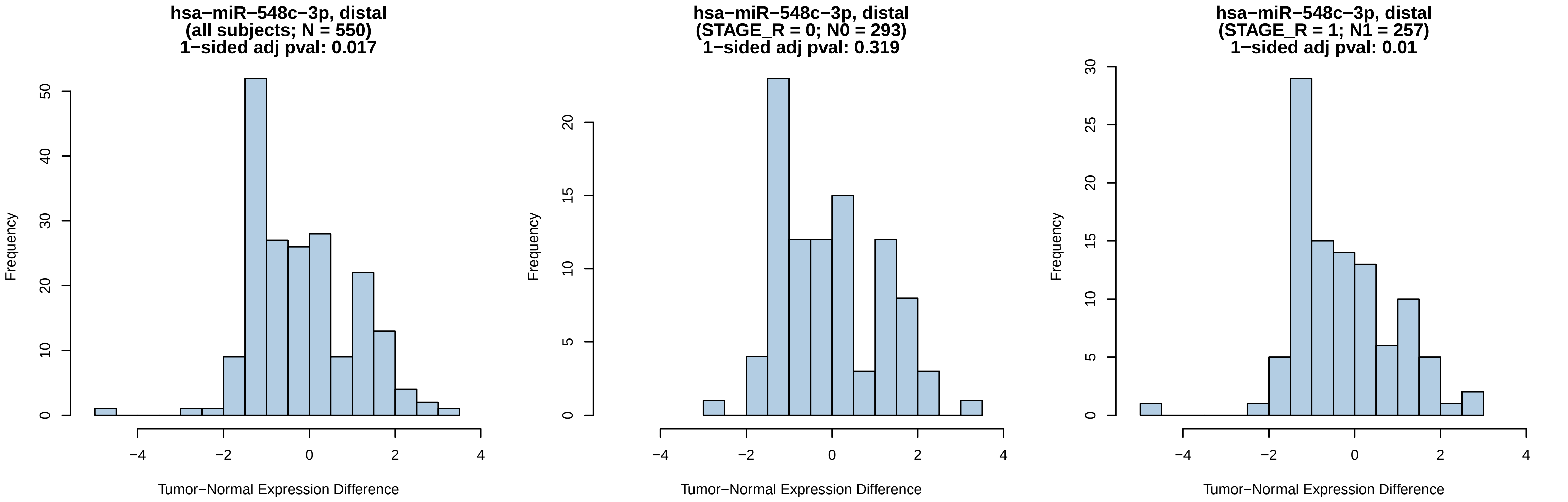
hsa−miR−548c−3p, distal
(all subjects; N = 550)
1−sided adj pval: 0.017
Frequency
0
10
20
30
40
50
−4
−2
0
2
4
Tumor−Normal Expression Difference
hsa−miR−548c−3p, distal
(STAGE_R = 0; N0 = 293)
1−sided adj pval: 0.319
Frequency
0
5
10
15
20
−4
−2
0
2
4
Tumor−Normal Expression Difference
hsa−miR−548c−3p, distal
(STAGE_R = 1; N1 = 257)
1−sided adj pval: 0.01
Frequency
0
5
10
15
20
25
30
−4
−2
0
2
4
Tumor−Normal Expression Difference

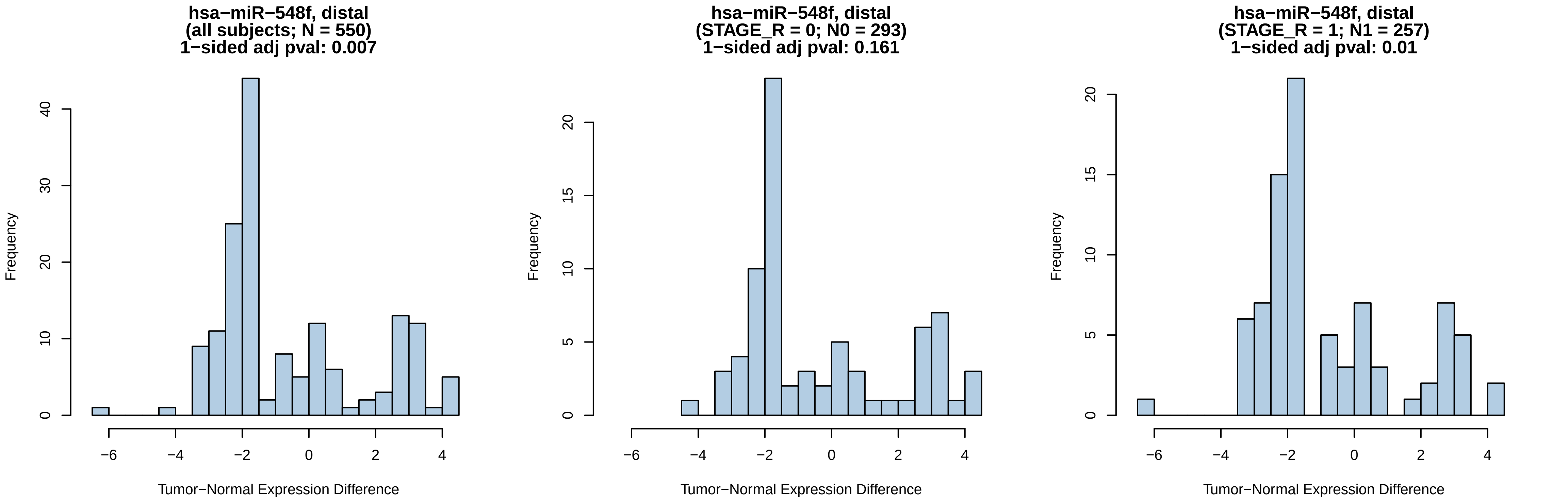
hsa−miR−548f, distal
(all subjects; N = 550)
1−sided adj pval: 0.007
Frequency
Tumor−Normal Expression Difference
hsa−miR−548f, distal
(STAGE_R = 0; N0 = 293)
1−sided adj pval: 0.161
Frequency
Tumor−Normal Expression Difference
hsa−miR−548f, distal
(STAGE_R = 1; N1 = 257)
1−sided adj pval: 0.01
Frequency
Tumor−Normal Expression Difference

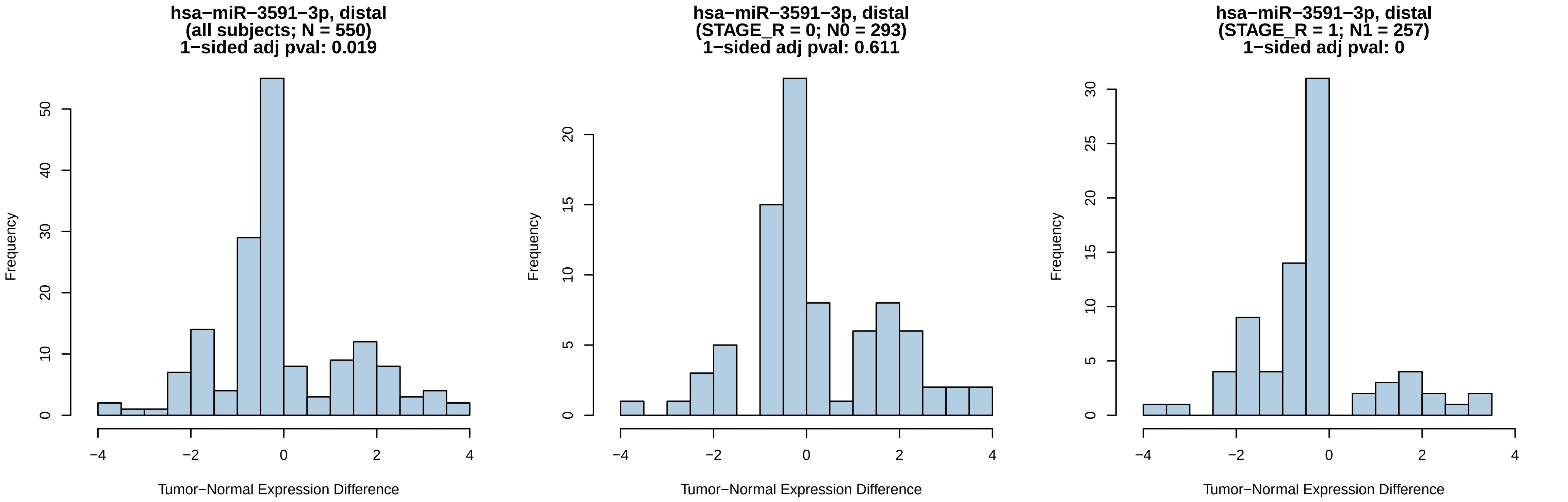

hsa−miR−3591−3p, distal
(all subjects; N = 550)
1−sided adj pval: 0.019
hsa−miR−3591−3p, distal
(STAGE_R = 0; N0 = 293)
1−sided adj pval: 0.611
hsa−miR−3591−3p, distal
(STAGE_R = 1; N1 = 257)
1−sided adj pval: 0
Frequency
Tumor−Normal Expression Difference

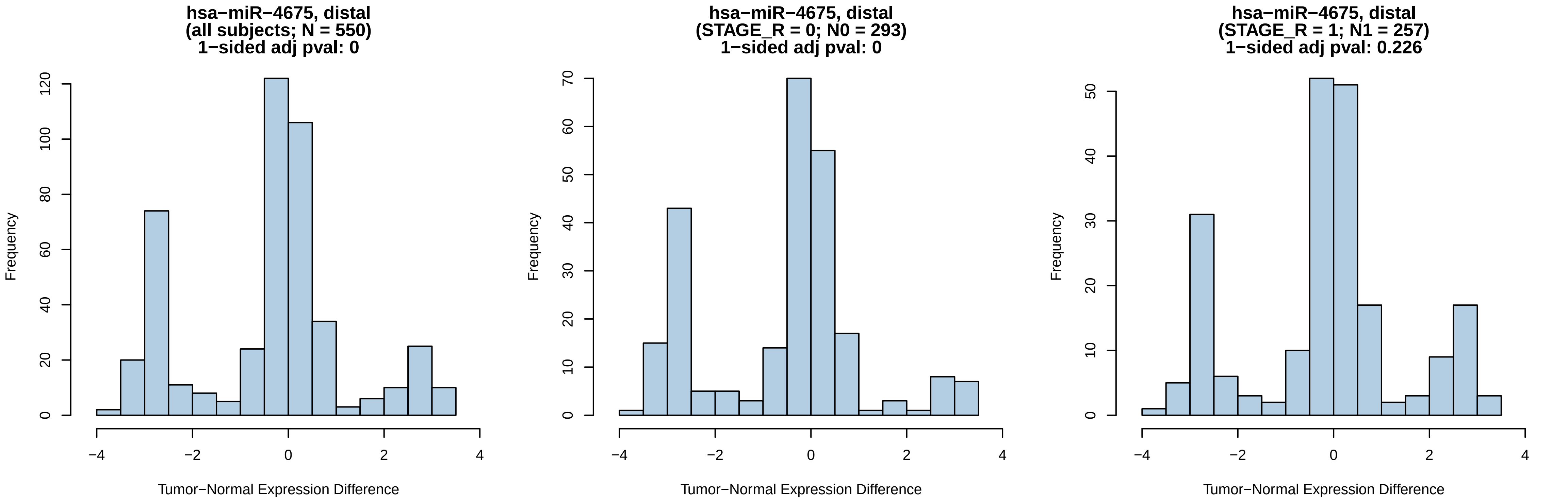

hsa−miR−4675, distal
(all subjects; N = 550)
1−sided adj pval: 0
Frequency
Tumor−Normal Expression Difference
hsa−miR−4675, distal
(STAGE_R = 0; N0 = 293)
1−sided adj pval: 0
Frequency
Tumor−Normal Expression Difference
hsa−miR−4675, distal
(STAGE_R = 1; N1 = 257)
1−sided adj pval: 0.226
Frequency
Tumor−Normal Expression Difference

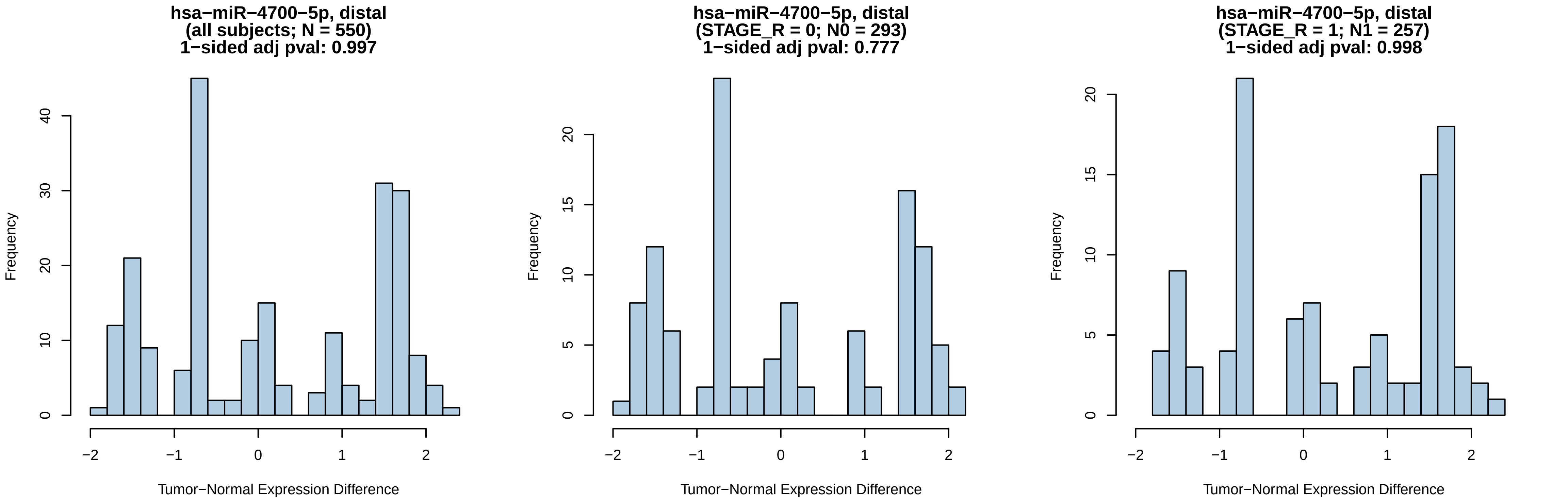

hsa−miR−4700−5p, distal
(all subjects; N = 550)
1−sided adj pval: 0.997
Frequency
0
10
20
30
40
−2
−1
0
1
2
Tumor−Normal Expression Difference
hsa−miR−4700−5p, distal
(STAGE_R = 0; N0 = 293)
1−sided adj pval: 0.777
Frequency
0
5
10
15
20
−2
−1
0
1
2
Tumor−Normal Expression Difference
hsa−miR−4700−5p, distal
(STAGE_R = 1; N1 = 257)
1−sided adj pval: 0.998
Frequency
0
5
10
15
20
−2
−1
0
1
2
Tumor−Normal Expression Difference

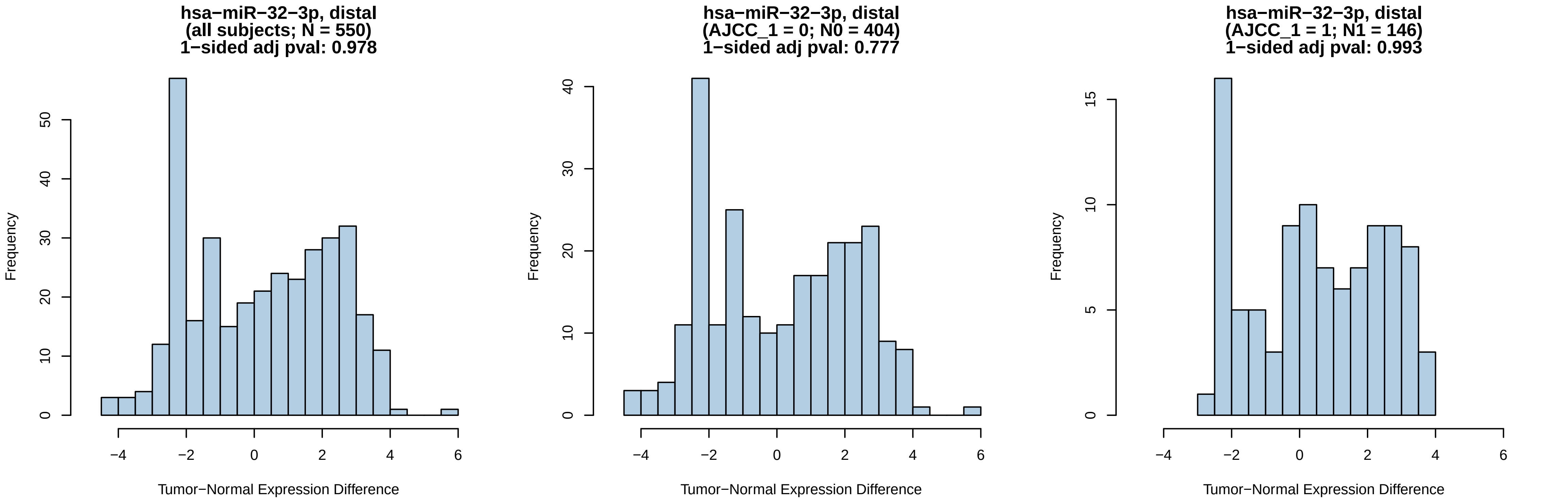
hsa−miR−32−3p, distal
(all subjects; N = 550)
1−sided adj pval: 0.978
Frequency
Tumor−Normal Expression Difference
hsa−miR−32−3p, distal
(AJCC_1 = 0; N0 = 404)
1−sided adj pval: 0.777
Frequency
Tumor−Normal Expression Difference
hsa−miR−32−3p, distal
(AJCC_1 = 1; N1 = 146)
1−sided adj pval: 0.993
Frequency
Tumor−Normal Expression Difference

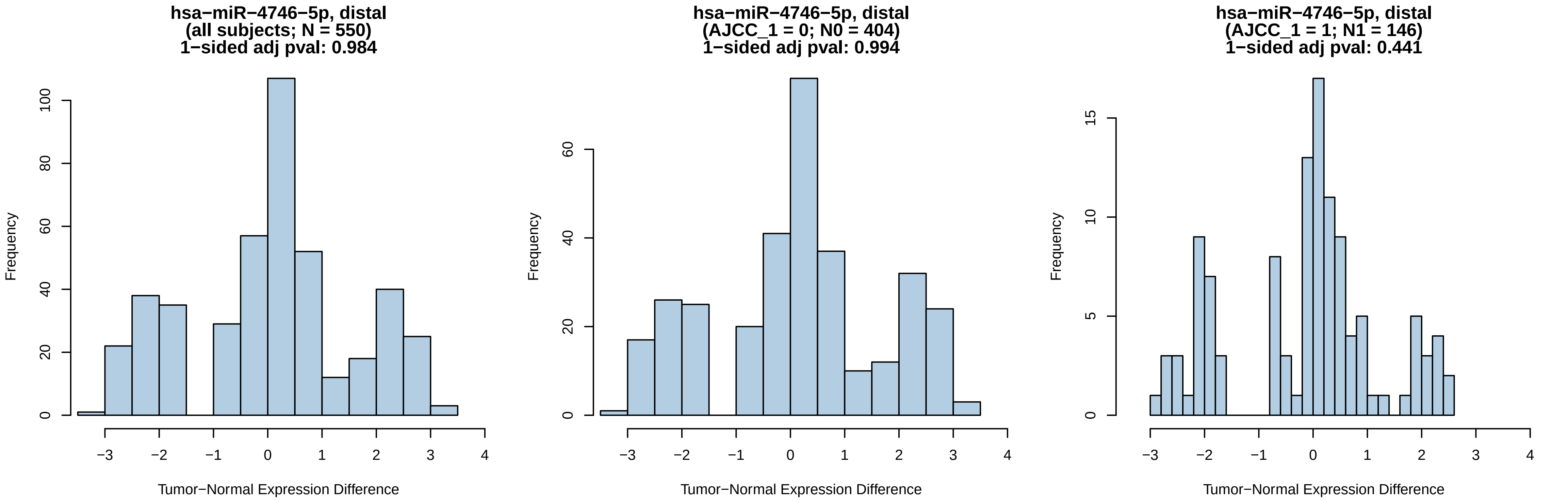
hsa−miR−4746−5p, distal
(all subjects; N = 550)
1−sided adj pval: 0.984
Frequency
0
20
40
60
80
100
−3
−2
−1
0
1
2
3
4
Tumor−Normal Expression Difference
hsa−miR−4746−5p, distal
(AJCC_1 = 0; N0 = 404)
1−sided adj pval: 0.994
Frequency
0
20
40
60
−3
−2
−1
0
1
2
3
4
Tumor−Normal Expression Difference
hsa−miR−4746−5p, distal
(AJCC_1 = 1; N1 = 146)
1−sided adj pval: 0.441
Frequency
0
5
10
15
−3
−2
−1
0
1
2
3
4
Tumor−Normal Expression Difference

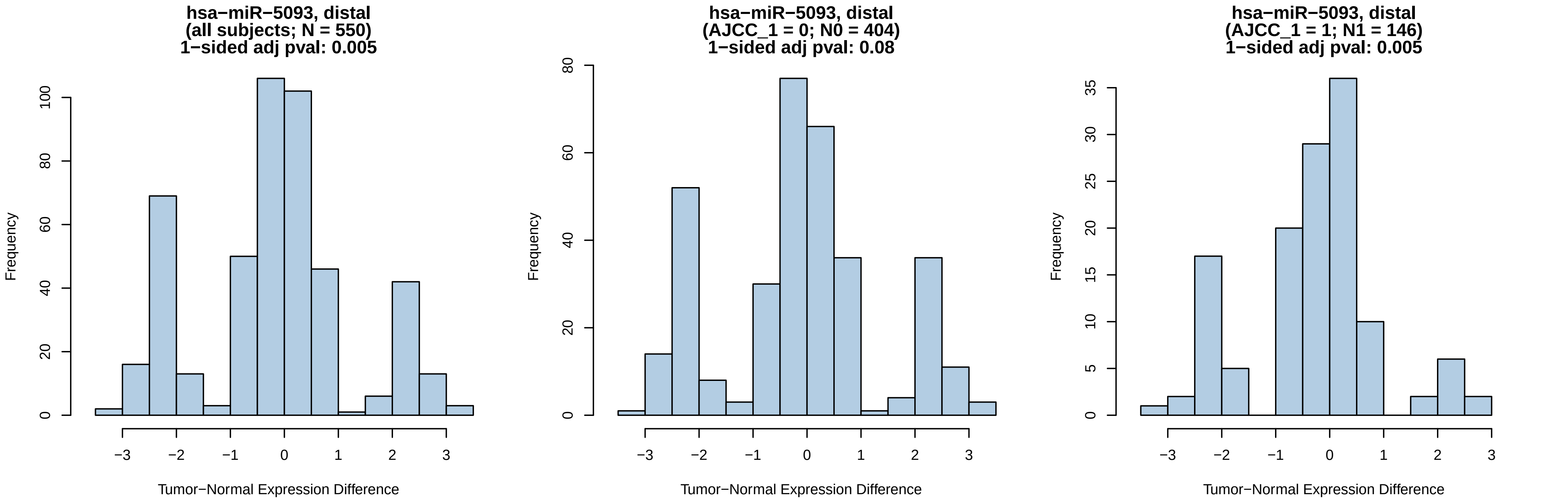
hsa−miR−5093, distal
(all subjects; N = 550)
1−sided adj pval: 0.005
Frequency
Tumor−Normal Expression Difference
hsa−miR−5093, distal
(AJCC_1 = 0; N0 = 404)
1−sided adj pval: 0.08
Frequency
Tumor−Normal Expression Difference
hsa−miR−5093, distal
(AJCC_1 = 1; N1 = 146)
1−sided adj pval: 0.005
Frequency
Tumor−Normal Expression Difference

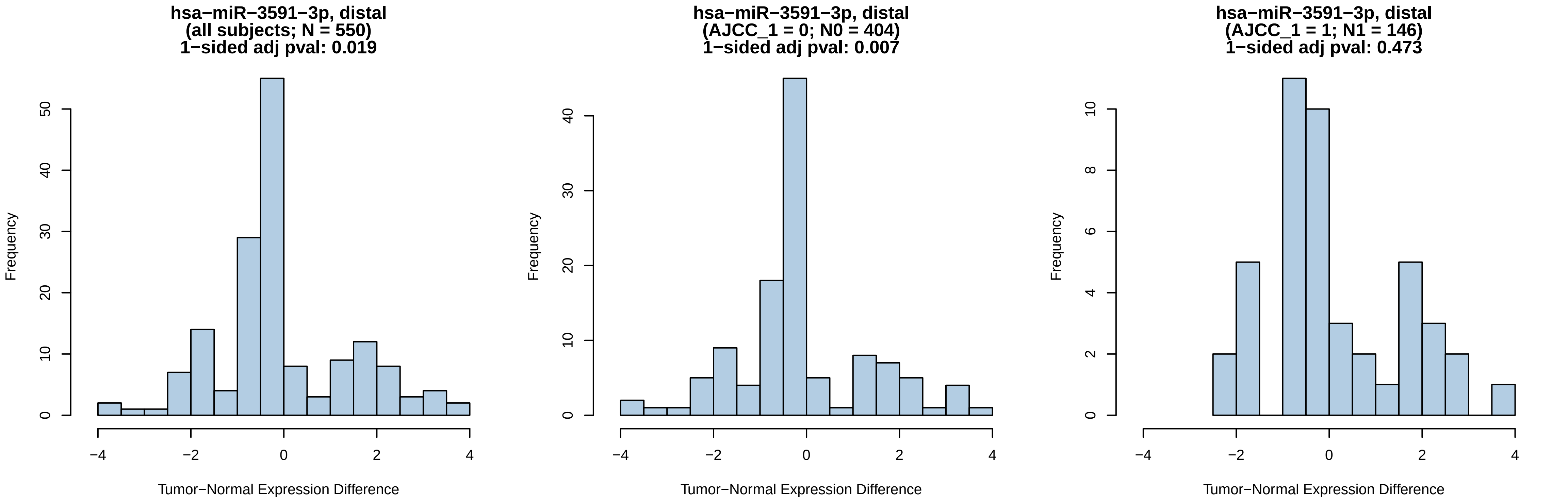
hsa−miR−3591−3p, distal
(all subjects; N = 550)
1−sided adj pval: 0.019
Frequency
Tumor−Normal Expression Difference
hsa−miR−3591−3p, distal
(AJCC_1 = 0; N0 = 404)
1−sided adj pval: 0.007
Frequency
Tumor−Normal Expression Difference
hsa−miR−3591−3p, distal
(AJCC_1 = 1; N1 = 146)
1−sided adj pval: 0.473
Frequency
Tumor−Normal Expression Difference

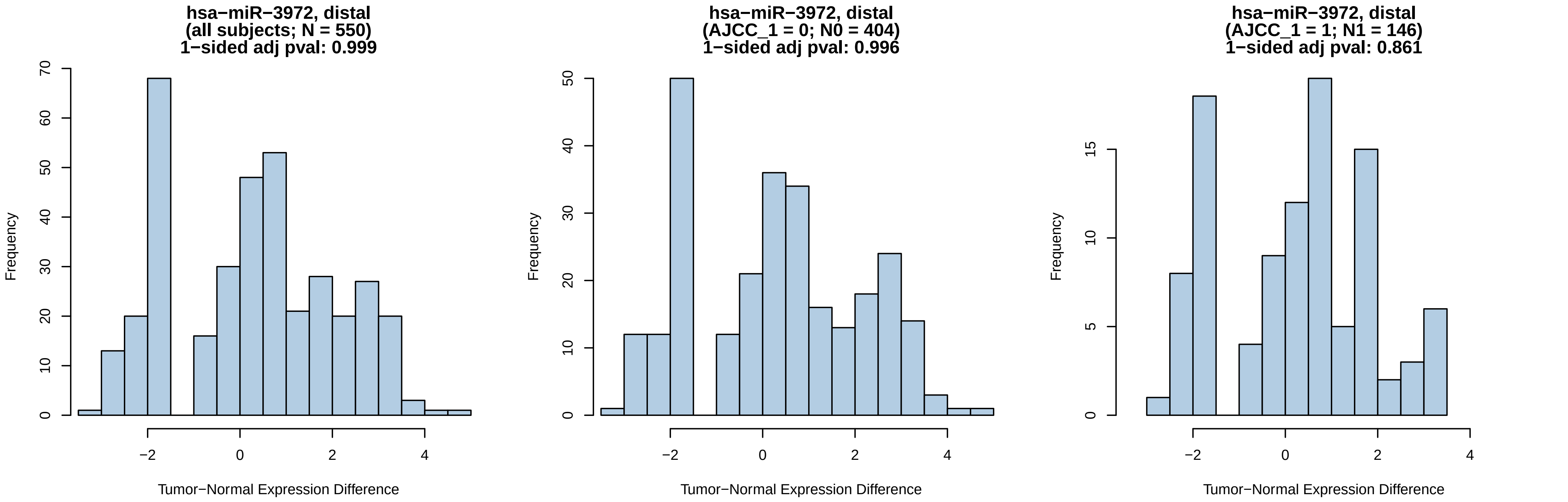
hsa−miR−3972, distal
(all subjects; N = 550)
1−sided adj pval: 0.999
Frequency
Tumor−Normal Expression Difference
0
10
20
30
40
50
60
70
−2
0
2
4
hsa−miR−3972, distal
(AJCC_1 = 0; N0 = 404)
1−sided adj pval: 0.996
Frequency
Tumor−Normal Expression Difference
0
10
20
30
40
50
−2
0
2
4
hsa−miR−3972, distal
(AJCC_1 = 1; N1 = 146)
1−sided adj pval: 0.861
Frequency
Tumor−Normal Expression Difference
0
5
10
15
−2
0
2
4

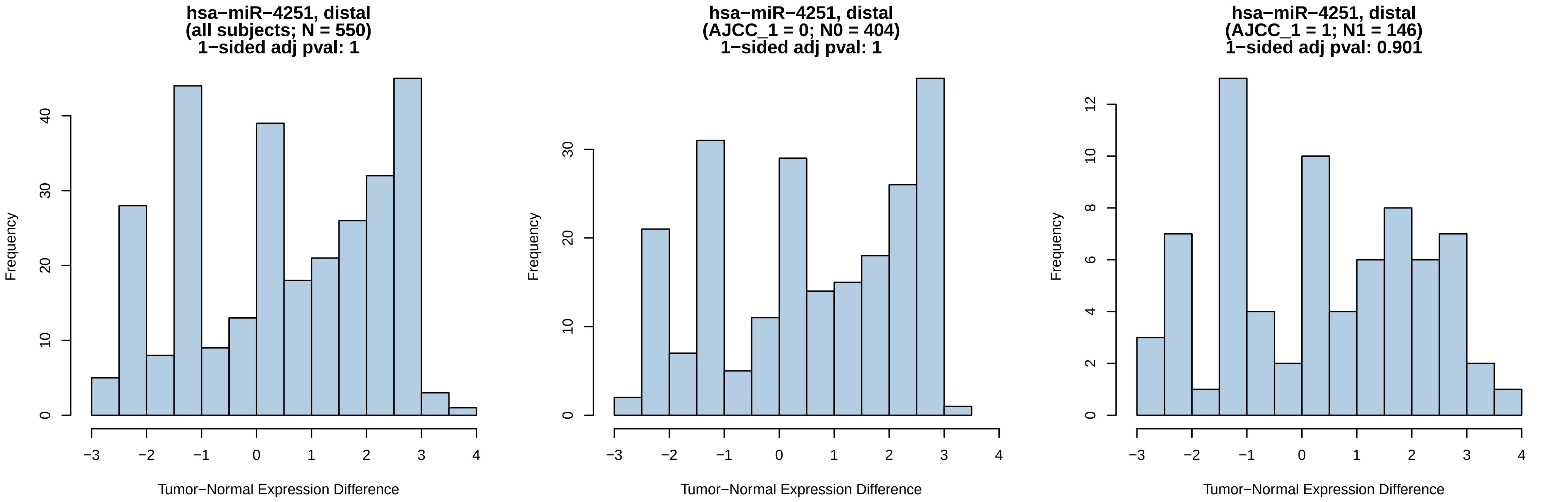
hsa−miR−4251, distal
(all subjects; N = 550)
1−sided adj pval: 1
hsa−miR−4251, distal
(AJCC_1 = 0; N0 = 404)
1−sided adj pval: 1
hsa−miR−4251, distal
(AJCC_1 = 1; N1 = 146)
1−sided adj pval: 0.901
Frequency
Tumor−Normal Expression Difference

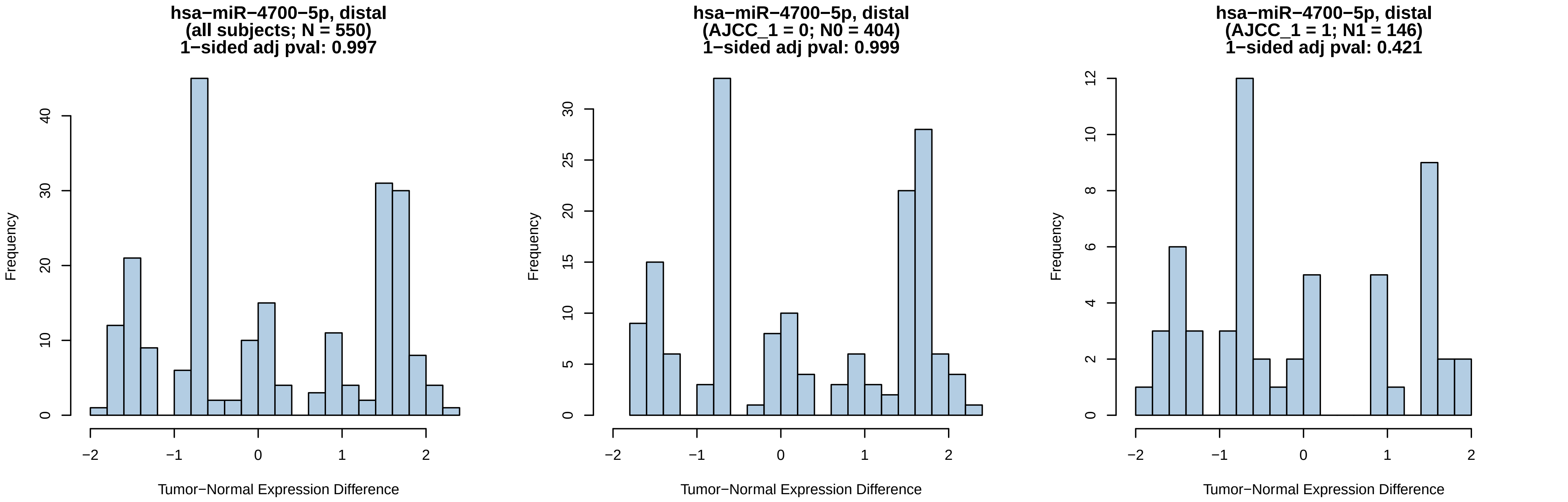

hsa−miR−4700−5p, distal
(all subjects; N = 550)
1−sided adj pval: 0.997
Frequency
Tumor−Normal Expression Difference
hsa−miR−4700−5p, distal
(AJCC_1 = 0; N0 = 404)
1−sided adj pval: 0.999
Frequency
Tumor−Normal Expression Difference
hsa−miR−4700−5p, distal
(AJCC_1 = 1; N1 = 146)
1−sided adj pval: 0.421
Frequency
Tumor−Normal Expression Difference

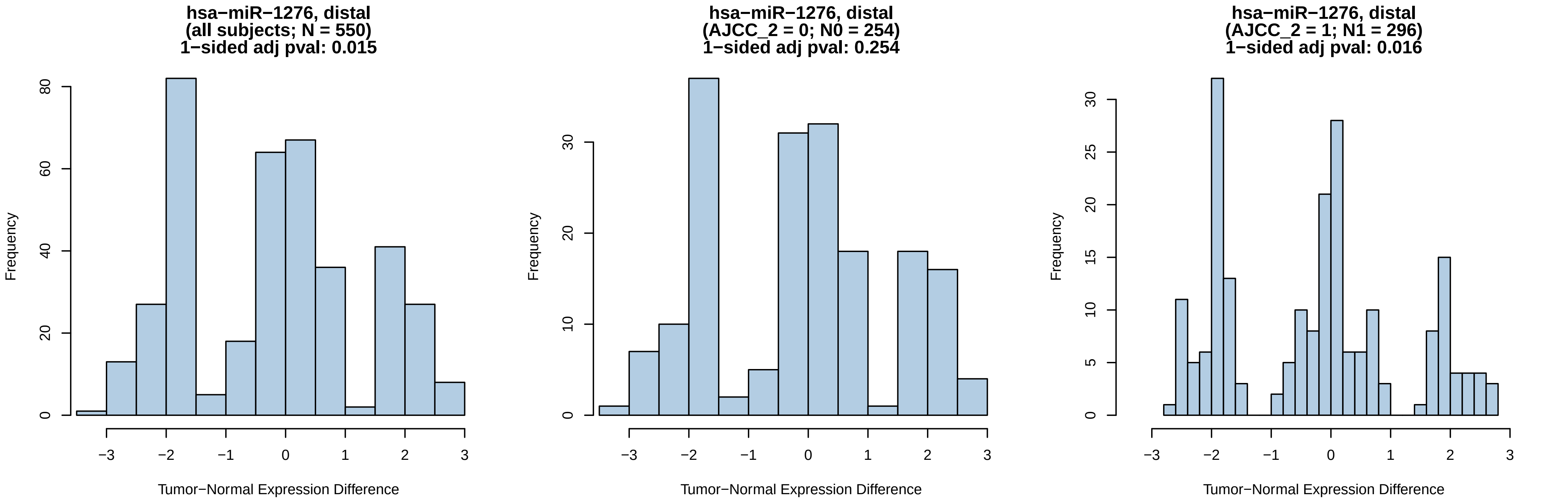
hsa−miR−1276, distal
(all subjects; N = 550)
1−sided adj pval: 0.015
Frequency
Tumor−Normal Expression Difference
hsa−miR−1276, distal
(AJCC_2 = 0; N0 = 254)
1−sided adj pval: 0.254
Frequency
Tumor−Normal Expression Difference
hsa−miR−1276, distal
(AJCC_2 = 1; N1 = 296)
1−sided adj pval: 0.016
Frequency
Tumor−Normal Expression Difference

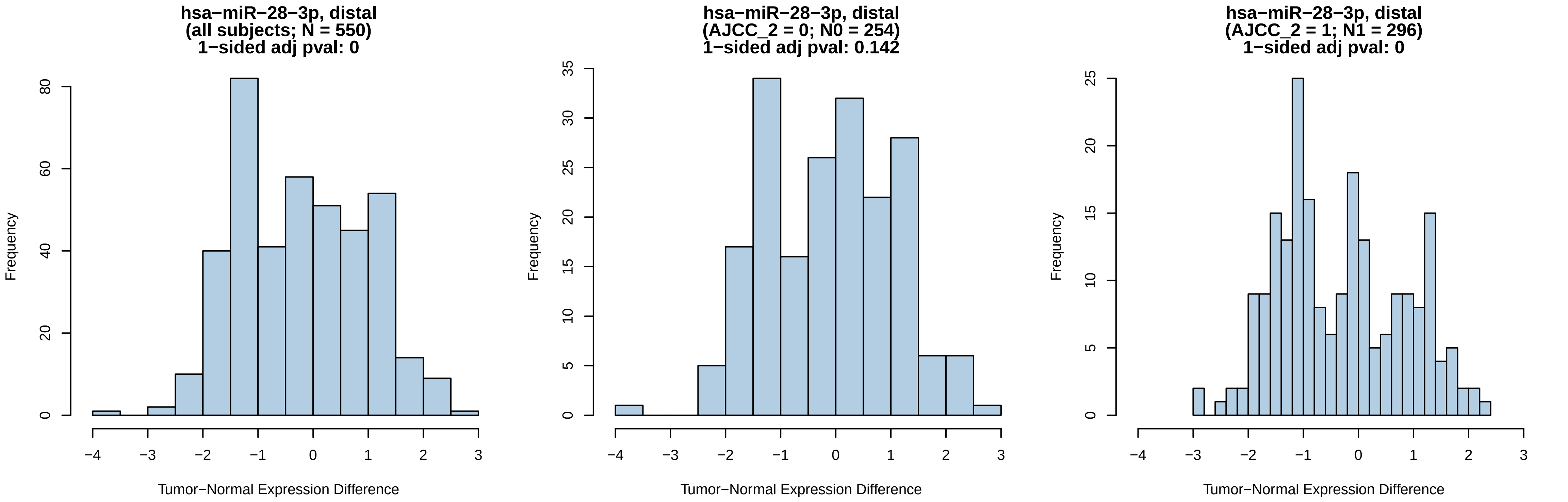
hsa−miR−28−3p, distal
(all subjects; N = 550)
1−sided adj pval: 0
hsa−miR−28−3p, distal
(AJCC_2 = 0; N0 = 254)
1−sided adj pval: 0.142
hsa−miR−28−3p, distal
(AJCC_2 = 1; N1 = 296)
1−sided adj pval: 0
Frequency
Tumor−Normal Expression Difference

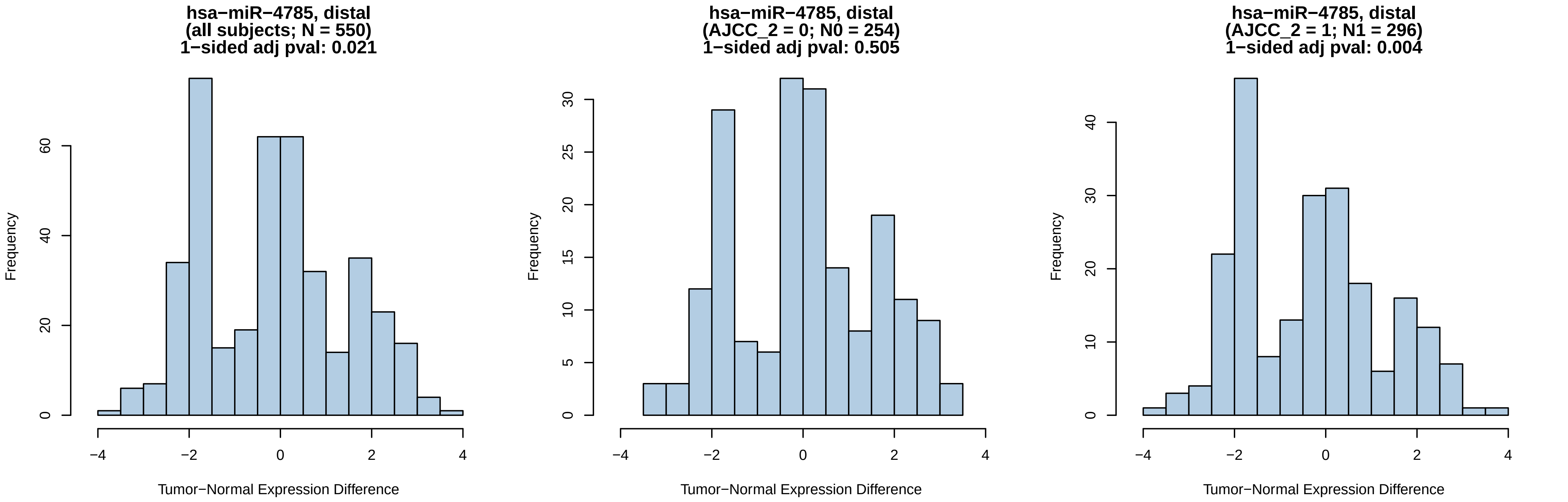

hsa−miR−4785, distal
(all subjects; N = 550)
1−sided adj pval: 0.021
Frequency
Tumor−Normal Expression Difference
hsa−miR−4785, distal
(AJCC_2 = 0; N0 = 254)
1−sided adj pval: 0.505
Frequency
Tumor−Normal Expression Difference
hsa−miR−4785, distal
(AJCC_2 = 1; N1 = 296)
1−sided adj pval: 0.004
Frequency
Tumor−Normal Expression Difference

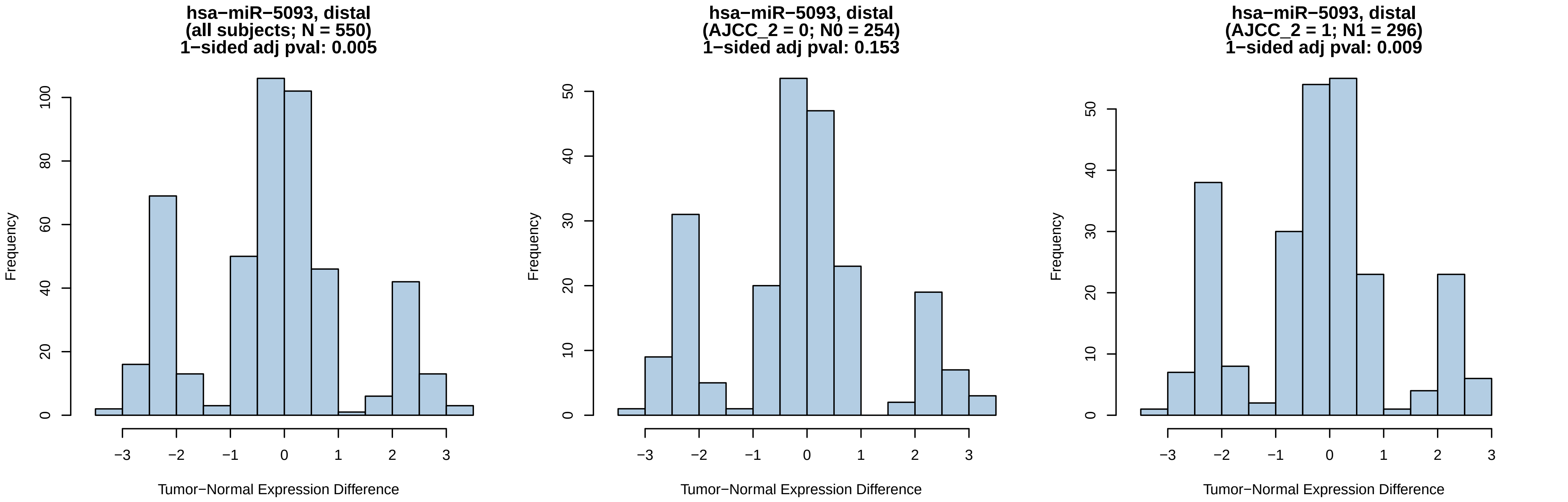
hsa−miR−5093, distal
(all subjects; N = 550)
1−sided adj pval: 0.005
Frequency
0
20
40
60
80
100
−3
−2
−1
0
1
2
3
Tumor−Normal Expression Difference
hsa−miR−5093, distal
(AJCC_2 = 0; N0 = 254)
1−sided adj pval: 0.153
Frequency
0
10
20
30
40
50
−3
−2
−1
0
1
2
3
Tumor−Normal Expression Difference
hsa−miR−5093, distal
(AJCC_2 = 1; N1 = 296)
1−sided adj pval: 0.009
Frequency
0
10
20
30
40
50
−3
−2
−1
0
1
2
3
Tumor−Normal Expression Difference

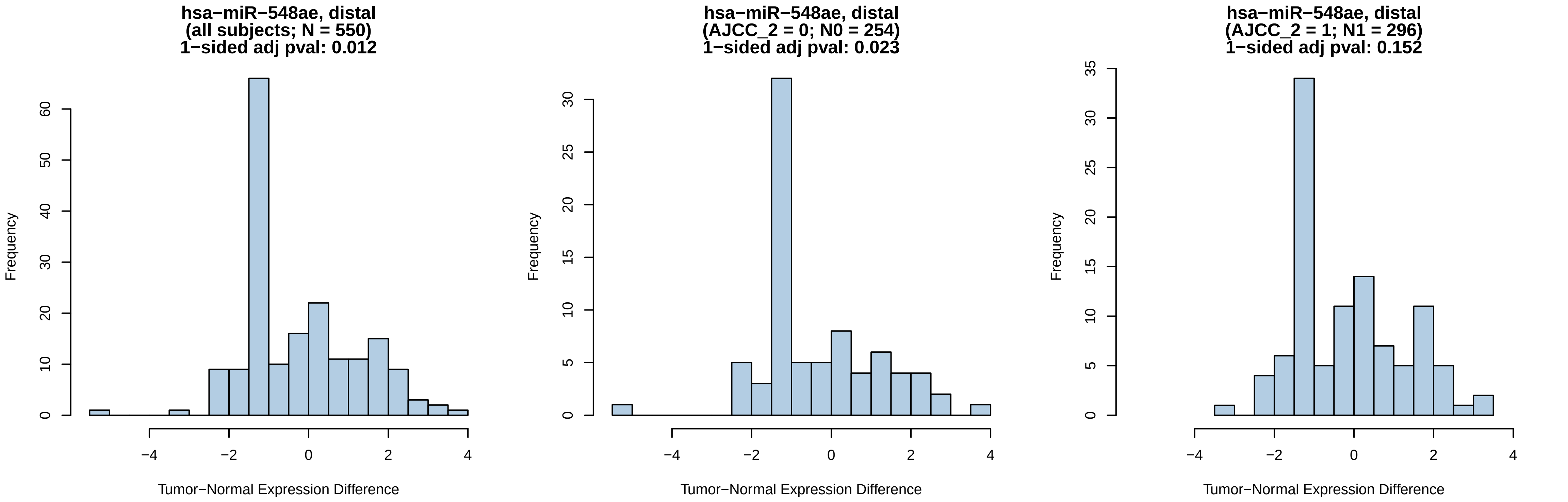
hsa−miR−548ae, distal
(all subjects; N = 550)
1−sided adj pval: 0.012
Frequency
0
10
20
30
40
50
60
−4
−2
0
2
4
Tumor−Normal Expression Difference
hsa−miR−548ae, distal
(AJCC_2 = 0; N0 = 254)
1−sided adj pval: 0.023
Frequency
0
5
10
15
20
25
30
−4
−2
0
2
4
Tumor−Normal Expression Difference
hsa−miR−548ae, distal
(AJCC_2 = 1; N1 = 296)
1−sided adj pval: 0.152
Frequency
0
5
10
15
20
25
30
35
−4
−2
0
2
4
Tumor−Normal Expression Difference

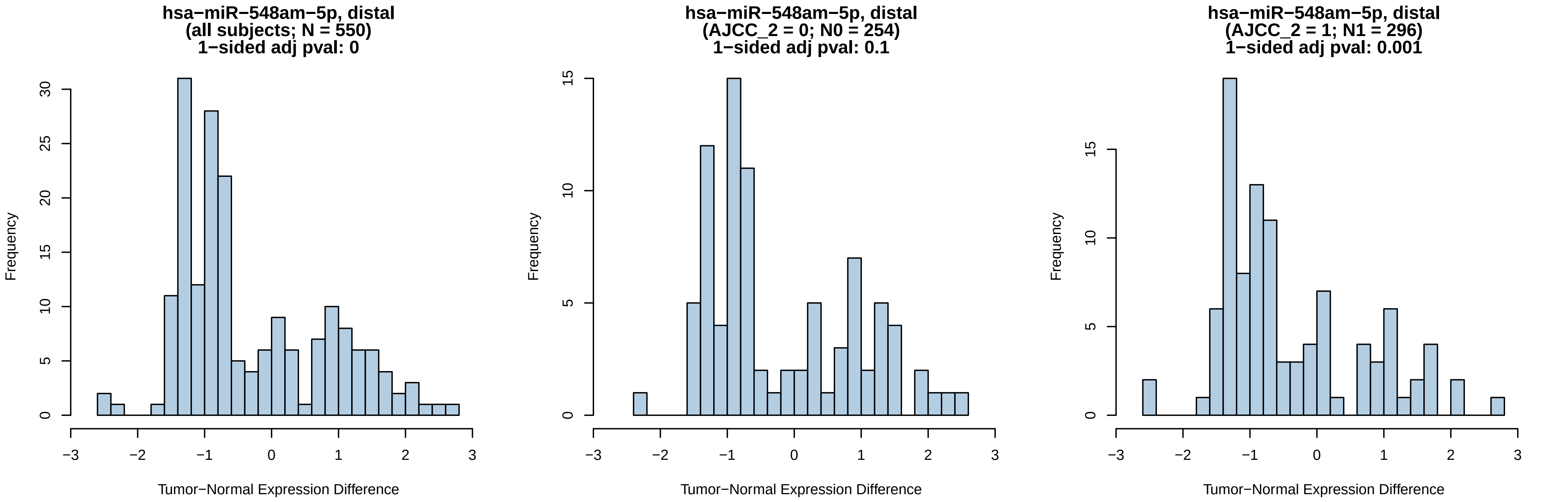
hsa−miR−548am−5p, distal
(all subjects; N = 550)
1−sided adj pval: 0
hsa−miR−548am−5p, distal
(AJCC_2 = 0; N0 = 254)
1−sided adj pval: 0.1
hsa−miR−548am−5p, distal
(AJCC_2 = 1; N1 = 296)
1−sided adj pval: 0.001
Frequency
Tumor−Normal Expression Difference

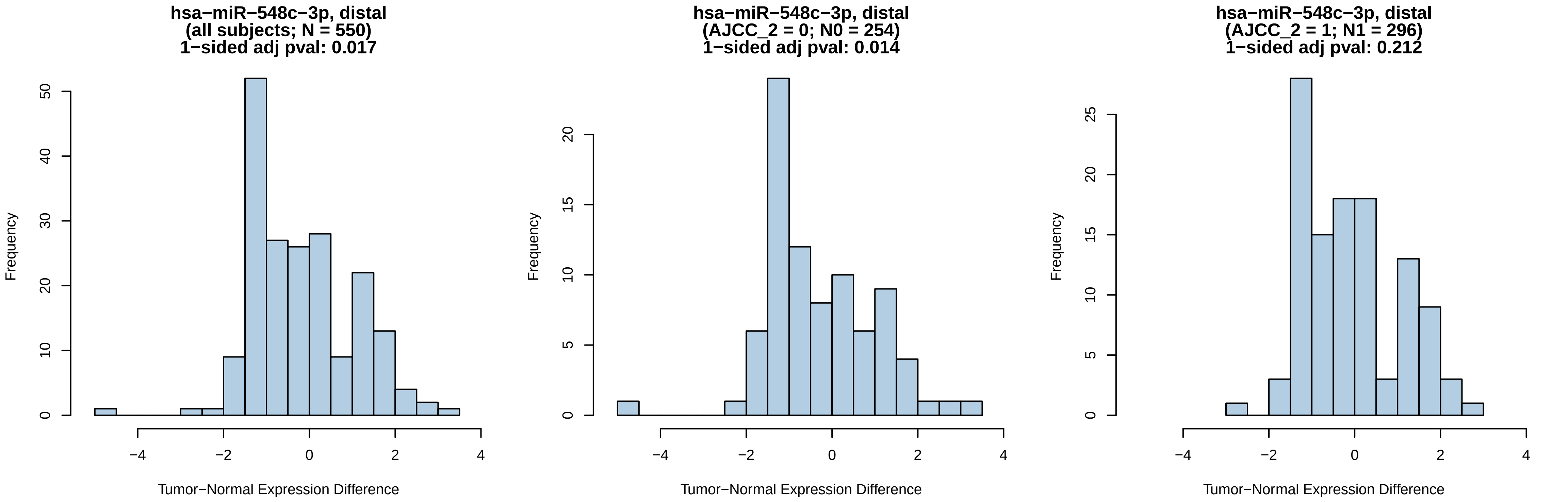
hsa−miR−548c−3p, distal
(all subjects; N = 550)
1−sided adj pval: 0.017
hsa−miR−548c−3p, distal
(AJCC_2 = 0; N0 = 254)
1−sided adj pval: 0.014
hsa−miR−548c−3p, distal
(AJCC_2 = 1; N1 = 296)
1−sided adj pval: 0.212
Frequency
Tumor−Normal Expression Difference

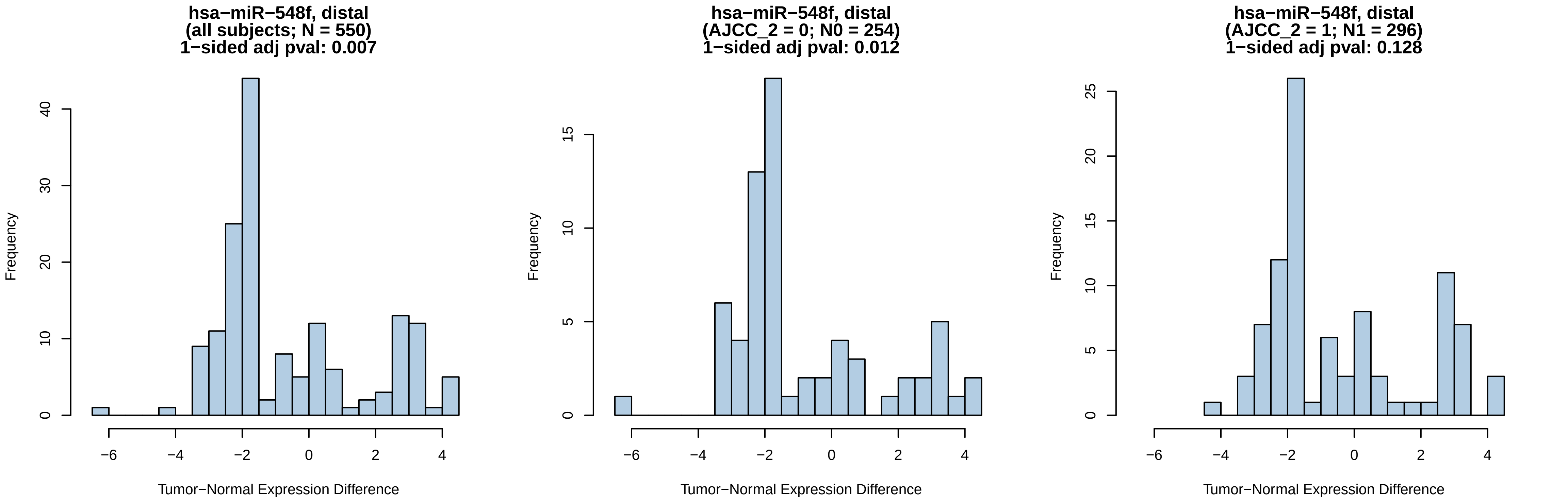
hsa−miR−548f, distal
(all subjects; N = 550)
1−sided adj pval: 0.007
Frequency
0
10
20
30
40
−6
−4
−2
0
2
4
Tumor−Normal Expression Difference
hsa−miR−548f, distal
(AJCC_2 = 0; N0 = 254)
1−sided adj pval: 0.012
Frequency
0
5
10
15
−6
−4
−2
0
2
4
Tumor−Normal Expression Difference
hsa−miR−548f, distal
(AJCC_2 = 1; N1 = 296)
1−sided adj pval: 0.128
Frequency
0
5
10
15
20
25
−6
−4
−2
0
2
4
Tumor−Normal Expression Difference

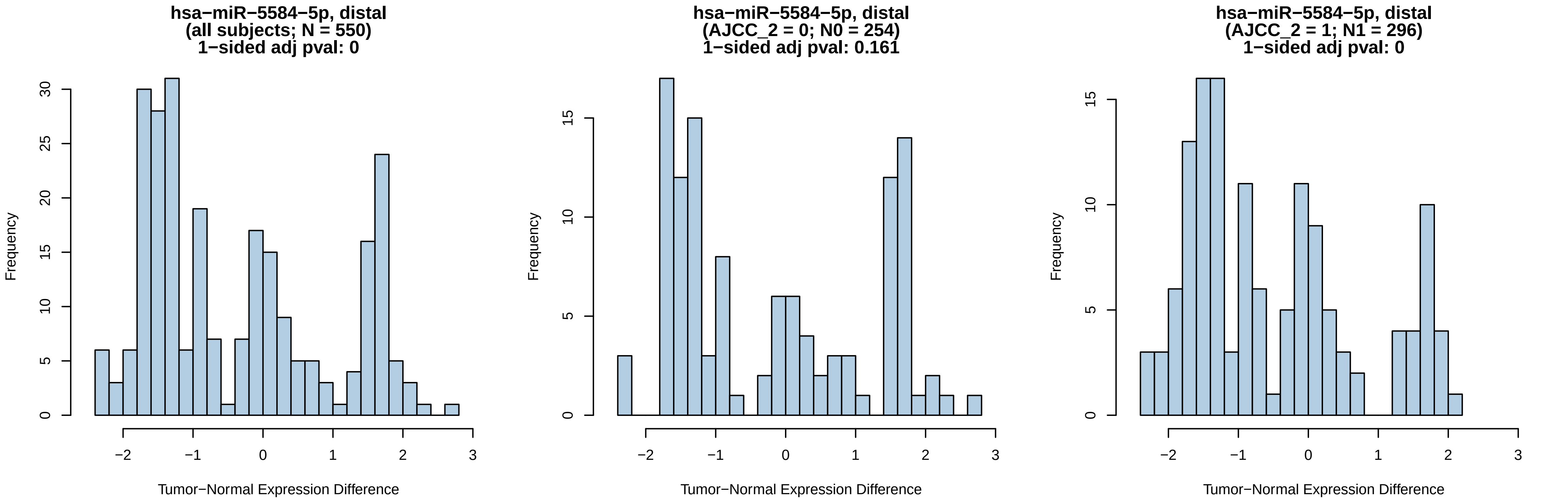

hsa−miR−5584−5p, distal
(all subjects; N = 550)
1−sided adj pval: 0
hsa−miR−5584−5p, distal
(AJCC_2 = 0; N0 = 254)
1−sided adj pval: 0.161
hsa−miR−5584−5p, distal
(AJCC_2 = 1; N1 = 296)
1−sided adj pval: 0
Frequency
Tumor−Normal Expression Difference

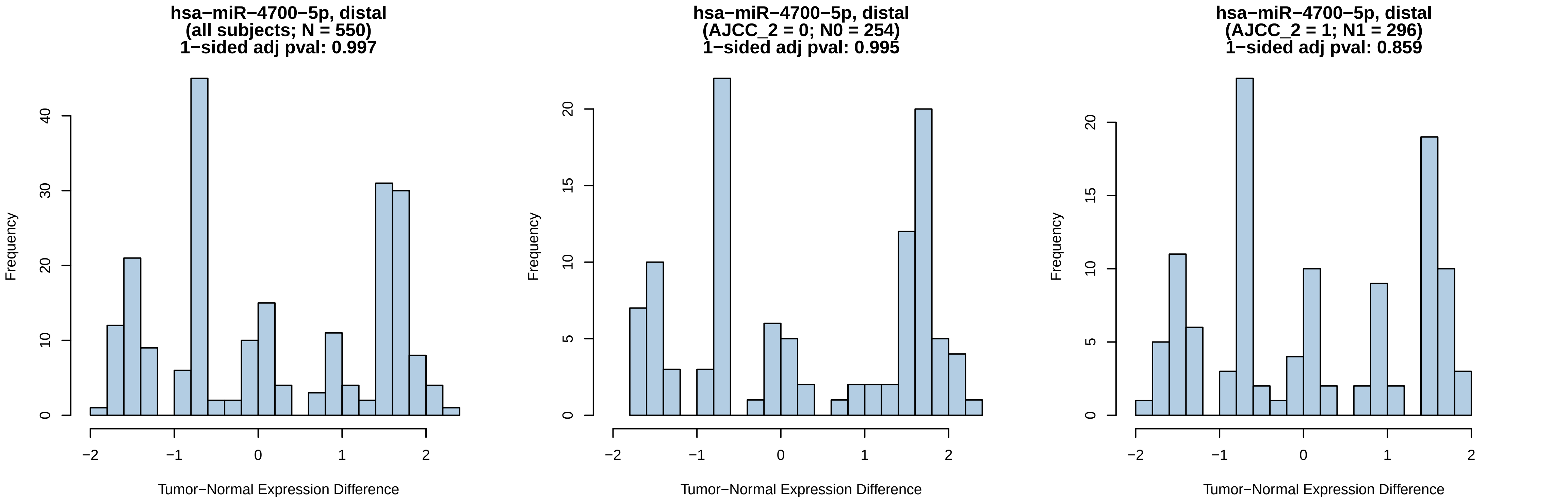
hsa−miR−4700−5p, distal
(all subjects; N = 550)
1−sided adj pval: 0.997
Frequency
Tumor−Normal Expression Difference
hsa−miR−4700−5p, distal
(AJCC_2 = 0; N0 = 254)
1−sided adj pval: 0.995
Frequency
Tumor−Normal Expression Difference
hsa−miR−4700−5p, distal
(AJCC_2 = 1; N1 = 296)
1−sided adj pval: 0.859
Frequency
Tumor−Normal Expression Difference

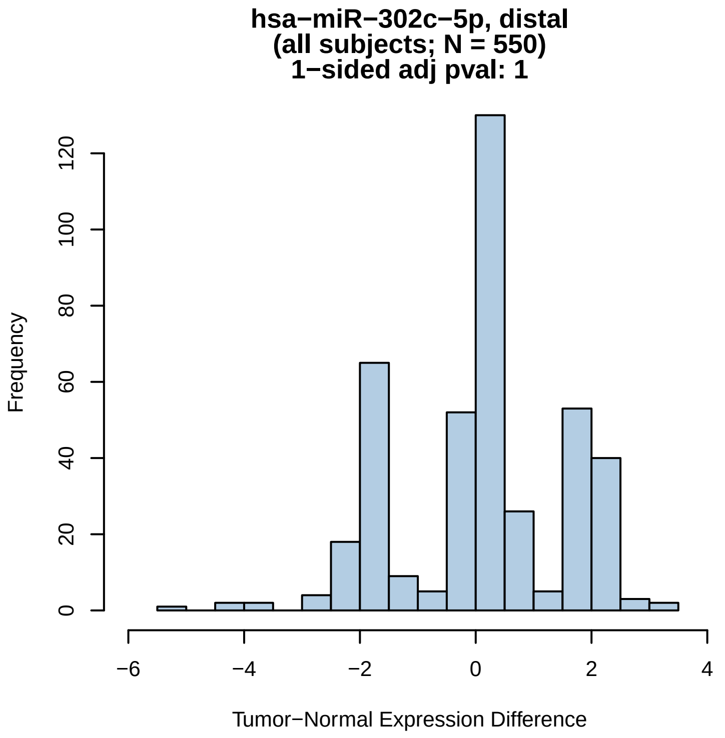
hsa−miR−302c−5p, distal
(all subjects; N = 550)
1−sided adj pval: 1
Frequency
0
20
40
60
80
100
120
−6
−4
−2
0
2
4
Tumor−Normal Expression Difference

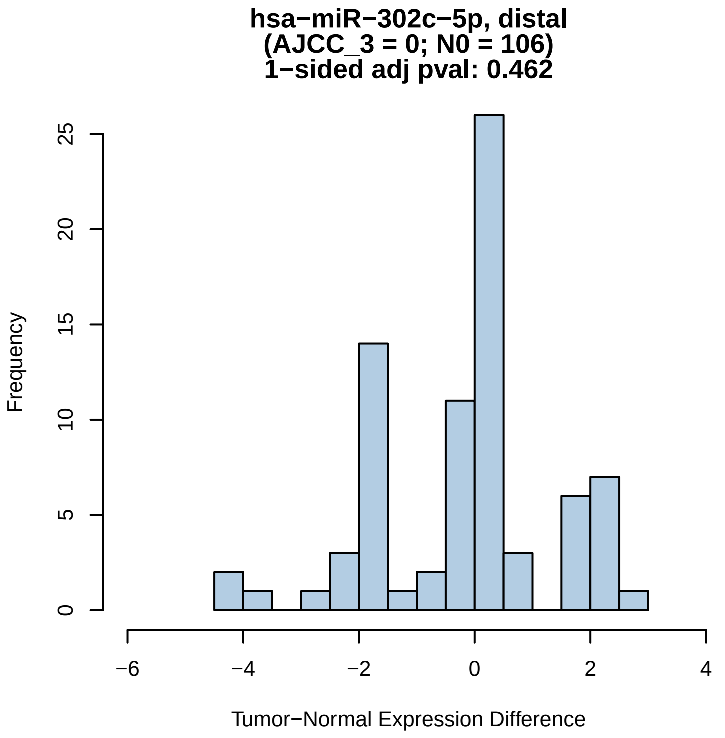
hsa−miR−302c−5p, distal
(AJCC_3 = 0; N0 = 106)
1−sided adj pval: 0.462
Frequency
0
5
10
15
20
25
−6
−4
−2
0
2
4
Tumor−Normal Expression Difference

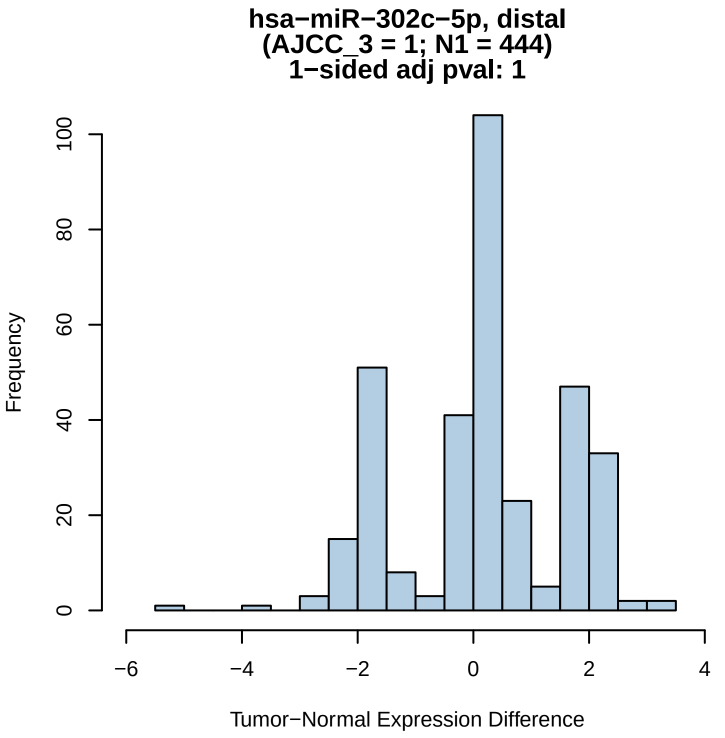
hsa−miR−302c−5p, distal
(AJCC_3 = 1; N1 = 444)
1−sided adj pval: 1
Frequency
0
20
40
60
80
100
−6
−4
−2
0
2
4
Tumor−Normal Expression Difference

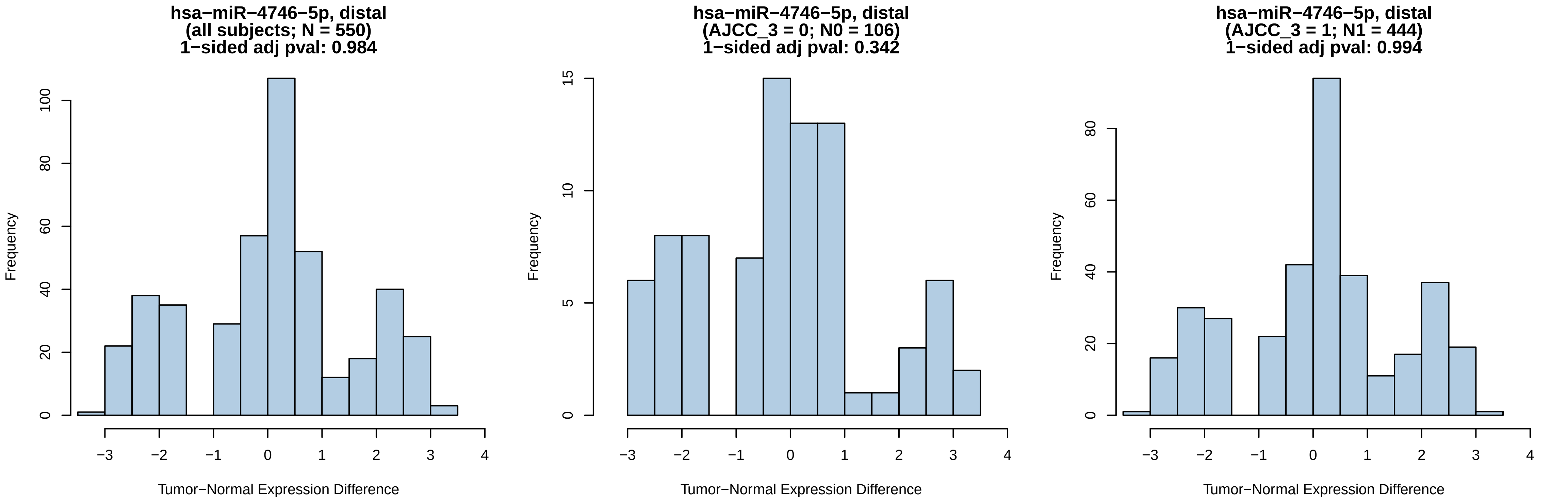
hsa−miR−4746−5p, distal
(all subjects; N = 550)
1−sided adj pval: 0.984
Frequency
Tumor−Normal Expression Difference
hsa−miR−4746−5p, distal
(AJCC_3 = 0; N0 = 106)
1−sided adj pval: 0.342
Frequency
Tumor−Normal Expression Difference
hsa−miR−4746−5p, distal
(AJCC_3 = 1; N1 = 444)
1−sided adj pval: 0.994
Frequency
Tumor−Normal Expression Difference

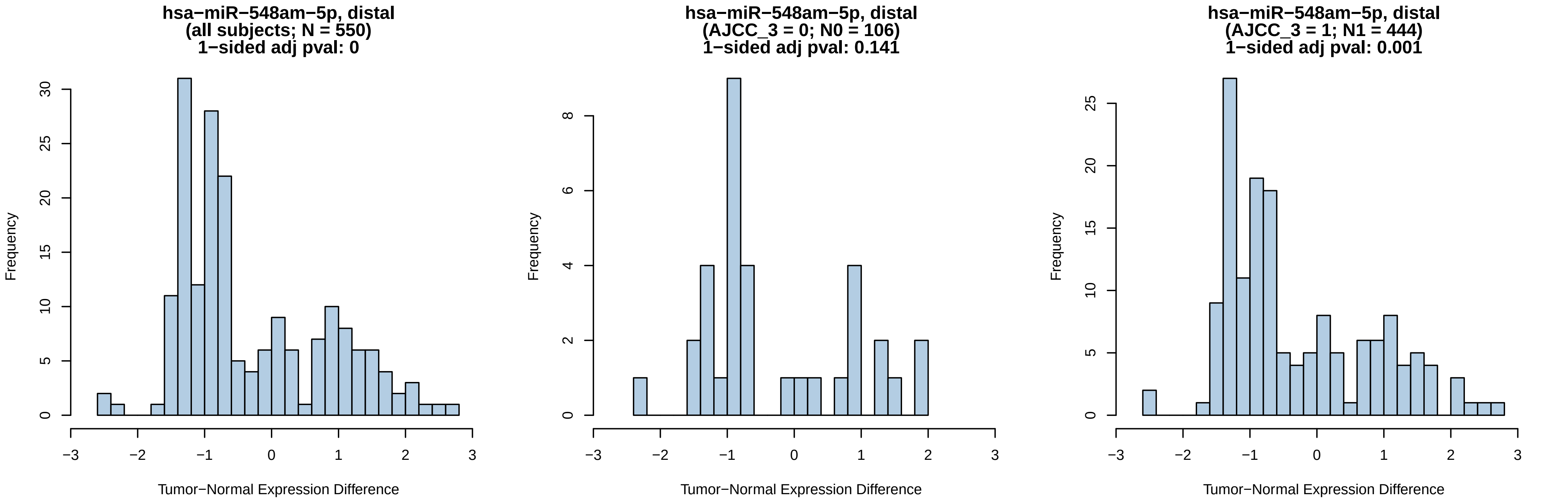
hsa−miR−548am−5p, distal
(all subjects; N = 550)
1−sided adj pval: 0
hsa−miR−548am−5p, distal
(AJCC_3 = 0; N0 = 106)
1−sided adj pval: 0.141
hsa−miR−548am−5p, distal
(AJCC_3 = 1; N1 = 444)
1−sided adj pval: 0.001
Frequency
Tumor−Normal Expression Difference
0
5
10
15
20
25
30
0
2
4
6
8
−3
−2
−1
0
1
2
3

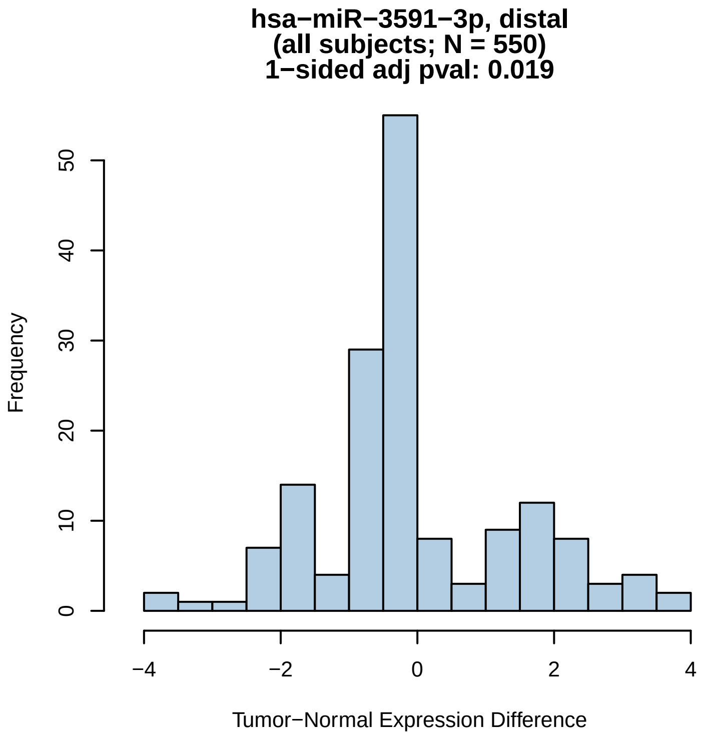
hsa−miR−3591−3p, distal
(all subjects; N = 550)
1−sided adj pval: 0.019
Frequency
Tumor−Normal Expression Difference

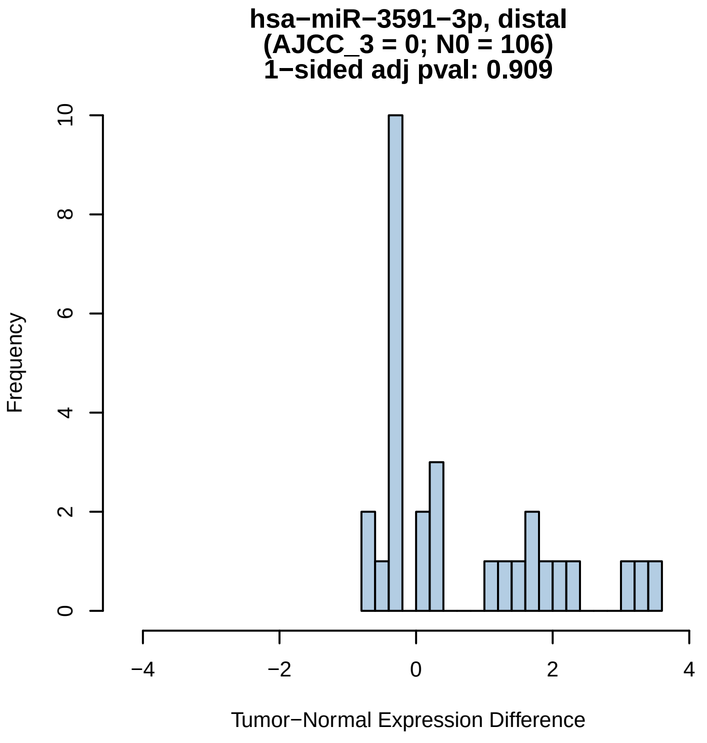
hsa−miR−3591−3p, distal
(AJCC_3 = 0; N0 = 106)
1−sided adj pval: 0.909
Frequency
Tumor−Normal Expression Difference

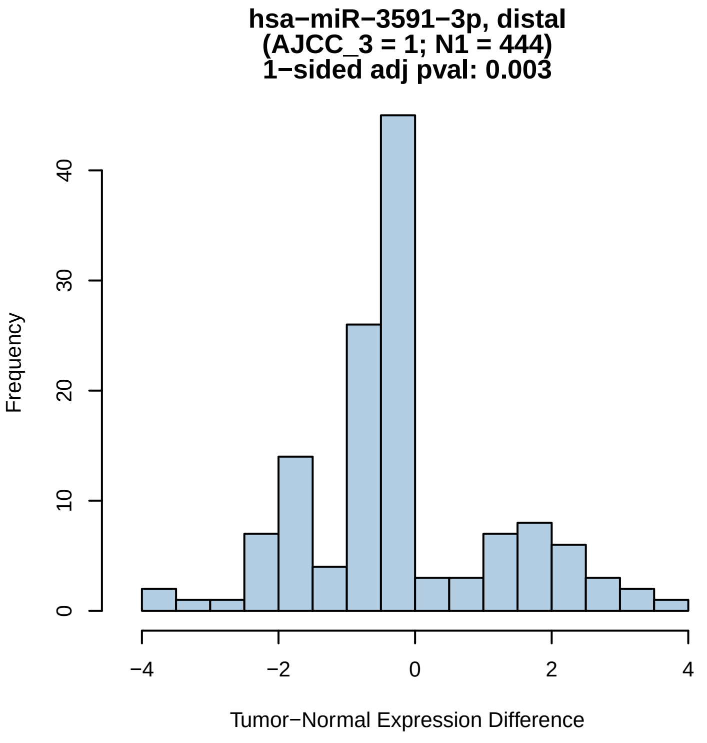
hsa−miR−3591−3p, distal
(AJCC_3 = 1; N1 = 444)
1−sided adj pval: 0.003
Frequency
Tumor−Normal Expression Difference

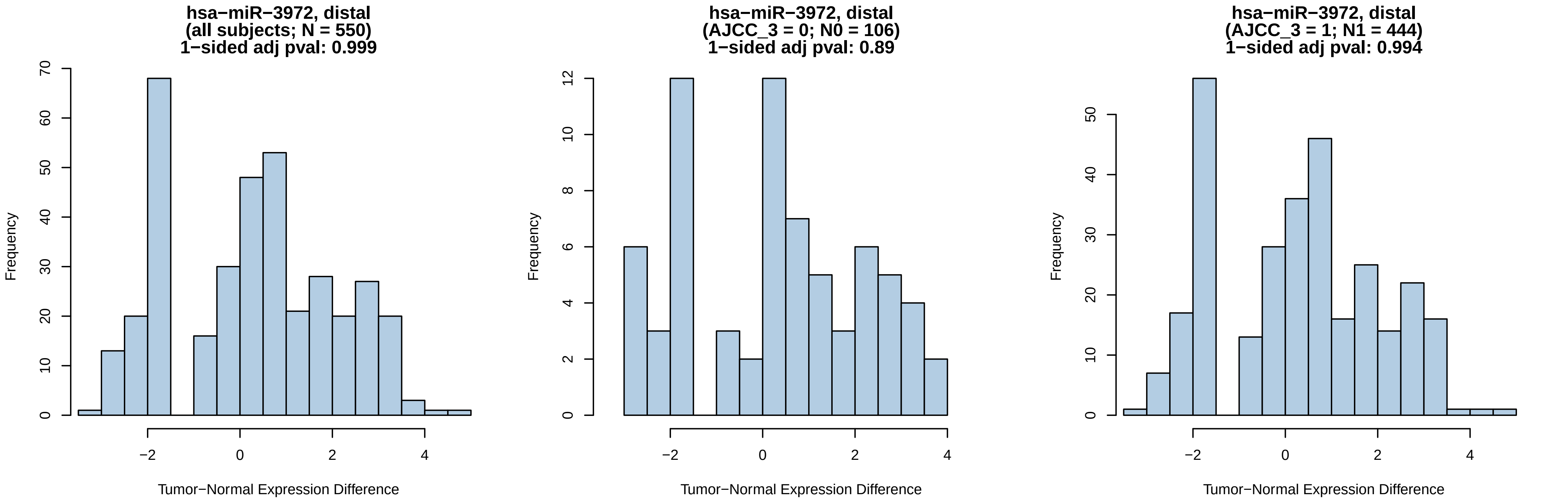

hsa−miR−3972, distal
(all subjects; N = 550)
1−sided adj pval: 0.999
Frequency
Tumor−Normal Expression Difference
hsa−miR−3972, distal
(AJCC_3 = 0; N0 = 106)
1−sided adj pval: 0.89
Frequency
Tumor−Normal Expression Difference
hsa−miR−3972, distal
(AJCC_3 = 1; N1 = 444)
1−sided adj pval: 0.994
Frequency
Tumor−Normal Expression Difference

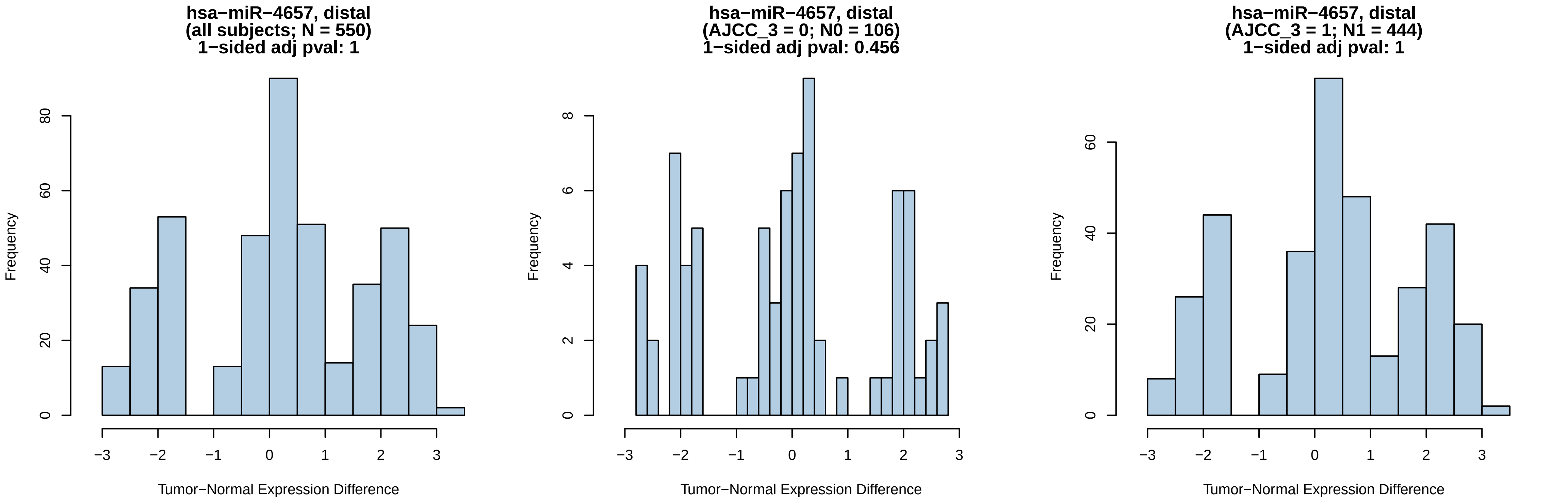

hsa−miR−4657, distal
(all subjects; N = 550)
1−sided adj pval: 1
Frequency
0
20
40
60
80
−3
−2
−1
0
1
2
3
Tumor−Normal Expression Difference
hsa−miR−4657, distal
(AJCC_3 = 0; N0 = 106)
1−sided adj pval: 0.456
Frequency
0
2
4
6
8
−3
−2
−1
0
1
2
3
Tumor−Normal Expression Difference
hsa−miR−4657, distal
(AJCC_3 = 1; N1 = 444)
1−sided adj pval: 1
Frequency
0
20
40
60
−3
−2
−1
0
1
2
3
Tumor−Normal Expression Difference

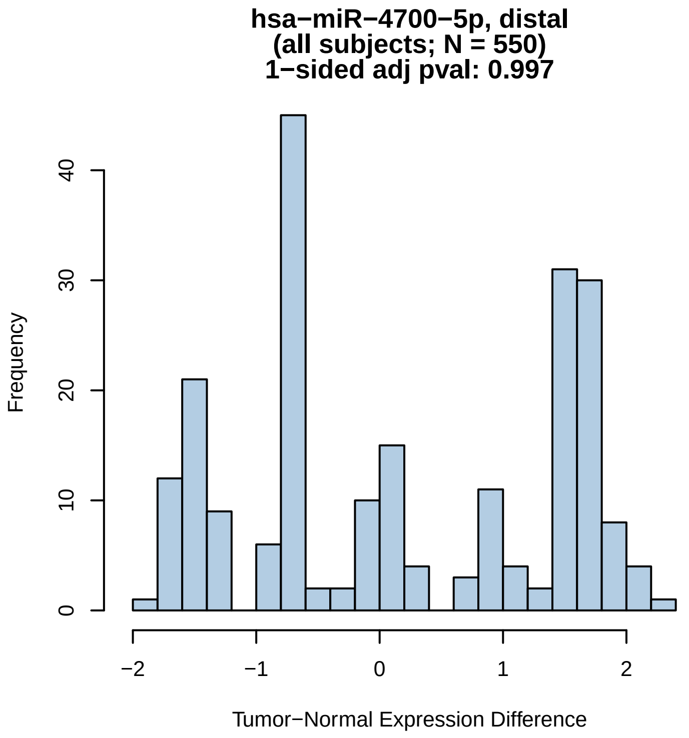
hsa−miR−4700−5p, distal
(all subjects; N = 550)
1−sided adj pval: 0.997
Frequency
0
10
20
30
40
−2
−1
0
1
2
Tumor−Normal Expression Difference

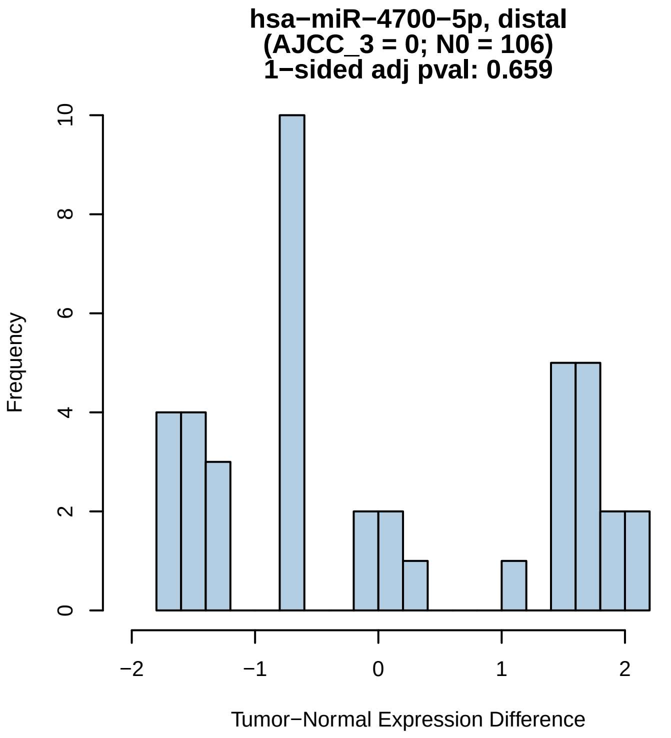
hsa−miR−4700−5p, distal
(AJCC_3 = 0; N0 = 106)
1−sided adj pval: 0.659
Frequency
0
2
4
6
8
10
−2
−1
0
1
2
Tumor−Normal Expression Difference

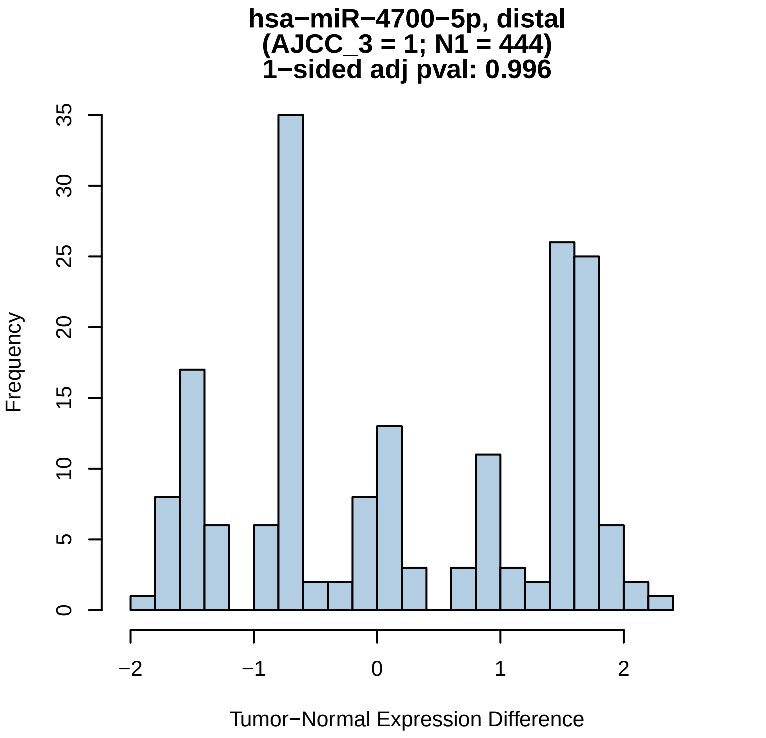
hsa−miR−4700−5p, distal
(AJCC_3 = 1; N1 = 444)
1−sided adj pval: 0.996
Frequency
0
5
10
15
20
25
30
35
−2
−1
0
1
2
Tumor−Normal Expression Difference

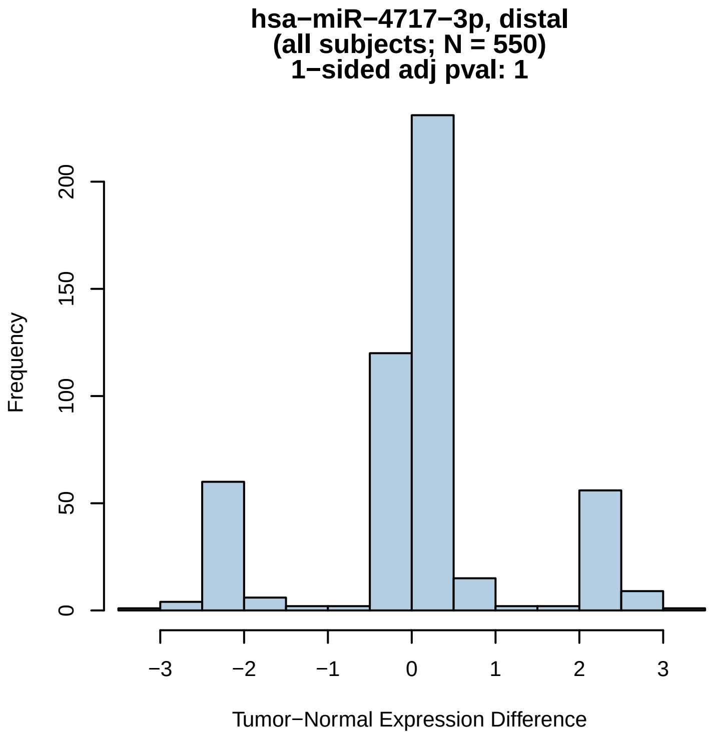
hsa−miR−4717−3p, distal
(all subjects; N = 550)
1−sided adj pval: 1
Frequency
Tumor−Normal Expression Difference

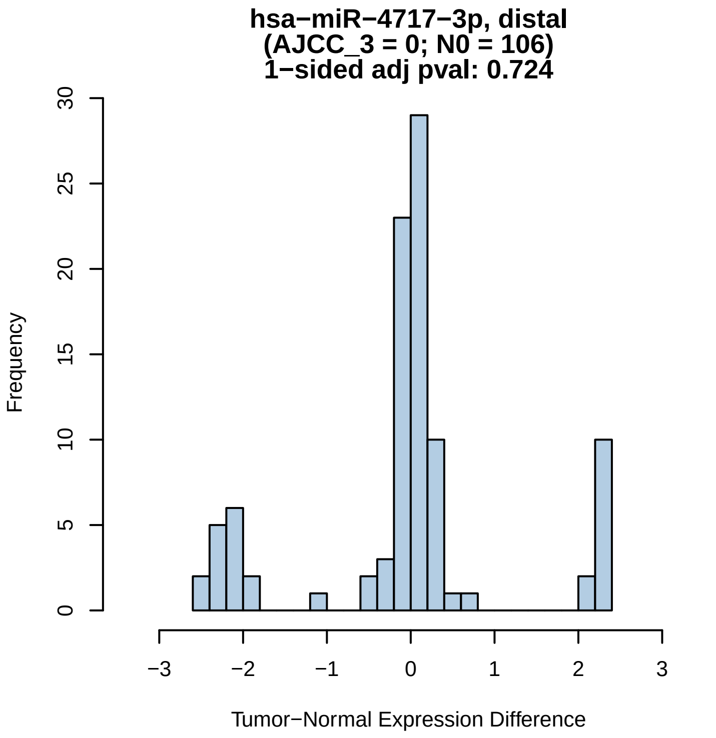
hsa−miR−4717−3p, distal
(AJCC_3 = 0; N0 = 106)
1−sided adj pval: 0.724
Frequency
Tumor−Normal Expression Difference

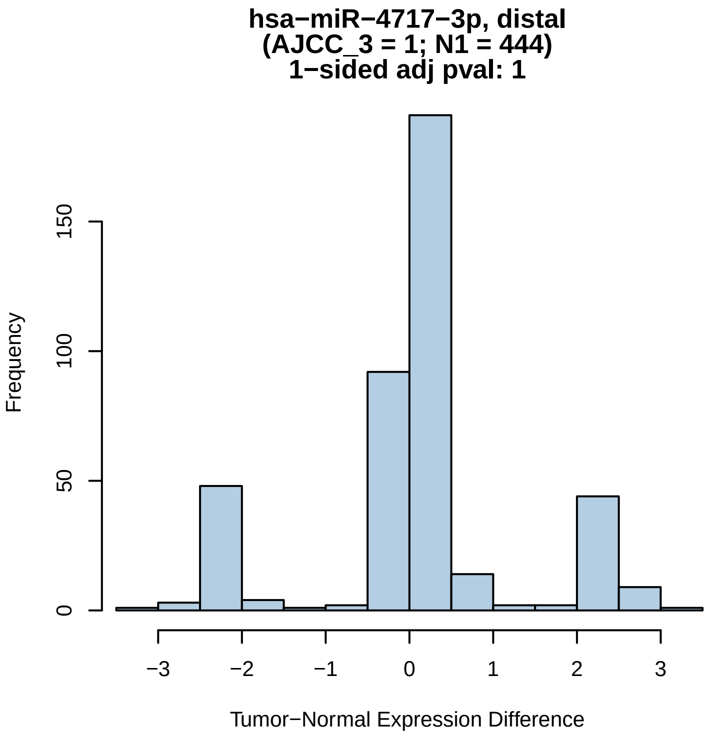
hsa−miR−4717−3p, distal
(AJCC_3 = 1; N1 = 444)
1−sided adj pval: 1
Frequency
Tumor−Normal Expression Difference

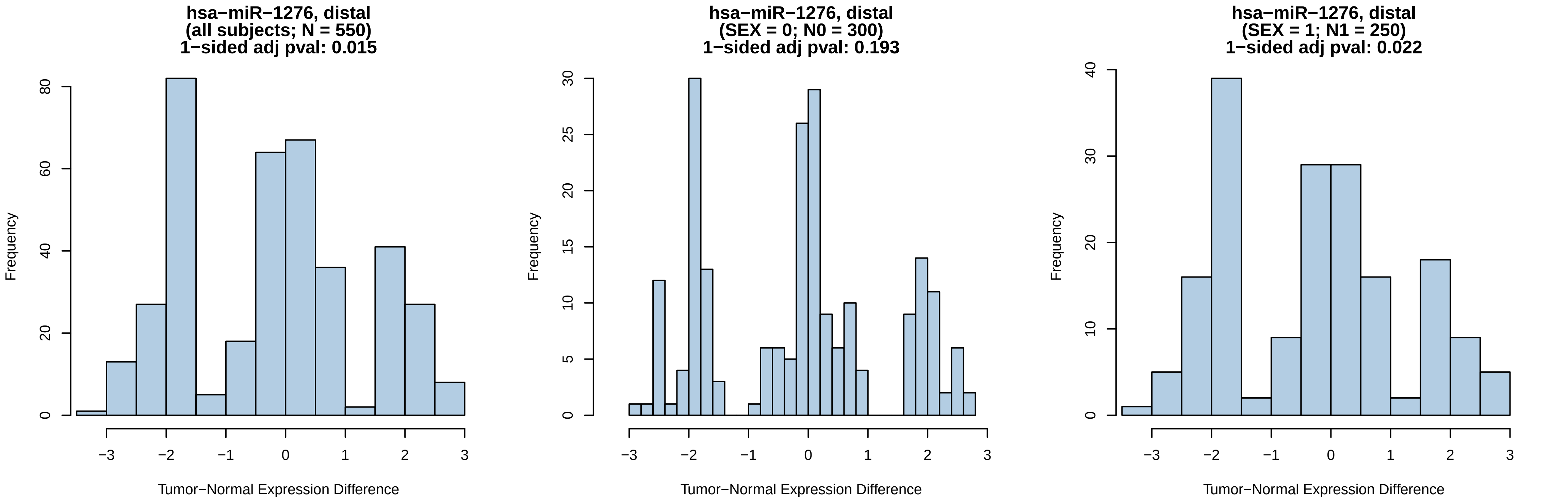
hsa−miR−1276, distal
(all subjects; N = 550)
1−sided adj pval: 0.015
Frequency
Tumor−Normal Expression Difference
hsa−miR−1276, distal
(SEX = 0; N0 = 300)
1−sided adj pval: 0.193
Frequency
Tumor−Normal Expression Difference
hsa−miR−1276, distal
(SEX = 1; N1 = 250)
1−sided adj pval: 0.022
Frequency
Tumor−Normal Expression Difference

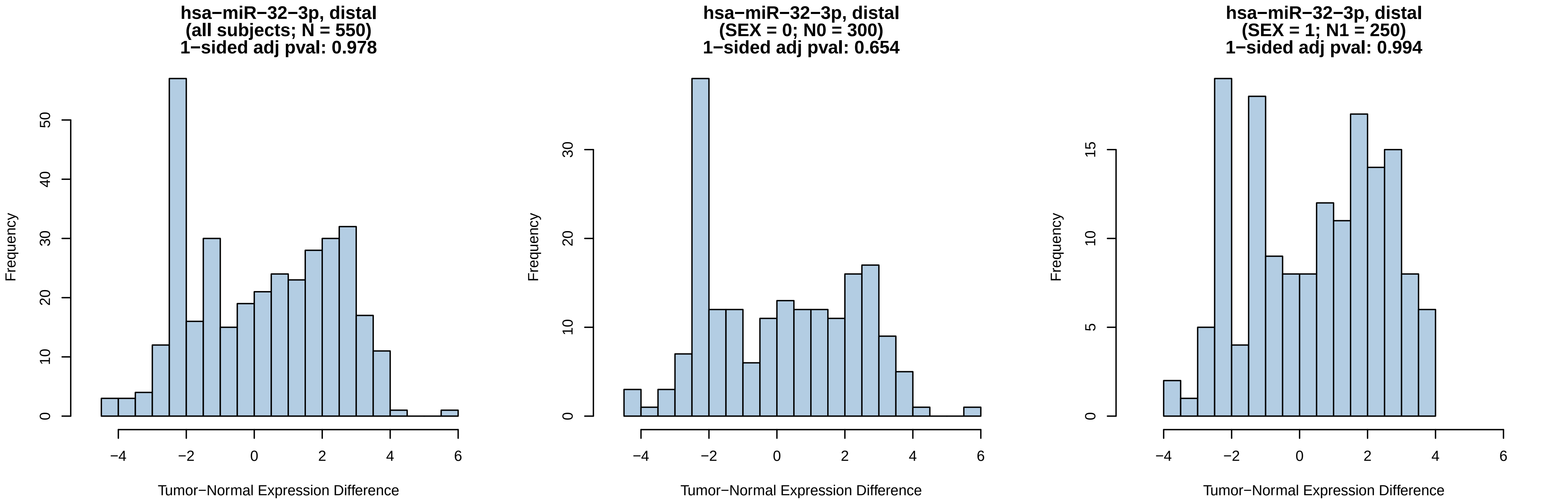

hsa−miR−32−3p, distal
(all subjects; N = 550)
1−sided adj pval: 0.978
Frequency
Tumor−Normal Expression Difference
hsa−miR−32−3p, distal
(SEX = 0; N0 = 300)
1−sided adj pval: 0.654
Frequency
Tumor−Normal Expression Difference
hsa−miR−32−3p, distal
(SEX = 1; N1 = 250)
1−sided adj pval: 0.994
Frequency
Tumor−Normal Expression Difference

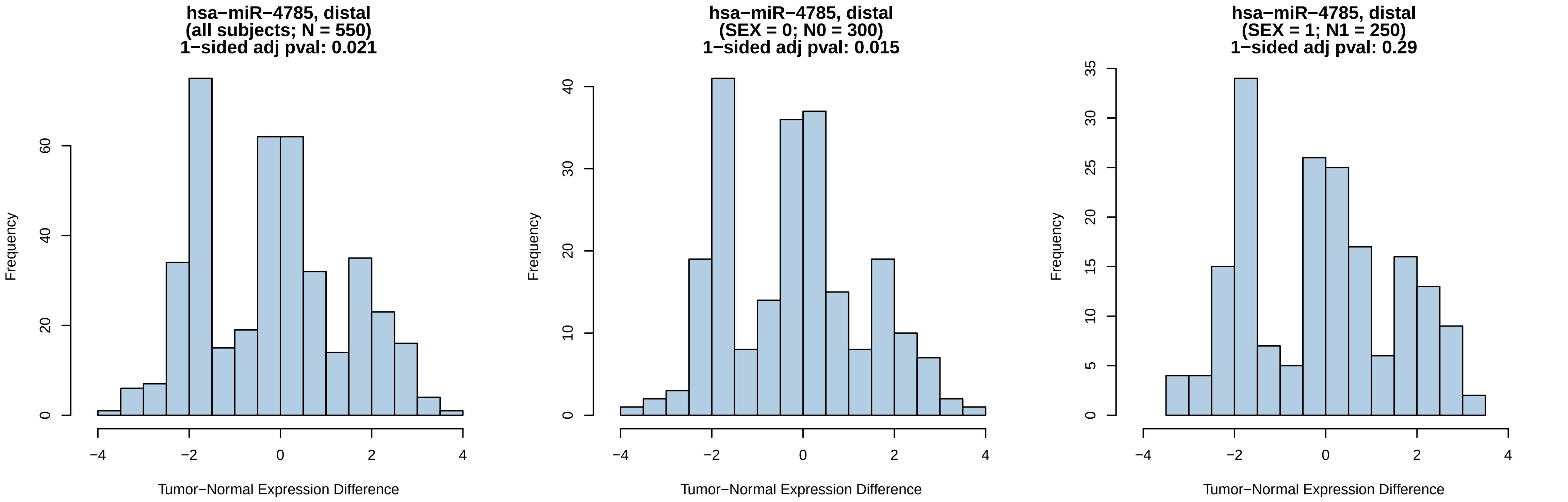

hsa−miR−4785, distal
(all subjects; N = 550)
1−sided adj pval: 0.021
Frequency
Tumor−Normal Expression Difference
hsa−miR−4785, distal
(SEX = 0; N0 = 300)
1−sided adj pval: 0.015
Frequency
Tumor−Normal Expression Difference
hsa−miR−4785, distal
(SEX = 1; N1 = 250)
1−sided adj pval: 0.29
Frequency
Tumor−Normal Expression Difference

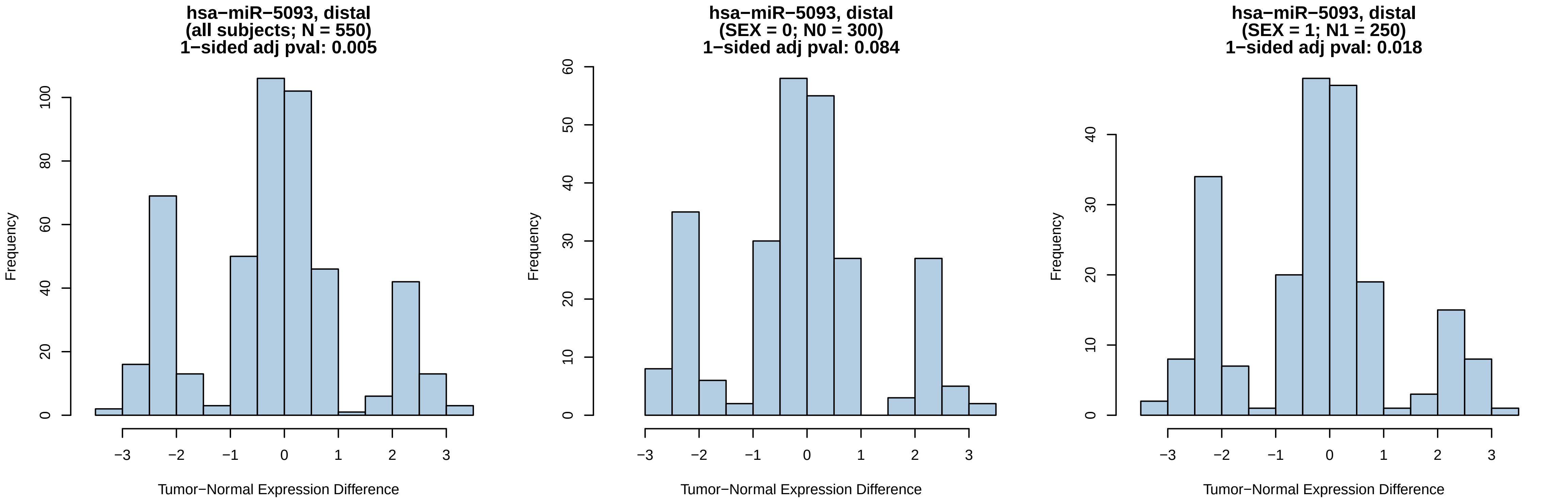
hsa−miR−5093, distal
(all subjects; N = 550)
1−sided adj pval: 0.005
Frequency
Tumor−Normal Expression Difference
hsa−miR−5093, distal
(SEX = 0; N0 = 300)
1−sided adj pval: 0.084
Frequency
Tumor−Normal Expression Difference
hsa−miR−5093, distal
(SEX = 1; N1 = 250)
1−sided adj pval: 0.018
Frequency
Tumor−Normal Expression Difference

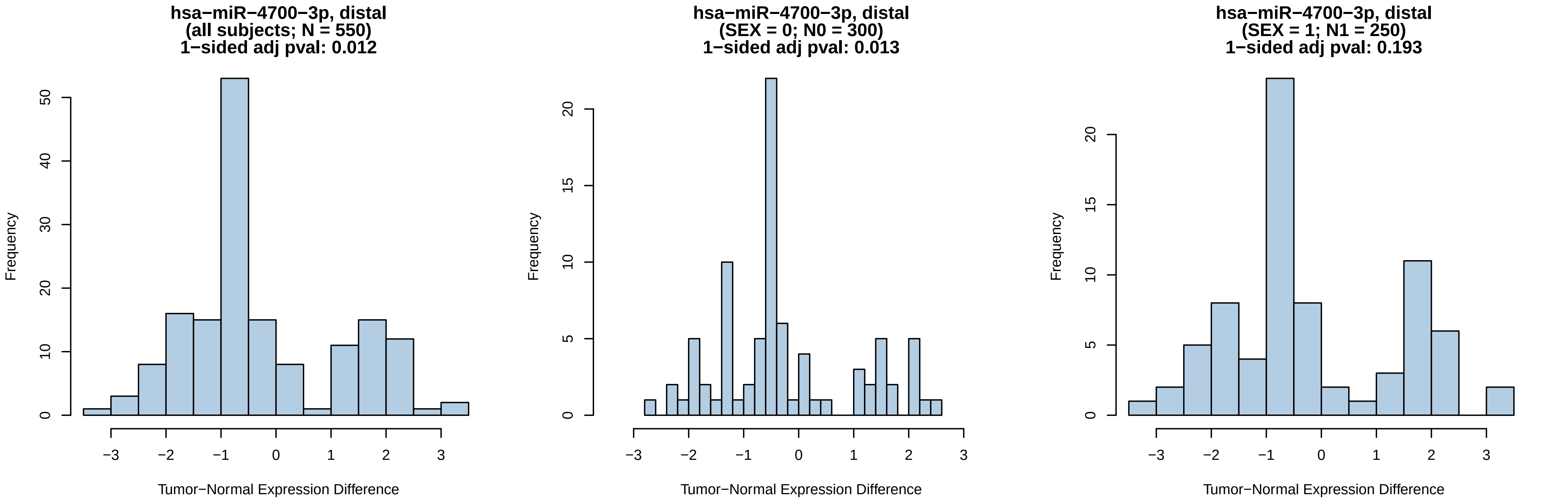
hsa−miR−4700−3p, distal
(all subjects; N = 550)
1−sided adj pval: 0.012
Frequency
Tumor−Normal Expression Difference
hsa−miR−4700−3p, distal
(SEX = 0; N0 = 300)
1−sided adj pval: 0.013
Frequency
Tumor−Normal Expression Difference
hsa−miR−4700−3p, distal
(SEX = 1; N1 = 250)
1−sided adj pval: 0.193
Frequency
Tumor−Normal Expression Difference

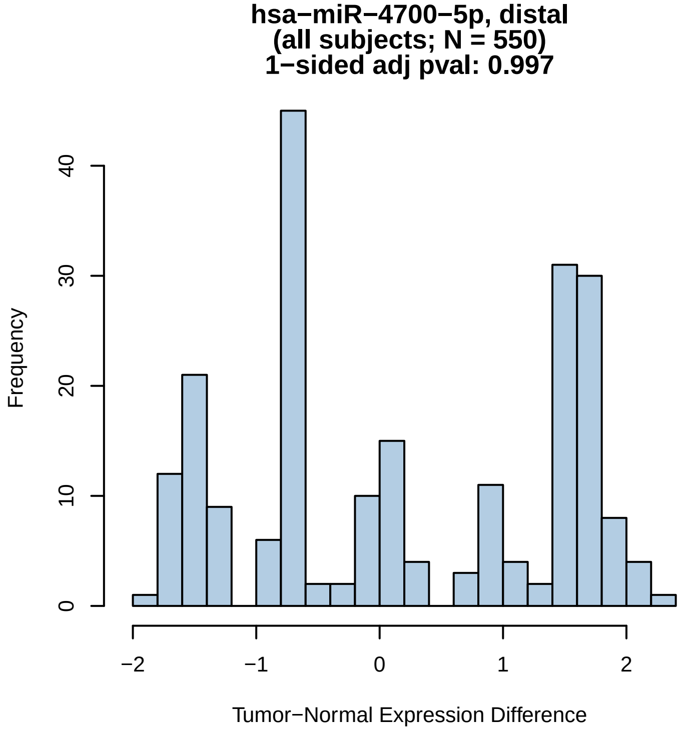
hsa−miR−4700−5p, distal
(all subjects; N = 550)
1−sided adj pval: 0.997
Frequency
Tumor−Normal Expression Difference

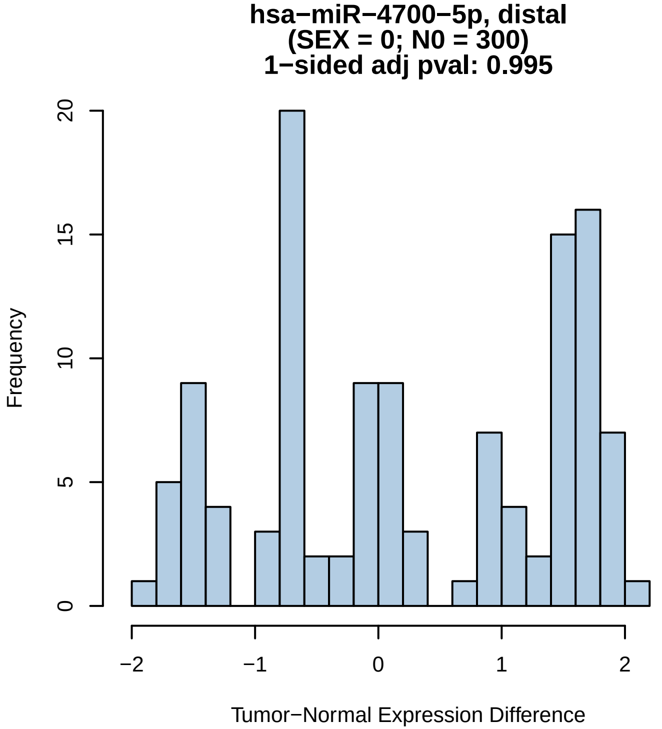
hsa−miR−4700−5p, distal
(SEX = 0; N0 = 300)
1−sided adj pval: 0.995
Frequency
Tumor−Normal Expression Difference

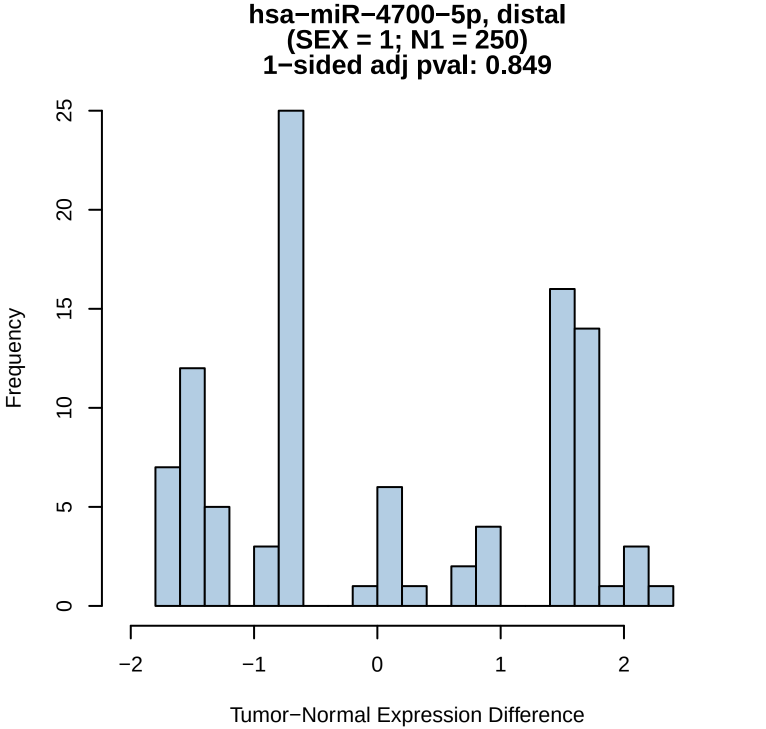
hsa−miR−4700−5p, distal
(SEX = 1; N1 = 250)
1−sided adj pval: 0.849
Frequency
Tumor−Normal Expression Difference

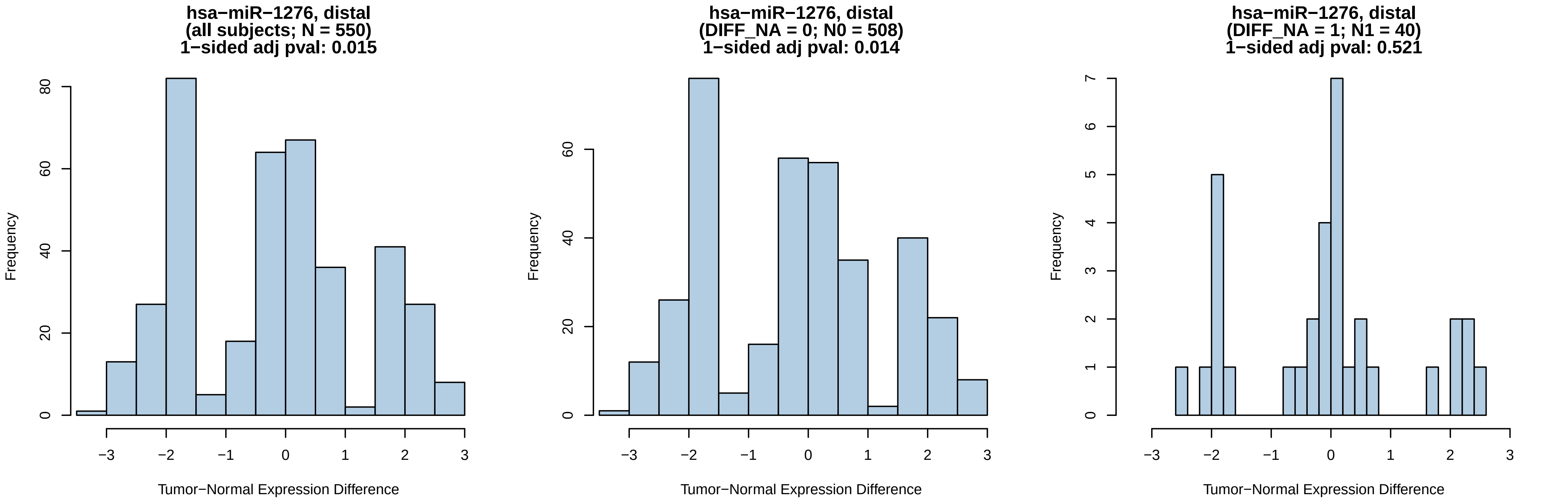
hsa−miR−1276, distal
(all subjects; N = 550)
1−sided adj pval: 0.015
hsa−miR−1276, distal
(DIFF_NA = 0; N0 = 508)
1−sided adj pval: 0.014
hsa−miR−1276, distal
(DIFF_NA = 1; N1 = 40)
1−sided adj pval: 0.521
Frequency
Tumor−Normal Expression Difference

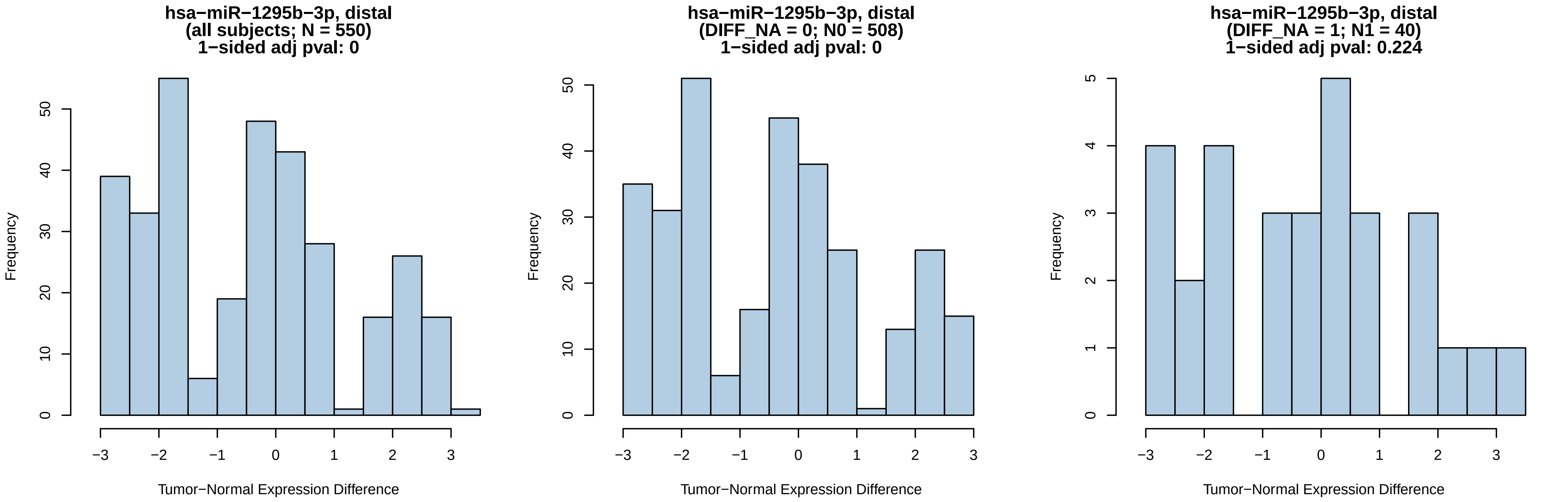
hsa−miR−1295b−3p, distal
(all subjects; N = 550)
1−sided adj pval: 0
Frequency
Tumor−Normal Expression Difference
hsa−miR−1295b−3p, distal
(DIFF_NA = 0; N0 = 508)
1−sided adj pval: 0
Frequency
Tumor−Normal Expression Difference
hsa−miR−1295b−3p, distal
(DIFF_NA = 1; N1 = 40)
1−sided adj pval: 0.224
Frequency
Tumor−Normal Expression Difference

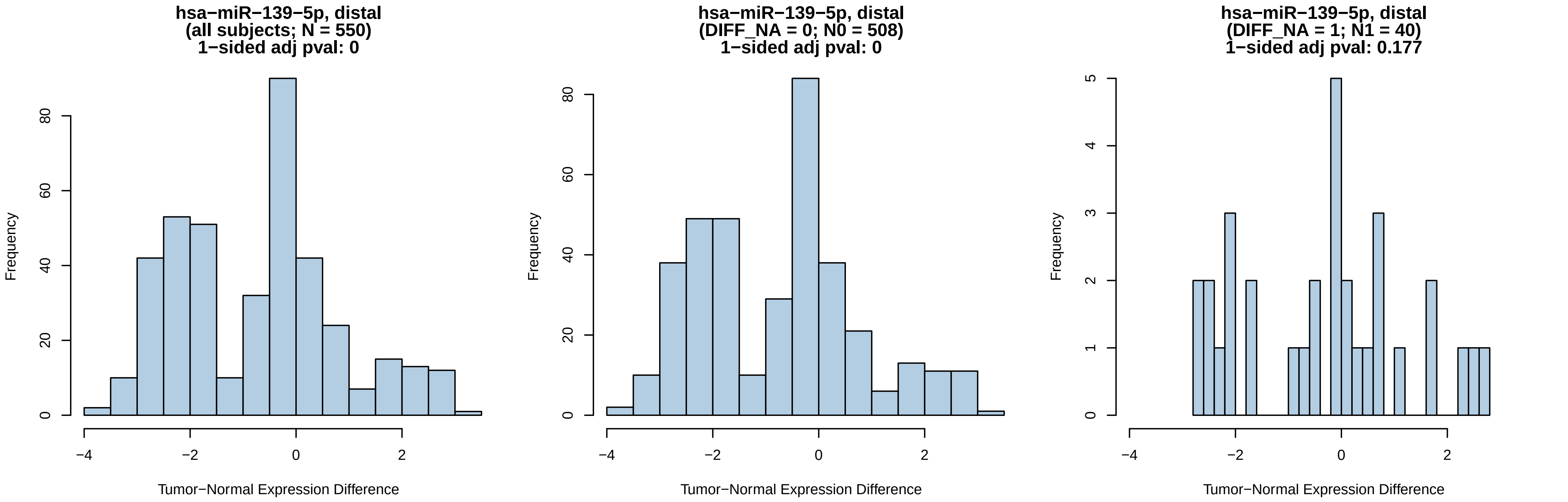
hsa−miR−139−5p, distal
(all subjects; N = 550)
1−sided adj pval: 0
Frequency
Tumor−Normal Expression Difference
0
20
40
60
80
−4
−2
0
2
hsa−miR−139−5p, distal
(DIFF_NA = 0; N0 = 508)
1−sided adj pval: 0
Frequency
Tumor−Normal Expression Difference
0
20
40
60
80
−4
−2
0
2
hsa−miR−139−5p, distal
(DIFF_NA = 1; N1 = 40)
1−sided adj pval: 0.177
Frequency
Tumor−Normal Expression Difference
0
1
2
3
4
5
−4
−2
0
2

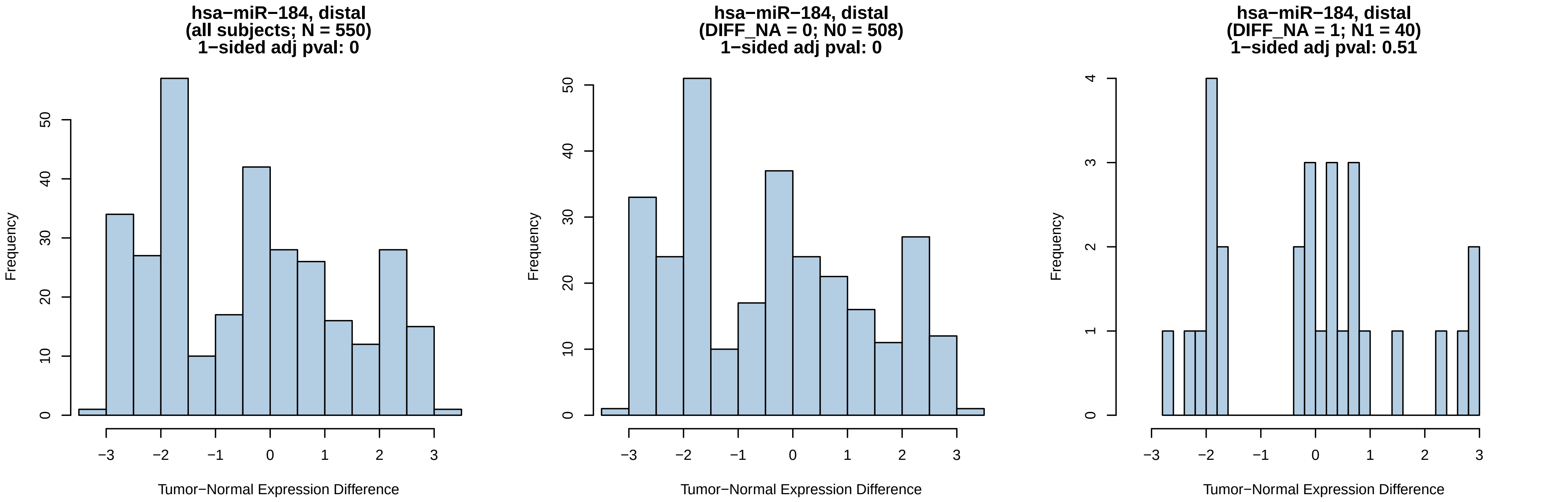

hsa−miR−184, distal
(all subjects; N = 550)
1−sided adj pval: 0
Frequency
0
10
20
30
40
50
−3
−2
−1
0
1
2
3
Tumor−Normal Expression Difference
hsa−miR−184, distal
(DIFF_NA = 0; N0 = 508)
1−sided adj pval: 0
Frequency
0
10
20
30
40
50
−3
−2
−1
0
1
2
3
Tumor−Normal Expression Difference
hsa−miR−184, distal
(DIFF_NA = 1; N1 = 40)
1−sided adj pval: 0.51
Frequency
0
1
2
3
4
−3
−2
−1
0
1
2
3
Tumor−Normal Expression Difference

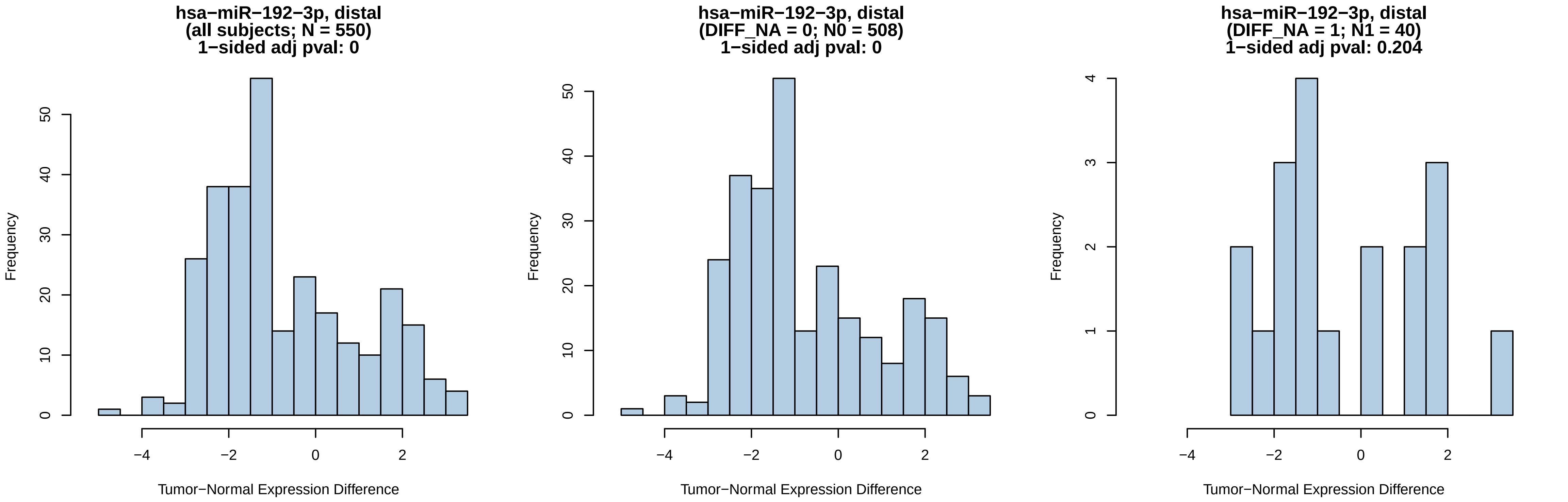
hsa−miR−192−3p, distal
(all subjects; N = 550)
1−sided adj pval: 0
Frequency
Tumor−Normal Expression Difference
hsa−miR−192−3p, distal
(DIFF_NA = 0; N0 = 508)
1−sided adj pval: 0
Frequency
Tumor−Normal Expression Difference
hsa−miR−192−3p, distal
(DIFF_NA = 1; N1 = 40)
1−sided adj pval: 0.204
Frequency
Tumor−Normal Expression Difference

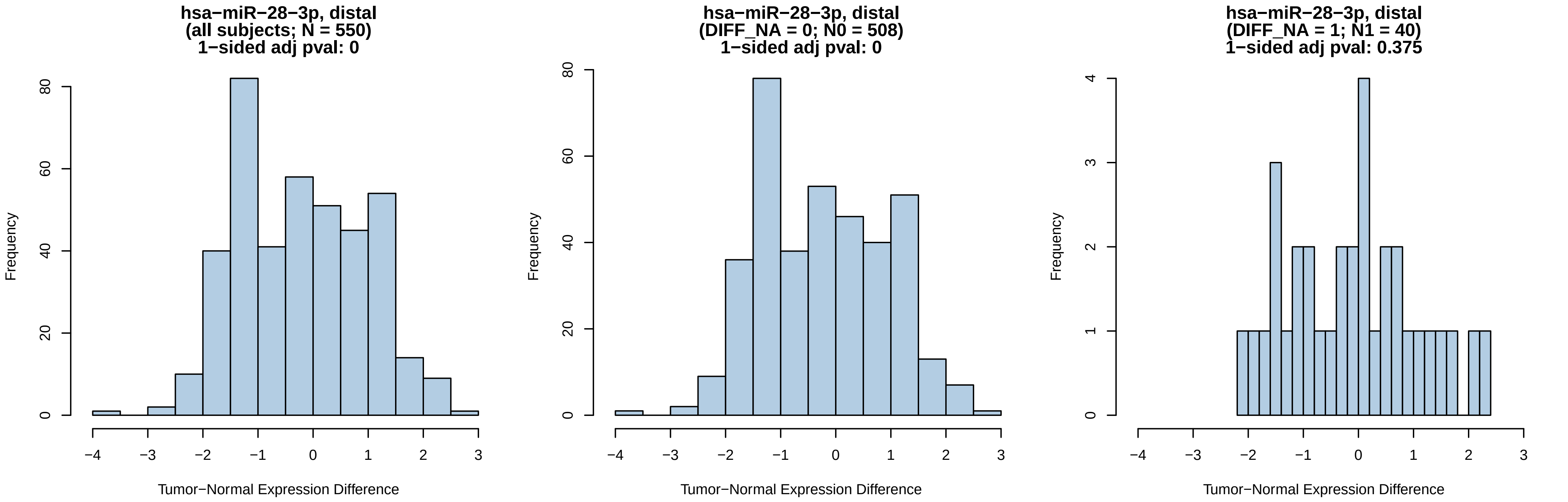
hsa−miR−28−3p, distal
(all subjects; N = 550)
1−sided adj pval: 0
Frequency
Tumor−Normal Expression Difference
hsa−miR−28−3p, distal
(DIFF_NA = 0; N0 = 508)
1−sided adj pval: 0
Frequency
Tumor−Normal Expression Difference
hsa−miR−28−3p, distal
(DIFF_NA = 1; N1 = 40)
1−sided adj pval: 0.375
Frequency
Tumor−Normal Expression Difference

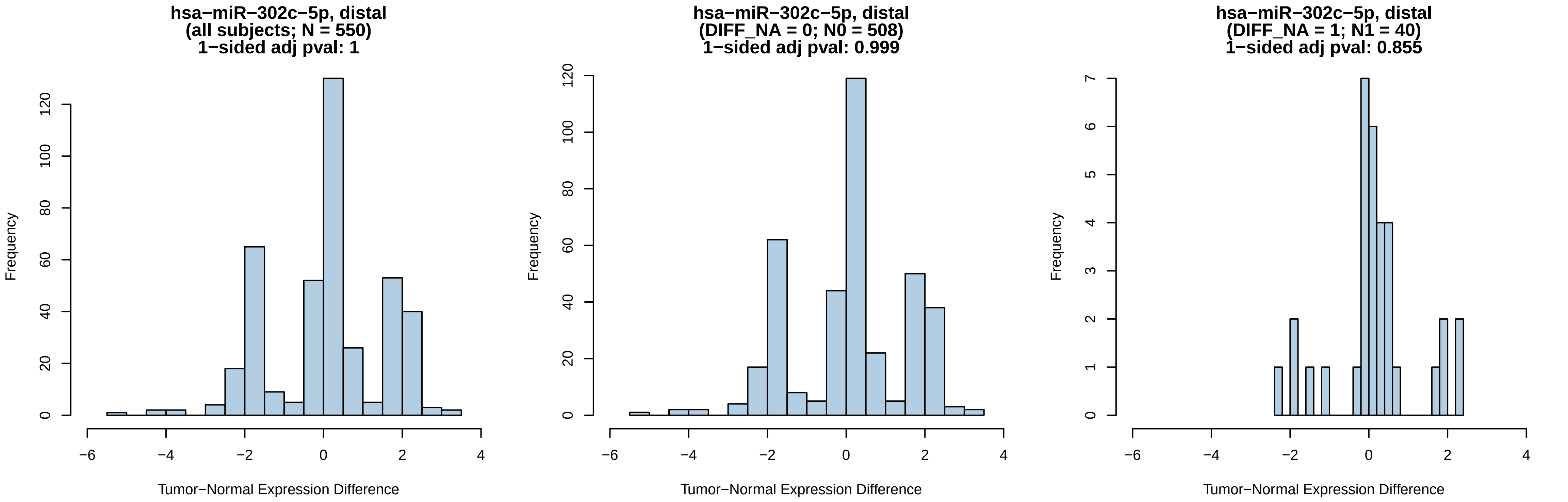

hsa−miR−302c−5p, distal
(all subjects; N = 550)
1−sided adj pval: 1
Frequency
Tumor−Normal Expression Difference
hsa−miR−302c−5p, distal
(DIFF_NA = 0; N0 = 508)
1−sided adj pval: 0.999
Frequency
Tumor−Normal Expression Difference
hsa−miR−302c−5p, distal
(DIFF_NA = 1; N1 = 40)
1−sided adj pval: 0.855
Frequency
Tumor−Normal Expression Difference

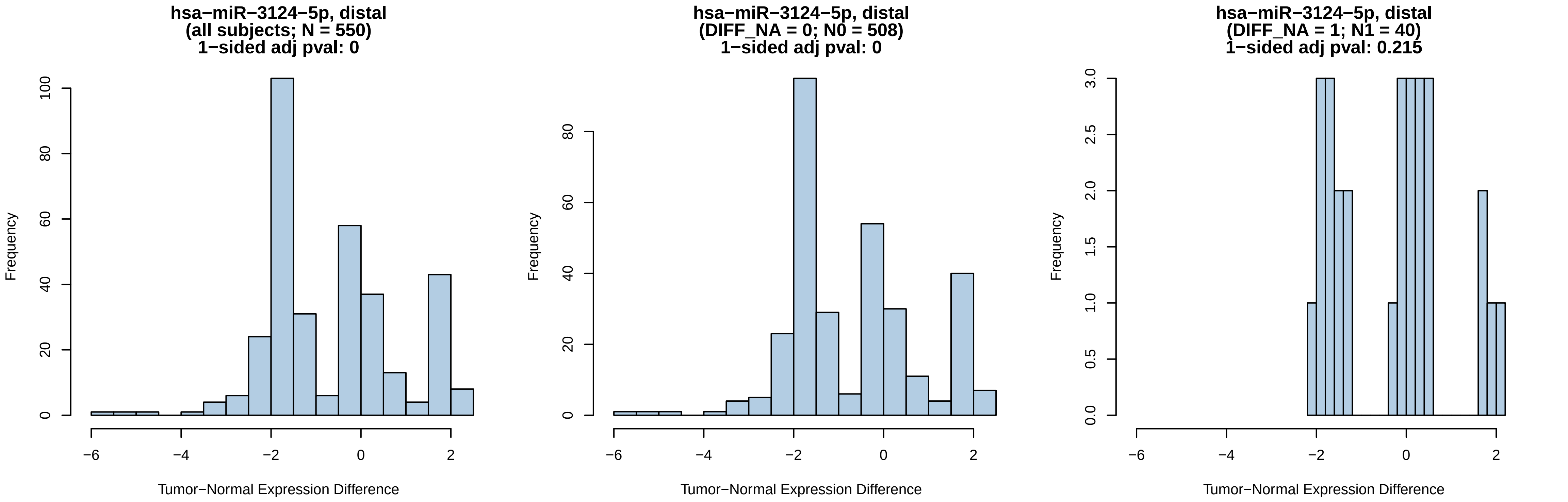
hsa−miR−3124−5p, distal
(all subjects; N = 550)
1−sided adj pval: 0
Frequency
Tumor−Normal Expression Difference
hsa−miR−3124−5p, distal
(DIFF_NA = 0; N0 = 508)
1−sided adj pval: 0
Frequency
Tumor−Normal Expression Difference
hsa−miR−3124−5p, distal
(DIFF_NA = 1; N1 = 40)
1−sided adj pval: 0.215
Frequency
Tumor−Normal Expression Difference

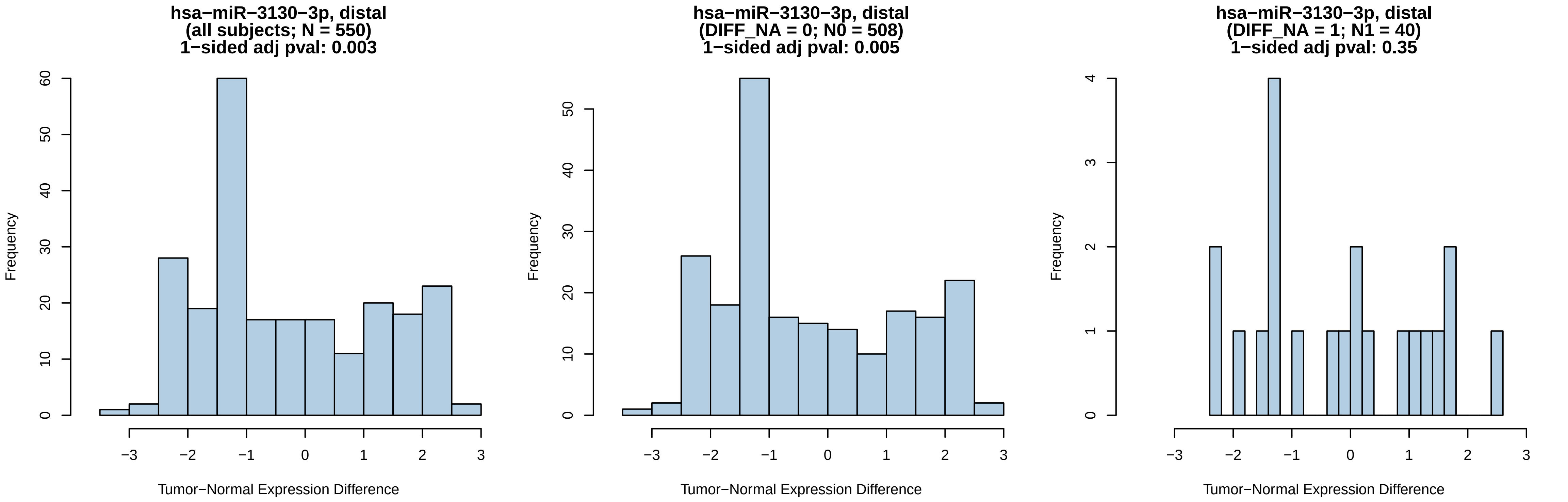
hsa−miR−3130−3p, distal
(all subjects; N = 550)
1−sided adj pval: 0.003
hsa−miR−3130−3p, distal
(DIFF_NA = 0; N0 = 508)
1−sided adj pval: 0.005
hsa−miR−3130−3p, distal
(DIFF_NA = 1; N1 = 40)
1−sided adj pval: 0.35
Frequency
Tumor−Normal Expression Difference

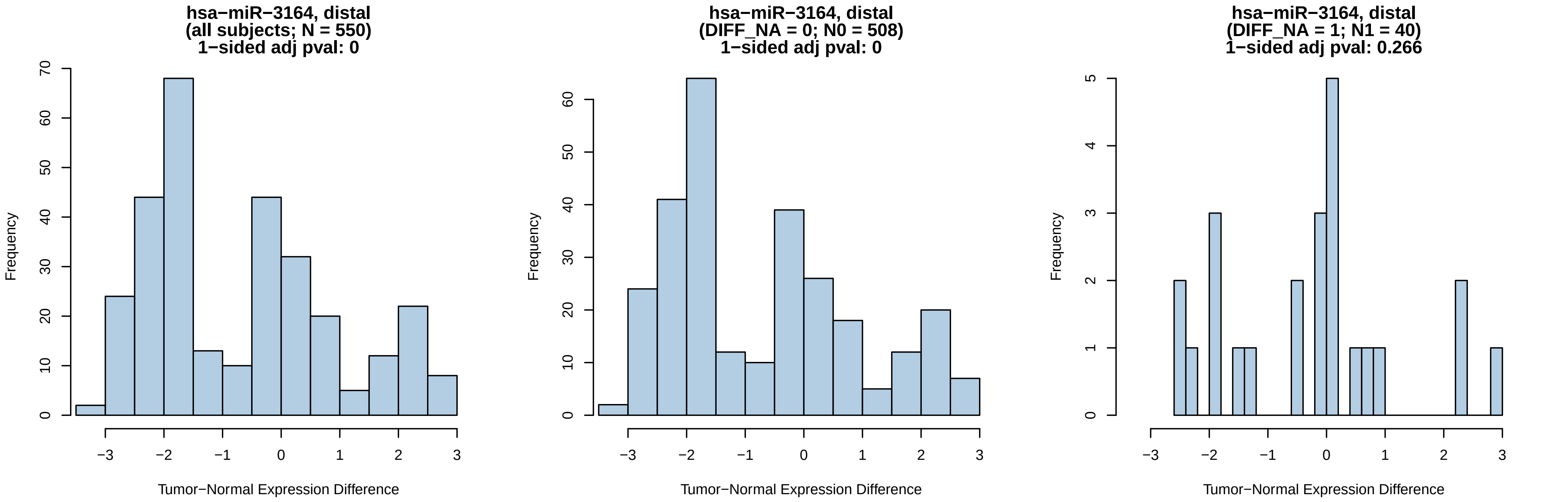

hsa−miR−3164, distal
(all subjects; N = 550)
1−sided adj pval: 0
Frequency
Tumor−Normal Expression Difference
hsa−miR−3164, distal
(DIFF_NA = 0; N0 = 508)
1−sided adj pval: 0
Frequency
Tumor−Normal Expression Difference
hsa−miR−3164, distal
(DIFF_NA = 1; N1 = 40)
1−sided adj pval: 0.266
Frequency
Tumor−Normal Expression Difference

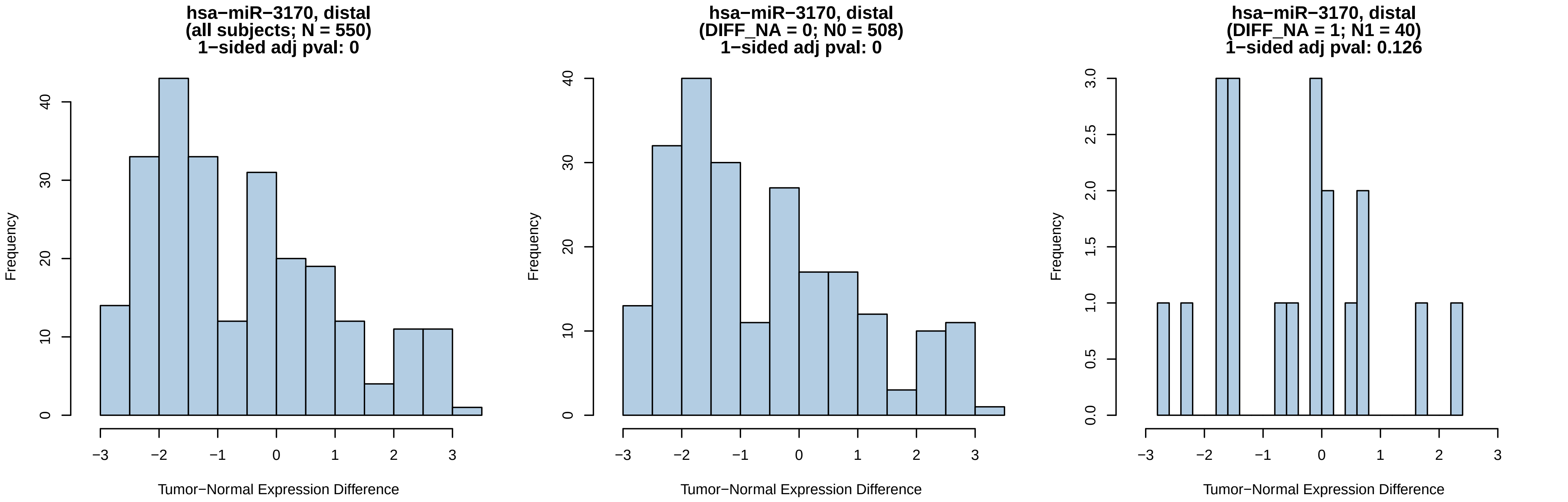
hsa−miR−3170, distal
(all subjects; N = 550)
1−sided adj pval: 0
hsa−miR−3170, distal
(DIFF_NA = 0; N0 = 508)
1−sided adj pval: 0
hsa−miR−3170, distal
(DIFF_NA = 1; N1 = 40)
1−sided adj pval: 0.126
Frequency
Tumor−Normal Expression Difference

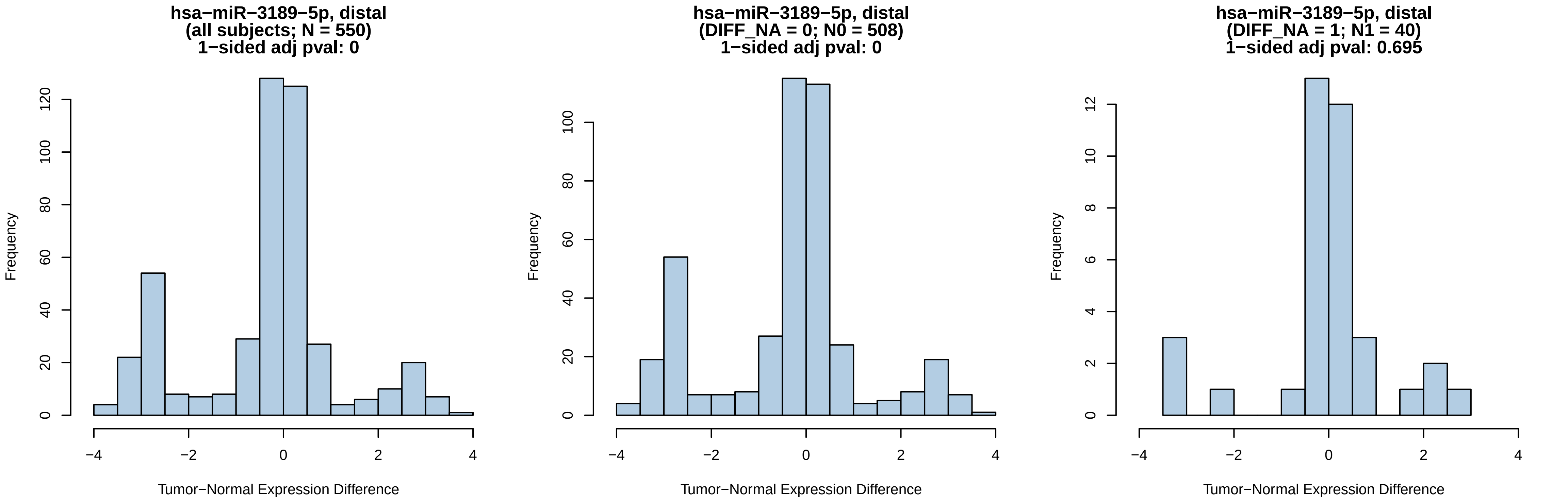
hsa−miR−3189−5p, distal
(all subjects; N = 550)
1−sided adj pval: 0
Frequency
0
20
40
60
80
100
120
−4
−2
0
2
4
Tumor−Normal Expression Difference
hsa−miR−3189−5p, distal
(DIFF_NA = 0; N0 = 508)
1−sided adj pval: 0
Frequency
0
20
40
60
80
100
−4
−2
0
2
4
Tumor−Normal Expression Difference
hsa−miR−3189−5p, distal
(DIFF_NA = 1; N1 = 40)
1−sided adj pval: 0.695
Frequency
0
2
4
6
8
10
12
−4
−2
0
2
4
Tumor−Normal Expression Difference

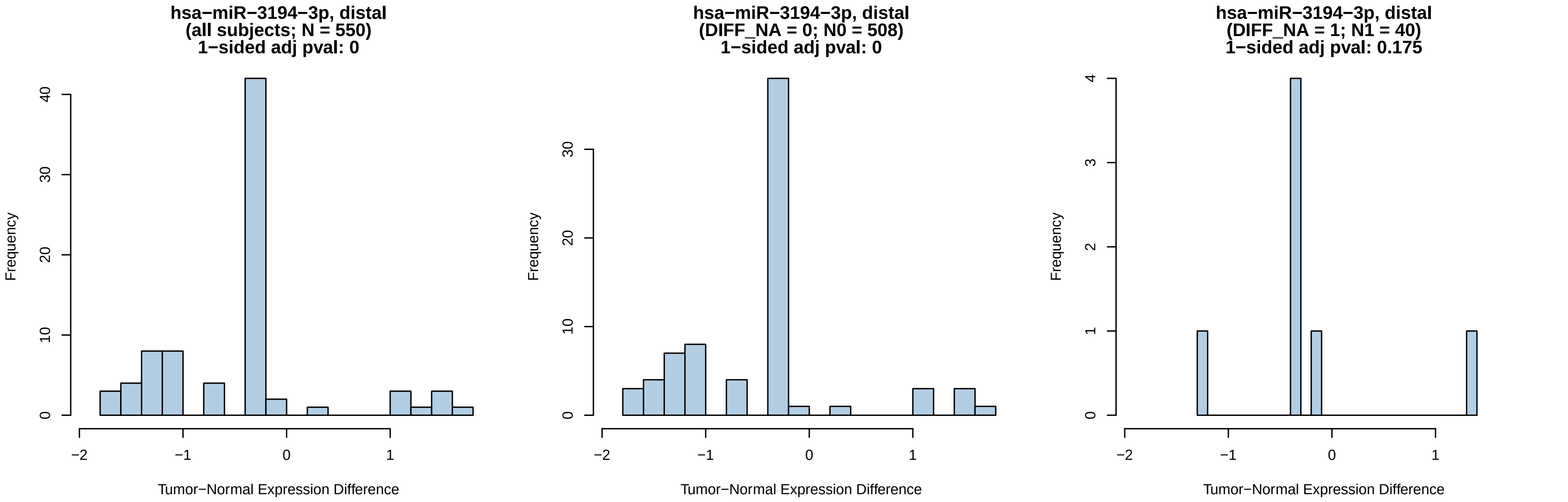
hsa−miR−3194−3p, distal
(all subjects; N = 550)
1−sided adj pval: 0
hsa−miR−3194−3p, distal
(DIFF_NA = 0; N0 = 508)
1−sided adj pval: 0
hsa−miR−3194−3p, distal
(DIFF_NA = 1; N1 = 40)
1−sided adj pval: 0.175
Frequency
Tumor−Normal Expression Difference

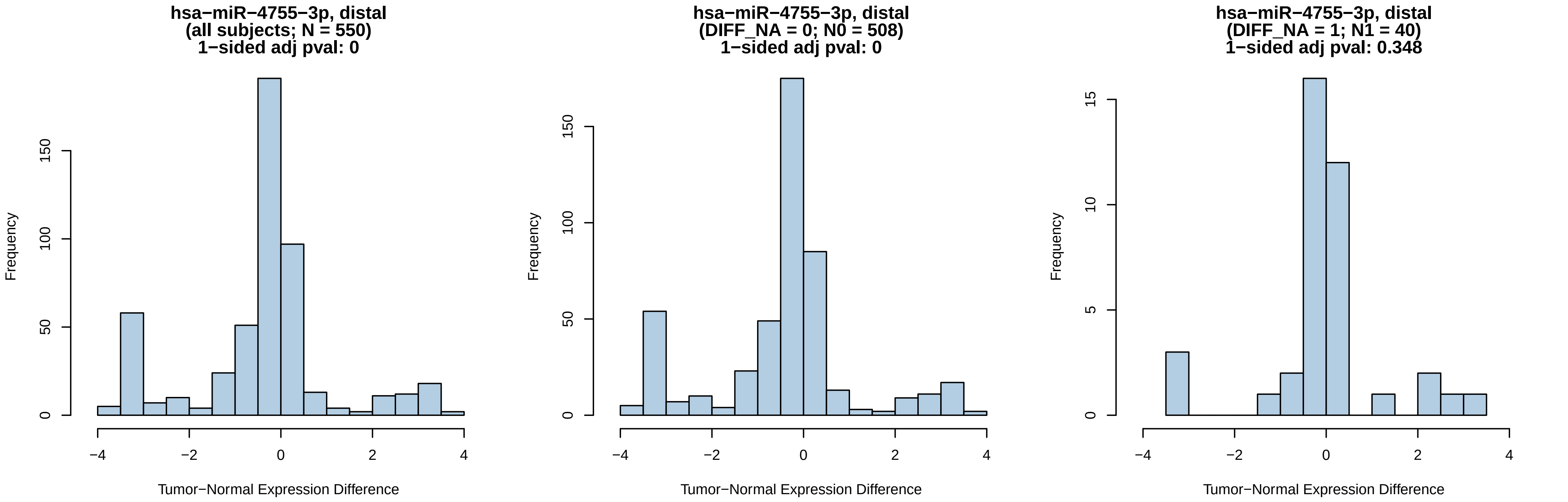

hsa−miR−4755−3p, distal
(all subjects; N = 550)
1−sided adj pval: 0
hsa−miR−4755−3p, distal
(DIFF_NA = 0; N0 = 508)
1−sided adj pval: 0
hsa−miR−4755−3p, distal
(DIFF_NA = 1; N1 = 40)
1−sided adj pval: 0.348
Frequency
Tumor−Normal Expression Difference

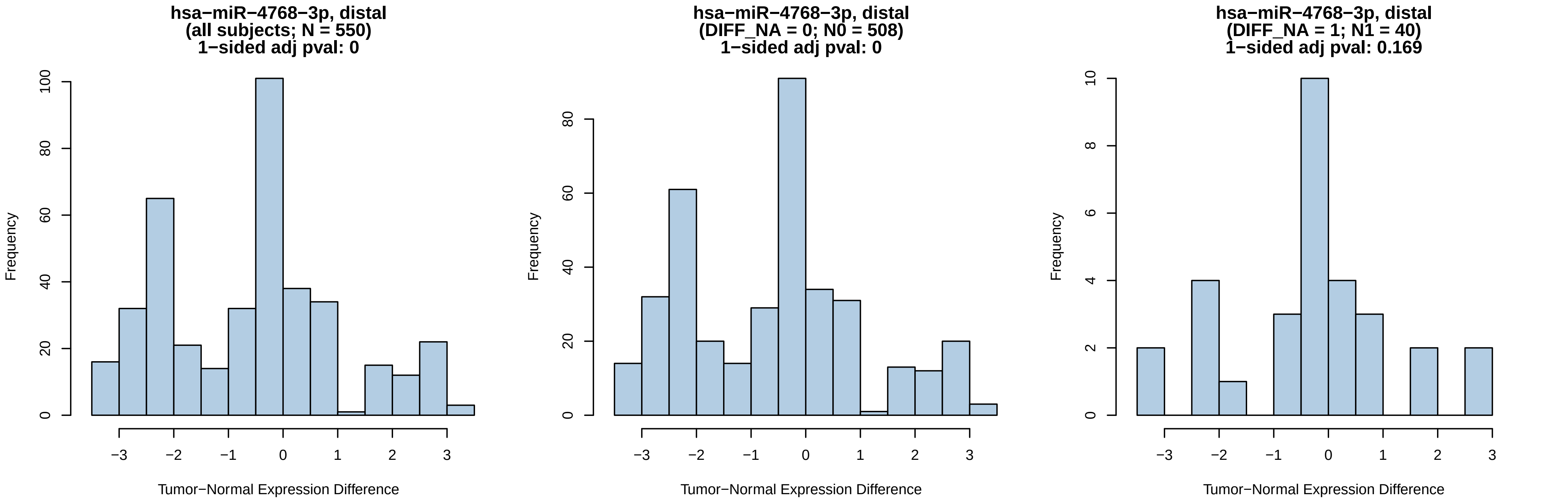

hsa−miR−4768−3p, distal
(all subjects; N = 550)
1−sided adj pval: 0
hsa−miR−4768−3p, distal
(DIFF_NA = 0; N0 = 508)
1−sided adj pval: 0
hsa−miR−4768−3p, distal
(DIFF_NA = 1; N1 = 40)
1−sided adj pval: 0.169
Frequency
Tumor−Normal Expression Difference

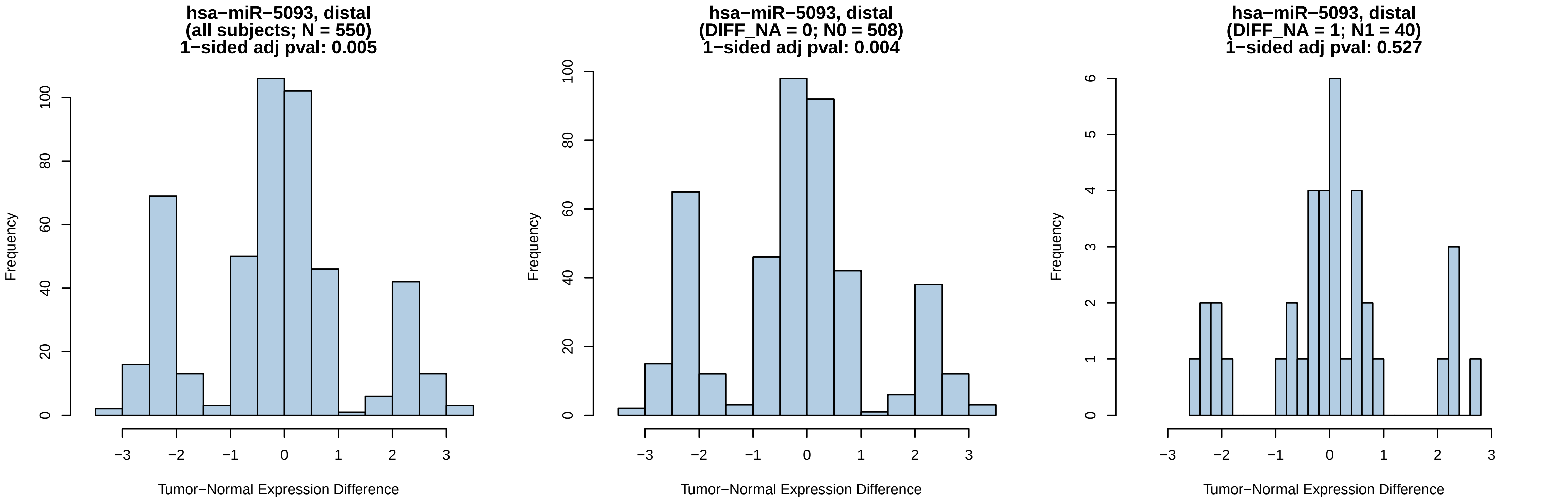
hsa−miR−5093, distal
(all subjects; N = 550)
1−sided adj pval: 0.005
Frequency
Tumor−Normal Expression Difference
hsa−miR−5093, distal
(DIFF_NA = 0; N0 = 508)
1−sided adj pval: 0.004
Frequency
Tumor−Normal Expression Difference
hsa−miR−5093, distal
(DIFF_NA = 1; N1 = 40)
1−sided adj pval: 0.527
Frequency
Tumor−Normal Expression Difference

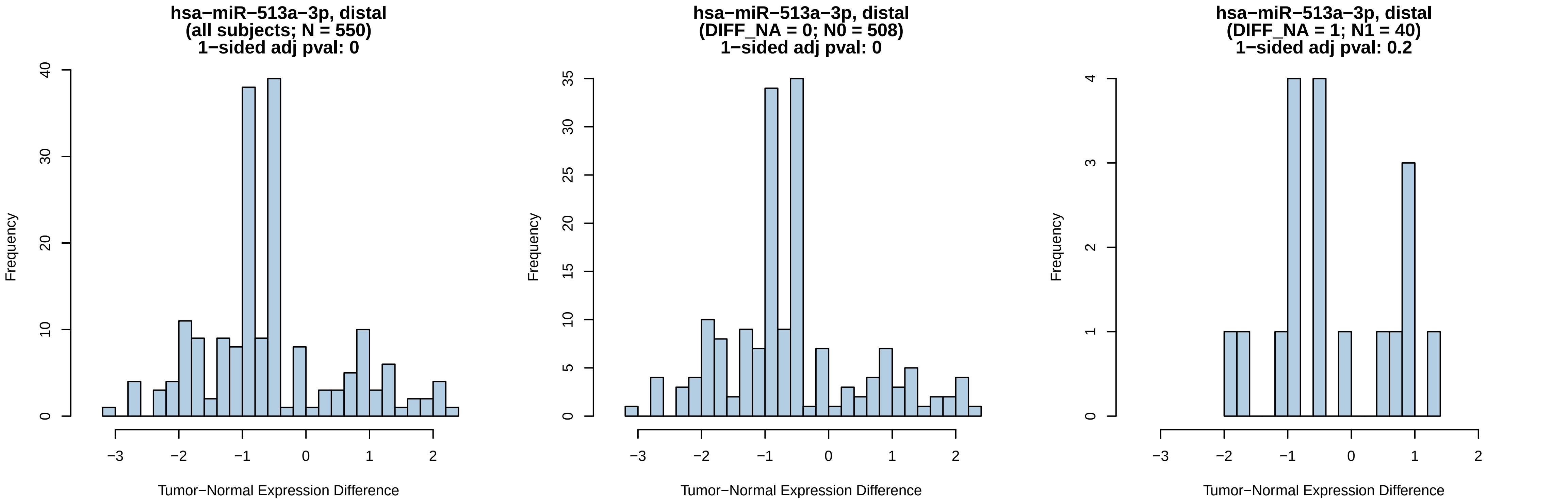
hsa−miR−513a−3p, distal
(all subjects; N = 550)
1−sided adj pval: 0
Frequency
Tumor−Normal Expression Difference
hsa−miR−513a−3p, distal
(DIFF_NA = 0; N0 = 508)
1−sided adj pval: 0
Frequency
Tumor−Normal Expression Difference
hsa−miR−513a−3p, distal
(DIFF_NA = 1; N1 = 40)
1−sided adj pval: 0.2
Frequency
Tumor−Normal Expression Difference

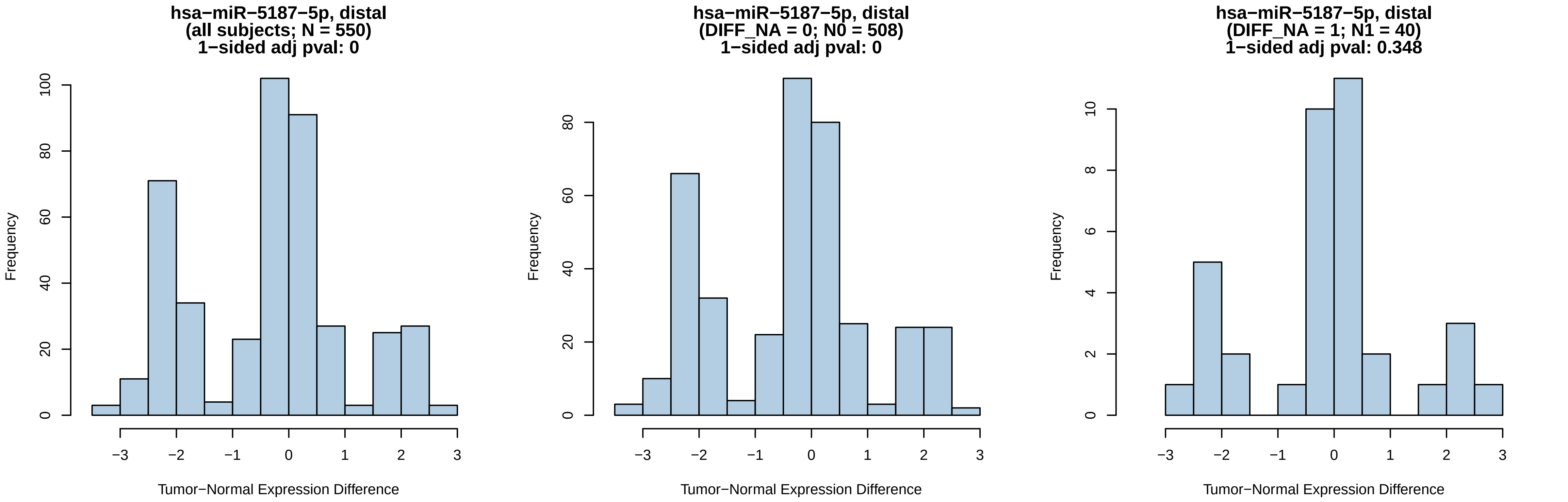

hsa−miR−5187−5p, distal
(all subjects; N = 550)
1−sided adj pval: 0
Frequency
Tumor−Normal Expression Difference
hsa−miR−5187−5p, distal
(DIFF_NA = 0; N0 = 508)
1−sided adj pval: 0
Frequency
Tumor−Normal Expression Difference
hsa−miR−5187−5p, distal
(DIFF_NA = 1; N1 = 40)
1−sided adj pval: 0.348
Frequency
Tumor−Normal Expression Difference

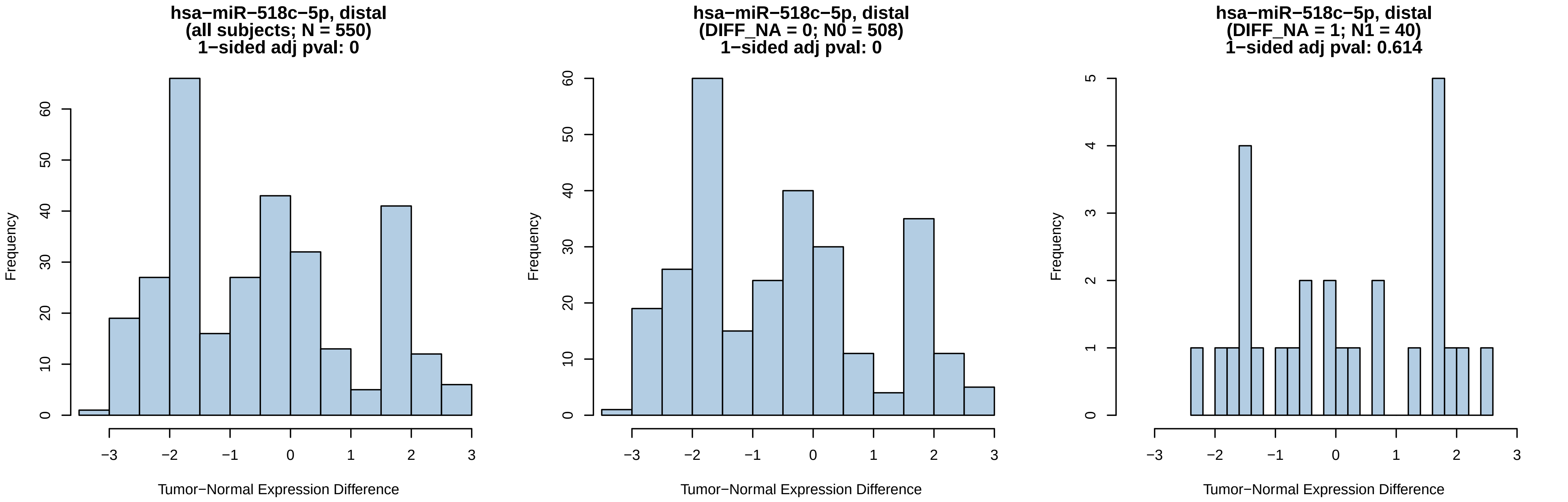
hsa−miR−518c−5p, distal
(all subjects; N = 550)
1−sided adj pval: 0
Frequency
0
10
20
30
40
50
60
−3
−2
−1
0
1
2
3
Tumor−Normal Expression Difference
hsa−miR−518c−5p, distal
(DIFF_NA = 0; N0 = 508)
1−sided adj pval: 0
Frequency
0
10
20
30
40
50
60
−3
−2
−1
0
1
2
3
Tumor−Normal Expression Difference
hsa−miR−518c−5p, distal
(DIFF_NA = 1; N1 = 40)
1−sided adj pval: 0.614
Frequency
0
1
2
3
4
5
−3
−2
−1
0
1
2
3
Tumor−Normal Expression Difference

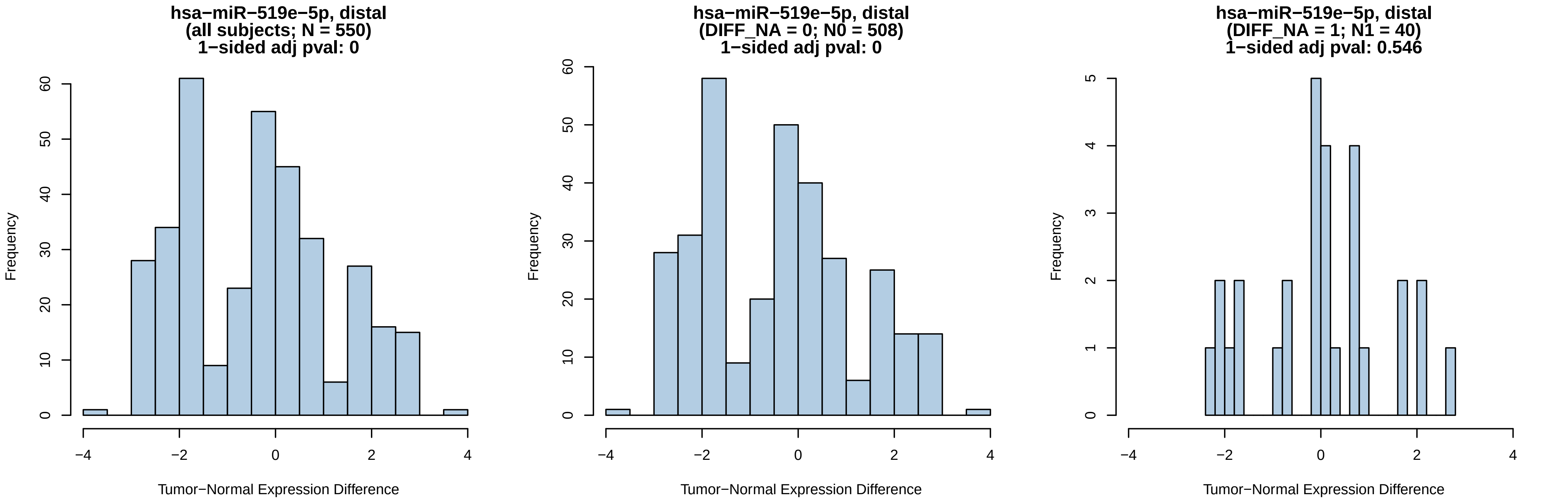
hsa−miR−519e−5p, distal
(all subjects; N = 550)
1−sided adj pval: 0
Frequency
Tumor−Normal Expression Difference
hsa−miR−519e−5p, distal
(DIFF_NA = 0; N0 = 508)
1−sided adj pval: 0
Frequency
Tumor−Normal Expression Difference
hsa−miR−519e−5p, distal
(DIFF_NA = 1; N1 = 40)
1−sided adj pval: 0.546
Frequency
Tumor−Normal Expression Difference

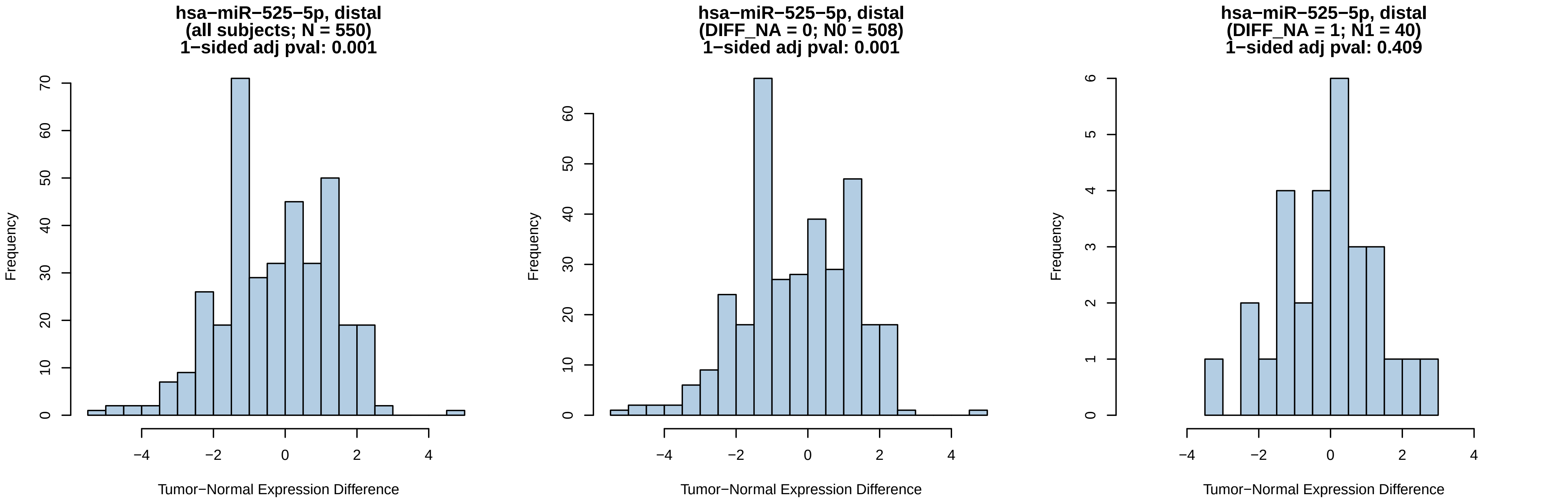

hsa−miR−525−5p, distal
(all subjects; N = 550)
1−sided adj pval: 0.001
Frequency
Tumor−Normal Expression Difference
hsa−miR−525−5p, distal
(DIFF_NA = 0; N0 = 508)
1−sided adj pval: 0.001
Frequency
Tumor−Normal Expression Difference
hsa−miR−525−5p, distal
(DIFF_NA = 1; N1 = 40)
1−sided adj pval: 0.409
Frequency
Tumor−Normal Expression Difference

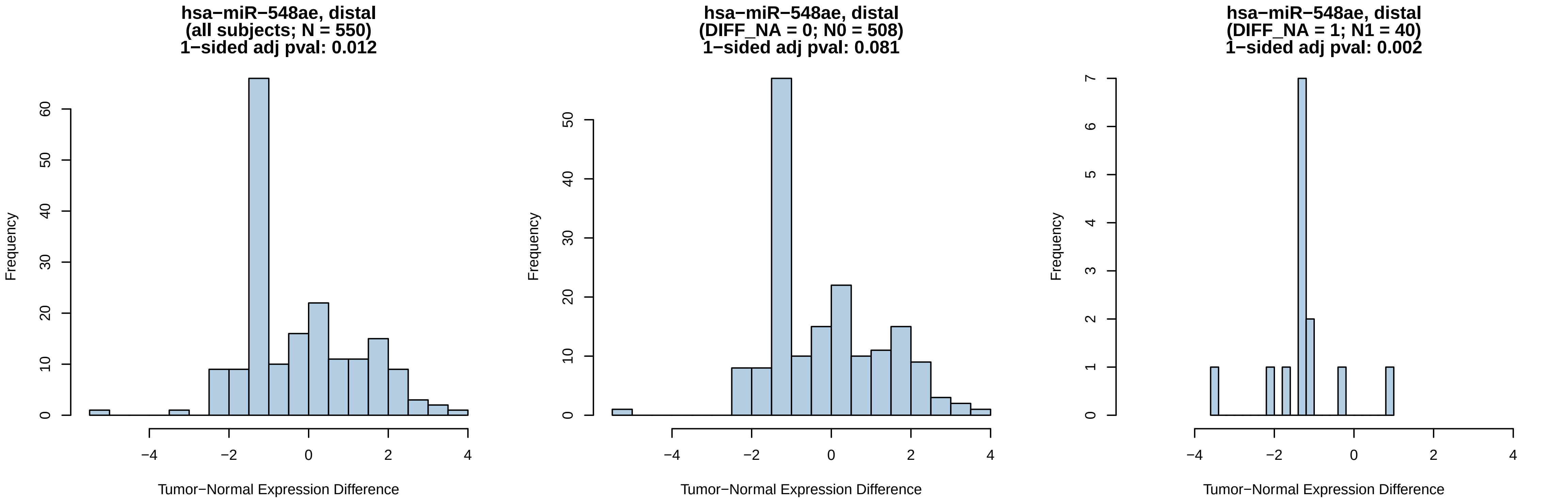
hsa−miR−548ae, distal
(all subjects; N = 550)
1−sided adj pval: 0.012
hsa−miR−548ae, distal
(DIFF_NA = 0; N0 = 508)
1−sided adj pval: 0.081
hsa−miR−548ae, distal
(DIFF_NA = 1; N1 = 40)
1−sided adj pval: 0.002
Frequency
Tumor−Normal Expression Difference

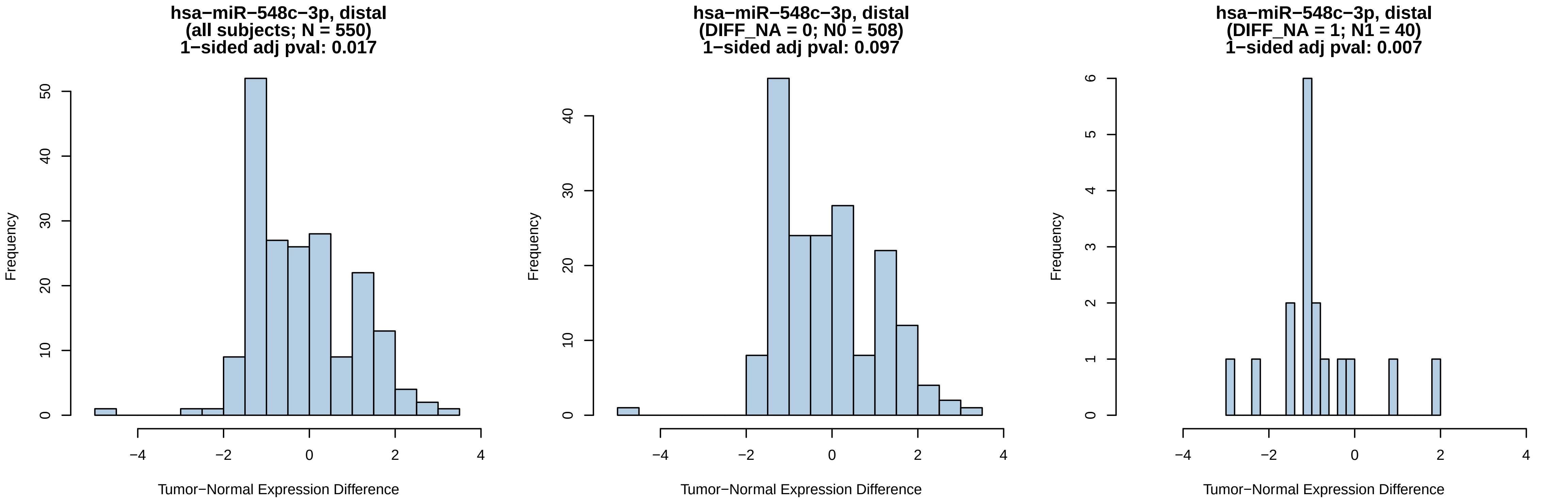
hsa−miR−548c−3p, distal
(all subjects; N = 550)
1−sided adj pval: 0.017
hsa−miR−548c−3p, distal
(DIFF_NA = 0; N0 = 508)
1−sided adj pval: 0.097
hsa−miR−548c−3p, distal
(DIFF_NA = 1; N1 = 40)
1−sided adj pval: 0.007
Frequency
Tumor−Normal Expression Difference

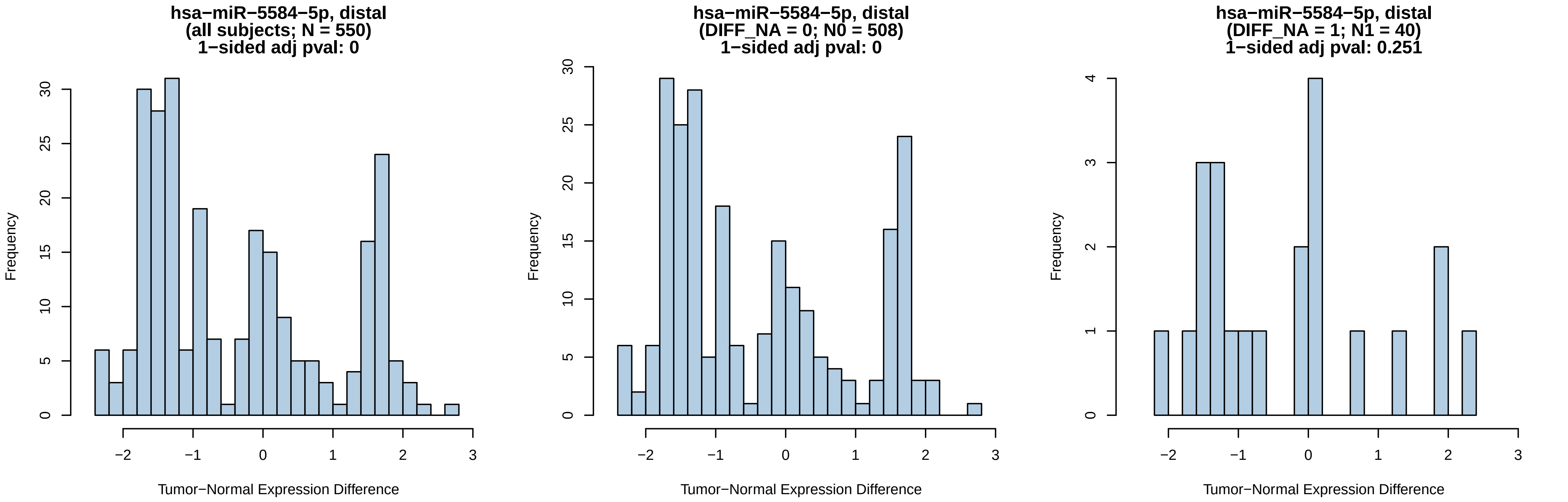
hsa−miR−5584−5p, distal
(all subjects; N = 550)
1−sided adj pval: 0
Frequency
Tumor−Normal Expression Difference
hsa−miR−5584−5p, distal
(DIFF_NA = 0; N0 = 508)
1−sided adj pval: 0
Frequency
Tumor−Normal Expression Difference
hsa−miR−5584−5p, distal
(DIFF_NA = 1; N1 = 40)
1−sided adj pval: 0.251
Frequency
Tumor−Normal Expression Difference

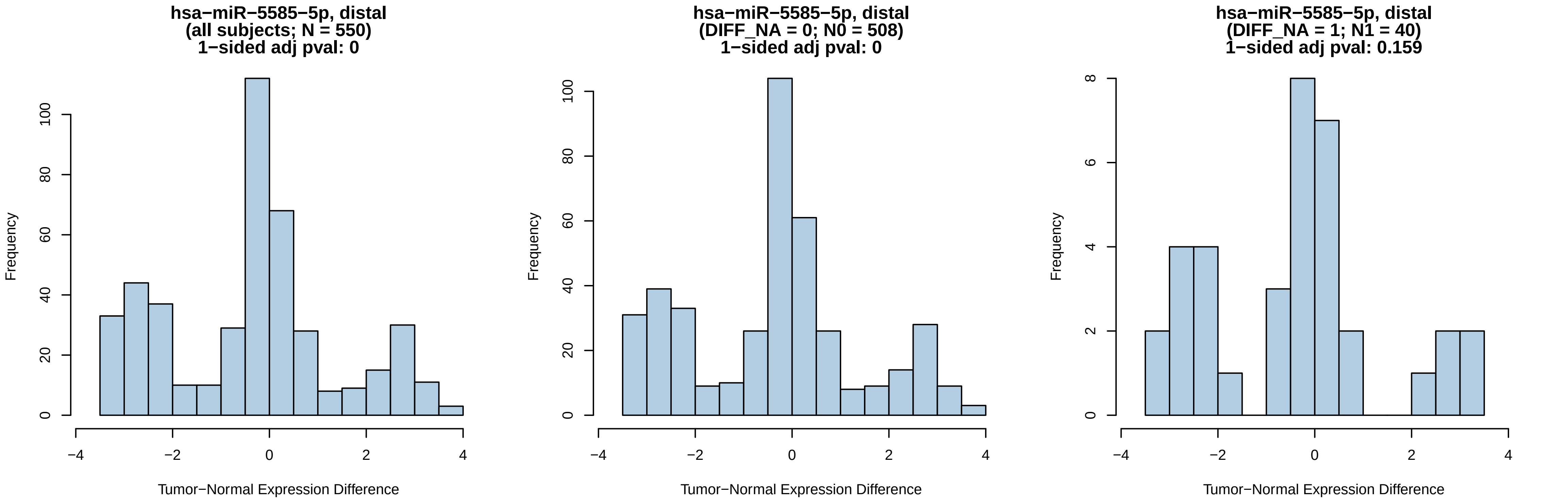
hsa−miR−5585−5p, distal
(all subjects; N = 550)
1−sided adj pval: 0
hsa−miR−5585−5p, distal
(DIFF_NA = 0; N0 = 508)
1−sided adj pval: 0
hsa−miR−5585−5p, distal
(DIFF_NA = 1; N1 = 40)
1−sided adj pval: 0.159
Frequency
Tumor−Normal Expression Difference

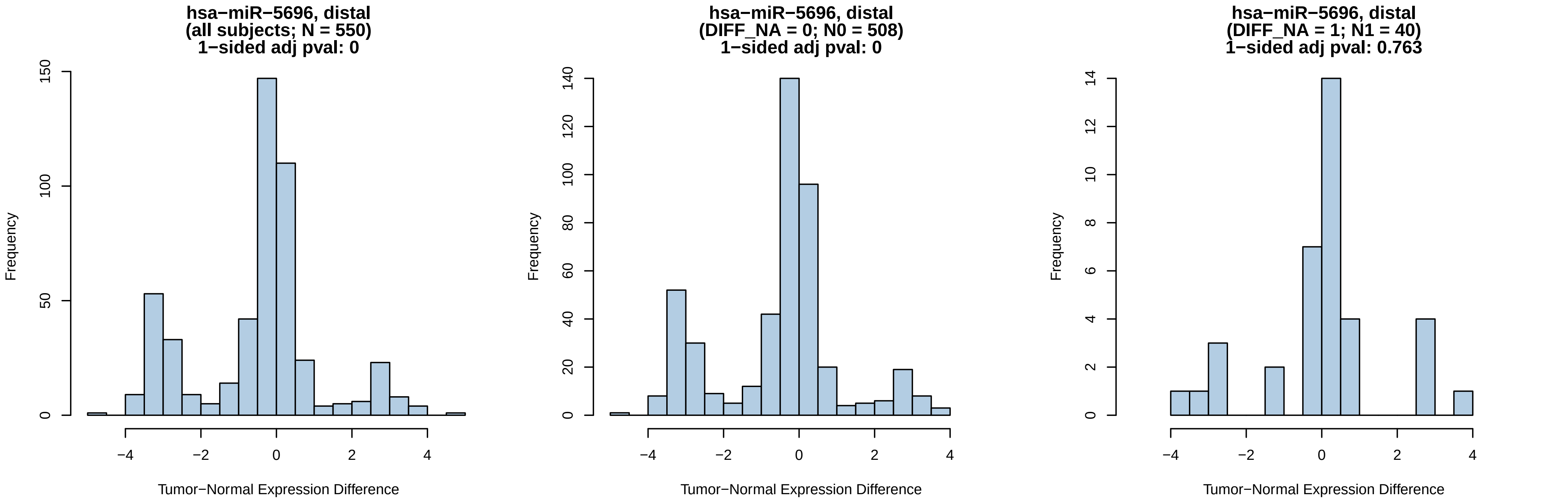

hsa−miR−5696, distal
(all subjects; N = 550)
1−sided adj pval: 0
Frequency
Tumor−Normal Expression Difference
hsa−miR−5696, distal
(DIFF_NA = 0; N0 = 508)
1−sided adj pval: 0
Frequency
Tumor−Normal Expression Difference
hsa−miR−5696, distal
(DIFF_NA = 1; N1 = 40)
1−sided adj pval: 0.763
Frequency
Tumor−Normal Expression Difference

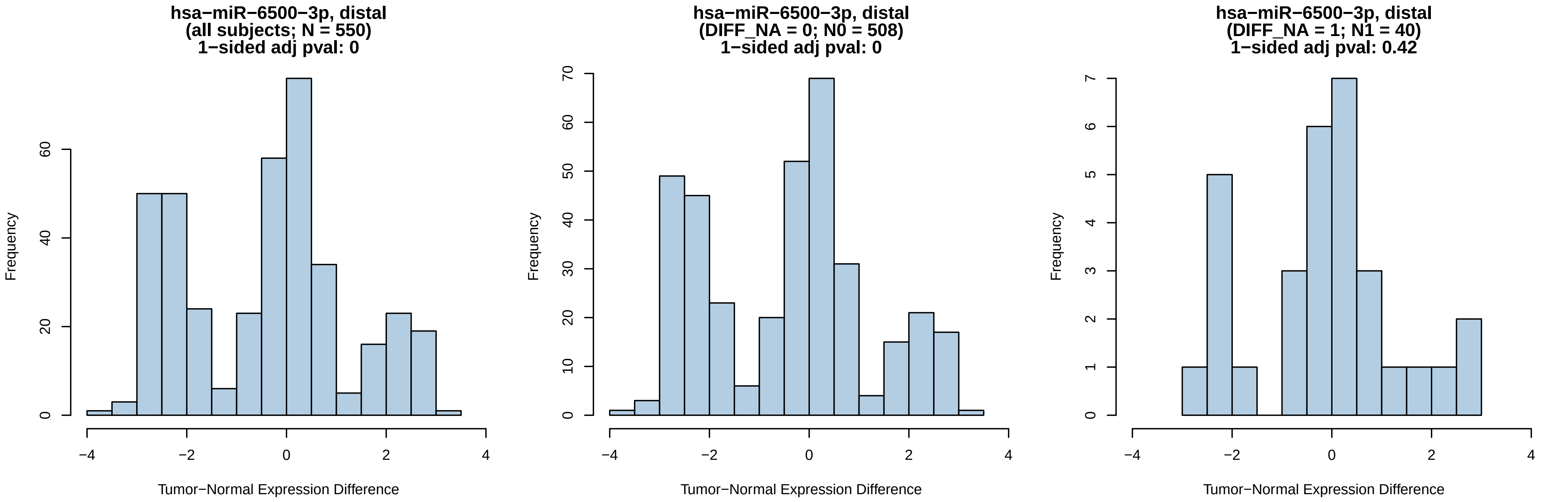
hsa−miR−6500−3p, distal
(all subjects; N = 550)
1−sided adj pval: 0
Frequency
Tumor−Normal Expression Difference
hsa−miR−6500−3p, distal
(DIFF_NA = 0; N0 = 508)
1−sided adj pval: 0
Frequency
Tumor−Normal Expression Difference
hsa−miR−6500−3p, distal
(DIFF_NA = 1; N1 = 40)
1−sided adj pval: 0.42
Frequency
Tumor−Normal Expression Difference

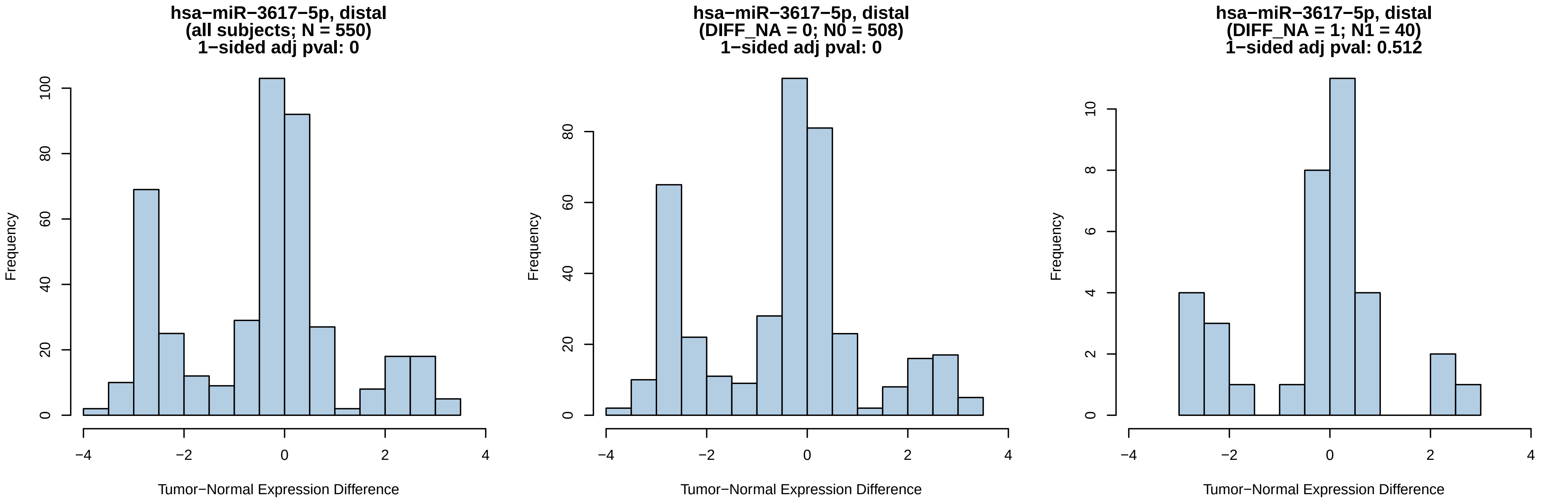
hsa−miR−3617−5p, distal
(all subjects; N = 550)
1−sided adj pval: 0
Frequency
Tumor−Normal Expression Difference
hsa−miR−3617−5p, distal
(DIFF_NA = 0; N0 = 508)
1−sided adj pval: 0
Frequency
Tumor−Normal Expression Difference
hsa−miR−3617−5p, distal
(DIFF_NA = 1; N1 = 40)
1−sided adj pval: 0.512
Frequency
Tumor−Normal Expression Difference

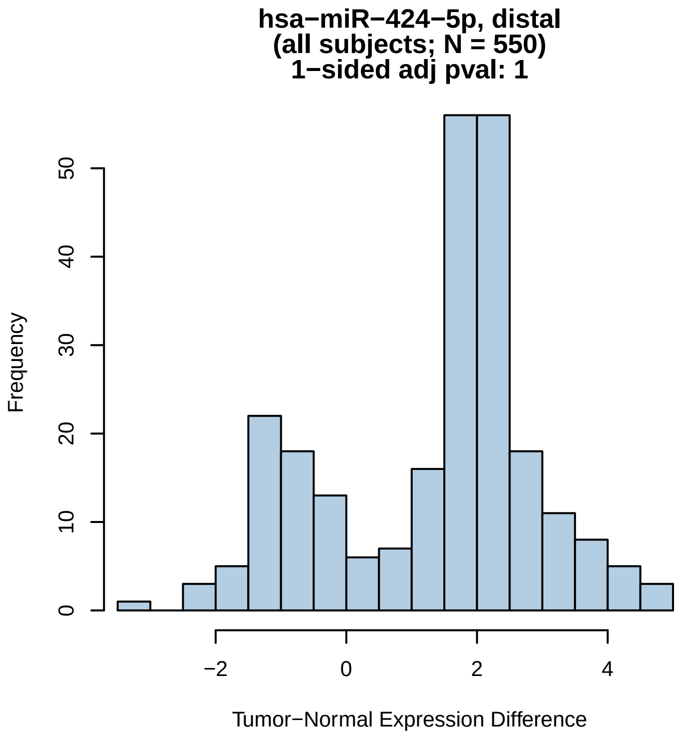
hsa−miR−424−5p, distal
(all subjects; N = 550)
1−sided adj pval: 1
Frequency
Tumor−Normal Expression Difference

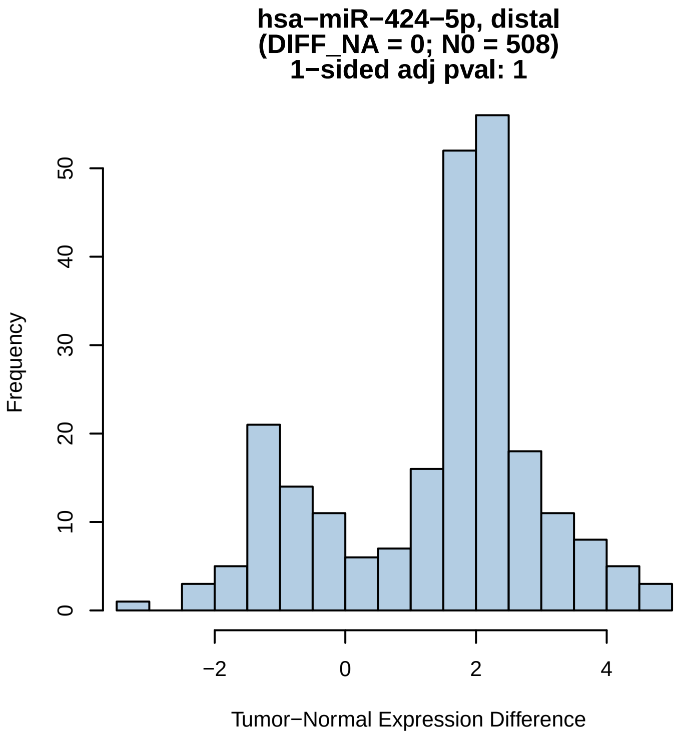
hsa−miR−424−5p, distal
(DIFF_NA = 0; N0 = 508)
1−sided adj pval: 1
Frequency
Tumor−Normal Expression Difference

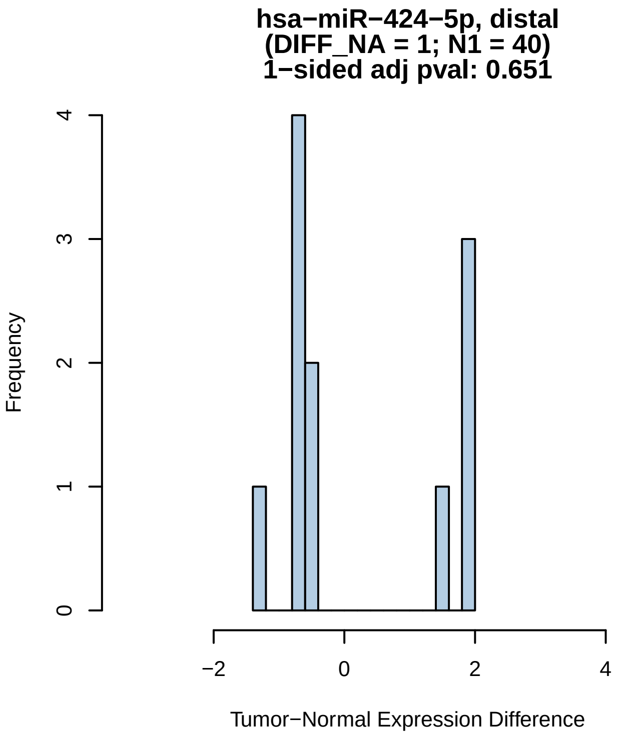
hsa−miR−424−5p, distal
(DIFF_NA = 1; N1 = 40)
1−sided adj pval: 0.651
Frequency
Tumor−Normal Expression Difference

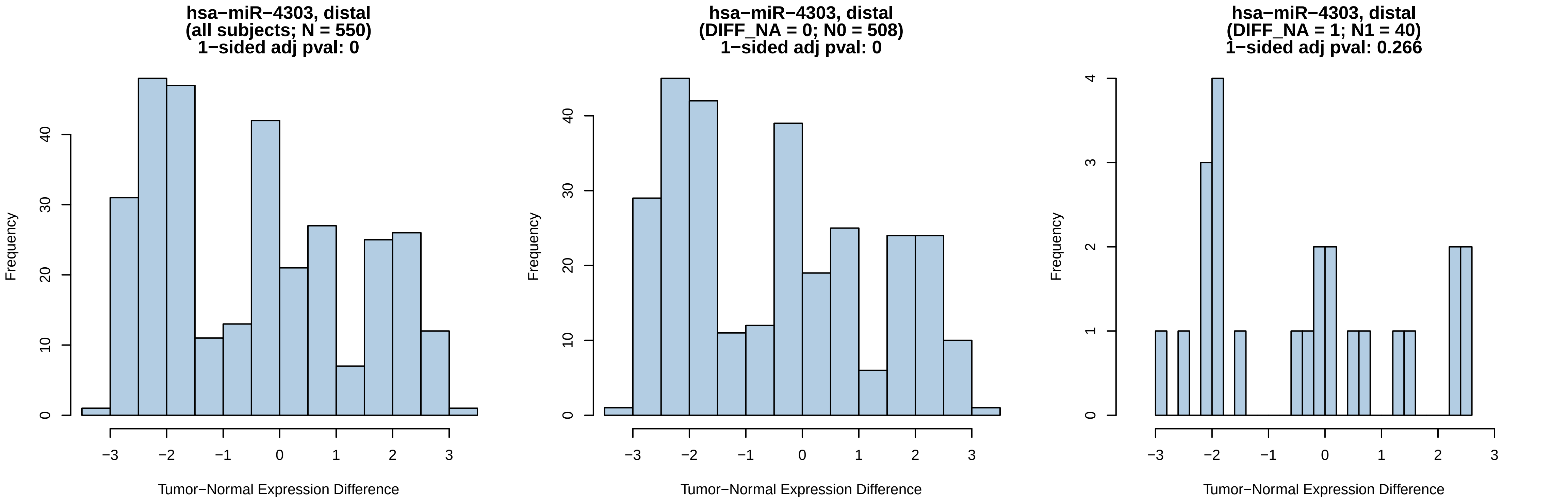
hsa−miR−4303, distal
(all subjects; N = 550)
1−sided adj pval: 0
Frequency
Tumor−Normal Expression Difference
hsa−miR−4303, distal
(DIFF_NA = 0; N0 = 508)
1−sided adj pval: 0
Frequency
Tumor−Normal Expression Difference
hsa−miR−4303, distal
(DIFF_NA = 1; N1 = 40)
1−sided adj pval: 0.266
Frequency
Tumor−Normal Expression Difference

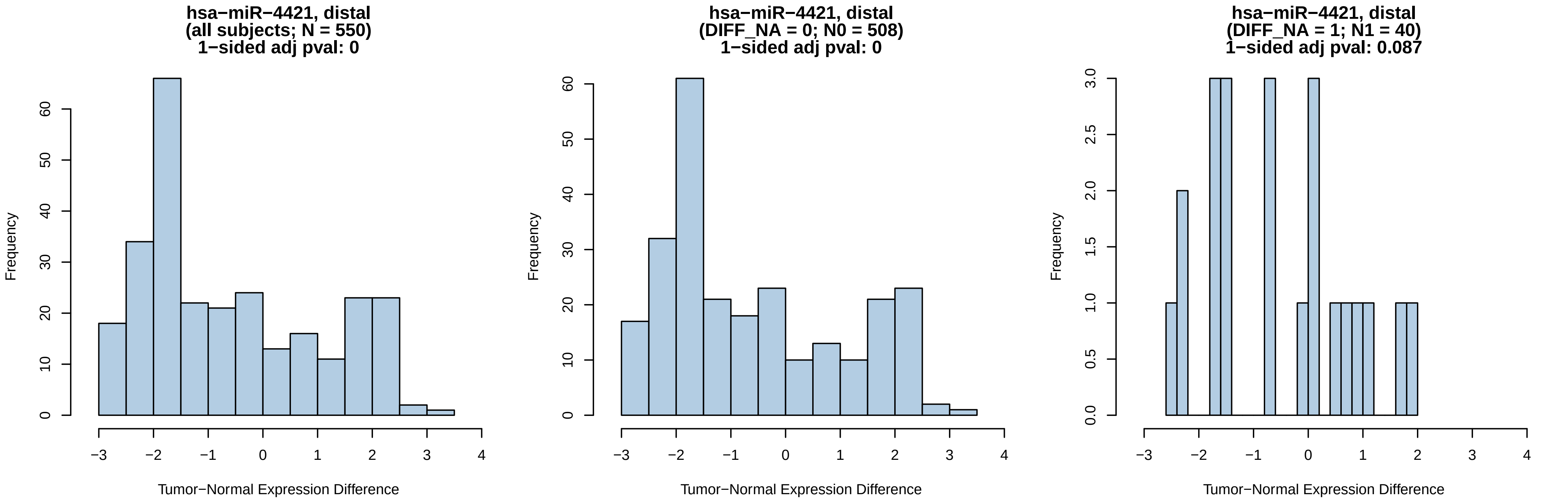

hsa−miR−4421, distal
(all subjects; N = 550)
1−sided adj pval: 0
Frequency
Tumor−Normal Expression Difference
hsa−miR−4421, distal
(DIFF_NA = 0; N0 = 508)
1−sided adj pval: 0
Frequency
Tumor−Normal Expression Difference
hsa−miR−4421, distal
(DIFF_NA = 1; N1 = 40)
1−sided adj pval: 0.087
Frequency
Tumor−Normal Expression Difference

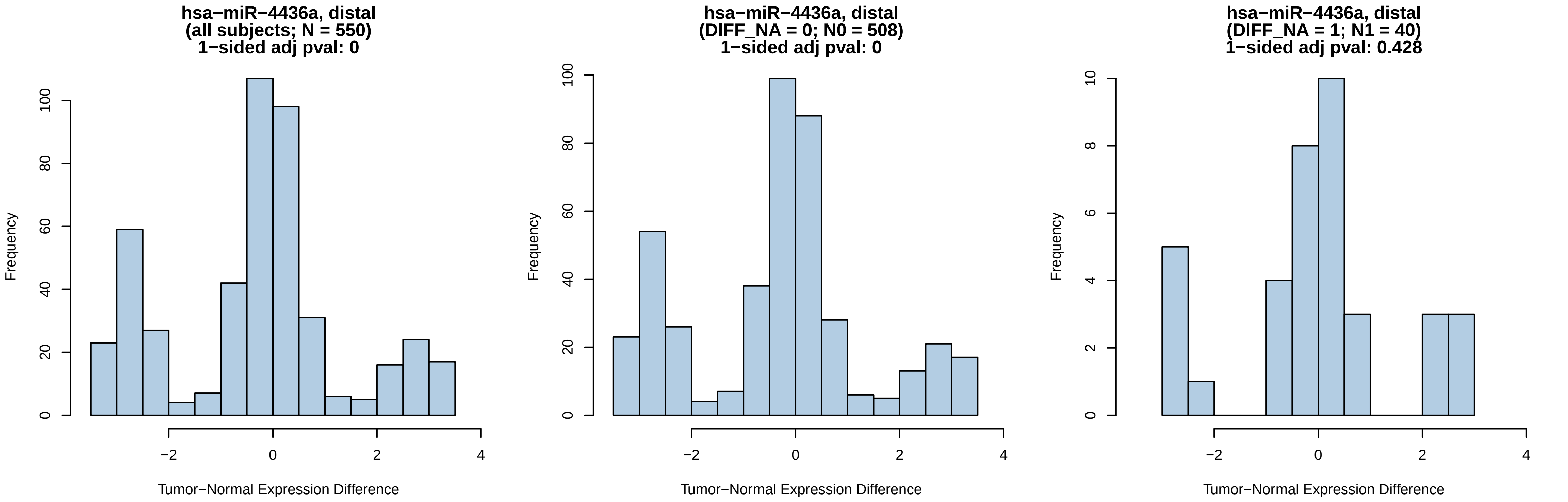
hsa−miR−4436a, distal
(all subjects; N = 550)
1−sided adj pval: 0
Frequency
0
20
40
60
80
100
−2
0
2
4
Tumor−Normal Expression Difference
hsa−miR−4436a, distal
(DIFF_NA = 0; N0 = 508)
1−sided adj pval: 0
Frequency
0
20
40
60
80
100
−2
0
2
4
Tumor−Normal Expression Difference
hsa−miR−4436a, distal
(DIFF_NA = 1; N1 = 40)
1−sided adj pval: 0.428
Frequency
0
2
4
6
8
10
−2
0
2
4
Tumor−Normal Expression Difference

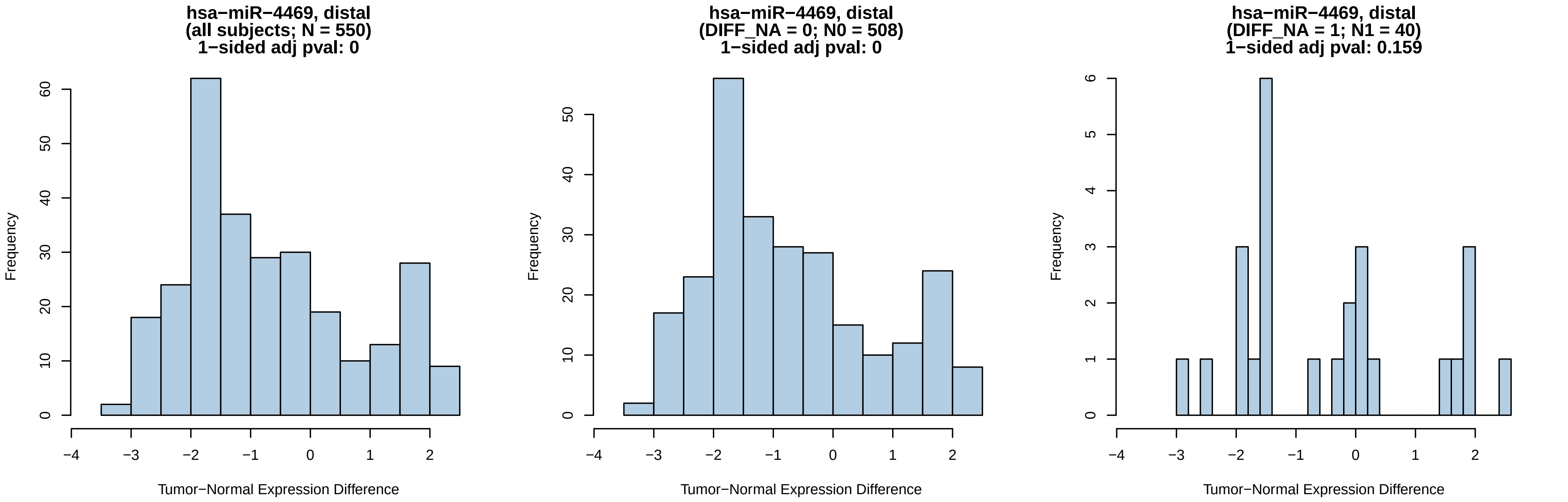

hsa−miR−4469, distal
(all subjects; N = 550)
1−sided adj pval: 0
Frequency
Tumor−Normal Expression Difference
hsa−miR−4469, distal
(DIFF_NA = 0; N0 = 508)
1−sided adj pval: 0
Frequency
Tumor−Normal Expression Difference
hsa−miR−4469, distal
(DIFF_NA = 1; N1 = 40)
1−sided adj pval: 0.159
Frequency
Tumor−Normal Expression Difference

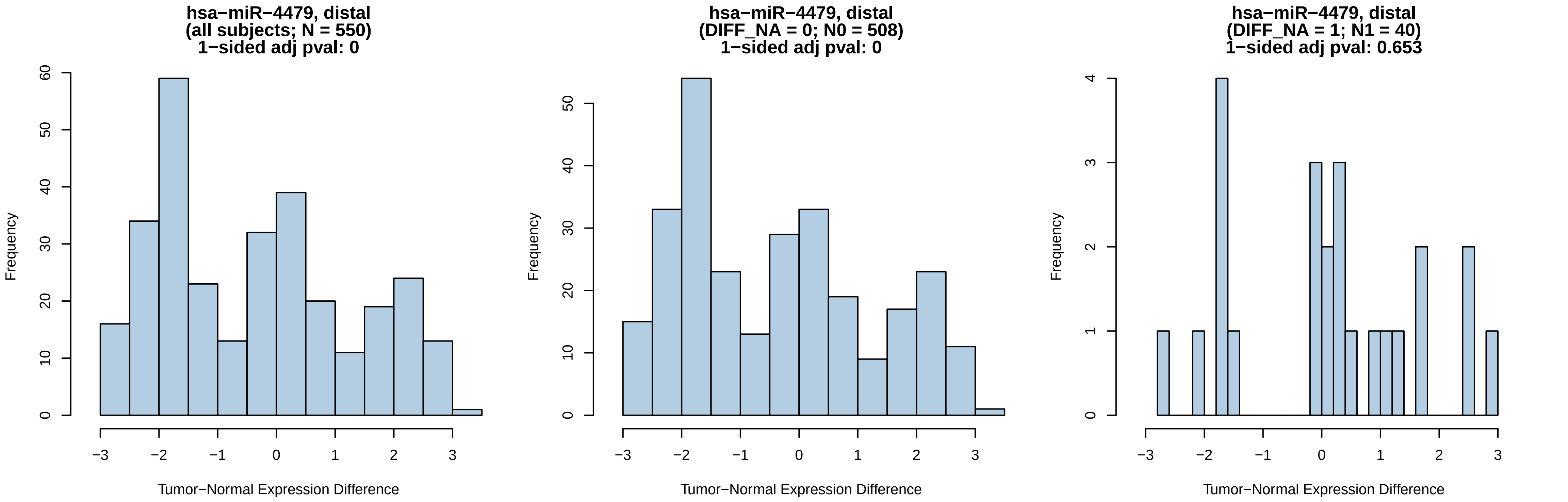
hsa−miR−4479, distal
(all subjects; N = 550)
1−sided adj pval: 0
hsa−miR−4479, distal
(DIFF_NA = 0; N0 = 508)
1−sided adj pval: 0
hsa−miR−4479, distal
(DIFF_NA = 1; N1 = 40)
1−sided adj pval: 0.653
Frequency
Tumor−Normal Expression Difference

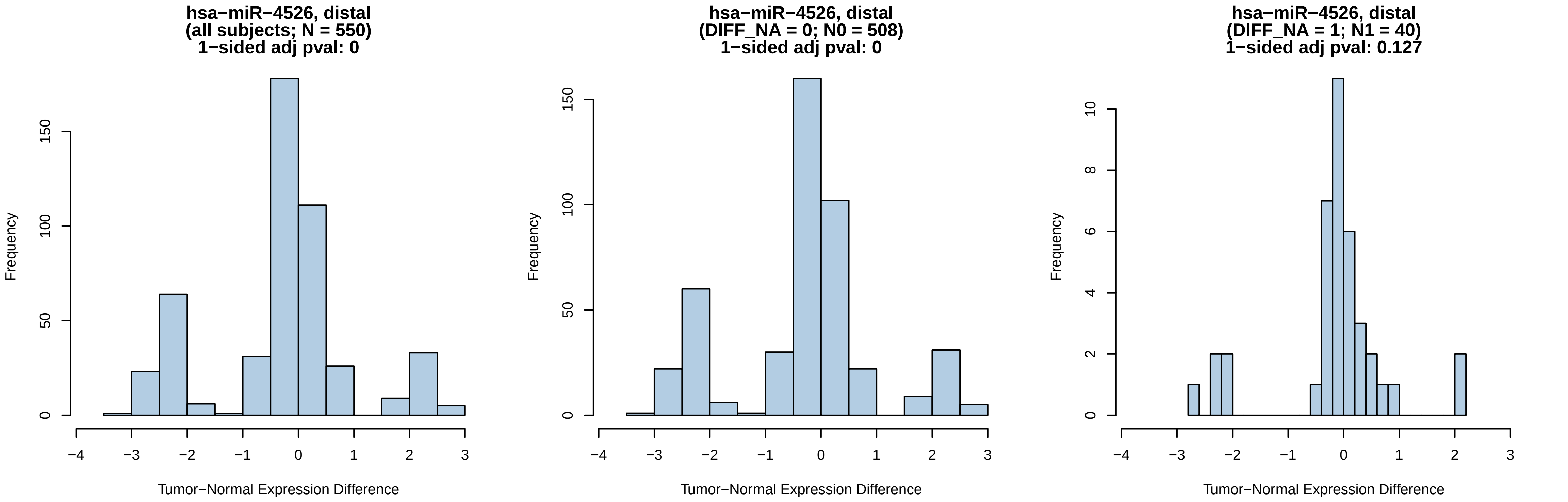
hsa−miR−4526, distal
(all subjects; N = 550)
1−sided adj pval: 0
Frequency
Tumor−Normal Expression Difference
hsa−miR−4526, distal
(DIFF_NA = 0; N0 = 508)
1−sided adj pval: 0
Frequency
Tumor−Normal Expression Difference
hsa−miR−4526, distal
(DIFF_NA = 1; N1 = 40)
1−sided adj pval: 0.127
Frequency
Tumor−Normal Expression Difference

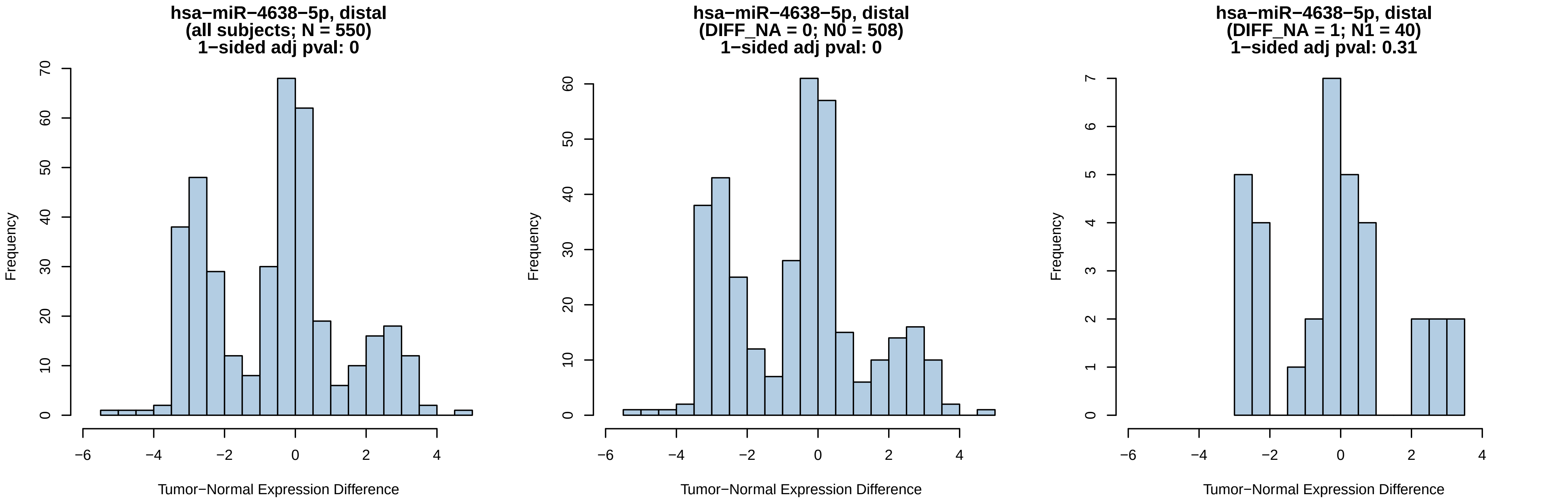

hsa−miR−4638−5p, distal
(all subjects; N = 550)
1−sided adj pval: 0
Frequency
Tumor−Normal Expression Difference
hsa−miR−4638−5p, distal
(DIFF_NA = 0; N0 = 508)
1−sided adj pval: 0
Frequency
Tumor−Normal Expression Difference
hsa−miR−4638−5p, distal
(DIFF_NA = 1; N1 = 40)
1−sided adj pval: 0.31
Frequency
Tumor−Normal Expression Difference

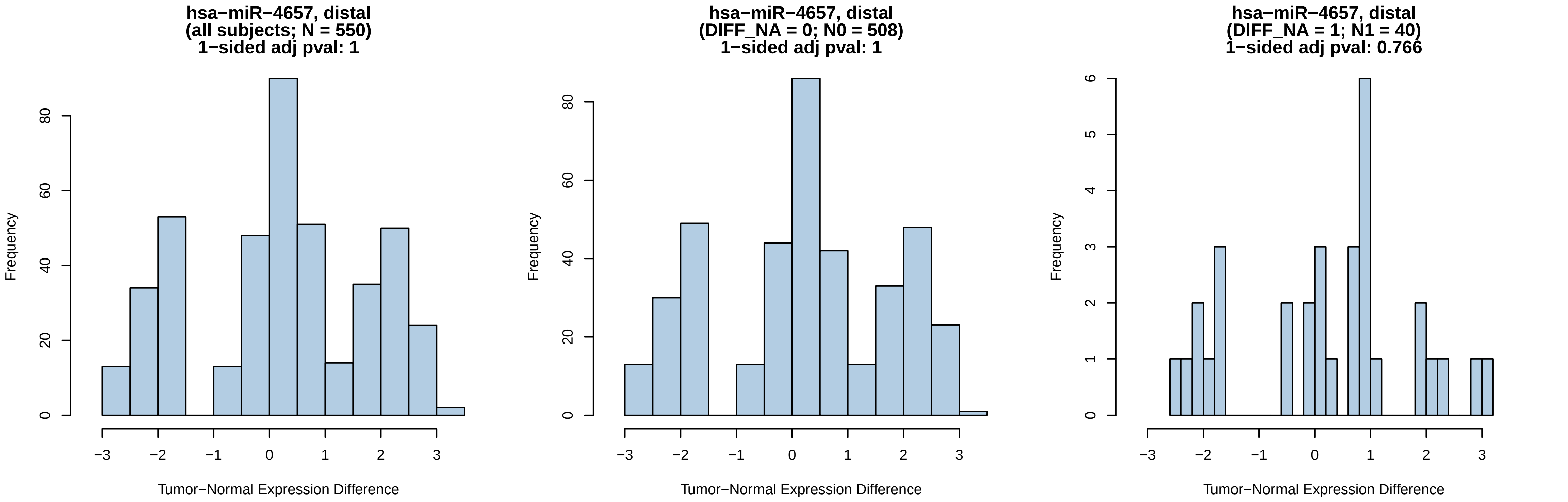

hsa−miR−4657, distal
(all subjects; N = 550)
1−sided adj pval: 1
Frequency
Tumor−Normal Expression Difference
hsa−miR−4657, distal
(DIFF_NA = 0; N0 = 508)
1−sided adj pval: 1
Frequency
Tumor−Normal Expression Difference
hsa−miR−4657, distal
(DIFF_NA = 1; N1 = 40)
1−sided adj pval: 0.766
Frequency
Tumor−Normal Expression Difference

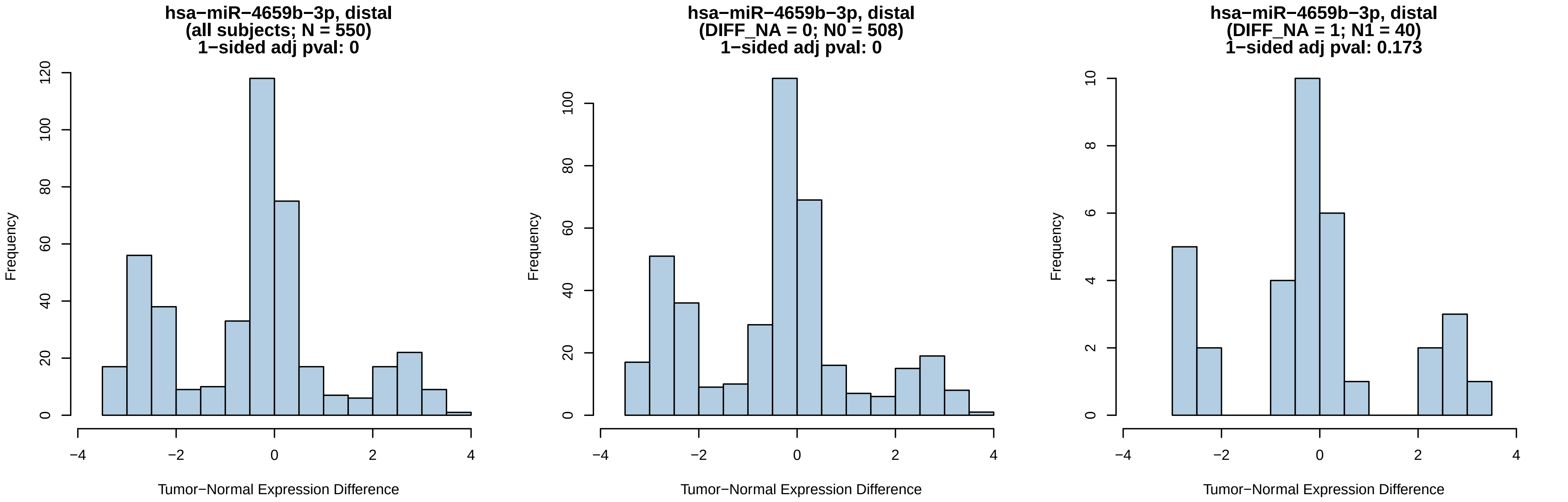
hsa−miR−4659b−3p, distal
(all subjects; N = 550)
1−sided adj pval: 0
hsa−miR−4659b−3p, distal
(DIFF_NA = 0; N0 = 508)
1−sided adj pval: 0
hsa−miR−4659b−3p, distal
(DIFF_NA = 1; N1 = 40)
1−sided adj pval: 0.173
Frequency
Tumor−Normal Expression Difference

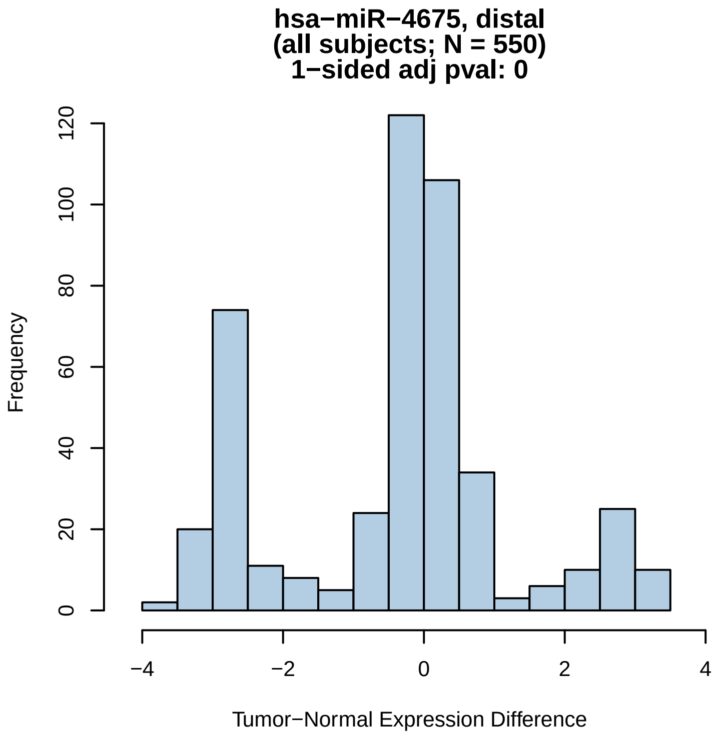

hsa−miR−4675, distal
(all subjects; N = 550)
1−sided adj pval: 0
Frequency
Tumor−Normal Expression Difference


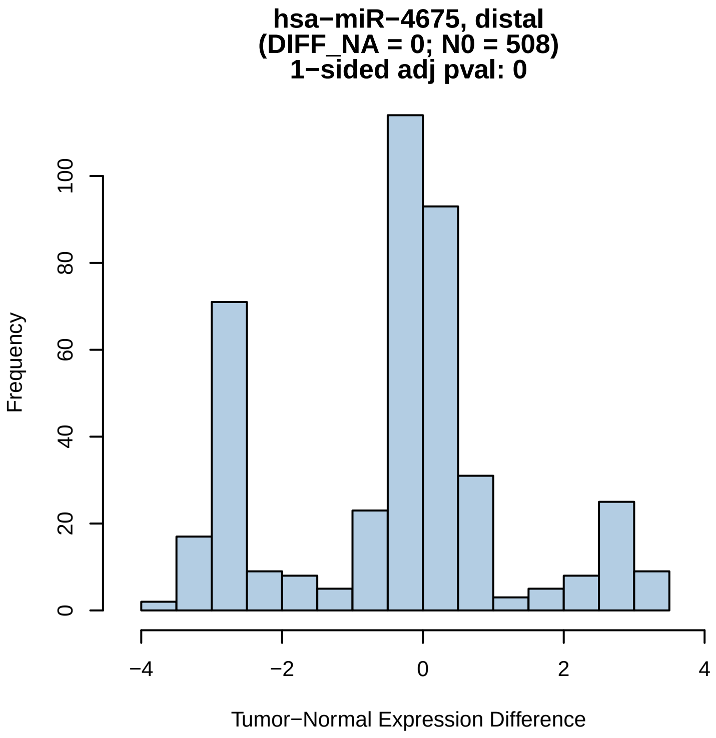

hsa−miR−4675, distal
(DIFF_NA = 0; N0 = 508)
1−sided adj pval: 0
Frequency
Tumor−Normal Expression Difference


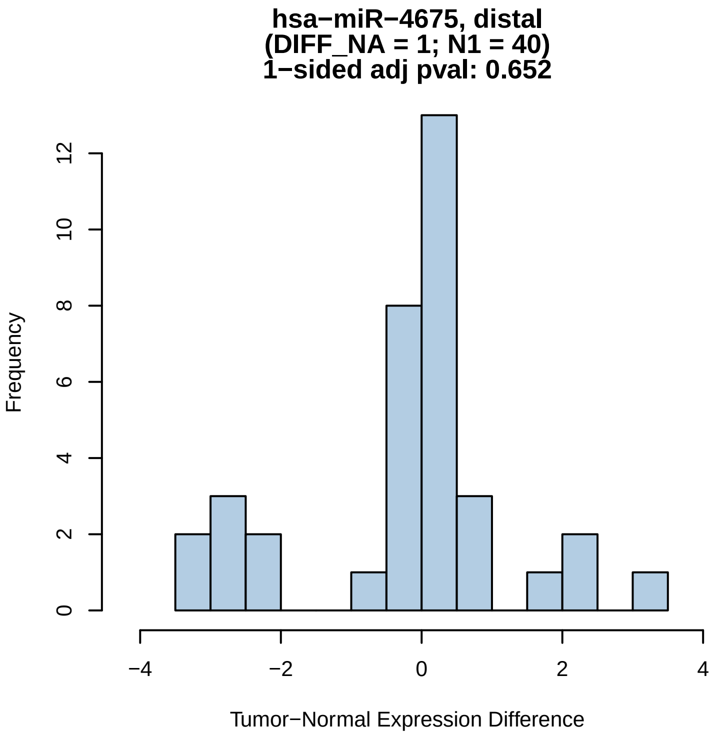

hsa−miR−4675, distal
(DIFF_NA = 1; N1 = 40)
1−sided adj pval: 0.652
Frequency
Tumor−Normal Expression Difference

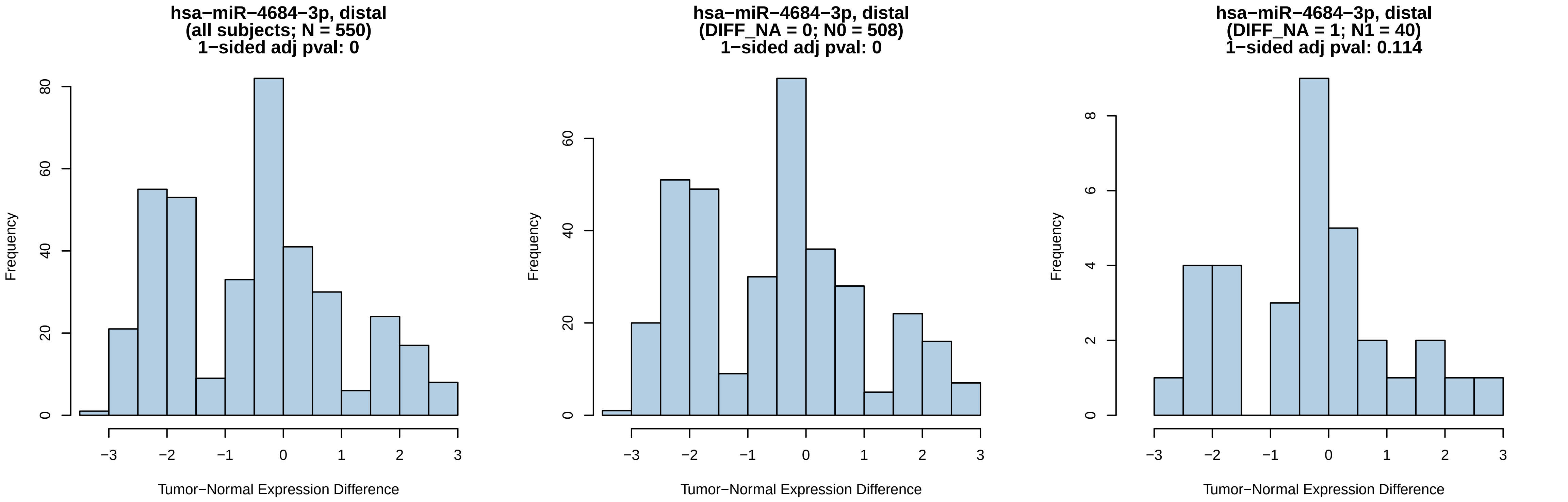
hsa−miR−4684−3p, distal
(all subjects; N = 550)
1−sided adj pval: 0
hsa−miR−4684−3p, distal
(DIFF_NA = 0; N0 = 508)
1−sided adj pval: 0
hsa−miR−4684−3p, distal
(DIFF_NA = 1; N1 = 40)
1−sided adj pval: 0.114
Frequency
Tumor−Normal Expression Difference

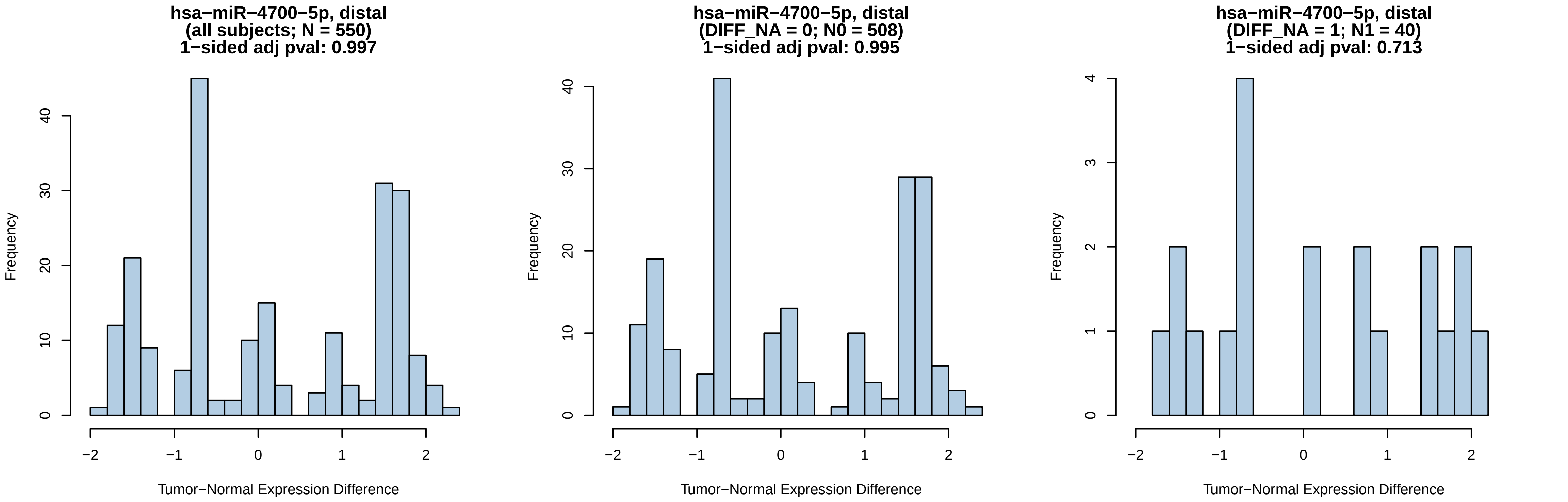

hsa−miR−4700−5p, distal
(all subjects; N = 550)
1−sided adj pval: 0.997
hsa−miR−4700−5p, distal
(DIFF_NA = 0; N0 = 508)
1−sided adj pval: 0.995
hsa−miR−4700−5p, distal
(DIFF_NA = 1; N1 = 40)
1−sided adj pval: 0.713
Frequency
Tumor−Normal Expression Difference

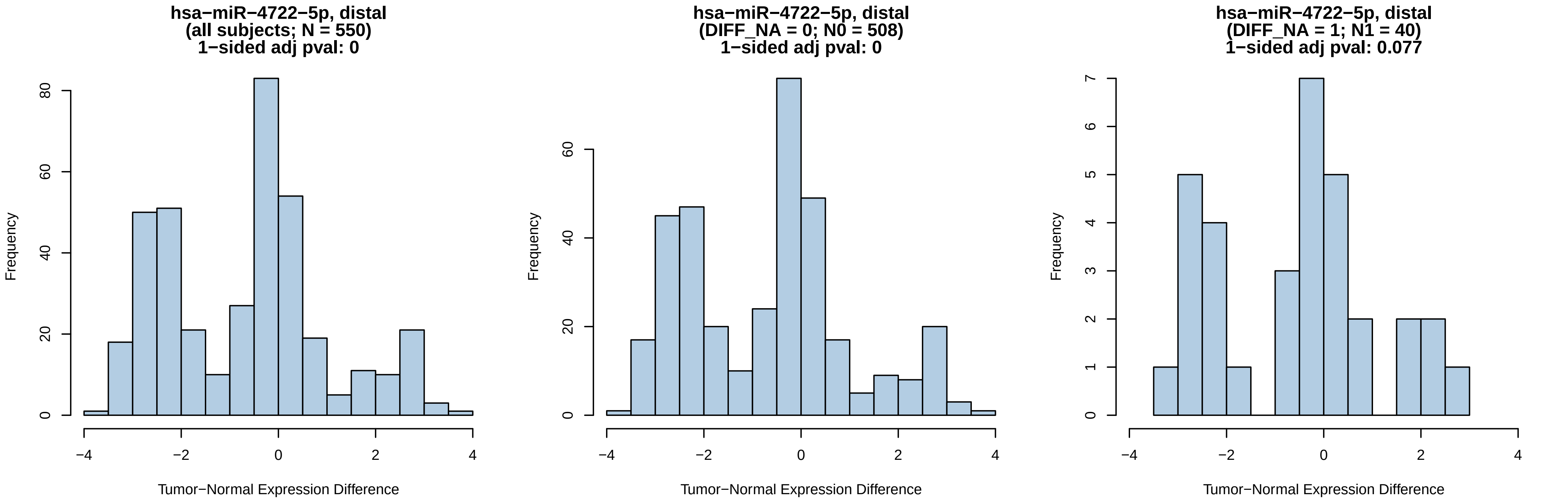

hsa−miR−4722−5p, distal
(all subjects; N = 550)
1−sided adj pval: 0
Frequency
Tumor−Normal Expression Difference
hsa−miR−4722−5p, distal
(DIFF_NA = 0; N0 = 508)
1−sided adj pval: 0
Frequency
Tumor−Normal Expression Difference
hsa−miR−4722−5p, distal
(DIFF_NA = 1; N1 = 40)
1−sided adj pval: 0.077
Frequency
Tumor−Normal Expression Difference

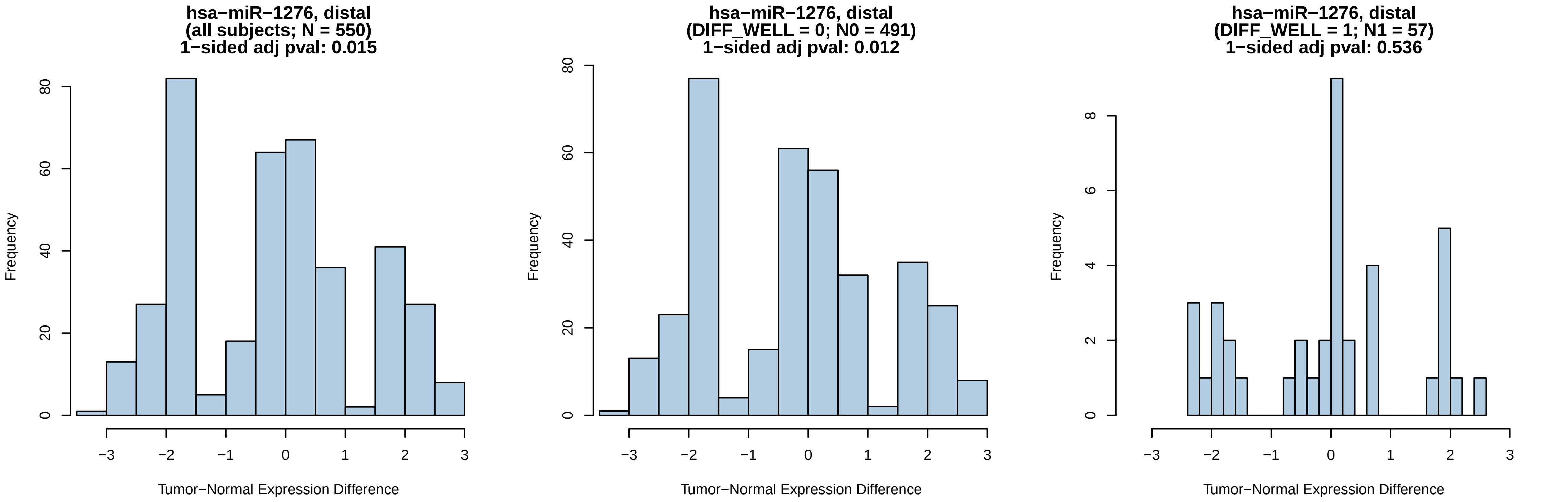
hsa−miR−1276, distal
(all subjects; N = 550)
1−sided adj pval: 0.015
hsa−miR−1276, distal
(DIFF_WELL = 0; N0 = 491)
1−sided adj pval: 0.012
hsa−miR−1276, distal
(DIFF_WELL = 1; N1 = 57)
1−sided adj pval: 0.536
Frequency
Tumor−Normal Expression Difference

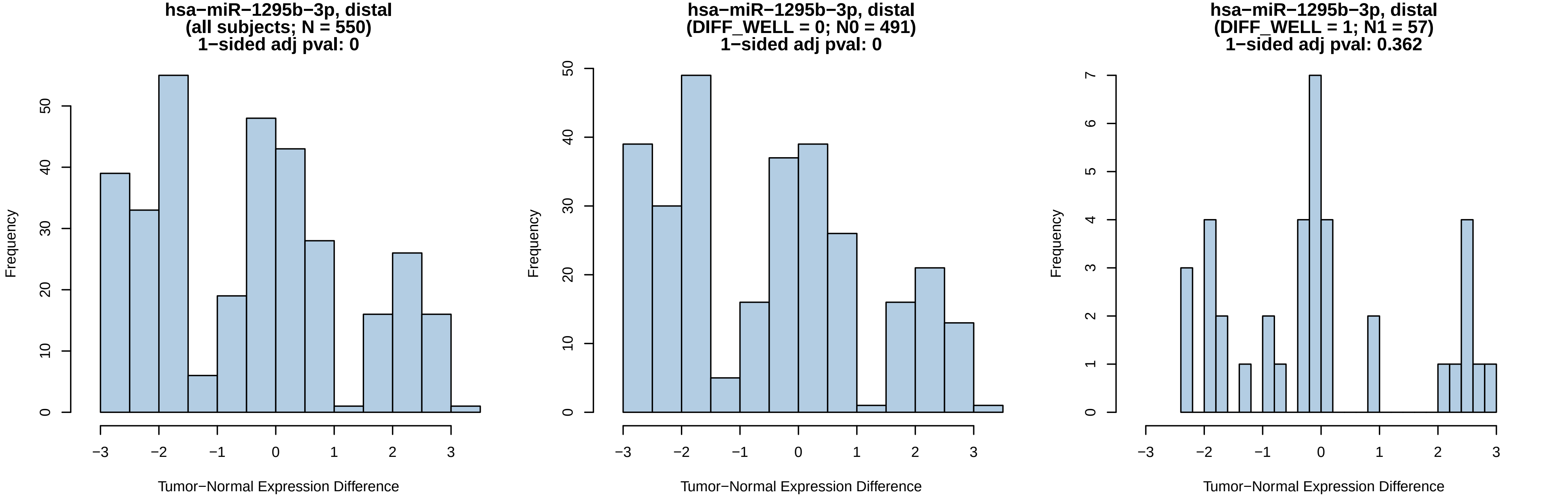
hsa−miR−1295b−3p, distal
(all subjects; N = 550)
1−sided adj pval: 0
Frequency
Tumor−Normal Expression Difference
hsa−miR−1295b−3p, distal
(DIFF_WELL = 0; N0 = 491)
1−sided adj pval: 0
Frequency
Tumor−Normal Expression Difference
hsa−miR−1295b−3p, distal
(DIFF_WELL = 1; N1 = 57)
1−sided adj pval: 0.362
Frequency
Tumor−Normal Expression Difference

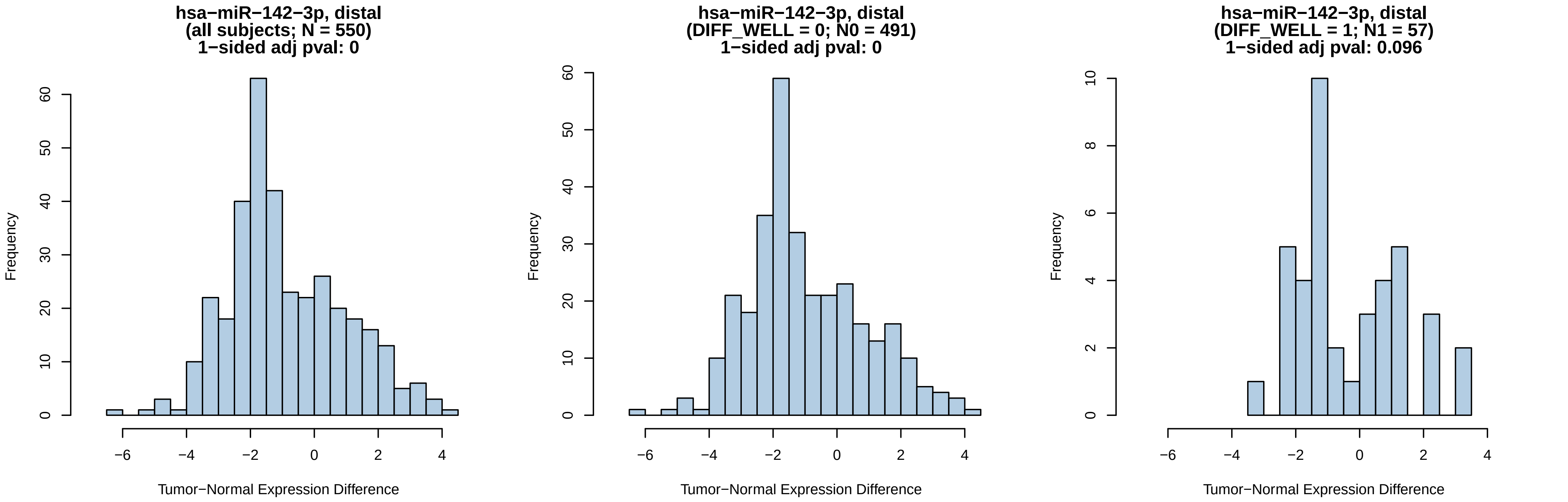

hsa−miR−142−3p, distal
(all subjects; N = 550)
1−sided adj pval: 0
Frequency
Tumor−Normal Expression Difference
hsa−miR−142−3p, distal
(DIFF_WELL = 0; N0 = 491)
1−sided adj pval: 0
Frequency
Tumor−Normal Expression Difference
hsa−miR−142−3p, distal
(DIFF_WELL = 1; N1 = 57)
1−sided adj pval: 0.096
Frequency
Tumor−Normal Expression Difference

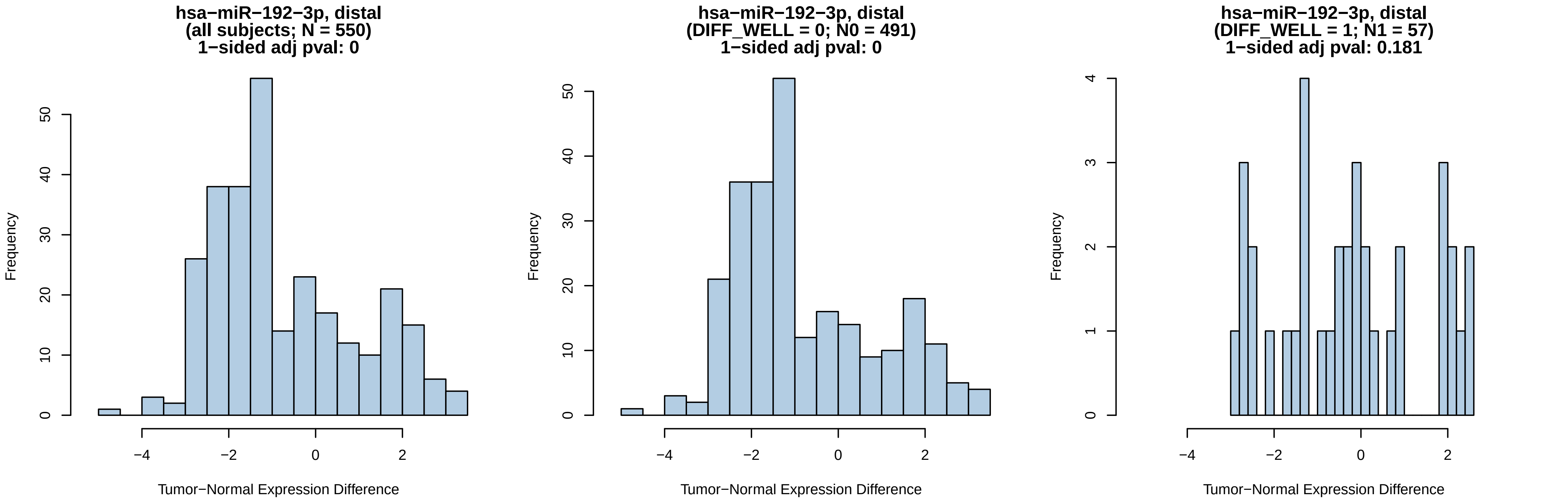

hsa−miR−192−3p, distal
(all subjects; N = 550)
1−sided adj pval: 0
Frequency
Tumor−Normal Expression Difference
hsa−miR−192−3p, distal
(DIFF_WELL = 0; N0 = 491)
1−sided adj pval: 0
Frequency
Tumor−Normal Expression Difference
hsa−miR−192−3p, distal
(DIFF_WELL = 1; N1 = 57)
1−sided adj pval: 0.181
Frequency
Tumor−Normal Expression Difference

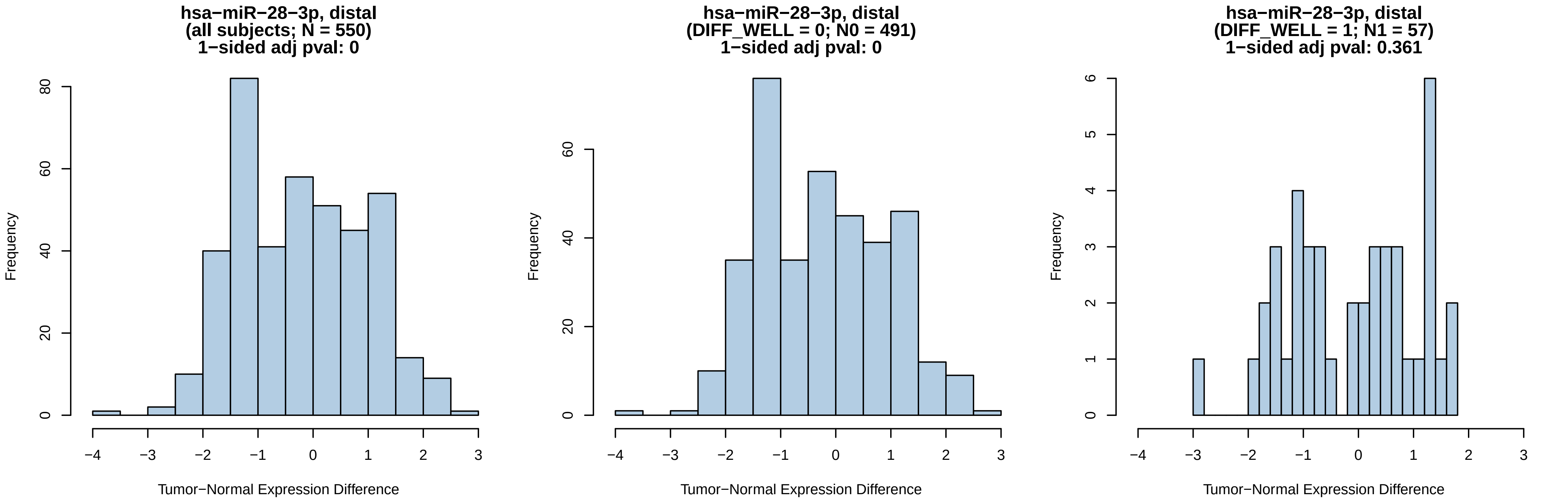
hsa−miR−28−3p, distal
(all subjects; N = 550)
1−sided adj pval: 0
Frequency
Tumor−Normal Expression Difference
hsa−miR−28−3p, distal
(DIFF_WELL = 0; N0 = 491)
1−sided adj pval: 0
Frequency
Tumor−Normal Expression Difference
hsa−miR−28−3p, distal
(DIFF_WELL = 1; N1 = 57)
1−sided adj pval: 0.361
Frequency
Tumor−Normal Expression Difference

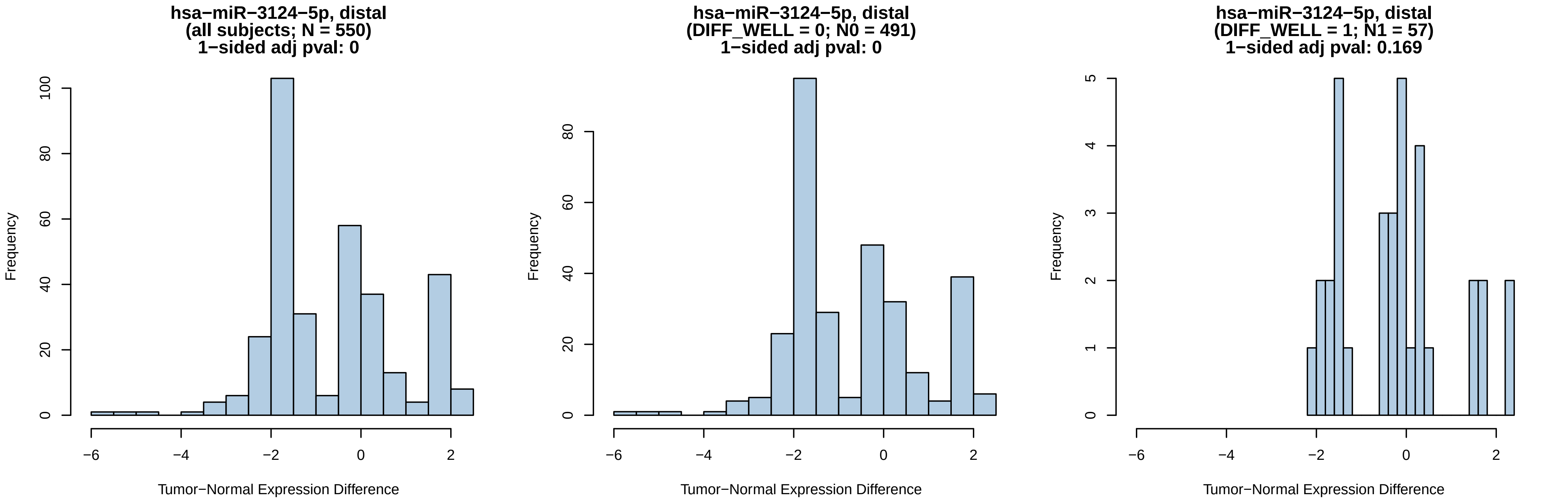
hsa−miR−3124−5p, distal
(all subjects; N = 550)
1−sided adj pval: 0
hsa−miR−3124−5p, distal
(DIFF_WELL = 0; N0 = 491)
1−sided adj pval: 0
hsa−miR−3124−5p, distal
(DIFF_WELL = 1; N1 = 57)
1−sided adj pval: 0.169
Frequency
Tumor−Normal Expression Difference
0
20
40
60
80
100
−6
−4
−2
0
2
1
2
3
4
5

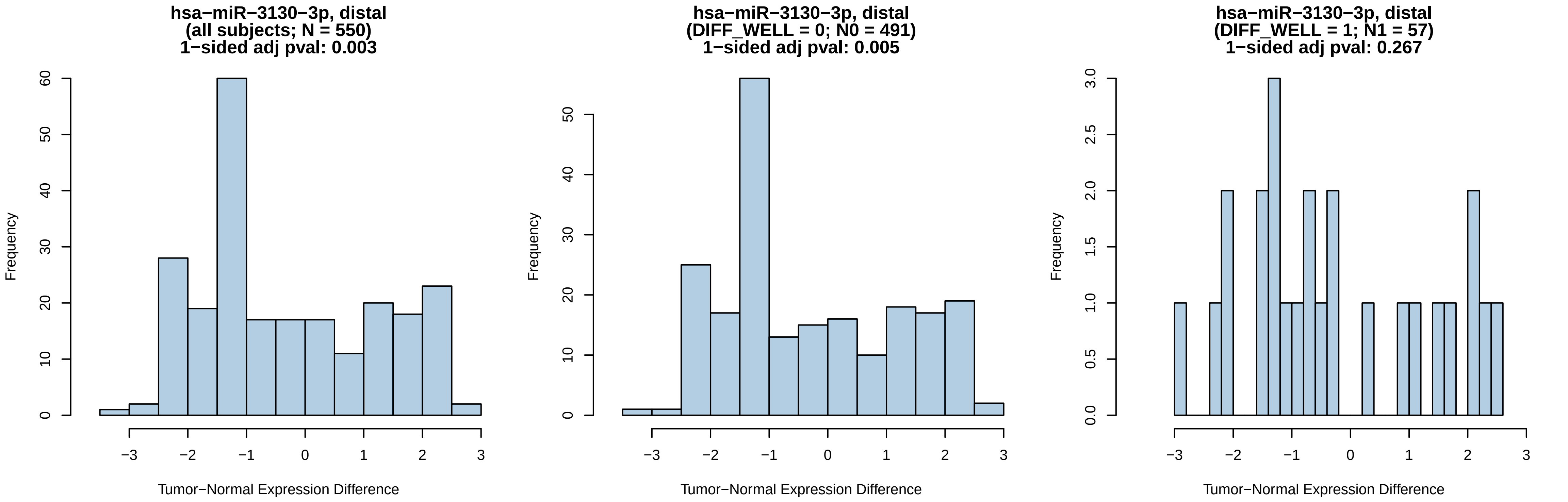
hsa−miR−3130−3p, distal
(all subjects; N = 550)
1−sided adj pval: 0.003
Frequency
Tumor−Normal Expression Difference
hsa−miR−3130−3p, distal
(DIFF_WELL = 0; N0 = 491)
1−sided adj pval: 0.005
Frequency
Tumor−Normal Expression Difference
hsa−miR−3130−3p, distal
(DIFF_WELL = 1; N1 = 57)
1−sided adj pval: 0.267
Frequency
Tumor−Normal Expression Difference

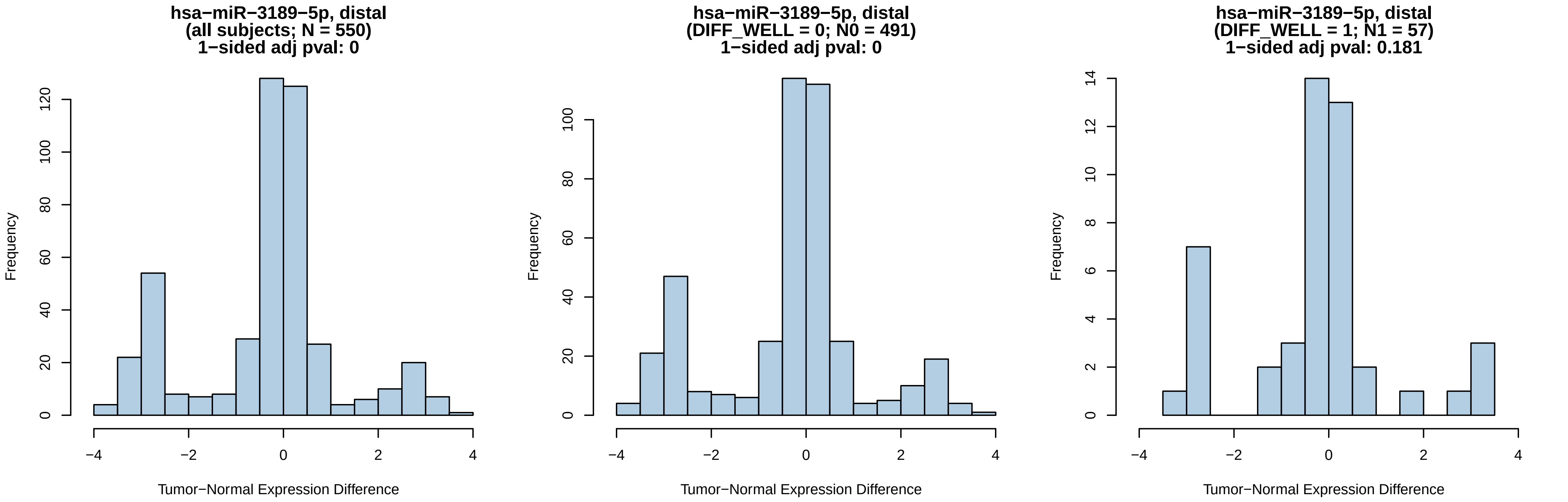
hsa−miR−3189−5p, distal
(all subjects; N = 550)
1−sided adj pval: 0
Frequency
Tumor−Normal Expression Difference
hsa−miR−3189−5p, distal
(DIFF_WELL = 0; N0 = 491)
1−sided adj pval: 0
Frequency
Tumor−Normal Expression Difference
hsa−miR−3189−5p, distal
(DIFF_WELL = 1; N1 = 57)
1−sided adj pval: 0.181
Frequency
Tumor−Normal Expression Difference

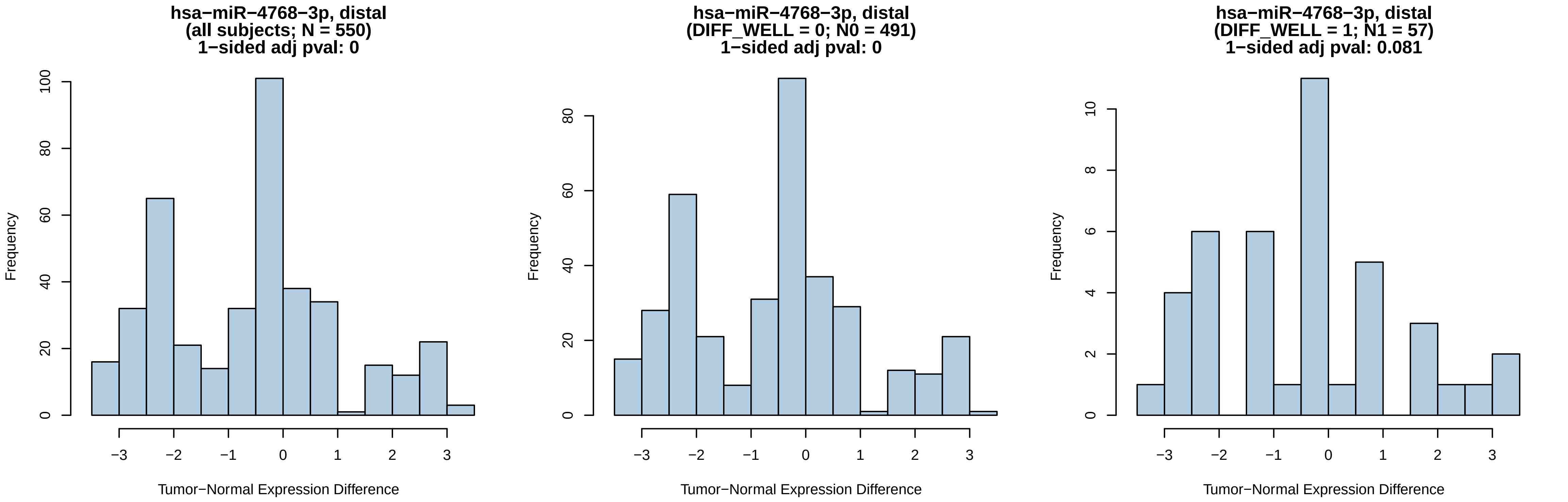
hsa−miR−4768−3p, distal
(all subjects; N = 550)
1−sided adj pval: 0
Frequency
Tumor−Normal Expression Difference
hsa−miR−4768−3p, distal
(DIFF_WELL = 0; N0 = 491)
1−sided adj pval: 0
Frequency
Tumor−Normal Expression Difference
hsa−miR−4768−3p, distal
(DIFF_WELL = 1; N1 = 57)
1−sided adj pval: 0.081
Frequency
Tumor−Normal Expression Difference

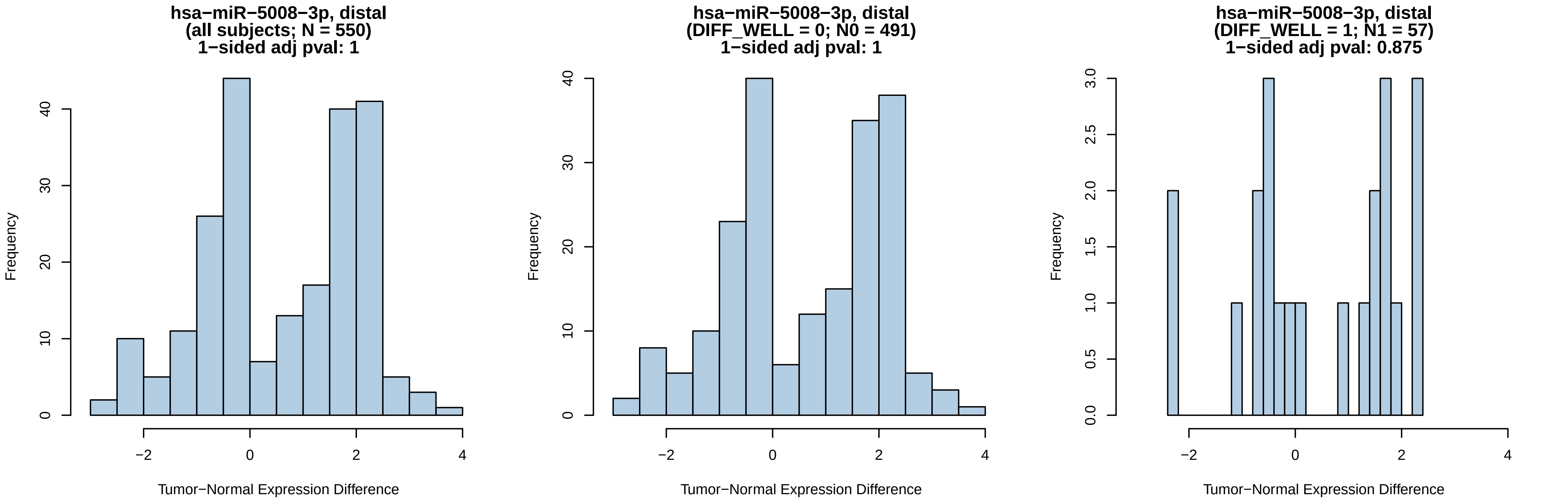
hsa−miR−5008−3p, distal
(all subjects; N = 550)
1−sided adj pval: 1
hsa−miR−5008−3p, distal
(DIFF_WELL = 0; N0 = 491)
1−sided adj pval: 1
hsa−miR−5008−3p, distal
(DIFF_WELL = 1; N1 = 57)
1−sided adj pval: 0.875
Frequency
Tumor−Normal Expression Difference

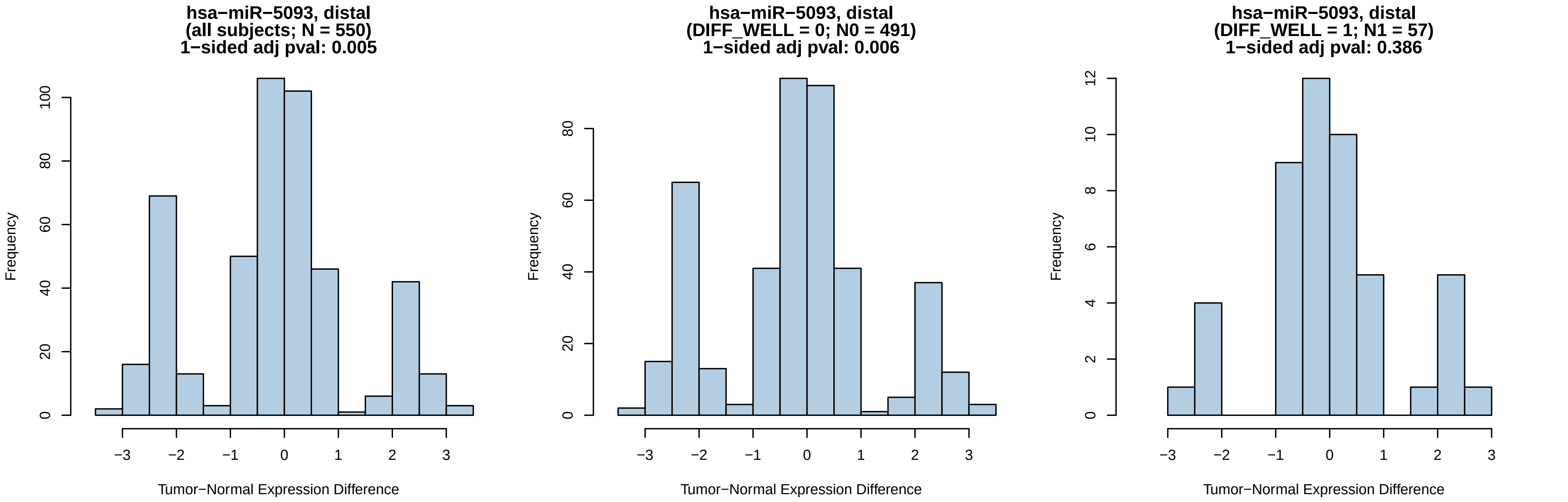

hsa−miR−5093, distal
(all subjects; N = 550)
1−sided adj pval: 0.005
Frequency
Tumor−Normal Expression Difference
hsa−miR−5093, distal
(DIFF_WELL = 0; N0 = 491)
1−sided adj pval: 0.006
Frequency
Tumor−Normal Expression Difference
hsa−miR−5093, distal
(DIFF_WELL = 1; N1 = 57)
1−sided adj pval: 0.386
Frequency
Tumor−Normal Expression Difference

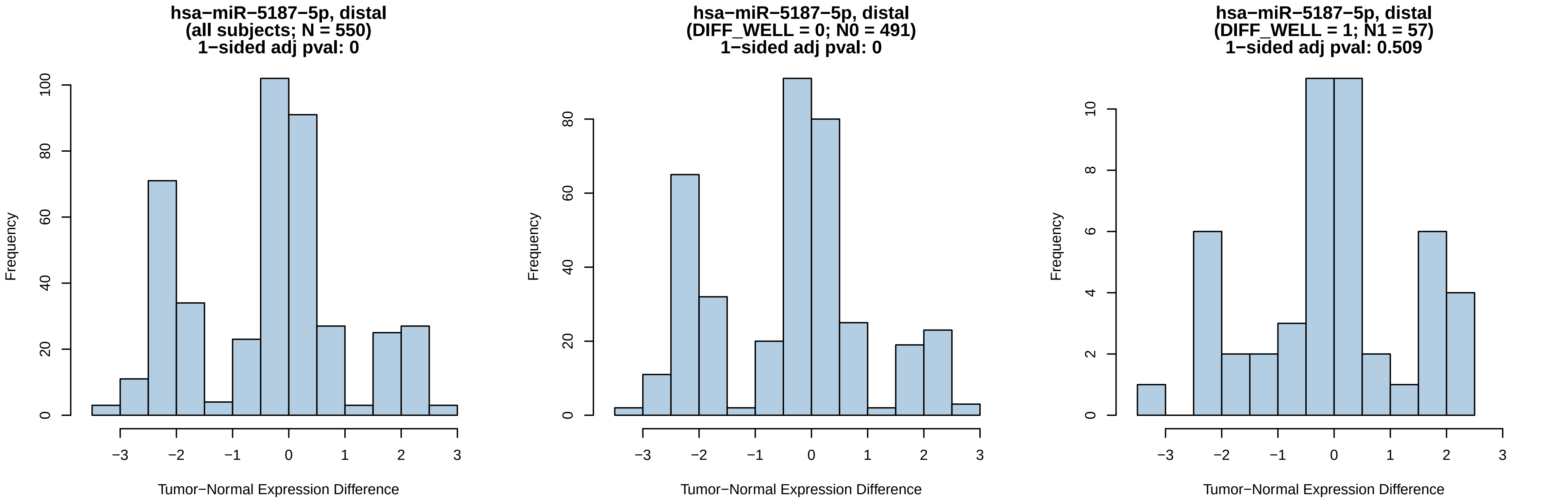
hsa−miR−5187−5p, distal
(all subjects; N = 550)
1−sided adj pval: 0
Frequency
Tumor−Normal Expression Difference
hsa−miR−5187−5p, distal
(DIFF_WELL = 0; N0 = 491)
1−sided adj pval: 0
Frequency
Tumor−Normal Expression Difference
hsa−miR−5187−5p, distal
(DIFF_WELL = 1; N1 = 57)
1−sided adj pval: 0.509
Frequency
Tumor−Normal Expression Difference

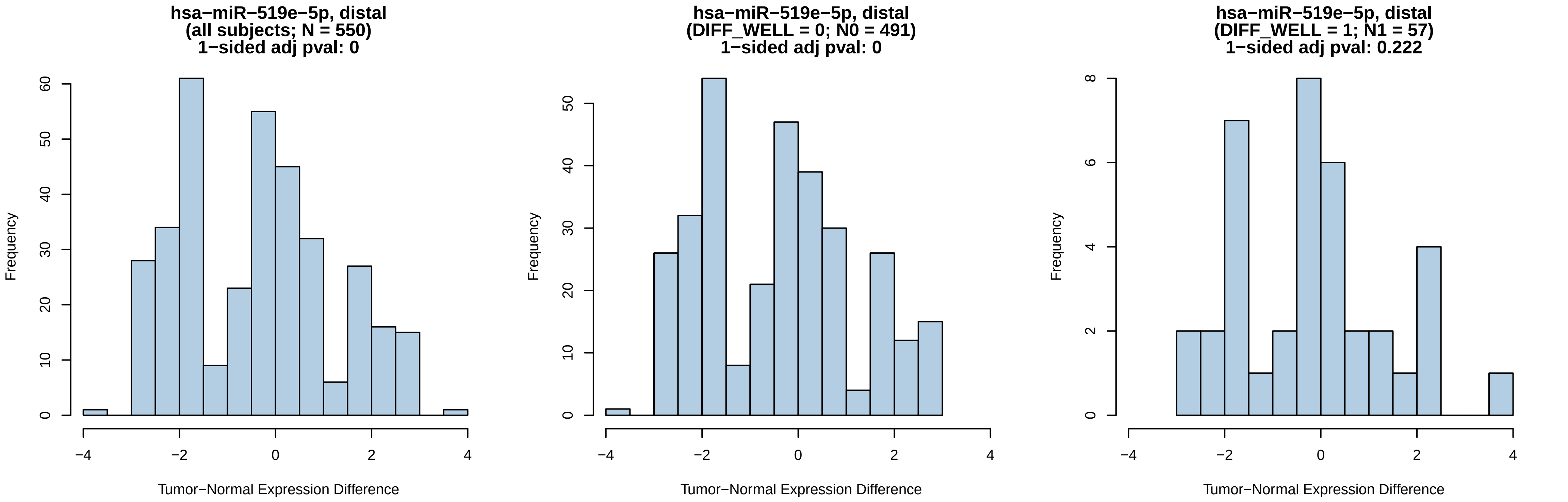
hsa−miR−519e−5p, distal
(all subjects; N = 550)
1−sided adj pval: 0
Frequency
0
10
20
30
40
50
60
−4
−2
0
2
4
Tumor−Normal Expression Difference
hsa−miR−519e−5p, distal
(DIFF_WELL = 0; N0 = 491)
1−sided adj pval: 0
Frequency
0
10
20
30
40
50
−4
−2
0
2
4
Tumor−Normal Expression Difference
hsa−miR−519e−5p, distal
(DIFF_WELL = 1; N1 = 57)
1−sided adj pval: 0.222
Frequency
0
2
4
6
8
−4
−2
0
2
4
Tumor−Normal Expression Difference

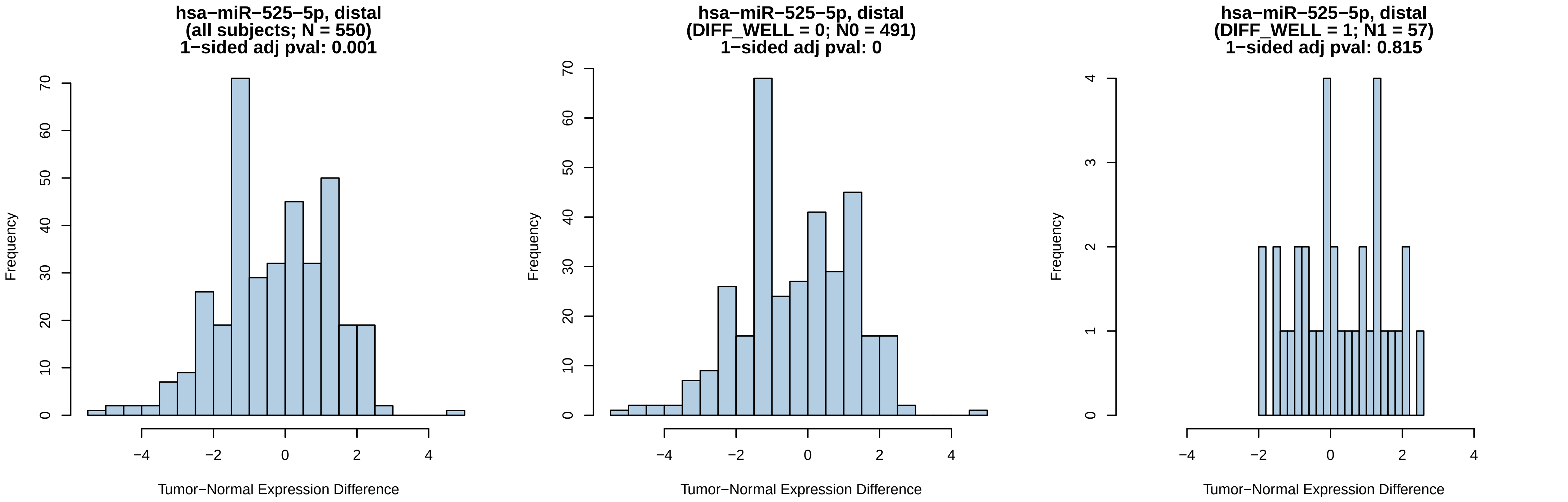
hsa−miR−525−5p, distal
(all subjects; N = 550)
1−sided adj pval: 0.001
Frequency
Tumor−Normal Expression Difference
hsa−miR−525−5p, distal
(DIFF_WELL = 0; N0 = 491)
1−sided adj pval: 0
Frequency
Tumor−Normal Expression Difference
hsa−miR−525−5p, distal
(DIFF_WELL = 1; N1 = 57)
1−sided adj pval: 0.815
Frequency
Tumor−Normal Expression Difference

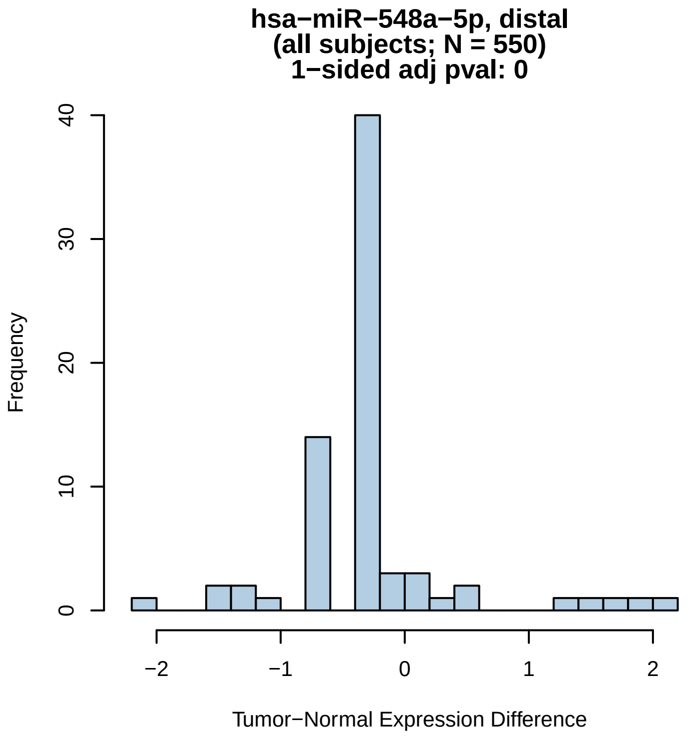
hsa−miR−548a−5p, distal
(all subjects; N = 550)
1−sided adj pval: 0
Frequency
0
10
20
30
40
−2
−1
0
1
2
Tumor−Normal Expression Difference

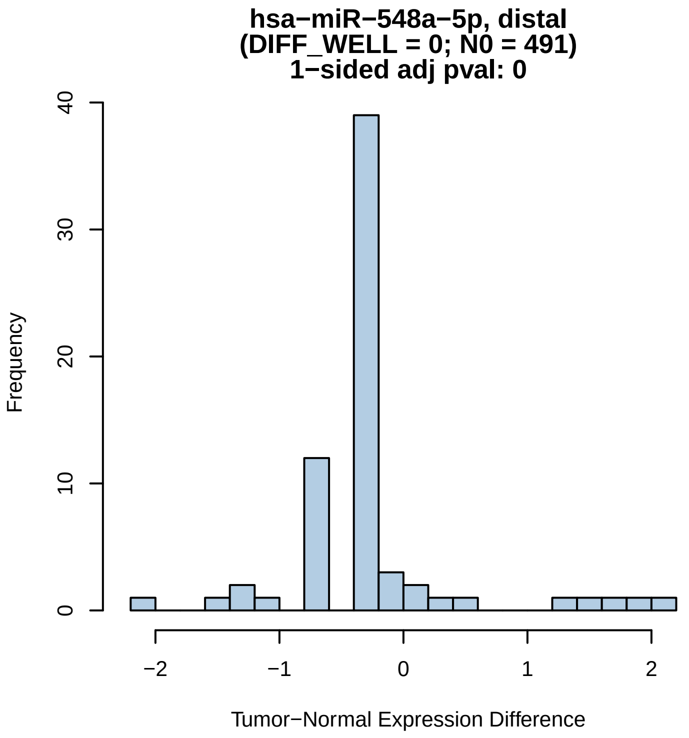
hsa−miR−548a−5p, distal
(DIFF_WELL = 0; N0 = 491)
1−sided adj pval: 0
Frequency
0
10
20
30
40
−2
−1
0
1
2
Tumor−Normal Expression Difference

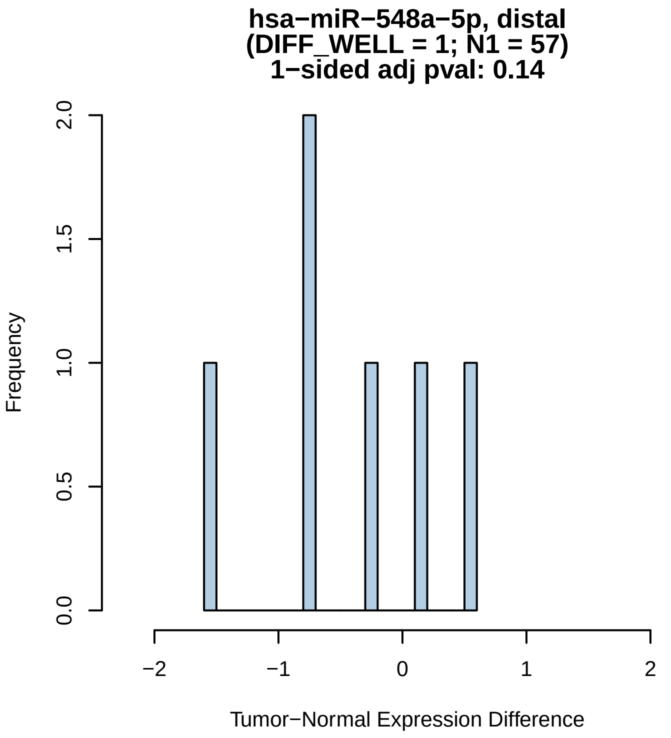
hsa−miR−548a−5p, distal
(DIFF_WELL = 1; N1 = 57)
1−sided adj pval: 0.14
Frequency
0.0
0.5
1.0
1.5
2.0
−2
−1
0
1
2
Tumor−Normal Expression Difference

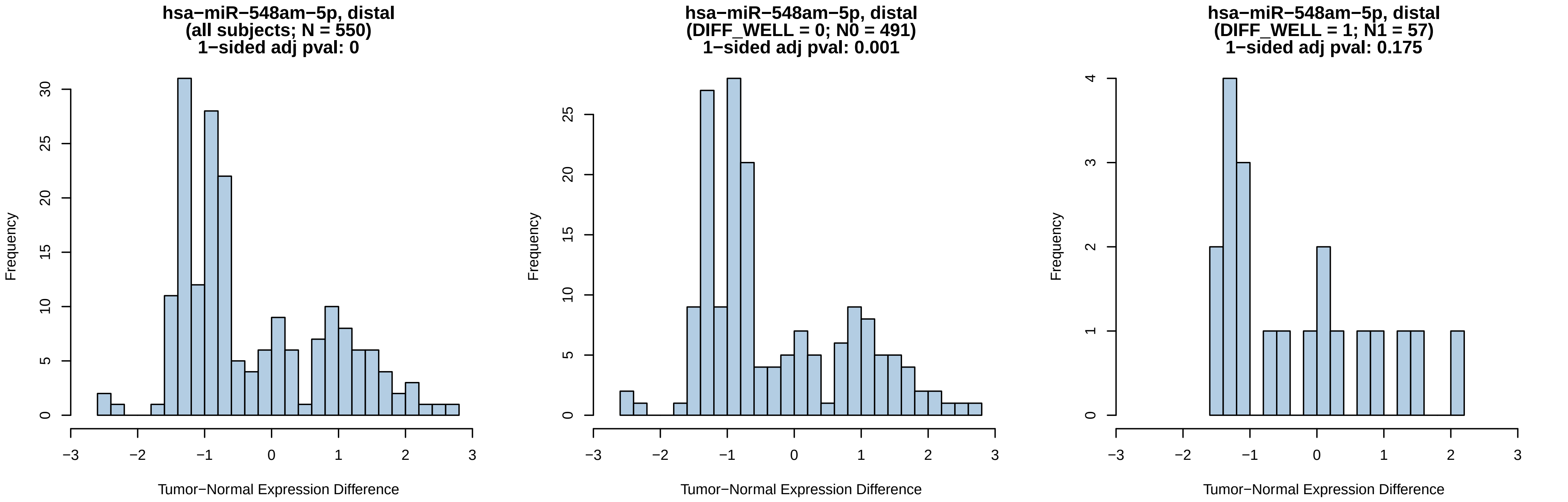
hsa−miR−548am−5p, distal
(all subjects; N = 550)
1−sided adj pval: 0
Frequency
Tumor−Normal Expression Difference
hsa−miR−548am−5p, distal
(DIFF_WELL = 0; N0 = 491)
1−sided adj pval: 0.001
Frequency
Tumor−Normal Expression Difference
hsa−miR−548am−5p, distal
(DIFF_WELL = 1; N1 = 57)
1−sided adj pval: 0.175
Frequency
Tumor−Normal Expression Difference

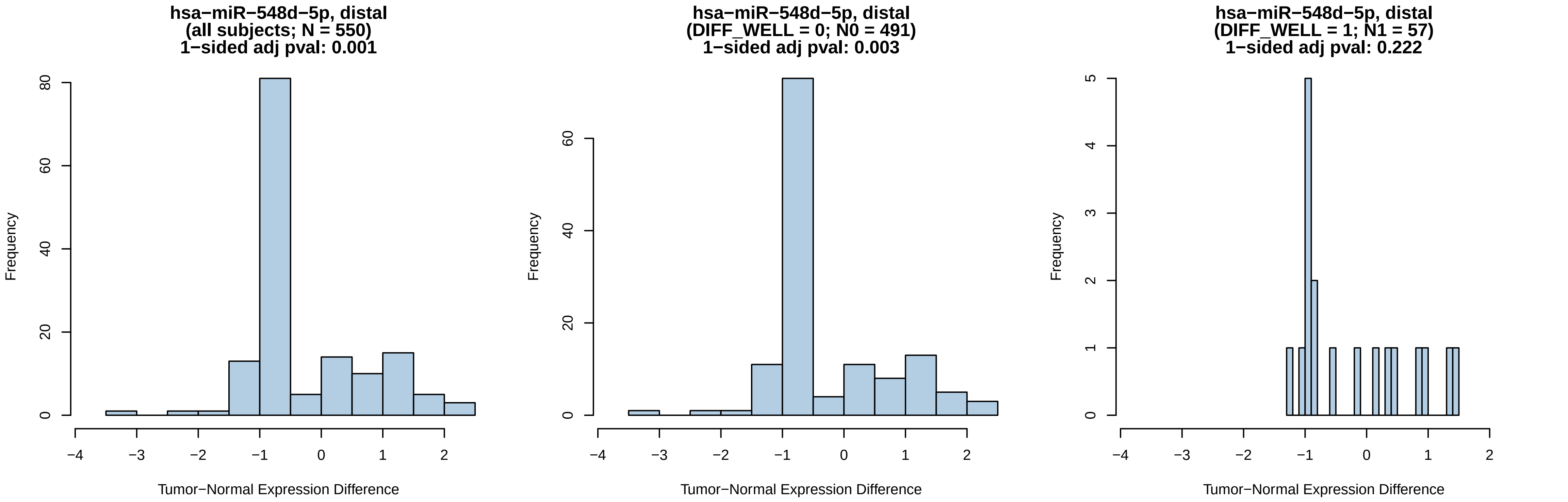
hsa−miR−548d−5p, distal
(all subjects; N = 550)
1−sided adj pval: 0.001
hsa−miR−548d−5p, distal
(DIFF_WELL = 0; N0 = 491)
1−sided adj pval: 0.003
hsa−miR−548d−5p, distal
(DIFF_WELL = 1; N1 = 57)
1−sided adj pval: 0.222
Frequency
Tumor−Normal Expression Difference

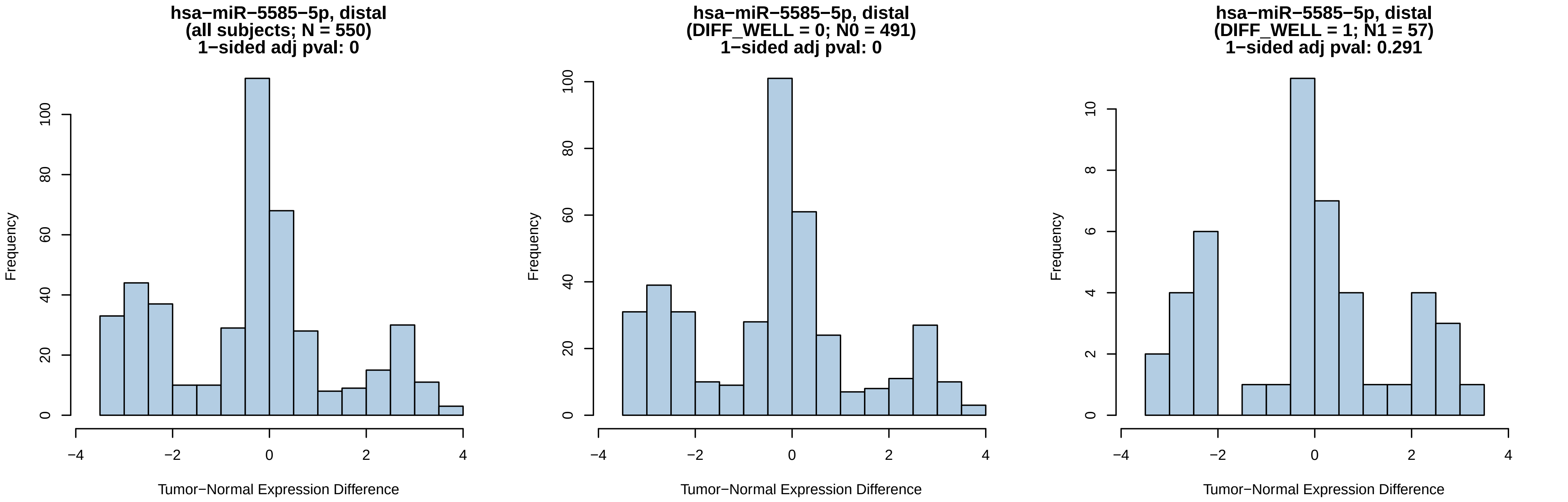
hsa−miR−5585−5p, distal
(all subjects; N = 550)
1−sided adj pval: 0
Frequency
0
20
40
60
80
100
−4
−2
0
2
4
Tumor−Normal Expression Difference
hsa−miR−5585−5p, distal
(DIFF_WELL = 0; N0 = 491)
1−sided adj pval: 0
Frequency
0
20
40
60
80
100
−4
−2
0
2
4
Tumor−Normal Expression Difference
hsa−miR−5585−5p, distal
(DIFF_WELL = 1; N1 = 57)
1−sided adj pval: 0.291
Frequency
0
2
4
6
8
10
−4
−2
0
2
4
Tumor−Normal Expression Difference

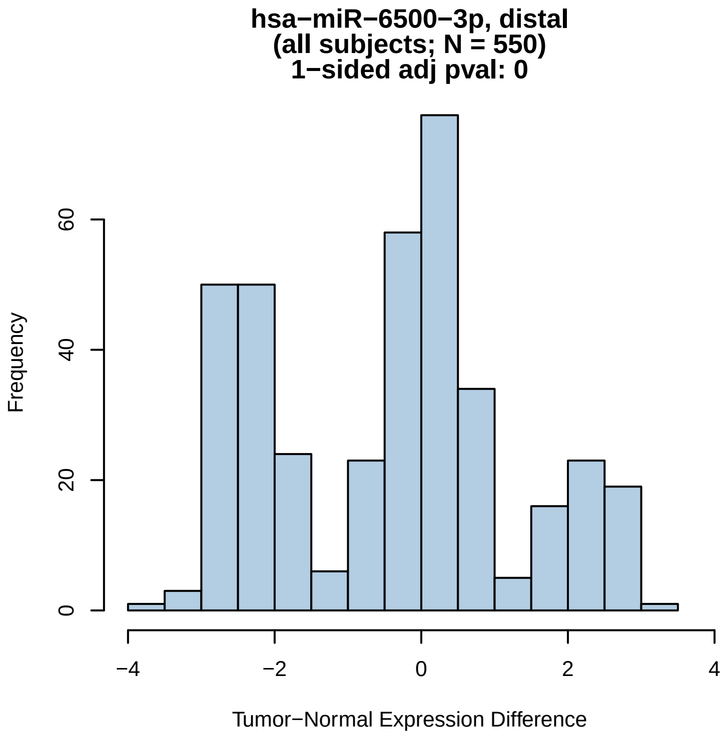
hsa−miR−6500−3p, distal
(all subjects; N = 550)
1−sided adj pval: 0
Frequency
0
20
40
60
−4
−2
0
2
4
Tumor−Normal Expression Difference

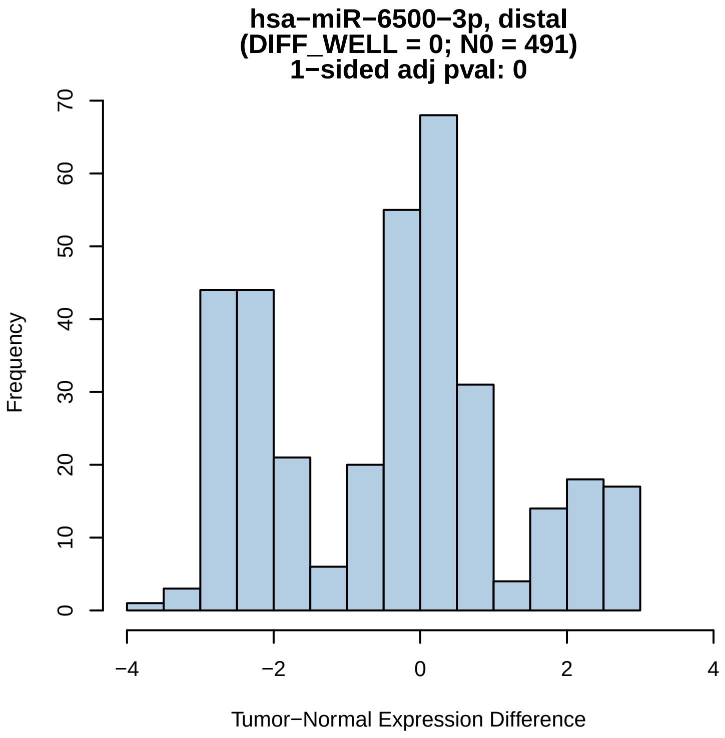
hsa−miR−6500−3p, distal
(DIFF_WELL = 0; N0 = 491)
1−sided adj pval: 0
Frequency
0
10
20
30
40
50
60
70
−4
−2
0
2
4
Tumor−Normal Expression Difference

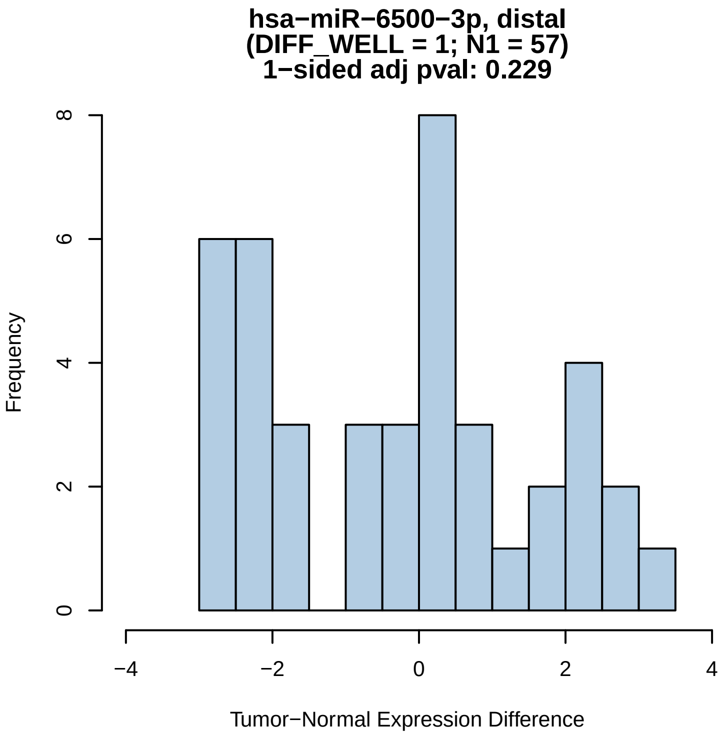
hsa−miR−6500−3p, distal
(DIFF_WELL = 1; N1 = 57)
1−sided adj pval: 0.229
Frequency
0
2
4
6
8
−4
−2
0
2
4
Tumor−Normal Expression Difference

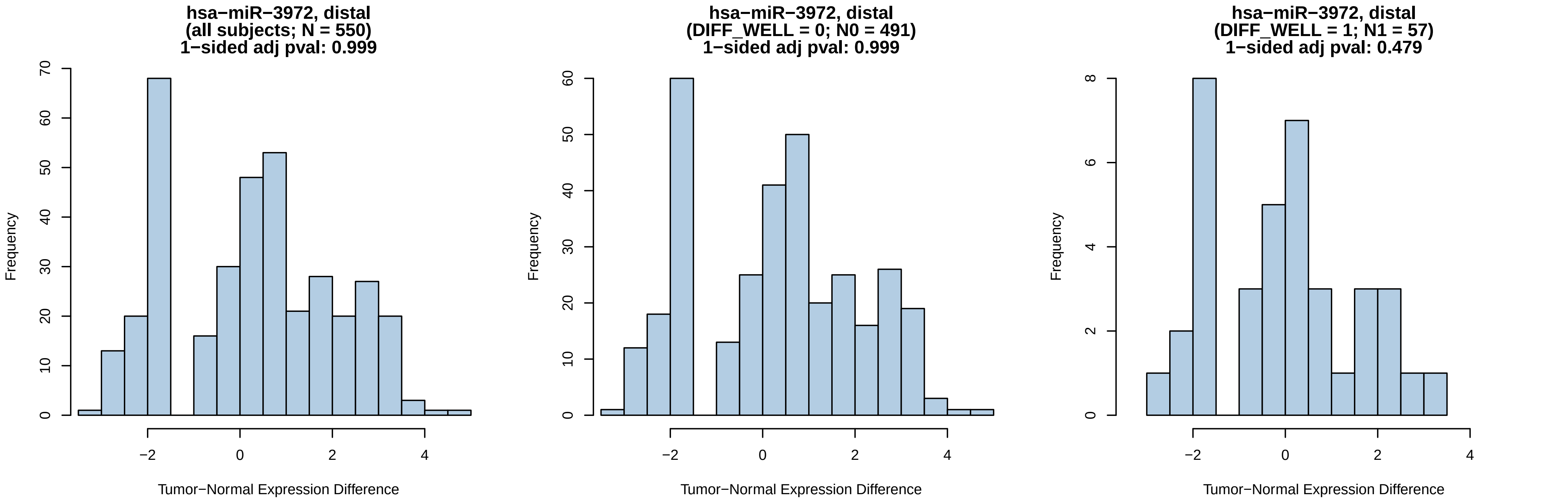

hsa−miR−3972, distal
(all subjects; N = 550)
1−sided adj pval: 0.999
Frequency
Tumor−Normal Expression Difference
hsa−miR−3972, distal
(DIFF_WELL = 0; N0 = 491)
1−sided adj pval: 0.999
Frequency
Tumor−Normal Expression Difference
hsa−miR−3972, distal
(DIFF_WELL = 1; N1 = 57)
1−sided adj pval: 0.479
Frequency
Tumor−Normal Expression Difference

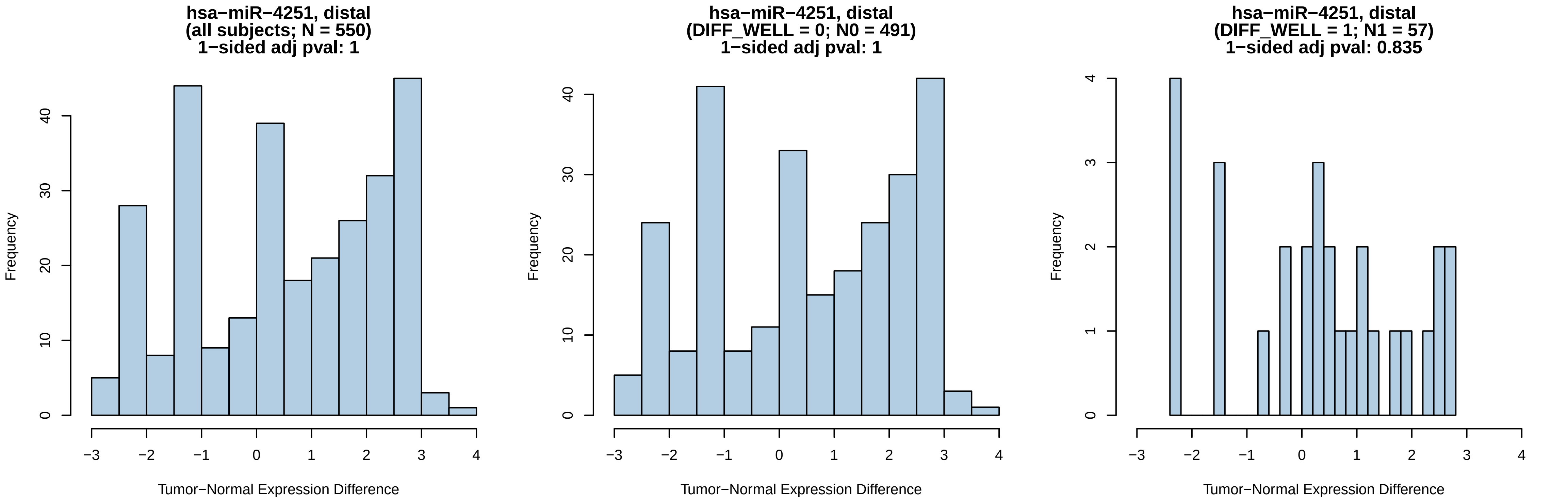

hsa−miR−4251, distal
(all subjects; N = 550)
1−sided adj pval: 1
Frequency
Tumor−Normal Expression Difference
hsa−miR−4251, distal
(DIFF_WELL = 0; N0 = 491)
1−sided adj pval: 1
Frequency
Tumor−Normal Expression Difference
hsa−miR−4251, distal
(DIFF_WELL = 1; N1 = 57)
1−sided adj pval: 0.835
Frequency
Tumor−Normal Expression Difference

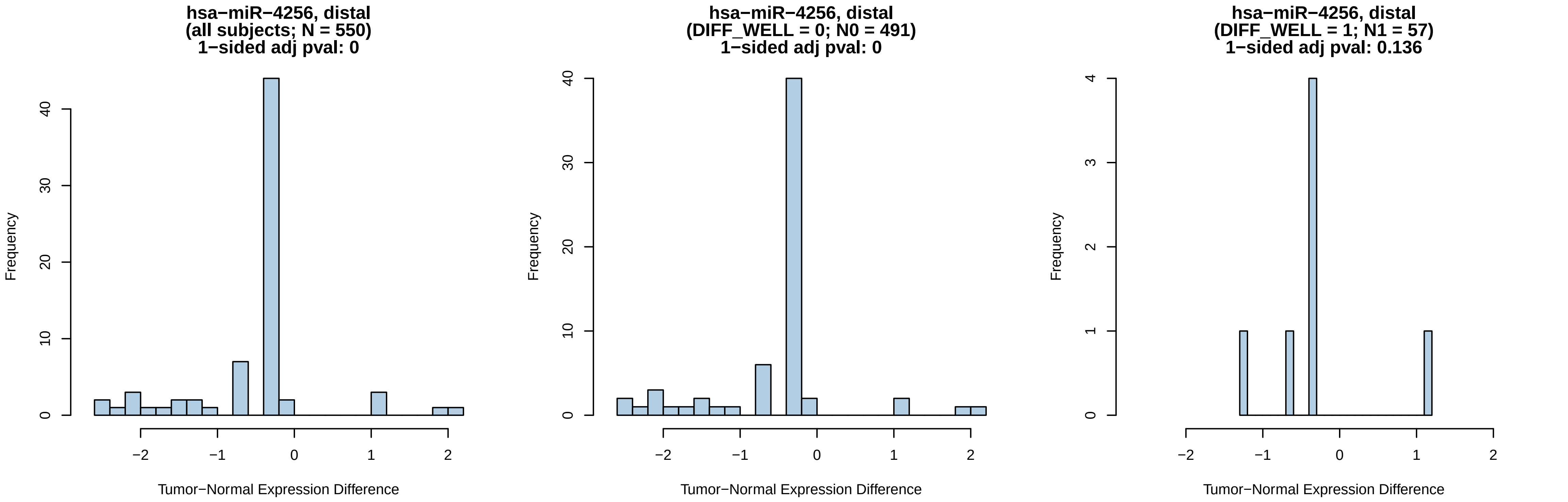
hsa−miR−4256, distal
(all subjects; N = 550)
1−sided adj pval: 0
hsa−miR−4256, distal
(DIFF_WELL = 0; N0 = 491)
1−sided adj pval: 0
hsa−miR−4256, distal
(DIFF_WELL = 1; N1 = 57)
1−sided adj pval: 0.136
Frequency
Tumor−Normal Expression Difference

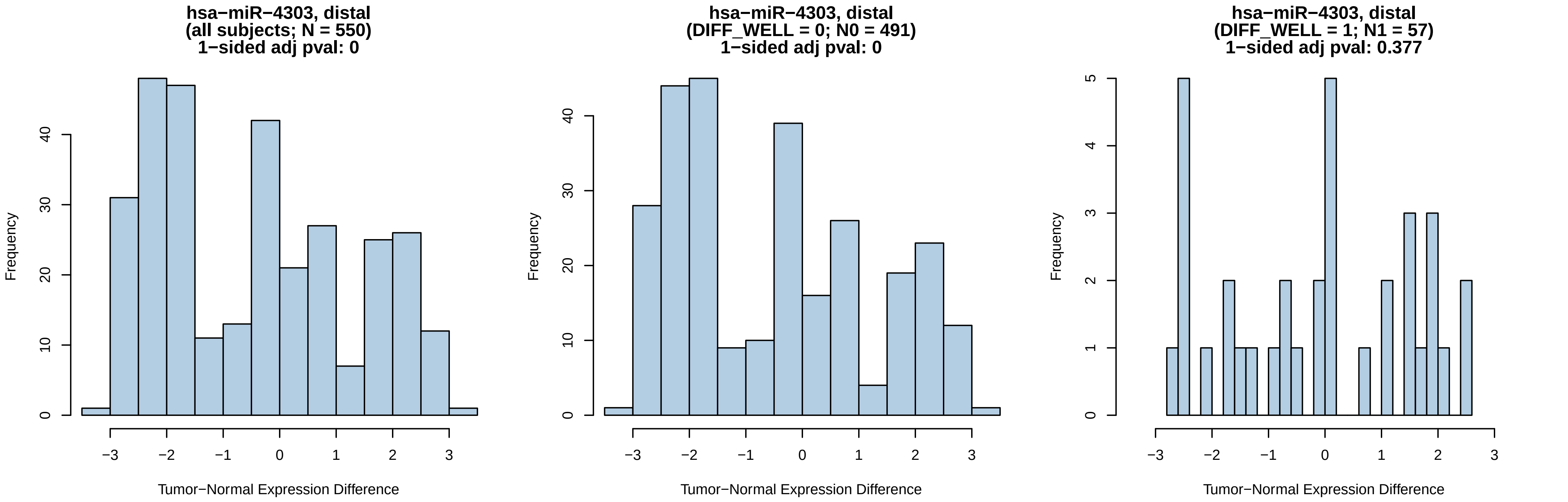

hsa−miR−4303, distal
(all subjects; N = 550)
1−sided adj pval: 0
Frequency
0
10
20
30
40
−3
−2
−1
0
1
2
3
Tumor−Normal Expression Difference
hsa−miR−4303, distal
(DIFF_WELL = 0; N0 = 491)
1−sided adj pval: 0
Frequency
0
10
20
30
40
−3
−2
−1
0
1
2
3
Tumor−Normal Expression Difference
hsa−miR−4303, distal
(DIFF_WELL = 1; N1 = 57)
1−sided adj pval: 0.377
Frequency
0
1
2
3
4
5
−3
−2
−1
0
1
2
3
Tumor−Normal Expression Difference

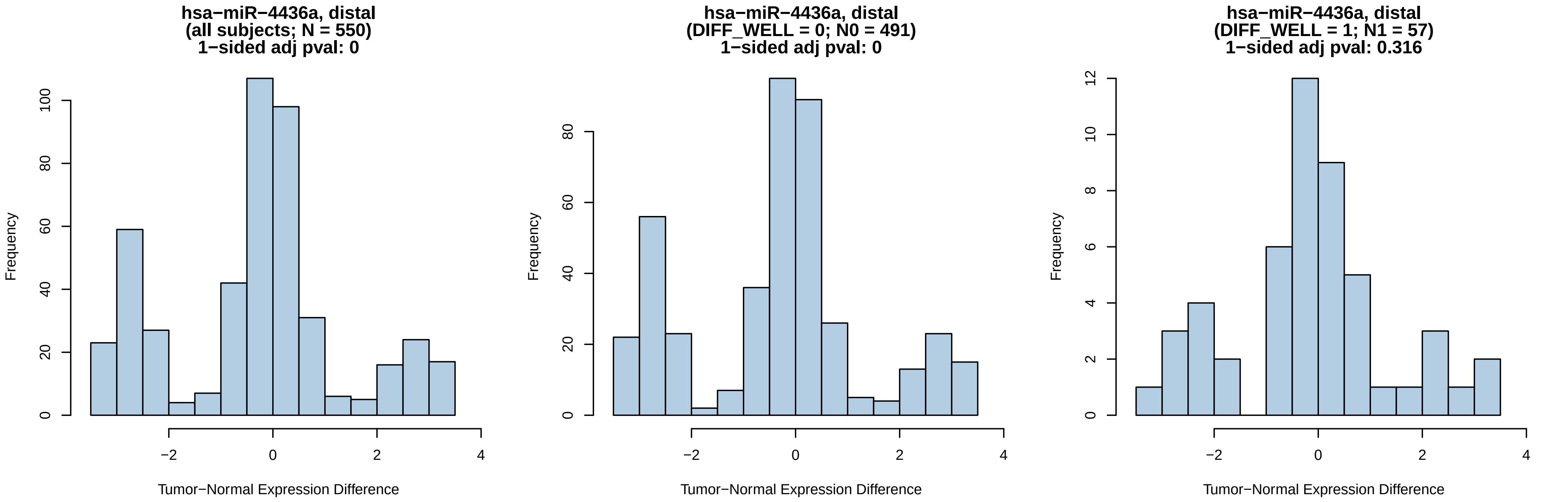
hsa−miR−4436a, distal
(all subjects; N = 550)
1−sided adj pval: 0
Frequency
0
20
40
60
80
100
−2
0
2
4
Tumor−Normal Expression Difference
hsa−miR−4436a, distal
(DIFF_WELL = 0; N0 = 491)
1−sided adj pval: 0
Frequency
0
20
40
60
80
−2
0
2
4
Tumor−Normal Expression Difference
hsa−miR−4436a, distal
(DIFF_WELL = 1; N1 = 57)
1−sided adj pval: 0.316
Frequency
0
2
4
6
8
10
12
−2
0
2
4
Tumor−Normal Expression Difference

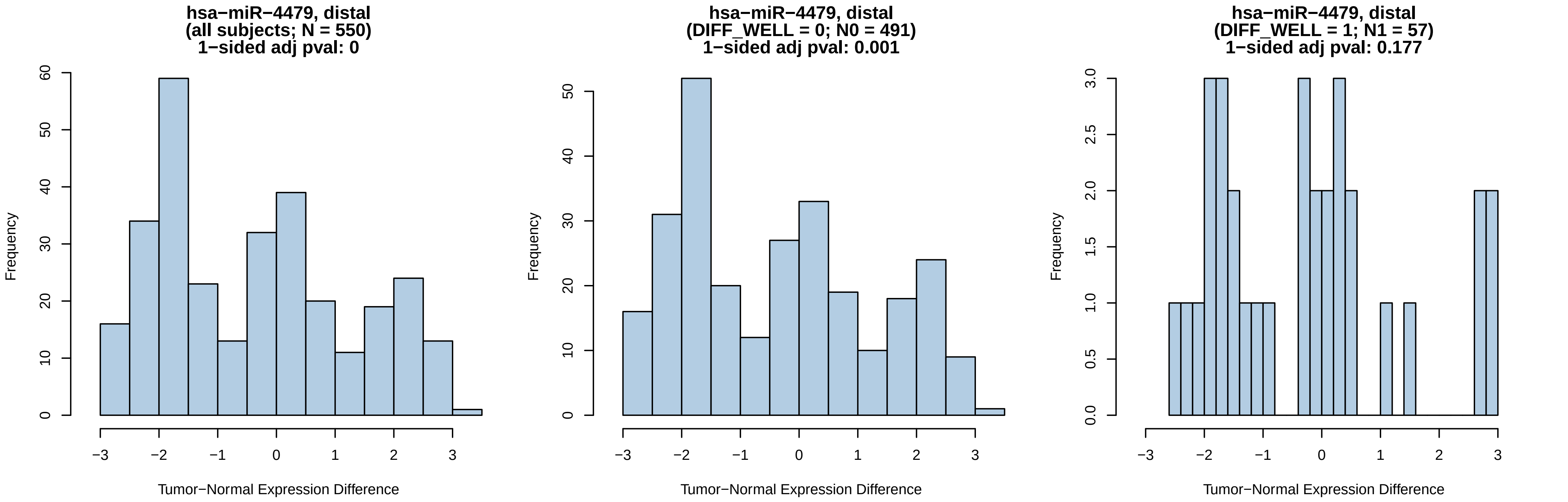

hsa−miR−4479, distal
(all subjects; N = 550)
1−sided adj pval: 0
Frequency
Tumor−Normal Expression Difference
hsa−miR−4479, distal
(DIFF_WELL = 0; N0 = 491)
1−sided adj pval: 0.001
Frequency
Tumor−Normal Expression Difference
hsa−miR−4479, distal
(DIFF_WELL = 1; N1 = 57)
1−sided adj pval: 0.177
Frequency
Tumor−Normal Expression Difference

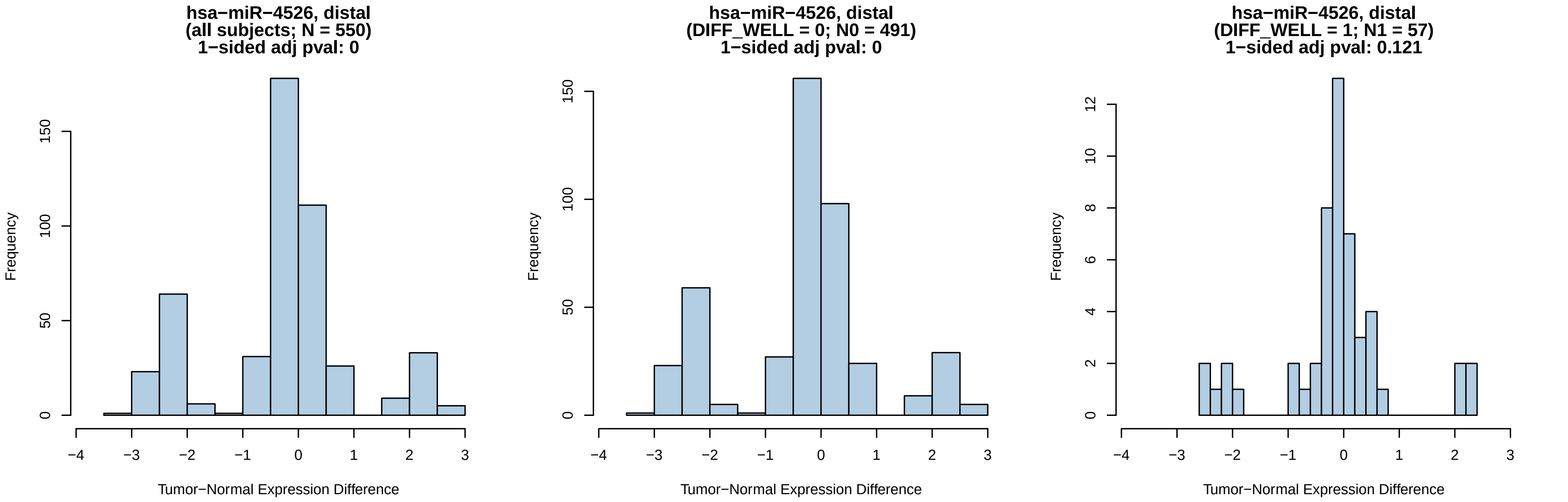
hsa−miR−4526, distal
(all subjects; N = 550)
1−sided adj pval: 0
hsa−miR−4526, distal
(DIFF_WELL = 0; N0 = 491)
1−sided adj pval: 0
hsa−miR−4526, distal
(DIFF_WELL = 1; N1 = 57)
1−sided adj pval: 0.121
Frequency
Tumor−Normal Expression Difference

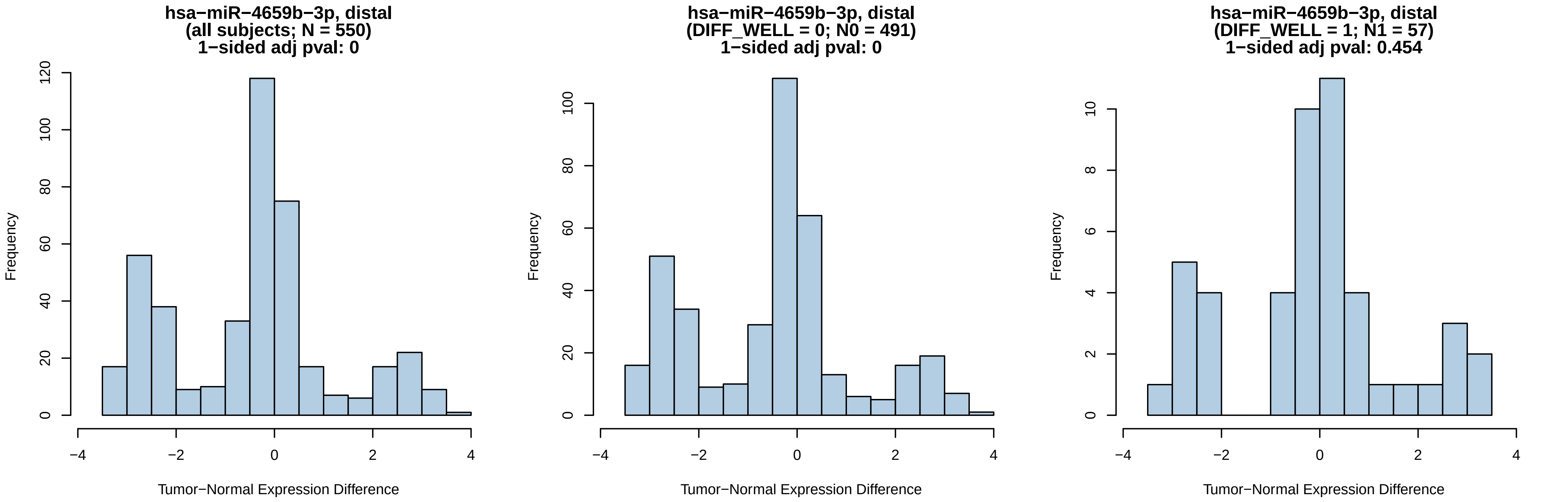
hsa−miR−4659b−3p, distal
(all subjects; N = 550)
1−sided adj pval: 0
hsa−miR−4659b−3p, distal
(DIFF_WELL = 0; N0 = 491)
1−sided adj pval: 0
hsa−miR−4659b−3p, distal
(DIFF_WELL = 1; N1 = 57)
1−sided adj pval: 0.454
Frequency
Tumor−Normal Expression Difference

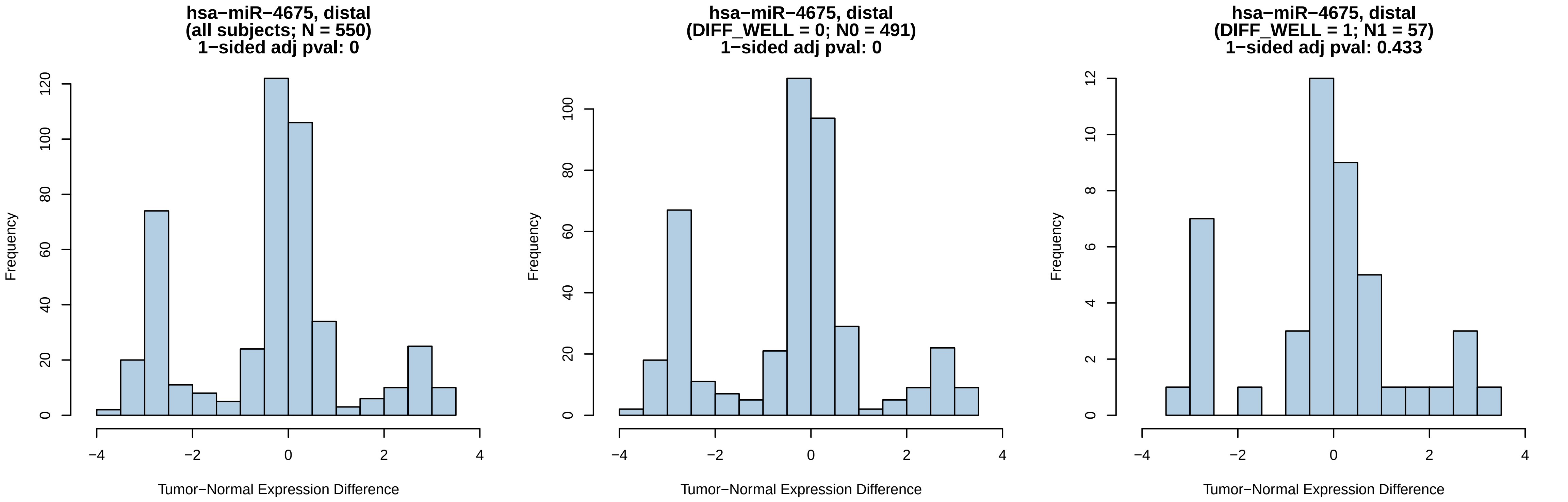
hsa−miR−4675, distal
(all subjects; N = 550)
1−sided adj pval: 0
Frequency
Tumor−Normal Expression Difference
hsa−miR−4675, distal
(DIFF_WELL = 0; N0 = 491)
1−sided adj pval: 0
Frequency
Tumor−Normal Expression Difference
hsa−miR−4675, distal
(DIFF_WELL = 1; N1 = 57)
1−sided adj pval: 0.433
Frequency
Tumor−Normal Expression Difference

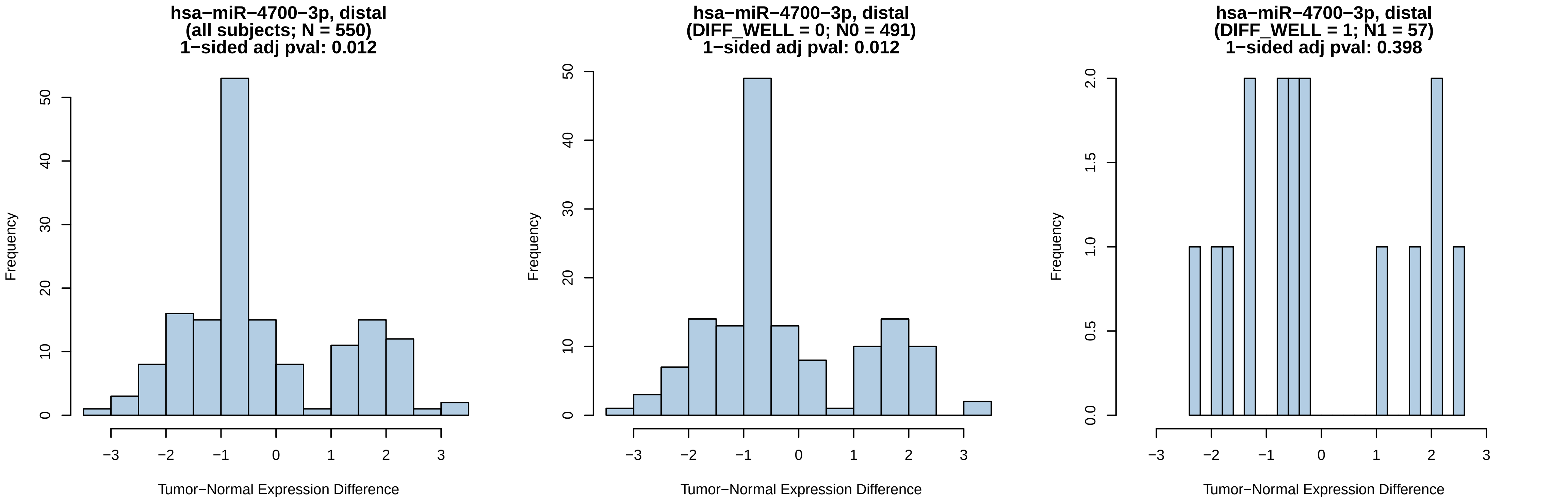
hsa−miR−4700−3p, distal
(all subjects; N = 550)
1−sided adj pval: 0.012
Frequency
Tumor−Normal Expression Difference
hsa−miR−4700−3p, distal
(DIFF_WELL = 0; N0 = 491)
1−sided adj pval: 0.012
Frequency
Tumor−Normal Expression Difference
hsa−miR−4700−3p, distal
(DIFF_WELL = 1; N1 = 57)
1−sided adj pval: 0.398
Frequency
Tumor−Normal Expression Difference

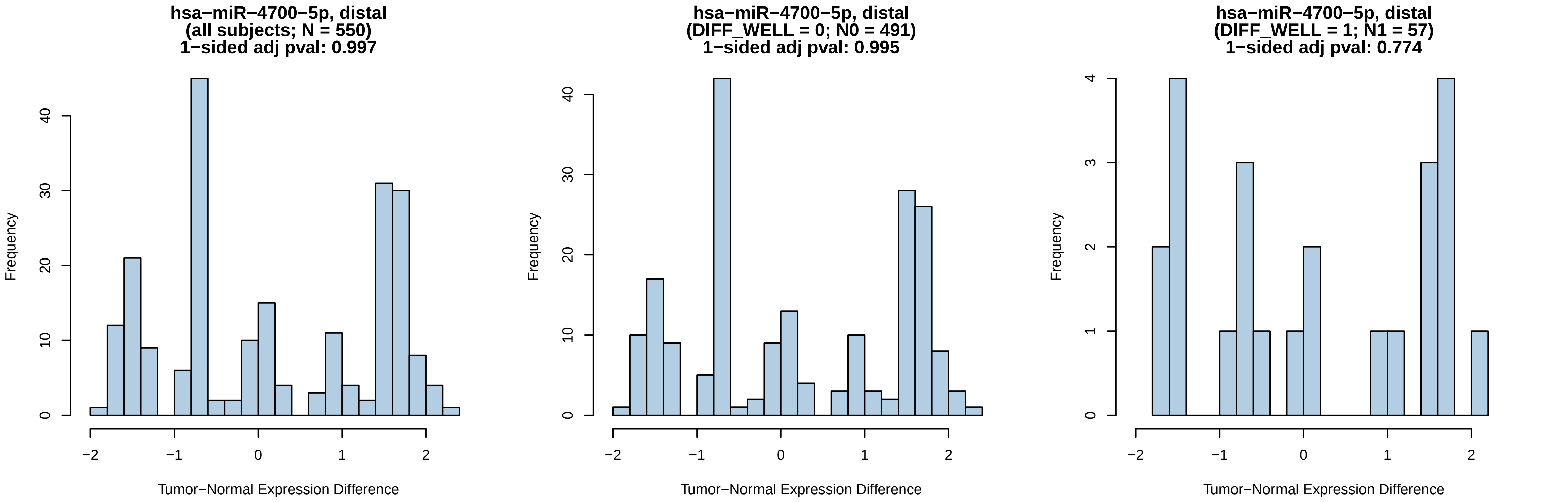

hsa−miR−4700−5p, distal
(all subjects; N = 550)
1−sided adj pval: 0.997
Frequency
Tumor−Normal Expression Difference
hsa−miR−4700−5p, distal
(DIFF_WELL = 0; N0 = 491)
1−sided adj pval: 0.995
Frequency
Tumor−Normal Expression Difference
hsa−miR−4700−5p, distal
(DIFF_WELL = 1; N1 = 57)
1−sided adj pval: 0.774
Frequency
Tumor−Normal Expression Difference

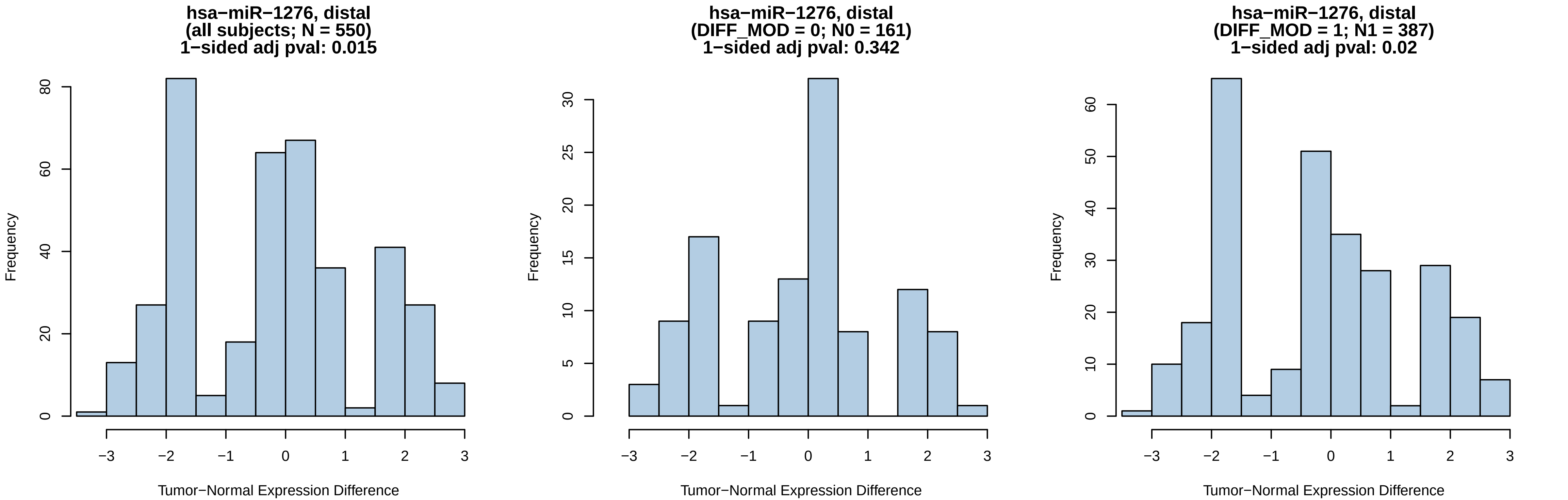
hsa−miR−1276, distal
(all subjects; N = 550)
1−sided adj pval: 0.015
Frequency
Tumor−Normal Expression Difference
hsa−miR−1276, distal
(DIFF_MOD = 0; N0 = 161)
1−sided adj pval: 0.342
Frequency
Tumor−Normal Expression Difference
hsa−miR−1276, distal
(DIFF_MOD = 1; N1 = 387)
1−sided adj pval: 0.02
Frequency
Tumor−Normal Expression Difference

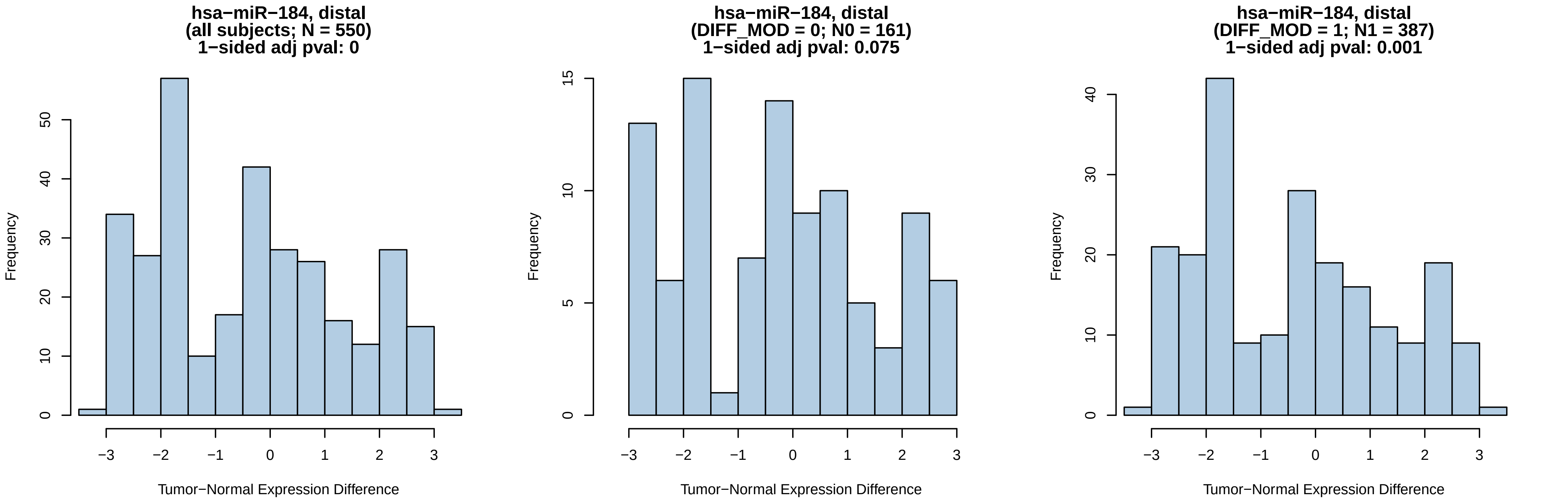
hsa−miR−184, distal
(all subjects; N = 550)
1−sided adj pval: 0
hsa−miR−184, distal
(DIFF_MOD = 0; N0 = 161)
1−sided adj pval: 0.075
hsa−miR−184, distal
(DIFF_MOD = 1; N1 = 387)
1−sided adj pval: 0.001
Frequency
Tumor−Normal Expression Difference

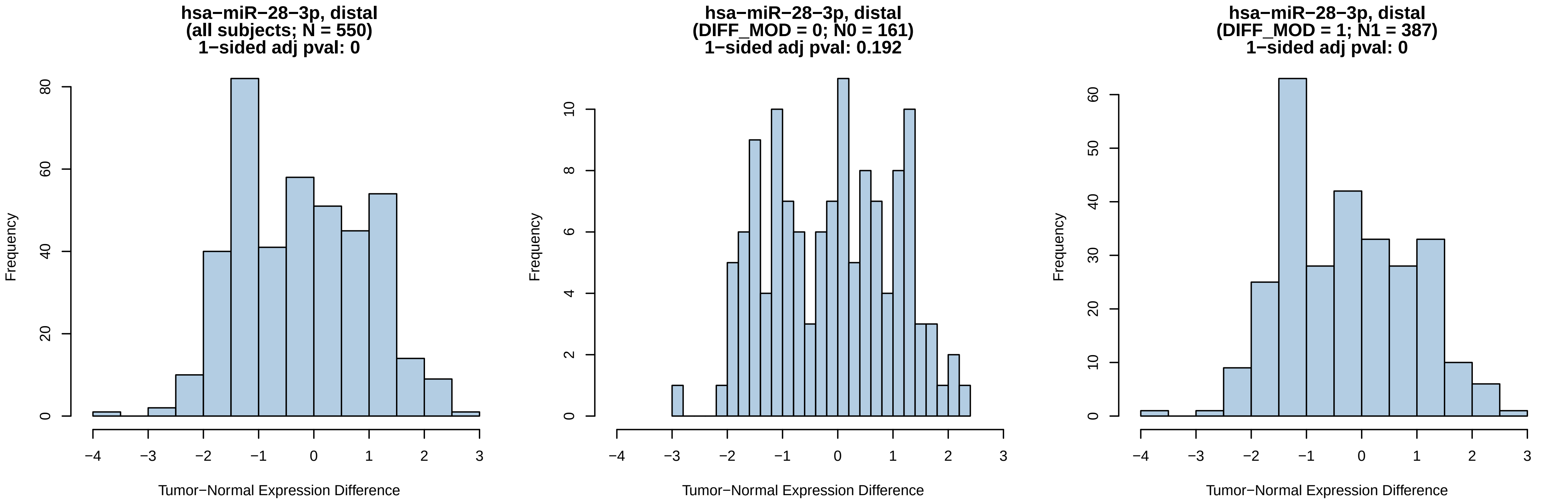

hsa−miR−28−3p, distal
(all subjects; N = 550)
1−sided adj pval: 0
Frequency
Tumor−Normal Expression Difference
hsa−miR−28−3p, distal
(DIFF_MOD = 0; N0 = 161)
1−sided adj pval: 0.192
Frequency
Tumor−Normal Expression Difference
hsa−miR−28−3p, distal
(DIFF_MOD = 1; N1 = 387)
1−sided adj pval: 0
Frequency
Tumor−Normal Expression Difference

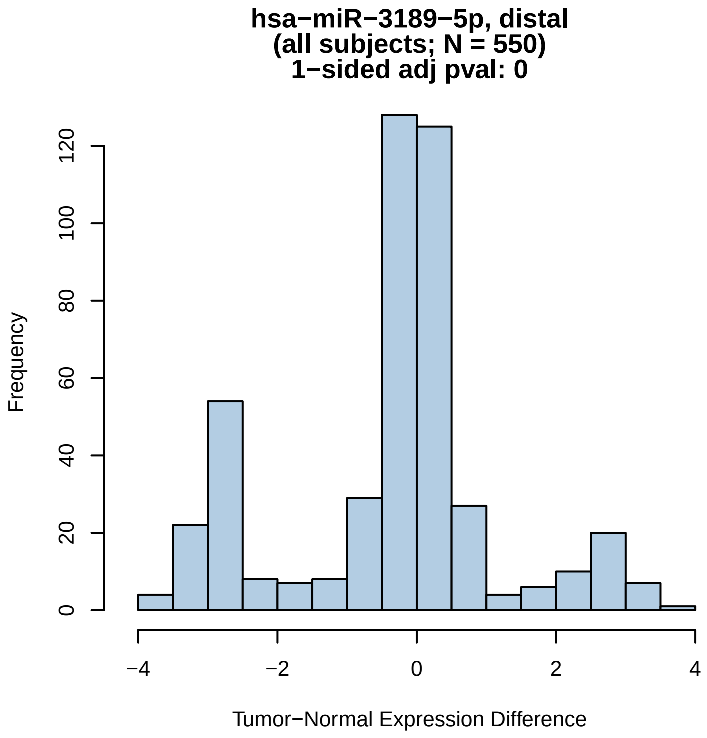

hsa−miR−3189−5p, distal
(all subjects; N = 550)
1−sided adj pval: 0
Frequency
Tumor−Normal Expression Difference


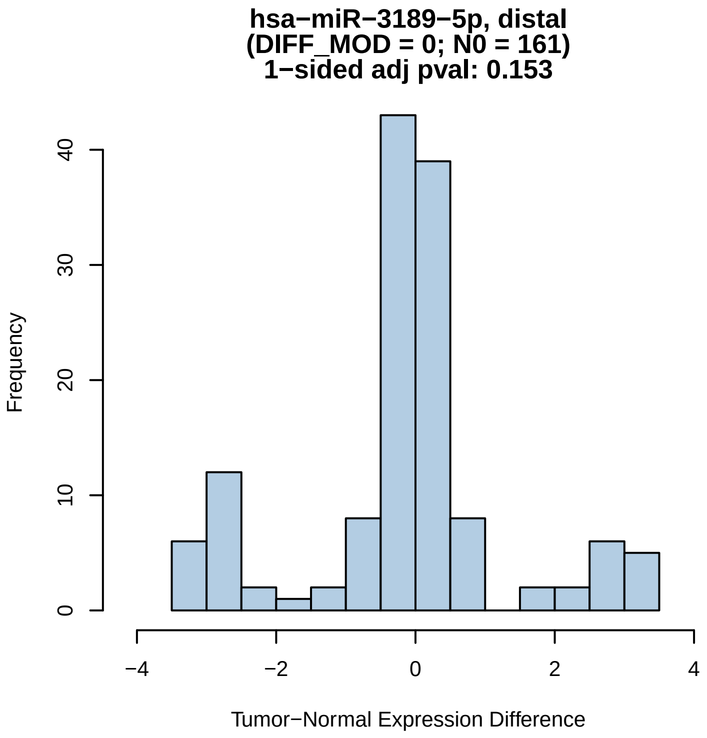

hsa−miR−3189−5p, distal
(DIFF_MOD = 0; N0 = 161)
1−sided adj pval: 0.153
Frequency
Tumor−Normal Expression Difference


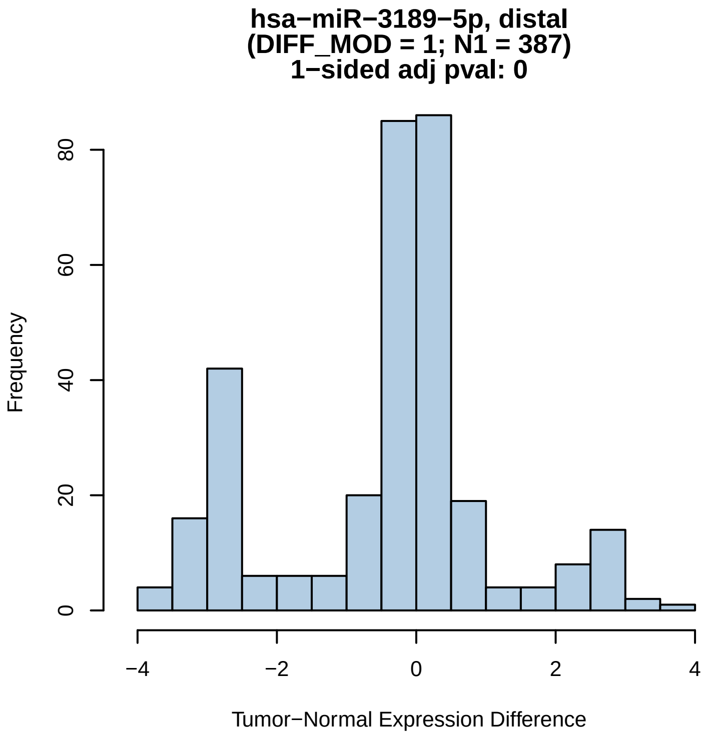

hsa−miR−3189−5p, distal
(DIFF_MOD = 1; N1 = 387)
1−sided adj pval: 0
Frequency
Tumor−Normal Expression Difference

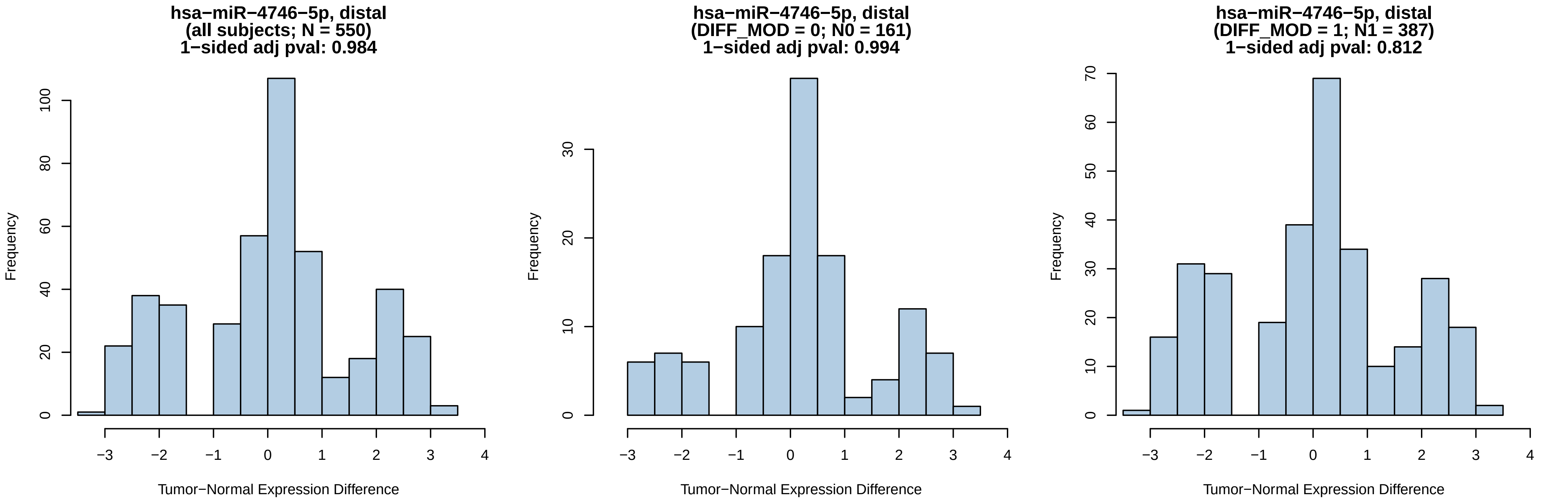

hsa−miR−4746−5p, distal
(all subjects; N = 550)
1−sided adj pval: 0.984
Frequency
0
20
40
60
80
100
−3
−2
−1
0
1
2
3
4
Tumor−Normal Expression Difference
hsa−miR−4746−5p, distal
(DIFF_MOD = 0; N0 = 161)
1−sided adj pval: 0.994
Frequency
0
10
20
30
−3
−2
−1
0
1
2
3
4
Tumor−Normal Expression Difference
hsa−miR−4746−5p, distal
(DIFF_MOD = 1; N1 = 387)
1−sided adj pval: 0.812
Frequency
0
10
20
30
40
50
60
70
−3
−2
−1
0
1
2
3
4
Tumor−Normal Expression Difference

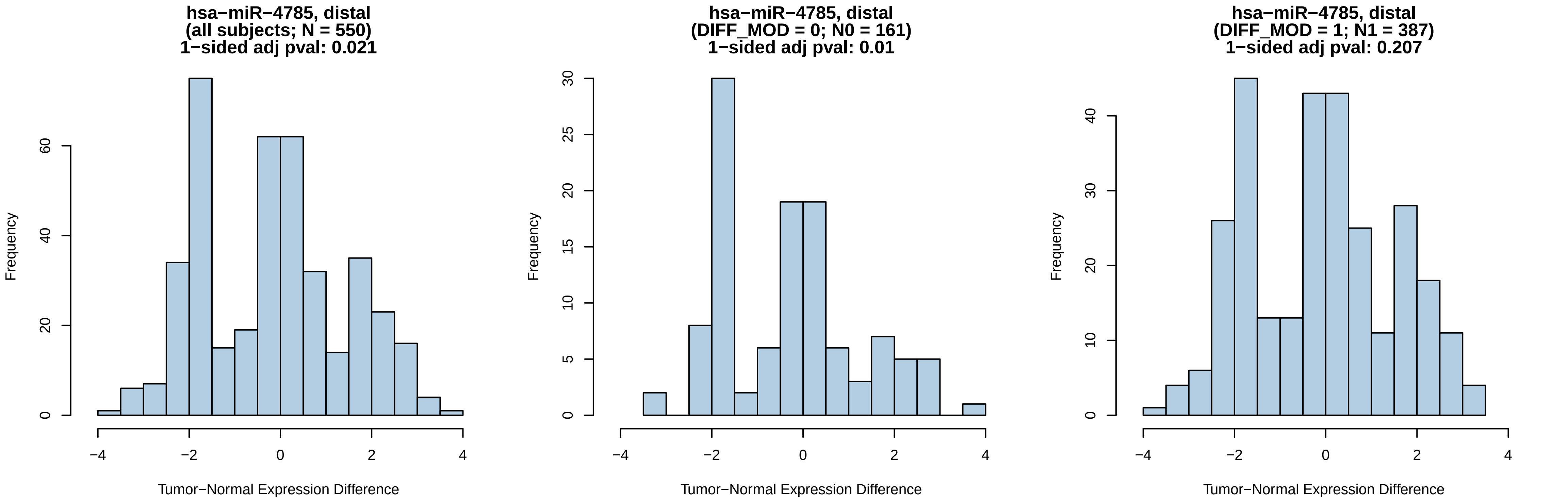
hsa−miR−4785, distal
(all subjects; N = 550)
1−sided adj pval: 0.021
Frequency
Tumor−Normal Expression Difference
hsa−miR−4785, distal
(DIFF_MOD = 0; N0 = 161)
1−sided adj pval: 0.01
Frequency
Tumor−Normal Expression Difference
hsa−miR−4785, distal
(DIFF_MOD = 1; N1 = 387)
1−sided adj pval: 0.207
Frequency
Tumor−Normal Expression Difference

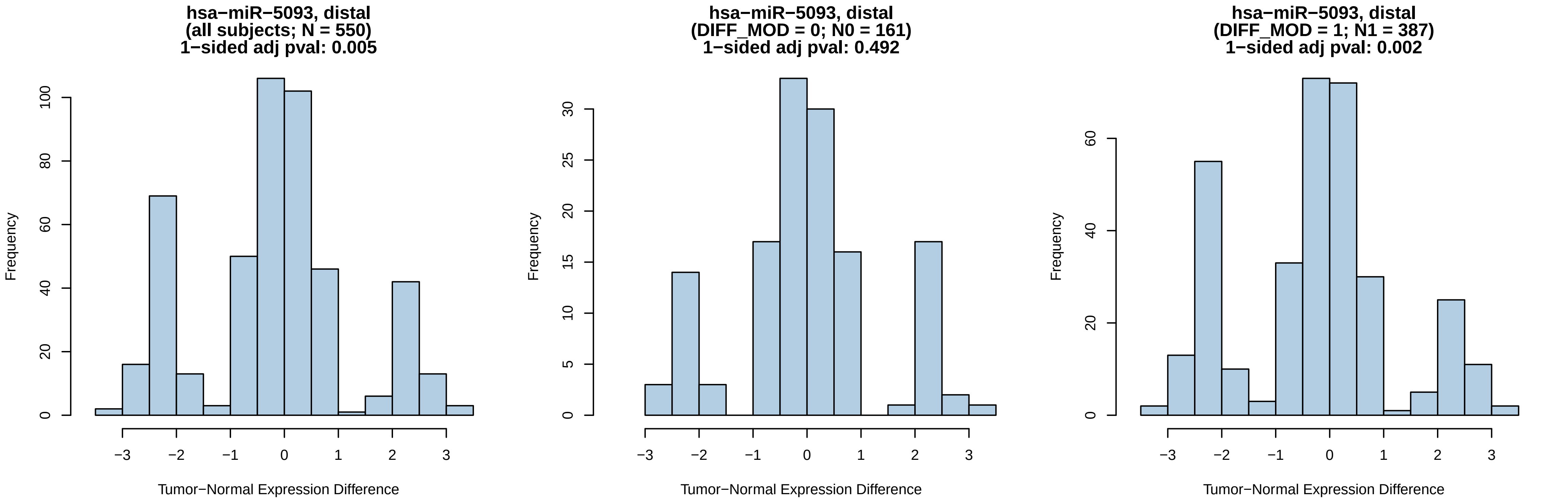

hsa−miR−5093, distal
(all subjects; N = 550)
1−sided adj pval: 0.005
Frequency
0
20
40
60
80
100
−3
−2
−1
0
1
2
3
Tumor−Normal Expression Difference
hsa−miR−5093, distal
(DIFF_MOD = 0; N0 = 161)
1−sided adj pval: 0.492
Frequency
0
5
10
15
20
25
30
−3
−2
−1
0
1
2
3
Tumor−Normal Expression Difference
hsa−miR−5093, distal
(DIFF_MOD = 1; N1 = 387)
1−sided adj pval: 0.002
Frequency
0
20
40
60
−3
−2
−1
0
1
2
3
Tumor−Normal Expression Difference

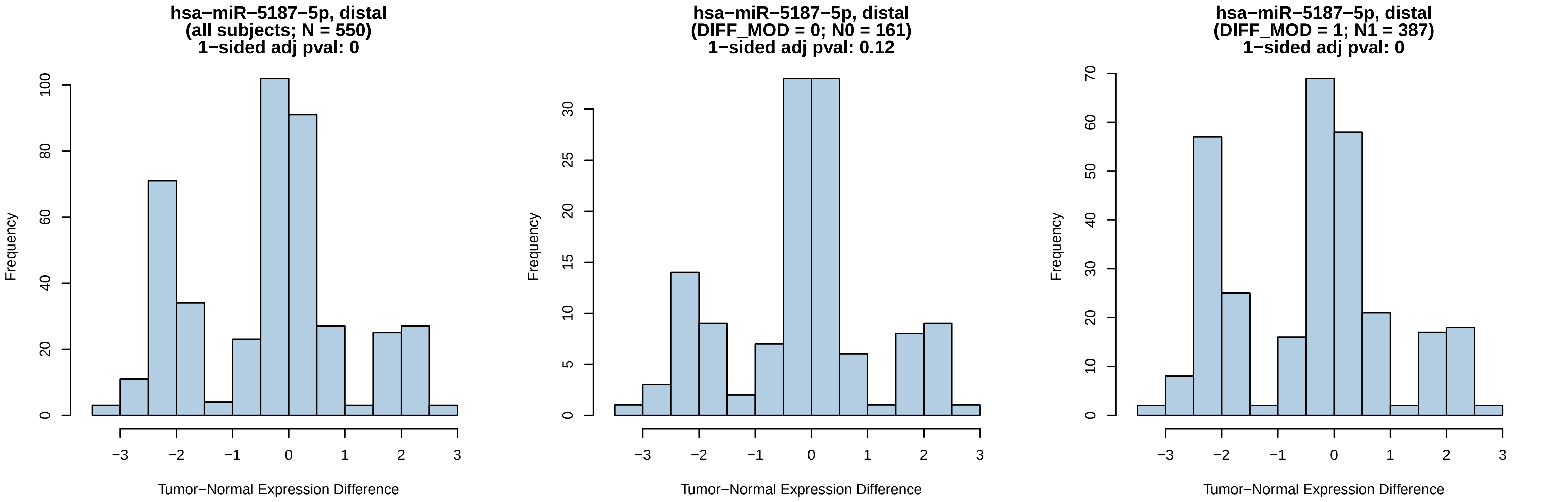

hsa−miR−5187−5p, distal
(all subjects; N = 550)
1−sided adj pval: 0
Frequency
Tumor−Normal Expression Difference
hsa−miR−5187−5p, distal
(DIFF_MOD = 0; N0 = 161)
1−sided adj pval: 0.12
Frequency
Tumor−Normal Expression Difference
hsa−miR−5187−5p, distal
(DIFF_MOD = 1; N1 = 387)
1−sided adj pval: 0
Frequency
Tumor−Normal Expression Difference

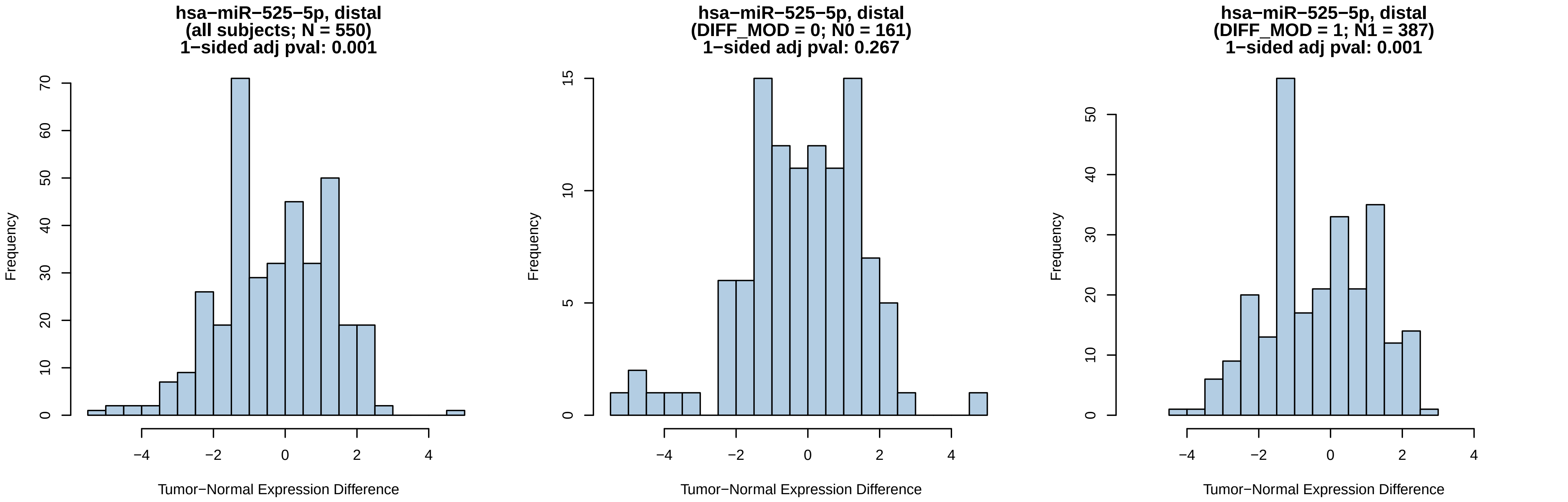

hsa−miR−525−5p, distal
(all subjects; N = 550)
1−sided adj pval: 0.001
Frequency
Tumor−Normal Expression Difference
hsa−miR−525−5p, distal
(DIFF_MOD = 0; N0 = 161)
1−sided adj pval: 0.267
Frequency
Tumor−Normal Expression Difference
hsa−miR−525−5p, distal
(DIFF_MOD = 1; N1 = 387)
1−sided adj pval: 0.001
Frequency
Tumor−Normal Expression Difference

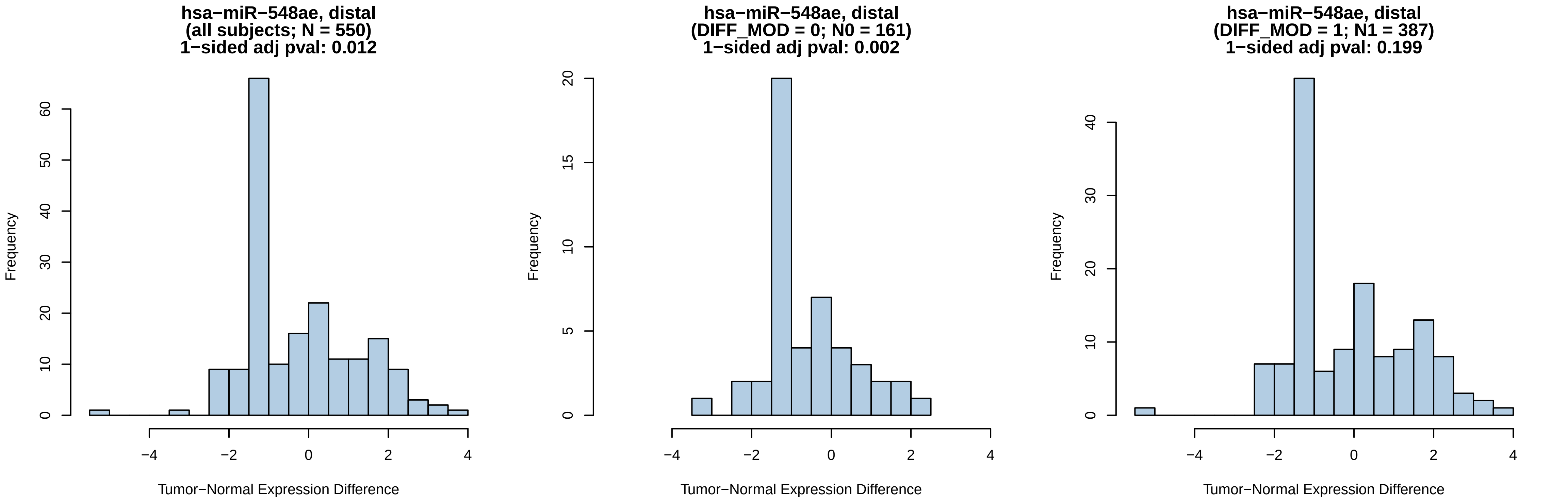

hsa−miR−548ae, distal
(all subjects; N = 550)
1−sided adj pval: 0.012
Frequency
0
10
20
30
40
50
60
−4
−2
0
2
4
Tumor−Normal Expression Difference
hsa−miR−548ae, distal
(DIFF_MOD = 0; N0 = 161)
1−sided adj pval: 0.002
Frequency
0
5
10
15
20
−4
−2
0
2
4
Tumor−Normal Expression Difference
hsa−miR−548ae, distal
(DIFF_MOD = 1; N1 = 387)
1−sided adj pval: 0.199
Frequency
0
10
20
30
40
−4
−2
0
2
4
Tumor−Normal Expression Difference

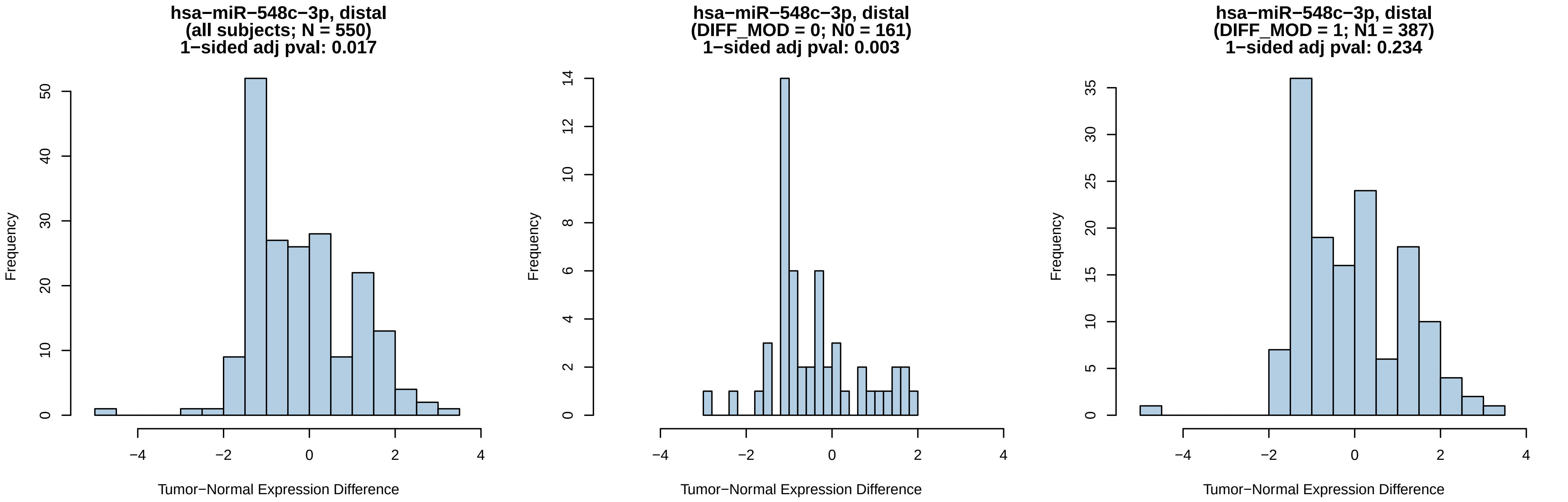
hsa−miR−548c−3p, distal
(all subjects; N = 550)
1−sided adj pval: 0.017
hsa−miR−548c−3p, distal
(DIFF_MOD = 0; N0 = 161)
1−sided adj pval: 0.003
hsa−miR−548c−3p, distal
(DIFF_MOD = 1; N1 = 387)
1−sided adj pval: 0.234
Frequency
Tumor−Normal Expression Difference

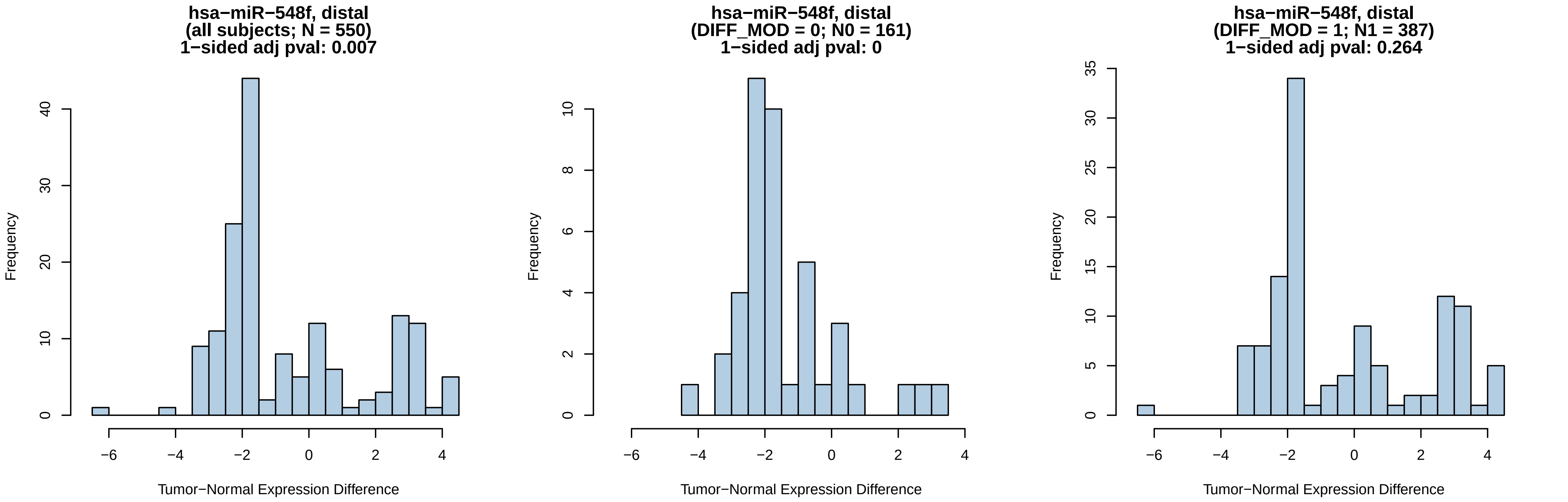
hsa−miR−548f, distal
(all subjects; N = 550)
1−sided adj pval: 0.007
Frequency
Tumor−Normal Expression Difference
hsa−miR−548f, distal
(DIFF_MOD = 0; N0 = 161)
1−sided adj pval: 0
Frequency
Tumor−Normal Expression Difference
hsa−miR−548f, distal
(DIFF_MOD = 1; N1 = 387)
1−sided adj pval: 0.264
Frequency
Tumor−Normal Expression Difference

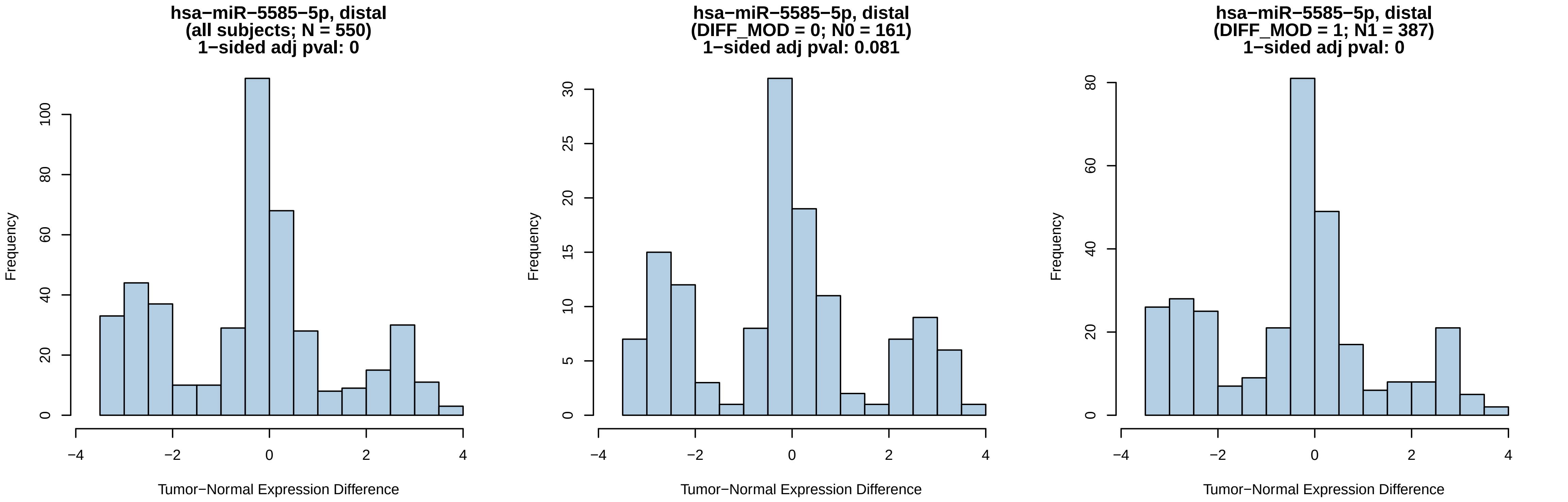

hsa−miR−5585−5p, distal
(all subjects; N = 550)
1−sided adj pval: 0
Frequency
Tumor−Normal Expression Difference
hsa−miR−5585−5p, distal
(DIFF_MOD = 0; N0 = 161)
1−sided adj pval: 0.081
Frequency
Tumor−Normal Expression Difference
hsa−miR−5585−5p, distal
(DIFF_MOD = 1; N1 = 387)
1−sided adj pval: 0
Frequency
Tumor−Normal Expression Difference

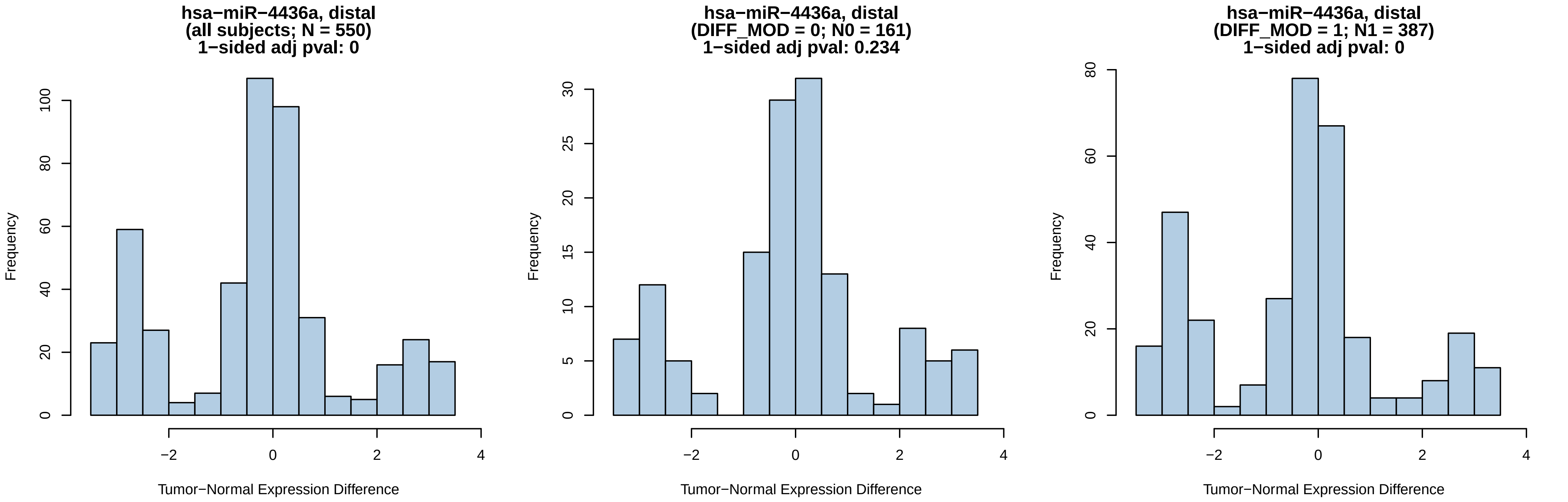
hsa−miR−4436a, distal
(all subjects; N = 550)
1−sided adj pval: 0
Frequency
Tumor−Normal Expression Difference
hsa−miR−4436a, distal
(DIFF_MOD = 0; N0 = 161)
1−sided adj pval: 0.234
Frequency
Tumor−Normal Expression Difference
hsa−miR−4436a, distal
(DIFF_MOD = 1; N1 = 387)
1−sided adj pval: 0
Frequency
Tumor−Normal Expression Difference

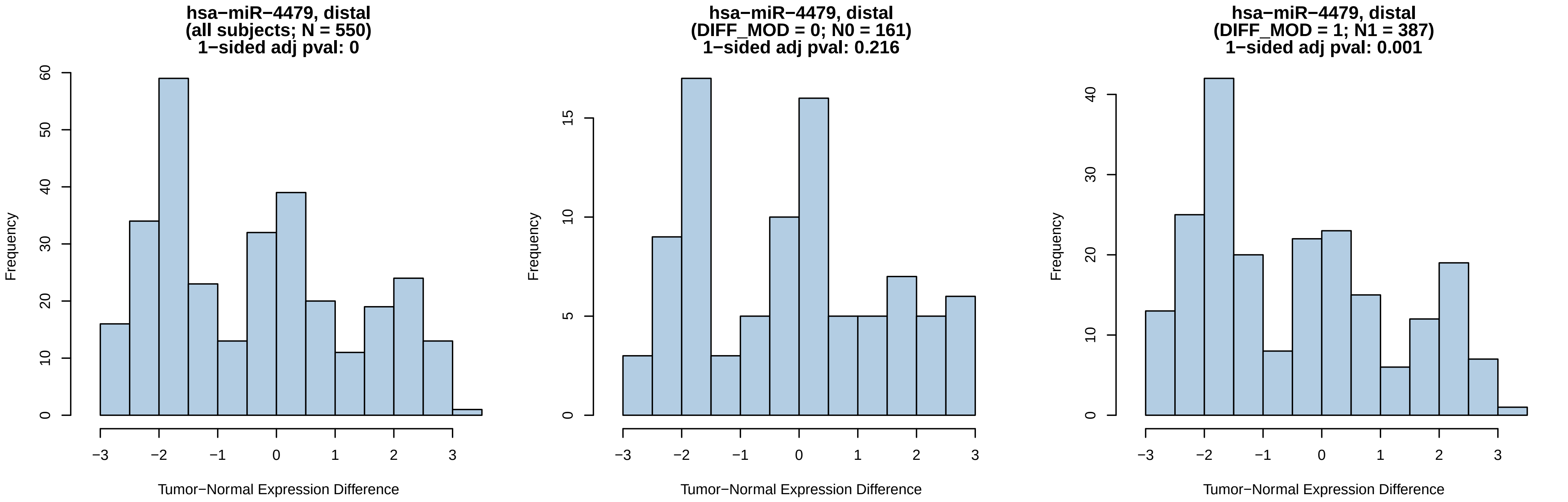
hsa−miR−4479, distal
(all subjects; N = 550)
1−sided adj pval: 0
Frequency
Tumor−Normal Expression Difference
hsa−miR−4479, distal
(DIFF_MOD = 0; N0 = 161)
1−sided adj pval: 0.216
Frequency
Tumor−Normal Expression Difference
hsa−miR−4479, distal
(DIFF_MOD = 1; N1 = 387)
1−sided adj pval: 0.001
Frequency
Tumor−Normal Expression Difference

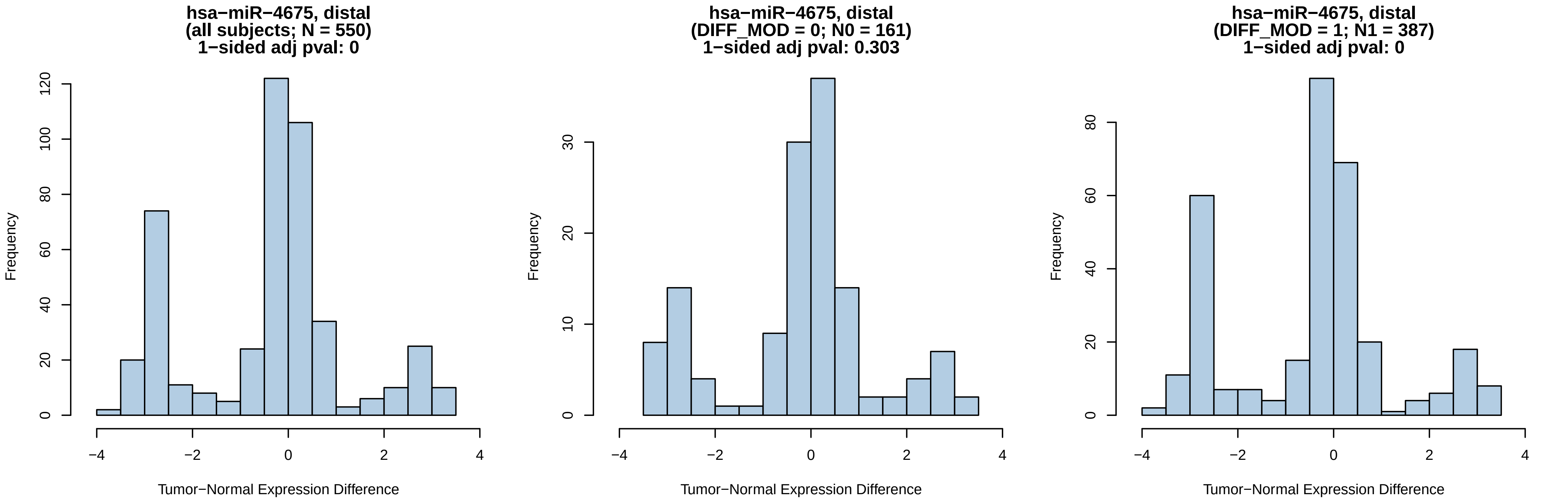

hsa−miR−4675, distal
(all subjects; N = 550)
1−sided adj pval: 0
Frequency
Tumor−Normal Expression Difference
hsa−miR−4675, distal
(DIFF_MOD = 0; N0 = 161)
1−sided adj pval: 0.303
Frequency
Tumor−Normal Expression Difference
hsa−miR−4675, distal
(DIFF_MOD = 1; N1 = 387)
1−sided adj pval: 0
Frequency
Tumor−Normal Expression Difference

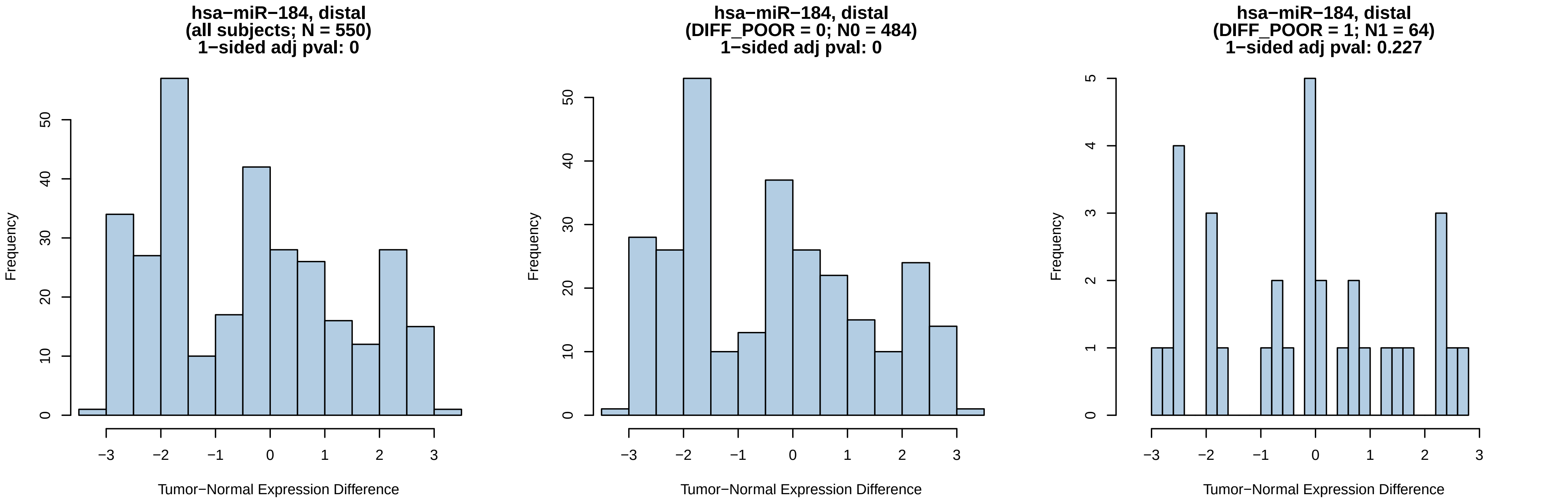

hsa−miR−184, distal
(all subjects; N = 550)
1−sided adj pval: 0
Frequency
Tumor−Normal Expression Difference
hsa−miR−184, distal
(DIFF_POOR = 0; N0 = 484)
1−sided adj pval: 0
Frequency
Tumor−Normal Expression Difference
hsa−miR−184, distal
(DIFF_POOR = 1; N1 = 64)
1−sided adj pval: 0.227
Frequency
Tumor−Normal Expression Difference

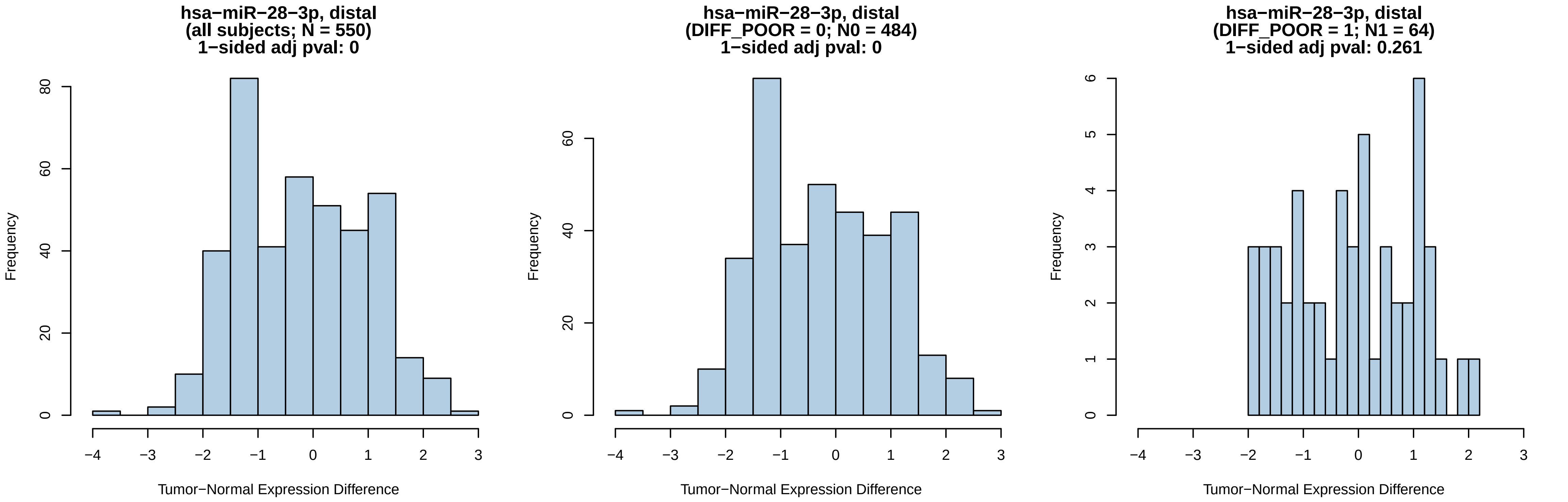
hsa−miR−28−3p, distal
(all subjects; N = 550)
1−sided adj pval: 0
Frequency
0
20
40
60
80
−4
−3
−2
−1
0
1
2
3
Tumor−Normal Expression Difference
hsa−miR−28−3p, distal
(DIFF_POOR = 0; N0 = 484)
1−sided adj pval: 0
Frequency
0
20
40
60
−4
−3
−2
−1
0
1
2
3
Tumor−Normal Expression Difference
hsa−miR−28−3p, distal
(DIFF_POOR = 1; N1 = 64)
1−sided adj pval: 0.261
Frequency
0
1
2
3
4
5
6
−4
−3
−2
−1
0
1
2
3
Tumor−Normal Expression Difference

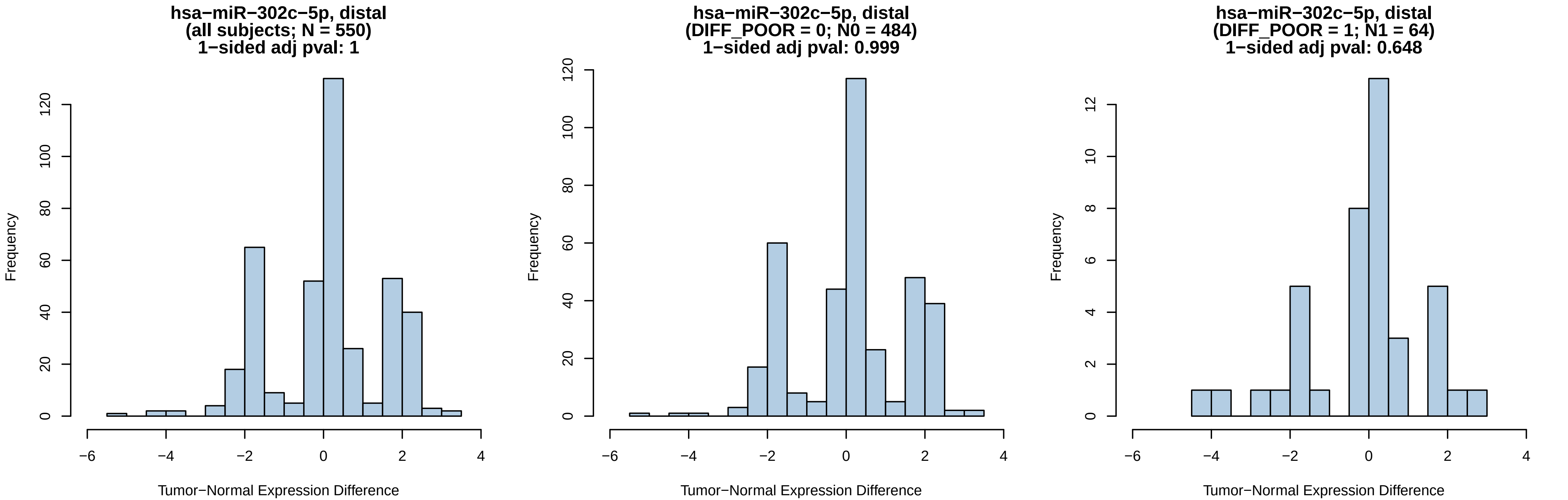

hsa−miR−302c−5p, distal
(all subjects; N = 550)
1−sided adj pval: 1
Frequency
Tumor−Normal Expression Difference
hsa−miR−302c−5p, distal
(DIFF_POOR = 0; N0 = 484)
1−sided adj pval: 0.999
Frequency
Tumor−Normal Expression Difference
hsa−miR−302c−5p, distal
(DIFF_POOR = 1; N1 = 64)
1−sided adj pval: 0.648
Frequency
Tumor−Normal Expression Difference

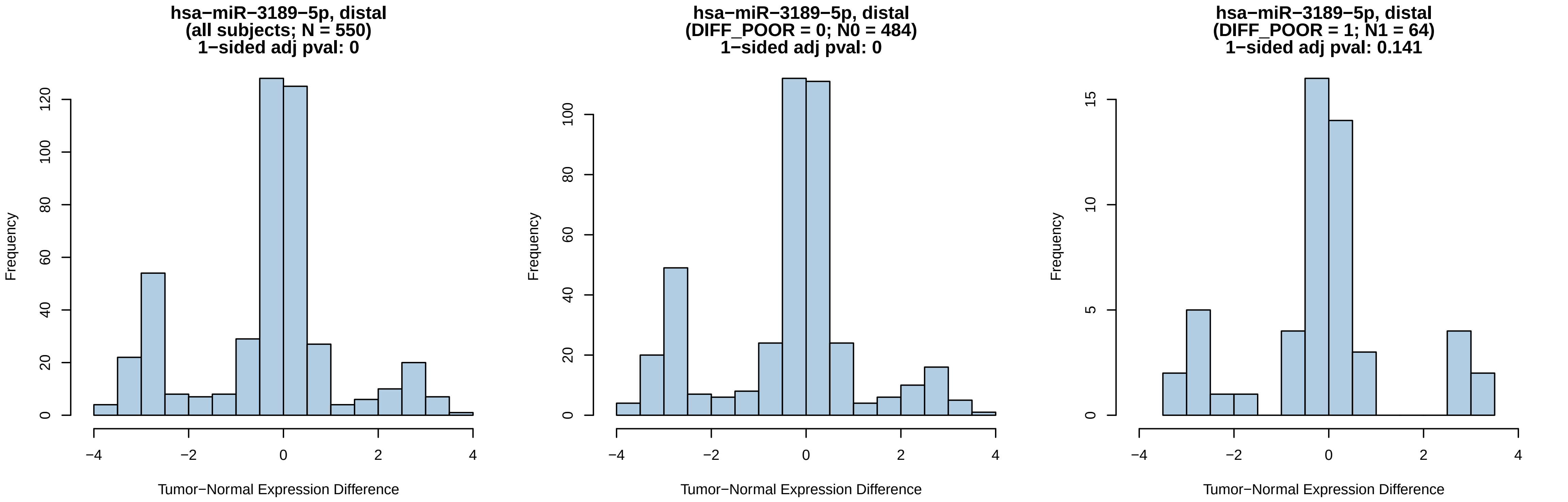
hsa−miR−3189−5p, distal
(all subjects; N = 550)
1−sided adj pval: 0
hsa−miR−3189−5p, distal
(DIFF_POOR = 0; N0 = 484)
1−sided adj pval: 0
hsa−miR−3189−5p, distal
(DIFF_POOR = 1; N1 = 64)
1−sided adj pval: 0.141
Frequency
Tumor−Normal Expression Difference

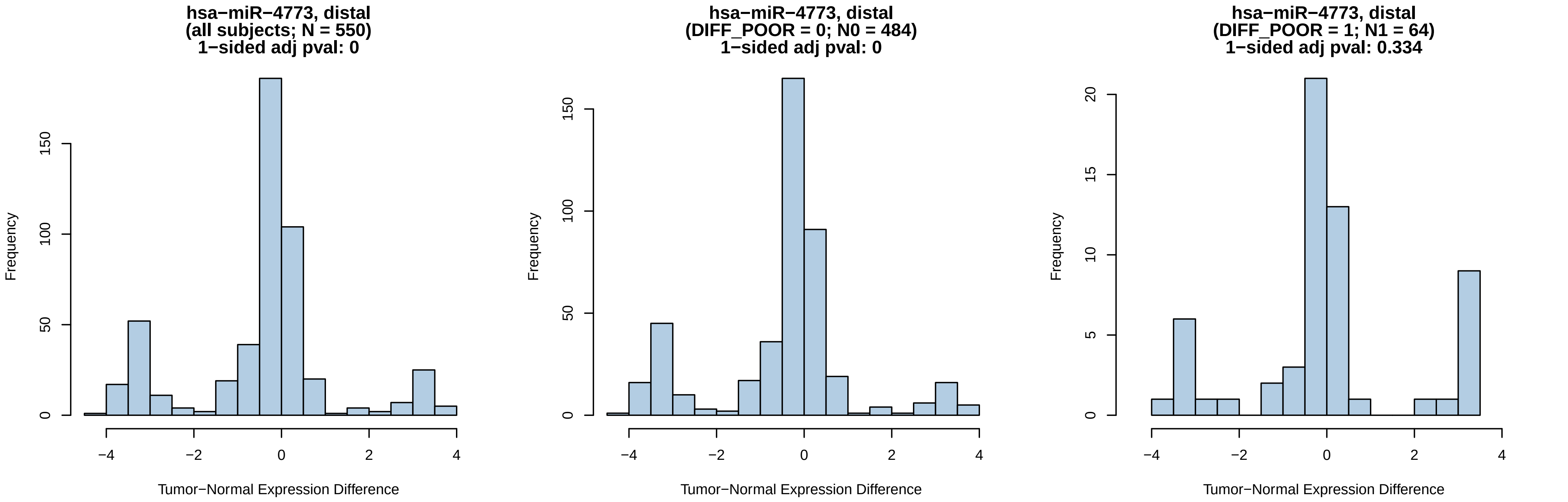
hsa−miR−4773, distal
(all subjects; N = 550)
1−sided adj pval: 0
Frequency
0
50
100
150
−4
−2
0
2
4
Tumor−Normal Expression Difference
hsa−miR−4773, distal
(DIFF_POOR = 0; N0 = 484)
1−sided adj pval: 0
Frequency
0
50
100
150
−4
−2
0
2
4
Tumor−Normal Expression Difference
hsa−miR−4773, distal
(DIFF_POOR = 1; N1 = 64)
1−sided adj pval: 0.334
Frequency
0
5
10
15
20
−4
−2
0
2
4
Tumor−Normal Expression Difference

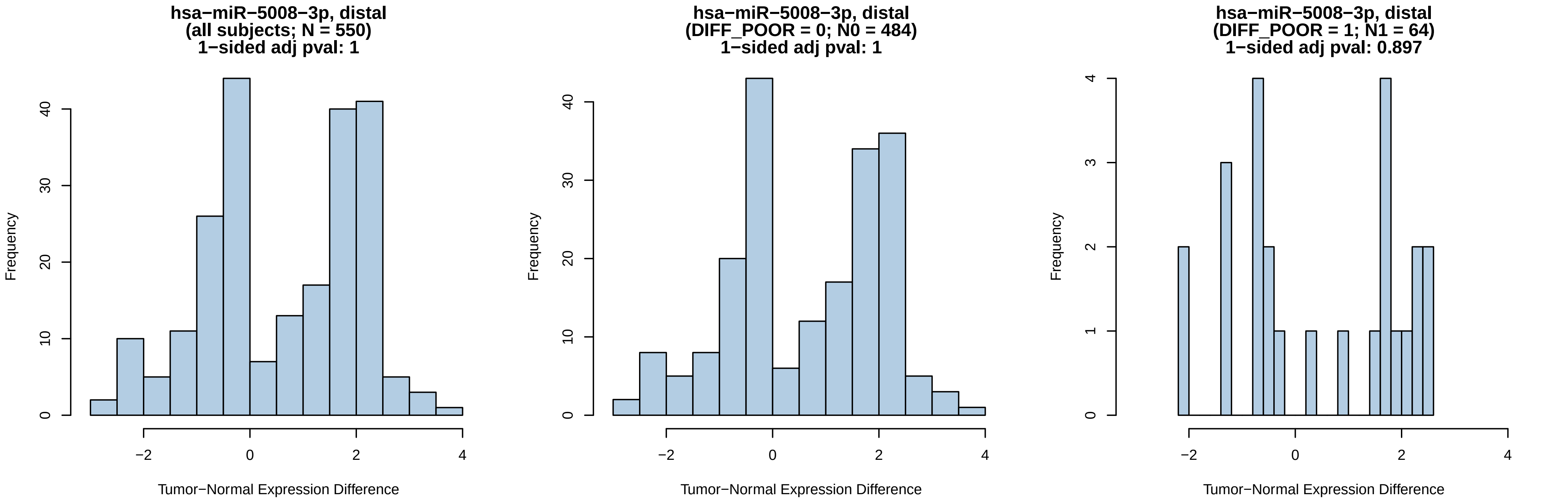

hsa−miR−5008−3p, distal
(all subjects; N = 550)
1−sided adj pval: 1
hsa−miR−5008−3p, distal
(DIFF_POOR = 0; N0 = 484)
1−sided adj pval: 1
hsa−miR−5008−3p, distal
(DIFF_POOR = 1; N1 = 64)
1−sided adj pval: 0.897
Frequency
Tumor−Normal Expression Difference

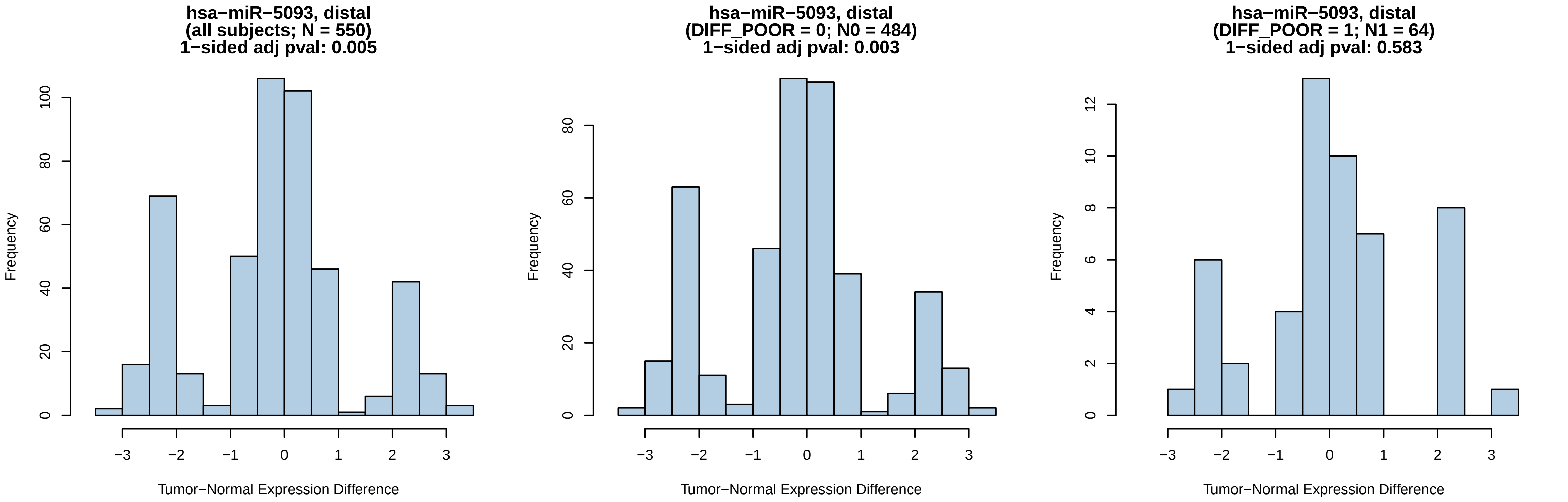

hsa−miR−5093, distal
(all subjects; N = 550)
1−sided adj pval: 0.005
hsa−miR−5093, distal
(DIFF_POOR = 0; N0 = 484)
1−sided adj pval: 0.003
hsa−miR−5093, distal
(DIFF_POOR = 1; N1 = 64)
1−sided adj pval: 0.583
Frequency
Tumor−Normal Expression Difference

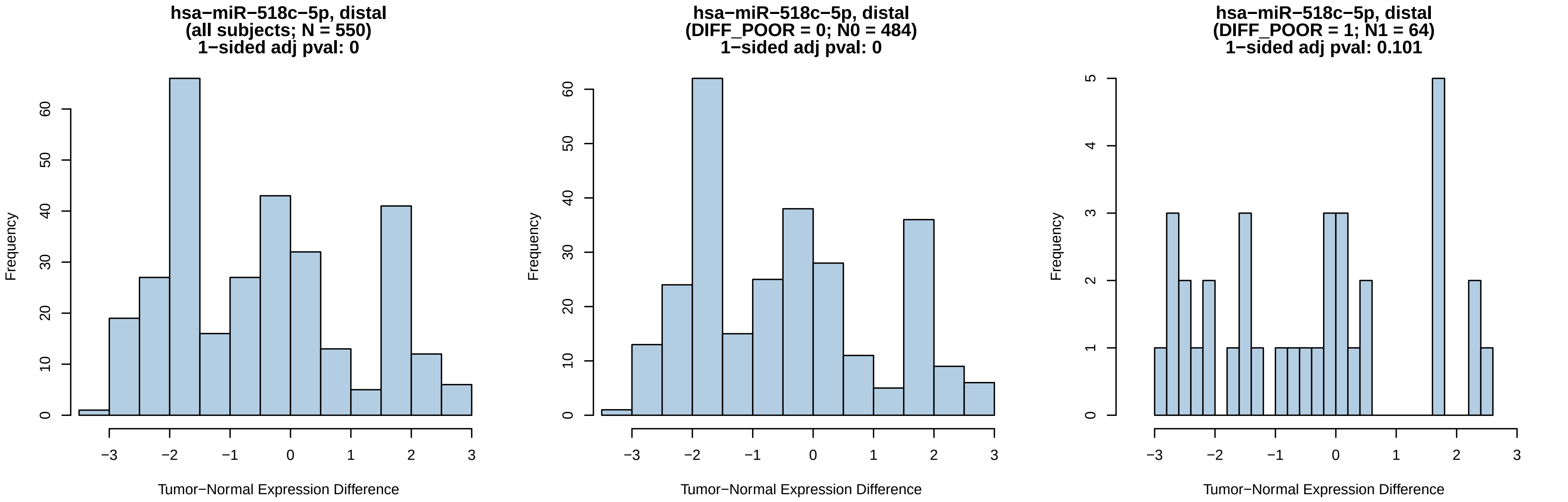

hsa−miR−518c−5p, distal
(all subjects; N = 550)
1−sided adj pval: 0
Frequency
0
10
20
30
40
50
60
−3
−2
−1
0
1
2
3
Tumor−Normal Expression Difference
hsa−miR−518c−5p, distal
(DIFF_POOR = 0; N0 = 484)
1−sided adj pval: 0
Frequency
0
10
20
30
40
50
60
−3
−2
−1
0
1
2
3
Tumor−Normal Expression Difference
hsa−miR−518c−5p, distal
(DIFF_POOR = 1; N1 = 64)
1−sided adj pval: 0.101
Frequency
0
1
2
3
4
5
−3
−2
−1
0
1
2
3
Tumor−Normal Expression Difference

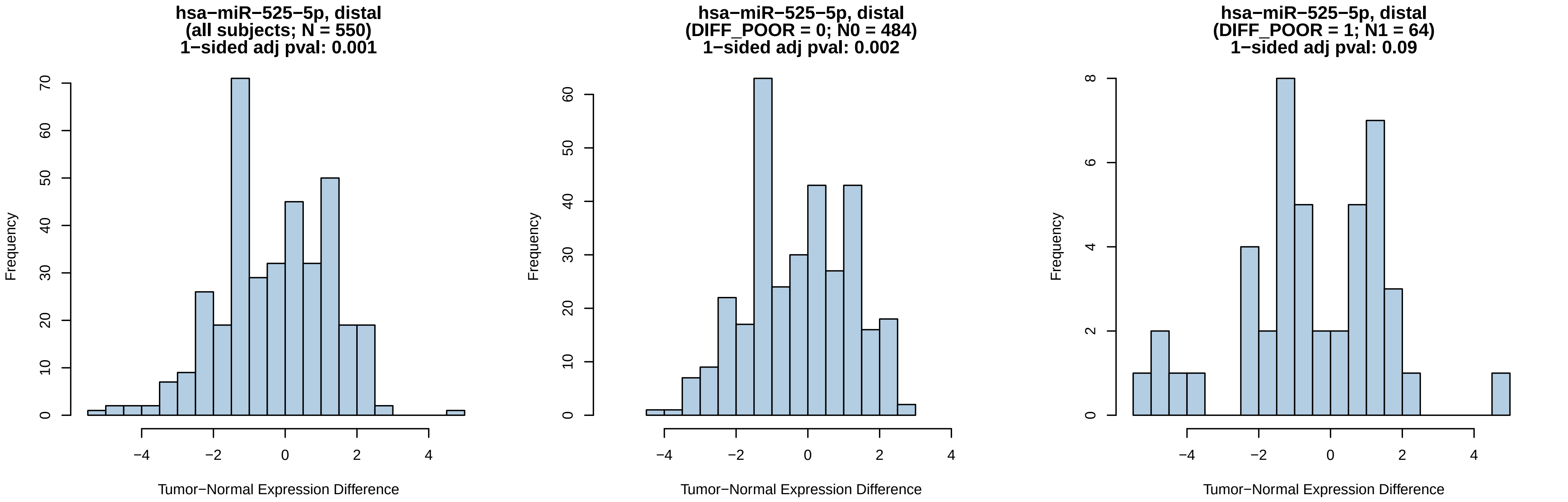
hsa−miR−525−5p, distal
(all subjects; N = 550)
1−sided adj pval: 0.001
hsa−miR−525−5p, distal
(DIFF_POOR = 0; N0 = 484)
1−sided adj pval: 0.002
hsa−miR−525−5p, distal
(DIFF_POOR = 1; N1 = 64)
1−sided adj pval: 0.09
Frequency
Tumor−Normal Expression Difference

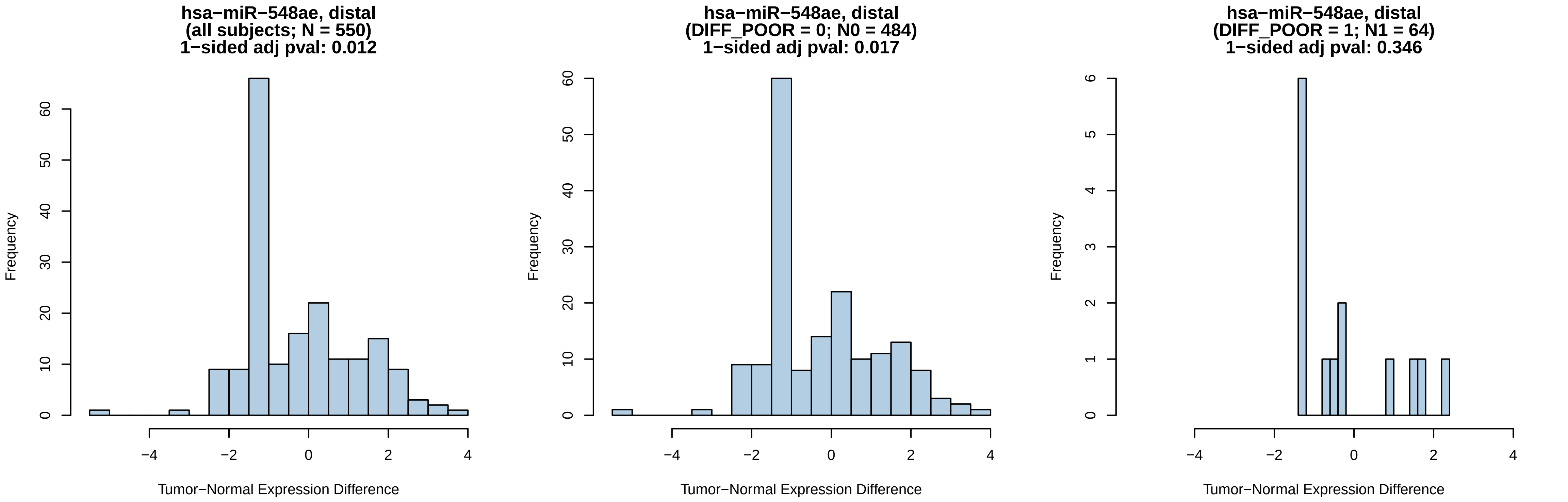
hsa−miR−548ae, distal
(all subjects; N = 550)
1−sided adj pval: 0.012
Frequency
Tumor−Normal Expression Difference
hsa−miR−548ae, distal
(DIFF_POOR = 0; N0 = 484)
1−sided adj pval: 0.017
Frequency
Tumor−Normal Expression Difference
hsa−miR−548ae, distal
(DIFF_POOR = 1; N1 = 64)
1−sided adj pval: 0.346
Frequency
Tumor−Normal Expression Difference

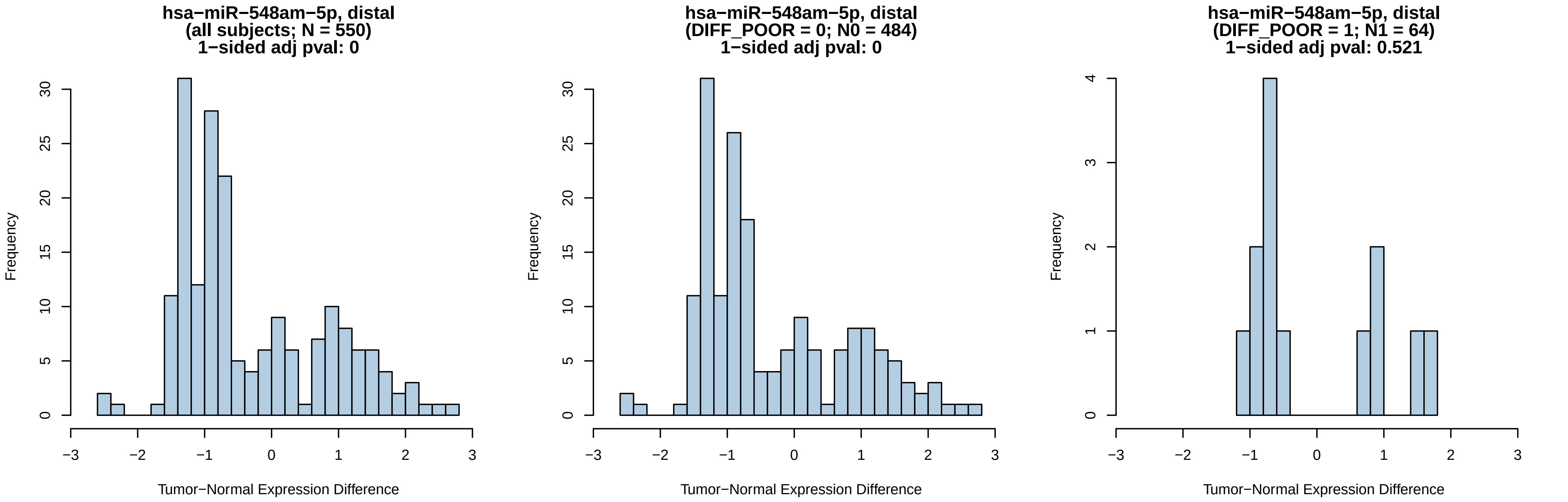

hsa−miR−548am−5p, distal
(all subjects; N = 550)
1−sided adj pval: 0
hsa−miR−548am−5p, distal
(DIFF_POOR = 0; N0 = 484)
1−sided adj pval: 0
hsa−miR−548am−5p, distal
(DIFF_POOR = 1; N1 = 64)
1−sided adj pval: 0.521
Frequency
Tumor−Normal Expression Difference

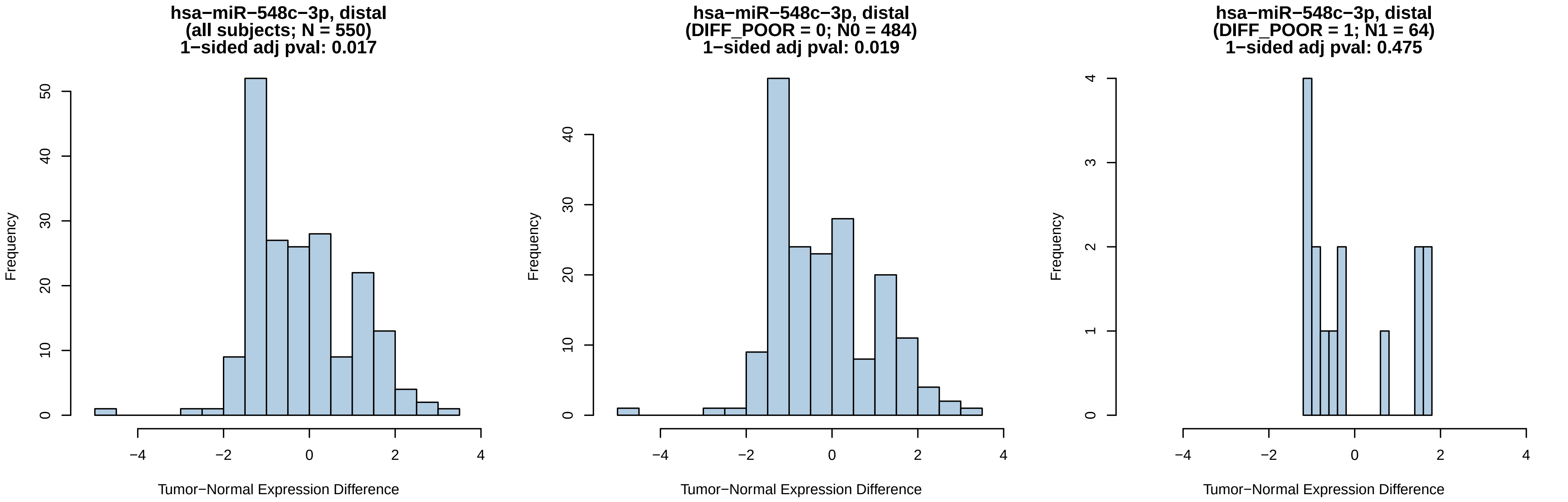
hsa−miR−548c−3p, distal
(all subjects; N = 550)
1−sided adj pval: 0.017
Frequency
Tumor−Normal Expression Difference
hsa−miR−548c−3p, distal
(DIFF_POOR = 0; N0 = 484)
1−sided adj pval: 0.019
Frequency
Tumor−Normal Expression Difference
hsa−miR−548c−3p, distal
(DIFF_POOR = 1; N1 = 64)
1−sided adj pval: 0.475
Frequency
Tumor−Normal Expression Difference

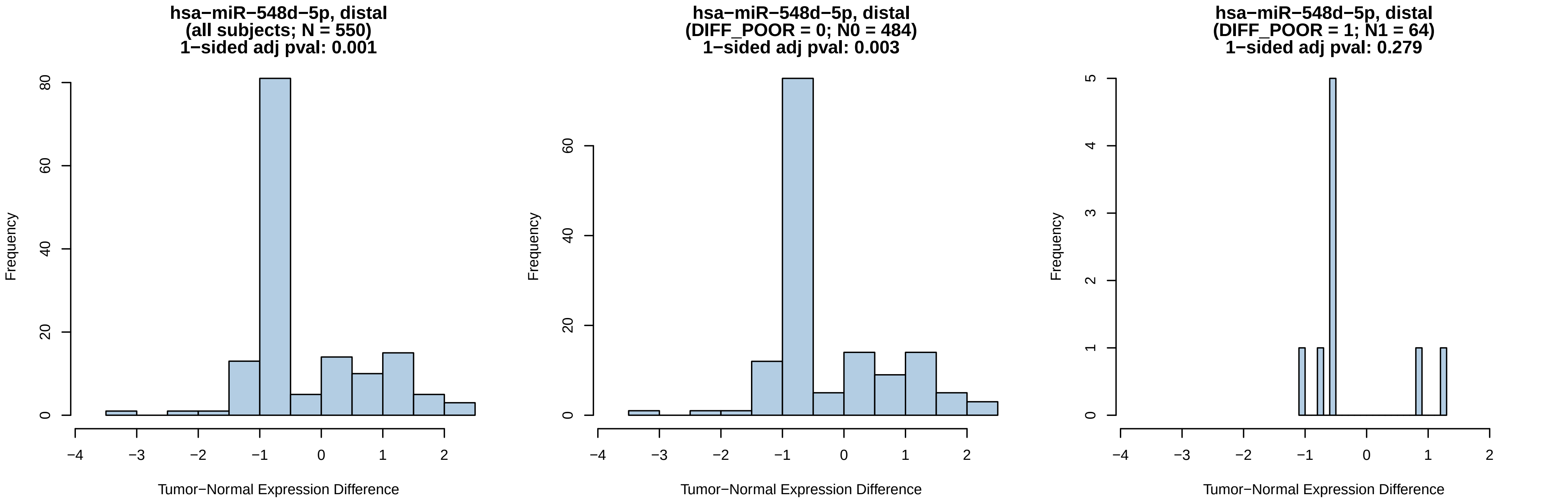
hsa−miR−548d−5p, distal
(all subjects; N = 550)
1−sided adj pval: 0.001
Frequency
Tumor−Normal Expression Difference
hsa−miR−548d−5p, distal
(DIFF_POOR = 0; N0 = 484)
1−sided adj pval: 0.003
Frequency
Tumor−Normal Expression Difference
hsa−miR−548d−5p, distal
(DIFF_POOR = 1; N1 = 64)
1−sided adj pval: 0.279
Frequency
Tumor−Normal Expression Difference

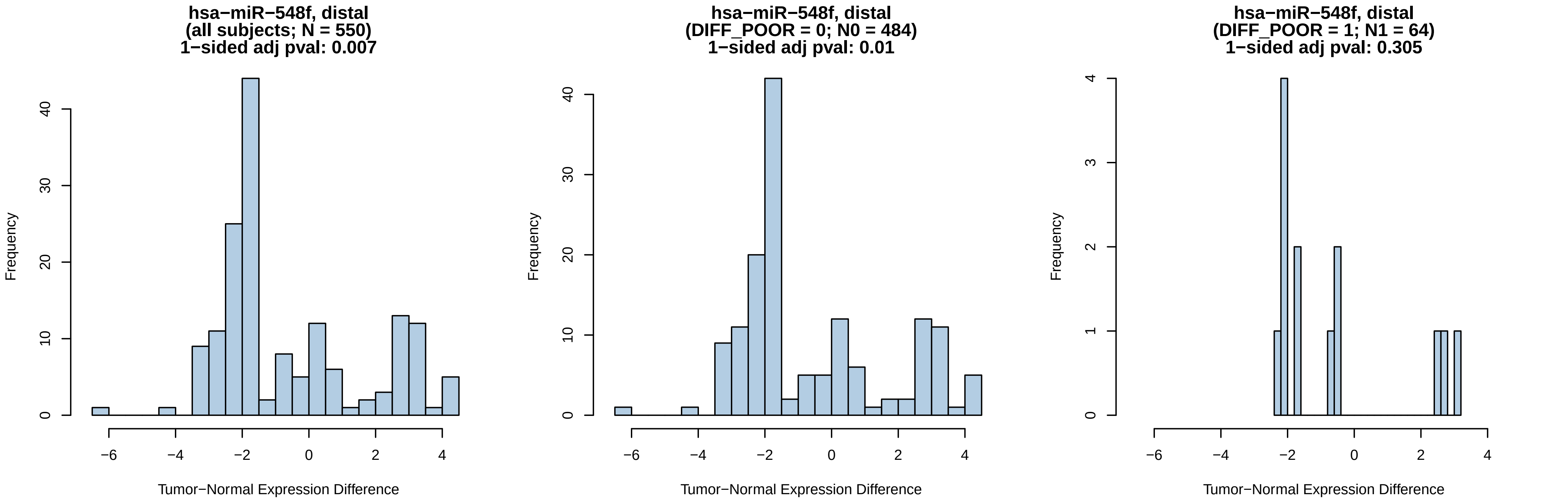

hsa−miR−548f, distal
(all subjects; N = 550)
1−sided adj pval: 0.007
Frequency
Tumor−Normal Expression Difference
hsa−miR−548f, distal
(DIFF_POOR = 0; N0 = 484)
1−sided adj pval: 0.01
Frequency
Tumor−Normal Expression Difference
hsa−miR−548f, distal
(DIFF_POOR = 1; N1 = 64)
1−sided adj pval: 0.305
Frequency
Tumor−Normal Expression Difference

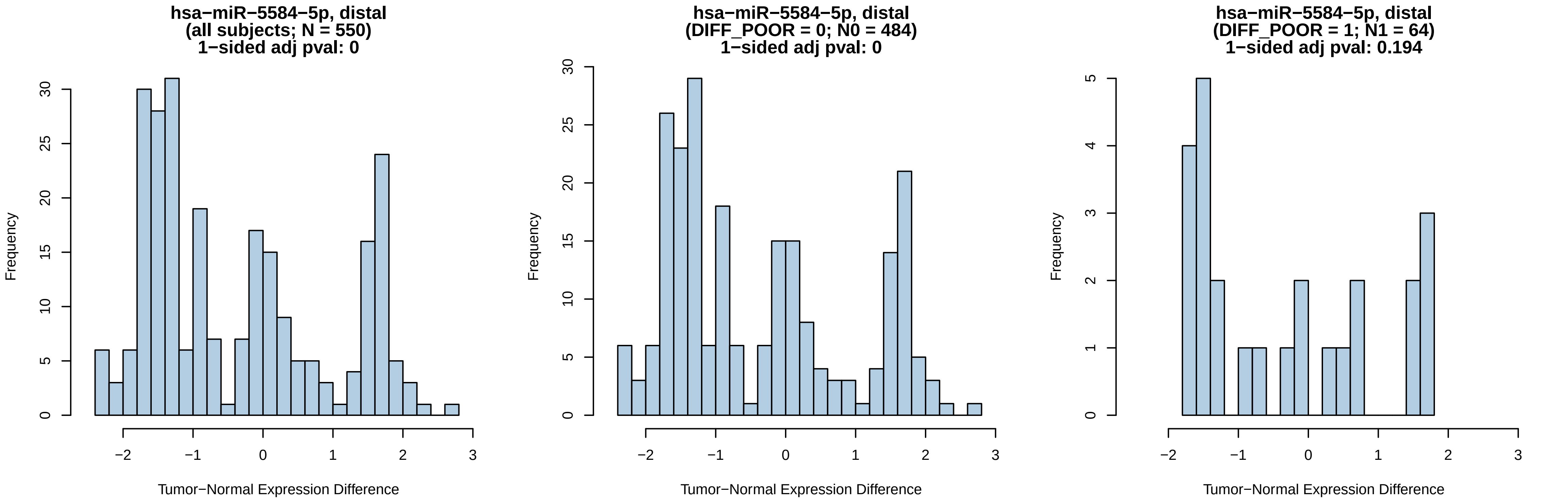
hsa−miR−5584−5p, distal
(all subjects; N = 550)
1−sided adj pval: 0
hsa−miR−5584−5p, distal
(DIFF_POOR = 0; N0 = 484)
1−sided adj pval: 0
hsa−miR−5584−5p, distal
(DIFF_POOR = 1; N1 = 64)
1−sided adj pval: 0.194
Frequency
Tumor−Normal Expression Difference

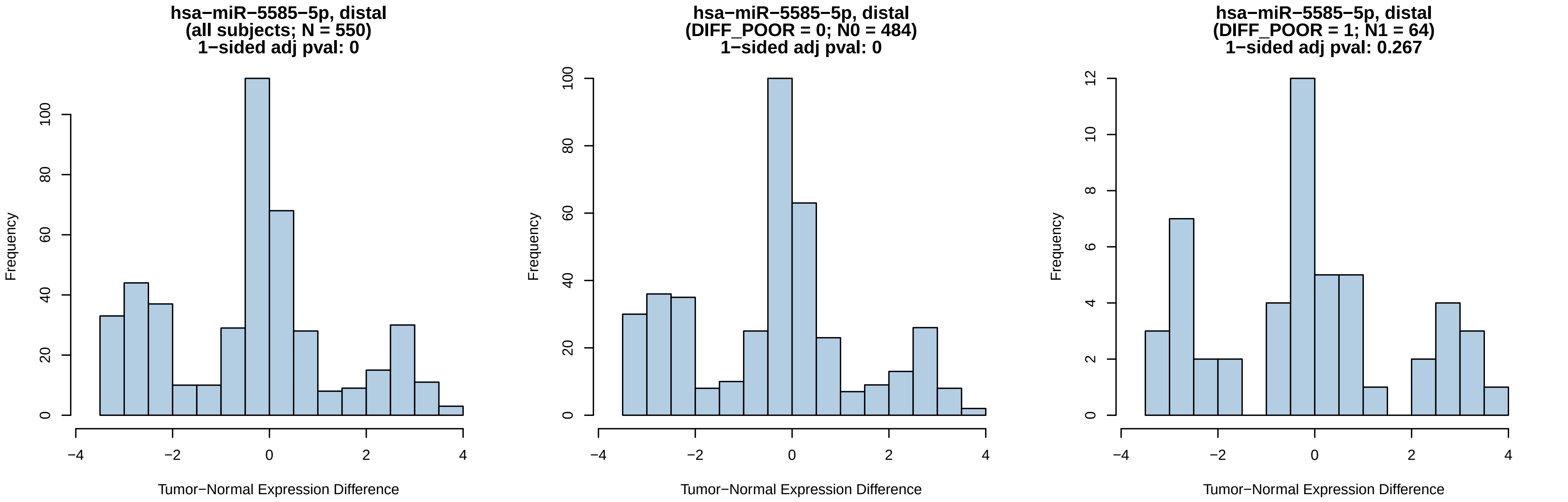
hsa−miR−5585−5p, distal
(all subjects; N = 550)
1−sided adj pval: 0
Frequency
Tumor−Normal Expression Difference
hsa−miR−5585−5p, distal
(DIFF_POOR = 0; N0 = 484)
1−sided adj pval: 0
Frequency
Tumor−Normal Expression Difference
hsa−miR−5585−5p, distal
(DIFF_POOR = 1; N1 = 64)
1−sided adj pval: 0.267
Frequency
Tumor−Normal Expression Difference

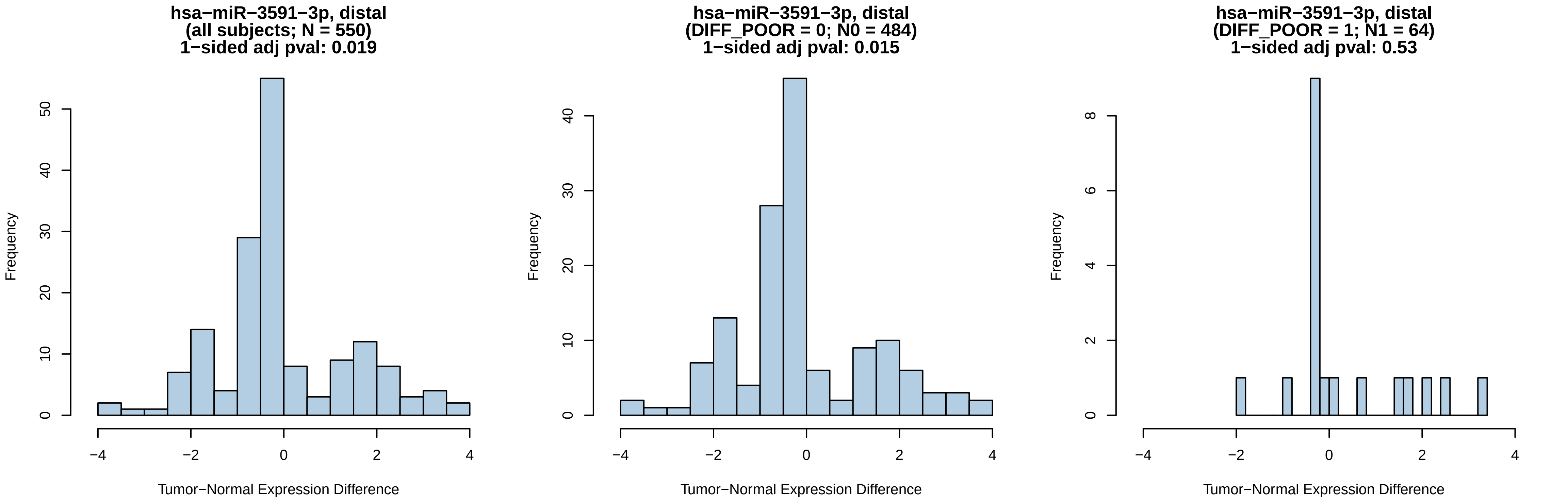
hsa−miR−3591−3p, distal
(all subjects; N = 550)
1−sided adj pval: 0.019
Frequency
Tumor−Normal Expression Difference
hsa−miR−3591−3p, distal
(DIFF_POOR = 0; N0 = 484)
1−sided adj pval: 0.015
Frequency
Tumor−Normal Expression Difference
hsa−miR−3591−3p, distal
(DIFF_POOR = 1; N1 = 64)
1−sided adj pval: 0.53
Frequency
Tumor−Normal Expression Difference

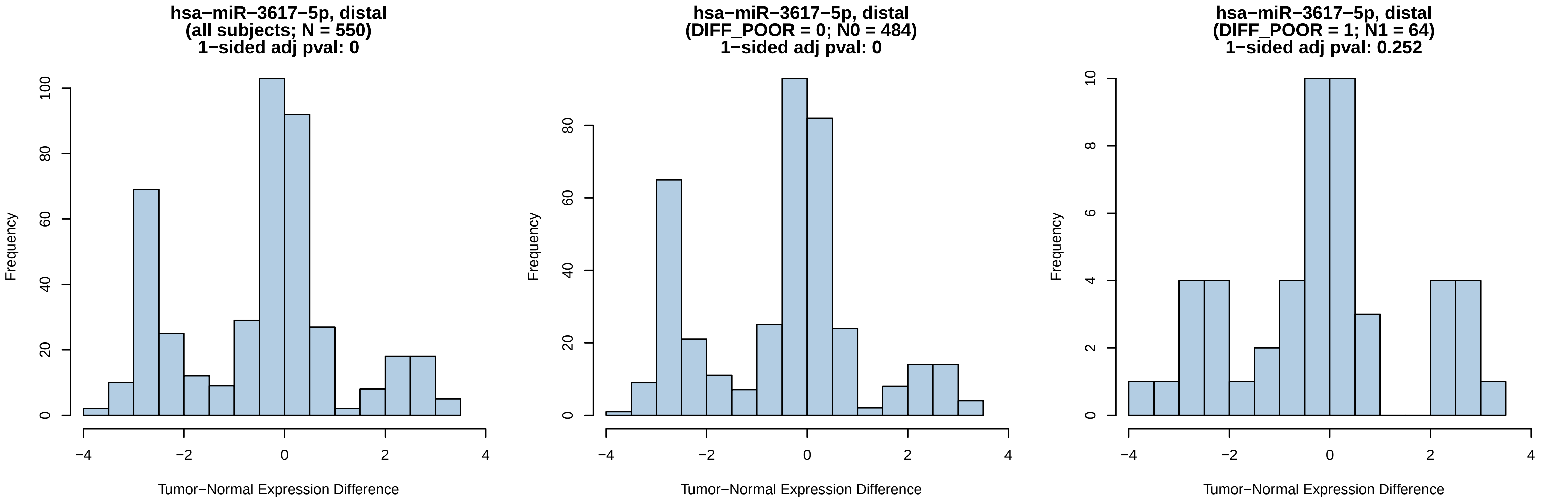
hsa−miR−3617−5p, distal
(all subjects; N = 550)
1−sided adj pval: 0
Frequency
Tumor−Normal Expression Difference
hsa−miR−3617−5p, distal
(DIFF_POOR = 0; N0 = 484)
1−sided adj pval: 0
Frequency
Tumor−Normal Expression Difference
hsa−miR−3617−5p, distal
(DIFF_POOR = 1; N1 = 64)
1−sided adj pval: 0.252
Frequency
Tumor−Normal Expression Difference

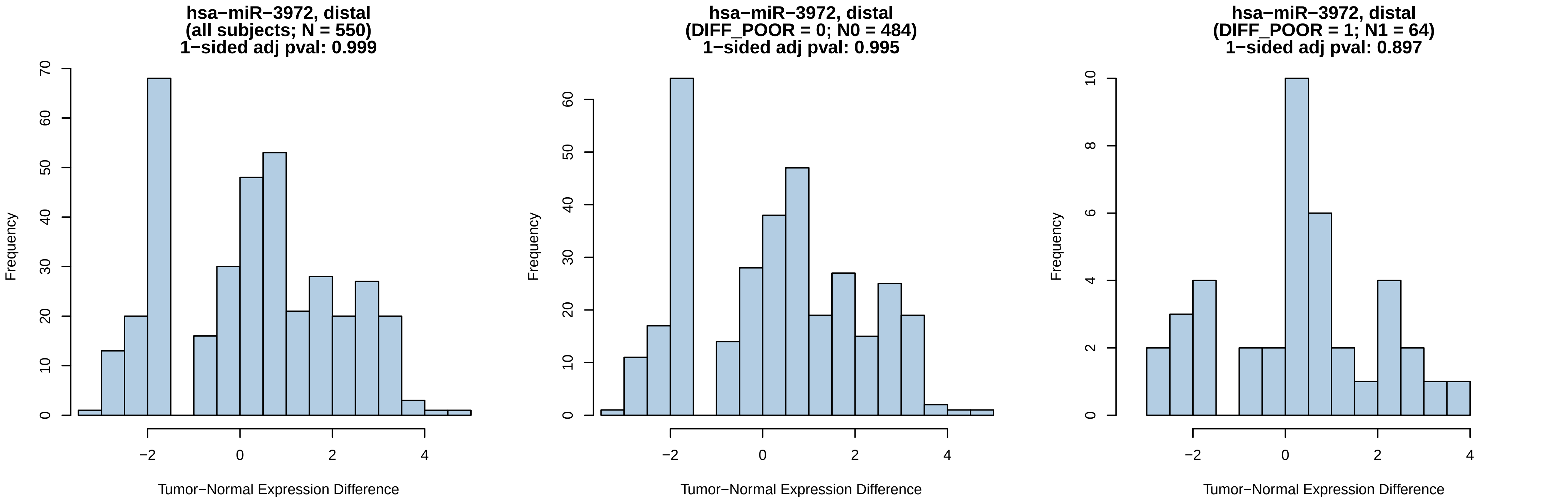
hsa−miR−3972, distal
(all subjects; N = 550)
1−sided adj pval: 0.999
Frequency
Tumor−Normal Expression Difference
hsa−miR−3972, distal
(DIFF_POOR = 0; N0 = 484)
1−sided adj pval: 0.995
Frequency
Tumor−Normal Expression Difference
hsa−miR−3972, distal
(DIFF_POOR = 1; N1 = 64)
1−sided adj pval: 0.897
Frequency
Tumor−Normal Expression Difference

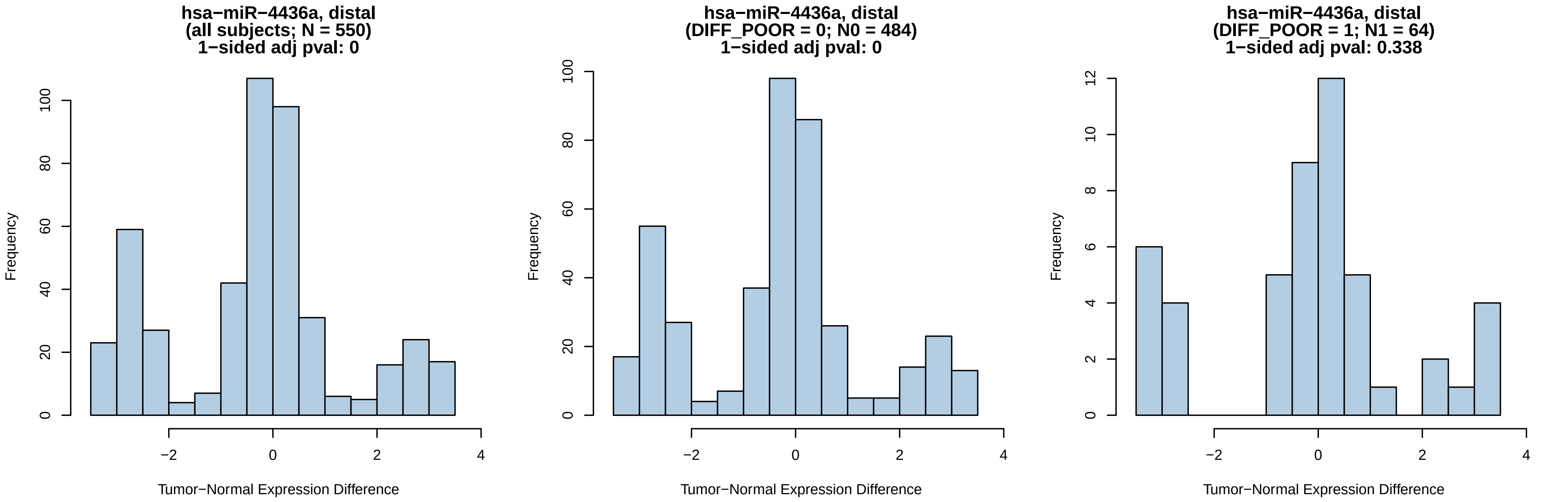

hsa−miR−4436a, distal
(all subjects; N = 550)
1−sided adj pval: 0
Frequency
0
20
40
60
80
100
−2
0
2
4
Tumor−Normal Expression Difference
hsa−miR−4436a, distal
(DIFF_POOR = 0; N0 = 484)
1−sided adj pval: 0
Frequency
0
20
40
60
80
100
−2
0
2
4
Tumor−Normal Expression Difference
hsa−miR−4436a, distal
(DIFF_POOR = 1; N1 = 64)
1−sided adj pval: 0.338
Frequency
0
2
4
6
8
10
12
−2
0
2
4
Tumor−Normal Expression Difference

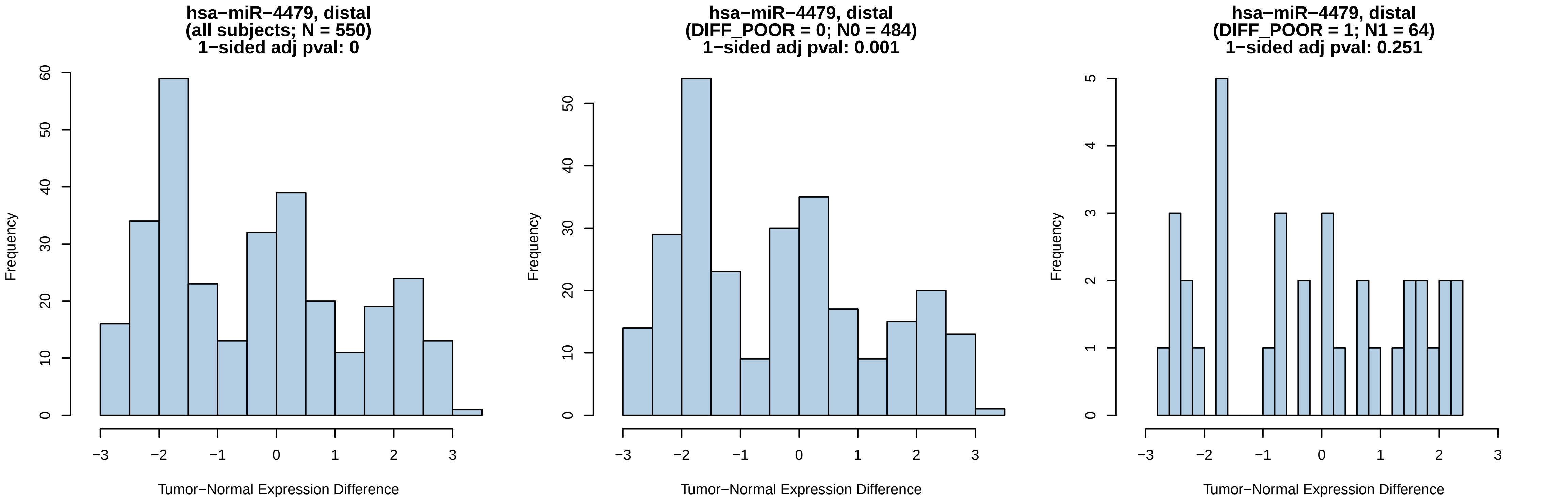

hsa−miR−4479, distal
(all subjects; N = 550)
1−sided adj pval: 0
Frequency
Tumor−Normal Expression Difference
hsa−miR−4479, distal
(DIFF_POOR = 0; N0 = 484)
1−sided adj pval: 0.001
Frequency
Tumor−Normal Expression Difference
hsa−miR−4479, distal
(DIFF_POOR = 1; N1 = 64)
1−sided adj pval: 0.251
Frequency
Tumor−Normal Expression Difference

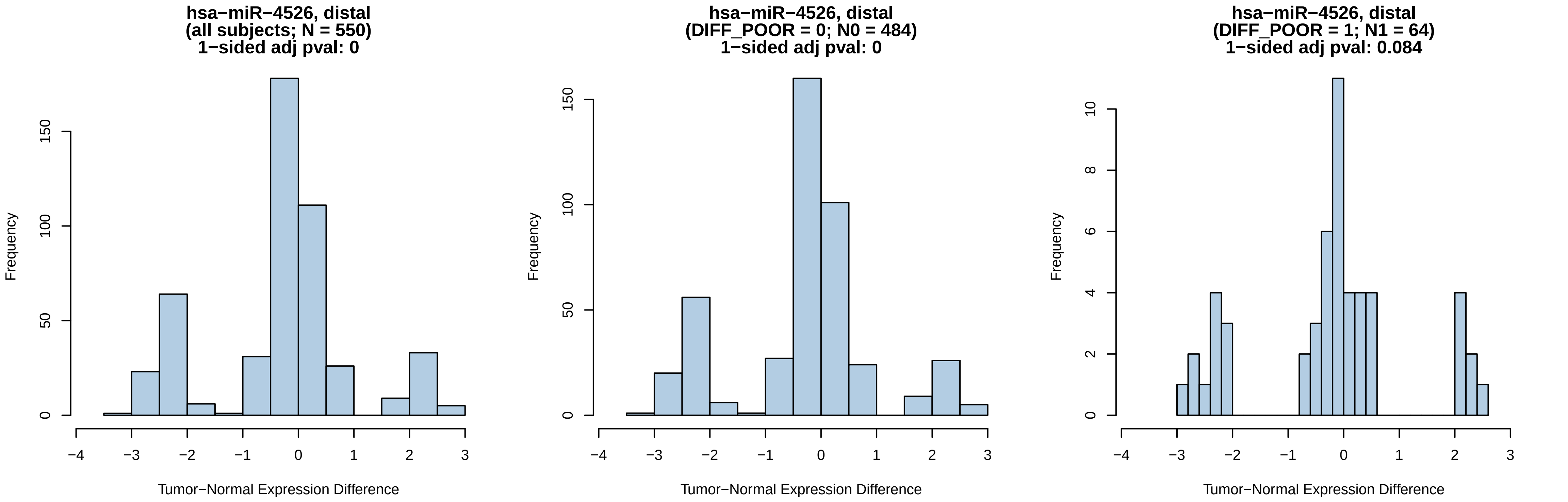
hsa−miR−4526, distal
(all subjects; N = 550)
1−sided adj pval: 0
hsa−miR−4526, distal
(DIFF_POOR = 0; N0 = 484)
1−sided adj pval: 0
hsa−miR−4526, distal
(DIFF_POOR = 1; N1 = 64)
1−sided adj pval: 0.084
Frequency
Tumor−Normal Expression Difference

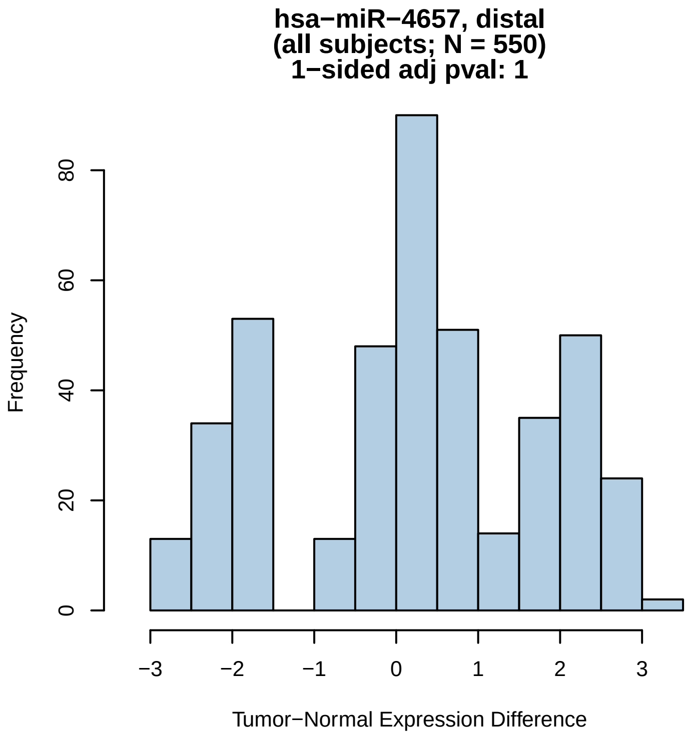

hsa−miR−4657, distal
(all subjects; N = 550)
1−sided adj pval: 1
Frequency
Tumor−Normal Expression Difference


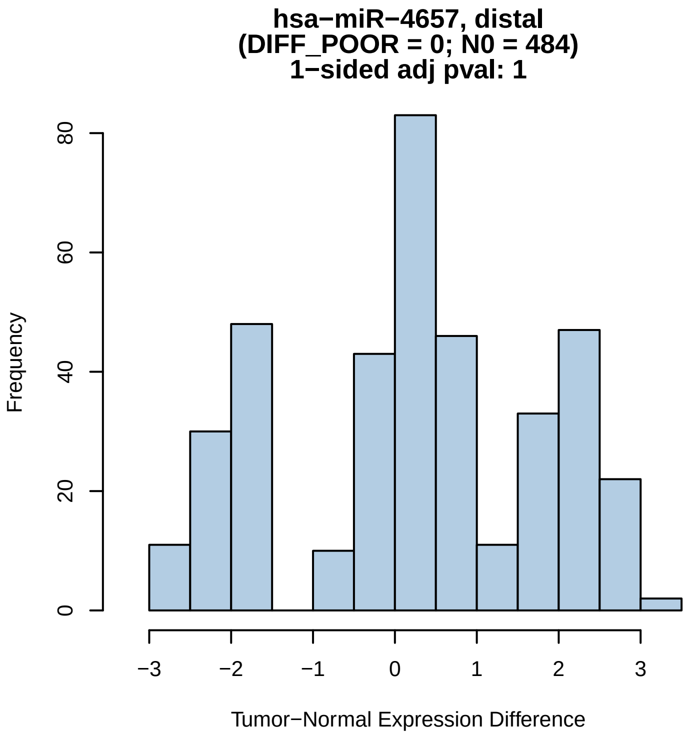

hsa−miR−4657, distal
(DIFF_POOR = 0; N0 = 484)
1−sided adj pval: 1
Frequency
Tumor−Normal Expression Difference


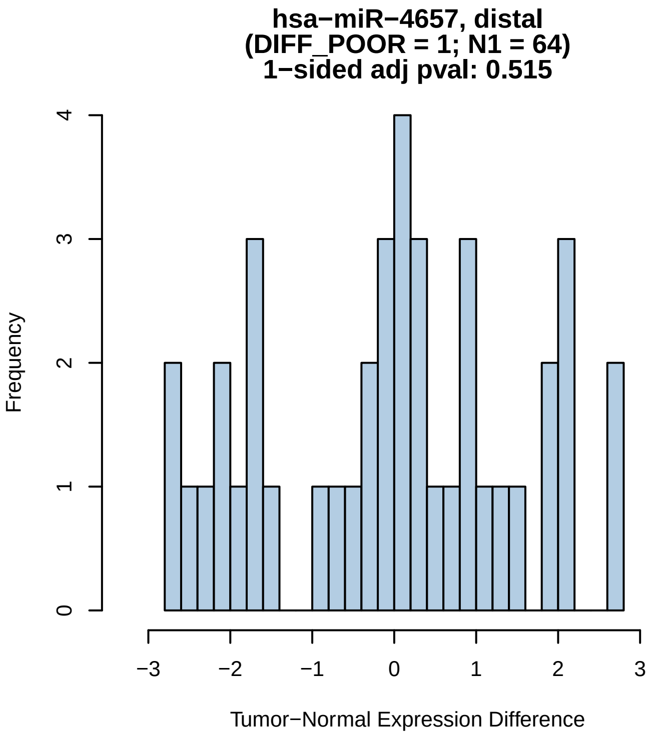

hsa−miR−4657, distal
(DIFF_POOR = 1; N1 = 64)
1−sided adj pval: 0.515
Frequency
Tumor−Normal Expression Difference

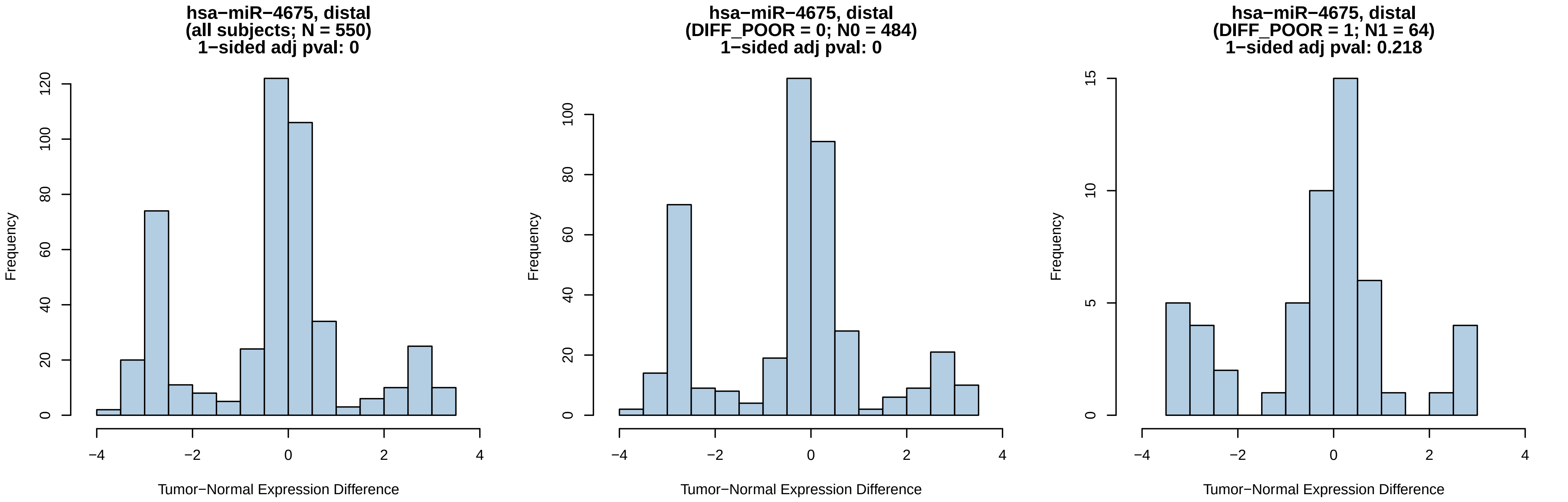

hsa−miR−4675, distal
(all subjects; N = 550)
1−sided adj pval: 0
Frequency
0
20
40
60
80
100
120
−4
−2
0
2
4
Tumor−Normal Expression Difference
hsa−miR−4675, distal
(DIFF_POOR = 0; N0 = 484)
1−sided adj pval: 0
Frequency
0
20
40
60
80
100
−4
−2
0
2
4
Tumor−Normal Expression Difference
hsa−miR−4675, distal
(DIFF_POOR = 1; N1 = 64)
1−sided adj pval: 0.218
Frequency
0
5
10
15
−4
−2
0
2
4
Tumor−Normal Expression Difference

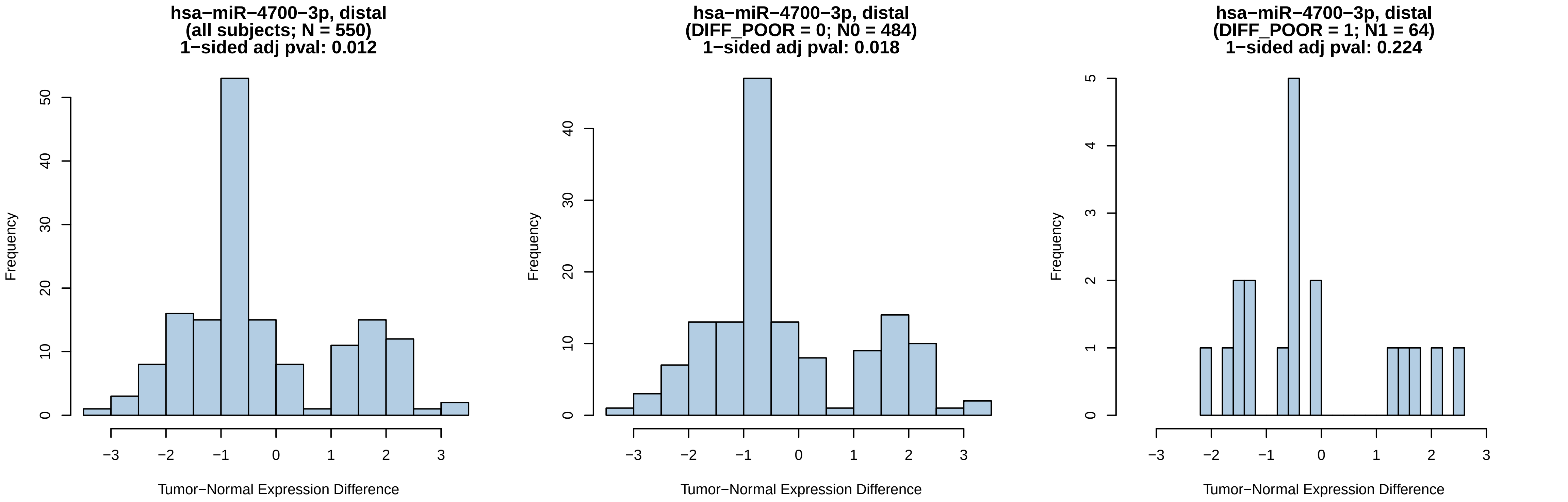

hsa−miR−4700−3p, distal
(all subjects; N = 550)
1−sided adj pval: 0.012
Frequency
Tumor−Normal Expression Difference
hsa−miR−4700−3p, distal
(DIFF_POOR = 0; N0 = 484)
1−sided adj pval: 0.018
Frequency
Tumor−Normal Expression Difference
hsa−miR−4700−3p, distal
(DIFF_POOR = 1; N1 = 64)
1−sided adj pval: 0.224
Frequency
Tumor−Normal Expression Difference

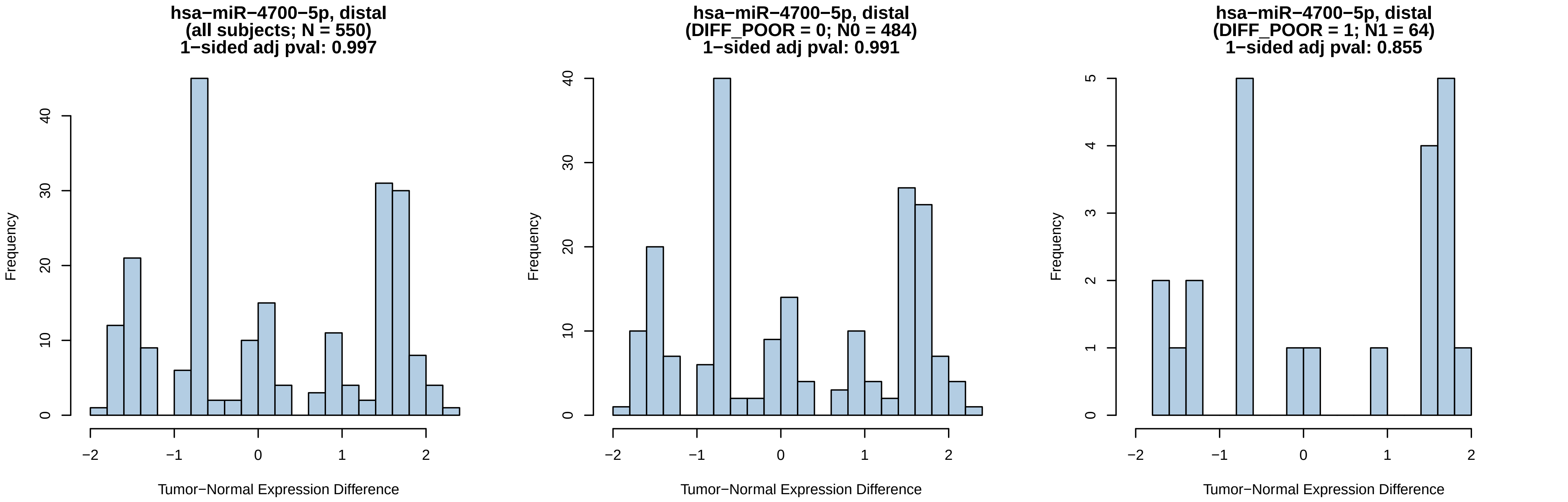
hsa−miR−4700−5p, distal
(all subjects; N = 550)
1−sided adj pval: 0.997
hsa−miR−4700−5p, distal
(DIFF_POOR = 0; N0 = 484)
1−sided adj pval: 0.991
hsa−miR−4700−5p, distal
(DIFF_POOR = 1; N1 = 64)
1−sided adj pval: 0.855
Frequency
Tumor−Normal Expression Difference

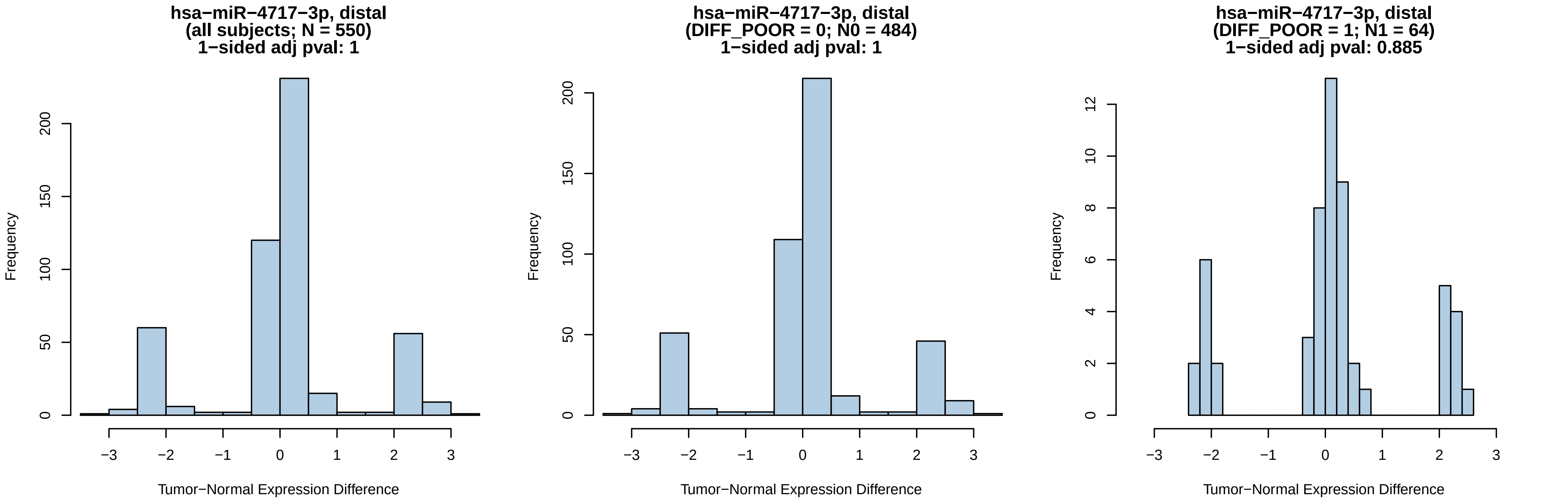
hsa−miR−4717−3p, distal
(all subjects; N = 550)
1−sided adj pval: 1
Frequency
Tumor−Normal Expression Difference
hsa−miR−4717−3p, distal
(DIFF_POOR = 0; N0 = 484)
1−sided adj pval: 1
Frequency
Tumor−Normal Expression Difference
hsa−miR−4717−3p, distal
(DIFF_POOR = 1; N1 = 64)
1−sided adj pval: 0.885
Frequency
Tumor−Normal Expression Difference

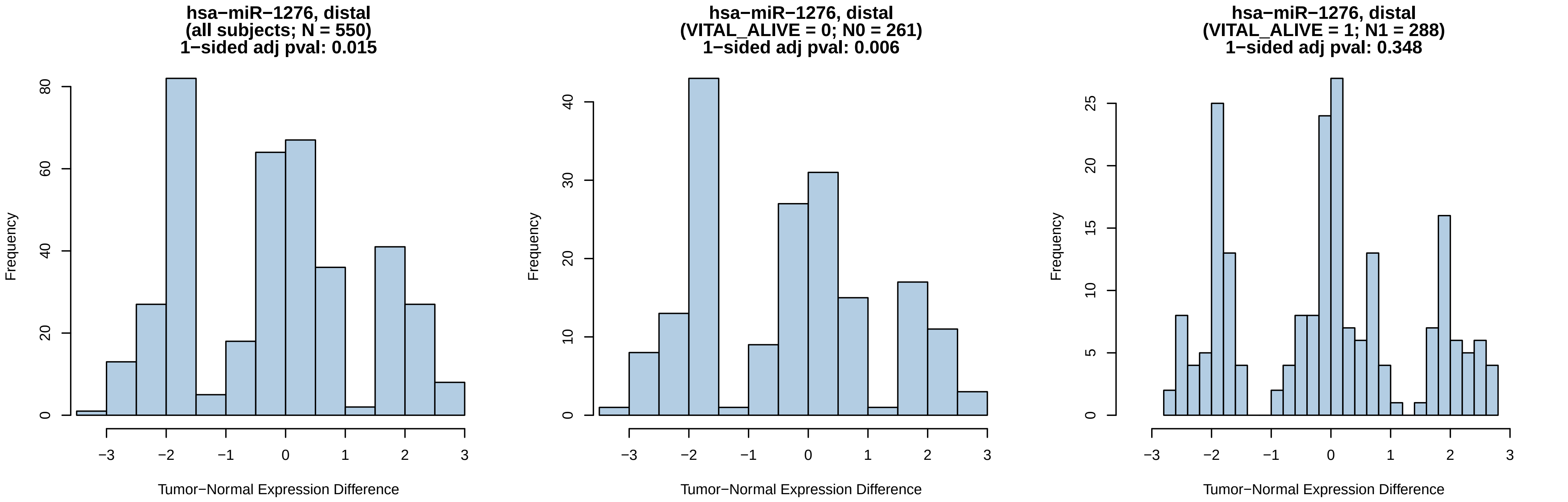
hsa−miR−1276, distal
(all subjects; N = 550)
1−sided adj pval: 0.015
Frequency
Tumor−Normal Expression Difference
hsa−miR−1276, distal
(VITAL_ALIVE = 0; N0 = 261)
1−sided adj pval: 0.006
Frequency
Tumor−Normal Expression Difference
hsa−miR−1276, distal
(VITAL_ALIVE = 1; N1 = 288)
1−sided adj pval: 0.348
Frequency
Tumor−Normal Expression Difference

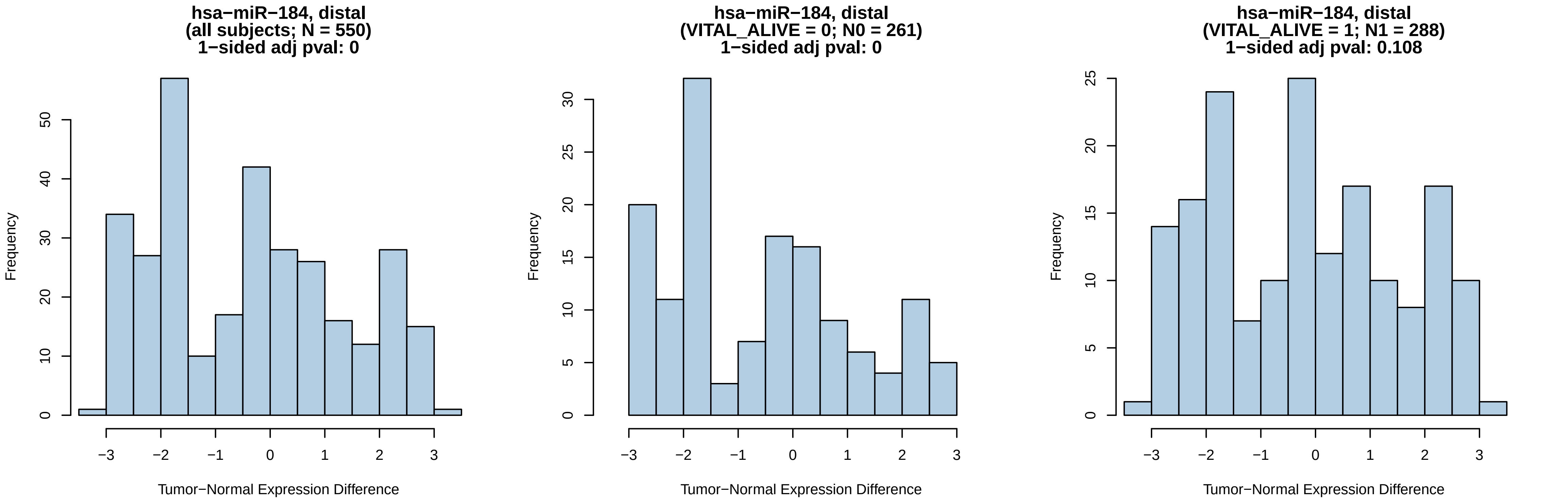

hsa−miR−184, distal
(all subjects; N = 550)
1−sided adj pval: 0
Frequency
0
10
20
30
40
50
−3
−2
−1
0
1
2
3
Tumor−Normal Expression Difference
hsa−miR−184, distal
(VITAL_ALIVE = 0; N0 = 261)
1−sided adj pval: 0
Frequency
0
5
10
15
20
25
30
−3
−2
−1
0
1
2
3
Tumor−Normal Expression Difference
hsa−miR−184, distal
(VITAL_ALIVE = 1; N1 = 288)
1−sided adj pval: 0.108
Frequency
0
5
10
15
20
25
−3
−2
−1
0
1
2
3
Tumor−Normal Expression Difference

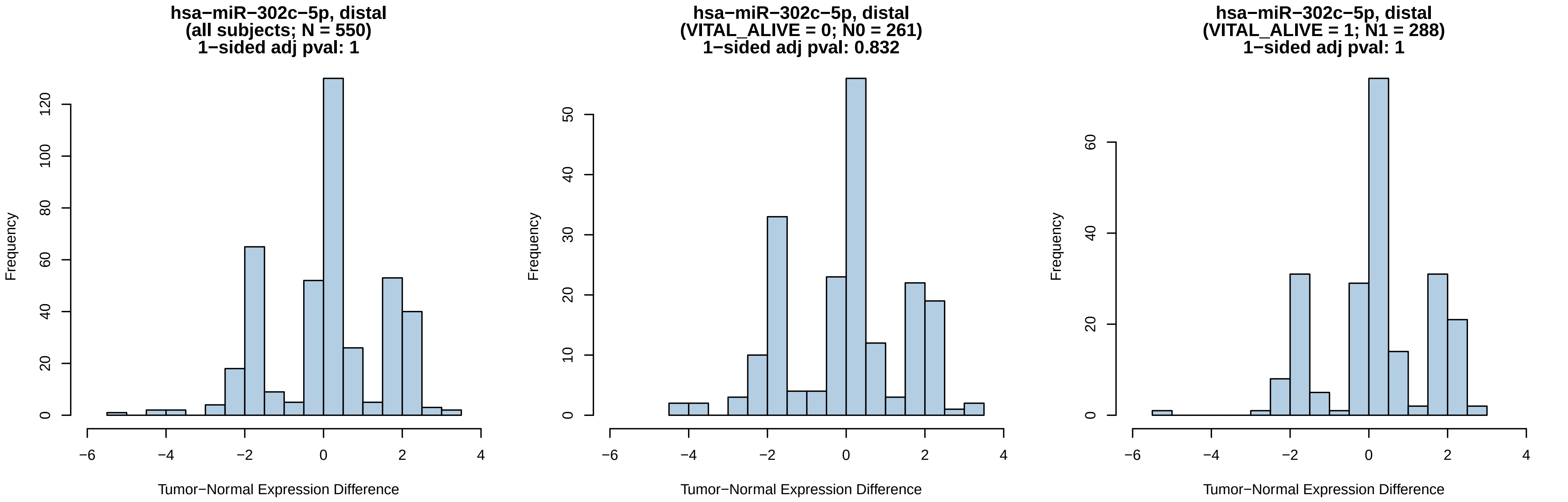
hsa−miR−302c−5p, distal
(all subjects; N = 550)
1−sided adj pval: 1
Frequency
Tumor−Normal Expression Difference
hsa−miR−302c−5p, distal
(VITAL_ALIVE = 0; N0 = 261)
1−sided adj pval: 0.832
Frequency
Tumor−Normal Expression Difference
hsa−miR−302c−5p, distal
(VITAL_ALIVE = 1; N1 = 288)
1−sided adj pval: 1
Frequency
Tumor−Normal Expression Difference

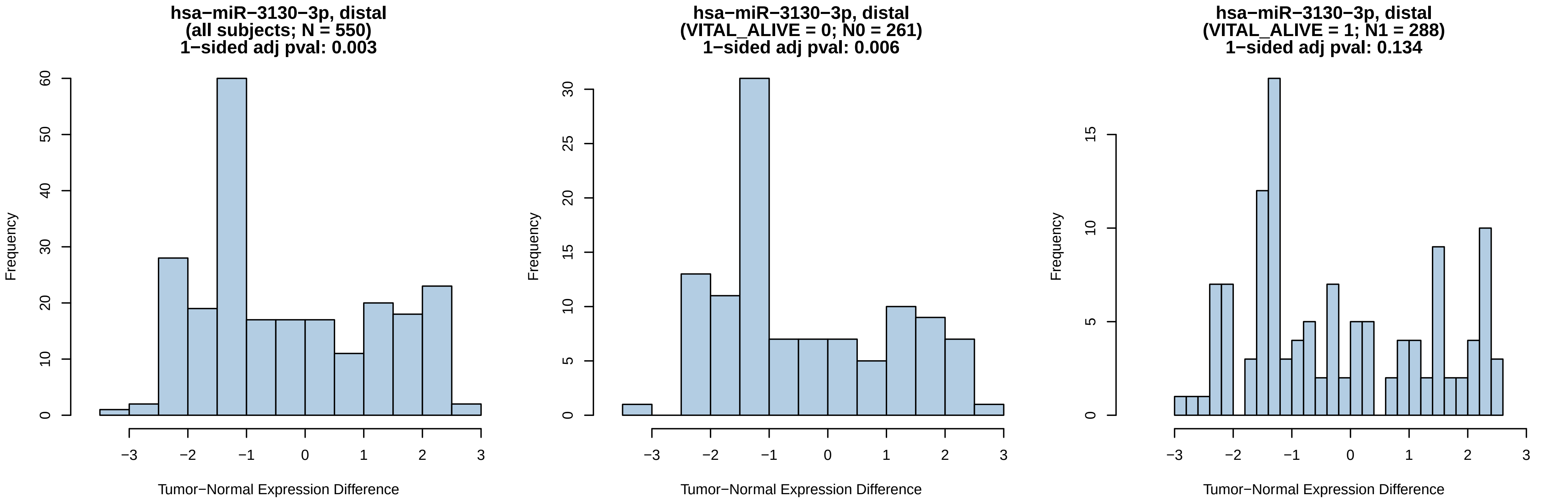
hsa−miR−3130−3p, distal
(all subjects; N = 550)
1−sided adj pval: 0.003
Frequency
Tumor−Normal Expression Difference
hsa−miR−3130−3p, distal
(VITAL_ALIVE = 0; N0 = 261)
1−sided adj pval: 0.006
Frequency
Tumor−Normal Expression Difference
hsa−miR−3130−3p, distal
(VITAL_ALIVE = 1; N1 = 288)
1−sided adj pval: 0.134
Frequency
Tumor−Normal Expression Difference

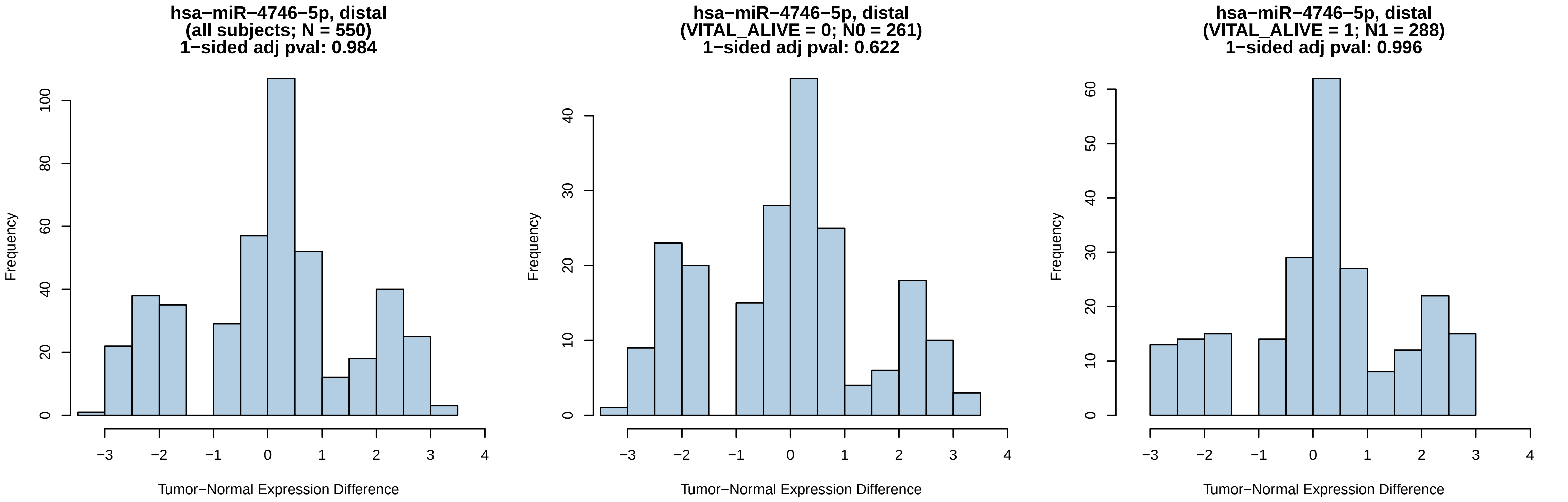

hsa−miR−4746−5p, distal
(all subjects; N = 550)
1−sided adj pval: 0.984
hsa−miR−4746−5p, distal
(VITAL_ALIVE = 0; N0 = 261)
1−sided adj pval: 0.622
hsa−miR−4746−5p, distal
(VITAL_ALIVE = 1; N1 = 288)
1−sided adj pval: 0.996
Frequency
Tumor−Normal Expression Difference

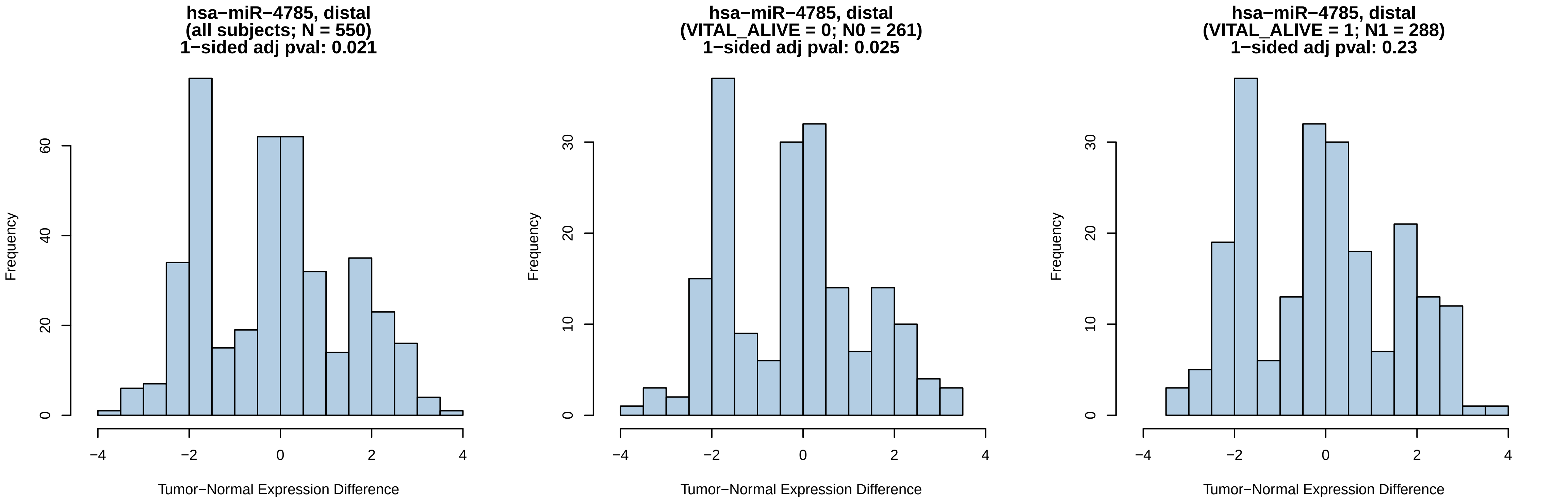
hsa−miR−4785, distal
(all subjects; N = 550)
1−sided adj pval: 0.021
hsa−miR−4785, distal
(VITAL_ALIVE = 0; N0 = 261)
1−sided adj pval: 0.025
hsa−miR−4785, distal
(VITAL_ALIVE = 1; N1 = 288)
1−sided adj pval: 0.23
Frequency
Tumor−Normal Expression Difference

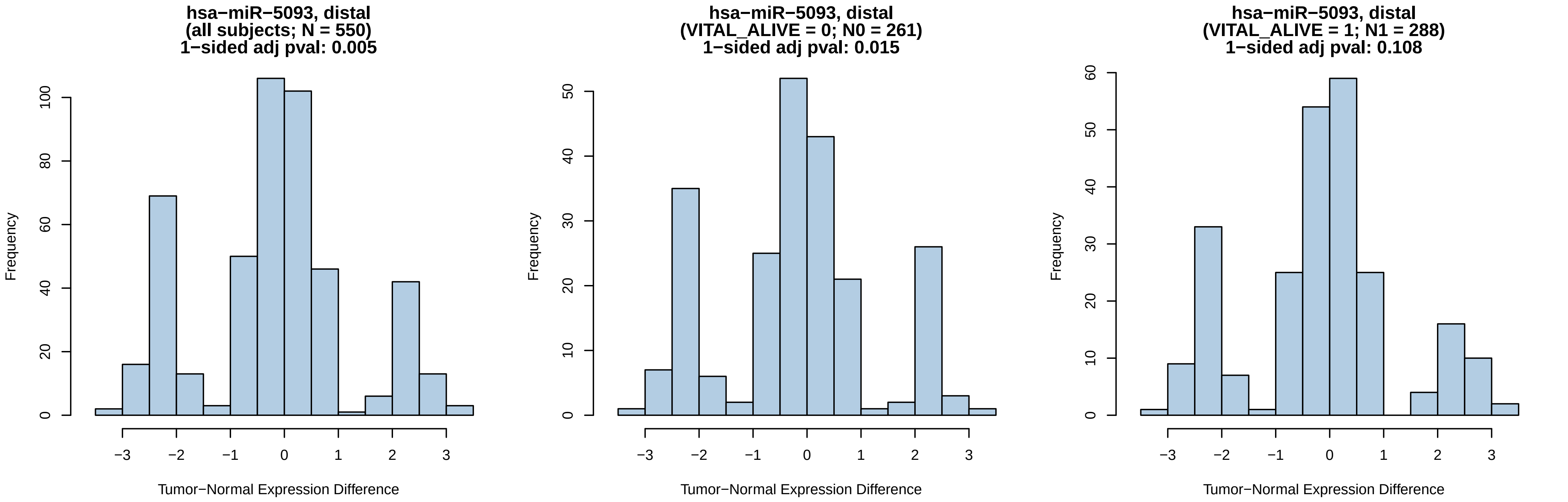

hsa−miR−5093, distal
(all subjects; N = 550)
1−sided adj pval: 0.005
Frequency
0
20
40
60
80
100
−3
−2
−1
0
1
2
3
Tumor−Normal Expression Difference
hsa−miR−5093, distal
(VITAL_ALIVE = 0; N0 = 261)
1−sided adj pval: 0.015
Frequency
0
10
20
30
40
50
−3
−2
−1
0
1
2
3
Tumor−Normal Expression Difference
hsa−miR−5093, distal
(VITAL_ALIVE = 1; N1 = 288)
1−sided adj pval: 0.108
Frequency
0
10
20
30
40
50
60
−3
−2
−1
0
1
2
3
Tumor−Normal Expression Difference

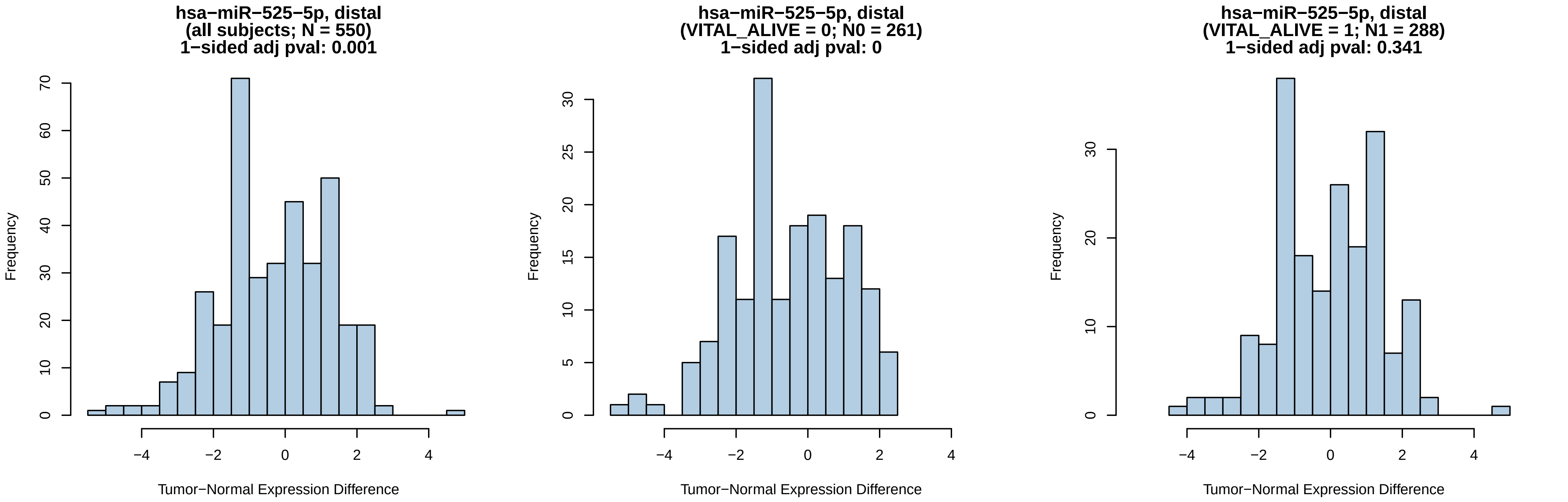
hsa−miR−525−5p, distal
(all subjects; N = 550)
1−sided adj pval: 0.001
Frequency
Tumor−Normal Expression Difference
hsa−miR−525−5p, distal
(VITAL_ALIVE = 0; N0 = 261)
1−sided adj pval: 0
Frequency
Tumor−Normal Expression Difference
hsa−miR−525−5p, distal
(VITAL_ALIVE = 1; N1 = 288)
1−sided adj pval: 0.341
Frequency
Tumor−Normal Expression Difference

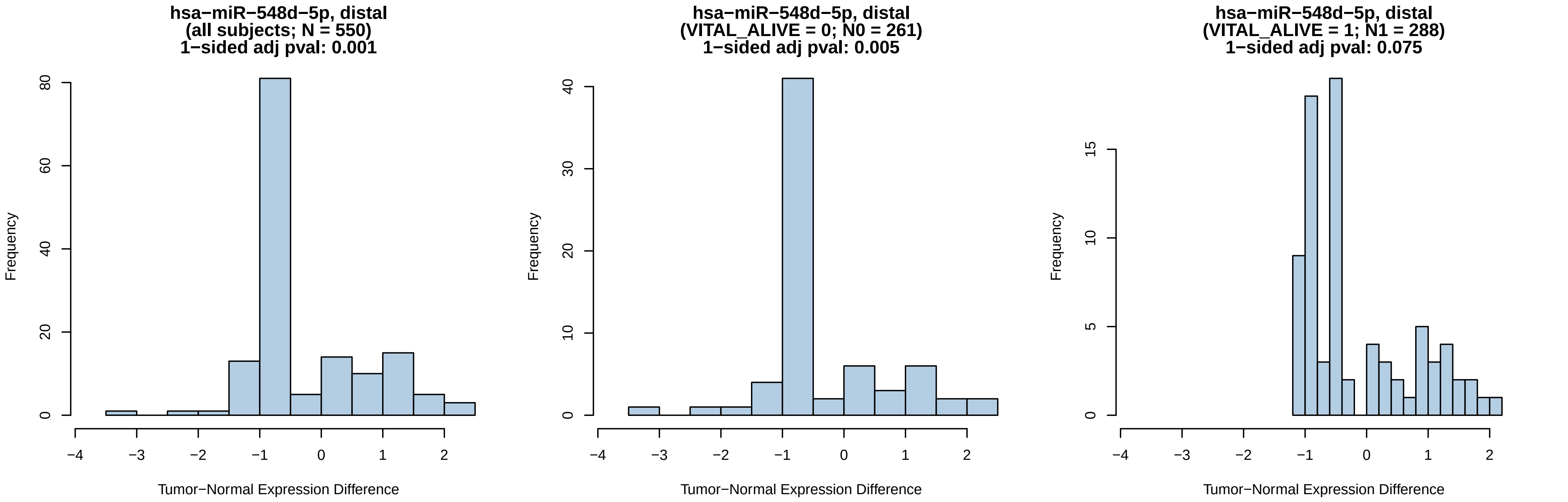
hsa−miR−548d−5p, distal
(all subjects; N = 550)
1−sided adj pval: 0.001
Frequency
Tumor−Normal Expression Difference
hsa−miR−548d−5p, distal
(VITAL_ALIVE = 0; N0 = 261)
1−sided adj pval: 0.005
Frequency
Tumor−Normal Expression Difference
hsa−miR−548d−5p, distal
(VITAL_ALIVE = 1; N1 = 288)
1−sided adj pval: 0.075
Frequency
Tumor−Normal Expression Difference

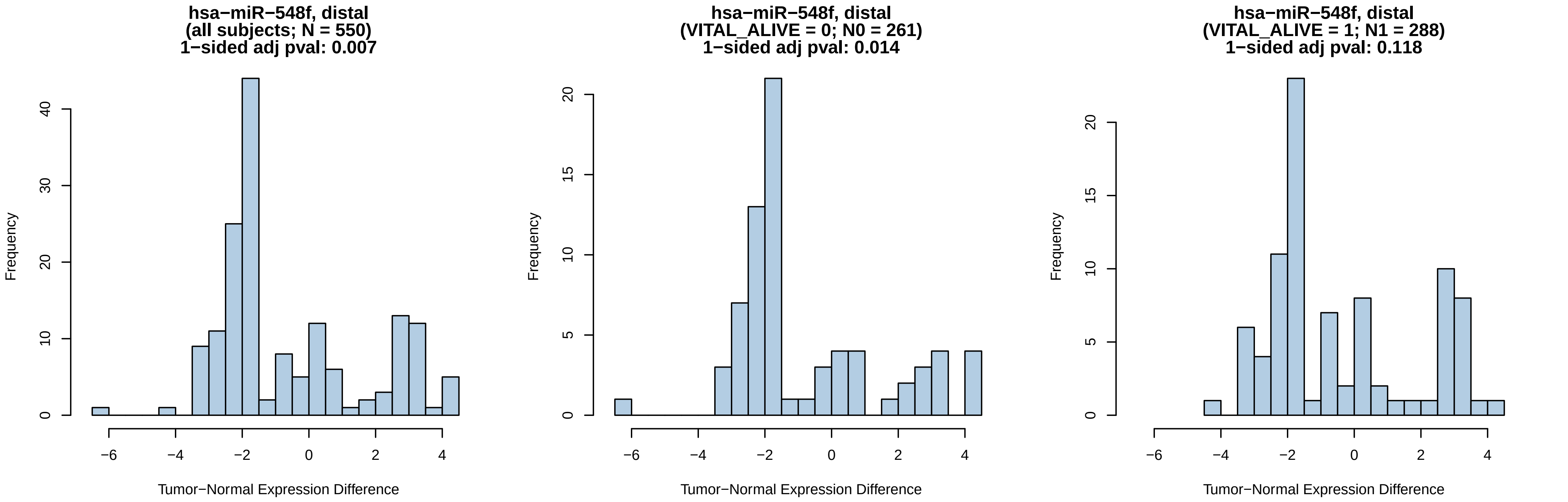
hsa−miR−548f, distal
(all subjects; N = 550)
1−sided adj pval: 0.007
Frequency
Tumor−Normal Expression Difference
hsa−miR−548f, distal
(VITAL_ALIVE = 0; N0 = 261)
1−sided adj pval: 0.014
Frequency
Tumor−Normal Expression Difference
hsa−miR−548f, distal
(VITAL_ALIVE = 1; N1 = 288)
1−sided adj pval: 0.118
Frequency
Tumor−Normal Expression Difference

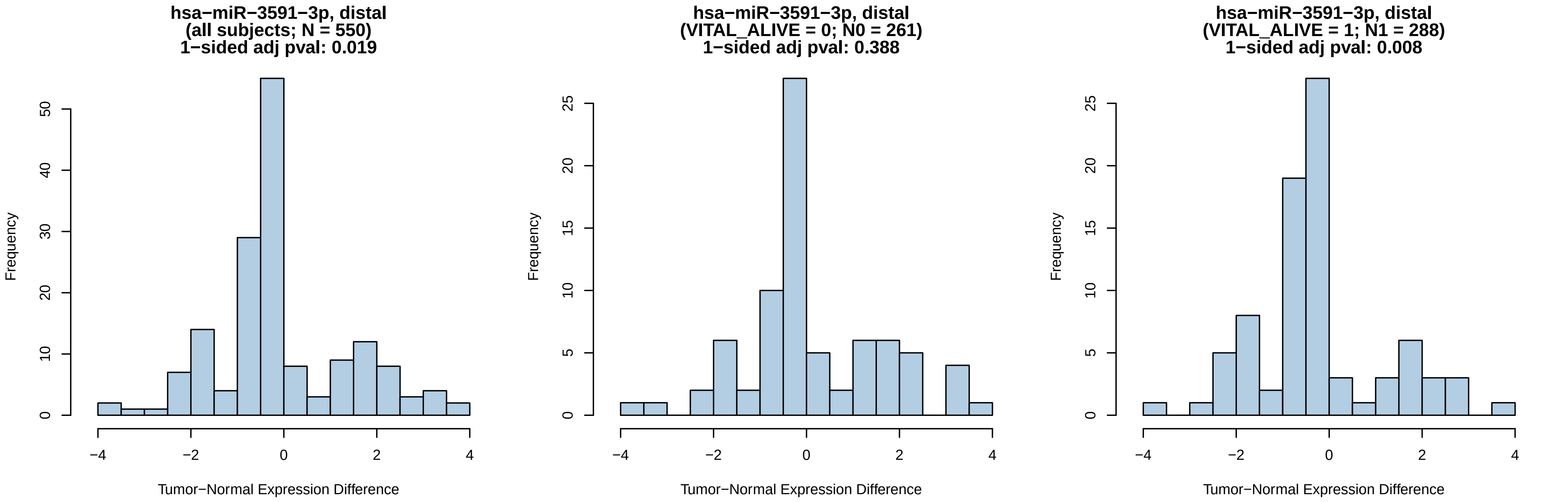
hsa−miR−3591−3p, distal
(all subjects; N = 550)
1−sided adj pval: 0.019
Frequency
Tumor−Normal Expression Difference
hsa−miR−3591−3p, distal
(VITAL_ALIVE = 0; N0 = 261)
1−sided adj pval: 0.388
Frequency
Tumor−Normal Expression Difference
hsa−miR−3591−3p, distal
(VITAL_ALIVE = 1; N1 = 288)
1−sided adj pval: 0.008
Frequency
Tumor−Normal Expression Difference

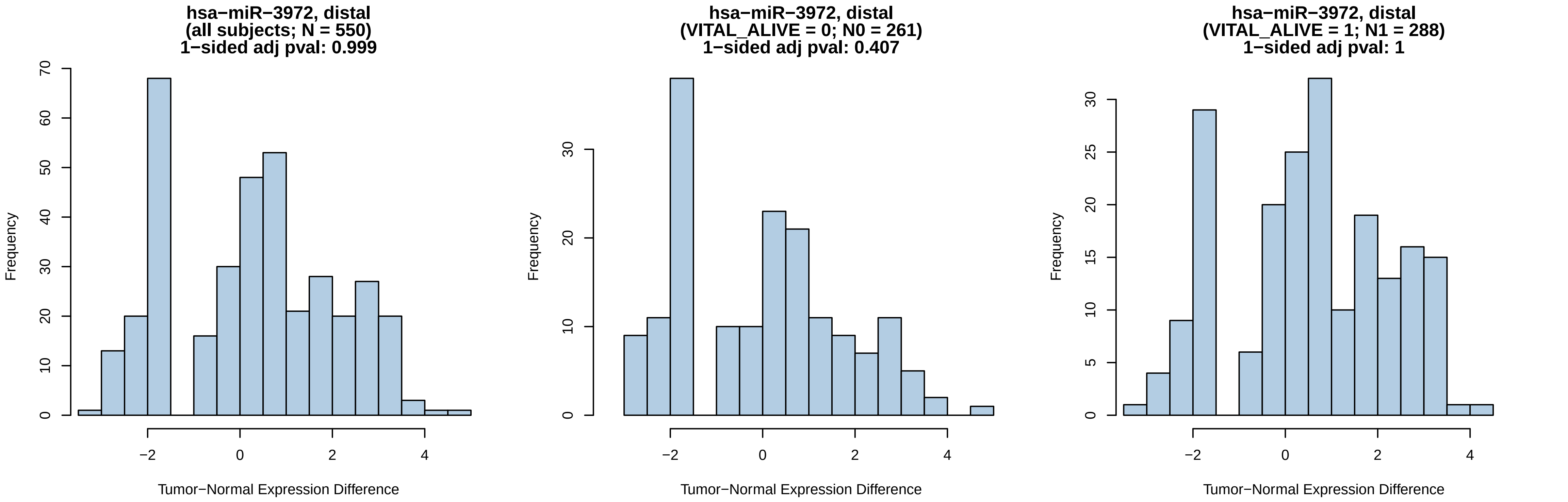

hsa−miR−3972, distal
(all subjects; N = 550)
1−sided adj pval: 0.999
Frequency
Tumor−Normal Expression Difference
hsa−miR−3972, distal
(VITAL_ALIVE = 0; N0 = 261)
1−sided adj pval: 0.407
Frequency
Tumor−Normal Expression Difference
hsa−miR−3972, distal
(VITAL_ALIVE = 1; N1 = 288)
1−sided adj pval: 1
Frequency
Tumor−Normal Expression Difference

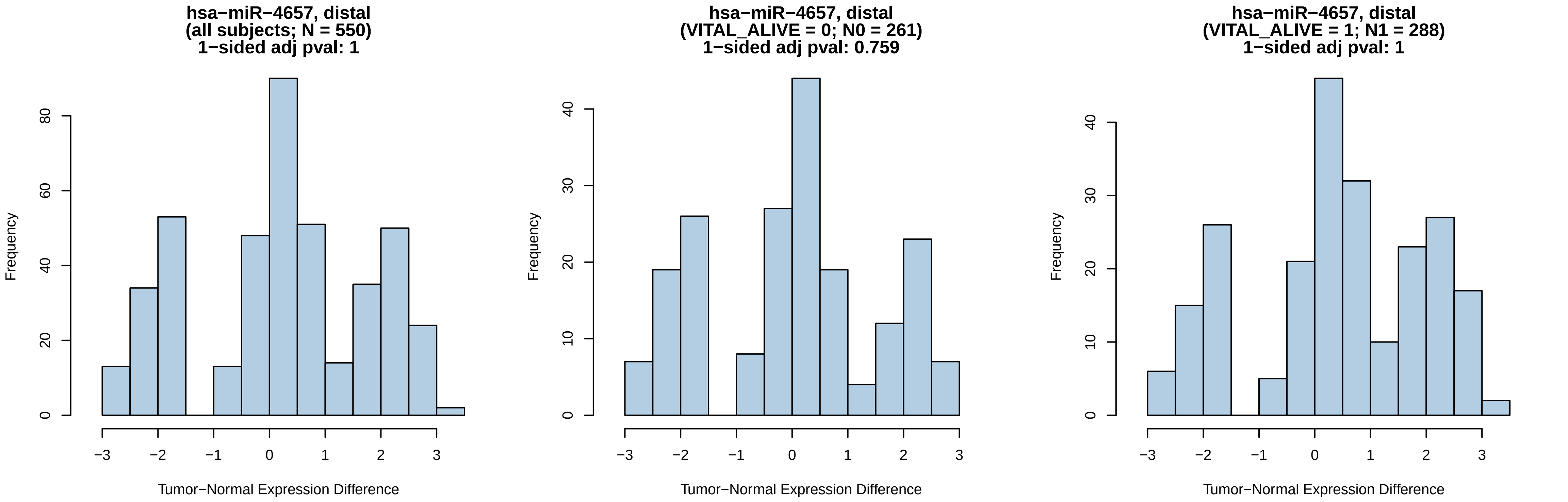

hsa−miR−4657, distal
(all subjects; N = 550)
1−sided adj pval: 1
Frequency
0
20
40
60
80
−3
−2
−1
0
1
2
3
Tumor−Normal Expression Difference
hsa−miR−4657, distal
(VITAL_ALIVE = 0; N0 = 261)
1−sided adj pval: 0.759
Frequency
0
10
20
30
40
−3
−2
−1
0
1
2
3
Tumor−Normal Expression Difference
hsa−miR−4657, distal
(VITAL_ALIVE = 1; N1 = 288)
1−sided adj pval: 1
Frequency
0
10
20
30
40
−3
−2
−1
0
1
2
3
Tumor−Normal Expression Difference

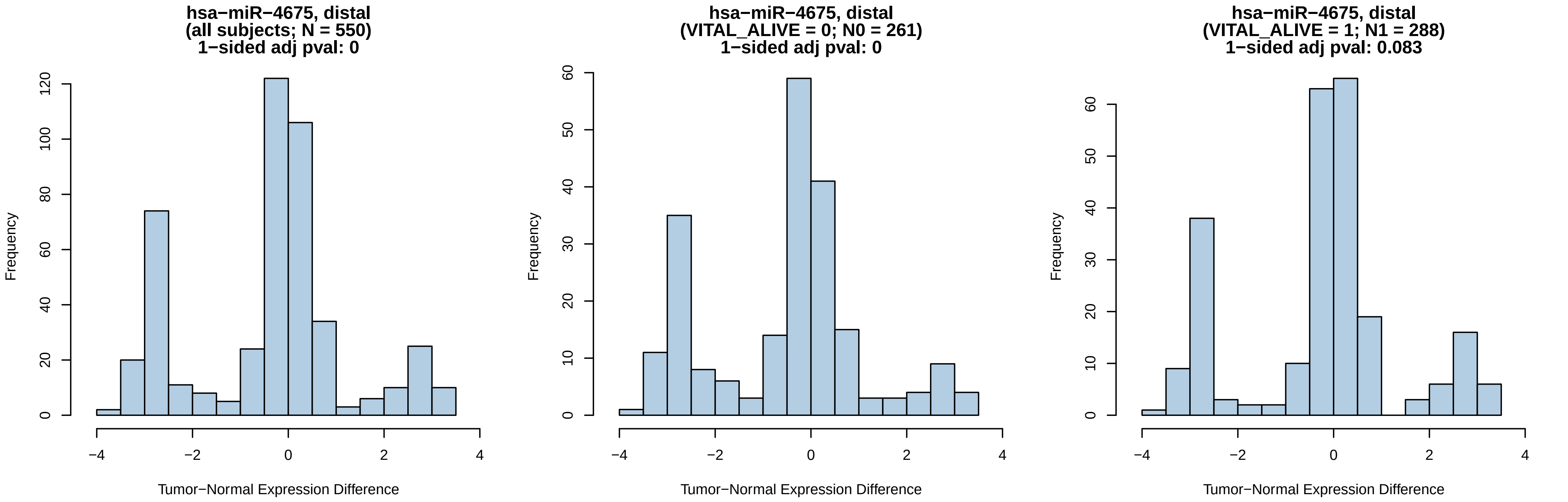
hsa−miR−4675, distal
(all subjects; N = 550)
1−sided adj pval: 0
Frequency
Tumor−Normal Expression Difference
hsa−miR−4675, distal
(VITAL_ALIVE = 0; N0 = 261)
1−sided adj pval: 0
Frequency
Tumor−Normal Expression Difference
hsa−miR−4675, distal
(VITAL_ALIVE = 1; N1 = 288)
1−sided adj pval: 0.083
Frequency
Tumor−Normal Expression Difference

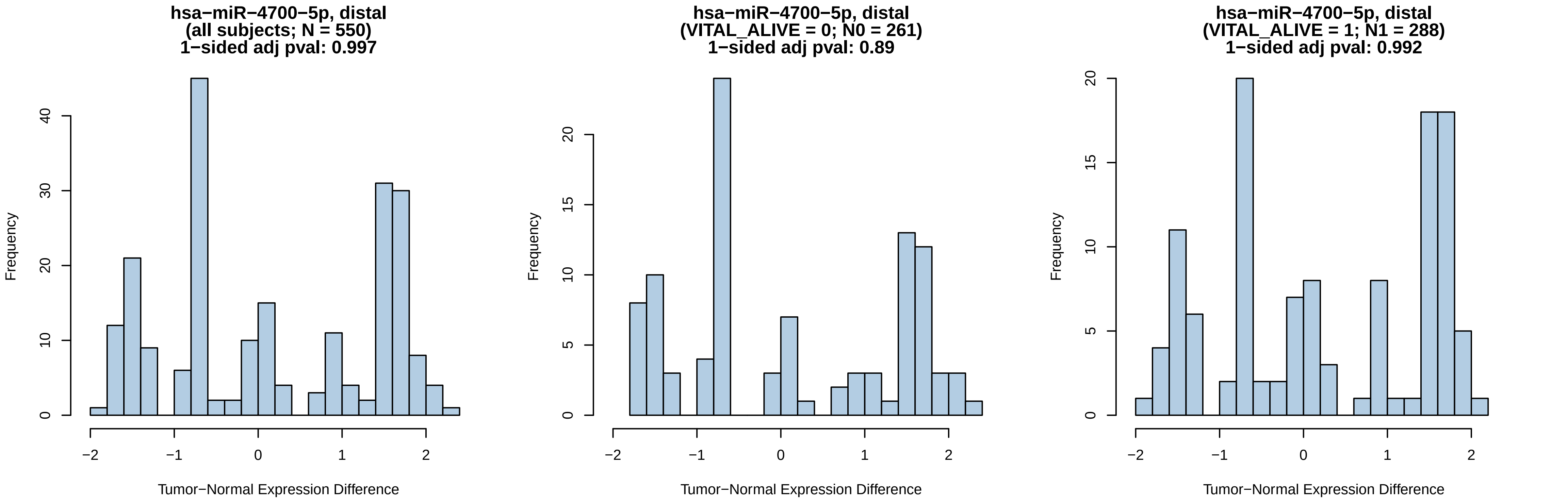

hsa−miR−4700−5p, distal
(all subjects; N = 550)
1−sided adj pval: 0.997
Frequency
Tumor−Normal Expression Difference
hsa−miR−4700−5p, distal
(VITAL_ALIVE = 0; N0 = 261)
1−sided adj pval: 0.89
Frequency
Tumor−Normal Expression Difference
hsa−miR−4700−5p, distal
(VITAL_ALIVE = 1; N1 = 288)
1−sided adj pval: 0.992
Frequency
Tumor−Normal Expression Difference

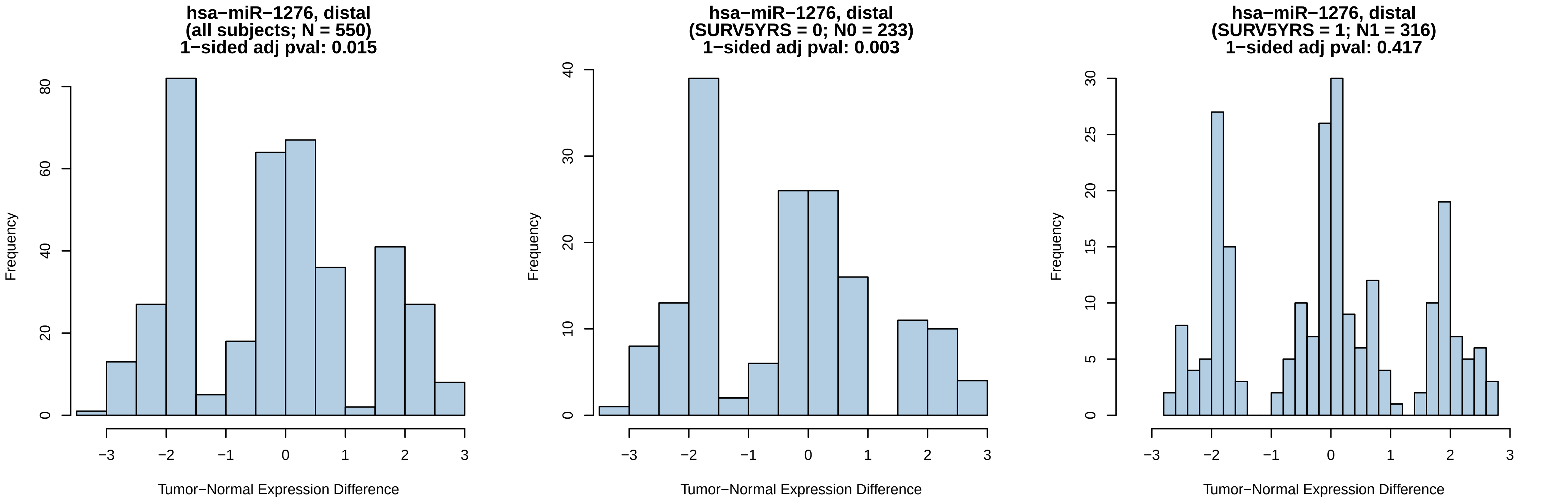
hsa−miR−1276, distal
(all subjects; N = 550)
1−sided adj pval: 0.015
Frequency
Tumor−Normal Expression Difference
hsa−miR−1276, distal
(SURV5YRS = 0; N0 = 233)
1−sided adj pval: 0.003
Frequency
Tumor−Normal Expression Difference
hsa−miR−1276, distal
(SURV5YRS = 1; N1 = 316)
1−sided adj pval: 0.417
Frequency
Tumor−Normal Expression Difference

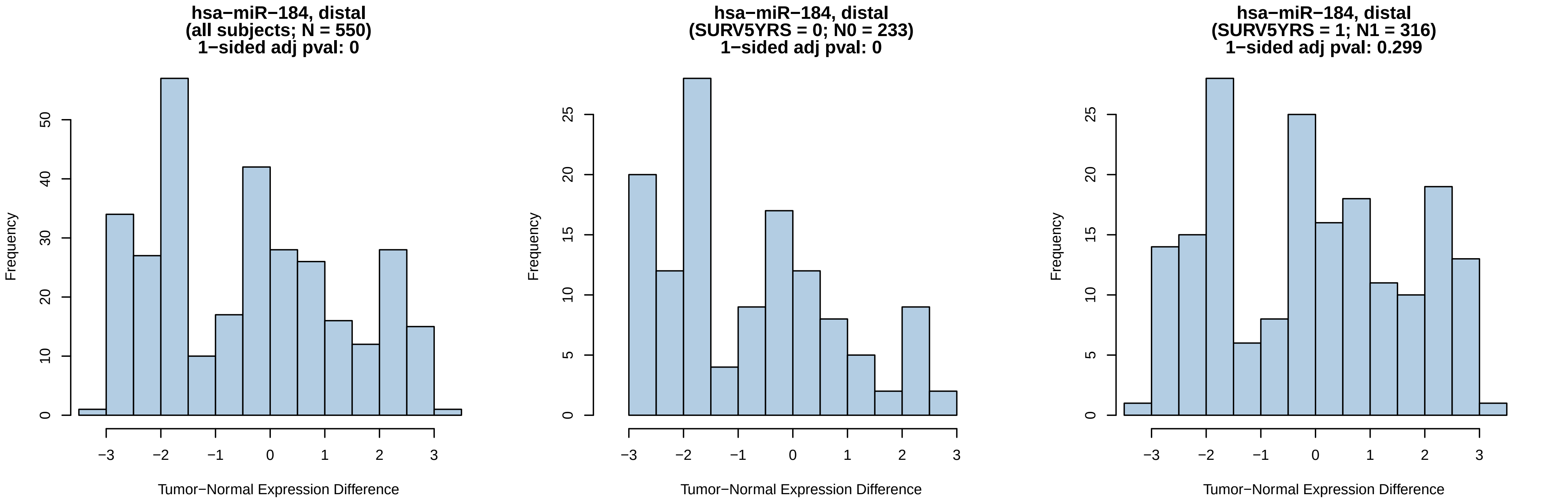
hsa−miR−184, distal
(all subjects; N = 550)
1−sided adj pval: 0
Frequency
Tumor−Normal Expression Difference
hsa−miR−184, distal
(SURV5YRS = 0; N0 = 233)
1−sided adj pval: 0
Frequency
Tumor−Normal Expression Difference
hsa−miR−184, distal
(SURV5YRS = 1; N1 = 316)
1−sided adj pval: 0.299
Frequency
Tumor−Normal Expression Difference

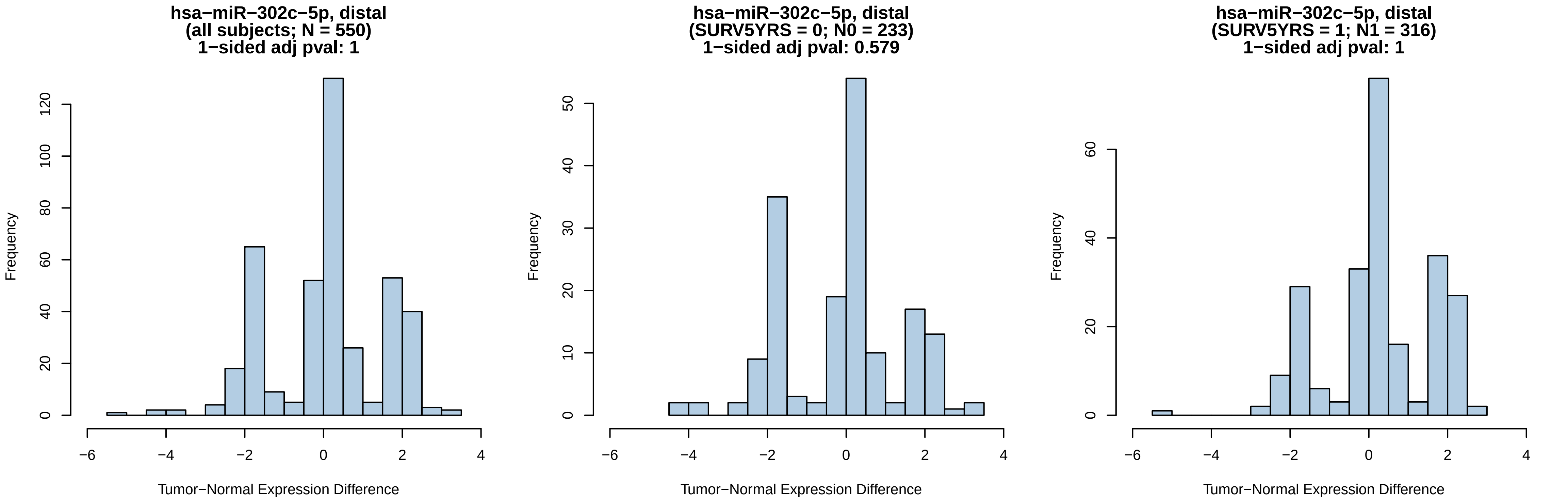

hsa−miR−302c−5p, distal
(all subjects; N = 550)
1−sided adj pval: 1
Frequency
Tumor−Normal Expression Difference
0
20
40
60
80
100
120
−6
−4
−2
0
2
4
hsa−miR−302c−5p, distal
(SURV5YRS = 0; N0 = 233)
1−sided adj pval: 0.579
Frequency
Tumor−Normal Expression Difference
0
10
20
30
40
50
hsa−miR−302c−5p, distal
(SURV5YRS = 1; N1 = 316)
1−sided adj pval: 1
Frequency
Tumor−Normal Expression Difference
0
20
40
60

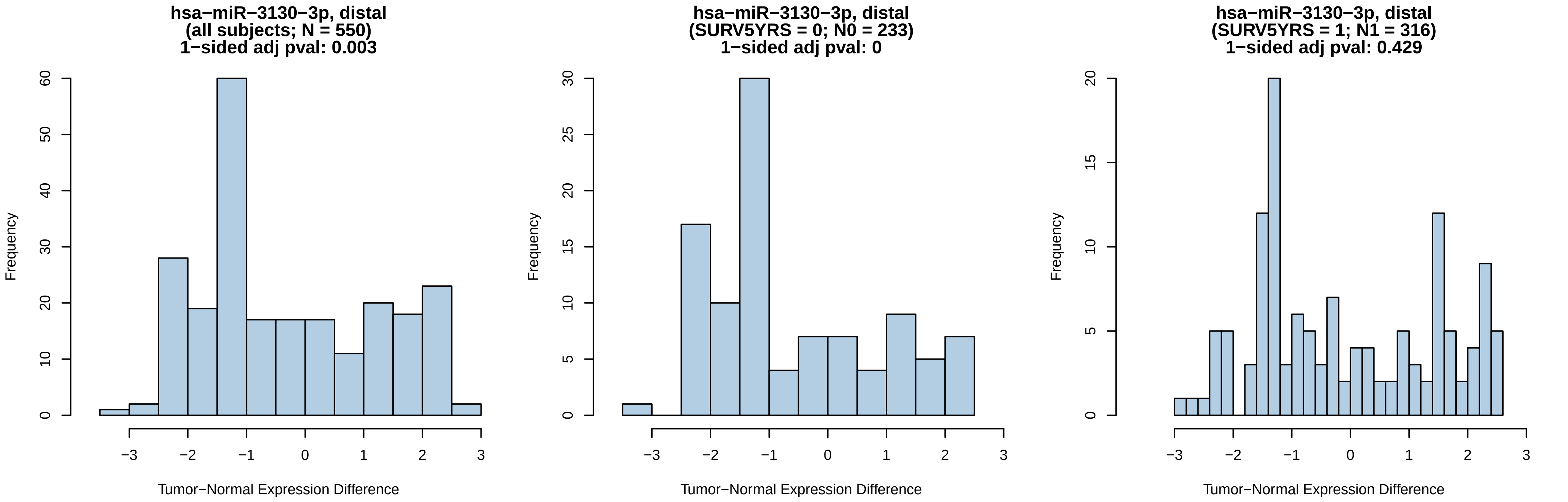

hsa−miR−3130−3p, distal
(all subjects; N = 550)
1−sided adj pval: 0.003
Frequency
Tumor−Normal Expression Difference
hsa−miR−3130−3p, distal
(SURV5YRS = 0; N0 = 233)
1−sided adj pval: 0
Frequency
Tumor−Normal Expression Difference
hsa−miR−3130−3p, distal
(SURV5YRS = 1; N1 = 316)
1−sided adj pval: 0.429
Frequency
Tumor−Normal Expression Difference

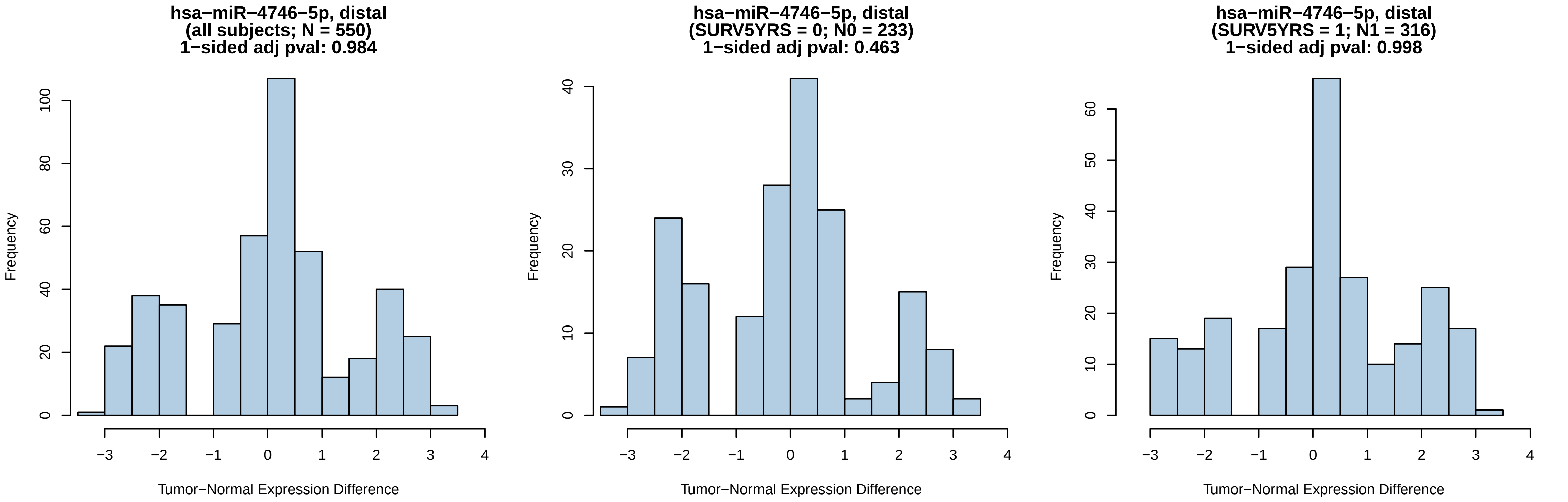

hsa−miR−4746−5p, distal
(all subjects; N = 550)
1−sided adj pval: 0.984
Frequency
Tumor−Normal Expression Difference
hsa−miR−4746−5p, distal
(SURV5YRS = 0; N0 = 233)
1−sided adj pval: 0.463
Frequency
Tumor−Normal Expression Difference
hsa−miR−4746−5p, distal
(SURV5YRS = 1; N1 = 316)
1−sided adj pval: 0.998
Frequency
Tumor−Normal Expression Difference

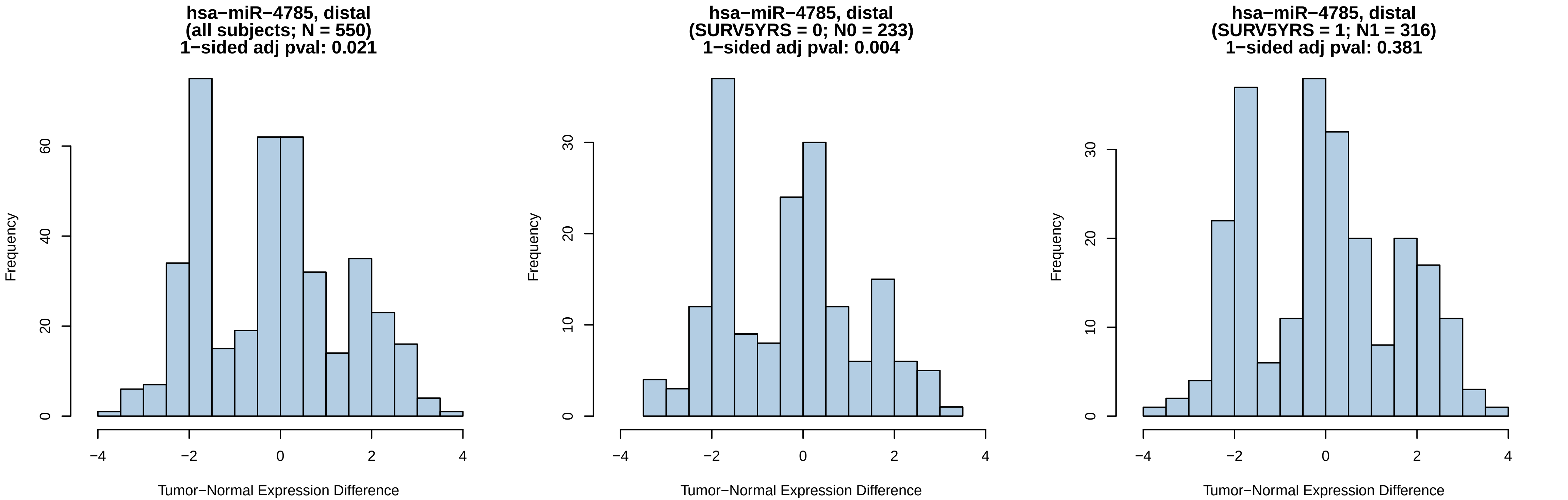
hsa−miR−4785, distal
(all subjects; N = 550)
1−sided adj pval: 0.021
Frequency
Tumor−Normal Expression Difference
hsa−miR−4785, distal
(SURV5YRS = 0; N0 = 233)
1−sided adj pval: 0.004
Frequency
Tumor−Normal Expression Difference
hsa−miR−4785, distal
(SURV5YRS = 1; N1 = 316)
1−sided adj pval: 0.381
Frequency
Tumor−Normal Expression Difference

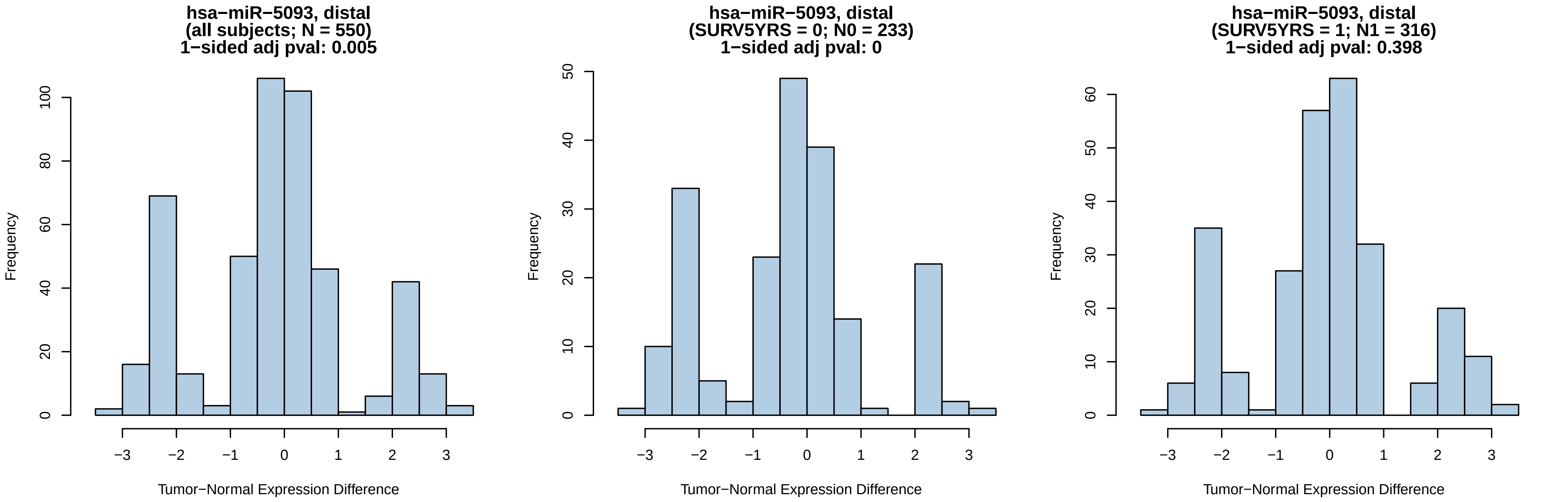
hsa−miR−5093, distal
(all subjects; N = 550)
1−sided adj pval: 0.005
Frequency
Tumor−Normal Expression Difference
0
20
40
60
80
100
−3
−2
−1
0
1
2
3
hsa−miR−5093, distal
(SURV5YRS = 0; N0 = 233)
1−sided adj pval: 0
Frequency
Tumor−Normal Expression Difference
0
10
20
30
40
50
hsa−miR−5093, distal
(SURV5YRS = 1; N1 = 316)
1−sided adj pval: 0.398
Frequency
Tumor−Normal Expression Difference
0
10
20
30
40
50
60

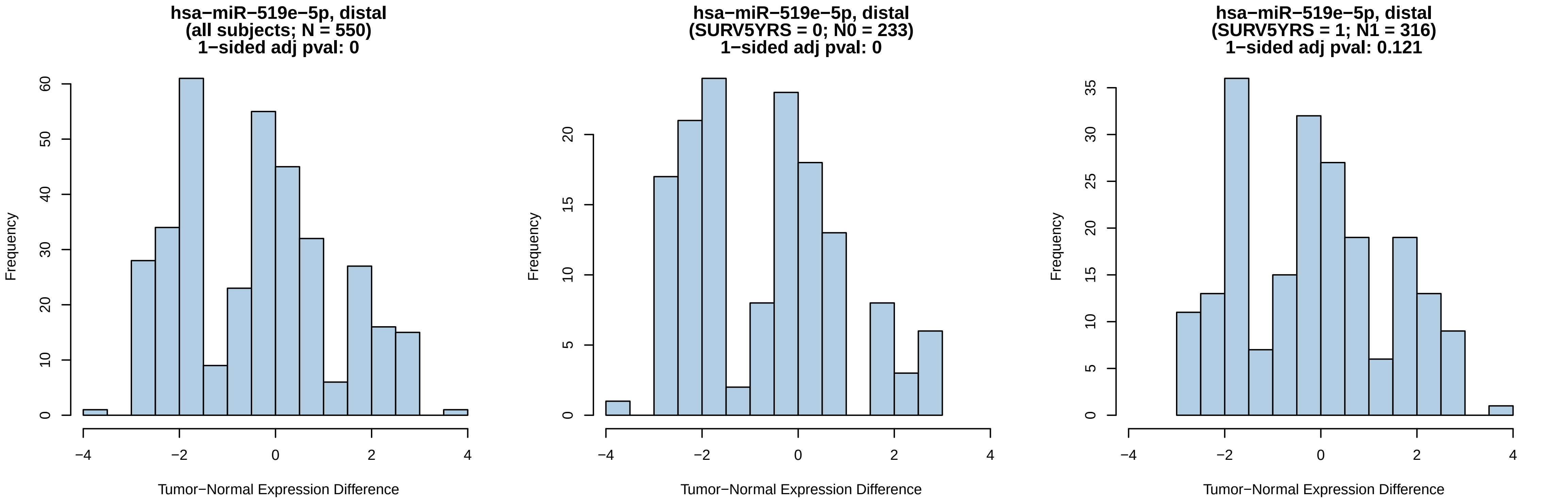

hsa−miR−519e−5p, distal
(all subjects; N = 550)
1−sided adj pval: 0
hsa−miR−519e−5p, distal
(SURV5YRS = 0; N0 = 233)
1−sided adj pval: 0
hsa−miR−519e−5p, distal
(SURV5YRS = 1; N1 = 316)
1−sided adj pval: 0.121
Frequency
Tumor−Normal Expression Difference

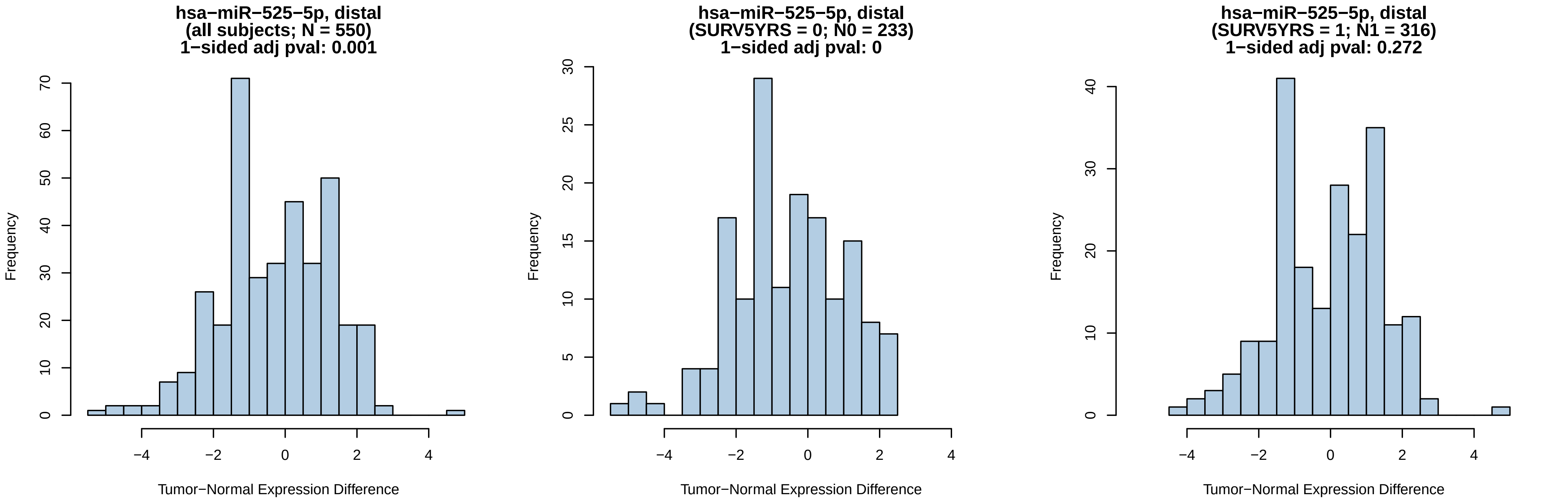
hsa−miR−525−5p, distal
(all subjects; N = 550)
1−sided adj pval: 0.001
Frequency
Tumor−Normal Expression Difference
hsa−miR−525−5p, distal
(SURV5YRS = 0; N0 = 233)
1−sided adj pval: 0
Frequency
Tumor−Normal Expression Difference
hsa−miR−525−5p, distal
(SURV5YRS = 1; N1 = 316)
1−sided adj pval: 0.272
Frequency
Tumor−Normal Expression Difference

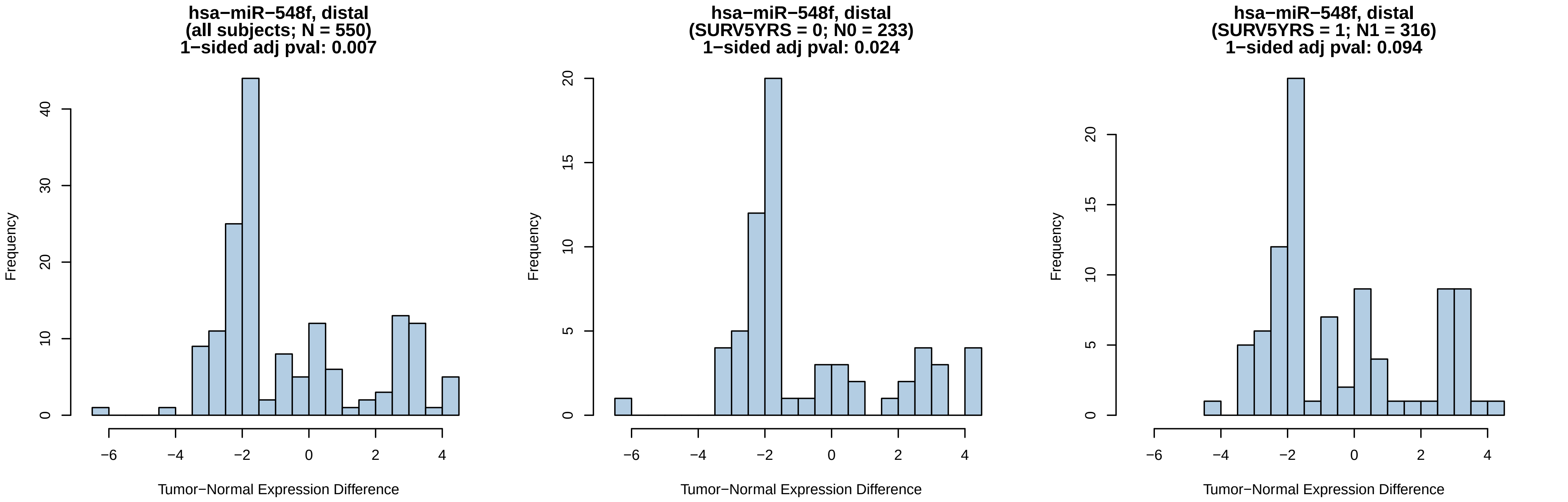

hsa−miR−548f, distal
(all subjects; N = 550)
1−sided adj pval: 0.007
Frequency
0
10
20
30
40
−6
−4
−2
0
2
4
Tumor−Normal Expression Difference
hsa−miR−548f, distal
(SURV5YRS = 0; N0 = 233)
1−sided adj pval: 0.024
Frequency
0
5
10
15
20
−6
−4
−2
0
2
4
Tumor−Normal Expression Difference
hsa−miR−548f, distal
(SURV5YRS = 1; N1 = 316)
1−sided adj pval: 0.094
Frequency
0
5
10
15
20
−6
−4
−2
0
2
4
Tumor−Normal Expression Difference

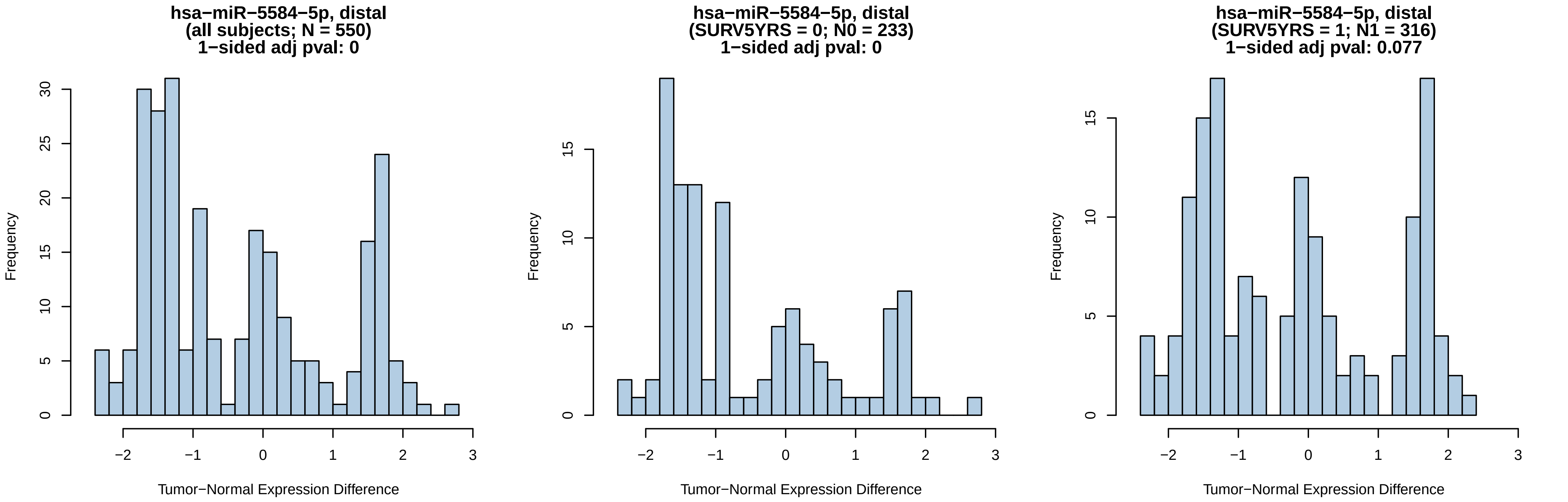
hsa−miR−5584−5p, distal
(all subjects; N = 550)
1−sided adj pval: 0
hsa−miR−5584−5p, distal
(SURV5YRS = 0; N0 = 233)
1−sided adj pval: 0
hsa−miR−5584−5p, distal
(SURV5YRS = 1; N1 = 316)
1−sided adj pval: 0.077
Frequency
Tumor−Normal Expression Difference
0
5
10
15
20
25
30
−2
−1
0
1
2
3

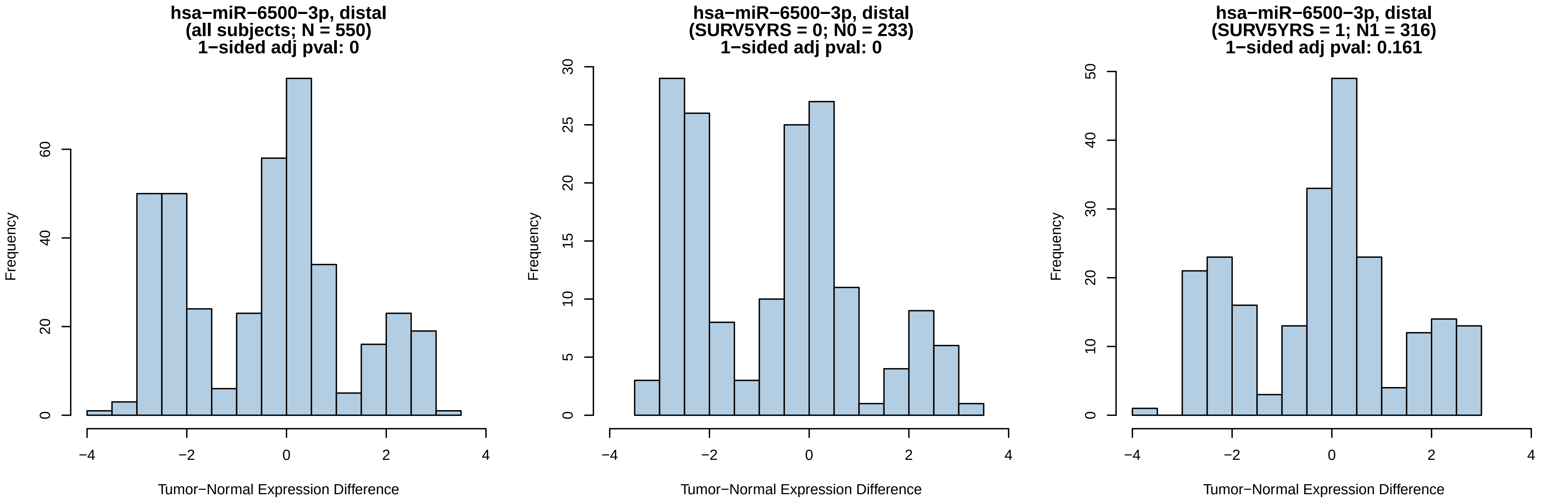
hsa−miR−6500−3p, distal
(all subjects; N = 550)
1−sided adj pval: 0
Frequency
Tumor−Normal Expression Difference
hsa−miR−6500−3p, distal
(SURV5YRS = 0; N0 = 233)
1−sided adj pval: 0
Frequency
Tumor−Normal Expression Difference
hsa−miR−6500−3p, distal
(SURV5YRS = 1; N1 = 316)
1−sided adj pval: 0.161
Frequency
Tumor−Normal Expression Difference

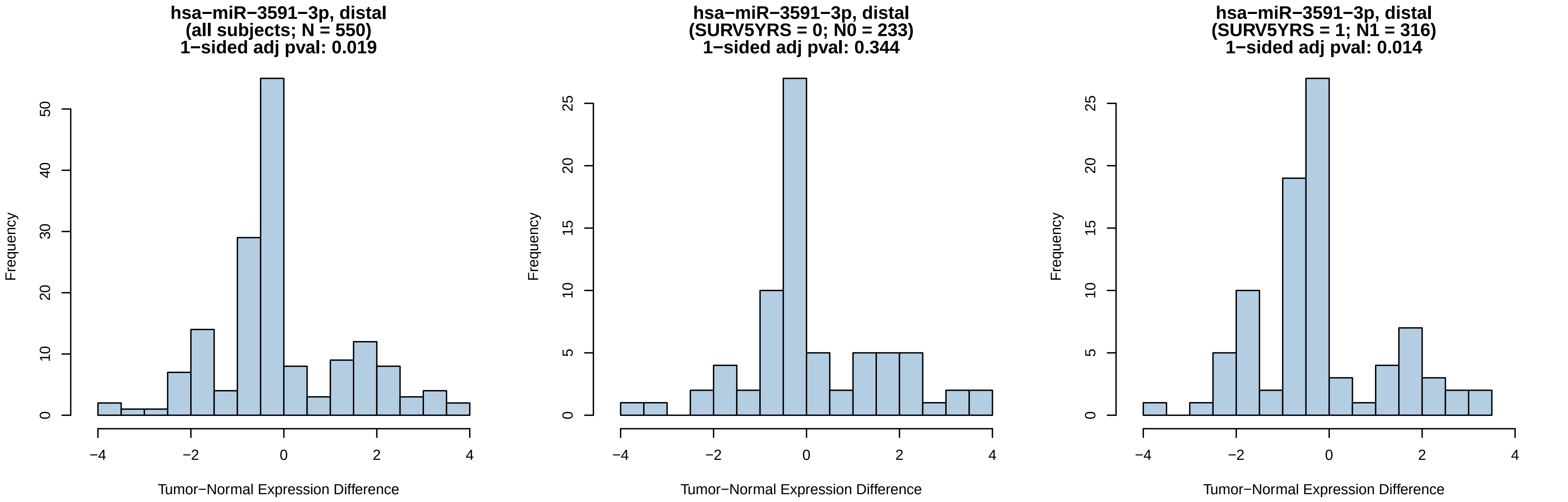
hsa−miR−3591−3p, distal
(all subjects; N = 550)
1−sided adj pval: 0.019
hsa−miR−3591−3p, distal
(SURV5YRS = 0; N0 = 233)
1−sided adj pval: 0.344
hsa−miR−3591−3p, distal
(SURV5YRS = 1; N1 = 316)
1−sided adj pval: 0.014
Frequency
Tumor−Normal Expression Difference

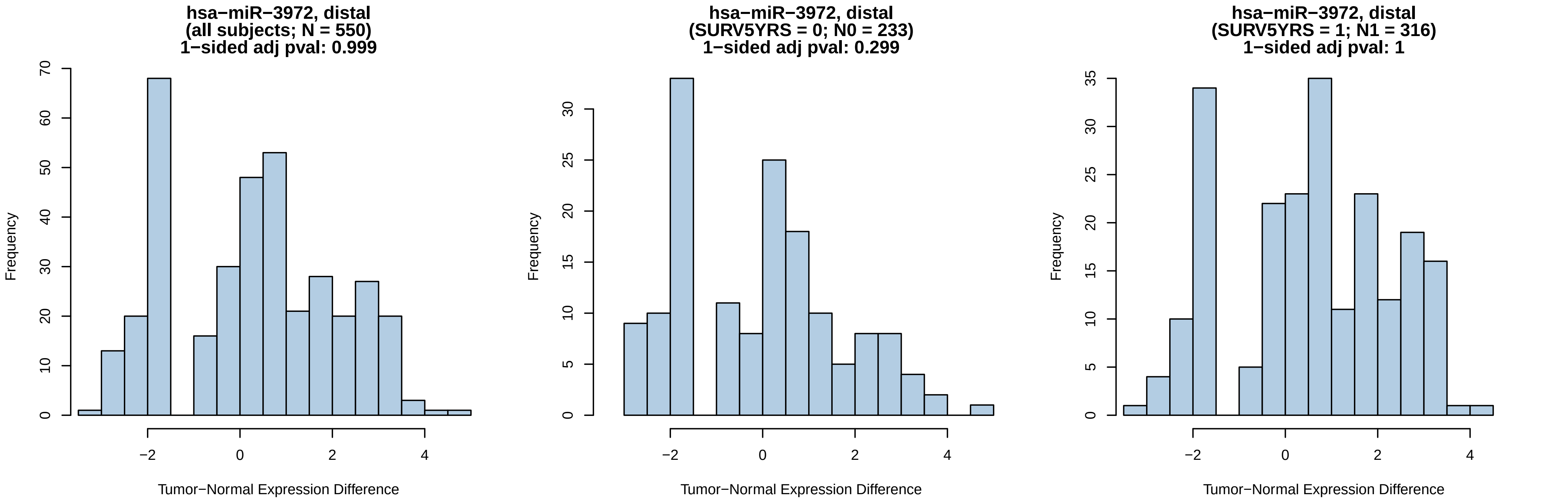

hsa−miR−3972, distal
(all subjects; N = 550)
1−sided adj pval: 0.999
Frequency
Tumor−Normal Expression Difference
hsa−miR−3972, distal
(SURV5YRS = 0; N0 = 233)
1−sided adj pval: 0.299
Frequency
Tumor−Normal Expression Difference
hsa−miR−3972, distal
(SURV5YRS = 1; N1 = 316)
1−sided adj pval: 1
Frequency
Tumor−Normal Expression Difference

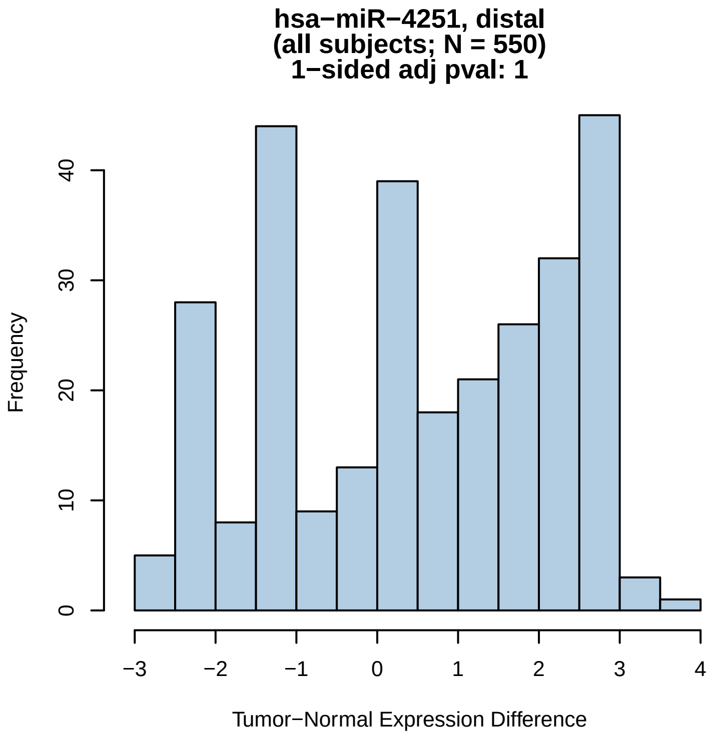
hsa−miR−4251, distal
(all subjects; N = 550)
1−sided adj pval: 1
Frequency
0
10
20
30
40
−3
−2
−1
0
1
2
3
4
Tumor−Normal Expression Difference

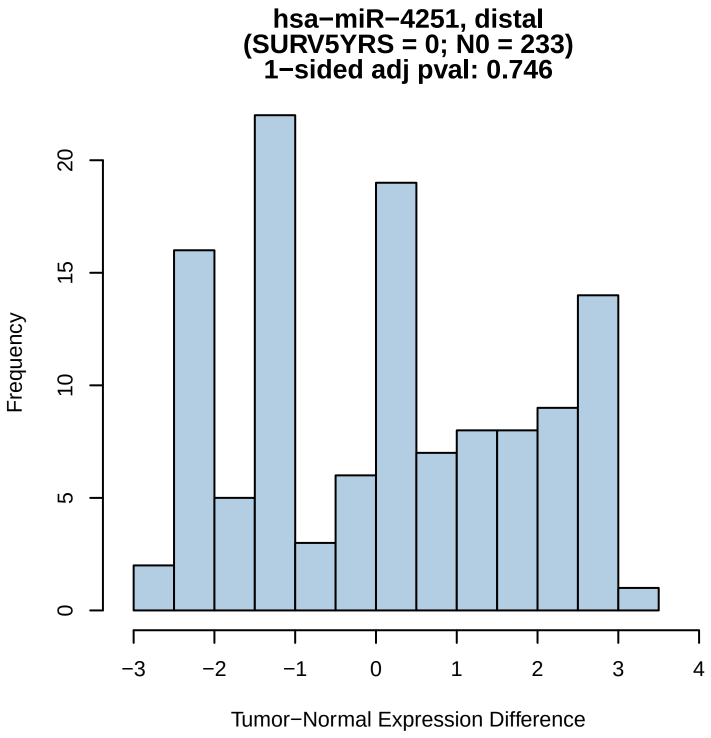
hsa−miR−4251, distal
(SURV5YRS = 0; N0 = 233)
1−sided adj pval: 0.746
Frequency
0
5
10
15
20
−3
−2
−1
0
1
2
3
4
Tumor−Normal Expression Difference

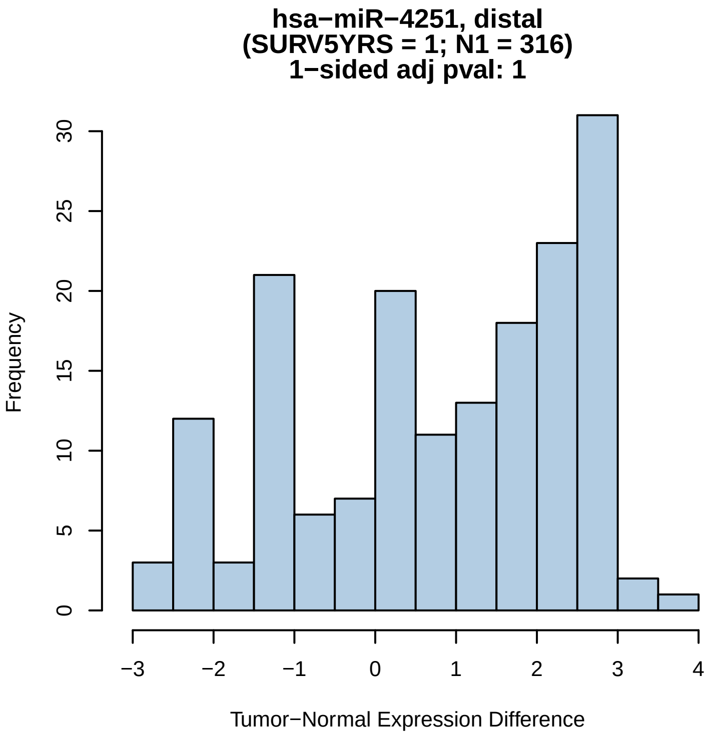
hsa−miR−4251, distal
(SURV5YRS = 1; N1 = 316)
1−sided adj pval: 1
Frequency
0
5
10
15
20
25
30
−3
−2
−1
0
1
2
3
4
Tumor−Normal Expression Difference

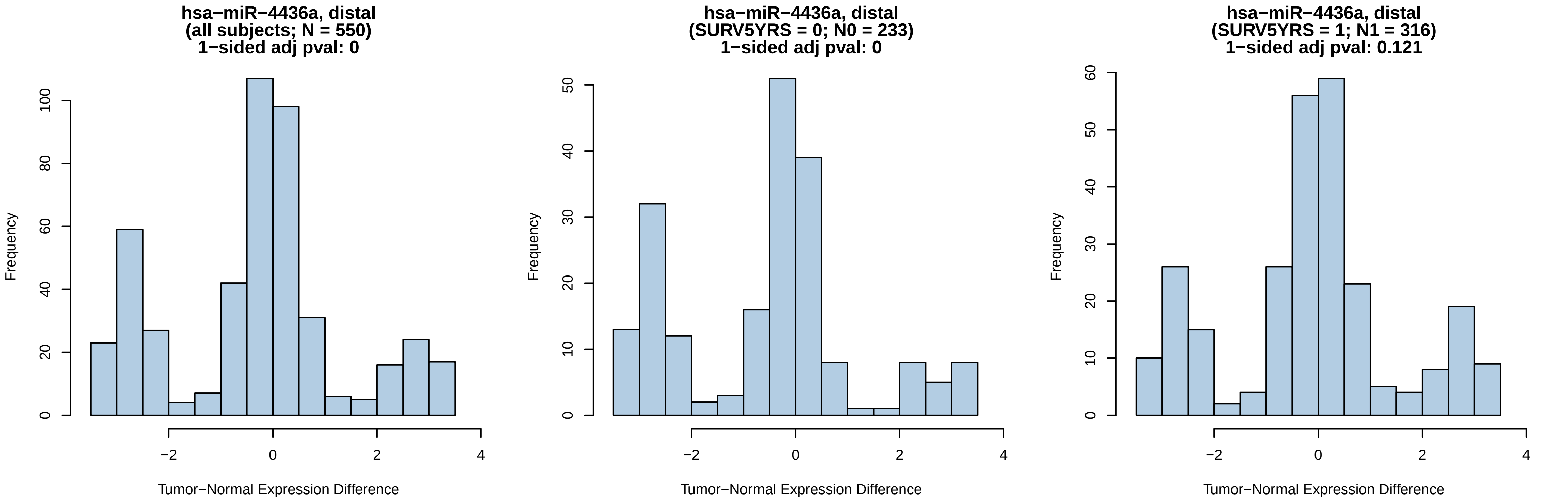
hsa−miR−4436a, distal
(all subjects; N = 550)
1−sided adj pval: 0
Frequency
Tumor−Normal Expression Difference
0
20
40
60
80
100
−2
0
2
4
hsa−miR−4436a, distal
(SURV5YRS = 0; N0 = 233)
1−sided adj pval: 0
Frequency
Tumor−Normal Expression Difference
0
10
20
30
40
50
−2
0
2
4
hsa−miR−4436a, distal
(SURV5YRS = 1; N1 = 316)
1−sided adj pval: 0.121
Frequency
Tumor−Normal Expression Difference
0
10
20
30
40
50
60
−2
0
2
4

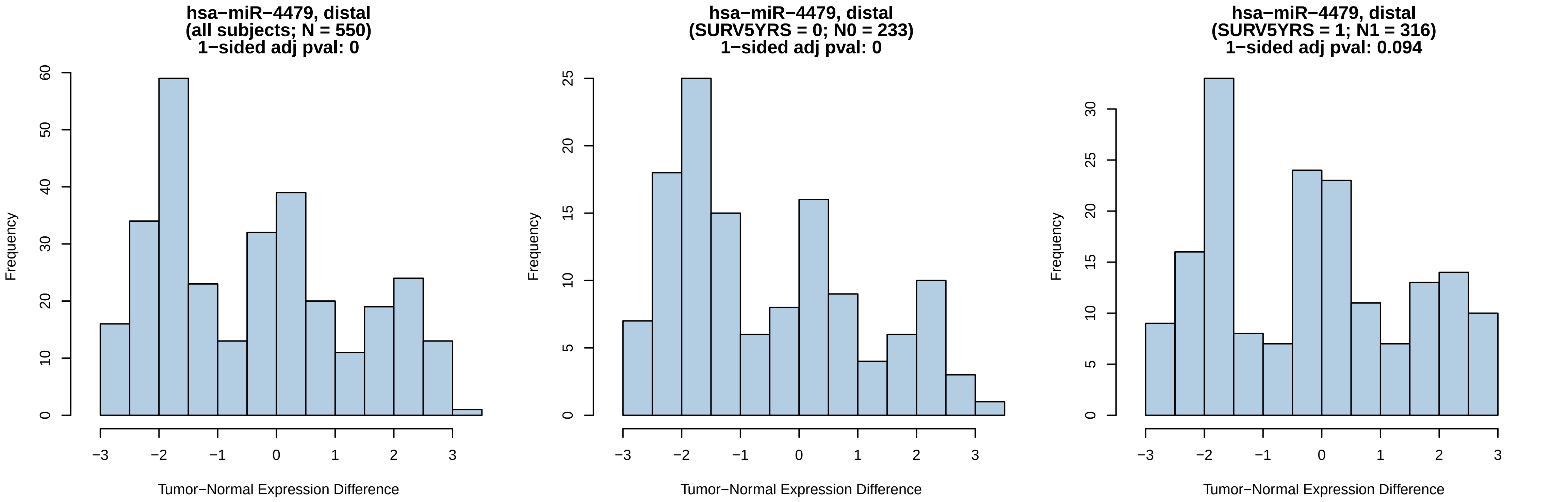
hsa−miR−4479, distal
(all subjects; N = 550)
1−sided adj pval: 0
Frequency
Tumor−Normal Expression Difference
hsa−miR−4479, distal
(SURV5YRS = 0; N0 = 233)
1−sided adj pval: 0
Frequency
Tumor−Normal Expression Difference
hsa−miR−4479, distal
(SURV5YRS = 1; N1 = 316)
1−sided adj pval: 0.094
Frequency
Tumor−Normal Expression Difference

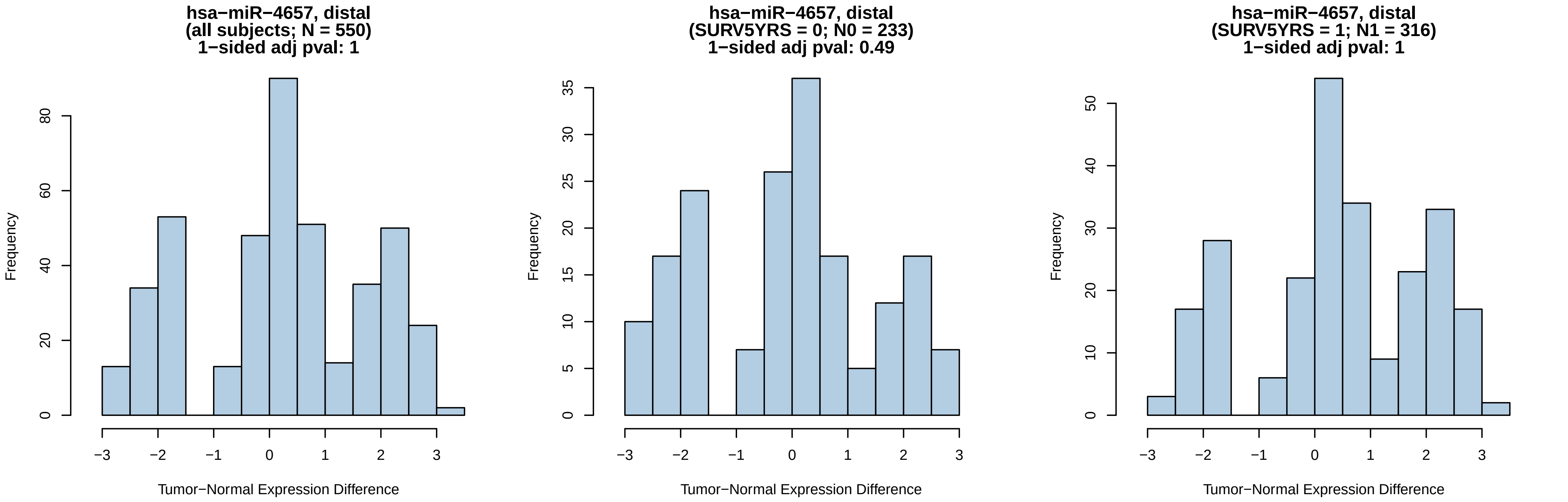

hsa−miR−4657, distal
(all subjects; N = 550)
1−sided adj pval: 1
Frequency
0
20
40
60
80
−3
−2
−1
0
1
2
3
Tumor−Normal Expression Difference
hsa−miR−4657, distal
(SURV5YRS = 0; N0 = 233)
1−sided adj pval: 0.49
Frequency
0
5
10
15
20
25
30
35
−3
−2
−1
0
1
2
3
Tumor−Normal Expression Difference
hsa−miR−4657, distal
(SURV5YRS = 1; N1 = 316)
1−sided adj pval: 1
Frequency
0
10
20
30
40
50
−3
−2
−1
0
1
2
3
Tumor−Normal Expression Difference

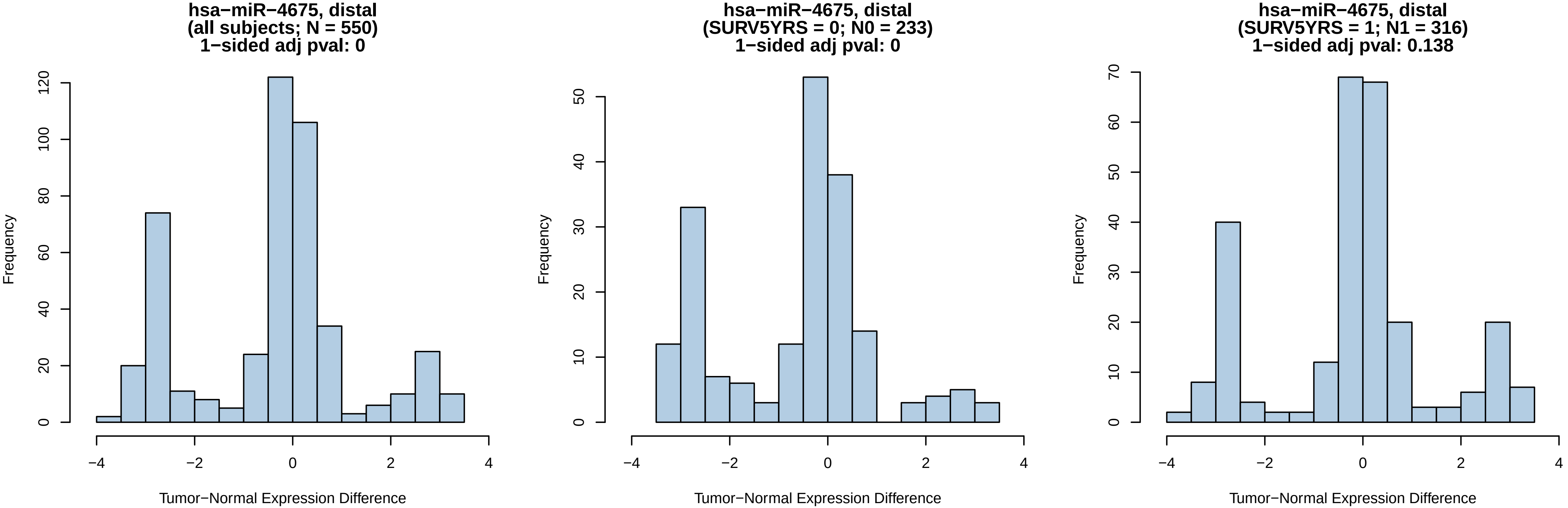
hsa−miR−4675, distal
(all subjects; N = 550)
1−sided adj pval: 0
Frequency
0
20
40
60
80
100
120
−4
−2
0
2
4
Tumor−Normal Expression Difference
hsa−miR−4675, distal
(SURV5YRS = 0; N0 = 233)
1−sided adj pval: 0
Frequency
0
10
20
30
40
50
−4
−2
0
2
4
Tumor−Normal Expression Difference
hsa−miR−4675, distal
(SURV5YRS = 1; N1 = 316)
1−sided adj pval: 0.138
Frequency
0
10
20
30
40
50
60
70
−4
−2
0
2
4
Tumor−Normal Expression Difference

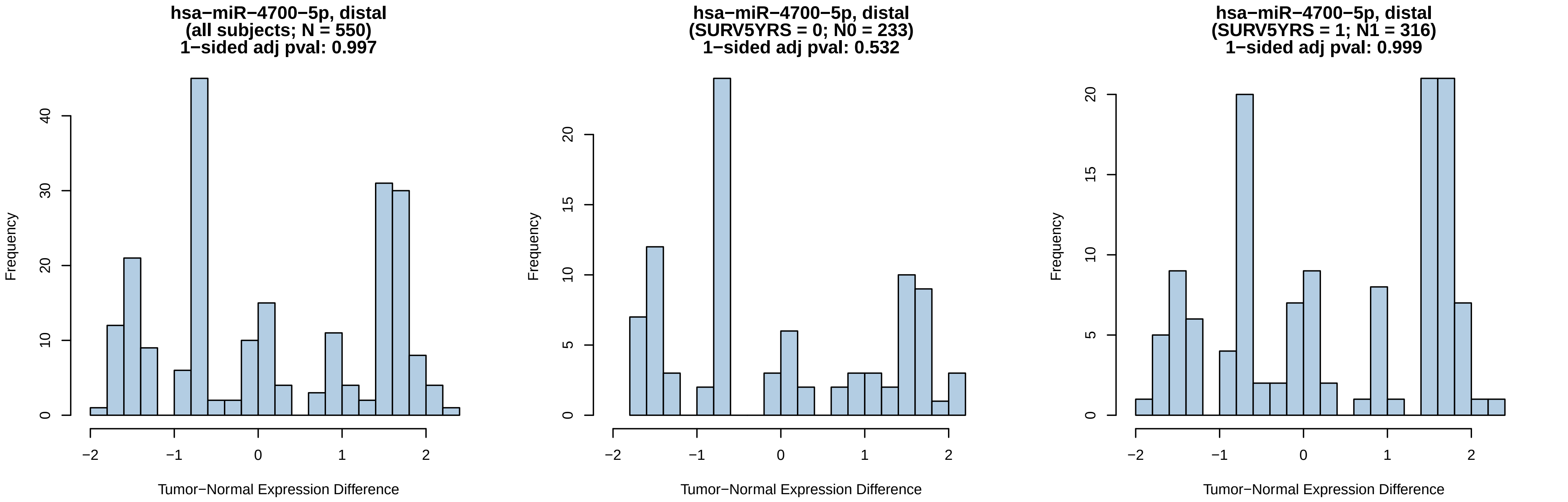

hsa−miR−4700−5p, distal
(all subjects; N = 550)
1−sided adj pval: 0.997
Frequency
0
10
20
30
40
−2
−1
0
1
2
Tumor−Normal Expression Difference
hsa−miR−4700−5p, distal
(SURV5YRS = 0; N0 = 233)
1−sided adj pval: 0.532
Frequency
0
5
10
15
20
−2
−1
0
1
2
Tumor−Normal Expression Difference
hsa−miR−4700−5p, distal
(SURV5YRS = 1; N1 = 316)
1−sided adj pval: 0.999
Frequency
0
5
10
15
20
−2
−1
0
1
2
Tumor−Normal Expression Difference

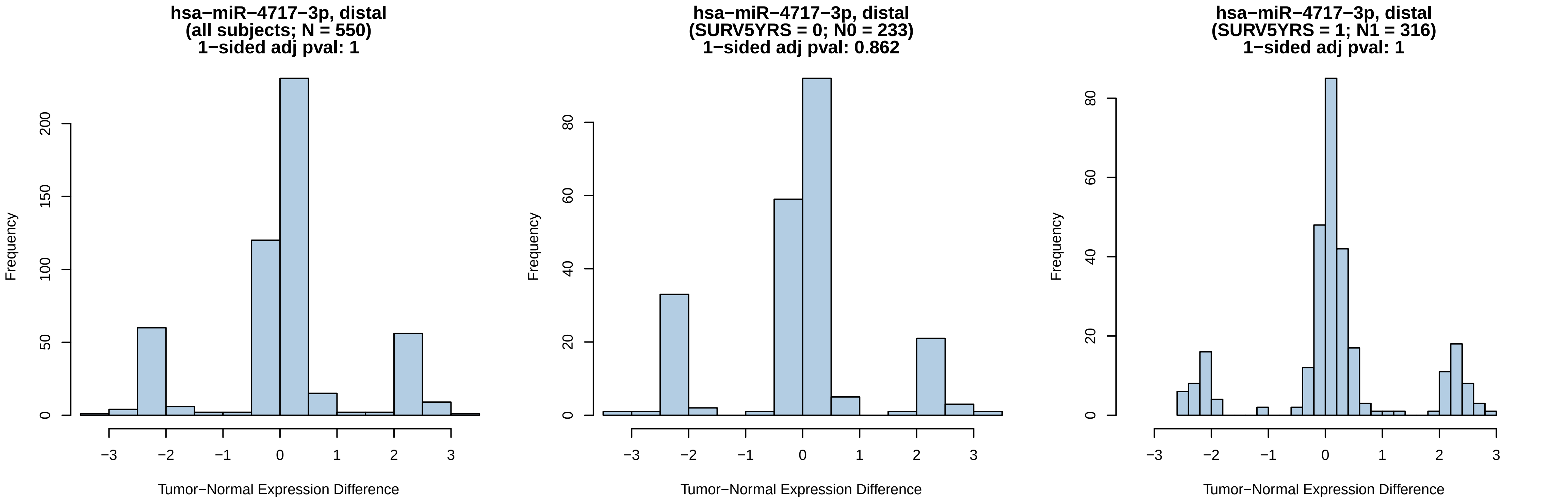
hsa−miR−4717−3p, distal
(all subjects; N = 550)
1−sided adj pval: 1
Frequency
0
50
100
150
200
−3
−2
−1
0
1
2
3
Tumor−Normal Expression Difference
hsa−miR−4717−3p, distal
(SURV5YRS = 0; N0 = 233)
1−sided adj pval: 0.862
Frequency
0
20
40
60
80
−3
−2
−1
0
1
2
3
Tumor−Normal Expression Difference
hsa−miR−4717−3p, distal
(SURV5YRS = 1; N1 = 316)
1−sided adj pval: 1
Frequency
0
20
40
60
80
−3
−2
−1
0
1
2
3
Tumor−Normal Expression Difference

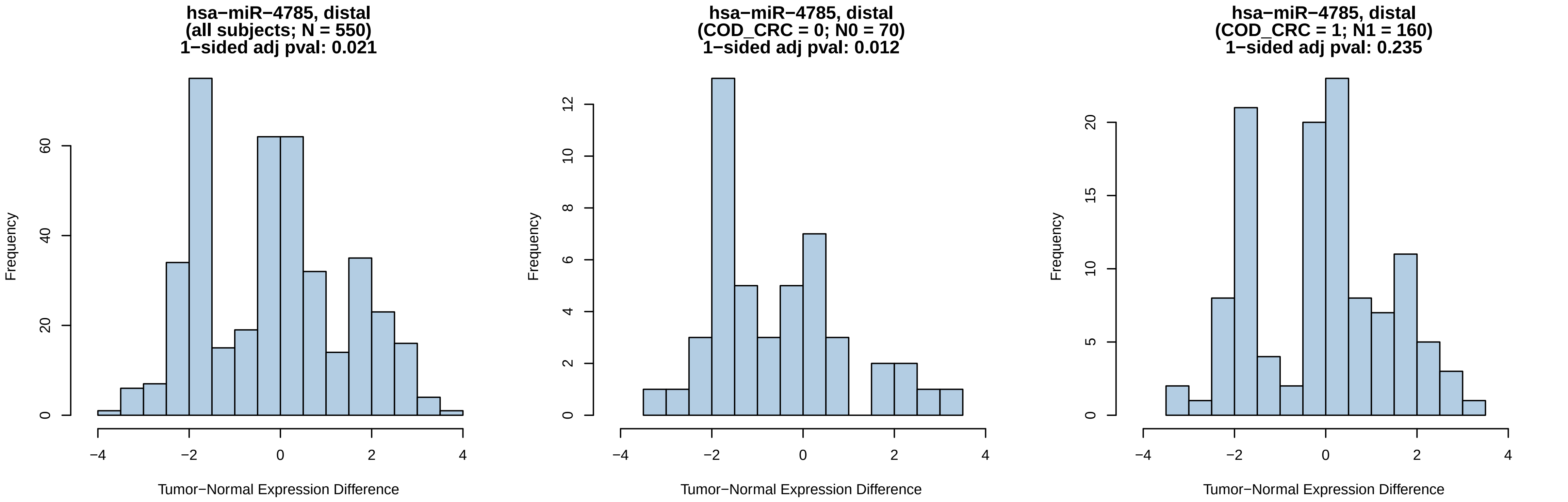
hsa−miR−4785, distal
(all subjects; N = 550)
1−sided adj pval: 0.021
Frequency
0
20
40
60
−4
−2
0
2
4
Tumor−Normal Expression Difference
hsa−miR−4785, distal
(COD_CRC = 0; N0 = 70)
1−sided adj pval: 0.012
Frequency
0
2
4
6
8
10
12
−4
−2
0
2
4
Tumor−Normal Expression Difference
hsa−miR−4785, distal
(COD_CRC = 1; N1 = 160)
1−sided adj pval: 0.235
Frequency
0
5
10
15
20
−4
−2
0
2
4
Tumor−Normal Expression Difference

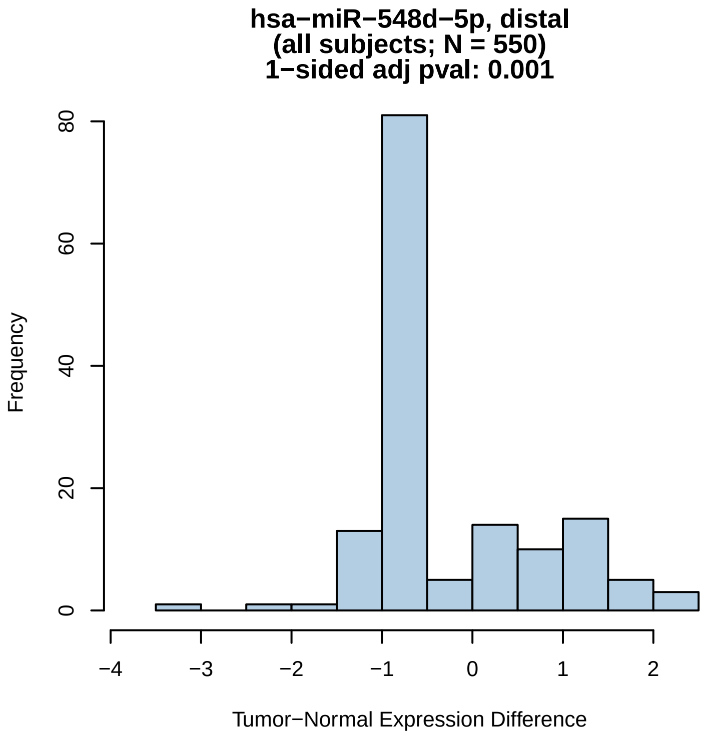

hsa−miR−548d−5p, distal
(all subjects; N = 550)
1−sided adj pval: 0.001
Frequency
Tumor−Normal Expression Difference


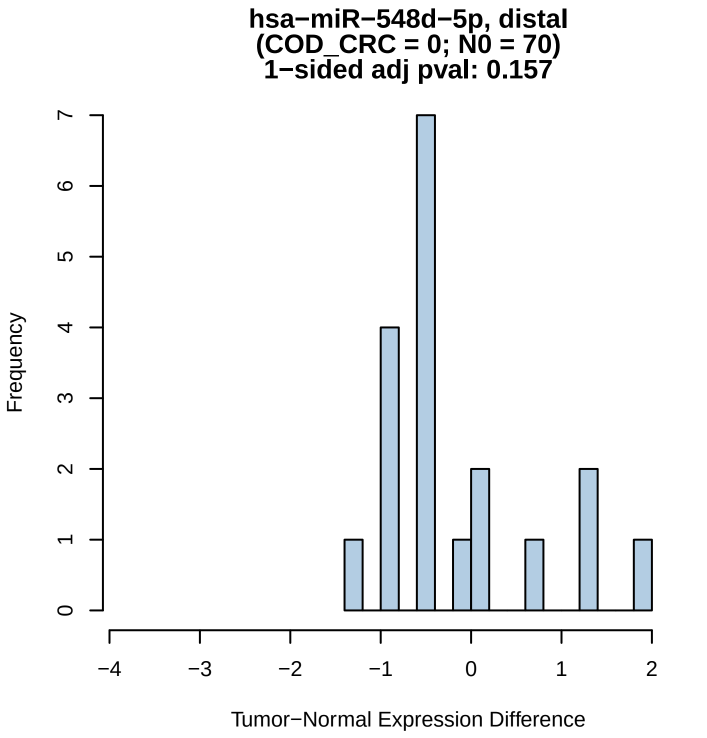

hsa−miR−548d−5p, distal
(COD_CRC = 0; N0 = 70)
1−sided adj pval: 0.157
Frequency
Tumor−Normal Expression Difference


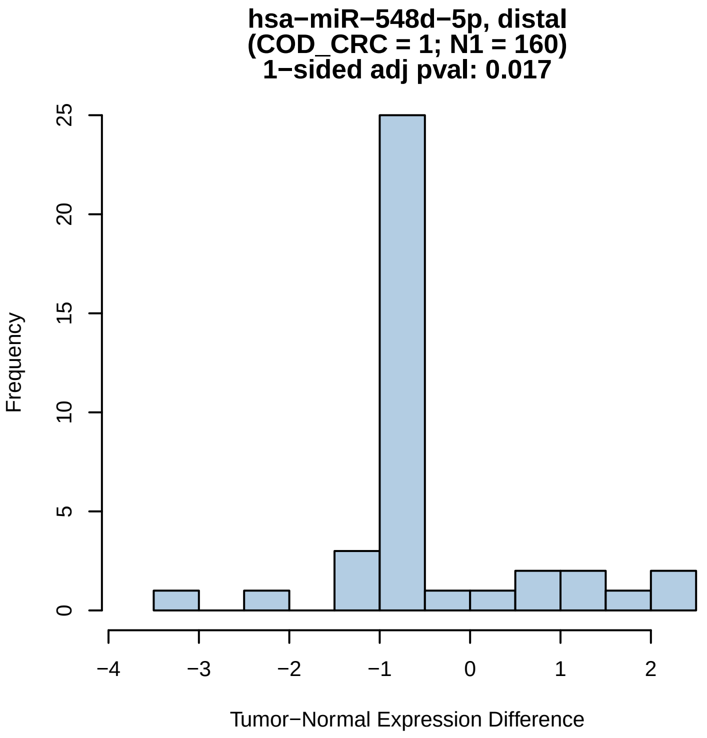

hsa−miR−548d−5p, distal
(COD_CRC = 1; N1 = 160)
1−sided adj pval: 0.017
Frequency
Tumor−Normal Expression Difference

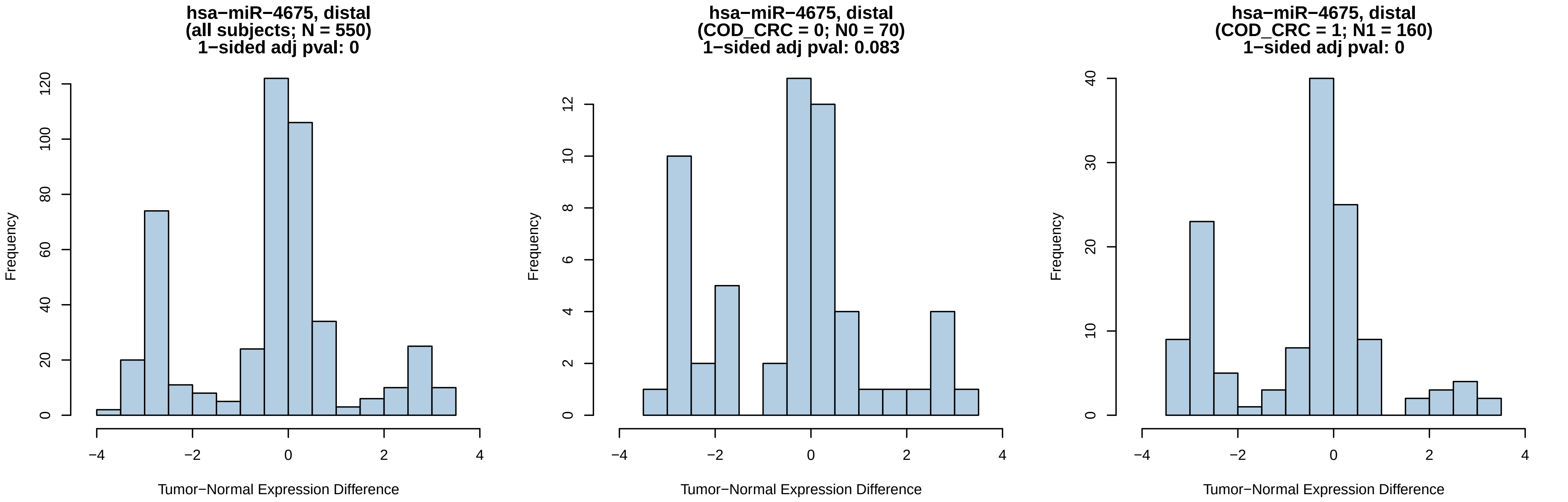
hsa−miR−4675, distal
(all subjects; N = 550)
1−sided adj pval: 0
Frequency
Tumor−Normal Expression Difference
hsa−miR−4675, distal
(COD_CRC = 0; N0 = 70)
1−sided adj pval: 0.083
Frequency
Tumor−Normal Expression Difference
hsa−miR−4675, distal
(COD_CRC = 1; N1 = 160)
1−sided adj pval: 0
Frequency
Tumor−Normal Expression Difference

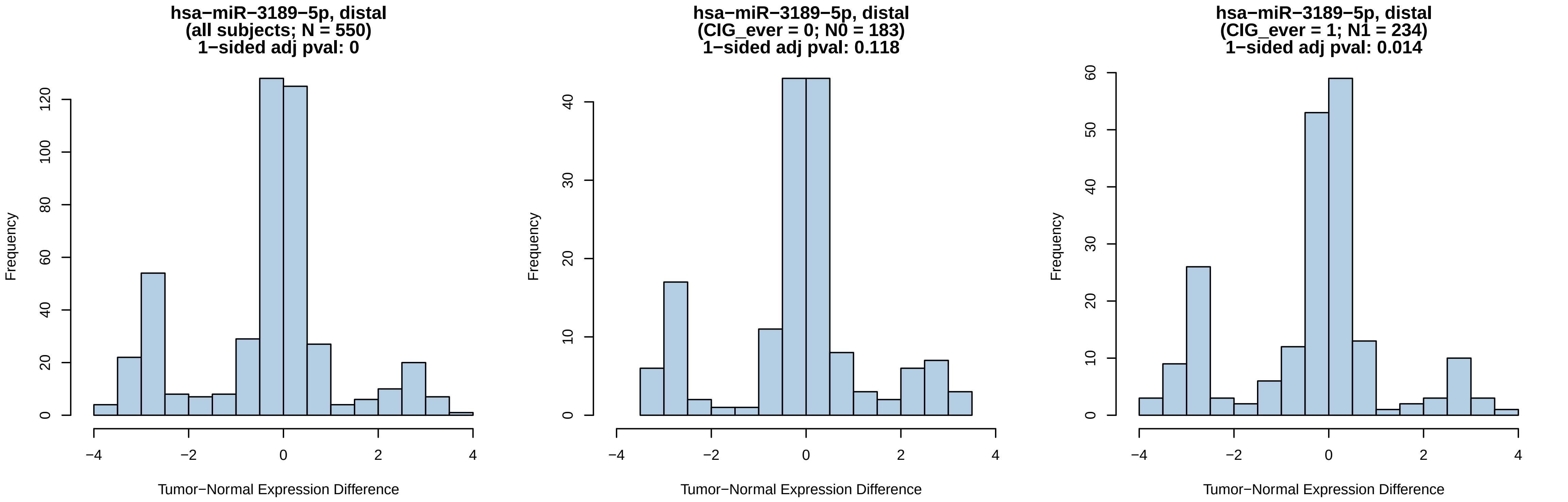
hsa−miR−3189−5p, distal
(all subjects; N = 550)
1−sided adj pval: 0
Frequency
0
20
40
60
80
100
120
−4
−2
0
2
4
Tumor−Normal Expression Difference
hsa−miR−3189−5p, distal
(CIG_ever = 0; N0 = 183)
1−sided adj pval: 0.118
Frequency
0
10
20
30
40
−4
−2
0
2
4
Tumor−Normal Expression Difference
hsa−miR−3189−5p, distal
(CIG_ever = 1; N1 = 234)
1−sided adj pval: 0.014
Frequency
0
10
20
30
40
50
60
−4
−2
0
2
4
Tumor−Normal Expression Difference

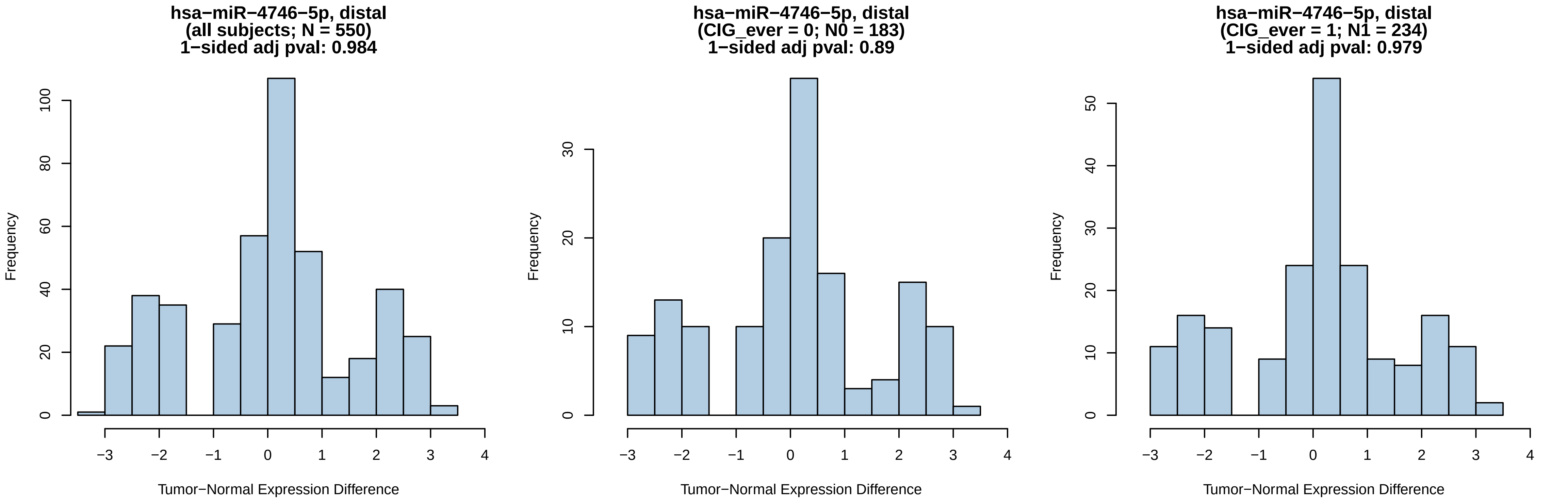
hsa−miR−4746−5p, distal
(all subjects; N = 550)
1−sided adj pval: 0.984
Frequency
Tumor−Normal Expression Difference
hsa−miR−4746−5p, distal
(CIG_ever = 0; N0 = 183)
1−sided adj pval: 0.89
Frequency
Tumor−Normal Expression Difference
hsa−miR−4746−5p, distal
(CIG_ever = 1; N1 = 234)
1−sided adj pval: 0.979
Frequency
Tumor−Normal Expression Difference

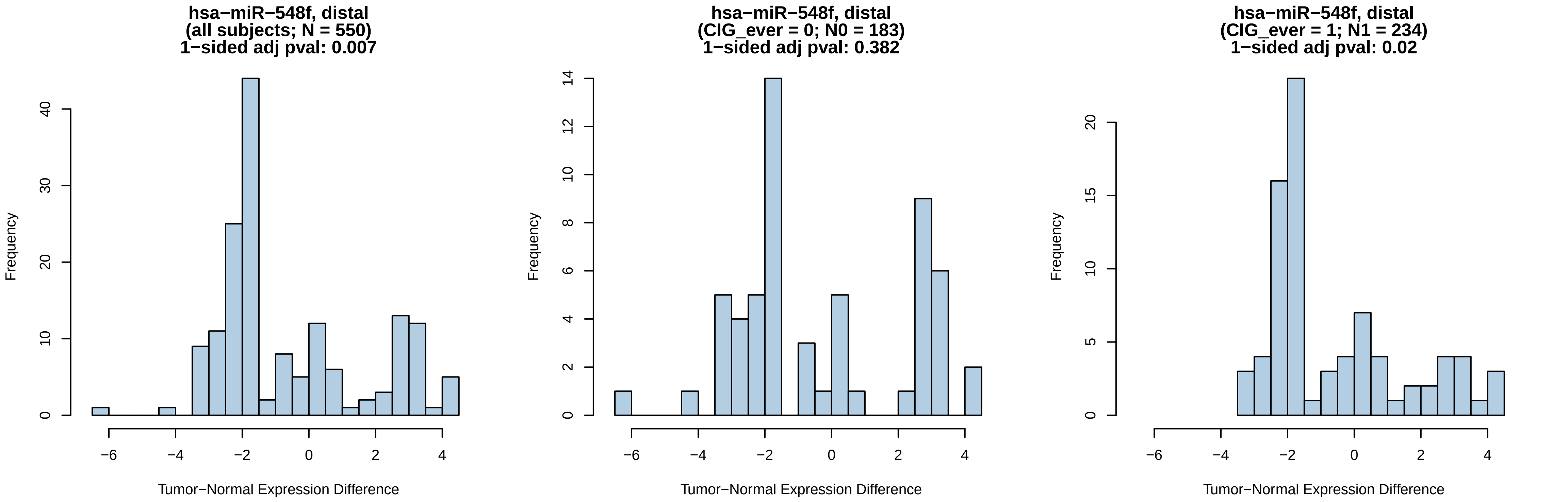
hsa−miR−548f, distal
(all subjects; N = 550)
1−sided adj pval: 0.007
Frequency
Tumor−Normal Expression Difference
hsa−miR−548f, distal
(CIG_ever = 0; N0 = 183)
1−sided adj pval: 0.382
Frequency
Tumor−Normal Expression Difference
hsa−miR−548f, distal
(CIG_ever = 1; N1 = 234)
1−sided adj pval: 0.02
Frequency
Tumor−Normal Expression Difference

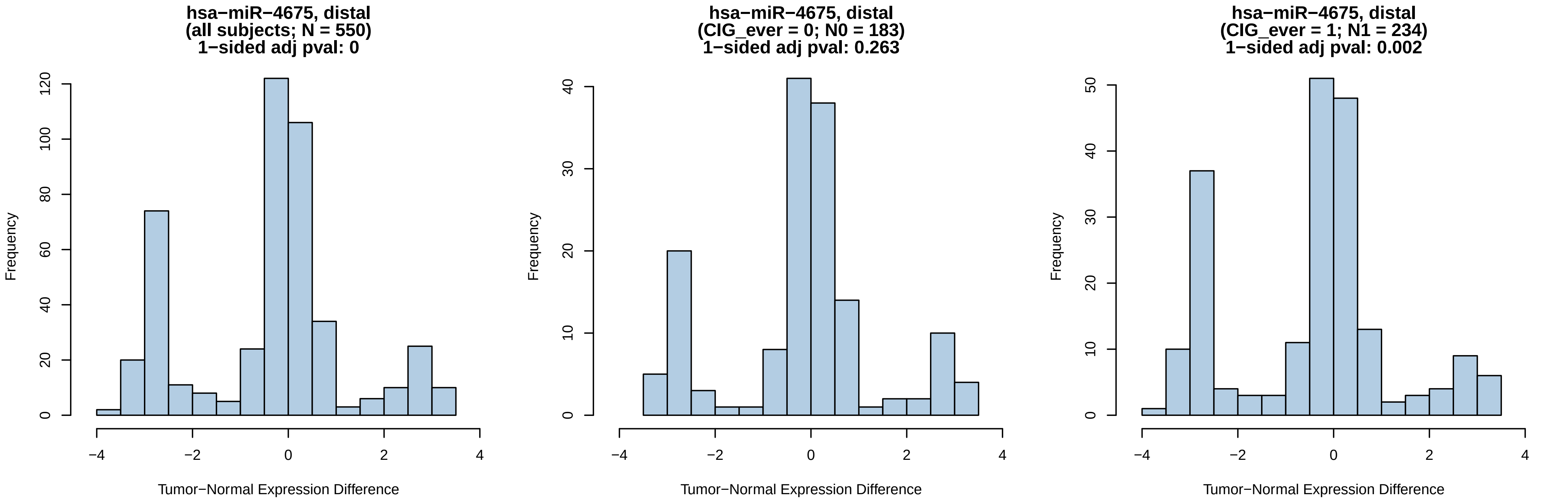
hsa−miR−4675, distal
(all subjects; N = 550)
1−sided adj pval: 0
Frequency
Tumor−Normal Expression Difference
0
20
40
60
80
100
120
−4
−2
0
2
4
hsa−miR−4675, distal
(CIG_ever = 0; N0 = 183)
1−sided adj pval: 0.263
Frequency
Tumor−Normal Expression Difference
0
10
20
30
40
hsa−miR−4675, distal
(CIG_ever = 1; N1 = 234)
1−sided adj pval: 0.002
Frequency
Tumor−Normal Expression Difference
0
10
20
30
40
50

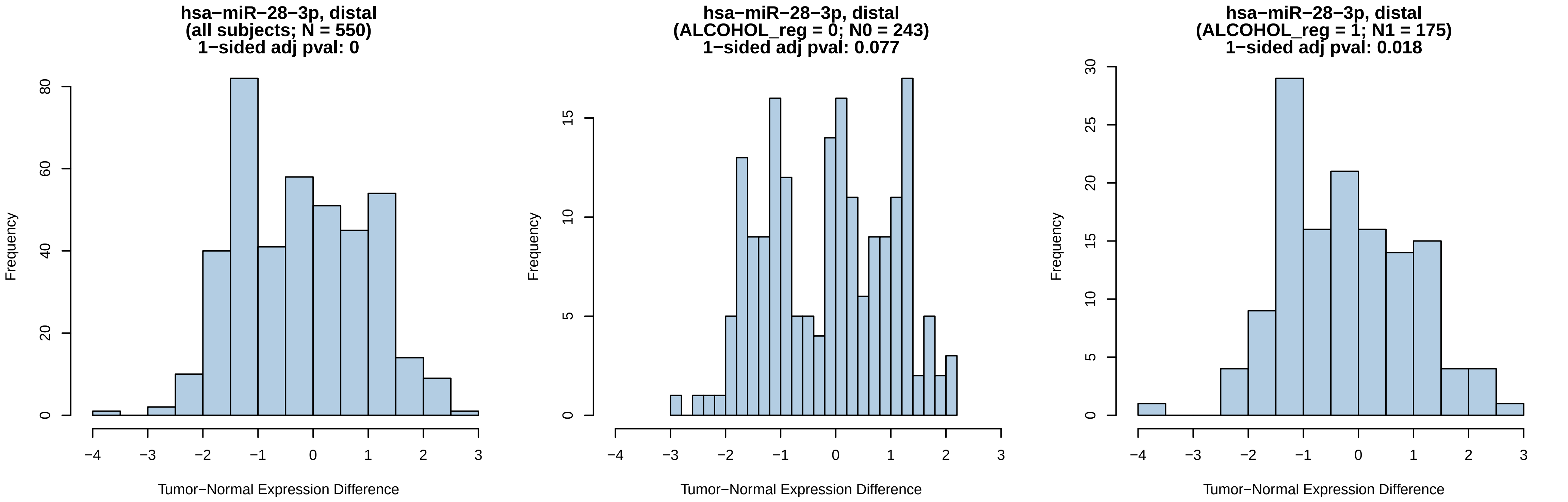
hsa−miR−28−3p, distal
(all subjects; N = 550)
1−sided adj pval: 0
Frequency
Tumor−Normal Expression Difference
hsa−miR−28−3p, distal
(ALCOHOL_reg = 0; N0 = 243)
1−sided adj pval: 0.077
Frequency
Tumor−Normal Expression Difference
hsa−miR−28−3p, distal
(ALCOHOL_reg = 1; N1 = 175)
1−sided adj pval: 0.018
Frequency
Tumor−Normal Expression Difference

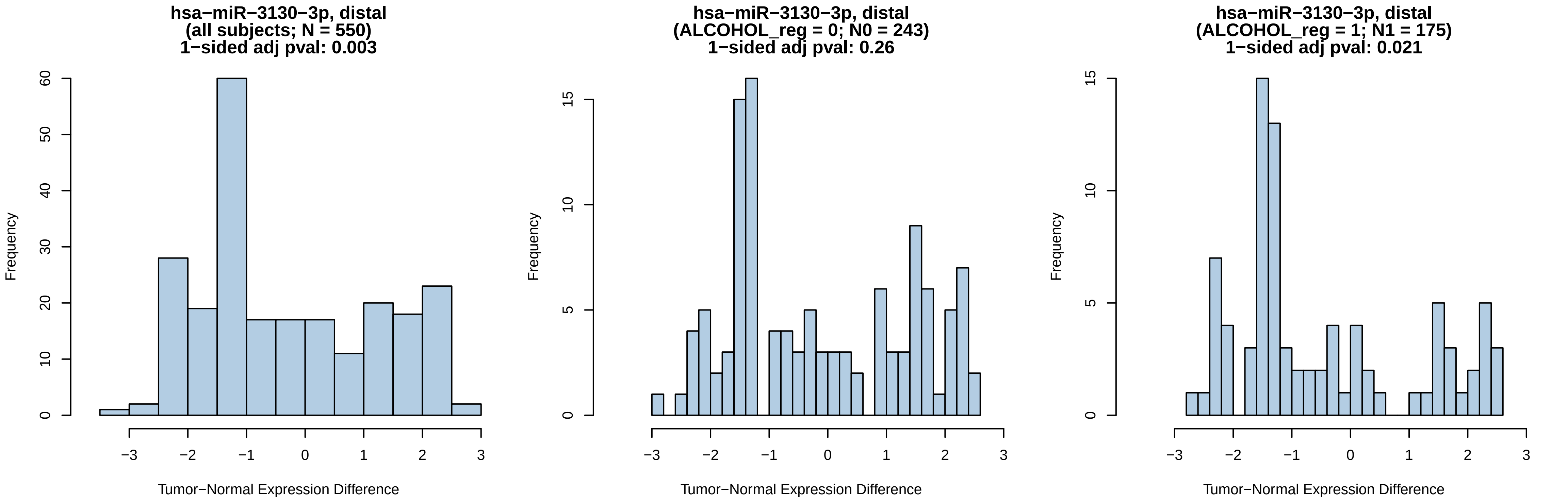
hsa−miR−3130−3p, distal
(all subjects; N = 550)
1−sided adj pval: 0.003
Frequency
Tumor−Normal Expression Difference
hsa−miR−3130−3p, distal
(ALCOHOL_reg = 0; N0 = 243)
1−sided adj pval: 0.26
Frequency
Tumor−Normal Expression Difference
hsa−miR−3130−3p, distal
(ALCOHOL_reg = 1; N1 = 175)
1−sided adj pval: 0.021
Frequency
Tumor−Normal Expression Difference

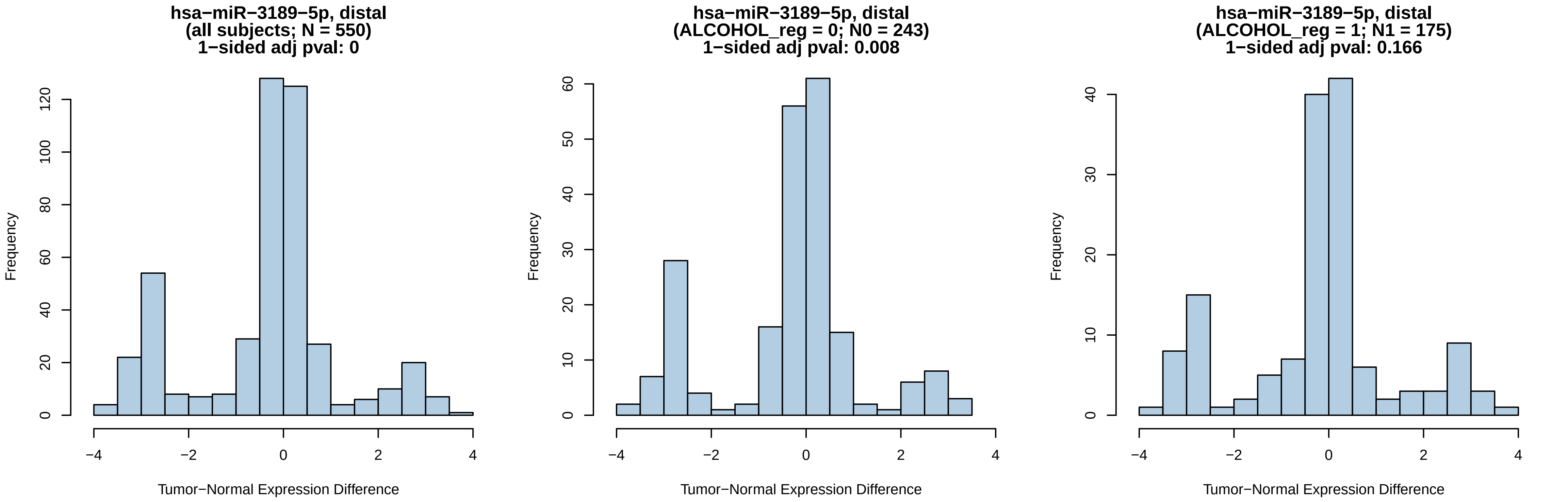
hsa−miR−3189−5p, distal
(all subjects; N = 550)
1−sided adj pval: 0
Frequency
0
20
40
60
80
100
120
−4
−2
0
2
4
Tumor−Normal Expression Difference
hsa−miR−3189−5p, distal
(ALCOHOL_reg = 0; N0 = 243)
1−sided adj pval: 0.008
Frequency
0
10
20
30
40
50
60
−4
−2
0
2
4
Tumor−Normal Expression Difference
hsa−miR−3189−5p, distal
(ALCOHOL_reg = 1; N1 = 175)
1−sided adj pval: 0.166
Frequency
0
10
20
30
40
−4
−2
0
2
4
Tumor−Normal Expression Difference

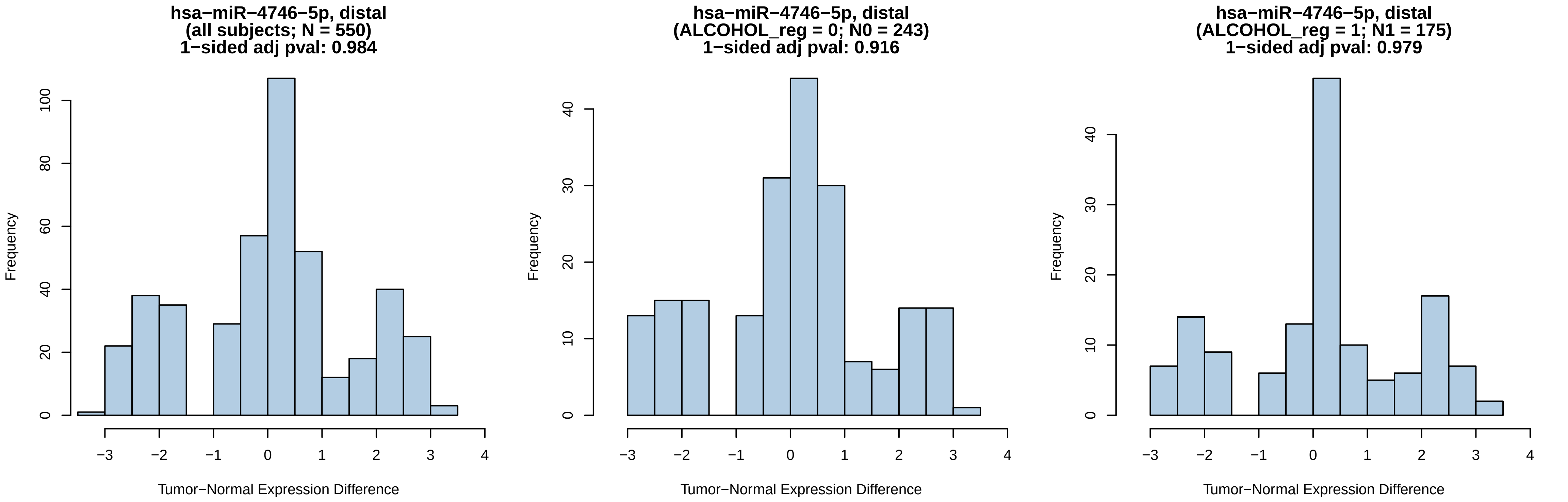
hsa−miR−4746−5p, distal
(all subjects; N = 550)
1−sided adj pval: 0.984
Frequency
Tumor−Normal Expression Difference
hsa−miR−4746−5p, distal
(ALCOHOL_reg = 0; N0 = 243)
1−sided adj pval: 0.916
Frequency
Tumor−Normal Expression Difference
hsa−miR−4746−5p, distal
(ALCOHOL_reg = 1; N1 = 175)
1−sided adj pval: 0.979
Frequency
Tumor−Normal Expression Difference

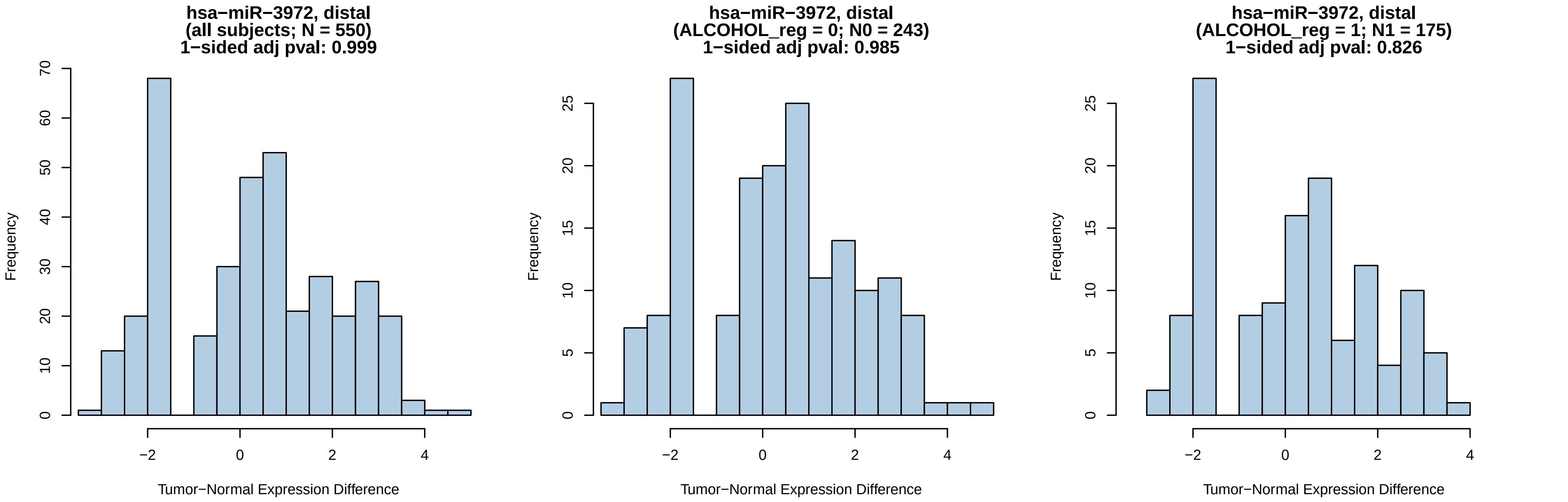
hsa−miR−3972, distal
(all subjects; N = 550)
1−sided adj pval: 0.999
Frequency
0
10
20
30
40
50
60
70
−2
0
2
4
Tumor−Normal Expression Difference
hsa−miR−3972, distal
(ALCOHOL_reg = 0; N0 = 243)
1−sided adj pval: 0.985
Frequency
0
5
10
15
20
25
−2
0
2
4
Tumor−Normal Expression Difference
hsa−miR−3972, distal
(ALCOHOL_reg = 1; N1 = 175)
1−sided adj pval: 0.826
Frequency
0
5
10
15
20
25
−2
0
2
4
Tumor−Normal Expression Difference

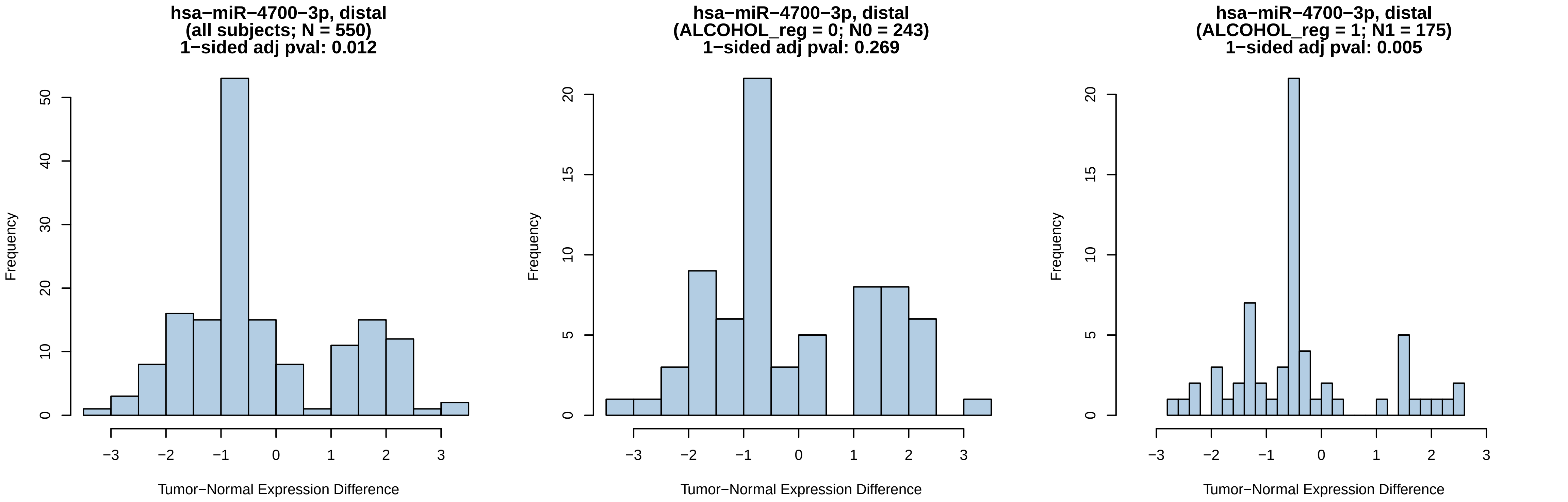

hsa−miR−4700−3p, distal
(all subjects; N = 550)
1−sided adj pval: 0.012
Frequency
0
10
20
30
40
50
−3
−2
−1
0
1
2
3
Tumor−Normal Expression Difference
hsa−miR−4700−3p, distal
(ALCOHOL_reg = 0; N0 = 243)
1−sided adj pval: 0.269
Frequency
0
5
10
15
20
−3
−2
−1
0
1
2
3
Tumor−Normal Expression Difference
hsa−miR−4700−3p, distal
(ALCOHOL_reg = 1; N1 = 175)
1−sided adj pval: 0.005
Frequency
0
5
10
15
20
−3
−2
−1
0
1
2
3
Tumor−Normal Expression Difference

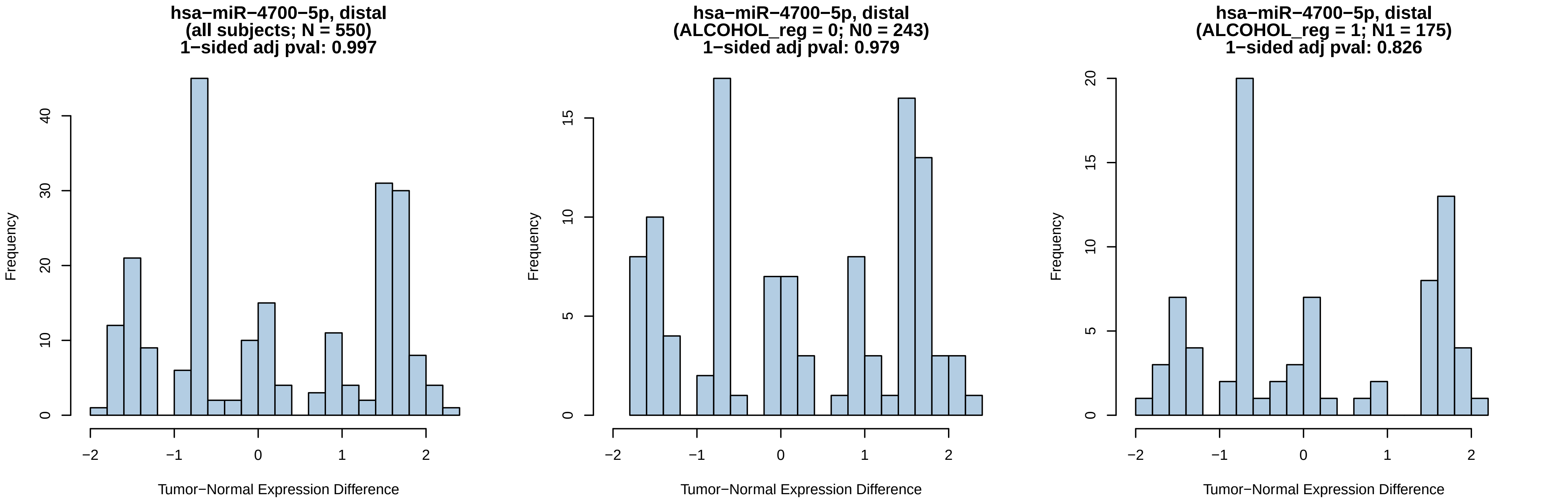
hsa−miR−4700−5p, distal
(all subjects; N = 550)
1−sided adj pval: 0.997
Frequency
Tumor−Normal Expression Difference
hsa−miR−4700−5p, distal
(ALCOHOL_reg = 0; N0 = 243)
1−sided adj pval: 0.979
Frequency
Tumor−Normal Expression Difference
hsa−miR−4700−5p, distal
(ALCOHOL_reg = 1; N1 = 175)
1−sided adj pval: 0.826
Frequency
Tumor−Normal Expression Difference

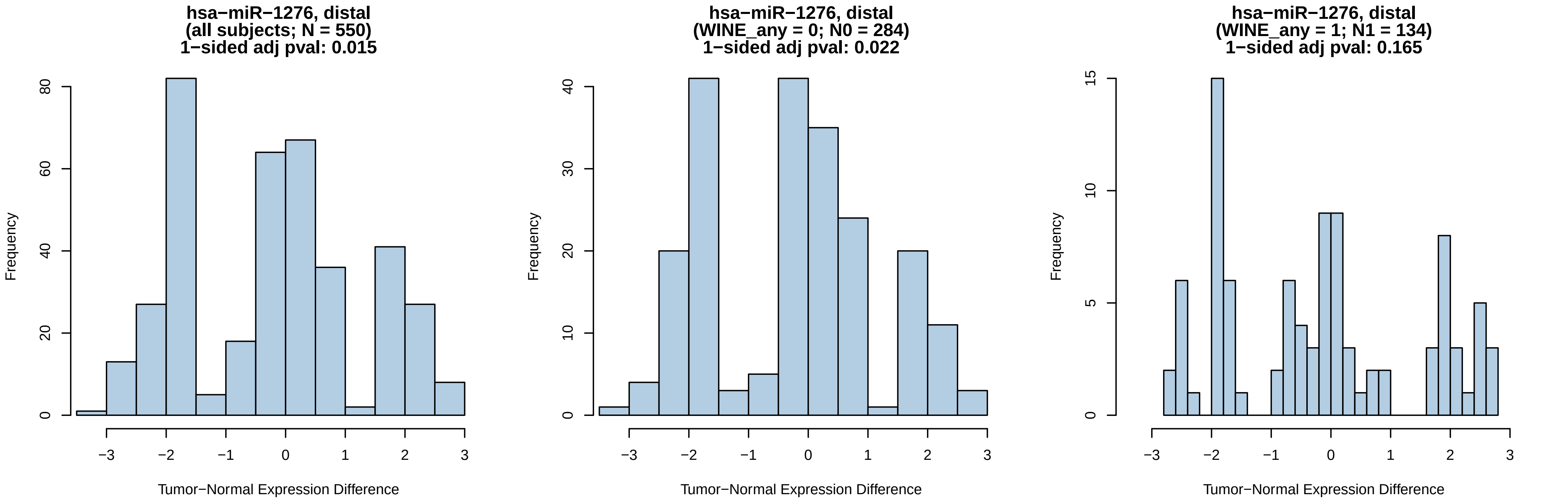
hsa−miR−1276, distal
(all subjects; N = 550)
1−sided adj pval: 0.015
hsa−miR−1276, distal
(WINE_any = 0; N0 = 284)
1−sided adj pval: 0.022
hsa−miR−1276, distal
(WINE_any = 1; N1 = 134)
1−sided adj pval: 0.165
Frequency
Tumor−Normal Expression Difference
0
20
40
60
80
10
30
5
15
−3
−2
−1
1
2
3

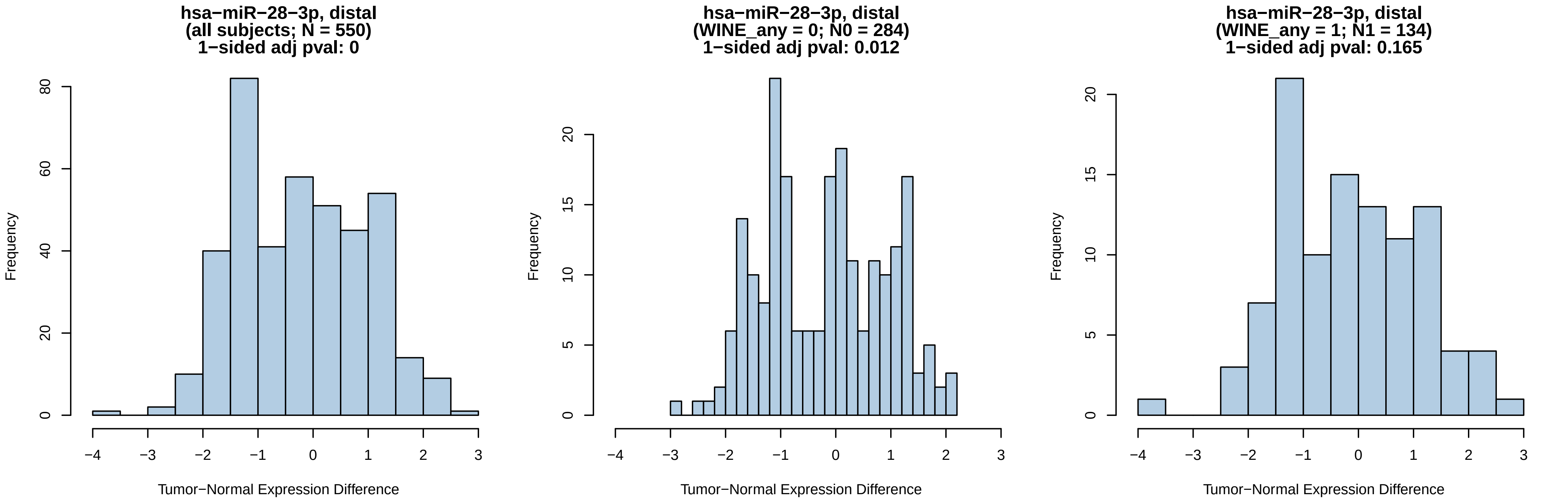

hsa−miR−28−3p, distal
(all subjects; N = 550)
1−sided adj pval: 0
Frequency
0
20
40
60
80
−4
−3
−2
−1
0
1
2
3
Tumor−Normal Expression Difference
hsa−miR−28−3p, distal
(WINE_any = 0; N0 = 284)
1−sided adj pval: 0.012
Frequency
0
5
10
15
20
−4
−3
−2
−1
0
1
2
3
Tumor−Normal Expression Difference
hsa−miR−28−3p, distal
(WINE_any = 1; N1 = 134)
1−sided adj pval: 0.165
Frequency
0
5
10
15
20
−4
−3
−2
−1
0
1
2
3
Tumor−Normal Expression Difference

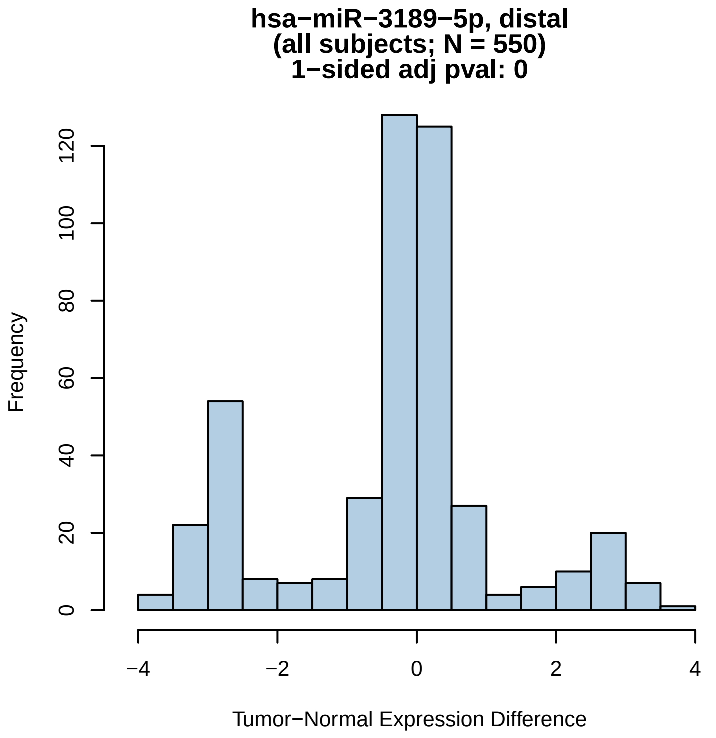
hsa−miR−3189−5p, distal
(all subjects; N = 550)
1−sided adj pval: 0
Frequency
0
20
40
60
80
100
120
−4
−2
0
2
4
Tumor−Normal Expression Difference

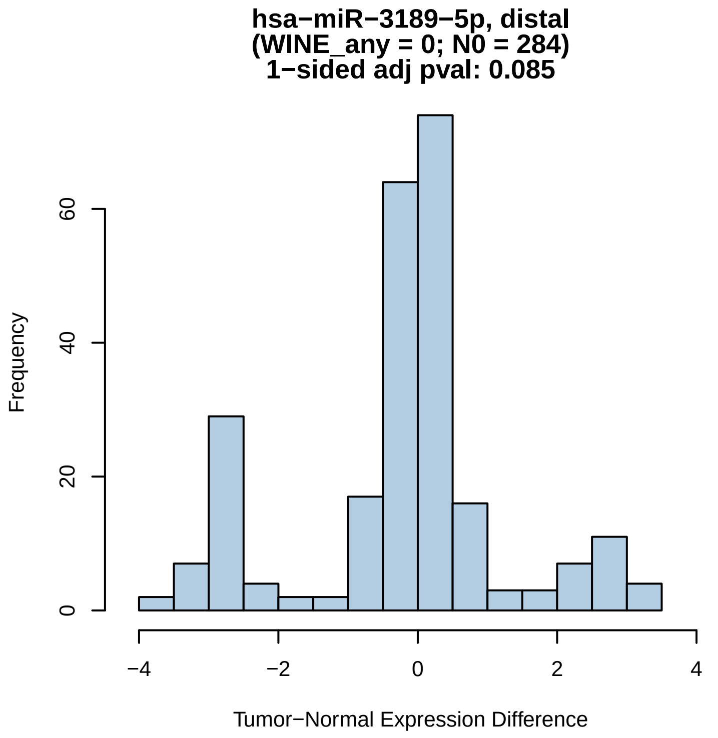
hsa−miR−3189−5p, distal
(WINE_any = 0; N0 = 284)
1−sided adj pval: 0.085
Frequency
0
20
40
60
−4
−2
0
2
4
Tumor−Normal Expression Difference

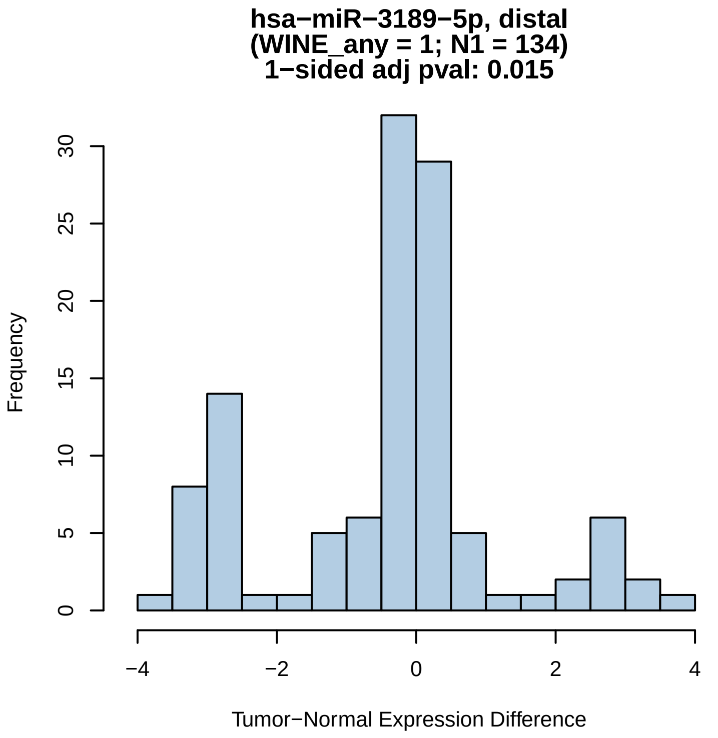
hsa−miR−3189−5p, distal
(WINE_any = 1; N1 = 134)
1−sided adj pval: 0.015
Frequency
0
5
10
15
20
25
30
−4
−2
0
2
4
Tumor−Normal Expression Difference

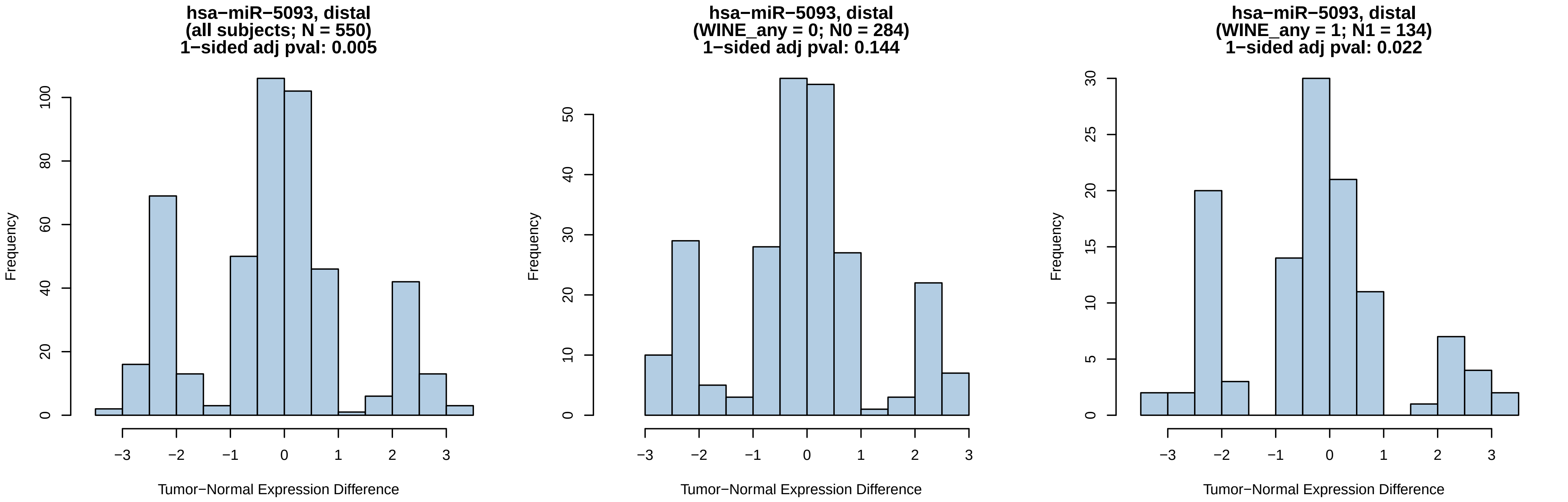

hsa−miR−5093, distal
(all subjects; N = 550)
1−sided adj pval: 0.005
Frequency
Tumor−Normal Expression Difference
hsa−miR−5093, distal
(WINE_any = 0; N0 = 284)
1−sided adj pval: 0.144
Frequency
Tumor−Normal Expression Difference
hsa−miR−5093, distal
(WINE_any = 1; N1 = 134)
1−sided adj pval: 0.022
Frequency
Tumor−Normal Expression Difference

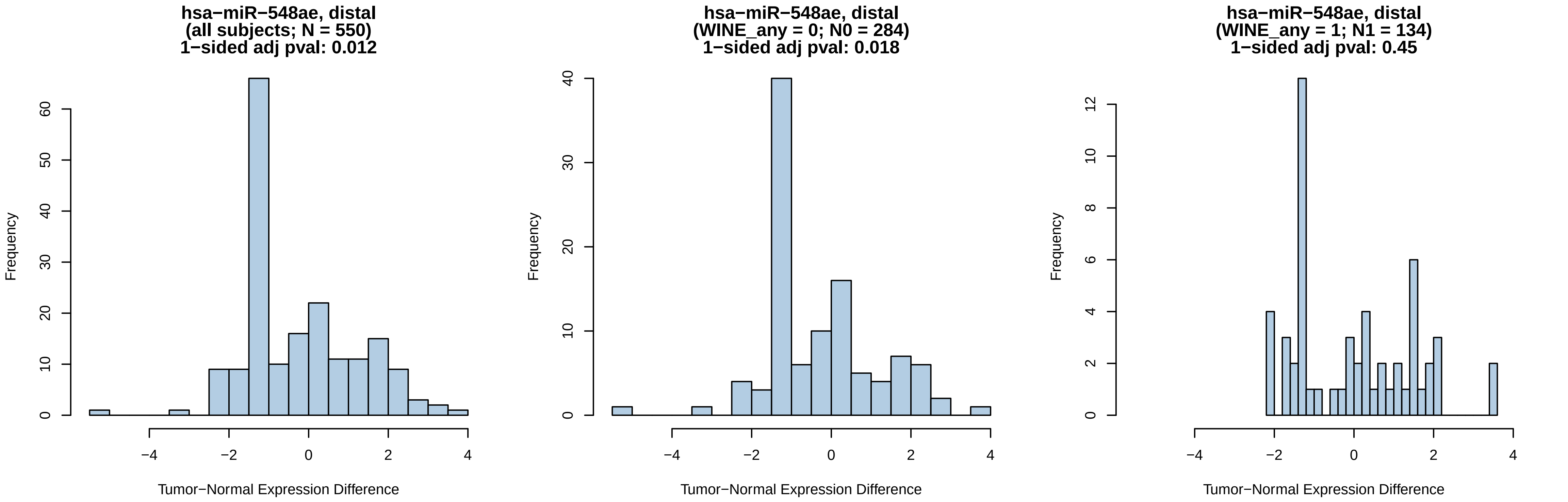
hsa−miR−548ae, distal
(all subjects; N = 550)
1−sided adj pval: 0.012
Frequency
Tumor−Normal Expression Difference
hsa−miR−548ae, distal
(WINE_any = 0; N0 = 284)
1−sided adj pval: 0.018
Frequency
Tumor−Normal Expression Difference
hsa−miR−548ae, distal
(WINE_any = 1; N1 = 134)
1−sided adj pval: 0.45
Frequency
Tumor−Normal Expression Difference

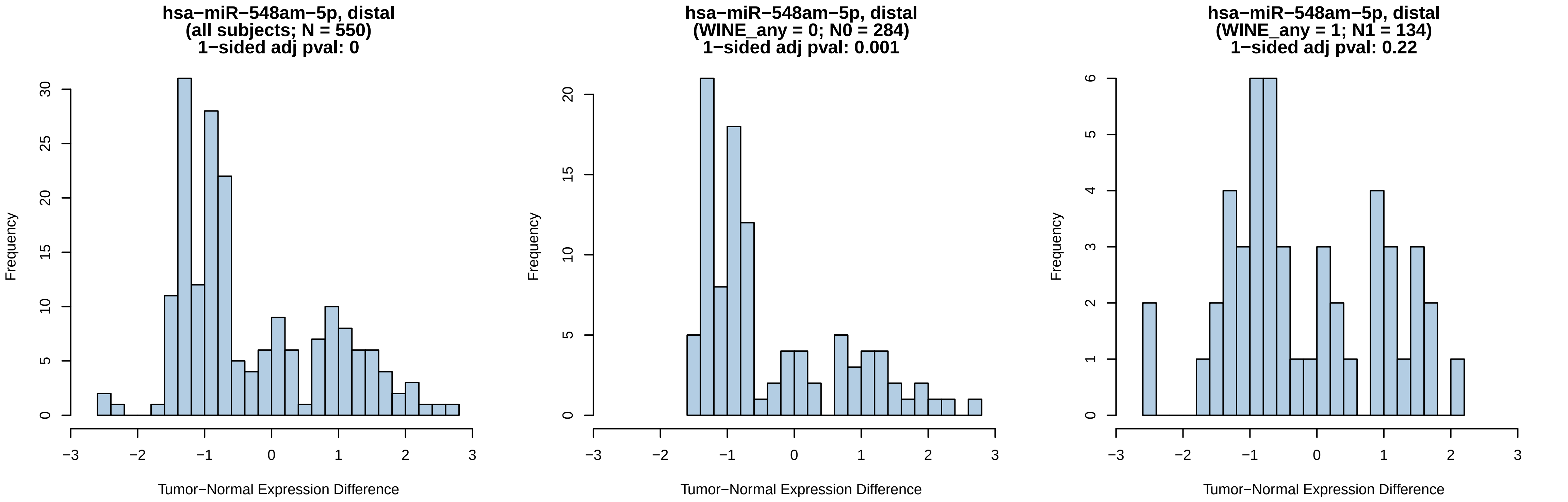
hsa−miR−548am−5p, distal
(all subjects; N = 550)
1−sided adj pval: 0
Frequency
Tumor−Normal Expression Difference
hsa−miR−548am−5p, distal
(WINE_any = 0; N0 = 284)
1−sided adj pval: 0.001
Frequency
Tumor−Normal Expression Difference
hsa−miR−548am−5p, distal
(WINE_any = 1; N1 = 134)
1−sided adj pval: 0.22
Frequency
Tumor−Normal Expression Difference

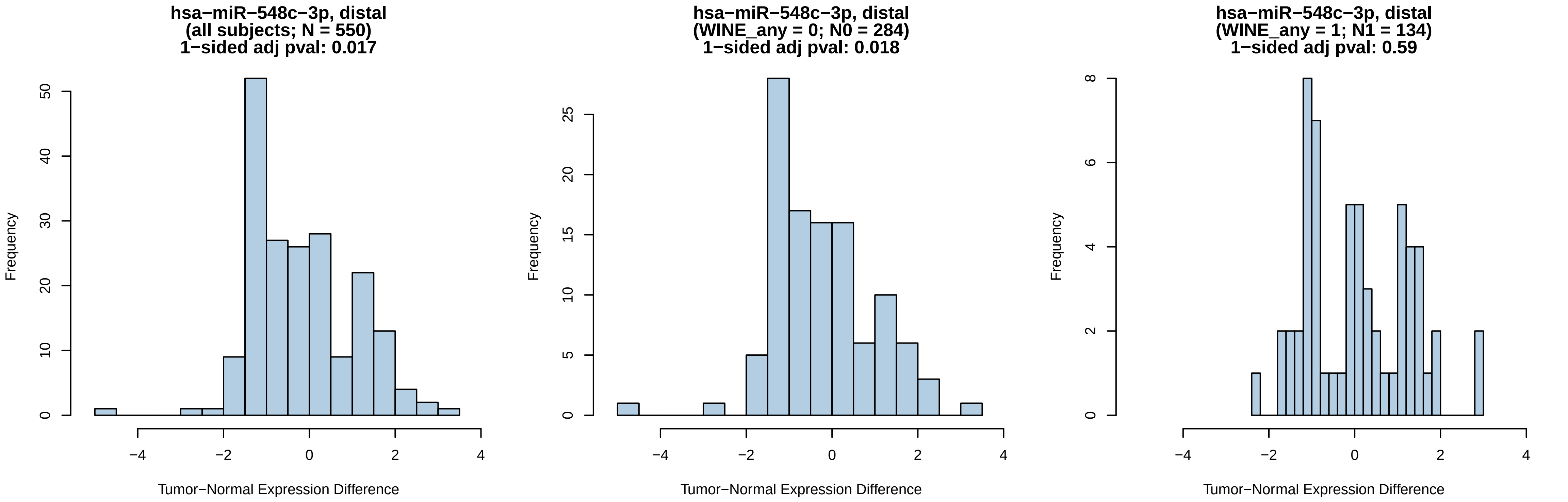
hsa−miR−548c−3p, distal
(all subjects; N = 550)
1−sided adj pval: 0.017
Frequency
0
10
20
30
40
50
−4
−2
0
2
4
Tumor−Normal Expression Difference
hsa−miR−548c−3p, distal
(WINE_any = 0; N0 = 284)
1−sided adj pval: 0.018
Frequency
0
5
10
15
20
25
−4
−2
0
2
4
Tumor−Normal Expression Difference
hsa−miR−548c−3p, distal
(WINE_any = 1; N1 = 134)
1−sided adj pval: 0.59
Frequency
0
2
4
6
8
−4
−2
0
2
4
Tumor−Normal Expression Difference

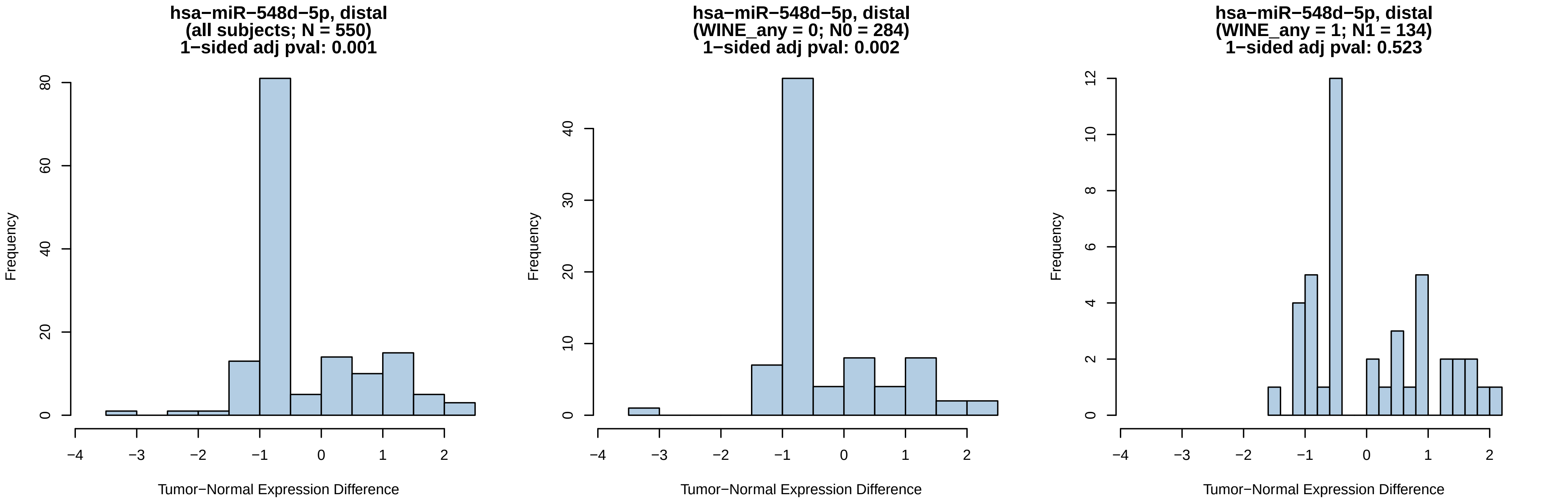
hsa−miR−548d−5p, distal
(all subjects; N = 550)
1−sided adj pval: 0.001
Frequency
Tumor−Normal Expression Difference
hsa−miR−548d−5p, distal
(WINE_any = 0; N0 = 284)
1−sided adj pval: 0.002
Frequency
Tumor−Normal Expression Difference
hsa−miR−548d−5p, distal
(WINE_any = 1; N1 = 134)
1−sided adj pval: 0.523
Frequency
Tumor−Normal Expression Difference

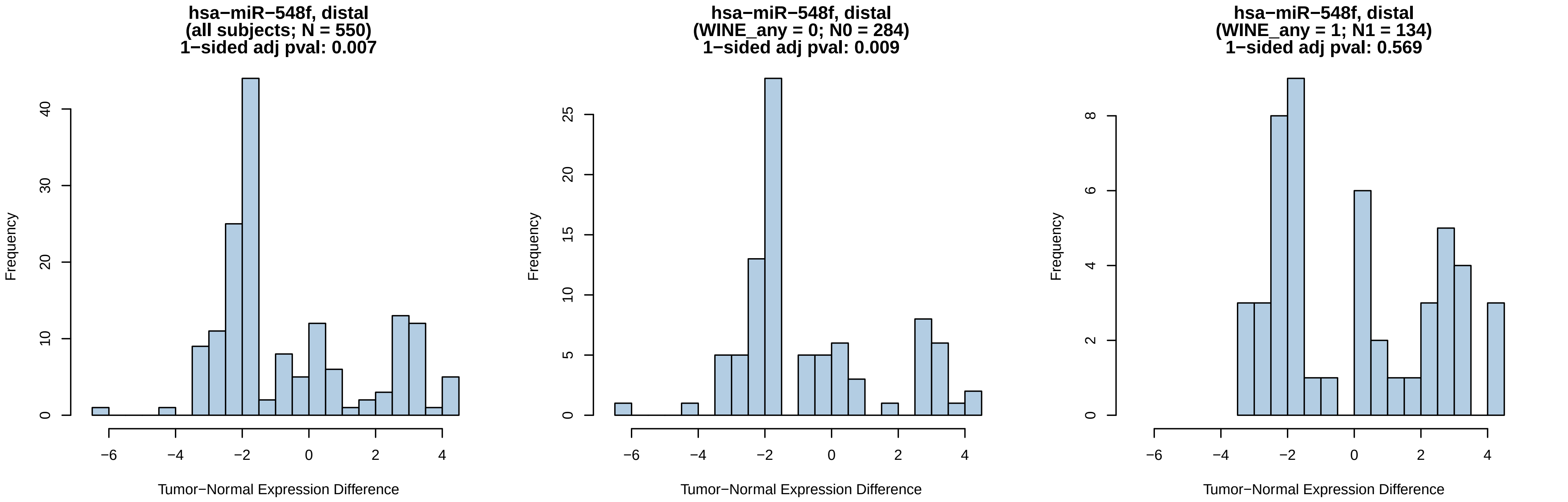

hsa−miR−548f, distal
(all subjects; N = 550)
1−sided adj pval: 0.007
Frequency
Tumor−Normal Expression Difference
hsa−miR−548f, distal
(WINE_any = 0; N0 = 284)
1−sided adj pval: 0.009
Frequency
Tumor−Normal Expression Difference
hsa−miR−548f, distal
(WINE_any = 1; N1 = 134)
1−sided adj pval: 0.569
Frequency
Tumor−Normal Expression Difference

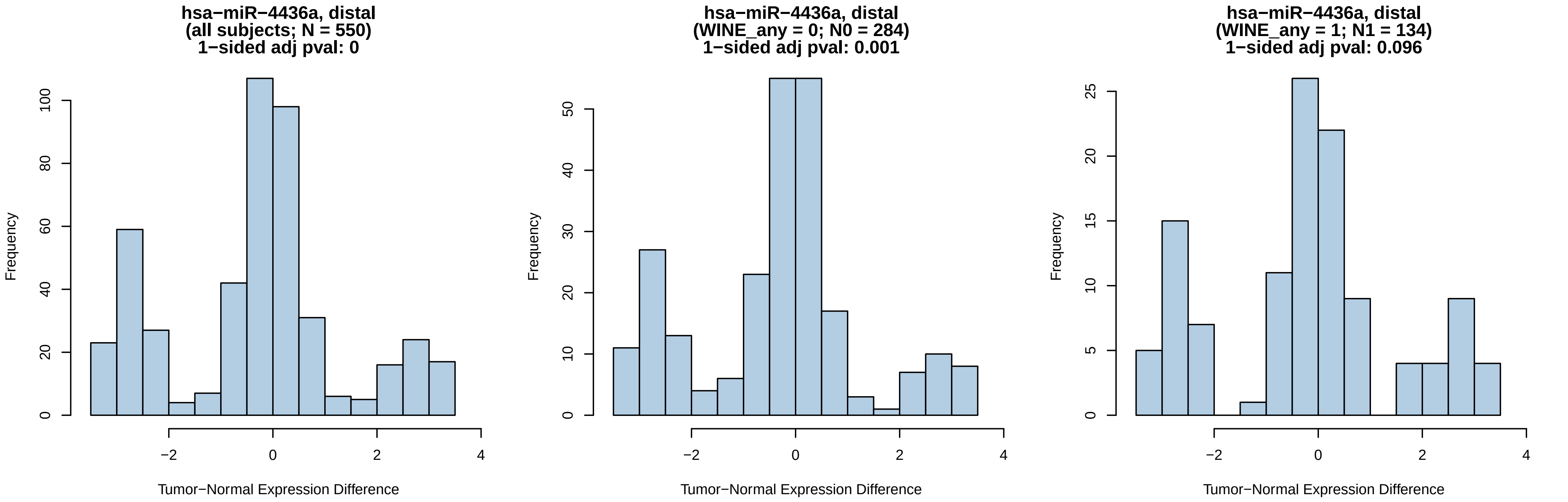

hsa−miR−4436a, distal
(all subjects; N = 550)
1−sided adj pval: 0
Frequency
0
20
40
60
80
100
−2
0
2
4
Tumor−Normal Expression Difference
hsa−miR−4436a, distal
(WINE_any = 0; N0 = 284)
1−sided adj pval: 0.001
Frequency
0
10
20
30
40
50
−2
0
2
4
Tumor−Normal Expression Difference
hsa−miR−4436a, distal
(WINE_any = 1; N1 = 134)
1−sided adj pval: 0.096
Frequency
0
5
10
15
20
25
−2
0
2
4
Tumor−Normal Expression Difference

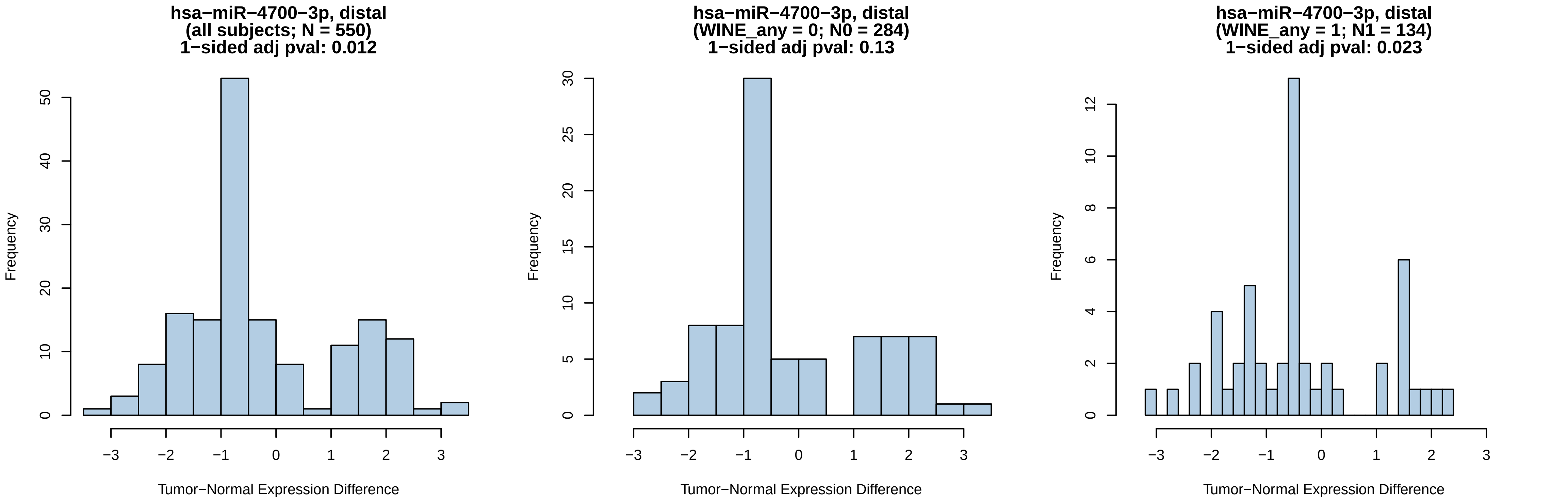
hsa−miR−4700−3p, distal
(all subjects; N = 550)
1−sided adj pval: 0.012
Frequency
Tumor−Normal Expression Difference
hsa−miR−4700−3p, distal
(WINE_any = 0; N0 = 284)
1−sided adj pval: 0.13
Frequency
Tumor−Normal Expression Difference
hsa−miR−4700−3p, distal
(WINE_any = 1; N1 = 134)
1−sided adj pval: 0.023
Frequency
Tumor−Normal Expression Difference

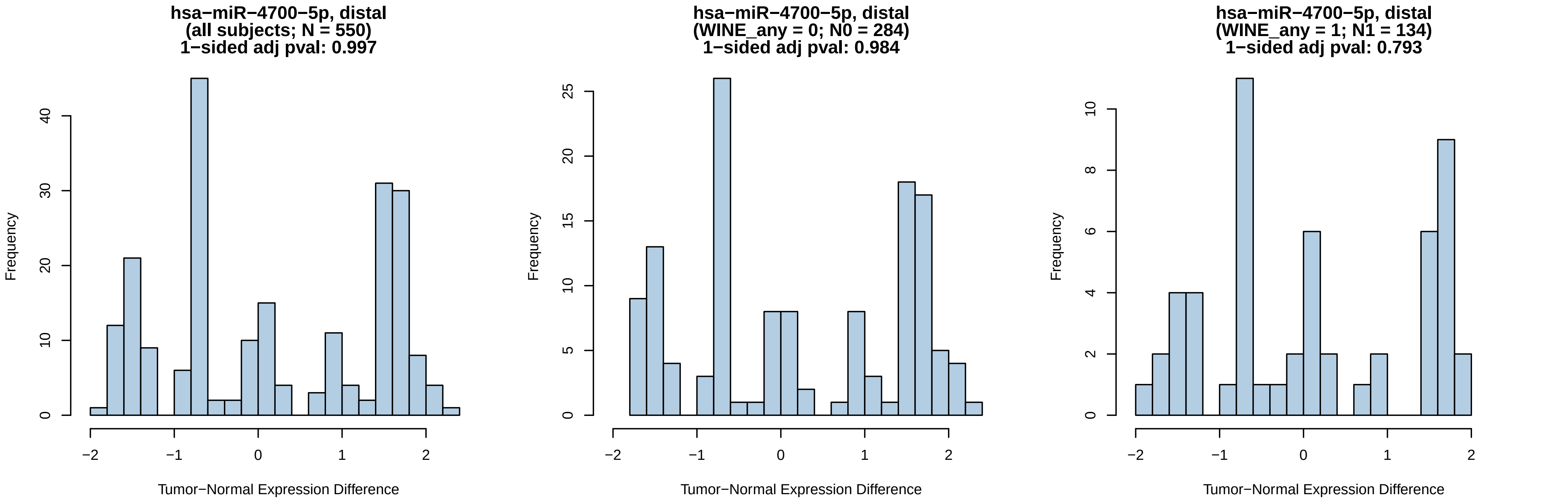

hsa−miR−4700−5p, distal
(all subjects; N = 550)
1−sided adj pval: 0.997
Frequency
Tumor−Normal Expression Difference
hsa−miR−4700−5p, distal
(WINE_any = 0; N0 = 284)
1−sided adj pval: 0.984
Frequency
Tumor−Normal Expression Difference
hsa−miR−4700−5p, distal
(WINE_any = 1; N1 = 134)
1−sided adj pval: 0.793
Frequency
Tumor−Normal Expression Difference

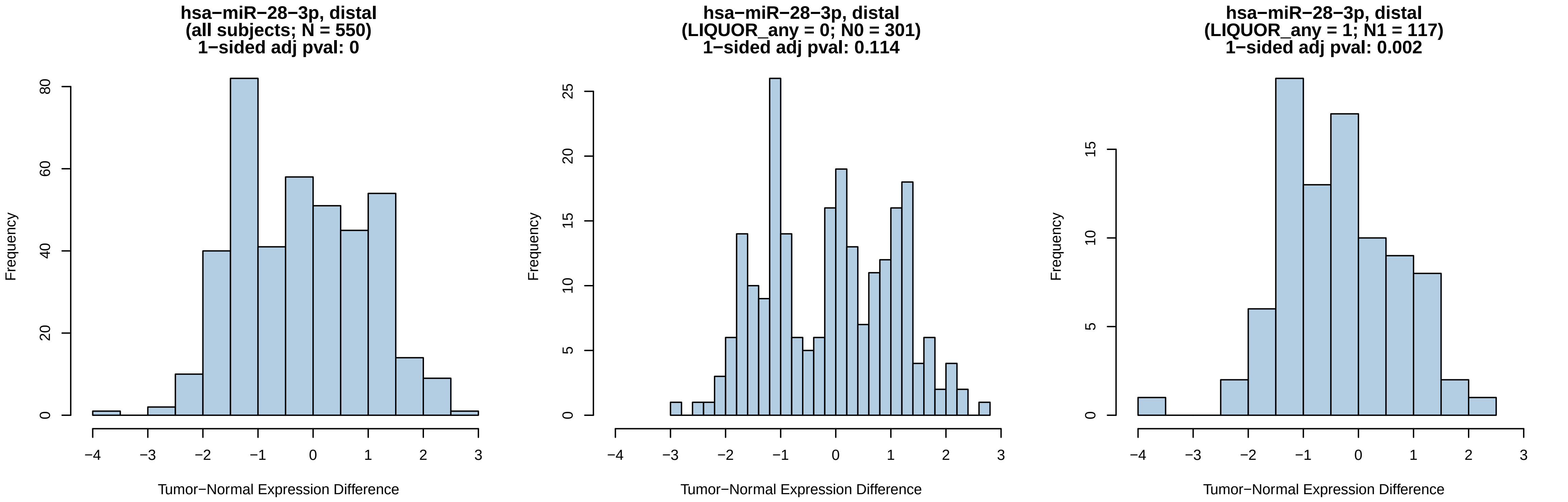
hsa−miR−28−3p, distal
(all subjects; N = 550)
1−sided adj pval: 0
Frequency
Tumor−Normal Expression Difference
hsa−miR−28−3p, distal
(LIQUOR_any = 0; N0 = 301)
1−sided adj pval: 0.114
Frequency
Tumor−Normal Expression Difference
hsa−miR−28−3p, distal
(LIQUOR_any = 1; N1 = 117)
1−sided adj pval: 0.002
Frequency
Tumor−Normal Expression Difference

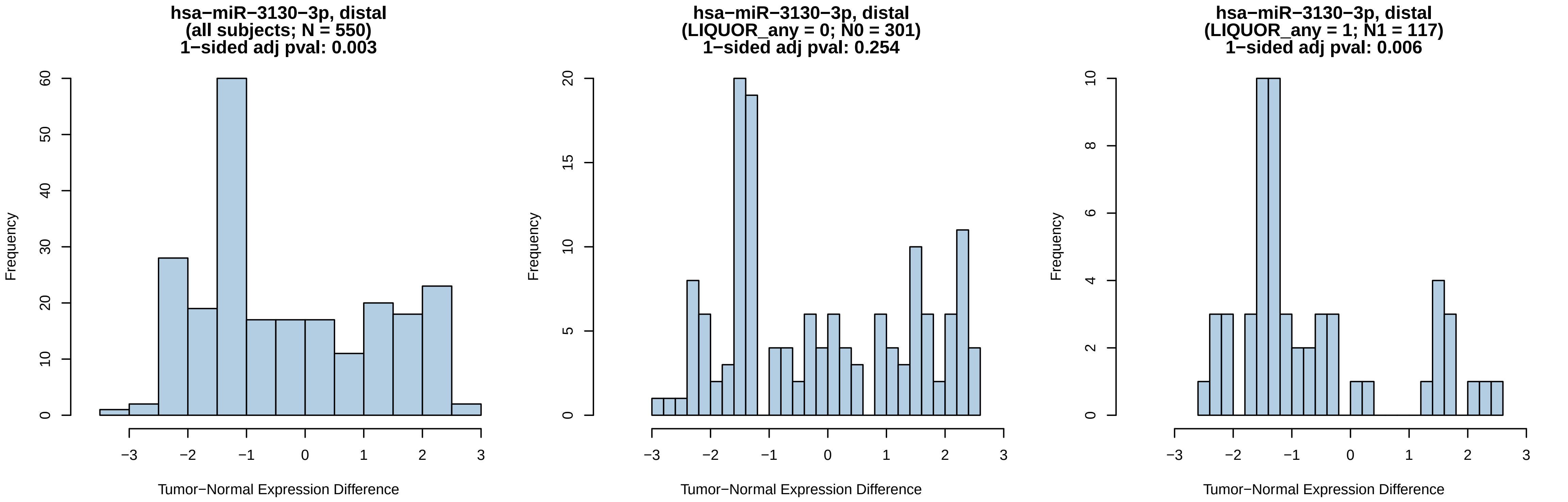

hsa−miR−3130−3p, distal
(all subjects; N = 550)
1−sided adj pval: 0.003
Frequency
Tumor−Normal Expression Difference
hsa−miR−3130−3p, distal
(LIQUOR_any = 0; N0 = 301)
1−sided adj pval: 0.254
Frequency
Tumor−Normal Expression Difference
hsa−miR−3130−3p, distal
(LIQUOR_any = 1; N1 = 117)
1−sided adj pval: 0.006
Frequency
Tumor−Normal Expression Difference

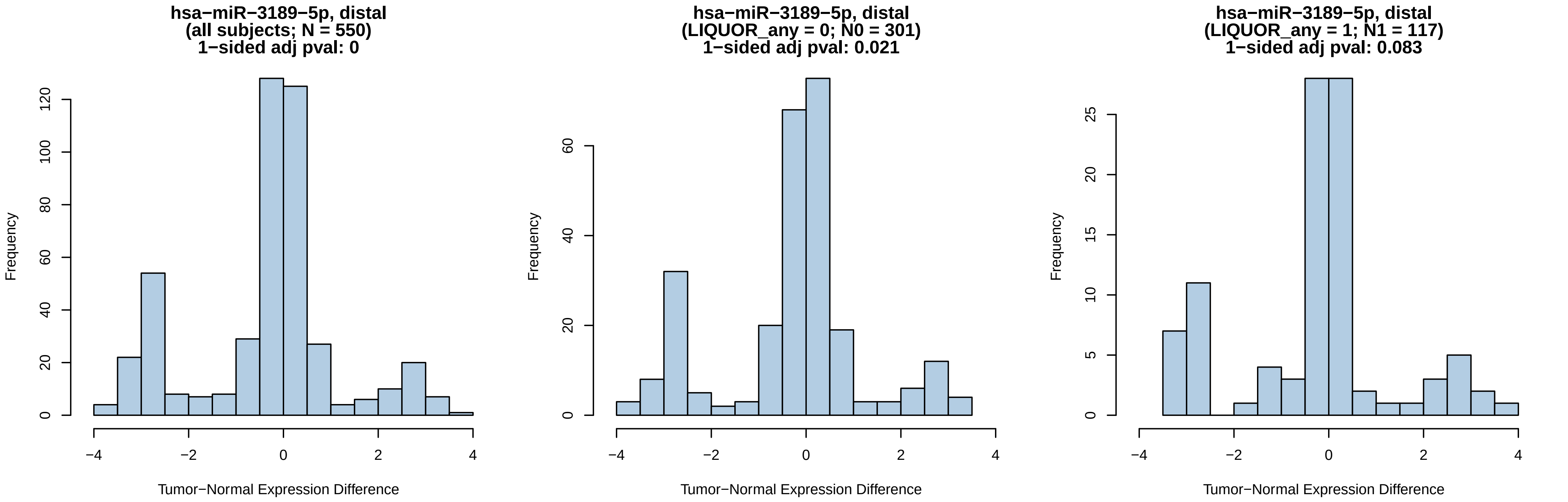
hsa−miR−3189−5p, distal
(all subjects; N = 550)
1−sided adj pval: 0
hsa−miR−3189−5p, distal
(LIQUOR_any = 0; N0 = 301)
1−sided adj pval: 0.021
hsa−miR−3189−5p, distal
(LIQUOR_any = 1; N1 = 117)
1−sided adj pval: 0.083
Frequency
Tumor−Normal Expression Difference
0
20
40
60
80
100
120
−4
−2
0
2
4
5
10
15
25

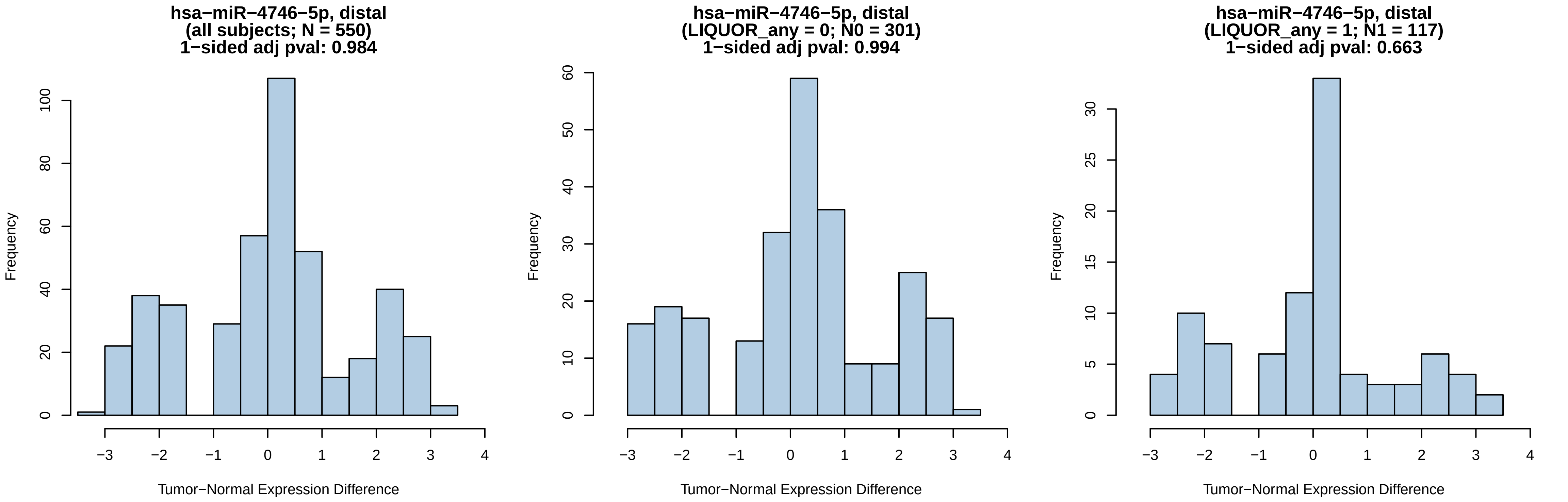
hsa−miR−4746−5p, distal
(all subjects; N = 550)
1−sided adj pval: 0.984
Frequency
Tumor−Normal Expression Difference
hsa−miR−4746−5p, distal
(LIQUOR_any = 0; N0 = 301)
1−sided adj pval: 0.994
Frequency
Tumor−Normal Expression Difference
hsa−miR−4746−5p, distal
(LIQUOR_any = 1; N1 = 117)
1−sided adj pval: 0.663
Frequency
Tumor−Normal Expression Difference

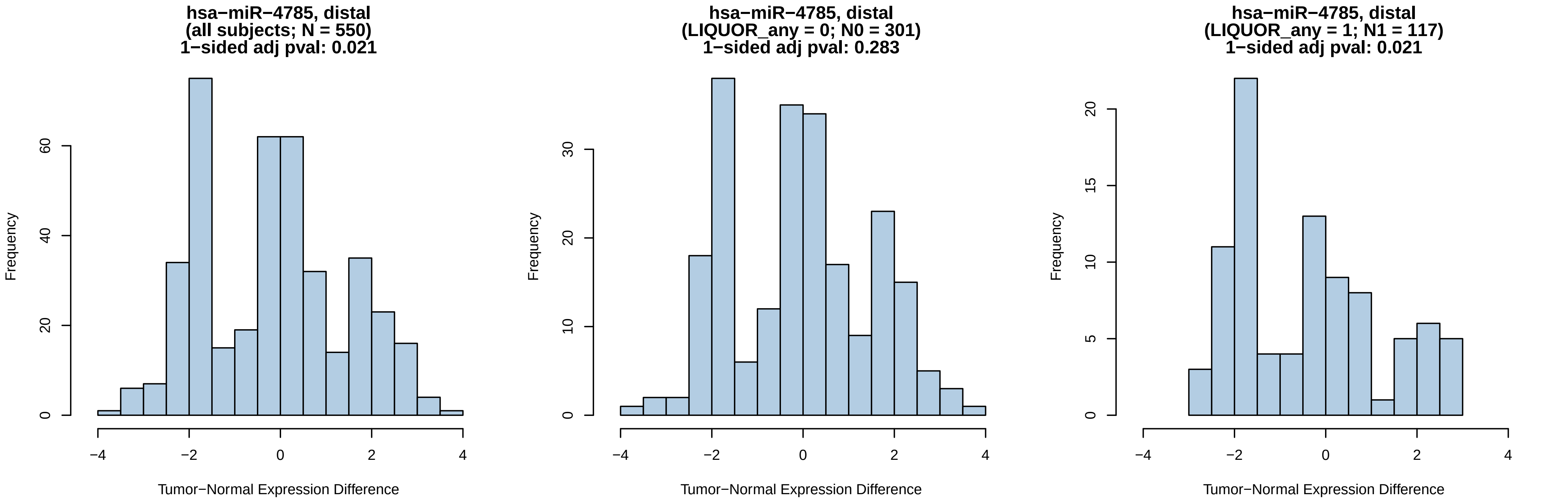

hsa−miR−4785, distal
(all subjects; N = 550)
1−sided adj pval: 0.021
Frequency
Tumor−Normal Expression Difference
hsa−miR−4785, distal
(LIQUOR_any = 0; N0 = 301)
1−sided adj pval: 0.283
Frequency
Tumor−Normal Expression Difference
hsa−miR−4785, distal
(LIQUOR_any = 1; N1 = 117)
1−sided adj pval: 0.021
Frequency
Tumor−Normal Expression Difference

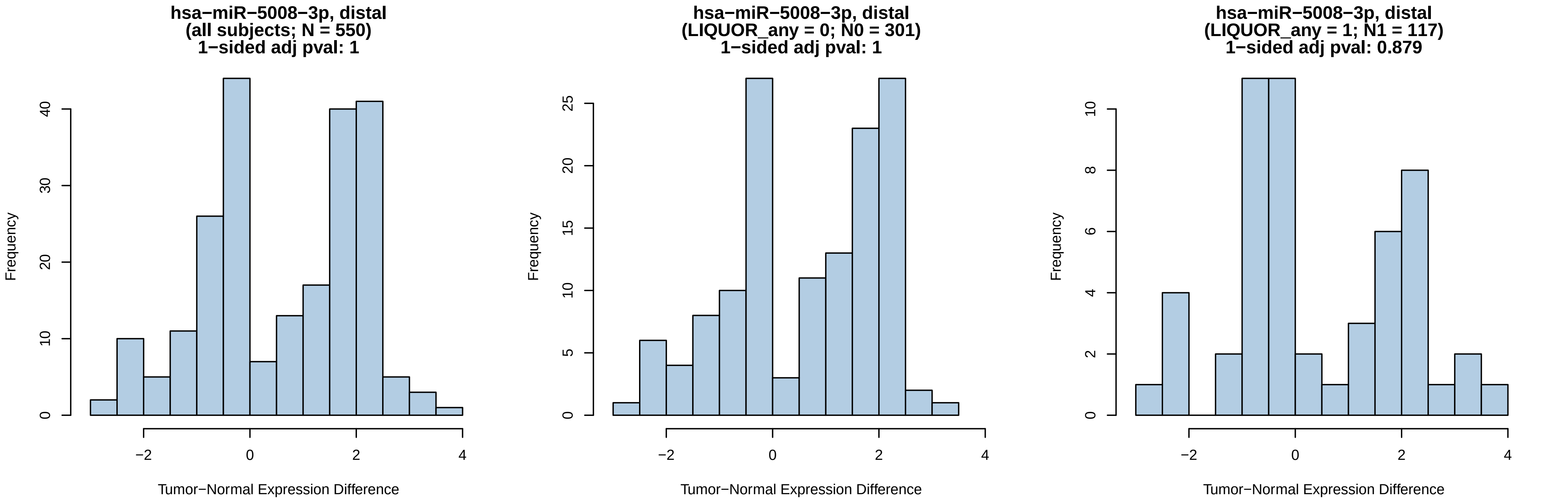
hsa−miR−5008−3p, distal
(all subjects; N = 550)
1−sided adj pval: 1
hsa−miR−5008−3p, distal
(LIQUOR_any = 0; N0 = 301)
1−sided adj pval: 1
hsa−miR−5008−3p, distal
(LIQUOR_any = 1; N1 = 117)
1−sided adj pval: 0.879
Frequency
Tumor−Normal Expression Difference
0
10
20
30
40
5
15
25
2
4
6
8
−2
0
2
4

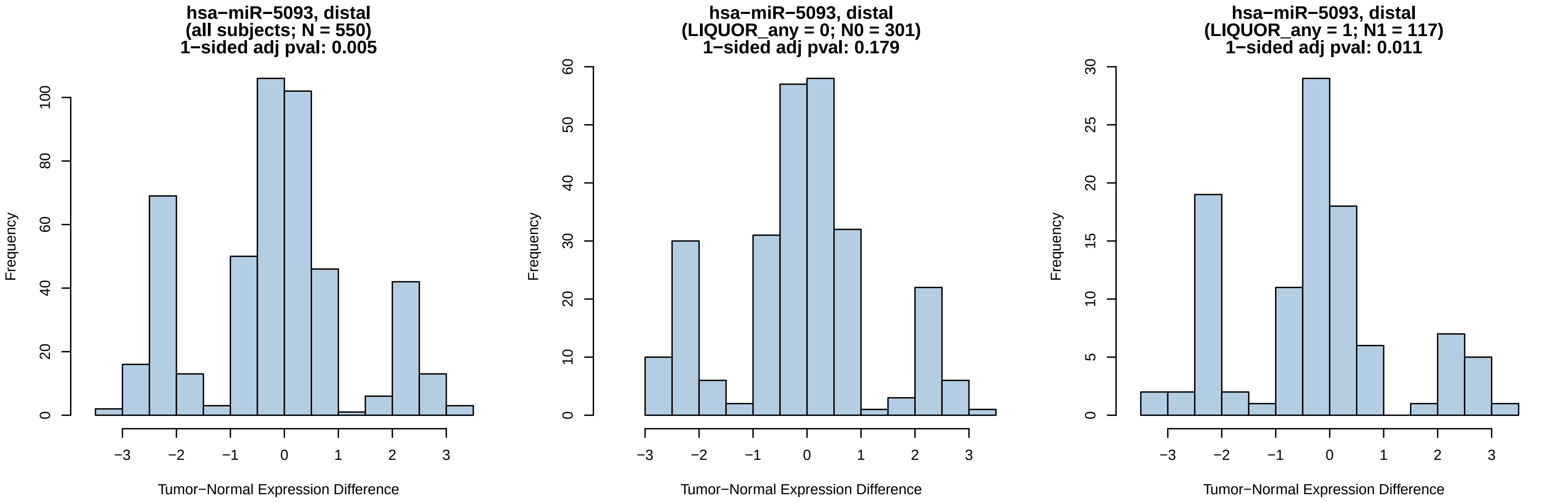
hsa−miR−5093, distal
(all subjects; N = 550)
1−sided adj pval: 0.005
Frequency
Tumor−Normal Expression Difference
hsa−miR−5093, distal
(LIQUOR_any = 0; N0 = 301)
1−sided adj pval: 0.179
Frequency
Tumor−Normal Expression Difference
hsa−miR−5093, distal
(LIQUOR_any = 1; N1 = 117)
1−sided adj pval: 0.011
Frequency
Tumor−Normal Expression Difference

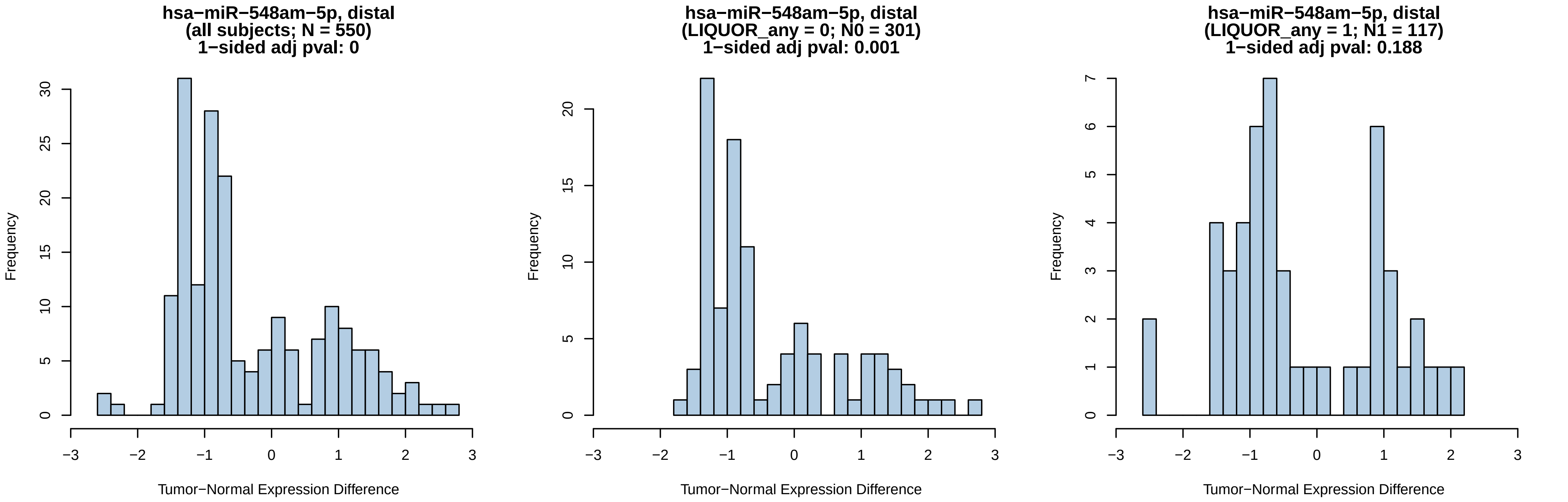
hsa−miR−548am−5p, distal
(all subjects; N = 550)
1−sided adj pval: 0
Frequency
Tumor−Normal Expression Difference
hsa−miR−548am−5p, distal
(LIQUOR_any = 0; N0 = 301)
1−sided adj pval: 0.001
Frequency
Tumor−Normal Expression Difference
hsa−miR−548am−5p, distal
(LIQUOR_any = 1; N1 = 117)
1−sided adj pval: 0.188
Frequency
Tumor−Normal Expression Difference

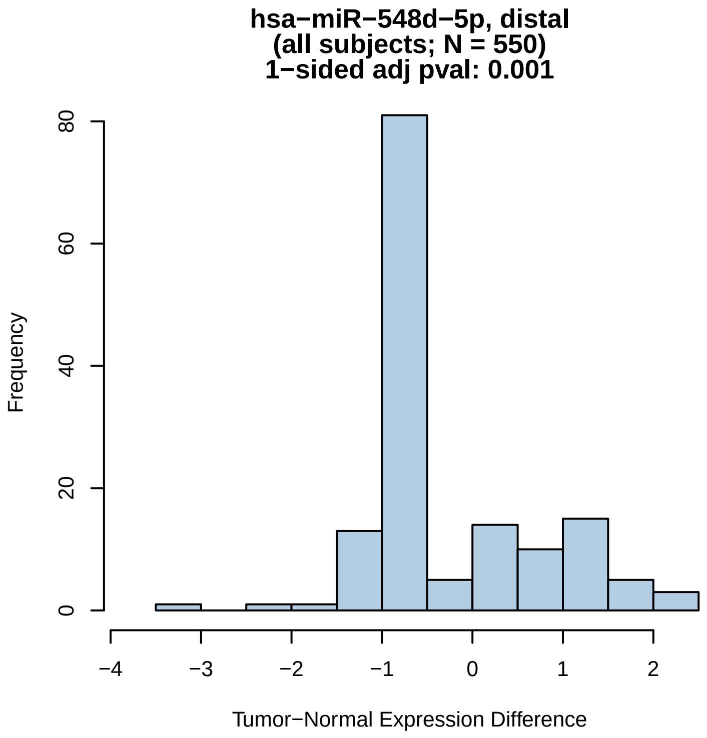

hsa−miR−548d−5p, distal
(all subjects; N = 550)
1−sided adj pval: 0.001
Frequency
Tumor−Normal Expression Difference


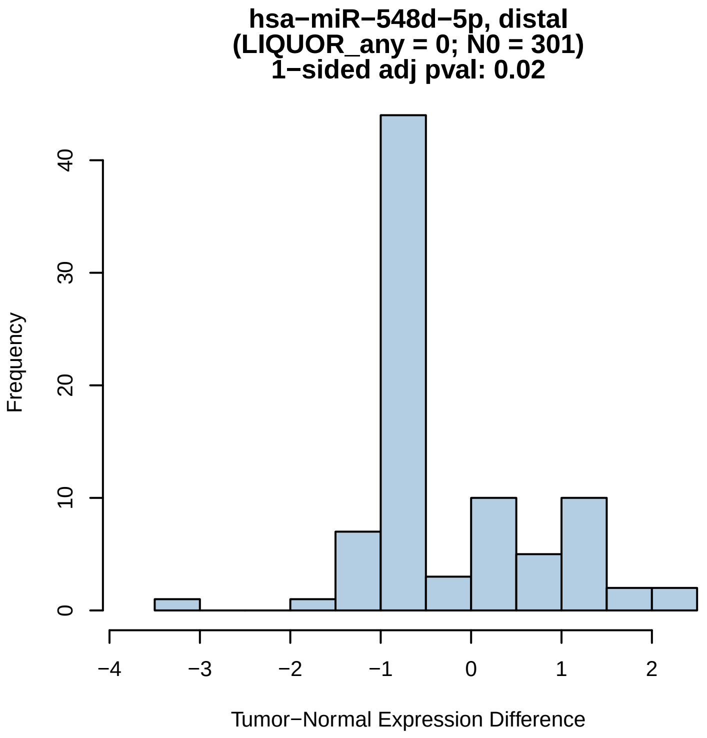

hsa−miR−548d−5p, distal
(LIQUOR_any = 0; N0 = 301)
1−sided adj pval: 0.02
Frequency
Tumor−Normal Expression Difference


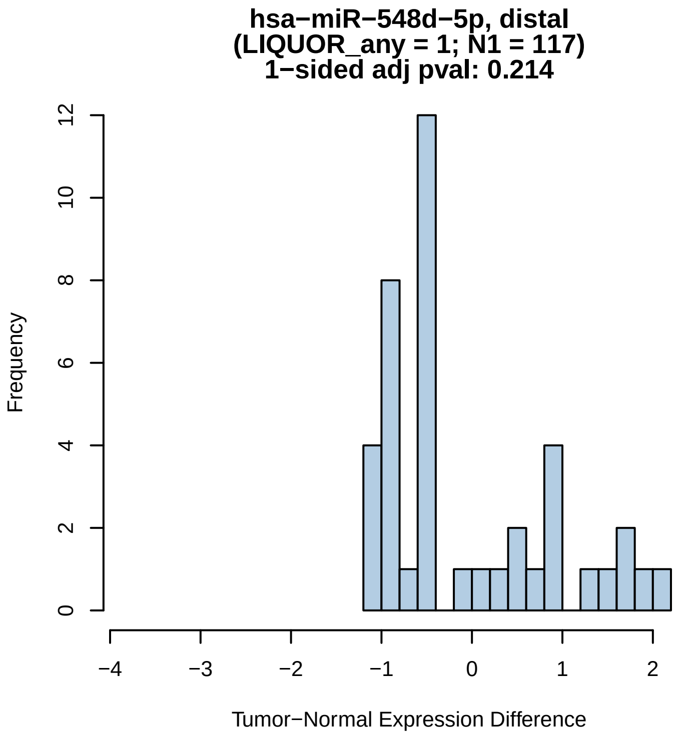

hsa−miR−548d−5p, distal
(LIQUOR_any = 1; N1 = 117)
1−sided adj pval: 0.214
Frequency
Tumor−Normal Expression Difference

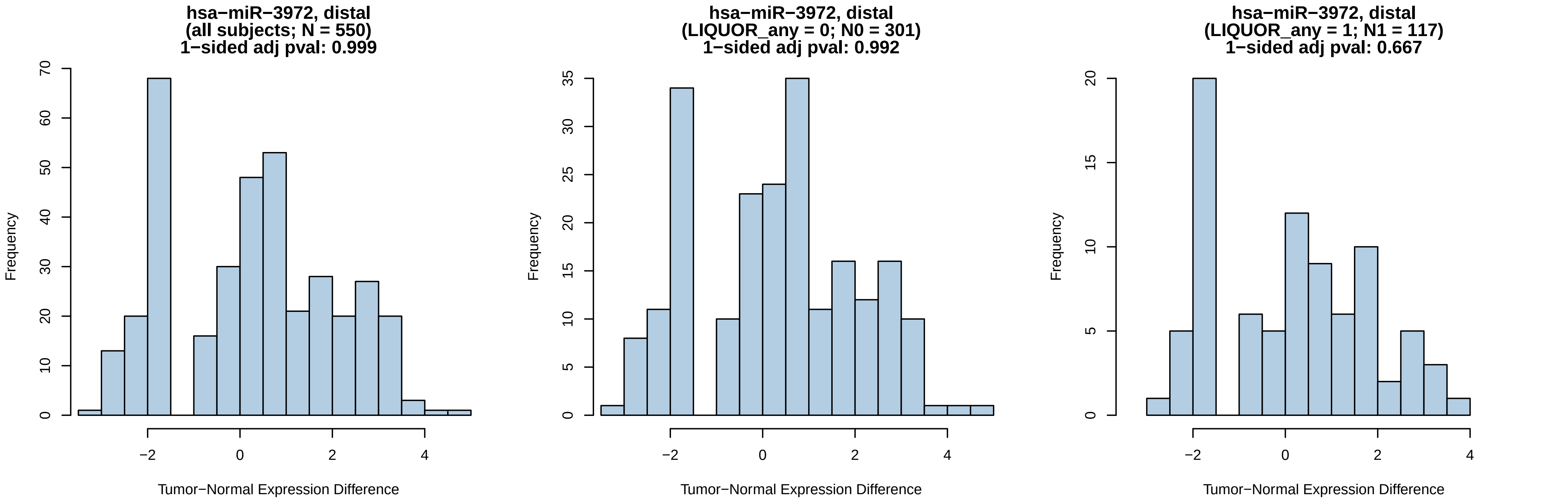
hsa−miR−3972, distal
(all subjects; N = 550)
1−sided adj pval: 0.999
Frequency
Tumor−Normal Expression Difference
hsa−miR−3972, distal
(LIQUOR_any = 0; N0 = 301)
1−sided adj pval: 0.992
Frequency
Tumor−Normal Expression Difference
hsa−miR−3972, distal
(LIQUOR_any = 1; N1 = 117)
1−sided adj pval: 0.667
Frequency
Tumor−Normal Expression Difference

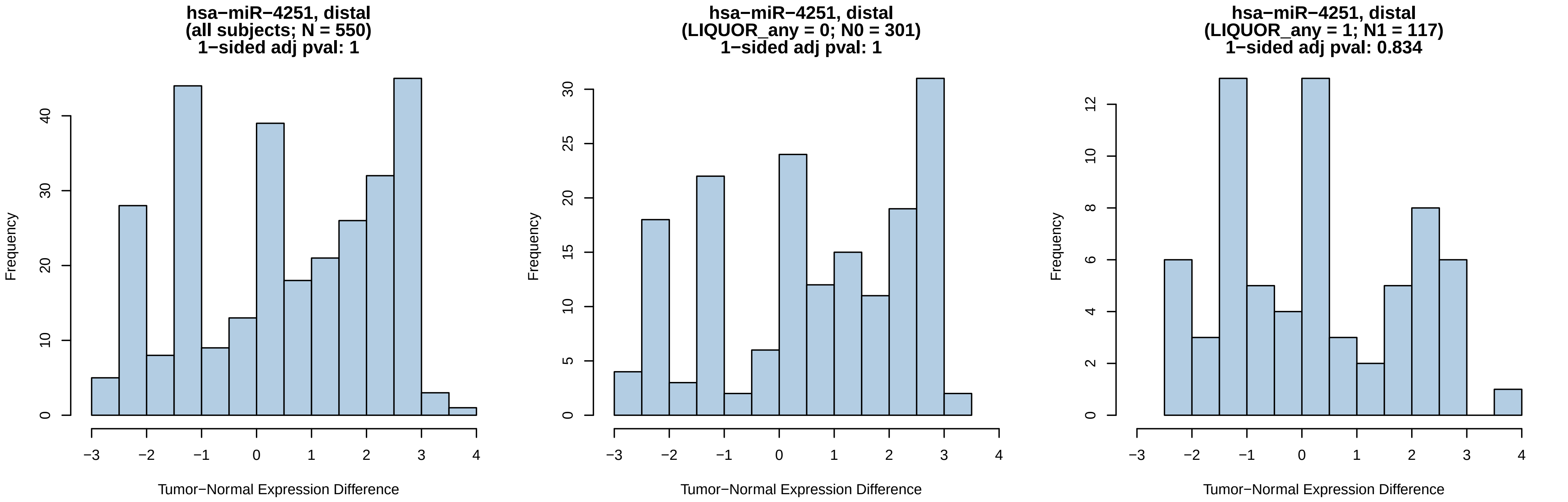
hsa−miR−4251, distal
(all subjects; N = 550)
1−sided adj pval: 1
Frequency
Tumor−Normal Expression Difference
hsa−miR−4251, distal
(LIQUOR_any = 0; N0 = 301)
1−sided adj pval: 1
Frequency
Tumor−Normal Expression Difference
hsa−miR−4251, distal
(LIQUOR_any = 1; N1 = 117)
1−sided adj pval: 0.834
Frequency
Tumor−Normal Expression Difference

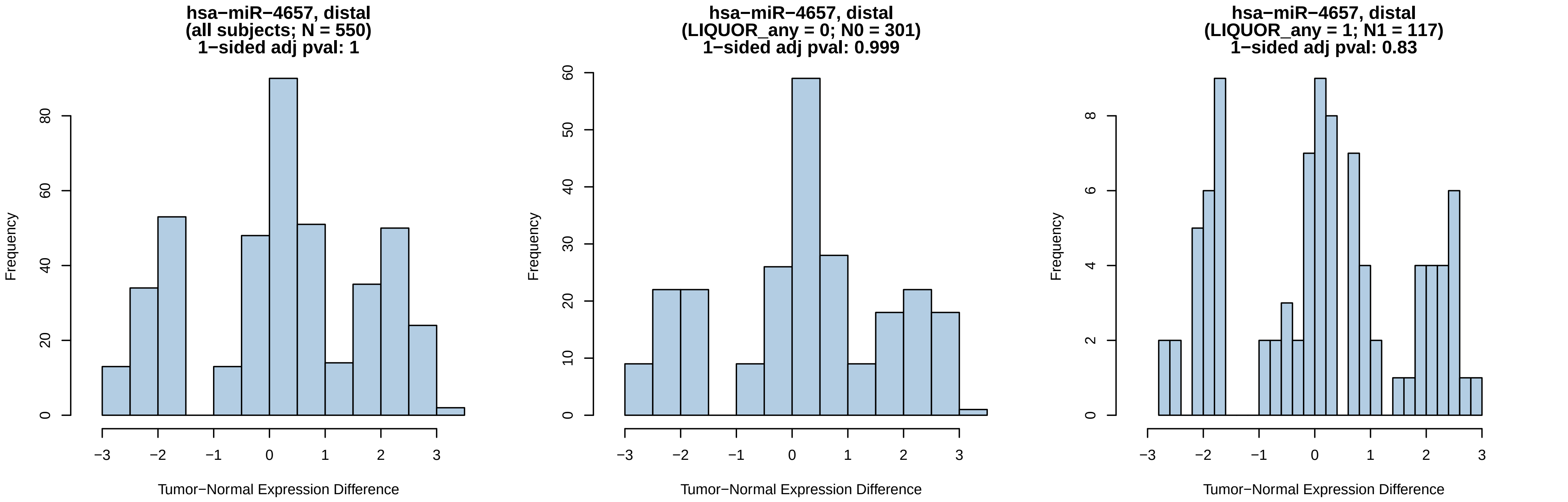
hsa−miR−4657, distal
(all subjects; N = 550)
1−sided adj pval: 1
Frequency
Tumor−Normal Expression Difference
hsa−miR−4657, distal
(LIQUOR_any = 0; N0 = 301)
1−sided adj pval: 0.999
Frequency
Tumor−Normal Expression Difference
hsa−miR−4657, distal
(LIQUOR_any = 1; N1 = 117)
1−sided adj pval: 0.83
Frequency
Tumor−Normal Expression Difference

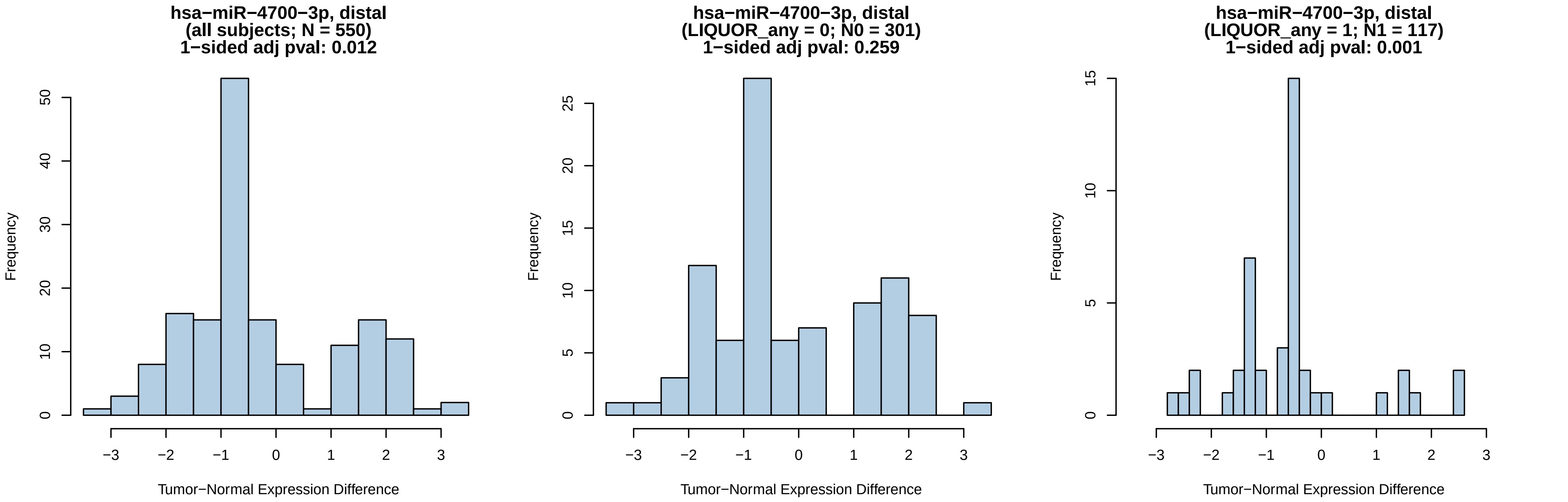
hsa−miR−4700−3p, distal
(all subjects; N = 550)
1−sided adj pval: 0.012
Frequency
0
10
20
30
40
50
−3
−2
−1
0
1
2
3
Tumor−Normal Expression Difference
hsa−miR−4700−3p, distal
(LIQUOR_any = 0; N0 = 301)
1−sided adj pval: 0.259
Frequency
0
5
10
15
20
25
−3
−2
−1
0
1
2
3
Tumor−Normal Expression Difference
hsa−miR−4700−3p, distal
(LIQUOR_any = 1; N1 = 117)
1−sided adj pval: 0.001
Frequency
0
5
10
15
−3
−2
−1
0
1
2
3
Tumor−Normal Expression Difference

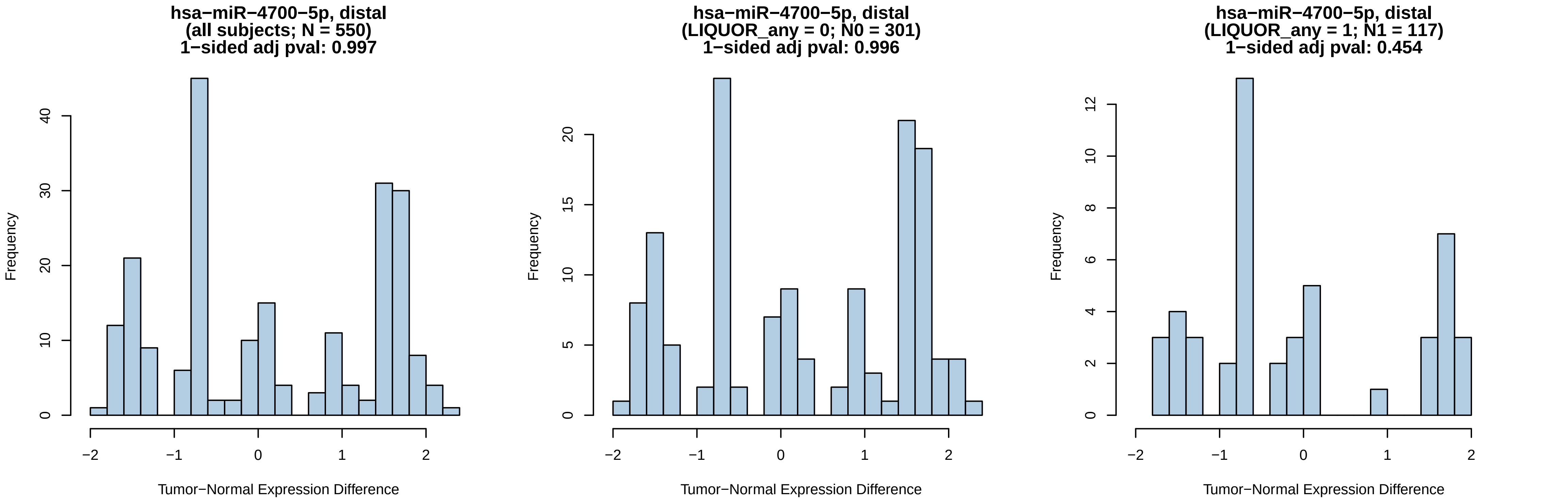
hsa−miR−4700−5p, distal
(all subjects; N = 550)
1−sided adj pval: 0.997
Frequency
Tumor−Normal Expression Difference
hsa−miR−4700−5p, distal
(LIQUOR_any = 0; N0 = 301)
1−sided adj pval: 0.996
Frequency
Tumor−Normal Expression Difference
hsa−miR−4700−5p, distal
(LIQUOR_any = 1; N1 = 117)
1−sided adj pval: 0.454
Frequency
Tumor−Normal Expression Difference

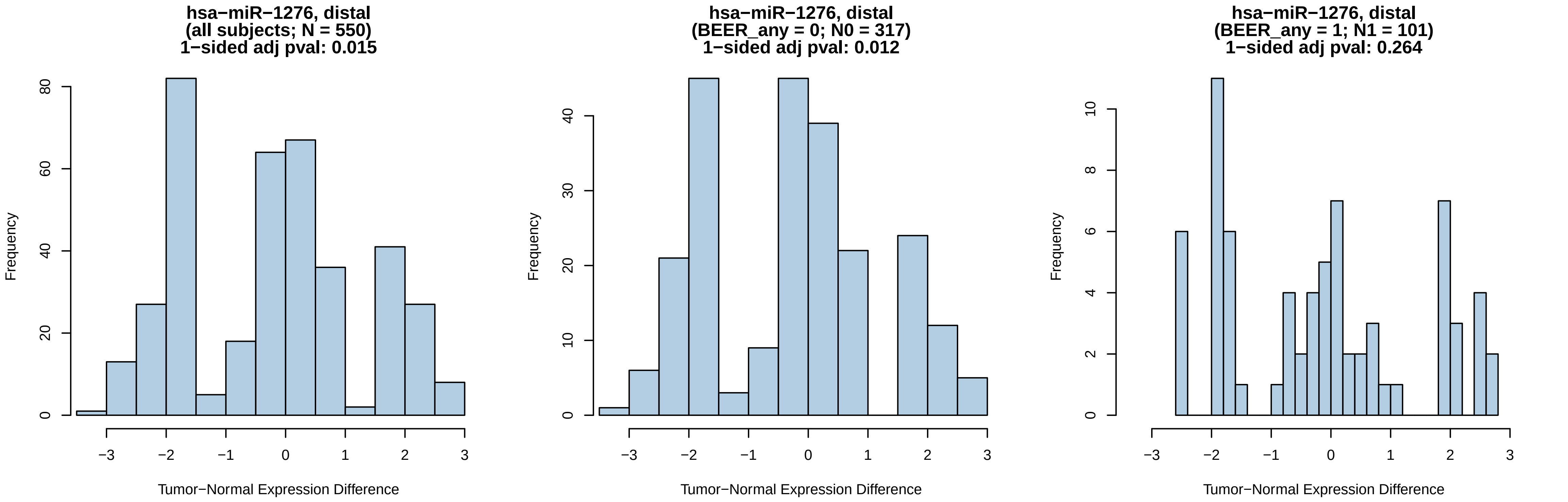
hsa−miR−1276, distal
(all subjects; N = 550)
1−sided adj pval: 0.015
Frequency
0
20
40
60
80
−3
−2
−1
0
1
2
3
Tumor−Normal Expression Difference
hsa−miR−1276, distal
(BEER_any = 0; N0 = 317)
1−sided adj pval: 0.012
Frequency
0
10
20
30
40
−3
−2
−1
0
1
2
3
Tumor−Normal Expression Difference
hsa−miR−1276, distal
(BEER_any = 1; N1 = 101)
1−sided adj pval: 0.264
Frequency
0
2
4
6
8
10
−3
−2
−1
0
1
2
3
Tumor−Normal Expression Difference

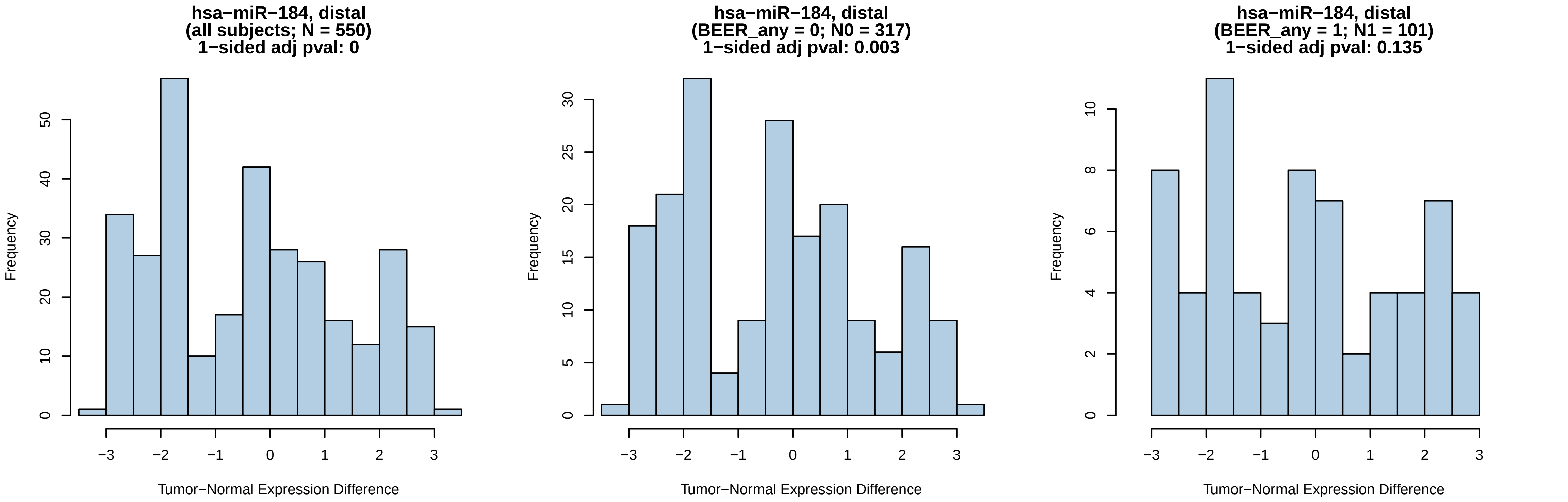
hsa−miR−184, distal
(all subjects; N = 550)
1−sided adj pval: 0
Frequency
0
10
20
30
40
50
−3
−2
−1
0
1
2
3
Tumor−Normal Expression Difference
hsa−miR−184, distal
(BEER_any = 0; N0 = 317)
1−sided adj pval: 0.003
Frequency
0
5
10
15
20
25
30
−3
−2
−1
0
1
2
3
Tumor−Normal Expression Difference
hsa−miR−184, distal
(BEER_any = 1; N1 = 101)
1−sided adj pval: 0.135
Frequency
0
2
4
6
8
10
−3
−2
−1
0
1
2
3
Tumor−Normal Expression Difference

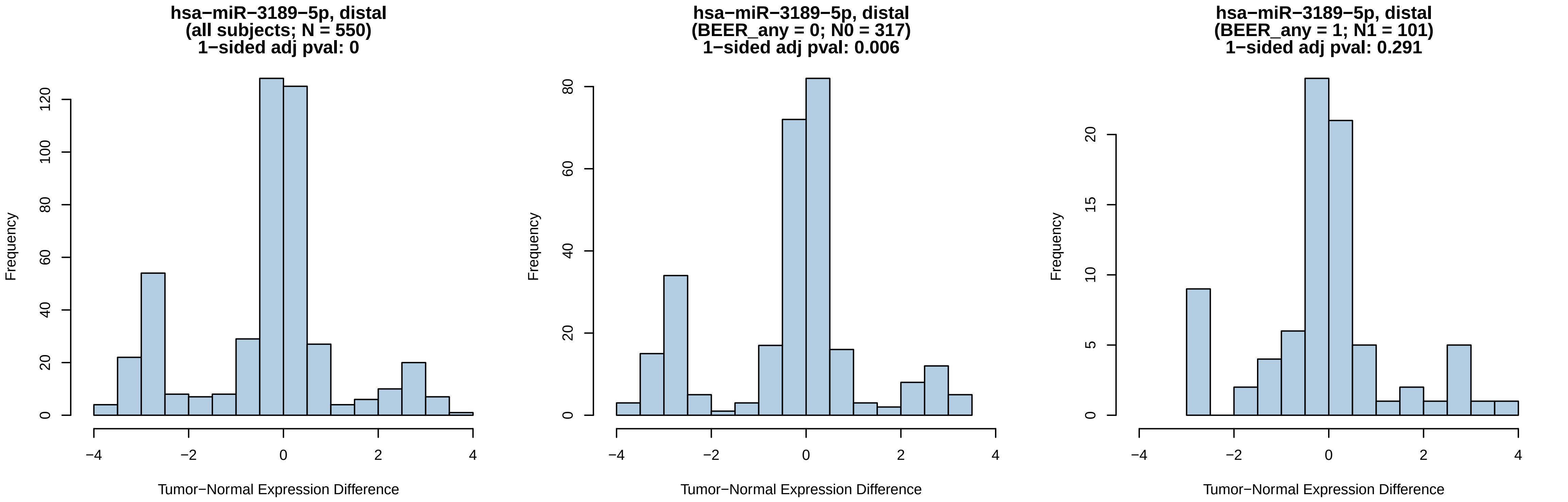

hsa−miR−3189−5p, distal
(all subjects; N = 550)
1−sided adj pval: 0
hsa−miR−3189−5p, distal
(BEER_any = 0; N0 = 317)
1−sided adj pval: 0.006
hsa−miR−3189−5p, distal
(BEER_any = 1; N1 = 101)
1−sided adj pval: 0.291
Frequency
Tumor−Normal Expression Difference

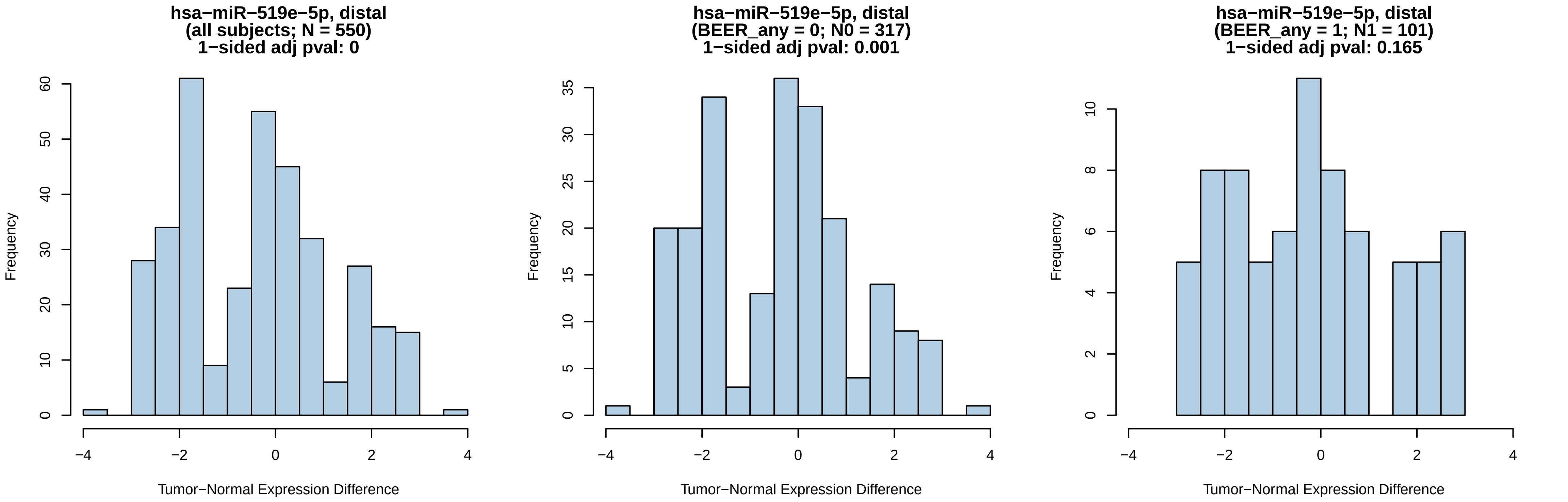

hsa−miR−519e−5p, distal
(all subjects; N = 550)
1−sided adj pval: 0
hsa−miR−519e−5p, distal
(BEER_any = 0; N0 = 317)
1−sided adj pval: 0.001
hsa−miR−519e−5p, distal
(BEER_any = 1; N1 = 101)
1−sided adj pval: 0.165
Frequency
Tumor−Normal Expression Difference

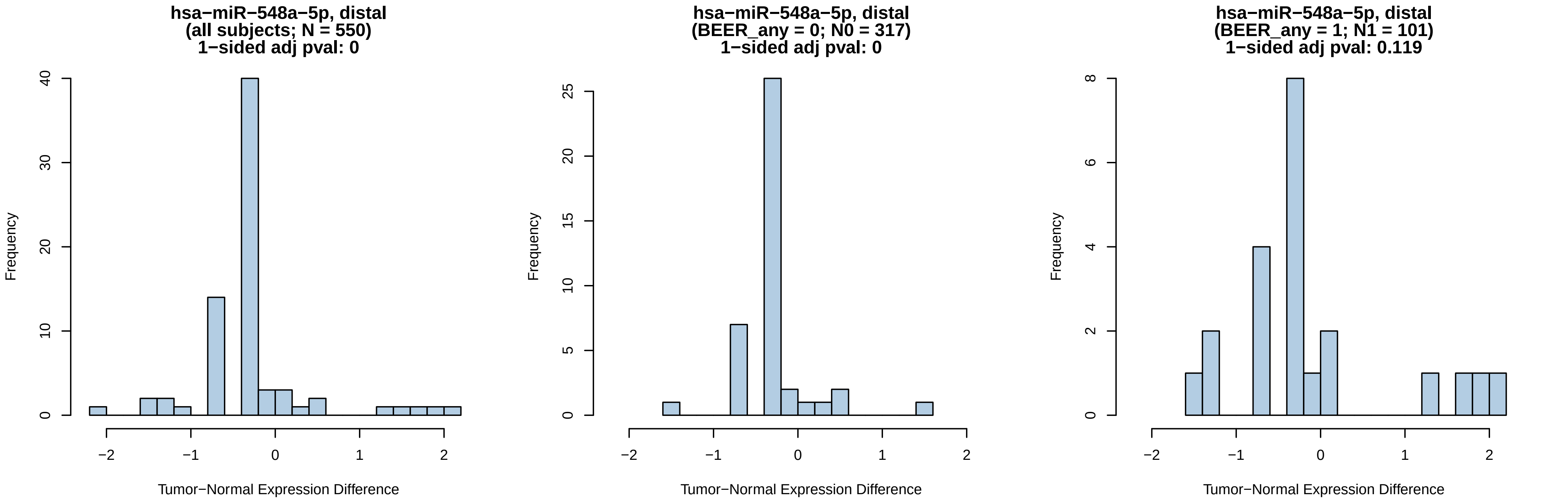
hsa−miR−548a−5p, distal
(all subjects; N = 550)
1−sided adj pval: 0
hsa−miR−548a−5p, distal
(BEER_any = 0; N0 = 317)
1−sided adj pval: 0
hsa−miR−548a−5p, distal
(BEER_any = 1; N1 = 101)
1−sided adj pval: 0.119
Frequency
Tumor−Normal Expression Difference
0
10
20
30
40
5
15
25
2
4
6
8
−2
−1
1

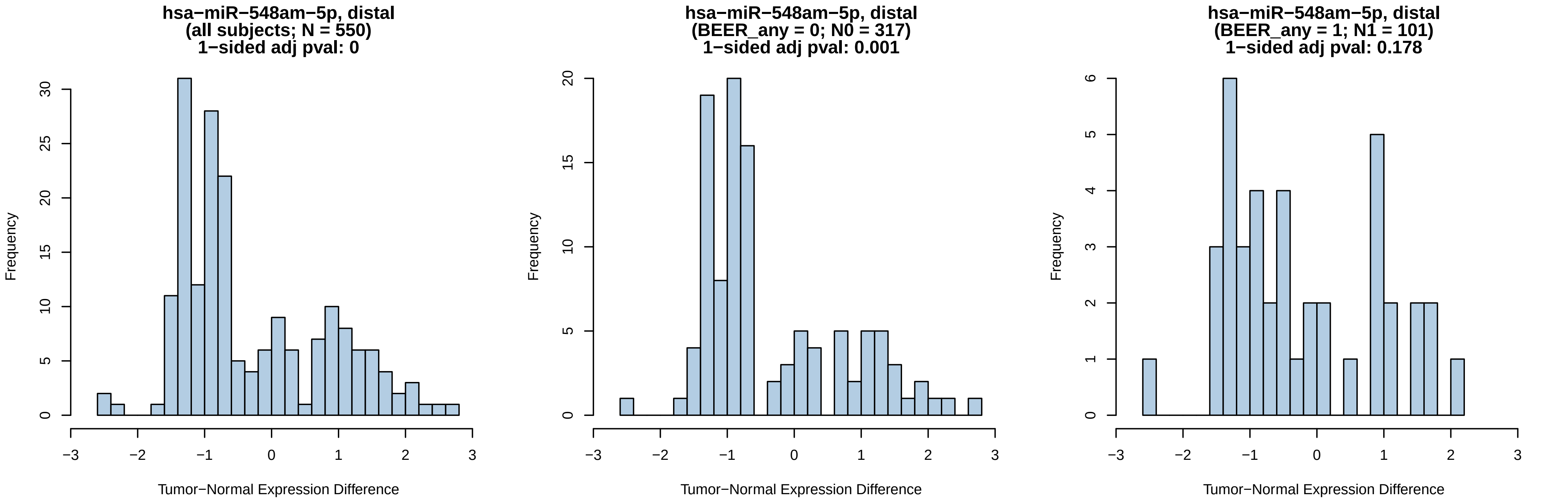
hsa−miR−548am−5p, distal
(all subjects; N = 550)
1−sided adj pval: 0
Frequency
Tumor−Normal Expression Difference
hsa−miR−548am−5p, distal
(BEER_any = 0; N0 = 317)
1−sided adj pval: 0.001
Frequency
Tumor−Normal Expression Difference
hsa−miR−548am−5p, distal
(BEER_any = 1; N1 = 101)
1−sided adj pval: 0.178
Frequency
Tumor−Normal Expression Difference

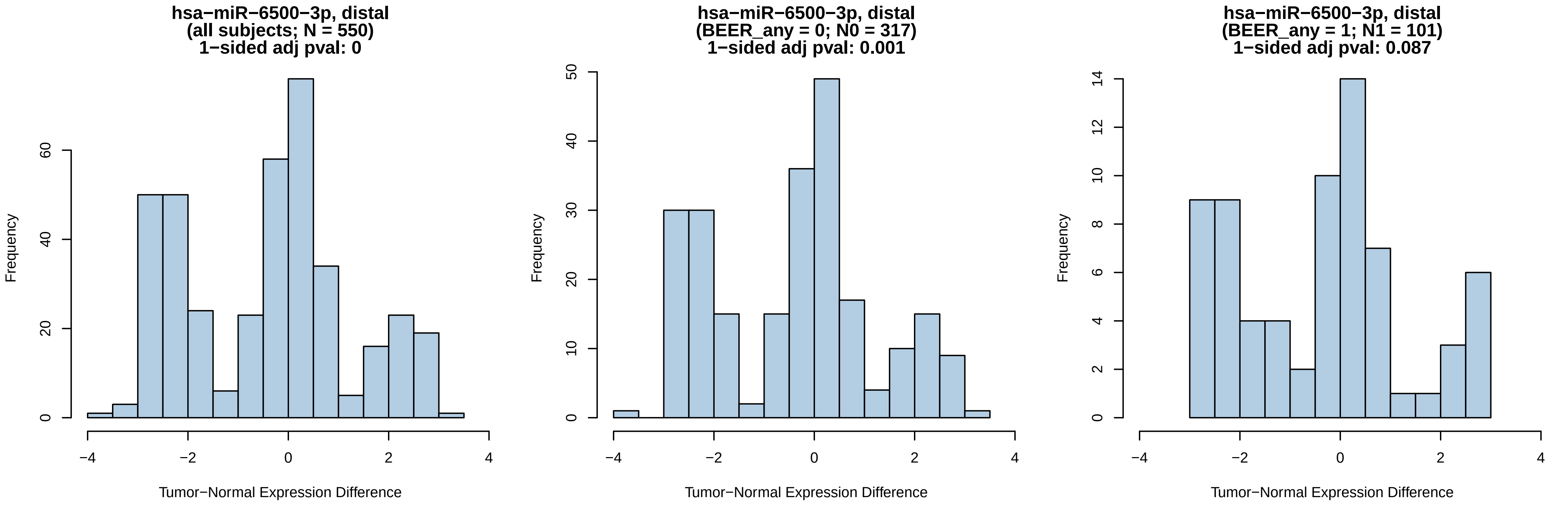

hsa−miR−6500−3p, distal
(all subjects; N = 550)
1−sided adj pval: 0
hsa−miR−6500−3p, distal
(BEER_any = 0; N0 = 317)
1−sided adj pval: 0.001
hsa−miR−6500−3p, distal
(BEER_any = 1; N1 = 101)
1−sided adj pval: 0.087
Frequency
Tumor−Normal Expression Difference

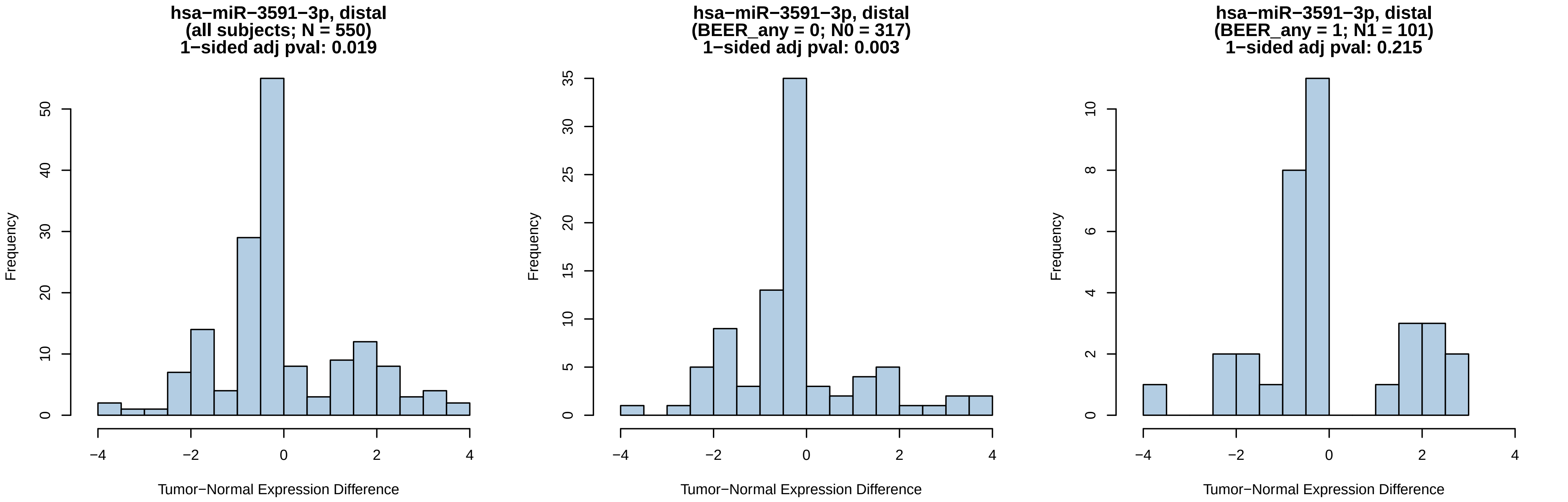
hsa−miR−3591−3p, distal
(all subjects; N = 550)
1−sided adj pval: 0.019
Frequency
0
10
20
30
40
50
−4
−2
0
2
4
Tumor−Normal Expression Difference
hsa−miR−3591−3p, distal
(BEER_any = 0; N0 = 317)
1−sided adj pval: 0.003
Frequency
0
5
10
15
20
25
30
35
−4
−2
0
2
4
Tumor−Normal Expression Difference
hsa−miR−3591−3p, distal
(BEER_any = 1; N1 = 101)
1−sided adj pval: 0.215
Frequency
0
2
4
6
8
10
−4
−2
0
2
4
Tumor−Normal Expression Difference

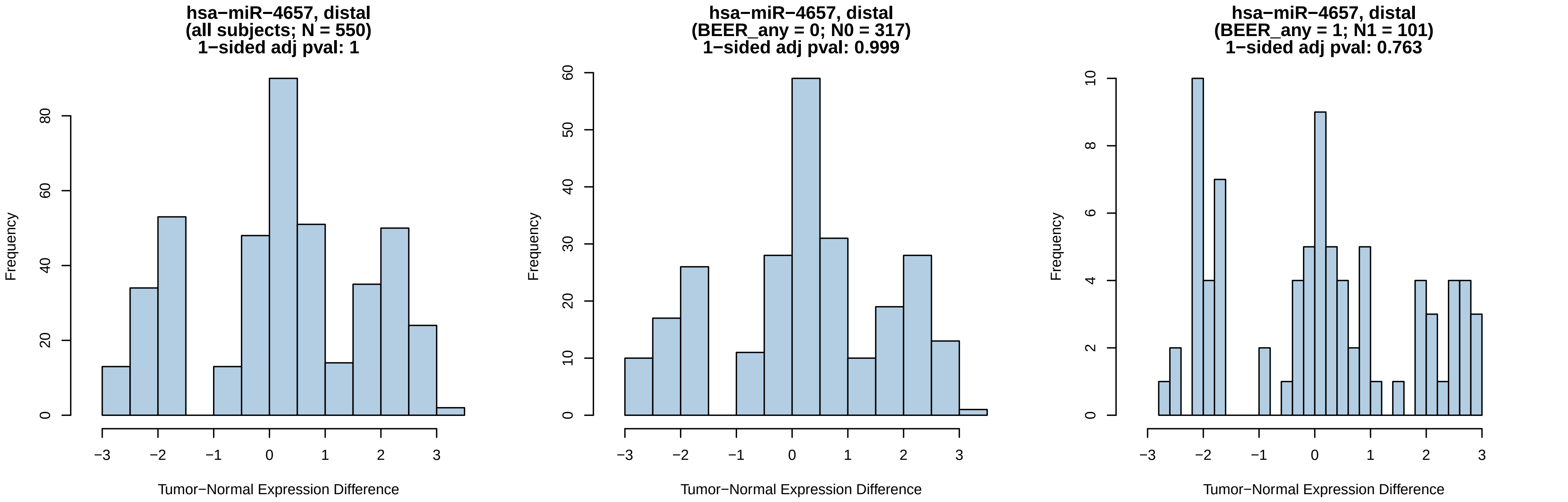
hsa−miR−4657, distal
(all subjects; N = 550)
1−sided adj pval: 1
Frequency
0
20
40
60
80
−3
−2
−1
0
1
2
3
Tumor−Normal Expression Difference
hsa−miR−4657, distal
(BEER_any = 0; N0 = 317)
1−sided adj pval: 0.999
Frequency
0
10
20
30
40
50
60
−3
−2
−1
0
1
2
3
Tumor−Normal Expression Difference
hsa−miR−4657, distal
(BEER_any = 1; N1 = 101)
1−sided adj pval: 0.763
Frequency
0
2
4
6
8
10
−3
−2
−1
0
1
2
3
Tumor−Normal Expression Difference

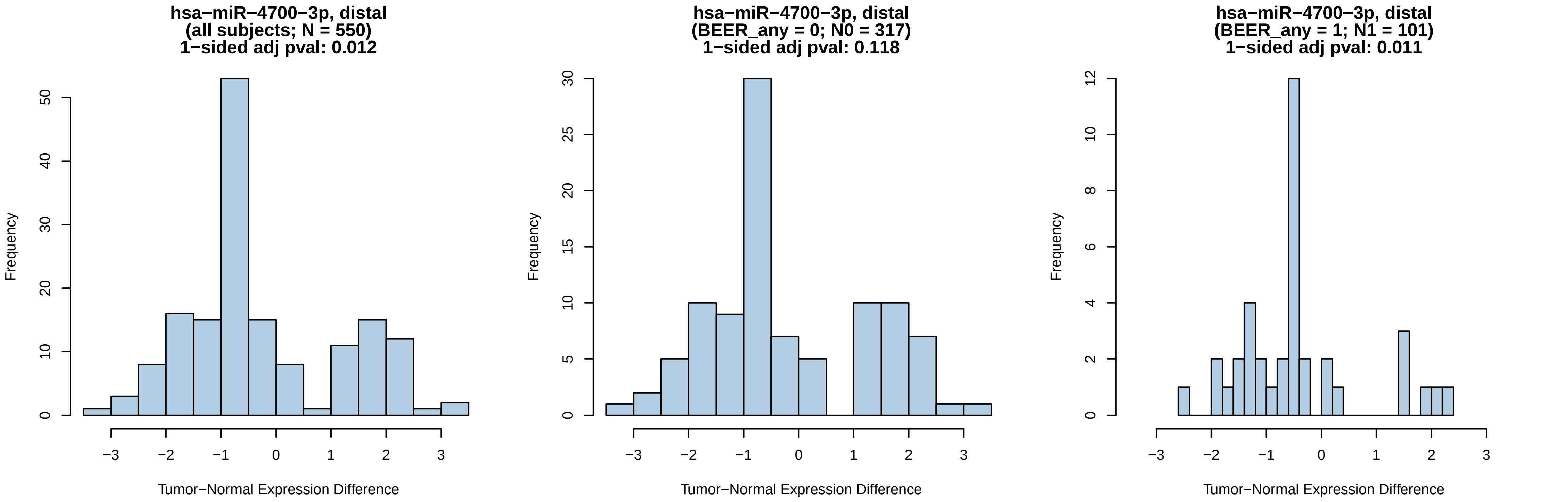

hsa−miR−4700−3p, distal
(all subjects; N = 550)
1−sided adj pval: 0.012
Frequency
0
10
20
30
40
50
−3
−2
−1
0
1
2
3
Tumor−Normal Expression Difference
hsa−miR−4700−3p, distal
(BEER_any = 0; N0 = 317)
1−sided adj pval: 0.118
Frequency
0
5
10
15
20
25
30
−3
−2
−1
0
1
2
3
Tumor−Normal Expression Difference
hsa−miR−4700−3p, distal
(BEER_any = 1; N1 = 101)
1−sided adj pval: 0.011
Frequency
0
2
4
6
8
10
12
−3
−2
−1
0
1
2
3
Tumor−Normal Expression Difference

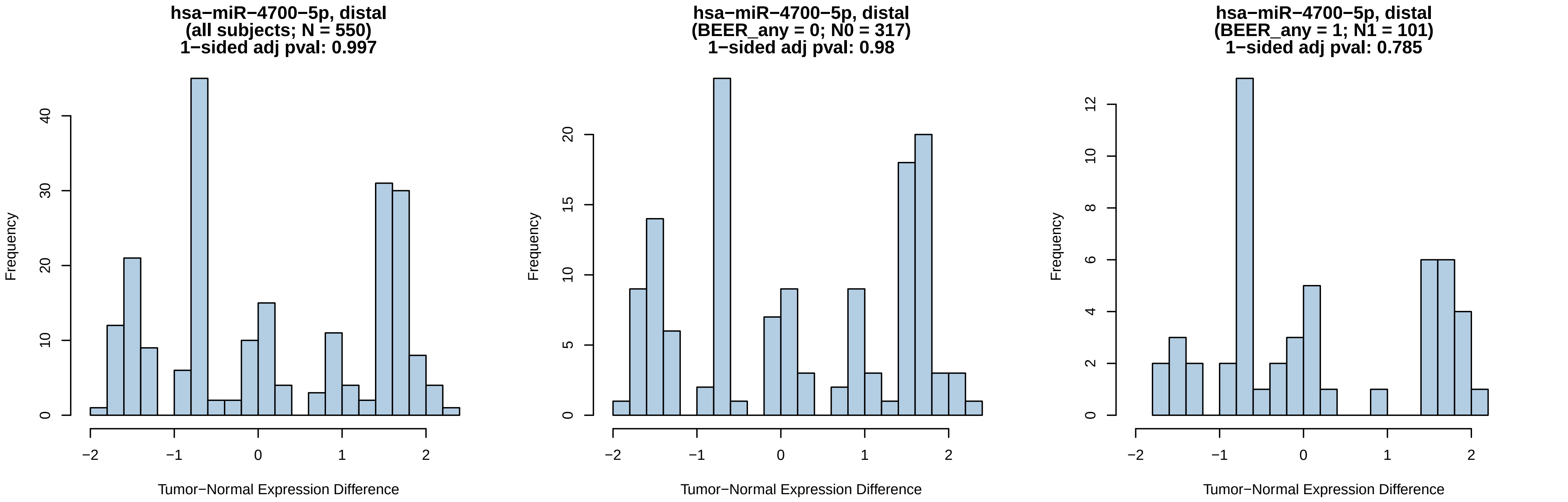
hsa−miR−4700−5p, distal
(all subjects; N = 550)
1−sided adj pval: 0.997
Frequency
Tumor−Normal Expression Difference
hsa−miR−4700−5p, distal
(BEER_any = 0; N0 = 317)
1−sided adj pval: 0.98
Frequency
Tumor−Normal Expression Difference
hsa−miR−4700−5p, distal
(BEER_any = 1; N1 = 101)
1−sided adj pval: 0.785
Frequency
Tumor−Normal Expression Difference

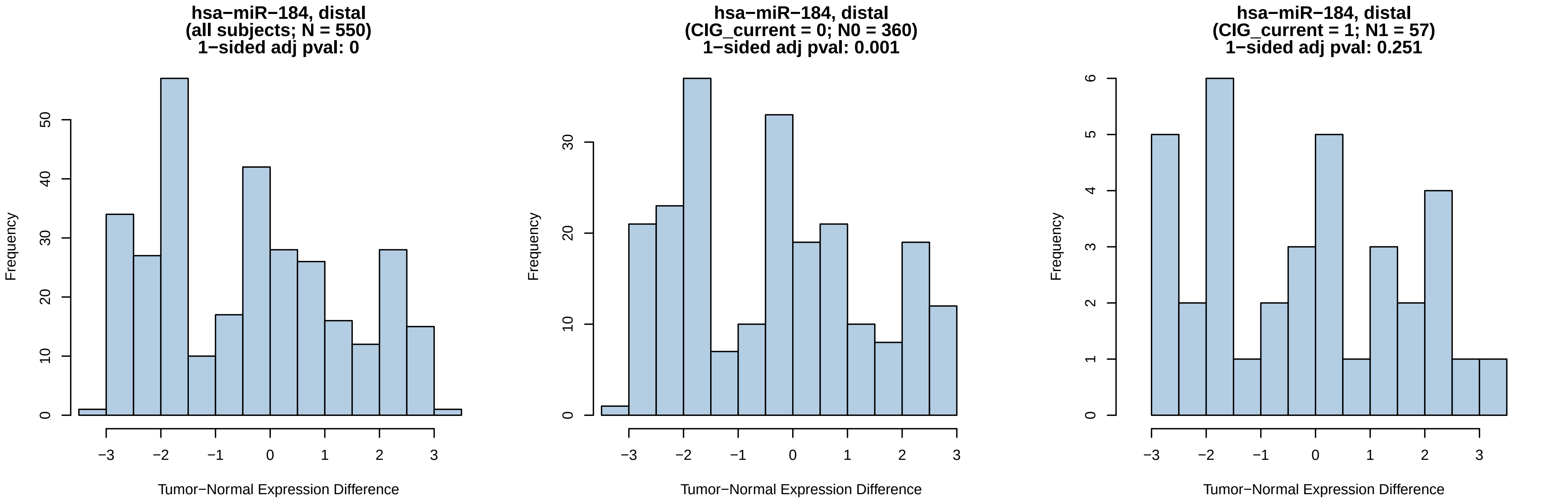

hsa−miR−184, distal
(all subjects; N = 550)
1−sided adj pval: 0
Frequency
Tumor−Normal Expression Difference
hsa−miR−184, distal
(CIG_current = 0; N0 = 360)
1−sided adj pval: 0.001
Frequency
Tumor−Normal Expression Difference
hsa−miR−184, distal
(CIG_current = 1; N1 = 57)
1−sided adj pval: 0.251
Frequency
Tumor−Normal Expression Difference

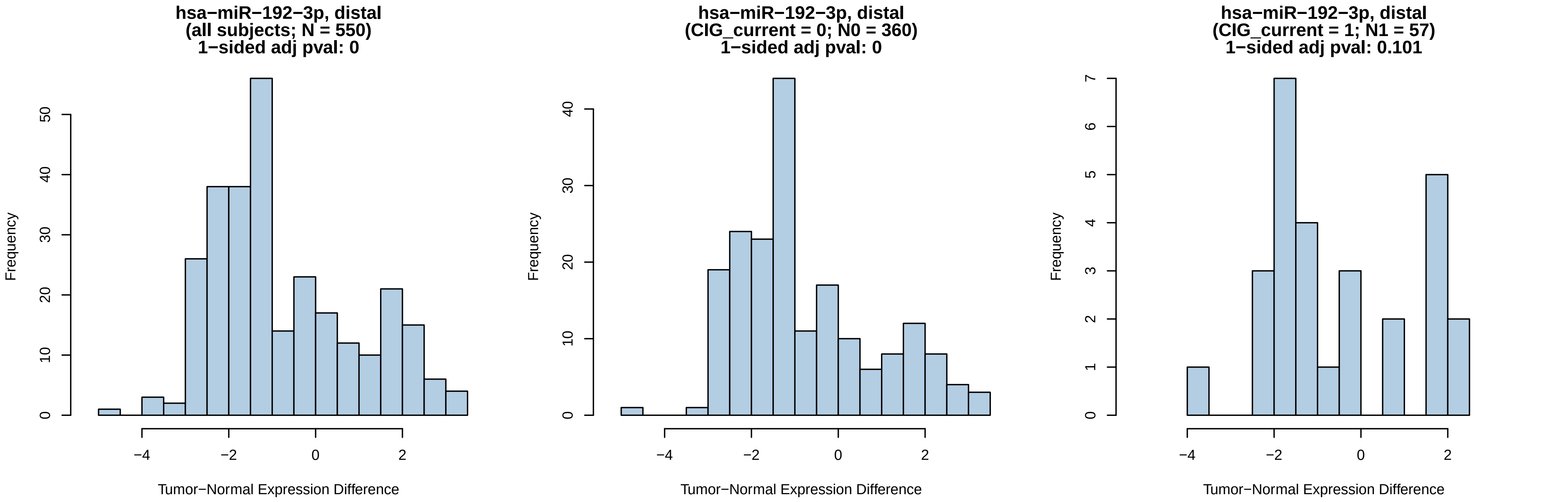
hsa−miR−192−3p, distal
(all subjects; N = 550)
1−sided adj pval: 0
Frequency
0
10
20
30
40
50
−4
−2
0
2
Tumor−Normal Expression Difference
hsa−miR−192−3p, distal
(CIG_current = 0; N0 = 360)
1−sided adj pval: 0
Frequency
0
10
20
30
40
−4
−2
0
2
Tumor−Normal Expression Difference
hsa−miR−192−3p, distal
(CIG_current = 1; N1 = 57)
1−sided adj pval: 0.101
Frequency
0
1
2
3
4
5
6
7
−4
−2
0
2
Tumor−Normal Expression Difference

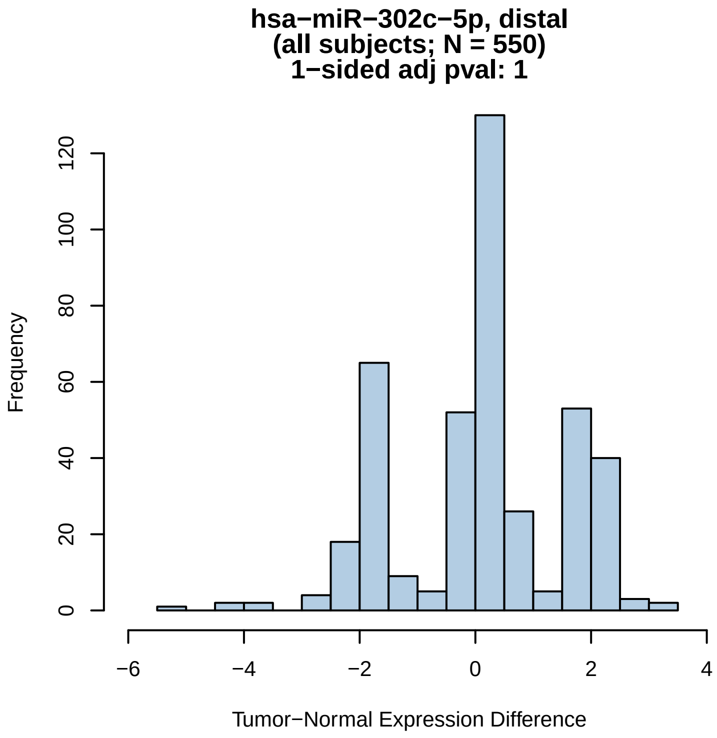

hsa−miR−302c−5p, distal
(all subjects; N = 550)
1−sided adj pval: 1
Frequency
Tumor−Normal Expression Difference


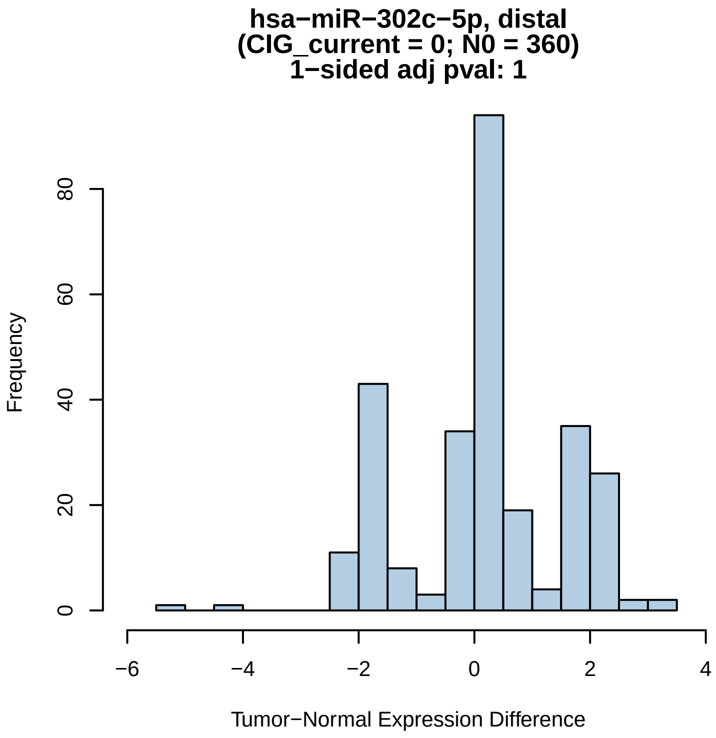

hsa−miR−302c−5p, distal
(CIG_current = 0; N0 = 360)
1−sided adj pval: 1
Frequency
Tumor−Normal Expression Difference


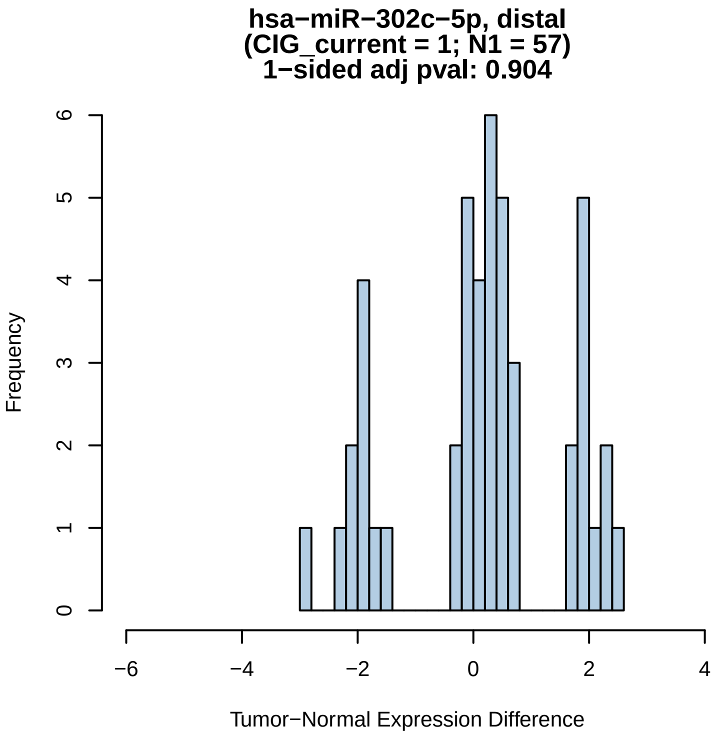

hsa−miR−302c−5p, distal
(CIG_current = 1; N1 = 57)
1−sided adj pval: 0.904
Frequency
Tumor−Normal Expression Difference

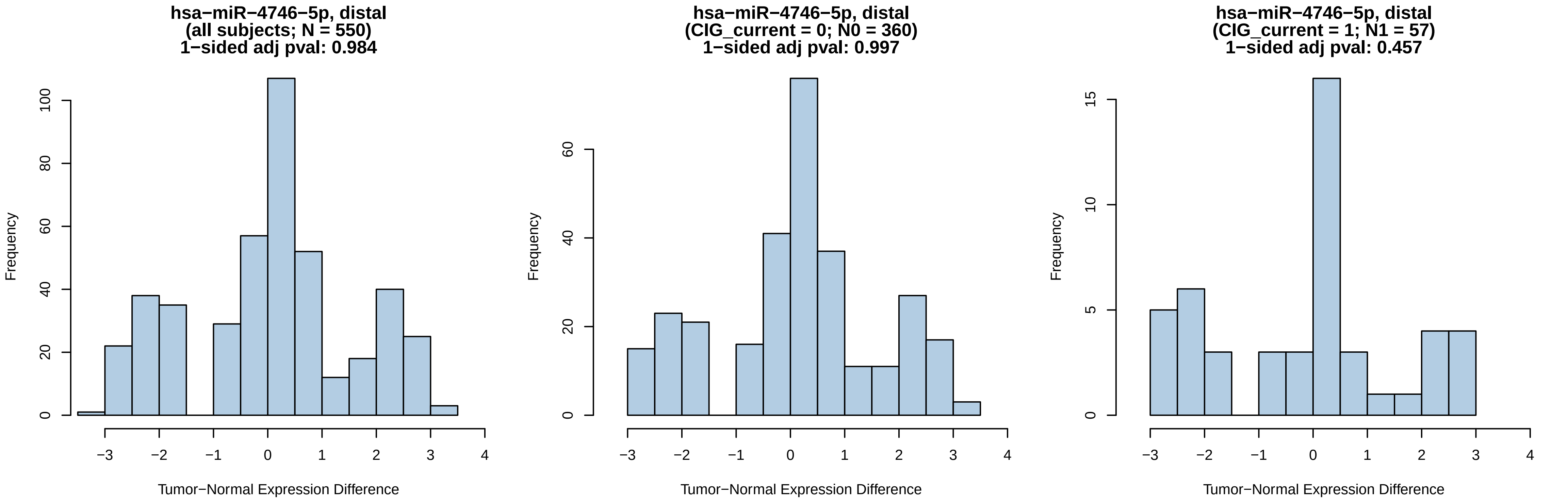

hsa−miR−4746−5p, distal
(all subjects; N = 550)
1−sided adj pval: 0.984
Frequency
Tumor−Normal Expression Difference
hsa−miR−4746−5p, distal
(CIG_current = 0; N0 = 360)
1−sided adj pval: 0.997
Frequency
Tumor−Normal Expression Difference
hsa−miR−4746−5p, distal
(CIG_current = 1; N1 = 57)
1−sided adj pval: 0.457
Frequency
Tumor−Normal Expression Difference

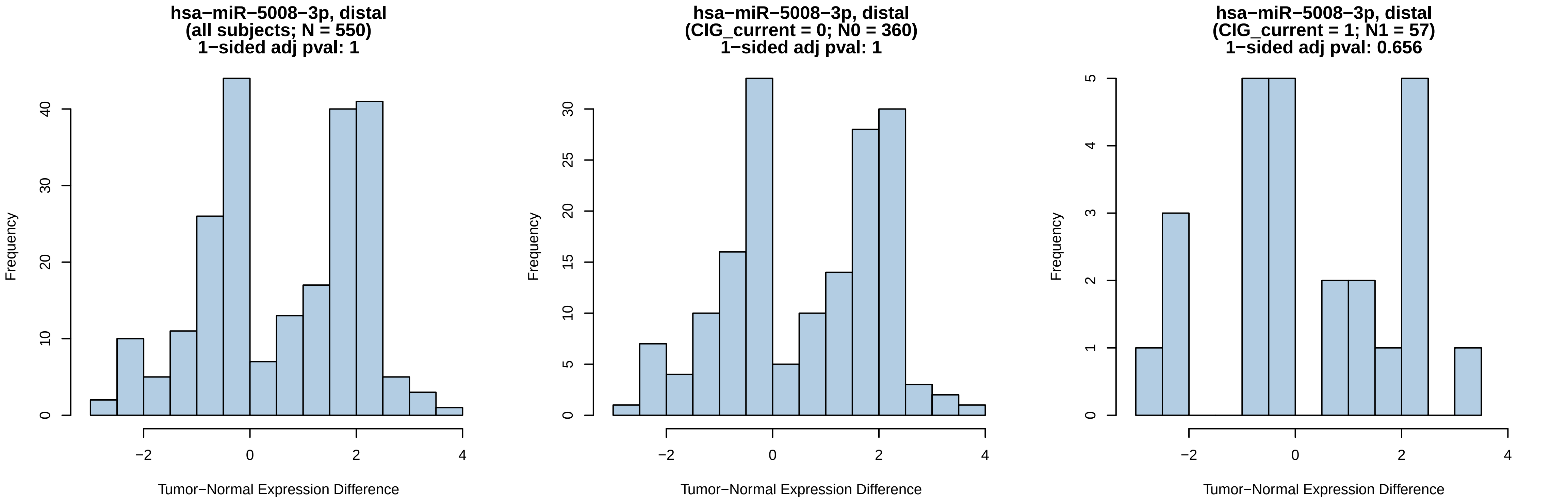

hsa−miR−5008−3p, distal
(all subjects; N = 550)
1−sided adj pval: 1
hsa−miR−5008−3p, distal
(CIG_current = 0; N0 = 360)
1−sided adj pval: 1
hsa−miR−5008−3p, distal
(CIG_current = 1; N1 = 57)
1−sided adj pval: 0.656
Frequency
Tumor−Normal Expression Difference

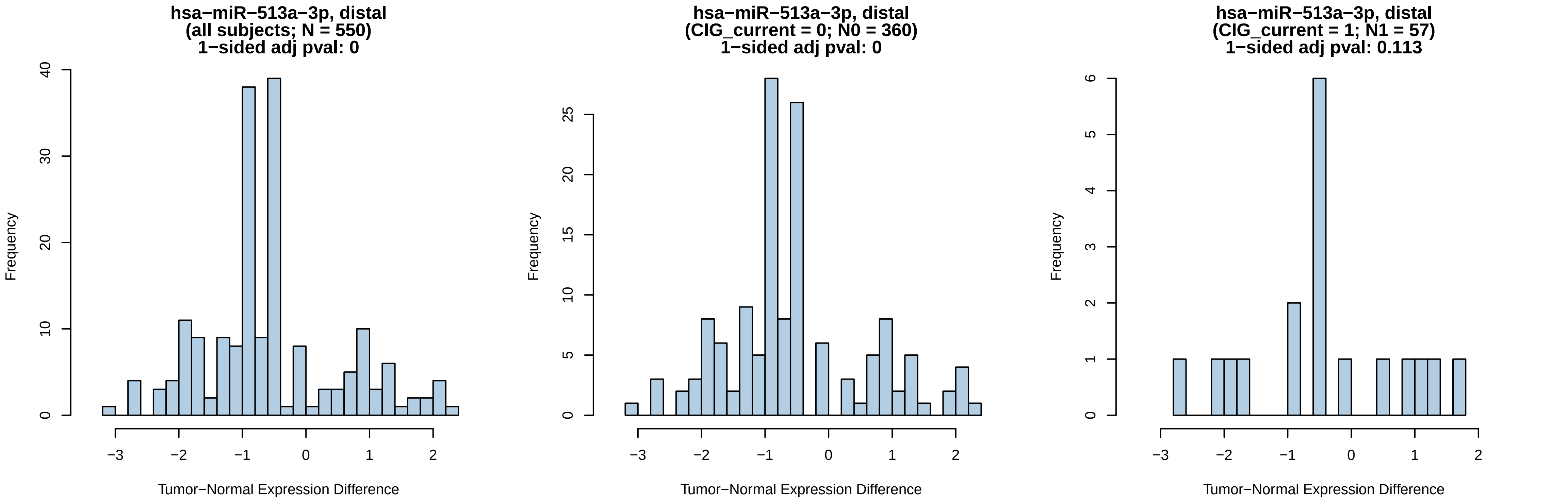
hsa−miR−513a−3p, distal
(all subjects; N = 550)
1−sided adj pval: 0
Frequency
Tumor−Normal Expression Difference
hsa−miR−513a−3p, distal
(CIG_current = 0; N0 = 360)
1−sided adj pval: 0
Frequency
Tumor−Normal Expression Difference
hsa−miR−513a−3p, distal
(CIG_current = 1; N1 = 57)
1−sided adj pval: 0.113
Frequency
Tumor−Normal Expression Difference

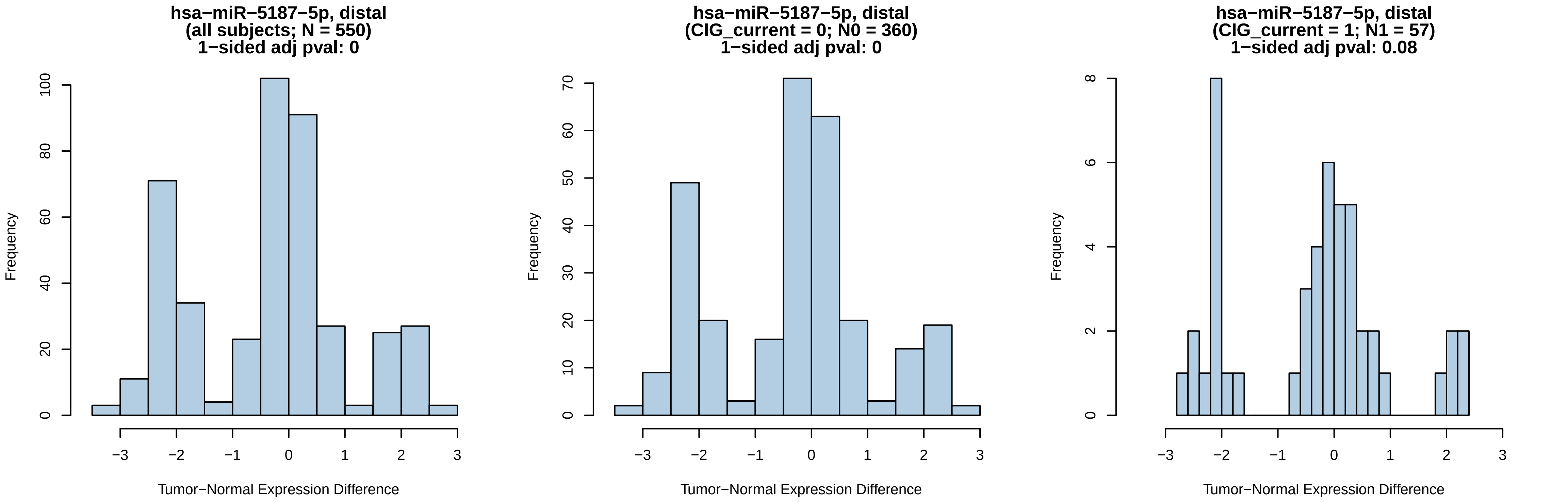
hsa−miR−5187−5p, distal
(all subjects; N = 550)
1−sided adj pval: 0
Frequency
Tumor−Normal Expression Difference
hsa−miR−5187−5p, distal
(CIG_current = 0; N0 = 360)
1−sided adj pval: 0
Frequency
Tumor−Normal Expression Difference
hsa−miR−5187−5p, distal
(CIG_current = 1; N1 = 57)
1−sided adj pval: 0.08
Frequency
Tumor−Normal Expression Difference

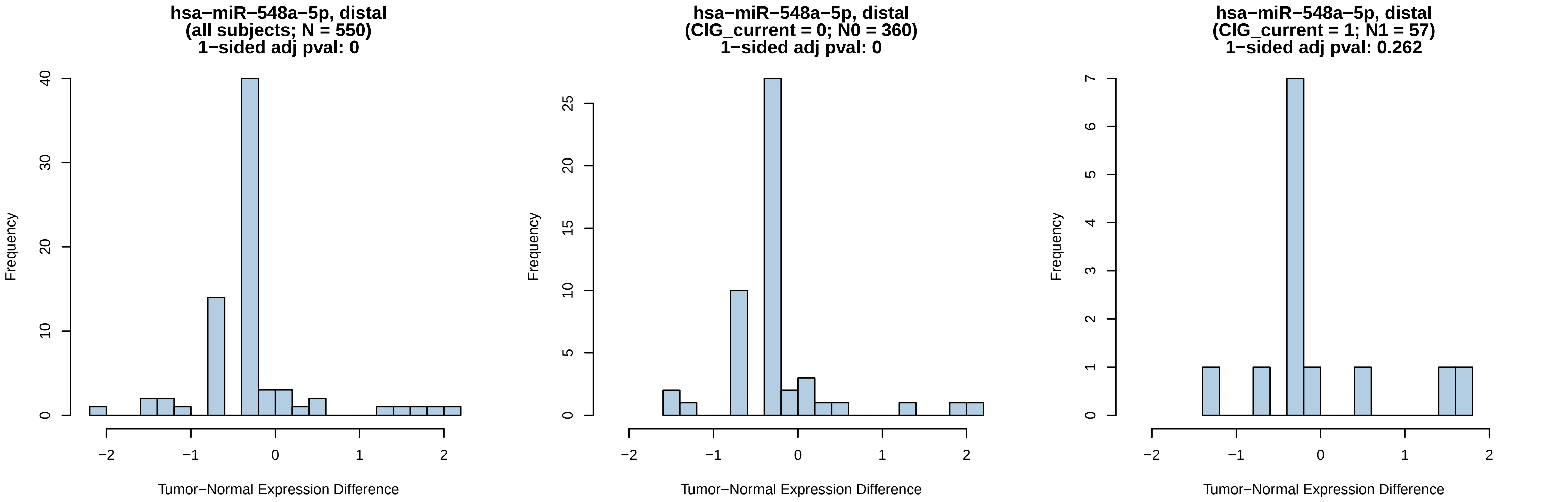
hsa−miR−548a−5p, distal
(all subjects; N = 550)
1−sided adj pval: 0
Frequency
Tumor−Normal Expression Difference
hsa−miR−548a−5p, distal
(CIG_current = 0; N0 = 360)
1−sided adj pval: 0
Frequency
Tumor−Normal Expression Difference
hsa−miR−548a−5p, distal
(CIG_current = 1; N1 = 57)
1−sided adj pval: 0.262
Frequency
Tumor−Normal Expression Difference

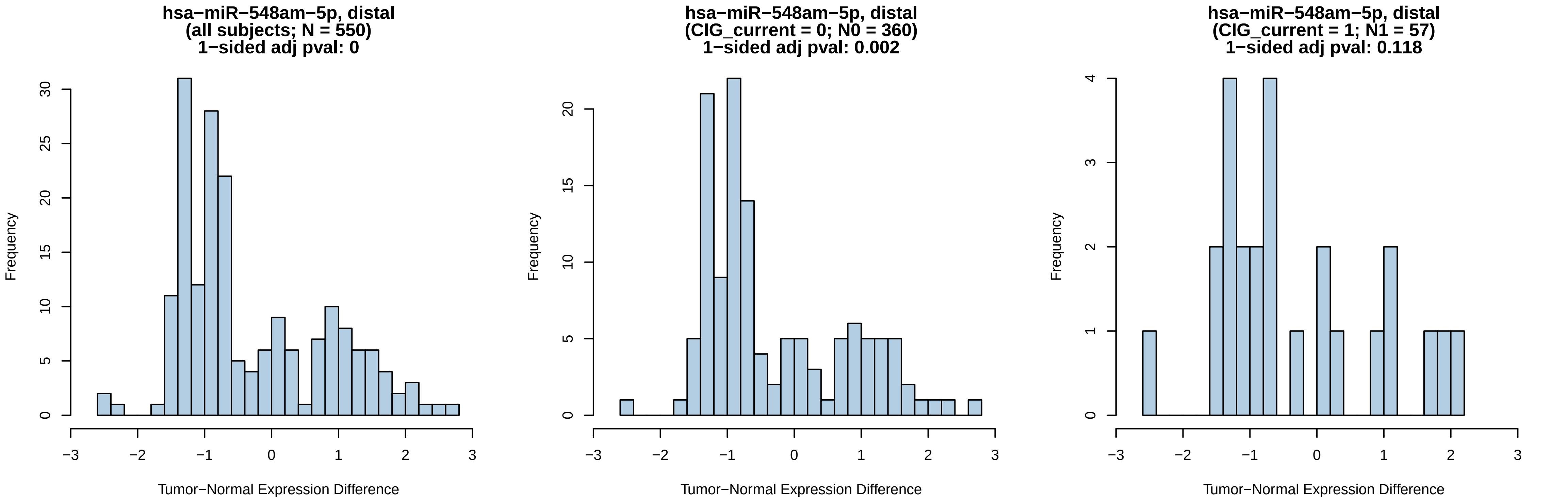
hsa−miR−548am−5p, distal
(all subjects; N = 550)
1−sided adj pval: 0
Frequency
Tumor−Normal Expression Difference
hsa−miR−548am−5p, distal
(CIG_current = 0; N0 = 360)
1−sided adj pval: 0.002
Frequency
Tumor−Normal Expression Difference
hsa−miR−548am−5p, distal
(CIG_current = 1; N1 = 57)
1−sided adj pval: 0.118
Frequency
Tumor−Normal Expression Difference

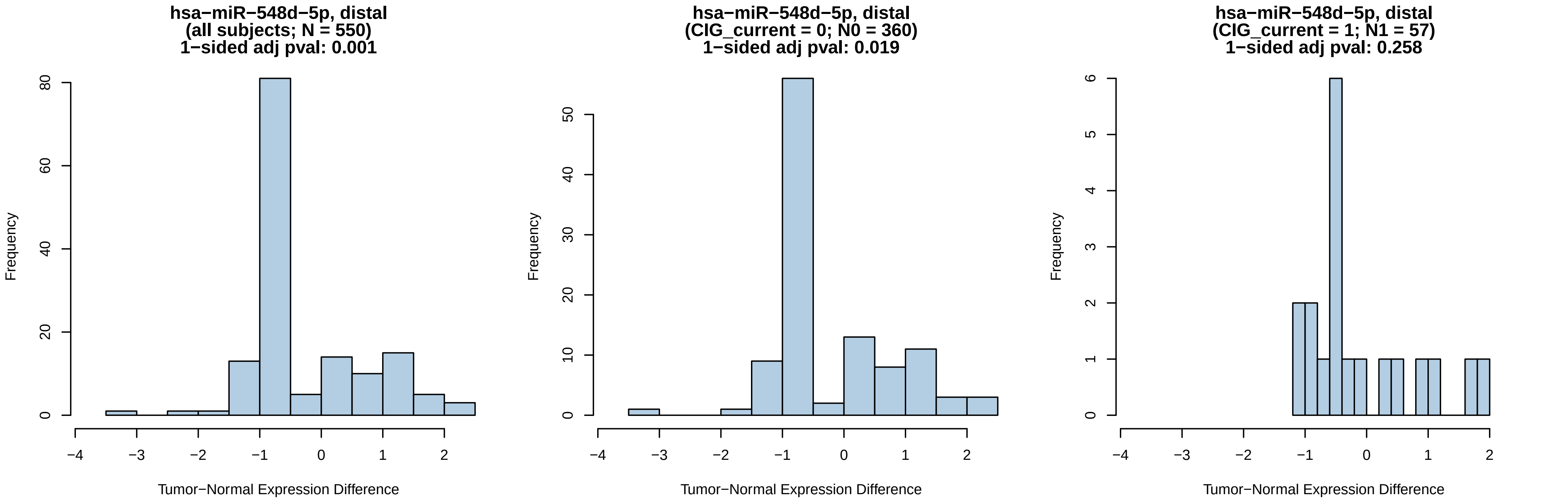
hsa−miR−548d−5p, distal
(all subjects; N = 550)
1−sided adj pval: 0.001
Frequency
Tumor−Normal Expression Difference
hsa−miR−548d−5p, distal
(CIG_current = 0; N0 = 360)
1−sided adj pval: 0.019
Frequency
Tumor−Normal Expression Difference
hsa−miR−548d−5p, distal
(CIG_current = 1; N1 = 57)
1−sided adj pval: 0.258
Frequency
Tumor−Normal Expression Difference

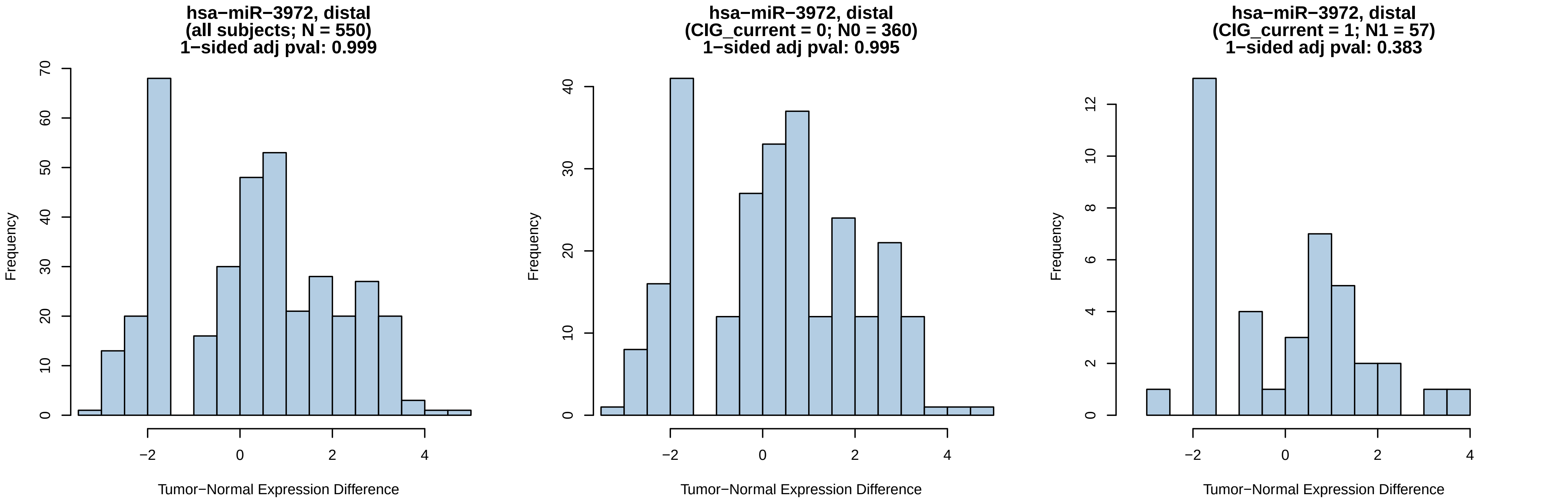
hsa−miR−3972, distal
(all subjects; N = 550)
1−sided adj pval: 0.999
Frequency
Tumor−Normal Expression Difference
hsa−miR−3972, distal
(CIG_current = 0; N0 = 360)
1−sided adj pval: 0.995
Frequency
Tumor−Normal Expression Difference
hsa−miR−3972, distal
(CIG_current = 1; N1 = 57)
1−sided adj pval: 0.383
Frequency
Tumor−Normal Expression Difference

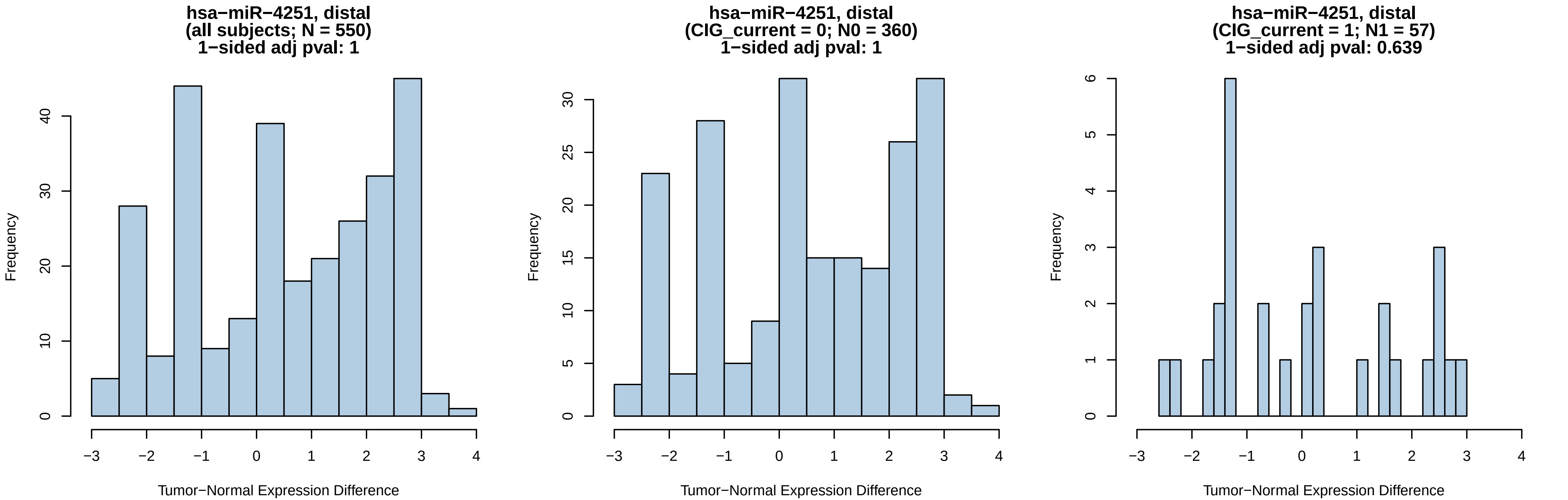
hsa−miR−4251, distal
(all subjects; N = 550)
1−sided adj pval: 1
hsa−miR−4251, distal
(CIG_current = 0; N0 = 360)
1−sided adj pval: 1
hsa−miR−4251, distal
(CIG_current = 1; N1 = 57)
1−sided adj pval: 0.639
Frequency
Tumor−Normal Expression Difference

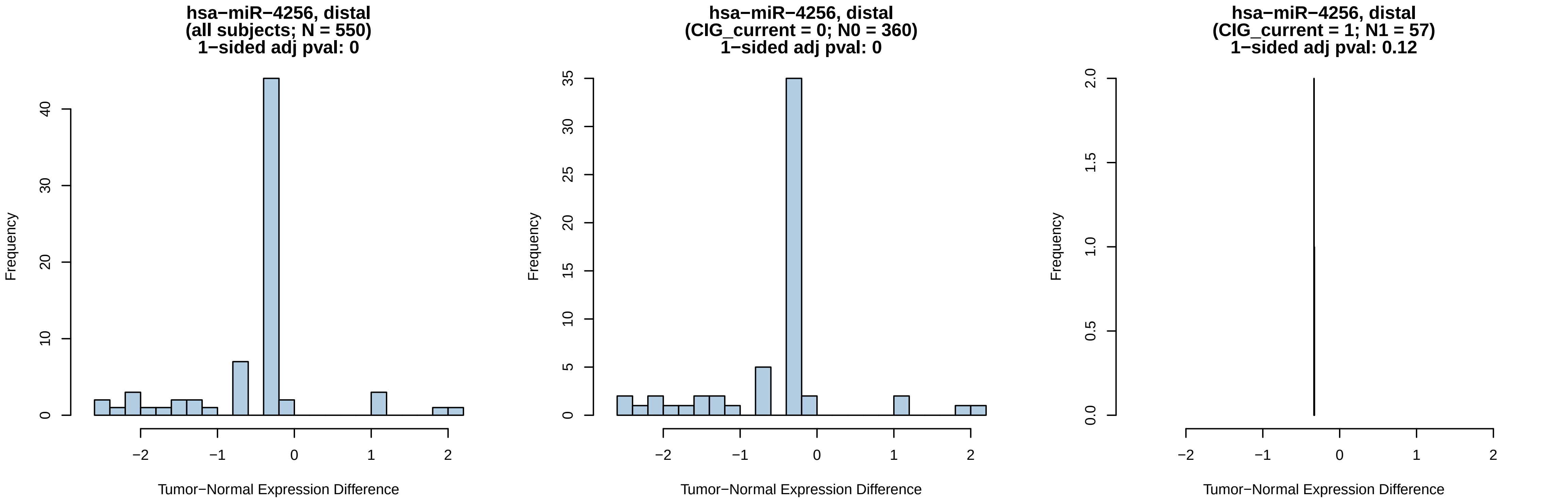
hsa−miR−4256, distal
(all subjects; N = 550)
1−sided adj pval: 0
Frequency
Tumor−Normal Expression Difference
hsa−miR−4256, distal
(CIG_current = 0; N0 = 360)
1−sided adj pval: 0
Frequency
Tumor−Normal Expression Difference
hsa−miR−4256, distal
(CIG_current = 1; N1 = 57)
1−sided adj pval: 0.12
Frequency
Tumor−Normal Expression Difference

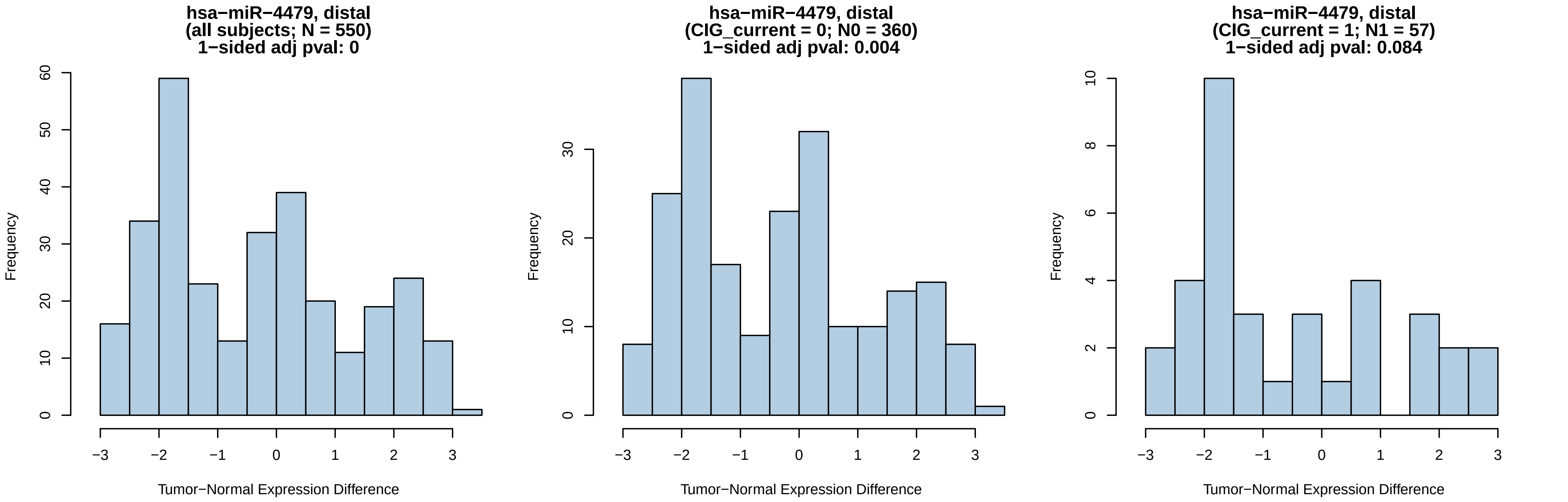
hsa−miR−4479, distal
(all subjects; N = 550)
1−sided adj pval: 0
Frequency
Tumor−Normal Expression Difference
hsa−miR−4479, distal
(CIG_current = 0; N0 = 360)
1−sided adj pval: 0.004
Frequency
Tumor−Normal Expression Difference
hsa−miR−4479, distal
(CIG_current = 1; N1 = 57)
1−sided adj pval: 0.084
Frequency
Tumor−Normal Expression Difference

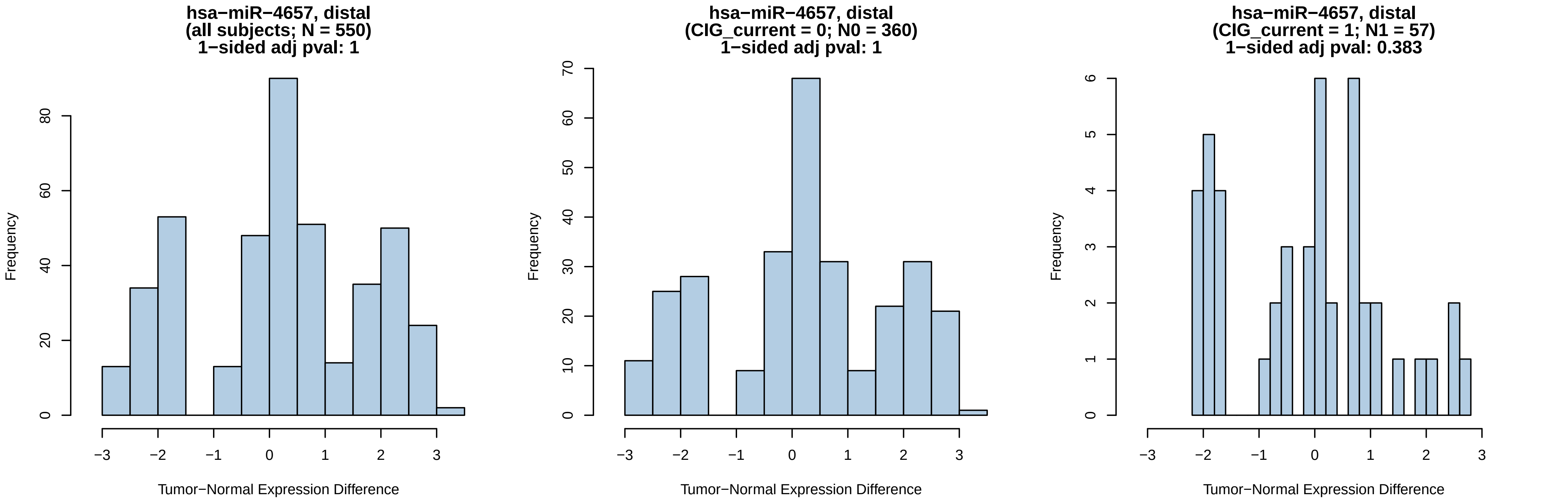

hsa−miR−4657, distal
(all subjects; N = 550)
1−sided adj pval: 1
Frequency
Tumor−Normal Expression Difference
hsa−miR−4657, distal
(CIG_current = 0; N0 = 360)
1−sided adj pval: 1
Frequency
Tumor−Normal Expression Difference
hsa−miR−4657, distal
(CIG_current = 1; N1 = 57)
1−sided adj pval: 0.383
Frequency
Tumor−Normal Expression Difference

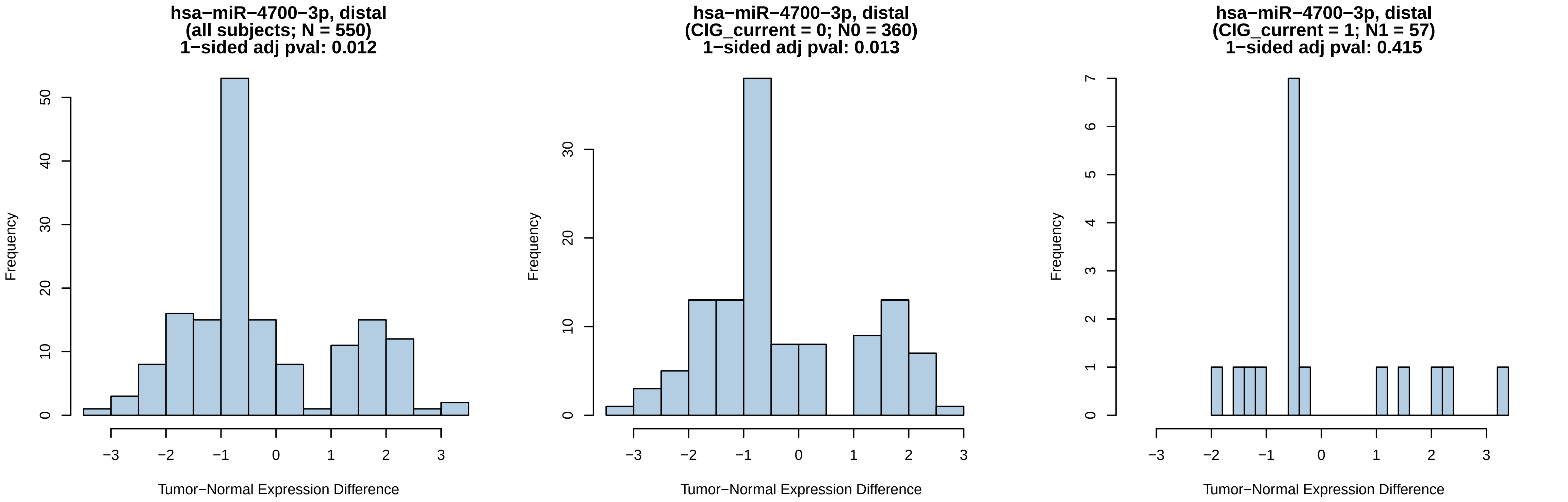
hsa−miR−4700−3p, distal
(all subjects; N = 550)
1−sided adj pval: 0.012
hsa−miR−4700−3p, distal
(CIG_current = 0; N0 = 360)
1−sided adj pval: 0.013
hsa−miR−4700−3p, distal
(CIG_current = 1; N1 = 57)
1−sided adj pval: 0.415
Frequency
Tumor−Normal Expression Difference

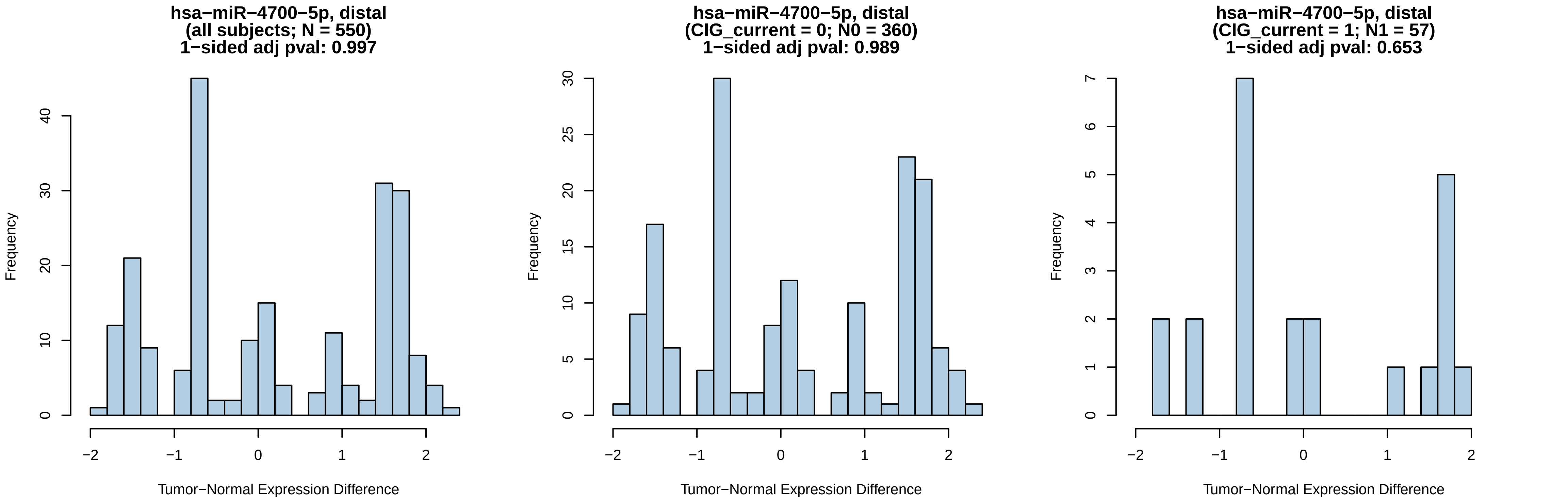

hsa−miR−4700−5p, distal
(all subjects; N = 550)
1−sided adj pval: 0.997
Frequency
Tumor−Normal Expression Difference
hsa−miR−4700−5p, distal
(CIG_current = 0; N0 = 360)
1−sided adj pval: 0.989
Frequency
Tumor−Normal Expression Difference
hsa−miR−4700−5p, distal
(CIG_current = 1; N1 = 57)
1−sided adj pval: 0.653
Frequency
Tumor−Normal Expression Difference

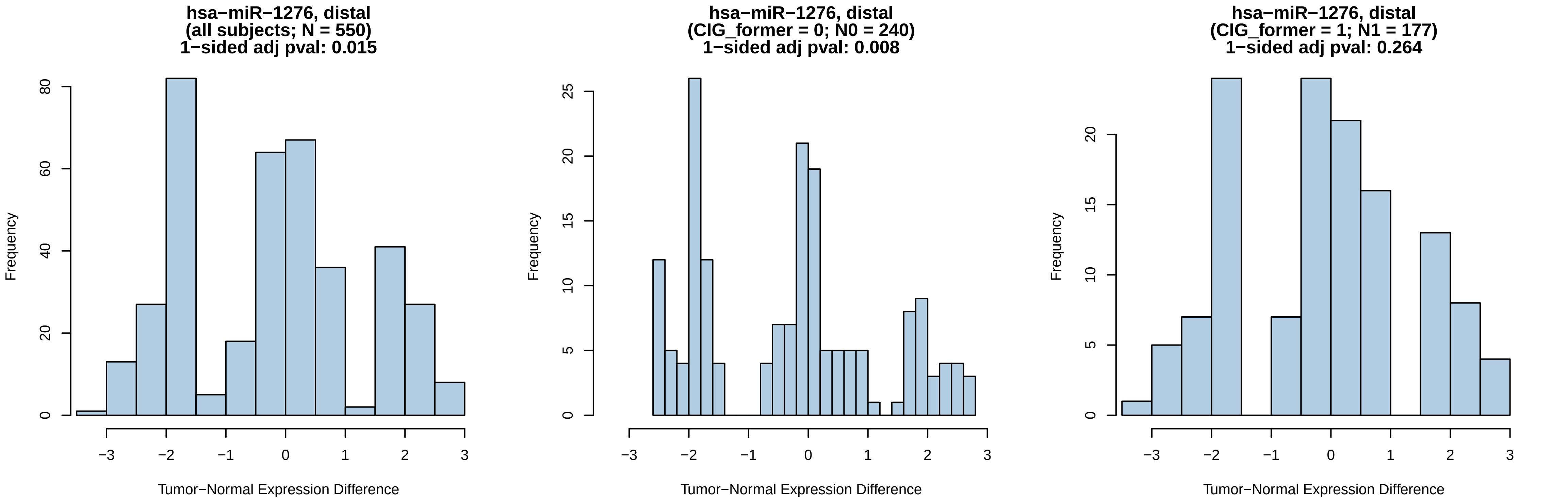
hsa−miR−1276, distal
(all subjects; N = 550)
1−sided adj pval: 0.015
Frequency
Tumor−Normal Expression Difference
hsa−miR−1276, distal
(CIG_former = 0; N0 = 240)
1−sided adj pval: 0.008
Frequency
Tumor−Normal Expression Difference
hsa−miR−1276, distal
(CIG_former = 1; N1 = 177)
1−sided adj pval: 0.264
Frequency
Tumor−Normal Expression Difference

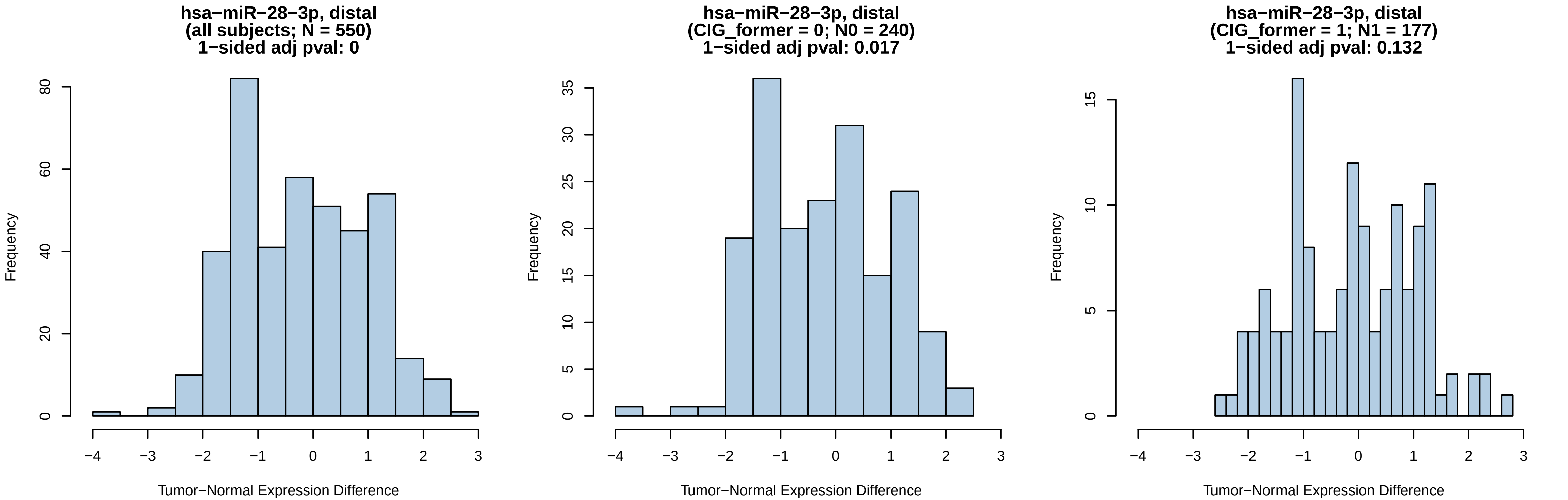

hsa−miR−28−3p, distal
(all subjects; N = 550)
1−sided adj pval: 0
hsa−miR−28−3p, distal
(CIG_former = 0; N0 = 240)
1−sided adj pval: 0.017
hsa−miR−28−3p, distal
(CIG_former = 1; N1 = 177)
1−sided adj pval: 0.132
Frequency
Tumor−Normal Expression Difference

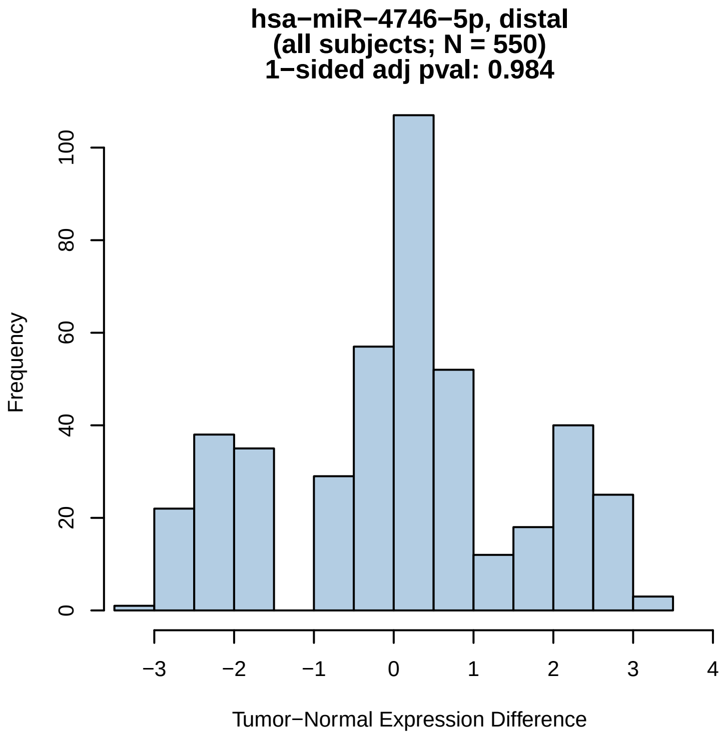
hsa−miR−4746−5p, distal
(all subjects; N = 550)
1−sided adj pval: 0.984
Frequency
0
20
40
60
80
100
−3
−2
−1
0
1
2
3
4
Tumor−Normal Expression Difference

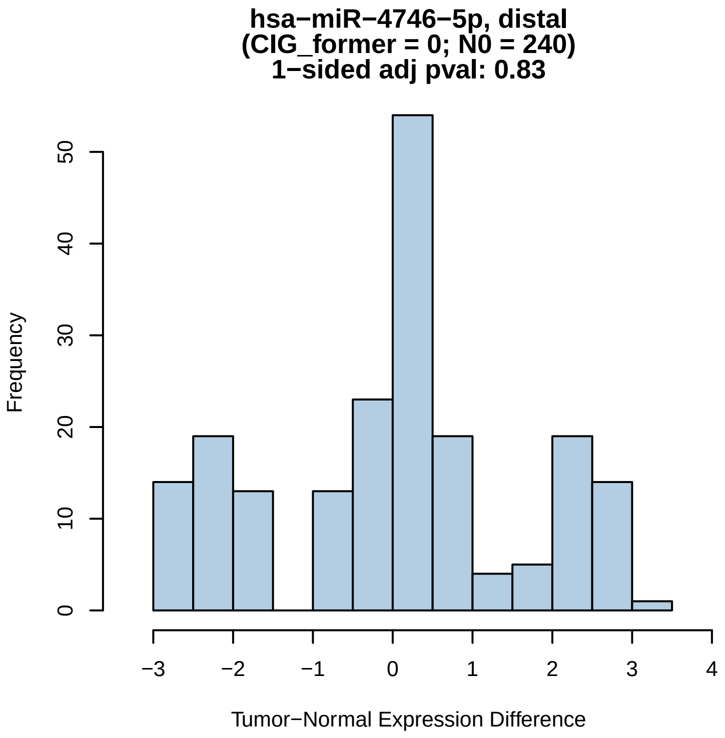
hsa−miR−4746−5p, distal
(CIG_former = 0; N0 = 240)
1−sided adj pval: 0.83
Frequency
0
10
20
30
40
50
−3
−2
−1
0
1
2
3
4
Tumor−Normal Expression Difference

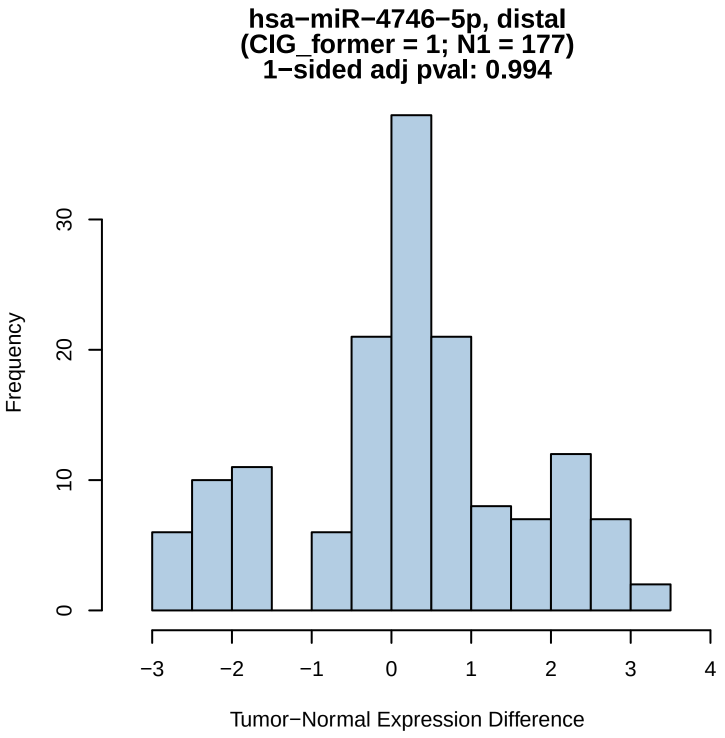
hsa−miR−4746−5p, distal
(CIG_former = 1; N1 = 177)
1−sided adj pval: 0.994
Frequency
0
10
20
30
−3
−2
−1
0
1
2
3
4
Tumor−Normal Expression Difference

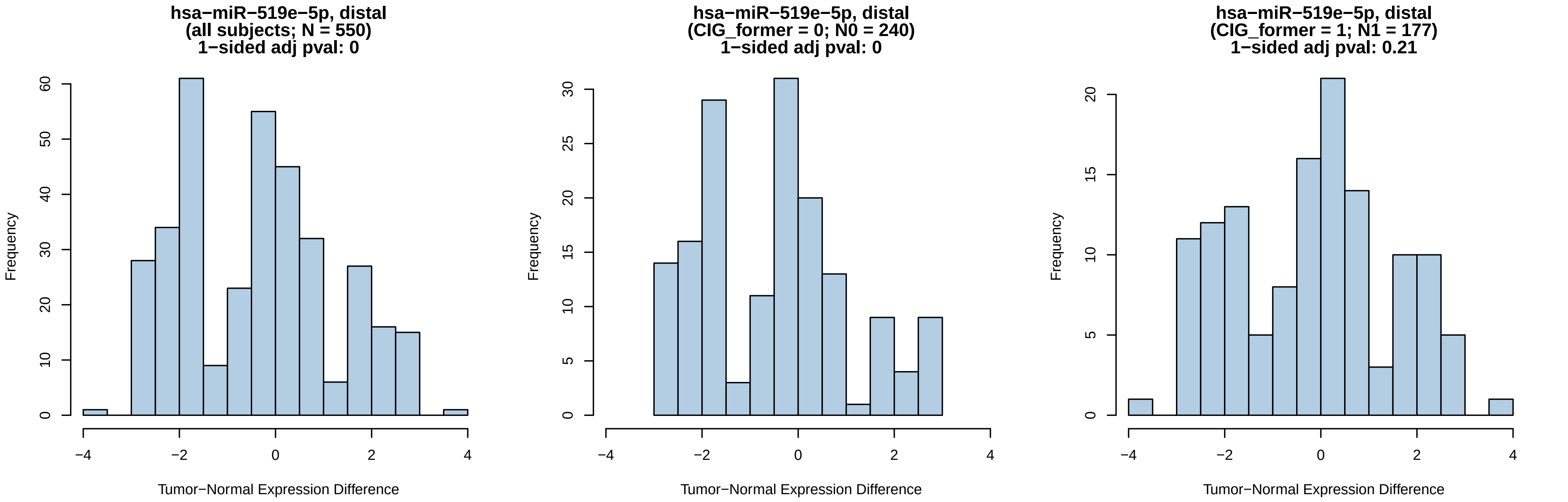
hsa−miR−519e−5p, distal
(all subjects; N = 550)
1−sided adj pval: 0
Frequency
Tumor−Normal Expression Difference
hsa−miR−519e−5p, distal
(CIG_former = 0; N0 = 240)
1−sided adj pval: 0
Frequency
Tumor−Normal Expression Difference
hsa−miR−519e−5p, distal
(CIG_former = 1; N1 = 177)
1−sided adj pval: 0.21
Frequency
Tumor−Normal Expression Difference

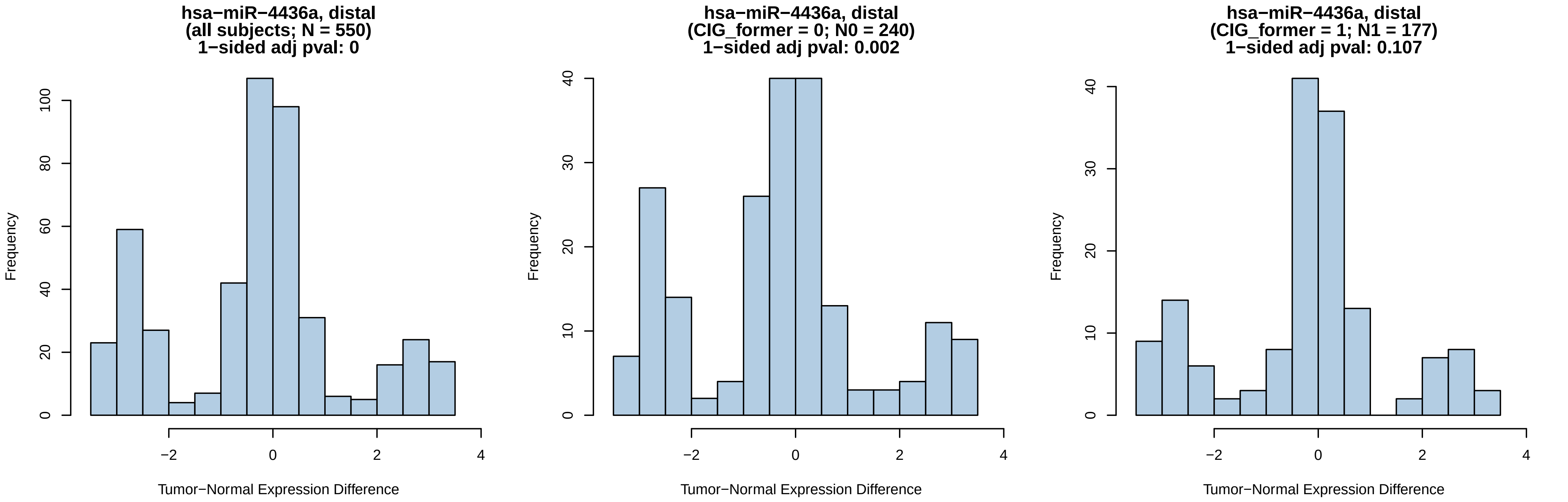
hsa−miR−4436a, distal
(all subjects; N = 550)
1−sided adj pval: 0
Frequency
0
20
40
60
80
100
−2
0
2
4
Tumor−Normal Expression Difference
hsa−miR−4436a, distal
(CIG_former = 0; N0 = 240)
1−sided adj pval: 0.002
Frequency
0
10
20
30
40
−2
0
2
4
Tumor−Normal Expression Difference
hsa−miR−4436a, distal
(CIG_former = 1; N1 = 177)
1−sided adj pval: 0.107
Frequency
0
10
20
30
40
−2
0
2
4
Tumor−Normal Expression Difference

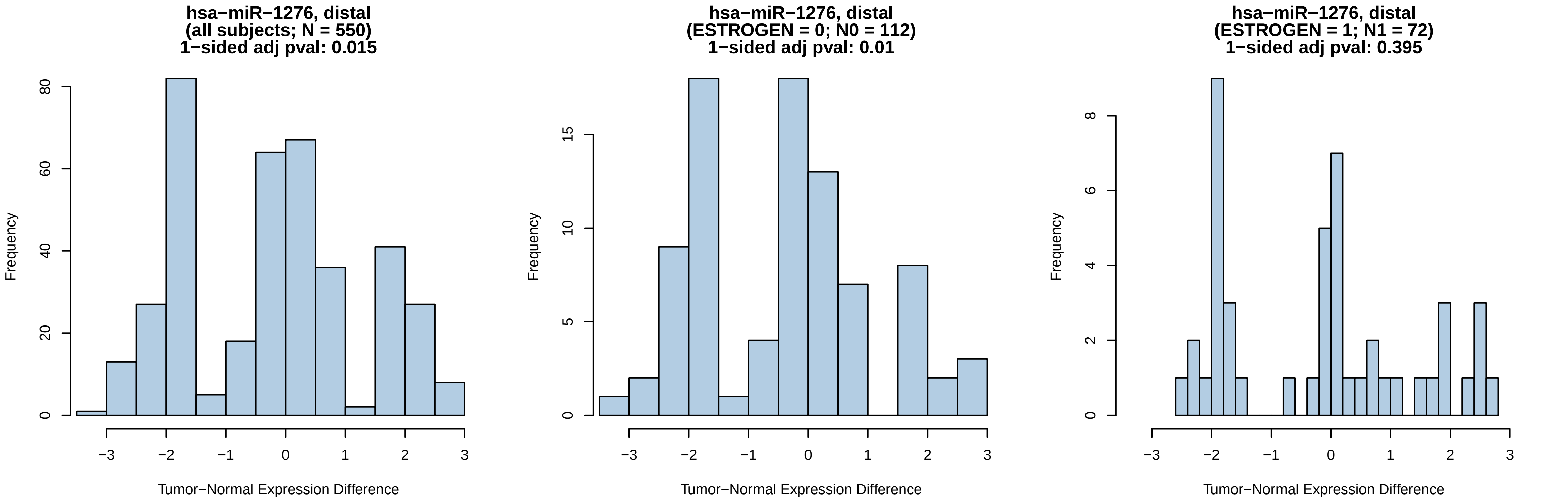
hsa−miR−1276, distal
(all subjects; N = 550)
1−sided adj pval: 0.015
Frequency
0
20
40
60
80
−3
−2
−1
0
1
2
3
Tumor−Normal Expression Difference
hsa−miR−1276, distal
(ESTROGEN = 0; N0 = 112)
1−sided adj pval: 0.01
Frequency
0
5
10
15
−3
−2
−1
0
1
2
3
Tumor−Normal Expression Difference
hsa−miR−1276, distal
(ESTROGEN = 1; N1 = 72)
1−sided adj pval: 0.395
Frequency
0
2
4
6
8
−3
−2
−1
0
1
2
3
Tumor−Normal Expression Difference

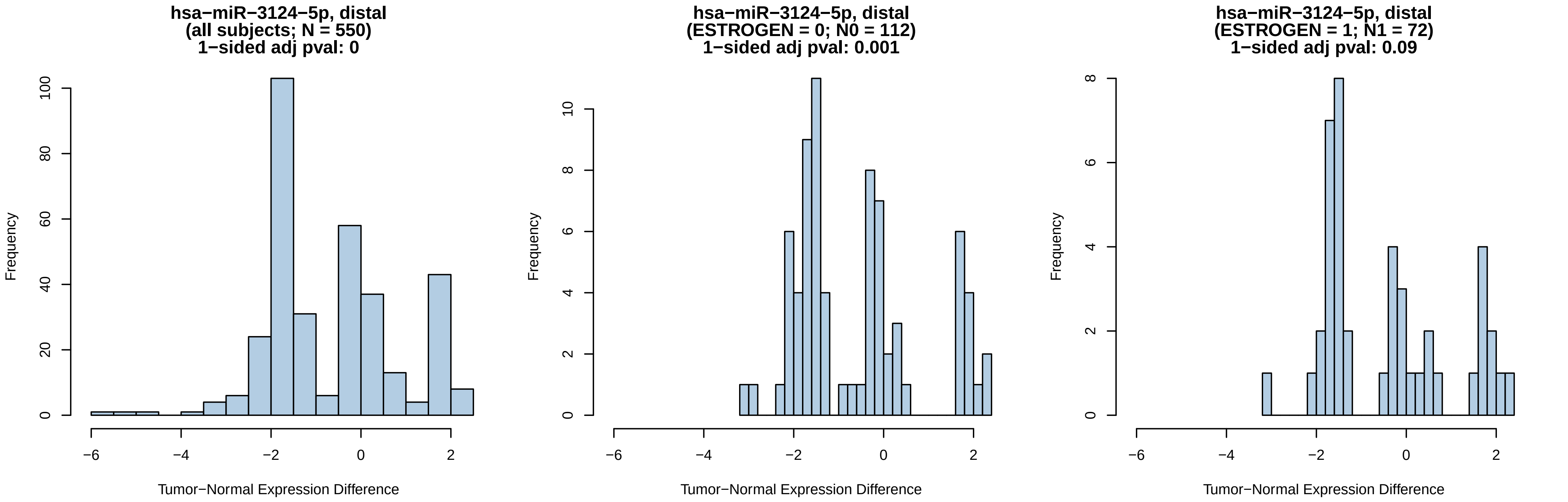

hsa−miR−3124−5p, distal
(all subjects; N = 550)
1−sided adj pval: 0
Frequency
Tumor−Normal Expression Difference
hsa−miR−3124−5p, distal
(ESTROGEN = 0; N0 = 112)
1−sided adj pval: 0.001
Frequency
Tumor−Normal Expression Difference
hsa−miR−3124−5p, distal
(ESTROGEN = 1; N1 = 72)
1−sided adj pval: 0.09
Frequency
Tumor−Normal Expression Difference

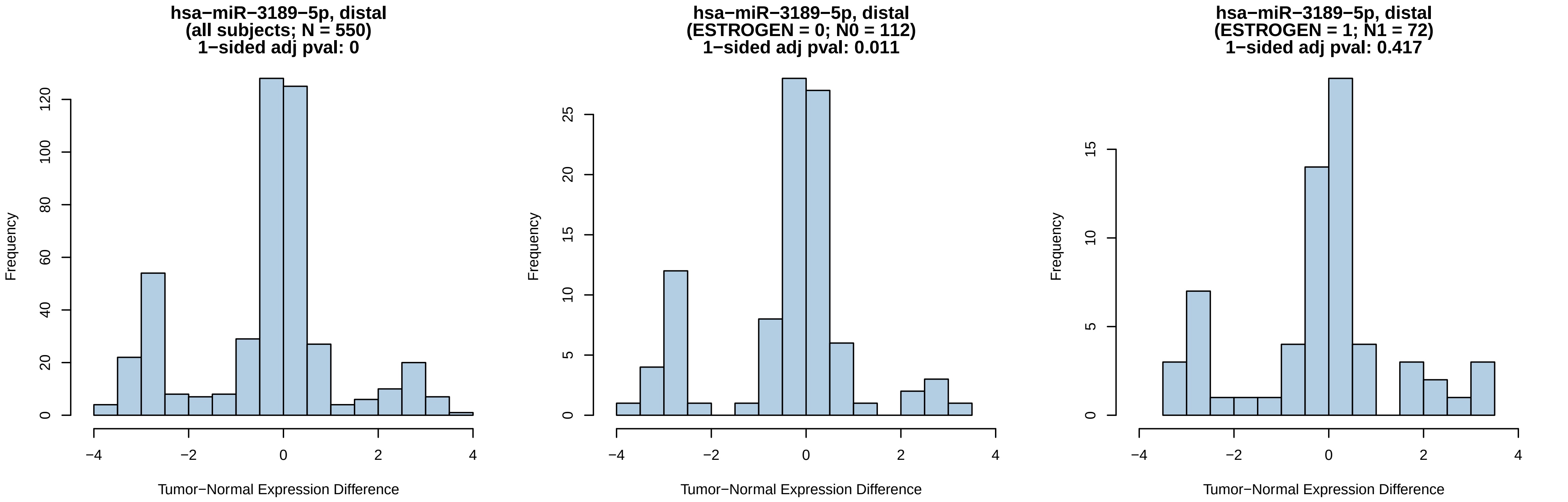
hsa−miR−3189−5p, distal
(all subjects; N = 550)
1−sided adj pval: 0
Frequency
Tumor−Normal Expression Difference
hsa−miR−3189−5p, distal
(ESTROGEN = 0; N0 = 112)
1−sided adj pval: 0.011
Frequency
Tumor−Normal Expression Difference
hsa−miR−3189−5p, distal
(ESTROGEN = 1; N1 = 72)
1−sided adj pval: 0.417
Frequency
Tumor−Normal Expression Difference

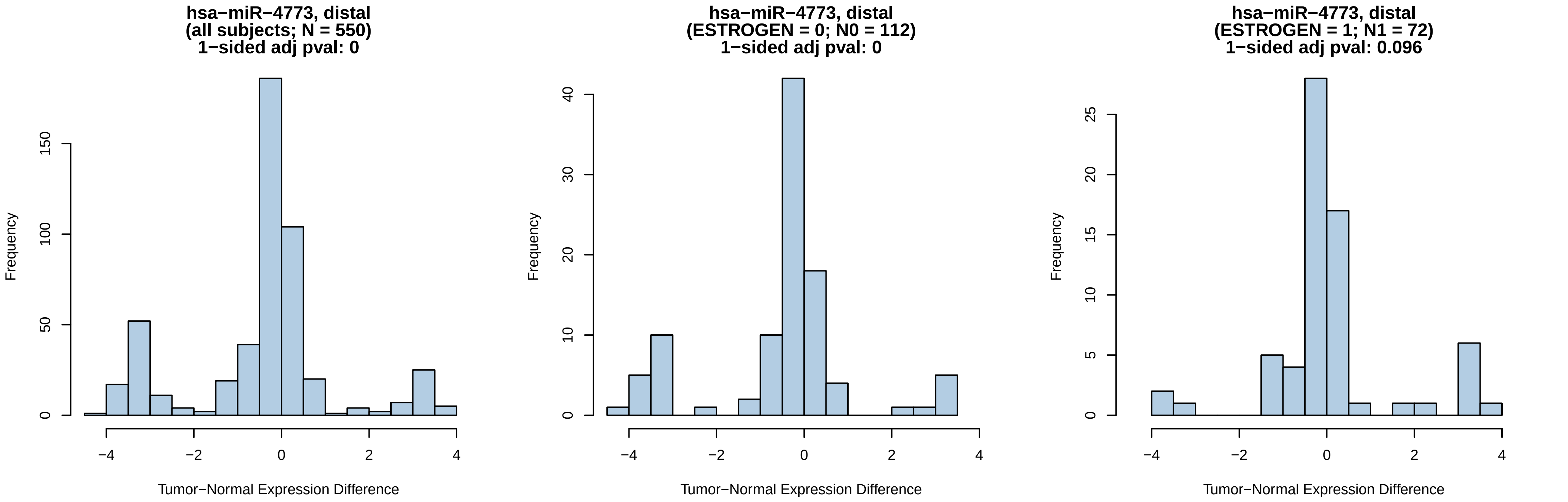
hsa−miR−4773, distal
(all subjects; N = 550)
1−sided adj pval: 0
Frequency
Tumor−Normal Expression Difference
hsa−miR−4773, distal
(ESTROGEN = 0; N0 = 112)
1−sided adj pval: 0
Frequency
Tumor−Normal Expression Difference
hsa−miR−4773, distal
(ESTROGEN = 1; N1 = 72)
1−sided adj pval: 0.096
Frequency
Tumor−Normal Expression Difference

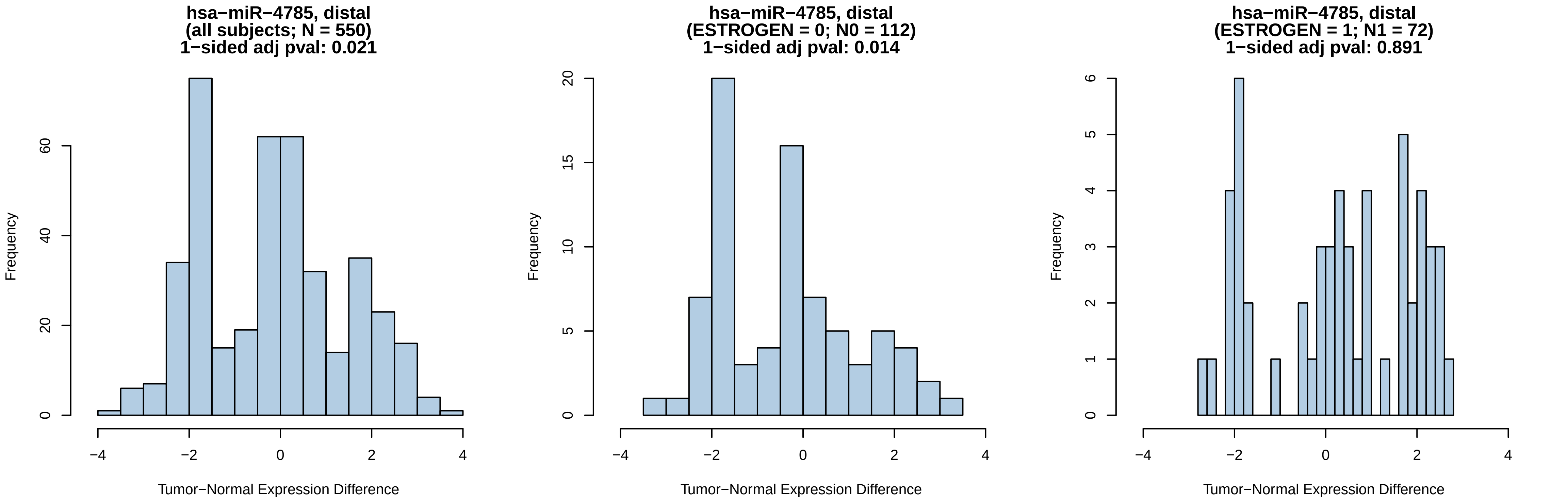
hsa−miR−4785, distal
(all subjects; N = 550)
1−sided adj pval: 0.021
hsa−miR−4785, distal
(ESTROGEN = 0; N0 = 112)
1−sided adj pval: 0.014
hsa−miR−4785, distal
(ESTROGEN = 1; N1 = 72)
1−sided adj pval: 0.891
Frequency
Tumor−Normal Expression Difference
0
20
40
60
5
10
15
1
2
3
4
6
−4
−2
2
4

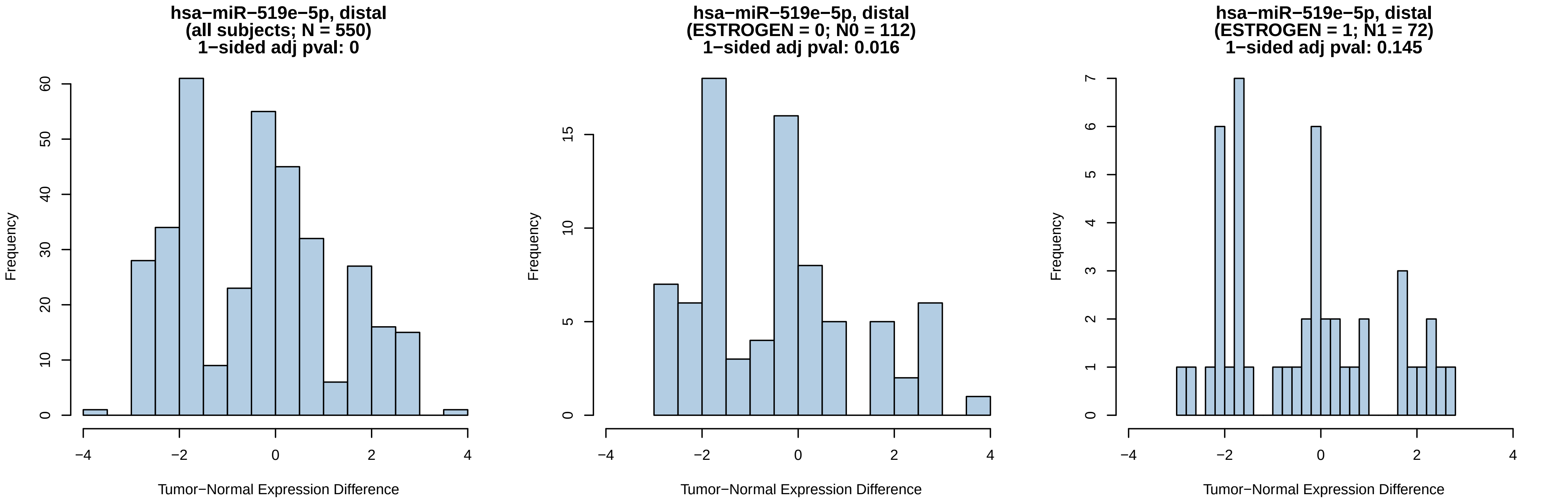
hsa−miR−519e−5p, distal
(all subjects; N = 550)
1−sided adj pval: 0
Frequency
0
10
20
30
40
50
60
−4
−2
0
2
4
Tumor−Normal Expression Difference
hsa−miR−519e−5p, distal
(ESTROGEN = 0; N0 = 112)
1−sided adj pval: 0.016
Frequency
0
5
10
15
−4
−2
0
2
4
Tumor−Normal Expression Difference
hsa−miR−519e−5p, distal
(ESTROGEN = 1; N1 = 72)
1−sided adj pval: 0.145
Frequency
0
1
2
3
4
5
6
7
−4
−2
0
2
4
Tumor−Normal Expression Difference

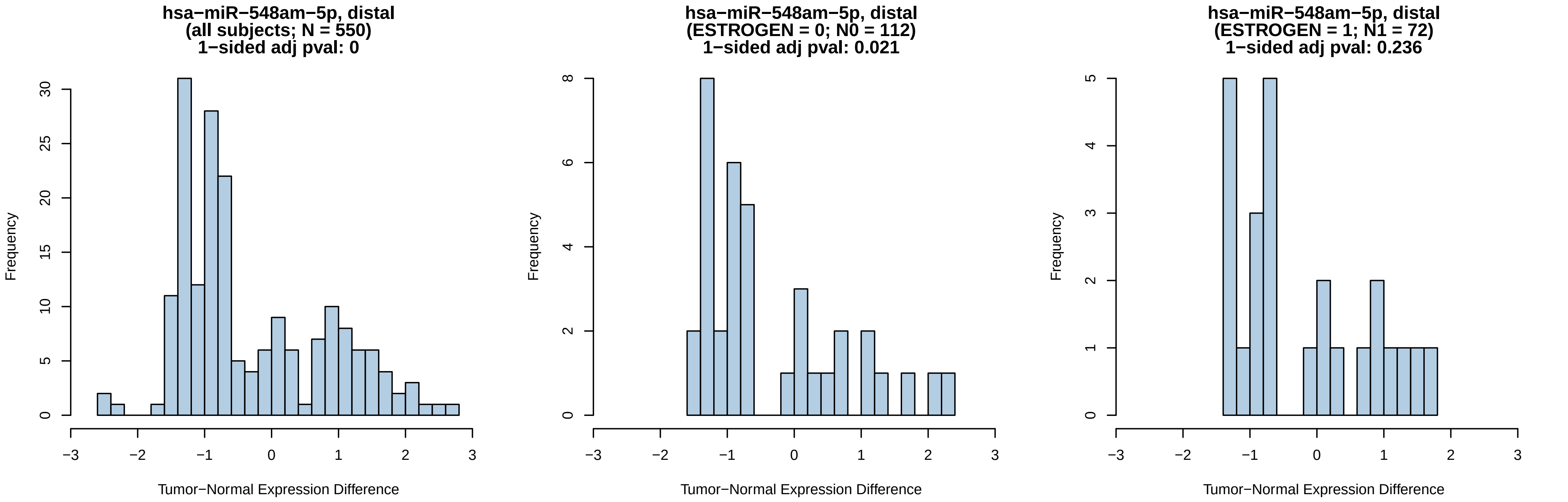

hsa−miR−548am−5p, distal
(all subjects; N = 550)
1−sided adj pval: 0
hsa−miR−548am−5p, distal
(ESTROGEN = 0; N0 = 112)
1−sided adj pval: 0.021
hsa−miR−548am−5p, distal
(ESTROGEN = 1; N1 = 72)
1−sided adj pval: 0.236
Frequency
Tumor−Normal Expression Difference

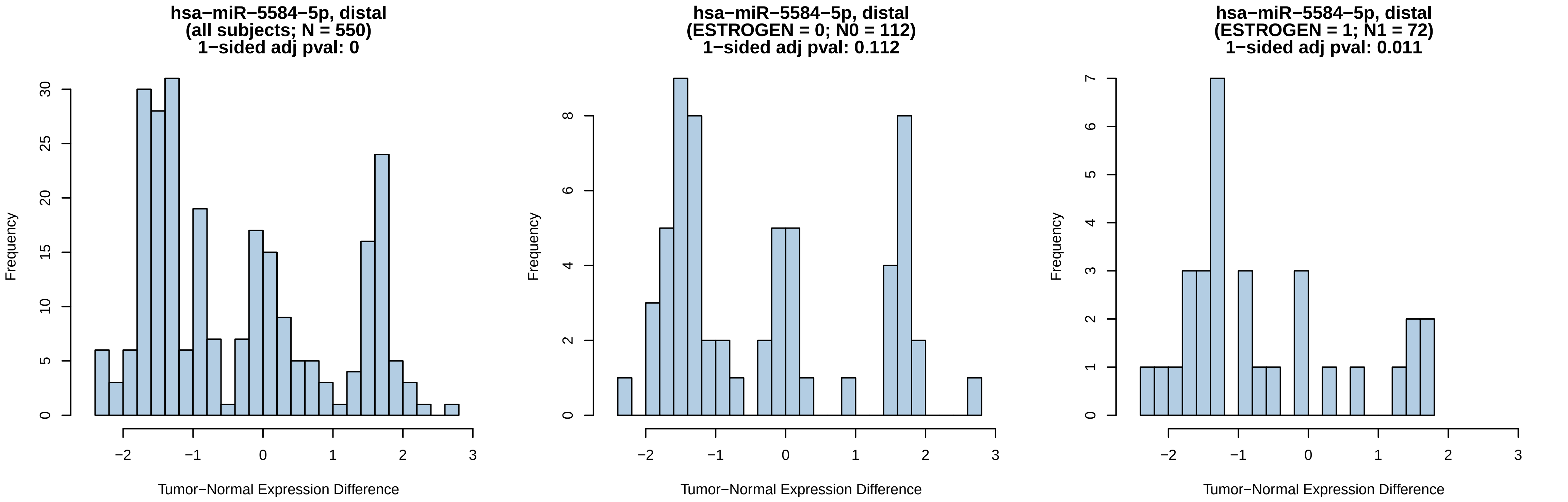
hsa−miR−5584−5p, distal
(all subjects; N = 550)
1−sided adj pval: 0
hsa−miR−5584−5p, distal
(ESTROGEN = 0; N0 = 112)
1−sided adj pval: 0.112
hsa−miR−5584−5p, distal
(ESTROGEN = 1; N1 = 72)
1−sided adj pval: 0.011
Frequency
Tumor−Normal Expression Difference

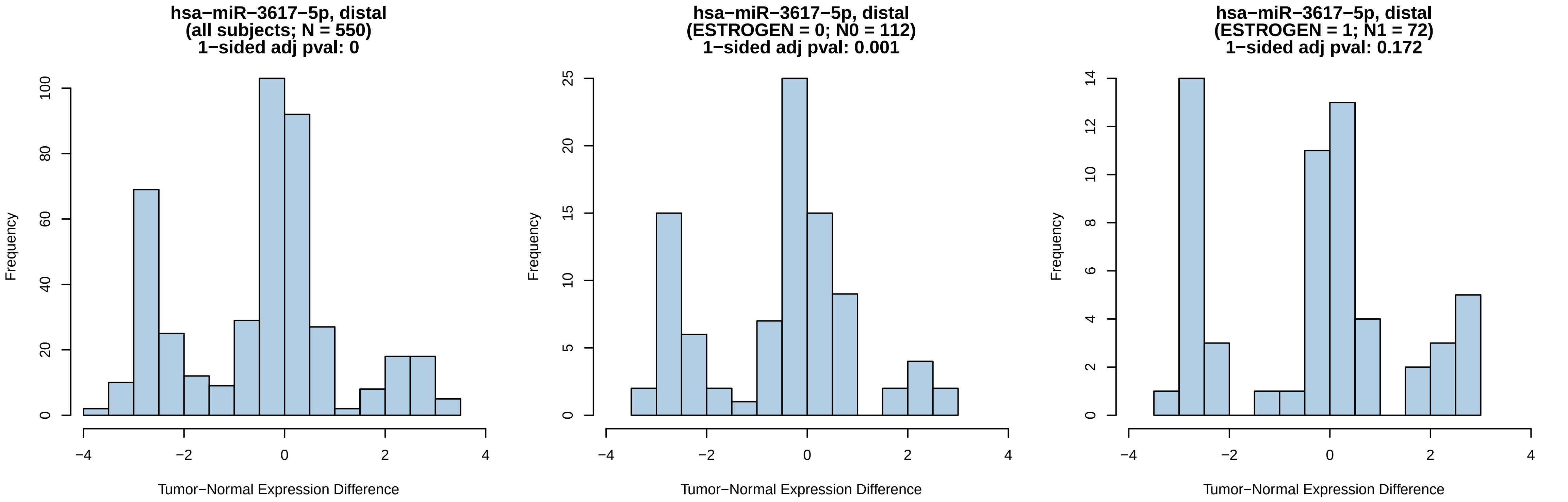

hsa−miR−3617−5p, distal
(all subjects; N = 550)
1−sided adj pval: 0
Frequency
Tumor−Normal Expression Difference
hsa−miR−3617−5p, distal
(ESTROGEN = 0; N0 = 112)
1−sided adj pval: 0.001
Frequency
Tumor−Normal Expression Difference
hsa−miR−3617−5p, distal
(ESTROGEN = 1; N1 = 72)
1−sided adj pval: 0.172
Frequency
Tumor−Normal Expression Difference

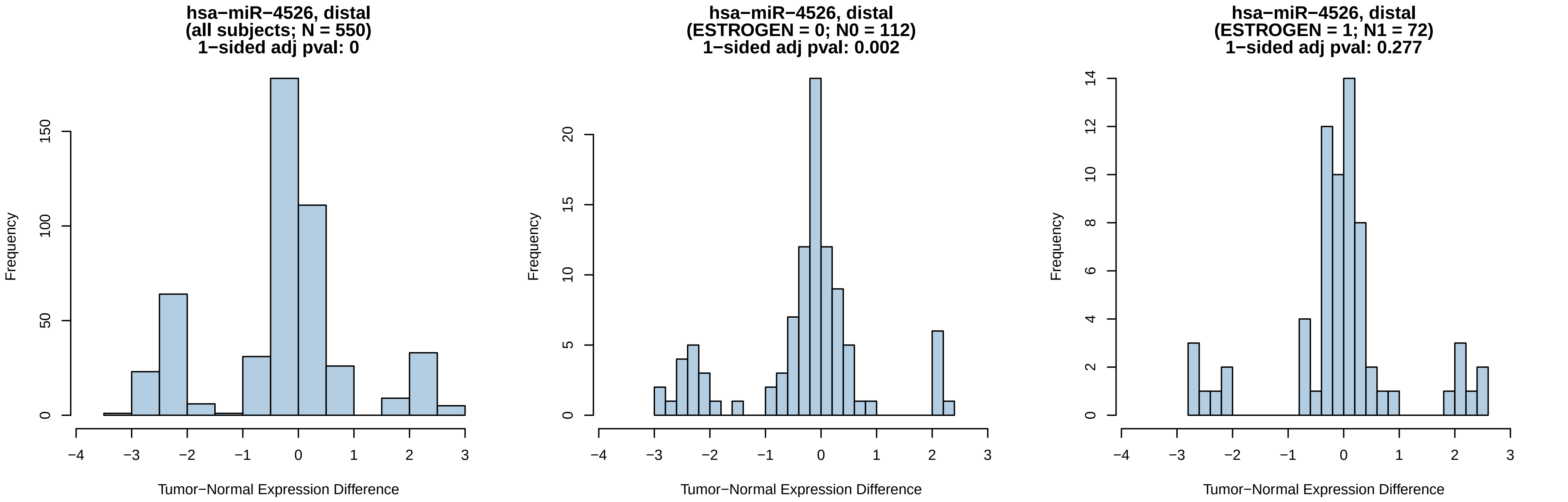

hsa−miR−4526, distal
(all subjects; N = 550)
1−sided adj pval: 0
Frequency
Tumor−Normal Expression Difference
hsa−miR−4526, distal
(ESTROGEN = 0; N0 = 112)
1−sided adj pval: 0.002
Frequency
Tumor−Normal Expression Difference
hsa−miR−4526, distal
(ESTROGEN = 1; N1 = 72)
1−sided adj pval: 0.277
Frequency
Tumor−Normal Expression Difference

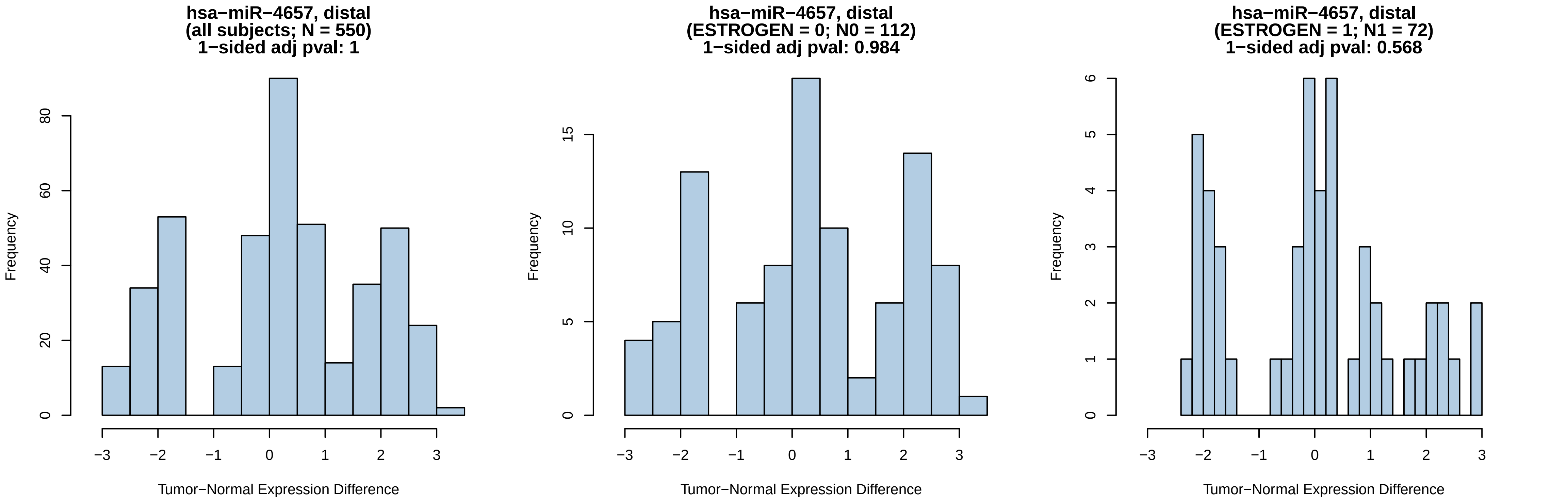
hsa−miR−4657, distal
(all subjects; N = 550)
1−sided adj pval: 1
Frequency
Tumor−Normal Expression Difference
hsa−miR−4657, distal
(ESTROGEN = 0; N0 = 112)
1−sided adj pval: 0.984
Frequency
Tumor−Normal Expression Difference
hsa−miR−4657, distal
(ESTROGEN = 1; N1 = 72)
1−sided adj pval: 0.568
Frequency
Tumor−Normal Expression Difference

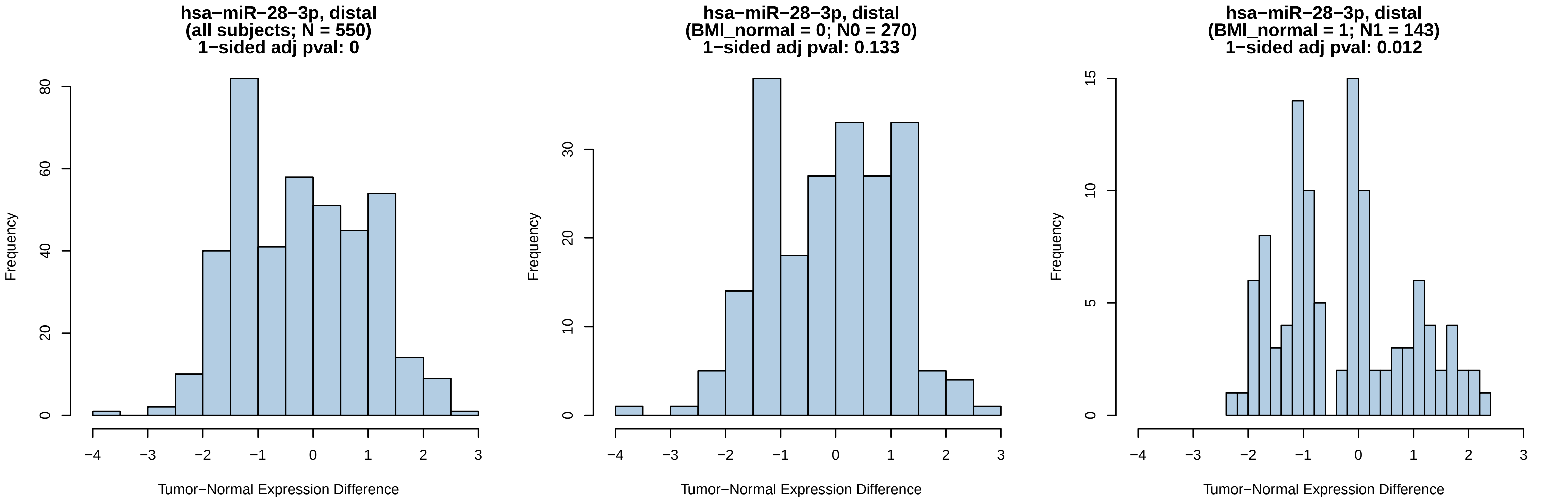

hsa−miR−28−3p, distal
(all subjects; N = 550)
1−sided adj pval: 0
Frequency
Tumor−Normal Expression Difference
hsa−miR−28−3p, distal
(BMI_normal = 0; N0 = 270)
1−sided adj pval: 0.133
Frequency
Tumor−Normal Expression Difference
hsa−miR−28−3p, distal
(BMI_normal = 1; N1 = 143)
1−sided adj pval: 0.012
Frequency
Tumor−Normal Expression Difference

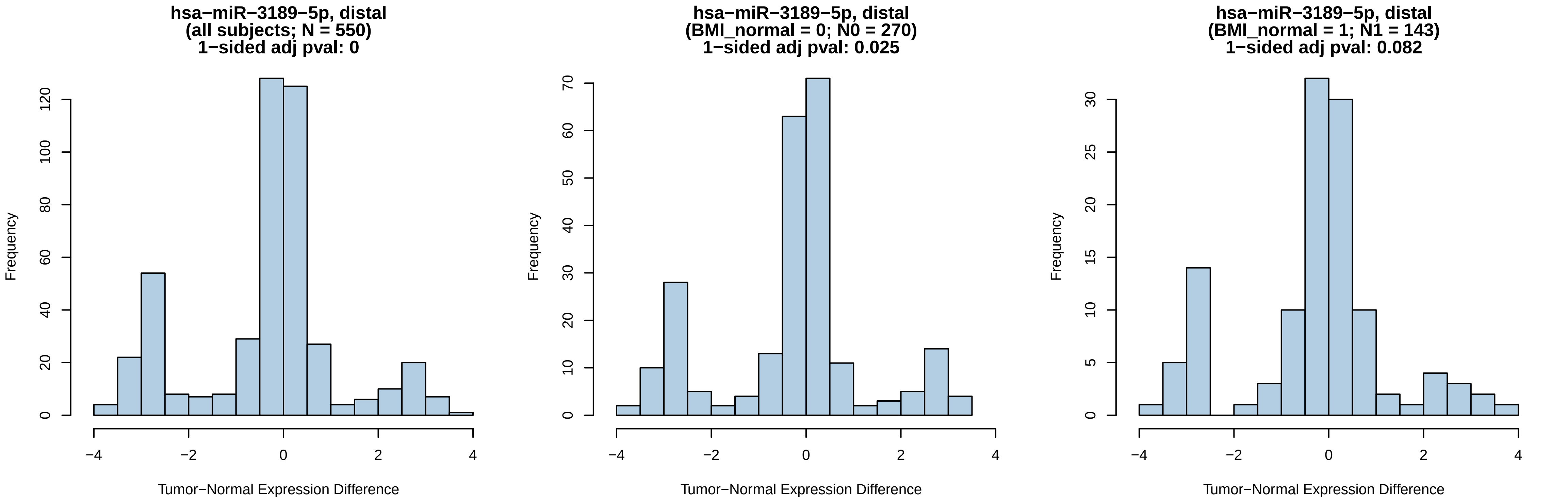

hsa−miR−3189−5p, distal
(all subjects; N = 550)
1−sided adj pval: 0
hsa−miR−3189−5p, distal
(BMI_normal = 0; N0 = 270)
1−sided adj pval: 0.025
hsa−miR−3189−5p, distal
(BMI_normal = 1; N1 = 143)
1−sided adj pval: 0.082
Frequency
Tumor−Normal Expression Difference

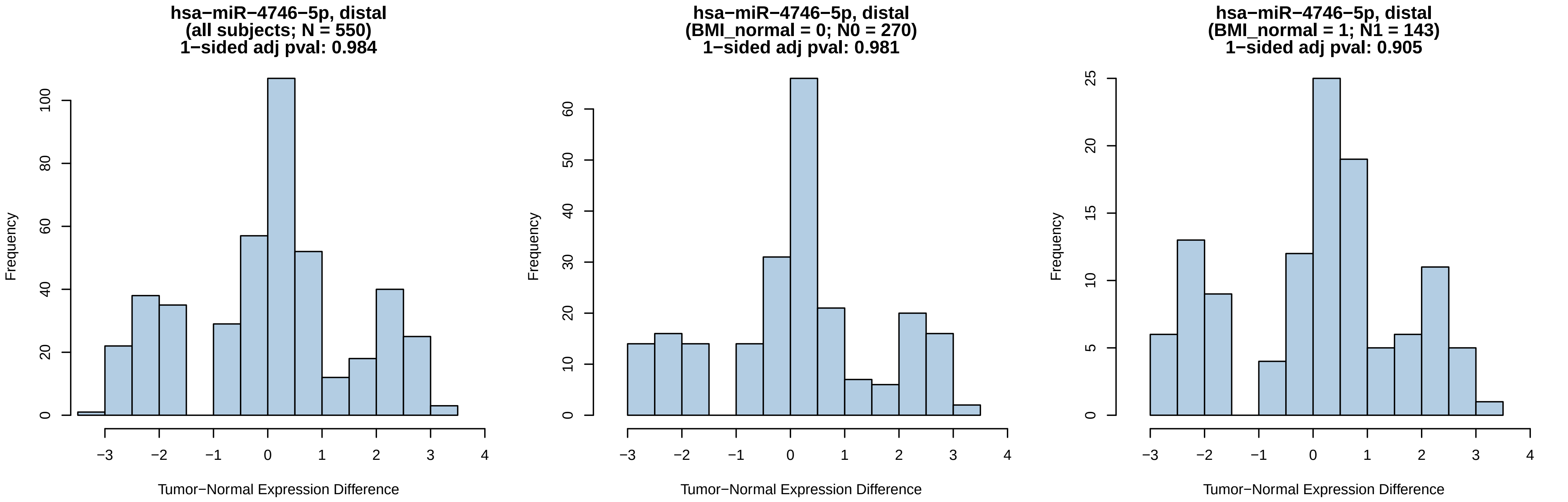
hsa−miR−4746−5p, distal
(all subjects; N = 550)
1−sided adj pval: 0.984
Frequency
Tumor−Normal Expression Difference
hsa−miR−4746−5p, distal
(BMI_normal = 0; N0 = 270)
1−sided adj pval: 0.981
Frequency
Tumor−Normal Expression Difference
hsa−miR−4746−5p, distal
(BMI_normal = 1; N1 = 143)
1−sided adj pval: 0.905
Frequency
Tumor−Normal Expression Difference

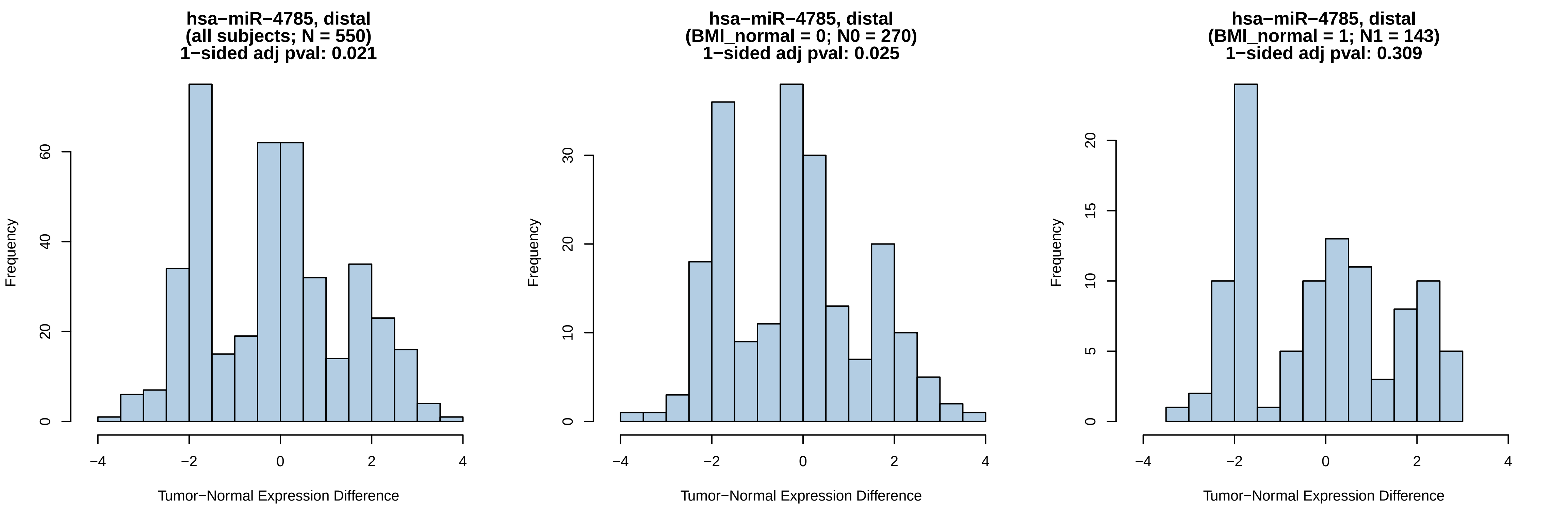
hsa−miR−4785, distal
(all subjects; N = 550)
1−sided adj pval: 0.021
Frequency
Tumor−Normal Expression Difference
hsa−miR−4785, distal
(BMI_normal = 0; N0 = 270)
1−sided adj pval: 0.025
Frequency
Tumor−Normal Expression Difference
hsa−miR−4785, distal
(BMI_normal = 1; N1 = 143)
1−sided adj pval: 0.309
Frequency
Tumor−Normal Expression Difference

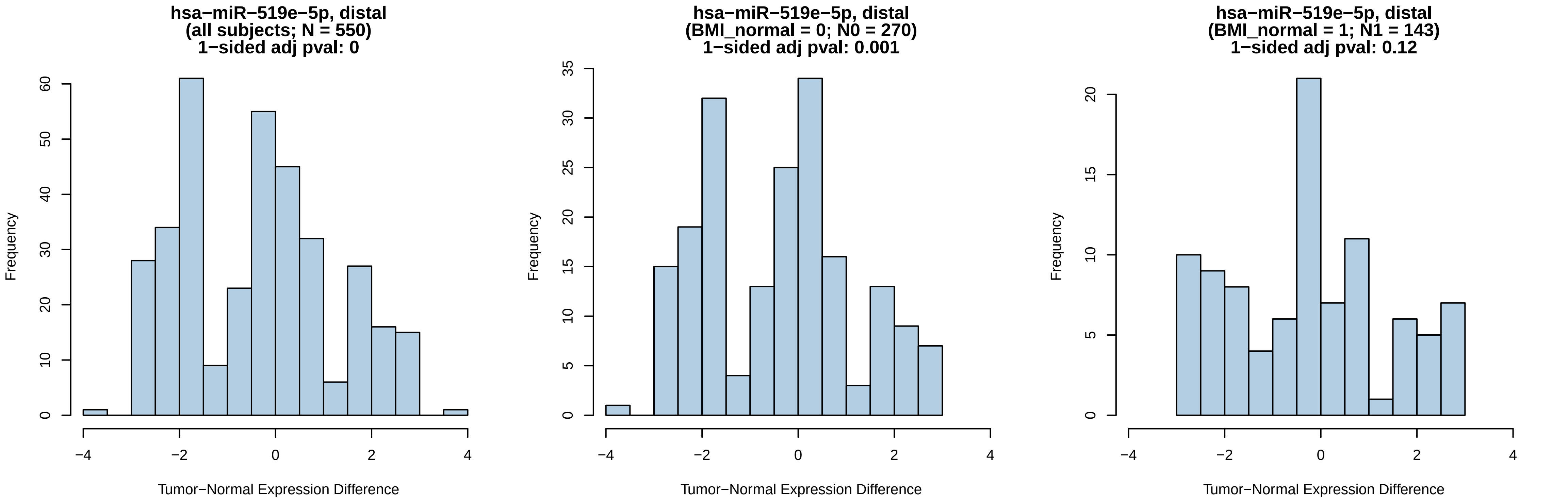

hsa−miR−519e−5p, distal
(all subjects; N = 550)
1−sided adj pval: 0
Frequency
Tumor−Normal Expression Difference
hsa−miR−519e−5p, distal
(BMI_normal = 0; N0 = 270)
1−sided adj pval: 0.001
Frequency
Tumor−Normal Expression Difference
hsa−miR−519e−5p, distal
(BMI_normal = 1; N1 = 143)
1−sided adj pval: 0.12
Frequency
Tumor−Normal Expression Difference

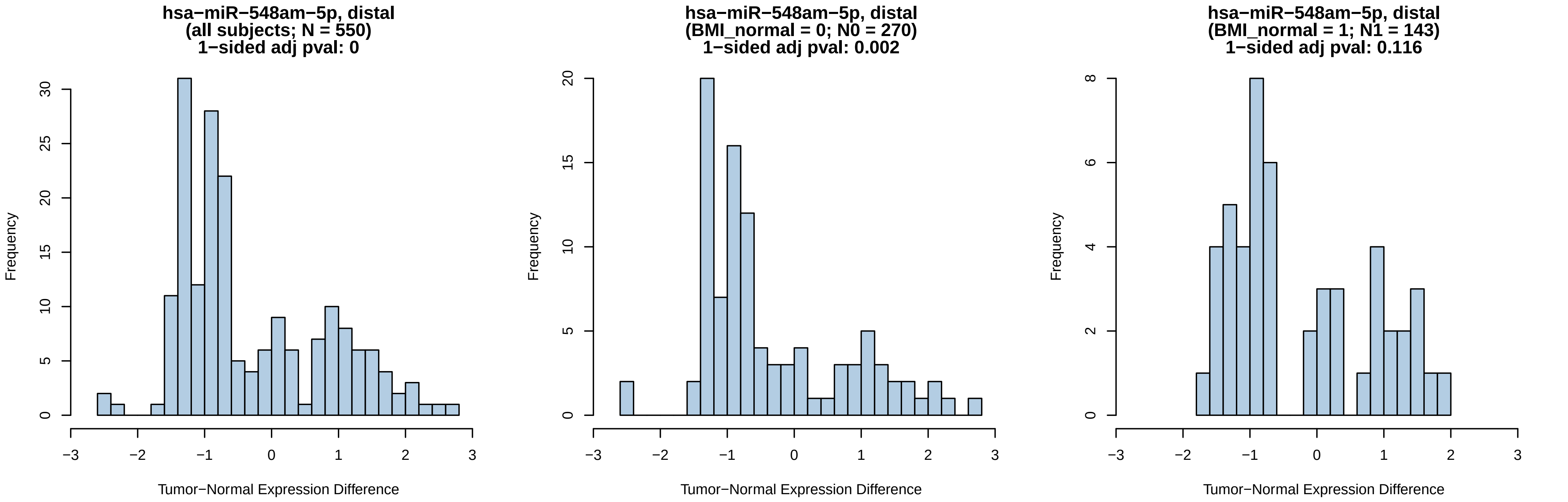

hsa−miR−548am−5p, distal
(all subjects; N = 550)
1−sided adj pval: 0
Frequency
Tumor−Normal Expression Difference
hsa−miR−548am−5p, distal
(BMI_normal = 0; N0 = 270)
1−sided adj pval: 0.002
Frequency
Tumor−Normal Expression Difference
hsa−miR−548am−5p, distal
(BMI_normal = 1; N1 = 143)
1−sided adj pval: 0.116
Frequency
Tumor−Normal Expression Difference

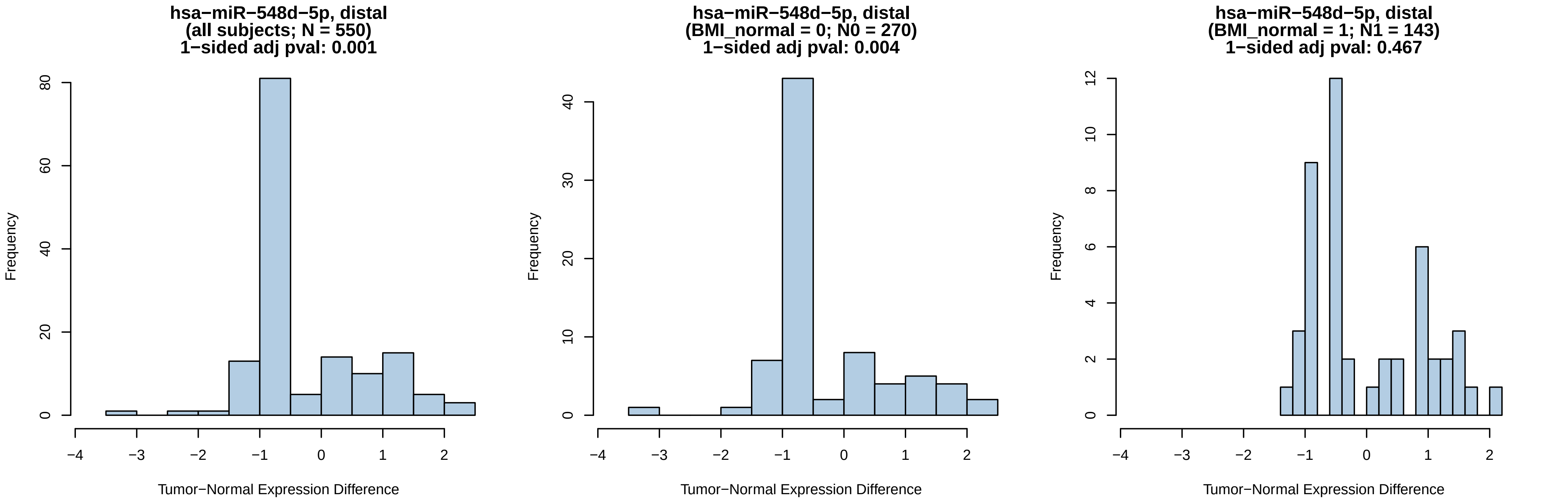
hsa−miR−548d−5p, distal
(all subjects; N = 550)
1−sided adj pval: 0.001
Frequency
Tumor−Normal Expression Difference
hsa−miR−548d−5p, distal
(BMI_normal = 0; N0 = 270)
1−sided adj pval: 0.004
Frequency
Tumor−Normal Expression Difference
hsa−miR−548d−5p, distal
(BMI_normal = 1; N1 = 143)
1−sided adj pval: 0.467
Frequency
Tumor−Normal Expression Difference

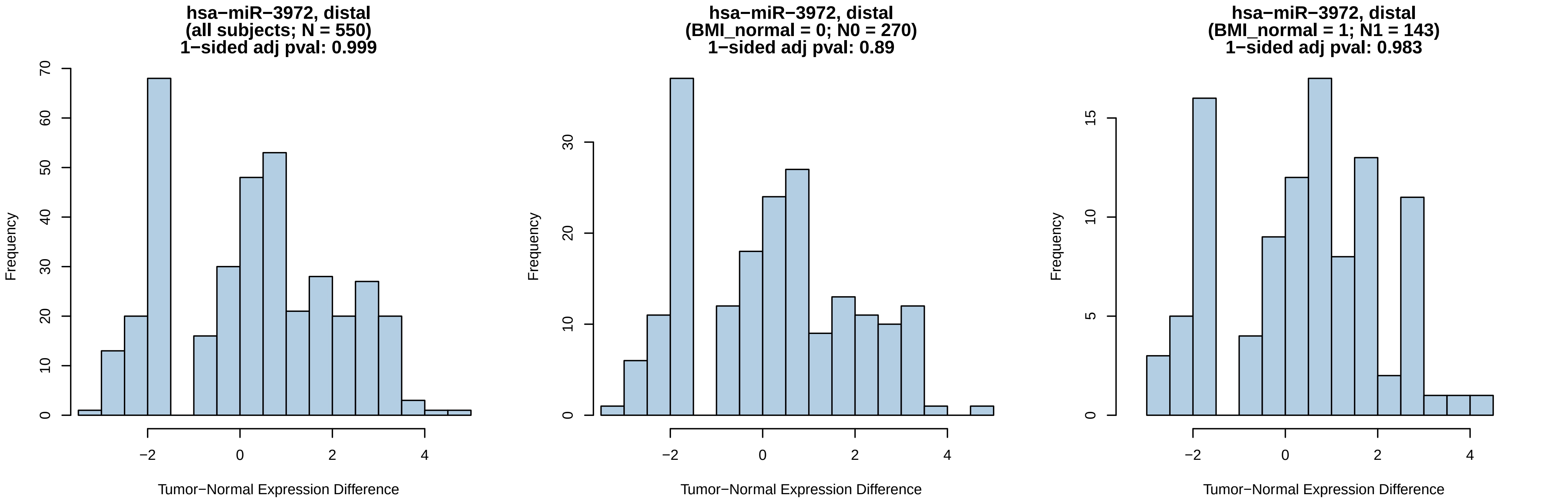
hsa−miR−3972, distal
(all subjects; N = 550)
1−sided adj pval: 0.999
Frequency
0
10
20
30
40
50
60
70
−2
0
2
4
Tumor−Normal Expression Difference
hsa−miR−3972, distal
(BMI_normal = 0; N0 = 270)
1−sided adj pval: 0.89
Frequency
0
10
20
30
−2
0
2
4
Tumor−Normal Expression Difference
hsa−miR−3972, distal
(BMI_normal = 1; N1 = 143)
1−sided adj pval: 0.983
Frequency
0
5
10
15
−2
0
2
4
Tumor−Normal Expression Difference

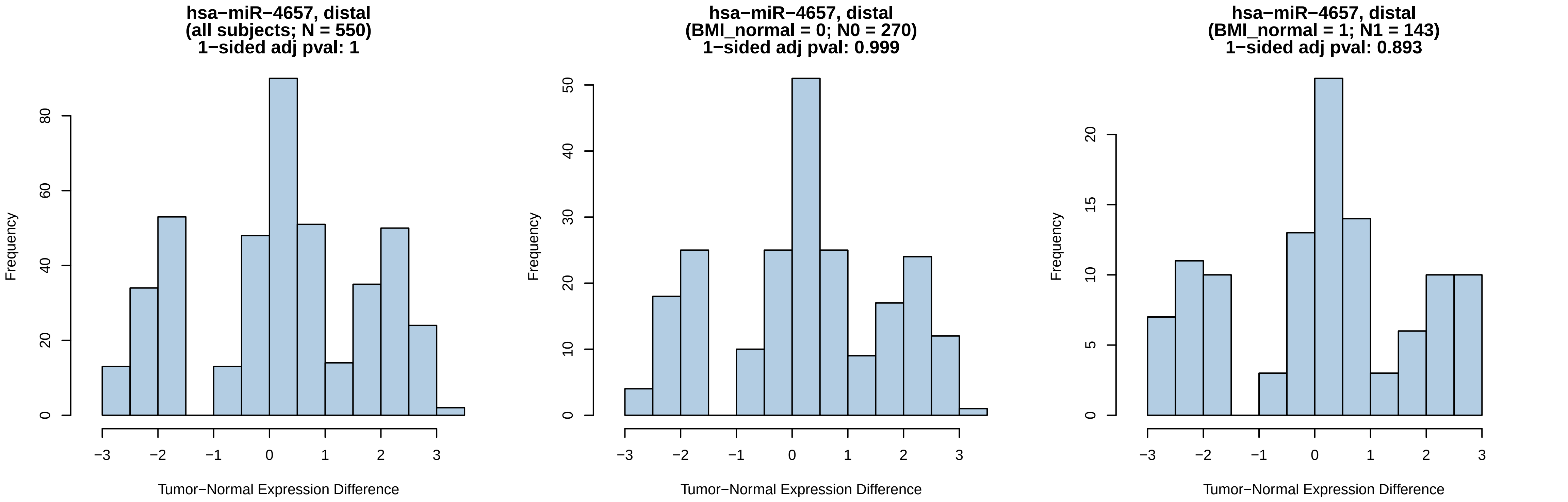
hsa−miR−4657, distal
(all subjects; N = 550)
1−sided adj pval: 1
Frequency
0
20
40
60
80
−3
−2
−1
0
1
2
3
Tumor−Normal Expression Difference
hsa−miR−4657, distal
(BMI_normal = 0; N0 = 270)
1−sided adj pval: 0.999
Frequency
0
10
20
30
40
50
−3
−2
−1
0
1
2
3
Tumor−Normal Expression Difference
hsa−miR−4657, distal
(BMI_normal = 1; N1 = 143)
1−sided adj pval: 0.893
Frequency
0
5
10
15
20
−3
−2
−1
0
1
2
3
Tumor−Normal Expression Difference

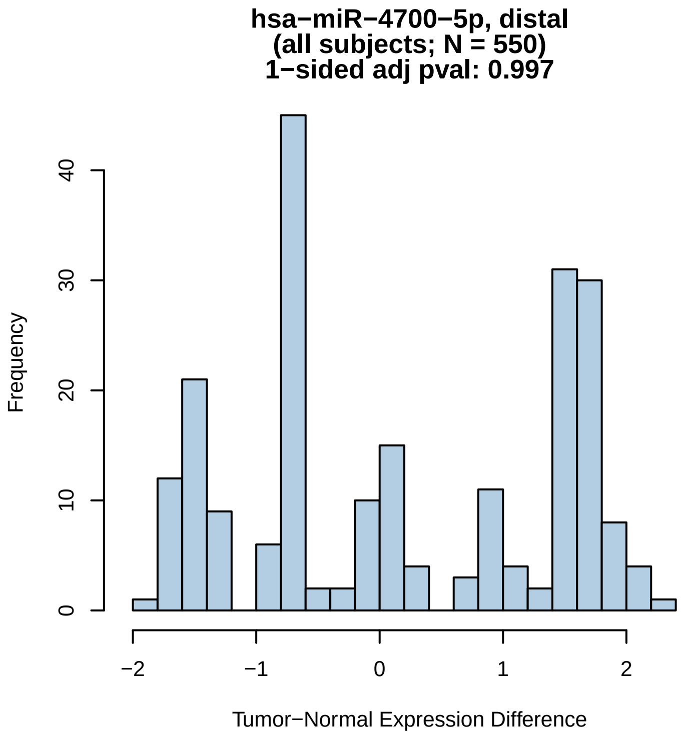
hsa−miR−4700−5p, distal
(all subjects; N = 550)
1−sided adj pval: 0.997
Frequency
Tumor−Normal Expression Difference

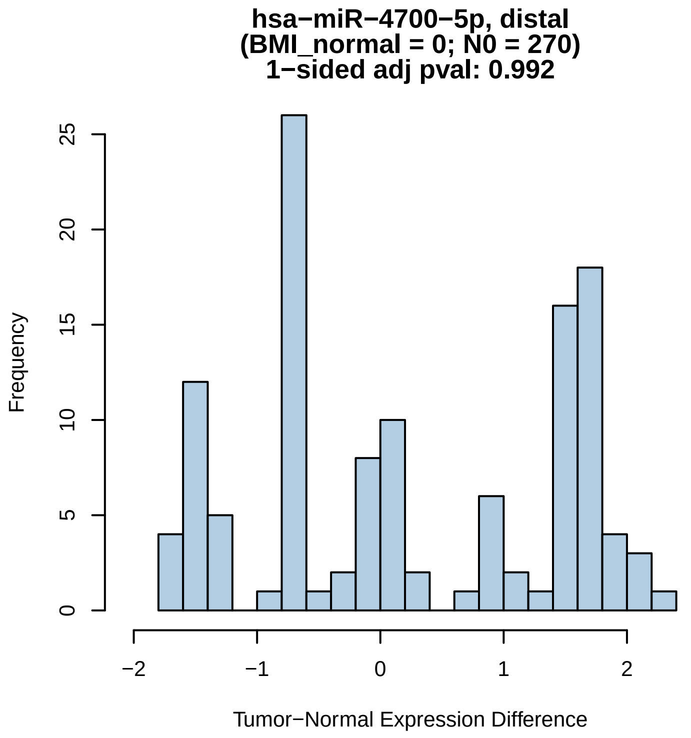
hsa−miR−4700−5p, distal
(BMI_normal = 0; N0 = 270)
1−sided adj pval: 0.992
Frequency
Tumor−Normal Expression Difference

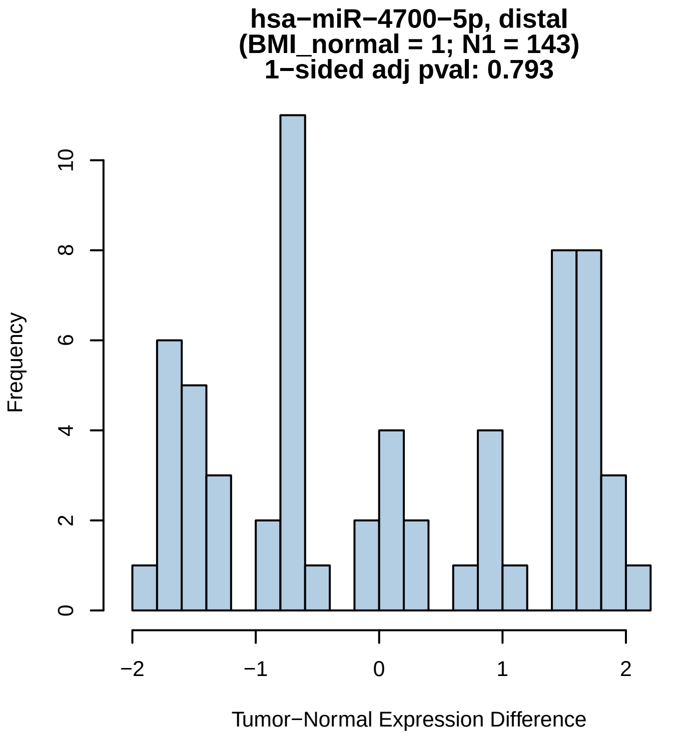
hsa−miR−4700−5p, distal
(BMI_normal = 1; N1 = 143)
1−sided adj pval: 0.793
Frequency
Tumor−Normal Expression Difference

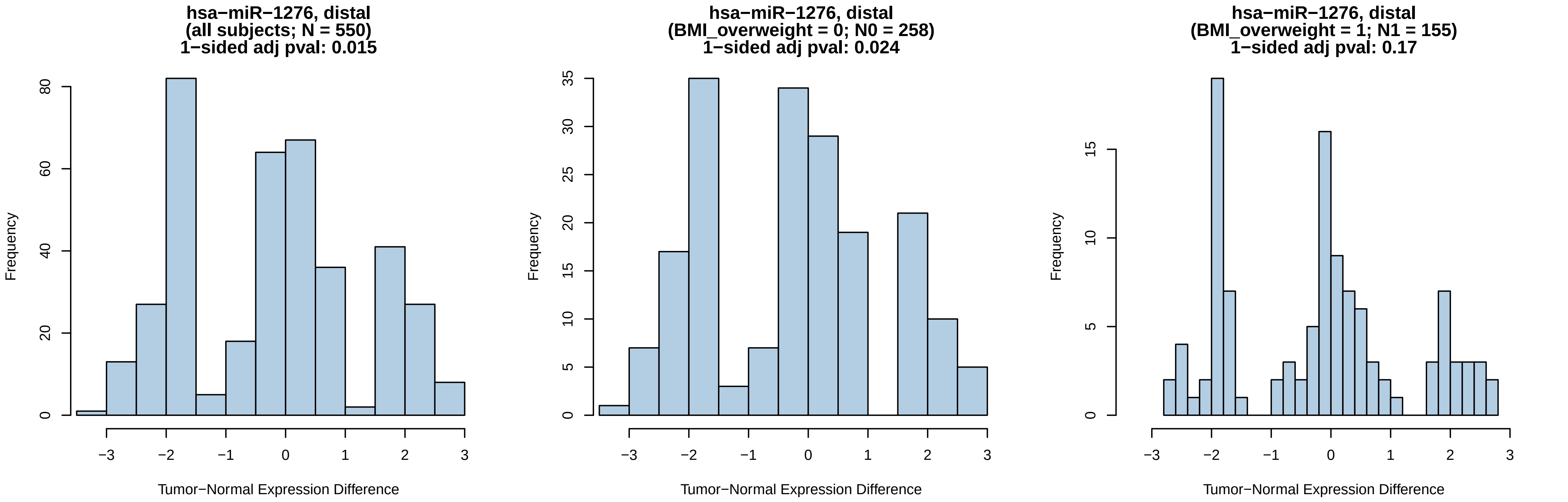

hsa−miR−1276, distal
(all subjects; N = 550)
1−sided adj pval: 0.015
Frequency
0
20
40
60
80
−3
−2
−1
0
1
2
3
Tumor−Normal Expression Difference
hsa−miR−1276, distal
(BMI_overweight = 0; N0 = 258)
1−sided adj pval: 0.024
Frequency
0
5
10
15
20
25
30
35
−3
−2
−1
0
1
2
3
Tumor−Normal Expression Difference
hsa−miR−1276, distal
(BMI_overweight = 1; N1 = 155)
1−sided adj pval: 0.17
Frequency
0
5
10
15
−3
−2
−1
0
1
2
3
Tumor−Normal Expression Difference

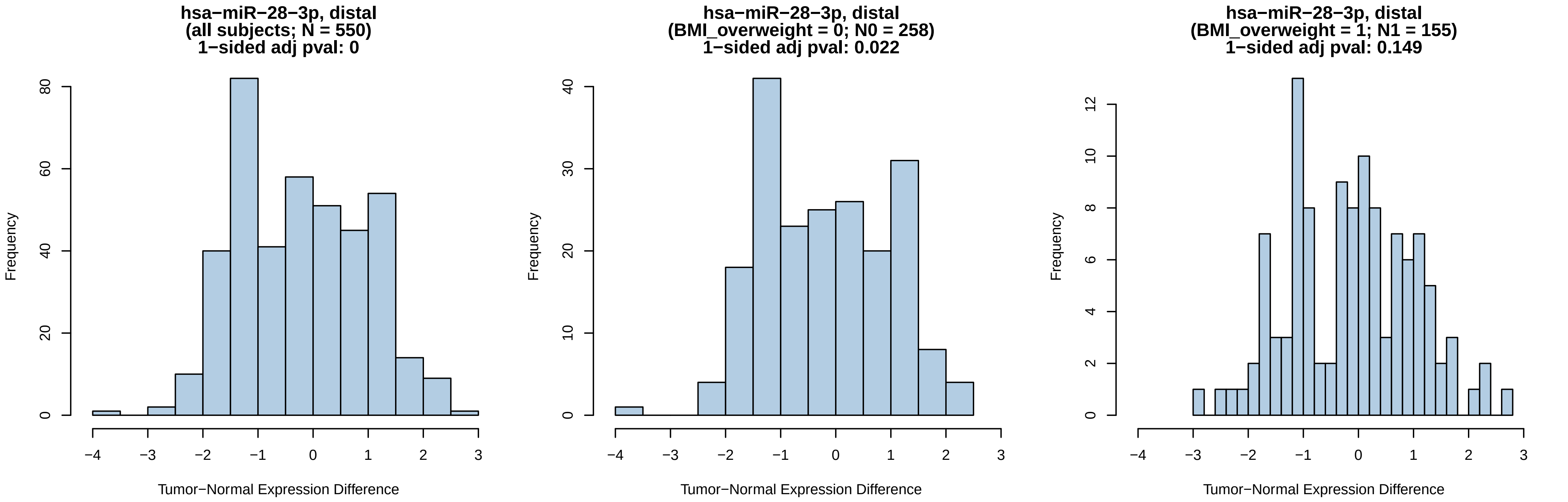

hsa−miR−28−3p, distal
(all subjects; N = 550)
1−sided adj pval: 0
Frequency
Tumor−Normal Expression Difference
hsa−miR−28−3p, distal
(BMI_overweight = 0; N0 = 258)
1−sided adj pval: 0.022
Frequency
Tumor−Normal Expression Difference
hsa−miR−28−3p, distal
(BMI_overweight = 1; N1 = 155)
1−sided adj pval: 0.149
Frequency
Tumor−Normal Expression Difference

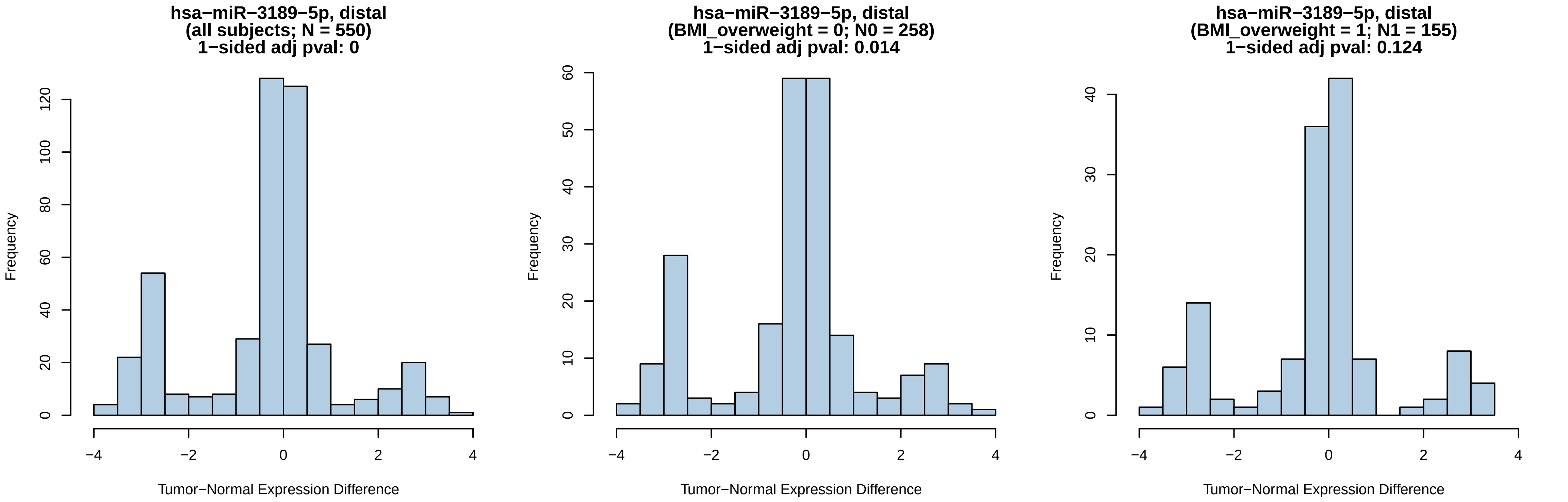

hsa−miR−3189−5p, distal
(all subjects; N = 550)
1−sided adj pval: 0
Frequency
Tumor−Normal Expression Difference
hsa−miR−3189−5p, distal
(BMI_overweight = 0; N0 = 258)
1−sided adj pval: 0.014
Frequency
Tumor−Normal Expression Difference
hsa−miR−3189−5p, distal
(BMI_overweight = 1; N1 = 155)
1−sided adj pval: 0.124
Frequency
Tumor−Normal Expression Difference

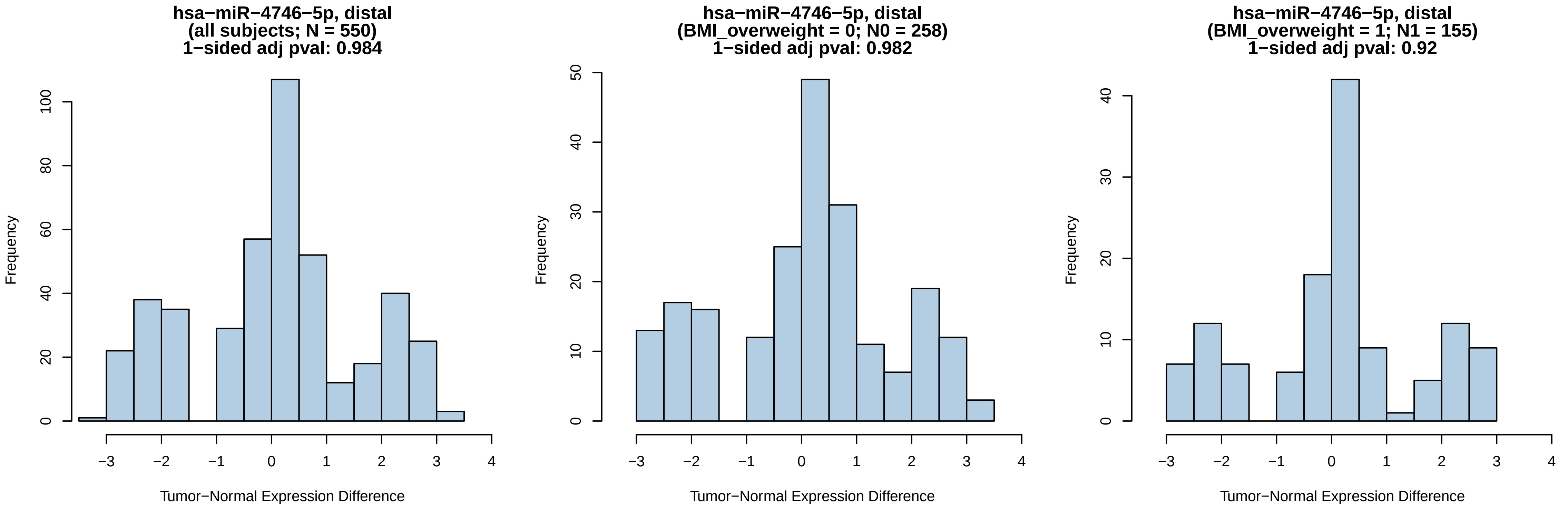
hsa−miR−4746−5p, distal
(all subjects; N = 550)
1−sided adj pval: 0.984
hsa−miR−4746−5p, distal
(BMI_overweight = 0; N0 = 258)
1−sided adj pval: 0.982
hsa−miR−4746−5p, distal
(BMI_overweight = 1; N1 = 155)
1−sided adj pval: 0.92
Frequency
Tumor−Normal Expression Difference

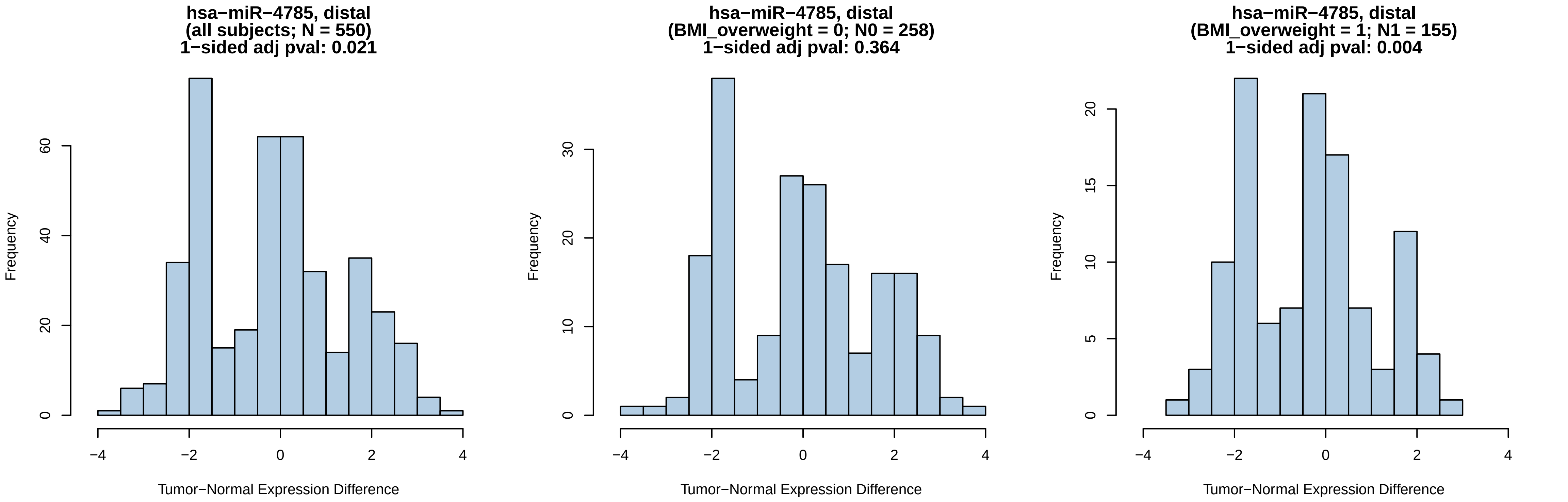
hsa−miR−4785, distal
(all subjects; N = 550)
1−sided adj pval: 0.021
Frequency
0
20
40
60
−4
−2
0
2
4
Tumor−Normal Expression Difference
hsa−miR−4785, distal
(BMI_overweight = 0; N0 = 258)
1−sided adj pval: 0.364
Frequency
0
10
20
30
−4
−2
0
2
4
Tumor−Normal Expression Difference
hsa−miR−4785, distal
(BMI_overweight = 1; N1 = 155)
1−sided adj pval: 0.004
Frequency
0
5
10
15
20
−4
−2
0
2
4
Tumor−Normal Expression Difference

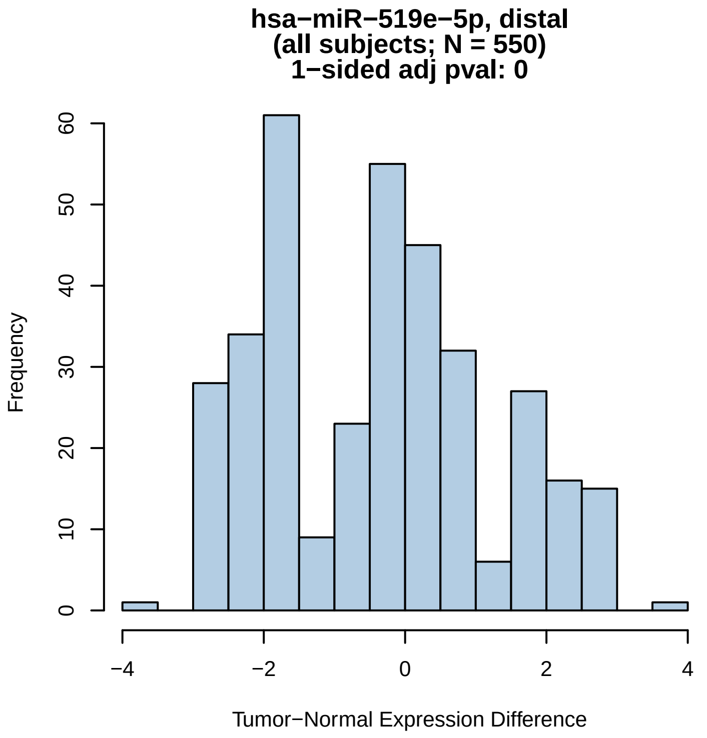
hsa−miR−519e−5p, distal
(all subjects; N = 550)
1−sided adj pval: 0
Frequency
0
10
20
30
40
50
60
−4
−2
0
2
4
Tumor−Normal Expression Difference

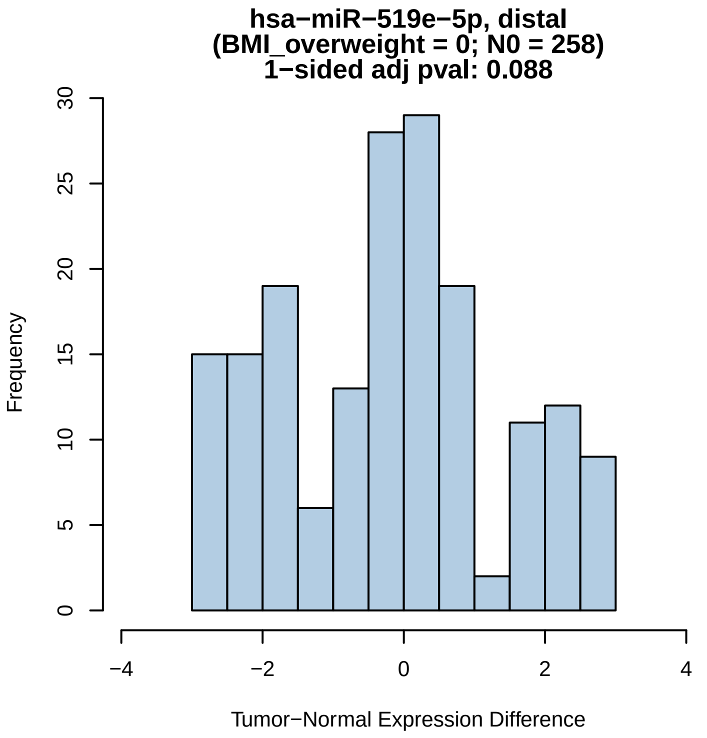
hsa−miR−519e−5p, distal
(BMI_overweight = 0; N0 = 258)
1−sided adj pval: 0.088
Frequency
0
5
10
15
20
25
30
−4
−2
0
2
4
Tumor−Normal Expression Difference

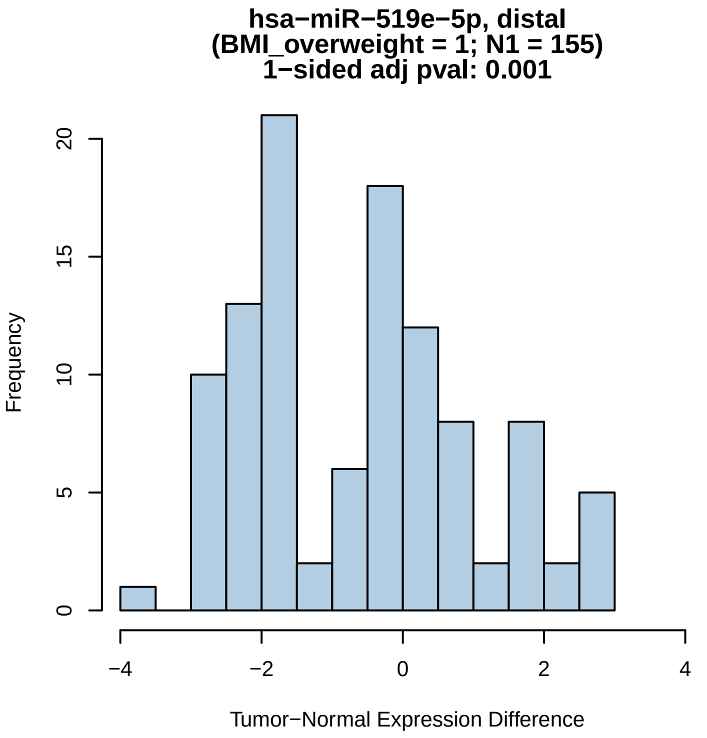
hsa−miR−519e−5p, distal
(BMI_overweight = 1; N1 = 155)
1−sided adj pval: 0.001
Frequency
0
5
10
15
20
−4
−2
0
2
4
Tumor−Normal Expression Difference

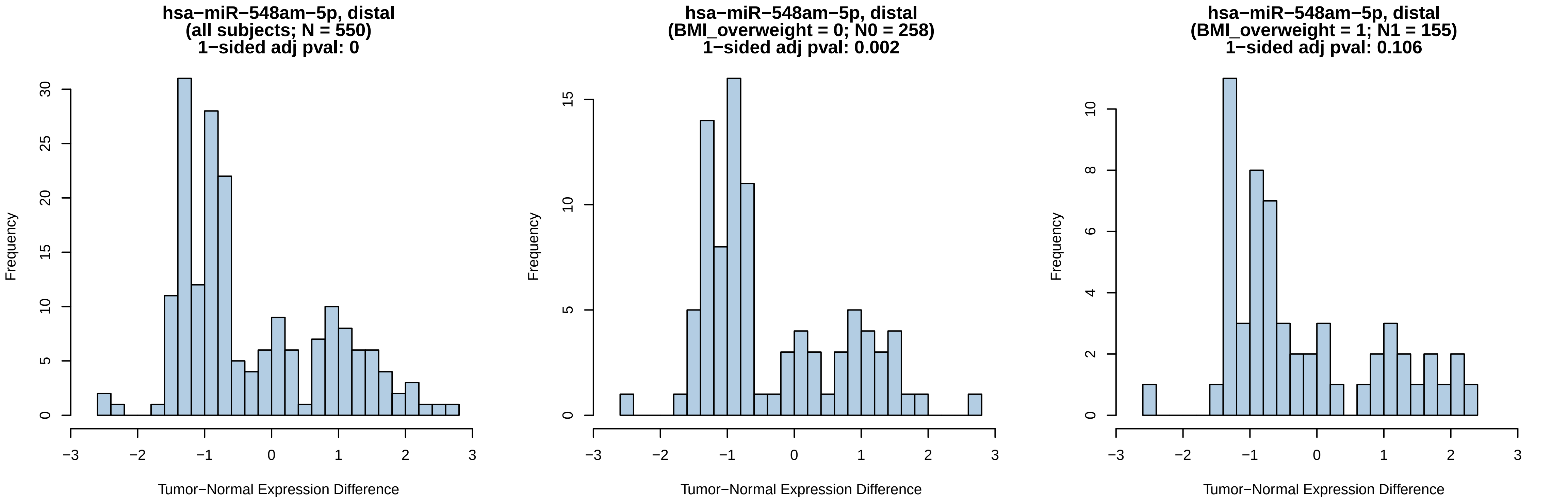

hsa−miR−548am−5p, distal
(all subjects; N = 550)
1−sided adj pval: 0
Frequency
Tumor−Normal Expression Difference
hsa−miR−548am−5p, distal
(BMI_overweight = 0; N0 = 258)
1−sided adj pval: 0.002
Frequency
Tumor−Normal Expression Difference
hsa−miR−548am−5p, distal
(BMI_overweight = 1; N1 = 155)
1−sided adj pval: 0.106
Frequency
Tumor−Normal Expression Difference

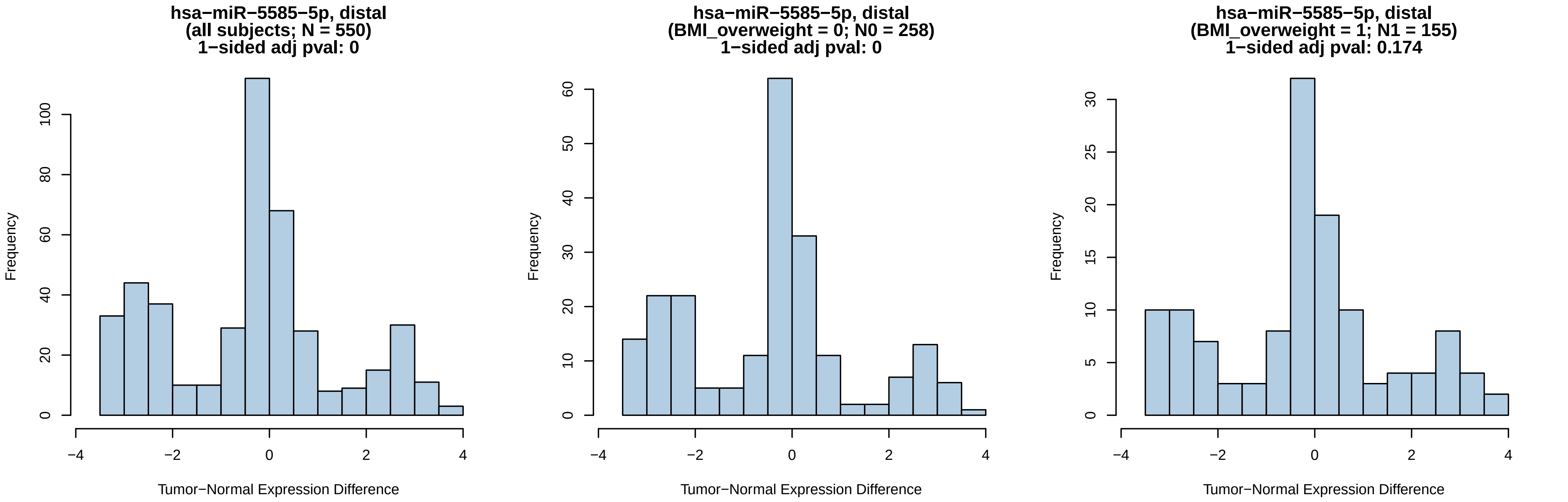

hsa−miR−5585−5p, distal
(all subjects; N = 550)
1−sided adj pval: 0
hsa−miR−5585−5p, distal
(BMI_overweight = 0; N0 = 258)
1−sided adj pval: 0
hsa−miR−5585−5p, distal
(BMI_overweight = 1; N1 = 155)
1−sided adj pval: 0.174
Frequency
Tumor−Normal Expression Difference

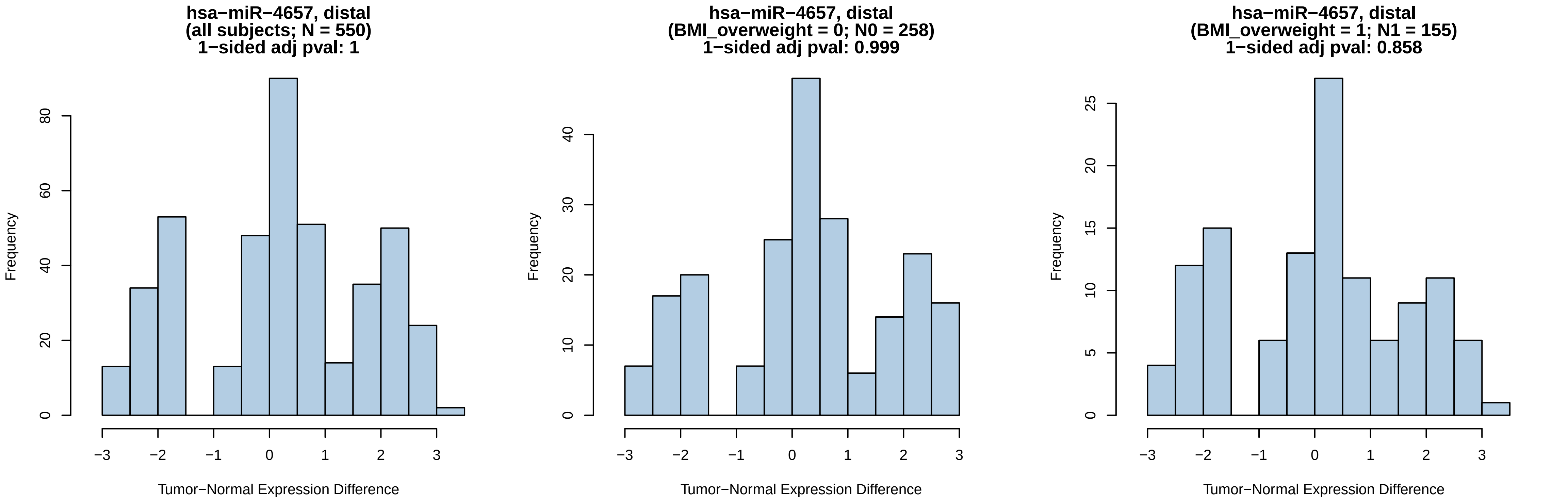
hsa−miR−4657, distal
(all subjects; N = 550)
1−sided adj pval: 1
hsa−miR−4657, distal
(BMI_overweight = 0; N0 = 258)
1−sided adj pval: 0.999
hsa−miR−4657, distal
(BMI_overweight = 1; N1 = 155)
1−sided adj pval: 0.858
Frequency
Tumor−Normal Expression Difference
0
20
40
60
80
0
10
20
30
40
0
5
10
15
20
25
−3
−2
−1
0
1
2
3

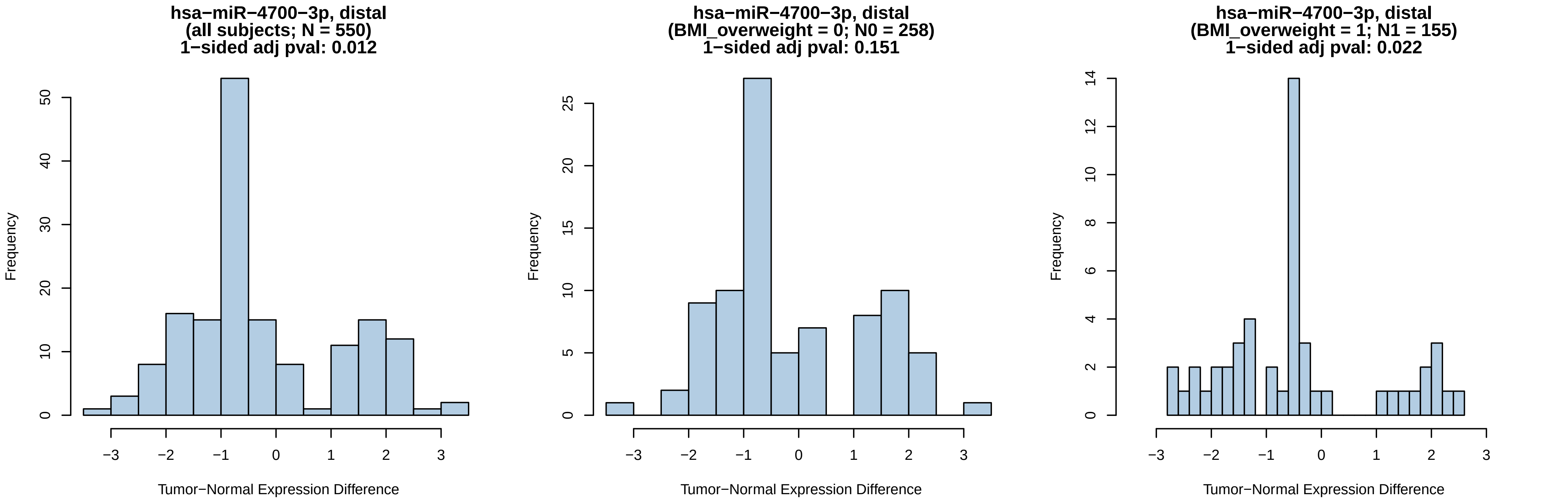

hsa−miR−4700−3p, distal
(all subjects; N = 550)
1−sided adj pval: 0.012
Frequency
0
10
20
30
40
50
−3
−2
−1
0
1
2
3
Tumor−Normal Expression Difference
hsa−miR−4700−3p, distal
(BMI_overweight = 0; N0 = 258)
1−sided adj pval: 0.151
Frequency
0
5
10
15
20
25
−3
−2
−1
0
1
2
3
Tumor−Normal Expression Difference
hsa−miR−4700−3p, distal
(BMI_overweight = 1; N1 = 155)
1−sided adj pval: 0.022
Frequency
0
2
4
6
8
10
12
14
−3
−2
−1
0
1
2
3
Tumor−Normal Expression Difference

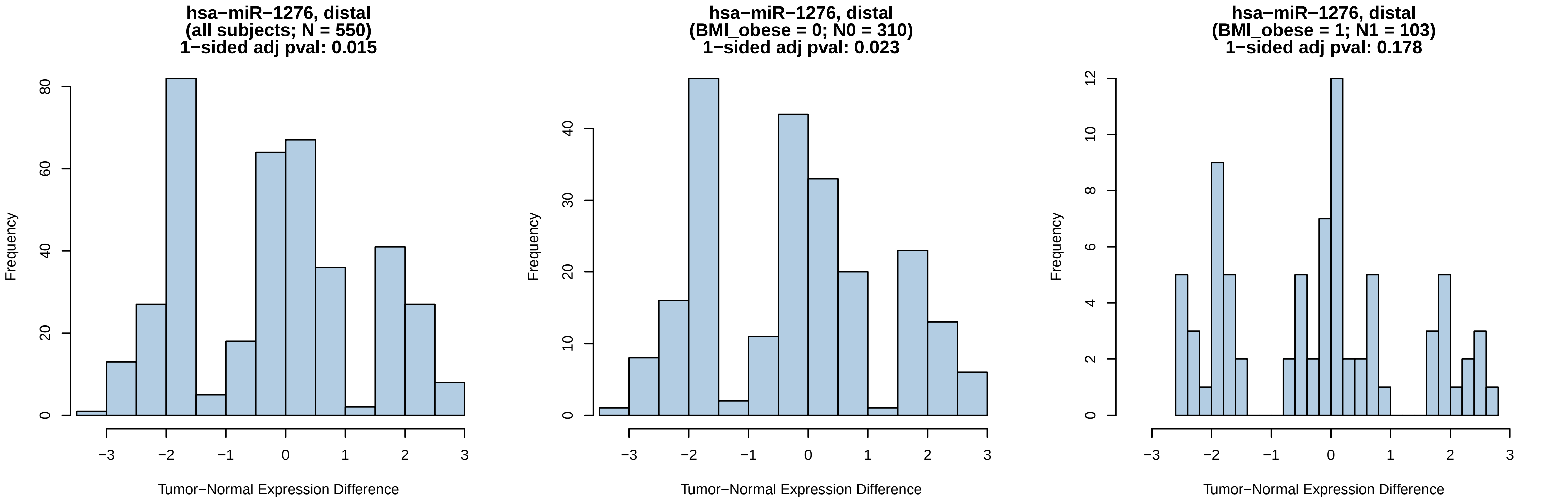
hsa−miR−1276, distal
(all subjects; N = 550)
1−sided adj pval: 0.015
Frequency
Tumor−Normal Expression Difference
hsa−miR−1276, distal
(BMI_obese = 0; N0 = 310)
1−sided adj pval: 0.023
Frequency
Tumor−Normal Expression Difference
hsa−miR−1276, distal
(BMI_obese = 1; N1 = 103)
1−sided adj pval: 0.178
Frequency
Tumor−Normal Expression Difference

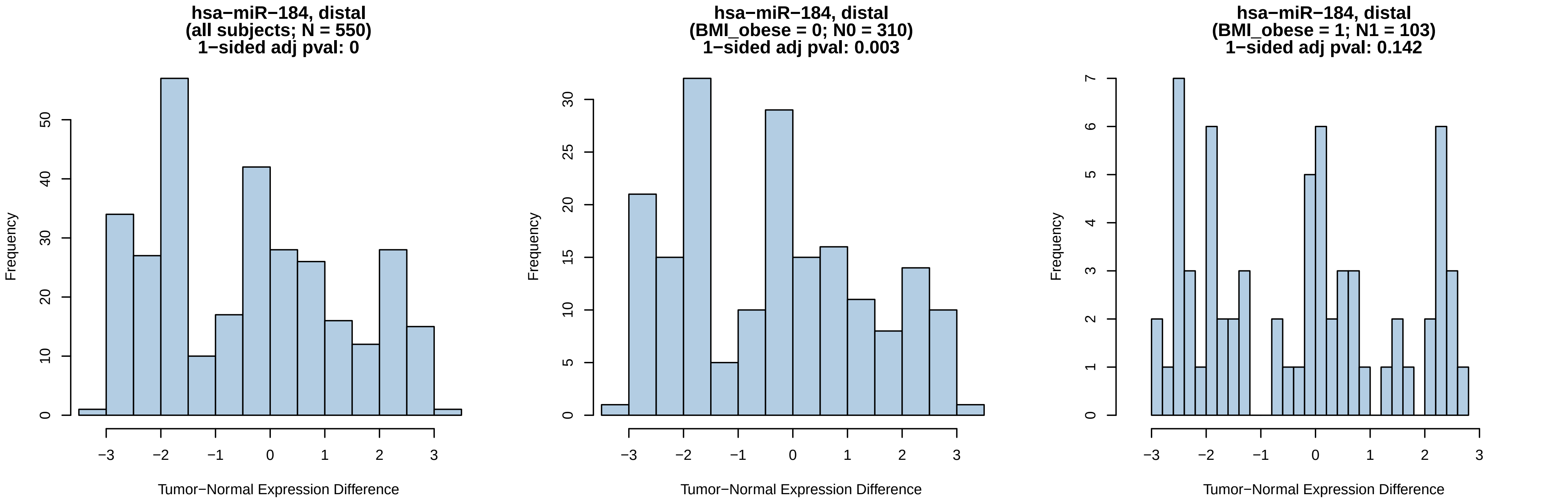

hsa−miR−184, distal
(all subjects; N = 550)
1−sided adj pval: 0
Frequency
Tumor−Normal Expression Difference
hsa−miR−184, distal
(BMI_obese = 0; N0 = 310)
1−sided adj pval: 0.003
Frequency
Tumor−Normal Expression Difference
hsa−miR−184, distal
(BMI_obese = 1; N1 = 103)
1−sided adj pval: 0.142
Frequency
Tumor−Normal Expression Difference

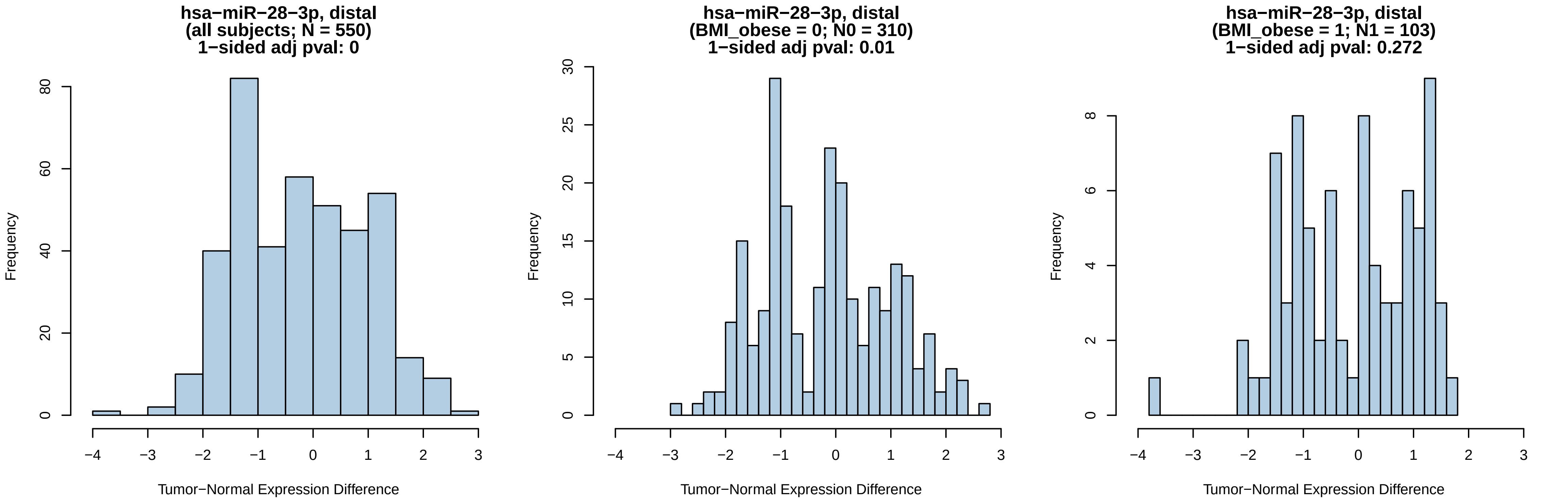
hsa−miR−28−3p, distal
(all subjects; N = 550)
1−sided adj pval: 0
Frequency
Tumor−Normal Expression Difference
hsa−miR−28−3p, distal
(BMI_obese = 0; N0 = 310)
1−sided adj pval: 0.01
Frequency
Tumor−Normal Expression Difference
hsa−miR−28−3p, distal
(BMI_obese = 1; N1 = 103)
1−sided adj pval: 0.272
Frequency
Tumor−Normal Expression Difference

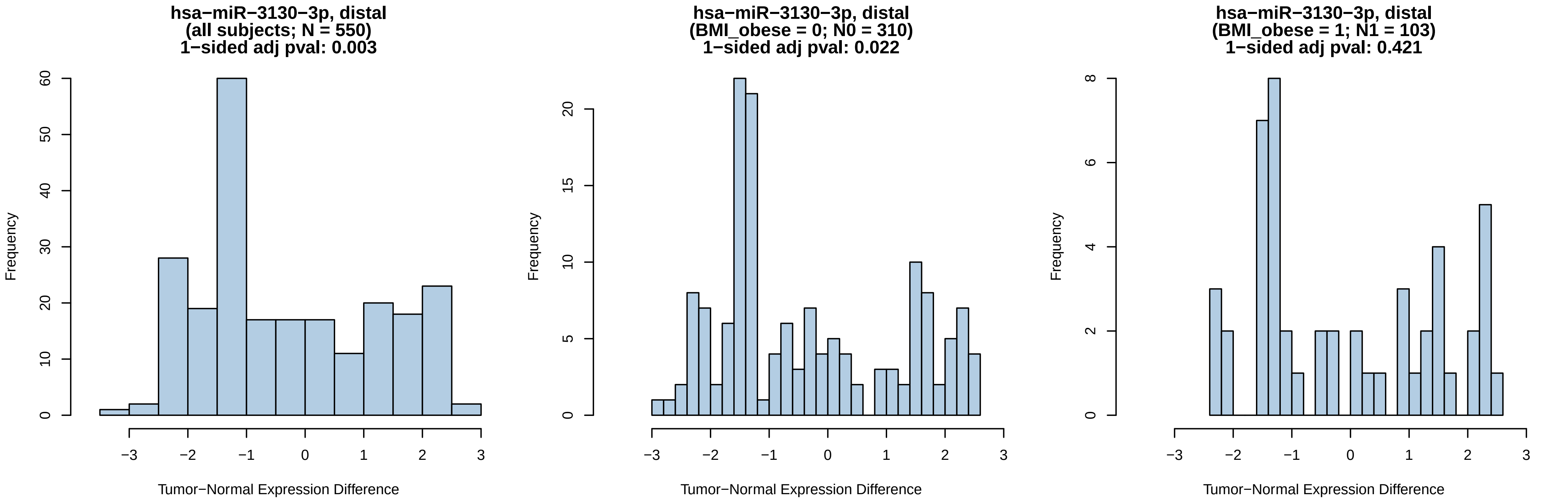
hsa−miR−3130−3p, distal
(all subjects; N = 550)
1−sided adj pval: 0.003
hsa−miR−3130−3p, distal
(BMI_obese = 0; N0 = 310)
1−sided adj pval: 0.022
hsa−miR−3130−3p, distal
(BMI_obese = 1; N1 = 103)
1−sided adj pval: 0.421
Frequency
Tumor−Normal Expression Difference
0
10
20
30
40
50
60
0
5
10
15
20
0
2
4
6
8
−3
−2
−1
0
1
2
3

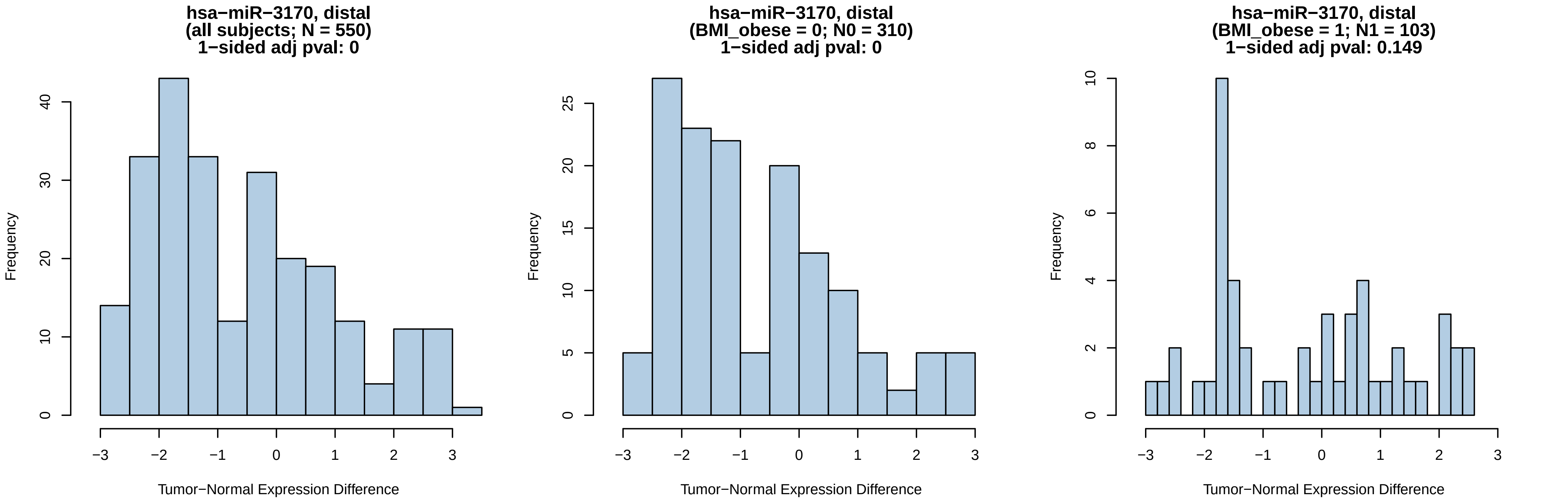
hsa−miR−3170, distal
(all subjects; N = 550)
1−sided adj pval: 0
Frequency
Tumor−Normal Expression Difference
hsa−miR−3170, distal
(BMI_obese = 0; N0 = 310)
1−sided adj pval: 0
Frequency
Tumor−Normal Expression Difference
hsa−miR−3170, distal
(BMI_obese = 1; N1 = 103)
1−sided adj pval: 0.149
Frequency
Tumor−Normal Expression Difference

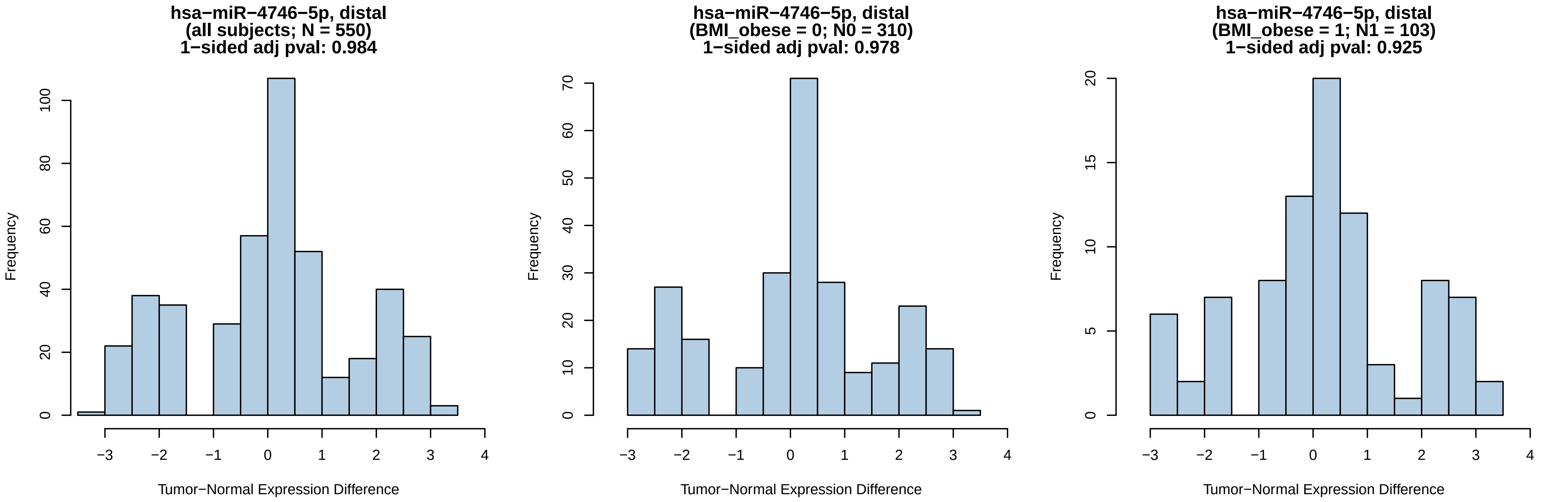
hsa−miR−4746−5p, distal
(all subjects; N = 550)
1−sided adj pval: 0.984
Frequency
Tumor−Normal Expression Difference
hsa−miR−4746−5p, distal
(BMI_obese = 0; N0 = 310)
1−sided adj pval: 0.978
Frequency
Tumor−Normal Expression Difference
hsa−miR−4746−5p, distal
(BMI_obese = 1; N1 = 103)
1−sided adj pval: 0.925
Frequency
Tumor−Normal Expression Difference

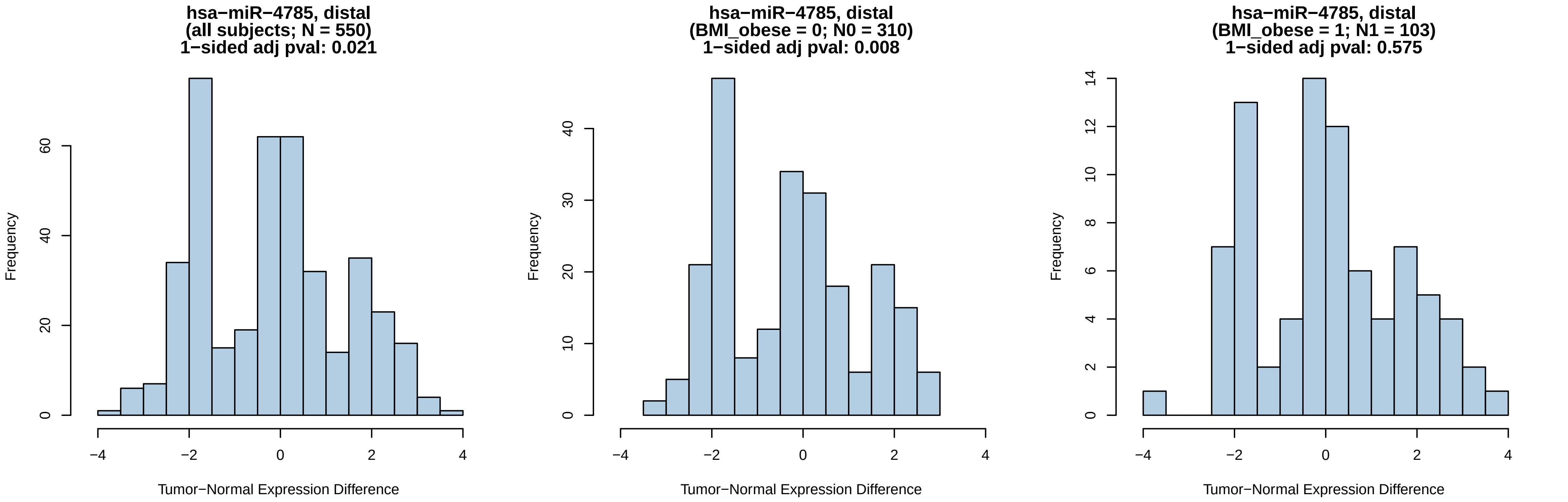
hsa−miR−4785, distal
(all subjects; N = 550)
1−sided adj pval: 0.021
Frequency
Tumor−Normal Expression Difference
hsa−miR−4785, distal
(BMI_obese = 0; N0 = 310)
1−sided adj pval: 0.008
Frequency
Tumor−Normal Expression Difference
hsa−miR−4785, distal
(BMI_obese = 1; N1 = 103)
1−sided adj pval: 0.575
Frequency
Tumor−Normal Expression Difference

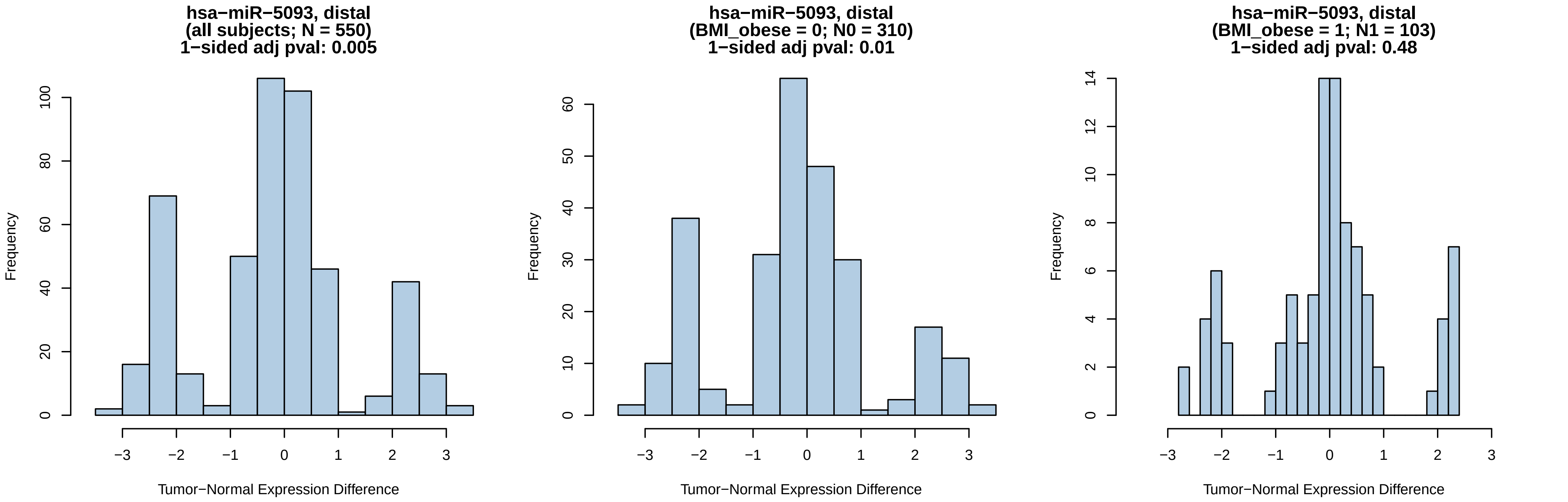
hsa−miR−5093, distal
(all subjects; N = 550)
1−sided adj pval: 0.005
Frequency
Tumor−Normal Expression Difference
hsa−miR−5093, distal
(BMI_obese = 0; N0 = 310)
1−sided adj pval: 0.01
Frequency
Tumor−Normal Expression Difference
hsa−miR−5093, distal
(BMI_obese = 1; N1 = 103)
1−sided adj pval: 0.48
Frequency
Tumor−Normal Expression Difference

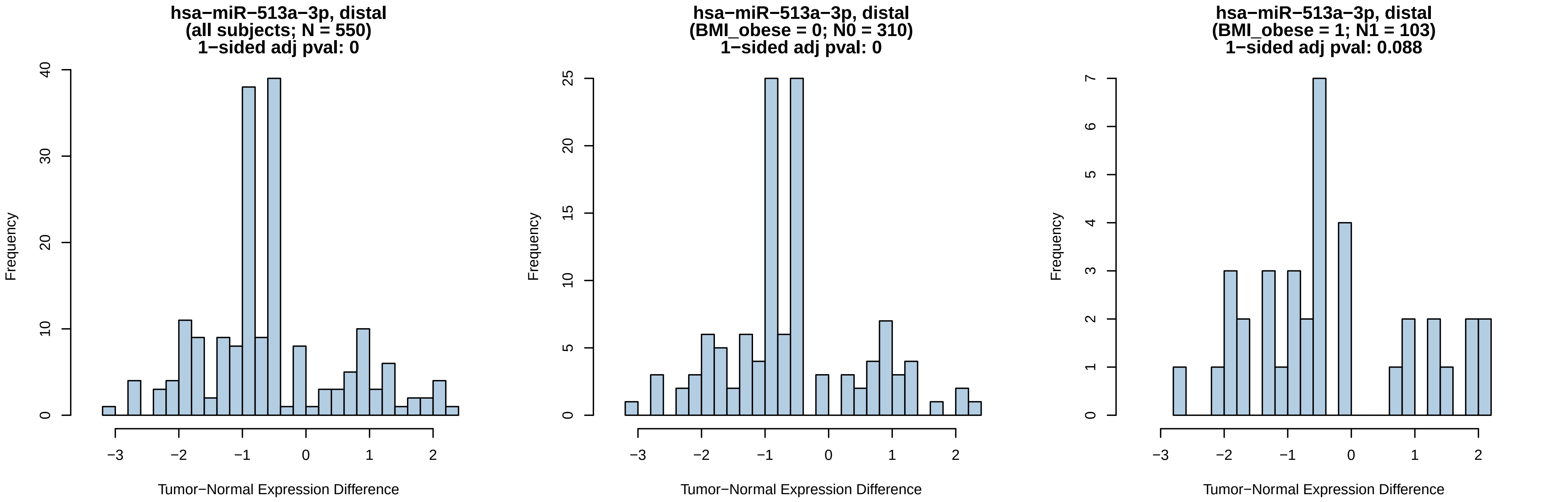

hsa−miR−513a−3p, distal
(all subjects; N = 550)
1−sided adj pval: 0
hsa−miR−513a−3p, distal
(BMI_obese = 0; N0 = 310)
1−sided adj pval: 0
hsa−miR−513a−3p, distal
(BMI_obese = 1; N1 = 103)
1−sided adj pval: 0.088
Frequency
Tumor−Normal Expression Difference

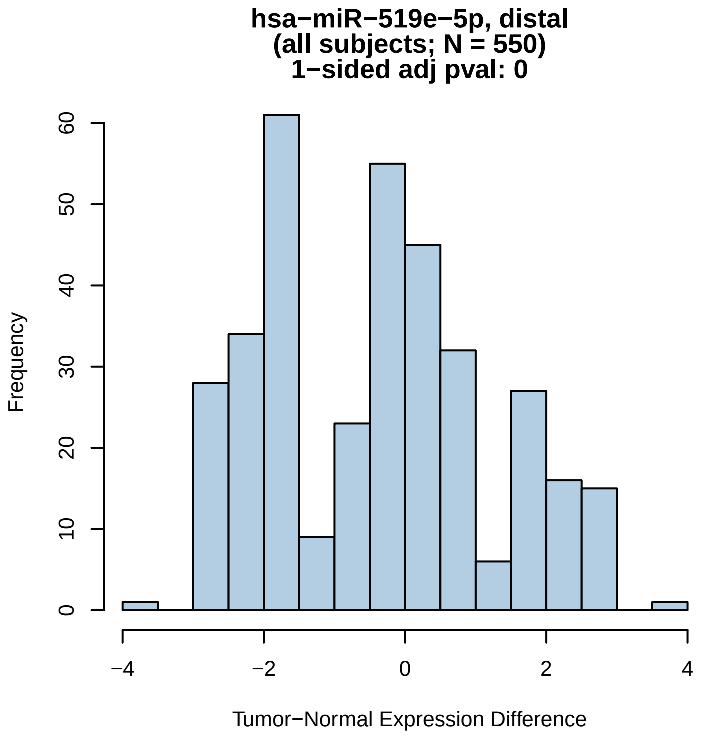

hsa−miR−519e−5p, distal
(all subjects; N = 550)
1−sided adj pval: 0
Frequency
Tumor−Normal Expression Difference


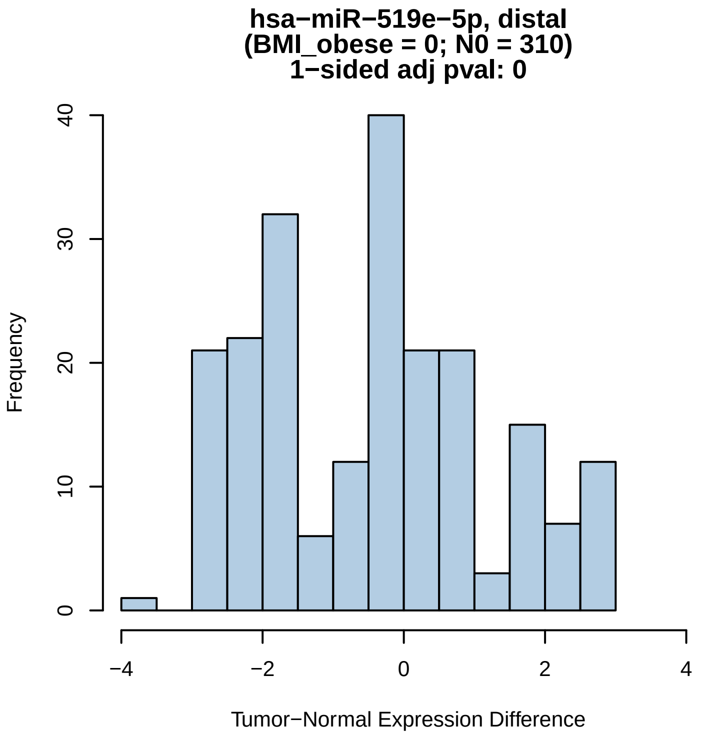

hsa−miR−519e−5p, distal
(BMI_obese = 0; N0 = 310)
1−sided adj pval: 0
Frequency
Tumor−Normal Expression Difference


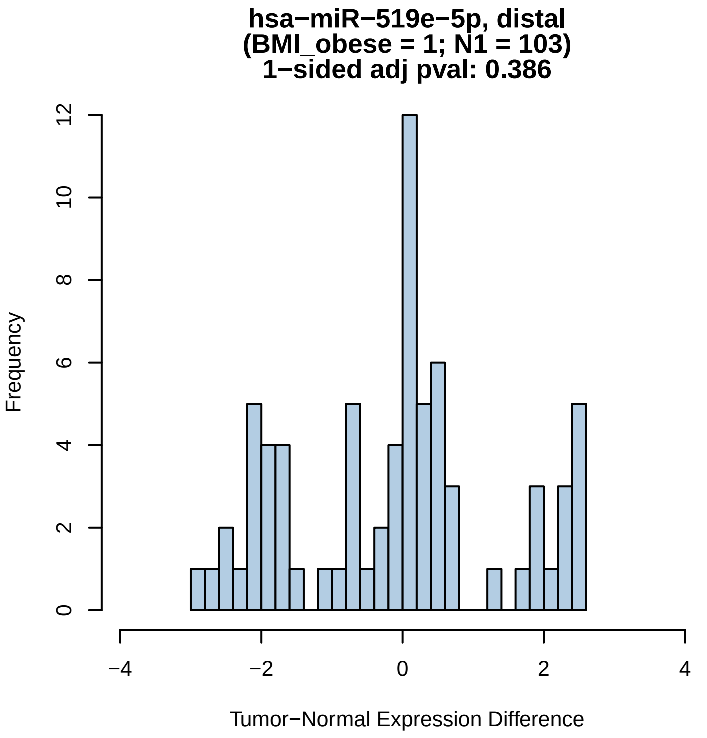

hsa−miR−519e−5p, distal
(BMI_obese = 1; N1 = 103)
1−sided adj pval: 0.386
Frequency
Tumor−Normal Expression Difference

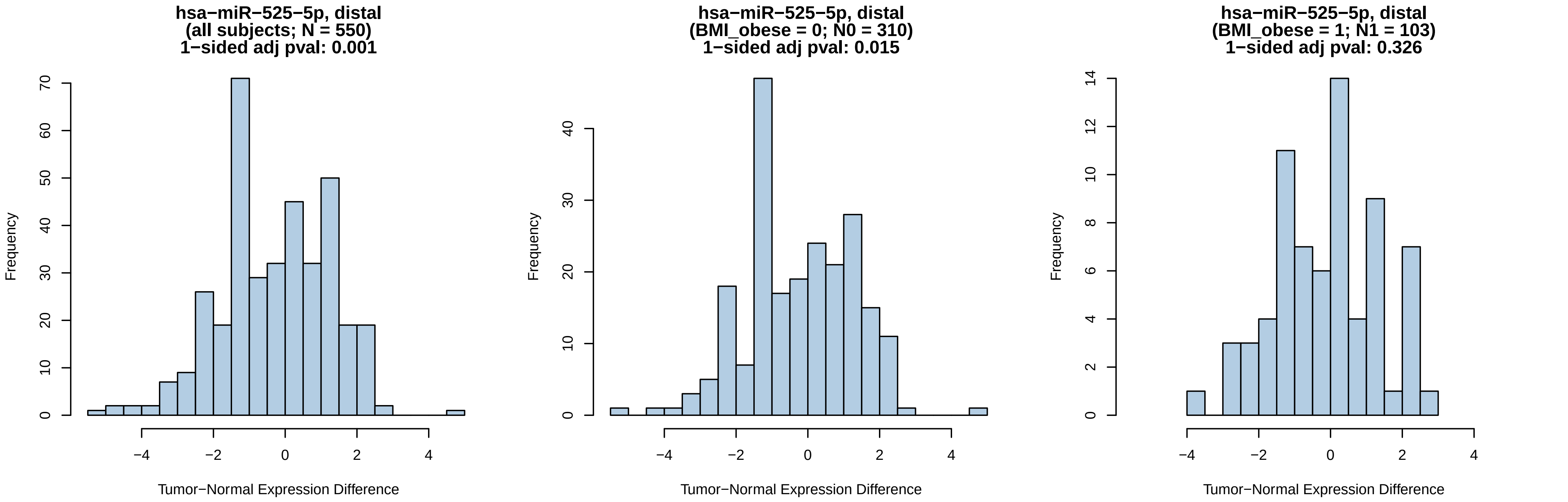
hsa−miR−525−5p, distal
(all subjects; N = 550)
1−sided adj pval: 0.001
hsa−miR−525−5p, distal
(BMI_obese = 0; N0 = 310)
1−sided adj pval: 0.015
hsa−miR−525−5p, distal
(BMI_obese = 1; N1 = 103)
1−sided adj pval: 0.326
Frequency
Tumor−Normal Expression Difference

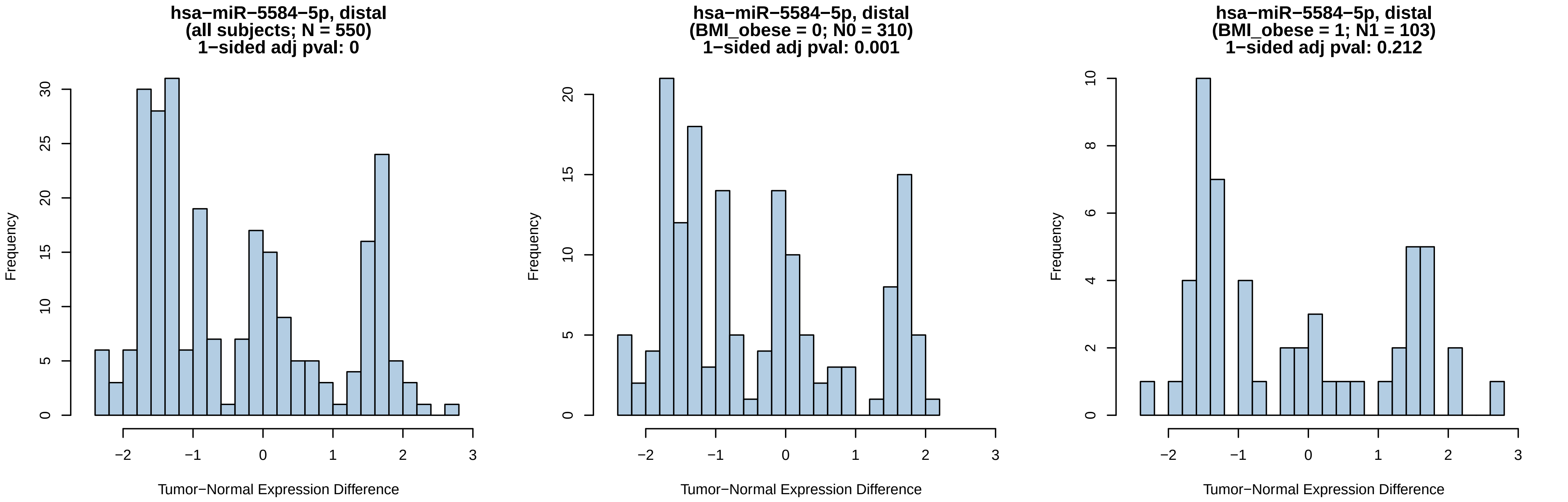

hsa−miR−5584−5p, distal
(all subjects; N = 550)
1−sided adj pval: 0
Frequency
Tumor−Normal Expression Difference
hsa−miR−5584−5p, distal
(BMI_obese = 0; N0 = 310)
1−sided adj pval: 0.001
Frequency
Tumor−Normal Expression Difference
hsa−miR−5584−5p, distal
(BMI_obese = 1; N1 = 103)
1−sided adj pval: 0.212
Frequency
Tumor−Normal Expression Difference

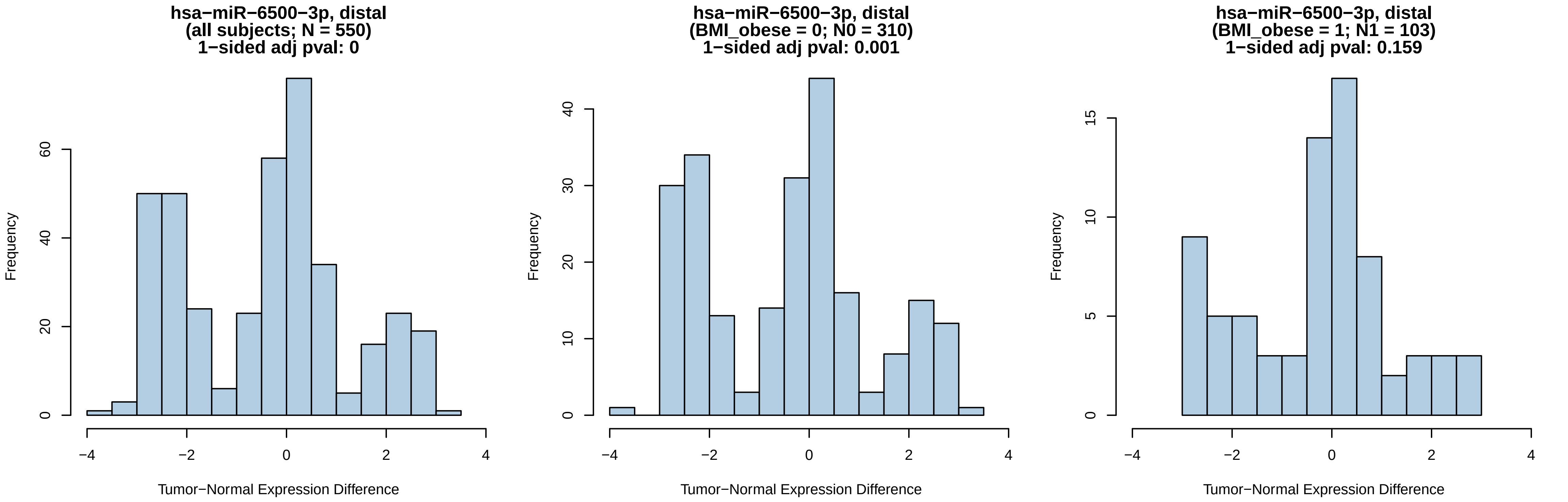

hsa−miR−6500−3p, distal
(all subjects; N = 550)
1−sided adj pval: 0
Frequency
Tumor−Normal Expression Difference
hsa−miR−6500−3p, distal
(BMI_obese = 0; N0 = 310)
1−sided adj pval: 0.001
Frequency
Tumor−Normal Expression Difference
hsa−miR−6500−3p, distal
(BMI_obese = 1; N1 = 103)
1−sided adj pval: 0.159
Frequency
Tumor−Normal Expression Difference

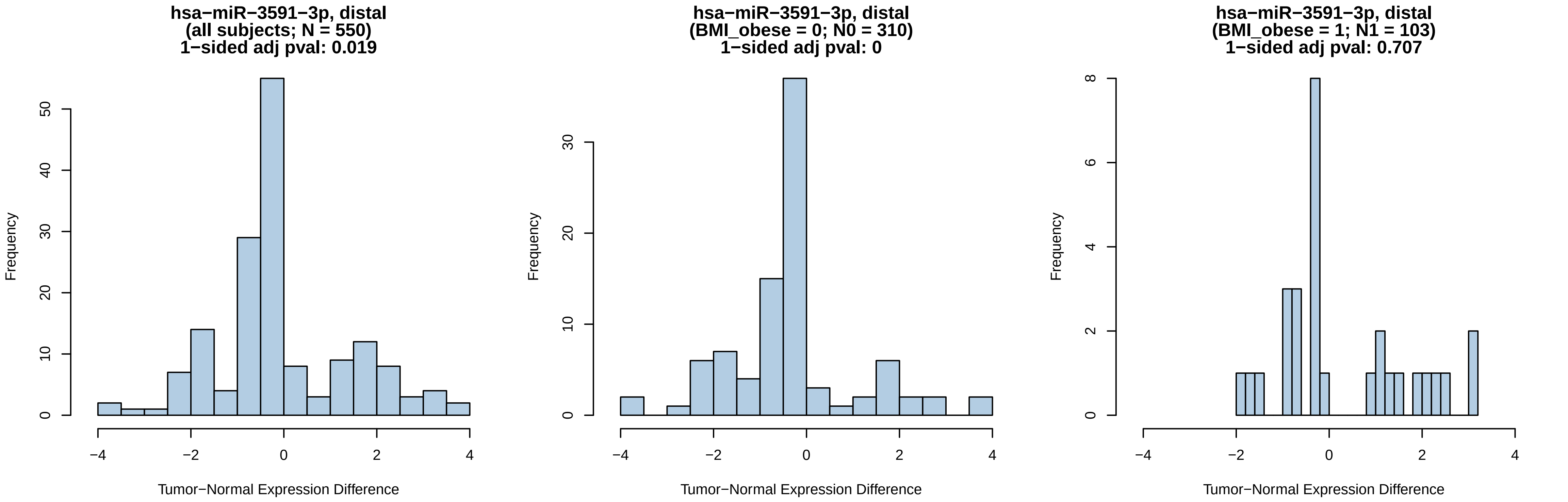
hsa−miR−3591−3p, distal
(all subjects; N = 550)
1−sided adj pval: 0.019
Frequency
Tumor−Normal Expression Difference
hsa−miR−3591−3p, distal
(BMI_obese = 0; N0 = 310)
1−sided adj pval: 0
Frequency
Tumor−Normal Expression Difference
hsa−miR−3591−3p, distal
(BMI_obese = 1; N1 = 103)
1−sided adj pval: 0.707
Frequency
Tumor−Normal Expression Difference

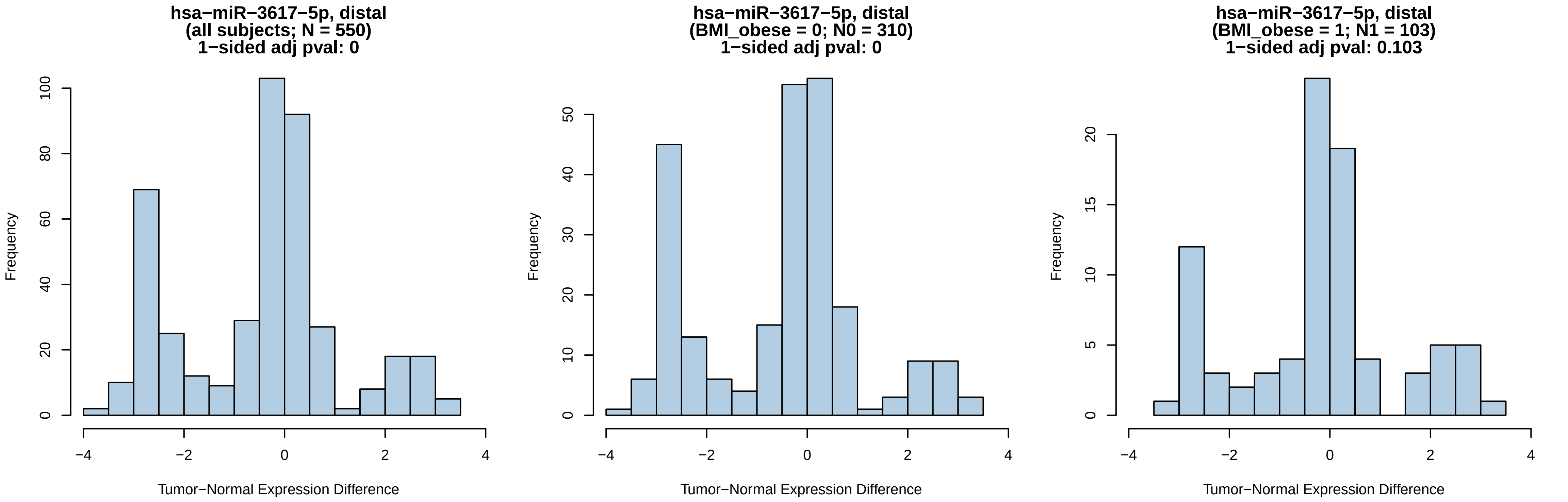
hsa−miR−3617−5p, distal
(all subjects; N = 550)
1−sided adj pval: 0
Frequency
Tumor−Normal Expression Difference
hsa−miR−3617−5p, distal
(BMI_obese = 0; N0 = 310)
1−sided adj pval: 0
Frequency
Tumor−Normal Expression Difference
hsa−miR−3617−5p, distal
(BMI_obese = 1; N1 = 103)
1−sided adj pval: 0.103
Frequency
Tumor−Normal Expression Difference

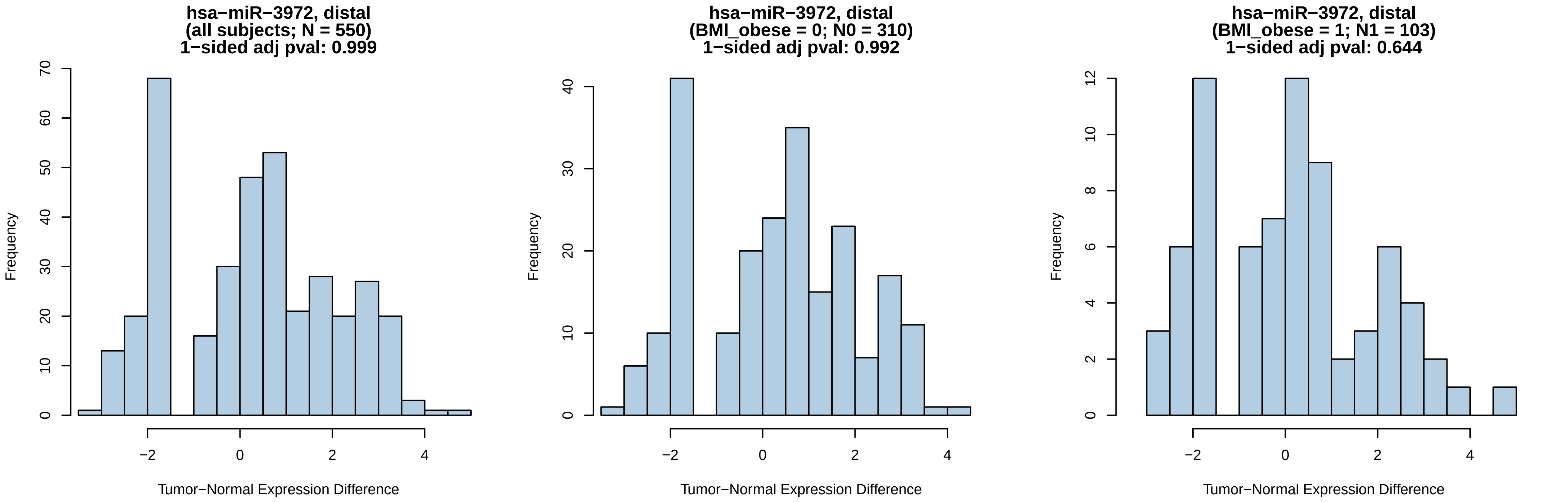
hsa−miR−3972, distal
(all subjects; N = 550)
1−sided adj pval: 0.999
Frequency
Tumor−Normal Expression Difference
hsa−miR−3972, distal
(BMI_obese = 0; N0 = 310)
1−sided adj pval: 0.992
Frequency
Tumor−Normal Expression Difference
hsa−miR−3972, distal
(BMI_obese = 1; N1 = 103)
1−sided adj pval: 0.644
Frequency
Tumor−Normal Expression Difference

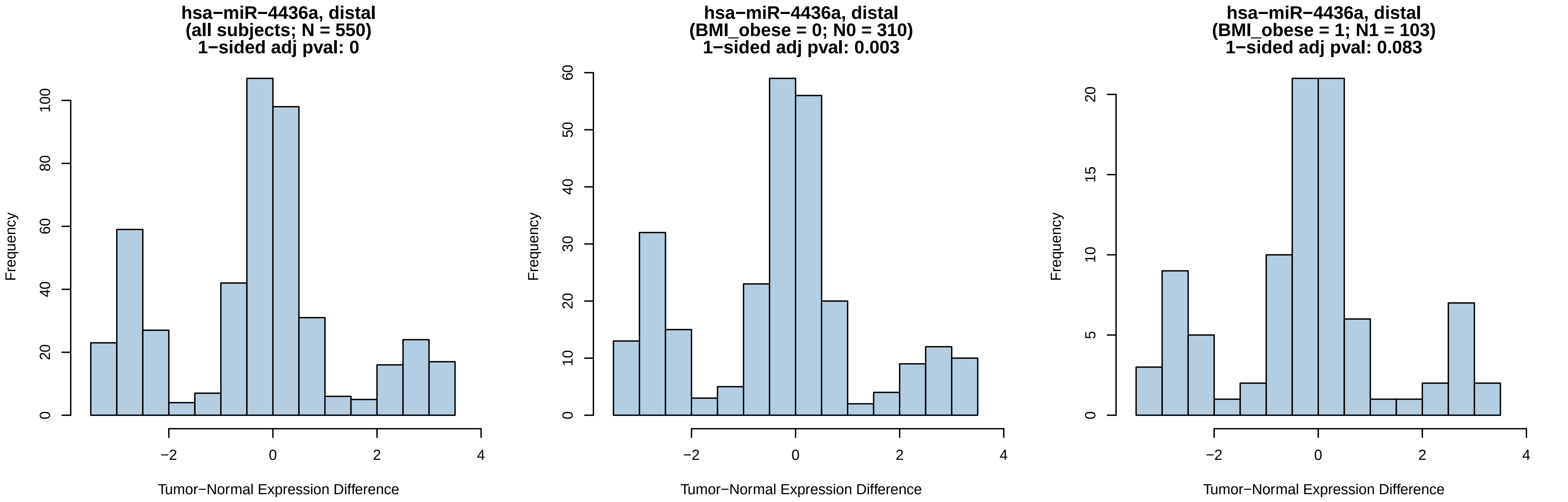

hsa−miR−4436a, distal
(all subjects; N = 550)
1−sided adj pval: 0
Frequency
Tumor−Normal Expression Difference
hsa−miR−4436a, distal
(BMI_obese = 0; N0 = 310)
1−sided adj pval: 0.003
Frequency
Tumor−Normal Expression Difference
hsa−miR−4436a, distal
(BMI_obese = 1; N1 = 103)
1−sided adj pval: 0.083
Frequency
Tumor−Normal Expression Difference

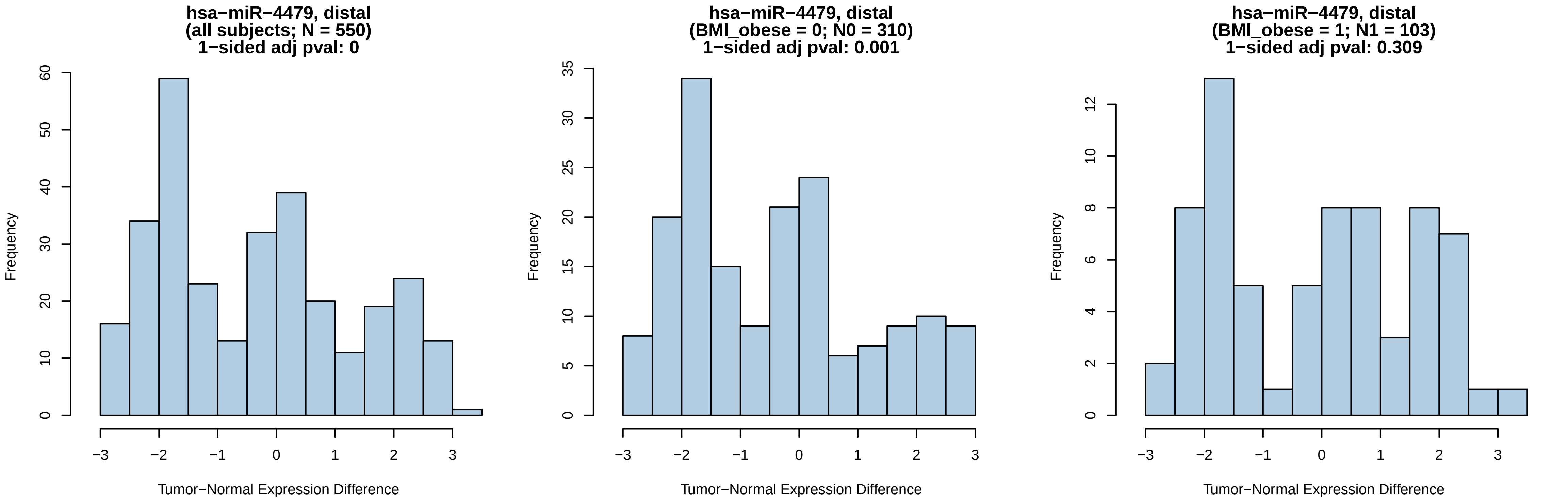
hsa−miR−4479, distal
(all subjects; N = 550)
1−sided adj pval: 0
Frequency
Tumor−Normal Expression Difference
hsa−miR−4479, distal
(BMI_obese = 0; N0 = 310)
1−sided adj pval: 0.001
Frequency
Tumor−Normal Expression Difference
hsa−miR−4479, distal
(BMI_obese = 1; N1 = 103)
1−sided adj pval: 0.309
Frequency
Tumor−Normal Expression Difference

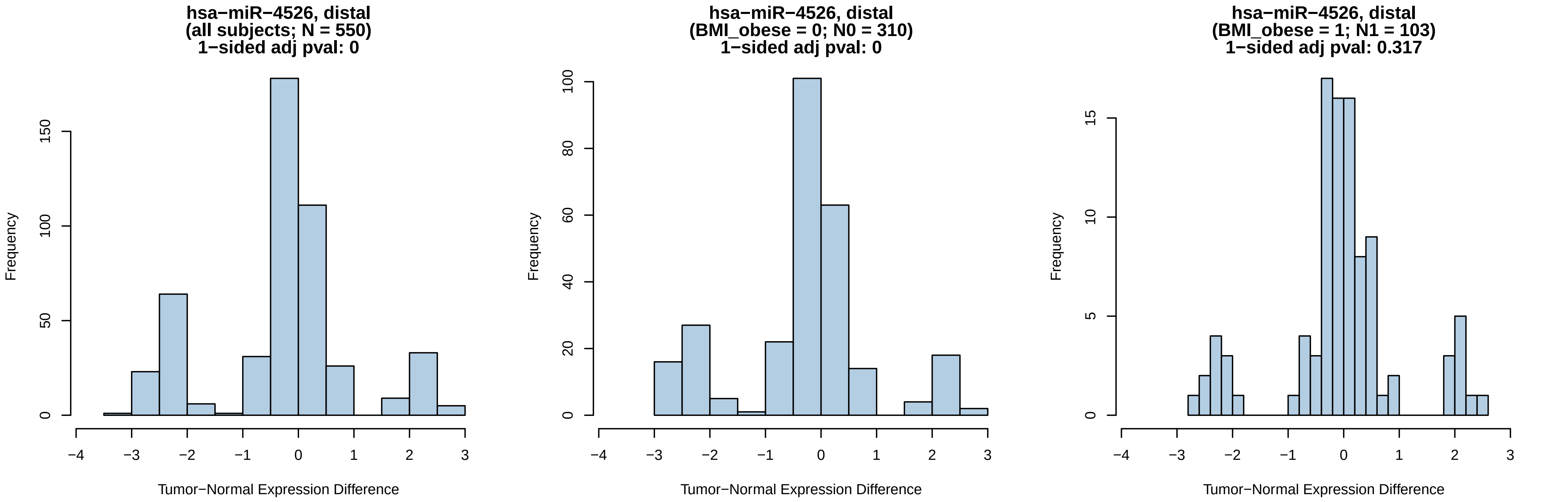
hsa−miR−4526, distal
(all subjects; N = 550)
1−sided adj pval: 0
Frequency
Tumor−Normal Expression Difference
hsa−miR−4526, distal
(BMI_obese = 0; N0 = 310)
1−sided adj pval: 0
Frequency
Tumor−Normal Expression Difference
hsa−miR−4526, distal
(BMI_obese = 1; N1 = 103)
1−sided adj pval: 0.317
Frequency
Tumor−Normal Expression Difference

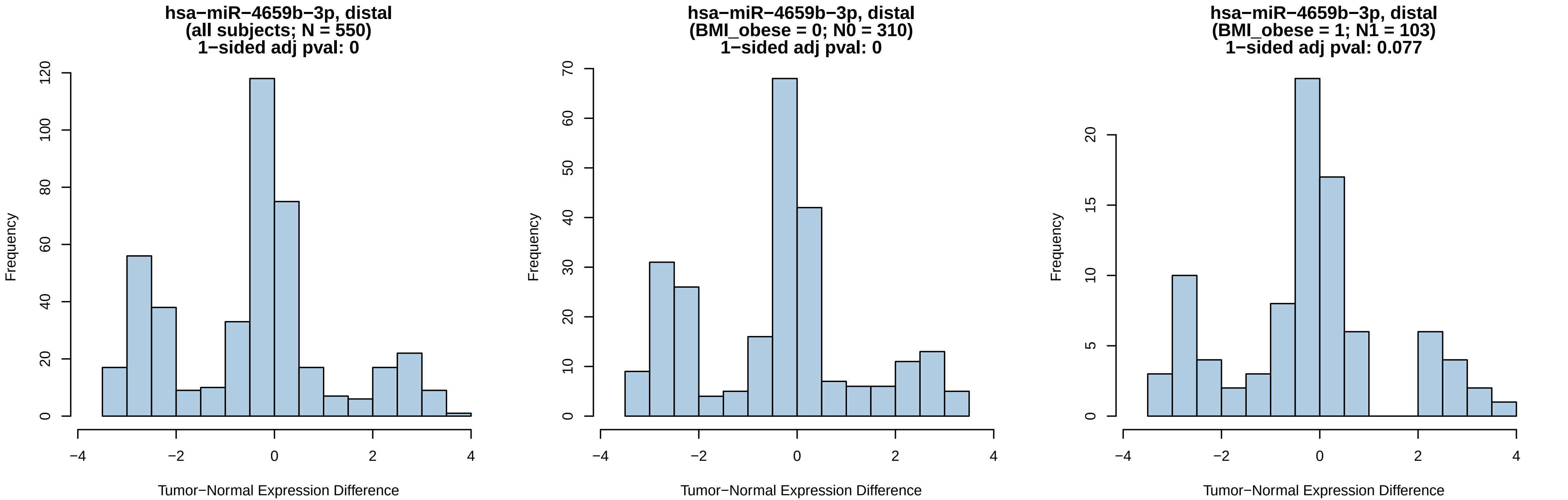

hsa−miR−4659b−3p, distal
(all subjects; N = 550)
1−sided adj pval: 0
Frequency
Tumor−Normal Expression Difference
hsa−miR−4659b−3p, distal
(BMI_obese = 0; N0 = 310)
1−sided adj pval: 0
Frequency
Tumor−Normal Expression Difference
hsa−miR−4659b−3p, distal
(BMI_obese = 1; N1 = 103)
1−sided adj pval: 0.077
Frequency
Tumor−Normal Expression Difference

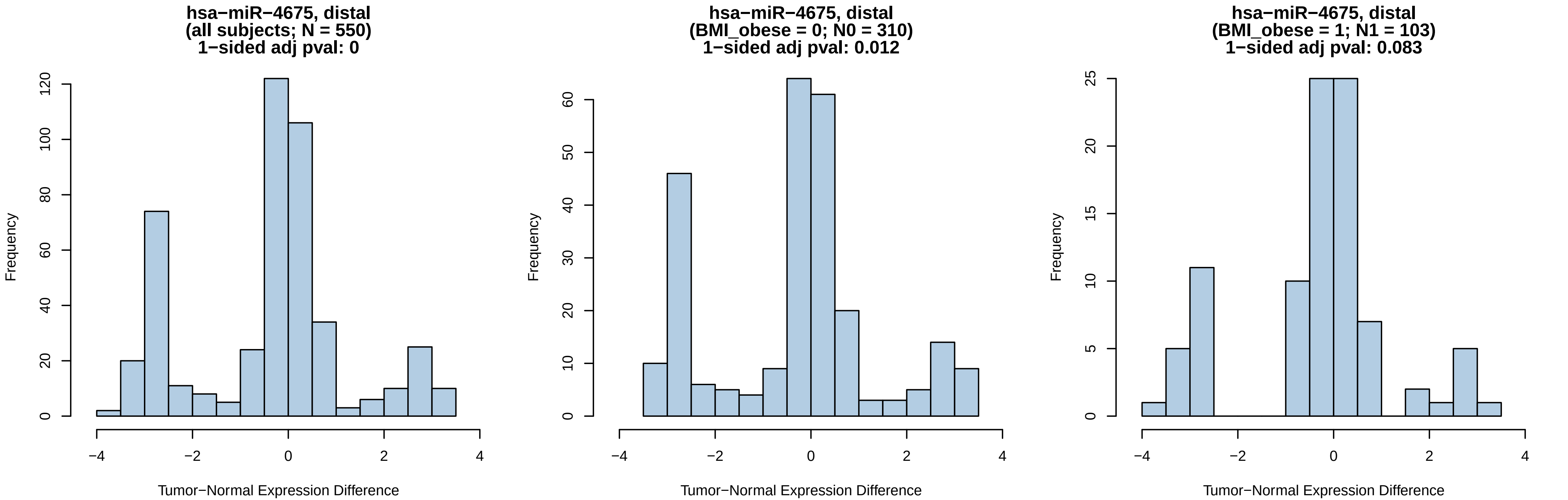
hsa−miR−4675, distal
(all subjects; N = 550)
1−sided adj pval: 0
hsa−miR−4675, distal
(BMI_obese = 0; N0 = 310)
1−sided adj pval: 0.012
hsa−miR−4675, distal
(BMI_obese = 1; N1 = 103)
1−sided adj pval: 0.083
Frequency
Tumor−Normal Expression Difference
0
20
40
60
80
100
120
10
30
50
5
15
25
−4
−2
2
4

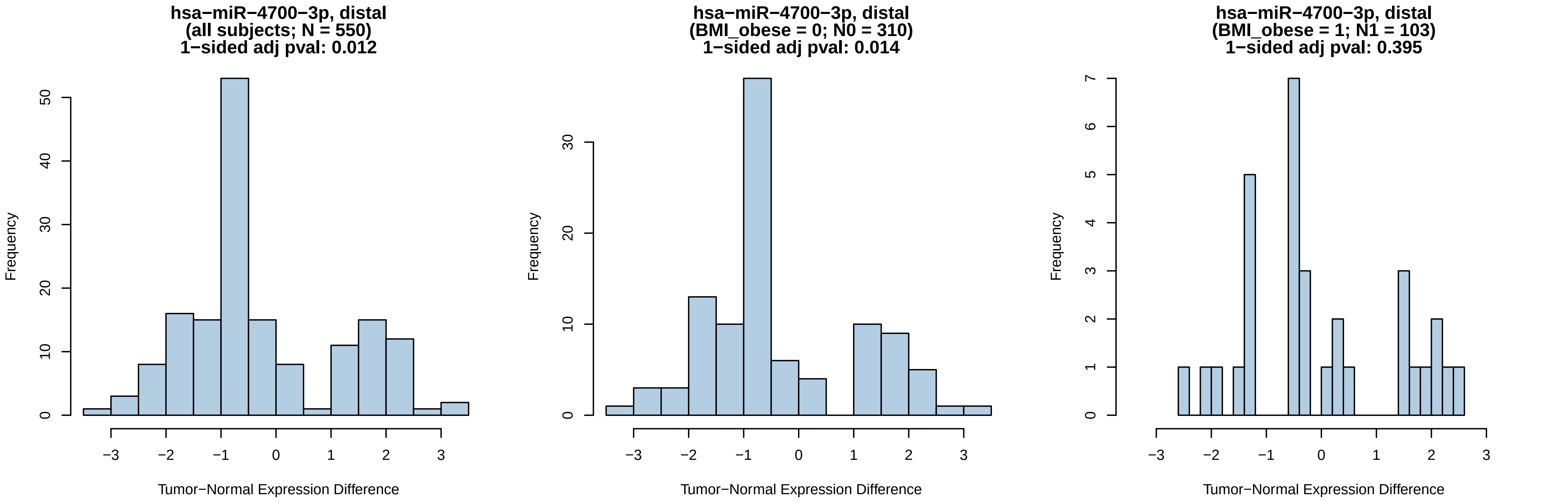
hsa−miR−4700−3p, distal
(all subjects; N = 550)
1−sided adj pval: 0.012
Frequency
Tumor−Normal Expression Difference
hsa−miR−4700−3p, distal
(BMI_obese = 0; N0 = 310)
1−sided adj pval: 0.014
Frequency
Tumor−Normal Expression Difference
hsa−miR−4700−3p, distal
(BMI_obese = 1; N1 = 103)
1−sided adj pval: 0.395
Frequency
Tumor−Normal Expression Difference

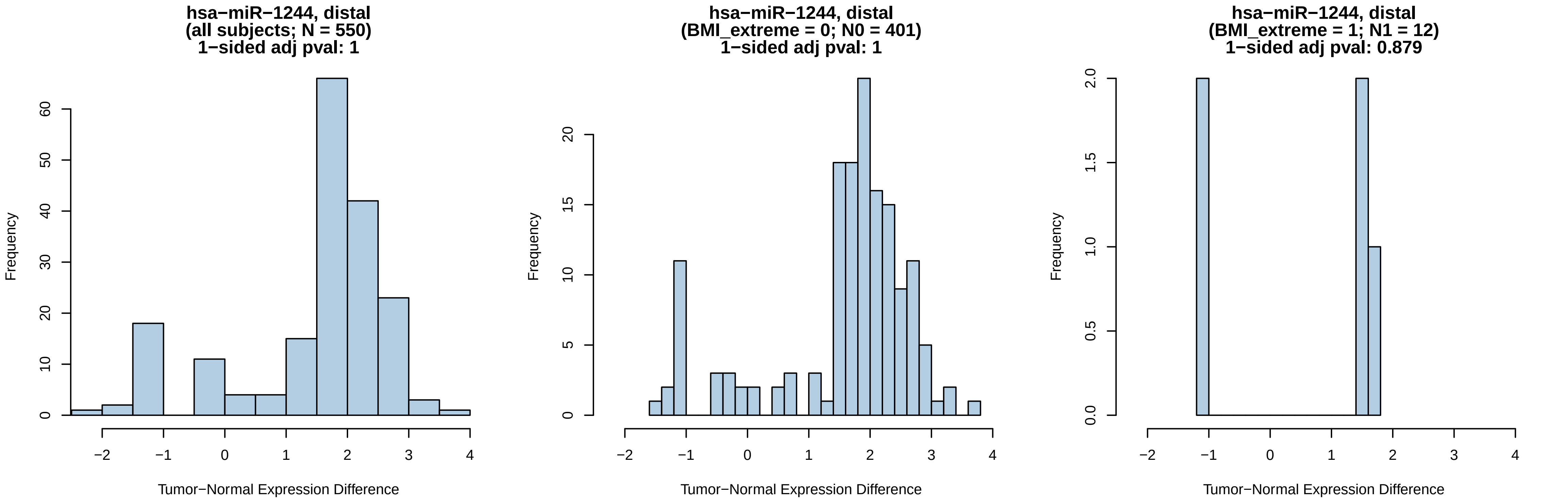
hsa−miR−1244, distal
(all subjects; N = 550)
1−sided adj pval: 1
Frequency
Tumor−Normal Expression Difference
hsa−miR−1244, distal
(BMI_extreme = 0; N0 = 401)
1−sided adj pval: 1
Frequency
Tumor−Normal Expression Difference
hsa−miR−1244, distal
(BMI_extreme = 1; N1 = 12)
1−sided adj pval: 0.879
Frequency
Tumor−Normal Expression Difference

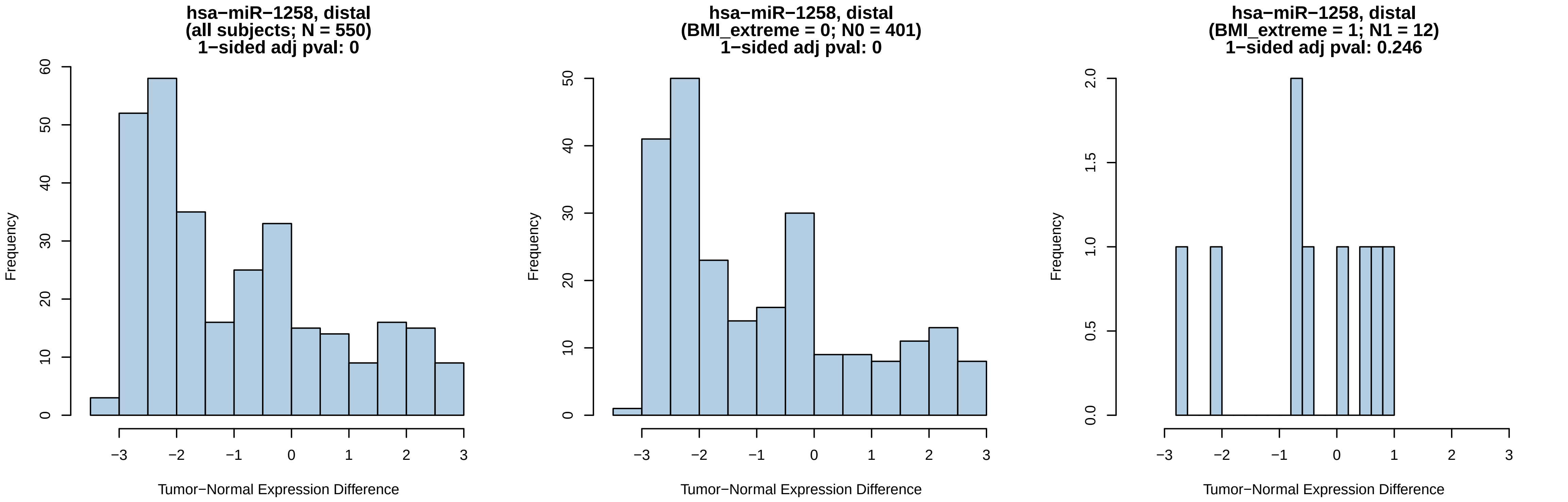
hsa−miR−1258, distal
(all subjects; N = 550)
1−sided adj pval: 0
Frequency
Tumor−Normal Expression Difference
hsa−miR−1258, distal
(BMI_extreme = 0; N0 = 401)
1−sided adj pval: 0
Frequency
Tumor−Normal Expression Difference
hsa−miR−1258, distal
(BMI_extreme = 1; N1 = 12)
1−sided adj pval: 0.246
Frequency
Tumor−Normal Expression Difference

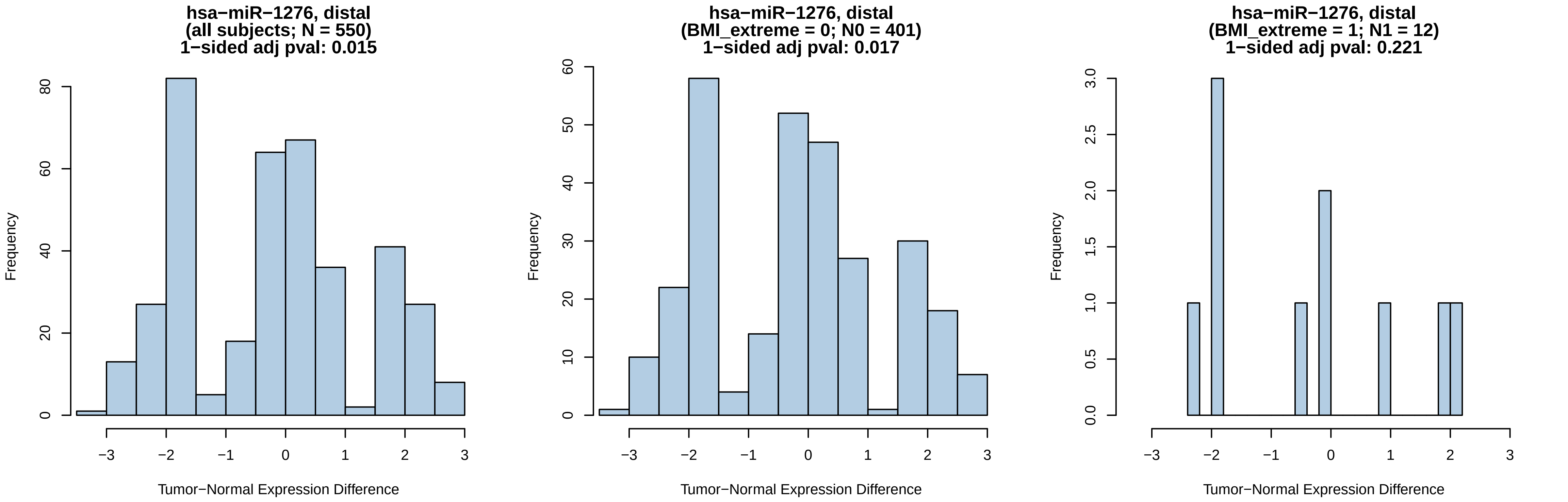
hsa−miR−1276, distal
(all subjects; N = 550)
1−sided adj pval: 0.015
Frequency
Tumor−Normal Expression Difference
0
20
40
60
80
−3
−2
−1
0
1
2
3
hsa−miR−1276, distal
(BMI_extreme = 0; N0 = 401)
1−sided adj pval: 0.017
Frequency
Tumor−Normal Expression Difference
0
10
20
30
40
50
60
−3
−2
−1
0
1
2
3
hsa−miR−1276, distal
(BMI_extreme = 1; N1 = 12)
1−sided adj pval: 0.221
Frequency
Tumor−Normal Expression Difference
0.0
0.5
1.0
1.5
2.0
2.5
3.0
−3
−2
−1
0
1
2
3

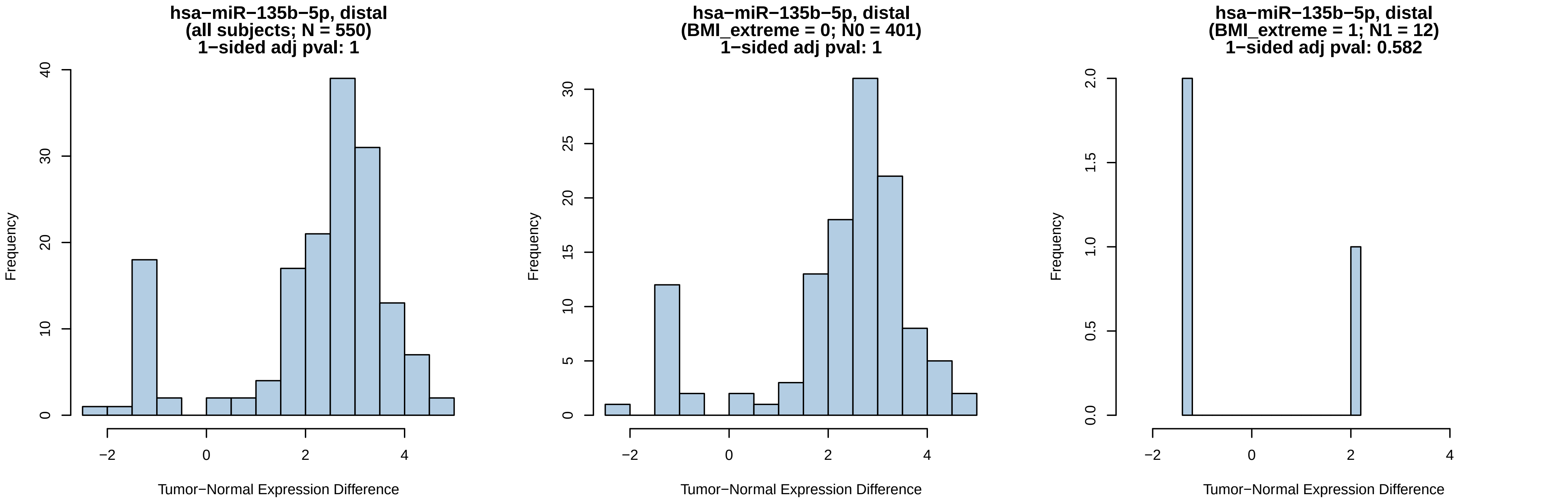

hsa−miR−135b−5p, distal
(all subjects; N = 550)
1−sided adj pval: 1
Frequency
Tumor−Normal Expression Difference
0
10
20
30
40
−2
0
2
4
hsa−miR−135b−5p, distal
(BMI_extreme = 0; N0 = 401)
1−sided adj pval: 1
Frequency
Tumor−Normal Expression Difference
0
5
10
15
20
25
30
−2
0
2
4
hsa−miR−135b−5p, distal
(BMI_extreme = 1; N1 = 12)
1−sided adj pval: 0.582
Frequency
Tumor−Normal Expression Difference
0.0
0.5
1.0
1.5
2.0
−2
0
2
4

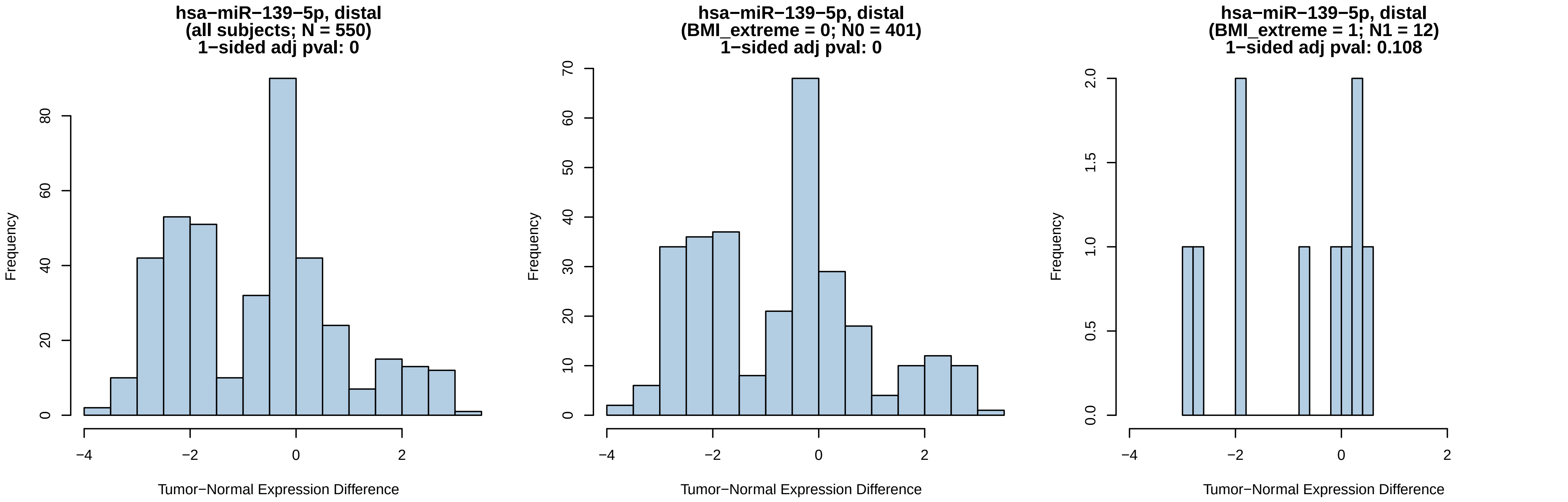
hsa−miR−139−5p, distal
(all subjects; N = 550)
1−sided adj pval: 0
Frequency
0
20
40
60
80
−4
−2
0
2
Tumor−Normal Expression Difference
hsa−miR−139−5p, distal
(BMI_extreme = 0; N0 = 401)
1−sided adj pval: 0
Frequency
0
10
20
30
40
50
60
70
−4
−2
0
2
Tumor−Normal Expression Difference
hsa−miR−139−5p, distal
(BMI_extreme = 1; N1 = 12)
1−sided adj pval: 0.108
Frequency
0.0
0.5
1.0
1.5
2.0
−4
−2
0
2
Tumor−Normal Expression Difference

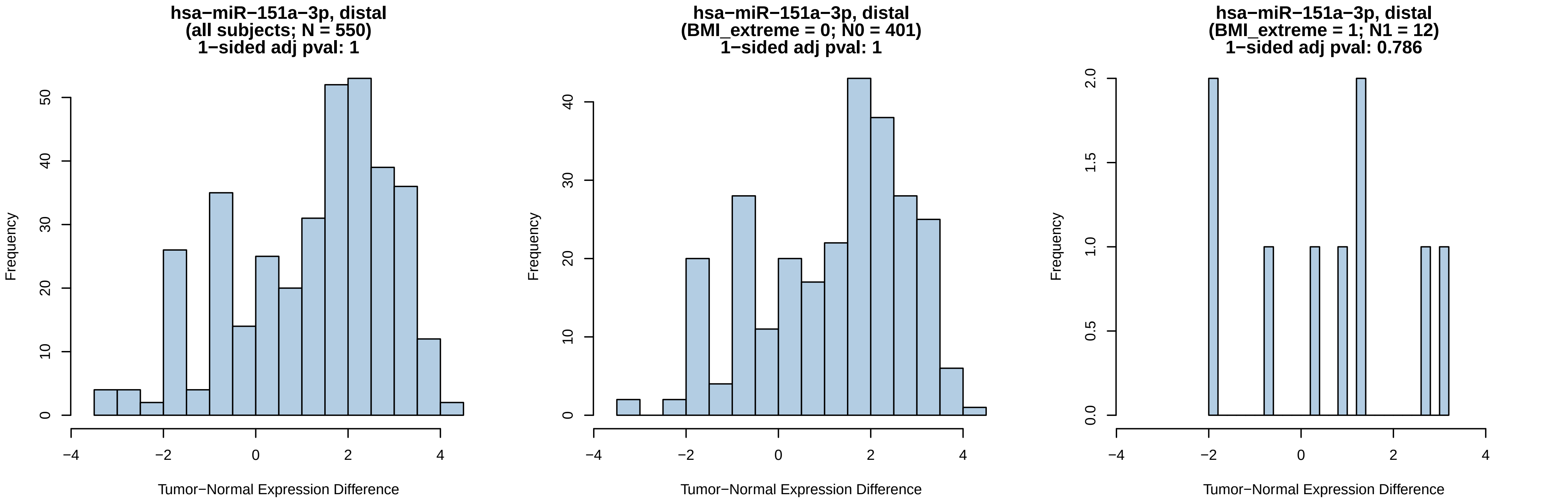

hsa−miR−151a−3p, distal
(all subjects; N = 550)
1−sided adj pval: 1
Frequency
Tumor−Normal Expression Difference
hsa−miR−151a−3p, distal
(BMI_extreme = 0; N0 = 401)
1−sided adj pval: 1
Frequency
Tumor−Normal Expression Difference
hsa−miR−151a−3p, distal
(BMI_extreme = 1; N1 = 12)
1−sided adj pval: 0.786
Frequency
Tumor−Normal Expression Difference

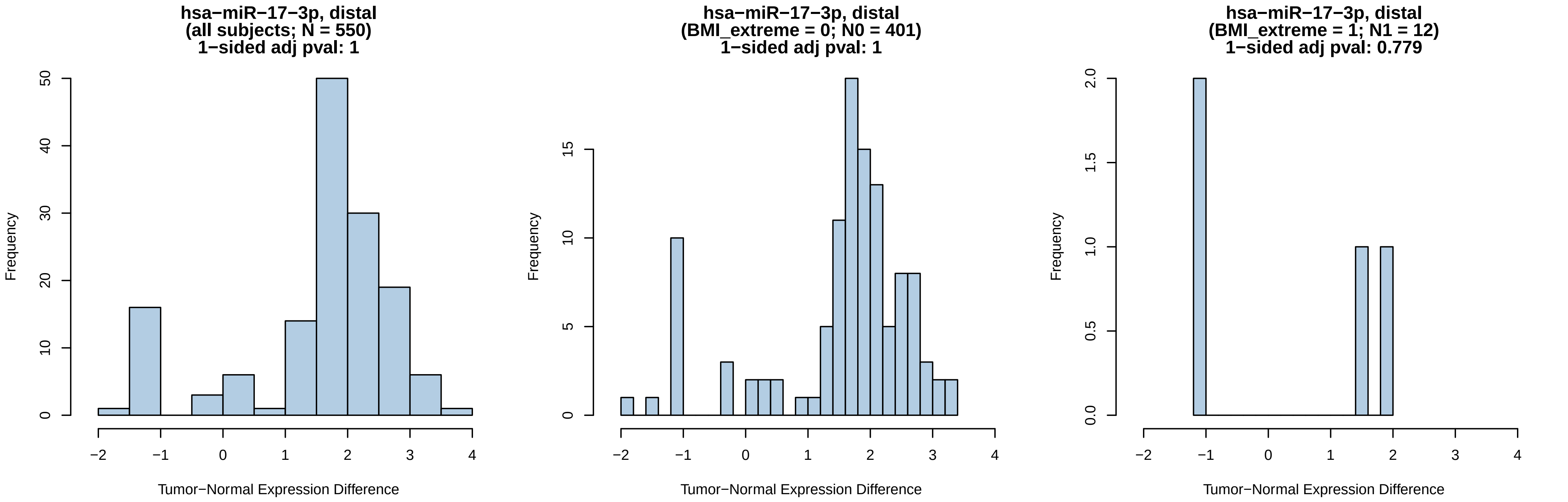
hsa−miR−17−3p, distal
(all subjects; N = 550)
1−sided adj pval: 1
Frequency
Tumor−Normal Expression Difference
hsa−miR−17−3p, distal
(BMI_extreme = 0; N0 = 401)
1−sided adj pval: 1
Frequency
Tumor−Normal Expression Difference
hsa−miR−17−3p, distal
(BMI_extreme = 1; N1 = 12)
1−sided adj pval: 0.779
Frequency
Tumor−Normal Expression Difference

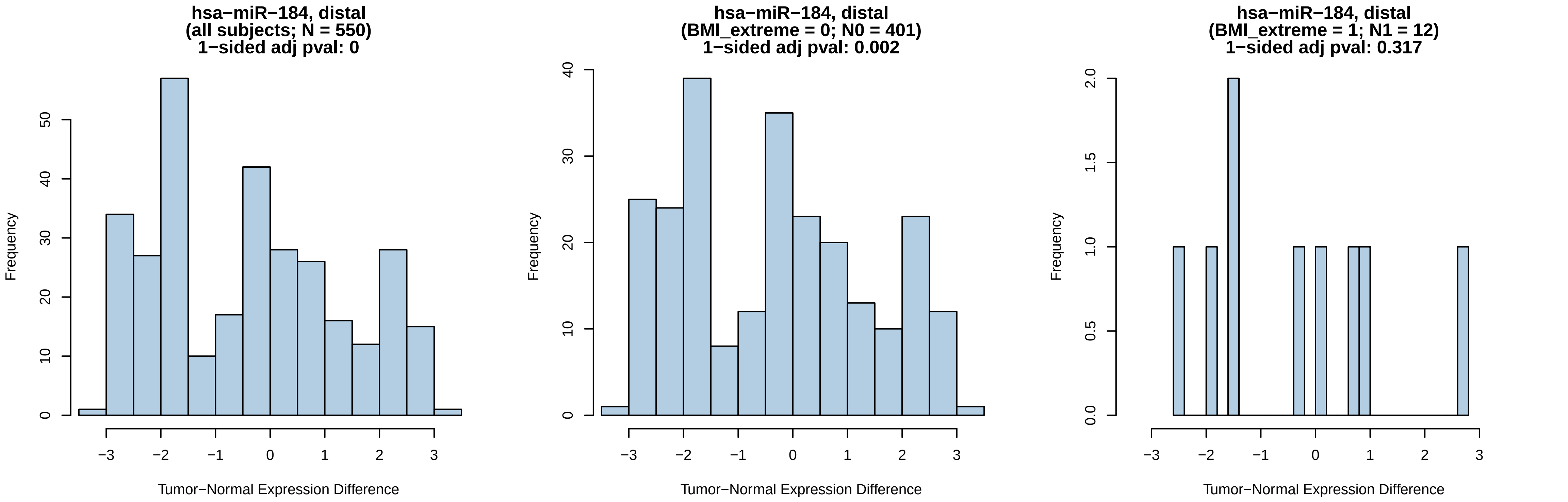
hsa−miR−184, distal
(all subjects; N = 550)
1−sided adj pval: 0
Frequency
Tumor−Normal Expression Difference
hsa−miR−184, distal
(BMI_extreme = 0; N0 = 401)
1−sided adj pval: 0.002
Frequency
Tumor−Normal Expression Difference
hsa−miR−184, distal
(BMI_extreme = 1; N1 = 12)
1−sided adj pval: 0.317
Frequency
Tumor−Normal Expression Difference

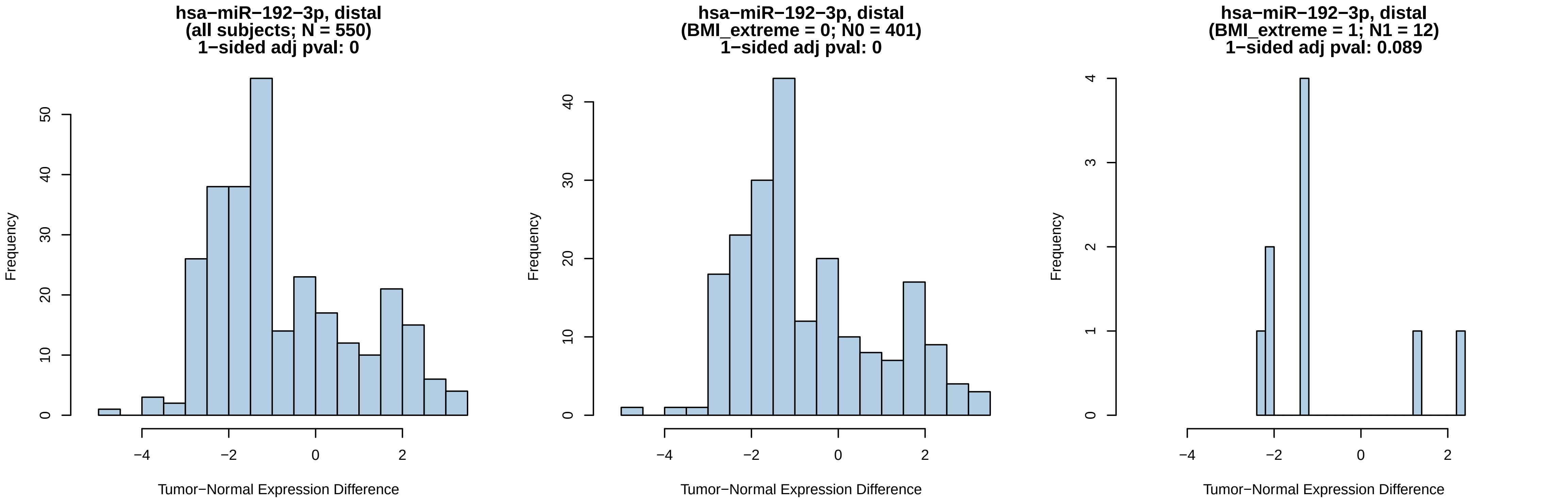
hsa−miR−192−3p, distal
(all subjects; N = 550)
1−sided adj pval: 0
Frequency
Tumor−Normal Expression Difference
hsa−miR−192−3p, distal
(BMI_extreme = 0; N0 = 401)
1−sided adj pval: 0
Frequency
Tumor−Normal Expression Difference
hsa−miR−192−3p, distal
(BMI_extreme = 1; N1 = 12)
1−sided adj pval: 0.089
Frequency
Tumor−Normal Expression Difference

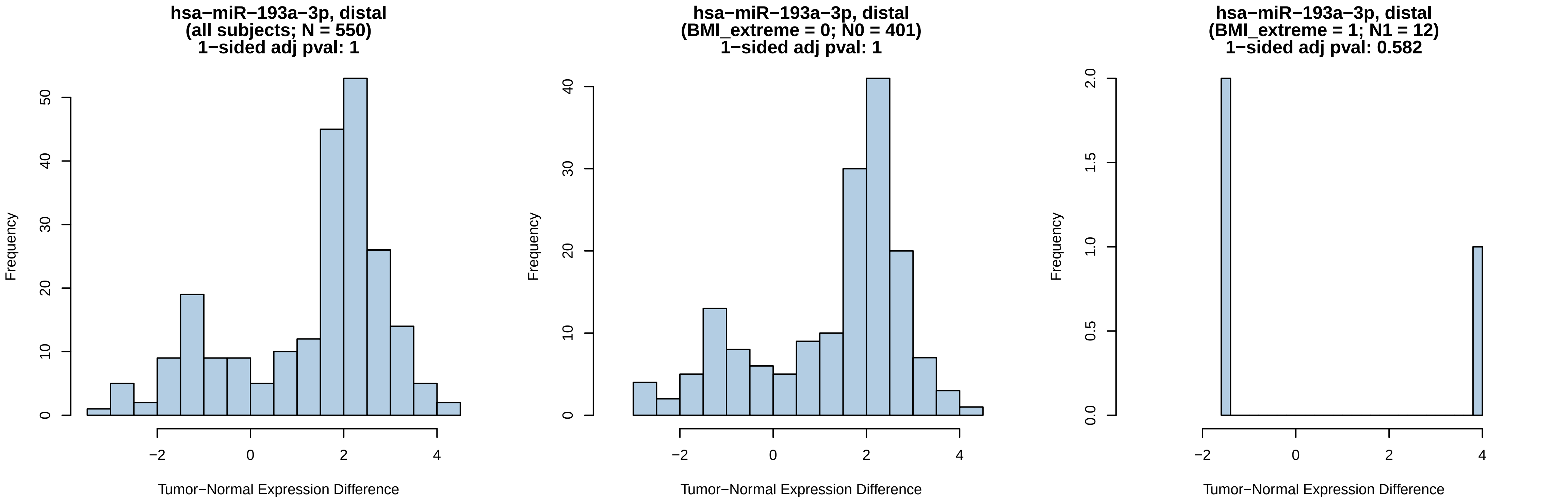
hsa−miR−193a−3p, distal
(all subjects; N = 550)
1−sided adj pval: 1
Frequency
Tumor−Normal Expression Difference
hsa−miR−193a−3p, distal
(BMI_extreme = 0; N0 = 401)
1−sided adj pval: 1
Frequency
Tumor−Normal Expression Difference
hsa−miR−193a−3p, distal
(BMI_extreme = 1; N1 = 12)
1−sided adj pval: 0.582
Frequency
Tumor−Normal Expression Difference

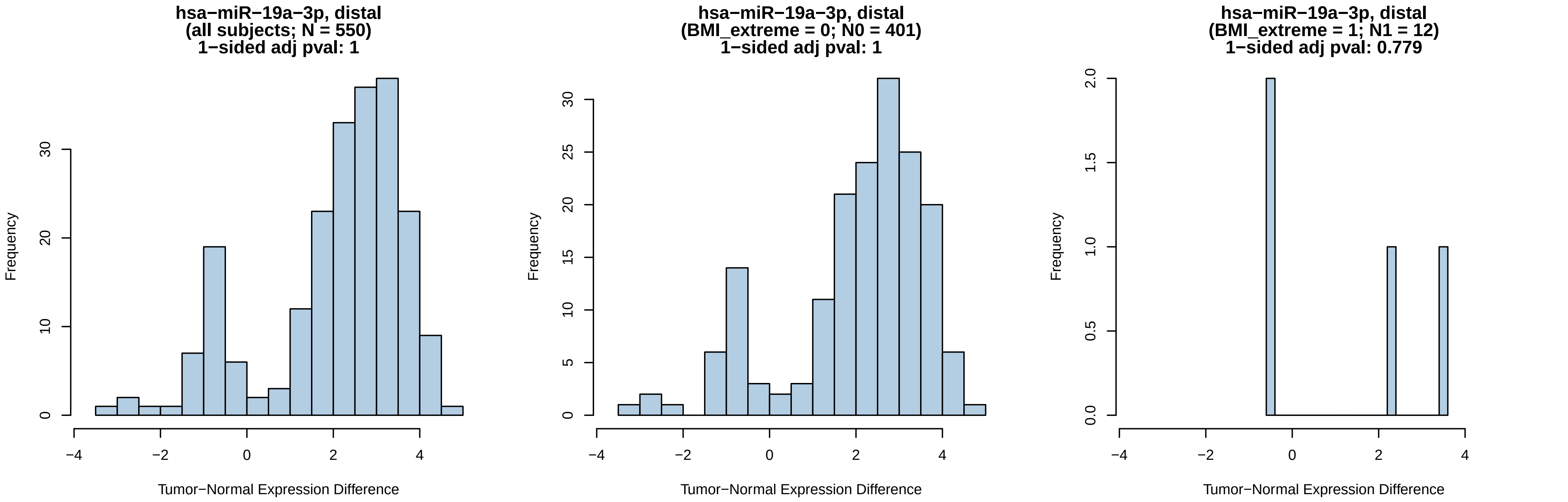
hsa−miR−19a−3p, distal
(all subjects; N = 550)
1−sided adj pval: 1
Frequency
Tumor−Normal Expression Difference
hsa−miR−19a−3p, distal
(BMI_extreme = 0; N0 = 401)
1−sided adj pval: 1
Frequency
Tumor−Normal Expression Difference
hsa−miR−19a−3p, distal
(BMI_extreme = 1; N1 = 12)
1−sided adj pval: 0.779
Frequency
Tumor−Normal Expression Difference

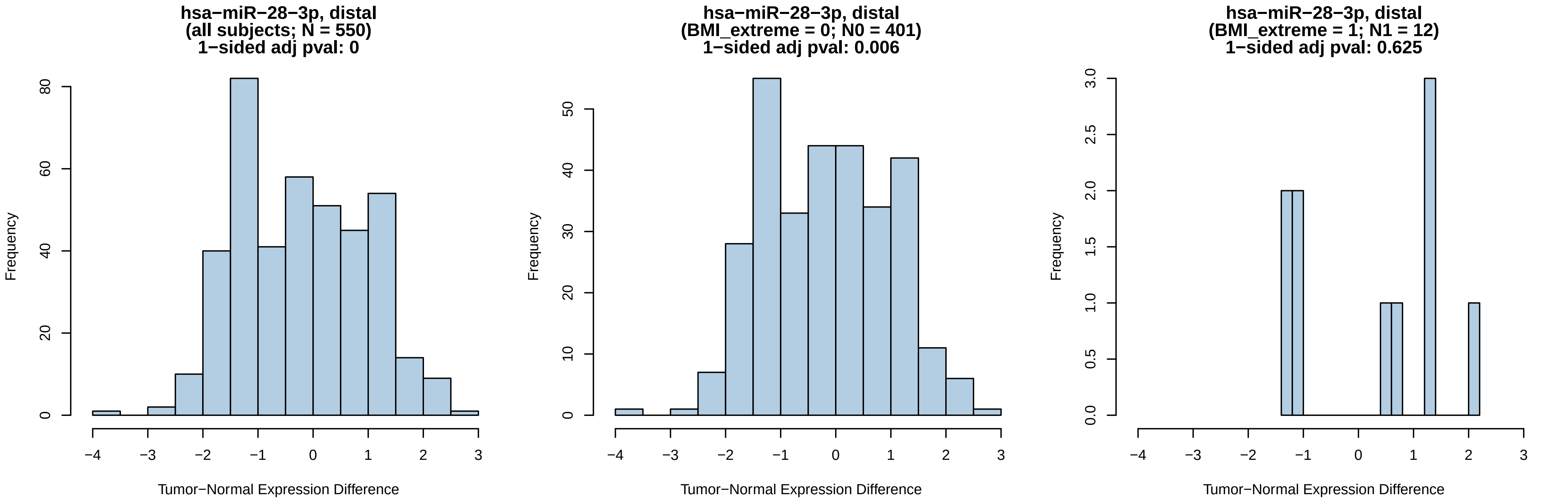
hsa−miR−28−3p, distal
(all subjects; N = 550)
1−sided adj pval: 0
Frequency
Tumor−Normal Expression Difference
hsa−miR−28−3p, distal
(BMI_extreme = 0; N0 = 401)
1−sided adj pval: 0.006
Frequency
Tumor−Normal Expression Difference
hsa−miR−28−3p, distal
(BMI_extreme = 1; N1 = 12)
1−sided adj pval: 0.625
Frequency
Tumor−Normal Expression Difference

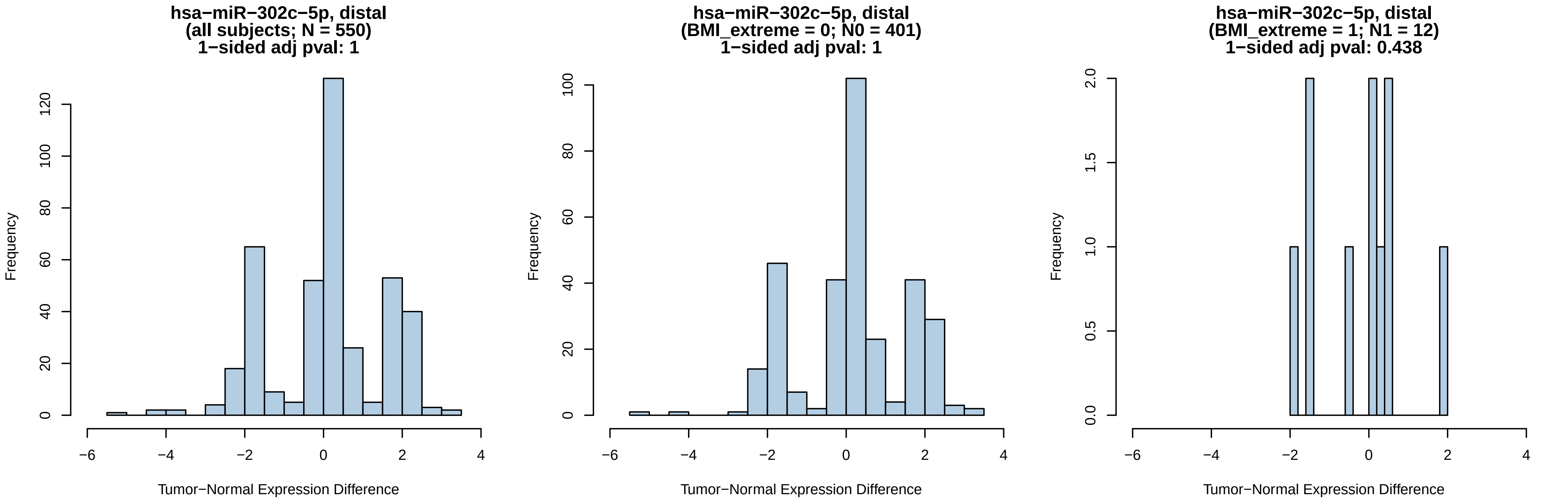

hsa−miR−302c−5p, distal
(all subjects; N = 550)
1−sided adj pval: 1
hsa−miR−302c−5p, distal
(BMI_extreme = 0; N0 = 401)
1−sided adj pval: 1
hsa−miR−302c−5p, distal
(BMI_extreme = 1; N1 = 12)
1−sided adj pval: 0.438
Frequency
Tumor−Normal Expression Difference

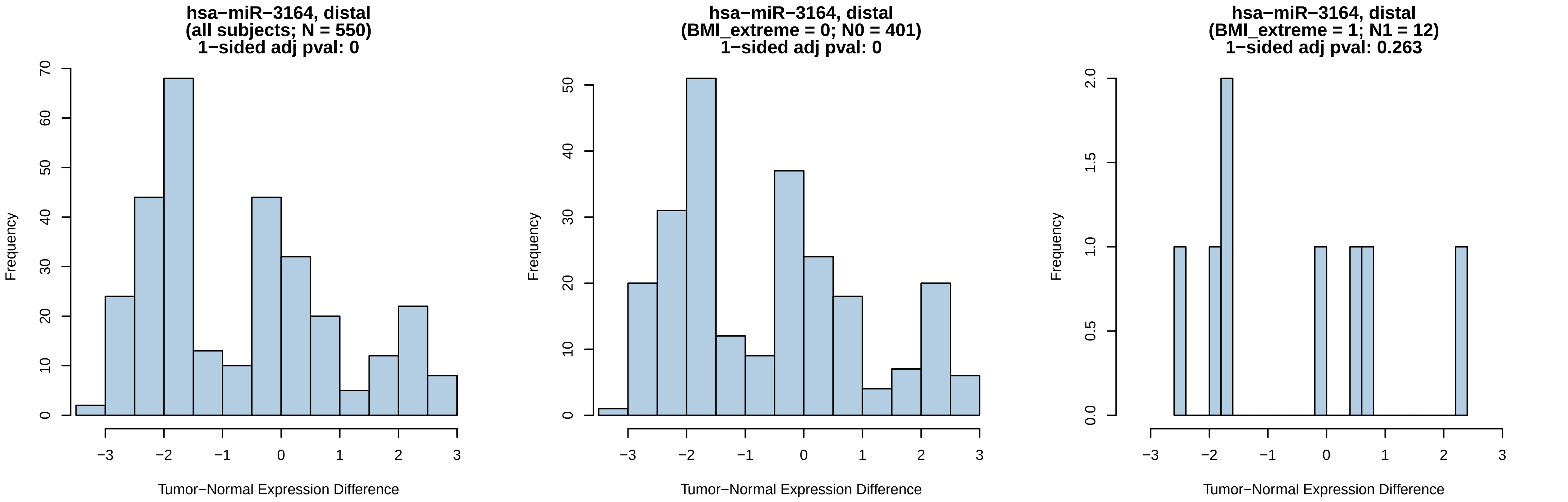
hsa−miR−3164, distal
(all subjects; N = 550)
1−sided adj pval: 0
Frequency
Tumor−Normal Expression Difference
hsa−miR−3164, distal
(BMI_extreme = 0; N0 = 401)
1−sided adj pval: 0
Frequency
Tumor−Normal Expression Difference
hsa−miR−3164, distal
(BMI_extreme = 1; N1 = 12)
1−sided adj pval: 0.263
Frequency
Tumor−Normal Expression Difference

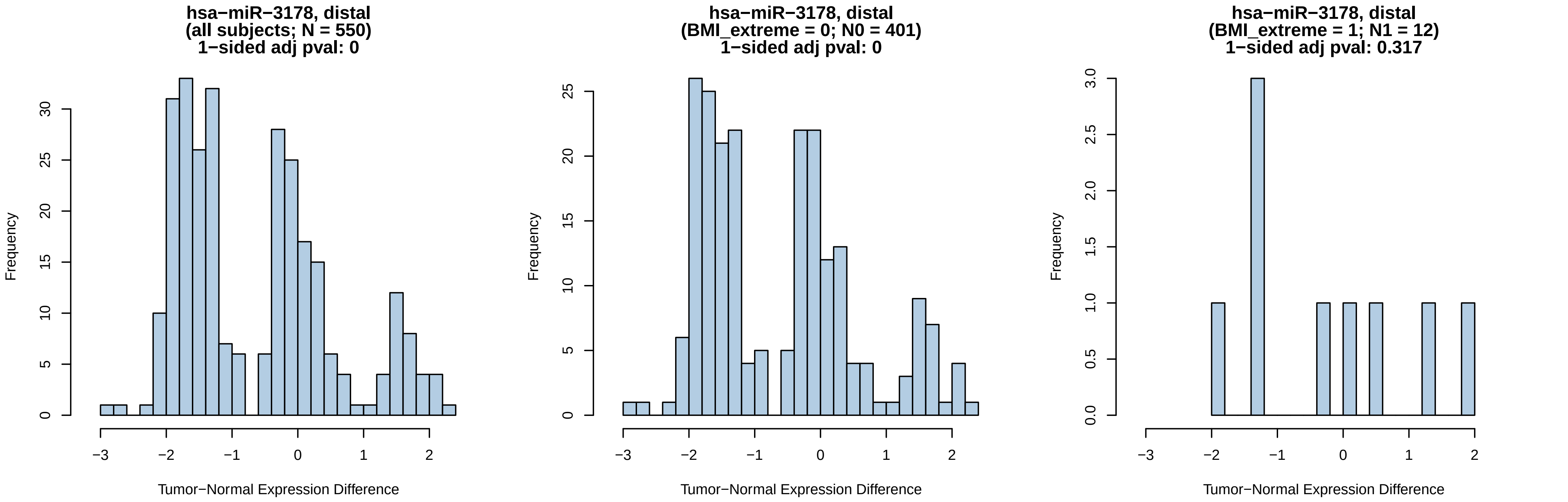
hsa−miR−3178, distal
(all subjects; N = 550)
1−sided adj pval: 0
hsa−miR−3178, distal
(BMI_extreme = 0; N0 = 401)
1−sided adj pval: 0
hsa−miR−3178, distal
(BMI_extreme = 1; N1 = 12)
1−sided adj pval: 0.317
Frequency
Tumor−Normal Expression Difference
−3
−2
−1
0
1
2
0
5
10
15
20
25
30
0.0
0.5
1.0
1.5
2.0
2.5
3.0

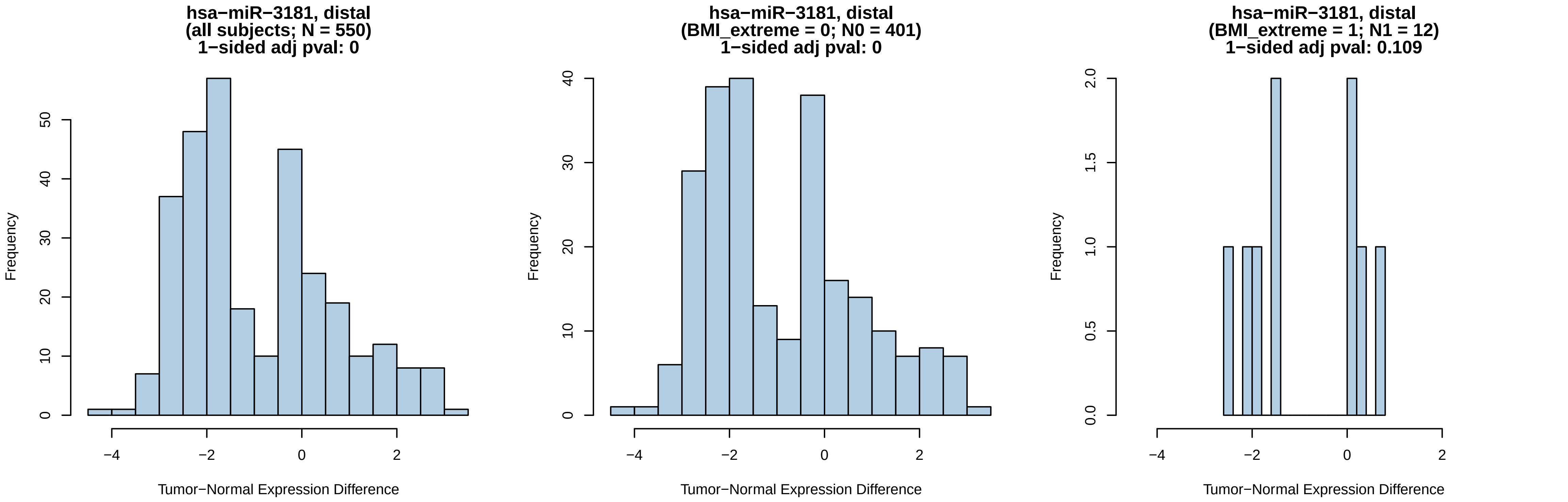

hsa−miR−3181, distal
(all subjects; N = 550)
1−sided adj pval: 0
hsa−miR−3181, distal
(BMI_extreme = 0; N0 = 401)
1−sided adj pval: 0
hsa−miR−3181, distal
(BMI_extreme = 1; N1 = 12)
1−sided adj pval: 0.109
Frequency
Tumor−Normal Expression Difference

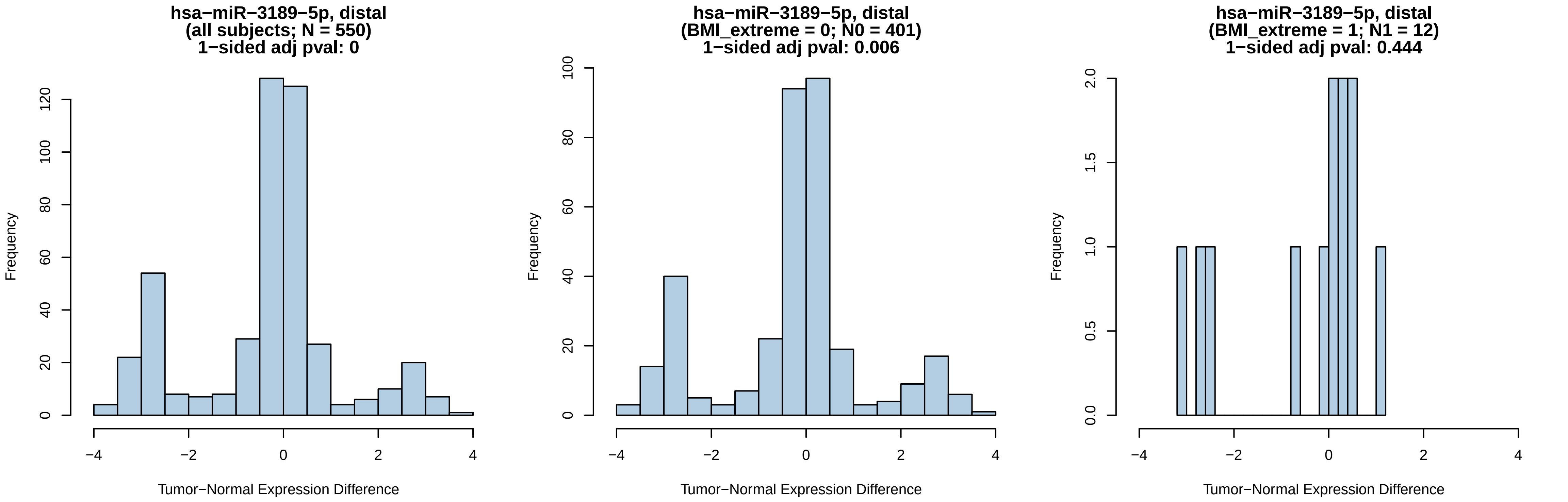
hsa−miR−3189−5p, distal
(all subjects; N = 550)
1−sided adj pval: 0
Frequency
Tumor−Normal Expression Difference
hsa−miR−3189−5p, distal
(BMI_extreme = 0; N0 = 401)
1−sided adj pval: 0.006
Frequency
Tumor−Normal Expression Difference
hsa−miR−3189−5p, distal
(BMI_extreme = 1; N1 = 12)
1−sided adj pval: 0.444
Frequency
Tumor−Normal Expression Difference

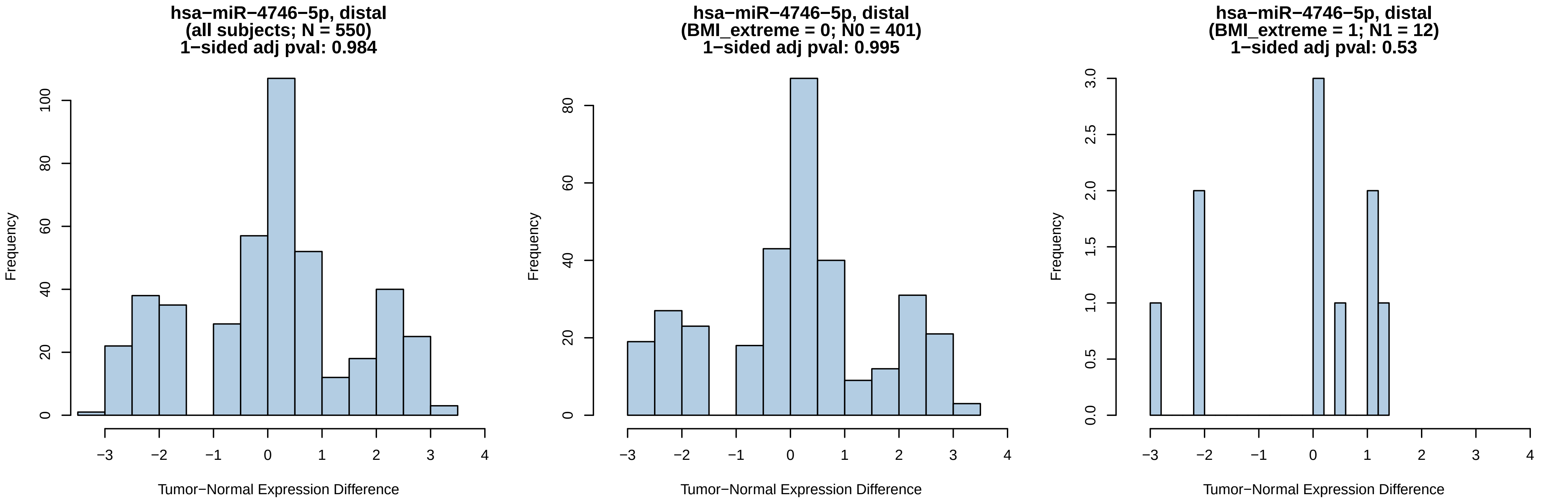

hsa−miR−4746−5p, distal
(all subjects; N = 550)
1−sided adj pval: 0.984
Frequency
Tumor−Normal Expression Difference
hsa−miR−4746−5p, distal
(BMI_extreme = 0; N0 = 401)
1−sided adj pval: 0.995
Frequency
Tumor−Normal Expression Difference
hsa−miR−4746−5p, distal
(BMI_extreme = 1; N1 = 12)
1−sided adj pval: 0.53
Frequency
Tumor−Normal Expression Difference

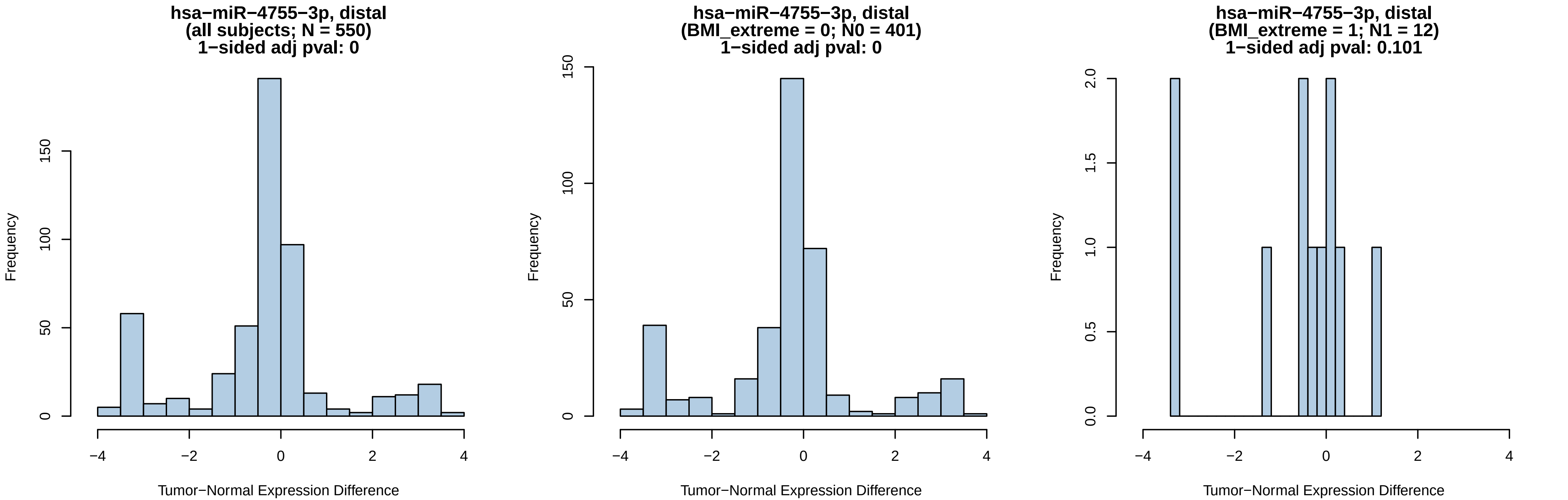

hsa−miR−4755−3p, distal
(all subjects; N = 550)
1−sided adj pval: 0
Frequency
0
50
100
150
−4
−2
0
2
4
Tumor−Normal Expression Difference
hsa−miR−4755−3p, distal
(BMI_extreme = 0; N0 = 401)
1−sided adj pval: 0
Frequency
0
50
100
150
−4
−2
0
2
4
Tumor−Normal Expression Difference
hsa−miR−4755−3p, distal
(BMI_extreme = 1; N1 = 12)
1−sided adj pval: 0.101
Frequency
0.0
0.5
1.0
1.5
2.0
−4
−2
0
2
4
Tumor−Normal Expression Difference

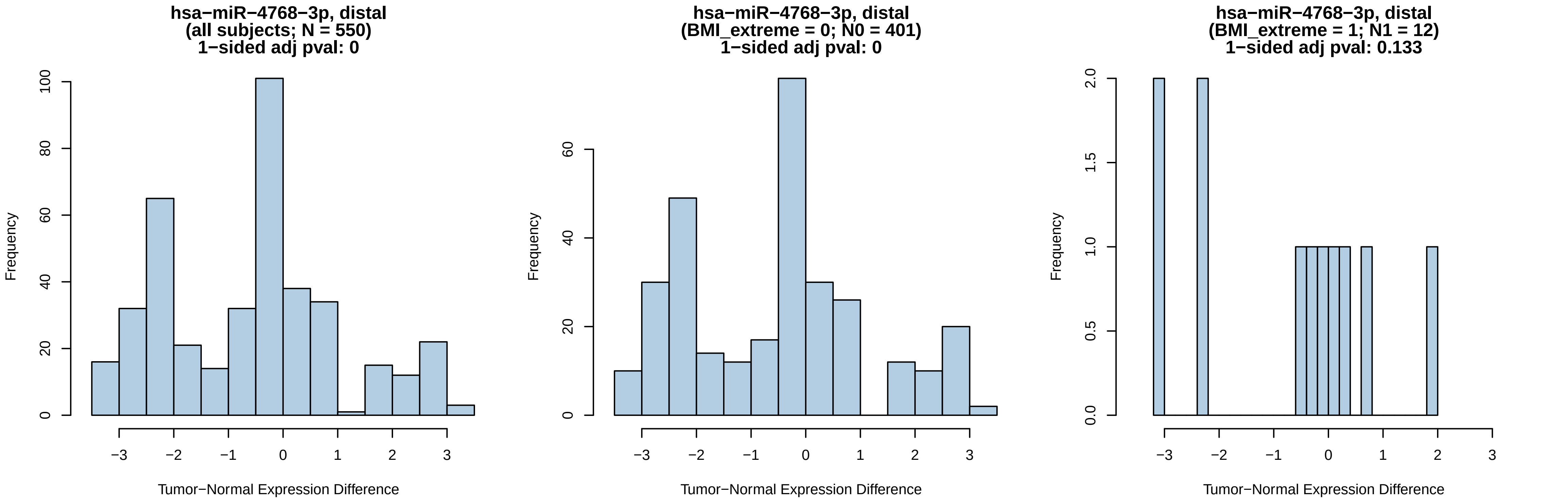

hsa−miR−4768−3p, distal
(all subjects; N = 550)
1−sided adj pval: 0
Frequency
Tumor−Normal Expression Difference
hsa−miR−4768−3p, distal
(BMI_extreme = 0; N0 = 401)
1−sided adj pval: 0
Frequency
Tumor−Normal Expression Difference
hsa−miR−4768−3p, distal
(BMI_extreme = 1; N1 = 12)
1−sided adj pval: 0.133
Frequency
Tumor−Normal Expression Difference

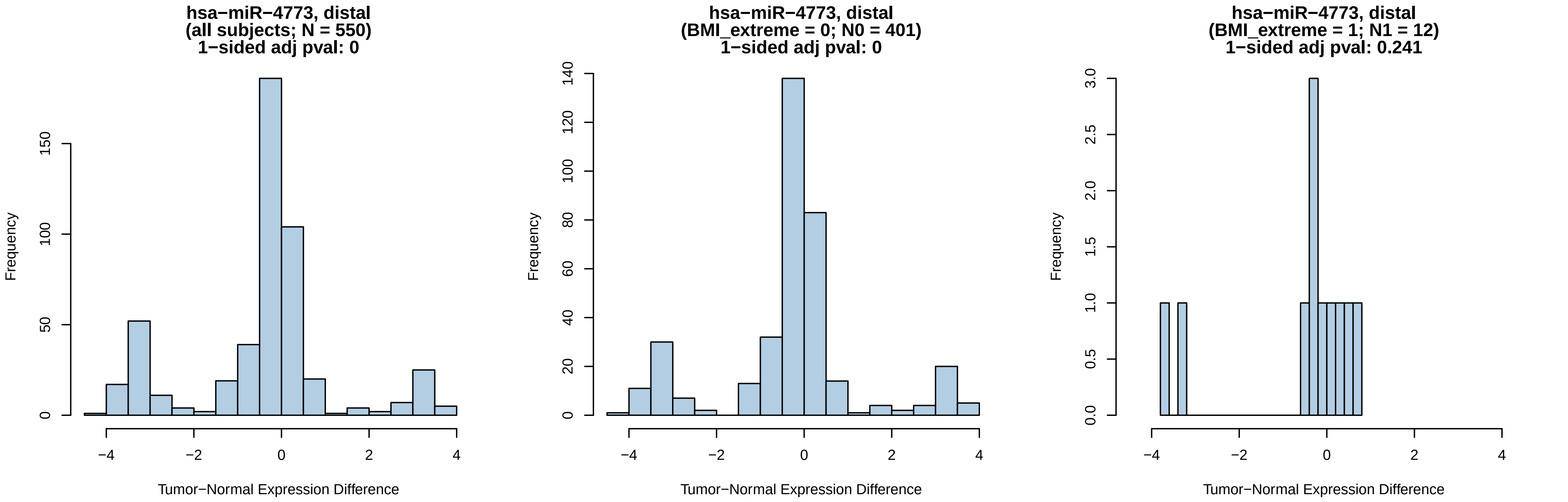
hsa−miR−4773, distal
(all subjects; N = 550)
1−sided adj pval: 0
Frequency
0
50
100
150
−4
−2
0
2
4
Tumor−Normal Expression Difference
hsa−miR−4773, distal
(BMI_extreme = 0; N0 = 401)
1−sided adj pval: 0
Frequency
0
20
40
60
80
100
120
140
−4
−2
0
2
4
Tumor−Normal Expression Difference
hsa−miR−4773, distal
(BMI_extreme = 1; N1 = 12)
1−sided adj pval: 0.241
Frequency
0.0
0.5
1.0
1.5
2.0
2.5
3.0
−4
−2
0
2
4
Tumor−Normal Expression Difference

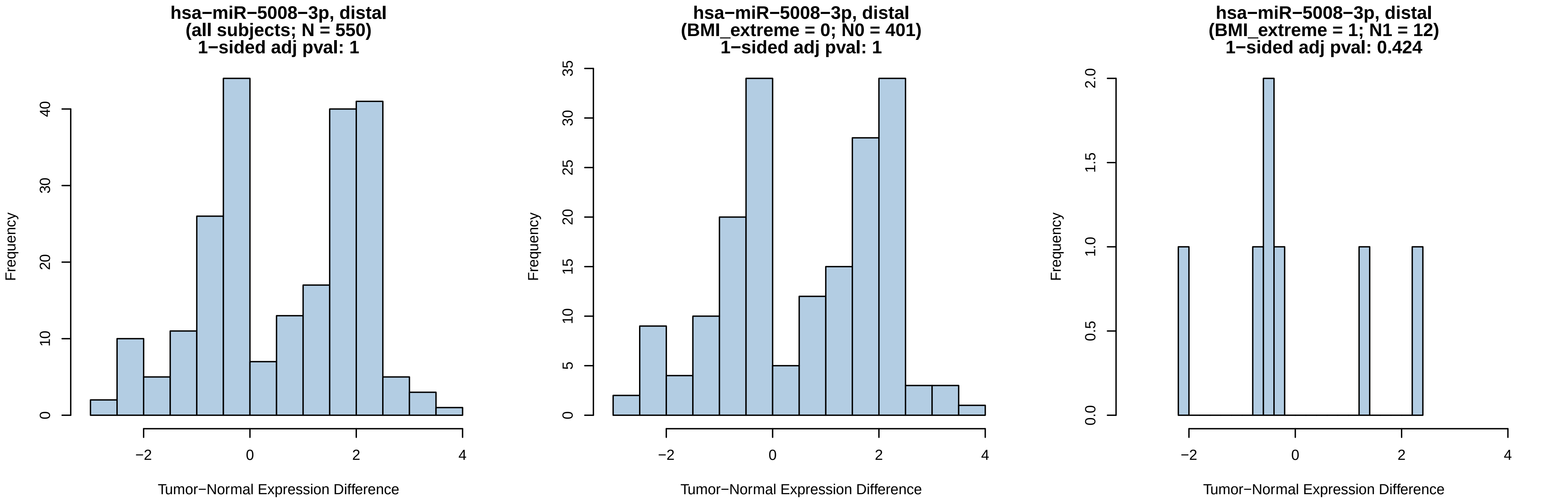
hsa−miR−5008−3p, distal
(all subjects; N = 550)
1−sided adj pval: 1
Frequency
Tumor−Normal Expression Difference
hsa−miR−5008−3p, distal
(BMI_extreme = 0; N0 = 401)
1−sided adj pval: 1
Frequency
Tumor−Normal Expression Difference
hsa−miR−5008−3p, distal
(BMI_extreme = 1; N1 = 12)
1−sided adj pval: 0.424
Frequency
Tumor−Normal Expression Difference

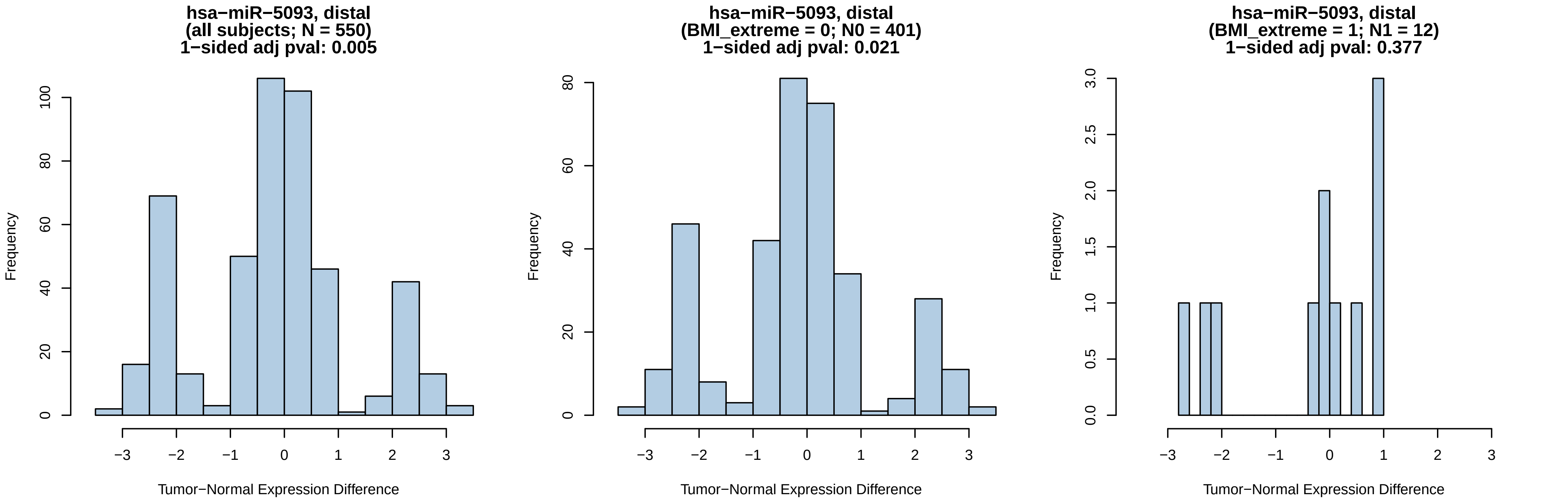
hsa−miR−5093, distal
(all subjects; N = 550)
1−sided adj pval: 0.005
Frequency
Tumor−Normal Expression Difference
hsa−miR−5093, distal
(BMI_extreme = 0; N0 = 401)
1−sided adj pval: 0.021
Frequency
Tumor−Normal Expression Difference
hsa−miR−5093, distal
(BMI_extreme = 1; N1 = 12)
1−sided adj pval: 0.377
Frequency
Tumor−Normal Expression Difference

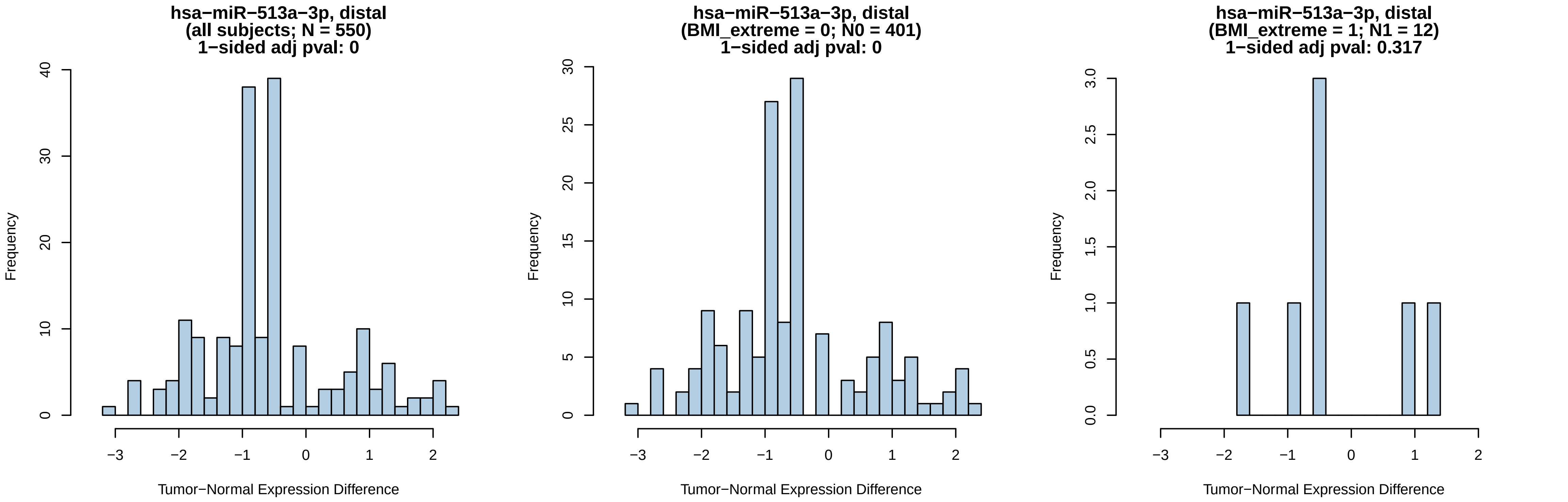

hsa−miR−513a−3p, distal
(all subjects; N = 550)
1−sided adj pval: 0
Frequency
Tumor−Normal Expression Difference
hsa−miR−513a−3p, distal
(BMI_extreme = 0; N0 = 401)
1−sided adj pval: 0
Frequency
Tumor−Normal Expression Difference
hsa−miR−513a−3p, distal
(BMI_extreme = 1; N1 = 12)
1−sided adj pval: 0.317
Frequency
Tumor−Normal Expression Difference

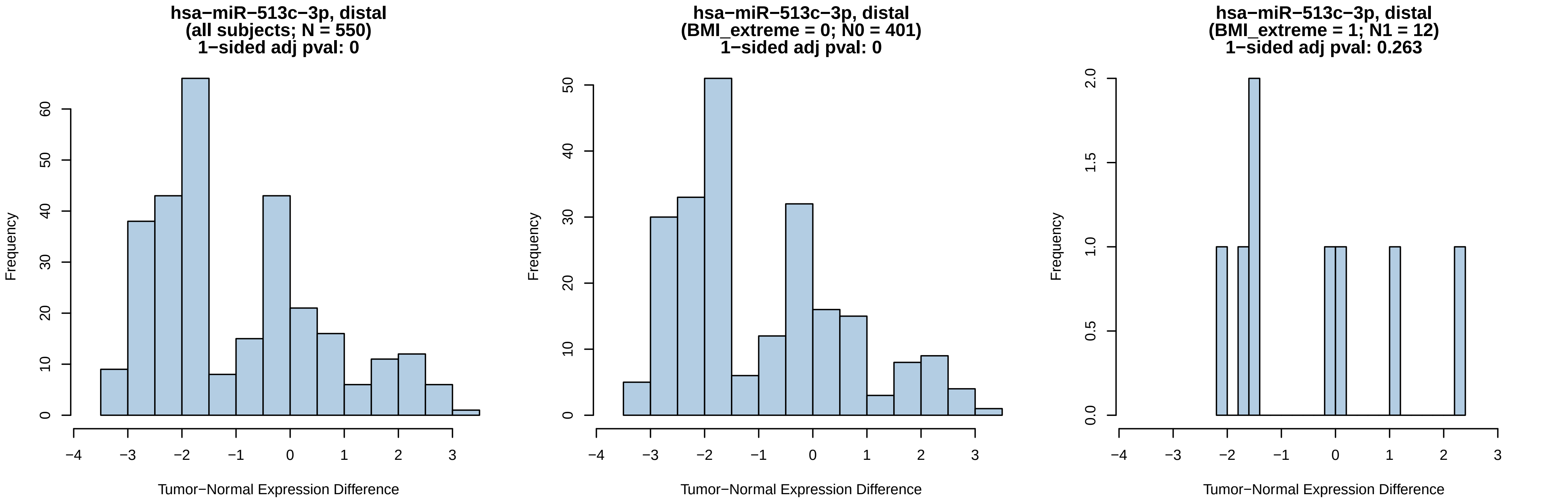
hsa−miR−513c−3p, distal
(all subjects; N = 550)
1−sided adj pval: 0
Frequency
Tumor−Normal Expression Difference
hsa−miR−513c−3p, distal
(BMI_extreme = 0; N0 = 401)
1−sided adj pval: 0
Frequency
Tumor−Normal Expression Difference
hsa−miR−513c−3p, distal
(BMI_extreme = 1; N1 = 12)
1−sided adj pval: 0.263
Frequency
Tumor−Normal Expression Difference

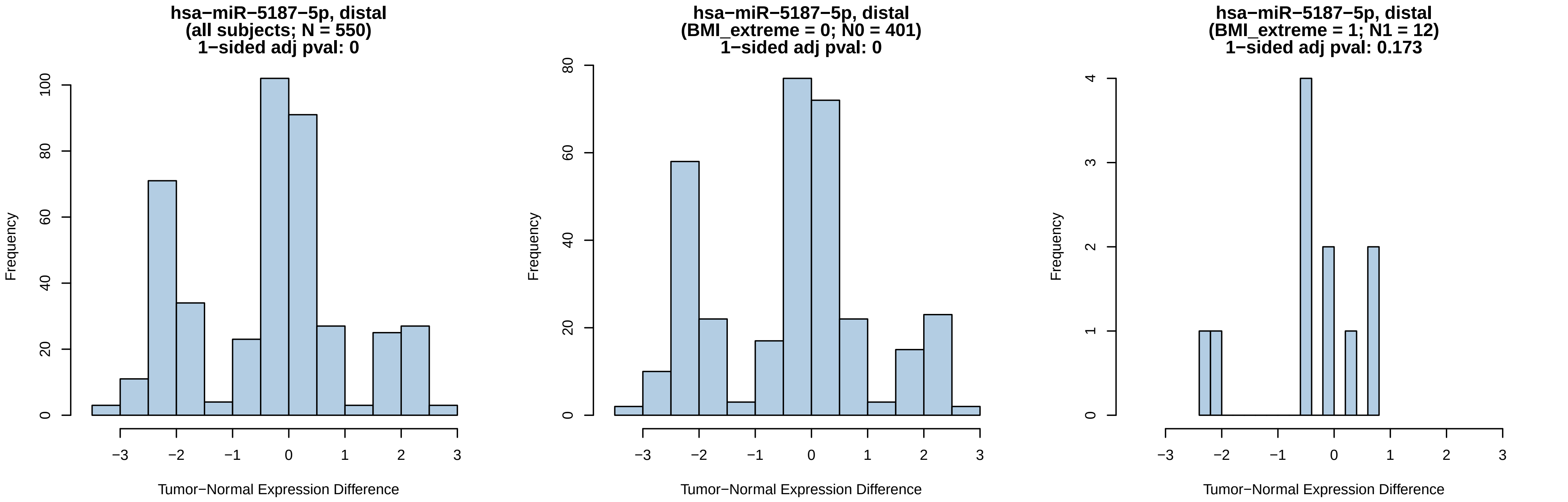
hsa−miR−5187−5p, distal
(all subjects; N = 550)
1−sided adj pval: 0
Frequency
0
20
40
60
80
100
−3
−2
−1
0
1
2
3
Tumor−Normal Expression Difference
hsa−miR−5187−5p, distal
(BMI_extreme = 0; N0 = 401)
1−sided adj pval: 0
Frequency
0
20
40
60
80
−3
−2
−1
0
1
2
3
Tumor−Normal Expression Difference
hsa−miR−5187−5p, distal
(BMI_extreme = 1; N1 = 12)
1−sided adj pval: 0.173
Frequency
0
1
2
3
4
−3
−2
−1
0
1
2
3
Tumor−Normal Expression Difference

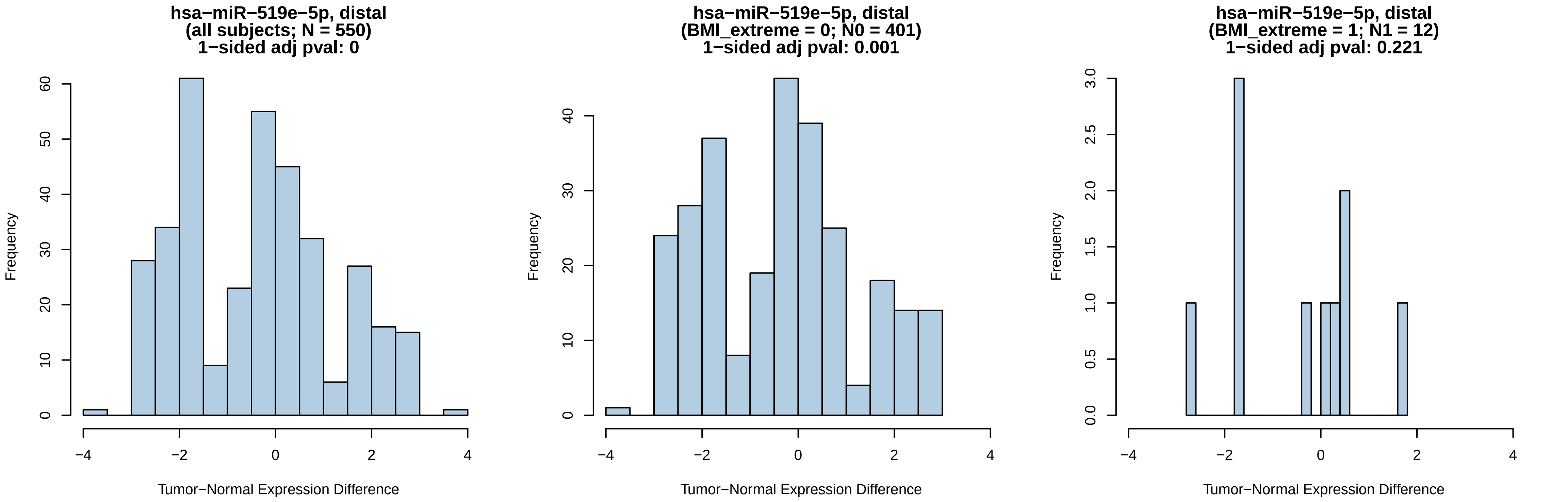
hsa−miR−519e−5p, distal
(all subjects; N = 550)
1−sided adj pval: 0
Frequency
Tumor−Normal Expression Difference
hsa−miR−519e−5p, distal
(BMI_extreme = 0; N0 = 401)
1−sided adj pval: 0.001
Frequency
Tumor−Normal Expression Difference
hsa−miR−519e−5p, distal
(BMI_extreme = 1; N1 = 12)
1−sided adj pval: 0.221
Frequency
Tumor−Normal Expression Difference

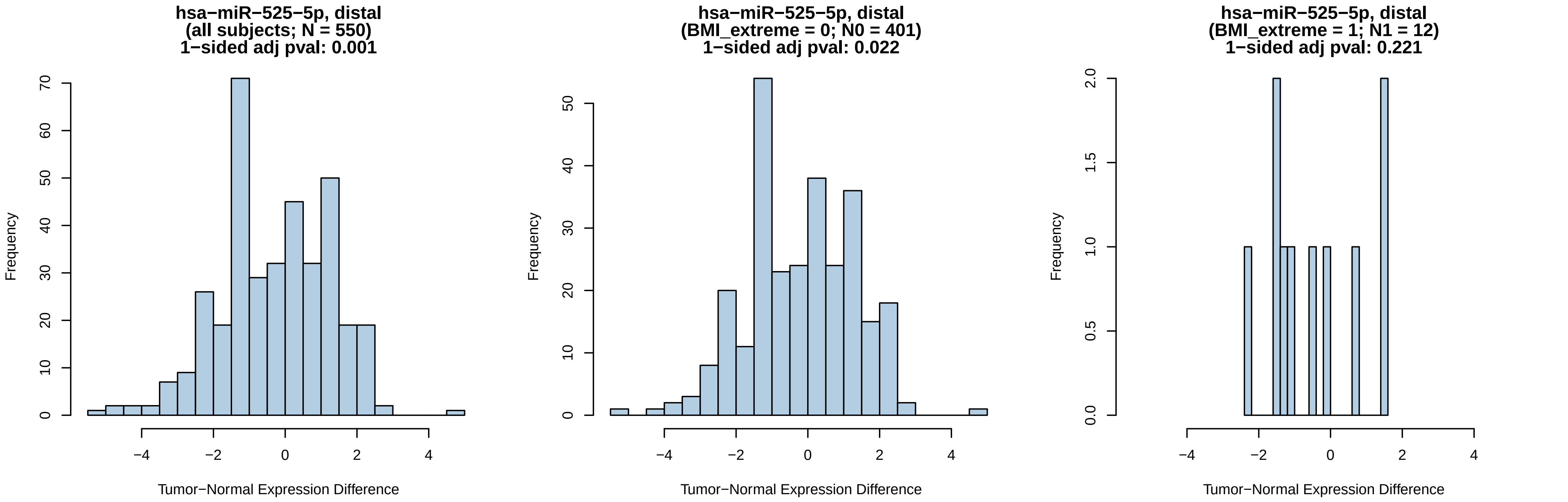
hsa−miR−525−5p, distal
(all subjects; N = 550)
1−sided adj pval: 0.001
hsa−miR−525−5p, distal
(BMI_extreme = 0; N0 = 401)
1−sided adj pval: 0.022
hsa−miR−525−5p, distal
(BMI_extreme = 1; N1 = 12)
1−sided adj pval: 0.221
Frequency
Tumor−Normal Expression Difference

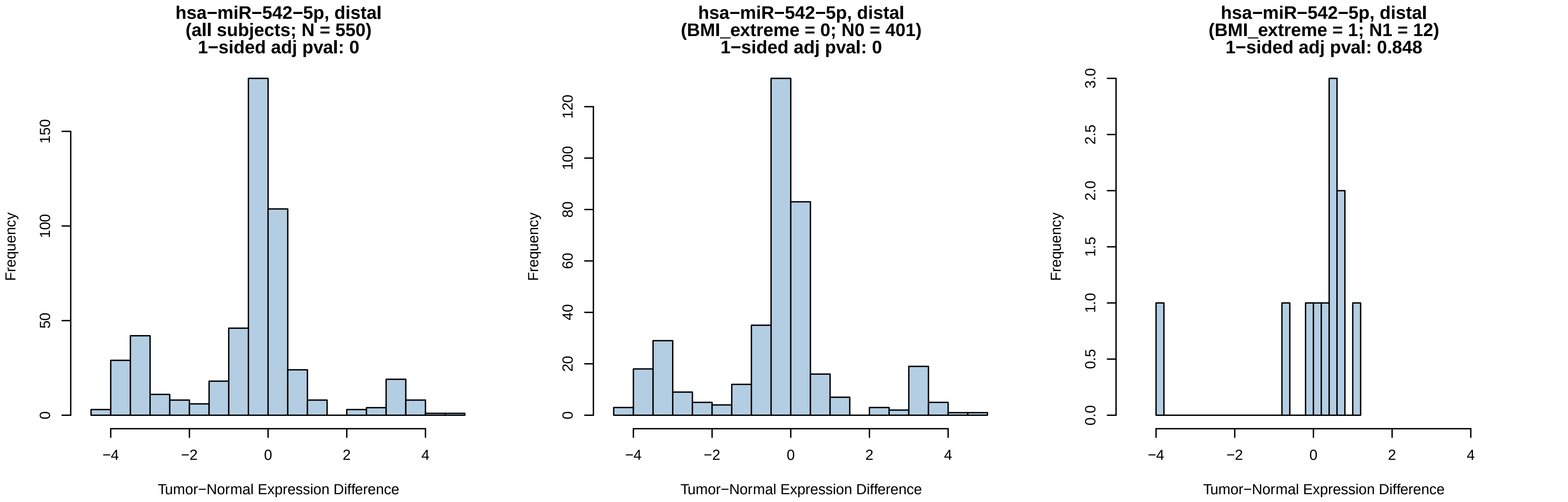

hsa−miR−542−5p, distal
(all subjects; N = 550)
1−sided adj pval: 0
hsa−miR−542−5p, distal
(BMI_extreme = 0; N0 = 401)
1−sided adj pval: 0
hsa−miR−542−5p, distal
(BMI_extreme = 1; N1 = 12)
1−sided adj pval: 0.848
Frequency
Tumor−Normal Expression Difference

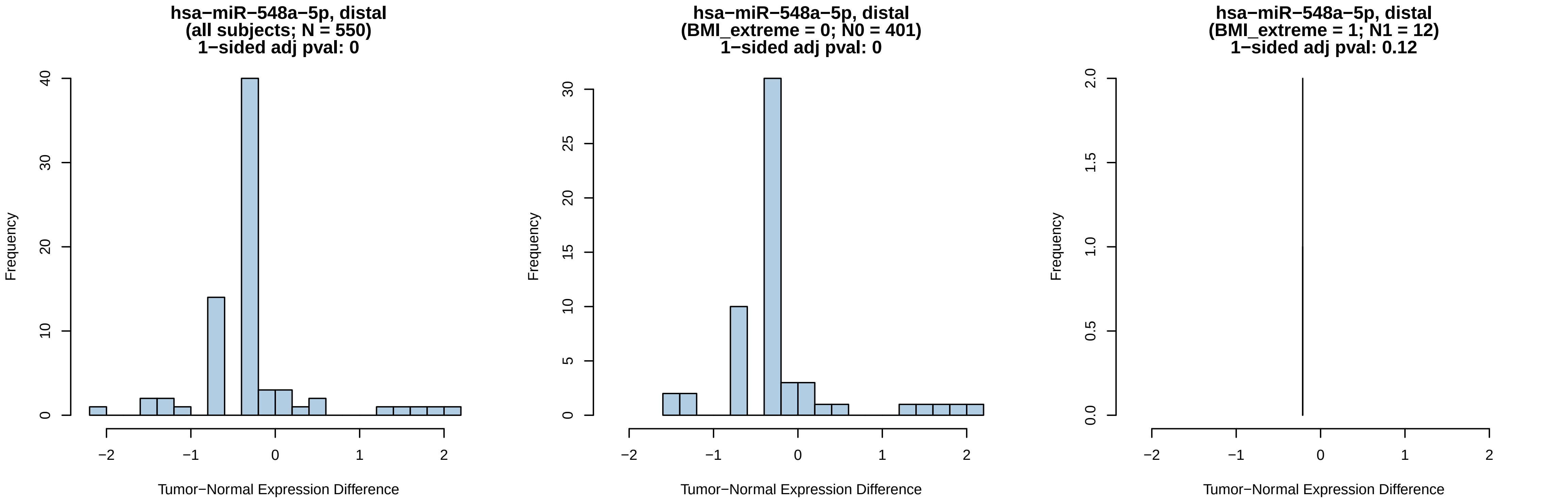
hsa−miR−548a−5p, distal
(all subjects; N = 550)
1−sided adj pval: 0
hsa−miR−548a−5p, distal
(BMI_extreme = 0; N0 = 401)
1−sided adj pval: 0
hsa−miR−548a−5p, distal
(BMI_extreme = 1; N1 = 12)
1−sided adj pval: 0.12
Frequency
Tumor−Normal Expression Difference
0
10
20
30
40
5
15
25
0.0
0.5
1.0
1.5
2.0
−2
−1
1
2

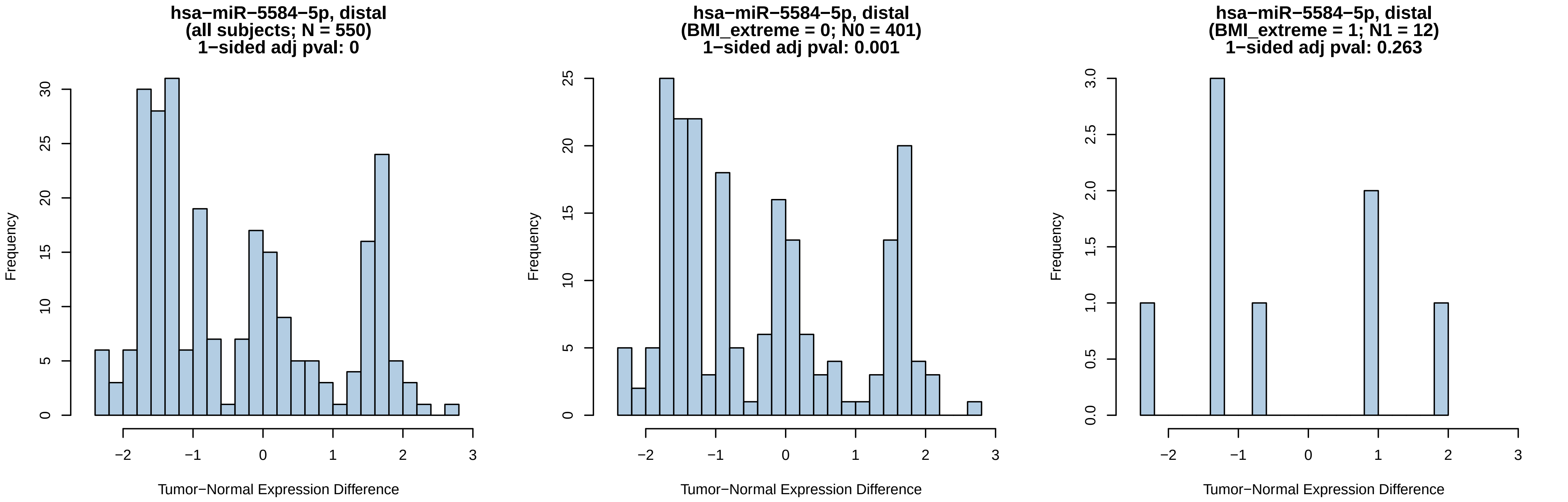
hsa−miR−5584−5p, distal
(all subjects; N = 550)
1−sided adj pval: 0
hsa−miR−5584−5p, distal
(BMI_extreme = 0; N0 = 401)
1−sided adj pval: 0.001
hsa−miR−5584−5p, distal
(BMI_extreme = 1; N1 = 12)
1−sided adj pval: 0.263
Frequency
Tumor−Normal Expression Difference

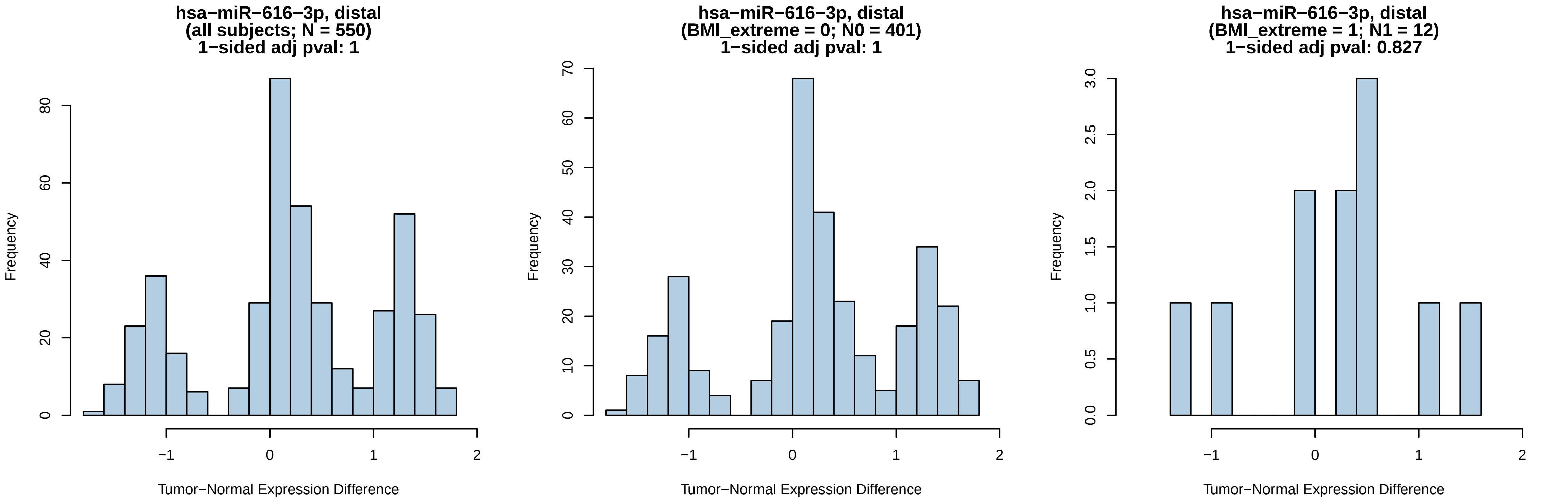
hsa−miR−616−3p, distal
(all subjects; N = 550)
1−sided adj pval: 1
Frequency
Tumor−Normal Expression Difference
hsa−miR−616−3p, distal
(BMI_extreme = 0; N0 = 401)
1−sided adj pval: 1
Frequency
Tumor−Normal Expression Difference
hsa−miR−616−3p, distal
(BMI_extreme = 1; N1 = 12)
1−sided adj pval: 0.827
Frequency
Tumor−Normal Expression Difference

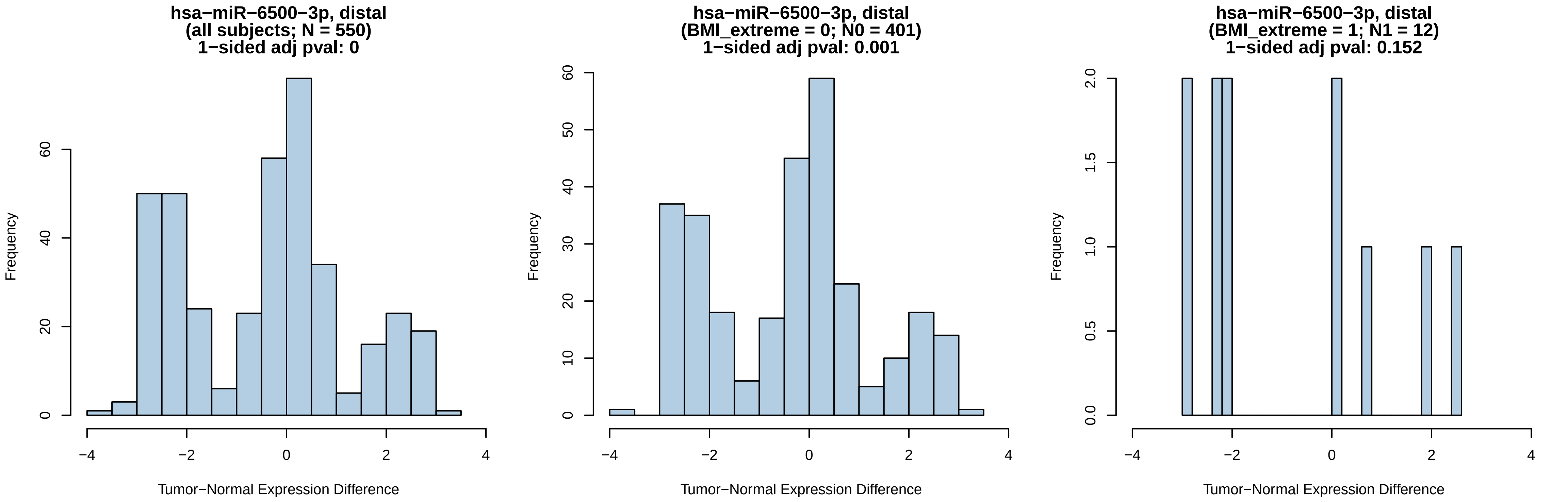
hsa−miR−6500−3p, distal
(all subjects; N = 550)
1−sided adj pval: 0
Frequency
Tumor−Normal Expression Difference
hsa−miR−6500−3p, distal
(BMI_extreme = 0; N0 = 401)
1−sided adj pval: 0.001
Frequency
Tumor−Normal Expression Difference
hsa−miR−6500−3p, distal
(BMI_extreme = 1; N1 = 12)
1−sided adj pval: 0.152
Frequency
Tumor−Normal Expression Difference

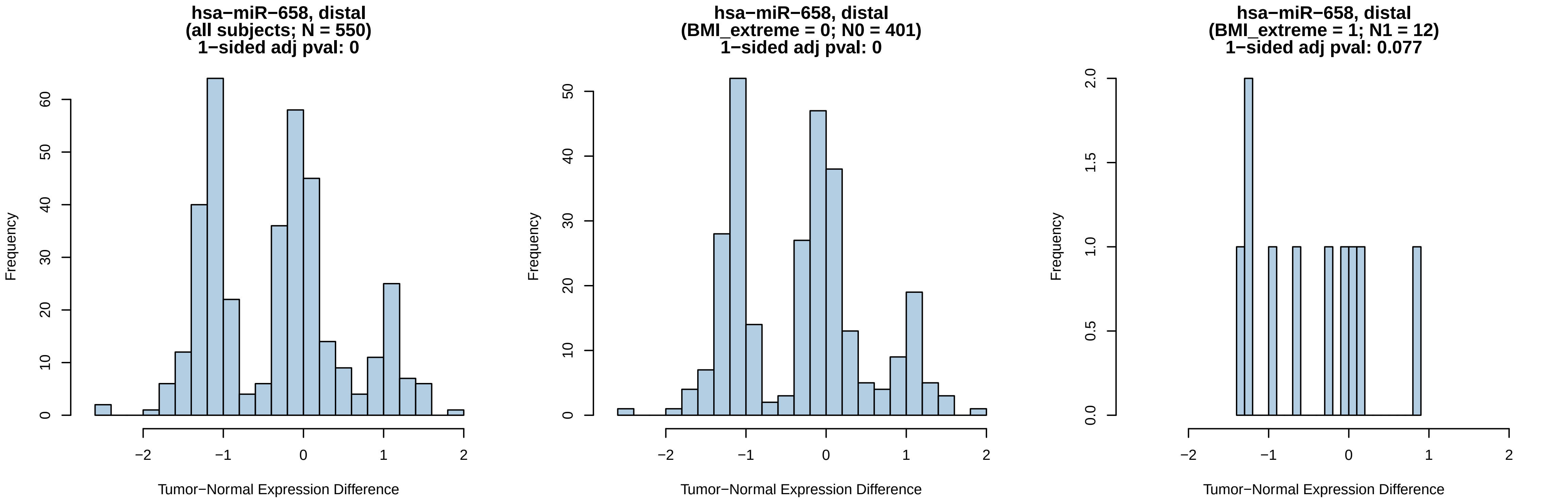
hsa−miR−658, distal
(all subjects; N = 550)
1−sided adj pval: 0
Frequency
Tumor−Normal Expression Difference
hsa−miR−658, distal
(BMI_extreme = 0; N0 = 401)
1−sided adj pval: 0
Frequency
Tumor−Normal Expression Difference
hsa−miR−658, distal
(BMI_extreme = 1; N1 = 12)
1−sided adj pval: 0.077
Frequency
Tumor−Normal Expression Difference

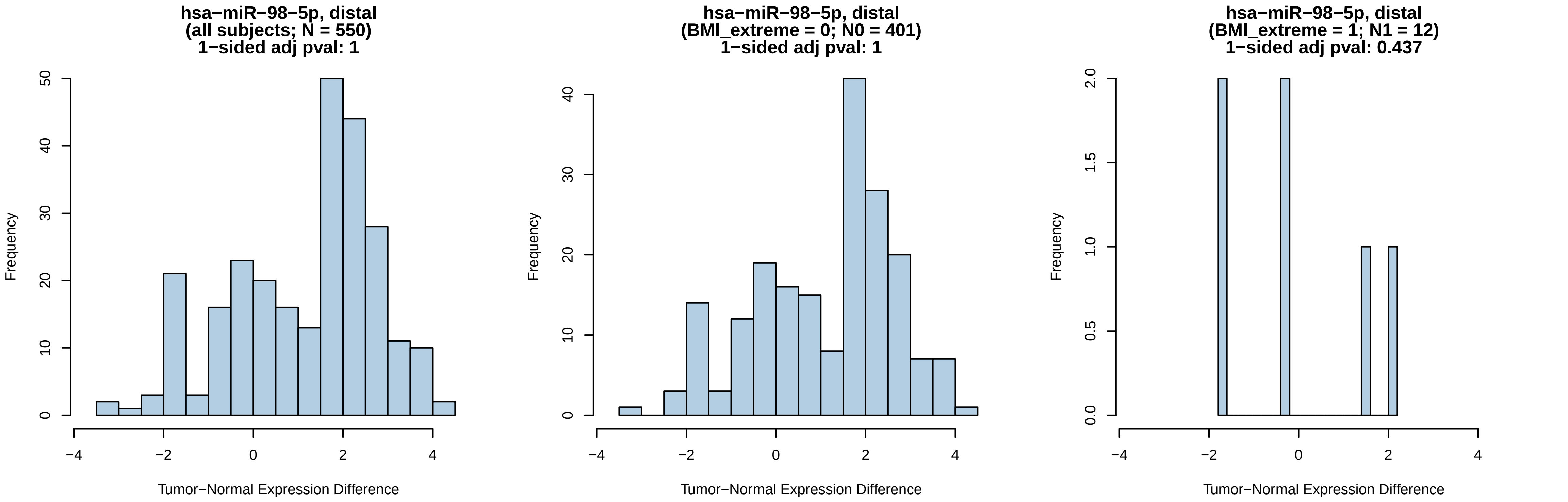
hsa−miR−98−5p, distal
(all subjects; N = 550)
1−sided adj pval: 1
Frequency
Tumor−Normal Expression Difference
hsa−miR−98−5p, distal
(BMI_extreme = 0; N0 = 401)
1−sided adj pval: 1
Frequency
Tumor−Normal Expression Difference
hsa−miR−98−5p, distal
(BMI_extreme = 1; N1 = 12)
1−sided adj pval: 0.437
Frequency
Tumor−Normal Expression Difference

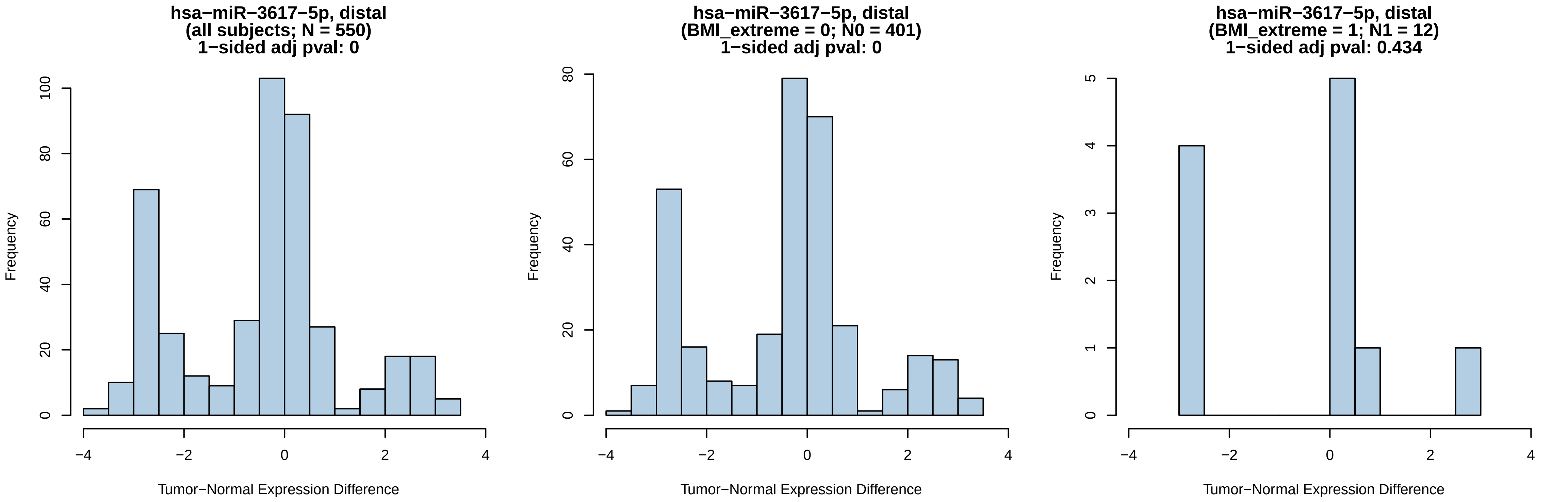

hsa−miR−3617−5p, distal
(all subjects; N = 550)
1−sided adj pval: 0
Frequency
Tumor−Normal Expression Difference
hsa−miR−3617−5p, distal
(BMI_extreme = 0; N0 = 401)
1−sided adj pval: 0
Frequency
Tumor−Normal Expression Difference
hsa−miR−3617−5p, distal
(BMI_extreme = 1; N1 = 12)
1−sided adj pval: 0.434
Frequency
Tumor−Normal Expression Difference

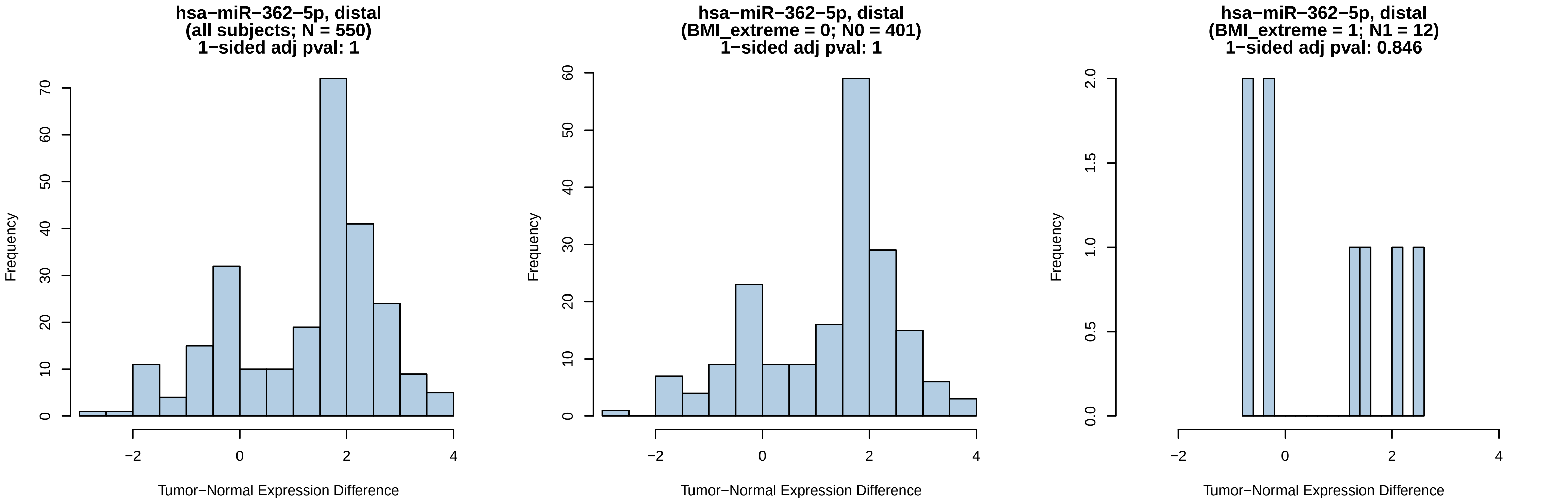
hsa−miR−362−5p, distal
(all subjects; N = 550)
1−sided adj pval: 1
Frequency
Tumor−Normal Expression Difference
hsa−miR−362−5p, distal
(BMI_extreme = 0; N0 = 401)
1−sided adj pval: 1
Frequency
Tumor−Normal Expression Difference
hsa−miR−362−5p, distal
(BMI_extreme = 1; N1 = 12)
1−sided adj pval: 0.846
Frequency
Tumor−Normal Expression Difference

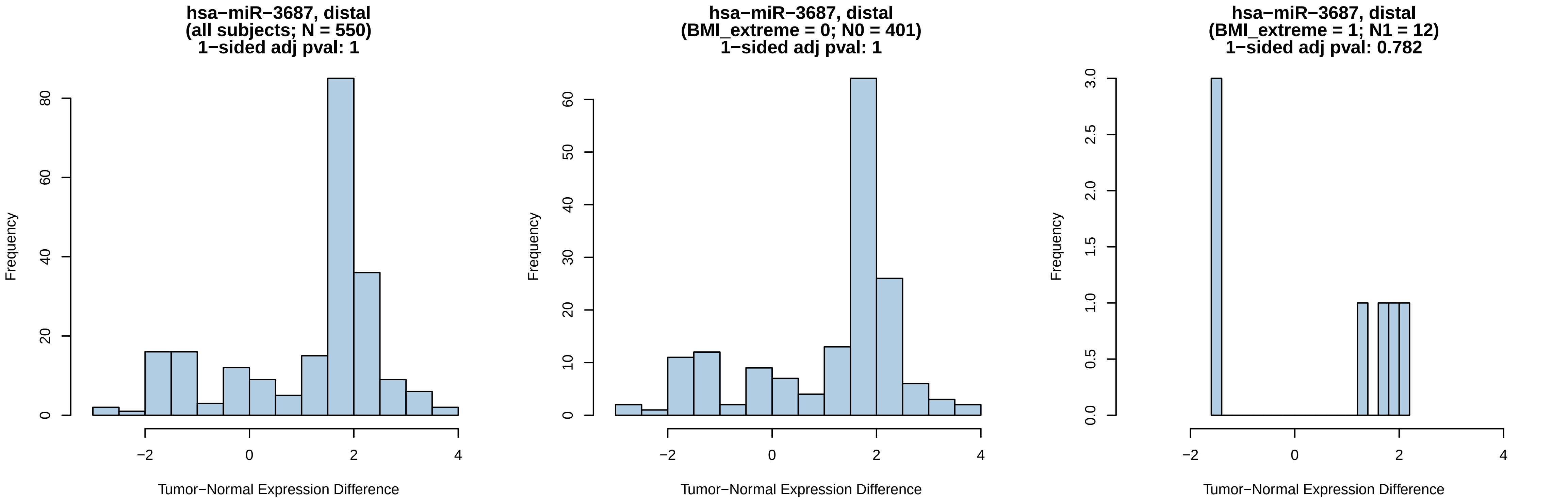
hsa−miR−3687, distal
(all subjects; N = 550)
1−sided adj pval: 1
Frequency
Tumor−Normal Expression Difference
hsa−miR−3687, distal
(BMI_extreme = 0; N0 = 401)
1−sided adj pval: 1
Frequency
Tumor−Normal Expression Difference
hsa−miR−3687, distal
(BMI_extreme = 1; N1 = 12)
1−sided adj pval: 0.782
Frequency
Tumor−Normal Expression Difference

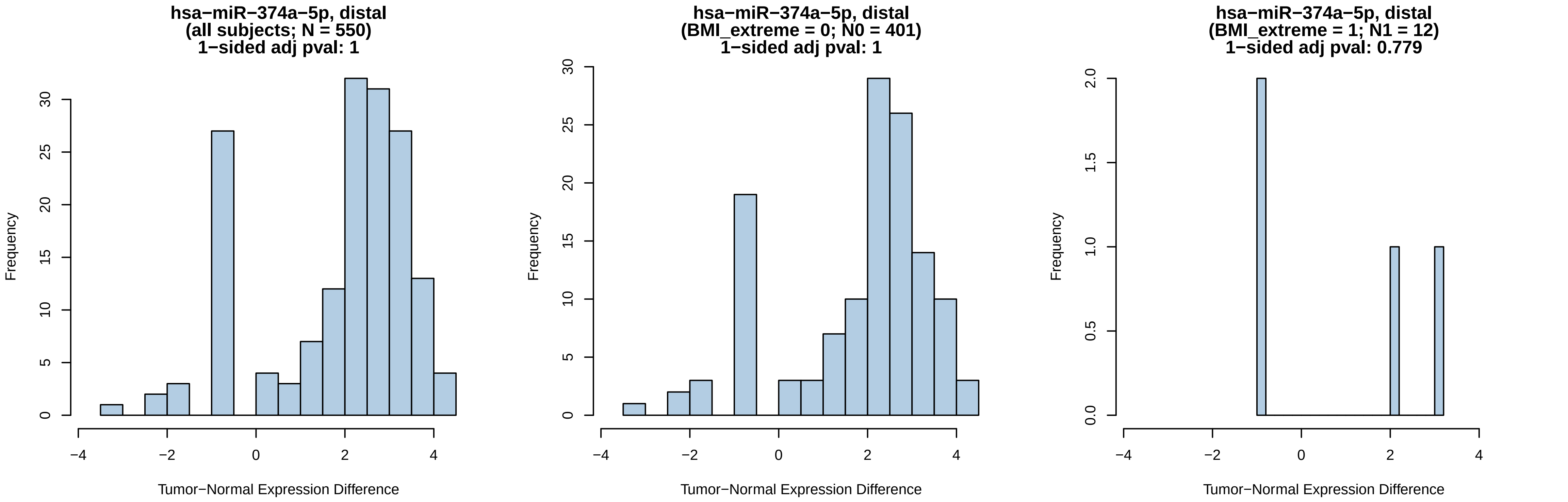

hsa−miR−374a−5p, distal
(all subjects; N = 550)
1−sided adj pval: 1
Frequency
Tumor−Normal Expression Difference
hsa−miR−374a−5p, distal
(BMI_extreme = 0; N0 = 401)
1−sided adj pval: 1
Frequency
Tumor−Normal Expression Difference
hsa−miR−374a−5p, distal
(BMI_extreme = 1; N1 = 12)
1−sided adj pval: 0.779
Frequency
Tumor−Normal Expression Difference

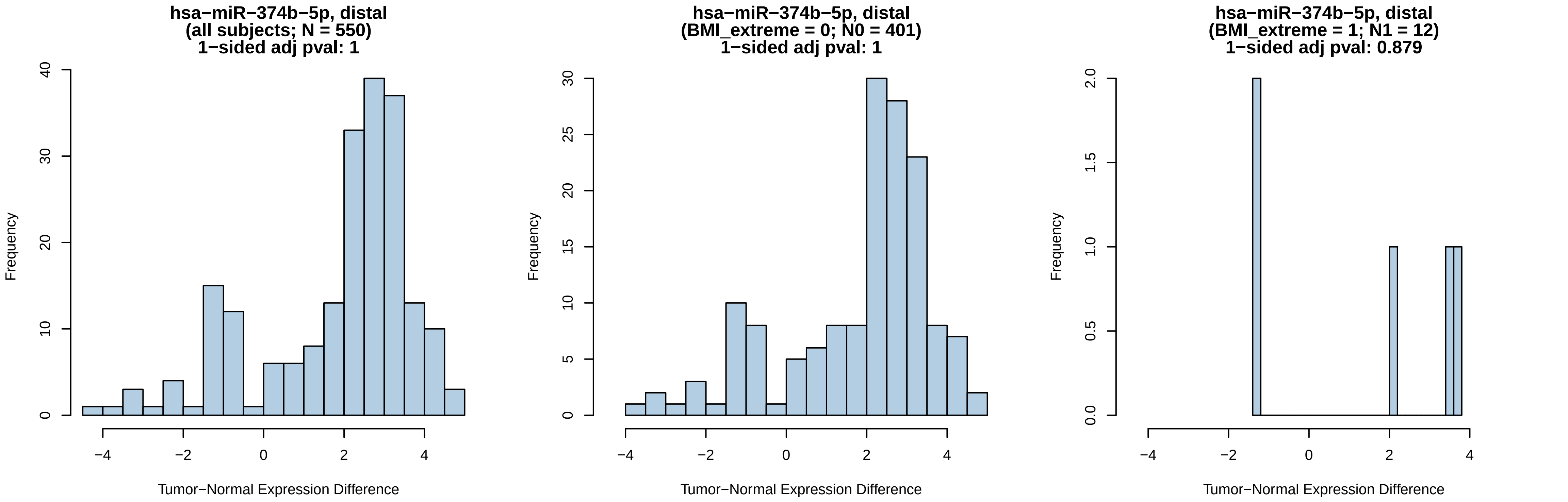

hsa−miR−374b−5p, distal
(all subjects; N = 550)
1−sided adj pval: 1
Frequency
Tumor−Normal Expression Difference
hsa−miR−374b−5p, distal
(BMI_extreme = 0; N0 = 401)
1−sided adj pval: 1
Frequency
Tumor−Normal Expression Difference
hsa−miR−374b−5p, distal
(BMI_extreme = 1; N1 = 12)
1−sided adj pval: 0.879
Frequency
Tumor−Normal Expression Difference

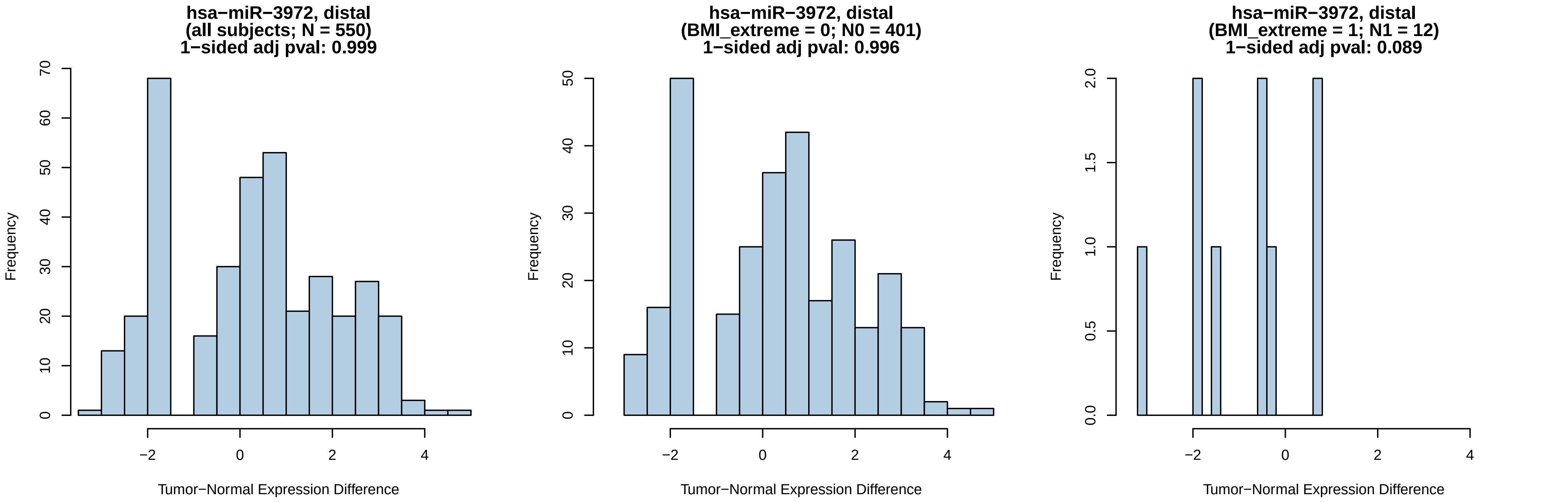

hsa−miR−3972, distal
(all subjects; N = 550)
1−sided adj pval: 0.999
Frequency
Tumor−Normal Expression Difference
hsa−miR−3972, distal
(BMI_extreme = 0; N0 = 401)
1−sided adj pval: 0.996
Frequency
Tumor−Normal Expression Difference
hsa−miR−3972, distal
(BMI_extreme = 1; N1 = 12)
1−sided adj pval: 0.089
Frequency
Tumor−Normal Expression Difference

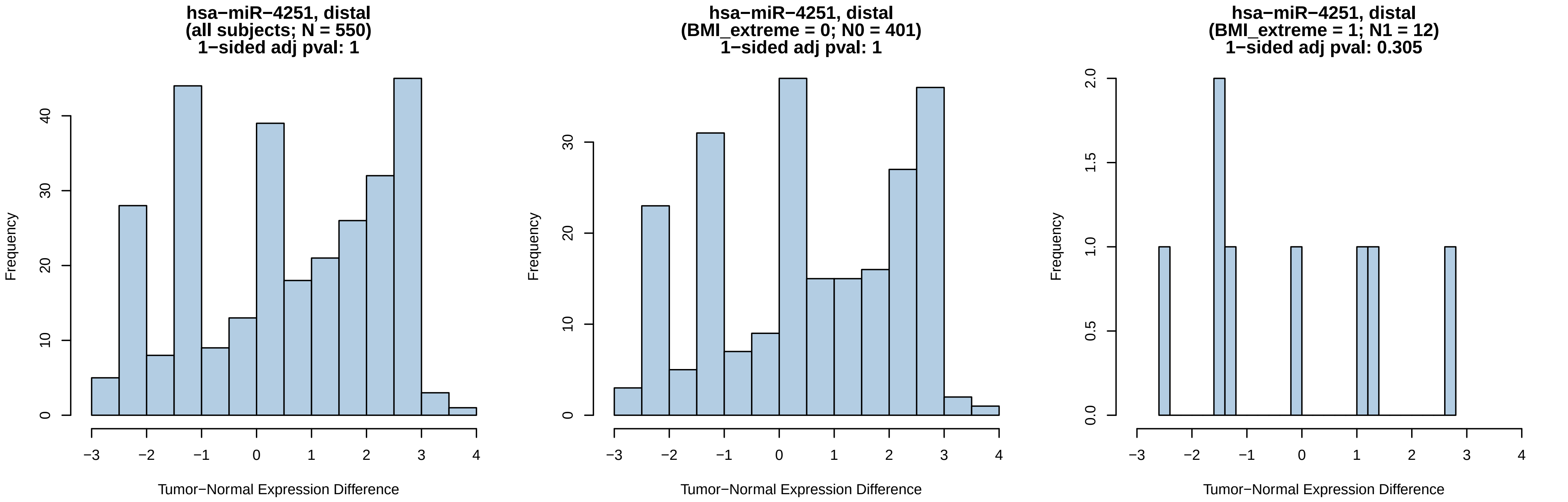

hsa−miR−4251, distal
(all subjects; N = 550)
1−sided adj pval: 1
Frequency
0
10
20
30
40
−3
−2
−1
0
1
2
3
4
Tumor−Normal Expression Difference
hsa−miR−4251, distal
(BMI_extreme = 0; N0 = 401)
1−sided adj pval: 1
Frequency
0
10
20
30
−3
−2
−1
0
1
2
3
4
Tumor−Normal Expression Difference
hsa−miR−4251, distal
(BMI_extreme = 1; N1 = 12)
1−sided adj pval: 0.305
Frequency
0.0
0.5
1.0
1.5
2.0
−3
−2
−1
0
1
2
3
4
Tumor−Normal Expression Difference

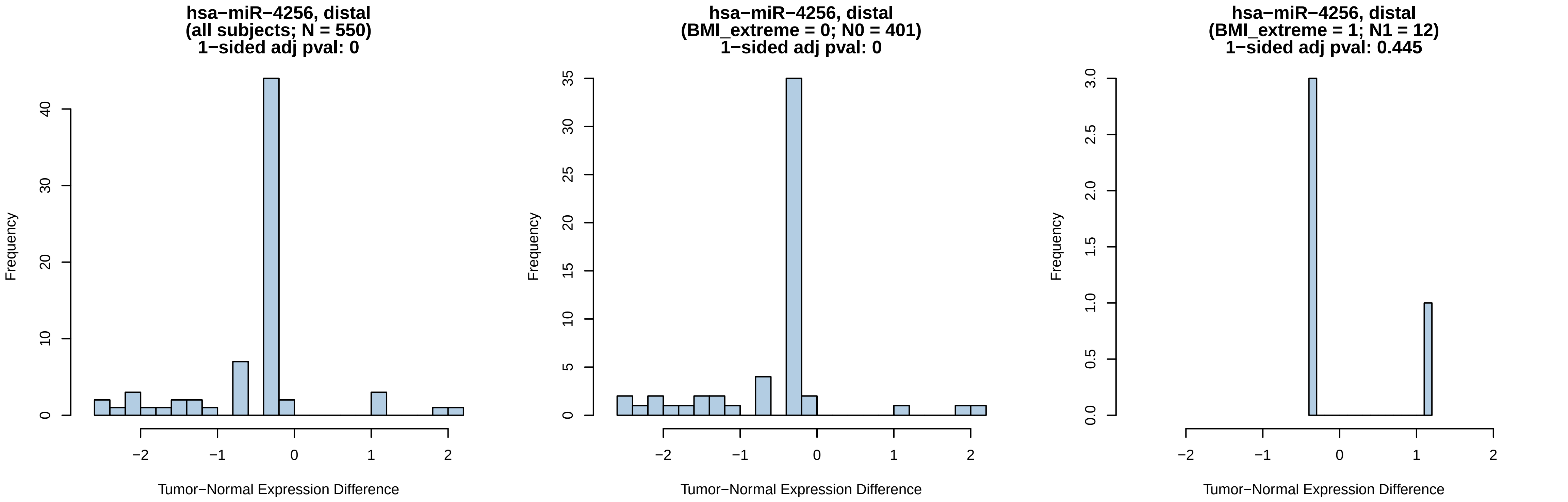
hsa−miR−4256, distal
(all subjects; N = 550)
1−sided adj pval: 0
Frequency
Tumor−Normal Expression Difference
hsa−miR−4256, distal
(BMI_extreme = 0; N0 = 401)
1−sided adj pval: 0
Frequency
Tumor−Normal Expression Difference
hsa−miR−4256, distal
(BMI_extreme = 1; N1 = 12)
1−sided adj pval: 0.445
Frequency
Tumor−Normal Expression Difference

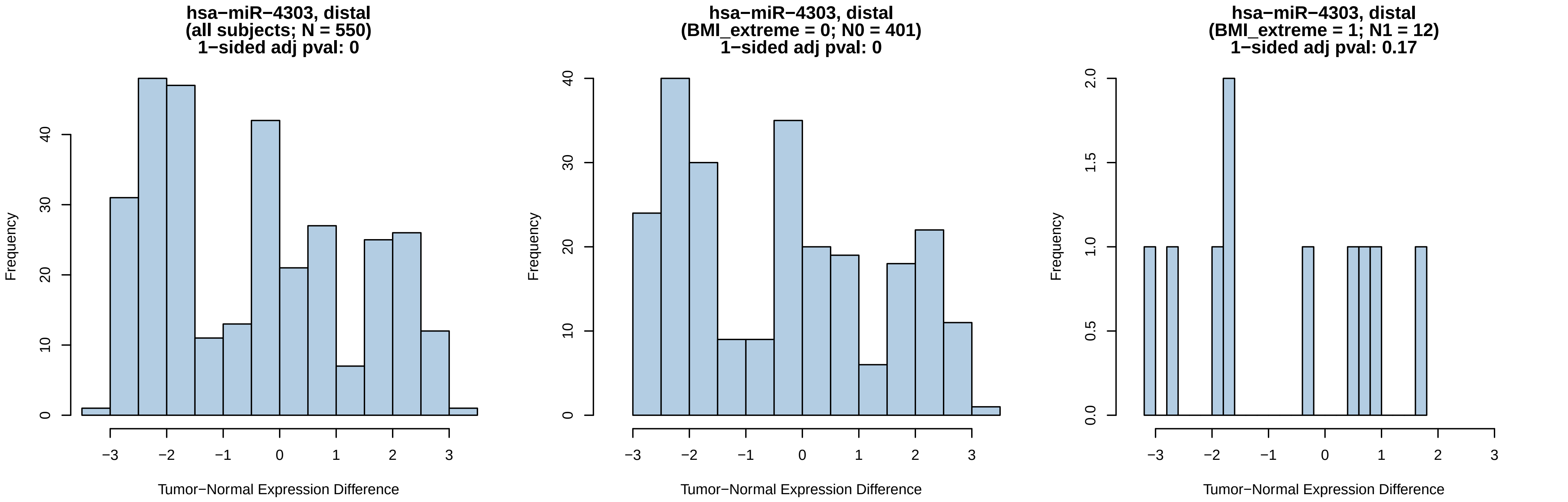
hsa−miR−4303, distal
(all subjects; N = 550)
1−sided adj pval: 0
Frequency
Tumor−Normal Expression Difference
hsa−miR−4303, distal
(BMI_extreme = 0; N0 = 401)
1−sided adj pval: 0
Frequency
Tumor−Normal Expression Difference
hsa−miR−4303, distal
(BMI_extreme = 1; N1 = 12)
1−sided adj pval: 0.17
Frequency
Tumor−Normal Expression Difference

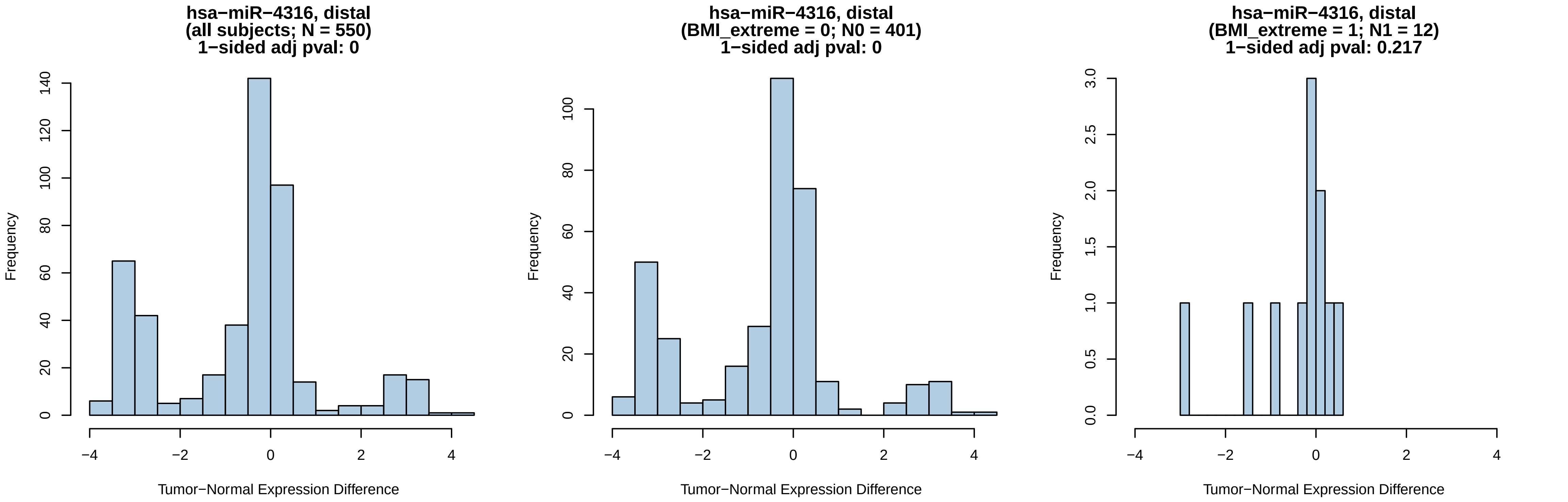

hsa−miR−4316, distal
(all subjects; N = 550)
1−sided adj pval: 0
Frequency
0
20
40
60
80
100
120
140
−4
−2
0
2
4
Tumor−Normal Expression Difference
hsa−miR−4316, distal
(BMI_extreme = 0; N0 = 401)
1−sided adj pval: 0
Frequency
0
20
40
60
80
100
−4
−2
0
2
4
Tumor−Normal Expression Difference
hsa−miR−4316, distal
(BMI_extreme = 1; N1 = 12)
1−sided adj pval: 0.217
Frequency
0.0
0.5
1.0
1.5
2.0
2.5
3.0
−4
−2
0
2
4
Tumor−Normal Expression Difference

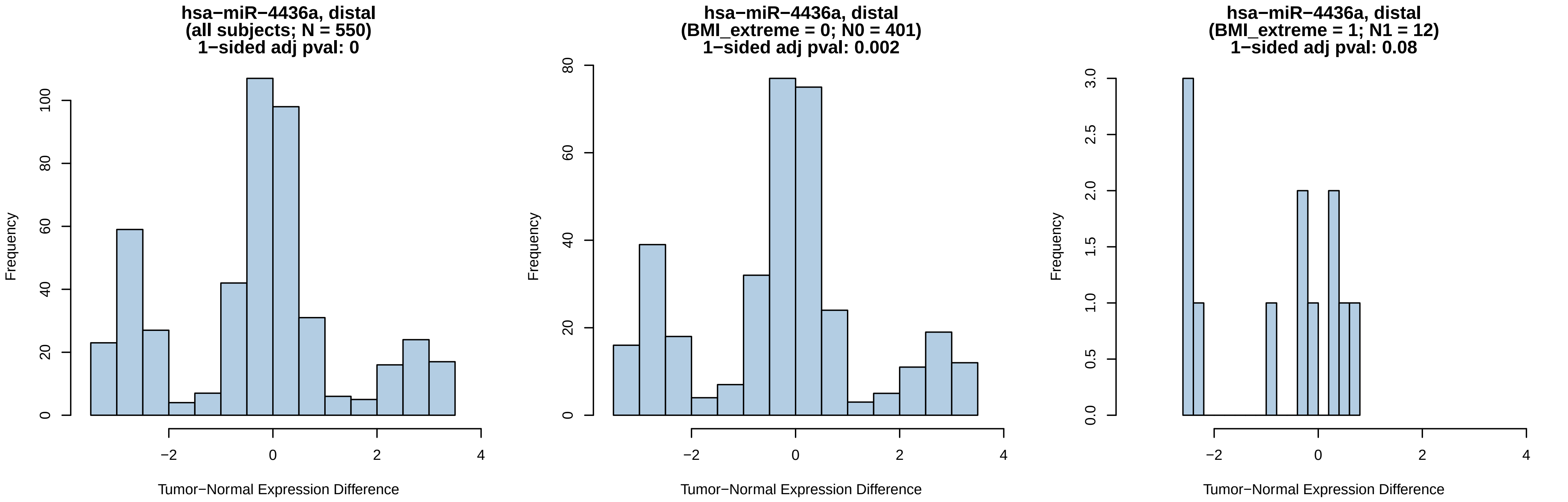
hsa−miR−4436a, distal
(all subjects; N = 550)
1−sided adj pval: 0
Frequency
Tumor−Normal Expression Difference
hsa−miR−4436a, distal
(BMI_extreme = 0; N0 = 401)
1−sided adj pval: 0.002
Frequency
Tumor−Normal Expression Difference
hsa−miR−4436a, distal
(BMI_extreme = 1; N1 = 12)
1−sided adj pval: 0.08
Frequency
Tumor−Normal Expression Difference

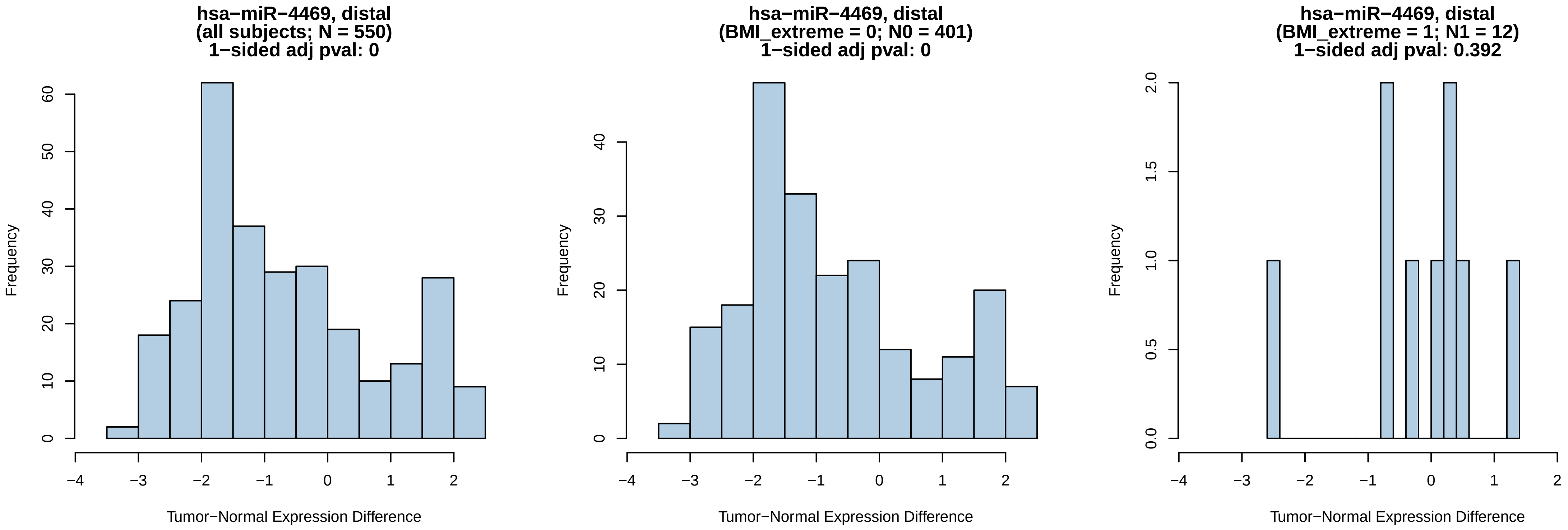

hsa−miR−4469, distal
(all subjects; N = 550)
1−sided adj pval: 0
Frequency
0
10
20
30
40
50
60
−4
−3
−2
−1
0
1
2
Tumor−Normal Expression Difference
hsa−miR−4469, distal
(BMI_extreme = 0; N0 = 401)
1−sided adj pval: 0
Frequency
0
10
20
30
40
−4
−3
−2
−1
0
1
2
Tumor−Normal Expression Difference
hsa−miR−4469, distal
(BMI_extreme = 1; N1 = 12)
1−sided adj pval: 0.392
Frequency
0.0
0.5
1.0
1.5
2.0
−4
−3
−2
−1
0
1
2
Tumor−Normal Expression Difference

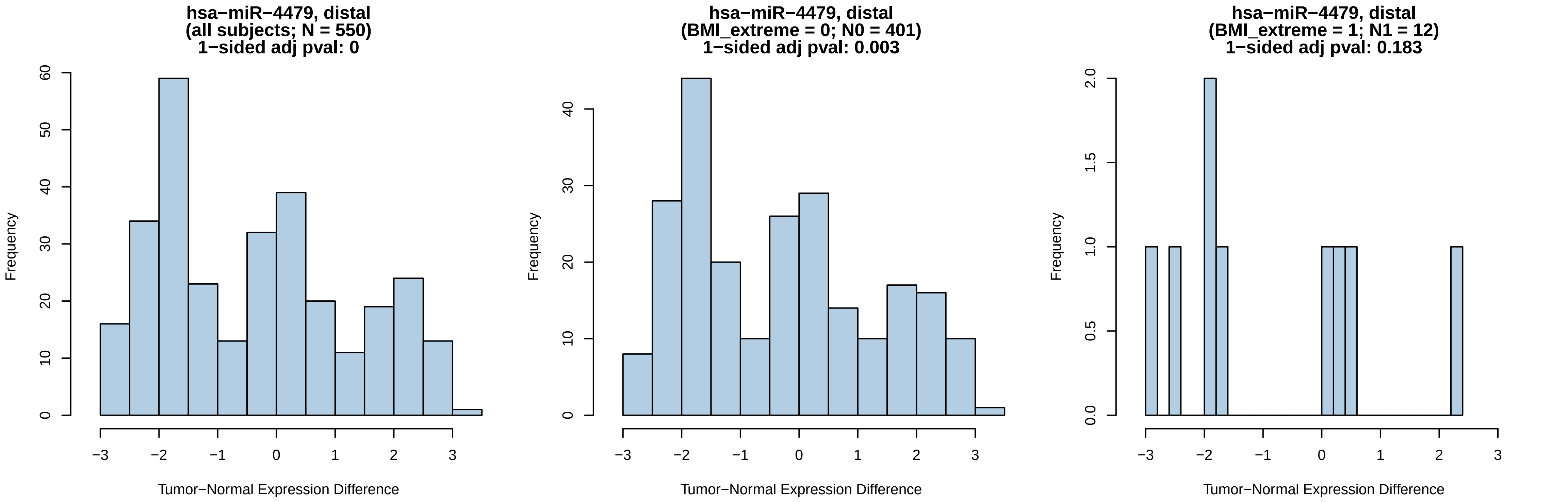
hsa−miR−4479, distal
(all subjects; N = 550)
1−sided adj pval: 0
Frequency
Tumor−Normal Expression Difference
hsa−miR−4479, distal
(BMI_extreme = 0; N0 = 401)
1−sided adj pval: 0.003
Frequency
Tumor−Normal Expression Difference
hsa−miR−4479, distal
(BMI_extreme = 1; N1 = 12)
1−sided adj pval: 0.183
Frequency
Tumor−Normal Expression Difference

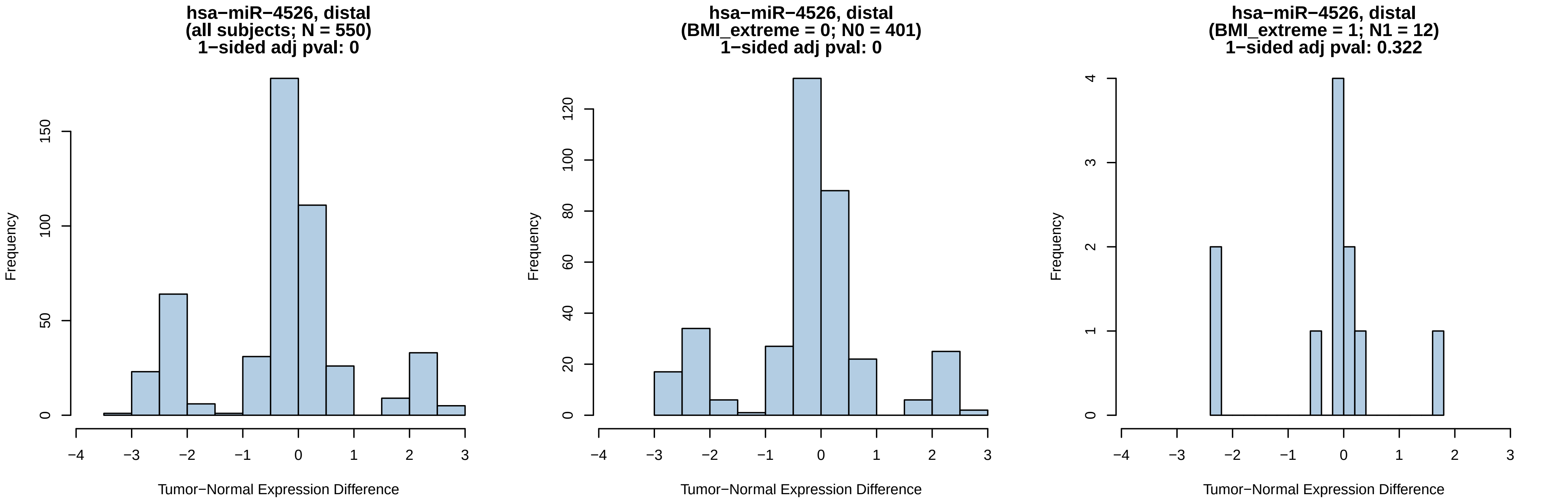
hsa−miR−4526, distal
(all subjects; N = 550)
1−sided adj pval: 0
Frequency
Tumor−Normal Expression Difference
hsa−miR−4526, distal
(BMI_extreme = 0; N0 = 401)
1−sided adj pval: 0
Frequency
Tumor−Normal Expression Difference
hsa−miR−4526, distal
(BMI_extreme = 1; N1 = 12)
1−sided adj pval: 0.322
Frequency
Tumor−Normal Expression Difference

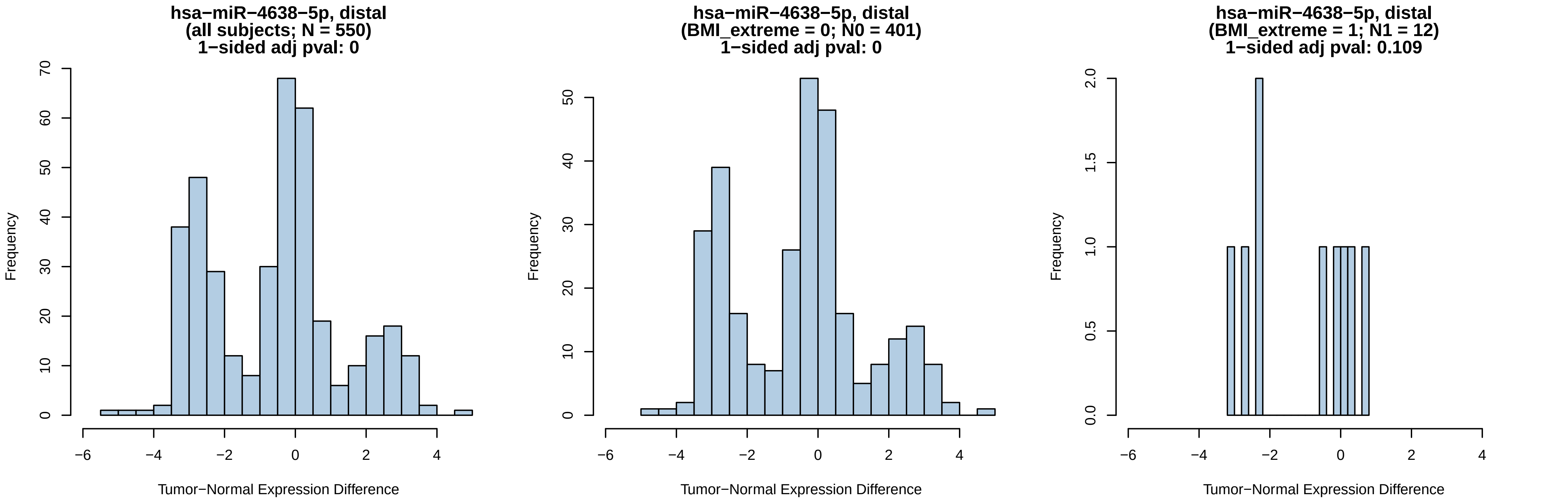
hsa−miR−4638−5p, distal
(all subjects; N = 550)
1−sided adj pval: 0
Frequency
Tumor−Normal Expression Difference
hsa−miR−4638−5p, distal
(BMI_extreme = 0; N0 = 401)
1−sided adj pval: 0
Frequency
Tumor−Normal Expression Difference
hsa−miR−4638−5p, distal
(BMI_extreme = 1; N1 = 12)
1−sided adj pval: 0.109
Frequency
Tumor−Normal Expression Difference

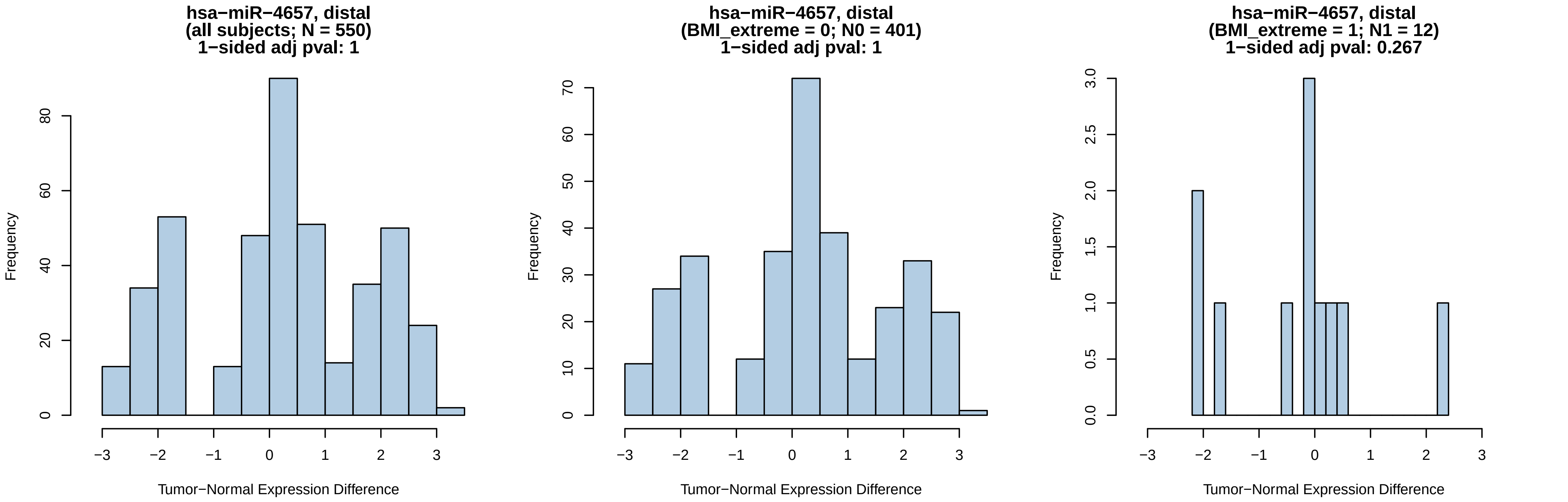

hsa−miR−4657, distal
(all subjects; N = 550)
1−sided adj pval: 1
Frequency
0
20
40
60
80
−3
−2
−1
0
1
2
3
Tumor−Normal Expression Difference
hsa−miR−4657, distal
(BMI_extreme = 0; N0 = 401)
1−sided adj pval: 1
Frequency
0
10
20
30
40
50
60
70
−3
−2
−1
0
1
2
3
Tumor−Normal Expression Difference
hsa−miR−4657, distal
(BMI_extreme = 1; N1 = 12)
1−sided adj pval: 0.267
Frequency
0.0
0.5
1.0
1.5
2.0
2.5
3.0
−3
−2
−1
0
1
2
3
Tumor−Normal Expression Difference

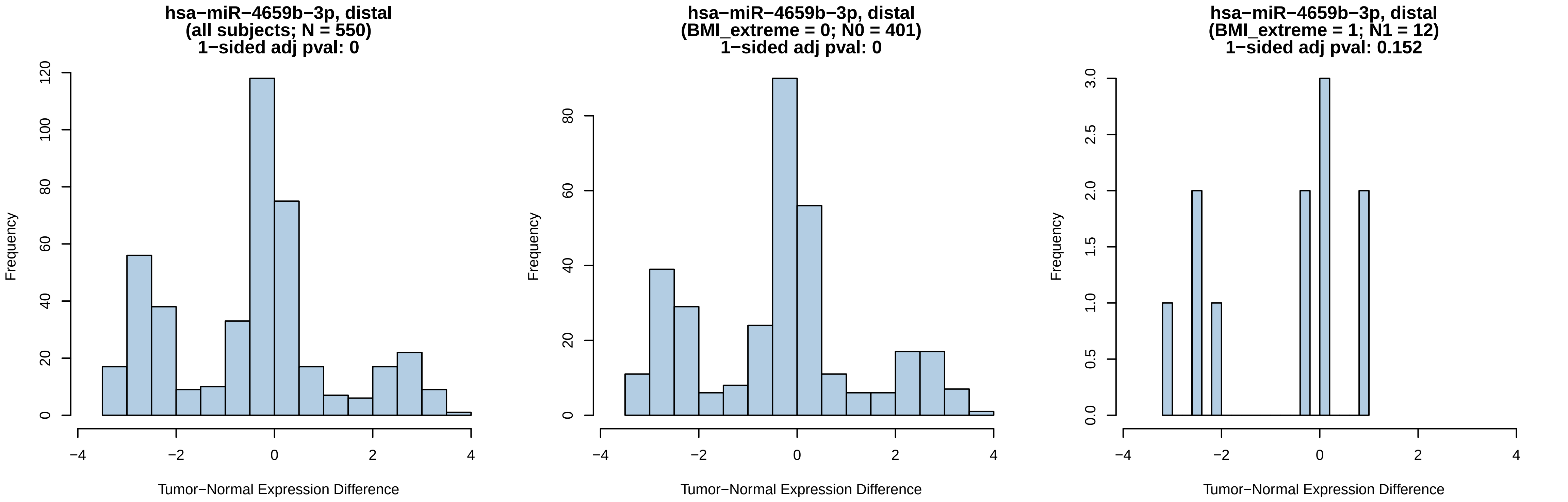
hsa−miR−4659b−3p, distal
(all subjects; N = 550)
1−sided adj pval: 0
hsa−miR−4659b−3p, distal
(BMI_extreme = 0; N0 = 401)
1−sided adj pval: 0
hsa−miR−4659b−3p, distal
(BMI_extreme = 1; N1 = 12)
1−sided adj pval: 0.152
Frequency
Tumor−Normal Expression Difference
0
20
40
60
80
100
120
0.0
0.5
1.0
1.5
2.0
2.5
3.0
−4
−2
0
2
4

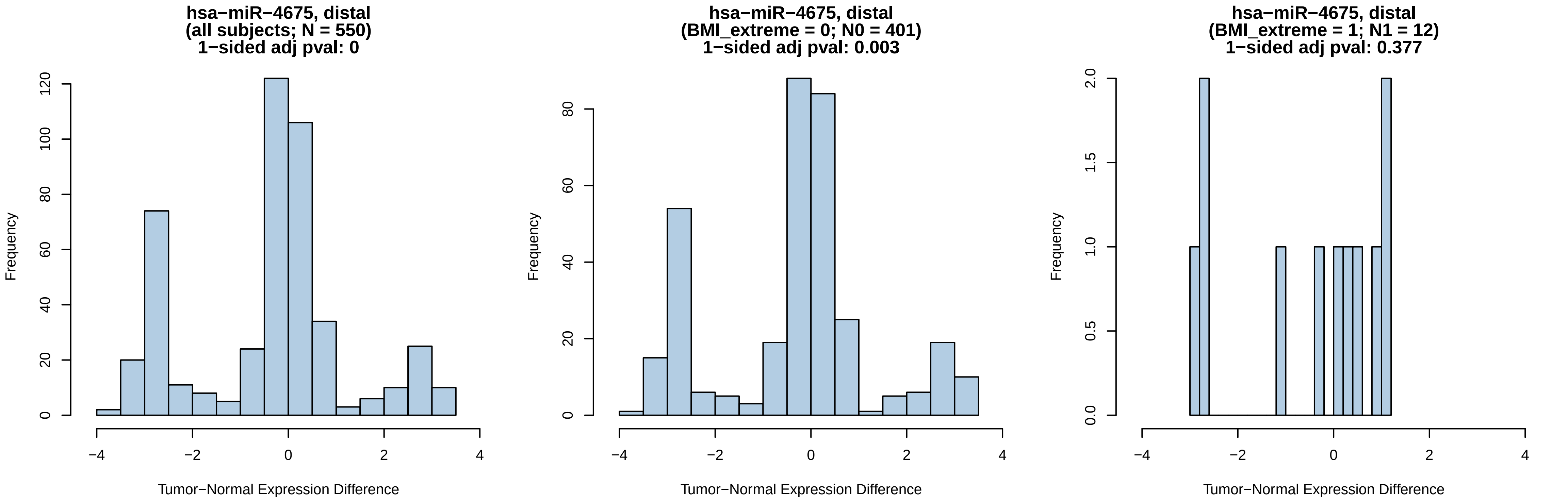
hsa−miR−4675, distal
(all subjects; N = 550)
1−sided adj pval: 0
Frequency
0
20
40
60
80
100
120
−4
−2
0
2
4
Tumor−Normal Expression Difference
hsa−miR−4675, distal
(BMI_extreme = 0; N0 = 401)
1−sided adj pval: 0.003
Frequency
0
20
40
60
80
−4
−2
0
2
4
Tumor−Normal Expression Difference
hsa−miR−4675, distal
(BMI_extreme = 1; N1 = 12)
1−sided adj pval: 0.377
Frequency
0.0
0.5
1.0
1.5
2.0
−4
−2
0
2
4
Tumor−Normal Expression Difference

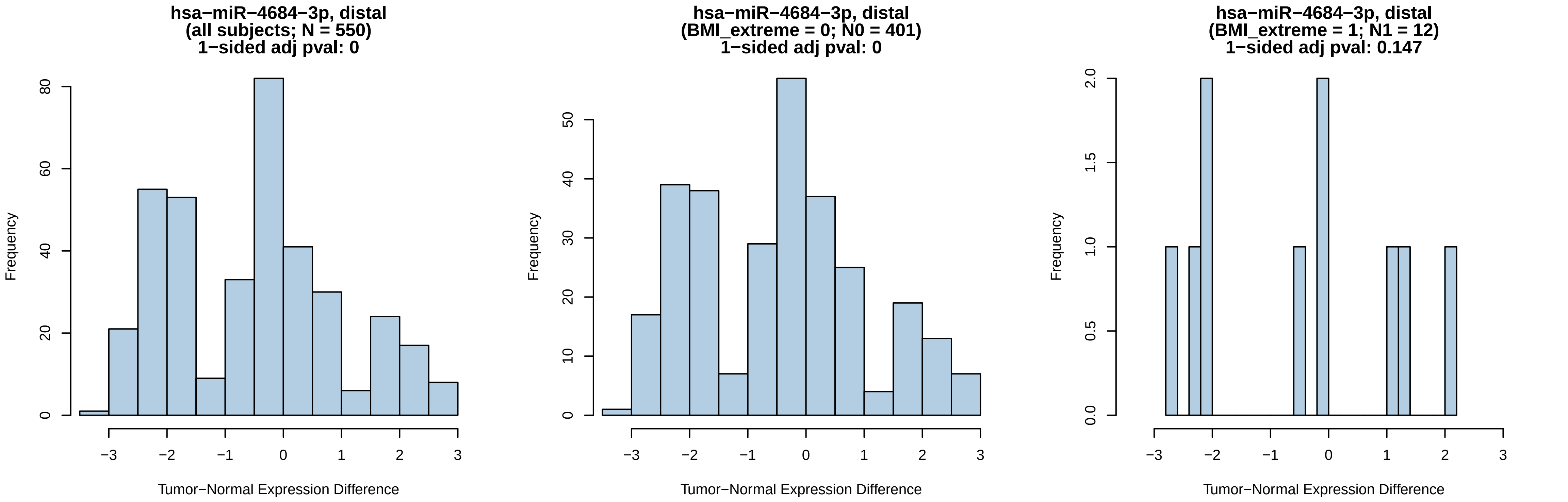

hsa−miR−4684−3p, distal
(all subjects; N = 550)
1−sided adj pval: 0
Frequency
Tumor−Normal Expression Difference
hsa−miR−4684−3p, distal
(BMI_extreme = 0; N0 = 401)
1−sided adj pval: 0
Frequency
Tumor−Normal Expression Difference
hsa−miR−4684−3p, distal
(BMI_extreme = 1; N1 = 12)
1−sided adj pval: 0.147
Frequency
Tumor−Normal Expression Difference

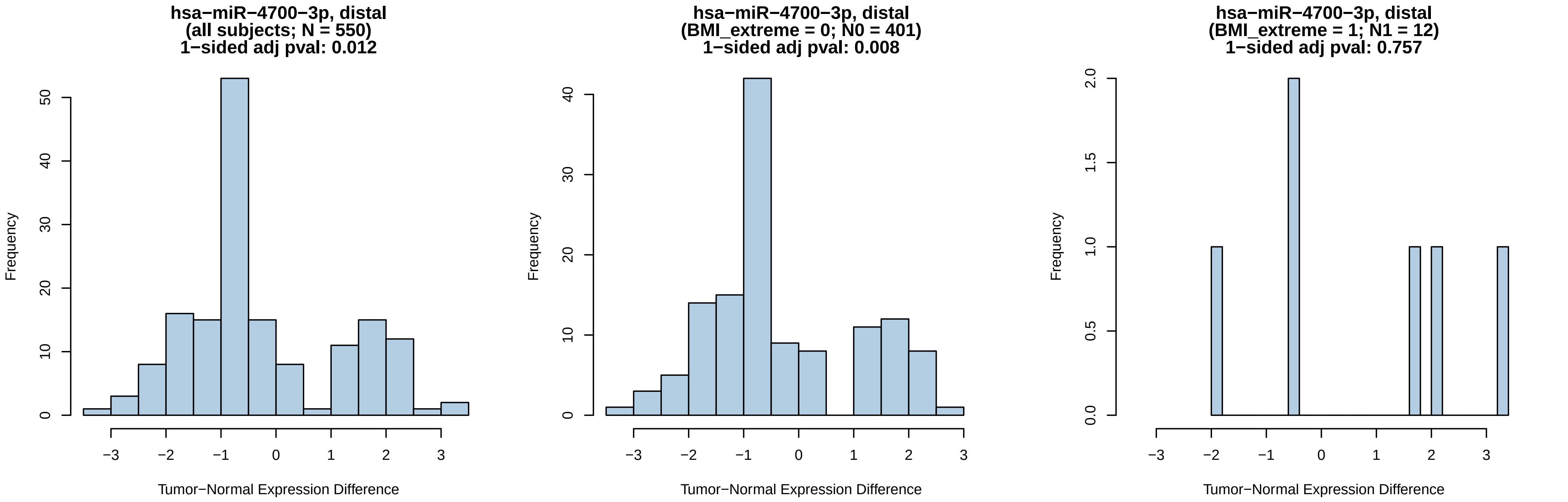
hsa−miR−4700−3p, distal
(all subjects; N = 550)
1−sided adj pval: 0.012
Frequency
Tumor−Normal Expression Difference
hsa−miR−4700−3p, distal
(BMI_extreme = 0; N0 = 401)
1−sided adj pval: 0.008
Frequency
Tumor−Normal Expression Difference
hsa−miR−4700−3p, distal
(BMI_extreme = 1; N1 = 12)
1−sided adj pval: 0.757
Frequency
Tumor−Normal Expression Difference

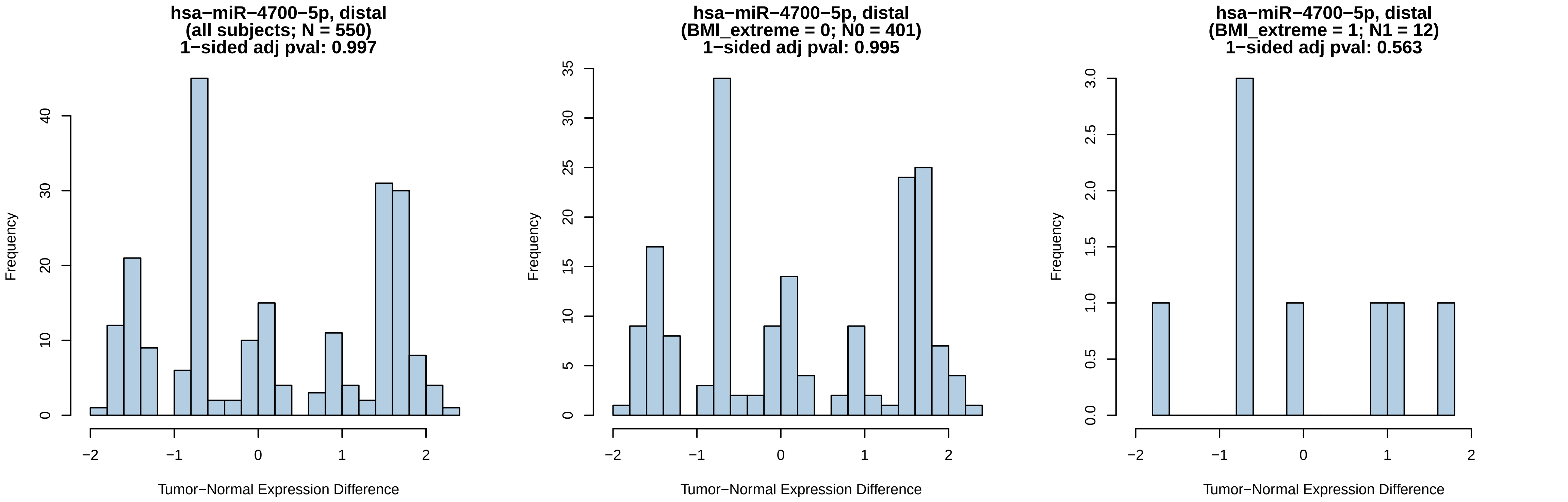
hsa−miR−4700−5p, distal
(all subjects; N = 550)
1−sided adj pval: 0.997
Frequency
Tumor−Normal Expression Difference
hsa−miR−4700−5p, distal
(BMI_extreme = 0; N0 = 401)
1−sided adj pval: 0.995
Frequency
Tumor−Normal Expression Difference
hsa−miR−4700−5p, distal
(BMI_extreme = 1; N1 = 12)
1−sided adj pval: 0.563
Frequency
Tumor−Normal Expression Difference

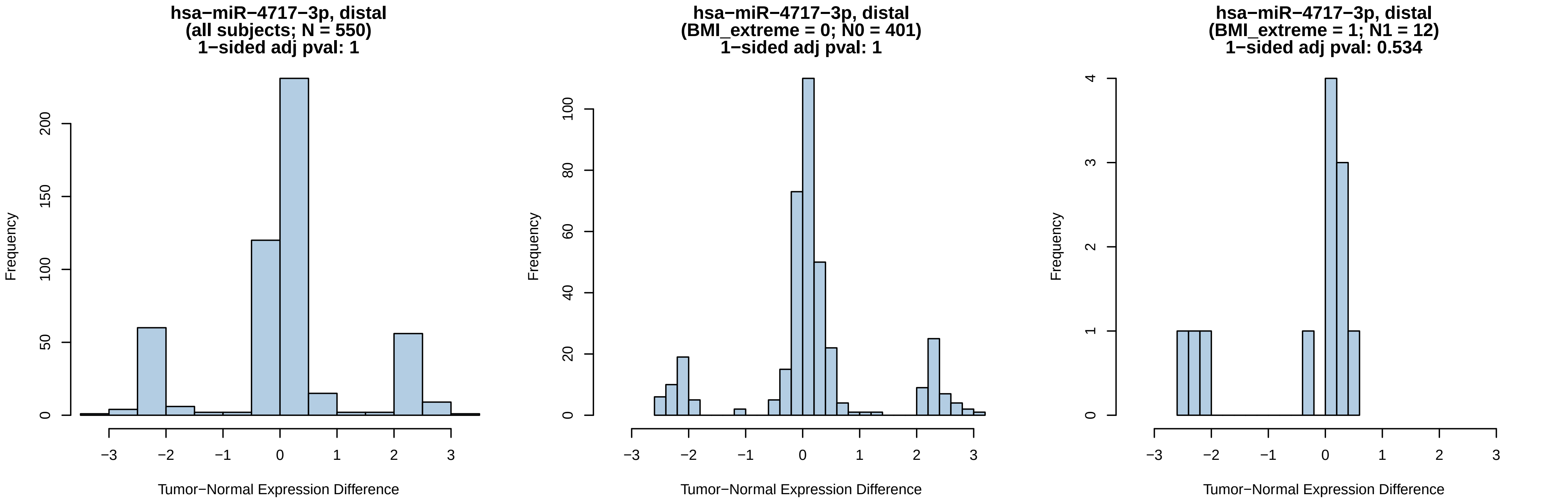

hsa−miR−4717−3p, distal
(all subjects; N = 550)
1−sided adj pval: 1
Frequency
Tumor−Normal Expression Difference
hsa−miR−4717−3p, distal
(BMI_extreme = 0; N0 = 401)
1−sided adj pval: 1
Frequency
Tumor−Normal Expression Difference
hsa−miR−4717−3p, distal
(BMI_extreme = 1; N1 = 12)
1−sided adj pval: 0.534
Frequency
Tumor−Normal Expression Difference

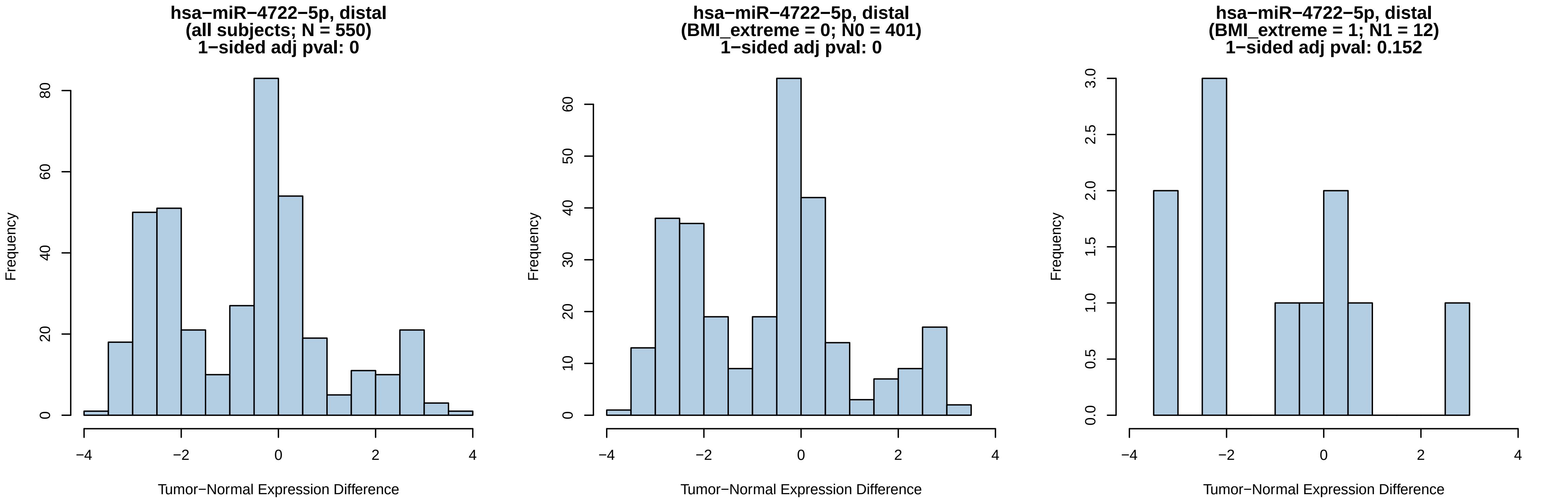
hsa−miR−4722−5p, distal
(all subjects; N = 550)
1−sided adj pval: 0
Frequency
Tumor−Normal Expression Difference
hsa−miR−4722−5p, distal
(BMI_extreme = 0; N0 = 401)
1−sided adj pval: 0
Frequency
Tumor−Normal Expression Difference
hsa−miR−4722−5p, distal
(BMI_extreme = 1; N1 = 12)
1−sided adj pval: 0.152
Frequency
Tumor−Normal Expression Difference

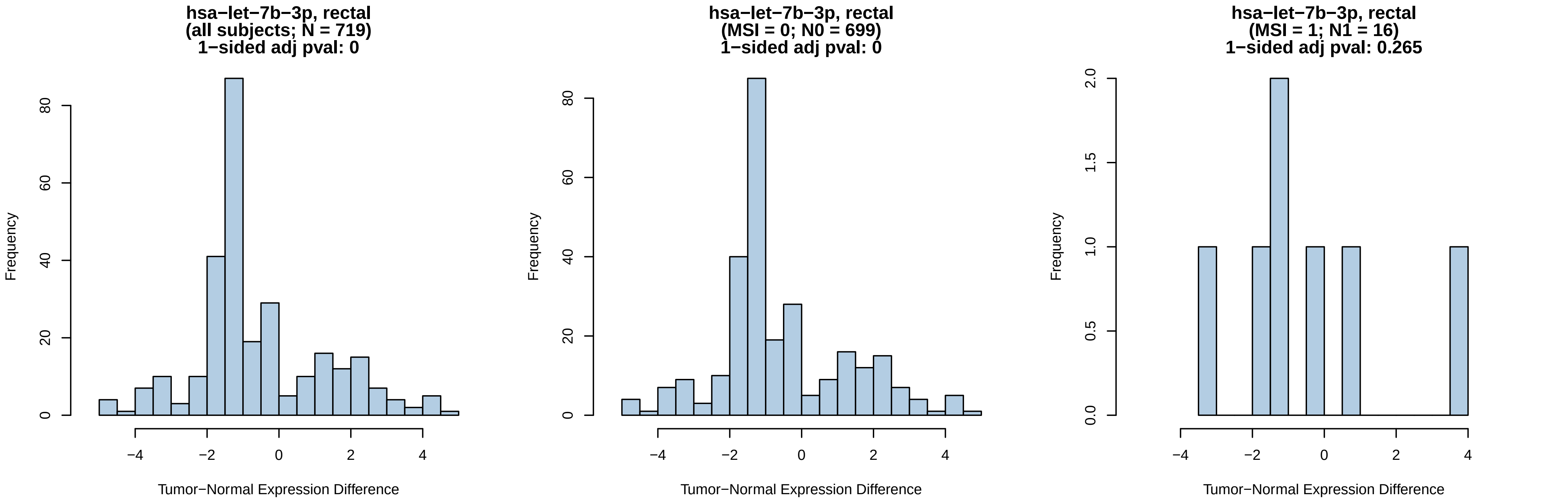
hsa−let−7b−3p, rectal
(all subjects; N = 719)
1−sided adj pval: 0
hsa−let−7b−3p, rectal
(MSI = 0; N0 = 699)
1−sided adj pval: 0
hsa−let−7b−3p, rectal
(MSI = 1; N1 = 16)
1−sided adj pval: 0.265
Frequency
Tumor−Normal Expression Difference
0
20
40
60
80
0.0
0.5
1.0
1.5
2.0
−4
−2
0
2
4

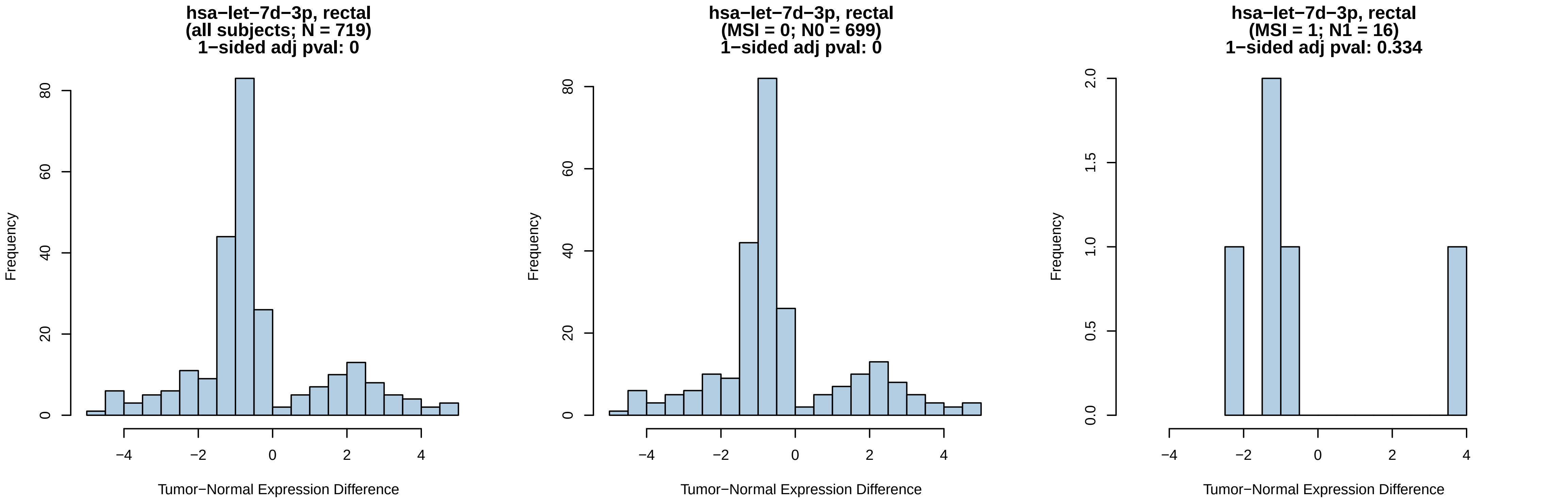

hsa−let−7d−3p, rectal
(all subjects; N = 719)
1−sided adj pval: 0
hsa−let−7d−3p, rectal
(MSI = 0; N0 = 699)
1−sided adj pval: 0
hsa−let−7d−3p, rectal
(MSI = 1; N1 = 16)
1−sided adj pval: 0.334
Frequency
Tumor−Normal Expression Difference

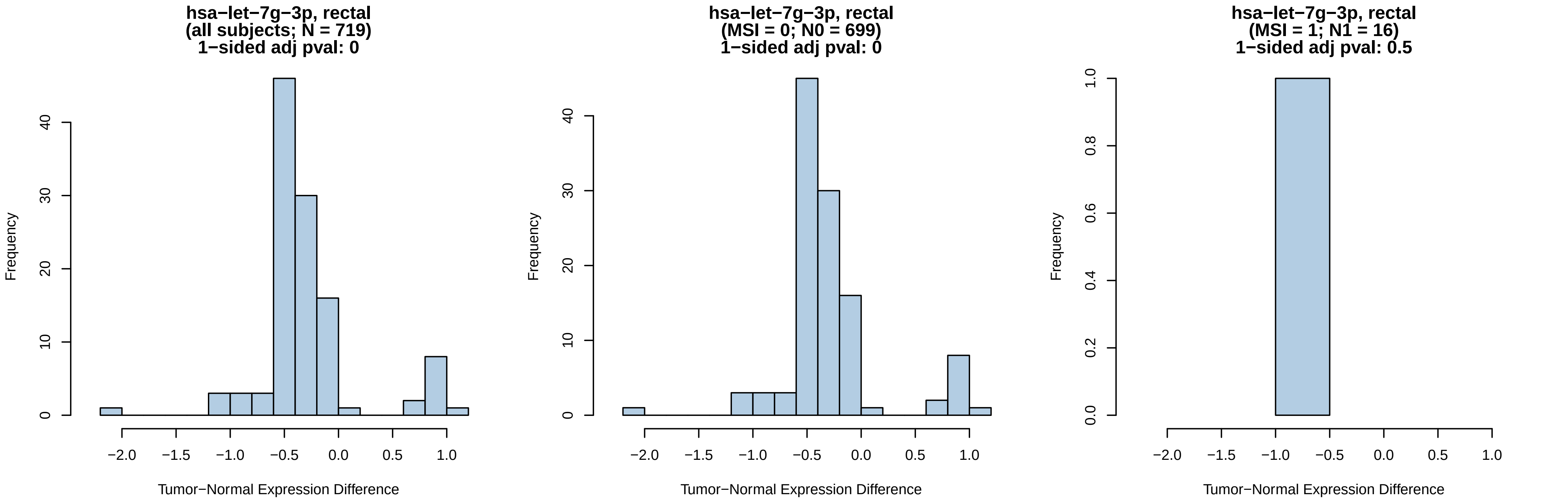

hsa−let−7g−3p, rectal
(all subjects; N = 719)
1−sided adj pval: 0
Frequency
0
10
20
30
40
−2.0
−1.5
−1.0
−0.5
0.0
0.5
1.0
Tumor−Normal Expression Difference
hsa−let−7g−3p, rectal
(MSI = 0; N0 = 699)
1−sided adj pval: 0
Frequency
0
10
20
30
40
−2.0
−1.5
−1.0
−0.5
0.0
0.5
1.0
Tumor−Normal Expression Difference
hsa−let−7g−3p, rectal
(MSI = 1; N1 = 16)
1−sided adj pval: 0.5
Frequency
0.0
0.2
0.4
0.6
0.8
1.0
−2.0
−1.5
−1.0
−0.5
0.0
0.5
1.0
Tumor−Normal Expression Difference

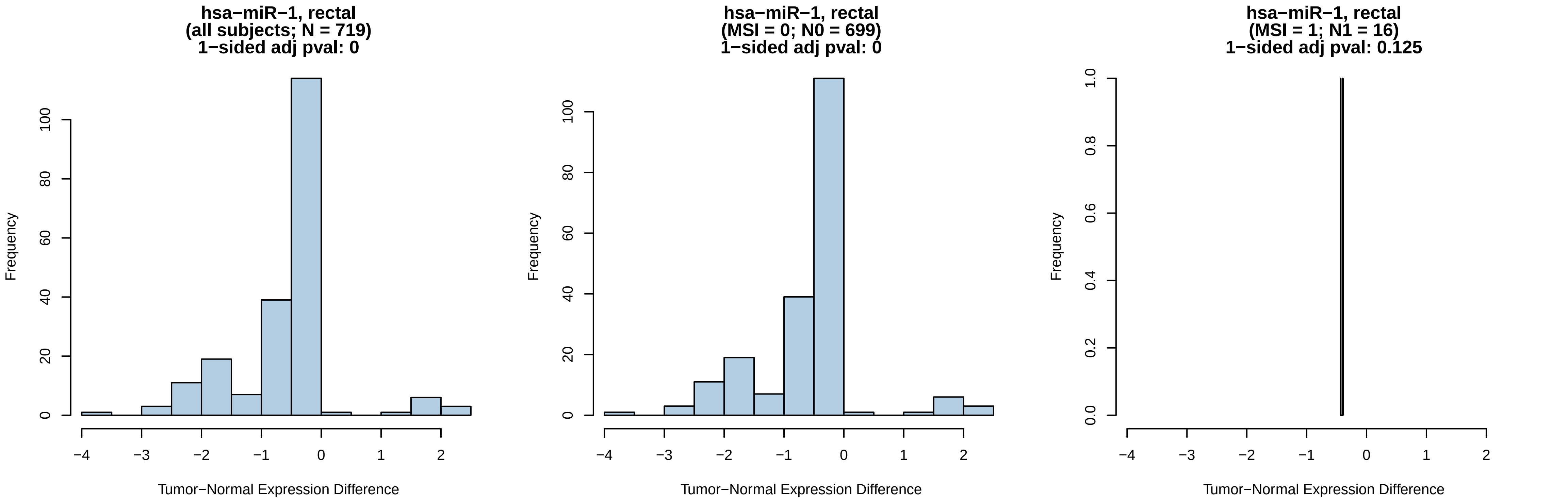
hsa−miR−1, rectal
(all subjects; N = 719)
1−sided adj pval: 0
Frequency
0
20
40
60
80
100
−4
−3
−2
−1
0
1
2
Tumor−Normal Expression Difference
hsa−miR−1, rectal
(MSI = 0; N0 = 699)
1−sided adj pval: 0
Frequency
0
20
40
60
80
100
−4
−3
−2
−1
0
1
2
Tumor−Normal Expression Difference
hsa−miR−1, rectal
(MSI = 1; N1 = 16)
1−sided adj pval: 0.125
Frequency
0.0
0.2
0.4
0.6
0.8
1.0
−4
−3
−2
−1
0
1
2
Tumor−Normal Expression Difference

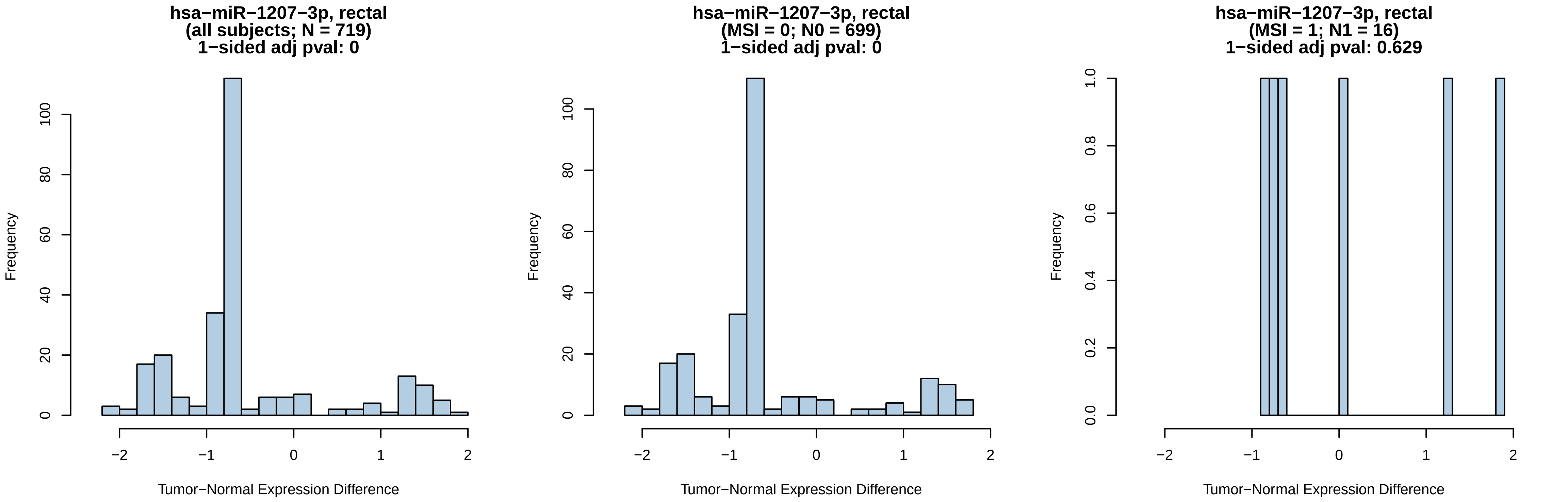

hsa−miR−1207−3p, rectal
(all subjects; N = 719)
1−sided adj pval: 0
Frequency
0
20
40
60
80
100
−2
−1
0
1
2
Tumor−Normal Expression Difference
hsa−miR−1207−3p, rectal
(MSI = 0; N0 = 699)
1−sided adj pval: 0
Frequency
0
20
40
60
80
100
−2
−1
0
1
2
Tumor−Normal Expression Difference
hsa−miR−1207−3p, rectal
(MSI = 1; N1 = 16)
1−sided adj pval: 0.629
Frequency
0.0
0.2
0.4
0.6
0.8
1.0
−2
−1
0
1
2
Tumor−Normal Expression Difference

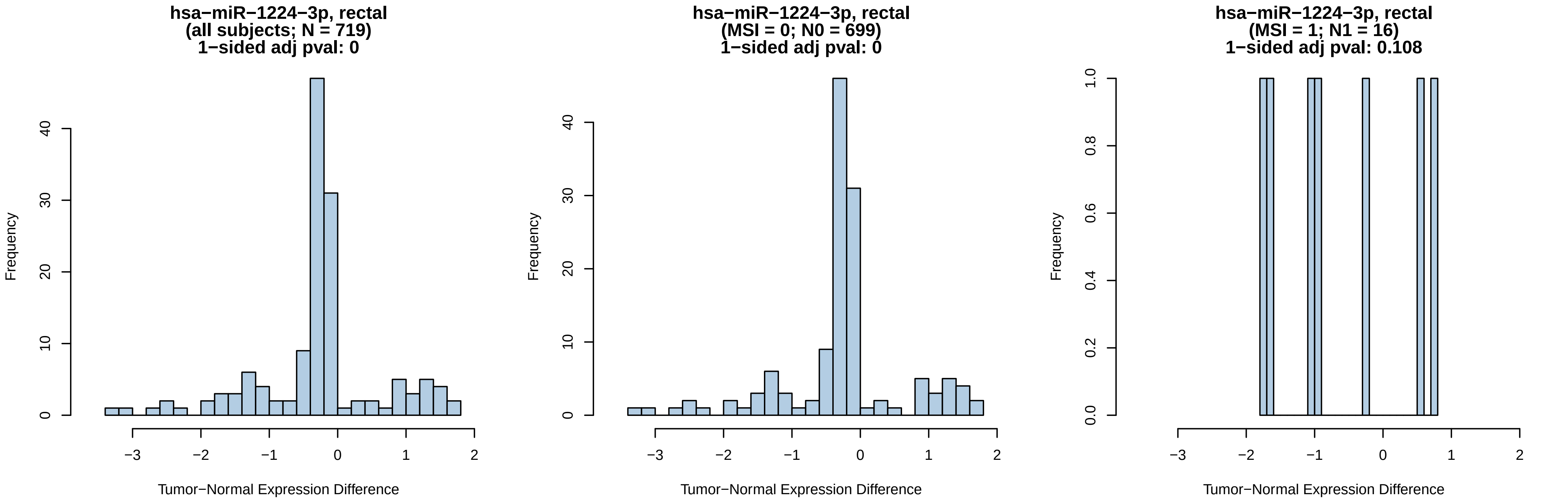
hsa−miR−1224−3p, rectal
(all subjects; N = 719)
1−sided adj pval: 0
Frequency
Tumor−Normal Expression Difference
hsa−miR−1224−3p, rectal
(MSI = 0; N0 = 699)
1−sided adj pval: 0
Frequency
Tumor−Normal Expression Difference
hsa−miR−1224−3p, rectal
(MSI = 1; N1 = 16)
1−sided adj pval: 0.108
Frequency
Tumor−Normal Expression Difference

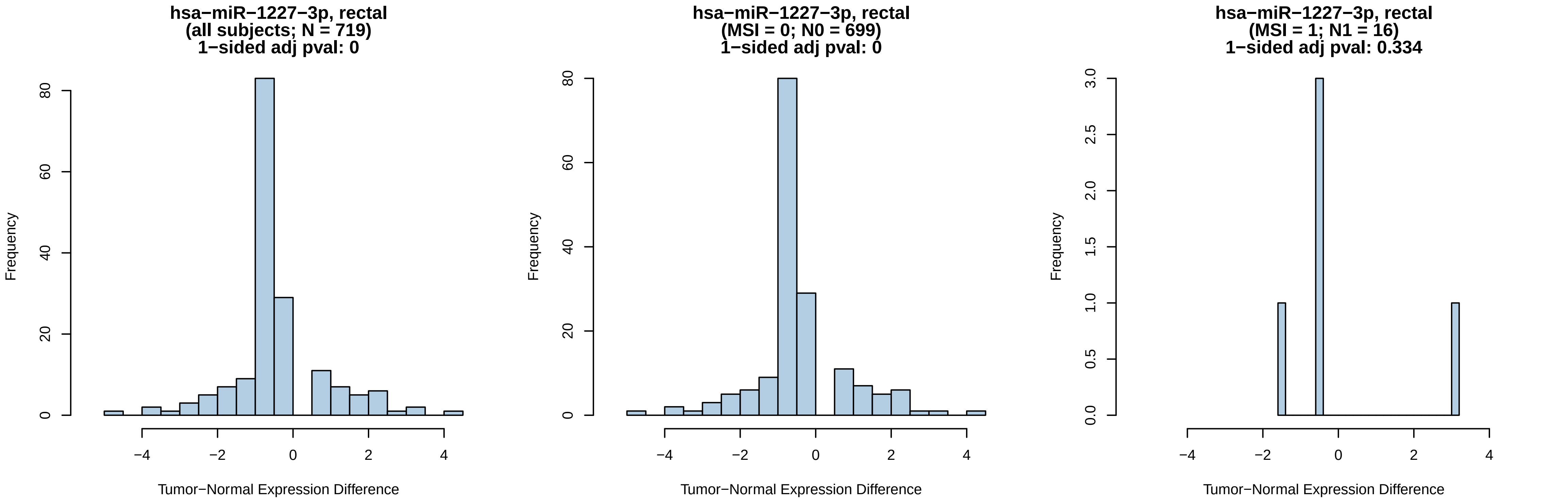
hsa−miR−1227−3p, rectal
(all subjects; N = 719)
1−sided adj pval: 0
Frequency
Tumor−Normal Expression Difference
hsa−miR−1227−3p, rectal
(MSI = 0; N0 = 699)
1−sided adj pval: 0
Frequency
Tumor−Normal Expression Difference
hsa−miR−1227−3p, rectal
(MSI = 1; N1 = 16)
1−sided adj pval: 0.334
Frequency
Tumor−Normal Expression Difference

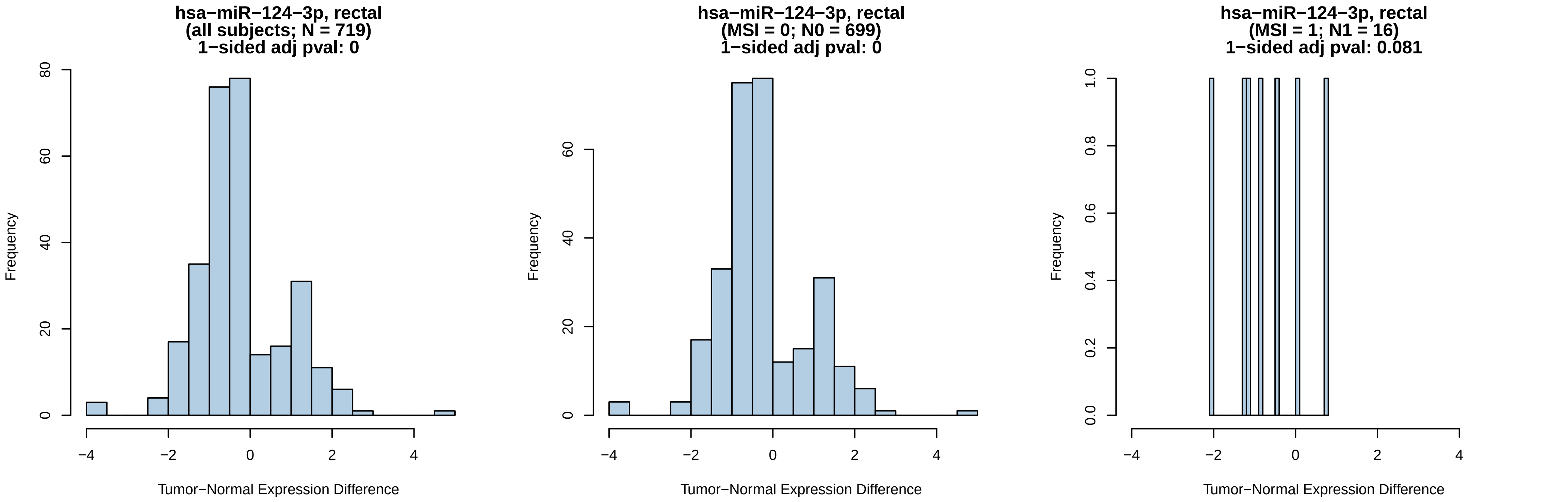
hsa−miR−124−3p, rectal
(all subjects; N = 719)
1−sided adj pval: 0
hsa−miR−124−3p, rectal
(MSI = 0; N0 = 699)
1−sided adj pval: 0
hsa−miR−124−3p, rectal
(MSI = 1; N1 = 16)
1−sided adj pval: 0.081
Frequency
Tumor−Normal Expression Difference
Frequency
Tumor−Normal Expression Difference
Frequency
Tumor−Normal Expression Difference

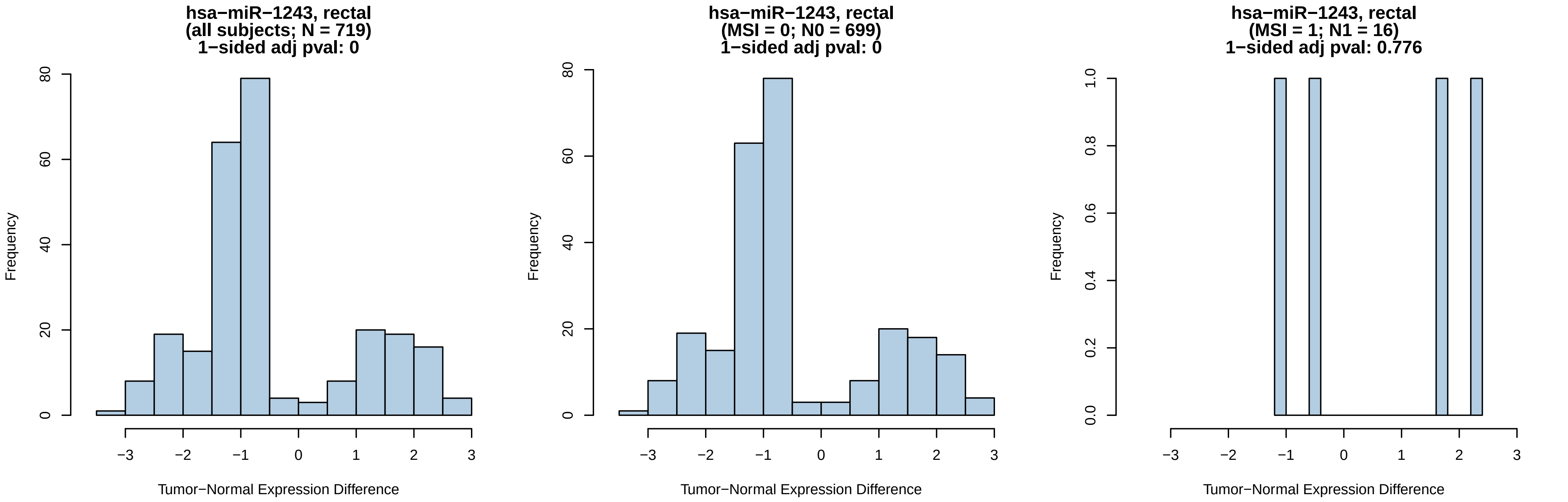

hsa−miR−1243, rectal
(all subjects; N = 719)
1−sided adj pval: 0
Frequency
0
20
40
60
80
−3
−2
−1
0
1
2
3
Tumor−Normal Expression Difference
hsa−miR−1243, rectal
(MSI = 0; N0 = 699)
1−sided adj pval: 0
Frequency
0
20
40
60
80
−3
−2
−1
0
1
2
3
Tumor−Normal Expression Difference
hsa−miR−1243, rectal
(MSI = 1; N1 = 16)
1−sided adj pval: 0.776
Frequency
0.0
0.2
0.4
0.6
0.8
1.0
−3
−2
−1
0
1
2
3
Tumor−Normal Expression Difference

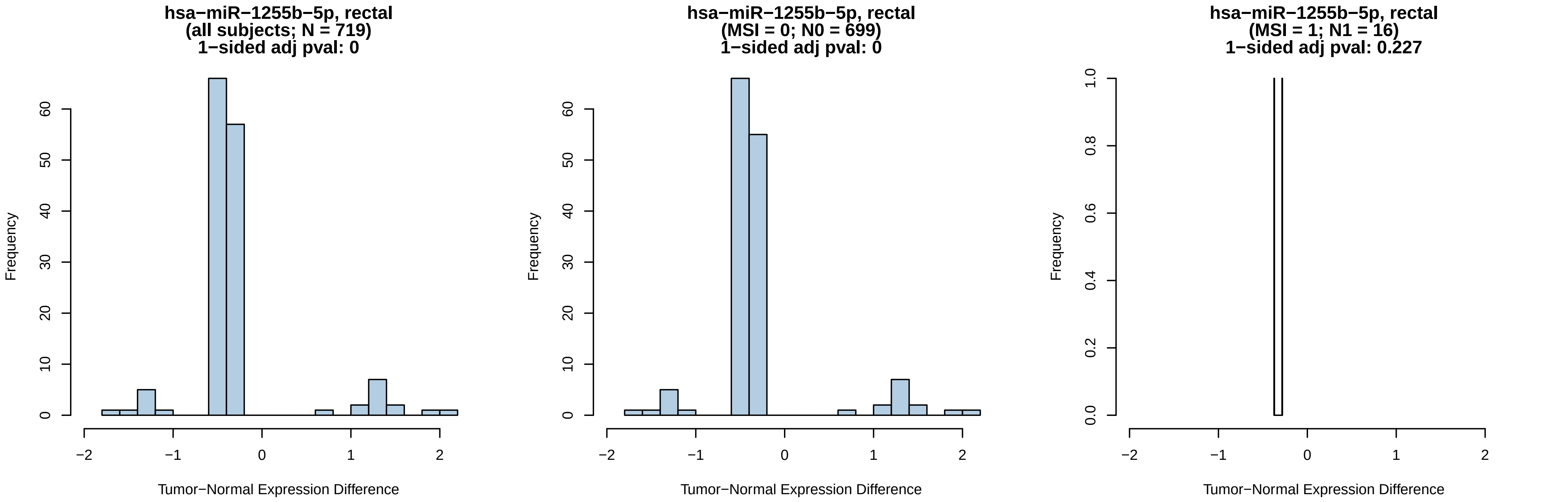

hsa−miR−1255b−5p, rectal
(all subjects; N = 719)
1−sided adj pval: 0
Frequency
Tumor−Normal Expression Difference
hsa−miR−1255b−5p, rectal
(MSI = 0; N0 = 699)
1−sided adj pval: 0
Frequency
Tumor−Normal Expression Difference
hsa−miR−1255b−5p, rectal
(MSI = 1; N1 = 16)
1−sided adj pval: 0.227
Frequency
Tumor−Normal Expression Difference

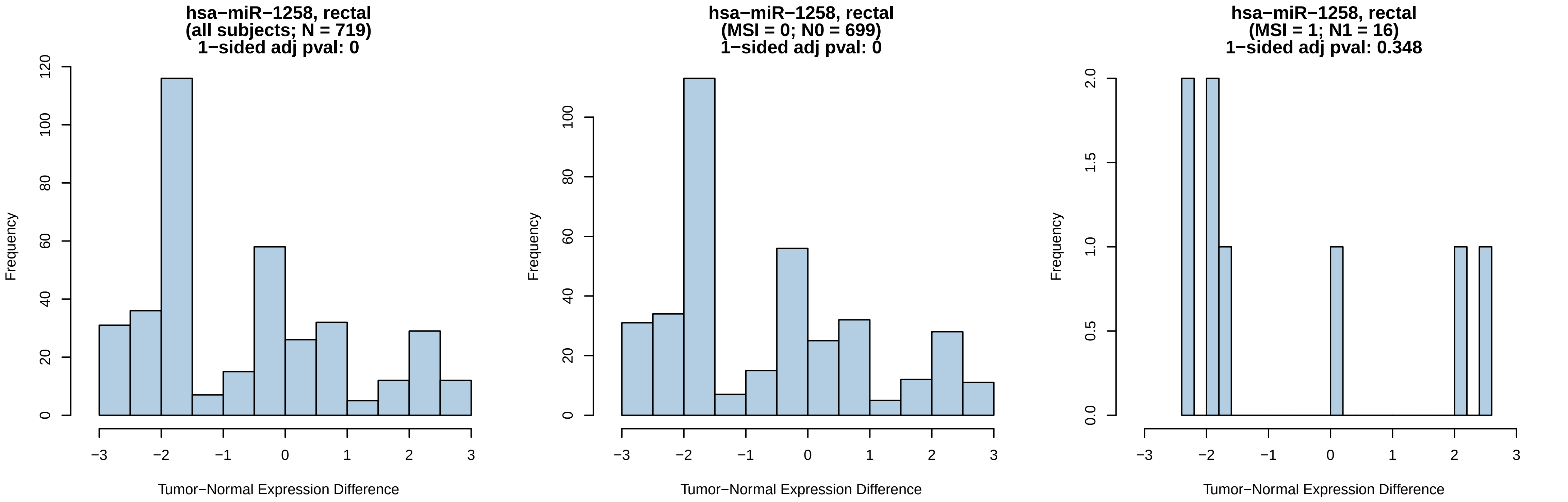
hsa−miR−1258, rectal
(all subjects; N = 719)
1−sided adj pval: 0
hsa−miR−1258, rectal
(MSI = 0; N0 = 699)
1−sided adj pval: 0
hsa−miR−1258, rectal
(MSI = 1; N1 = 16)
1−sided adj pval: 0.348
Frequency
Tumor−Normal Expression Difference

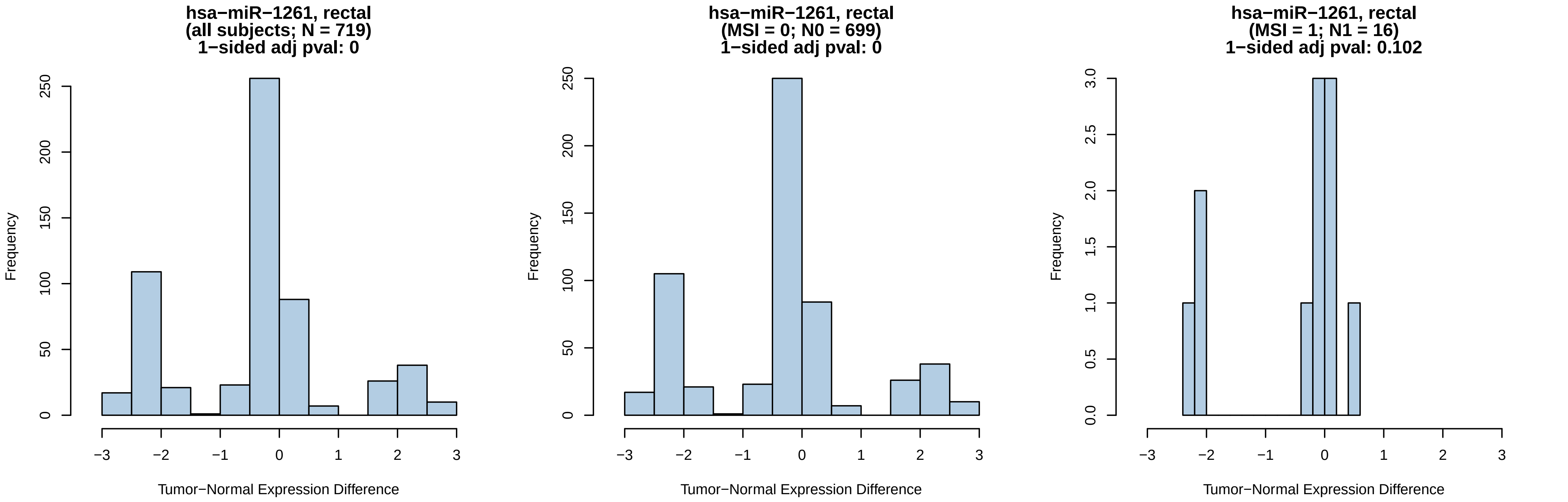
hsa−miR−1261, rectal
(all subjects; N = 719)
1−sided adj pval: 0
Frequency
Tumor−Normal Expression Difference
hsa−miR−1261, rectal
(MSI = 0; N0 = 699)
1−sided adj pval: 0
Frequency
Tumor−Normal Expression Difference
hsa−miR−1261, rectal
(MSI = 1; N1 = 16)
1−sided adj pval: 0.102
Frequency
Tumor−Normal Expression Difference

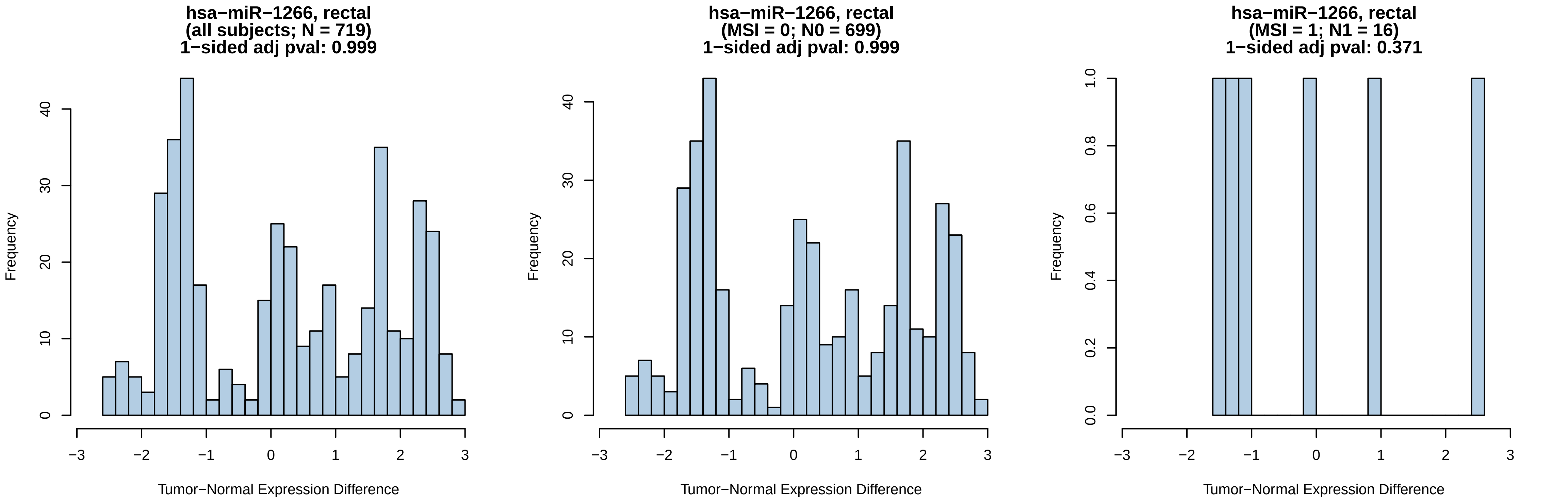
hsa−miR−1266, rectal
(all subjects; N = 719)
1−sided adj pval: 0.999
hsa−miR−1266, rectal
(MSI = 0; N0 = 699)
1−sided adj pval: 0.999
hsa−miR−1266, rectal
(MSI = 1; N1 = 16)
1−sided adj pval: 0.371
Frequency
Tumor−Normal Expression Difference
0
10
20
30
40
0.0
0.2
0.4
0.6
0.8
1.0
−3
−2
−1
0
1
2
3

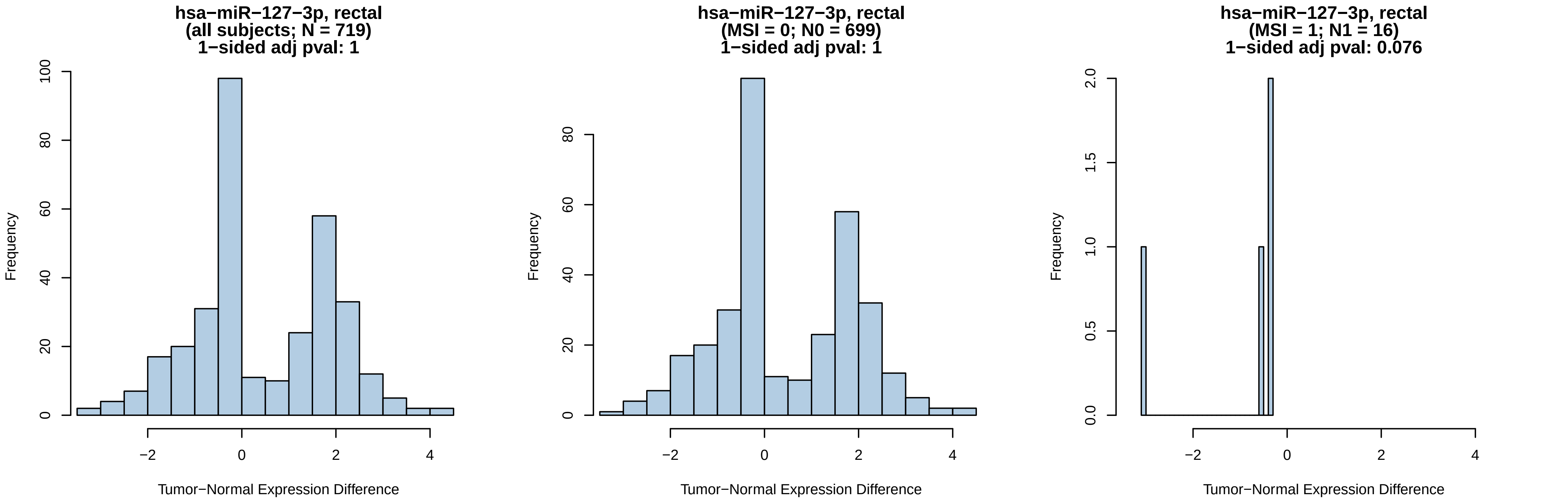
hsa−miR−127−3p, rectal
(all subjects; N = 719)
1−sided adj pval: 1
Frequency
Tumor−Normal Expression Difference
hsa−miR−127−3p, rectal
(MSI = 0; N0 = 699)
1−sided adj pval: 1
Frequency
Tumor−Normal Expression Difference
hsa−miR−127−3p, rectal
(MSI = 1; N1 = 16)
1−sided adj pval: 0.076
Frequency
Tumor−Normal Expression Difference

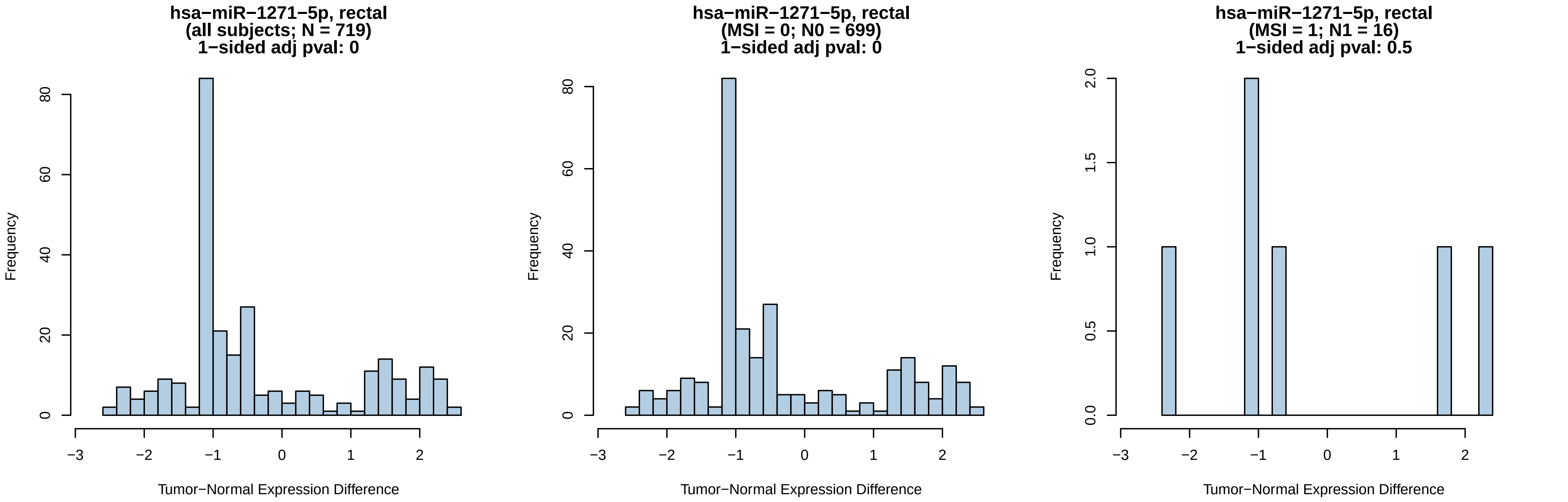

hsa−miR−1271−5p, rectal
(all subjects; N = 719)
1−sided adj pval: 0
Frequency
Tumor−Normal Expression Difference
hsa−miR−1271−5p, rectal
(MSI = 0; N0 = 699)
1−sided adj pval: 0
Frequency
Tumor−Normal Expression Difference
hsa−miR−1271−5p, rectal
(MSI = 1; N1 = 16)
1−sided adj pval: 0.5
Frequency
Tumor−Normal Expression Difference

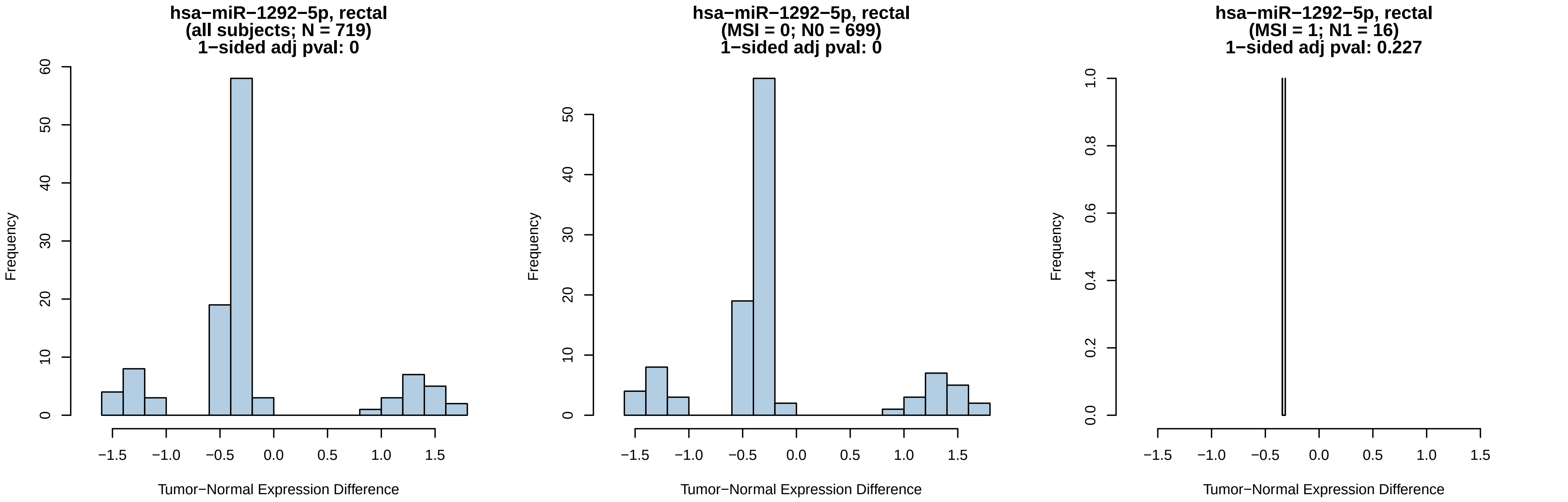
hsa−miR−1292−5p, rectal
(all subjects; N = 719)
1−sided adj pval: 0
Frequency
Tumor−Normal Expression Difference
hsa−miR−1292−5p, rectal
(MSI = 0; N0 = 699)
1−sided adj pval: 0
Frequency
Tumor−Normal Expression Difference
hsa−miR−1292−5p, rectal
(MSI = 1; N1 = 16)
1−sided adj pval: 0.227
Frequency
Tumor−Normal Expression Difference

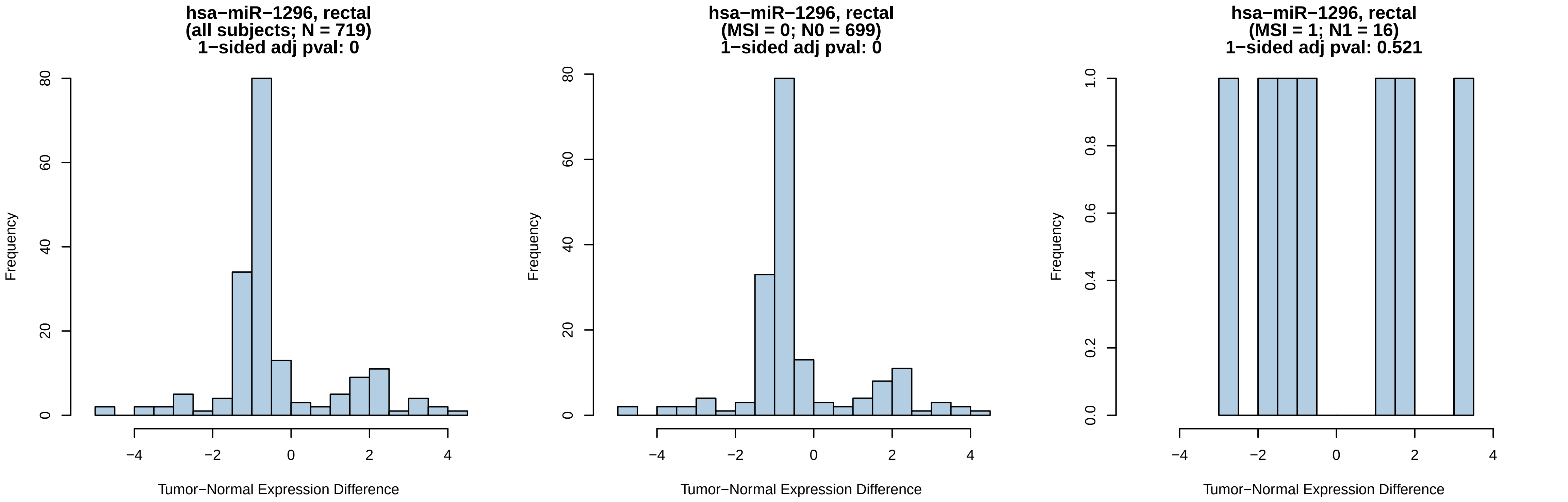
hsa−miR−1296, rectal
(all subjects; N = 719)
1−sided adj pval: 0
Frequency
Tumor−Normal Expression Difference
hsa−miR−1296, rectal
(MSI = 0; N0 = 699)
1−sided adj pval: 0
Frequency
Tumor−Normal Expression Difference
hsa−miR−1296, rectal
(MSI = 1; N1 = 16)
1−sided adj pval: 0.521
Frequency
Tumor−Normal Expression Difference

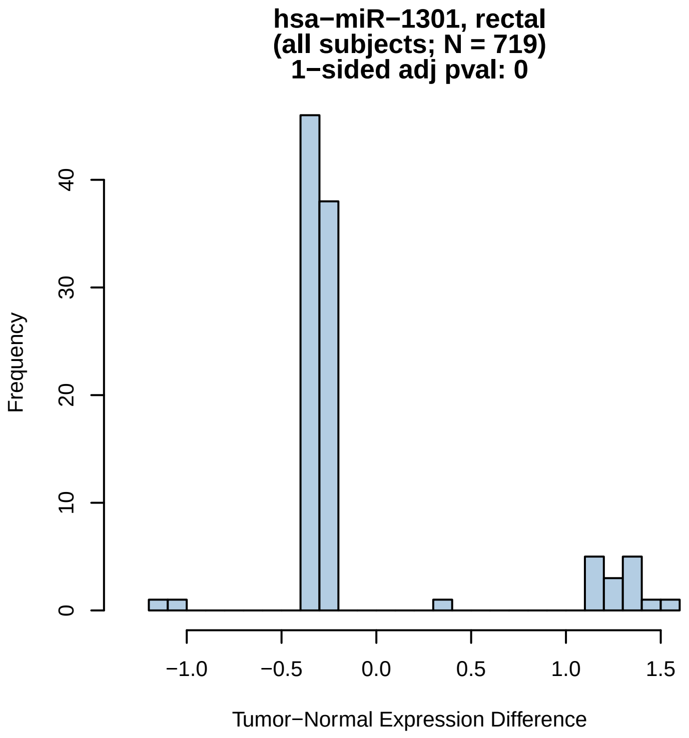

hsa−miR−1301, rectal
(all subjects; N = 719)
1−sided adj pval: 0
Frequency
Tumor−Normal Expression Difference


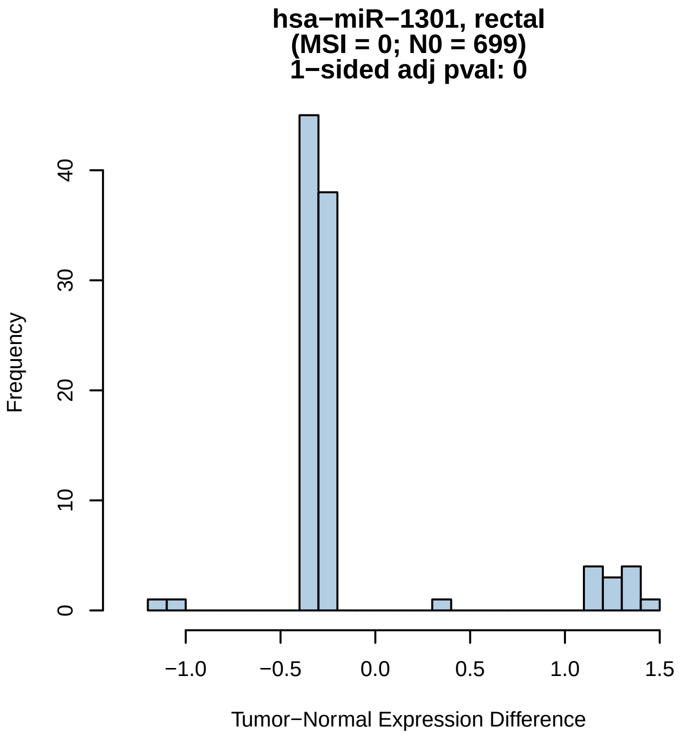

hsa−miR−1301, rectal
(MSI = 0; N0 = 699)
1−sided adj pval: 0
Frequency
Tumor−Normal Expression Difference


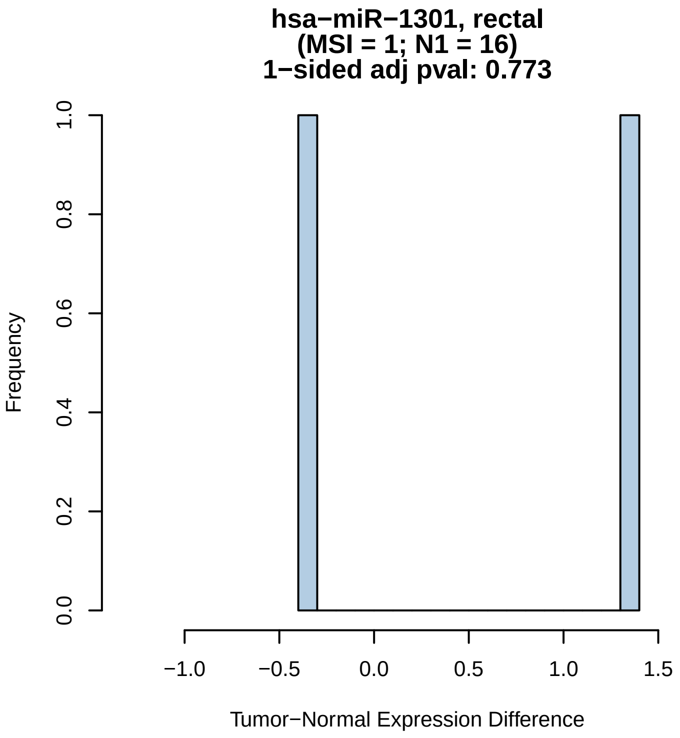

hsa−miR−1301, rectal
(MSI = 1; N1 = 16)
1−sided adj pval: 0.773
Frequency
Tumor−Normal Expression Difference

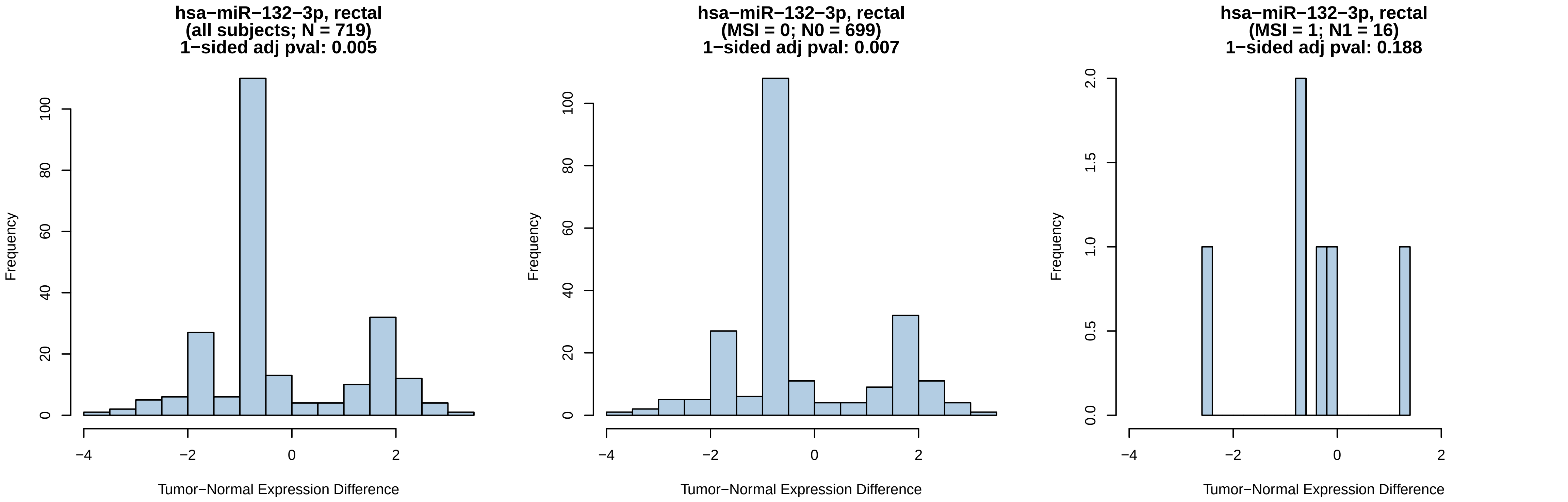

hsa−miR−132−3p, rectal
(all subjects; N = 719)
1−sided adj pval: 0.005
Frequency
Tumor−Normal Expression Difference
hsa−miR−132−3p, rectal
(MSI = 0; N0 = 699)
1−sided adj pval: 0.007
Frequency
Tumor−Normal Expression Difference
hsa−miR−132−3p, rectal
(MSI = 1; N1 = 16)
1−sided adj pval: 0.188
Frequency
Tumor−Normal Expression Difference

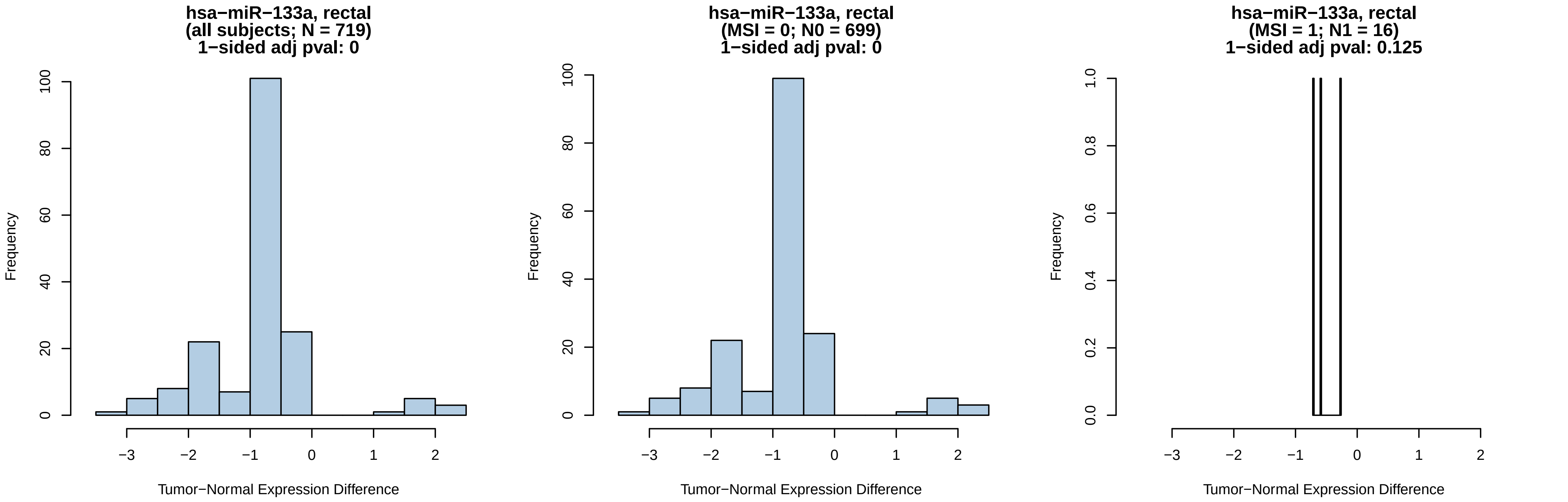
hsa−miR−133a, rectal
(all subjects; N = 719)
1−sided adj pval: 0
Frequency
Tumor−Normal Expression Difference
hsa−miR−133a, rectal
(MSI = 0; N0 = 699)
1−sided adj pval: 0
Frequency
Tumor−Normal Expression Difference
hsa−miR−133a, rectal
(MSI = 1; N1 = 16)
1−sided adj pval: 0.125
Frequency
Tumor−Normal Expression Difference

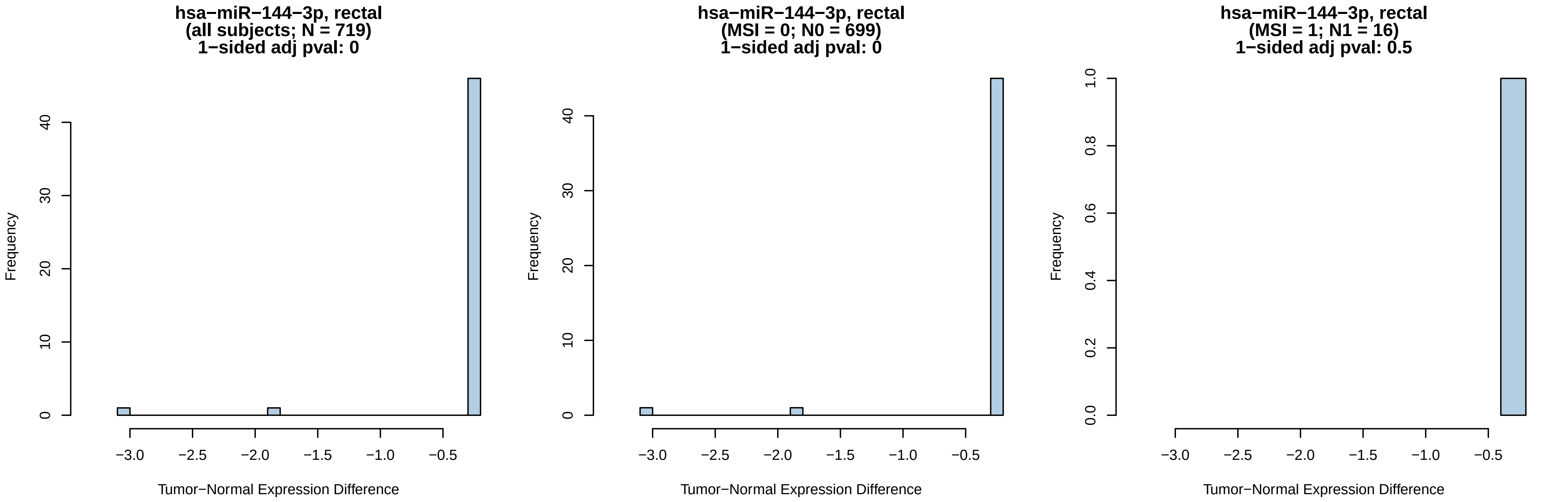

hsa−miR−144−3p, rectal
(all subjects; N = 719)
1−sided adj pval: 0
Frequency
0
10
20
30
40
−3.0
−2.5
−2.0
−1.5
−1.0
−0.5
Tumor−Normal Expression Difference
hsa−miR−144−3p, rectal
(MSI = 0; N0 = 699)
1−sided adj pval: 0
Frequency
0
10
20
30
40
−3.0
−2.5
−2.0
−1.5
−1.0
−0.5
Tumor−Normal Expression Difference
hsa−miR−144−3p, rectal
(MSI = 1; N1 = 16)
1−sided adj pval: 0.5
Frequency
0.0
0.2
0.4
0.6
0.8
1.0
−3.0
−2.5
−2.0
−1.5
−1.0
−0.5
Tumor−Normal Expression Difference

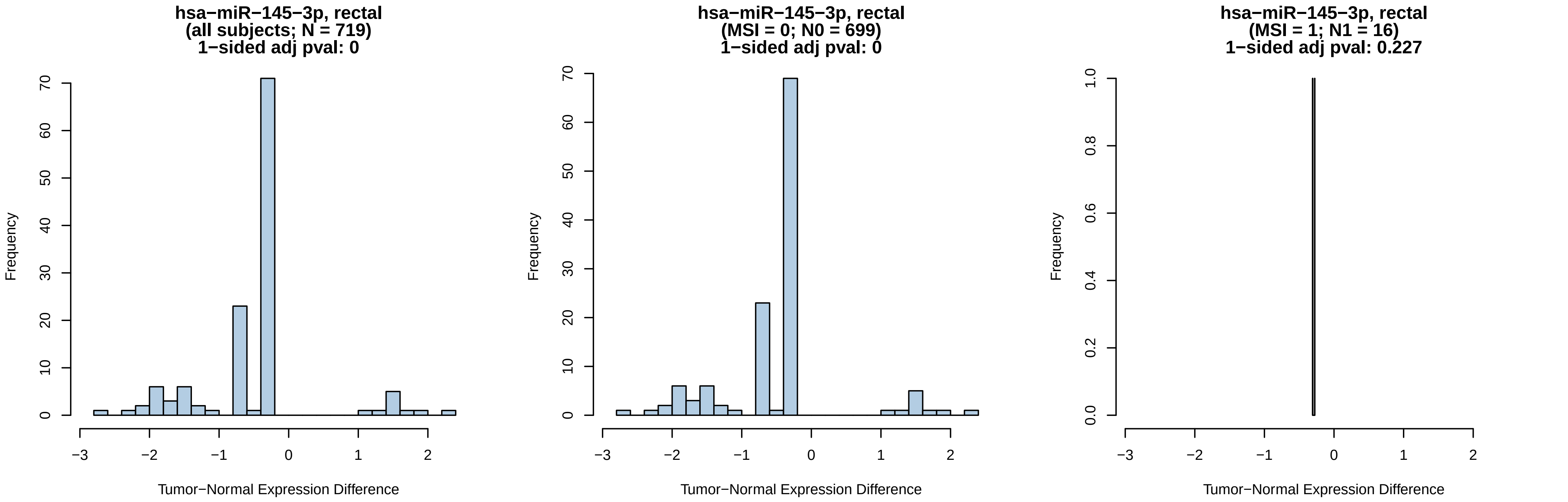

hsa−miR−145−3p, rectal
(all subjects; N = 719)
1−sided adj pval: 0
Frequency
Tumor−Normal Expression Difference
hsa−miR−145−3p, rectal
(MSI = 0; N0 = 699)
1−sided adj pval: 0
Frequency
Tumor−Normal Expression Difference
hsa−miR−145−3p, rectal
(MSI = 1; N1 = 16)
1−sided adj pval: 0.227
Frequency
Tumor−Normal Expression Difference

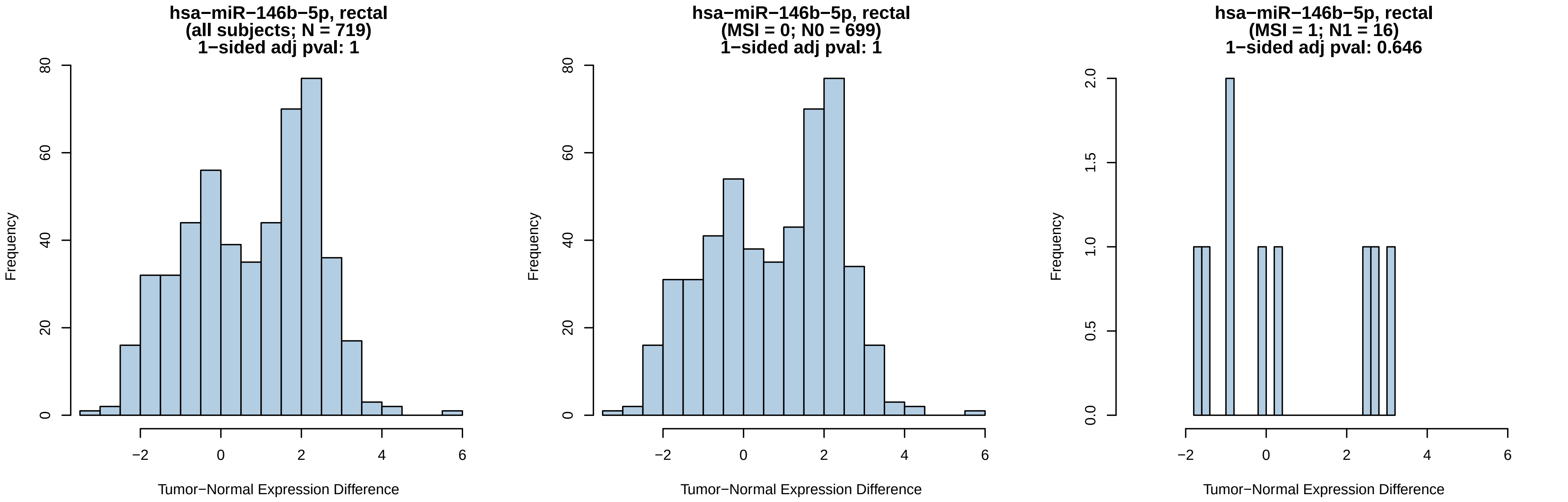
hsa−miR−146b−5p, rectal
(all subjects; N = 719)
1−sided adj pval: 1
hsa−miR−146b−5p, rectal
(MSI = 0; N0 = 699)
1−sided adj pval: 1
hsa−miR−146b−5p, rectal
(MSI = 1; N1 = 16)
1−sided adj pval: 0.646
Frequency
Tumor−Normal Expression Difference

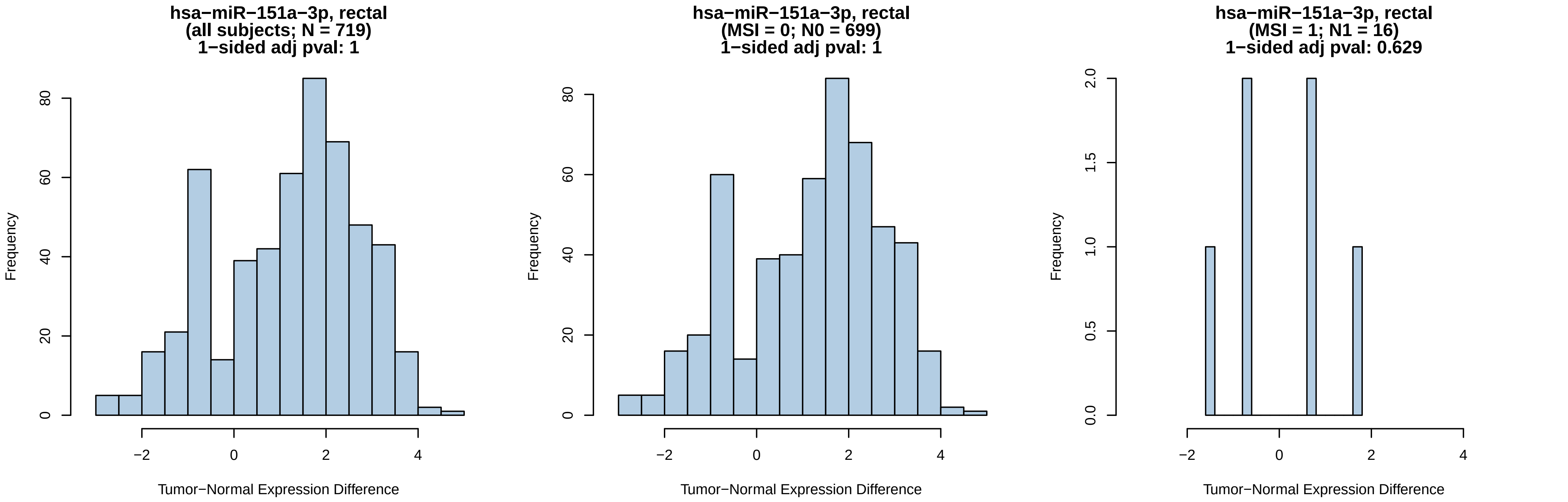

hsa−miR−151a−3p, rectal
(all subjects; N = 719)
1−sided adj pval: 1
Frequency
Tumor−Normal Expression Difference
hsa−miR−151a−3p, rectal
(MSI = 0; N0 = 699)
1−sided adj pval: 1
Frequency
Tumor−Normal Expression Difference
hsa−miR−151a−3p, rectal
(MSI = 1; N1 = 16)
1−sided adj pval: 0.629
Frequency
Tumor−Normal Expression Difference

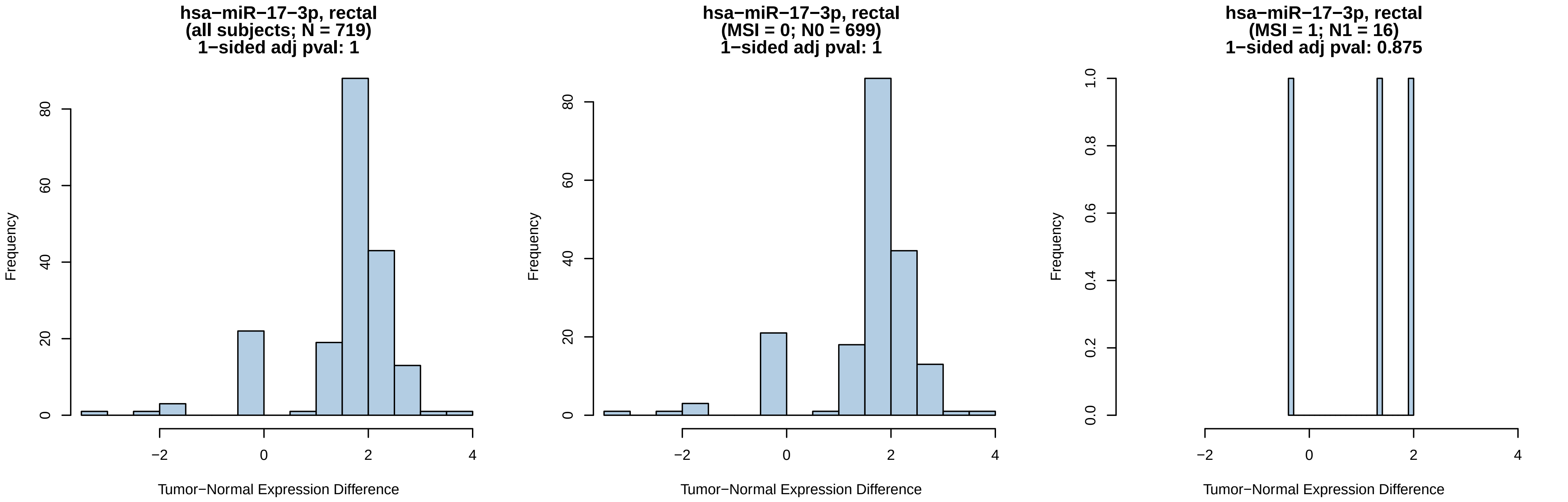

hsa−miR−17−3p, rectal
(all subjects; N = 719)
1−sided adj pval: 1
Frequency
0
20
40
60
80
−2
0
2
4
Tumor−Normal Expression Difference
hsa−miR−17−3p, rectal
(MSI = 0; N0 = 699)
1−sided adj pval: 1
Frequency
0
20
40
60
80
−2
0
2
4
Tumor−Normal Expression Difference
hsa−miR−17−3p, rectal
(MSI = 1; N1 = 16)
1−sided adj pval: 0.875
Frequency
0.0
0.2
0.4
0.6
0.8
1.0
−2
0
2
4
Tumor−Normal Expression Difference

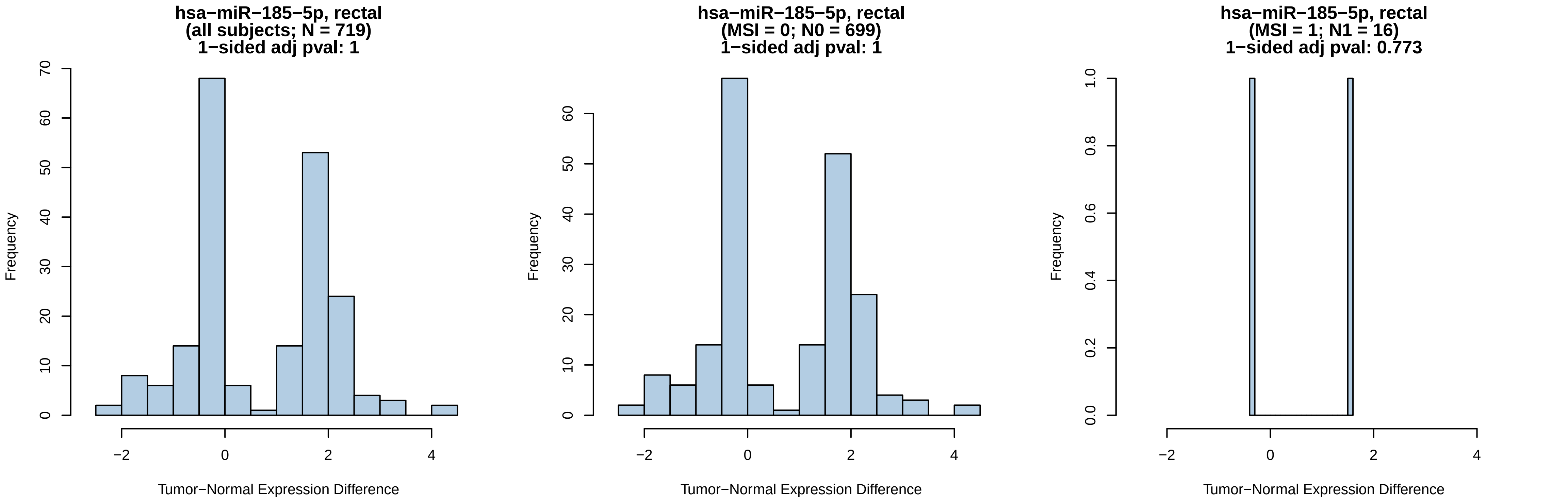

hsa−miR−185−5p, rectal
(all subjects; N = 719)
1−sided adj pval: 1
Frequency
Tumor−Normal Expression Difference
hsa−miR−185−5p, rectal
(MSI = 0; N0 = 699)
1−sided adj pval: 1
Frequency
Tumor−Normal Expression Difference
hsa−miR−185−5p, rectal
(MSI = 1; N1 = 16)
1−sided adj pval: 0.773
Frequency
Tumor−Normal Expression Difference

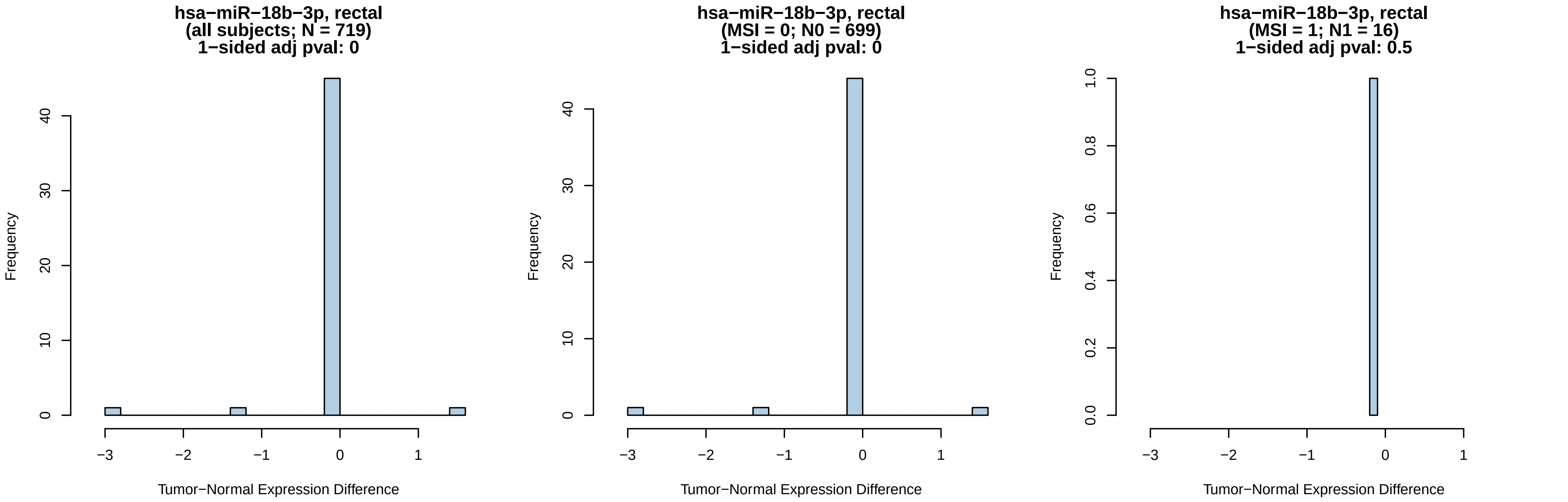
hsa−miR−18b−3p, rectal
(all subjects; N = 719)
1−sided adj pval: 0
Frequency
Tumor−Normal Expression Difference
hsa−miR−18b−3p, rectal
(MSI = 0; N0 = 699)
1−sided adj pval: 0
Frequency
Tumor−Normal Expression Difference
hsa−miR−18b−3p, rectal
(MSI = 1; N1 = 16)
1−sided adj pval: 0.5
Frequency
Tumor−Normal Expression Difference

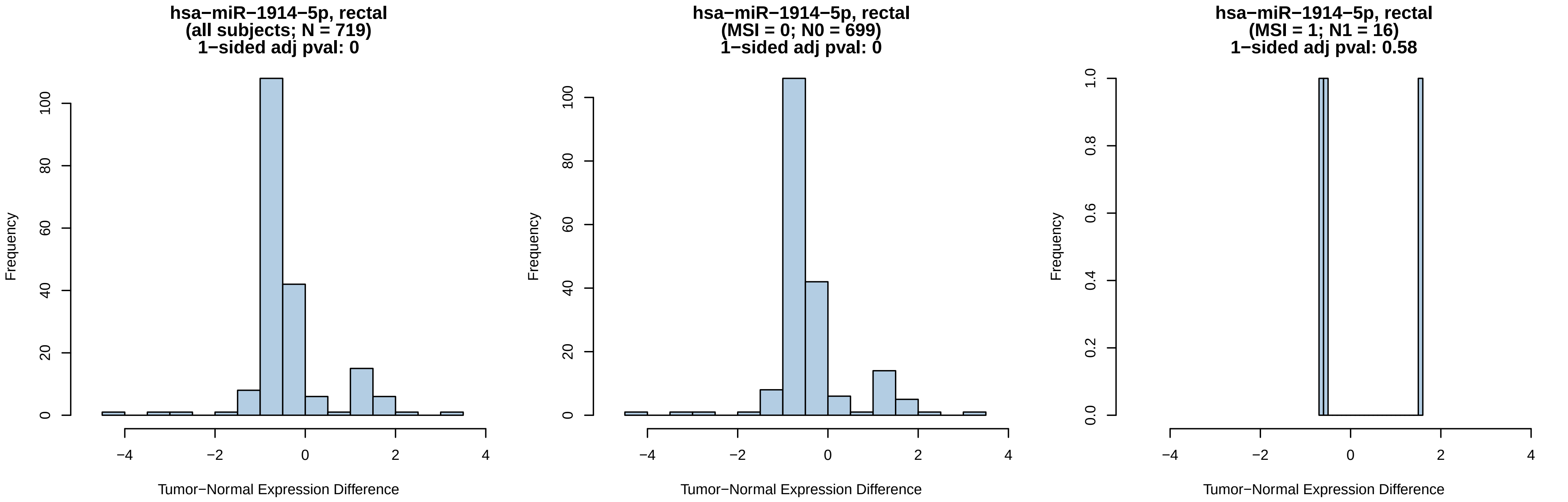
hsa−miR−1914−5p, rectal
(all subjects; N = 719)
1−sided adj pval: 0
Frequency
0
20
40
60
80
100
−4
−2
0
2
4
Tumor−Normal Expression Difference
hsa−miR−1914−5p, rectal
(MSI = 0; N0 = 699)
1−sided adj pval: 0
Frequency
0
20
40
60
80
100
−4
−2
0
2
4
Tumor−Normal Expression Difference
hsa−miR−1914−5p, rectal
(MSI = 1; N1 = 16)
1−sided adj pval: 0.58
Frequency
0.0
0.2
0.4
0.6
0.8
1.0
−4
−2
0
2
4
Tumor−Normal Expression Difference

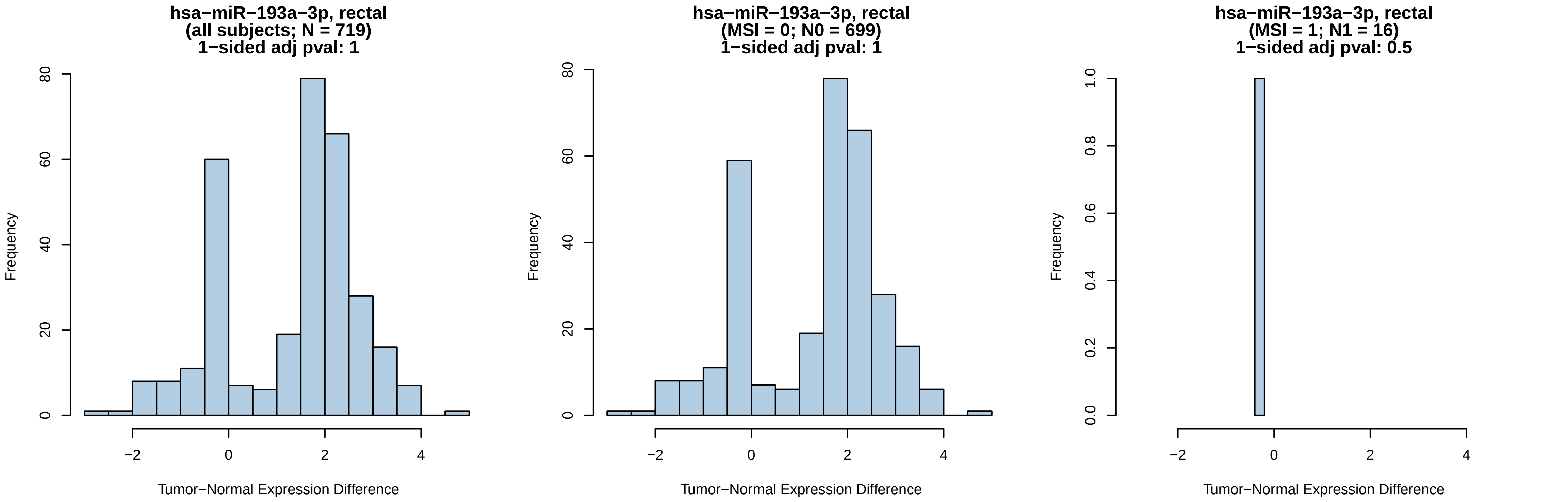
hsa−miR−193a−3p, rectal
(all subjects; N = 719)
1−sided adj pval: 1
Frequency
Tumor−Normal Expression Difference
hsa−miR−193a−3p, rectal
(MSI = 0; N0 = 699)
1−sided adj pval: 1
Frequency
Tumor−Normal Expression Difference
hsa−miR−193a−3p, rectal
(MSI = 1; N1 = 16)
1−sided adj pval: 0.5
Frequency
Tumor−Normal Expression Difference

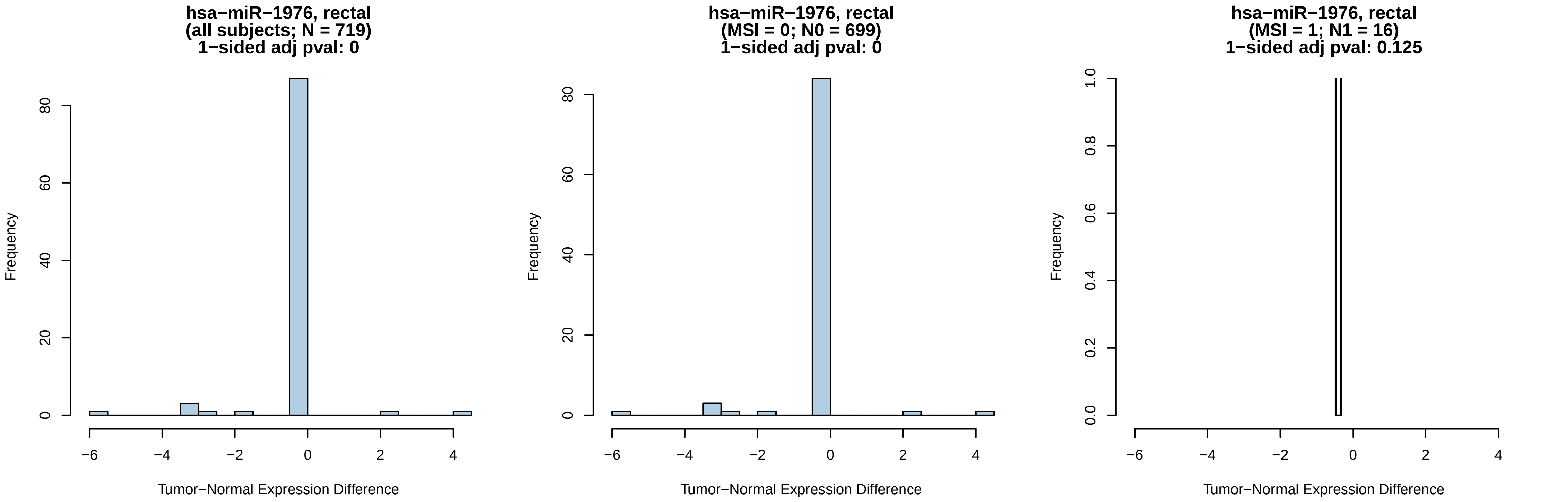

hsa−miR−1976, rectal
(all subjects; N = 719)
1−sided adj pval: 0
Frequency
Tumor−Normal Expression Difference
hsa−miR−1976, rectal
(MSI = 0; N0 = 699)
1−sided adj pval: 0
Frequency
Tumor−Normal Expression Difference
hsa−miR−1976, rectal
(MSI = 1; N1 = 16)
1−sided adj pval: 0.125
Frequency
Tumor−Normal Expression Difference

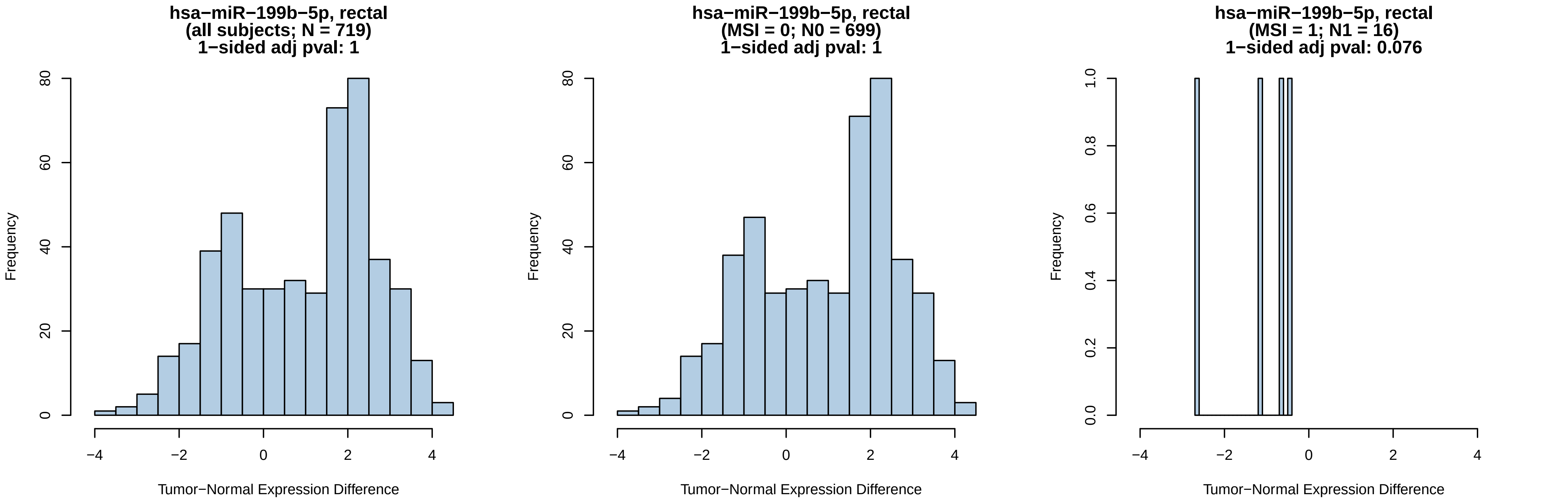
hsa−miR−199b−5p, rectal
(all subjects; N = 719)
1−sided adj pval: 1
Frequency
Tumor−Normal Expression Difference
hsa−miR−199b−5p, rectal
(MSI = 0; N0 = 699)
1−sided adj pval: 1
Frequency
Tumor−Normal Expression Difference
hsa−miR−199b−5p, rectal
(MSI = 1; N1 = 16)
1−sided adj pval: 0.076
Frequency
Tumor−Normal Expression Difference

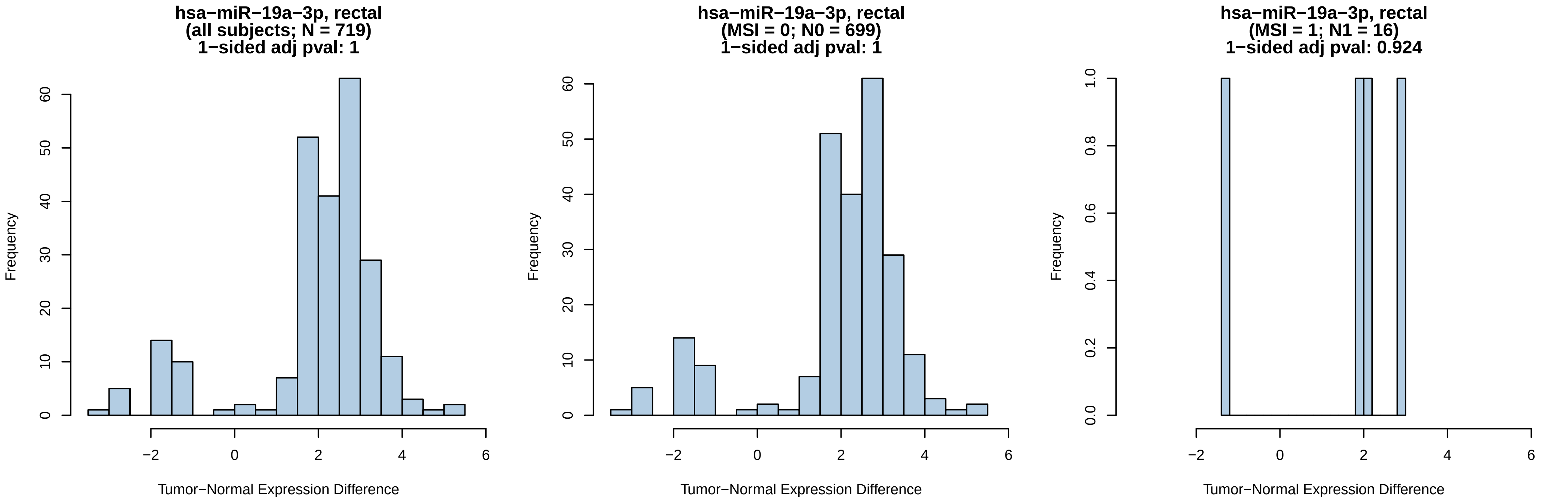
hsa−miR−19a−3p, rectal
(all subjects; N = 719)
1−sided adj pval: 1
Frequency
Tumor−Normal Expression Difference
hsa−miR−19a−3p, rectal
(MSI = 0; N0 = 699)
1−sided adj pval: 1
Frequency
Tumor−Normal Expression Difference
hsa−miR−19a−3p, rectal
(MSI = 1; N1 = 16)
1−sided adj pval: 0.924
Frequency
Tumor−Normal Expression Difference

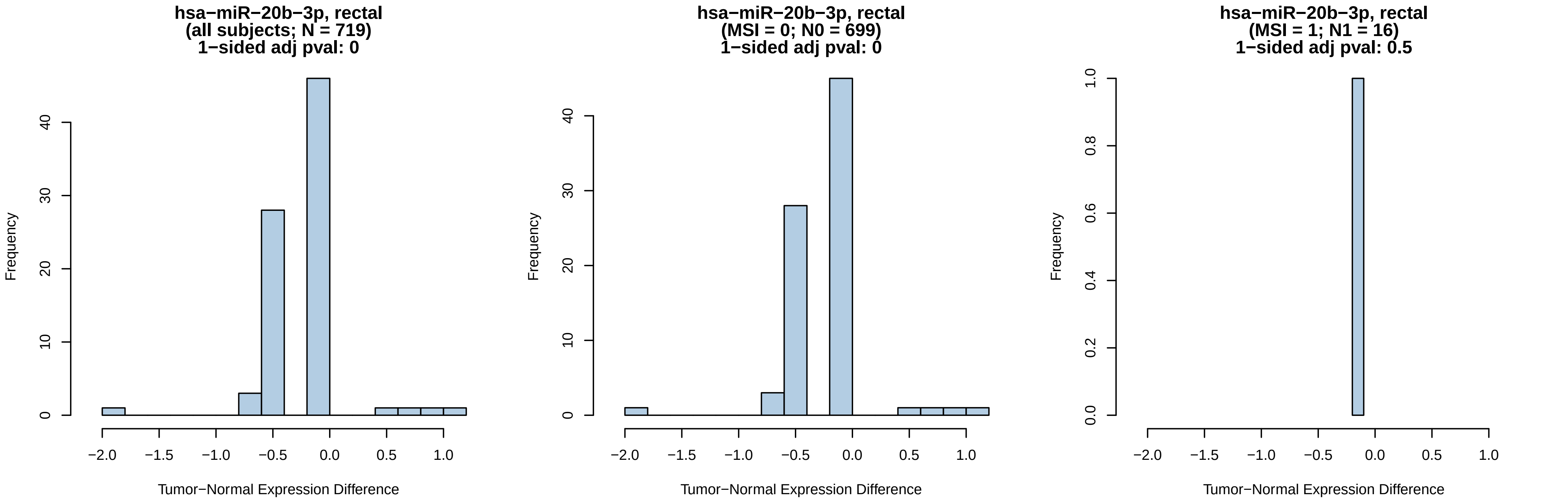

hsa−miR−20b−3p, rectal
(all subjects; N = 719)
1−sided adj pval: 0
Frequency
Tumor−Normal Expression Difference
hsa−miR−20b−3p, rectal
(MSI = 0; N0 = 699)
1−sided adj pval: 0
Frequency
Tumor−Normal Expression Difference
hsa−miR−20b−3p, rectal
(MSI = 1; N1 = 16)
1−sided adj pval: 0.5
Frequency
Tumor−Normal Expression Difference

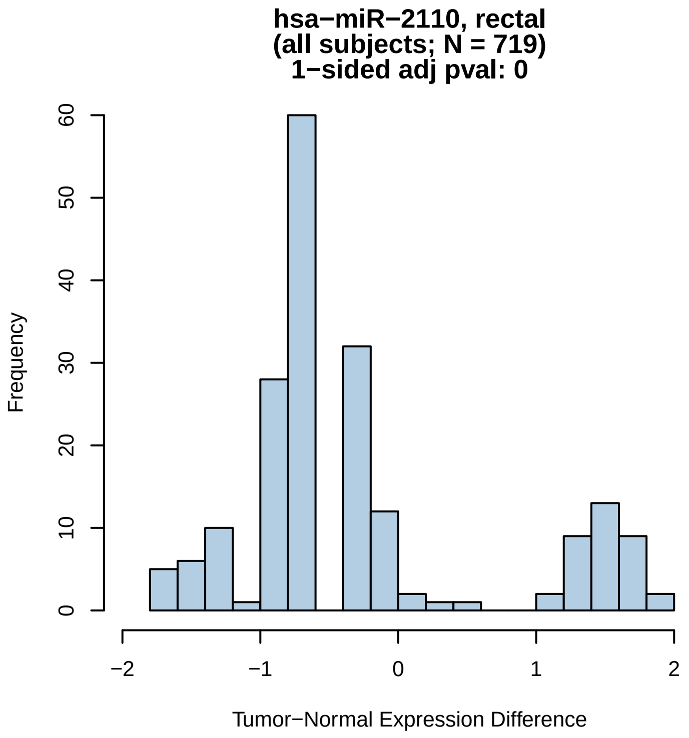

hsa−miR−2110, rectal
(all subjects; N = 719)
1−sided adj pval: 0
Frequency
Tumor−Normal Expression Difference
0
10
20
30
40
50
60
−2
−1
0
1
2


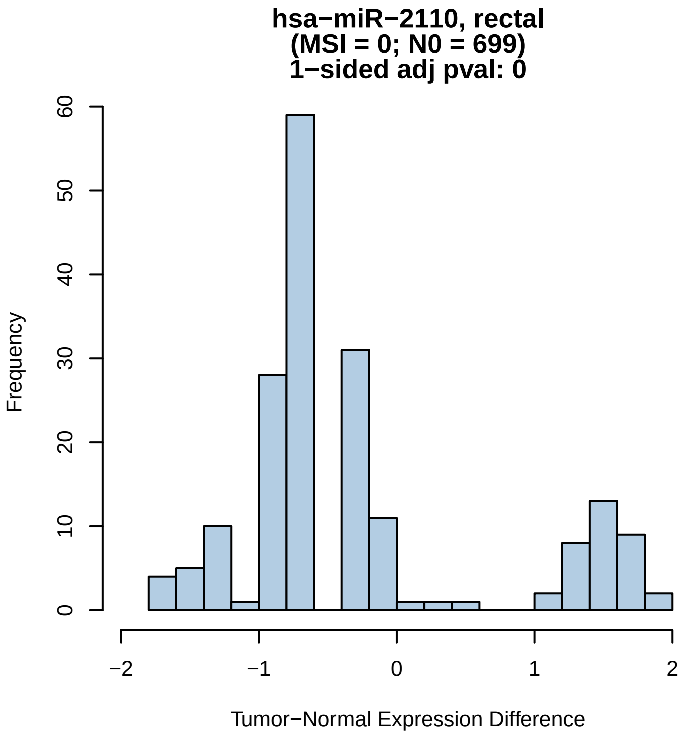

hsa−miR−2110, rectal
(MSI = 0; N0 = 699)
1−sided adj pval: 0
Frequency
Tumor−Normal Expression Difference
0
10
20
30
40
50
60
−2
−1
0
1
2


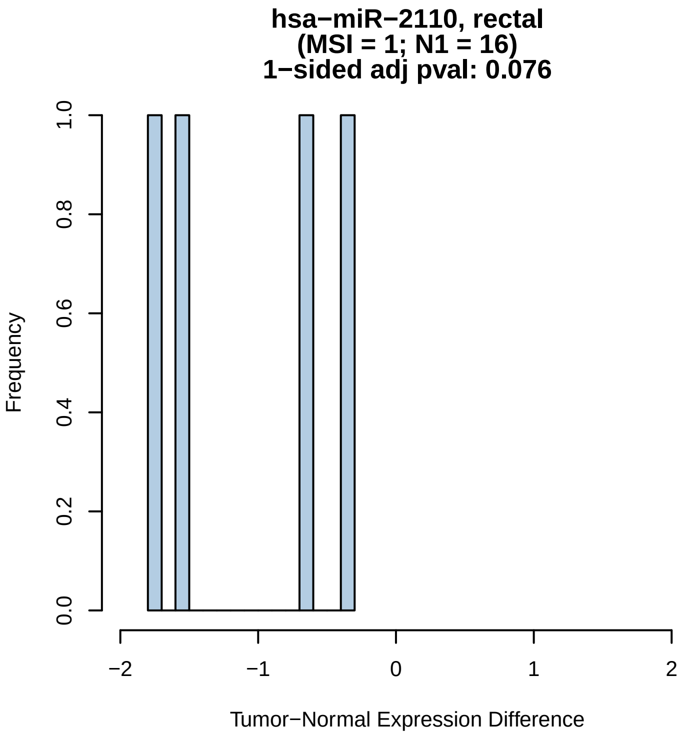

hsa−miR−2110, rectal
(MSI = 1; N1 = 16)
1−sided adj pval: 0.076
Frequency
Tumor−Normal Expression Difference
0.0
0.2
0.4
0.6
0.8
1.0
−2
−1
0
1
2

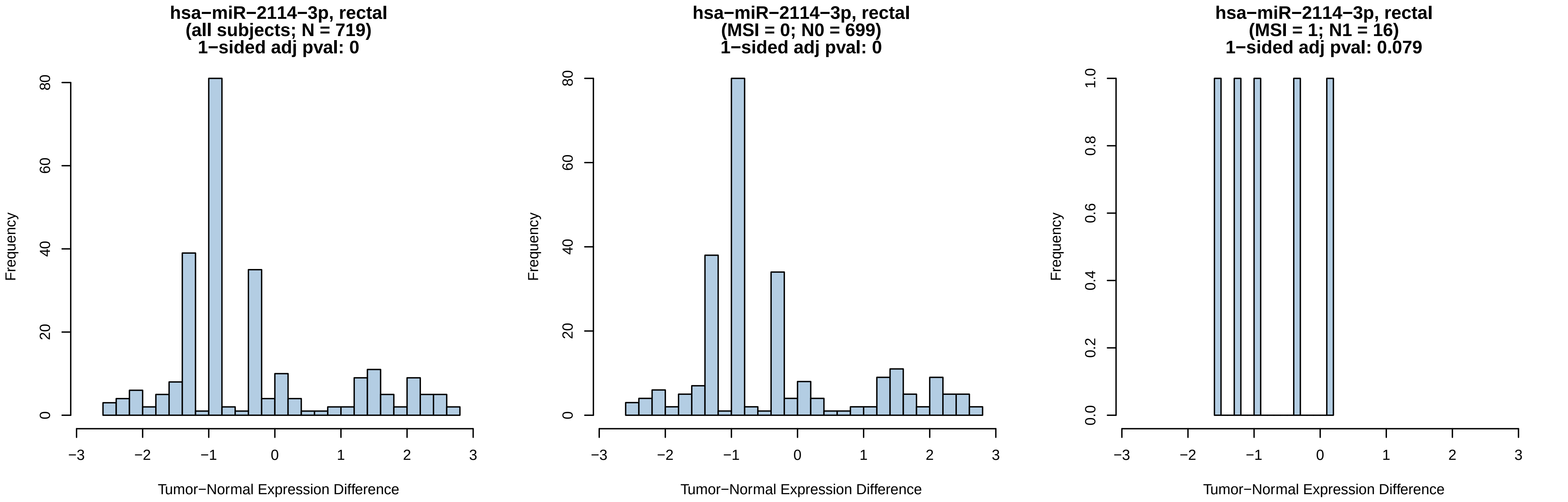
hsa−miR−2114−3p, rectal
(all subjects; N = 719)
1−sided adj pval: 0
hsa−miR−2114−3p, rectal
(MSI = 0; N0 = 699)
1−sided adj pval: 0
hsa−miR−2114−3p, rectal
(MSI = 1; N1 = 16)
1−sided adj pval: 0.079
Frequency
Tumor−Normal Expression Difference
0
20
40
60
80
0.0
0.2
0.4
0.6
0.8
1.0
−3
−2
−1
0
1
2
3

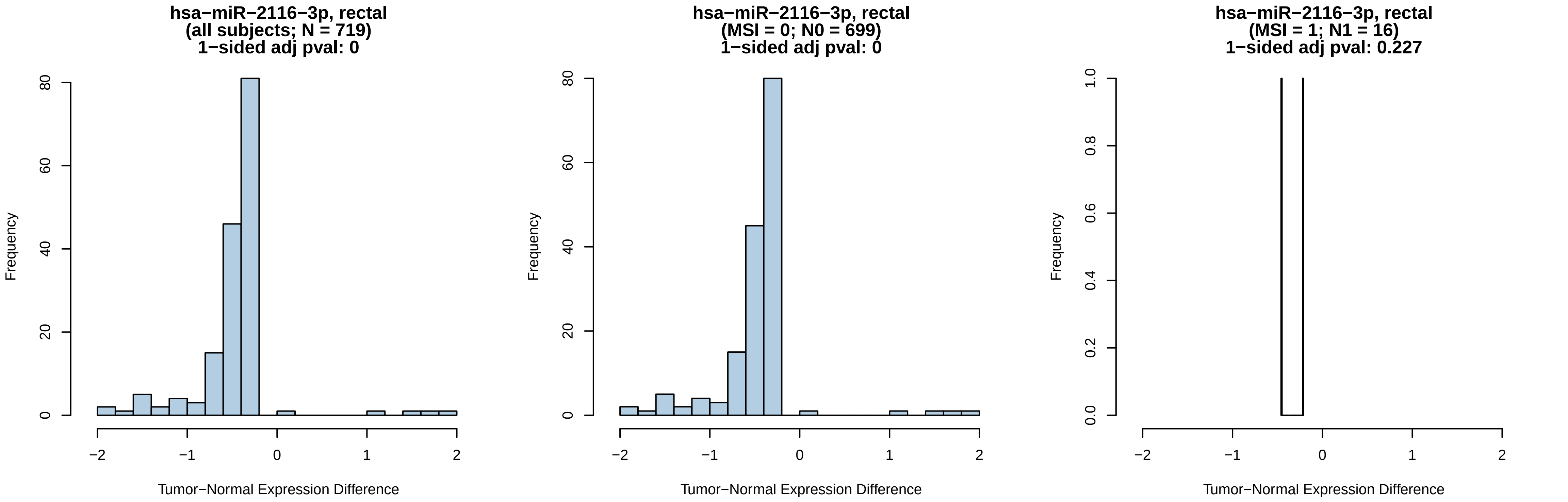

hsa−miR−2116−3p, rectal
(all subjects; N = 719)
1−sided adj pval: 0
Frequency
Tumor−Normal Expression Difference
hsa−miR−2116−3p, rectal
(MSI = 0; N0 = 699)
1−sided adj pval: 0
Frequency
Tumor−Normal Expression Difference
hsa−miR−2116−3p, rectal
(MSI = 1; N1 = 16)
1−sided adj pval: 0.227
Frequency
Tumor−Normal Expression Difference

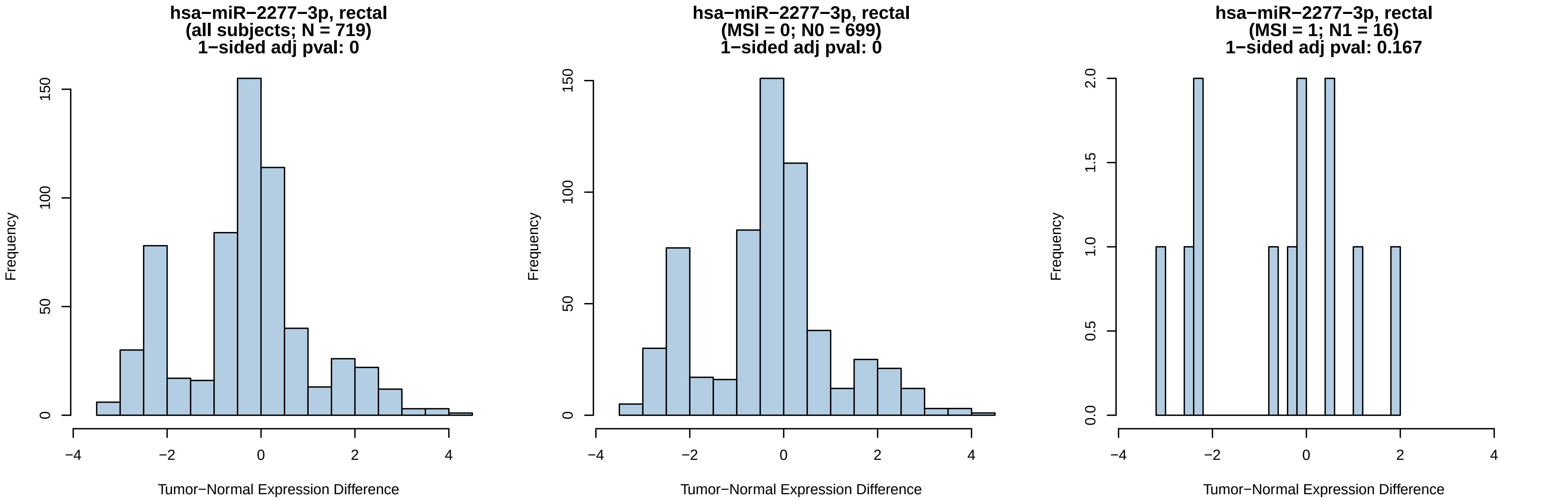

hsa−miR−2277−3p, rectal
(all subjects; N = 719)
1−sided adj pval: 0
Frequency
Tumor−Normal Expression Difference
hsa−miR−2277−3p, rectal
(MSI = 0; N0 = 699)
1−sided adj pval: 0
Frequency
Tumor−Normal Expression Difference
hsa−miR−2277−3p, rectal
(MSI = 1; N1 = 16)
1−sided adj pval: 0.167
Frequency
Tumor−Normal Expression Difference

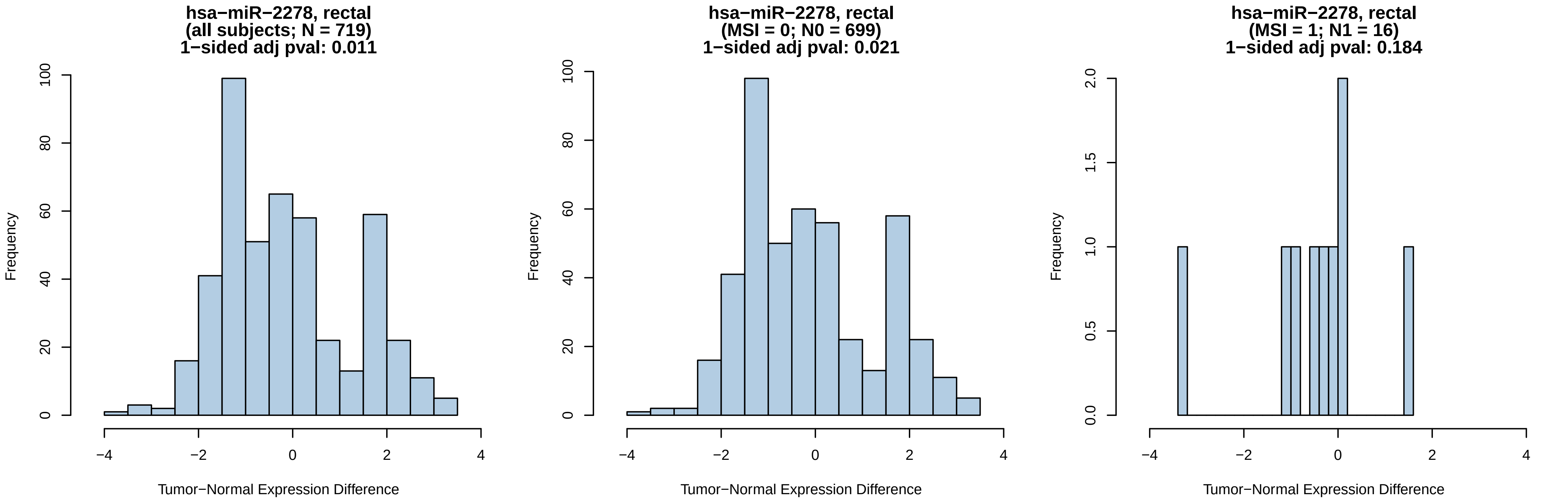
hsa−miR−2278, rectal
(all subjects; N = 719)
1−sided adj pval: 0.011
Frequency
Tumor−Normal Expression Difference
hsa−miR−2278, rectal
(MSI = 0; N0 = 699)
1−sided adj pval: 0.021
Frequency
Tumor−Normal Expression Difference
hsa−miR−2278, rectal
(MSI = 1; N1 = 16)
1−sided adj pval: 0.184
Frequency
Tumor−Normal Expression Difference

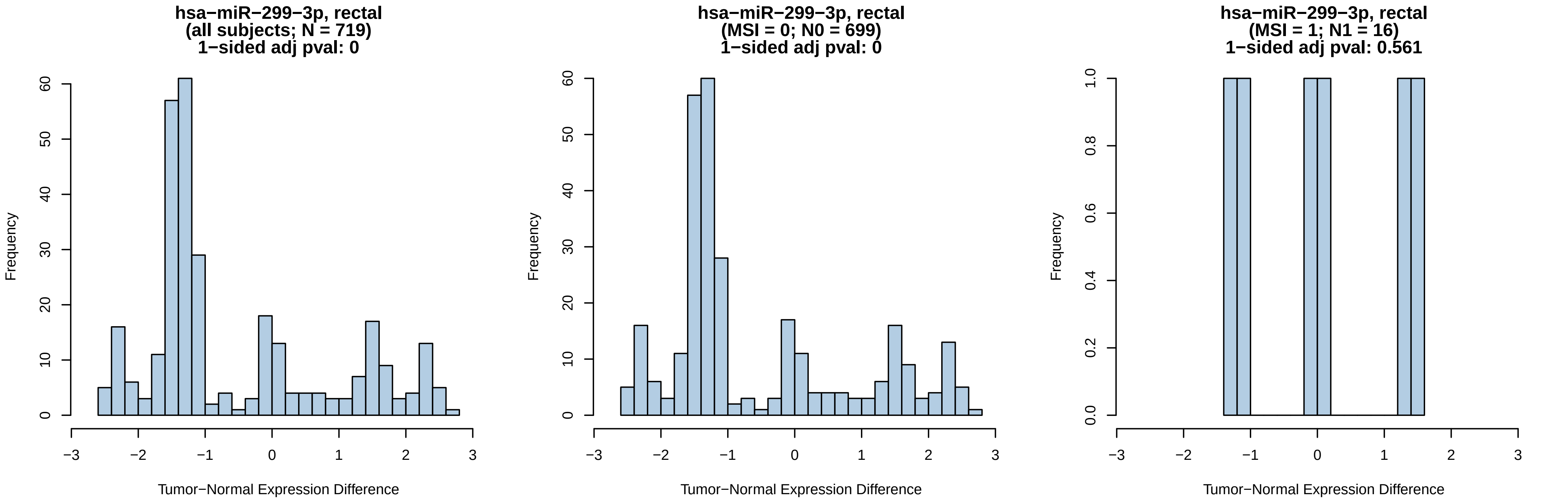

hsa−miR−299−3p, rectal
(all subjects; N = 719)
1−sided adj pval: 0
Frequency
Tumor−Normal Expression Difference
hsa−miR−299−3p, rectal
(MSI = 0; N0 = 699)
1−sided adj pval: 0
Frequency
Tumor−Normal Expression Difference
hsa−miR−299−3p, rectal
(MSI = 1; N1 = 16)
1−sided adj pval: 0.561
Frequency
Tumor−Normal Expression Difference

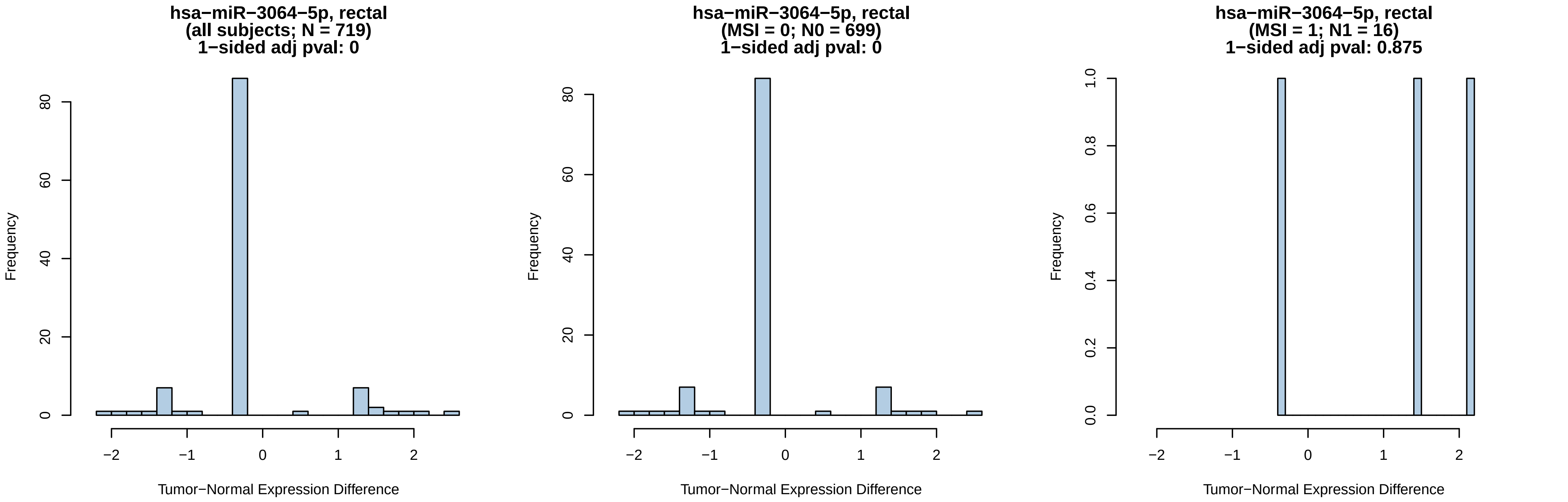
hsa−miR−3064−5p, rectal
(all subjects; N = 719)
1−sided adj pval: 0
Frequency
0
20
40
60
80
−2
−1
0
1
2
Tumor−Normal Expression Difference
hsa−miR−3064−5p, rectal
(MSI = 0; N0 = 699)
1−sided adj pval: 0
Frequency
0
20
40
60
80
−2
−1
0
1
2
Tumor−Normal Expression Difference
hsa−miR−3064−5p, rectal
(MSI = 1; N1 = 16)
1−sided adj pval: 0.875
Frequency
0.0
0.2
0.4
0.6
0.8
1.0
−2
−1
0
1
2
Tumor−Normal Expression Difference

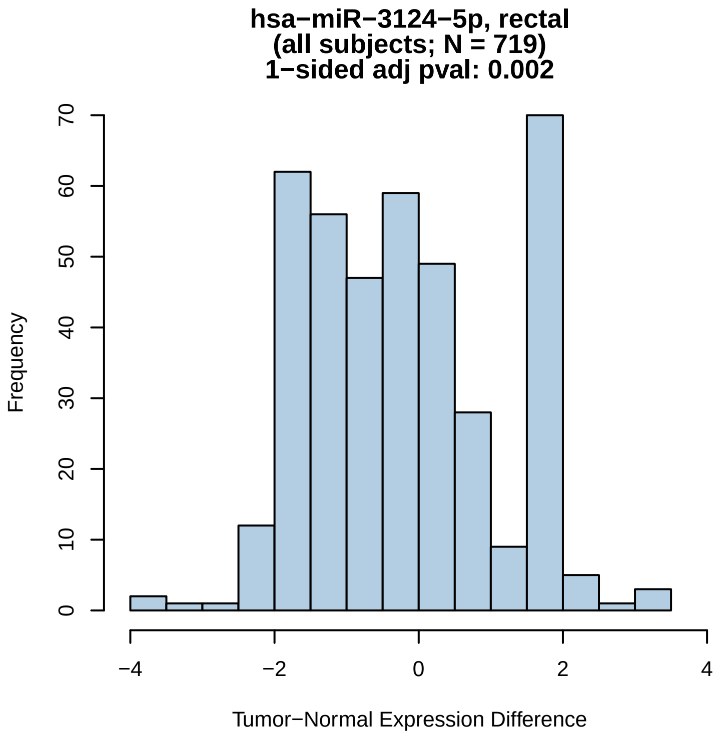

hsa−miR−3124−5p, rectal
(all subjects; N = 719)
1−sided adj pval: 0.002
Frequency
Tumor−Normal Expression Difference


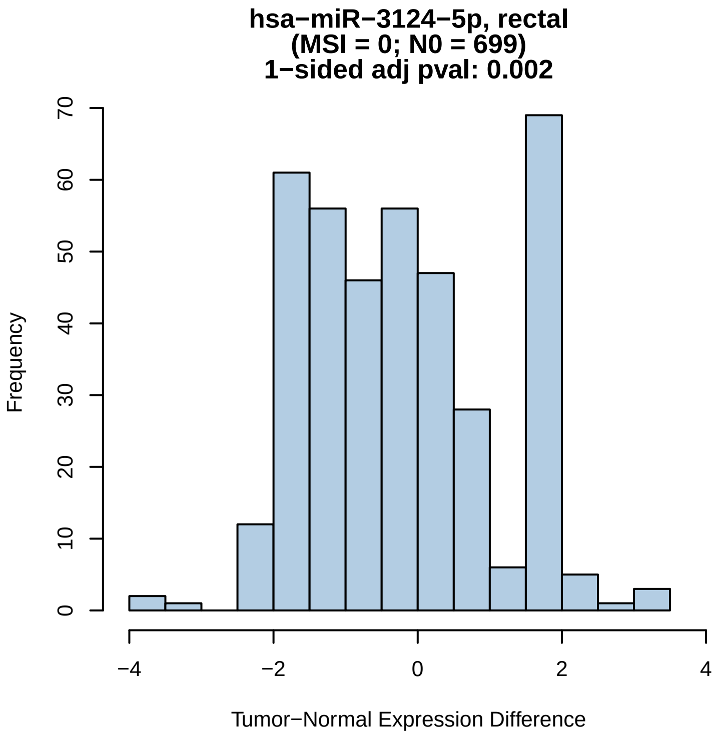

hsa−miR−3124−5p, rectal
(MSI = 0; N0 = 699)
1−sided adj pval: 0.002
Frequency
Tumor−Normal Expression Difference


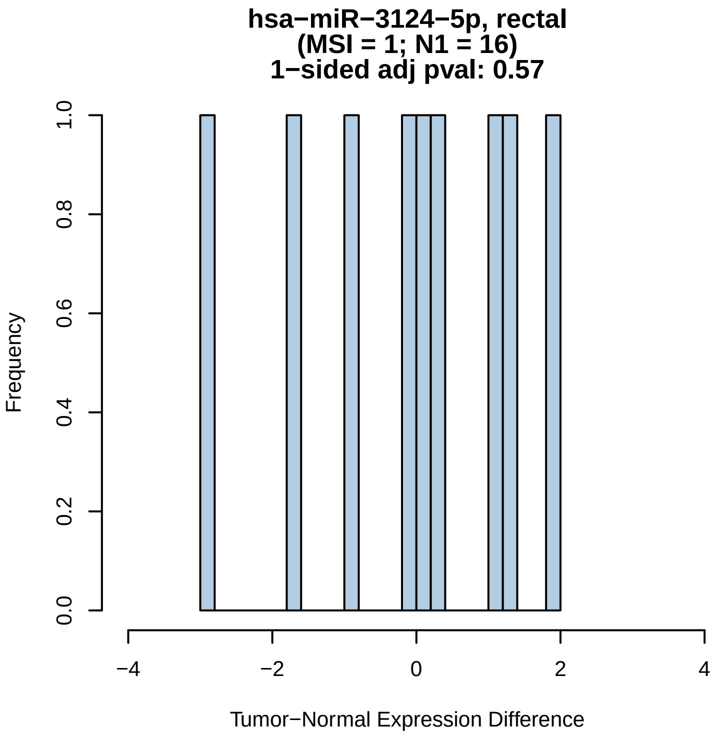

hsa−miR−3124−5p, rectal
(MSI = 1; N1 = 16)
1−sided adj pval: 0.57
Frequency
Tumor−Normal Expression Difference

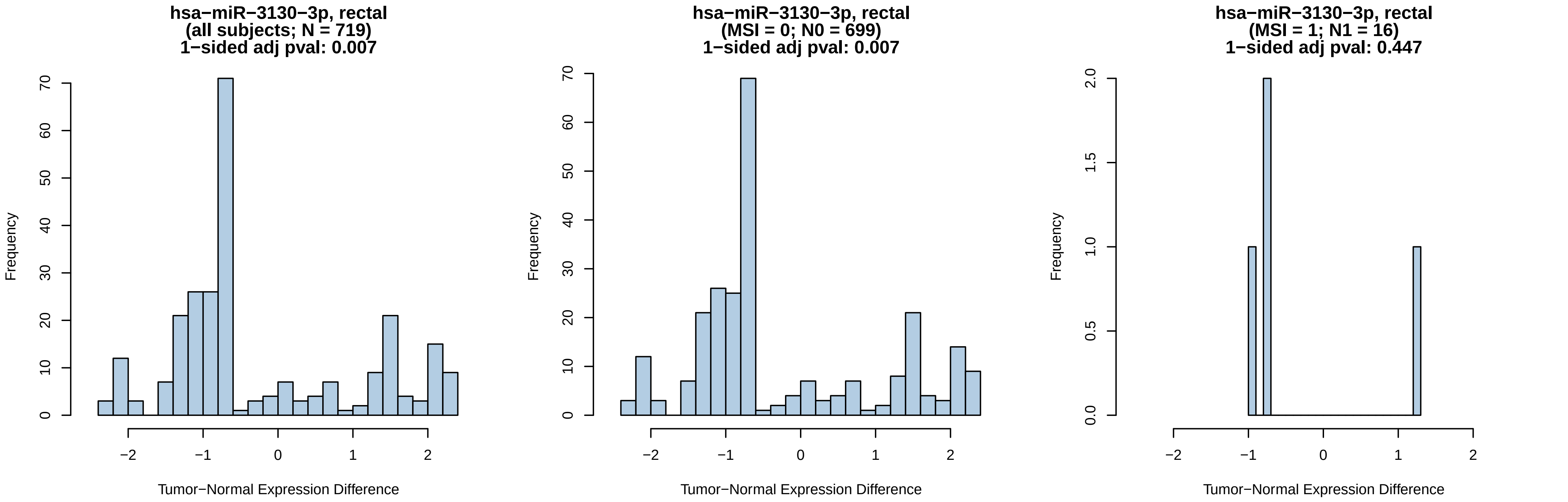

hsa−miR−3130−3p, rectal
(all subjects; N = 719)
1−sided adj pval: 0.007
Frequency
Tumor−Normal Expression Difference
hsa−miR−3130−3p, rectal
(MSI = 0; N0 = 699)
1−sided adj pval: 0.007
Frequency
Tumor−Normal Expression Difference
hsa−miR−3130−3p, rectal
(MSI = 1; N1 = 16)
1−sided adj pval: 0.447
Frequency
Tumor−Normal Expression Difference

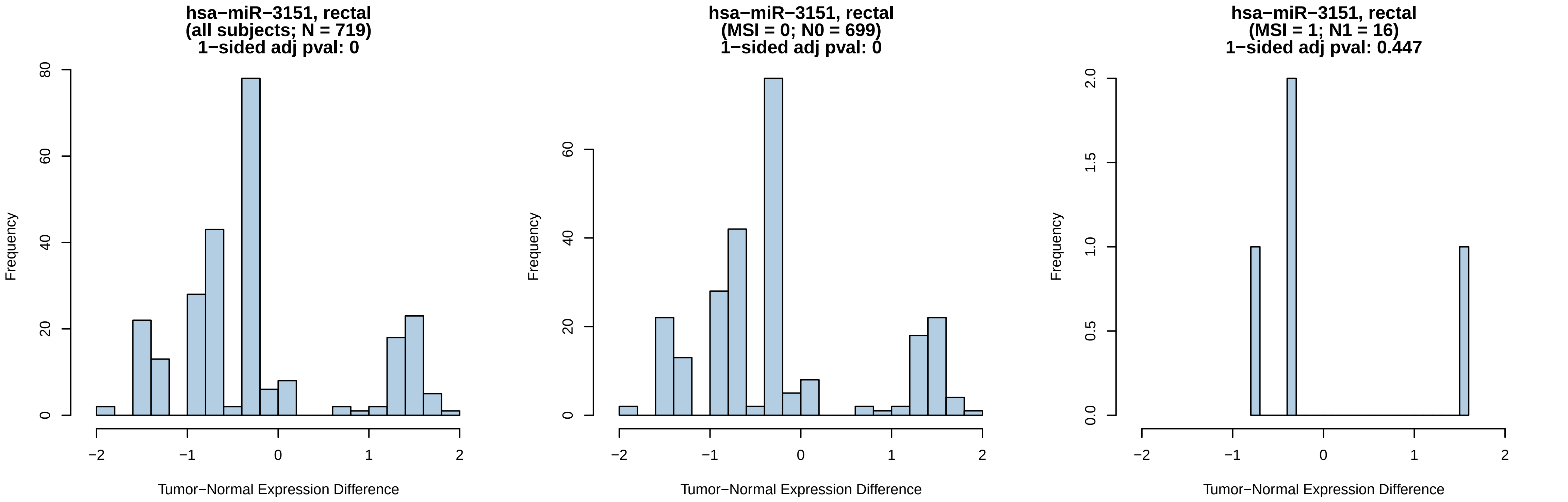
hsa−miR−3151, rectal
(all subjects; N = 719)
1−sided adj pval: 0
Frequency
Tumor−Normal Expression Difference
hsa−miR−3151, rectal
(MSI = 0; N0 = 699)
1−sided adj pval: 0
Frequency
Tumor−Normal Expression Difference
hsa−miR−3151, rectal
(MSI = 1; N1 = 16)
1−sided adj pval: 0.447
Frequency
Tumor−Normal Expression Difference

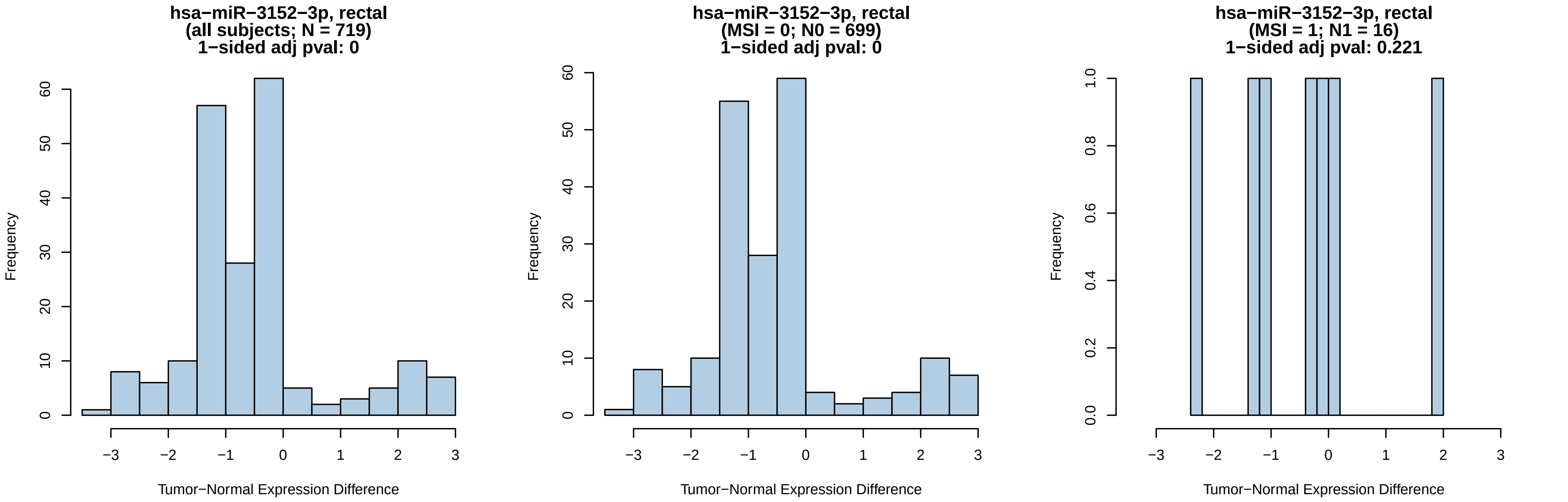

hsa−miR−3152−3p, rectal
(all subjects; N = 719)
1−sided adj pval: 0
hsa−miR−3152−3p, rectal
(MSI = 0; N0 = 699)
1−sided adj pval: 0
hsa−miR−3152−3p, rectal
(MSI = 1; N1 = 16)
1−sided adj pval: 0.221
Frequency
Tumor−Normal Expression Difference

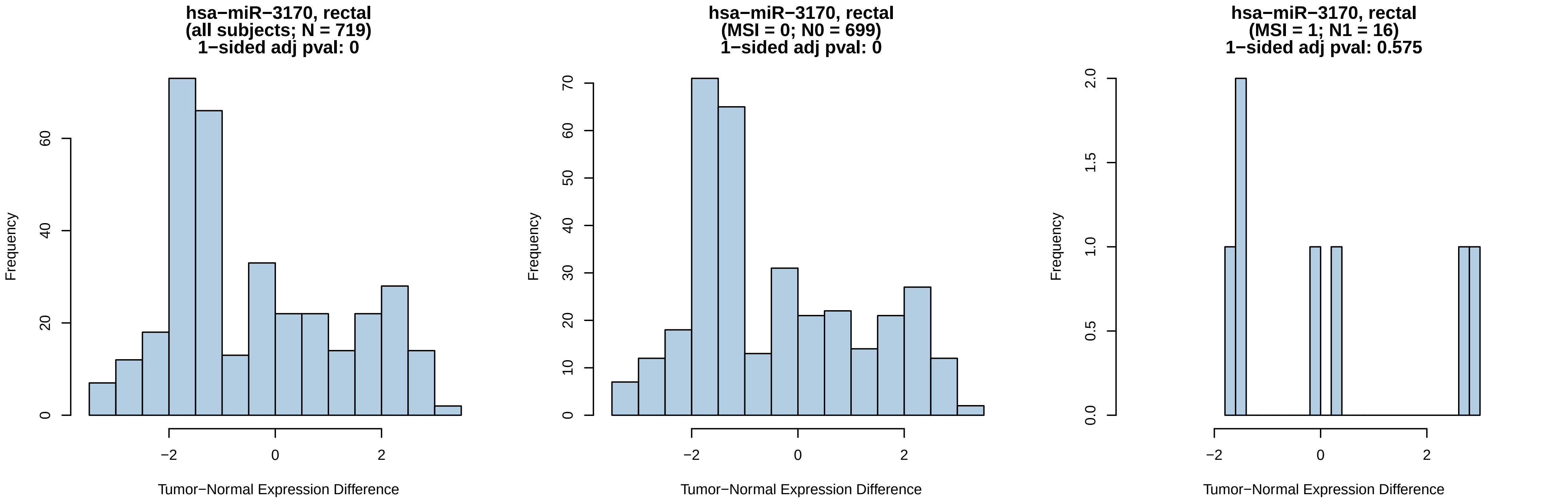
hsa−miR−3170, rectal
(all subjects; N = 719)
1−sided adj pval: 0
Frequency
Tumor−Normal Expression Difference
hsa−miR−3170, rectal
(MSI = 0; N0 = 699)
1−sided adj pval: 0
Frequency
Tumor−Normal Expression Difference
hsa−miR−3170, rectal
(MSI = 1; N1 = 16)
1−sided adj pval: 0.575
Frequency
Tumor−Normal Expression Difference

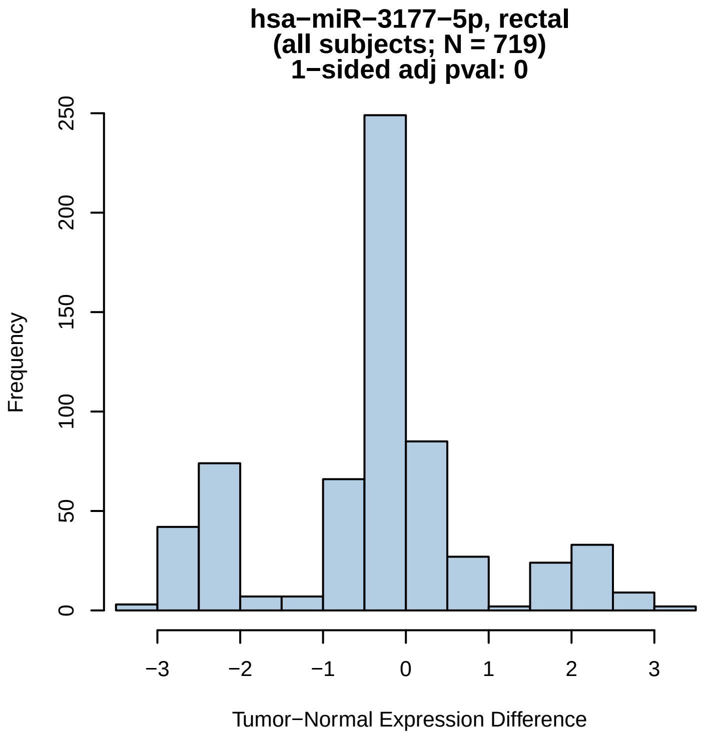

hsa−miR−3177−5p, rectal
(all subjects; N = 719)
1−sided adj pval: 0
Frequency
0
50
100
150
200
250
−3
−2
−1
0
1
2
3
Tumor−Normal Expression Difference


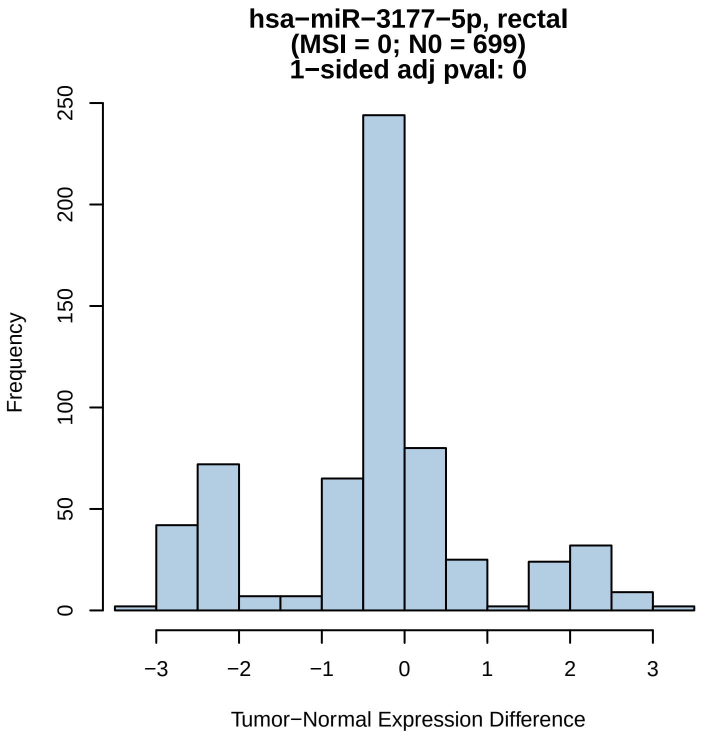

hsa−miR−3177−5p, rectal
(MSI = 0; N0 = 699)
1−sided adj pval: 0
Frequency
0
50
100
150
200
250
−3
−2
−1
0
1
2
3
Tumor−Normal Expression Difference


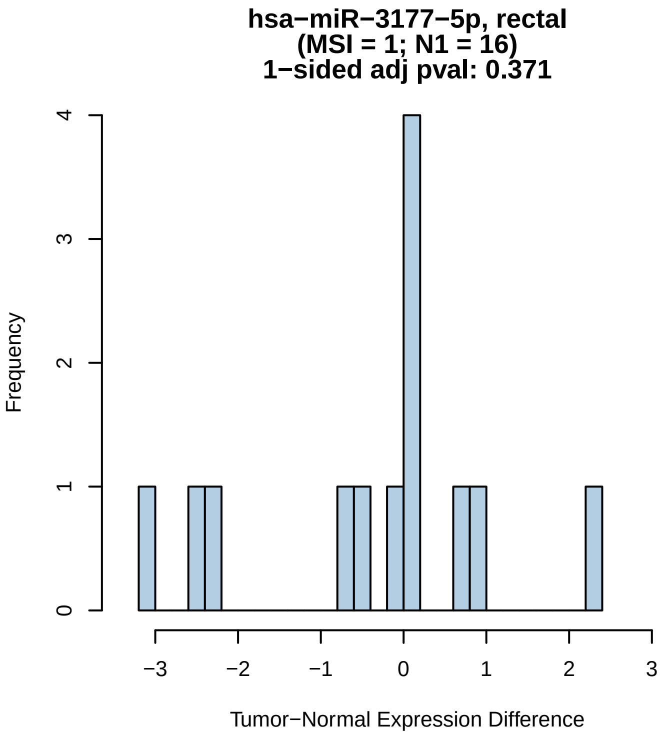

hsa−miR−3177−5p, rectal
(MSI = 1; N1 = 16)
1−sided adj pval: 0.371
Frequency
0
1
2
3
4
−3
−2
−1
0
1
2
3
Tumor−Normal Expression Difference

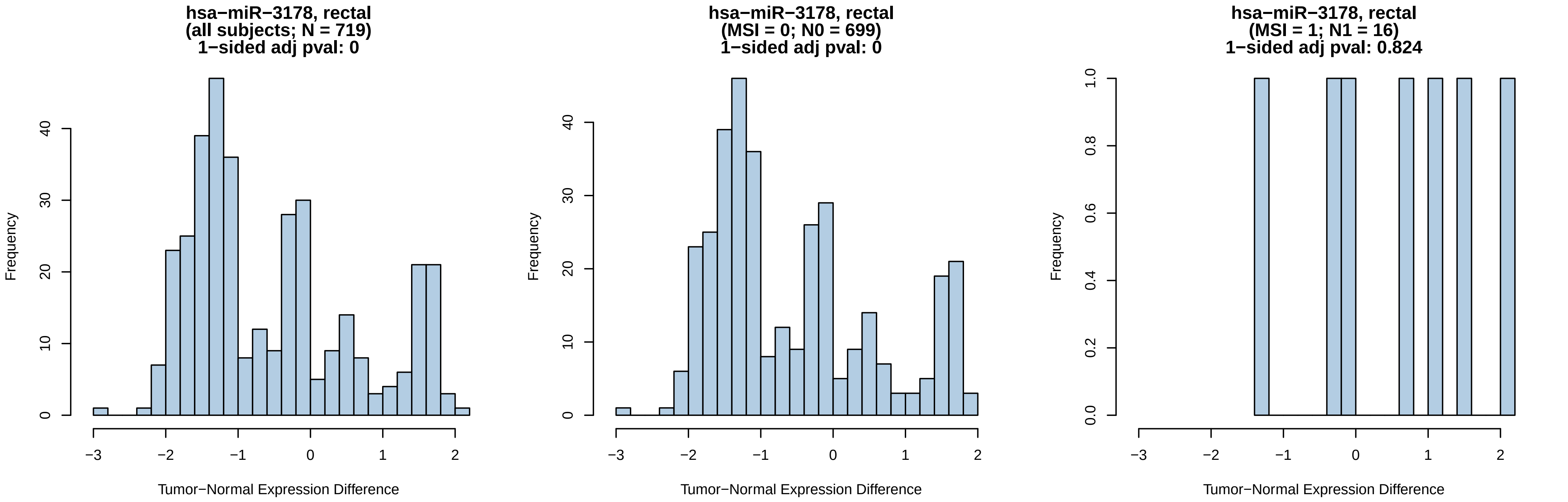
hsa−miR−3178, rectal
(all subjects; N = 719)
1−sided adj pval: 0
Frequency
Tumor−Normal Expression Difference
hsa−miR−3178, rectal
(MSI = 0; N0 = 699)
1−sided adj pval: 0
Frequency
Tumor−Normal Expression Difference
hsa−miR−3178, rectal
(MSI = 1; N1 = 16)
1−sided adj pval: 0.824
Frequency
Tumor−Normal Expression Difference

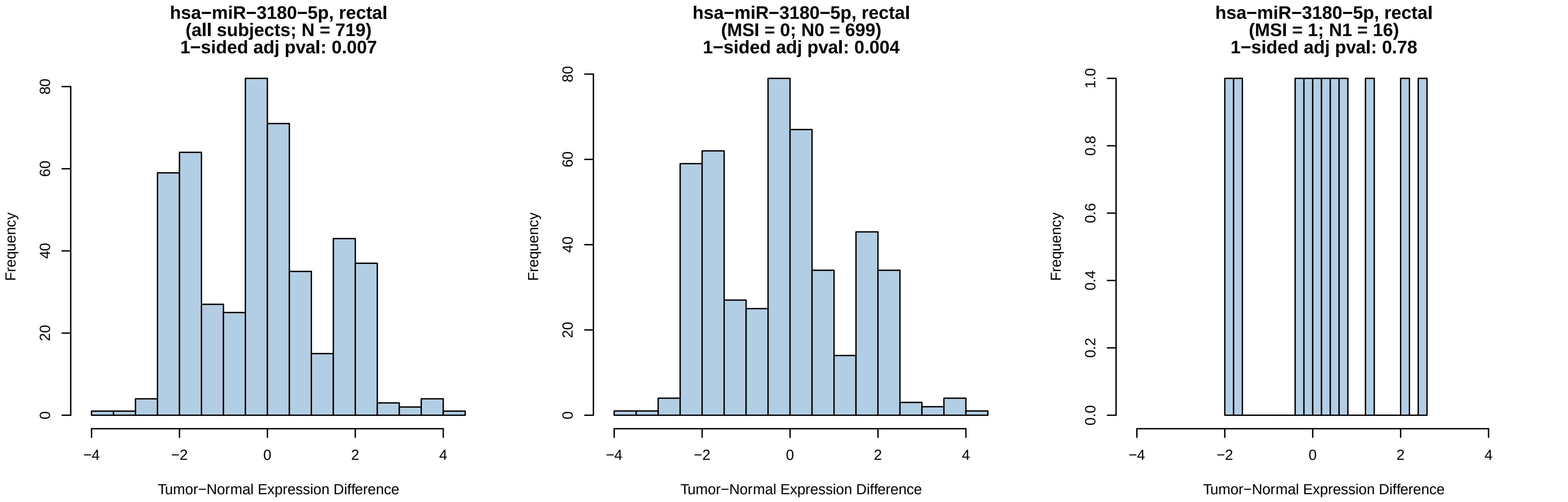

hsa−miR−3180−5p, rectal
(all subjects; N = 719)
1−sided adj pval: 0.007
Frequency
Tumor−Normal Expression Difference
hsa−miR−3180−5p, rectal
(MSI = 0; N0 = 699)
1−sided adj pval: 0.004
Frequency
Tumor−Normal Expression Difference
hsa−miR−3180−5p, rectal
(MSI = 1; N1 = 16)
1−sided adj pval: 0.78
Frequency
Tumor−Normal Expression Difference

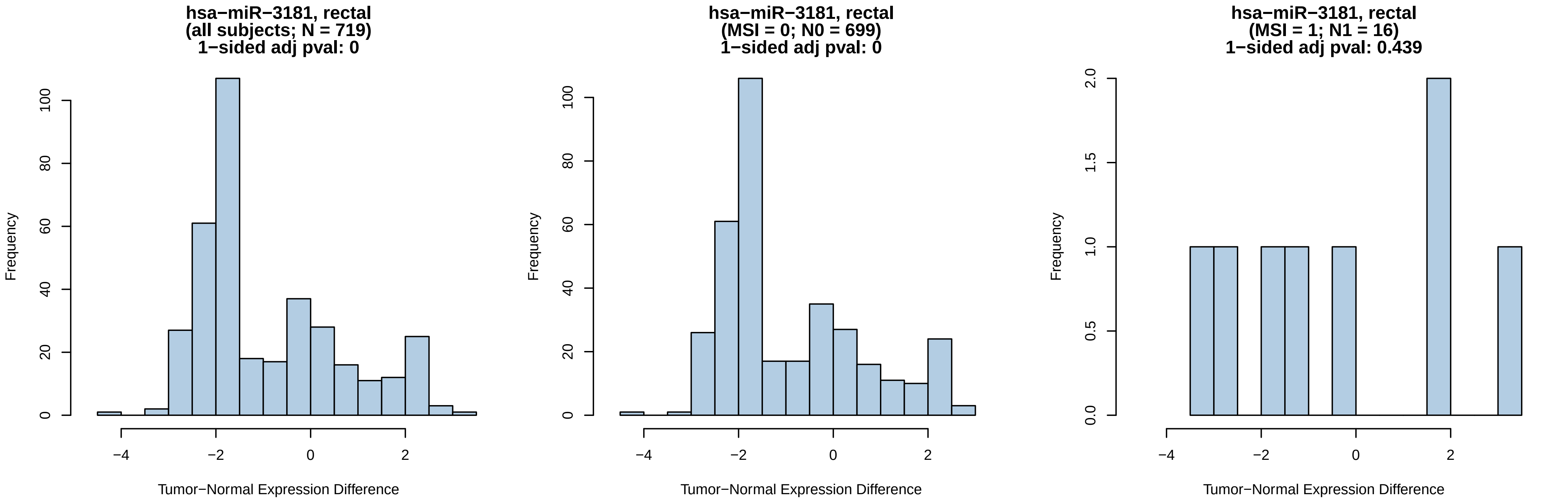

hsa−miR−3181, rectal
(all subjects; N = 719)
1−sided adj pval: 0
hsa−miR−3181, rectal
(MSI = 0; N0 = 699)
1−sided adj pval: 0
hsa−miR−3181, rectal
(MSI = 1; N1 = 16)
1−sided adj pval: 0.439
Frequency
Tumor−Normal Expression Difference

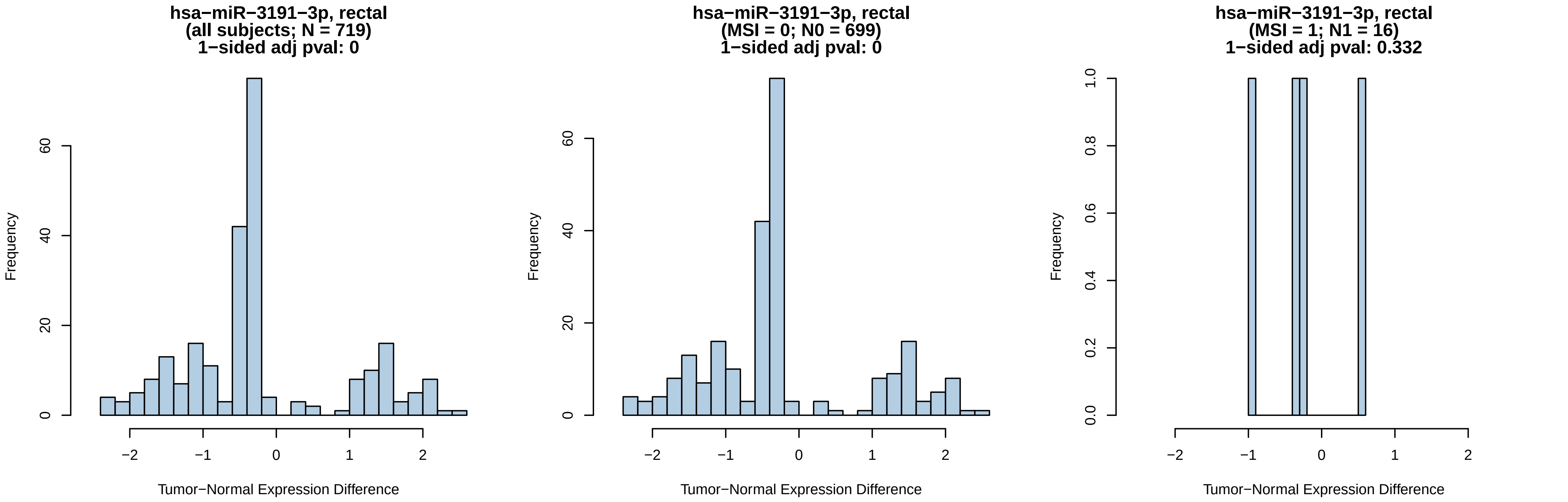

hsa−miR−3191−3p, rectal
(all subjects; N = 719)
1−sided adj pval: 0
Frequency
Tumor−Normal Expression Difference
hsa−miR−3191−3p, rectal
(MSI = 0; N0 = 699)
1−sided adj pval: 0
Frequency
Tumor−Normal Expression Difference
hsa−miR−3191−3p, rectal
(MSI = 1; N1 = 16)
1−sided adj pval: 0.332
Frequency
Tumor−Normal Expression Difference

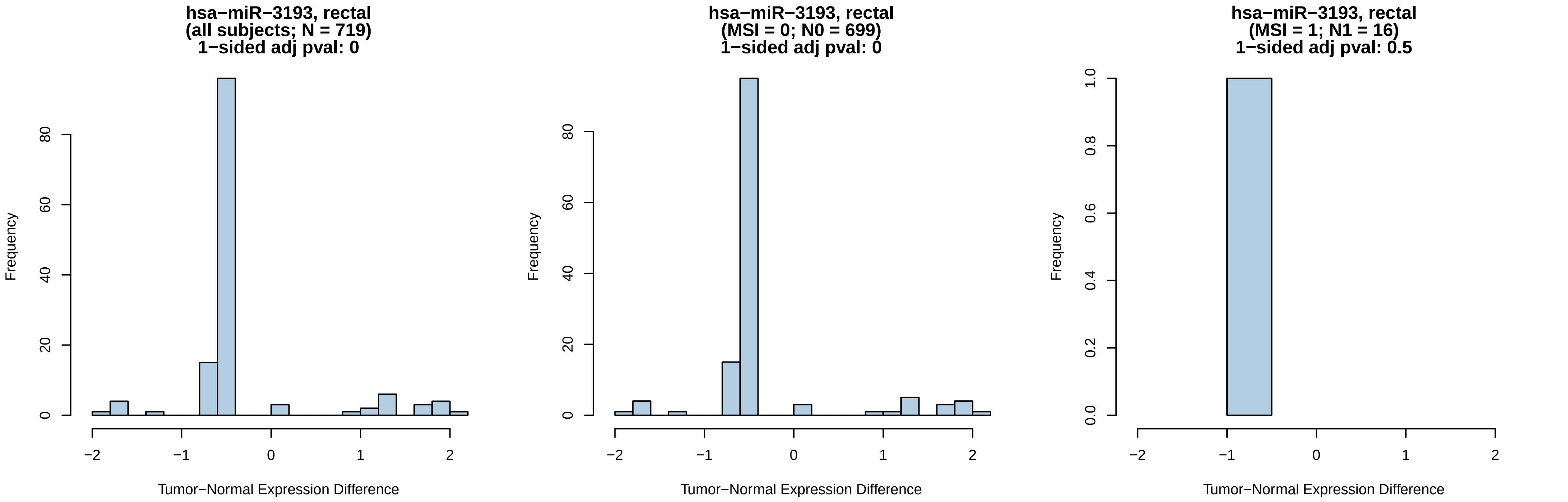
hsa−miR−3193, rectal
(all subjects; N = 719)
1−sided adj pval: 0
hsa−miR−3193, rectal
(MSI = 0; N0 = 699)
1−sided adj pval: 0
hsa−miR−3193, rectal
(MSI = 1; N1 = 16)
1−sided adj pval: 0.5
Frequency
Tumor−Normal Expression Difference

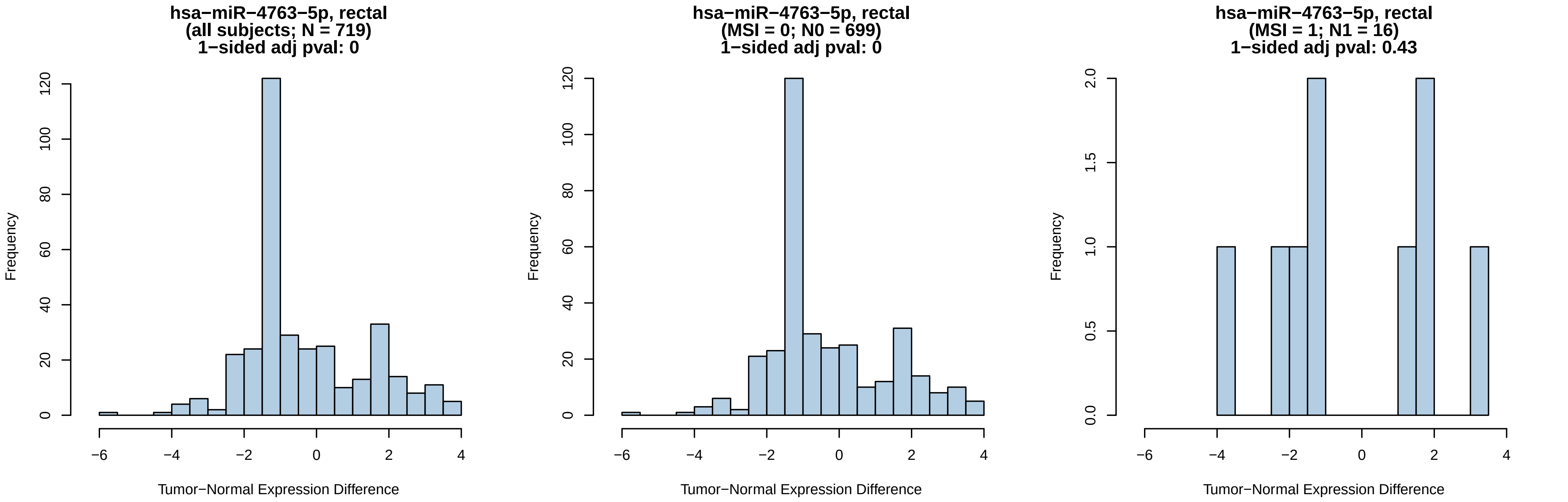
hsa−miR−4763−5p, rectal
(all subjects; N = 719)
1−sided adj pval: 0
Frequency
Tumor−Normal Expression Difference
hsa−miR−4763−5p, rectal
(MSI = 0; N0 = 699)
1−sided adj pval: 0
Frequency
Tumor−Normal Expression Difference
hsa−miR−4763−5p, rectal
(MSI = 1; N1 = 16)
1−sided adj pval: 0.43
Frequency
Tumor−Normal Expression Difference

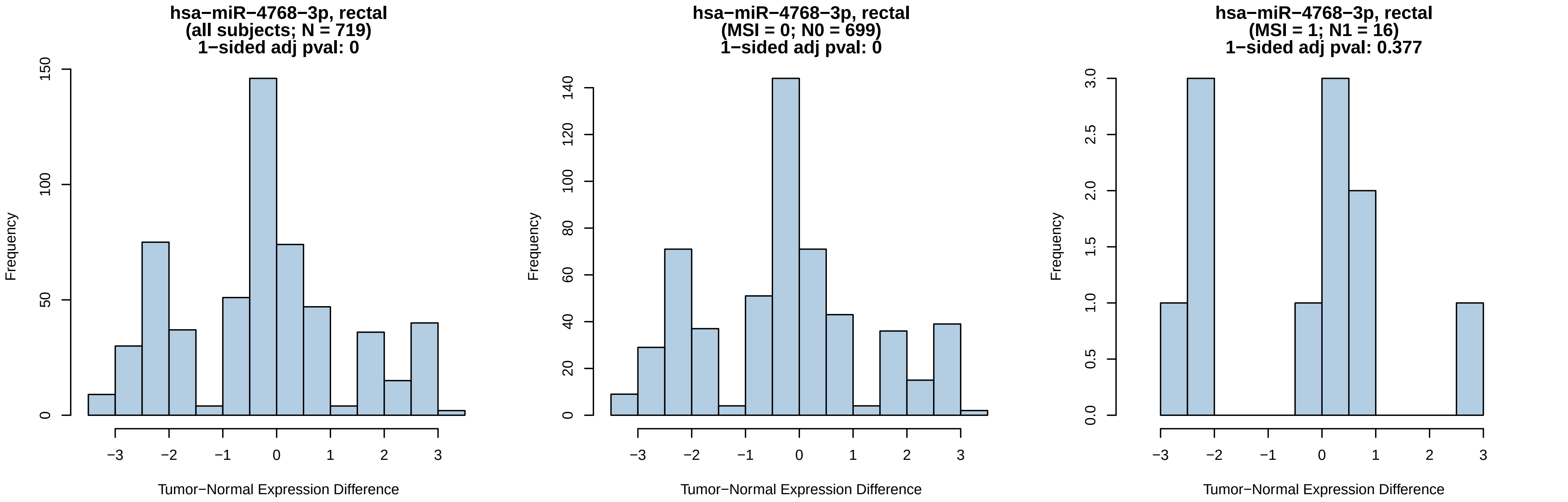
hsa−miR−4768−3p, rectal
(all subjects; N = 719)
1−sided adj pval: 0
hsa−miR−4768−3p, rectal
(MSI = 0; N0 = 699)
1−sided adj pval: 0
hsa−miR−4768−3p, rectal
(MSI = 1; N1 = 16)
1−sided adj pval: 0.377
Frequency
Tumor−Normal Expression Difference

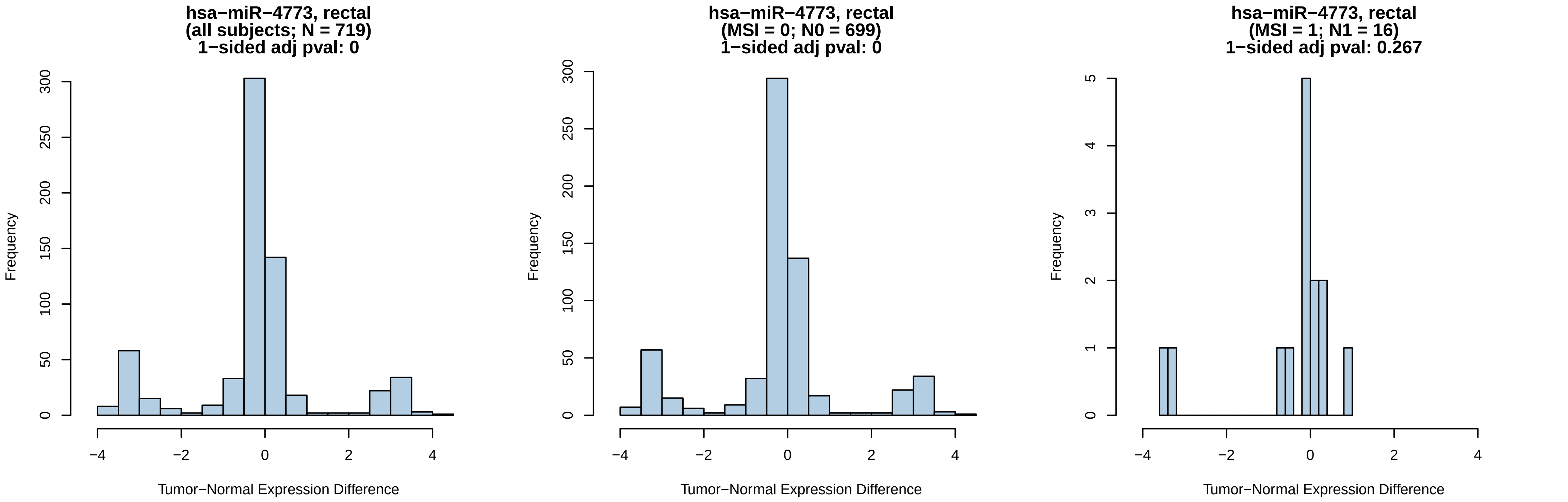
hsa−miR−4773, rectal
(all subjects; N = 719)
1−sided adj pval: 0
hsa−miR−4773, rectal
(MSI = 0; N0 = 699)
1−sided adj pval: 0
hsa−miR−4773, rectal
(MSI = 1; N1 = 16)
1−sided adj pval: 0.267
Frequency
Tumor−Normal Expression Difference

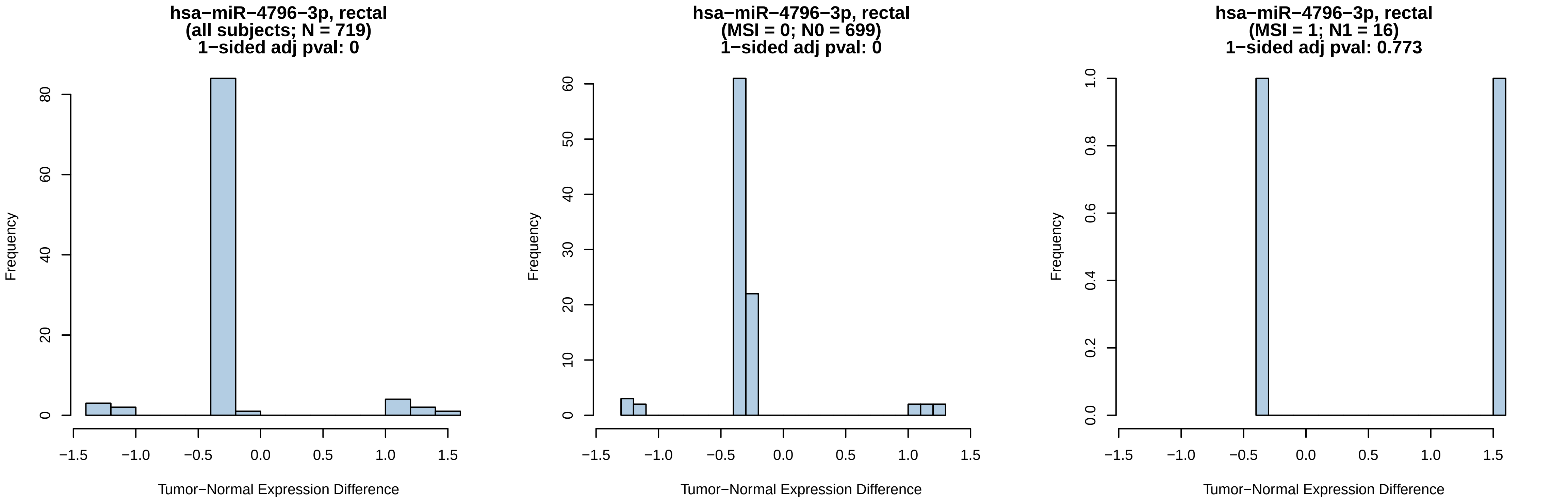
hsa−miR−4796−3p, rectal
(all subjects; N = 719)
1−sided adj pval: 0
Frequency
Tumor−Normal Expression Difference
hsa−miR−4796−3p, rectal
(MSI = 0; N0 = 699)
1−sided adj pval: 0
Frequency
Tumor−Normal Expression Difference
hsa−miR−4796−3p, rectal
(MSI = 1; N1 = 16)
1−sided adj pval: 0.773
Frequency
Tumor−Normal Expression Difference

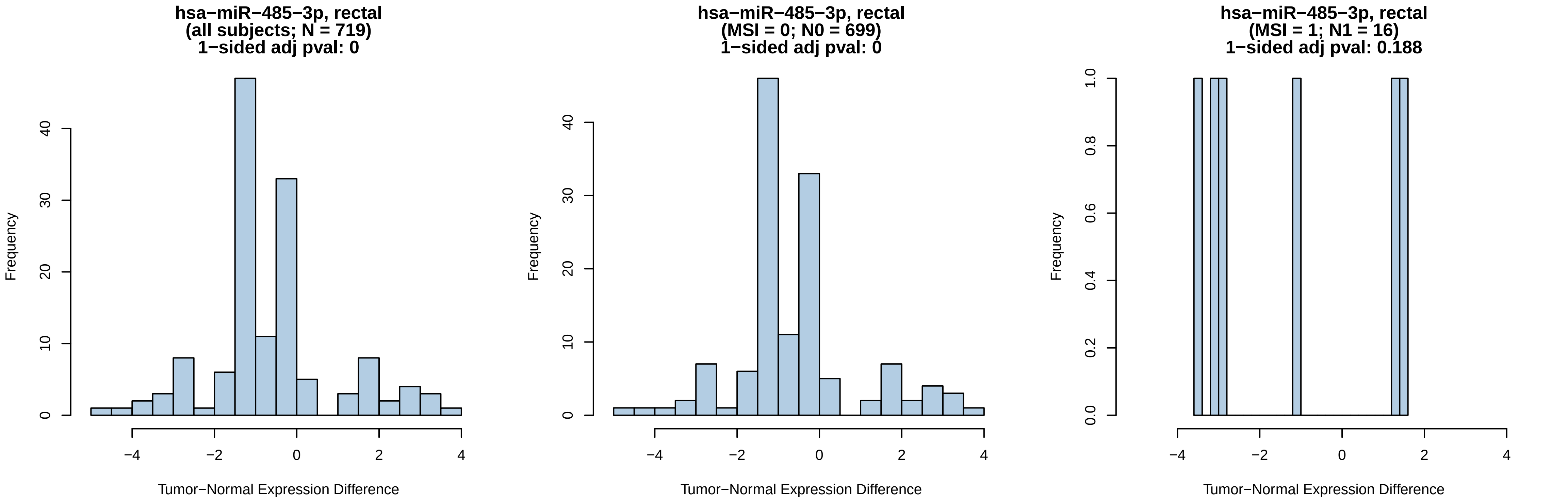

hsa−miR−485−3p, rectal
(all subjects; N = 719)
1−sided adj pval: 0
Frequency
Tumor−Normal Expression Difference
hsa−miR−485−3p, rectal
(MSI = 0; N0 = 699)
1−sided adj pval: 0
Frequency
Tumor−Normal Expression Difference
hsa−miR−485−3p, rectal
(MSI = 1; N1 = 16)
1−sided adj pval: 0.188
Frequency
Tumor−Normal Expression Difference

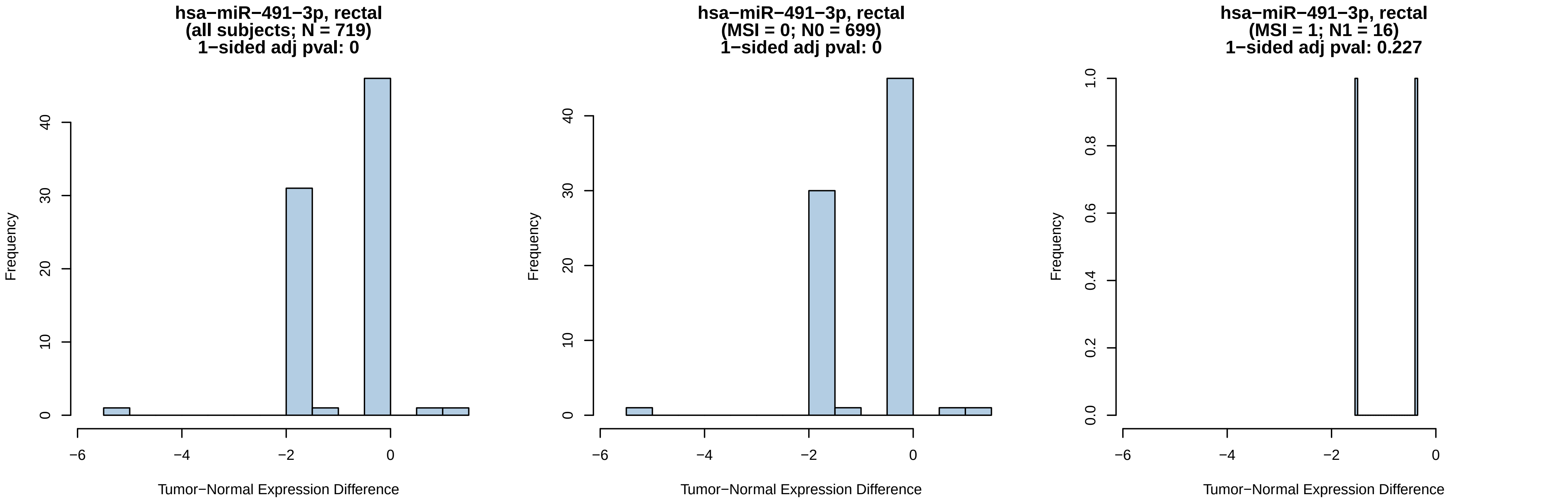
hsa−miR−491−3p, rectal
(all subjects; N = 719)
1−sided adj pval: 0
Frequency
0
10
20
30
40
−6
−4
−2
0
Tumor−Normal Expression Difference
hsa−miR−491−3p, rectal
(MSI = 0; N0 = 699)
1−sided adj pval: 0
Frequency
0
10
20
30
40
−6
−4
−2
0
Tumor−Normal Expression Difference
hsa−miR−491−3p, rectal
(MSI = 1; N1 = 16)
1−sided adj pval: 0.227
Frequency
0.0
0.2
0.4
0.6
0.8
1.0
−6
−4
−2
0
Tumor−Normal Expression Difference

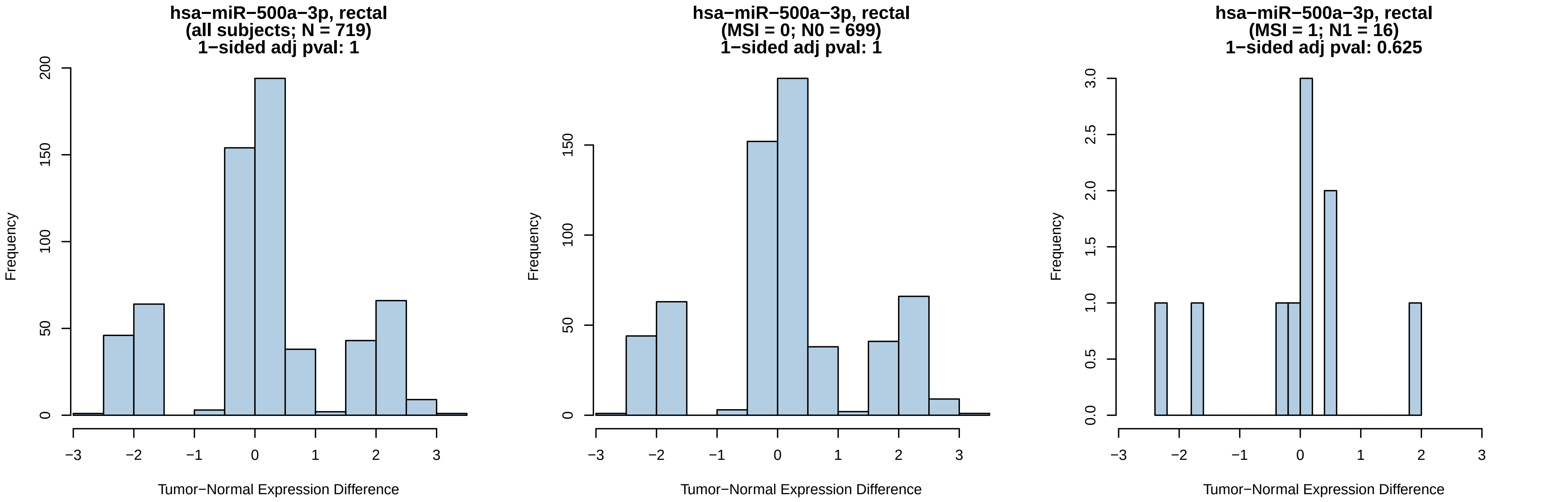

hsa−miR−500a−3p, rectal
(all subjects; N = 719)
1−sided adj pval: 1
Frequency
Tumor−Normal Expression Difference
hsa−miR−500a−3p, rectal
(MSI = 0; N0 = 699)
1−sided adj pval: 1
Frequency
Tumor−Normal Expression Difference
hsa−miR−500a−3p, rectal
(MSI = 1; N1 = 16)
1−sided adj pval: 0.625
Frequency
Tumor−Normal Expression Difference

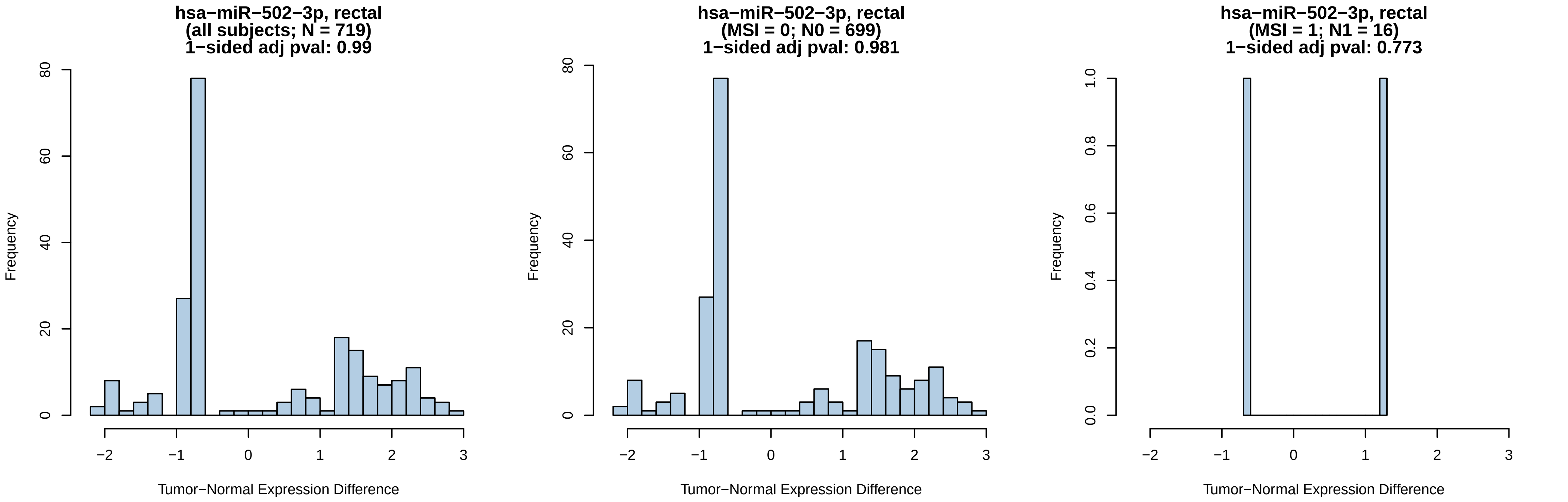
hsa−miR−502−3p, rectal
(all subjects; N = 719)
1−sided adj pval: 0.99
Frequency
Tumor−Normal Expression Difference
hsa−miR−502−3p, rectal
(MSI = 0; N0 = 699)
1−sided adj pval: 0.981
Frequency
Tumor−Normal Expression Difference
hsa−miR−502−3p, rectal
(MSI = 1; N1 = 16)
1−sided adj pval: 0.773
Frequency
Tumor−Normal Expression Difference

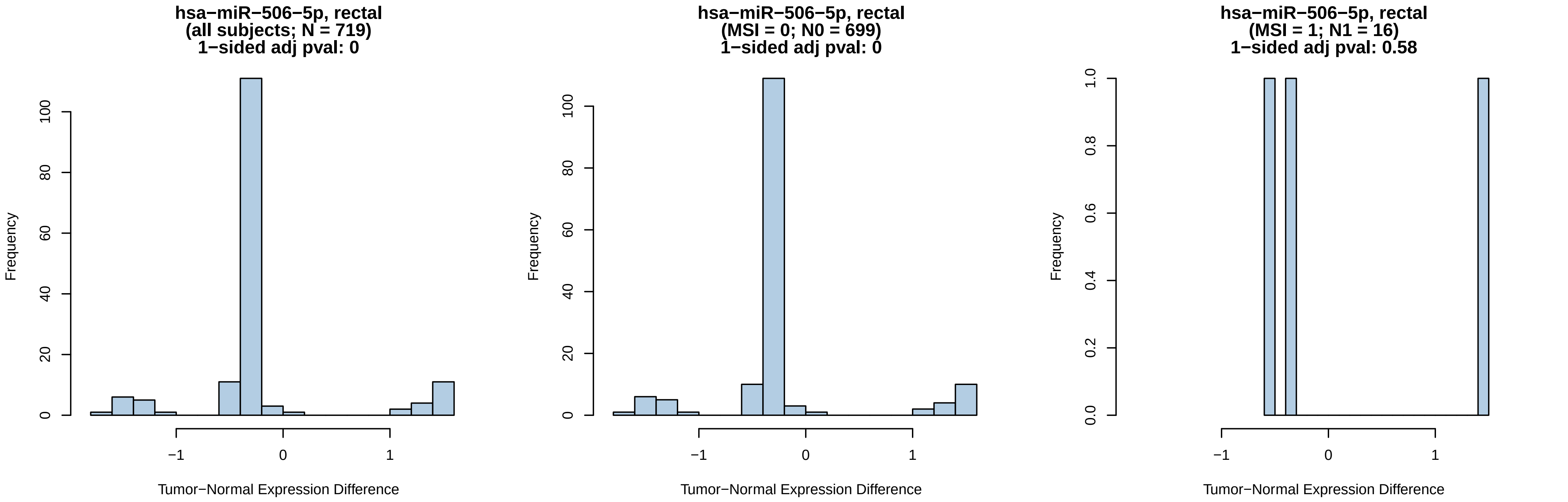

hsa−miR−506−5p, rectal
(all subjects; N = 719)
1−sided adj pval: 0
Frequency
0
20
40
60
80
100
−1
0
1
Tumor−Normal Expression Difference
hsa−miR−506−5p, rectal
(MSI = 0; N0 = 699)
1−sided adj pval: 0
Frequency
0
20
40
60
80
100
−1
0
1
Tumor−Normal Expression Difference
hsa−miR−506−5p, rectal
(MSI = 1; N1 = 16)
1−sided adj pval: 0.58
Frequency
0.0
0.2
0.4
0.6
0.8
1.0
−1
0
1
Tumor−Normal Expression Difference

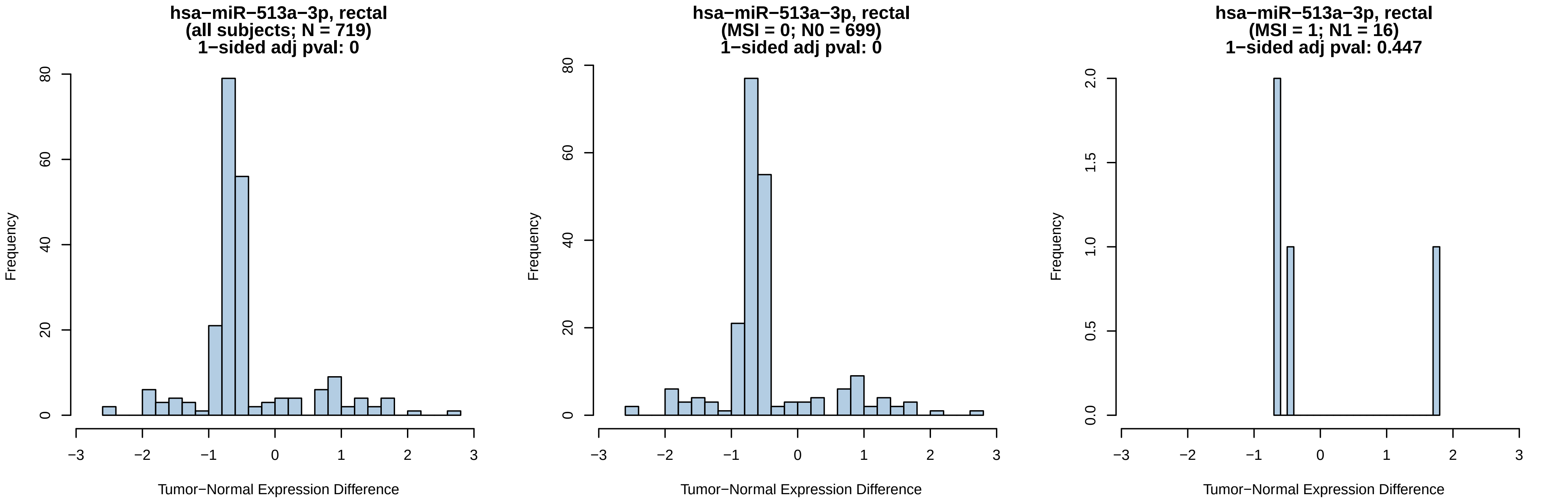
hsa−miR−513a−3p, rectal
(all subjects; N = 719)
1−sided adj pval: 0
Frequency
Tumor−Normal Expression Difference
hsa−miR−513a−3p, rectal
(MSI = 0; N0 = 699)
1−sided adj pval: 0
Frequency
Tumor−Normal Expression Difference
hsa−miR−513a−3p, rectal
(MSI = 1; N1 = 16)
1−sided adj pval: 0.447
Frequency
Tumor−Normal Expression Difference

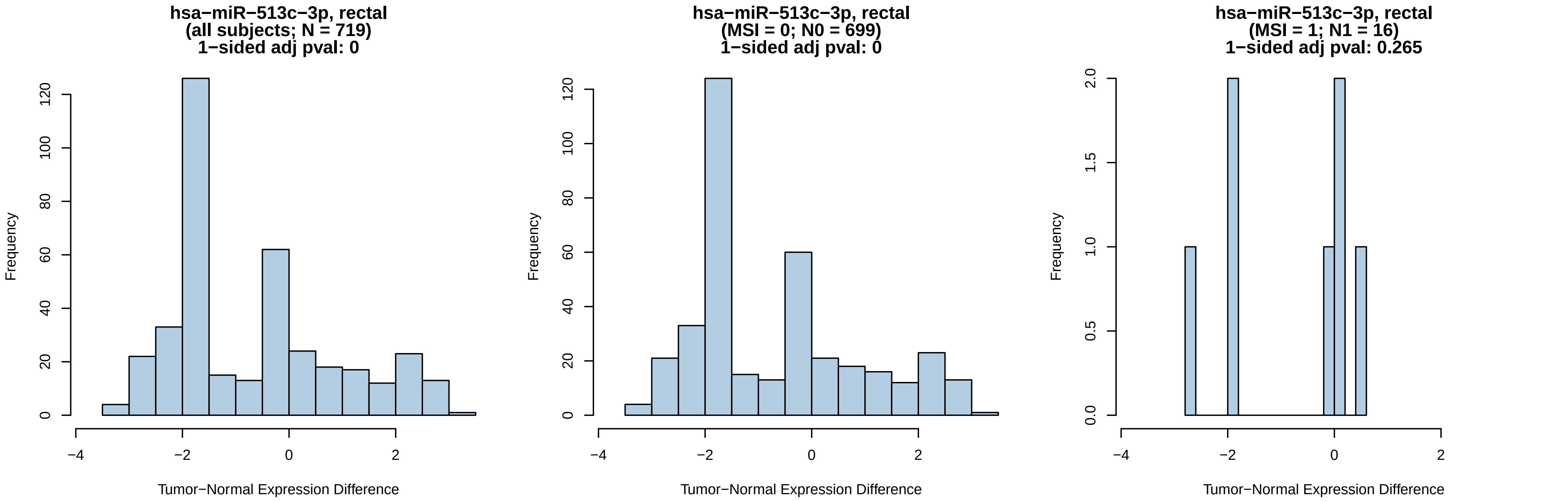

hsa−miR−513c−3p, rectal
(all subjects; N = 719)
1−sided adj pval: 0
Frequency
Tumor−Normal Expression Difference
hsa−miR−513c−3p, rectal
(MSI = 0; N0 = 699)
1−sided adj pval: 0
Frequency
Tumor−Normal Expression Difference
hsa−miR−513c−3p, rectal
(MSI = 1; N1 = 16)
1−sided adj pval: 0.265
Frequency
Tumor−Normal Expression Difference

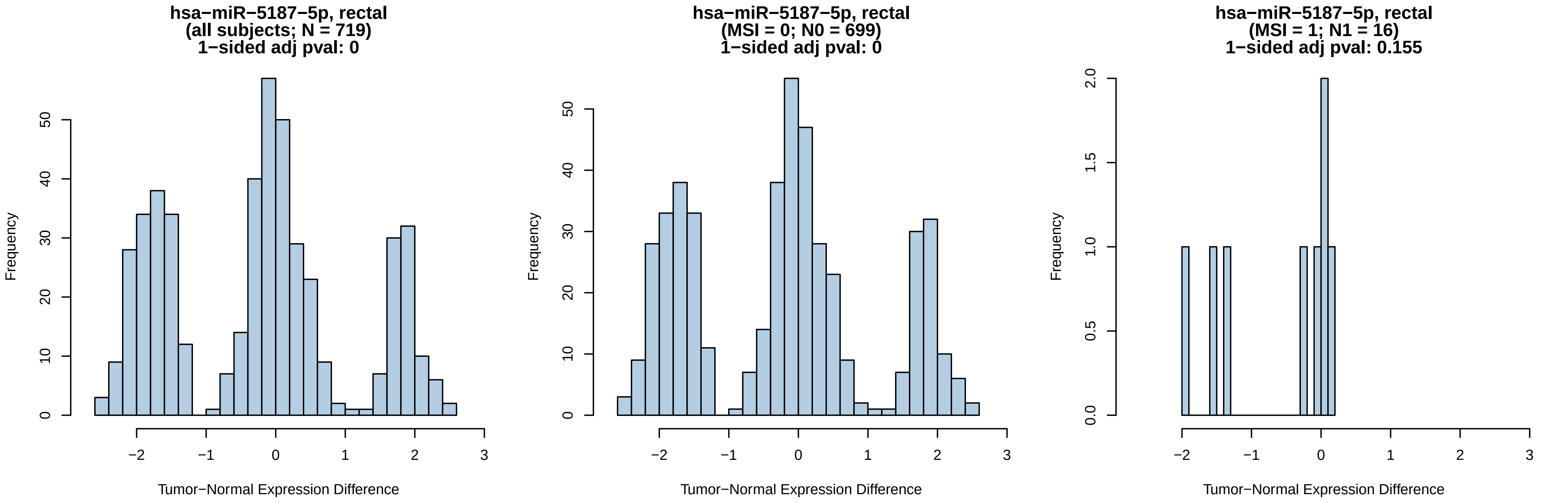

hsa−miR−5187−5p, rectal
(all subjects; N = 719)
1−sided adj pval: 0
Frequency
Tumor−Normal Expression Difference
hsa−miR−5187−5p, rectal
(MSI = 0; N0 = 699)
1−sided adj pval: 0
Frequency
Tumor−Normal Expression Difference
hsa−miR−5187−5p, rectal
(MSI = 1; N1 = 16)
1−sided adj pval: 0.155
Frequency
Tumor−Normal Expression Difference

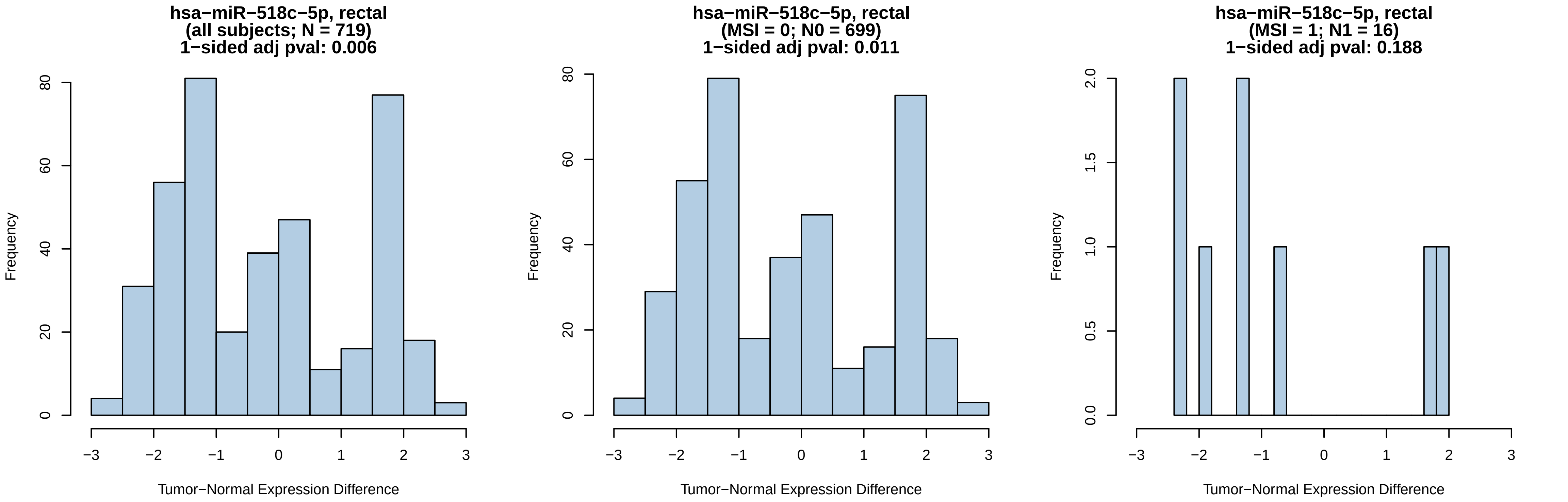

hsa−miR−518c−5p, rectal
(all subjects; N = 719)
1−sided adj pval: 0.006
Frequency
Tumor−Normal Expression Difference
hsa−miR−518c−5p, rectal
(MSI = 0; N0 = 699)
1−sided adj pval: 0.011
Frequency
Tumor−Normal Expression Difference
hsa−miR−518c−5p, rectal
(MSI = 1; N1 = 16)
1−sided adj pval: 0.188
Frequency
Tumor−Normal Expression Difference

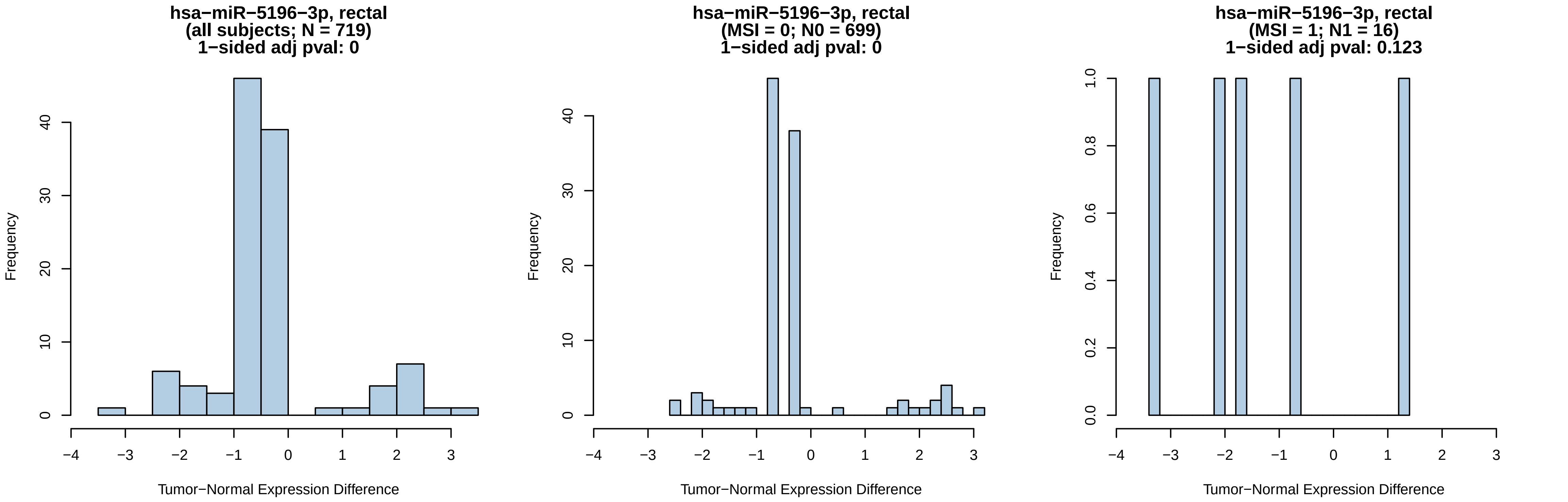
hsa−miR−5196−3p, rectal
(all subjects; N = 719)
1−sided adj pval: 0
Frequency
0
10
20
30
40
−4
−3
−2
−1
0
1
2
3
Tumor−Normal Expression Difference
hsa−miR−5196−3p, rectal
(MSI = 0; N0 = 699)
1−sided adj pval: 0
Frequency
0
10
20
30
40
−4
−3
−2
−1
0
1
2
3
Tumor−Normal Expression Difference
hsa−miR−5196−3p, rectal
(MSI = 1; N1 = 16)
1−sided adj pval: 0.123
Frequency
0.0
0.2
0.4
0.6
0.8
1.0
−4
−3
−2
−1
0
1
2
3
Tumor−Normal Expression Difference

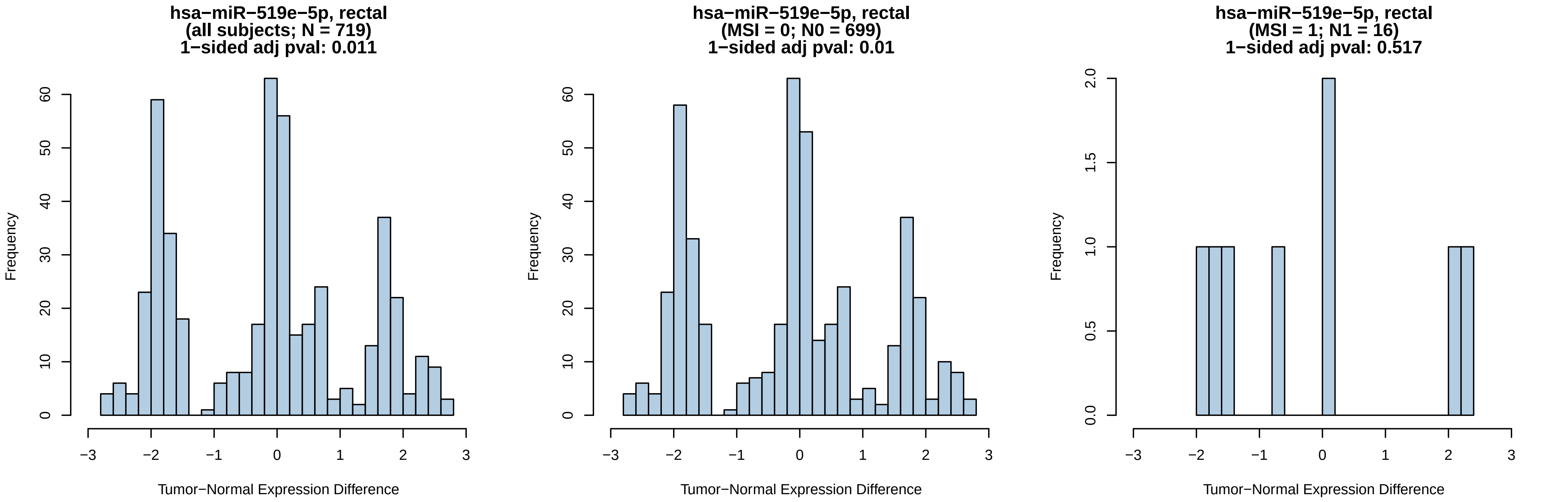
hsa−miR−519e−5p, rectal
(all subjects; N = 719)
1−sided adj pval: 0.011
Frequency
Tumor−Normal Expression Difference
hsa−miR−519e−5p, rectal
(MSI = 0; N0 = 699)
1−sided adj pval: 0.01
Frequency
Tumor−Normal Expression Difference
hsa−miR−519e−5p, rectal
(MSI = 1; N1 = 16)
1−sided adj pval: 0.517
Frequency
Tumor−Normal Expression Difference

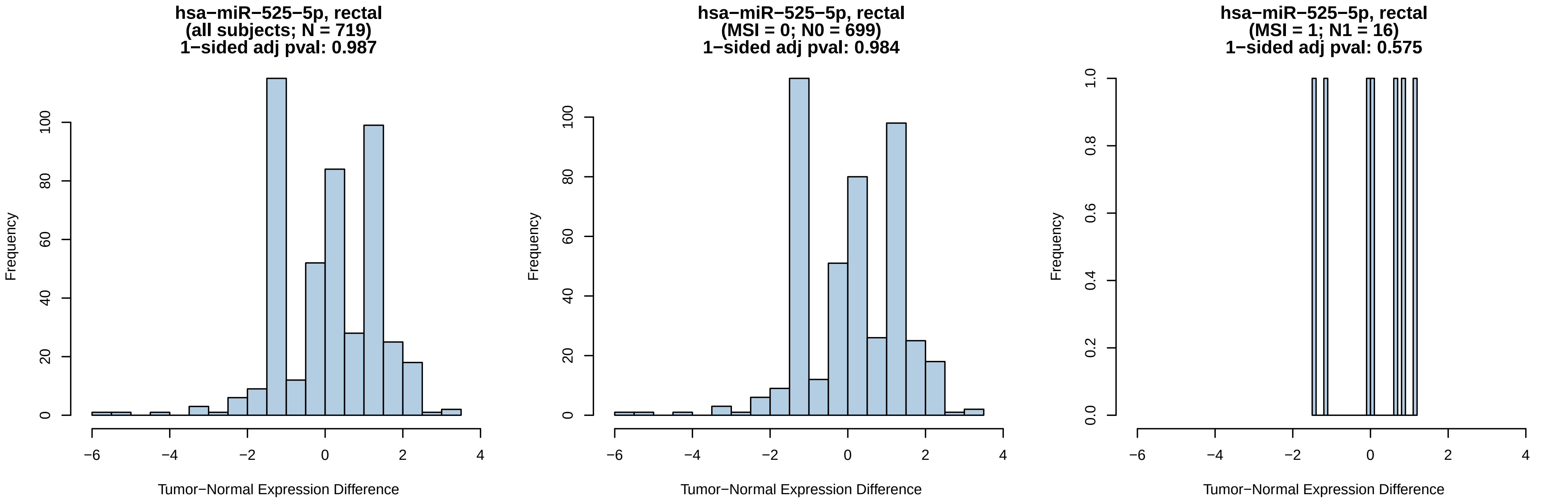
hsa−miR−525−5p, rectal
(all subjects; N = 719)
1−sided adj pval: 0.987
hsa−miR−525−5p, rectal
(MSI = 0; N0 = 699)
1−sided adj pval: 0.984
hsa−miR−525−5p, rectal
(MSI = 1; N1 = 16)
1−sided adj pval: 0.575
Frequency
Tumor−Normal Expression Difference
−6
−4
−2
0
2
4
0
20
40
60
80
100
0.0
0.2
0.4
0.6
0.8
1.0

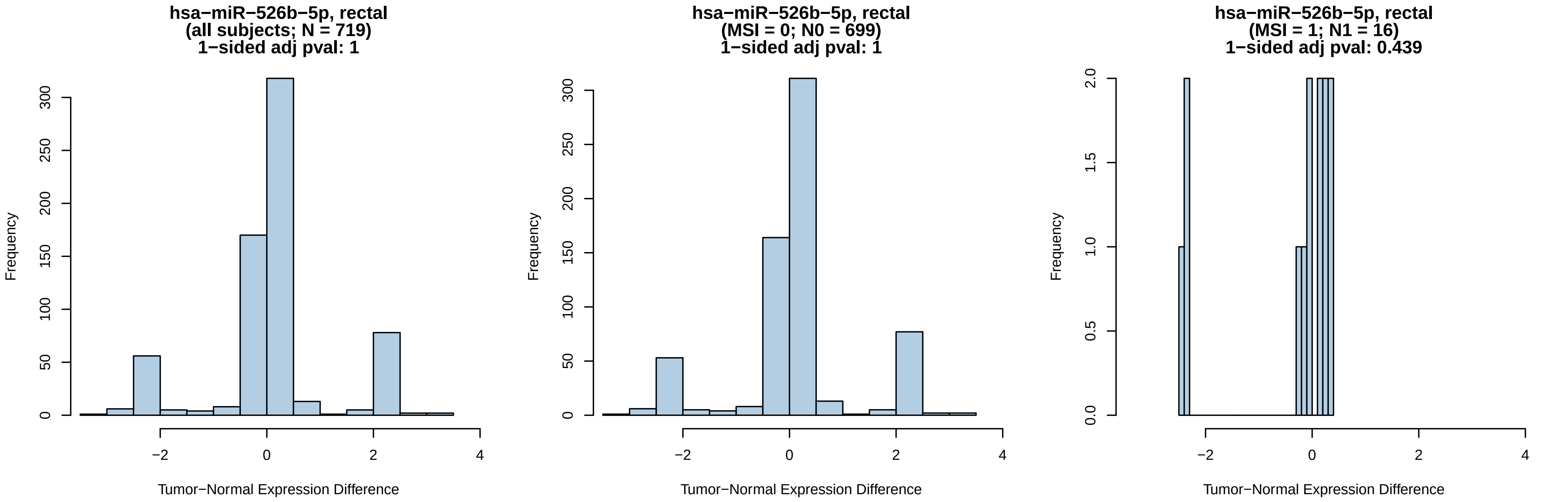
hsa−miR−526b−5p, rectal
(all subjects; N = 719)
1−sided adj pval: 1
hsa−miR−526b−5p, rectal
(MSI = 0; N0 = 699)
1−sided adj pval: 1
hsa−miR−526b−5p, rectal
(MSI = 1; N1 = 16)
1−sided adj pval: 0.439
Frequency
Tumor−Normal Expression Difference
0
50
100
150
200
250
300
0.0
0.5
1.0
1.5
2.0
−2
0
2
4

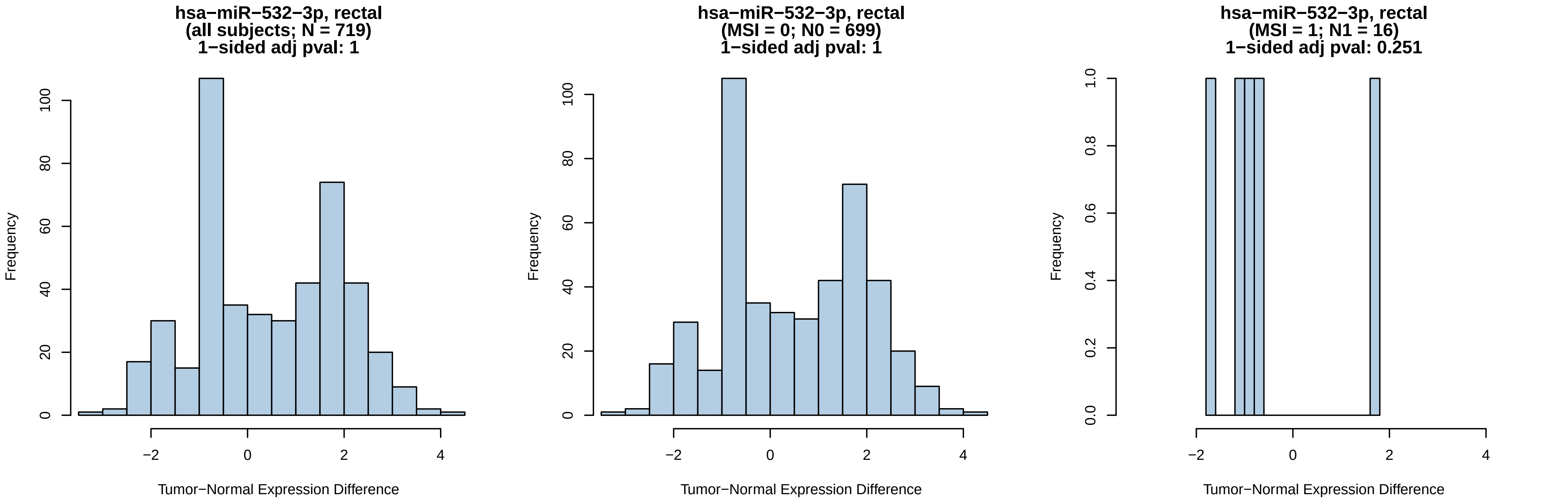

hsa−miR−532−3p, rectal
(all subjects; N = 719)
1−sided adj pval: 1
Frequency
Tumor−Normal Expression Difference
hsa−miR−532−3p, rectal
(MSI = 0; N0 = 699)
1−sided adj pval: 1
Frequency
Tumor−Normal Expression Difference
hsa−miR−532−3p, rectal
(MSI = 1; N1 = 16)
1−sided adj pval: 0.251
Frequency
Tumor−Normal Expression Difference

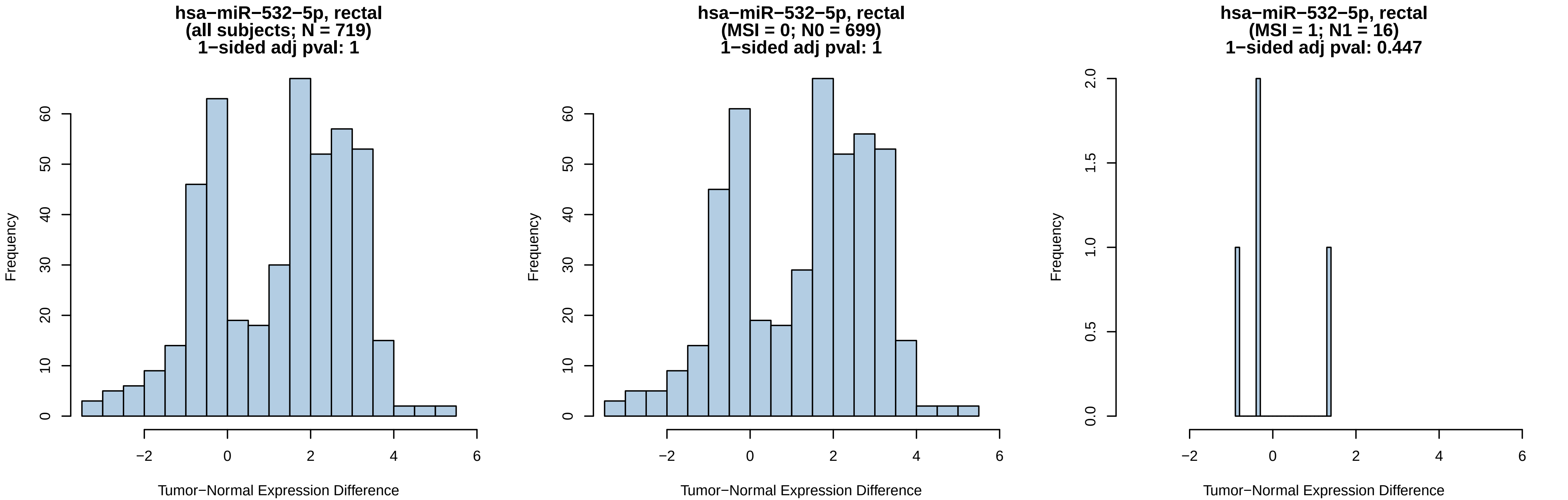

hsa−miR−532−5p, rectal
(all subjects; N = 719)
1−sided adj pval: 1
hsa−miR−532−5p, rectal
(MSI = 0; N0 = 699)
1−sided adj pval: 1
hsa−miR−532−5p, rectal
(MSI = 1; N1 = 16)
1−sided adj pval: 0.447
Frequency
Tumor−Normal Expression Difference

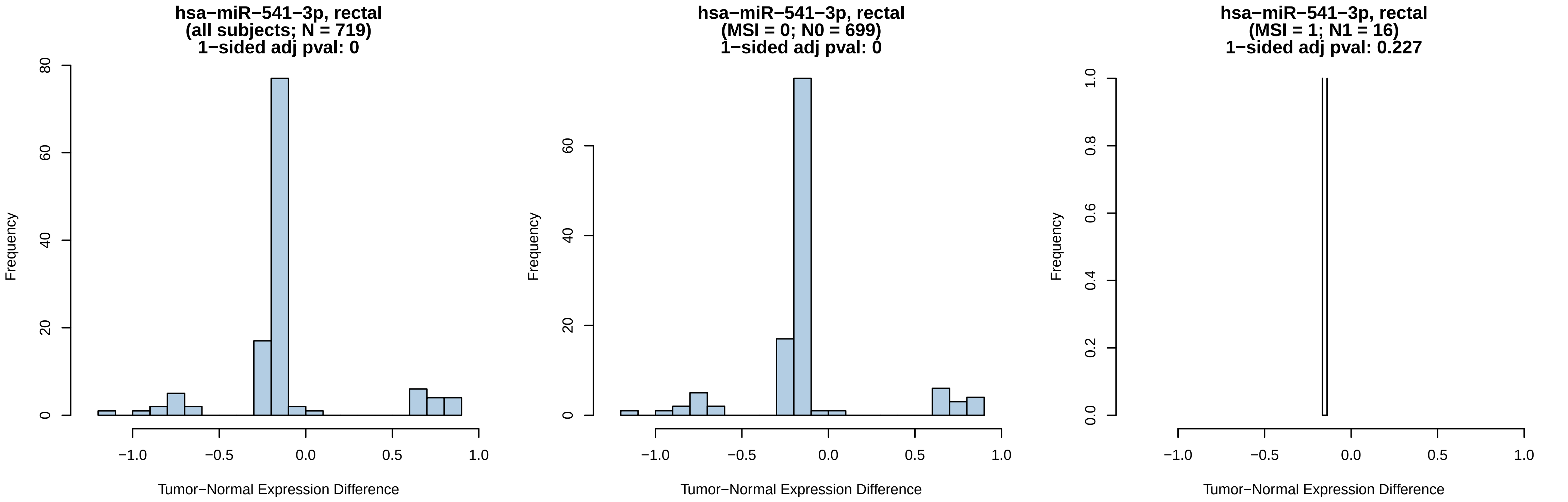
hsa−miR−541−3p, rectal
(all subjects; N = 719)
1−sided adj pval: 0
hsa−miR−541−3p, rectal
(MSI = 0; N0 = 699)
1−sided adj pval: 0
hsa−miR−541−3p, rectal
(MSI = 1; N1 = 16)
1−sided adj pval: 0.227
Frequency
Tumor−Normal Expression Difference
0
20
40
60
80
0.0
0.2
0.4
0.6
0.8
1.0
−1.0
−0.5
0.5

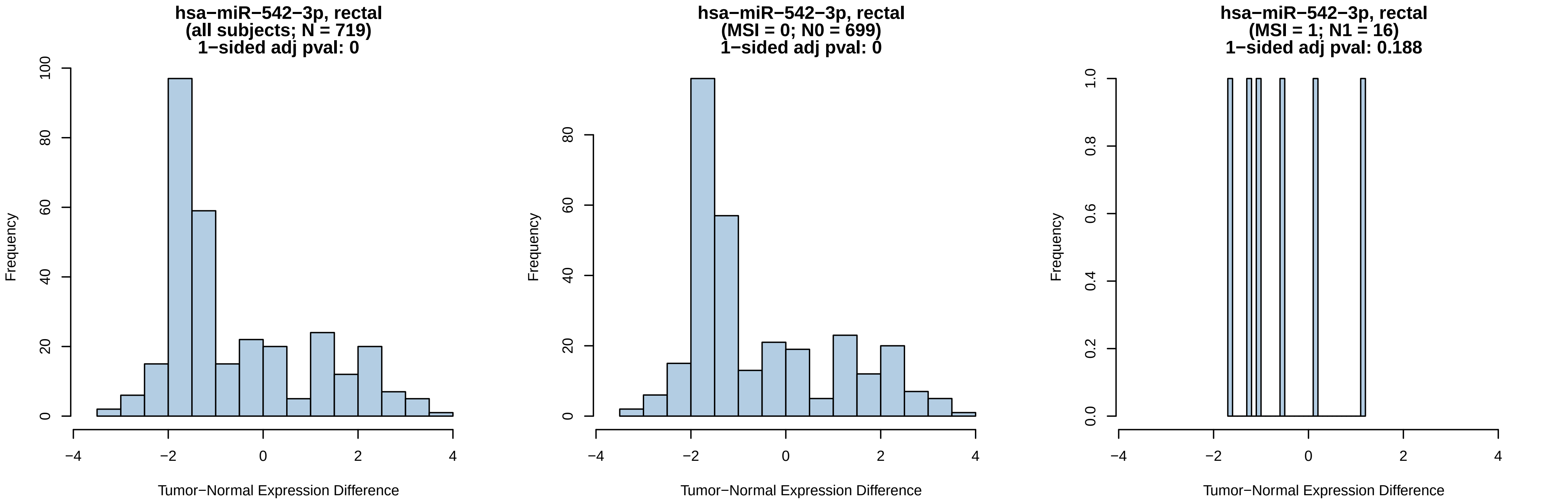
hsa−miR−542−3p, rectal
(all subjects; N = 719)
1−sided adj pval: 0
Frequency
0
20
40
60
80
100
−4
−2
0
2
4
Tumor−Normal Expression Difference
hsa−miR−542−3p, rectal
(MSI = 0; N0 = 699)
1−sided adj pval: 0
Frequency
0
20
40
60
80
−4
−2
0
2
4
Tumor−Normal Expression Difference
hsa−miR−542−3p, rectal
(MSI = 1; N1 = 16)
1−sided adj pval: 0.188
Frequency
0.0
0.2
0.4
0.6
0.8
1.0
−4
−2
0
2
4
Tumor−Normal Expression Difference

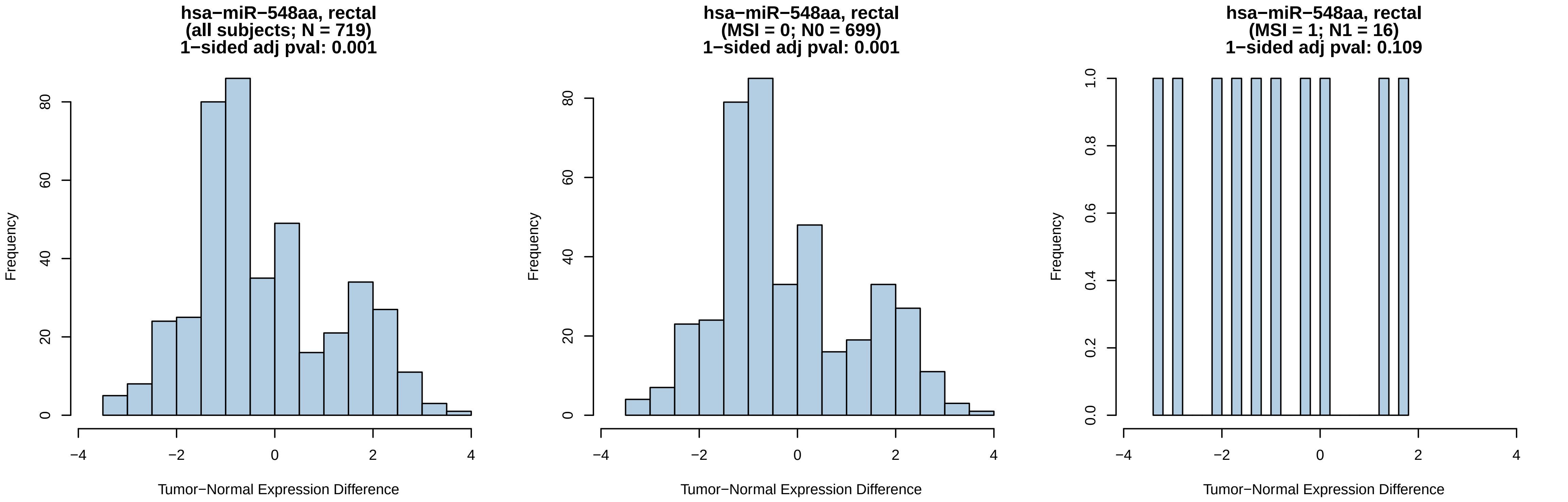
hsa−miR−548aa, rectal
(all subjects; N = 719)
1−sided adj pval: 0.001
Frequency
Tumor−Normal Expression Difference
hsa−miR−548aa, rectal
(MSI = 0; N0 = 699)
1−sided adj pval: 0.001
Frequency
Tumor−Normal Expression Difference
hsa−miR−548aa, rectal
(MSI = 1; N1 = 16)
1−sided adj pval: 0.109
Frequency
Tumor−Normal Expression Difference

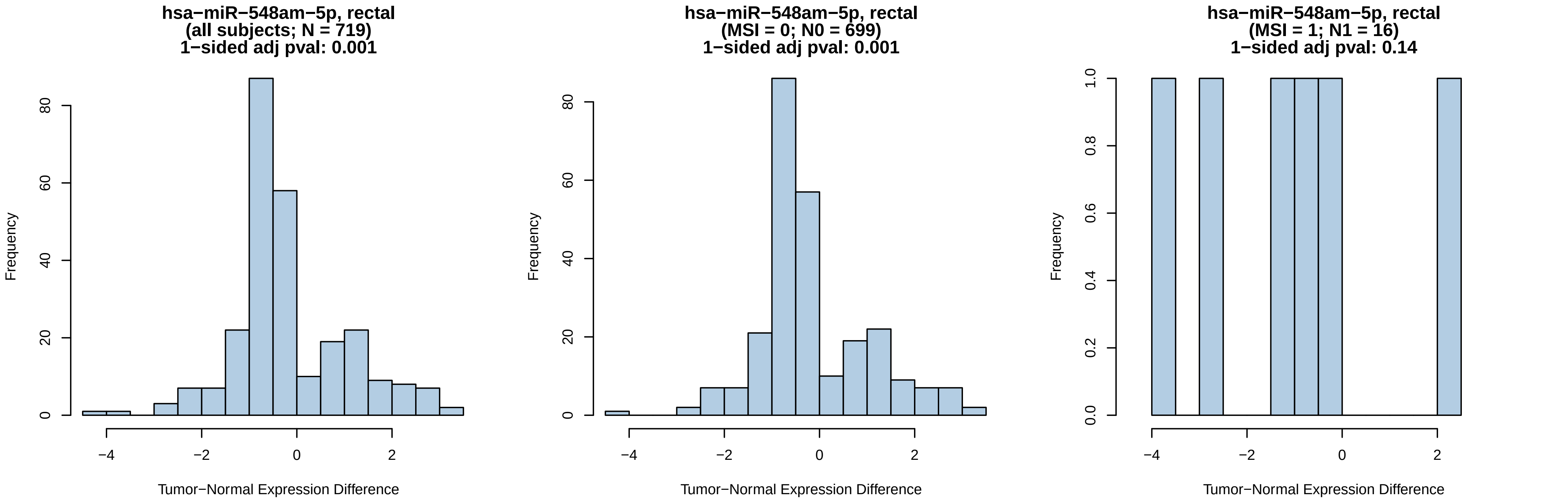
hsa−miR−548am−5p, rectal
(all subjects; N = 719)
1−sided adj pval: 0.001
Frequency
Tumor−Normal Expression Difference
hsa−miR−548am−5p, rectal
(MSI = 0; N0 = 699)
1−sided adj pval: 0.001
Frequency
Tumor−Normal Expression Difference
hsa−miR−548am−5p, rectal
(MSI = 1; N1 = 16)
1−sided adj pval: 0.14
Frequency
Tumor−Normal Expression Difference

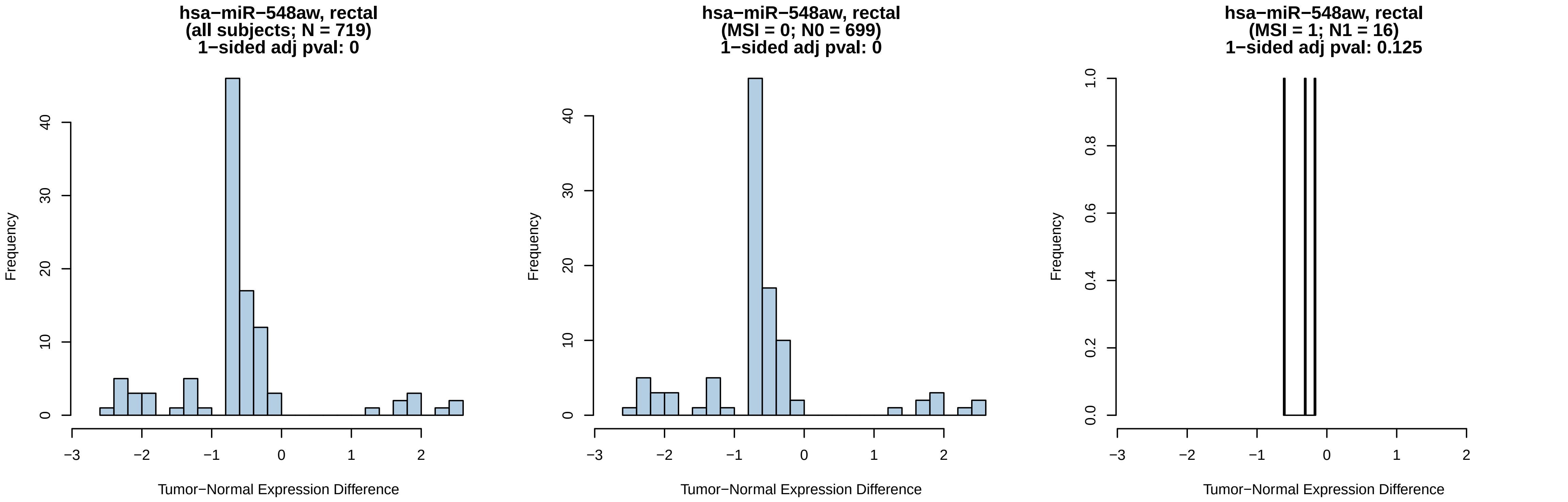

hsa−miR−548aw, rectal
(all subjects; N = 719)
1−sided adj pval: 0
Frequency
Tumor−Normal Expression Difference
hsa−miR−548aw, rectal
(MSI = 0; N0 = 699)
1−sided adj pval: 0
Frequency
Tumor−Normal Expression Difference
hsa−miR−548aw, rectal
(MSI = 1; N1 = 16)
1−sided adj pval: 0.125
Frequency
Tumor−Normal Expression Difference

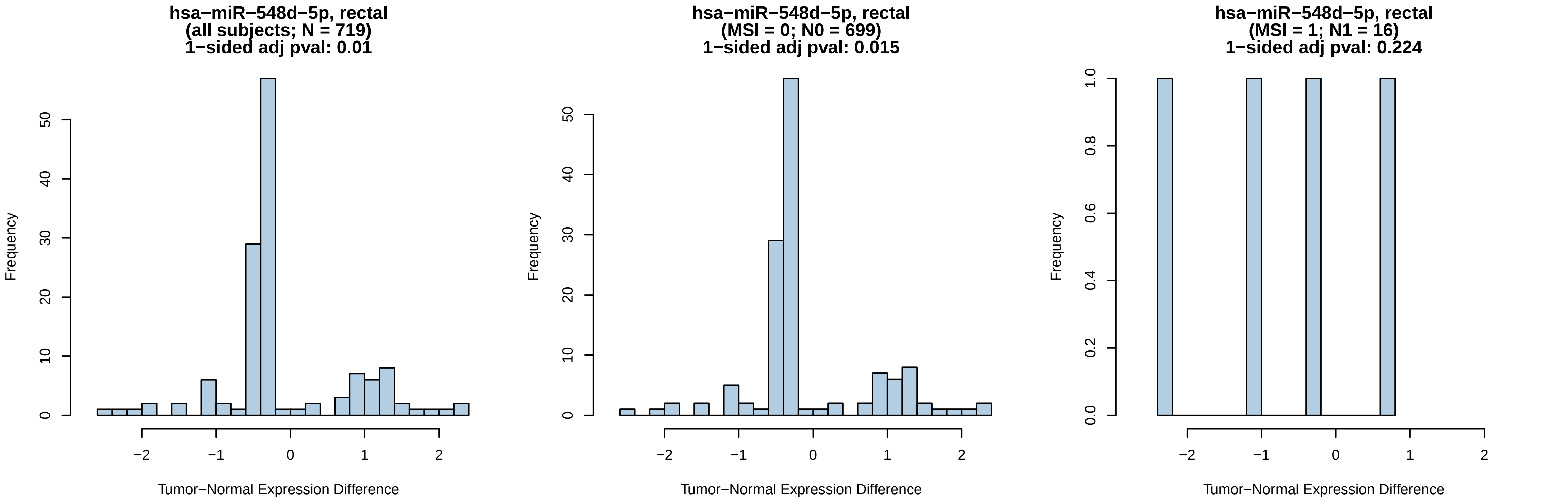
hsa−miR−548d−5p, rectal
(all subjects; N = 719)
1−sided adj pval: 0.01
Frequency
Tumor−Normal Expression Difference
hsa−miR−548d−5p, rectal
(MSI = 0; N0 = 699)
1−sided adj pval: 0.015
Frequency
Tumor−Normal Expression Difference
hsa−miR−548d−5p, rectal
(MSI = 1; N1 = 16)
1−sided adj pval: 0.224
Frequency
Tumor−Normal Expression Difference

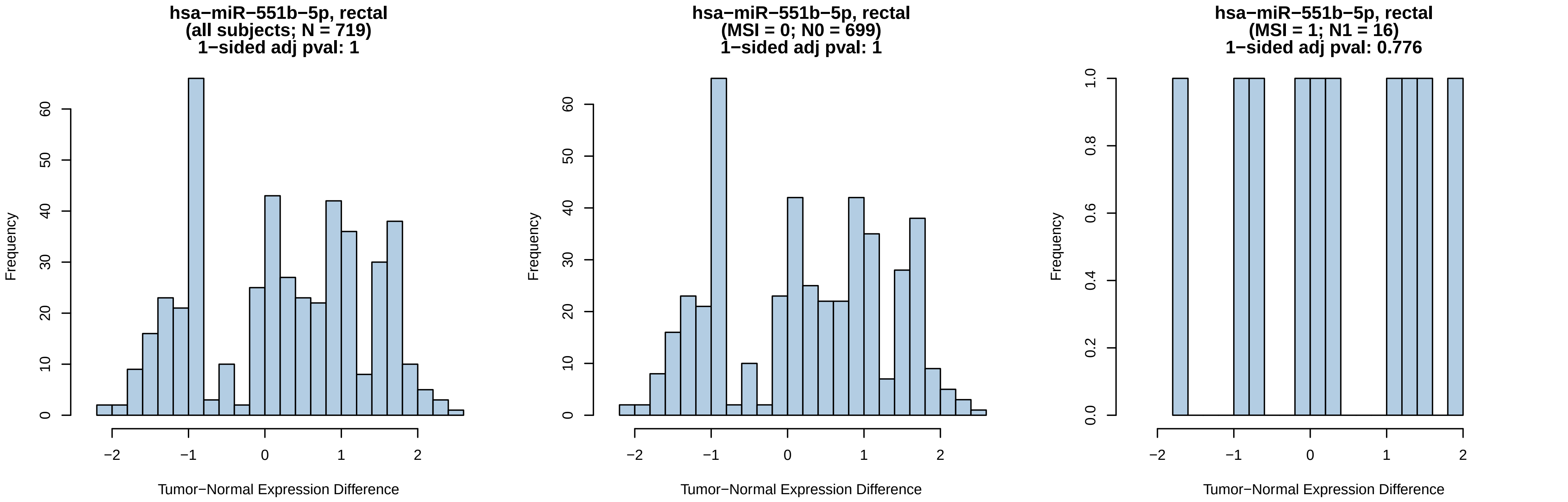
hsa−miR−551b−5p, rectal
(all subjects; N = 719)
1−sided adj pval: 1
hsa−miR−551b−5p, rectal
(MSI = 0; N0 = 699)
1−sided adj pval: 1
hsa−miR−551b−5p, rectal
(MSI = 1; N1 = 16)
1−sided adj pval: 0.776
Frequency
Tumor−Normal Expression Difference

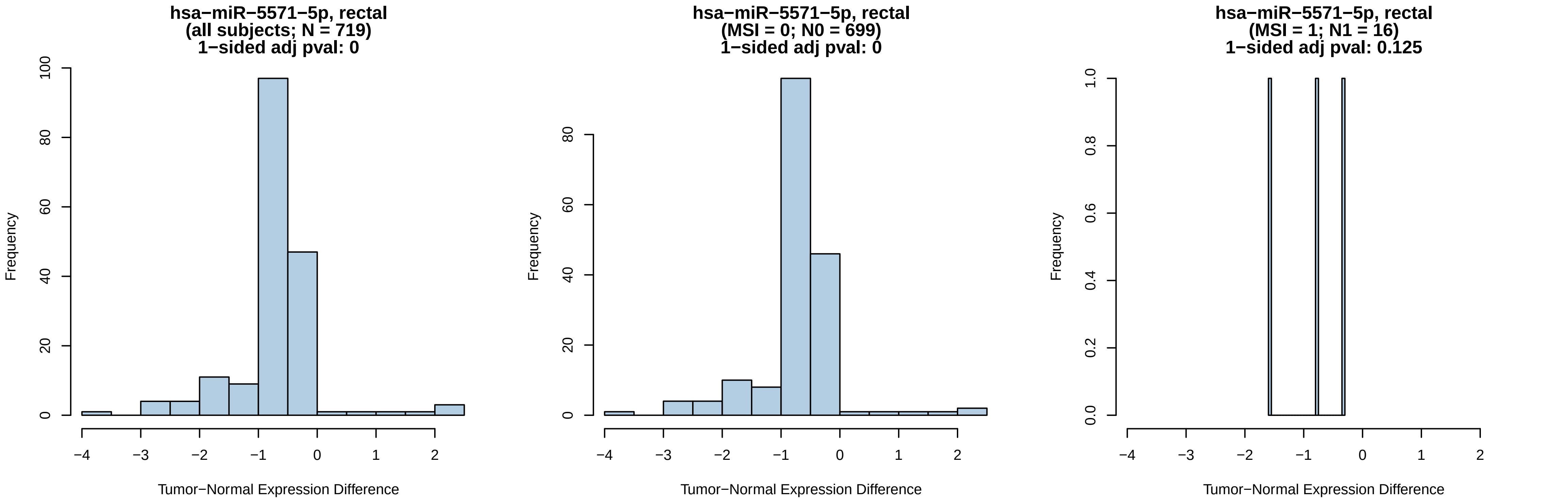

hsa−miR−5571−5p, rectal
(all subjects; N = 719)
1−sided adj pval: 0
Frequency
0
20
40
60
80
100
−4
−3
−2
−1
0
1
2
Tumor−Normal Expression Difference
hsa−miR−5571−5p, rectal
(MSI = 0; N0 = 699)
1−sided adj pval: 0
Frequency
0
20
40
60
80
−4
−3
−2
−1
0
1
2
Tumor−Normal Expression Difference
hsa−miR−5571−5p, rectal
(MSI = 1; N1 = 16)
1−sided adj pval: 0.125
Frequency
0.0
0.2
0.4
0.6
0.8
1.0
−4
−3
−2
−1
0
1
2
Tumor−Normal Expression Difference

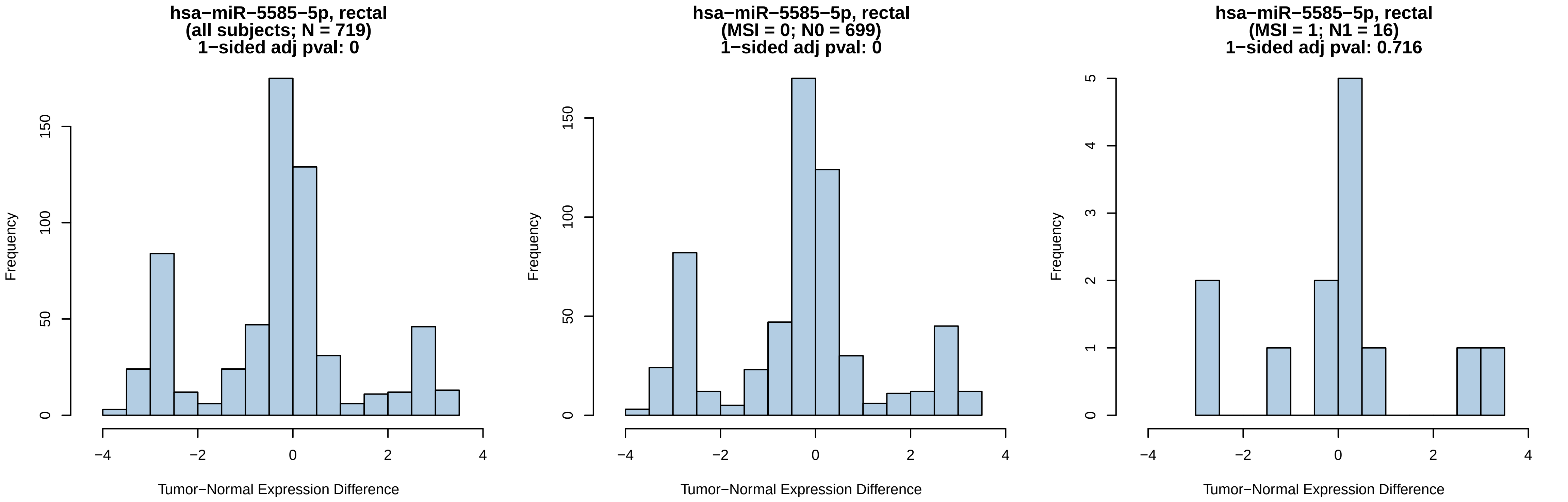
hsa−miR−5585−5p, rectal
(all subjects; N = 719)
1−sided adj pval: 0
Frequency
Tumor−Normal Expression Difference
hsa−miR−5585−5p, rectal
(MSI = 0; N0 = 699)
1−sided adj pval: 0
Frequency
Tumor−Normal Expression Difference
hsa−miR−5585−5p, rectal
(MSI = 1; N1 = 16)
1−sided adj pval: 0.716
Frequency
Tumor−Normal Expression Difference

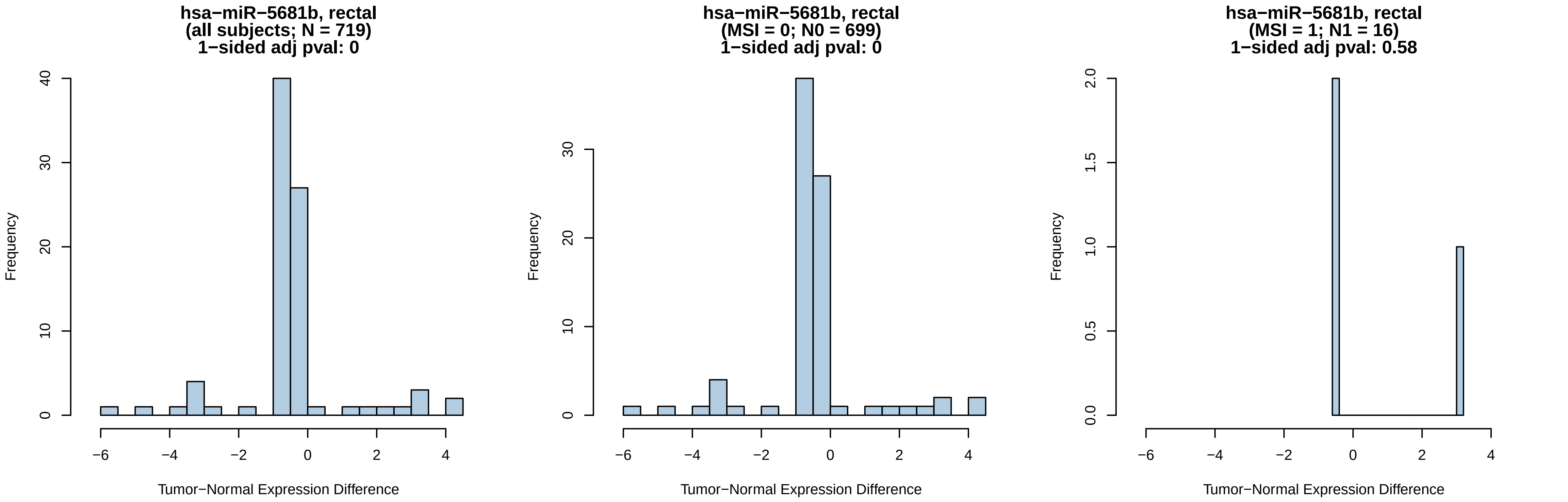
hsa−miR−5681b, rectal
(all subjects; N = 719)
1−sided adj pval: 0
hsa−miR−5681b, rectal
(MSI = 0; N0 = 699)
1−sided adj pval: 0
hsa−miR−5681b, rectal
(MSI = 1; N1 = 16)
1−sided adj pval: 0.58
Frequency
Tumor−Normal Expression Difference
0
10
20
30
40
0.0
0.5
1.0
1.5
2.0
−6
−4
−2
0
2
4

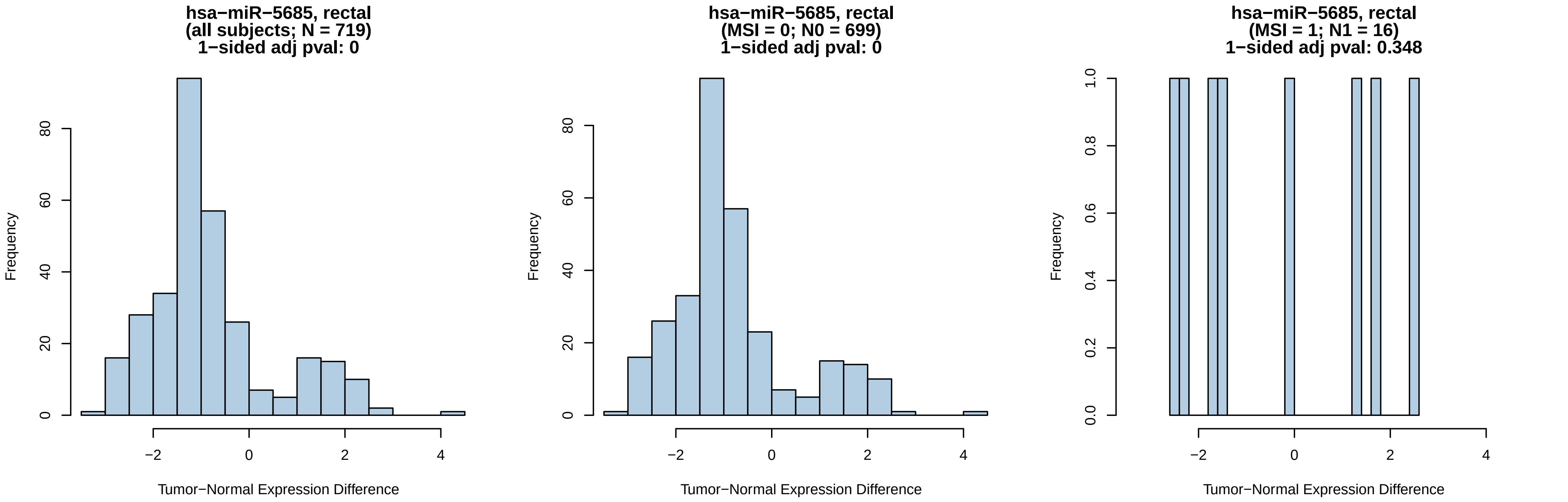

hsa−miR−5685, rectal
(all subjects; N = 719)
1−sided adj pval: 0
Frequency
0
20
40
60
80
−2
0
2
4
Tumor−Normal Expression Difference
hsa−miR−5685, rectal
(MSI = 0; N0 = 699)
1−sided adj pval: 0
Frequency
0
20
40
60
80
−2
0
2
4
Tumor−Normal Expression Difference
hsa−miR−5685, rectal
(MSI = 1; N1 = 16)
1−sided adj pval: 0.348
Frequency
0.0
0.2
0.4
0.6
0.8
1.0
−2
0
2
4
Tumor−Normal Expression Difference

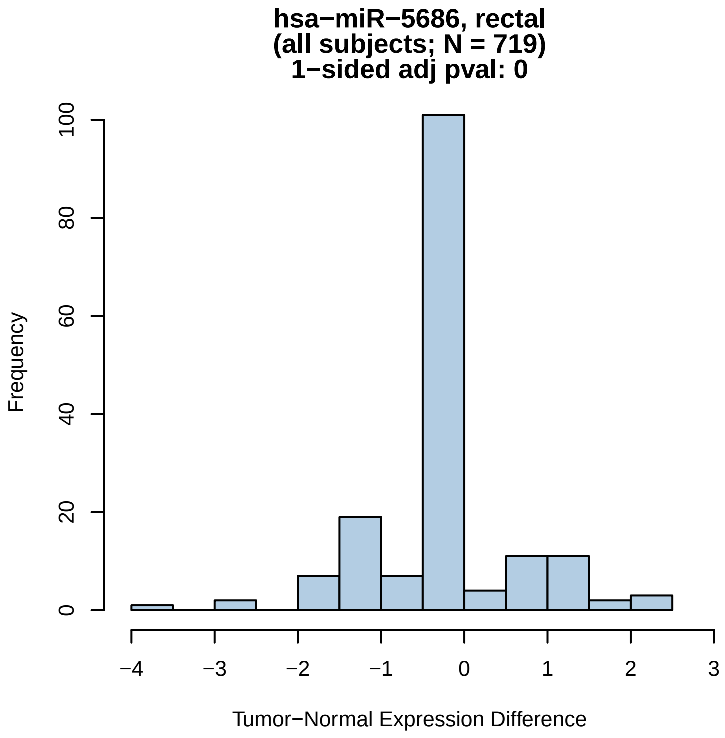
hsa−miR−5686, rectal
(all subjects; N = 719)
1−sided adj pval: 0
Frequency
Tumor−Normal Expression Difference

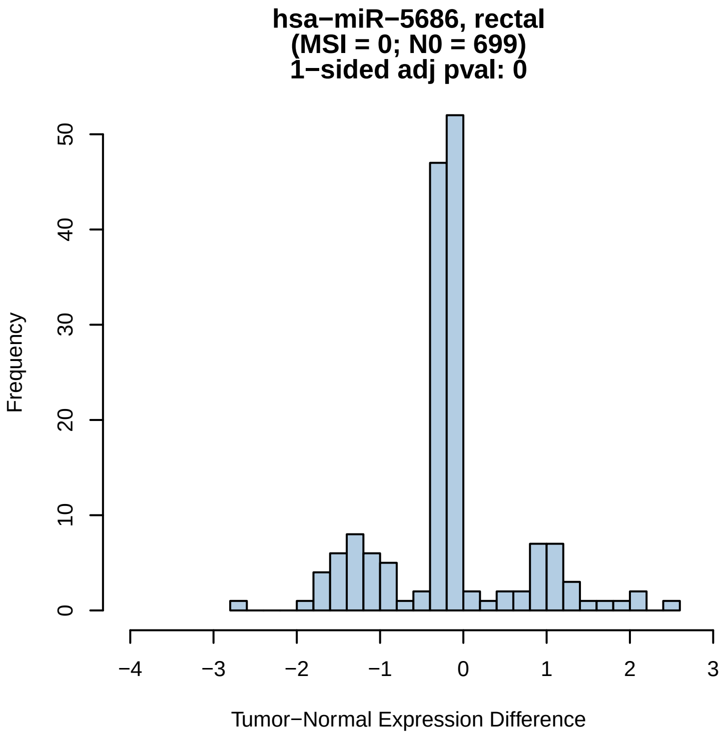
hsa−miR−5686, rectal
(MSI = 0; N0 = 699)
1−sided adj pval: 0
Frequency
Tumor−Normal Expression Difference

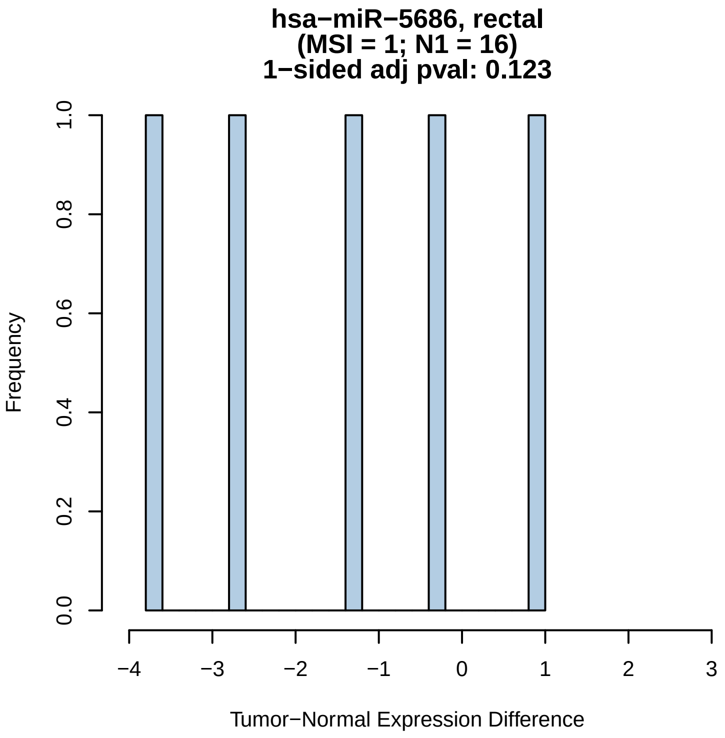
hsa−miR−5686, rectal
(MSI = 1; N1 = 16)
1−sided adj pval: 0.123
Frequency
Tumor−Normal Expression Difference

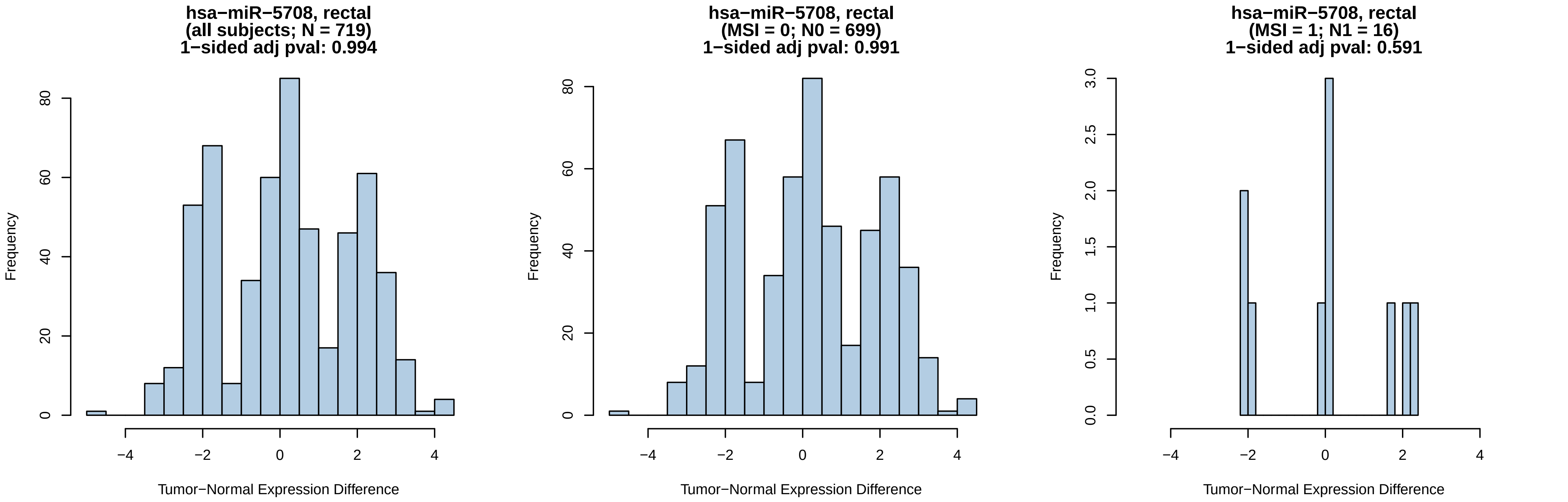
hsa−miR−5708, rectal
(all subjects; N = 719)
1−sided adj pval: 0.994
Frequency
Tumor−Normal Expression Difference
hsa−miR−5708, rectal
(MSI = 0; N0 = 699)
1−sided adj pval: 0.991
Frequency
Tumor−Normal Expression Difference
hsa−miR−5708, rectal
(MSI = 1; N1 = 16)
1−sided adj pval: 0.591
Frequency
Tumor−Normal Expression Difference

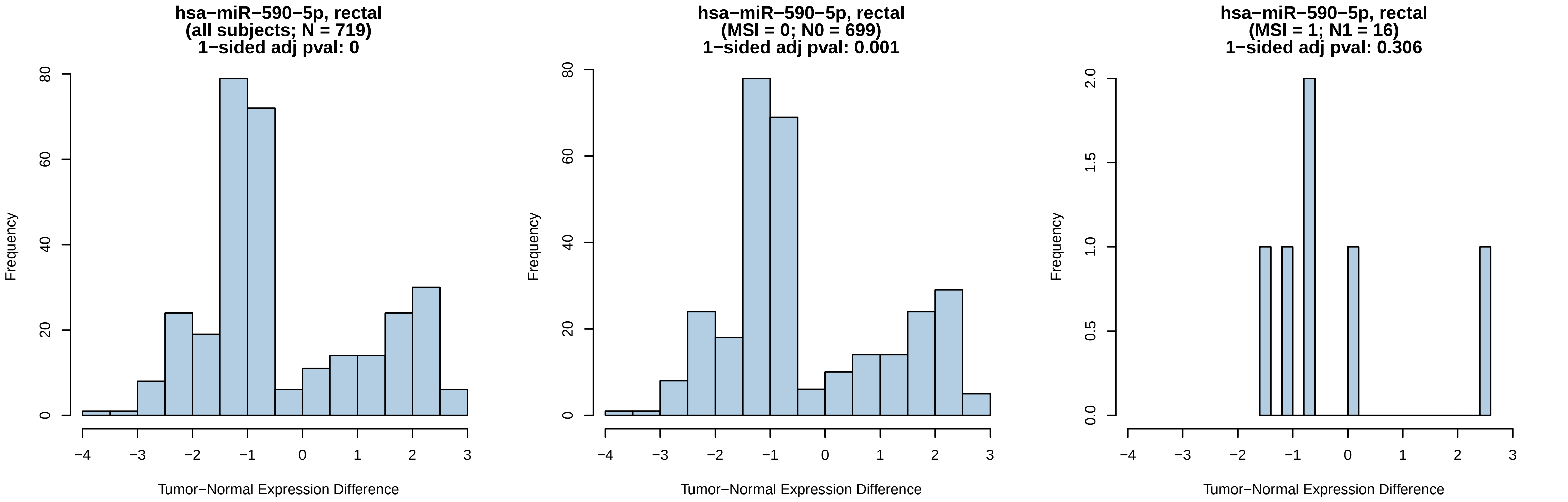

hsa−miR−590−5p, rectal
(all subjects; N = 719)
1−sided adj pval: 0
Frequency
Tumor−Normal Expression Difference
hsa−miR−590−5p, rectal
(MSI = 0; N0 = 699)
1−sided adj pval: 0.001
Frequency
Tumor−Normal Expression Difference
hsa−miR−590−5p, rectal
(MSI = 1; N1 = 16)
1−sided adj pval: 0.306
Frequency
Tumor−Normal Expression Difference

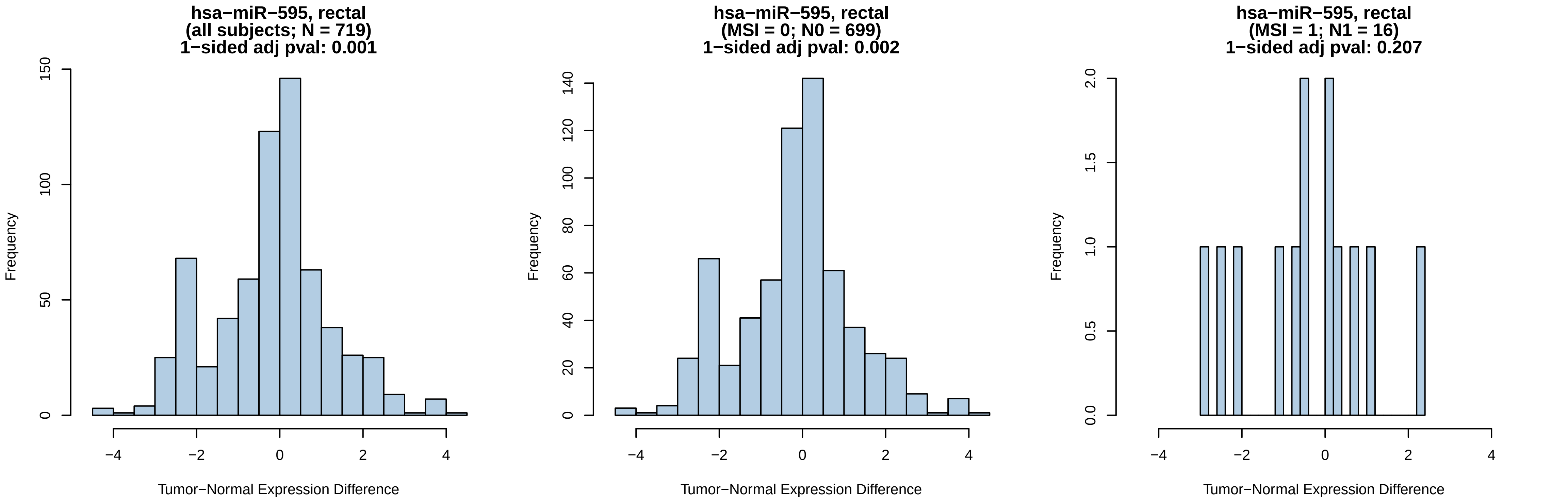

hsa−miR−595, rectal
(all subjects; N = 719)
1−sided adj pval: 0.001
Frequency
0
50
100
150
−4
−2
0
2
4
Tumor−Normal Expression Difference
hsa−miR−595, rectal
(MSI = 0; N0 = 699)
1−sided adj pval: 0.002
Frequency
0
20
40
60
80
100
120
140
−4
−2
0
2
4
Tumor−Normal Expression Difference
hsa−miR−595, rectal
(MSI = 1; N1 = 16)
1−sided adj pval: 0.207
Frequency
0.0
0.5
1.0
1.5
2.0
−4
−2
0
2
4
Tumor−Normal Expression Difference

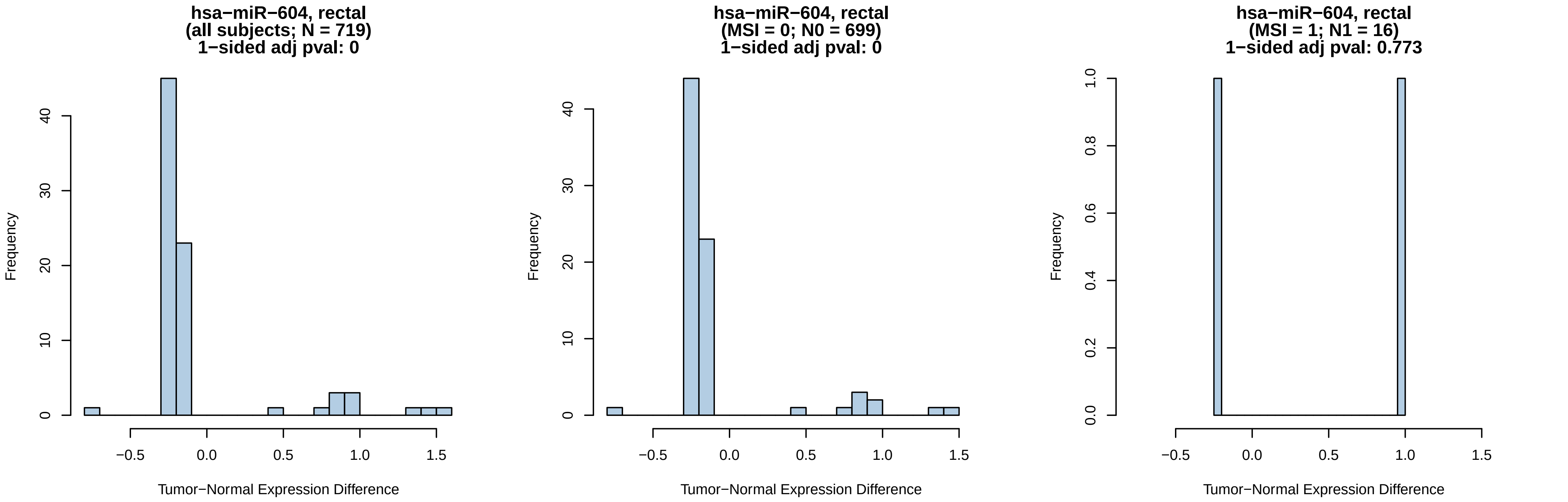
hsa−miR−604, rectal
(all subjects; N = 719)
1−sided adj pval: 0
Frequency
Tumor−Normal Expression Difference
hsa−miR−604, rectal
(MSI = 0; N0 = 699)
1−sided adj pval: 0
Frequency
Tumor−Normal Expression Difference
hsa−miR−604, rectal
(MSI = 1; N1 = 16)
1−sided adj pval: 0.773
Frequency
Tumor−Normal Expression Difference

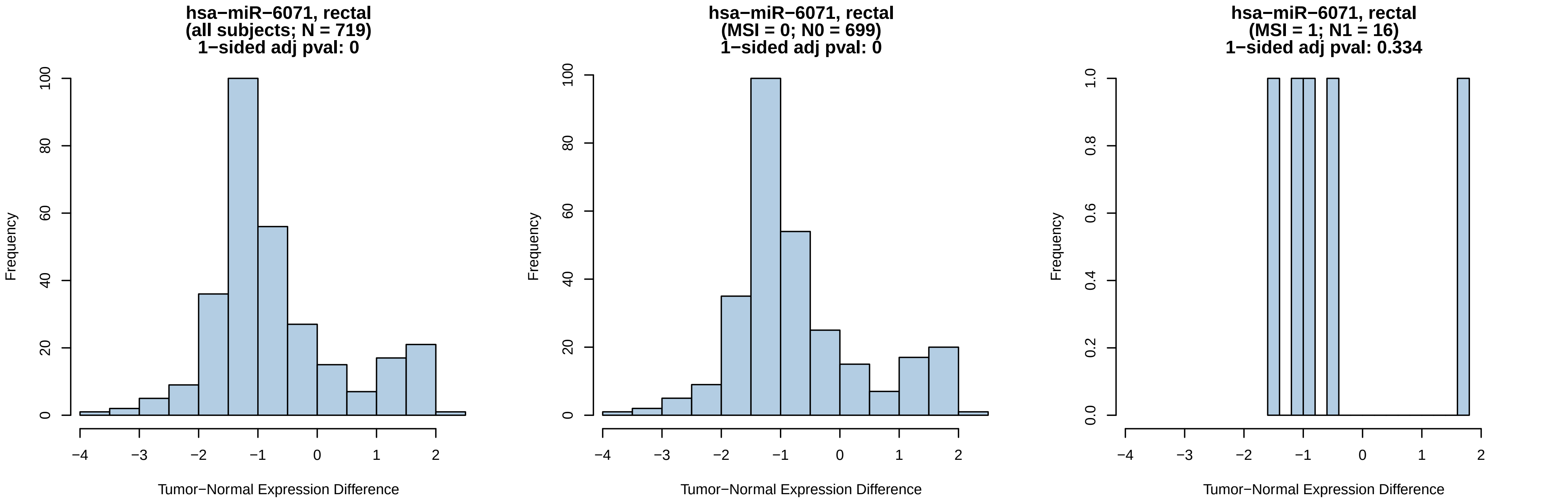

hsa−miR−6071, rectal
(all subjects; N = 719)
1−sided adj pval: 0
Frequency
Tumor−Normal Expression Difference
hsa−miR−6071, rectal
(MSI = 0; N0 = 699)
1−sided adj pval: 0
Frequency
Tumor−Normal Expression Difference
hsa−miR−6071, rectal
(MSI = 1; N1 = 16)
1−sided adj pval: 0.334
Frequency
Tumor−Normal Expression Difference

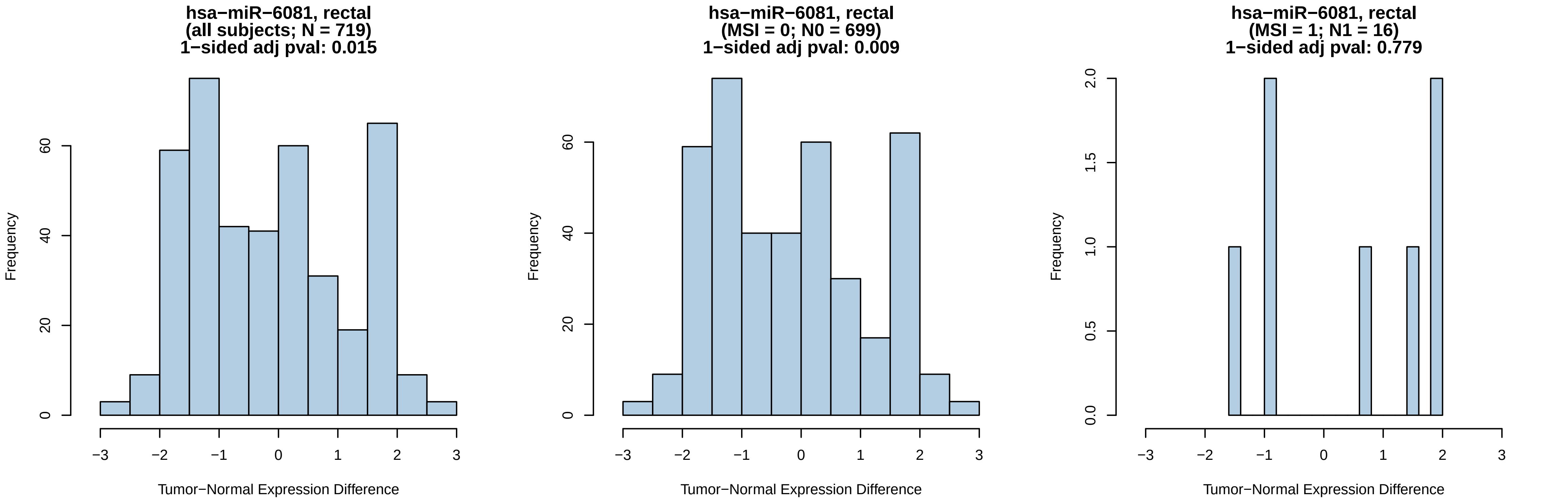

hsa−miR−6081, rectal
(all subjects; N = 719)
1−sided adj pval: 0.015
Frequency
0
20
40
60
−3
−2
−1
0
1
2
3
Tumor−Normal Expression Difference
hsa−miR−6081, rectal
(MSI = 0; N0 = 699)
1−sided adj pval: 0.009
Frequency
0
20
40
60
−3
−2
−1
0
1
2
3
Tumor−Normal Expression Difference
hsa−miR−6081, rectal
(MSI = 1; N1 = 16)
1−sided adj pval: 0.779
Frequency
0.0
0.5
1.0
1.5
2.0
−3
−2
−1
0
1
2
3
Tumor−Normal Expression Difference

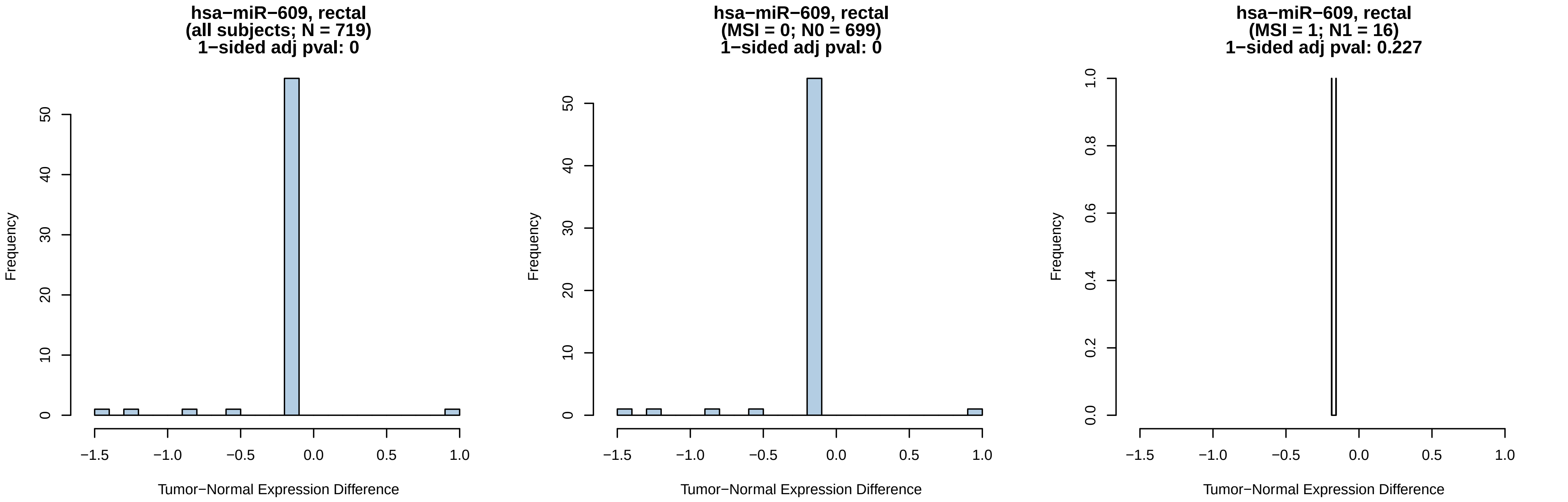
hsa−miR−609, rectal
(all subjects; N = 719)
1−sided adj pval: 0
Frequency
0
10
20
30
40
50
−1.5
−1.0
−0.5
0.0
0.5
1.0
Tumor−Normal Expression Difference
hsa−miR−609, rectal
(MSI = 0; N0 = 699)
1−sided adj pval: 0
Frequency
0
10
20
30
40
50
−1.5
−1.0
−0.5
0.0
0.5
1.0
Tumor−Normal Expression Difference
hsa−miR−609, rectal
(MSI = 1; N1 = 16)
1−sided adj pval: 0.227
Frequency
0.0
0.2
0.4
0.6
0.8
1.0
−1.5
−1.0
−0.5
0.0
0.5
1.0
Tumor−Normal Expression Difference

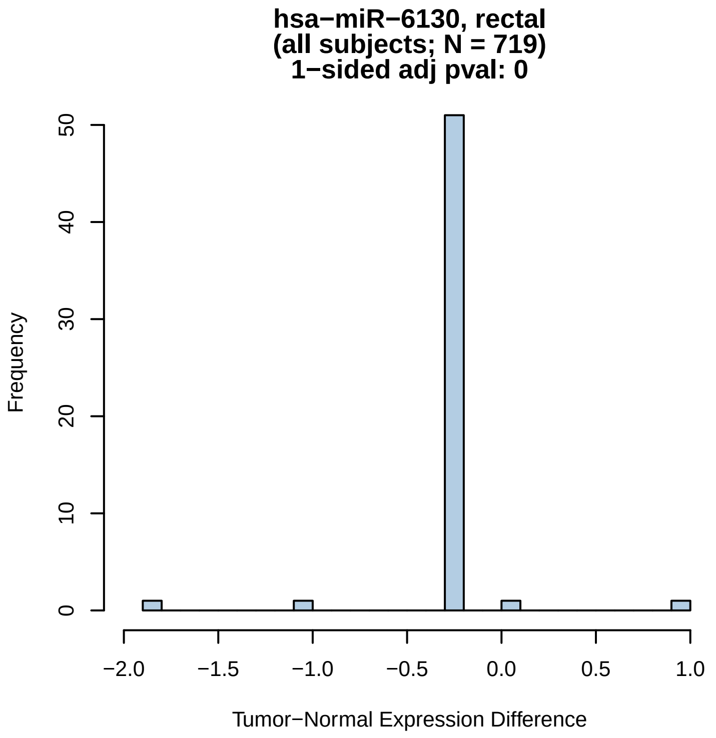
hsa−miR−6130, rectal
(all subjects; N = 719)
1−sided adj pval: 0
Frequency
0
10
20
30
40
50
−2.0
−1.5
−1.0
−0.5
0.0
0.5
1.0
Tumor−Normal Expression Difference

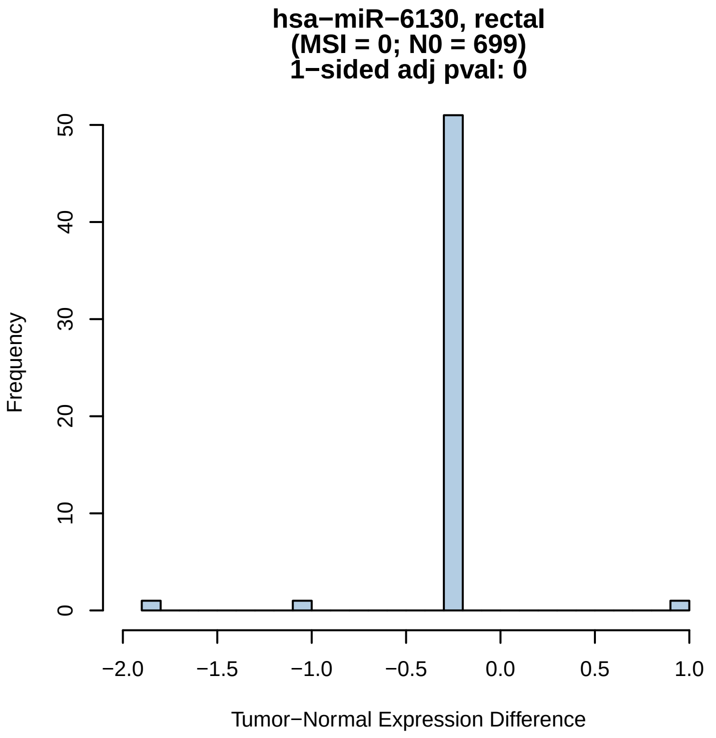
hsa−miR−6130, rectal
(MSI = 0; N0 = 699)
1−sided adj pval: 0
Frequency
0
10
20
30
40
50
−2.0
−1.5
−1.0
−0.5
0.0
0.5
1.0
Tumor−Normal Expression Difference

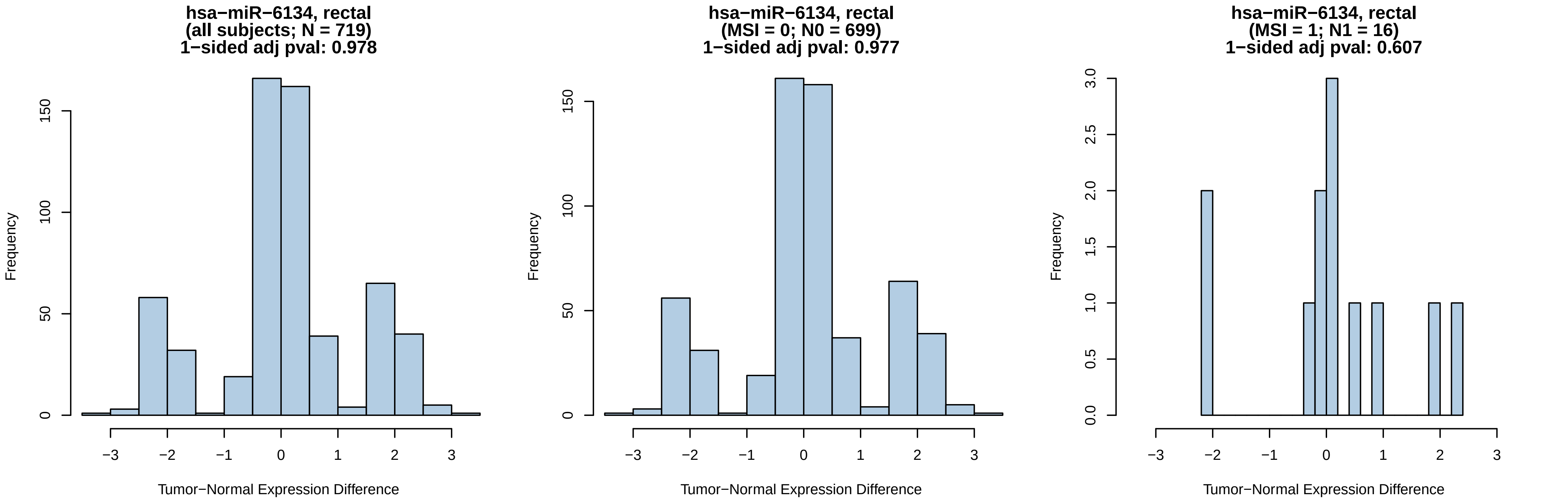

hsa−miR−6134, rectal
(all subjects; N = 719)
1−sided adj pval: 0.978
Frequency
0
50
100
150
−3
−2
−1
0
1
2
3
Tumor−Normal Expression Difference
hsa−miR−6134, rectal
(MSI = 0; N0 = 699)
1−sided adj pval: 0.977
Frequency
0
50
100
150
−3
−2
−1
0
1
2
3
Tumor−Normal Expression Difference
hsa−miR−6134, rectal
(MSI = 1; N1 = 16)
1−sided adj pval: 0.607
Frequency
0.0
0.5
1.0
1.5
2.0
2.5
3.0
−3
−2
−1
0
1
2
3
Tumor−Normal Expression Difference

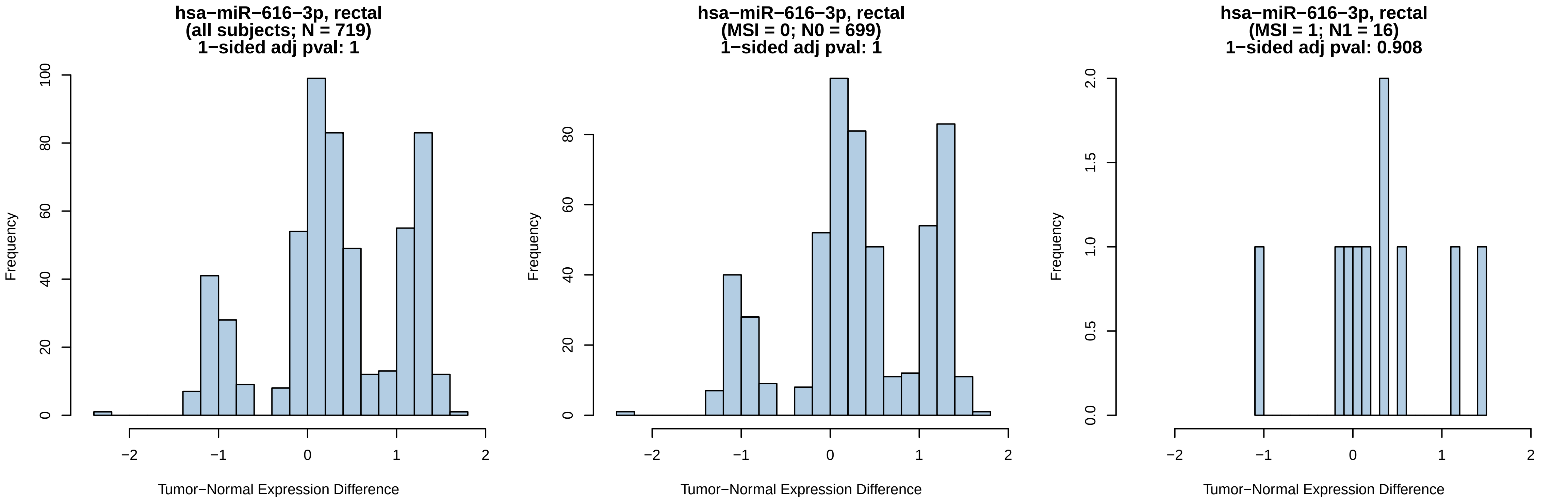
hsa−miR−616−3p, rectal
(all subjects; N = 719)
1−sided adj pval: 1
hsa−miR−616−3p, rectal
(MSI = 0; N0 = 699)
1−sided adj pval: 1
hsa−miR−616−3p, rectal
(MSI = 1; N1 = 16)
1−sided adj pval: 0.908
Frequency
Tumor−Normal Expression Difference

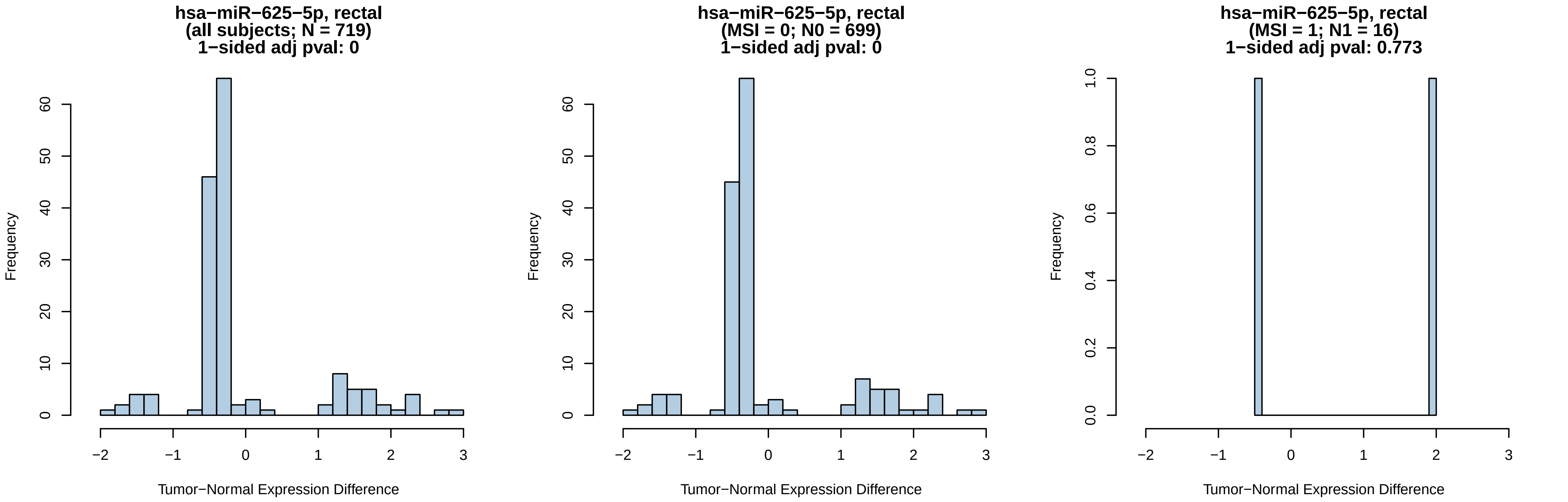

hsa−miR−625−5p, rectal
(all subjects; N = 719)
1−sided adj pval: 0
hsa−miR−625−5p, rectal
(MSI = 0; N0 = 699)
1−sided adj pval: 0
hsa−miR−625−5p, rectal
(MSI = 1; N1 = 16)
1−sided adj pval: 0.773
Frequency
Tumor−Normal Expression Difference

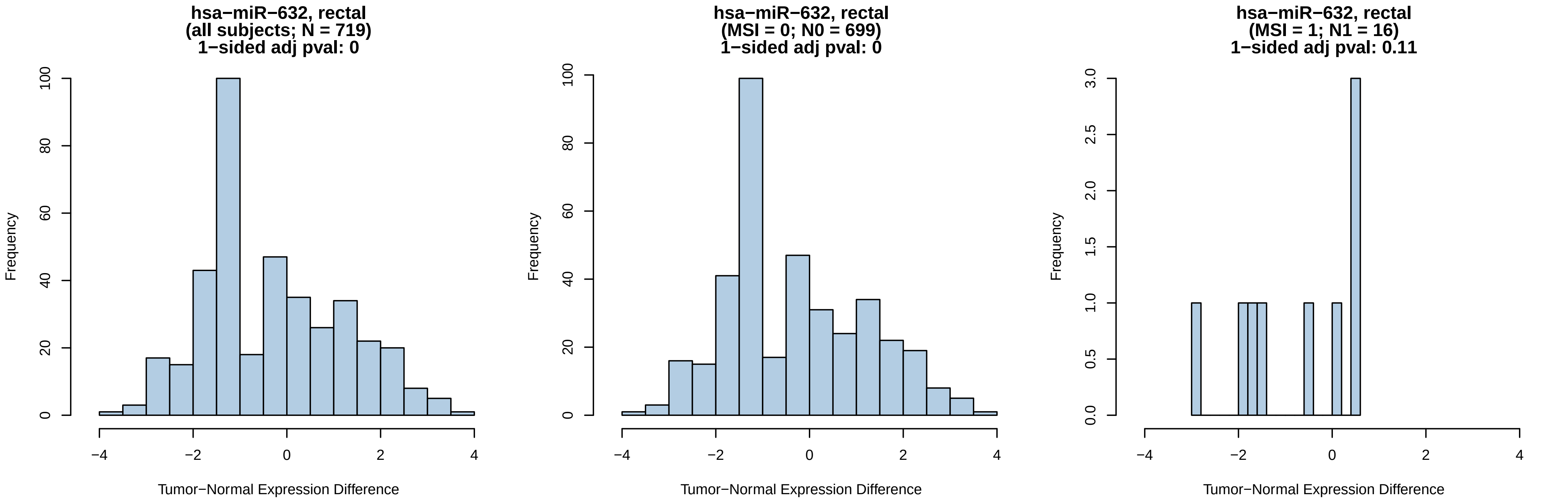

hsa−miR−632, rectal
(all subjects; N = 719)
1−sided adj pval: 0
Frequency
Tumor−Normal Expression Difference
hsa−miR−632, rectal
(MSI = 0; N0 = 699)
1−sided adj pval: 0
Frequency
Tumor−Normal Expression Difference
hsa−miR−632, rectal
(MSI = 1; N1 = 16)
1−sided adj pval: 0.11
Frequency
Tumor−Normal Expression Difference

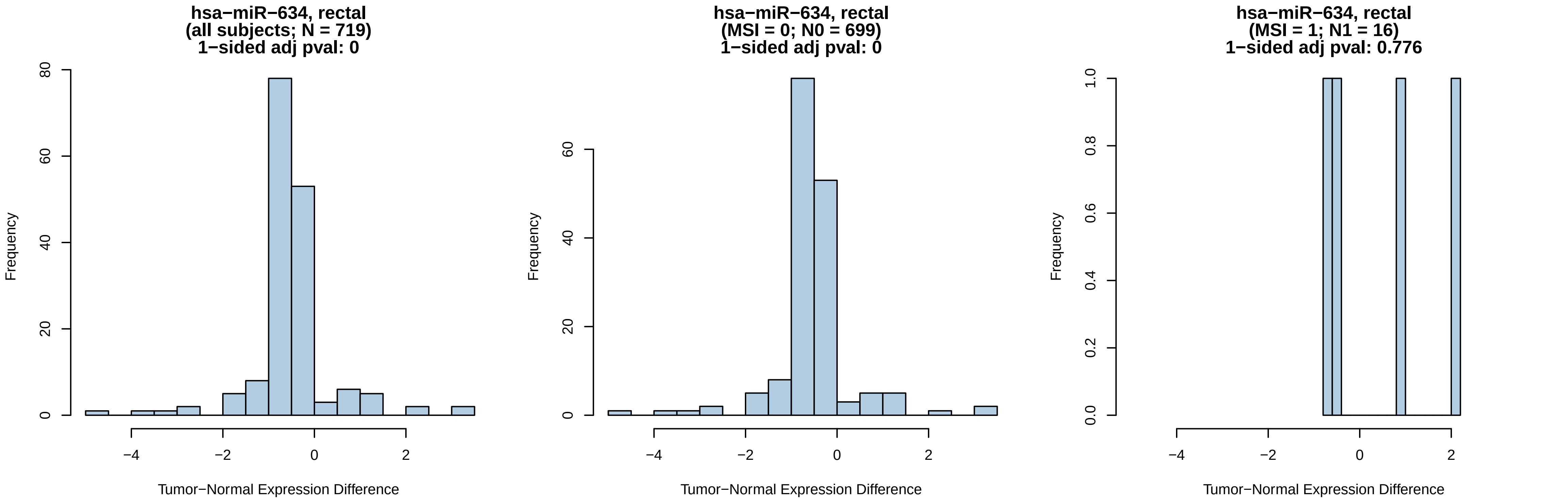

hsa−miR−634, rectal
(all subjects; N = 719)
1−sided adj pval: 0
Frequency
0
20
40
60
80
−4
−2
0
2
Tumor−Normal Expression Difference
hsa−miR−634, rectal
(MSI = 0; N0 = 699)
1−sided adj pval: 0
Frequency
0
20
40
60
−4
−2
0
2
Tumor−Normal Expression Difference
hsa−miR−634, rectal
(MSI = 1; N1 = 16)
1−sided adj pval: 0.776
Frequency
0.0
0.2
0.4
0.6
0.8
1.0
−4
−2
0
2
Tumor−Normal Expression Difference

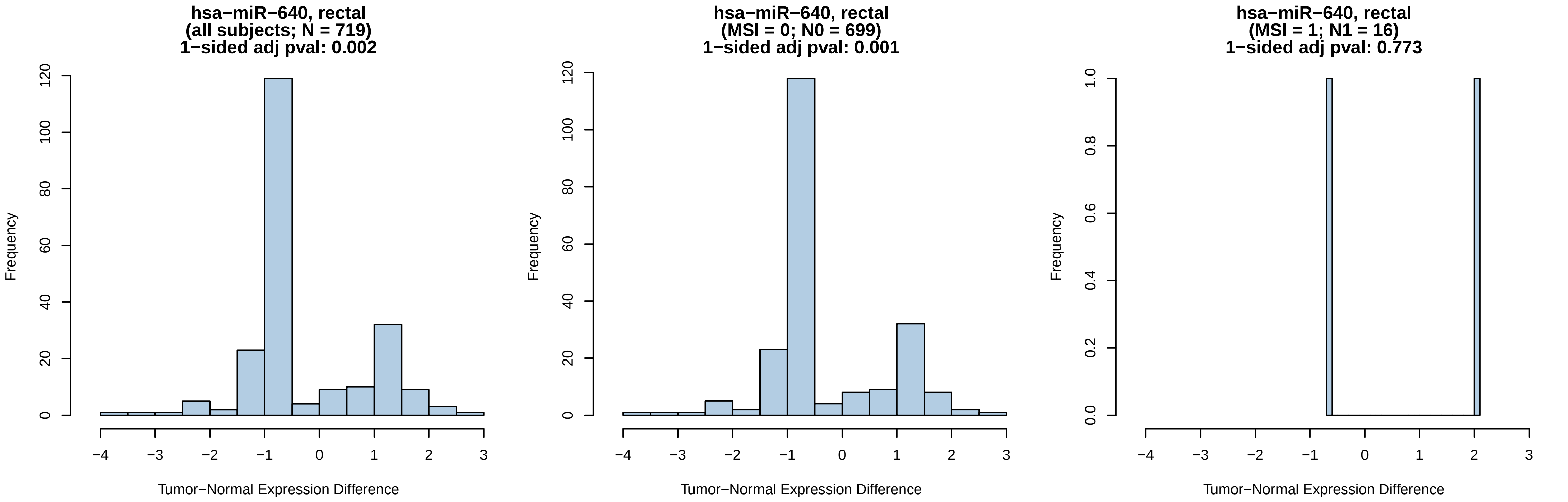
hsa−miR−640, rectal
(all subjects; N = 719)
1−sided adj pval: 0.002
Frequency
Tumor−Normal Expression Difference
hsa−miR−640, rectal
(MSI = 0; N0 = 699)
1−sided adj pval: 0.001
Frequency
Tumor−Normal Expression Difference
hsa−miR−640, rectal
(MSI = 1; N1 = 16)
1−sided adj pval: 0.773
Frequency
Tumor−Normal Expression Difference

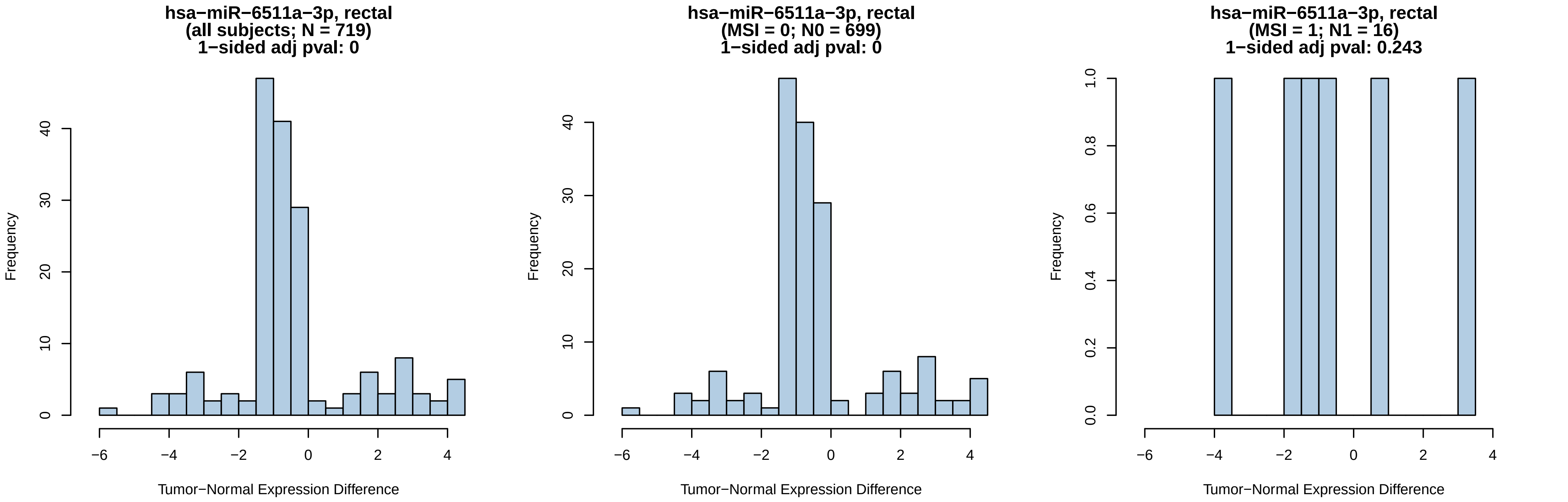
hsa−miR−6511a−3p, rectal
(all subjects; N = 719)
1−sided adj pval: 0
Frequency
Tumor−Normal Expression Difference
hsa−miR−6511a−3p, rectal
(MSI = 0; N0 = 699)
1−sided adj pval: 0
Frequency
Tumor−Normal Expression Difference
hsa−miR−6511a−3p, rectal
(MSI = 1; N1 = 16)
1−sided adj pval: 0.243
Frequency
Tumor−Normal Expression Difference

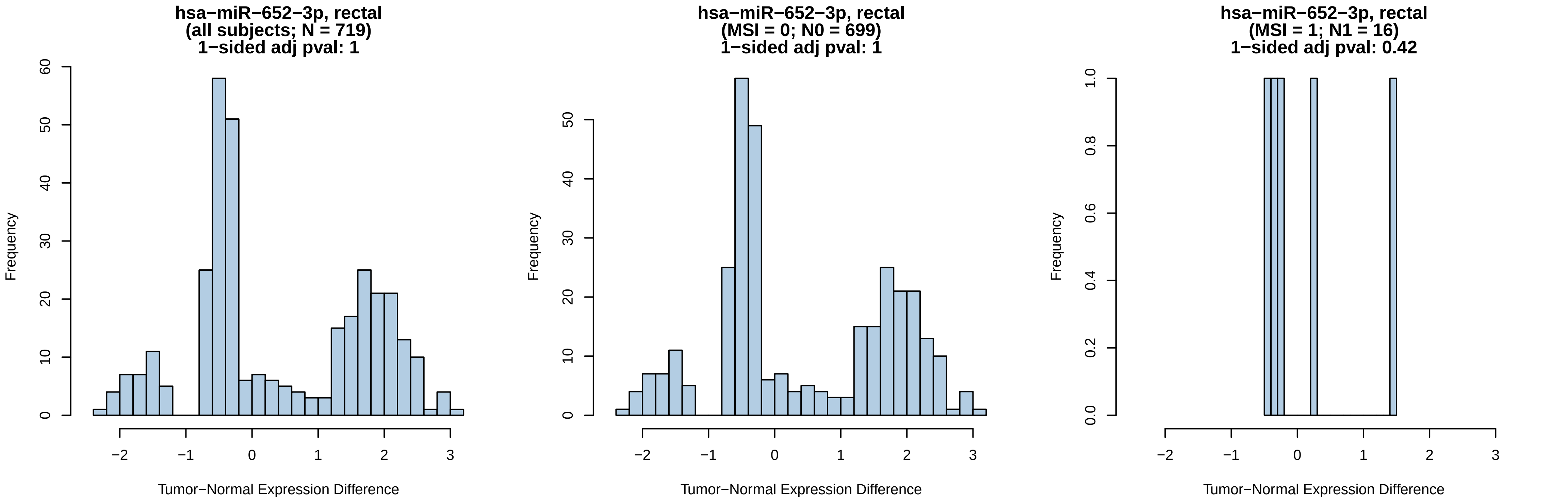

hsa−miR−652−3p, rectal
(all subjects; N = 719)
1−sided adj pval: 1
Frequency
Tumor−Normal Expression Difference
hsa−miR−652−3p, rectal
(MSI = 0; N0 = 699)
1−sided adj pval: 1
Frequency
Tumor−Normal Expression Difference
hsa−miR−652−3p, rectal
(MSI = 1; N1 = 16)
1−sided adj pval: 0.42
Frequency
Tumor−Normal Expression Difference

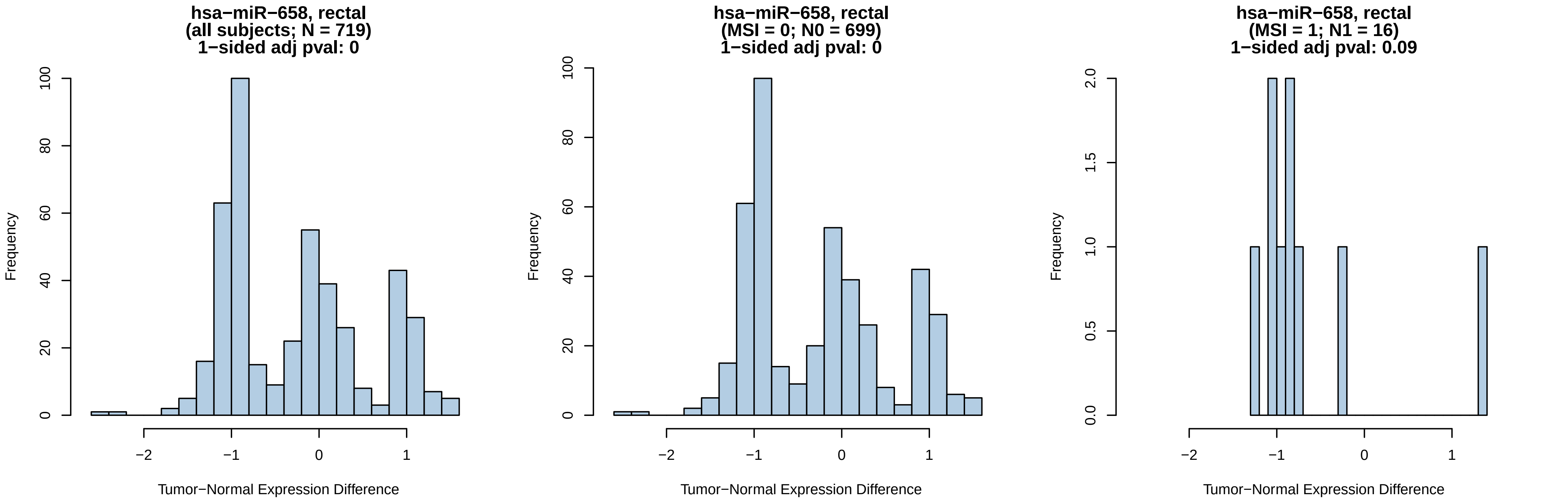
hsa−miR−658, rectal
(all subjects; N = 719)
1−sided adj pval: 0
Frequency
Tumor−Normal Expression Difference
hsa−miR−658, rectal
(MSI = 0; N0 = 699)
1−sided adj pval: 0
Frequency
Tumor−Normal Expression Difference
hsa−miR−658, rectal
(MSI = 1; N1 = 16)
1−sided adj pval: 0.09
Frequency
Tumor−Normal Expression Difference

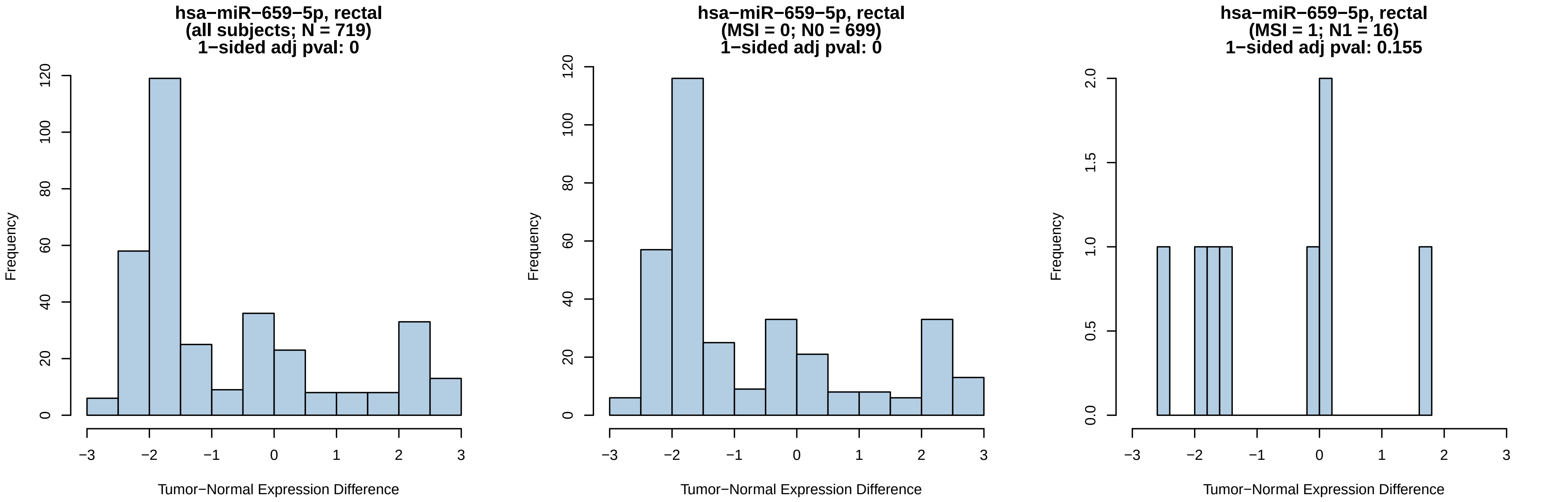

hsa−miR−659−5p, rectal
(all subjects; N = 719)
1−sided adj pval: 0
hsa−miR−659−5p, rectal
(MSI = 0; N0 = 699)
1−sided adj pval: 0
hsa−miR−659−5p, rectal
(MSI = 1; N1 = 16)
1−sided adj pval: 0.155
Frequency
Tumor−Normal Expression Difference

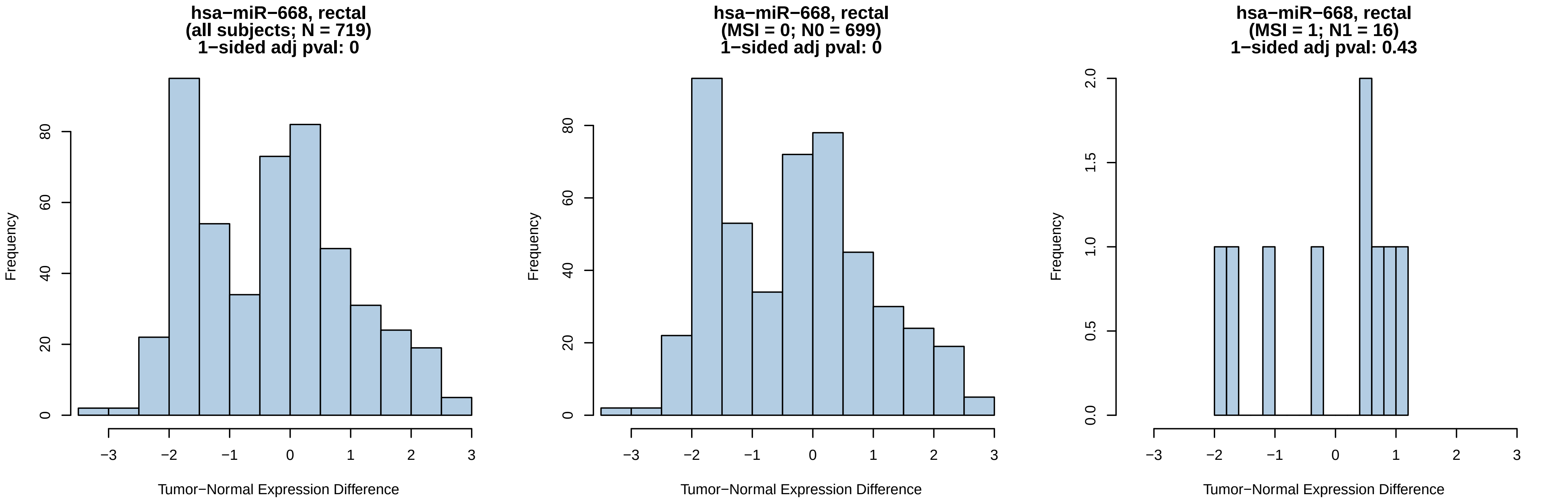
hsa−miR−668, rectal
(all subjects; N = 719)
1−sided adj pval: 0
Frequency
Tumor−Normal Expression Difference
hsa−miR−668, rectal
(MSI = 0; N0 = 699)
1−sided adj pval: 0
Frequency
Tumor−Normal Expression Difference
hsa−miR−668, rectal
(MSI = 1; N1 = 16)
1−sided adj pval: 0.43
Frequency
Tumor−Normal Expression Difference

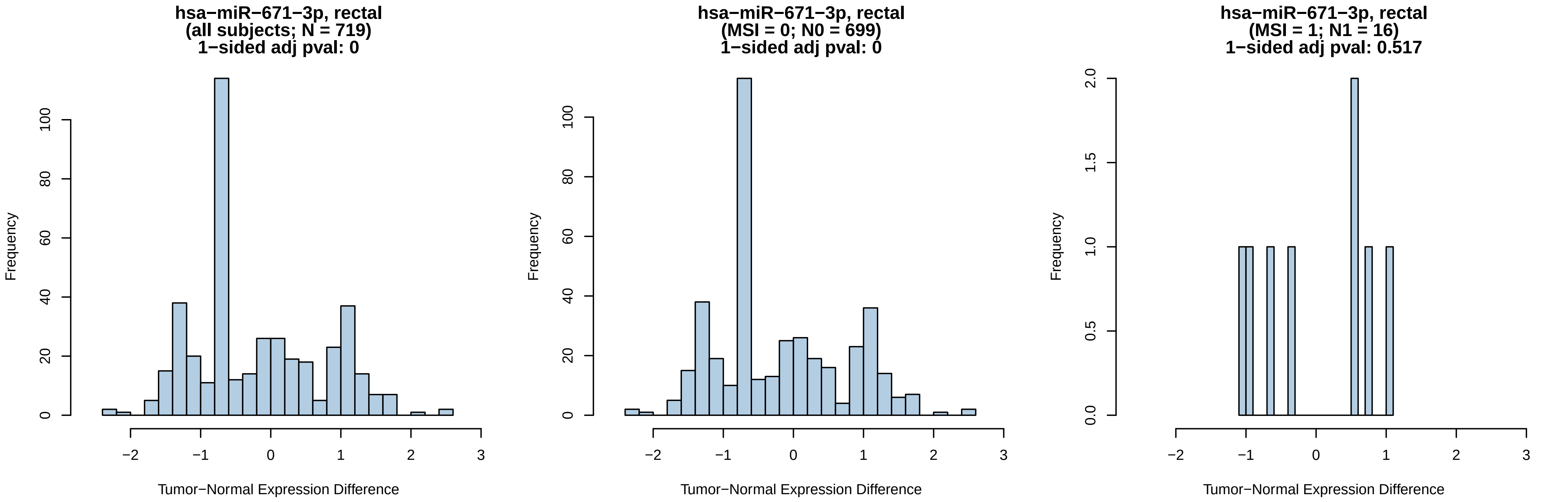
hsa−miR−671−3p, rectal
(all subjects; N = 719)
1−sided adj pval: 0
Frequency
Tumor−Normal Expression Difference
hsa−miR−671−3p, rectal
(MSI = 0; N0 = 699)
1−sided adj pval: 0
Frequency
Tumor−Normal Expression Difference
hsa−miR−671−3p, rectal
(MSI = 1; N1 = 16)
1−sided adj pval: 0.517
Frequency
Tumor−Normal Expression Difference

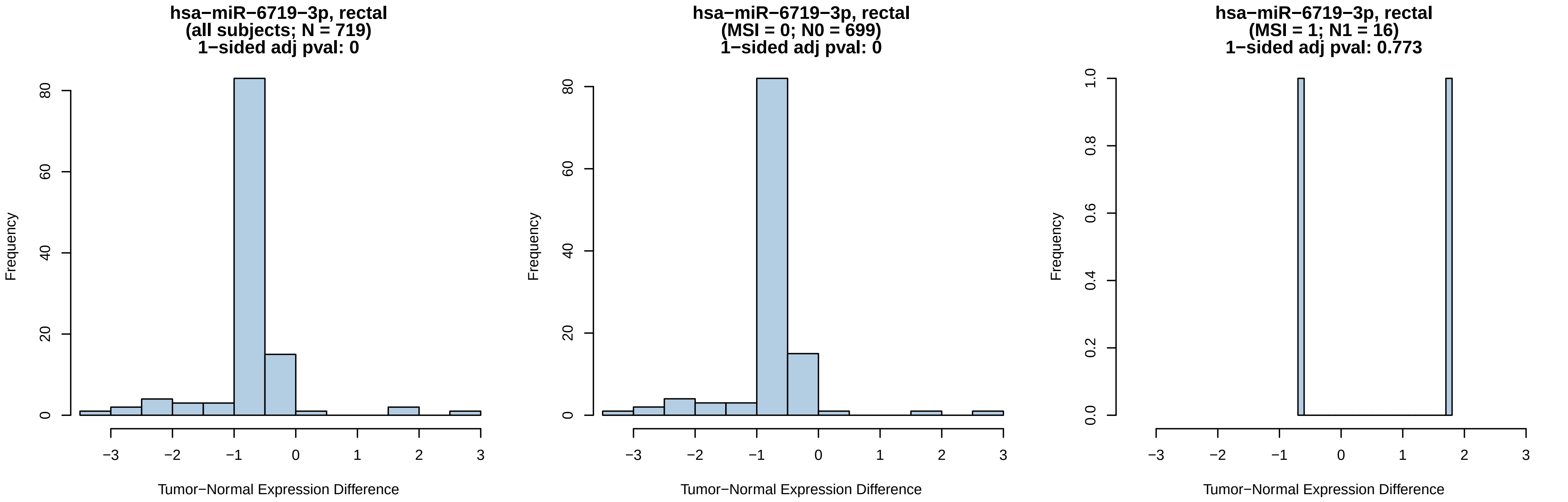

hsa−miR−6719−3p, rectal
(all subjects; N = 719)
1−sided adj pval: 0
Frequency
Tumor−Normal Expression Difference
hsa−miR−6719−3p, rectal
(MSI = 0; N0 = 699)
1−sided adj pval: 0
Frequency
Tumor−Normal Expression Difference
hsa−miR−6719−3p, rectal
(MSI = 1; N1 = 16)
1−sided adj pval: 0.773
Frequency
Tumor−Normal Expression Difference

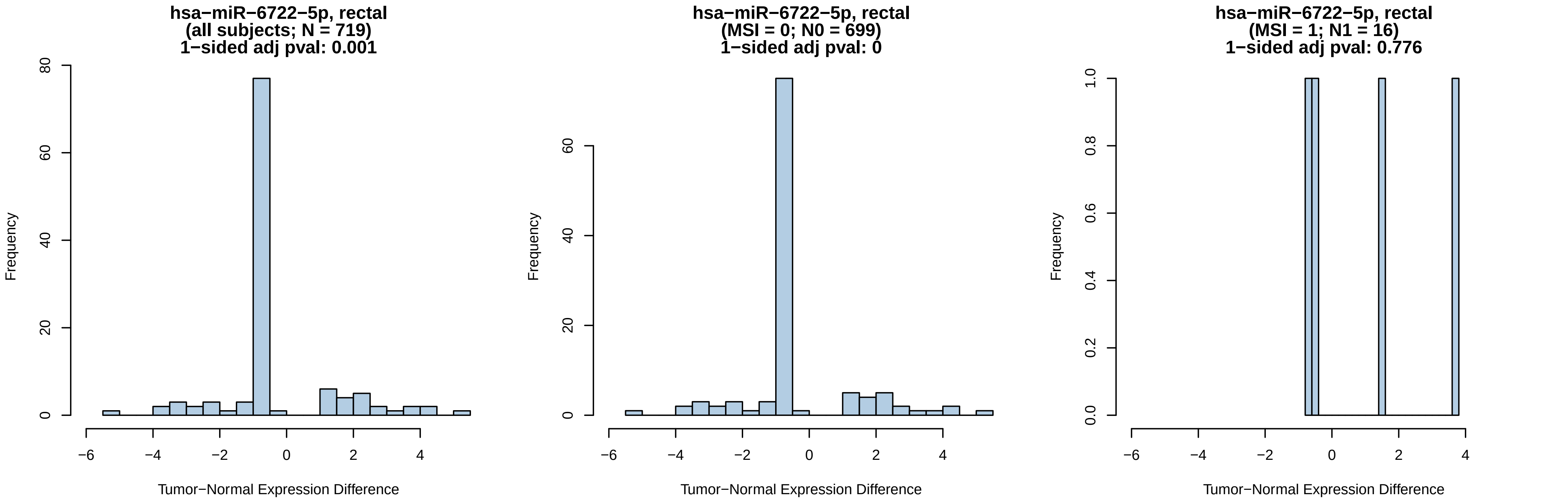
hsa−miR−6722−5p, rectal
(all subjects; N = 719)
1−sided adj pval: 0.001
Frequency
Tumor−Normal Expression Difference
hsa−miR−6722−5p, rectal
(MSI = 0; N0 = 699)
1−sided adj pval: 0
Frequency
Tumor−Normal Expression Difference
hsa−miR−6722−5p, rectal
(MSI = 1; N1 = 16)
1−sided adj pval: 0.776
Frequency
Tumor−Normal Expression Difference

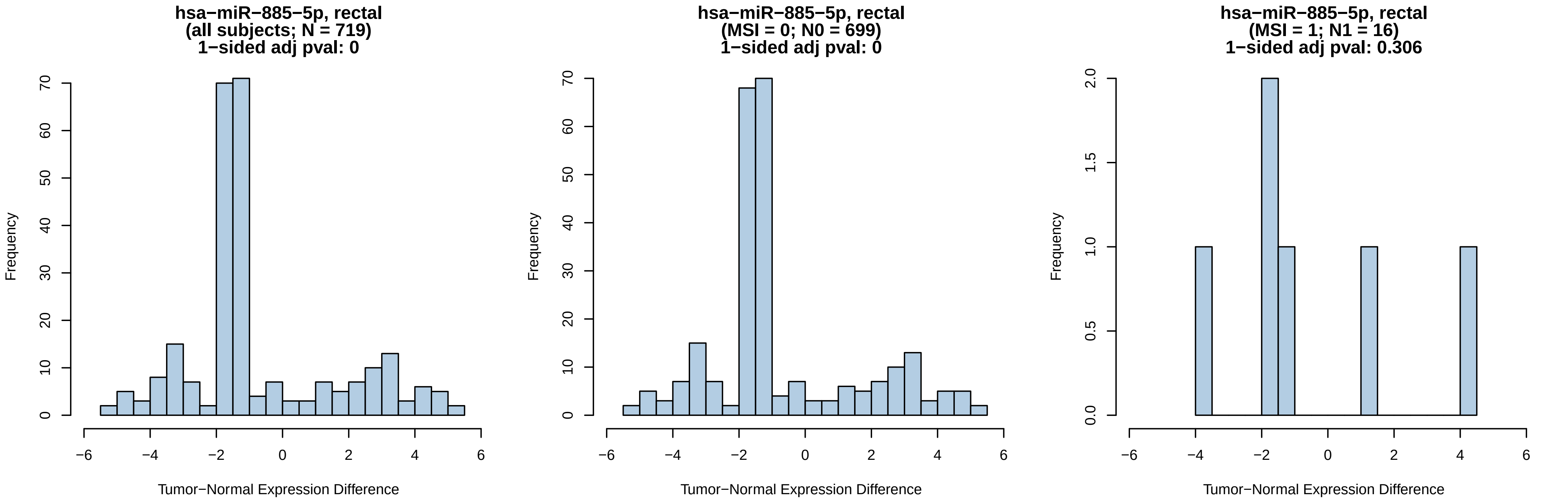
hsa−miR−885−5p, rectal
(all subjects; N = 719)
1−sided adj pval: 0
Frequency
Tumor−Normal Expression Difference
hsa−miR−885−5p, rectal
(MSI = 0; N0 = 699)
1−sided adj pval: 0
Frequency
Tumor−Normal Expression Difference
hsa−miR−885−5p, rectal
(MSI = 1; N1 = 16)
1−sided adj pval: 0.306
Frequency
Tumor−Normal Expression Difference

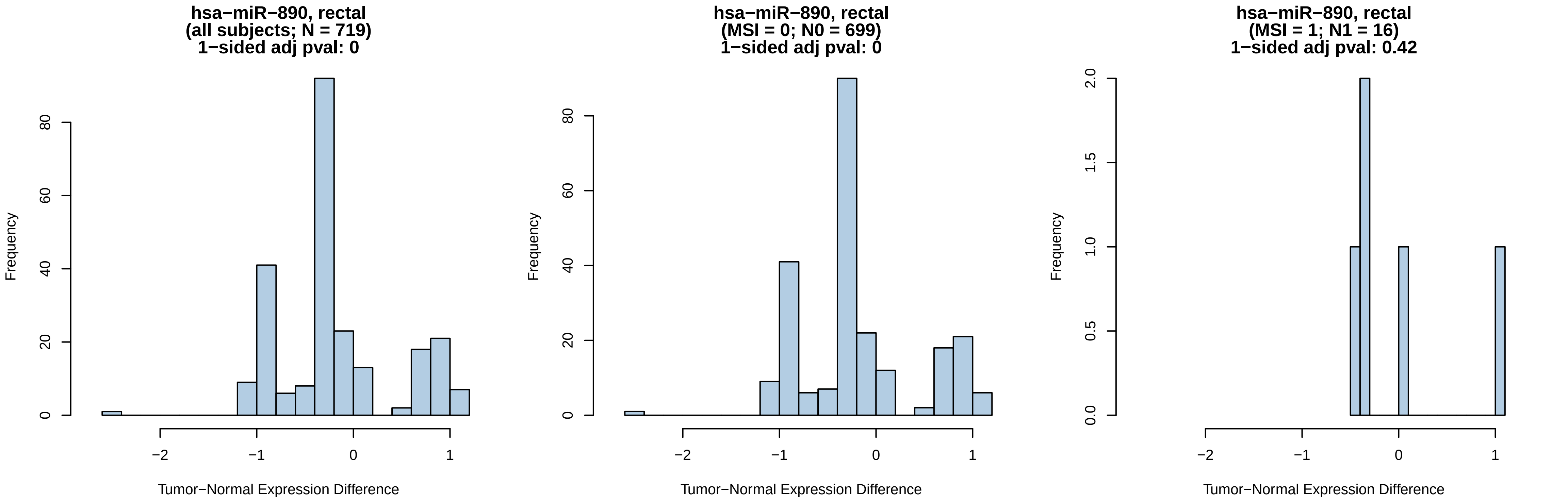

hsa−miR−890, rectal
(all subjects; N = 719)
1−sided adj pval: 0
Frequency
0
20
40
60
80
−2
−1
0
1
Tumor−Normal Expression Difference
hsa−miR−890, rectal
(MSI = 0; N0 = 699)
1−sided adj pval: 0
Frequency
0
20
40
60
80
−2
−1
0
1
Tumor−Normal Expression Difference
hsa−miR−890, rectal
(MSI = 1; N1 = 16)
1−sided adj pval: 0.42
Frequency
0.0
0.5
1.0
1.5
2.0
−2
−1
0
1
Tumor−Normal Expression Difference

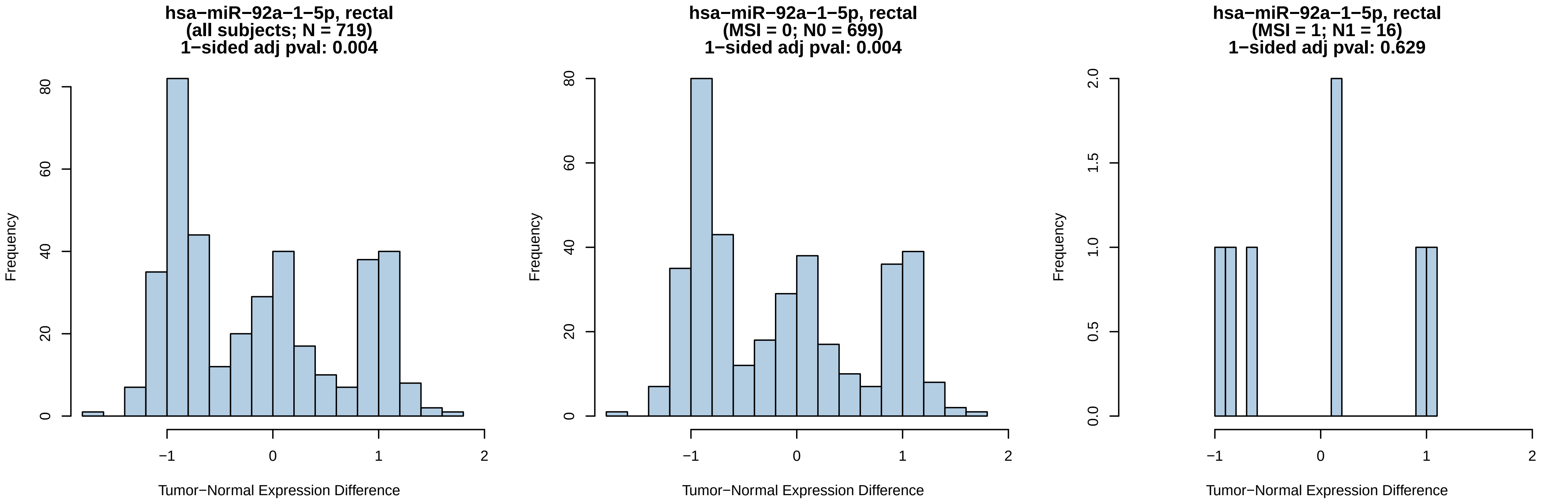

hsa−miR−92a−1−5p, rectal
(all subjects; N = 719)
1−sided adj pval: 0.004
Frequency
0
20
40
60
80
−1
0
1
2
Tumor−Normal Expression Difference
hsa−miR−92a−1−5p, rectal
(MSI = 0; N0 = 699)
1−sided adj pval: 0.004
Frequency
0
20
40
60
80
−1
0
1
2
Tumor−Normal Expression Difference
hsa−miR−92a−1−5p, rectal
(MSI = 1; N1 = 16)
1−sided adj pval: 0.629
Frequency
0.0
0.5
1.0
1.5
2.0
−1
0
1
2
Tumor−Normal Expression Difference

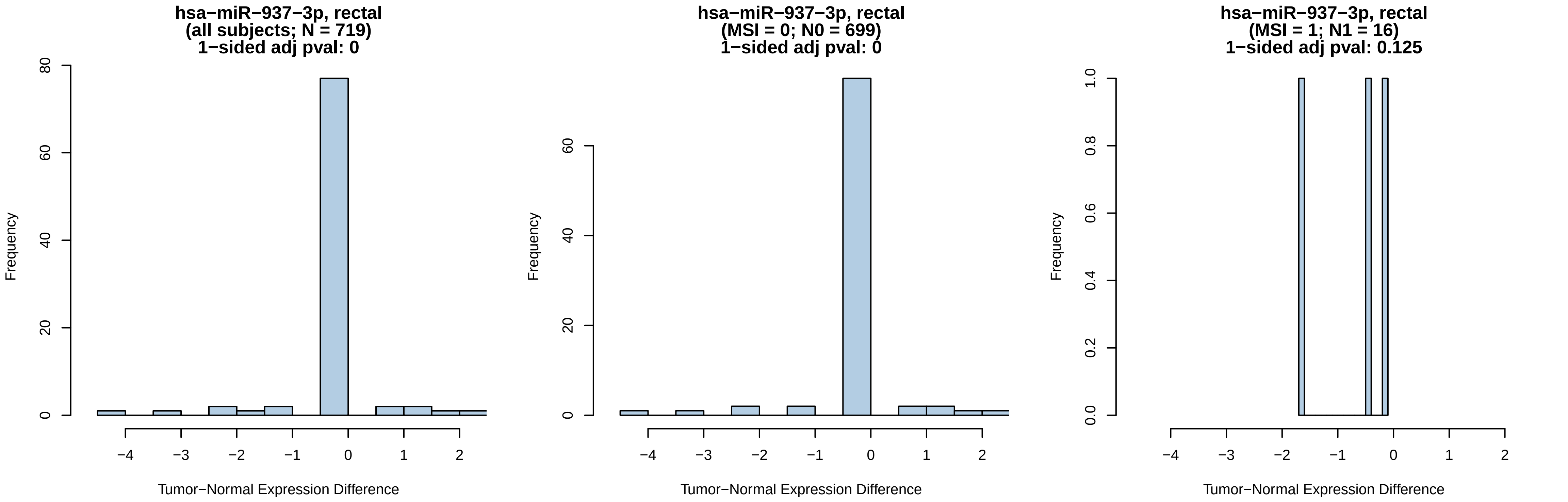
hsa−miR−937−3p, rectal
(all subjects; N = 719)
1−sided adj pval: 0
Frequency
Tumor−Normal Expression Difference
hsa−miR−937−3p, rectal
(MSI = 0; N0 = 699)
1−sided adj pval: 0
Frequency
Tumor−Normal Expression Difference
hsa−miR−937−3p, rectal
(MSI = 1; N1 = 16)
1−sided adj pval: 0.125
Frequency
Tumor−Normal Expression Difference

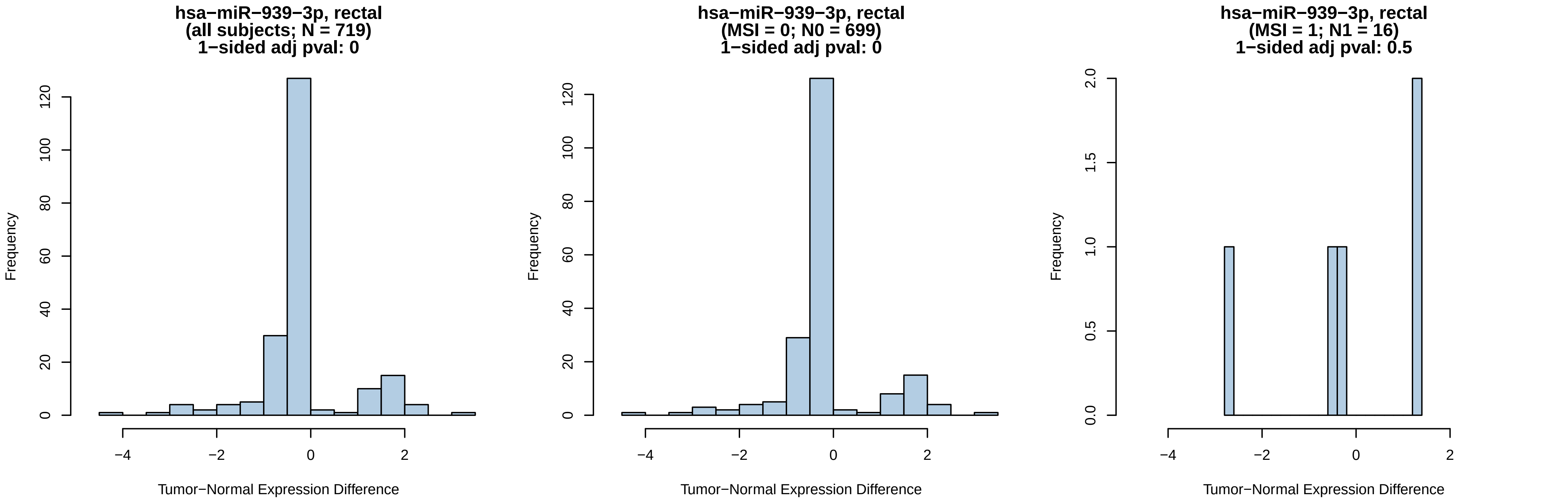
hsa−miR−939−3p, rectal
(all subjects; N = 719)
1−sided adj pval: 0
Frequency
Tumor−Normal Expression Difference
hsa−miR−939−3p, rectal
(MSI = 0; N0 = 699)
1−sided adj pval: 0
Frequency
Tumor−Normal Expression Difference
hsa−miR−939−3p, rectal
(MSI = 1; N1 = 16)
1−sided adj pval: 0.5
Frequency
Tumor−Normal Expression Difference

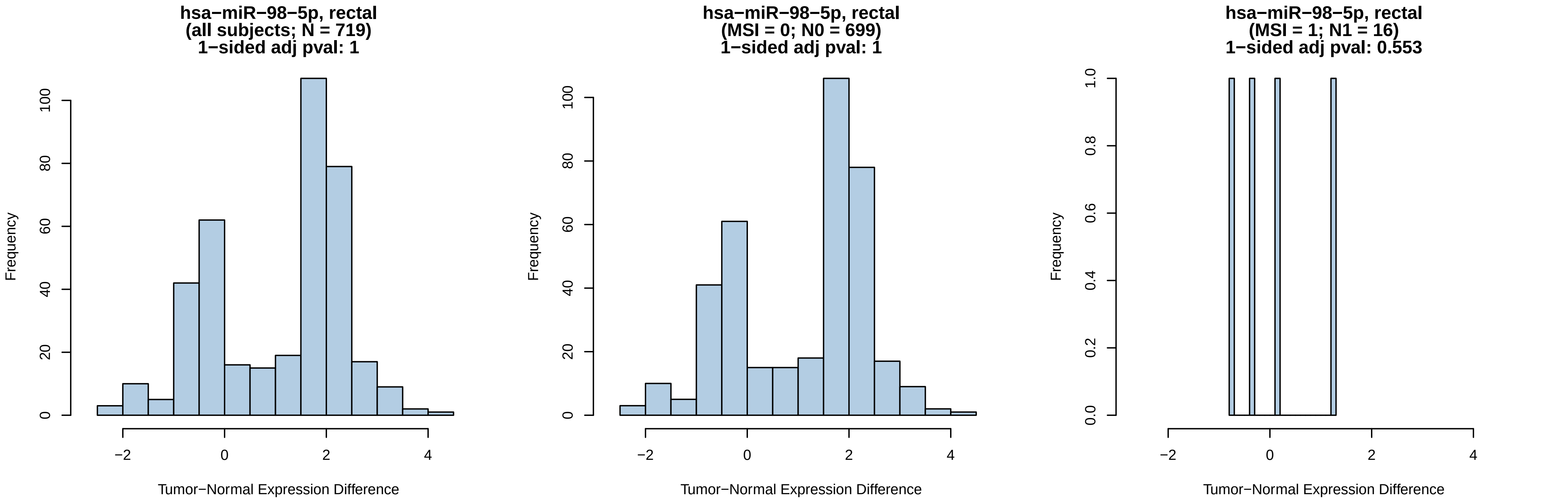

hsa−miR−98−5p, rectal
(all subjects; N = 719)
1−sided adj pval: 1
Frequency
0
20
40
60
80
100
−2
0
2
4
Tumor−Normal Expression Difference
hsa−miR−98−5p, rectal
(MSI = 0; N0 = 699)
1−sided adj pval: 1
Frequency
0
20
40
60
80
100
−2
0
2
4
Tumor−Normal Expression Difference
hsa−miR−98−5p, rectal
(MSI = 1; N1 = 16)
1−sided adj pval: 0.553
Frequency
0.0
0.2
0.4
0.6
0.8
1.0
−2
0
2
4
Tumor−Normal Expression Difference

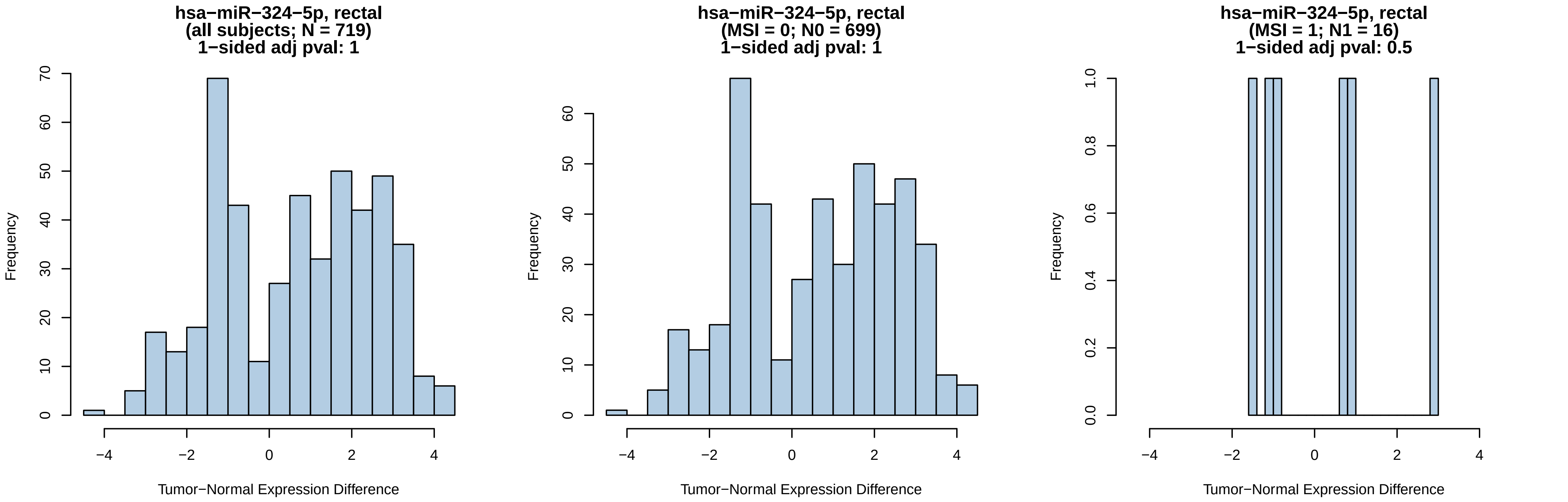
hsa−miR−324−5p, rectal
(all subjects; N = 719)
1−sided adj pval: 1
Frequency
Tumor−Normal Expression Difference
hsa−miR−324−5p, rectal
(MSI = 0; N0 = 699)
1−sided adj pval: 1
Frequency
Tumor−Normal Expression Difference
hsa−miR−324−5p, rectal
(MSI = 1; N1 = 16)
1−sided adj pval: 0.5
Frequency
Tumor−Normal Expression Difference

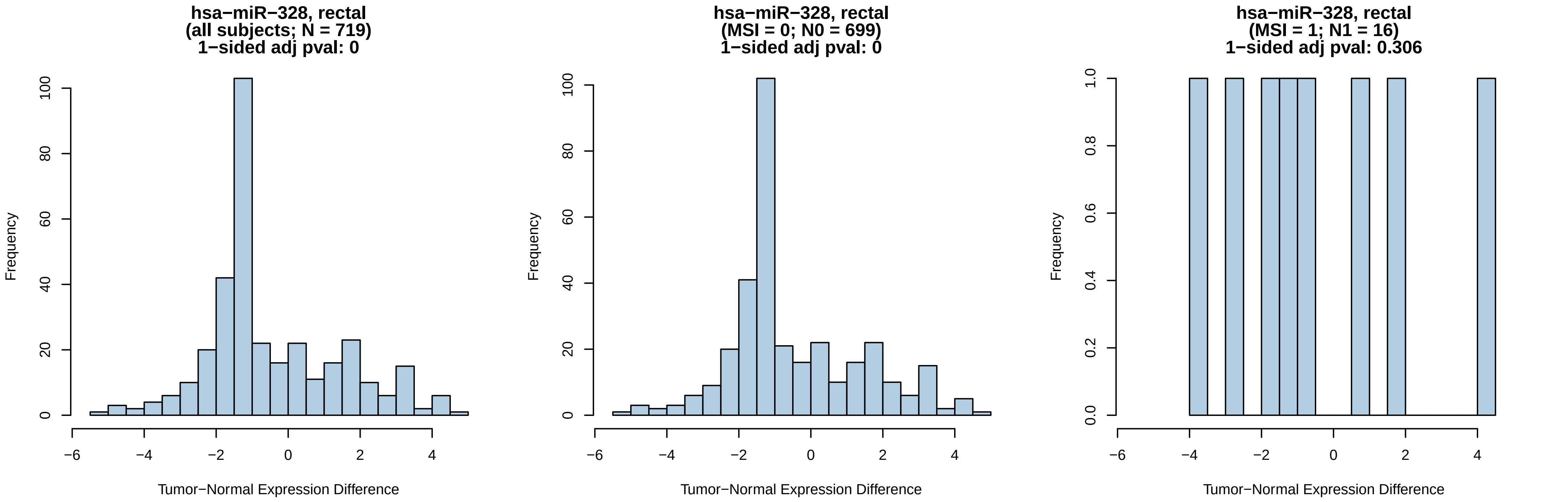
hsa−miR−328, rectal
(all subjects; N = 719)
1−sided adj pval: 0
Frequency
Tumor−Normal Expression Difference
hsa−miR−328, rectal
(MSI = 0; N0 = 699)
1−sided adj pval: 0
Frequency
Tumor−Normal Expression Difference
hsa−miR−328, rectal
(MSI = 1; N1 = 16)
1−sided adj pval: 0.306
Frequency
Tumor−Normal Expression Difference

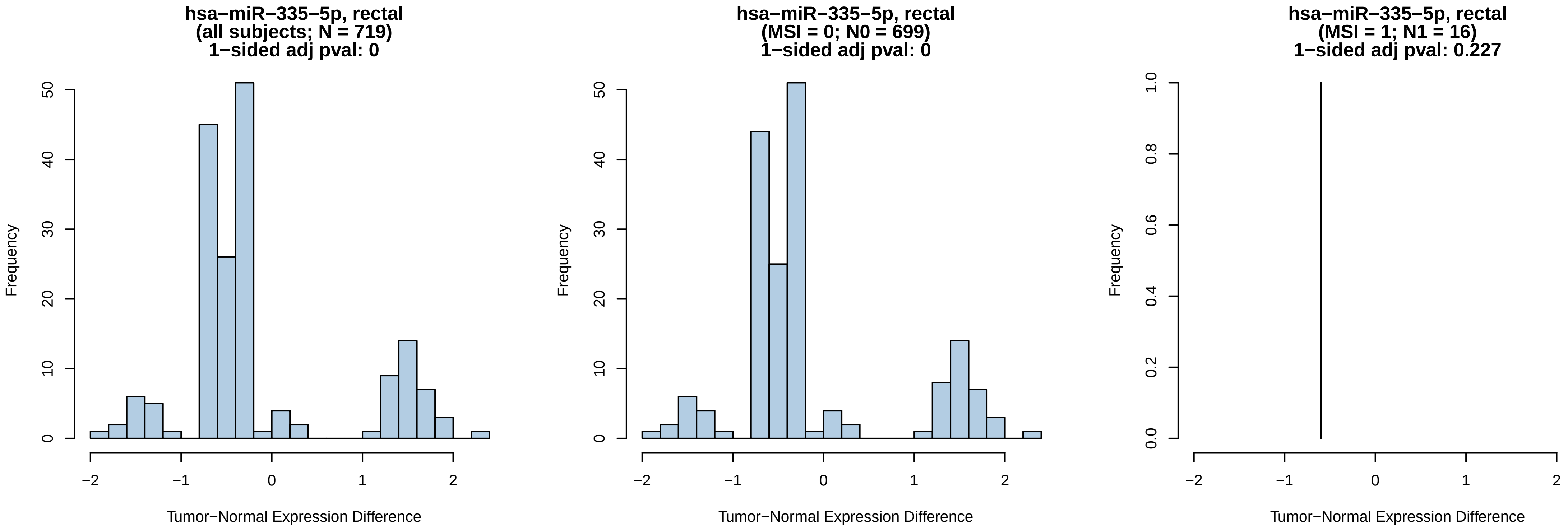
hsa−miR−335−5p, rectal
(all subjects; N = 719)
1−sided adj pval: 0
Frequency
Tumor−Normal Expression Difference
hsa−miR−335−5p, rectal
(MSI = 0; N0 = 699)
1−sided adj pval: 0
Frequency
Tumor−Normal Expression Difference
hsa−miR−335−5p, rectal
(MSI = 1; N1 = 16)
1−sided adj pval: 0.227
Frequency
Tumor−Normal Expression Difference

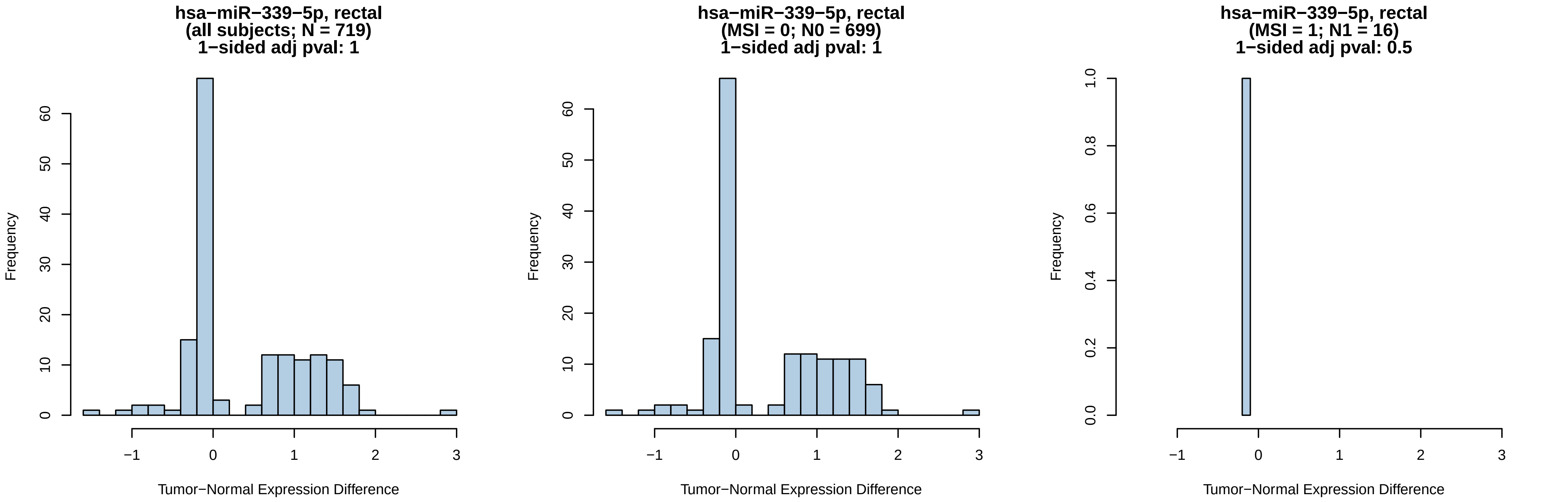
hsa−miR−339−5p, rectal
(all subjects; N = 719)
1−sided adj pval: 1
hsa−miR−339−5p, rectal
(MSI = 0; N0 = 699)
1−sided adj pval: 1
hsa−miR−339−5p, rectal
(MSI = 1; N1 = 16)
1−sided adj pval: 0.5
Frequency
Tumor−Normal Expression Difference

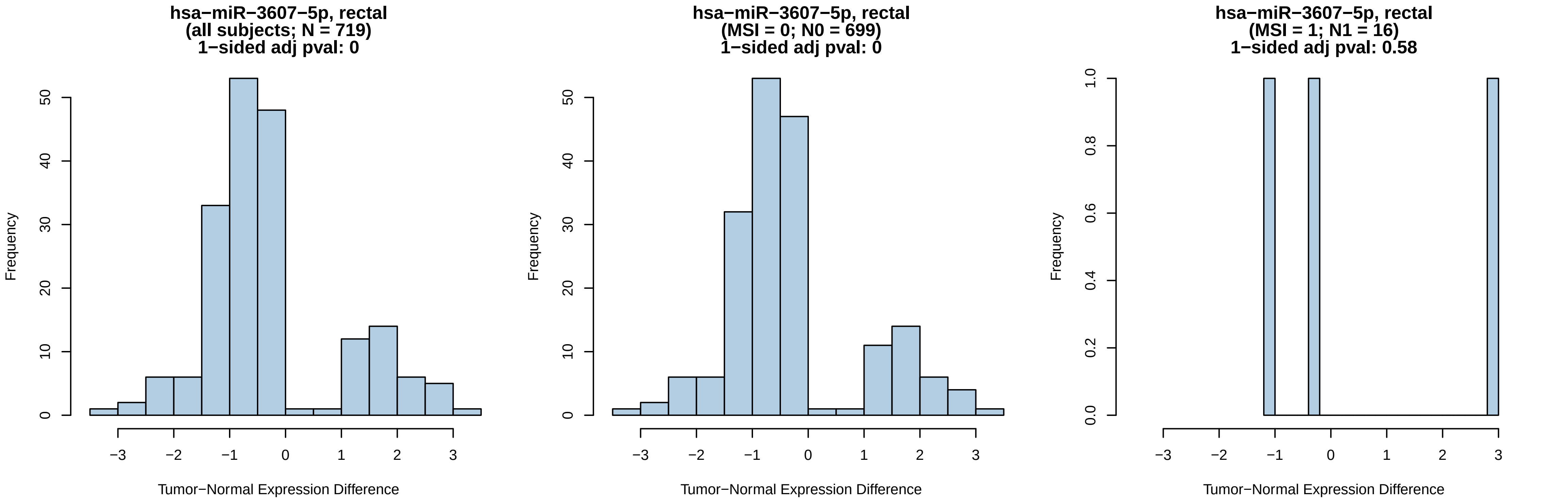
hsa−miR−3607−5p, rectal
(all subjects; N = 719)
1−sided adj pval: 0
Frequency
Tumor−Normal Expression Difference
hsa−miR−3607−5p, rectal
(MSI = 0; N0 = 699)
1−sided adj pval: 0
Frequency
Tumor−Normal Expression Difference
hsa−miR−3607−5p, rectal
(MSI = 1; N1 = 16)
1−sided adj pval: 0.58
Frequency
Tumor−Normal Expression Difference

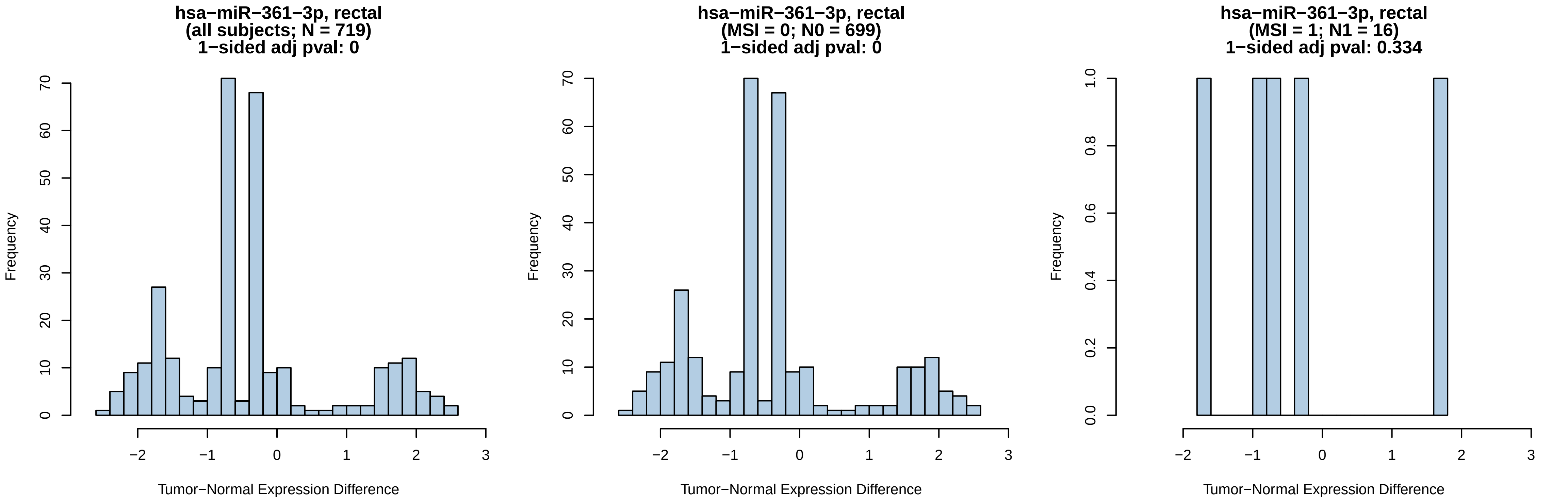
hsa−miR−361−3p, rectal
(all subjects; N = 719)
1−sided adj pval: 0
hsa−miR−361−3p, rectal
(MSI = 0; N0 = 699)
1−sided adj pval: 0
hsa−miR−361−3p, rectal
(MSI = 1; N1 = 16)
1−sided adj pval: 0.334
Frequency
Tumor−Normal Expression Difference
0
10
20
30
40
50
60
70
0.0
0.2
0.4
0.6
0.8
1.0
−2
−1
0
1
2
3

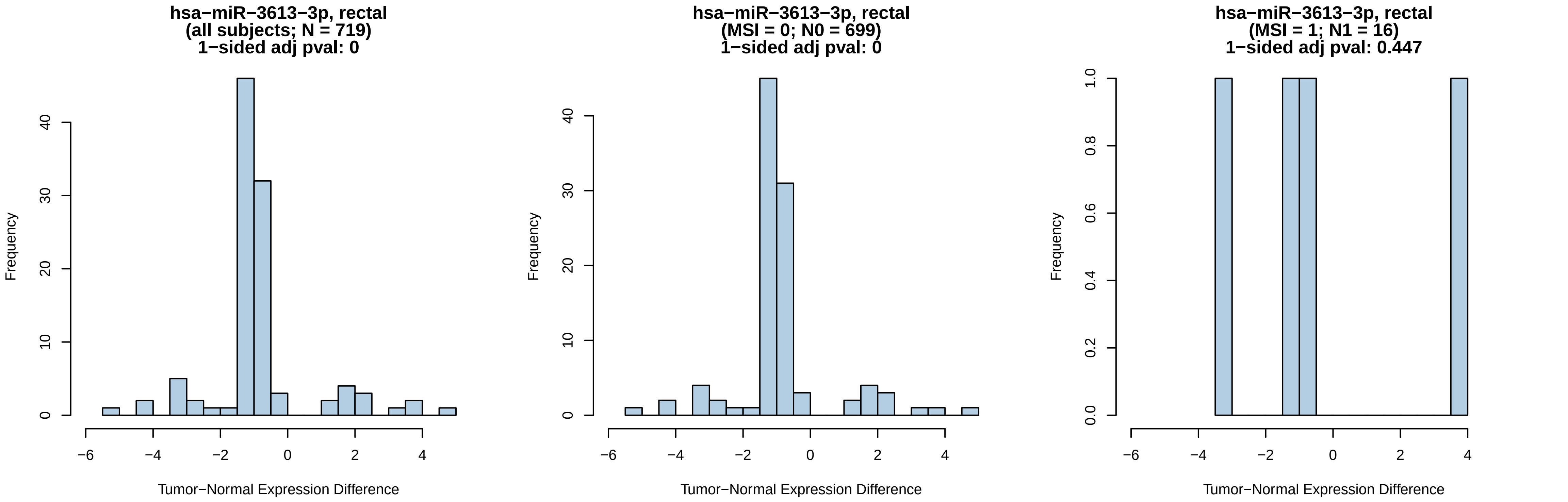

hsa−miR−3613−3p, rectal
(all subjects; N = 719)
1−sided adj pval: 0
Frequency
Tumor−Normal Expression Difference
hsa−miR−3613−3p, rectal
(MSI = 0; N0 = 699)
1−sided adj pval: 0
Frequency
Tumor−Normal Expression Difference
hsa−miR−3613−3p, rectal
(MSI = 1; N1 = 16)
1−sided adj pval: 0.447
Frequency
Tumor−Normal Expression Difference

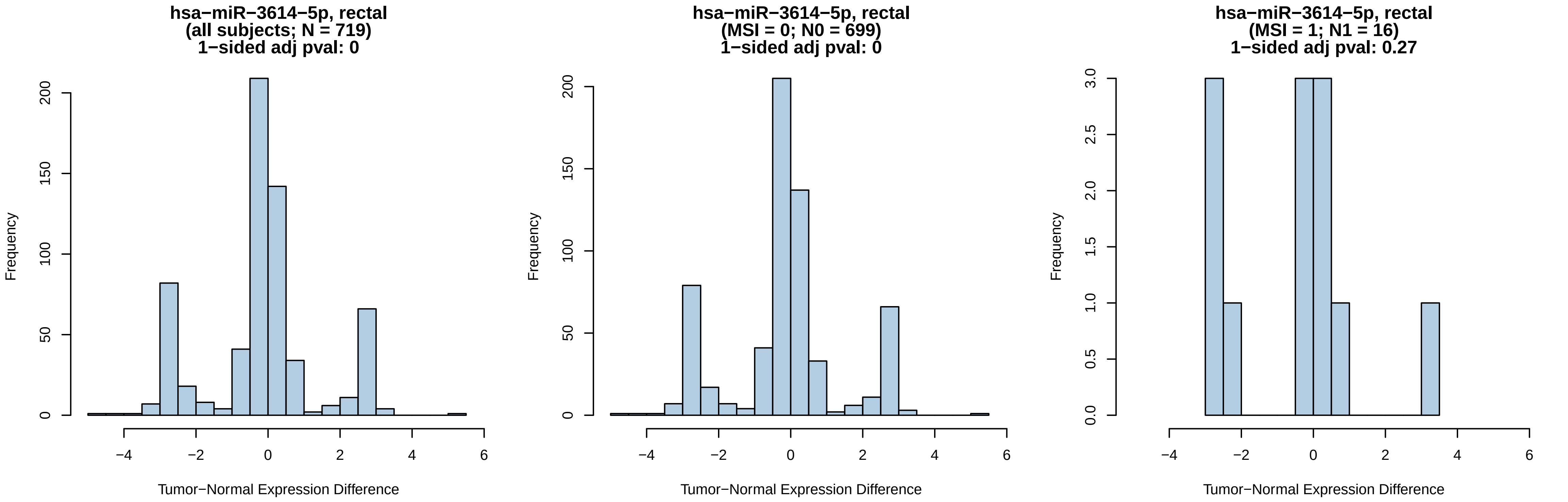
hsa−miR−3614−5p, rectal
(all subjects; N = 719)
1−sided adj pval: 0
Frequency
Tumor−Normal Expression Difference
hsa−miR−3614−5p, rectal
(MSI = 0; N0 = 699)
1−sided adj pval: 0
Frequency
Tumor−Normal Expression Difference
hsa−miR−3614−5p, rectal
(MSI = 1; N1 = 16)
1−sided adj pval: 0.27
Frequency
Tumor−Normal Expression Difference

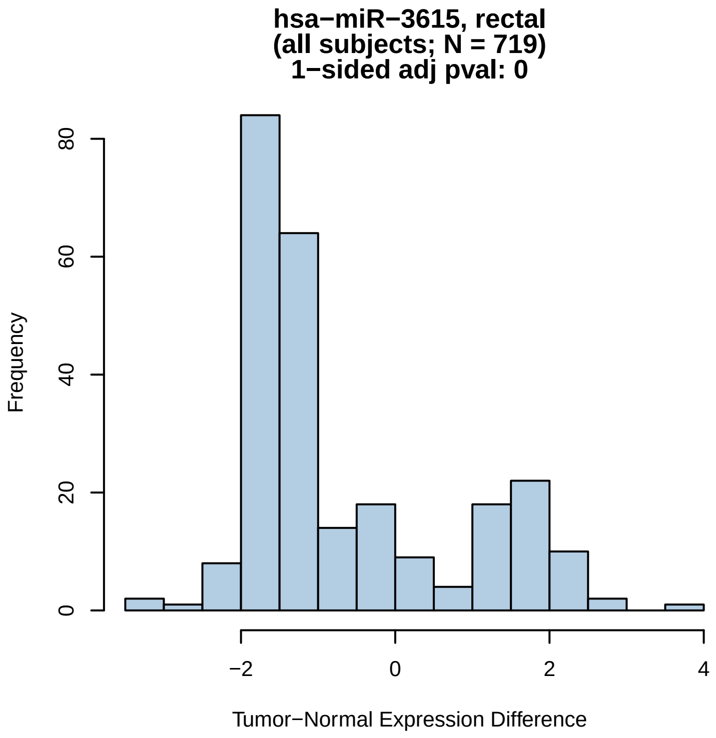
hsa−miR−3615, rectal
(all subjects; N = 719)
1−sided adj pval: 0
Frequency
Tumor−Normal Expression Difference
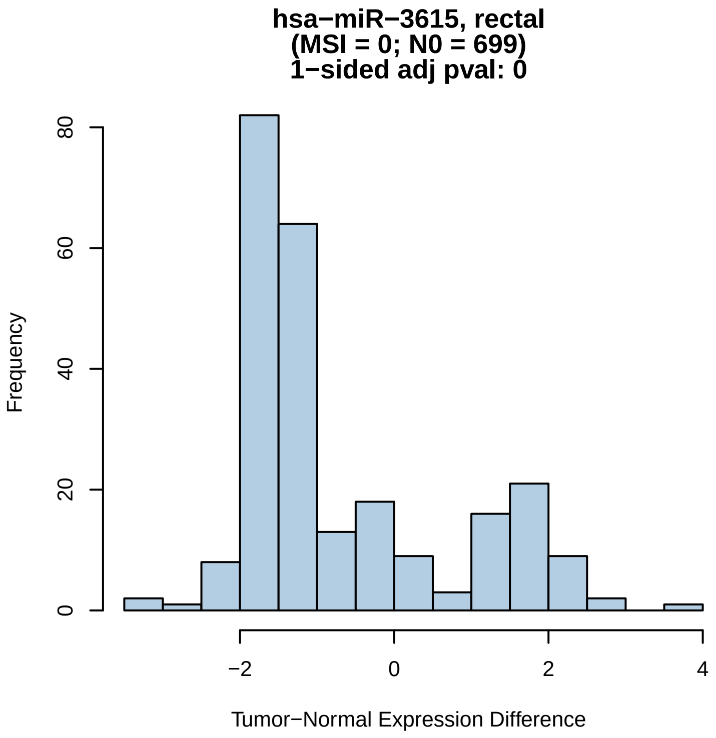
hsa−miR−3615, rectal
(MSI = 0; N0 = 699)
1−sided adj pval: 0
Frequency
Tumor−Normal Expression Difference
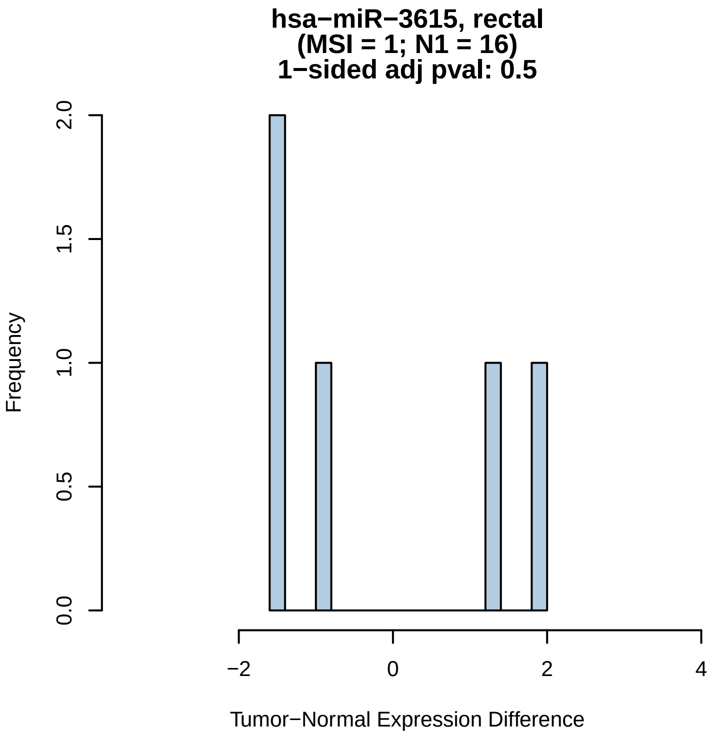
hsa−miR−3615, rectal
(MSI = 1; N1 = 16)
1−sided adj pval: 0.5
Frequency
Tumor−Normal Expression Difference

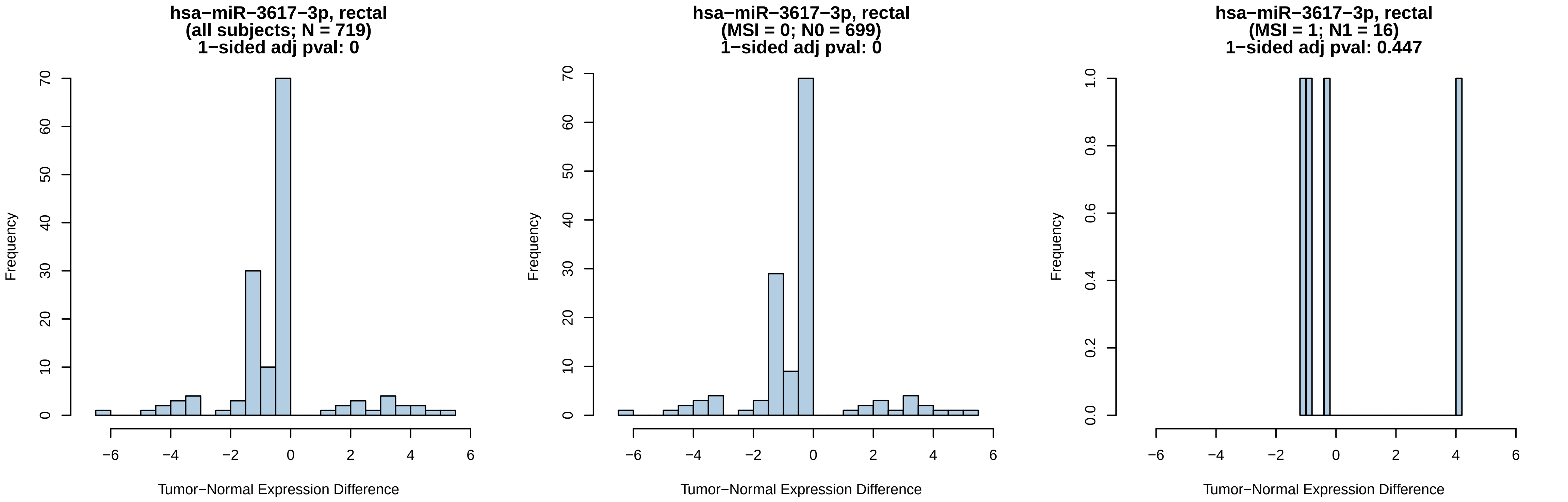
hsa−miR−3617−3p, rectal
(all subjects; N = 719)
1−sided adj pval: 0
Frequency
Tumor−Normal Expression Difference
hsa−miR−3617−3p, rectal
(MSI = 0; N0 = 699)
1−sided adj pval: 0
Frequency
Tumor−Normal Expression Difference
hsa−miR−3617−3p, rectal
(MSI = 1; N1 = 16)
1−sided adj pval: 0.447
Frequency
Tumor−Normal Expression Difference

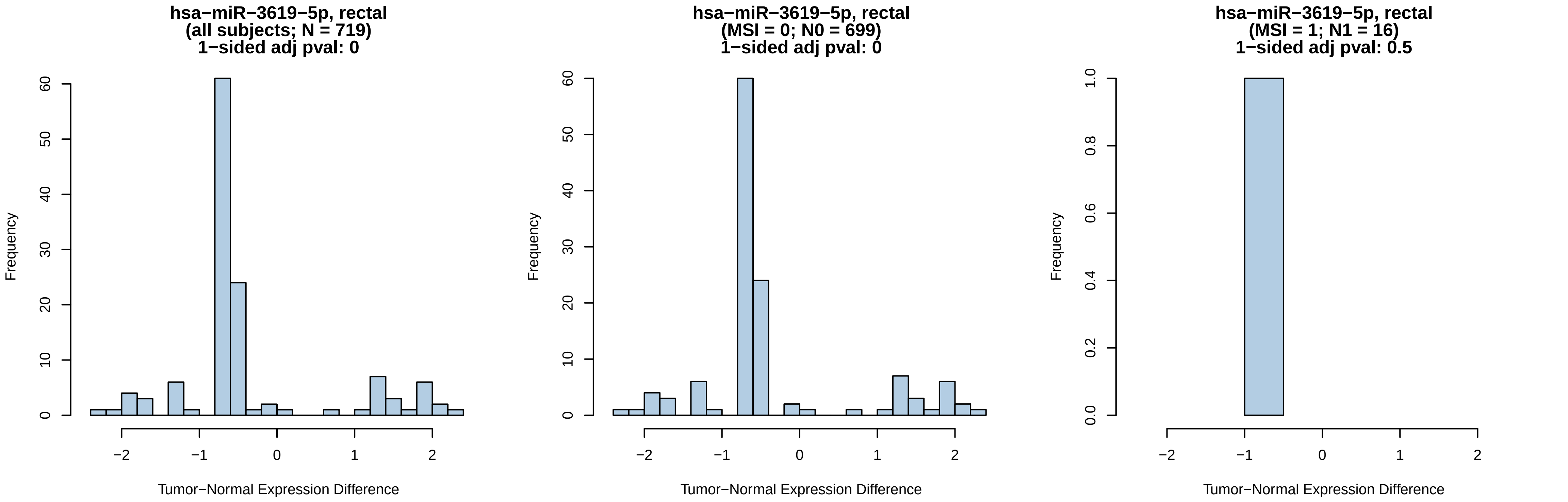
hsa−miR−3619−5p, rectal
(all subjects; N = 719)
1−sided adj pval: 0
hsa−miR−3619−5p, rectal
(MSI = 0; N0 = 699)
1−sided adj pval: 0
hsa−miR−3619−5p, rectal
(MSI = 1; N1 = 16)
1−sided adj pval: 0.5
Frequency
Tumor−Normal Expression Difference

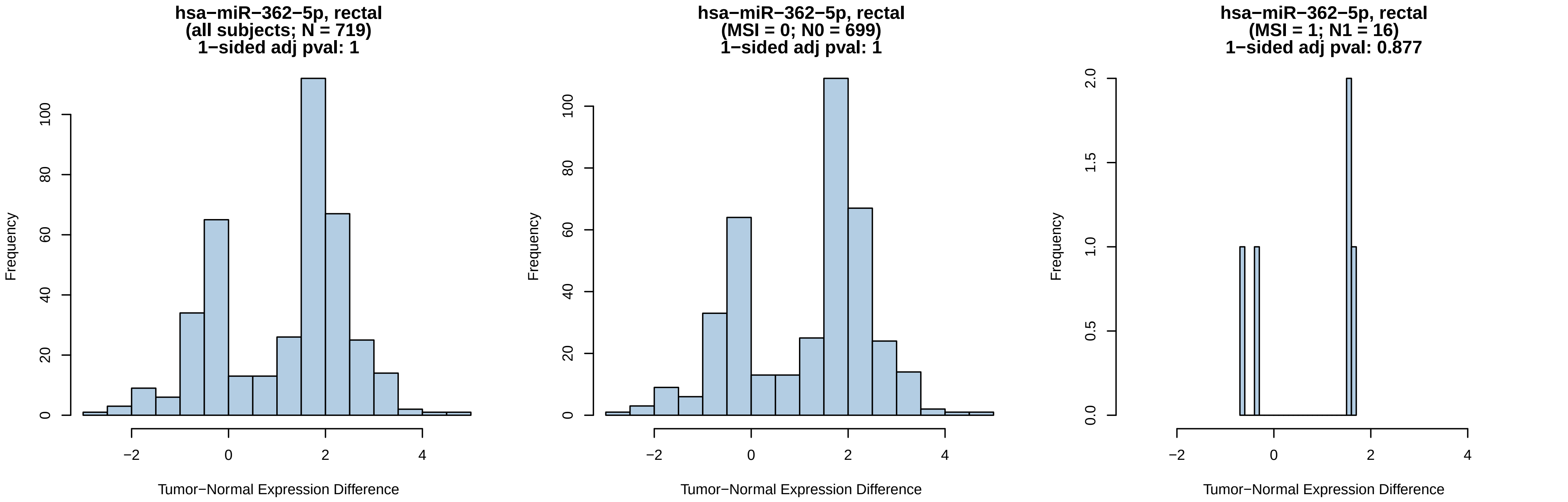
hsa−miR−362−5p, rectal
(all subjects; N = 719)
1−sided adj pval: 1
Frequency
Tumor−Normal Expression Difference
0
20
40
60
80
100
−2
0
2
4
hsa−miR−362−5p, rectal
(MSI = 0; N0 = 699)
1−sided adj pval: 1
Frequency
Tumor−Normal Expression Difference
0
20
40
60
80
100
−2
0
2
4
hsa−miR−362−5p, rectal
(MSI = 1; N1 = 16)
1−sided adj pval: 0.877
Frequency
Tumor−Normal Expression Difference
0.0
0.5
1.0
1.5
2.0
−2
0
2
4

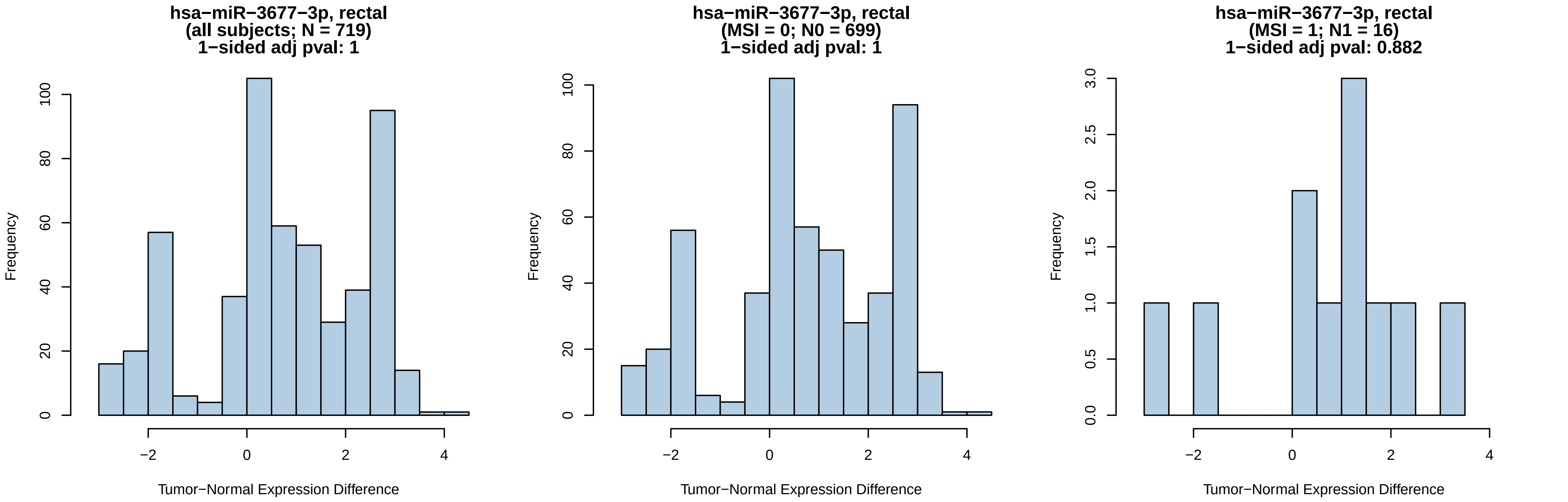

hsa−miR−3677−3p, rectal
(all subjects; N = 719)
1−sided adj pval: 1
Frequency
Tumor−Normal Expression Difference
hsa−miR−3677−3p, rectal
(MSI = 0; N0 = 699)
1−sided adj pval: 1
Frequency
Tumor−Normal Expression Difference
hsa−miR−3677−3p, rectal
(MSI = 1; N1 = 16)
1−sided adj pval: 0.882
Frequency
Tumor−Normal Expression Difference

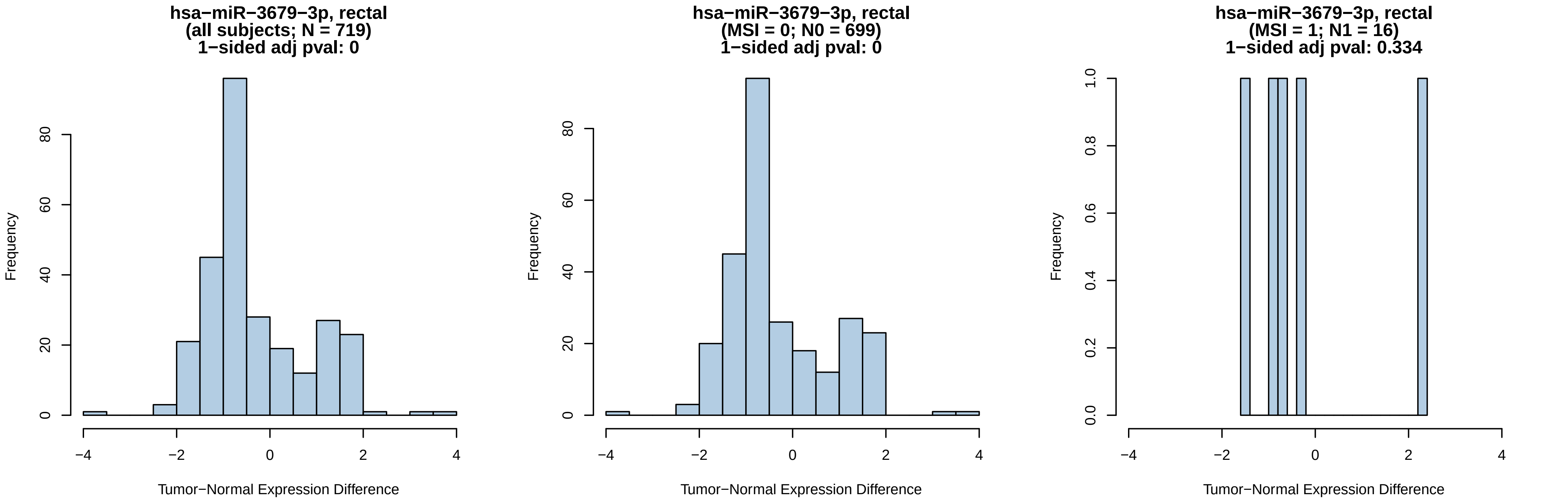
hsa−miR−3679−3p, rectal
(all subjects; N = 719)
1−sided adj pval: 0
hsa−miR−3679−3p, rectal
(MSI = 0; N0 = 699)
1−sided adj pval: 0
hsa−miR−3679−3p, rectal
(MSI = 1; N1 = 16)
1−sided adj pval: 0.334
Frequency
Tumor−Normal Expression Difference

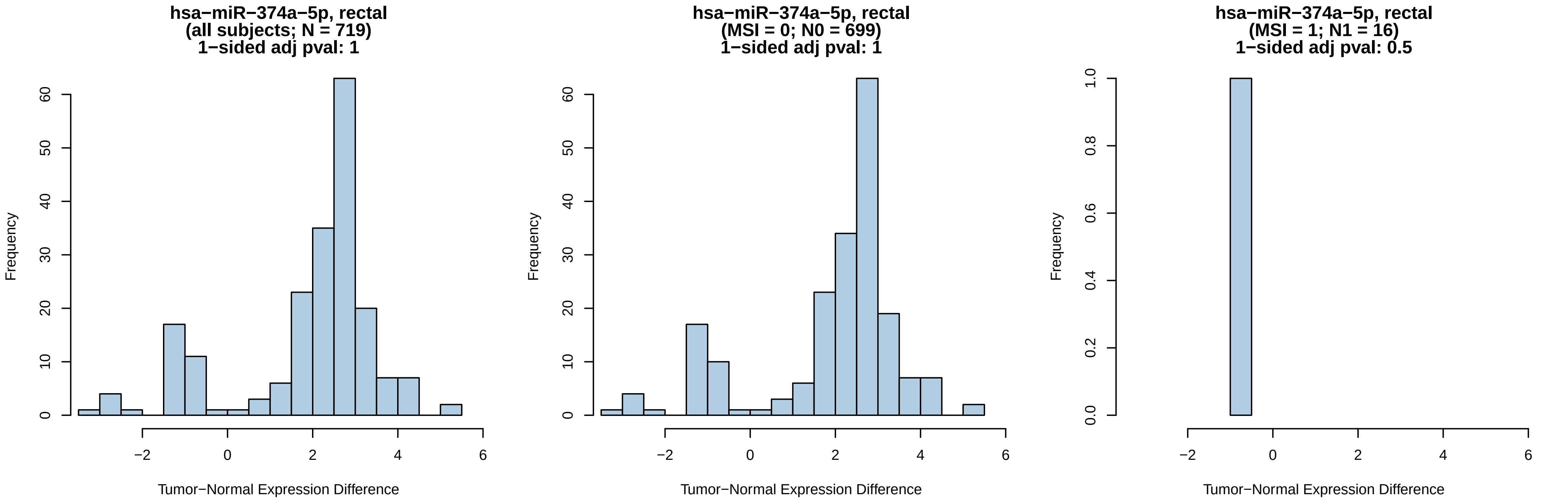

hsa−miR−374a−5p, rectal
(all subjects; N = 719)
1−sided adj pval: 1
Frequency
Tumor−Normal Expression Difference
hsa−miR−374a−5p, rectal
(MSI = 0; N0 = 699)
1−sided adj pval: 1
Frequency
Tumor−Normal Expression Difference
hsa−miR−374a−5p, rectal
(MSI = 1; N1 = 16)
1−sided adj pval: 0.5
Frequency
Tumor−Normal Expression Difference

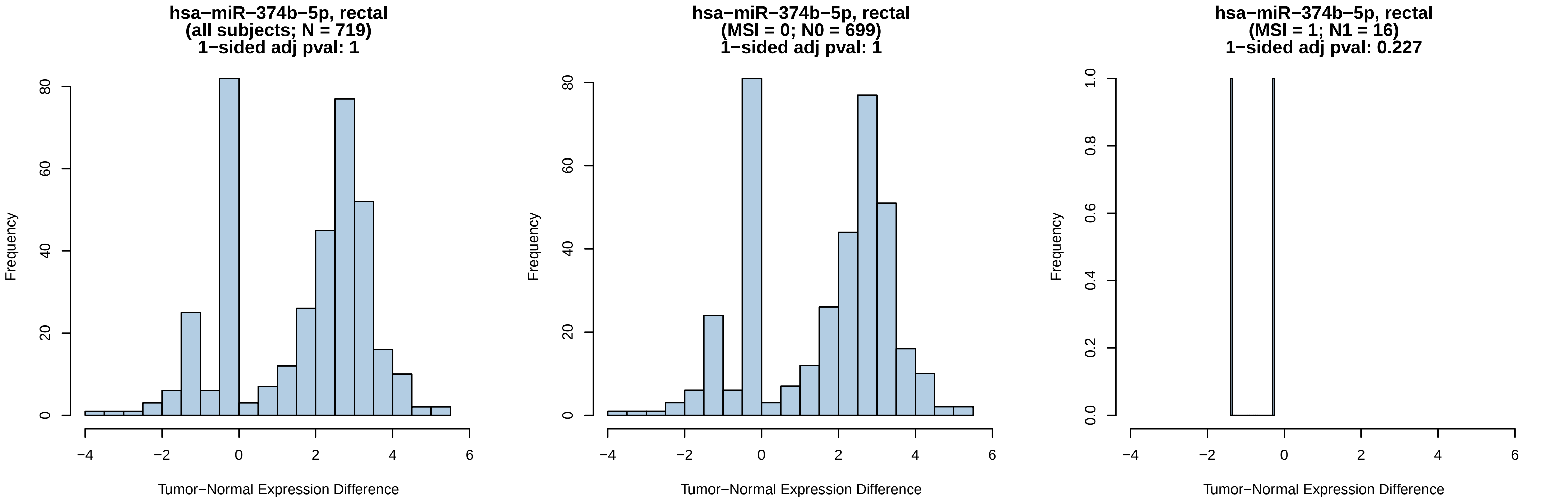
hsa−miR−374b−5p, rectal
(all subjects; N = 719)
1−sided adj pval: 1
Frequency
Tumor−Normal Expression Difference
hsa−miR−374b−5p, rectal
(MSI = 0; N0 = 699)
1−sided adj pval: 1
Frequency
Tumor−Normal Expression Difference
hsa−miR−374b−5p, rectal
(MSI = 1; N1 = 16)
1−sided adj pval: 0.227
Frequency
Tumor−Normal Expression Difference

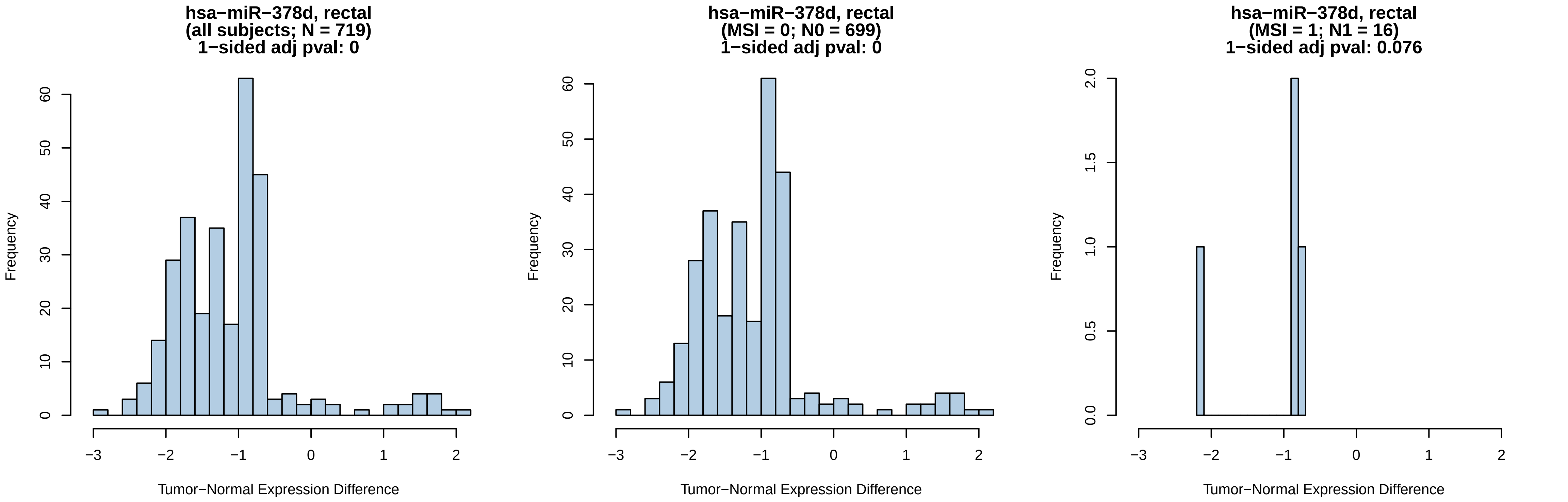

hsa−miR−378d, rectal
(all subjects; N = 719)
1−sided adj pval: 0
hsa−miR−378d, rectal
(MSI = 0; N0 = 699)
1−sided adj pval: 0
hsa−miR−378d, rectal
(MSI = 1; N1 = 16)
1−sided adj pval: 0.076
Frequency
Tumor−Normal Expression Difference

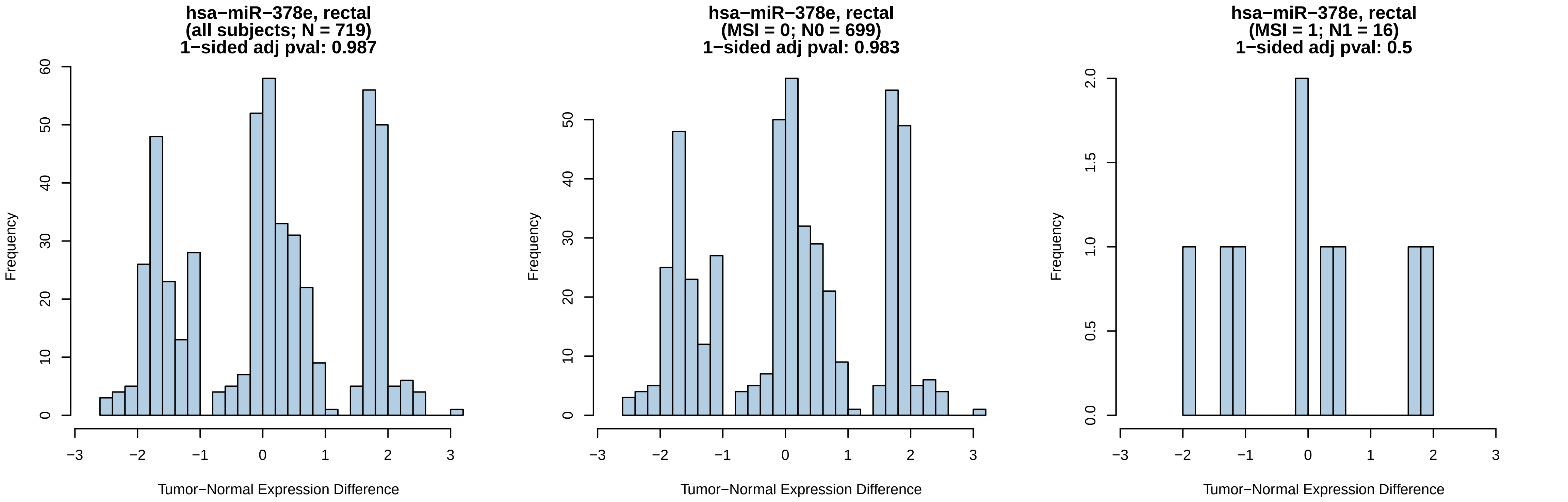
hsa−miR−378e, rectal
(all subjects; N = 719)
1−sided adj pval: 0.987
hsa−miR−378e, rectal
(MSI = 0; N0 = 699)
1−sided adj pval: 0.983
hsa−miR−378e, rectal
(MSI = 1; N1 = 16)
1−sided adj pval: 0.5
Frequency
Tumor−Normal Expression Difference
0
10
20
30
40
50
60
0.0
0.5
1.0
1.5
2.0
−3
−2
−1
0
1
2
3

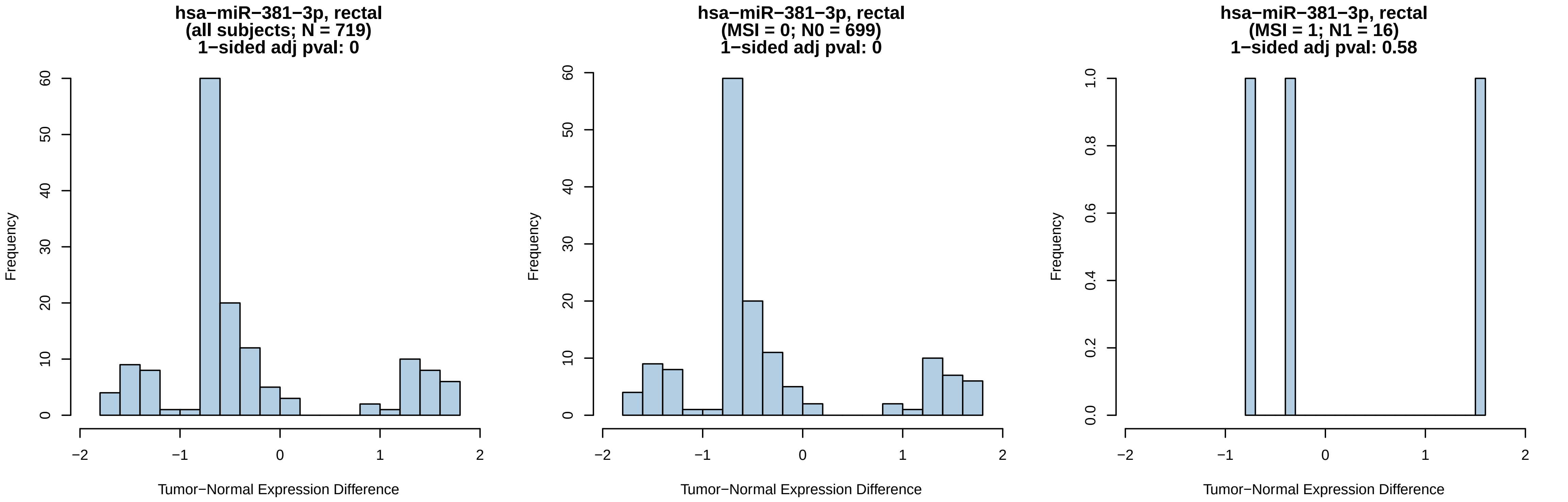
hsa−miR−381−3p, rectal
(all subjects; N = 719)
1−sided adj pval: 0
Frequency
Tumor−Normal Expression Difference
hsa−miR−381−3p, rectal
(MSI = 0; N0 = 699)
1−sided adj pval: 0
Frequency
Tumor−Normal Expression Difference
hsa−miR−381−3p, rectal
(MSI = 1; N1 = 16)
1−sided adj pval: 0.58
Frequency
Tumor−Normal Expression Difference

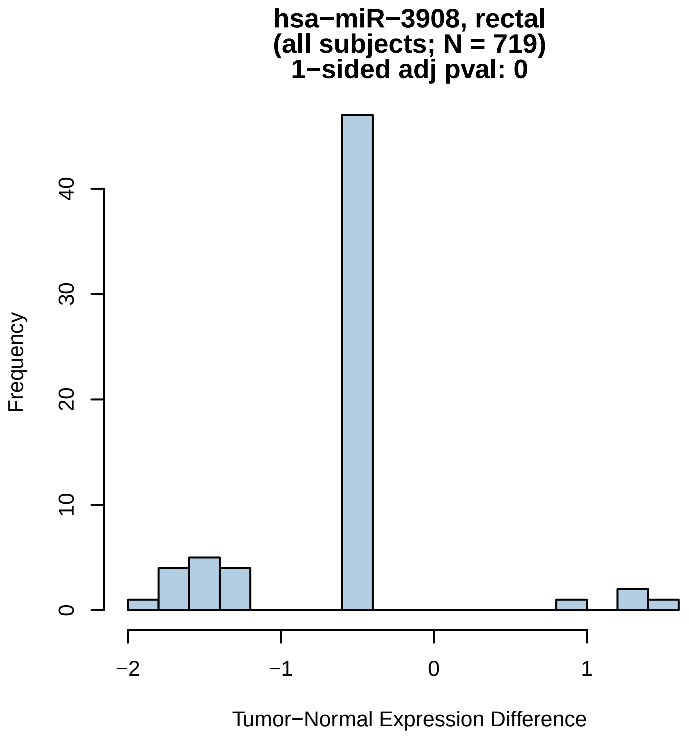

hsa−miR−3908, rectal
(all subjects; N = 719)
1−sided adj pval: 0
Frequency
Tumor−Normal Expression Difference


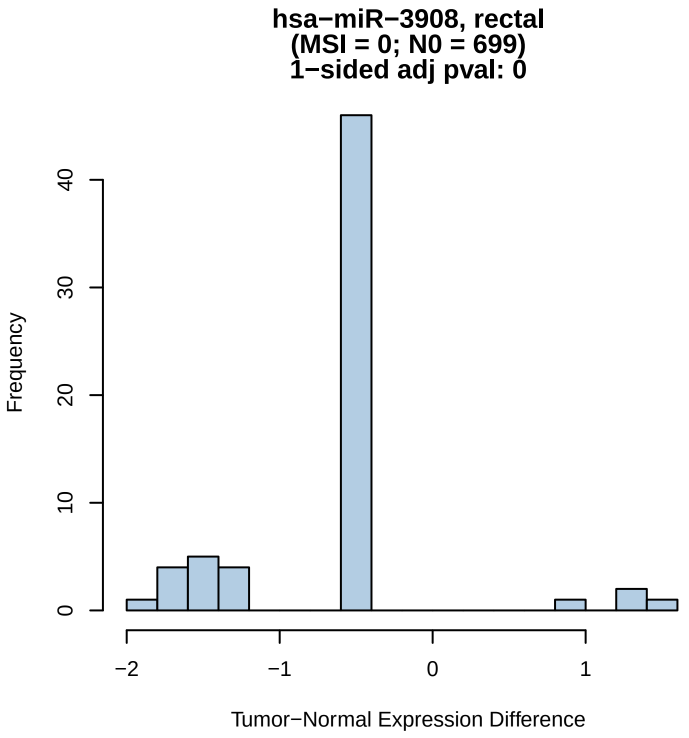

hsa−miR−3908, rectal
(MSI = 0; N0 = 699)
1−sided adj pval: 0
Frequency
Tumor−Normal Expression Difference


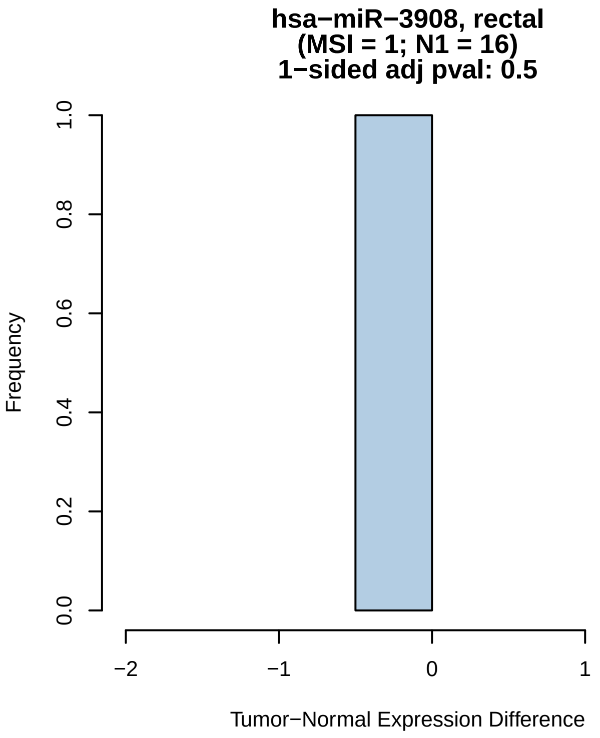

hsa−miR−3908, rectal
(MSI = 1; N1 = 16)
1−sided adj pval: 0.5
Frequency
Tumor−Normal Expression Difference

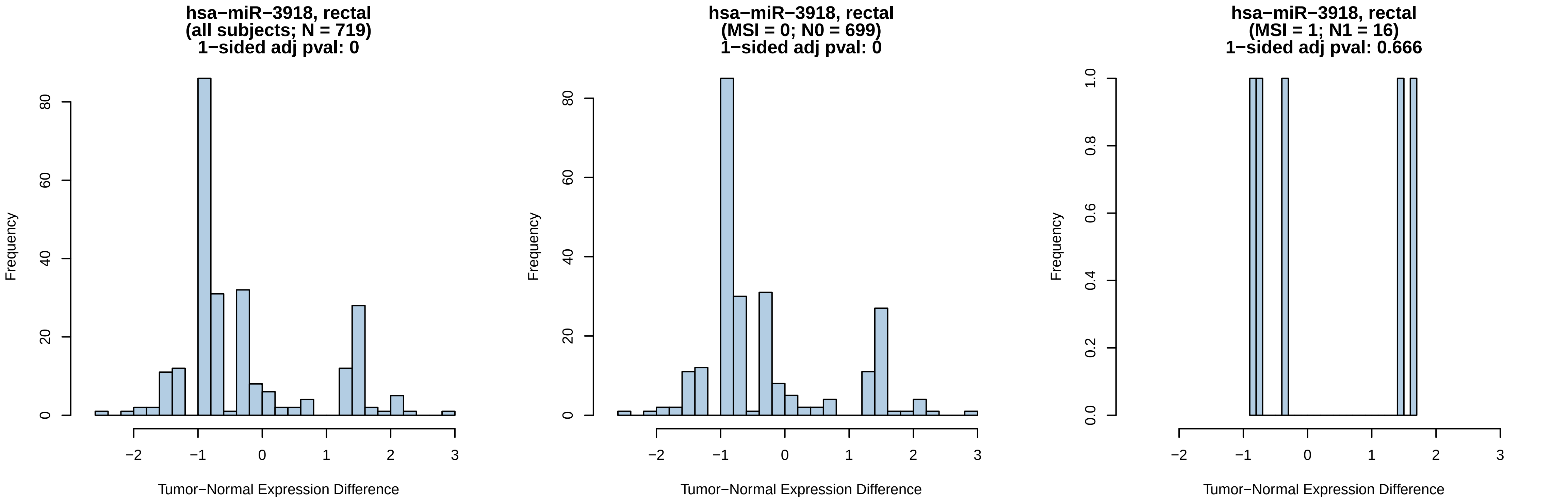

hsa−miR−3918, rectal
(all subjects; N = 719)
1−sided adj pval: 0
Frequency
Tumor−Normal Expression Difference
hsa−miR−3918, rectal
(MSI = 0; N0 = 699)
1−sided adj pval: 0
Frequency
Tumor−Normal Expression Difference
hsa−miR−3918, rectal
(MSI = 1; N1 = 16)
1−sided adj pval: 0.666
Frequency
Tumor−Normal Expression Difference

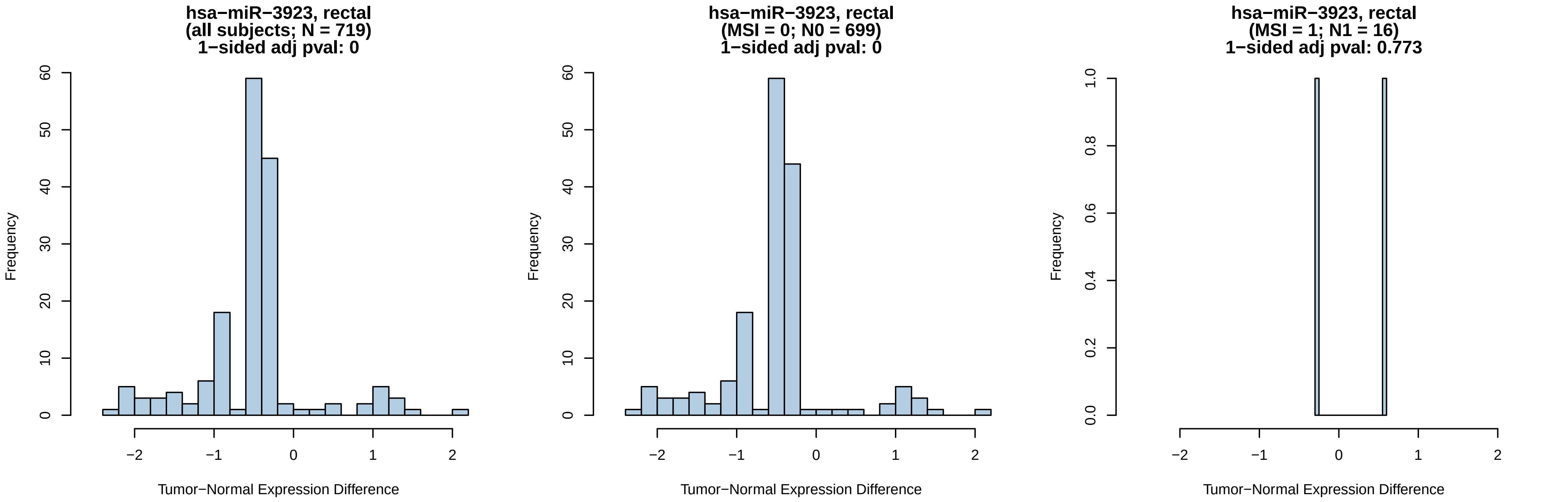
hsa−miR−3923, rectal
(all subjects; N = 719)
1−sided adj pval: 0
Frequency
Tumor−Normal Expression Difference
hsa−miR−3923, rectal
(MSI = 0; N0 = 699)
1−sided adj pval: 0
Frequency
Tumor−Normal Expression Difference
hsa−miR−3923, rectal
(MSI = 1; N1 = 16)
1−sided adj pval: 0.773
Frequency
Tumor−Normal Expression Difference

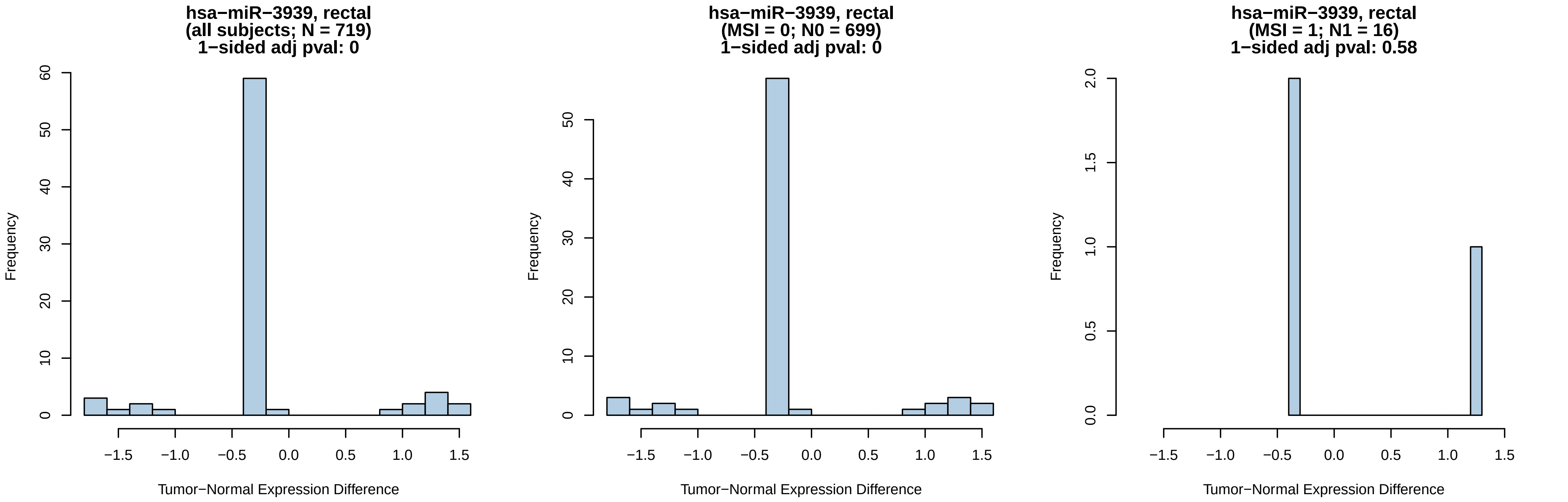
hsa−miR−3939, rectal
(all subjects; N = 719)
1−sided adj pval: 0
Frequency
Tumor−Normal Expression Difference
hsa−miR−3939, rectal
(MSI = 0; N0 = 699)
1−sided adj pval: 0
Frequency
Tumor−Normal Expression Difference
hsa−miR−3939, rectal
(MSI = 1; N1 = 16)
1−sided adj pval: 0.58
Frequency
Tumor−Normal Expression Difference

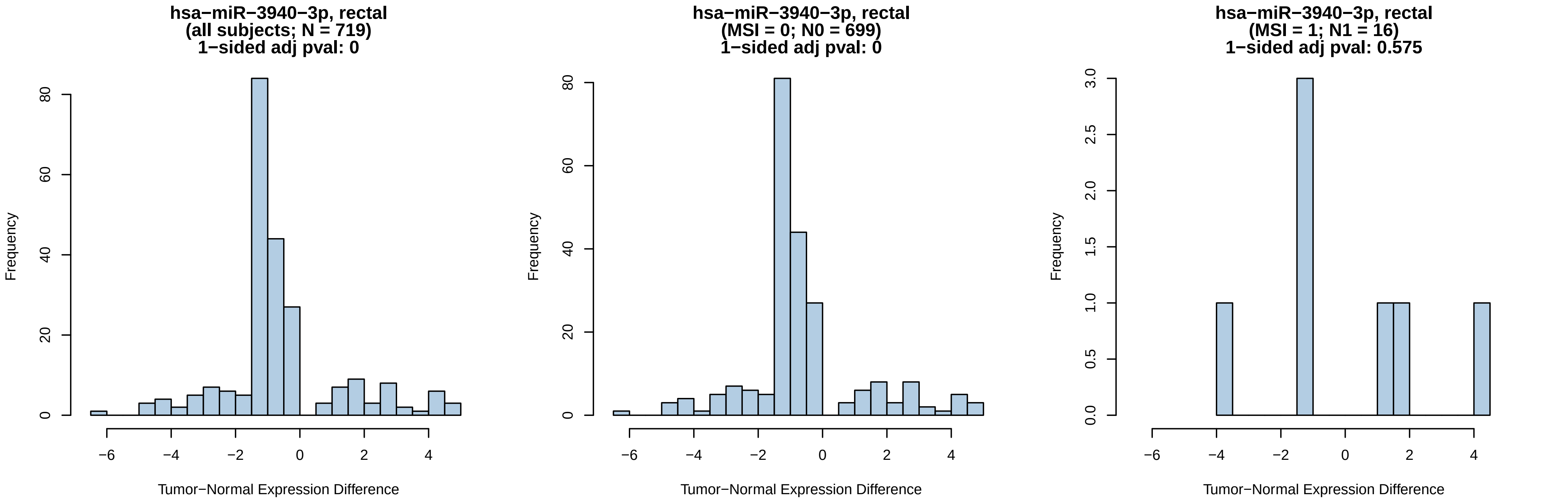
hsa−miR−3940−3p, rectal
(all subjects; N = 719)
1−sided adj pval: 0
Frequency
Tumor−Normal Expression Difference
hsa−miR−3940−3p, rectal
(MSI = 0; N0 = 699)
1−sided adj pval: 0
Frequency
Tumor−Normal Expression Difference
hsa−miR−3940−3p, rectal
(MSI = 1; N1 = 16)
1−sided adj pval: 0.575
Frequency
Tumor−Normal Expression Difference

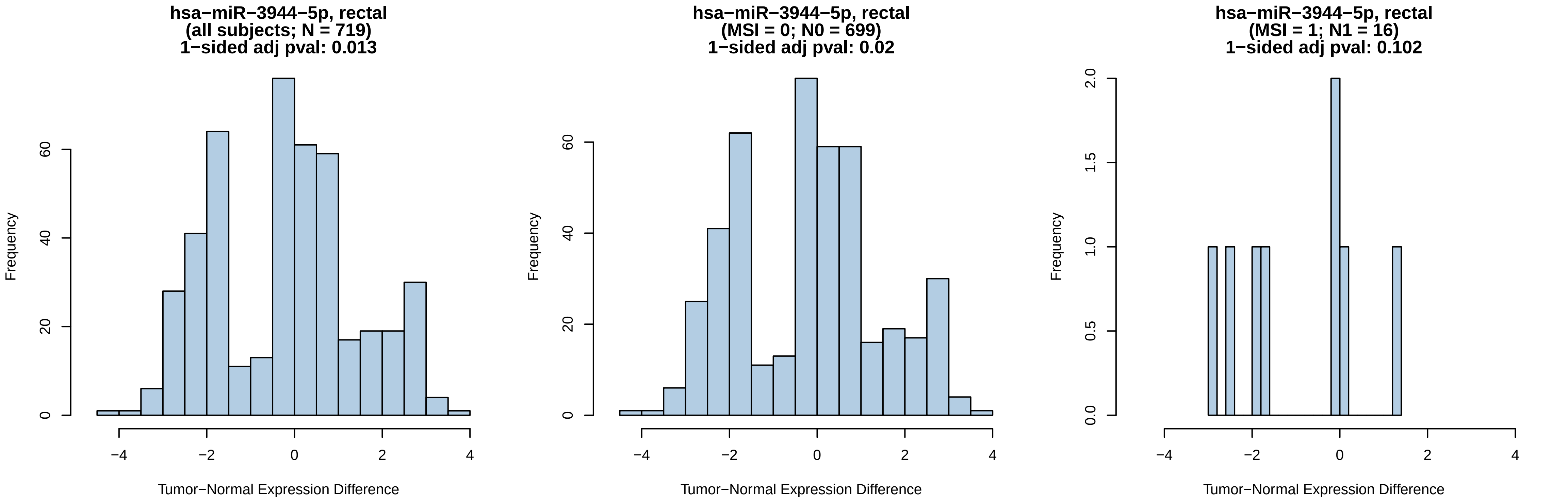
hsa−miR−3944−5p, rectal
(all subjects; N = 719)
1−sided adj pval: 0.013
Frequency
0
20
40
60
−4
−2
0
2
4
Tumor−Normal Expression Difference
hsa−miR−3944−5p, rectal
(MSI = 0; N0 = 699)
1−sided adj pval: 0.02
Frequency
0
20
40
60
−4
−2
0
2
4
Tumor−Normal Expression Difference
hsa−miR−3944−5p, rectal
(MSI = 1; N1 = 16)
1−sided adj pval: 0.102
Frequency
0.0
0.5
1.0
1.5
2.0
−4
−2
0
2
4
Tumor−Normal Expression Difference

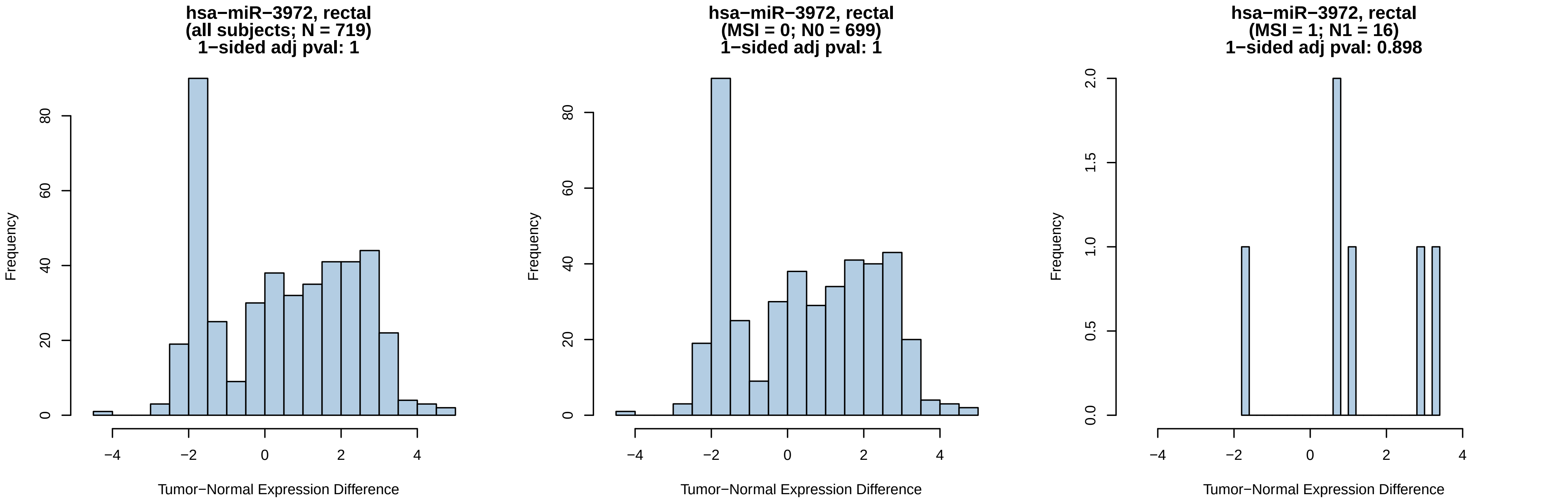

hsa−miR−3972, rectal
(all subjects; N = 719)
1−sided adj pval: 1
Frequency
Tumor−Normal Expression Difference
hsa−miR−3972, rectal
(MSI = 0; N0 = 699)
1−sided adj pval: 1
Frequency
Tumor−Normal Expression Difference
hsa−miR−3972, rectal
(MSI = 1; N1 = 16)
1−sided adj pval: 0.898
Frequency
Tumor−Normal Expression Difference

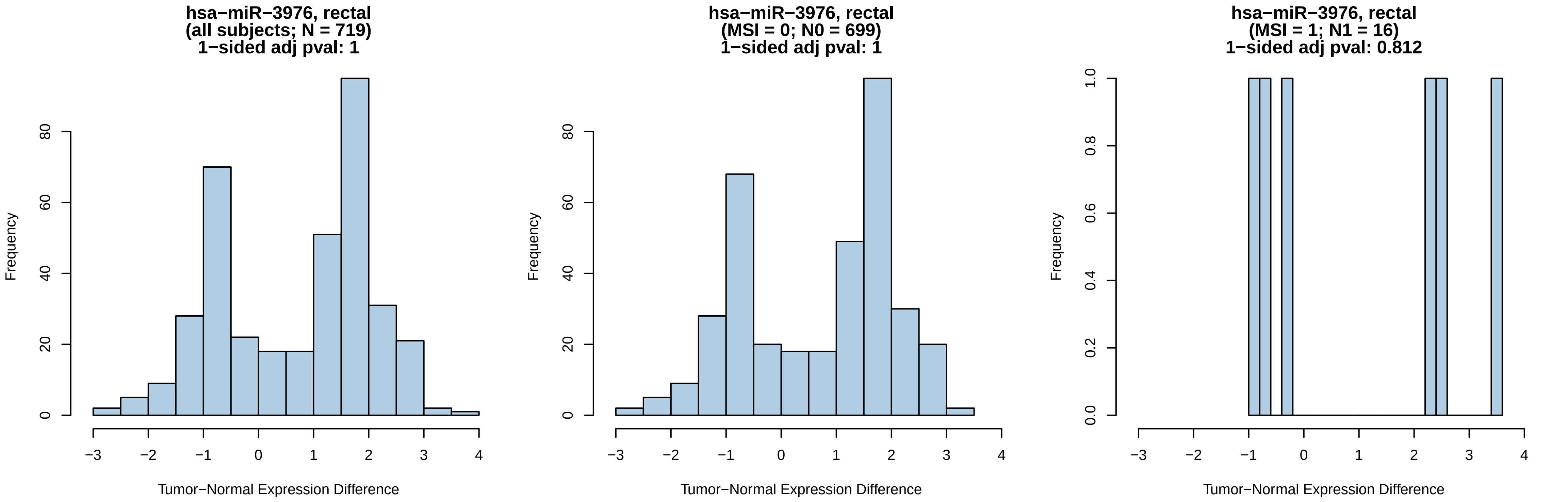

hsa−miR−3976, rectal
(all subjects; N = 719)
1−sided adj pval: 1
hsa−miR−3976, rectal
(MSI = 0; N0 = 699)
1−sided adj pval: 1
hsa−miR−3976, rectal
(MSI = 1; N1 = 16)
1−sided adj pval: 0.812
Frequency
Tumor−Normal Expression Difference

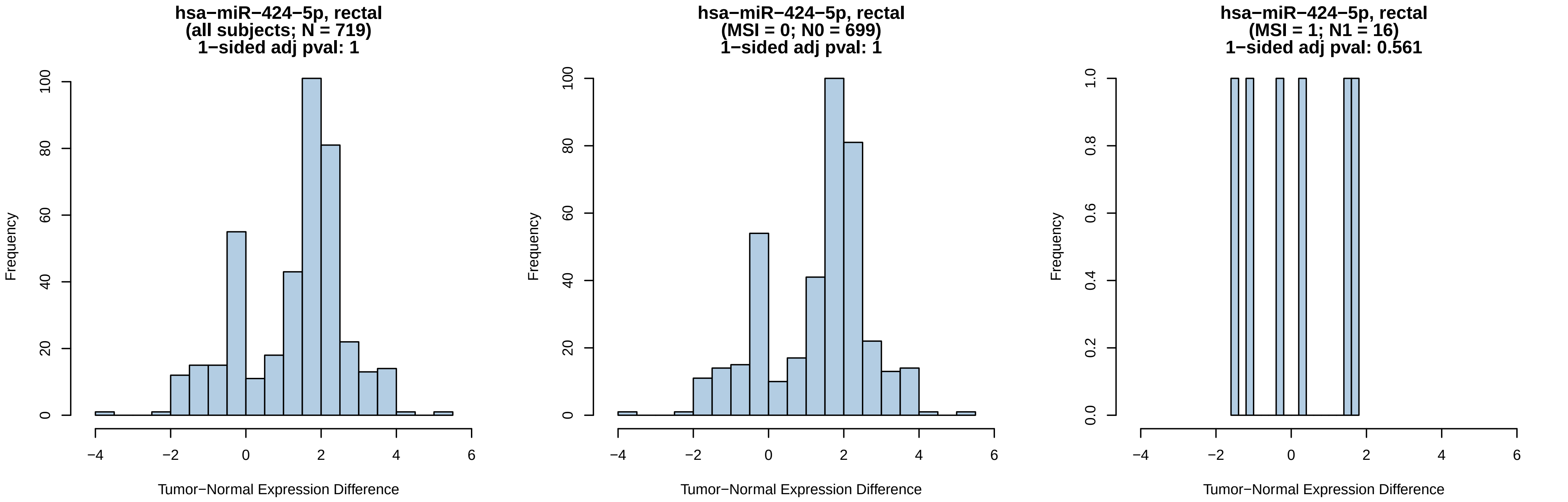
hsa−miR−424−5p, rectal
(all subjects; N = 719)
1−sided adj pval: 1
Frequency
Tumor−Normal Expression Difference
hsa−miR−424−5p, rectal
(MSI = 0; N0 = 699)
1−sided adj pval: 1
Frequency
Tumor−Normal Expression Difference
hsa−miR−424−5p, rectal
(MSI = 1; N1 = 16)
1−sided adj pval: 0.561
Frequency
Tumor−Normal Expression Difference

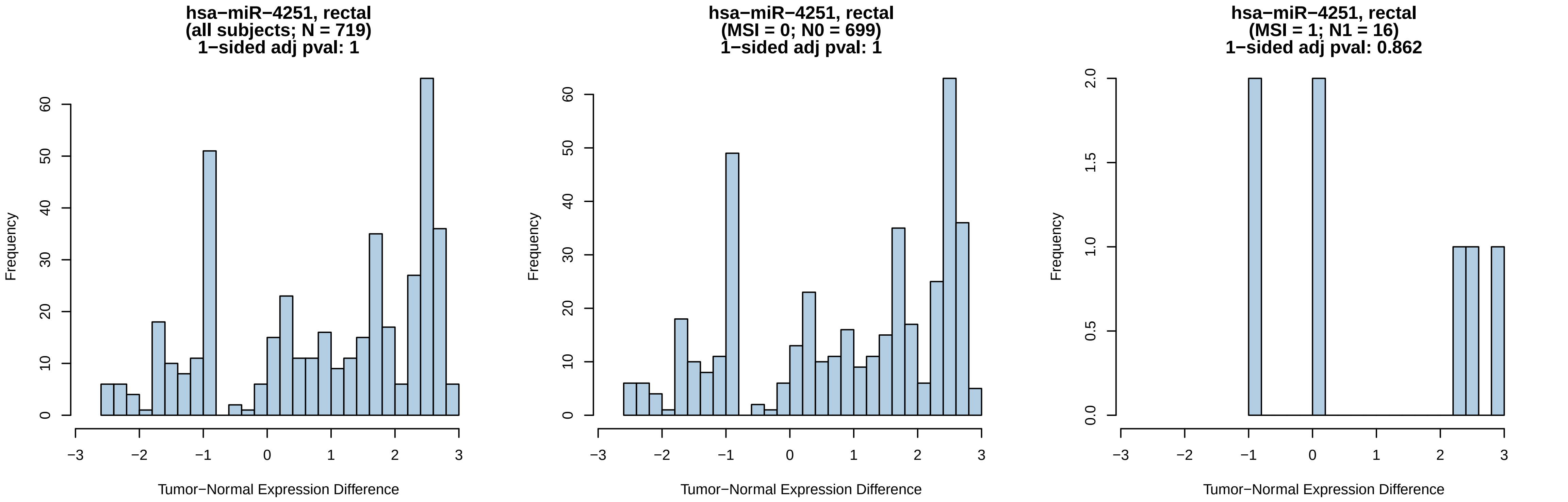

hsa−miR−4251, rectal
(all subjects; N = 719)
1−sided adj pval: 1
Frequency
0
10
20
30
40
50
60
−3
−2
−1
0
1
2
3
Tumor−Normal Expression Difference
hsa−miR−4251, rectal
(MSI = 0; N0 = 699)
1−sided adj pval: 1
Frequency
0
10
20
30
40
50
60
−3
−2
−1
0
1
2
3
Tumor−Normal Expression Difference
hsa−miR−4251, rectal
(MSI = 1; N1 = 16)
1−sided adj pval: 0.862
Frequency
0.0
0.5
1.0
1.5
2.0
−3
−2
−1
0
1
2
3
Tumor−Normal Expression Difference

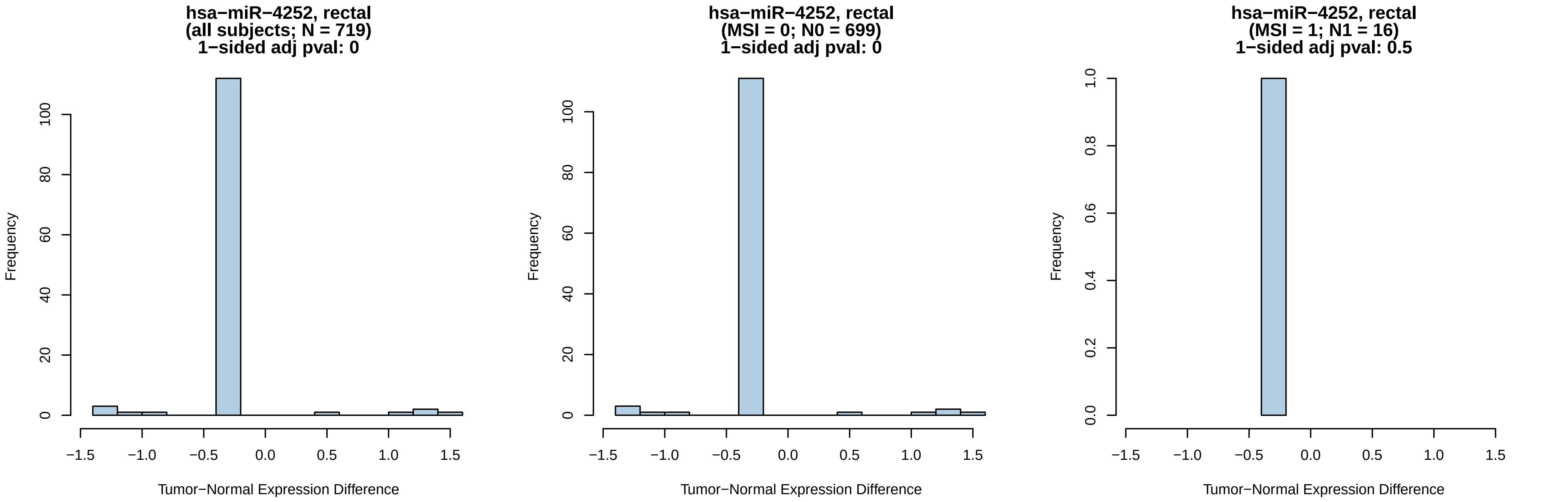

hsa−miR−4252, rectal
(all subjects; N = 719)
1−sided adj pval: 0
Frequency
Tumor−Normal Expression Difference
hsa−miR−4252, rectal
(MSI = 0; N0 = 699)
1−sided adj pval: 0
Frequency
Tumor−Normal Expression Difference
hsa−miR−4252, rectal
(MSI = 1; N1 = 16)
1−sided adj pval: 0.5
Frequency
Tumor−Normal Expression Difference

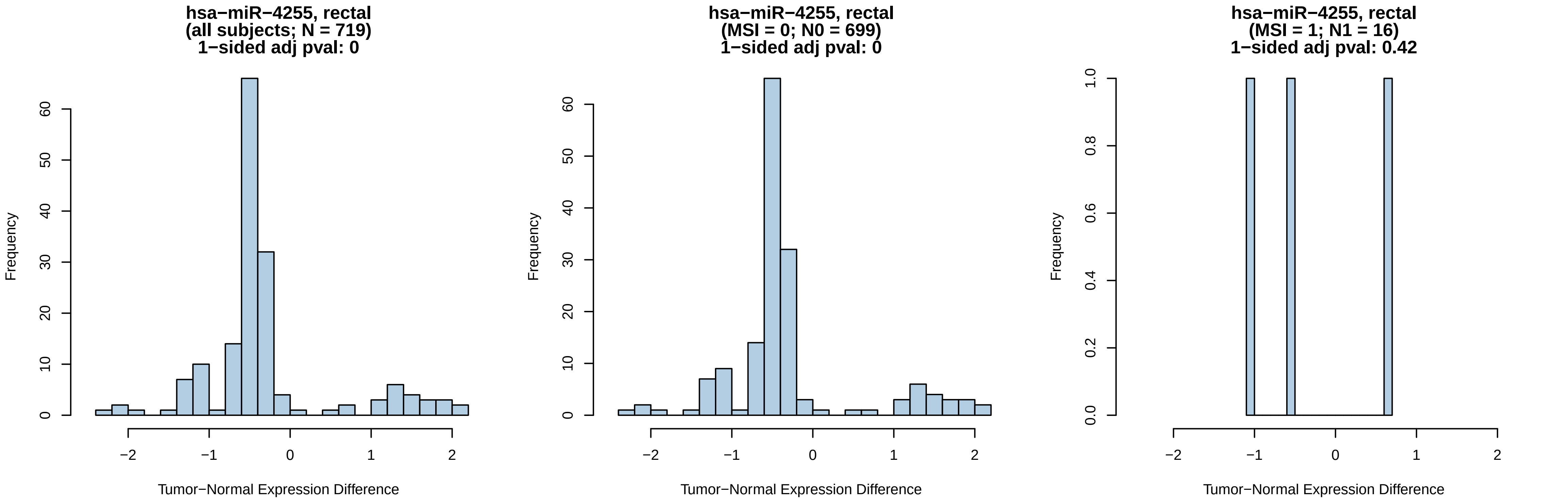
hsa−miR−4255, rectal
(all subjects; N = 719)
1−sided adj pval: 0
Frequency
Tumor−Normal Expression Difference
hsa−miR−4255, rectal
(MSI = 0; N0 = 699)
1−sided adj pval: 0
Frequency
Tumor−Normal Expression Difference
hsa−miR−4255, rectal
(MSI = 1; N1 = 16)
1−sided adj pval: 0.42
Frequency
Tumor−Normal Expression Difference

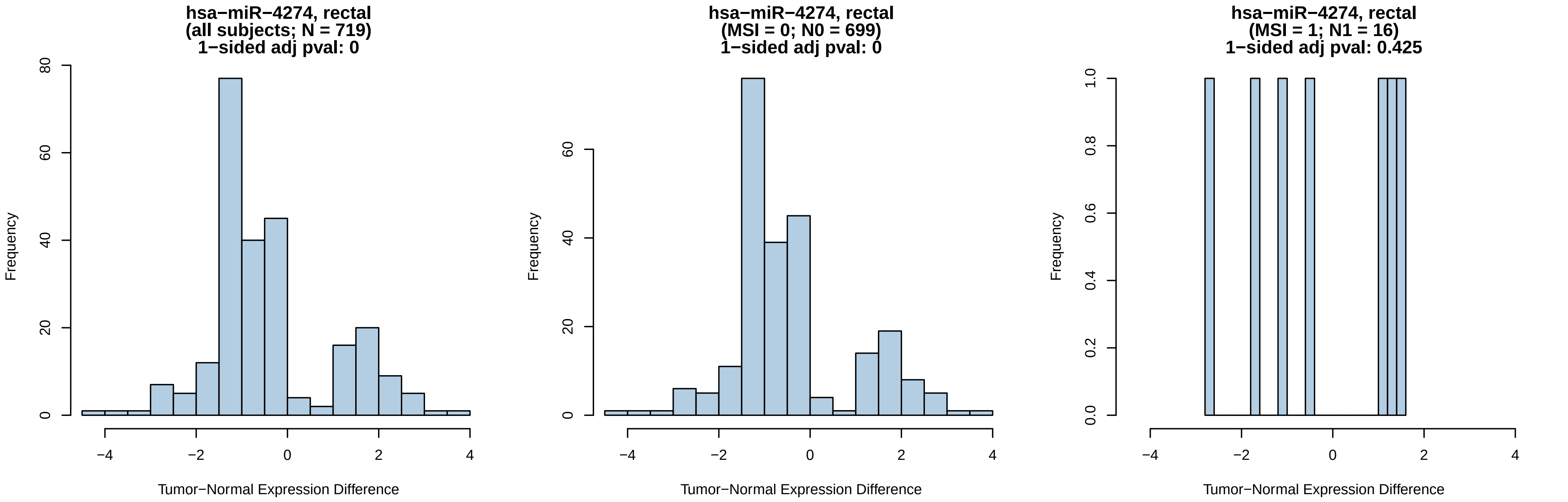

hsa−miR−4274, rectal
(all subjects; N = 719)
1−sided adj pval: 0
Frequency
0
20
40
60
80
−4
−2
0
2
4
Tumor−Normal Expression Difference
hsa−miR−4274, rectal
(MSI = 0; N0 = 699)
1−sided adj pval: 0
Frequency
0
20
40
60
−4
−2
0
2
4
Tumor−Normal Expression Difference
hsa−miR−4274, rectal
(MSI = 1; N1 = 16)
1−sided adj pval: 0.425
Frequency
0.0
0.2
0.4
0.6
0.8
1.0
−4
−2
0
2
4
Tumor−Normal Expression Difference

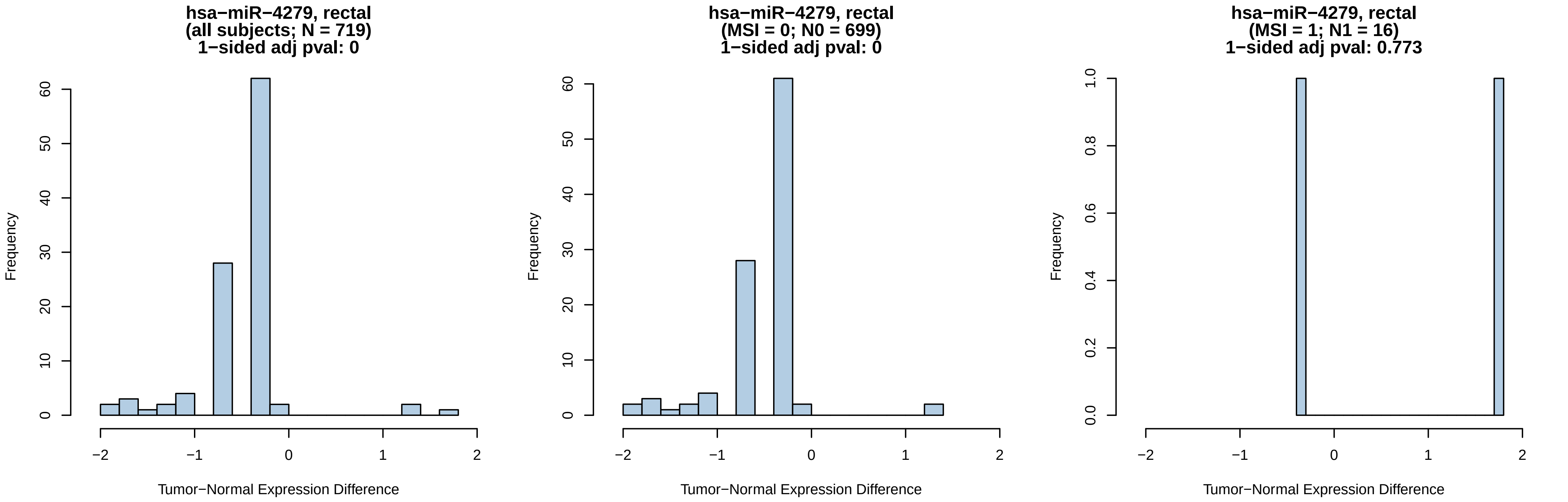

hsa−miR−4279, rectal
(all subjects; N = 719)
1−sided adj pval: 0
Frequency
Tumor−Normal Expression Difference
hsa−miR−4279, rectal
(MSI = 0; N0 = 699)
1−sided adj pval: 0
Frequency
Tumor−Normal Expression Difference
hsa−miR−4279, rectal
(MSI = 1; N1 = 16)
1−sided adj pval: 0.773
Frequency
Tumor−Normal Expression Difference

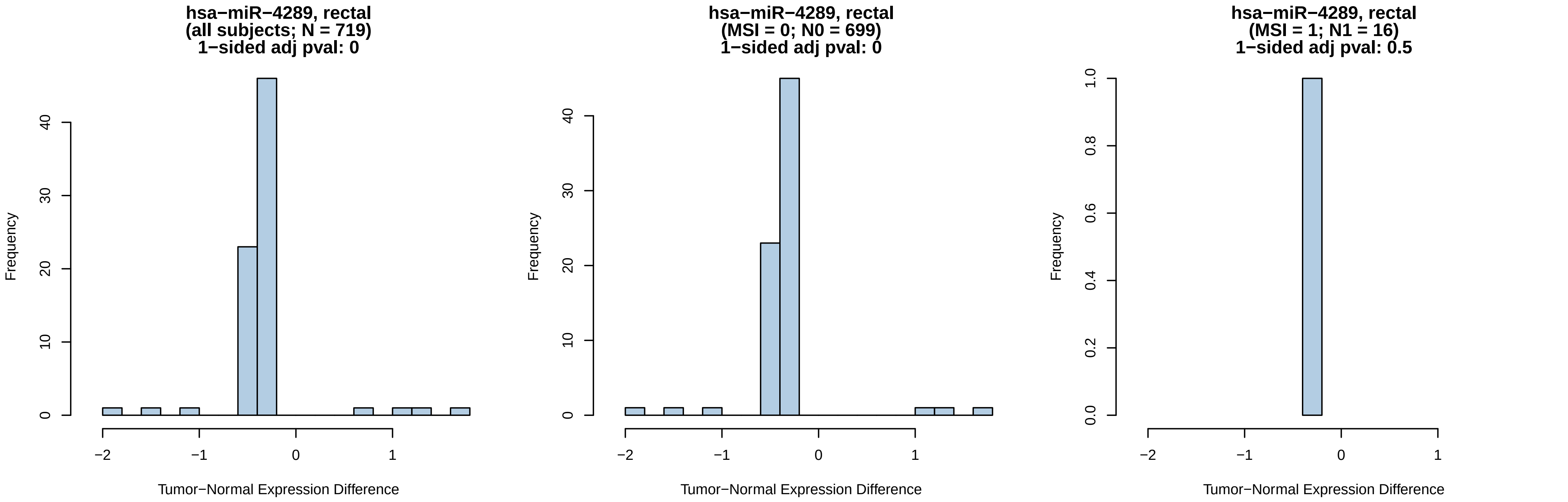
hsa−miR−4289, rectal
(all subjects; N = 719)
1−sided adj pval: 0
Frequency
0
10
20
30
40
−2
−1
0
1
Tumor−Normal Expression Difference
hsa−miR−4289, rectal
(MSI = 0; N0 = 699)
1−sided adj pval: 0
Frequency
0
10
20
30
40
−2
−1
0
1
Tumor−Normal Expression Difference
hsa−miR−4289, rectal
(MSI = 1; N1 = 16)
1−sided adj pval: 0.5
Frequency
0.0
0.2
0.4
0.6
0.8
1.0
−2
−1
0
1
Tumor−Normal Expression Difference

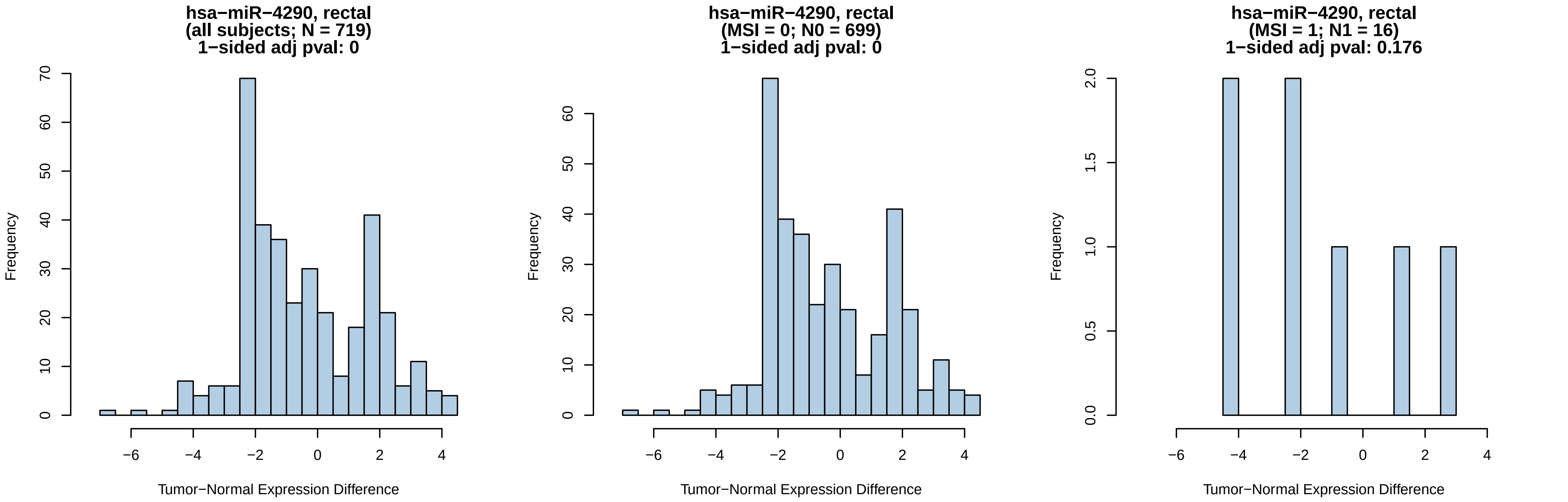
hsa−miR−4290, rectal
(all subjects; N = 719)
1−sided adj pval: 0
Frequency
Tumor−Normal Expression Difference
hsa−miR−4290, rectal
(MSI = 0; N0 = 699)
1−sided adj pval: 0
Frequency
Tumor−Normal Expression Difference
hsa−miR−4290, rectal
(MSI = 1; N1 = 16)
1−sided adj pval: 0.176
Frequency
Tumor−Normal Expression Difference

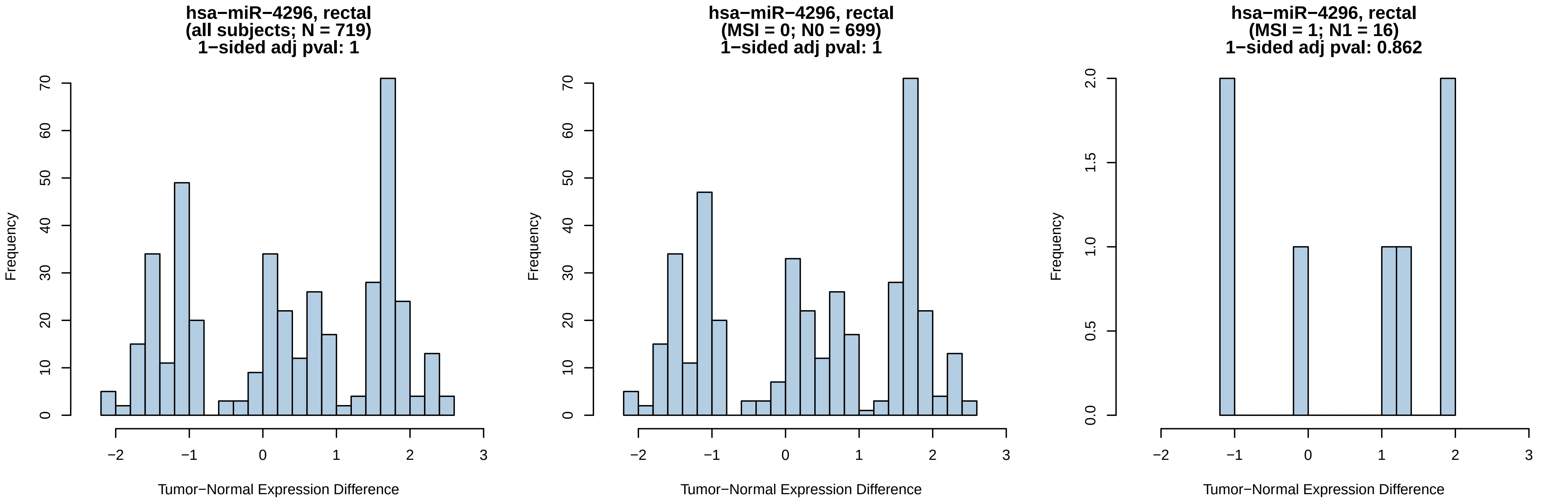

hsa−miR−4296, rectal
(all subjects; N = 719)
1−sided adj pval: 1
Frequency
Tumor−Normal Expression Difference
hsa−miR−4296, rectal
(MSI = 0; N0 = 699)
1−sided adj pval: 1
Frequency
Tumor−Normal Expression Difference
hsa−miR−4296, rectal
(MSI = 1; N1 = 16)
1−sided adj pval: 0.862
Frequency
Tumor−Normal Expression Difference

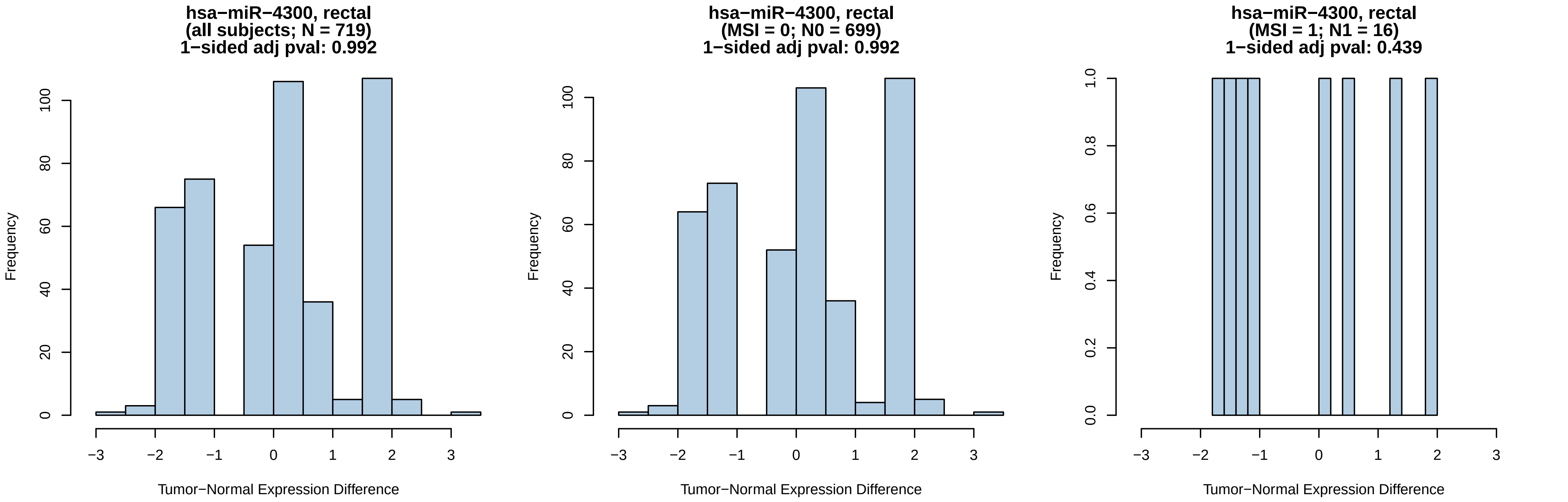
hsa−miR−4300, rectal
(all subjects; N = 719)
1−sided adj pval: 0.992
Frequency
Tumor−Normal Expression Difference
hsa−miR−4300, rectal
(MSI = 0; N0 = 699)
1−sided adj pval: 0.992
Frequency
Tumor−Normal Expression Difference
hsa−miR−4300, rectal
(MSI = 1; N1 = 16)
1−sided adj pval: 0.439
Frequency
Tumor−Normal Expression Difference

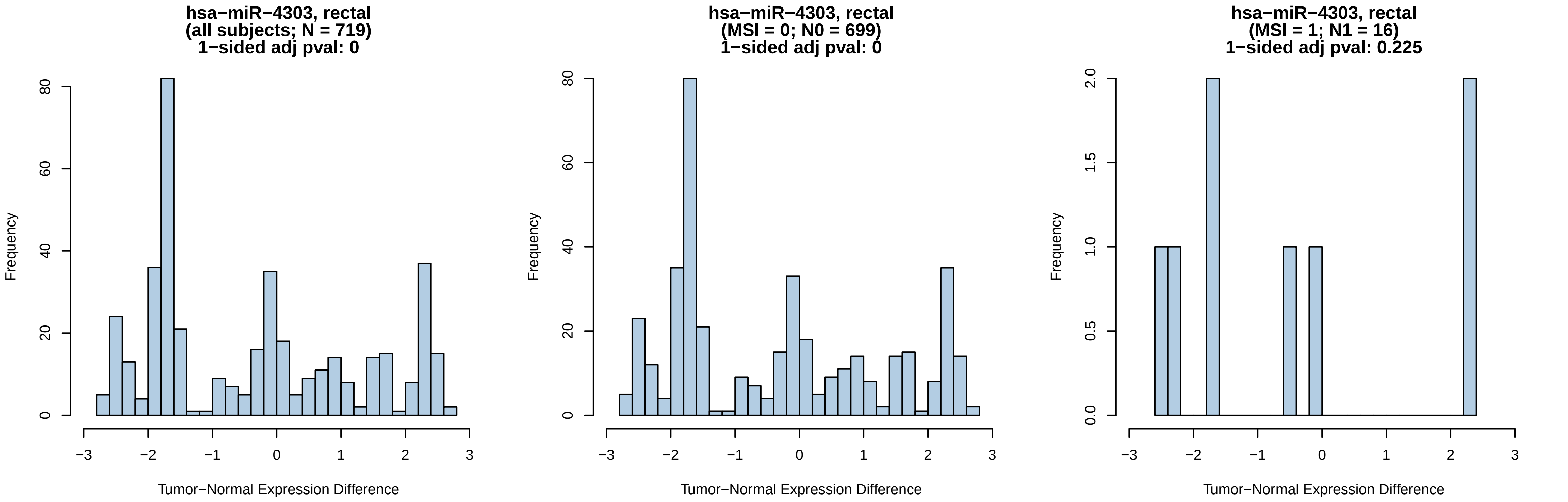

hsa−miR−4303, rectal
(all subjects; N = 719)
1−sided adj pval: 0
Frequency
0
20
40
60
80
−3
−2
−1
0
1
2
3
Tumor−Normal Expression Difference
hsa−miR−4303, rectal
(MSI = 0; N0 = 699)
1−sided adj pval: 0
Frequency
0
20
40
60
80
−3
−2
−1
0
1
2
3
Tumor−Normal Expression Difference
hsa−miR−4303, rectal
(MSI = 1; N1 = 16)
1−sided adj pval: 0.225
Frequency
0.0
0.5
1.0
1.5
2.0
−3
−2
−1
0
1
2
3
Tumor−Normal Expression Difference

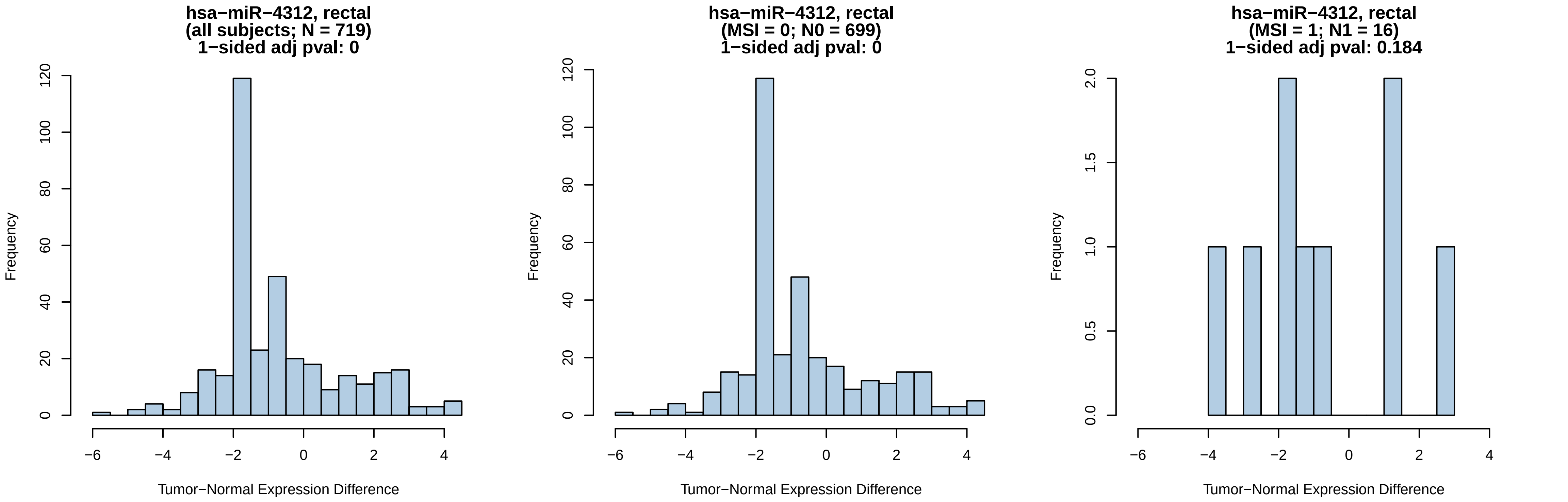
hsa−miR−4312, rectal
(all subjects; N = 719)
1−sided adj pval: 0
Frequency
Tumor−Normal Expression Difference
hsa−miR−4312, rectal
(MSI = 0; N0 = 699)
1−sided adj pval: 0
Frequency
Tumor−Normal Expression Difference
hsa−miR−4312, rectal
(MSI = 1; N1 = 16)
1−sided adj pval: 0.184
Frequency
Tumor−Normal Expression Difference

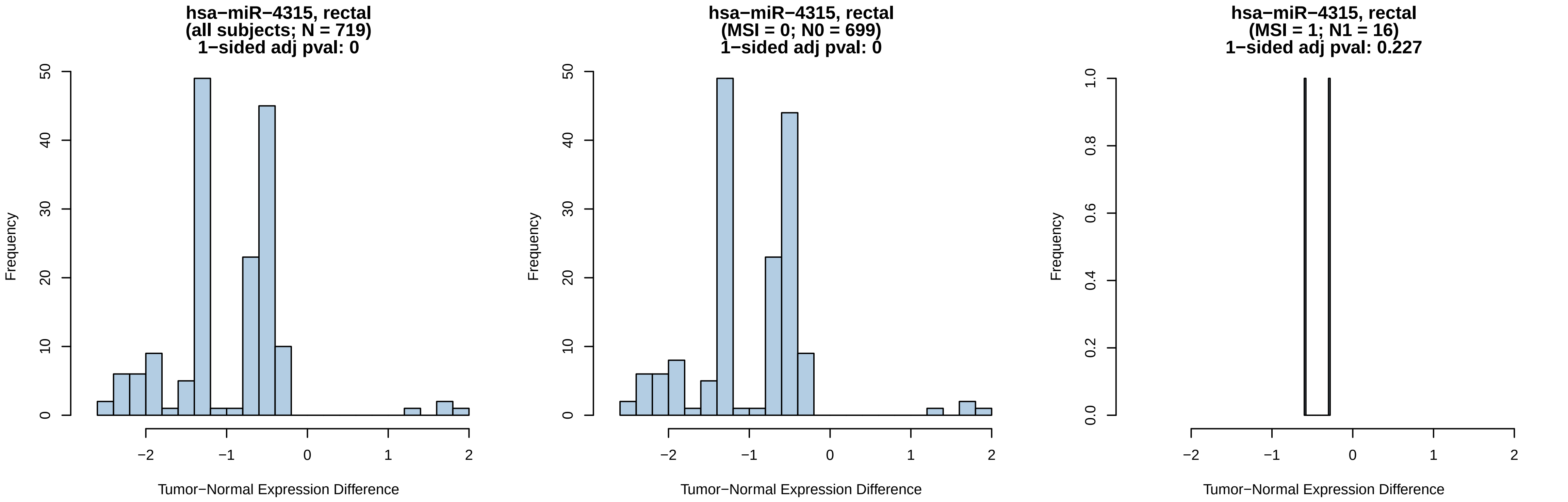

hsa−miR−4315, rectal
(all subjects; N = 719)
1−sided adj pval: 0
Frequency
Tumor−Normal Expression Difference
hsa−miR−4315, rectal
(MSI = 0; N0 = 699)
1−sided adj pval: 0
Frequency
Tumor−Normal Expression Difference
hsa−miR−4315, rectal
(MSI = 1; N1 = 16)
1−sided adj pval: 0.227
Frequency
Tumor−Normal Expression Difference

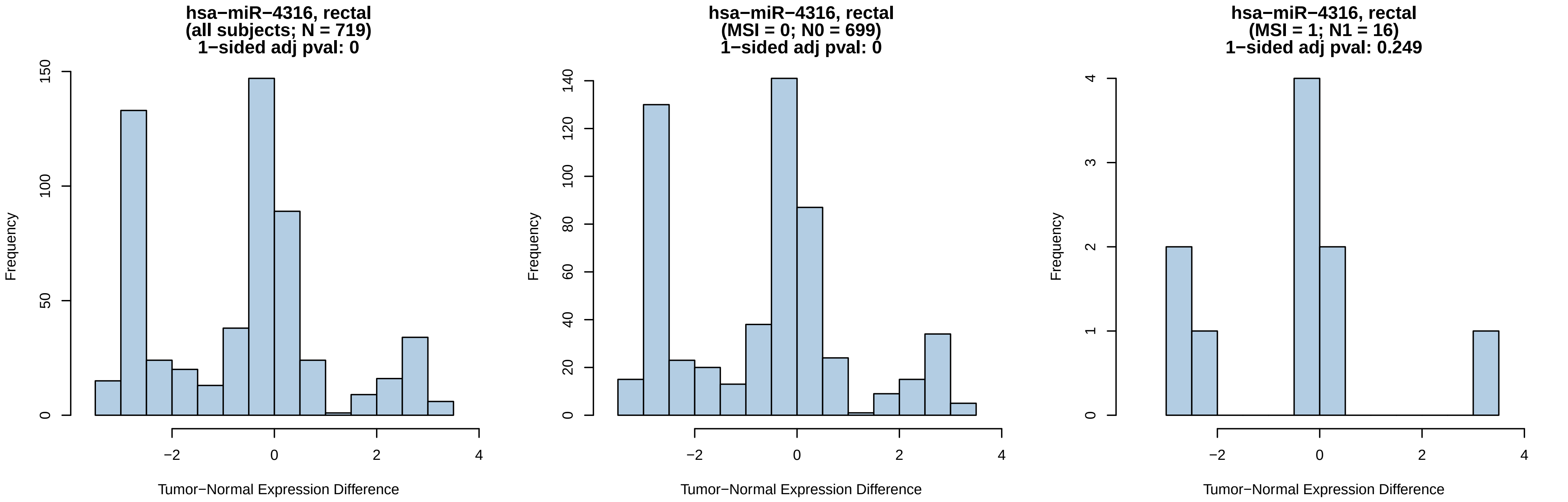
hsa−miR−4316, rectal
(all subjects; N = 719)
1−sided adj pval: 0
Frequency
0
50
100
150
−2
0
2
4
Tumor−Normal Expression Difference
hsa−miR−4316, rectal
(MSI = 0; N0 = 699)
1−sided adj pval: 0
Frequency
0
20
40
60
80
100
120
140
−2
0
2
4
Tumor−Normal Expression Difference
hsa−miR−4316, rectal
(MSI = 1; N1 = 16)
1−sided adj pval: 0.249
Frequency
0
1
2
3
4
−2
0
2
4
Tumor−Normal Expression Difference

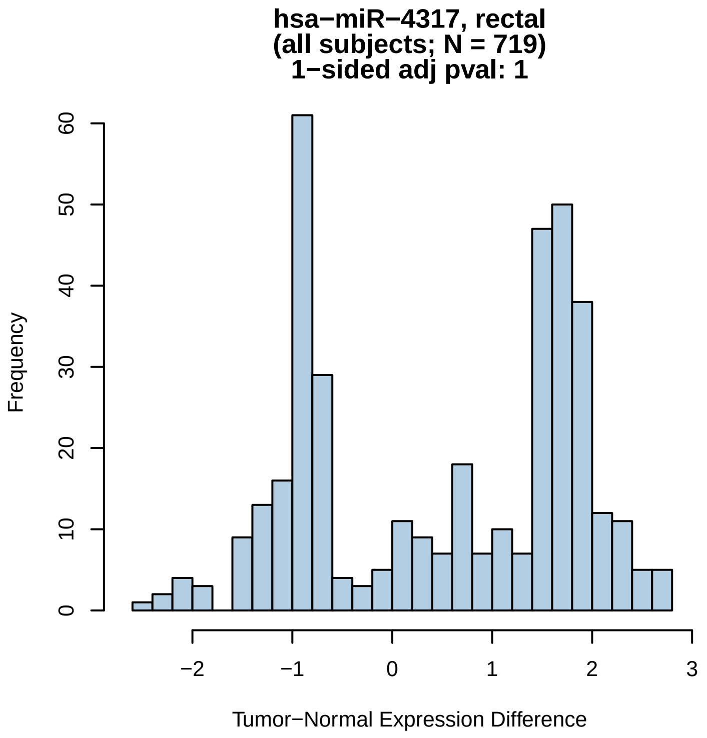
hsa−miR−4317, rectal
(all subjects; N = 719)
1−sided adj pval: 1
Frequency
Tumor−Normal Expression Difference
0
10
20
30
40
50
60
−2
−1
0
1
2
3

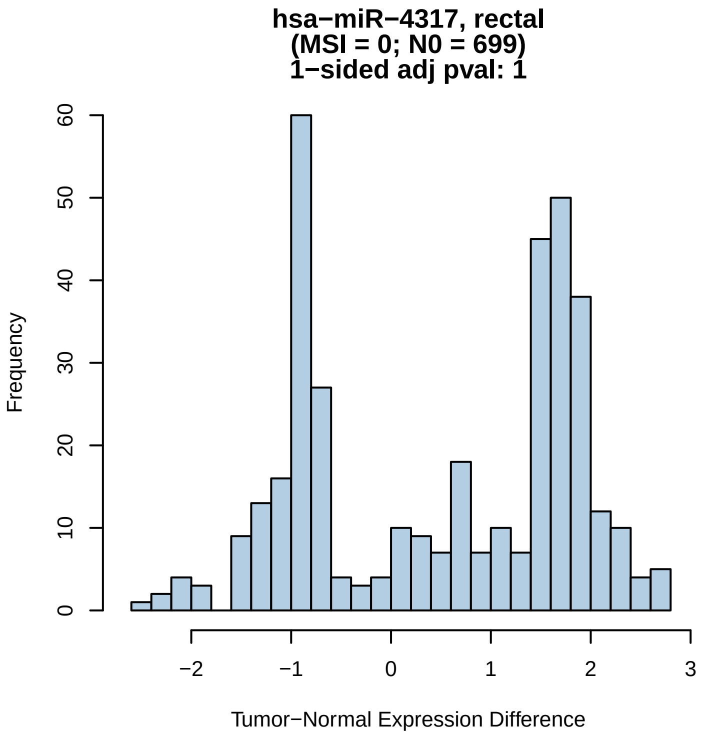
hsa−miR−4317, rectal
(MSI = 0; N0 = 699)
1−sided adj pval: 1
Frequency
Tumor−Normal Expression Difference
0
10
20
30
40
50
60
−2
−1
0
1
2
3

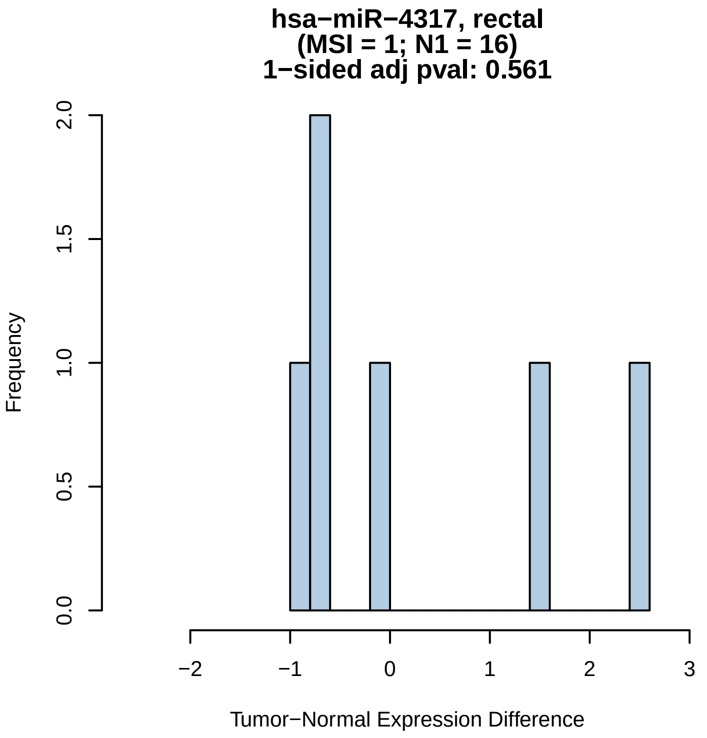
hsa−miR−4317, rectal
(MSI = 1; N1 = 16)
1−sided adj pval: 0.561
Frequency
Tumor−Normal Expression Difference
0.0
0.5
1.0
1.5
2.0
−2
−1
0
1
2
3

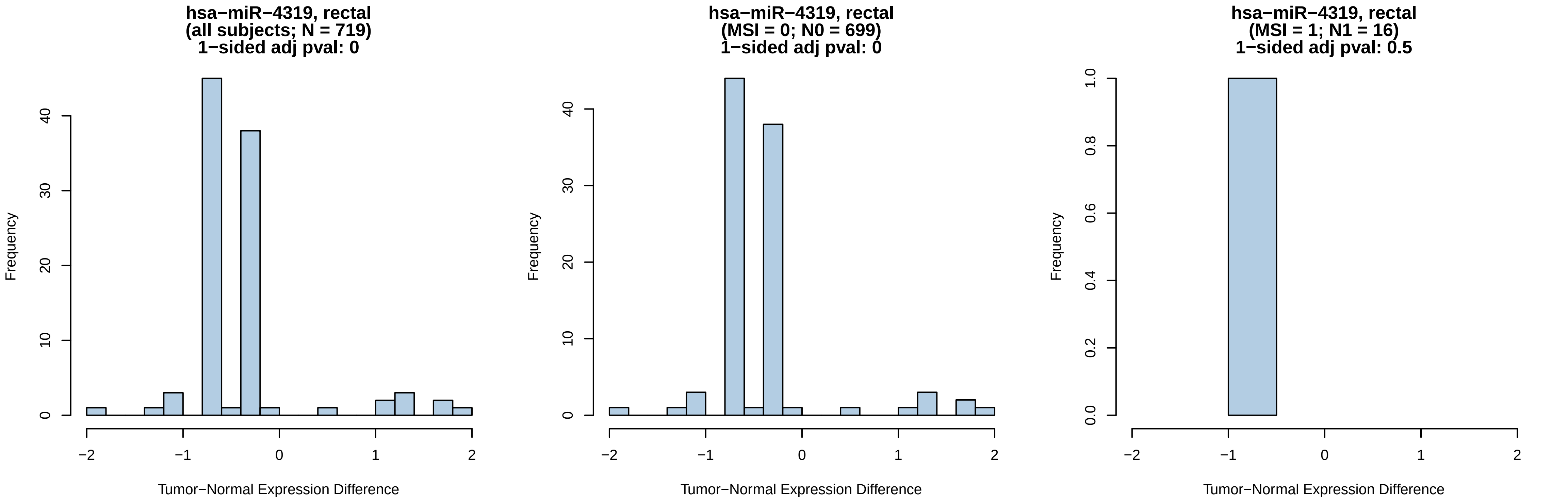

hsa−miR−4319, rectal
(all subjects; N = 719)
1−sided adj pval: 0
Frequency
Tumor−Normal Expression Difference
hsa−miR−4319, rectal
(MSI = 0; N0 = 699)
1−sided adj pval: 0
Frequency
Tumor−Normal Expression Difference
hsa−miR−4319, rectal
(MSI = 1; N1 = 16)
1−sided adj pval: 0.5
Frequency
Tumor−Normal Expression Difference

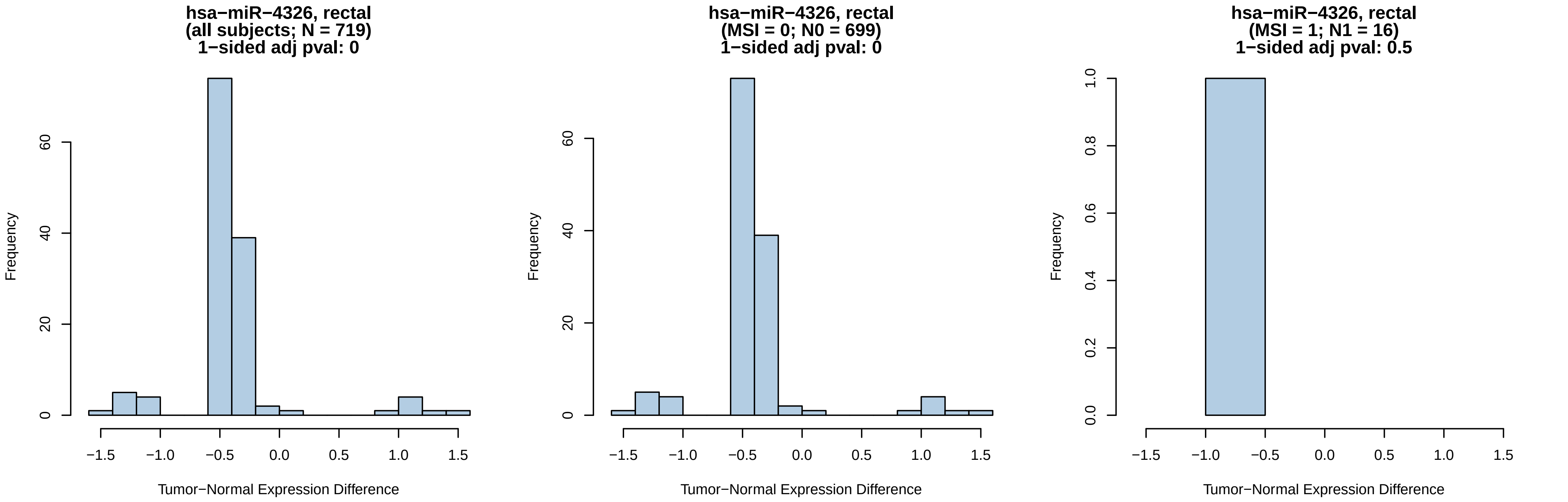
hsa−miR−4326, rectal
(all subjects; N = 719)
1−sided adj pval: 0
Frequency
0
20
40
60
−1.5
−1.0
−0.5
0.0
0.5
1.0
1.5
Tumor−Normal Expression Difference
hsa−miR−4326, rectal
(MSI = 0; N0 = 699)
1−sided adj pval: 0
Frequency
0
20
40
60
−1.5
−1.0
−0.5
0.0
0.5
1.0
1.5
Tumor−Normal Expression Difference
hsa−miR−4326, rectal
(MSI = 1; N1 = 16)
1−sided adj pval: 0.5
Frequency
0.0
0.2
0.4
0.6
0.8
1.0
−1.5
−1.0
−0.5
0.0
0.5
1.0
1.5
Tumor−Normal Expression Difference

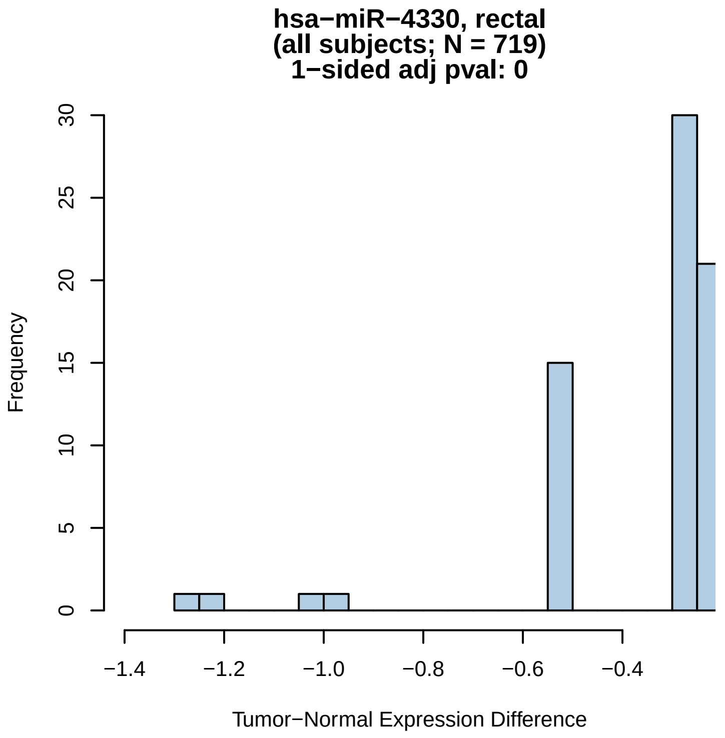
hsa−miR−4330, rectal
(all subjects; N = 719)
1−sided adj pval: 0
Frequency
0
5
10
15
20
25
30
−1.4
−1.2
−1.0
−0.8
−0.6
−0.4
Tumor−Normal Expression Difference

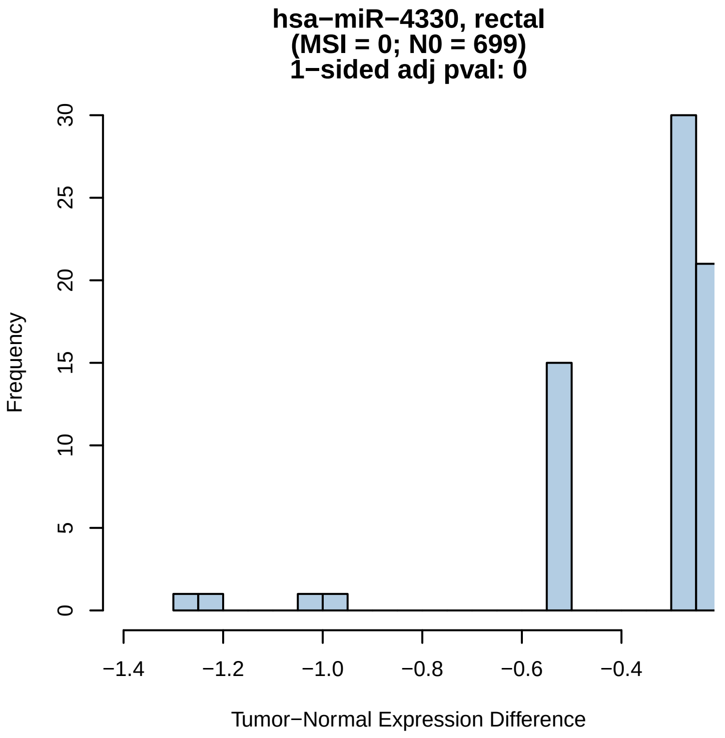
hsa−miR−4330, rectal
(MSI = 0; N0 = 699)
1−sided adj pval: 0
Frequency
0
5
10
15
20
25
30
−1.4
−1.2
−1.0
−0.8
−0.6
−0.4
Tumor−Normal Expression Difference

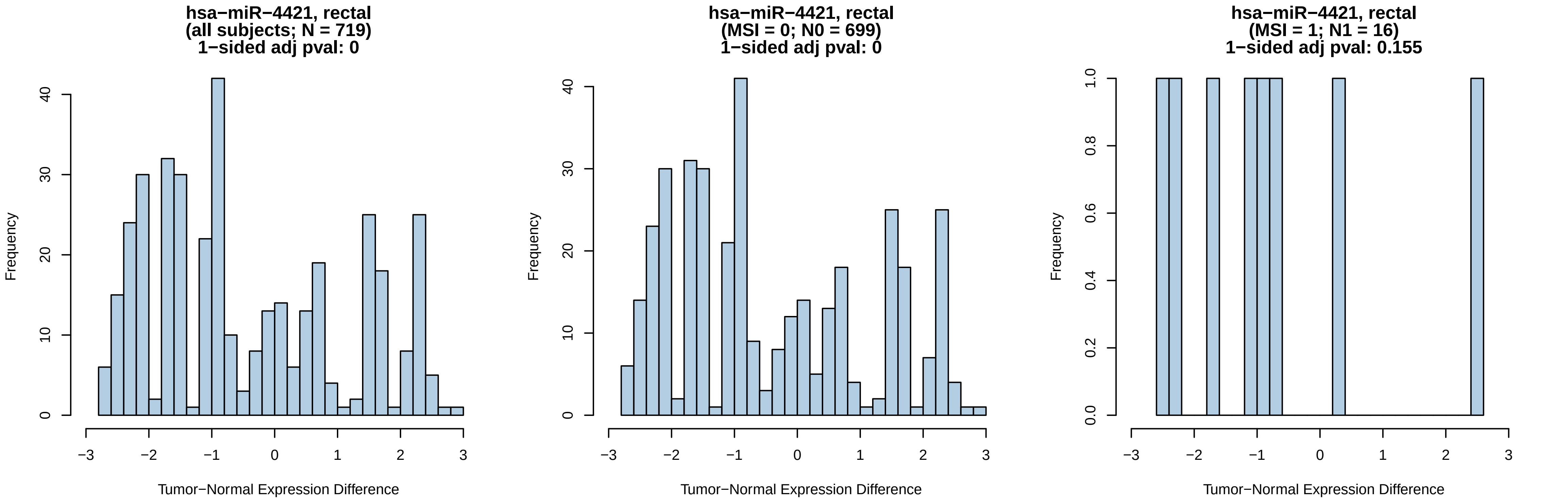

hsa−miR−4421, rectal
(all subjects; N = 719)
1−sided adj pval: 0
hsa−miR−4421, rectal
(MSI = 0; N0 = 699)
1−sided adj pval: 0
hsa−miR−4421, rectal
(MSI = 1; N1 = 16)
1−sided adj pval: 0.155
Frequency
Tumor−Normal Expression Difference

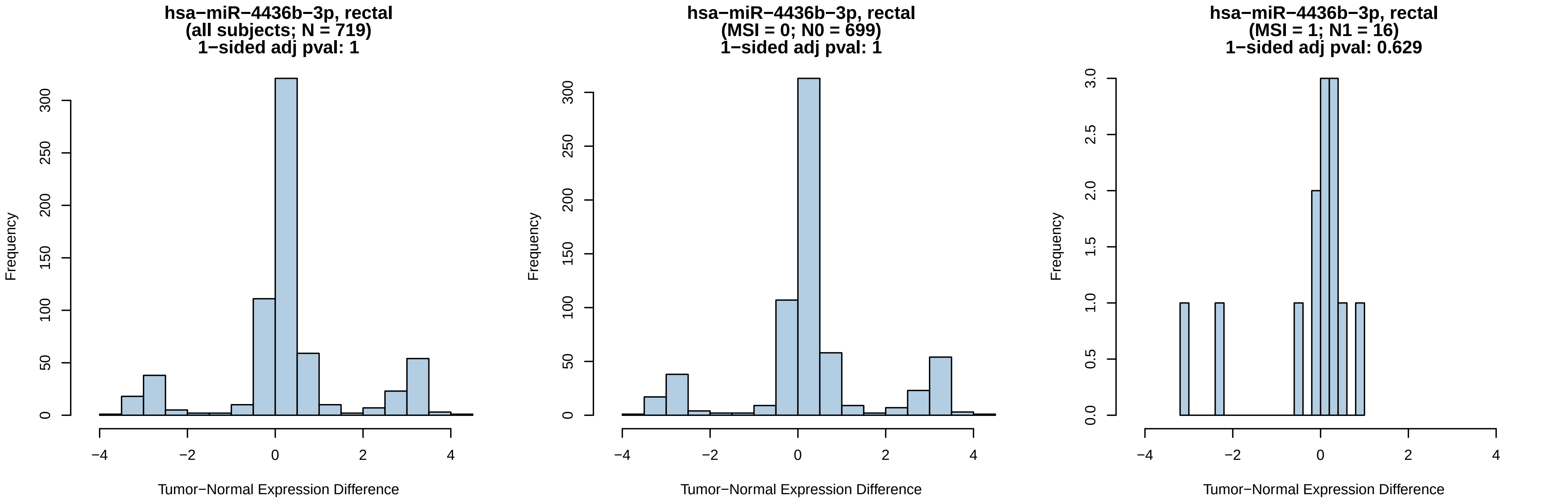
hsa−miR−4436b−3p, rectal
(all subjects; N = 719)
1−sided adj pval: 1
Frequency
Tumor−Normal Expression Difference
hsa−miR−4436b−3p, rectal
(MSI = 0; N0 = 699)
1−sided adj pval: 1
Frequency
Tumor−Normal Expression Difference
hsa−miR−4436b−3p, rectal
(MSI = 1; N1 = 16)
1−sided adj pval: 0.629
Frequency
Tumor−Normal Expression Difference

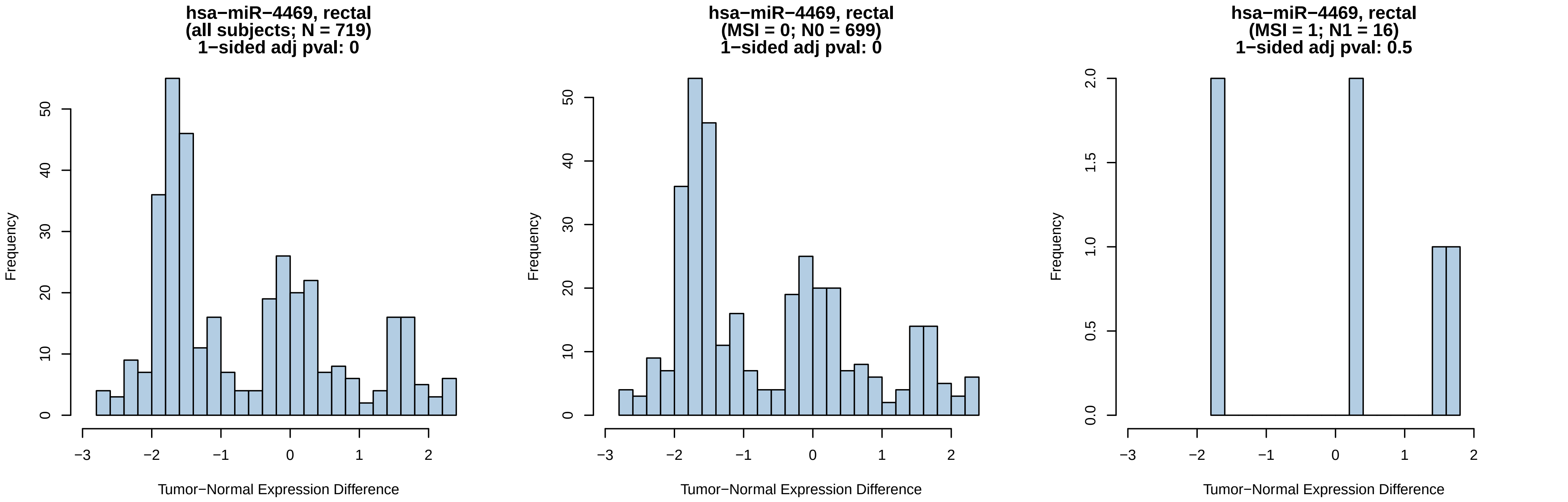
hsa−miR−4469, rectal
(all subjects; N = 719)
1−sided adj pval: 0
Frequency
Tumor−Normal Expression Difference
hsa−miR−4469, rectal
(MSI = 0; N0 = 699)
1−sided adj pval: 0
Frequency
Tumor−Normal Expression Difference
hsa−miR−4469, rectal
(MSI = 1; N1 = 16)
1−sided adj pval: 0.5
Frequency
Tumor−Normal Expression Difference

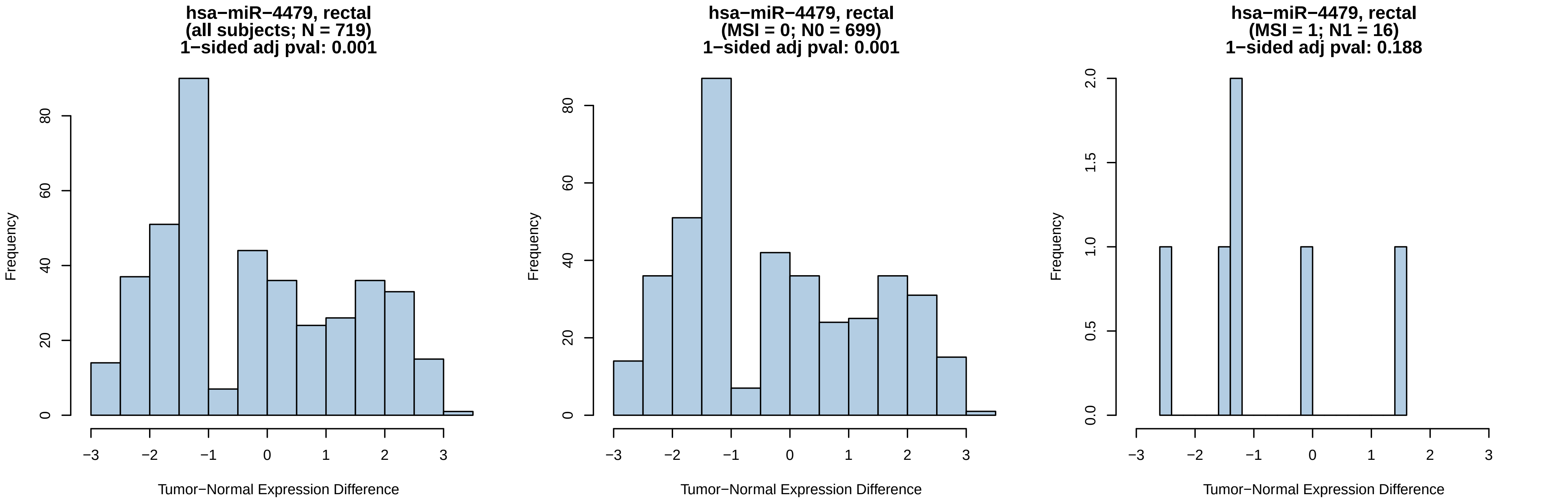
hsa−miR−4479, rectal
(all subjects; N = 719)
1−sided adj pval: 0.001
hsa−miR−4479, rectal
(MSI = 0; N0 = 699)
1−sided adj pval: 0.001
hsa−miR−4479, rectal
(MSI = 1; N1 = 16)
1−sided adj pval: 0.188
Frequency
Tumor−Normal Expression Difference
0
20
40
60
80
0.0
0.5
1.0
1.5
2.0
−3
−2
−1
0
1
2
3

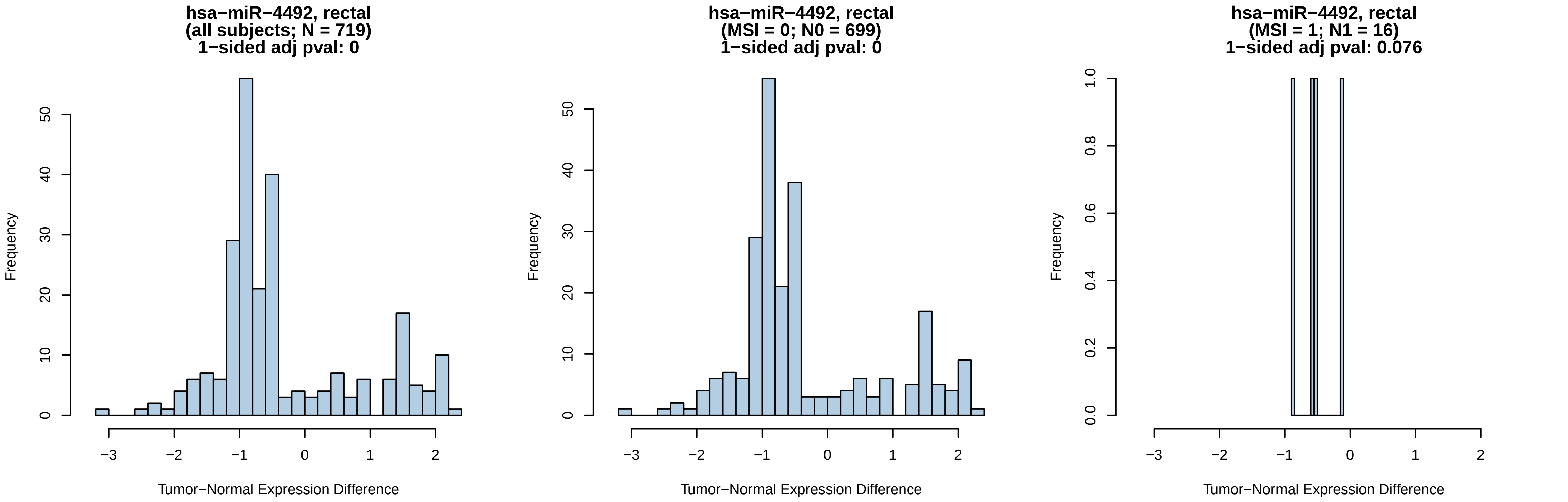
hsa−miR−4492, rectal
(all subjects; N = 719)
1−sided adj pval: 0
hsa−miR−4492, rectal
(MSI = 0; N0 = 699)
1−sided adj pval: 0
hsa−miR−4492, rectal
(MSI = 1; N1 = 16)
1−sided adj pval: 0.076
Frequency
Tumor−Normal Expression Difference

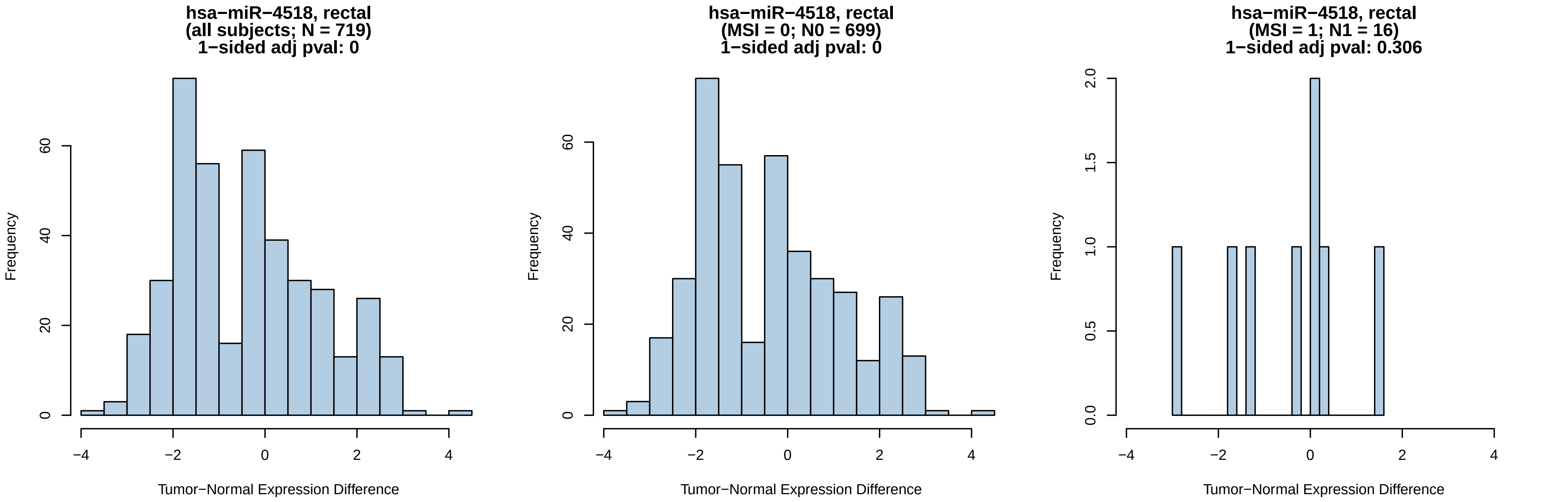

hsa−miR−4518, rectal
(all subjects; N = 719)
1−sided adj pval: 0
Frequency
Tumor−Normal Expression Difference
hsa−miR−4518, rectal
(MSI = 0; N0 = 699)
1−sided adj pval: 0
Frequency
Tumor−Normal Expression Difference
hsa−miR−4518, rectal
(MSI = 1; N1 = 16)
1−sided adj pval: 0.306
Frequency
Tumor−Normal Expression Difference

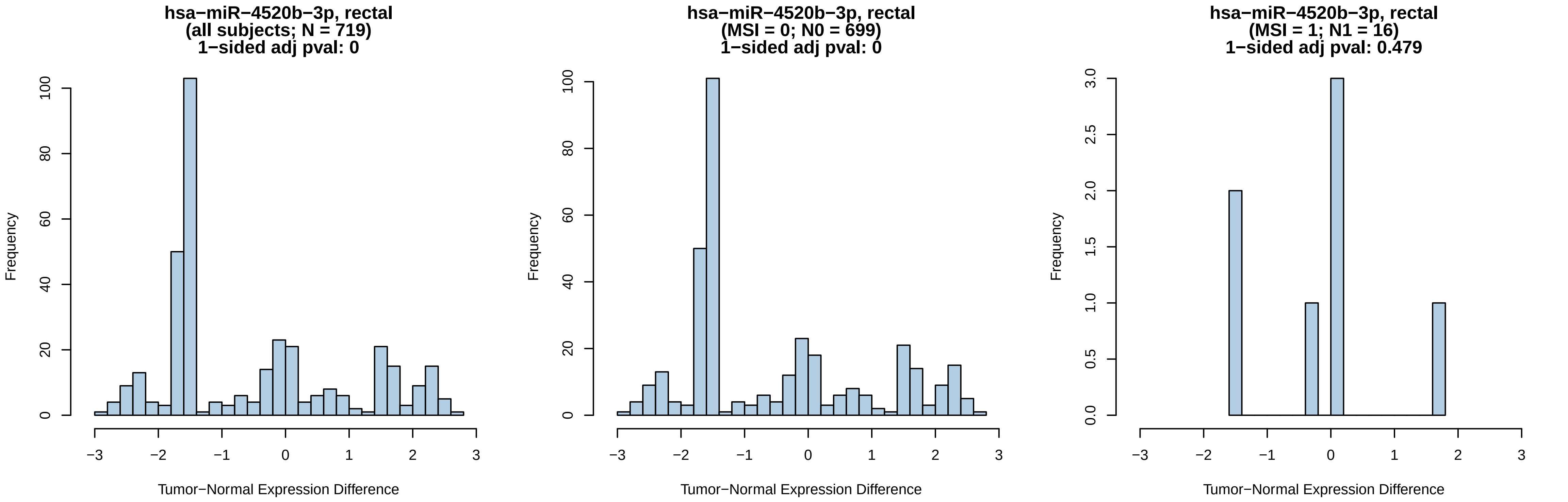

hsa−miR−4520b−3p, rectal
(all subjects; N = 719)
1−sided adj pval: 0
hsa−miR−4520b−3p, rectal
(MSI = 0; N0 = 699)
1−sided adj pval: 0
hsa−miR−4520b−3p, rectal
(MSI = 1; N1 = 16)
1−sided adj pval: 0.479
Frequency
Tumor−Normal Expression Difference

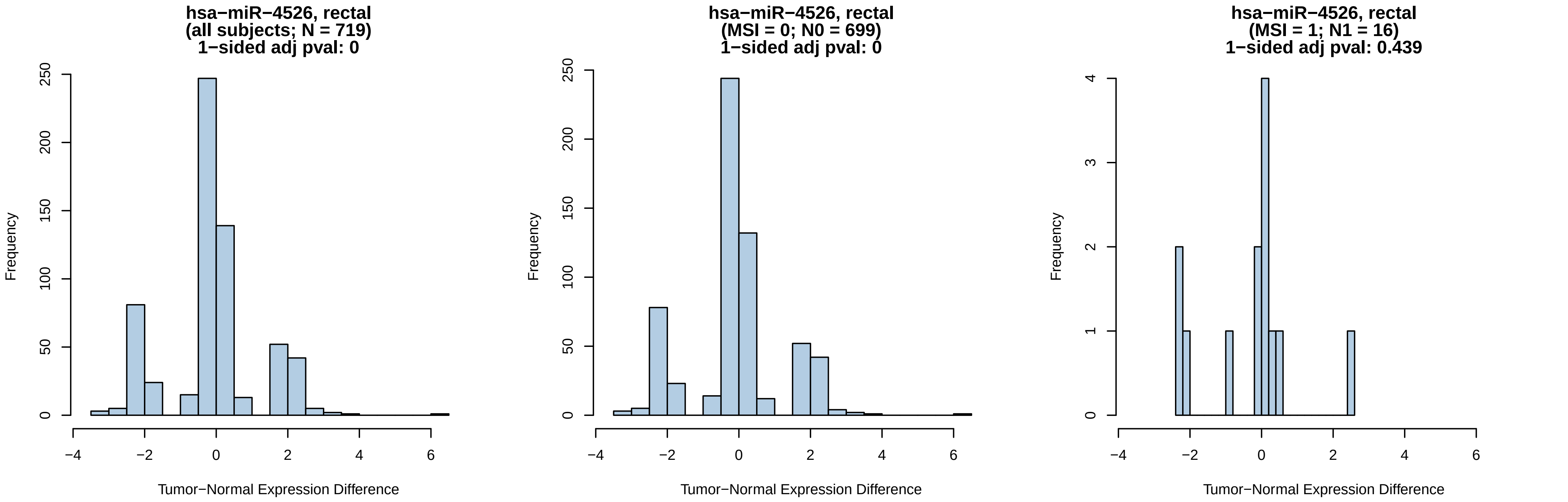
hsa−miR−4526, rectal
(all subjects; N = 719)
1−sided adj pval: 0
hsa−miR−4526, rectal
(MSI = 0; N0 = 699)
1−sided adj pval: 0
hsa−miR−4526, rectal
(MSI = 1; N1 = 16)
1−sided adj pval: 0.439
Frequency
Tumor−Normal Expression Difference
0
50
100
150
200
250
0
1
2
3
4
−4
−2
0
2
4
6

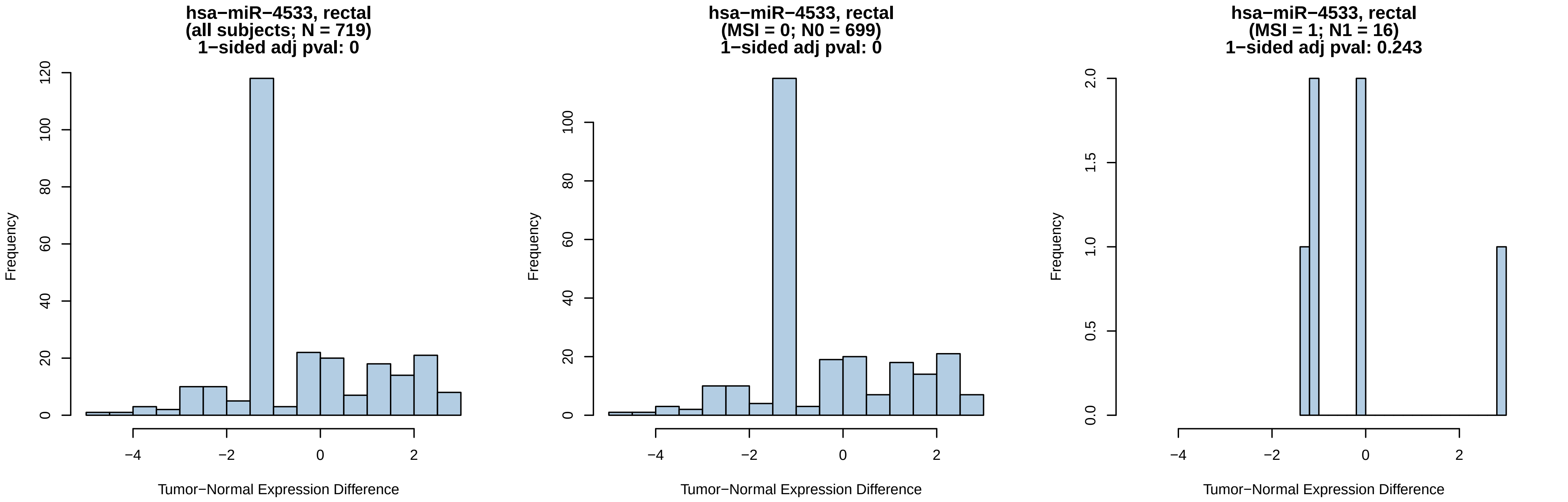

hsa−miR−4533, rectal
(all subjects; N = 719)
1−sided adj pval: 0
Frequency
Tumor−Normal Expression Difference
hsa−miR−4533, rectal
(MSI = 0; N0 = 699)
1−sided adj pval: 0
Frequency
Tumor−Normal Expression Difference
hsa−miR−4533, rectal
(MSI = 1; N1 = 16)
1−sided adj pval: 0.243
Frequency
Tumor−Normal Expression Difference

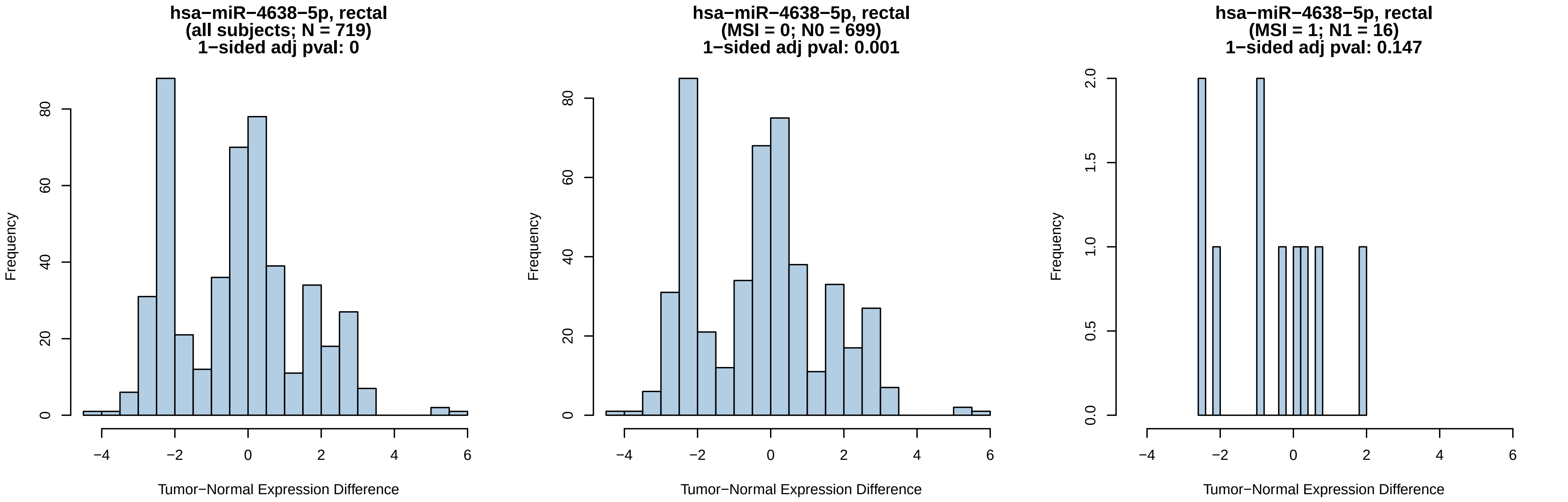

hsa−miR−4638−5p, rectal
(all subjects; N = 719)
1−sided adj pval: 0
Frequency
Tumor−Normal Expression Difference
hsa−miR−4638−5p, rectal
(MSI = 0; N0 = 699)
1−sided adj pval: 0.001
Frequency
Tumor−Normal Expression Difference
hsa−miR−4638−5p, rectal
(MSI = 1; N1 = 16)
1−sided adj pval: 0.147
Frequency
Tumor−Normal Expression Difference

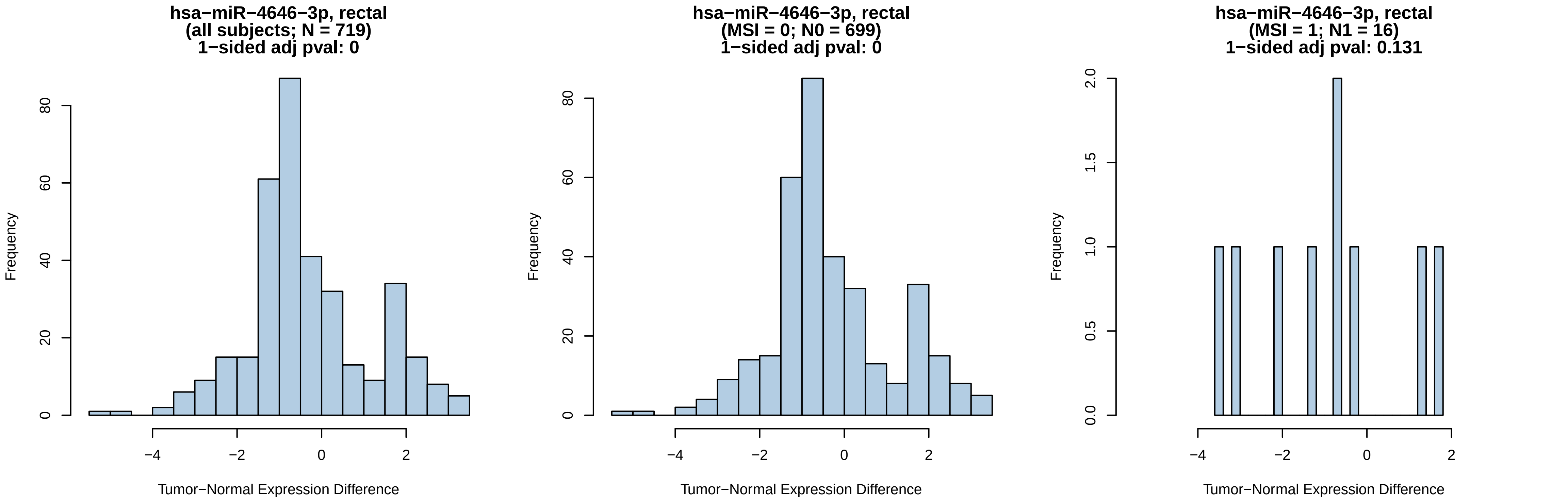
hsa−miR−4646−3p, rectal
(all subjects; N = 719)
1−sided adj pval: 0
Frequency
Tumor−Normal Expression Difference
hsa−miR−4646−3p, rectal
(MSI = 0; N0 = 699)
1−sided adj pval: 0
Frequency
Tumor−Normal Expression Difference
hsa−miR−4646−3p, rectal
(MSI = 1; N1 = 16)
1−sided adj pval: 0.131
Frequency
Tumor−Normal Expression Difference

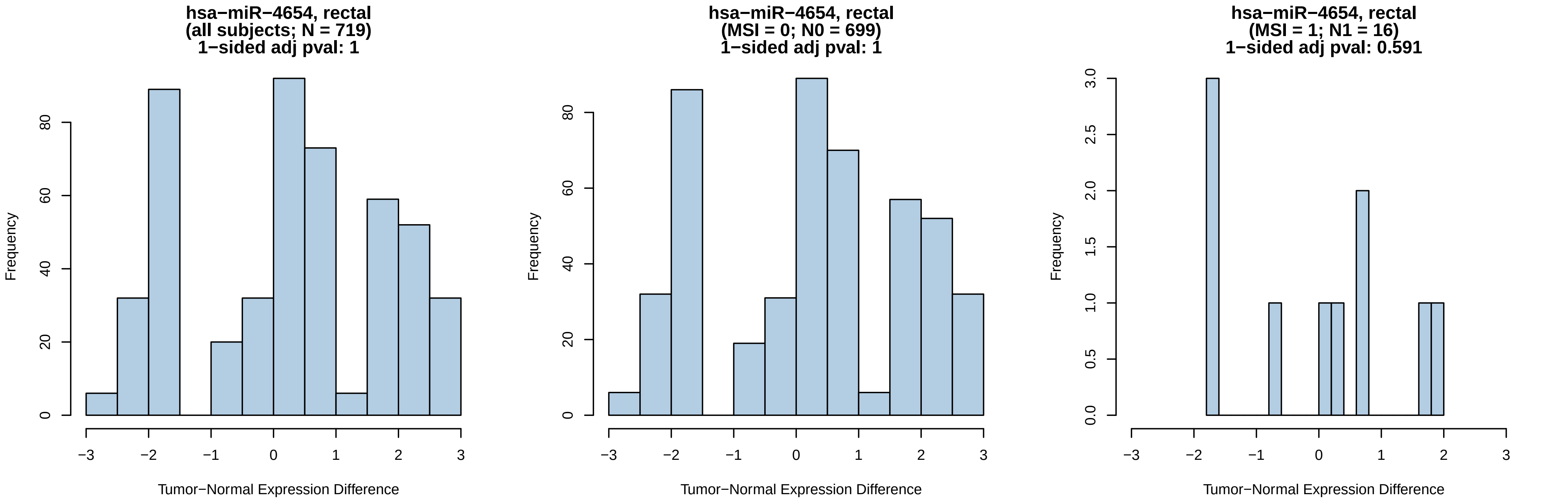
hsa−miR−4654, rectal
(all subjects; N = 719)
1−sided adj pval: 1
Frequency
Tumor−Normal Expression Difference
hsa−miR−4654, rectal
(MSI = 0; N0 = 699)
1−sided adj pval: 1
Frequency
Tumor−Normal Expression Difference
hsa−miR−4654, rectal
(MSI = 1; N1 = 16)
1−sided adj pval: 0.591
Frequency
Tumor−Normal Expression Difference

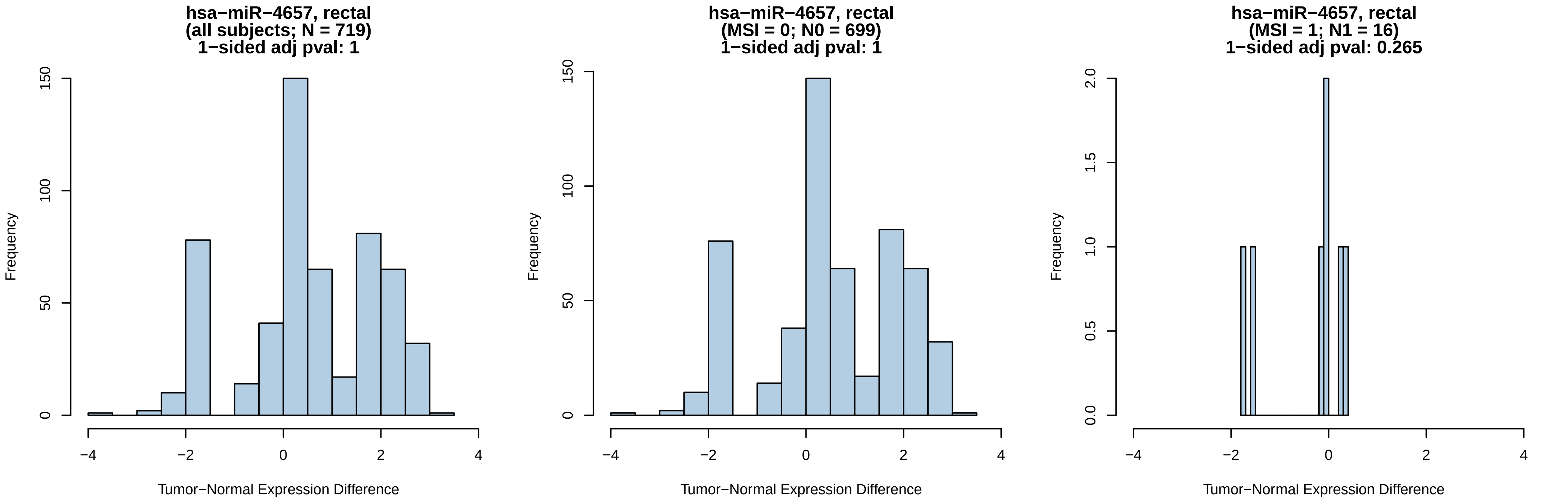
hsa−miR−4657, rectal
(all subjects; N = 719)
1−sided adj pval: 1
Frequency
Tumor−Normal Expression Difference
hsa−miR−4657, rectal
(MSI = 0; N0 = 699)
1−sided adj pval: 1
Frequency
Tumor−Normal Expression Difference
hsa−miR−4657, rectal
(MSI = 1; N1 = 16)
1−sided adj pval: 0.265
Frequency
Tumor−Normal Expression Difference

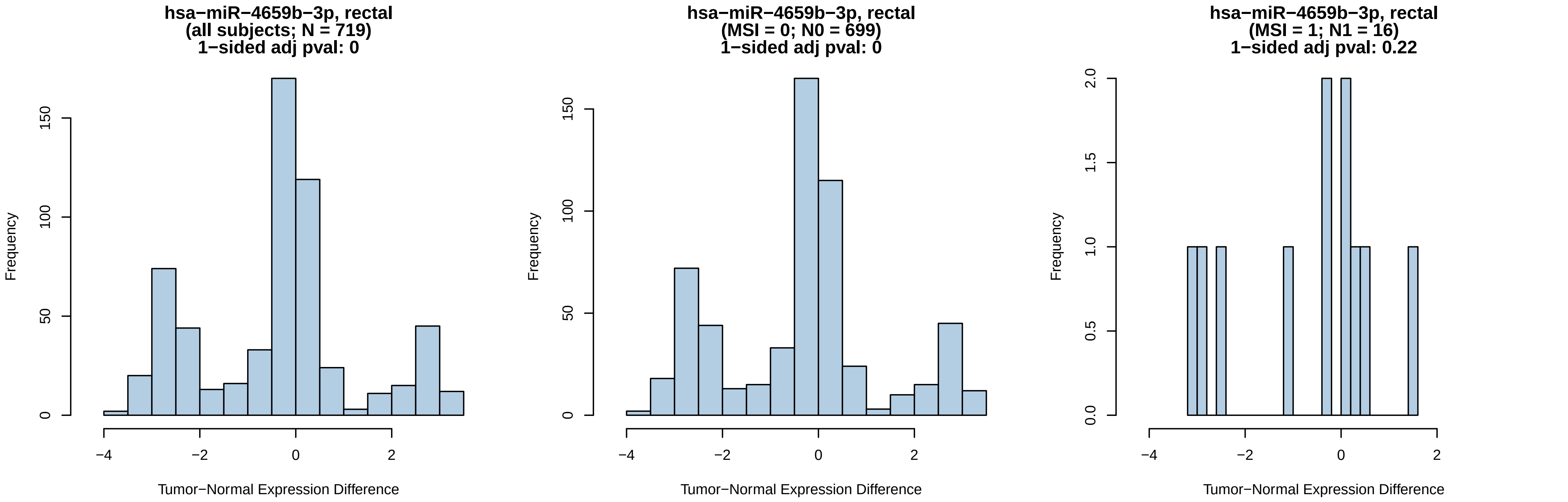
hsa−miR−4659b−3p, rectal
(all subjects; N = 719)
1−sided adj pval: 0
Frequency
0
50
100
150
−4
−2
0
2
Tumor−Normal Expression Difference
hsa−miR−4659b−3p, rectal
(MSI = 0; N0 = 699)
1−sided adj pval: 0
Frequency
0
50
100
150
−4
−2
0
2
Tumor−Normal Expression Difference
hsa−miR−4659b−3p, rectal
(MSI = 1; N1 = 16)
1−sided adj pval: 0.22
Frequency
0.0
0.5
1.0
1.5
2.0
−4
−2
0
2
Tumor−Normal Expression Difference

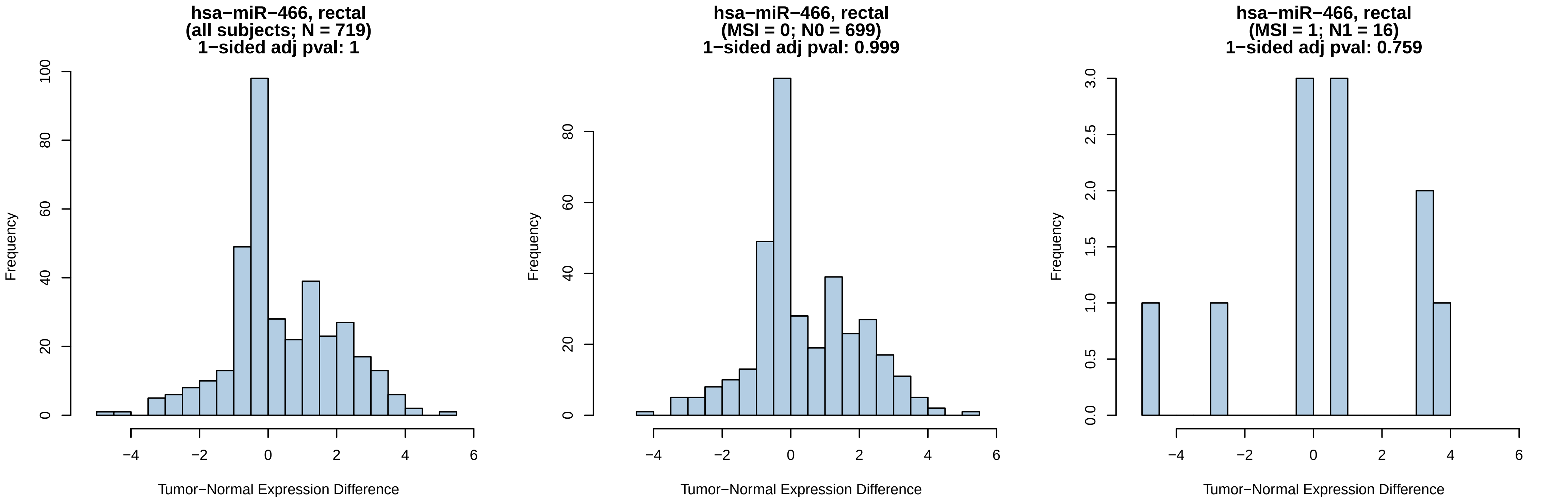
hsa−miR−466, rectal
(all subjects; N = 719)
1−sided adj pval: 1
Frequency
Tumor−Normal Expression Difference
hsa−miR−466, rectal
(MSI = 0; N0 = 699)
1−sided adj pval: 0.999
Frequency
Tumor−Normal Expression Difference
hsa−miR−466, rectal
(MSI = 1; N1 = 16)
1−sided adj pval: 0.759
Frequency
Tumor−Normal Expression Difference

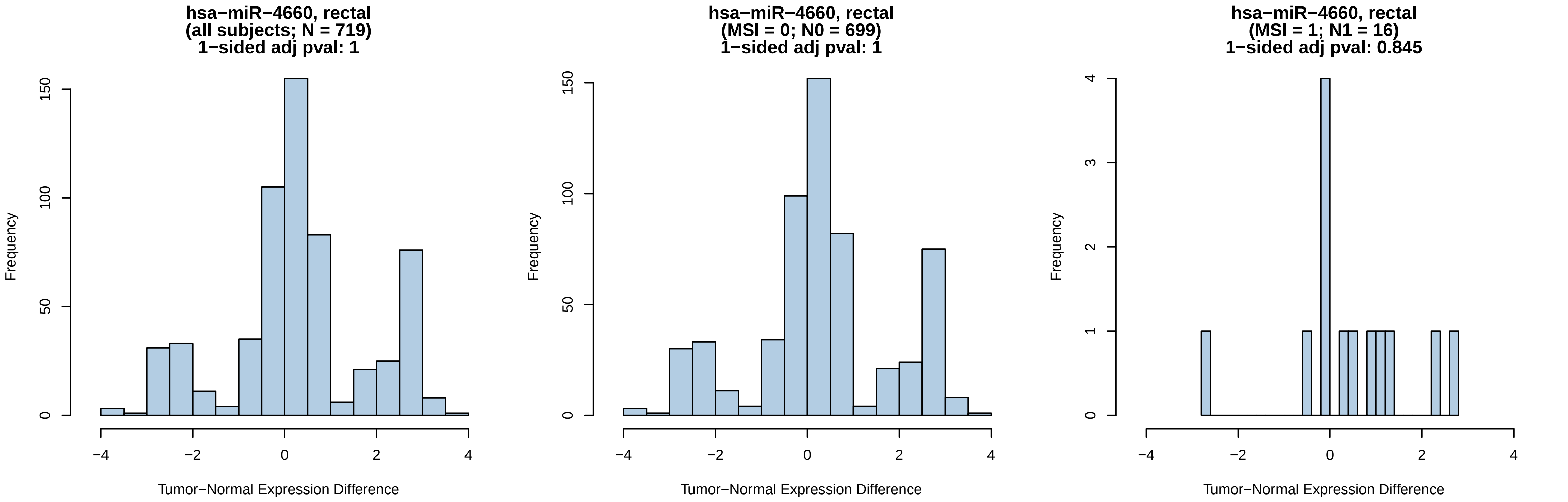

hsa−miR−4660, rectal
(all subjects; N = 719)
1−sided adj pval: 1
hsa−miR−4660, rectal
(MSI = 0; N0 = 699)
1−sided adj pval: 1
hsa−miR−4660, rectal
(MSI = 1; N1 = 16)
1−sided adj pval: 0.845
Frequency
Tumor−Normal Expression Difference

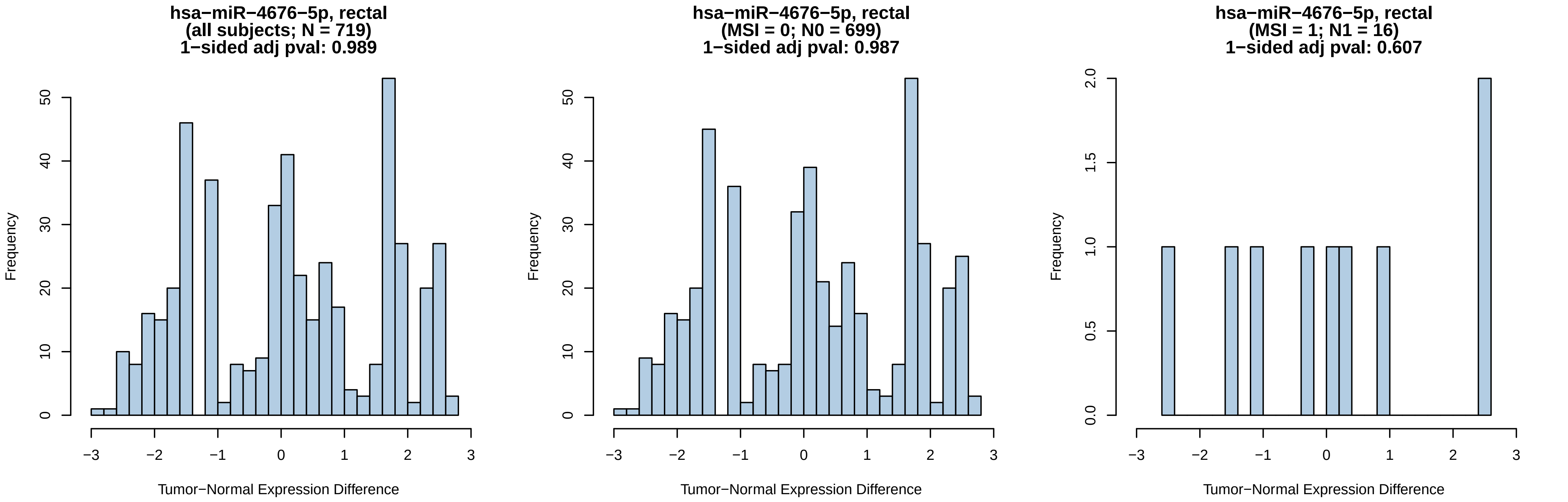
hsa−miR−4676−5p, rectal
(all subjects; N = 719)
1−sided adj pval: 0.989
Frequency
Tumor−Normal Expression Difference
hsa−miR−4676−5p, rectal
(MSI = 0; N0 = 699)
1−sided adj pval: 0.987
Frequency
Tumor−Normal Expression Difference
hsa−miR−4676−5p, rectal
(MSI = 1; N1 = 16)
1−sided adj pval: 0.607
Frequency
Tumor−Normal Expression Difference

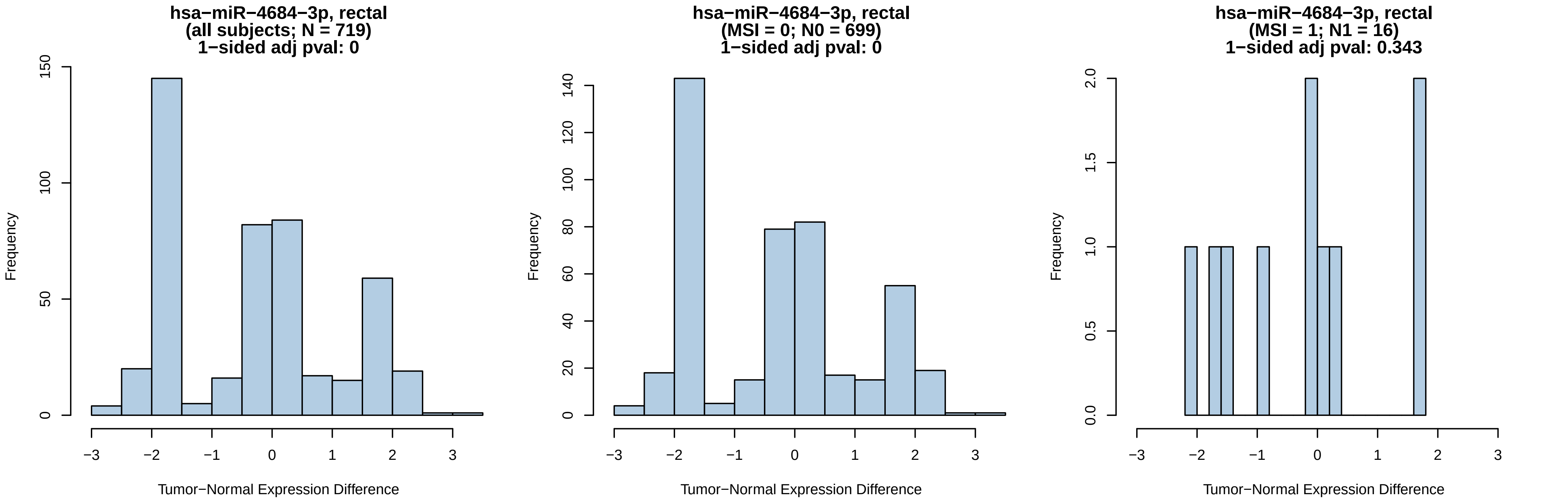
hsa−miR−4684−3p, rectal
(all subjects; N = 719)
1−sided adj pval: 0
Frequency
Tumor−Normal Expression Difference
hsa−miR−4684−3p, rectal
(MSI = 0; N0 = 699)
1−sided adj pval: 0
Frequency
Tumor−Normal Expression Difference
hsa−miR−4684−3p, rectal
(MSI = 1; N1 = 16)
1−sided adj pval: 0.343
Frequency
Tumor−Normal Expression Difference

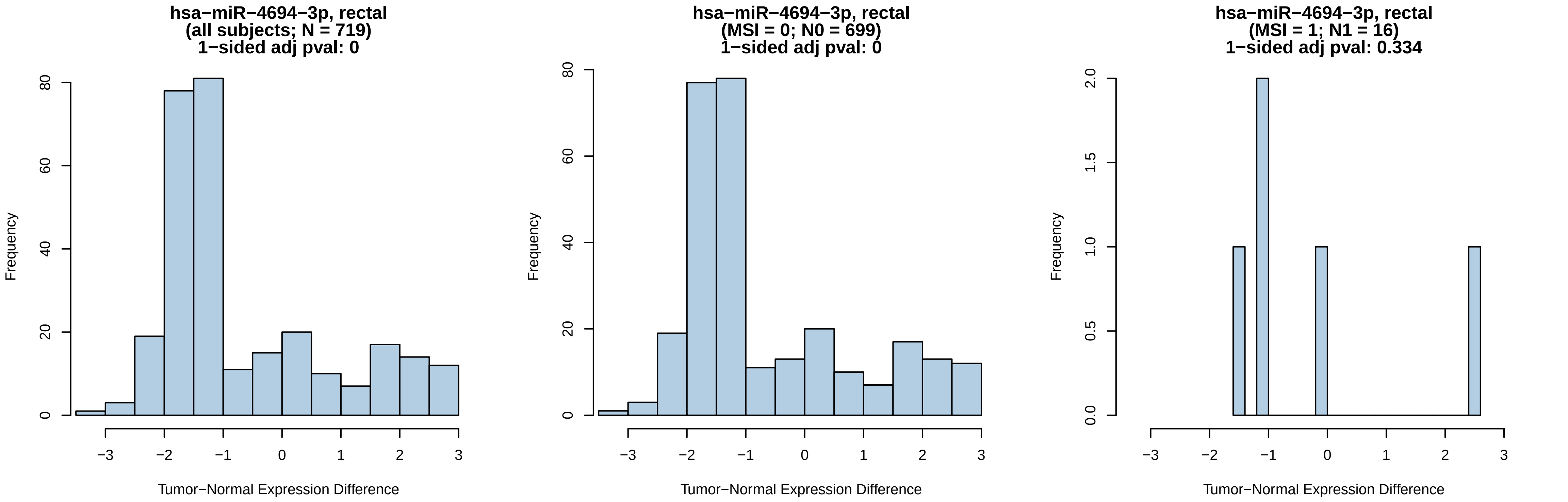

hsa−miR−4694−3p, rectal
(all subjects; N = 719)
1−sided adj pval: 0
Frequency
0
20
40
60
80
−3
−2
−1
0
1
2
3
Tumor−Normal Expression Difference
hsa−miR−4694−3p, rectal
(MSI = 0; N0 = 699)
1−sided adj pval: 0
Frequency
0
20
40
60
80
−3
−2
−1
0
1
2
3
Tumor−Normal Expression Difference
hsa−miR−4694−3p, rectal
(MSI = 1; N1 = 16)
1−sided adj pval: 0.334
Frequency
0.0
0.5
1.0
1.5
2.0
−3
−2
−1
0
1
2
3
Tumor−Normal Expression Difference

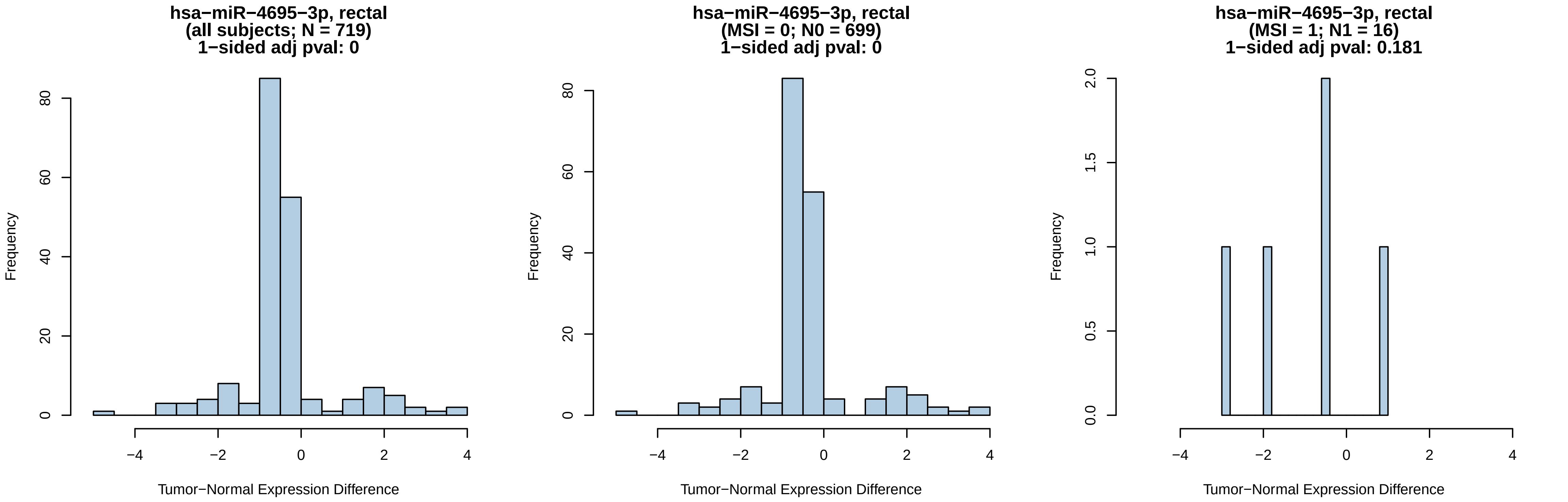
hsa−miR−4695−3p, rectal
(all subjects; N = 719)
1−sided adj pval: 0
Frequency
Tumor−Normal Expression Difference
hsa−miR−4695−3p, rectal
(MSI = 0; N0 = 699)
1−sided adj pval: 0
Frequency
Tumor−Normal Expression Difference
hsa−miR−4695−3p, rectal
(MSI = 1; N1 = 16)
1−sided adj pval: 0.181
Frequency
Tumor−Normal Expression Difference

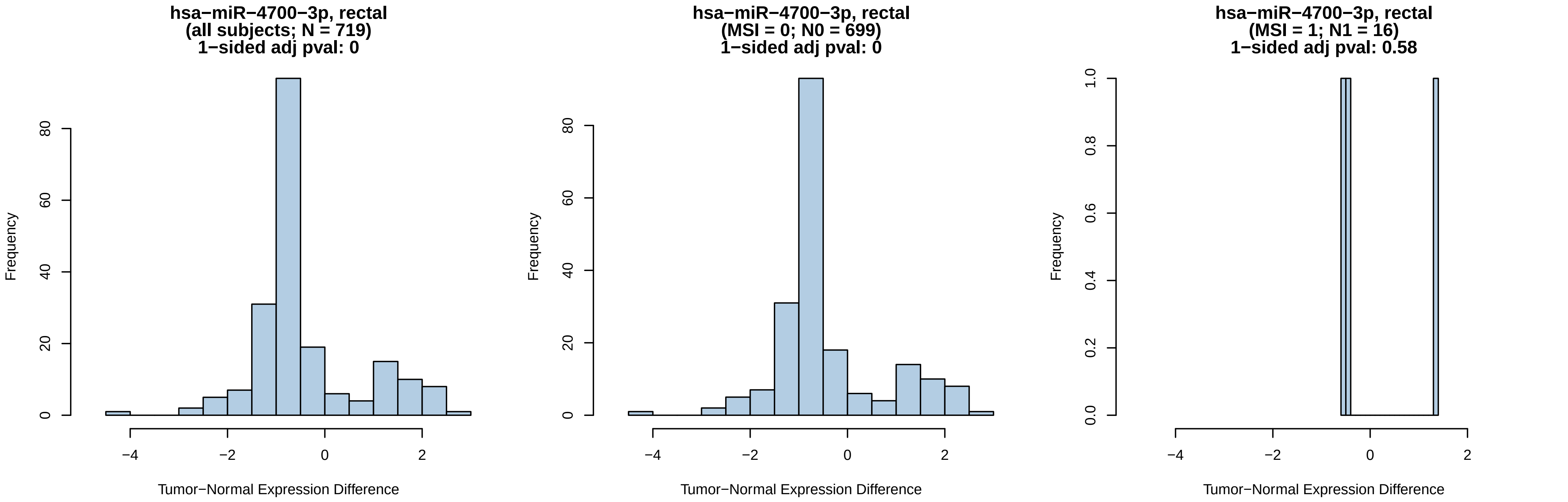
hsa−miR−4700−3p, rectal
(all subjects; N = 719)
1−sided adj pval: 0
Frequency
Tumor−Normal Expression Difference
hsa−miR−4700−3p, rectal
(MSI = 0; N0 = 699)
1−sided adj pval: 0
Frequency
Tumor−Normal Expression Difference
hsa−miR−4700−3p, rectal
(MSI = 1; N1 = 16)
1−sided adj pval: 0.58
Frequency
Tumor−Normal Expression Difference

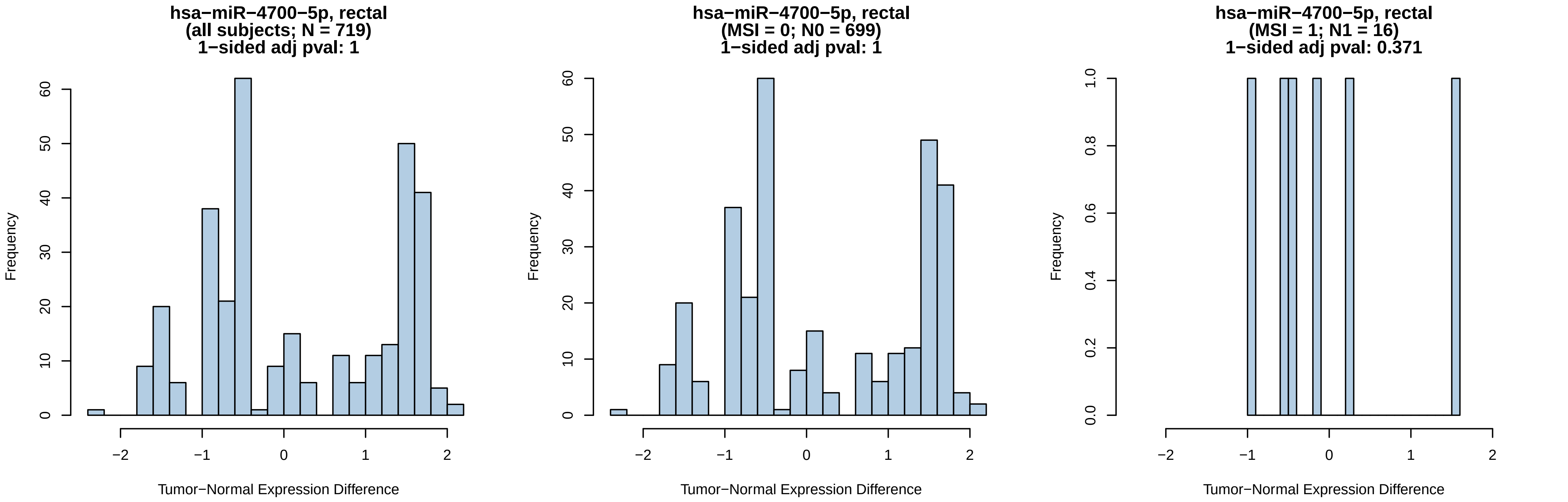
hsa−miR−4700−5p, rectal
(all subjects; N = 719)
1−sided adj pval: 1
Frequency
Tumor−Normal Expression Difference
hsa−miR−4700−5p, rectal
(MSI = 0; N0 = 699)
1−sided adj pval: 1
Frequency
Tumor−Normal Expression Difference
hsa−miR−4700−5p, rectal
(MSI = 1; N1 = 16)
1−sided adj pval: 0.371
Frequency
Tumor−Normal Expression Difference

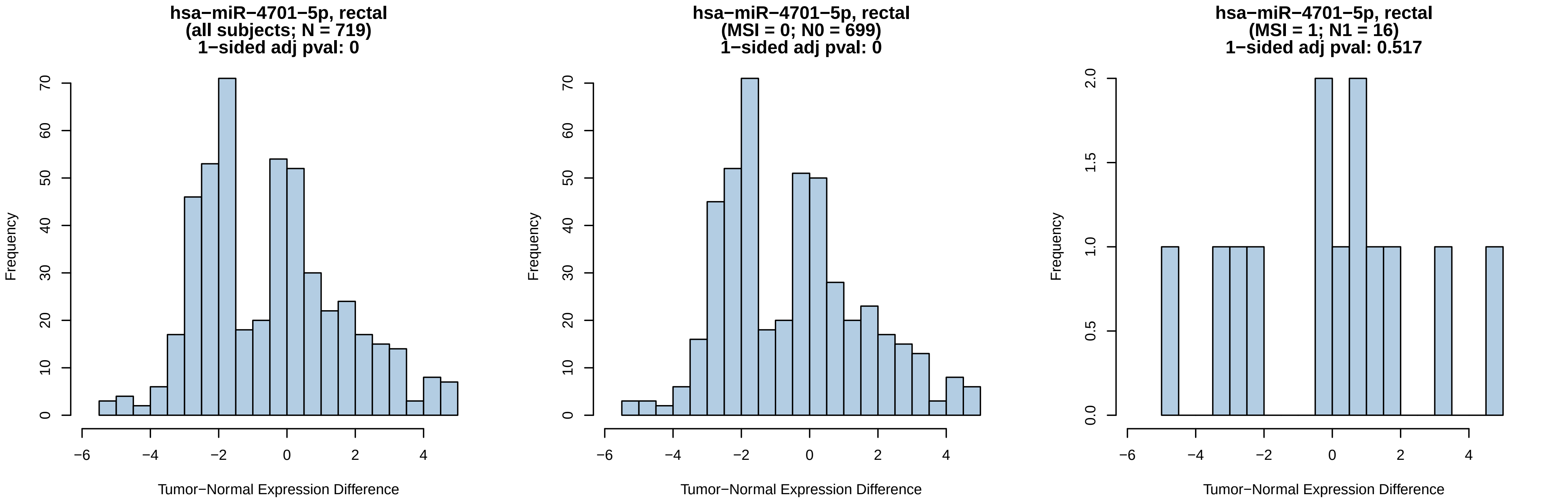

hsa−miR−4701−5p, rectal
(all subjects; N = 719)
1−sided adj pval: 0
Frequency
Tumor−Normal Expression Difference
hsa−miR−4701−5p, rectal
(MSI = 0; N0 = 699)
1−sided adj pval: 0
Frequency
Tumor−Normal Expression Difference
hsa−miR−4701−5p, rectal
(MSI = 1; N1 = 16)
1−sided adj pval: 0.517
Frequency
Tumor−Normal Expression Difference

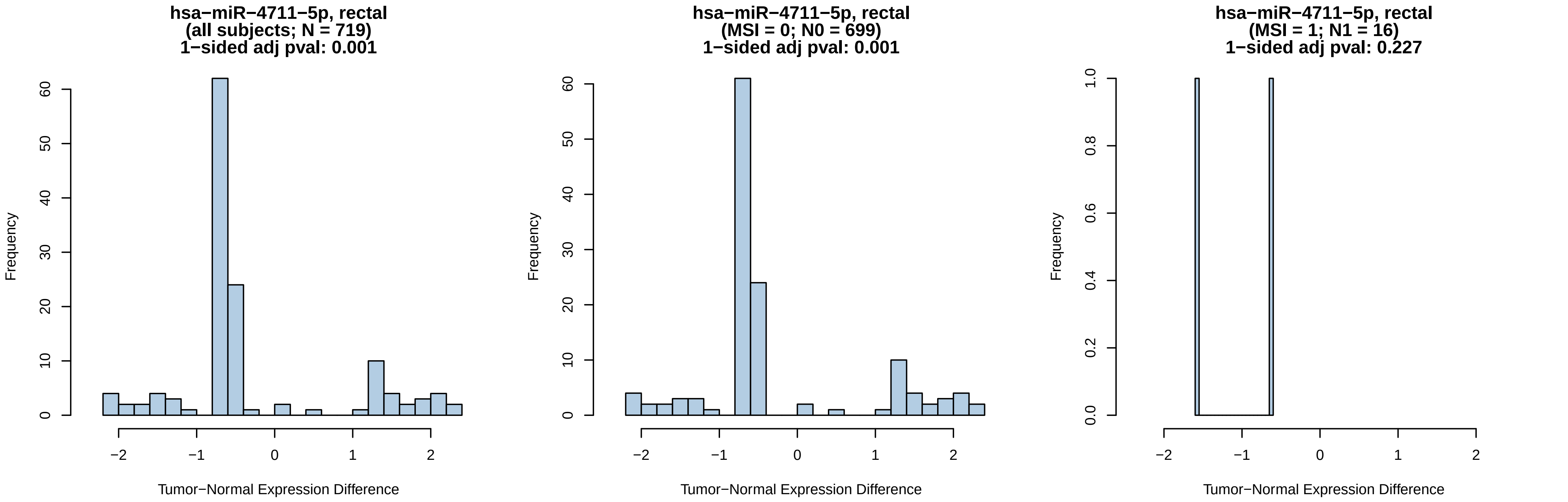

hsa−miR−4711−5p, rectal
(all subjects; N = 719)
1−sided adj pval: 0.001
hsa−miR−4711−5p, rectal
(MSI = 0; N0 = 699)
1−sided adj pval: 0.001
hsa−miR−4711−5p, rectal
(MSI = 1; N1 = 16)
1−sided adj pval: 0.227
Frequency
Tumor−Normal Expression Difference
0
10
20
30
40
50
60
0.0
0.2
0.4
0.6
0.8
1.0
−2
−1
0
1
2

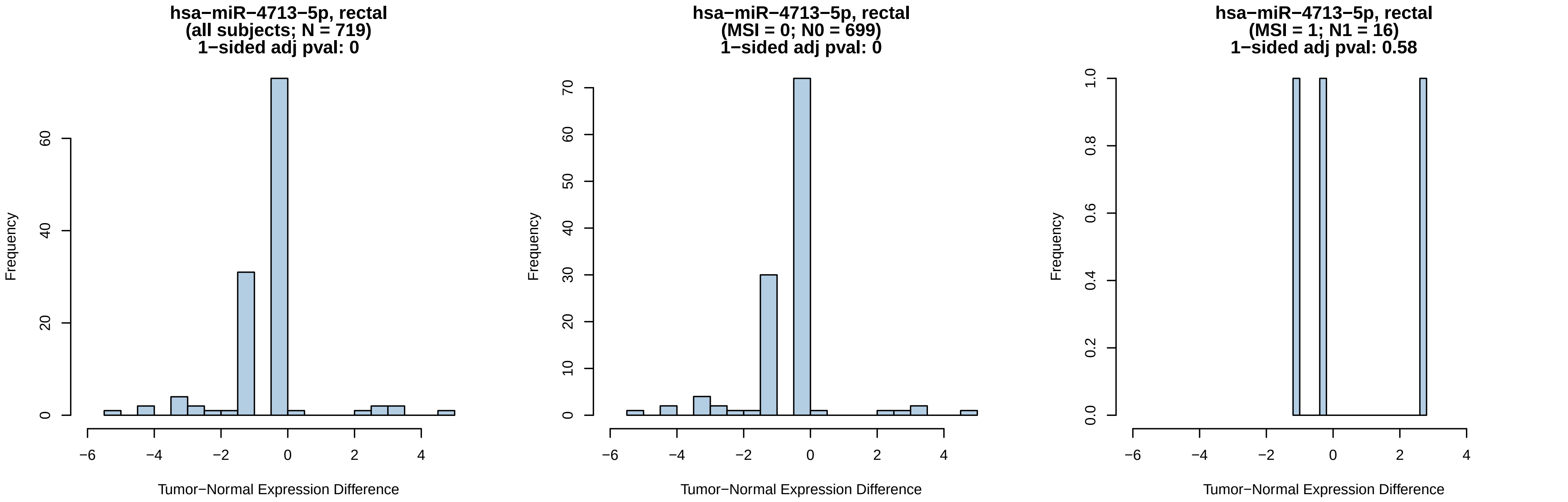
hsa−miR−4713−5p, rectal
(all subjects; N = 719)
1−sided adj pval: 0
hsa−miR−4713−5p, rectal
(MSI = 0; N0 = 699)
1−sided adj pval: 0
hsa−miR−4713−5p, rectal
(MSI = 1; N1 = 16)
1−sided adj pval: 0.58
Frequency
Tumor−Normal Expression Difference

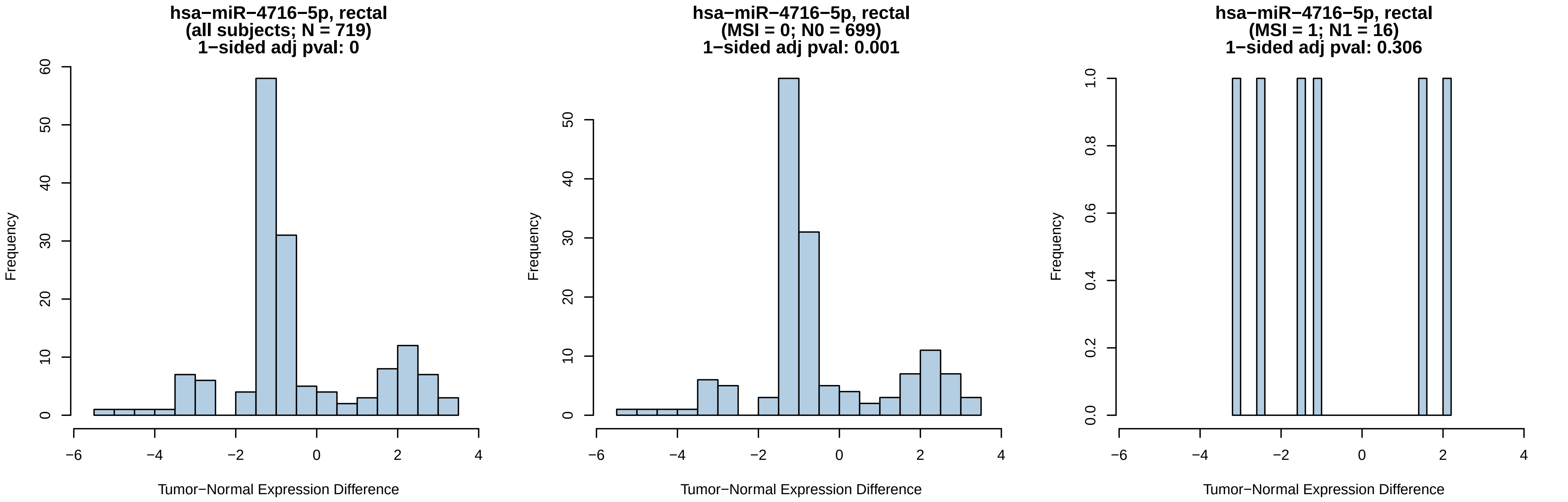
hsa−miR−4716−5p, rectal
(all subjects; N = 719)
1−sided adj pval: 0
Frequency
Tumor−Normal Expression Difference
hsa−miR−4716−5p, rectal
(MSI = 0; N0 = 699)
1−sided adj pval: 0.001
Frequency
Tumor−Normal Expression Difference
hsa−miR−4716−5p, rectal
(MSI = 1; N1 = 16)
1−sided adj pval: 0.306
Frequency
Tumor−Normal Expression Difference

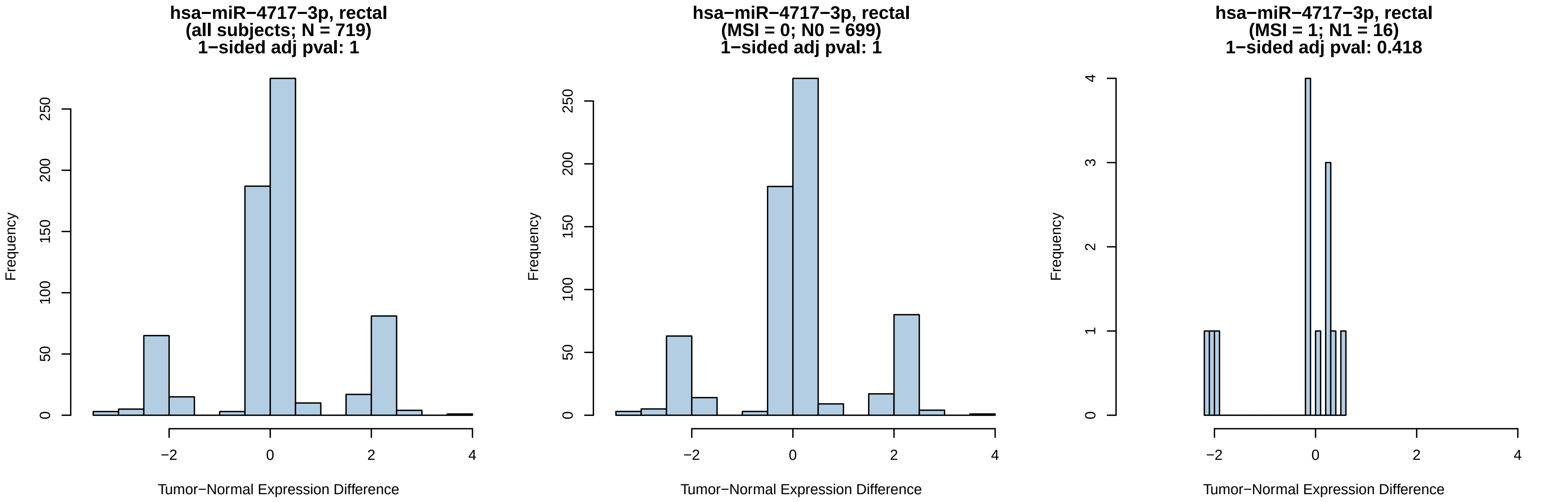
hsa−miR−4717−3p, rectal
(all subjects; N = 719)
1−sided adj pval: 1
Frequency
Tumor−Normal Expression Difference
hsa−miR−4717−3p, rectal
(MSI = 0; N0 = 699)
1−sided adj pval: 1
Frequency
Tumor−Normal Expression Difference
hsa−miR−4717−3p, rectal
(MSI = 1; N1 = 16)
1−sided adj pval: 0.418
Frequency
Tumor−Normal Expression Difference

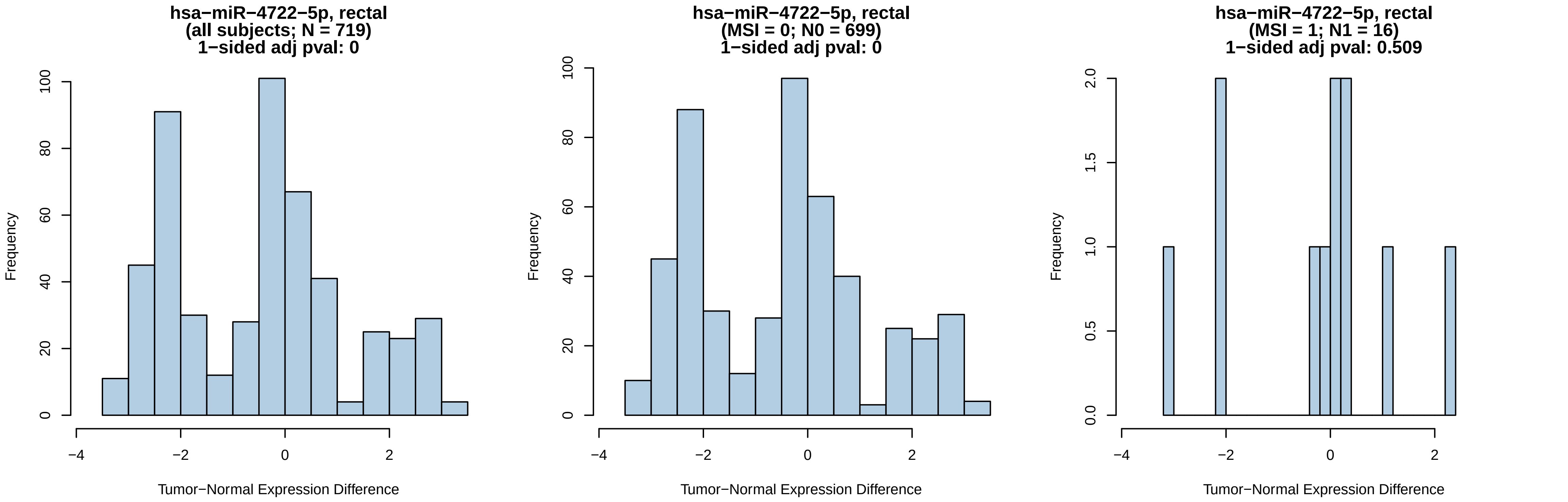

hsa−miR−4722−5p, rectal
(all subjects; N = 719)
1−sided adj pval: 0
Frequency
Tumor−Normal Expression Difference
0
20
40
60
80
100
−4
−2
0
2
hsa−miR−4722−5p, rectal
(MSI = 0; N0 = 699)
1−sided adj pval: 0
Frequency
Tumor−Normal Expression Difference
0
20
40
60
80
100
−4
−2
0
2
hsa−miR−4722−5p, rectal
(MSI = 1; N1 = 16)
1−sided adj pval: 0.509
Frequency
Tumor−Normal Expression Difference
0.0
0.5
1.0
1.5
2.0
−4
−2
0
2

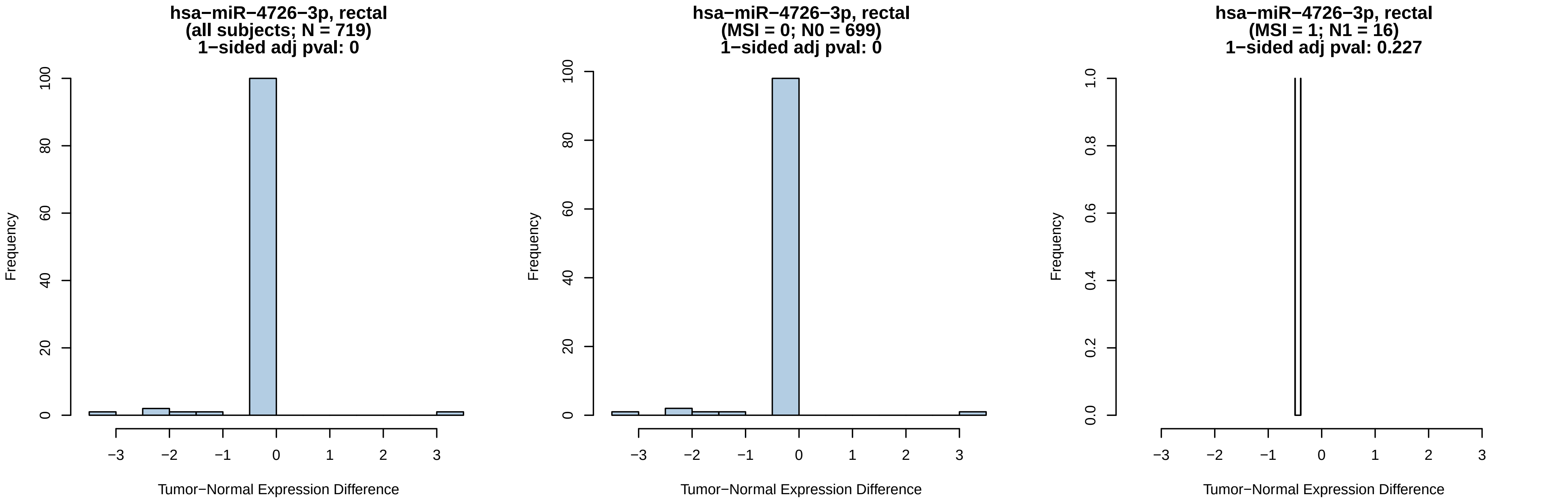
hsa−miR−4726−3p, rectal
(all subjects; N = 719)
1−sided adj pval: 0
Frequency
0
20
40
60
80
100
−3
−2
−1
0
1
2
3
Tumor−Normal Expression Difference
hsa−miR−4726−3p, rectal
(MSI = 0; N0 = 699)
1−sided adj pval: 0
Frequency
0
20
40
60
80
100
−3
−2
−1
0
1
2
3
Tumor−Normal Expression Difference
hsa−miR−4726−3p, rectal
(MSI = 1; N1 = 16)
1−sided adj pval: 0.227
Frequency
0.0
0.2
0.4
0.6
0.8
1.0
−3
−2
−1
0
1
2
3
Tumor−Normal Expression Difference

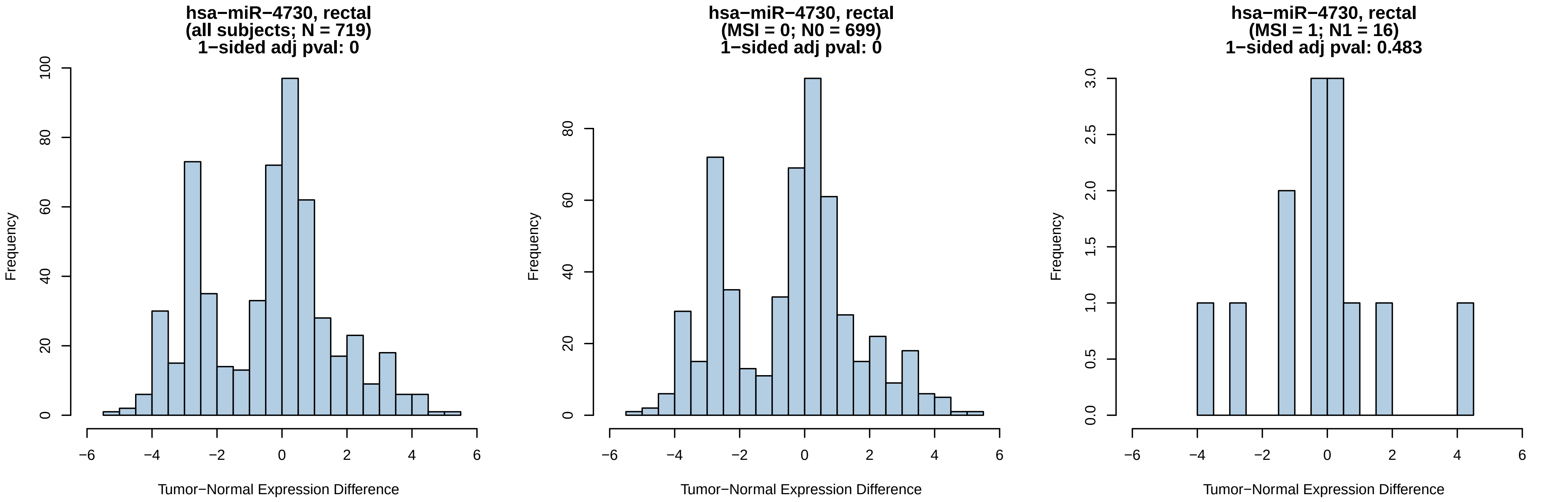
hsa−miR−4730, rectal
(all subjects; N = 719)
1−sided adj pval: 0
Frequency
0
20
40
60
80
100
−6
−4
−2
0
2
4
6
Tumor−Normal Expression Difference
hsa−miR−4730, rectal
(MSI = 0; N0 = 699)
1−sided adj pval: 0
Frequency
0
20
40
60
80
−6
−4
−2
0
2
4
6
Tumor−Normal Expression Difference
hsa−miR−4730, rectal
(MSI = 1; N1 = 16)
1−sided adj pval: 0.483
Frequency
0.0
0.5
1.0
1.5
2.0
2.5
3.0
−6
−4
−2
0
2
4
6
Tumor−Normal Expression Difference

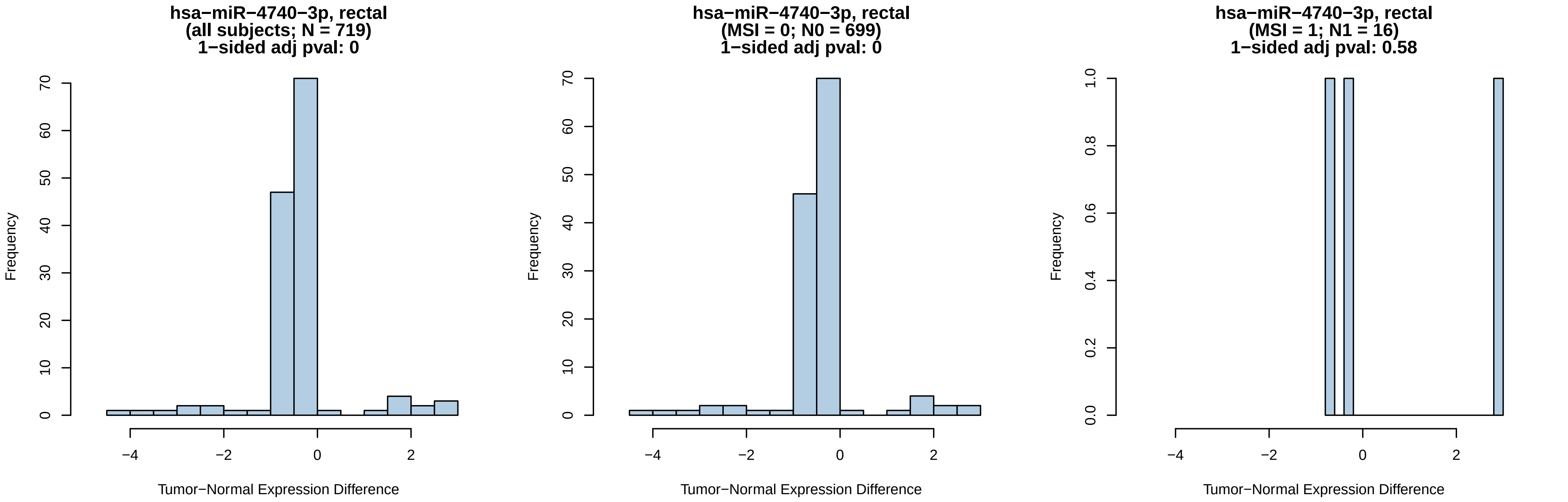
hsa−miR−4740−3p, rectal
(all subjects; N = 719)
1−sided adj pval: 0
Frequency
Tumor−Normal Expression Difference
0
10
20
30
40
50
60
70
−4
−2
0
2
hsa−miR−4740−3p, rectal
(MSI = 0; N0 = 699)
1−sided adj pval: 0
Frequency
Tumor−Normal Expression Difference
hsa−miR−4740−3p, rectal
(MSI = 1; N1 = 16)
1−sided adj pval: 0.58
Frequency
0.0
0.2
0.4
0.6
0.8
1.0
Tumor−Normal Expression Difference

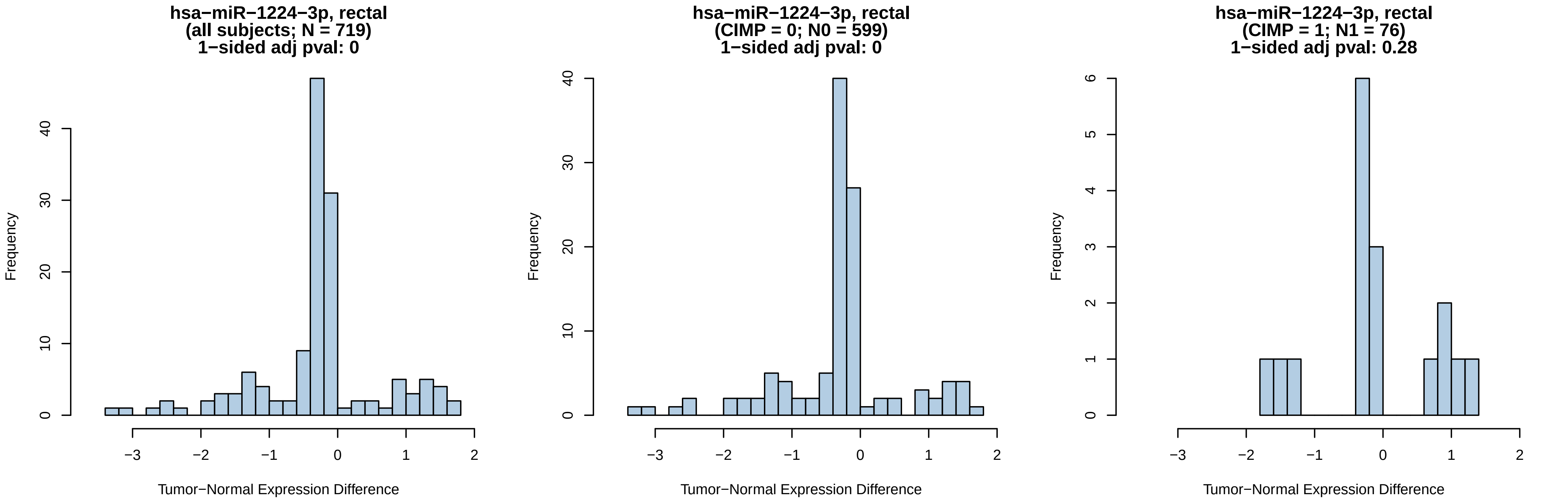
hsa−miR−1224−3p, rectal
(all subjects; N = 719)
1−sided adj pval: 0
Frequency
Tumor−Normal Expression Difference
hsa−miR−1224−3p, rectal
(CIMP = 0; N0 = 599)
1−sided adj pval: 0
Frequency
Tumor−Normal Expression Difference
hsa−miR−1224−3p, rectal
(CIMP = 1; N1 = 76)
1−sided adj pval: 0.28
Frequency
Tumor−Normal Expression Difference

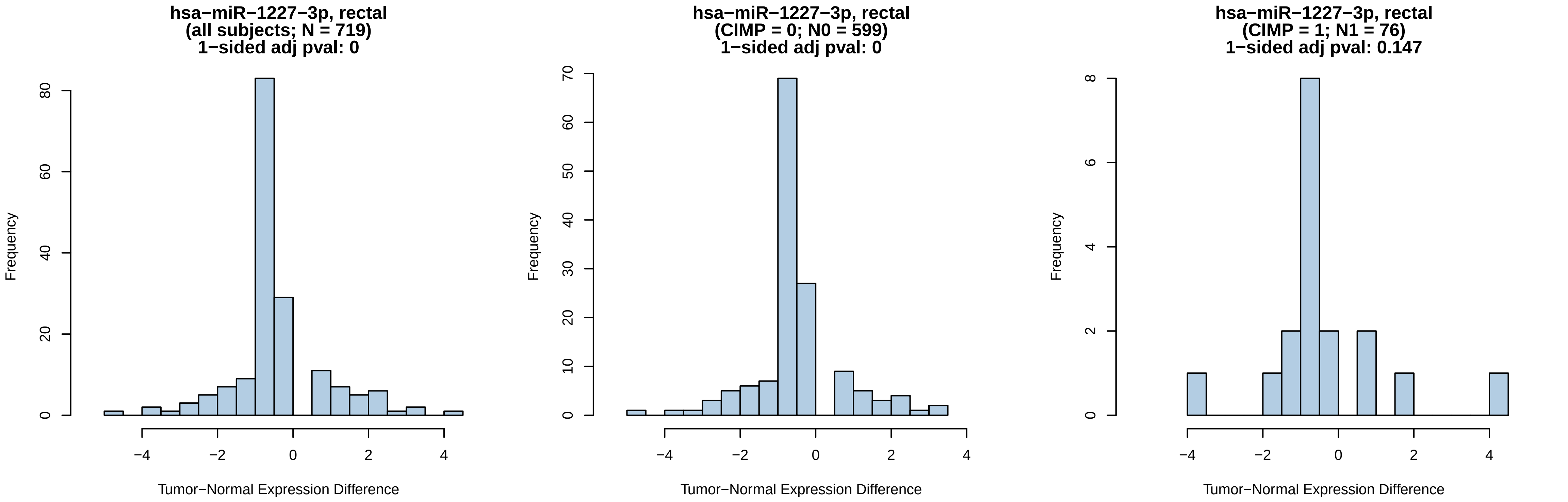
hsa−miR−1227−3p, rectal
(all subjects; N = 719)
1−sided adj pval: 0
Frequency
Tumor−Normal Expression Difference
hsa−miR−1227−3p, rectal
(CIMP = 0; N0 = 599)
1−sided adj pval: 0
Frequency
Tumor−Normal Expression Difference
hsa−miR−1227−3p, rectal
(CIMP = 1; N1 = 76)
1−sided adj pval: 0.147
Frequency
Tumor−Normal Expression Difference

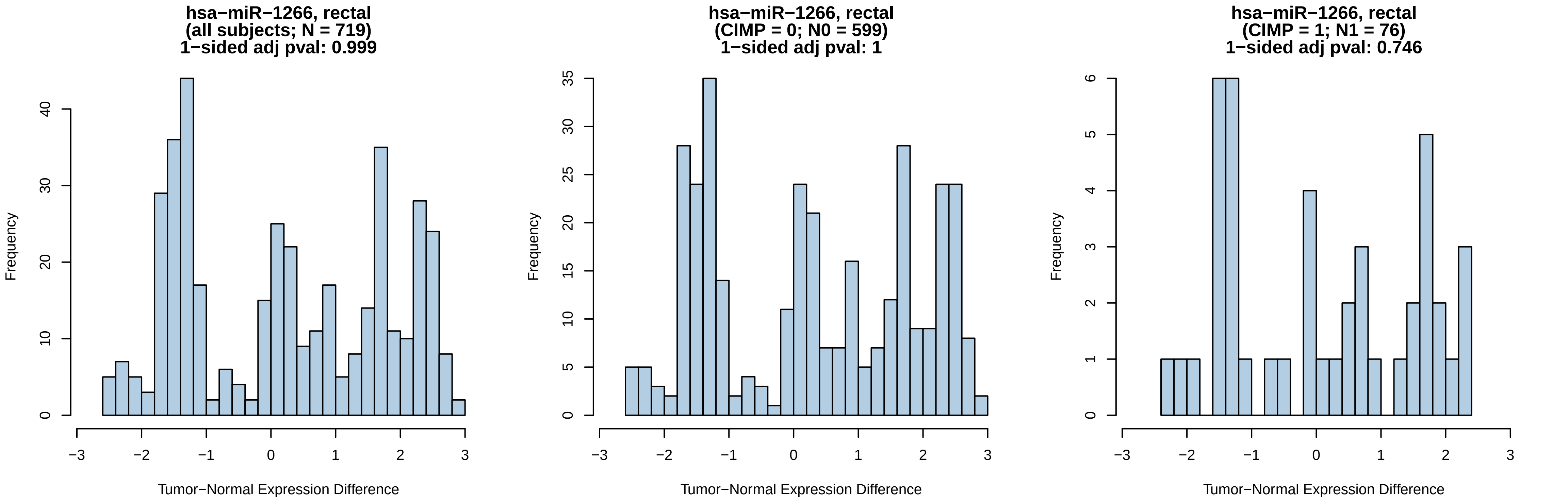

hsa−miR−1266, rectal
(all subjects; N = 719)
1−sided adj pval: 0.999
hsa−miR−1266, rectal
(CIMP = 0; N0 = 599)
1−sided adj pval: 1
hsa−miR−1266, rectal
(CIMP = 1; N1 = 76)
1−sided adj pval: 0.746
Frequency
Tumor−Normal Expression Difference

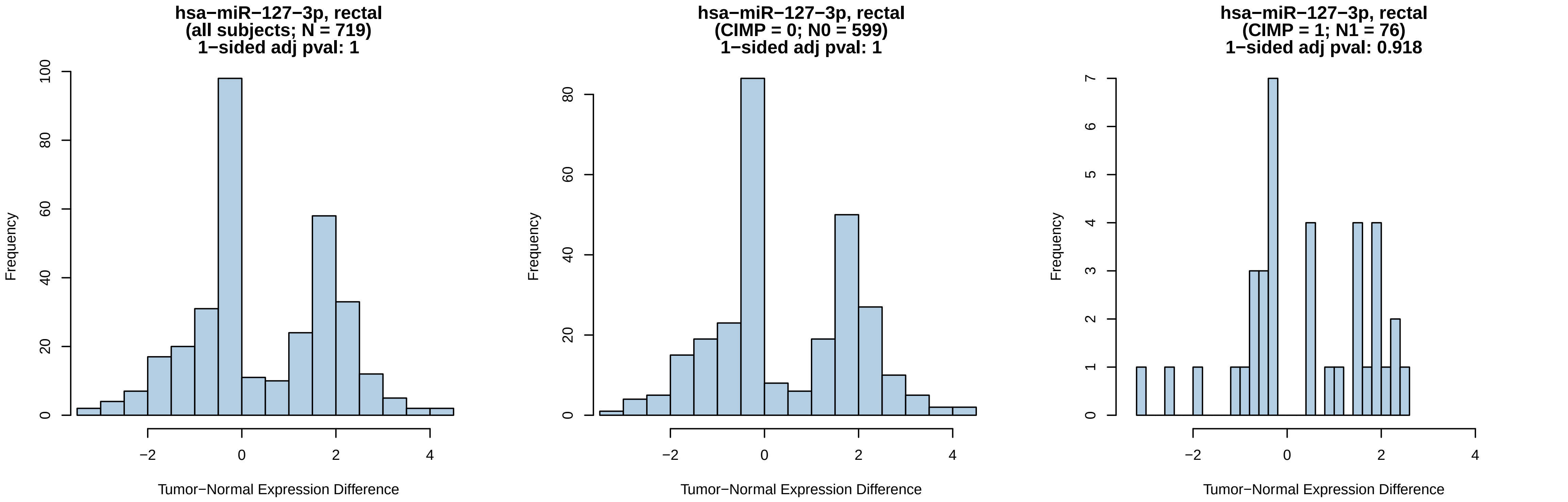

hsa−miR−127−3p, rectal
(all subjects; N = 719)
1−sided adj pval: 1
Frequency
Tumor−Normal Expression Difference
hsa−miR−127−3p, rectal
(CIMP = 0; N0 = 599)
1−sided adj pval: 1
Frequency
Tumor−Normal Expression Difference
hsa−miR−127−3p, rectal
(CIMP = 1; N1 = 76)
1−sided adj pval: 0.918
Frequency
Tumor−Normal Expression Difference

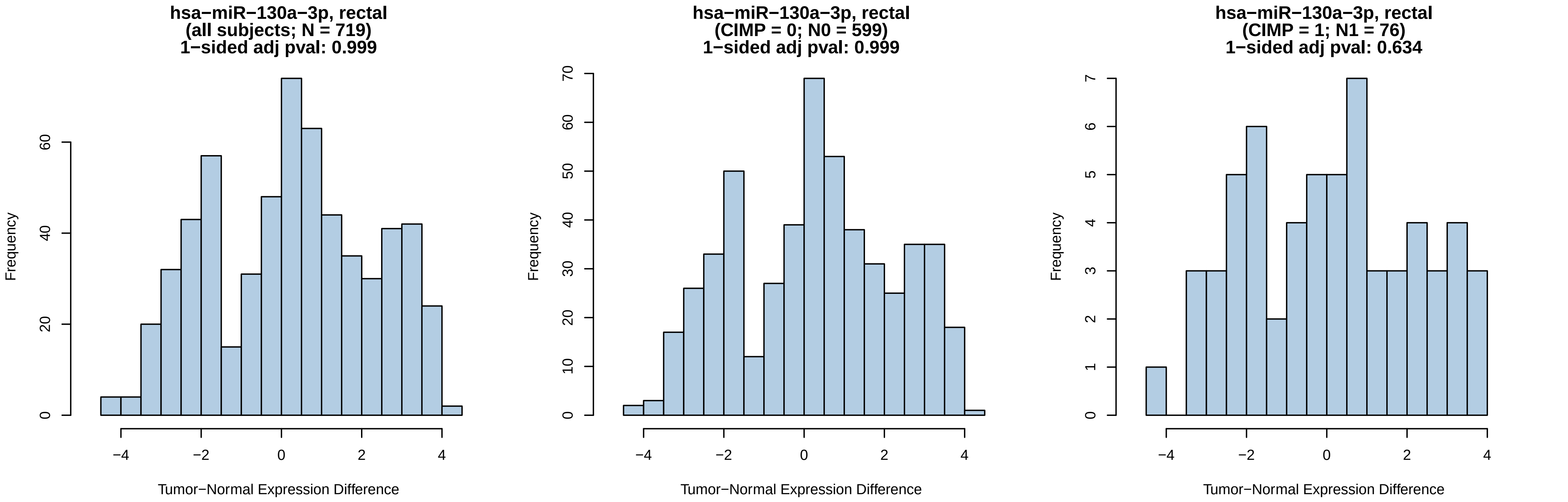

hsa−miR−130a−3p, rectal
(all subjects; N = 719)
1−sided adj pval: 0.999
Frequency
0
20
40
60
−4
−2
0
2
4
Tumor−Normal Expression Difference
hsa−miR−130a−3p, rectal
(CIMP = 0; N0 = 599)
1−sided adj pval: 0.999
Frequency
0
10
20
30
40
50
60
70
−4
−2
0
2
4
Tumor−Normal Expression Difference
hsa−miR−130a−3p, rectal
(CIMP = 1; N1 = 76)
1−sided adj pval: 0.634
Frequency
0
1
2
3
4
5
6
7
−4
−2
0
2
4
Tumor−Normal Expression Difference

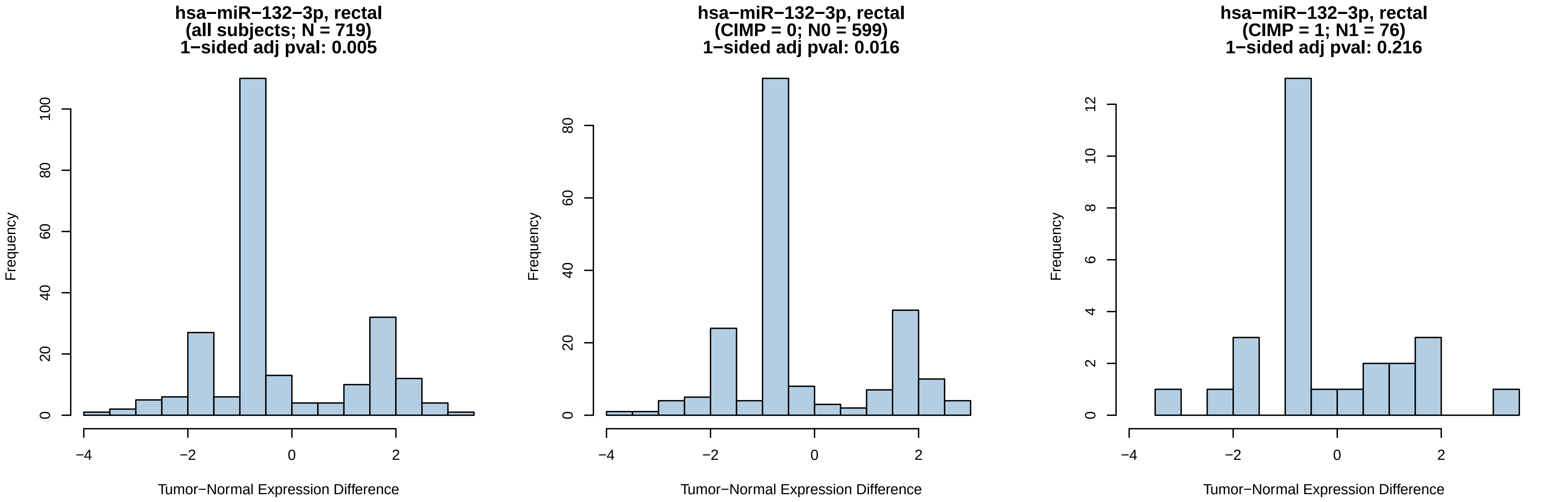
hsa−miR−132−3p, rectal
(all subjects; N = 719)
1−sided adj pval: 0.005
hsa−miR−132−3p, rectal
(CIMP = 0; N0 = 599)
1−sided adj pval: 0.016
hsa−miR−132−3p, rectal
(CIMP = 1; N1 = 76)
1−sided adj pval: 0.216
Frequency
Tumor−Normal Expression Difference

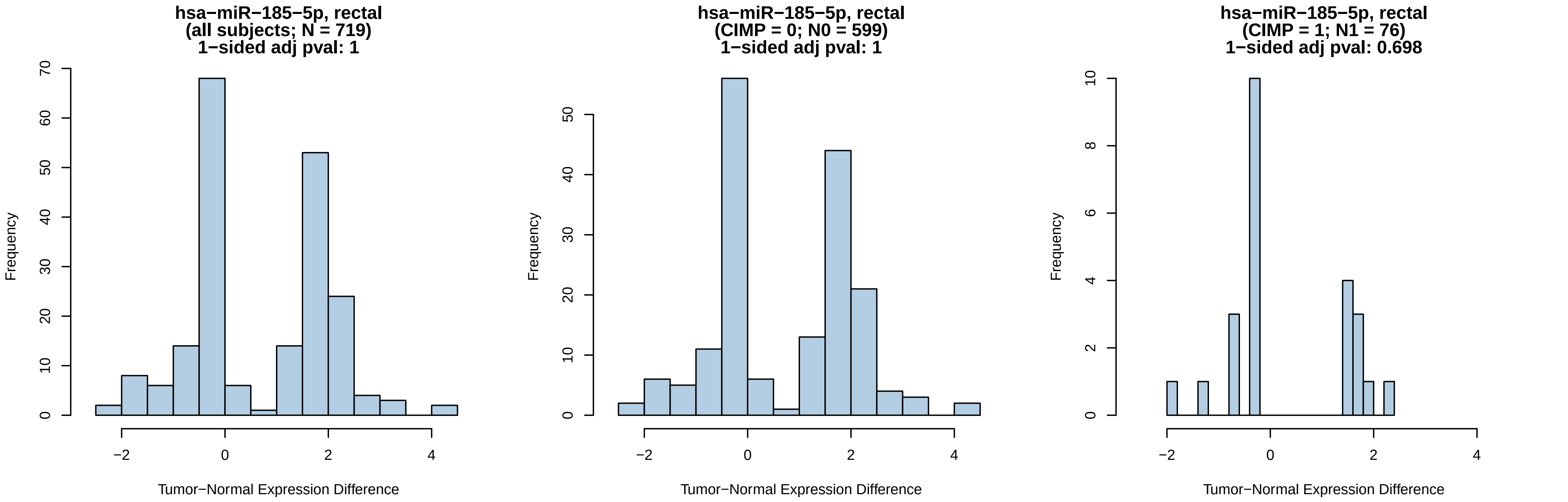

hsa−miR−185−5p, rectal
(all subjects; N = 719)
1−sided adj pval: 1
Frequency
Tumor−Normal Expression Difference
hsa−miR−185−5p, rectal
(CIMP = 0; N0 = 599)
1−sided adj pval: 1
Frequency
Tumor−Normal Expression Difference
hsa−miR−185−5p, rectal
(CIMP = 1; N1 = 76)
1−sided adj pval: 0.698
Frequency
Tumor−Normal Expression Difference

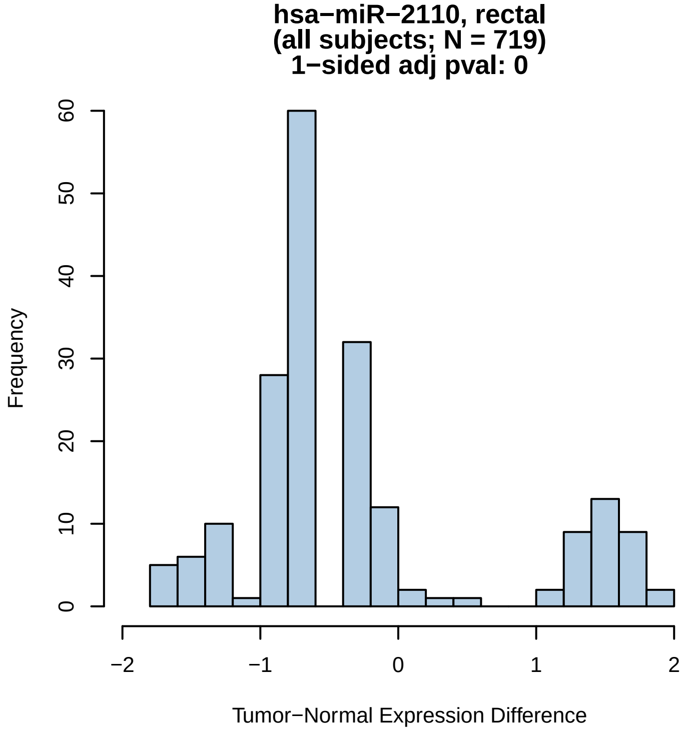
hsa−miR−2110, rectal
(all subjects; N = 719)
1−sided adj pval: 0
Frequency
0
10
20
30
40
50
60
−2
−1
0
1
2
Tumor−Normal Expression Difference

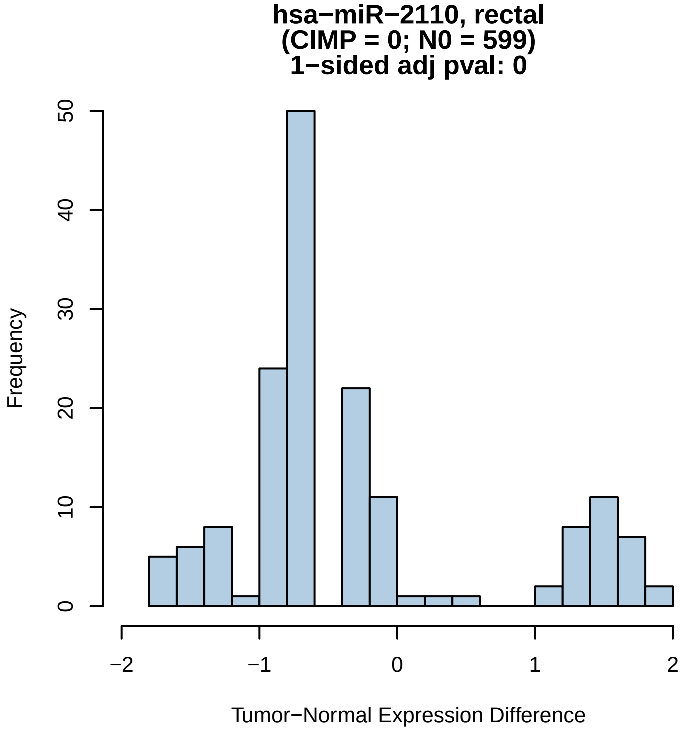
hsa−miR−2110, rectal
(CIMP = 0; N0 = 599)
1−sided adj pval: 0
Frequency
0
10
20
30
40
50
−2
−1
0
1
2
Tumor−Normal Expression Difference

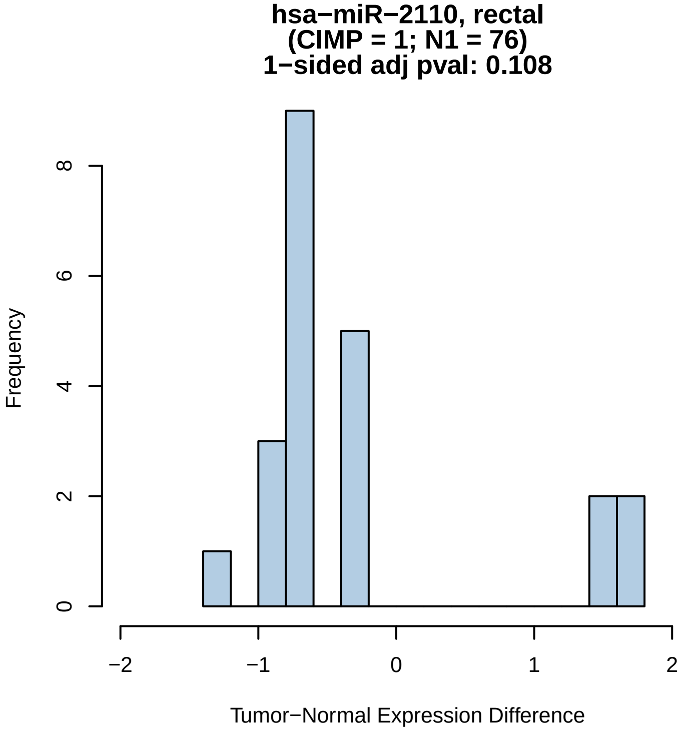
hsa−miR−2110, rectal
(CIMP = 1; N1 = 76)
1−sided adj pval: 0.108
Frequency
0
2
4
6
8
−2
−1
0
1
2
Tumor−Normal Expression Difference

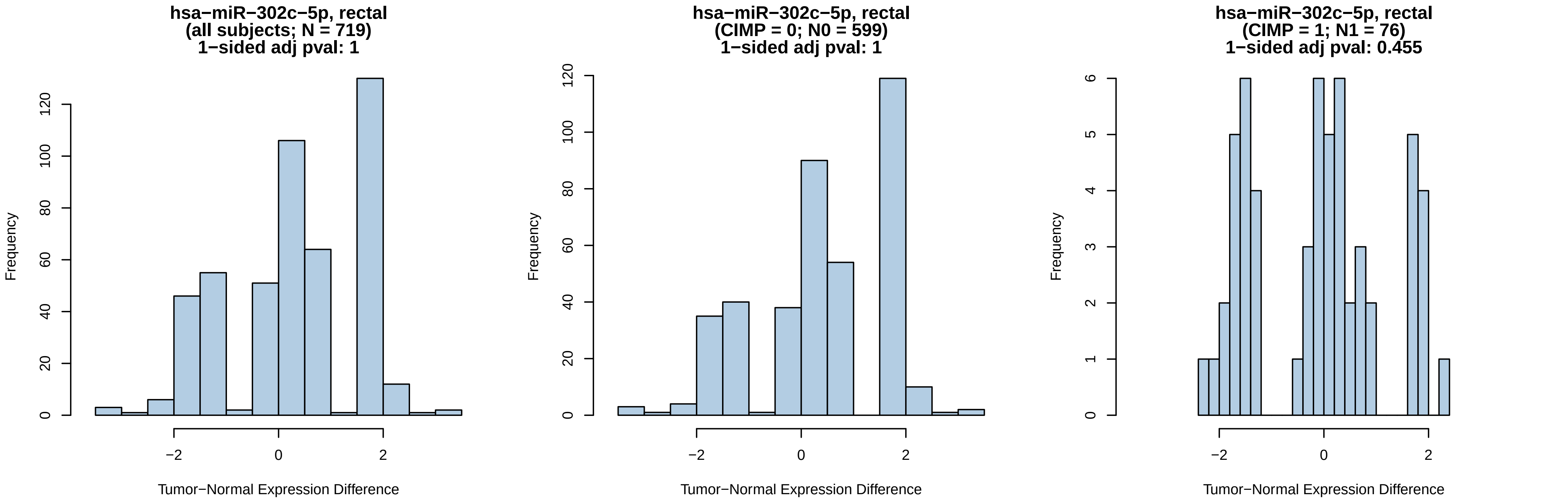

hsa−miR−302c−5p, rectal
(all subjects; N = 719)
1−sided adj pval: 1
Frequency
Tumor−Normal Expression Difference
hsa−miR−302c−5p, rectal
(CIMP = 0; N0 = 599)
1−sided adj pval: 1
Frequency
Tumor−Normal Expression Difference
hsa−miR−302c−5p, rectal
(CIMP = 1; N1 = 76)
1−sided adj pval: 0.455
Frequency
Tumor−Normal Expression Difference

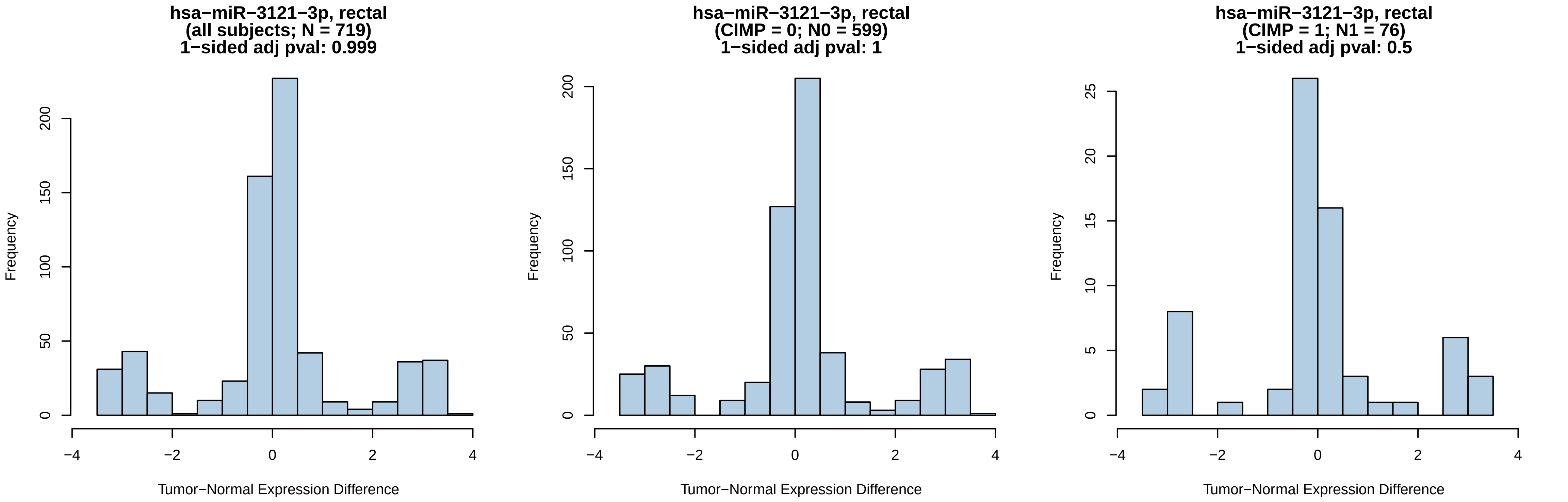
hsa−miR−3121−3p, rectal
(all subjects; N = 719)
1−sided adj pval: 0.999
Frequency
Tumor−Normal Expression Difference
hsa−miR−3121−3p, rectal
(CIMP = 0; N0 = 599)
1−sided adj pval: 1
Frequency
Tumor−Normal Expression Difference
hsa−miR−3121−3p, rectal
(CIMP = 1; N1 = 76)
1−sided adj pval: 0.5
Frequency
Tumor−Normal Expression Difference

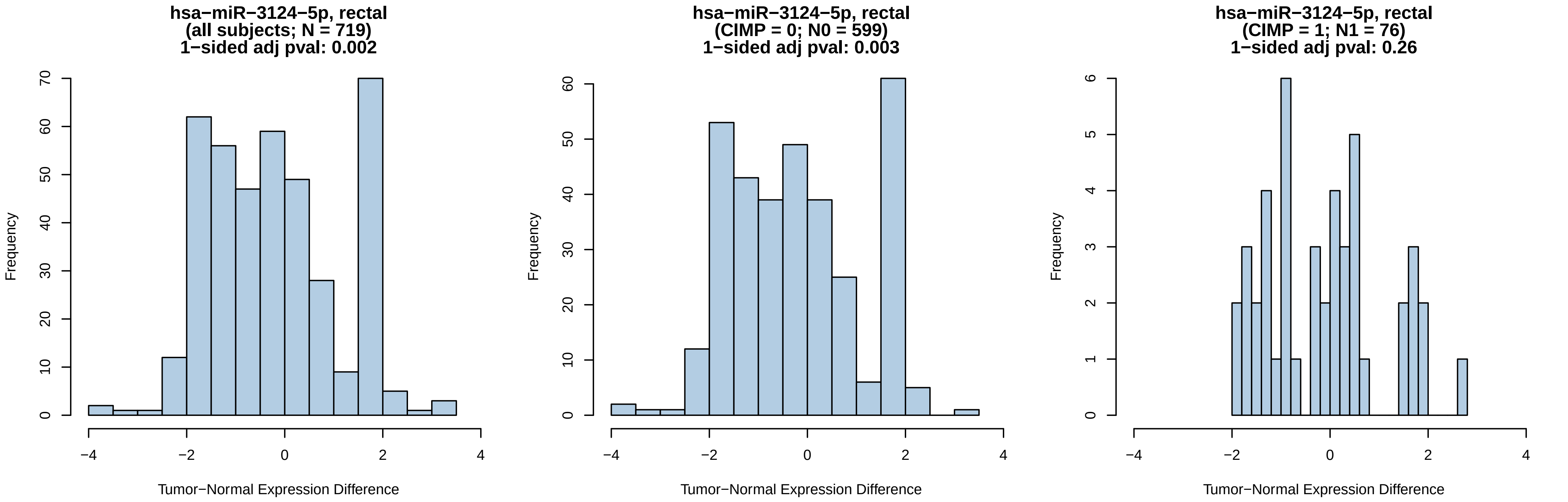

hsa−miR−3124−5p, rectal
(all subjects; N = 719)
1−sided adj pval: 0.002
Frequency
0
10
20
30
40
50
60
70
−4
−2
0
2
4
Tumor−Normal Expression Difference
hsa−miR−3124−5p, rectal
(CIMP = 0; N0 = 599)
1−sided adj pval: 0.003
Frequency
0
10
20
30
40
50
60
−4
−2
0
2
4
Tumor−Normal Expression Difference
hsa−miR−3124−5p, rectal
(CIMP = 1; N1 = 76)
1−sided adj pval: 0.26
Frequency
0
1
2
3
4
5
6
−4
−2
0
2
4
Tumor−Normal Expression Difference

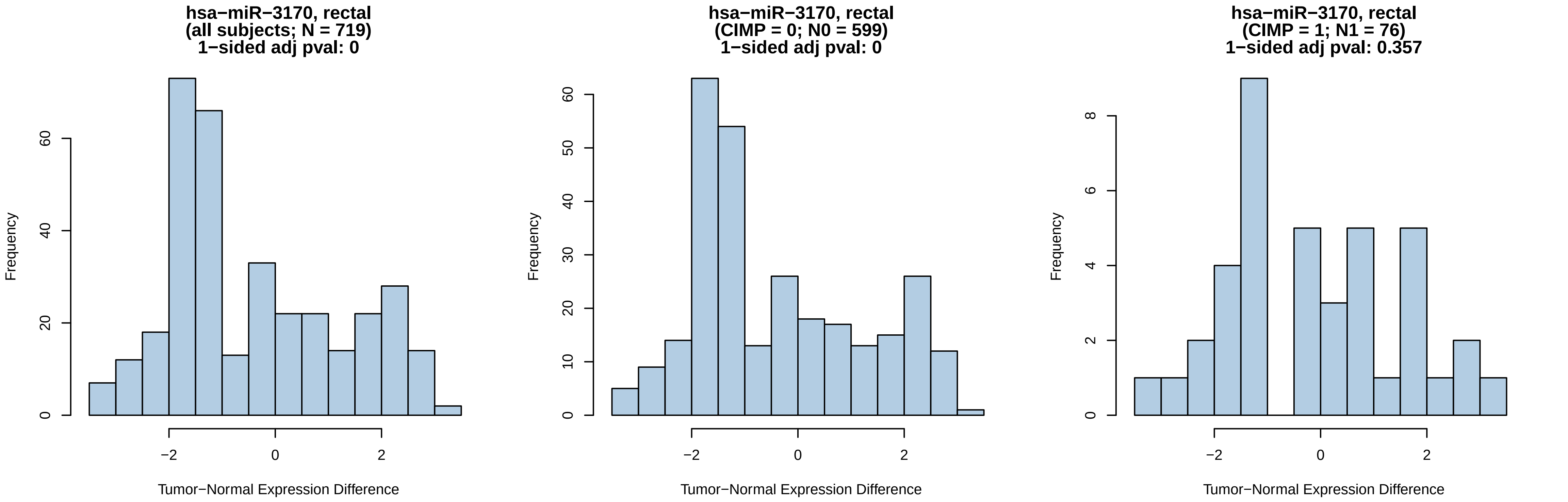

hsa−miR−3170, rectal
(all subjects; N = 719)
1−sided adj pval: 0
Frequency
Tumor−Normal Expression Difference
hsa−miR−3170, rectal
(CIMP = 0; N0 = 599)
1−sided adj pval: 0
Frequency
Tumor−Normal Expression Difference
hsa−miR−3170, rectal
(CIMP = 1; N1 = 76)
1−sided adj pval: 0.357
Frequency
Tumor−Normal Expression Difference

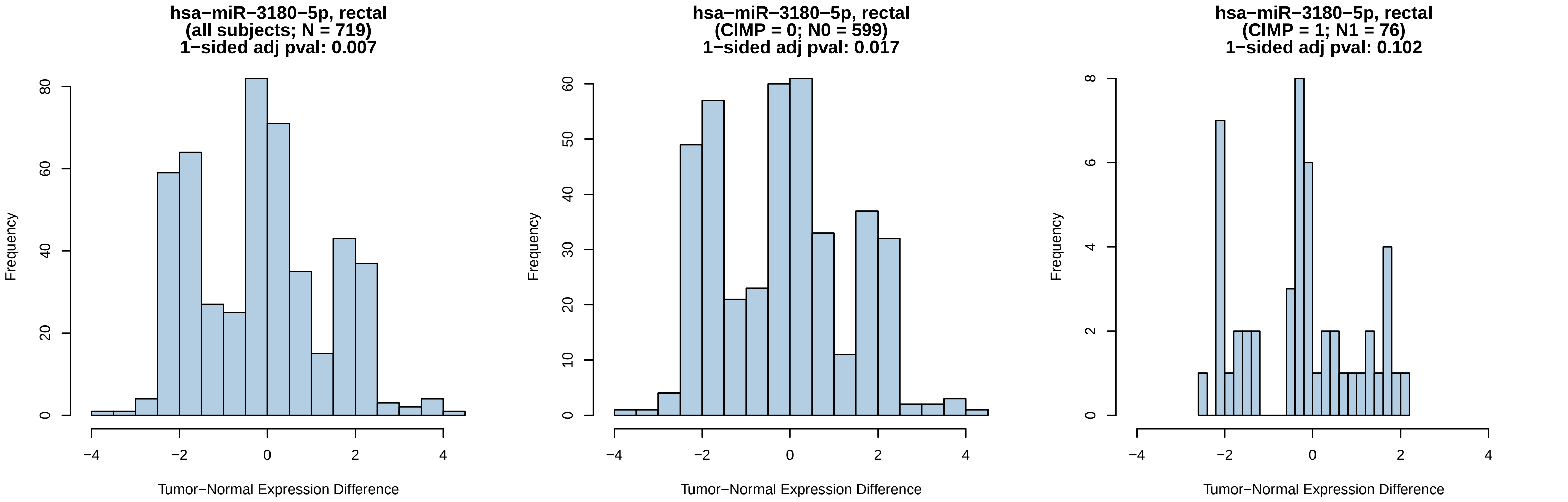

hsa−miR−3180−5p, rectal
(all subjects; N = 719)
1−sided adj pval: 0.007
Frequency
0
20
40
60
80
−4
−2
0
2
4
Tumor−Normal Expression Difference
hsa−miR−3180−5p, rectal
(CIMP = 0; N0 = 599)
1−sided adj pval: 0.017
Frequency
0
10
20
30
40
50
60
−4
−2
0
2
4
Tumor−Normal Expression Difference
hsa−miR−3180−5p, rectal
(CIMP = 1; N1 = 76)
1−sided adj pval: 0.102
Frequency
0
2
4
6
8
−4
−2
0
2
4
Tumor−Normal Expression Difference

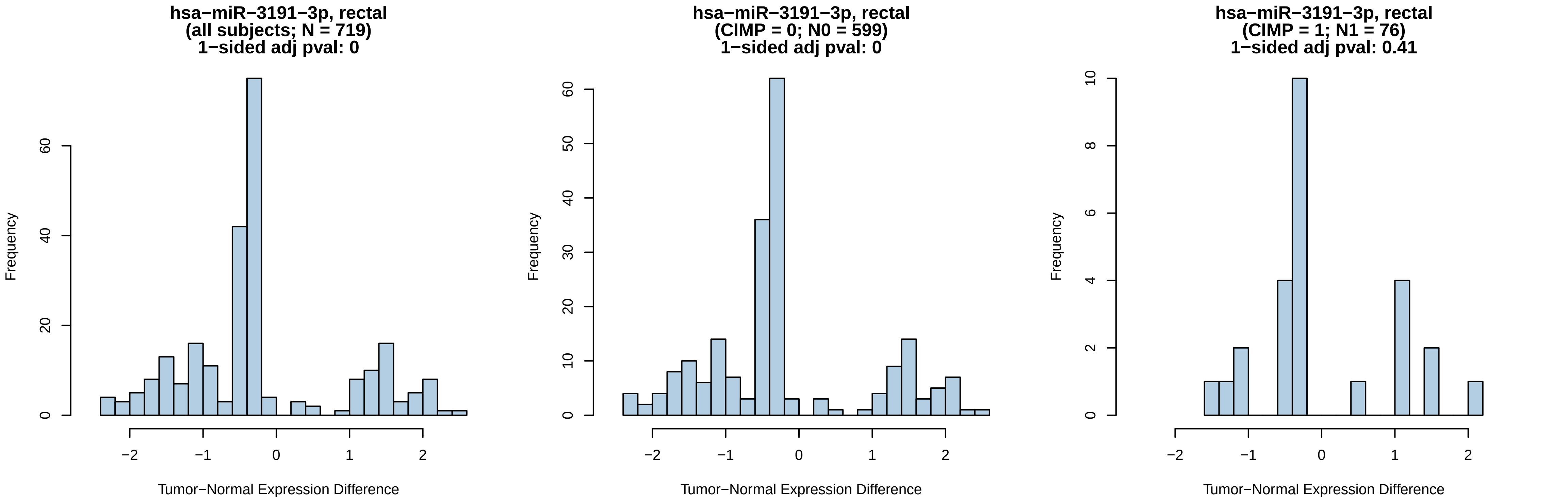
hsa−miR−3191−3p, rectal
(all subjects; N = 719)
1−sided adj pval: 0
Frequency
Tumor−Normal Expression Difference
hsa−miR−3191−3p, rectal
(CIMP = 0; N0 = 599)
1−sided adj pval: 0
Frequency
Tumor−Normal Expression Difference
hsa−miR−3191−3p, rectal
(CIMP = 1; N1 = 76)
1−sided adj pval: 0.41
Frequency
Tumor−Normal Expression Difference

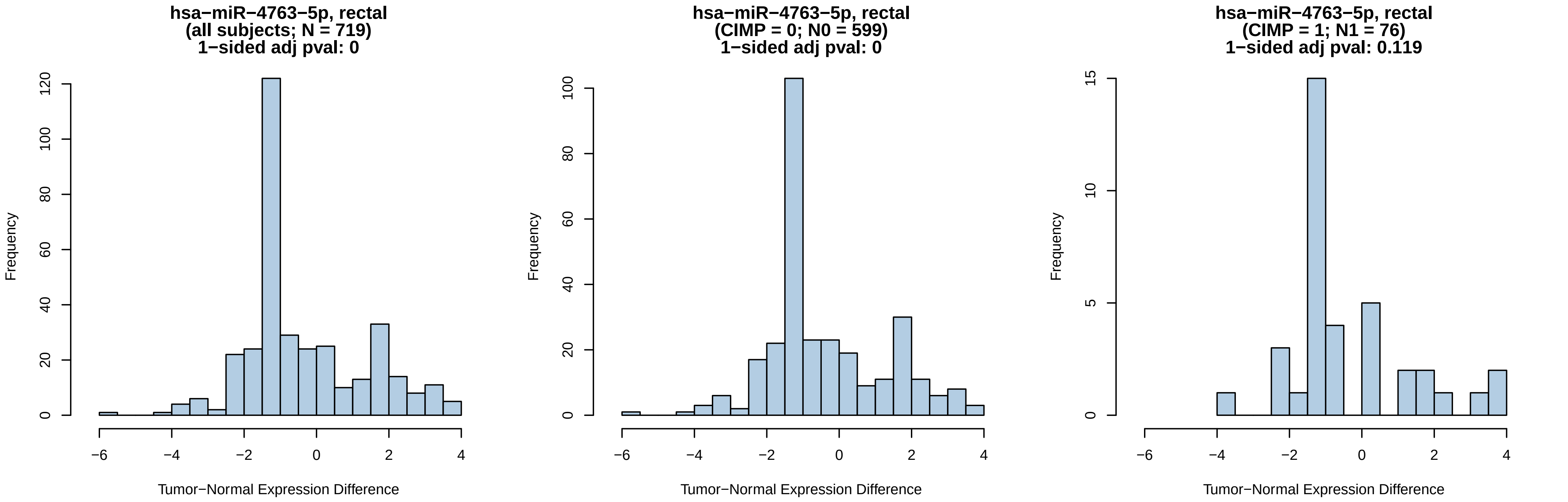

hsa−miR−4763−5p, rectal
(all subjects; N = 719)
1−sided adj pval: 0
Frequency
Tumor−Normal Expression Difference
hsa−miR−4763−5p, rectal
(CIMP = 0; N0 = 599)
1−sided adj pval: 0
Frequency
Tumor−Normal Expression Difference
hsa−miR−4763−5p, rectal
(CIMP = 1; N1 = 76)
1−sided adj pval: 0.119
Frequency
Tumor−Normal Expression Difference

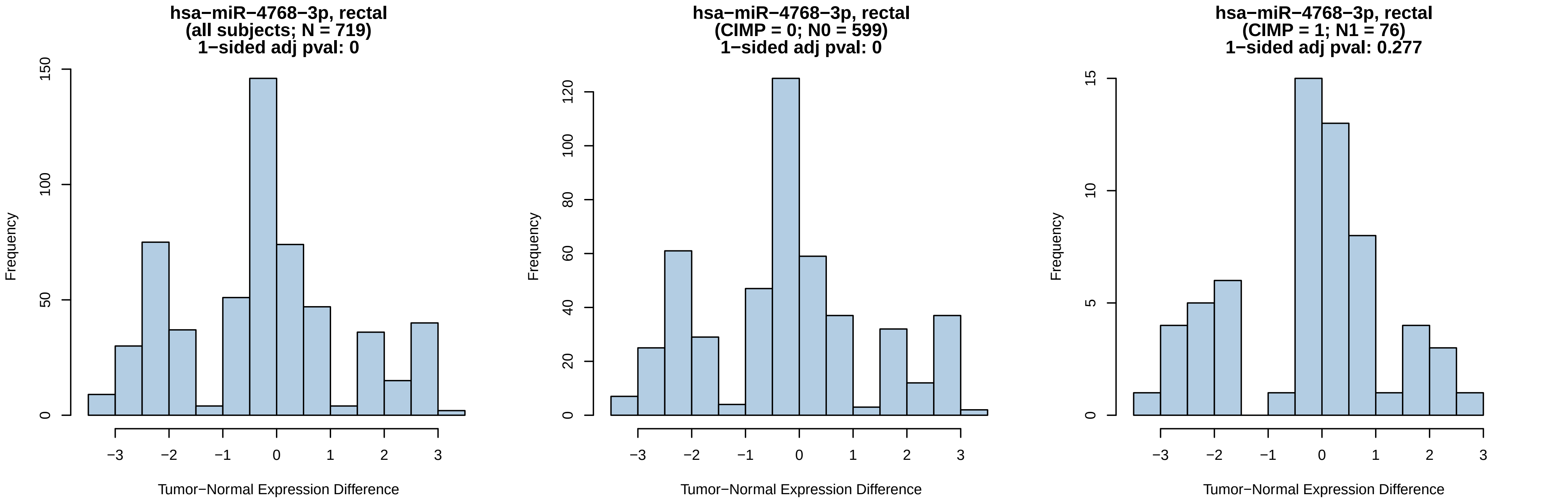
hsa−miR−4768−3p, rectal
(all subjects; N = 719)
1−sided adj pval: 0
Frequency
0
50
100
150
−3
−2
−1
0
1
2
3
Tumor−Normal Expression Difference
hsa−miR−4768−3p, rectal
(CIMP = 0; N0 = 599)
1−sided adj pval: 0
Frequency
0
20
40
60
80
100
120
−3
−2
−1
0
1
2
3
Tumor−Normal Expression Difference
hsa−miR−4768−3p, rectal
(CIMP = 1; N1 = 76)
1−sided adj pval: 0.277
Frequency
0
5
10
15
−3
−2
−1
0
1
2
3
Tumor−Normal Expression Difference

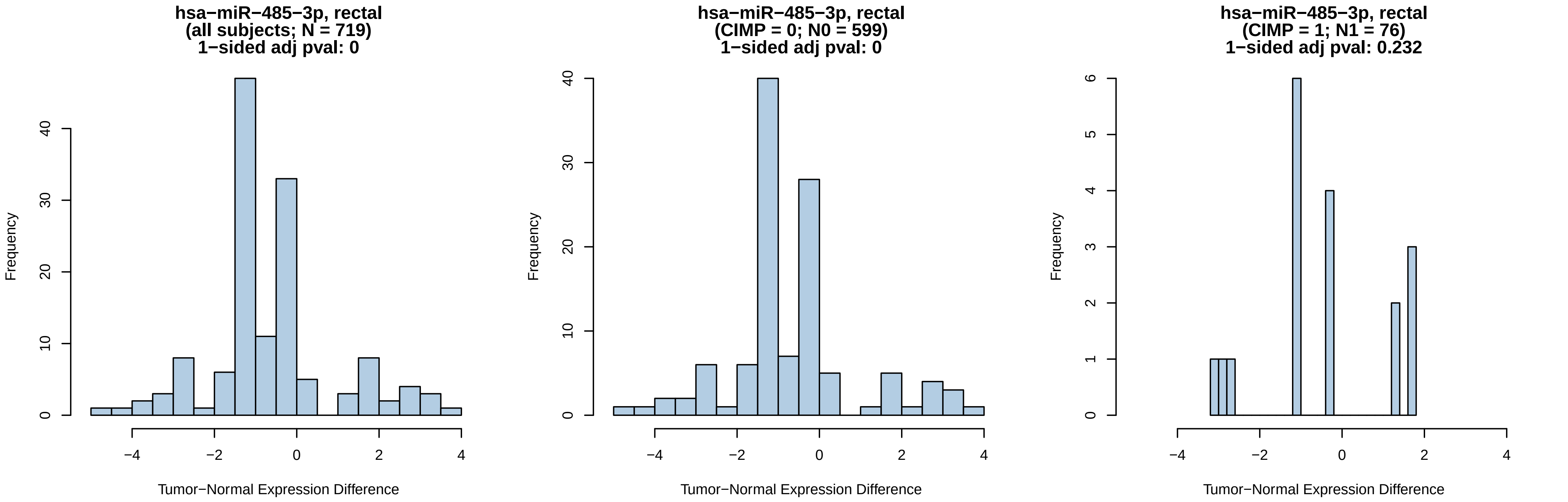

hsa−miR−485−3p, rectal
(all subjects; N = 719)
1−sided adj pval: 0
Frequency
Tumor−Normal Expression Difference
hsa−miR−485−3p, rectal
(CIMP = 0; N0 = 599)
1−sided adj pval: 0
Frequency
Tumor−Normal Expression Difference
hsa−miR−485−3p, rectal
(CIMP = 1; N1 = 76)
1−sided adj pval: 0.232
Frequency
Tumor−Normal Expression Difference

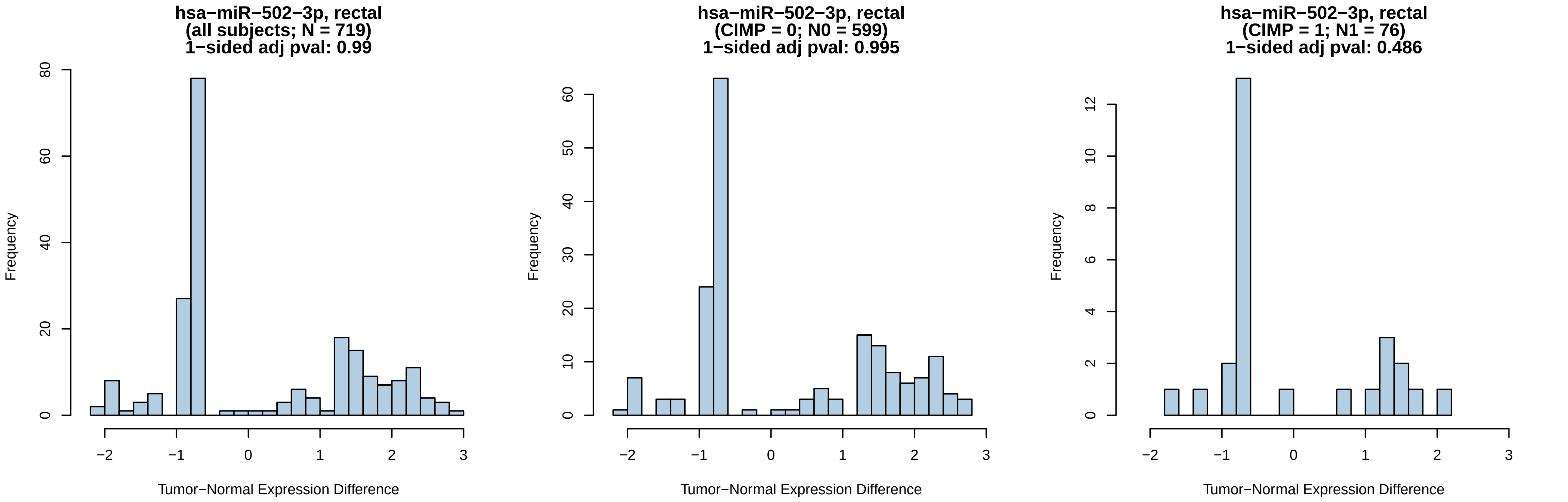
hsa−miR−502−3p, rectal
(all subjects; N = 719)
1−sided adj pval: 0.99
Frequency
Tumor−Normal Expression Difference
hsa−miR−502−3p, rectal
(CIMP = 0; N0 = 599)
1−sided adj pval: 0.995
Frequency
Tumor−Normal Expression Difference
hsa−miR−502−3p, rectal
(CIMP = 1; N1 = 76)
1−sided adj pval: 0.486
Frequency
Tumor−Normal Expression Difference

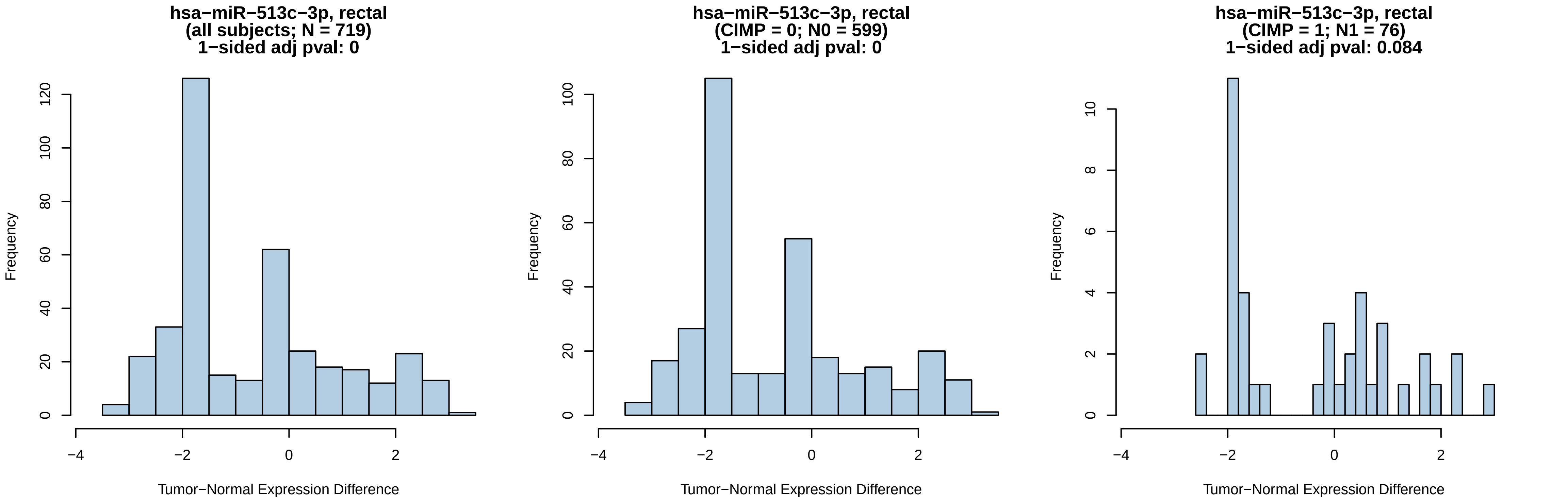
hsa−miR−513c−3p, rectal
(all subjects; N = 719)
1−sided adj pval: 0
Frequency
0
20
40
60
80
100
120
−4
−2
0
2
Tumor−Normal Expression Difference
hsa−miR−513c−3p, rectal
(CIMP = 0; N0 = 599)
1−sided adj pval: 0
Frequency
0
20
40
60
80
100
−4
−2
0
2
Tumor−Normal Expression Difference
hsa−miR−513c−3p, rectal
(CIMP = 1; N1 = 76)
1−sided adj pval: 0.084
Frequency
0
2
4
6
8
10
−4
−2
0
2
Tumor−Normal Expression Difference

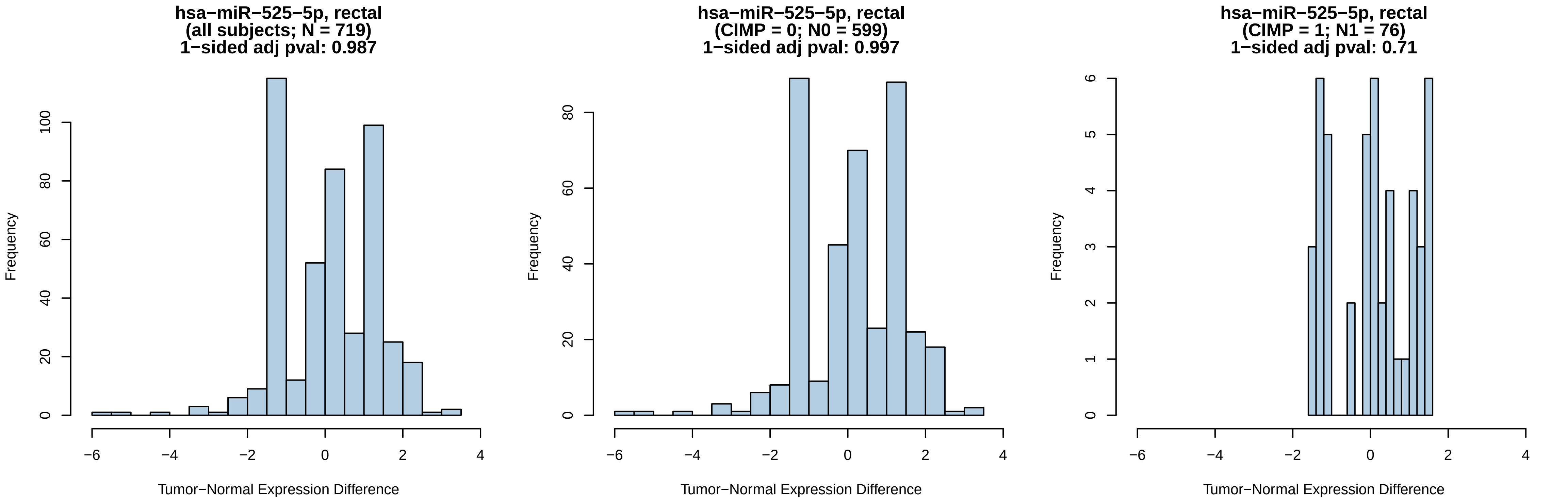
hsa−miR−525−5p, rectal
(all subjects; N = 719)
1−sided adj pval: 0.987
Frequency
Tumor−Normal Expression Difference
hsa−miR−525−5p, rectal
(CIMP = 0; N0 = 599)
1−sided adj pval: 0.997
Frequency
Tumor−Normal Expression Difference
hsa−miR−525−5p, rectal
(CIMP = 1; N1 = 76)
1−sided adj pval: 0.71
Frequency
Tumor−Normal Expression Difference

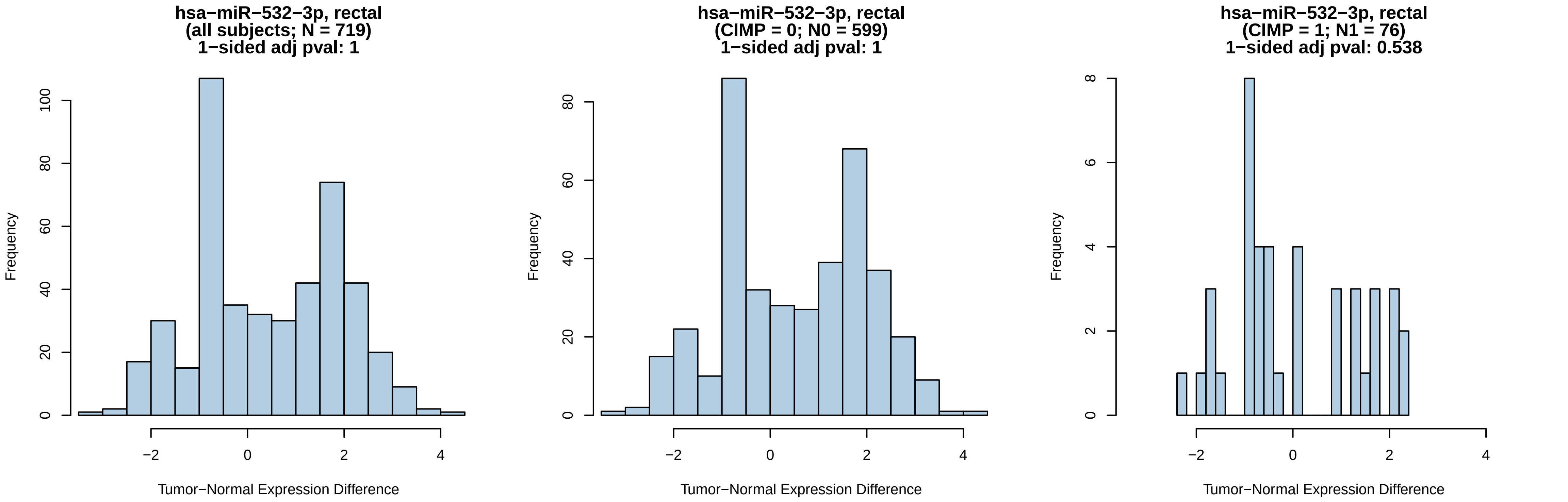

hsa−miR−532−3p, rectal
(all subjects; N = 719)
1−sided adj pval: 1
Frequency
Tumor−Normal Expression Difference
hsa−miR−532−3p, rectal
(CIMP = 0; N0 = 599)
1−sided adj pval: 1
Frequency
Tumor−Normal Expression Difference
hsa−miR−532−3p, rectal
(CIMP = 1; N1 = 76)
1−sided adj pval: 0.538
Frequency
Tumor−Normal Expression Difference

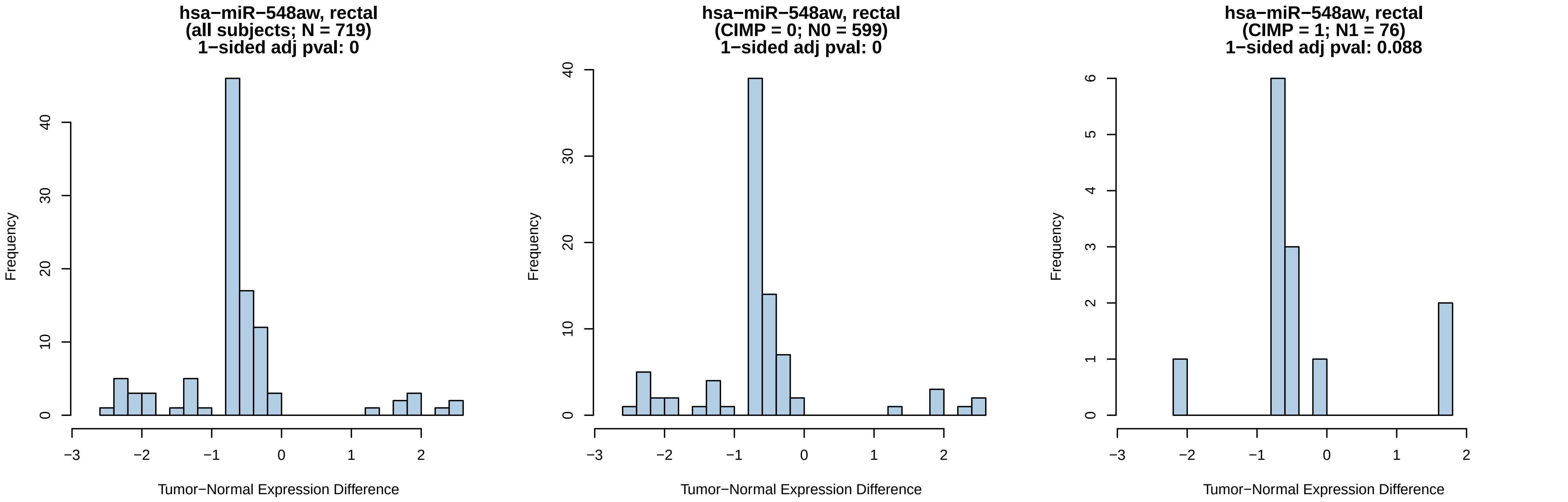
hsa−miR−548aw, rectal
(all subjects; N = 719)
1−sided adj pval: 0
Frequency
Tumor−Normal Expression Difference
hsa−miR−548aw, rectal
(CIMP = 0; N0 = 599)
1−sided adj pval: 0
Frequency
Tumor−Normal Expression Difference
hsa−miR−548aw, rectal
(CIMP = 1; N1 = 76)
1−sided adj pval: 0.088
Frequency
Tumor−Normal Expression Difference

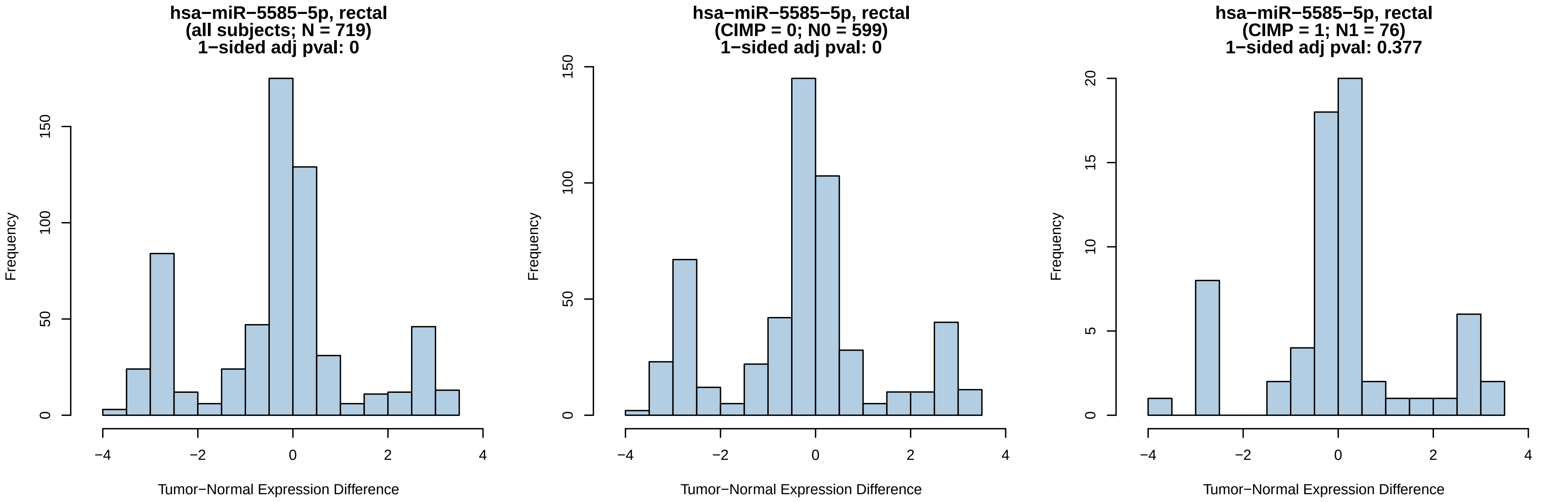
hsa−miR−5585−5p, rectal
(all subjects; N = 719)
1−sided adj pval: 0
hsa−miR−5585−5p, rectal
(CIMP = 0; N0 = 599)
1−sided adj pval: 0
hsa−miR−5585−5p, rectal
(CIMP = 1; N1 = 76)
1−sided adj pval: 0.377
Frequency
Tumor−Normal Expression Difference

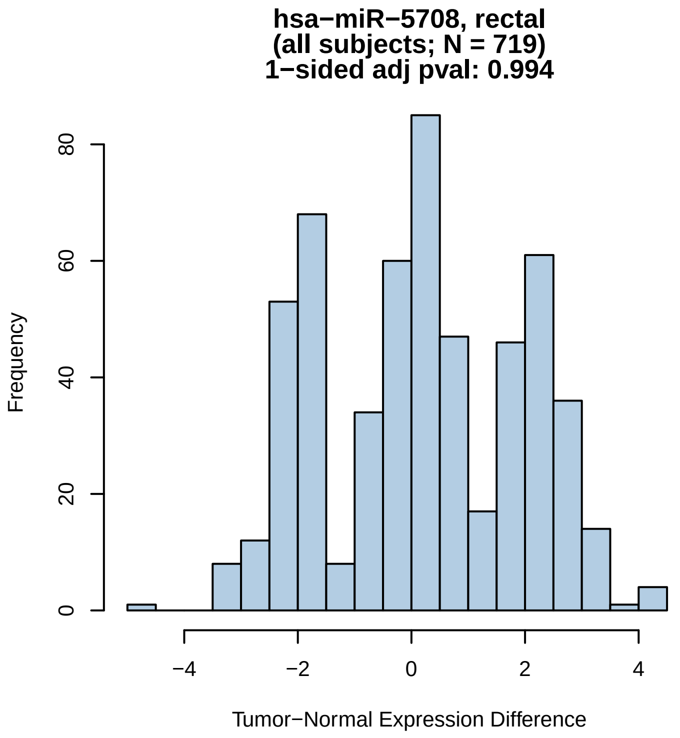

hsa−miR−5708, rectal
(all subjects; N = 719)
1−sided adj pval: 0.994
Frequency
Tumor−Normal Expression Difference


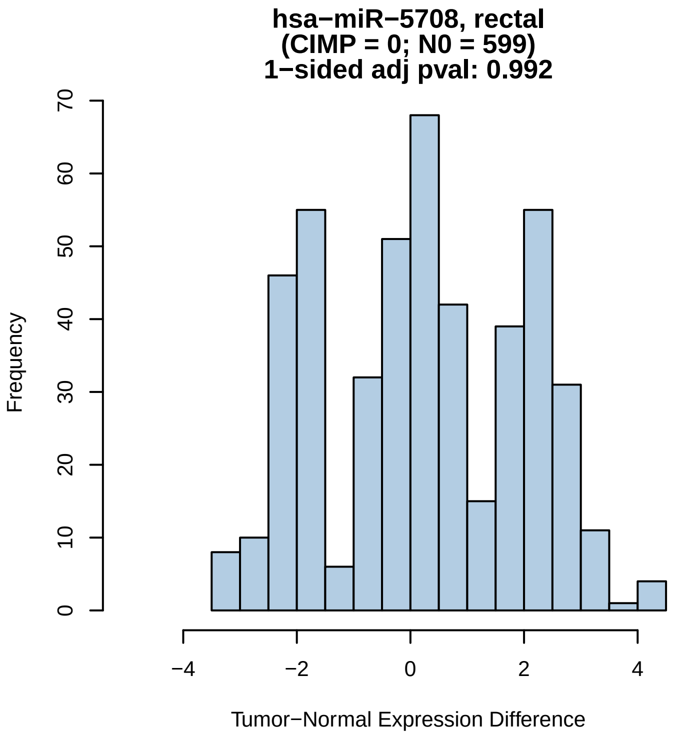

hsa−miR−5708, rectal
(CIMP = 0; N0 = 599)
1−sided adj pval: 0.992
Frequency
Tumor−Normal Expression Difference


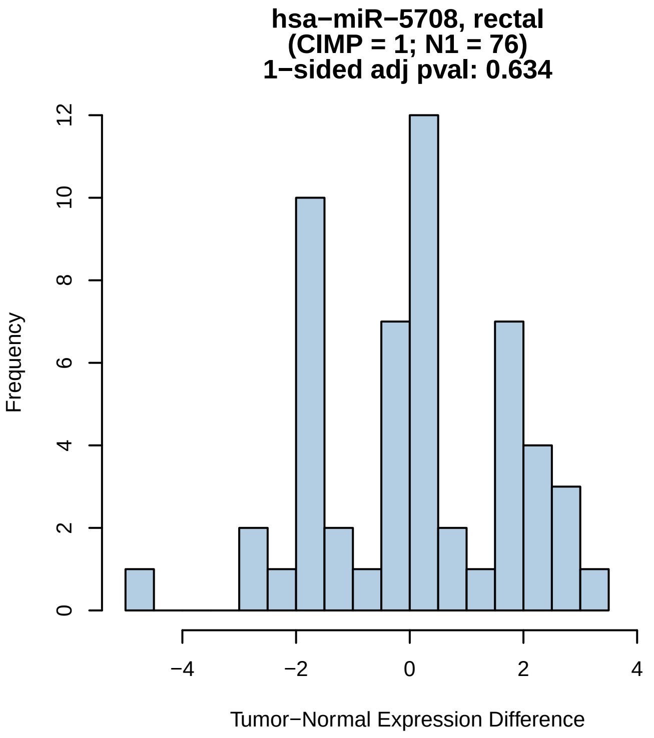

hsa−miR−5708, rectal
(CIMP = 1; N1 = 76)
1−sided adj pval: 0.634
Frequency
Tumor−Normal Expression Difference

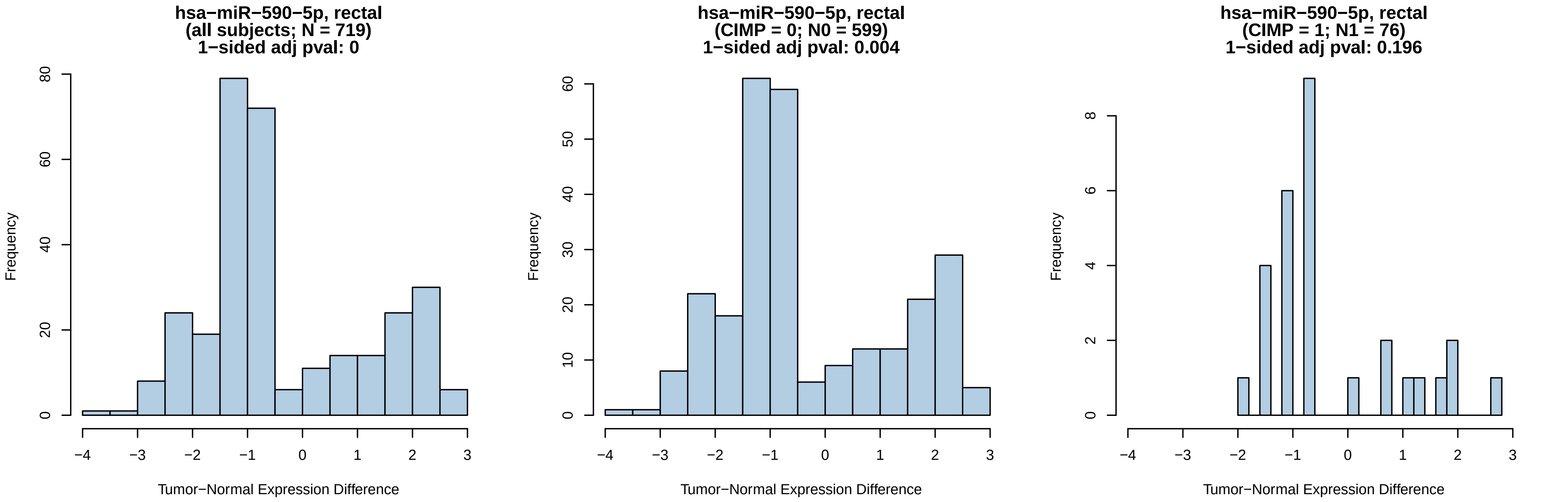
hsa−miR−590−5p, rectal
(all subjects; N = 719)
1−sided adj pval: 0
Frequency
Tumor−Normal Expression Difference
hsa−miR−590−5p, rectal
(CIMP = 0; N0 = 599)
1−sided adj pval: 0.004
Frequency
Tumor−Normal Expression Difference
hsa−miR−590−5p, rectal
(CIMP = 1; N1 = 76)
1−sided adj pval: 0.196
Frequency
Tumor−Normal Expression Difference

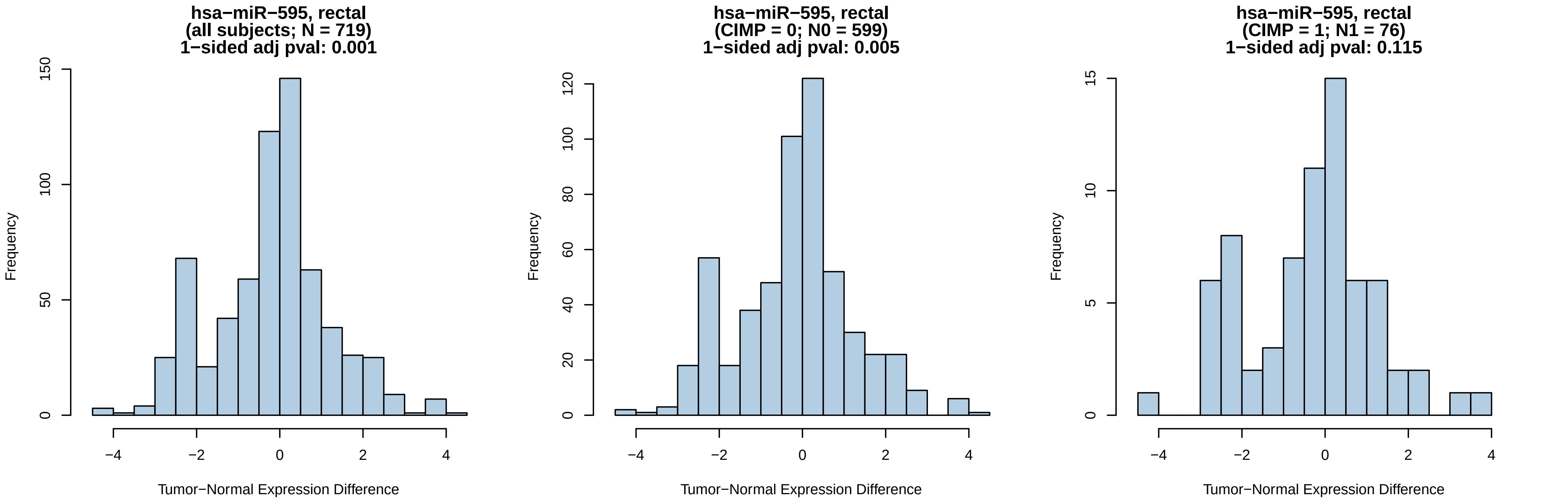

hsa−miR−595, rectal
(all subjects; N = 719)
1−sided adj pval: 0.001
hsa−miR−595, rectal
(CIMP = 0; N0 = 599)
1−sided adj pval: 0.005
hsa−miR−595, rectal
(CIMP = 1; N1 = 76)
1−sided adj pval: 0.115
Frequency
Tumor−Normal Expression Difference
0
50
100
150
0
20
40
60
80
100
120
0
5
10
15
−4
−2
0
2
4

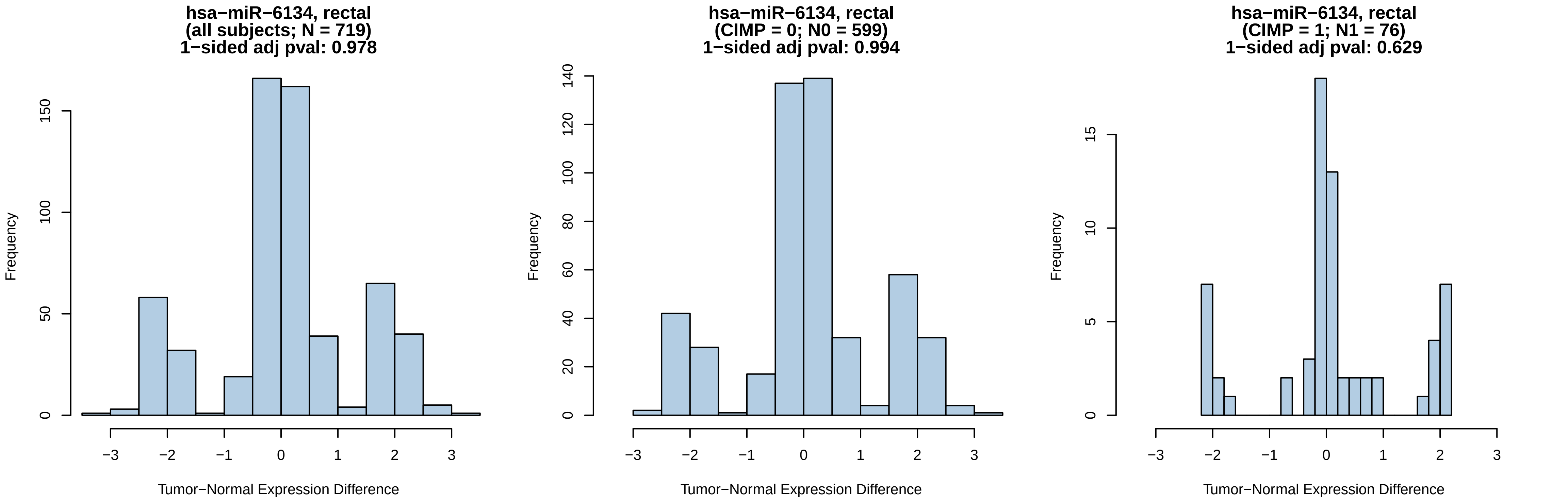

hsa−miR−6134, rectal
(all subjects; N = 719)
1−sided adj pval: 0.978
Frequency
Tumor−Normal Expression Difference
hsa−miR−6134, rectal
(CIMP = 0; N0 = 599)
1−sided adj pval: 0.994
Frequency
Tumor−Normal Expression Difference
hsa−miR−6134, rectal
(CIMP = 1; N1 = 76)
1−sided adj pval: 0.629
Frequency
Tumor−Normal Expression Difference

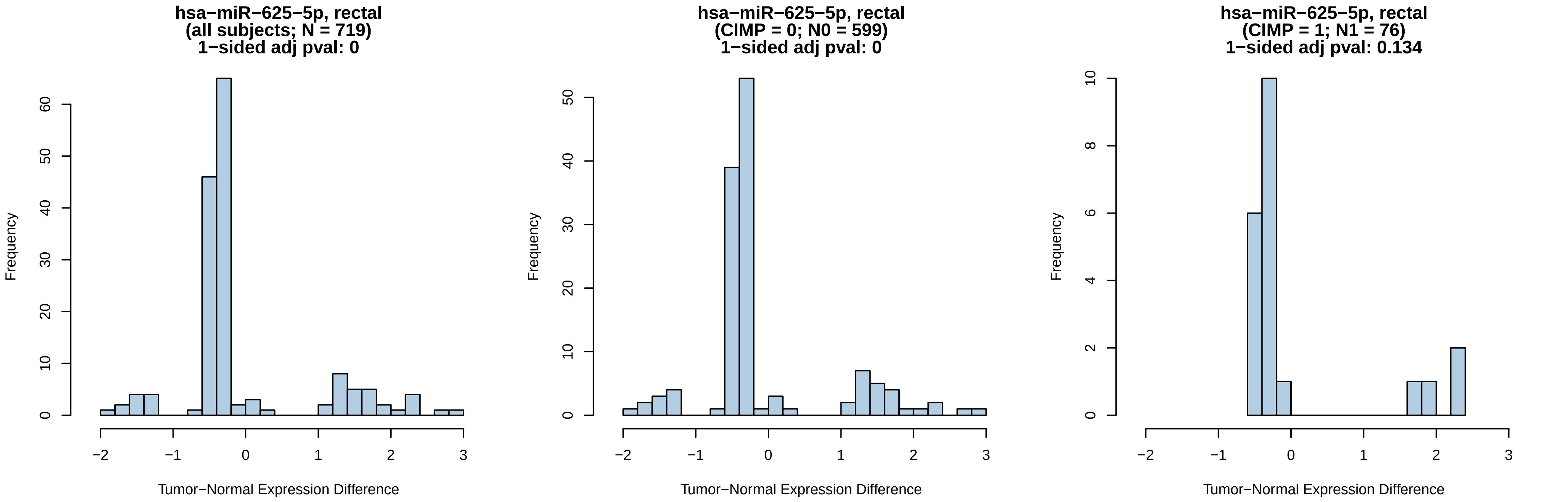
hsa−miR−625−5p, rectal
(all subjects; N = 719)
1−sided adj pval: 0
hsa−miR−625−5p, rectal
(CIMP = 0; N0 = 599)
1−sided adj pval: 0
hsa−miR−625−5p, rectal
(CIMP = 1; N1 = 76)
1−sided adj pval: 0.134
Frequency
Tumor−Normal Expression Difference

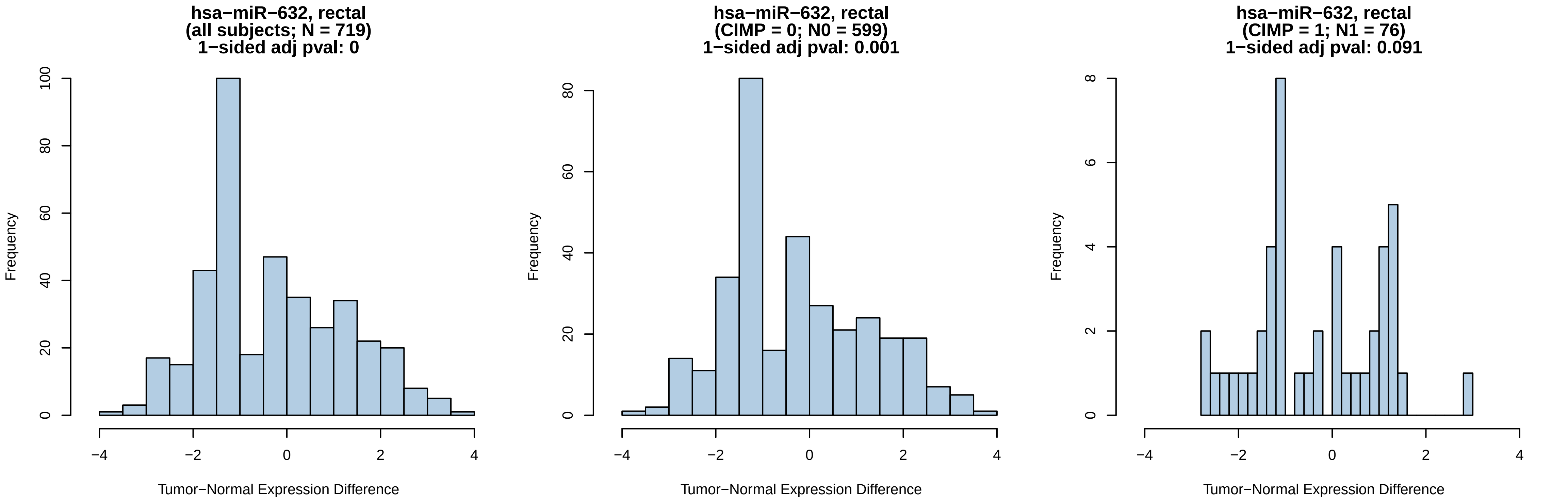

hsa−miR−632, rectal
(all subjects; N = 719)
1−sided adj pval: 0
Frequency
Tumor−Normal Expression Difference
hsa−miR−632, rectal
(CIMP = 0; N0 = 599)
1−sided adj pval: 0.001
Frequency
Tumor−Normal Expression Difference
hsa−miR−632, rectal
(CIMP = 1; N1 = 76)
1−sided adj pval: 0.091
Frequency
Tumor−Normal Expression Difference

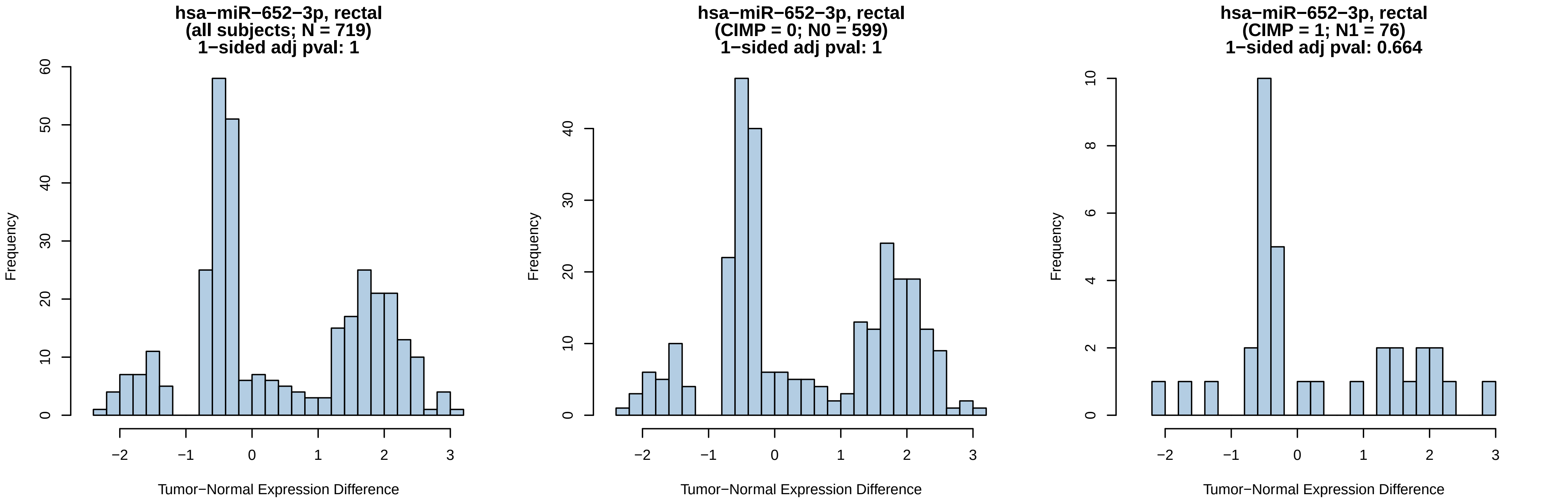

hsa−miR−652−3p, rectal
(all subjects; N = 719)
1−sided adj pval: 1
Frequency
Tumor−Normal Expression Difference
hsa−miR−652−3p, rectal
(CIMP = 0; N0 = 599)
1−sided adj pval: 1
Frequency
Tumor−Normal Expression Difference
hsa−miR−652−3p, rectal
(CIMP = 1; N1 = 76)
1−sided adj pval: 0.664
Frequency
Tumor−Normal Expression Difference

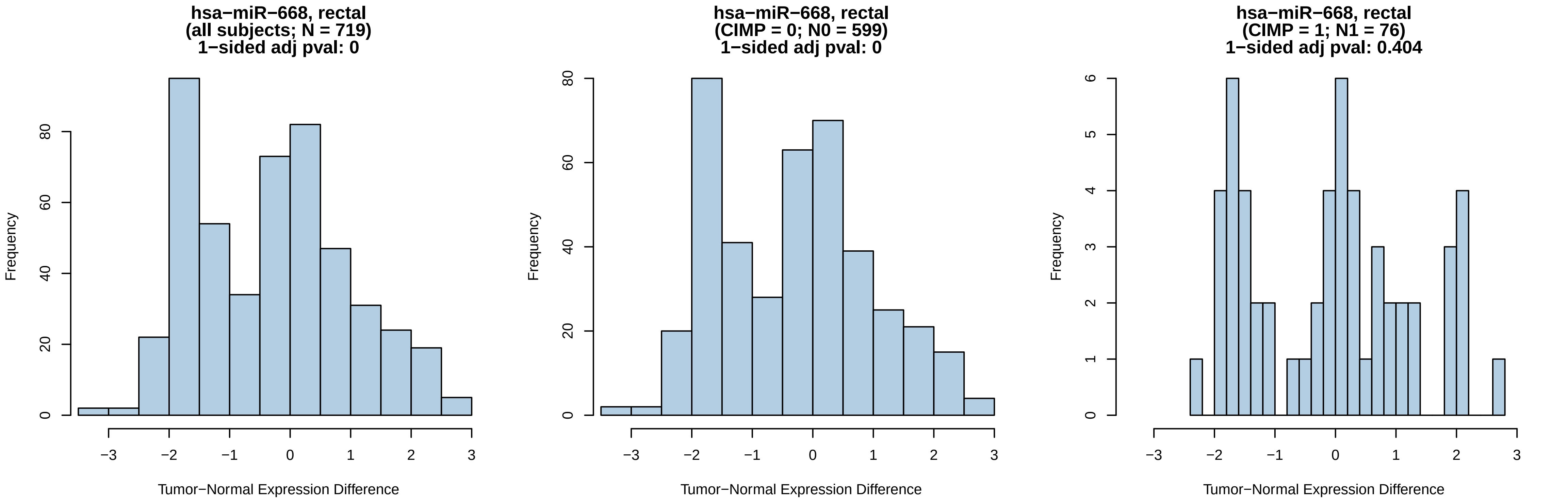
hsa−miR−668, rectal
(all subjects; N = 719)
1−sided adj pval: 0
Frequency
Tumor−Normal Expression Difference
hsa−miR−668, rectal
(CIMP = 0; N0 = 599)
1−sided adj pval: 0
Frequency
Tumor−Normal Expression Difference
hsa−miR−668, rectal
(CIMP = 1; N1 = 76)
1−sided adj pval: 0.404
Frequency
Tumor−Normal Expression Difference

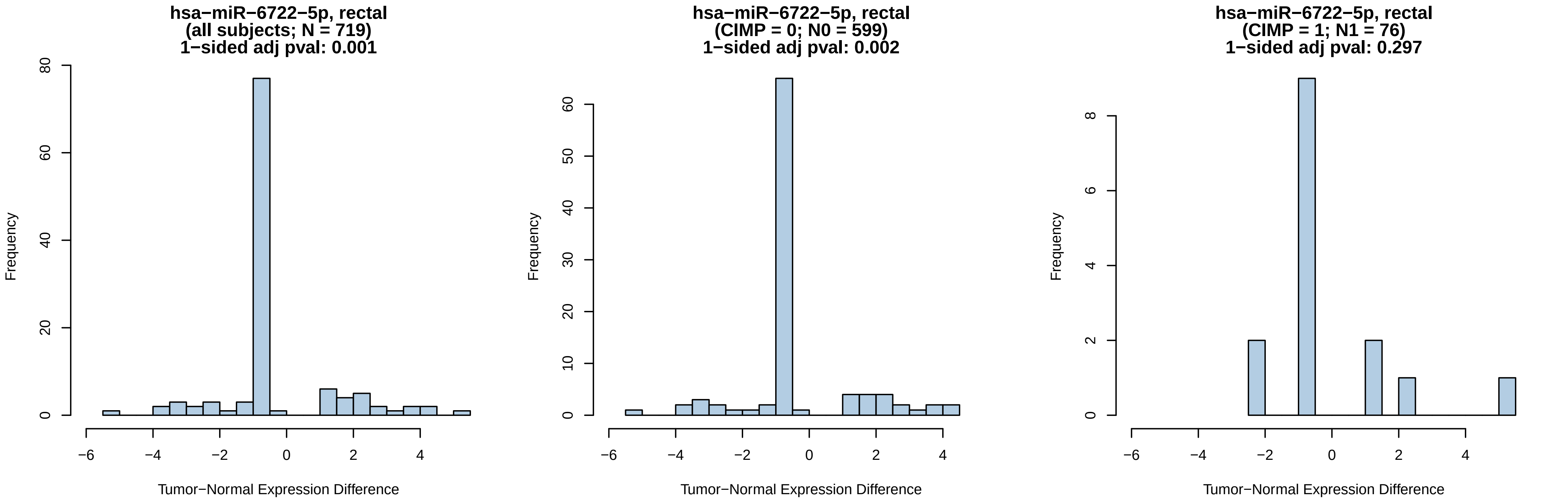
hsa−miR−6722−5p, rectal
(all subjects; N = 719)
1−sided adj pval: 0.001
hsa−miR−6722−5p, rectal
(CIMP = 0; N0 = 599)
1−sided adj pval: 0.002
hsa−miR−6722−5p, rectal
(CIMP = 1; N1 = 76)
1−sided adj pval: 0.297
Frequency
Tumor−Normal Expression Difference

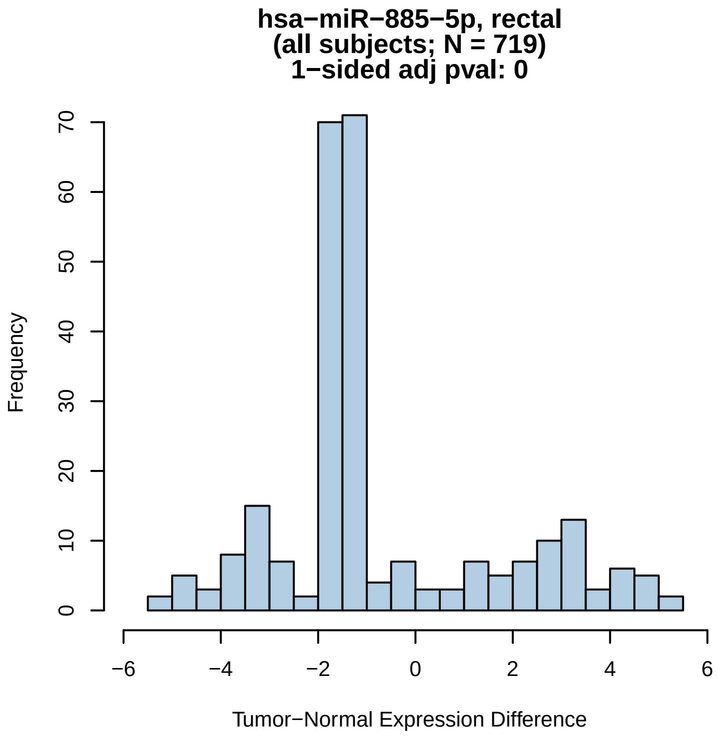

hsa−miR−885−5p, rectal
(all subjects; N = 719)
1−sided adj pval: 0
Frequency
0
10
20
30
40
50
60
70
−6
−4
−2
0
2
4
6
Tumor−Normal Expression Difference


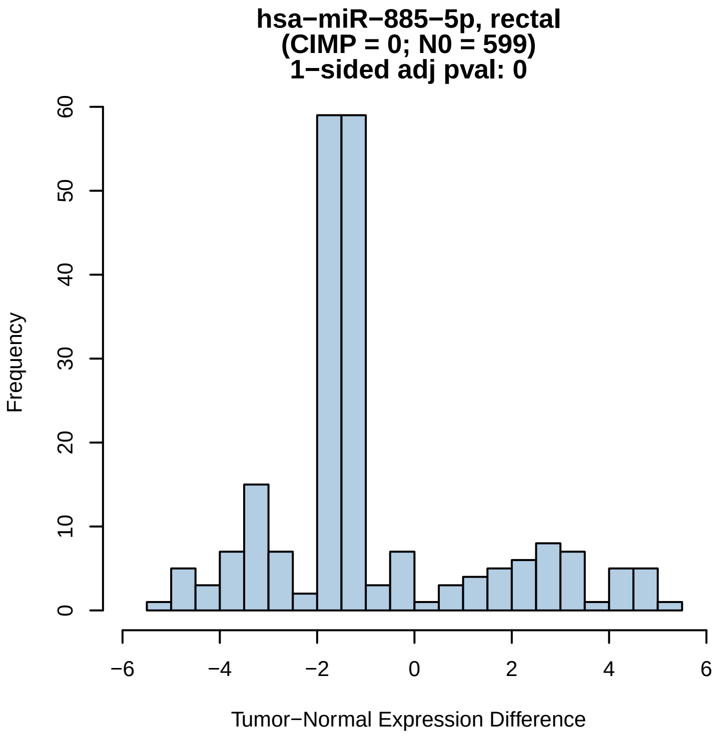

hsa−miR−885−5p, rectal
(CIMP = 0; N0 = 599)
1−sided adj pval: 0
Frequency
0
10
20
30
40
50
60
−6
−4
−2
0
2
4
6
Tumor−Normal Expression Difference


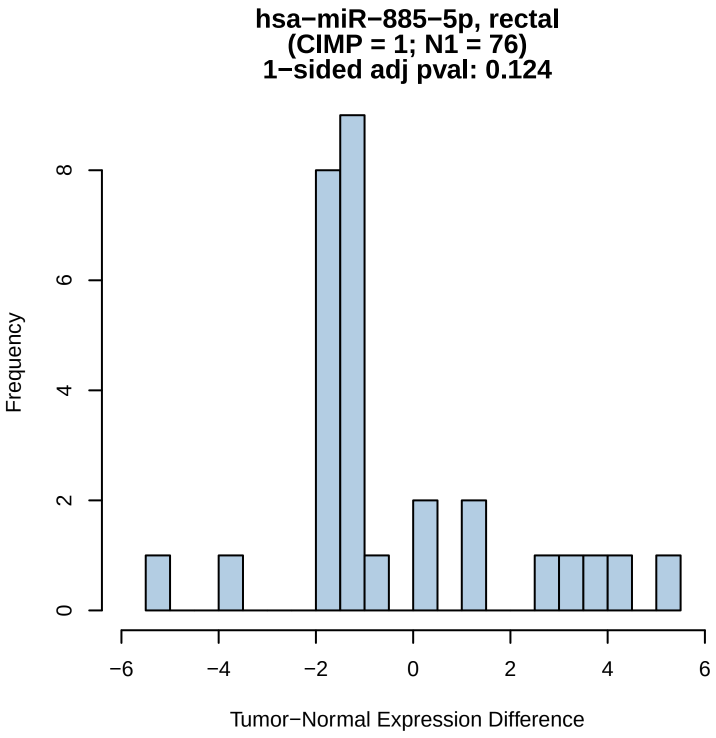

hsa−miR−885−5p, rectal
(CIMP = 1; N1 = 76)
1−sided adj pval: 0.124
Frequency
0
2
4
6
8
−6
−4
−2
0
2
4
6
Tumor−Normal Expression Difference

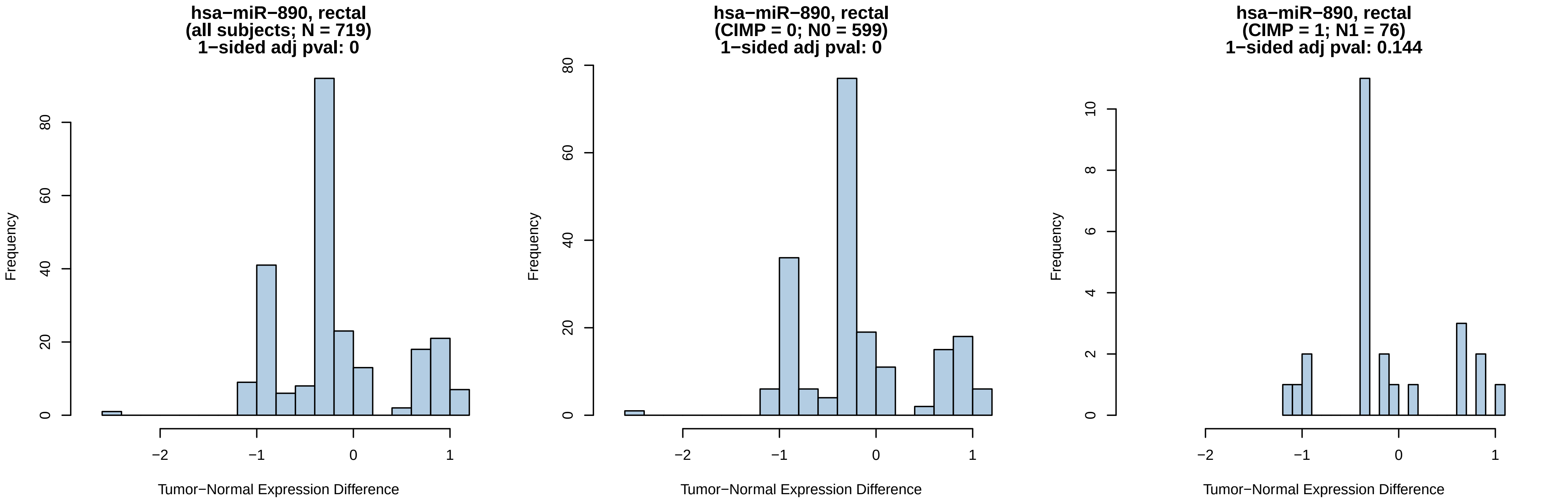

hsa−miR−890, rectal
(all subjects; N = 719)
1−sided adj pval: 0
Frequency
0
20
40
60
80
−2
−1
0
1
Tumor−Normal Expression Difference
hsa−miR−890, rectal
(CIMP = 0; N0 = 599)
1−sided adj pval: 0
Frequency
0
20
40
60
80
−2
−1
0
1
Tumor−Normal Expression Difference
hsa−miR−890, rectal
(CIMP = 1; N1 = 76)
1−sided adj pval: 0.144
Frequency
0
2
4
6
8
10
−2
−1
0
1
Tumor−Normal Expression Difference

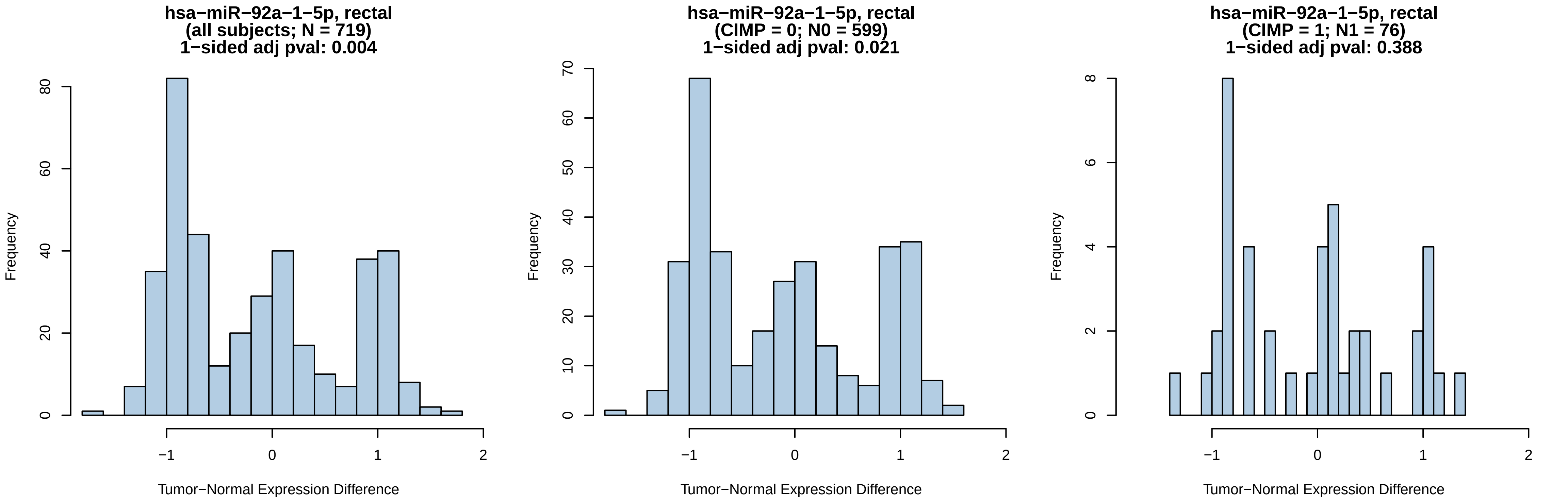
hsa−miR−92a−1−5p, rectal
(all subjects; N = 719)
1−sided adj pval: 0.004
Frequency
Tumor−Normal Expression Difference
hsa−miR−92a−1−5p, rectal
(CIMP = 0; N0 = 599)
1−sided adj pval: 0.021
Frequency
Tumor−Normal Expression Difference
hsa−miR−92a−1−5p, rectal
(CIMP = 1; N1 = 76)
1−sided adj pval: 0.388
Frequency
Tumor−Normal Expression Difference

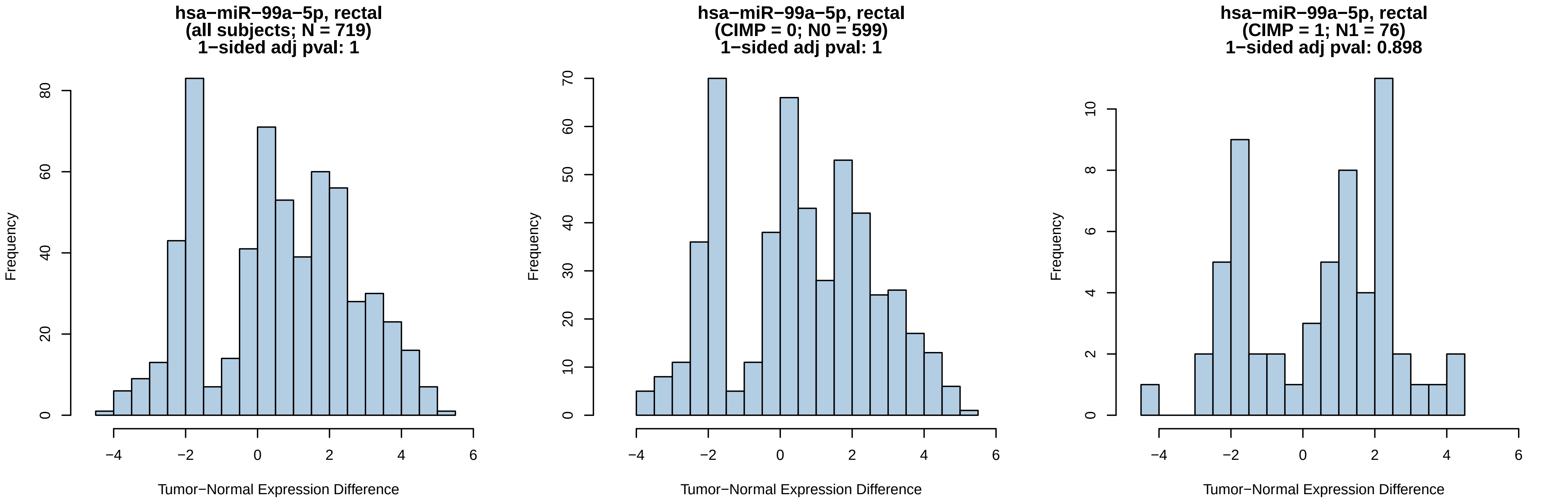
hsa−miR−99a−5p, rectal
(all subjects; N = 719)
1−sided adj pval: 1
Frequency
Tumor−Normal Expression Difference
hsa−miR−99a−5p, rectal
(CIMP = 0; N0 = 599)
1−sided adj pval: 1
Frequency
Tumor−Normal Expression Difference
hsa−miR−99a−5p, rectal
(CIMP = 1; N1 = 76)
1−sided adj pval: 0.898
Frequency
Tumor−Normal Expression Difference

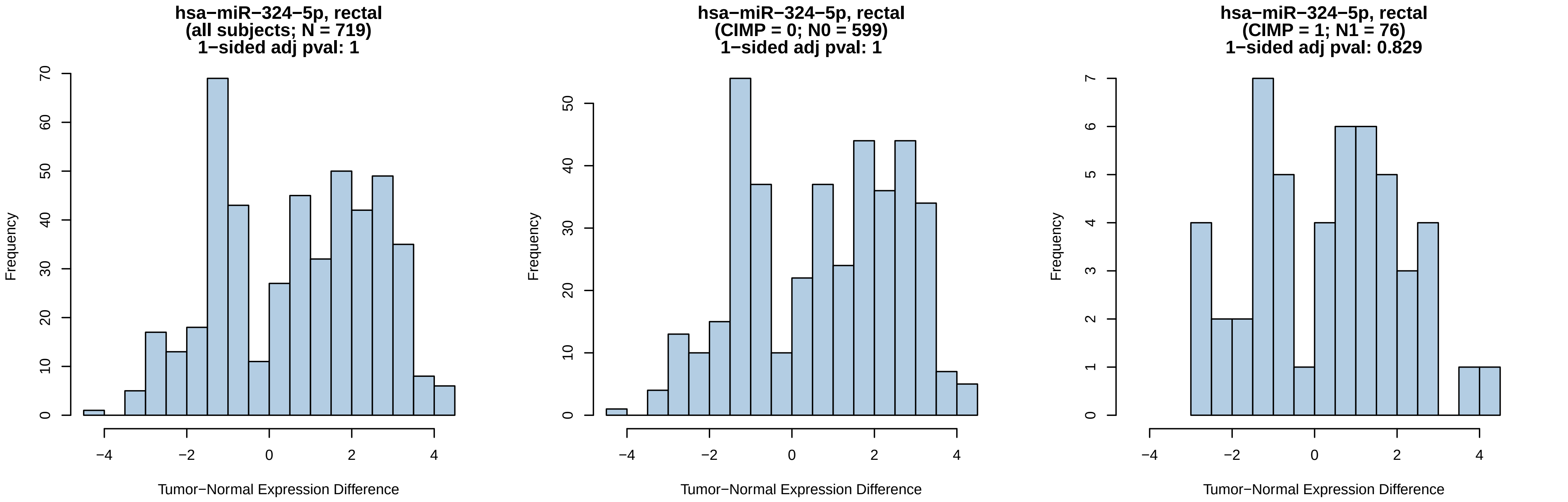

hsa−miR−324−5p, rectal
(all subjects; N = 719)
1−sided adj pval: 1
Frequency
Tumor−Normal Expression Difference
hsa−miR−324−5p, rectal
(CIMP = 0; N0 = 599)
1−sided adj pval: 1
Frequency
Tumor−Normal Expression Difference
hsa−miR−324−5p, rectal
(CIMP = 1; N1 = 76)
1−sided adj pval: 0.829
Frequency
Tumor−Normal Expression Difference

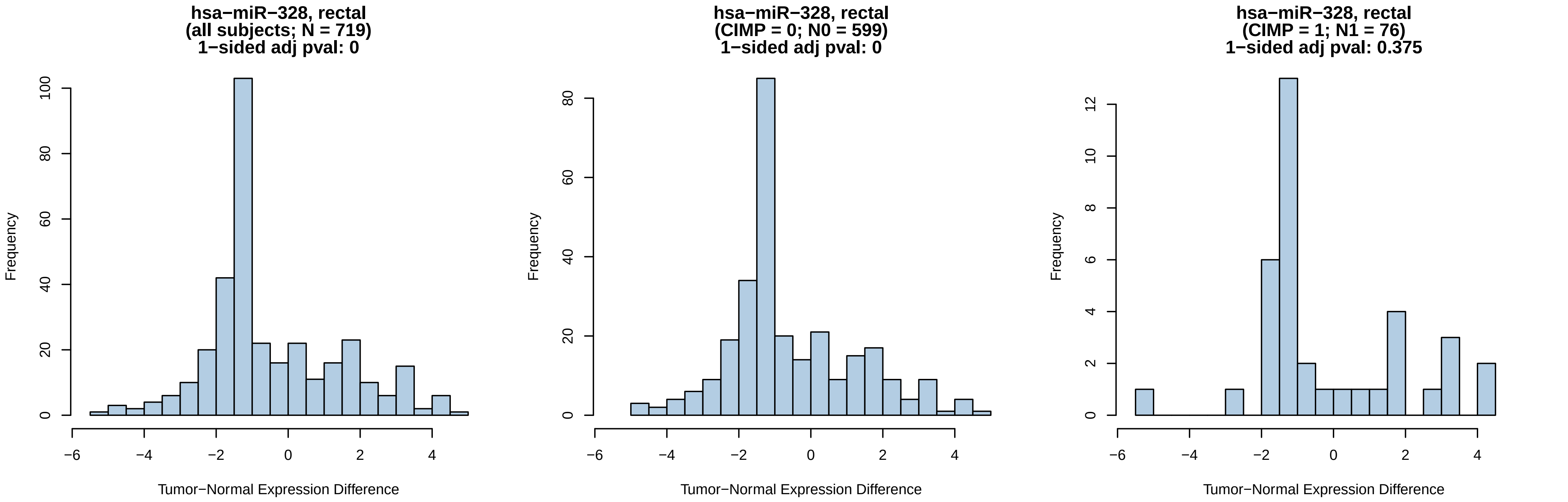

hsa−miR−328, rectal
(all subjects; N = 719)
1−sided adj pval: 0
Frequency
Tumor−Normal Expression Difference
hsa−miR−328, rectal
(CIMP = 0; N0 = 599)
1−sided adj pval: 0
Frequency
Tumor−Normal Expression Difference
hsa−miR−328, rectal
(CIMP = 1; N1 = 76)
1−sided adj pval: 0.375
Frequency
Tumor−Normal Expression Difference

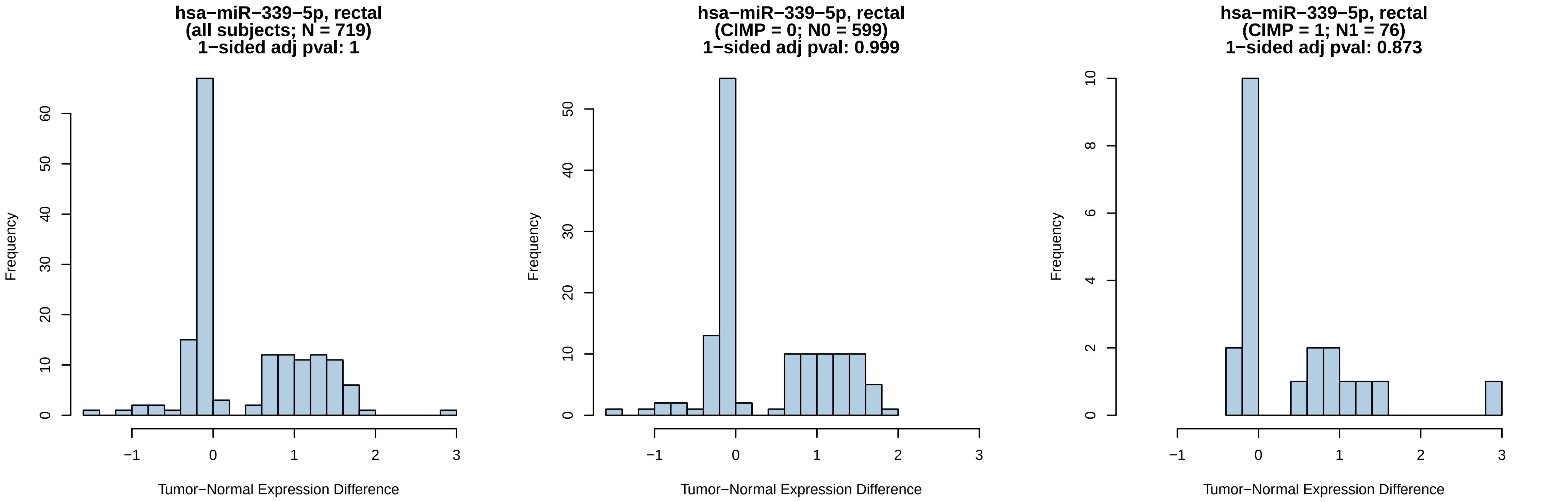
hsa−miR−339−5p, rectal
(all subjects; N = 719)
1−sided adj pval: 1
Frequency
0
10
20
30
40
50
60
−1
0
1
2
3
Tumor−Normal Expression Difference
hsa−miR−339−5p, rectal
(CIMP = 0; N0 = 599)
1−sided adj pval: 0.999
Frequency
0
10
20
30
40
50
−1
0
1
2
3
Tumor−Normal Expression Difference
hsa−miR−339−5p, rectal
(CIMP = 1; N1 = 76)
1−sided adj pval: 0.873
Frequency
0
2
4
6
8
10
−1
0
1
2
3
Tumor−Normal Expression Difference

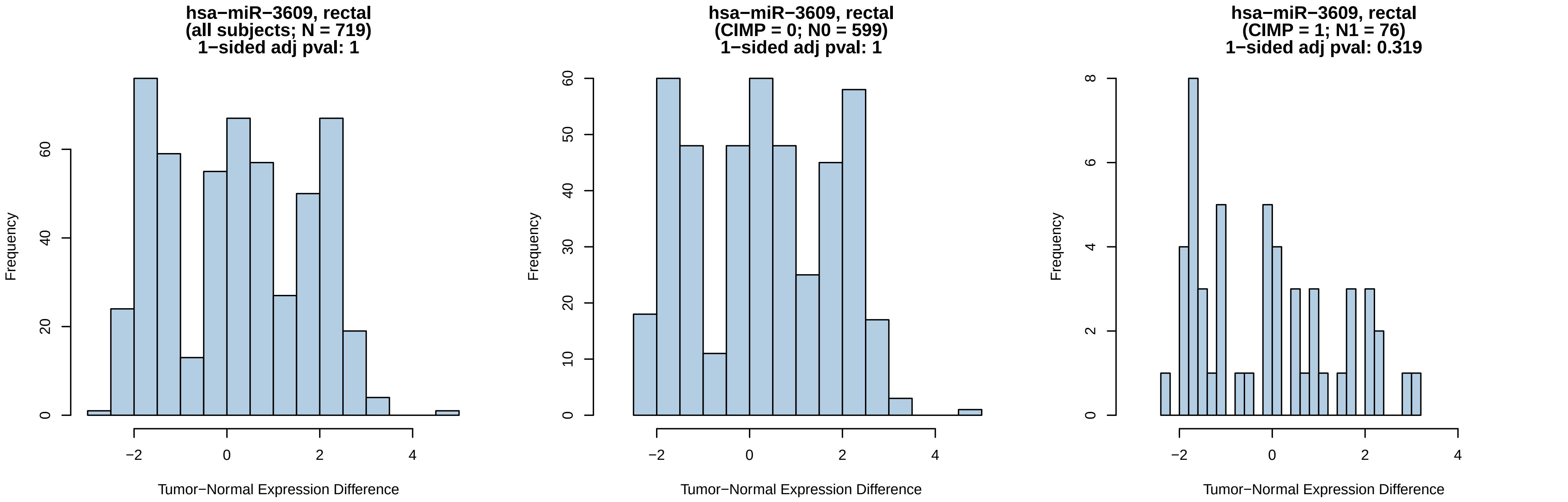

hsa−miR−3609, rectal
(all subjects; N = 719)
1−sided adj pval: 1
Frequency
0
20
40
60
−2
0
2
4
Tumor−Normal Expression Difference
hsa−miR−3609, rectal
(CIMP = 0; N0 = 599)
1−sided adj pval: 1
Frequency
0
10
20
30
40
50
60
−2
0
2
4
Tumor−Normal Expression Difference
hsa−miR−3609, rectal
(CIMP = 1; N1 = 76)
1−sided adj pval: 0.319
Frequency
0
2
4
6
8
−2
0
2
4
Tumor−Normal Expression Difference

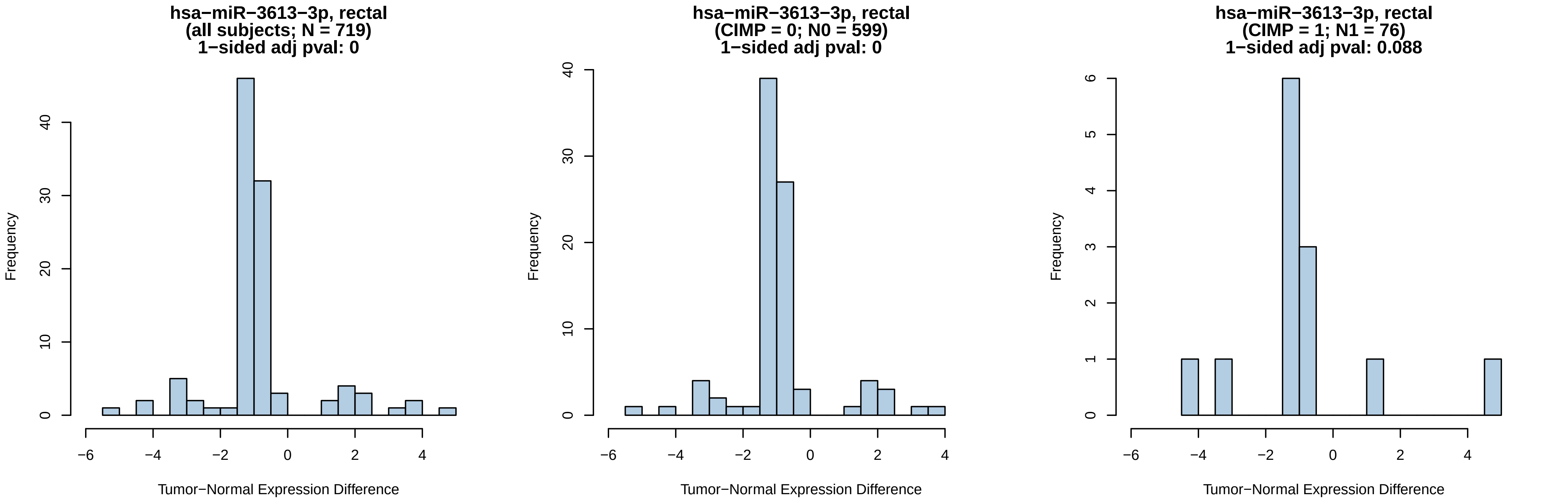

hsa−miR−3613−3p, rectal
(all subjects; N = 719)
1−sided adj pval: 0
Frequency
Tumor−Normal Expression Difference
hsa−miR−3613−3p, rectal
(CIMP = 0; N0 = 599)
1−sided adj pval: 0
Frequency
Tumor−Normal Expression Difference
hsa−miR−3613−3p, rectal
(CIMP = 1; N1 = 76)
1−sided adj pval: 0.088
Frequency
Tumor−Normal Expression Difference

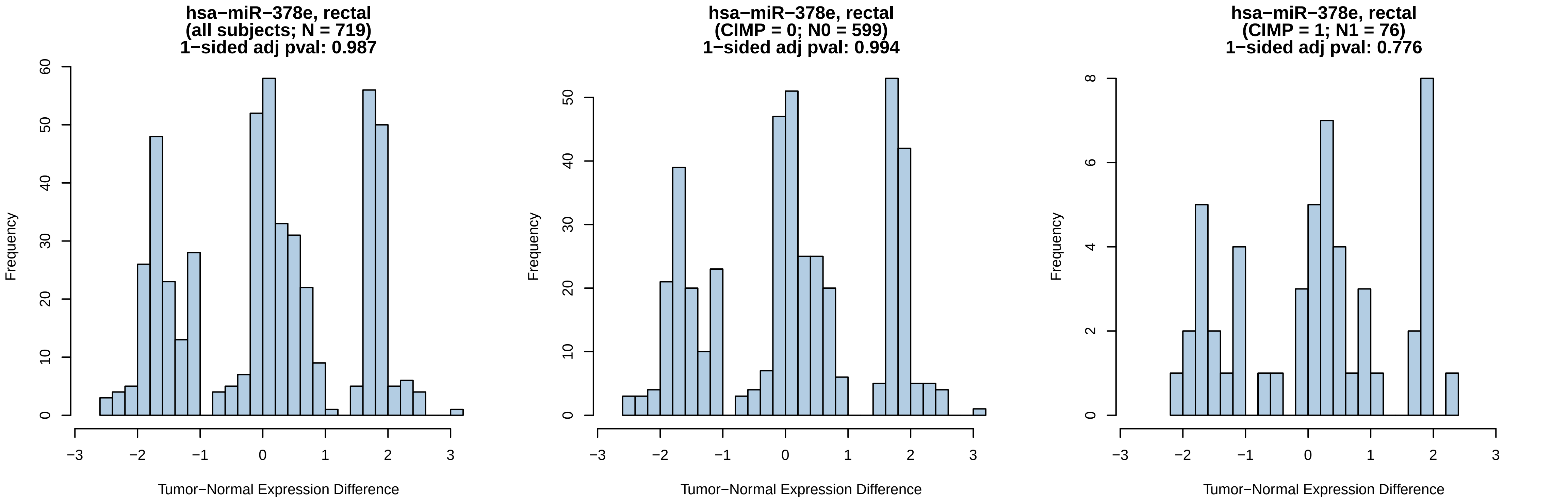
hsa−miR−378e, rectal
(all subjects; N = 719)
1−sided adj pval: 0.987
hsa−miR−378e, rectal
(CIMP = 0; N0 = 599)
1−sided adj pval: 0.994
hsa−miR−378e, rectal
(CIMP = 1; N1 = 76)
1−sided adj pval: 0.776
Frequency
Tumor−Normal Expression Difference

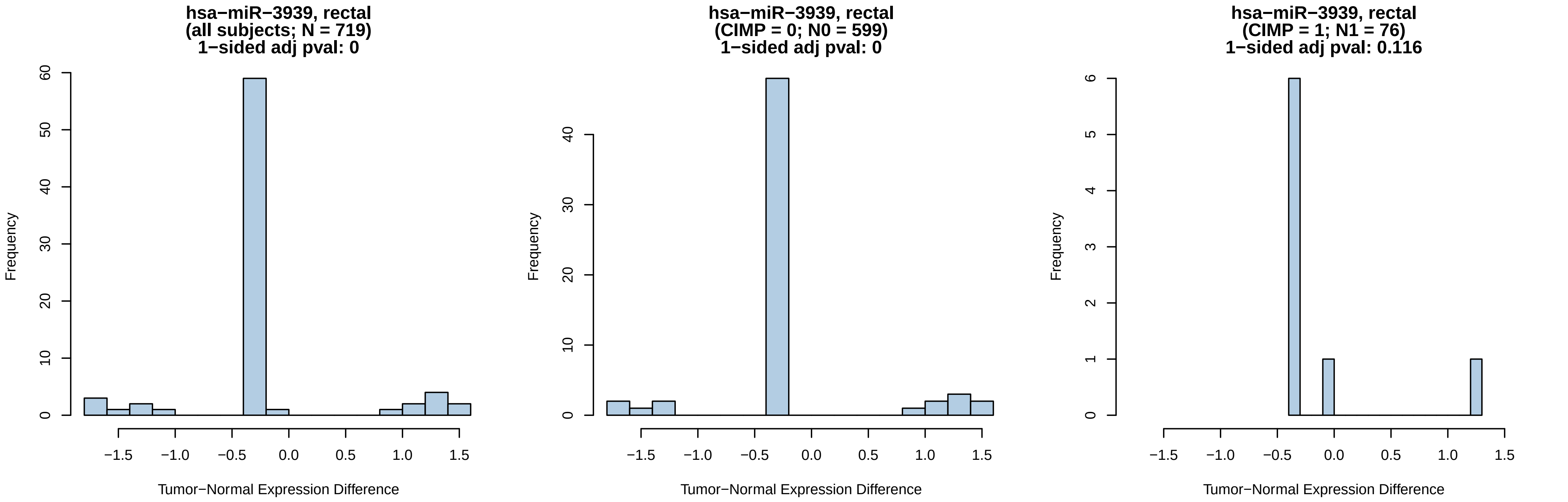

hsa−miR−3939, rectal
(all subjects; N = 719)
1−sided adj pval: 0
Frequency
Tumor−Normal Expression Difference
hsa−miR−3939, rectal
(CIMP = 0; N0 = 599)
1−sided adj pval: 0
Frequency
Tumor−Normal Expression Difference
hsa−miR−3939, rectal
(CIMP = 1; N1 = 76)
1−sided adj pval: 0.116
Frequency
Tumor−Normal Expression Difference

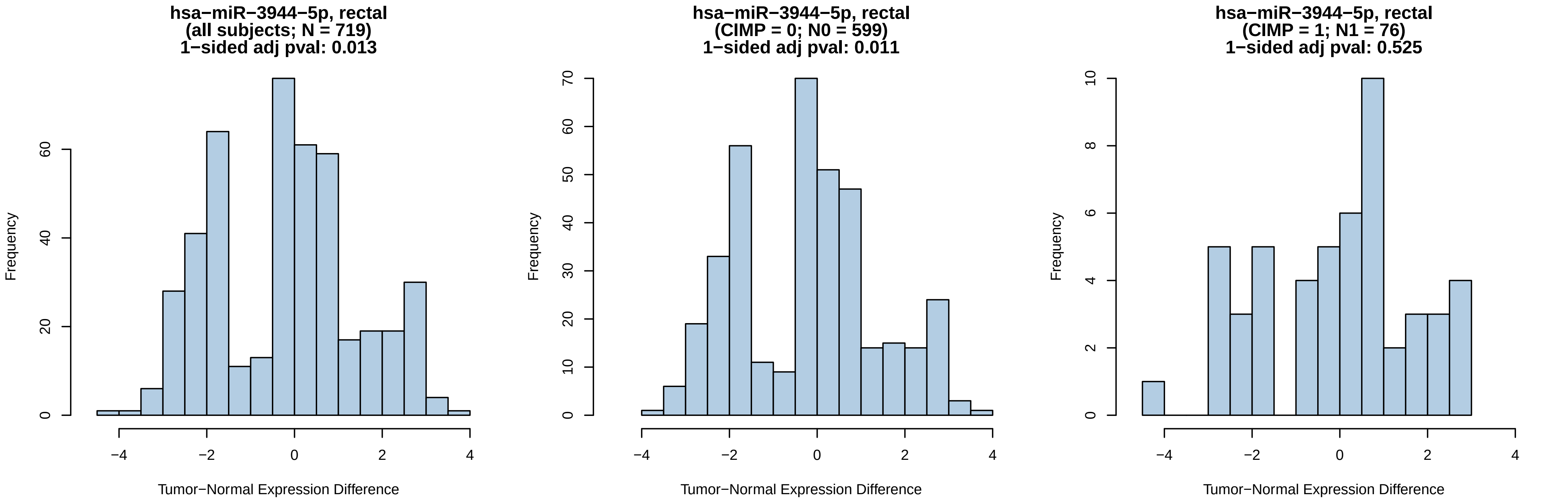
hsa−miR−3944−5p, rectal
(all subjects; N = 719)
1−sided adj pval: 0.013
hsa−miR−3944−5p, rectal
(CIMP = 0; N0 = 599)
1−sided adj pval: 0.011
hsa−miR−3944−5p, rectal
(CIMP = 1; N1 = 76)
1−sided adj pval: 0.525
Frequency
Tumor−Normal Expression Difference

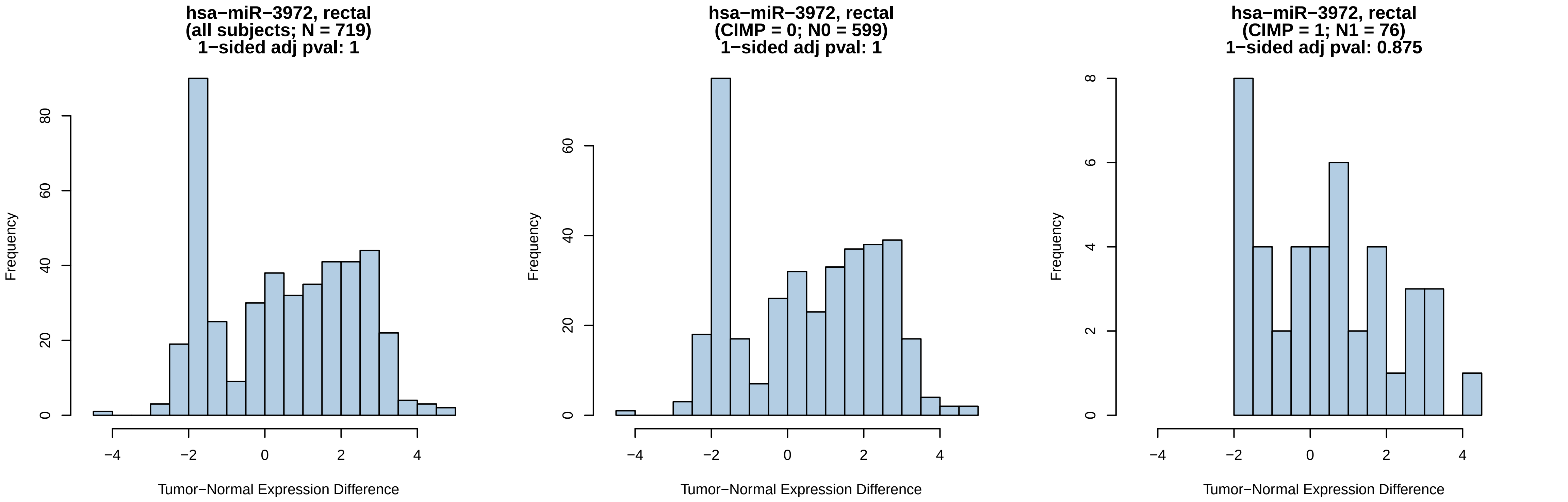
hsa−miR−3972, rectal
(all subjects; N = 719)
1−sided adj pval: 1
hsa−miR−3972, rectal
(CIMP = 0; N0 = 599)
1−sided adj pval: 1
hsa−miR−3972, rectal
(CIMP = 1; N1 = 76)
1−sided adj pval: 0.875
Frequency
Tumor−Normal Expression Difference

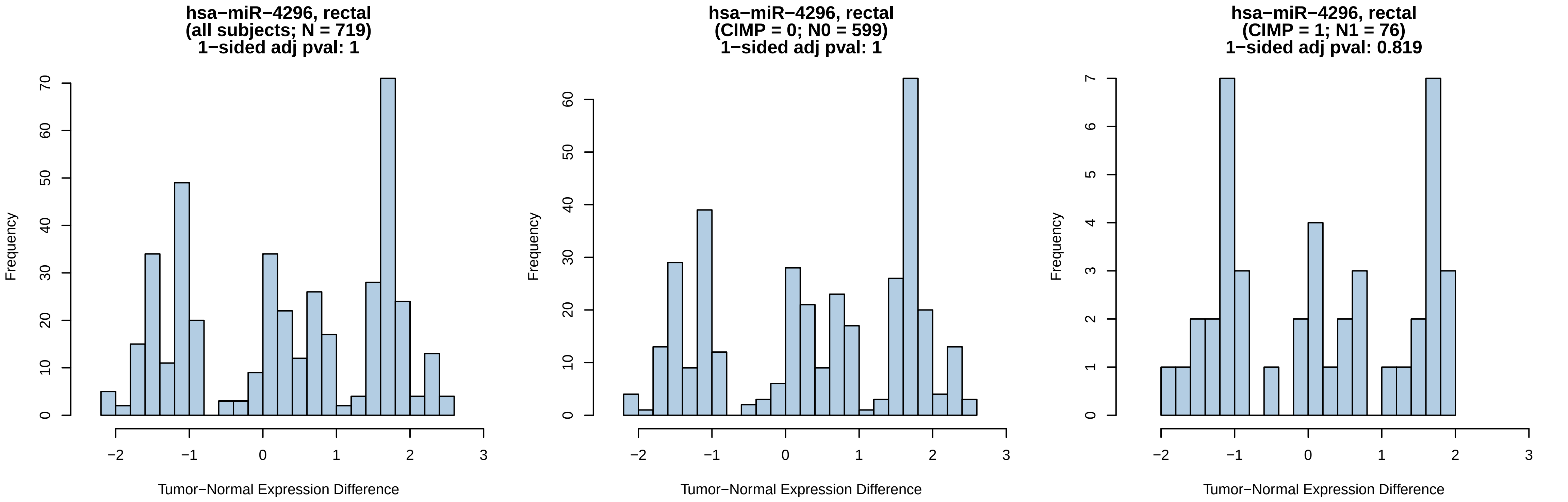
hsa−miR−4296, rectal
(all subjects; N = 719)
1−sided adj pval: 1
hsa−miR−4296, rectal
(CIMP = 0; N0 = 599)
1−sided adj pval: 1
hsa−miR−4296, rectal
(CIMP = 1; N1 = 76)
1−sided adj pval: 0.819
Frequency
Tumor−Normal Expression Difference

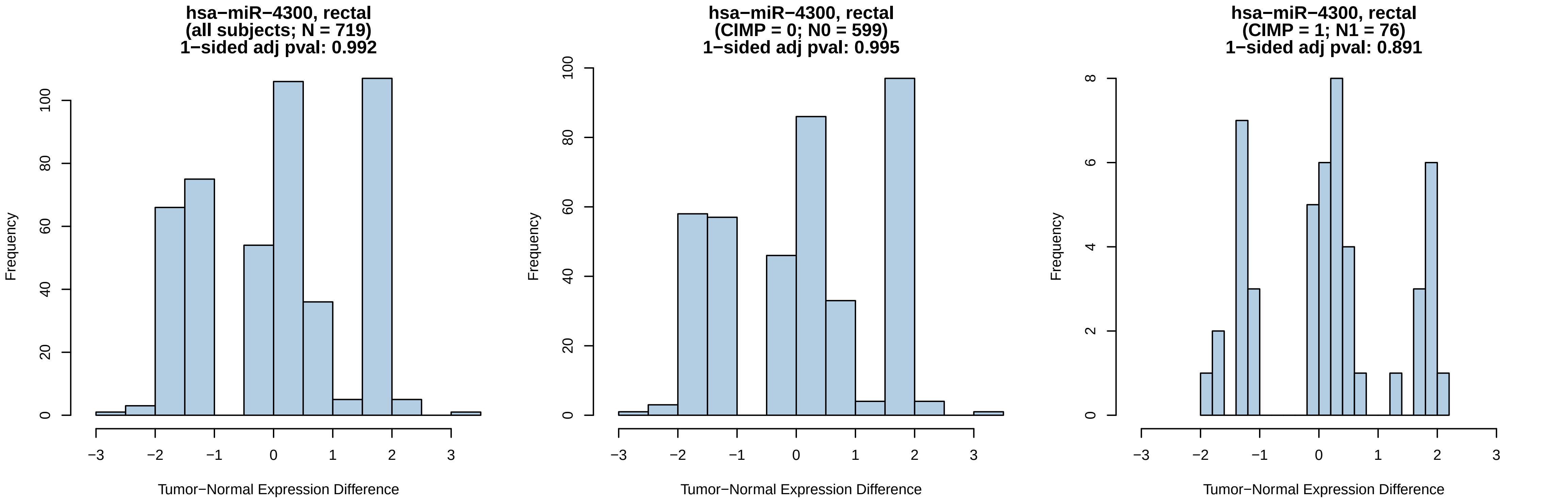
hsa−miR−4300, rectal
(all subjects; N = 719)
1−sided adj pval: 0.992
hsa−miR−4300, rectal
(CIMP = 0; N0 = 599)
1−sided adj pval: 0.995
hsa−miR−4300, rectal
(CIMP = 1; N1 = 76)
1−sided adj pval: 0.891
Frequency
Tumor−Normal Expression Difference

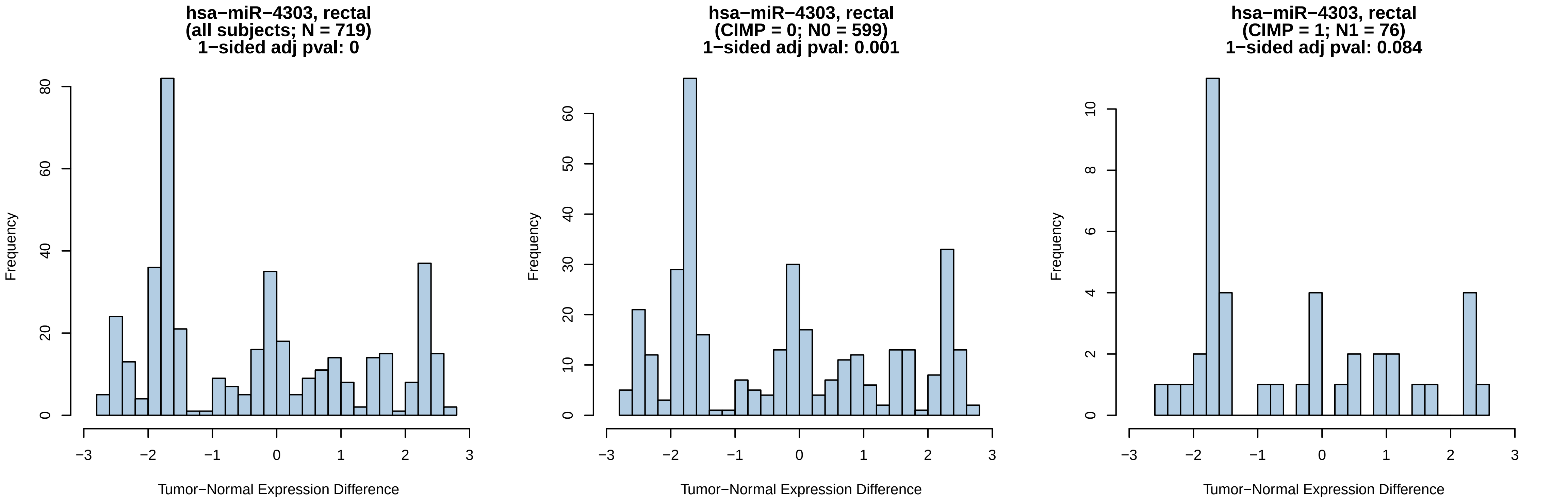
hsa−miR−4303, rectal
(all subjects; N = 719)
1−sided adj pval: 0
Frequency
0
20
40
60
80
−3
−2
−1
0
1
2
3
Tumor−Normal Expression Difference
hsa−miR−4303, rectal
(CIMP = 0; N0 = 599)
1−sided adj pval: 0.001
Frequency
0
10
20
30
40
50
60
Tumor−Normal Expression Difference
hsa−miR−4303, rectal
(CIMP = 1; N1 = 76)
1−sided adj pval: 0.084
Frequency
0
2
4
6
8
10
Tumor−Normal Expression Difference

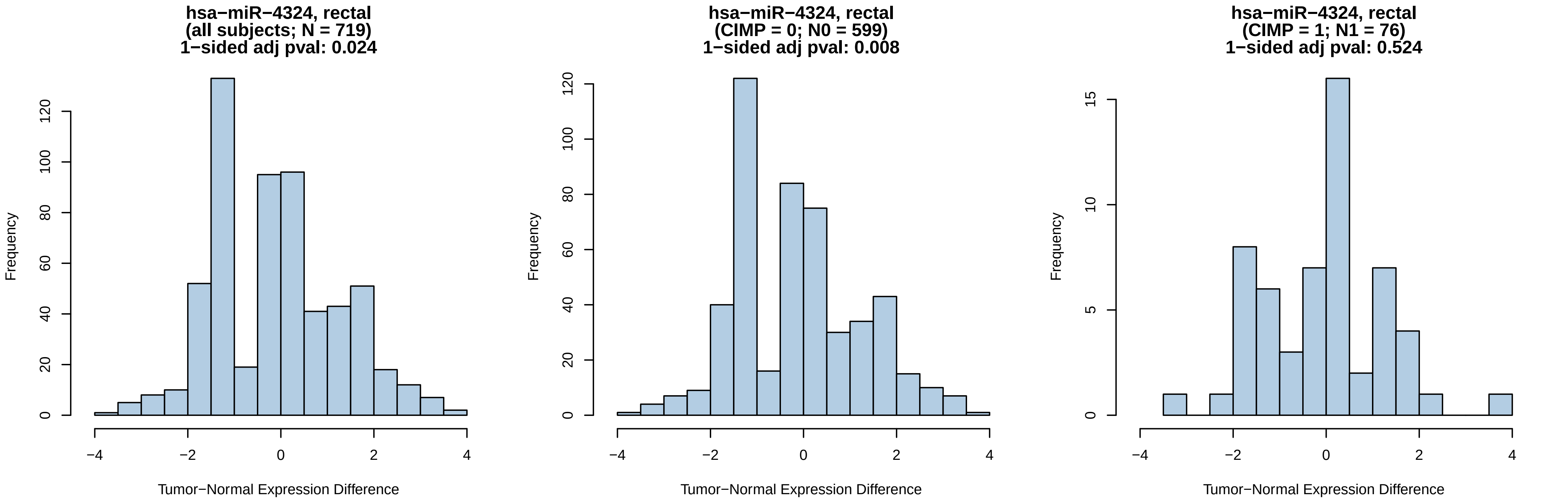

hsa−miR−4324, rectal
(all subjects; N = 719)
1−sided adj pval: 0.024
Frequency
Tumor−Normal Expression Difference
hsa−miR−4324, rectal
(CIMP = 0; N0 = 599)
1−sided adj pval: 0.008
Frequency
Tumor−Normal Expression Difference
hsa−miR−4324, rectal
(CIMP = 1; N1 = 76)
1−sided adj pval: 0.524
Frequency
Tumor−Normal Expression Difference

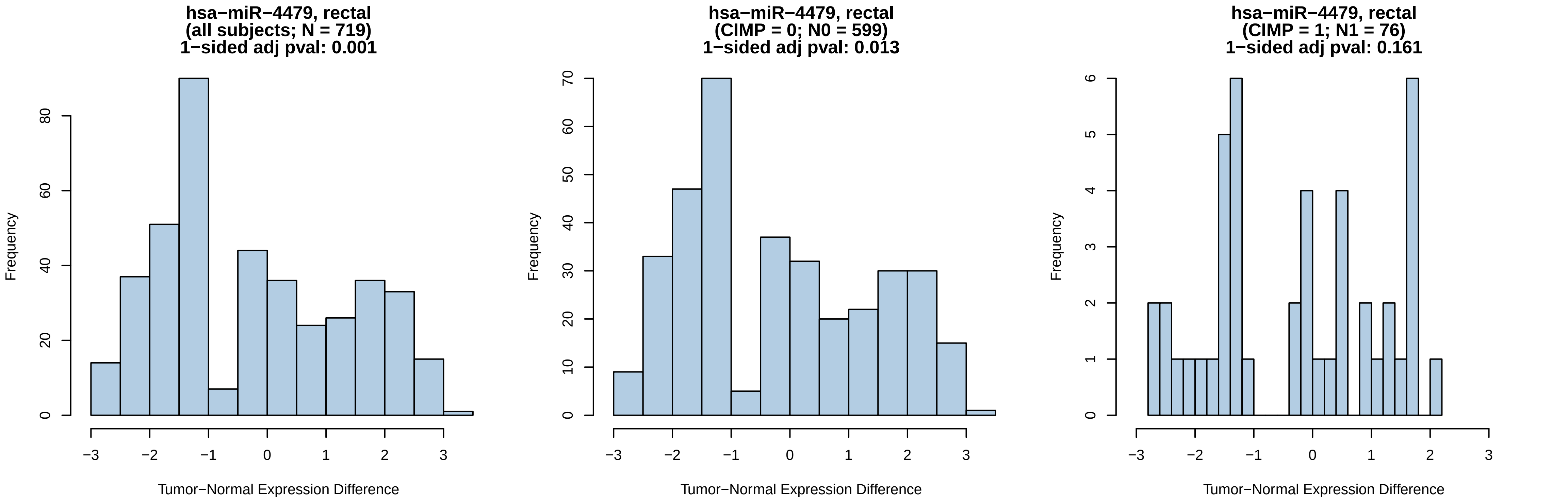

hsa−miR−4479, rectal
(all subjects; N = 719)
1−sided adj pval: 0.001
Frequency
0
20
40
60
80
−3
−2
−1
0
1
2
3
Tumor−Normal Expression Difference
hsa−miR−4479, rectal
(CIMP = 0; N0 = 599)
1−sided adj pval: 0.013
Frequency
0
10
20
30
40
50
60
70
−3
−2
−1
0
1
2
3
Tumor−Normal Expression Difference
hsa−miR−4479, rectal
(CIMP = 1; N1 = 76)
1−sided adj pval: 0.161
Frequency
0
1
2
3
4
5
6
−3
−2
−1
0
1
2
3
Tumor−Normal Expression Difference

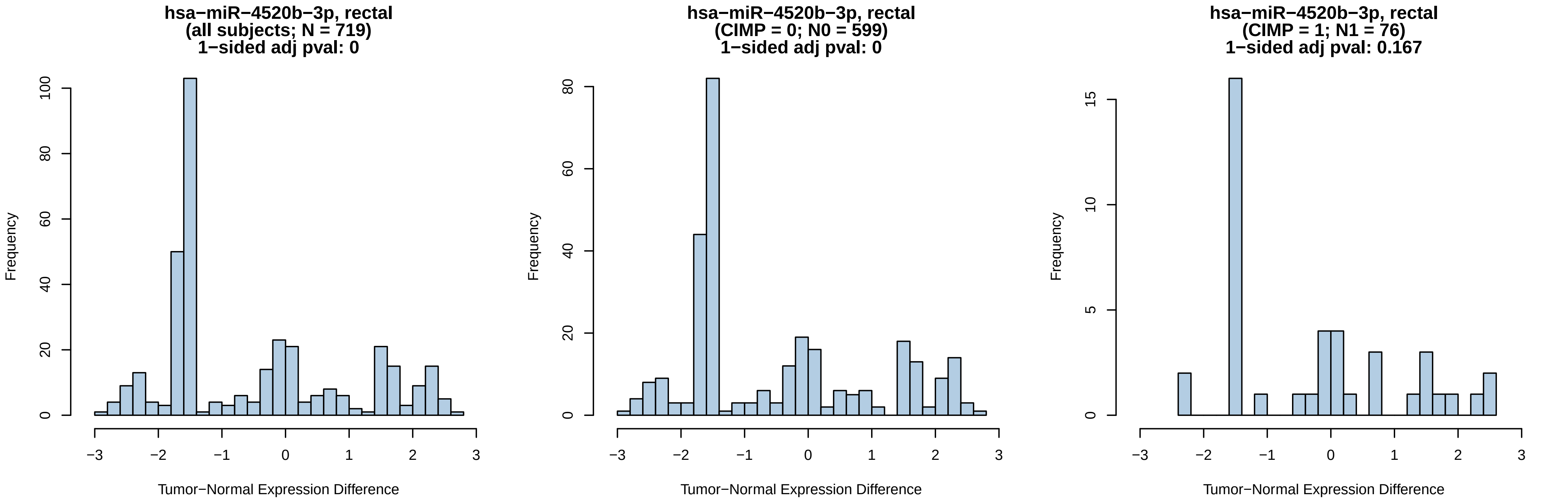
hsa−miR−4520b−3p, rectal
(all subjects; N = 719)
1−sided adj pval: 0
Frequency
Tumor−Normal Expression Difference
hsa−miR−4520b−3p, rectal
(CIMP = 0; N0 = 599)
1−sided adj pval: 0
Frequency
Tumor−Normal Expression Difference
hsa−miR−4520b−3p, rectal
(CIMP = 1; N1 = 76)
1−sided adj pval: 0.167
Frequency
Tumor−Normal Expression Difference

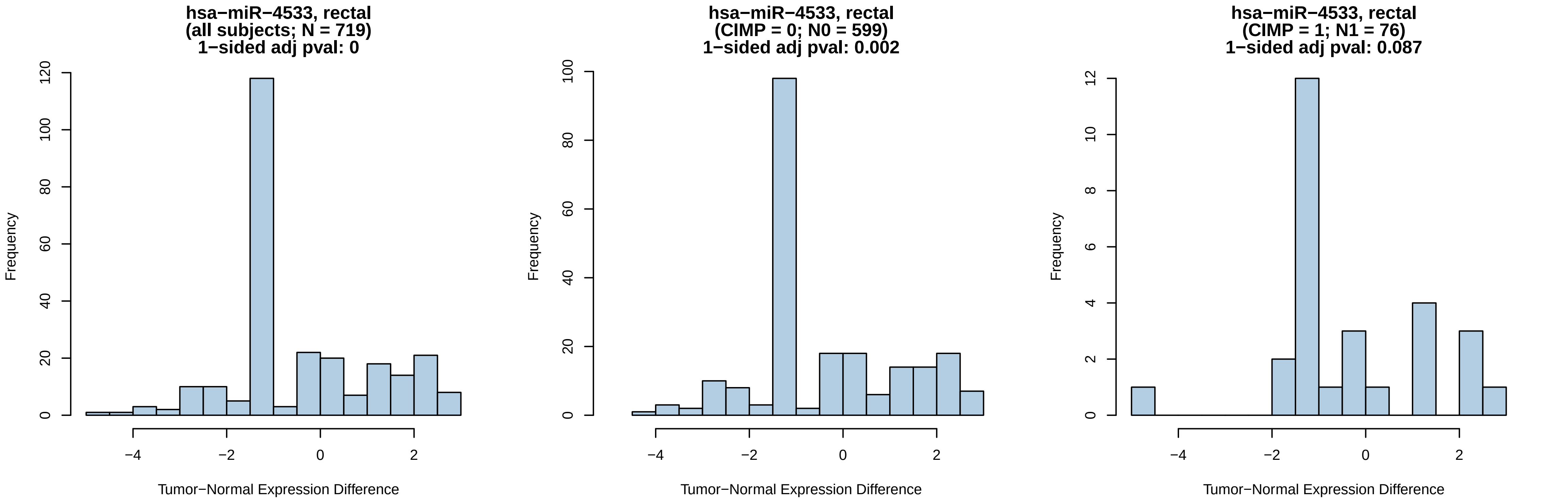
hsa−miR−4533, rectal
(all subjects; N = 719)
1−sided adj pval: 0
hsa−miR−4533, rectal
(CIMP = 0; N0 = 599)
1−sided adj pval: 0.002
hsa−miR−4533, rectal
(CIMP = 1; N1 = 76)
1−sided adj pval: 0.087
Frequency
Tumor−Normal Expression Difference
0
20
40
60
80
100
120
0
2
4
6
8
10
12
−4
−2
0
2

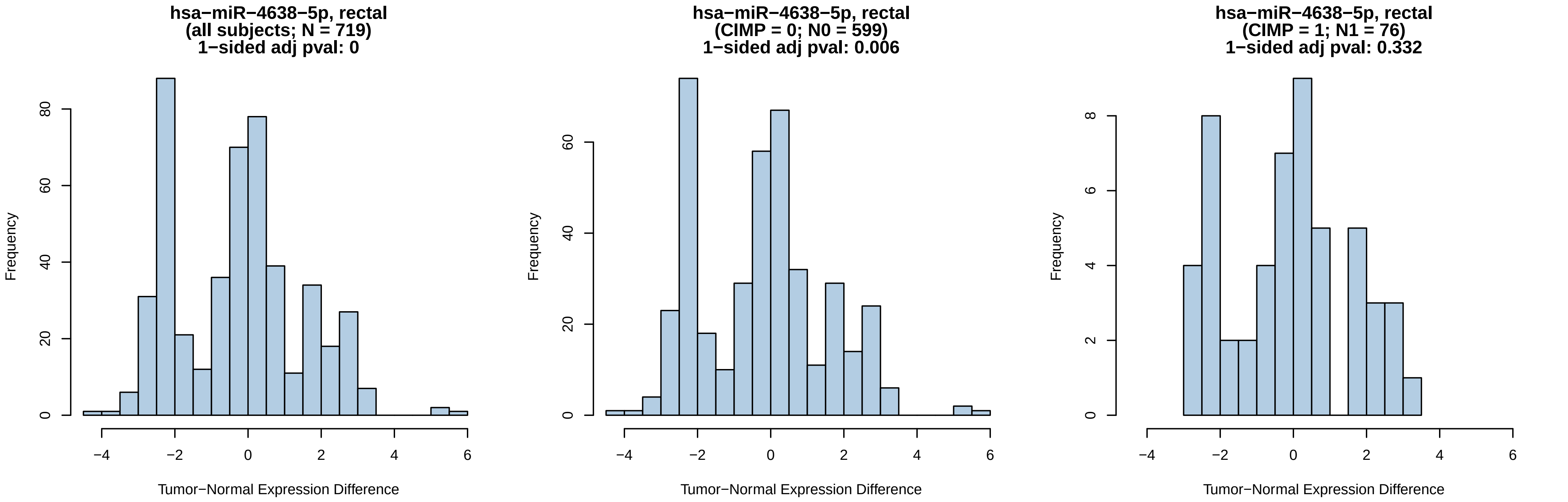
hsa−miR−4638−5p, rectal
(all subjects; N = 719)
1−sided adj pval: 0
hsa−miR−4638−5p, rectal
(CIMP = 0; N0 = 599)
1−sided adj pval: 0.006
hsa−miR−4638−5p, rectal
(CIMP = 1; N1 = 76)
1−sided adj pval: 0.332
Frequency
Tumor−Normal Expression Difference

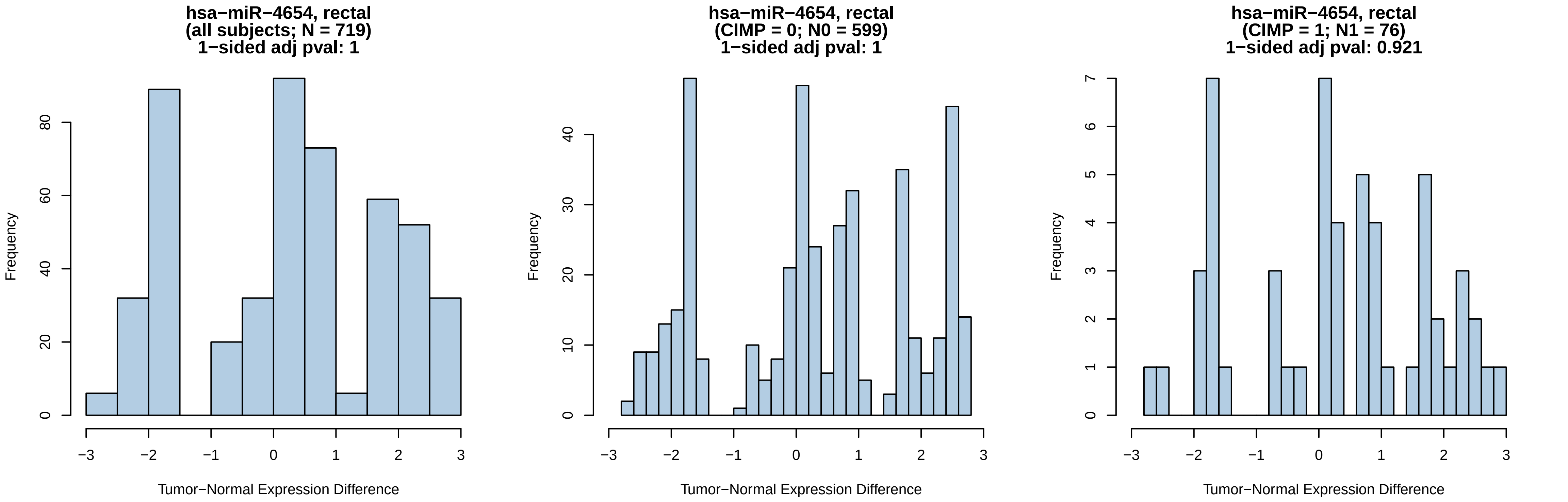
hsa−miR−4654, rectal
(all subjects; N = 719)
1−sided adj pval: 1
Frequency
Tumor−Normal Expression Difference
hsa−miR−4654, rectal
(CIMP = 0; N0 = 599)
1−sided adj pval: 1
Frequency
Tumor−Normal Expression Difference
hsa−miR−4654, rectal
(CIMP = 1; N1 = 76)
1−sided adj pval: 0.921
Frequency
Tumor−Normal Expression Difference

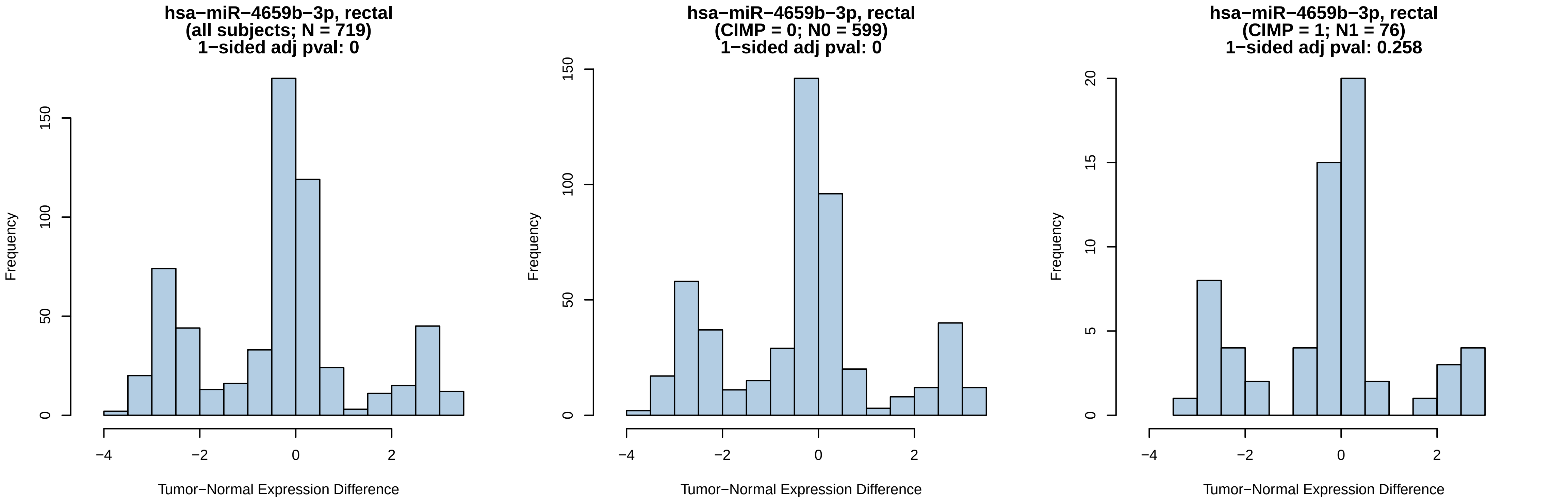
hsa−miR−4659b−3p, rectal
(all subjects; N = 719)
1−sided adj pval: 0
Frequency
Tumor−Normal Expression Difference
hsa−miR−4659b−3p, rectal
(CIMP = 0; N0 = 599)
1−sided adj pval: 0
Frequency
Tumor−Normal Expression Difference
hsa−miR−4659b−3p, rectal
(CIMP = 1; N1 = 76)
1−sided adj pval: 0.258
Frequency
Tumor−Normal Expression Difference

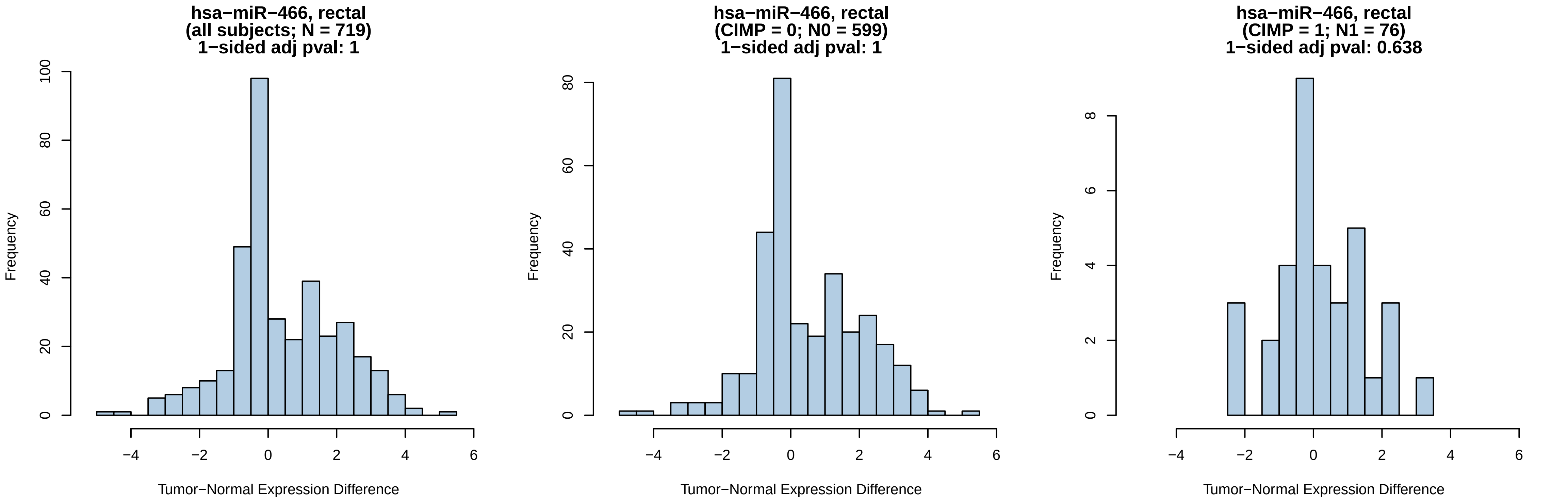

hsa−miR−466, rectal
(all subjects; N = 719)
1−sided adj pval: 1
Frequency
Tumor−Normal Expression Difference
hsa−miR−466, rectal
(CIMP = 0; N0 = 599)
1−sided adj pval: 1
Frequency
Tumor−Normal Expression Difference
hsa−miR−466, rectal
(CIMP = 1; N1 = 76)
1−sided adj pval: 0.638
Frequency
Tumor−Normal Expression Difference

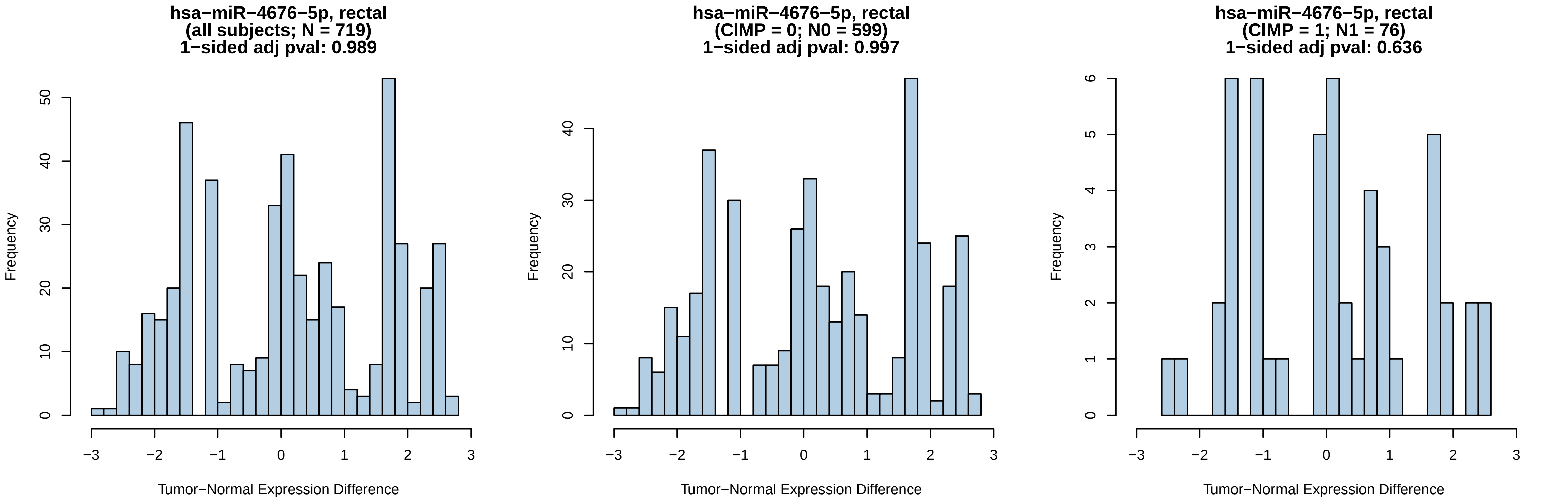
hsa−miR−4676−5p, rectal
(all subjects; N = 719)
1−sided adj pval: 0.989
hsa−miR−4676−5p, rectal
(CIMP = 0; N0 = 599)
1−sided adj pval: 0.997
hsa−miR−4676−5p, rectal
(CIMP = 1; N1 = 76)
1−sided adj pval: 0.636
Frequency
Tumor−Normal Expression Difference

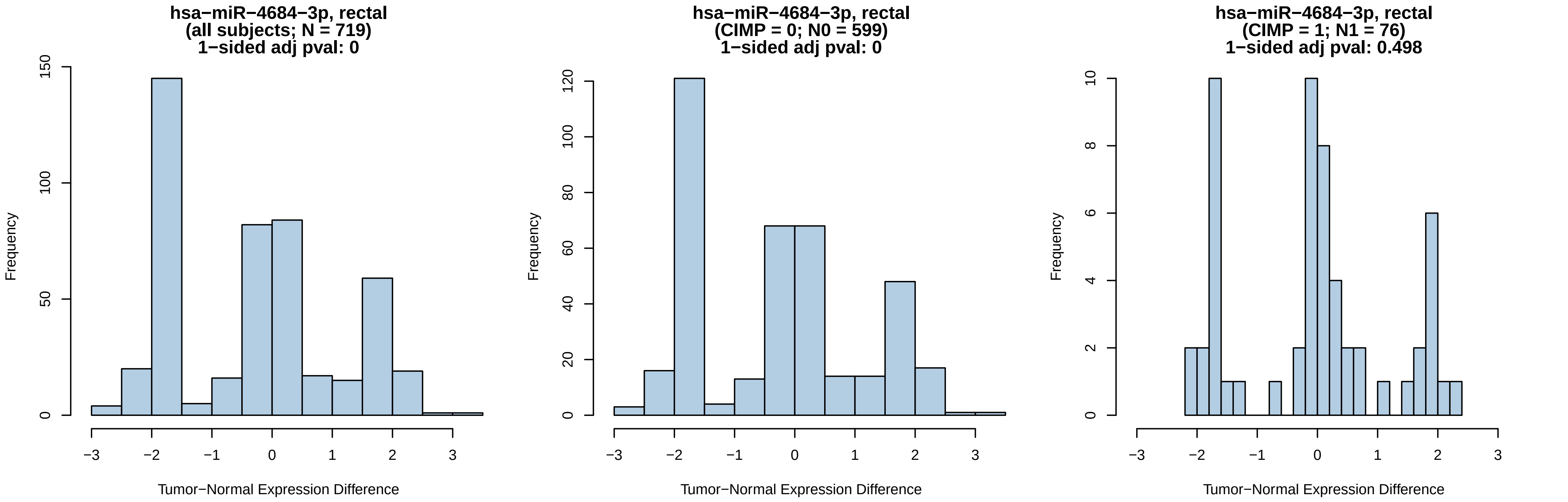

hsa−miR−4684−3p, rectal
(all subjects; N = 719)
1−sided adj pval: 0
Frequency
0
50
100
150
−3
−2
−1
0
1
2
3
Tumor−Normal Expression Difference
hsa−miR−4684−3p, rectal
(CIMP = 0; N0 = 599)
1−sided adj pval: 0
Frequency
0
20
40
60
80
100
120
−3
−2
−1
0
1
2
3
Tumor−Normal Expression Difference
hsa−miR−4684−3p, rectal
(CIMP = 1; N1 = 76)
1−sided adj pval: 0.498
Frequency
0
2
4
6
8
10
−3
−2
−1
0
1
2
3
Tumor−Normal Expression Difference

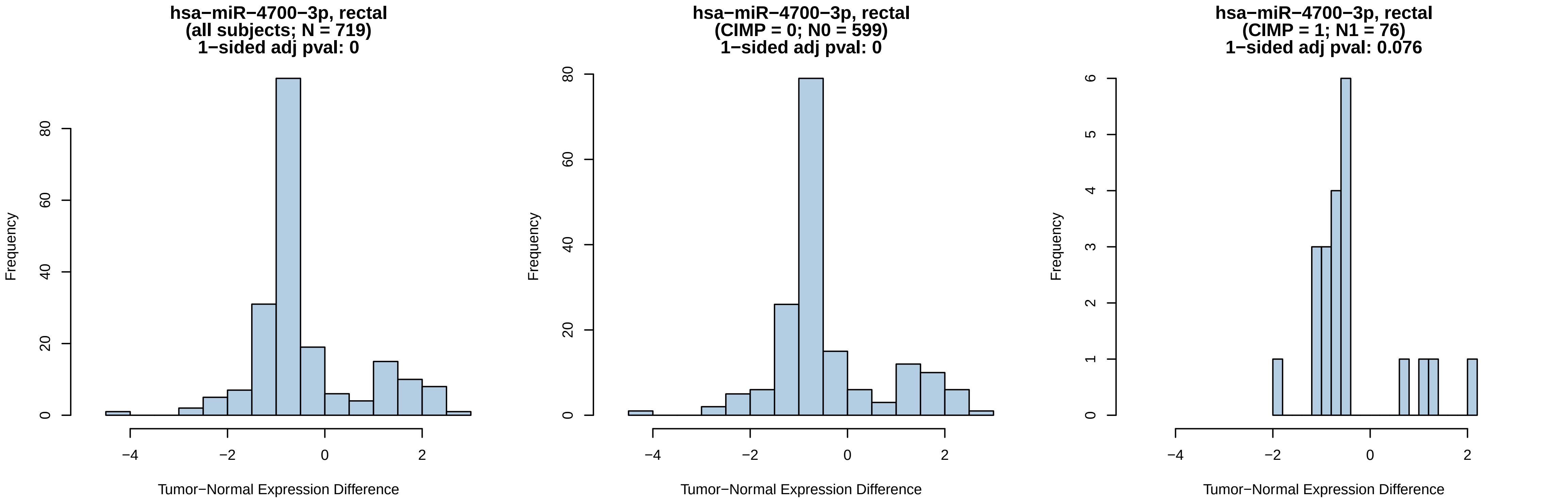
hsa−miR−4700−3p, rectal
(all subjects; N = 719)
1−sided adj pval: 0
Frequency
Tumor−Normal Expression Difference
hsa−miR−4700−3p, rectal
(CIMP = 0; N0 = 599)
1−sided adj pval: 0
Frequency
Tumor−Normal Expression Difference
hsa−miR−4700−3p, rectal
(CIMP = 1; N1 = 76)
1−sided adj pval: 0.076
Frequency
Tumor−Normal Expression Difference

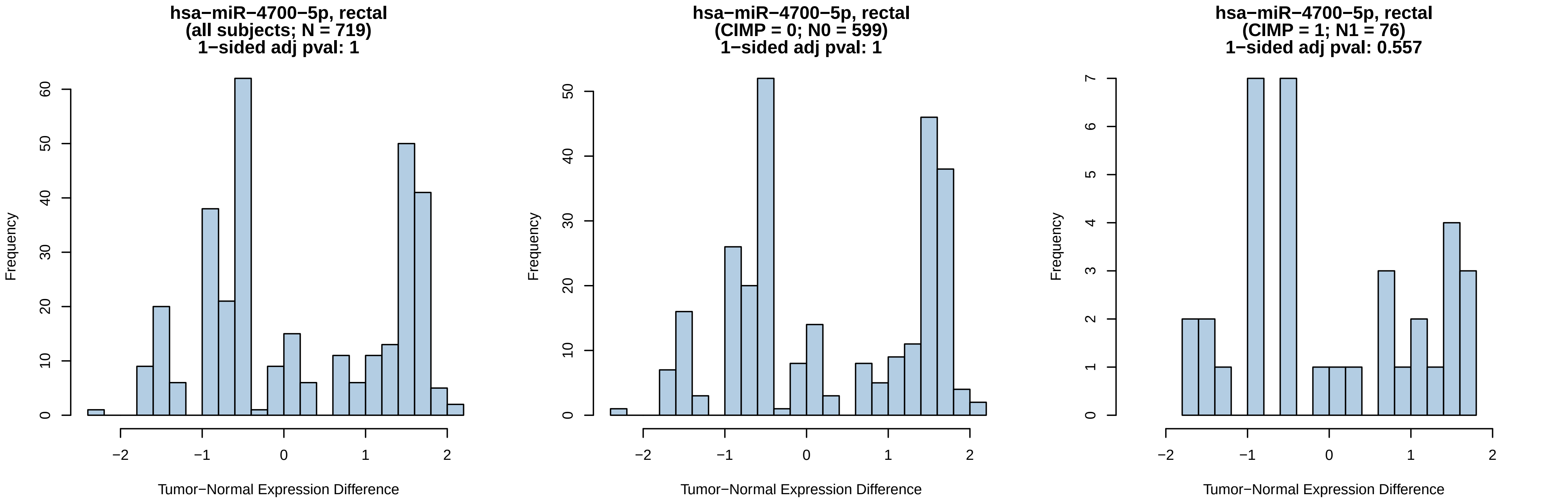
hsa−miR−4700−5p, rectal
(all subjects; N = 719)
1−sided adj pval: 1
Frequency
Tumor−Normal Expression Difference
hsa−miR−4700−5p, rectal
(CIMP = 0; N0 = 599)
1−sided adj pval: 1
Frequency
Tumor−Normal Expression Difference
hsa−miR−4700−5p, rectal
(CIMP = 1; N1 = 76)
1−sided adj pval: 0.557
Frequency
Tumor−Normal Expression Difference

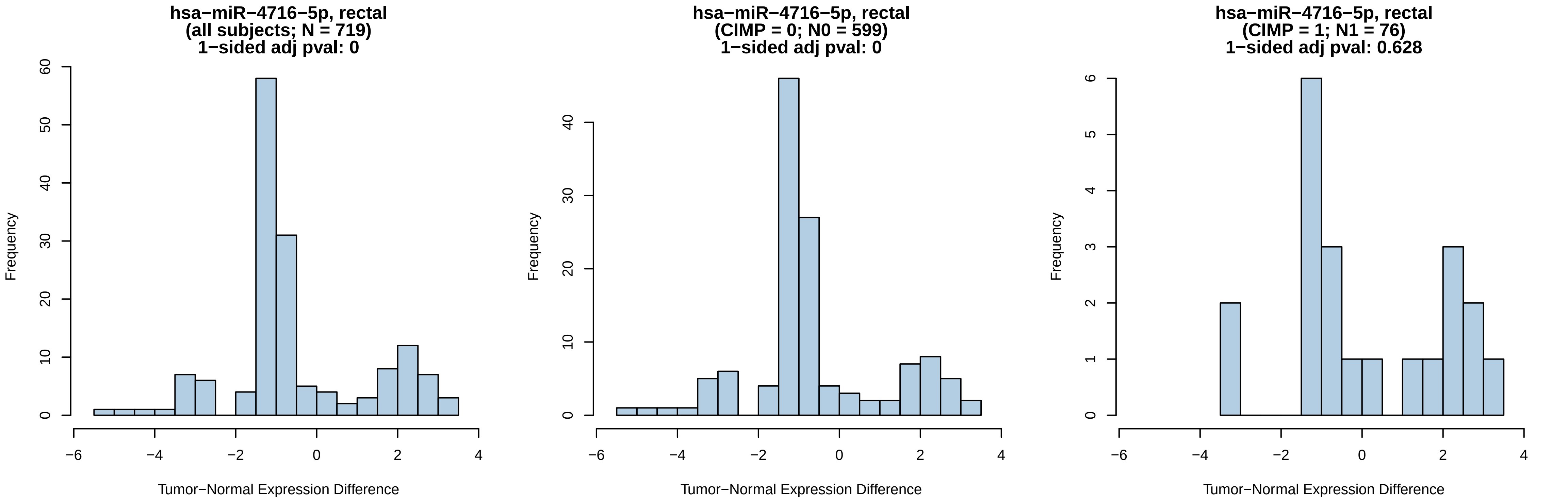

hsa−miR−4716−5p, rectal
(all subjects; N = 719)
1−sided adj pval: 0
Frequency
Tumor−Normal Expression Difference
hsa−miR−4716−5p, rectal
(CIMP = 0; N0 = 599)
1−sided adj pval: 0
Frequency
Tumor−Normal Expression Difference
hsa−miR−4716−5p, rectal
(CIMP = 1; N1 = 76)
1−sided adj pval: 0.628
Frequency
Tumor−Normal Expression Difference

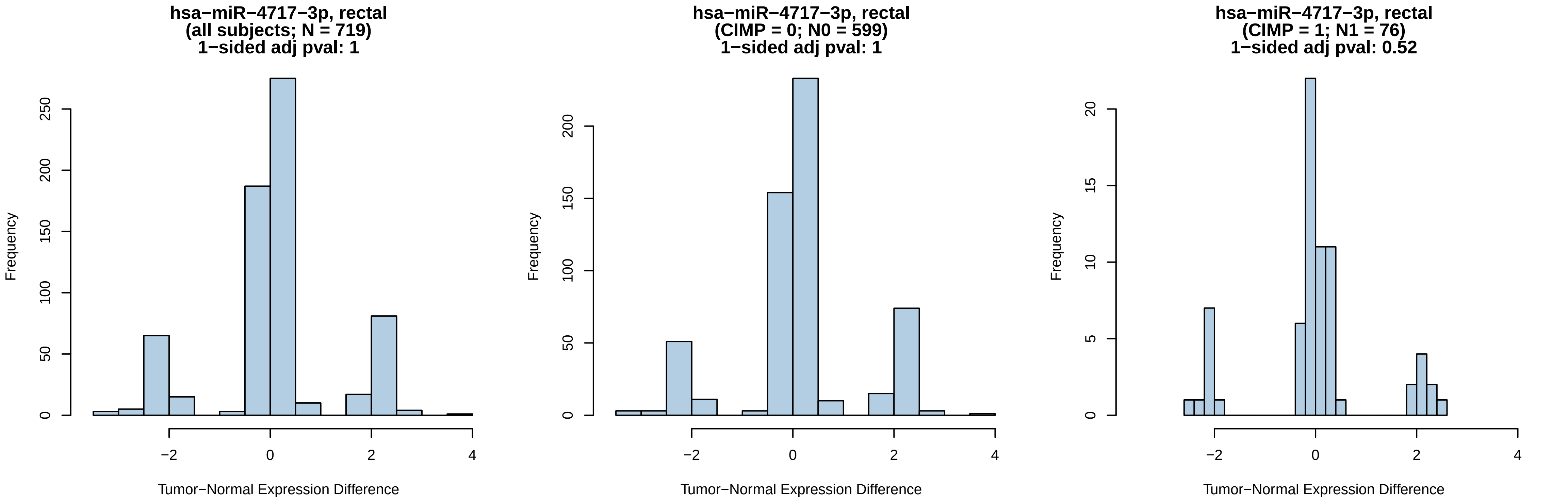
hsa−miR−4717−3p, rectal
(all subjects; N = 719)
1−sided adj pval: 1
hsa−miR−4717−3p, rectal
(CIMP = 0; N0 = 599)
1−sided adj pval: 1
hsa−miR−4717−3p, rectal
(CIMP = 1; N1 = 76)
1−sided adj pval: 0.52
Frequency
Tumor−Normal Expression Difference
0
50
100
150
200
250
5
10
15
20
−2
2
4

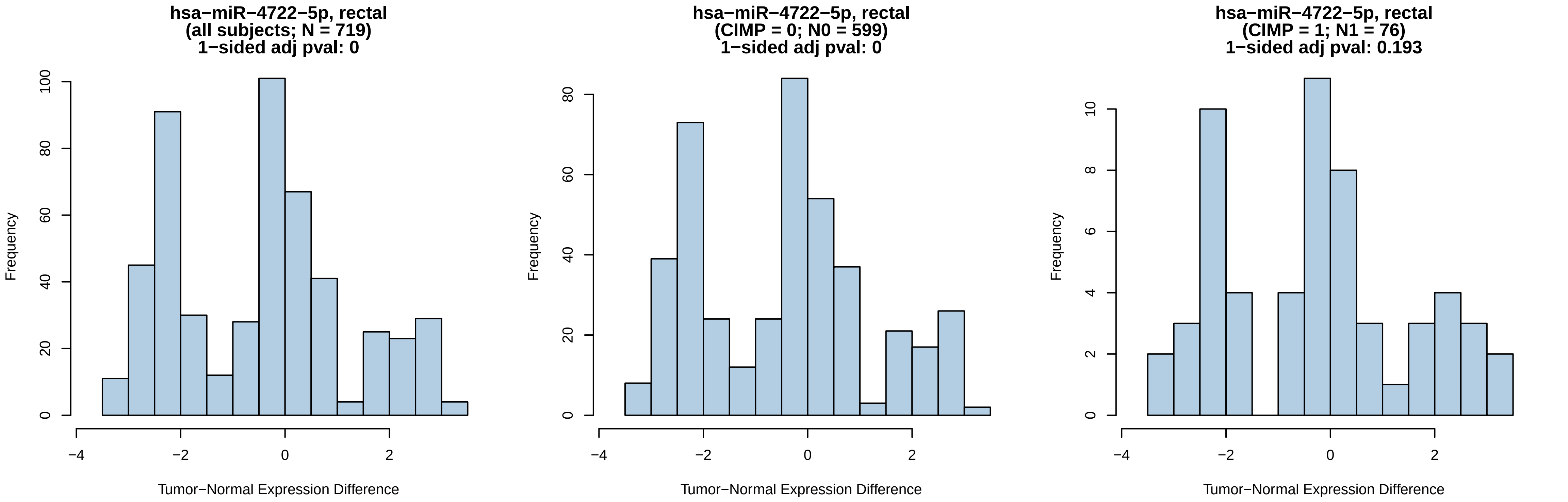
hsa−miR−4722−5p, rectal
(all subjects; N = 719)
1−sided adj pval: 0
hsa−miR−4722−5p, rectal
(CIMP = 0; N0 = 599)
1−sided adj pval: 0
hsa−miR−4722−5p, rectal
(CIMP = 1; N1 = 76)
1−sided adj pval: 0.193
Frequency
Tumor−Normal Expression Difference
−4
−2
0
2
0
20
40
60
80
100
0
2
4
6
8
10

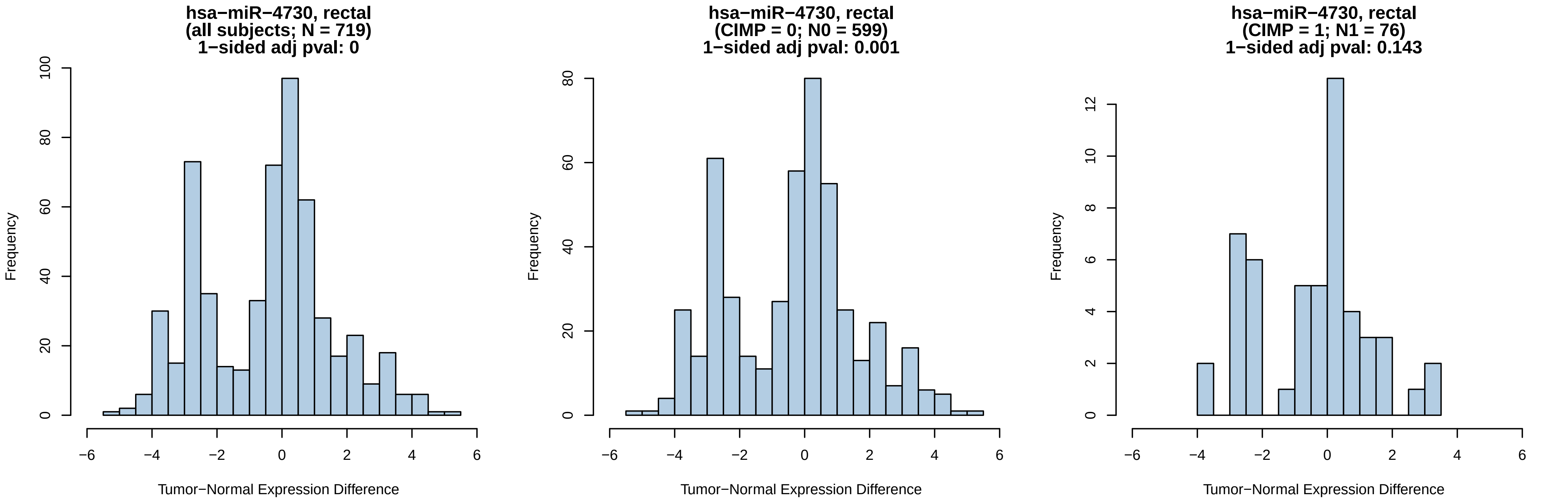
hsa−miR−4730, rectal
(all subjects; N = 719)
1−sided adj pval: 0
Frequency
0
20
40
60
80
100
−6
−4
−2
0
2
4
6
Tumor−Normal Expression Difference
hsa−miR−4730, rectal
(CIMP = 0; N0 = 599)
1−sided adj pval: 0.001
Frequency
0
20
40
60
80
−6
−4
−2
0
2
4
6
Tumor−Normal Expression Difference
hsa−miR−4730, rectal
(CIMP = 1; N1 = 76)
1−sided adj pval: 0.143
Frequency
0
2
4
6
8
10
12
−6
−4
−2
0
2
4
6
Tumor−Normal Expression Difference

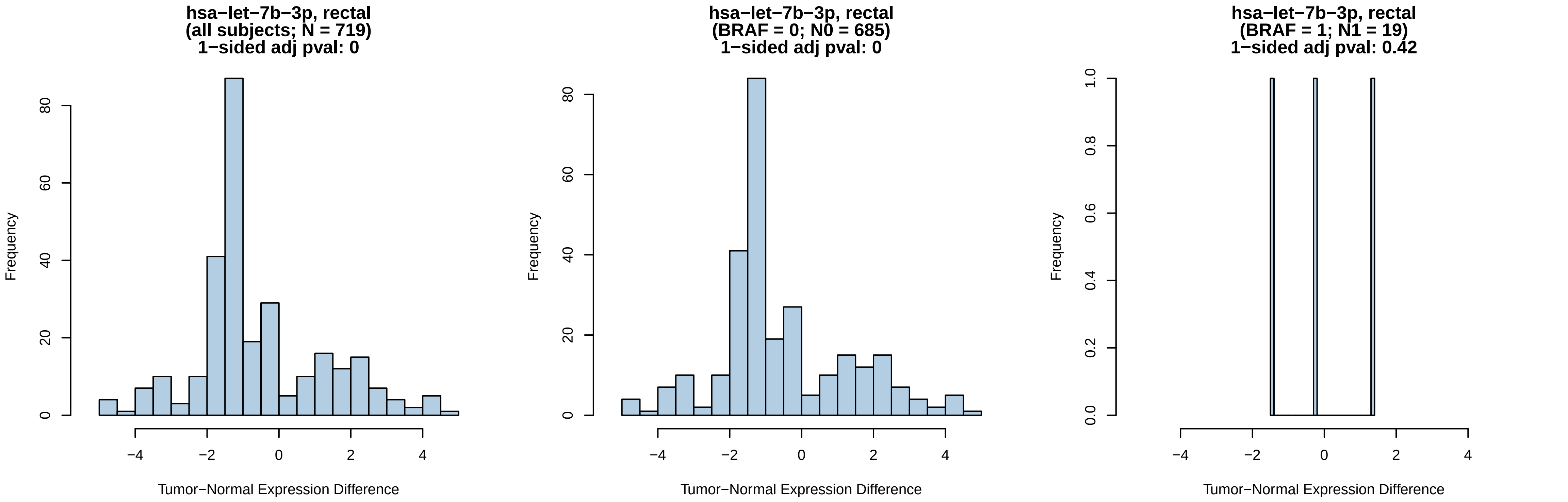
hsa−let−7b−3p, rectal
(all subjects; N = 719)
1−sided adj pval: 0
Frequency
0
20
40
60
80
−4
−2
0
2
4
Tumor−Normal Expression Difference
hsa−let−7b−3p, rectal
(BRAF = 0; N0 = 685)
1−sided adj pval: 0
Frequency
0
20
40
60
80
−4
−2
0
2
4
Tumor−Normal Expression Difference
hsa−let−7b−3p, rectal
(BRAF = 1; N1 = 19)
1−sided adj pval: 0.42
Frequency
0.0
0.2
0.4
0.6
0.8
1.0
−4
−2
0
2
4
Tumor−Normal Expression Difference

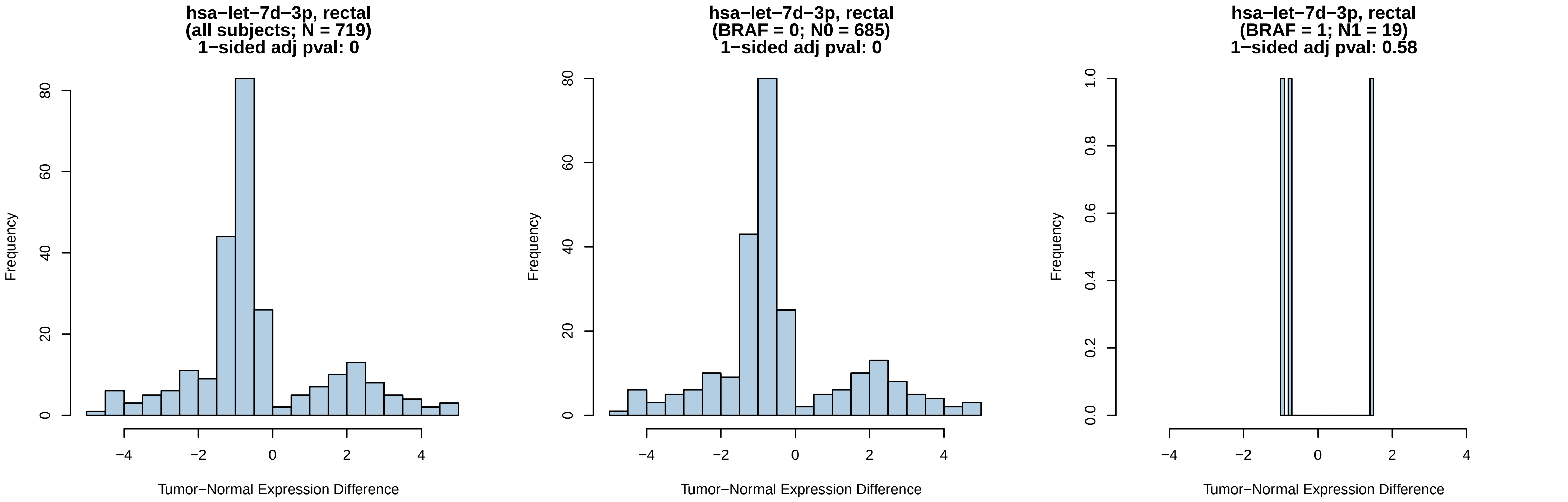

hsa−let−7d−3p, rectal
(all subjects; N = 719)
1−sided adj pval: 0
hsa−let−7d−3p, rectal
(BRAF = 0; N0 = 685)
1−sided adj pval: 0
hsa−let−7d−3p, rectal
(BRAF = 1; N1 = 19)
1−sided adj pval: 0.58
Frequency
Tumor−Normal Expression Difference

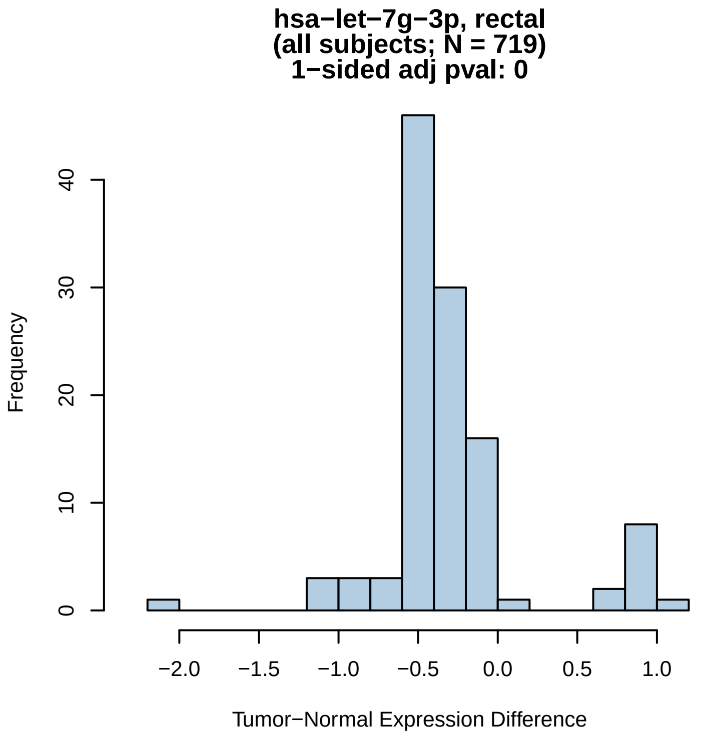

hsa−let−7g−3p, rectal
(all subjects; N = 719)
1−sided adj pval: 0
Frequency
Tumor−Normal Expression Difference
0
10
20
30
40
−2.0
−1.5
−1.0
−0.5
0.0
0.5
1.0


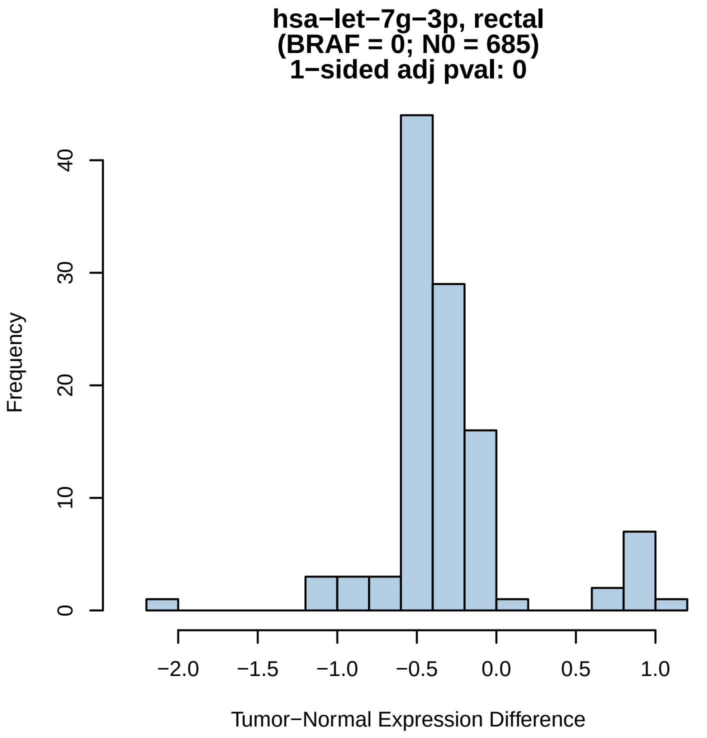

hsa−let−7g−3p, rectal
(BRAF = 0; N0 = 685)
1−sided adj pval: 0
Frequency
Tumor−Normal Expression Difference
0
10
20
30
40
−2.0
−1.5
−1.0
−0.5
0.0
0.5
1.0


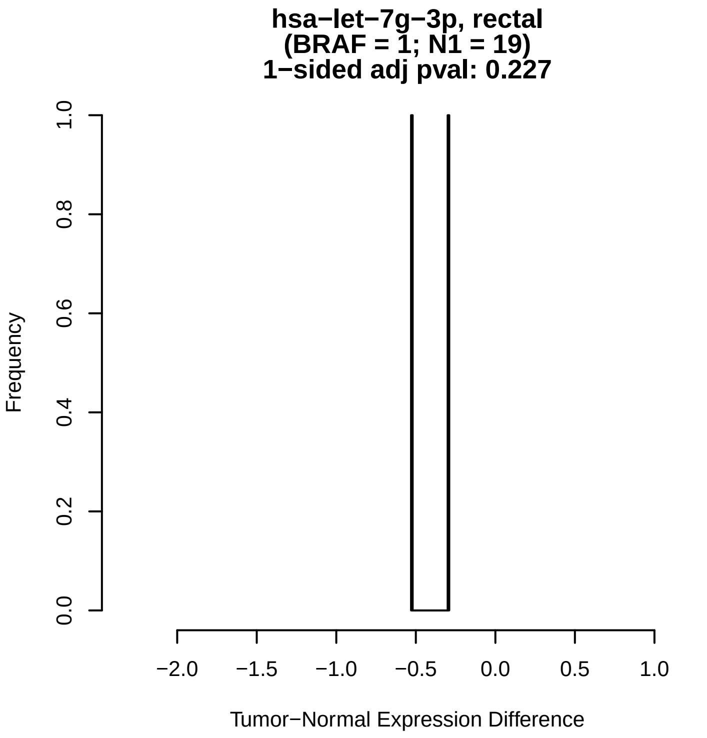

hsa−let−7g−3p, rectal
(BRAF = 1; N1 = 19)
1−sided adj pval: 0.227
Frequency
Tumor−Normal Expression Difference
0.0
0.2
0.4
0.6
0.8
1.0
−2.0
−1.5
−1.0
−0.5
0.0
0.5
1.0

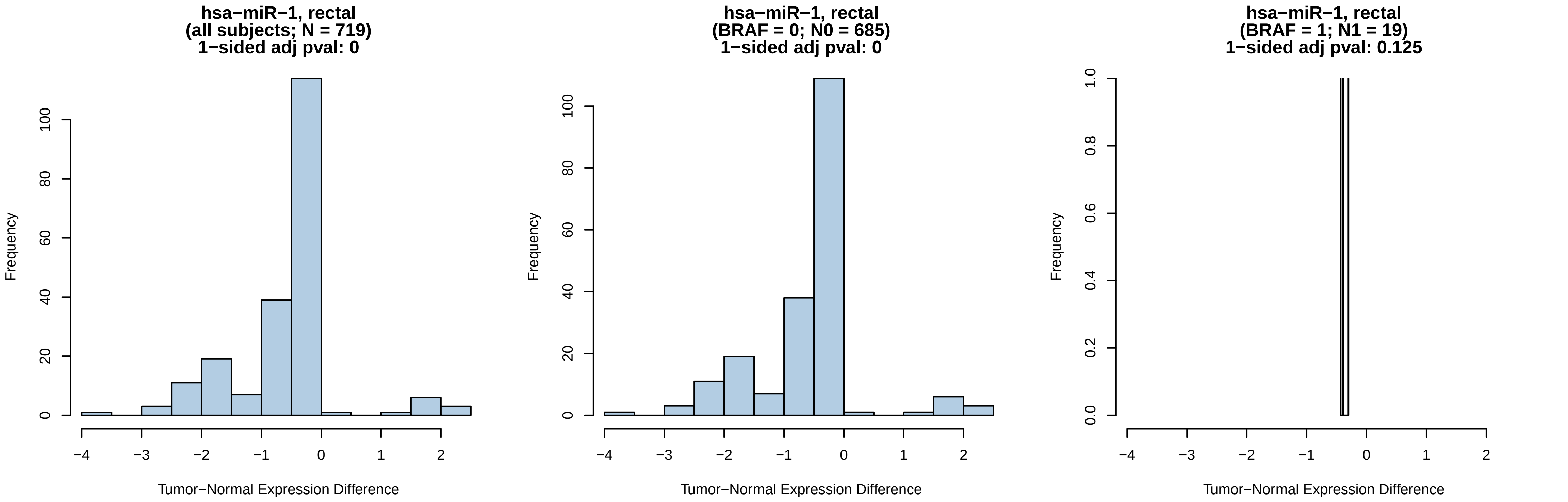
hsa−miR−1, rectal
(all subjects; N = 719)
1−sided adj pval: 0
hsa−miR−1, rectal
(BRAF = 0; N0 = 685)
1−sided adj pval: 0
hsa−miR−1, rectal
(BRAF = 1; N1 = 19)
1−sided adj pval: 0.125
Frequency
Tumor−Normal Expression Difference

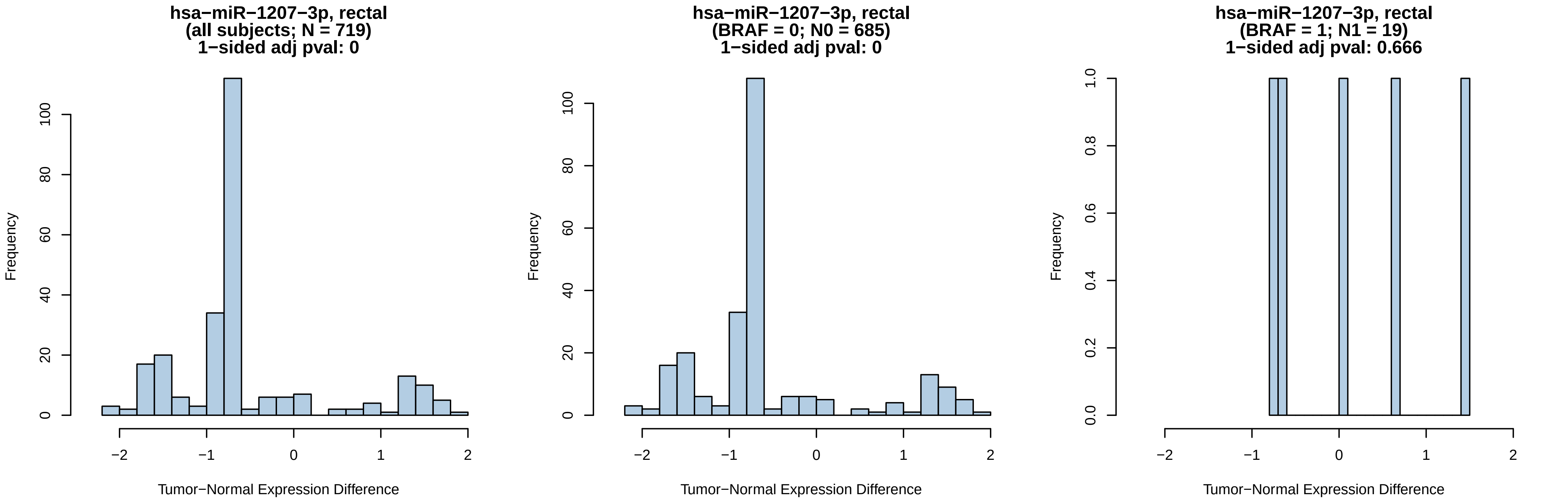

hsa−miR−1207−3p, rectal
(all subjects; N = 719)
1−sided adj pval: 0
Frequency
0
20
40
60
80
100
−2
−1
0
1
2
Tumor−Normal Expression Difference
hsa−miR−1207−3p, rectal
(BRAF = 0; N0 = 685)
1−sided adj pval: 0
Frequency
0
20
40
60
80
100
−2
−1
0
1
2
Tumor−Normal Expression Difference
hsa−miR−1207−3p, rectal
(BRAF = 1; N1 = 19)
1−sided adj pval: 0.666
Frequency
0.0
0.2
0.4
0.6
0.8
1.0
−2
−1
0
1
2
Tumor−Normal Expression Difference

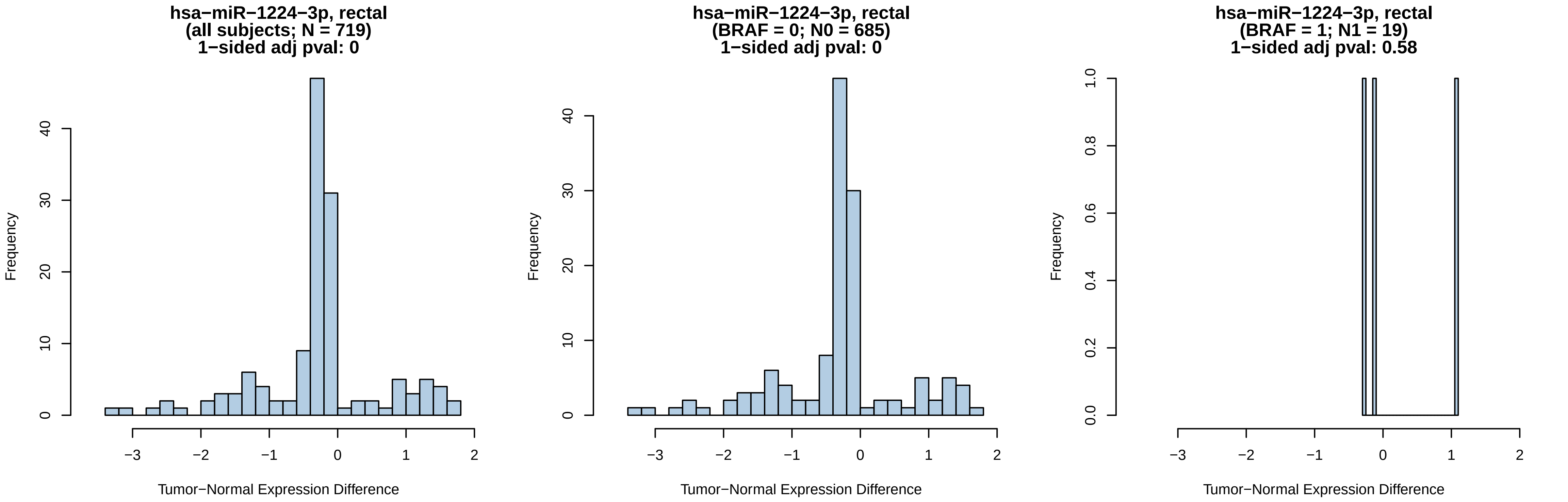
hsa−miR−1224−3p, rectal
(all subjects; N = 719)
1−sided adj pval: 0
Frequency
Tumor−Normal Expression Difference
hsa−miR−1224−3p, rectal
(BRAF = 0; N0 = 685)
1−sided adj pval: 0
Frequency
Tumor−Normal Expression Difference
hsa−miR−1224−3p, rectal
(BRAF = 1; N1 = 19)
1−sided adj pval: 0.58
Frequency
Tumor−Normal Expression Difference

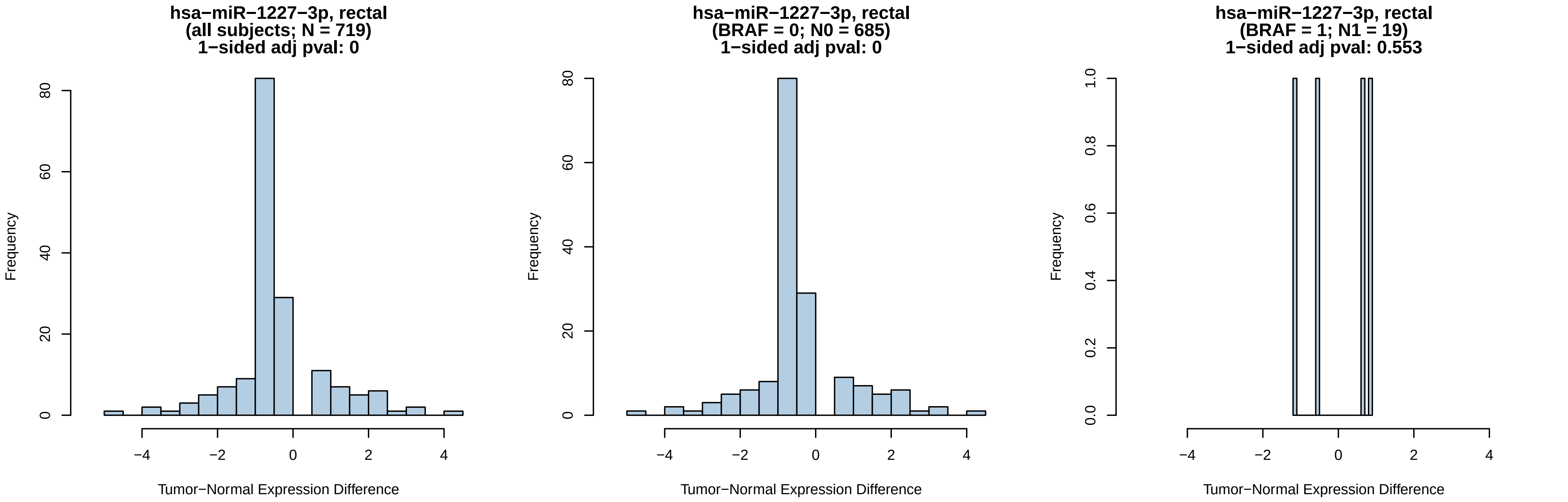
hsa−miR−1227−3p, rectal
(all subjects; N = 719)
1−sided adj pval: 0
Frequency
0
20
40
60
80
−4
−2
0
2
4
Tumor−Normal Expression Difference
hsa−miR−1227−3p, rectal
(BRAF = 0; N0 = 685)
1−sided adj pval: 0
Frequency
0
20
40
60
80
−4
−2
0
2
4
Tumor−Normal Expression Difference
hsa−miR−1227−3p, rectal
(BRAF = 1; N1 = 19)
1−sided adj pval: 0.553
Frequency
0.0
0.2
0.4
0.6
0.8
1.0
−4
−2
0
2
4
Tumor−Normal Expression Difference

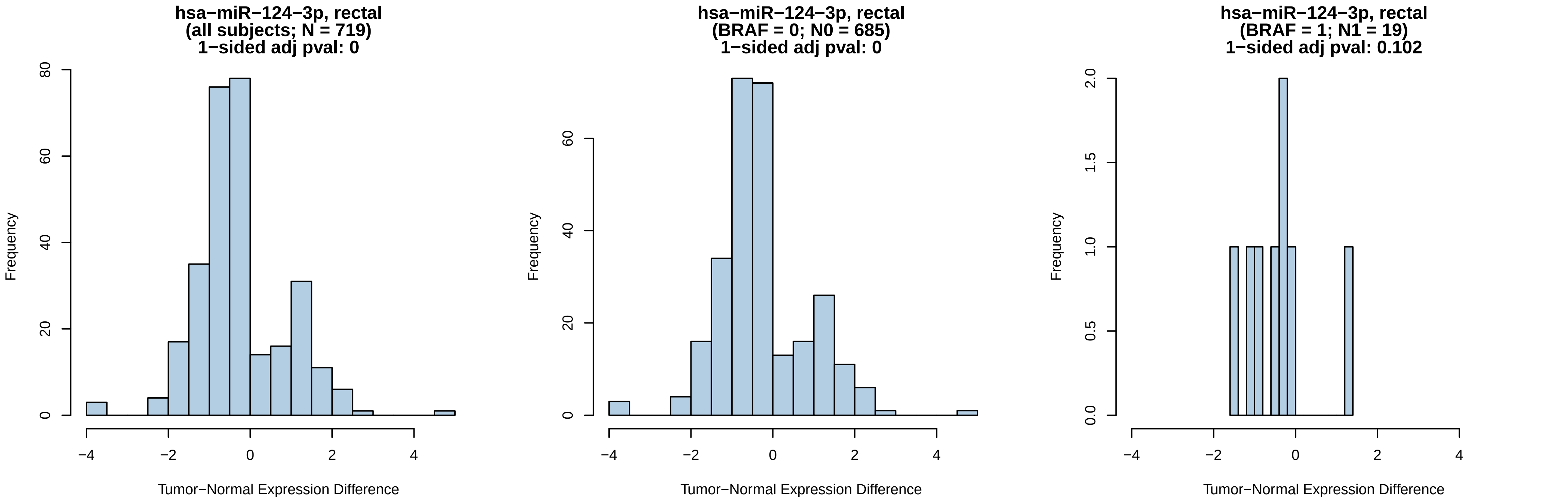
hsa−miR−124−3p, rectal
(all subjects; N = 719)
1−sided adj pval: 0
Frequency
Tumor−Normal Expression Difference
hsa−miR−124−3p, rectal
(BRAF = 0; N0 = 685)
1−sided adj pval: 0
Frequency
Tumor−Normal Expression Difference
hsa−miR−124−3p, rectal
(BRAF = 1; N1 = 19)
1−sided adj pval: 0.102
Frequency
Tumor−Normal Expression Difference

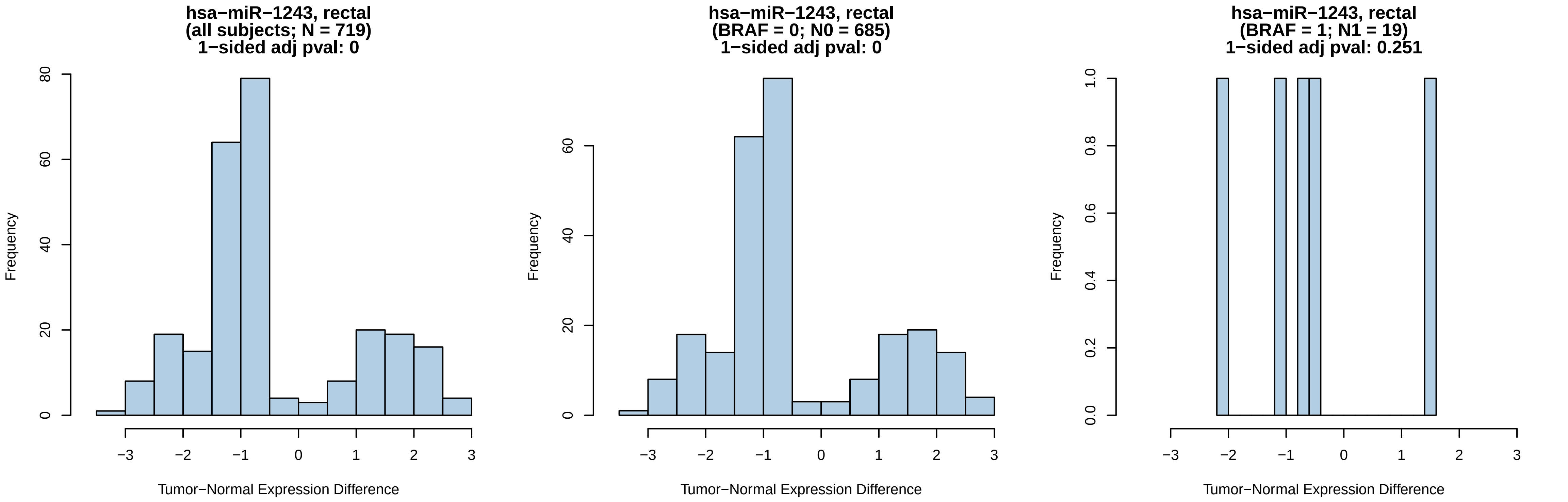

hsa−miR−1243, rectal
(all subjects; N = 719)
1−sided adj pval: 0
Frequency
Tumor−Normal Expression Difference
hsa−miR−1243, rectal
(BRAF = 0; N0 = 685)
1−sided adj pval: 0
Frequency
Tumor−Normal Expression Difference
hsa−miR−1243, rectal
(BRAF = 1; N1 = 19)
1−sided adj pval: 0.251
Frequency
Tumor−Normal Expression Difference

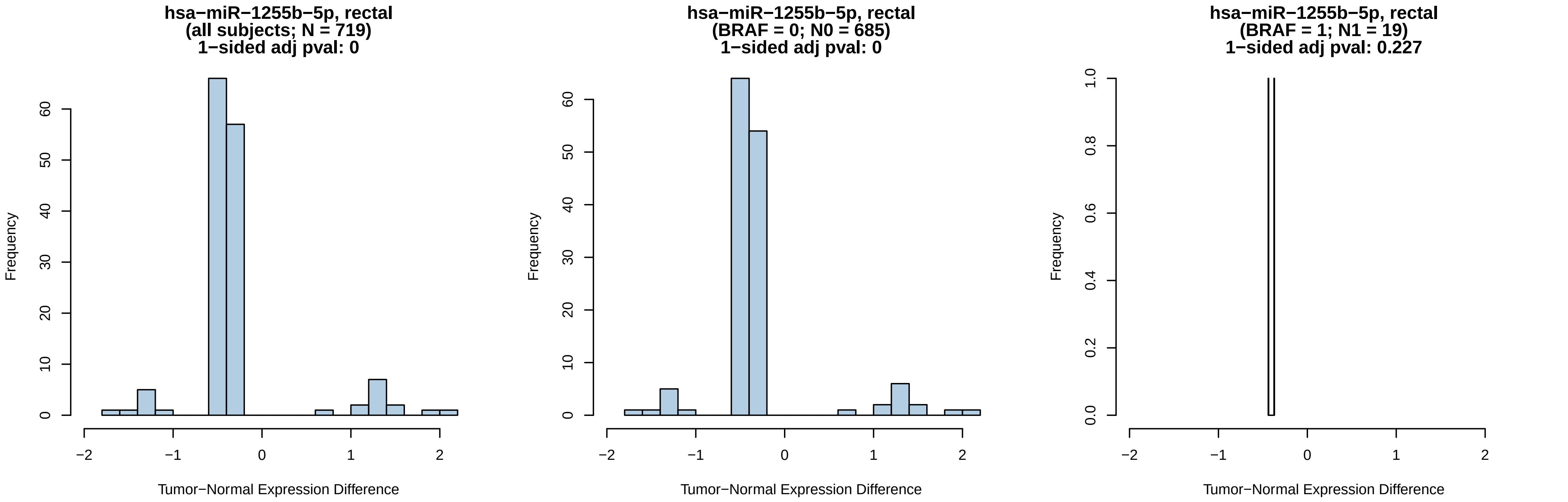

hsa−miR−1255b−5p, rectal
(all subjects; N = 719)
1−sided adj pval: 0
Frequency
Tumor−Normal Expression Difference
hsa−miR−1255b−5p, rectal
(BRAF = 0; N0 = 685)
1−sided adj pval: 0
Frequency
Tumor−Normal Expression Difference
hsa−miR−1255b−5p, rectal
(BRAF = 1; N1 = 19)
1−sided adj pval: 0.227
Frequency
Tumor−Normal Expression Difference

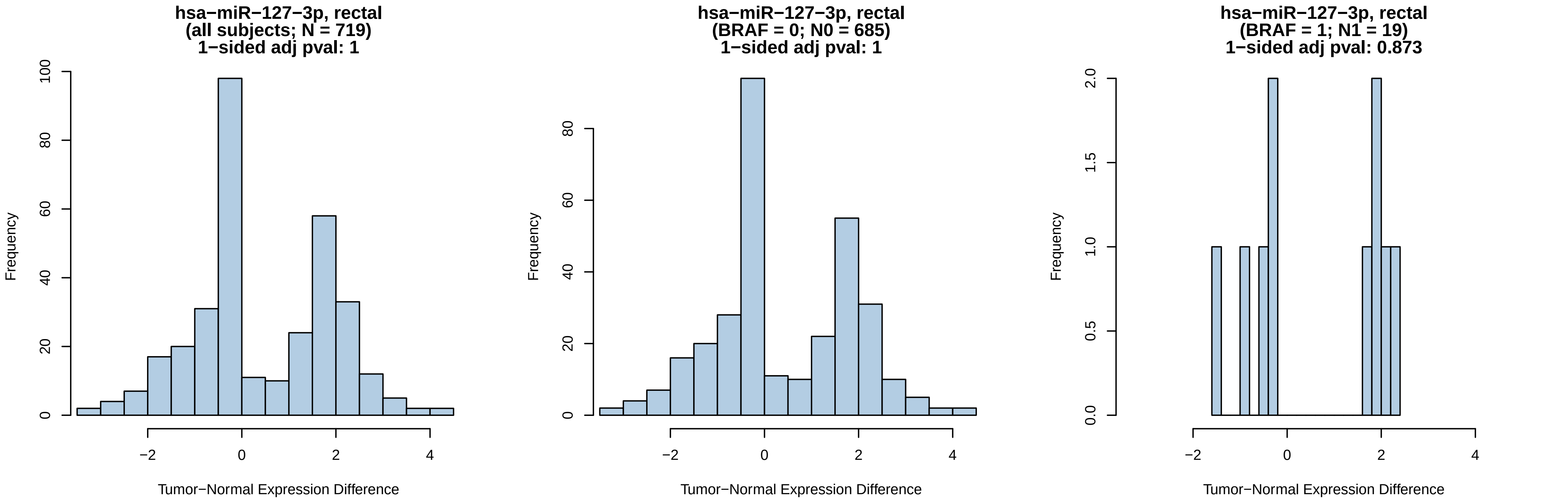
hsa−miR−127−3p, rectal
(all subjects; N = 719)
1−sided adj pval: 1
Frequency
Tumor−Normal Expression Difference
hsa−miR−127−3p, rectal
(BRAF = 0; N0 = 685)
1−sided adj pval: 1
Frequency
Tumor−Normal Expression Difference
hsa−miR−127−3p, rectal
(BRAF = 1; N1 = 19)
1−sided adj pval: 0.873
Frequency
Tumor−Normal Expression Difference

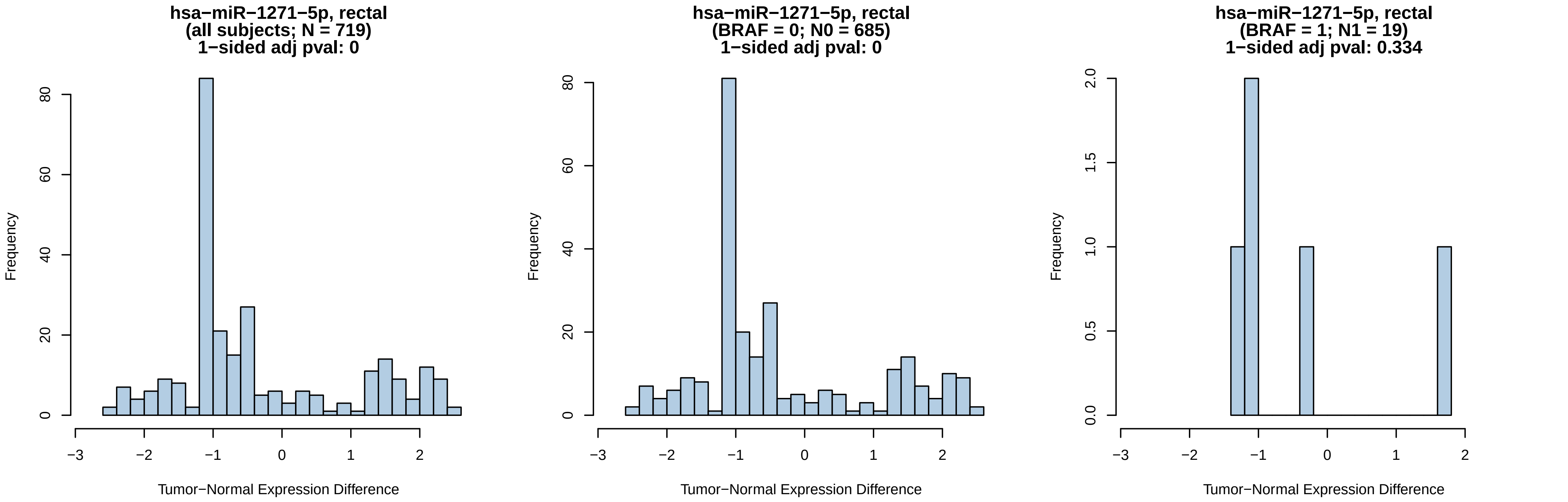

hsa−miR−1271−5p, rectal
(all subjects; N = 719)
1−sided adj pval: 0
Frequency
Tumor−Normal Expression Difference
hsa−miR−1271−5p, rectal
(BRAF = 0; N0 = 685)
1−sided adj pval: 0
Frequency
Tumor−Normal Expression Difference
hsa−miR−1271−5p, rectal
(BRAF = 1; N1 = 19)
1−sided adj pval: 0.334
Frequency
Tumor−Normal Expression Difference

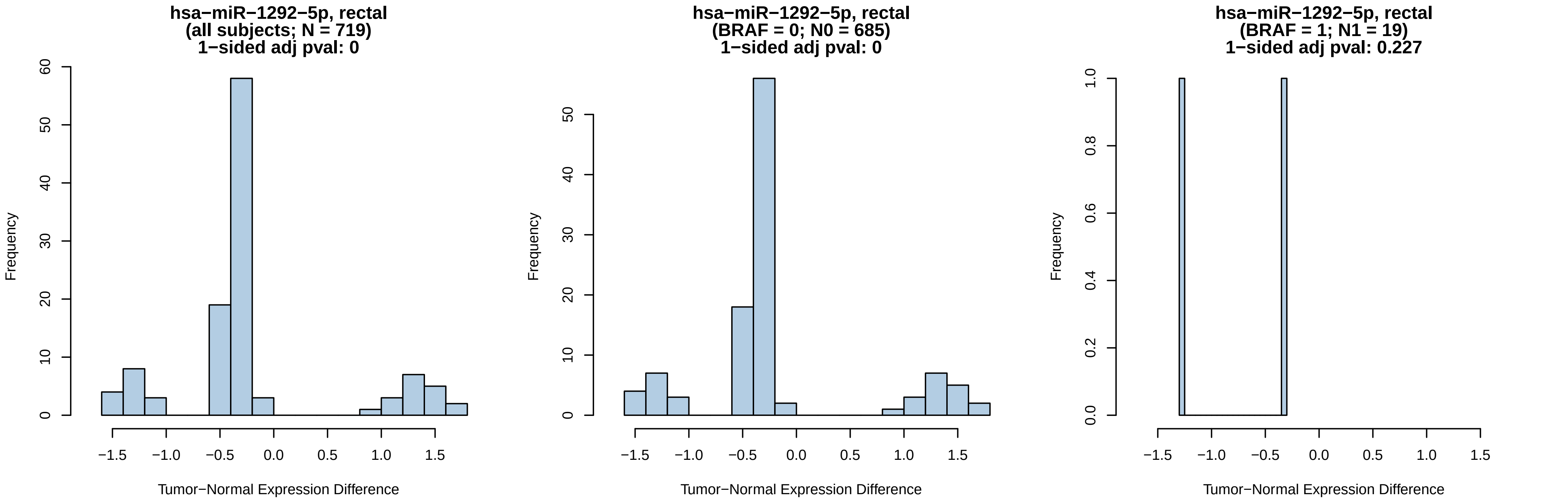

hsa−miR−1292−5p, rectal
(all subjects; N = 719)
1−sided adj pval: 0
Frequency
Tumor−Normal Expression Difference
hsa−miR−1292−5p, rectal
(BRAF = 0; N0 = 685)
1−sided adj pval: 0
Frequency
Tumor−Normal Expression Difference
hsa−miR−1292−5p, rectal
(BRAF = 1; N1 = 19)
1−sided adj pval: 0.227
Frequency
Tumor−Normal Expression Difference

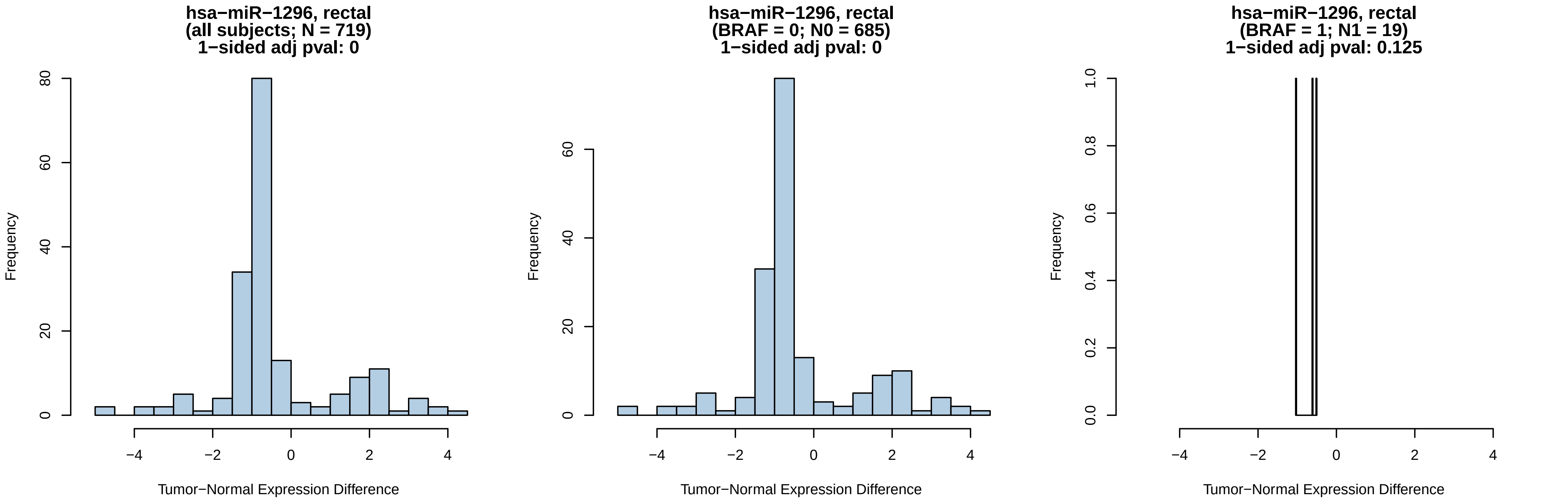

hsa−miR−1296, rectal
(all subjects; N = 719)
1−sided adj pval: 0
hsa−miR−1296, rectal
(BRAF = 0; N0 = 685)
1−sided adj pval: 0
hsa−miR−1296, rectal
(BRAF = 1; N1 = 19)
1−sided adj pval: 0.125
Frequency
Tumor−Normal Expression Difference

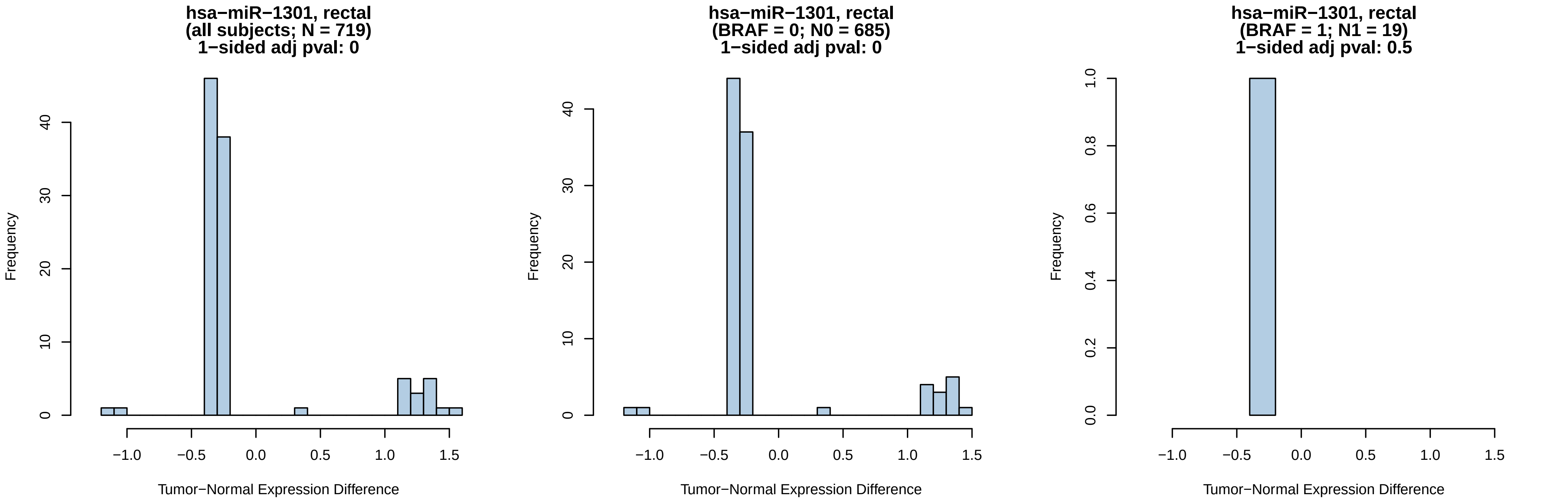
hsa−miR−1301, rectal
(all subjects; N = 719)
1−sided adj pval: 0
Frequency
Tumor−Normal Expression Difference
hsa−miR−1301, rectal
(BRAF = 0; N0 = 685)
1−sided adj pval: 0
Frequency
Tumor−Normal Expression Difference
hsa−miR−1301, rectal
(BRAF = 1; N1 = 19)
1−sided adj pval: 0.5
Frequency
Tumor−Normal Expression Difference

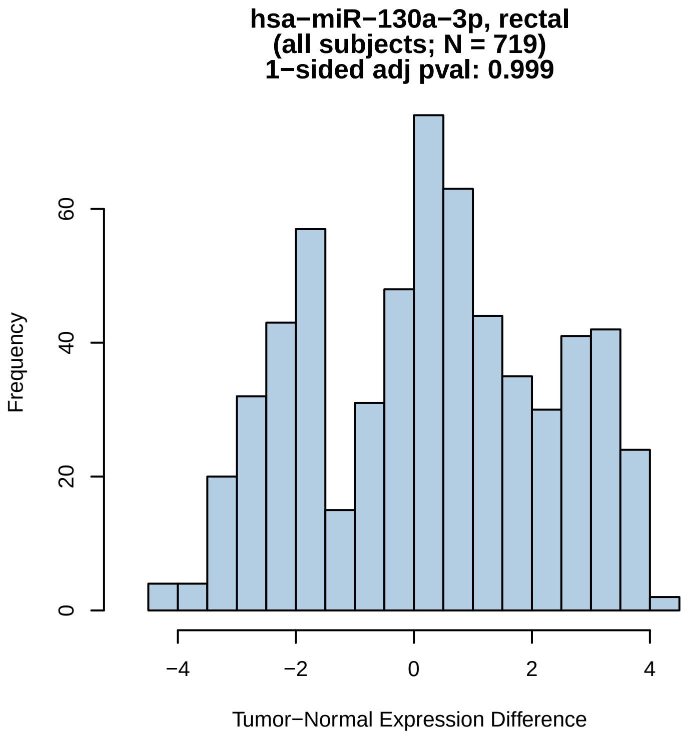

hsa−miR−130a−3p, rectal
(all subjects; N = 719)
1−sided adj pval: 0.999
Frequency
0
20
40
60
−4
−2
0
2
4
Tumor−Normal Expression Difference


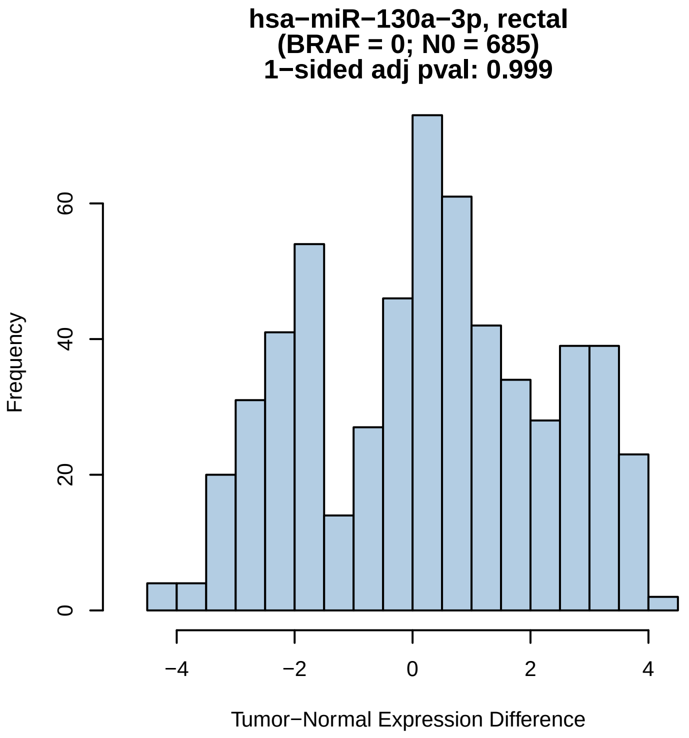

hsa−miR−130a−3p, rectal
(BRAF = 0; N0 = 685)
1−sided adj pval: 0.999
Frequency
0
20
40
60
−4
−2
0
2
4
Tumor−Normal Expression Difference


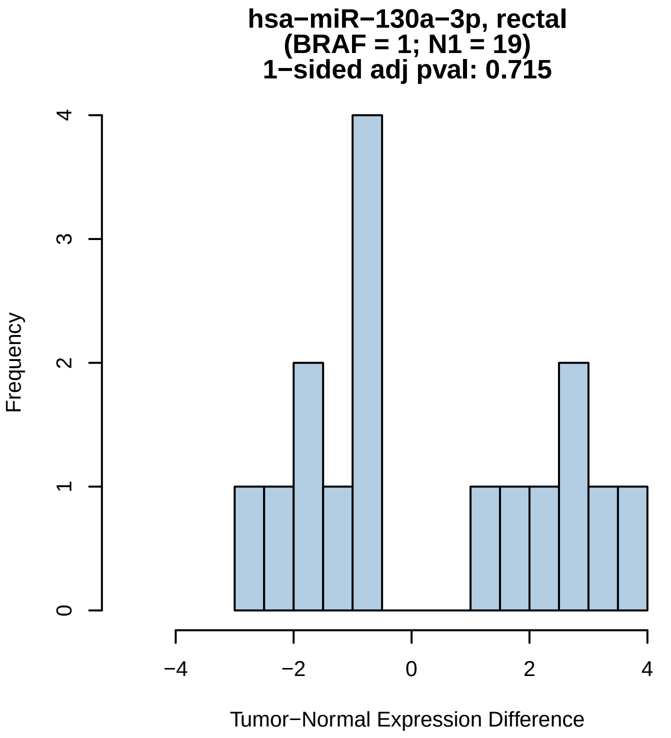

hsa−miR−130a−3p, rectal
(BRAF = 1; N1 = 19)
1−sided adj pval: 0.715
Frequency
0
1
2
3
4
−4
−2
0
2
4
Tumor−Normal Expression Difference

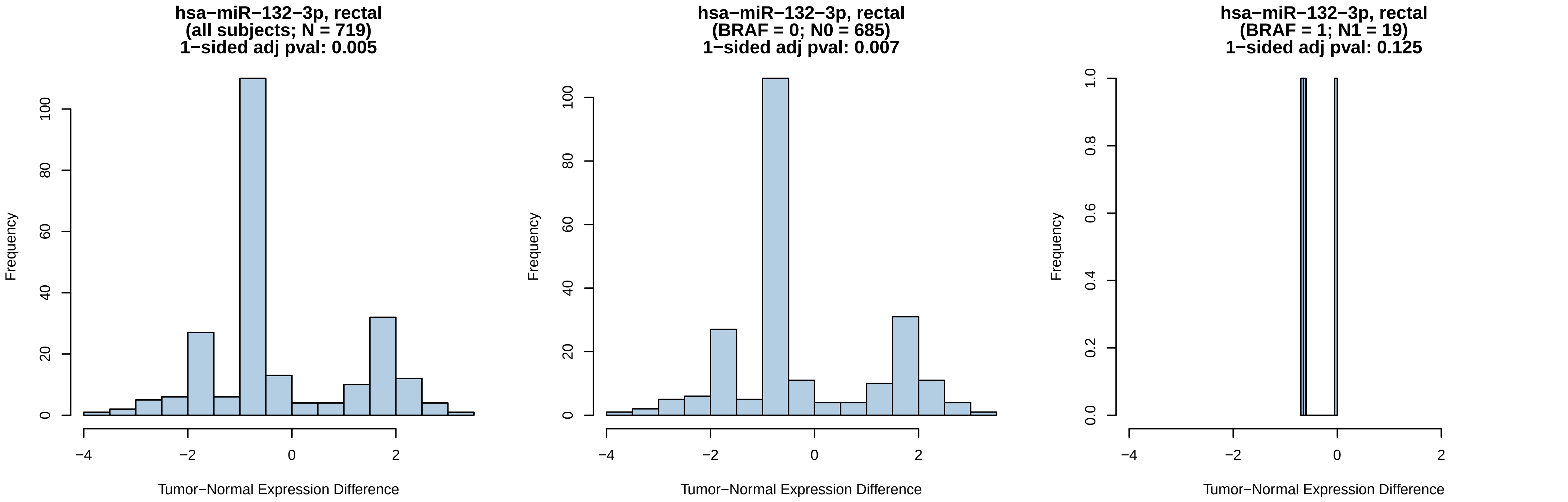
hsa−miR−132−3p, rectal
(all subjects; N = 719)
1−sided adj pval: 0.005
Frequency
Tumor−Normal Expression Difference
hsa−miR−132−3p, rectal
(BRAF = 0; N0 = 685)
1−sided adj pval: 0.007
Frequency
Tumor−Normal Expression Difference
hsa−miR−132−3p, rectal
(BRAF = 1; N1 = 19)
1−sided adj pval: 0.125
Frequency
Tumor−Normal Expression Difference

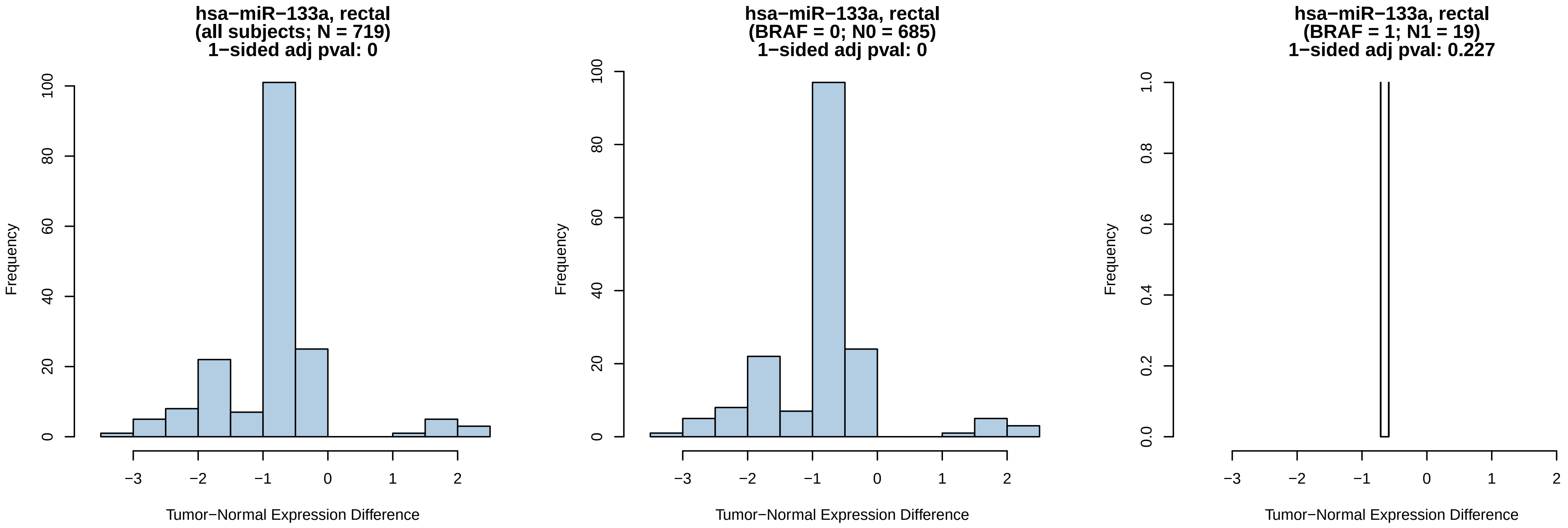
hsa−miR−133a, rectal
(all subjects; N = 719)
1−sided adj pval: 0
Frequency
0
20
40
60
80
100
−3
−2
−1
0
1
2
Tumor−Normal Expression Difference
hsa−miR−133a, rectal
(BRAF = 0; N0 = 685)
1−sided adj pval: 0
Frequency
0
20
40
60
80
100
−3
−2
−1
0
1
2
Tumor−Normal Expression Difference
hsa−miR−133a, rectal
(BRAF = 1; N1 = 19)
1−sided adj pval: 0.227
Frequency
0.0
0.2
0.4
0.6
0.8
1.0
−3
−2
−1
0
1
2
Tumor−Normal Expression Difference

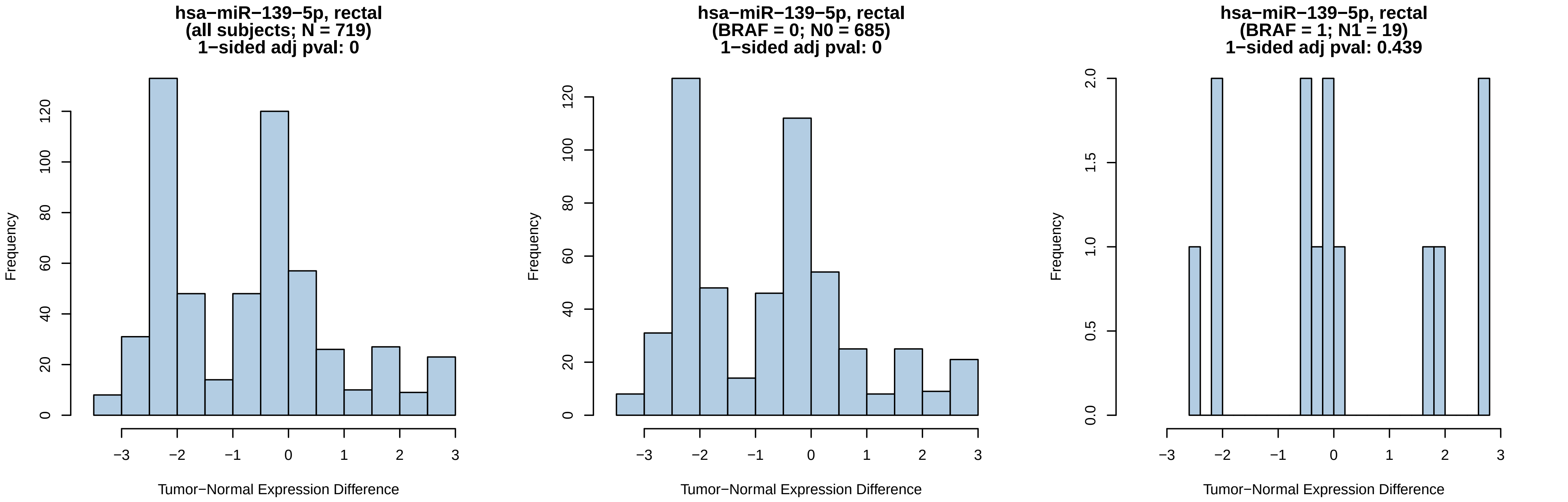
hsa−miR−139−5p, rectal
(all subjects; N = 719)
1−sided adj pval: 0
Frequency
Tumor−Normal Expression Difference
hsa−miR−139−5p, rectal
(BRAF = 0; N0 = 685)
1−sided adj pval: 0
Frequency
Tumor−Normal Expression Difference
hsa−miR−139−5p, rectal
(BRAF = 1; N1 = 19)
1−sided adj pval: 0.439
Frequency
Tumor−Normal Expression Difference

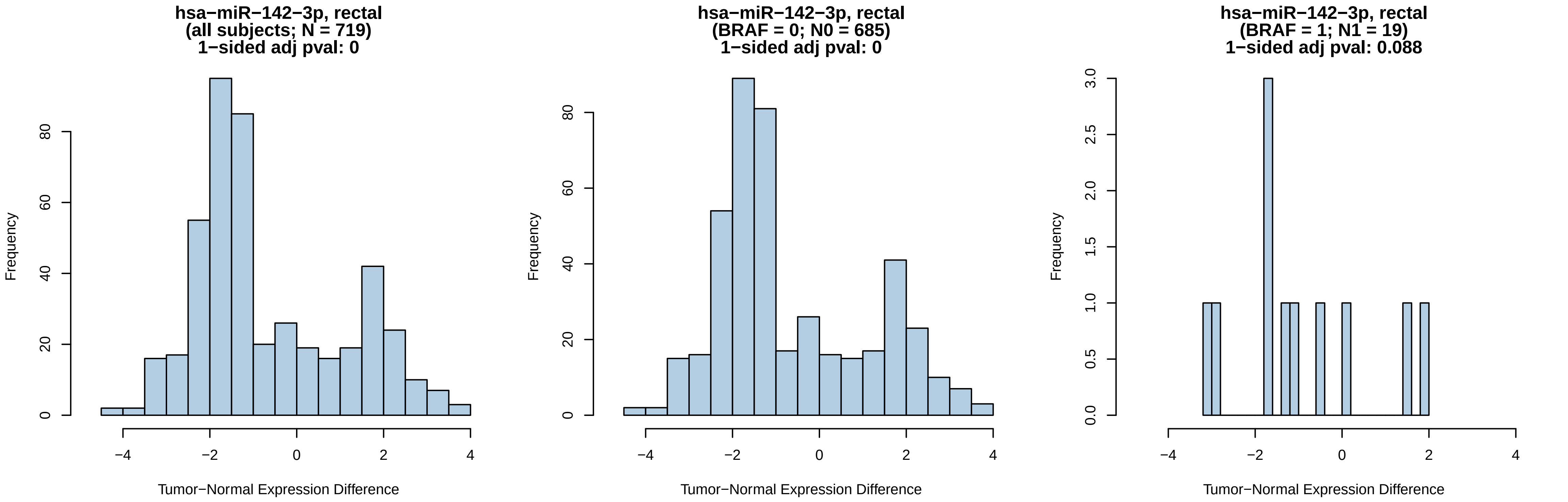
hsa−miR−142−3p, rectal
(all subjects; N = 719)
1−sided adj pval: 0
Frequency
Tumor−Normal Expression Difference
hsa−miR−142−3p, rectal
(BRAF = 0; N0 = 685)
1−sided adj pval: 0
Frequency
Tumor−Normal Expression Difference
hsa−miR−142−3p, rectal
(BRAF = 1; N1 = 19)
1−sided adj pval: 0.088
Frequency
Tumor−Normal Expression Difference

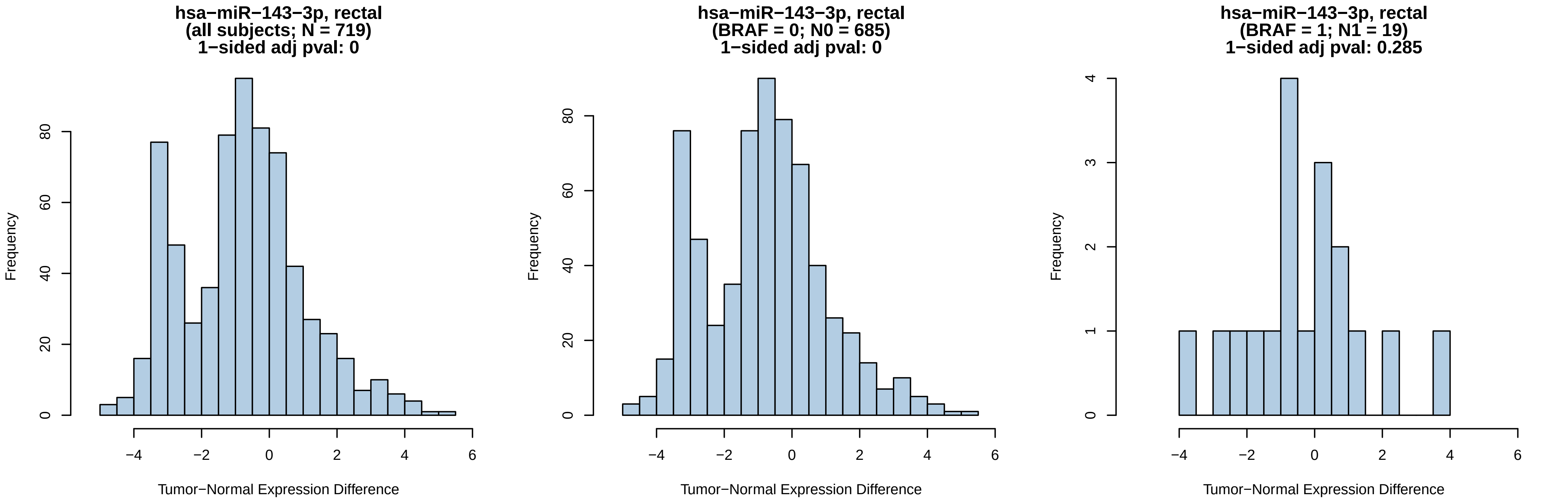
hsa−miR−143−3p, rectal
(all subjects; N = 719)
1−sided adj pval: 0
Frequency
Tumor−Normal Expression Difference
hsa−miR−143−3p, rectal
(BRAF = 0; N0 = 685)
1−sided adj pval: 0
Frequency
Tumor−Normal Expression Difference
hsa−miR−143−3p, rectal
(BRAF = 1; N1 = 19)
1−sided adj pval: 0.285
Frequency
Tumor−Normal Expression Difference

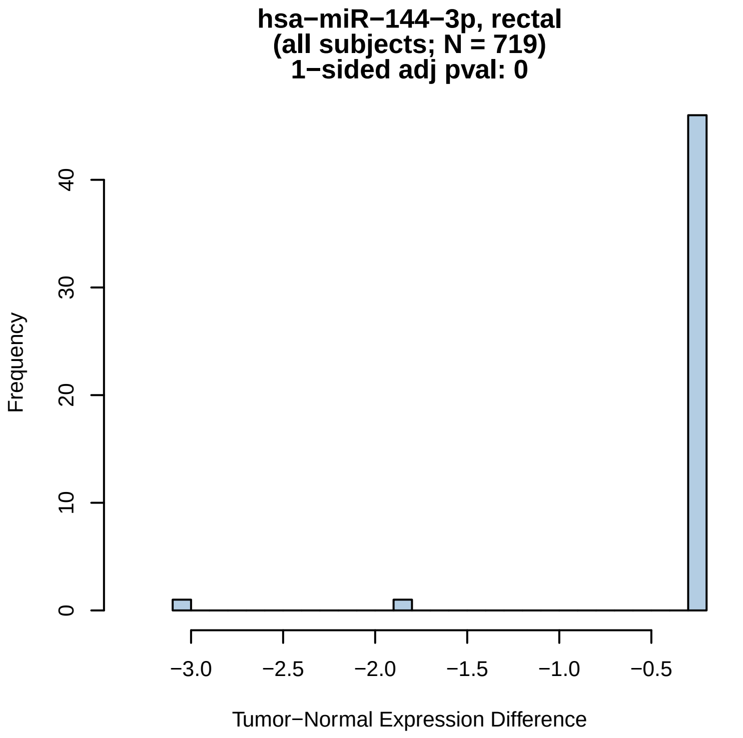
hsa−miR−144−3p, rectal
(all subjects; N = 719)
1−sided adj pval: 0
Frequency
0
10
20
30
40
−3.0
−2.5
−2.0
−1.5
−1.0
−0.5
Tumor−Normal Expression Difference

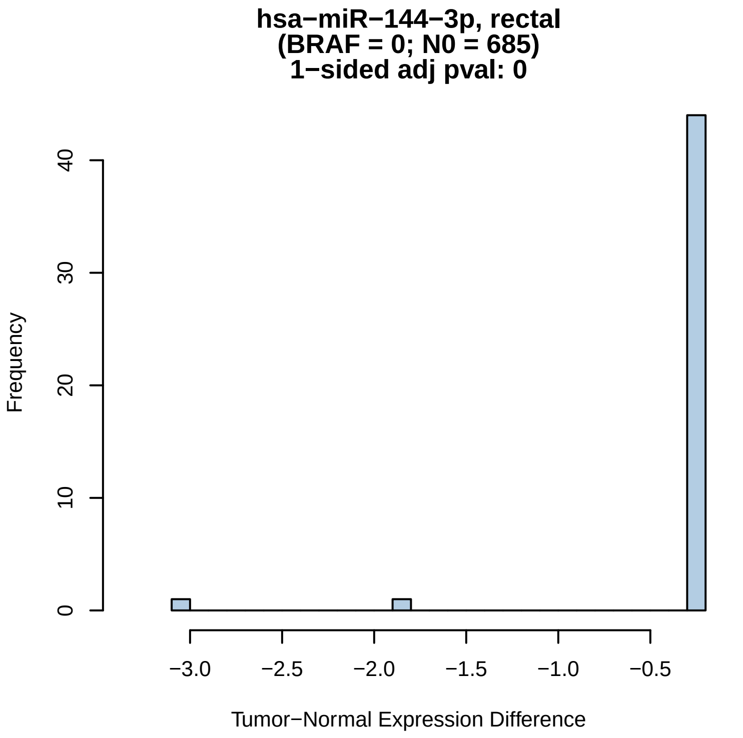
hsa−miR−144−3p, rectal
(BRAF = 0; N0 = 685)
1−sided adj pval: 0
Frequency
0
10
20
30
40
−3.0
−2.5
−2.0
−1.5
−1.0
−0.5
Tumor−Normal Expression Difference

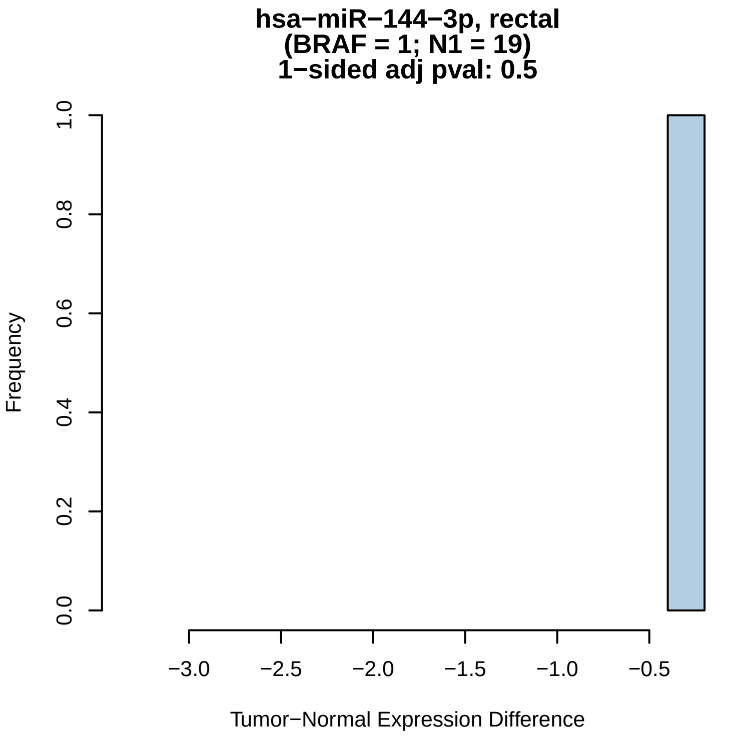
hsa−miR−144−3p, rectal
(BRAF = 1; N1 = 19)
1−sided adj pval: 0.5
Frequency
0.0
0.2
0.4
0.6
0.8
1.0
−3.0
−2.5
−2.0
−1.5
−1.0
−0.5
Tumor−Normal Expression Difference

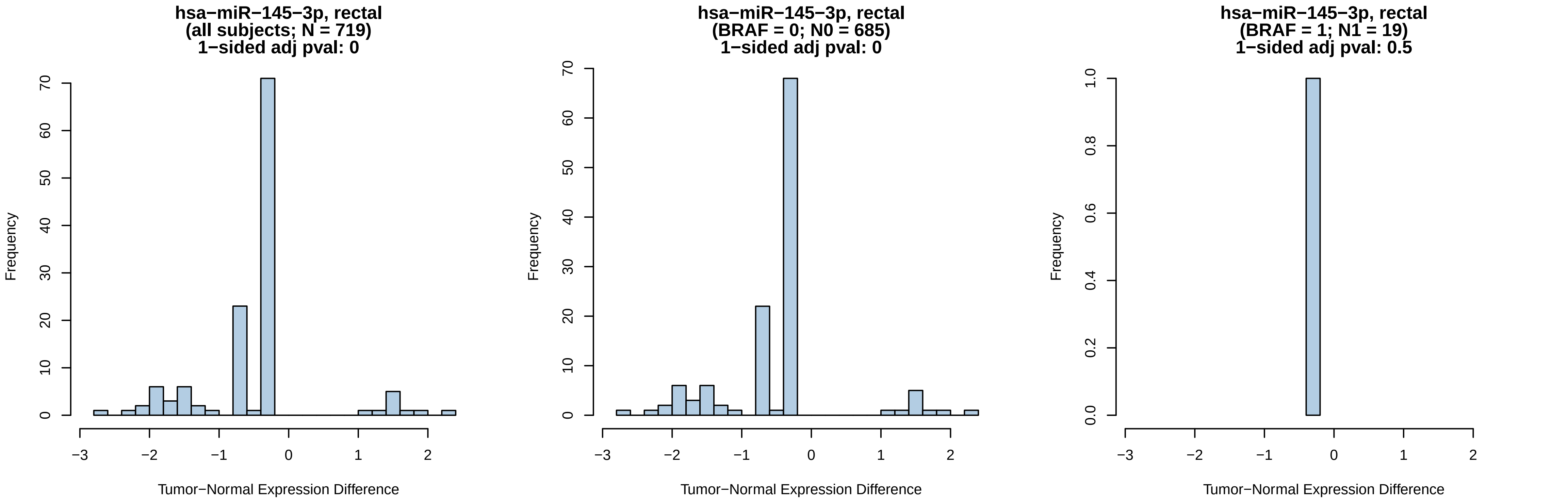
hsa−miR−145−3p, rectal
(all subjects; N = 719)
1−sided adj pval: 0
Frequency
0
10
20
30
40
50
60
70
−3
−2
−1
0
1
2
Tumor−Normal Expression Difference
hsa−miR−145−3p, rectal
(BRAF = 0; N0 = 685)
1−sided adj pval: 0
Frequency
0
10
20
30
40
50
60
70
−3
−2
−1
0
1
2
Tumor−Normal Expression Difference
hsa−miR−145−3p, rectal
(BRAF = 1; N1 = 19)
1−sided adj pval: 0.5
Frequency
0.0
0.2
0.4
0.6
0.8
1.0
−3
−2
−1
0
1
2
Tumor−Normal Expression Difference

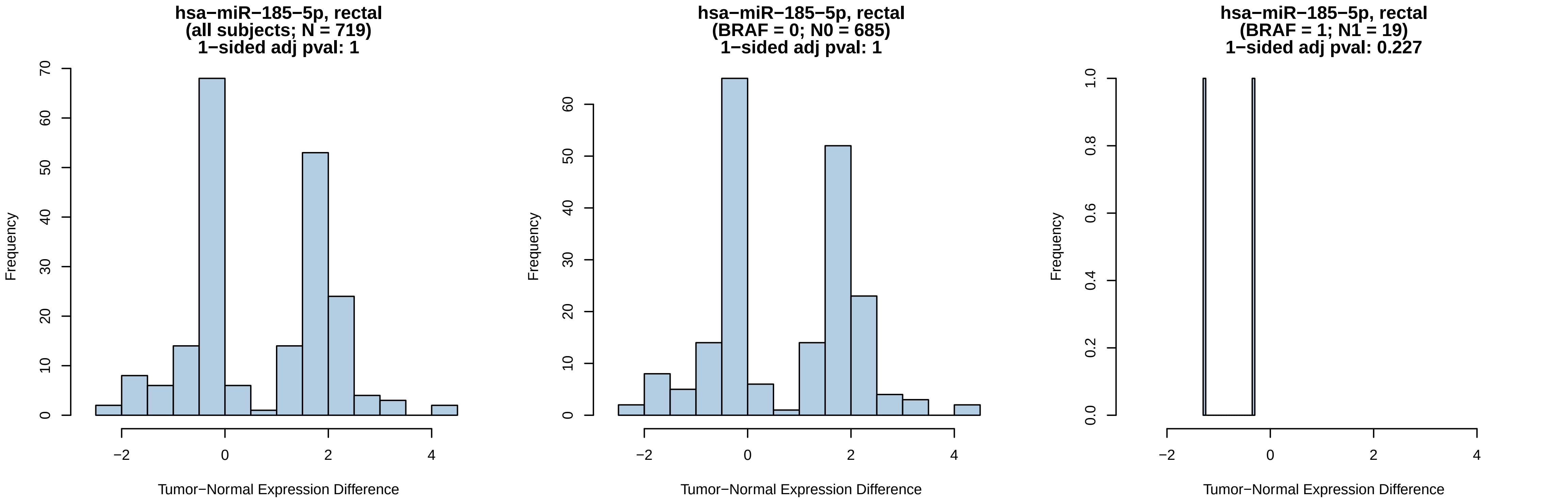

hsa−miR−185−5p, rectal
(all subjects; N = 719)
1−sided adj pval: 1
hsa−miR−185−5p, rectal
(BRAF = 0; N0 = 685)
1−sided adj pval: 1
hsa−miR−185−5p, rectal
(BRAF = 1; N1 = 19)
1−sided adj pval: 0.227
Frequency
Tumor−Normal Expression Difference

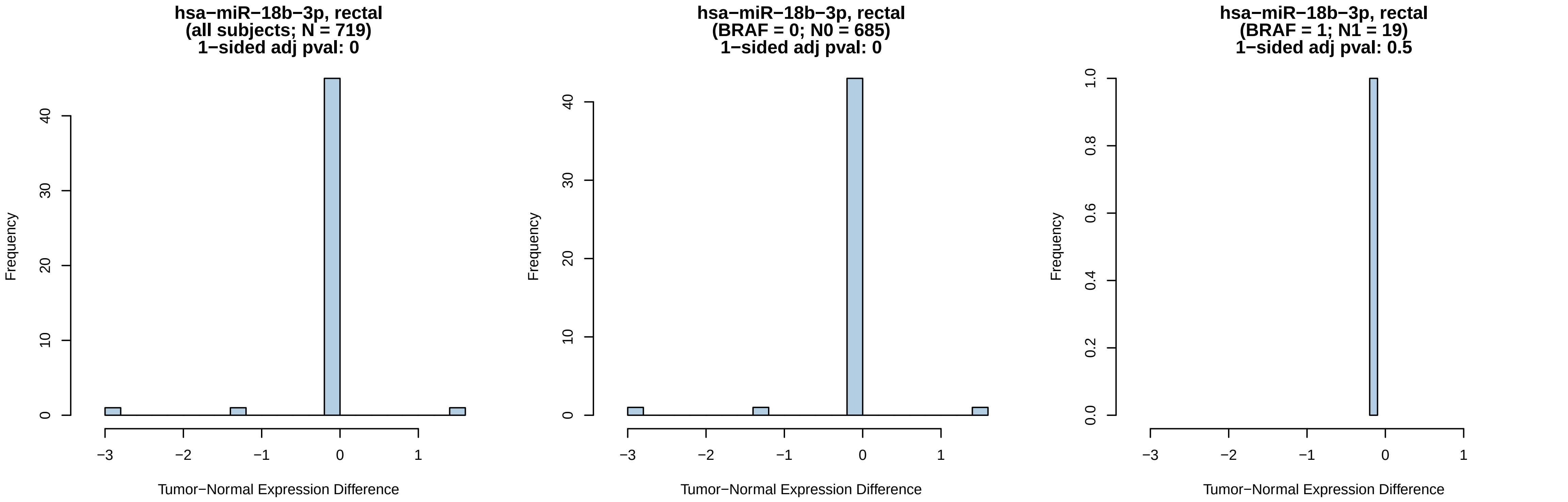

hsa−miR−18b−3p, rectal
(all subjects; N = 719)
1−sided adj pval: 0
Frequency
0
10
20
30
40
−3
−2
−1
0
1
Tumor−Normal Expression Difference
hsa−miR−18b−3p, rectal
(BRAF = 0; N0 = 685)
1−sided adj pval: 0
Frequency
0
10
20
30
40
−3
−2
−1
0
1
Tumor−Normal Expression Difference
hsa−miR−18b−3p, rectal
(BRAF = 1; N1 = 19)
1−sided adj pval: 0.5
Frequency
0.0
0.2
0.4
0.6
0.8
1.0
−3
−2
−1
0
1
Tumor−Normal Expression Difference

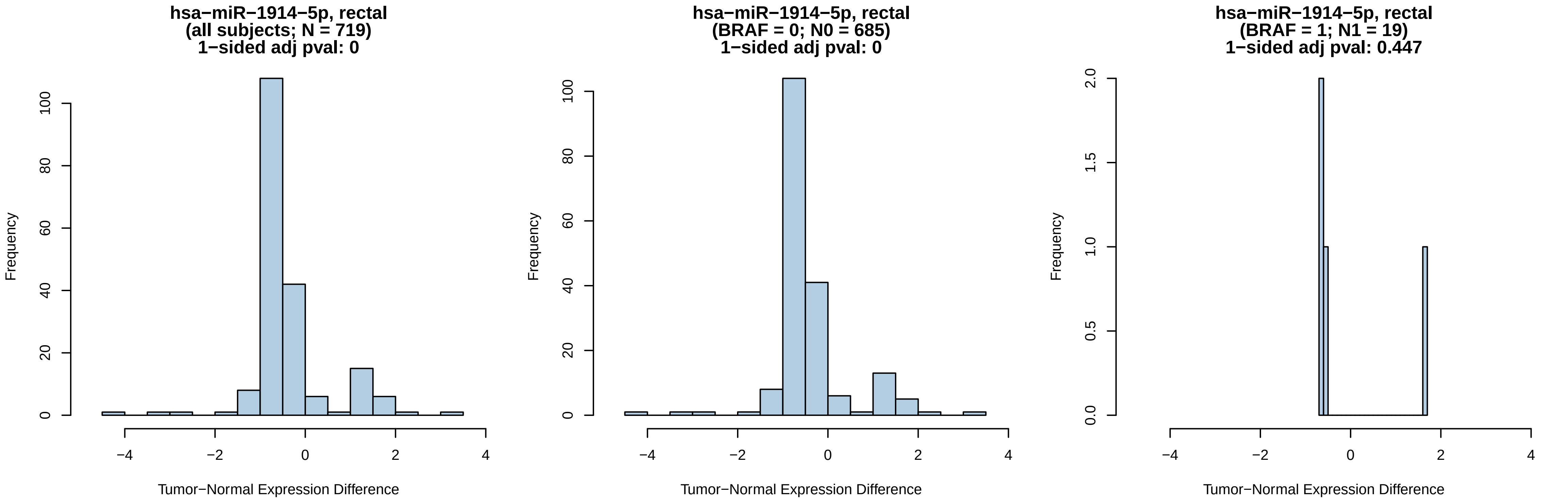
hsa−miR−1914−5p, rectal
(all subjects; N = 719)
1−sided adj pval: 0
Frequency
Tumor−Normal Expression Difference
hsa−miR−1914−5p, rectal
(BRAF = 0; N0 = 685)
1−sided adj pval: 0
Frequency
Tumor−Normal Expression Difference
hsa−miR−1914−5p, rectal
(BRAF = 1; N1 = 19)
1−sided adj pval: 0.447
Frequency
Tumor−Normal Expression Difference

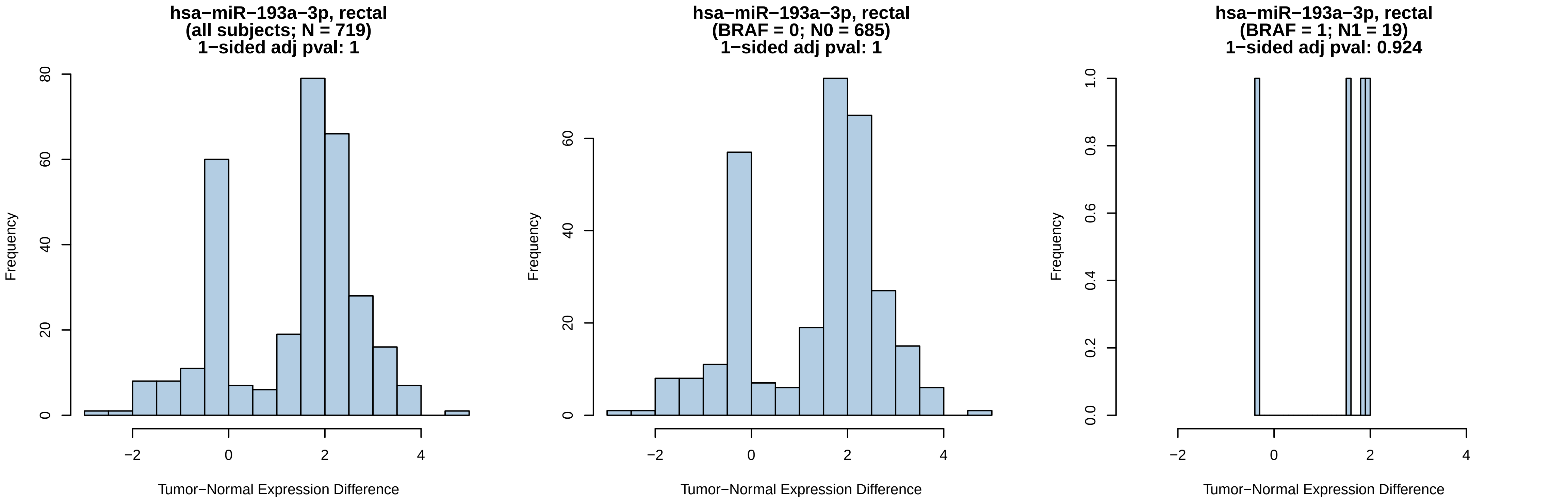
hsa−miR−193a−3p, rectal
(all subjects; N = 719)
1−sided adj pval: 1
hsa−miR−193a−3p, rectal
(BRAF = 0; N0 = 685)
1−sided adj pval: 1
hsa−miR−193a−3p, rectal
(BRAF = 1; N1 = 19)
1−sided adj pval: 0.924
Frequency
Tumor−Normal Expression Difference

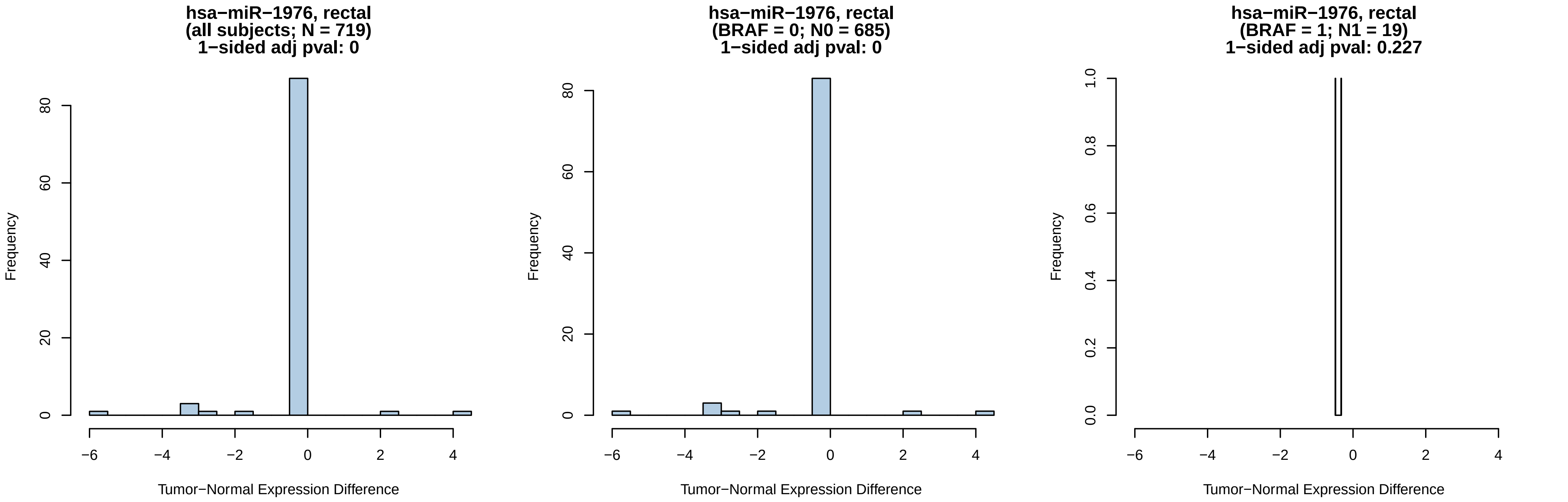

hsa−miR−1976, rectal
(all subjects; N = 719)
1−sided adj pval: 0
Frequency
0
20
40
60
80
−6
−4
−2
0
2
4
Tumor−Normal Expression Difference
hsa−miR−1976, rectal
(BRAF = 0; N0 = 685)
1−sided adj pval: 0
Frequency
0
20
40
60
80
−6
−4
−2
0
2
4
Tumor−Normal Expression Difference
hsa−miR−1976, rectal
(BRAF = 1; N1 = 19)
1−sided adj pval: 0.227
Frequency
0.0
0.2
0.4
0.6
0.8
1.0
−6
−4
−2
0
2
4
Tumor−Normal Expression Difference

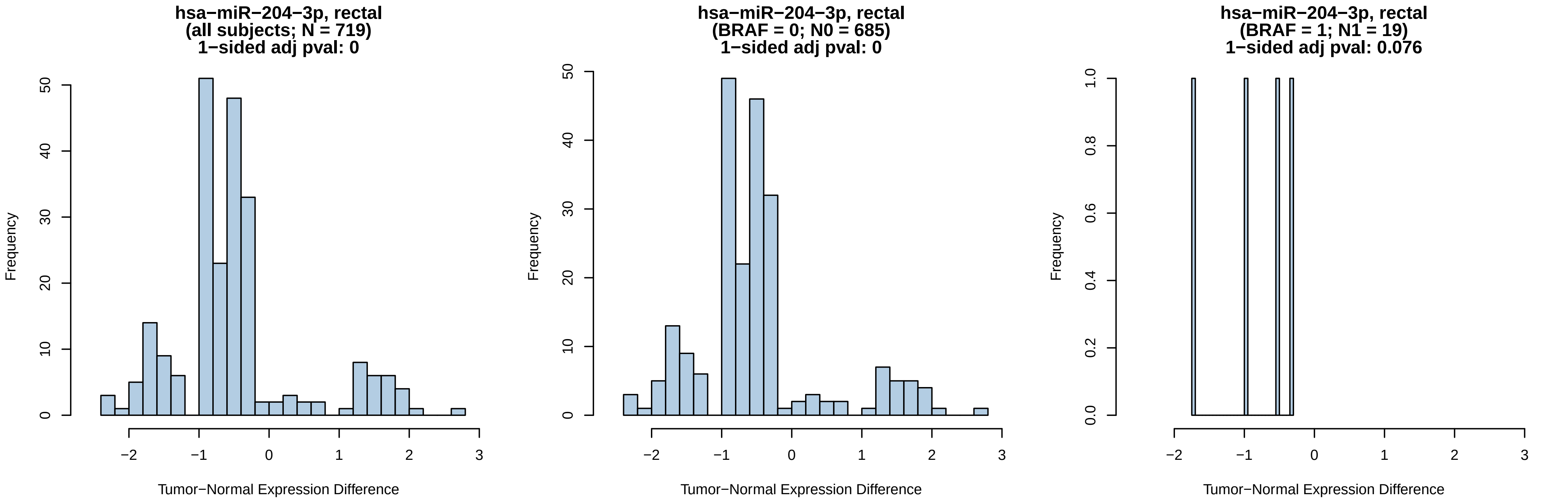

hsa−miR−204−3p, rectal
(all subjects; N = 719)
1−sided adj pval: 0
hsa−miR−204−3p, rectal
(BRAF = 0; N0 = 685)
1−sided adj pval: 0
hsa−miR−204−3p, rectal
(BRAF = 1; N1 = 19)
1−sided adj pval: 0.076
Frequency
Tumor−Normal Expression Difference

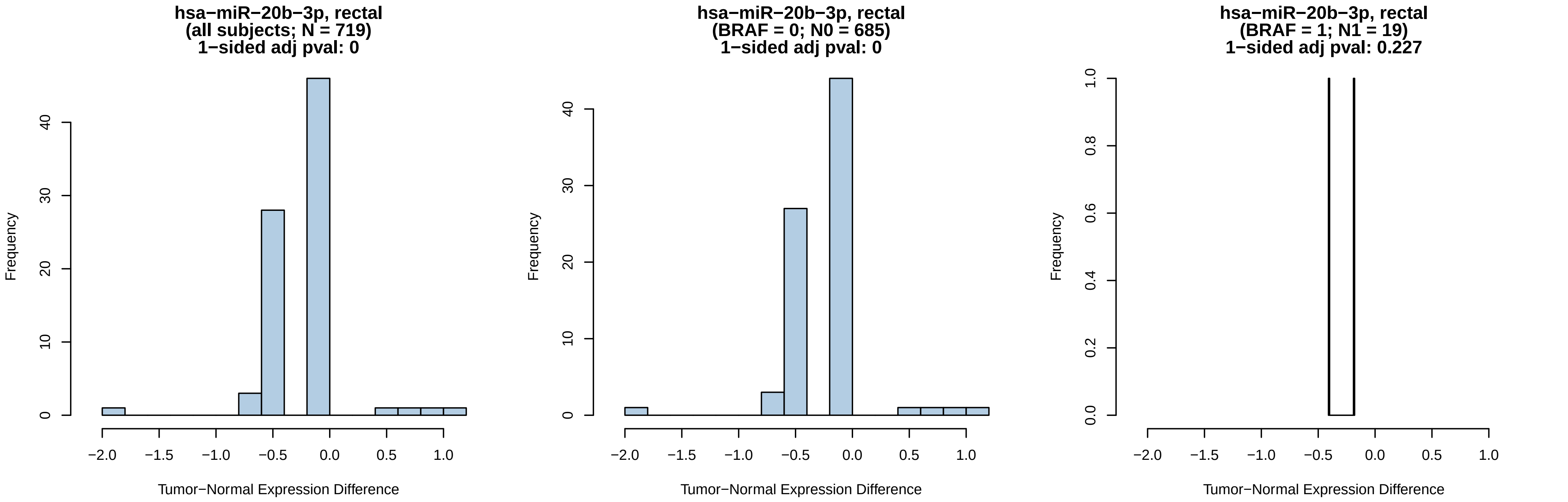
hsa−miR−20b−3p, rectal
(all subjects; N = 719)
1−sided adj pval: 0
Frequency
Tumor−Normal Expression Difference
hsa−miR−20b−3p, rectal
(BRAF = 0; N0 = 685)
1−sided adj pval: 0
Frequency
Tumor−Normal Expression Difference
hsa−miR−20b−3p, rectal
(BRAF = 1; N1 = 19)
1−sided adj pval: 0.227
Frequency
Tumor−Normal Expression Difference

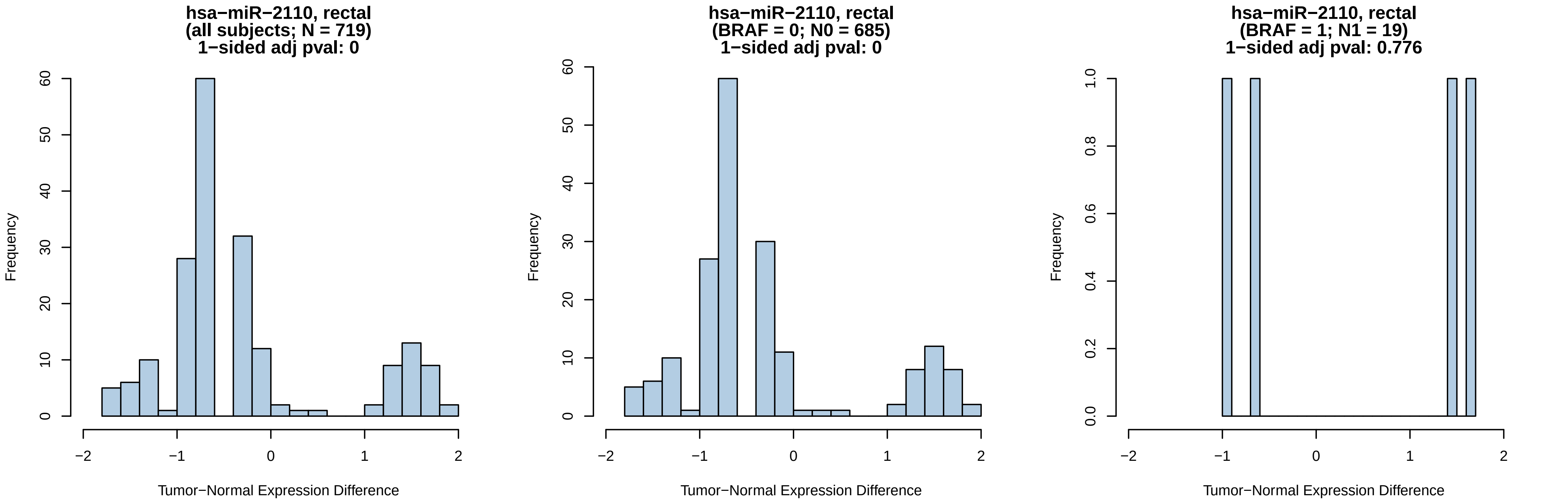
hsa−miR−2110, rectal
(all subjects; N = 719)
1−sided adj pval: 0
Frequency
Tumor−Normal Expression Difference
hsa−miR−2110, rectal
(BRAF = 0; N0 = 685)
1−sided adj pval: 0
Frequency
Tumor−Normal Expression Difference
hsa−miR−2110, rectal
(BRAF = 1; N1 = 19)
1−sided adj pval: 0.776
Frequency
Tumor−Normal Expression Difference

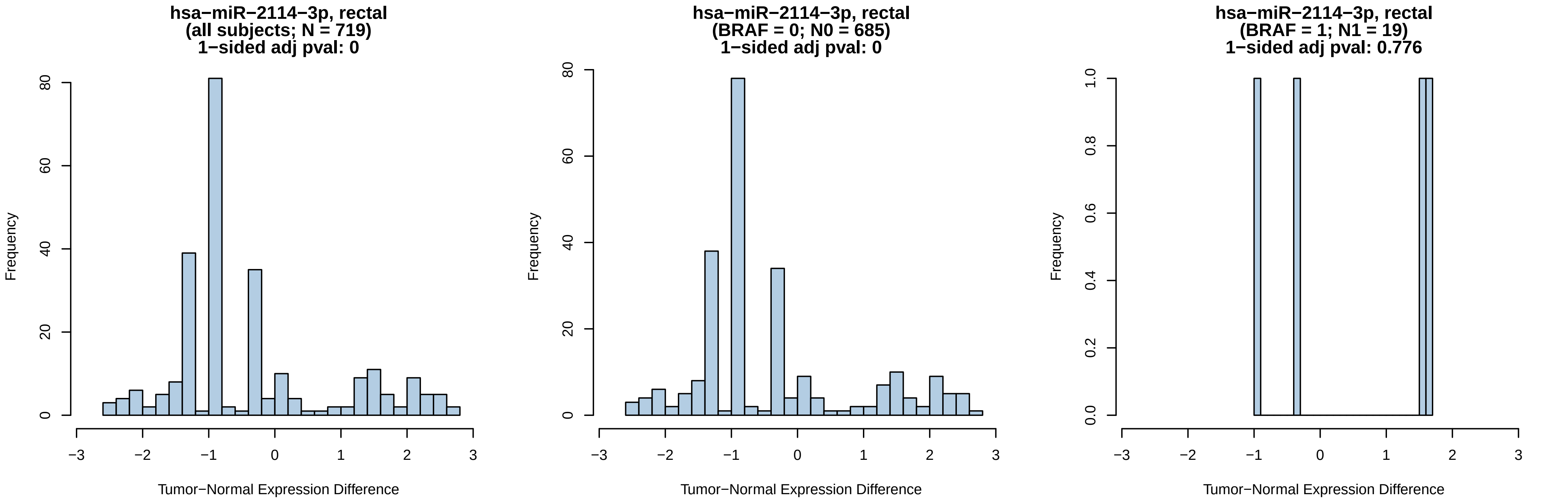
hsa−miR−2114−3p, rectal
(all subjects; N = 719)
1−sided adj pval: 0
Frequency
Tumor−Normal Expression Difference
hsa−miR−2114−3p, rectal
(BRAF = 0; N0 = 685)
1−sided adj pval: 0
Frequency
Tumor−Normal Expression Difference
hsa−miR−2114−3p, rectal
(BRAF = 1; N1 = 19)
1−sided adj pval: 0.776
Frequency
Tumor−Normal Expression Difference

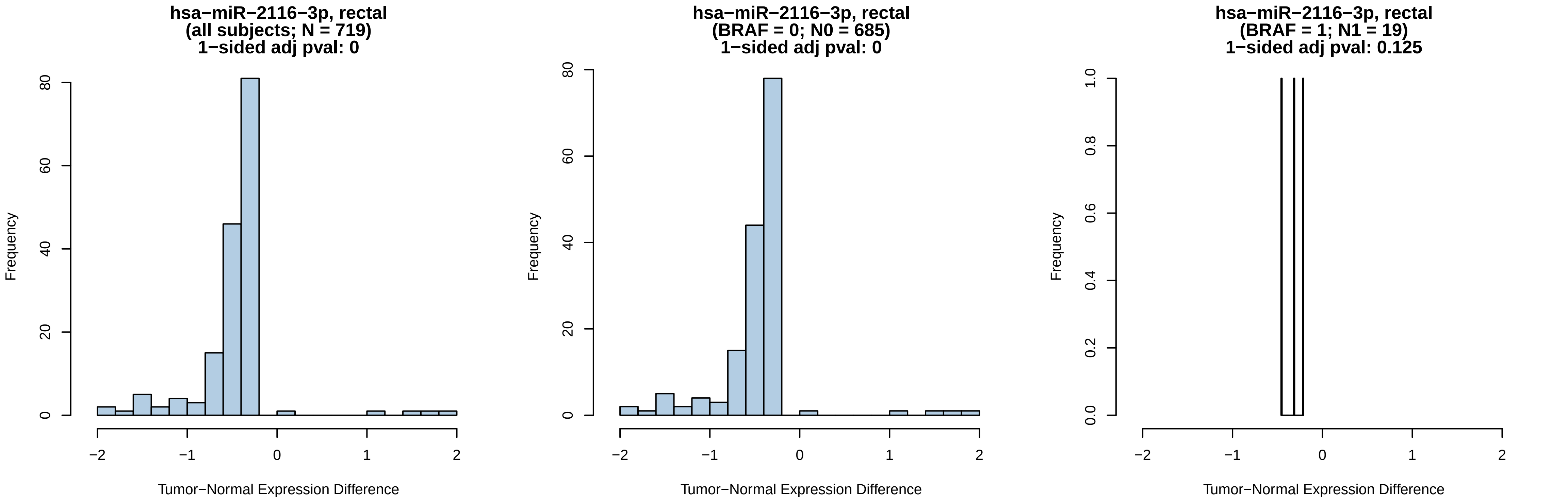

hsa−miR−2116−3p, rectal
(all subjects; N = 719)
1−sided adj pval: 0
Frequency
Tumor−Normal Expression Difference
hsa−miR−2116−3p, rectal
(BRAF = 0; N0 = 685)
1−sided adj pval: 0
Frequency
Tumor−Normal Expression Difference
hsa−miR−2116−3p, rectal
(BRAF = 1; N1 = 19)
1−sided adj pval: 0.125
Frequency
Tumor−Normal Expression Difference

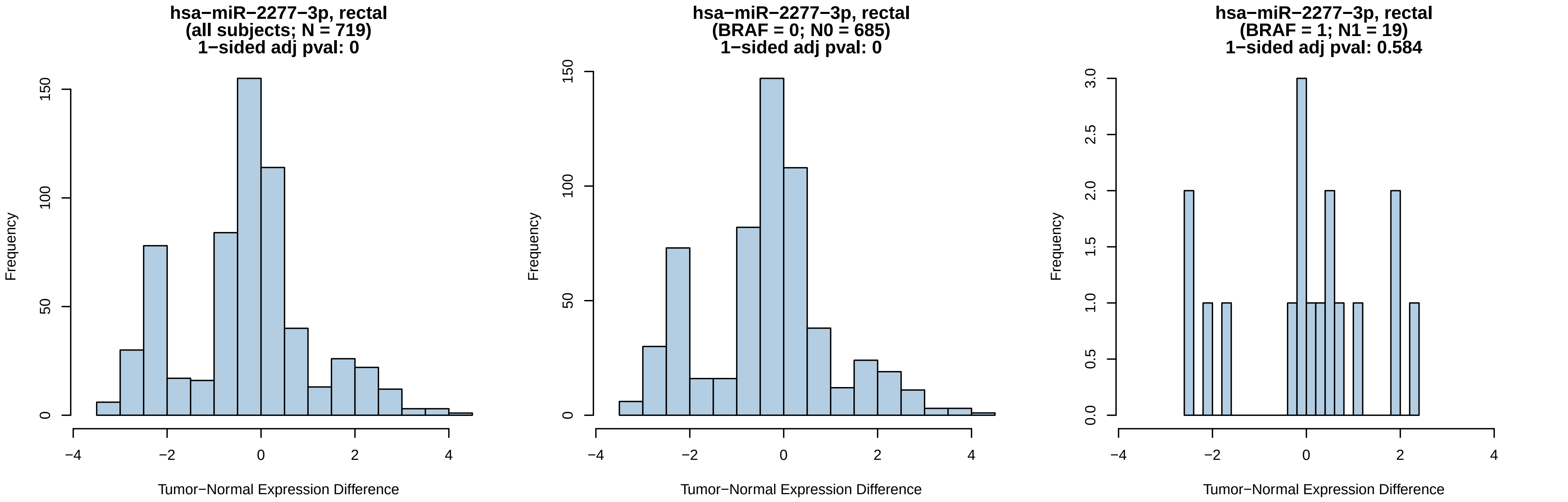
hsa−miR−2277−3p, rectal
(all subjects; N = 719)
1−sided adj pval: 0
hsa−miR−2277−3p, rectal
(BRAF = 0; N0 = 685)
1−sided adj pval: 0
hsa−miR−2277−3p, rectal
(BRAF = 1; N1 = 19)
1−sided adj pval: 0.584
Frequency
Tumor−Normal Expression Difference
0
50
100
150
0.0
0.5
1.0
1.5
2.0
2.5
3.0
−4
−2
0
2
4

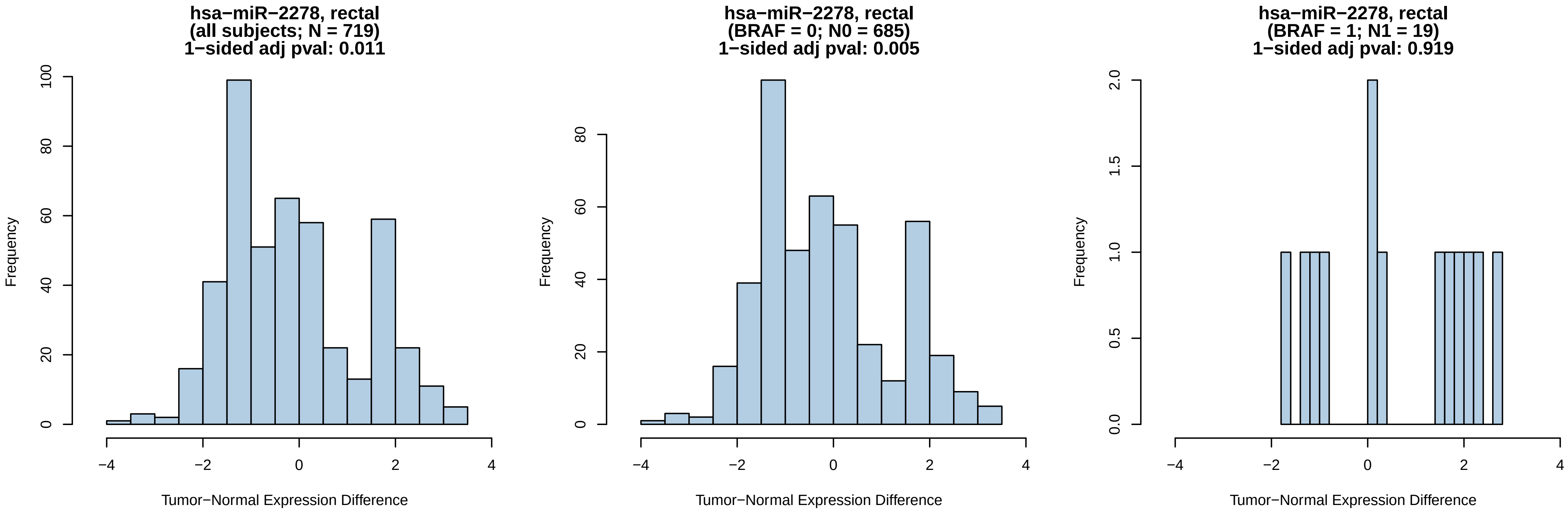
hsa−miR−2278, rectal
(all subjects; N = 719)
1−sided adj pval: 0.011
hsa−miR−2278, rectal
(BRAF = 0; N0 = 685)
1−sided adj pval: 0.005
hsa−miR−2278, rectal
(BRAF = 1; N1 = 19)
1−sided adj pval: 0.919
Frequency
Tumor−Normal Expression Difference

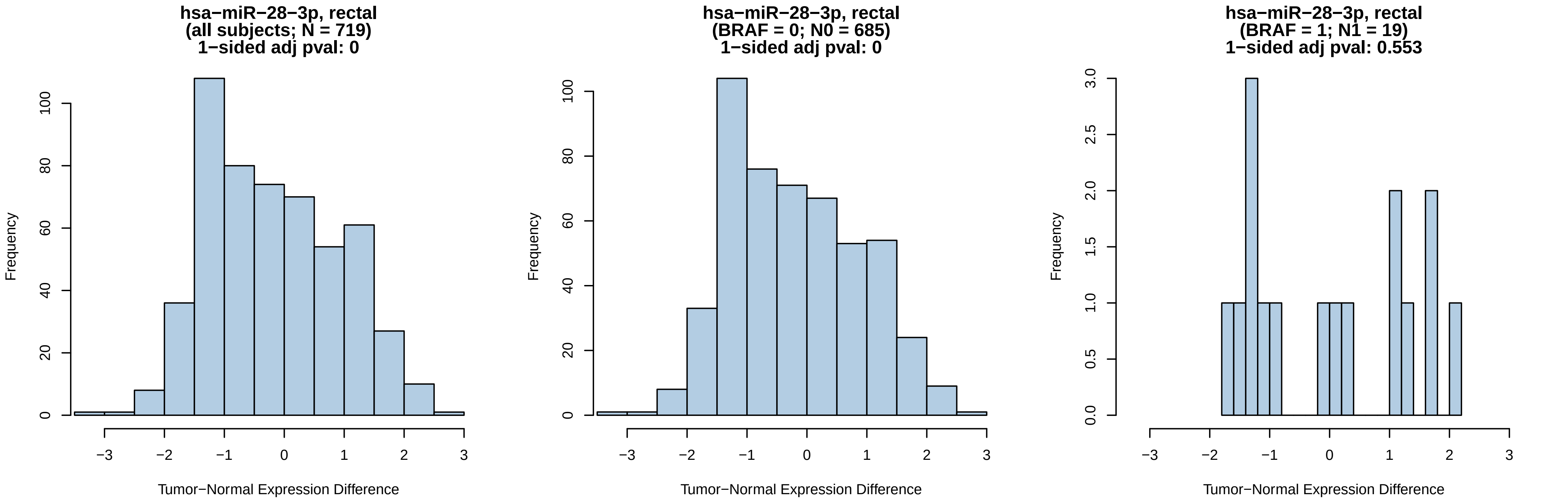
hsa−miR−28−3p, rectal
(all subjects; N = 719)
1−sided adj pval: 0
Frequency
Tumor−Normal Expression Difference
hsa−miR−28−3p, rectal
(BRAF = 0; N0 = 685)
1−sided adj pval: 0
Frequency
Tumor−Normal Expression Difference
hsa−miR−28−3p, rectal
(BRAF = 1; N1 = 19)
1−sided adj pval: 0.553
Frequency
Tumor−Normal Expression Difference

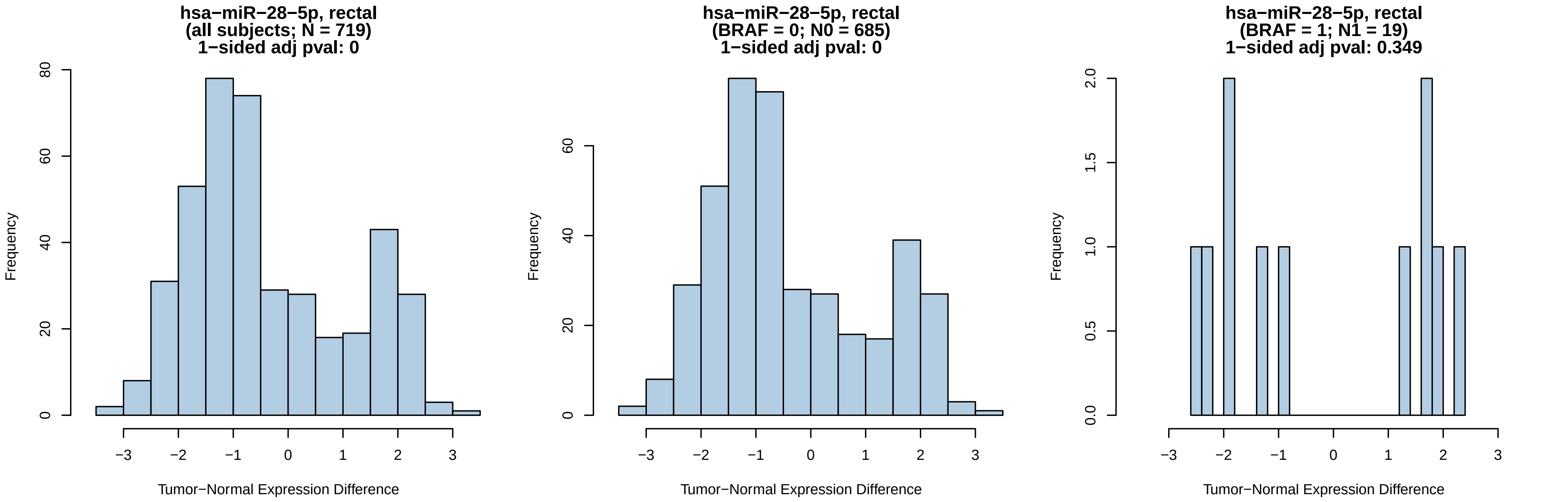

hsa−miR−28−5p, rectal
(all subjects; N = 719)
1−sided adj pval: 0
Frequency
Tumor−Normal Expression Difference
hsa−miR−28−5p, rectal
(BRAF = 0; N0 = 685)
1−sided adj pval: 0
Frequency
Tumor−Normal Expression Difference
hsa−miR−28−5p, rectal
(BRAF = 1; N1 = 19)
1−sided adj pval: 0.349
Frequency
Tumor−Normal Expression Difference

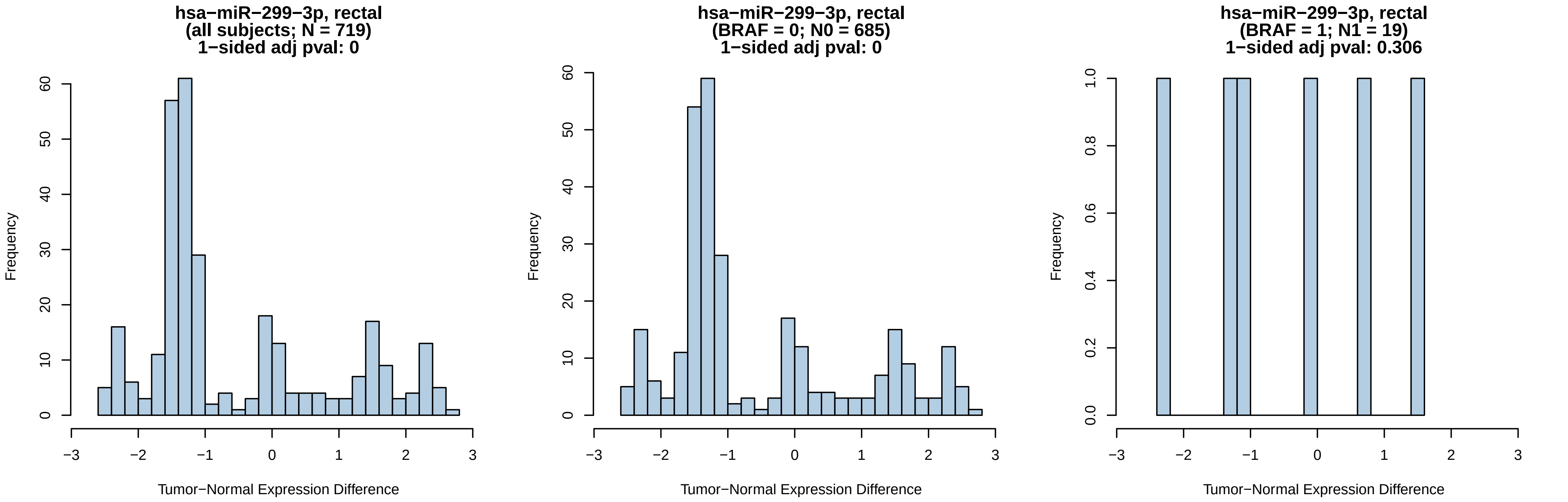

hsa−miR−299−3p, rectal
(all subjects; N = 719)
1−sided adj pval: 0
Frequency
Tumor−Normal Expression Difference
hsa−miR−299−3p, rectal
(BRAF = 0; N0 = 685)
1−sided adj pval: 0
Frequency
Tumor−Normal Expression Difference
hsa−miR−299−3p, rectal
(BRAF = 1; N1 = 19)
1−sided adj pval: 0.306
Frequency
Tumor−Normal Expression Difference

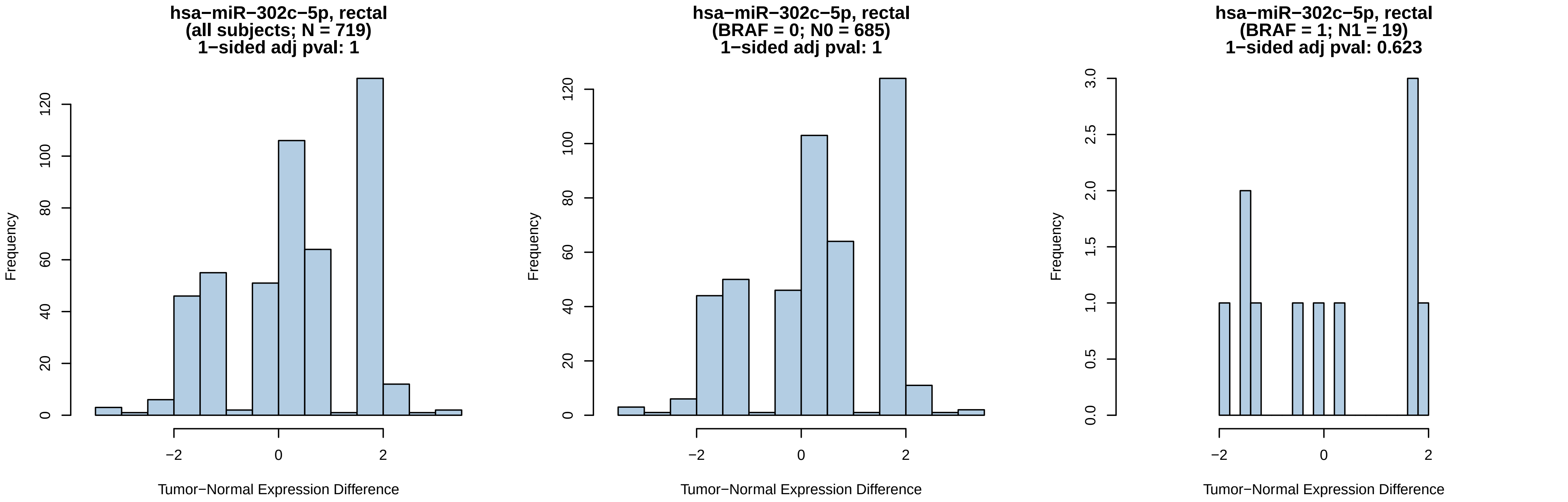

hsa−miR−302c−5p, rectal
(all subjects; N = 719)
1−sided adj pval: 1
Frequency
Tumor−Normal Expression Difference
hsa−miR−302c−5p, rectal
(BRAF = 0; N0 = 685)
1−sided adj pval: 1
Frequency
Tumor−Normal Expression Difference
hsa−miR−302c−5p, rectal
(BRAF = 1; N1 = 19)
1−sided adj pval: 0.623
Frequency
Tumor−Normal Expression Difference

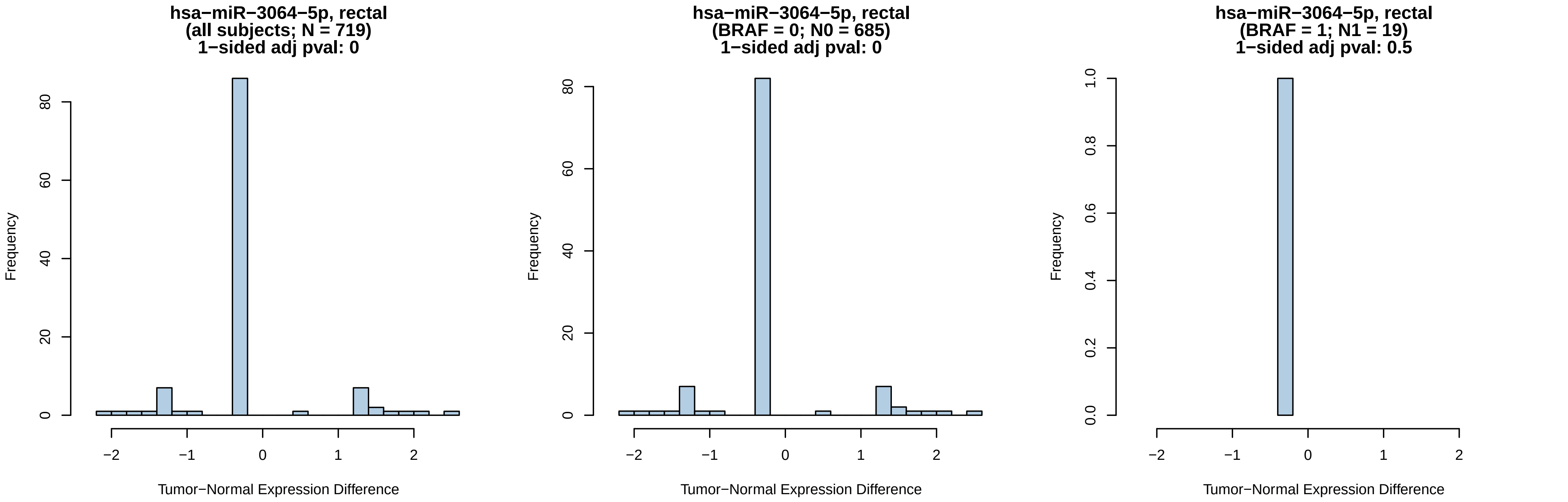

hsa−miR−3064−5p, rectal
(all subjects; N = 719)
1−sided adj pval: 0
Frequency
0
20
40
60
80
−2
−1
0
1
2
Tumor−Normal Expression Difference
hsa−miR−3064−5p, rectal
(BRAF = 0; N0 = 685)
1−sided adj pval: 0
Frequency
0
20
40
60
80
−2
−1
0
1
2
Tumor−Normal Expression Difference
hsa−miR−3064−5p, rectal
(BRAF = 1; N1 = 19)
1−sided adj pval: 0.5
Frequency
0.0
0.2
0.4
0.6
0.8
1.0
−2
−1
0
1
2
Tumor−Normal Expression Difference

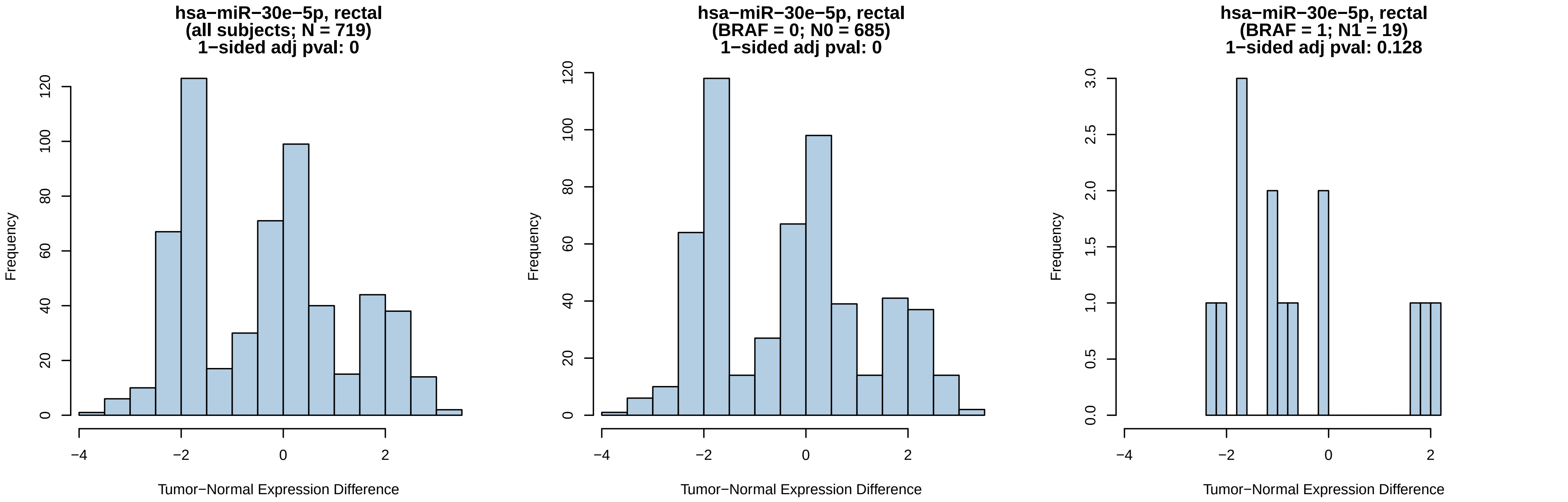

hsa−miR−30e−5p, rectal
(all subjects; N = 719)
1−sided adj pval: 0
Frequency
Tumor−Normal Expression Difference
0
20
40
60
80
100
120
−4
−2
0
2
hsa−miR−30e−5p, rectal
(BRAF = 0; N0 = 685)
1−sided adj pval: 0
Frequency
Tumor−Normal Expression Difference
0
20
40
60
80
100
120
−4
−2
0
2
hsa−miR−30e−5p, rectal
(BRAF = 1; N1 = 19)
1−sided adj pval: 0.128
Frequency
Tumor−Normal Expression Difference
0.0
0.5
1.0
1.5
2.0
2.5
3.0
−4
−2
0
2

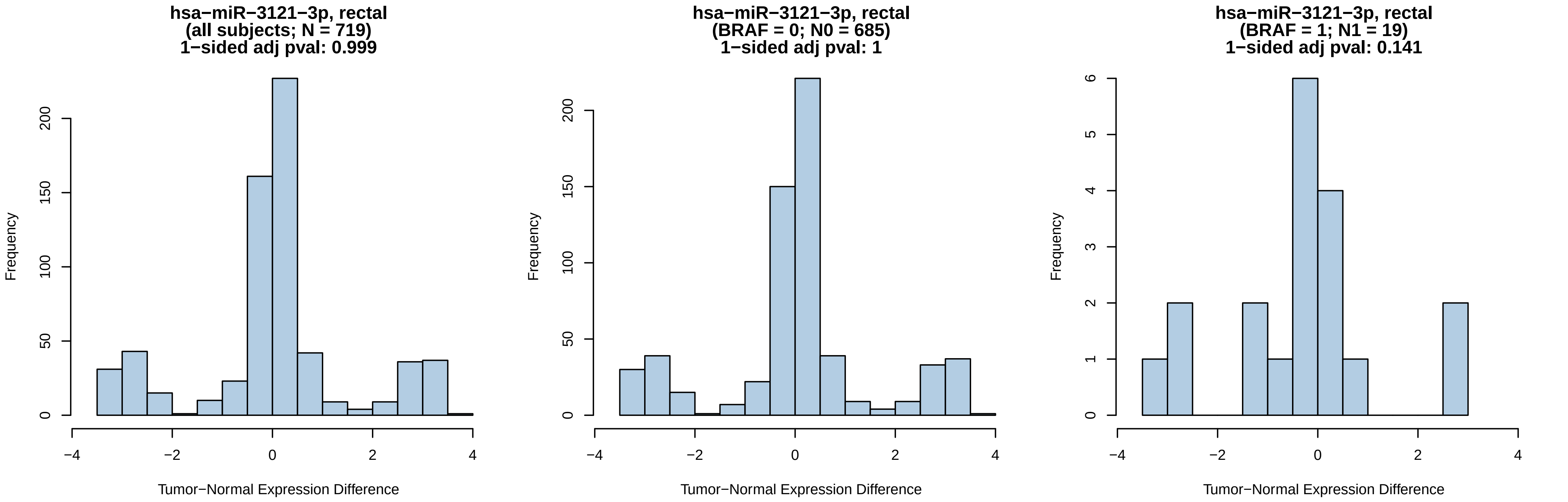

hsa−miR−3121−3p, rectal
(all subjects; N = 719)
1−sided adj pval: 0.999
Frequency
Tumor−Normal Expression Difference
hsa−miR−3121−3p, rectal
(BRAF = 0; N0 = 685)
1−sided adj pval: 1
Frequency
Tumor−Normal Expression Difference
hsa−miR−3121−3p, rectal
(BRAF = 1; N1 = 19)
1−sided adj pval: 0.141
Frequency
Tumor−Normal Expression Difference

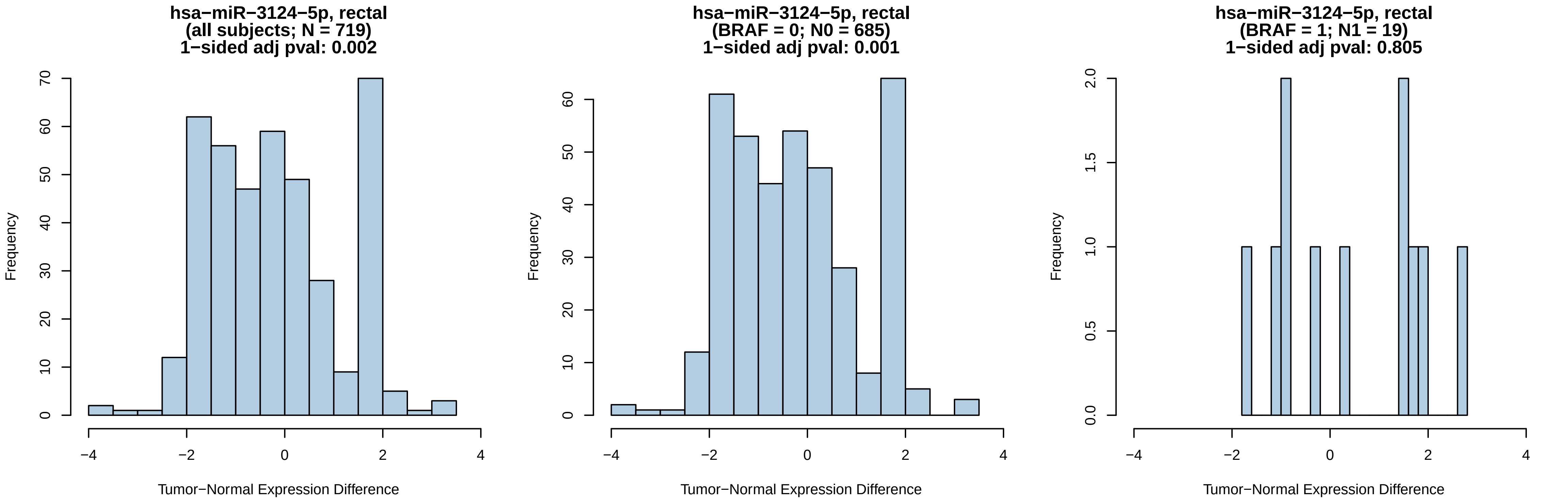

hsa−miR−3124−5p, rectal
(all subjects; N = 719)
1−sided adj pval: 0.002
Frequency
Tumor−Normal Expression Difference
hsa−miR−3124−5p, rectal
(BRAF = 0; N0 = 685)
1−sided adj pval: 0.001
Frequency
Tumor−Normal Expression Difference
hsa−miR−3124−5p, rectal
(BRAF = 1; N1 = 19)
1−sided adj pval: 0.805
Frequency
Tumor−Normal Expression Difference

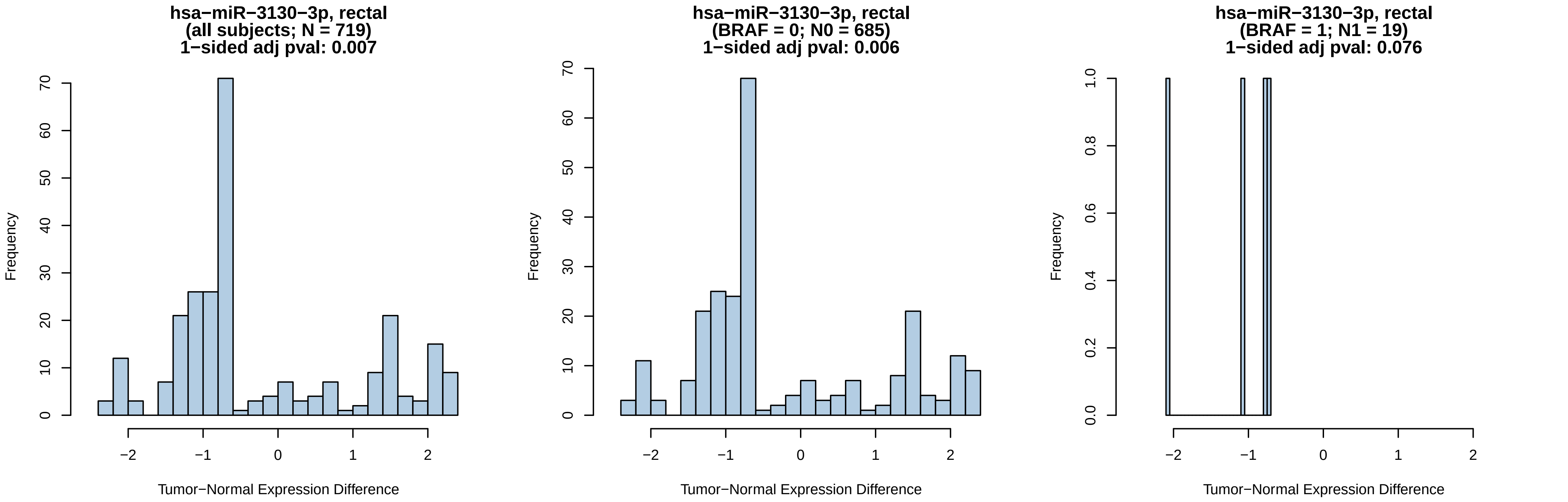

hsa−miR−3130−3p, rectal
(all subjects; N = 719)
1−sided adj pval: 0.007
hsa−miR−3130−3p, rectal
(BRAF = 0; N0 = 685)
1−sided adj pval: 0.006
hsa−miR−3130−3p, rectal
(BRAF = 1; N1 = 19)
1−sided adj pval: 0.076
Frequency
Tumor−Normal Expression Difference

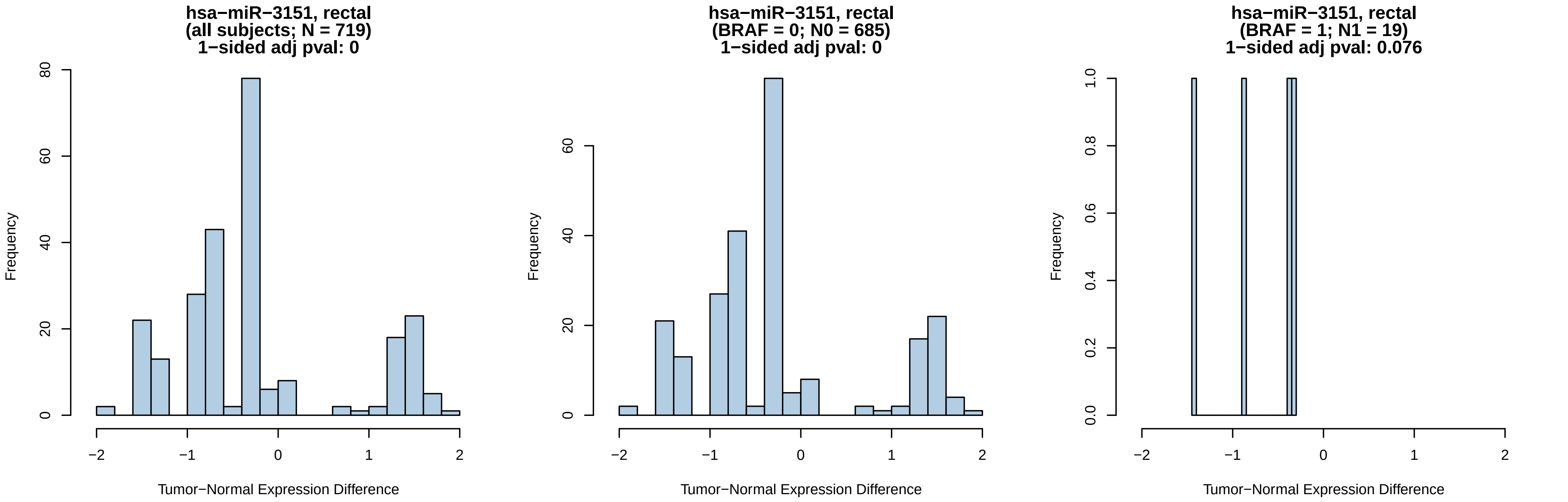
hsa−miR−3151, rectal
(all subjects; N = 719)
1−sided adj pval: 0
Frequency
Tumor−Normal Expression Difference
hsa−miR−3151, rectal
(BRAF = 0; N0 = 685)
1−sided adj pval: 0
Frequency
Tumor−Normal Expression Difference
hsa−miR−3151, rectal
(BRAF = 1; N1 = 19)
1−sided adj pval: 0.076
Frequency
Tumor−Normal Expression Difference

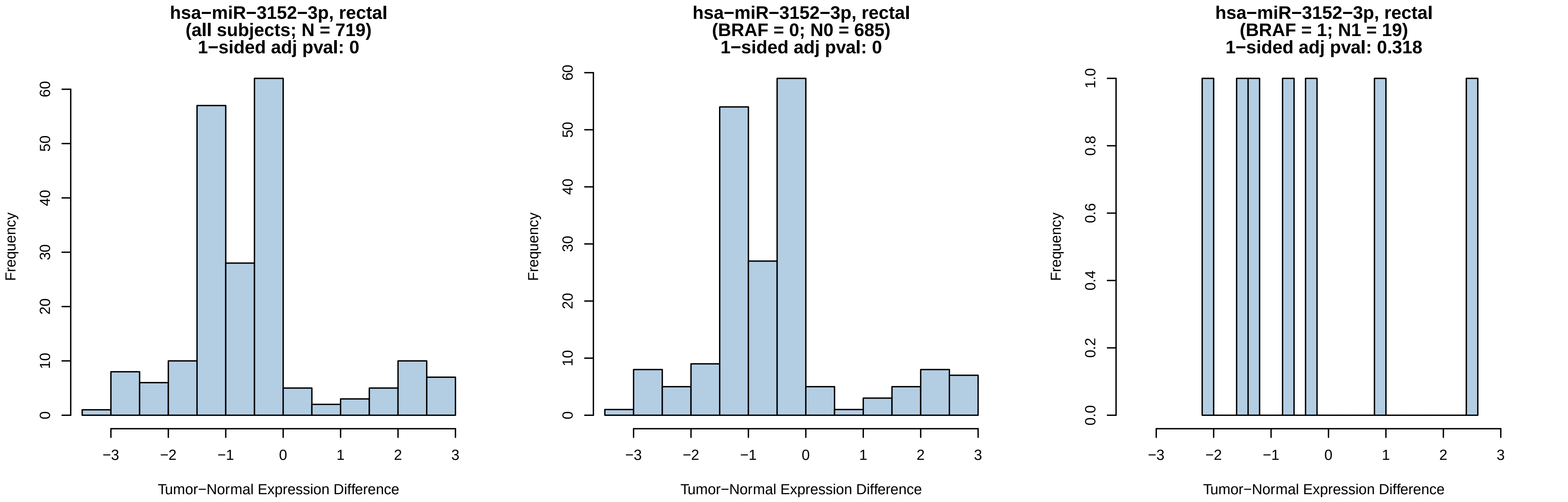

hsa−miR−3152−3p, rectal
(all subjects; N = 719)
1−sided adj pval: 0
Frequency
Tumor−Normal Expression Difference
hsa−miR−3152−3p, rectal
(BRAF = 0; N0 = 685)
1−sided adj pval: 0
Frequency
Tumor−Normal Expression Difference
hsa−miR−3152−3p, rectal
(BRAF = 1; N1 = 19)
1−sided adj pval: 0.318
Frequency
Tumor−Normal Expression Difference

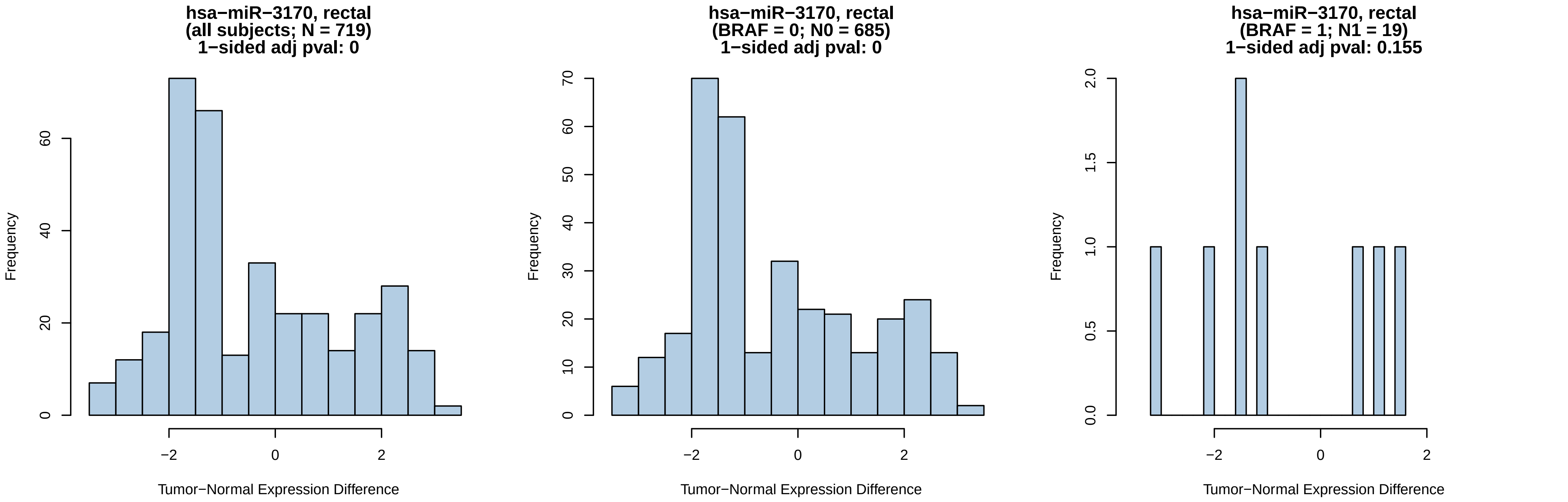
hsa−miR−3170, rectal
(all subjects; N = 719)
1−sided adj pval: 0
Frequency
Tumor−Normal Expression Difference
hsa−miR−3170, rectal
(BRAF = 0; N0 = 685)
1−sided adj pval: 0
Frequency
Tumor−Normal Expression Difference
hsa−miR−3170, rectal
(BRAF = 1; N1 = 19)
1−sided adj pval: 0.155
Frequency
Tumor−Normal Expression Difference

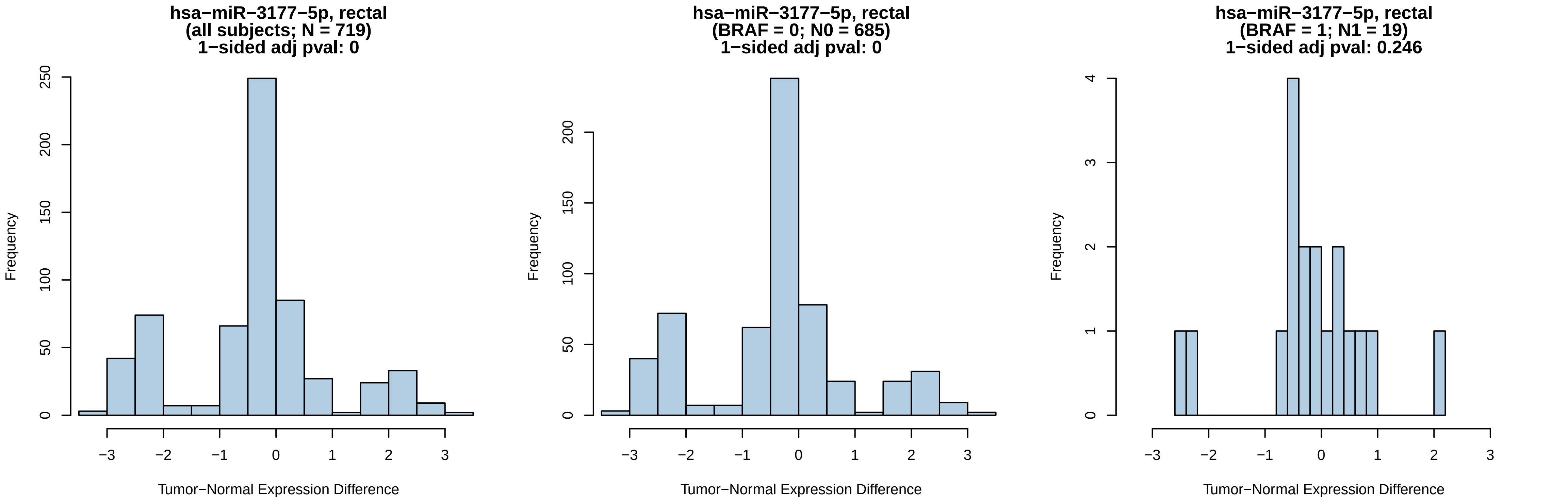

hsa−miR−3177−5p, rectal
(all subjects; N = 719)
1−sided adj pval: 0
hsa−miR−3177−5p, rectal
(BRAF = 0; N0 = 685)
1−sided adj pval: 0
hsa−miR−3177−5p, rectal
(BRAF = 1; N1 = 19)
1−sided adj pval: 0.246
Frequency
Tumor−Normal Expression Difference
0
50
100
150
200
250
0
1
2
3
4
−3
−2
−1
0
1
2
3

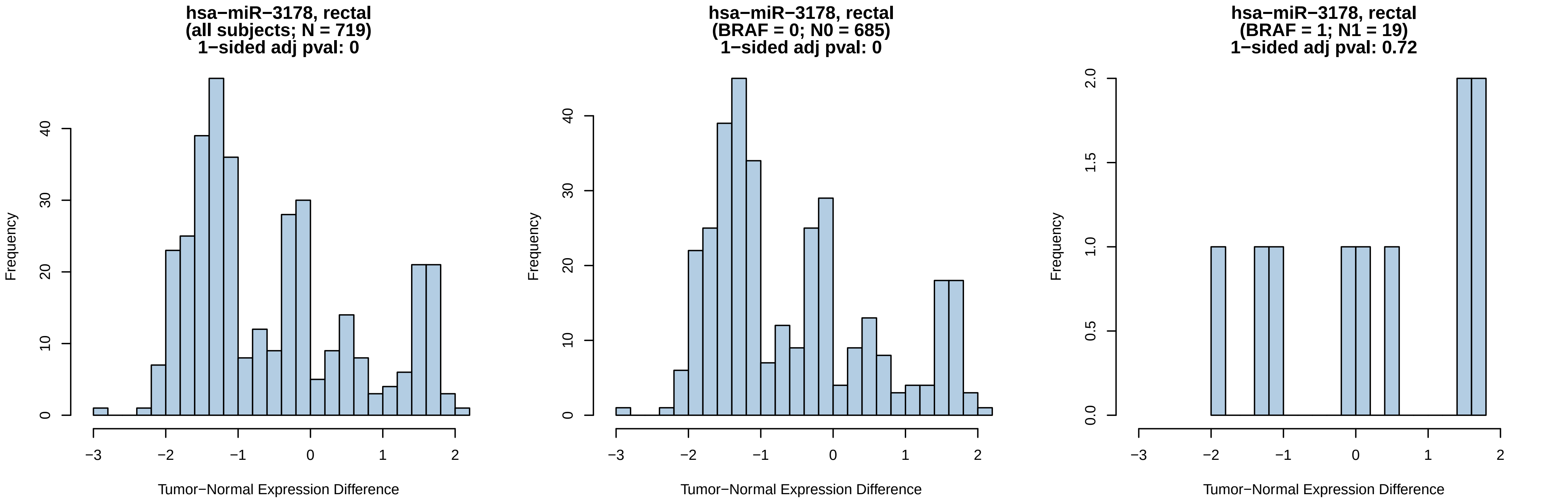

hsa−miR−3178, rectal
(all subjects; N = 719)
1−sided adj pval: 0
hsa−miR−3178, rectal
(BRAF = 0; N0 = 685)
1−sided adj pval: 0
hsa−miR−3178, rectal
(BRAF = 1; N1 = 19)
1−sided adj pval: 0.72
Frequency
Tumor−Normal Expression Difference

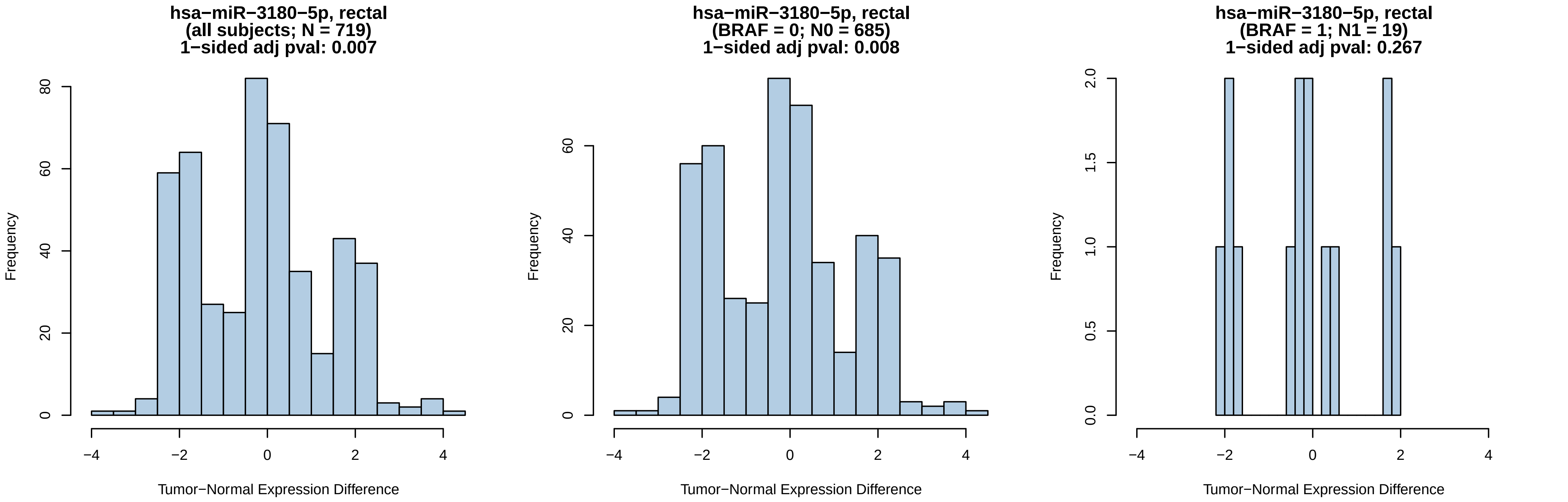
hsa−miR−3180−5p, rectal
(all subjects; N = 719)
1−sided adj pval: 0.007
Frequency
Tumor−Normal Expression Difference
hsa−miR−3180−5p, rectal
(BRAF = 0; N0 = 685)
1−sided adj pval: 0.008
Frequency
Tumor−Normal Expression Difference
hsa−miR−3180−5p, rectal
(BRAF = 1; N1 = 19)
1−sided adj pval: 0.267
Frequency
Tumor−Normal Expression Difference

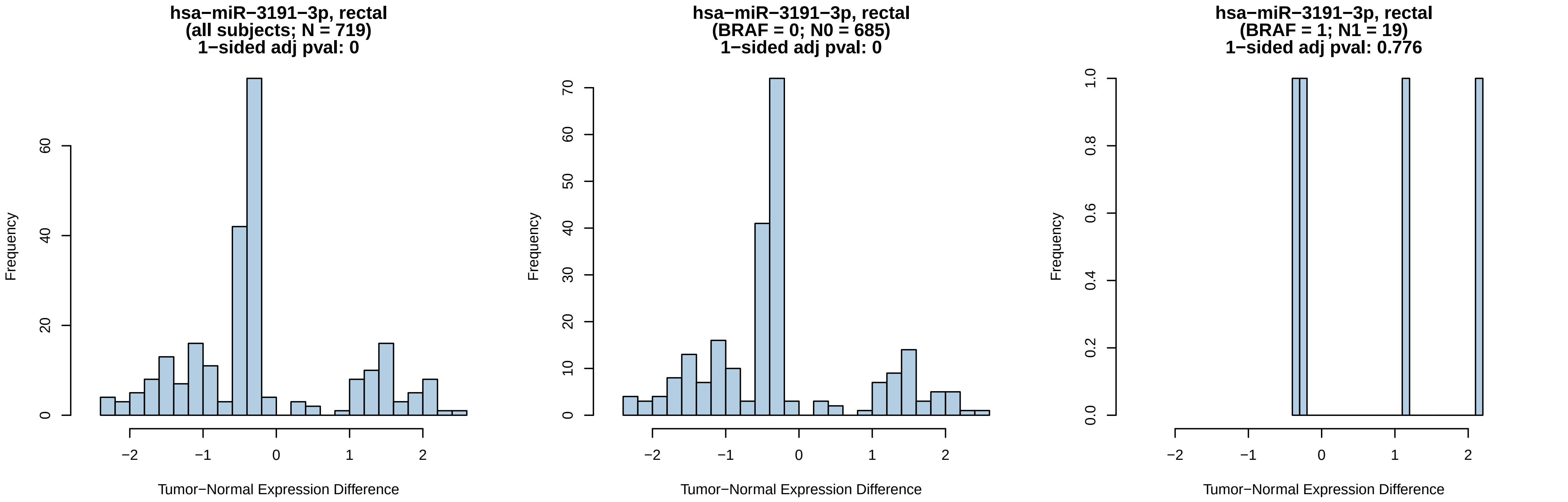
hsa−miR−3191−3p, rectal
(all subjects; N = 719)
1−sided adj pval: 0
Frequency
Tumor−Normal Expression Difference
hsa−miR−3191−3p, rectal
(BRAF = 0; N0 = 685)
1−sided adj pval: 0
Frequency
Tumor−Normal Expression Difference
hsa−miR−3191−3p, rectal
(BRAF = 1; N1 = 19)
1−sided adj pval: 0.776
Frequency
Tumor−Normal Expression Difference

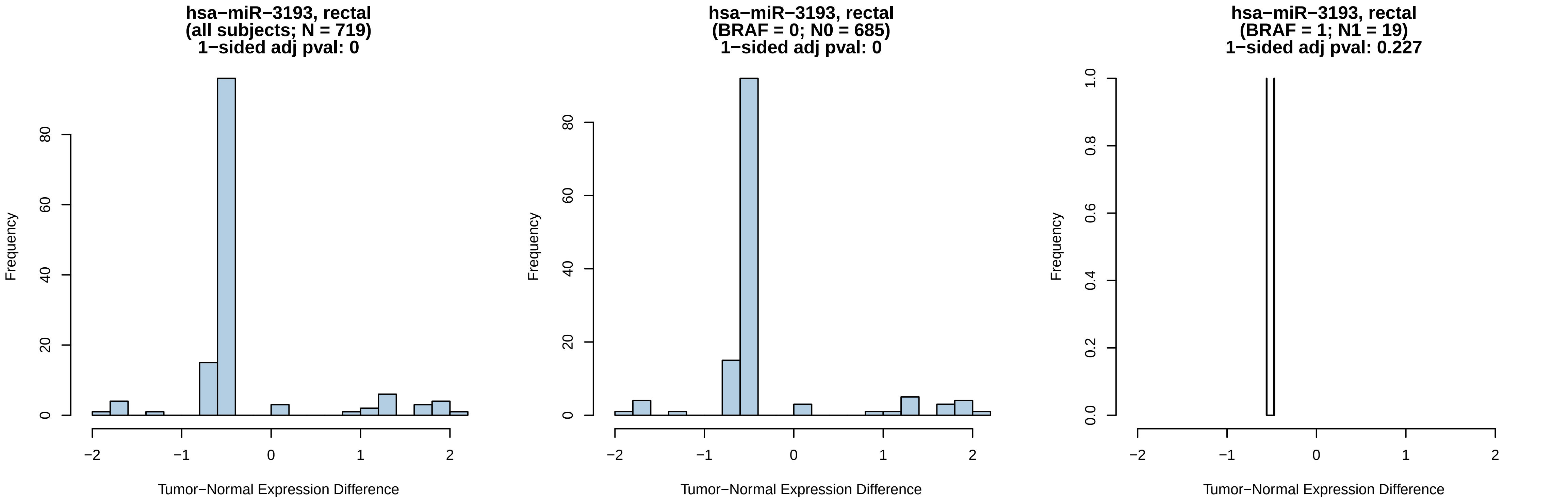

hsa−miR−3193, rectal
(all subjects; N = 719)
1−sided adj pval: 0
Frequency
0
20
40
60
80
−2
−1
0
1
2
Tumor−Normal Expression Difference
hsa−miR−3193, rectal
(BRAF = 0; N0 = 685)
1−sided adj pval: 0
Frequency
0
20
40
60
80
−2
−1
0
1
2
Tumor−Normal Expression Difference
hsa−miR−3193, rectal
(BRAF = 1; N1 = 19)
1−sided adj pval: 0.227
Frequency
0.0
0.2
0.4
0.6
0.8
1.0
−2
−1
0
1
2
Tumor−Normal Expression Difference

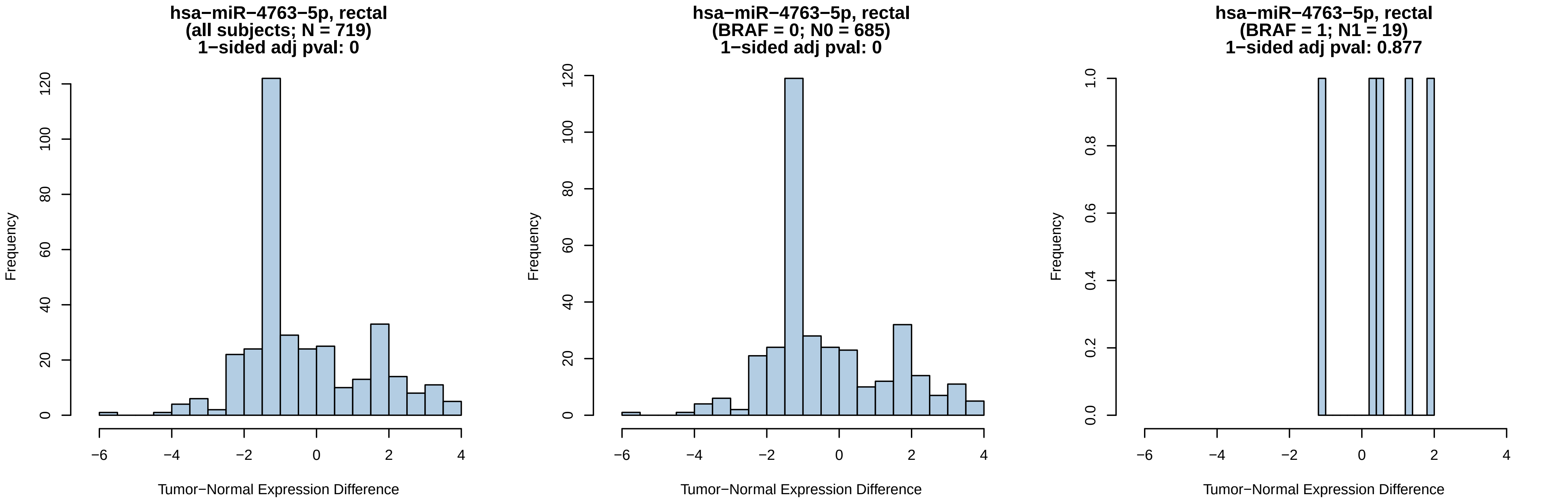

hsa−miR−4763−5p, rectal
(all subjects; N = 719)
1−sided adj pval: 0
hsa−miR−4763−5p, rectal
(BRAF = 0; N0 = 685)
1−sided adj pval: 0
hsa−miR−4763−5p, rectal
(BRAF = 1; N1 = 19)
1−sided adj pval: 0.877
Frequency
Tumor−Normal Expression Difference

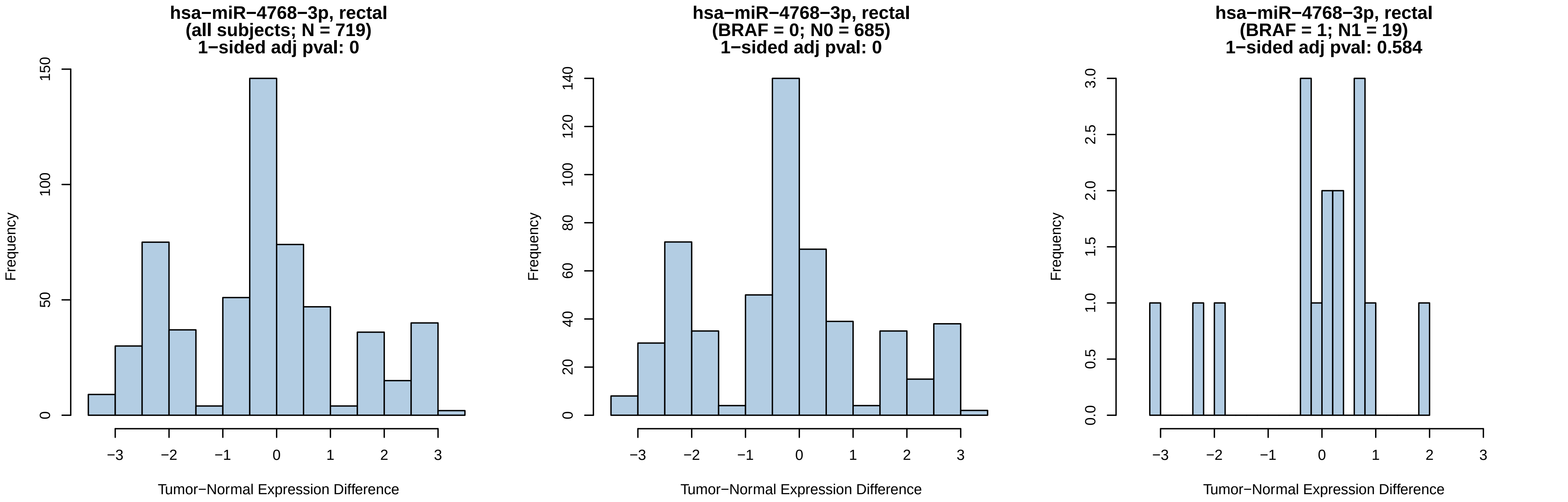
hsa−miR−4768−3p, rectal
(all subjects; N = 719)
1−sided adj pval: 0
Frequency
0
50
100
150
−3
−2
−1
0
1
2
3
Tumor−Normal Expression Difference
hsa−miR−4768−3p, rectal
(BRAF = 0; N0 = 685)
1−sided adj pval: 0
Frequency
0
20
40
60
80
100
120
140
−3
−2
−1
0
1
2
3
Tumor−Normal Expression Difference
hsa−miR−4768−3p, rectal
(BRAF = 1; N1 = 19)
1−sided adj pval: 0.584
Frequency
0.0
0.5
1.0
1.5
2.0
2.5
3.0
−3
−2
−1
0
1
2
3
Tumor−Normal Expression Difference

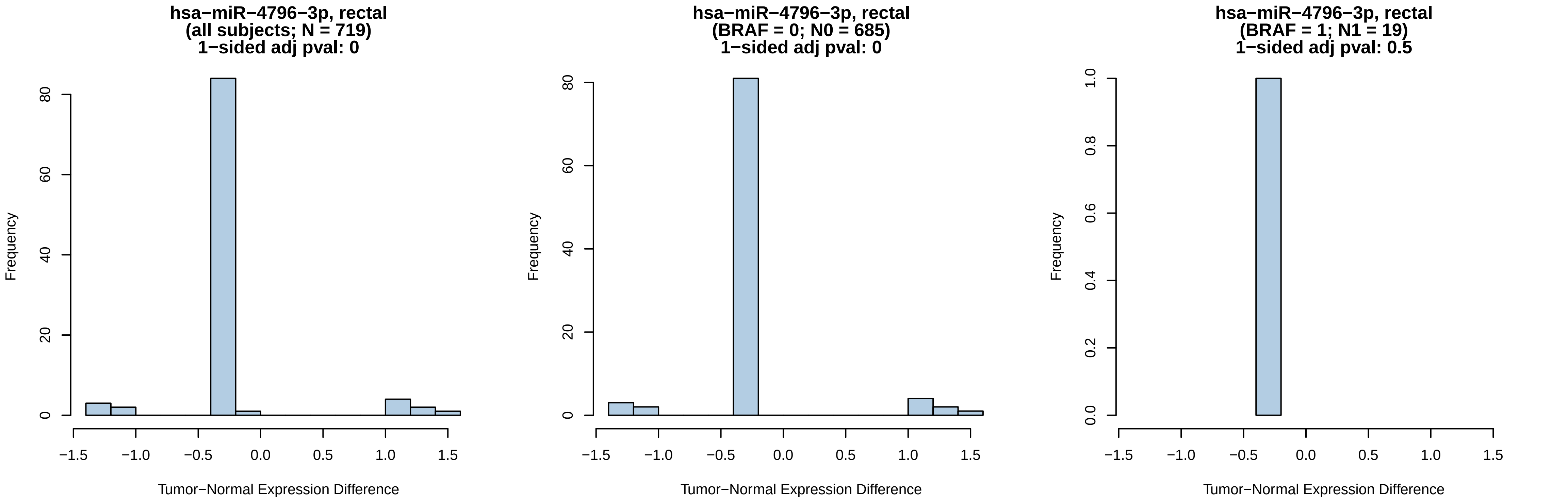
hsa−miR−4796−3p, rectal
(all subjects; N = 719)
1−sided adj pval: 0
Frequency
Tumor−Normal Expression Difference
hsa−miR−4796−3p, rectal
(BRAF = 0; N0 = 685)
1−sided adj pval: 0
Frequency
Tumor−Normal Expression Difference
hsa−miR−4796−3p, rectal
(BRAF = 1; N1 = 19)
1−sided adj pval: 0.5
Frequency
Tumor−Normal Expression Difference

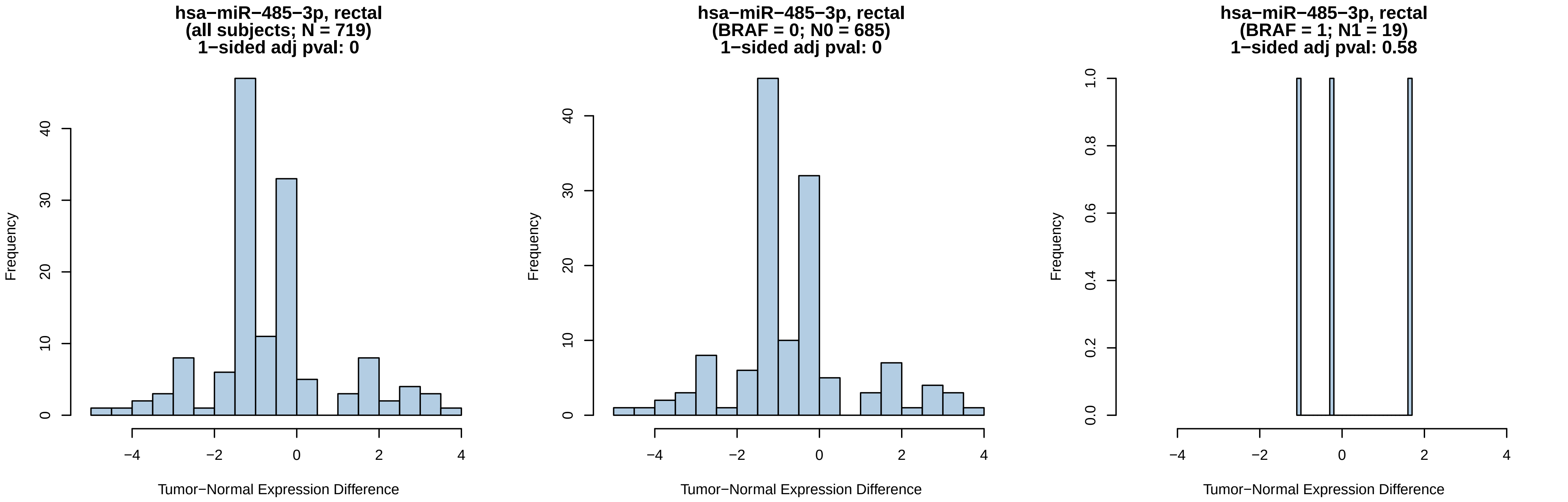

hsa−miR−485−3p, rectal
(all subjects; N = 719)
1−sided adj pval: 0
Frequency
Tumor−Normal Expression Difference
hsa−miR−485−3p, rectal
(BRAF = 0; N0 = 685)
1−sided adj pval: 0
Frequency
Tumor−Normal Expression Difference
hsa−miR−485−3p, rectal
(BRAF = 1; N1 = 19)
1−sided adj pval: 0.58
Frequency
Tumor−Normal Expression Difference

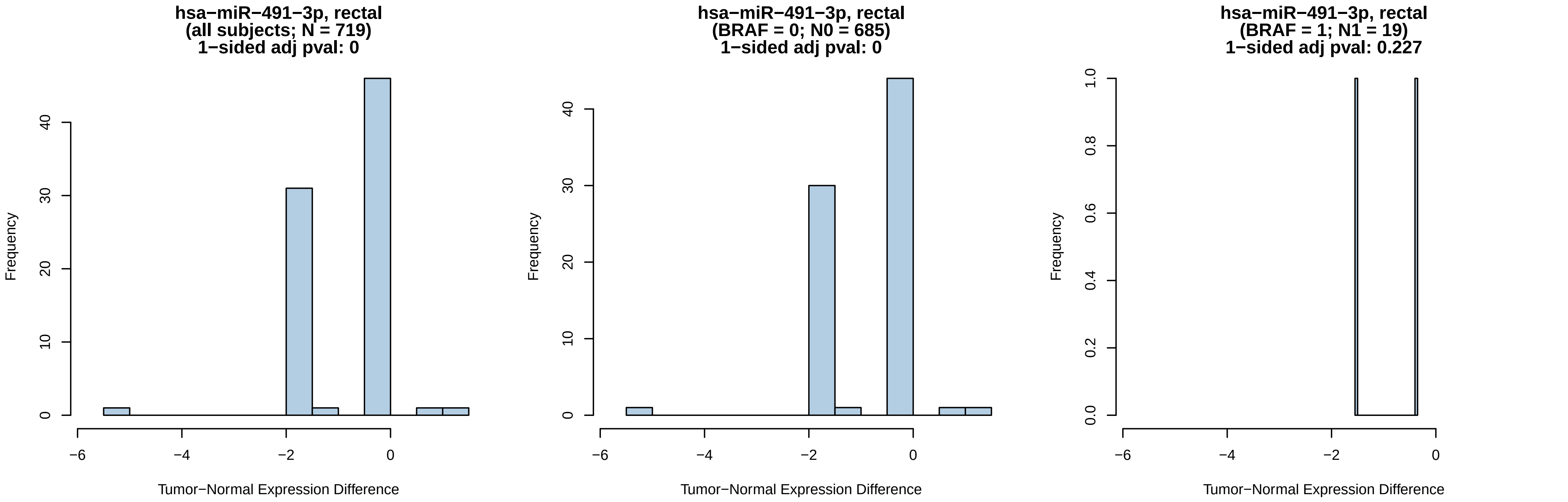
hsa−miR−491−3p, rectal
(all subjects; N = 719)
1−sided adj pval: 0
Frequency
Tumor−Normal Expression Difference
hsa−miR−491−3p, rectal
(BRAF = 0; N0 = 685)
1−sided adj pval: 0
Frequency
Tumor−Normal Expression Difference
hsa−miR−491−3p, rectal
(BRAF = 1; N1 = 19)
1−sided adj pval: 0.227
Frequency
Tumor−Normal Expression Difference

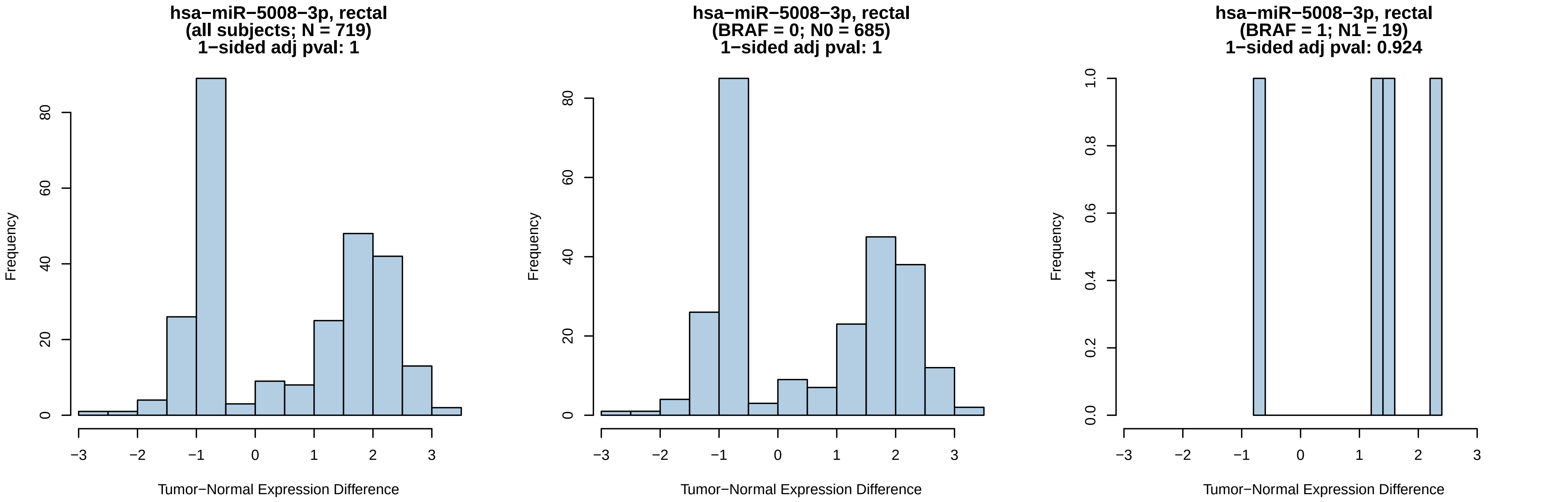
hsa−miR−5008−3p, rectal
(all subjects; N = 719)
1−sided adj pval: 1
Frequency
Tumor−Normal Expression Difference
hsa−miR−5008−3p, rectal
(BRAF = 0; N0 = 685)
1−sided adj pval: 1
Frequency
Tumor−Normal Expression Difference
hsa−miR−5008−3p, rectal
(BRAF = 1; N1 = 19)
1−sided adj pval: 0.924
Frequency
Tumor−Normal Expression Difference

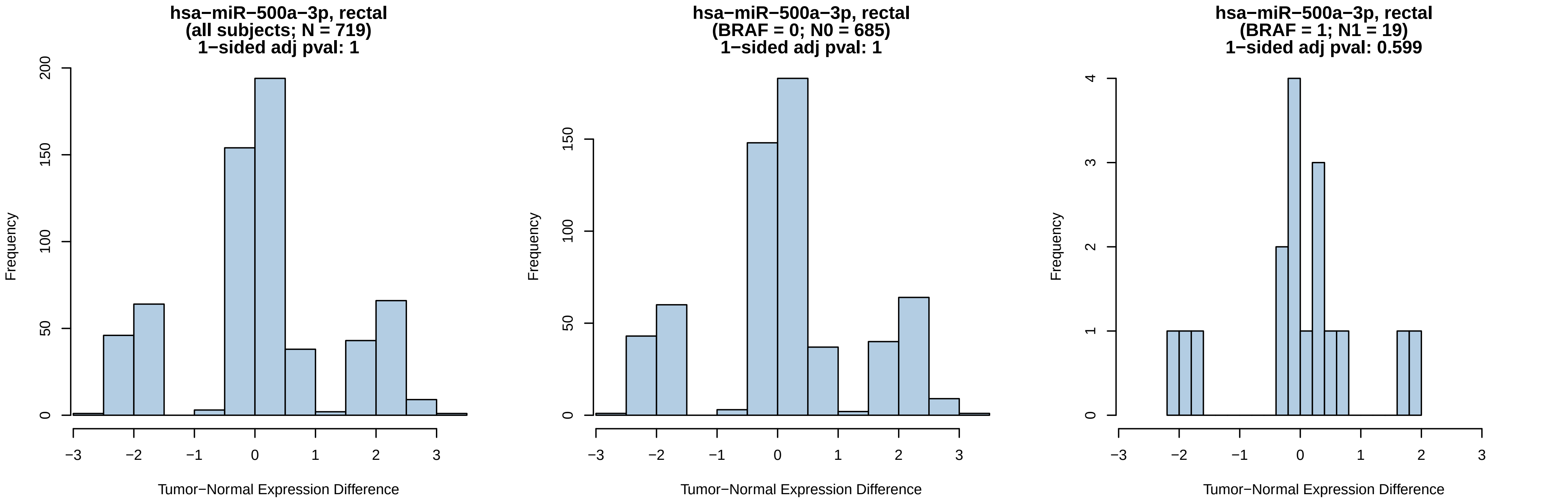

hsa−miR−500a−3p, rectal
(all subjects; N = 719)
1−sided adj pval: 1
Frequency
Tumor−Normal Expression Difference
hsa−miR−500a−3p, rectal
(BRAF = 0; N0 = 685)
1−sided adj pval: 1
Frequency
Tumor−Normal Expression Difference
hsa−miR−500a−3p, rectal
(BRAF = 1; N1 = 19)
1−sided adj pval: 0.599
Frequency
Tumor−Normal Expression Difference

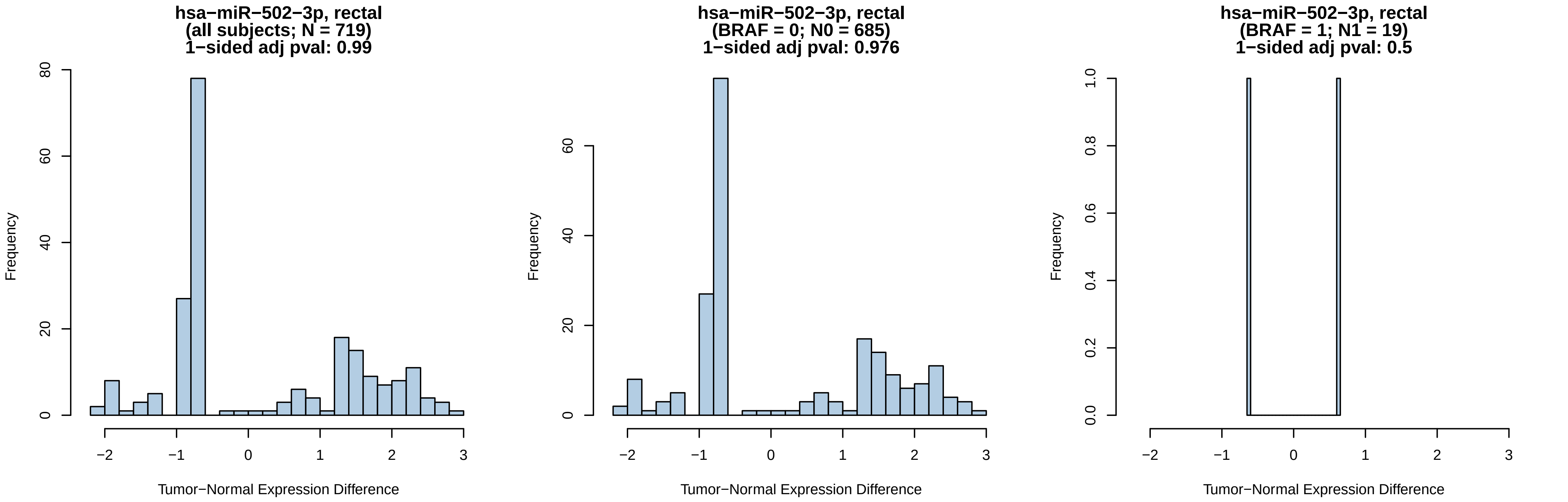
hsa−miR−502−3p, rectal
(all subjects; N = 719)
1−sided adj pval: 0.99
hsa−miR−502−3p, rectal
(BRAF = 0; N0 = 685)
1−sided adj pval: 0.976
hsa−miR−502−3p, rectal
(BRAF = 1; N1 = 19)
1−sided adj pval: 0.5
Frequency
Tumor−Normal Expression Difference

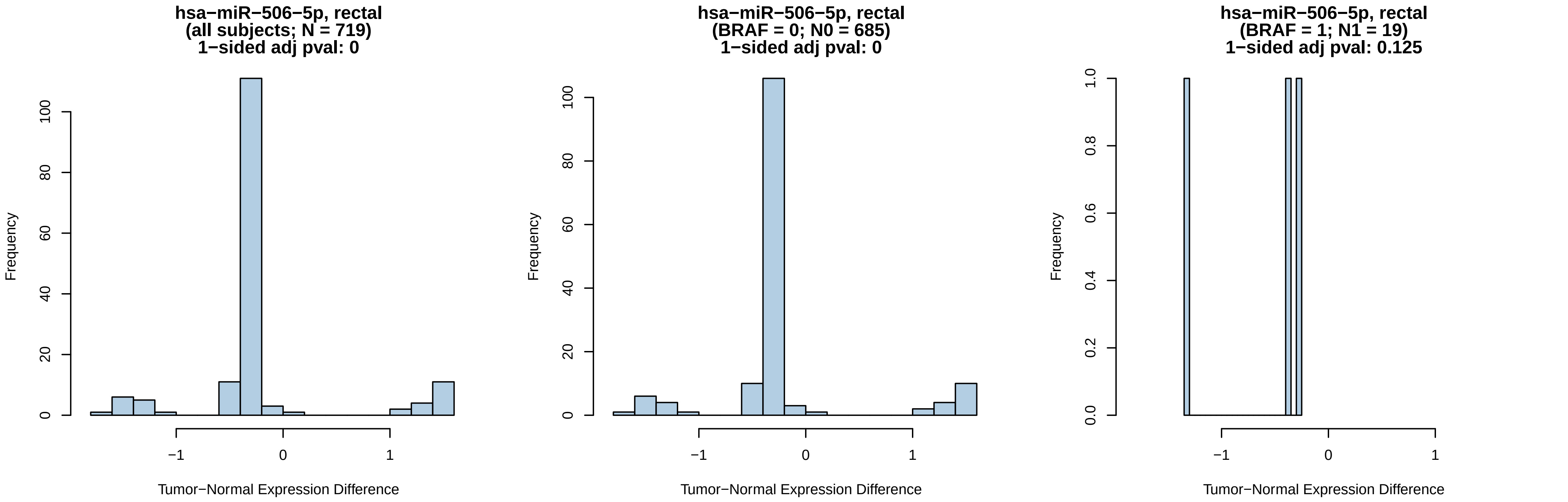

hsa−miR−506−5p, rectal
(all subjects; N = 719)
1−sided adj pval: 0
hsa−miR−506−5p, rectal
(BRAF = 0; N0 = 685)
1−sided adj pval: 0
hsa−miR−506−5p, rectal
(BRAF = 1; N1 = 19)
1−sided adj pval: 0.125
Frequency
Tumor−Normal Expression Difference

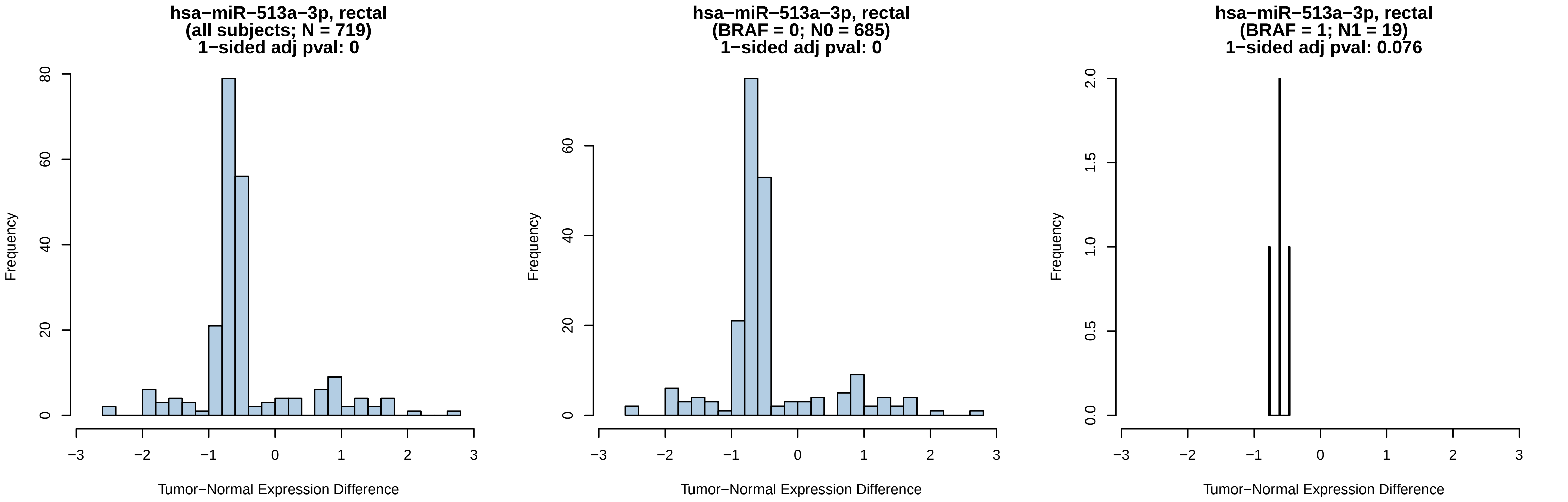

hsa−miR−513a−3p, rectal
(all subjects; N = 719)
1−sided adj pval: 0
hsa−miR−513a−3p, rectal
(BRAF = 0; N0 = 685)
1−sided adj pval: 0
hsa−miR−513a−3p, rectal
(BRAF = 1; N1 = 19)
1−sided adj pval: 0.076
Frequency
Tumor−Normal Expression Difference

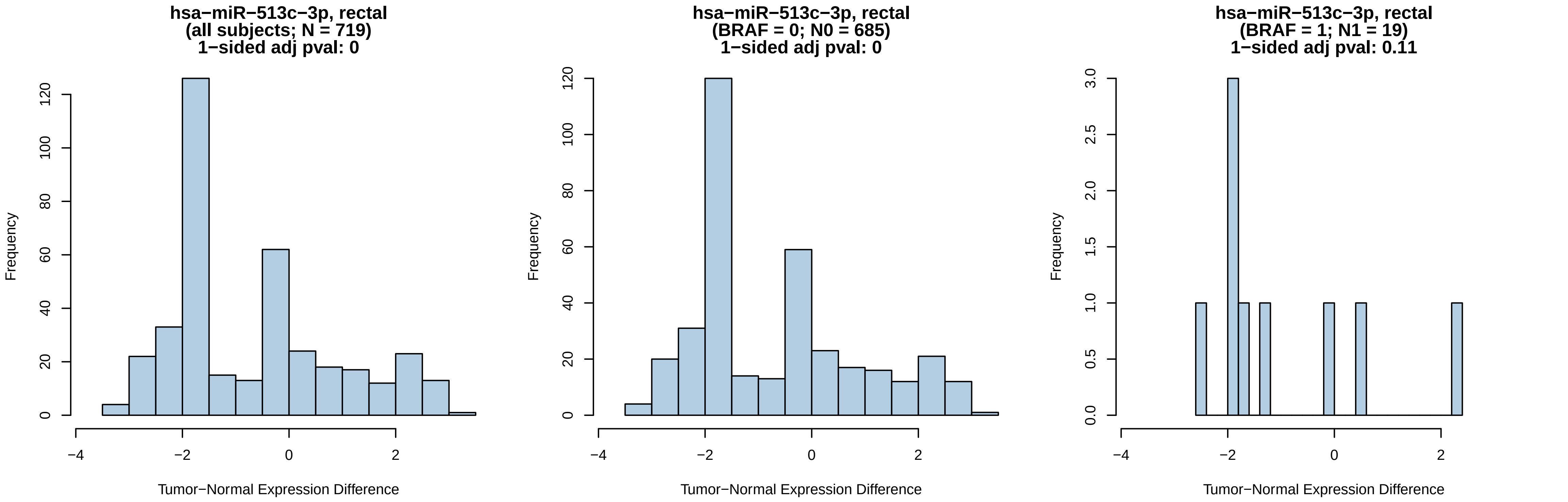

hsa−miR−513c−3p, rectal
(all subjects; N = 719)
1−sided adj pval: 0
Frequency
Tumor−Normal Expression Difference
hsa−miR−513c−3p, rectal
(BRAF = 0; N0 = 685)
1−sided adj pval: 0
Frequency
Tumor−Normal Expression Difference
hsa−miR−513c−3p, rectal
(BRAF = 1; N1 = 19)
1−sided adj pval: 0.11
Frequency
Tumor−Normal Expression Difference

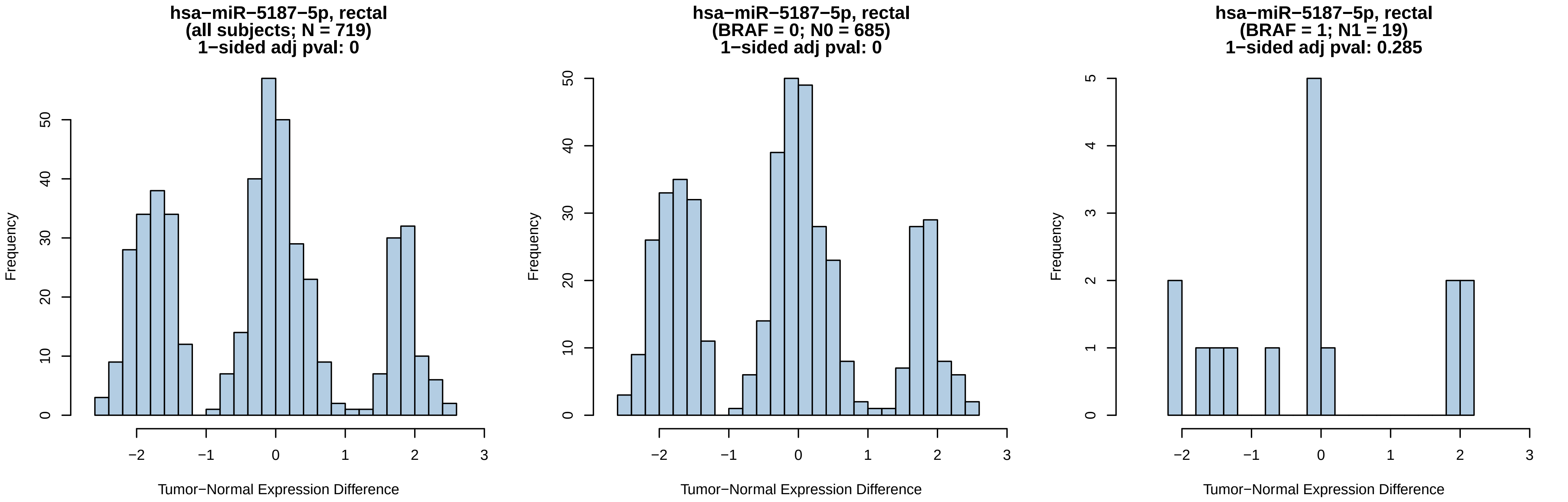
hsa−miR−5187−5p, rectal
(all subjects; N = 719)
1−sided adj pval: 0
Frequency
Tumor−Normal Expression Difference
hsa−miR−5187−5p, rectal
(BRAF = 0; N0 = 685)
1−sided adj pval: 0
Frequency
Tumor−Normal Expression Difference
hsa−miR−5187−5p, rectal
(BRAF = 1; N1 = 19)
1−sided adj pval: 0.285
Frequency
Tumor−Normal Expression Difference

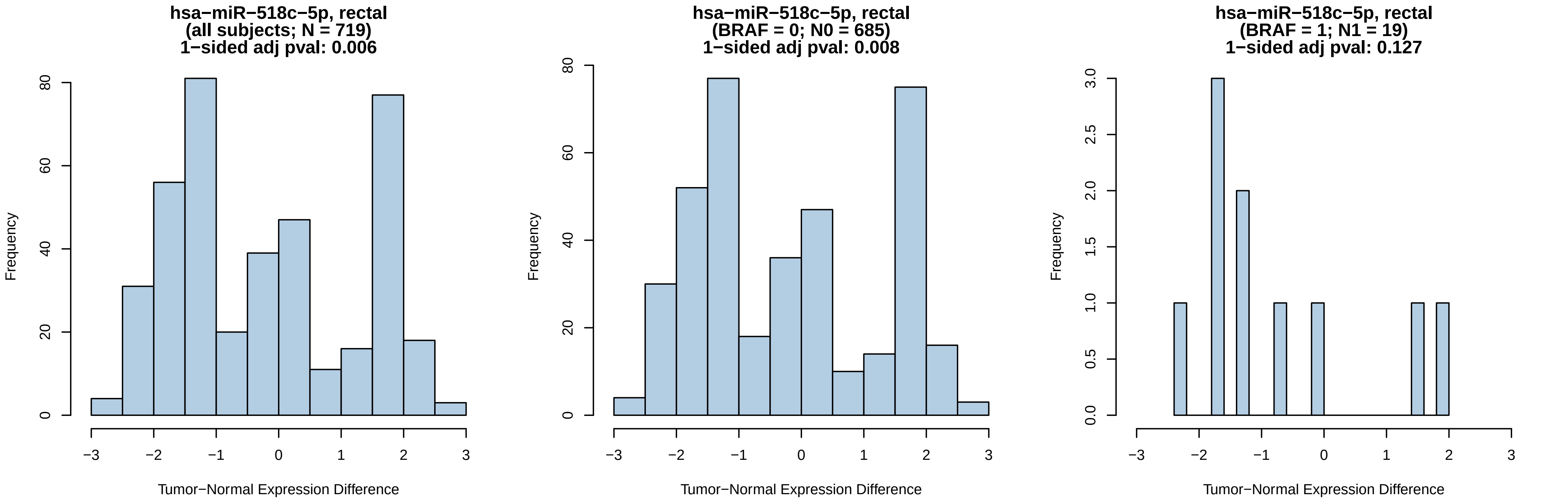
hsa−miR−518c−5p, rectal
(all subjects; N = 719)
1−sided adj pval: 0.006
Frequency
Tumor−Normal Expression Difference
hsa−miR−518c−5p, rectal
(BRAF = 0; N0 = 685)
1−sided adj pval: 0.008
Frequency
Tumor−Normal Expression Difference
hsa−miR−518c−5p, rectal
(BRAF = 1; N1 = 19)
1−sided adj pval: 0.127
Frequency
Tumor−Normal Expression Difference

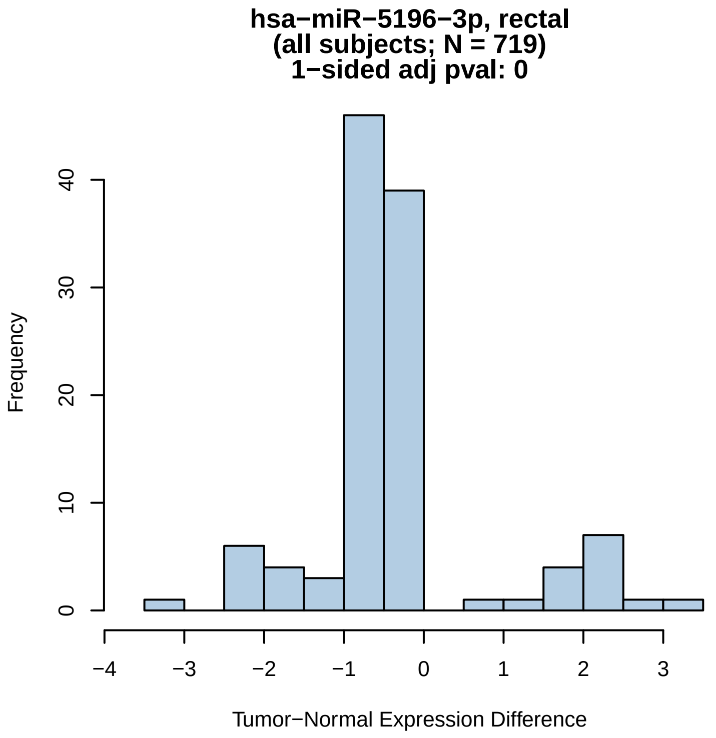

hsa−miR−5196−3p, rectal
(all subjects; N = 719)
1−sided adj pval: 0
Frequency
Tumor−Normal Expression Difference


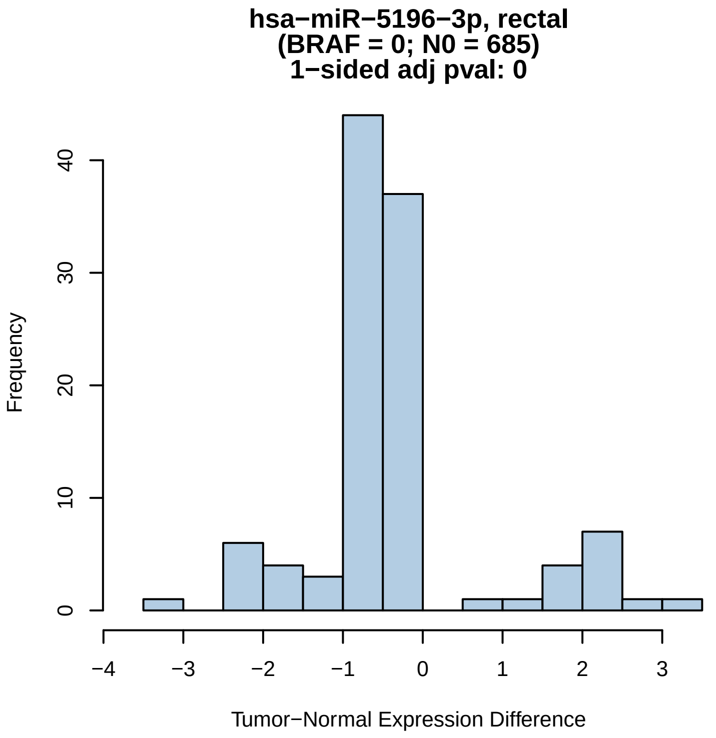

hsa−miR−5196−3p, rectal
(BRAF = 0; N0 = 685)
1−sided adj pval: 0
Frequency
Tumor−Normal Expression Difference


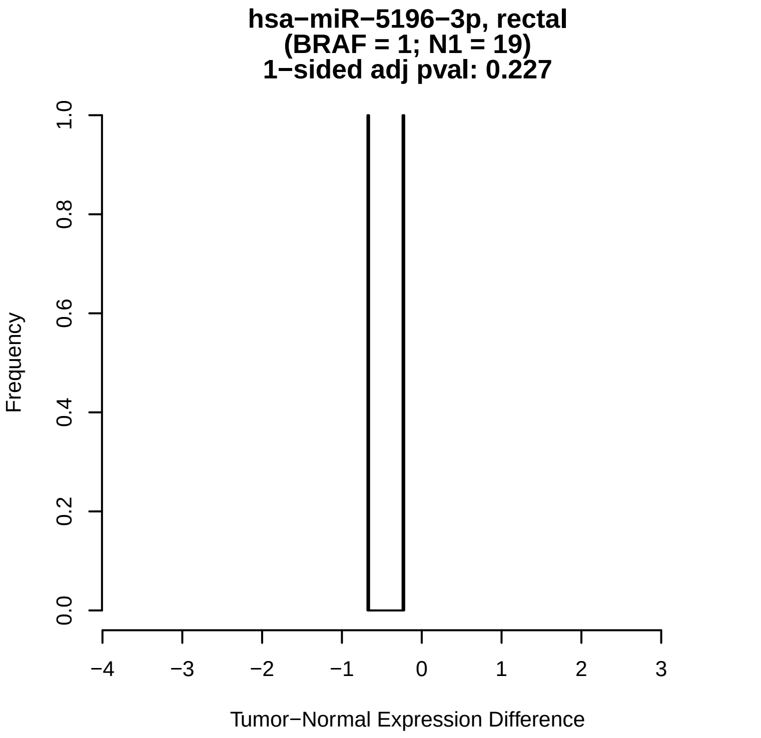

hsa−miR−5196−3p, rectal
(BRAF = 1; N1 = 19)
1−sided adj pval: 0.227
Frequency
Tumor−Normal Expression Difference

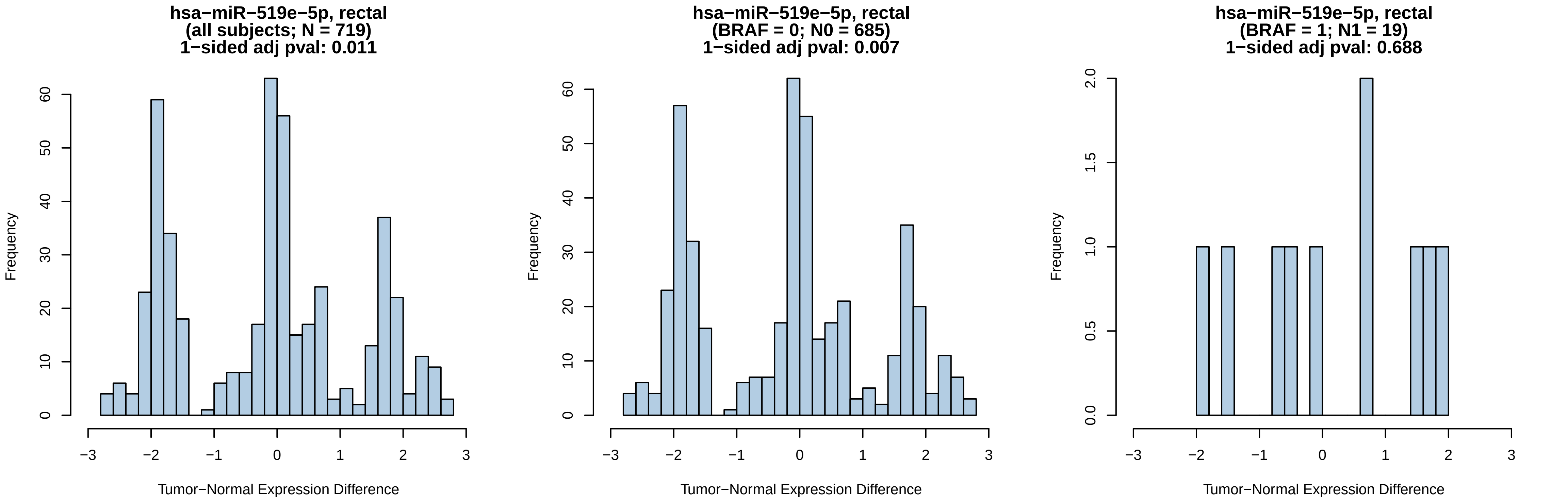

hsa−miR−519e−5p, rectal
(all subjects; N = 719)
1−sided adj pval: 0.011
Frequency
Tumor−Normal Expression Difference
hsa−miR−519e−5p, rectal
(BRAF = 0; N0 = 685)
1−sided adj pval: 0.007
Frequency
Tumor−Normal Expression Difference
hsa−miR−519e−5p, rectal
(BRAF = 1; N1 = 19)
1−sided adj pval: 0.688
Frequency
Tumor−Normal Expression Difference

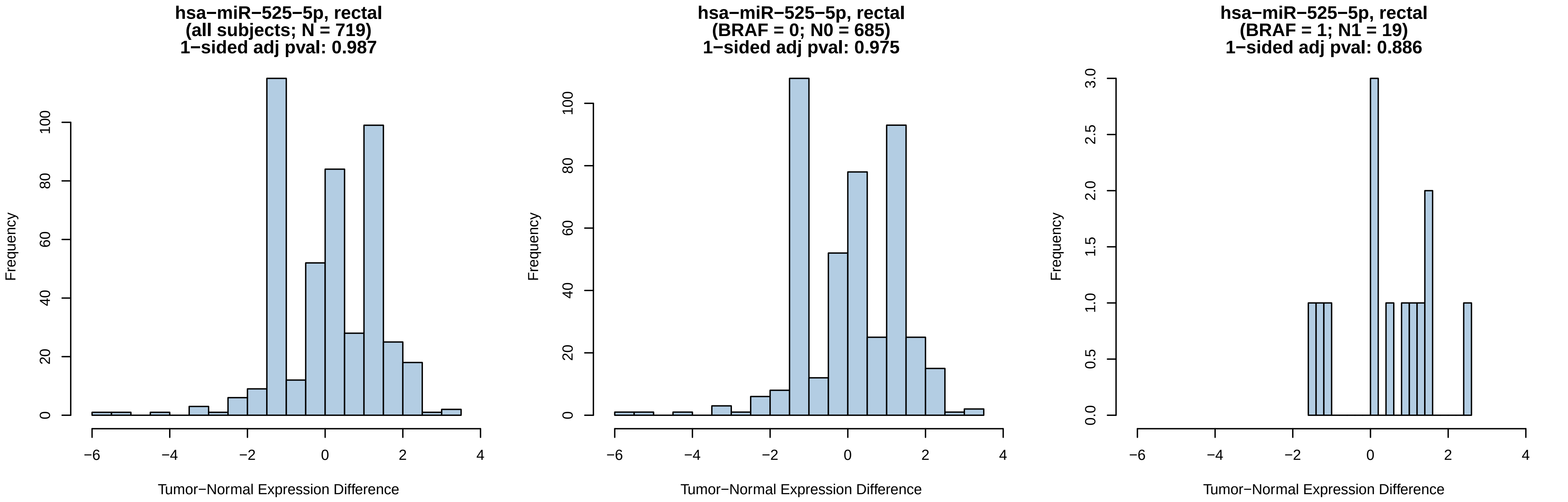
hsa−miR−525−5p, rectal
(all subjects; N = 719)
1−sided adj pval: 0.987
Frequency
Tumor−Normal Expression Difference
hsa−miR−525−5p, rectal
(BRAF = 0; N0 = 685)
1−sided adj pval: 0.975
Frequency
Tumor−Normal Expression Difference
hsa−miR−525−5p, rectal
(BRAF = 1; N1 = 19)
1−sided adj pval: 0.886
Frequency
Tumor−Normal Expression Difference

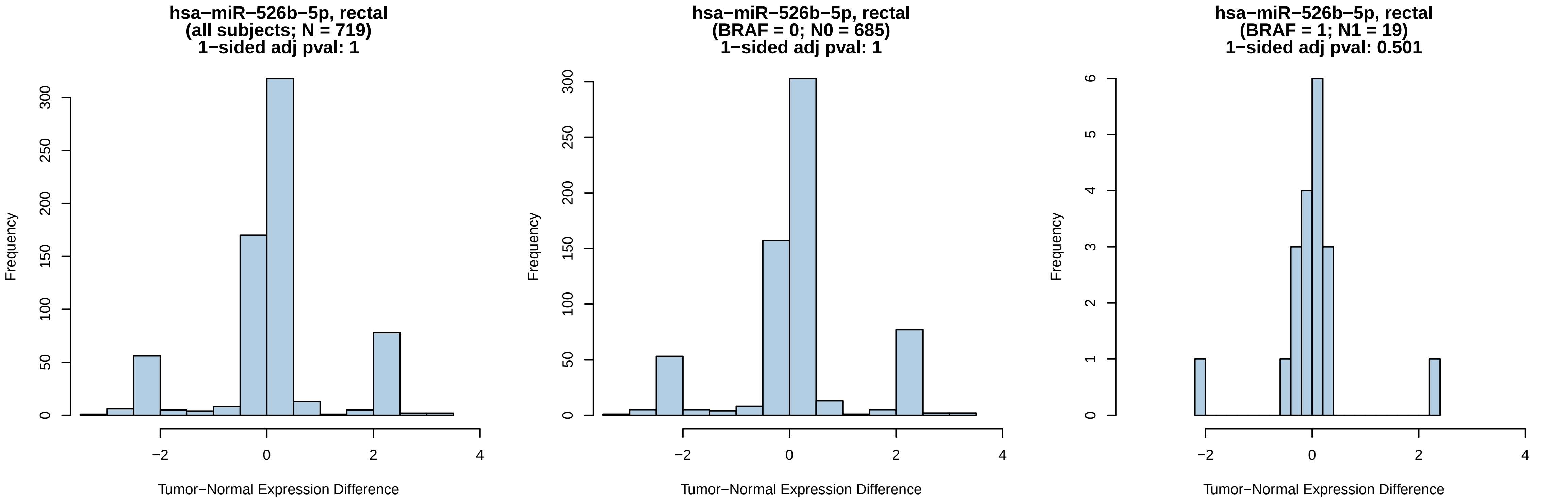

hsa−miR−526b−5p, rectal
(all subjects; N = 719)
1−sided adj pval: 1
Frequency
Tumor−Normal Expression Difference
hsa−miR−526b−5p, rectal
(BRAF = 0; N0 = 685)
1−sided adj pval: 1
Frequency
Tumor−Normal Expression Difference
hsa−miR−526b−5p, rectal
(BRAF = 1; N1 = 19)
1−sided adj pval: 0.501
Frequency
Tumor−Normal Expression Difference

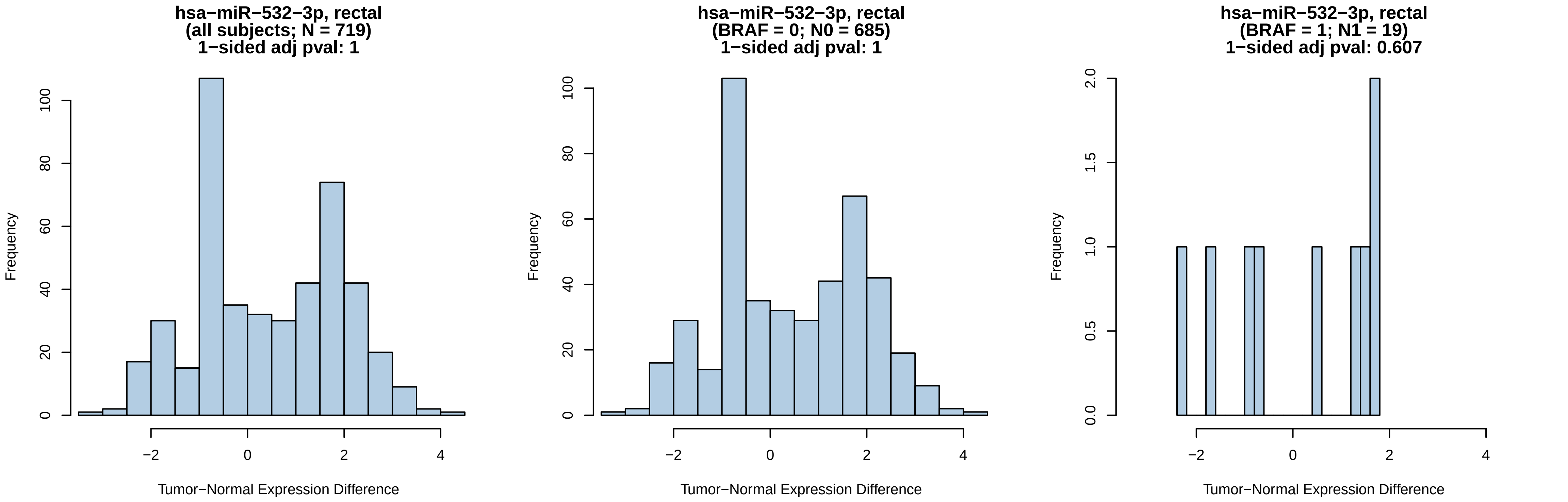
hsa−miR−532−3p, rectal
(all subjects; N = 719)
1−sided adj pval: 1
Frequency
Tumor−Normal Expression Difference
0
20
40
60
80
100
−2
0
2
4
hsa−miR−532−3p, rectal
(BRAF = 0; N0 = 685)
1−sided adj pval: 1
Frequency
Tumor−Normal Expression Difference
0
20
40
60
80
100
−2
0
2
4
hsa−miR−532−3p, rectal
(BRAF = 1; N1 = 19)
1−sided adj pval: 0.607
Frequency
Tumor−Normal Expression Difference
0.0
0.5
1.0
1.5
2.0
−2
0
2
4

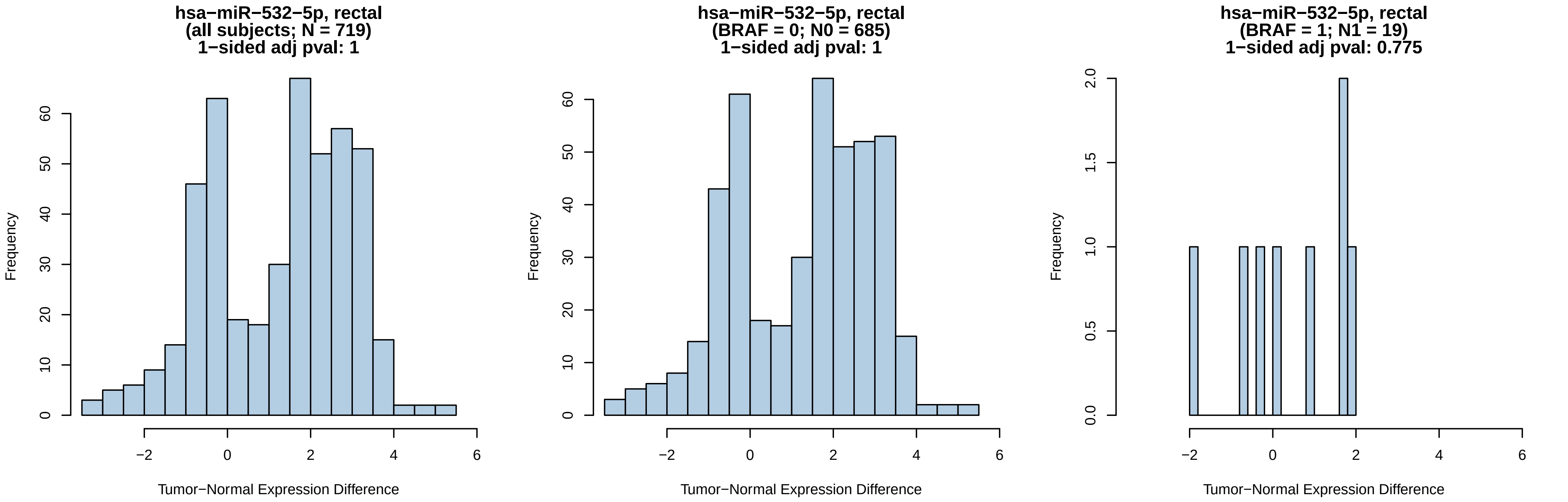

hsa−miR−532−5p, rectal
(all subjects; N = 719)
1−sided adj pval: 1
Frequency
Tumor−Normal Expression Difference
hsa−miR−532−5p, rectal
(BRAF = 0; N0 = 685)
1−sided adj pval: 1
Frequency
Tumor−Normal Expression Difference
hsa−miR−532−5p, rectal
(BRAF = 1; N1 = 19)
1−sided adj pval: 0.775
Frequency
Tumor−Normal Expression Difference

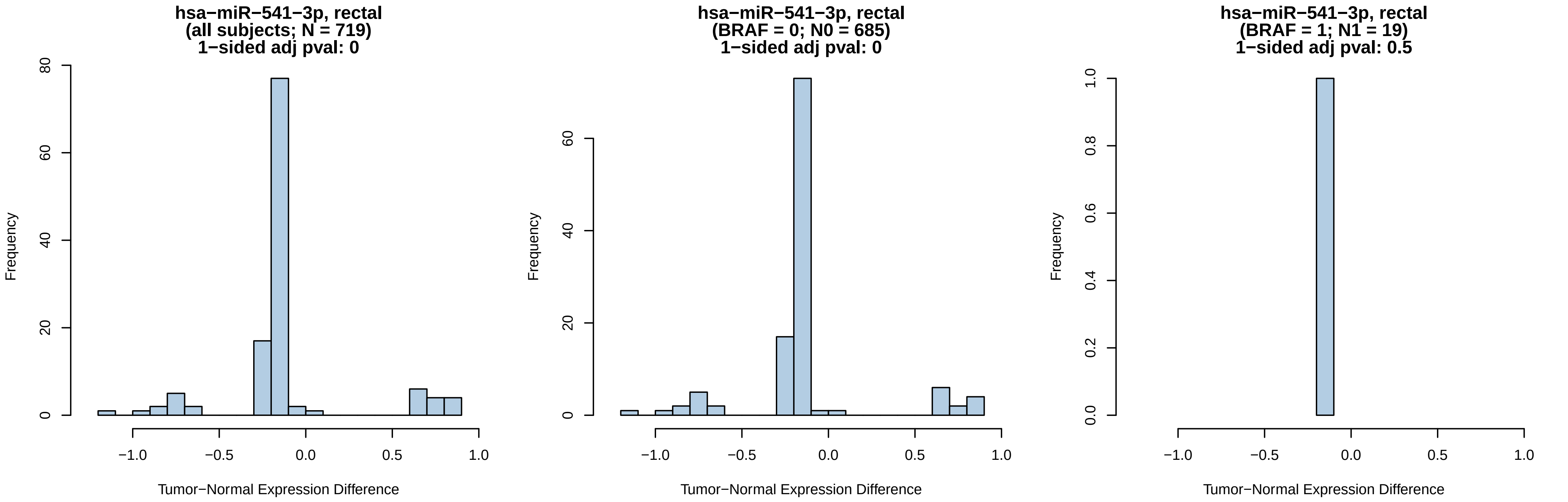
hsa−miR−541−3p, rectal
(all subjects; N = 719)
1−sided adj pval: 0
Frequency
Tumor−Normal Expression Difference
hsa−miR−541−3p, rectal
(BRAF = 0; N0 = 685)
1−sided adj pval: 0
Frequency
Tumor−Normal Expression Difference
hsa−miR−541−3p, rectal
(BRAF = 1; N1 = 19)
1−sided adj pval: 0.5
Frequency
Tumor−Normal Expression Difference

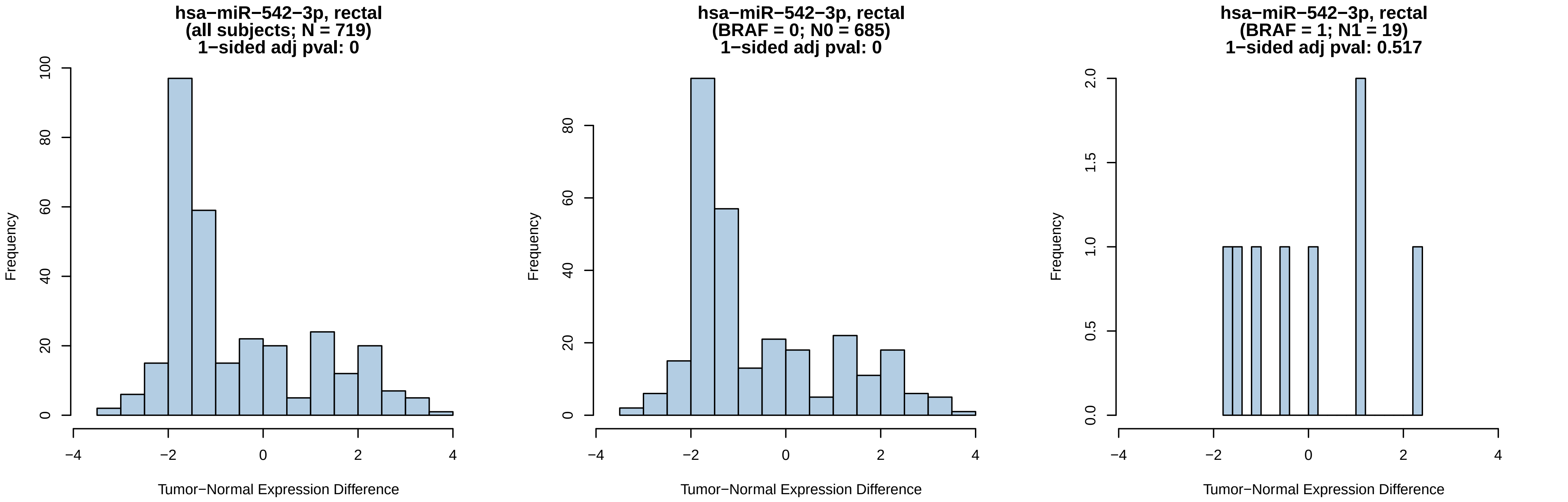
hsa−miR−542−3p, rectal
(all subjects; N = 719)
1−sided adj pval: 0
Frequency
Tumor−Normal Expression Difference
hsa−miR−542−3p, rectal
(BRAF = 0; N0 = 685)
1−sided adj pval: 0
Frequency
Tumor−Normal Expression Difference
hsa−miR−542−3p, rectal
(BRAF = 1; N1 = 19)
1−sided adj pval: 0.517
Frequency
Tumor−Normal Expression Difference

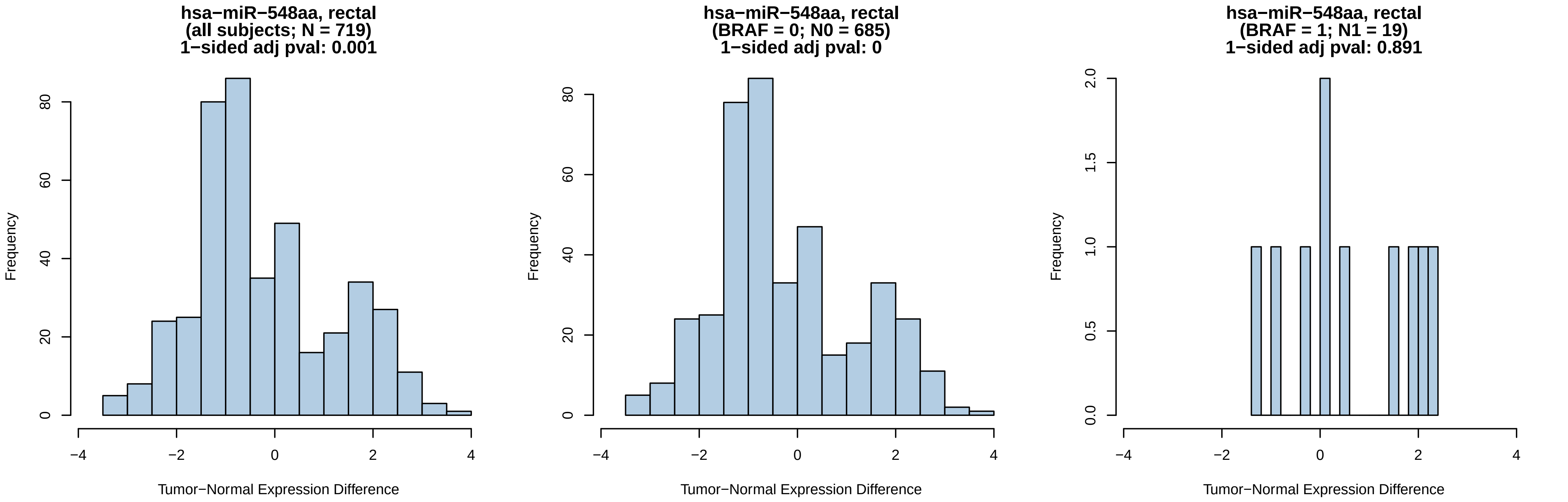
hsa−miR−548aa, rectal
(all subjects; N = 719)
1−sided adj pval: 0.001
Frequency
0
20
40
60
80
−4
−2
0
2
4
Tumor−Normal Expression Difference
hsa−miR−548aa, rectal
(BRAF = 0; N0 = 685)
1−sided adj pval: 0
Frequency
0
20
40
60
80
−4
−2
0
2
4
Tumor−Normal Expression Difference
hsa−miR−548aa, rectal
(BRAF = 1; N1 = 19)
1−sided adj pval: 0.891
Frequency
0.0
0.5
1.0
1.5
2.0
−4
−2
0
2
4
Tumor−Normal Expression Difference

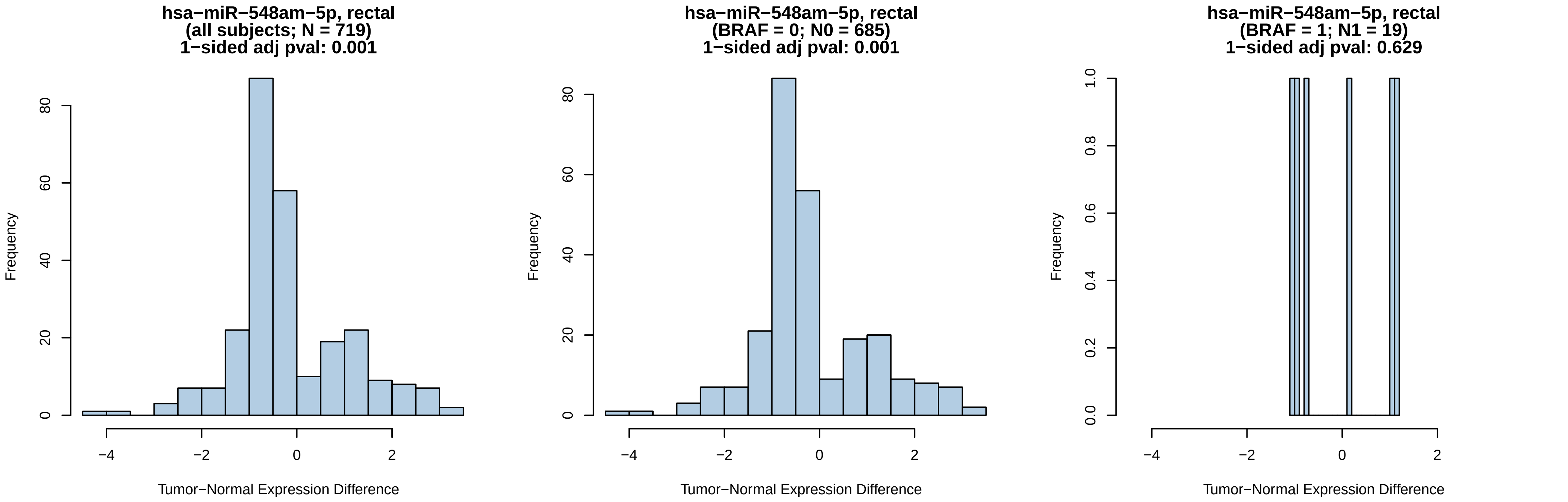
hsa−miR−548am−5p, rectal
(all subjects; N = 719)
1−sided adj pval: 0.001
Frequency
Tumor−Normal Expression Difference
hsa−miR−548am−5p, rectal
(BRAF = 0; N0 = 685)
1−sided adj pval: 0.001
Frequency
Tumor−Normal Expression Difference
hsa−miR−548am−5p, rectal
(BRAF = 1; N1 = 19)
1−sided adj pval: 0.629
Frequency
Tumor−Normal Expression Difference

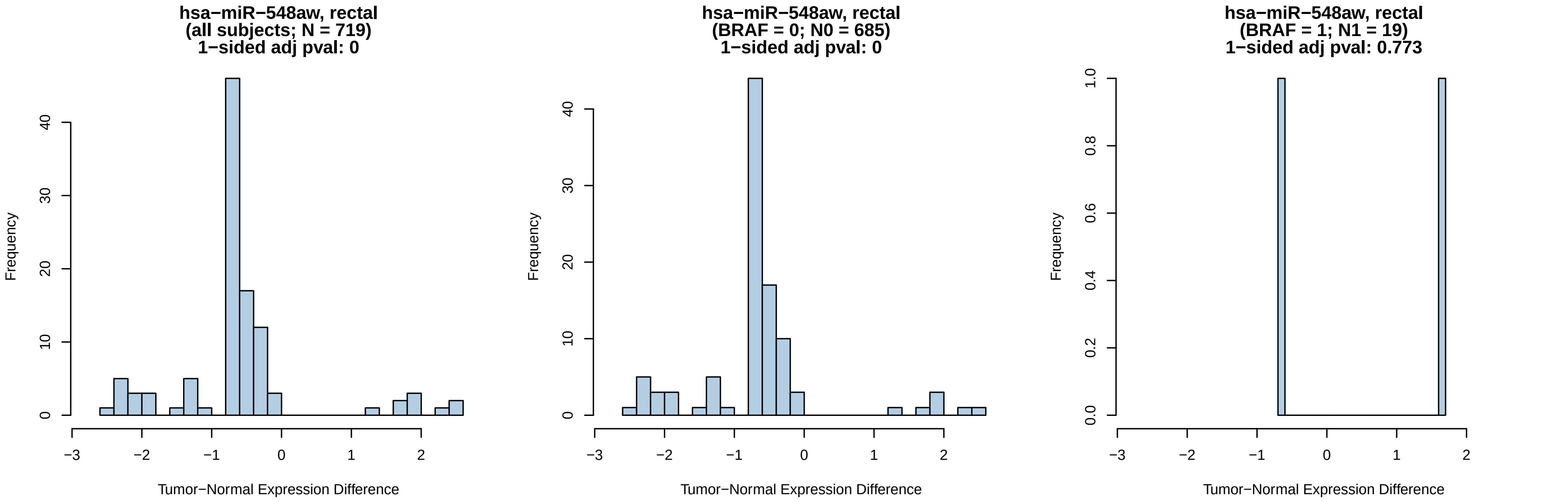
hsa−miR−548aw, rectal
(all subjects; N = 719)
1−sided adj pval: 0
hsa−miR−548aw, rectal
(BRAF = 0; N0 = 685)
1−sided adj pval: 0
hsa−miR−548aw, rectal
(BRAF = 1; N1 = 19)
1−sided adj pval: 0.773
Frequency
Tumor−Normal Expression Difference

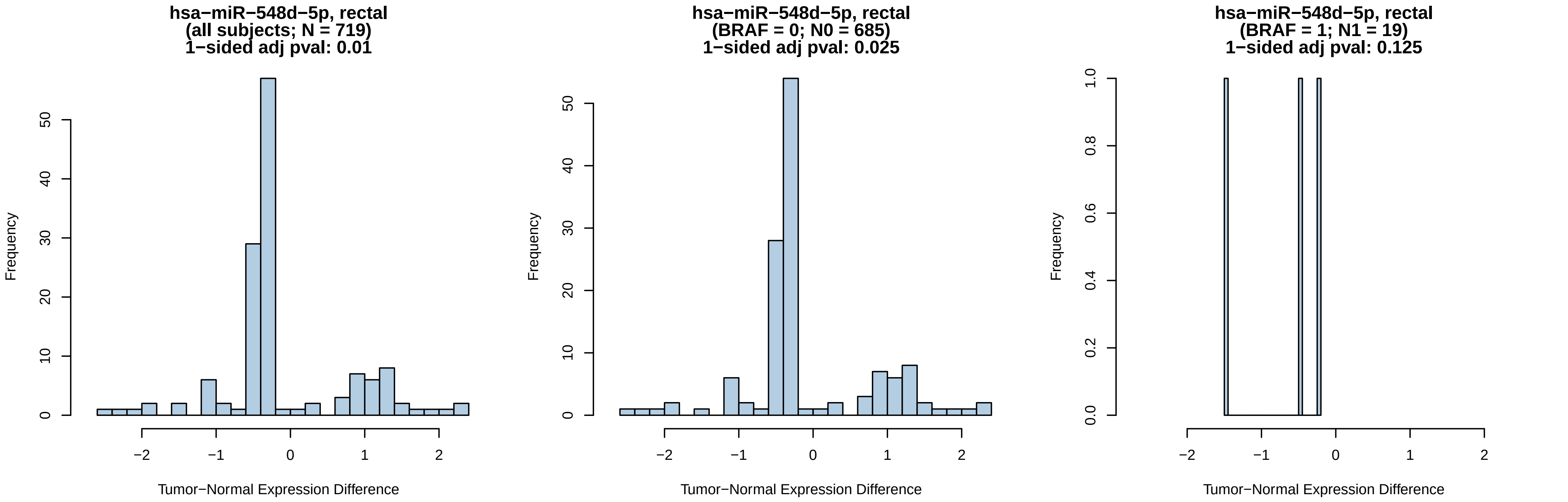
hsa−miR−548d−5p, rectal
(all subjects; N = 719)
1−sided adj pval: 0.01
hsa−miR−548d−5p, rectal
(BRAF = 0; N0 = 685)
1−sided adj pval: 0.025
hsa−miR−548d−5p, rectal
(BRAF = 1; N1 = 19)
1−sided adj pval: 0.125
Frequency
Tumor−Normal Expression Difference
0
10
20
30
40
50
0.0
0.2
0.4
0.6
0.8
1.0
−2
−1
0
1
2

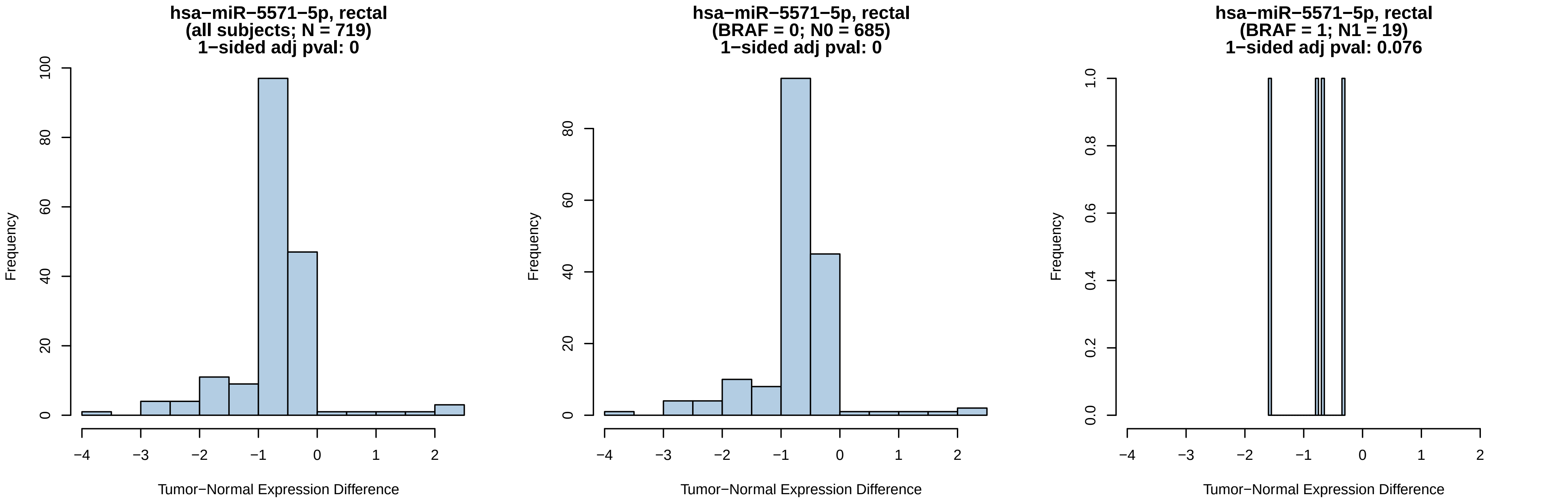
hsa−miR−5571−5p, rectal
(all subjects; N = 719)
1−sided adj pval: 0
Frequency
Tumor−Normal Expression Difference
hsa−miR−5571−5p, rectal
(BRAF = 0; N0 = 685)
1−sided adj pval: 0
Frequency
Tumor−Normal Expression Difference
hsa−miR−5571−5p, rectal
(BRAF = 1; N1 = 19)
1−sided adj pval: 0.076
Frequency
Tumor−Normal Expression Difference

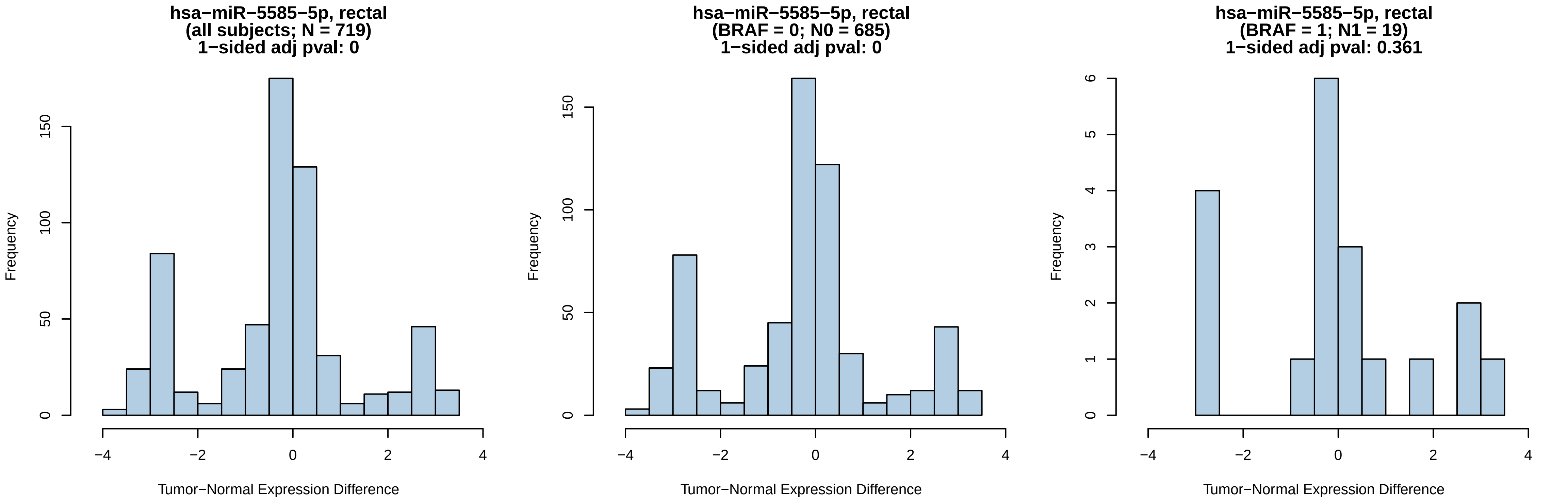

hsa−miR−5585−5p, rectal
(all subjects; N = 719)
1−sided adj pval: 0
Frequency
0
50
100
150
−4
−2
0
2
4
Tumor−Normal Expression Difference
hsa−miR−5585−5p, rectal
(BRAF = 0; N0 = 685)
1−sided adj pval: 0
Frequency
0
50
100
150
−4
−2
0
2
4
Tumor−Normal Expression Difference
hsa−miR−5585−5p, rectal
(BRAF = 1; N1 = 19)
1−sided adj pval: 0.361
Frequency
0
1
2
3
4
5
6
−4
−2
0
2
4
Tumor−Normal Expression Difference

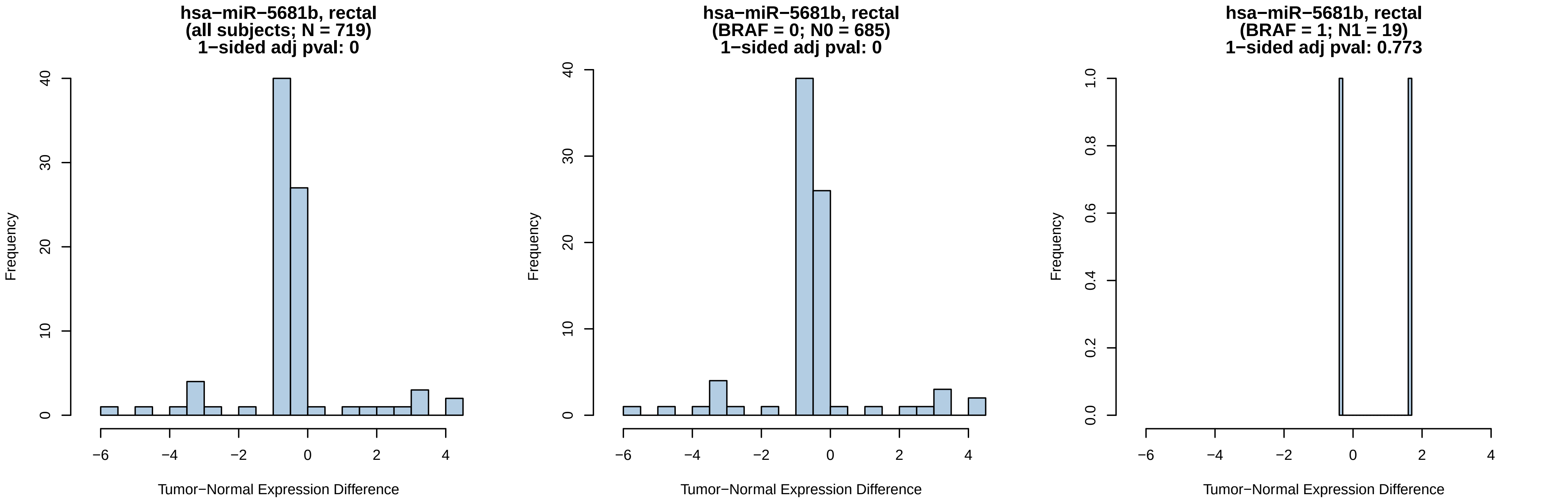
hsa−miR−5681b, rectal
(all subjects; N = 719)
1−sided adj pval: 0
Frequency
0
10
20
30
40
−6
−4
−2
0
2
4
Tumor−Normal Expression Difference
hsa−miR−5681b, rectal
(BRAF = 0; N0 = 685)
1−sided adj pval: 0
Frequency
0
10
20
30
40
−6
−4
−2
0
2
4
Tumor−Normal Expression Difference
hsa−miR−5681b, rectal
(BRAF = 1; N1 = 19)
1−sided adj pval: 0.773
Frequency
0.0
0.2
0.4
0.6
0.8
1.0
−6
−4
−2
0
2
4
Tumor−Normal Expression Difference

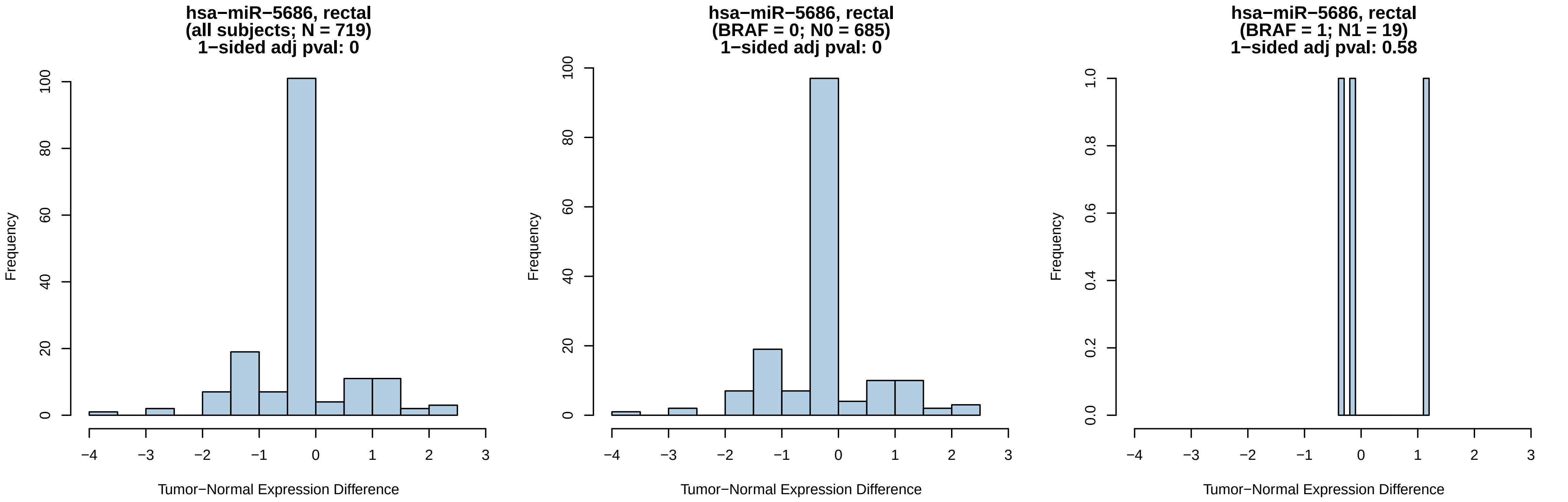

hsa−miR−5686, rectal
(all subjects; N = 719)
1−sided adj pval: 0
hsa−miR−5686, rectal
(BRAF = 0; N0 = 685)
1−sided adj pval: 0
hsa−miR−5686, rectal
(BRAF = 1; N1 = 19)
1−sided adj pval: 0.58
Frequency
Tumor−Normal Expression Difference

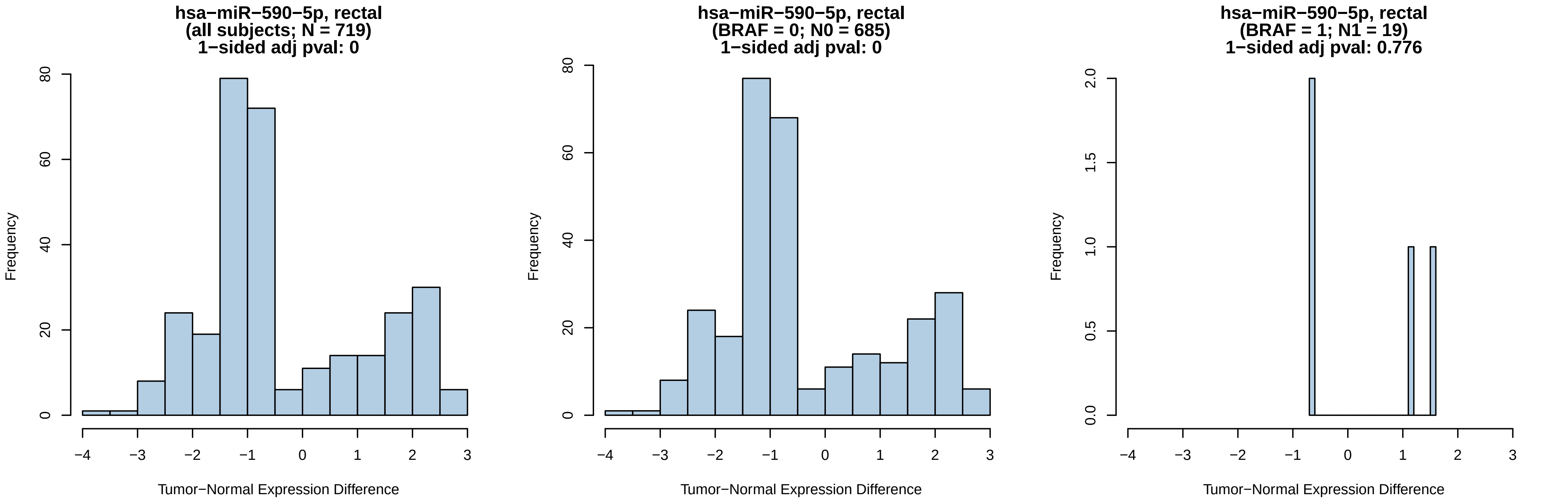
hsa−miR−590−5p, rectal
(all subjects; N = 719)
1−sided adj pval: 0
Frequency
Tumor−Normal Expression Difference
hsa−miR−590−5p, rectal
(BRAF = 0; N0 = 685)
1−sided adj pval: 0
Frequency
Tumor−Normal Expression Difference
hsa−miR−590−5p, rectal
(BRAF = 1; N1 = 19)
1−sided adj pval: 0.776
Frequency
Tumor−Normal Expression Difference

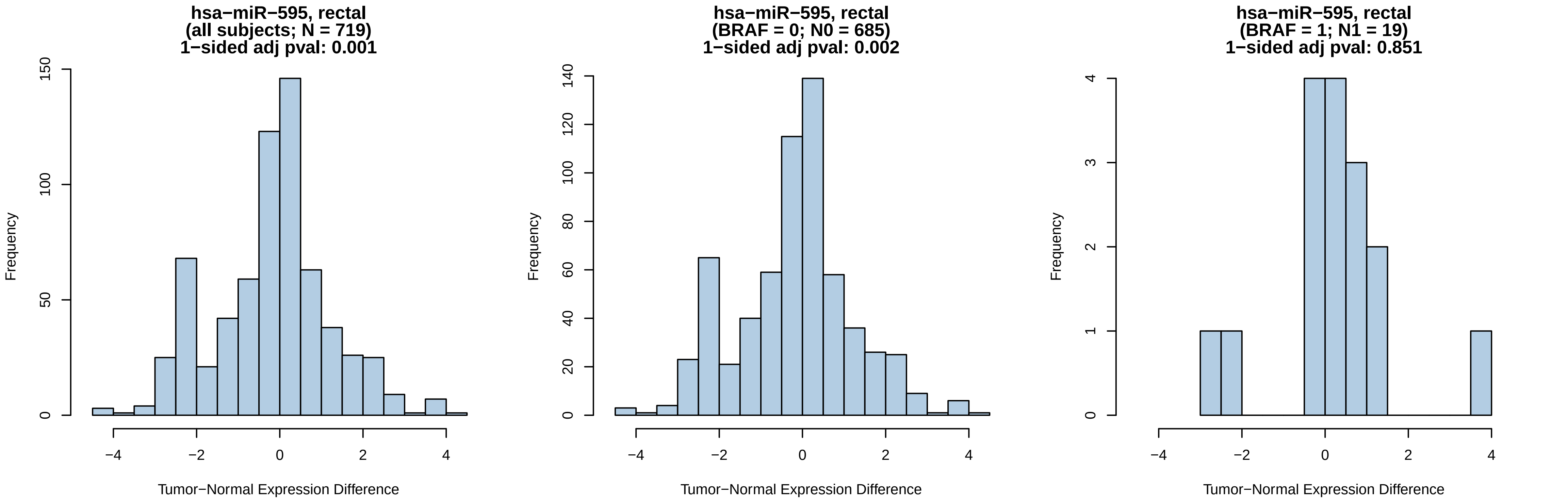

hsa−miR−595, rectal
(all subjects; N = 719)
1−sided adj pval: 0.001
Frequency
0
50
100
150
−4
−2
0
2
4
Tumor−Normal Expression Difference
hsa−miR−595, rectal
(BRAF = 0; N0 = 685)
1−sided adj pval: 0.002
Frequency
0
20
40
60
80
100
120
140
−4
−2
0
2
4
Tumor−Normal Expression Difference
hsa−miR−595, rectal
(BRAF = 1; N1 = 19)
1−sided adj pval: 0.851
Frequency
0
1
2
3
4
−4
−2
0
2
4
Tumor−Normal Expression Difference

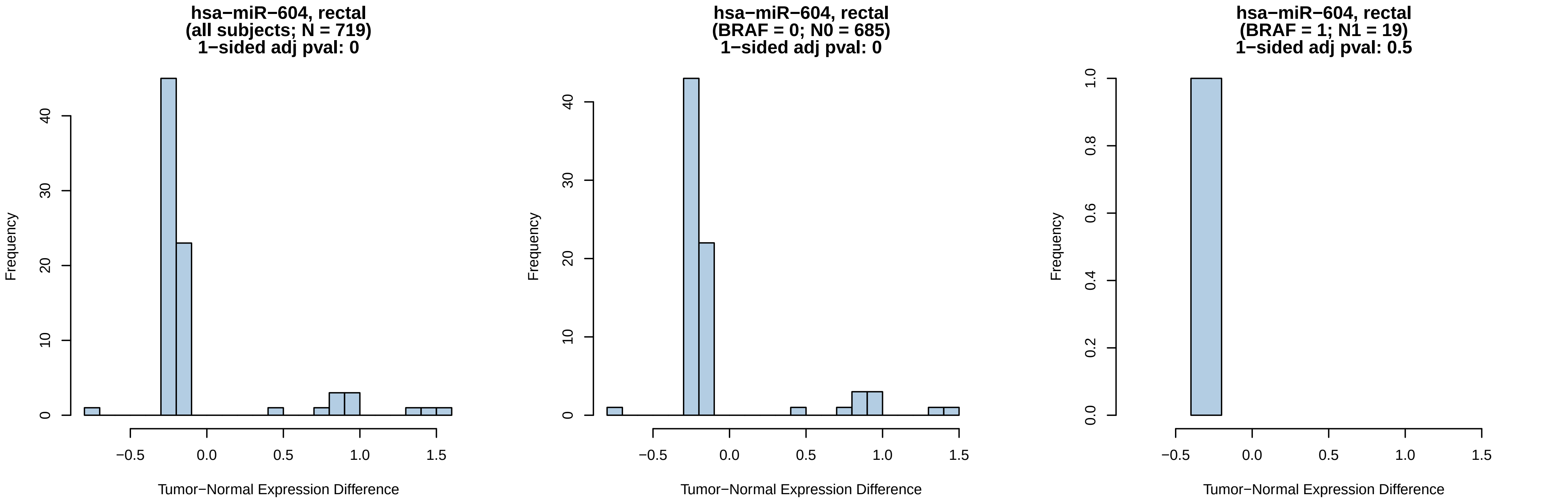

hsa−miR−604, rectal
(all subjects; N = 719)
1−sided adj pval: 0
Frequency
Tumor−Normal Expression Difference
hsa−miR−604, rectal
(BRAF = 0; N0 = 685)
1−sided adj pval: 0
Frequency
Tumor−Normal Expression Difference
hsa−miR−604, rectal
(BRAF = 1; N1 = 19)
1−sided adj pval: 0.5
Frequency
Tumor−Normal Expression Difference

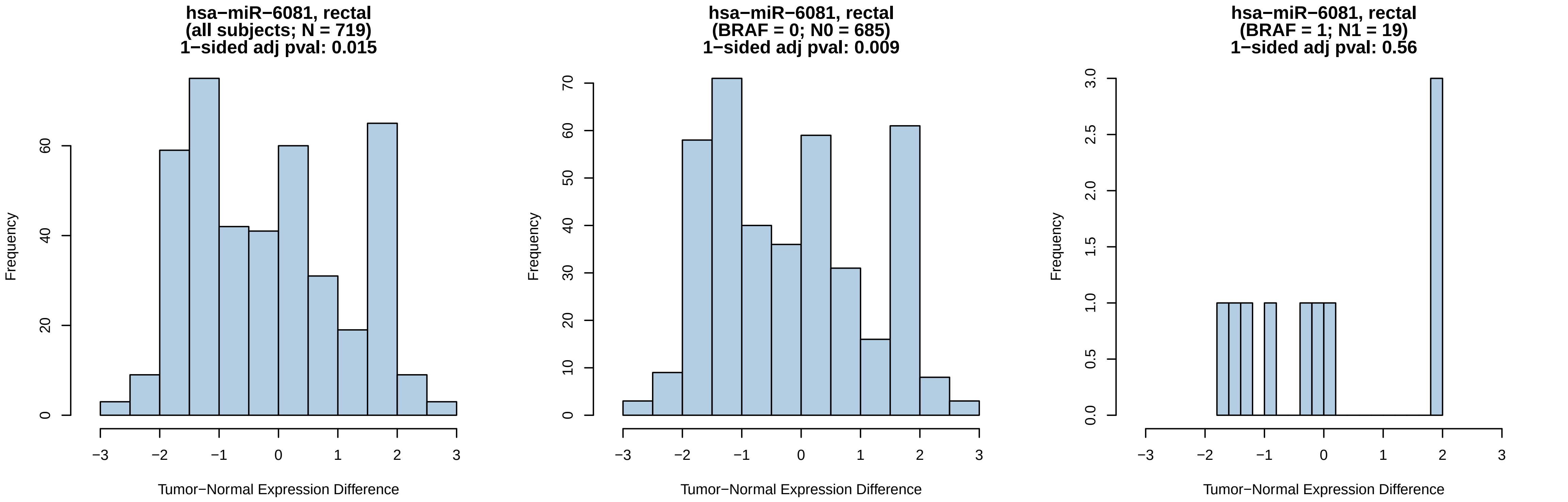
hsa−miR−6081, rectal
(all subjects; N = 719)
1−sided adj pval: 0.015
Frequency
Tumor−Normal Expression Difference
hsa−miR−6081, rectal
(BRAF = 0; N0 = 685)
1−sided adj pval: 0.009
Frequency
Tumor−Normal Expression Difference
hsa−miR−6081, rectal
(BRAF = 1; N1 = 19)
1−sided adj pval: 0.56
Frequency
Tumor−Normal Expression Difference

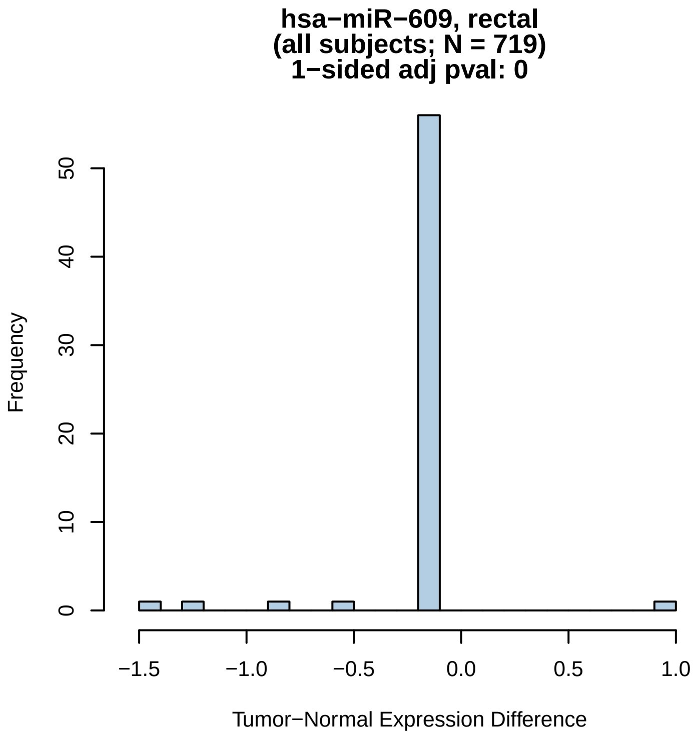

hsa−miR−609, rectal
(all subjects; N = 719)
1−sided adj pval: 0
Frequency
Tumor−Normal Expression Difference


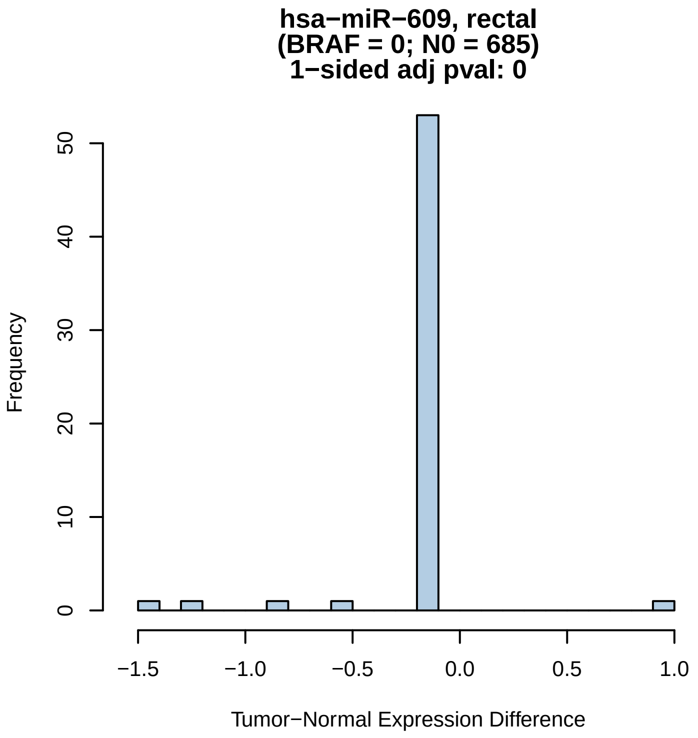

hsa−miR−609, rectal
(BRAF = 0; N0 = 685)
1−sided adj pval: 0
Frequency
Tumor−Normal Expression Difference


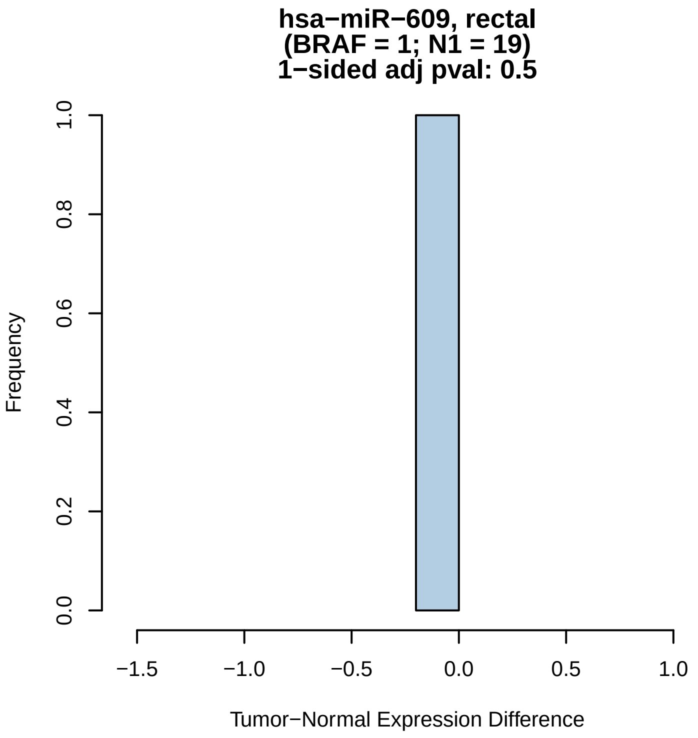

hsa−miR−609, rectal
(BRAF = 1; N1 = 19)
1−sided adj pval: 0.5
Frequency
Tumor−Normal Expression Difference

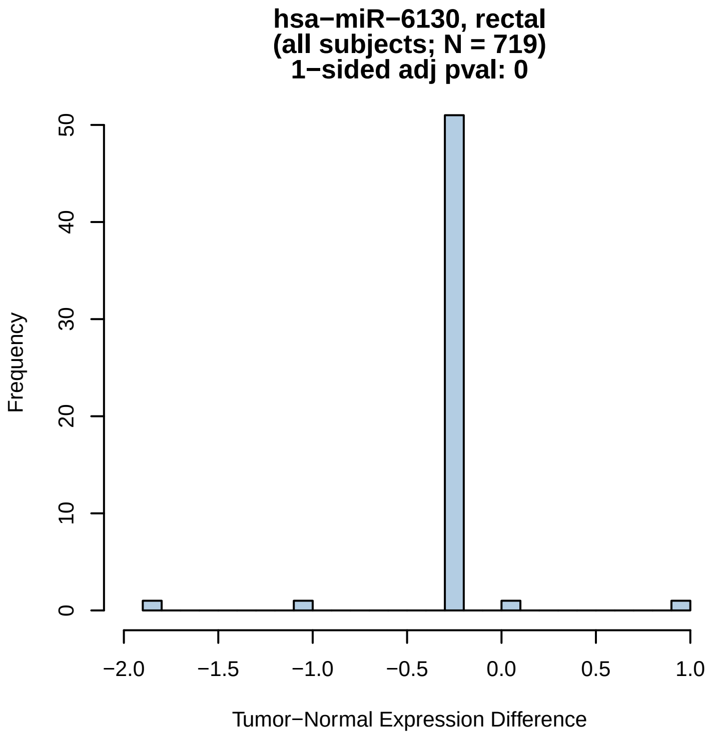

hsa−miR−6130, rectal
(all subjects; N = 719)
1−sided adj pval: 0
Frequency
0
10
20
30
40
50
−2.0
−1.5
−1.0
−0.5
0.0
0.5
1.0
Tumor−Normal Expression Difference


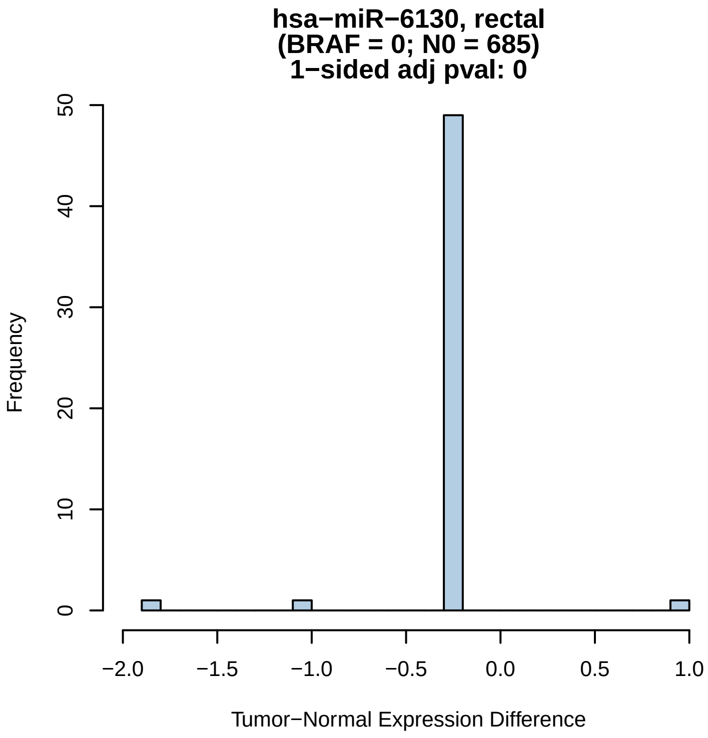

hsa−miR−6130, rectal
(BRAF = 0; N0 = 685)
1−sided adj pval: 0
Frequency
0
10
20
30
40
50
−2.0
−1.5
−1.0
−0.5
0.0
0.5
1.0
Tumor−Normal Expression Difference


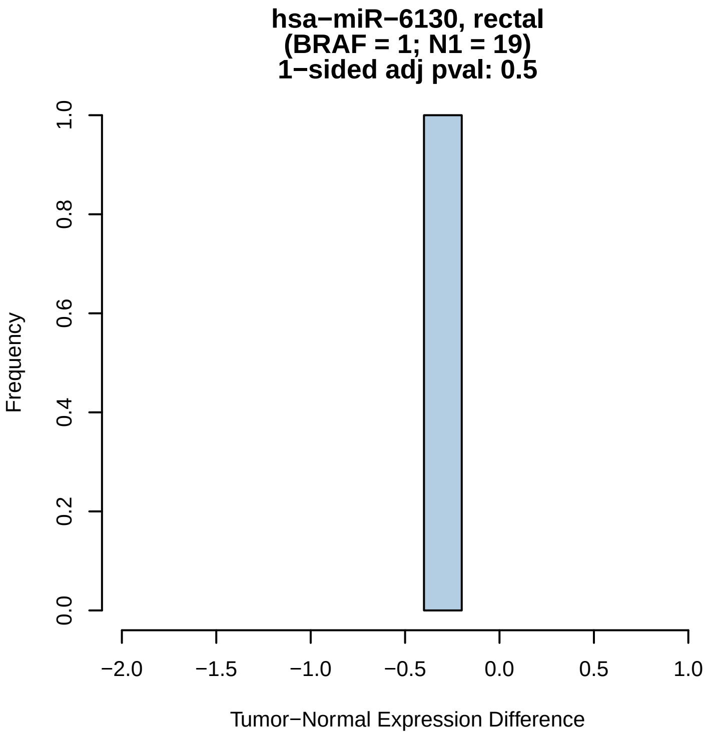

hsa−miR−6130, rectal
(BRAF = 1; N1 = 19)
1−sided adj pval: 0.5
Frequency
0.0
0.2
0.4
0.6
0.8
1.0
−2.0
−1.5
−1.0
−0.5
0.0
0.5
1.0
Tumor−Normal Expression Difference

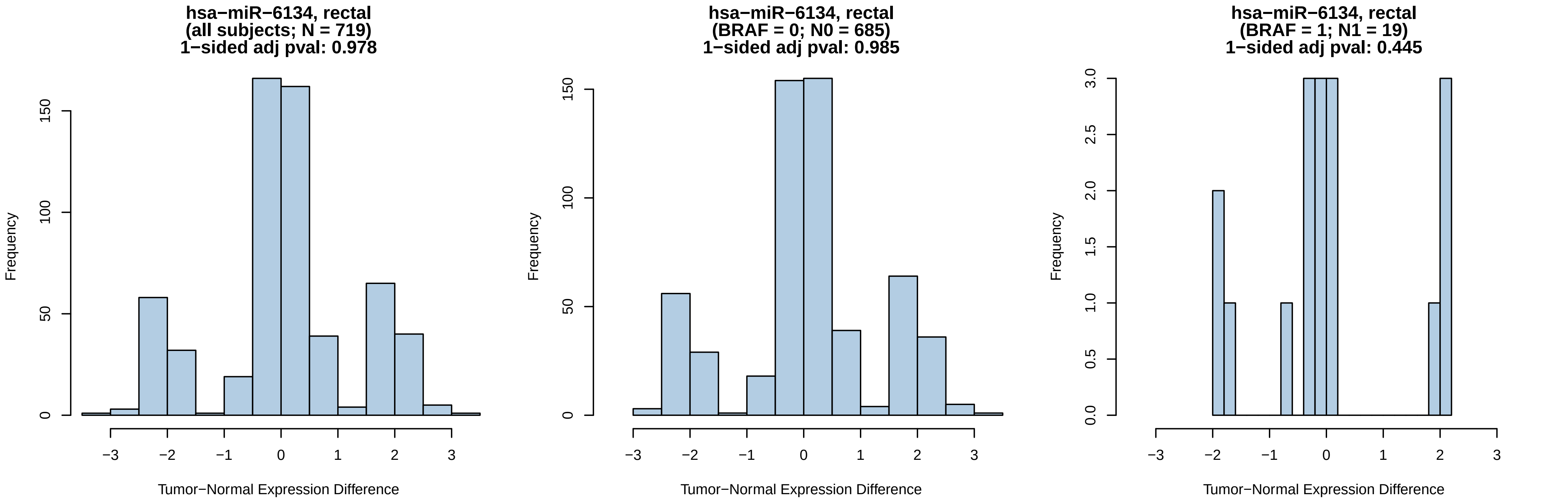

hsa−miR−6134, rectal
(all subjects; N = 719)
1−sided adj pval: 0.978
hsa−miR−6134, rectal
(BRAF = 0; N0 = 685)
1−sided adj pval: 0.985
hsa−miR−6134, rectal
(BRAF = 1; N1 = 19)
1−sided adj pval: 0.445
Frequency
Tumor−Normal Expression Difference

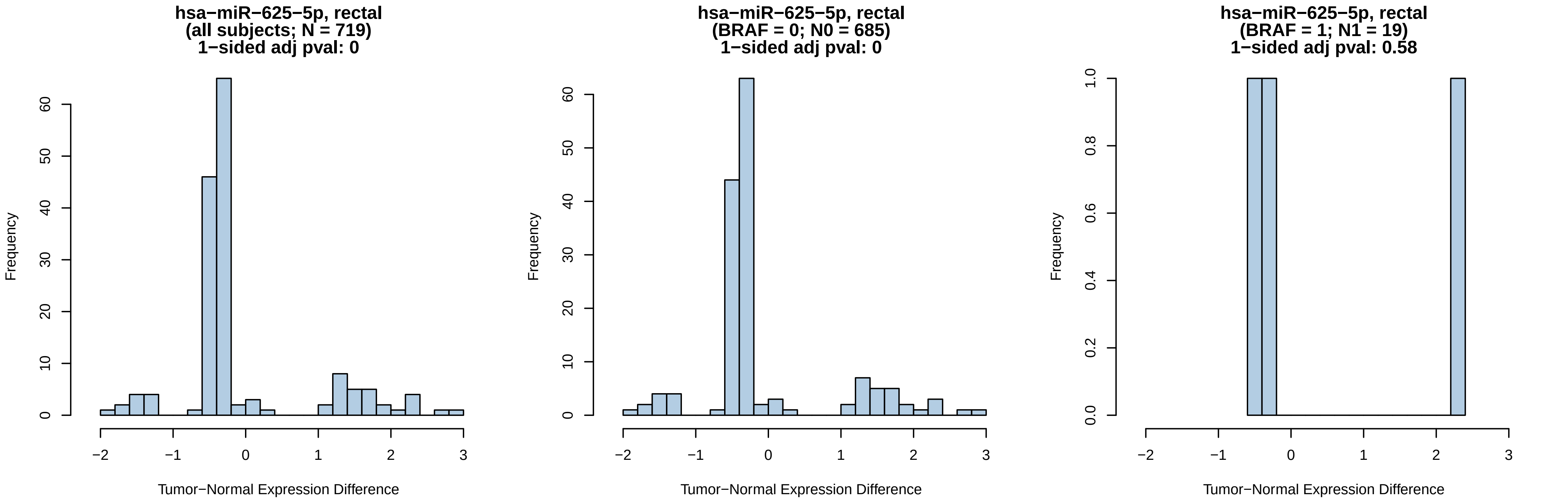
hsa−miR−625−5p, rectal
(all subjects; N = 719)
1−sided adj pval: 0
Frequency
Tumor−Normal Expression Difference
hsa−miR−625−5p, rectal
(BRAF = 0; N0 = 685)
1−sided adj pval: 0
Frequency
Tumor−Normal Expression Difference
hsa−miR−625−5p, rectal
(BRAF = 1; N1 = 19)
1−sided adj pval: 0.58
Frequency
Tumor−Normal Expression Difference

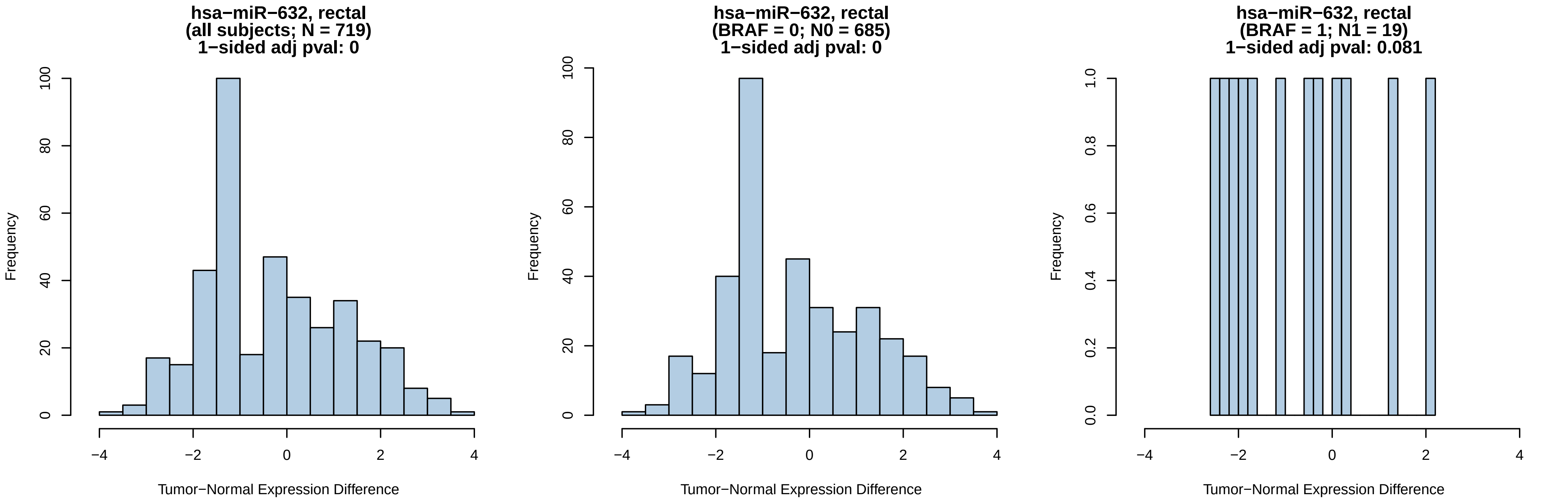
hsa−miR−632, rectal
(all subjects; N = 719)
1−sided adj pval: 0
hsa−miR−632, rectal
(BRAF = 0; N0 = 685)
1−sided adj pval: 0
hsa−miR−632, rectal
(BRAF = 1; N1 = 19)
1−sided adj pval: 0.081
Frequency
Tumor−Normal Expression Difference
0
20
40
60
80
100
0.0
0.2
0.4
0.6
0.8
1.0
−4
−2
0
2
4

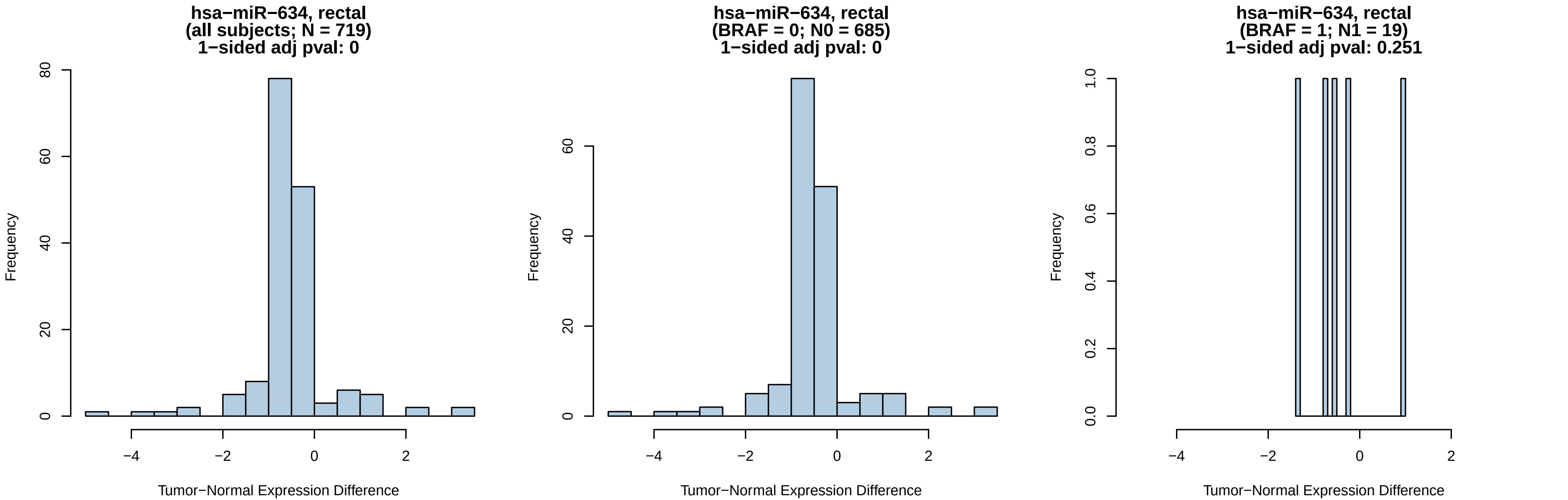
hsa−miR−634, rectal
(all subjects; N = 719)
1−sided adj pval: 0
Frequency
0
20
40
60
80
−4
−2
0
2
Tumor−Normal Expression Difference
hsa−miR−634, rectal
(BRAF = 0; N0 = 685)
1−sided adj pval: 0
Frequency
0
20
40
60
−4
−2
0
2
Tumor−Normal Expression Difference
hsa−miR−634, rectal
(BRAF = 1; N1 = 19)
1−sided adj pval: 0.251
Frequency
0.0
0.2
0.4
0.6
0.8
1.0
−4
−2
0
2
Tumor−Normal Expression Difference

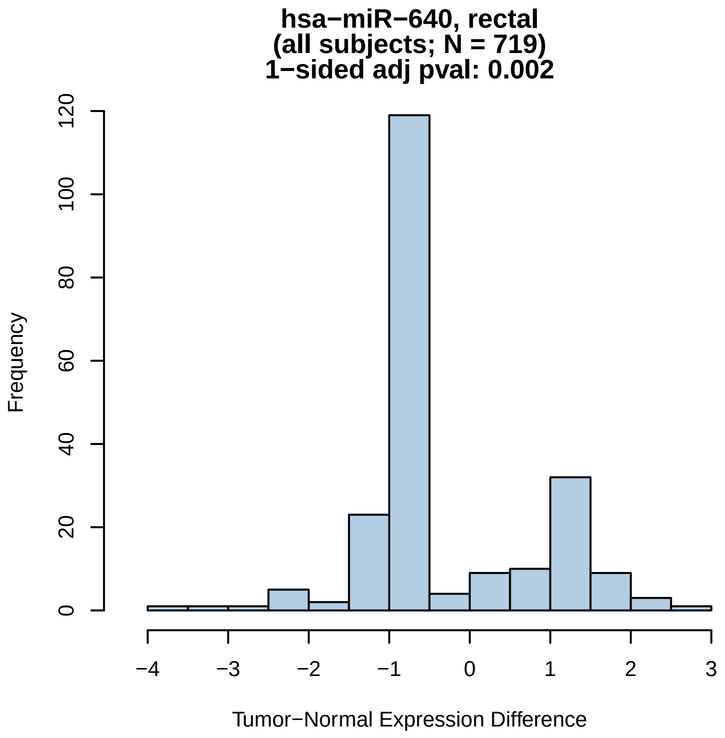

hsa−miR−640, rectal
(all subjects; N = 719)
1−sided adj pval: 0.002
Frequency
0
20
40
60
80
100
120
−4
−3
−2
−1
0
1
2
3
Tumor−Normal Expression Difference


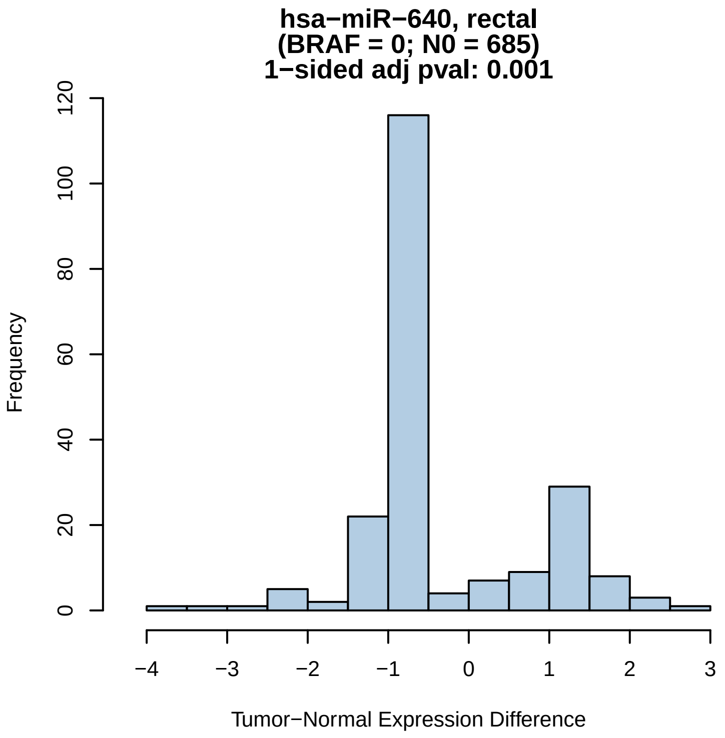

hsa−miR−640, rectal
(BRAF = 0; N0 = 685)
1−sided adj pval: 0.001
Frequency
0
20
40
60
80
100
120
−4
−3
−2
−1
0
1
2
3
Tumor−Normal Expression Difference


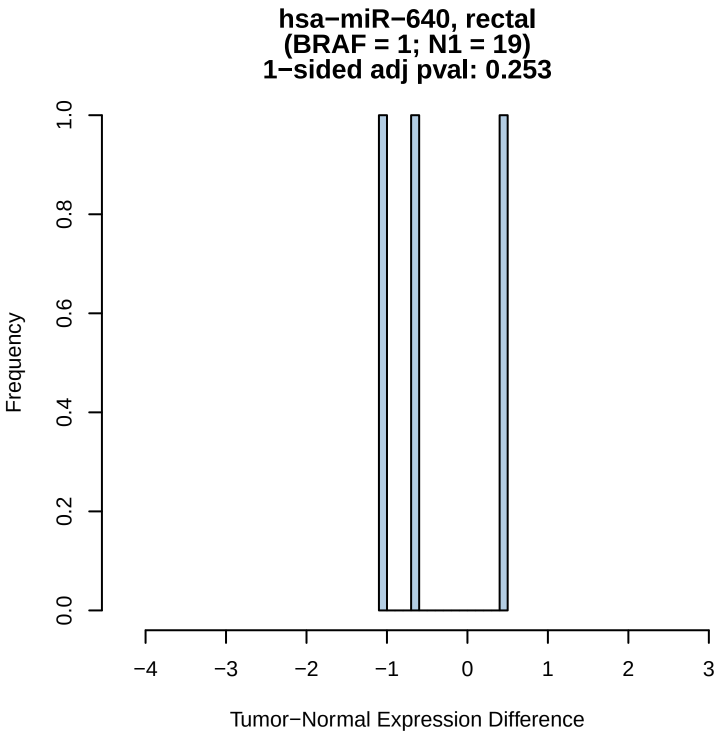

hsa−miR−640, rectal
(BRAF = 1; N1 = 19)
1−sided adj pval: 0.253
Frequency
0.0
0.2
0.4
0.6
0.8
1.0
−4
−3
−2
−1
0
1
2
3
Tumor−Normal Expression Difference

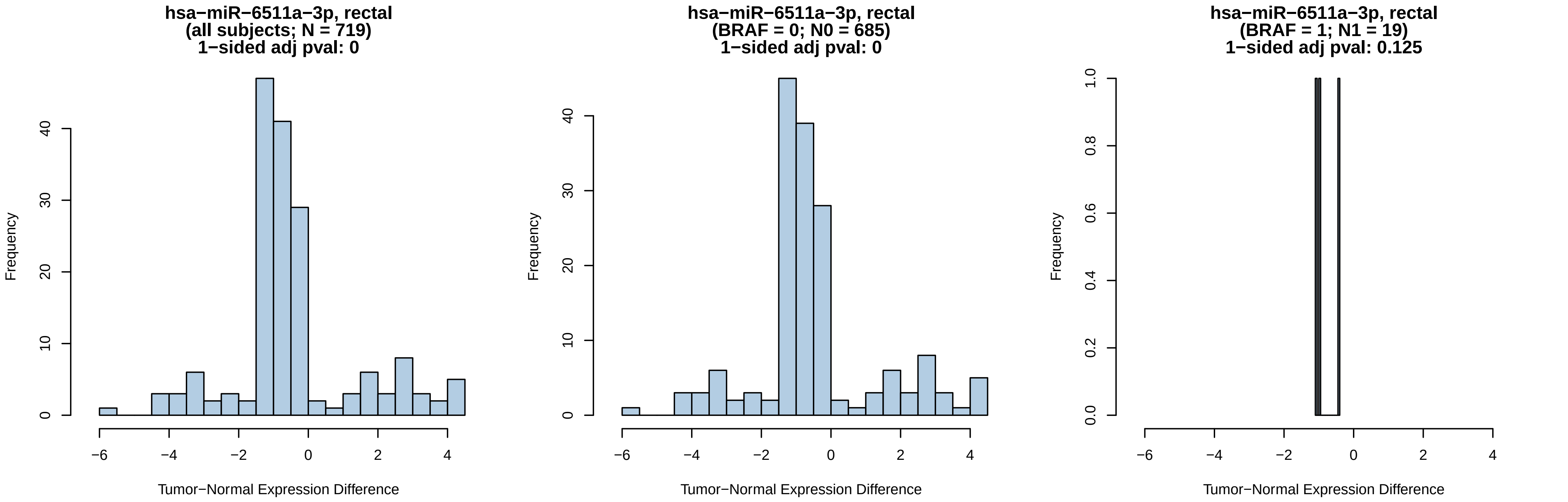

hsa−miR−6511a−3p, rectal
(all subjects; N = 719)
1−sided adj pval: 0
hsa−miR−6511a−3p, rectal
(BRAF = 0; N0 = 685)
1−sided adj pval: 0
hsa−miR−6511a−3p, rectal
(BRAF = 1; N1 = 19)
1−sided adj pval: 0.125
Frequency
Tumor−Normal Expression Difference

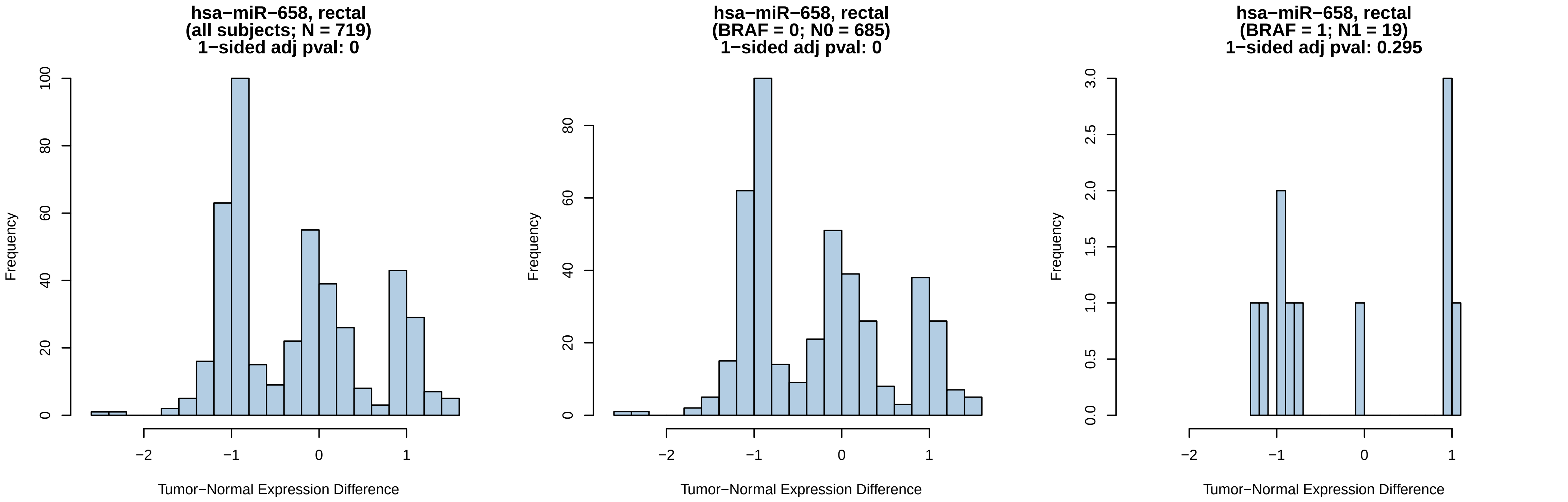

hsa−miR−658, rectal
(all subjects; N = 719)
1−sided adj pval: 0
Frequency
0
20
40
60
80
100
−2
−1
0
1
Tumor−Normal Expression Difference
hsa−miR−658, rectal
(BRAF = 0; N0 = 685)
1−sided adj pval: 0
Frequency
0
20
40
60
80
−2
−1
0
1
Tumor−Normal Expression Difference
hsa−miR−658, rectal
(BRAF = 1; N1 = 19)
1−sided adj pval: 0.295
Frequency
0.0
0.5
1.0
1.5
2.0
2.5
3.0
−2
−1
0
1
Tumor−Normal Expression Difference

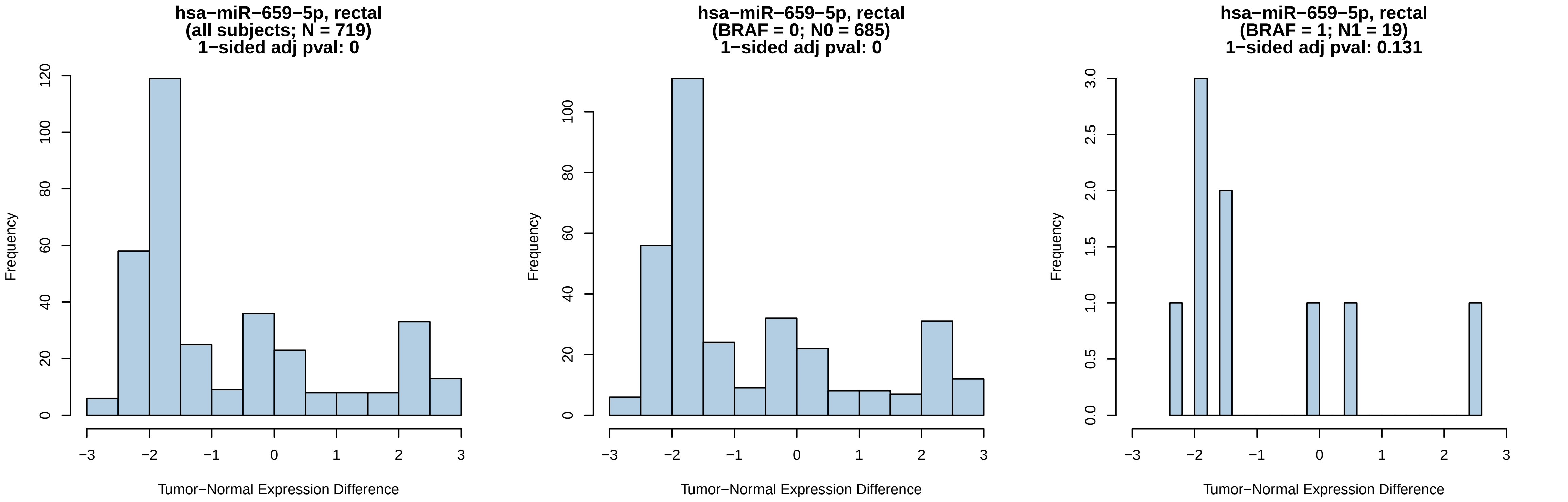
hsa−miR−659−5p, rectal
(all subjects; N = 719)
1−sided adj pval: 0
Frequency
Tumor−Normal Expression Difference
hsa−miR−659−5p, rectal
(BRAF = 0; N0 = 685)
1−sided adj pval: 0
Frequency
Tumor−Normal Expression Difference
hsa−miR−659−5p, rectal
(BRAF = 1; N1 = 19)
1−sided adj pval: 0.131
Frequency
Tumor−Normal Expression Difference

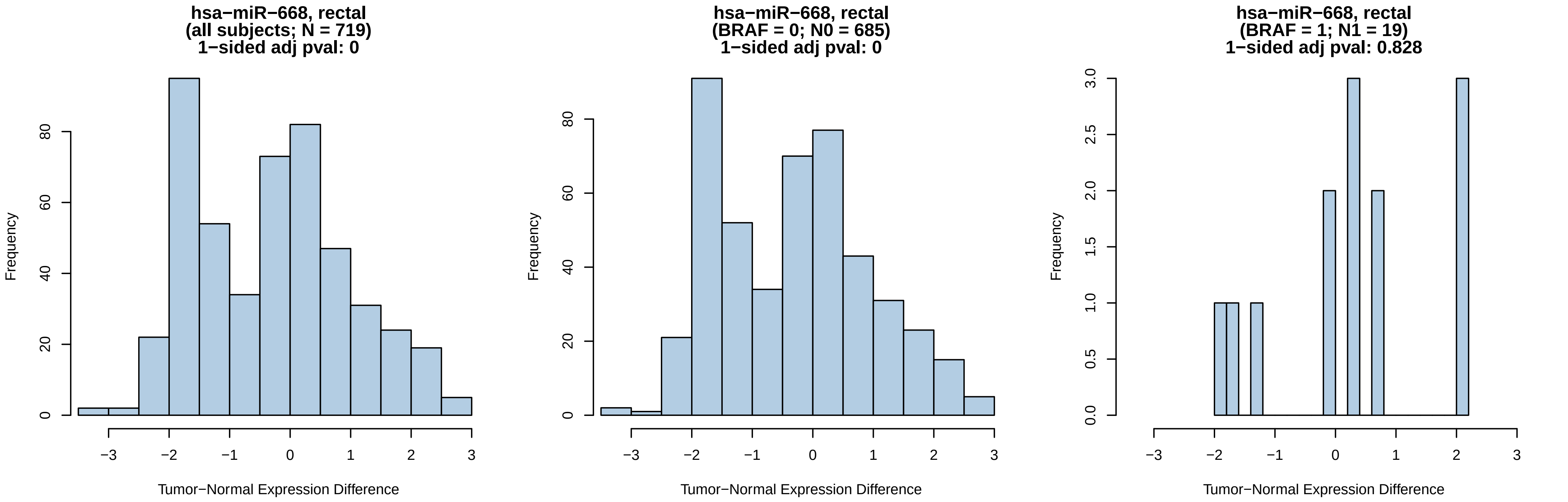

hsa−miR−668, rectal
(all subjects; N = 719)
1−sided adj pval: 0
Frequency
0
20
40
60
80
−3
−2
−1
0
1
2
3
Tumor−Normal Expression Difference
hsa−miR−668, rectal
(BRAF = 0; N0 = 685)
1−sided adj pval: 0
Frequency
0
20
40
60
80
−3
−2
−1
0
1
2
3
Tumor−Normal Expression Difference
hsa−miR−668, rectal
(BRAF = 1; N1 = 19)
1−sided adj pval: 0.828
Frequency
0.0
0.5
1.0
1.5
2.0
2.5
3.0
−3
−2
−1
0
1
2
3
Tumor−Normal Expression Difference

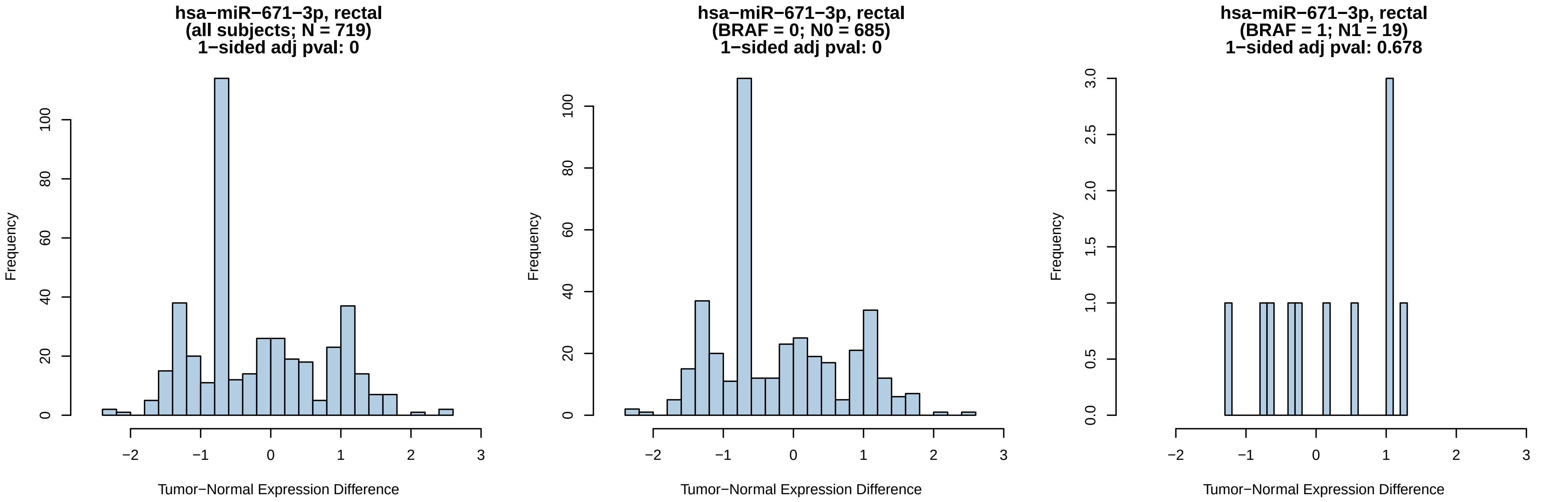

hsa−miR−671−3p, rectal
(all subjects; N = 719)
1−sided adj pval: 0
hsa−miR−671−3p, rectal
(BRAF = 0; N0 = 685)
1−sided adj pval: 0
hsa−miR−671−3p, rectal
(BRAF = 1; N1 = 19)
1−sided adj pval: 0.678
Frequency
Tumor−Normal Expression Difference
0
20
40
60
80
100
0.0
0.5
1.0
1.5
2.0
2.5
3.0
−2
−1
0
1
2
3

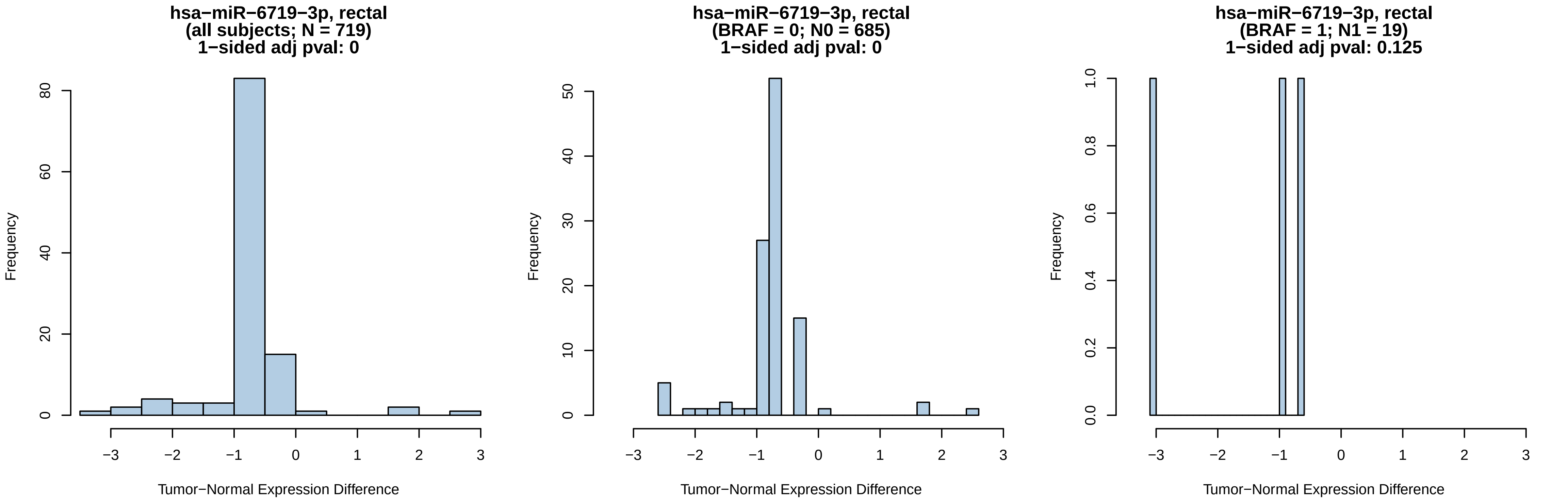
hsa−miR−6719−3p, rectal
(all subjects; N = 719)
1−sided adj pval: 0
Frequency
Tumor−Normal Expression Difference
hsa−miR−6719−3p, rectal
(BRAF = 0; N0 = 685)
1−sided adj pval: 0
Frequency
Tumor−Normal Expression Difference
hsa−miR−6719−3p, rectal
(BRAF = 1; N1 = 19)
1−sided adj pval: 0.125
Frequency
Tumor−Normal Expression Difference

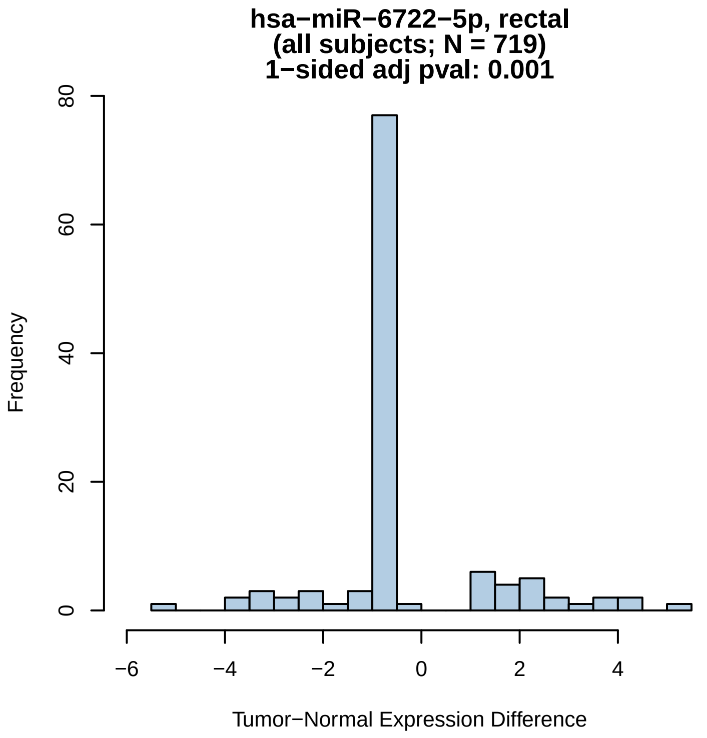
hsa−miR−6722−5p, rectal
(all subjects; N = 719)
1−sided adj pval: 0.001
Frequency
Tumor−Normal Expression Difference

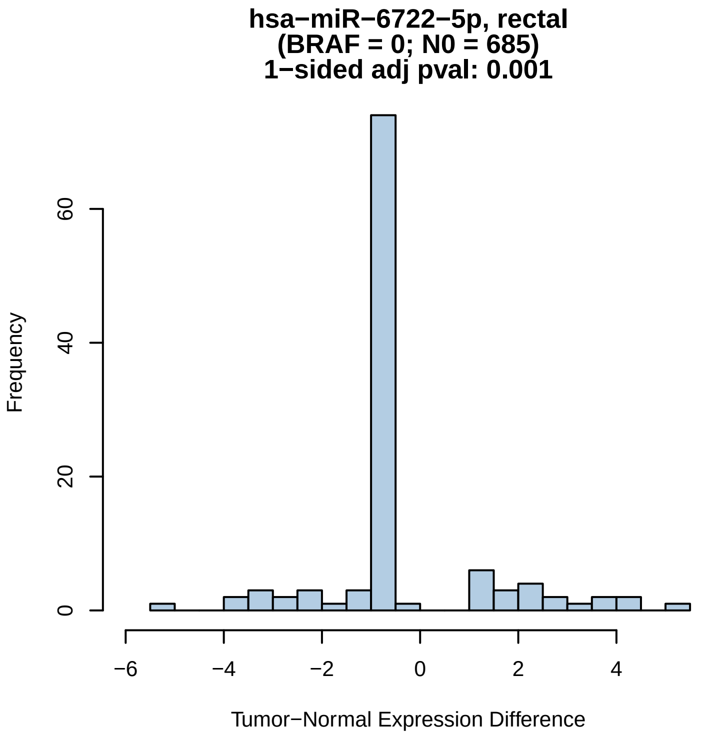
hsa−miR−6722−5p, rectal
(BRAF = 0; N0 = 685)
1−sided adj pval: 0.001
Frequency
Tumor−Normal Expression Difference

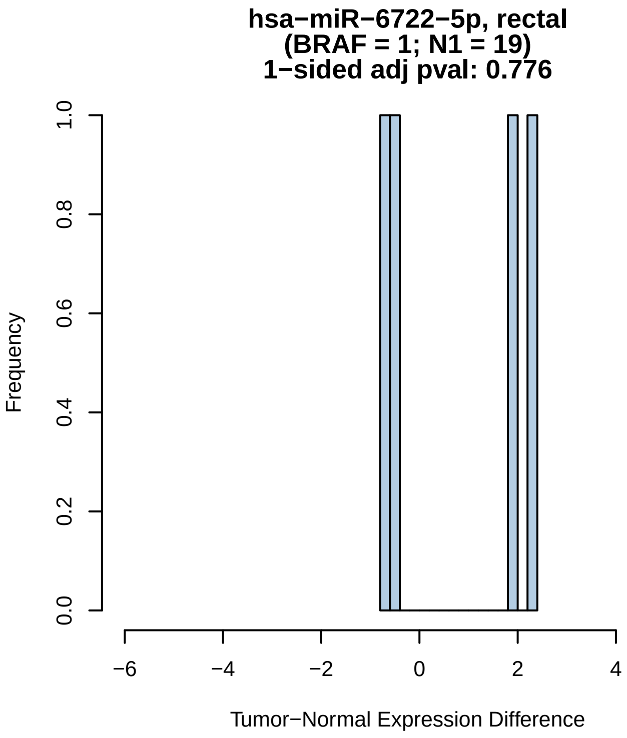
hsa−miR−6722−5p, rectal
(BRAF = 1; N1 = 19)
1−sided adj pval: 0.776
Frequency
Tumor−Normal Expression Difference

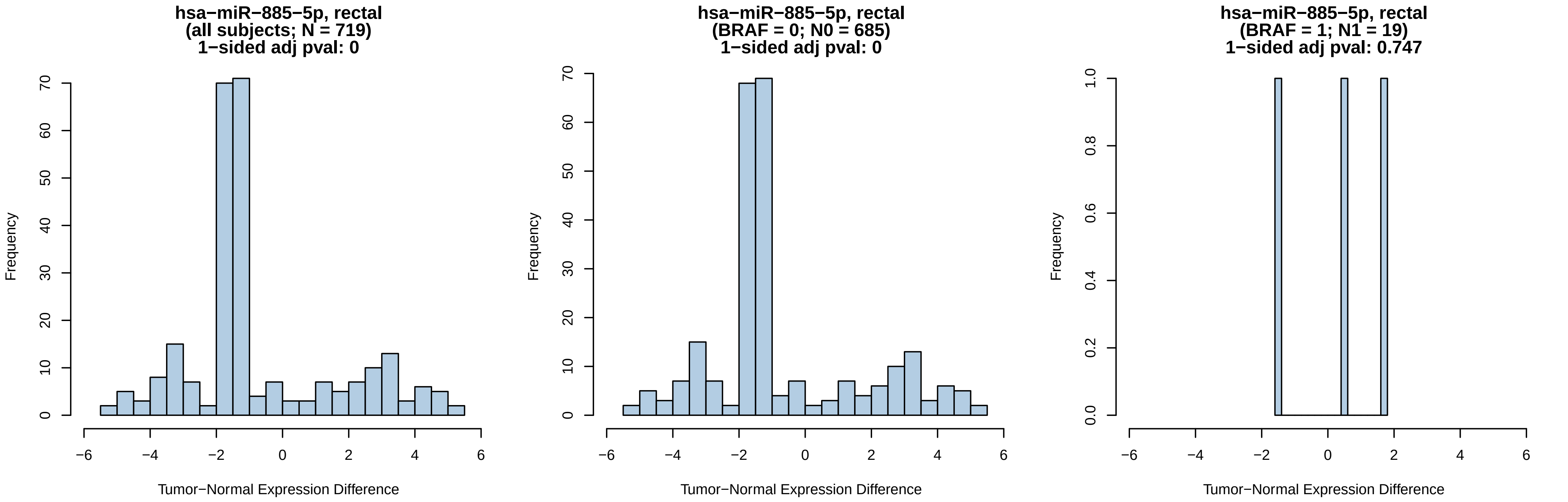

hsa−miR−885−5p, rectal
(all subjects; N = 719)
1−sided adj pval: 0
hsa−miR−885−5p, rectal
(BRAF = 0; N0 = 685)
1−sided adj pval: 0
hsa−miR−885−5p, rectal
(BRAF = 1; N1 = 19)
1−sided adj pval: 0.747
Frequency
Tumor−Normal Expression Difference

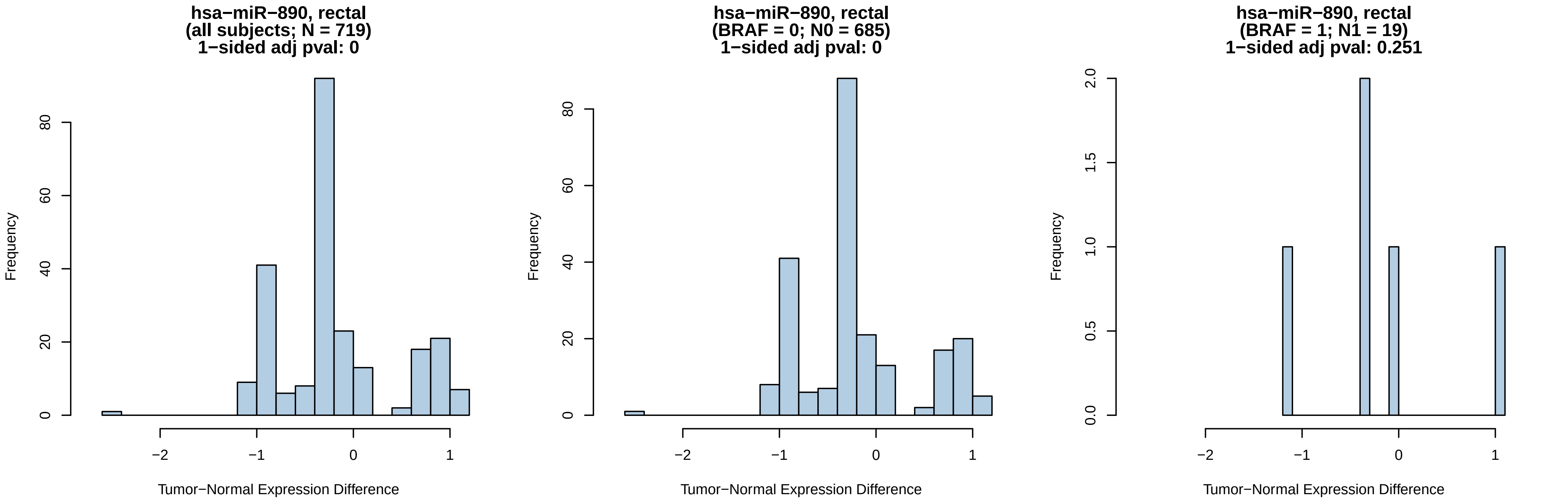

hsa−miR−890, rectal
(all subjects; N = 719)
1−sided adj pval: 0
hsa−miR−890, rectal
(BRAF = 0; N0 = 685)
1−sided adj pval: 0
hsa−miR−890, rectal
(BRAF = 1; N1 = 19)
1−sided adj pval: 0.251
Frequency
Tumor−Normal Expression Difference

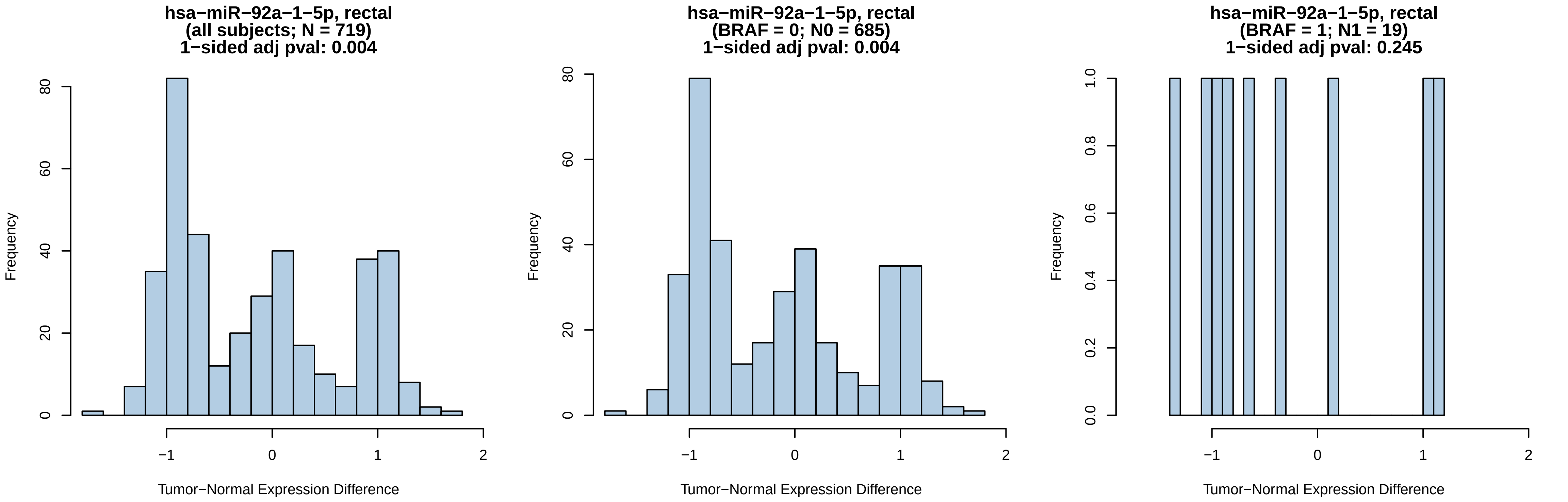
hsa−miR−92a−1−5p, rectal
(all subjects; N = 719)
1−sided adj pval: 0.004
Frequency
0
20
40
60
80
−1
0
1
2
Tumor−Normal Expression Difference
hsa−miR−92a−1−5p, rectal
(BRAF = 0; N0 = 685)
1−sided adj pval: 0.004
Frequency
0
20
40
60
80
−1
0
1
2
Tumor−Normal Expression Difference
hsa−miR−92a−1−5p, rectal
(BRAF = 1; N1 = 19)
1−sided adj pval: 0.245
Frequency
0.0
0.2
0.4
0.6
0.8
1.0
−1
0
1
2
Tumor−Normal Expression Difference

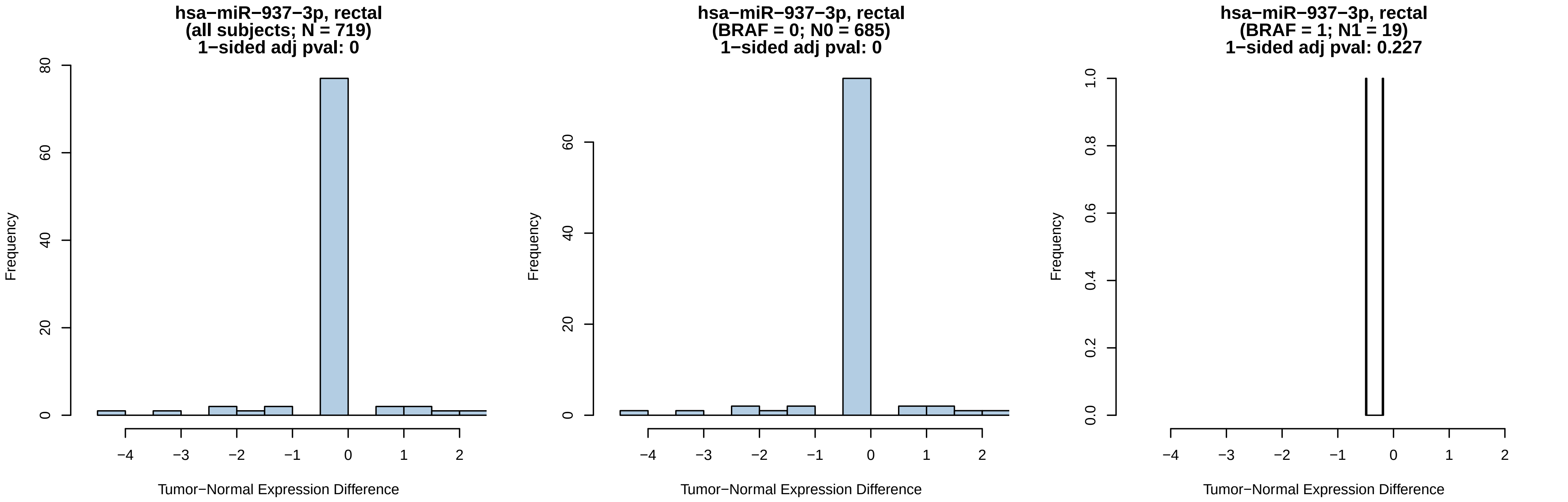

hsa−miR−937−3p, rectal
(all subjects; N = 719)
1−sided adj pval: 0
Frequency
Tumor−Normal Expression Difference
hsa−miR−937−3p, rectal
(BRAF = 0; N0 = 685)
1−sided adj pval: 0
Frequency
Tumor−Normal Expression Difference
hsa−miR−937−3p, rectal
(BRAF = 1; N1 = 19)
1−sided adj pval: 0.227
Frequency
Tumor−Normal Expression Difference

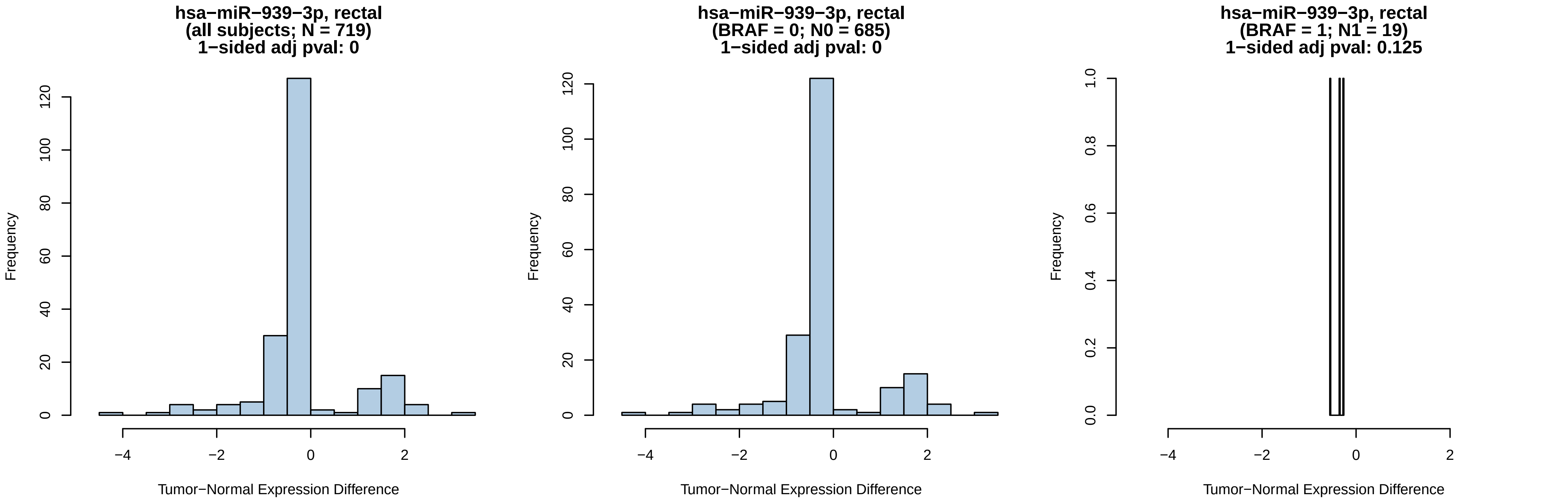

hsa−miR−939−3p, rectal
(all subjects; N = 719)
1−sided adj pval: 0
Frequency
0
20
40
60
80
100
120
−4
−2
0
2
Tumor−Normal Expression Difference
hsa−miR−939−3p, rectal
(BRAF = 0; N0 = 685)
1−sided adj pval: 0
Frequency
0
20
40
60
80
100
120
−4
−2
0
2
Tumor−Normal Expression Difference
hsa−miR−939−3p, rectal
(BRAF = 1; N1 = 19)
1−sided adj pval: 0.125
Frequency
0.0
0.2
0.4
0.6
0.8
1.0
−4
−2
0
2
Tumor−Normal Expression Difference

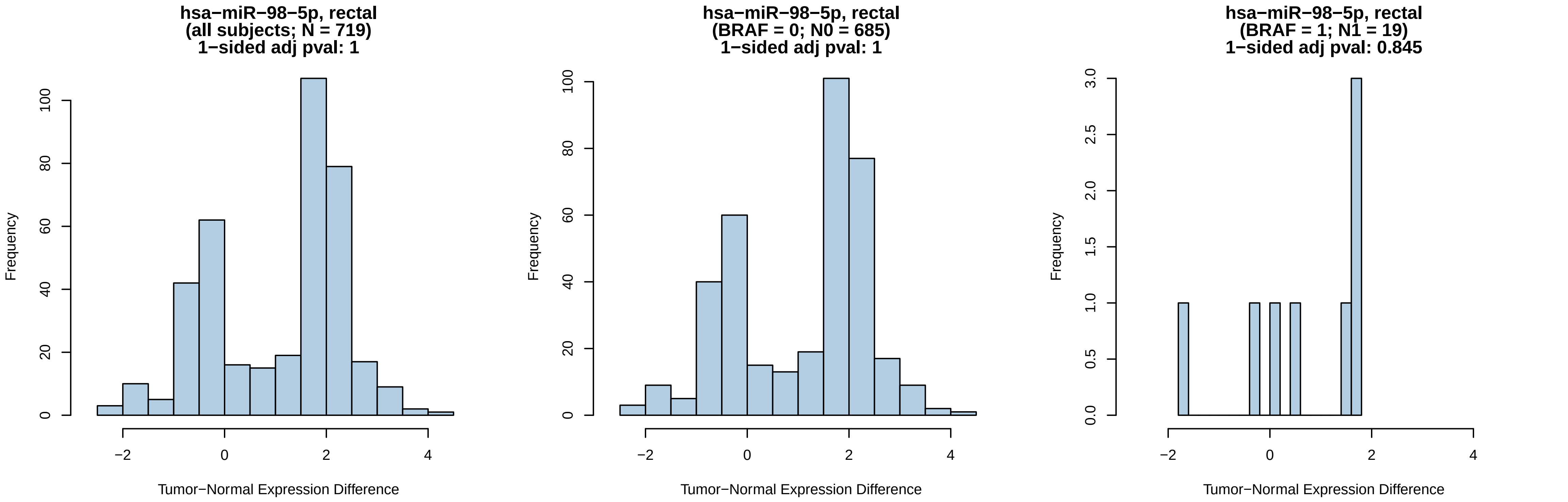

hsa−miR−98−5p, rectal
(all subjects; N = 719)
1−sided adj pval: 1
Frequency
Tumor−Normal Expression Difference
hsa−miR−98−5p, rectal
(BRAF = 0; N0 = 685)
1−sided adj pval: 1
Frequency
Tumor−Normal Expression Difference
hsa−miR−98−5p, rectal
(BRAF = 1; N1 = 19)
1−sided adj pval: 0.845
Frequency
Tumor−Normal Expression Difference

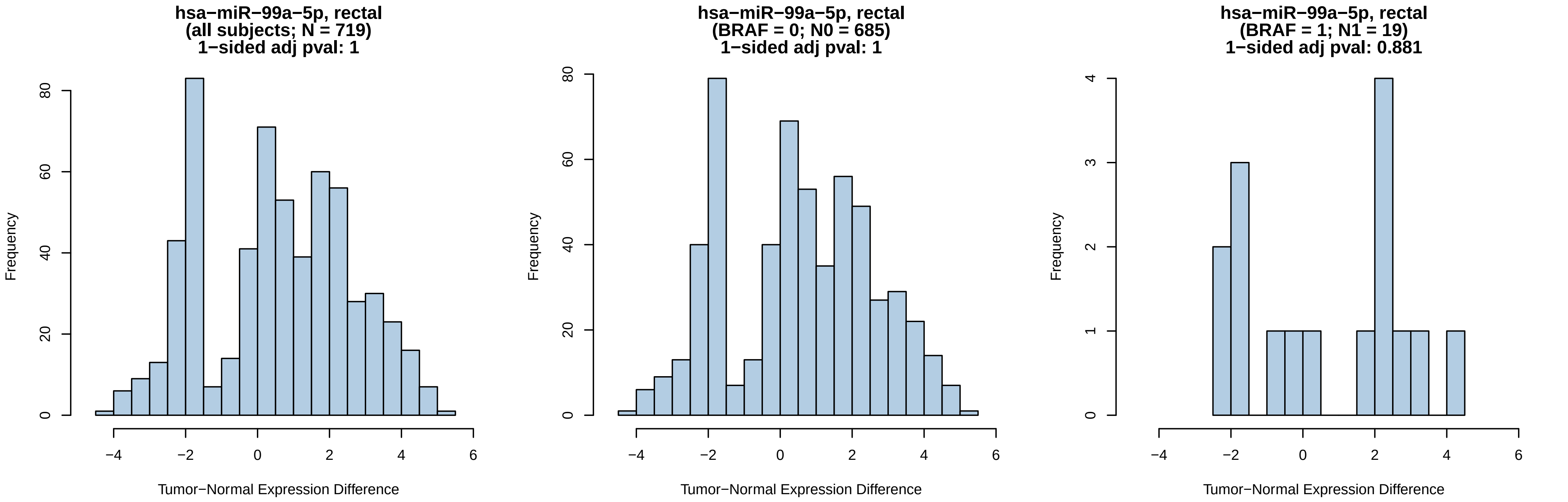
hsa−miR−99a−5p, rectal
(all subjects; N = 719)
1−sided adj pval: 1
hsa−miR−99a−5p, rectal
(BRAF = 0; N0 = 685)
1−sided adj pval: 1
hsa−miR−99a−5p, rectal
(BRAF = 1; N1 = 19)
1−sided adj pval: 0.881
Frequency
Tumor−Normal Expression Difference
0
20
40
60
80
1
2
3
4
−4
−2
0
2
4
6

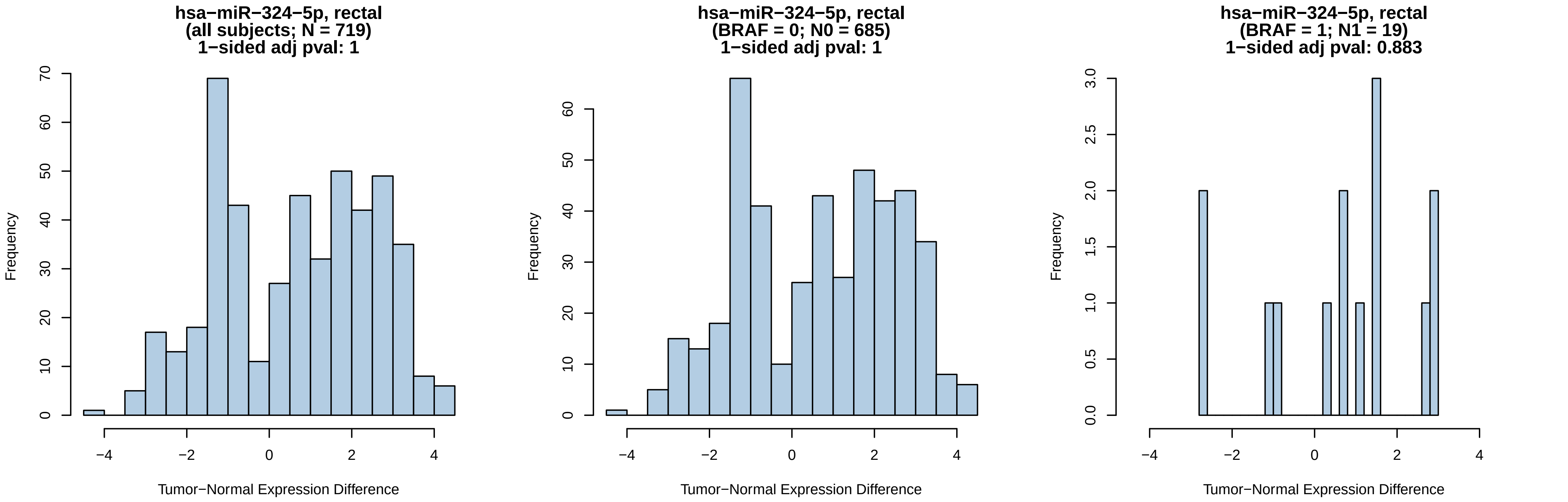
hsa−miR−324−5p, rectal
(all subjects; N = 719)
1−sided adj pval: 1
Frequency
Tumor−Normal Expression Difference
hsa−miR−324−5p, rectal
(BRAF = 0; N0 = 685)
1−sided adj pval: 1
Frequency
Tumor−Normal Expression Difference
hsa−miR−324−5p, rectal
(BRAF = 1; N1 = 19)
1−sided adj pval: 0.883
Frequency
Tumor−Normal Expression Difference

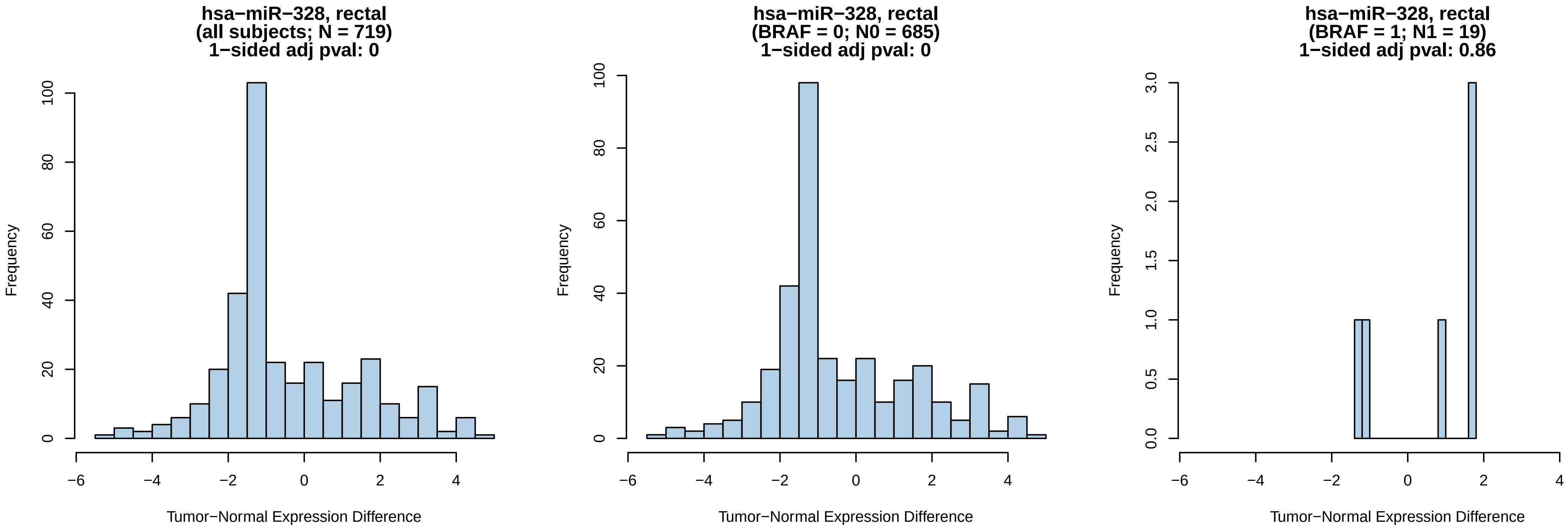
hsa−miR−328, rectal
(all subjects; N = 719)
1−sided adj pval: 0
Frequency
Tumor−Normal Expression Difference
hsa−miR−328, rectal
(BRAF = 0; N0 = 685)
1−sided adj pval: 0
Frequency
Tumor−Normal Expression Difference
hsa−miR−328, rectal
(BRAF = 1; N1 = 19)
1−sided adj pval: 0.86
Frequency
Tumor−Normal Expression Difference

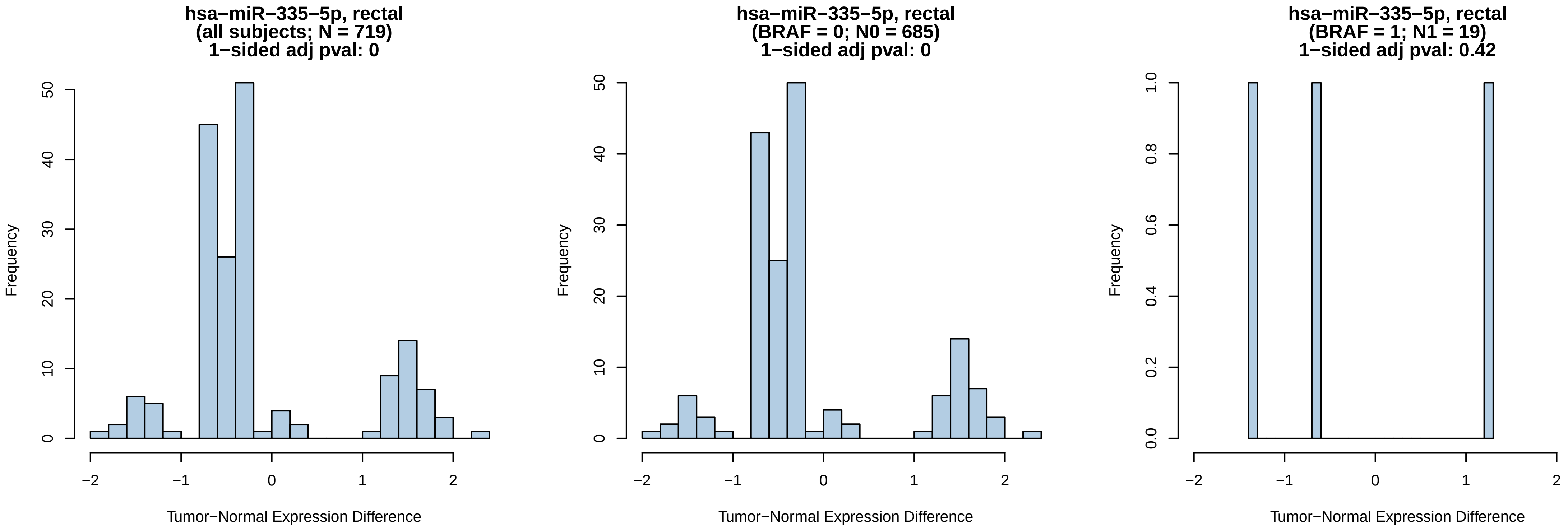

hsa−miR−335−5p, rectal
(all subjects; N = 719)
1−sided adj pval: 0
Frequency
Tumor−Normal Expression Difference
hsa−miR−335−5p, rectal
(BRAF = 0; N0 = 685)
1−sided adj pval: 0
Frequency
Tumor−Normal Expression Difference
hsa−miR−335−5p, rectal
(BRAF = 1; N1 = 19)
1−sided adj pval: 0.42
Frequency
Tumor−Normal Expression Difference

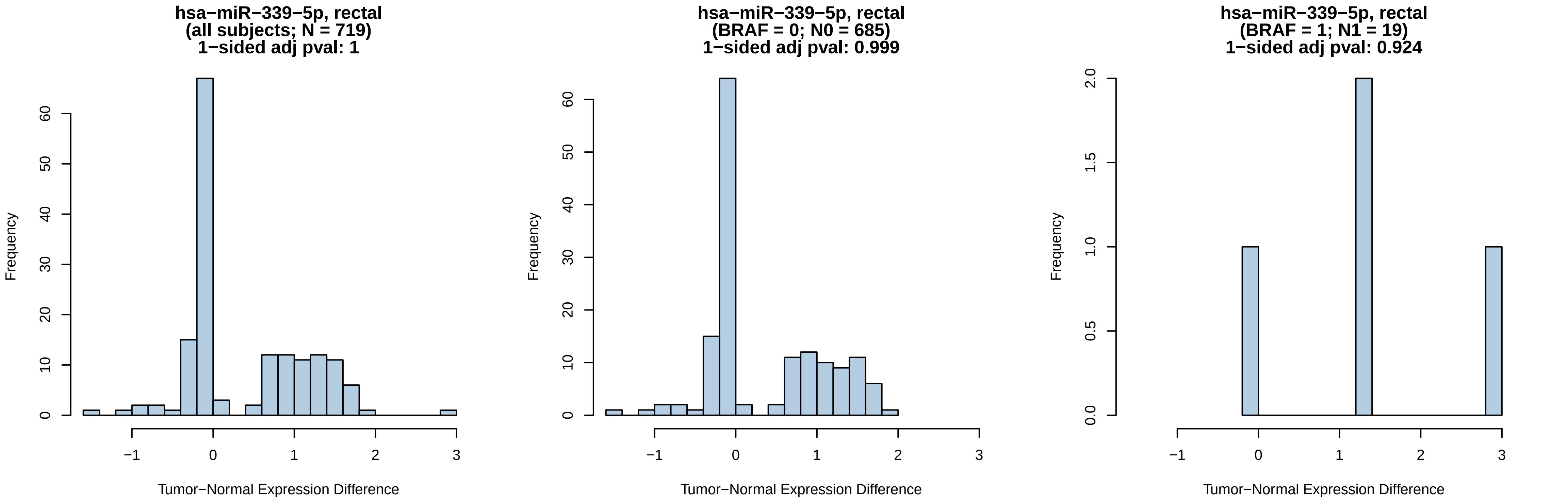
hsa−miR−339−5p, rectal
(all subjects; N = 719)
1−sided adj pval: 1
hsa−miR−339−5p, rectal
(BRAF = 0; N0 = 685)
1−sided adj pval: 0.999
hsa−miR−339−5p, rectal
(BRAF = 1; N1 = 19)
1−sided adj pval: 0.924
Frequency
Tumor−Normal Expression Difference

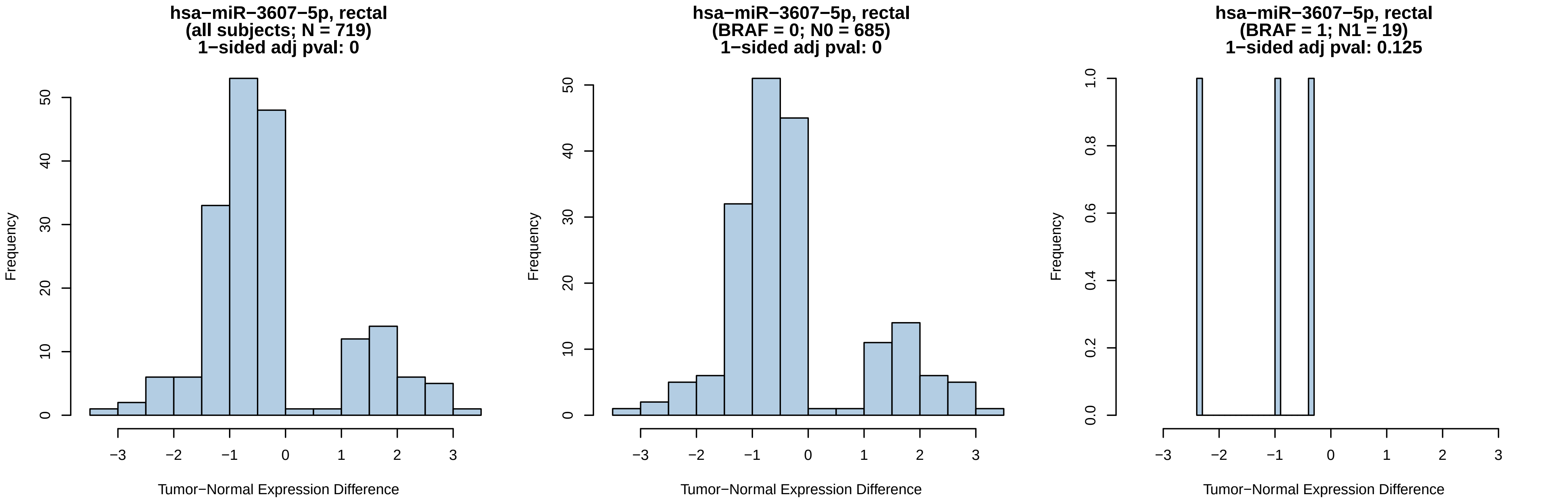
hsa−miR−3607−5p, rectal
(all subjects; N = 719)
1−sided adj pval: 0
Frequency
Tumor−Normal Expression Difference
hsa−miR−3607−5p, rectal
(BRAF = 0; N0 = 685)
1−sided adj pval: 0
Frequency
Tumor−Normal Expression Difference
hsa−miR−3607−5p, rectal
(BRAF = 1; N1 = 19)
1−sided adj pval: 0.125
Frequency
Tumor−Normal Expression Difference

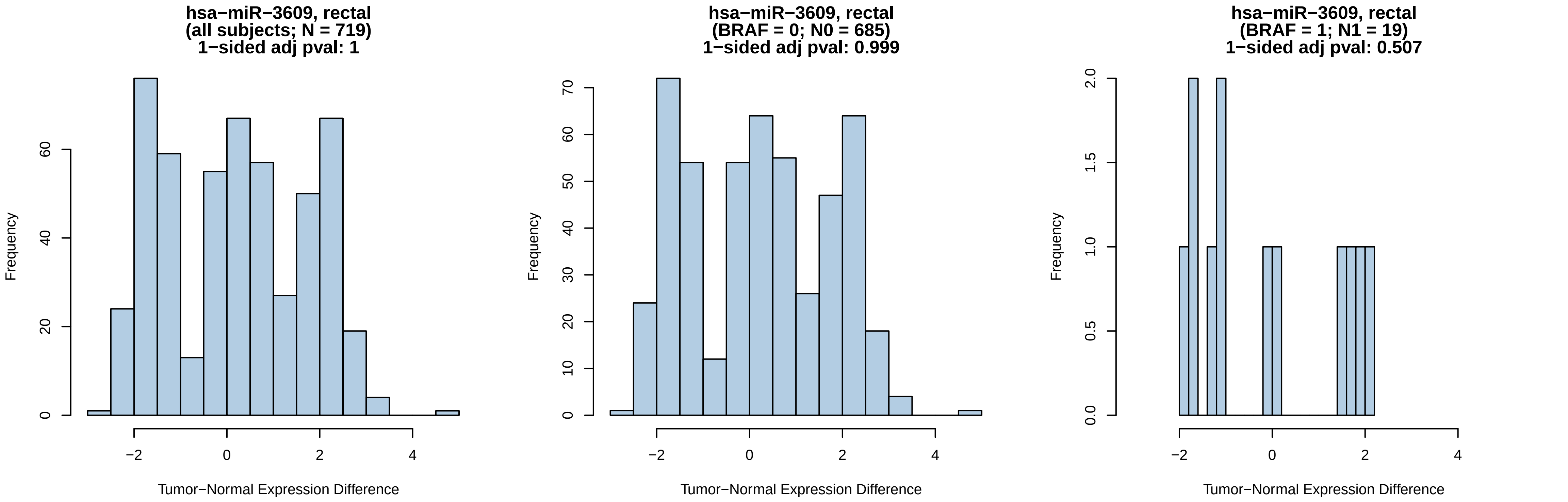
hsa−miR−3609, rectal
(all subjects; N = 719)
1−sided adj pval: 1
Frequency
Tumor−Normal Expression Difference
hsa−miR−3609, rectal
(BRAF = 0; N0 = 685)
1−sided adj pval: 0.999
Frequency
Tumor−Normal Expression Difference
hsa−miR−3609, rectal
(BRAF = 1; N1 = 19)
1−sided adj pval: 0.507
Frequency
Tumor−Normal Expression Difference

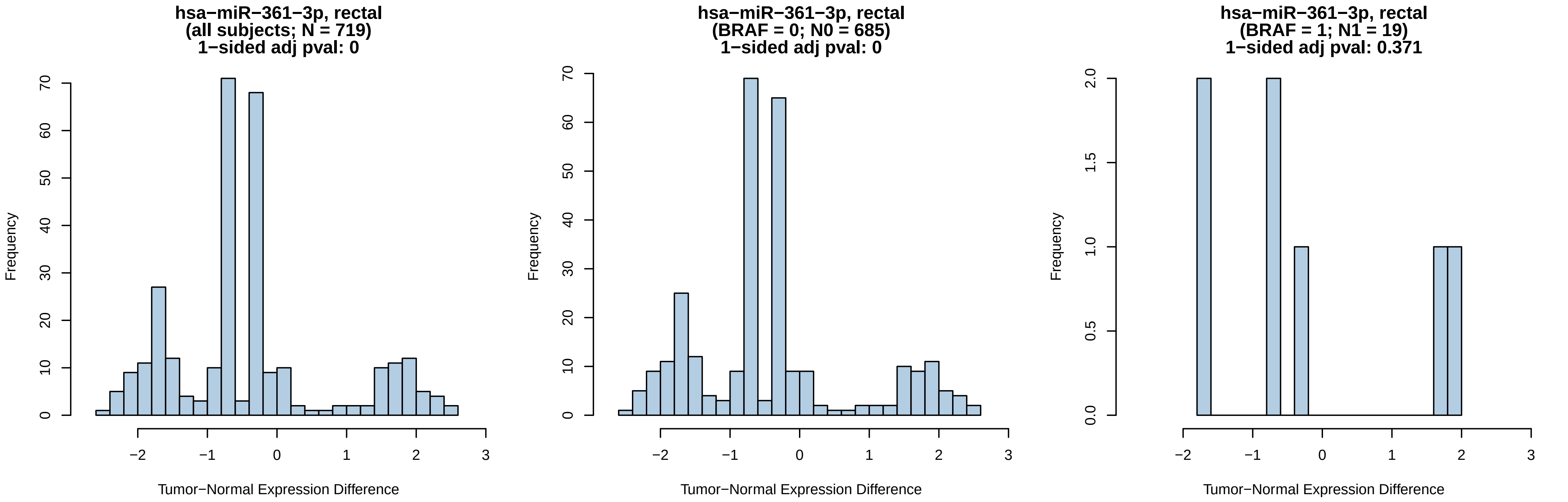

hsa−miR−361−3p, rectal
(all subjects; N = 719)
1−sided adj pval: 0
Frequency
0
10
20
30
40
50
60
70
−2
−1
0
1
2
3
Tumor−Normal Expression Difference
hsa−miR−361−3p, rectal
(BRAF = 0; N0 = 685)
1−sided adj pval: 0
Frequency
0
10
20
30
40
50
60
70
−2
−1
0
1
2
3
Tumor−Normal Expression Difference
hsa−miR−361−3p, rectal
(BRAF = 1; N1 = 19)
1−sided adj pval: 0.371
Frequency
0.0
0.5
1.0
1.5
2.0
−2
−1
0
1
2
3
Tumor−Normal Expression Difference

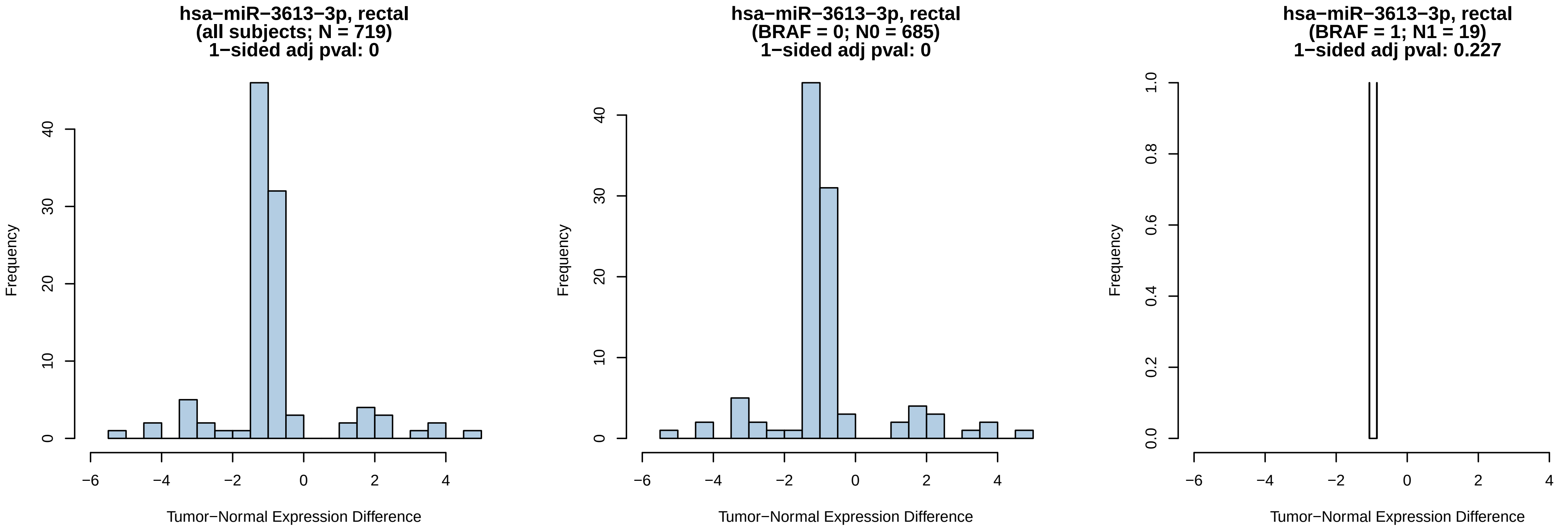
hsa−miR−3613−3p, rectal
(all subjects; N = 719)
1−sided adj pval: 0
hsa−miR−3613−3p, rectal
(BRAF = 0; N0 = 685)
1−sided adj pval: 0
hsa−miR−3613−3p, rectal
(BRAF = 1; N1 = 19)
1−sided adj pval: 0.227
Frequency
Tumor−Normal Expression Difference

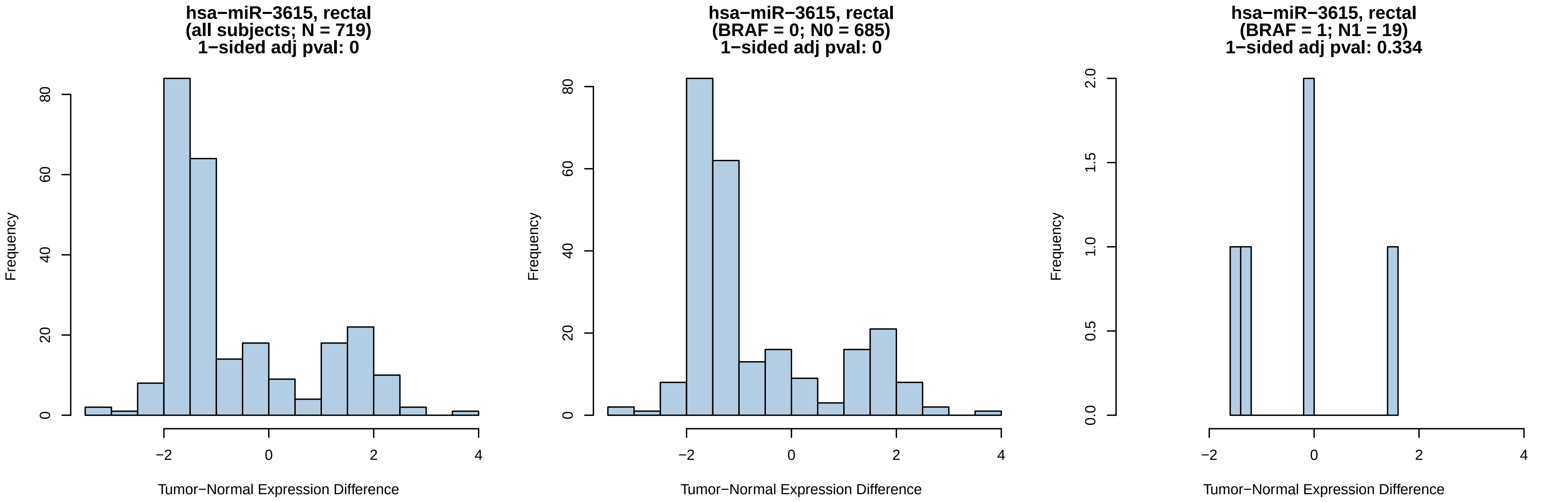

hsa−miR−3615, rectal
(all subjects; N = 719)
1−sided adj pval: 0
Frequency
Tumor−Normal Expression Difference
hsa−miR−3615, rectal
(BRAF = 0; N0 = 685)
1−sided adj pval: 0
Frequency
Tumor−Normal Expression Difference
hsa−miR−3615, rectal
(BRAF = 1; N1 = 19)
1−sided adj pval: 0.334
Frequency
Tumor−Normal Expression Difference

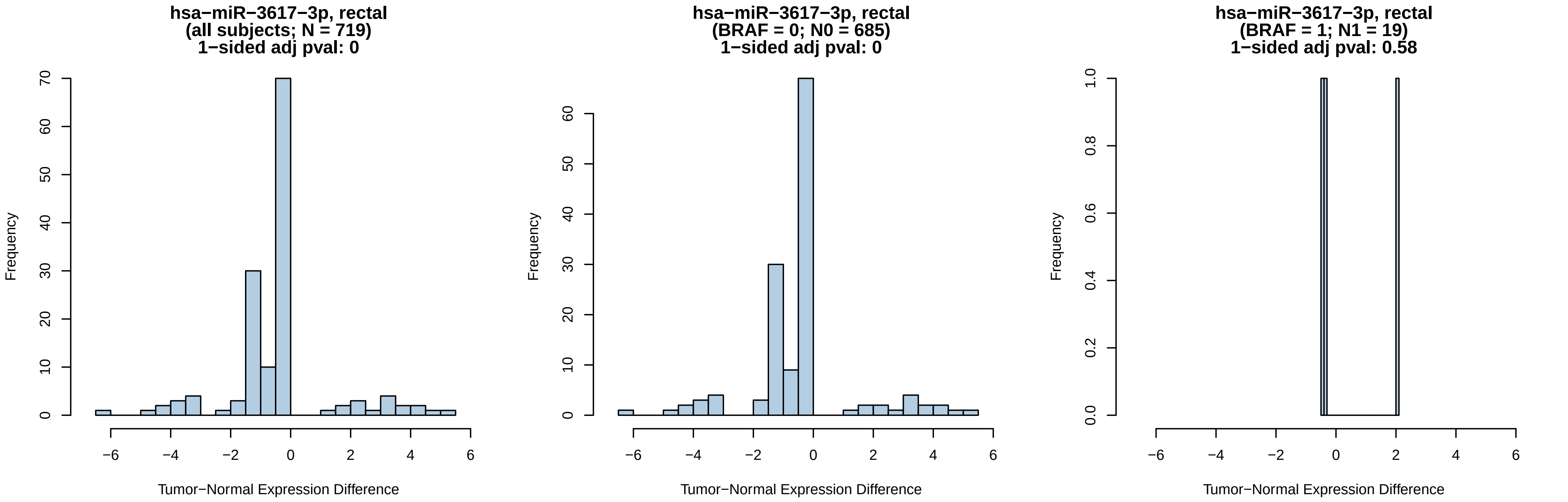

hsa−miR−3617−3p, rectal
(all subjects; N = 719)
1−sided adj pval: 0
Frequency
Tumor−Normal Expression Difference
hsa−miR−3617−3p, rectal
(BRAF = 0; N0 = 685)
1−sided adj pval: 0
Frequency
Tumor−Normal Expression Difference
hsa−miR−3617−3p, rectal
(BRAF = 1; N1 = 19)
1−sided adj pval: 0.58
Frequency
Tumor−Normal Expression Difference

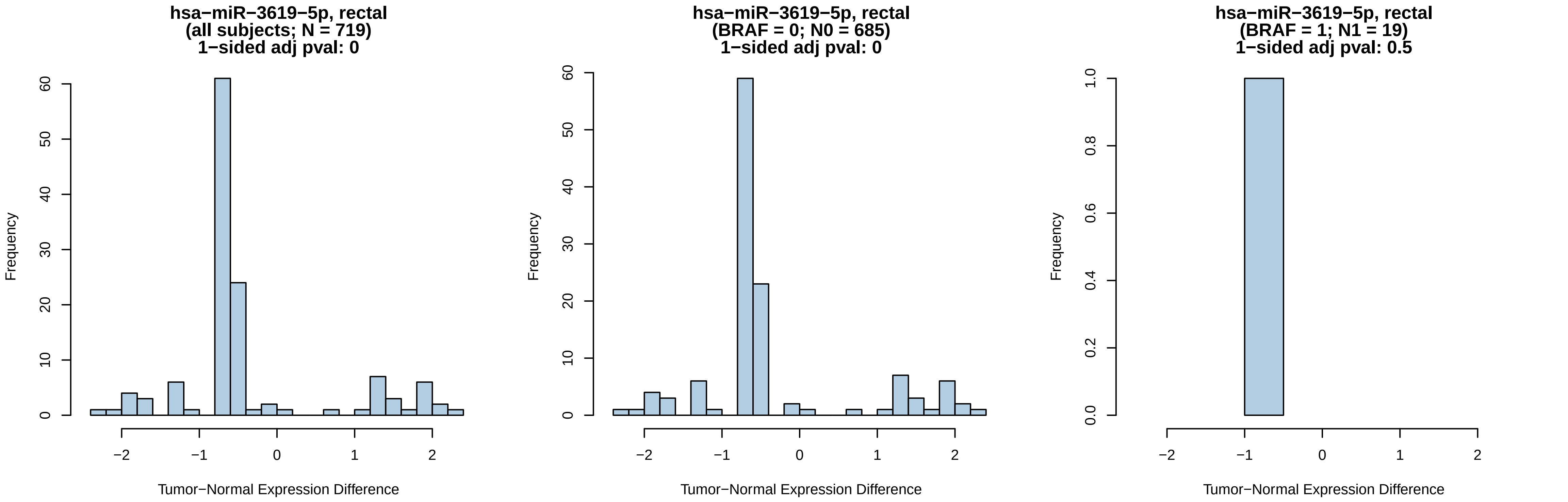

hsa−miR−3619−5p, rectal
(all subjects; N = 719)
1−sided adj pval: 0
Frequency
Tumor−Normal Expression Difference
hsa−miR−3619−5p, rectal
(BRAF = 0; N0 = 685)
1−sided adj pval: 0
Frequency
Tumor−Normal Expression Difference
hsa−miR−3619−5p, rectal
(BRAF = 1; N1 = 19)
1−sided adj pval: 0.5
Frequency
Tumor−Normal Expression Difference

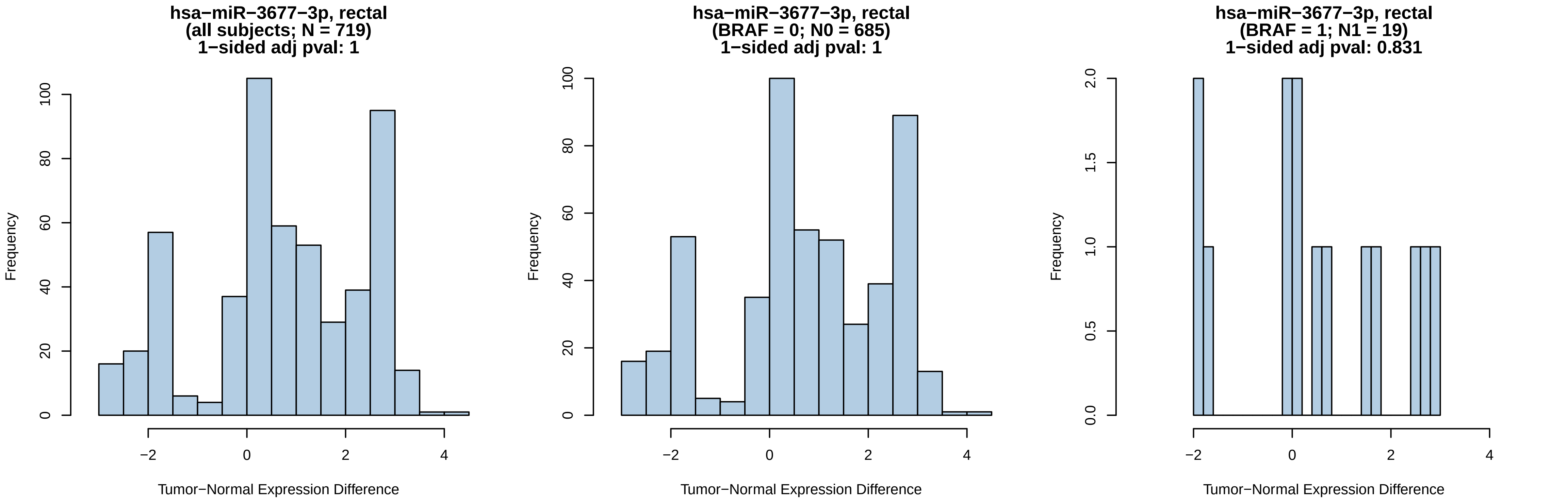
hsa−miR−3677−3p, rectal
(all subjects; N = 719)
1−sided adj pval: 1
Frequency
Tumor−Normal Expression Difference
hsa−miR−3677−3p, rectal
(BRAF = 0; N0 = 685)
1−sided adj pval: 1
Frequency
Tumor−Normal Expression Difference
hsa−miR−3677−3p, rectal
(BRAF = 1; N1 = 19)
1−sided adj pval: 0.831
Frequency
Tumor−Normal Expression Difference

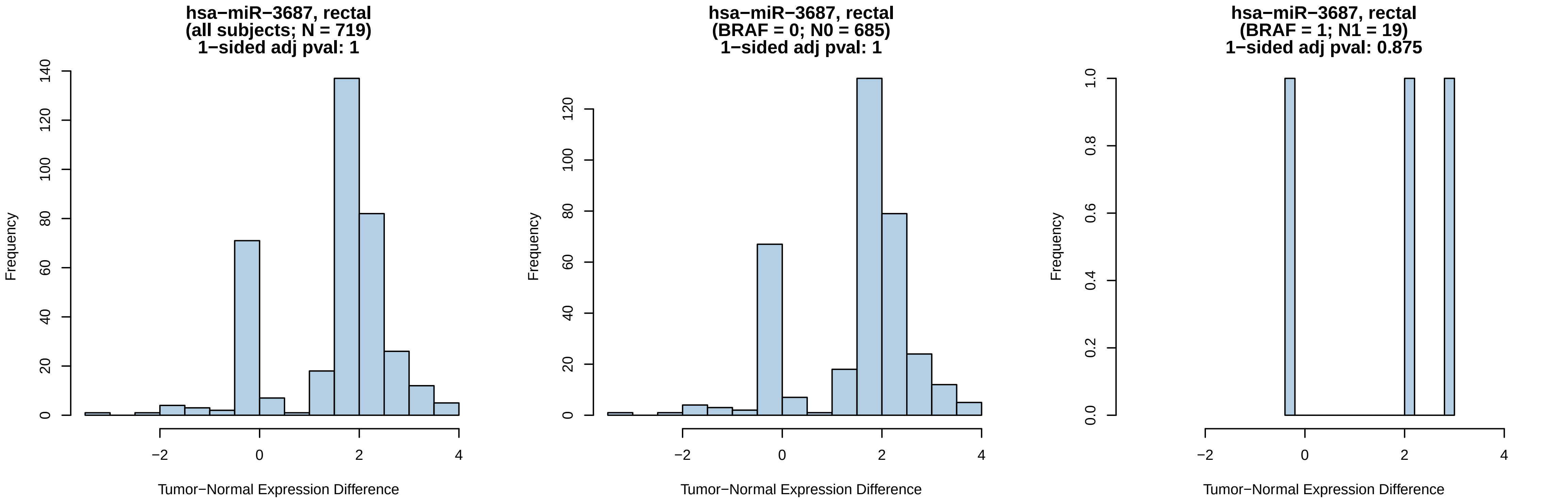
hsa−miR−3687, rectal
(all subjects; N = 719)
1−sided adj pval: 1
Frequency
Tumor−Normal Expression Difference
hsa−miR−3687, rectal
(BRAF = 0; N0 = 685)
1−sided adj pval: 1
Frequency
Tumor−Normal Expression Difference
hsa−miR−3687, rectal
(BRAF = 1; N1 = 19)
1−sided adj pval: 0.875
Frequency
Tumor−Normal Expression Difference

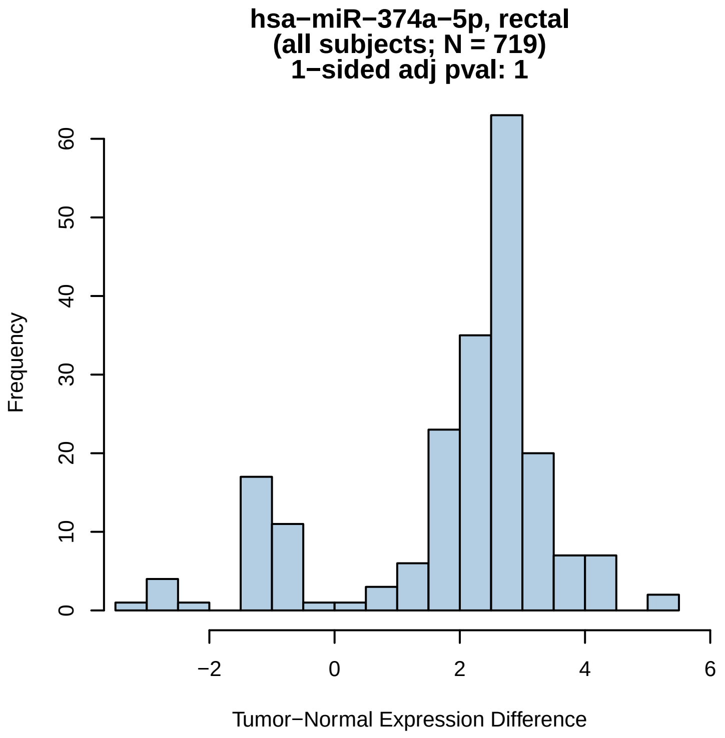

hsa−miR−374a−5p, rectal
(all subjects; N = 719)
1−sided adj pval: 1
Frequency
0
10
20
30
40
50
60
−2
0
2
4
6
Tumor−Normal Expression Difference


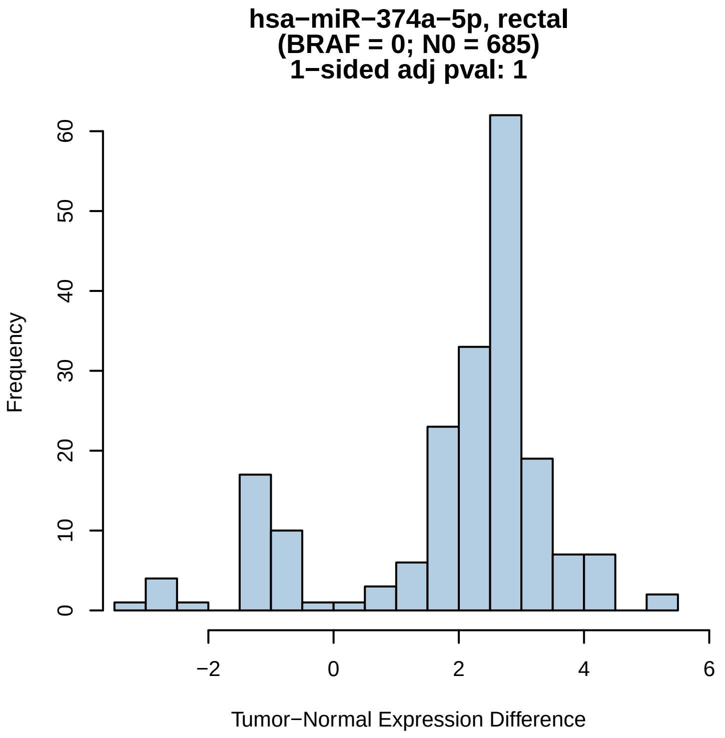

hsa−miR−374a−5p, rectal
(BRAF = 0; N0 = 685)
1−sided adj pval: 1
Frequency
0
10
20
30
40
50
60
−2
0
2
4
6
Tumor−Normal Expression Difference

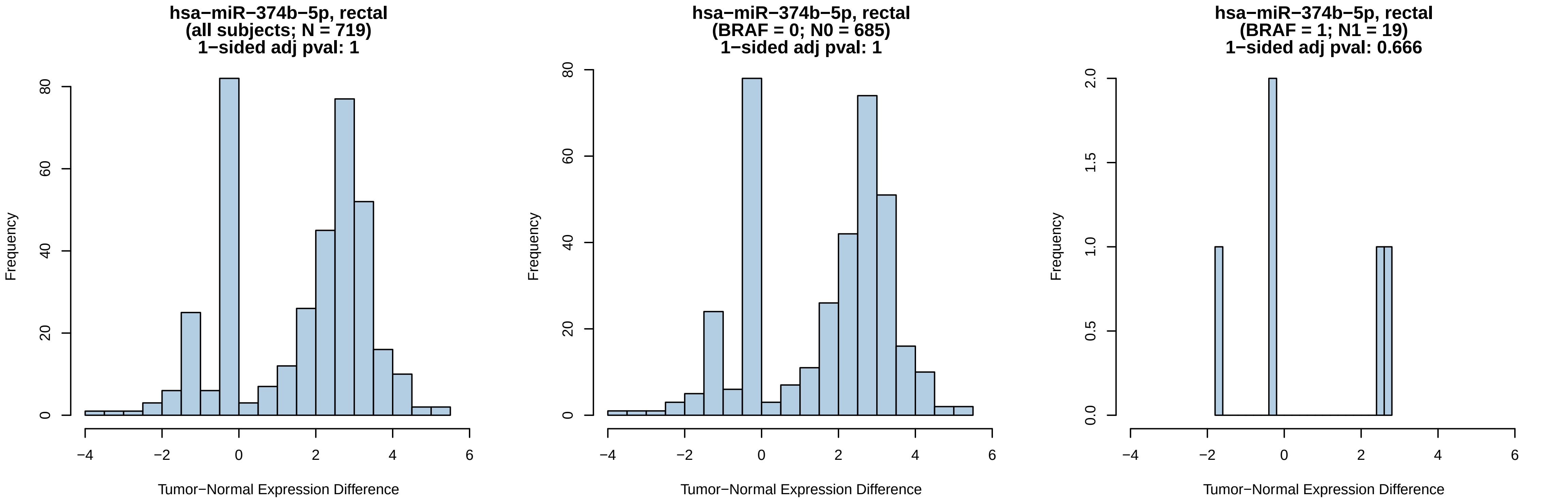
hsa−miR−374b−5p, rectal
(all subjects; N = 719)
1−sided adj pval: 1
Frequency
Tumor−Normal Expression Difference
hsa−miR−374b−5p, rectal
(BRAF = 0; N0 = 685)
1−sided adj pval: 1
Frequency
Tumor−Normal Expression Difference
hsa−miR−374b−5p, rectal
(BRAF = 1; N1 = 19)
1−sided adj pval: 0.666
Frequency
Tumor−Normal Expression Difference

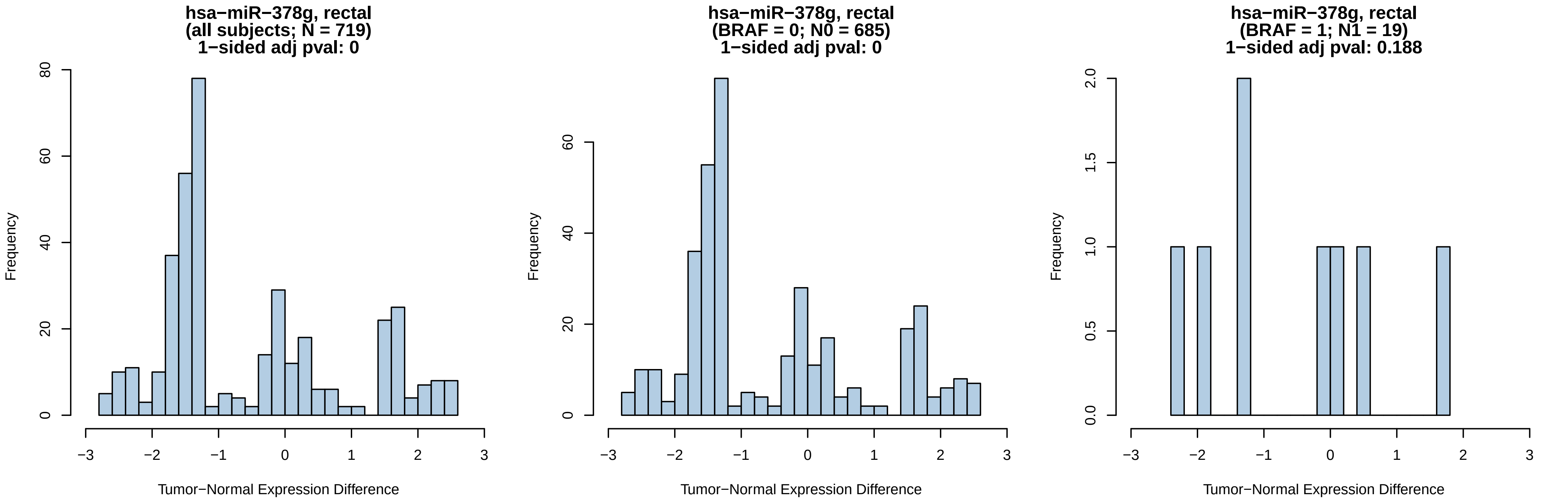

hsa−miR−378g, rectal
(all subjects; N = 719)
1−sided adj pval: 0
Frequency
Tumor−Normal Expression Difference
hsa−miR−378g, rectal
(BRAF = 0; N0 = 685)
1−sided adj pval: 0
Frequency
Tumor−Normal Expression Difference
hsa−miR−378g, rectal
(BRAF = 1; N1 = 19)
1−sided adj pval: 0.188
Frequency
Tumor−Normal Expression Difference

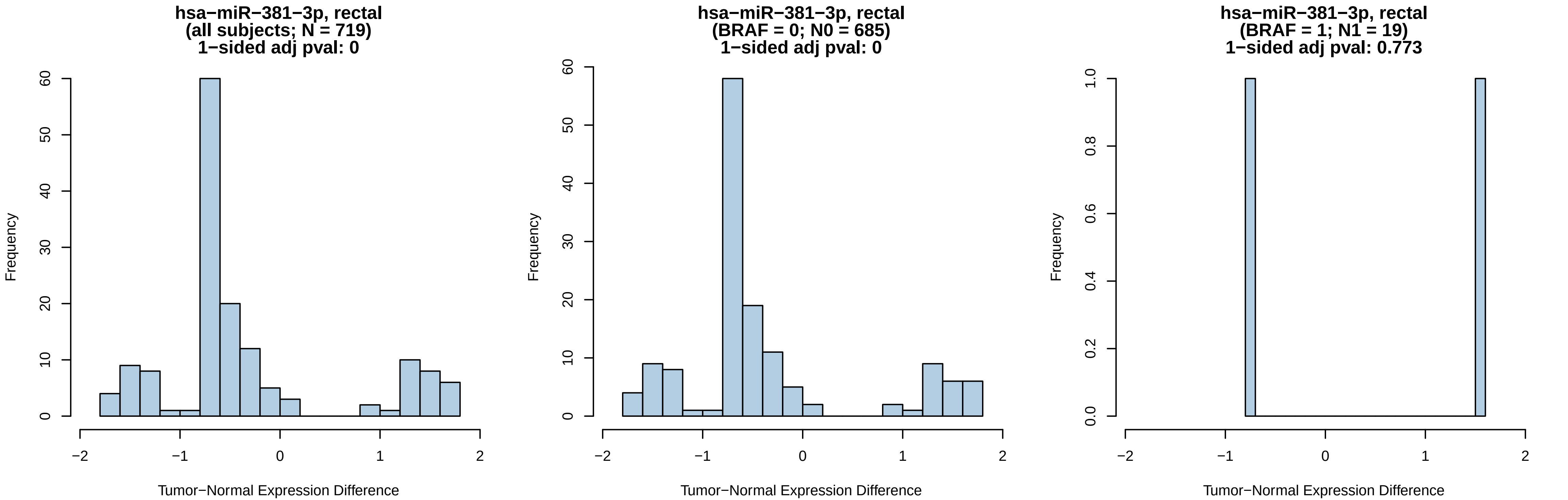

hsa−miR−381−3p, rectal
(all subjects; N = 719)
1−sided adj pval: 0
hsa−miR−381−3p, rectal
(BRAF = 0; N0 = 685)
1−sided adj pval: 0
hsa−miR−381−3p, rectal
(BRAF = 1; N1 = 19)
1−sided adj pval: 0.773
Frequency
Tumor−Normal Expression Difference

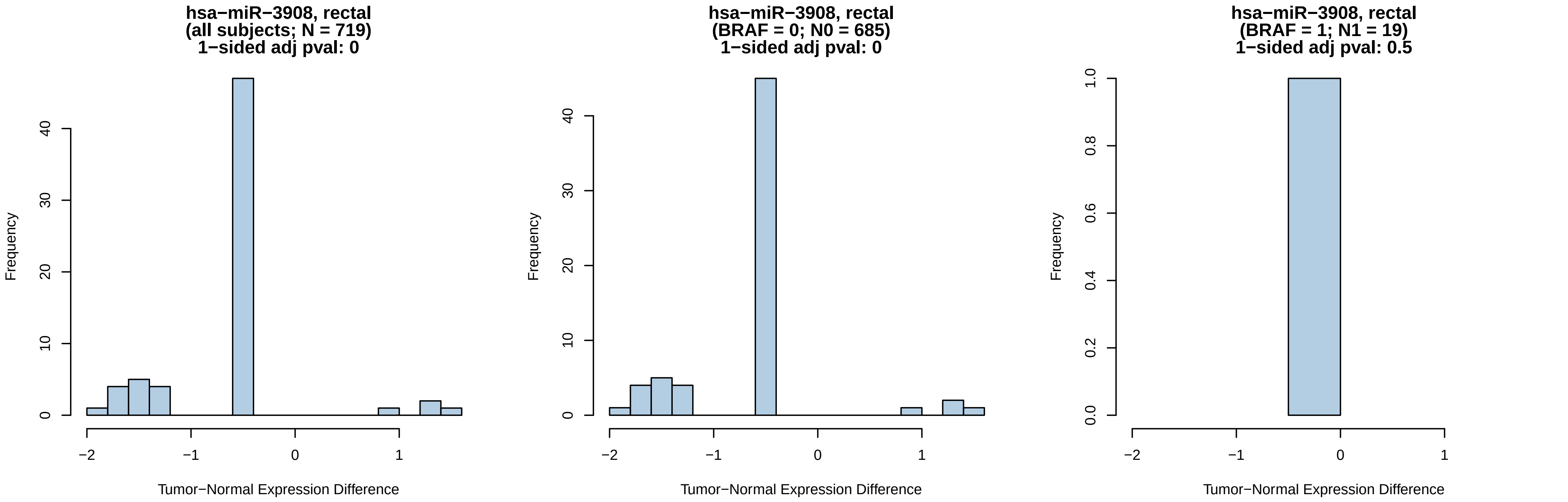
hsa−miR−3908, rectal
(all subjects; N = 719)
1−sided adj pval: 0
Frequency
Tumor−Normal Expression Difference
hsa−miR−3908, rectal
(BRAF = 0; N0 = 685)
1−sided adj pval: 0
Frequency
Tumor−Normal Expression Difference
hsa−miR−3908, rectal
(BRAF = 1; N1 = 19)
1−sided adj pval: 0.5
Frequency
Tumor−Normal Expression Difference

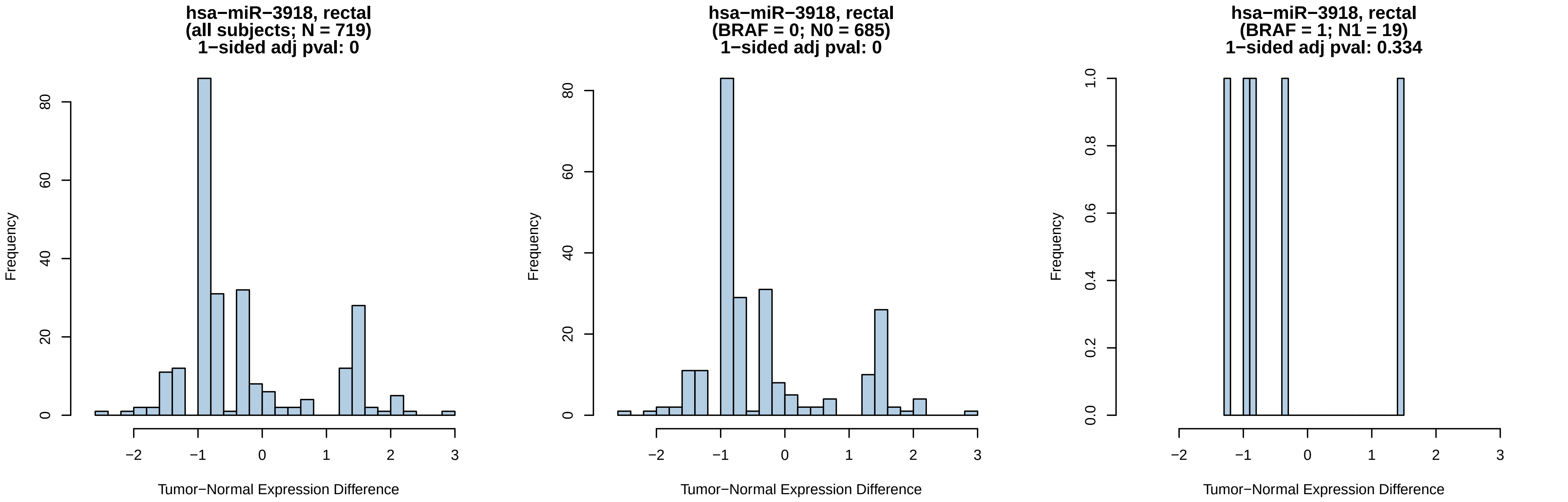
hsa−miR−3918, rectal
(all subjects; N = 719)
1−sided adj pval: 0
Frequency
Tumor−Normal Expression Difference
hsa−miR−3918, rectal
(BRAF = 0; N0 = 685)
1−sided adj pval: 0
Frequency
Tumor−Normal Expression Difference
hsa−miR−3918, rectal
(BRAF = 1; N1 = 19)
1−sided adj pval: 0.334
Frequency
Tumor−Normal Expression Difference

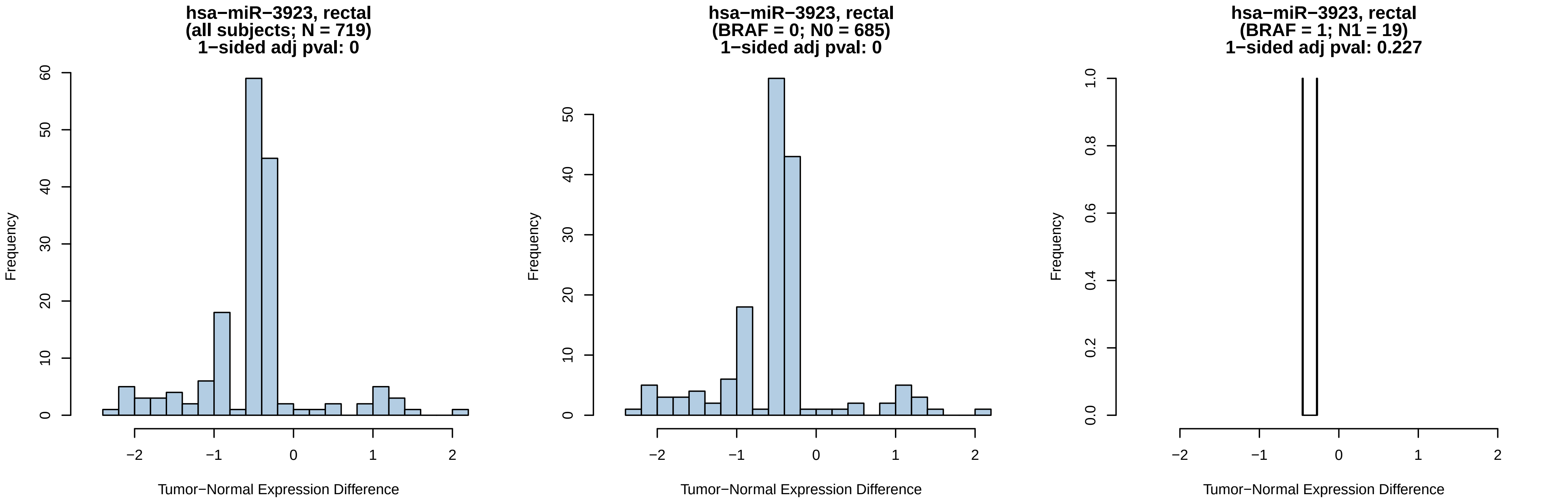
hsa−miR−3923, rectal
(all subjects; N = 719)
1−sided adj pval: 0
hsa−miR−3923, rectal
(BRAF = 0; N0 = 685)
1−sided adj pval: 0
hsa−miR−3923, rectal
(BRAF = 1; N1 = 19)
1−sided adj pval: 0.227
Frequency
Tumor−Normal Expression Difference

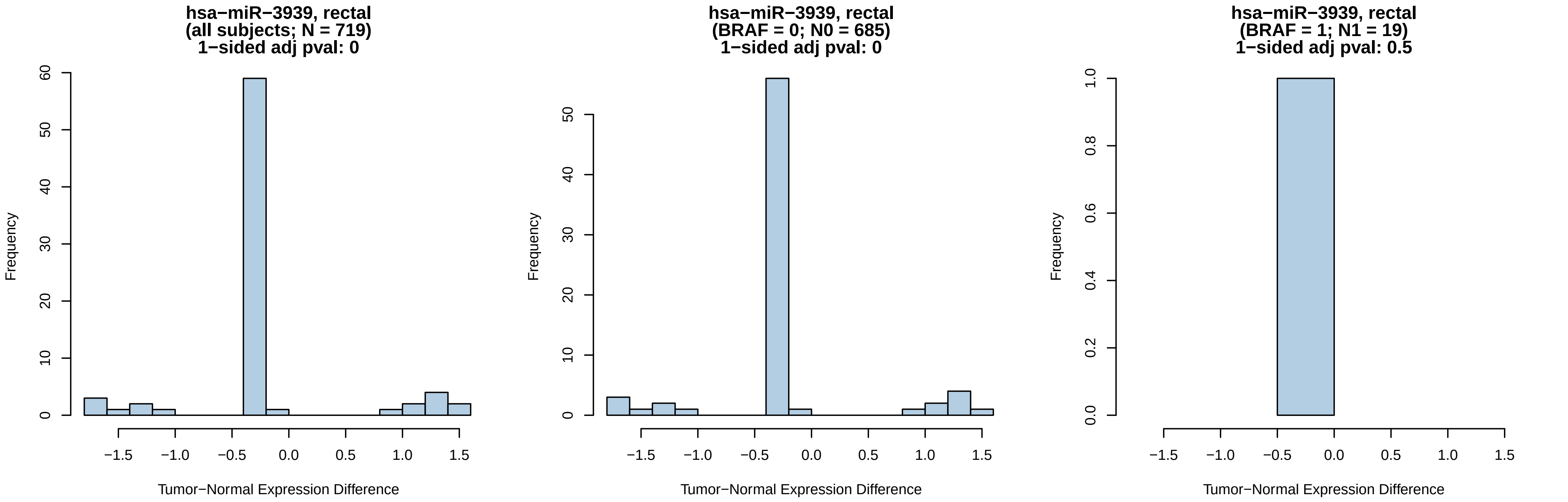

hsa−miR−3939, rectal
(all subjects; N = 719)
1−sided adj pval: 0
Frequency
0
10
20
30
40
50
60
−1.5
−1.0
−0.5
0.0
0.5
1.0
1.5
Tumor−Normal Expression Difference
hsa−miR−3939, rectal
(BRAF = 0; N0 = 685)
1−sided adj pval: 0
Frequency
0
10
20
30
40
50
−1.5
−1.0
−0.5
0.0
0.5
1.0
1.5
Tumor−Normal Expression Difference
hsa−miR−3939, rectal
(BRAF = 1; N1 = 19)
1−sided adj pval: 0.5
Frequency
0.0
0.2
0.4
0.6
0.8
1.0
−1.5
−1.0
−0.5
0.0
0.5
1.0
1.5
Tumor−Normal Expression Difference

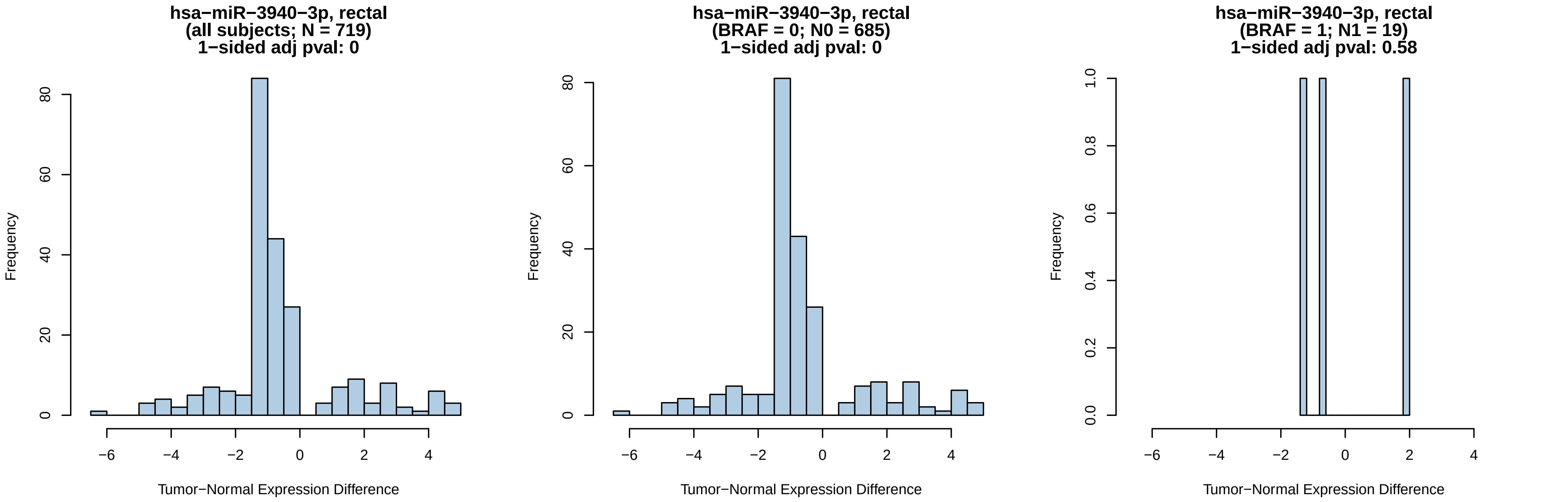

hsa−miR−3940−3p, rectal
(all subjects; N = 719)
1−sided adj pval: 0
Frequency
Tumor−Normal Expression Difference
hsa−miR−3940−3p, rectal
(BRAF = 0; N0 = 685)
1−sided adj pval: 0
Frequency
Tumor−Normal Expression Difference
hsa−miR−3940−3p, rectal
(BRAF = 1; N1 = 19)
1−sided adj pval: 0.58
Frequency
Tumor−Normal Expression Difference

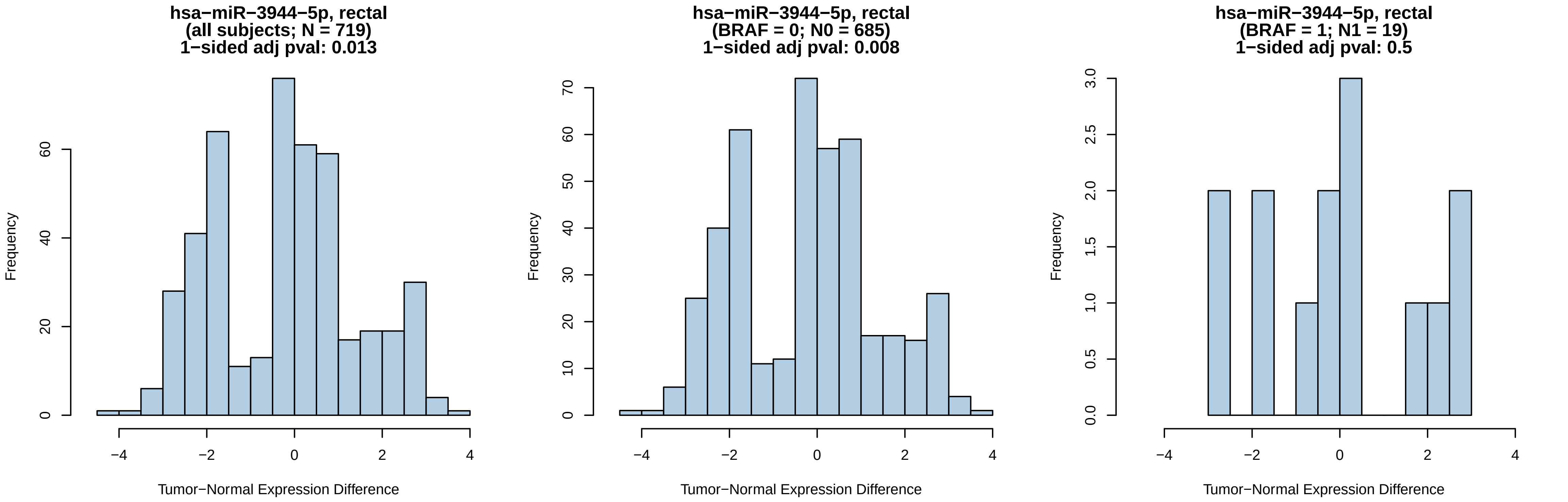

hsa−miR−3944−5p, rectal
(all subjects; N = 719)
1−sided adj pval: 0.013
Frequency
Tumor−Normal Expression Difference
hsa−miR−3944−5p, rectal
(BRAF = 0; N0 = 685)
1−sided adj pval: 0.008
Frequency
Tumor−Normal Expression Difference
hsa−miR−3944−5p, rectal
(BRAF = 1; N1 = 19)
1−sided adj pval: 0.5
Frequency
Tumor−Normal Expression Difference

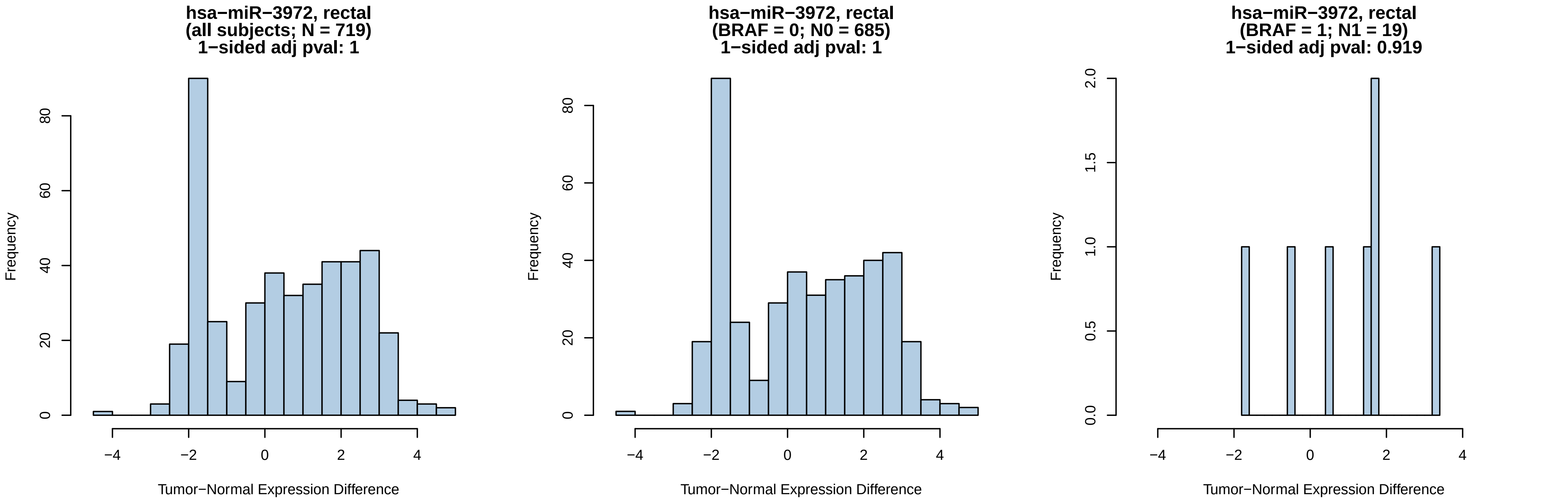
hsa−miR−3972, rectal
(all subjects; N = 719)
1−sided adj pval: 1
Frequency
0
20
40
60
80
−4
−2
0
2
4
Tumor−Normal Expression Difference
hsa−miR−3972, rectal
(BRAF = 0; N0 = 685)
1−sided adj pval: 1
Frequency
0
20
40
60
80
−4
−2
0
2
4
Tumor−Normal Expression Difference
hsa−miR−3972, rectal
(BRAF = 1; N1 = 19)
1−sided adj pval: 0.919
Frequency
0.0
0.5
1.0
1.5
2.0
−4
−2
0
2
4
Tumor−Normal Expression Difference

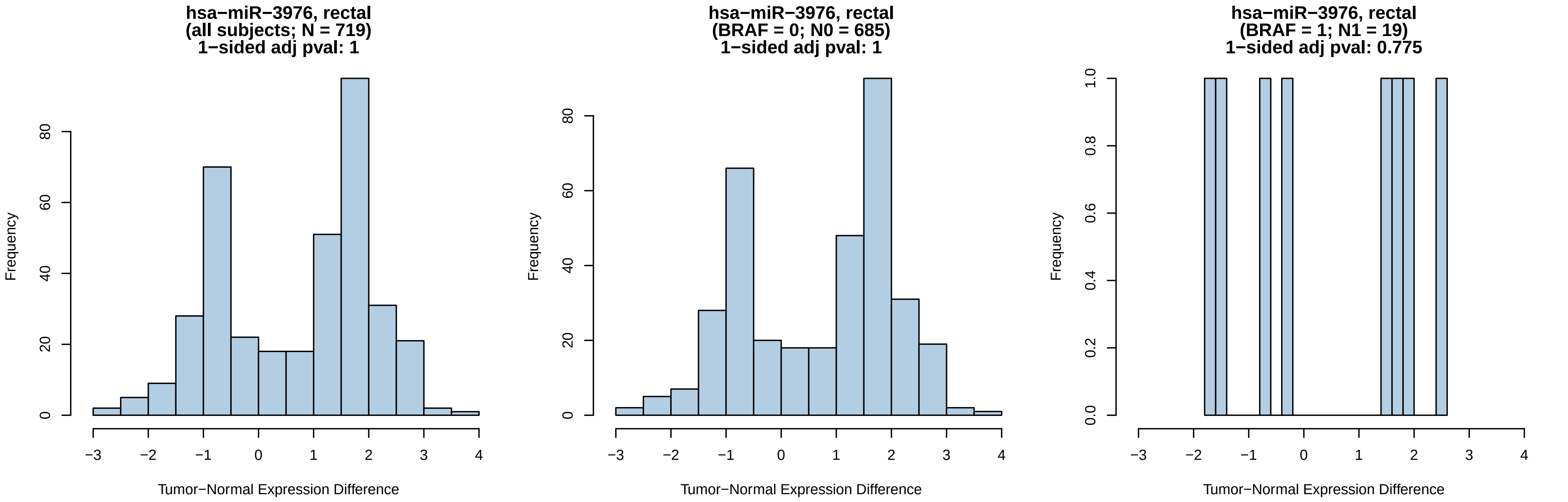
hsa−miR−3976, rectal
(all subjects; N = 719)
1−sided adj pval: 1
Frequency
Tumor−Normal Expression Difference
hsa−miR−3976, rectal
(BRAF = 0; N0 = 685)
1−sided adj pval: 1
Frequency
Tumor−Normal Expression Difference
hsa−miR−3976, rectal
(BRAF = 1; N1 = 19)
1−sided adj pval: 0.775
Frequency
Tumor−Normal Expression Difference

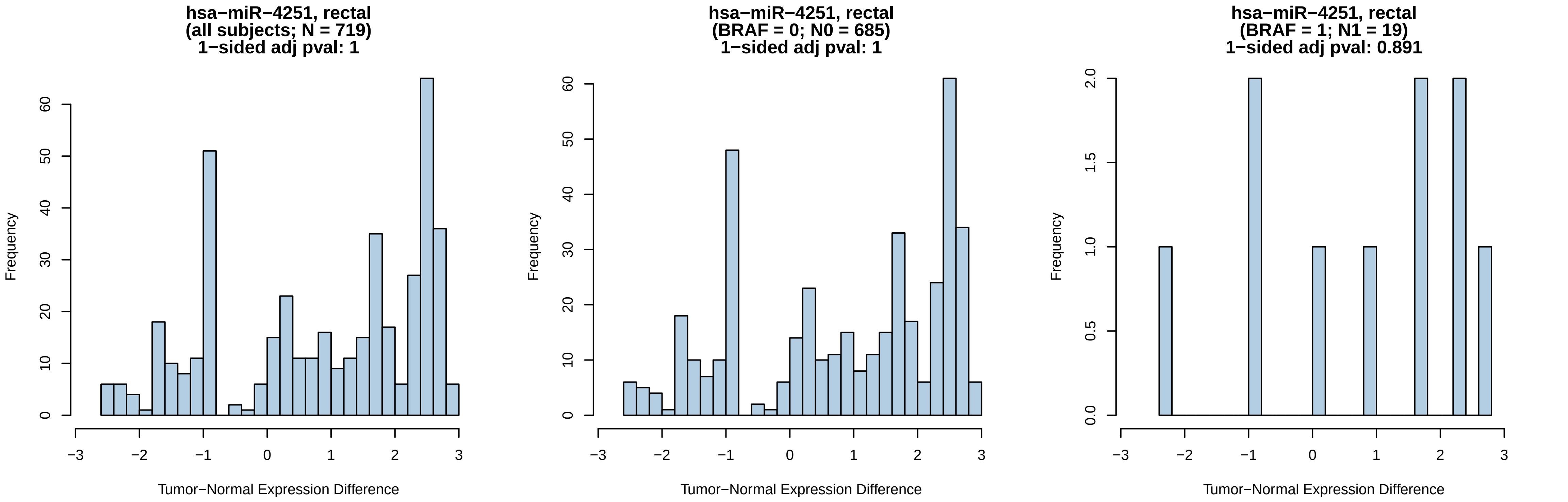

hsa−miR−4251, rectal
(all subjects; N = 719)
1−sided adj pval: 1
Frequency
Tumor−Normal Expression Difference
hsa−miR−4251, rectal
(BRAF = 0; N0 = 685)
1−sided adj pval: 1
Frequency
Tumor−Normal Expression Difference
hsa−miR−4251, rectal
(BRAF = 1; N1 = 19)
1−sided adj pval: 0.891
Frequency
Tumor−Normal Expression Difference

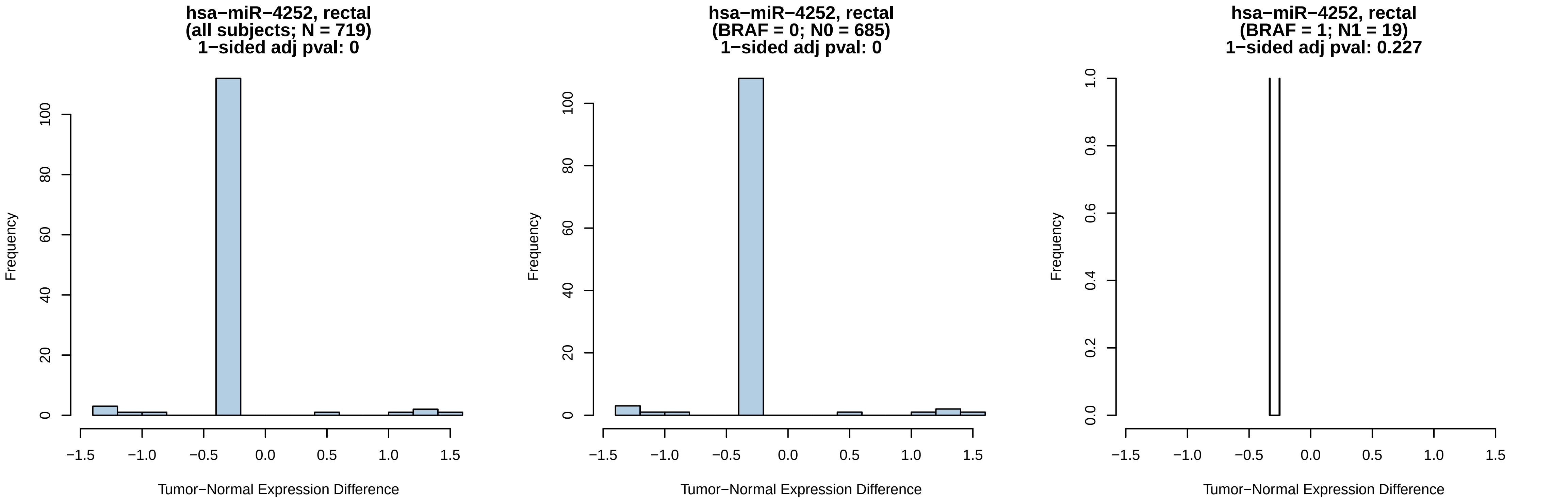
hsa−miR−4252, rectal
(all subjects; N = 719)
1−sided adj pval: 0
Frequency
Tumor−Normal Expression Difference
hsa−miR−4252, rectal
(BRAF = 0; N0 = 685)
1−sided adj pval: 0
Frequency
Tumor−Normal Expression Difference
hsa−miR−4252, rectal
(BRAF = 1; N1 = 19)
1−sided adj pval: 0.227
Frequency
Tumor−Normal Expression Difference

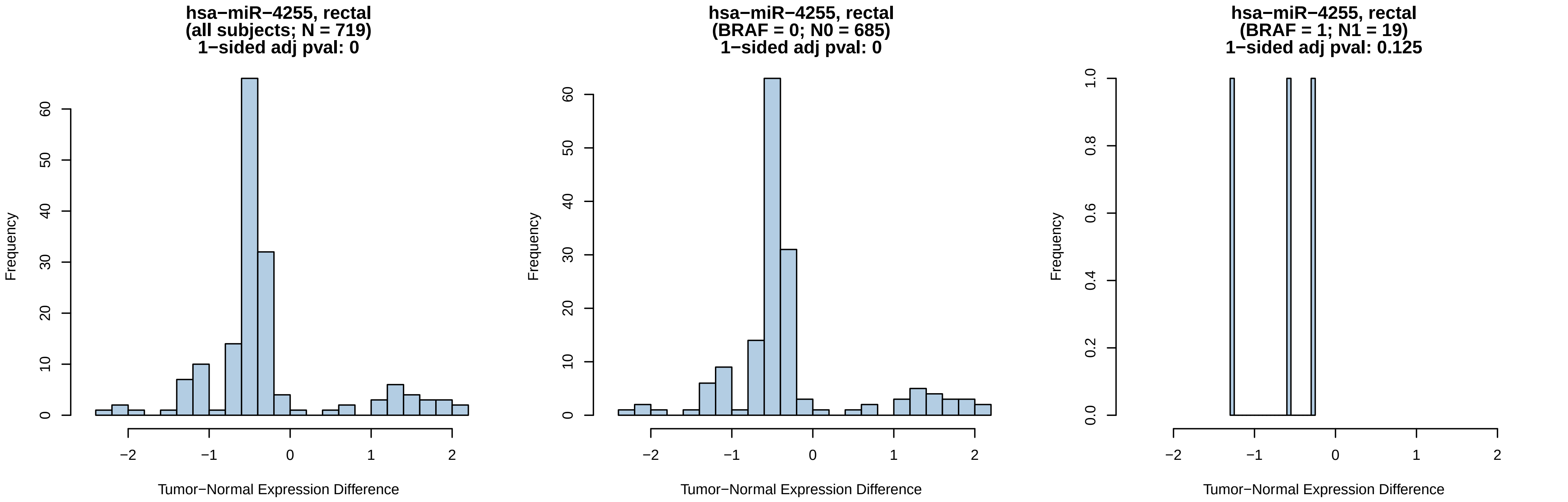
hsa−miR−4255, rectal
(all subjects; N = 719)
1−sided adj pval: 0
hsa−miR−4255, rectal
(BRAF = 0; N0 = 685)
1−sided adj pval: 0
hsa−miR−4255, rectal
(BRAF = 1; N1 = 19)
1−sided adj pval: 0.125
Frequency
Tumor−Normal Expression Difference
0
10
20
30
40
50
60
0.0
0.2
0.4
0.6
0.8
1.0
−2
−1
0
1
2

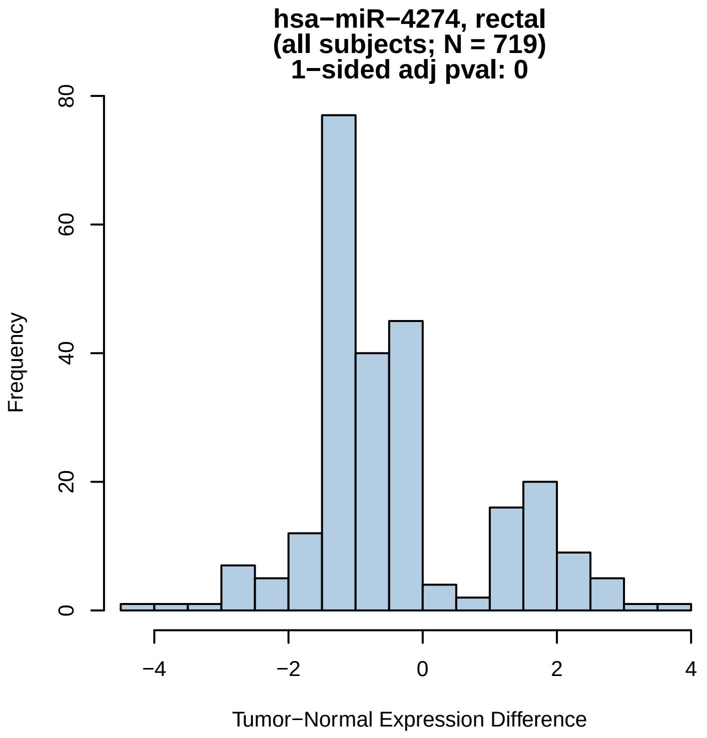

hsa−miR−4274, rectal
(all subjects; N = 719)
1−sided adj pval: 0
Frequency
Tumor−Normal Expression Difference


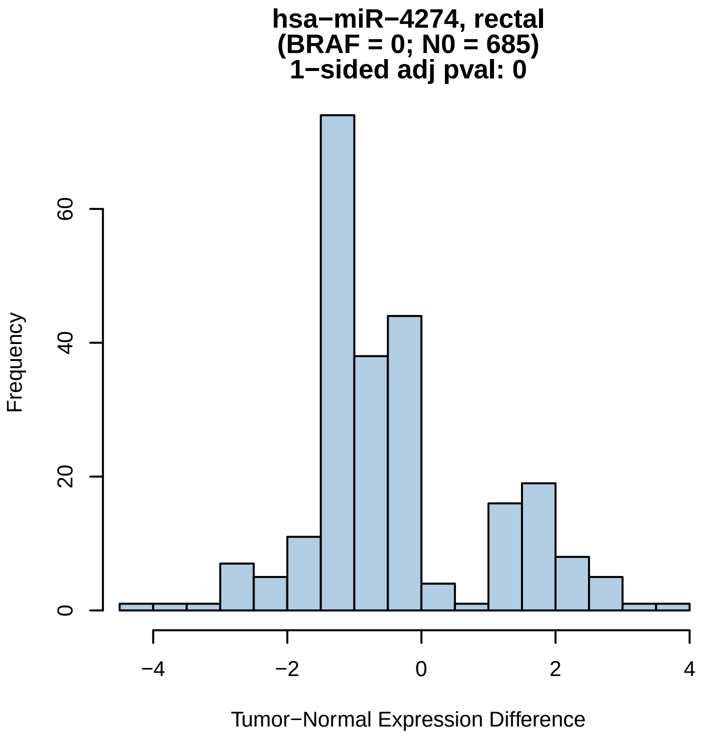

hsa−miR−4274, rectal
(BRAF = 0; N0 = 685)
1−sided adj pval: 0
Frequency
Tumor−Normal Expression Difference


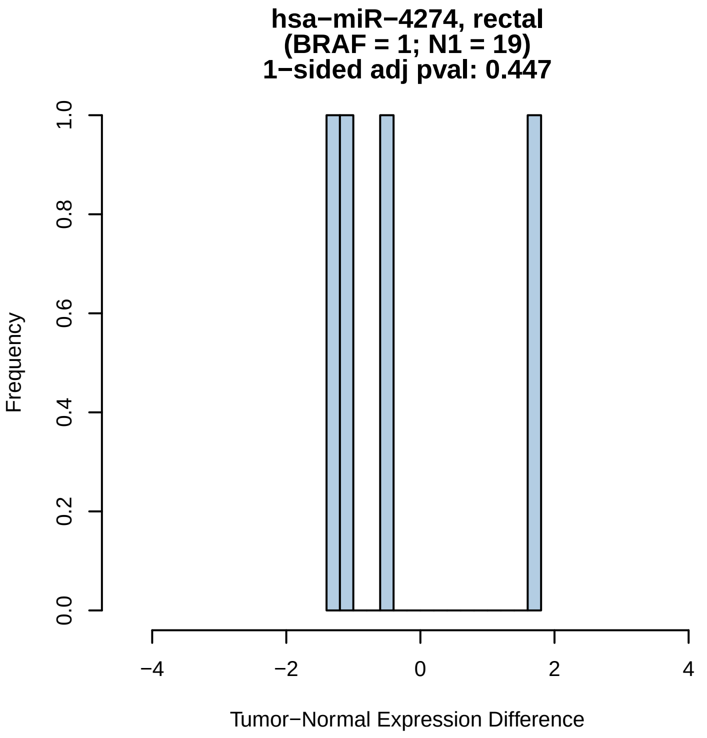

hsa−miR−4274, rectal
(BRAF = 1; N1 = 19)
1−sided adj pval: 0.447
Frequency
Tumor−Normal Expression Difference

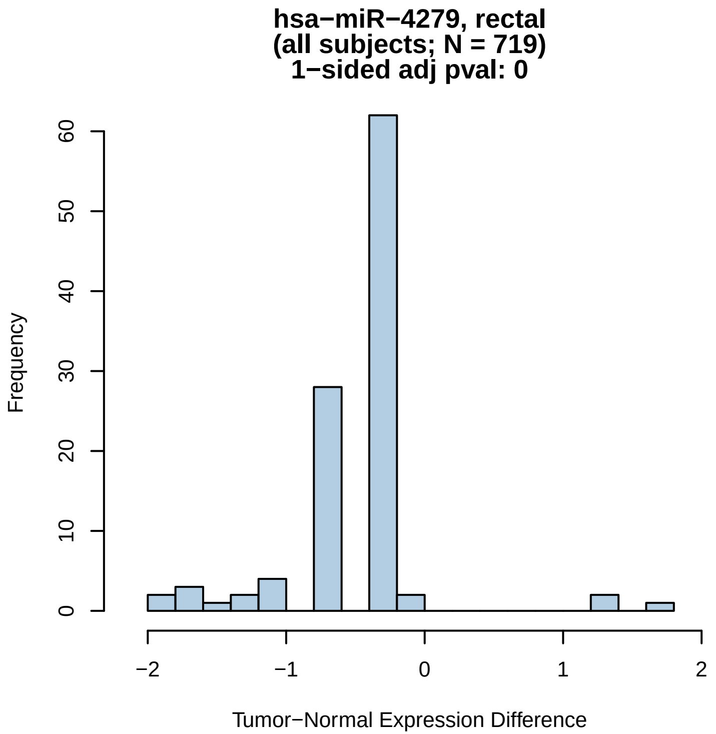
hsa−miR−4279, rectal
(all subjects; N = 719)
1−sided adj pval: 0
Frequency
Tumor−Normal Expression Difference

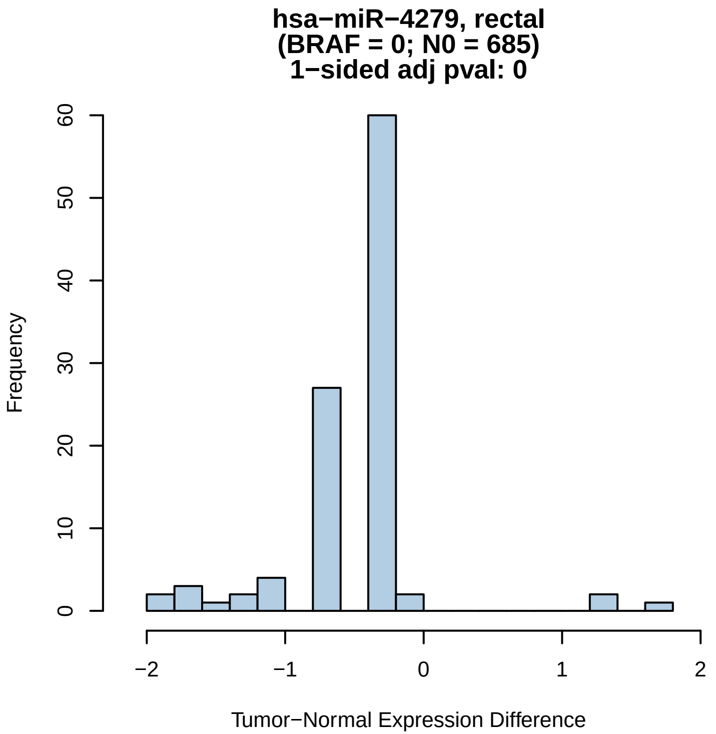
hsa−miR−4279, rectal
(BRAF = 0; N0 = 685)
1−sided adj pval: 0
Frequency
Tumor−Normal Expression Difference

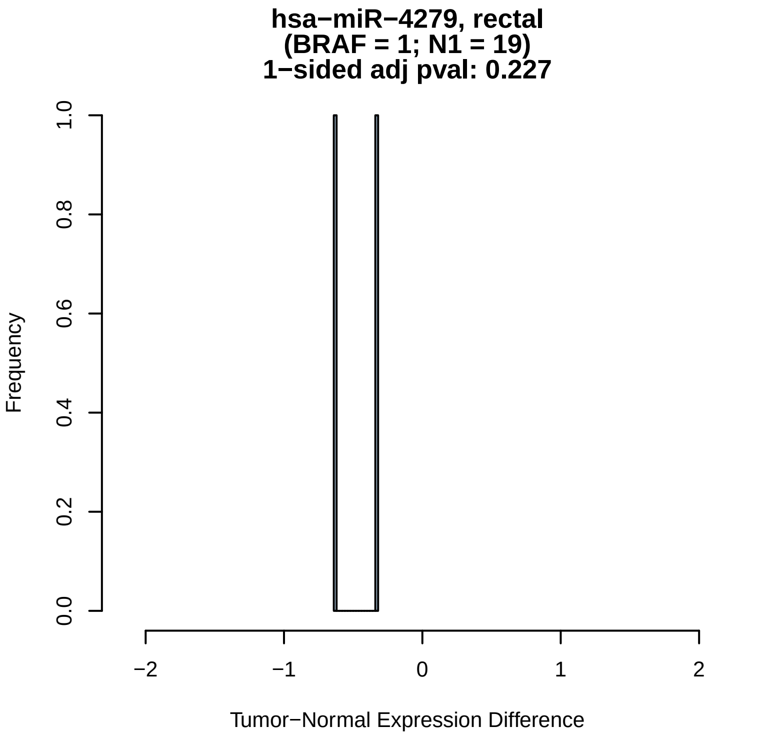
hsa−miR−4279, rectal
(BRAF = 1; N1 = 19)
1−sided adj pval: 0.227
Frequency
Tumor−Normal Expression Difference

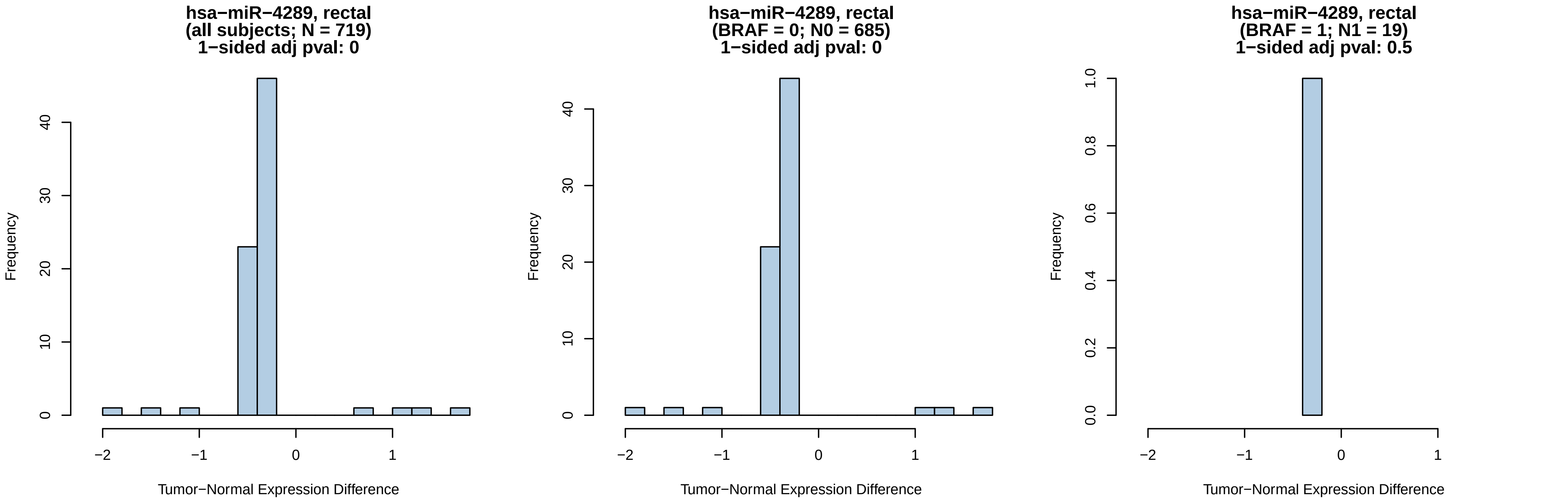

hsa−miR−4289, rectal
(all subjects; N = 719)
1−sided adj pval: 0
Frequency
Tumor−Normal Expression Difference
hsa−miR−4289, rectal
(BRAF = 0; N0 = 685)
1−sided adj pval: 0
Frequency
Tumor−Normal Expression Difference
hsa−miR−4289, rectal
(BRAF = 1; N1 = 19)
1−sided adj pval: 0.5
Frequency
Tumor−Normal Expression Difference

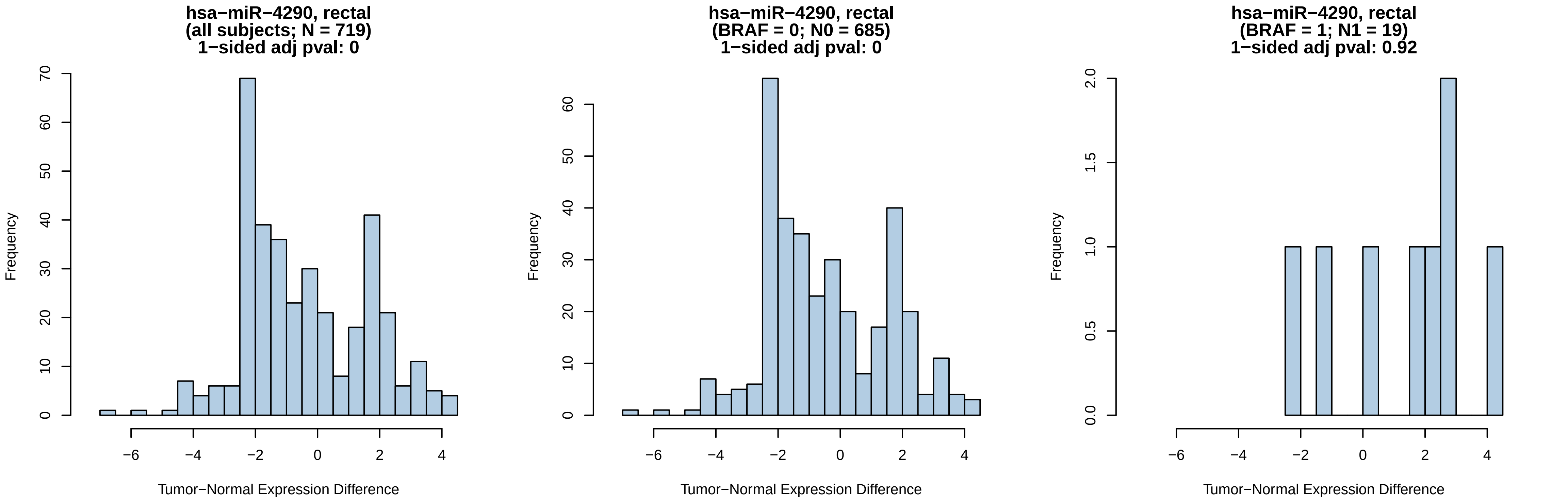

hsa−miR−4290, rectal
(all subjects; N = 719)
1−sided adj pval: 0
Frequency
Tumor−Normal Expression Difference
hsa−miR−4290, rectal
(BRAF = 0; N0 = 685)
1−sided adj pval: 0
Frequency
Tumor−Normal Expression Difference
hsa−miR−4290, rectal
(BRAF = 1; N1 = 19)
1−sided adj pval: 0.92
Frequency
Tumor−Normal Expression Difference

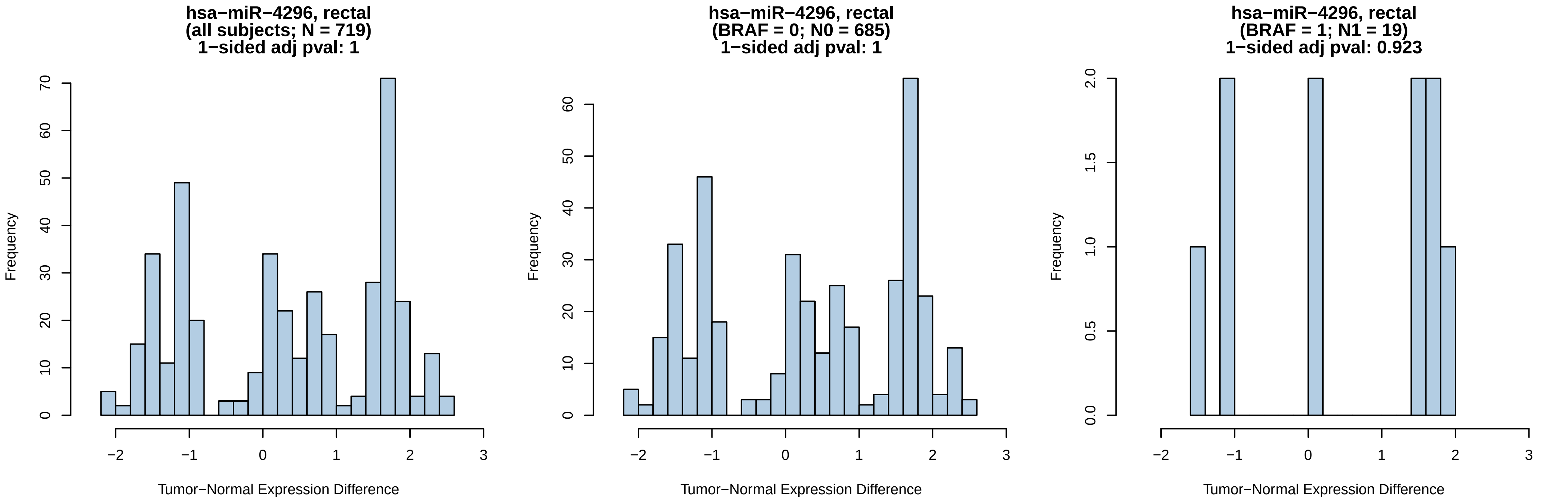

hsa−miR−4296, rectal
(all subjects; N = 719)
1−sided adj pval: 1
Frequency
Tumor−Normal Expression Difference
hsa−miR−4296, rectal
(BRAF = 0; N0 = 685)
1−sided adj pval: 1
Frequency
Tumor−Normal Expression Difference
hsa−miR−4296, rectal
(BRAF = 1; N1 = 19)
1−sided adj pval: 0.923
Frequency
Tumor−Normal Expression Difference

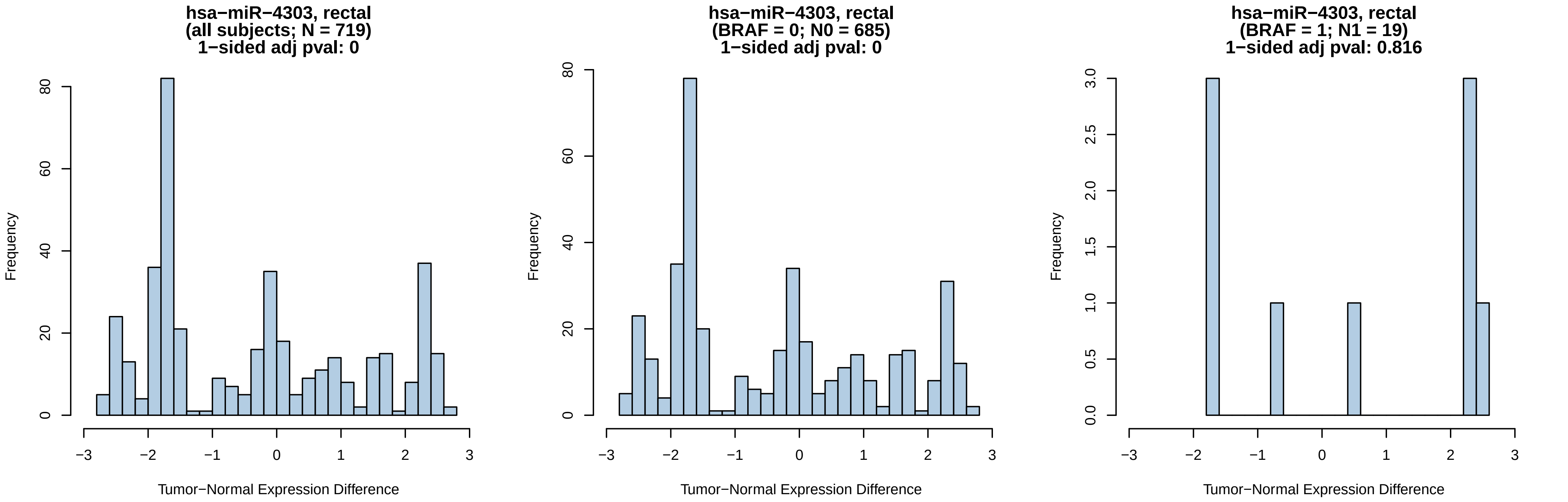
hsa−miR−4303, rectal
(all subjects; N = 719)
1−sided adj pval: 0
hsa−miR−4303, rectal
(BRAF = 0; N0 = 685)
1−sided adj pval: 0
hsa−miR−4303, rectal
(BRAF = 1; N1 = 19)
1−sided adj pval: 0.816
Frequency
Tumor−Normal Expression Difference
0
20
40
60
80
0.0
0.5
1.0
1.5
2.0
2.5
3.0
−3
−2
−1
0
1
2
3

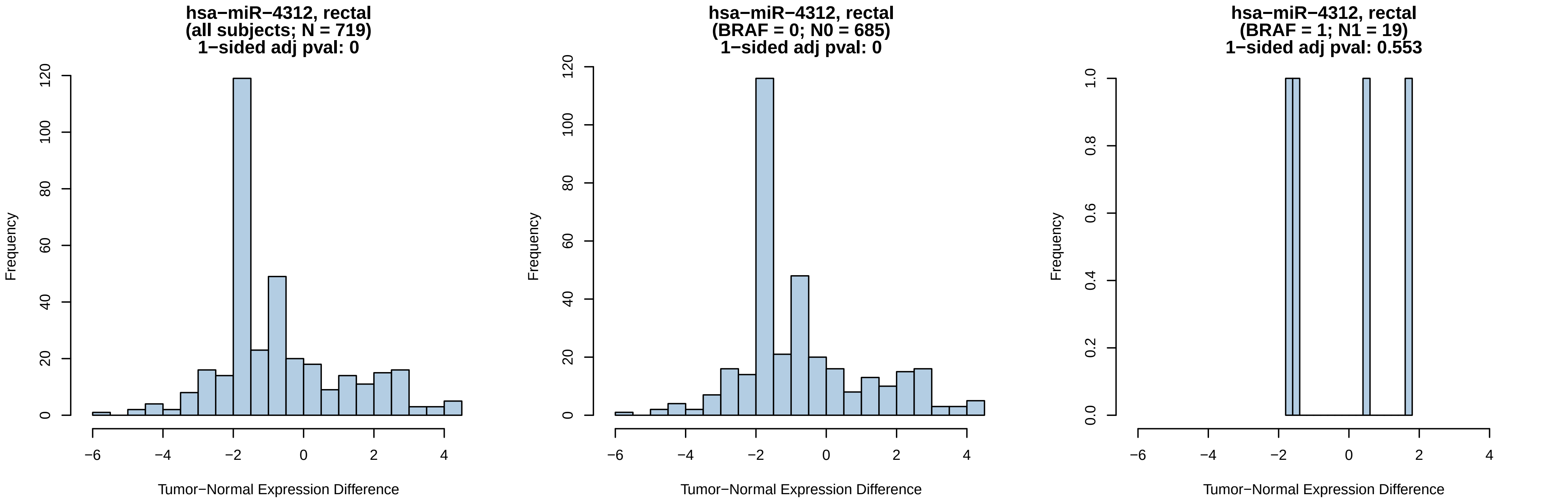
hsa−miR−4312, rectal
(all subjects; N = 719)
1−sided adj pval: 0
hsa−miR−4312, rectal
(BRAF = 0; N0 = 685)
1−sided adj pval: 0
hsa−miR−4312, rectal
(BRAF = 1; N1 = 19)
1−sided adj pval: 0.553
Frequency
Tumor−Normal Expression Difference

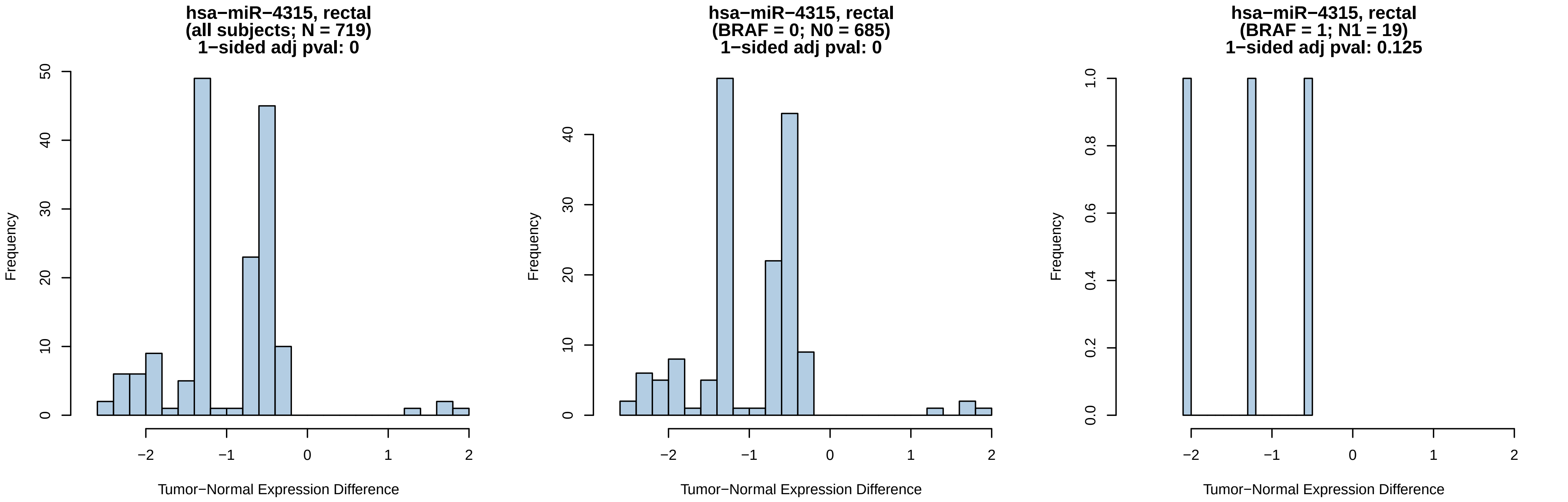

hsa−miR−4315, rectal
(all subjects; N = 719)
1−sided adj pval: 0
Frequency
Tumor−Normal Expression Difference
hsa−miR−4315, rectal
(BRAF = 0; N0 = 685)
1−sided adj pval: 0
Frequency
Tumor−Normal Expression Difference
hsa−miR−4315, rectal
(BRAF = 1; N1 = 19)
1−sided adj pval: 0.125
Frequency
Tumor−Normal Expression Difference

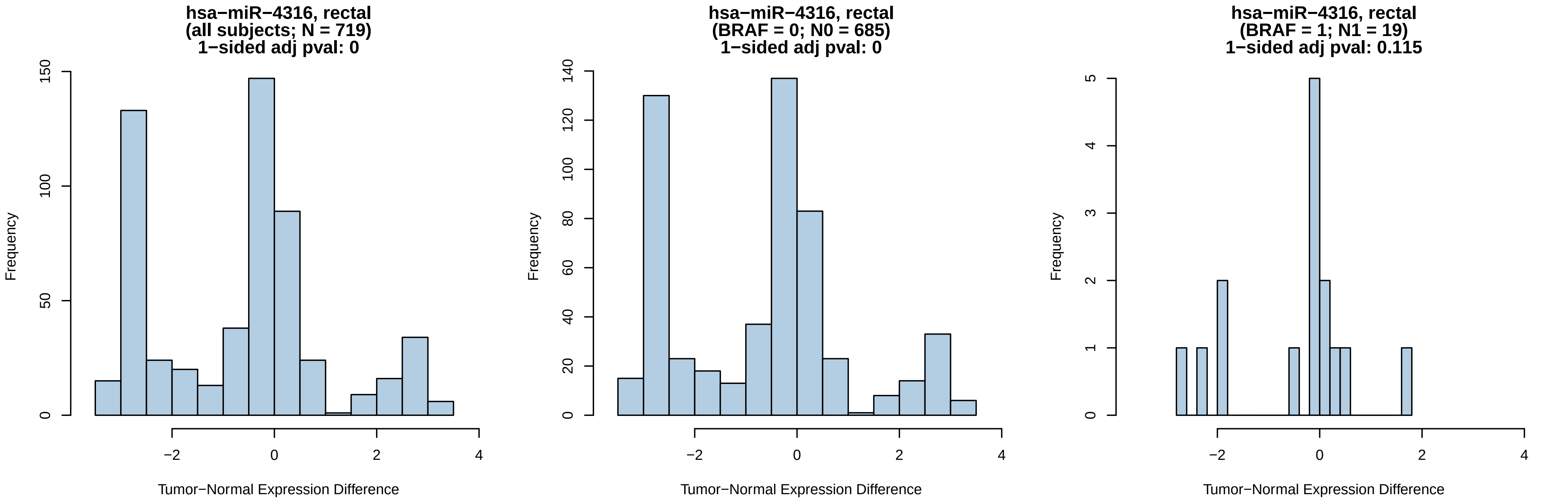

hsa−miR−4316, rectal
(all subjects; N = 719)
1−sided adj pval: 0
hsa−miR−4316, rectal
(BRAF = 0; N0 = 685)
1−sided adj pval: 0
hsa−miR−4316, rectal
(BRAF = 1; N1 = 19)
1−sided adj pval: 0.115
Frequency
Tumor−Normal Expression Difference

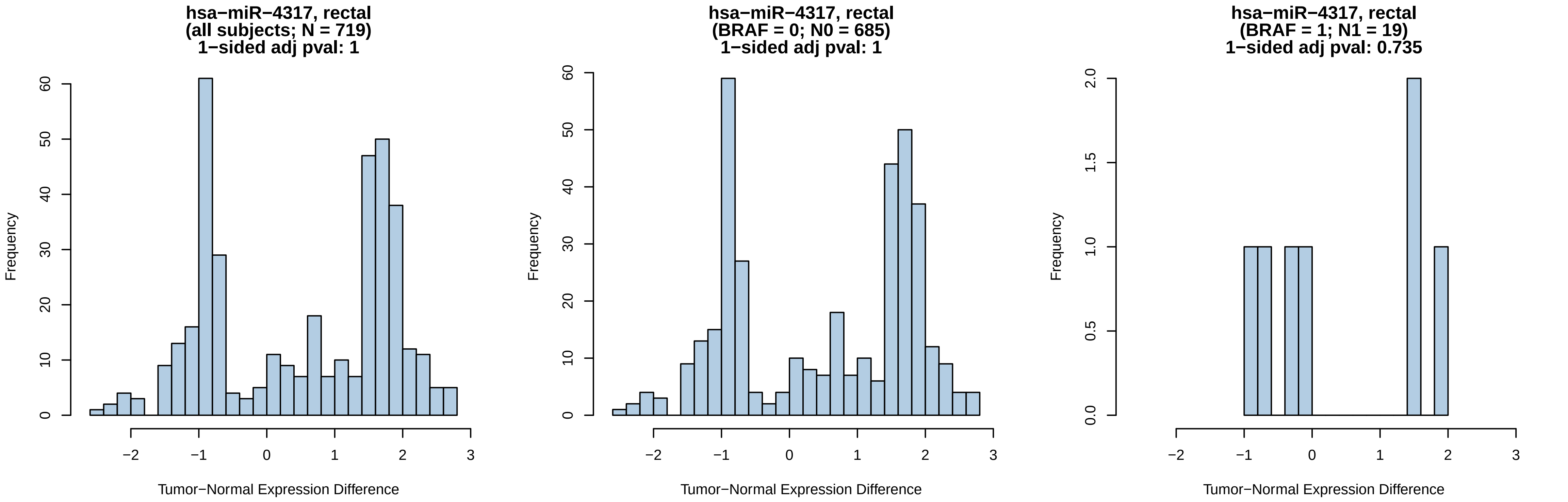

hsa−miR−4317, rectal
(all subjects; N = 719)
1−sided adj pval: 1
Frequency
Tumor−Normal Expression Difference
hsa−miR−4317, rectal
(BRAF = 0; N0 = 685)
1−sided adj pval: 1
Frequency
Tumor−Normal Expression Difference
hsa−miR−4317, rectal
(BRAF = 1; N1 = 19)
1−sided adj pval: 0.735
Frequency
Tumor−Normal Expression Difference

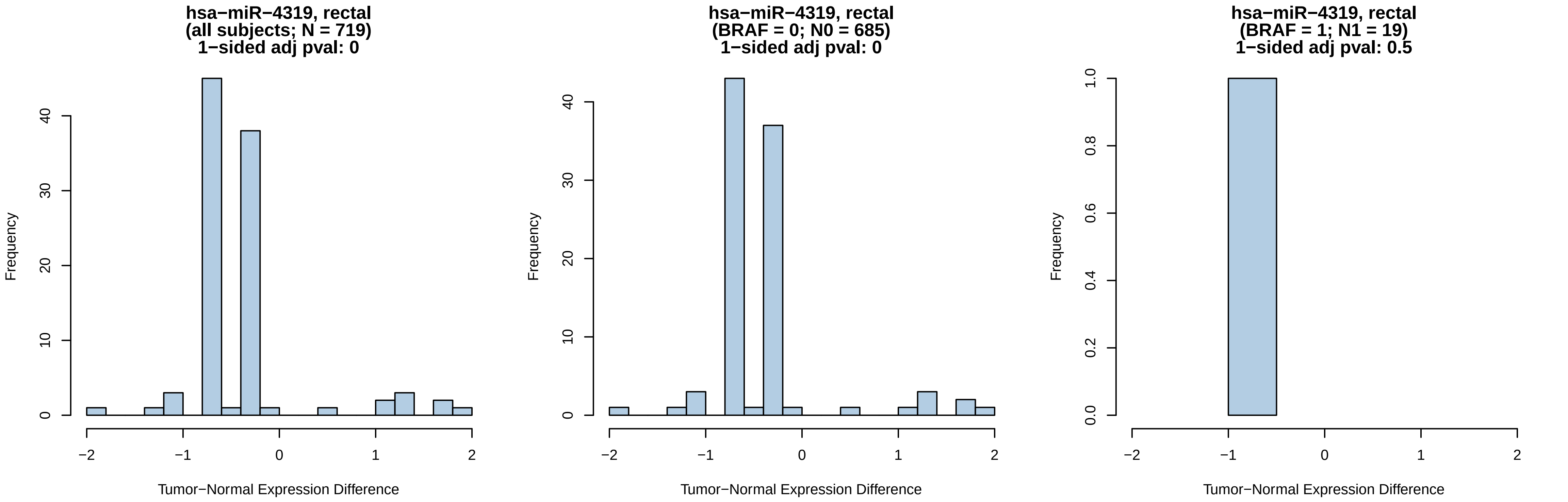
hsa−miR−4319, rectal
(all subjects; N = 719)
1−sided adj pval: 0
Frequency
Tumor−Normal Expression Difference
hsa−miR−4319, rectal
(BRAF = 0; N0 = 685)
1−sided adj pval: 0
Frequency
Tumor−Normal Expression Difference
hsa−miR−4319, rectal
(BRAF = 1; N1 = 19)
1−sided adj pval: 0.5
Frequency
Tumor−Normal Expression Difference

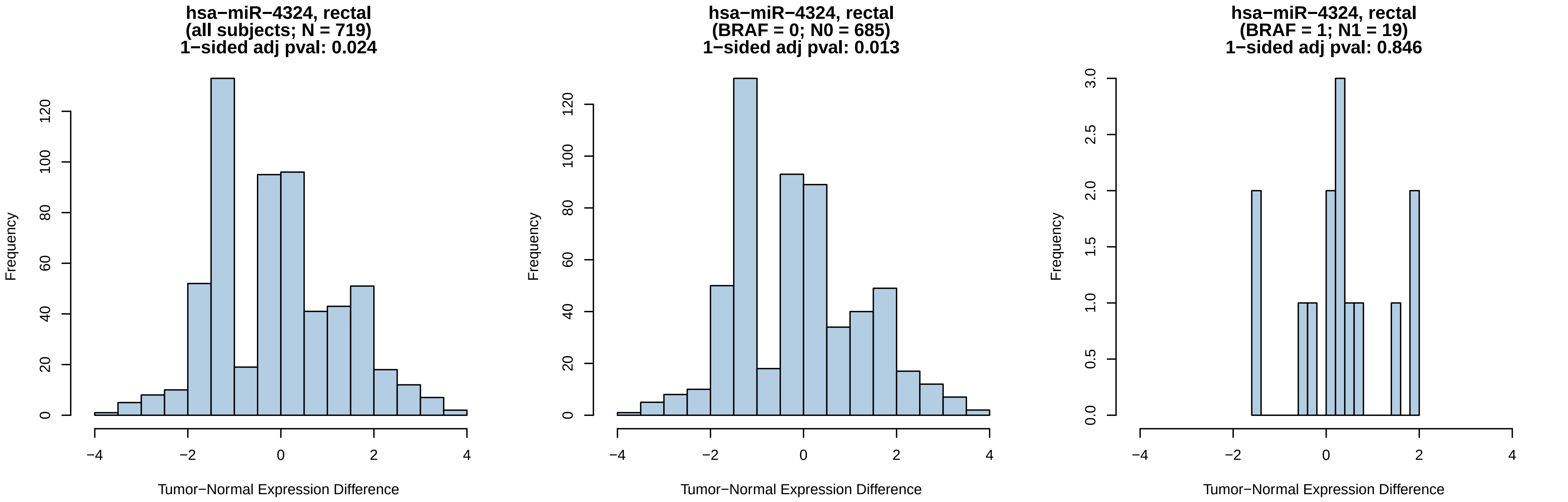

hsa−miR−4324, rectal
(all subjects; N = 719)
1−sided adj pval: 0.024
hsa−miR−4324, rectal
(BRAF = 0; N0 = 685)
1−sided adj pval: 0.013
hsa−miR−4324, rectal
(BRAF = 1; N1 = 19)
1−sided adj pval: 0.846
Frequency
Tumor−Normal Expression Difference

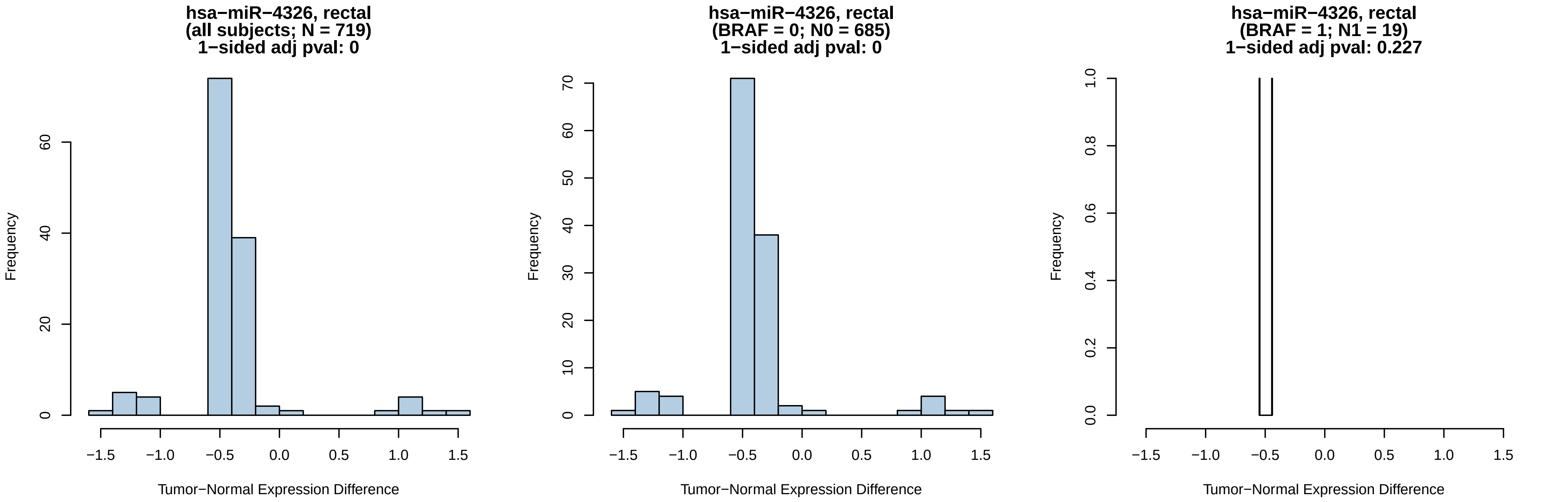

hsa−miR−4326, rectal
(all subjects; N = 719)
1−sided adj pval: 0
hsa−miR−4326, rectal
(BRAF = 0; N0 = 685)
1−sided adj pval: 0
hsa−miR−4326, rectal
(BRAF = 1; N1 = 19)
1−sided adj pval: 0.227
Frequency
Tumor−Normal Expression Difference

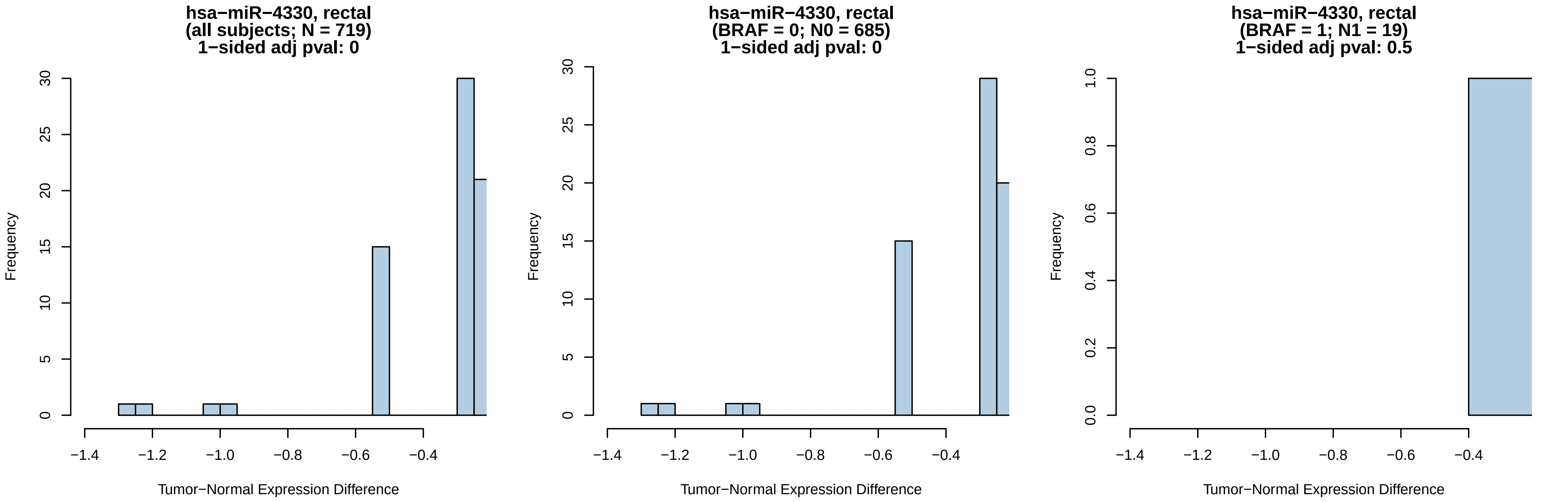
hsa−miR−4330, rectal
(all subjects; N = 719)
1−sided adj pval: 0
Frequency
Tumor−Normal Expression Difference
hsa−miR−4330, rectal
(BRAF = 0; N0 = 685)
1−sided adj pval: 0
Frequency
Tumor−Normal Expression Difference
hsa−miR−4330, rectal
(BRAF = 1; N1 = 19)
1−sided adj pval: 0.5
Frequency
Tumor−Normal Expression Difference

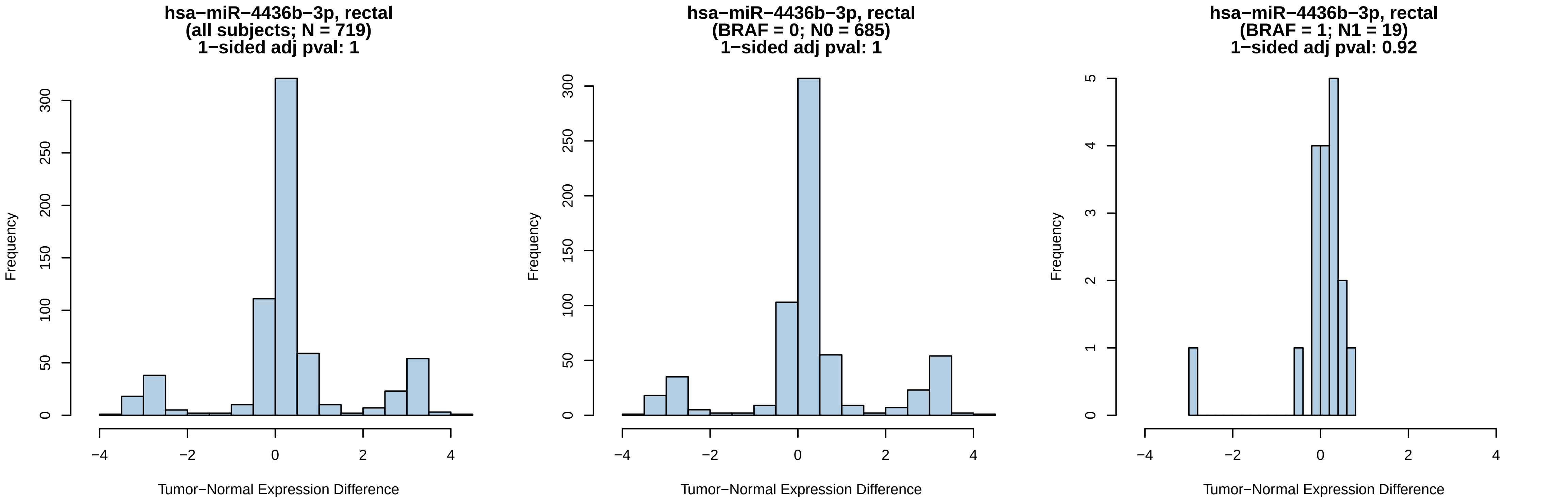
hsa−miR−4436b−3p, rectal
(all subjects; N = 719)
1−sided adj pval: 1
hsa−miR−4436b−3p, rectal
(BRAF = 0; N0 = 685)
1−sided adj pval: 1
hsa−miR−4436b−3p, rectal
(BRAF = 1; N1 = 19)
1−sided adj pval: 0.92
Frequency
Tumor−Normal Expression Difference

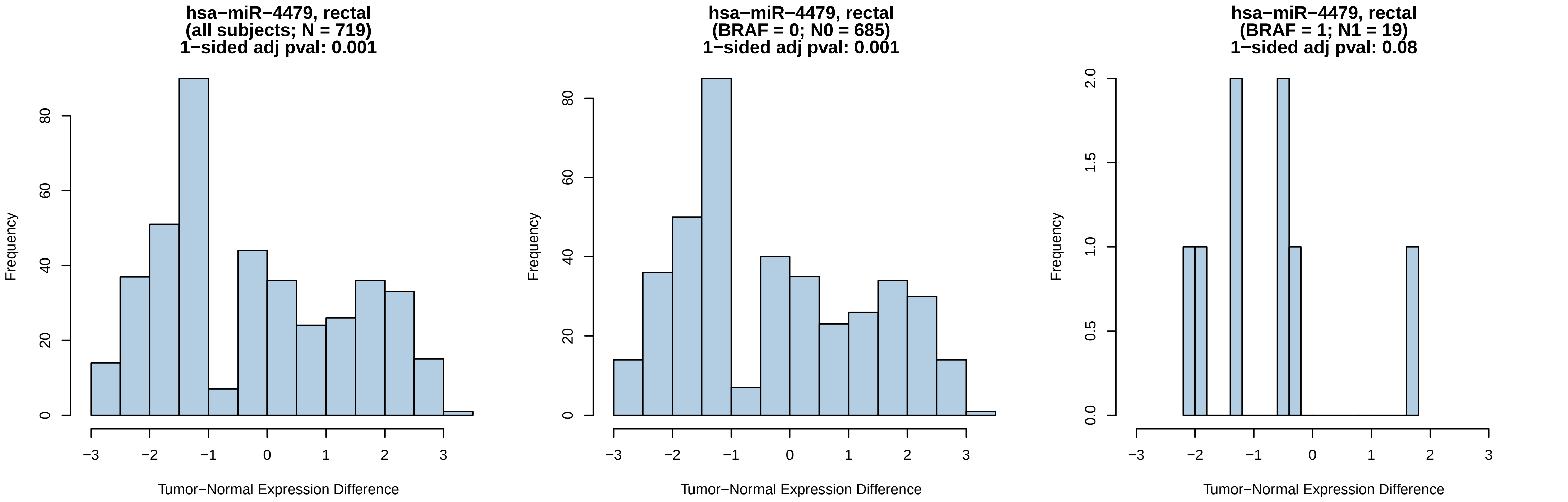
hsa−miR−4479, rectal
(all subjects; N = 719)
1−sided adj pval: 0.001
Frequency
Tumor−Normal Expression Difference
hsa−miR−4479, rectal
(BRAF = 0; N0 = 685)
1−sided adj pval: 0.001
Frequency
Tumor−Normal Expression Difference
hsa−miR−4479, rectal
(BRAF = 1; N1 = 19)
1−sided adj pval: 0.08
Frequency
Tumor−Normal Expression Difference

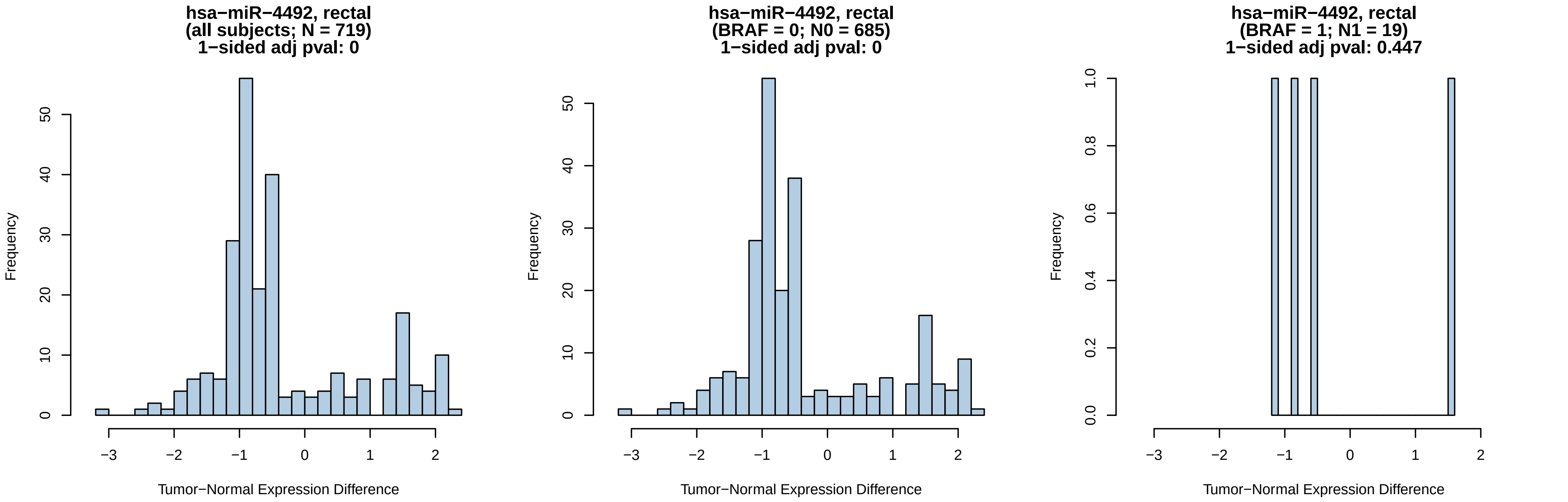
hsa−miR−4492, rectal
(all subjects; N = 719)
1−sided adj pval: 0
hsa−miR−4492, rectal
(BRAF = 0; N0 = 685)
1−sided adj pval: 0
hsa−miR−4492, rectal
(BRAF = 1; N1 = 19)
1−sided adj pval: 0.447
Frequency
Tumor−Normal Expression Difference
0
10
20
30
40
50
−3
−2
−1
0
1
2
0.0
0.2
0.4
0.6
0.8
1.0

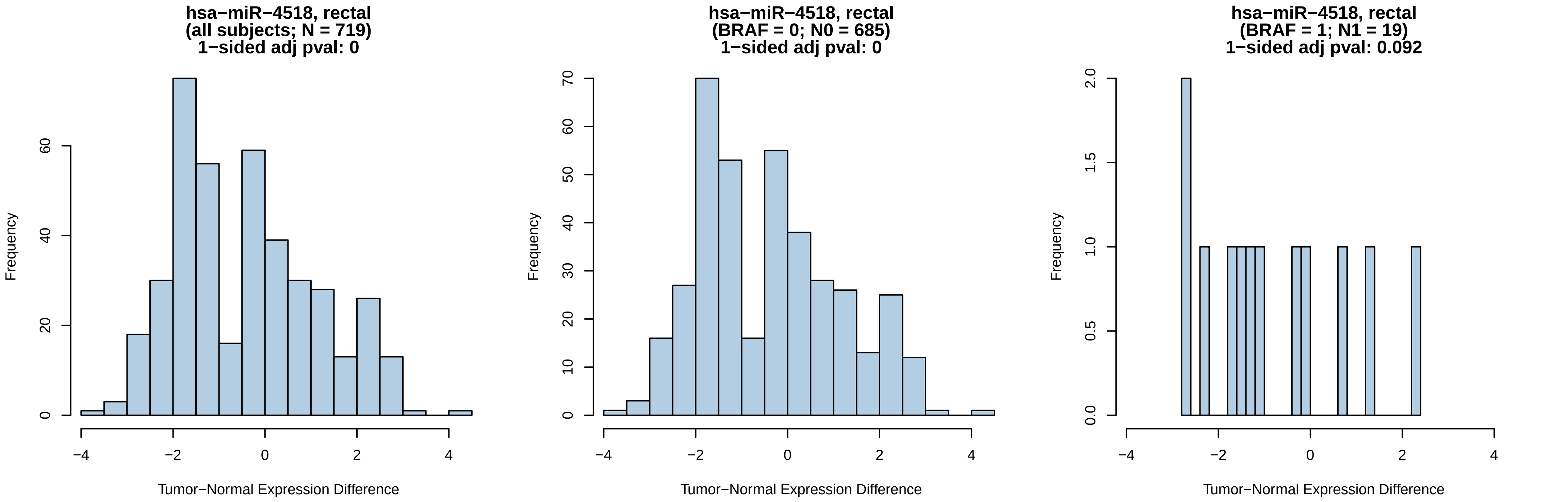
hsa−miR−4518, rectal
(all subjects; N = 719)
1−sided adj pval: 0
hsa−miR−4518, rectal
(BRAF = 0; N0 = 685)
1−sided adj pval: 0
hsa−miR−4518, rectal
(BRAF = 1; N1 = 19)
1−sided adj pval: 0.092
Frequency
Tumor−Normal Expression Difference
0
20
40
60
10
30
50
70
0.0
0.5
1.0
1.5
2.0
−4
−2
0
2
4

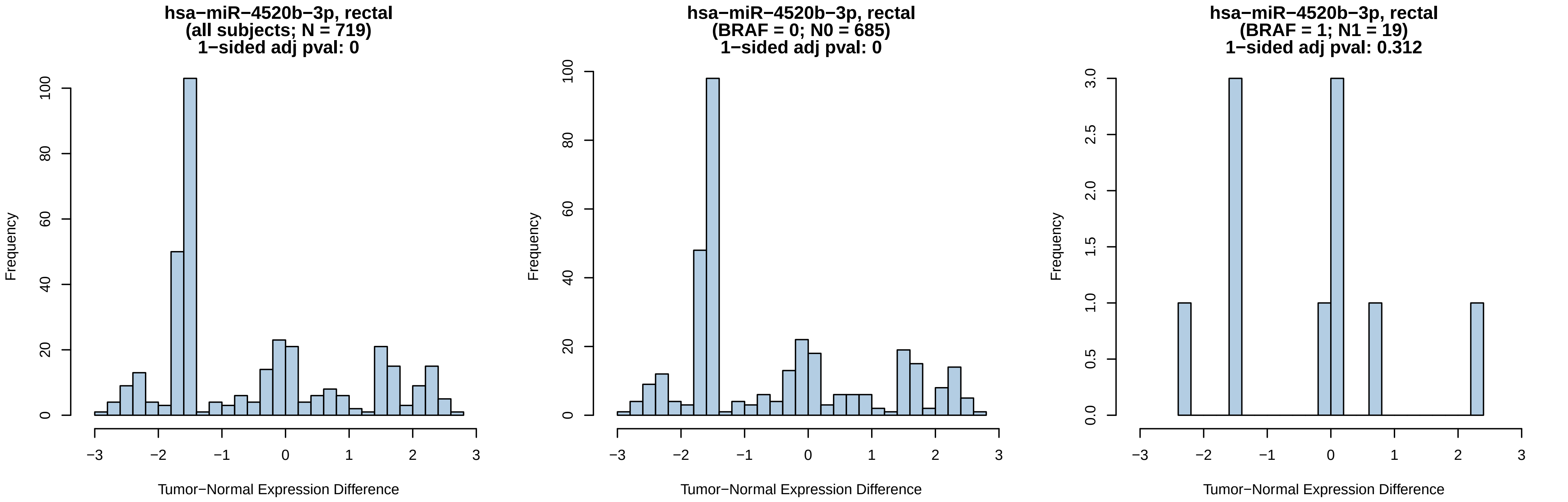

hsa−miR−4520b−3p, rectal
(all subjects; N = 719)
1−sided adj pval: 0
Frequency
Tumor−Normal Expression Difference
hsa−miR−4520b−3p, rectal
(BRAF = 0; N0 = 685)
1−sided adj pval: 0
Frequency
Tumor−Normal Expression Difference
hsa−miR−4520b−3p, rectal
(BRAF = 1; N1 = 19)
1−sided adj pval: 0.312
Frequency
Tumor−Normal Expression Difference

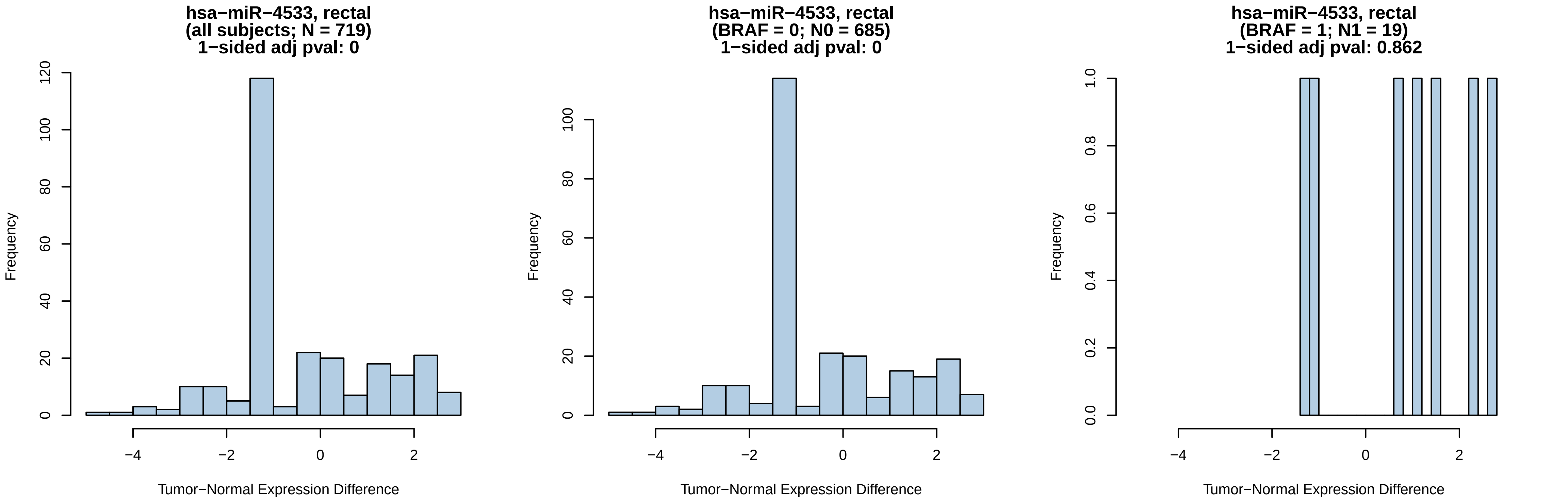
hsa−miR−4533, rectal
(all subjects; N = 719)
1−sided adj pval: 0
hsa−miR−4533, rectal
(BRAF = 0; N0 = 685)
1−sided adj pval: 0
hsa−miR−4533, rectal
(BRAF = 1; N1 = 19)
1−sided adj pval: 0.862
Frequency
Tumor−Normal Expression Difference

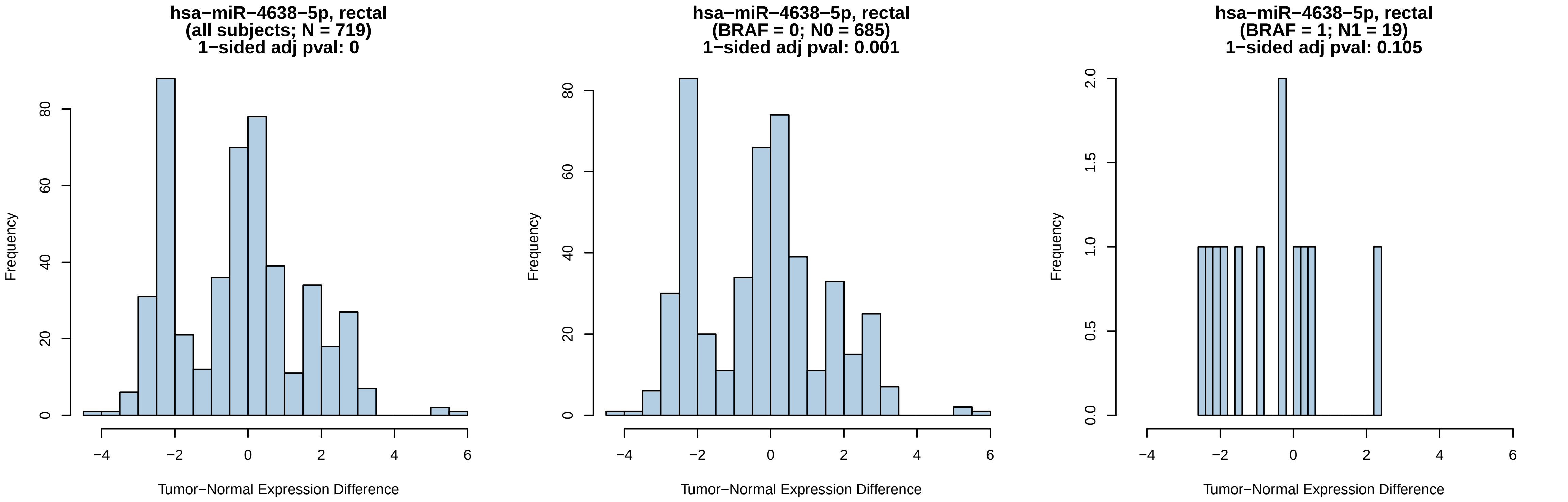

hsa−miR−4638−5p, rectal
(all subjects; N = 719)
1−sided adj pval: 0
Frequency
Tumor−Normal Expression Difference
hsa−miR−4638−5p, rectal
(BRAF = 0; N0 = 685)
1−sided adj pval: 0.001
Frequency
Tumor−Normal Expression Difference
hsa−miR−4638−5p, rectal
(BRAF = 1; N1 = 19)
1−sided adj pval: 0.105
Frequency
Tumor−Normal Expression Difference

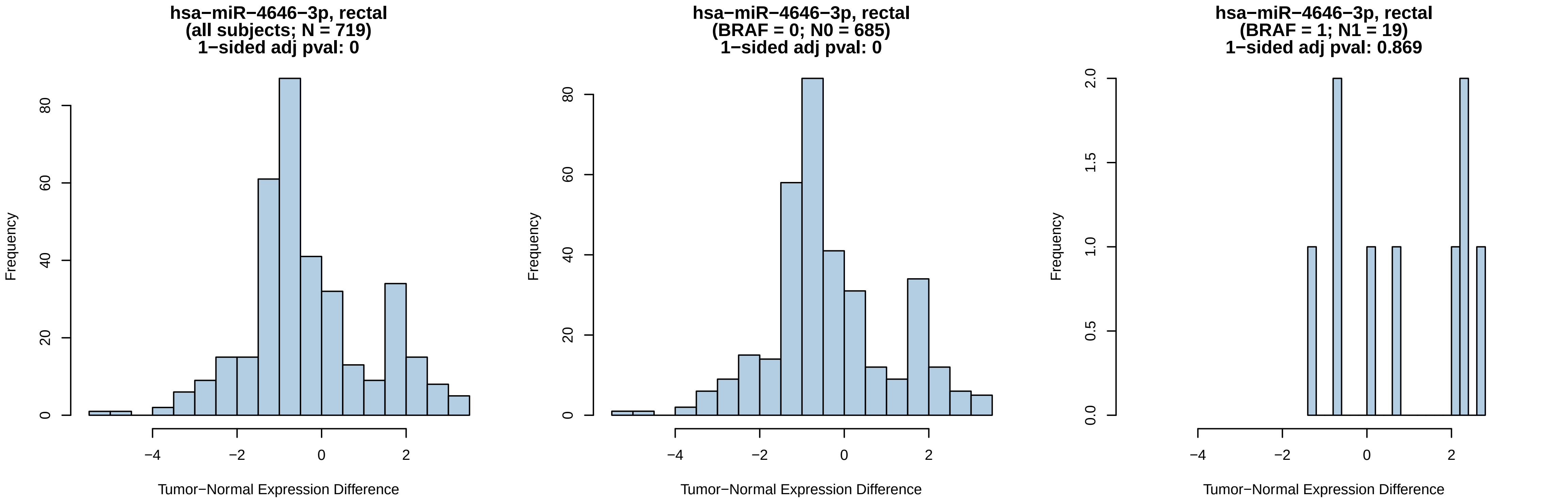
hsa−miR−4646−3p, rectal
(all subjects; N = 719)
1−sided adj pval: 0
hsa−miR−4646−3p, rectal
(BRAF = 0; N0 = 685)
1−sided adj pval: 0
hsa−miR−4646−3p, rectal
(BRAF = 1; N1 = 19)
1−sided adj pval: 0.869
Frequency
Tumor−Normal Expression Difference

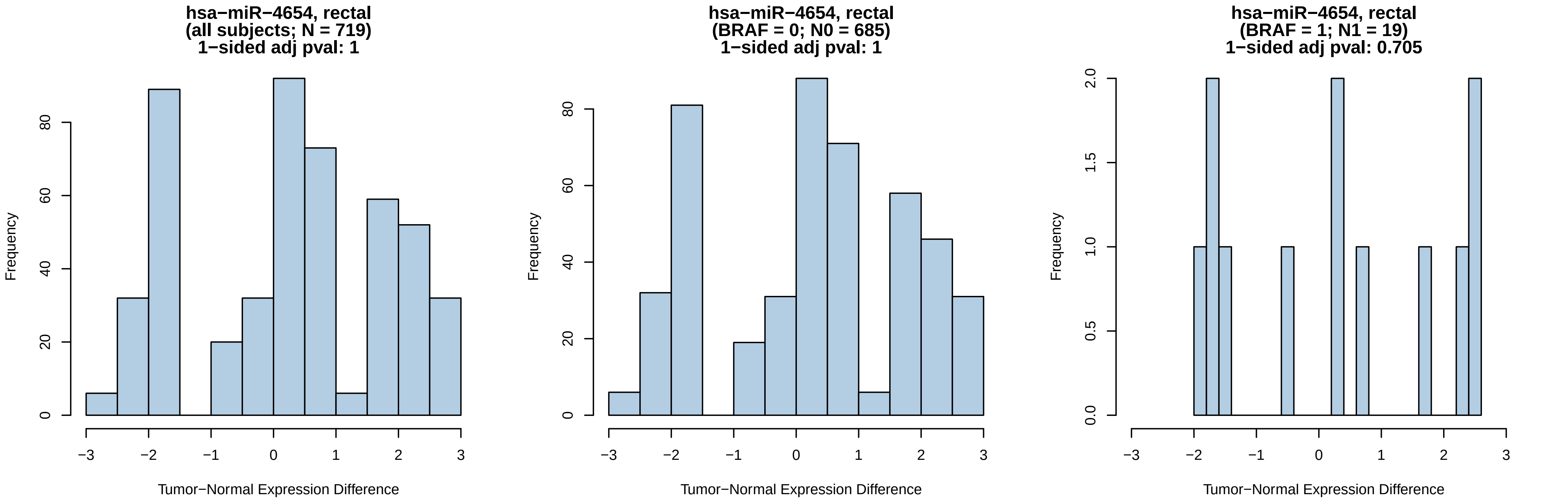

hsa−miR−4654, rectal
(all subjects; N = 719)
1−sided adj pval: 1
Frequency
0
20
40
60
80
−3
−2
−1
0
1
2
3
Tumor−Normal Expression Difference
hsa−miR−4654, rectal
(BRAF = 0; N0 = 685)
1−sided adj pval: 1
Frequency
0
20
40
60
80
−3
−2
−1
0
1
2
3
Tumor−Normal Expression Difference
hsa−miR−4654, rectal
(BRAF = 1; N1 = 19)
1−sided adj pval: 0.705
Frequency
0.0
0.5
1.0
1.5
2.0
−3
−2
−1
0
1
2
3
Tumor−Normal Expression Difference

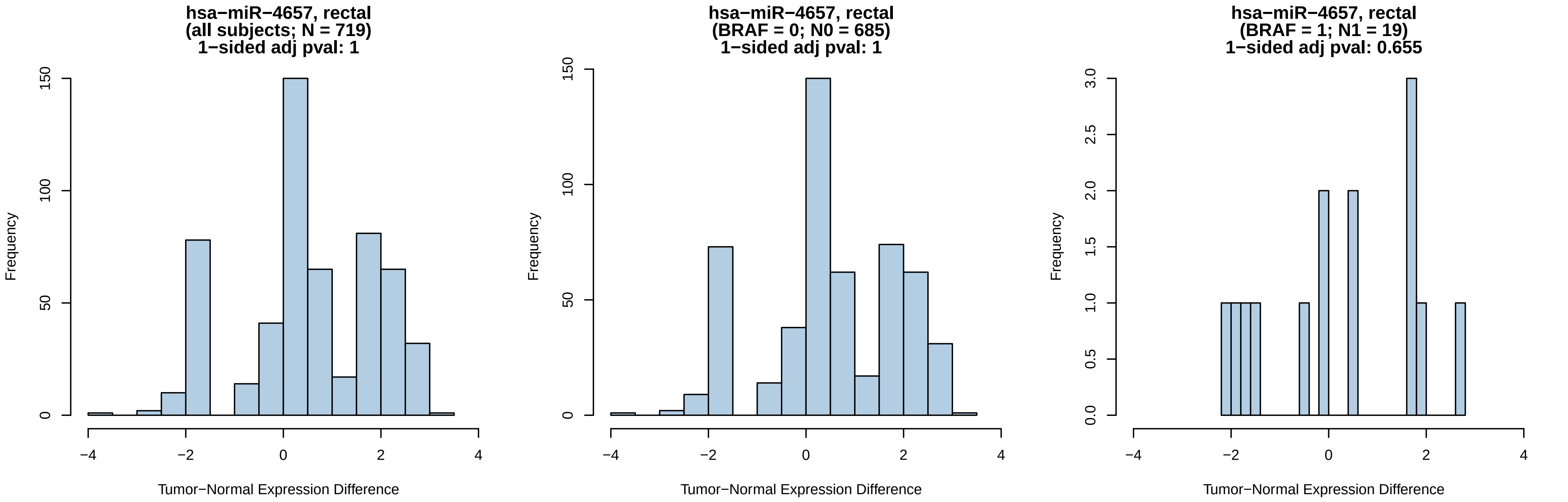

hsa−miR−4657, rectal
(all subjects; N = 719)
1−sided adj pval: 1
hsa−miR−4657, rectal
(BRAF = 0; N0 = 685)
1−sided adj pval: 1
hsa−miR−4657, rectal
(BRAF = 1; N1 = 19)
1−sided adj pval: 0.655
Frequency
Tumor−Normal Expression Difference

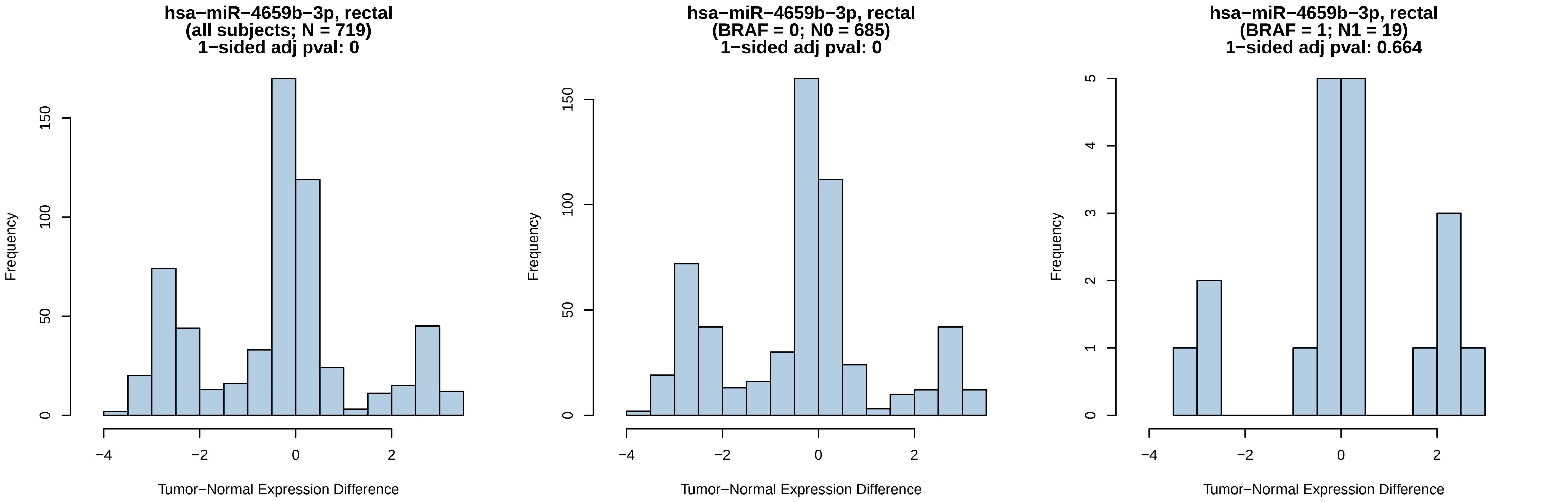

hsa−miR−4659b−3p, rectal
(all subjects; N = 719)
1−sided adj pval: 0
Frequency
Tumor−Normal Expression Difference
hsa−miR−4659b−3p, rectal
(BRAF = 0; N0 = 685)
1−sided adj pval: 0
Frequency
Tumor−Normal Expression Difference
hsa−miR−4659b−3p, rectal
(BRAF = 1; N1 = 19)
1−sided adj pval: 0.664
Frequency
Tumor−Normal Expression Difference

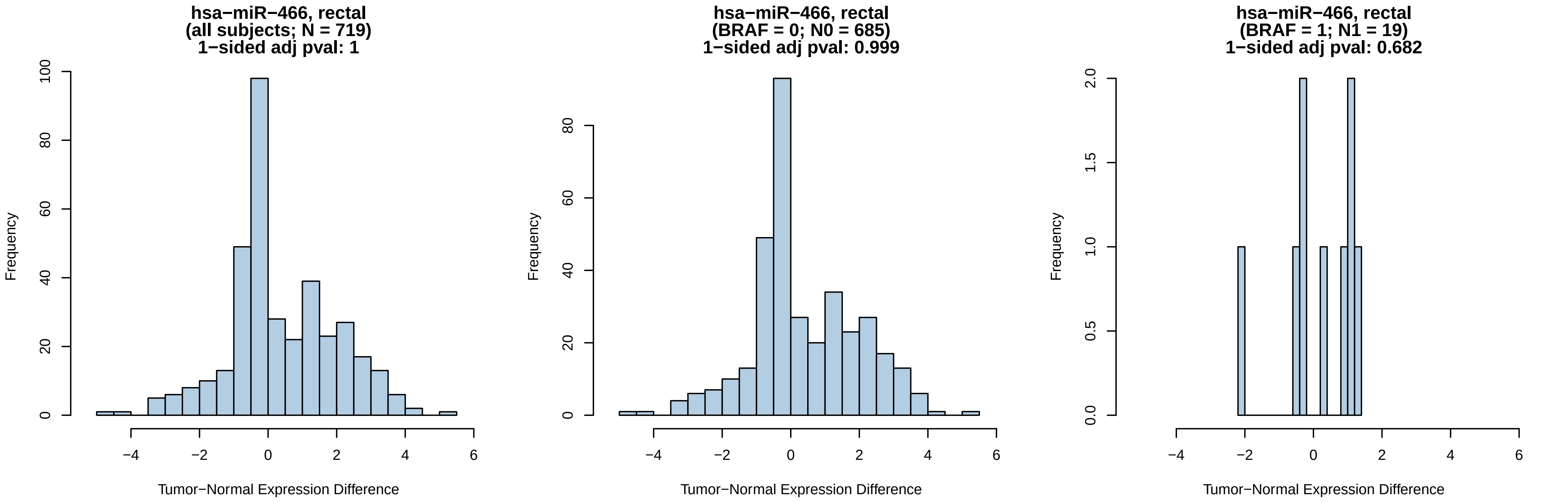
hsa−miR−466, rectal
(all subjects; N = 719)
1−sided adj pval: 1
Frequency
Tumor−Normal Expression Difference
hsa−miR−466, rectal
(BRAF = 0; N0 = 685)
1−sided adj pval: 0.999
Frequency
Tumor−Normal Expression Difference
hsa−miR−466, rectal
(BRAF = 1; N1 = 19)
1−sided adj pval: 0.682
Frequency
Tumor−Normal Expression Difference

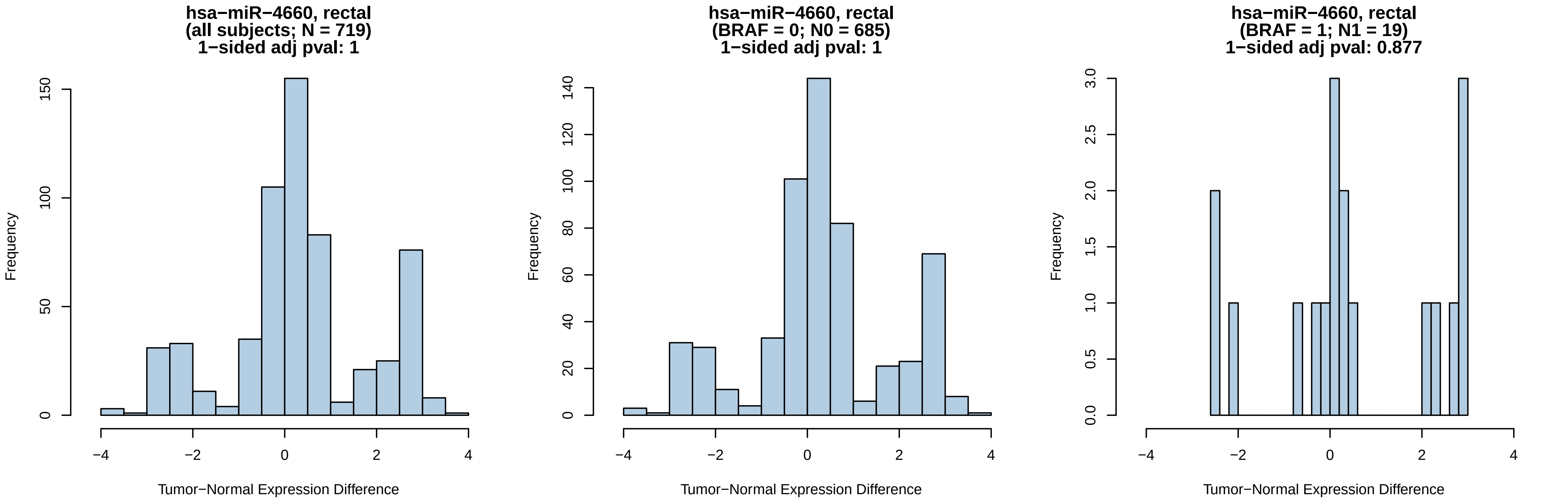

hsa−miR−4660, rectal
(all subjects; N = 719)
1−sided adj pval: 1
Frequency
Tumor−Normal Expression Difference
hsa−miR−4660, rectal
(BRAF = 0; N0 = 685)
1−sided adj pval: 1
Frequency
Tumor−Normal Expression Difference
hsa−miR−4660, rectal
(BRAF = 1; N1 = 19)
1−sided adj pval: 0.877
Frequency
Tumor−Normal Expression Difference

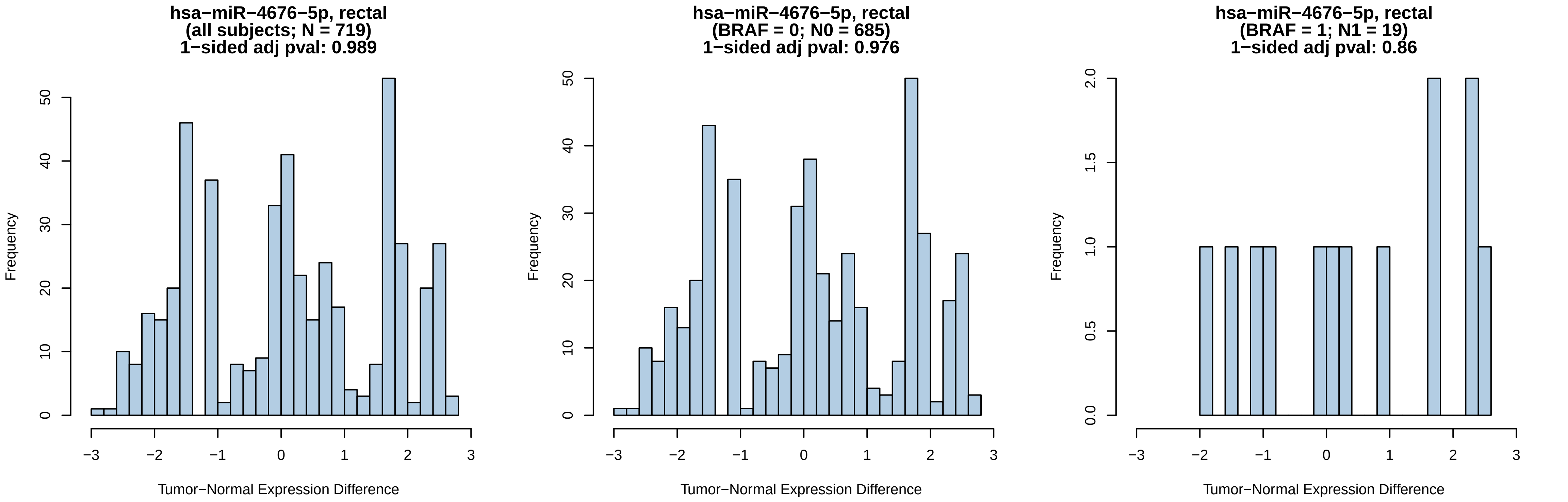
hsa−miR−4676−5p, rectal
(all subjects; N = 719)
1−sided adj pval: 0.989
Frequency
Tumor−Normal Expression Difference
hsa−miR−4676−5p, rectal
(BRAF = 0; N0 = 685)
1−sided adj pval: 0.976
Frequency
Tumor−Normal Expression Difference
hsa−miR−4676−5p, rectal
(BRAF = 1; N1 = 19)
1−sided adj pval: 0.86
Frequency
Tumor−Normal Expression Difference

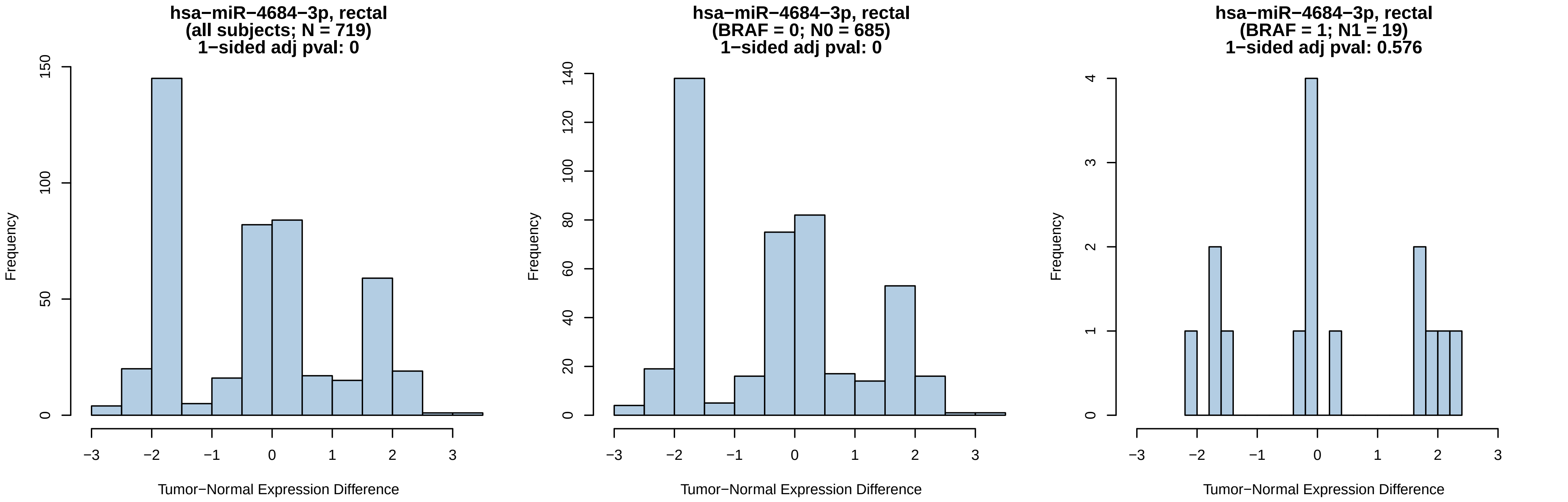
hsa−miR−4684−3p, rectal
(all subjects; N = 719)
1−sided adj pval: 0
Frequency
Tumor−Normal Expression Difference
hsa−miR−4684−3p, rectal
(BRAF = 0; N0 = 685)
1−sided adj pval: 0
Frequency
Tumor−Normal Expression Difference
hsa−miR−4684−3p, rectal
(BRAF = 1; N1 = 19)
1−sided adj pval: 0.576
Frequency
Tumor−Normal Expression Difference

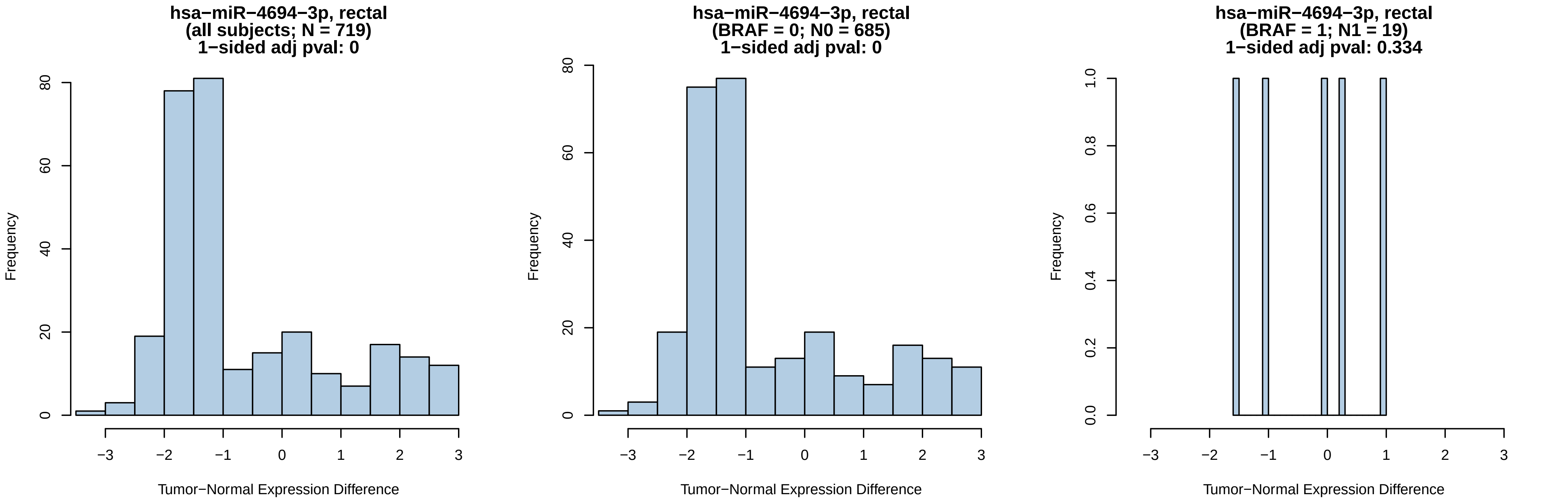
hsa−miR−4694−3p, rectal
(all subjects; N = 719)
1−sided adj pval: 0
Frequency
Tumor−Normal Expression Difference
hsa−miR−4694−3p, rectal
(BRAF = 0; N0 = 685)
1−sided adj pval: 0
Frequency
Tumor−Normal Expression Difference
hsa−miR−4694−3p, rectal
(BRAF = 1; N1 = 19)
1−sided adj pval: 0.334
Frequency
Tumor−Normal Expression Difference

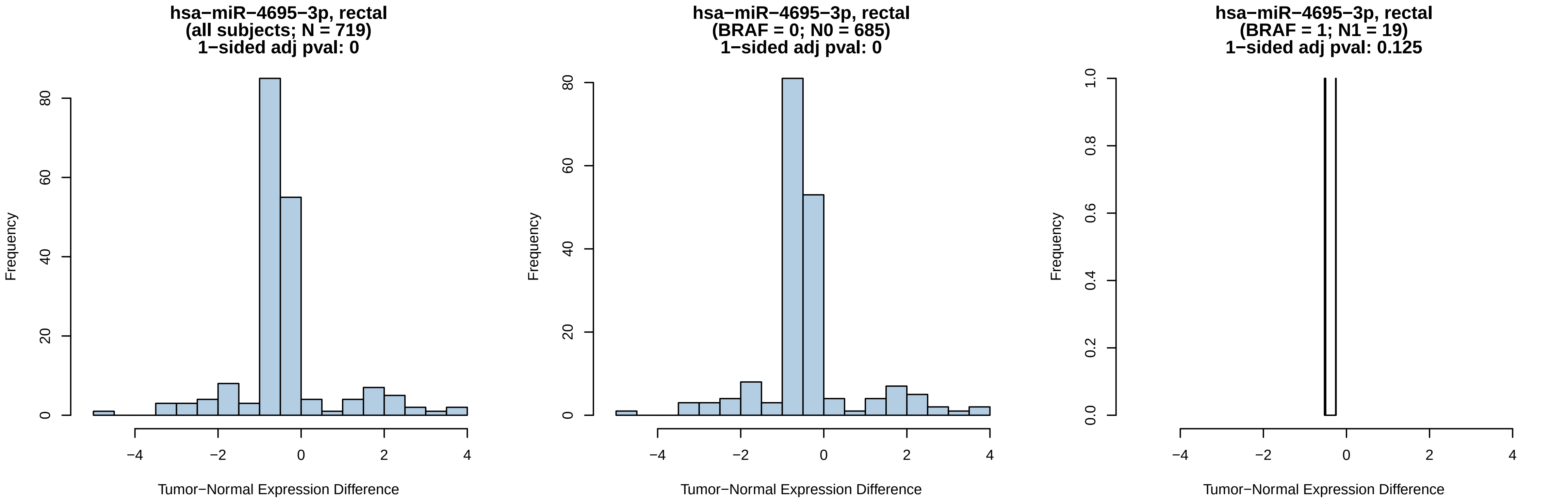

hsa−miR−4695−3p, rectal
(all subjects; N = 719)
1−sided adj pval: 0
Frequency
0
20
40
60
80
−4
−2
0
2
4
Tumor−Normal Expression Difference
hsa−miR−4695−3p, rectal
(BRAF = 0; N0 = 685)
1−sided adj pval: 0
Frequency
0
20
40
60
80
−4
−2
0
2
4
Tumor−Normal Expression Difference
hsa−miR−4695−3p, rectal
(BRAF = 1; N1 = 19)
1−sided adj pval: 0.125
Frequency
0.0
0.2
0.4
0.6
0.8
1.0
−4
−2
0
2
4
Tumor−Normal Expression Difference

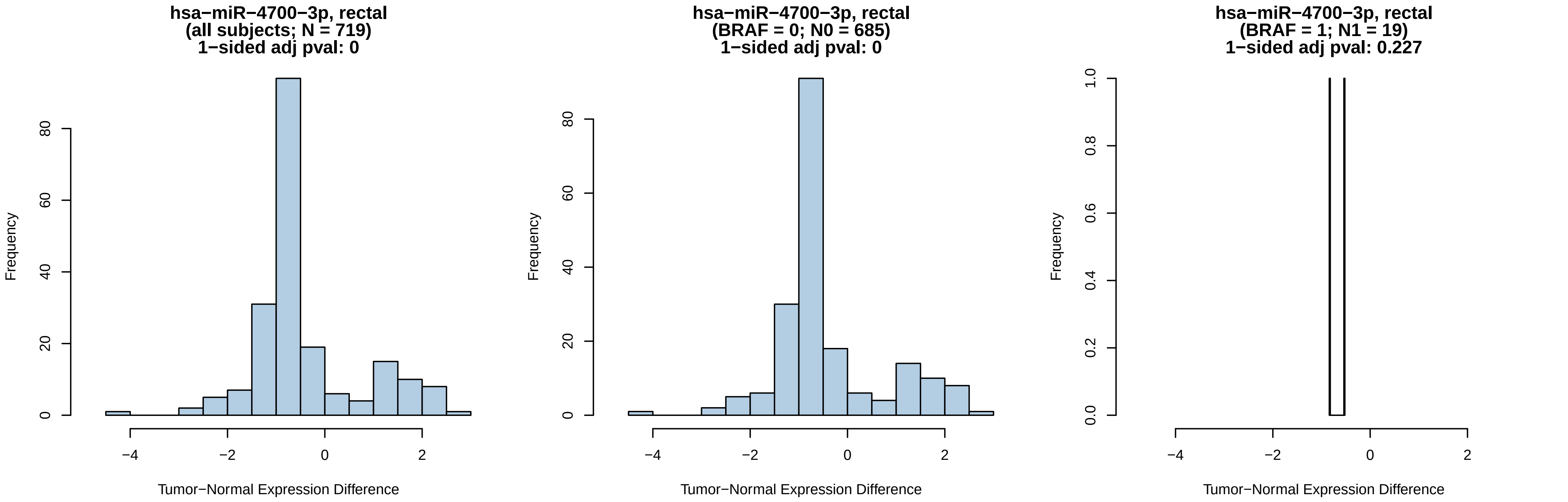

hsa−miR−4700−3p, rectal
(all subjects; N = 719)
1−sided adj pval: 0
Frequency
Tumor−Normal Expression Difference
hsa−miR−4700−3p, rectal
(BRAF = 0; N0 = 685)
1−sided adj pval: 0
Frequency
Tumor−Normal Expression Difference
hsa−miR−4700−3p, rectal
(BRAF = 1; N1 = 19)
1−sided adj pval: 0.227
Frequency
Tumor−Normal Expression Difference

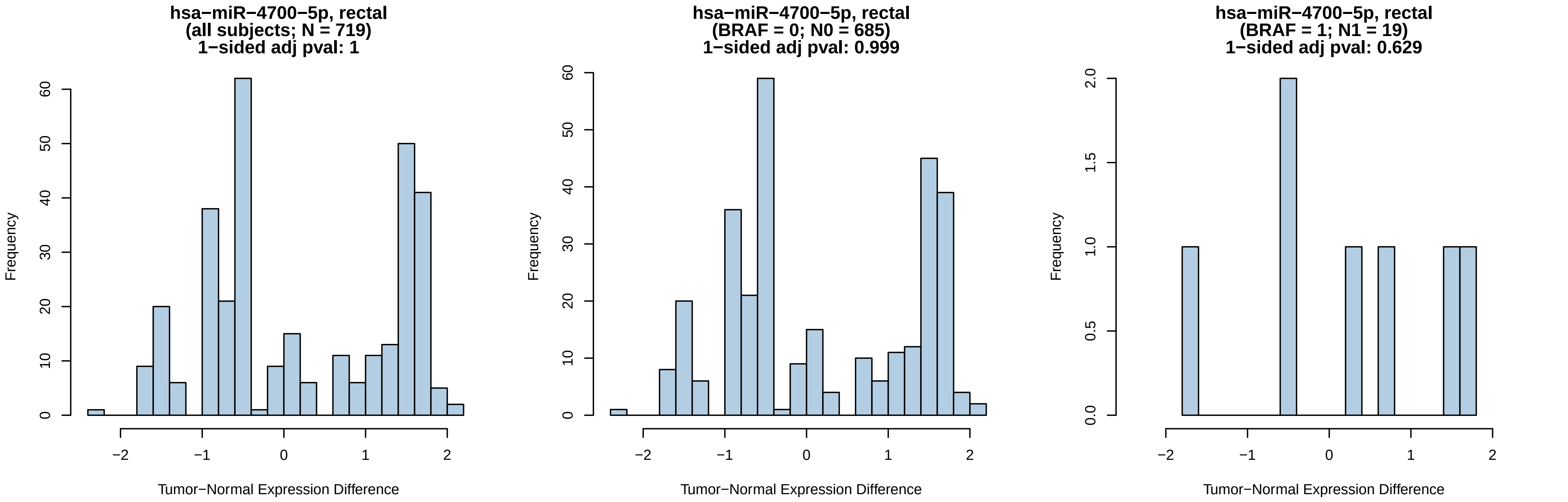
hsa−miR−4700−5p, rectal
(all subjects; N = 719)
1−sided adj pval: 1
hsa−miR−4700−5p, rectal
(BRAF = 0; N0 = 685)
1−sided adj pval: 0.999
hsa−miR−4700−5p, rectal
(BRAF = 1; N1 = 19)
1−sided adj pval: 0.629
Frequency
Tumor−Normal Expression Difference

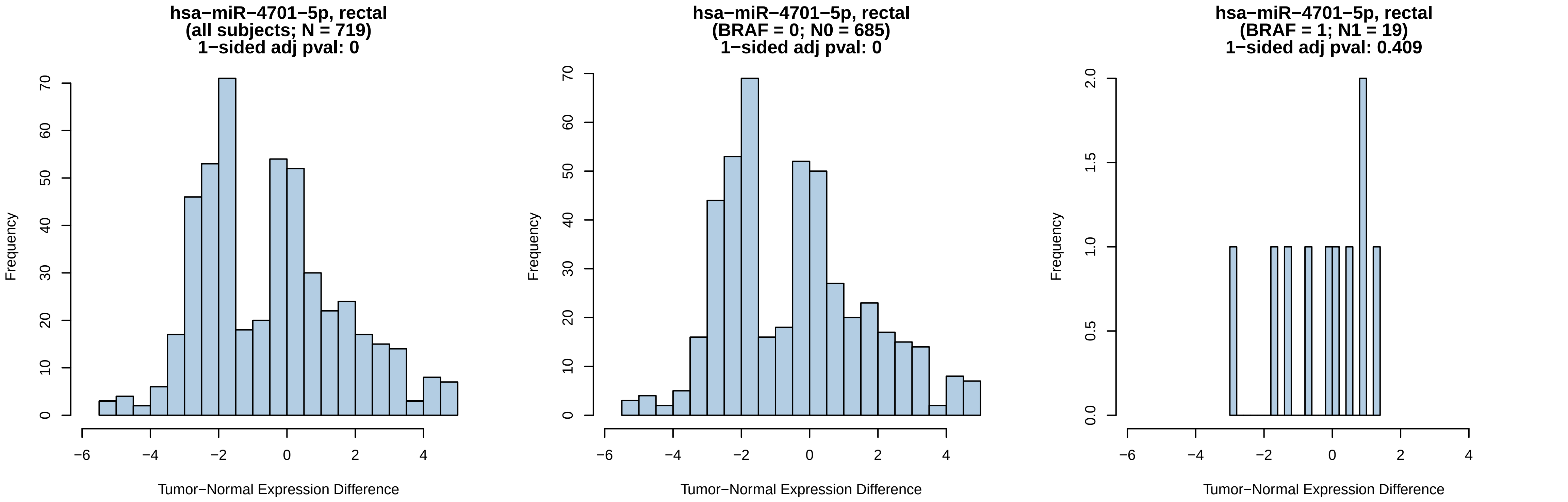
hsa−miR−4701−5p, rectal
(all subjects; N = 719)
1−sided adj pval: 0
Frequency
Tumor−Normal Expression Difference
hsa−miR−4701−5p, rectal
(BRAF = 0; N0 = 685)
1−sided adj pval: 0
Frequency
Tumor−Normal Expression Difference
hsa−miR−4701−5p, rectal
(BRAF = 1; N1 = 19)
1−sided adj pval: 0.409
Frequency
Tumor−Normal Expression Difference

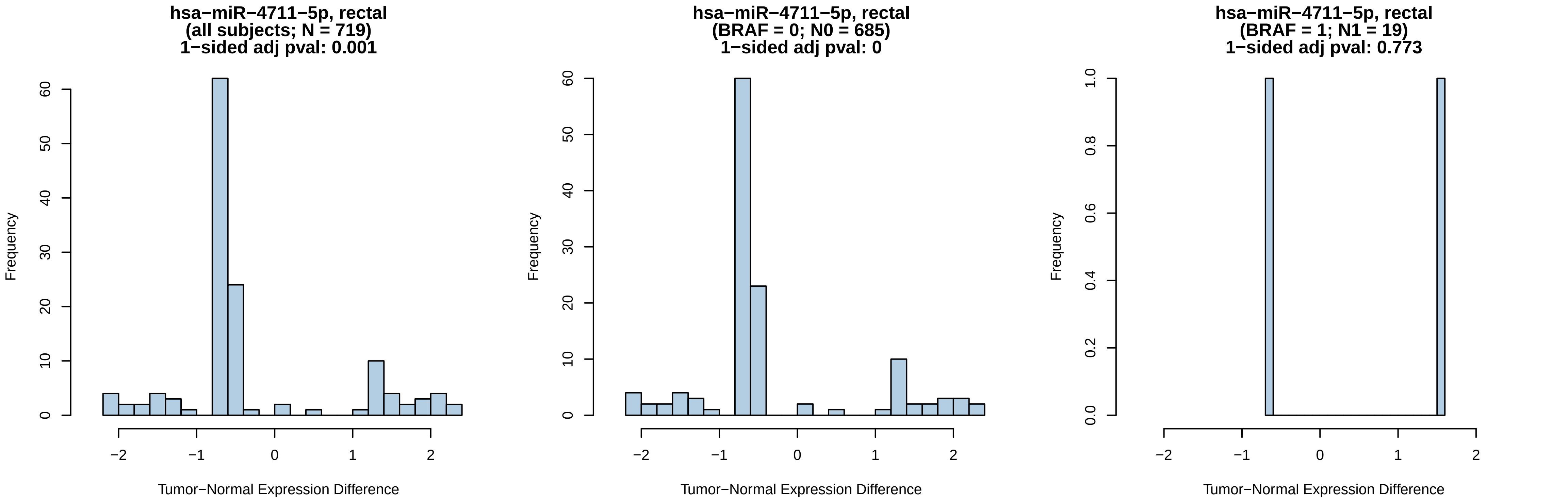

hsa−miR−4711−5p, rectal
(all subjects; N = 719)
1−sided adj pval: 0.001
Frequency
Tumor−Normal Expression Difference
hsa−miR−4711−5p, rectal
(BRAF = 0; N0 = 685)
1−sided adj pval: 0
Frequency
Tumor−Normal Expression Difference
hsa−miR−4711−5p, rectal
(BRAF = 1; N1 = 19)
1−sided adj pval: 0.773
Frequency
Tumor−Normal Expression Difference

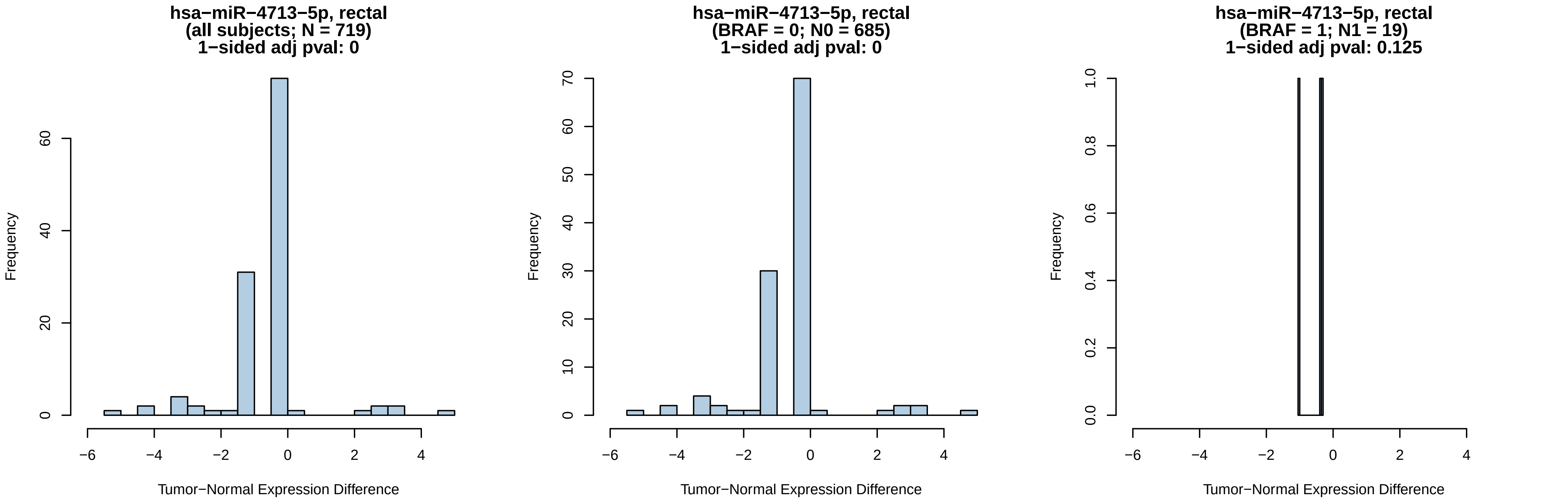
hsa−miR−4713−5p, rectal
(all subjects; N = 719)
1−sided adj pval: 0
Frequency
Tumor−Normal Expression Difference
hsa−miR−4713−5p, rectal
(BRAF = 0; N0 = 685)
1−sided adj pval: 0
Frequency
Tumor−Normal Expression Difference
hsa−miR−4713−5p, rectal
(BRAF = 1; N1 = 19)
1−sided adj pval: 0.125
Frequency
Tumor−Normal Expression Difference

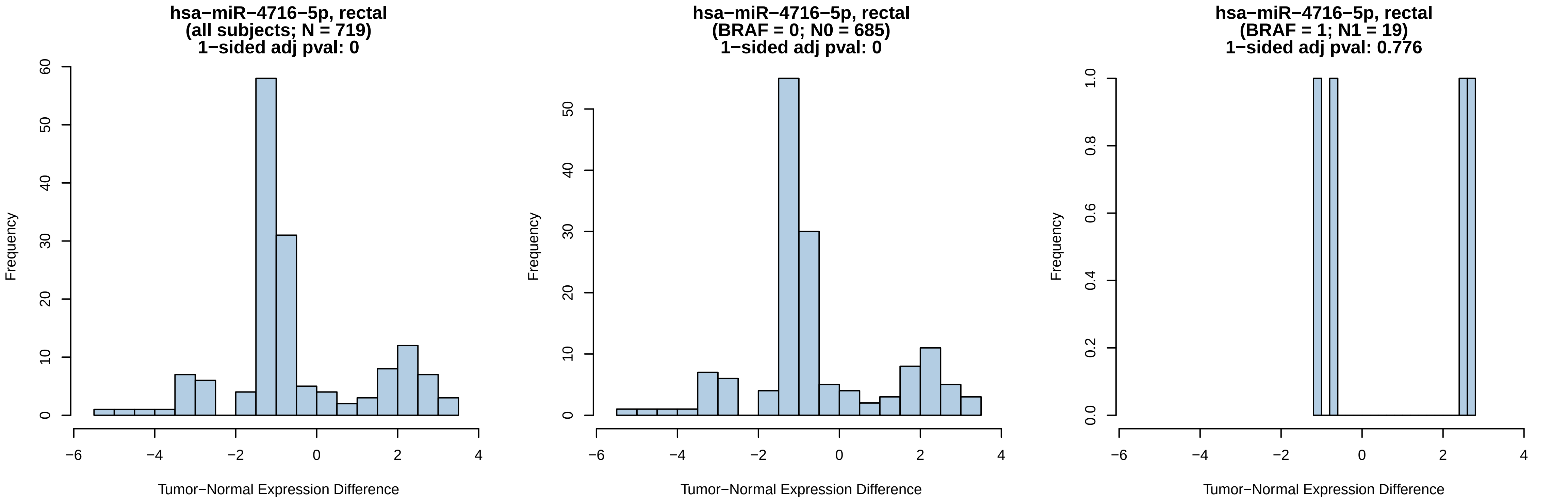
hsa−miR−4716−5p, rectal
(all subjects; N = 719)
1−sided adj pval: 0
hsa−miR−4716−5p, rectal
(BRAF = 0; N0 = 685)
1−sided adj pval: 0
hsa−miR−4716−5p, rectal
(BRAF = 1; N1 = 19)
1−sided adj pval: 0.776
Frequency
Tumor−Normal Expression Difference

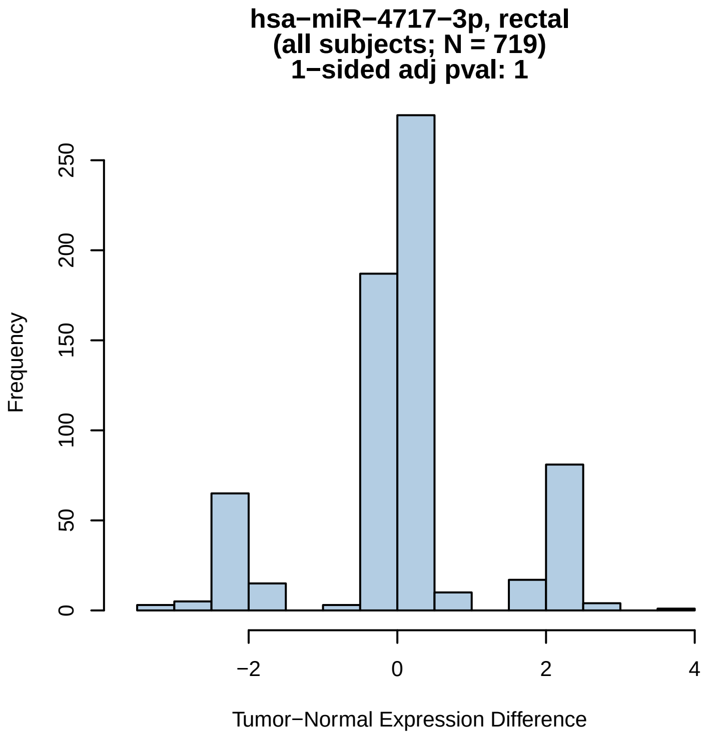
hsa−miR−4717−3p, rectal
(all subjects; N = 719)
1−sided adj pval: 1
Frequency
0
50
100
150
200
250
−2
0
2
4
Tumor−Normal Expression Difference

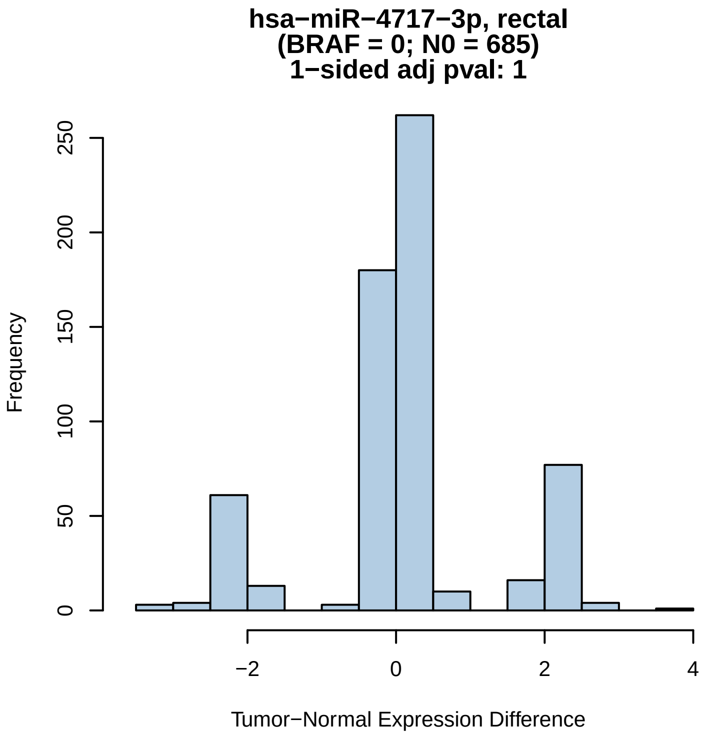
hsa−miR−4717−3p, rectal
(BRAF = 0; N0 = 685)
1−sided adj pval: 1
Frequency
0
50
100
150
200
250
−2
0
2
4
Tumor−Normal Expression Difference

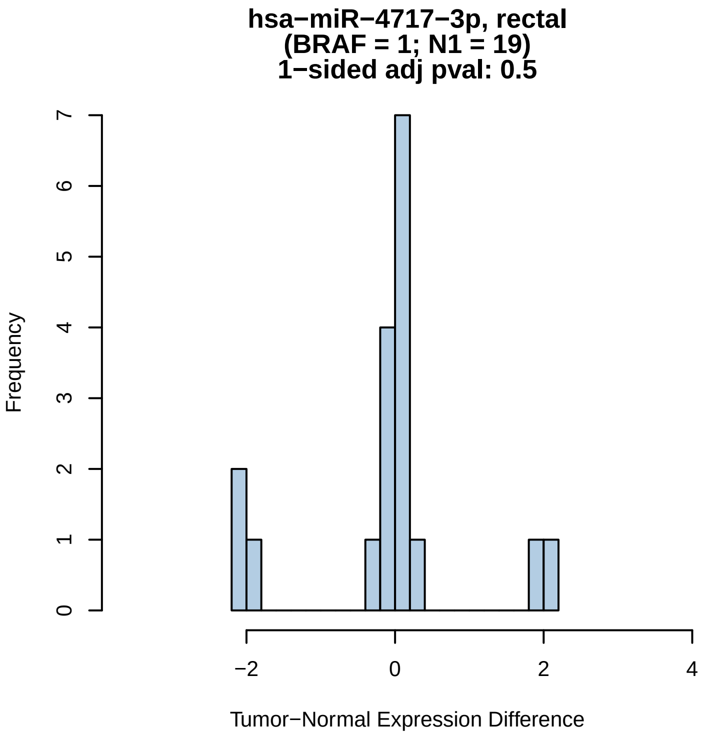
hsa−miR−4717−3p, rectal
(BRAF = 1; N1 = 19)
1−sided adj pval: 0.5
Frequency
0
1
2
3
4
5
6
7
−2
0
2
4
Tumor−Normal Expression Difference

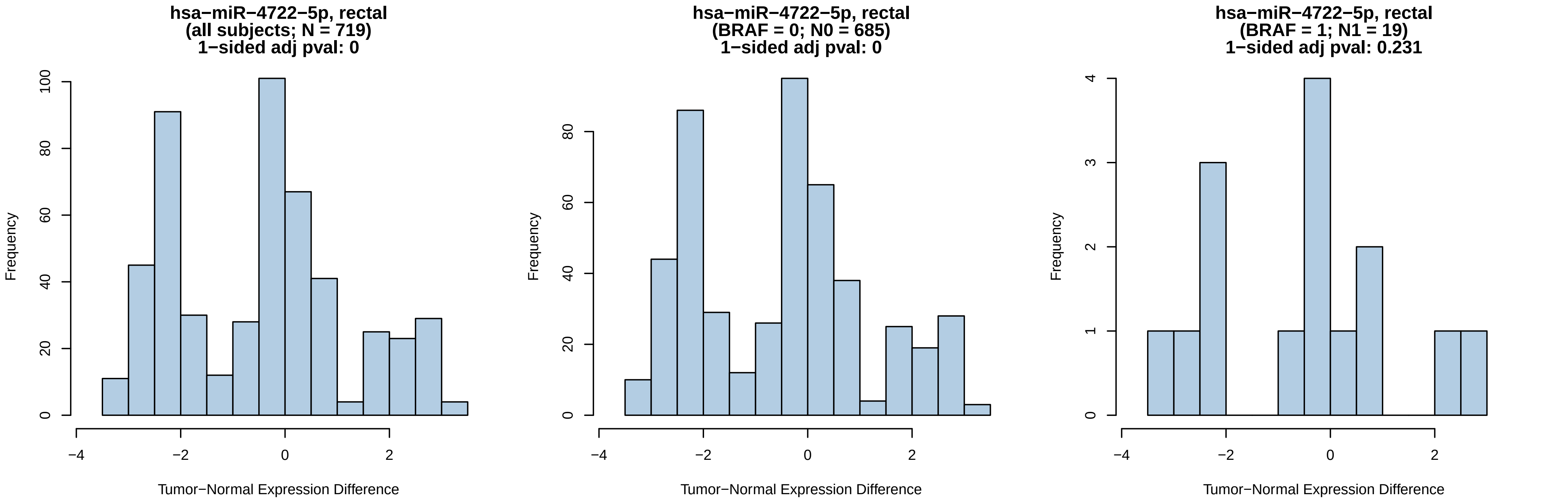
hsa−miR−4722−5p, rectal
(all subjects; N = 719)
1−sided adj pval: 0
Frequency
Tumor−Normal Expression Difference
hsa−miR−4722−5p, rectal
(BRAF = 0; N0 = 685)
1−sided adj pval: 0
Frequency
Tumor−Normal Expression Difference
hsa−miR−4722−5p, rectal
(BRAF = 1; N1 = 19)
1−sided adj pval: 0.231
Frequency
Tumor−Normal Expression Difference

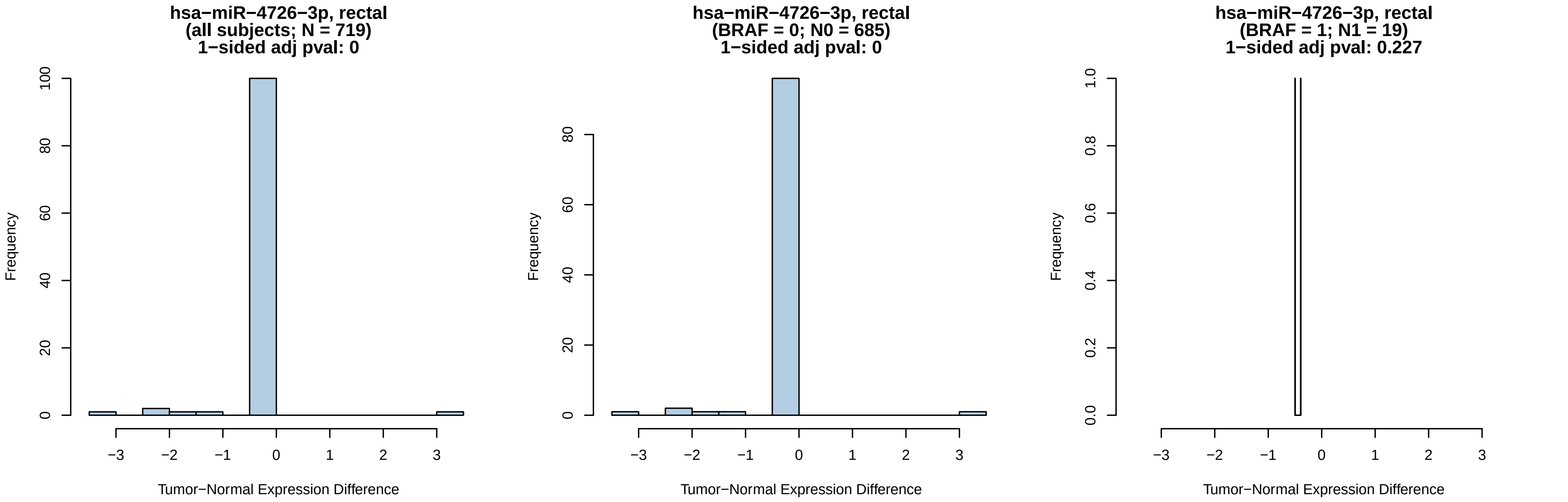

hsa−miR−4726−3p, rectal
(all subjects; N = 719)
1−sided adj pval: 0
Frequency
0
20
40
60
80
100
−3
−2
−1
0
1
2
3
Tumor−Normal Expression Difference
hsa−miR−4726−3p, rectal
(BRAF = 0; N0 = 685)
1−sided adj pval: 0
Frequency
0
20
40
60
80
−3
−2
−1
0
1
2
3
Tumor−Normal Expression Difference
hsa−miR−4726−3p, rectal
(BRAF = 1; N1 = 19)
1−sided adj pval: 0.227
Frequency
0.0
0.2
0.4
0.6
0.8
1.0
−3
−2
−1
0
1
2
3
Tumor−Normal Expression Difference

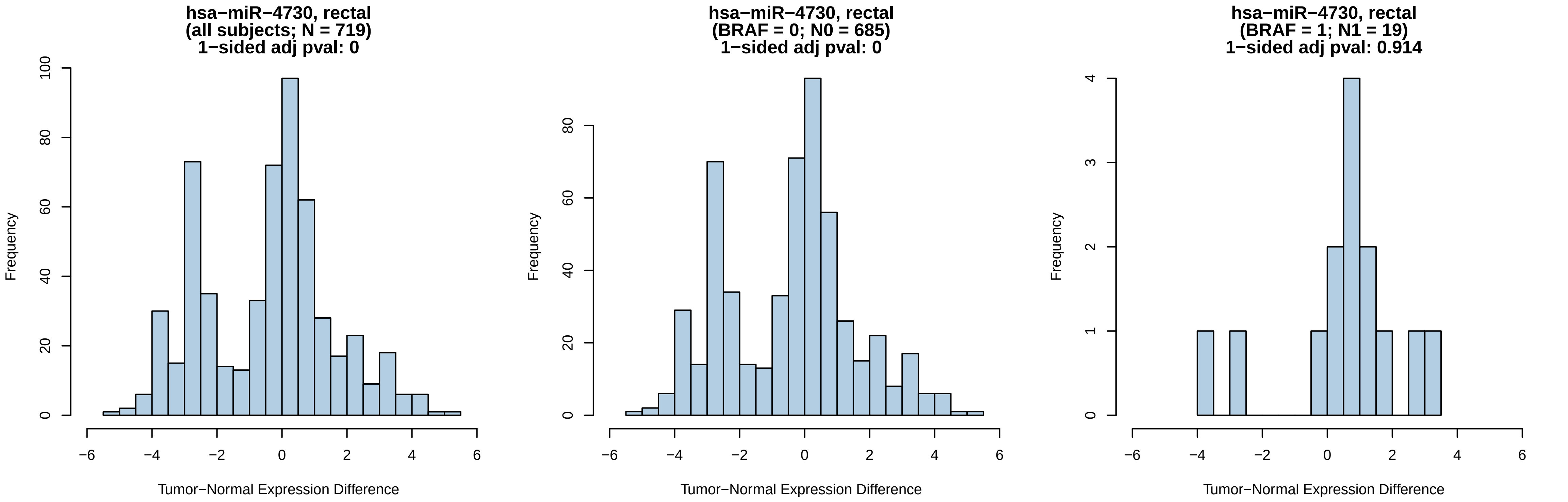
hsa−miR−4730, rectal
(all subjects; N = 719)
1−sided adj pval: 0
Frequency
0
20
40
60
80
100
−6
−4
−2
0
2
4
6
Tumor−Normal Expression Difference
hsa−miR−4730, rectal
(BRAF = 0; N0 = 685)
1−sided adj pval: 0
Frequency
0
20
40
60
80
−6
−4
−2
0
2
4
6
Tumor−Normal Expression Difference
hsa−miR−4730, rectal
(BRAF = 1; N1 = 19)
1−sided adj pval: 0.914
Frequency
0
1
2
3
4
−6
−4
−2
0
2
4
6
Tumor−Normal Expression Difference

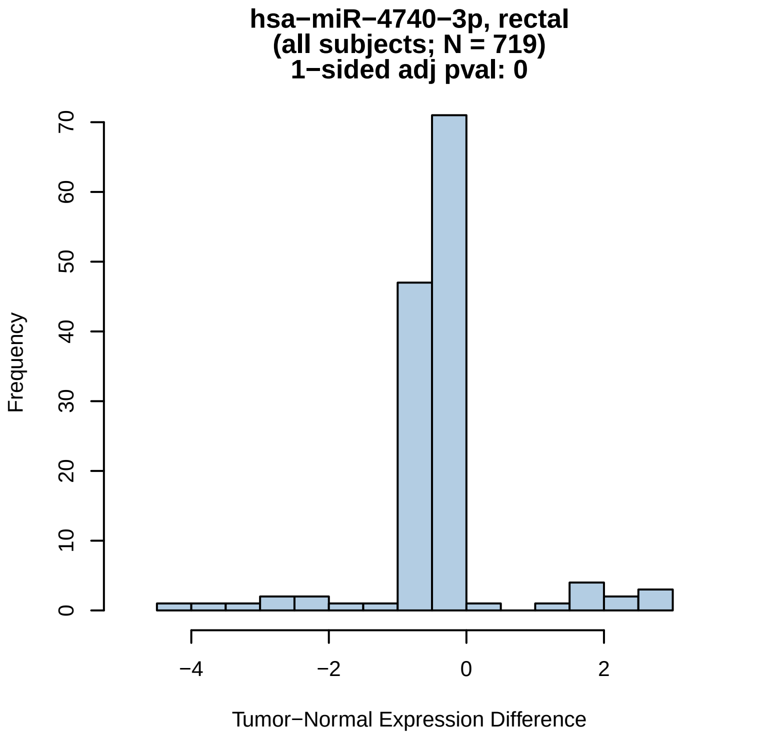
hsa−miR−4740−3p, rectal
(all subjects; N = 719)
1−sided adj pval: 0
Frequency
Tumor−Normal Expression Difference

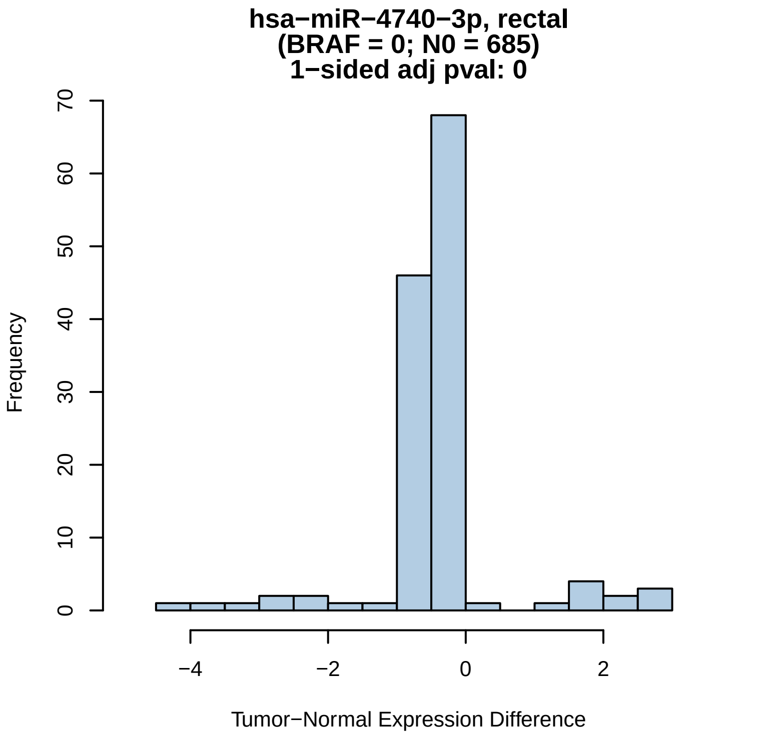
hsa−miR−4740−3p, rectal
(BRAF = 0; N0 = 685)
1−sided adj pval: 0
Frequency
Tumor−Normal Expression Difference

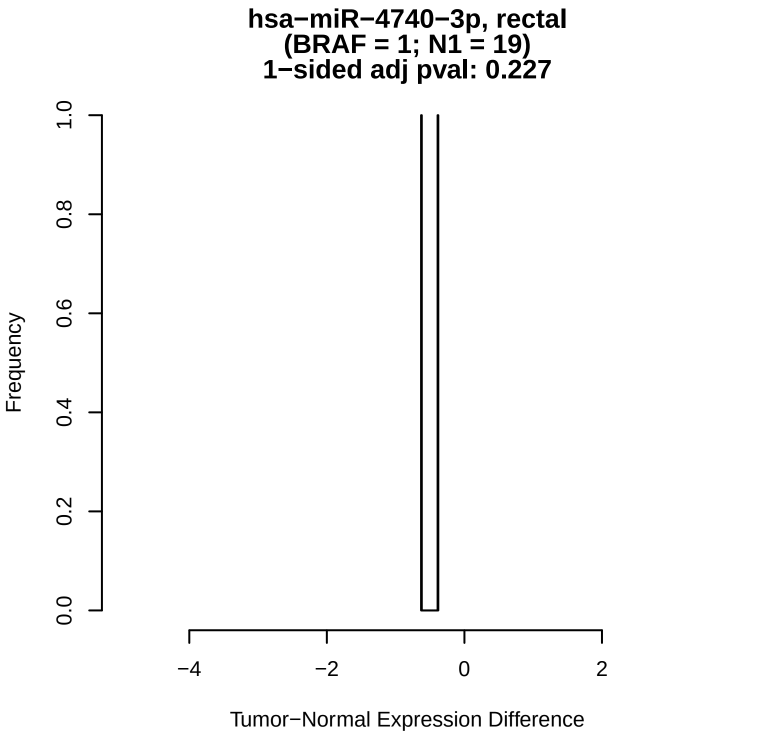
hsa−miR−4740−3p, rectal
(BRAF = 1; N1 = 19)
1−sided adj pval: 0.227
Frequency
Tumor−Normal Expression Difference

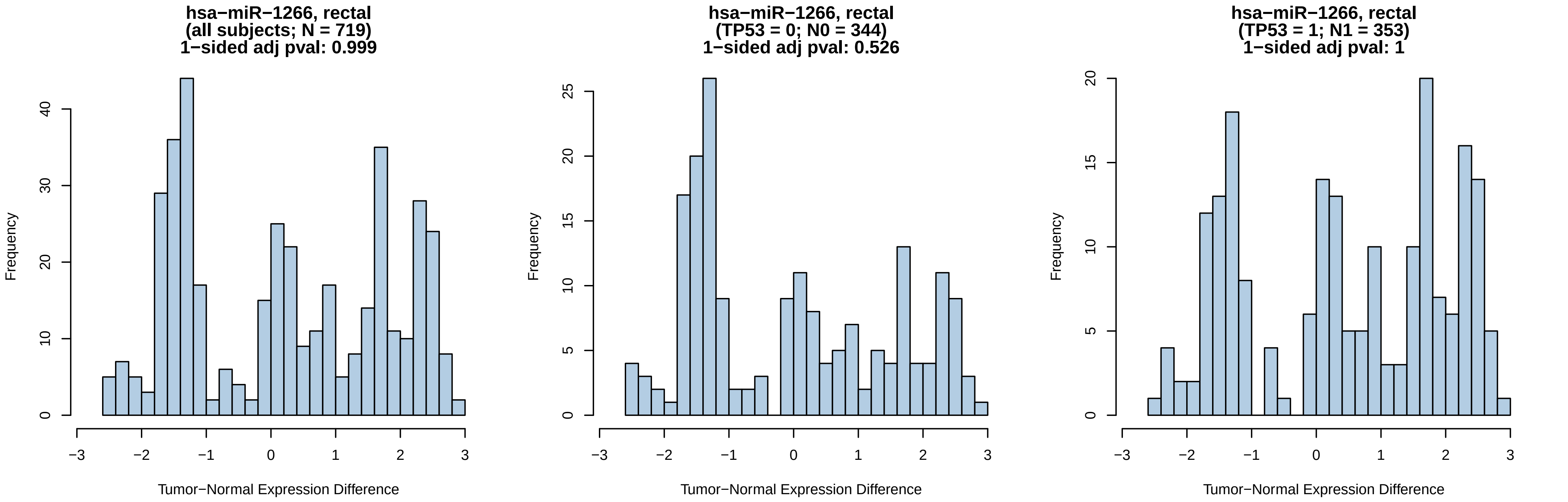
hsa−miR−1266, rectal
(all subjects; N = 719)
1−sided adj pval: 0.999
hsa−miR−1266, rectal
(TP53 = 0; N0 = 344)
1−sided adj pval: 0.526
hsa−miR−1266, rectal
(TP53 = 1; N1 = 353)
1−sided adj pval: 1
Frequency
Tumor−Normal Expression Difference

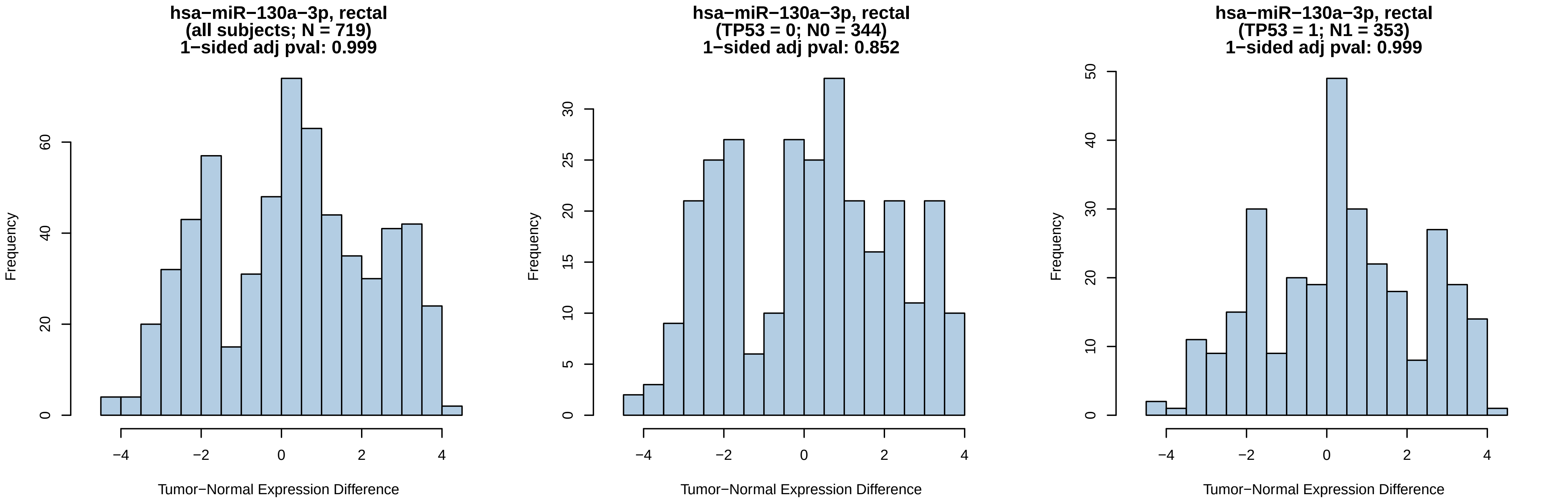
hsa−miR−130a−3p, rectal
(all subjects; N = 719)
1−sided adj pval: 0.999
hsa−miR−130a−3p, rectal
(TP53 = 0; N0 = 344)
1−sided adj pval: 0.852
hsa−miR−130a−3p, rectal
(TP53 = 1; N1 = 353)
1−sided adj pval: 0.999
Frequency
Tumor−Normal Expression Difference
0
20
40
60
5
10
15
25
30
50
−4
−2
2
4

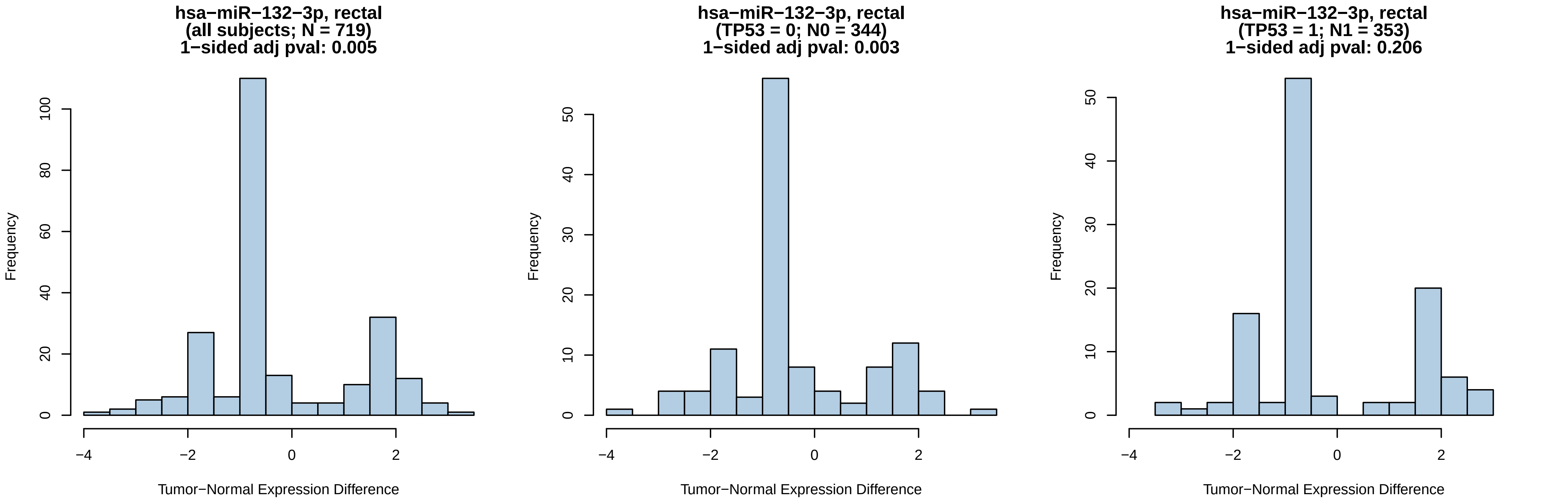
hsa−miR−132−3p, rectal
(all subjects; N = 719)
1−sided adj pval: 0.005
Frequency
Tumor−Normal Expression Difference
hsa−miR−132−3p, rectal
(TP53 = 0; N0 = 344)
1−sided adj pval: 0.003
Frequency
Tumor−Normal Expression Difference
hsa−miR−132−3p, rectal
(TP53 = 1; N1 = 353)
1−sided adj pval: 0.206
Frequency
Tumor−Normal Expression Difference

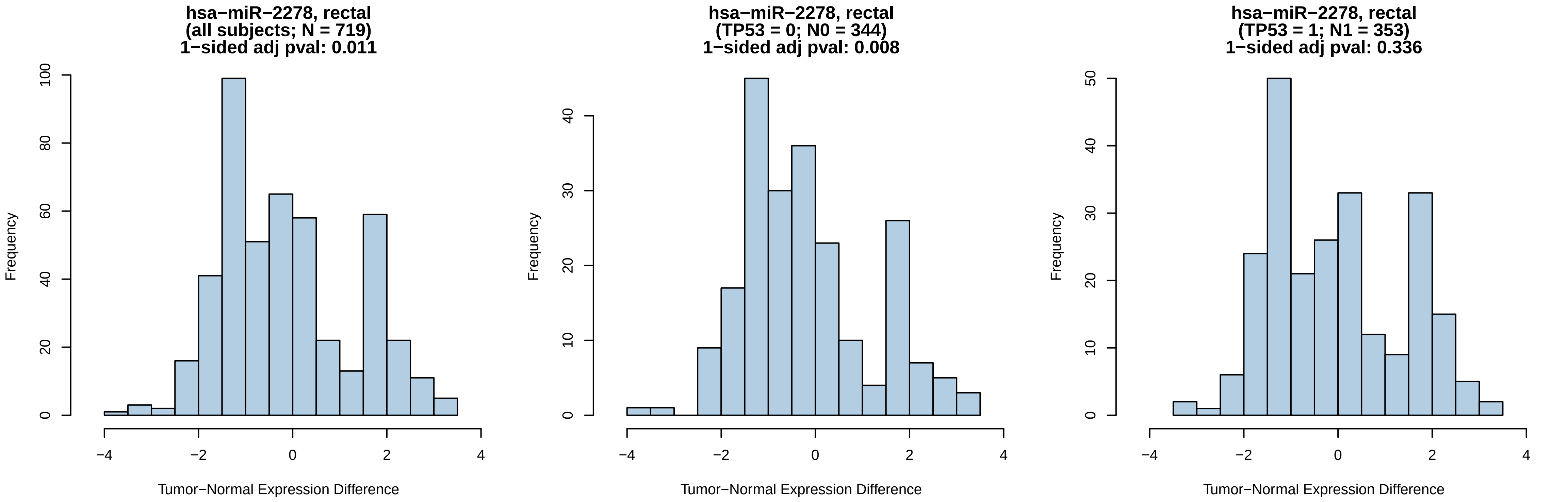
hsa−miR−2278, rectal
(all subjects; N = 719)
1−sided adj pval: 0.011
Frequency
0
20
40
60
80
100
−4
−2
0
2
4
Tumor−Normal Expression Difference
hsa−miR−2278, rectal
(TP53 = 0; N0 = 344)
1−sided adj pval: 0.008
Frequency
0
10
20
30
40
−4
−2
0
2
4
Tumor−Normal Expression Difference
hsa−miR−2278, rectal
(TP53 = 1; N1 = 353)
1−sided adj pval: 0.336
Frequency
0
10
20
30
40
50
−4
−2
0
2
4
Tumor−Normal Expression Difference

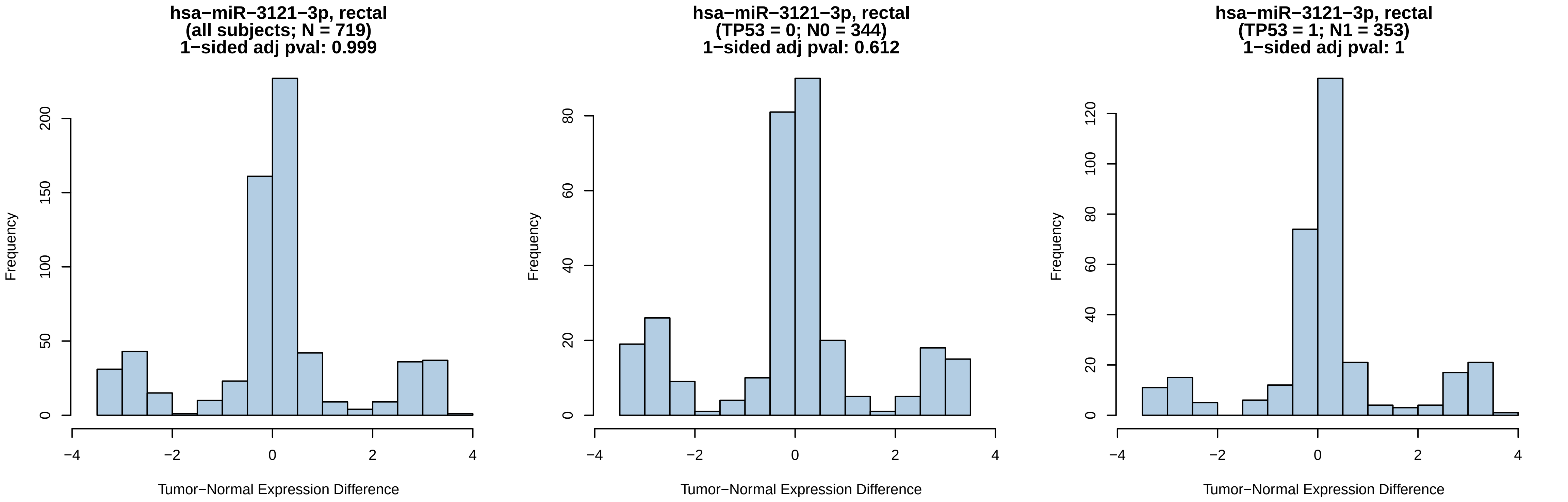

hsa−miR−3121−3p, rectal
(all subjects; N = 719)
1−sided adj pval: 0.999
Frequency
0
50
100
150
200
−4
−2
0
2
4
Tumor−Normal Expression Difference
hsa−miR−3121−3p, rectal
(TP53 = 0; N0 = 344)
1−sided adj pval: 0.612
Frequency
0
20
40
60
80
−4
−2
0
2
4
Tumor−Normal Expression Difference
hsa−miR−3121−3p, rectal
(TP53 = 1; N1 = 353)
1−sided adj pval: 1
Frequency
0
20
40
60
80
100
120
−4
−2
0
2
4
Tumor−Normal Expression Difference

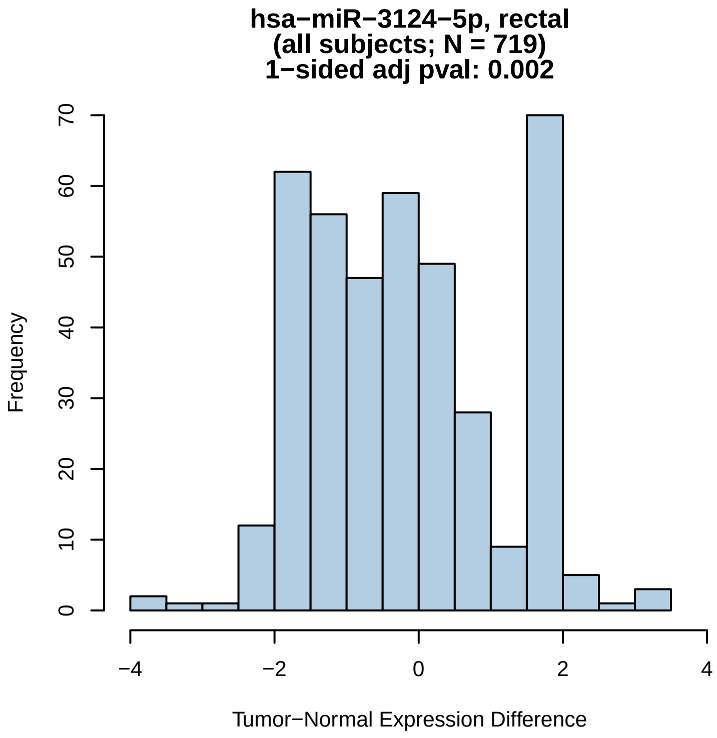
hsa−miR−3124−5p, rectal
(all subjects; N = 719)
1−sided adj pval: 0.002
Frequency
0
10
20
30
40
50
60
70
−4
−2
0
2
4
Tumor−Normal Expression Difference

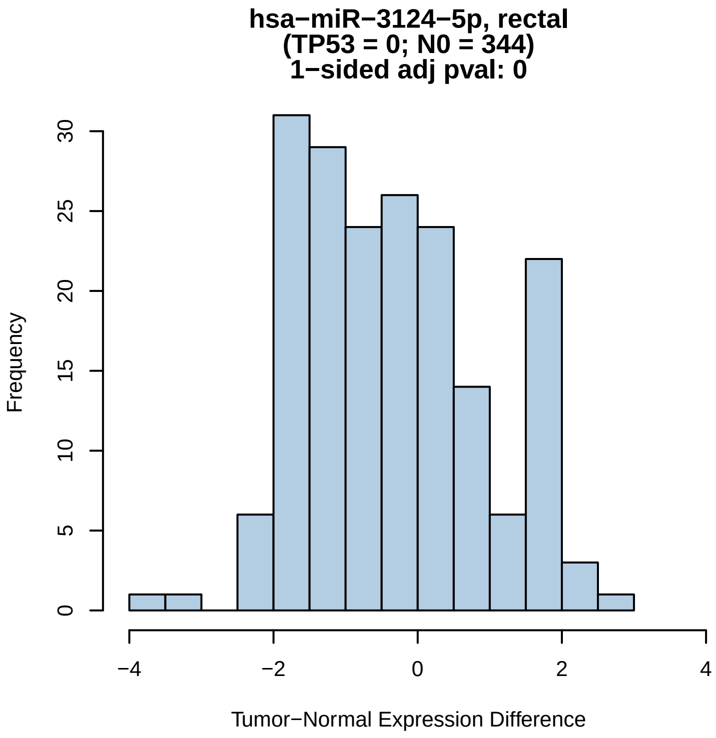
hsa−miR−3124−5p, rectal
(TP53 = 0; N0 = 344)
1−sided adj pval: 0
Frequency
0
5
10
15
20
25
30
−4
−2
0
2
4
Tumor−Normal Expression Difference

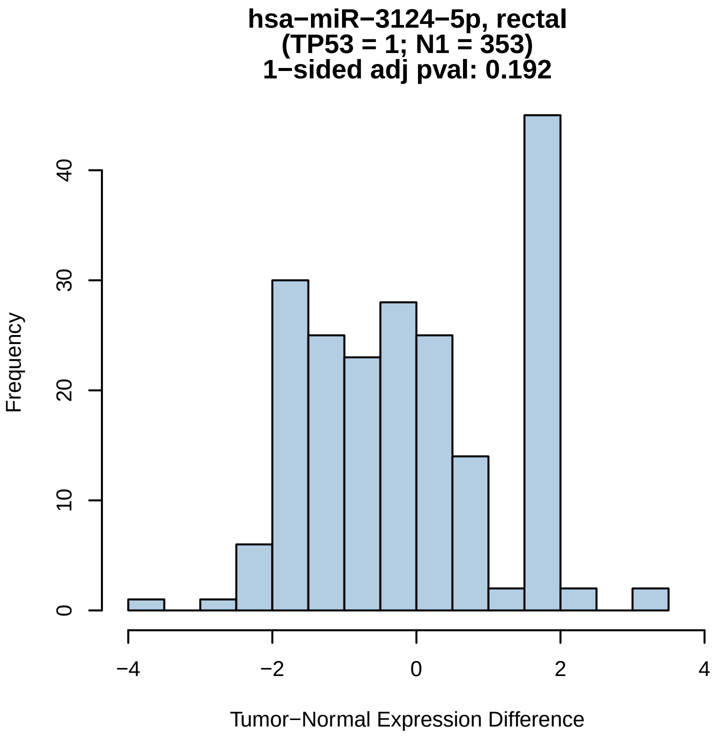
hsa−miR−3124−5p, rectal
(TP53 = 1; N1 = 353)
1−sided adj pval: 0.192
Frequency
0
10
20
30
40
−4
−2
0
2
4
Tumor−Normal Expression Difference

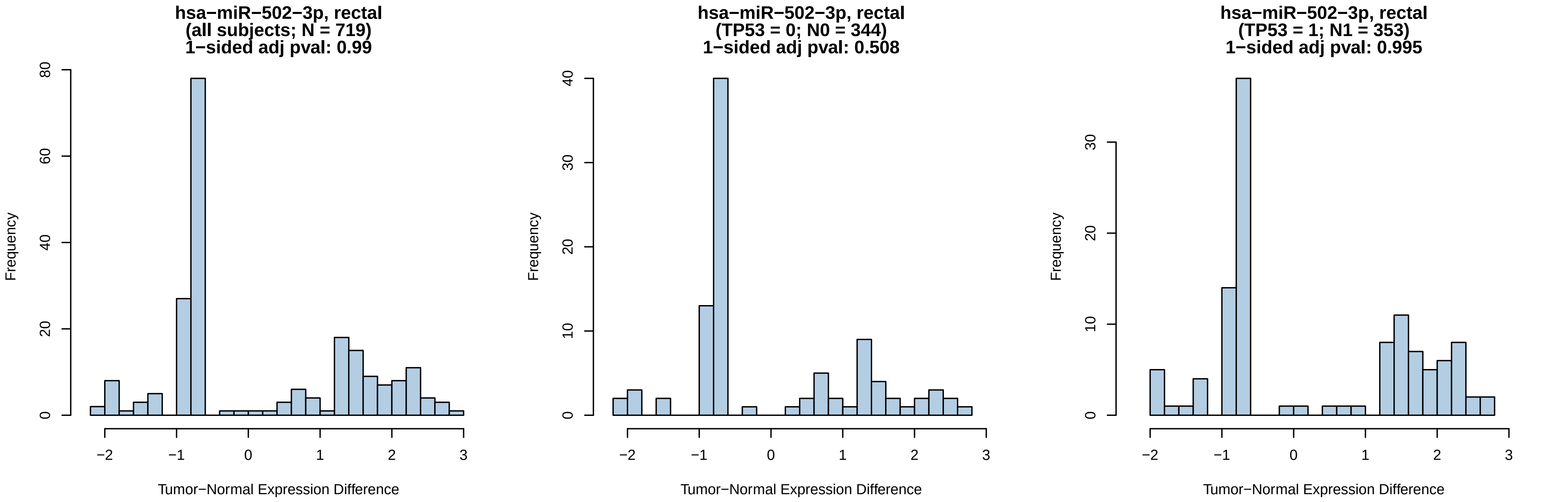
hsa−miR−502−3p, rectal
(all subjects; N = 719)
1−sided adj pval: 0.99
Frequency
Tumor−Normal Expression Difference
hsa−miR−502−3p, rectal
(TP53 = 0; N0 = 344)
1−sided adj pval: 0.508
Frequency
Tumor−Normal Expression Difference
hsa−miR−502−3p, rectal
(TP53 = 1; N1 = 353)
1−sided adj pval: 0.995
Frequency
Tumor−Normal Expression Difference

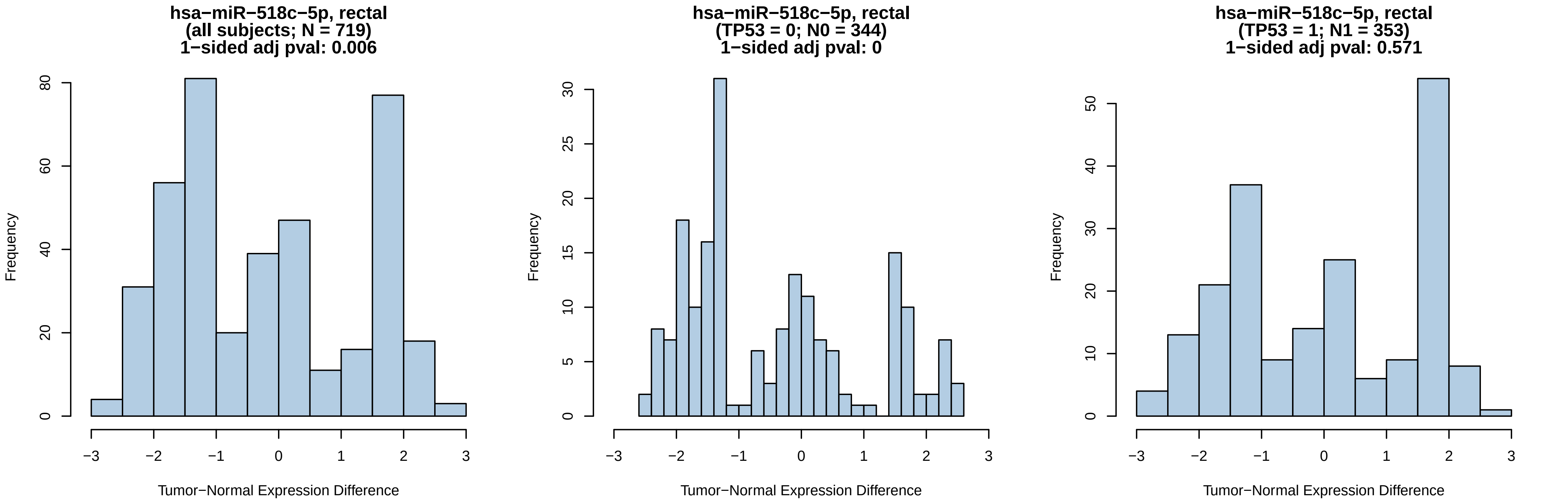

hsa−miR−518c−5p, rectal
(all subjects; N = 719)
1−sided adj pval: 0.006
Frequency
Tumor−Normal Expression Difference
hsa−miR−518c−5p, rectal
(TP53 = 0; N0 = 344)
1−sided adj pval: 0
Frequency
Tumor−Normal Expression Difference
hsa−miR−518c−5p, rectal
(TP53 = 1; N1 = 353)
1−sided adj pval: 0.571
Frequency
Tumor−Normal Expression Difference

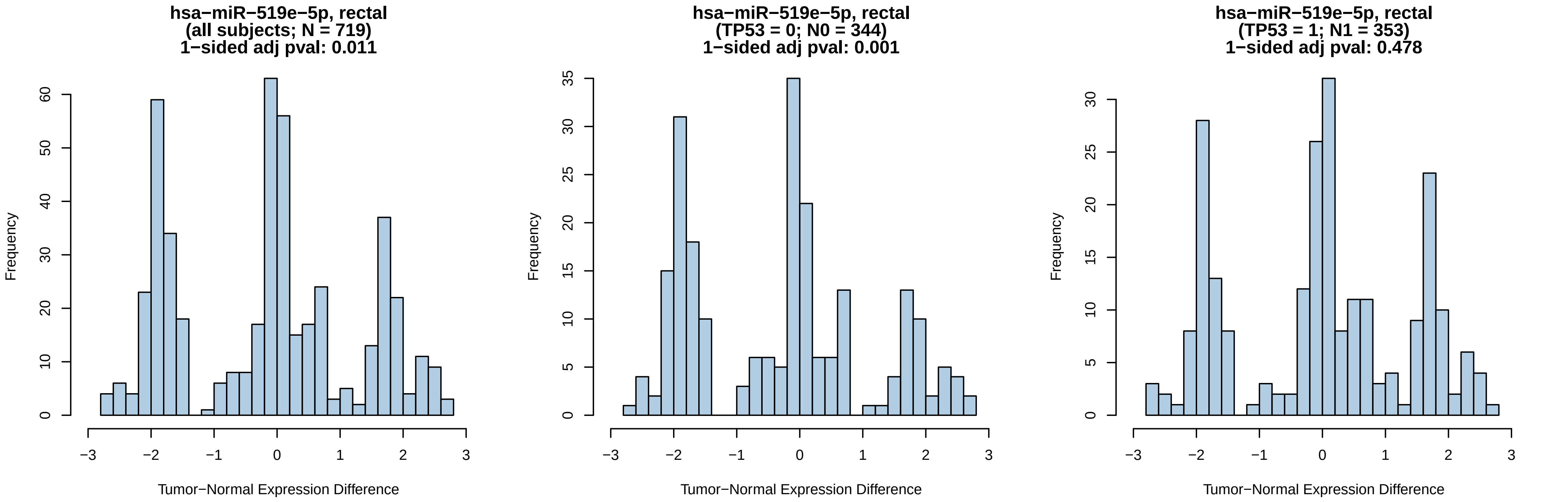
hsa−miR−519e−5p, rectal
(all subjects; N = 719)
1−sided adj pval: 0.011
hsa−miR−519e−5p, rectal
(TP53 = 0; N0 = 344)
1−sided adj pval: 0.001
hsa−miR−519e−5p, rectal
(TP53 = 1; N1 = 353)
1−sided adj pval: 0.478
Frequency
Tumor−Normal Expression Difference

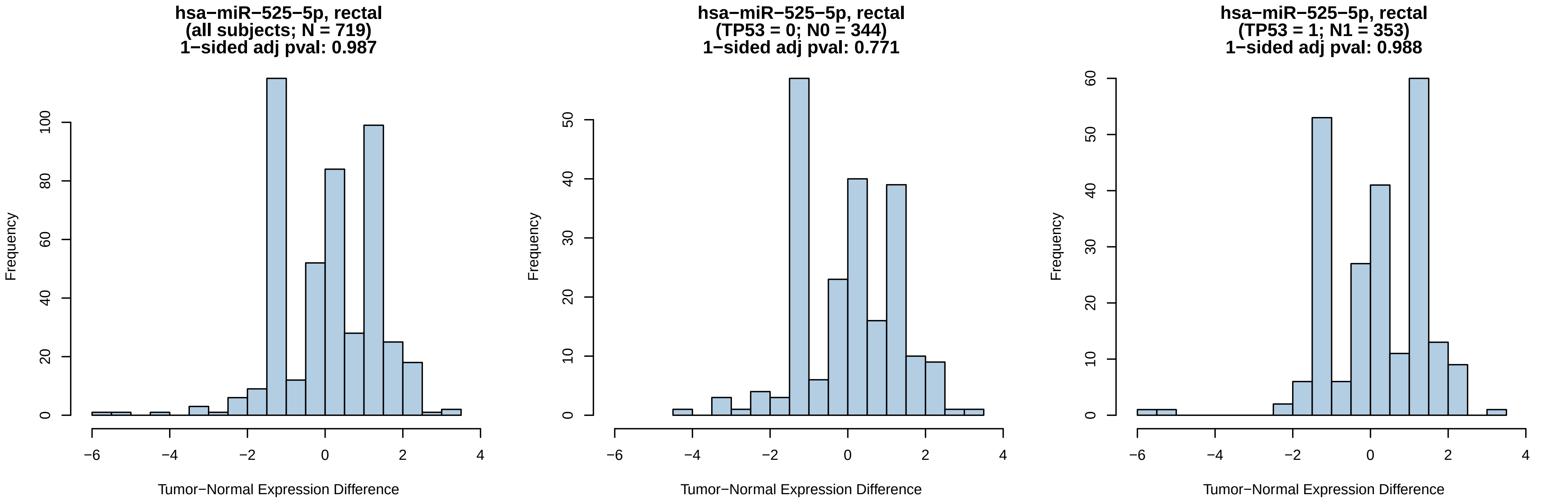

hsa−miR−525−5p, rectal
(all subjects; N = 719)
1−sided adj pval: 0.987
Frequency
Tumor−Normal Expression Difference
hsa−miR−525−5p, rectal
(TP53 = 0; N0 = 344)
1−sided adj pval: 0.771
Frequency
Tumor−Normal Expression Difference
hsa−miR−525−5p, rectal
(TP53 = 1; N1 = 353)
1−sided adj pval: 0.988
Frequency
Tumor−Normal Expression Difference

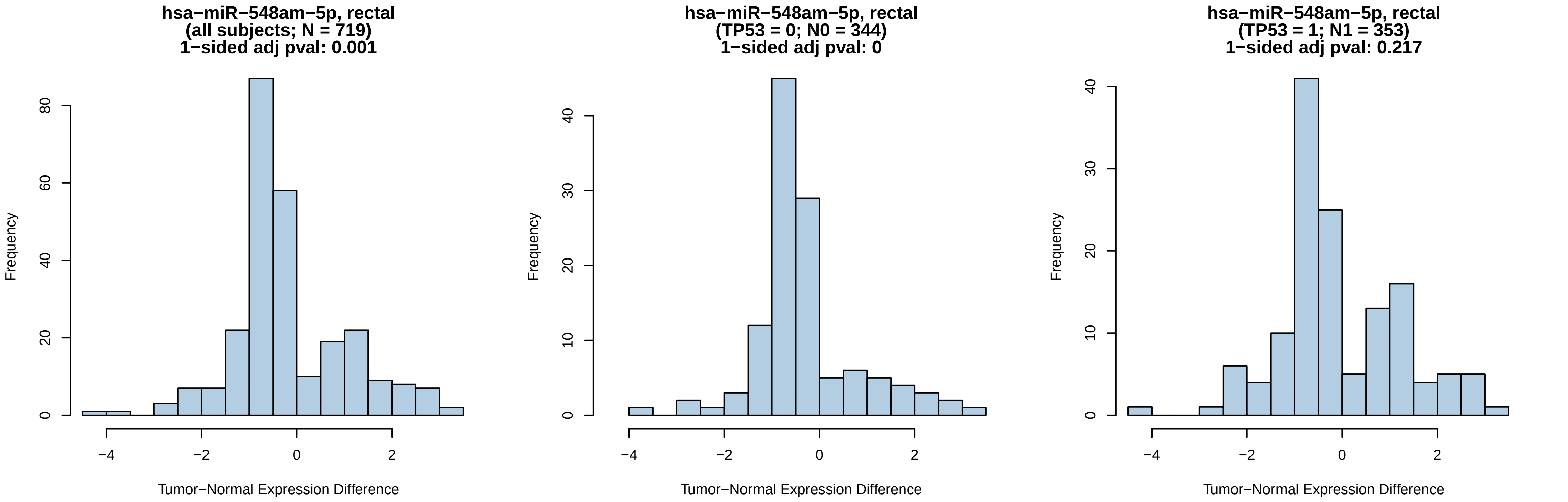

hsa−miR−548am−5p, rectal
(all subjects; N = 719)
1−sided adj pval: 0.001
Frequency
0
20
40
60
80
−4
−2
0
2
Tumor−Normal Expression Difference
hsa−miR−548am−5p, rectal
(TP53 = 0; N0 = 344)
1−sided adj pval: 0
Frequency
0
10
20
30
40
−4
−2
0
2
Tumor−Normal Expression Difference
hsa−miR−548am−5p, rectal
(TP53 = 1; N1 = 353)
1−sided adj pval: 0.217
Frequency
0
10
20
30
40
−4
−2
0
2
Tumor−Normal Expression Difference

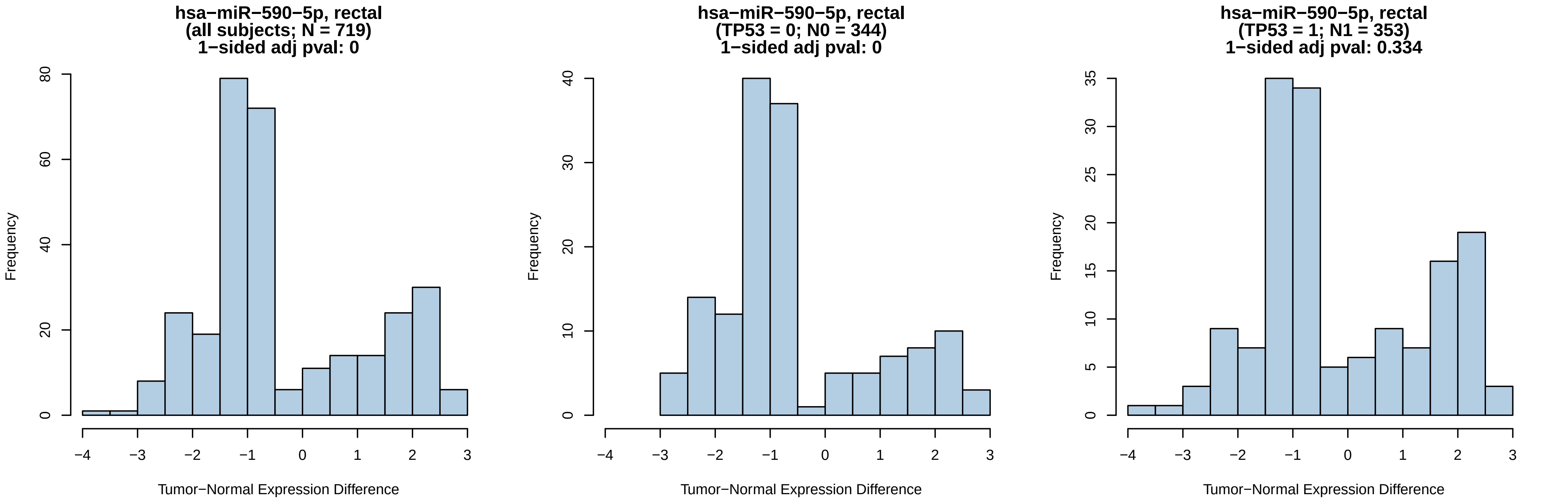

hsa−miR−590−5p, rectal
(all subjects; N = 719)
1−sided adj pval: 0
Frequency
Tumor−Normal Expression Difference
hsa−miR−590−5p, rectal
(TP53 = 0; N0 = 344)
1−sided adj pval: 0
Frequency
Tumor−Normal Expression Difference
hsa−miR−590−5p, rectal
(TP53 = 1; N1 = 353)
1−sided adj pval: 0.334
Frequency
Tumor−Normal Expression Difference

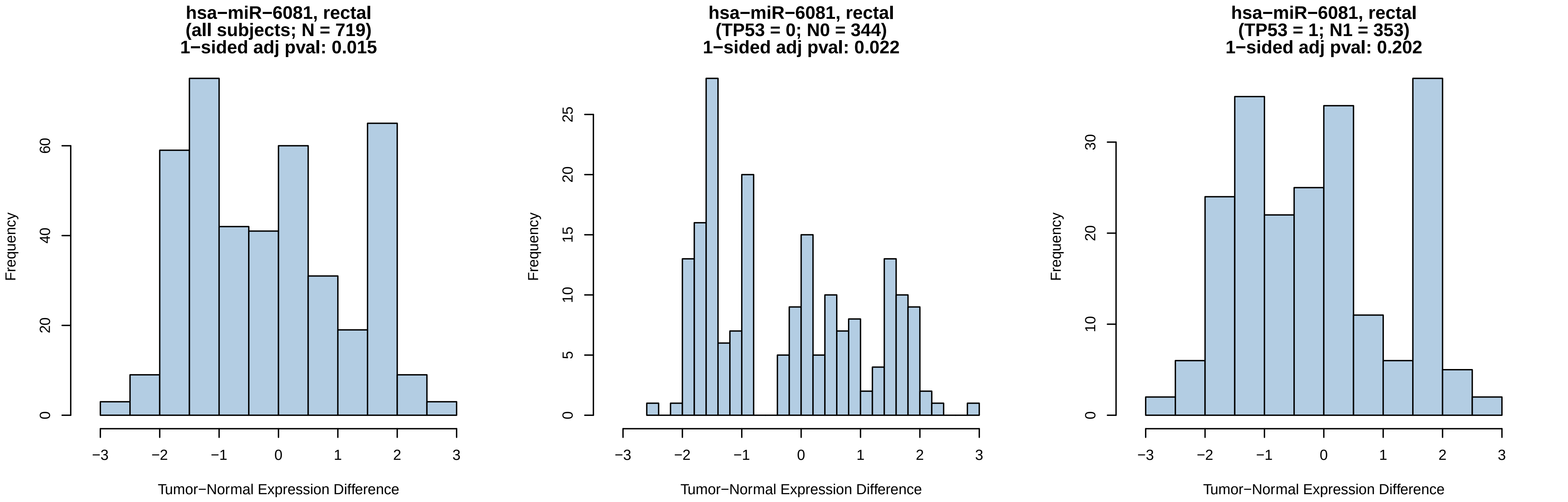

hsa−miR−6081, rectal
(all subjects; N = 719)
1−sided adj pval: 0.015
Frequency
Tumor−Normal Expression Difference
hsa−miR−6081, rectal
(TP53 = 0; N0 = 344)
1−sided adj pval: 0.022
Frequency
Tumor−Normal Expression Difference
hsa−miR−6081, rectal
(TP53 = 1; N1 = 353)
1−sided adj pval: 0.202
Frequency
Tumor−Normal Expression Difference

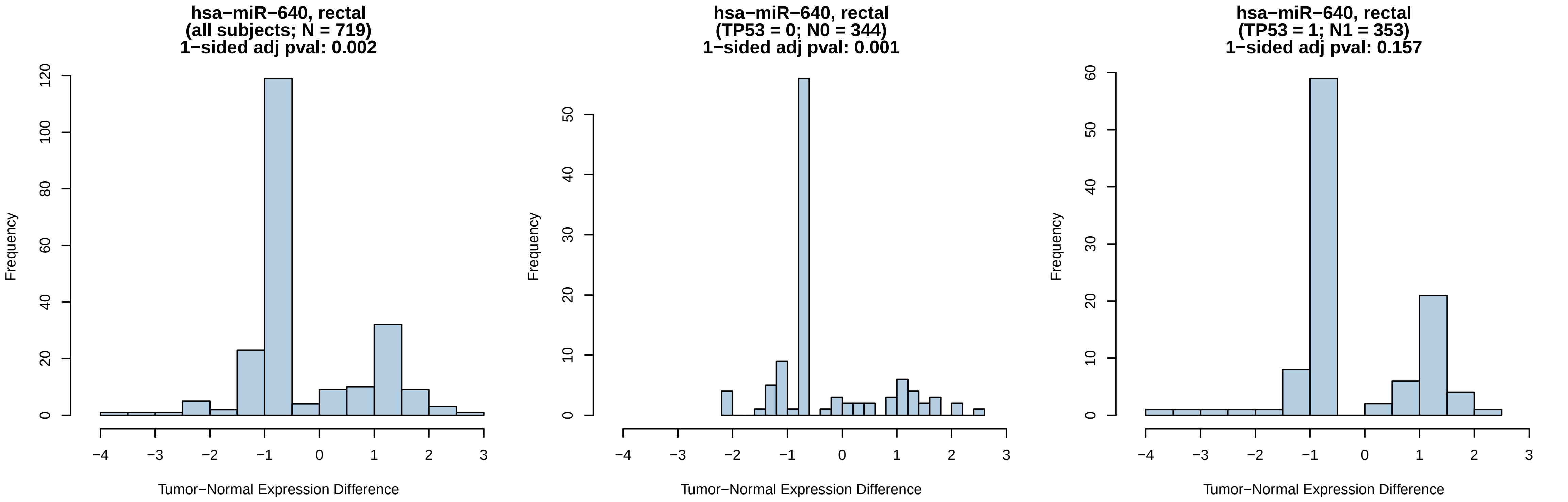
hsa−miR−640, rectal
(all subjects; N = 719)
1−sided adj pval: 0.002
hsa−miR−640, rectal
(TP53 = 0; N0 = 344)
1−sided adj pval: 0.001
hsa−miR−640, rectal
(TP53 = 1; N1 = 353)
1−sided adj pval: 0.157
Frequency
Tumor−Normal Expression Difference

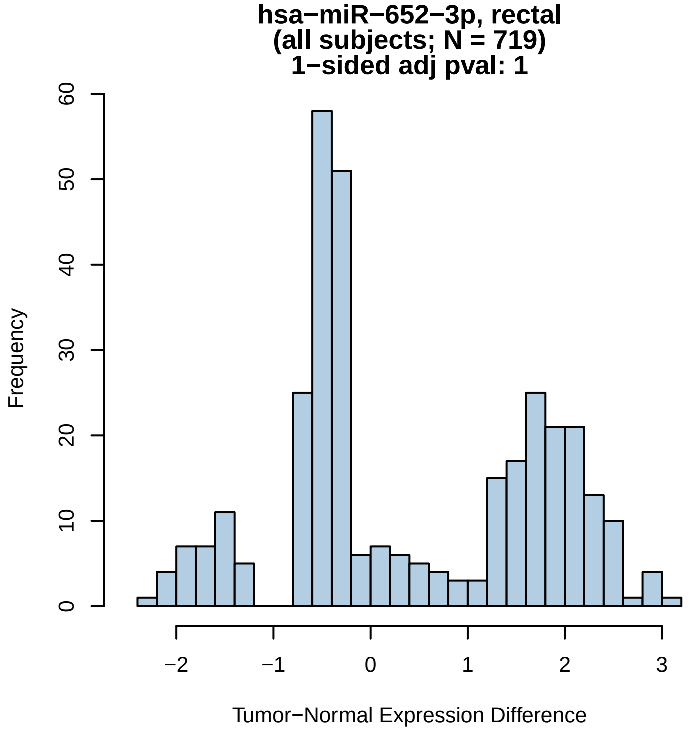

hsa−miR−652−3p, rectal
(all subjects; N = 719)
1−sided adj pval: 1
Frequency
Tumor−Normal Expression Difference

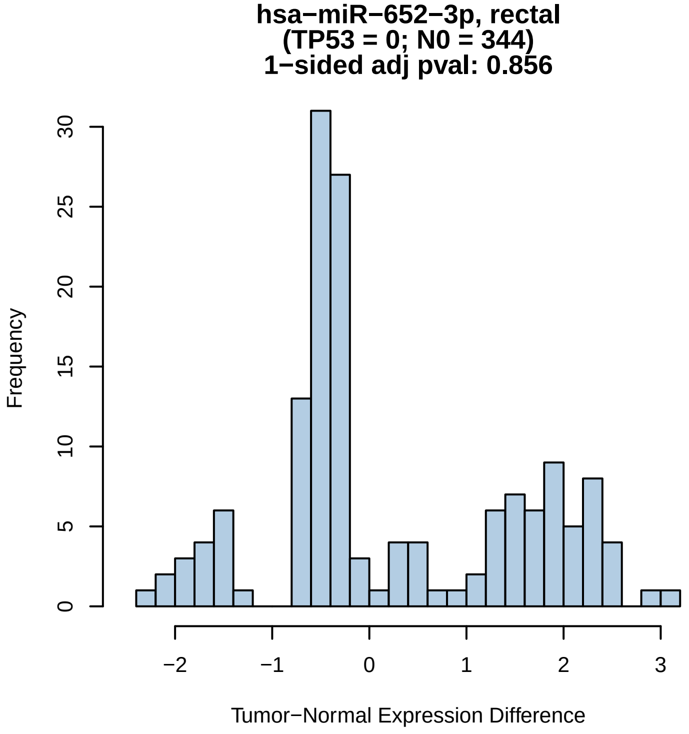

hsa−miR−652−3p, rectal
(TP53 = 0; N0 = 344)
1−sided adj pval: 0.856
Frequency
Tumor−Normal Expression Difference

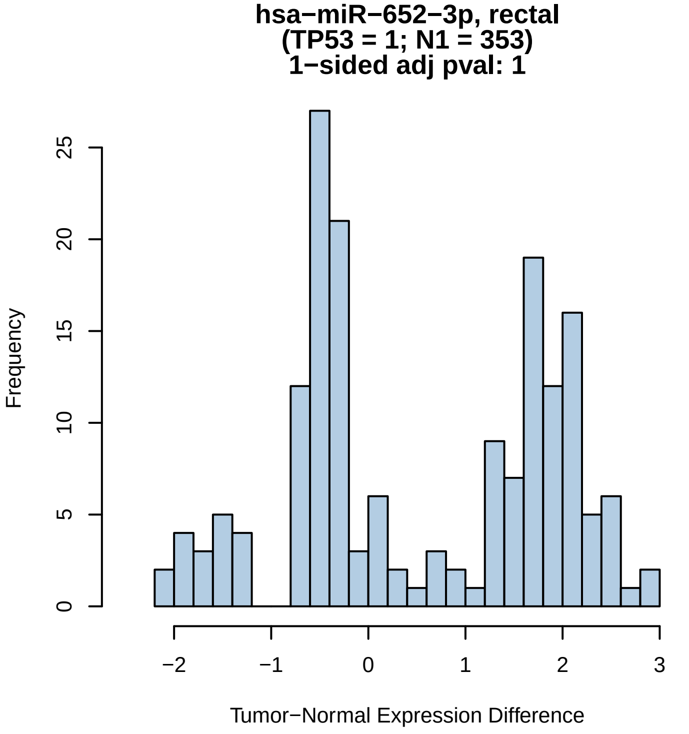

hsa−miR−652−3p, rectal
(TP53 = 1; N1 = 353)
1−sided adj pval: 1
Frequency
Tumor−Normal Expression Difference

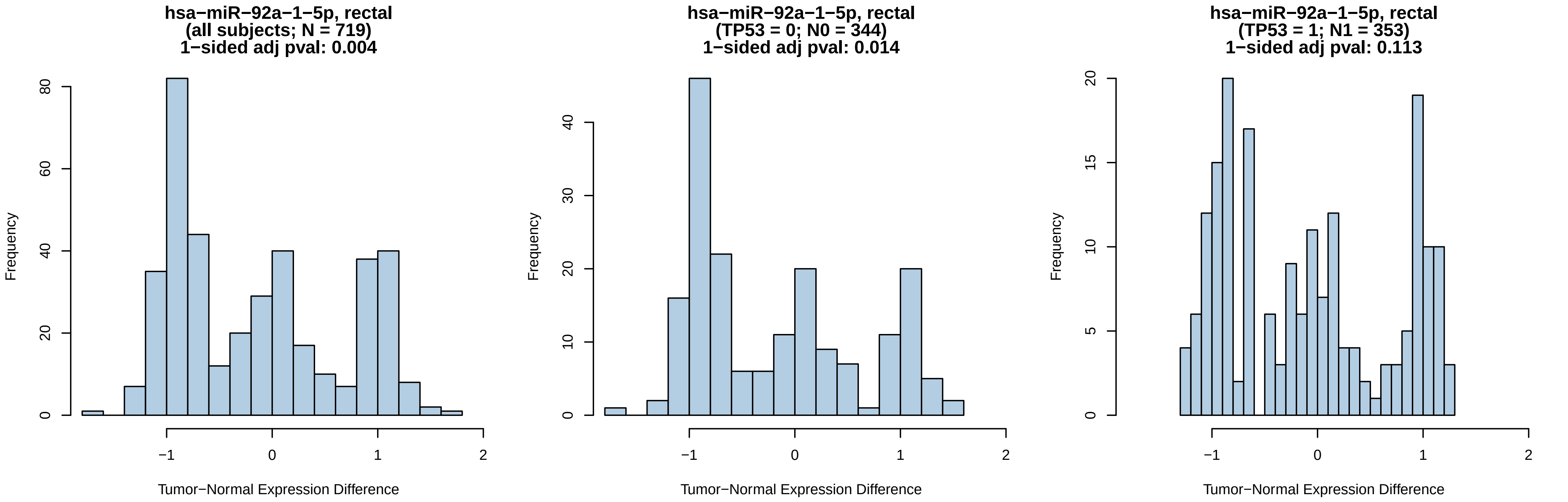

hsa−miR−92a−1−5p, rectal
(all subjects; N = 719)
1−sided adj pval: 0.004
Frequency
Tumor−Normal Expression Difference
hsa−miR−92a−1−5p, rectal
(TP53 = 0; N0 = 344)
1−sided adj pval: 0.014
Frequency
Tumor−Normal Expression Difference
hsa−miR−92a−1−5p, rectal
(TP53 = 1; N1 = 353)
1−sided adj pval: 0.113
Frequency
Tumor−Normal Expression Difference

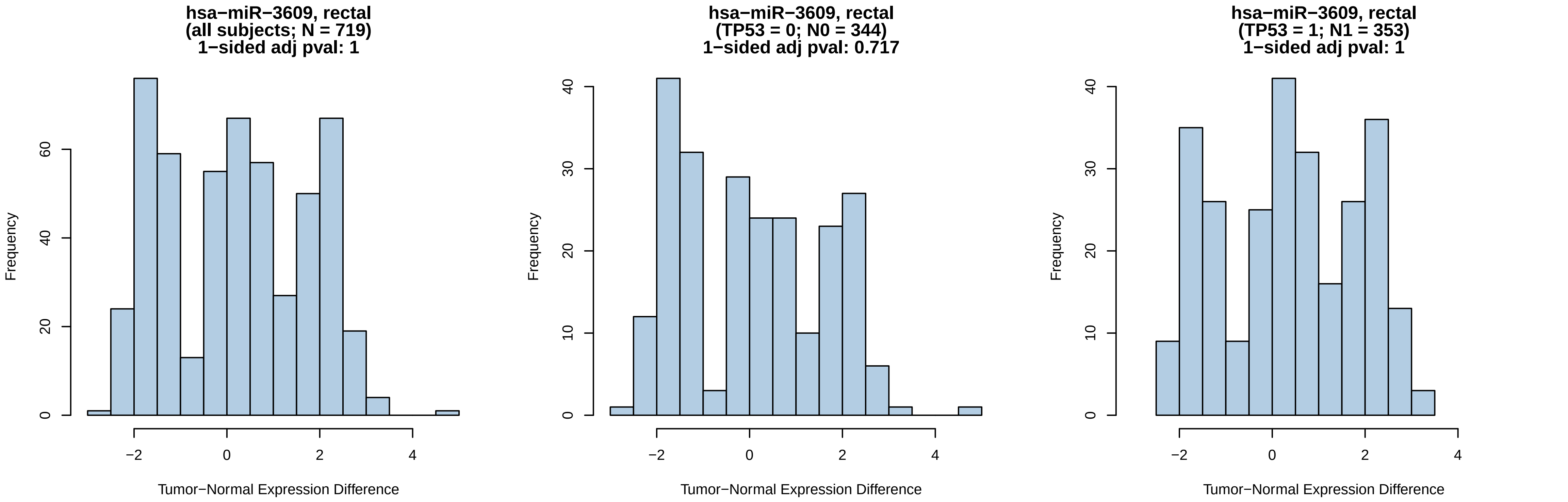

hsa−miR−3609, rectal
(all subjects; N = 719)
1−sided adj pval: 1
Frequency
0
20
40
60
−2
0
2
4
Tumor−Normal Expression Difference
hsa−miR−3609, rectal
(TP53 = 0; N0 = 344)
1−sided adj pval: 0.717
Frequency
0
10
20
30
40
−2
0
2
4
Tumor−Normal Expression Difference
hsa−miR−3609, rectal
(TP53 = 1; N1 = 353)
1−sided adj pval: 1
Frequency
0
10
20
30
40
−2
0
2
4
Tumor−Normal Expression Difference

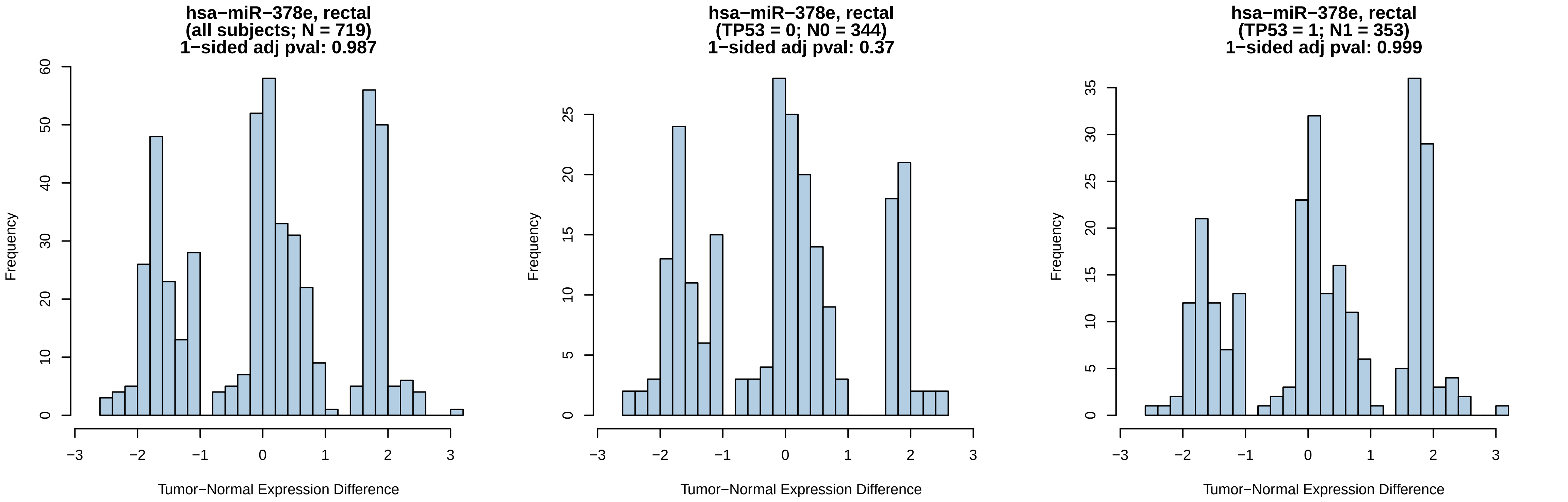
hsa−miR−378e, rectal
(all subjects; N = 719)
1−sided adj pval: 0.987
Frequency
Tumor−Normal Expression Difference
hsa−miR−378e, rectal
(TP53 = 0; N0 = 344)
1−sided adj pval: 0.37
Frequency
Tumor−Normal Expression Difference
hsa−miR−378e, rectal
(TP53 = 1; N1 = 353)
1−sided adj pval: 0.999
Frequency
Tumor−Normal Expression Difference

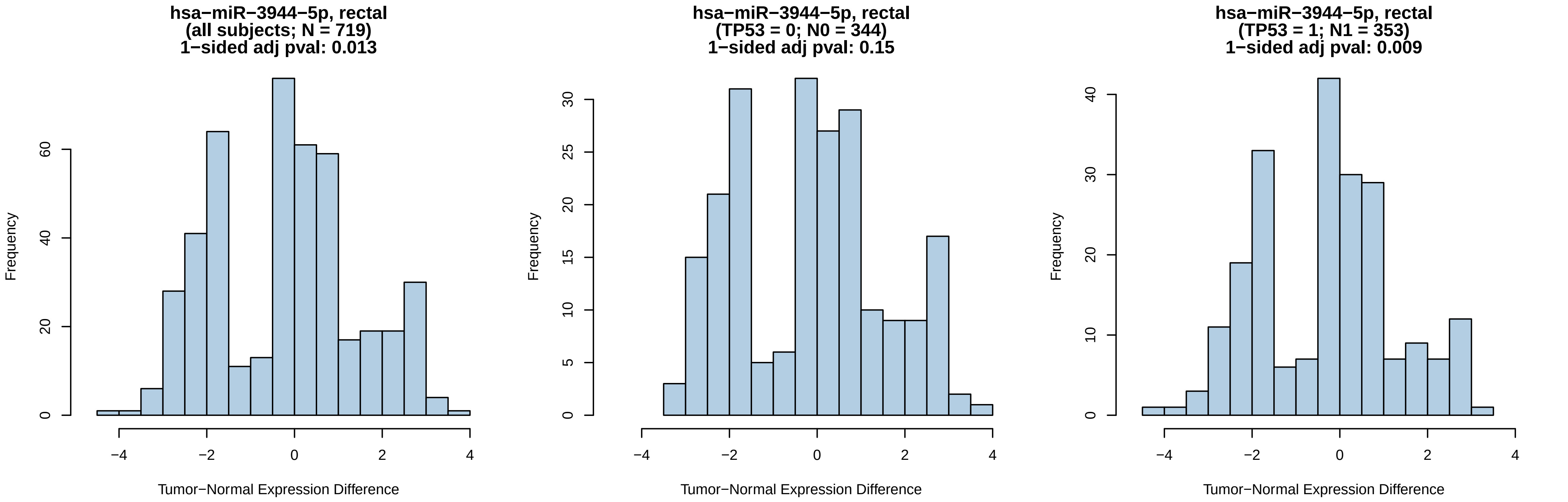

hsa−miR−3944−5p, rectal
(all subjects; N = 719)
1−sided adj pval: 0.013
Frequency
Tumor−Normal Expression Difference
hsa−miR−3944−5p, rectal
(TP53 = 0; N0 = 344)
1−sided adj pval: 0.15
Frequency
Tumor−Normal Expression Difference
hsa−miR−3944−5p, rectal
(TP53 = 1; N1 = 353)
1−sided adj pval: 0.009
Frequency
Tumor−Normal Expression Difference

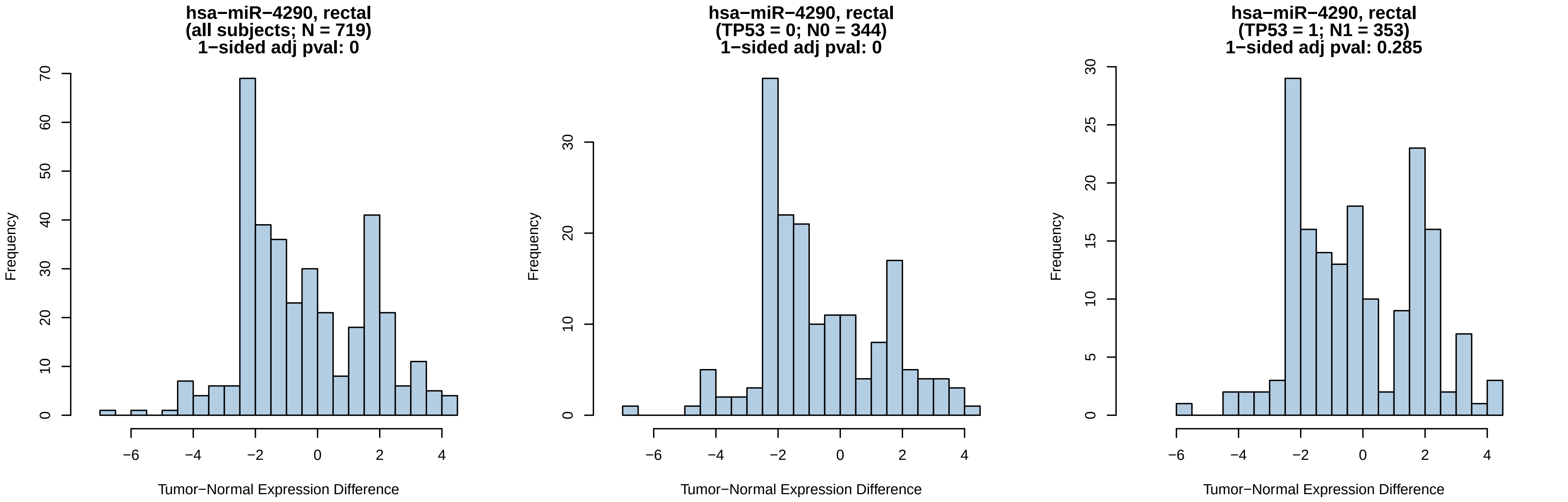

hsa−miR−4290, rectal
(all subjects; N = 719)
1−sided adj pval: 0
Frequency
Tumor−Normal Expression Difference
hsa−miR−4290, rectal
(TP53 = 0; N0 = 344)
1−sided adj pval: 0
Frequency
Tumor−Normal Expression Difference
hsa−miR−4290, rectal
(TP53 = 1; N1 = 353)
1−sided adj pval: 0.285
Frequency
Tumor−Normal Expression Difference

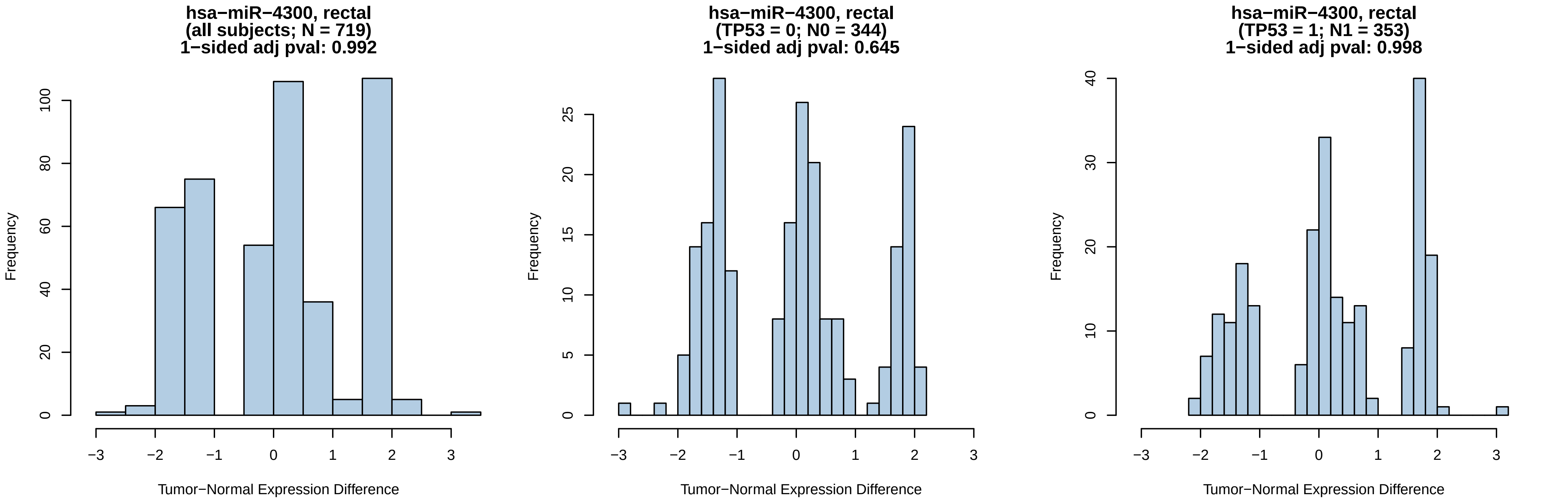

hsa−miR−4300, rectal
(all subjects; N = 719)
1−sided adj pval: 0.992
Frequency
Tumor−Normal Expression Difference
hsa−miR−4300, rectal
(TP53 = 0; N0 = 344)
1−sided adj pval: 0.645
Frequency
Tumor−Normal Expression Difference
hsa−miR−4300, rectal
(TP53 = 1; N1 = 353)
1−sided adj pval: 0.998
Frequency
Tumor−Normal Expression Difference

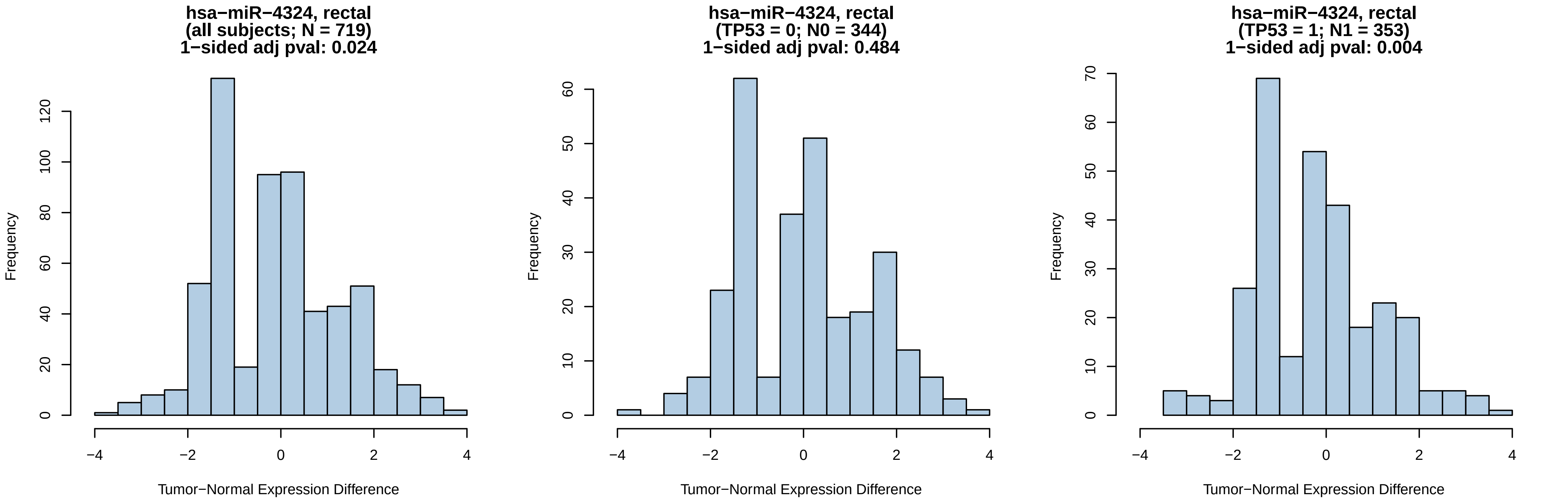
hsa−miR−4324, rectal
(all subjects; N = 719)
1−sided adj pval: 0.024
Frequency
0
20
40
60
80
100
120
−4
−2
0
2
4
Tumor−Normal Expression Difference
hsa−miR−4324, rectal
(TP53 = 0; N0 = 344)
1−sided adj pval: 0.484
Frequency
0
10
20
30
40
50
60
−4
−2
0
2
4
Tumor−Normal Expression Difference
hsa−miR−4324, rectal
(TP53 = 1; N1 = 353)
1−sided adj pval: 0.004
Frequency
0
10
20
30
40
50
60
70
−4
−2
0
2
4
Tumor−Normal Expression Difference

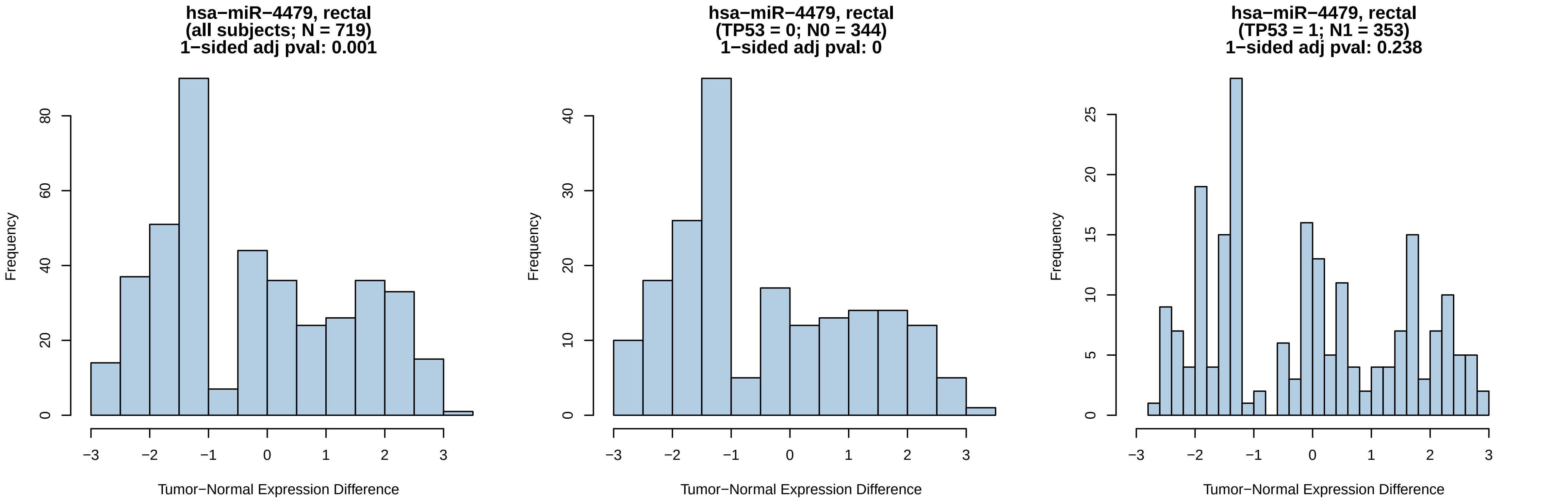

hsa−miR−4479, rectal
(all subjects; N = 719)
1−sided adj pval: 0.001
hsa−miR−4479, rectal
(TP53 = 0; N0 = 344)
1−sided adj pval: 0
hsa−miR−4479, rectal
(TP53 = 1; N1 = 353)
1−sided adj pval: 0.238
Frequency
Tumor−Normal Expression Difference

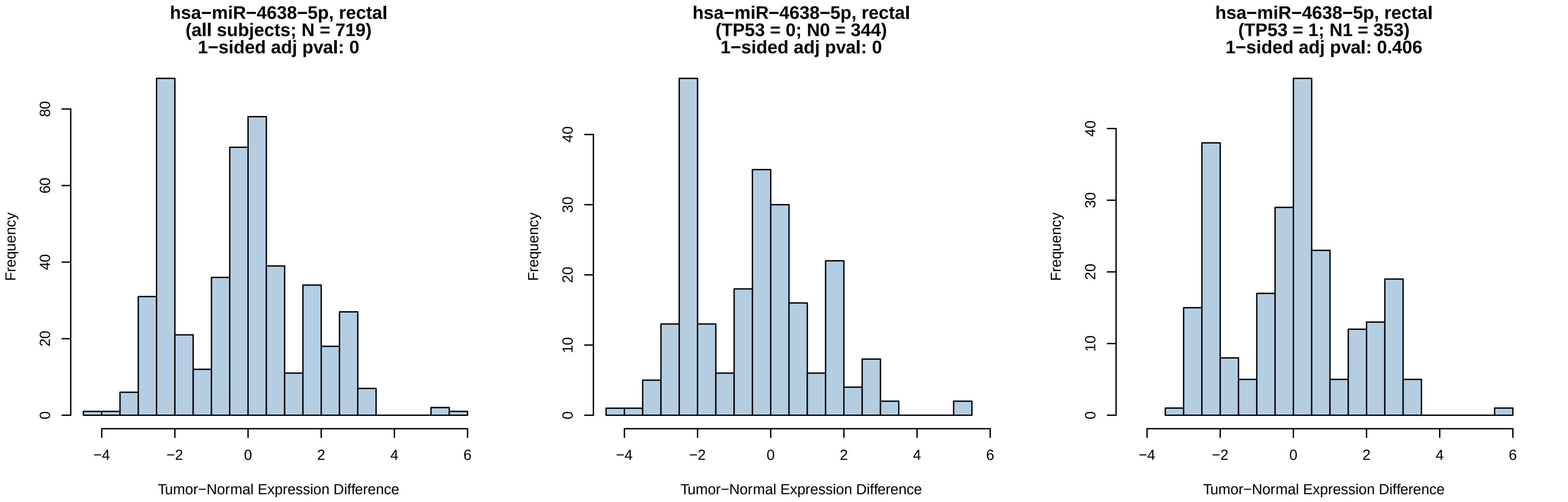
hsa−miR−4638−5p, rectal
(all subjects; N = 719)
1−sided adj pval: 0
hsa−miR−4638−5p, rectal
(TP53 = 0; N0 = 344)
1−sided adj pval: 0
hsa−miR−4638−5p, rectal
(TP53 = 1; N1 = 353)
1−sided adj pval: 0.406
Frequency
Tumor−Normal Expression Difference
0
20
40
60
80
10
30
−4
−2
2
4
6

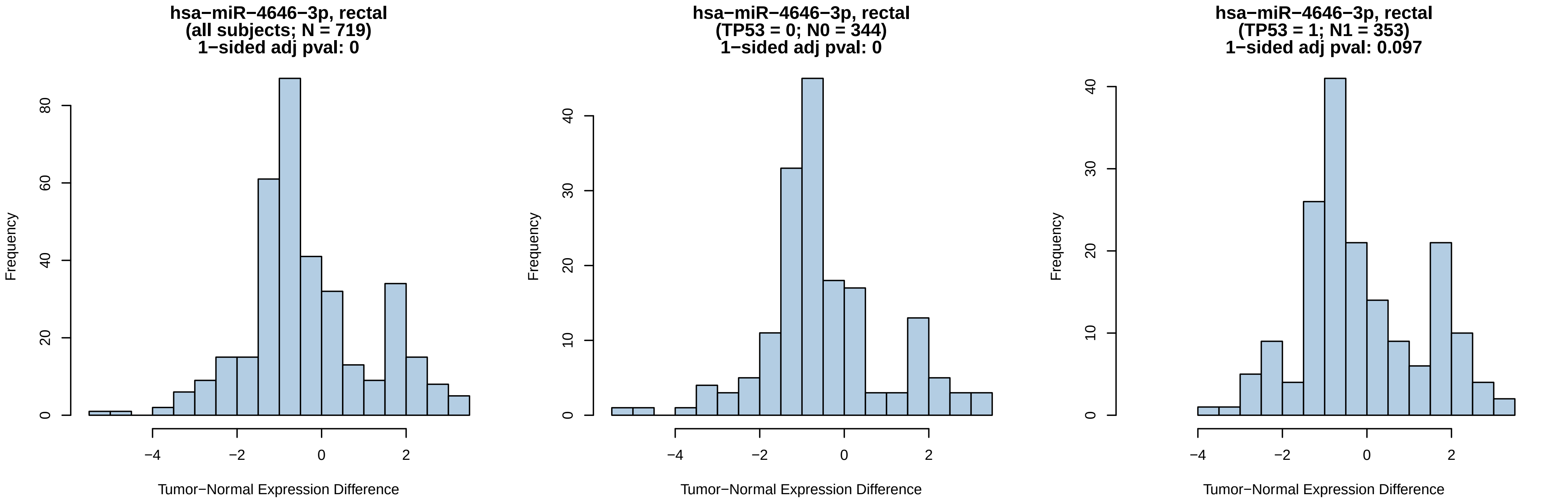
hsa−miR−4646−3p, rectal
(all subjects; N = 719)
1−sided adj pval: 0
Frequency
0
20
40
60
80
−4
−2
0
2
Tumor−Normal Expression Difference
hsa−miR−4646−3p, rectal
(TP53 = 0; N0 = 344)
1−sided adj pval: 0
Frequency
0
10
20
30
40
−4
−2
0
2
Tumor−Normal Expression Difference
hsa−miR−4646−3p, rectal
(TP53 = 1; N1 = 353)
1−sided adj pval: 0.097
Frequency
0
10
20
30
40
−4
−2
0
2
Tumor−Normal Expression Difference

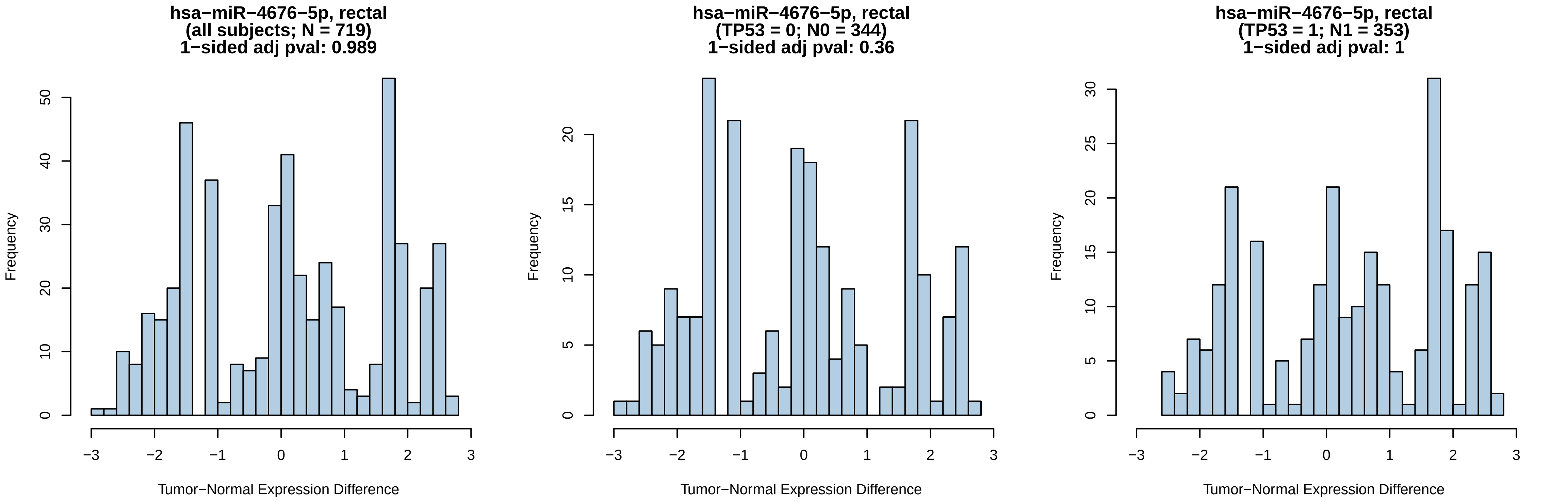

hsa−miR−4676−5p, rectal
(all subjects; N = 719)
1−sided adj pval: 0.989
Frequency
0
10
20
30
40
50
−3
−2
−1
0
1
2
3
Tumor−Normal Expression Difference
hsa−miR−4676−5p, rectal
(TP53 = 0; N0 = 344)
1−sided adj pval: 0.36
Frequency
0
5
10
15
20
Tumor−Normal Expression Difference
hsa−miR−4676−5p, rectal
(TP53 = 1; N1 = 353)
1−sided adj pval: 1
Frequency
0
5
10
15
20
25
30
Tumor−Normal Expression Difference

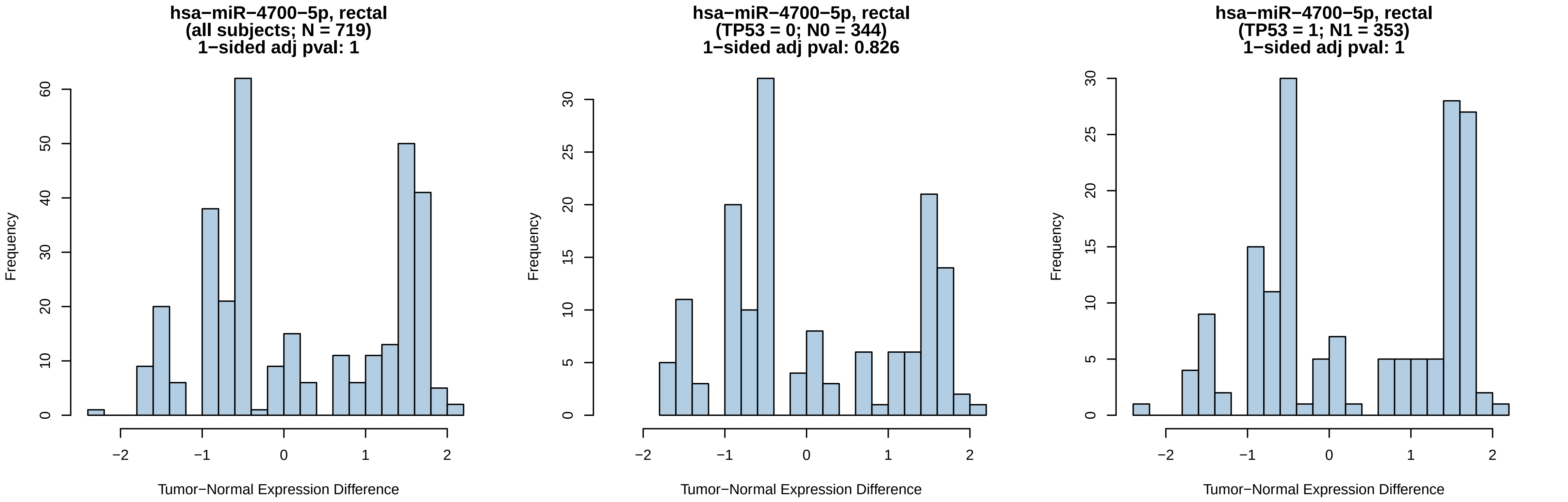

hsa−miR−4700−5p, rectal
(all subjects; N = 719)
1−sided adj pval: 1
Frequency
Tumor−Normal Expression Difference
hsa−miR−4700−5p, rectal
(TP53 = 0; N0 = 344)
1−sided adj pval: 0.826
Frequency
Tumor−Normal Expression Difference
hsa−miR−4700−5p, rectal
(TP53 = 1; N1 = 353)
1−sided adj pval: 1
Frequency
Tumor−Normal Expression Difference

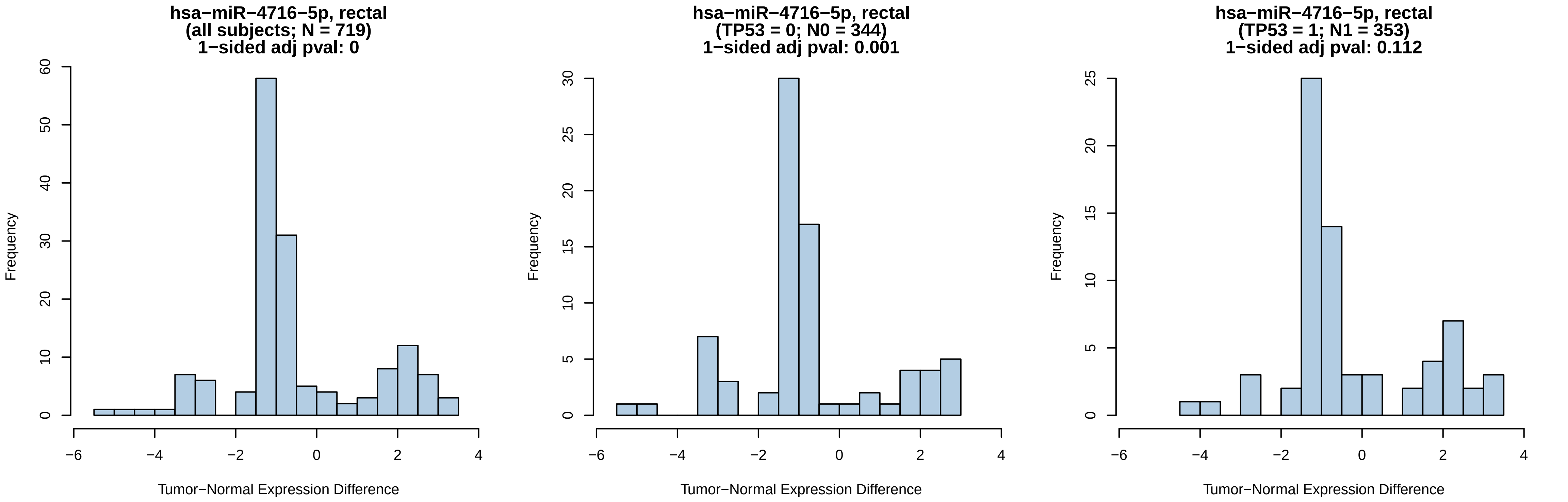
hsa−miR−4716−5p, rectal
(all subjects; N = 719)
1−sided adj pval: 0
Frequency
0
10
20
30
40
50
60
−6
−4
−2
0
2
4
Tumor−Normal Expression Difference
hsa−miR−4716−5p, rectal
(TP53 = 0; N0 = 344)
1−sided adj pval: 0.001
Frequency
0
5
10
15
20
25
30
−6
−4
−2
0
2
4
Tumor−Normal Expression Difference
hsa−miR−4716−5p, rectal
(TP53 = 1; N1 = 353)
1−sided adj pval: 0.112
Frequency
0
5
10
15
20
25
−6
−4
−2
0
2
4
Tumor−Normal Expression Difference

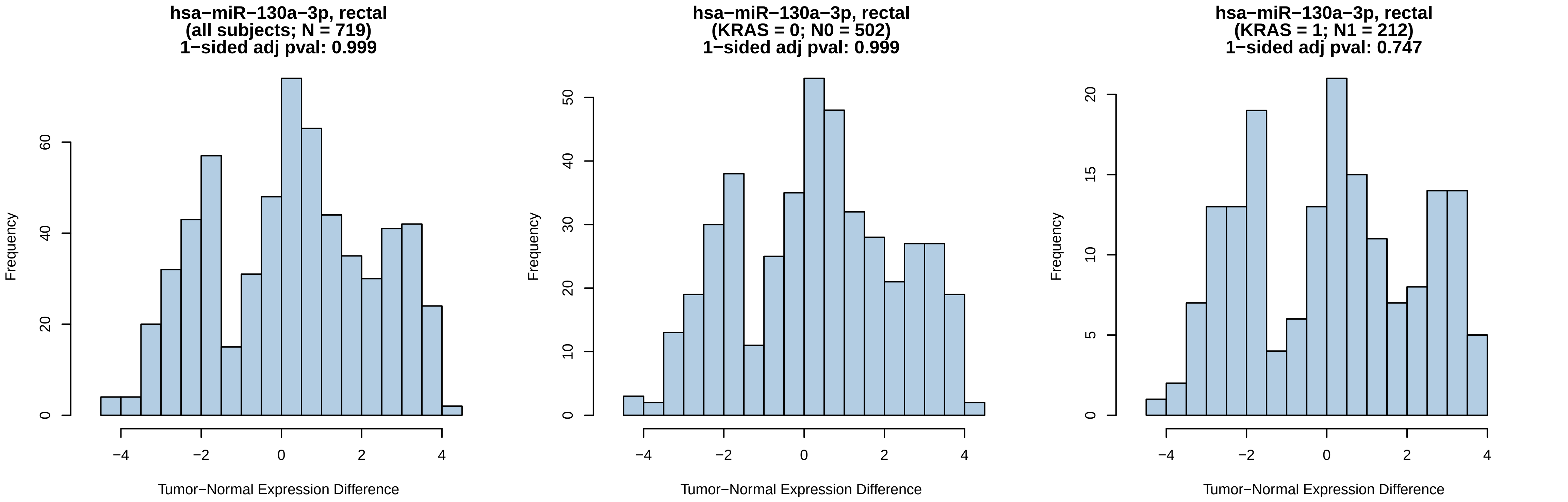

hsa−miR−130a−3p, rectal
(all subjects; N = 719)
1−sided adj pval: 0.999
Frequency
0
20
40
60
−4
−2
0
2
4
Tumor−Normal Expression Difference
hsa−miR−130a−3p, rectal
(KRAS = 0; N0 = 502)
1−sided adj pval: 0.999
Frequency
0
10
20
30
40
50
−4
−2
0
2
4
Tumor−Normal Expression Difference
hsa−miR−130a−3p, rectal
(KRAS = 1; N1 = 212)
1−sided adj pval: 0.747
Frequency
0
5
10
15
20
−4
−2
0
2
4
Tumor−Normal Expression Difference

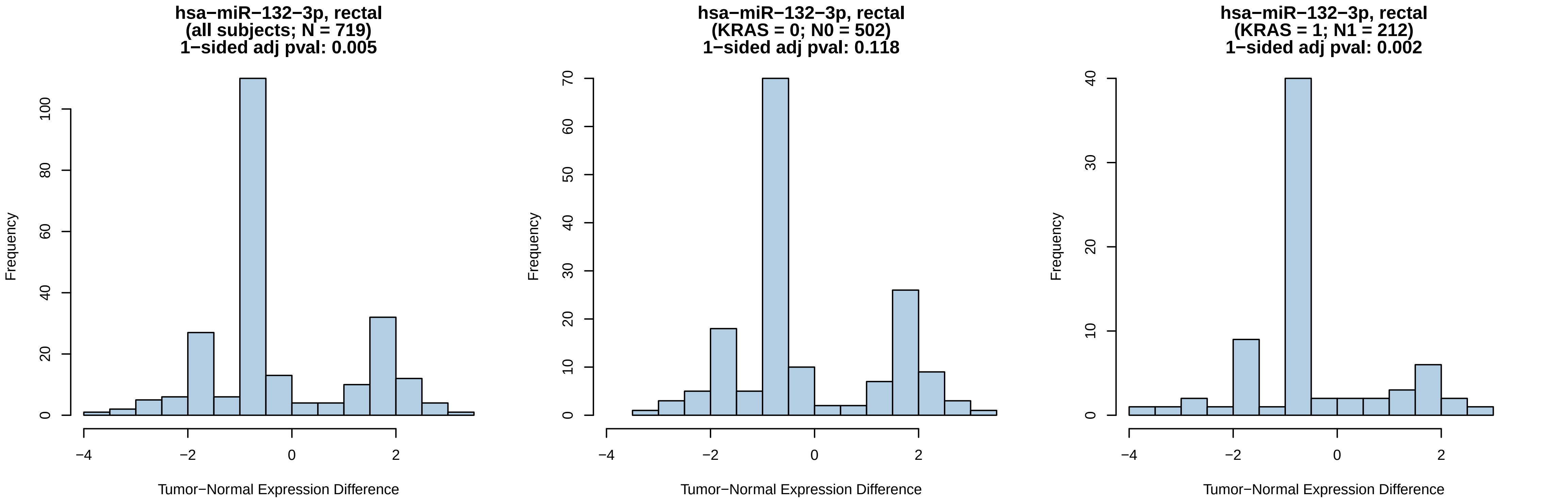

hsa−miR−132−3p, rectal
(all subjects; N = 719)
1−sided adj pval: 0.005
Frequency
0
20
40
60
80
100
−4
−2
0
2
Tumor−Normal Expression Difference
hsa−miR−132−3p, rectal
(KRAS = 0; N0 = 502)
1−sided adj pval: 0.118
Frequency
0
10
20
30
40
50
60
70
−4
−2
0
2
Tumor−Normal Expression Difference
hsa−miR−132−3p, rectal
(KRAS = 1; N1 = 212)
1−sided adj pval: 0.002
Frequency
0
10
20
30
40
−4
−2
0
2
Tumor−Normal Expression Difference

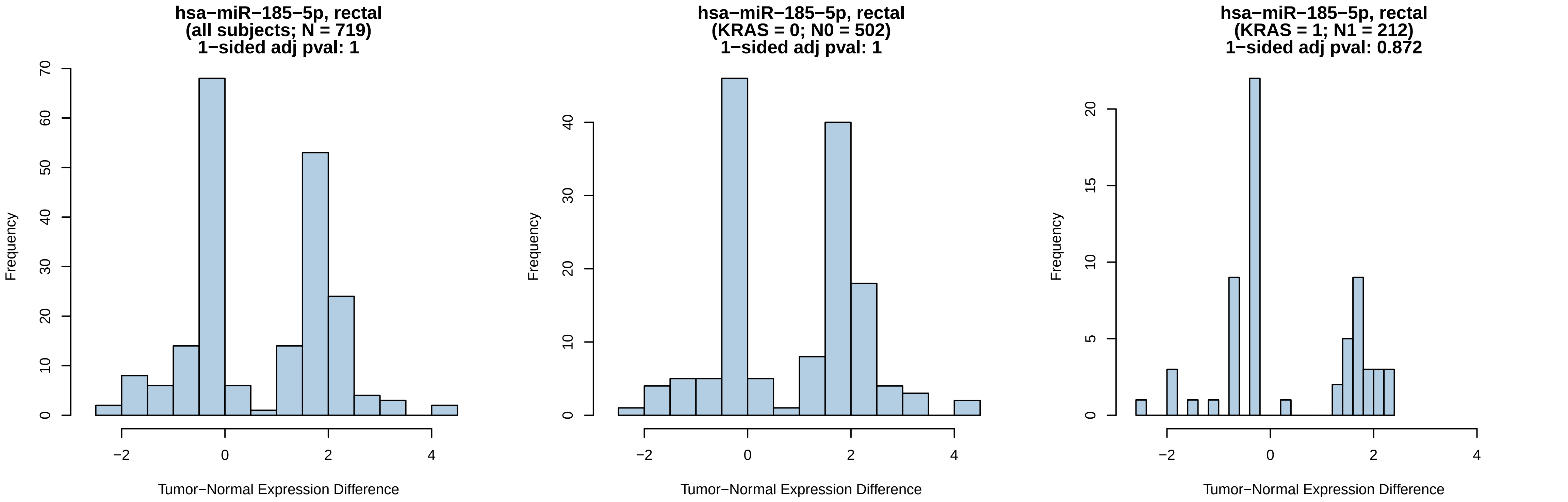

hsa−miR−185−5p, rectal
(all subjects; N = 719)
1−sided adj pval: 1
Frequency
Tumor−Normal Expression Difference
hsa−miR−185−5p, rectal
(KRAS = 0; N0 = 502)
1−sided adj pval: 1
Frequency
Tumor−Normal Expression Difference
hsa−miR−185−5p, rectal
(KRAS = 1; N1 = 212)
1−sided adj pval: 0.872
Frequency
Tumor−Normal Expression Difference

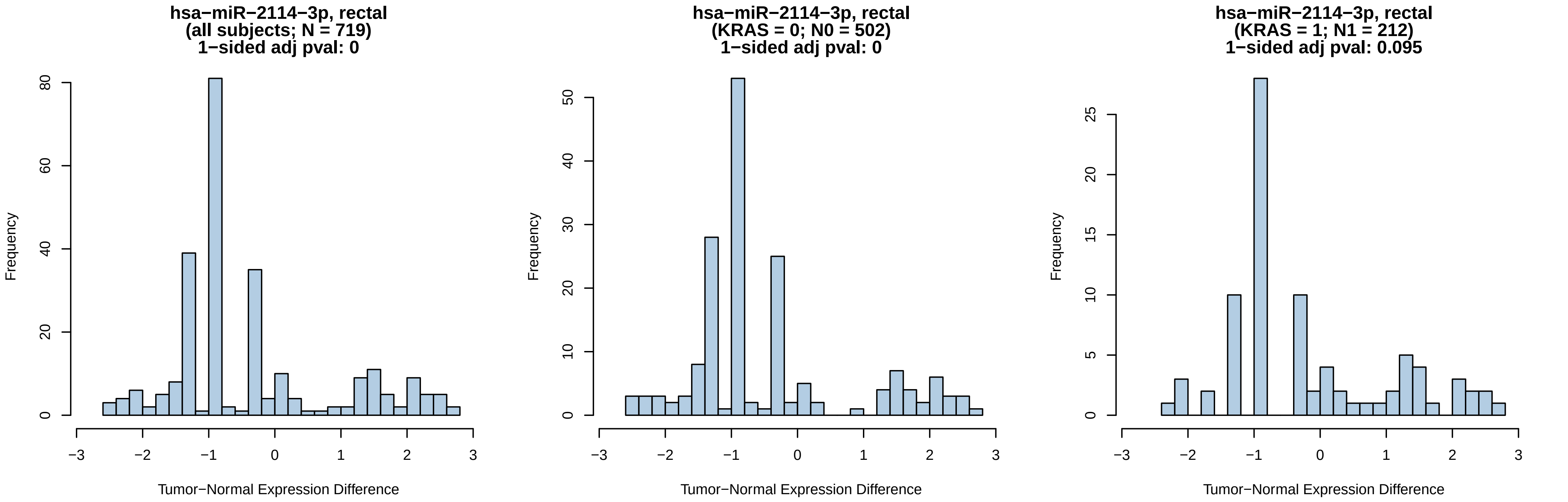
hsa−miR−2114−3p, rectal
(all subjects; N = 719)
1−sided adj pval: 0
hsa−miR−2114−3p, rectal
(KRAS = 0; N0 = 502)
1−sided adj pval: 0
hsa−miR−2114−3p, rectal
(KRAS = 1; N1 = 212)
1−sided adj pval: 0.095
Frequency
Tumor−Normal Expression Difference

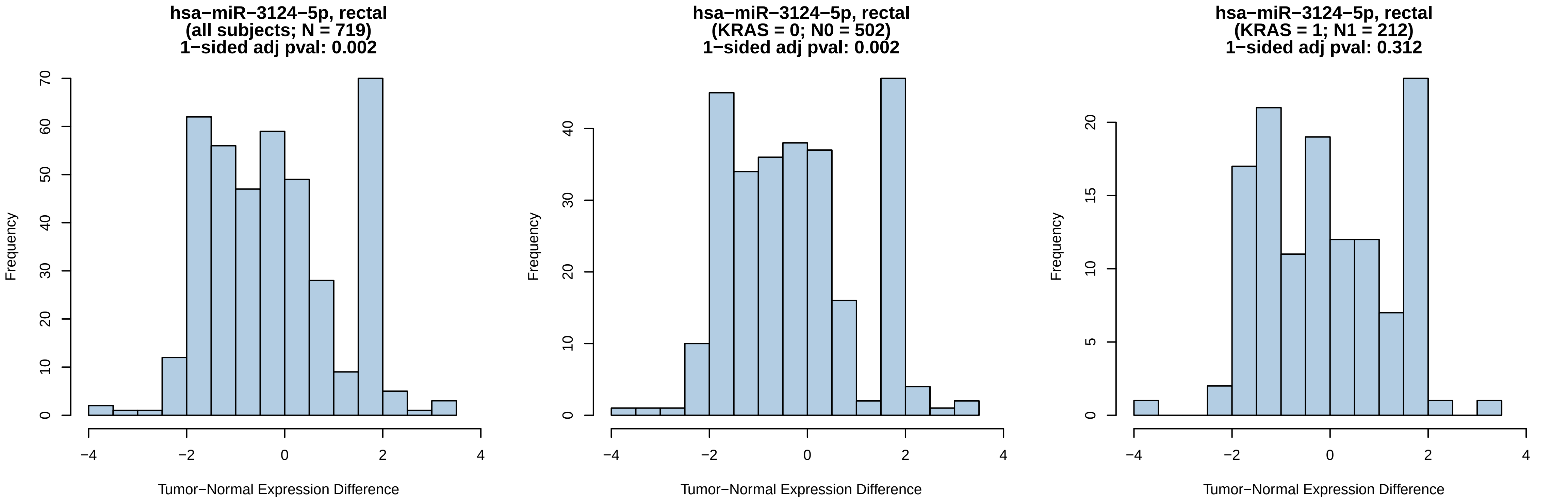

hsa−miR−3124−5p, rectal
(all subjects; N = 719)
1−sided adj pval: 0.002
hsa−miR−3124−5p, rectal
(KRAS = 0; N0 = 502)
1−sided adj pval: 0.002
hsa−miR−3124−5p, rectal
(KRAS = 1; N1 = 212)
1−sided adj pval: 0.312
Frequency
Tumor−Normal Expression Difference

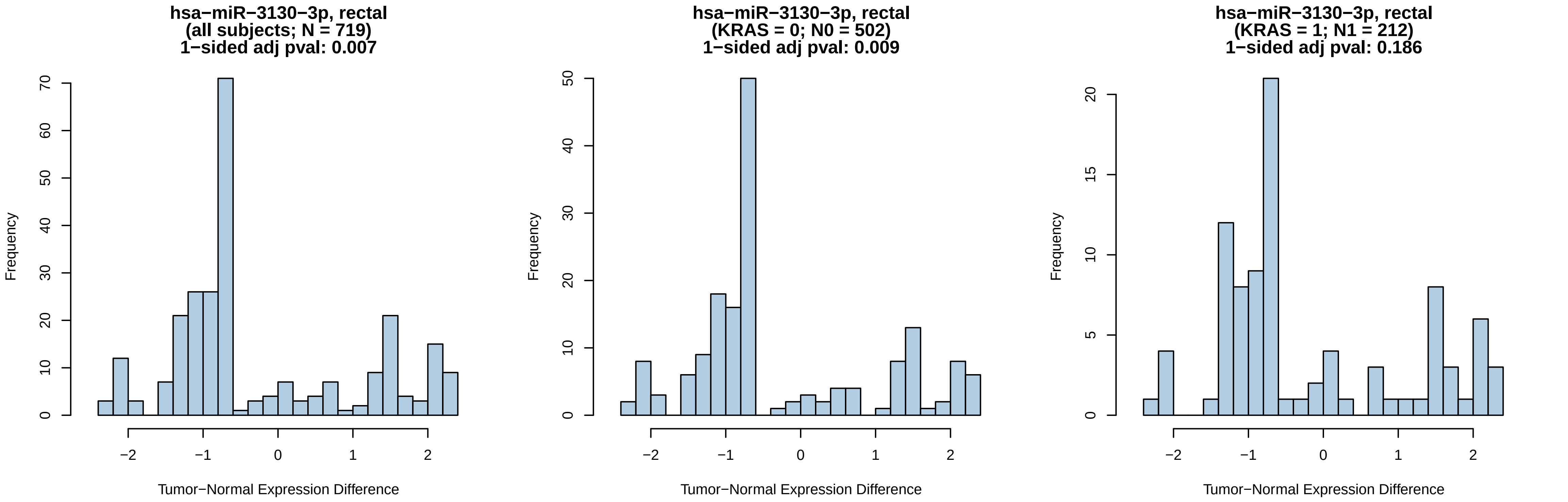

hsa−miR−3130−3p, rectal
(all subjects; N = 719)
1−sided adj pval: 0.007
Frequency
Tumor−Normal Expression Difference
hsa−miR−3130−3p, rectal
(KRAS = 0; N0 = 502)
1−sided adj pval: 0.009
Frequency
Tumor−Normal Expression Difference
hsa−miR−3130−3p, rectal
(KRAS = 1; N1 = 212)
1−sided adj pval: 0.186
Frequency
Tumor−Normal Expression Difference

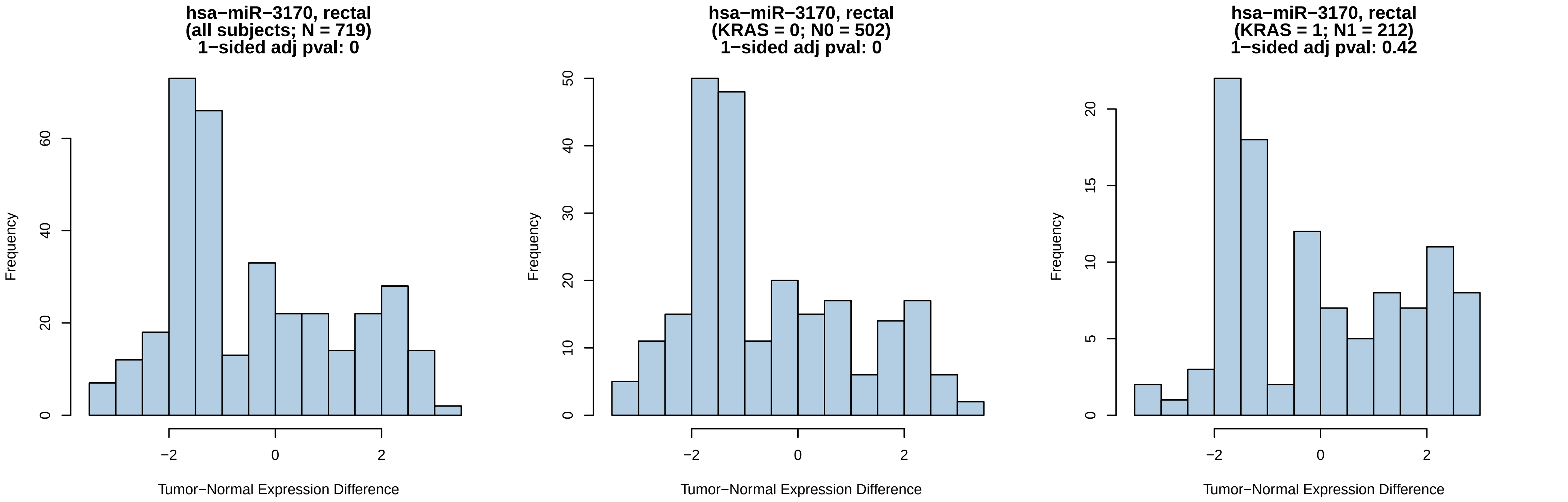

hsa−miR−3170, rectal
(all subjects; N = 719)
1−sided adj pval: 0
Frequency
0
20
40
60
−2
0
2
Tumor−Normal Expression Difference
hsa−miR−3170, rectal
(KRAS = 0; N0 = 502)
1−sided adj pval: 0
Frequency
0
10
20
30
40
50
−2
0
2
Tumor−Normal Expression Difference
hsa−miR−3170, rectal
(KRAS = 1; N1 = 212)
1−sided adj pval: 0.42
Frequency
0
5
10
15
20
−2
0
2
Tumor−Normal Expression Difference

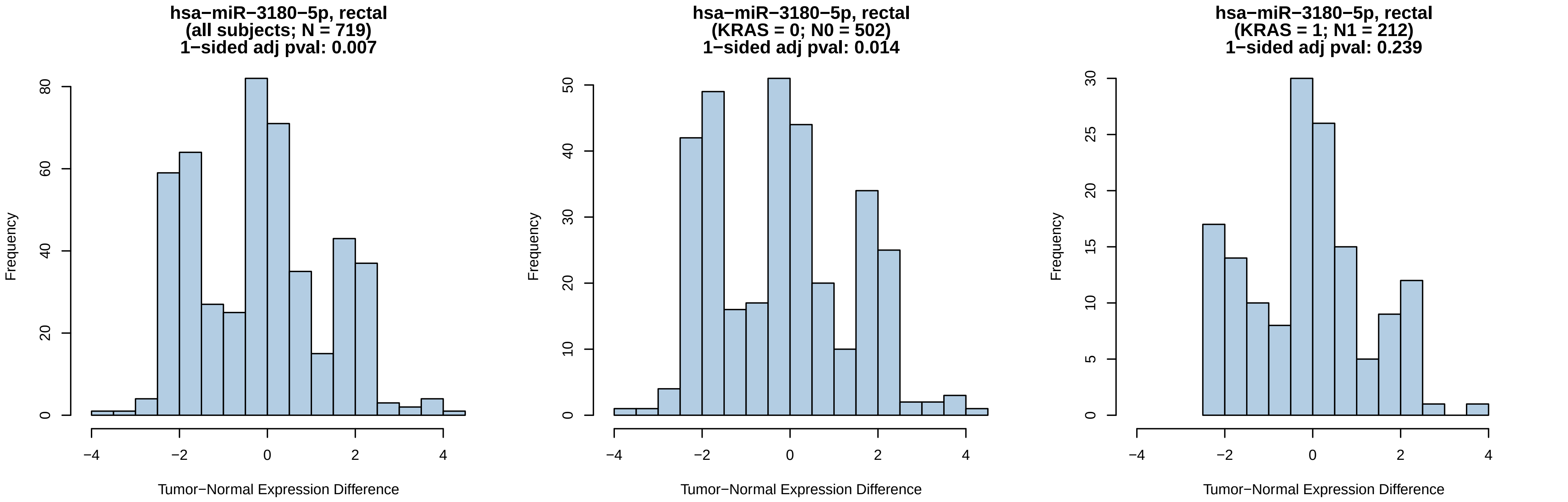
hsa−miR−3180−5p, rectal
(all subjects; N = 719)
1−sided adj pval: 0.007
Frequency
0
20
40
60
80
−4
−2
0
2
4
Tumor−Normal Expression Difference
hsa−miR−3180−5p, rectal
(KRAS = 0; N0 = 502)
1−sided adj pval: 0.014
Frequency
0
10
20
30
40
50
−4
−2
0
2
4
Tumor−Normal Expression Difference
hsa−miR−3180−5p, rectal
(KRAS = 1; N1 = 212)
1−sided adj pval: 0.239
Frequency
0
5
10
15
20
25
30
−4
−2
0
2
4
Tumor−Normal Expression Difference

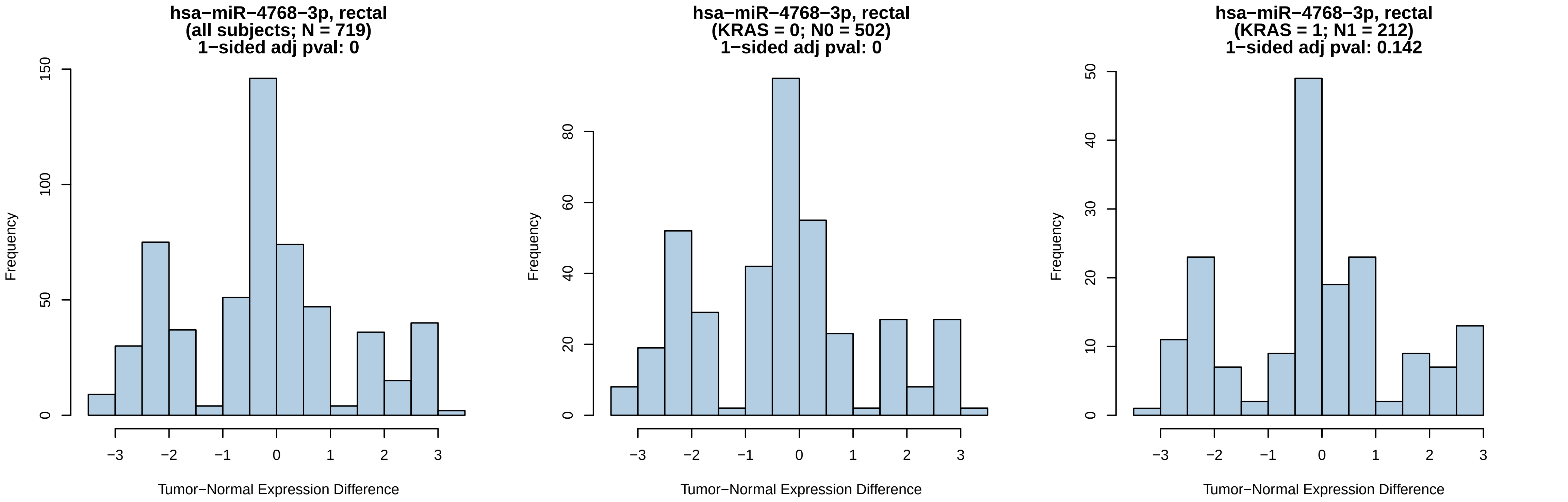
hsa−miR−4768−3p, rectal
(all subjects; N = 719)
1−sided adj pval: 0
Frequency
0
50
100
150
−3
−2
−1
0
1
2
3
Tumor−Normal Expression Difference
hsa−miR−4768−3p, rectal
(KRAS = 0; N0 = 502)
1−sided adj pval: 0
Frequency
0
20
40
60
80
−3
−2
−1
0
1
2
3
Tumor−Normal Expression Difference
hsa−miR−4768−3p, rectal
(KRAS = 1; N1 = 212)
1−sided adj pval: 0.142
Frequency
0
10
20
30
40
50
−3
−2
−1
0
1
2
3
Tumor−Normal Expression Difference

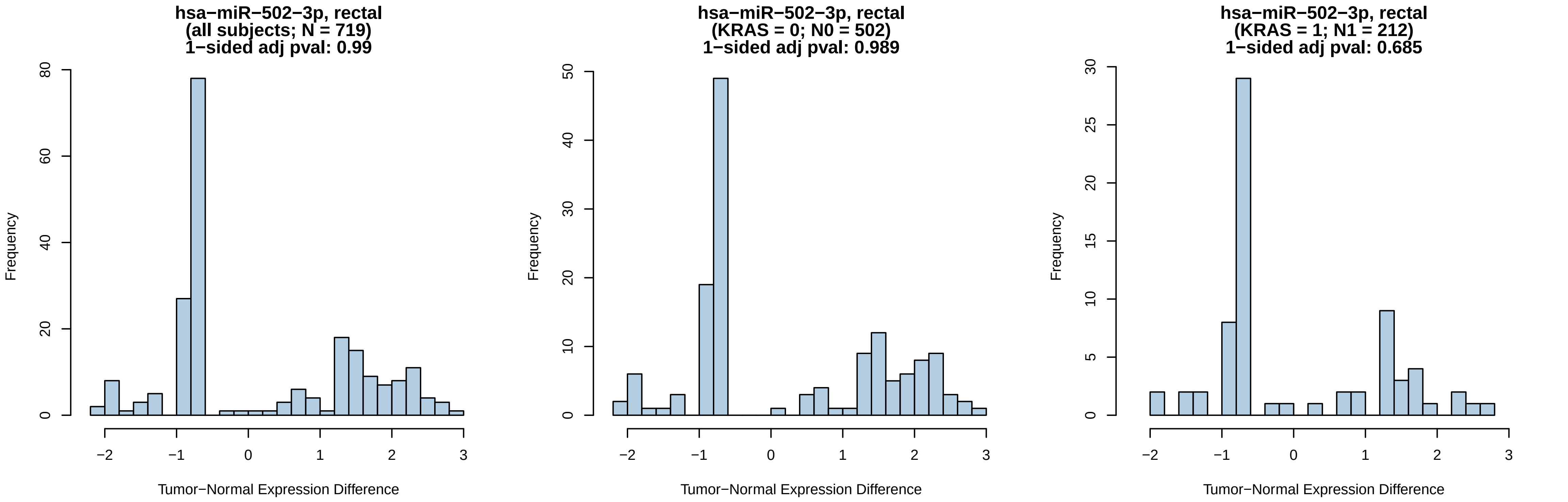

hsa−miR−502−3p, rectal
(all subjects; N = 719)
1−sided adj pval: 0.99
Frequency
Tumor−Normal Expression Difference
hsa−miR−502−3p, rectal
(KRAS = 0; N0 = 502)
1−sided adj pval: 0.989
Frequency
Tumor−Normal Expression Difference
hsa−miR−502−3p, rectal
(KRAS = 1; N1 = 212)
1−sided adj pval: 0.685
Frequency
Tumor−Normal Expression Difference

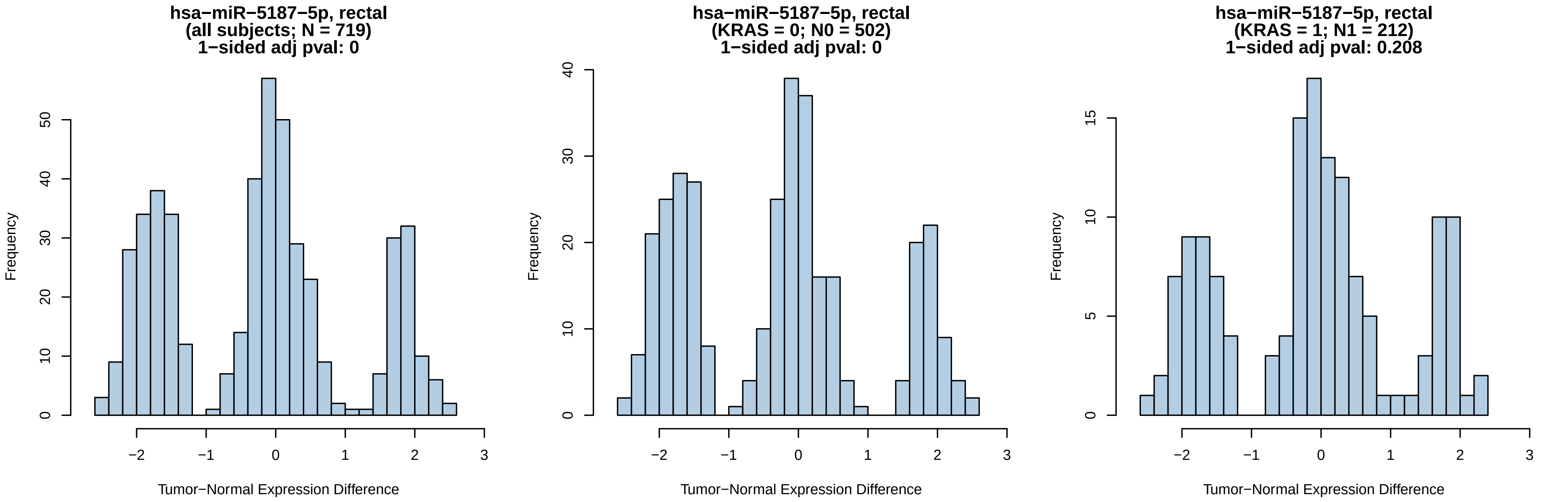
hsa−miR−5187−5p, rectal
(all subjects; N = 719)
1−sided adj pval: 0
Frequency
Tumor−Normal Expression Difference
hsa−miR−5187−5p, rectal
(KRAS = 0; N0 = 502)
1−sided adj pval: 0
Frequency
Tumor−Normal Expression Difference
hsa−miR−5187−5p, rectal
(KRAS = 1; N1 = 212)
1−sided adj pval: 0.208
Frequency
Tumor−Normal Expression Difference

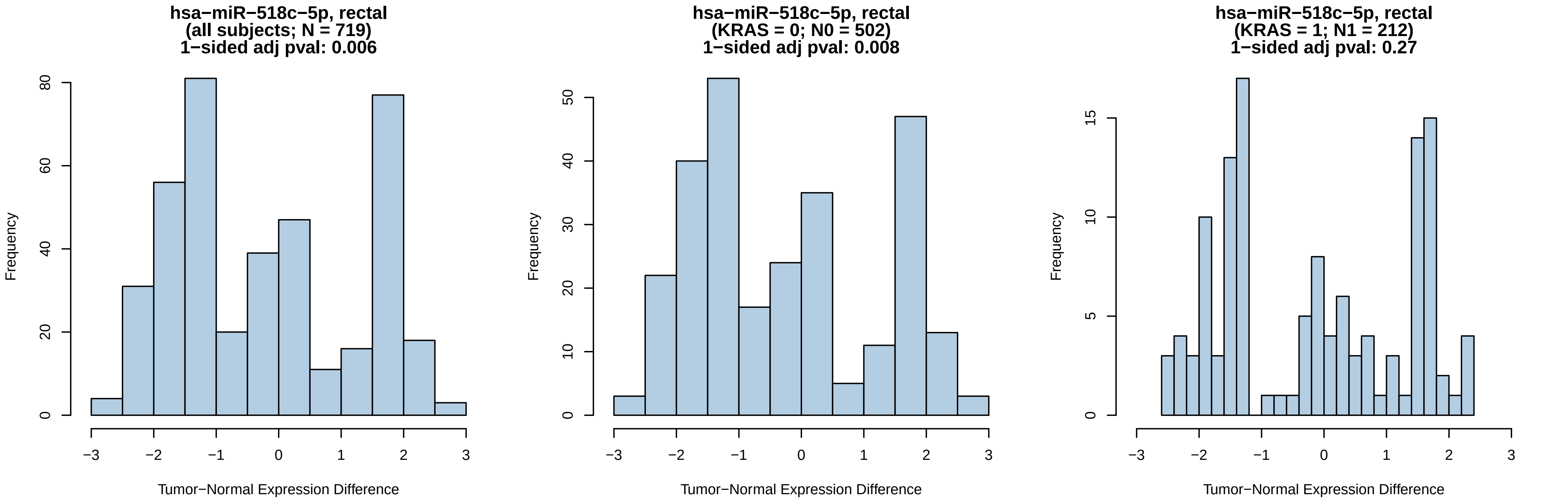
hsa−miR−518c−5p, rectal
(all subjects; N = 719)
1−sided adj pval: 0.006
Frequency
Tumor−Normal Expression Difference
hsa−miR−518c−5p, rectal
(KRAS = 0; N0 = 502)
1−sided adj pval: 0.008
Frequency
Tumor−Normal Expression Difference
hsa−miR−518c−5p, rectal
(KRAS = 1; N1 = 212)
1−sided adj pval: 0.27
Frequency
Tumor−Normal Expression Difference

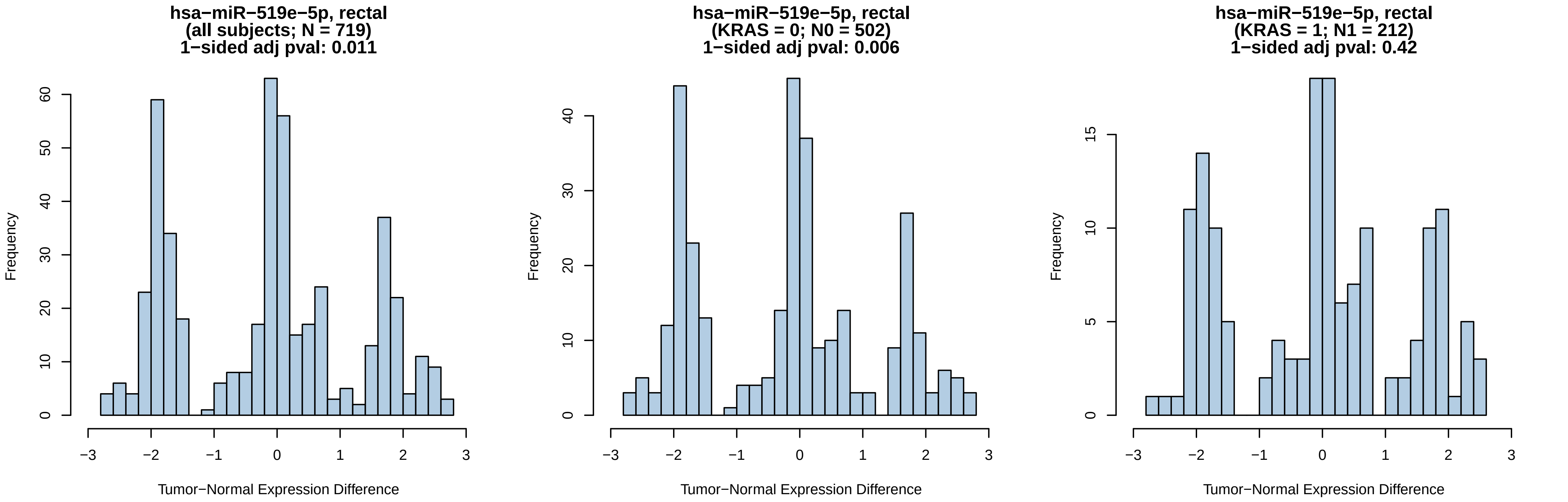
hsa−miR−519e−5p, rectal
(all subjects; N = 719)
1−sided adj pval: 0.011
Frequency
Tumor−Normal Expression Difference
hsa−miR−519e−5p, rectal
(KRAS = 0; N0 = 502)
1−sided adj pval: 0.006
Frequency
Tumor−Normal Expression Difference
hsa−miR−519e−5p, rectal
(KRAS = 1; N1 = 212)
1−sided adj pval: 0.42
Frequency
Tumor−Normal Expression Difference

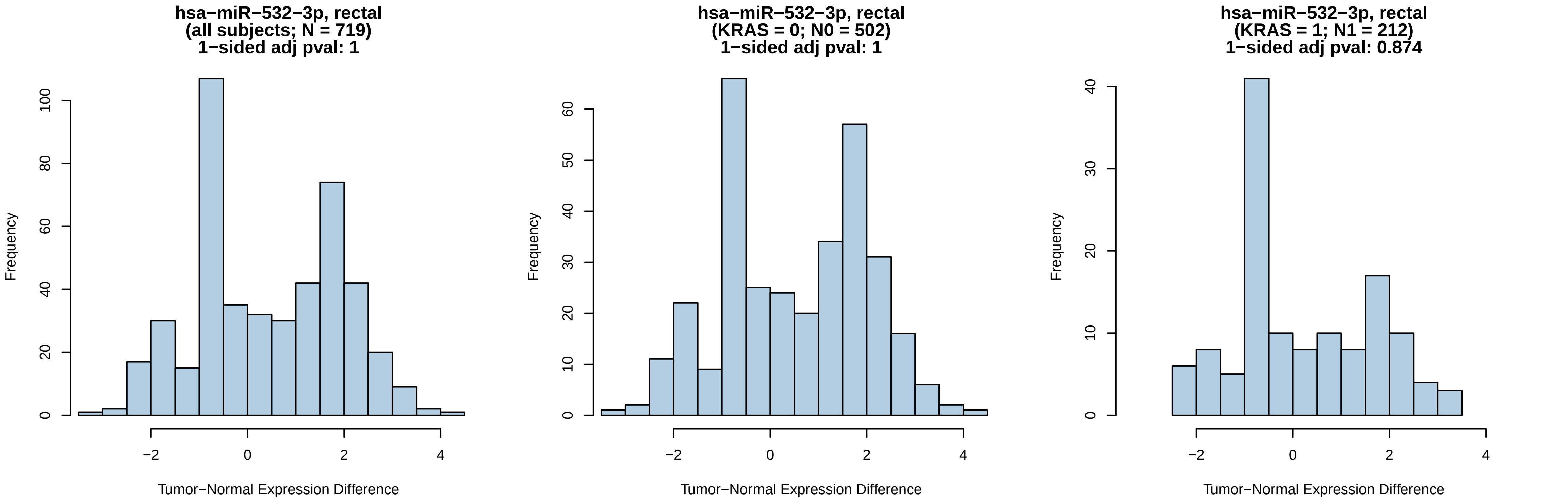

hsa−miR−532−3p, rectal
(all subjects; N = 719)
1−sided adj pval: 1
Frequency
0
20
40
60
80
100
−2
0
2
4
Tumor−Normal Expression Difference
hsa−miR−532−3p, rectal
(KRAS = 0; N0 = 502)
1−sided adj pval: 1
Frequency
0
10
20
30
40
50
60
−2
0
2
4
Tumor−Normal Expression Difference
hsa−miR−532−3p, rectal
(KRAS = 1; N1 = 212)
1−sided adj pval: 0.874
Frequency
0
10
20
30
40
−2
0
2
4
Tumor−Normal Expression Difference

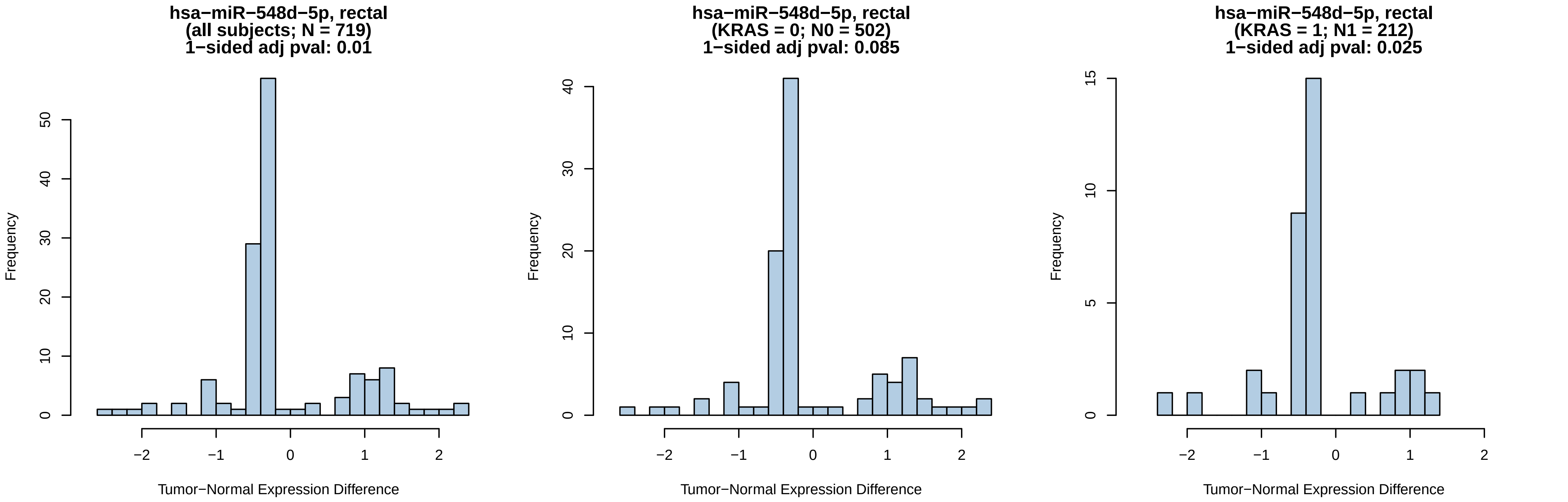
hsa−miR−548d−5p, rectal
(all subjects; N = 719)
1−sided adj pval: 0.01
Frequency
0
10
20
30
40
50
−2
−1
0
1
2
Tumor−Normal Expression Difference
hsa−miR−548d−5p, rectal
(KRAS = 0; N0 = 502)
1−sided adj pval: 0.085
Frequency
0
10
20
30
40
−2
−1
0
1
2
Tumor−Normal Expression Difference
hsa−miR−548d−5p, rectal
(KRAS = 1; N1 = 212)
1−sided adj pval: 0.025
Frequency
0
5
10
15
−2
−1
0
1
2
Tumor−Normal Expression Difference

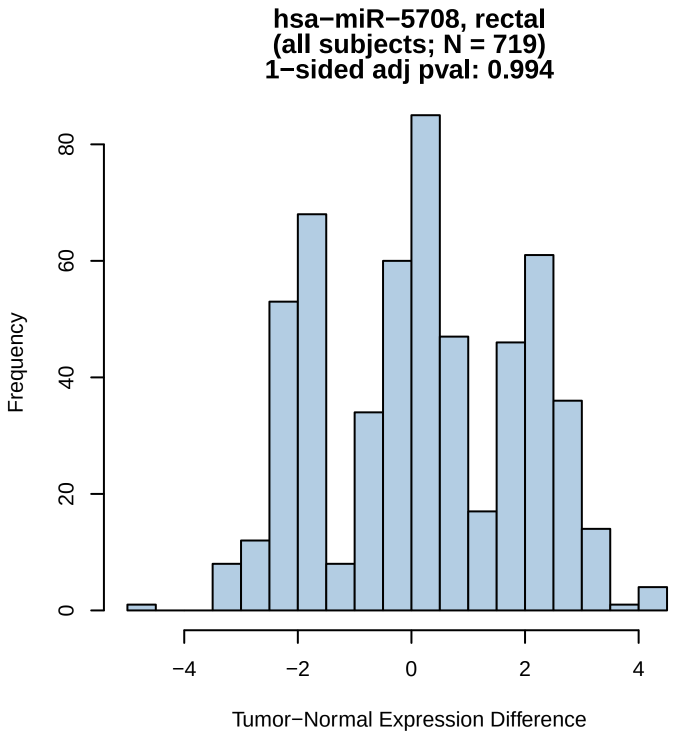
hsa−miR−5708, rectal
(all subjects; N = 719)
1−sided adj pval: 0.994
Frequency
Tumor−Normal Expression Difference
0
20
40
60
80
−4
−2
0
2
4

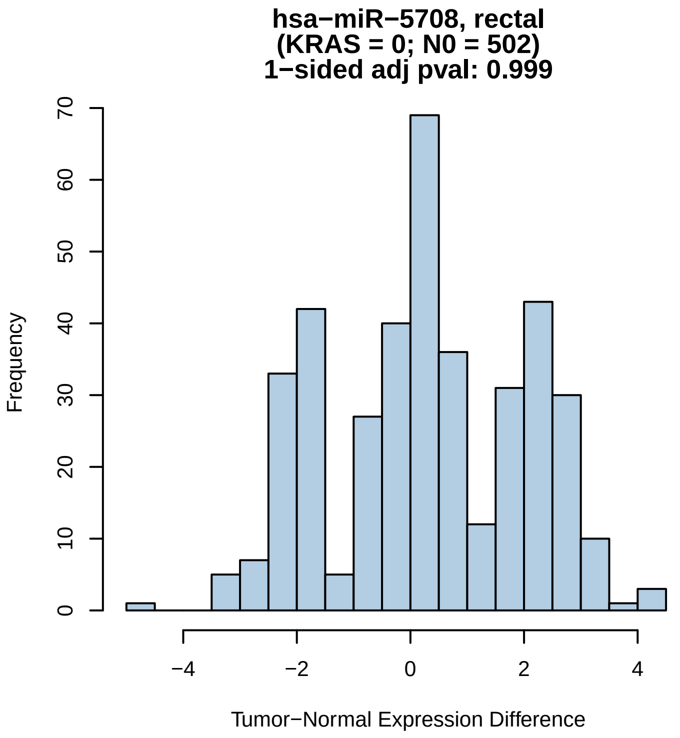
hsa−miR−5708, rectal
(KRAS = 0; N0 = 502)
1−sided adj pval: 0.999
Frequency
Tumor−Normal Expression Difference
0
10
20
30
40
50
60
70
−4
−2
0
2
4

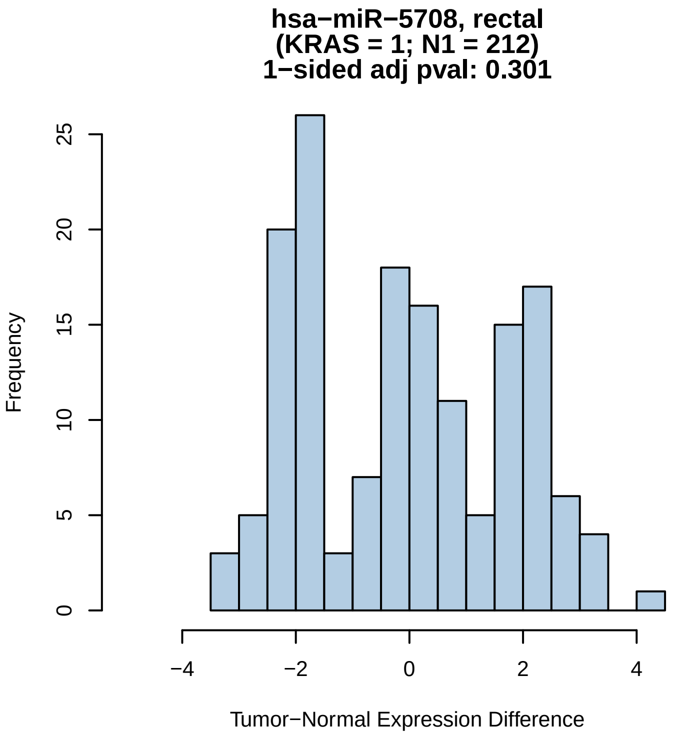
hsa−miR−5708, rectal
(KRAS = 1; N1 = 212)
1−sided adj pval: 0.301
Frequency
Tumor−Normal Expression Difference
0
5
10
15
20
25
−4
−2
0
2
4

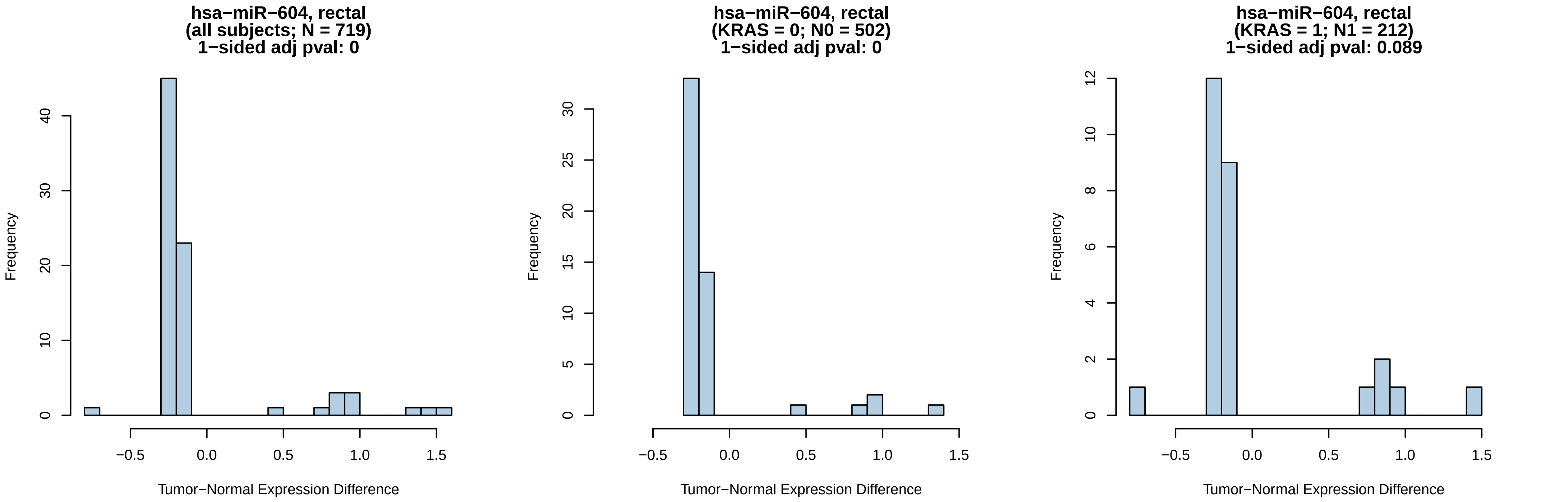
hsa−miR−604, rectal
(all subjects; N = 719)
1−sided adj pval: 0
Frequency
0
10
20
30
40
−0.5
0.0
0.5
1.0
1.5
Tumor−Normal Expression Difference
hsa−miR−604, rectal
(KRAS = 0; N0 = 502)
1−sided adj pval: 0
Frequency
0
5
10
15
20
25
30
−0.5
0.0
0.5
1.0
1.5
Tumor−Normal Expression Difference
hsa−miR−604, rectal
(KRAS = 1; N1 = 212)
1−sided adj pval: 0.089
Frequency
0
2
4
6
8
10
12
−0.5
0.0
0.5
1.0
1.5
Tumor−Normal Expression Difference

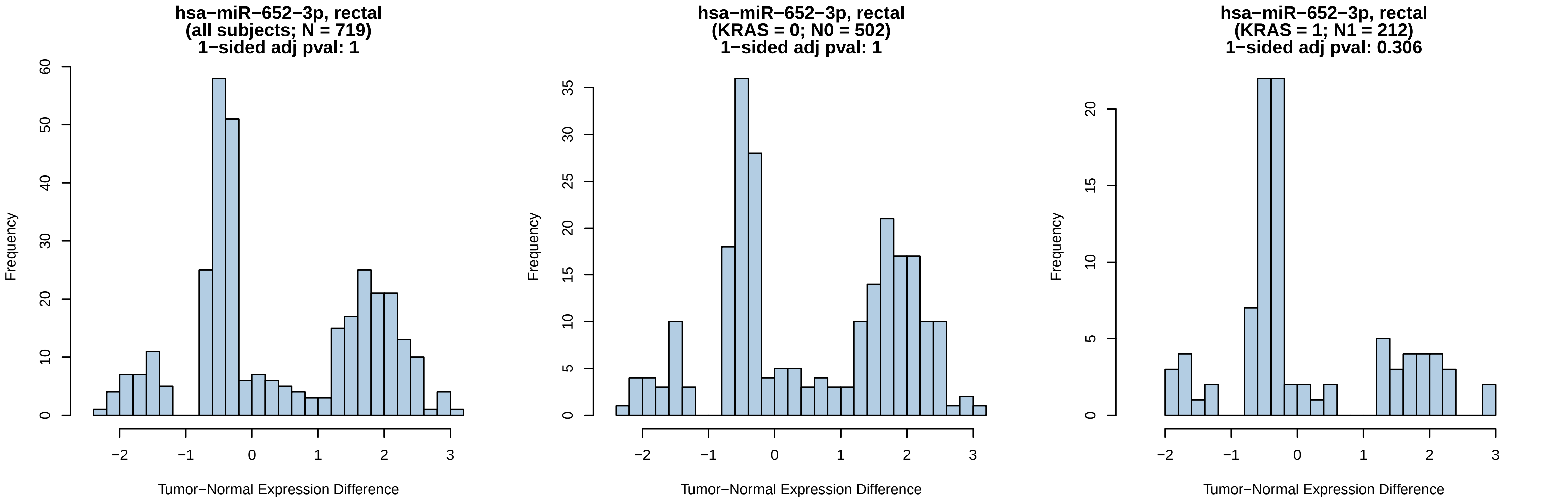
hsa−miR−652−3p, rectal
(all subjects; N = 719)
1−sided adj pval: 1
Frequency
Tumor−Normal Expression Difference
hsa−miR−652−3p, rectal
(KRAS = 0; N0 = 502)
1−sided adj pval: 1
Frequency
Tumor−Normal Expression Difference
hsa−miR−652−3p, rectal
(KRAS = 1; N1 = 212)
1−sided adj pval: 0.306
Frequency
Tumor−Normal Expression Difference

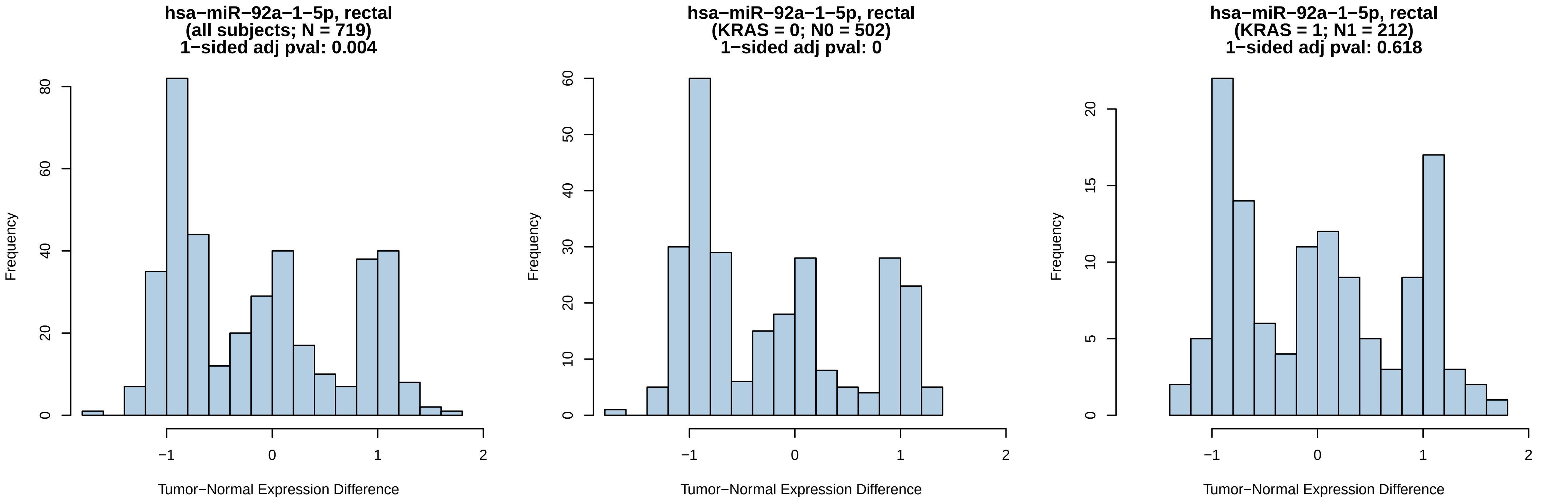

hsa−miR−92a−1−5p, rectal
(all subjects; N = 719)
1−sided adj pval: 0.004
Frequency
Tumor−Normal Expression Difference
hsa−miR−92a−1−5p, rectal
(KRAS = 0; N0 = 502)
1−sided adj pval: 0
Frequency
Tumor−Normal Expression Difference
hsa−miR−92a−1−5p, rectal
(KRAS = 1; N1 = 212)
1−sided adj pval: 0.618
Frequency
Tumor−Normal Expression Difference

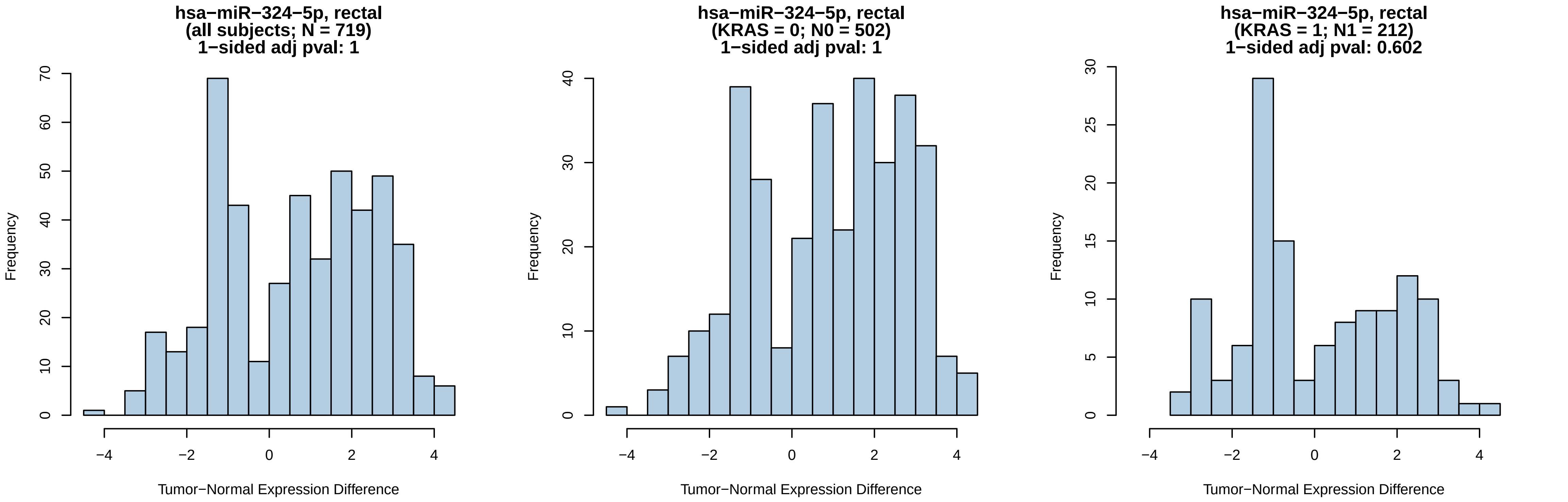
hsa−miR−324−5p, rectal
(all subjects; N = 719)
1−sided adj pval: 1
Frequency
Tumor−Normal Expression Difference
hsa−miR−324−5p, rectal
(KRAS = 0; N0 = 502)
1−sided adj pval: 1
Frequency
Tumor−Normal Expression Difference
hsa−miR−324−5p, rectal
(KRAS = 1; N1 = 212)
1−sided adj pval: 0.602
Frequency
Tumor−Normal Expression Difference

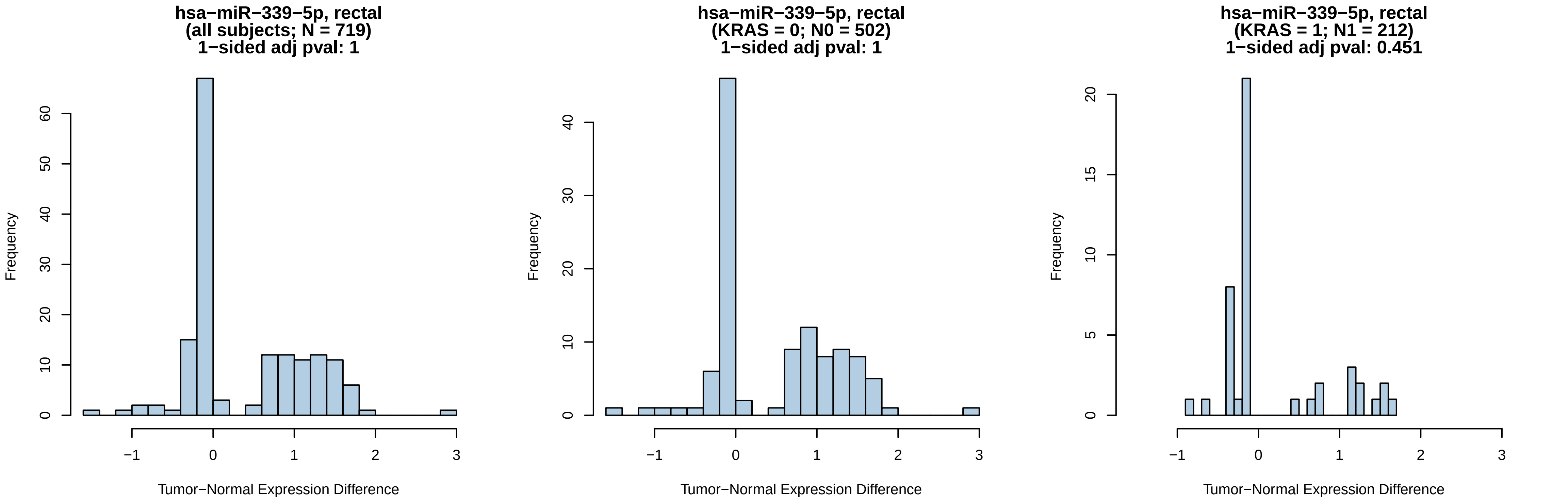

hsa−miR−339−5p, rectal
(all subjects; N = 719)
1−sided adj pval: 1
Frequency
Tumor−Normal Expression Difference
hsa−miR−339−5p, rectal
(KRAS = 0; N0 = 502)
1−sided adj pval: 1
Frequency
Tumor−Normal Expression Difference
hsa−miR−339−5p, rectal
(KRAS = 1; N1 = 212)
1−sided adj pval: 0.451
Frequency
Tumor−Normal Expression Difference

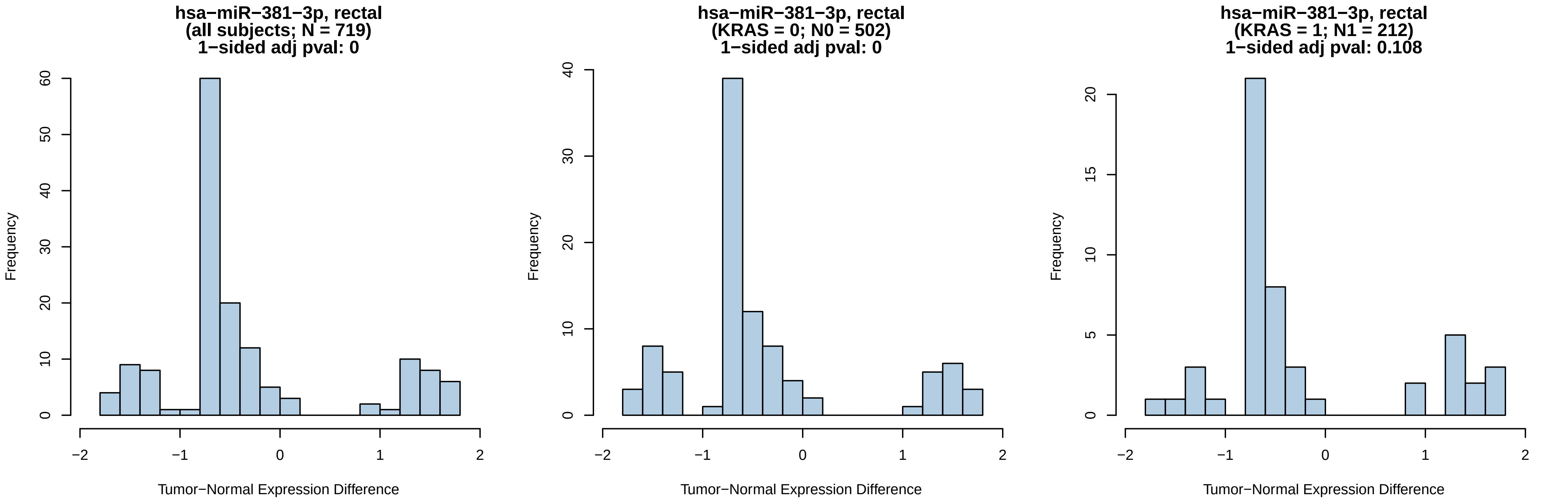
hsa−miR−381−3p, rectal
(all subjects; N = 719)
1−sided adj pval: 0
Frequency
Tumor−Normal Expression Difference
hsa−miR−381−3p, rectal
(KRAS = 0; N0 = 502)
1−sided adj pval: 0
Frequency
Tumor−Normal Expression Difference
hsa−miR−381−3p, rectal
(KRAS = 1; N1 = 212)
1−sided adj pval: 0.108
Frequency
Tumor−Normal Expression Difference

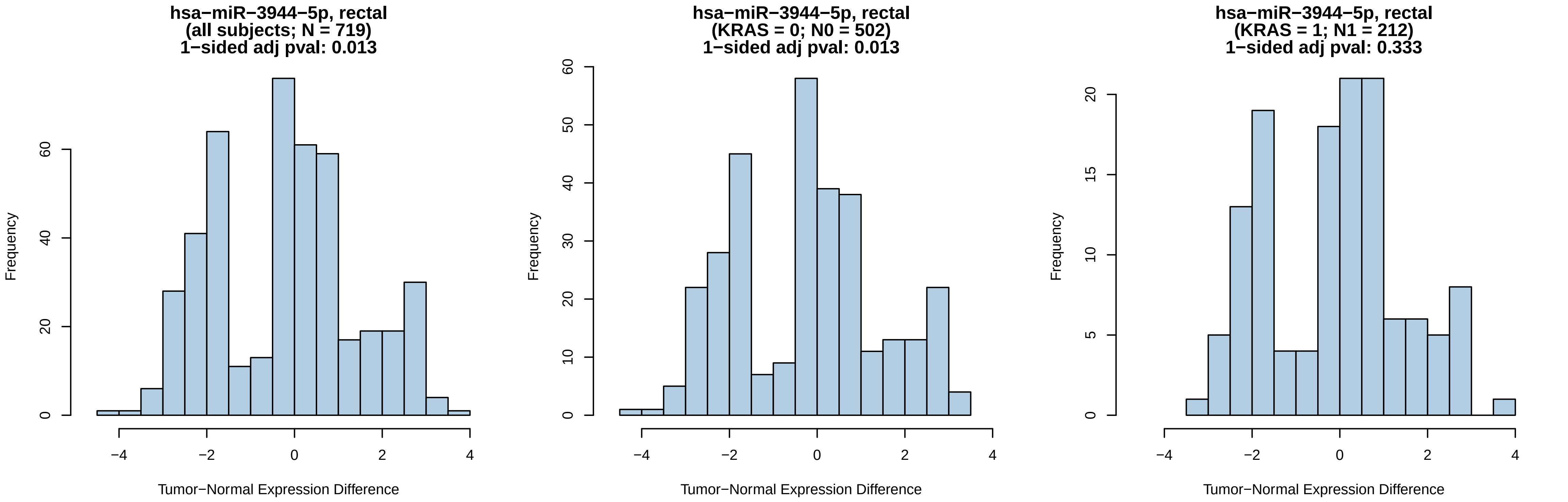
hsa−miR−3944−5p, rectal
(all subjects; N = 719)
1−sided adj pval: 0.013
Frequency
Tumor−Normal Expression Difference
hsa−miR−3944−5p, rectal
(KRAS = 0; N0 = 502)
1−sided adj pval: 0.013
Frequency
Tumor−Normal Expression Difference
hsa−miR−3944−5p, rectal
(KRAS = 1; N1 = 212)
1−sided adj pval: 0.333
Frequency
Tumor−Normal Expression Difference

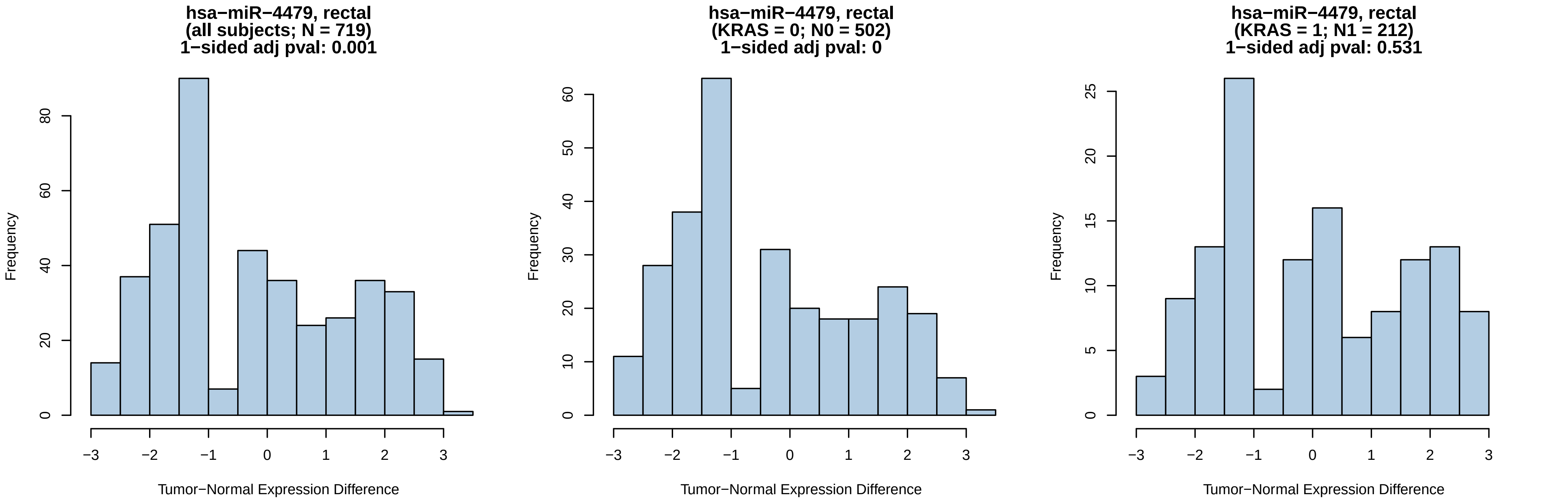

hsa−miR−4479, rectal
(all subjects; N = 719)
1−sided adj pval: 0.001
Frequency
Tumor−Normal Expression Difference
hsa−miR−4479, rectal
(KRAS = 0; N0 = 502)
1−sided adj pval: 0
Frequency
Tumor−Normal Expression Difference
hsa−miR−4479, rectal
(KRAS = 1; N1 = 212)
1−sided adj pval: 0.531
Frequency
Tumor−Normal Expression Difference

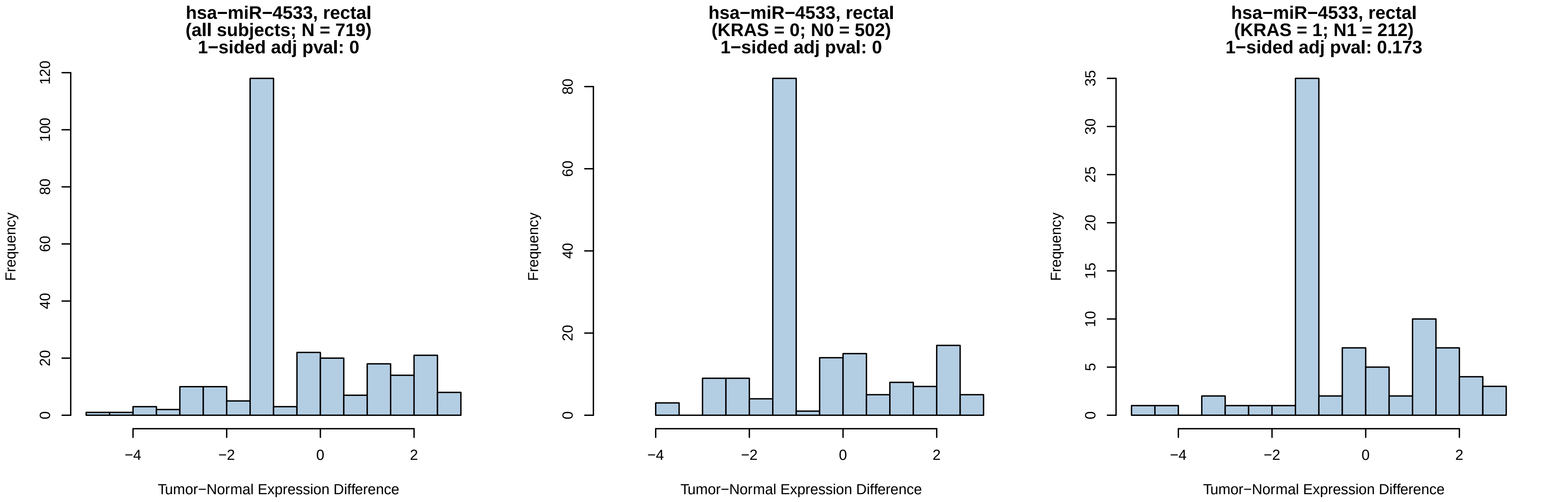
hsa−miR−4533, rectal
(all subjects; N = 719)
1−sided adj pval: 0
Frequency
0
20
40
60
80
100
120
−4
−2
0
2
Tumor−Normal Expression Difference
hsa−miR−4533, rectal
(KRAS = 0; N0 = 502)
1−sided adj pval: 0
Frequency
0
20
40
60
80
−4
−2
0
2
Tumor−Normal Expression Difference
hsa−miR−4533, rectal
(KRAS = 1; N1 = 212)
1−sided adj pval: 0.173
Frequency
0
5
10
15
20
25
30
35
−4
−2
0
2
Tumor−Normal Expression Difference

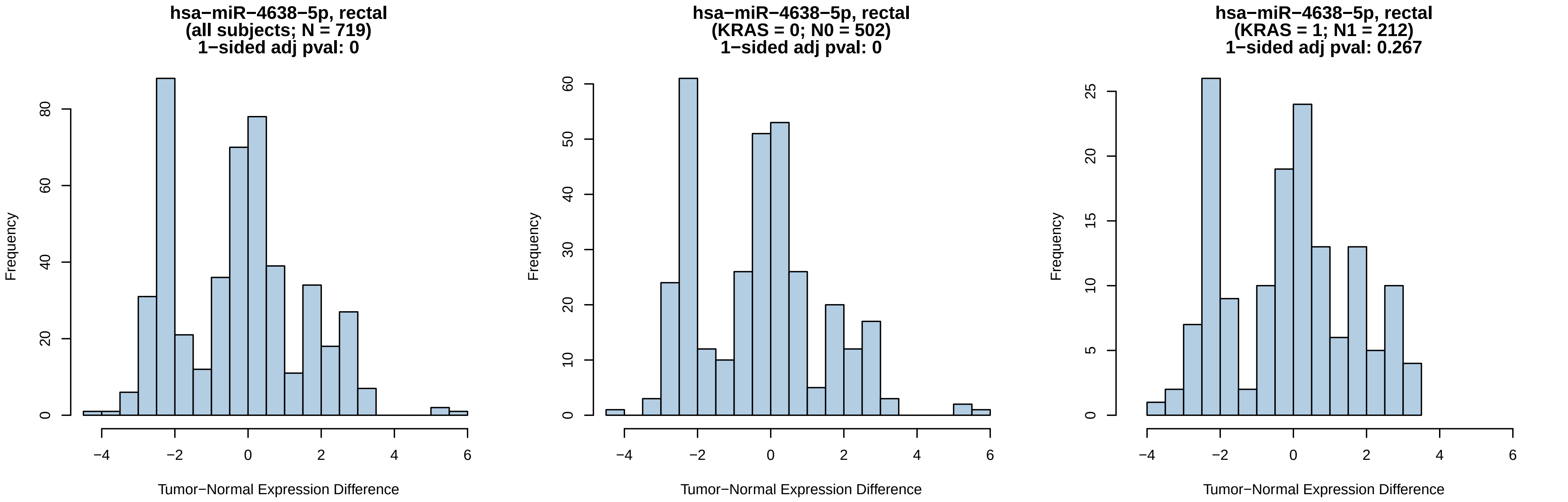

hsa−miR−4638−5p, rectal
(all subjects; N = 719)
1−sided adj pval: 0
Frequency
Tumor−Normal Expression Difference
hsa−miR−4638−5p, rectal
(KRAS = 0; N0 = 502)
1−sided adj pval: 0
Frequency
Tumor−Normal Expression Difference
hsa−miR−4638−5p, rectal
(KRAS = 1; N1 = 212)
1−sided adj pval: 0.267
Frequency
Tumor−Normal Expression Difference

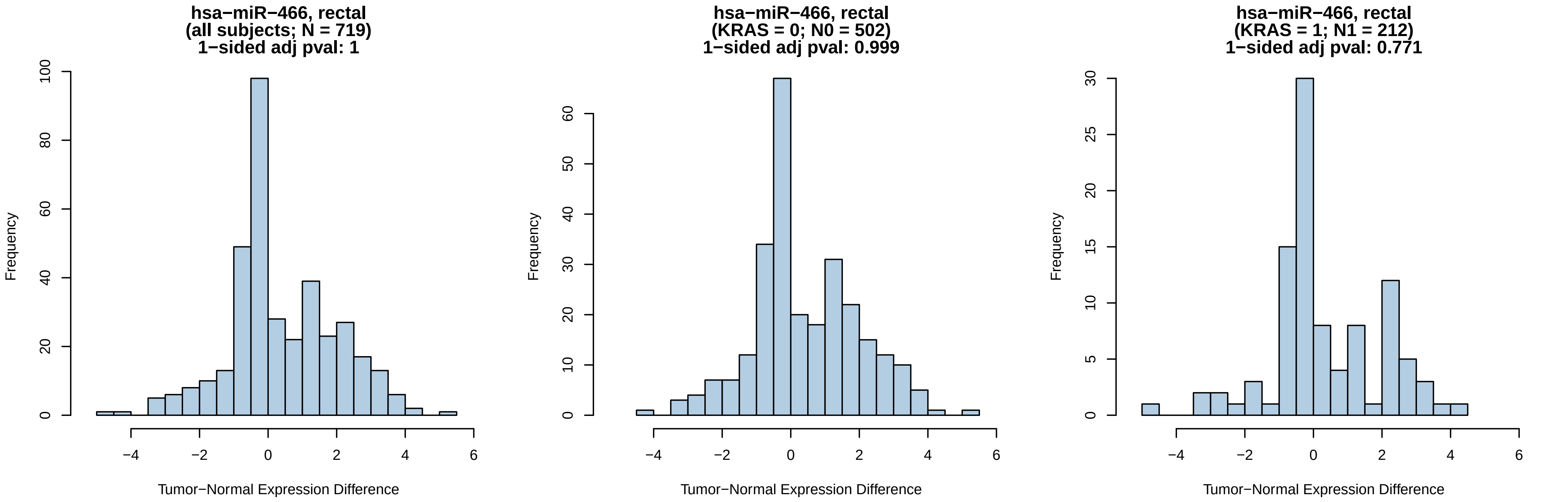

hsa−miR−466, rectal
(all subjects; N = 719)
1−sided adj pval: 1
Frequency
Tumor−Normal Expression Difference
hsa−miR−466, rectal
(KRAS = 0; N0 = 502)
1−sided adj pval: 0.999
Frequency
Tumor−Normal Expression Difference
hsa−miR−466, rectal
(KRAS = 1; N1 = 212)
1−sided adj pval: 0.771
Frequency
Tumor−Normal Expression Difference

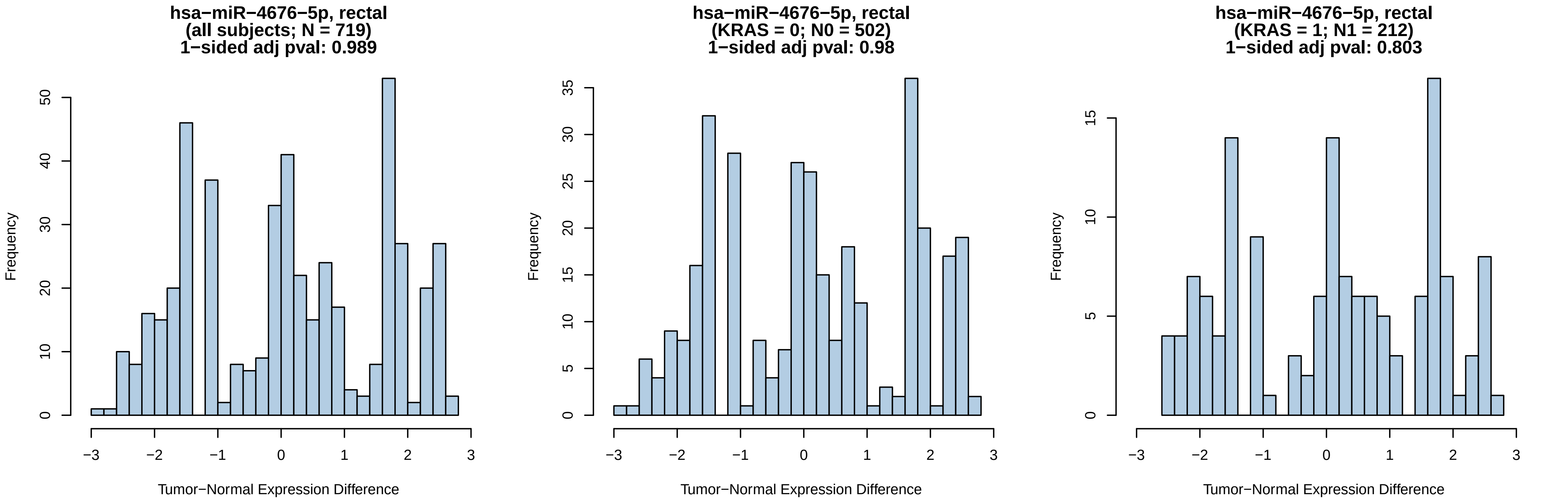
hsa−miR−4676−5p, rectal
(all subjects; N = 719)
1−sided adj pval: 0.989
Frequency
Tumor−Normal Expression Difference
hsa−miR−4676−5p, rectal
(KRAS = 0; N0 = 502)
1−sided adj pval: 0.98
Frequency
Tumor−Normal Expression Difference
hsa−miR−4676−5p, rectal
(KRAS = 1; N1 = 212)
1−sided adj pval: 0.803
Frequency
Tumor−Normal Expression Difference

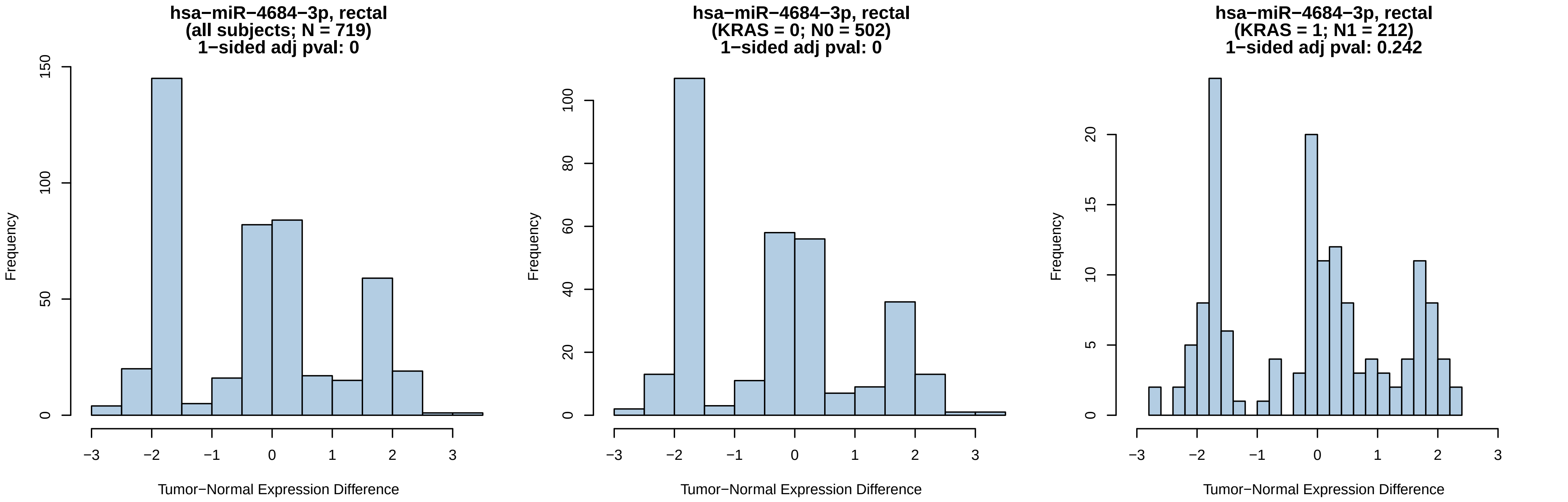
hsa−miR−4684−3p, rectal
(all subjects; N = 719)
1−sided adj pval: 0
Frequency
Tumor−Normal Expression Difference
hsa−miR−4684−3p, rectal
(KRAS = 0; N0 = 502)
1−sided adj pval: 0
Frequency
Tumor−Normal Expression Difference
hsa−miR−4684−3p, rectal
(KRAS = 1; N1 = 212)
1−sided adj pval: 0.242
Frequency
Tumor−Normal Expression Difference

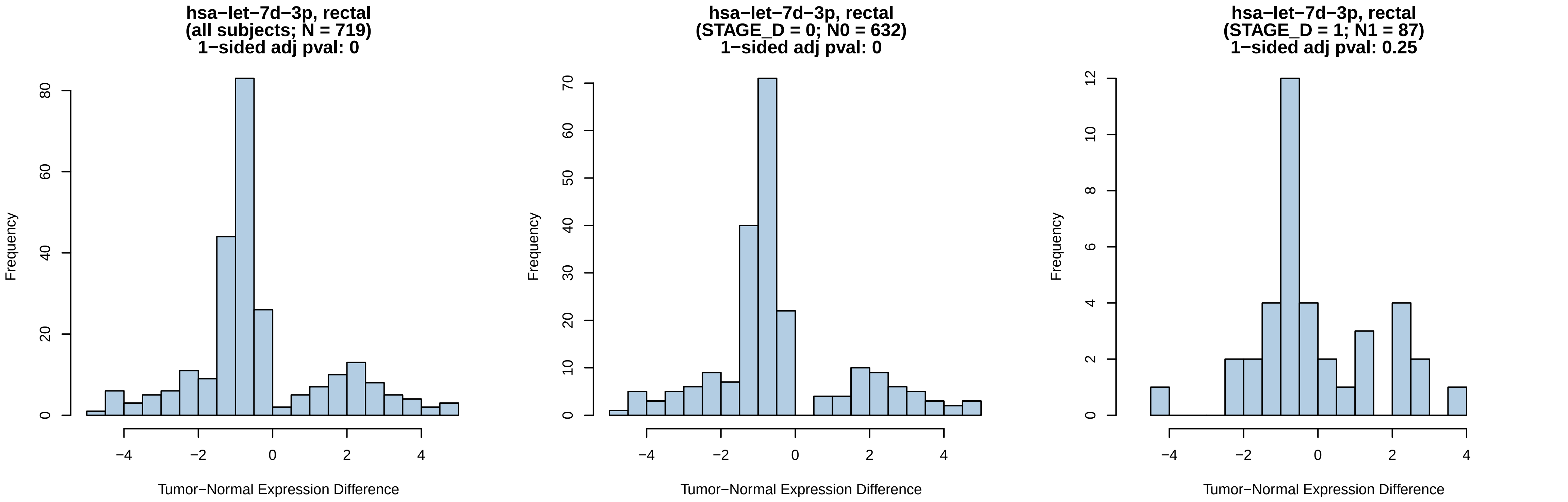
hsa−let−7d−3p, rectal
(all subjects; N = 719)
1−sided adj pval: 0
Frequency
Tumor−Normal Expression Difference
hsa−let−7d−3p, rectal
(STAGE_D = 0; N0 = 632)
1−sided adj pval: 0
Frequency
Tumor−Normal Expression Difference
hsa−let−7d−3p, rectal
(STAGE_D = 1; N1 = 87)
1−sided adj pval: 0.25
Frequency
Tumor−Normal Expression Difference

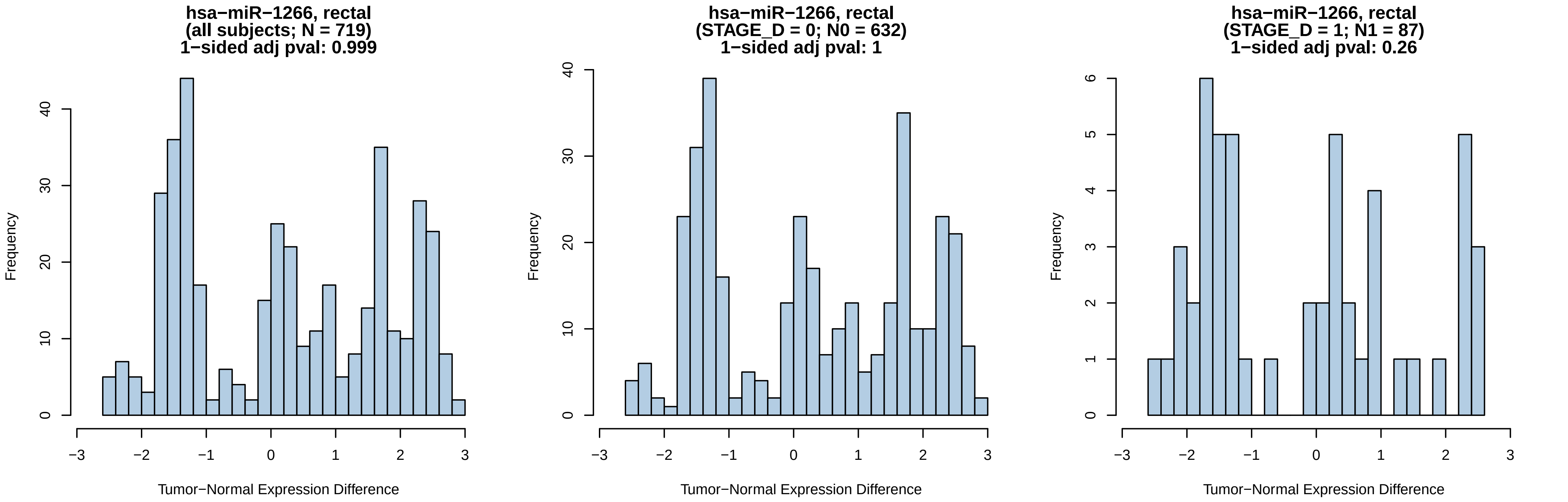
hsa−miR−1266, rectal
(all subjects; N = 719)
1−sided adj pval: 0.999
Frequency
Tumor−Normal Expression Difference
hsa−miR−1266, rectal
(STAGE_D = 0; N0 = 632)
1−sided adj pval: 1
Frequency
Tumor−Normal Expression Difference
hsa−miR−1266, rectal
(STAGE_D = 1; N1 = 87)
1−sided adj pval: 0.26
Frequency
Tumor−Normal Expression Difference

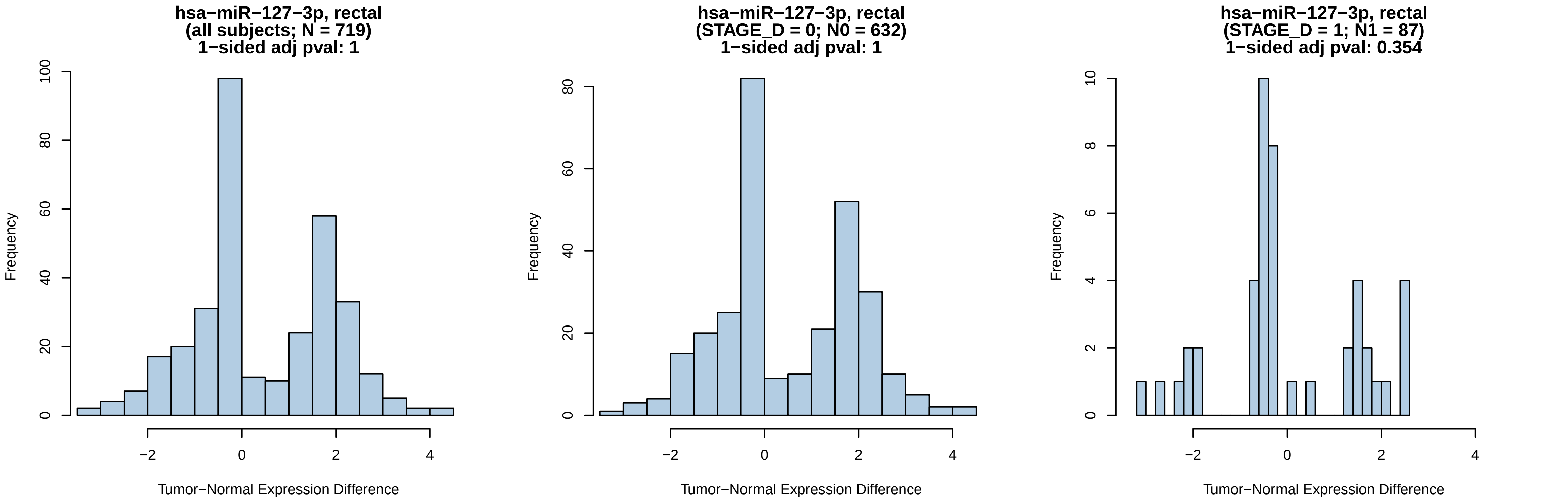

hsa−miR−127−3p, rectal
(all subjects; N = 719)
1−sided adj pval: 1
Frequency
0
20
40
60
80
100
−2
0
2
4
Tumor−Normal Expression Difference
hsa−miR−127−3p, rectal
(STAGE_D = 0; N0 = 632)
1−sided adj pval: 1
Frequency
0
20
40
60
80
−2
0
2
4
Tumor−Normal Expression Difference
hsa−miR−127−3p, rectal
(STAGE_D = 1; N1 = 87)
1−sided adj pval: 0.354
Frequency
0
2
4
6
8
10
−2
0
2
4
Tumor−Normal Expression Difference

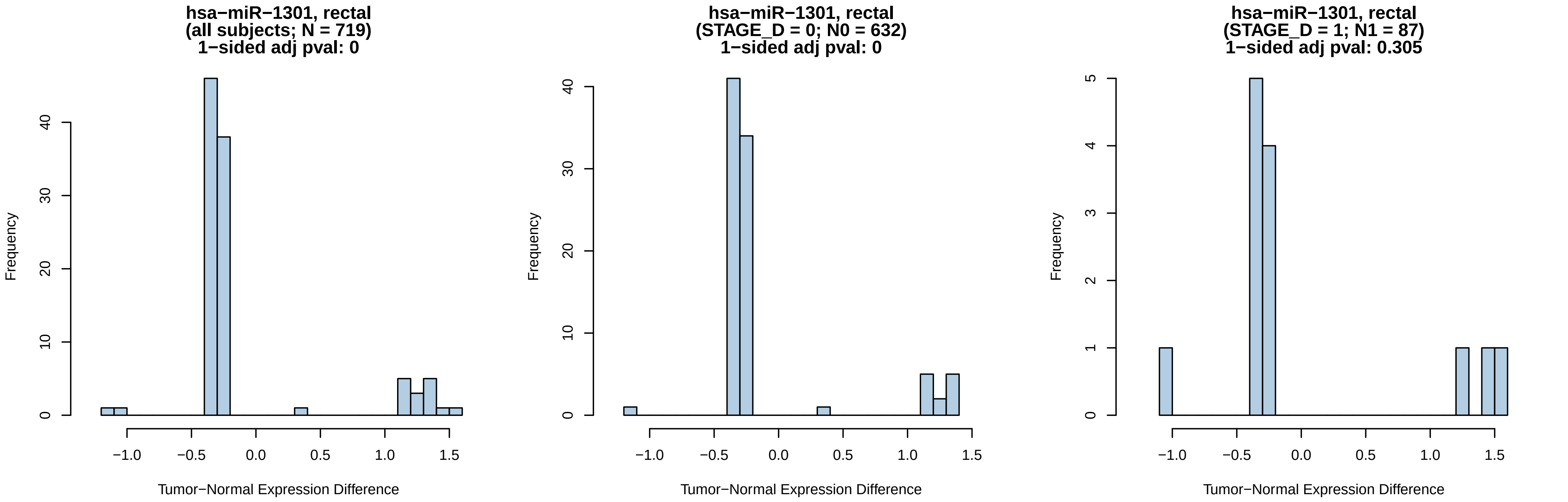
hsa−miR−1301, rectal
(all subjects; N = 719)
1−sided adj pval: 0
Frequency
0
10
20
30
40
−1.0
−0.5
0.0
0.5
1.0
1.5
Tumor−Normal Expression Difference
hsa−miR−1301, rectal
(STAGE_D = 0; N0 = 632)
1−sided adj pval: 0
Frequency
0
10
20
30
40
−1.0
−0.5
0.0
0.5
1.0
1.5
Tumor−Normal Expression Difference
hsa−miR−1301, rectal
(STAGE_D = 1; N1 = 87)
1−sided adj pval: 0.305
Frequency
0
1
2
3
4
5
−1.0
−0.5
0.0
0.5
1.0
1.5
Tumor−Normal Expression Difference

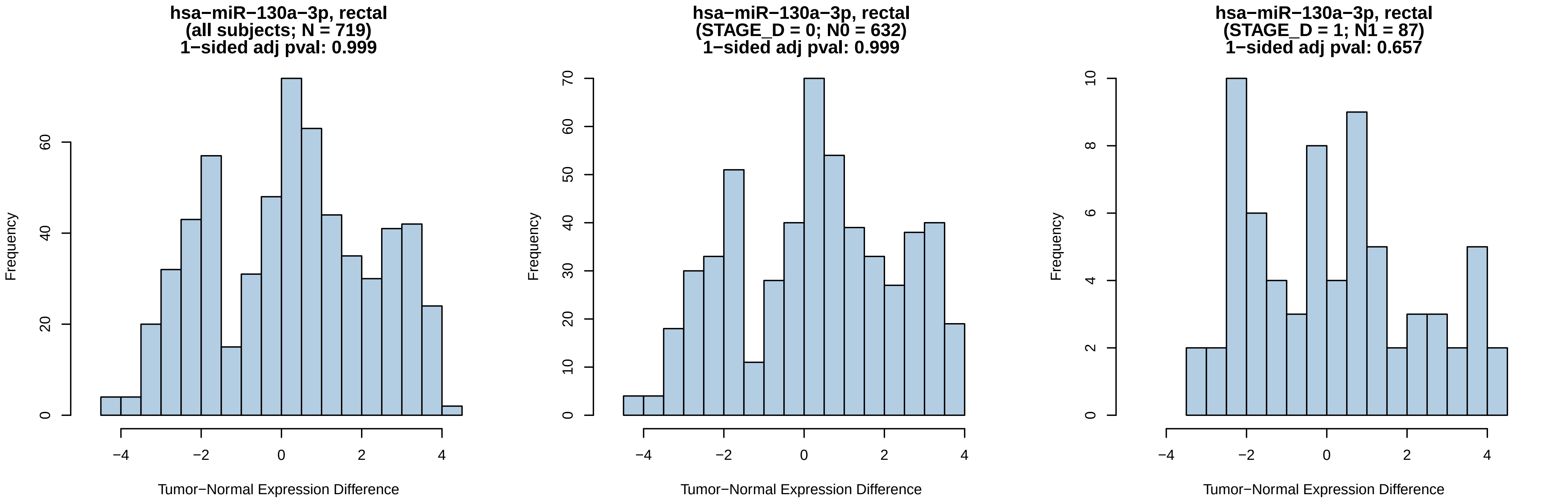
hsa−miR−130a−3p, rectal
(all subjects; N = 719)
1−sided adj pval: 0.999
hsa−miR−130a−3p, rectal
(STAGE_D = 0; N0 = 632)
1−sided adj pval: 0.999
hsa−miR−130a−3p, rectal
(STAGE_D = 1; N1 = 87)
1−sided adj pval: 0.657
Frequency
Tumor−Normal Expression Difference

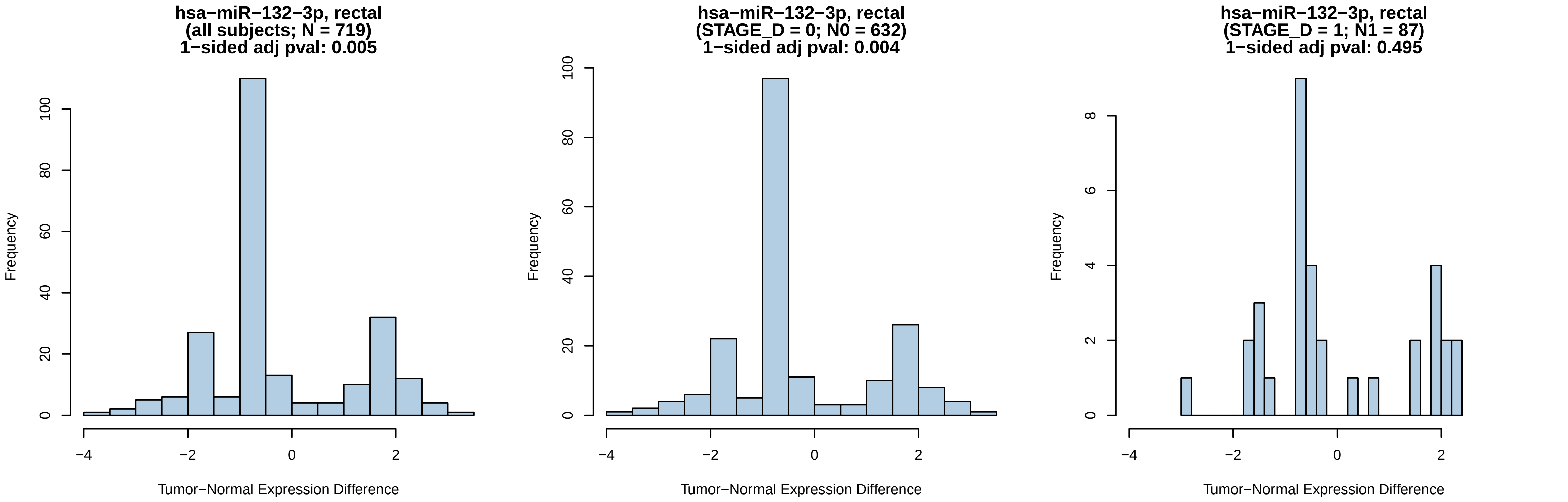

hsa−miR−132−3p, rectal
(all subjects; N = 719)
1−sided adj pval: 0.005
hsa−miR−132−3p, rectal
(STAGE_D = 0; N0 = 632)
1−sided adj pval: 0.004
hsa−miR−132−3p, rectal
(STAGE_D = 1; N1 = 87)
1−sided adj pval: 0.495
Frequency
Tumor−Normal Expression Difference

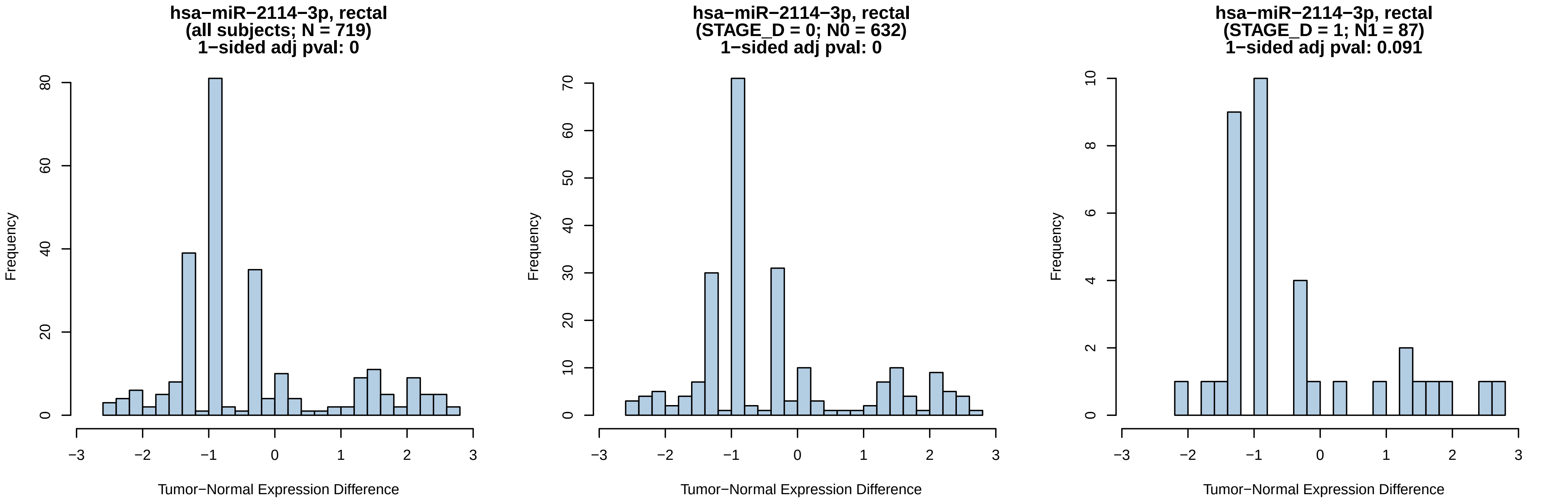
hsa−miR−2114−3p, rectal
(all subjects; N = 719)
1−sided adj pval: 0
Frequency
Tumor−Normal Expression Difference
hsa−miR−2114−3p, rectal
(STAGE_D = 0; N0 = 632)
1−sided adj pval: 0
Frequency
Tumor−Normal Expression Difference
hsa−miR−2114−3p, rectal
(STAGE_D = 1; N1 = 87)
1−sided adj pval: 0.091
Frequency
Tumor−Normal Expression Difference

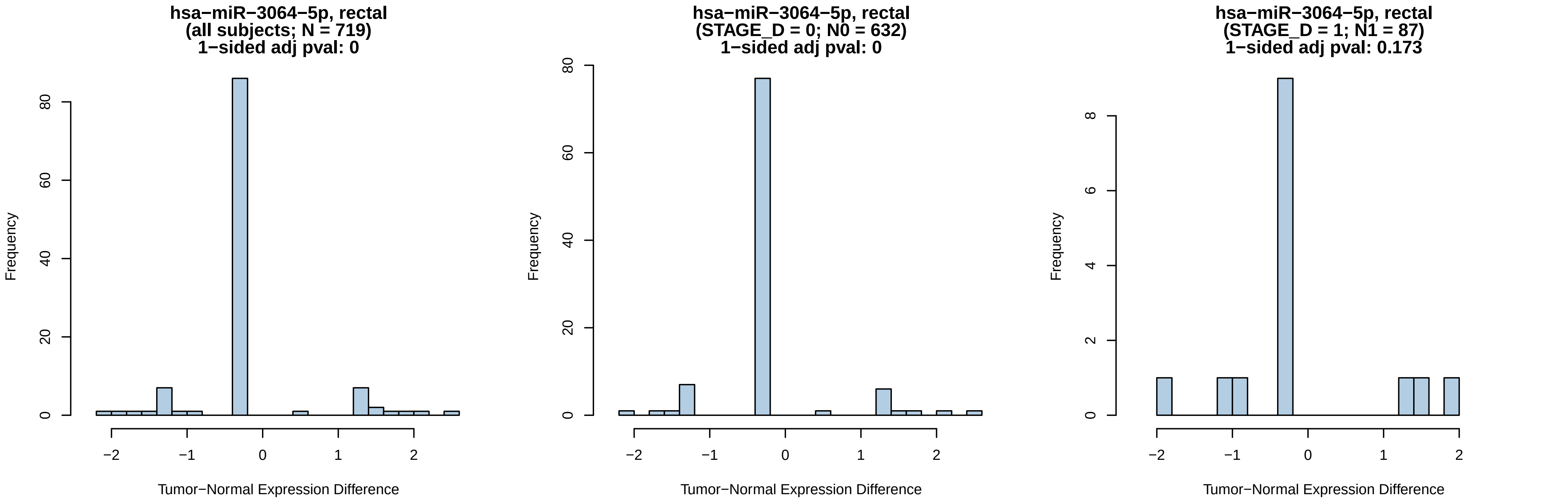
hsa−miR−3064−5p, rectal
(all subjects; N = 719)
1−sided adj pval: 0
Frequency
Tumor−Normal Expression Difference
hsa−miR−3064−5p, rectal
(STAGE_D = 0; N0 = 632)
1−sided adj pval: 0
Frequency
Tumor−Normal Expression Difference
hsa−miR−3064−5p, rectal
(STAGE_D = 1; N1 = 87)
1−sided adj pval: 0.173
Frequency
Tumor−Normal Expression Difference

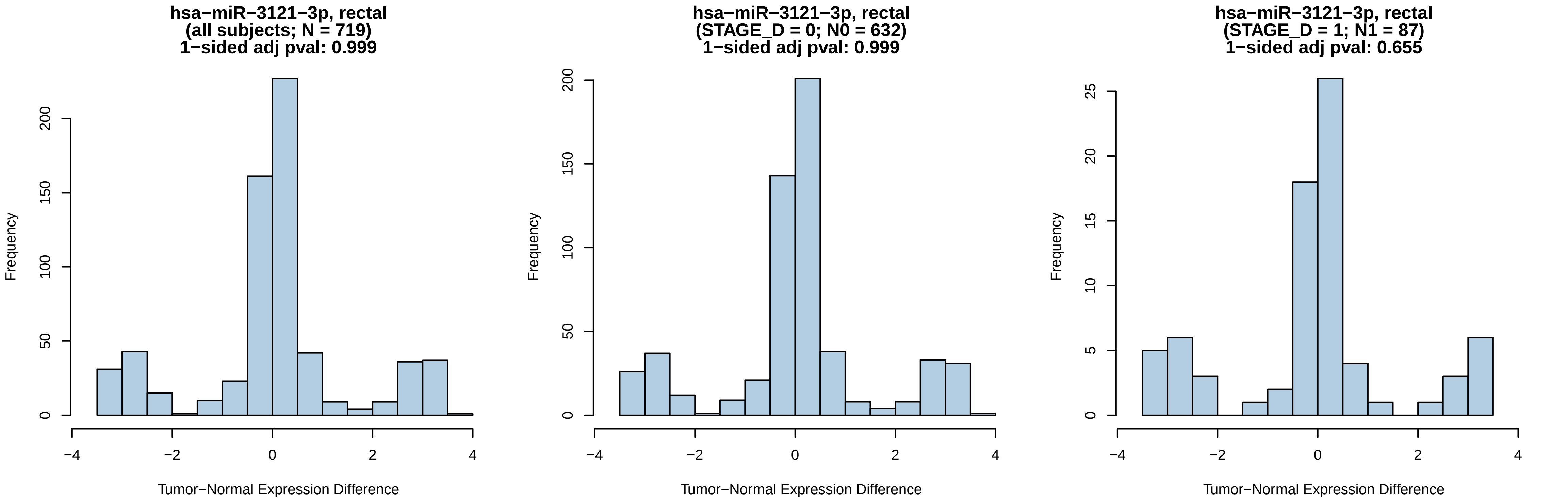
hsa−miR−3121−3p, rectal
(all subjects; N = 719)
1−sided adj pval: 0.999
Frequency
Tumor−Normal Expression Difference
hsa−miR−3121−3p, rectal
(STAGE_D = 0; N0 = 632)
1−sided adj pval: 0.999
Frequency
Tumor−Normal Expression Difference
hsa−miR−3121−3p, rectal
(STAGE_D = 1; N1 = 87)
1−sided adj pval: 0.655
Frequency
Tumor−Normal Expression Difference

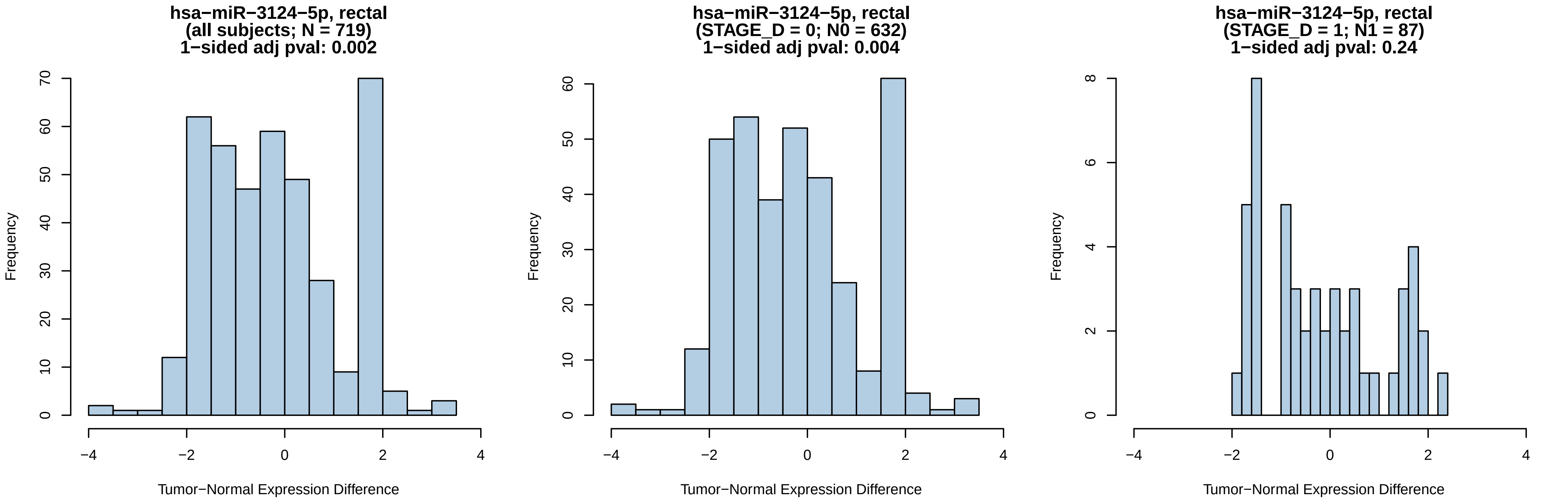

hsa−miR−3124−5p, rectal
(all subjects; N = 719)
1−sided adj pval: 0.002
Frequency
Tumor−Normal Expression Difference
hsa−miR−3124−5p, rectal
(STAGE_D = 0; N0 = 632)
1−sided adj pval: 0.004
Frequency
Tumor−Normal Expression Difference
hsa−miR−3124−5p, rectal
(STAGE_D = 1; N1 = 87)
1−sided adj pval: 0.24
Frequency
Tumor−Normal Expression Difference

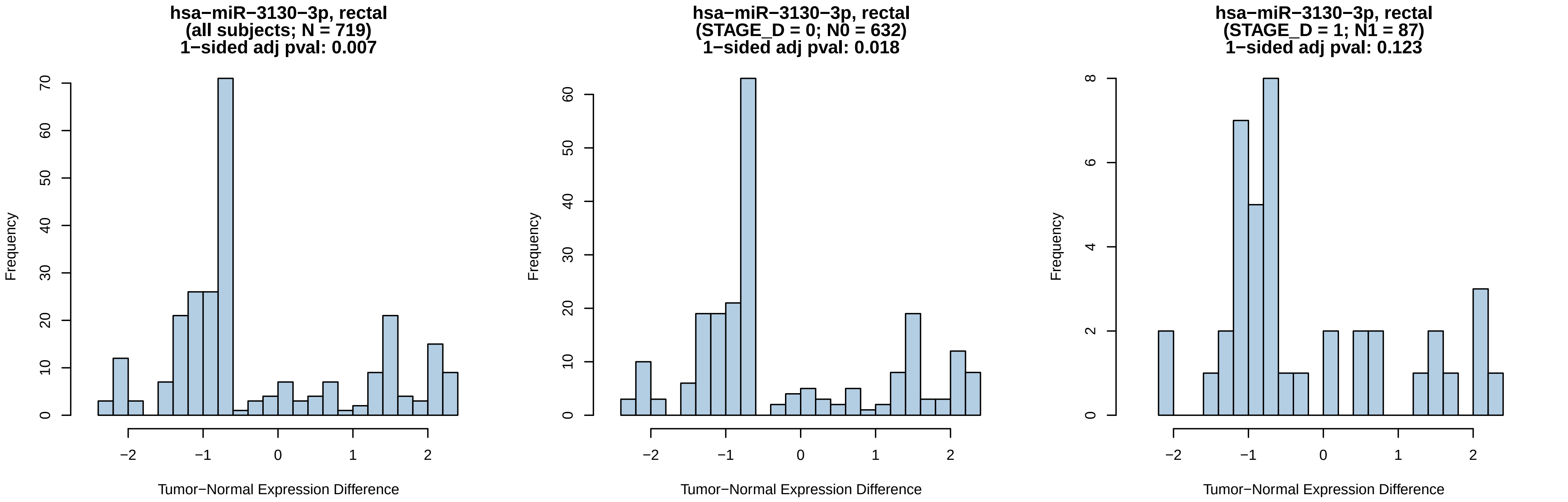
hsa−miR−3130−3p, rectal
(all subjects; N = 719)
1−sided adj pval: 0.007
hsa−miR−3130−3p, rectal
(STAGE_D = 0; N0 = 632)
1−sided adj pval: 0.018
hsa−miR−3130−3p, rectal
(STAGE_D = 1; N1 = 87)
1−sided adj pval: 0.123
Frequency
Tumor−Normal Expression Difference

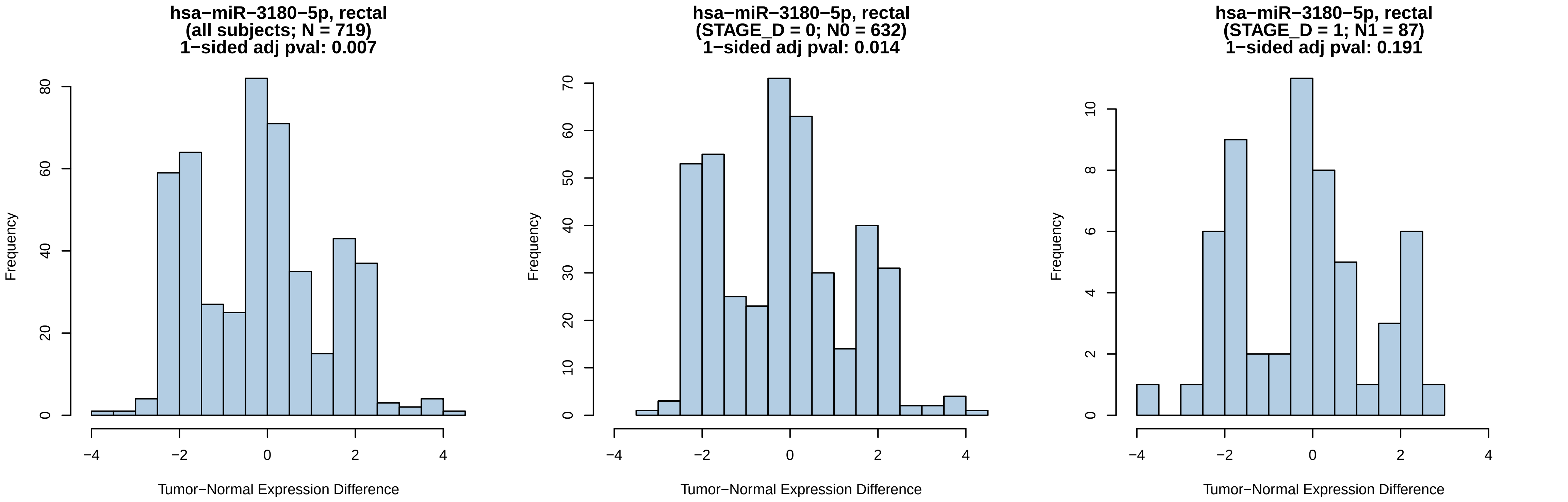
hsa−miR−3180−5p, rectal
(all subjects; N = 719)
1−sided adj pval: 0.007
Frequency
0
20
40
60
80
−4
−2
0
2
4
Tumor−Normal Expression Difference
hsa−miR−3180−5p, rectal
(STAGE_D = 0; N0 = 632)
1−sided adj pval: 0.014
Frequency
0
10
20
30
40
50
60
70
−4
−2
0
2
4
Tumor−Normal Expression Difference
hsa−miR−3180−5p, rectal
(STAGE_D = 1; N1 = 87)
1−sided adj pval: 0.191
Frequency
0
2
4
6
8
10
−4
−2
0
2
4
Tumor−Normal Expression Difference

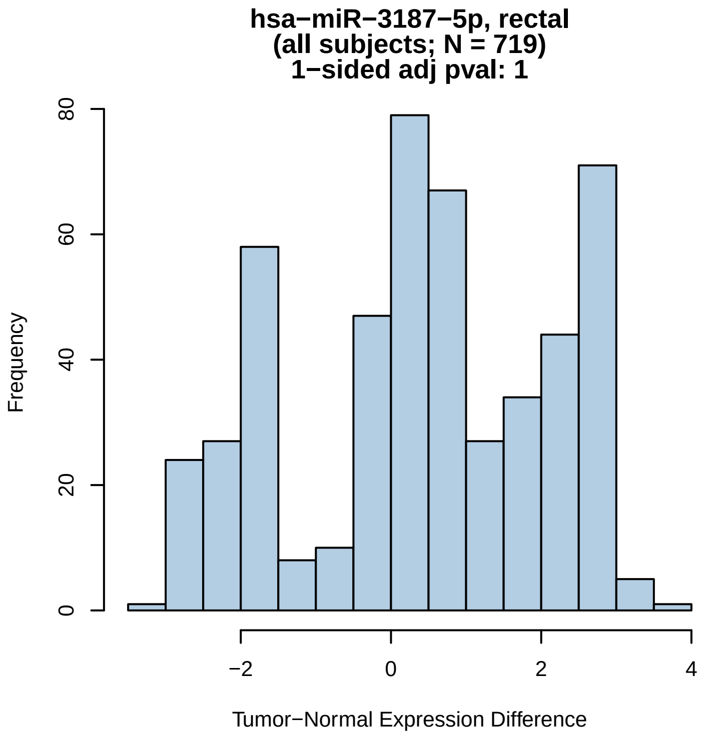

hsa−miR−3187−5p, rectal
(all subjects; N = 719)
1−sided adj pval: 1
Frequency
0
20
40
60
80
−2
0
2
4
Tumor−Normal Expression Difference


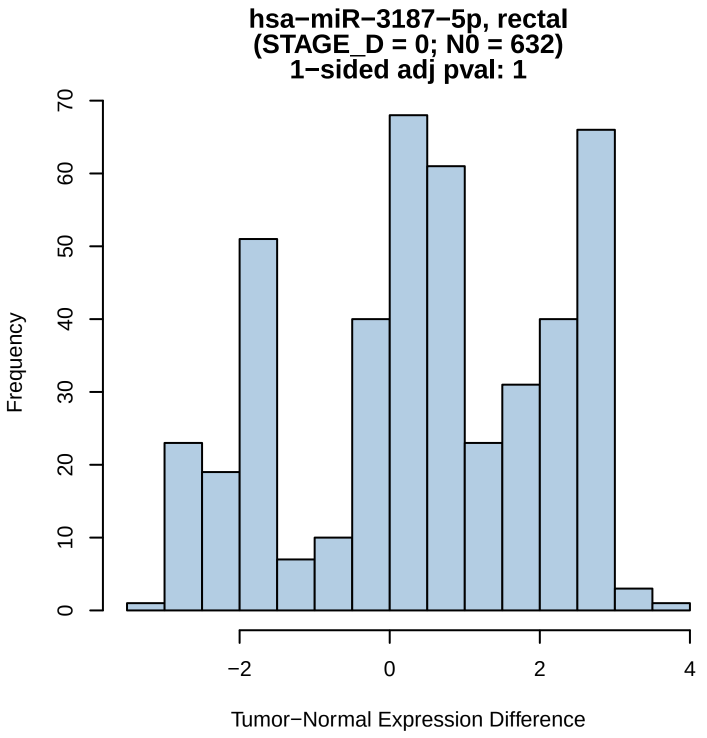

hsa−miR−3187−5p, rectal
(STAGE_D = 0; N0 = 632)
1−sided adj pval: 1
Frequency
0
10
20
30
40
50
60
70
−2
0
2
4
Tumor−Normal Expression Difference


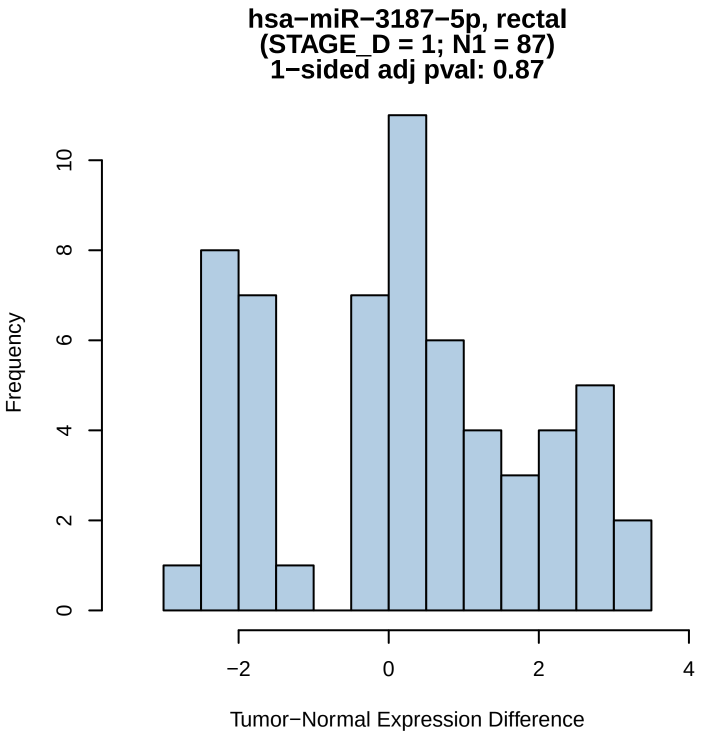

hsa−miR−3187−5p, rectal
(STAGE_D = 1; N1 = 87)
1−sided adj pval: 0.87
Frequency
0
2
4
6
8
10
−2
0
2
4
Tumor−Normal Expression Difference

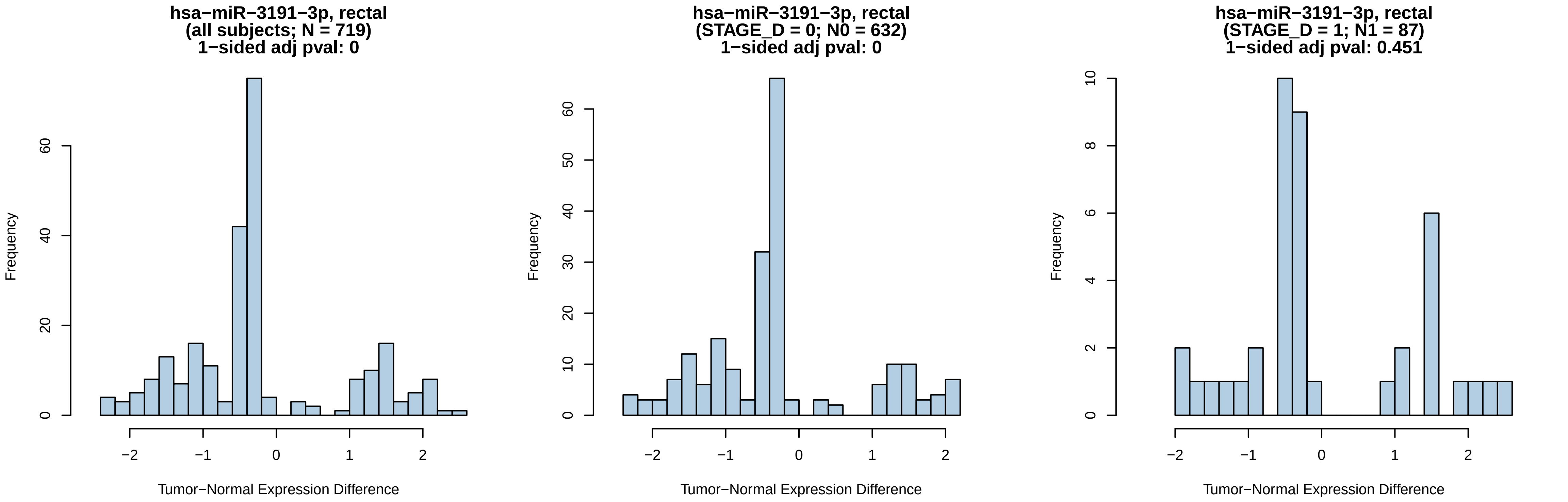
hsa−miR−3191−3p, rectal
(all subjects; N = 719)
1−sided adj pval: 0
Frequency
Tumor−Normal Expression Difference
hsa−miR−3191−3p, rectal
(STAGE_D = 0; N0 = 632)
1−sided adj pval: 0
Frequency
Tumor−Normal Expression Difference
hsa−miR−3191−3p, rectal
(STAGE_D = 1; N1 = 87)
1−sided adj pval: 0.451
Frequency
Tumor−Normal Expression Difference

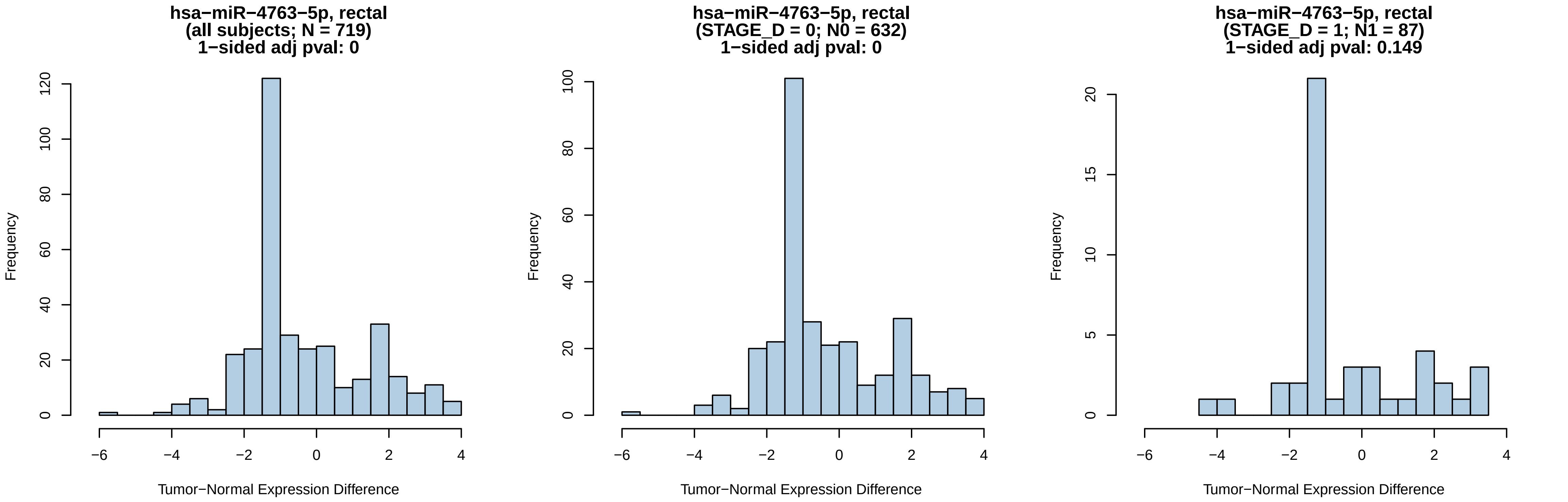

hsa−miR−4763−5p, rectal
(all subjects; N = 719)
1−sided adj pval: 0
hsa−miR−4763−5p, rectal
(STAGE_D = 0; N0 = 632)
1−sided adj pval: 0
hsa−miR−4763−5p, rectal
(STAGE_D = 1; N1 = 87)
1−sided adj pval: 0.149
Frequency
Tumor−Normal Expression Difference

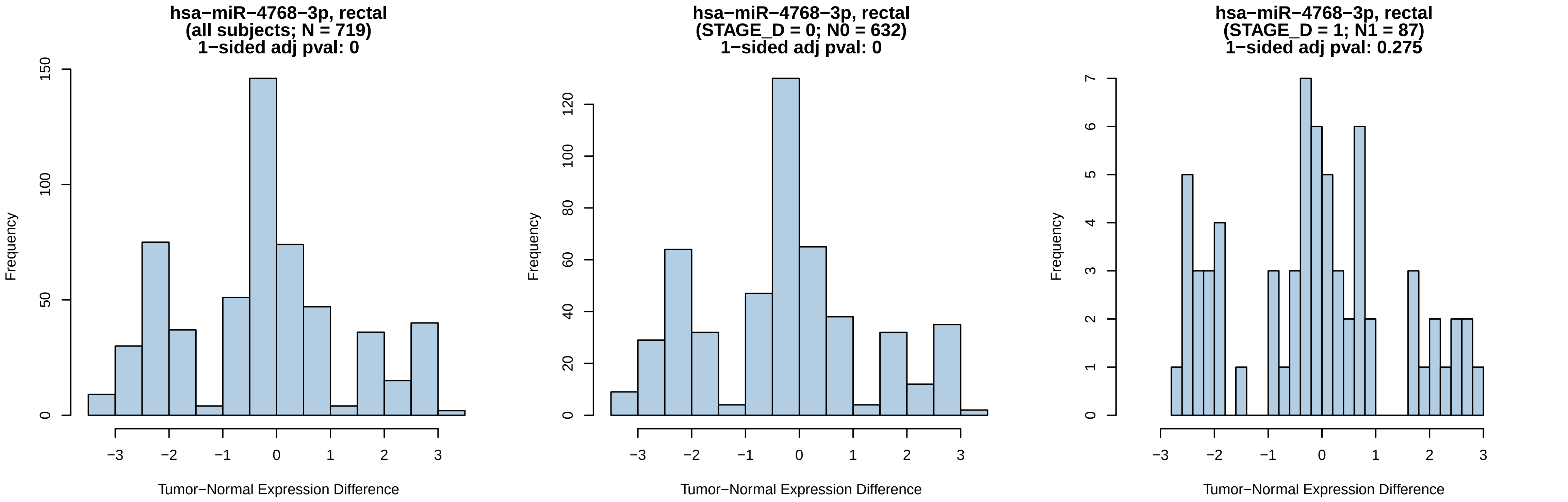

hsa−miR−4768−3p, rectal
(all subjects; N = 719)
1−sided adj pval: 0
hsa−miR−4768−3p, rectal
(STAGE_D = 0; N0 = 632)
1−sided adj pval: 0
hsa−miR−4768−3p, rectal
(STAGE_D = 1; N1 = 87)
1−sided adj pval: 0.275
Frequency
Tumor−Normal Expression Difference

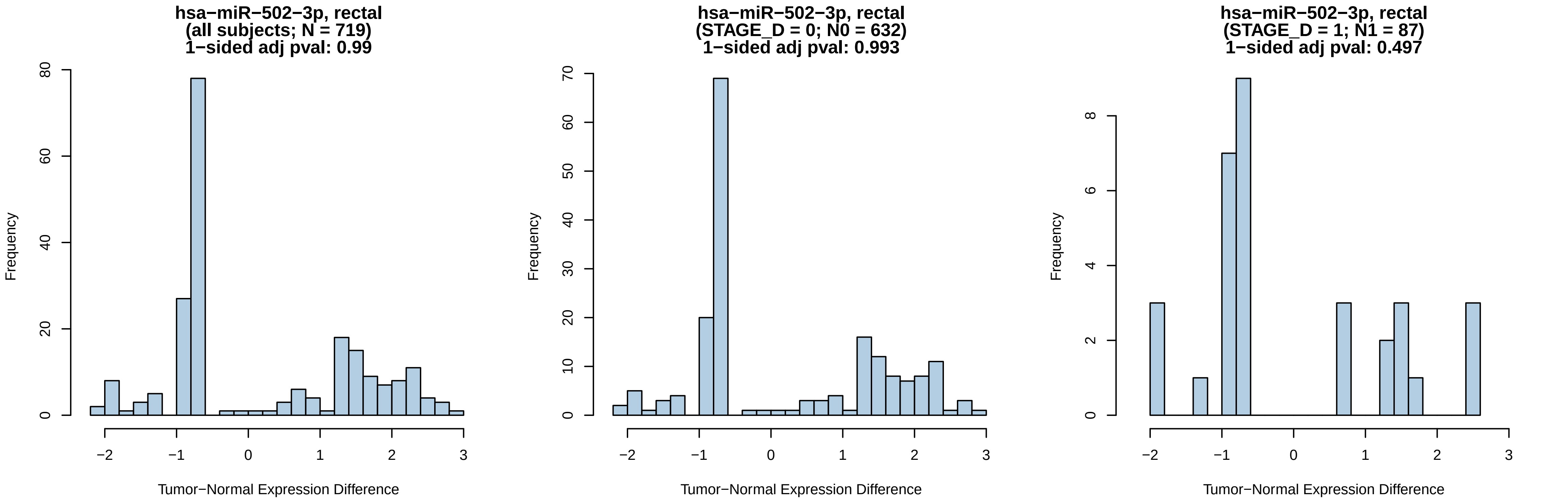
hsa−miR−502−3p, rectal
(all subjects; N = 719)
1−sided adj pval: 0.99
Frequency
0
20
40
60
80
−2
−1
0
1
2
3
Tumor−Normal Expression Difference
hsa−miR−502−3p, rectal
(STAGE_D = 0; N0 = 632)
1−sided adj pval: 0.993
Frequency
0
10
20
30
40
50
60
70
−2
−1
0
1
2
3
Tumor−Normal Expression Difference
hsa−miR−502−3p, rectal
(STAGE_D = 1; N1 = 87)
1−sided adj pval: 0.497
Frequency
0
2
4
6
8
−2
−1
0
1
2
3
Tumor−Normal Expression Difference

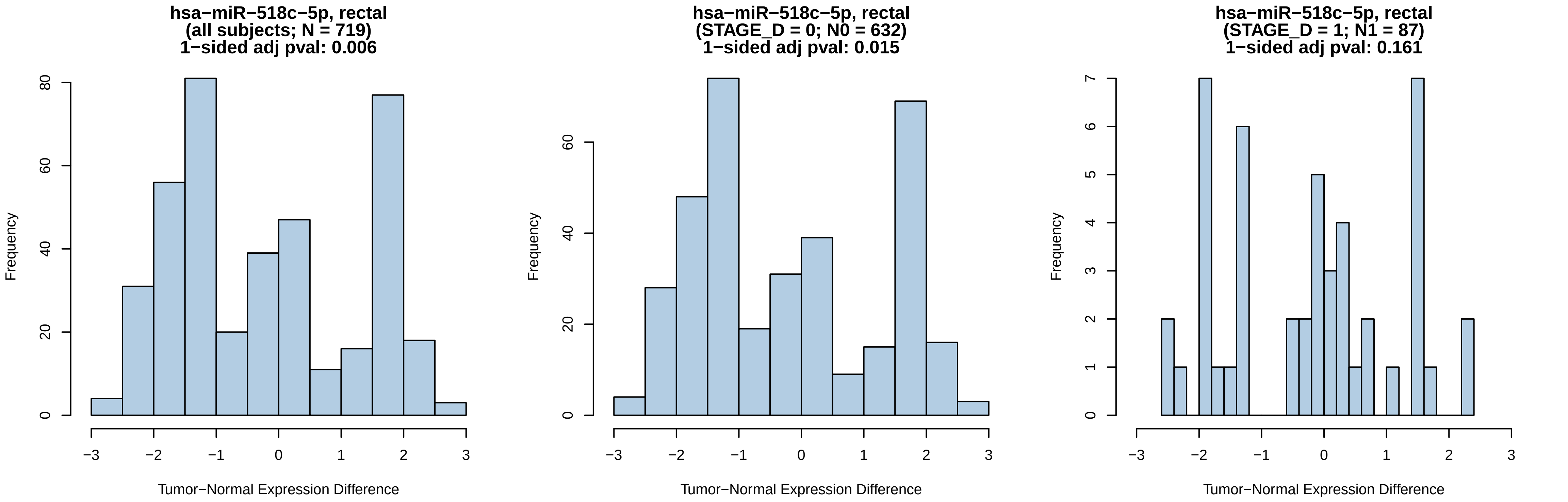
hsa−miR−518c−5p, rectal
(all subjects; N = 719)
1−sided adj pval: 0.006
hsa−miR−518c−5p, rectal
(STAGE_D = 0; N0 = 632)
1−sided adj pval: 0.015
hsa−miR−518c−5p, rectal
(STAGE_D = 1; N1 = 87)
1−sided adj pval: 0.161
Frequency
Tumor−Normal Expression Difference

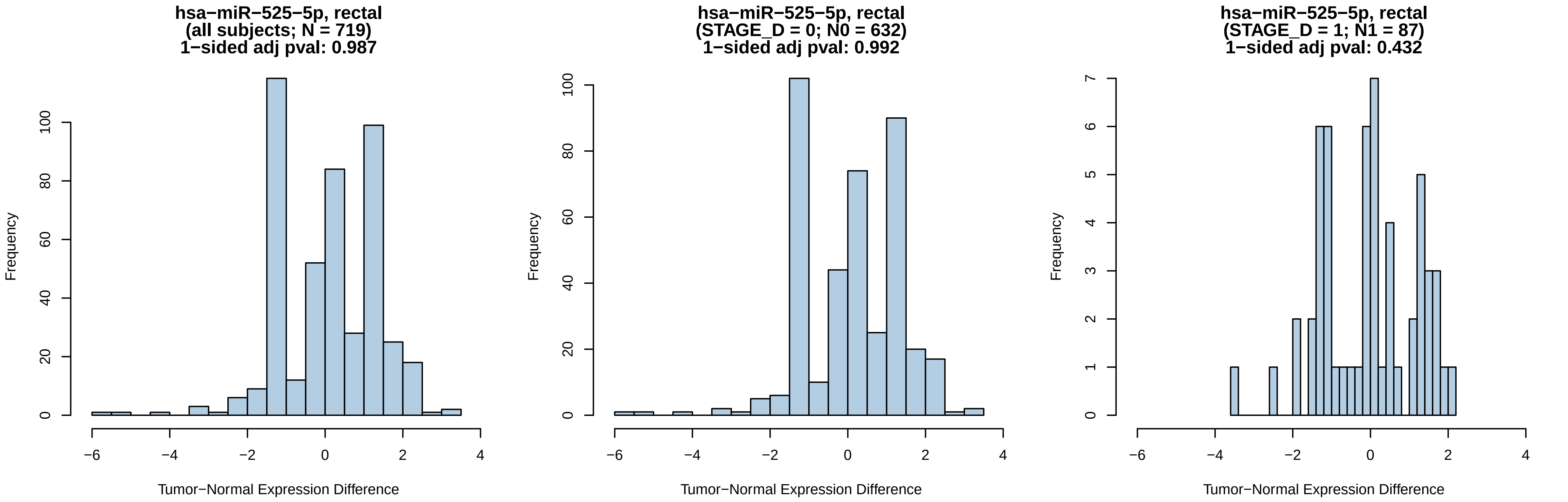

hsa−miR−525−5p, rectal
(all subjects; N = 719)
1−sided adj pval: 0.987
Frequency
Tumor−Normal Expression Difference
hsa−miR−525−5p, rectal
(STAGE_D = 0; N0 = 632)
1−sided adj pval: 0.992
Frequency
Tumor−Normal Expression Difference
hsa−miR−525−5p, rectal
(STAGE_D = 1; N1 = 87)
1−sided adj pval: 0.432
Frequency
Tumor−Normal Expression Difference

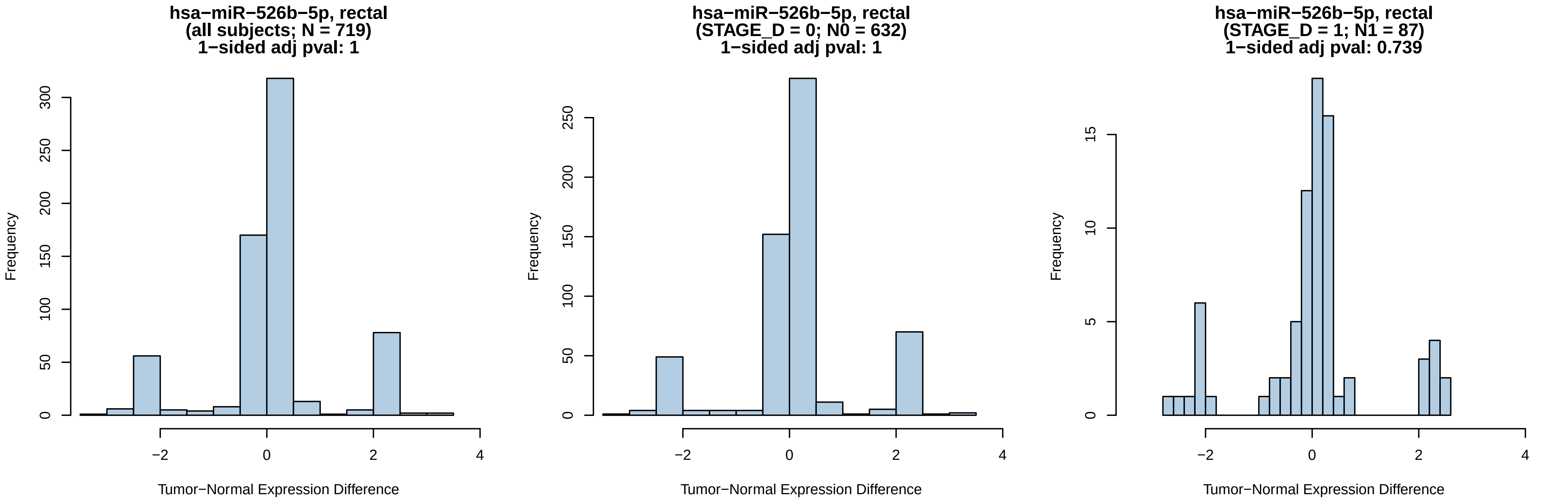
hsa−miR−526b−5p, rectal
(all subjects; N = 719)
1−sided adj pval: 1
Frequency
Tumor−Normal Expression Difference
hsa−miR−526b−5p, rectal
(STAGE_D = 0; N0 = 632)
1−sided adj pval: 1
Frequency
Tumor−Normal Expression Difference
hsa−miR−526b−5p, rectal
(STAGE_D = 1; N1 = 87)
1−sided adj pval: 0.739
Frequency
Tumor−Normal Expression Difference

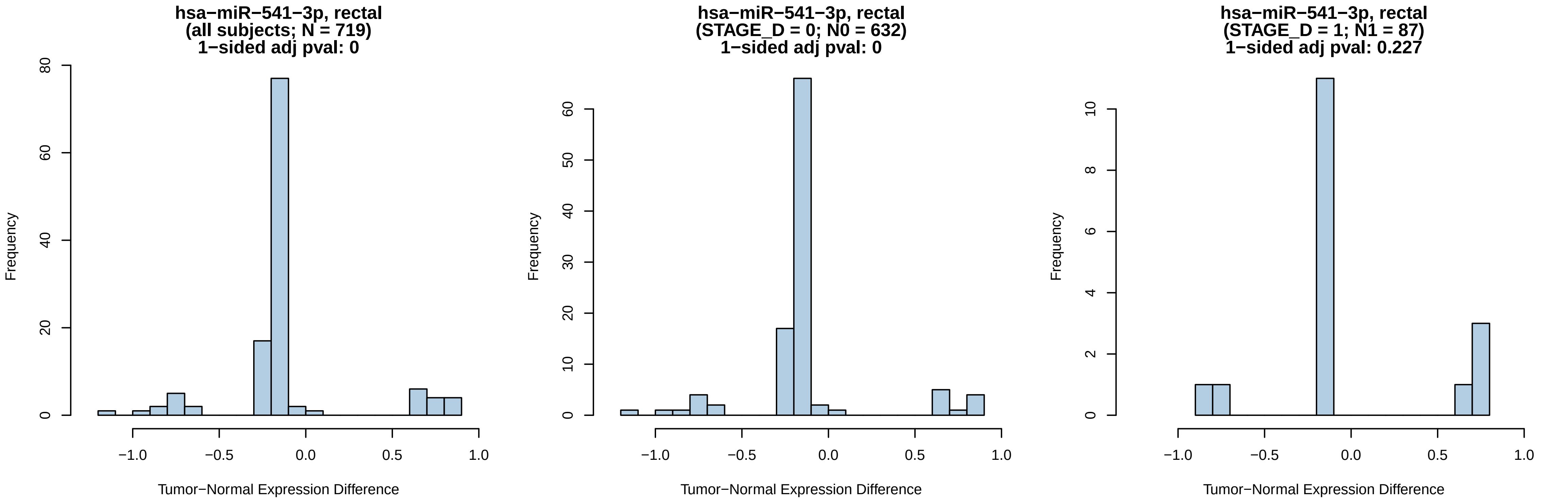

hsa−miR−541−3p, rectal
(all subjects; N = 719)
1−sided adj pval: 0
hsa−miR−541−3p, rectal
(STAGE_D = 0; N0 = 632)
1−sided adj pval: 0
hsa−miR−541−3p, rectal
(STAGE_D = 1; N1 = 87)
1−sided adj pval: 0.227
Frequency
Tumor−Normal Expression Difference

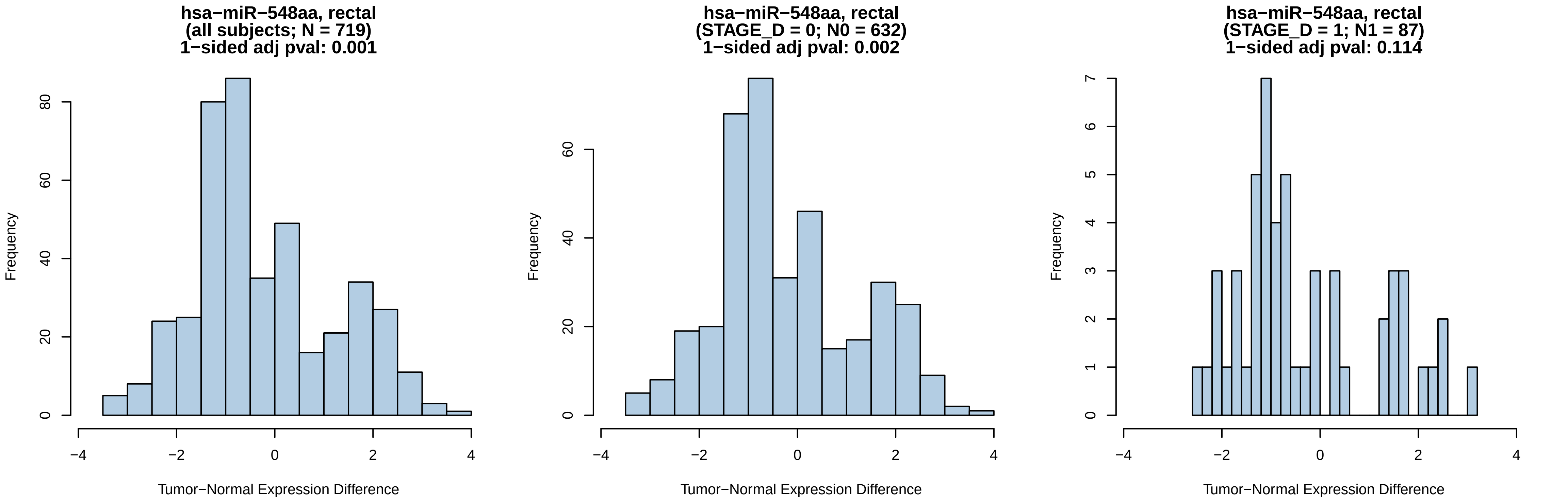

hsa−miR−548aa, rectal
(all subjects; N = 719)
1−sided adj pval: 0.001
Frequency
0
20
40
60
80
−4
−2
0
2
4
Tumor−Normal Expression Difference
hsa−miR−548aa, rectal
(STAGE_D = 0; N0 = 632)
1−sided adj pval: 0.002
Frequency
0
20
40
60
−4
−2
0
2
4
Tumor−Normal Expression Difference
hsa−miR−548aa, rectal
(STAGE_D = 1; N1 = 87)
1−sided adj pval: 0.114
Frequency
0
1
2
3
4
5
6
7
−4
−2
0
2
4
Tumor−Normal Expression Difference

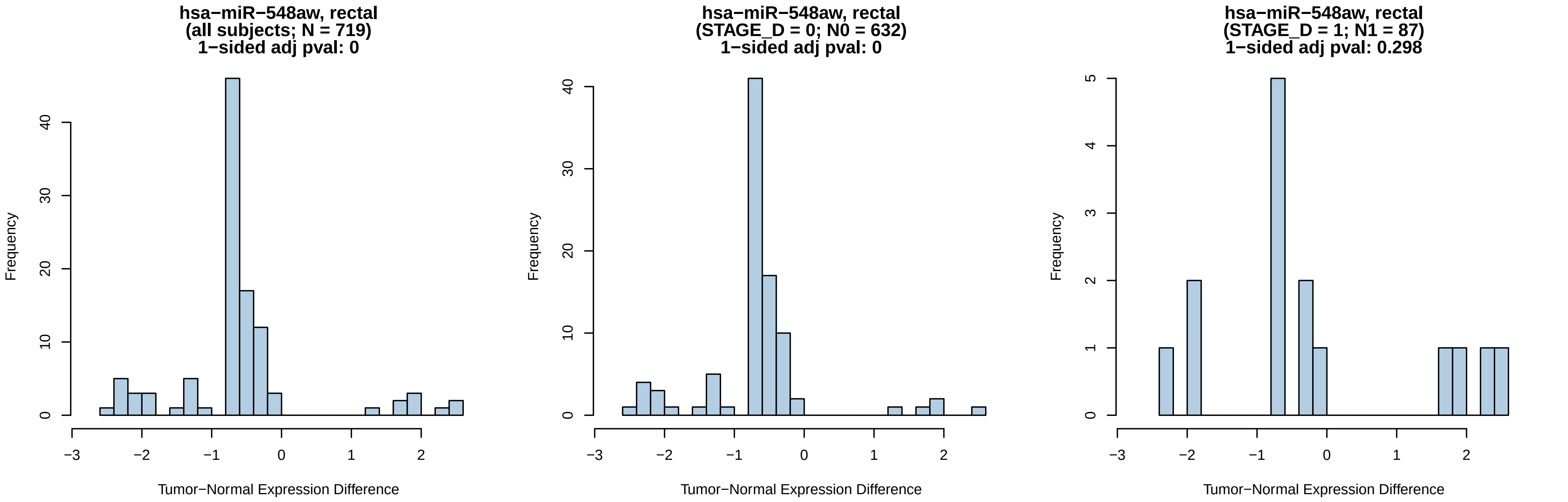
hsa−miR−548aw, rectal
(all subjects; N = 719)
1−sided adj pval: 0
hsa−miR−548aw, rectal
(STAGE_D = 0; N0 = 632)
1−sided adj pval: 0
hsa−miR−548aw, rectal
(STAGE_D = 1; N1 = 87)
1−sided adj pval: 0.298
Frequency
Tumor−Normal Expression Difference

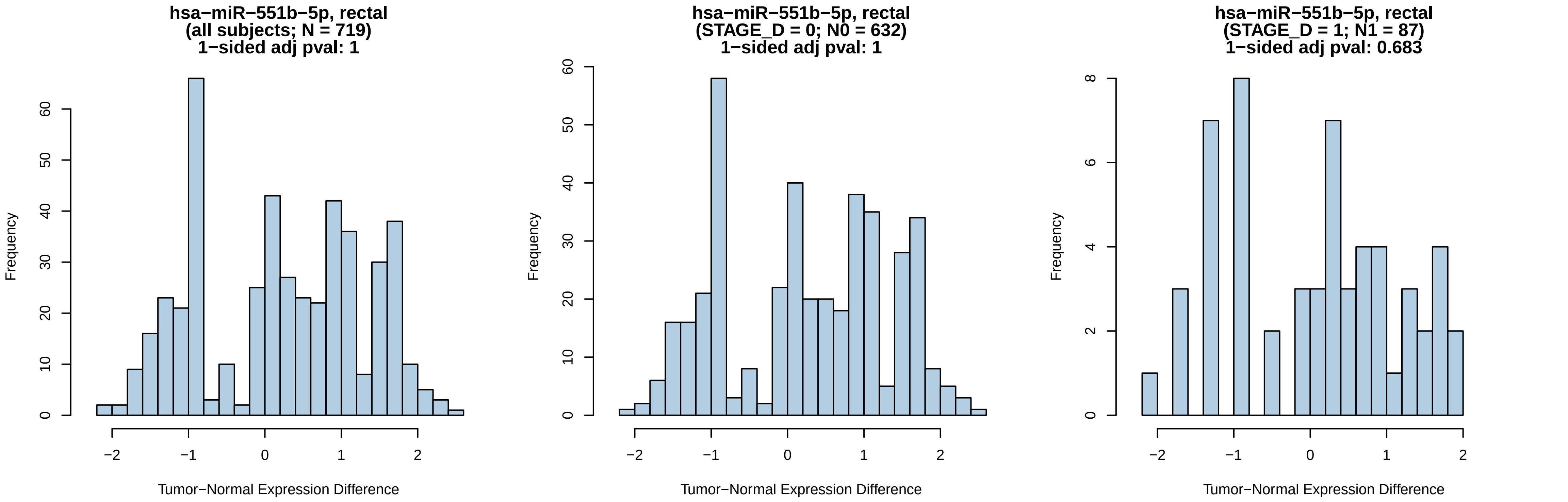

hsa−miR−551b−5p, rectal
(all subjects; N = 719)
1−sided adj pval: 1
Frequency
Tumor−Normal Expression Difference
hsa−miR−551b−5p, rectal
(STAGE_D = 0; N0 = 632)
1−sided adj pval: 1
Frequency
Tumor−Normal Expression Difference
hsa−miR−551b−5p, rectal
(STAGE_D = 1; N1 = 87)
1−sided adj pval: 0.683
Frequency
Tumor−Normal Expression Difference

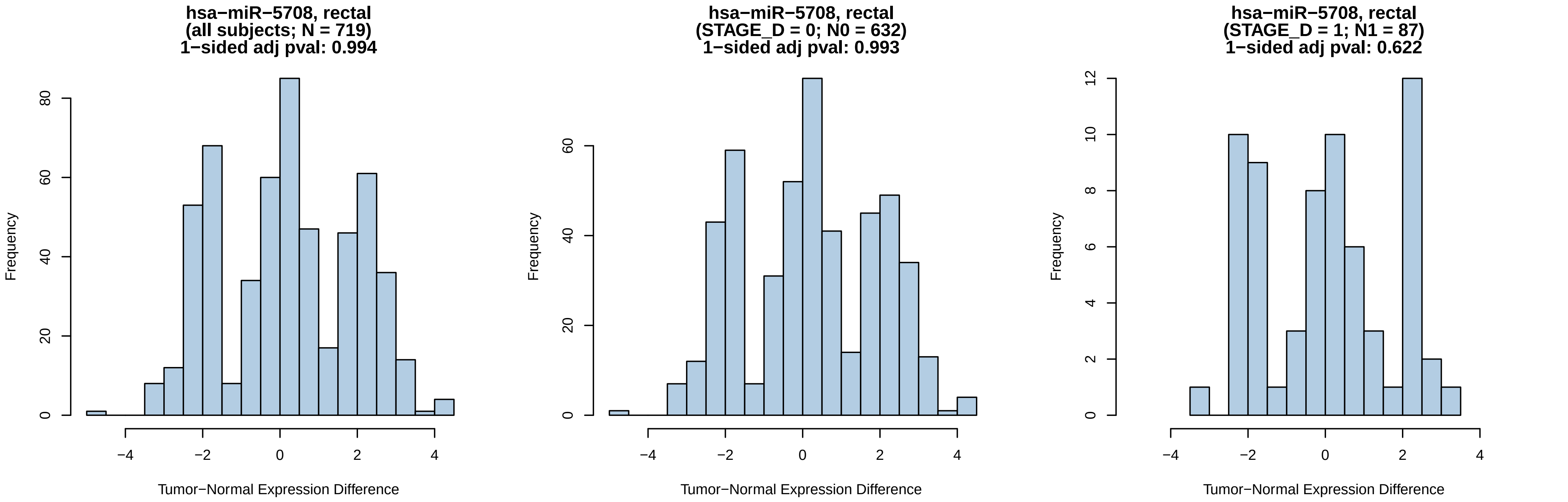
hsa−miR−5708, rectal
(all subjects; N = 719)
1−sided adj pval: 0.994
Frequency
Tumor−Normal Expression Difference
hsa−miR−5708, rectal
(STAGE_D = 0; N0 = 632)
1−sided adj pval: 0.993
Frequency
Tumor−Normal Expression Difference
hsa−miR−5708, rectal
(STAGE_D = 1; N1 = 87)
1−sided adj pval: 0.622
Frequency
Tumor−Normal Expression Difference

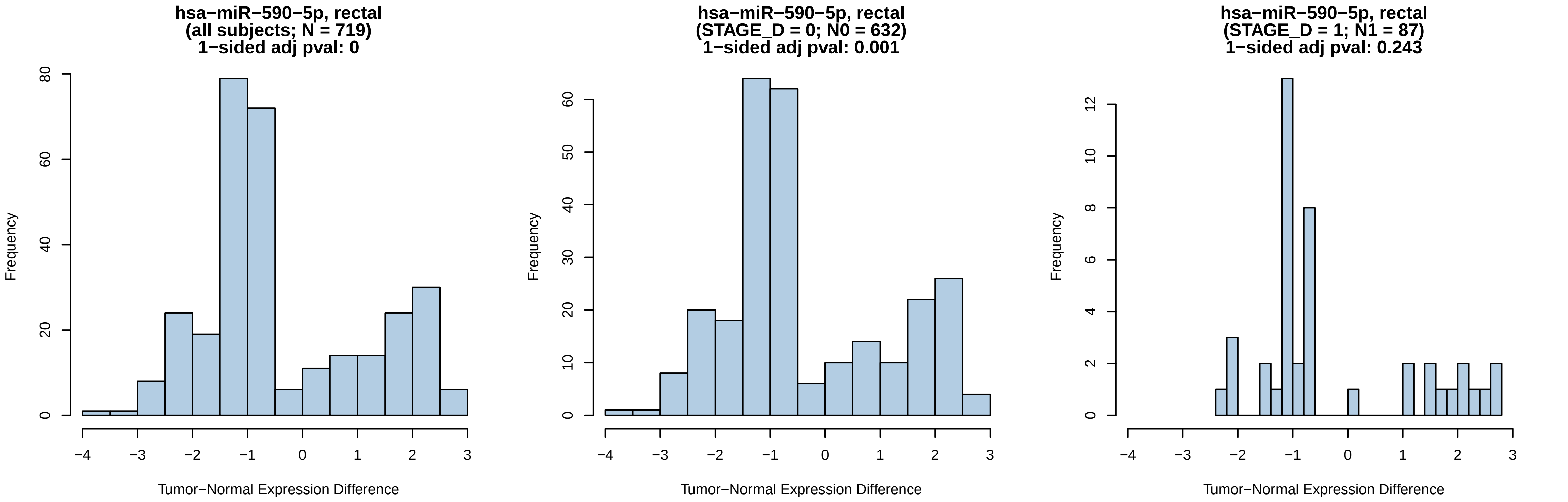
hsa−miR−590−5p, rectal
(all subjects; N = 719)
1−sided adj pval: 0
Frequency
0
20
40
60
80
−4
−3
−2
−1
0
1
2
3
Tumor−Normal Expression Difference
hsa−miR−590−5p, rectal
(STAGE_D = 0; N0 = 632)
1−sided adj pval: 0.001
Frequency
0
10
20
30
40
50
60
−4
−3
−2
−1
0
1
2
3
Tumor−Normal Expression Difference
hsa−miR−590−5p, rectal
(STAGE_D = 1; N1 = 87)
1−sided adj pval: 0.243
Frequency
0
2
4
6
8
10
12
−4
−3
−2
−1
0
1
2
3
Tumor−Normal Expression Difference

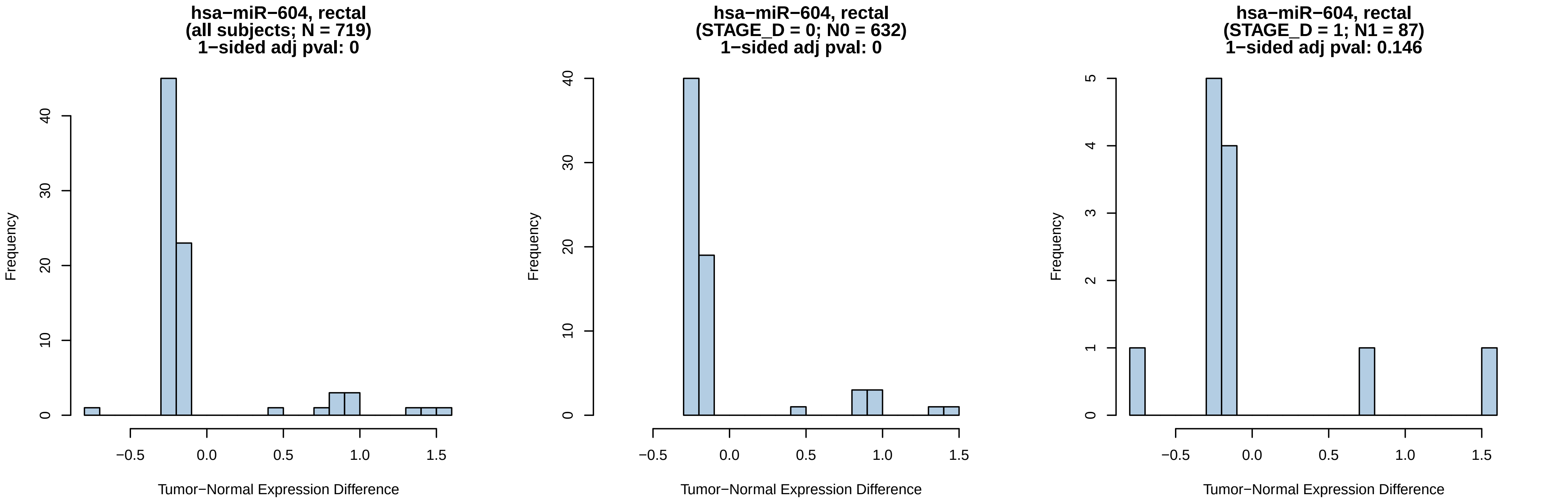

hsa−miR−604, rectal
(all subjects; N = 719)
1−sided adj pval: 0
Frequency
0
10
20
30
40
−0.5
0.0
0.5
1.0
1.5
Tumor−Normal Expression Difference
hsa−miR−604, rectal
(STAGE_D = 0; N0 = 632)
1−sided adj pval: 0
Frequency
0
10
20
30
40
−0.5
0.0
0.5
1.0
1.5
Tumor−Normal Expression Difference
hsa−miR−604, rectal
(STAGE_D = 1; N1 = 87)
1−sided adj pval: 0.146
Frequency
0
1
2
3
4
5
−0.5
0.0
0.5
1.0
1.5
Tumor−Normal Expression Difference

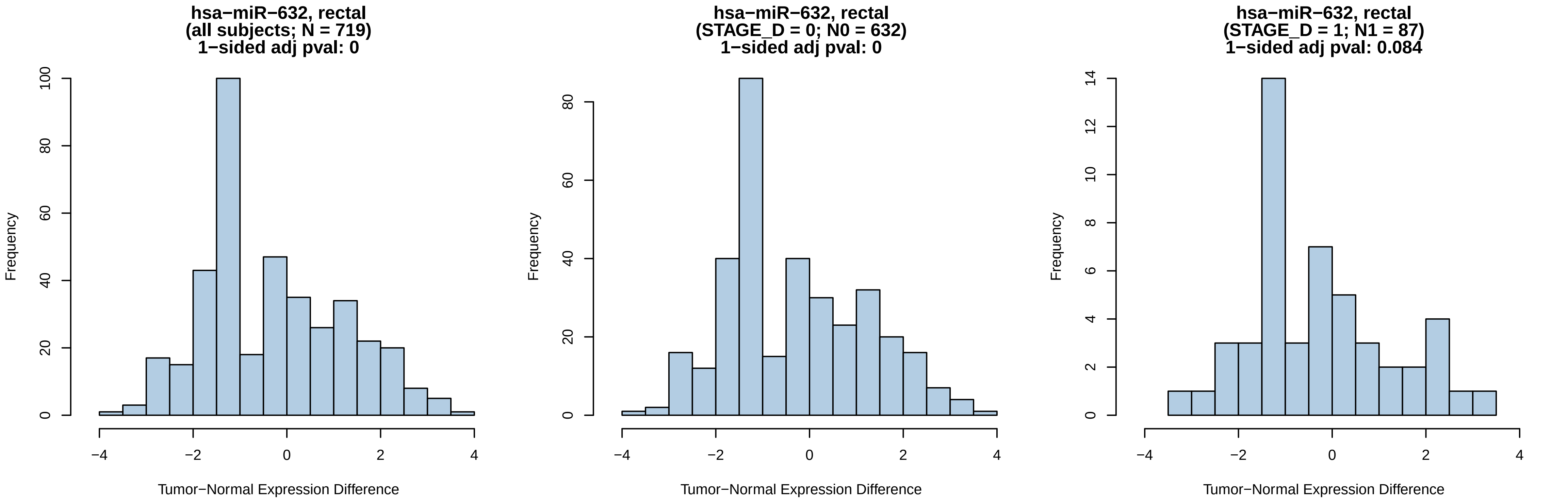

hsa−miR−632, rectal
(all subjects; N = 719)
1−sided adj pval: 0
Frequency
0
20
40
60
80
100
−4
−2
0
2
4
Tumor−Normal Expression Difference
hsa−miR−632, rectal
(STAGE_D = 0; N0 = 632)
1−sided adj pval: 0
Frequency
0
20
40
60
80
−4
−2
0
2
4
Tumor−Normal Expression Difference
hsa−miR−632, rectal
(STAGE_D = 1; N1 = 87)
1−sided adj pval: 0.084
Frequency
0
2
4
6
8
10
12
14
−4
−2
0
2
4
Tumor−Normal Expression Difference

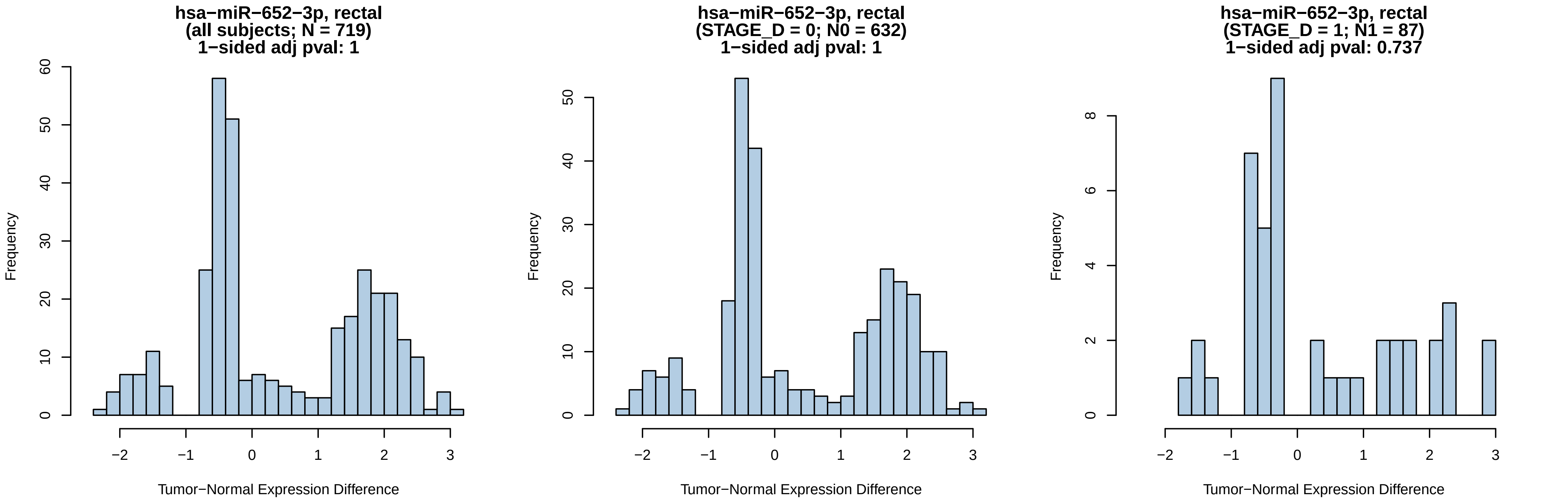

hsa−miR−652−3p, rectal
(all subjects; N = 719)
1−sided adj pval: 1
hsa−miR−652−3p, rectal
(STAGE_D = 0; N0 = 632)
1−sided adj pval: 1
hsa−miR−652−3p, rectal
(STAGE_D = 1; N1 = 87)
1−sided adj pval: 0.737
Frequency
Tumor−Normal Expression Difference

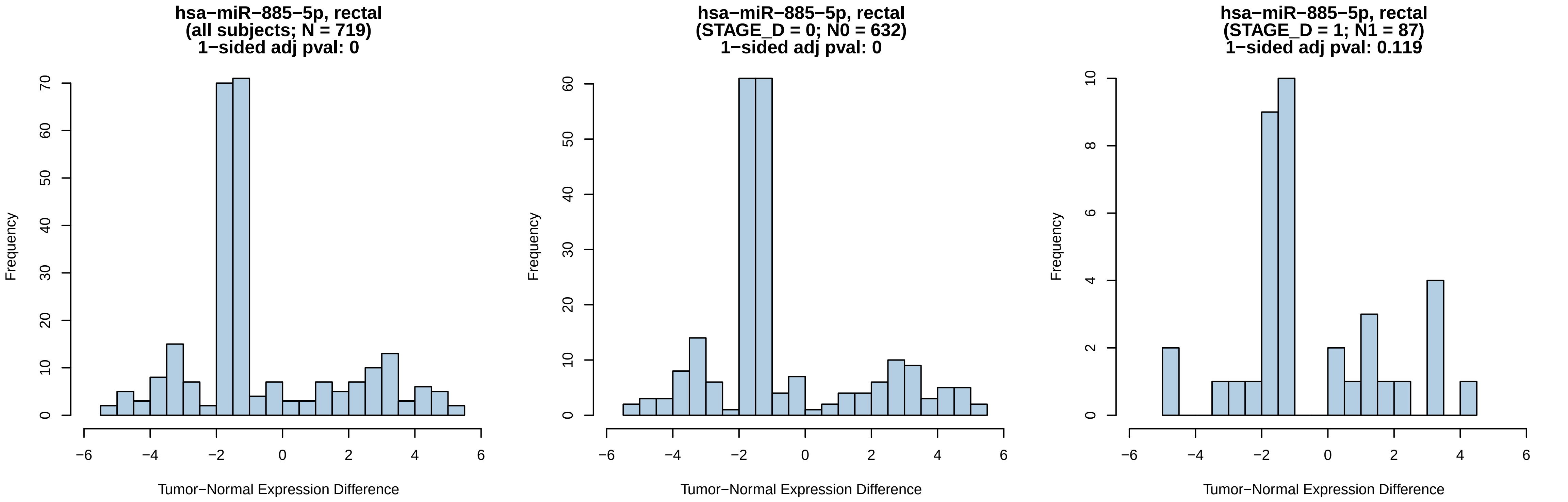

hsa−miR−885−5p, rectal
(all subjects; N = 719)
1−sided adj pval: 0
Frequency
Tumor−Normal Expression Difference
hsa−miR−885−5p, rectal
(STAGE_D = 0; N0 = 632)
1−sided adj pval: 0
Frequency
Tumor−Normal Expression Difference
hsa−miR−885−5p, rectal
(STAGE_D = 1; N1 = 87)
1−sided adj pval: 0.119
Frequency
Tumor−Normal Expression Difference

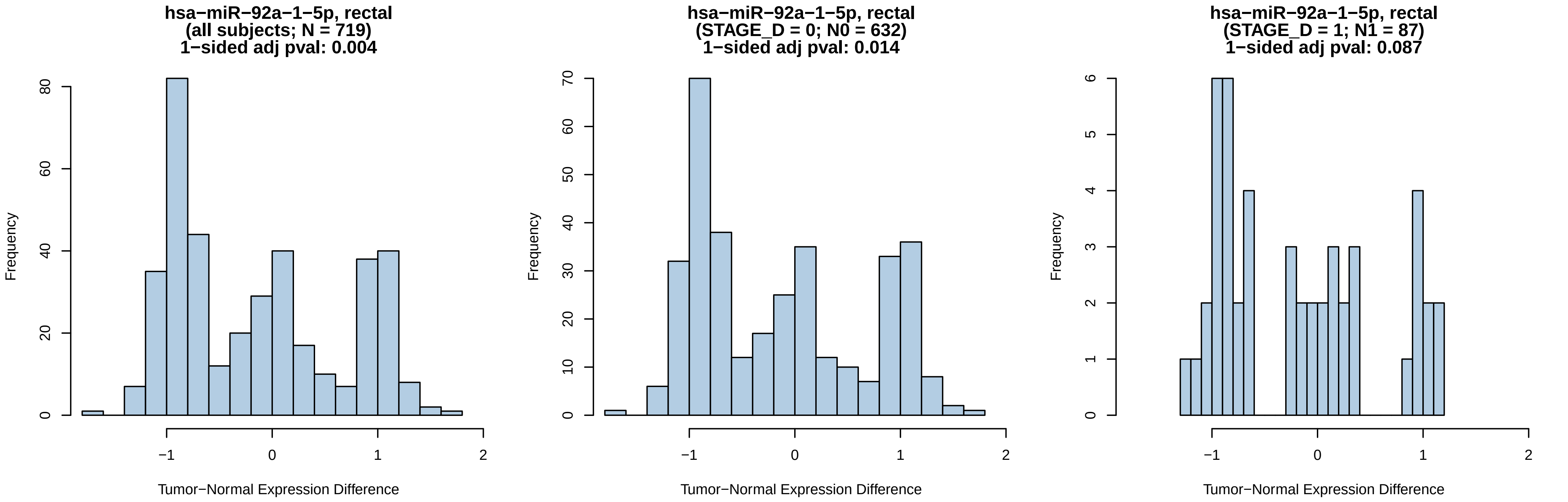

hsa−miR−92a−1−5p, rectal
(all subjects; N = 719)
1−sided adj pval: 0.004
Frequency
Tumor−Normal Expression Difference
hsa−miR−92a−1−5p, rectal
(STAGE_D = 0; N0 = 632)
1−sided adj pval: 0.014
Frequency
Tumor−Normal Expression Difference
hsa−miR−92a−1−5p, rectal
(STAGE_D = 1; N1 = 87)
1−sided adj pval: 0.087
Frequency
Tumor−Normal Expression Difference

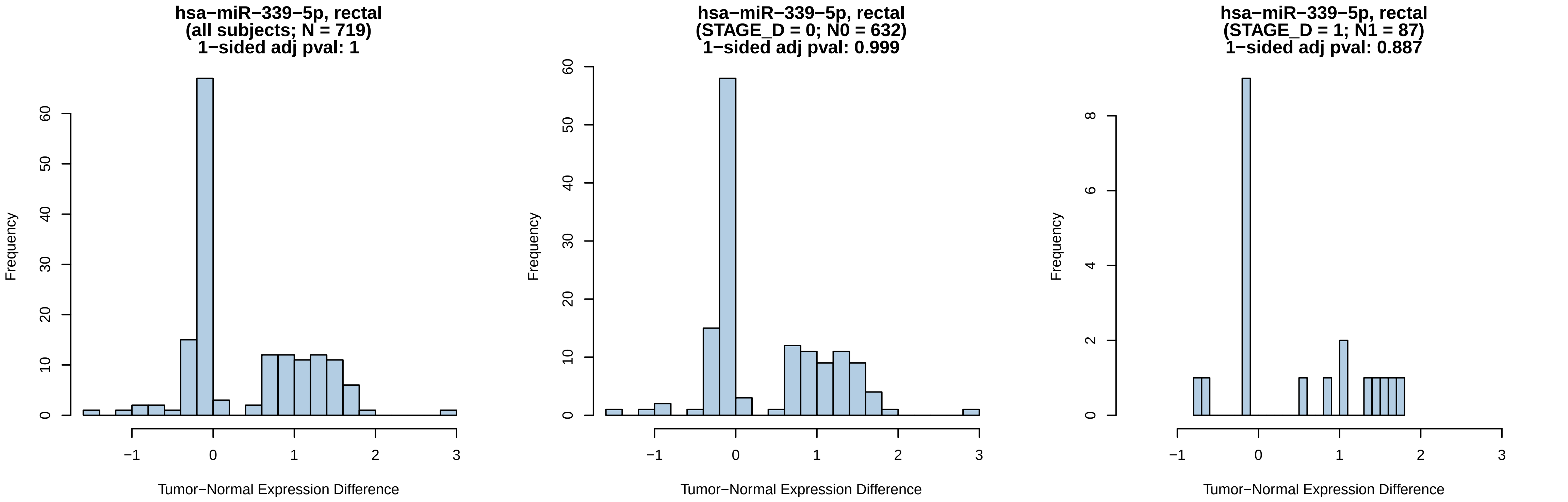
hsa−miR−339−5p, rectal
(all subjects; N = 719)
1−sided adj pval: 1
Frequency
Tumor−Normal Expression Difference
hsa−miR−339−5p, rectal
(STAGE_D = 0; N0 = 632)
1−sided adj pval: 0.999
Frequency
Tumor−Normal Expression Difference
hsa−miR−339−5p, rectal
(STAGE_D = 1; N1 = 87)
1−sided adj pval: 0.887
Frequency
Tumor−Normal Expression Difference

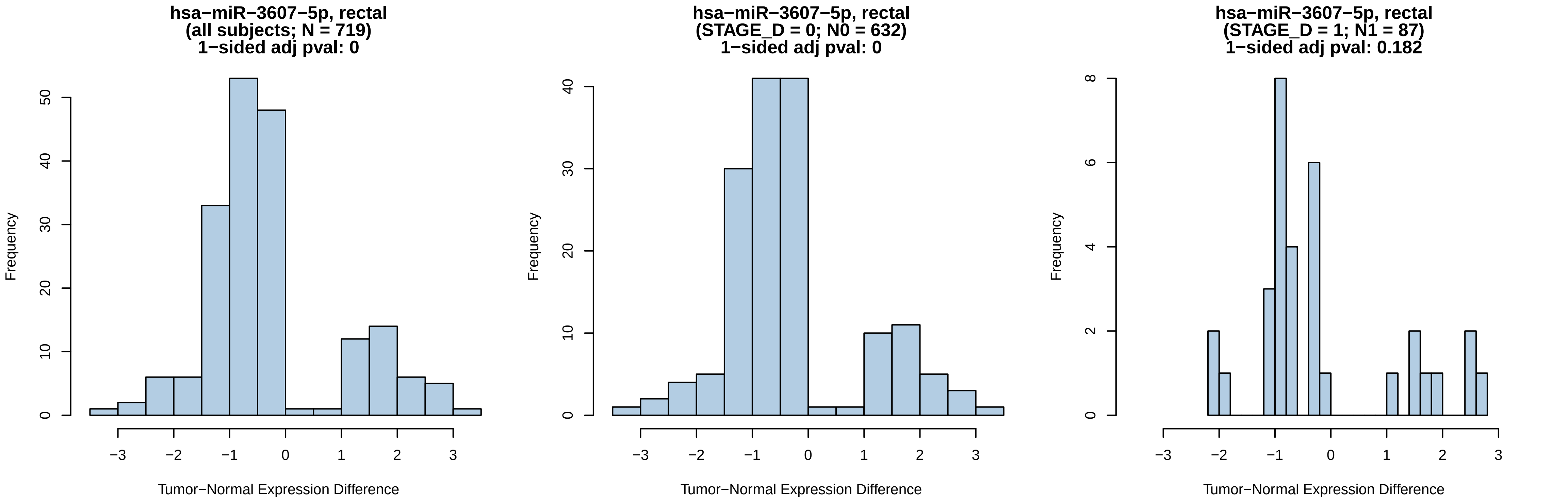

hsa−miR−3607−5p, rectal
(all subjects; N = 719)
1−sided adj pval: 0
Frequency
Tumor−Normal Expression Difference
hsa−miR−3607−5p, rectal
(STAGE_D = 0; N0 = 632)
1−sided adj pval: 0
Frequency
Tumor−Normal Expression Difference
hsa−miR−3607−5p, rectal
(STAGE_D = 1; N1 = 87)
1−sided adj pval: 0.182
Frequency
Tumor−Normal Expression Difference

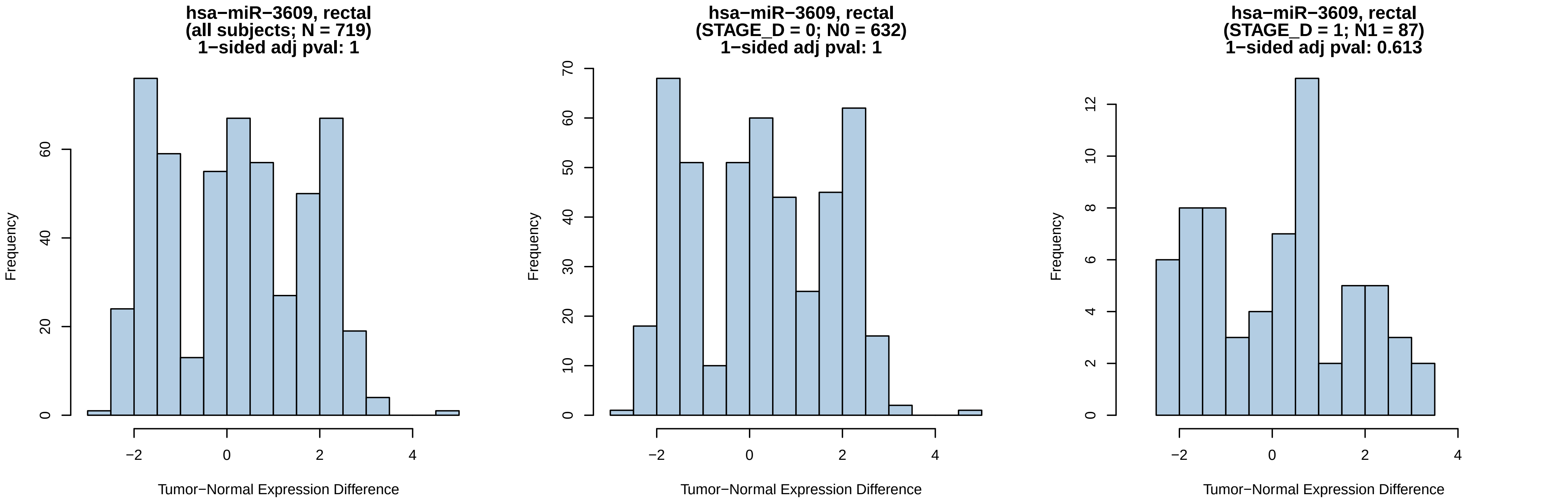
hsa−miR−3609, rectal
(all subjects; N = 719)
1−sided adj pval: 1
hsa−miR−3609, rectal
(STAGE_D = 0; N0 = 632)
1−sided adj pval: 1
hsa−miR−3609, rectal
(STAGE_D = 1; N1 = 87)
1−sided adj pval: 0.613
Frequency
Tumor−Normal Expression Difference

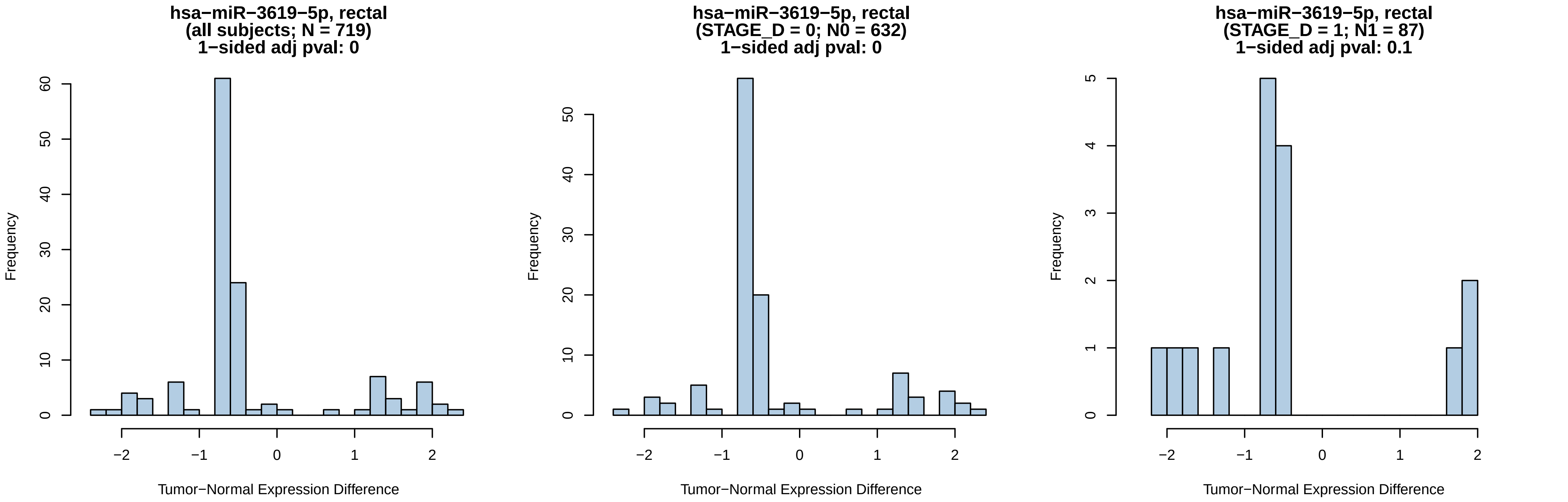
hsa−miR−3619−5p, rectal
(all subjects; N = 719)
1−sided adj pval: 0
Frequency
Tumor−Normal Expression Difference
hsa−miR−3619−5p, rectal
(STAGE_D = 0; N0 = 632)
1−sided adj pval: 0
Frequency
Tumor−Normal Expression Difference
hsa−miR−3619−5p, rectal
(STAGE_D = 1; N1 = 87)
1−sided adj pval: 0.1
Frequency
Tumor−Normal Expression Difference

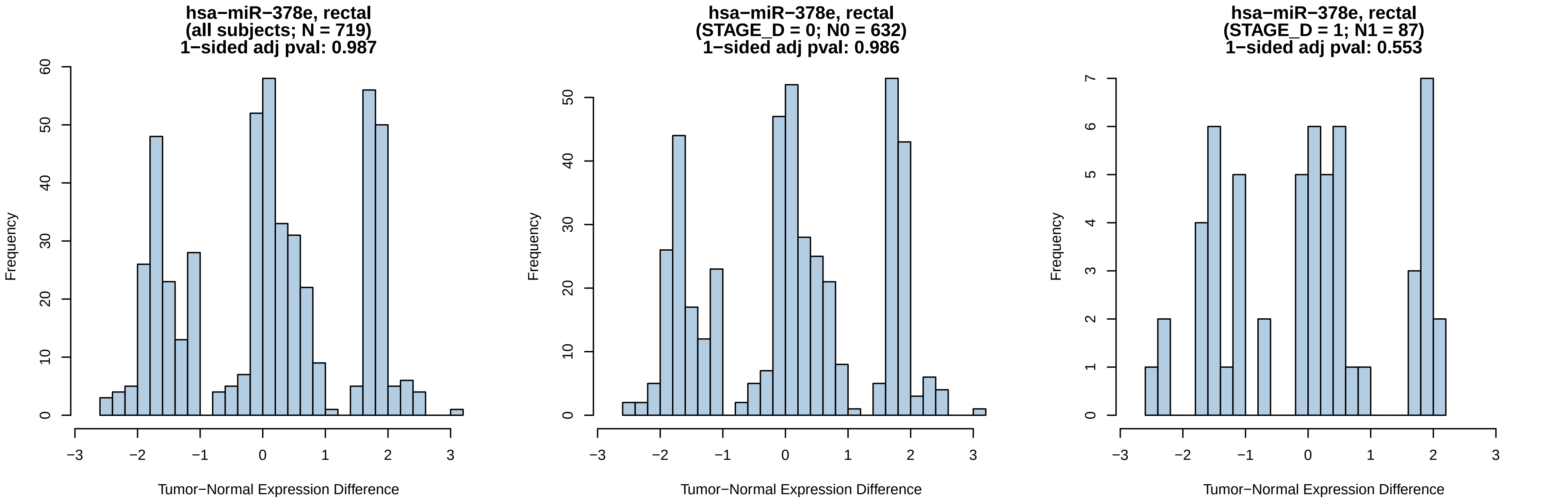
hsa−miR−378e, rectal
(all subjects; N = 719)
1−sided adj pval: 0.987
Frequency
Tumor−Normal Expression Difference
hsa−miR−378e, rectal
(STAGE_D = 0; N0 = 632)
1−sided adj pval: 0.986
Frequency
Tumor−Normal Expression Difference
hsa−miR−378e, rectal
(STAGE_D = 1; N1 = 87)
1−sided adj pval: 0.553
Frequency
Tumor−Normal Expression Difference

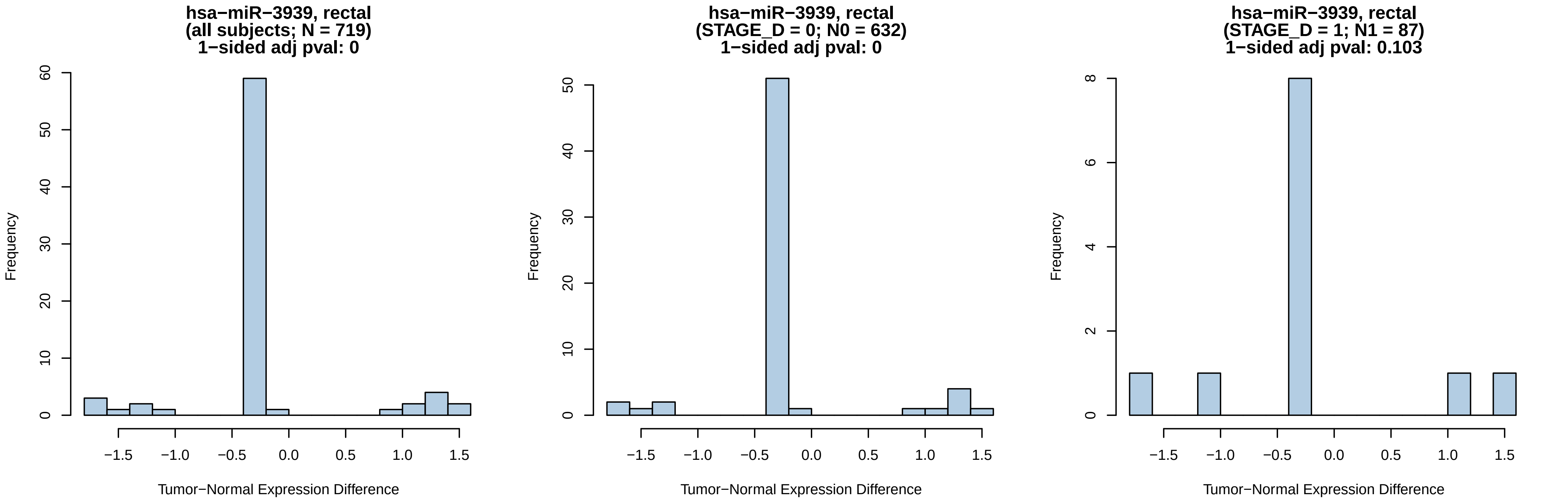

hsa−miR−3939, rectal
(all subjects; N = 719)
1−sided adj pval: 0
Frequency
Tumor−Normal Expression Difference
hsa−miR−3939, rectal
(STAGE_D = 0; N0 = 632)
1−sided adj pval: 0
Frequency
Tumor−Normal Expression Difference
hsa−miR−3939, rectal
(STAGE_D = 1; N1 = 87)
1−sided adj pval: 0.103
Frequency
Tumor−Normal Expression Difference

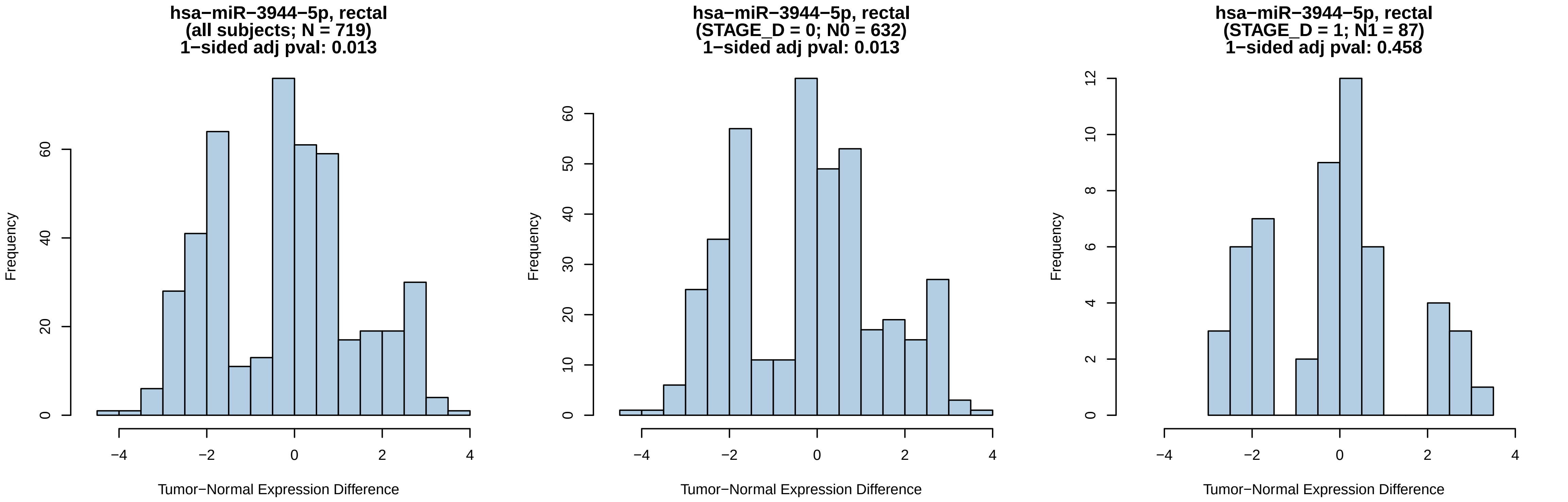
hsa−miR−3944−5p, rectal
(all subjects; N = 719)
1−sided adj pval: 0.013
Frequency
Tumor−Normal Expression Difference
hsa−miR−3944−5p, rectal
(STAGE_D = 0; N0 = 632)
1−sided adj pval: 0.013
Frequency
Tumor−Normal Expression Difference
hsa−miR−3944−5p, rectal
(STAGE_D = 1; N1 = 87)
1−sided adj pval: 0.458
Frequency
Tumor−Normal Expression Difference

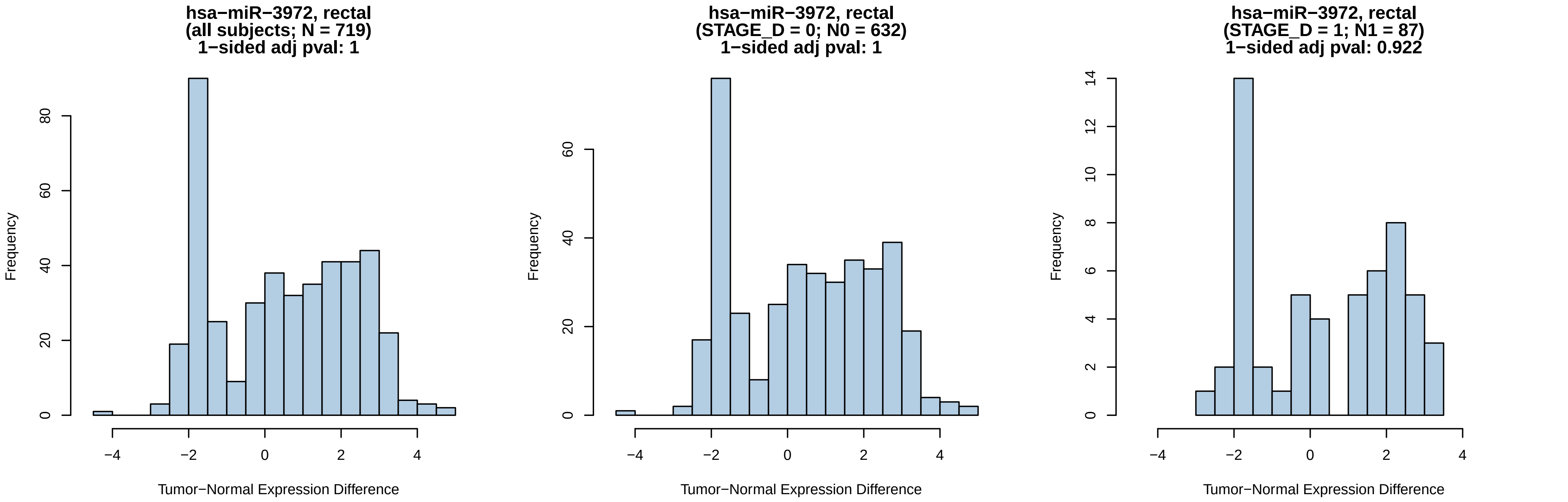
hsa−miR−3972, rectal
(all subjects; N = 719)
1−sided adj pval: 1
hsa−miR−3972, rectal
(STAGE_D = 0; N0 = 632)
1−sided adj pval: 1
hsa−miR−3972, rectal
(STAGE_D = 1; N1 = 87)
1−sided adj pval: 0.922
Frequency
Tumor−Normal Expression Difference

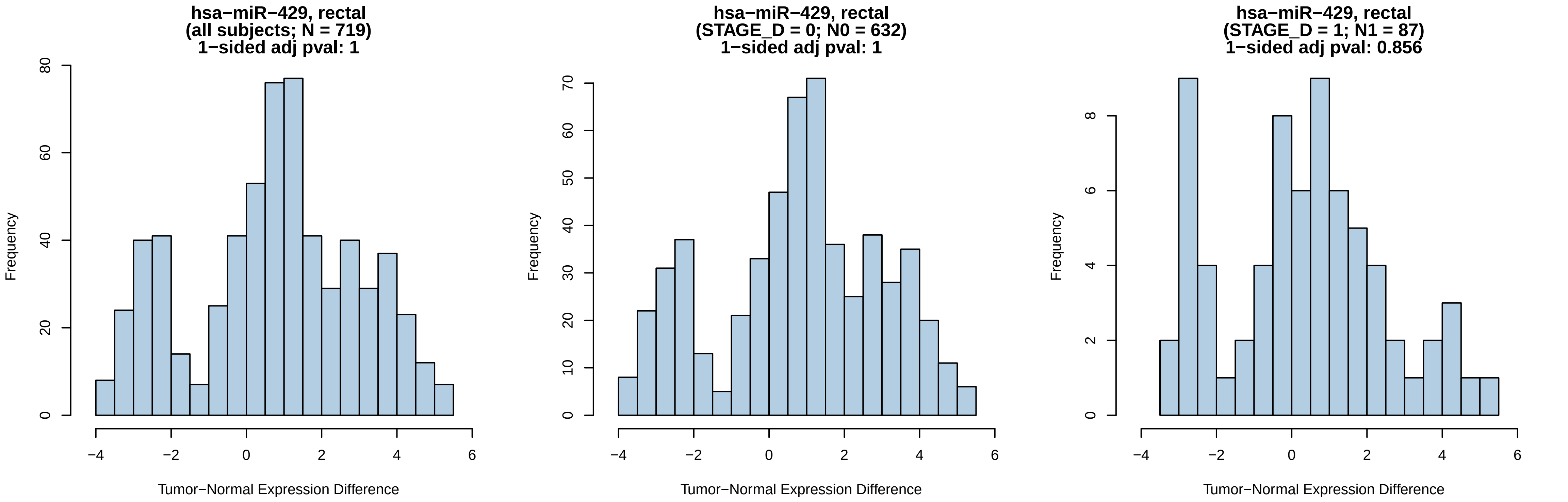
hsa−miR−429, rectal
(all subjects; N = 719)
1−sided adj pval: 1
hsa−miR−429, rectal
(STAGE_D = 0; N0 = 632)
1−sided adj pval: 1
hsa−miR−429, rectal
(STAGE_D = 1; N1 = 87)
1−sided adj pval: 0.856
Frequency
Tumor−Normal Expression Difference

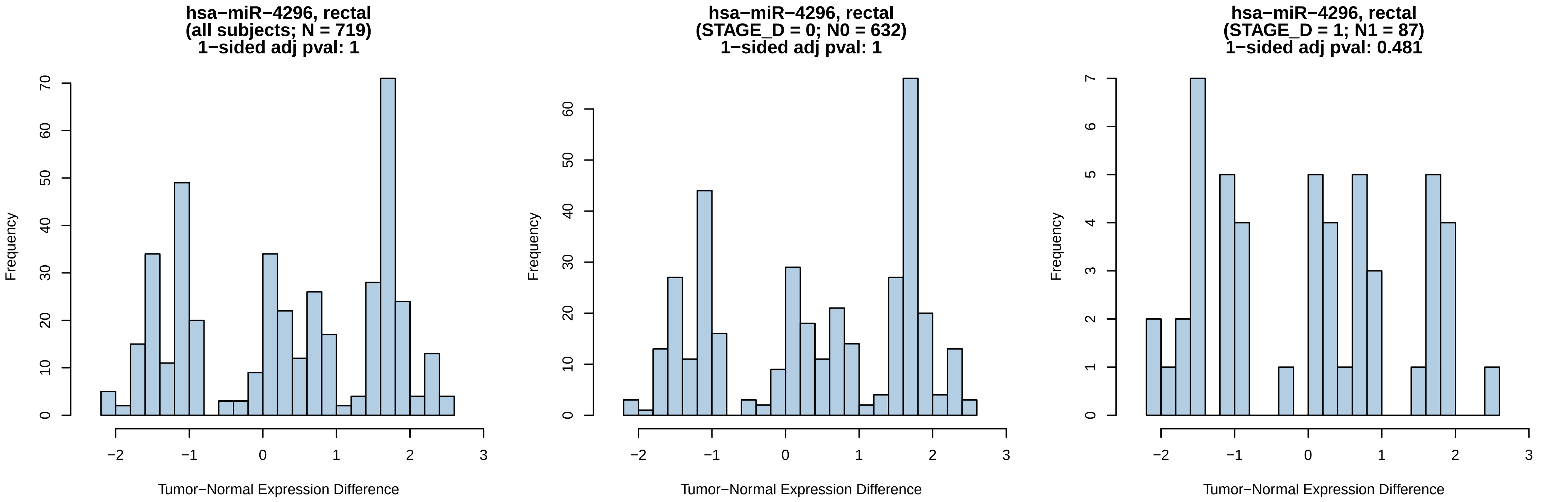

hsa−miR−4296, rectal
(all subjects; N = 719)
1−sided adj pval: 1
hsa−miR−4296, rectal
(STAGE_D = 0; N0 = 632)
1−sided adj pval: 1
hsa−miR−4296, rectal
(STAGE_D = 1; N1 = 87)
1−sided adj pval: 0.481
Frequency
Tumor−Normal Expression Difference

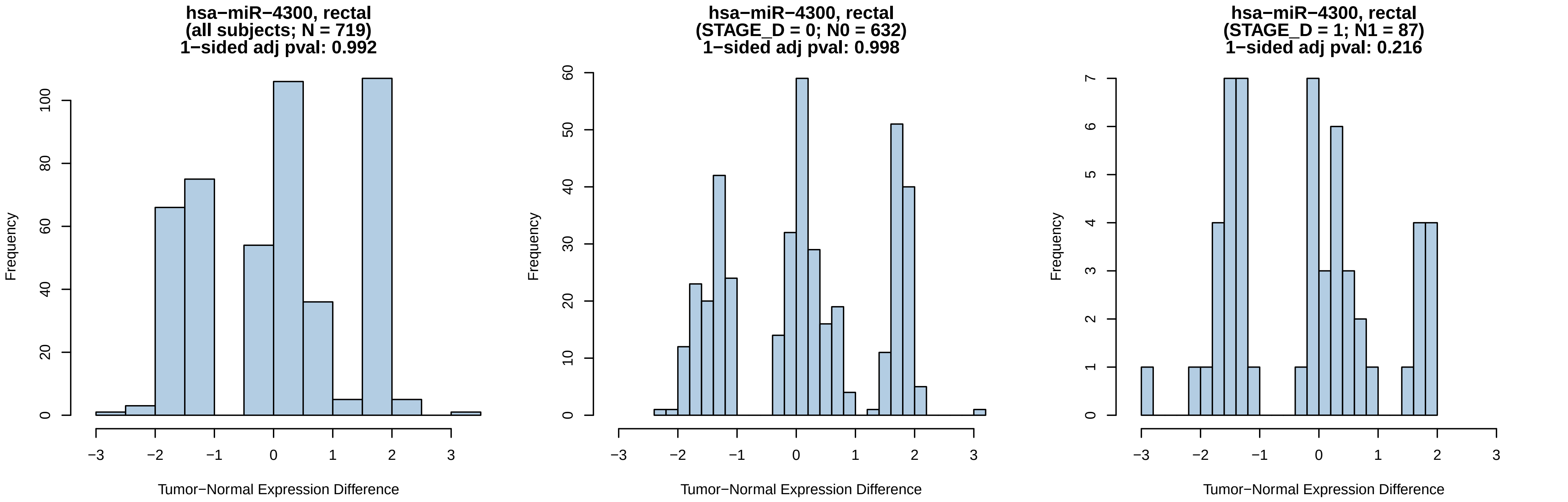
hsa−miR−4300, rectal
(all subjects; N = 719)
1−sided adj pval: 0.992
Frequency
Tumor−Normal Expression Difference
hsa−miR−4300, rectal
(STAGE_D = 0; N0 = 632)
1−sided adj pval: 0.998
Frequency
Tumor−Normal Expression Difference
hsa−miR−4300, rectal
(STAGE_D = 1; N1 = 87)
1−sided adj pval: 0.216
Frequency
Tumor−Normal Expression Difference

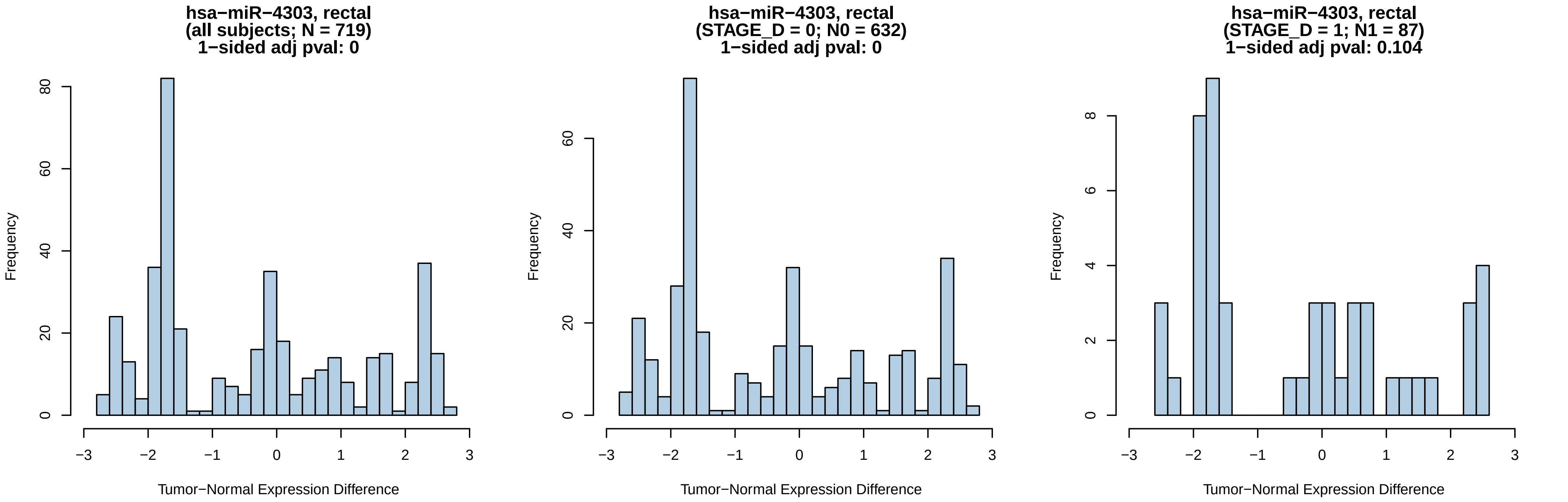
hsa−miR−4303, rectal
(all subjects; N = 719)
1−sided adj pval: 0
Frequency
Tumor−Normal Expression Difference
hsa−miR−4303, rectal
(STAGE_D = 0; N0 = 632)
1−sided adj pval: 0
Frequency
Tumor−Normal Expression Difference
hsa−miR−4303, rectal
(STAGE_D = 1; N1 = 87)
1−sided adj pval: 0.104
Frequency
Tumor−Normal Expression Difference

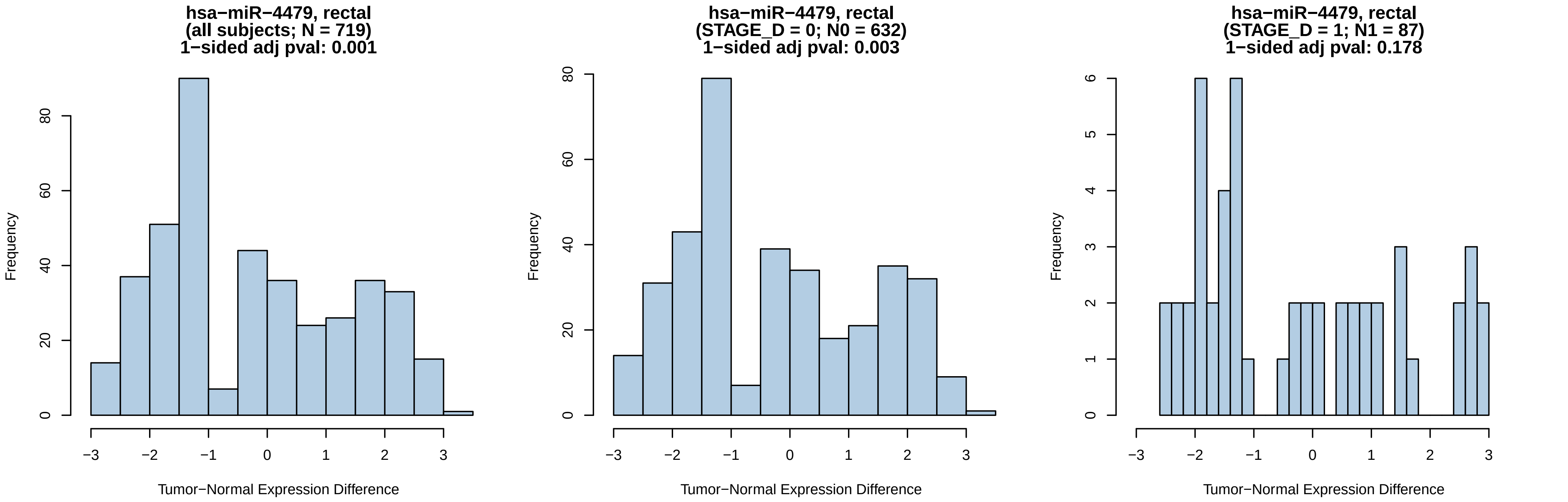
hsa−miR−4479, rectal
(all subjects; N = 719)
1−sided adj pval: 0.001
Frequency
Tumor−Normal Expression Difference
hsa−miR−4479, rectal
(STAGE_D = 0; N0 = 632)
1−sided adj pval: 0.003
Frequency
Tumor−Normal Expression Difference
hsa−miR−4479, rectal
(STAGE_D = 1; N1 = 87)
1−sided adj pval: 0.178
Frequency
Tumor−Normal Expression Difference

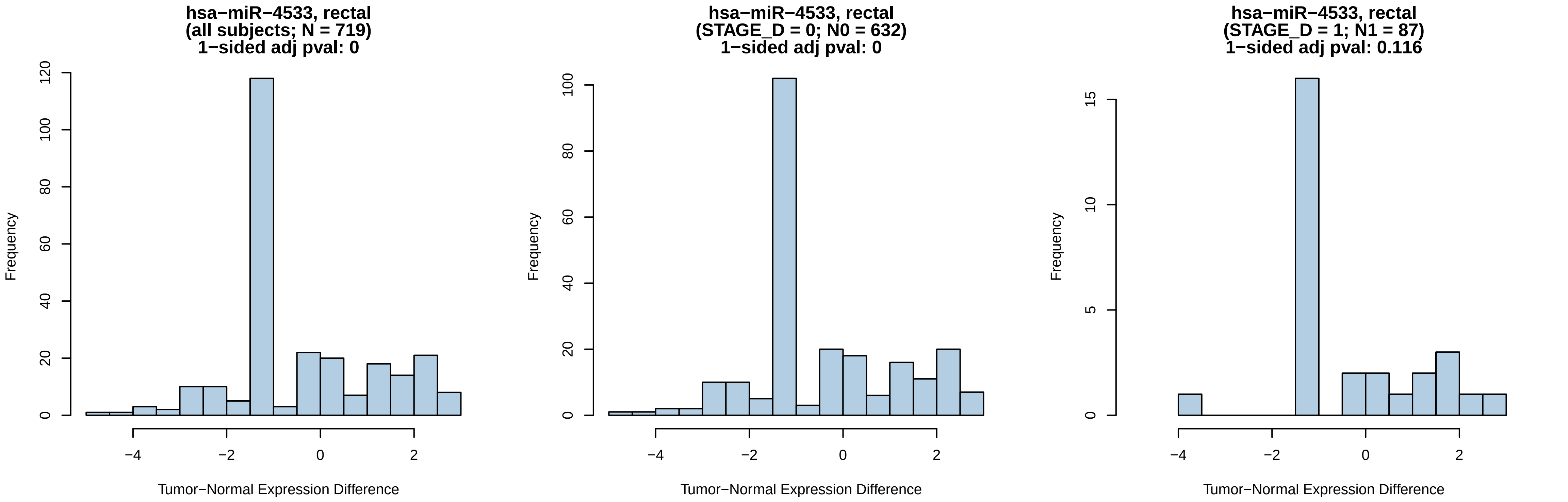

hsa−miR−4533, rectal
(all subjects; N = 719)
1−sided adj pval: 0
Frequency
0
20
40
60
80
100
120
−4
−2
0
2
Tumor−Normal Expression Difference
hsa−miR−4533, rectal
(STAGE_D = 0; N0 = 632)
1−sided adj pval: 0
Frequency
0
20
40
60
80
100
−4
−2
0
2
Tumor−Normal Expression Difference
hsa−miR−4533, rectal
(STAGE_D = 1; N1 = 87)
1−sided adj pval: 0.116
Frequency
0
5
10
15
−4
−2
0
2
Tumor−Normal Expression Difference

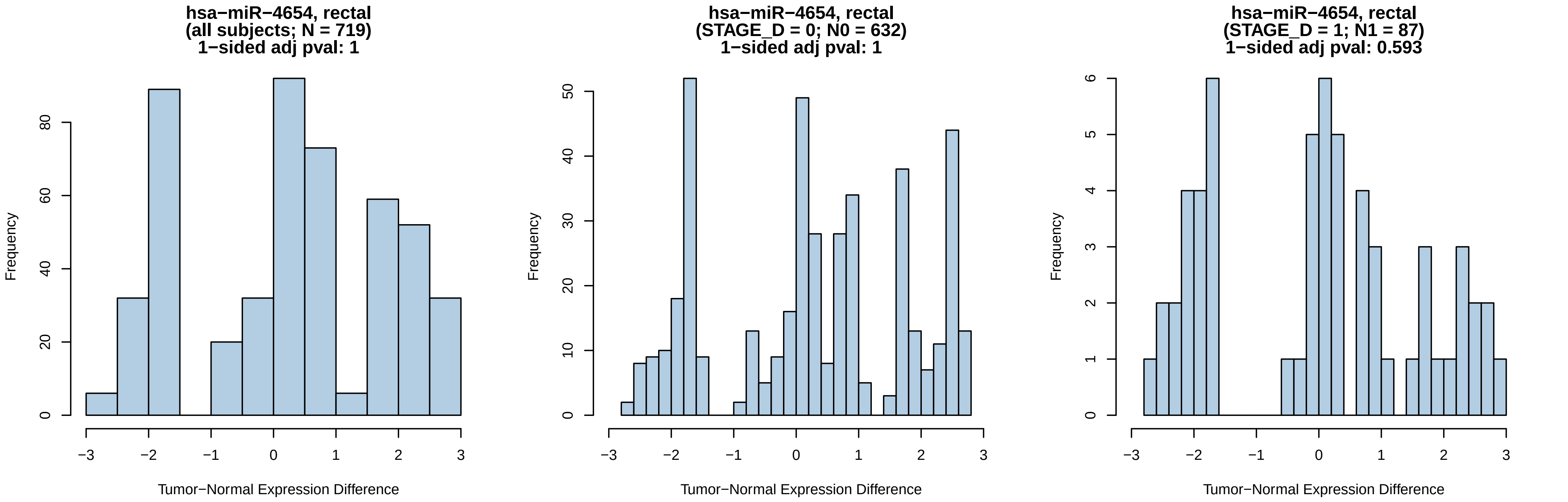
hsa−miR−4654, rectal
(all subjects; N = 719)
1−sided adj pval: 1
hsa−miR−4654, rectal
(STAGE_D = 0; N0 = 632)
1−sided adj pval: 1
hsa−miR−4654, rectal
(STAGE_D = 1; N1 = 87)
1−sided adj pval: 0.593
Frequency
Tumor−Normal Expression Difference

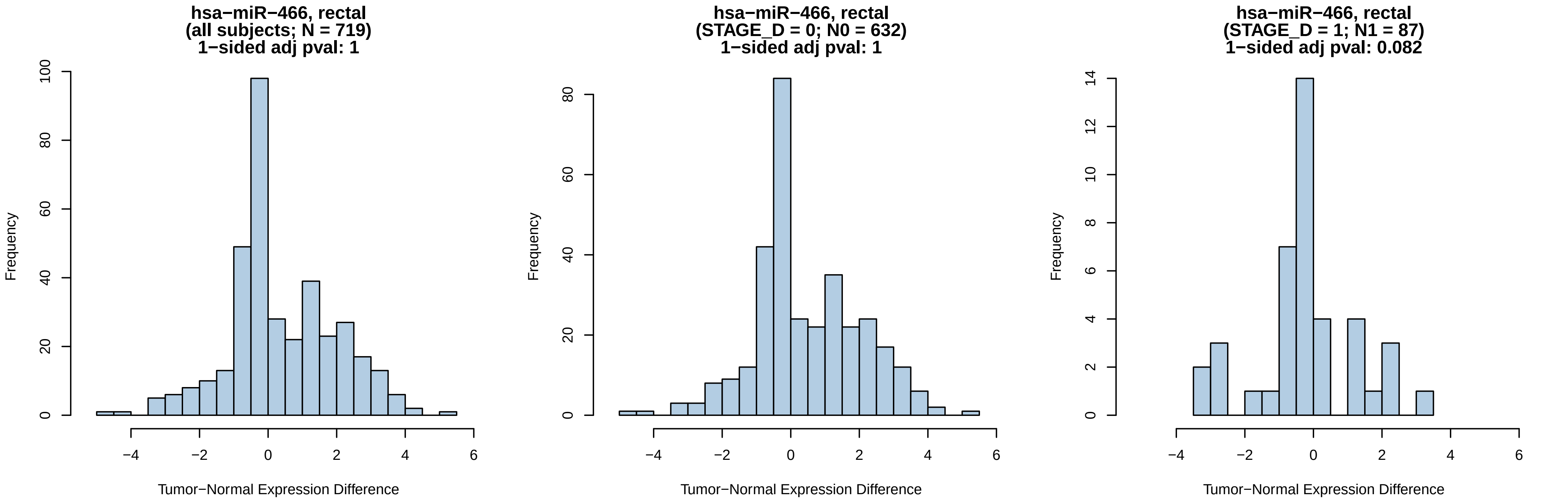

hsa−miR−466, rectal
(all subjects; N = 719)
1−sided adj pval: 1
Frequency
Tumor−Normal Expression Difference
hsa−miR−466, rectal
(STAGE_D = 0; N0 = 632)
1−sided adj pval: 1
Frequency
Tumor−Normal Expression Difference
hsa−miR−466, rectal
(STAGE_D = 1; N1 = 87)
1−sided adj pval: 0.082
Frequency
Tumor−Normal Expression Difference

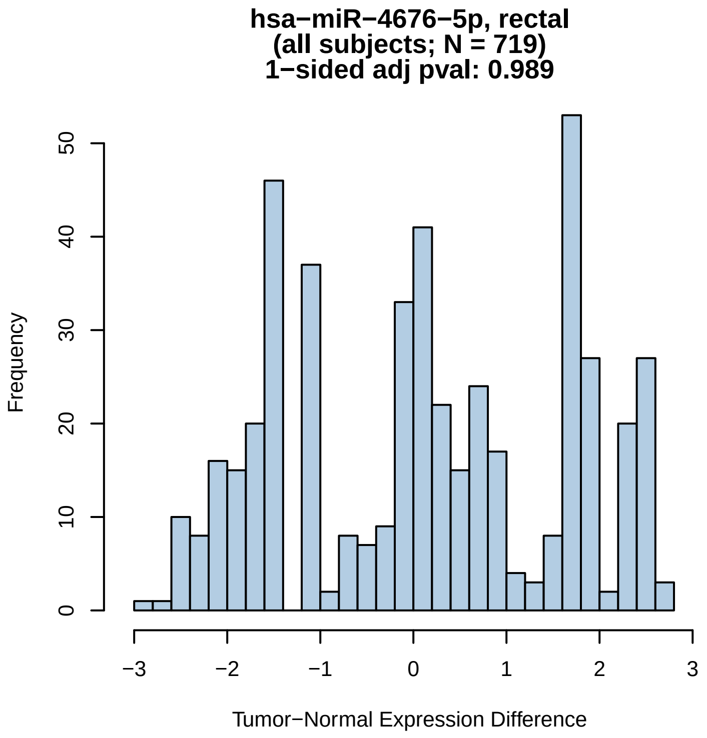

hsa−miR−4676−5p, rectal
(all subjects; N = 719)
1−sided adj pval: 0.989
Frequency
Tumor−Normal Expression Difference


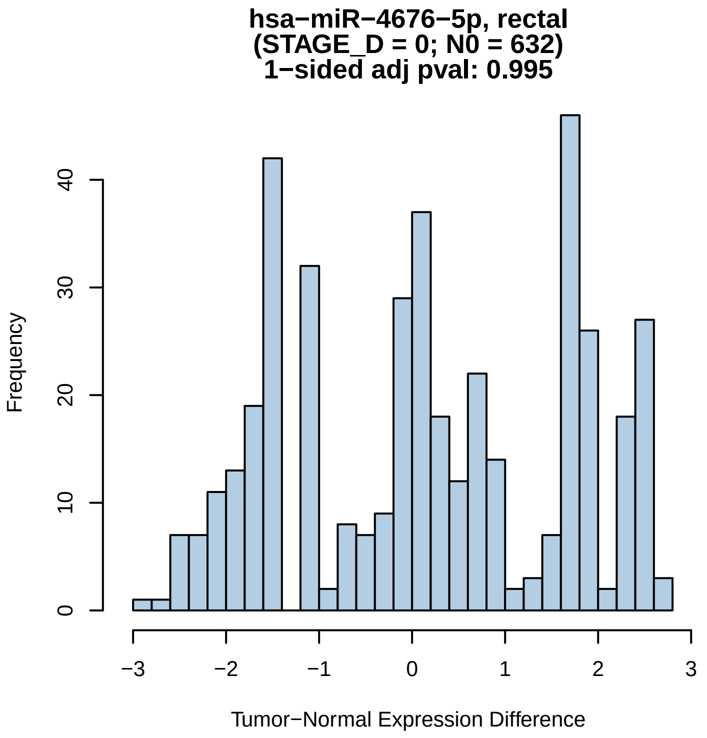

hsa−miR−4676−5p, rectal
(STAGE_D = 0; N0 = 632)
1−sided adj pval: 0.995
Frequency
Tumor−Normal Expression Difference


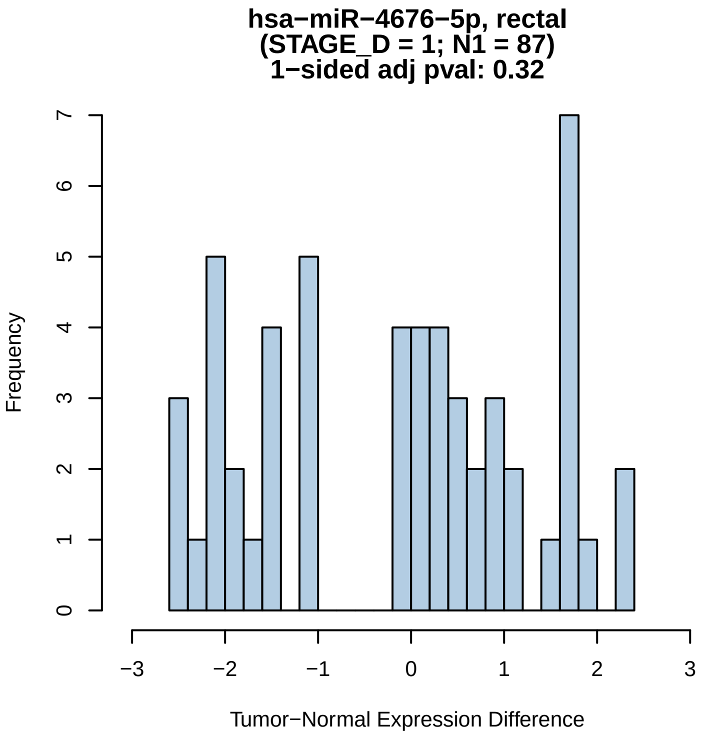

hsa−miR−4676−5p, rectal
(STAGE_D = 1; N1 = 87)
1−sided adj pval: 0.32
Frequency
Tumor−Normal Expression Difference

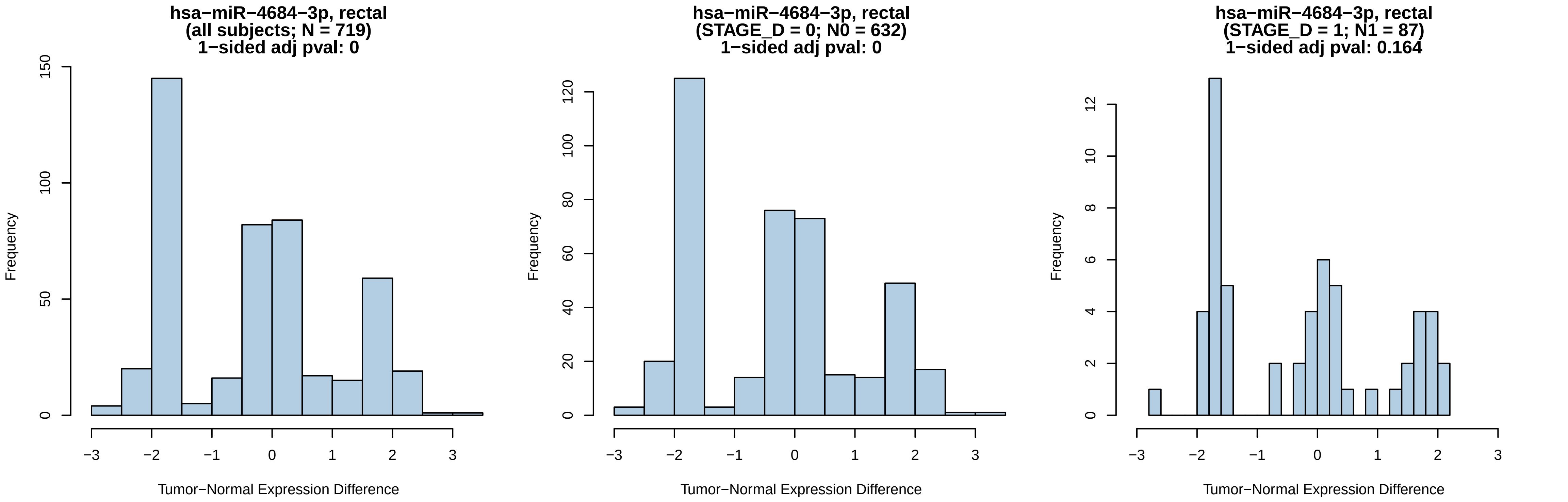
hsa−miR−4684−3p, rectal
(all subjects; N = 719)
1−sided adj pval: 0
hsa−miR−4684−3p, rectal
(STAGE_D = 0; N0 = 632)
1−sided adj pval: 0
hsa−miR−4684−3p, rectal
(STAGE_D = 1; N1 = 87)
1−sided adj pval: 0.164
Frequency
Tumor−Normal Expression Difference

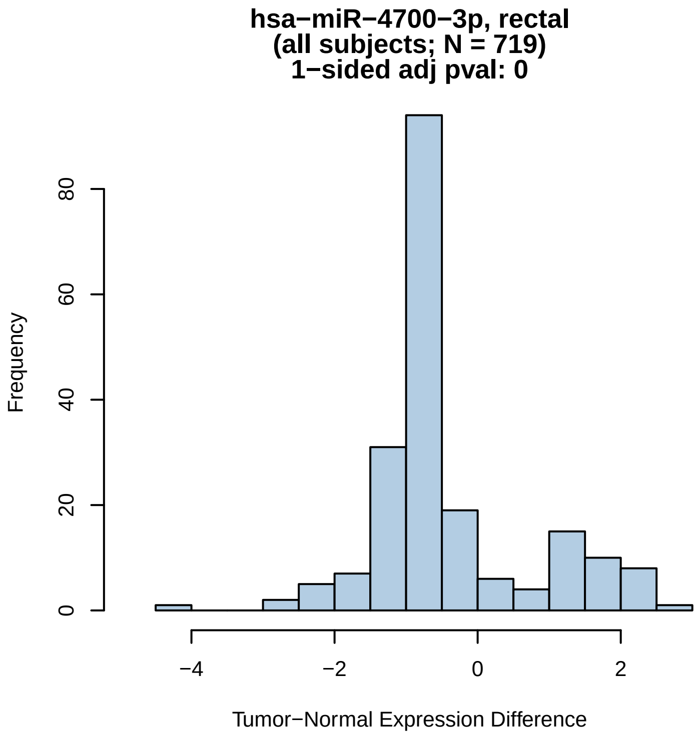

hsa−miR−4700−3p, rectal
(all subjects; N = 719)
1−sided adj pval: 0
Frequency
Tumor−Normal Expression Difference


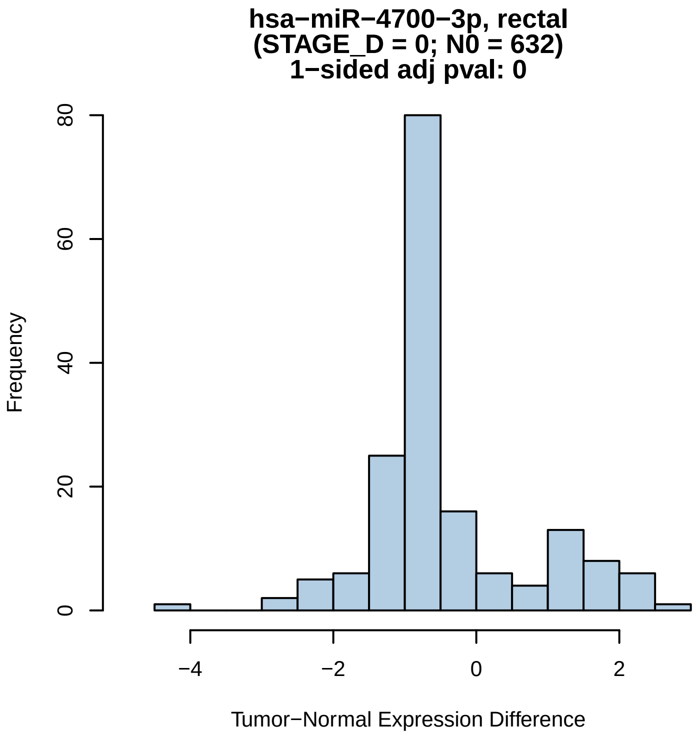

hsa−miR−4700−3p, rectal
(STAGE_D = 0; N0 = 632)
1−sided adj pval: 0
Frequency
Tumor−Normal Expression Difference


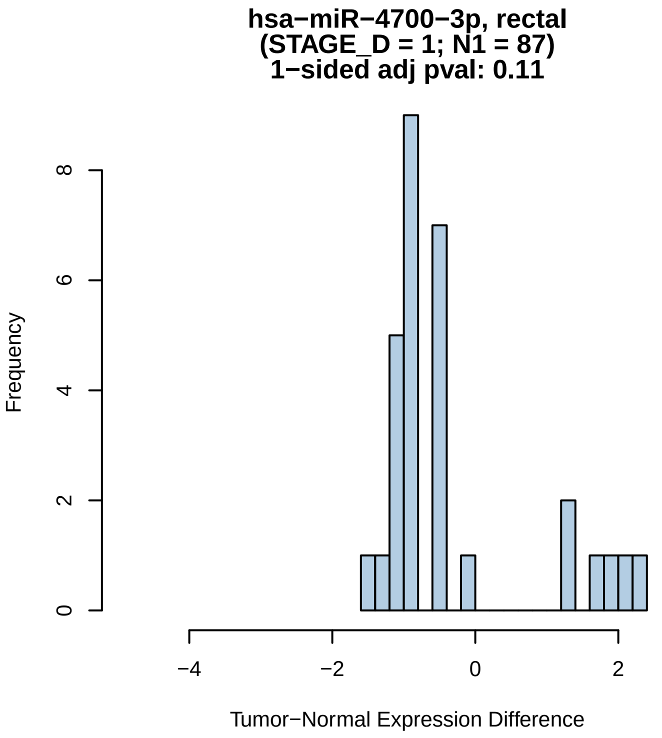

hsa−miR−4700−3p, rectal
(STAGE_D = 1; N1 = 87)
1−sided adj pval: 0.11
Frequency
Tumor−Normal Expression Difference

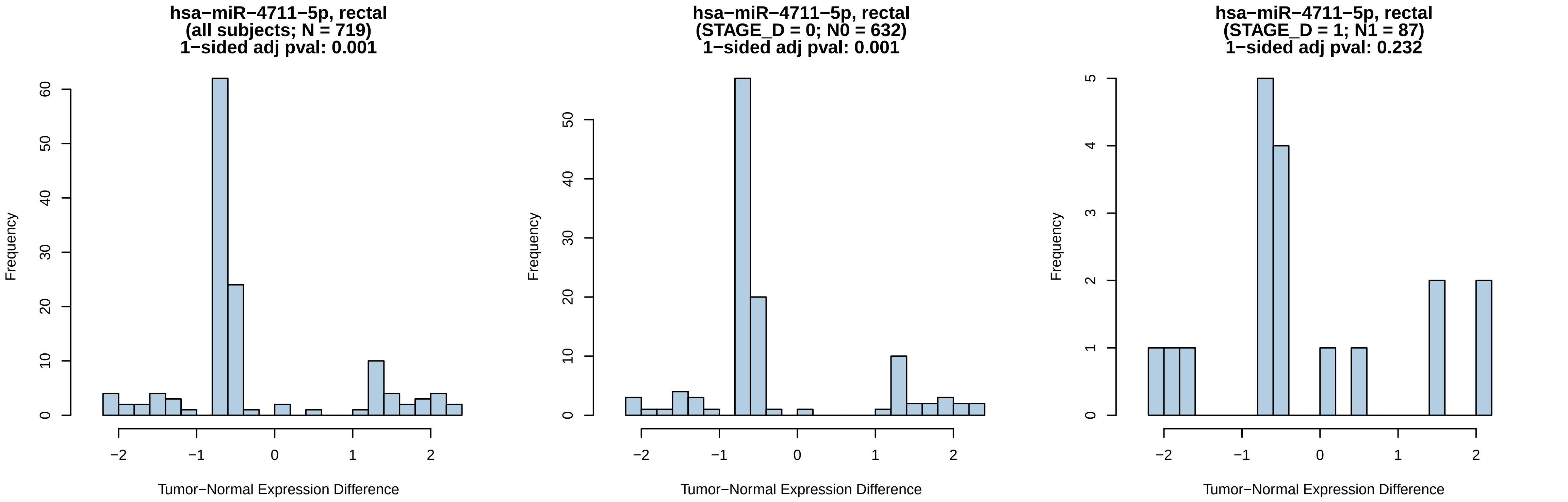

hsa−miR−4711−5p, rectal
(all subjects; N = 719)
1−sided adj pval: 0.001
hsa−miR−4711−5p, rectal
(STAGE_D = 0; N0 = 632)
1−sided adj pval: 0.001
hsa−miR−4711−5p, rectal
(STAGE_D = 1; N1 = 87)
1−sided adj pval: 0.232
Frequency
Tumor−Normal Expression Difference

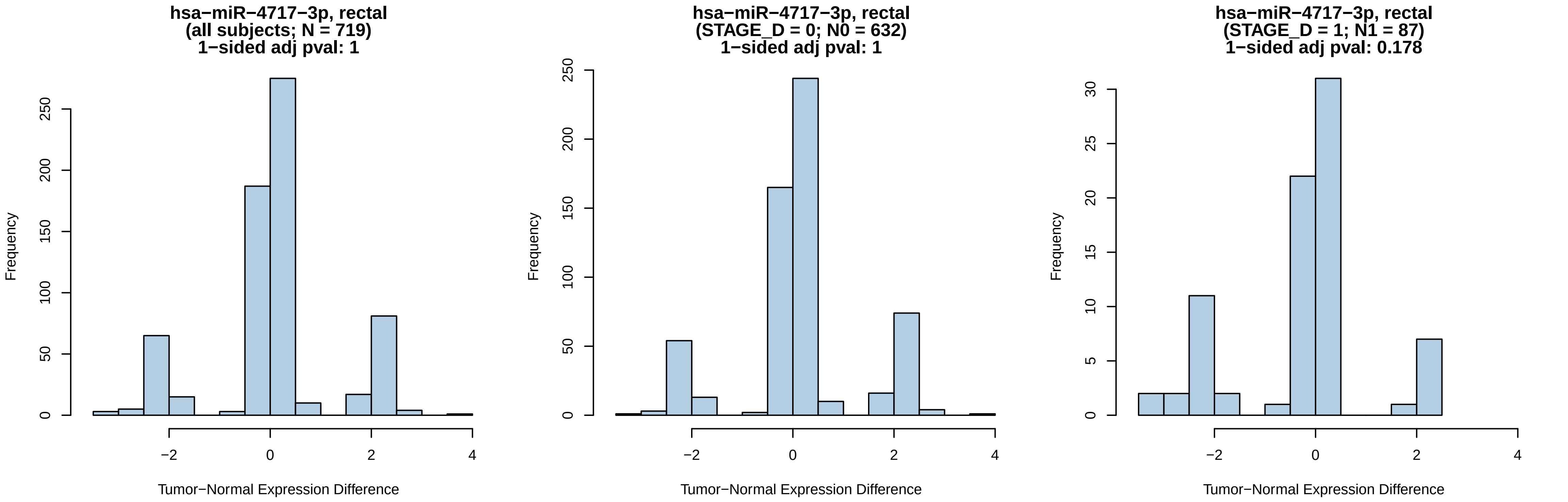

hsa−miR−4717−3p, rectal
(all subjects; N = 719)
1−sided adj pval: 1
Frequency
Tumor−Normal Expression Difference
hsa−miR−4717−3p, rectal
(STAGE_D = 0; N0 = 632)
1−sided adj pval: 1
Frequency
Tumor−Normal Expression Difference
hsa−miR−4717−3p, rectal
(STAGE_D = 1; N1 = 87)
1−sided adj pval: 0.178
Frequency
Tumor−Normal Expression Difference

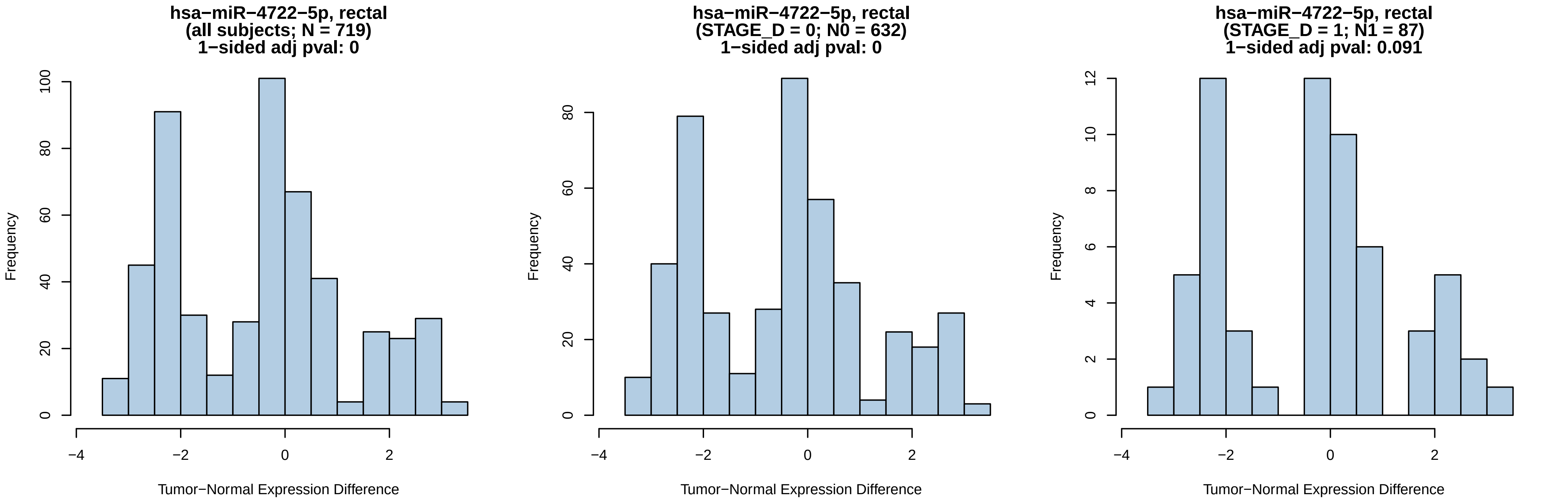

hsa−miR−4722−5p, rectal
(all subjects; N = 719)
1−sided adj pval: 0
Frequency
0
20
40
60
80
100
−4
−2
0
2
Tumor−Normal Expression Difference
hsa−miR−4722−5p, rectal
(STAGE_D = 0; N0 = 632)
1−sided adj pval: 0
Frequency
0
20
40
60
80
−4
−2
0
2
Tumor−Normal Expression Difference
hsa−miR−4722−5p, rectal
(STAGE_D = 1; N1 = 87)
1−sided adj pval: 0.091
Frequency
0
2
4
6
8
10
12
−4
−2
0
2
Tumor−Normal Expression Difference

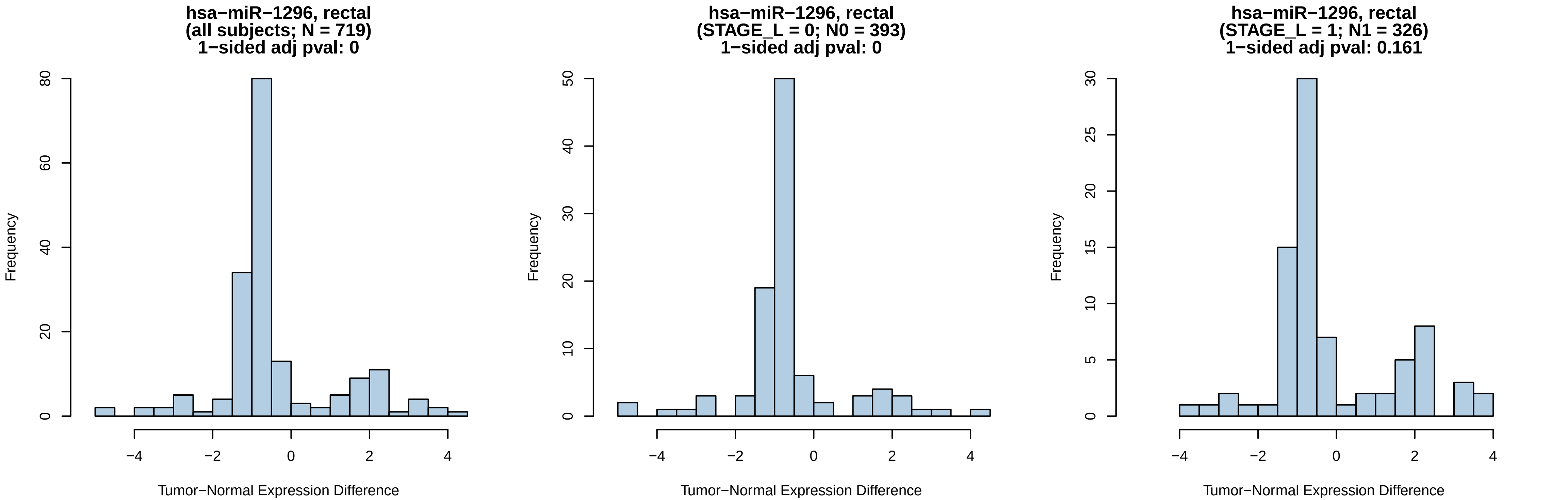
hsa−miR−1296, rectal
(all subjects; N = 719)
1−sided adj pval: 0
Frequency
0
20
40
60
80
−4
−2
0
2
4
Tumor−Normal Expression Difference
hsa−miR−1296, rectal
(STAGE_L = 0; N0 = 393)
1−sided adj pval: 0
Frequency
0
10
20
30
40
50
−4
−2
0
2
4
Tumor−Normal Expression Difference
hsa−miR−1296, rectal
(STAGE_L = 1; N1 = 326)
1−sided adj pval: 0.161
Frequency
0
5
10
15
20
25
30
−4
−2
0
2
4
Tumor−Normal Expression Difference

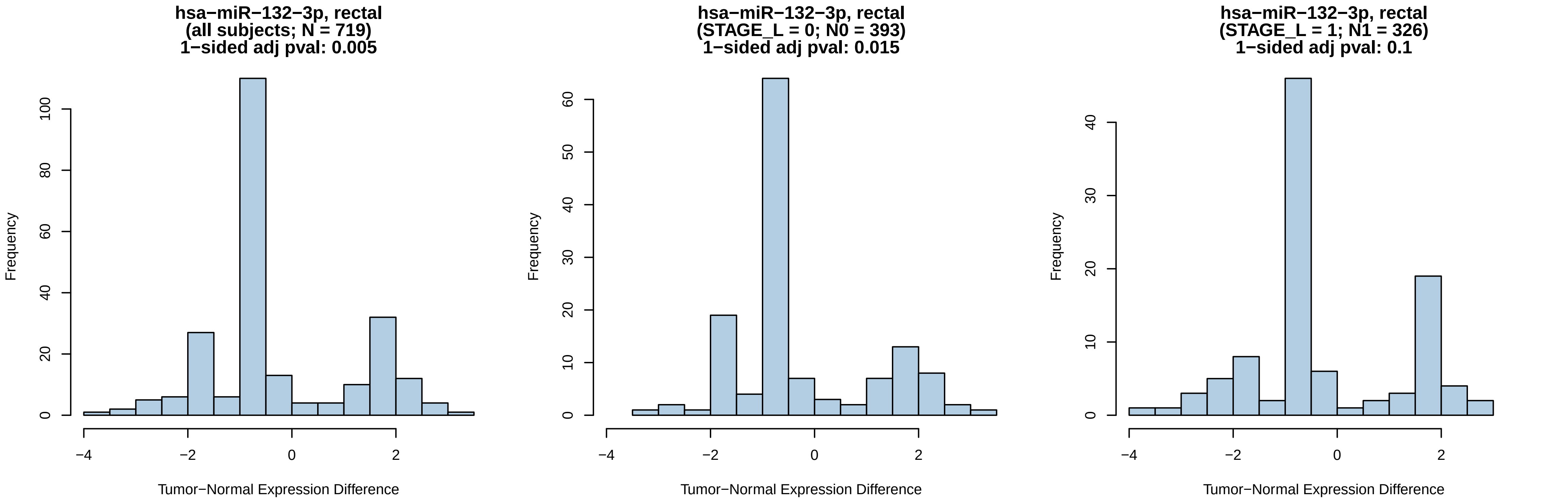
hsa−miR−132−3p, rectal
(all subjects; N = 719)
1−sided adj pval: 0.005
Frequency
Tumor−Normal Expression Difference
hsa−miR−132−3p, rectal
(STAGE_L = 0; N0 = 393)
1−sided adj pval: 0.015
Frequency
Tumor−Normal Expression Difference
hsa−miR−132−3p, rectal
(STAGE_L = 1; N1 = 326)
1−sided adj pval: 0.1
Frequency
Tumor−Normal Expression Difference

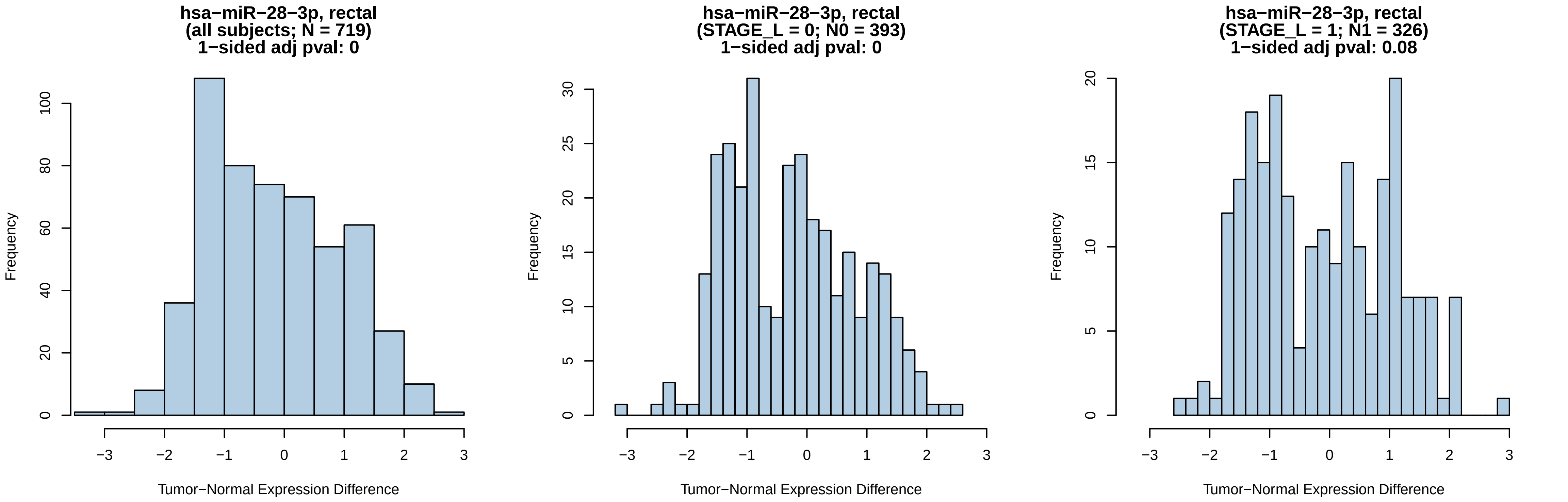
hsa−miR−28−3p, rectal
(all subjects; N = 719)
1−sided adj pval: 0
Frequency
Tumor−Normal Expression Difference
hsa−miR−28−3p, rectal
(STAGE_L = 0; N0 = 393)
1−sided adj pval: 0
Frequency
Tumor−Normal Expression Difference
hsa−miR−28−3p, rectal
(STAGE_L = 1; N1 = 326)
1−sided adj pval: 0.08
Frequency
Tumor−Normal Expression Difference

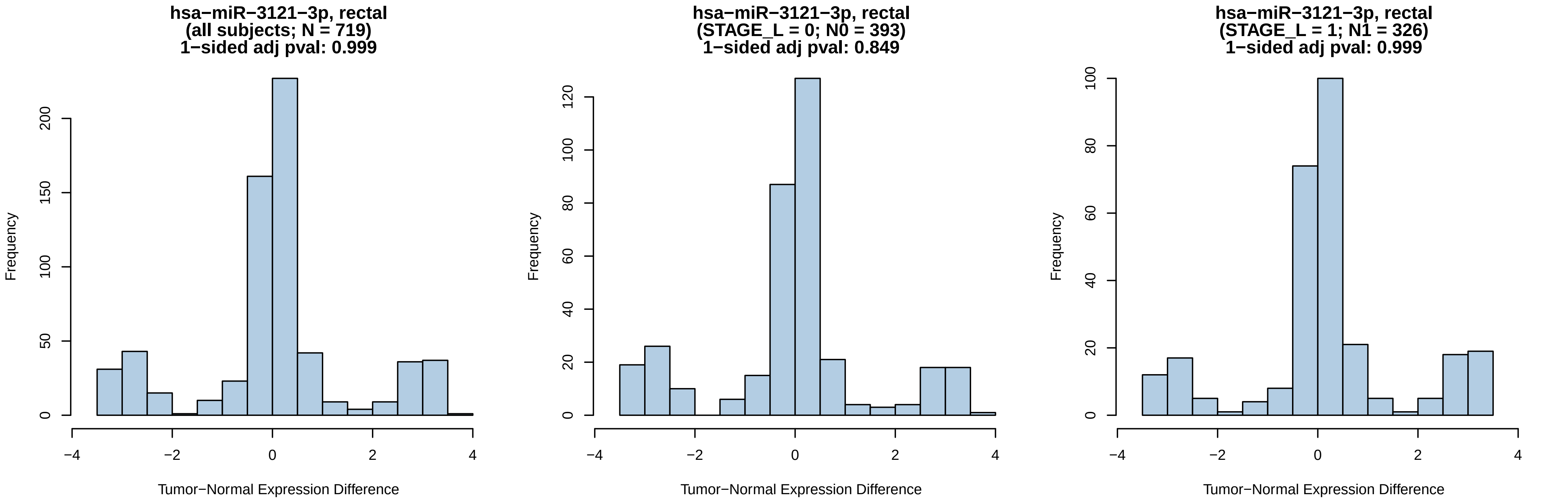

hsa−miR−3121−3p, rectal
(all subjects; N = 719)
1−sided adj pval: 0.999
Frequency
Tumor−Normal Expression Difference
hsa−miR−3121−3p, rectal
(STAGE_L = 0; N0 = 393)
1−sided adj pval: 0.849
Frequency
Tumor−Normal Expression Difference
hsa−miR−3121−3p, rectal
(STAGE_L = 1; N1 = 326)
1−sided adj pval: 0.999
Frequency
Tumor−Normal Expression Difference

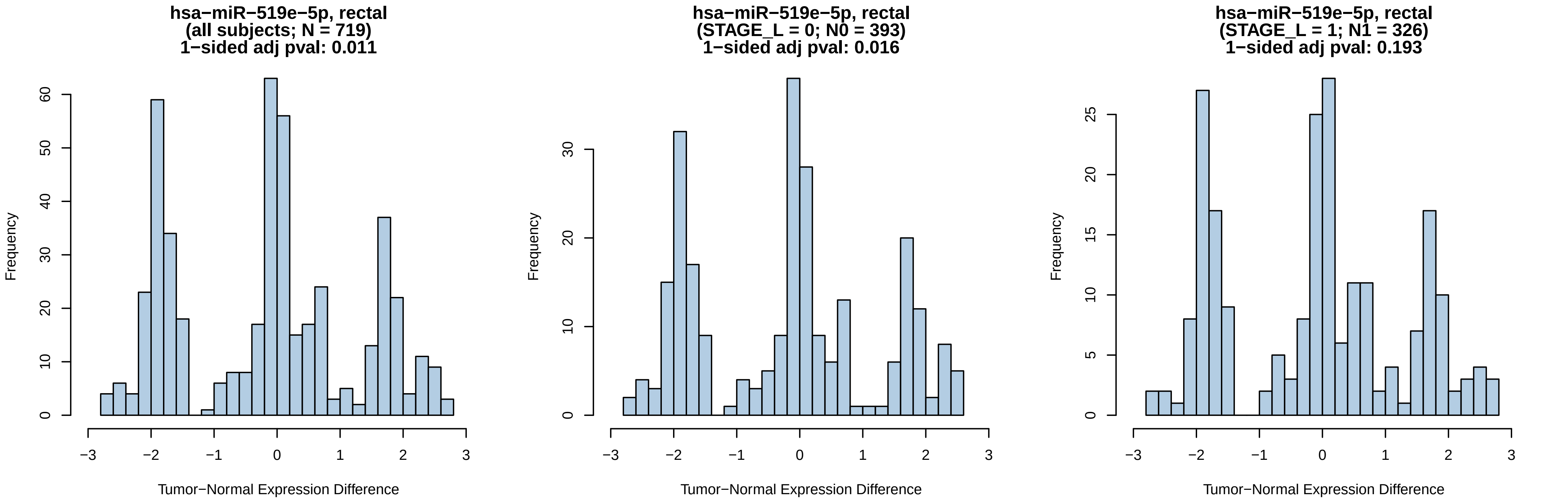
hsa−miR−519e−5p, rectal
(all subjects; N = 719)
1−sided adj pval: 0.011
Frequency
Tumor−Normal Expression Difference
hsa−miR−519e−5p, rectal
(STAGE_L = 0; N0 = 393)
1−sided adj pval: 0.016
Frequency
Tumor−Normal Expression Difference
hsa−miR−519e−5p, rectal
(STAGE_L = 1; N1 = 326)
1−sided adj pval: 0.193
Frequency
Tumor−Normal Expression Difference

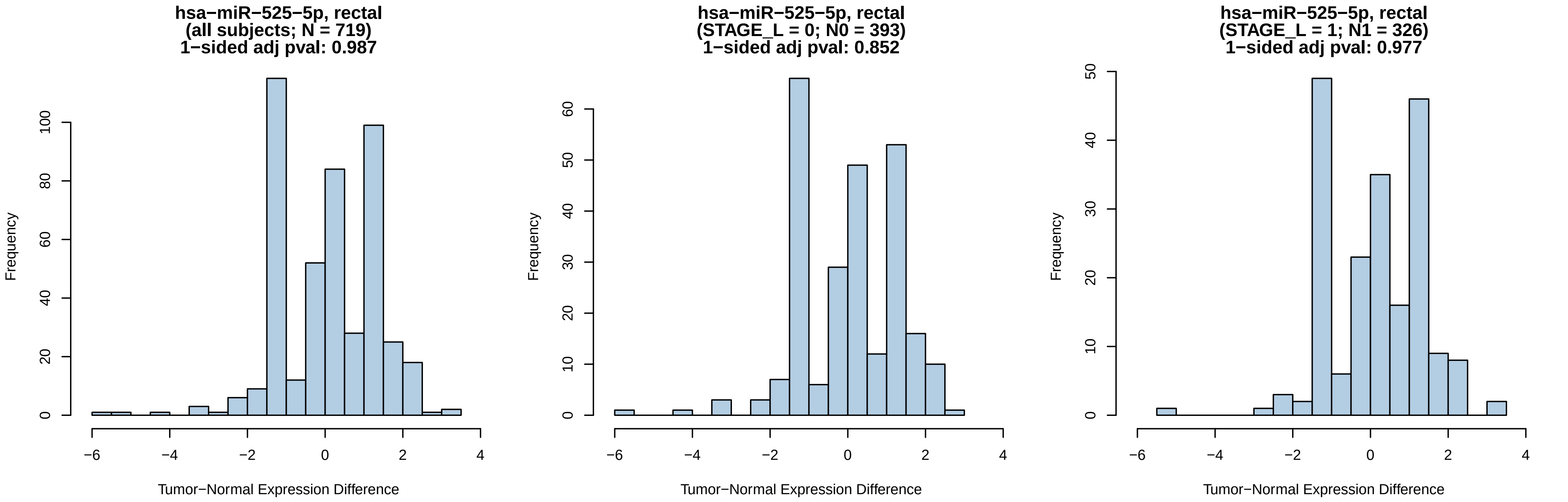
hsa−miR−525−5p, rectal
(all subjects; N = 719)
1−sided adj pval: 0.987
Frequency
0
20
40
60
80
100
−6
−4
−2
0
2
4
Tumor−Normal Expression Difference
hsa−miR−525−5p, rectal
(STAGE_L = 0; N0 = 393)
1−sided adj pval: 0.852
Frequency
0
10
20
30
40
50
60
−6
−4
−2
0
2
4
Tumor−Normal Expression Difference
hsa−miR−525−5p, rectal
(STAGE_L = 1; N1 = 326)
1−sided adj pval: 0.977
Frequency
0
10
20
30
40
50
−6
−4
−2
0
2
4
Tumor−Normal Expression Difference

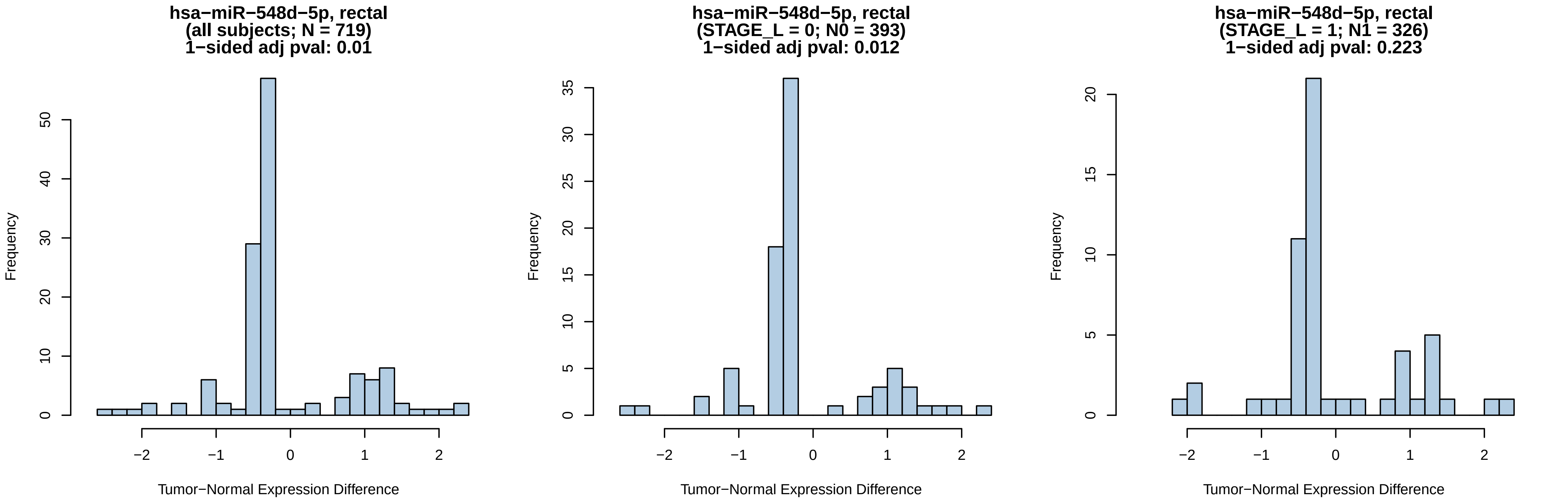
hsa−miR−548d−5p, rectal
(all subjects; N = 719)
1−sided adj pval: 0.01
Frequency
Tumor−Normal Expression Difference
hsa−miR−548d−5p, rectal
(STAGE_L = 0; N0 = 393)
1−sided adj pval: 0.012
Frequency
Tumor−Normal Expression Difference
hsa−miR−548d−5p, rectal
(STAGE_L = 1; N1 = 326)
1−sided adj pval: 0.223
Frequency
Tumor−Normal Expression Difference

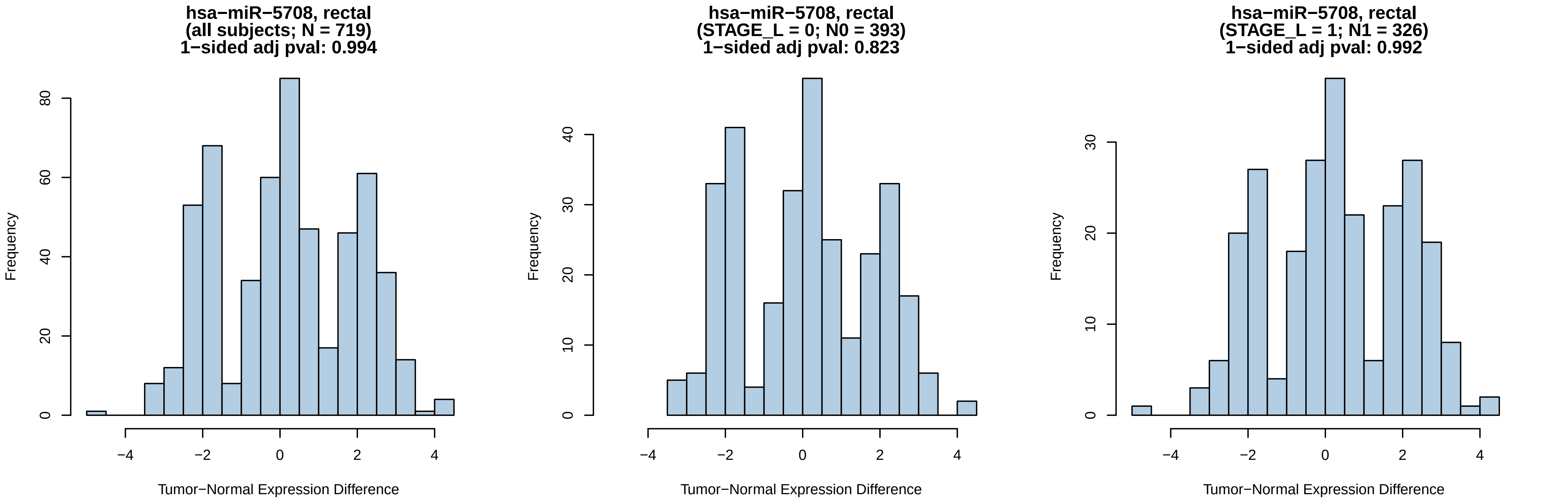
hsa−miR−5708, rectal
(all subjects; N = 719)
1−sided adj pval: 0.994
Frequency
Tumor−Normal Expression Difference
hsa−miR−5708, rectal
(STAGE_L = 0; N0 = 393)
1−sided adj pval: 0.823
Frequency
Tumor−Normal Expression Difference
hsa−miR−5708, rectal
(STAGE_L = 1; N1 = 326)
1−sided adj pval: 0.992
Frequency
Tumor−Normal Expression Difference

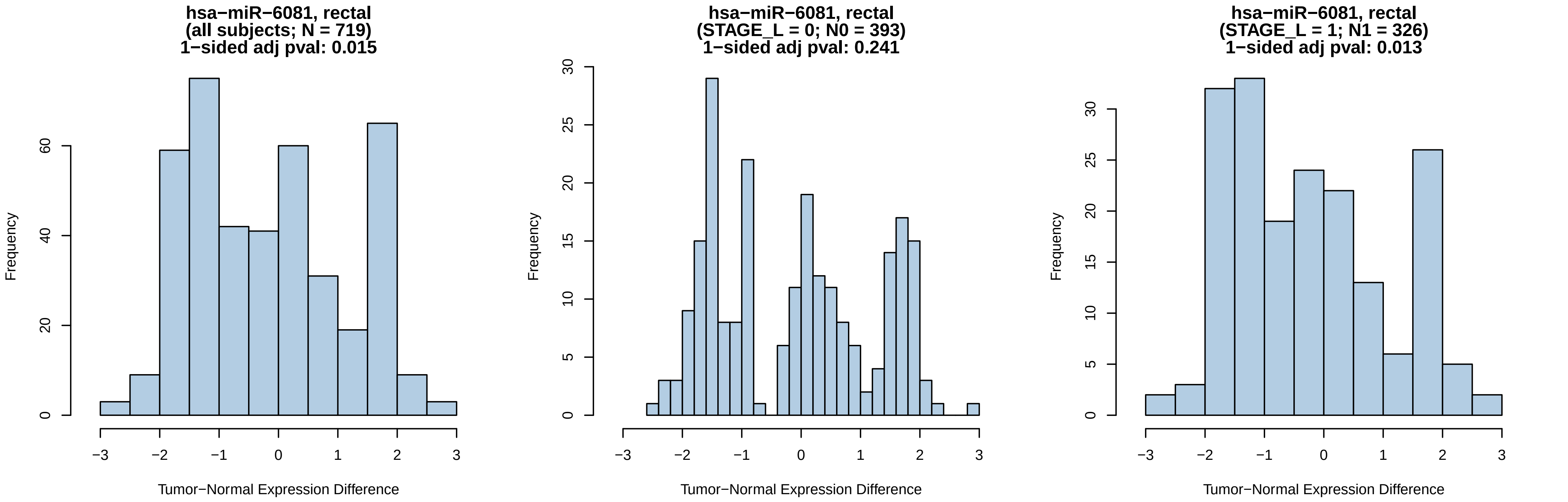
hsa−miR−6081, rectal
(all subjects; N = 719)
1−sided adj pval: 0.015
hsa−miR−6081, rectal
(STAGE_L = 0; N0 = 393)
1−sided adj pval: 0.241
hsa−miR−6081, rectal
(STAGE_L = 1; N1 = 326)
1−sided adj pval: 0.013
Frequency
Tumor−Normal Expression Difference

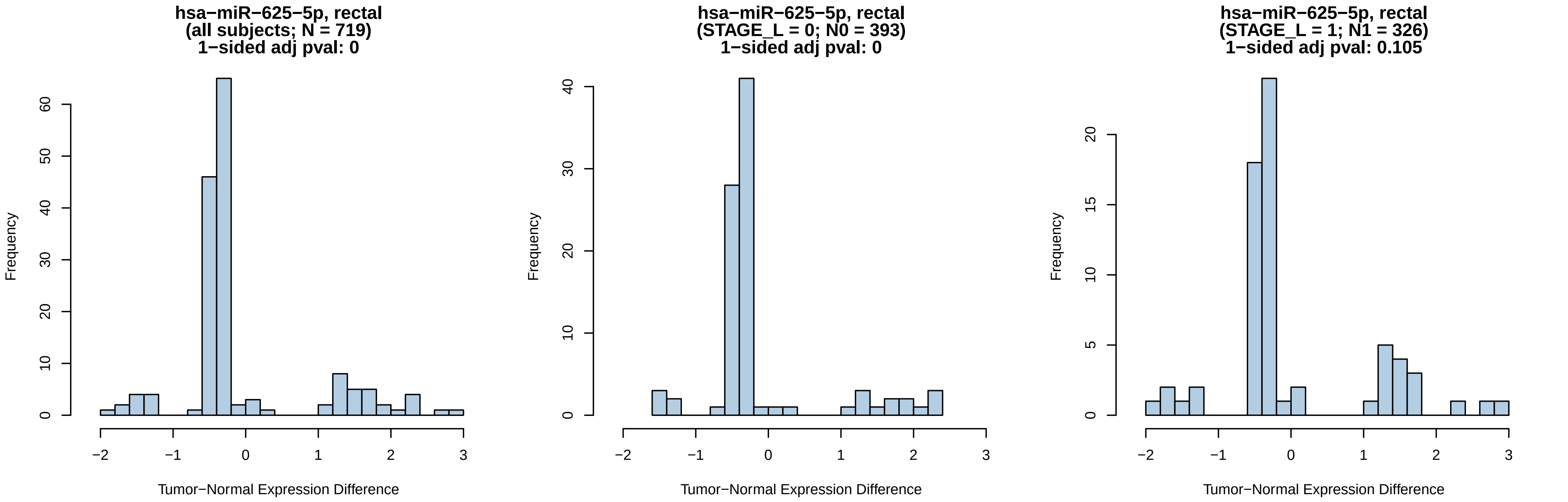
hsa−miR−625−5p, rectal
(all subjects; N = 719)
1−sided adj pval: 0
Frequency
0
10
20
30
40
50
60
−2
−1
0
1
2
3
Tumor−Normal Expression Difference
hsa−miR−625−5p, rectal
(STAGE_L = 0; N0 = 393)
1−sided adj pval: 0
Frequency
0
10
20
30
40
−2
−1
0
1
2
3
Tumor−Normal Expression Difference
hsa−miR−625−5p, rectal
(STAGE_L = 1; N1 = 326)
1−sided adj pval: 0.105
Frequency
0
5
10
15
20
−2
−1
0
1
2
3
Tumor−Normal Expression Difference

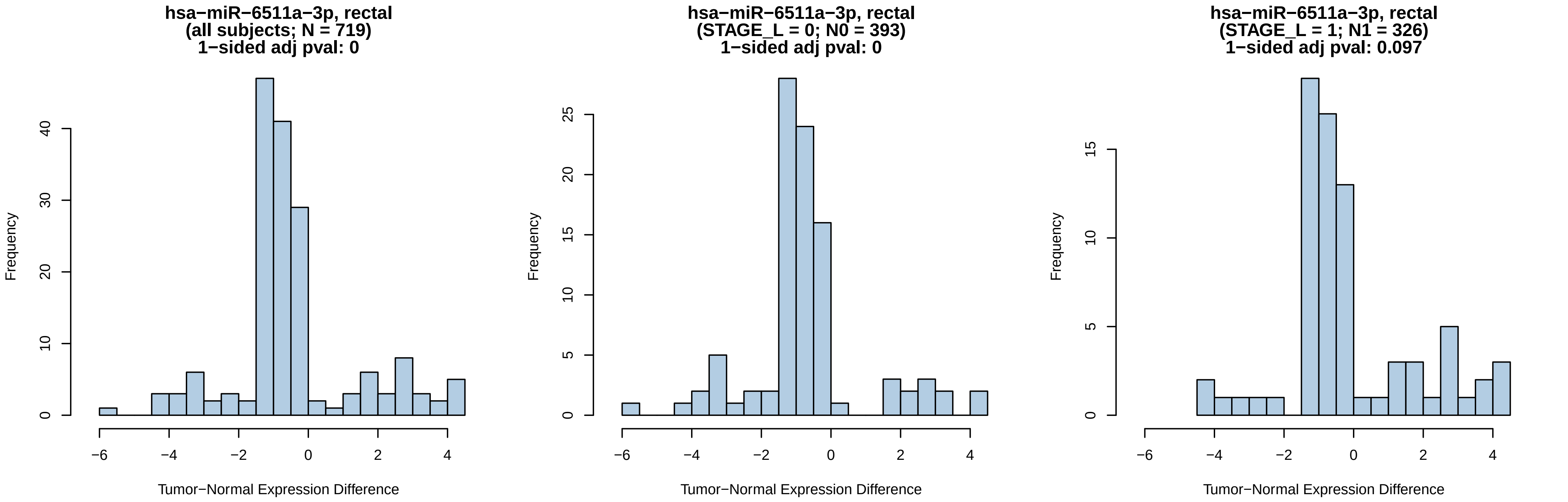

hsa−miR−6511a−3p, rectal
(all subjects; N = 719)
1−sided adj pval: 0
Frequency
Tumor−Normal Expression Difference
hsa−miR−6511a−3p, rectal
(STAGE_L = 0; N0 = 393)
1−sided adj pval: 0
Frequency
Tumor−Normal Expression Difference
hsa−miR−6511a−3p, rectal
(STAGE_L = 1; N1 = 326)
1−sided adj pval: 0.097
Frequency
Tumor−Normal Expression Difference

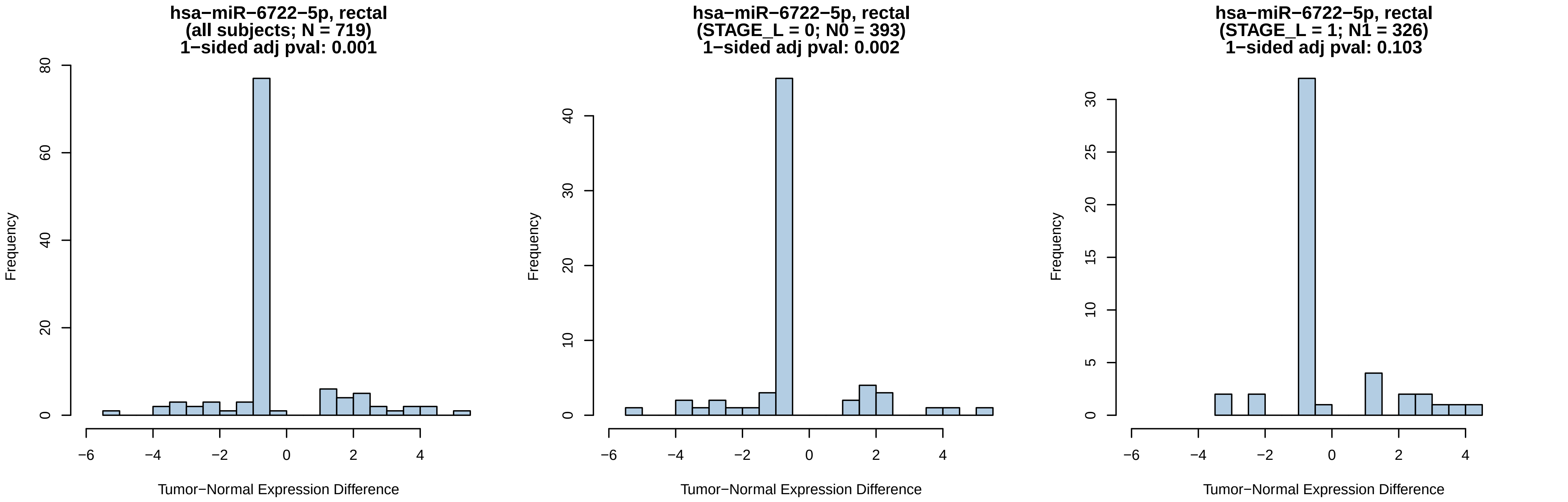
hsa−miR−6722−5p, rectal
(all subjects; N = 719)
1−sided adj pval: 0.001
Frequency
Tumor−Normal Expression Difference
hsa−miR−6722−5p, rectal
(STAGE_L = 0; N0 = 393)
1−sided adj pval: 0.002
Frequency
Tumor−Normal Expression Difference
hsa−miR−6722−5p, rectal
(STAGE_L = 1; N1 = 326)
1−sided adj pval: 0.103
Frequency
Tumor−Normal Expression Difference

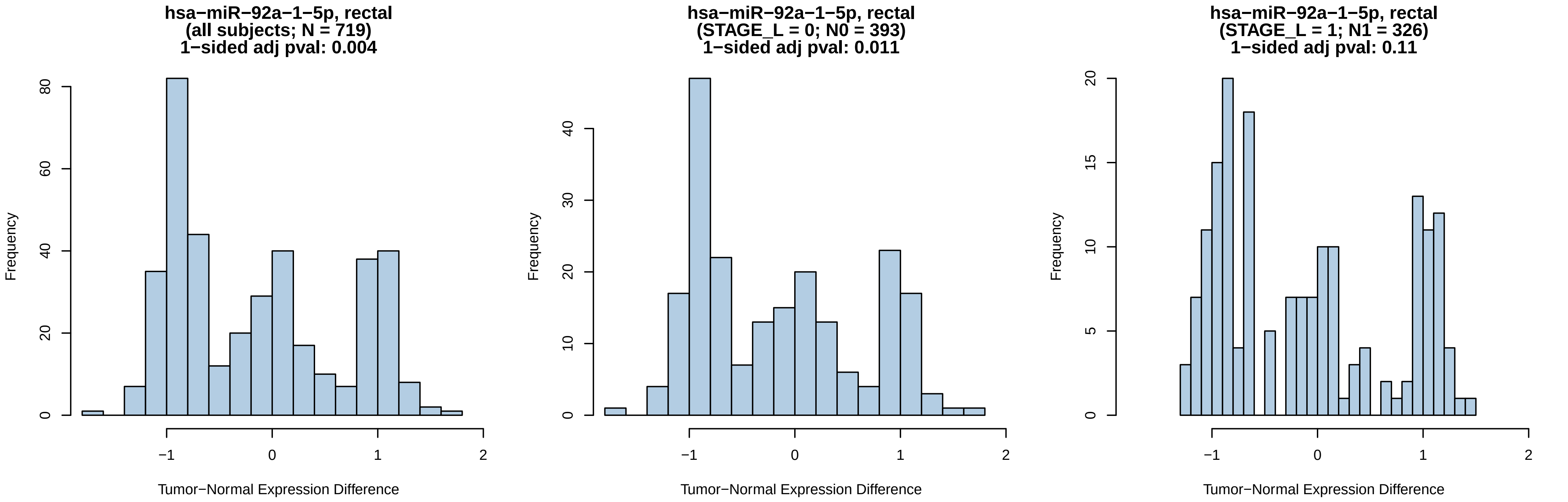
hsa−miR−92a−1−5p, rectal
(all subjects; N = 719)
1−sided adj pval: 0.004
Frequency
Tumor−Normal Expression Difference
hsa−miR−92a−1−5p, rectal
(STAGE_L = 0; N0 = 393)
1−sided adj pval: 0.011
Frequency
Tumor−Normal Expression Difference
hsa−miR−92a−1−5p, rectal
(STAGE_L = 1; N1 = 326)
1−sided adj pval: 0.11
Frequency
Tumor−Normal Expression Difference

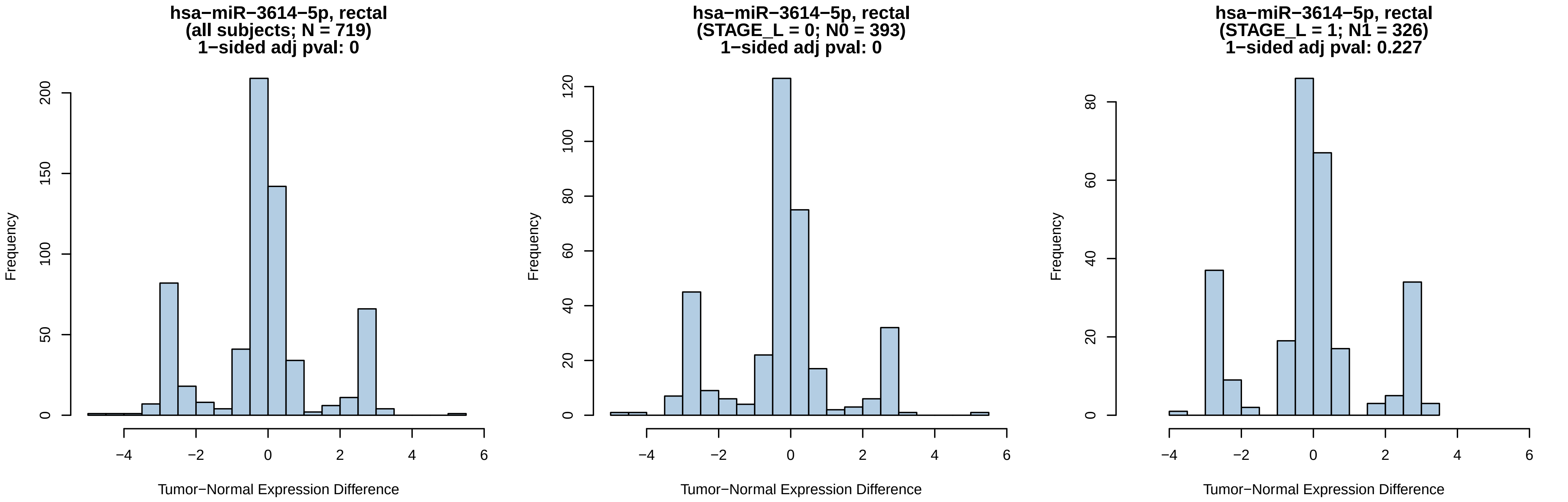
hsa−miR−3614−5p, rectal
(all subjects; N = 719)
1−sided adj pval: 0
Frequency
Tumor−Normal Expression Difference
hsa−miR−3614−5p, rectal
(STAGE_L = 0; N0 = 393)
1−sided adj pval: 0
Frequency
Tumor−Normal Expression Difference
hsa−miR−3614−5p, rectal
(STAGE_L = 1; N1 = 326)
1−sided adj pval: 0.227
Frequency
Tumor−Normal Expression Difference

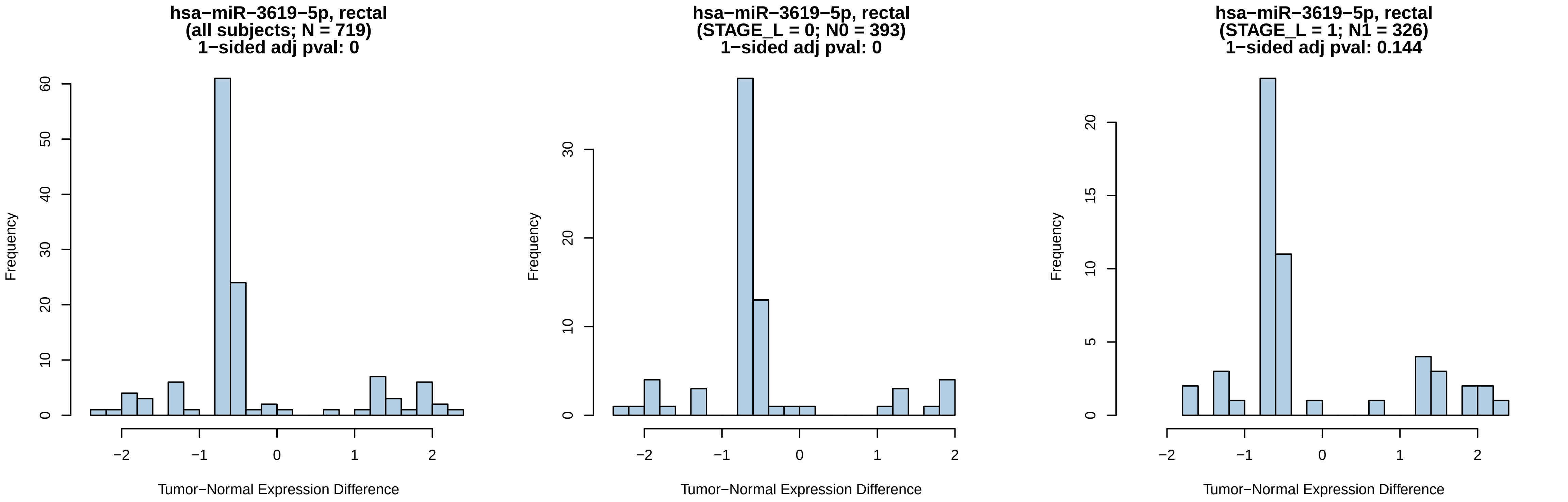

hsa−miR−3619−5p, rectal
(all subjects; N = 719)
1−sided adj pval: 0
Frequency
Tumor−Normal Expression Difference
hsa−miR−3619−5p, rectal
(STAGE_L = 0; N0 = 393)
1−sided adj pval: 0
Frequency
Tumor−Normal Expression Difference
hsa−miR−3619−5p, rectal
(STAGE_L = 1; N1 = 326)
1−sided adj pval: 0.144
Frequency
Tumor−Normal Expression Difference

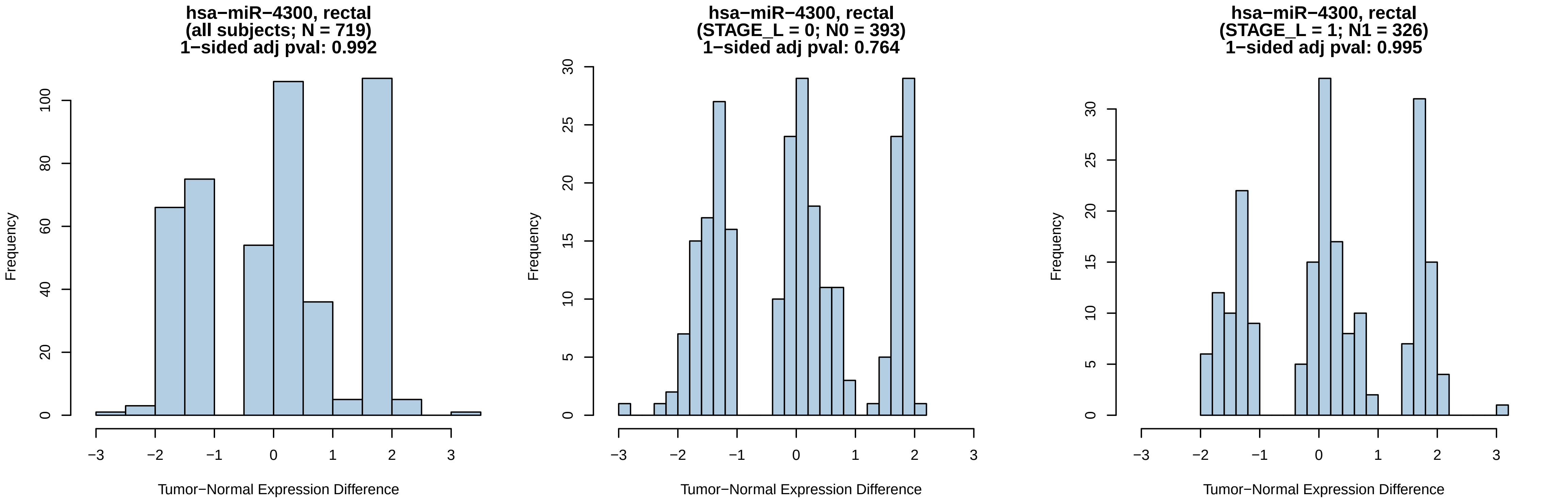
hsa−miR−4300, rectal
(all subjects; N = 719)
1−sided adj pval: 0.992
hsa−miR−4300, rectal
(STAGE_L = 0; N0 = 393)
1−sided adj pval: 0.764
hsa−miR−4300, rectal
(STAGE_L = 1; N1 = 326)
1−sided adj pval: 0.995
Frequency
Tumor−Normal Expression Difference

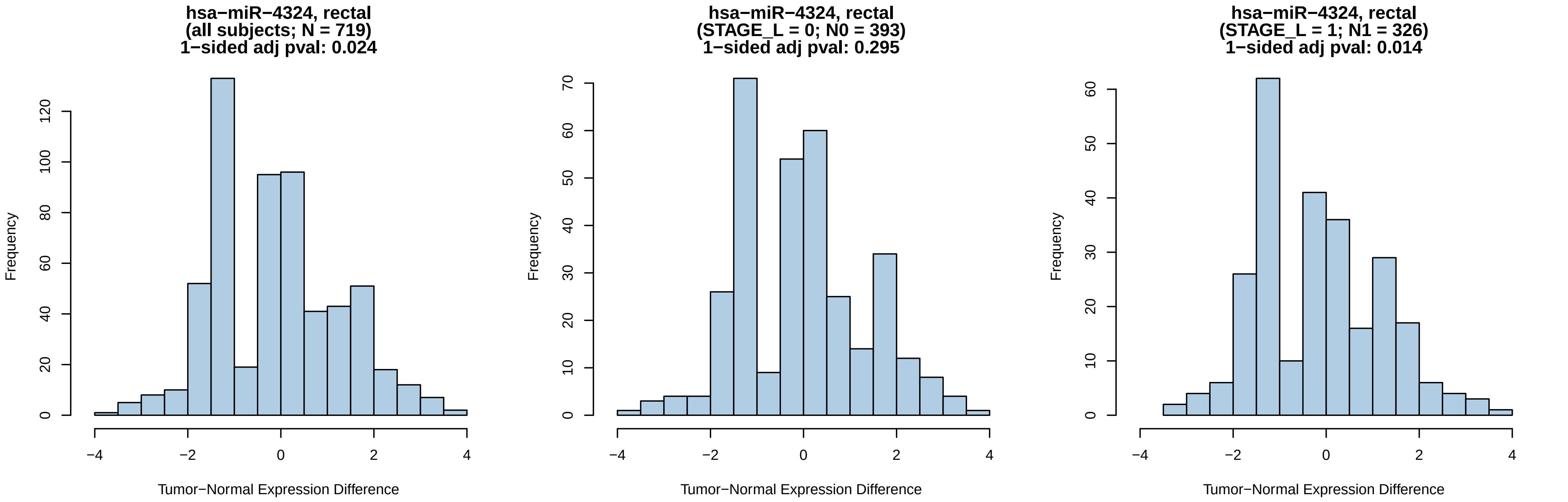

hsa−miR−4324, rectal
(all subjects; N = 719)
1−sided adj pval: 0.024
hsa−miR−4324, rectal
(STAGE_L = 0; N0 = 393)
1−sided adj pval: 0.295
hsa−miR−4324, rectal
(STAGE_L = 1; N1 = 326)
1−sided adj pval: 0.014
Frequency
Tumor−Normal Expression Difference

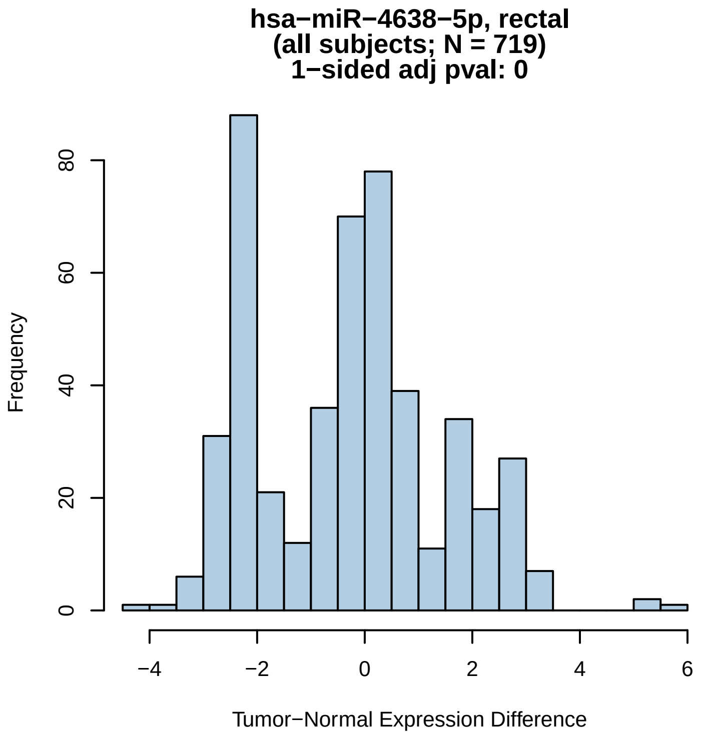

hsa−miR−4638−5p, rectal
(all subjects; N = 719)
1−sided adj pval: 0
Frequency
Tumor−Normal Expression Difference


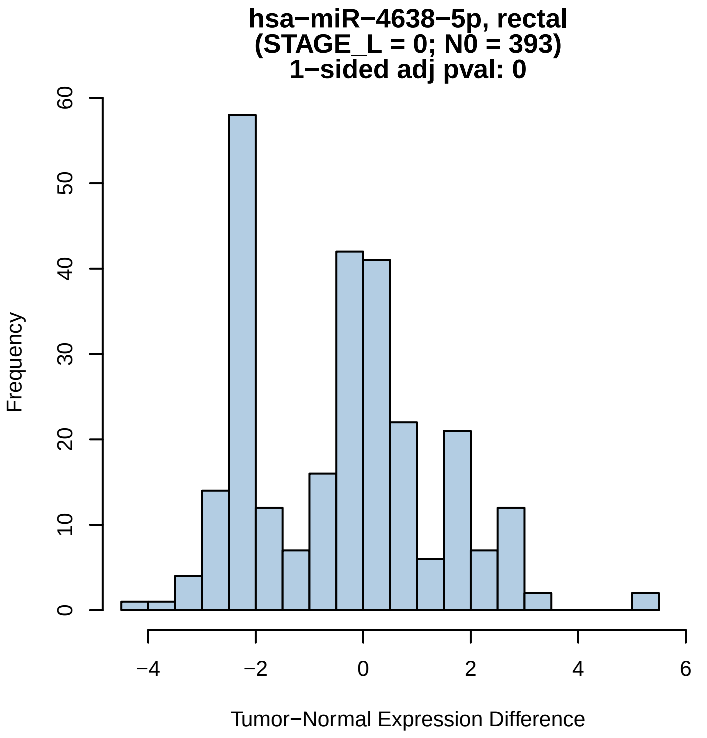

hsa−miR−4638−5p, rectal
(STAGE_L = 0; N0 = 393)
1−sided adj pval: 0
Frequency
Tumor−Normal Expression Difference


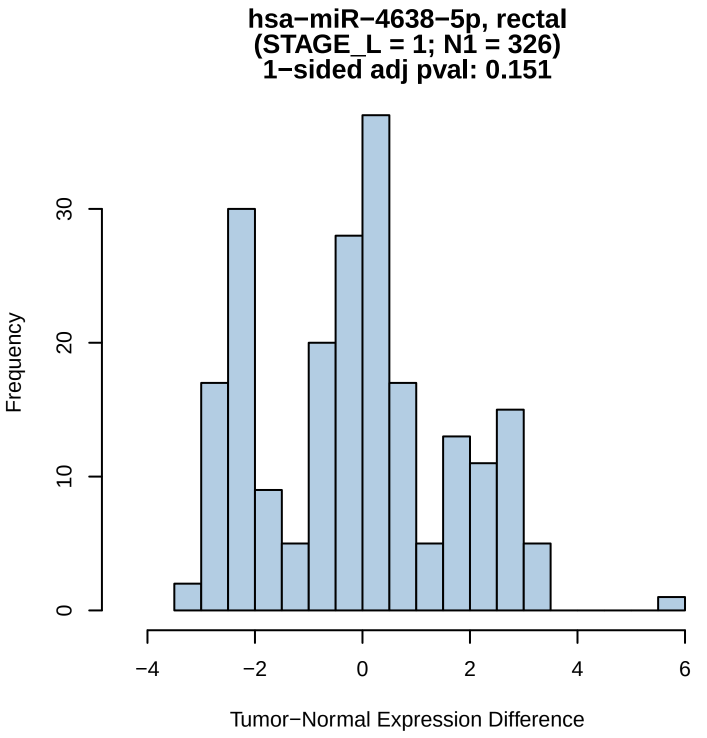

hsa−miR−4638−5p, rectal
(STAGE_L = 1; N1 = 326)
1−sided adj pval: 0.151
Frequency
Tumor−Normal Expression Difference

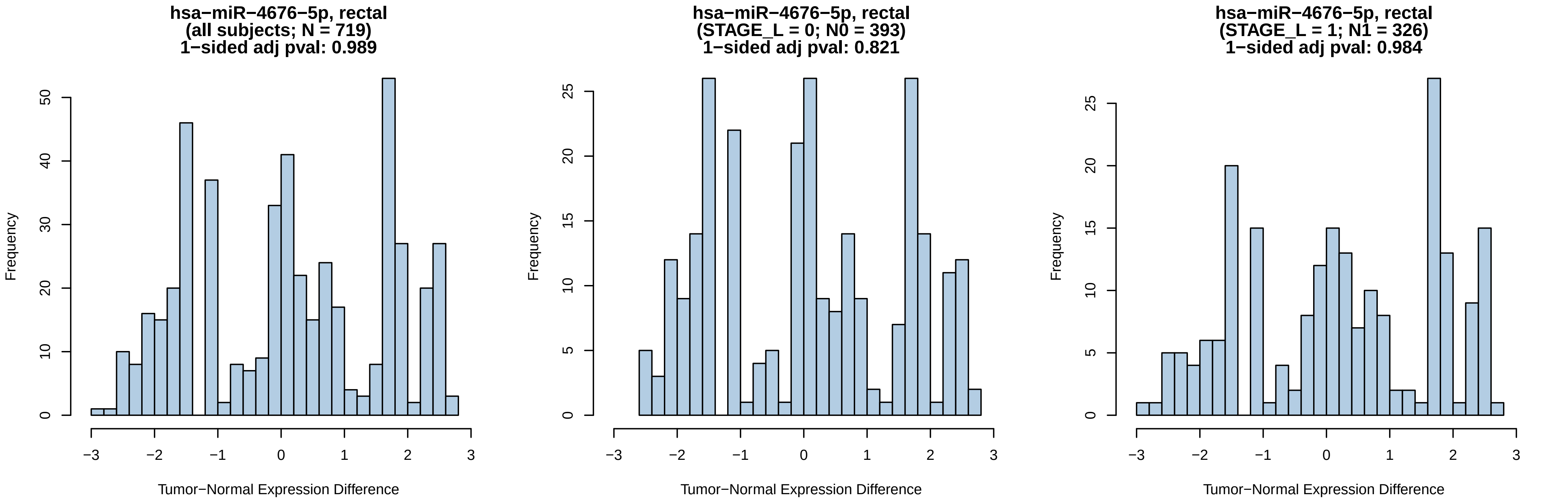
hsa−miR−4676−5p, rectal
(all subjects; N = 719)
1−sided adj pval: 0.989
Frequency
Tumor−Normal Expression Difference
hsa−miR−4676−5p, rectal
(STAGE_L = 0; N0 = 393)
1−sided adj pval: 0.821
Frequency
Tumor−Normal Expression Difference
hsa−miR−4676−5p, rectal
(STAGE_L = 1; N1 = 326)
1−sided adj pval: 0.984
Frequency
Tumor−Normal Expression Difference

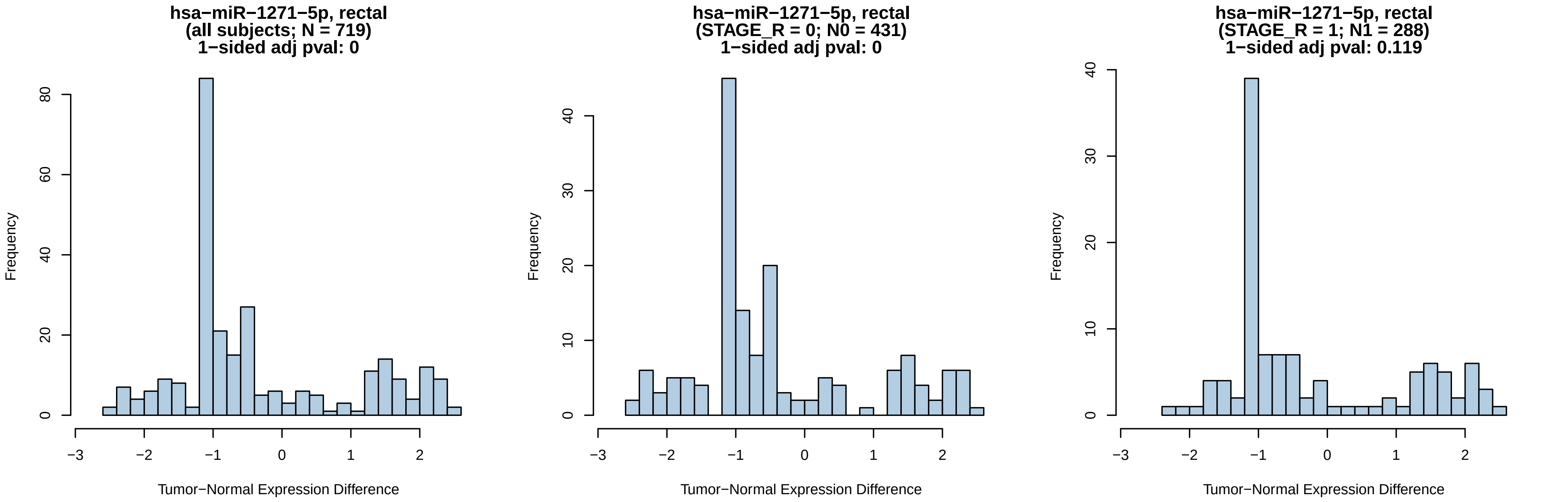

hsa−miR−1271−5p, rectal
(all subjects; N = 719)
1−sided adj pval: 0
Frequency
0
20
40
60
80
−3
−2
−1
0
1
2
Tumor−Normal Expression Difference
hsa−miR−1271−5p, rectal
(STAGE_R = 0; N0 = 431)
1−sided adj pval: 0
Frequency
0
10
20
30
40
−3
−2
−1
0
1
2
Tumor−Normal Expression Difference
hsa−miR−1271−5p, rectal
(STAGE_R = 1; N1 = 288)
1−sided adj pval: 0.119
Frequency
0
10
20
30
40
−3
−2
−1
0
1
2
Tumor−Normal Expression Difference

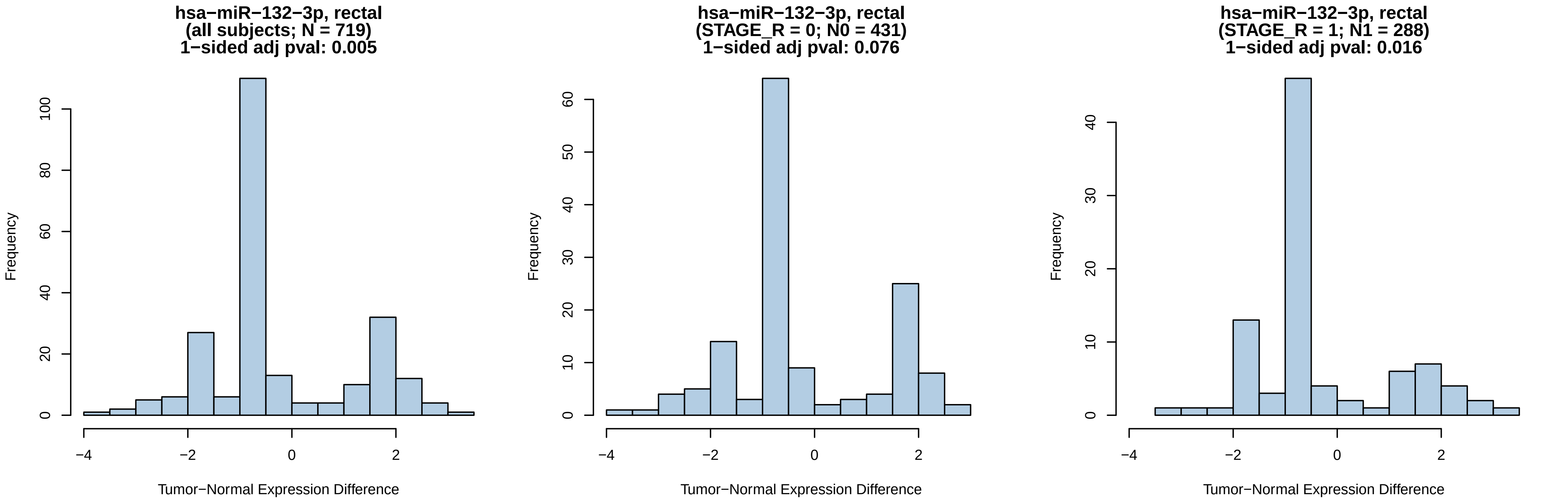

hsa−miR−132−3p, rectal
(all subjects; N = 719)
1−sided adj pval: 0.005
Frequency
Tumor−Normal Expression Difference
hsa−miR−132−3p, rectal
(STAGE_R = 0; N0 = 431)
1−sided adj pval: 0.076
Frequency
Tumor−Normal Expression Difference
hsa−miR−132−3p, rectal
(STAGE_R = 1; N1 = 288)
1−sided adj pval: 0.016
Frequency
Tumor−Normal Expression Difference

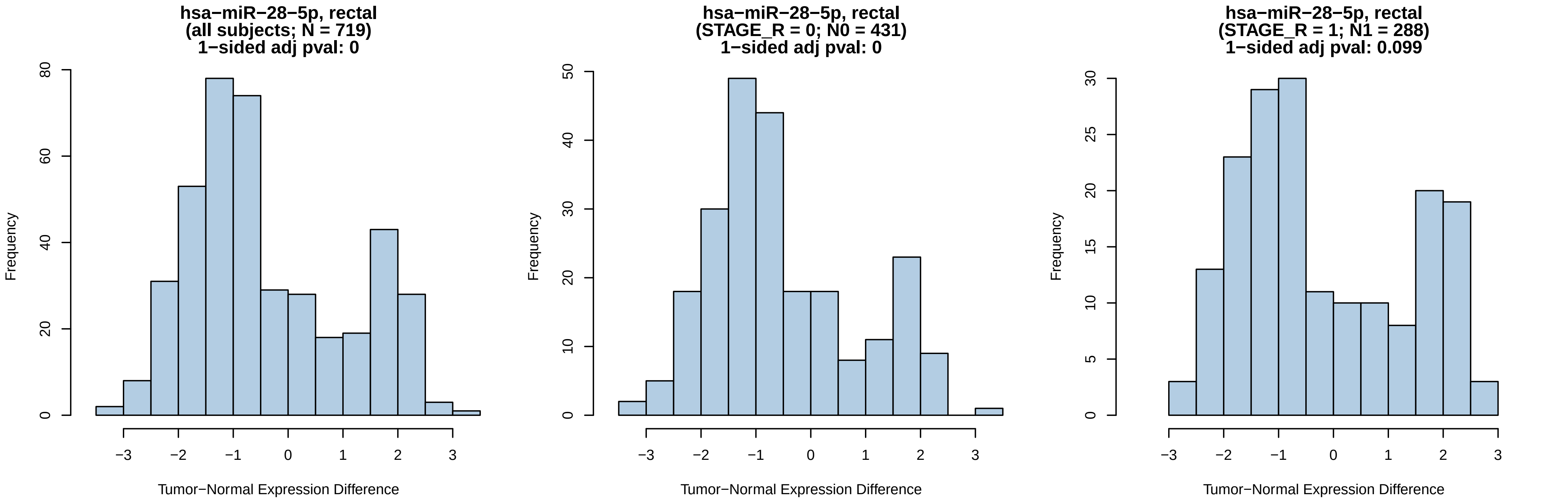

hsa−miR−28−5p, rectal
(all subjects; N = 719)
1−sided adj pval: 0
Frequency
Tumor−Normal Expression Difference
hsa−miR−28−5p, rectal
(STAGE_R = 0; N0 = 431)
1−sided adj pval: 0
Frequency
Tumor−Normal Expression Difference
hsa−miR−28−5p, rectal
(STAGE_R = 1; N1 = 288)
1−sided adj pval: 0.099
Frequency
Tumor−Normal Expression Difference

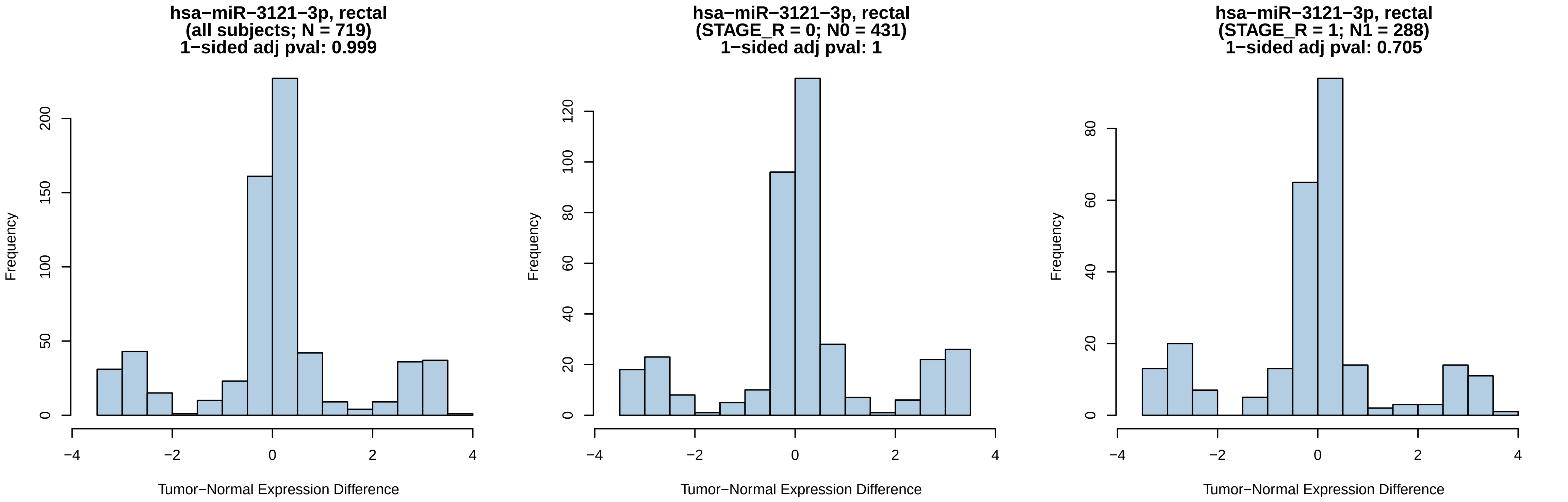

hsa−miR−3121−3p, rectal
(all subjects; N = 719)
1−sided adj pval: 0.999
Frequency
0
50
100
150
200
−4
−2
0
2
4
Tumor−Normal Expression Difference
hsa−miR−3121−3p, rectal
(STAGE_R = 0; N0 = 431)
1−sided adj pval: 1
Frequency
0
20
40
60
80
100
120
−4
−2
0
2
4
Tumor−Normal Expression Difference
hsa−miR−3121−3p, rectal
(STAGE_R = 1; N1 = 288)
1−sided adj pval: 0.705
Frequency
0
20
40
60
80
−4
−2
0
2
4
Tumor−Normal Expression Difference

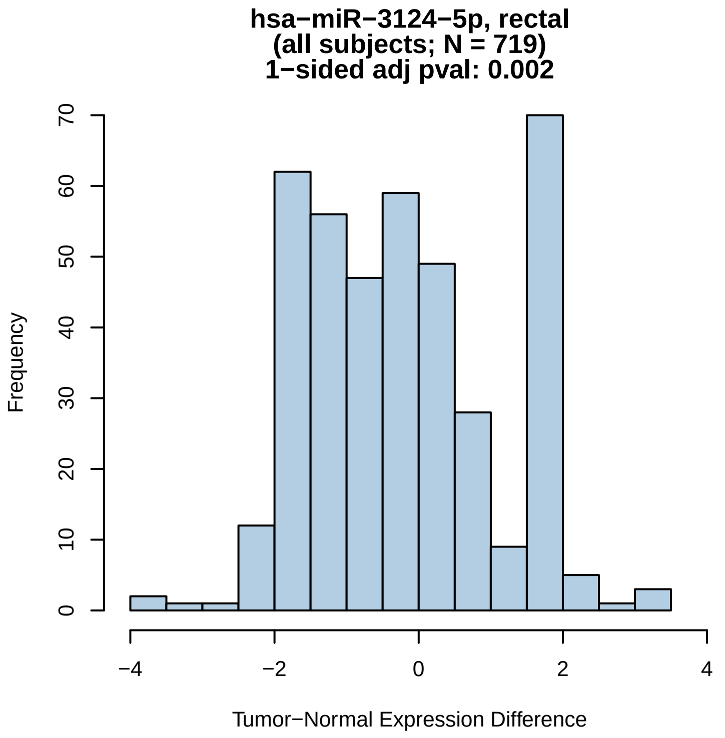

hsa−miR−3124−5p, rectal
(all subjects; N = 719)
1−sided adj pval: 0.002
Frequency
Tumor−Normal Expression Difference


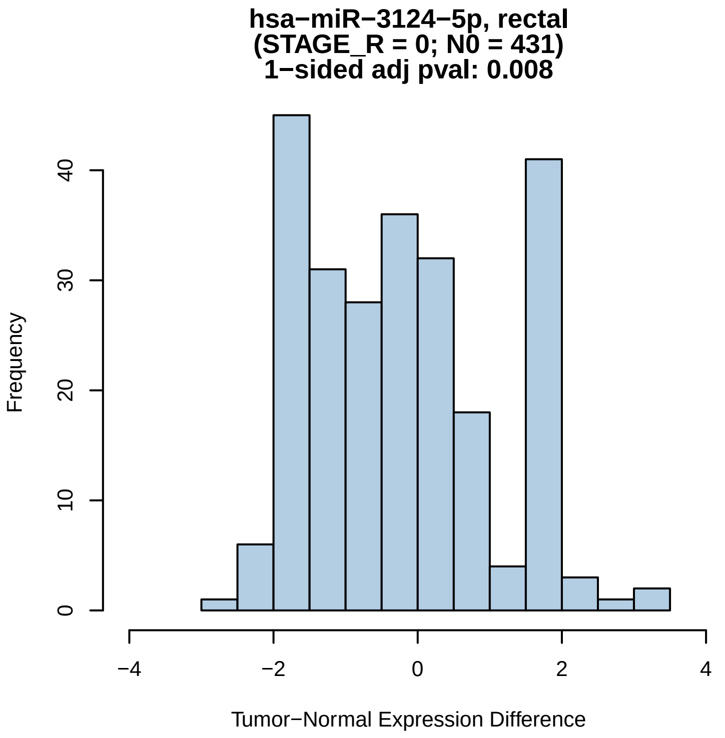

hsa−miR−3124−5p, rectal
(STAGE_R = 0; N0 = 431)
1−sided adj pval: 0.008
Frequency
Tumor−Normal Expression Difference


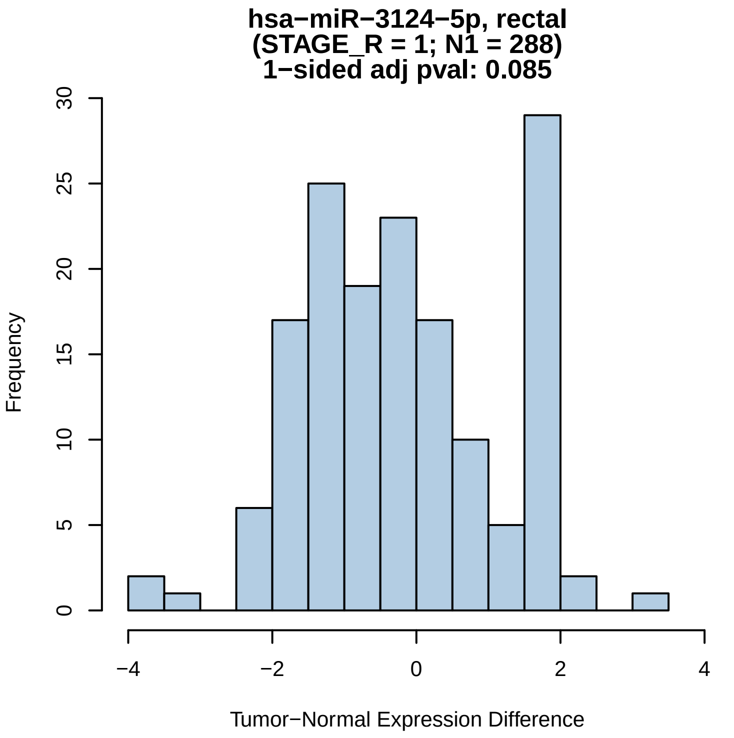

hsa−miR−3124−5p, rectal
(STAGE_R = 1; N1 = 288)
1−sided adj pval: 0.085
Frequency
Tumor−Normal Expression Difference

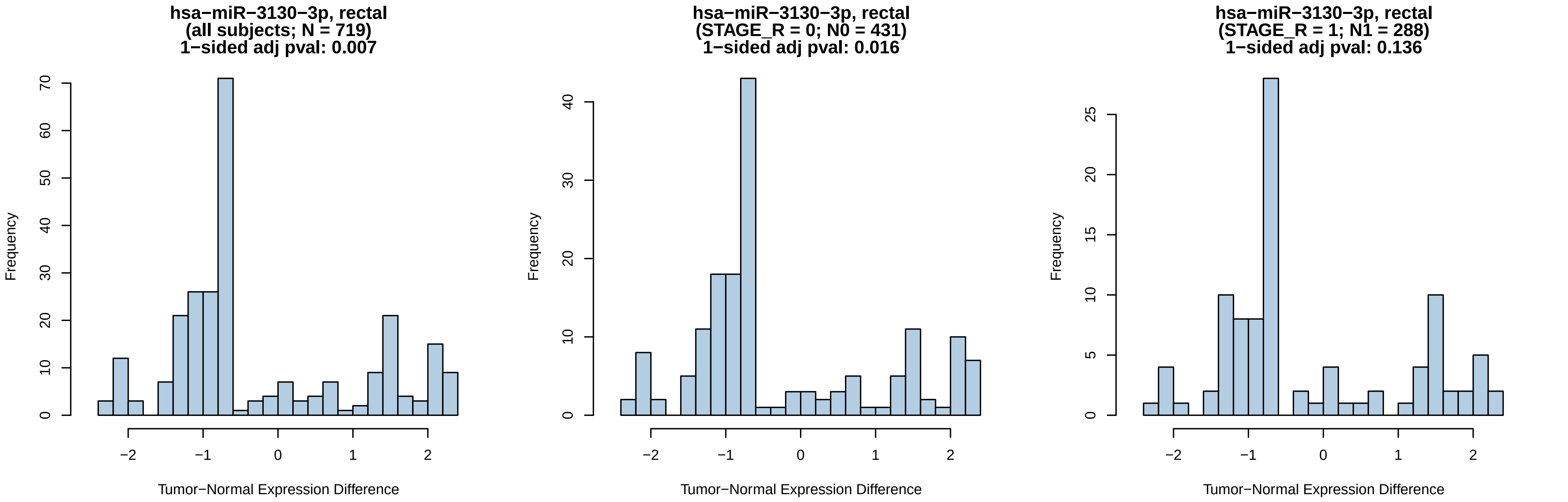
hsa−miR−3130−3p, rectal
(all subjects; N = 719)
1−sided adj pval: 0.007
hsa−miR−3130−3p, rectal
(STAGE_R = 0; N0 = 431)
1−sided adj pval: 0.016
hsa−miR−3130−3p, rectal
(STAGE_R = 1; N1 = 288)
1−sided adj pval: 0.136
Frequency
Tumor−Normal Expression Difference

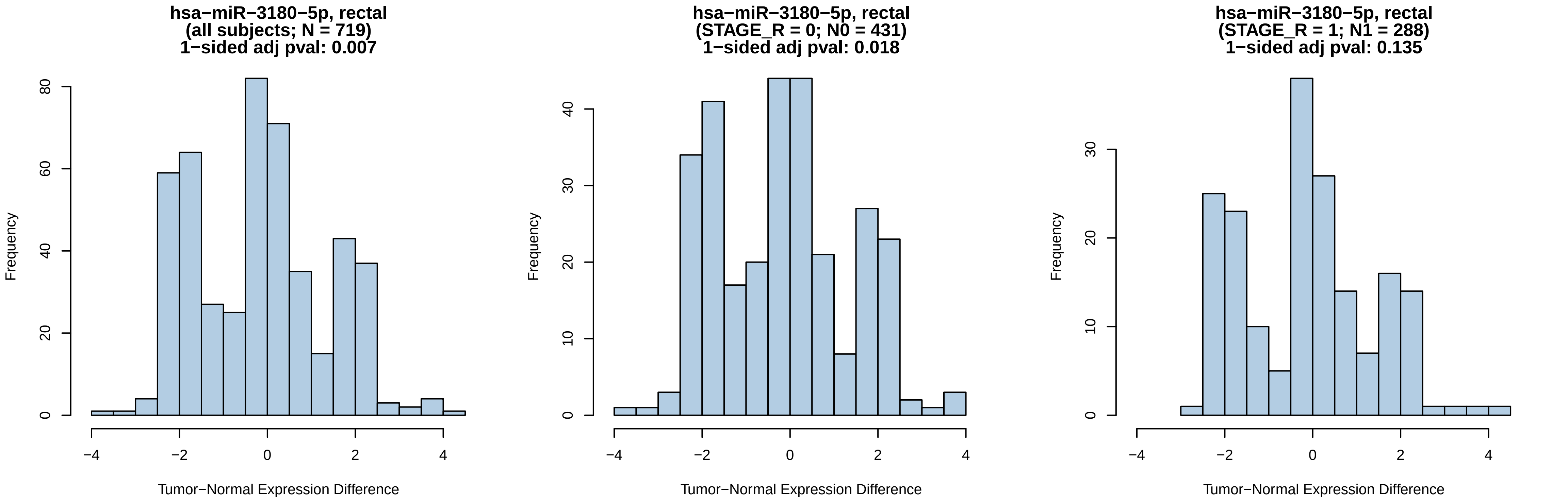

hsa−miR−3180−5p, rectal
(all subjects; N = 719)
1−sided adj pval: 0.007
Frequency
Tumor−Normal Expression Difference
hsa−miR−3180−5p, rectal
(STAGE_R = 0; N0 = 431)
1−sided adj pval: 0.018
Frequency
Tumor−Normal Expression Difference
hsa−miR−3180−5p, rectal
(STAGE_R = 1; N1 = 288)
1−sided adj pval: 0.135
Frequency
Tumor−Normal Expression Difference

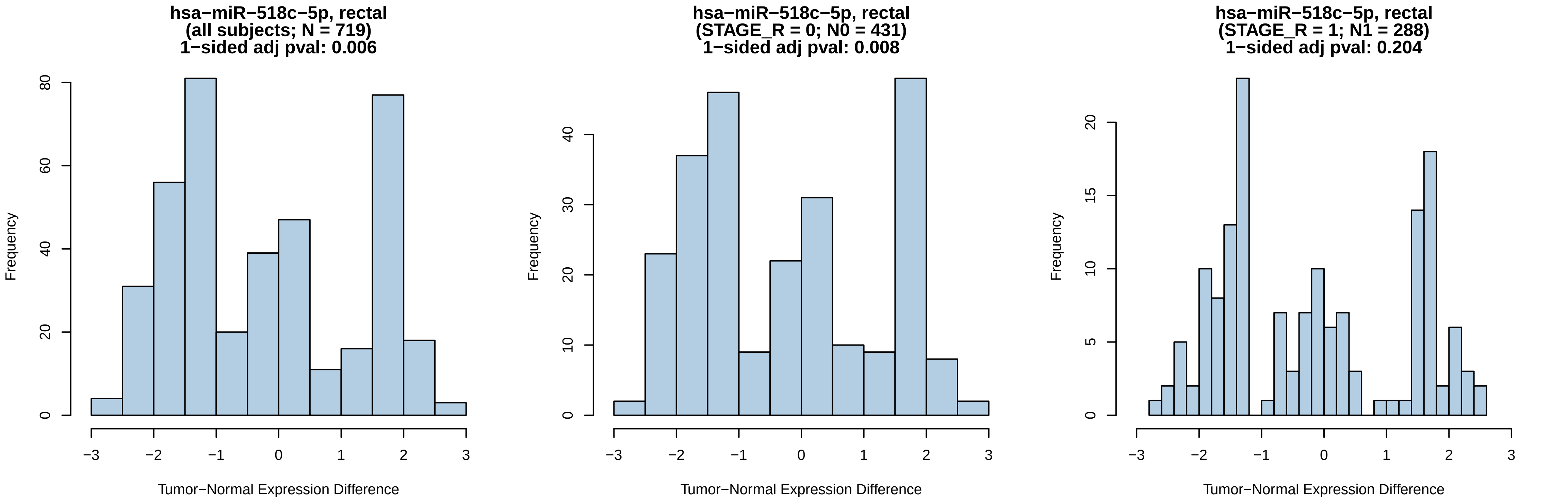

hsa−miR−518c−5p, rectal
(all subjects; N = 719)
1−sided adj pval: 0.006
Frequency
0
20
40
60
80
−3
−2
−1
0
1
2
3
Tumor−Normal Expression Difference
hsa−miR−518c−5p, rectal
(STAGE_R = 0; N0 = 431)
1−sided adj pval: 0.008
Frequency
0
10
20
30
40
−3
−2
−1
0
1
2
3
Tumor−Normal Expression Difference
hsa−miR−518c−5p, rectal
(STAGE_R = 1; N1 = 288)
1−sided adj pval: 0.204
Frequency
0
5
10
15
20
−3
−2
−1
0
1
2
3
Tumor−Normal Expression Difference

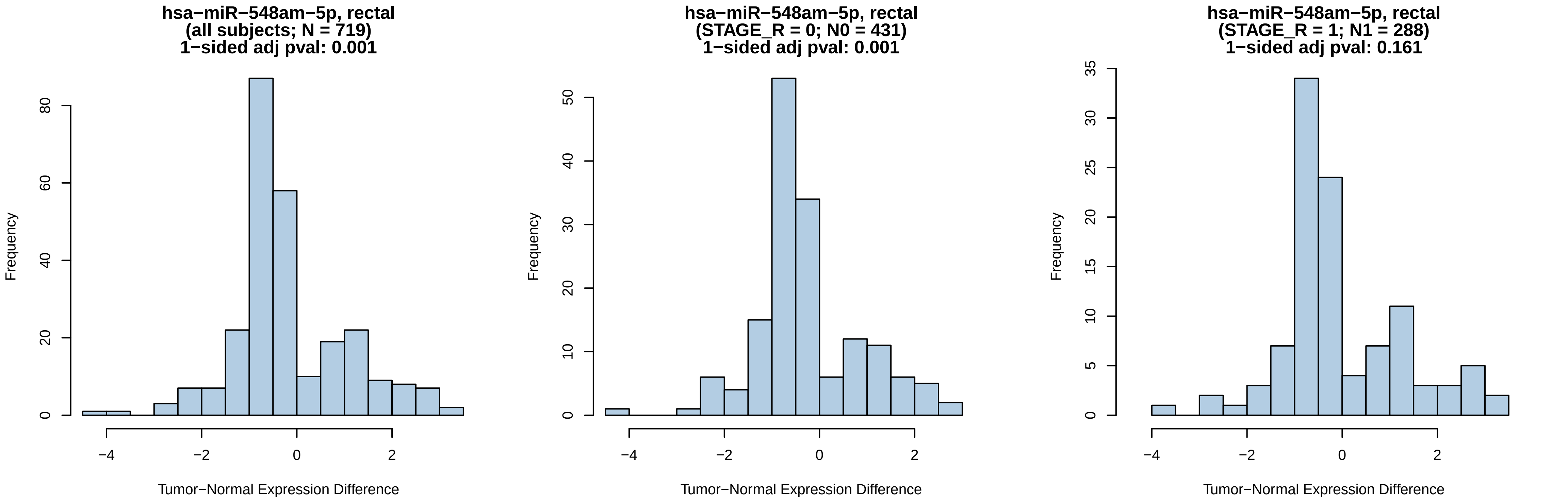

hsa−miR−548am−5p, rectal
(all subjects; N = 719)
1−sided adj pval: 0.001
Frequency
0
20
40
60
80
−4
−2
0
2
Tumor−Normal Expression Difference
hsa−miR−548am−5p, rectal
(STAGE_R = 0; N0 = 431)
1−sided adj pval: 0.001
Frequency
0
10
20
30
40
50
−4
−2
0
2
Tumor−Normal Expression Difference
hsa−miR−548am−5p, rectal
(STAGE_R = 1; N1 = 288)
1−sided adj pval: 0.161
Frequency
0
5
10
15
20
25
30
35
−4
−2
0
2
Tumor−Normal Expression Difference

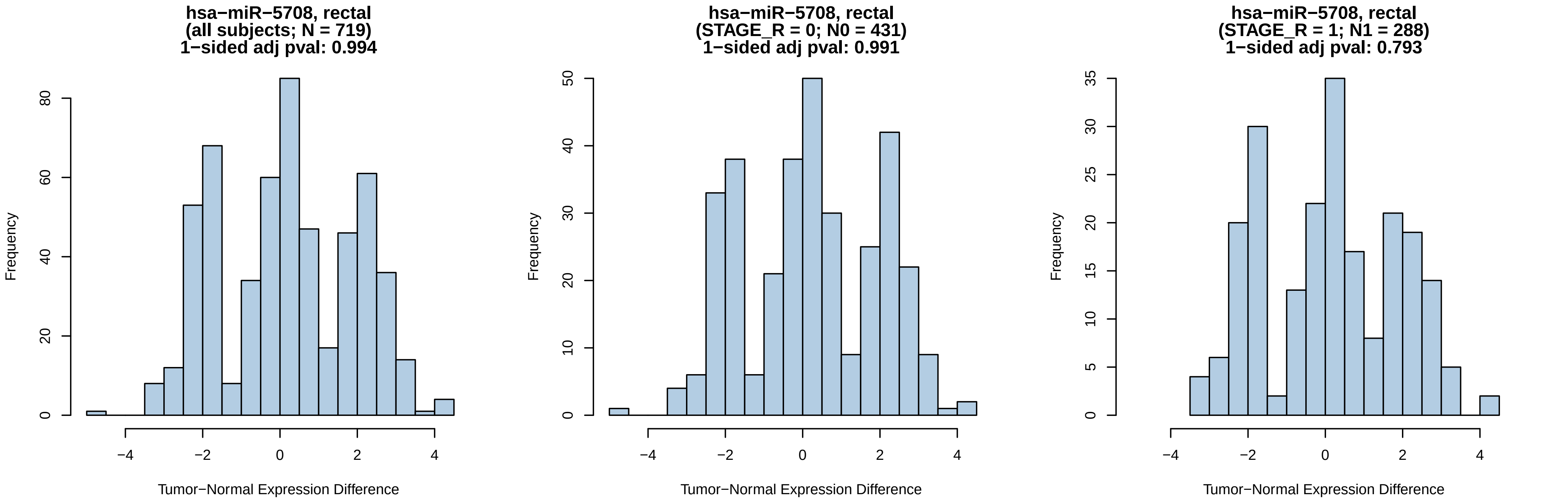
hsa−miR−5708, rectal
(all subjects; N = 719)
1−sided adj pval: 0.994
Frequency
Tumor−Normal Expression Difference
hsa−miR−5708, rectal
(STAGE_R = 0; N0 = 431)
1−sided adj pval: 0.991
Frequency
Tumor−Normal Expression Difference
hsa−miR−5708, rectal
(STAGE_R = 1; N1 = 288)
1−sided adj pval: 0.793
Frequency
Tumor−Normal Expression Difference

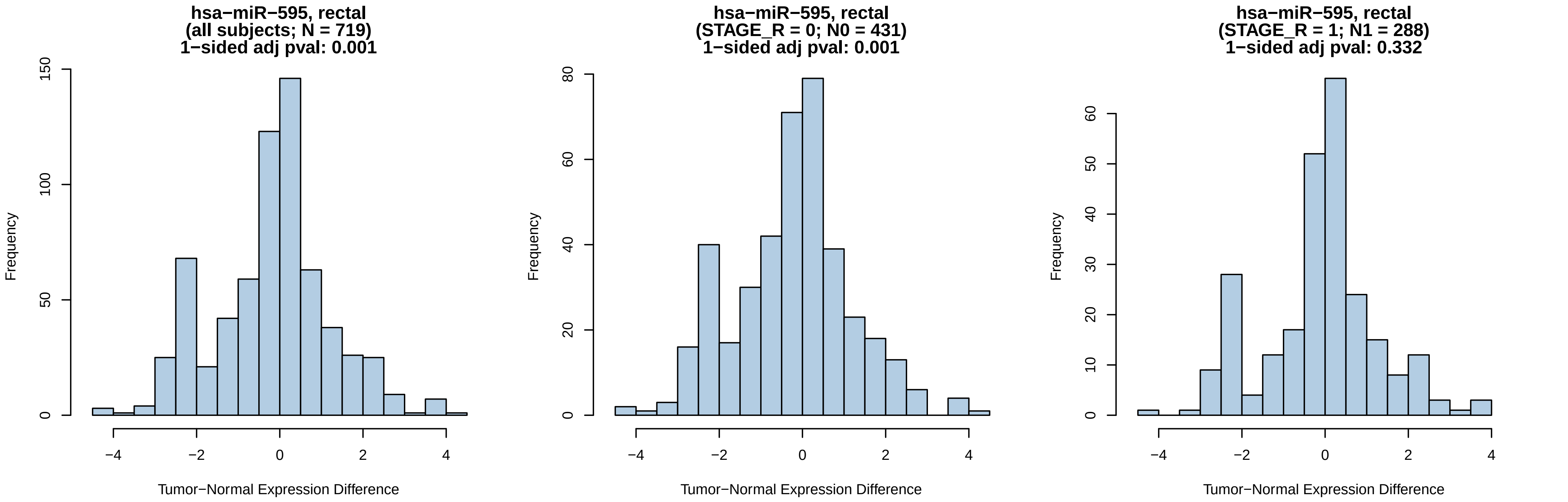

hsa−miR−595, rectal
(all subjects; N = 719)
1−sided adj pval: 0.001
Frequency
Tumor−Normal Expression Difference
hsa−miR−595, rectal
(STAGE_R = 0; N0 = 431)
1−sided adj pval: 0.001
Frequency
Tumor−Normal Expression Difference
hsa−miR−595, rectal
(STAGE_R = 1; N1 = 288)
1−sided adj pval: 0.332
Frequency
Tumor−Normal Expression Difference

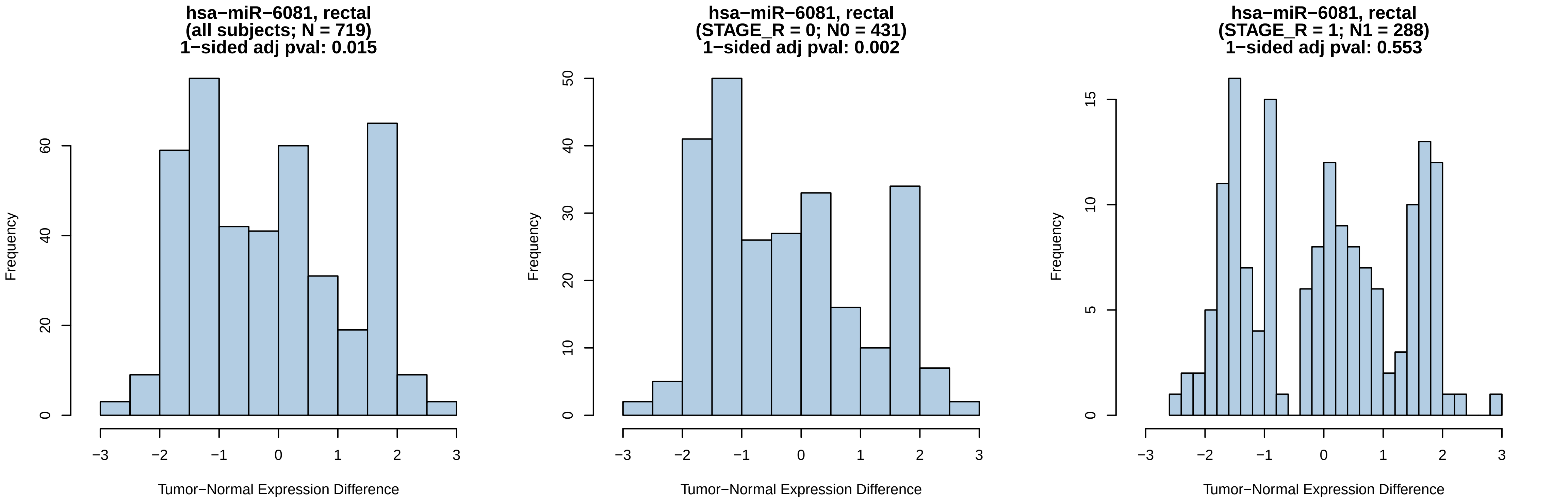
hsa−miR−6081, rectal
(all subjects; N = 719)
1−sided adj pval: 0.015
Frequency
0
20
40
60
−3
−2
−1
0
1
2
3
Tumor−Normal Expression Difference
hsa−miR−6081, rectal
(STAGE_R = 0; N0 = 431)
1−sided adj pval: 0.002
Frequency
0
10
20
30
40
50
−3
−2
−1
0
1
2
3
Tumor−Normal Expression Difference
hsa−miR−6081, rectal
(STAGE_R = 1; N1 = 288)
1−sided adj pval: 0.553
Frequency
0
5
10
15
−3
−2
−1
0
1
2
3
Tumor−Normal Expression Difference

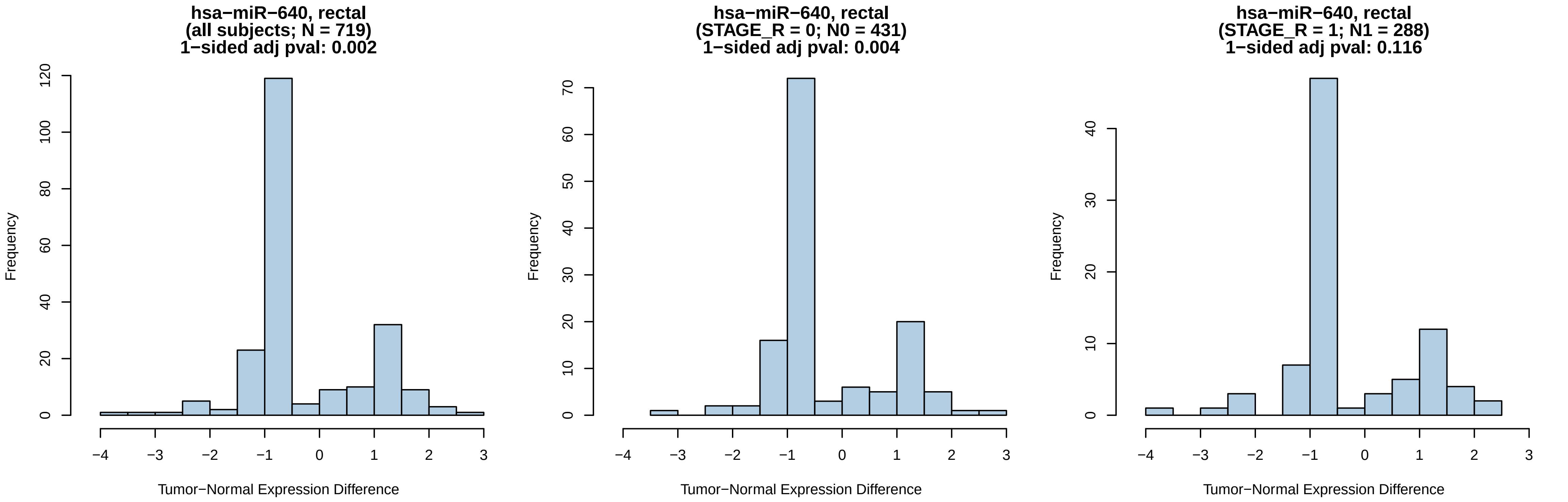
hsa−miR−640, rectal
(all subjects; N = 719)
1−sided adj pval: 0.002
hsa−miR−640, rectal
(STAGE_R = 0; N0 = 431)
1−sided adj pval: 0.004
hsa−miR−640, rectal
(STAGE_R = 1; N1 = 288)
1−sided adj pval: 0.116
Frequency
Tumor−Normal Expression Difference

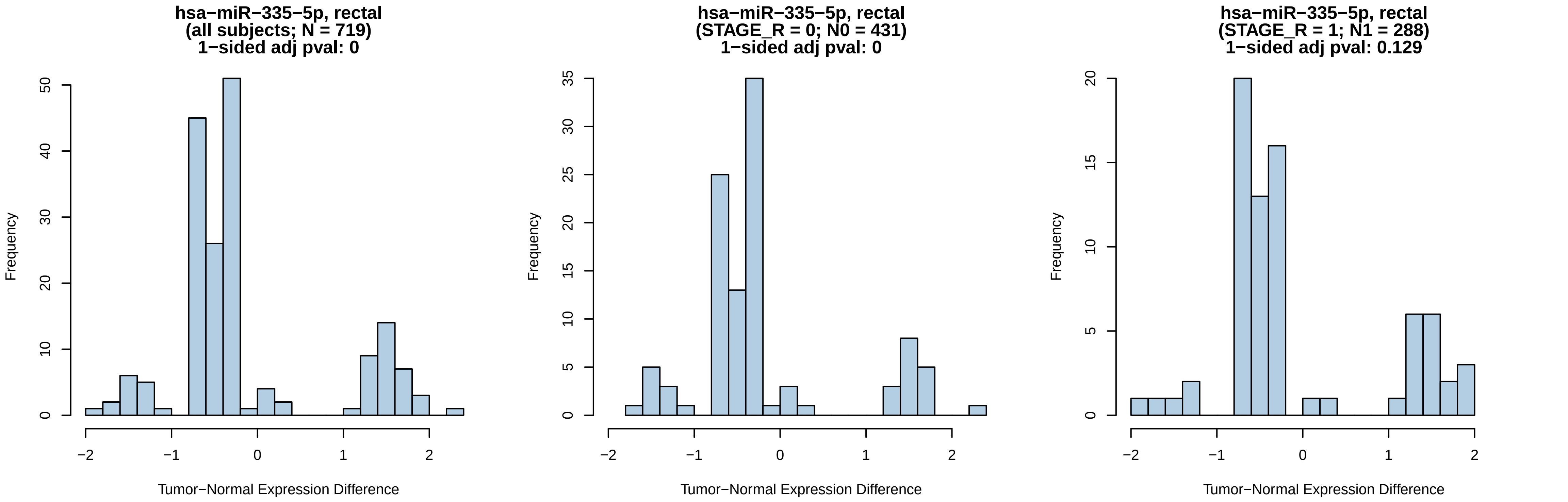
hsa−miR−335−5p, rectal
(all subjects; N = 719)
1−sided adj pval: 0
Frequency
Tumor−Normal Expression Difference
hsa−miR−335−5p, rectal
(STAGE_R = 0; N0 = 431)
1−sided adj pval: 0
Frequency
Tumor−Normal Expression Difference
hsa−miR−335−5p, rectal
(STAGE_R = 1; N1 = 288)
1−sided adj pval: 0.129
Frequency
Tumor−Normal Expression Difference

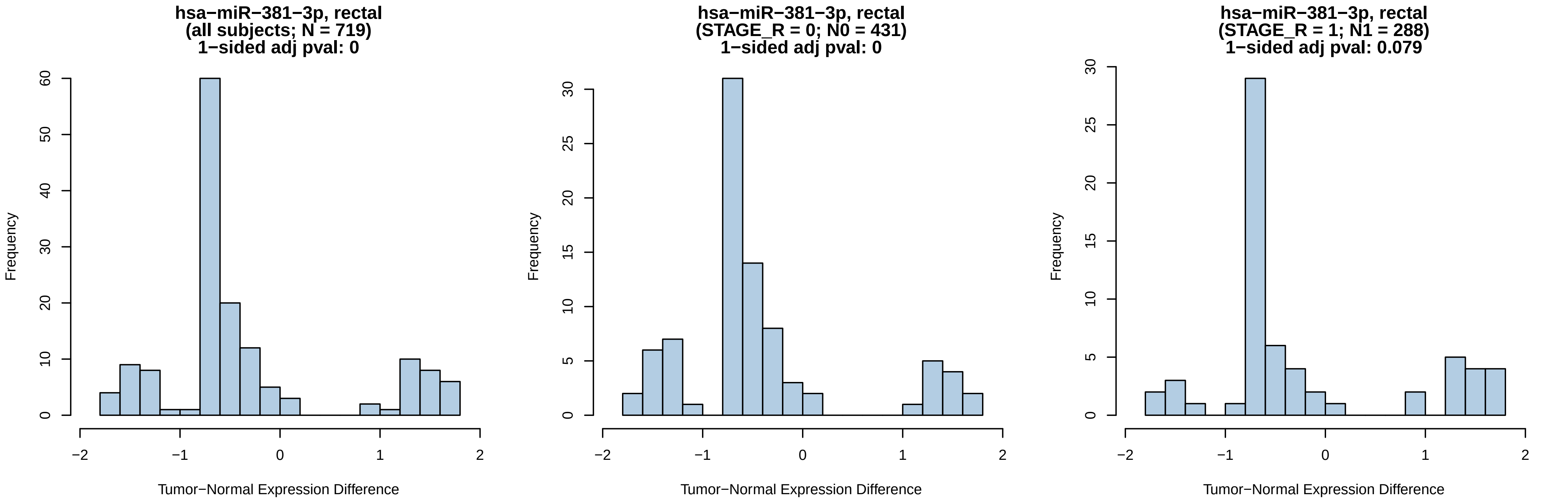

hsa−miR−381−3p, rectal
(all subjects; N = 719)
1−sided adj pval: 0
Frequency
Tumor−Normal Expression Difference
hsa−miR−381−3p, rectal
(STAGE_R = 0; N0 = 431)
1−sided adj pval: 0
Frequency
Tumor−Normal Expression Difference
hsa−miR−381−3p, rectal
(STAGE_R = 1; N1 = 288)
1−sided adj pval: 0.079
Frequency
Tumor−Normal Expression Difference

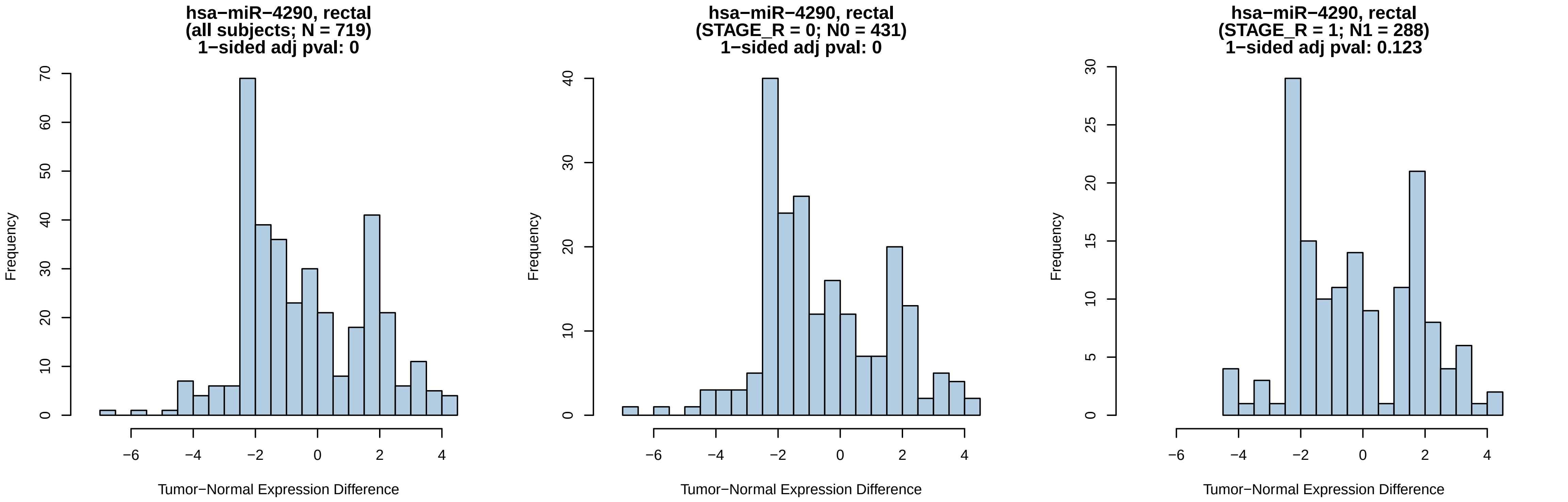

hsa−miR−4290, rectal
(all subjects; N = 719)
1−sided adj pval: 0
hsa−miR−4290, rectal
(STAGE_R = 0; N0 = 431)
1−sided adj pval: 0
hsa−miR−4290, rectal
(STAGE_R = 1; N1 = 288)
1−sided adj pval: 0.123
Frequency
Tumor−Normal Expression Difference

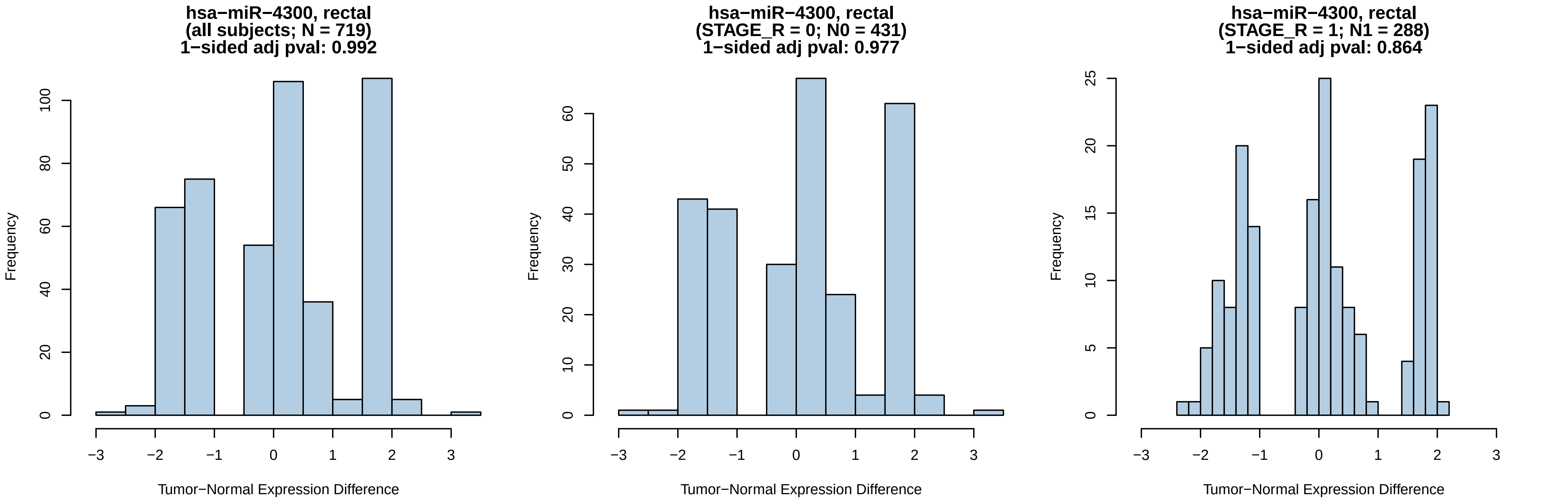
hsa−miR−4300, rectal
(all subjects; N = 719)
1−sided adj pval: 0.992
Frequency
Tumor−Normal Expression Difference
hsa−miR−4300, rectal
(STAGE_R = 0; N0 = 431)
1−sided adj pval: 0.977
Frequency
Tumor−Normal Expression Difference
hsa−miR−4300, rectal
(STAGE_R = 1; N1 = 288)
1−sided adj pval: 0.864
Frequency
Tumor−Normal Expression Difference

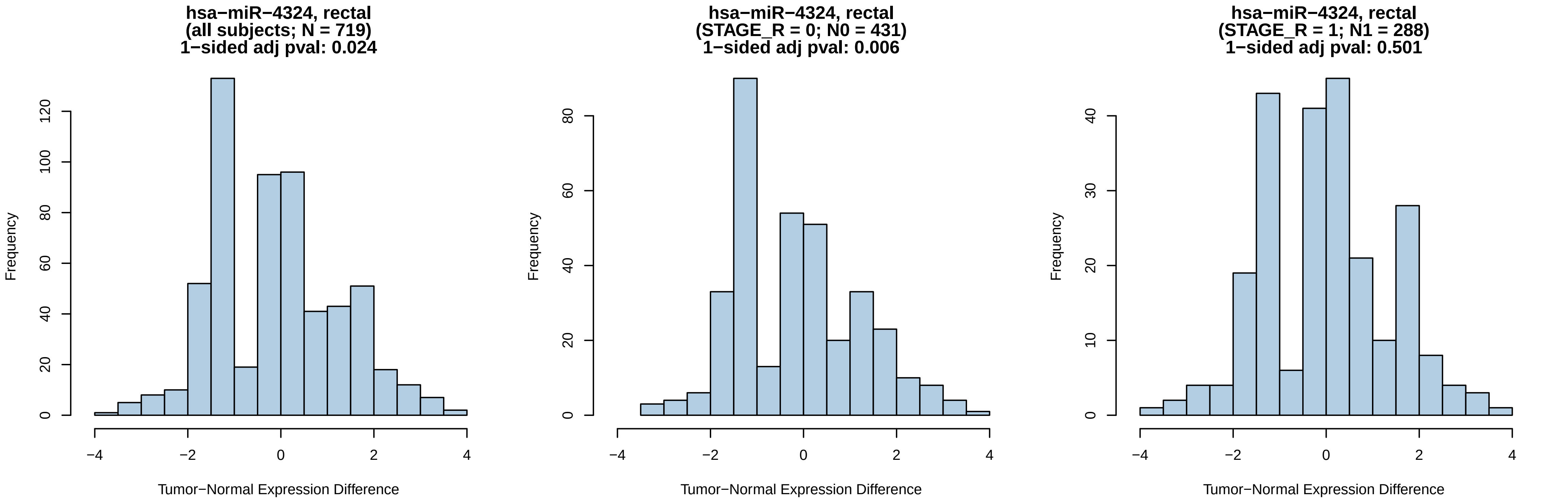
hsa−miR−4324, rectal
(all subjects; N = 719)
1−sided adj pval: 0.024
Frequency
Tumor−Normal Expression Difference
hsa−miR−4324, rectal
(STAGE_R = 0; N0 = 431)
1−sided adj pval: 0.006
Frequency
Tumor−Normal Expression Difference
hsa−miR−4324, rectal
(STAGE_R = 1; N1 = 288)
1−sided adj pval: 0.501
Frequency
Tumor−Normal Expression Difference

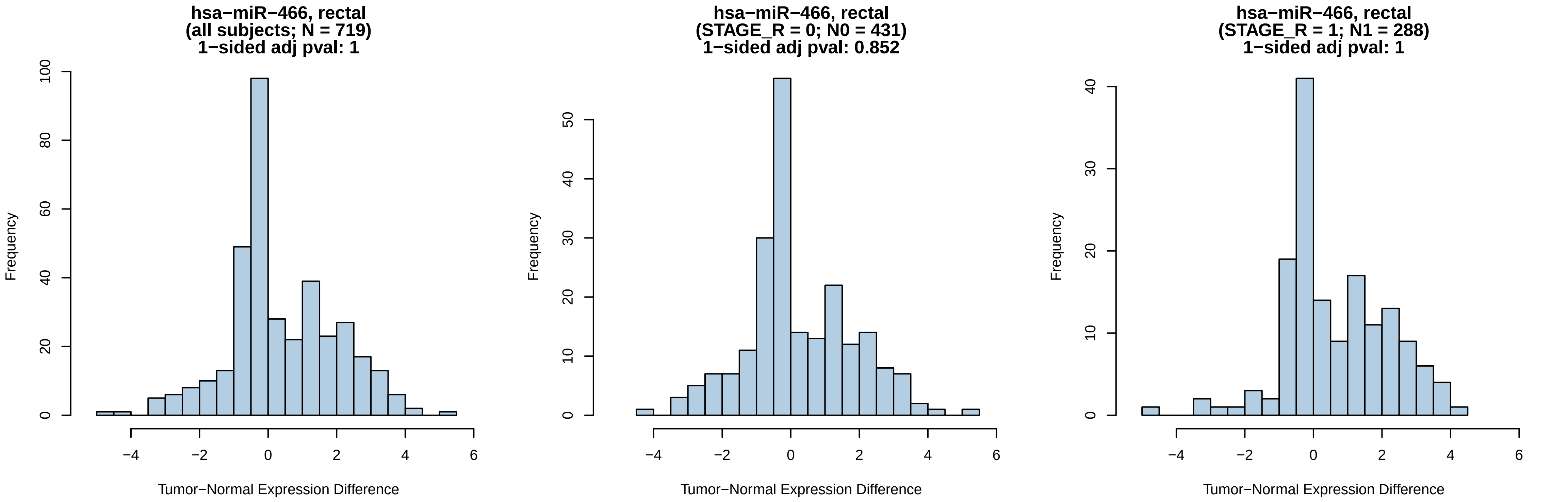
hsa−miR−466, rectal
(all subjects; N = 719)
1−sided adj pval: 1
Frequency
Tumor−Normal Expression Difference
hsa−miR−466, rectal
(STAGE_R = 0; N0 = 431)
1−sided adj pval: 0.852
Frequency
Tumor−Normal Expression Difference
hsa−miR−466, rectal
(STAGE_R = 1; N1 = 288)
1−sided adj pval: 1
Frequency
Tumor−Normal Expression Difference

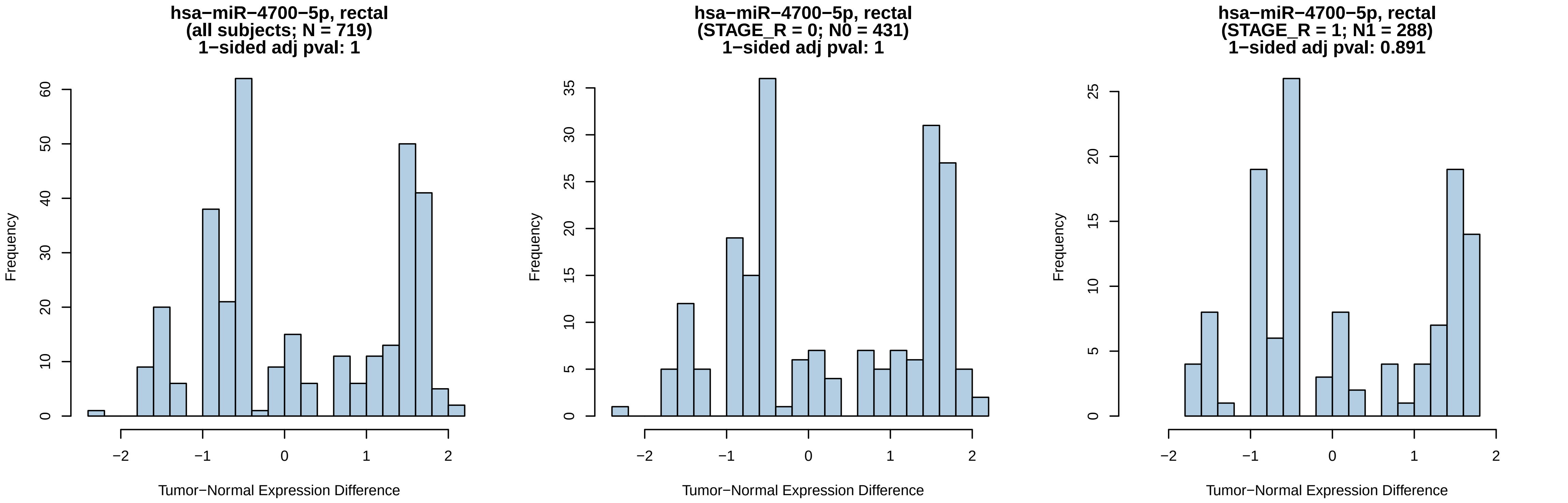
hsa−miR−4700−5p, rectal
(all subjects; N = 719)
1−sided adj pval: 1
Frequency
0
10
20
30
40
50
60
−2
−1
0
1
2
Tumor−Normal Expression Difference
hsa−miR−4700−5p, rectal
(STAGE_R = 0; N0 = 431)
1−sided adj pval: 1
Frequency
0
5
10
15
20
25
30
35
−2
−1
0
1
2
Tumor−Normal Expression Difference
hsa−miR−4700−5p, rectal
(STAGE_R = 1; N1 = 288)
1−sided adj pval: 0.891
Frequency
0
5
10
15
20
25
−2
−1
0
1
2
Tumor−Normal Expression Difference

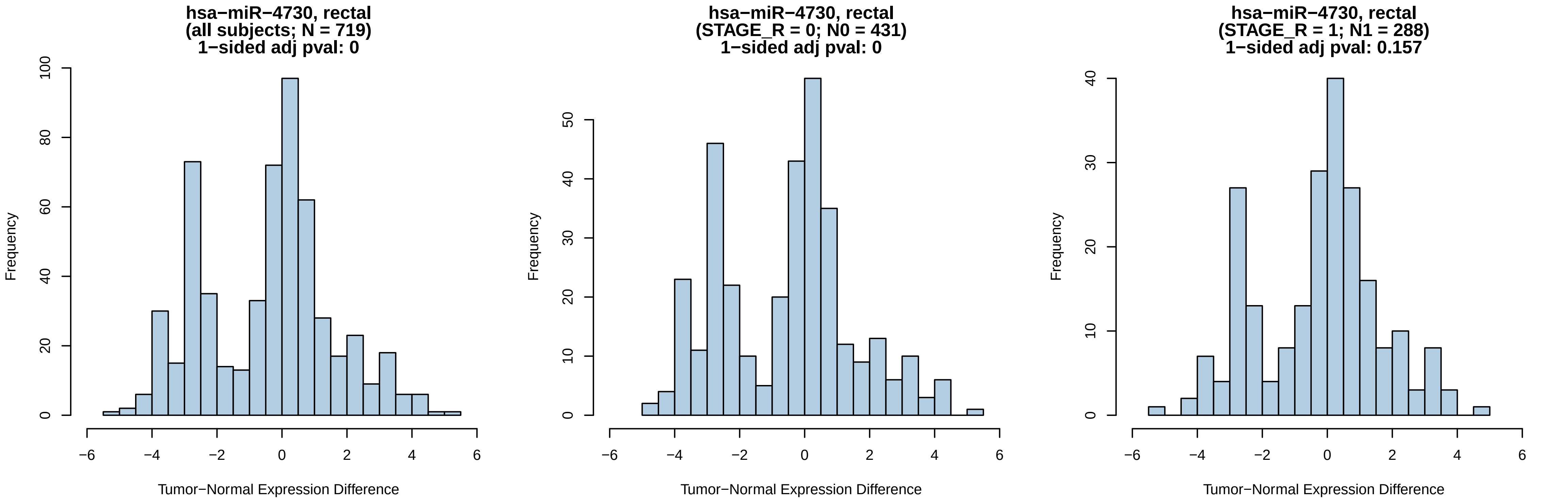
hsa−miR−4730, rectal
(all subjects; N = 719)
1−sided adj pval: 0
hsa−miR−4730, rectal
(STAGE_R = 0; N0 = 431)
1−sided adj pval: 0
hsa−miR−4730, rectal
(STAGE_R = 1; N1 = 288)
1−sided adj pval: 0.157
Frequency
Tumor−Normal Expression Difference

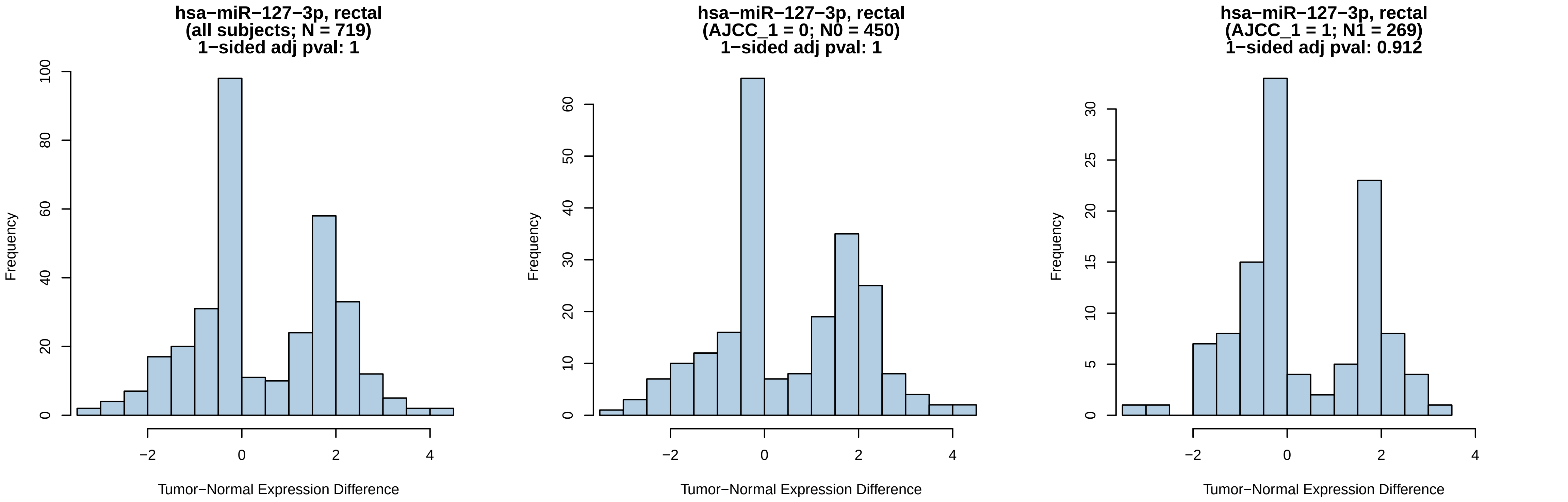
hsa−miR−127−3p, rectal
(all subjects; N = 719)
1−sided adj pval: 1
Frequency
Tumor−Normal Expression Difference
hsa−miR−127−3p, rectal
(AJCC_1 = 0; N0 = 450)
1−sided adj pval: 1
Frequency
Tumor−Normal Expression Difference
hsa−miR−127−3p, rectal
(AJCC_1 = 1; N1 = 269)
1−sided adj pval: 0.912
Frequency
Tumor−Normal Expression Difference

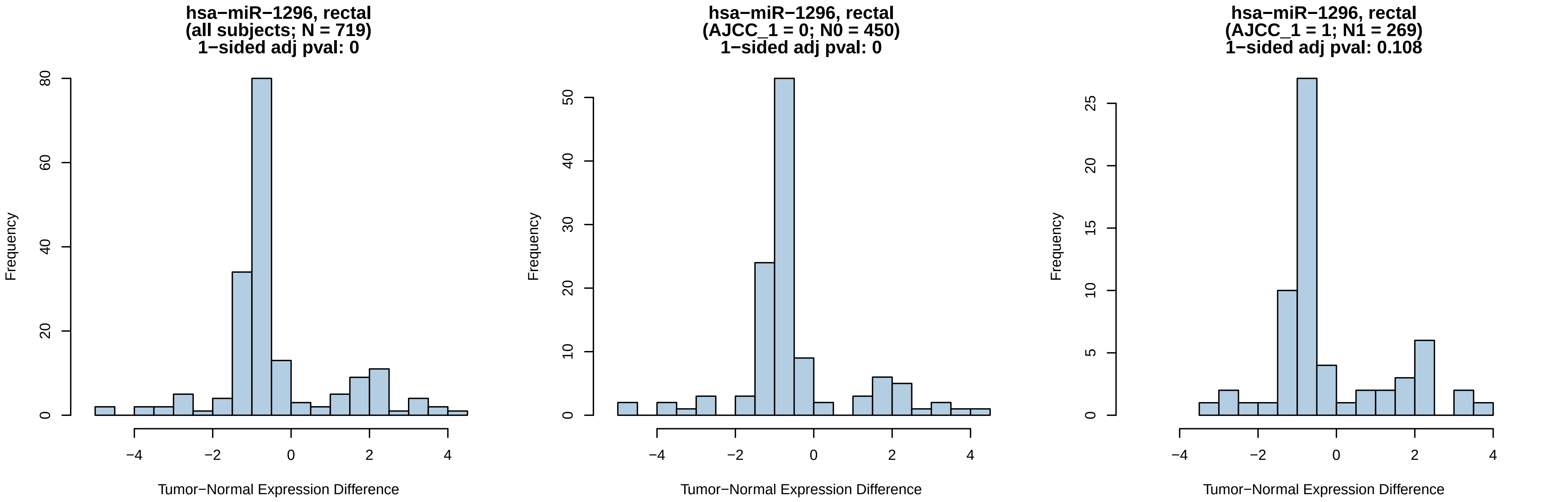
hsa−miR−1296, rectal
(all subjects; N = 719)
1−sided adj pval: 0
Frequency
Tumor−Normal Expression Difference
hsa−miR−1296, rectal
(AJCC_1 = 0; N0 = 450)
1−sided adj pval: 0
Frequency
Tumor−Normal Expression Difference
hsa−miR−1296, rectal
(AJCC_1 = 1; N1 = 269)
1−sided adj pval: 0.108
Frequency
Tumor−Normal Expression Difference

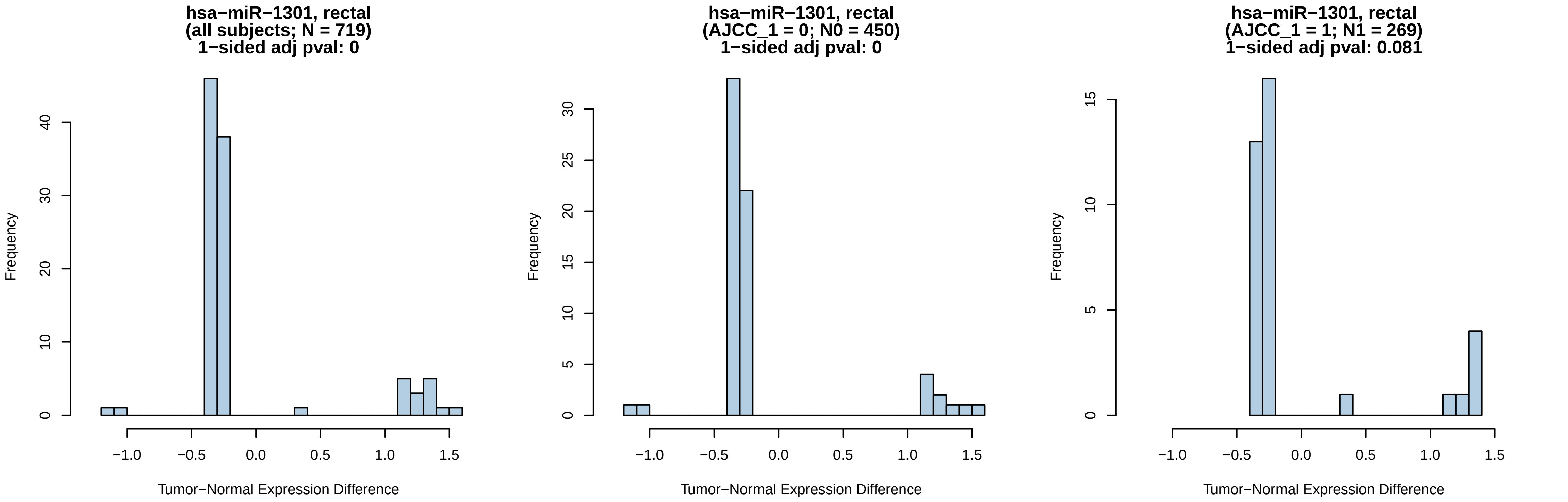

hsa−miR−1301, rectal
(all subjects; N = 719)
1−sided adj pval: 0
Frequency
Tumor−Normal Expression Difference
hsa−miR−1301, rectal
(AJCC_1 = 0; N0 = 450)
1−sided adj pval: 0
Frequency
Tumor−Normal Expression Difference
hsa−miR−1301, rectal
(AJCC_1 = 1; N1 = 269)
1−sided adj pval: 0.081
Frequency
Tumor−Normal Expression Difference

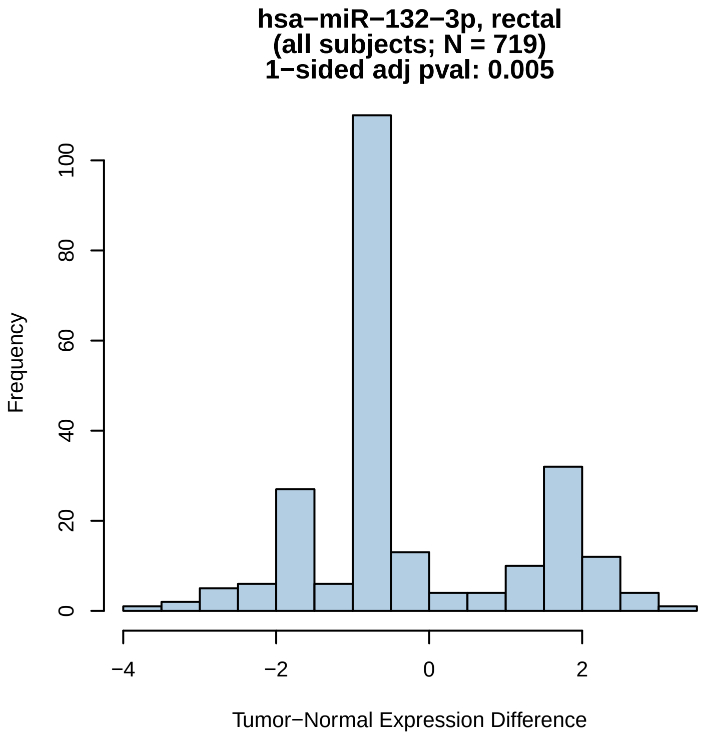
hsa−miR−132−3p, rectal
(all subjects; N = 719)
1−sided adj pval: 0.005
Frequency
0
20
40
60
80
100
−4
−2
0
2
Tumor−Normal Expression Difference

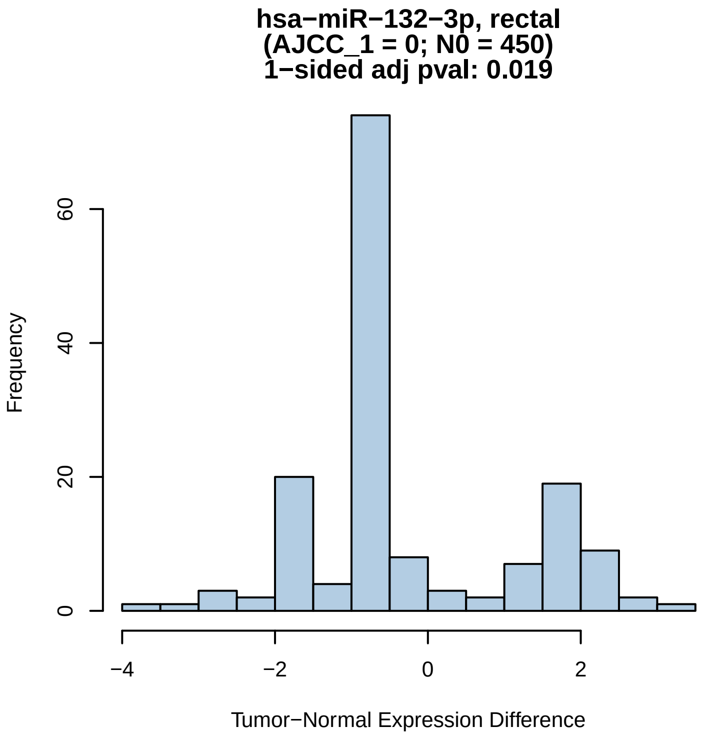
hsa−miR−132−3p, rectal
(AJCC_1 = 0; N0 = 450)
1−sided adj pval: 0.019
Frequency
0
20
40
60
−4
−2
0
2
Tumor−Normal Expression Difference

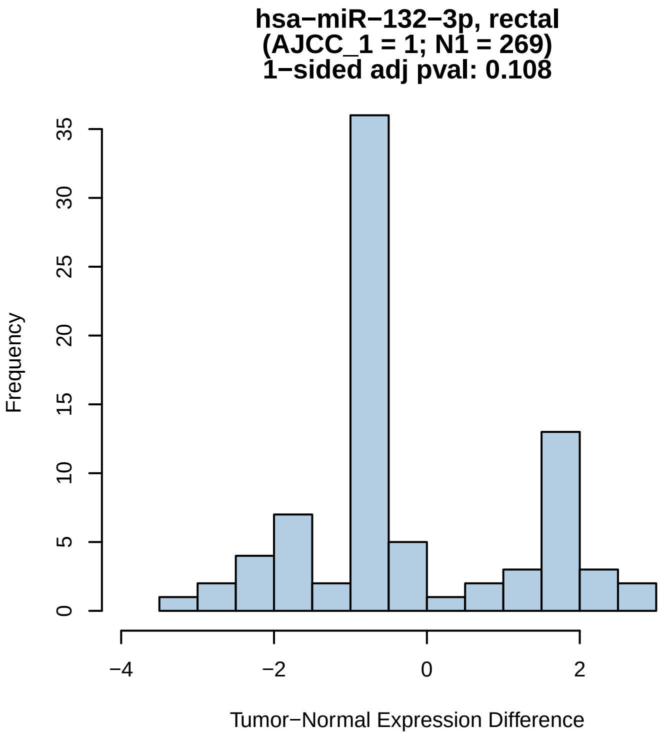
hsa−miR−132−3p, rectal
(AJCC_1 = 1; N1 = 269)
1−sided adj pval: 0.108
Frequency
0
5
10
15
20
25
30
35
−4
−2
0
2
Tumor−Normal Expression Difference

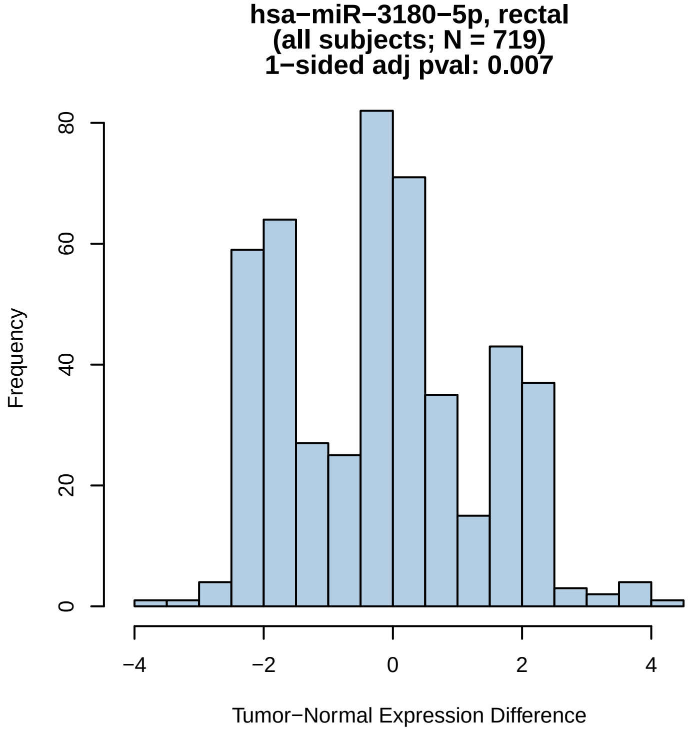
hsa−miR−3180−5p, rectal
(all subjects; N = 719)
1−sided adj pval: 0.007
Frequency
0
20
40
60
80
−4
−2
0
2
4
Tumor−Normal Expression Difference

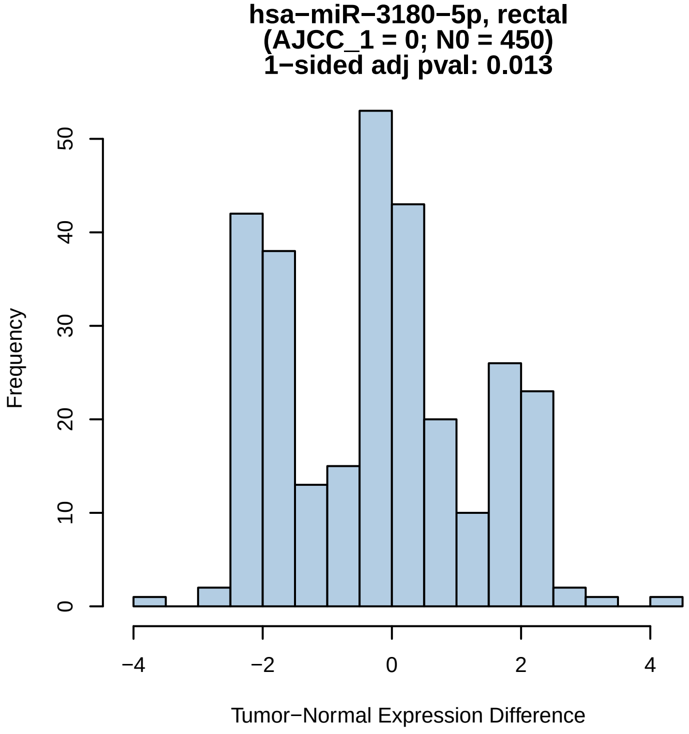
hsa−miR−3180−5p, rectal
(AJCC_1 = 0; N0 = 450)
1−sided adj pval: 0.013
Frequency
0
10
20
30
40
50
−4
−2
0
2
4
Tumor−Normal Expression Difference

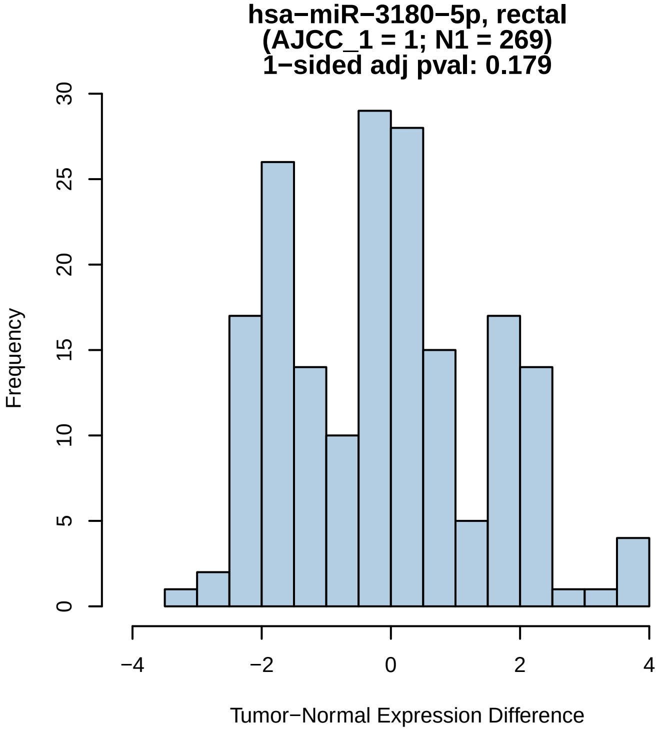
hsa−miR−3180−5p, rectal
(AJCC_1 = 1; N1 = 269)
1−sided adj pval: 0.179
Frequency
0
5
10
15
20
25
30
−4
−2
0
2
4
Tumor−Normal Expression Difference

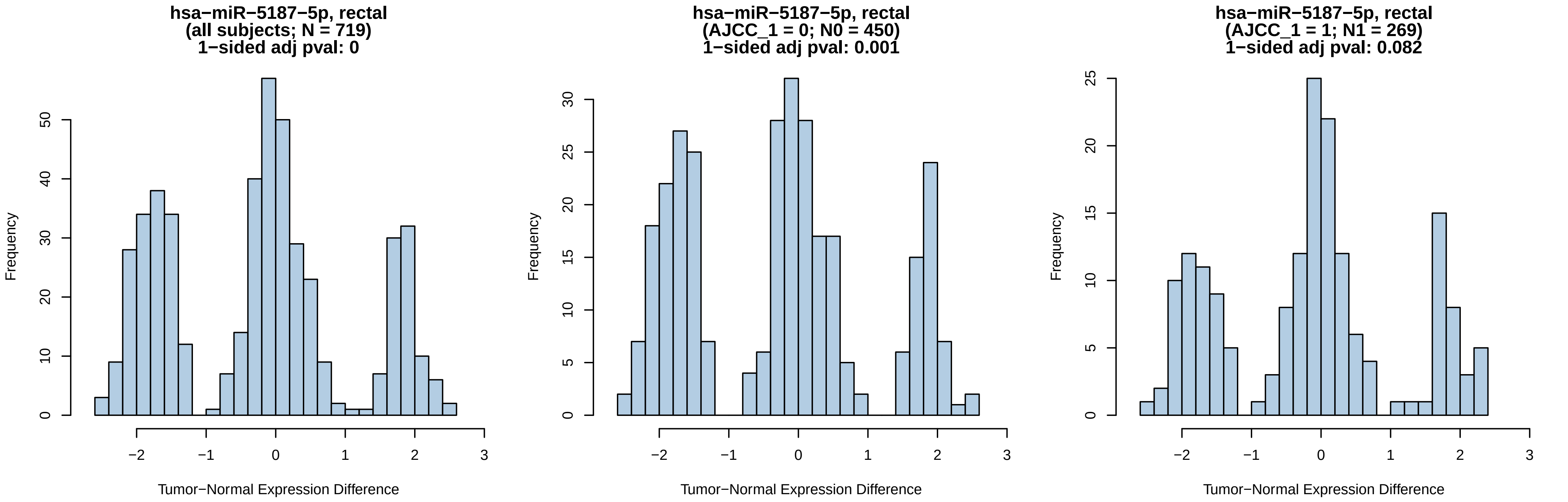
hsa−miR−5187−5p, rectal
(all subjects; N = 719)
1−sided adj pval: 0
Frequency
Tumor−Normal Expression Difference
hsa−miR−5187−5p, rectal
(AJCC_1 = 0; N0 = 450)
1−sided adj pval: 0.001
Frequency
Tumor−Normal Expression Difference
hsa−miR−5187−5p, rectal
(AJCC_1 = 1; N1 = 269)
1−sided adj pval: 0.082
Frequency
Tumor−Normal Expression Difference

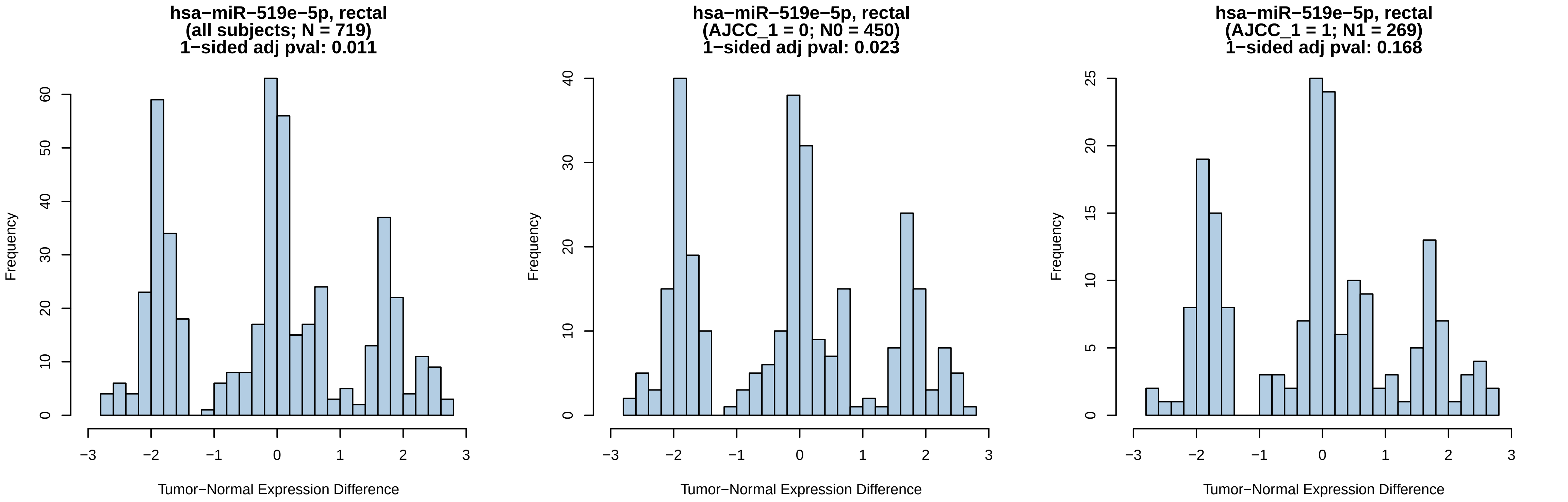
hsa−miR−519e−5p, rectal
(all subjects; N = 719)
1−sided adj pval: 0.011
hsa−miR−519e−5p, rectal
(AJCC_1 = 0; N0 = 450)
1−sided adj pval: 0.023
hsa−miR−519e−5p, rectal
(AJCC_1 = 1; N1 = 269)
1−sided adj pval: 0.168
Frequency
Tumor−Normal Expression Difference

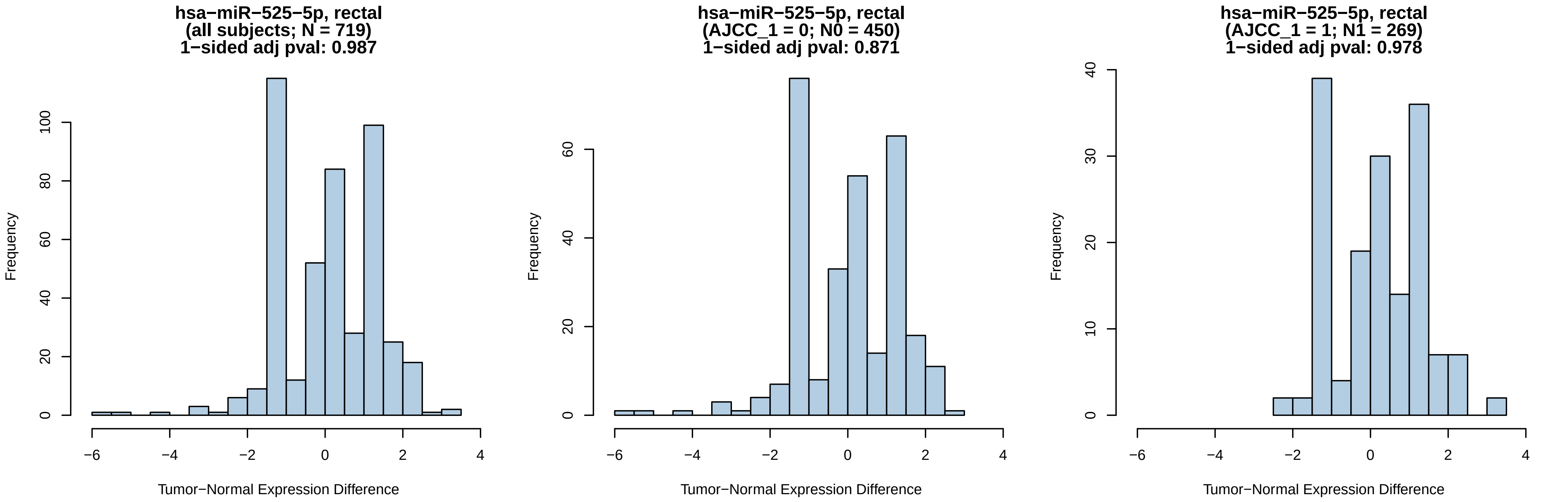
hsa−miR−525−5p, rectal
(all subjects; N = 719)
1−sided adj pval: 0.987
Frequency
Tumor−Normal Expression Difference
0
20
40
60
80
100
−6
−4
−2
0
2
4
hsa−miR−525−5p, rectal
(AJCC_1 = 0; N0 = 450)
1−sided adj pval: 0.871
Frequency
Tumor−Normal Expression Difference
0
20
40
60
−6
−4
−2
0
2
4
hsa−miR−525−5p, rectal
(AJCC_1 = 1; N1 = 269)
1−sided adj pval: 0.978
Frequency
Tumor−Normal Expression Difference
0
10
20
30
40
−6
−4
−2
0
2
4

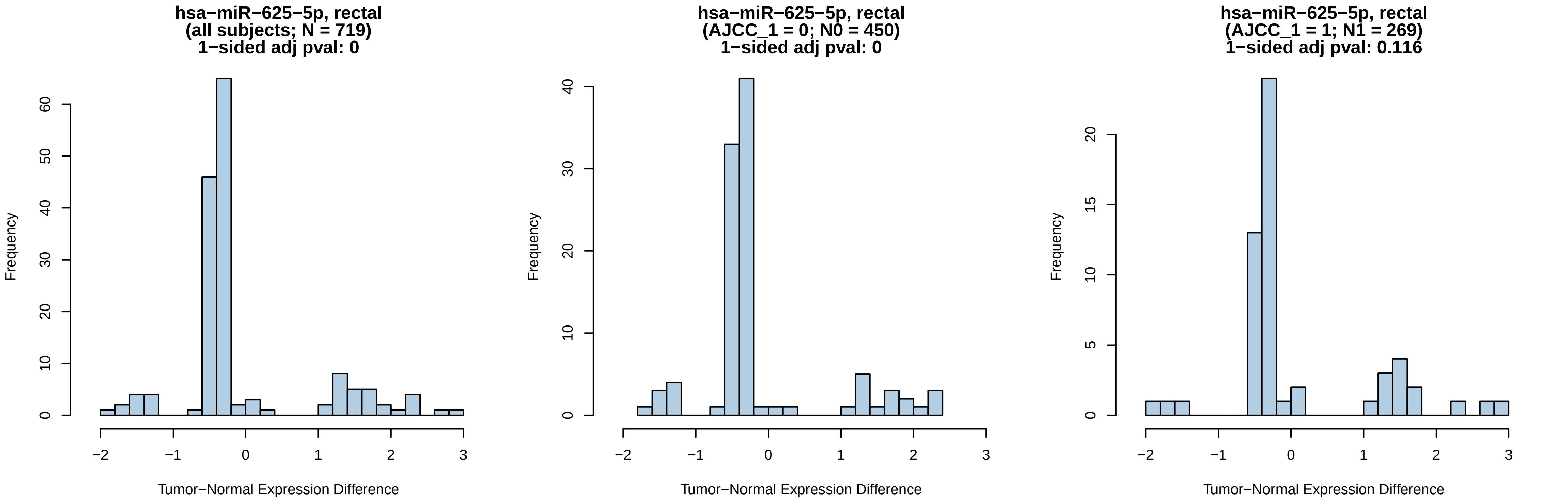
hsa−miR−625−5p, rectal
(all subjects; N = 719)
1−sided adj pval: 0
hsa−miR−625−5p, rectal
(AJCC_1 = 0; N0 = 450)
1−sided adj pval: 0
hsa−miR−625−5p, rectal
(AJCC_1 = 1; N1 = 269)
1−sided adj pval: 0.116
Frequency
Tumor−Normal Expression Difference
0
10
20
30
40
50
60
5
15
−2
−1
1
2
3

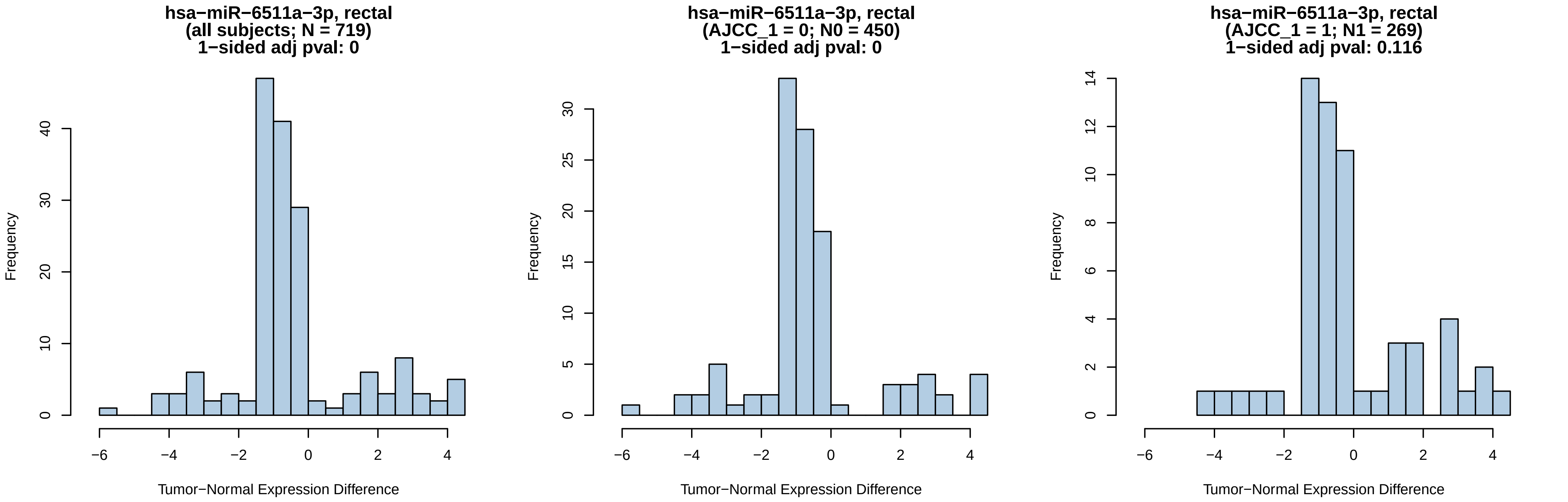
hsa−miR−6511a−3p, rectal
(all subjects; N = 719)
1−sided adj pval: 0
hsa−miR−6511a−3p, rectal
(AJCC_1 = 0; N0 = 450)
1−sided adj pval: 0
hsa−miR−6511a−3p, rectal
(AJCC_1 = 1; N1 = 269)
1−sided adj pval: 0.116
Frequency
Tumor−Normal Expression Difference

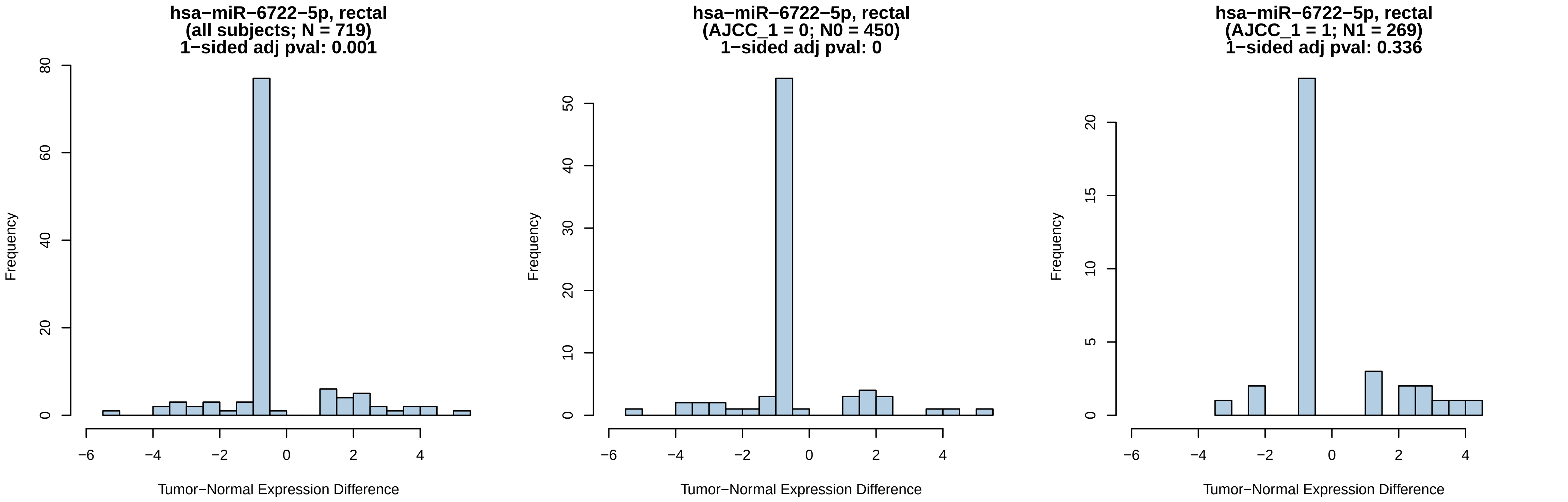

hsa−miR−6722−5p, rectal
(all subjects; N = 719)
1−sided adj pval: 0.001
Frequency
Tumor−Normal Expression Difference
hsa−miR−6722−5p, rectal
(AJCC_1 = 0; N0 = 450)
1−sided adj pval: 0
Frequency
Tumor−Normal Expression Difference
hsa−miR−6722−5p, rectal
(AJCC_1 = 1; N1 = 269)
1−sided adj pval: 0.336
Frequency
Tumor−Normal Expression Difference

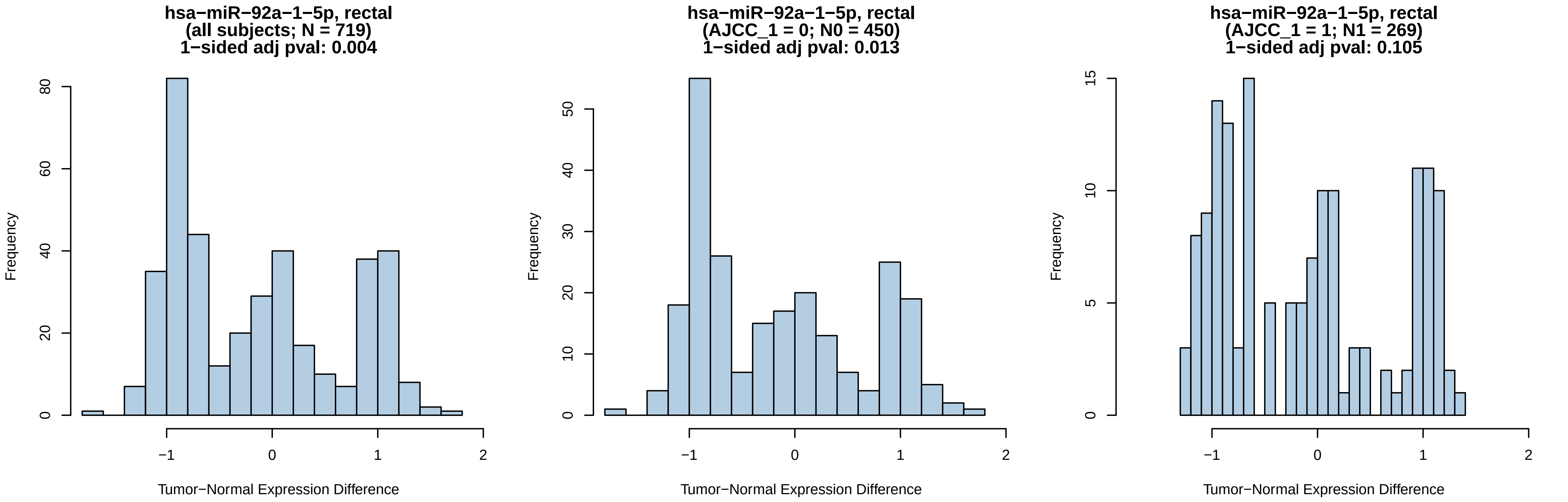
hsa−miR−92a−1−5p, rectal
(all subjects; N = 719)
1−sided adj pval: 0.004
Frequency
0
20
40
60
80
−1
0
1
2
Tumor−Normal Expression Difference
hsa−miR−92a−1−5p, rectal
(AJCC_1 = 0; N0 = 450)
1−sided adj pval: 0.013
Frequency
0
10
20
30
40
50
−1
0
1
2
Tumor−Normal Expression Difference
hsa−miR−92a−1−5p, rectal
(AJCC_1 = 1; N1 = 269)
1−sided adj pval: 0.105
Frequency
0
5
10
15
−1
0
1
2
Tumor−Normal Expression Difference

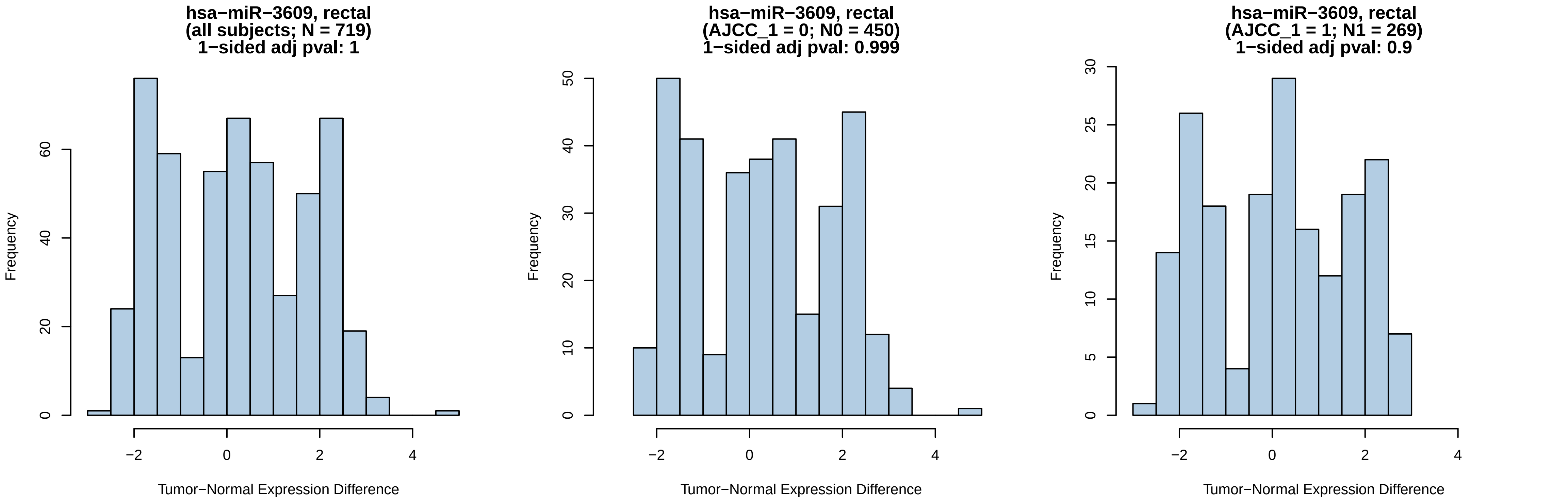
hsa−miR−3609, rectal
(all subjects; N = 719)
1−sided adj pval: 1
Frequency
0
20
40
60
−2
0
2
4
Tumor−Normal Expression Difference
hsa−miR−3609, rectal
(AJCC_1 = 0; N0 = 450)
1−sided adj pval: 0.999
Frequency
0
10
20
30
40
50
−2
0
2
4
Tumor−Normal Expression Difference
hsa−miR−3609, rectal
(AJCC_1 = 1; N1 = 269)
1−sided adj pval: 0.9
Frequency
0
5
10
15
20
25
30
−2
0
2
4
Tumor−Normal Expression Difference

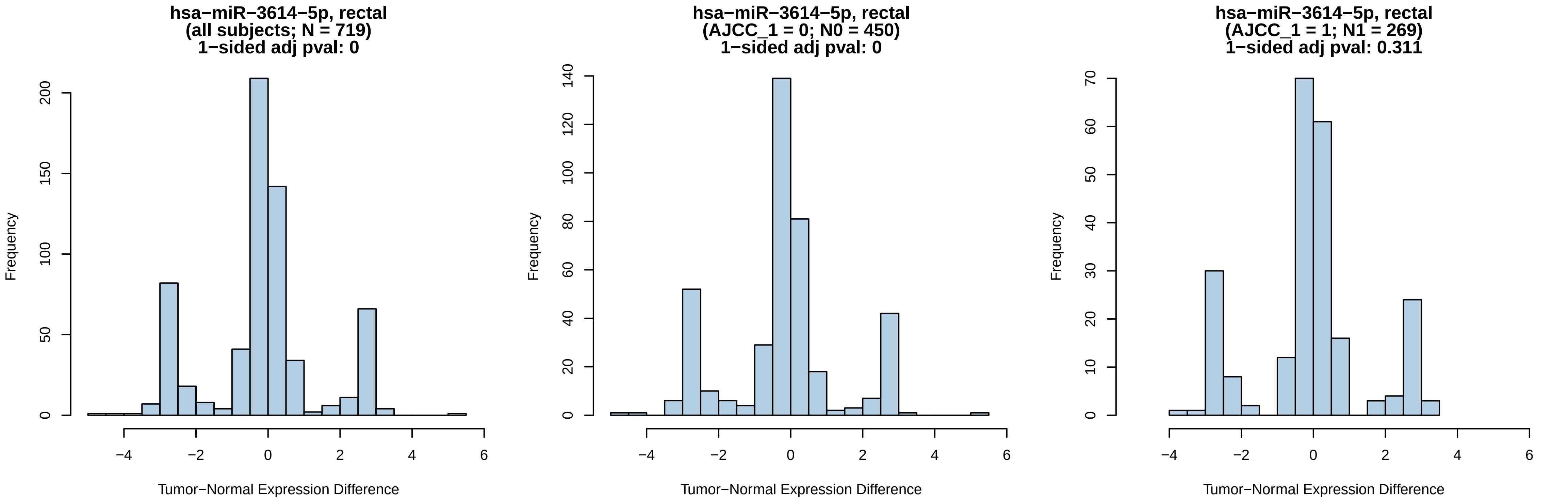
hsa−miR−3614−5p, rectal
(all subjects; N = 719)
1−sided adj pval: 0
Frequency
Tumor−Normal Expression Difference
hsa−miR−3614−5p, rectal
(AJCC_1 = 0; N0 = 450)
1−sided adj pval: 0
Frequency
Tumor−Normal Expression Difference
hsa−miR−3614−5p, rectal
(AJCC_1 = 1; N1 = 269)
1−sided adj pval: 0.311
Frequency
Tumor−Normal Expression Difference

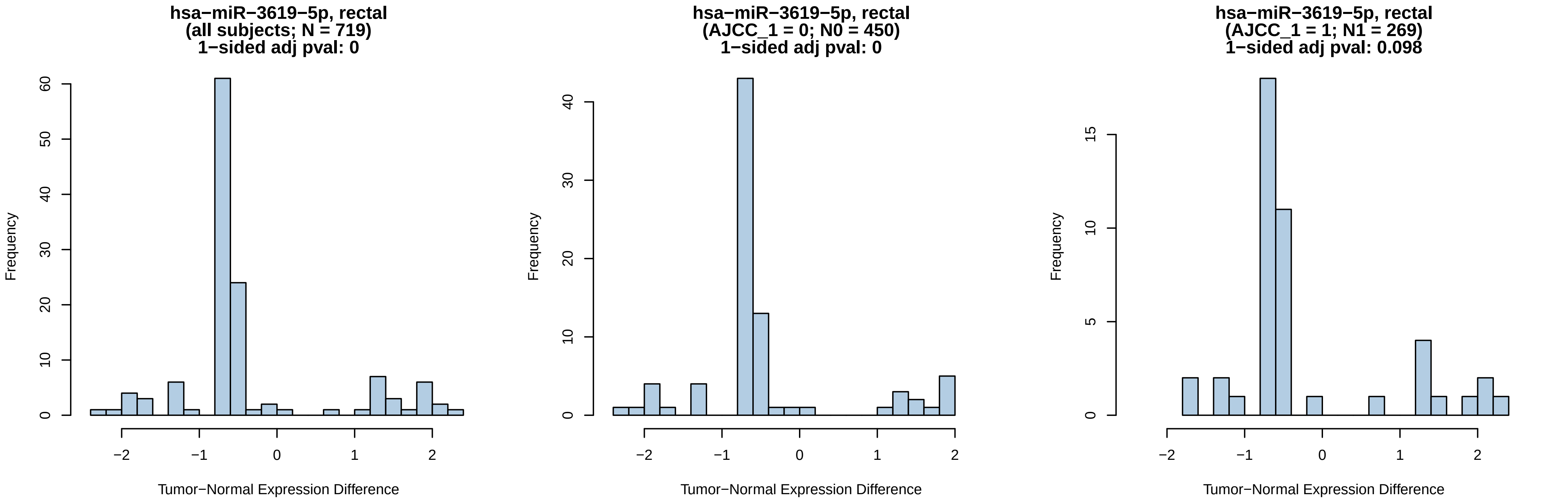

hsa−miR−3619−5p, rectal
(all subjects; N = 719)
1−sided adj pval: 0
Frequency
Tumor−Normal Expression Difference
hsa−miR−3619−5p, rectal
(AJCC_1 = 0; N0 = 450)
1−sided adj pval: 0
Frequency
Tumor−Normal Expression Difference
hsa−miR−3619−5p, rectal
(AJCC_1 = 1; N1 = 269)
1−sided adj pval: 0.098
Frequency
Tumor−Normal Expression Difference

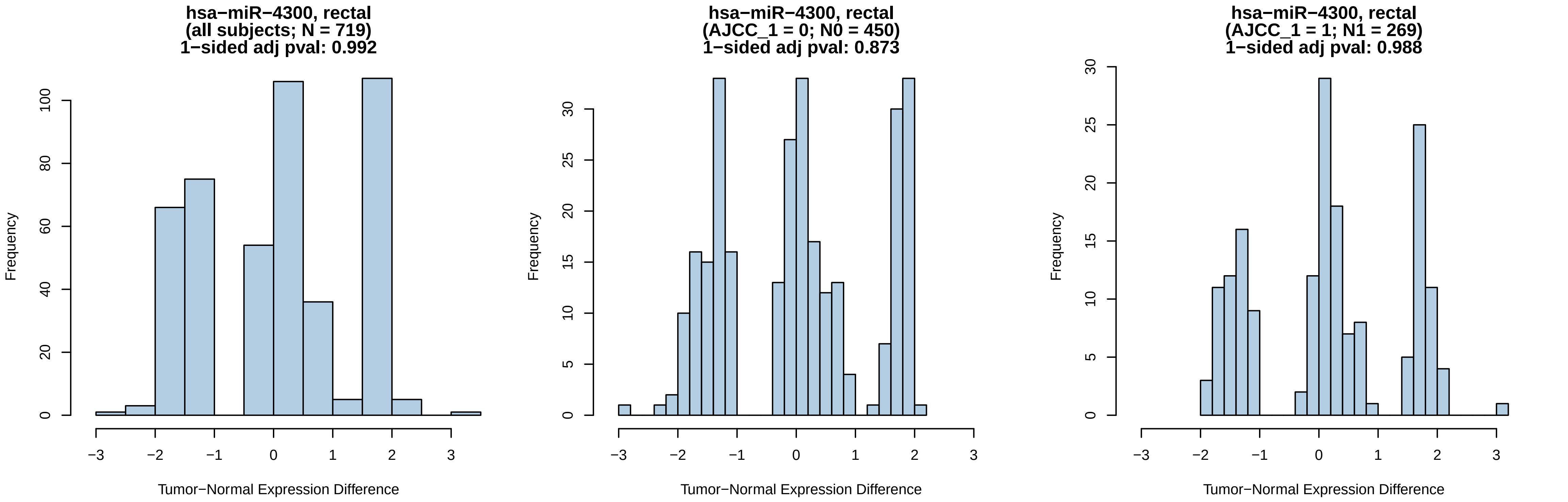

hsa−miR−4300, rectal
(all subjects; N = 719)
1−sided adj pval: 0.992
Frequency
0
20
40
60
80
100
−3
−2
−1
0
1
2
3
Tumor−Normal Expression Difference
hsa−miR−4300, rectal
(AJCC_1 = 0; N0 = 450)
1−sided adj pval: 0.873
Frequency
0
5
10
15
20
25
30
−3
−2
−1
0
1
2
3
Tumor−Normal Expression Difference
hsa−miR−4300, rectal
(AJCC_1 = 1; N1 = 269)
1−sided adj pval: 0.988
Frequency
0
5
10
15
20
25
30
−3
−2
−1
0
1
2
3
Tumor−Normal Expression Difference

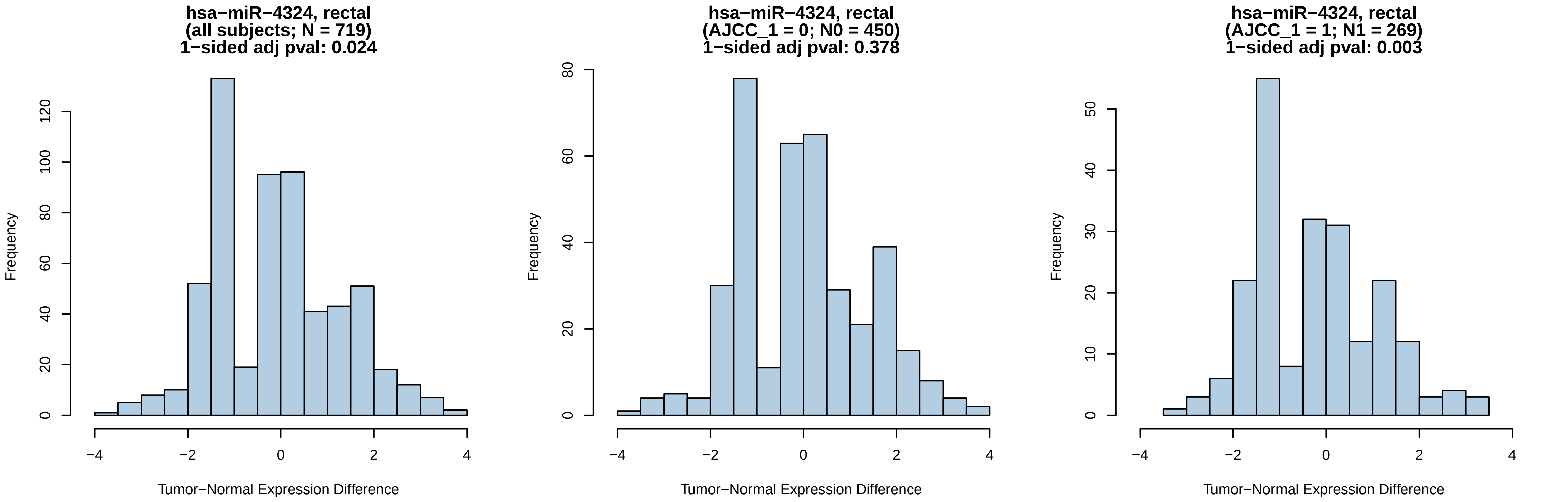
hsa−miR−4324, rectal
(all subjects; N = 719)
1−sided adj pval: 0.024
Frequency
Tumor−Normal Expression Difference
hsa−miR−4324, rectal
(AJCC_1 = 0; N0 = 450)
1−sided adj pval: 0.378
Frequency
Tumor−Normal Expression Difference
hsa−miR−4324, rectal
(AJCC_1 = 1; N1 = 269)
1−sided adj pval: 0.003
Frequency
Tumor−Normal Expression Difference

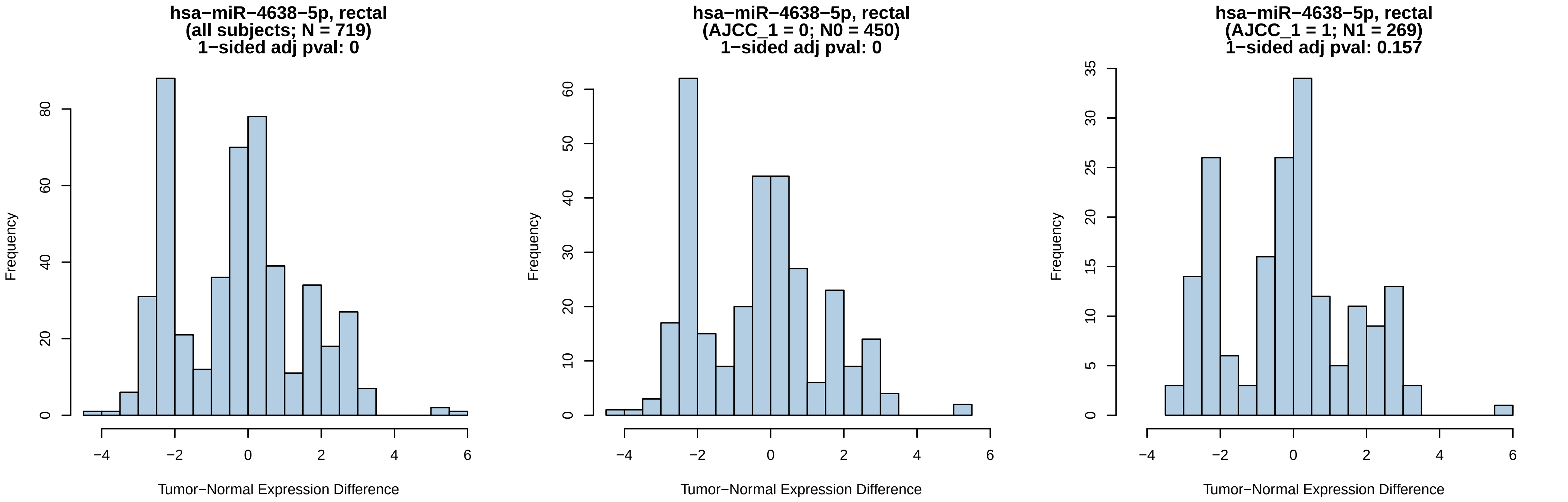
hsa−miR−4638−5p, rectal
(all subjects; N = 719)
1−sided adj pval: 0
Frequency
Tumor−Normal Expression Difference
hsa−miR−4638−5p, rectal
(AJCC_1 = 0; N0 = 450)
1−sided adj pval: 0
Frequency
Tumor−Normal Expression Difference
hsa−miR−4638−5p, rectal
(AJCC_1 = 1; N1 = 269)
1−sided adj pval: 0.157
Frequency
Tumor−Normal Expression Difference

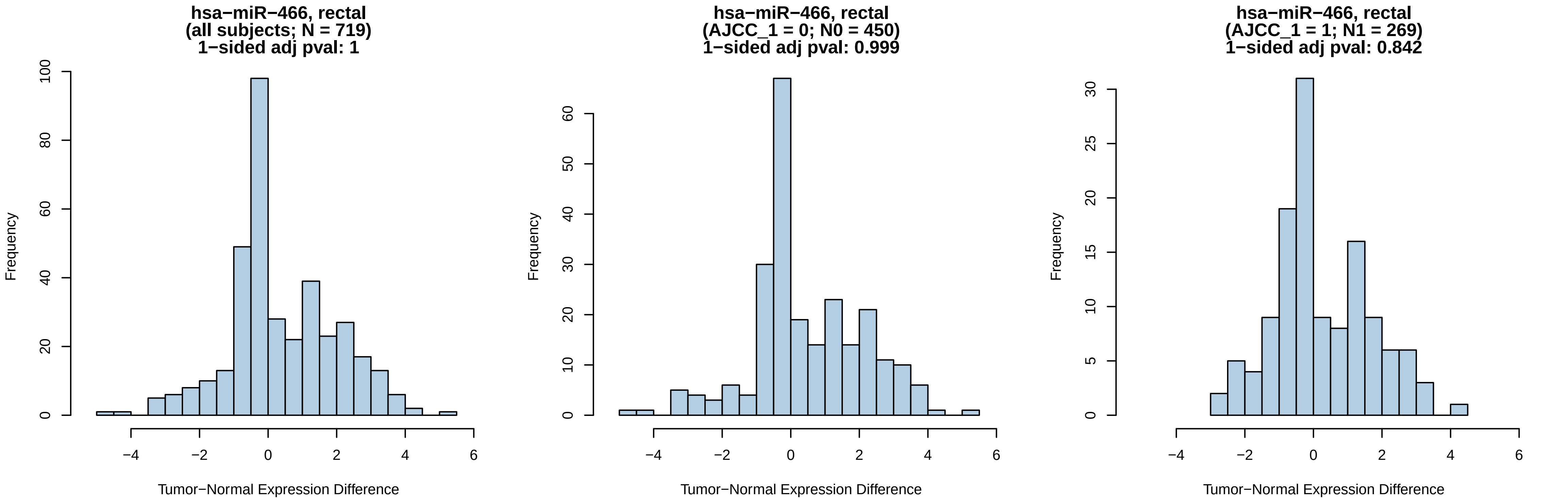
hsa−miR−466, rectal
(all subjects; N = 719)
1−sided adj pval: 1
Frequency
0
20
40
60
80
100
−4
−2
0
2
4
6
Tumor−Normal Expression Difference
hsa−miR−466, rectal
(AJCC_1 = 0; N0 = 450)
1−sided adj pval: 0.999
Frequency
0
10
20
30
40
50
60
−4
−2
0
2
4
6
Tumor−Normal Expression Difference
hsa−miR−466, rectal
(AJCC_1 = 1; N1 = 269)
1−sided adj pval: 0.842
Frequency
0
5
10
15
20
25
30
−4
−2
0
2
4
6
Tumor−Normal Expression Difference

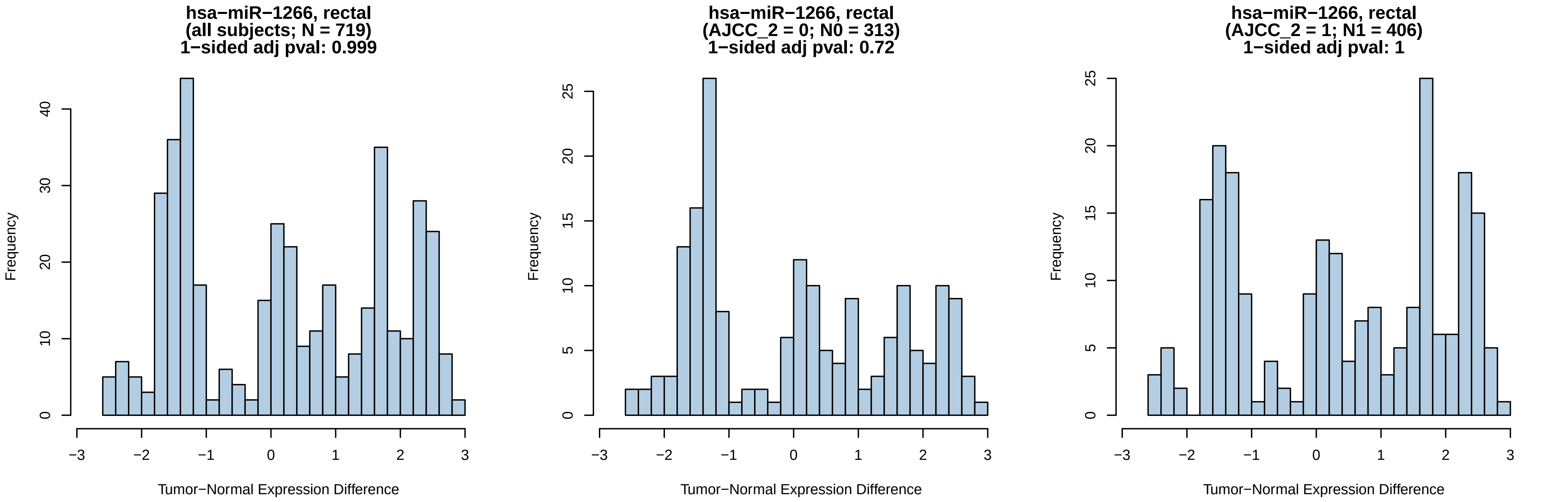
hsa−miR−1266, rectal
(all subjects; N = 719)
1−sided adj pval: 0.999
hsa−miR−1266, rectal
(AJCC_2 = 0; N0 = 313)
1−sided adj pval: 0.72
hsa−miR−1266, rectal
(AJCC_2 = 1; N1 = 406)
1−sided adj pval: 1
Frequency
Tumor−Normal Expression Difference

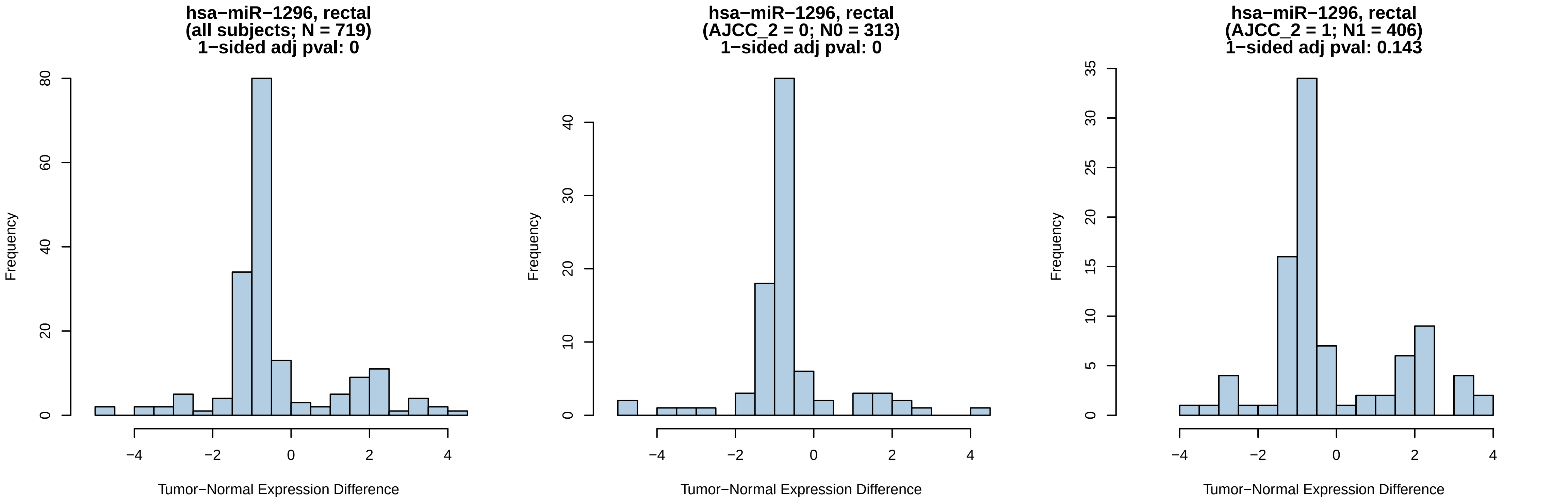

hsa−miR−1296, rectal
(all subjects; N = 719)
1−sided adj pval: 0
Frequency
Tumor−Normal Expression Difference
hsa−miR−1296, rectal
(AJCC_2 = 0; N0 = 313)
1−sided adj pval: 0
Frequency
Tumor−Normal Expression Difference
hsa−miR−1296, rectal
(AJCC_2 = 1; N1 = 406)
1−sided adj pval: 0.143
Frequency
Tumor−Normal Expression Difference

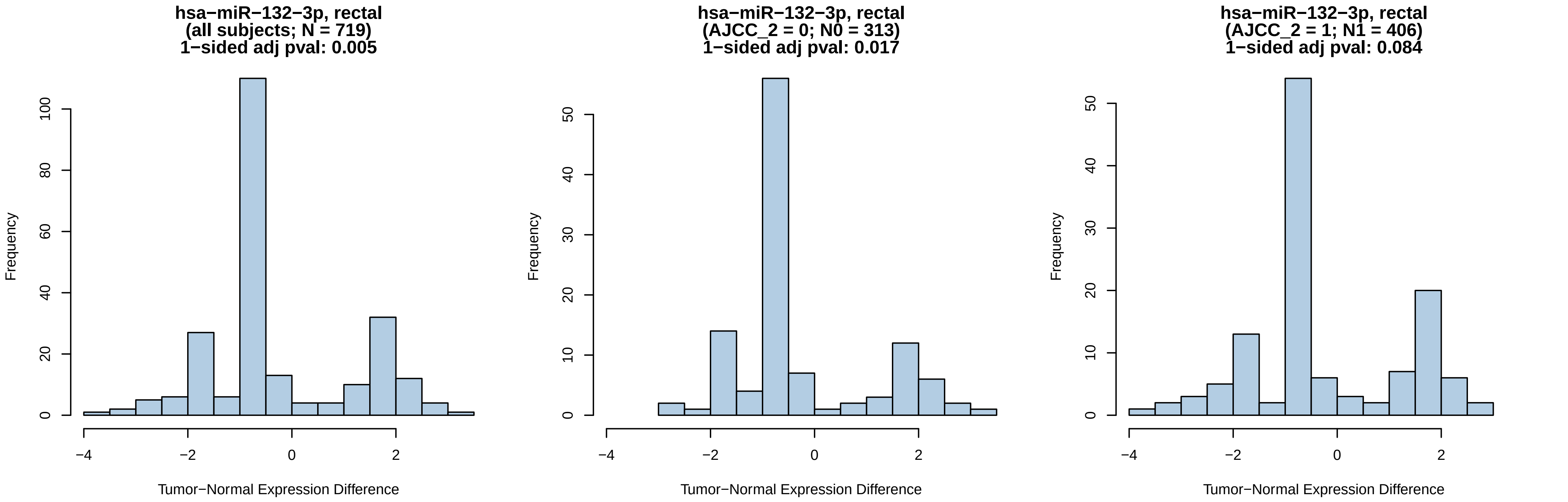

hsa−miR−132−3p, rectal
(all subjects; N = 719)
1−sided adj pval: 0.005
Frequency
Tumor−Normal Expression Difference
hsa−miR−132−3p, rectal
(AJCC_2 = 0; N0 = 313)
1−sided adj pval: 0.017
Frequency
Tumor−Normal Expression Difference
hsa−miR−132−3p, rectal
(AJCC_2 = 1; N1 = 406)
1−sided adj pval: 0.084
Frequency
Tumor−Normal Expression Difference

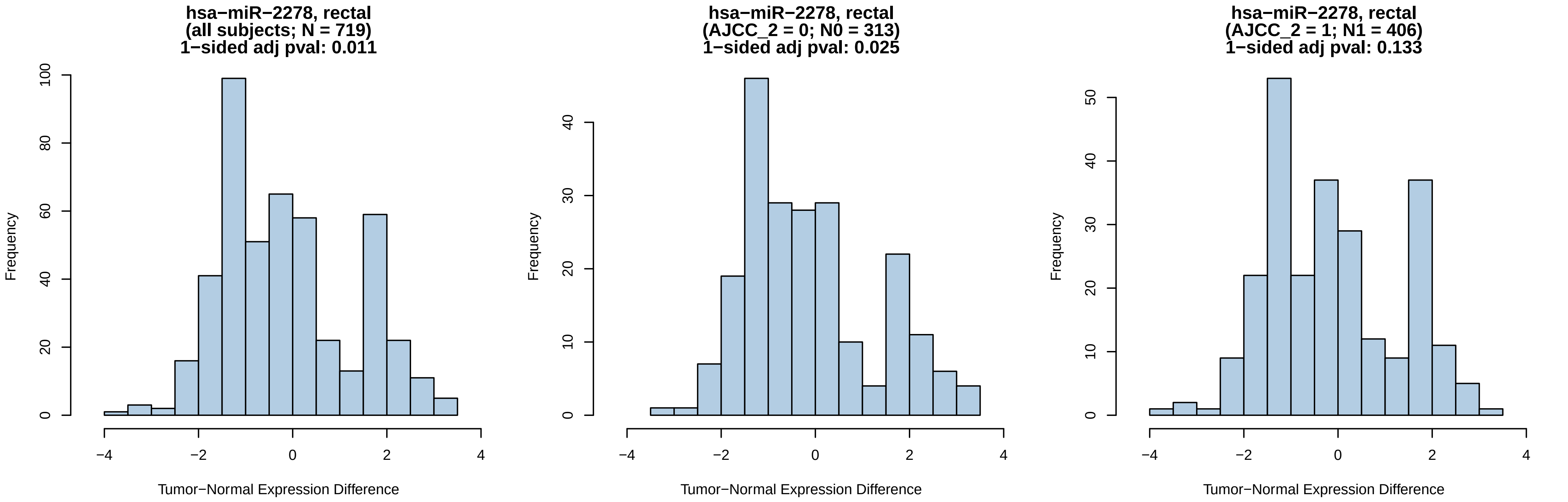

hsa−miR−2278, rectal
(all subjects; N = 719)
1−sided adj pval: 0.011
Frequency
Tumor−Normal Expression Difference
hsa−miR−2278, rectal
(AJCC_2 = 0; N0 = 313)
1−sided adj pval: 0.025
Frequency
Tumor−Normal Expression Difference
hsa−miR−2278, rectal
(AJCC_2 = 1; N1 = 406)
1−sided adj pval: 0.133
Frequency
Tumor−Normal Expression Difference

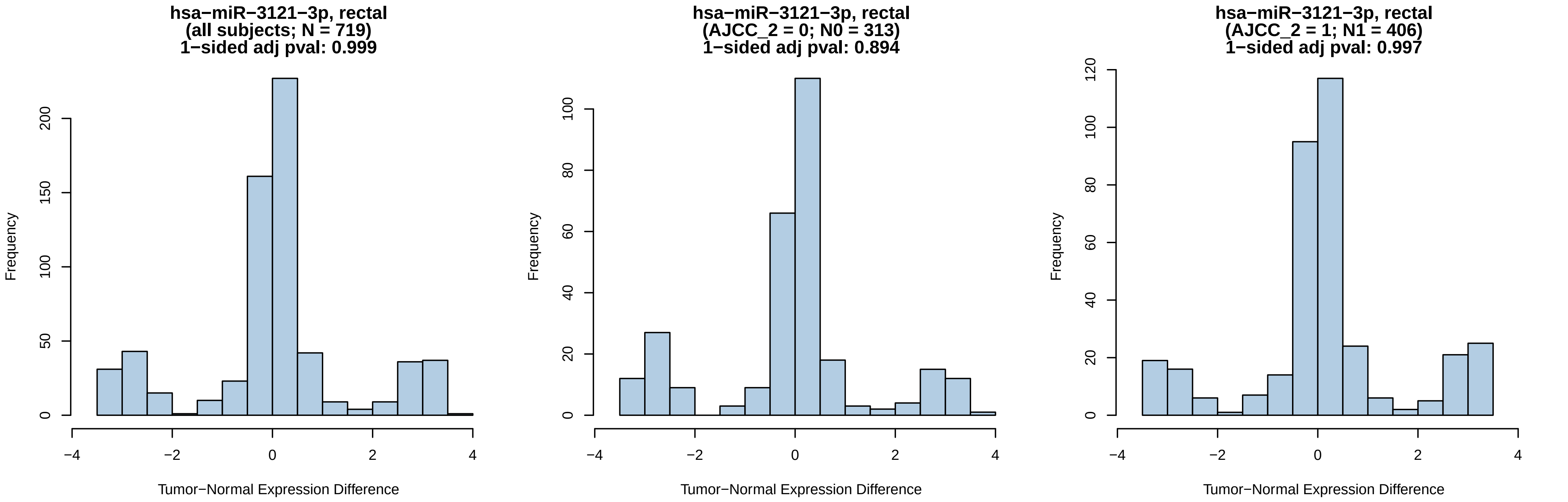
hsa−miR−3121−3p, rectal
(all subjects; N = 719)
1−sided adj pval: 0.999
Frequency
0
50
100
150
200
−4
−2
0
2
4
Tumor−Normal Expression Difference
hsa−miR−3121−3p, rectal
(AJCC_2 = 0; N0 = 313)
1−sided adj pval: 0.894
Frequency
0
20
40
60
80
100
−4
−2
0
2
4
Tumor−Normal Expression Difference
hsa−miR−3121−3p, rectal
(AJCC_2 = 1; N1 = 406)
1−sided adj pval: 0.997
Frequency
0
20
40
60
80
100
120
−4
−2
0
2
4
Tumor−Normal Expression Difference

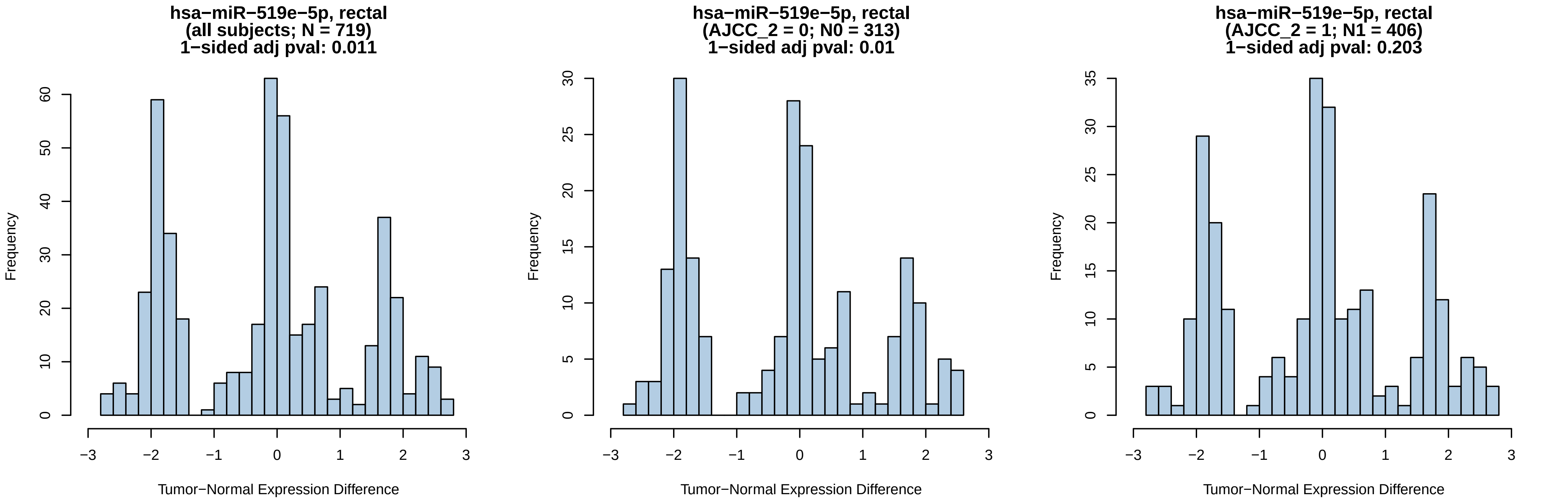
hsa−miR−519e−5p, rectal
(all subjects; N = 719)
1−sided adj pval: 0.011
Frequency
Tumor−Normal Expression Difference
hsa−miR−519e−5p, rectal
(AJCC_2 = 0; N0 = 313)
1−sided adj pval: 0.01
Frequency
Tumor−Normal Expression Difference
hsa−miR−519e−5p, rectal
(AJCC_2 = 1; N1 = 406)
1−sided adj pval: 0.203
Frequency
Tumor−Normal Expression Difference

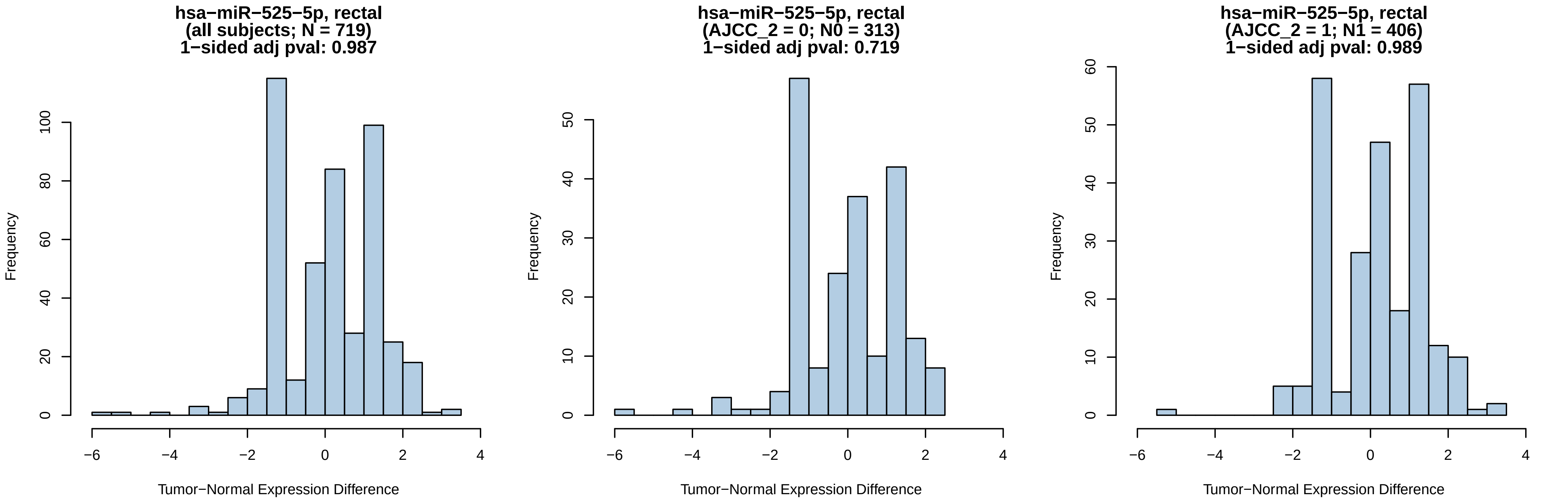

hsa−miR−525−5p, rectal
(all subjects; N = 719)
1−sided adj pval: 0.987
Frequency
Tumor−Normal Expression Difference
hsa−miR−525−5p, rectal
(AJCC_2 = 0; N0 = 313)
1−sided adj pval: 0.719
Frequency
Tumor−Normal Expression Difference
hsa−miR−525−5p, rectal
(AJCC_2 = 1; N1 = 406)
1−sided adj pval: 0.989
Frequency
Tumor−Normal Expression Difference

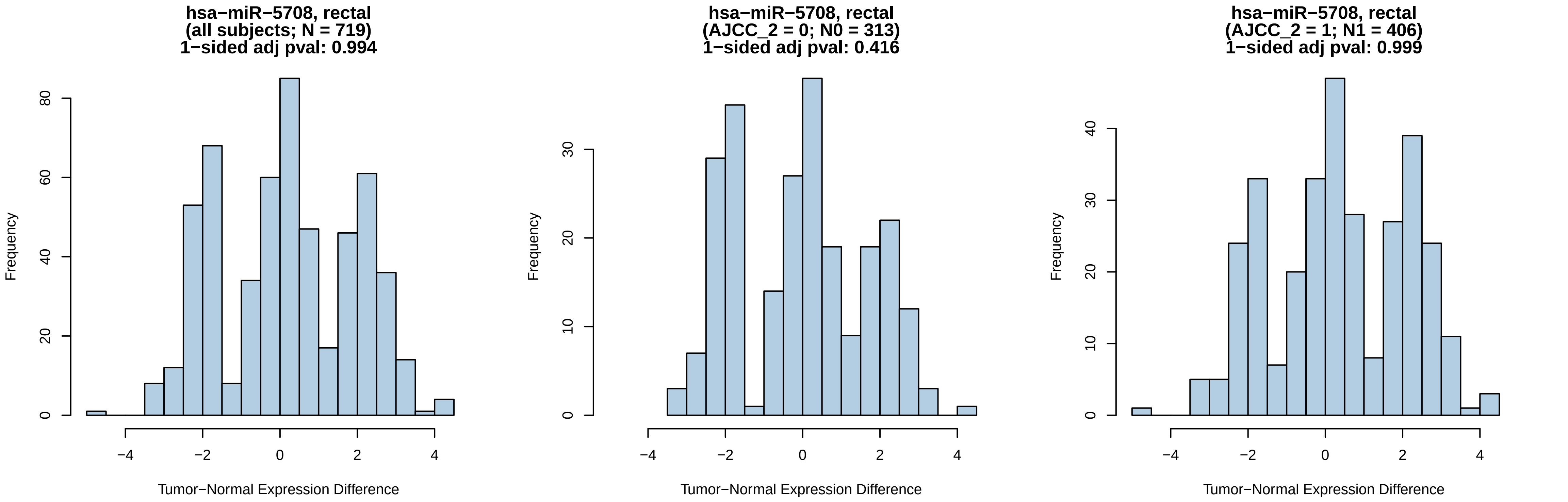
hsa−miR−5708, rectal
(all subjects; N = 719)
1−sided adj pval: 0.994
Frequency
Tumor−Normal Expression Difference
hsa−miR−5708, rectal
(AJCC_2 = 0; N0 = 313)
1−sided adj pval: 0.416
Frequency
Tumor−Normal Expression Difference
hsa−miR−5708, rectal
(AJCC_2 = 1; N1 = 406)
1−sided adj pval: 0.999
Frequency
Tumor−Normal Expression Difference

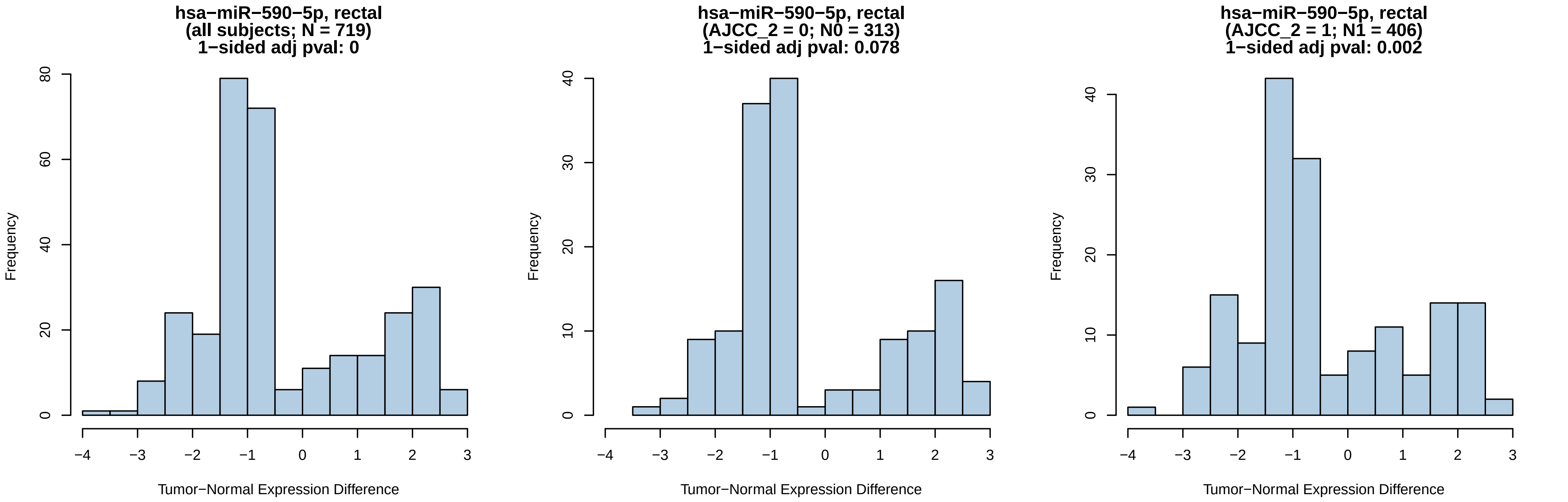
hsa−miR−590−5p, rectal
(all subjects; N = 719)
1−sided adj pval: 0
Frequency
Tumor−Normal Expression Difference
hsa−miR−590−5p, rectal
(AJCC_2 = 0; N0 = 313)
1−sided adj pval: 0.078
Frequency
Tumor−Normal Expression Difference
hsa−miR−590−5p, rectal
(AJCC_2 = 1; N1 = 406)
1−sided adj pval: 0.002
Frequency
Tumor−Normal Expression Difference

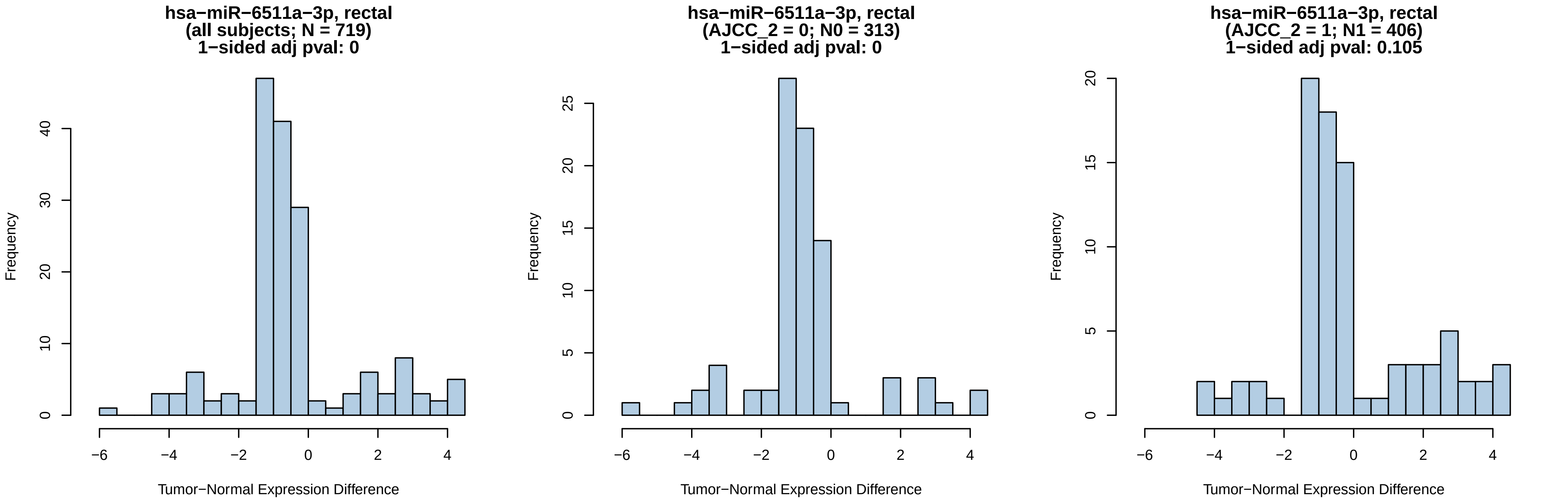
hsa−miR−6511a−3p, rectal
(all subjects; N = 719)
1−sided adj pval: 0
Frequency
Tumor−Normal Expression Difference
hsa−miR−6511a−3p, rectal
(AJCC_2 = 0; N0 = 313)
1−sided adj pval: 0
Frequency
Tumor−Normal Expression Difference
hsa−miR−6511a−3p, rectal
(AJCC_2 = 1; N1 = 406)
1−sided adj pval: 0.105
Frequency
Tumor−Normal Expression Difference

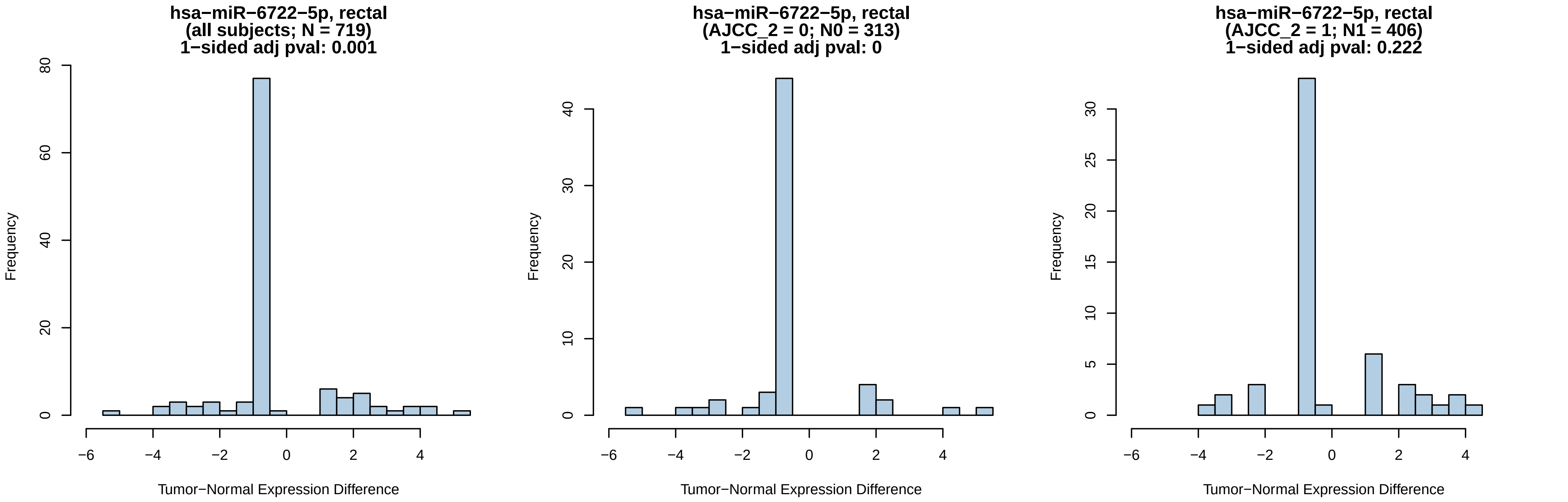
hsa−miR−6722−5p, rectal
(all subjects; N = 719)
1−sided adj pval: 0.001
hsa−miR−6722−5p, rectal
(AJCC_2 = 0; N0 = 313)
1−sided adj pval: 0
hsa−miR−6722−5p, rectal
(AJCC_2 = 1; N1 = 406)
1−sided adj pval: 0.222
Frequency
Tumor−Normal Expression Difference
0
20
40
60
80
10
30
5
15
25
−6
−4
−2
2
4

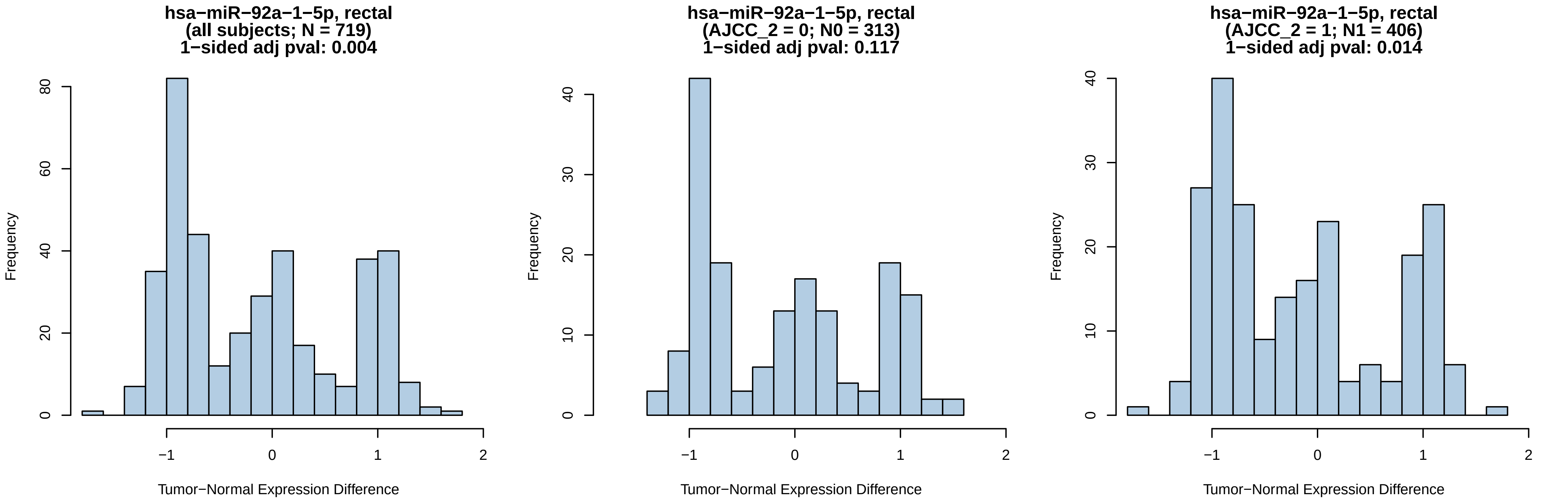

hsa−miR−92a−1−5p, rectal
(all subjects; N = 719)
1−sided adj pval: 0.004
Frequency
0
20
40
60
80
−1
0
1
2
Tumor−Normal Expression Difference
hsa−miR−92a−1−5p, rectal
(AJCC_2 = 0; N0 = 313)
1−sided adj pval: 0.117
Frequency
0
10
20
30
40
−1
0
1
2
Tumor−Normal Expression Difference
hsa−miR−92a−1−5p, rectal
(AJCC_2 = 1; N1 = 406)
1−sided adj pval: 0.014
Frequency
0
10
20
30
40
−1
0
1
2
Tumor−Normal Expression Difference

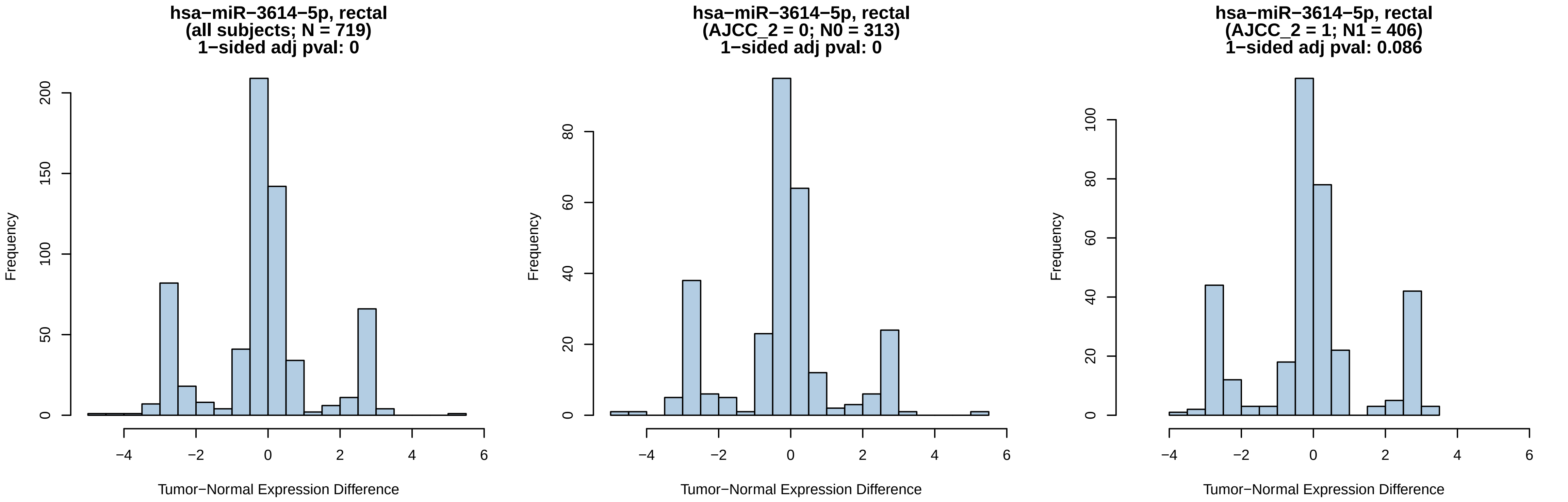
hsa−miR−3614−5p, rectal
(all subjects; N = 719)
1−sided adj pval: 0
Frequency
Tumor−Normal Expression Difference
hsa−miR−3614−5p, rectal
(AJCC_2 = 0; N0 = 313)
1−sided adj pval: 0
Frequency
Tumor−Normal Expression Difference
hsa−miR−3614−5p, rectal
(AJCC_2 = 1; N1 = 406)
1−sided adj pval: 0.086
Frequency
Tumor−Normal Expression Difference

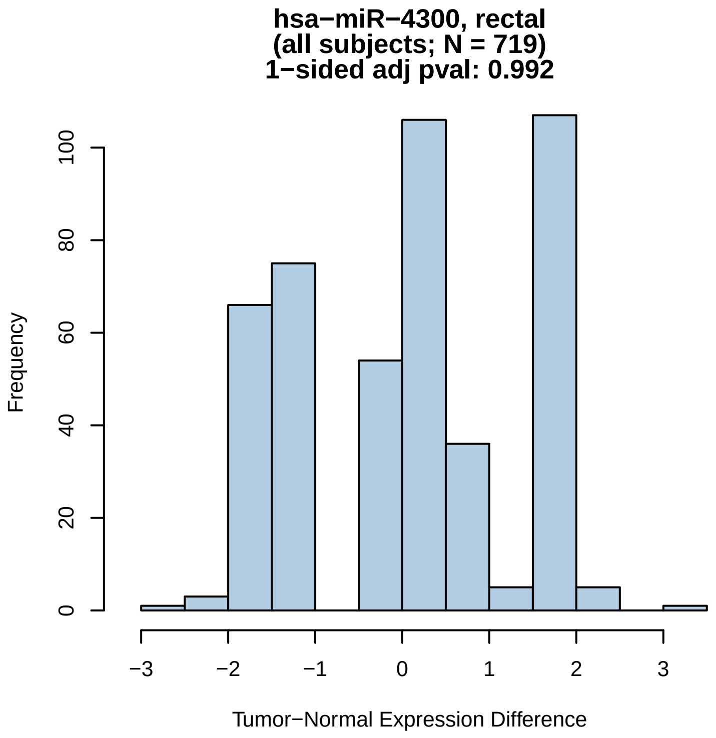

hsa−miR−4300, rectal
(all subjects; N = 719)
1−sided adj pval: 0.992
Frequency
Tumor−Normal Expression Difference


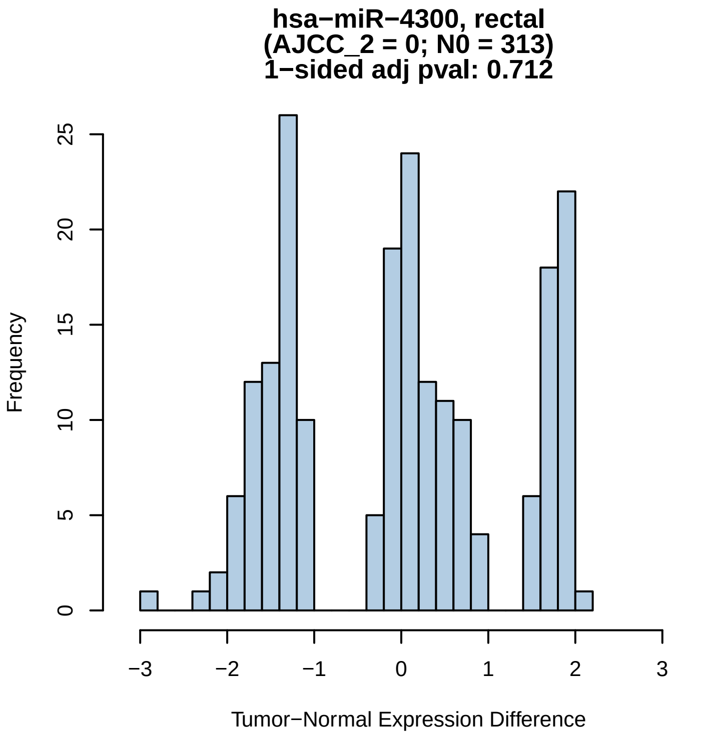

hsa−miR−4300, rectal
(AJCC_2 = 0; N0 = 313)
1−sided adj pval: 0.712
Frequency
Tumor−Normal Expression Difference


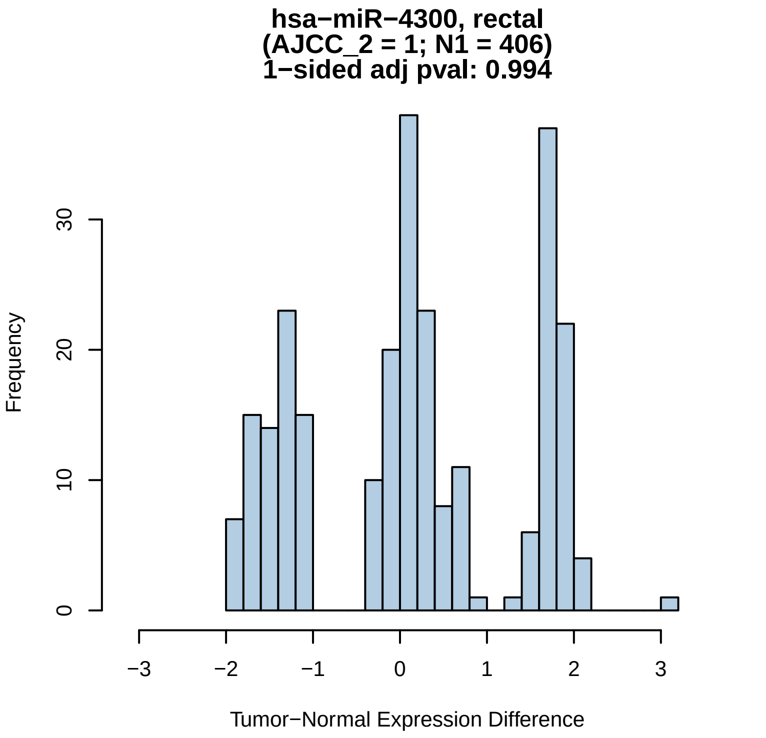

hsa−miR−4300, rectal
(AJCC_2 = 1; N1 = 406)
1−sided adj pval: 0.994
Frequency
Tumor−Normal Expression Difference

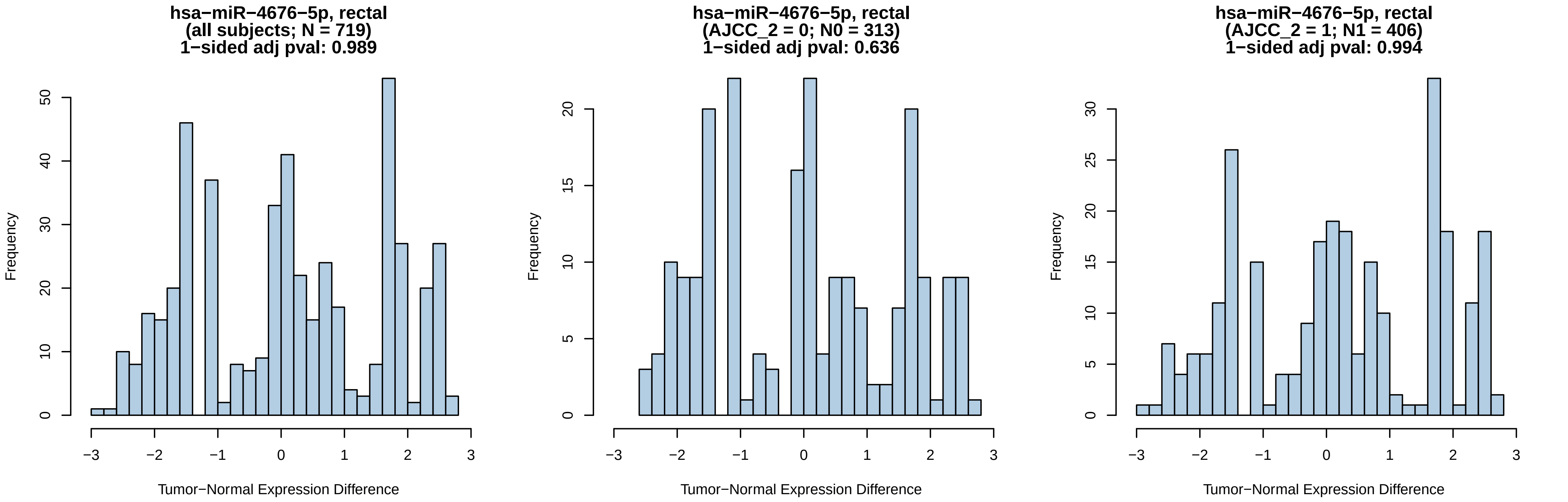

hsa−miR−4676−5p, rectal
(all subjects; N = 719)
1−sided adj pval: 0.989
hsa−miR−4676−5p, rectal
(AJCC_2 = 0; N0 = 313)
1−sided adj pval: 0.636
hsa−miR−4676−5p, rectal
(AJCC_2 = 1; N1 = 406)
1−sided adj pval: 0.994
Frequency
Tumor−Normal Expression Difference

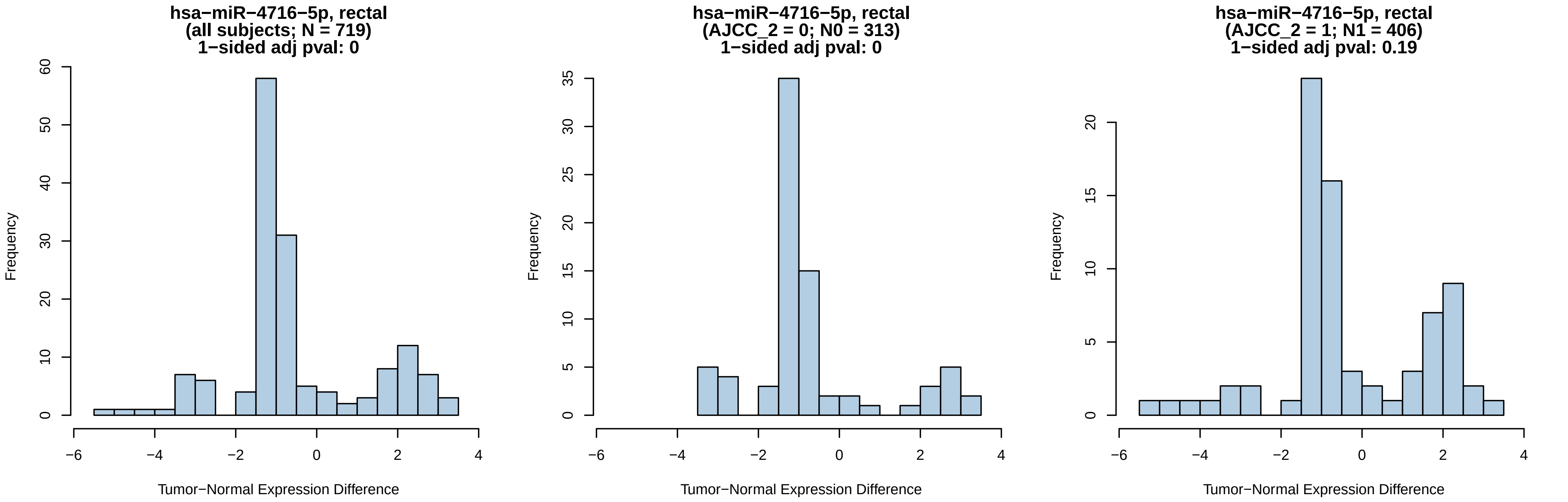

hsa−miR−4716−5p, rectal
(all subjects; N = 719)
1−sided adj pval: 0
Frequency
Tumor−Normal Expression Difference
hsa−miR−4716−5p, rectal
(AJCC_2 = 0; N0 = 313)
1−sided adj pval: 0
Frequency
Tumor−Normal Expression Difference
hsa−miR−4716−5p, rectal
(AJCC_2 = 1; N1 = 406)
1−sided adj pval: 0.19
Frequency
Tumor−Normal Expression Difference

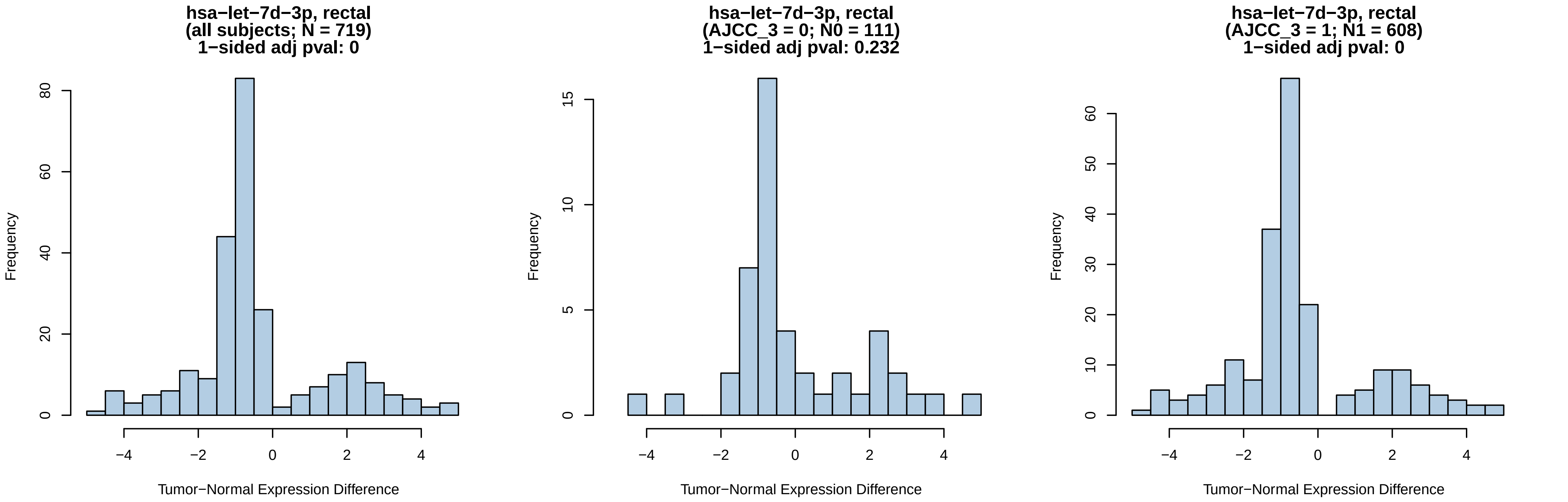
hsa−let−7d−3p, rectal
(all subjects; N = 719)
1−sided adj pval: 0
hsa−let−7d−3p, rectal
(AJCC_3 = 0; N0 = 111)
1−sided adj pval: 0.232
hsa−let−7d−3p, rectal
(AJCC_3 = 1; N1 = 608)
1−sided adj pval: 0
Frequency
Tumor−Normal Expression Difference

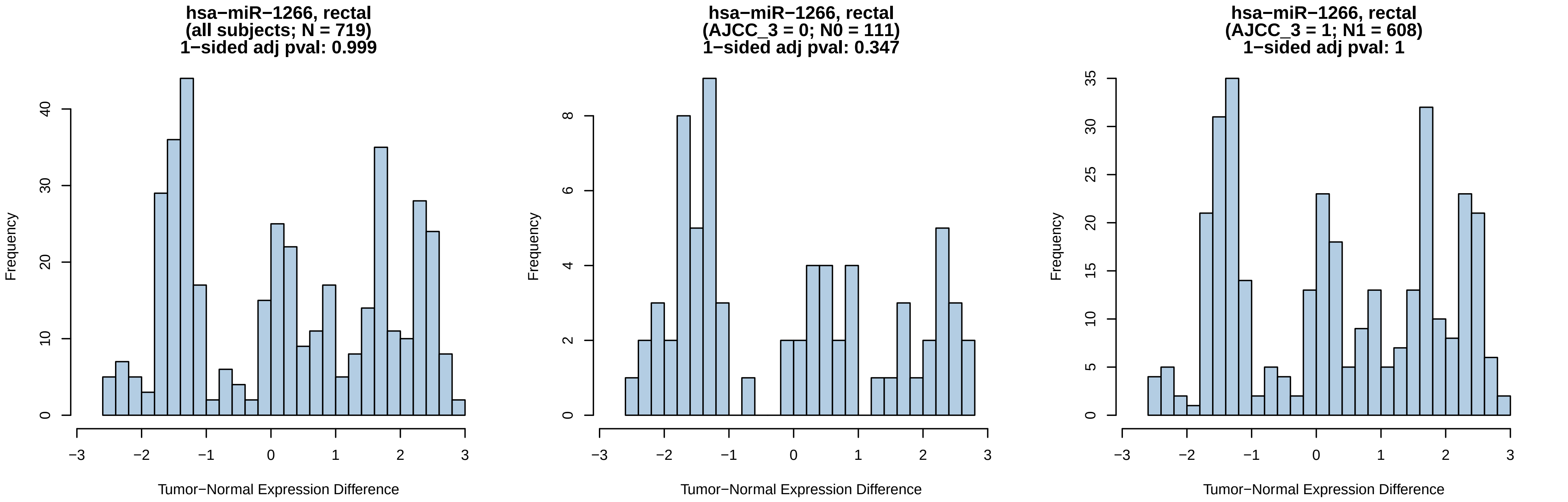
hsa−miR−1266, rectal
(all subjects; N = 719)
1−sided adj pval: 0.999
Frequency
Tumor−Normal Expression Difference
hsa−miR−1266, rectal
(AJCC_3 = 0; N0 = 111)
1−sided adj pval: 0.347
Frequency
Tumor−Normal Expression Difference
hsa−miR−1266, rectal
(AJCC_3 = 1; N1 = 608)
1−sided adj pval: 1
Frequency
Tumor−Normal Expression Difference

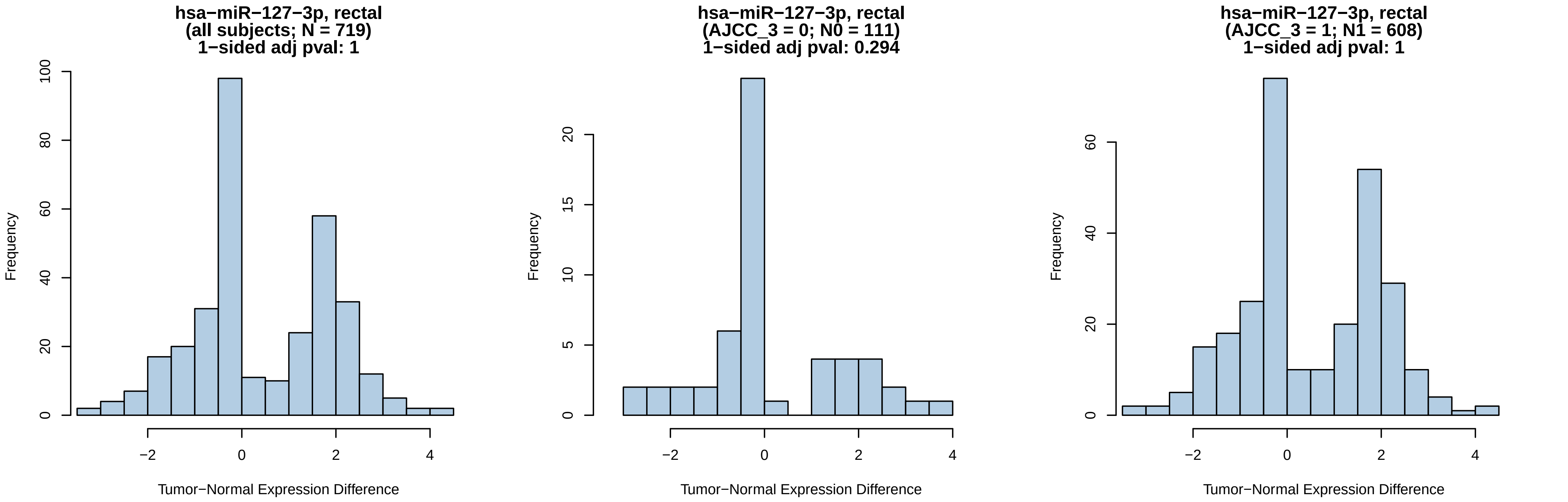

hsa−miR−127−3p, rectal
(all subjects; N = 719)
1−sided adj pval: 1
Frequency
Tumor−Normal Expression Difference
hsa−miR−127−3p, rectal
(AJCC_3 = 0; N0 = 111)
1−sided adj pval: 0.294
Frequency
Tumor−Normal Expression Difference
hsa−miR−127−3p, rectal
(AJCC_3 = 1; N1 = 608)
1−sided adj pval: 1
Frequency
Tumor−Normal Expression Difference

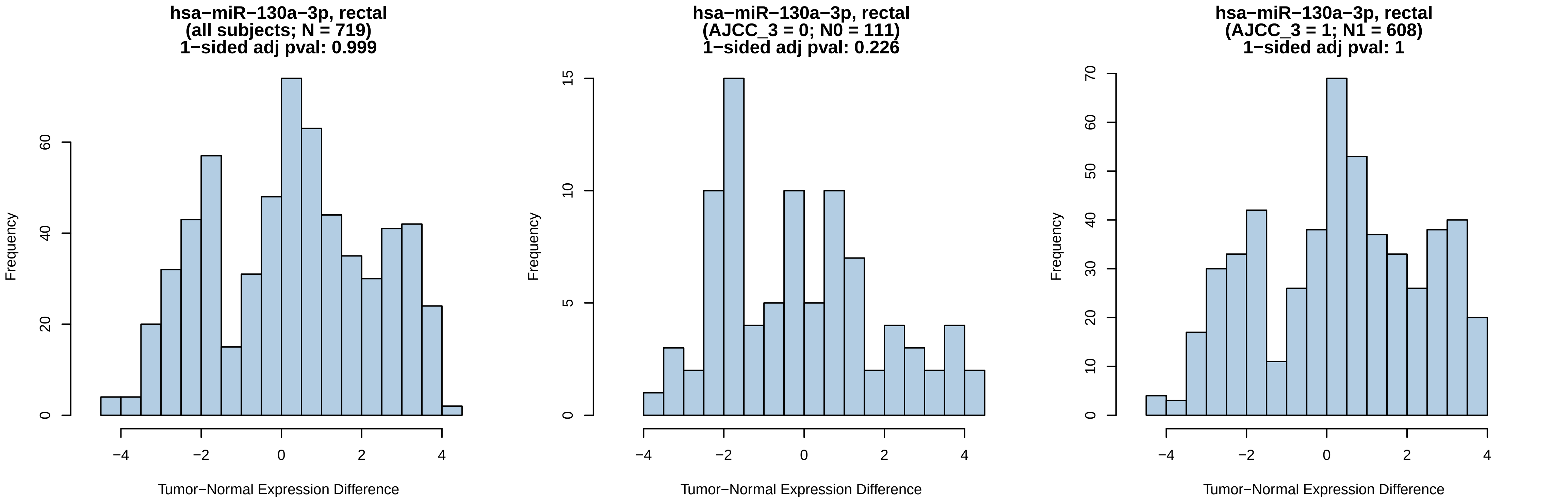

hsa−miR−130a−3p, rectal
(all subjects; N = 719)
1−sided adj pval: 0.999
hsa−miR−130a−3p, rectal
(AJCC_3 = 0; N0 = 111)
1−sided adj pval: 0.226
hsa−miR−130a−3p, rectal
(AJCC_3 = 1; N1 = 608)
1−sided adj pval: 1
Frequency
Tumor−Normal Expression Difference

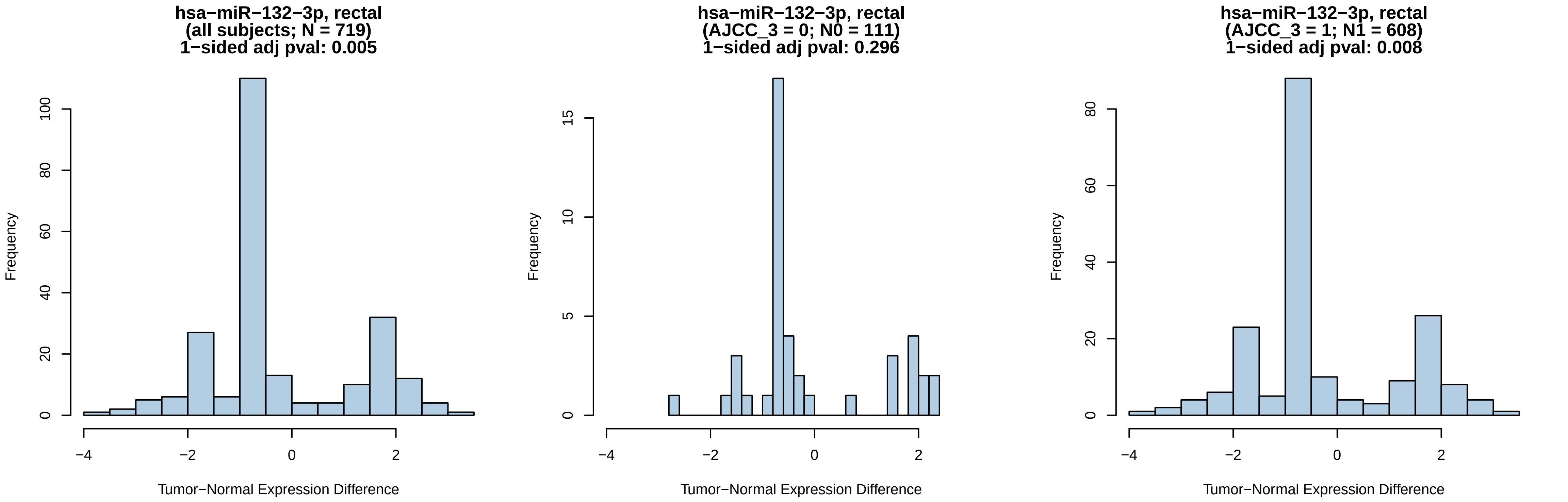

hsa−miR−132−3p, rectal
(all subjects; N = 719)
1−sided adj pval: 0.005
Frequency
0
20
40
60
80
100
−4
−2
0
2
Tumor−Normal Expression Difference
hsa−miR−132−3p, rectal
(AJCC_3 = 0; N0 = 111)
1−sided adj pval: 0.296
Frequency
0
5
10
15
−4
−2
0
2
Tumor−Normal Expression Difference
hsa−miR−132−3p, rectal
(AJCC_3 = 1; N1 = 608)
1−sided adj pval: 0.008
Frequency
0
20
40
60
80
−4
−2
0
2
Tumor−Normal Expression Difference

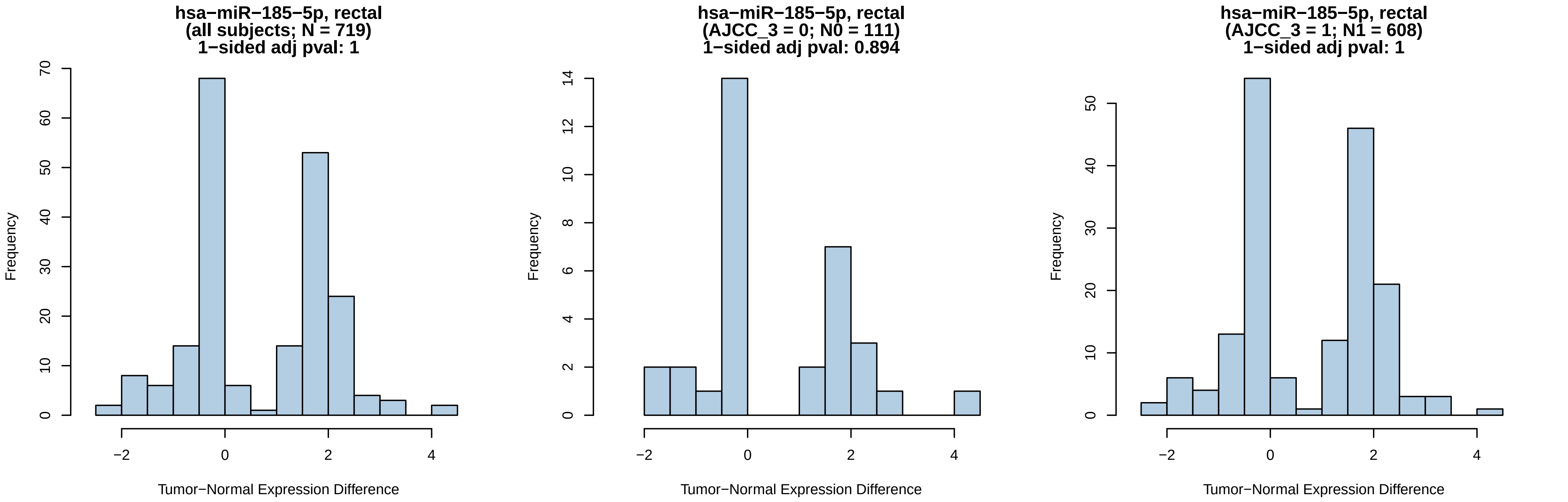

hsa−miR−185−5p, rectal
(all subjects; N = 719)
1−sided adj pval: 1
Frequency
Tumor−Normal Expression Difference
hsa−miR−185−5p, rectal
(AJCC_3 = 0; N0 = 111)
1−sided adj pval: 0.894
Frequency
Tumor−Normal Expression Difference
hsa−miR−185−5p, rectal
(AJCC_3 = 1; N1 = 608)
1−sided adj pval: 1
Frequency
Tumor−Normal Expression Difference

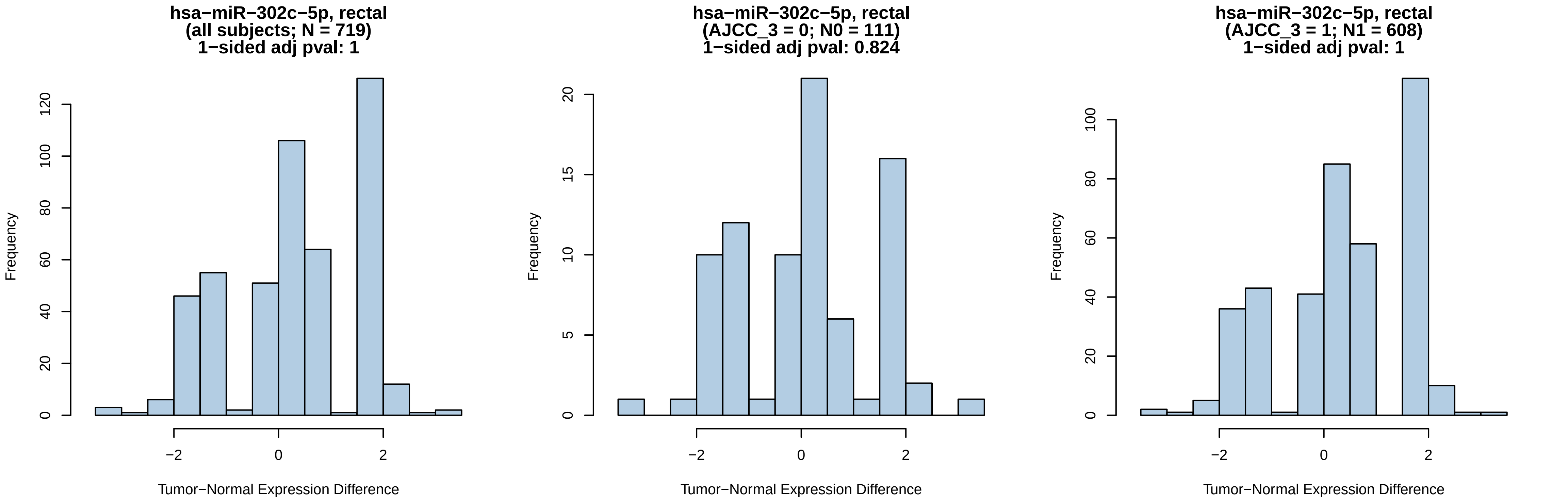
hsa−miR−302c−5p, rectal
(all subjects; N = 719)
1−sided adj pval: 1
Frequency
Tumor−Normal Expression Difference
hsa−miR−302c−5p, rectal
(AJCC_3 = 0; N0 = 111)
1−sided adj pval: 0.824
Frequency
Tumor−Normal Expression Difference
hsa−miR−302c−5p, rectal
(AJCC_3 = 1; N1 = 608)
1−sided adj pval: 1
Frequency
Tumor−Normal Expression Difference

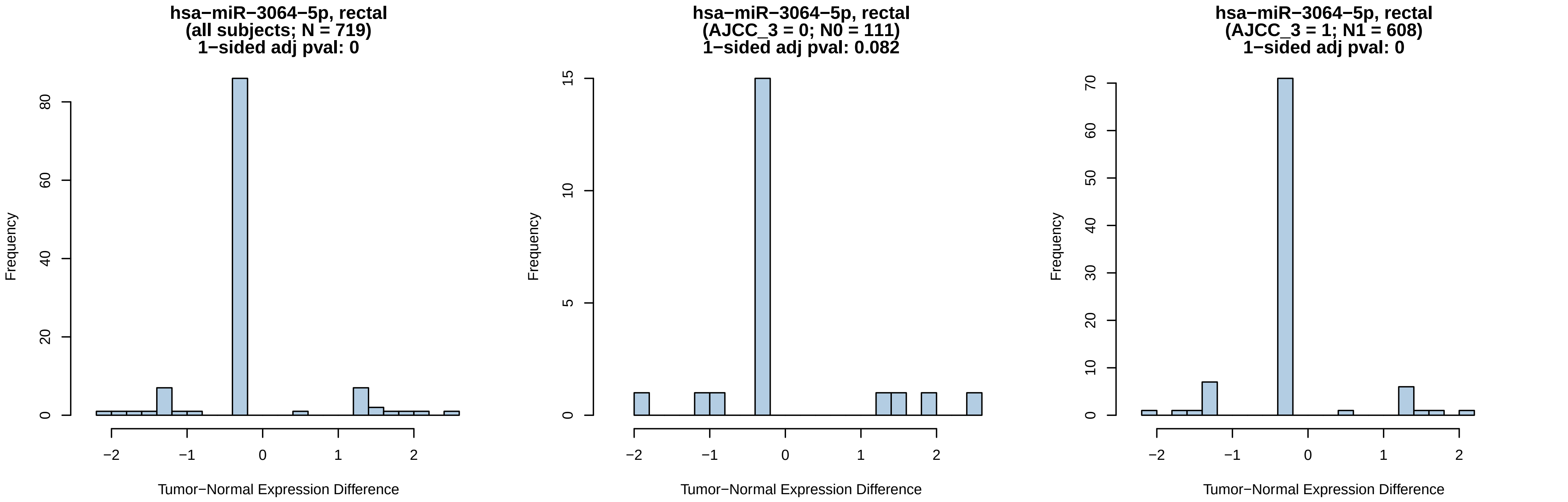
hsa−miR−3064−5p, rectal
(all subjects; N = 719)
1−sided adj pval: 0
Frequency
Tumor−Normal Expression Difference
hsa−miR−3064−5p, rectal
(AJCC_3 = 0; N0 = 111)
1−sided adj pval: 0.082
Frequency
Tumor−Normal Expression Difference
hsa−miR−3064−5p, rectal
(AJCC_3 = 1; N1 = 608)
1−sided adj pval: 0
Frequency
Tumor−Normal Expression Difference

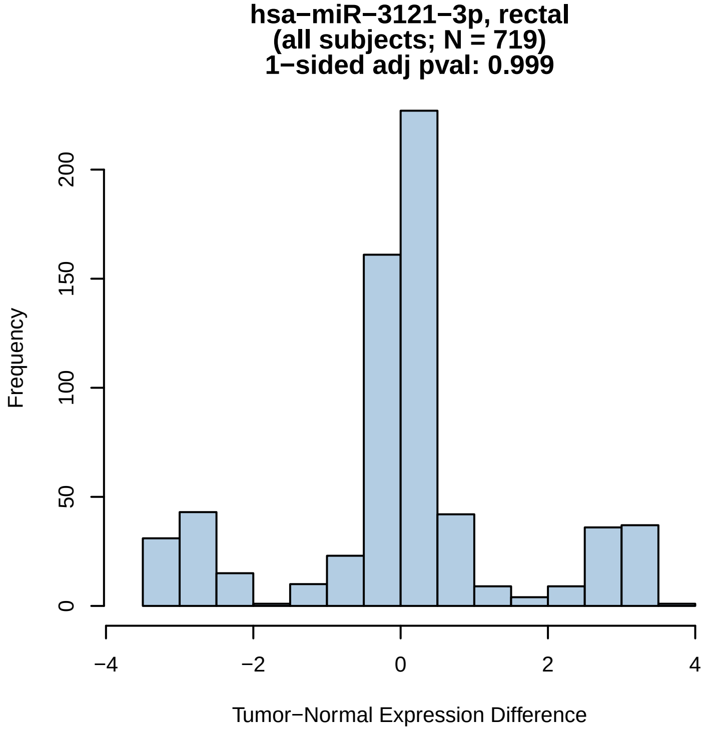
hsa−miR−3121−3p, rectal
(all subjects; N = 719)
1−sided adj pval: 0.999
Frequency
0
50
100
150
200
−4
−2
0
2
4
Tumor−Normal Expression Difference

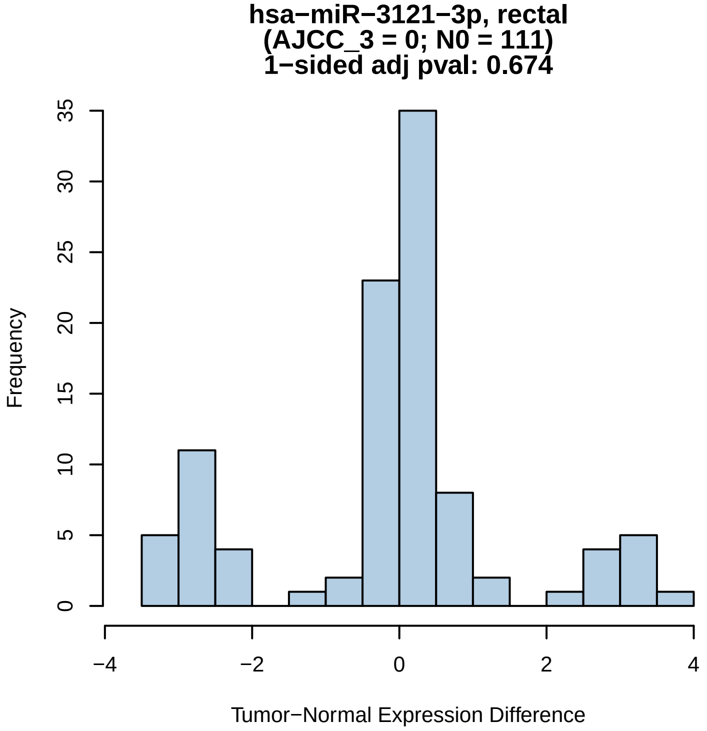
hsa−miR−3121−3p, rectal
(AJCC_3 = 0; N0 = 111)
1−sided adj pval: 0.674
Frequency
0
5
10
15
20
25
30
35
−4
−2
0
2
4
Tumor−Normal Expression Difference

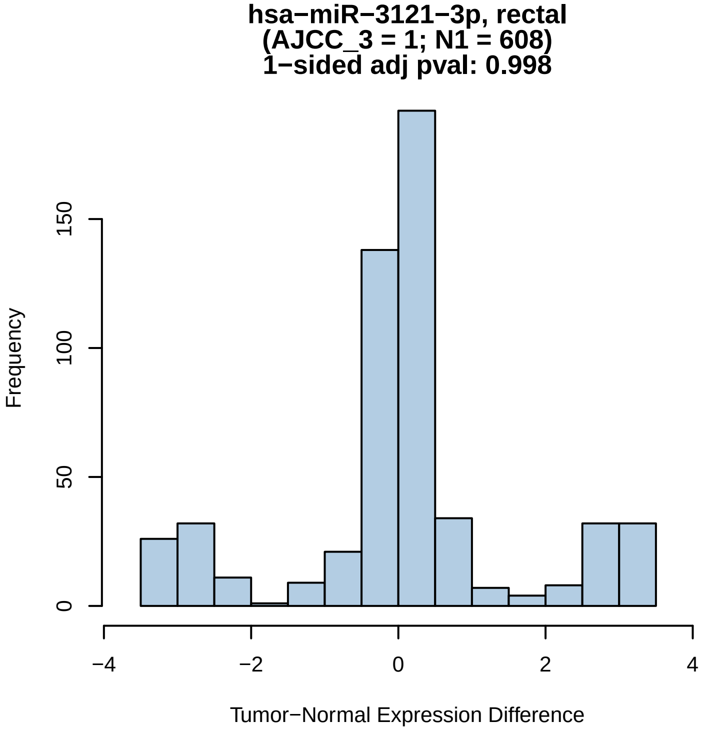
hsa−miR−3121−3p, rectal
(AJCC_3 = 1; N1 = 608)
1−sided adj pval: 0.998
Frequency
0
50
100
150
−4
−2
0
2
4
Tumor−Normal Expression Difference

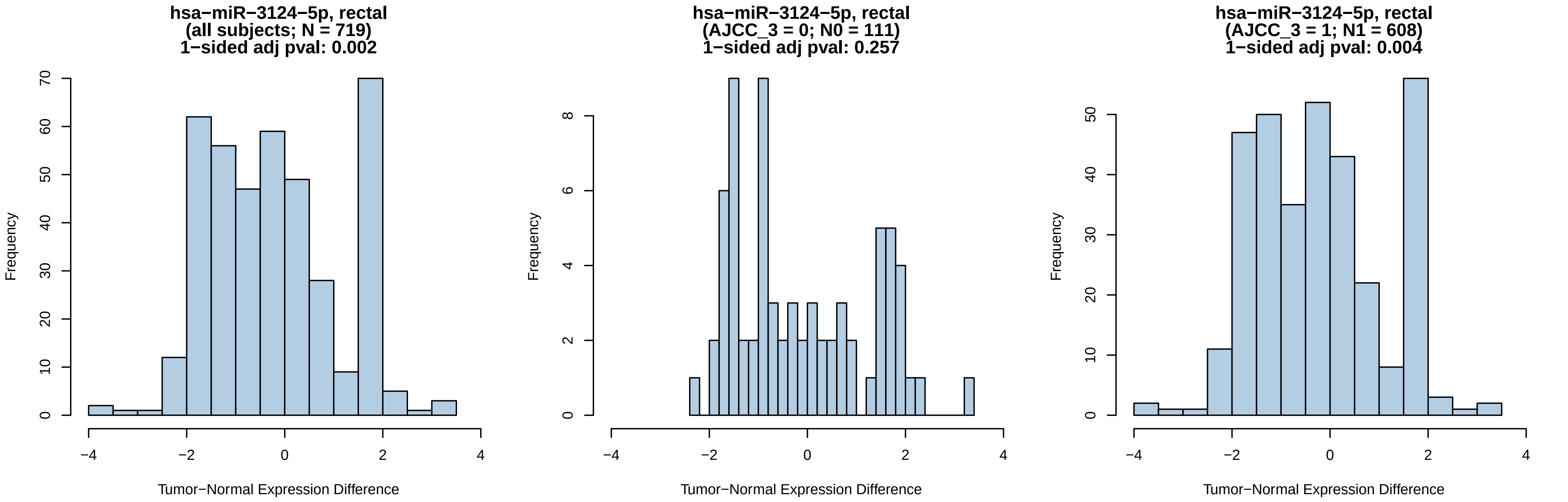
hsa−miR−3124−5p, rectal
(all subjects; N = 719)
1−sided adj pval: 0.002
Frequency
Tumor−Normal Expression Difference
hsa−miR−3124−5p, rectal
(AJCC_3 = 0; N0 = 111)
1−sided adj pval: 0.257
Frequency
Tumor−Normal Expression Difference
hsa−miR−3124−5p, rectal
(AJCC_3 = 1; N1 = 608)
1−sided adj pval: 0.004
Frequency
Tumor−Normal Expression Difference

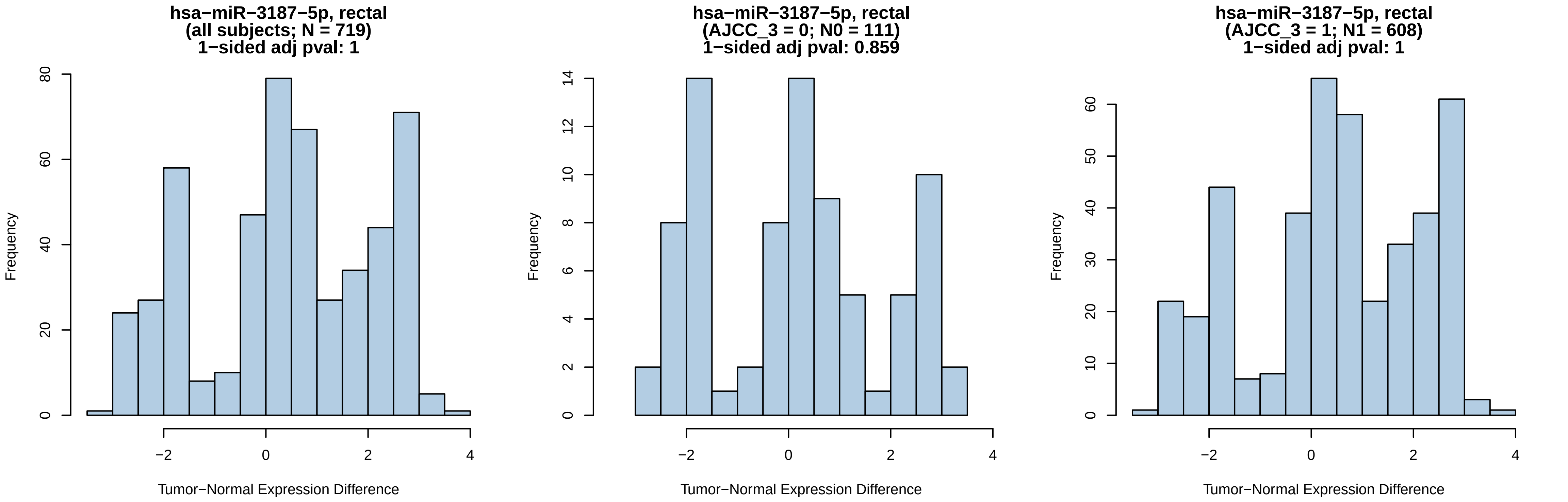

hsa−miR−3187−5p, rectal
(all subjects; N = 719)
1−sided adj pval: 1
Frequency
Tumor−Normal Expression Difference
hsa−miR−3187−5p, rectal
(AJCC_3 = 0; N0 = 111)
1−sided adj pval: 0.859
Frequency
Tumor−Normal Expression Difference
hsa−miR−3187−5p, rectal
(AJCC_3 = 1; N1 = 608)
1−sided adj pval: 1
Frequency
Tumor−Normal Expression Difference

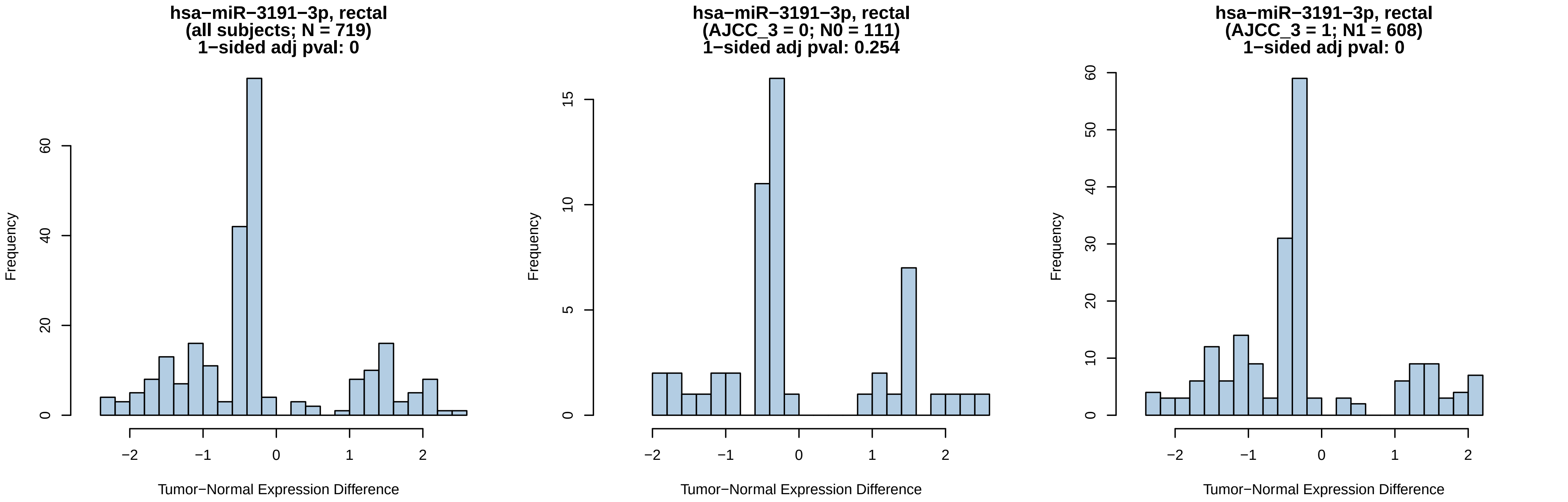
hsa−miR−3191−3p, rectal
(all subjects; N = 719)
1−sided adj pval: 0
hsa−miR−3191−3p, rectal
(AJCC_3 = 0; N0 = 111)
1−sided adj pval: 0.254
hsa−miR−3191−3p, rectal
(AJCC_3 = 1; N1 = 608)
1−sided adj pval: 0
Frequency
Tumor−Normal Expression Difference

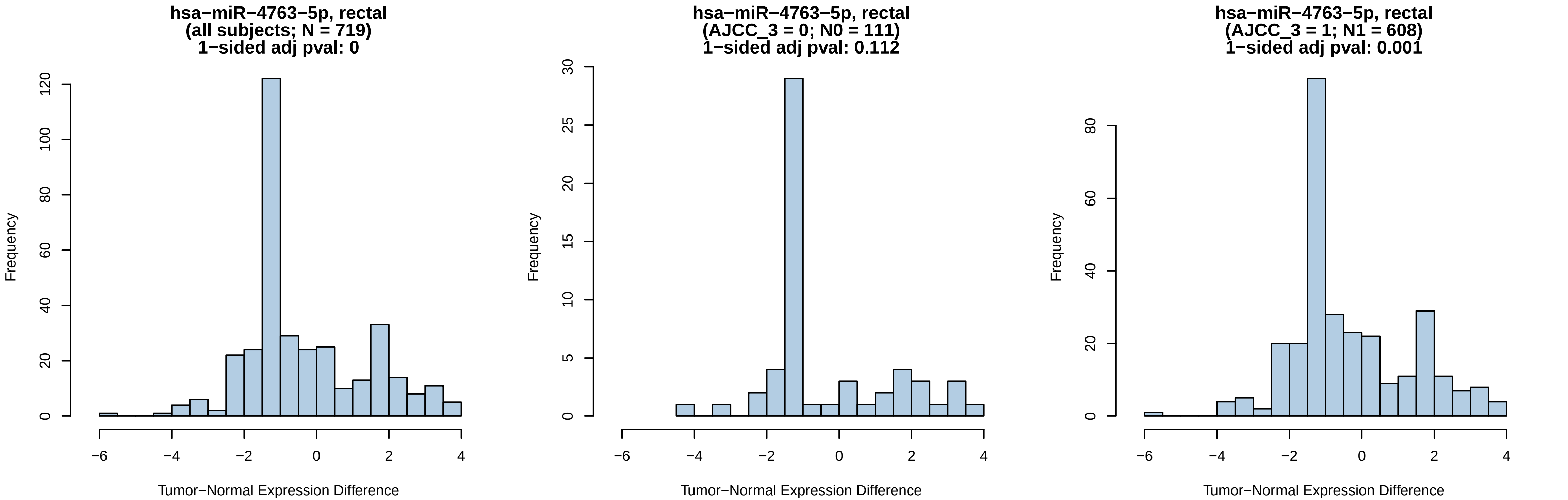
hsa−miR−4763−5p, rectal
(all subjects; N = 719)
1−sided adj pval: 0
Frequency
Tumor−Normal Expression Difference
hsa−miR−4763−5p, rectal
(AJCC_3 = 0; N0 = 111)
1−sided adj pval: 0.112
Frequency
Tumor−Normal Expression Difference
hsa−miR−4763−5p, rectal
(AJCC_3 = 1; N1 = 608)
1−sided adj pval: 0.001
Frequency
Tumor−Normal Expression Difference

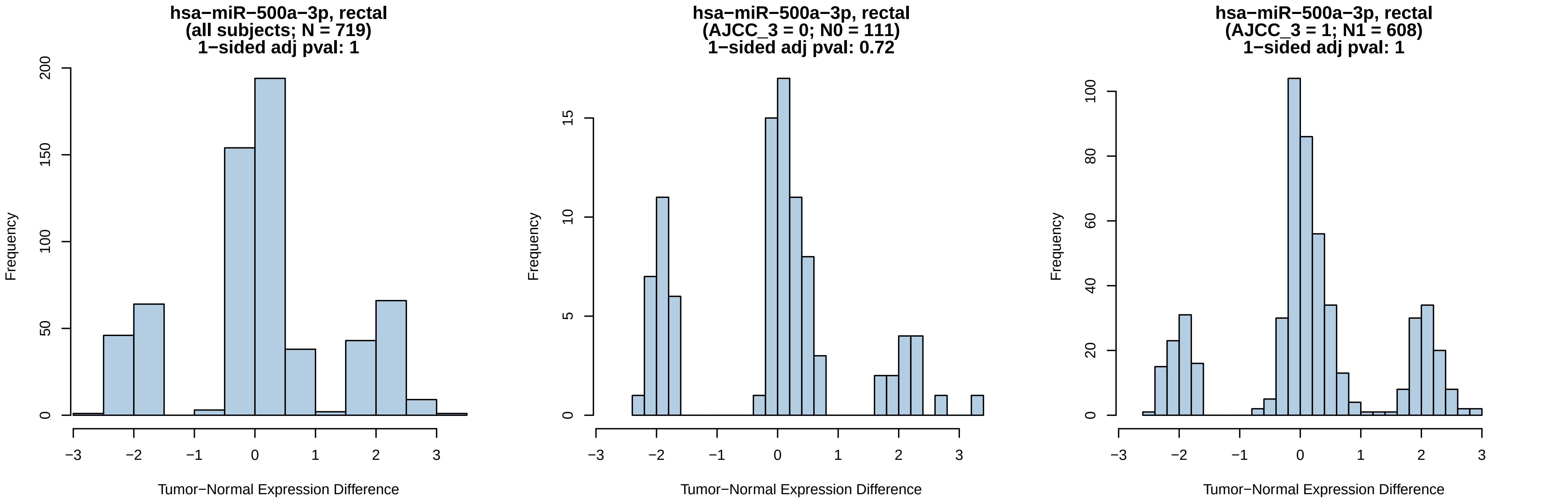
hsa−miR−500a−3p, rectal
(all subjects; N = 719)
1−sided adj pval: 1
hsa−miR−500a−3p, rectal
(AJCC_3 = 0; N0 = 111)
1−sided adj pval: 0.72
hsa−miR−500a−3p, rectal
(AJCC_3 = 1; N1 = 608)
1−sided adj pval: 1
Frequency
Tumor−Normal Expression Difference

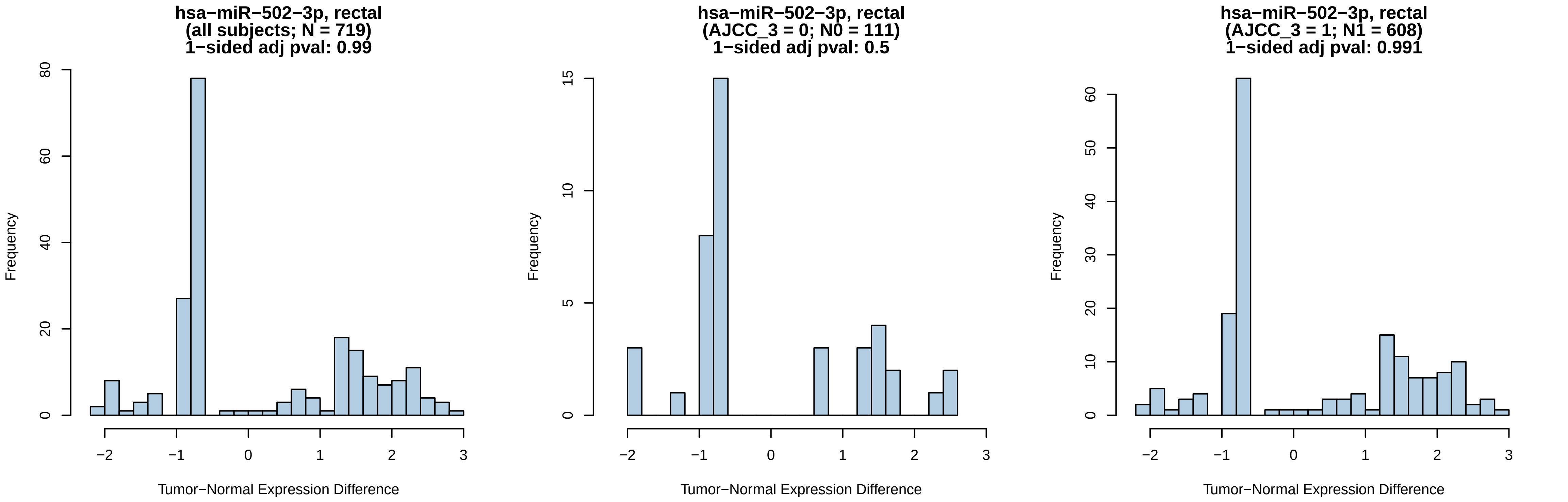
hsa−miR−502−3p, rectal
(all subjects; N = 719)
1−sided adj pval: 0.99
Frequency
Tumor−Normal Expression Difference
hsa−miR−502−3p, rectal
(AJCC_3 = 0; N0 = 111)
1−sided adj pval: 0.5
Frequency
Tumor−Normal Expression Difference
hsa−miR−502−3p, rectal
(AJCC_3 = 1; N1 = 608)
1−sided adj pval: 0.991
Frequency
Tumor−Normal Expression Difference

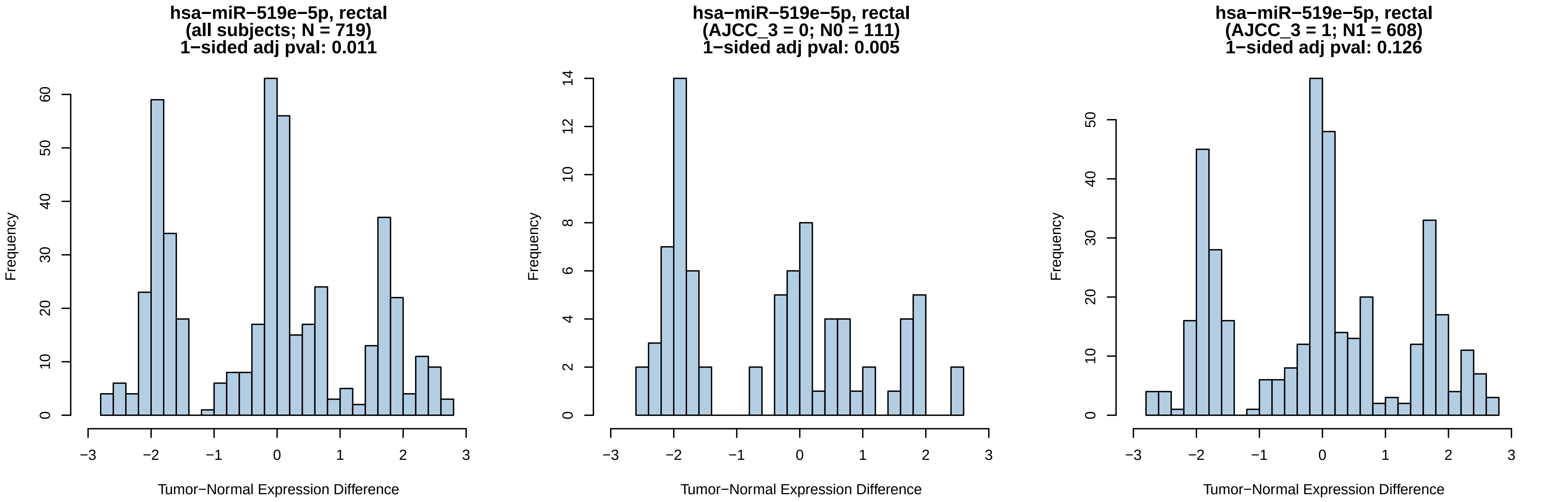

hsa−miR−519e−5p, rectal
(all subjects; N = 719)
1−sided adj pval: 0.011
hsa−miR−519e−5p, rectal
(AJCC_3 = 0; N0 = 111)
1−sided adj pval: 0.005
hsa−miR−519e−5p, rectal
(AJCC_3 = 1; N1 = 608)
1−sided adj pval: 0.126
Frequency
Tumor−Normal Expression Difference

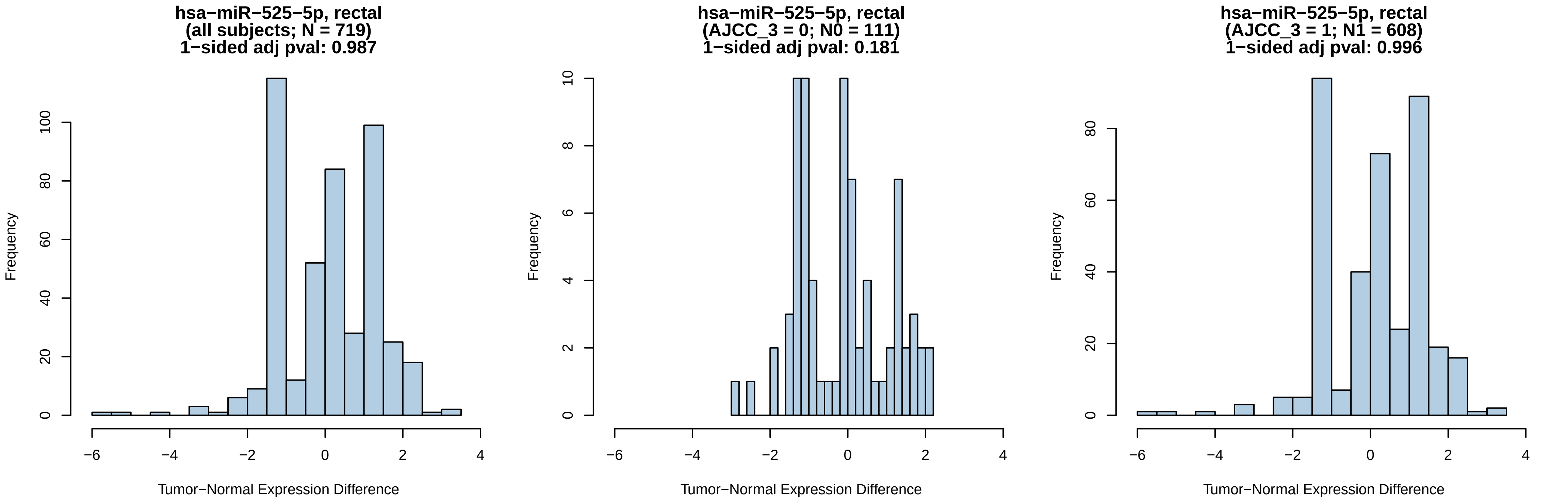
hsa−miR−525−5p, rectal
(all subjects; N = 719)
1−sided adj pval: 0.987
hsa−miR−525−5p, rectal
(AJCC_3 = 0; N0 = 111)
1−sided adj pval: 0.181
hsa−miR−525−5p, rectal
(AJCC_3 = 1; N1 = 608)
1−sided adj pval: 0.996
Frequency
Tumor−Normal Expression Difference

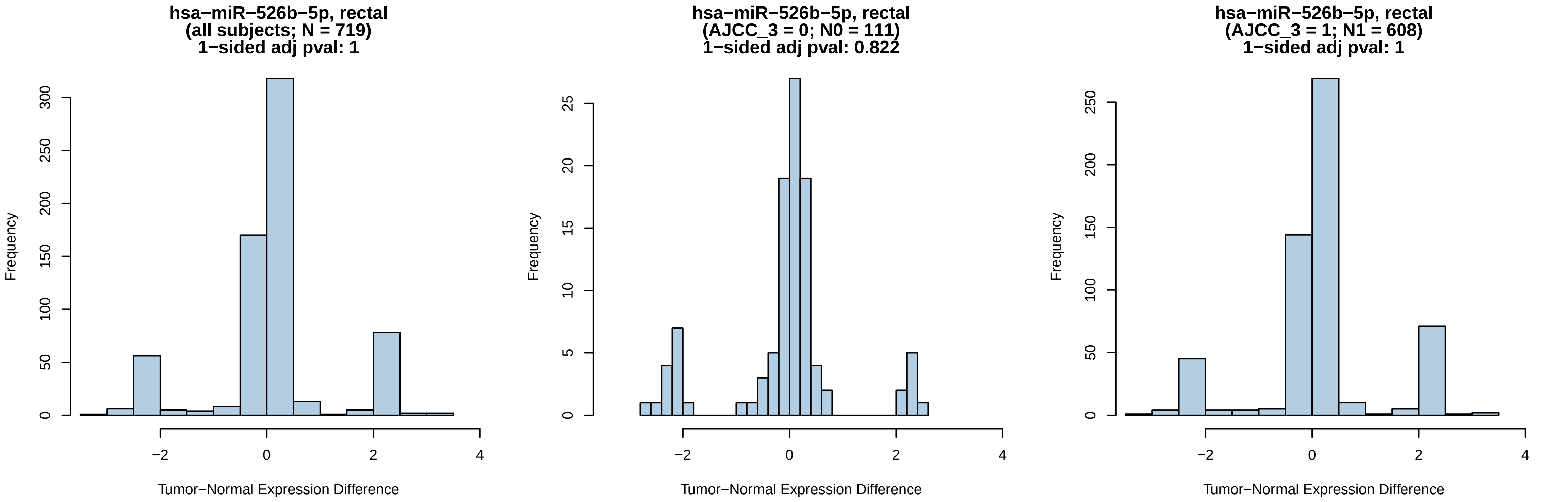
hsa−miR−526b−5p, rectal
(all subjects; N = 719)
1−sided adj pval: 1
Frequency
0
50
100
150
200
250
300
−2
0
2
4
Tumor−Normal Expression Difference
hsa−miR−526b−5p, rectal
(AJCC_3 = 0; N0 = 111)
1−sided adj pval: 0.822
Frequency
0
5
10
15
20
25
−2
0
2
4
Tumor−Normal Expression Difference
hsa−miR−526b−5p, rectal
(AJCC_3 = 1; N1 = 608)
1−sided adj pval: 1
Frequency
0
50
100
150
200
250
−2
0
2
4
Tumor−Normal Expression Difference

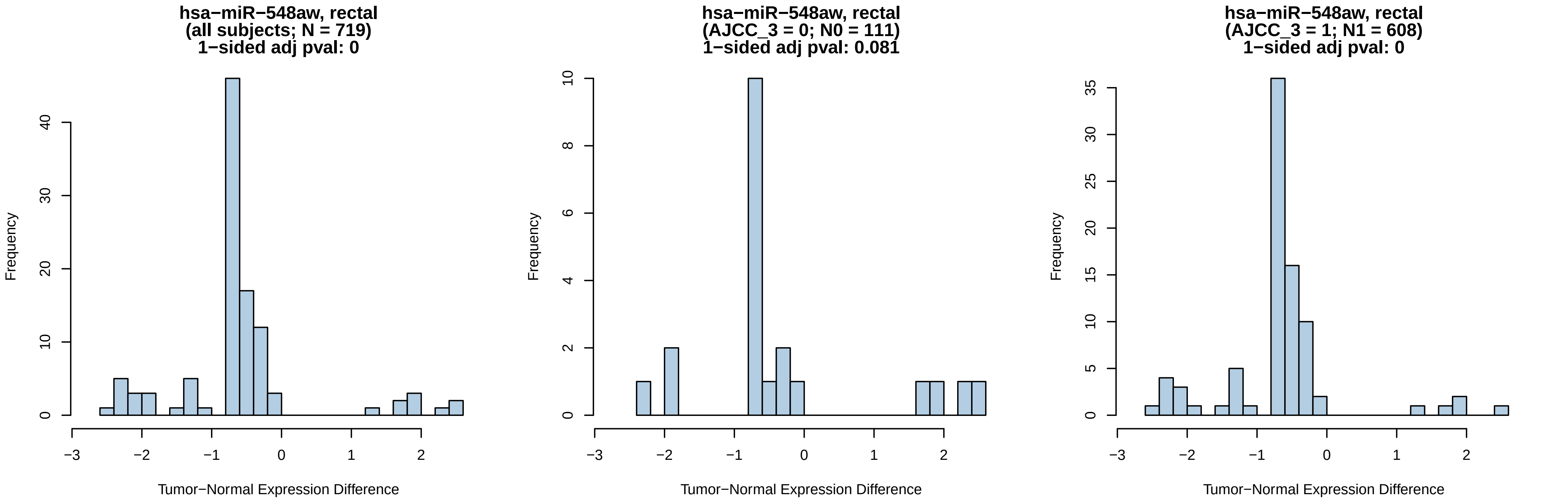
hsa−miR−548aw, rectal
(all subjects; N = 719)
1−sided adj pval: 0
Frequency
Tumor−Normal Expression Difference
hsa−miR−548aw, rectal
(AJCC_3 = 0; N0 = 111)
1−sided adj pval: 0.081
Frequency
Tumor−Normal Expression Difference
hsa−miR−548aw, rectal
(AJCC_3 = 1; N1 = 608)
1−sided adj pval: 0
Frequency
Tumor−Normal Expression Difference

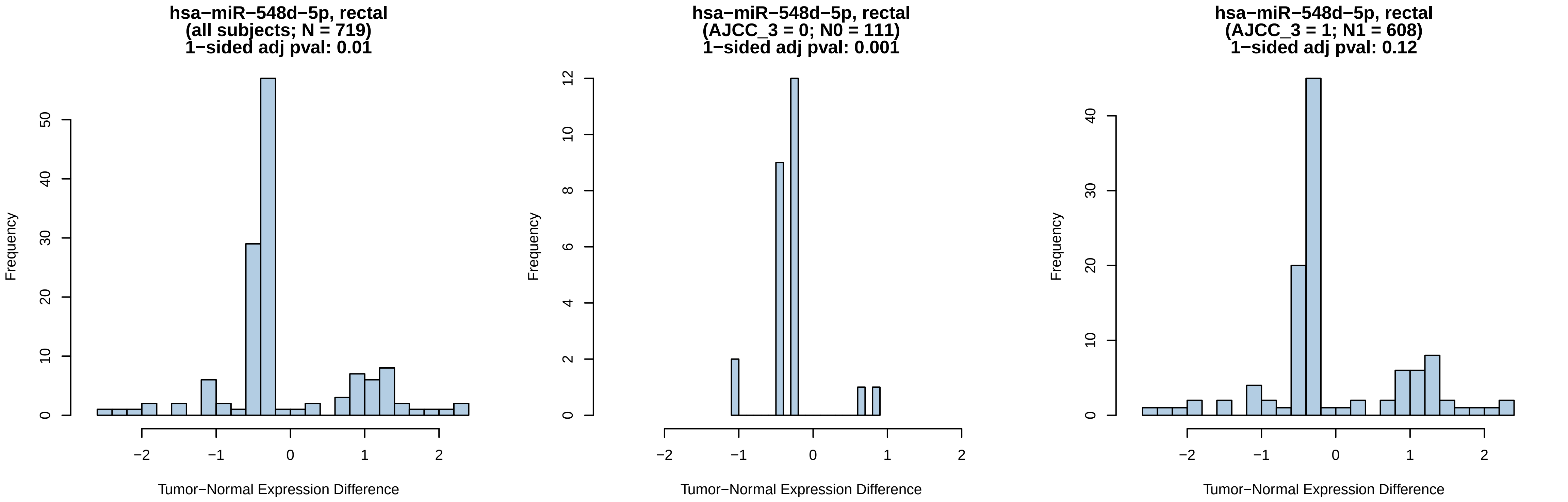
hsa−miR−548d−5p, rectal
(all subjects; N = 719)
1−sided adj pval: 0.01
Frequency
Tumor−Normal Expression Difference
hsa−miR−548d−5p, rectal
(AJCC_3 = 0; N0 = 111)
1−sided adj pval: 0.001
Frequency
Tumor−Normal Expression Difference
hsa−miR−548d−5p, rectal
(AJCC_3 = 1; N1 = 608)
1−sided adj pval: 0.12
Frequency
Tumor−Normal Expression Difference

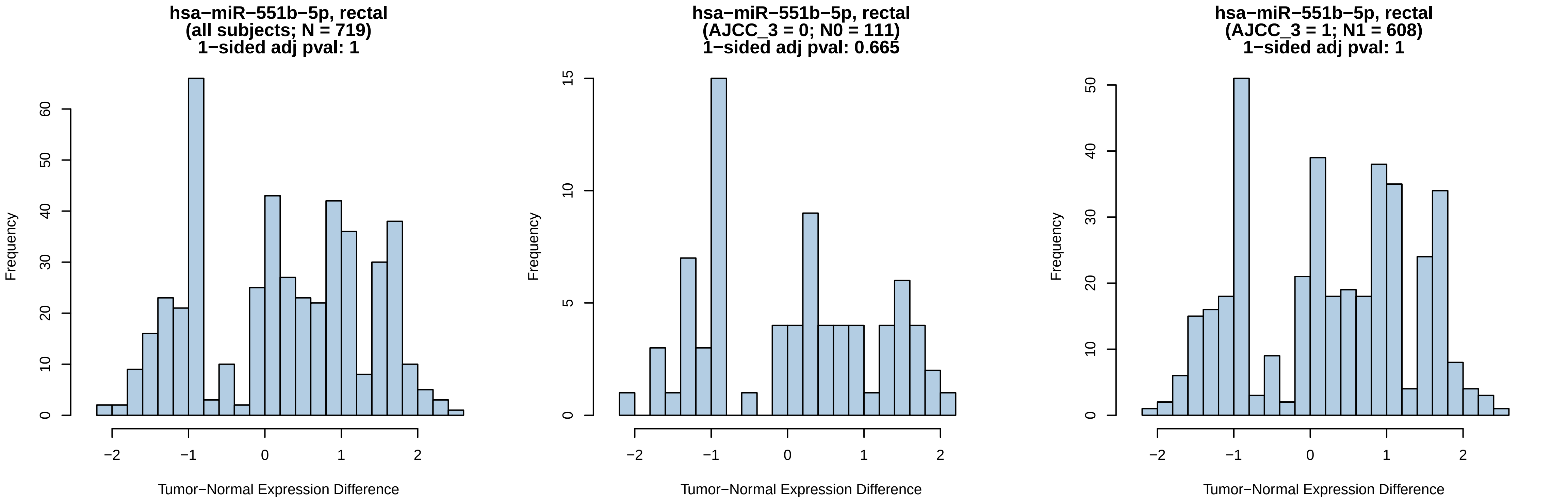

hsa−miR−551b−5p, rectal
(all subjects; N = 719)
1−sided adj pval: 1
Frequency
Tumor−Normal Expression Difference
hsa−miR−551b−5p, rectal
(AJCC_3 = 0; N0 = 111)
1−sided adj pval: 0.665
Frequency
Tumor−Normal Expression Difference
hsa−miR−551b−5p, rectal
(AJCC_3 = 1; N1 = 608)
1−sided adj pval: 1
Frequency
Tumor−Normal Expression Difference

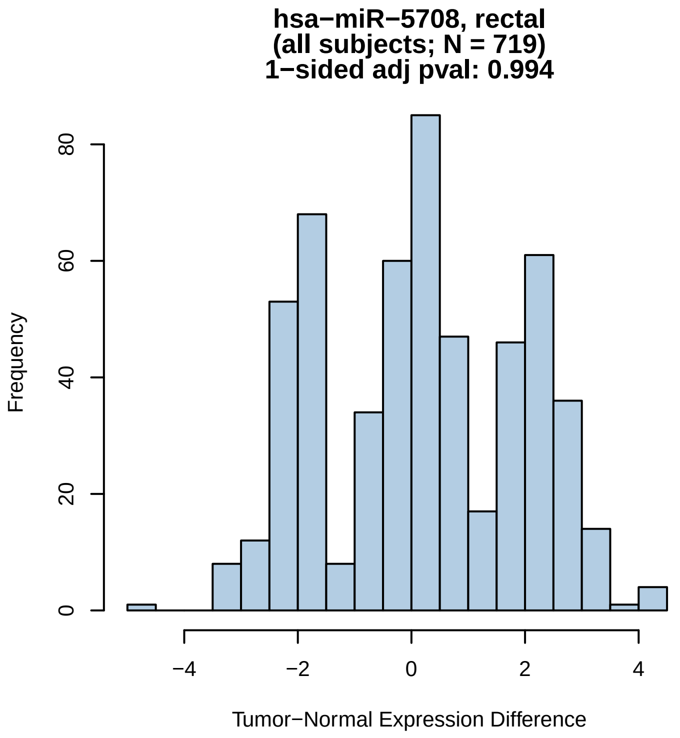

hsa−miR−5708, rectal
(all subjects; N = 719)
1−sided adj pval: 0.994
Frequency
Tumor−Normal Expression Difference
0
20
40
60
80
−4
−2
0
2
4


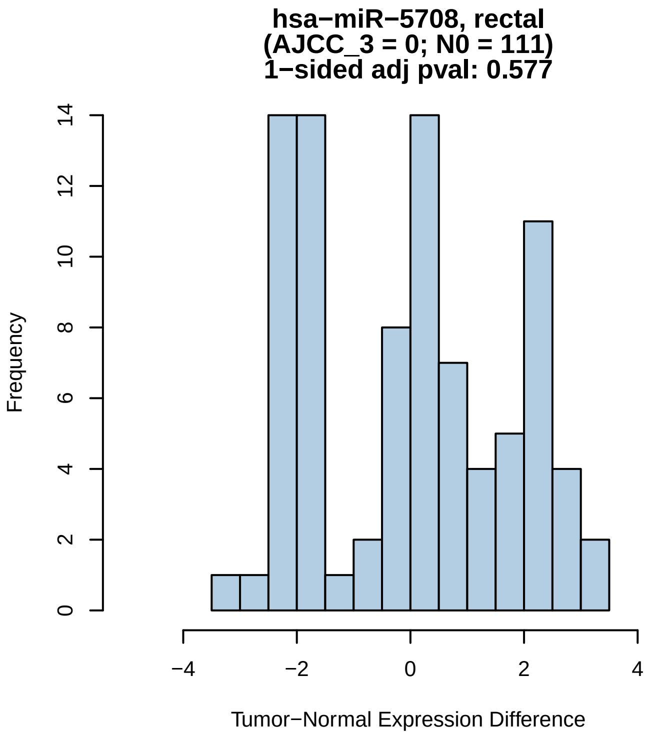

hsa−miR−5708, rectal
(AJCC_3 = 0; N0 = 111)
1−sided adj pval: 0.577
Frequency
Tumor−Normal Expression Difference
0
2
4
6
8
10
12
14
−4
−2
0
2
4


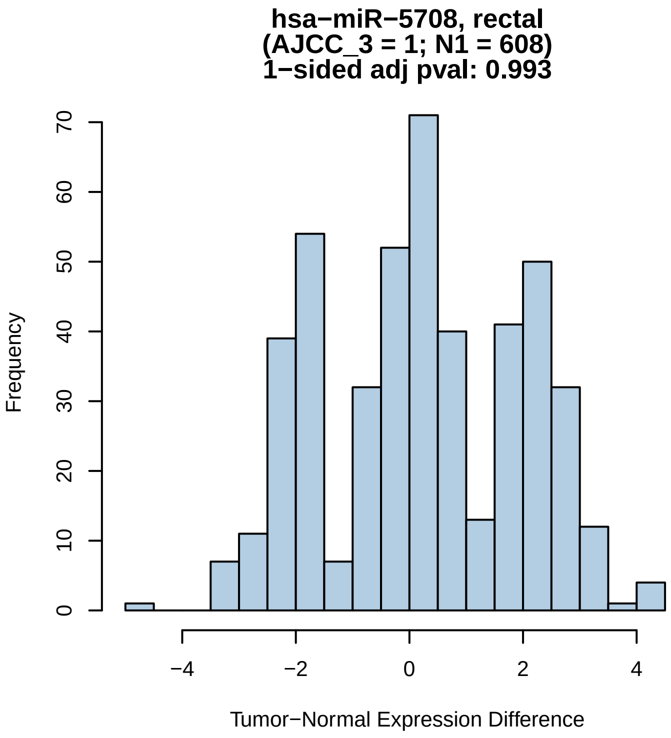

hsa−miR−5708, rectal
(AJCC_3 = 1; N1 = 608)
1−sided adj pval: 0.993
Frequency
Tumor−Normal Expression Difference
0
10
20
30
40
50
60
70
−4
−2
0
2
4

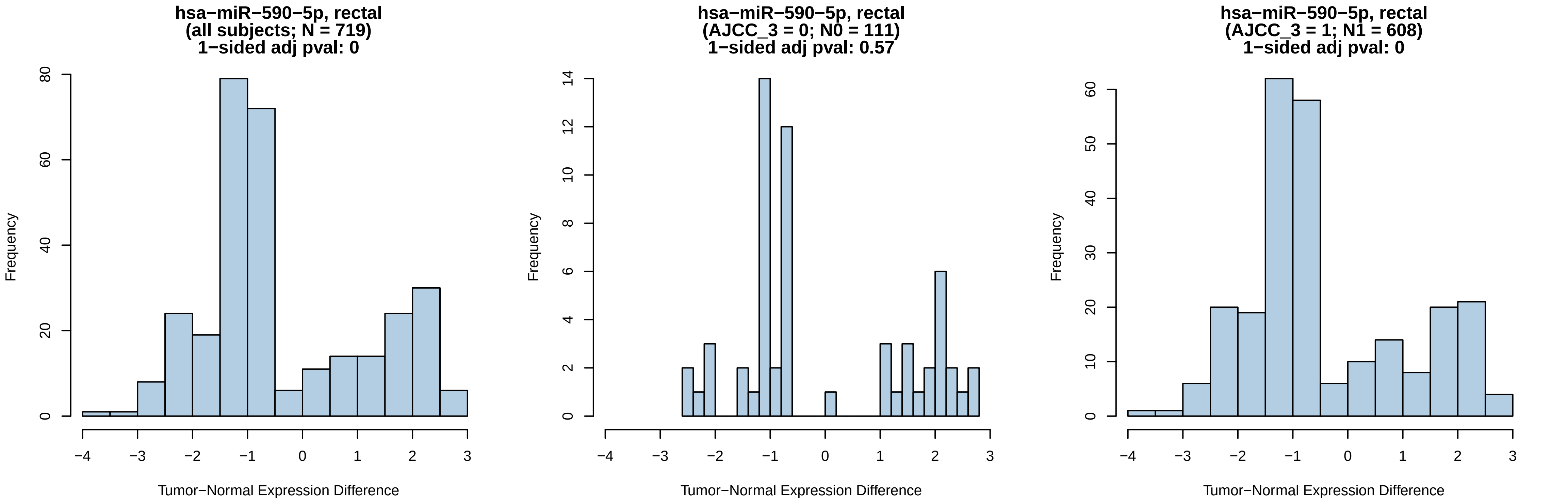
hsa−miR−590−5p, rectal
(all subjects; N = 719)
1−sided adj pval: 0
Frequency
Tumor−Normal Expression Difference
hsa−miR−590−5p, rectal
(AJCC_3 = 0; N0 = 111)
1−sided adj pval: 0.57
Frequency
Tumor−Normal Expression Difference
hsa−miR−590−5p, rectal
(AJCC_3 = 1; N1 = 608)
1−sided adj pval: 0
Frequency
Tumor−Normal Expression Difference

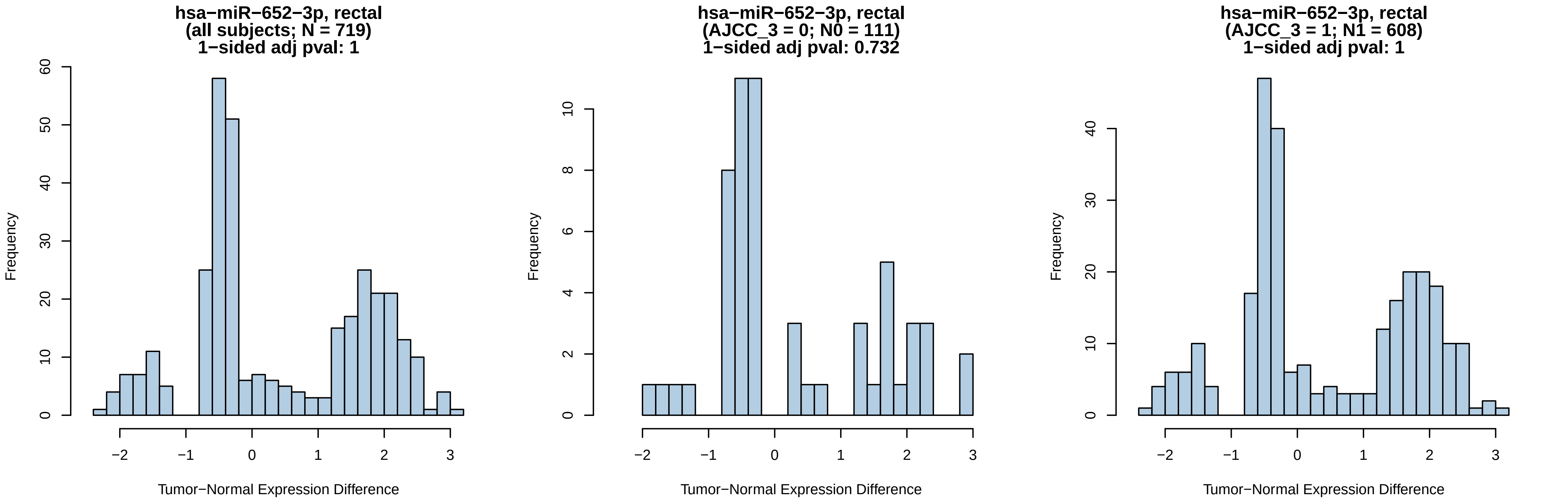

hsa−miR−652−3p, rectal
(all subjects; N = 719)
1−sided adj pval: 1
Frequency
Tumor−Normal Expression Difference
hsa−miR−652−3p, rectal
(AJCC_3 = 0; N0 = 111)
1−sided adj pval: 0.732
Frequency
Tumor−Normal Expression Difference
hsa−miR−652−3p, rectal
(AJCC_3 = 1; N1 = 608)
1−sided adj pval: 1
Frequency
Tumor−Normal Expression Difference

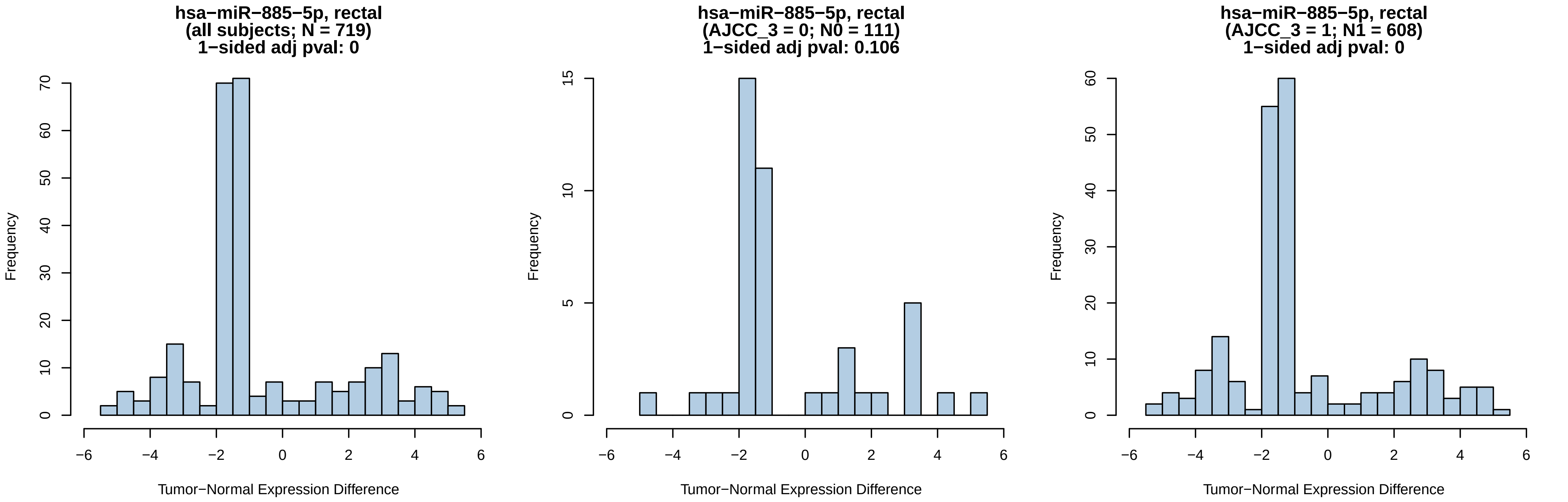
hsa−miR−885−5p, rectal
(all subjects; N = 719)
1−sided adj pval: 0
Frequency
Tumor−Normal Expression Difference
hsa−miR−885−5p, rectal
(AJCC_3 = 0; N0 = 111)
1−sided adj pval: 0.106
Frequency
Tumor−Normal Expression Difference
hsa−miR−885−5p, rectal
(AJCC_3 = 1; N1 = 608)
1−sided adj pval: 0
Frequency
Tumor−Normal Expression Difference

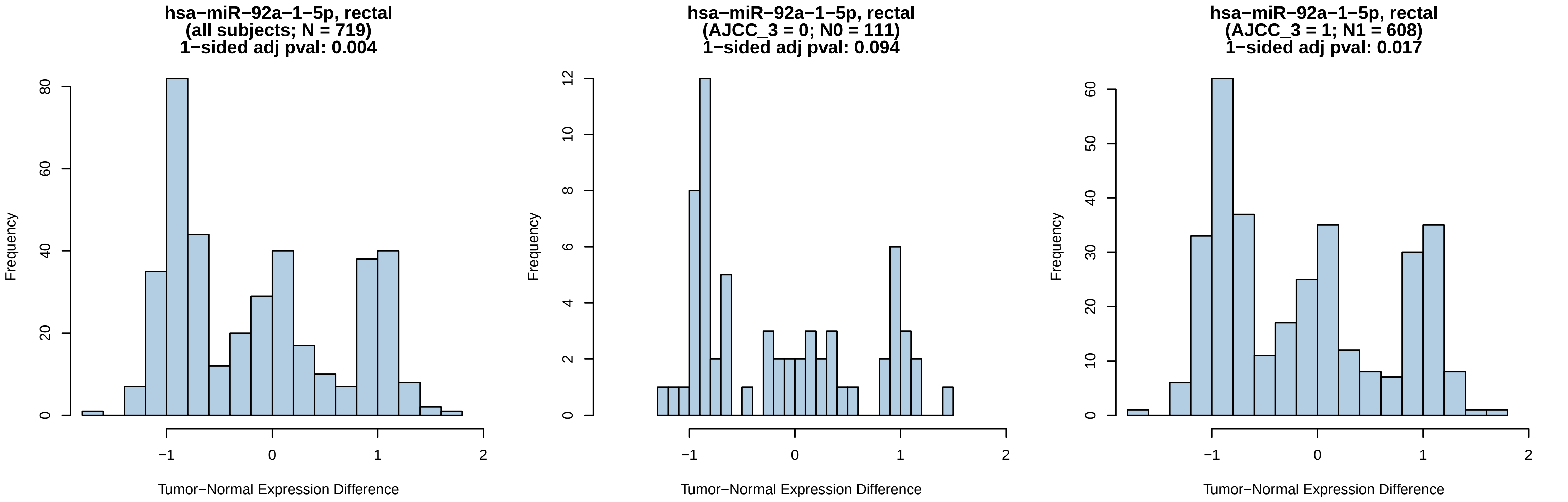

hsa−miR−92a−1−5p, rectal
(all subjects; N = 719)
1−sided adj pval: 0.004
Frequency
Tumor−Normal Expression Difference
hsa−miR−92a−1−5p, rectal
(AJCC_3 = 0; N0 = 111)
1−sided adj pval: 0.094
Frequency
Tumor−Normal Expression Difference
hsa−miR−92a−1−5p, rectal
(AJCC_3 = 1; N1 = 608)
1−sided adj pval: 0.017
Frequency
Tumor−Normal Expression Difference

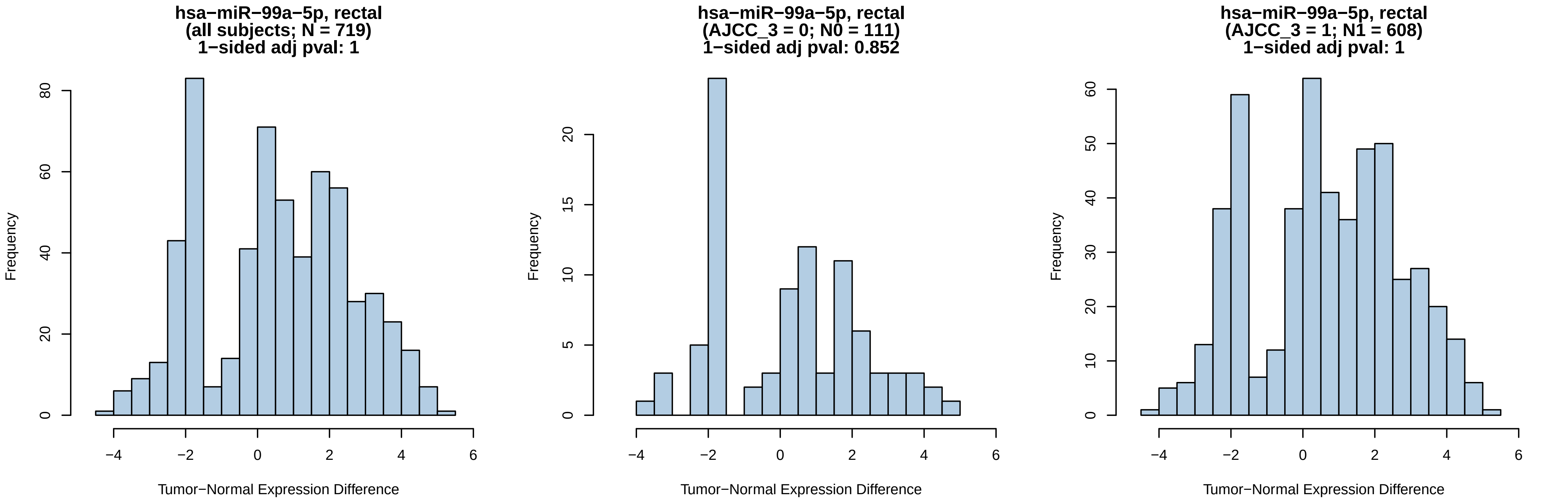

hsa−miR−99a−5p, rectal
(all subjects; N = 719)
1−sided adj pval: 1
Frequency
Tumor−Normal Expression Difference
hsa−miR−99a−5p, rectal
(AJCC_3 = 0; N0 = 111)
1−sided adj pval: 0.852
Frequency
Tumor−Normal Expression Difference
hsa−miR−99a−5p, rectal
(AJCC_3 = 1; N1 = 608)
1−sided adj pval: 1
Frequency
Tumor−Normal Expression Difference

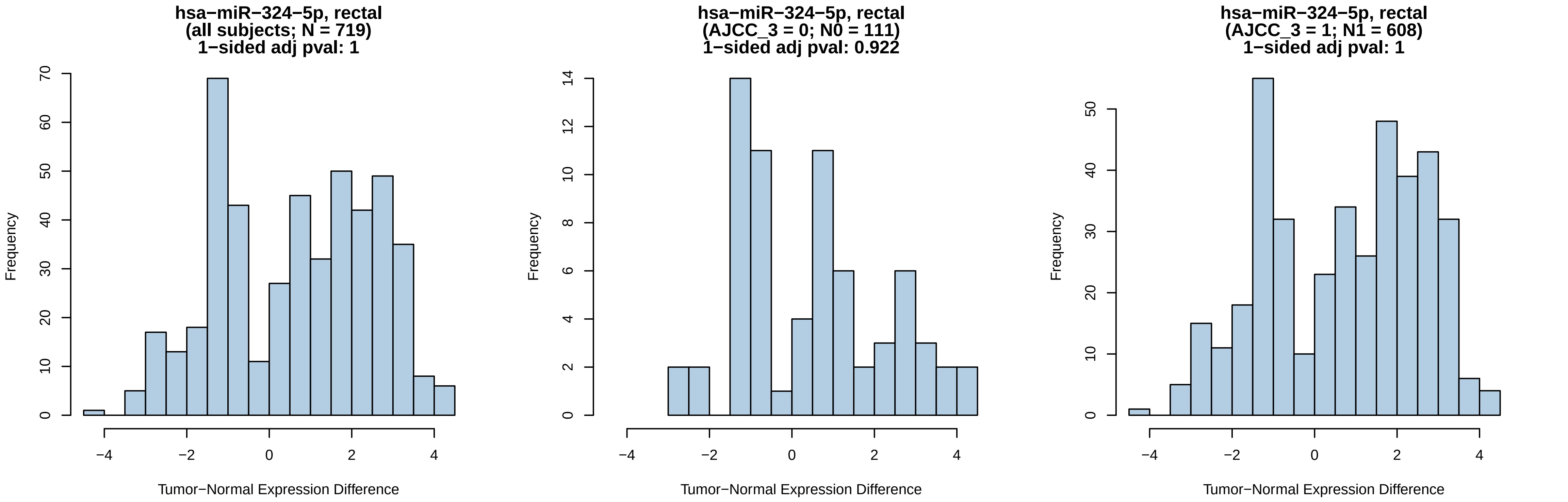
hsa−miR−324−5p, rectal
(all subjects; N = 719)
1−sided adj pval: 1
Frequency
Tumor−Normal Expression Difference
hsa−miR−324−5p, rectal
(AJCC_3 = 0; N0 = 111)
1−sided adj pval: 0.922
Frequency
Tumor−Normal Expression Difference
hsa−miR−324−5p, rectal
(AJCC_3 = 1; N1 = 608)
1−sided adj pval: 1
Frequency
Tumor−Normal Expression Difference

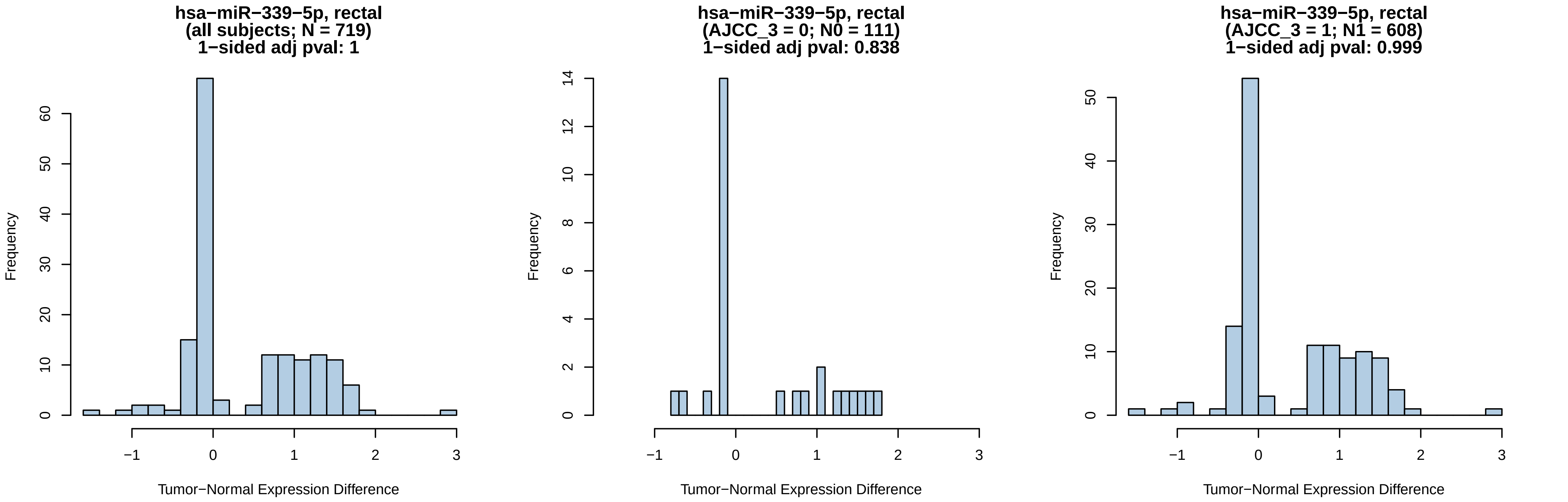

hsa−miR−339−5p, rectal
(all subjects; N = 719)
1−sided adj pval: 1
hsa−miR−339−5p, rectal
(AJCC_3 = 0; N0 = 111)
1−sided adj pval: 0.838
hsa−miR−339−5p, rectal
(AJCC_3 = 1; N1 = 608)
1−sided adj pval: 0.999
Frequency
Tumor−Normal Expression Difference

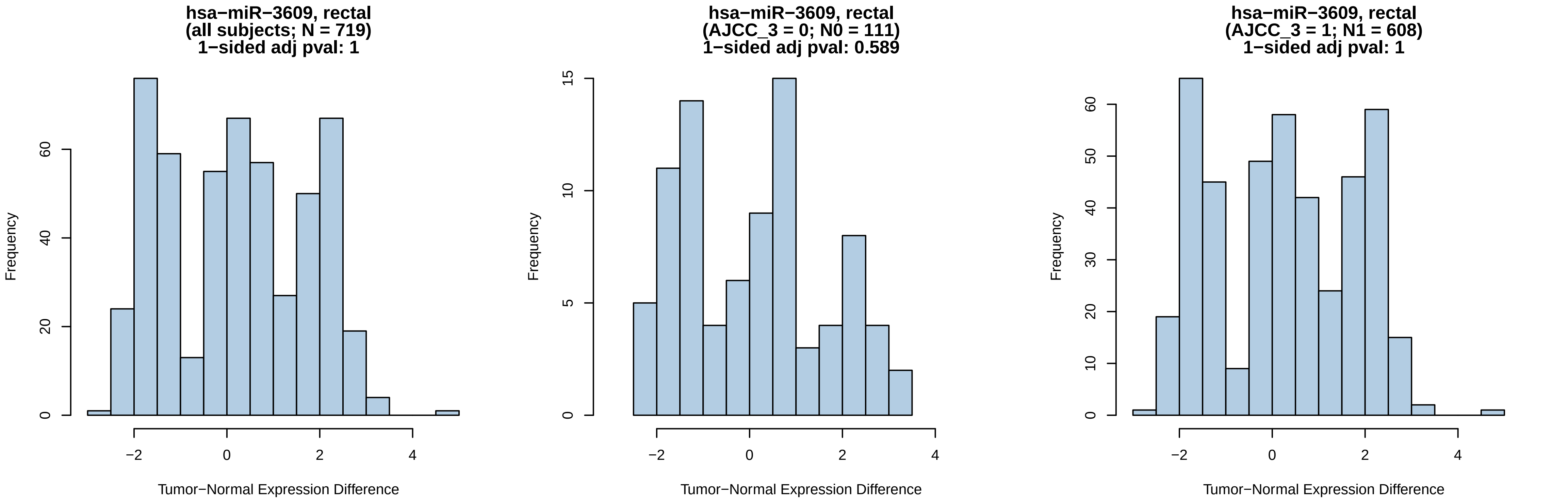

hsa−miR−3609, rectal
(all subjects; N = 719)
1−sided adj pval: 1
Frequency
Tumor−Normal Expression Difference
hsa−miR−3609, rectal
(AJCC_3 = 0; N0 = 111)
1−sided adj pval: 0.589
Frequency
Tumor−Normal Expression Difference
hsa−miR−3609, rectal
(AJCC_3 = 1; N1 = 608)
1−sided adj pval: 1
Frequency
Tumor−Normal Expression Difference

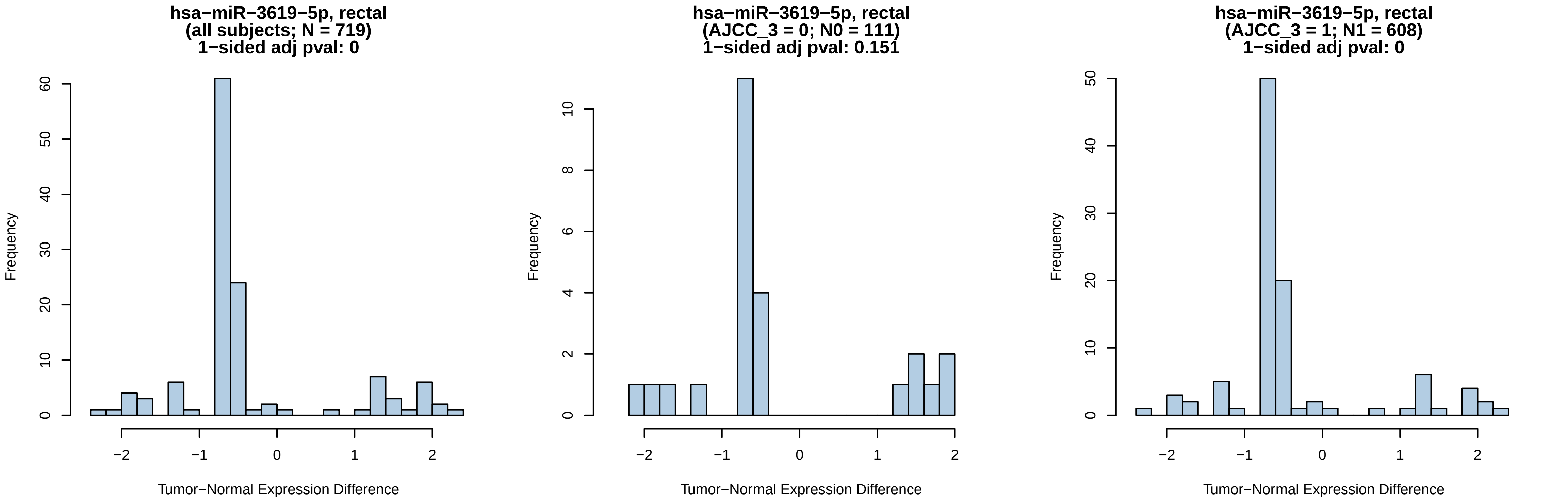
hsa−miR−3619−5p, rectal
(all subjects; N = 719)
1−sided adj pval: 0
Frequency
Tumor−Normal Expression Difference
hsa−miR−3619−5p, rectal
(AJCC_3 = 0; N0 = 111)
1−sided adj pval: 0.151
Frequency
Tumor−Normal Expression Difference
hsa−miR−3619−5p, rectal
(AJCC_3 = 1; N1 = 608)
1−sided adj pval: 0
Frequency
Tumor−Normal Expression Difference

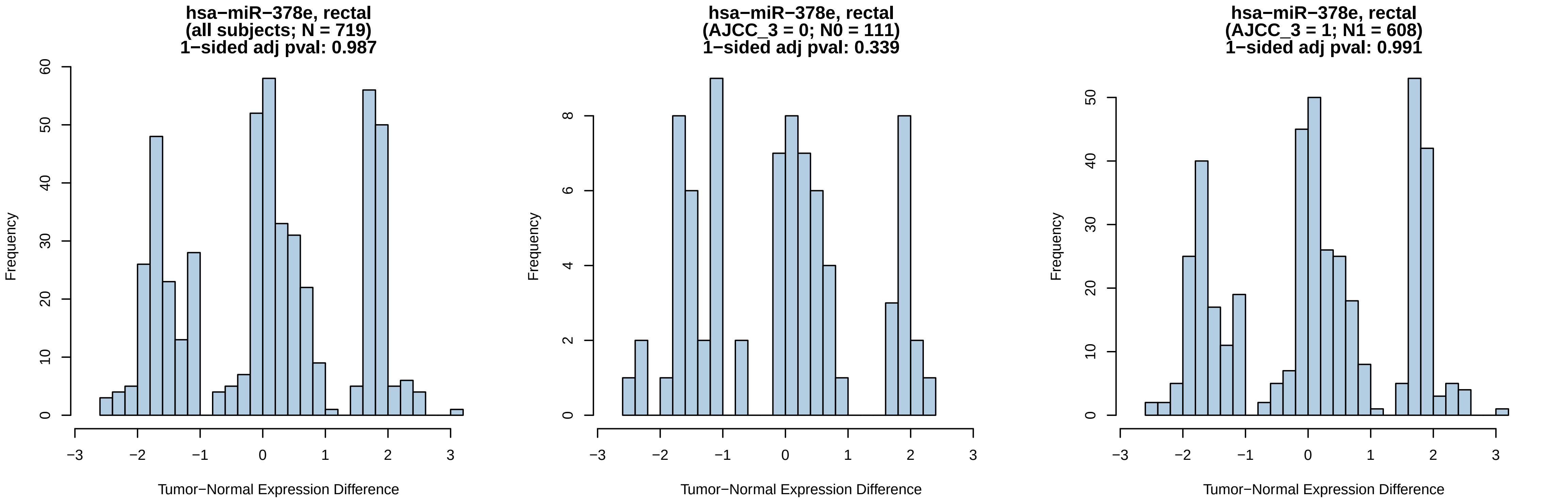

hsa−miR−378e, rectal
(all subjects; N = 719)
1−sided adj pval: 0.987
Frequency
Tumor−Normal Expression Difference
hsa−miR−378e, rectal
(AJCC_3 = 0; N0 = 111)
1−sided adj pval: 0.339
Frequency
Tumor−Normal Expression Difference
hsa−miR−378e, rectal
(AJCC_3 = 1; N1 = 608)
1−sided adj pval: 0.991
Frequency
Tumor−Normal Expression Difference

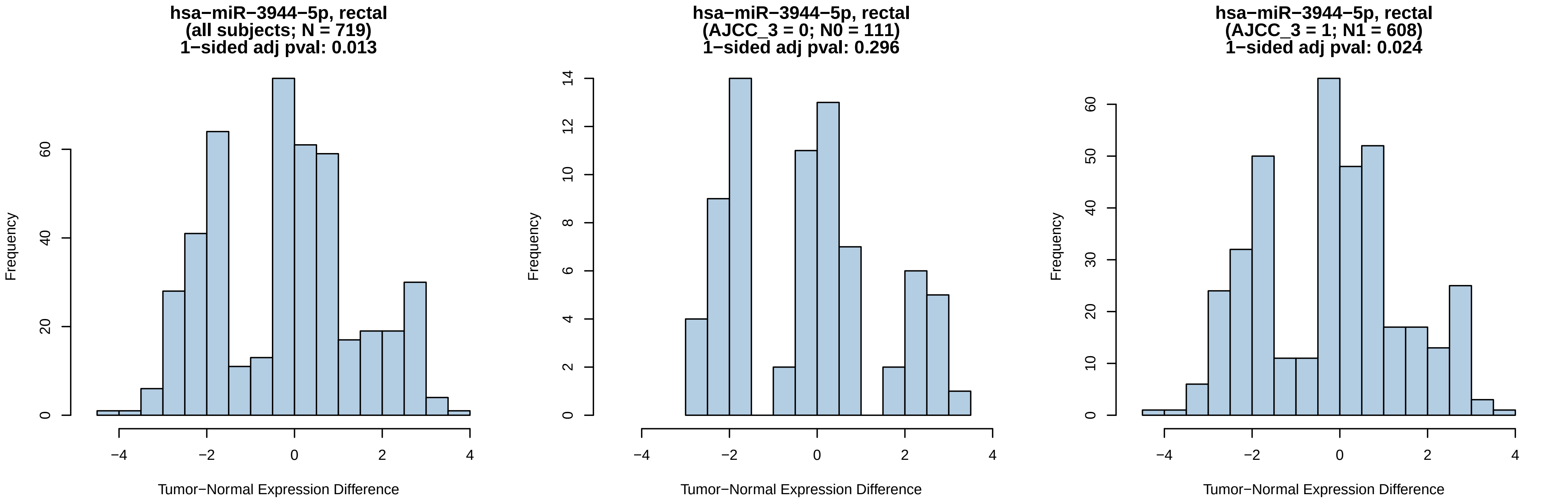
hsa−miR−3944−5p, rectal
(all subjects; N = 719)
1−sided adj pval: 0.013
Frequency
0
20
40
60
−4
−2
0
2
4
Tumor−Normal Expression Difference
hsa−miR−3944−5p, rectal
(AJCC_3 = 0; N0 = 111)
1−sided adj pval: 0.296
Frequency
0
2
4
6
8
10
12
14
−4
−2
0
2
4
Tumor−Normal Expression Difference
hsa−miR−3944−5p, rectal
(AJCC_3 = 1; N1 = 608)
1−sided adj pval: 0.024
Frequency
0
10
20
30
40
50
60
−4
−2
0
2
4
Tumor−Normal Expression Difference

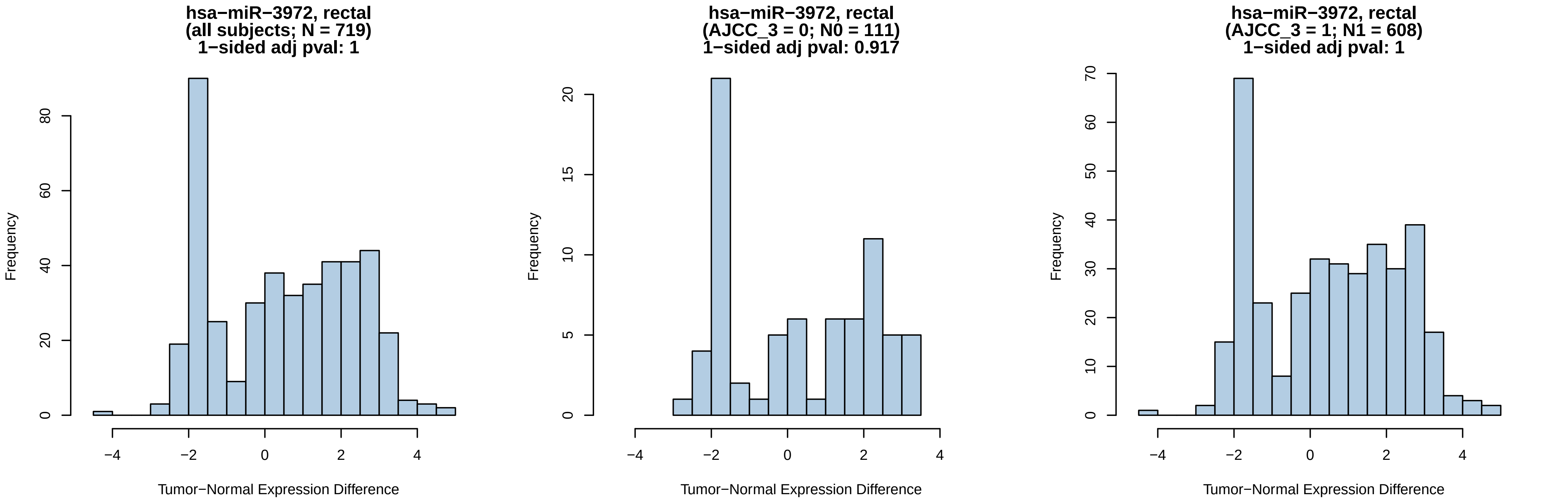
hsa−miR−3972, rectal
(all subjects; N = 719)
1−sided adj pval: 1
Frequency
Tumor−Normal Expression Difference
hsa−miR−3972, rectal
(AJCC_3 = 0; N0 = 111)
1−sided adj pval: 0.917
Frequency
Tumor−Normal Expression Difference
hsa−miR−3972, rectal
(AJCC_3 = 1; N1 = 608)
1−sided adj pval: 1
Frequency
Tumor−Normal Expression Difference

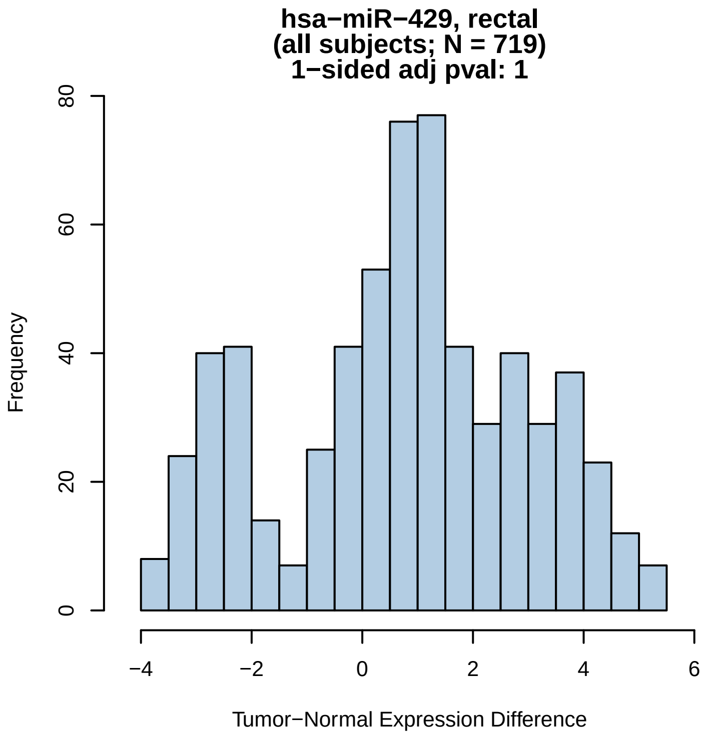
hsa−miR−429, rectal
(all subjects; N = 719)
1−sided adj pval: 1
Frequency
Tumor−Normal Expression Difference
0
20
40
60
80
−4
−2
0
2
4
6

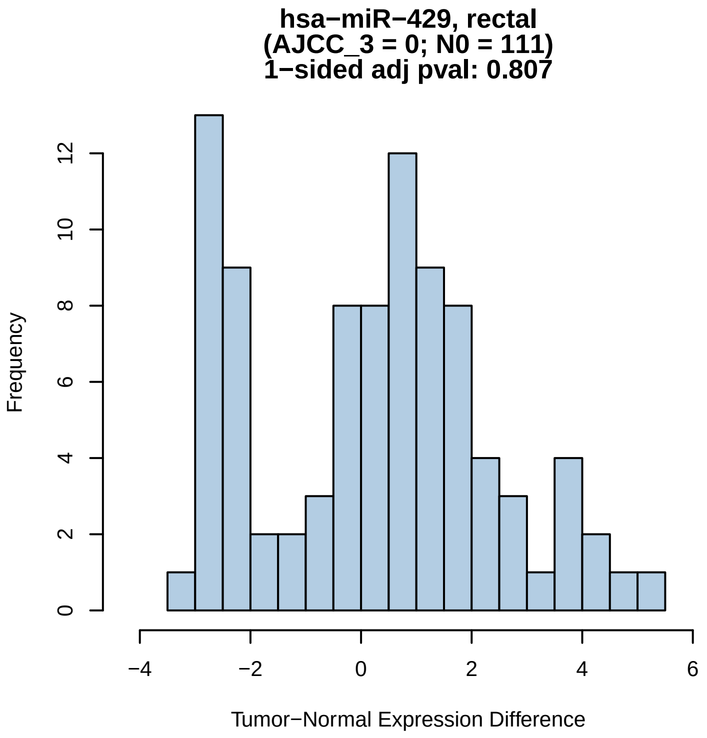
hsa−miR−429, rectal
(AJCC_3 = 0; N0 = 111)
1−sided adj pval: 0.807
Frequency
Tumor−Normal Expression Difference
0
2
4
6
8
10
12
−4
−2
0
2
4
6

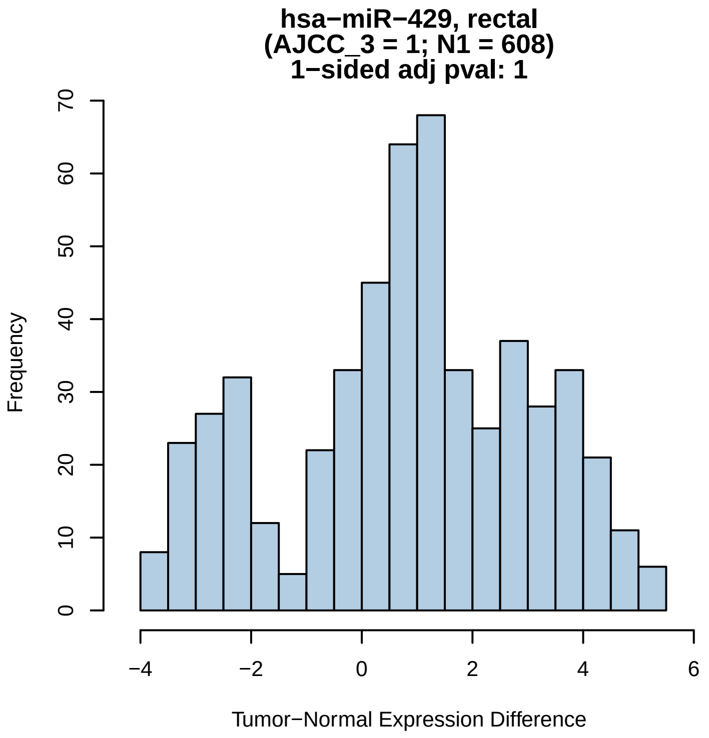
hsa−miR−429, rectal
(AJCC_3 = 1; N1 = 608)
1−sided adj pval: 1
Frequency
Tumor−Normal Expression Difference
0
10
20
30
40
50
60
70
−4
−2
0
2
4
6

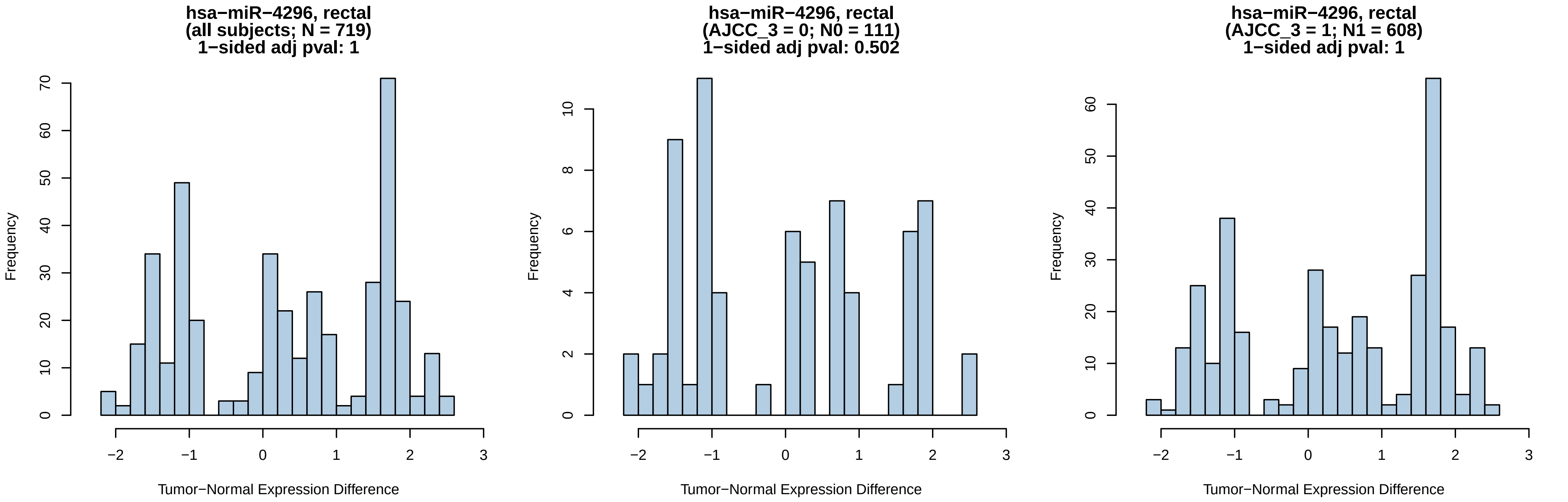
hsa−miR−4296, rectal
(all subjects; N = 719)
1−sided adj pval: 1
hsa−miR−4296, rectal
(AJCC_3 = 0; N0 = 111)
1−sided adj pval: 0.502
hsa−miR−4296, rectal
(AJCC_3 = 1; N1 = 608)
1−sided adj pval: 1
Frequency
Tumor−Normal Expression Difference

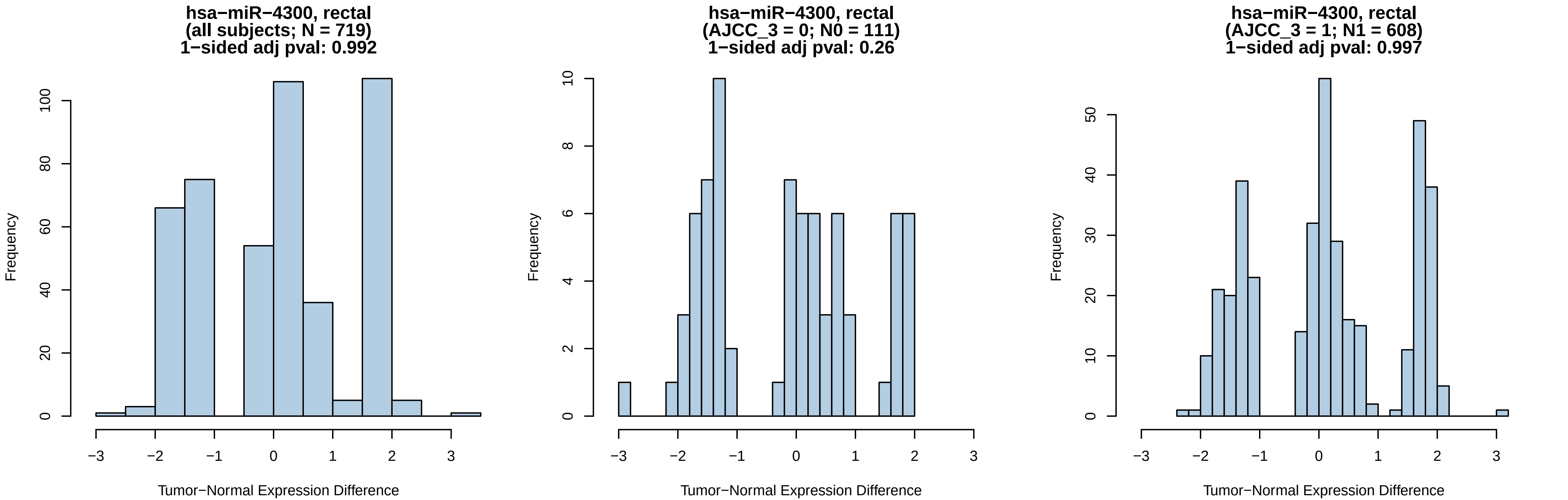

hsa−miR−4300, rectal
(all subjects; N = 719)
1−sided adj pval: 0.992
hsa−miR−4300, rectal
(AJCC_3 = 0; N0 = 111)
1−sided adj pval: 0.26
hsa−miR−4300, rectal
(AJCC_3 = 1; N1 = 608)
1−sided adj pval: 0.997
Frequency
Tumor−Normal Expression Difference

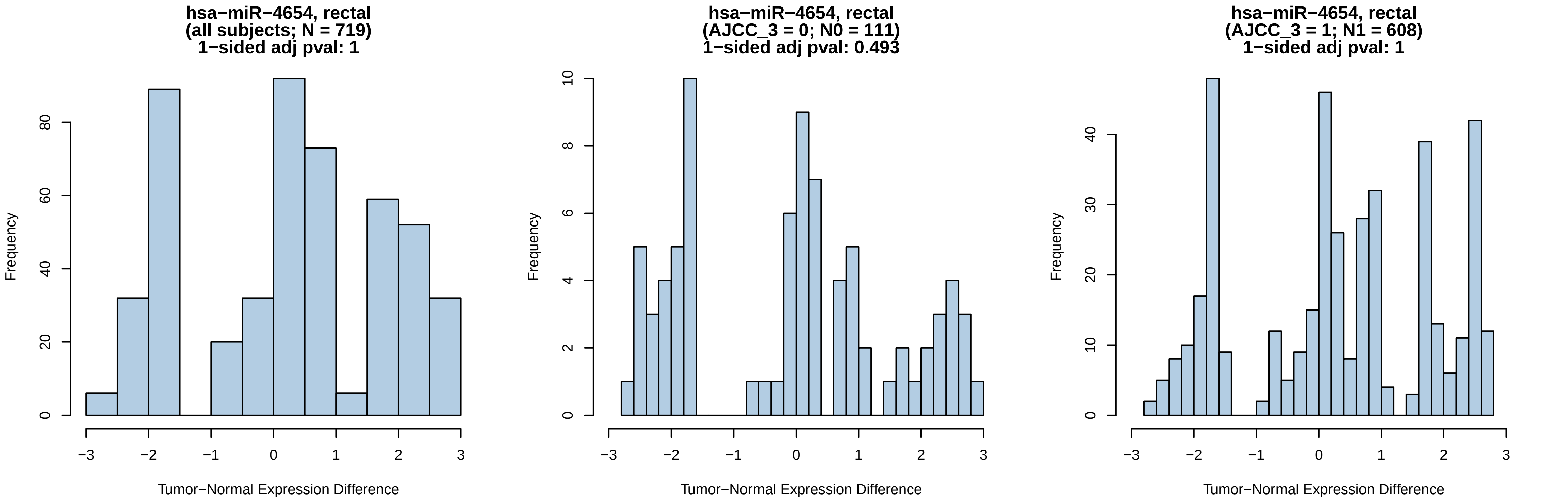

hsa−miR−4654, rectal
(all subjects; N = 719)
1−sided adj pval: 1
hsa−miR−4654, rectal
(AJCC_3 = 0; N0 = 111)
1−sided adj pval: 0.493
hsa−miR−4654, rectal
(AJCC_3 = 1; N1 = 608)
1−sided adj pval: 1
Frequency
Tumor−Normal Expression Difference

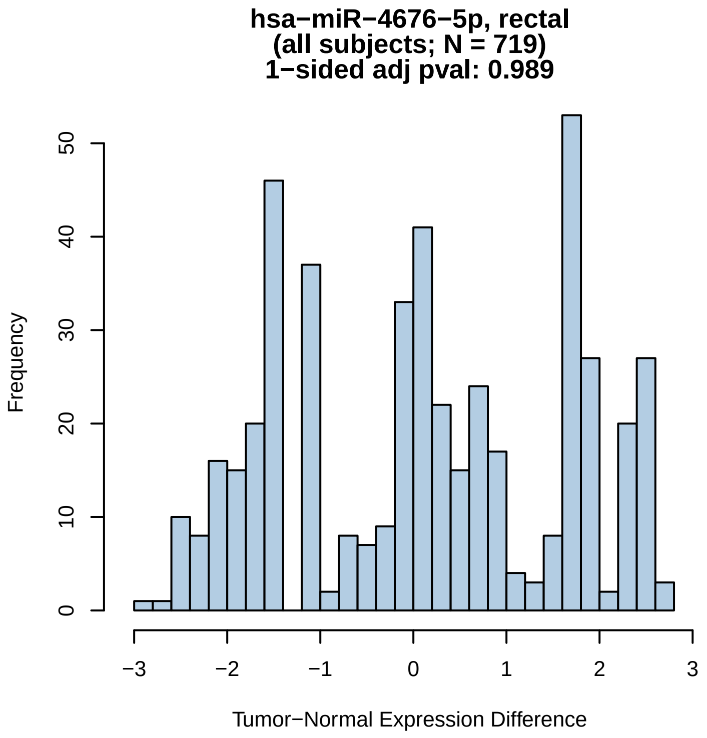
hsa−miR−4676−5p, rectal
(all subjects; N = 719)
1−sided adj pval: 0.989
Frequency
Tumor−Normal Expression Difference

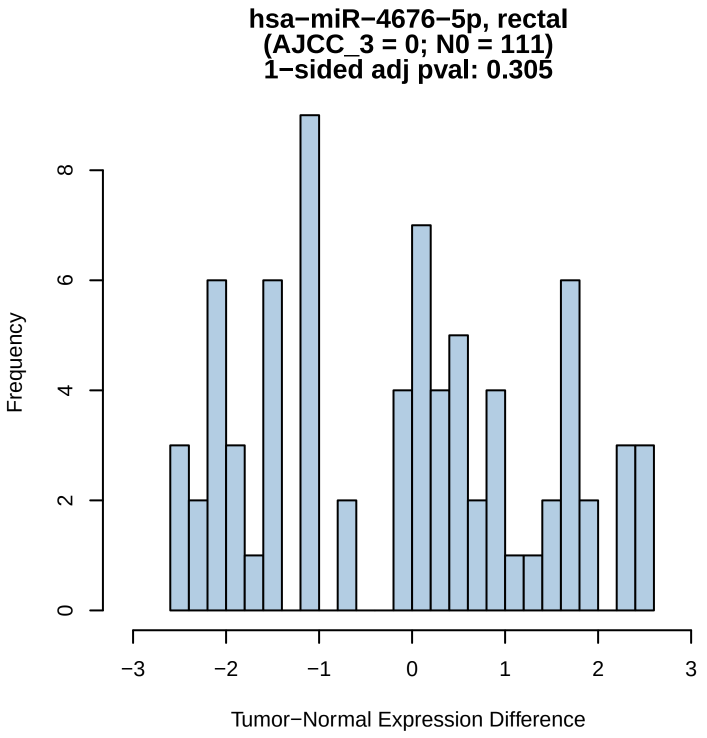
hsa−miR−4676−5p, rectal
(AJCC_3 = 0; N0 = 111)
1−sided adj pval: 0.305
Frequency
Tumor−Normal Expression Difference

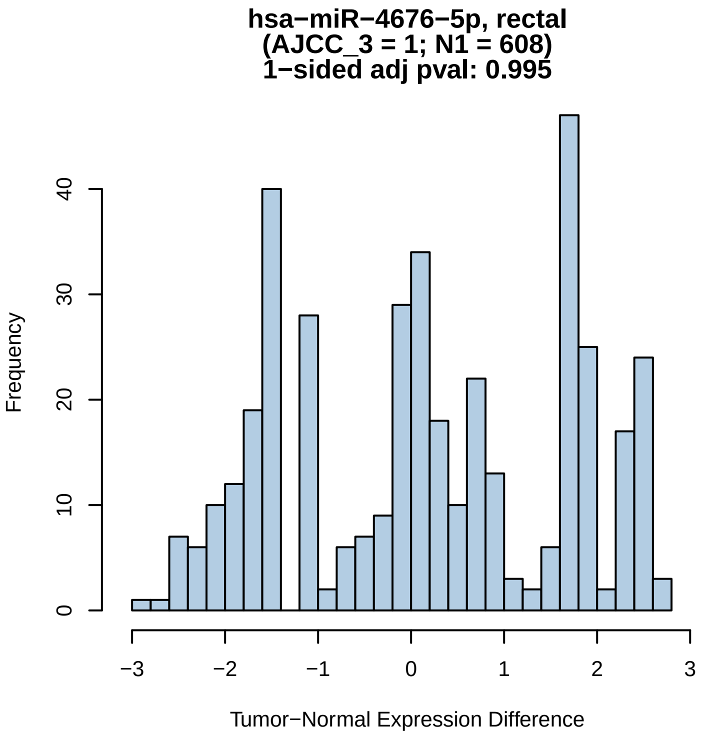
hsa−miR−4676−5p, rectal
(AJCC_3 = 1; N1 = 608)
1−sided adj pval: 0.995
Frequency
Tumor−Normal Expression Difference

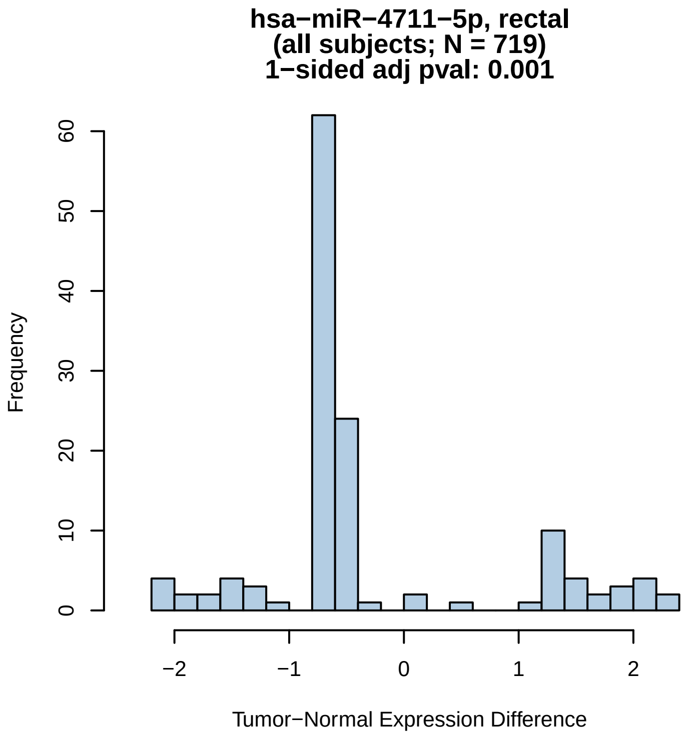

hsa−miR−4711−5p, rectal
(all subjects; N = 719)
1−sided adj pval: 0.001
Frequency
0
10
20
30
40
50
60
−2
−1
0
1
2
Tumor−Normal Expression Difference


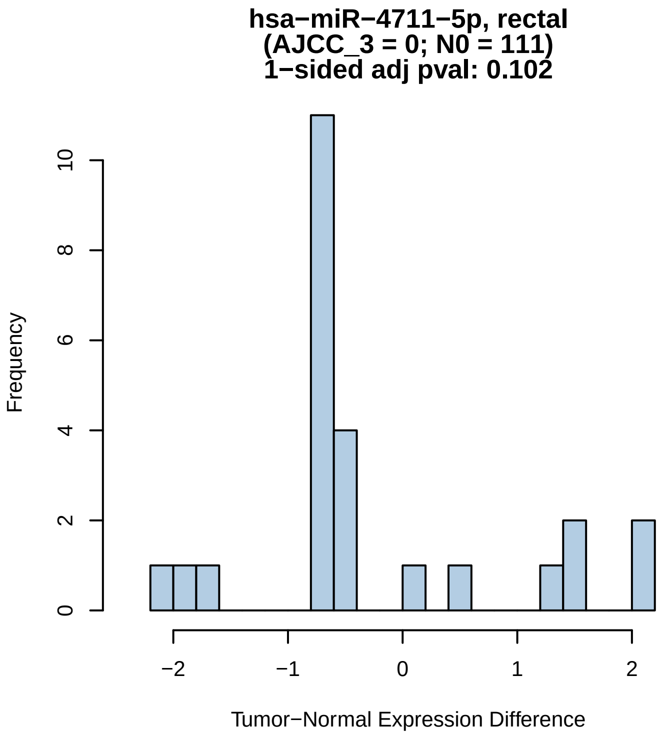

hsa−miR−4711−5p, rectal
(AJCC_3 = 0; N0 = 111)
1−sided adj pval: 0.102
Frequency
0
2
4
6
8
10
−2
−1
0
1
2
Tumor−Normal Expression Difference


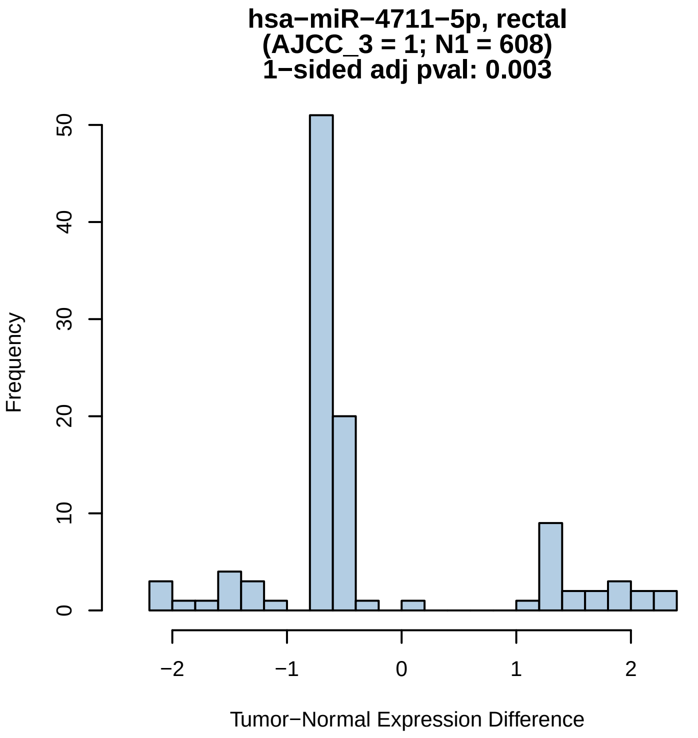

hsa−miR−4711−5p, rectal
(AJCC_3 = 1; N1 = 608)
1−sided adj pval: 0.003
Frequency
0
10
20
30
40
50
−2
−1
0
1
2
Tumor−Normal Expression Difference

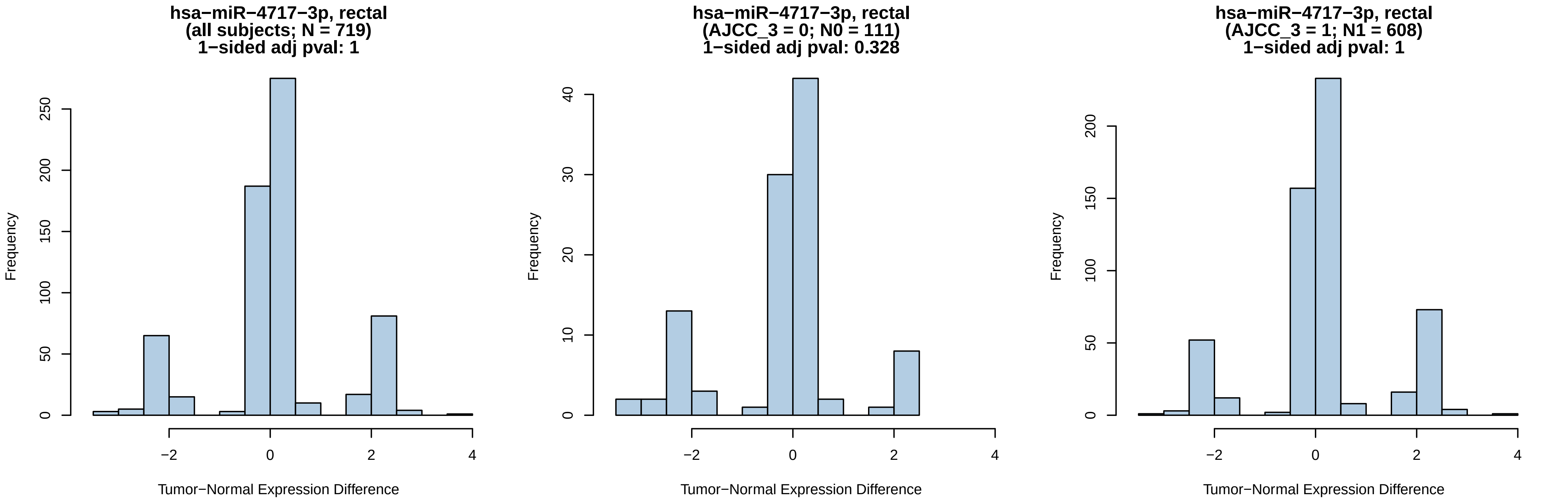
hsa−miR−4717−3p, rectal
(all subjects; N = 719)
1−sided adj pval: 1
Frequency
0
50
100
150
200
250
−2
0
2
4
Tumor−Normal Expression Difference
hsa−miR−4717−3p, rectal
(AJCC_3 = 0; N0 = 111)
1−sided adj pval: 0.328
Frequency
0
10
20
30
40
−2
0
2
4
Tumor−Normal Expression Difference
hsa−miR−4717−3p, rectal
(AJCC_3 = 1; N1 = 608)
1−sided adj pval: 1
Frequency
0
50
100
150
200
−2
0
2
4
Tumor−Normal Expression Difference

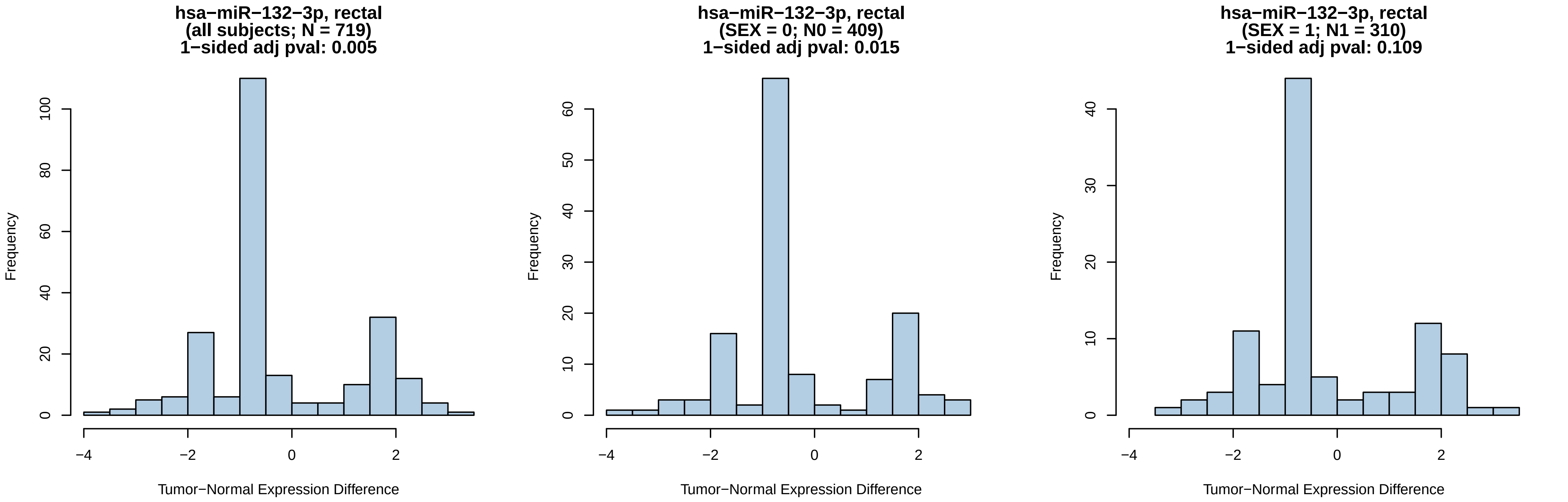

hsa−miR−132−3p, rectal
(all subjects; N = 719)
1−sided adj pval: 0.005
Frequency
0
20
40
60
80
100
−4
−2
0
2
Tumor−Normal Expression Difference
hsa−miR−132−3p, rectal
(SEX = 0; N0 = 409)
1−sided adj pval: 0.015
Frequency
0
10
20
30
40
50
60
−4
−2
0
2
Tumor−Normal Expression Difference
hsa−miR−132−3p, rectal
(SEX = 1; N1 = 310)
1−sided adj pval: 0.109
Frequency
0
10
20
30
40
−4
−2
0
2
Tumor−Normal Expression Difference

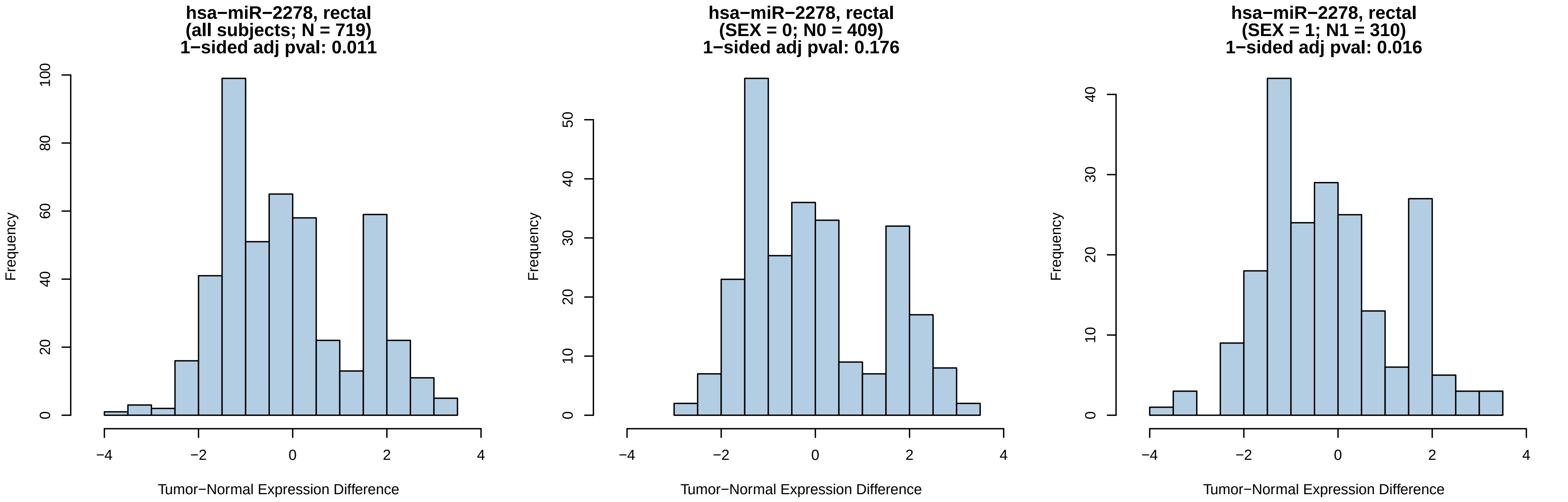
hsa−miR−2278, rectal
(all subjects; N = 719)
1−sided adj pval: 0.011
Frequency
Tumor−Normal Expression Difference
hsa−miR−2278, rectal
(SEX = 0; N0 = 409)
1−sided adj pval: 0.176
Frequency
Tumor−Normal Expression Difference
hsa−miR−2278, rectal
(SEX = 1; N1 = 310)
1−sided adj pval: 0.016
Frequency
Tumor−Normal Expression Difference

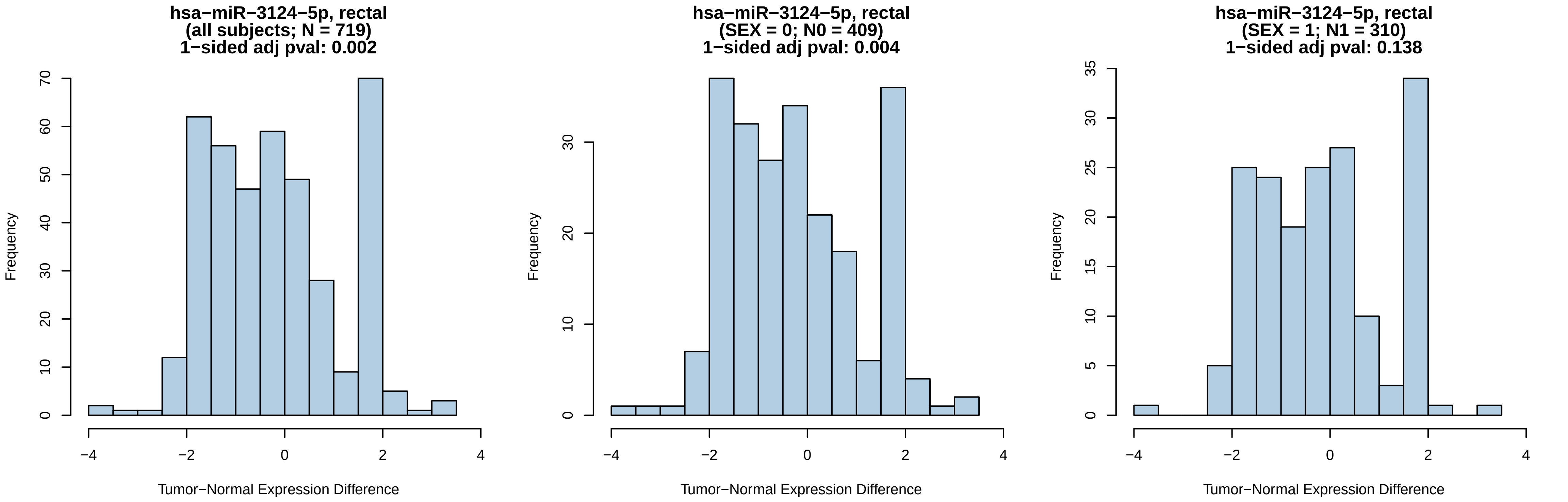
hsa−miR−3124−5p, rectal
(all subjects; N = 719)
1−sided adj pval: 0.002
Frequency
Tumor−Normal Expression Difference
hsa−miR−3124−5p, rectal
(SEX = 0; N0 = 409)
1−sided adj pval: 0.004
Frequency
Tumor−Normal Expression Difference
hsa−miR−3124−5p, rectal
(SEX = 1; N1 = 310)
1−sided adj pval: 0.138
Frequency
Tumor−Normal Expression Difference

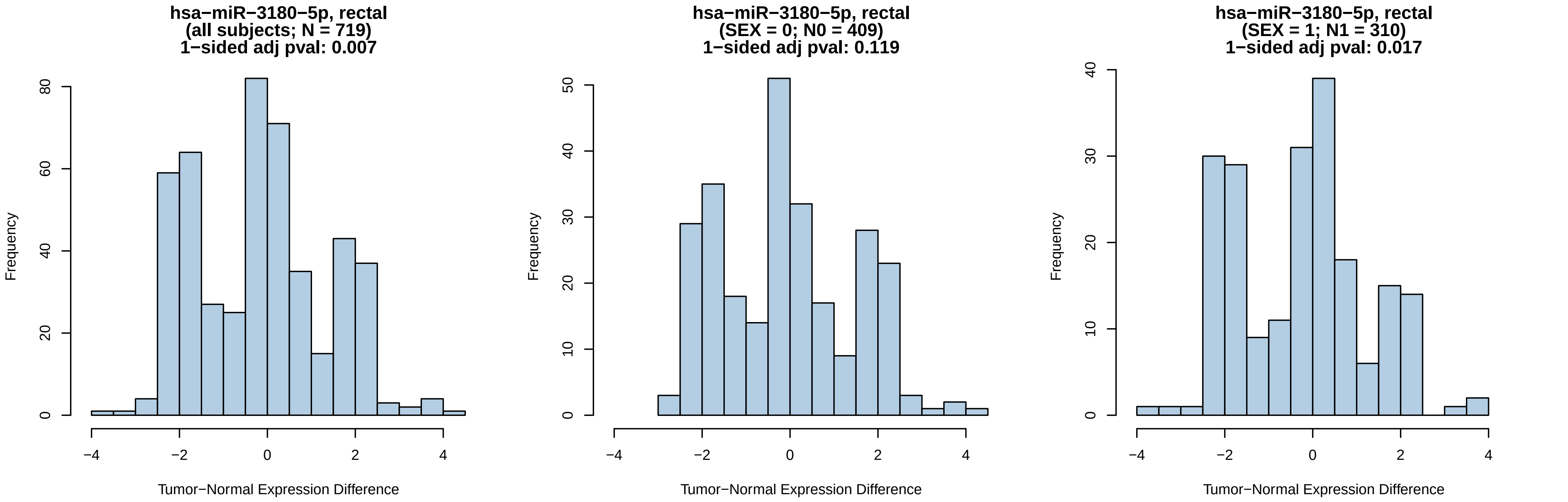

hsa−miR−3180−5p, rectal
(all subjects; N = 719)
1−sided adj pval: 0.007
Frequency
0
20
40
60
80
−4
−2
0
2
4
Tumor−Normal Expression Difference
hsa−miR−3180−5p, rectal
(SEX = 0; N0 = 409)
1−sided adj pval: 0.119
Frequency
0
10
20
30
40
50
−4
−2
0
2
4
Tumor−Normal Expression Difference
hsa−miR−3180−5p, rectal
(SEX = 1; N1 = 310)
1−sided adj pval: 0.017
Frequency
0
10
20
30
40
−4
−2
0
2
4
Tumor−Normal Expression Difference

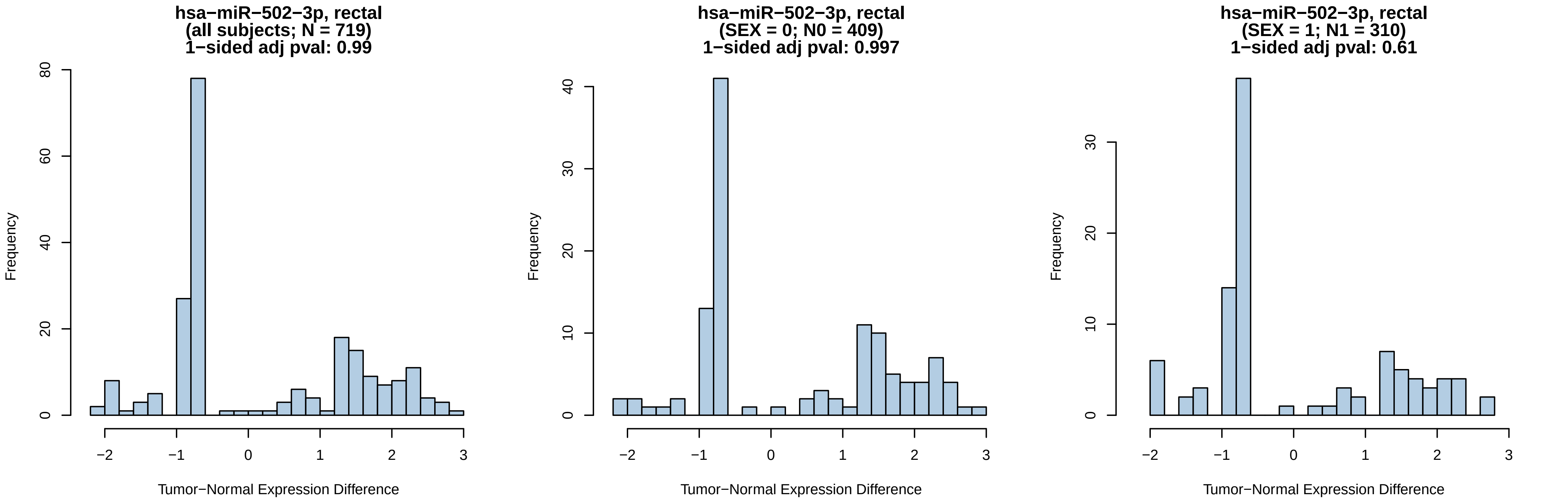
hsa−miR−502−3p, rectal
(all subjects; N = 719)
1−sided adj pval: 0.99
Frequency
Tumor−Normal Expression Difference
hsa−miR−502−3p, rectal
(SEX = 0; N0 = 409)
1−sided adj pval: 0.997
Frequency
Tumor−Normal Expression Difference
hsa−miR−502−3p, rectal
(SEX = 1; N1 = 310)
1−sided adj pval: 0.61
Frequency
Tumor−Normal Expression Difference

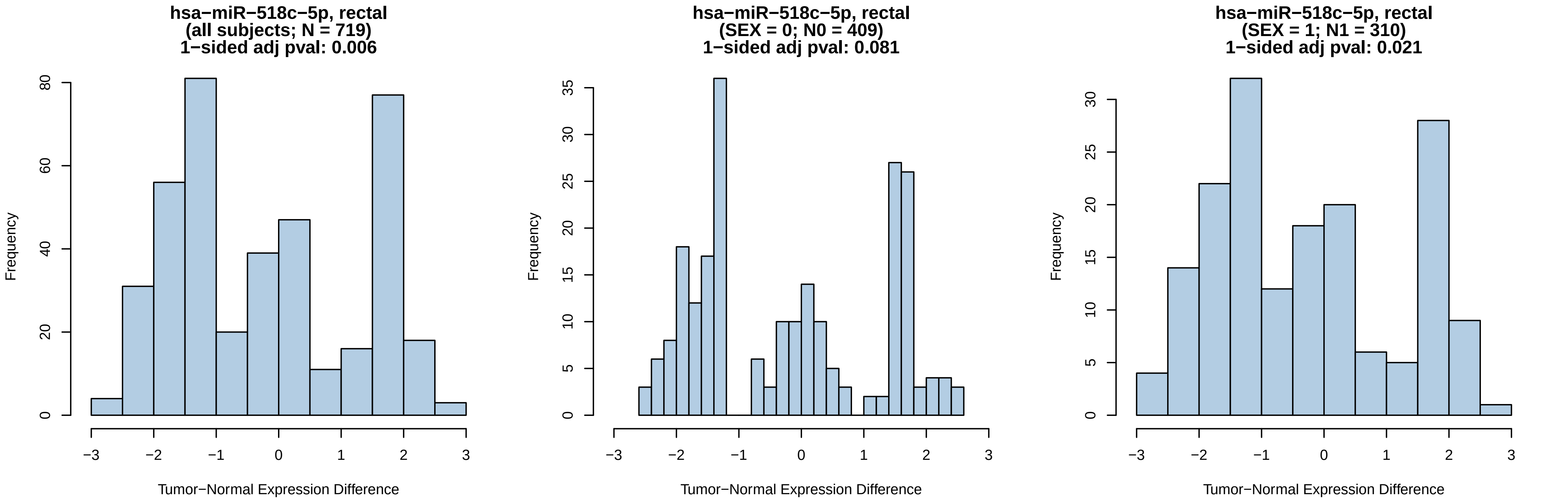
hsa−miR−518c−5p, rectal
(all subjects; N = 719)
1−sided adj pval: 0.006
hsa−miR−518c−5p, rectal
(SEX = 0; N0 = 409)
1−sided adj pval: 0.081
hsa−miR−518c−5p, rectal
(SEX = 1; N1 = 310)
1−sided adj pval: 0.021
Frequency
Tumor−Normal Expression Difference

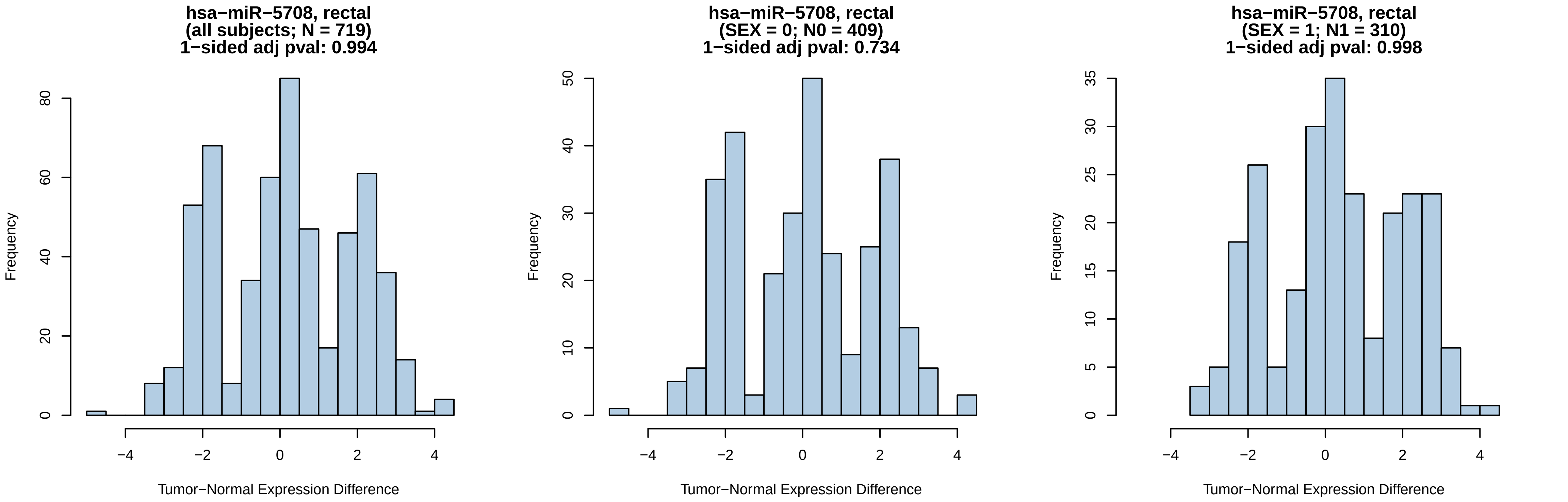
hsa−miR−5708, rectal
(all subjects; N = 719)
1−sided adj pval: 0.994
hsa−miR−5708, rectal
(SEX = 0; N0 = 409)
1−sided adj pval: 0.734
hsa−miR−5708, rectal
(SEX = 1; N1 = 310)
1−sided adj pval: 0.998
Frequency
Tumor−Normal Expression Difference
0
20
40
60
80
0
10
20
30
40
50
0
5
10
15
20
25
30
35
−4
−2
0
2
4

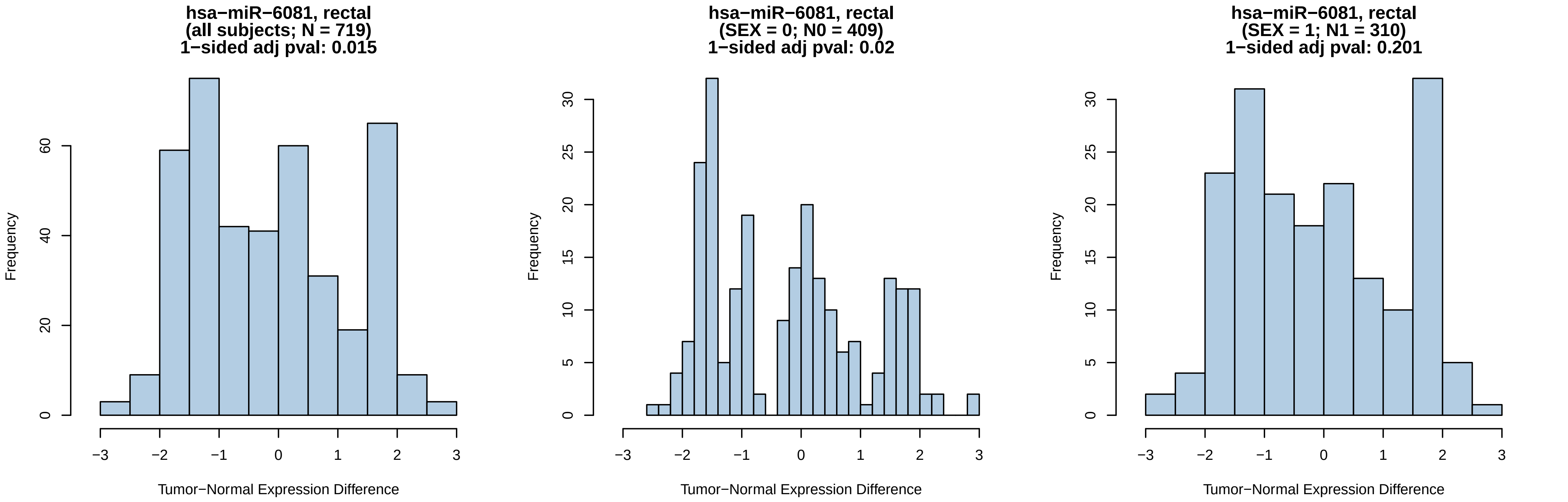

hsa−miR−6081, rectal
(all subjects; N = 719)
1−sided adj pval: 0.015
Frequency
0
20
40
60
−3
−2
−1
0
1
2
3
Tumor−Normal Expression Difference
hsa−miR−6081, rectal
(SEX = 0; N0 = 409)
1−sided adj pval: 0.02
Frequency
0
5
10
15
20
25
30
−3
−2
−1
0
1
2
3
Tumor−Normal Expression Difference
hsa−miR−6081, rectal
(SEX = 1; N1 = 310)
1−sided adj pval: 0.201
Frequency
0
5
10
15
20
25
30
−3
−2
−1
0
1
2
3
Tumor−Normal Expression Difference

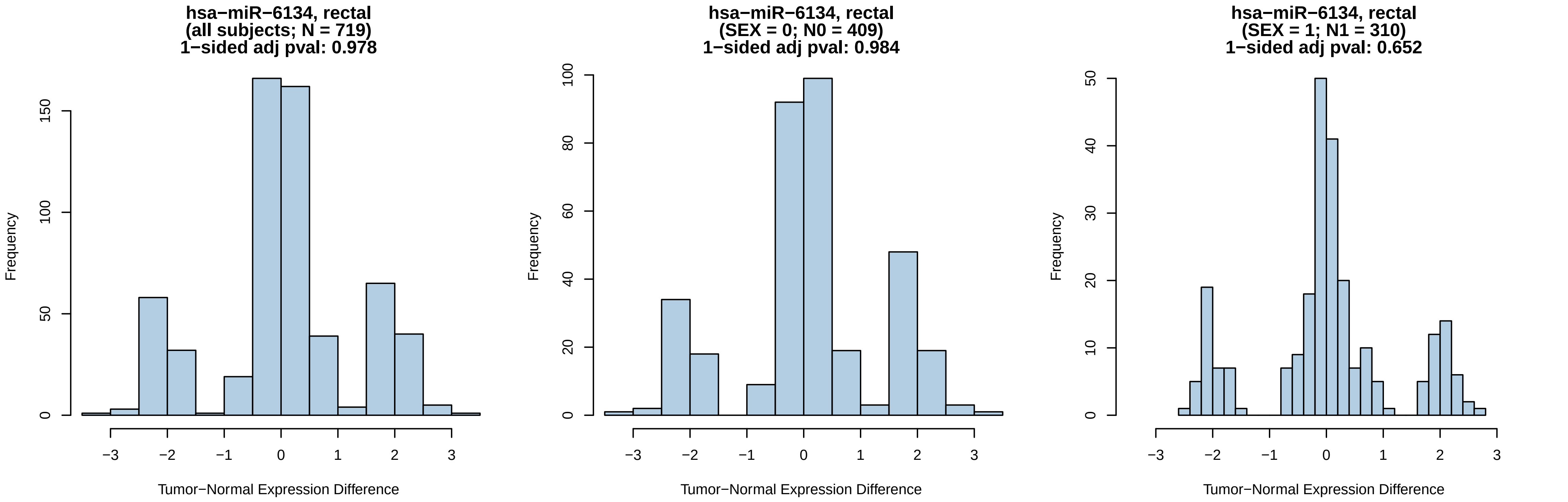

hsa−miR−6134, rectal
(all subjects; N = 719)
1−sided adj pval: 0.978
Frequency
0
50
100
150
−3
−2
−1
0
1
2
3
Tumor−Normal Expression Difference
hsa−miR−6134, rectal
(SEX = 0; N0 = 409)
1−sided adj pval: 0.984
Frequency
0
20
40
60
80
100
−3
−2
−1
0
1
2
3
Tumor−Normal Expression Difference
hsa−miR−6134, rectal
(SEX = 1; N1 = 310)
1−sided adj pval: 0.652
Frequency
0
10
20
30
40
50
−3
−2
−1
0
1
2
3
Tumor−Normal Expression Difference

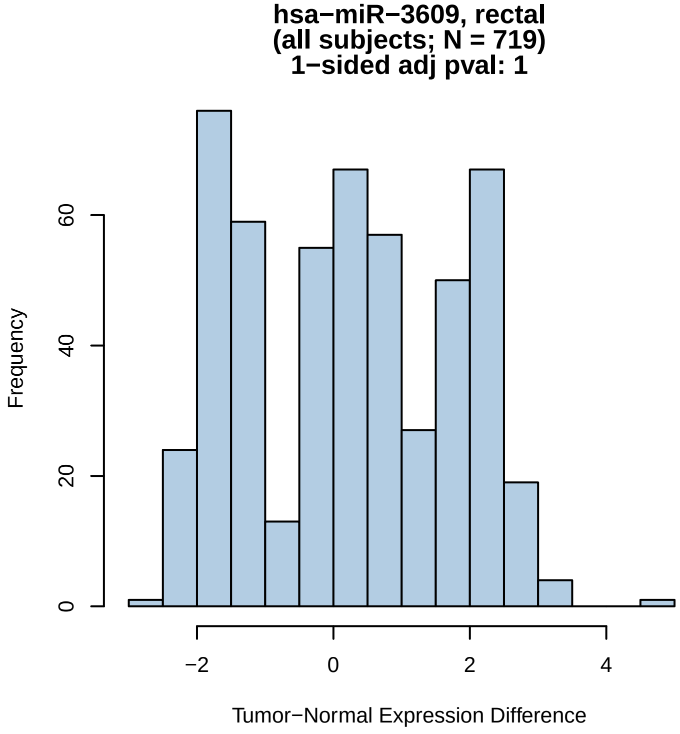

hsa−miR−3609, rectal
(all subjects; N = 719)
1−sided adj pval: 1
Frequency
Tumor−Normal Expression Difference


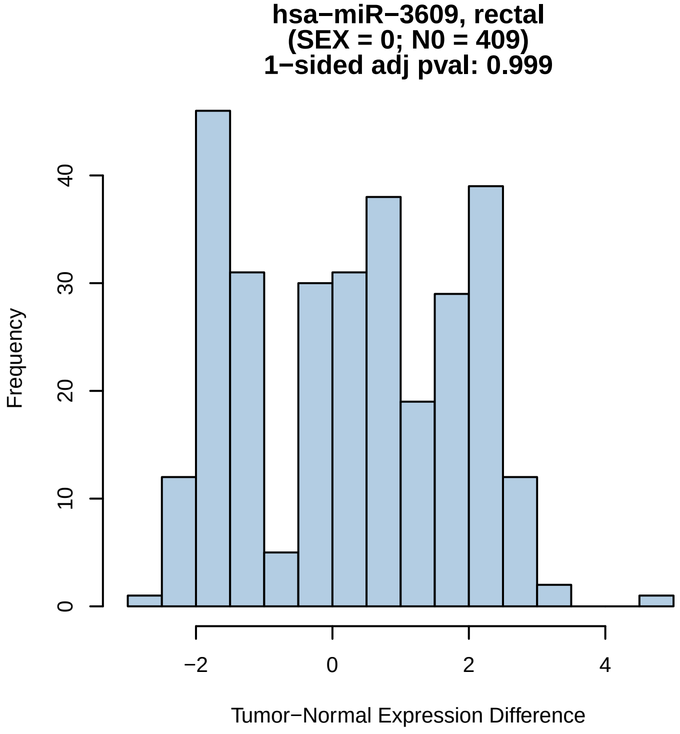

hsa−miR−3609, rectal
(SEX = 0; N0 = 409)
1−sided adj pval: 0.999
Frequency
Tumor−Normal Expression Difference


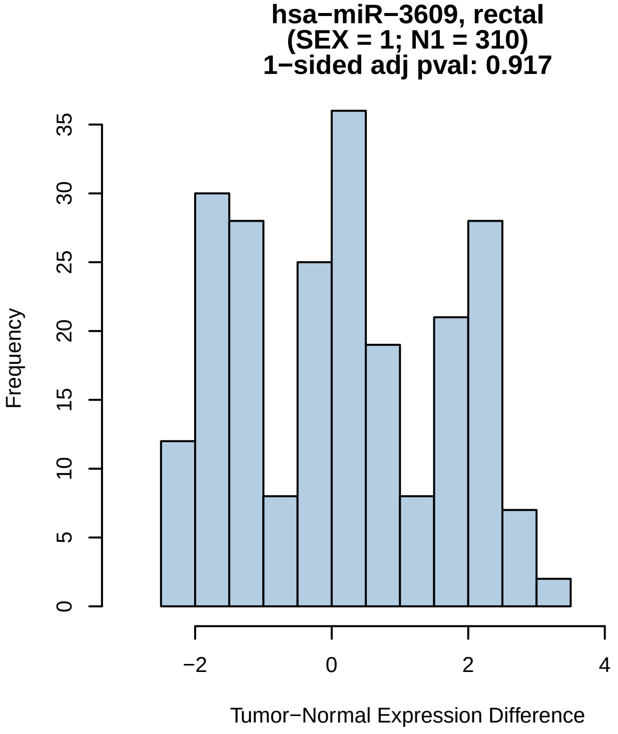

hsa−miR−3609, rectal
(SEX = 1; N1 = 310)
1−sided adj pval: 0.917
Frequency
Tumor−Normal Expression Difference

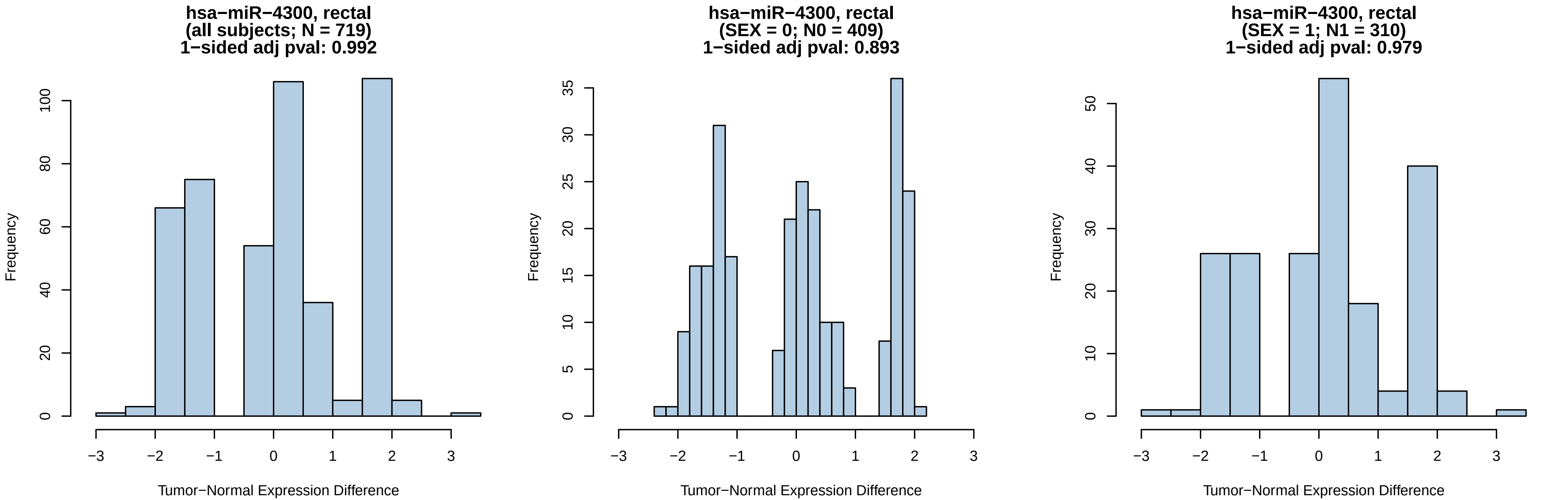

hsa−miR−4300, rectal
(all subjects; N = 719)
1−sided adj pval: 0.992
Frequency
Tumor−Normal Expression Difference
hsa−miR−4300, rectal
(SEX = 0; N0 = 409)
1−sided adj pval: 0.893
Frequency
Tumor−Normal Expression Difference
hsa−miR−4300, rectal
(SEX = 1; N1 = 310)
1−sided adj pval: 0.979
Frequency
Tumor−Normal Expression Difference

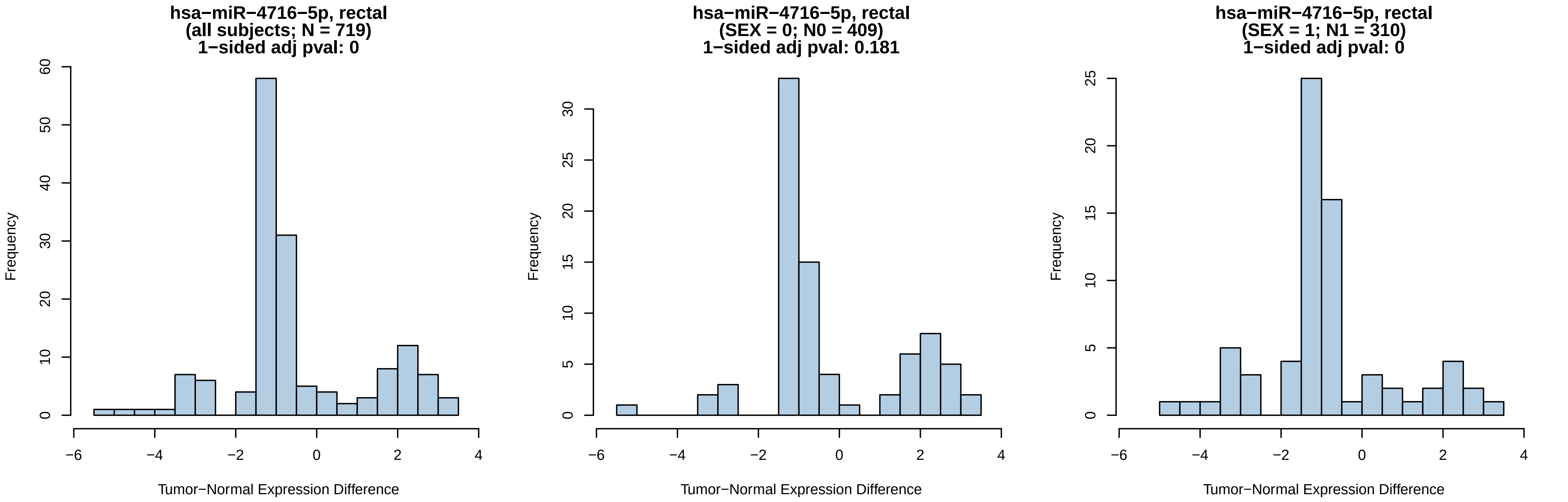
hsa−miR−4716−5p, rectal
(all subjects; N = 719)
1−sided adj pval: 0
Frequency
Tumor−Normal Expression Difference
hsa−miR−4716−5p, rectal
(SEX = 0; N0 = 409)
1−sided adj pval: 0.181
Frequency
Tumor−Normal Expression Difference
hsa−miR−4716−5p, rectal
(SEX = 1; N1 = 310)
1−sided adj pval: 0
Frequency
Tumor−Normal Expression Difference

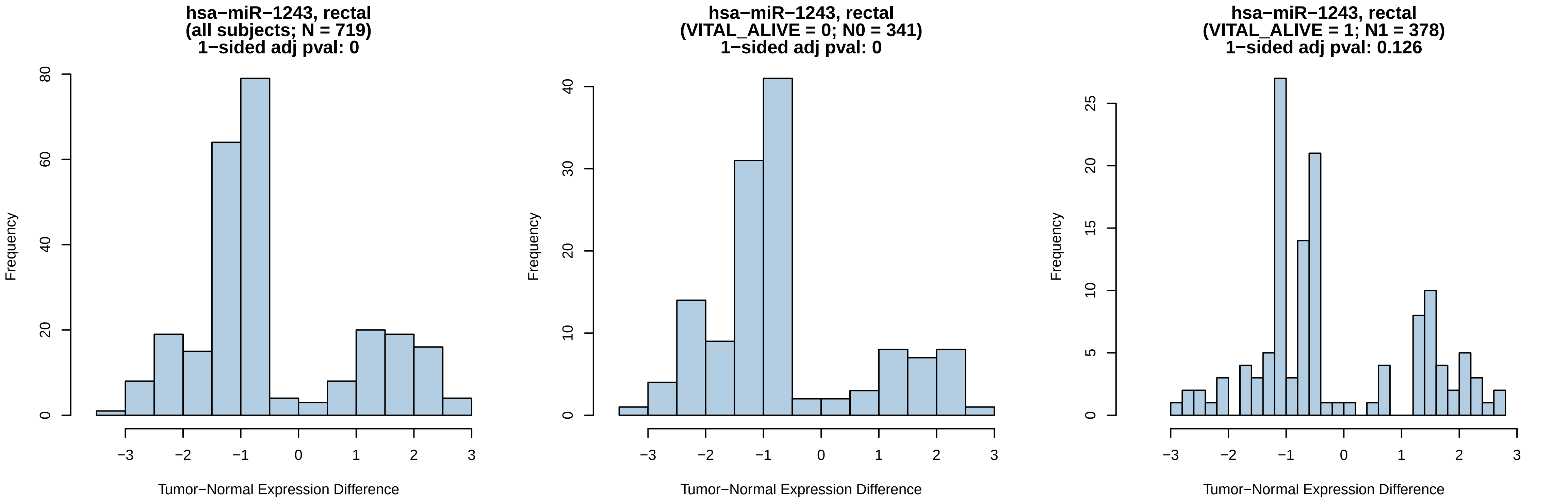
hsa−miR−1243, rectal
(all subjects; N = 719)
1−sided adj pval: 0
Frequency
Tumor−Normal Expression Difference
hsa−miR−1243, rectal
(VITAL_ALIVE = 0; N0 = 341)
1−sided adj pval: 0
Frequency
Tumor−Normal Expression Difference
hsa−miR−1243, rectal
(VITAL_ALIVE = 1; N1 = 378)
1−sided adj pval: 0.126
Frequency
Tumor−Normal Expression Difference

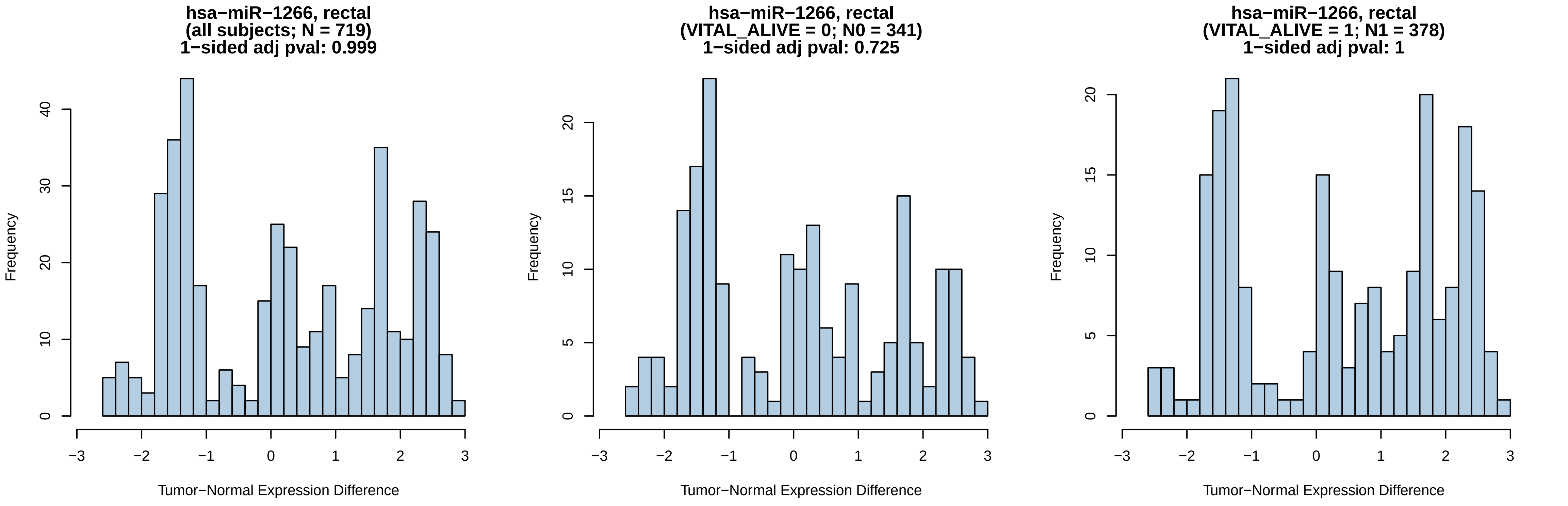

hsa−miR−1266, rectal
(all subjects; N = 719)
1−sided adj pval: 0.999
Frequency
Tumor−Normal Expression Difference
hsa−miR−1266, rectal
(VITAL_ALIVE = 0; N0 = 341)
1−sided adj pval: 0.725
Frequency
Tumor−Normal Expression Difference
hsa−miR−1266, rectal
(VITAL_ALIVE = 1; N1 = 378)
1−sided adj pval: 1
Frequency
Tumor−Normal Expression Difference

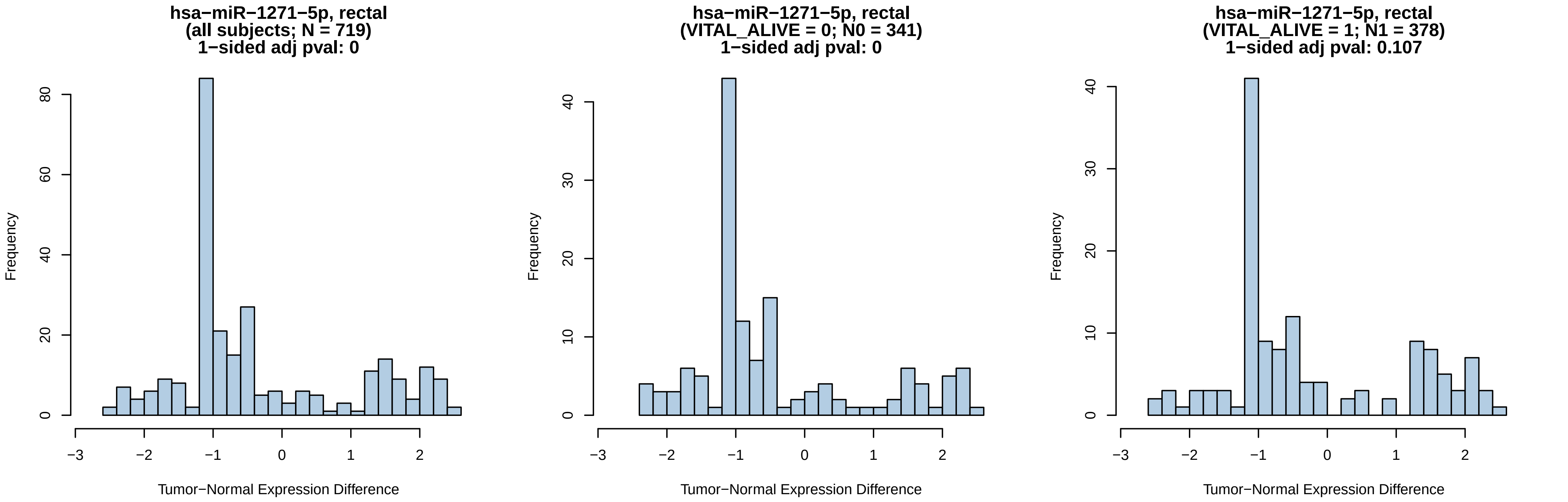
hsa−miR−1271−5p, rectal
(all subjects; N = 719)
1−sided adj pval: 0
hsa−miR−1271−5p, rectal
(VITAL_ALIVE = 0; N0 = 341)
1−sided adj pval: 0
hsa−miR−1271−5p, rectal
(VITAL_ALIVE = 1; N1 = 378)
1−sided adj pval: 0.107
Frequency
Tumor−Normal Expression Difference
Frequency
Tumor−Normal Expression Difference
Frequency
Tumor−Normal Expression Difference

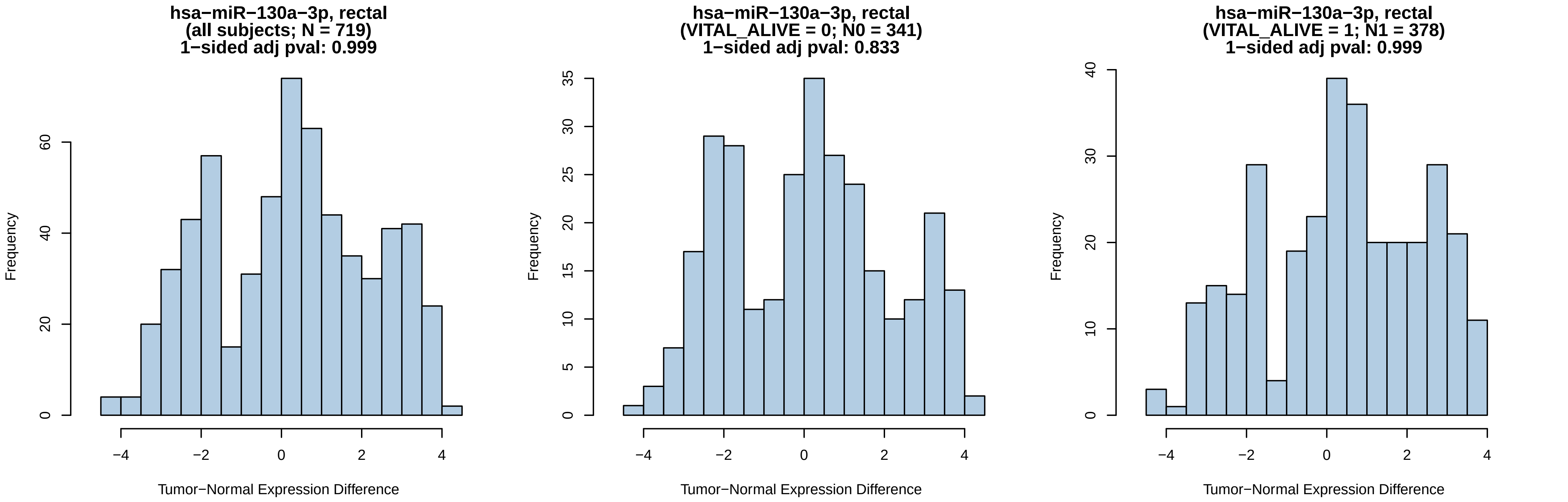
hsa−miR−130a−3p, rectal
(all subjects; N = 719)
1−sided adj pval: 0.999
Frequency
Tumor−Normal Expression Difference
hsa−miR−130a−3p, rectal
(VITAL_ALIVE = 0; N0 = 341)
1−sided adj pval: 0.833
Frequency
Tumor−Normal Expression Difference
hsa−miR−130a−3p, rectal
(VITAL_ALIVE = 1; N1 = 378)
1−sided adj pval: 0.999
Frequency
Tumor−Normal Expression Difference

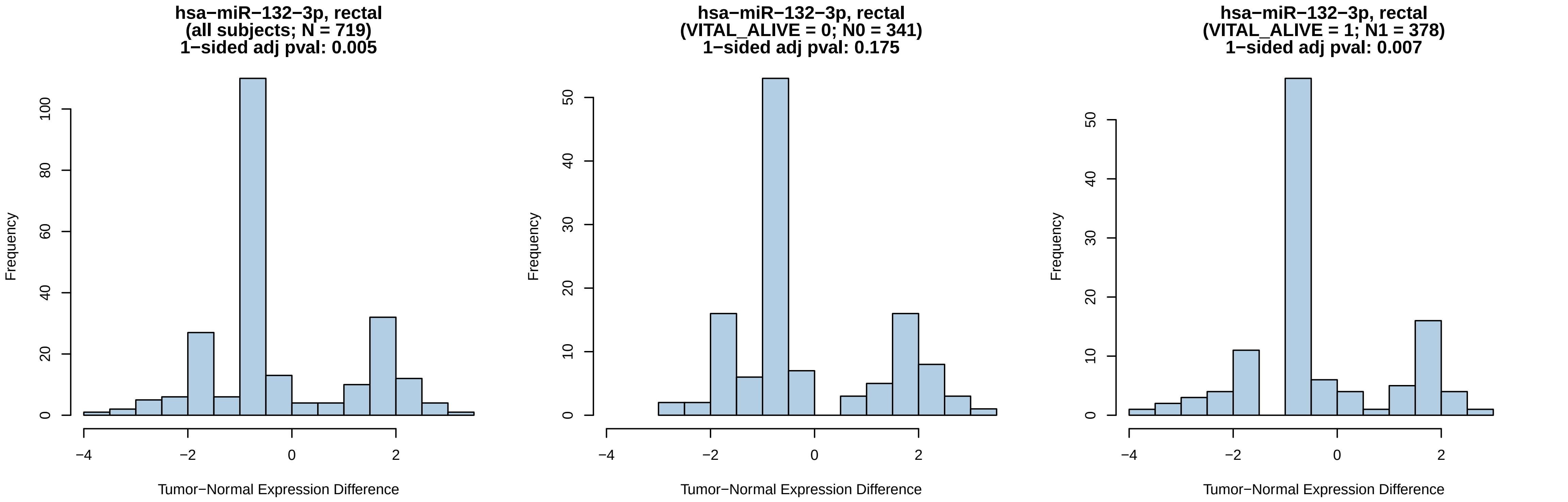
hsa−miR−132−3p, rectal
(all subjects; N = 719)
1−sided adj pval: 0.005
hsa−miR−132−3p, rectal
(VITAL_ALIVE = 0; N0 = 341)
1−sided adj pval: 0.175
hsa−miR−132−3p, rectal
(VITAL_ALIVE = 1; N1 = 378)
1−sided adj pval: 0.007
Frequency
Tumor−Normal Expression Difference
0
20
40
60
80
100
10
30
50
−4
−2
0
2

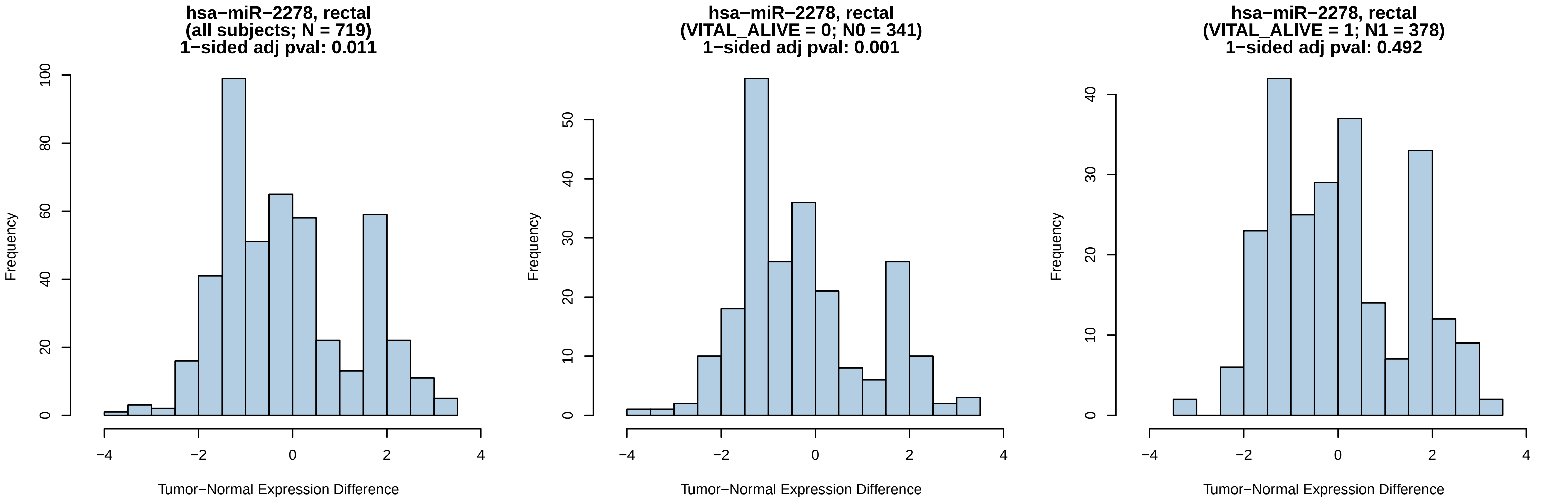

hsa−miR−2278, rectal
(all subjects; N = 719)
1−sided adj pval: 0.011
Frequency
Tumor−Normal Expression Difference
hsa−miR−2278, rectal
(VITAL_ALIVE = 0; N0 = 341)
1−sided adj pval: 0.001
Frequency
Tumor−Normal Expression Difference
hsa−miR−2278, rectal
(VITAL_ALIVE = 1; N1 = 378)
1−sided adj pval: 0.492
Frequency
Tumor−Normal Expression Difference

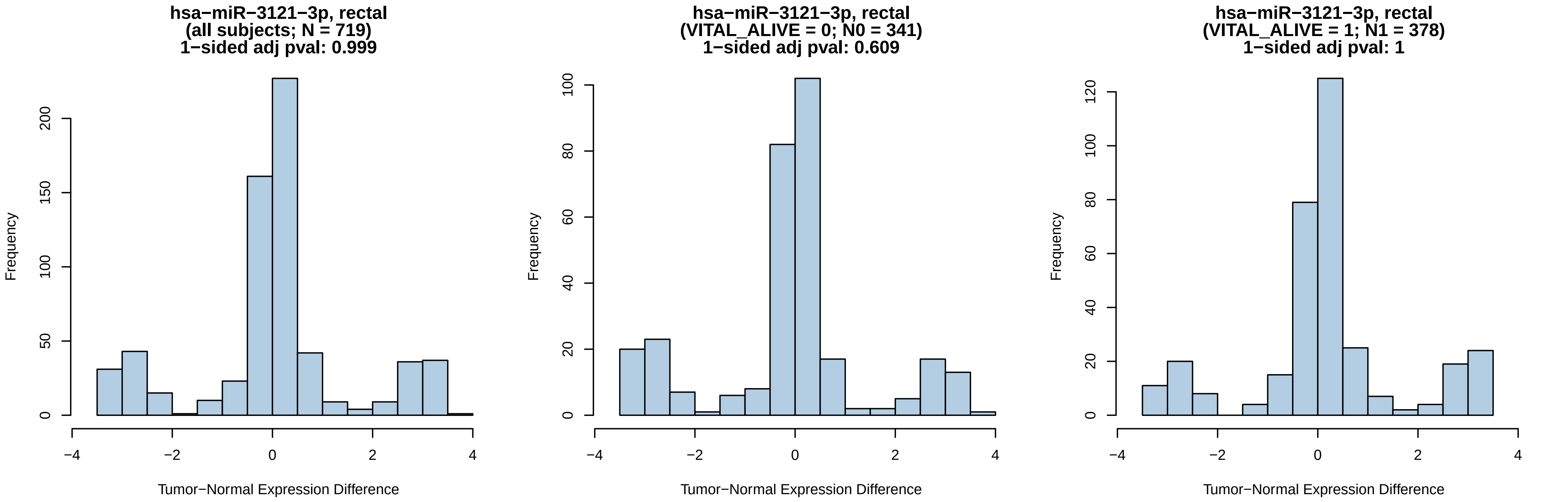

hsa−miR−3121−3p, rectal
(all subjects; N = 719)
1−sided adj pval: 0.999
Frequency
0
50
100
150
200
−4
−2
0
2
4
Tumor−Normal Expression Difference
hsa−miR−3121−3p, rectal
(VITAL_ALIVE = 0; N0 = 341)
1−sided adj pval: 0.609
Frequency
0
20
40
60
80
100
−4
−2
0
2
4
Tumor−Normal Expression Difference
hsa−miR−3121−3p, rectal
(VITAL_ALIVE = 1; N1 = 378)
1−sided adj pval: 1
Frequency
0
20
40
60
80
100
120
−4
−2
0
2
4
Tumor−Normal Expression Difference

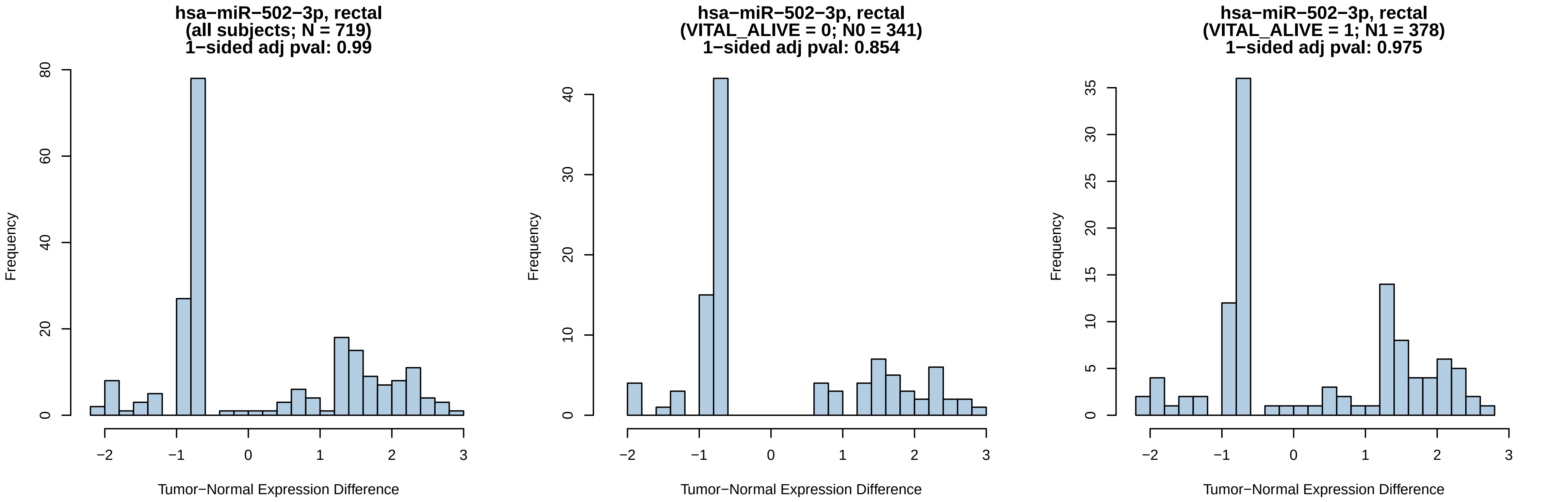

hsa−miR−502−3p, rectal
(all subjects; N = 719)
1−sided adj pval: 0.99
hsa−miR−502−3p, rectal
(VITAL_ALIVE = 0; N0 = 341)
1−sided adj pval: 0.854
hsa−miR−502−3p, rectal
(VITAL_ALIVE = 1; N1 = 378)
1−sided adj pval: 0.975
Frequency
Tumor−Normal Expression Difference

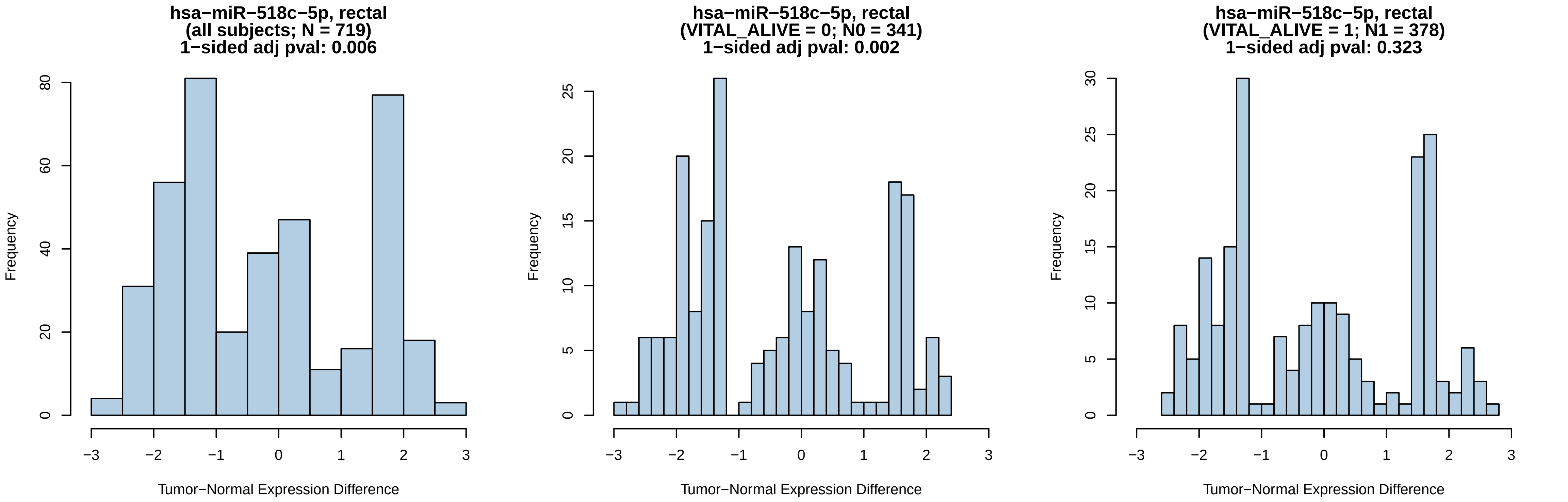
hsa−miR−518c−5p, rectal
(all subjects; N = 719)
1−sided adj pval: 0.006
Frequency
0
20
40
60
80
−3
−2
−1
0
1
2
3
Tumor−Normal Expression Difference
hsa−miR−518c−5p, rectal
(VITAL_ALIVE = 0; N0 = 341)
1−sided adj pval: 0.002
Frequency
0
5
10
15
20
25
−3
−2
−1
0
1
2
3
Tumor−Normal Expression Difference
hsa−miR−518c−5p, rectal
(VITAL_ALIVE = 1; N1 = 378)
1−sided adj pval: 0.323
Frequency
0
5
10
15
20
25
30
−3
−2
−1
0
1
2
3
Tumor−Normal Expression Difference

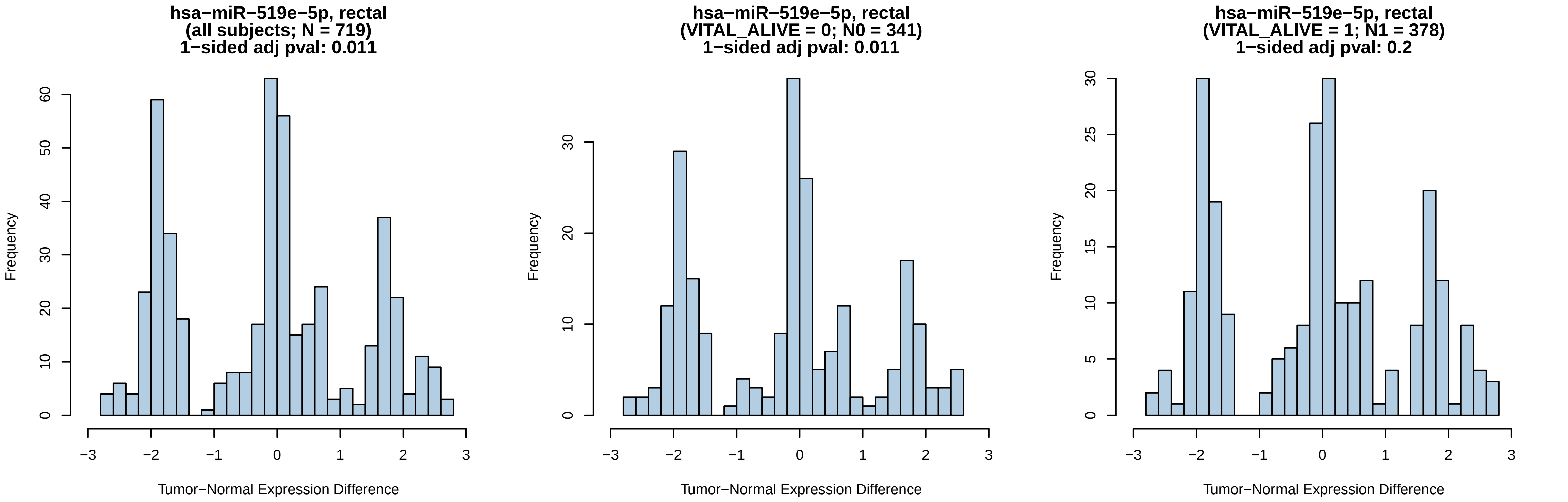

hsa−miR−519e−5p, rectal
(all subjects; N = 719)
1−sided adj pval: 0.011
Frequency
Tumor−Normal Expression Difference
hsa−miR−519e−5p, rectal
(VITAL_ALIVE = 0; N0 = 341)
1−sided adj pval: 0.011
Frequency
Tumor−Normal Expression Difference
hsa−miR−519e−5p, rectal
(VITAL_ALIVE = 1; N1 = 378)
1−sided adj pval: 0.2
Frequency
Tumor−Normal Expression Difference

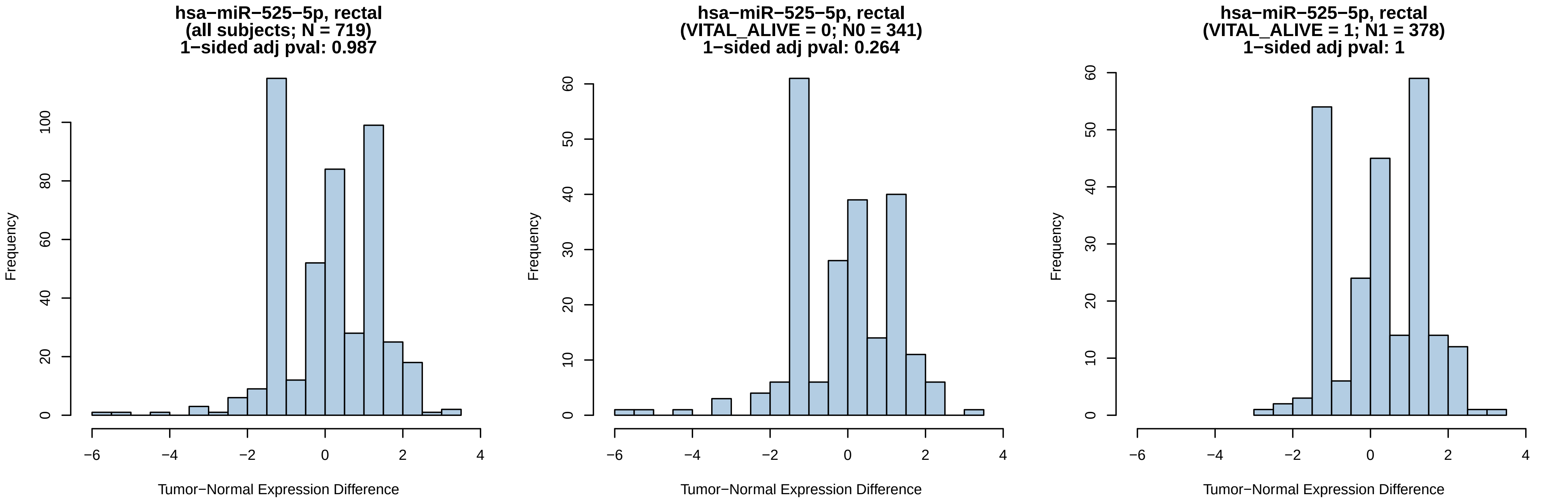
hsa−miR−525−5p, rectal
(all subjects; N = 719)
1−sided adj pval: 0.987
Frequency
Tumor−Normal Expression Difference
hsa−miR−525−5p, rectal
(VITAL_ALIVE = 0; N0 = 341)
1−sided adj pval: 0.264
Frequency
Tumor−Normal Expression Difference
hsa−miR−525−5p, rectal
(VITAL_ALIVE = 1; N1 = 378)
1−sided adj pval: 1
Frequency
Tumor−Normal Expression Difference

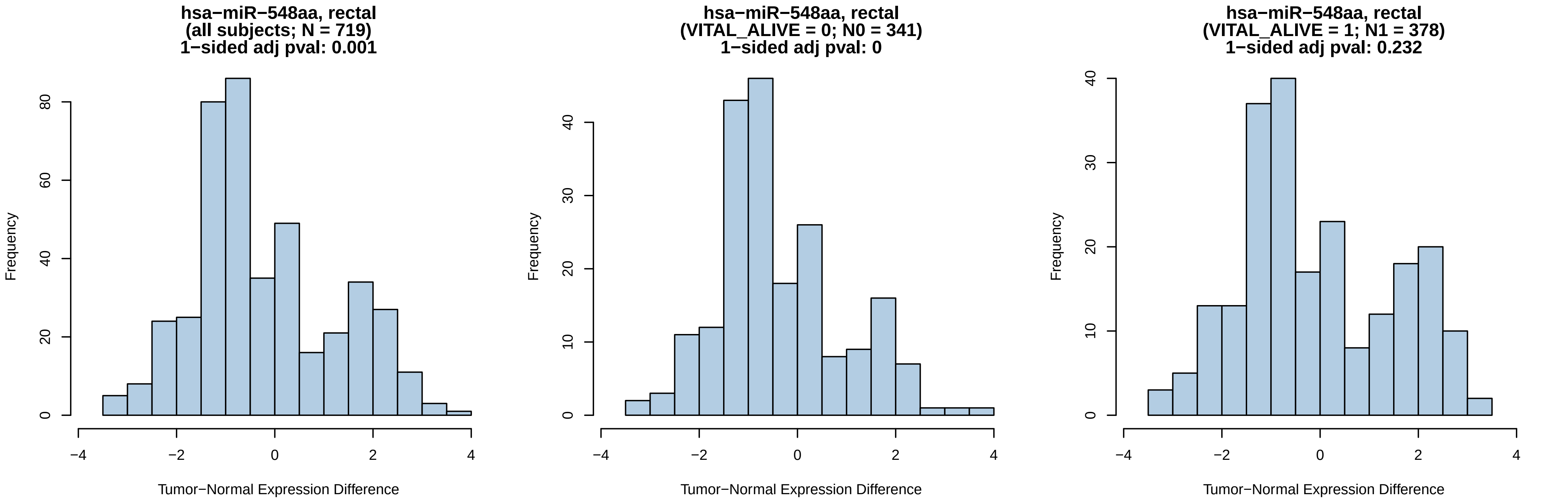
hsa−miR−548aa, rectal
(all subjects; N = 719)
1−sided adj pval: 0.001
Frequency
0
20
40
60
80
−4
−2
0
2
4
Tumor−Normal Expression Difference
hsa−miR−548aa, rectal
(VITAL_ALIVE = 0; N0 = 341)
1−sided adj pval: 0
Frequency
0
10
20
30
40
−4
−2
0
2
4
Tumor−Normal Expression Difference
hsa−miR−548aa, rectal
(VITAL_ALIVE = 1; N1 = 378)
1−sided adj pval: 0.232
Frequency
0
10
20
30
40
−4
−2
0
2
4
Tumor−Normal Expression Difference

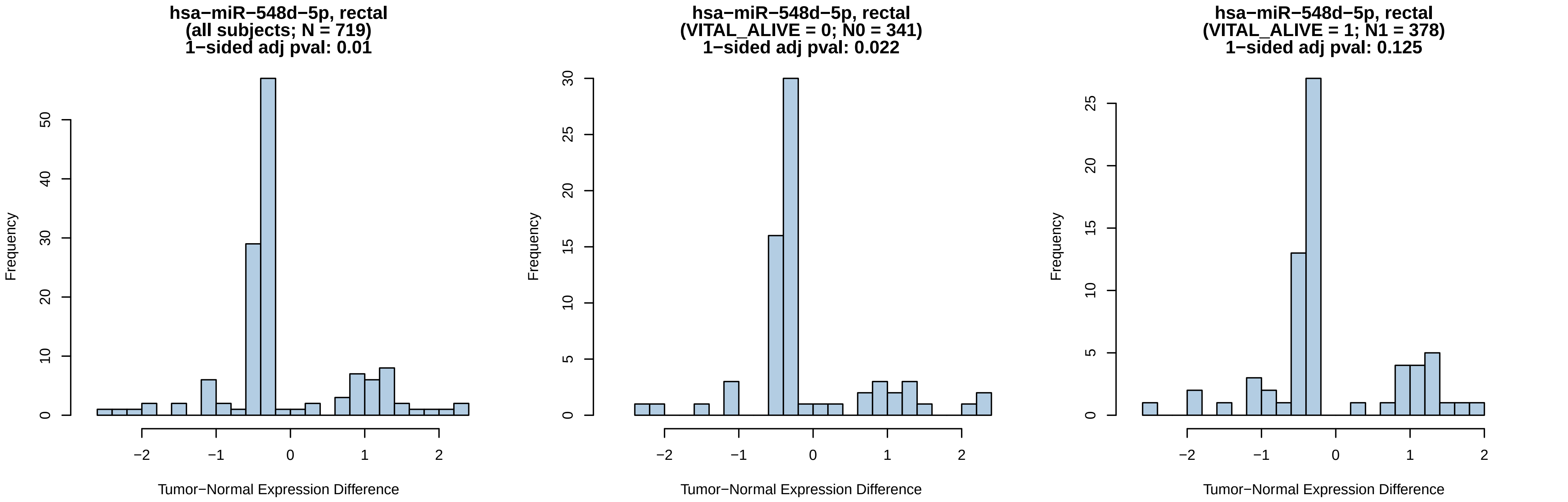

hsa−miR−548d−5p, rectal
(all subjects; N = 719)
1−sided adj pval: 0.01
Frequency
Tumor−Normal Expression Difference
hsa−miR−548d−5p, rectal
(VITAL_ALIVE = 0; N0 = 341)
1−sided adj pval: 0.022
Frequency
Tumor−Normal Expression Difference
hsa−miR−548d−5p, rectal
(VITAL_ALIVE = 1; N1 = 378)
1−sided adj pval: 0.125
Frequency
Tumor−Normal Expression Difference

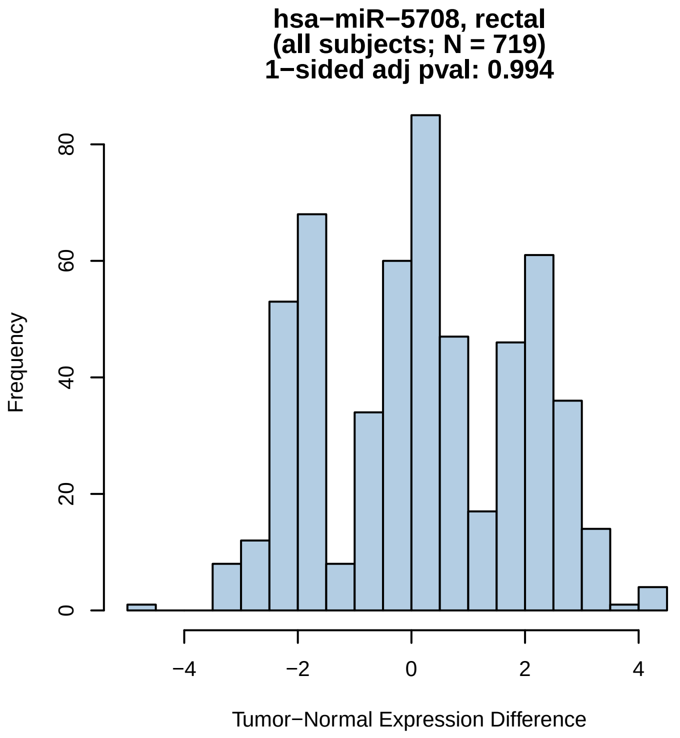
hsa−miR−5708, rectal
(all subjects; N = 719)
1−sided adj pval: 0.994
Frequency
0
20
40
60
80
−4
−2
0
2
4
Tumor−Normal Expression Difference
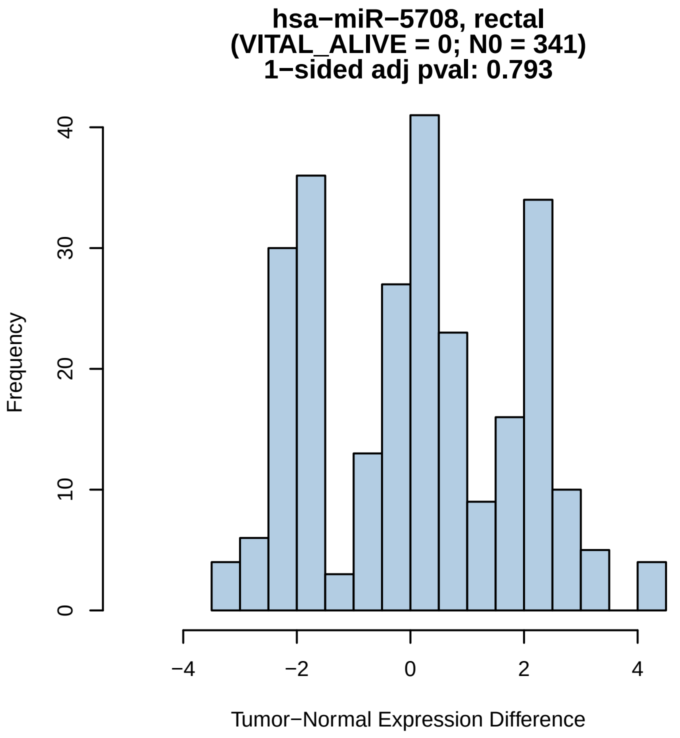
hsa−miR−5708, rectal
(VITAL_ALIVE = 0; N0 = 341)
1−sided adj pval: 0.793
Frequency
0
10
20
30
40
−4
−2
0
2
4
Tumor−Normal Expression Difference
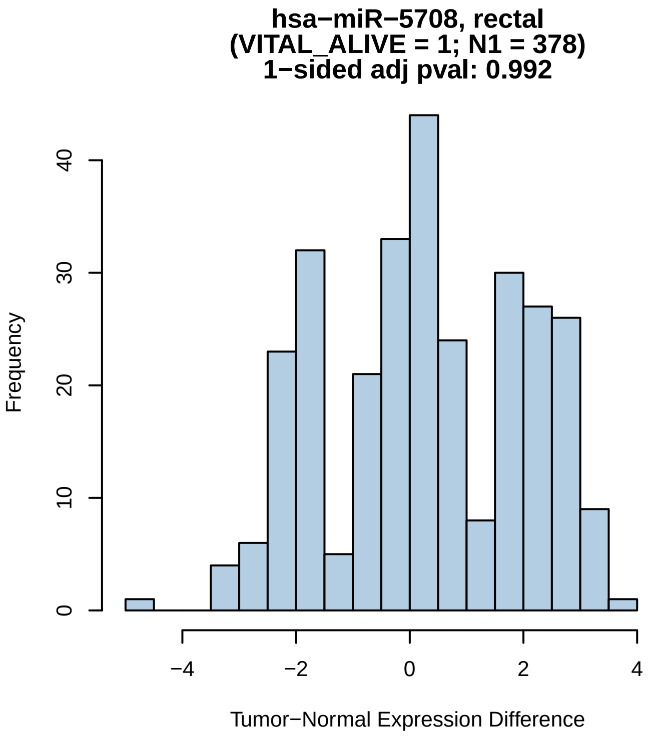
hsa−miR−5708, rectal
(VITAL_ALIVE = 1; N1 = 378)
1−sided adj pval: 0.992
Frequency
0
10
20
30
40
−4
−2
0
2
4
Tumor−Normal Expression Difference

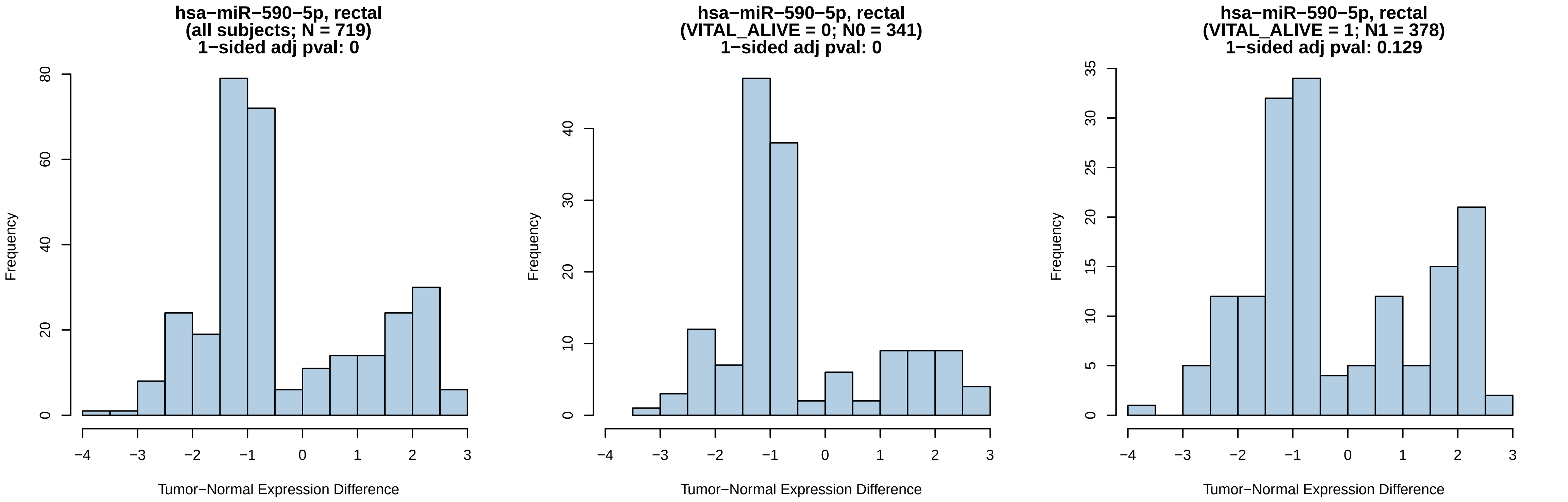

hsa−miR−590−5p, rectal
(all subjects; N = 719)
1−sided adj pval: 0
Frequency
Tumor−Normal Expression Difference
hsa−miR−590−5p, rectal
(VITAL_ALIVE = 0; N0 = 341)
1−sided adj pval: 0
Frequency
Tumor−Normal Expression Difference
hsa−miR−590−5p, rectal
(VITAL_ALIVE = 1; N1 = 378)
1−sided adj pval: 0.129
Frequency
Tumor−Normal Expression Difference

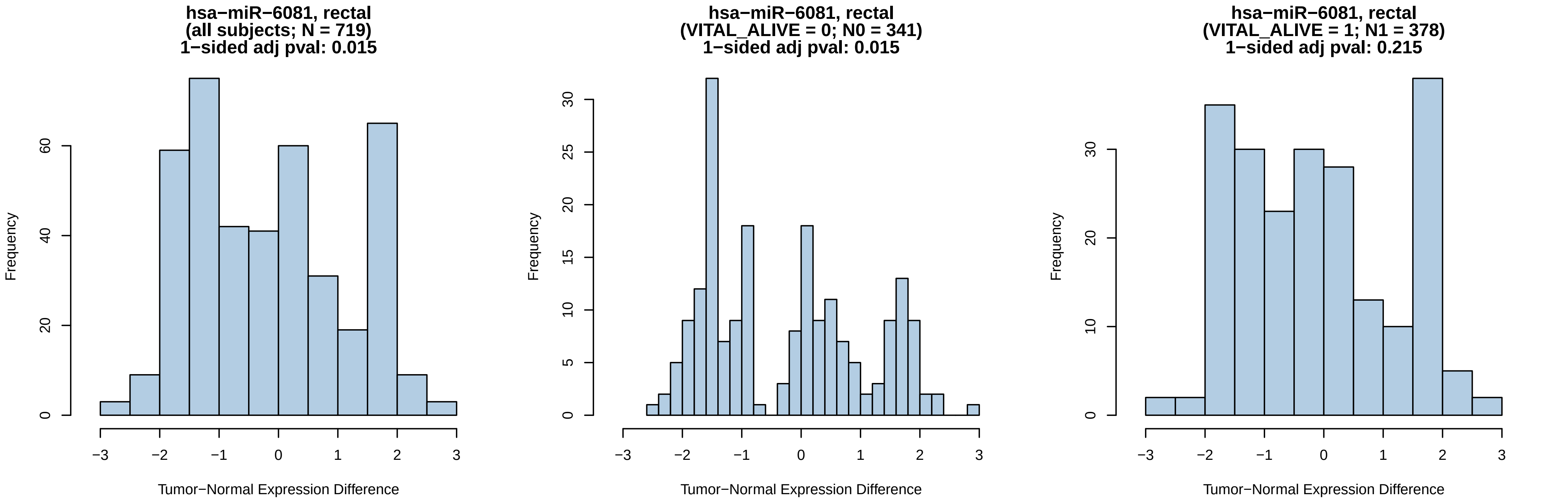

hsa−miR−6081, rectal
(all subjects; N = 719)
1−sided adj pval: 0.015
Frequency
0
20
40
60
−3
−2
−1
0
1
2
3
Tumor−Normal Expression Difference
hsa−miR−6081, rectal
(VITAL_ALIVE = 0; N0 = 341)
1−sided adj pval: 0.015
Frequency
0
5
10
15
20
25
30
−3
−2
−1
0
1
2
3
Tumor−Normal Expression Difference
hsa−miR−6081, rectal
(VITAL_ALIVE = 1; N1 = 378)
1−sided adj pval: 0.215
Frequency
0
10
20
30
−3
−2
−1
0
1
2
3
Tumor−Normal Expression Difference

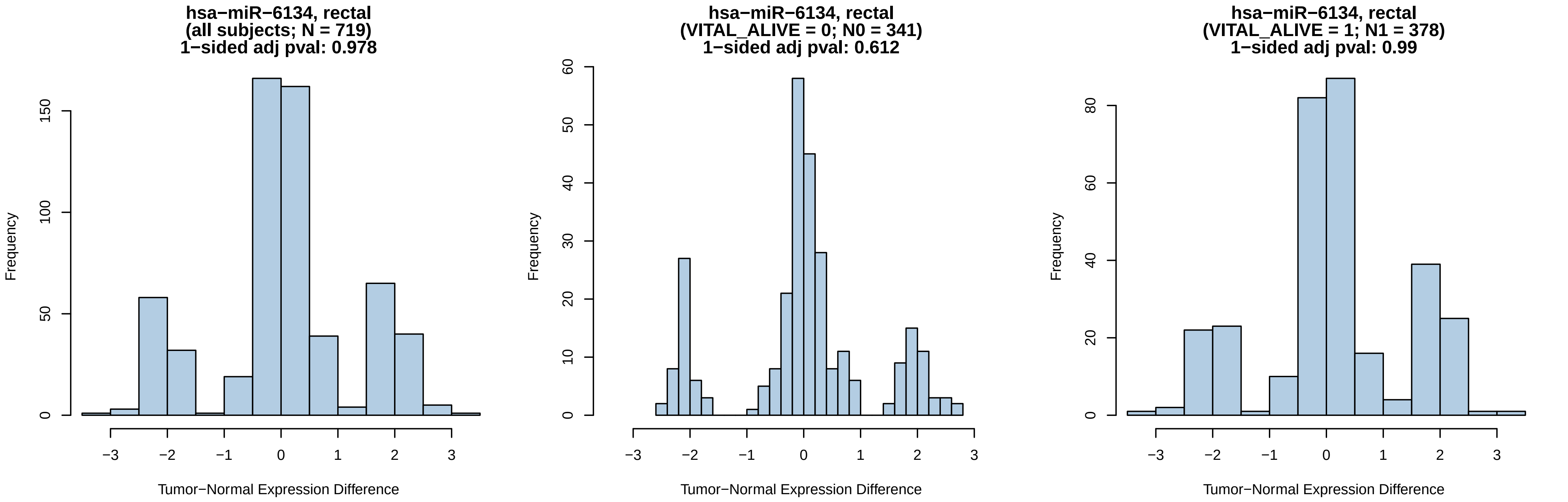

hsa−miR−6134, rectal
(all subjects; N = 719)
1−sided adj pval: 0.978
hsa−miR−6134, rectal
(VITAL_ALIVE = 0; N0 = 341)
1−sided adj pval: 0.612
hsa−miR−6134, rectal
(VITAL_ALIVE = 1; N1 = 378)
1−sided adj pval: 0.99
Frequency
Tumor−Normal Expression Difference

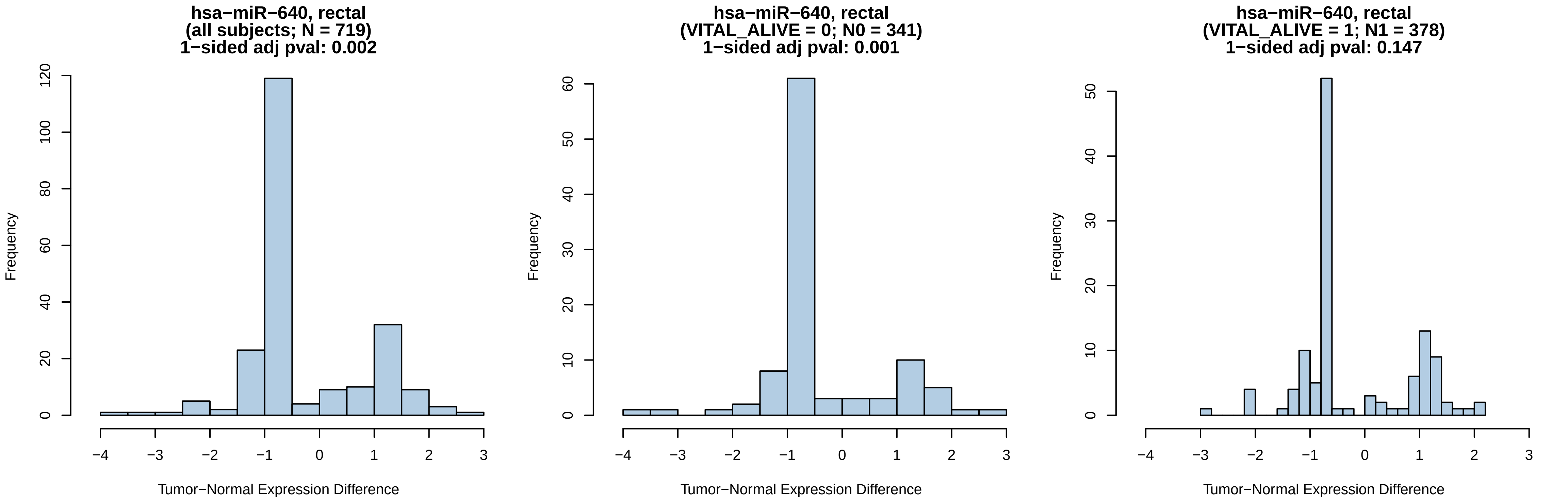

hsa−miR−640, rectal
(all subjects; N = 719)
1−sided adj pval: 0.002
Frequency
Tumor−Normal Expression Difference
hsa−miR−640, rectal
(VITAL_ALIVE = 0; N0 = 341)
1−sided adj pval: 0.001
Frequency
Tumor−Normal Expression Difference
hsa−miR−640, rectal
(VITAL_ALIVE = 1; N1 = 378)
1−sided adj pval: 0.147
Frequency
Tumor−Normal Expression Difference

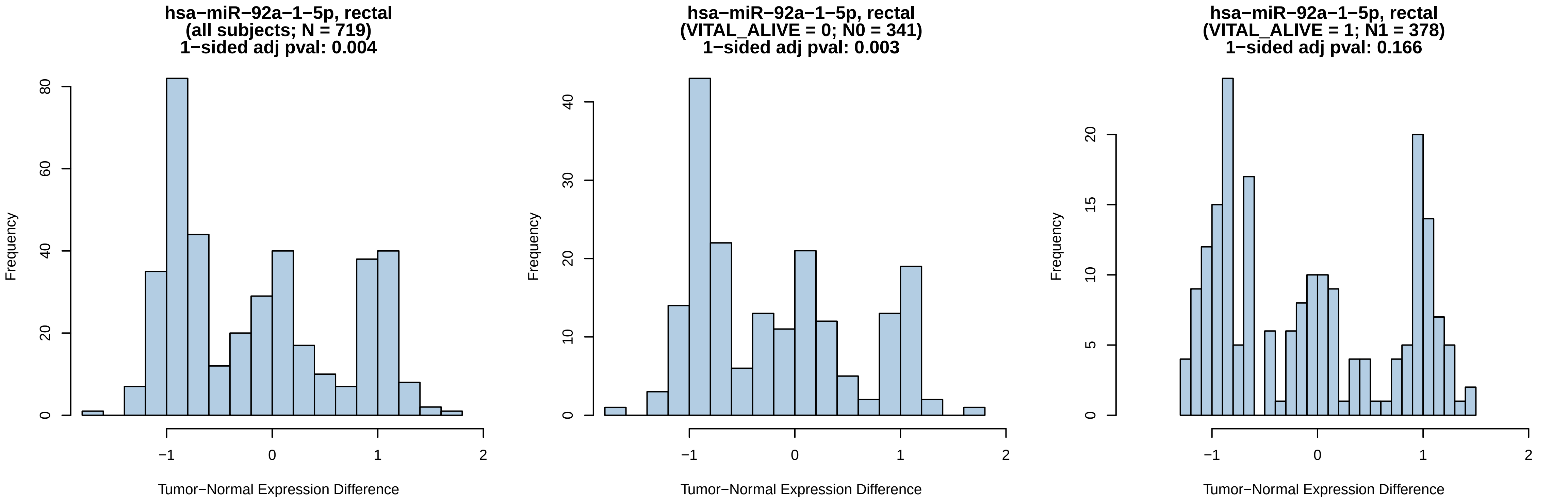
hsa−miR−92a−1−5p, rectal
(all subjects; N = 719)
1−sided adj pval: 0.004
Frequency
Tumor−Normal Expression Difference
hsa−miR−92a−1−5p, rectal
(VITAL_ALIVE = 0; N0 = 341)
1−sided adj pval: 0.003
Frequency
Tumor−Normal Expression Difference
hsa−miR−92a−1−5p, rectal
(VITAL_ALIVE = 1; N1 = 378)
1−sided adj pval: 0.166
Frequency
Tumor−Normal Expression Difference

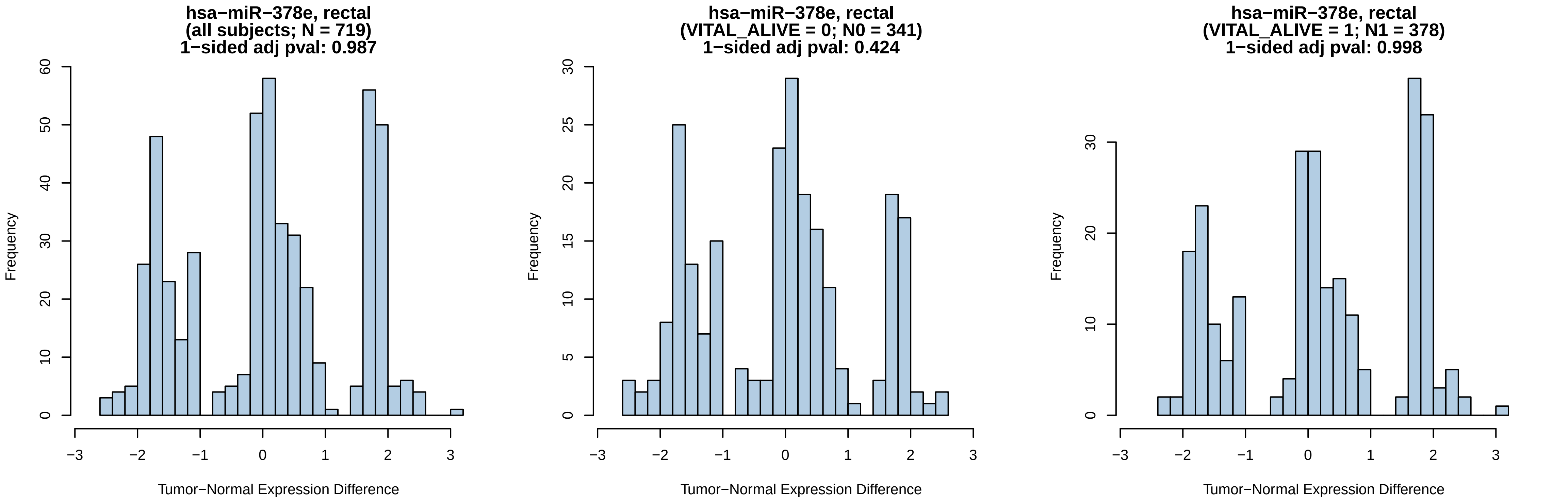

hsa−miR−378e, rectal
(all subjects; N = 719)
1−sided adj pval: 0.987
Frequency
Tumor−Normal Expression Difference
hsa−miR−378e, rectal
(VITAL_ALIVE = 0; N0 = 341)
1−sided adj pval: 0.424
Frequency
Tumor−Normal Expression Difference
hsa−miR−378e, rectal
(VITAL_ALIVE = 1; N1 = 378)
1−sided adj pval: 0.998
Frequency
Tumor−Normal Expression Difference

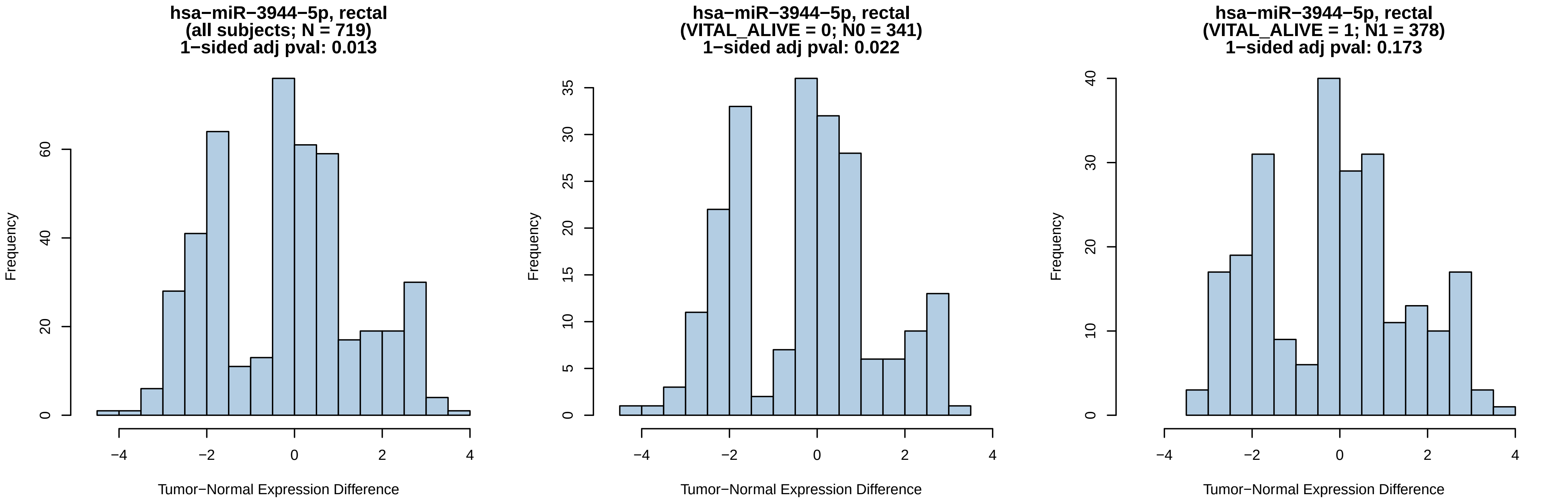
hsa−miR−3944−5p, rectal
(all subjects; N = 719)
1−sided adj pval: 0.013
Frequency
Tumor−Normal Expression Difference
hsa−miR−3944−5p, rectal
(VITAL_ALIVE = 0; N0 = 341)
1−sided adj pval: 0.022
Frequency
Tumor−Normal Expression Difference
hsa−miR−3944−5p, rectal
(VITAL_ALIVE = 1; N1 = 378)
1−sided adj pval: 0.173
Frequency
Tumor−Normal Expression Difference

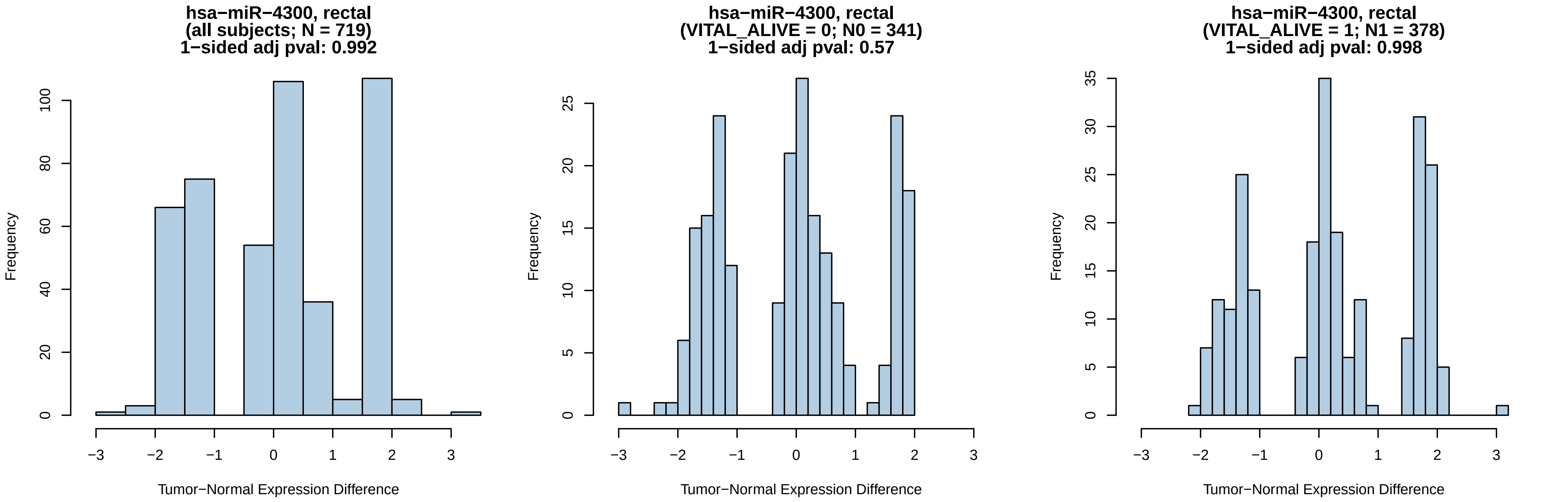
hsa−miR−4300, rectal
(all subjects; N = 719)
1−sided adj pval: 0.992
Frequency
Tumor−Normal Expression Difference
hsa−miR−4300, rectal
(VITAL_ALIVE = 0; N0 = 341)
1−sided adj pval: 0.57
Frequency
Tumor−Normal Expression Difference
hsa−miR−4300, rectal
(VITAL_ALIVE = 1; N1 = 378)
1−sided adj pval: 0.998
Frequency
Tumor−Normal Expression Difference

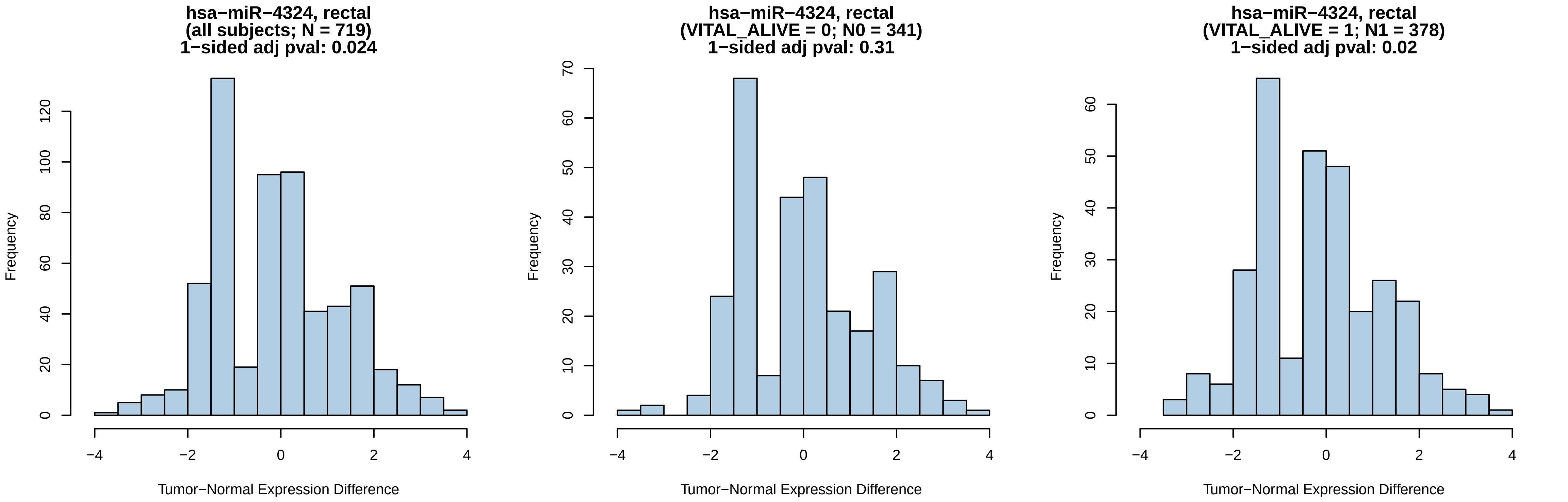
hsa−miR−4324, rectal
(all subjects; N = 719)
1−sided adj pval: 0.024
Frequency
0
20
40
60
80
100
120
−4
−2
0
2
4
Tumor−Normal Expression Difference
hsa−miR−4324, rectal
(VITAL_ALIVE = 0; N0 = 341)
1−sided adj pval: 0.31
Frequency
0
10
20
30
40
50
60
70
−4
−2
0
2
4
Tumor−Normal Expression Difference
hsa−miR−4324, rectal
(VITAL_ALIVE = 1; N1 = 378)
1−sided adj pval: 0.02
Frequency
0
10
20
30
40
50
60
−4
−2
0
2
4
Tumor−Normal Expression Difference

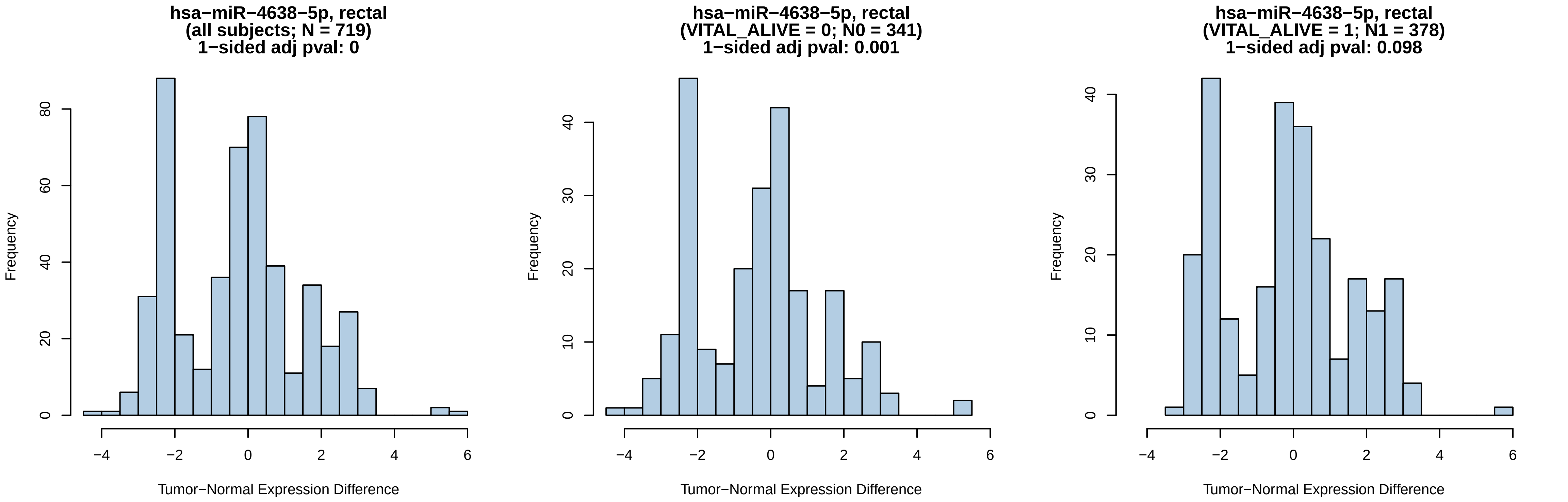
hsa−miR−4638−5p, rectal
(all subjects; N = 719)
1−sided adj pval: 0
Frequency
Tumor−Normal Expression Difference
hsa−miR−4638−5p, rectal
(VITAL_ALIVE = 0; N0 = 341)
1−sided adj pval: 0.001
Frequency
Tumor−Normal Expression Difference
hsa−miR−4638−5p, rectal
(VITAL_ALIVE = 1; N1 = 378)
1−sided adj pval: 0.098
Frequency
Tumor−Normal Expression Difference

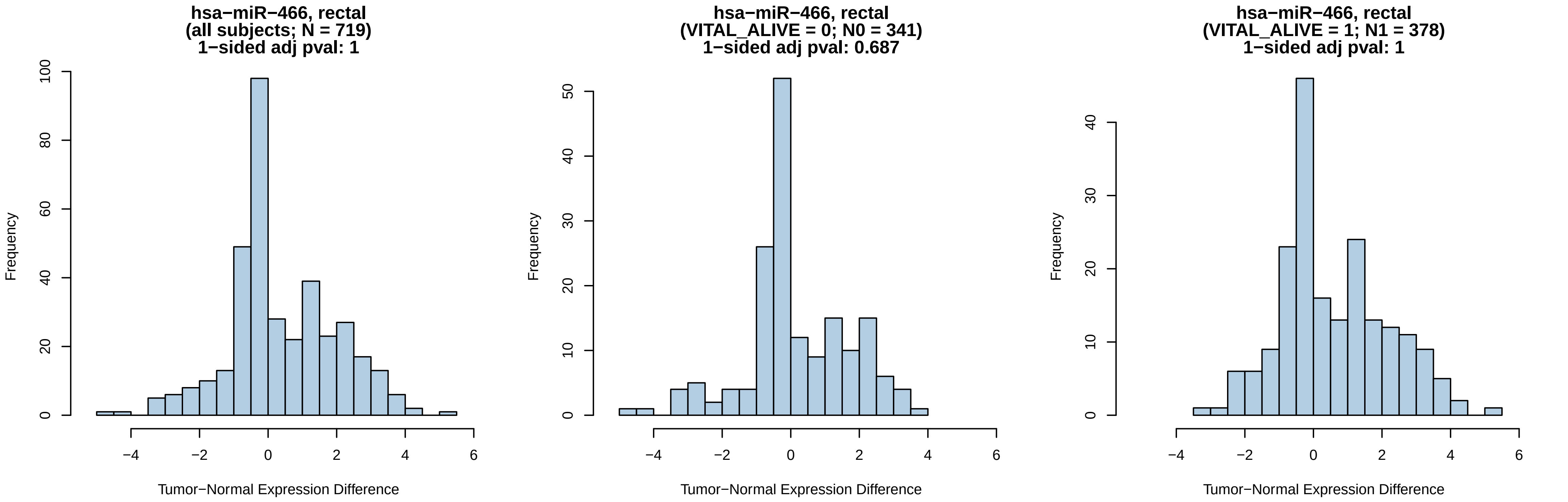
hsa−miR−466, rectal
(all subjects; N = 719)
1−sided adj pval: 1
hsa−miR−466, rectal
(VITAL_ALIVE = 0; N0 = 341)
1−sided adj pval: 0.687
hsa−miR−466, rectal
(VITAL_ALIVE = 1; N1 = 378)
1−sided adj pval: 1
Frequency
Tumor−Normal Expression Difference

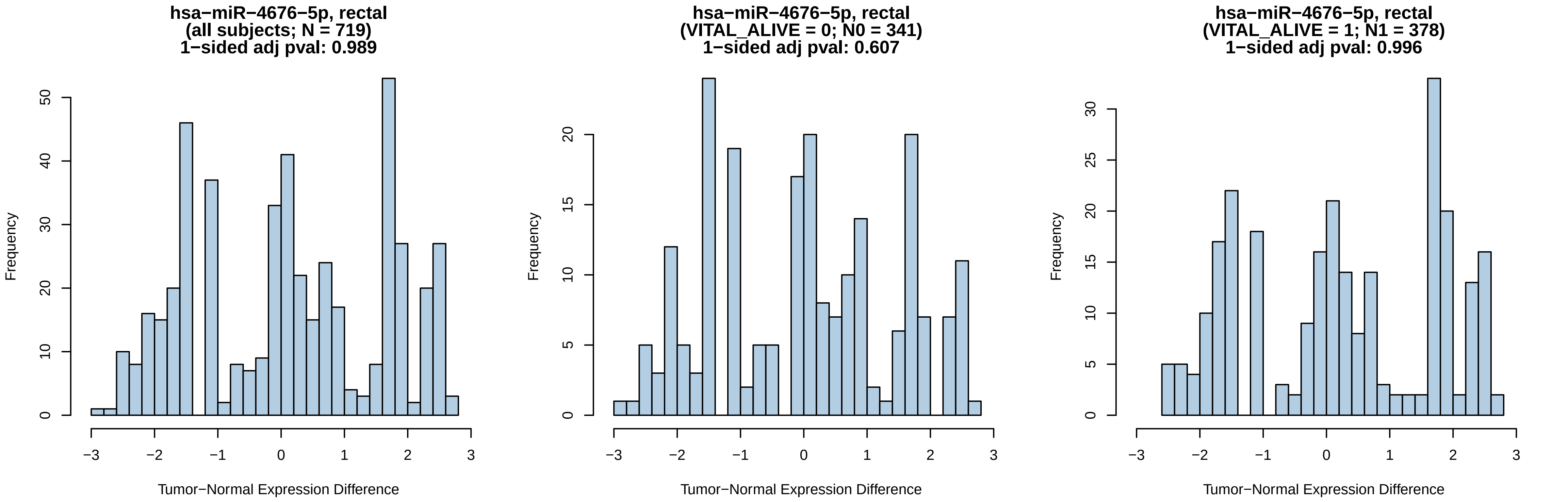

hsa−miR−4676−5p, rectal
(all subjects; N = 719)
1−sided adj pval: 0.989
hsa−miR−4676−5p, rectal
(VITAL_ALIVE = 0; N0 = 341)
1−sided adj pval: 0.607
hsa−miR−4676−5p, rectal
(VITAL_ALIVE = 1; N1 = 378)
1−sided adj pval: 0.996
Frequency
Tumor−Normal Expression Difference

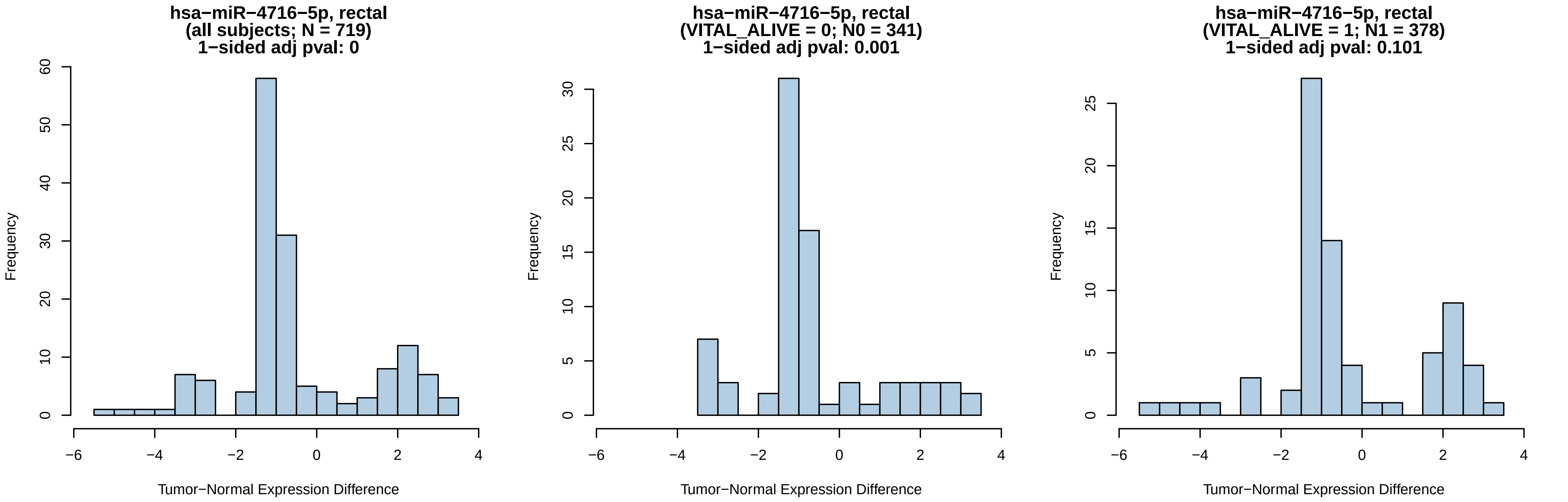
hsa−miR−4716−5p, rectal
(all subjects; N = 719)
1−sided adj pval: 0
hsa−miR−4716−5p, rectal
(VITAL_ALIVE = 0; N0 = 341)
1−sided adj pval: 0.001
hsa−miR−4716−5p, rectal
(VITAL_ALIVE = 1; N1 = 378)
1−sided adj pval: 0.101
Frequency
Tumor−Normal Expression Difference

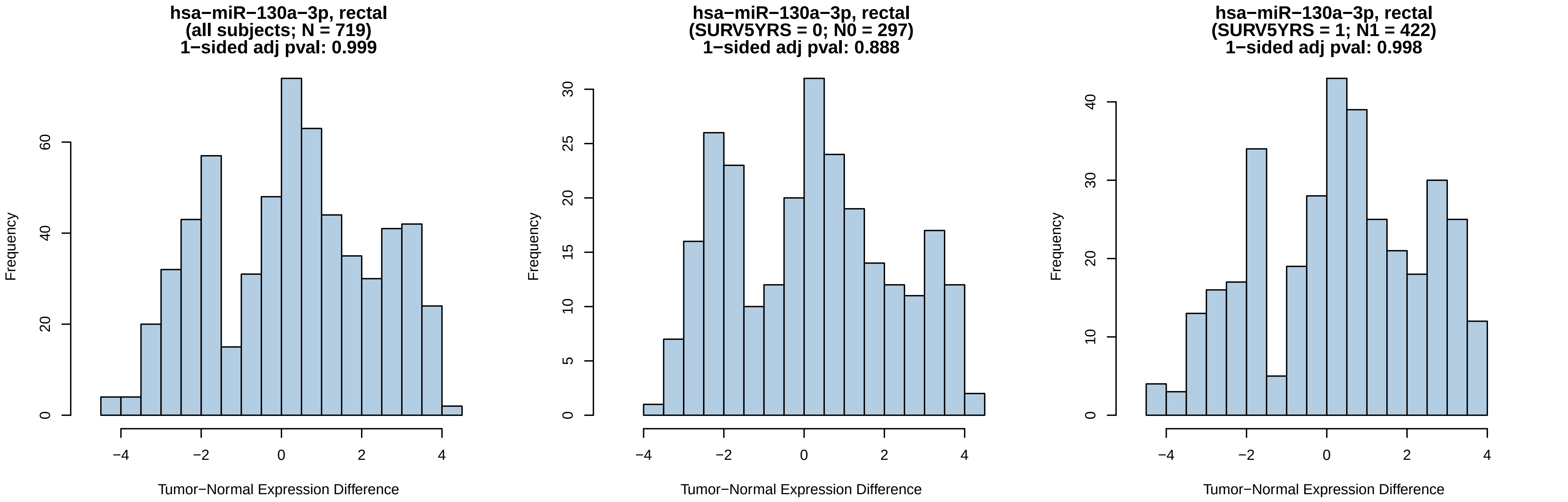

hsa−miR−130a−3p, rectal
(all subjects; N = 719)
1−sided adj pval: 0.999
Frequency
Tumor−Normal Expression Difference
hsa−miR−130a−3p, rectal
(SURV5YRS = 0; N0 = 297)
1−sided adj pval: 0.888
Frequency
Tumor−Normal Expression Difference
hsa−miR−130a−3p, rectal
(SURV5YRS = 1; N1 = 422)
1−sided adj pval: 0.998
Frequency
Tumor−Normal Expression Difference

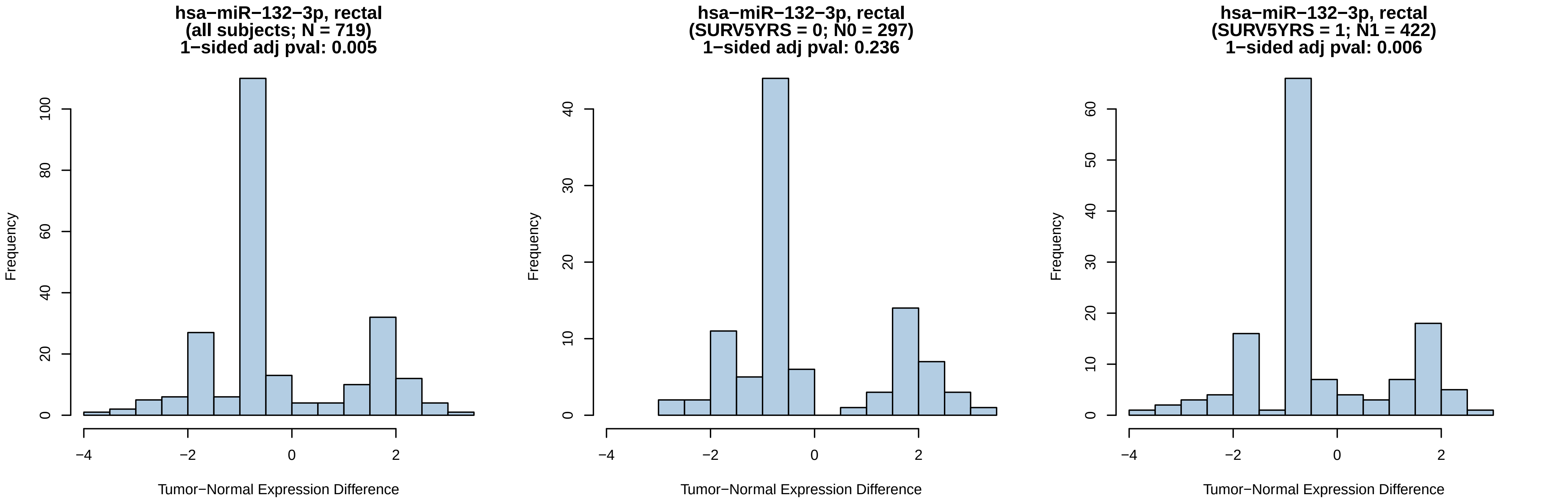
hsa−miR−132−3p, rectal
(all subjects; N = 719)
1−sided adj pval: 0.005
Frequency
Tumor−Normal Expression Difference
hsa−miR−132−3p, rectal
(SURV5YRS = 0; N0 = 297)
1−sided adj pval: 0.236
Frequency
Tumor−Normal Expression Difference
hsa−miR−132−3p, rectal
(SURV5YRS = 1; N1 = 422)
1−sided adj pval: 0.006
Frequency
Tumor−Normal Expression Difference

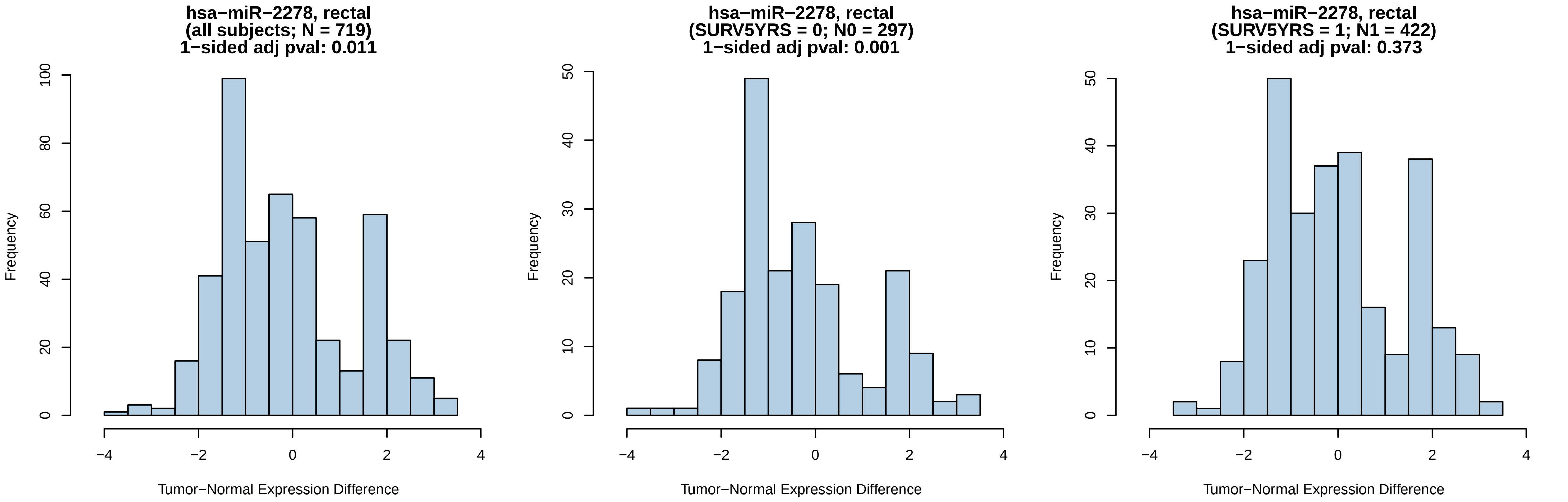
hsa−miR−2278, rectal
(all subjects; N = 719)
1−sided adj pval: 0.011
Frequency
Tumor−Normal Expression Difference
hsa−miR−2278, rectal
(SURV5YRS = 0; N0 = 297)
1−sided adj pval: 0.001
Frequency
Tumor−Normal Expression Difference
hsa−miR−2278, rectal
(SURV5YRS = 1; N1 = 422)
1−sided adj pval: 0.373
Frequency
Tumor−Normal Expression Difference

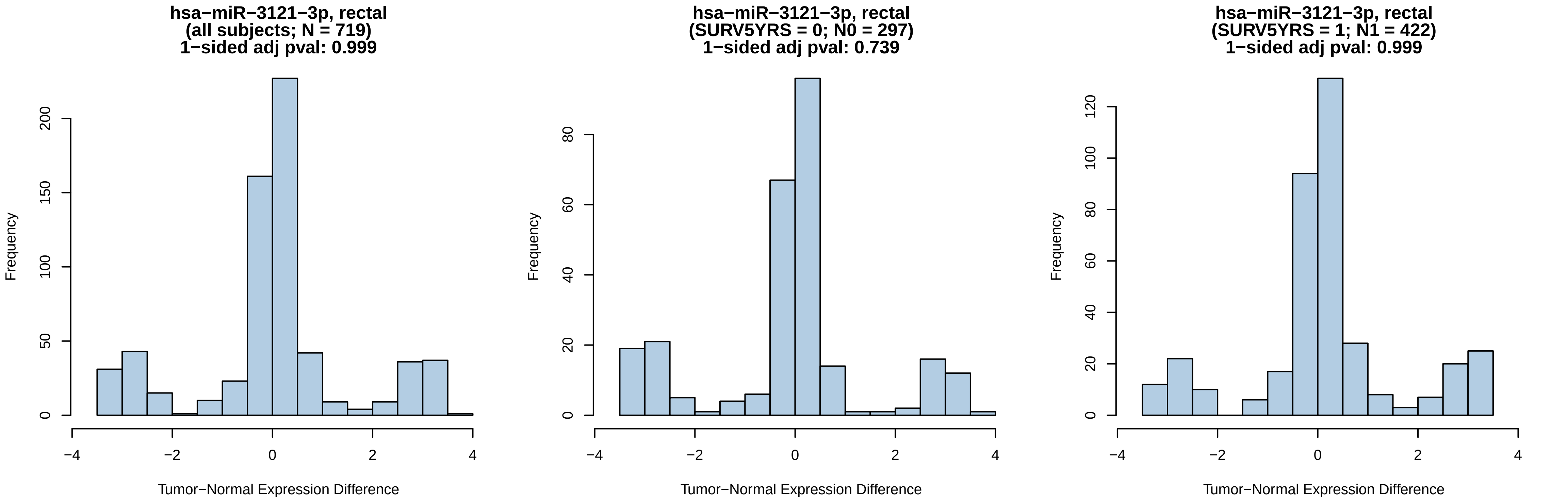
hsa−miR−3121−3p, rectal
(all subjects; N = 719)
1−sided adj pval: 0.999
Frequency
0
50
100
150
200
−4
−2
0
2
4
Tumor−Normal Expression Difference
hsa−miR−3121−3p, rectal
(SURV5YRS = 0; N0 = 297)
1−sided adj pval: 0.739
Frequency
0
20
40
60
80
−4
−2
0
2
4
Tumor−Normal Expression Difference
hsa−miR−3121−3p, rectal
(SURV5YRS = 1; N1 = 422)
1−sided adj pval: 0.999
Frequency
0
20
40
60
80
100
120
−4
−2
0
2
4
Tumor−Normal Expression Difference

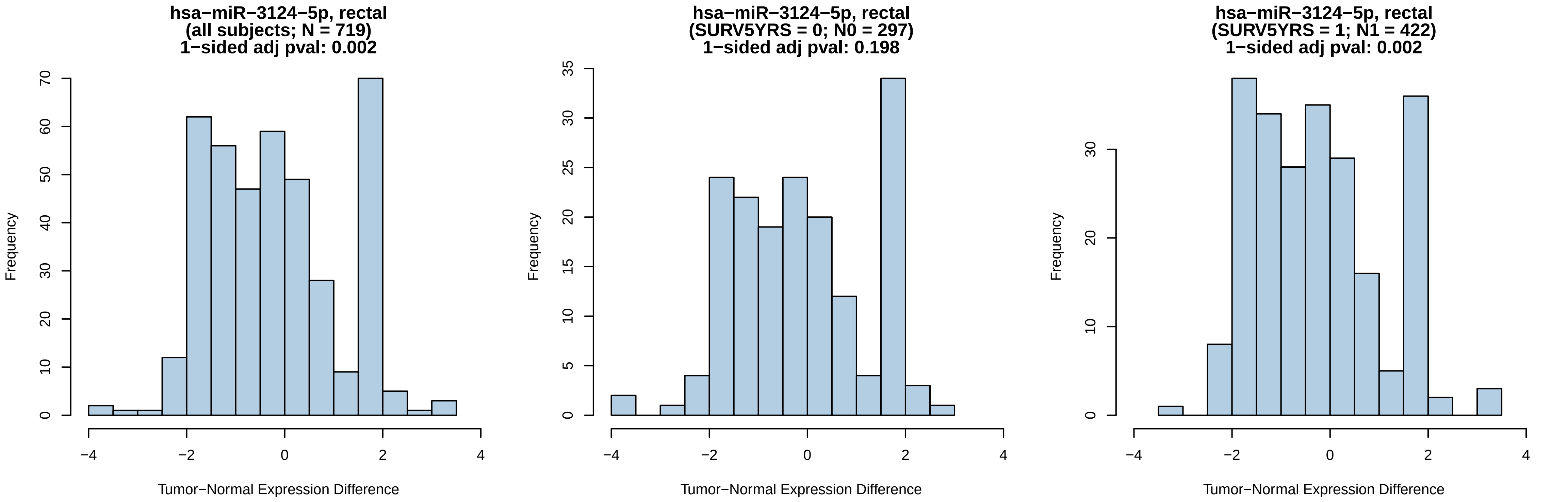
hsa−miR−3124−5p, rectal
(all subjects; N = 719)
1−sided adj pval: 0.002
Frequency
Tumor−Normal Expression Difference
hsa−miR−3124−5p, rectal
(SURV5YRS = 0; N0 = 297)
1−sided adj pval: 0.198
Frequency
Tumor−Normal Expression Difference
hsa−miR−3124−5p, rectal
(SURV5YRS = 1; N1 = 422)
1−sided adj pval: 0.002
Frequency
Tumor−Normal Expression Difference

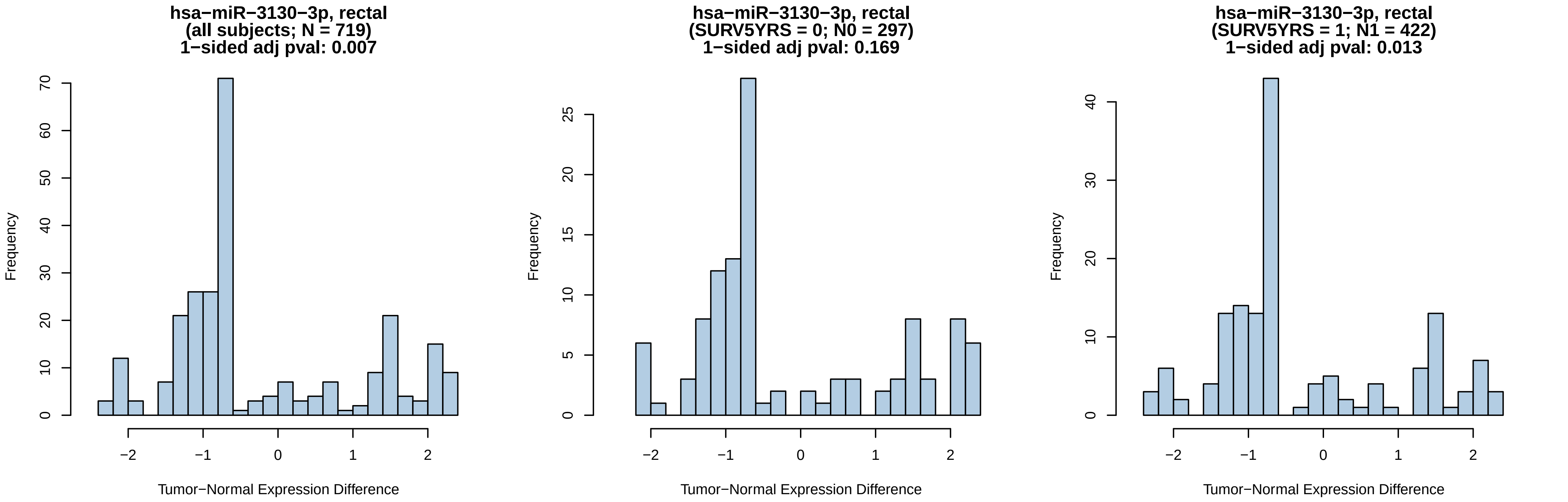

hsa−miR−3130−3p, rectal
(all subjects; N = 719)
1−sided adj pval: 0.007
Frequency
0
10
20
30
40
50
60
70
−2
−1
0
1
2
Tumor−Normal Expression Difference
hsa−miR−3130−3p, rectal
(SURV5YRS = 0; N0 = 297)
1−sided adj pval: 0.169
Frequency
0
5
10
15
20
25
−2
−1
0
1
2
Tumor−Normal Expression Difference
hsa−miR−3130−3p, rectal
(SURV5YRS = 1; N1 = 422)
1−sided adj pval: 0.013
Frequency
0
10
20
30
40
−2
−1
0
1
2
Tumor−Normal Expression Difference

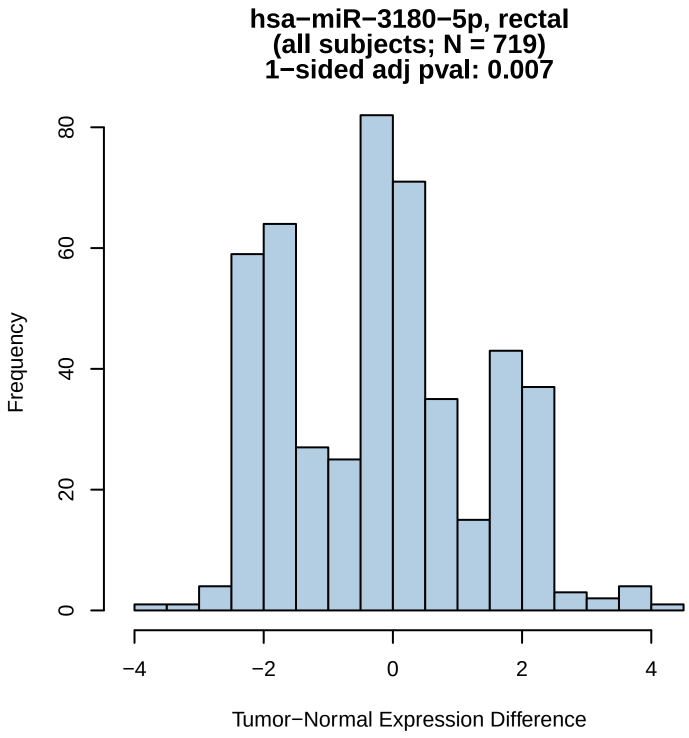
hsa−miR−3180−5p, rectal
(all subjects; N = 719)
1−sided adj pval: 0.007
Frequency
0
20
40
60
80
−4
−2
0
2
4
Tumor−Normal Expression Difference

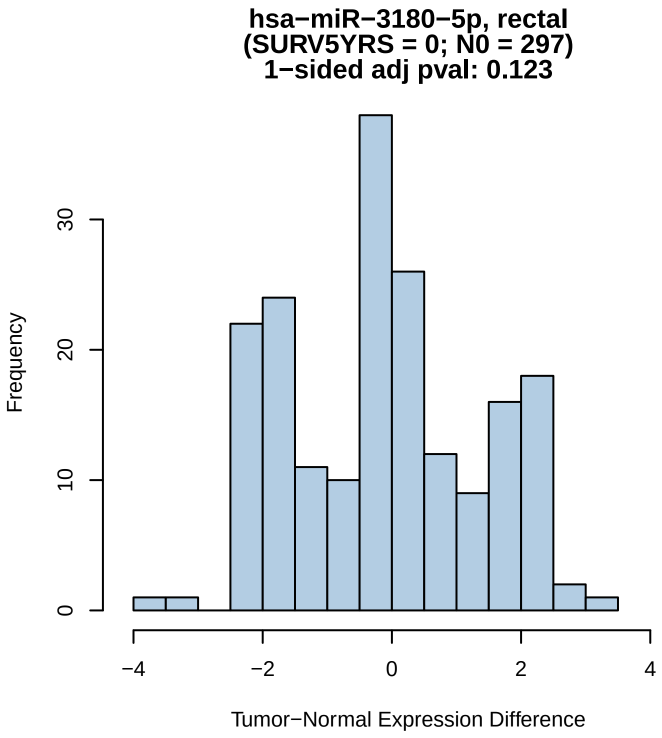
hsa−miR−3180−5p, rectal
(SURV5YRS = 0; N0 = 297)
1−sided adj pval: 0.123
Frequency
0
10
20
30
−4
−2
0
2
4
Tumor−Normal Expression Difference

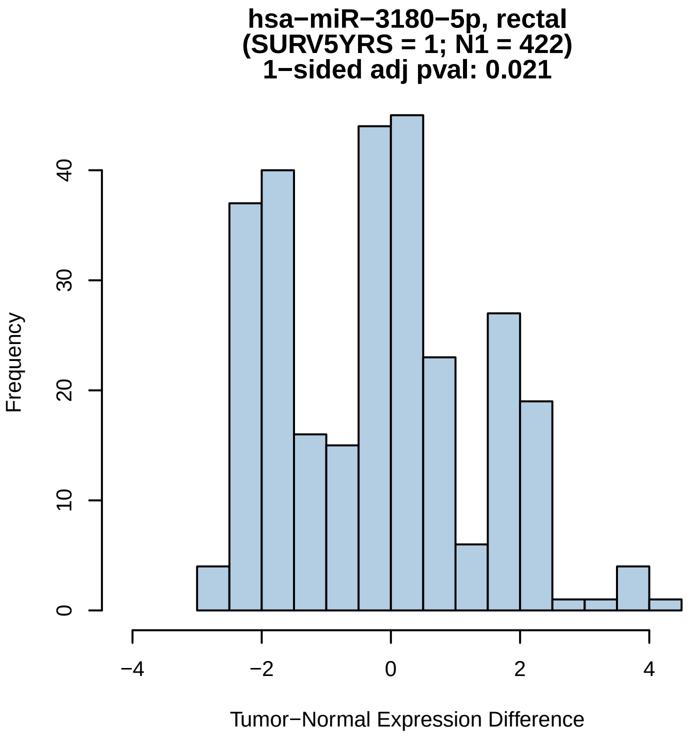
hsa−miR−3180−5p, rectal
(SURV5YRS = 1; N1 = 422)
1−sided adj pval: 0.021
Frequency
0
10
20
30
40
−4
−2
0
2
4
Tumor−Normal Expression Difference

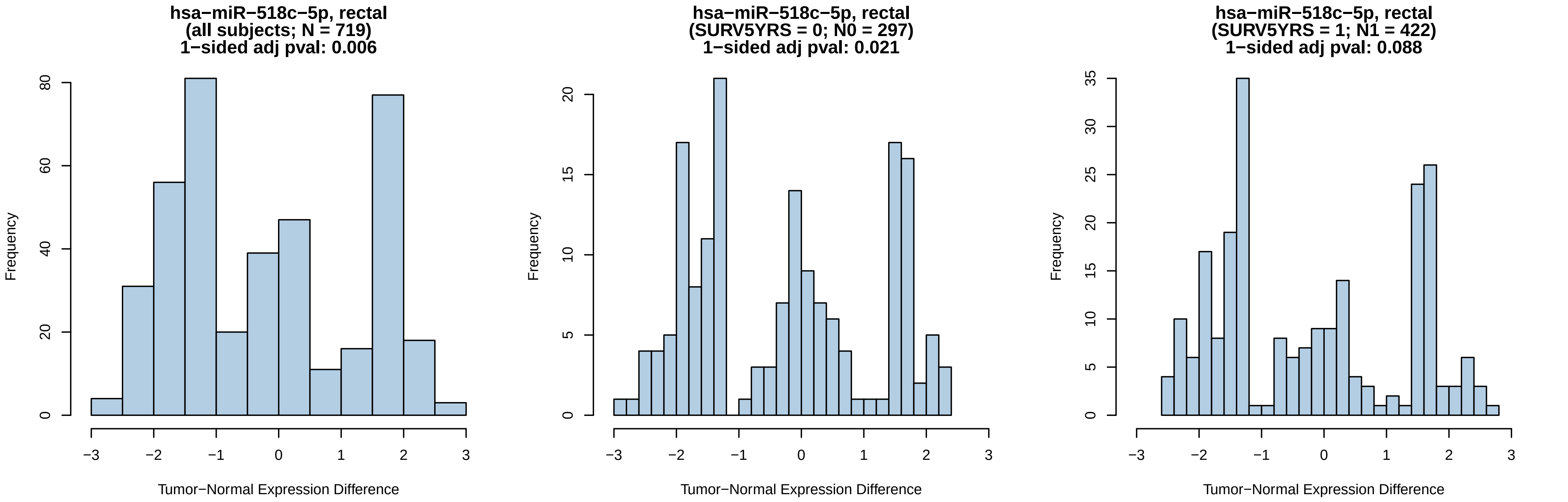

hsa−miR−518c−5p, rectal
(all subjects; N = 719)
1−sided adj pval: 0.006
Frequency
Tumor−Normal Expression Difference
hsa−miR−518c−5p, rectal
(SURV5YRS = 0; N0 = 297)
1−sided adj pval: 0.021
Frequency
Tumor−Normal Expression Difference
hsa−miR−518c−5p, rectal
(SURV5YRS = 1; N1 = 422)
1−sided adj pval: 0.088
Frequency
Tumor−Normal Expression Difference

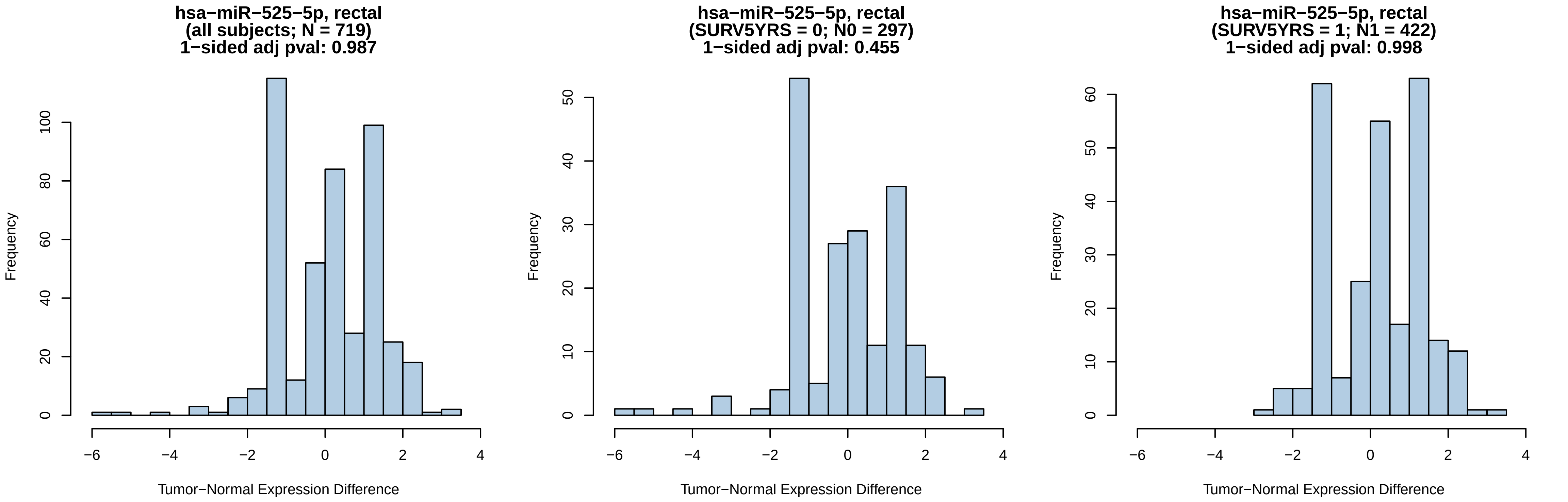
hsa−miR−525−5p, rectal
(all subjects; N = 719)
1−sided adj pval: 0.987
Frequency
Tumor−Normal Expression Difference
hsa−miR−525−5p, rectal
(SURV5YRS = 0; N0 = 297)
1−sided adj pval: 0.455
Frequency
Tumor−Normal Expression Difference
hsa−miR−525−5p, rectal
(SURV5YRS = 1; N1 = 422)
1−sided adj pval: 0.998
Frequency
Tumor−Normal Expression Difference

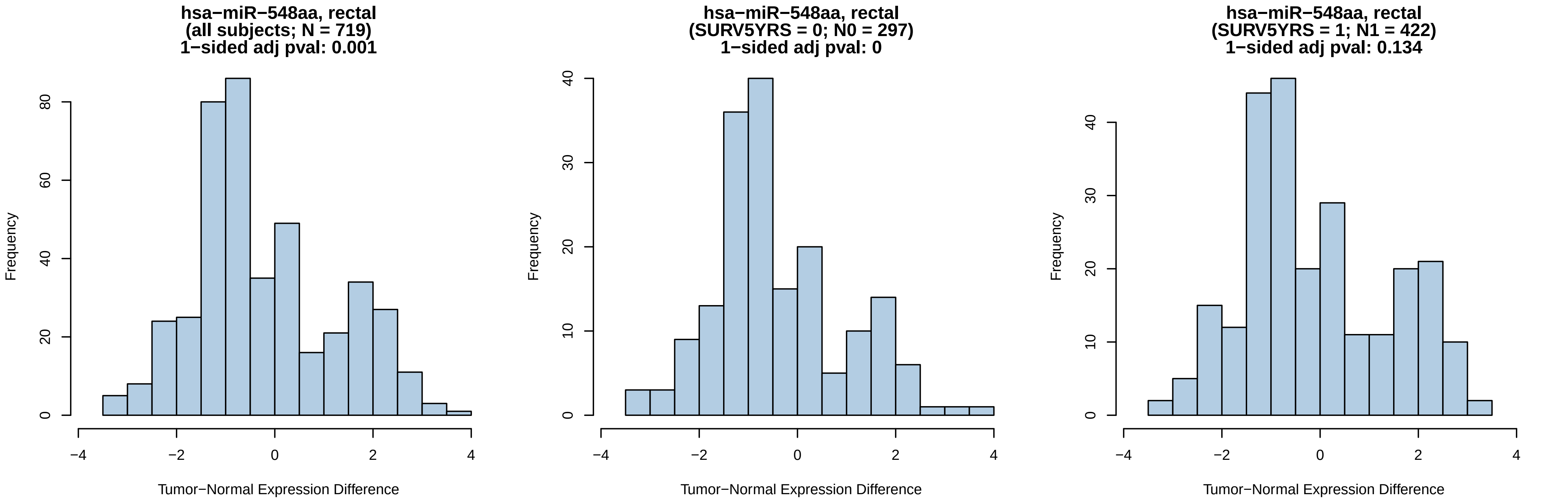

hsa−miR−548aa, rectal
(all subjects; N = 719)
1−sided adj pval: 0.001
Frequency
Tumor−Normal Expression Difference
hsa−miR−548aa, rectal
(SURV5YRS = 0; N0 = 297)
1−sided adj pval: 0
Frequency
Tumor−Normal Expression Difference
hsa−miR−548aa, rectal
(SURV5YRS = 1; N1 = 422)
1−sided adj pval: 0.134
Frequency
Tumor−Normal Expression Difference

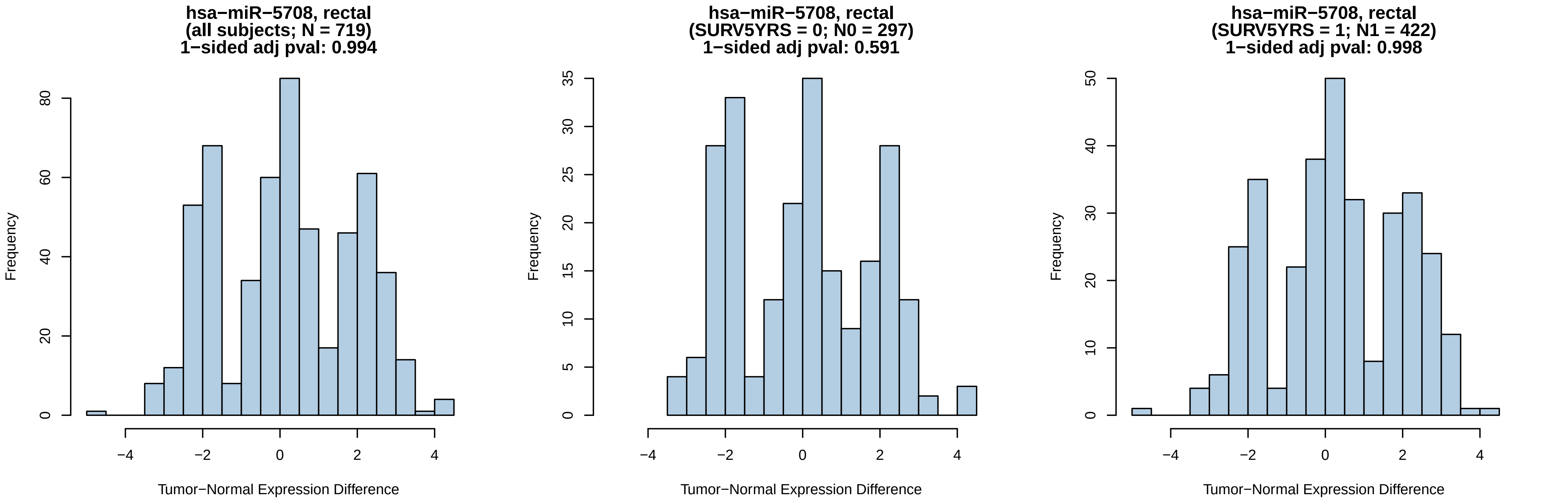

hsa−miR−5708, rectal
(all subjects; N = 719)
1−sided adj pval: 0.994
Frequency
Tumor−Normal Expression Difference
hsa−miR−5708, rectal
(SURV5YRS = 0; N0 = 297)
1−sided adj pval: 0.591
Frequency
Tumor−Normal Expression Difference
hsa−miR−5708, rectal
(SURV5YRS = 1; N1 = 422)
1−sided adj pval: 0.998
Frequency
Tumor−Normal Expression Difference

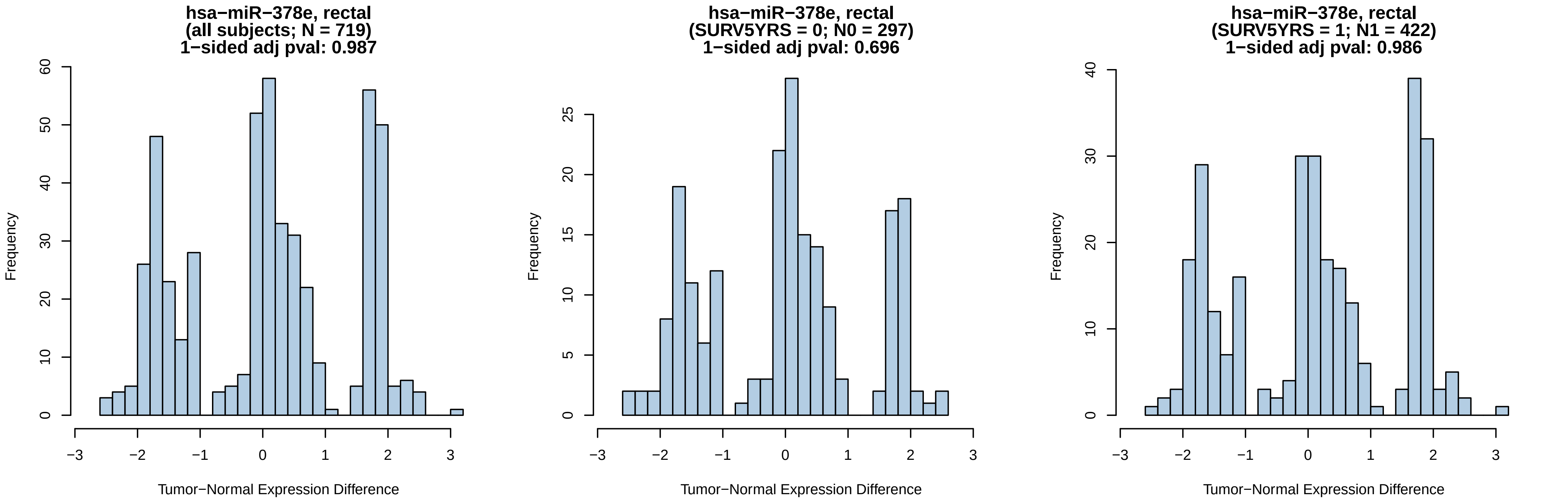
hsa−miR−378e, rectal
(all subjects; N = 719)
1−sided adj pval: 0.987
Frequency
Tumor−Normal Expression Difference
hsa−miR−378e, rectal
(SURV5YRS = 0; N0 = 297)
1−sided adj pval: 0.696
Frequency
Tumor−Normal Expression Difference
hsa−miR−378e, rectal
(SURV5YRS = 1; N1 = 422)
1−sided adj pval: 0.986
Frequency
Tumor−Normal Expression Difference

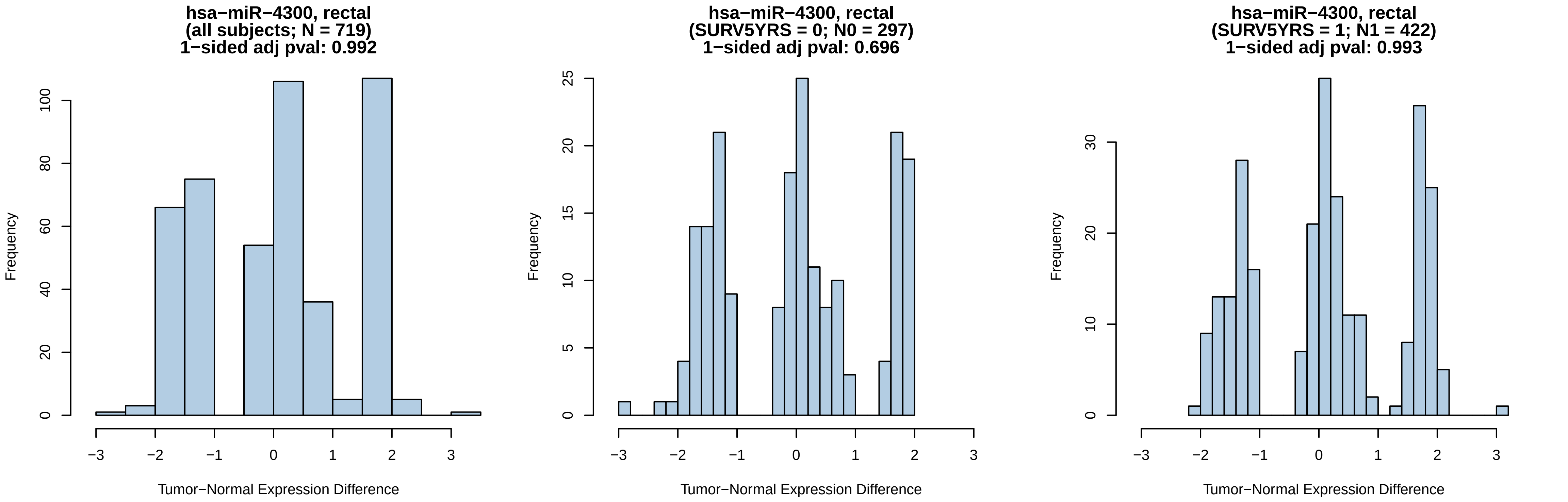

hsa−miR−4300, rectal
(all subjects; N = 719)
1−sided adj pval: 0.992
Frequency
0
20
40
60
80
100
−3
−2
−1
0
1
2
3
Tumor−Normal Expression Difference
hsa−miR−4300, rectal
(SURV5YRS = 0; N0 = 297)
1−sided adj pval: 0.696
Frequency
0
5
10
15
20
25
−3
−2
−1
0
1
2
3
Tumor−Normal Expression Difference
hsa−miR−4300, rectal
(SURV5YRS = 1; N1 = 422)
1−sided adj pval: 0.993
Frequency
0
10
20
30
−3
−2
−1
0
1
2
3
Tumor−Normal Expression Difference

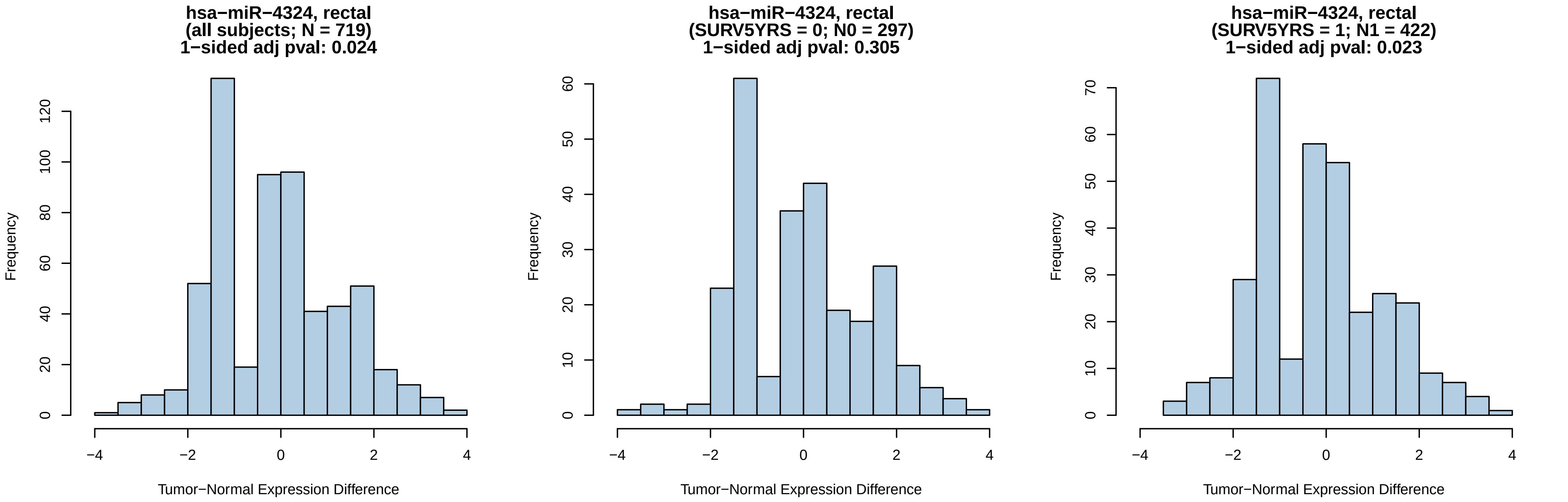

hsa−miR−4324, rectal
(all subjects; N = 719)
1−sided adj pval: 0.024
Frequency
0
20
40
60
80
100
120
−4
−2
0
2
4
Tumor−Normal Expression Difference
hsa−miR−4324, rectal
(SURV5YRS = 0; N0 = 297)
1−sided adj pval: 0.305
Frequency
0
10
20
30
40
50
60
−4
−2
0
2
4
Tumor−Normal Expression Difference
hsa−miR−4324, rectal
(SURV5YRS = 1; N1 = 422)
1−sided adj pval: 0.023
Frequency
0
10
20
30
40
50
60
70
−4
−2
0
2
4
Tumor−Normal Expression Difference

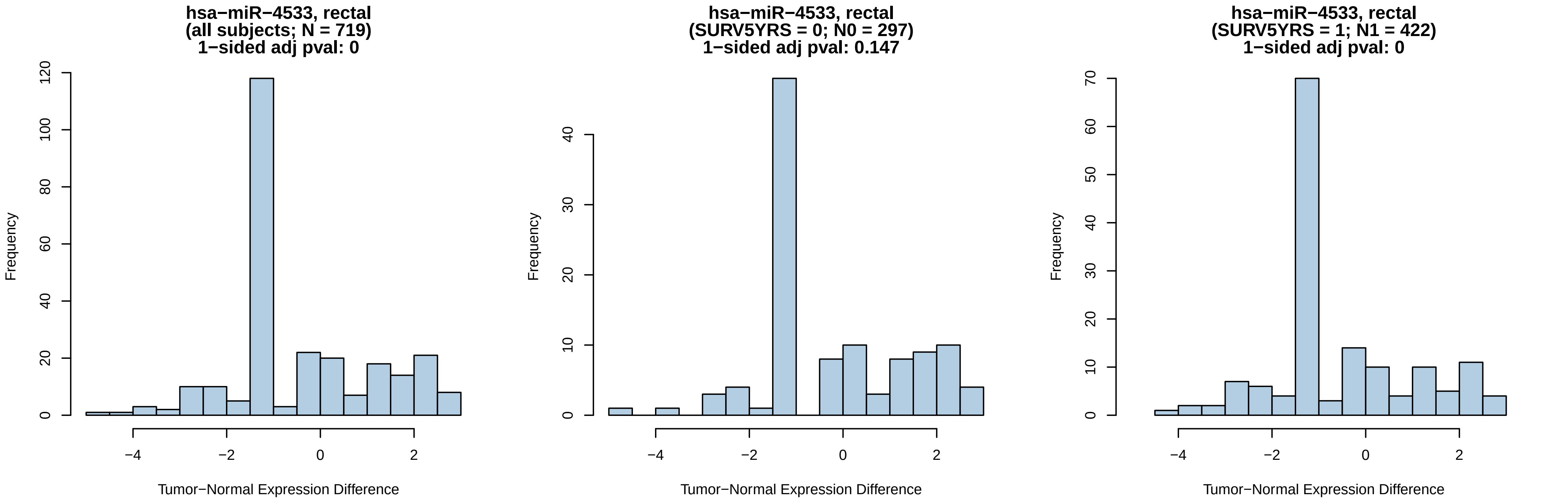
hsa−miR−4533, rectal
(all subjects; N = 719)
1−sided adj pval: 0
Frequency
Tumor−Normal Expression Difference
hsa−miR−4533, rectal
(SURV5YRS = 0; N0 = 297)
1−sided adj pval: 0.147
Frequency
Tumor−Normal Expression Difference
hsa−miR−4533, rectal
(SURV5YRS = 1; N1 = 422)
1−sided adj pval: 0
Frequency
Tumor−Normal Expression Difference

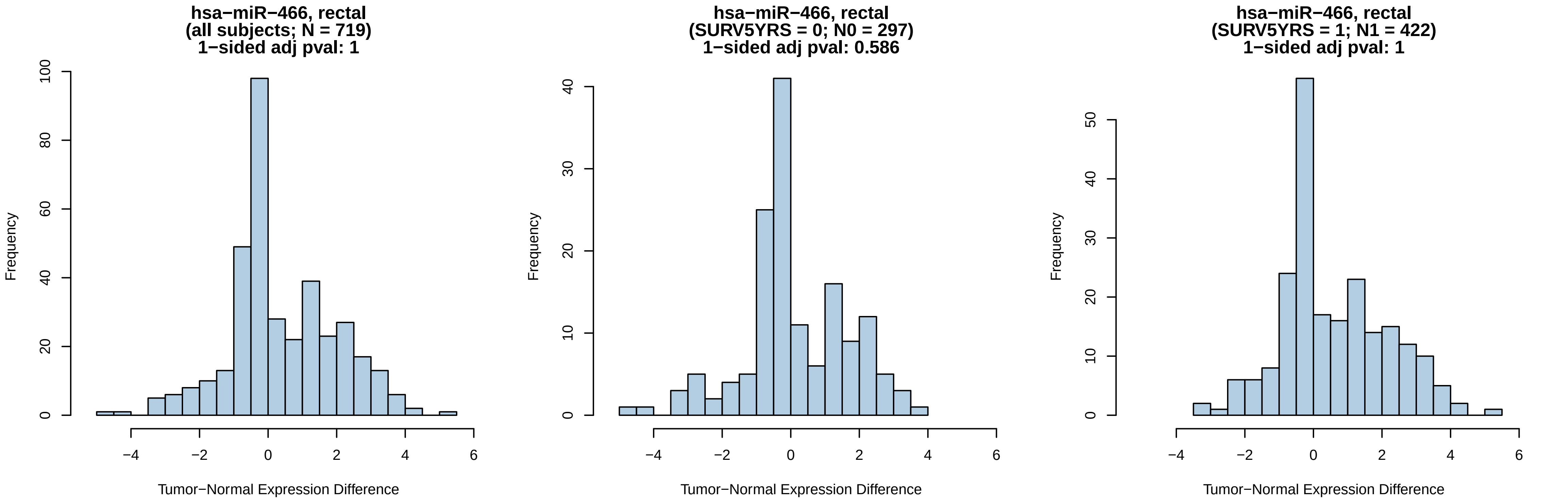
hsa−miR−466, rectal
(all subjects; N = 719)
1−sided adj pval: 1
Frequency
Tumor−Normal Expression Difference
hsa−miR−466, rectal
(SURV5YRS = 0; N0 = 297)
1−sided adj pval: 0.586
Frequency
Tumor−Normal Expression Difference
hsa−miR−466, rectal
(SURV5YRS = 1; N1 = 422)
1−sided adj pval: 1
Frequency
Tumor−Normal Expression Difference

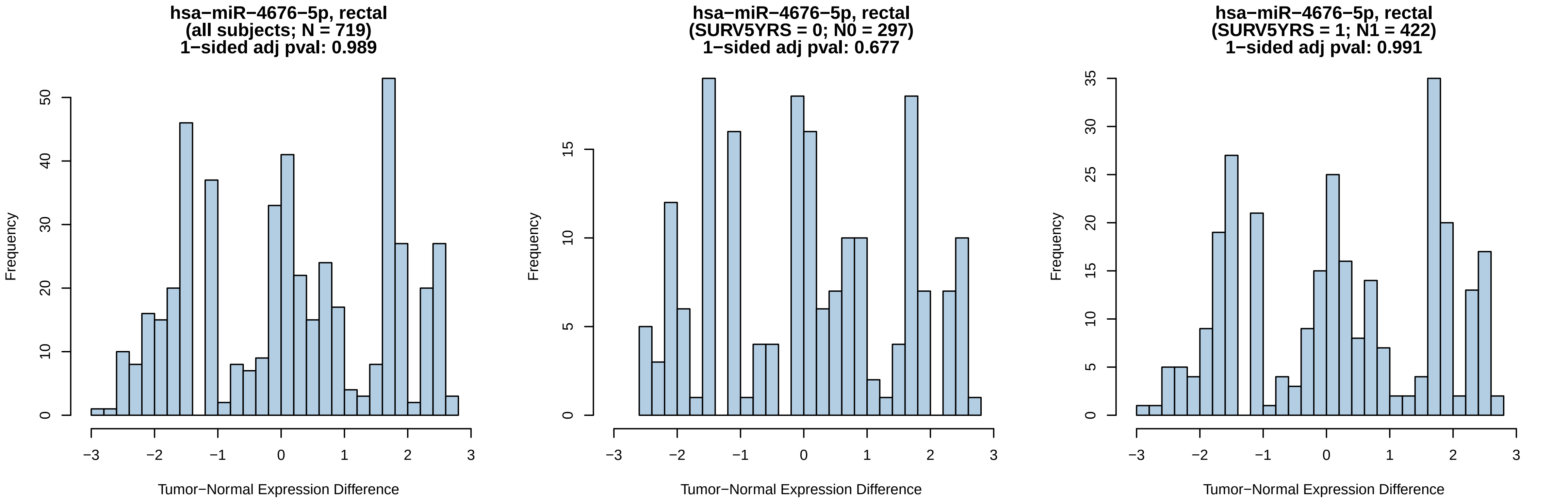

hsa−miR−4676−5p, rectal
(all subjects; N = 719)
1−sided adj pval: 0.989
Frequency
Tumor−Normal Expression Difference
hsa−miR−4676−5p, rectal
(SURV5YRS = 0; N0 = 297)
1−sided adj pval: 0.677
Frequency
Tumor−Normal Expression Difference
hsa−miR−4676−5p, rectal
(SURV5YRS = 1; N1 = 422)
1−sided adj pval: 0.991
Frequency
Tumor−Normal Expression Difference

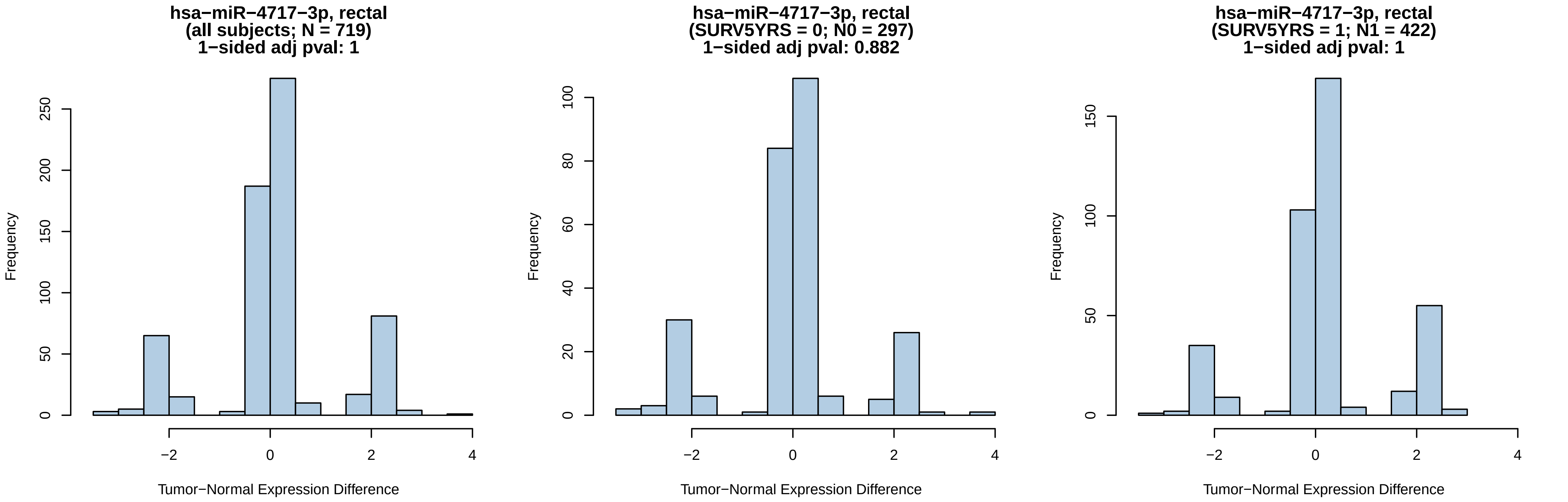
hsa−miR−4717−3p, rectal
(all subjects; N = 719)
1−sided adj pval: 1
Frequency
Tumor−Normal Expression Difference
hsa−miR−4717−3p, rectal
(SURV5YRS = 0; N0 = 297)
1−sided adj pval: 0.882
Frequency
Tumor−Normal Expression Difference
hsa−miR−4717−3p, rectal
(SURV5YRS = 1; N1 = 422)
1−sided adj pval: 1
Frequency
Tumor−Normal Expression Difference

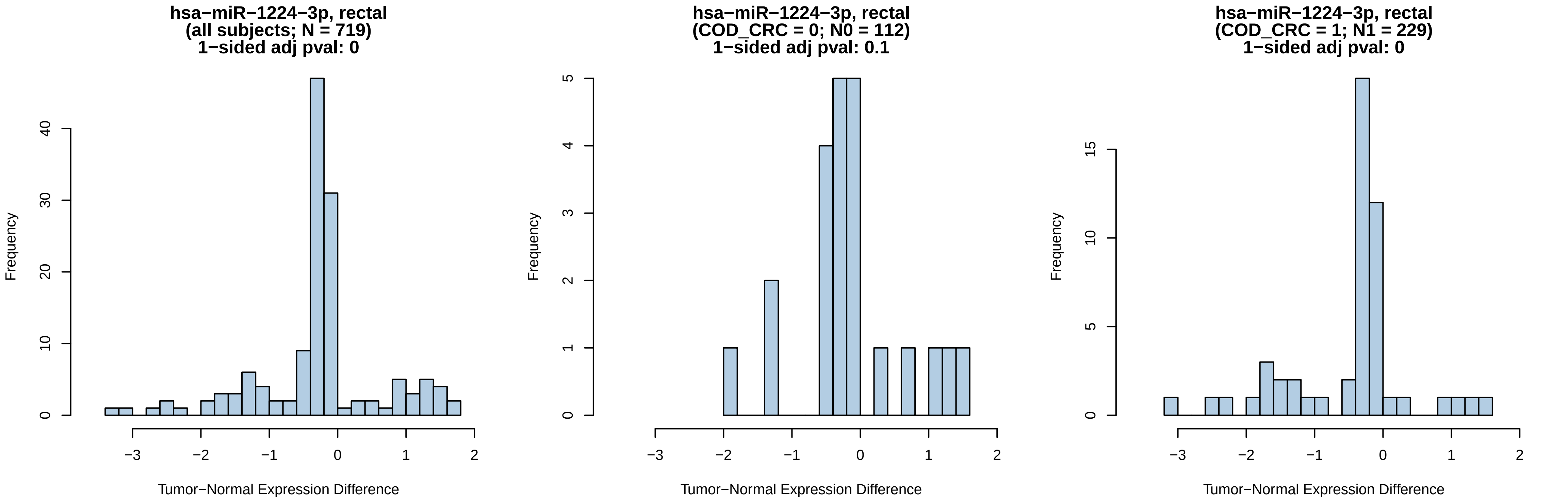

hsa−miR−1224−3p, rectal
(all subjects; N = 719)
1−sided adj pval: 0
Frequency
0
10
20
30
40
−3
−2
−1
0
1
2
Tumor−Normal Expression Difference
hsa−miR−1224−3p, rectal
(COD_CRC = 0; N0 = 112)
1−sided adj pval: 0.1
Frequency
0
1
2
3
4
5
−3
−2
−1
0
1
2
Tumor−Normal Expression Difference
hsa−miR−1224−3p, rectal
(COD_CRC = 1; N1 = 229)
1−sided adj pval: 0
Frequency
0
5
10
15
−3
−2
−1
0
1
2
Tumor−Normal Expression Difference

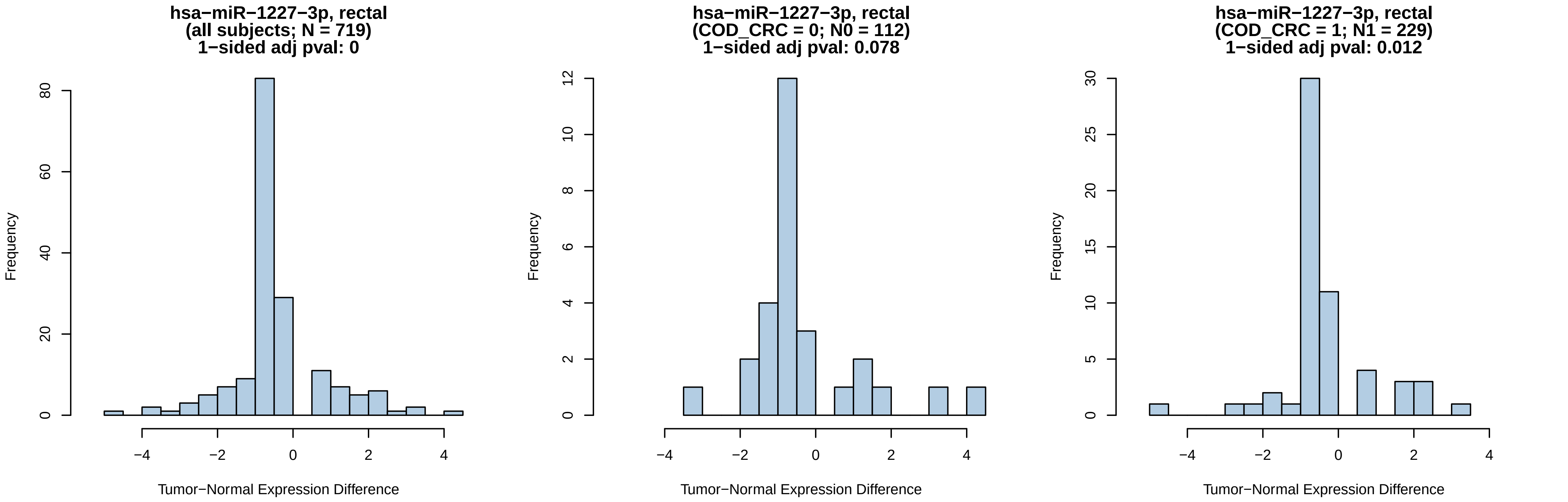
hsa−miR−1227−3p, rectal
(all subjects; N = 719)
1−sided adj pval: 0
Frequency
0
20
40
60
80
−4
−2
0
2
4
Tumor−Normal Expression Difference
hsa−miR−1227−3p, rectal
(COD_CRC = 0; N0 = 112)
1−sided adj pval: 0.078
Frequency
0
2
4
6
8
10
12
−4
−2
0
2
4
Tumor−Normal Expression Difference
hsa−miR−1227−3p, rectal
(COD_CRC = 1; N1 = 229)
1−sided adj pval: 0.012
Frequency
0
5
10
15
20
25
30
−4
−2
0
2
4
Tumor−Normal Expression Difference

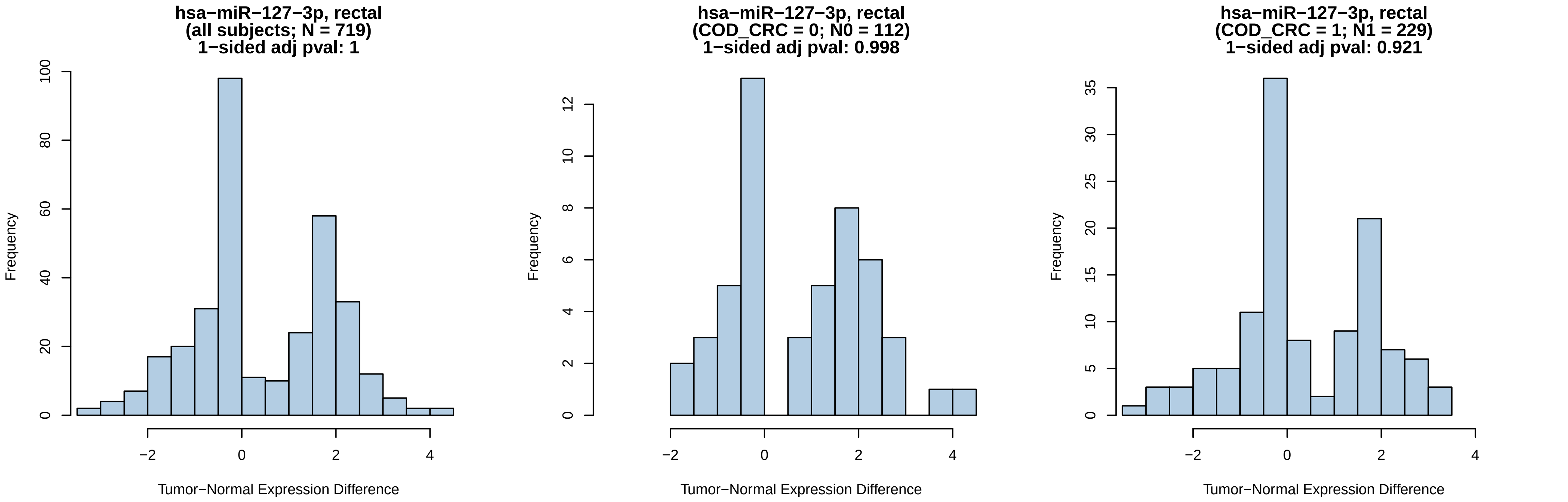
hsa−miR−127−3p, rectal
(all subjects; N = 719)
1−sided adj pval: 1
hsa−miR−127−3p, rectal
(COD_CRC = 0; N0 = 112)
1−sided adj pval: 0.998
hsa−miR−127−3p, rectal
(COD_CRC = 1; N1 = 229)
1−sided adj pval: 0.921
Frequency
Tumor−Normal Expression Difference

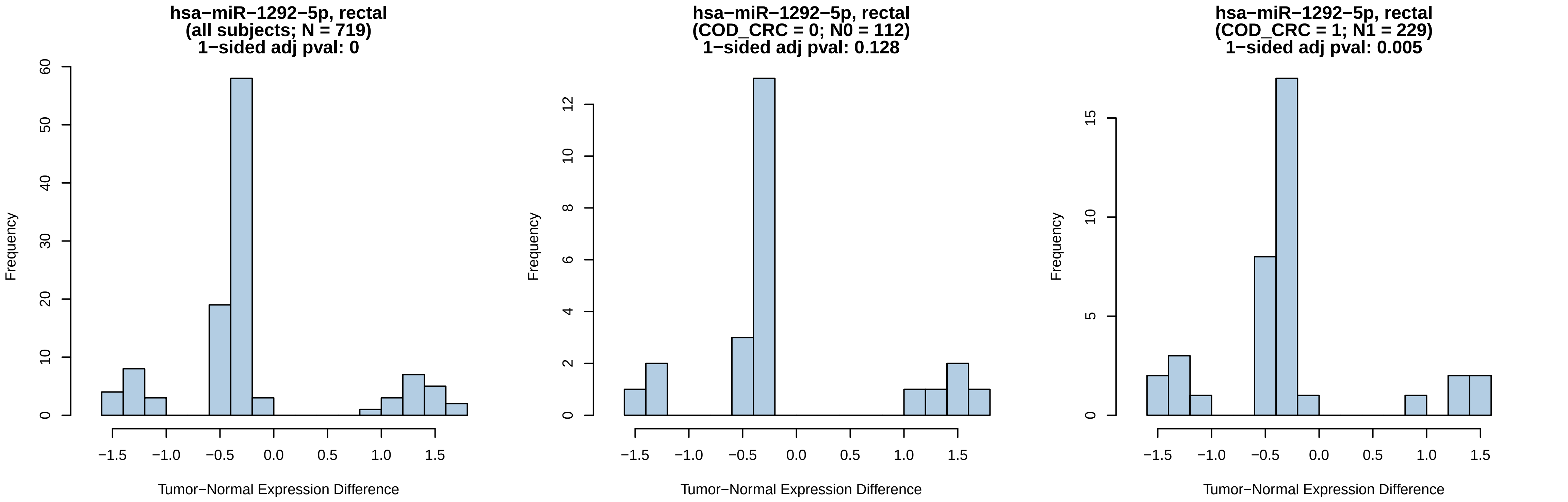

hsa−miR−1292−5p, rectal
(all subjects; N = 719)
1−sided adj pval: 0
hsa−miR−1292−5p, rectal
(COD_CRC = 0; N0 = 112)
1−sided adj pval: 0.128
hsa−miR−1292−5p, rectal
(COD_CRC = 1; N1 = 229)
1−sided adj pval: 0.005
Frequency
Tumor−Normal Expression Difference
0
10
20
30
40
50
60
2
4
6
8
10
12
5
15
−1.5
−1.0
−0.5
0.0
0.5
1.0
1.5

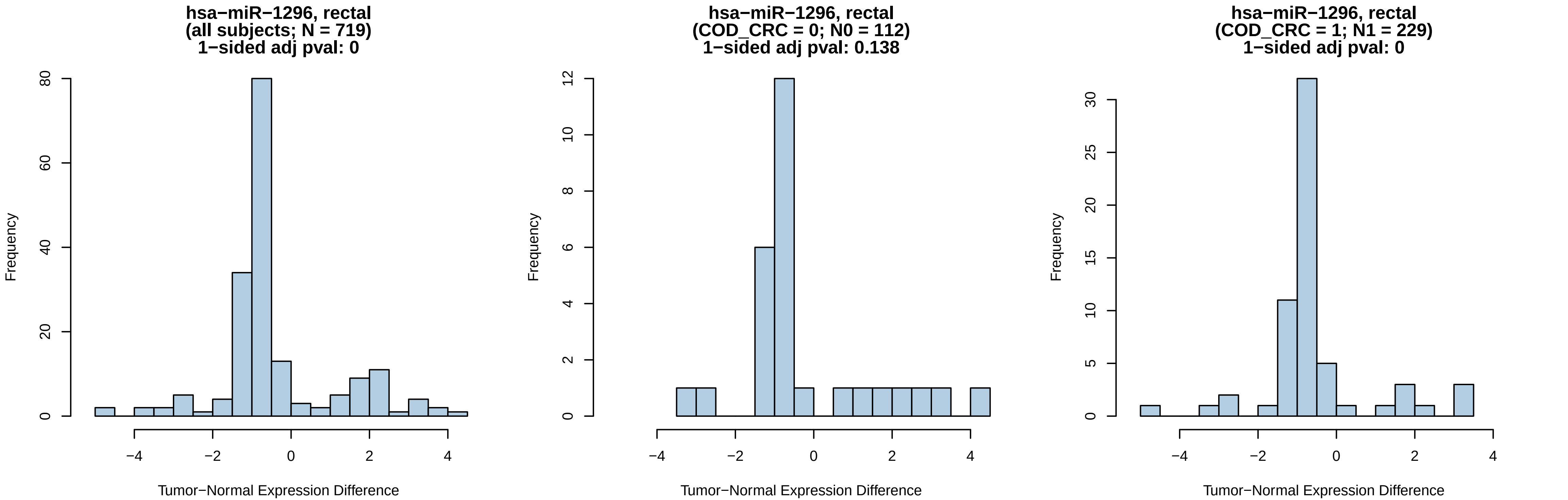
hsa−miR−1296, rectal
(all subjects; N = 719)
1−sided adj pval: 0
Frequency
Tumor−Normal Expression Difference
hsa−miR−1296, rectal
(COD_CRC = 0; N0 = 112)
1−sided adj pval: 0.138
Frequency
Tumor−Normal Expression Difference
hsa−miR−1296, rectal
(COD_CRC = 1; N1 = 229)
1−sided adj pval: 0
Frequency
Tumor−Normal Expression Difference

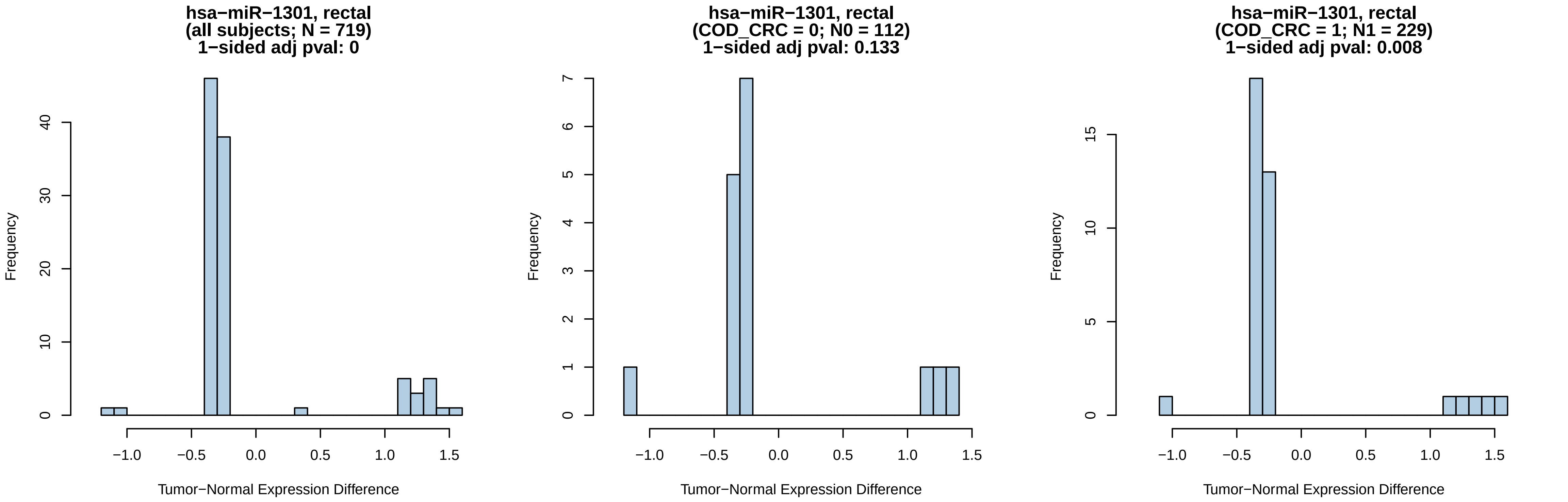

hsa−miR−1301, rectal
(all subjects; N = 719)
1−sided adj pval: 0
Frequency
Tumor−Normal Expression Difference
hsa−miR−1301, rectal
(COD_CRC = 0; N0 = 112)
1−sided adj pval: 0.133
Frequency
Tumor−Normal Expression Difference
hsa−miR−1301, rectal
(COD_CRC = 1; N1 = 229)
1−sided adj pval: 0.008
Frequency
Tumor−Normal Expression Difference

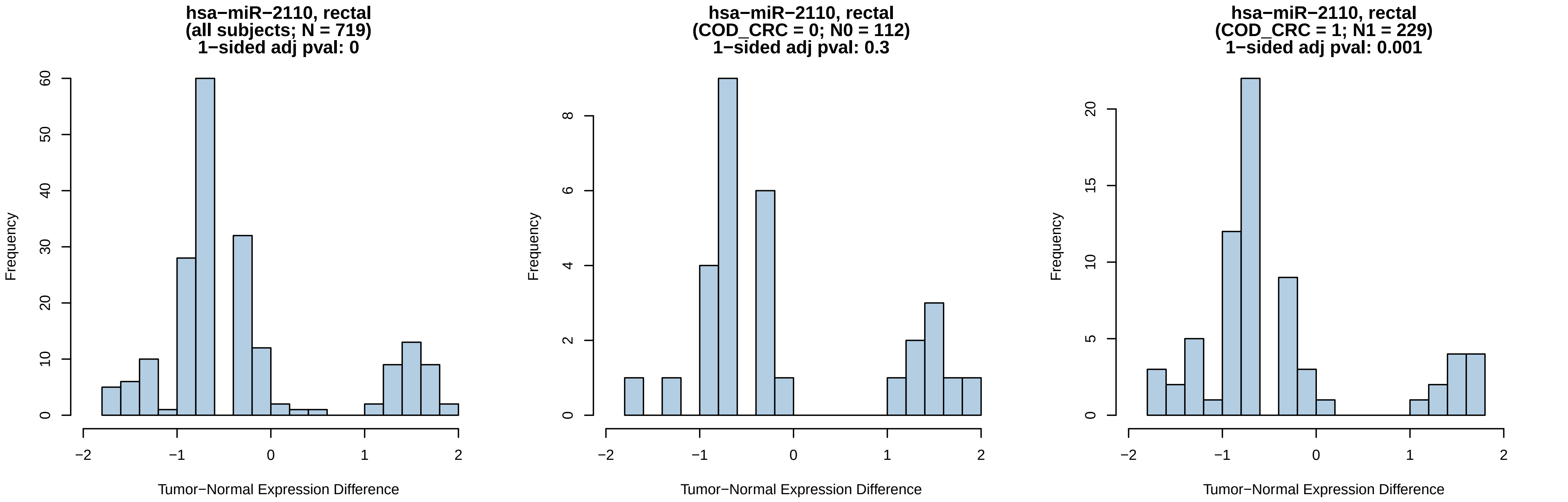

hsa−miR−2110, rectal
(all subjects; N = 719)
1−sided adj pval: 0
hsa−miR−2110, rectal
(COD_CRC = 0; N0 = 112)
1−sided adj pval: 0.3
hsa−miR−2110, rectal
(COD_CRC = 1; N1 = 229)
1−sided adj pval: 0.001
Frequency
Tumor−Normal Expression Difference

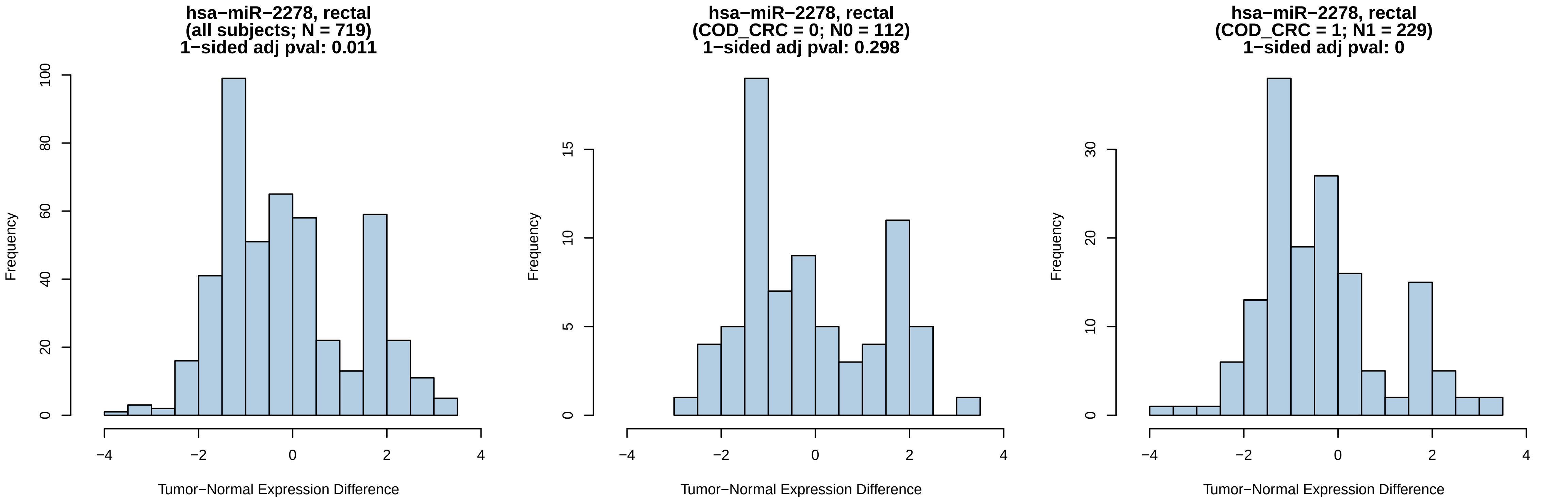
hsa−miR−2278, rectal
(all subjects; N = 719)
1−sided adj pval: 0.011
hsa−miR−2278, rectal
(COD_CRC = 0; N0 = 112)
1−sided adj pval: 0.298
hsa−miR−2278, rectal
(COD_CRC = 1; N1 = 229)
1−sided adj pval: 0
Frequency
Tumor−Normal Expression Difference

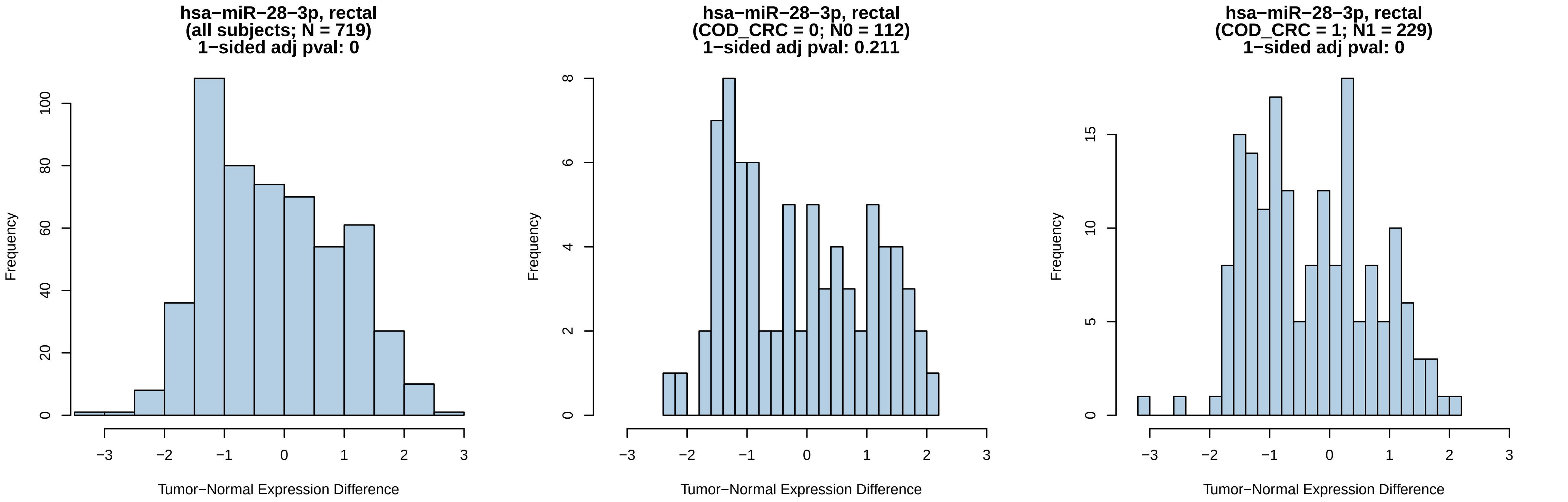
hsa−miR−28−3p, rectal
(all subjects; N = 719)
1−sided adj pval: 0
Frequency
Tumor−Normal Expression Difference
hsa−miR−28−3p, rectal
(COD_CRC = 0; N0 = 112)
1−sided adj pval: 0.211
Frequency
Tumor−Normal Expression Difference
hsa−miR−28−3p, rectal
(COD_CRC = 1; N1 = 229)
1−sided adj pval: 0
Frequency
Tumor−Normal Expression Difference

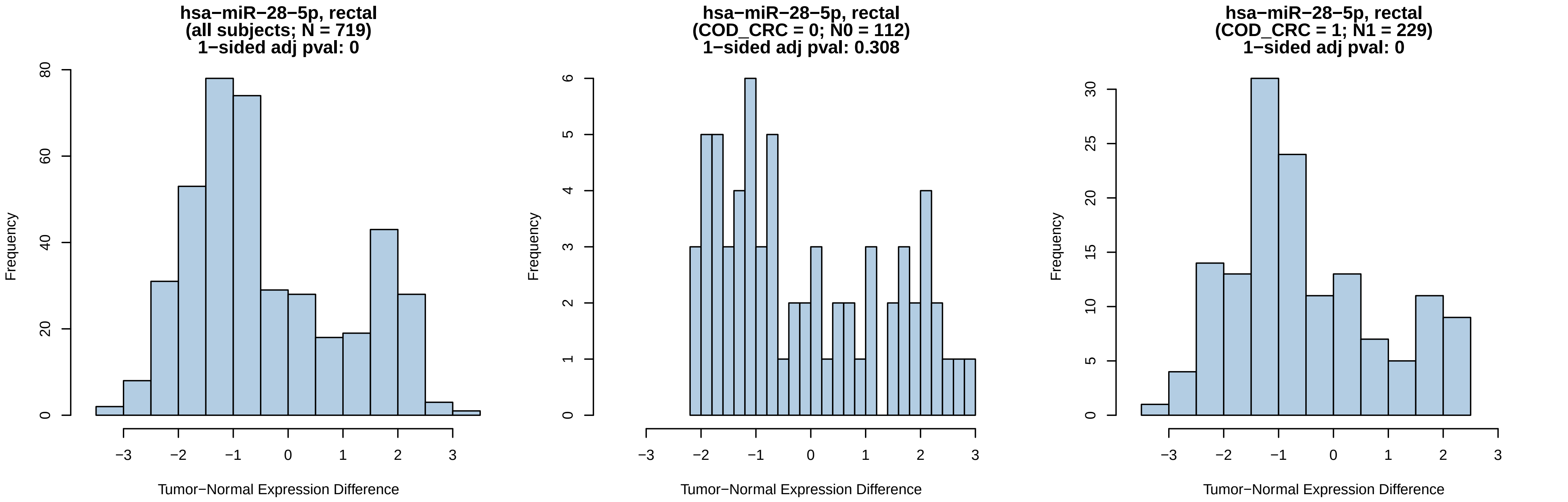
hsa−miR−28−5p, rectal
(all subjects; N = 719)
1−sided adj pval: 0
hsa−miR−28−5p, rectal
(COD_CRC = 0; N0 = 112)
1−sided adj pval: 0.308
hsa−miR−28−5p, rectal
(COD_CRC = 1; N1 = 229)
1−sided adj pval: 0
Frequency
Tumor−Normal Expression Difference

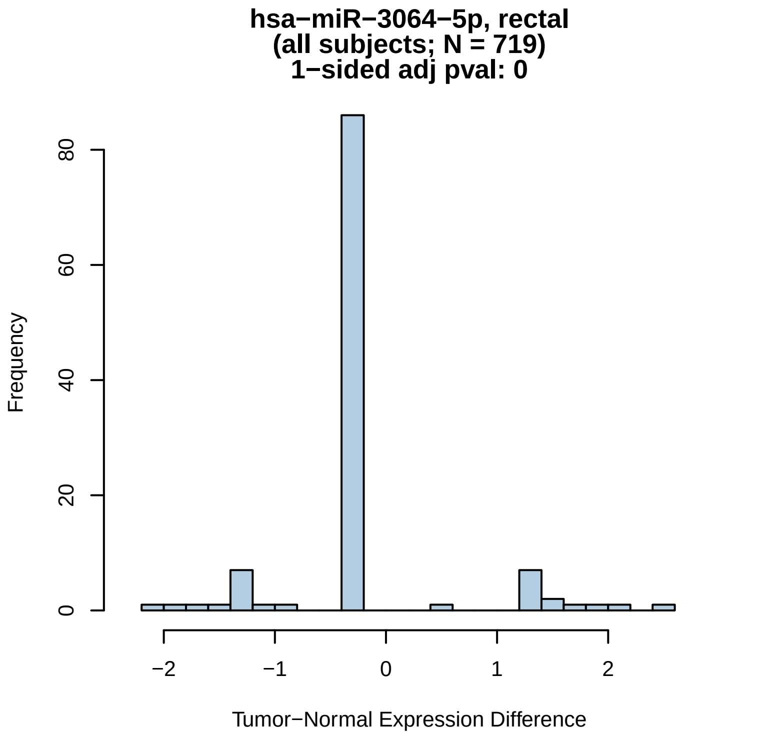
hsa−miR−3064−5p, rectal
(all subjects; N = 719)
1−sided adj pval: 0
Frequency
0
20
40
60
80
−2
−1
0
1
2
Tumor−Normal Expression Difference

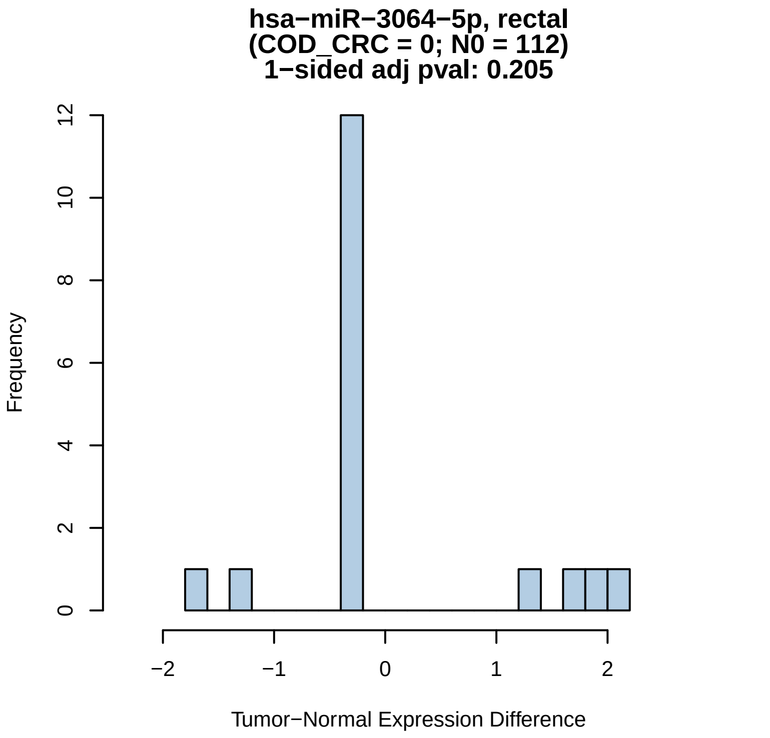
hsa−miR−3064−5p, rectal
(COD_CRC = 0; N0 = 112)
1−sided adj pval: 0.205
Frequency
0
2
4
6
8
10
12
−2
−1
0
1
2
Tumor−Normal Expression Difference

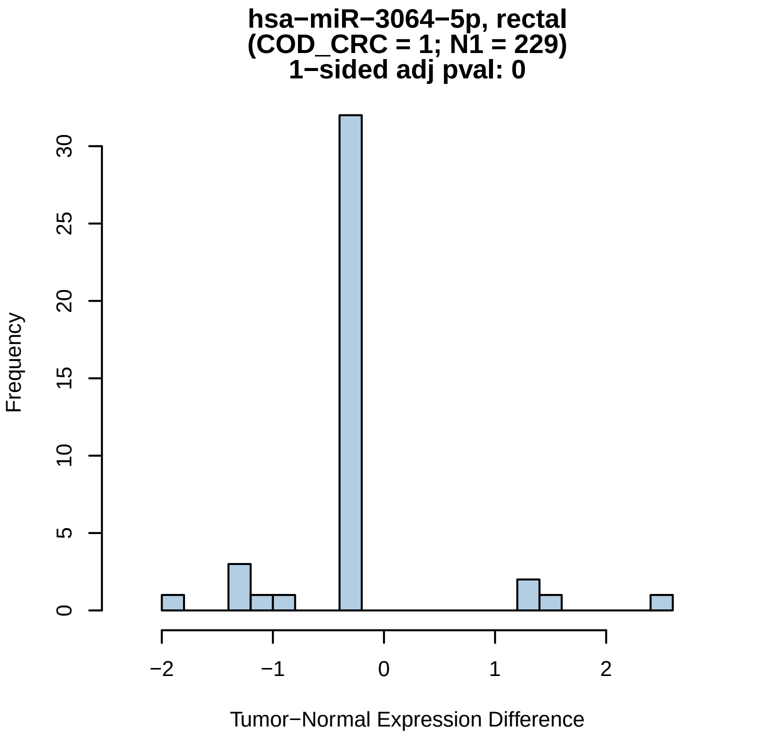
hsa−miR−3064−5p, rectal
(COD_CRC = 1; N1 = 229)
1−sided adj pval: 0
Frequency
0
5
10
15
20
25
30
−2
−1
0
1
2
Tumor−Normal Expression Difference

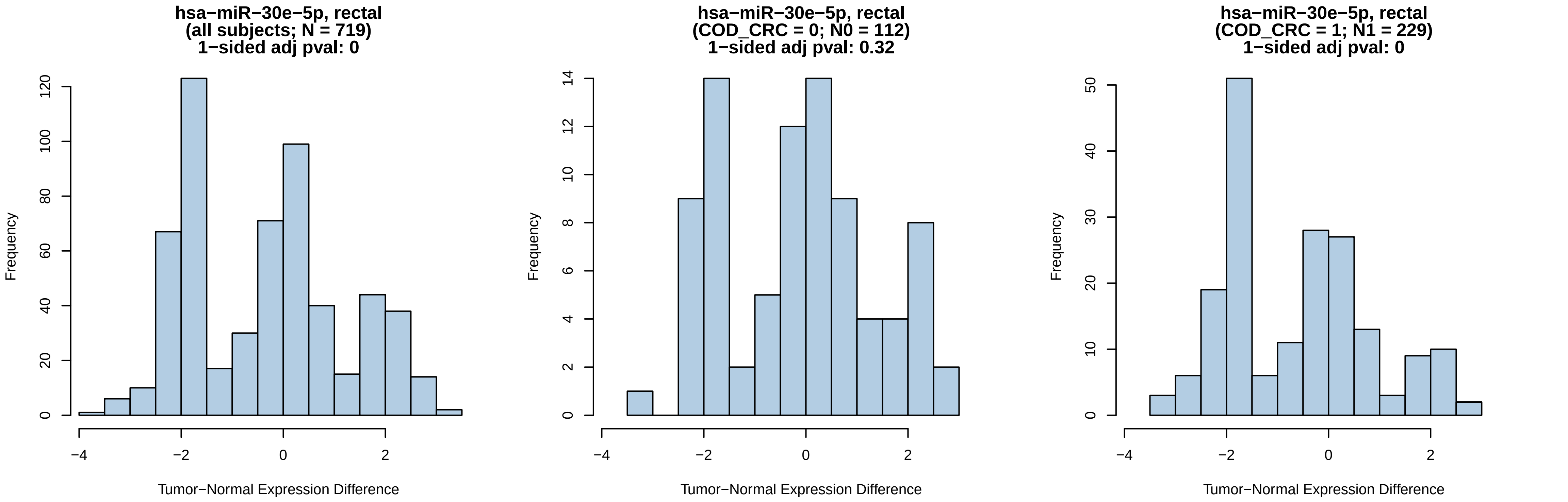
hsa−miR−30e−5p, rectal
(all subjects; N = 719)
1−sided adj pval: 0
Frequency
Tumor−Normal Expression Difference
hsa−miR−30e−5p, rectal
(COD_CRC = 0; N0 = 112)
1−sided adj pval: 0.32
Frequency
Tumor−Normal Expression Difference
hsa−miR−30e−5p, rectal
(COD_CRC = 1; N1 = 229)
1−sided adj pval: 0
Frequency
Tumor−Normal Expression Difference

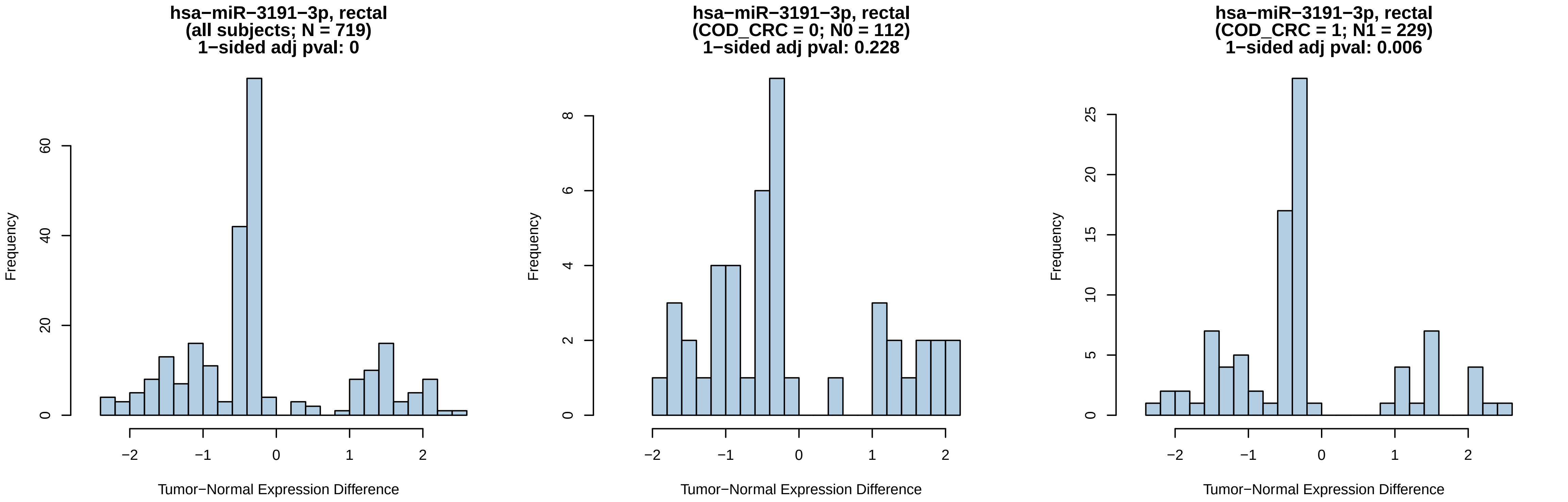
hsa−miR−3191−3p, rectal
(all subjects; N = 719)
1−sided adj pval: 0
Frequency
0
20
40
60
−2
−1
0
1
2
Tumor−Normal Expression Difference
hsa−miR−3191−3p, rectal
(COD_CRC = 0; N0 = 112)
1−sided adj pval: 0.228
Frequency
0
2
4
6
8
−2
−1
0
1
2
Tumor−Normal Expression Difference
hsa−miR−3191−3p, rectal
(COD_CRC = 1; N1 = 229)
1−sided adj pval: 0.006
Frequency
0
5
10
15
20
25
−2
−1
0
1
2
Tumor−Normal Expression Difference

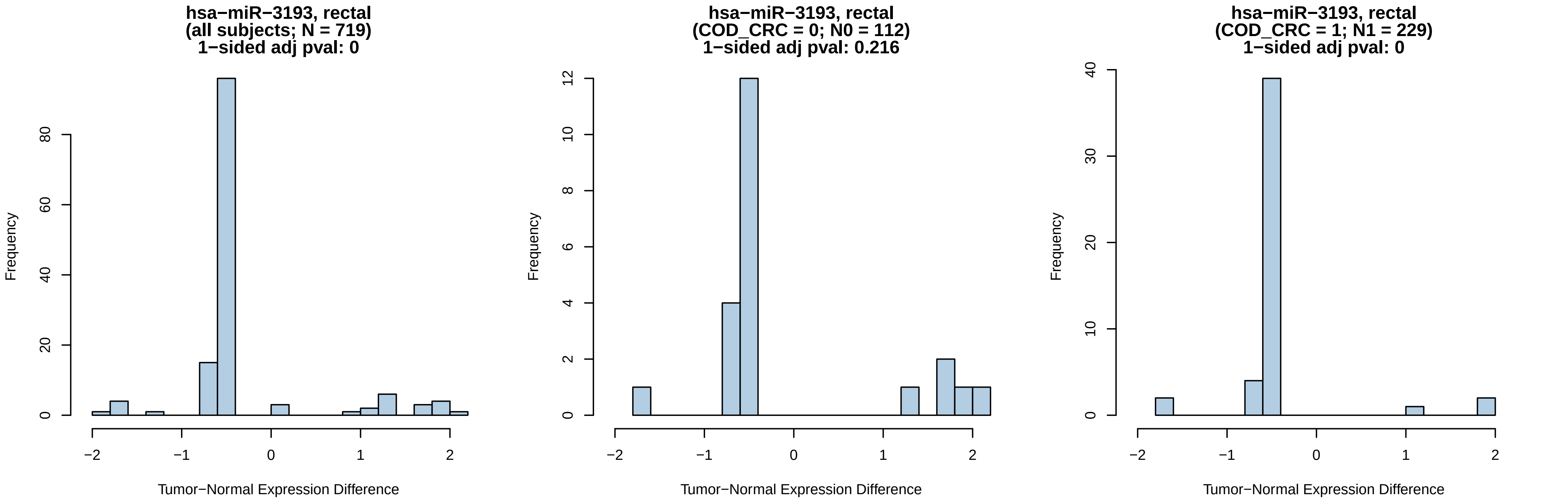

hsa−miR−3193, rectal
(all subjects; N = 719)
1−sided adj pval: 0
Frequency
Tumor−Normal Expression Difference
hsa−miR−3193, rectal
(COD_CRC = 0; N0 = 112)
1−sided adj pval: 0.216
Frequency
Tumor−Normal Expression Difference
hsa−miR−3193, rectal
(COD_CRC = 1; N1 = 229)
1−sided adj pval: 0
Frequency
Tumor−Normal Expression Difference

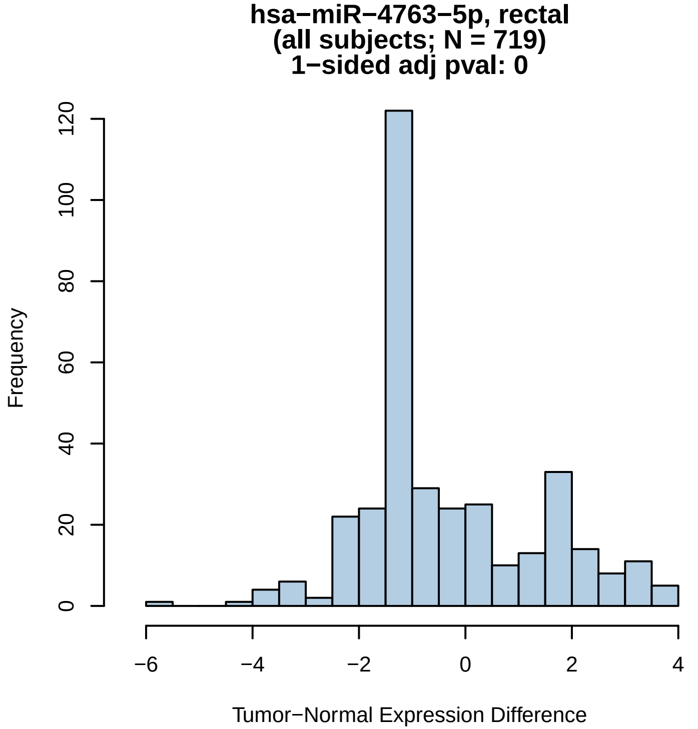
hsa−miR−4763−5p, rectal
(all subjects; N = 719)
1−sided adj pval: 0
Frequency
Tumor−Normal Expression Difference
0
20
40
60
80
100
120
−6
−4
−2
0
2
4

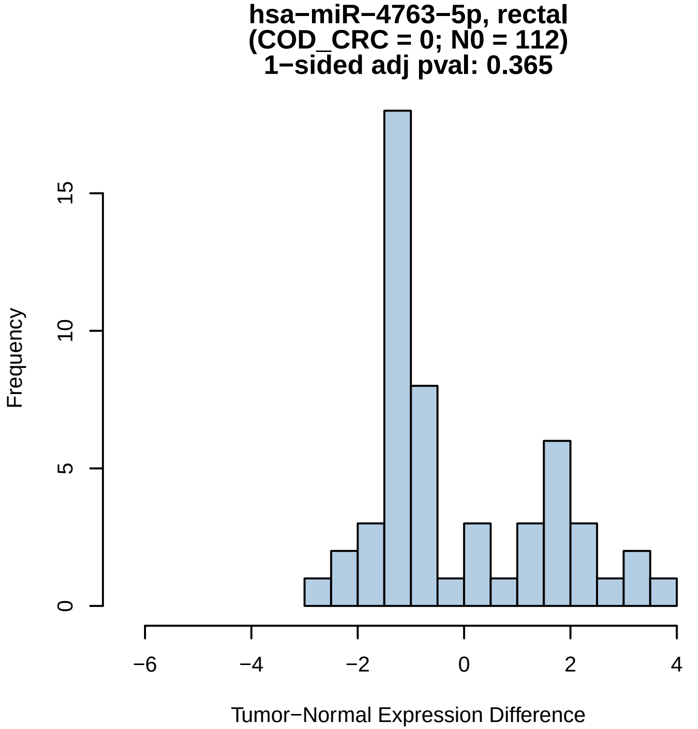
hsa−miR−4763−5p, rectal
(COD_CRC = 0; N0 = 112)
1−sided adj pval: 0.365
Frequency
Tumor−Normal Expression Difference
0
5
10
15
−6
−4
−2
0
2
4

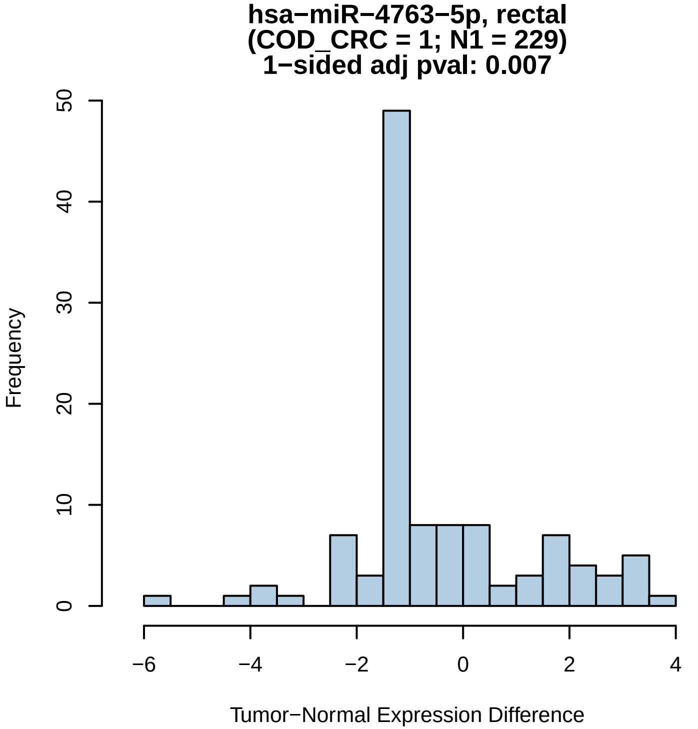
hsa−miR−4763−5p, rectal
(COD_CRC = 1; N1 = 229)
1−sided adj pval: 0.007
Frequency
Tumor−Normal Expression Difference
0
10
20
30
40
50
−6
−4
−2
0
2
4

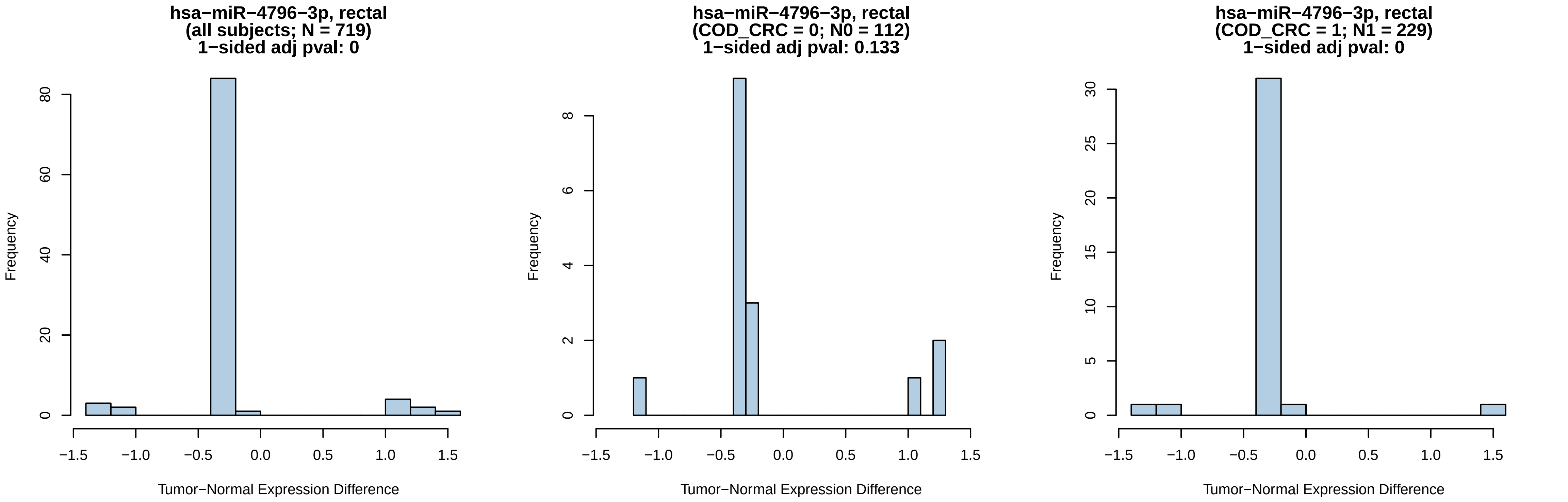

hsa−miR−4796−3p, rectal
(all subjects; N = 719)
1−sided adj pval: 0
Frequency
Tumor−Normal Expression Difference
hsa−miR−4796−3p, rectal
(COD_CRC = 0; N0 = 112)
1−sided adj pval: 0.133
Frequency
Tumor−Normal Expression Difference
hsa−miR−4796−3p, rectal
(COD_CRC = 1; N1 = 229)
1−sided adj pval: 0
Frequency
Tumor−Normal Expression Difference

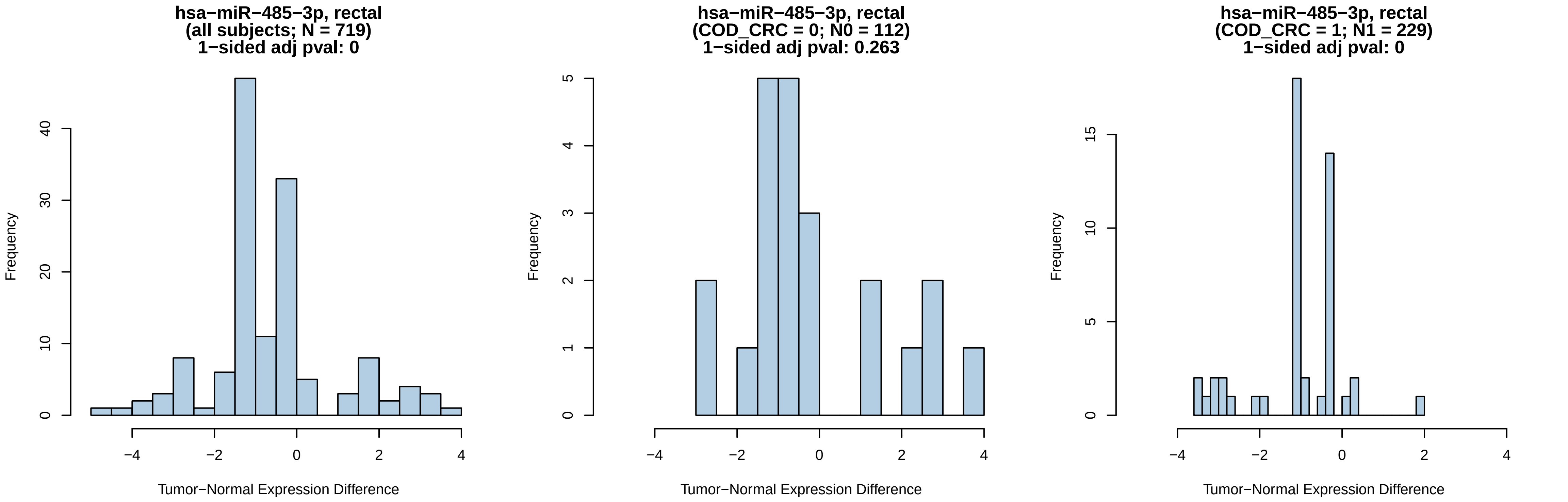
hsa−miR−485−3p, rectal
(all subjects; N = 719)
1−sided adj pval: 0
Frequency
Tumor−Normal Expression Difference
hsa−miR−485−3p, rectal
(COD_CRC = 0; N0 = 112)
1−sided adj pval: 0.263
Frequency
Tumor−Normal Expression Difference
hsa−miR−485−3p, rectal
(COD_CRC = 1; N1 = 229)
1−sided adj pval: 0
Frequency
Tumor−Normal Expression Difference

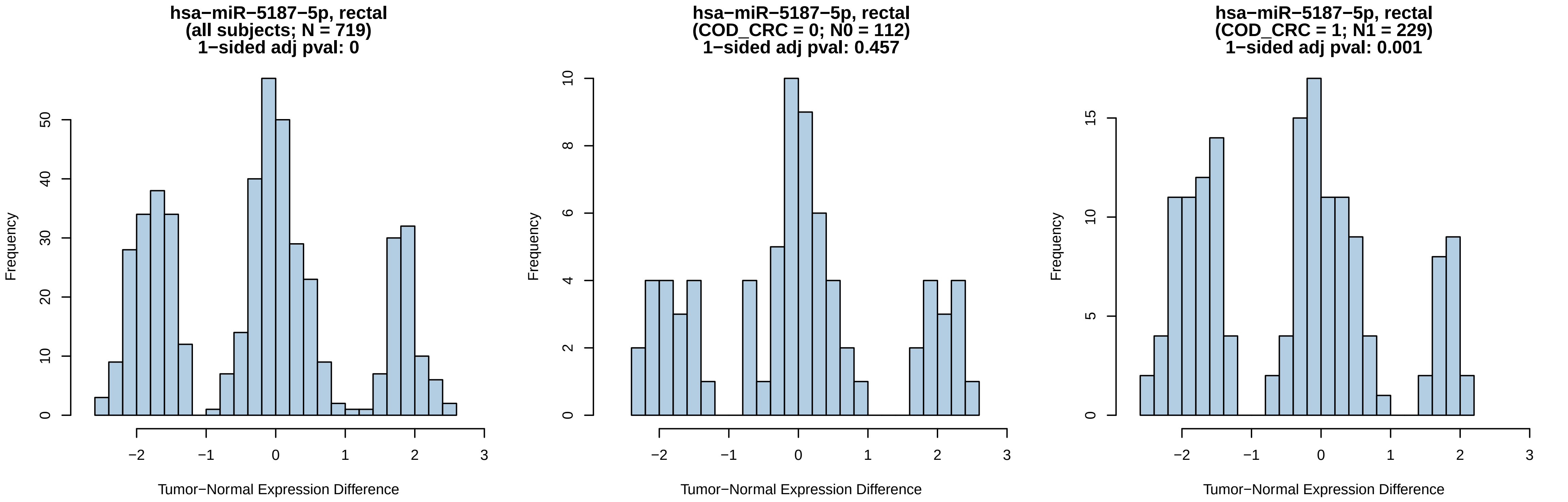

hsa−miR−5187−5p, rectal
(all subjects; N = 719)
1−sided adj pval: 0
Frequency
0
10
20
30
40
50
−2
−1
0
1
2
3
Tumor−Normal Expression Difference
hsa−miR−5187−5p, rectal
(COD_CRC = 0; N0 = 112)
1−sided adj pval: 0.457
Frequency
0
2
4
6
8
10
−2
−1
0
1
2
3
Tumor−Normal Expression Difference
hsa−miR−5187−5p, rectal
(COD_CRC = 1; N1 = 229)
1−sided adj pval: 0.001
Frequency
0
5
10
15
−2
−1
0
1
2
3
Tumor−Normal Expression Difference

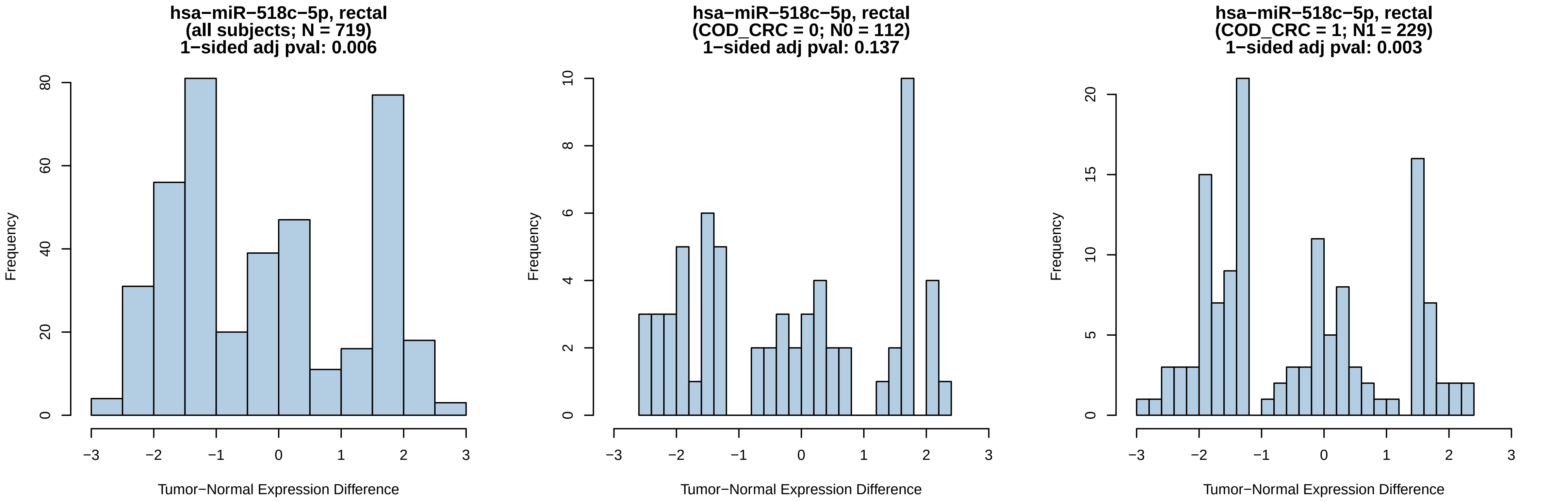

hsa−miR−518c−5p, rectal
(all subjects; N = 719)
1−sided adj pval: 0.006
Frequency
Tumor−Normal Expression Difference
hsa−miR−518c−5p, rectal
(COD_CRC = 0; N0 = 112)
1−sided adj pval: 0.137
Frequency
Tumor−Normal Expression Difference
hsa−miR−518c−5p, rectal
(COD_CRC = 1; N1 = 229)
1−sided adj pval: 0.003
Frequency
Tumor−Normal Expression Difference

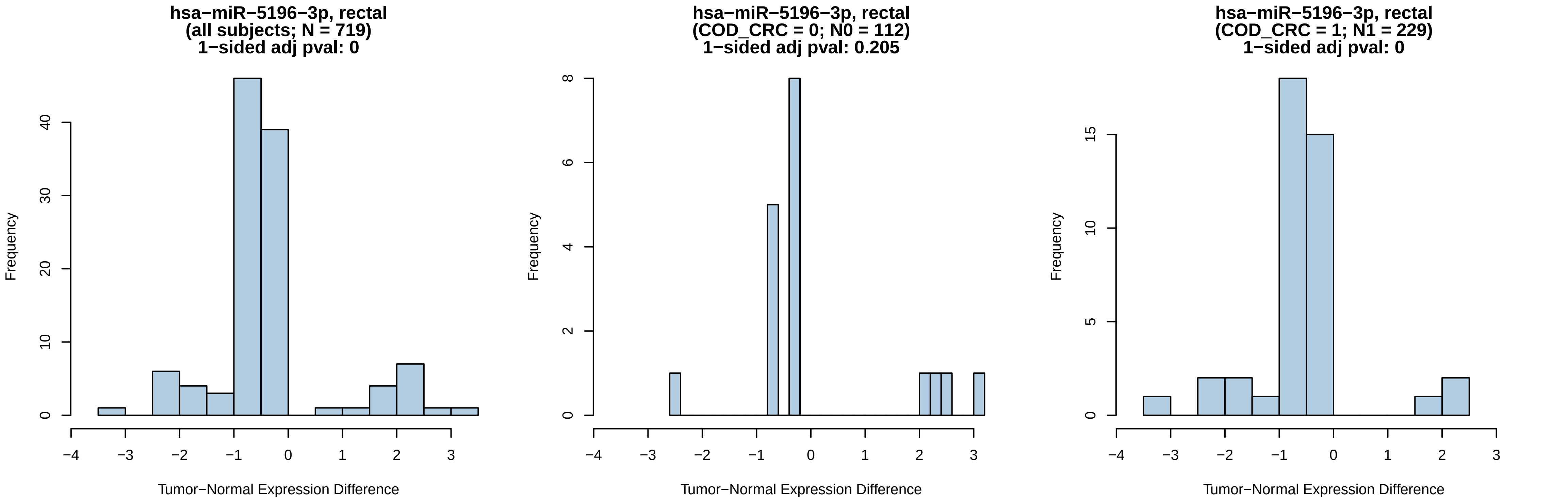
hsa−miR−5196−3p, rectal
(all subjects; N = 719)
1−sided adj pval: 0
Frequency
Tumor−Normal Expression Difference
hsa−miR−5196−3p, rectal
(COD_CRC = 0; N0 = 112)
1−sided adj pval: 0.205
Frequency
Tumor−Normal Expression Difference
hsa−miR−5196−3p, rectal
(COD_CRC = 1; N1 = 229)
1−sided adj pval: 0
Frequency
Tumor−Normal Expression Difference

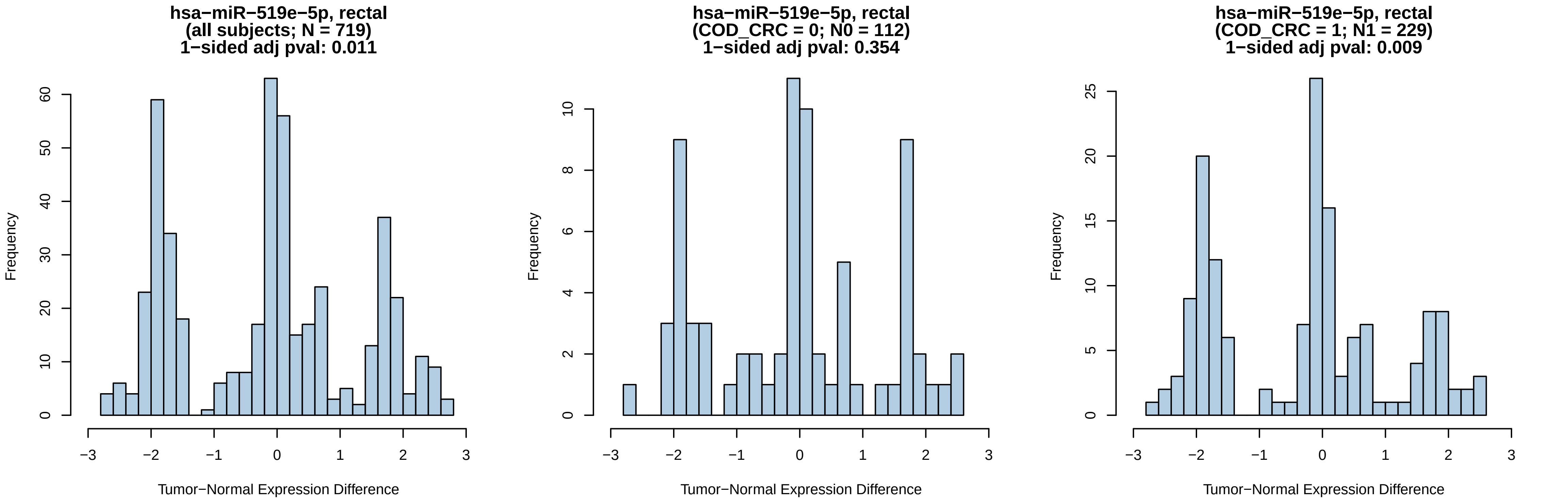

hsa−miR−519e−5p, rectal
(all subjects; N = 719)
1−sided adj pval: 0.011
Frequency
Tumor−Normal Expression Difference
hsa−miR−519e−5p, rectal
(COD_CRC = 0; N0 = 112)
1−sided adj pval: 0.354
Frequency
Tumor−Normal Expression Difference
hsa−miR−519e−5p, rectal
(COD_CRC = 1; N1 = 229)
1−sided adj pval: 0.009
Frequency
Tumor−Normal Expression Difference

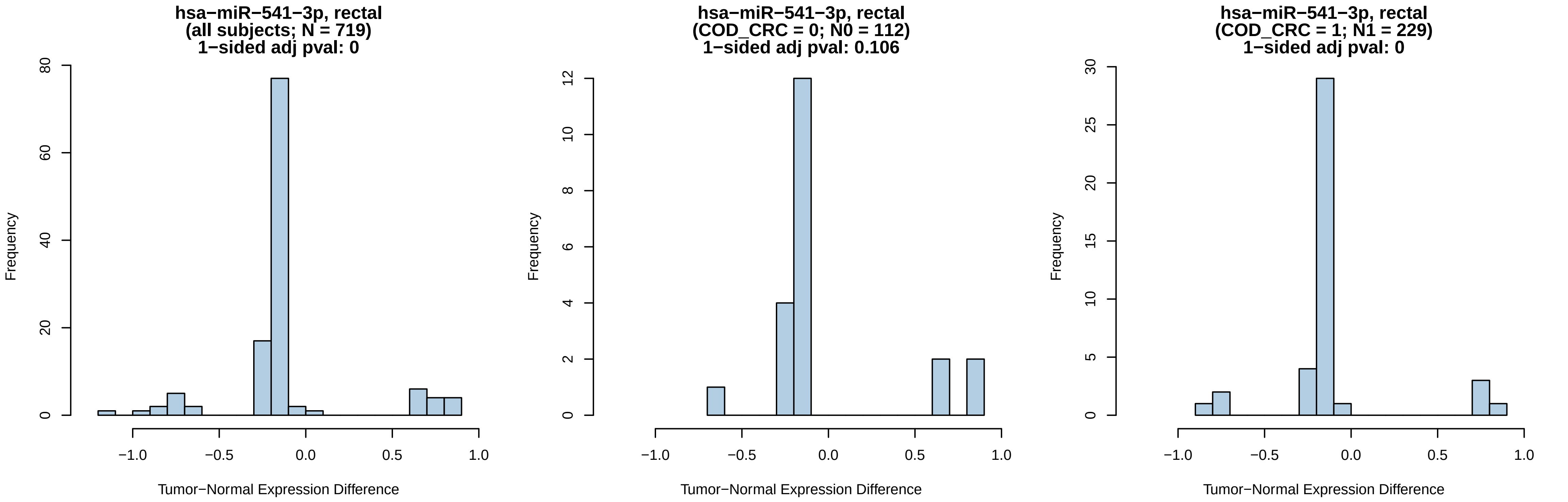
hsa−miR−541−3p, rectal
(all subjects; N = 719)
1−sided adj pval: 0
hsa−miR−541−3p, rectal
(COD_CRC = 0; N0 = 112)
1−sided adj pval: 0.106
hsa−miR−541−3p, rectal
(COD_CRC = 1; N1 = 229)
1−sided adj pval: 0
Frequency
Tumor−Normal Expression Difference

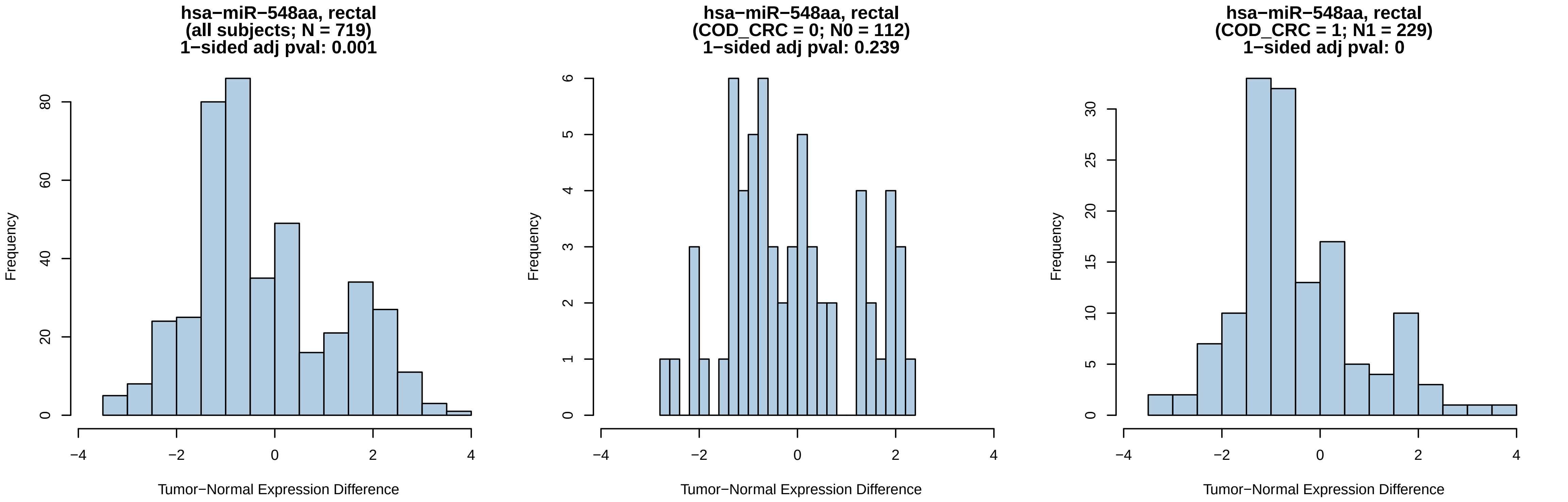
hsa−miR−548aa, rectal
(all subjects; N = 719)
1−sided adj pval: 0.001
Frequency
Tumor−Normal Expression Difference
hsa−miR−548aa, rectal
(COD_CRC = 0; N0 = 112)
1−sided adj pval: 0.239
Frequency
Tumor−Normal Expression Difference
hsa−miR−548aa, rectal
(COD_CRC = 1; N1 = 229)
1−sided adj pval: 0
Frequency
Tumor−Normal Expression Difference

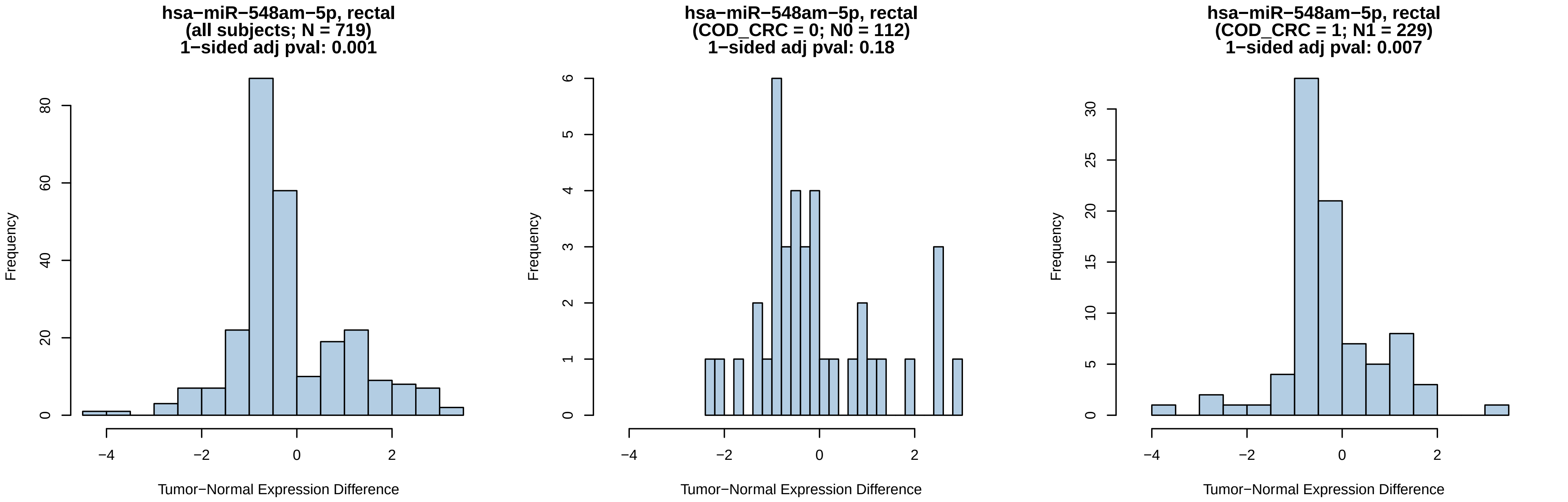

hsa−miR−548am−5p, rectal
(all subjects; N = 719)
1−sided adj pval: 0.001
Frequency
Tumor−Normal Expression Difference
hsa−miR−548am−5p, rectal
(COD_CRC = 0; N0 = 112)
1−sided adj pval: 0.18
Frequency
Tumor−Normal Expression Difference
hsa−miR−548am−5p, rectal
(COD_CRC = 1; N1 = 229)
1−sided adj pval: 0.007
Frequency
Tumor−Normal Expression Difference

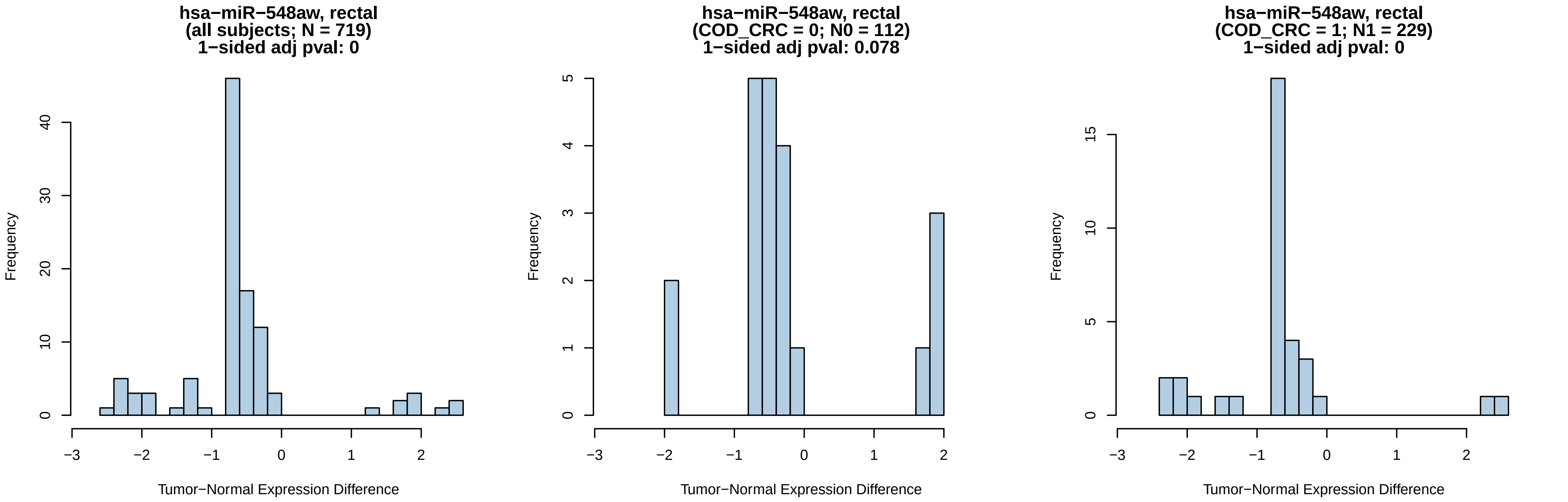
hsa−miR−548aw, rectal
(all subjects; N = 719)
1−sided adj pval: 0
hsa−miR−548aw, rectal
(COD_CRC = 0; N0 = 112)
1−sided adj pval: 0.078
hsa−miR−548aw, rectal
(COD_CRC = 1; N1 = 229)
1−sided adj pval: 0
Frequency
Tumor−Normal Expression Difference

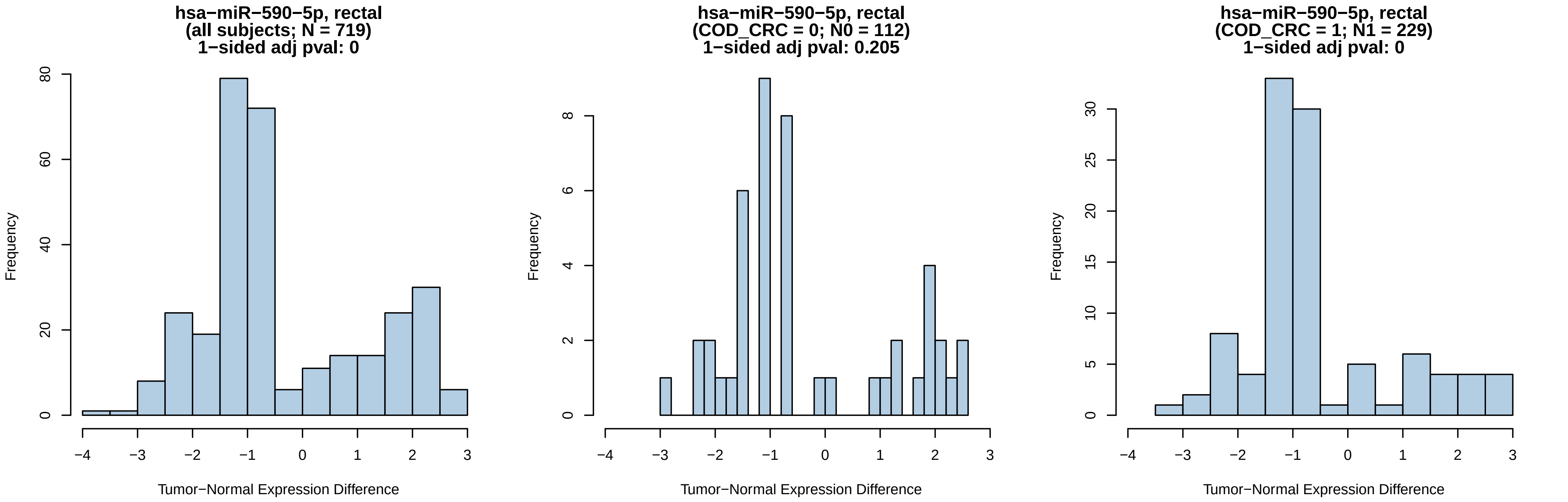
hsa−miR−590−5p, rectal
(all subjects; N = 719)
1−sided adj pval: 0
Frequency
Tumor−Normal Expression Difference
hsa−miR−590−5p, rectal
(COD_CRC = 0; N0 = 112)
1−sided adj pval: 0.205
Frequency
Tumor−Normal Expression Difference
hsa−miR−590−5p, rectal
(COD_CRC = 1; N1 = 229)
1−sided adj pval: 0
Frequency
Tumor−Normal Expression Difference

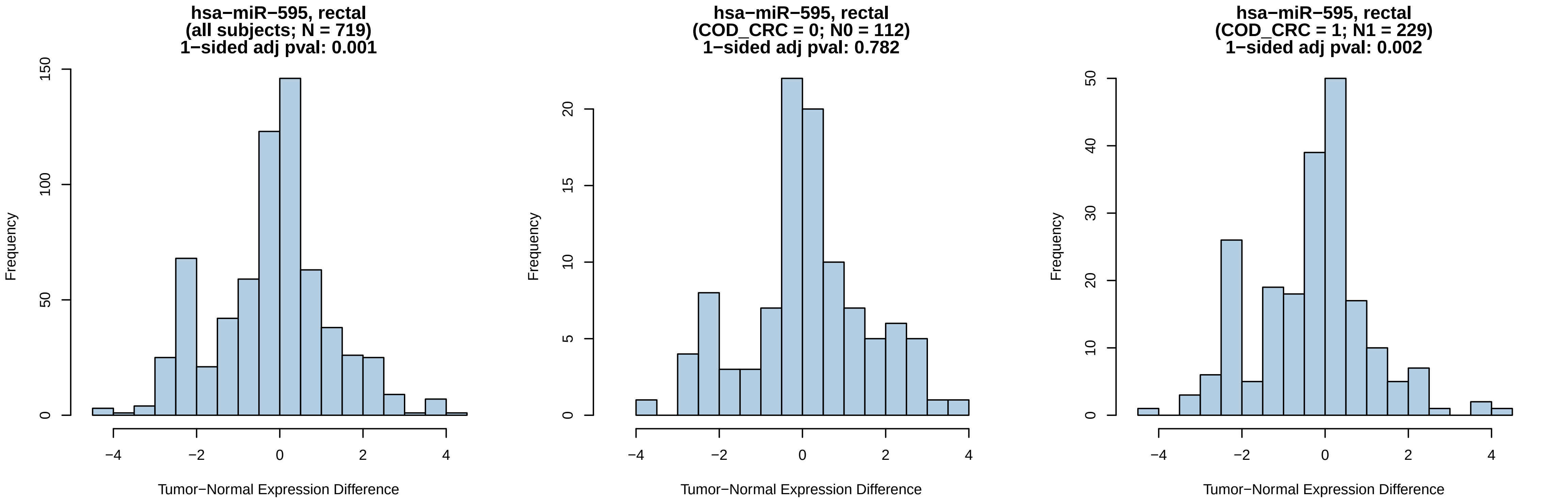
hsa−miR−595, rectal
(all subjects; N = 719)
1−sided adj pval: 0.001
Frequency
Tumor−Normal Expression Difference
hsa−miR−595, rectal
(COD_CRC = 0; N0 = 112)
1−sided adj pval: 0.782
Frequency
Tumor−Normal Expression Difference
hsa−miR−595, rectal
(COD_CRC = 1; N1 = 229)
1−sided adj pval: 0.002
Frequency
Tumor−Normal Expression Difference

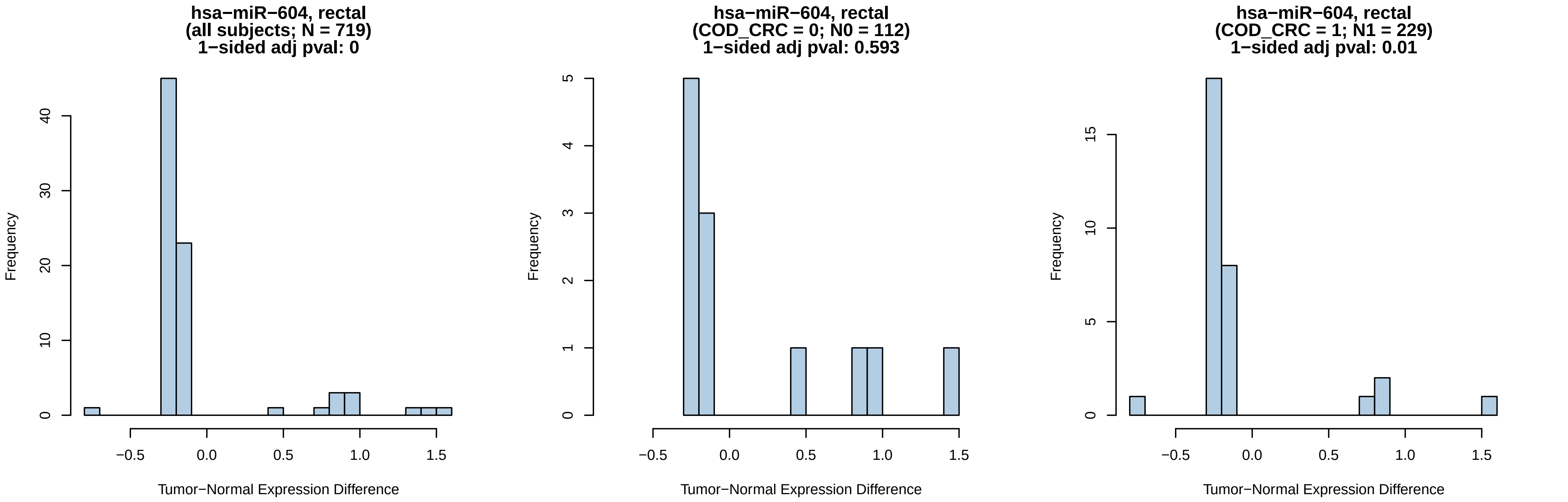

hsa−miR−604, rectal
(all subjects; N = 719)
1−sided adj pval: 0
Frequency
0
10
20
30
40
−0.5
0.0
0.5
1.0
1.5
Tumor−Normal Expression Difference
hsa−miR−604, rectal
(COD_CRC = 0; N0 = 112)
1−sided adj pval: 0.593
Frequency
0
1
2
3
4
5
−0.5
0.0
0.5
1.0
1.5
Tumor−Normal Expression Difference
hsa−miR−604, rectal
(COD_CRC = 1; N1 = 229)
1−sided adj pval: 0.01
Frequency
0
5
10
15
−0.5
0.0
0.5
1.0
1.5
Tumor−Normal Expression Difference

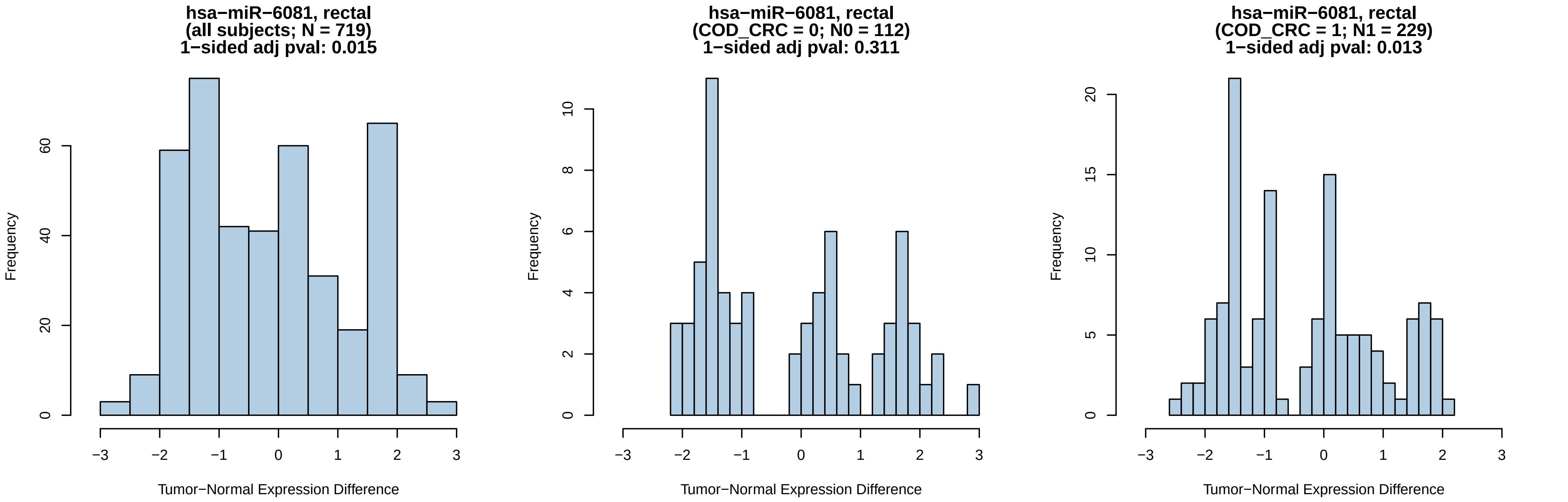
hsa−miR−6081, rectal
(all subjects; N = 719)
1−sided adj pval: 0.015
Frequency
Tumor−Normal Expression Difference
hsa−miR−6081, rectal
(COD_CRC = 0; N0 = 112)
1−sided adj pval: 0.311
Frequency
Tumor−Normal Expression Difference
hsa−miR−6081, rectal
(COD_CRC = 1; N1 = 229)
1−sided adj pval: 0.013
Frequency
Tumor−Normal Expression Difference

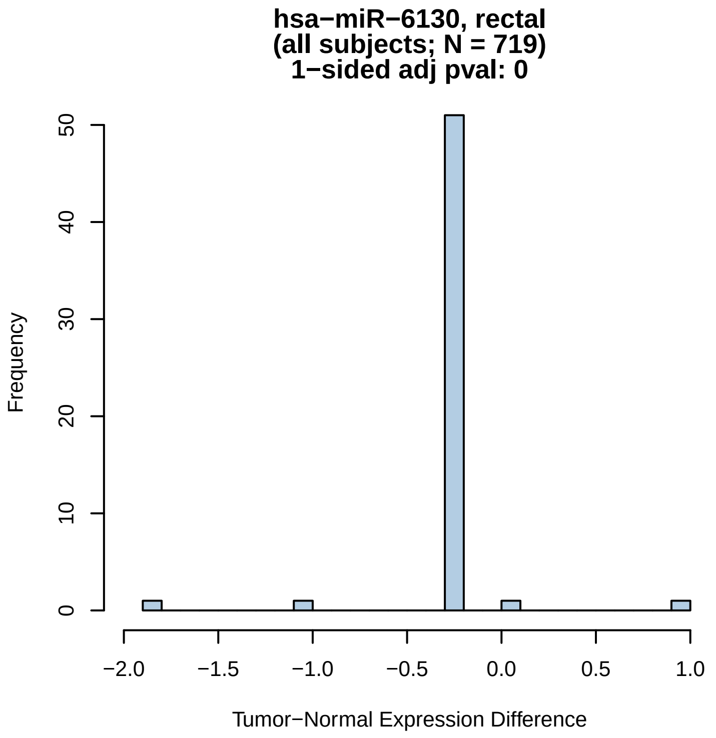

hsa−miR−6130, rectal
(all subjects; N = 719)
1−sided adj pval: 0
Frequency
Tumor−Normal Expression Difference


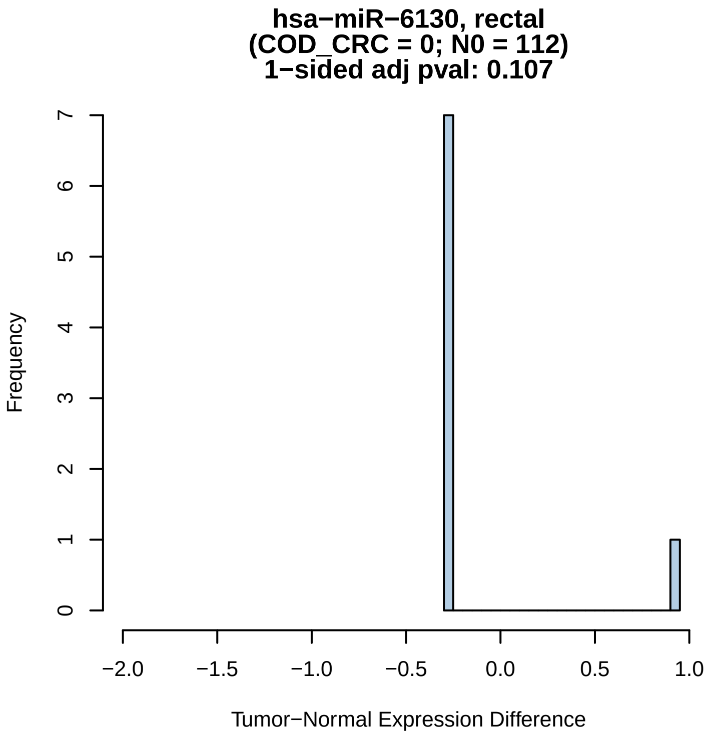

hsa−miR−6130, rectal
(COD_CRC = 0; N0 = 112)
1−sided adj pval: 0.107
Frequency
Tumor−Normal Expression Difference


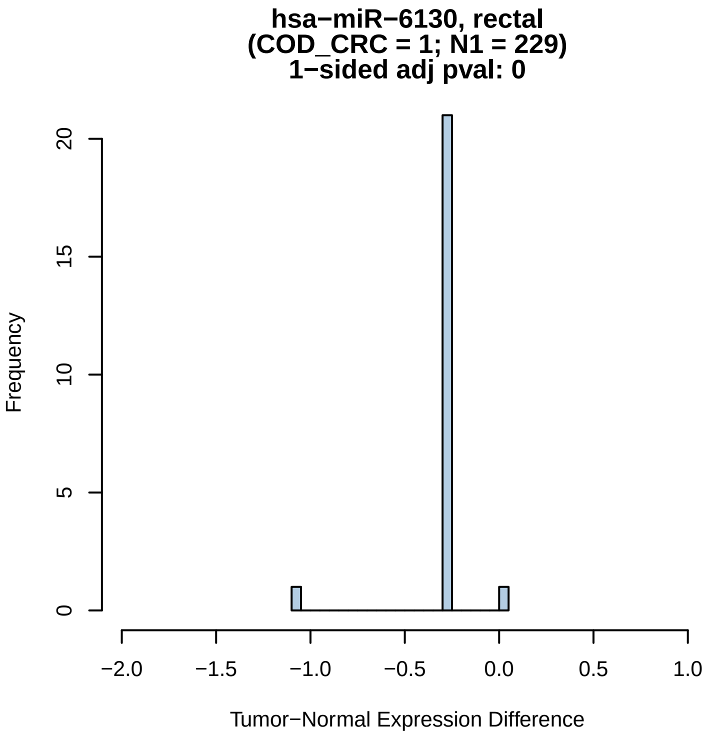

hsa−miR−6130, rectal
(COD_CRC = 1; N1 = 229)
1−sided adj pval: 0
Frequency
Tumor−Normal Expression Difference

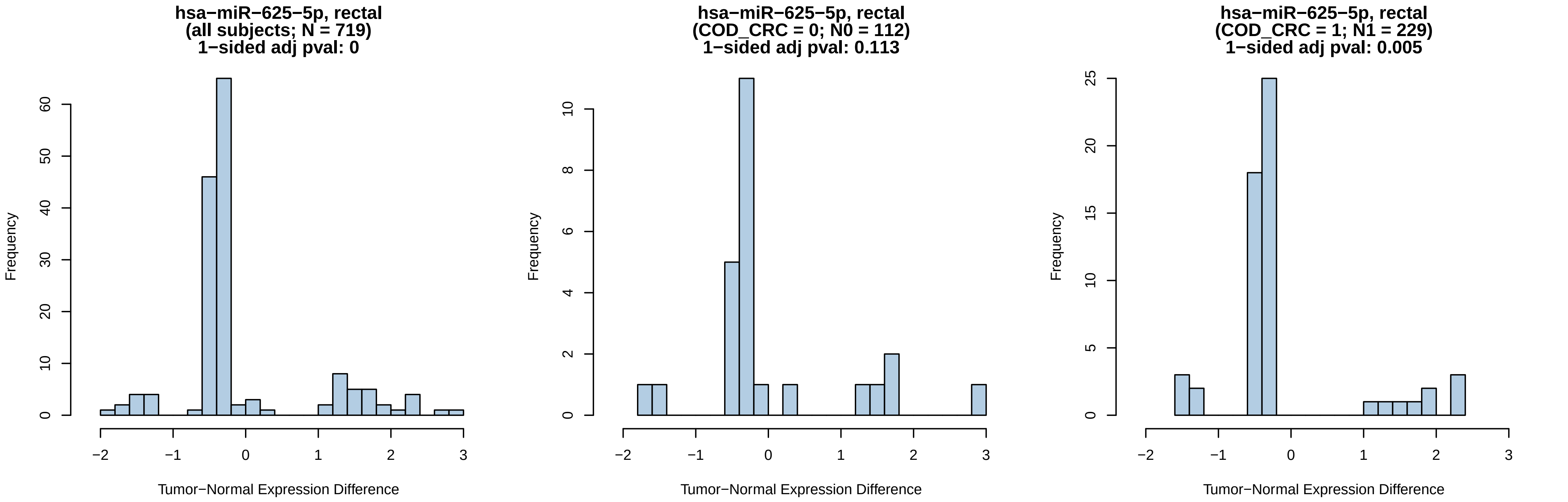
hsa−miR−625−5p, rectal
(all subjects; N = 719)
1−sided adj pval: 0
Frequency
Tumor−Normal Expression Difference
hsa−miR−625−5p, rectal
(COD_CRC = 0; N0 = 112)
1−sided adj pval: 0.113
Frequency
Tumor−Normal Expression Difference
hsa−miR−625−5p, rectal
(COD_CRC = 1; N1 = 229)
1−sided adj pval: 0.005
Frequency
Tumor−Normal Expression Difference

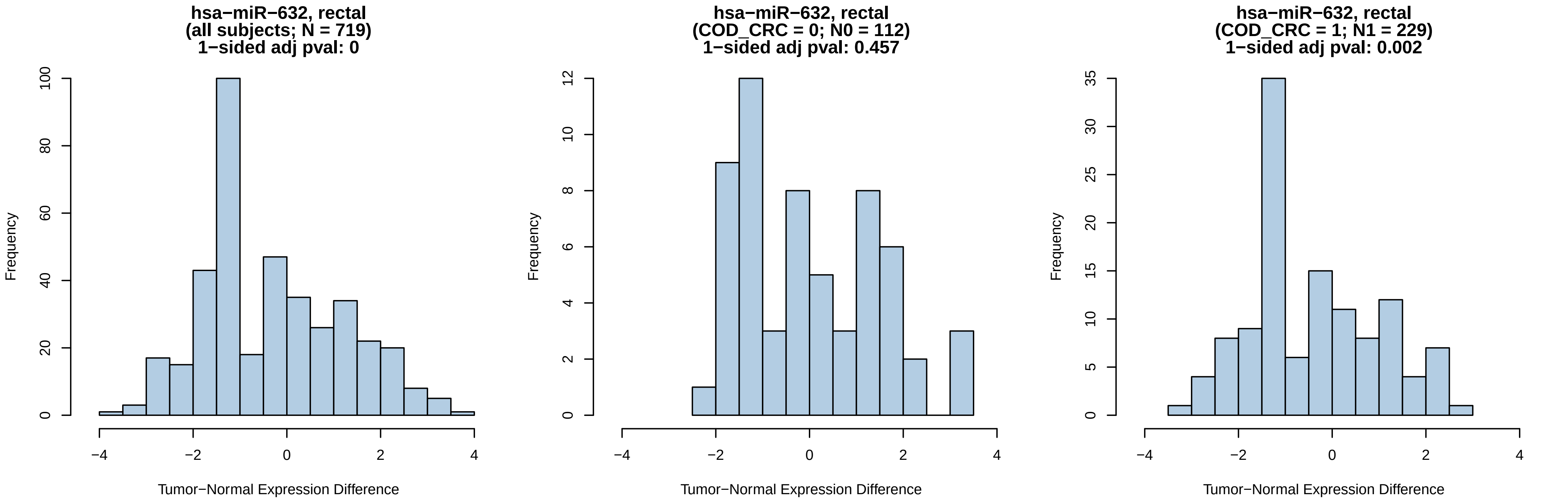
hsa−miR−632, rectal
(all subjects; N = 719)
1−sided adj pval: 0
hsa−miR−632, rectal
(COD_CRC = 0; N0 = 112)
1−sided adj pval: 0.457
hsa−miR−632, rectal
(COD_CRC = 1; N1 = 229)
1−sided adj pval: 0.002
Frequency
Tumor−Normal Expression Difference

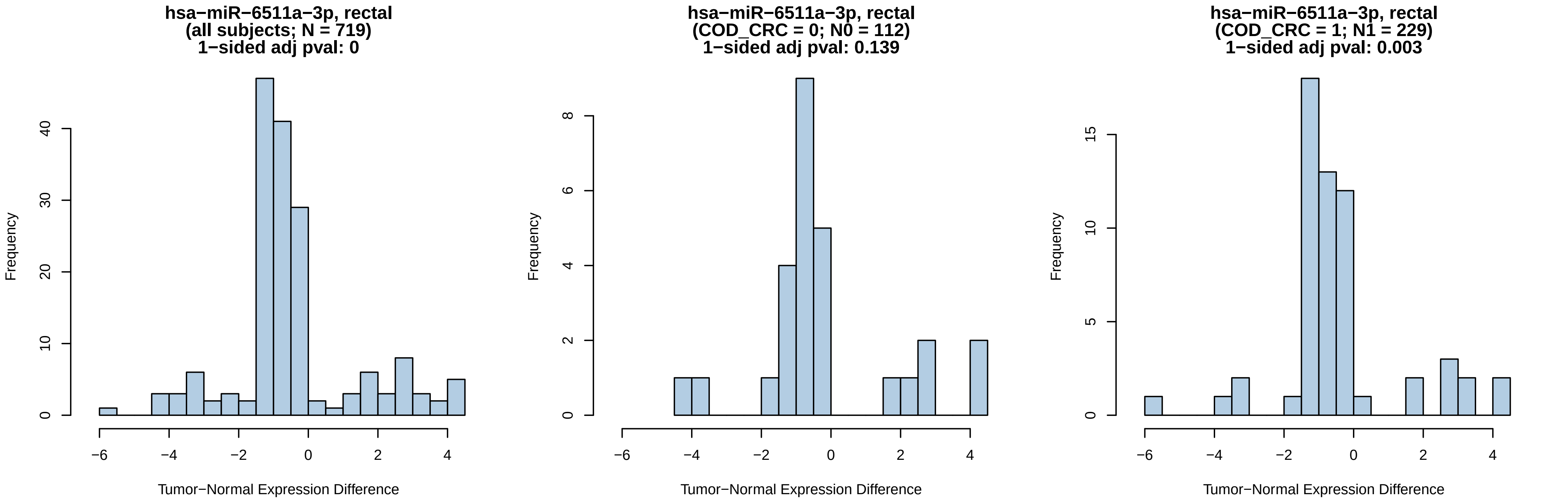

hsa−miR−6511a−3p, rectal
(all subjects; N = 719)
1−sided adj pval: 0
hsa−miR−6511a−3p, rectal
(COD_CRC = 0; N0 = 112)
1−sided adj pval: 0.139
hsa−miR−6511a−3p, rectal
(COD_CRC = 1; N1 = 229)
1−sided adj pval: 0.003
Frequency
Tumor−Normal Expression Difference

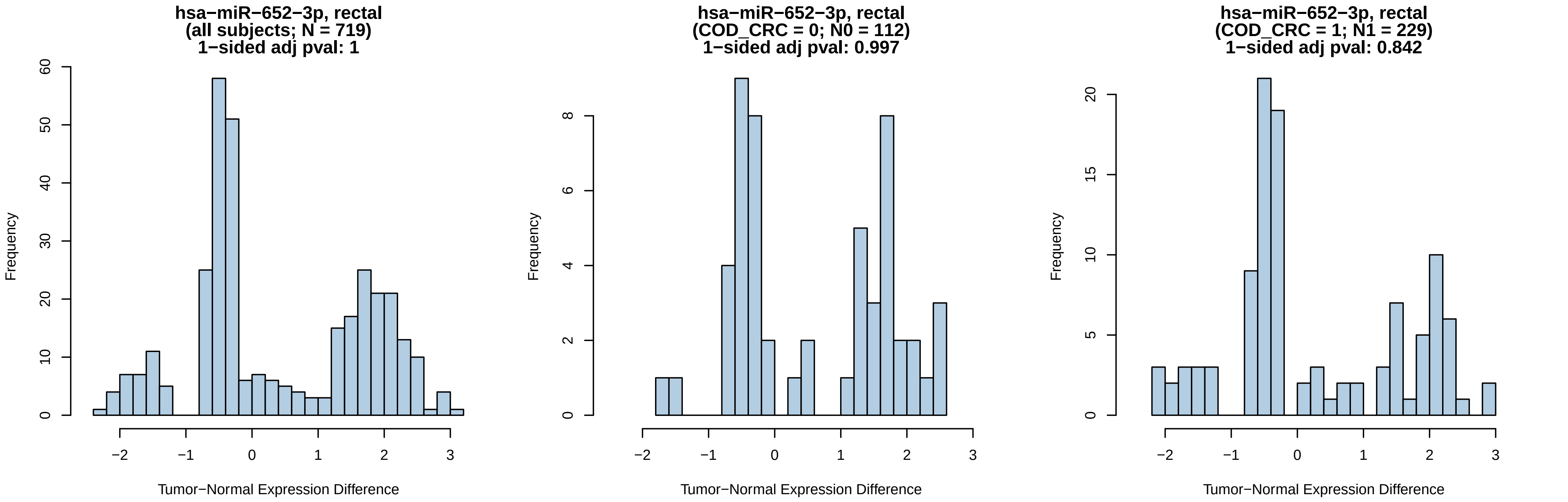

hsa−miR−652−3p, rectal
(all subjects; N = 719)
1−sided adj pval: 1
Frequency
Tumor−Normal Expression Difference
hsa−miR−652−3p, rectal
(COD_CRC = 0; N0 = 112)
1−sided adj pval: 0.997
Frequency
Tumor−Normal Expression Difference
hsa−miR−652−3p, rectal
(COD_CRC = 1; N1 = 229)
1−sided adj pval: 0.842
Frequency
Tumor−Normal Expression Difference

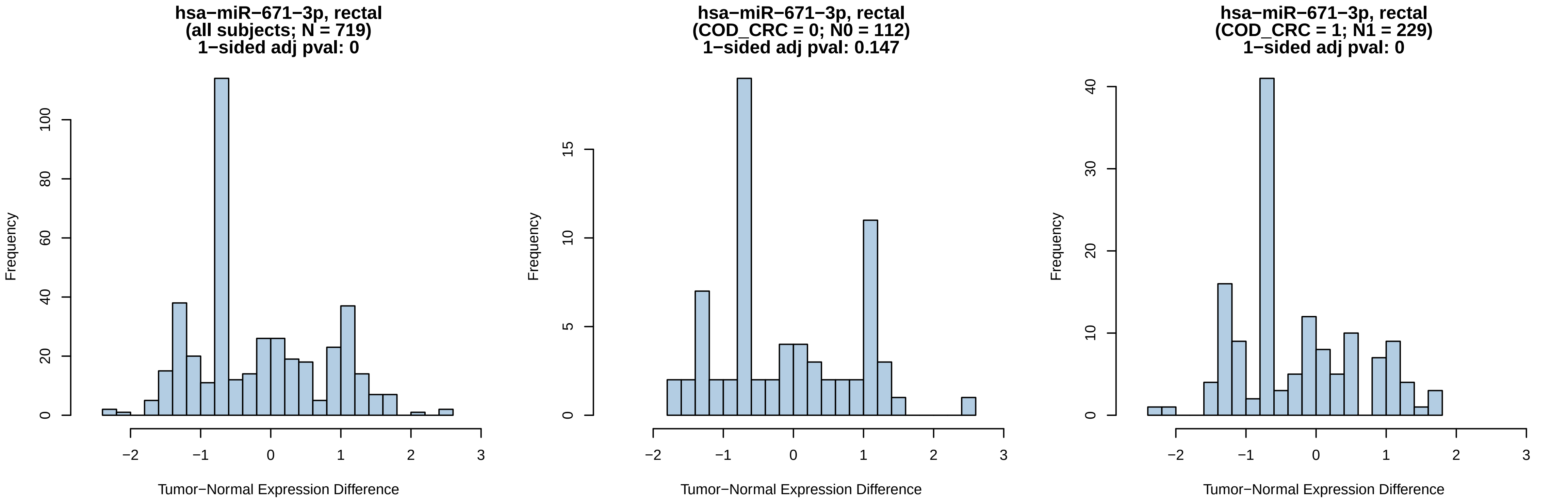
hsa−miR−671−3p, rectal
(all subjects; N = 719)
1−sided adj pval: 0
Frequency
Tumor−Normal Expression Difference
hsa−miR−671−3p, rectal
(COD_CRC = 0; N0 = 112)
1−sided adj pval: 0.147
Frequency
Tumor−Normal Expression Difference
hsa−miR−671−3p, rectal
(COD_CRC = 1; N1 = 229)
1−sided adj pval: 0
Frequency
Tumor−Normal Expression Difference

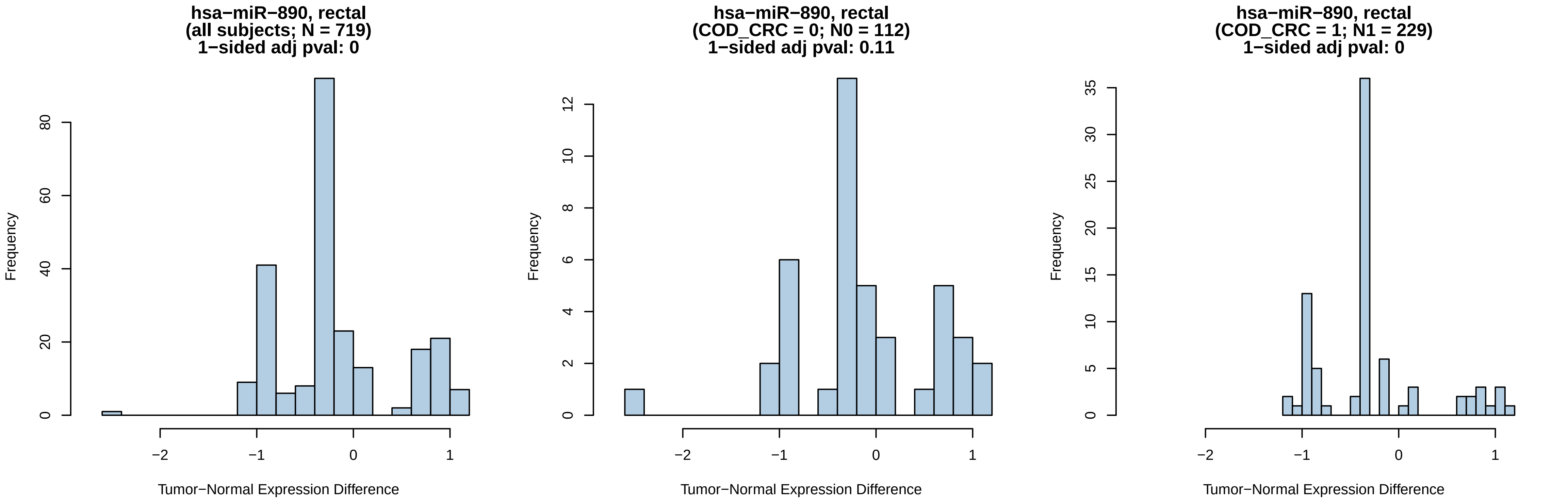
hsa−miR−890, rectal
(all subjects; N = 719)
1−sided adj pval: 0
hsa−miR−890, rectal
(COD_CRC = 0; N0 = 112)
1−sided adj pval: 0.11
hsa−miR−890, rectal
(COD_CRC = 1; N1 = 229)
1−sided adj pval: 0
Frequency
Tumor−Normal Expression Difference

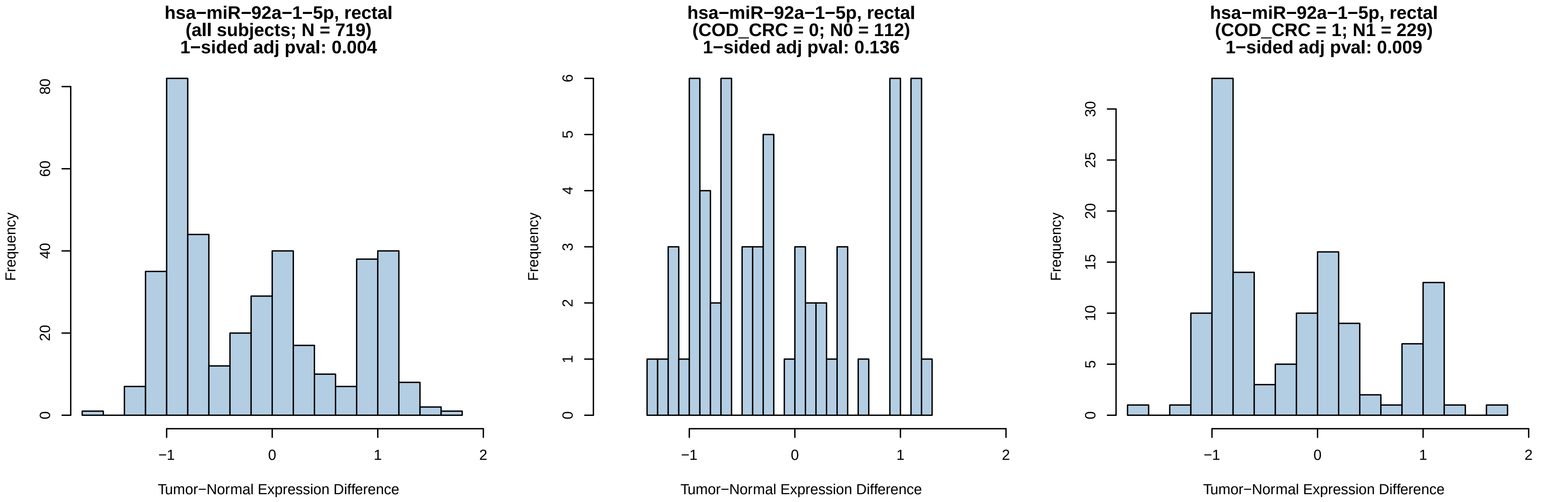
hsa−miR−92a−1−5p, rectal
(all subjects; N = 719)
1−sided adj pval: 0.004
Frequency
Tumor−Normal Expression Difference
hsa−miR−92a−1−5p, rectal
(COD_CRC = 0; N0 = 112)
1−sided adj pval: 0.136
Frequency
Tumor−Normal Expression Difference
hsa−miR−92a−1−5p, rectal
(COD_CRC = 1; N1 = 229)
1−sided adj pval: 0.009
Frequency
Tumor−Normal Expression Difference

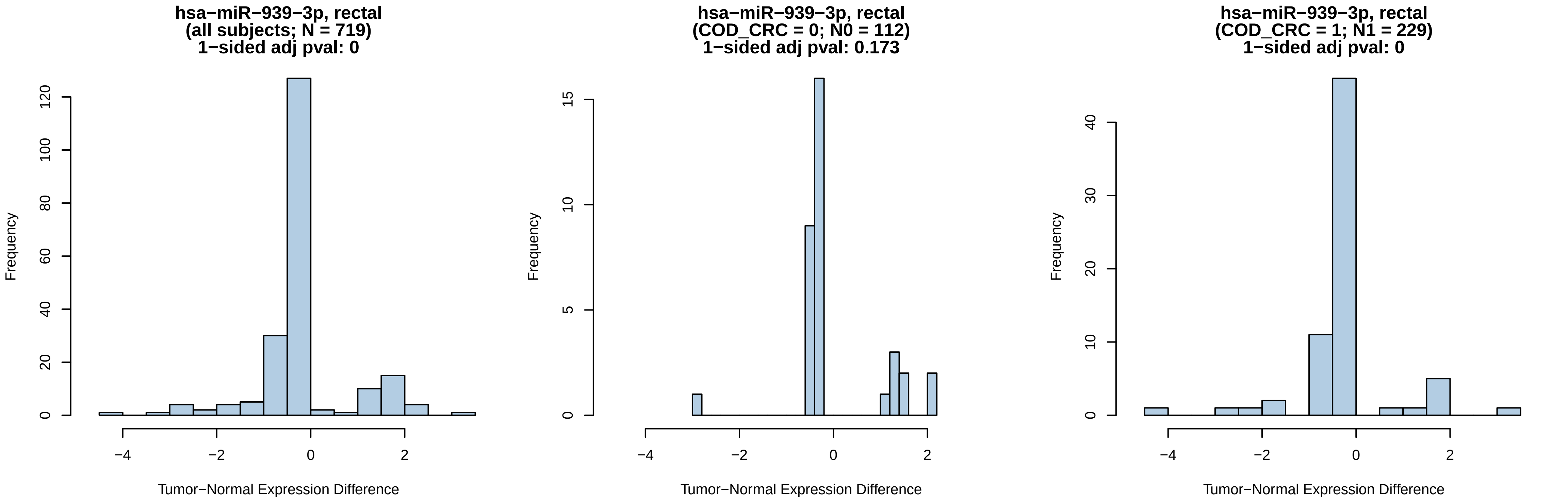

hsa−miR−939−3p, rectal
(all subjects; N = 719)
1−sided adj pval: 0
Frequency
0
20
40
60
80
100
120
−4
−2
0
2
Tumor−Normal Expression Difference
hsa−miR−939−3p, rectal
(COD_CRC = 0; N0 = 112)
1−sided adj pval: 0.173
Frequency
0
5
10
15
−4
−2
0
2
Tumor−Normal Expression Difference
hsa−miR−939−3p, rectal
(COD_CRC = 1; N1 = 229)
1−sided adj pval: 0
Frequency
0
10
20
30
40
−4
−2
0
2
Tumor−Normal Expression Difference

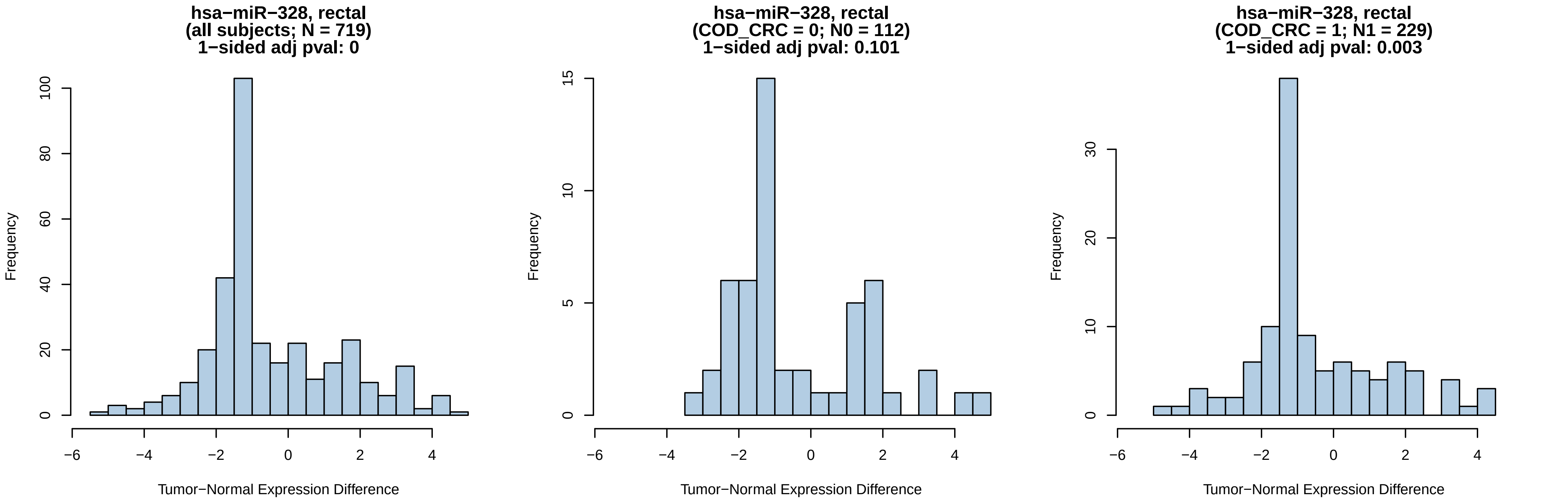

hsa−miR−328, rectal
(all subjects; N = 719)
1−sided adj pval: 0
hsa−miR−328, rectal
(COD_CRC = 0; N0 = 112)
1−sided adj pval: 0.101
hsa−miR−328, rectal
(COD_CRC = 1; N1 = 229)
1−sided adj pval: 0.003
Frequency
Tumor−Normal Expression Difference

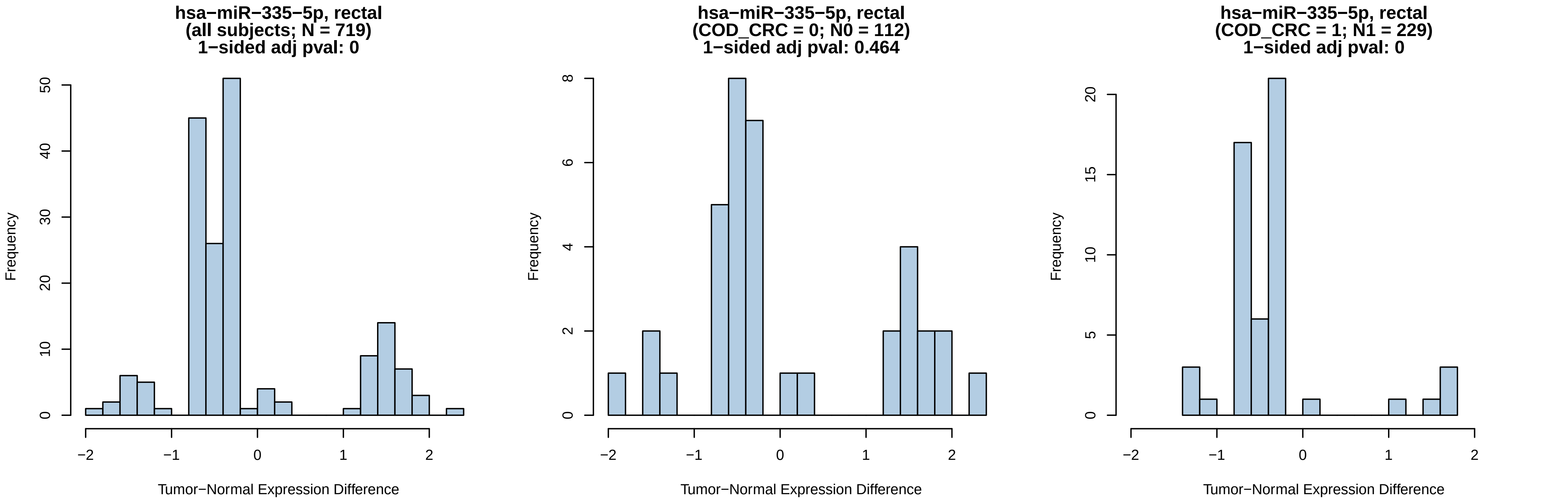
hsa−miR−335−5p, rectal
(all subjects; N = 719)
1−sided adj pval: 0
Frequency
Tumor−Normal Expression Difference
hsa−miR−335−5p, rectal
(COD_CRC = 0; N0 = 112)
1−sided adj pval: 0.464
Frequency
Tumor−Normal Expression Difference
hsa−miR−335−5p, rectal
(COD_CRC = 1; N1 = 229)
1−sided adj pval: 0
Frequency
Tumor−Normal Expression Difference

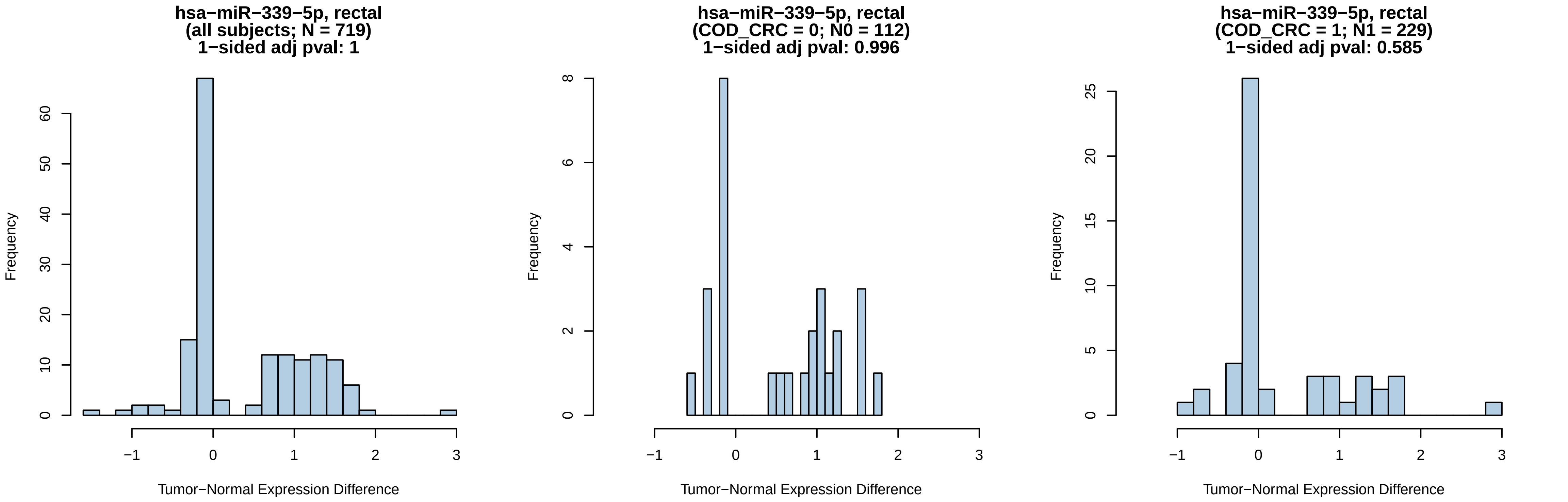
hsa−miR−339−5p, rectal
(all subjects; N = 719)
1−sided adj pval: 1
Frequency
0
10
20
30
40
50
60
−1
0
1
2
3
Tumor−Normal Expression Difference
hsa−miR−339−5p, rectal
(COD_CRC = 0; N0 = 112)
1−sided adj pval: 0.996
Frequency
0
2
4
6
8
−1
0
1
2
3
Tumor−Normal Expression Difference
hsa−miR−339−5p, rectal
(COD_CRC = 1; N1 = 229)
1−sided adj pval: 0.585
Frequency
0
5
10
15
20
25
−1
0
1
2
3
Tumor−Normal Expression Difference

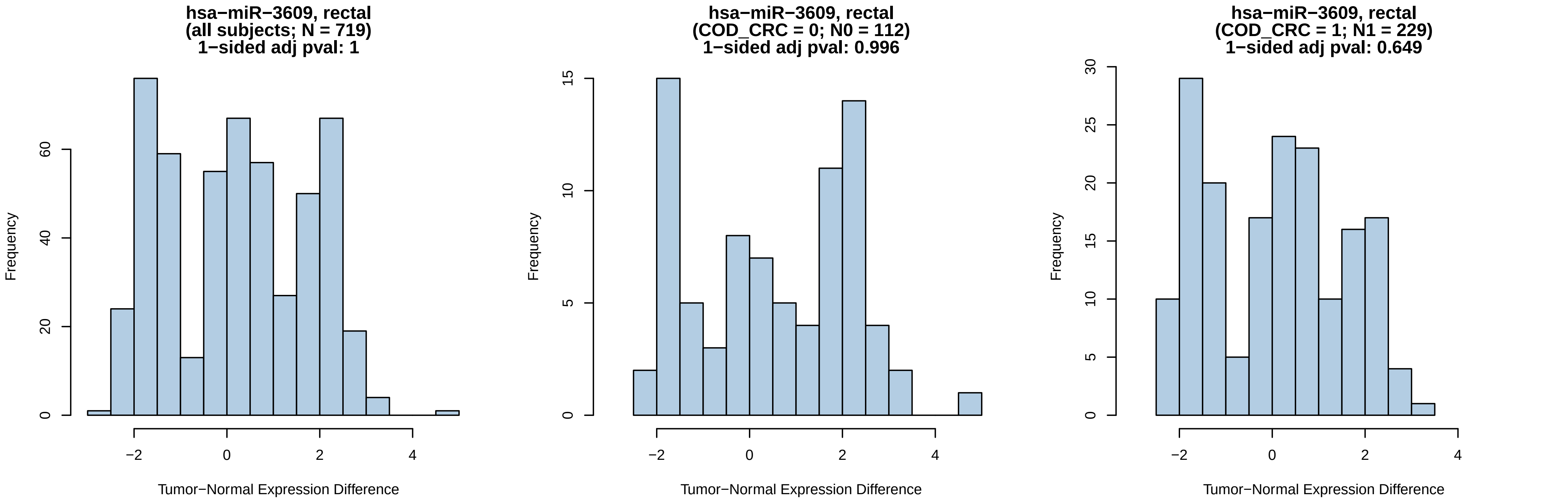

hsa−miR−3609, rectal
(all subjects; N = 719)
1−sided adj pval: 1
Frequency
0
20
40
60
−2
0
2
4
Tumor−Normal Expression Difference
hsa−miR−3609, rectal
(COD_CRC = 0; N0 = 112)
1−sided adj pval: 0.996
Frequency
0
5
10
15
−2
0
2
4
Tumor−Normal Expression Difference
hsa−miR−3609, rectal
(COD_CRC = 1; N1 = 229)
1−sided adj pval: 0.649
Frequency
0
5
10
15
20
25
30
−2
0
2
4
Tumor−Normal Expression Difference

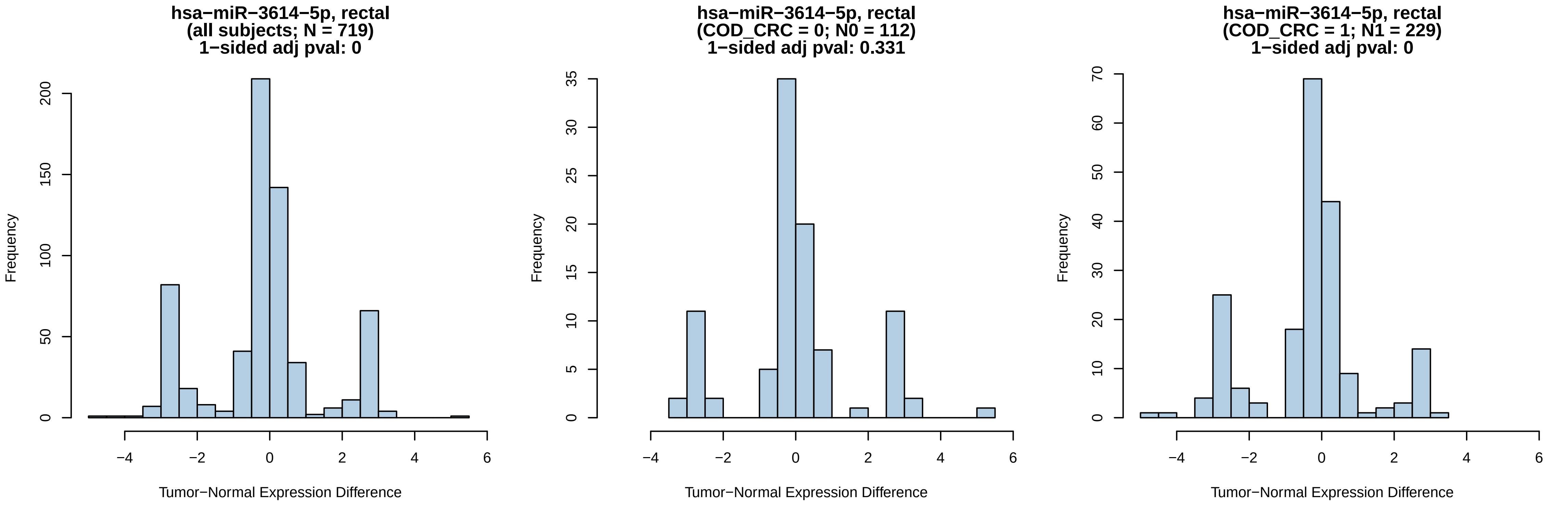
hsa−miR−3614−5p, rectal
(all subjects; N = 719)
1−sided adj pval: 0
Frequency
0
50
100
150
200
−4
−2
0
2
4
6
Tumor−Normal Expression Difference
hsa−miR−3614−5p, rectal
(COD_CRC = 0; N0 = 112)
1−sided adj pval: 0.331
Frequency
0
5
10
15
20
25
30
35
−4
−2
0
2
4
6
Tumor−Normal Expression Difference
hsa−miR−3614−5p, rectal
(COD_CRC = 1; N1 = 229)
1−sided adj pval: 0
Frequency
0
10
20
30
40
50
60
70
−4
−2
0
2
4
6
Tumor−Normal Expression Difference

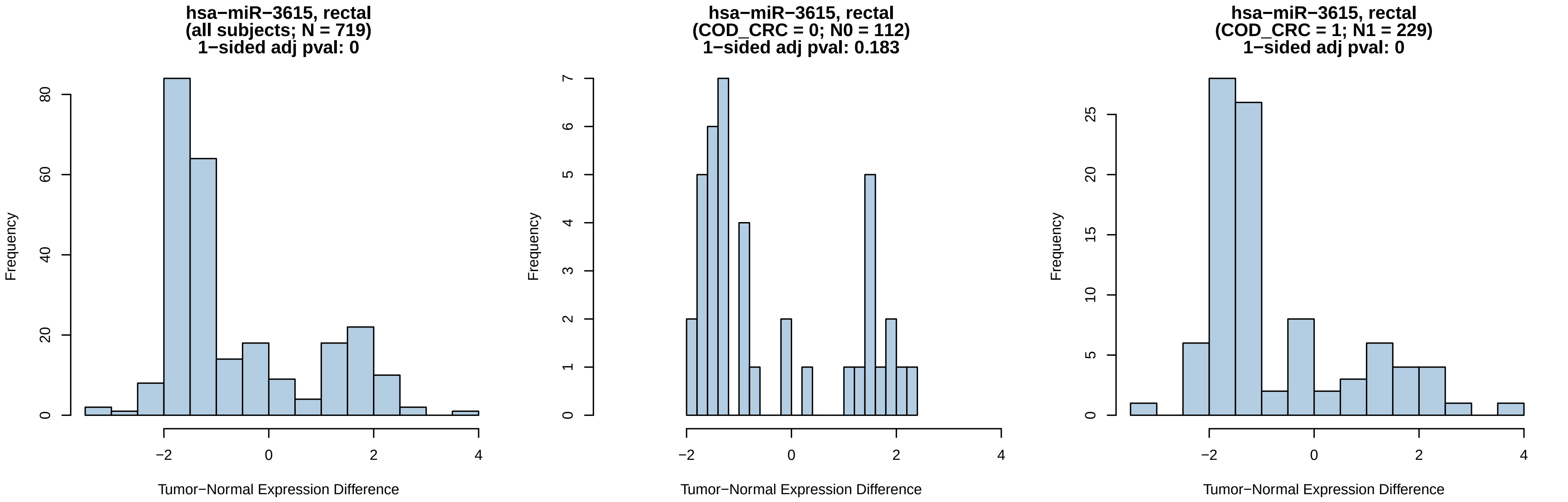

hsa−miR−3615, rectal
(all subjects; N = 719)
1−sided adj pval: 0
Frequency
Tumor−Normal Expression Difference
hsa−miR−3615, rectal
(COD_CRC = 0; N0 = 112)
1−sided adj pval: 0.183
Frequency
Tumor−Normal Expression Difference
hsa−miR−3615, rectal
(COD_CRC = 1; N1 = 229)
1−sided adj pval: 0
Frequency
Tumor−Normal Expression Difference

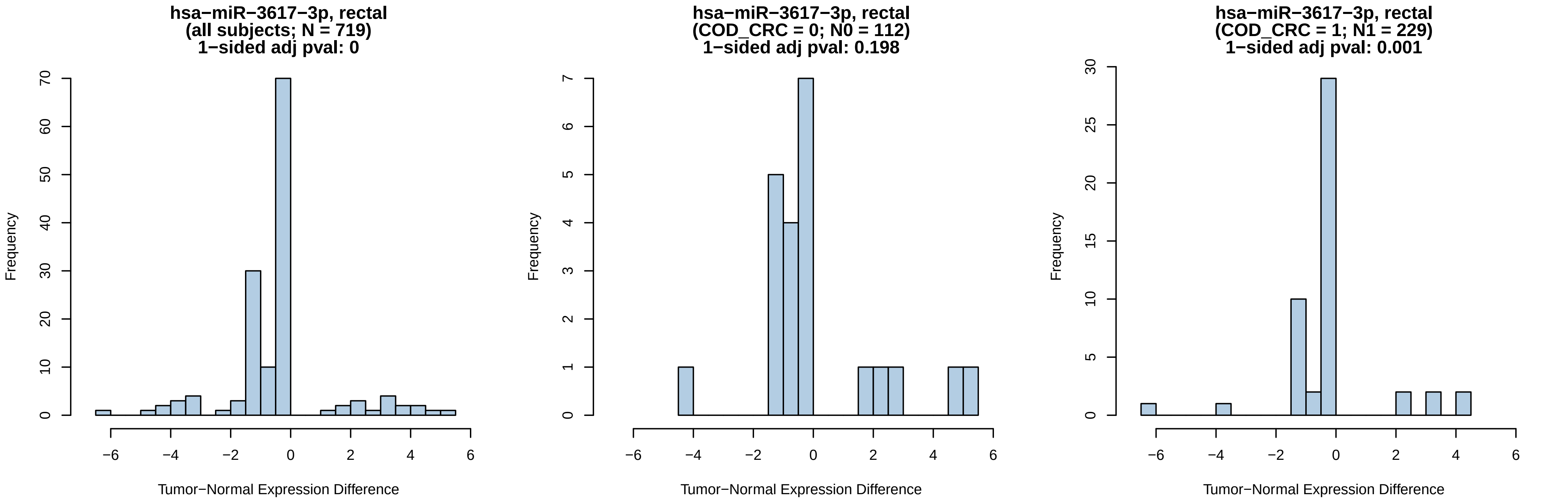

hsa−miR−3617−3p, rectal
(all subjects; N = 719)
1−sided adj pval: 0
Frequency
Tumor−Normal Expression Difference
hsa−miR−3617−3p, rectal
(COD_CRC = 0; N0 = 112)
1−sided adj pval: 0.198
Frequency
Tumor−Normal Expression Difference
hsa−miR−3617−3p, rectal
(COD_CRC = 1; N1 = 229)
1−sided adj pval: 0.001
Frequency
Tumor−Normal Expression Difference

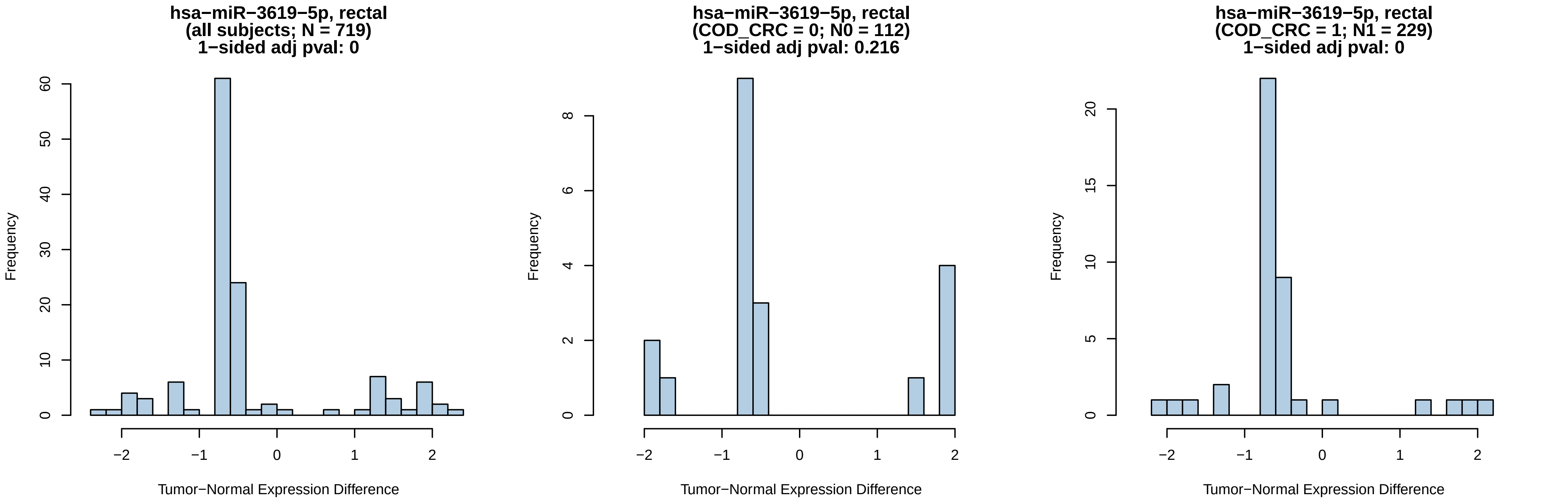
hsa−miR−3619−5p, rectal
(all subjects; N = 719)
1−sided adj pval: 0
Frequency
Tumor−Normal Expression Difference
hsa−miR−3619−5p, rectal
(COD_CRC = 0; N0 = 112)
1−sided adj pval: 0.216
Frequency
Tumor−Normal Expression Difference
hsa−miR−3619−5p, rectal
(COD_CRC = 1; N1 = 229)
1−sided adj pval: 0
Frequency
Tumor−Normal Expression Difference

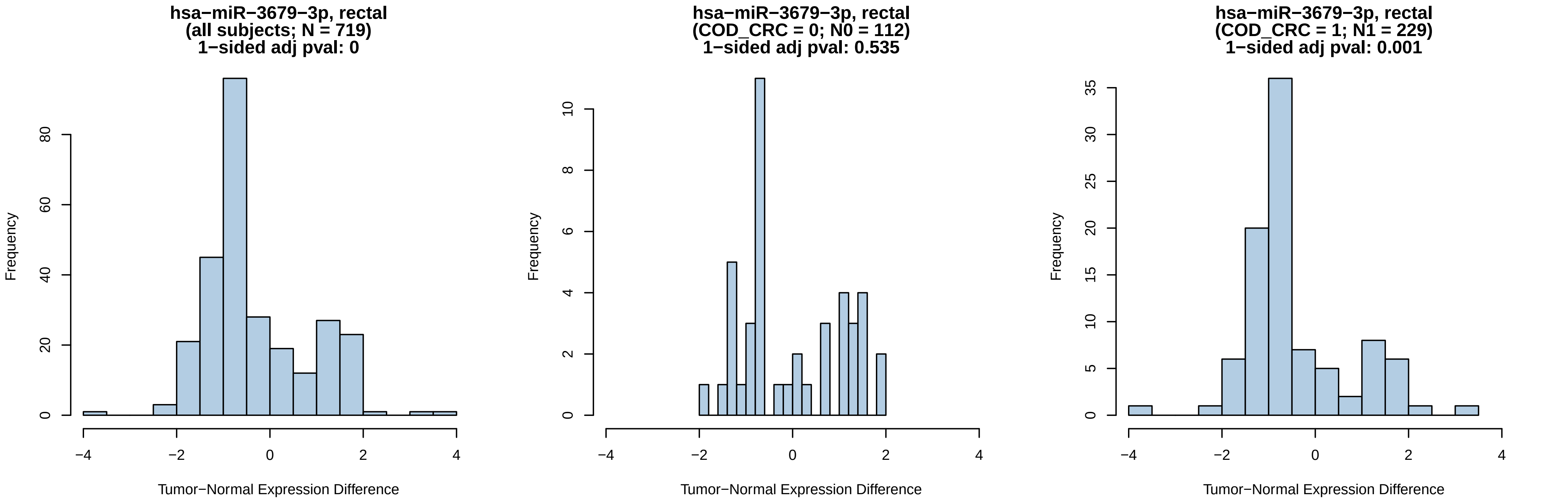
hsa−miR−3679−3p, rectal
(all subjects; N = 719)
1−sided adj pval: 0
Frequency
Tumor−Normal Expression Difference
hsa−miR−3679−3p, rectal
(COD_CRC = 0; N0 = 112)
1−sided adj pval: 0.535
Frequency
Tumor−Normal Expression Difference
hsa−miR−3679−3p, rectal
(COD_CRC = 1; N1 = 229)
1−sided adj pval: 0.001
Frequency
Tumor−Normal Expression Difference

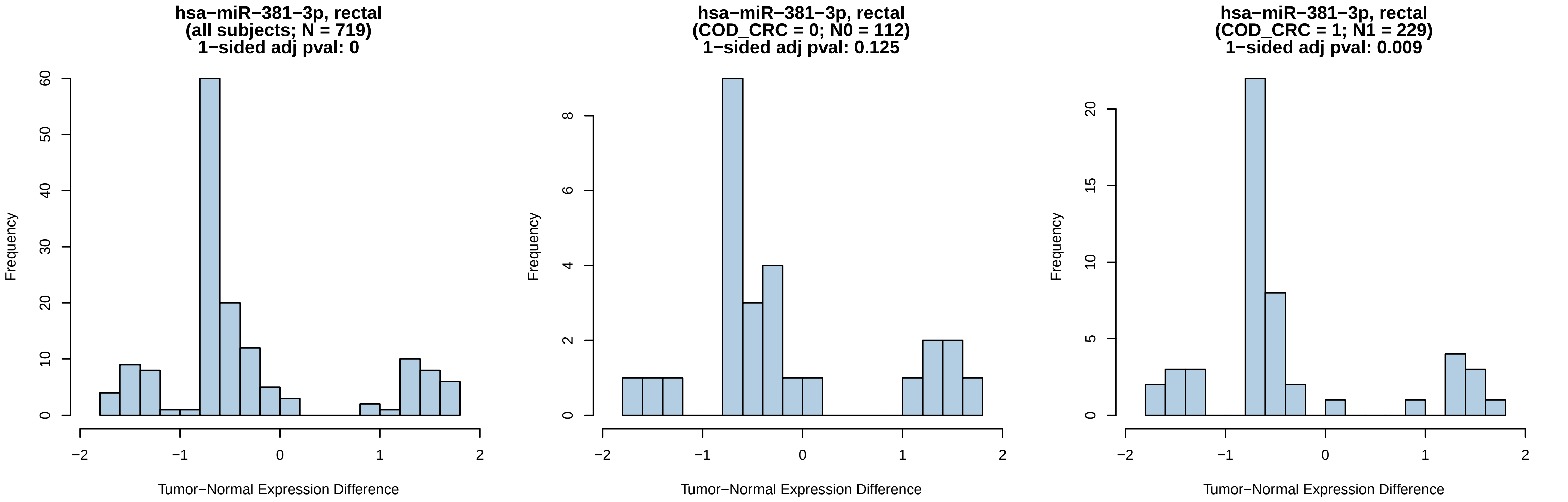

hsa−miR−381−3p, rectal
(all subjects; N = 719)
1−sided adj pval: 0
Frequency
Tumor−Normal Expression Difference
hsa−miR−381−3p, rectal
(COD_CRC = 0; N0 = 112)
1−sided adj pval: 0.125
Frequency
Tumor−Normal Expression Difference
hsa−miR−381−3p, rectal
(COD_CRC = 1; N1 = 229)
1−sided adj pval: 0.009
Frequency
Tumor−Normal Expression Difference

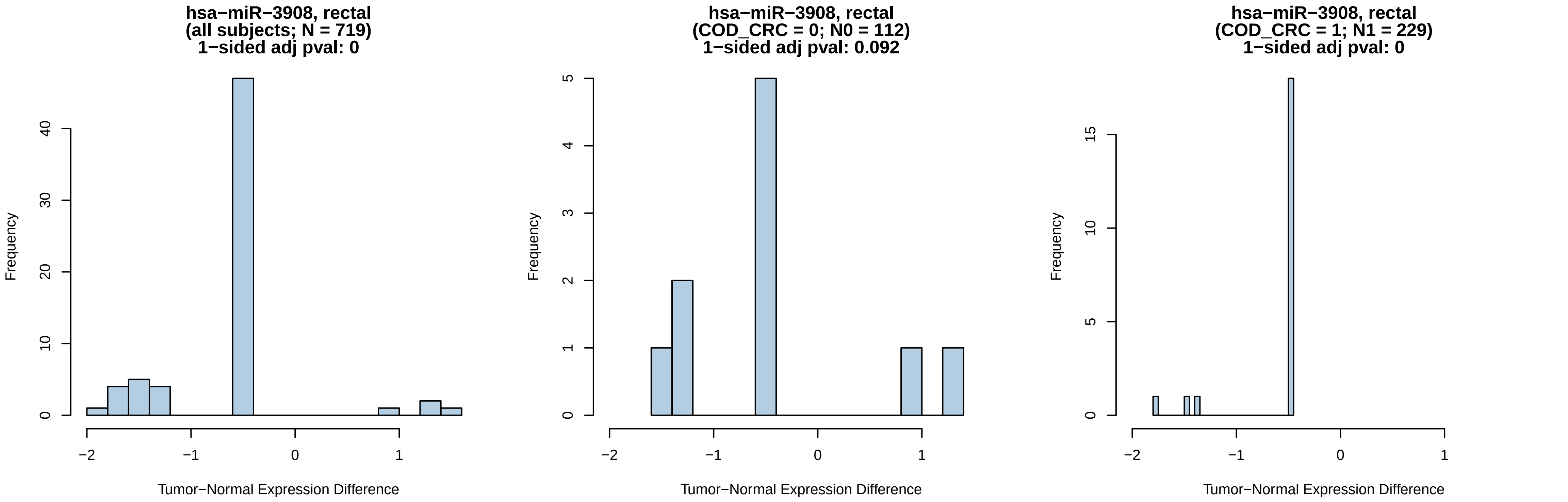

hsa−miR−3908, rectal
(all subjects; N = 719)
1−sided adj pval: 0
Frequency
0
10
20
30
40
−2
−1
0
1
Tumor−Normal Expression Difference
hsa−miR−3908, rectal
(COD_CRC = 0; N0 = 112)
1−sided adj pval: 0.092
Frequency
0
1
2
3
4
5
−2
−1
0
1
Tumor−Normal Expression Difference
hsa−miR−3908, rectal
(COD_CRC = 1; N1 = 229)
1−sided adj pval: 0
Frequency
0
5
10
15
−2
−1
0
1
Tumor−Normal Expression Difference

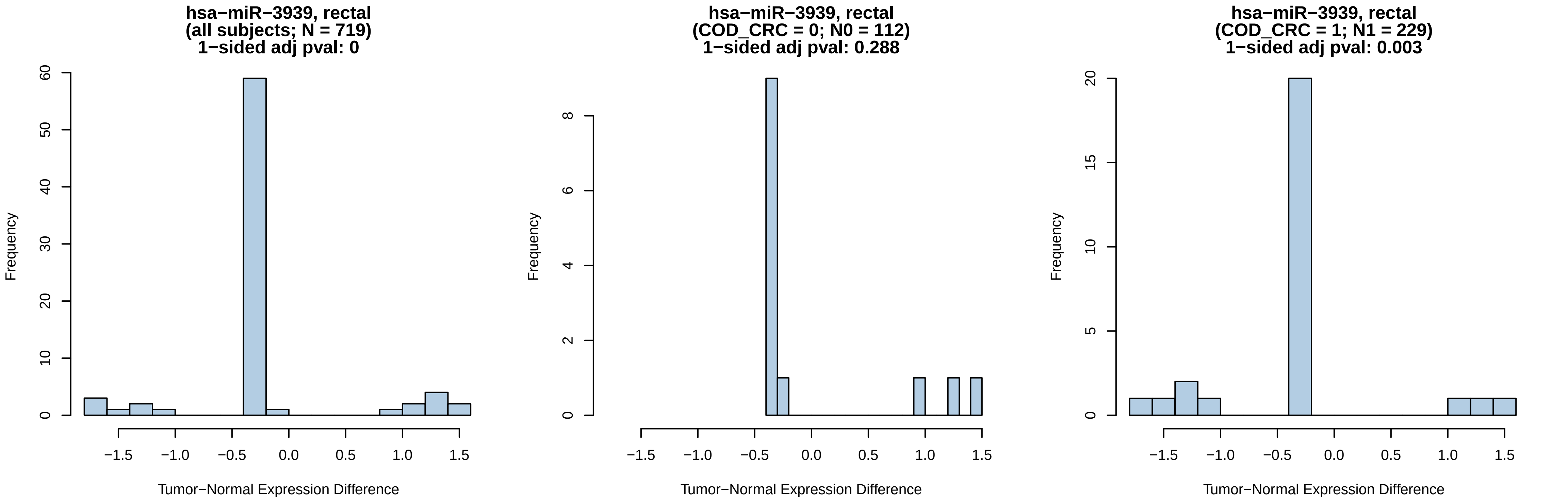

hsa−miR−3939, rectal
(all subjects; N = 719)
1−sided adj pval: 0
hsa−miR−3939, rectal
(COD_CRC = 0; N0 = 112)
1−sided adj pval: 0.288
hsa−miR−3939, rectal
(COD_CRC = 1; N1 = 229)
1−sided adj pval: 0.003
Frequency
Tumor−Normal Expression Difference

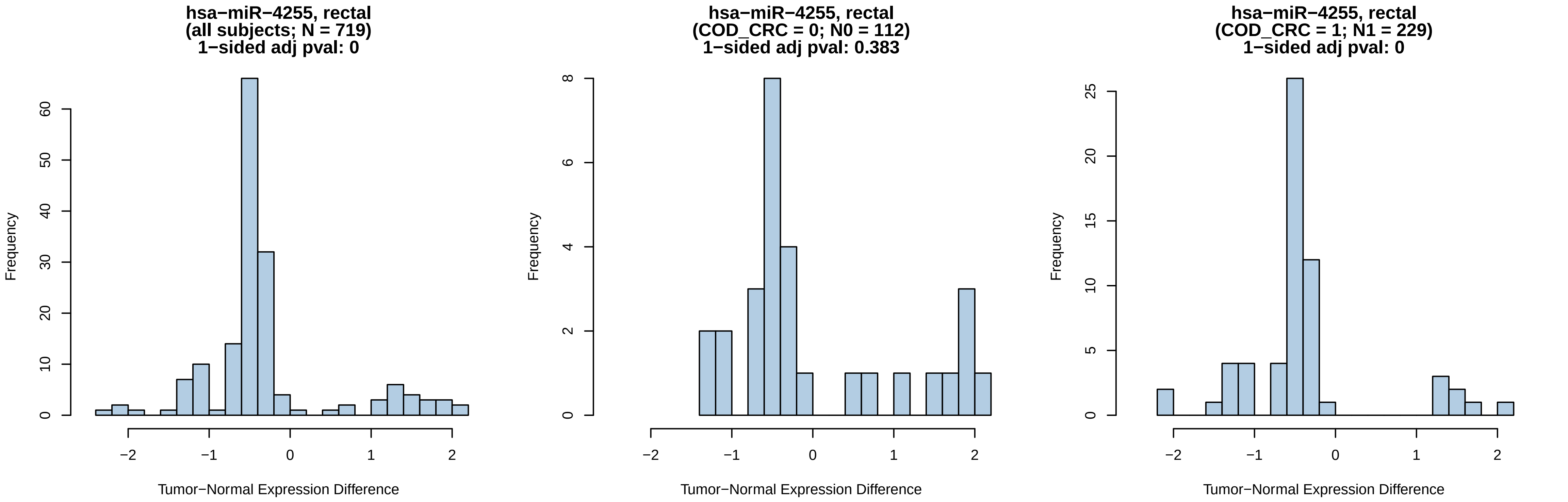
hsa−miR−4255, rectal
(all subjects; N = 719)
1−sided adj pval: 0
Frequency
Tumor−Normal Expression Difference
hsa−miR−4255, rectal
(COD_CRC = 0; N0 = 112)
1−sided adj pval: 0.383
Frequency
Tumor−Normal Expression Difference
hsa−miR−4255, rectal
(COD_CRC = 1; N1 = 229)
1−sided adj pval: 0
Frequency
Tumor−Normal Expression Difference

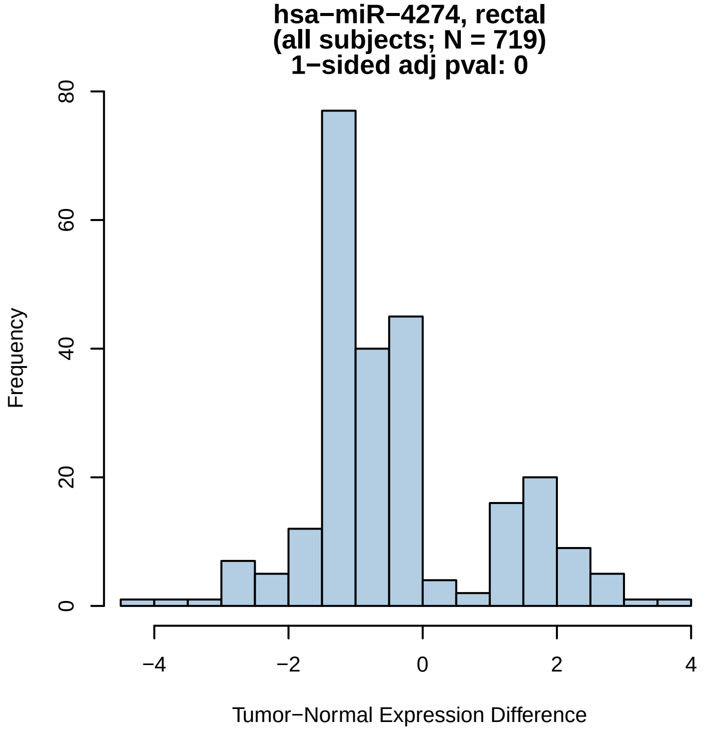
hsa−miR−4274, rectal
(all subjects; N = 719)
1−sided adj pval: 0
Frequency
0
20
40
60
80
−4
−2
0
2
4
Tumor−Normal Expression Difference

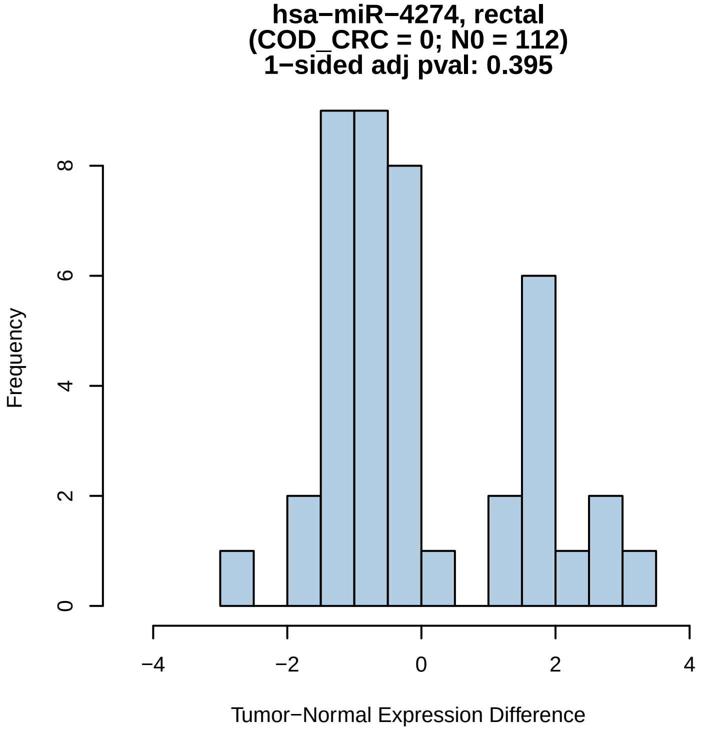
hsa−miR−4274, rectal
(COD_CRC = 0; N0 = 112)
1−sided adj pval: 0.395
Frequency
0
2
4
6
8
−4
−2
0
2
4
Tumor−Normal Expression Difference

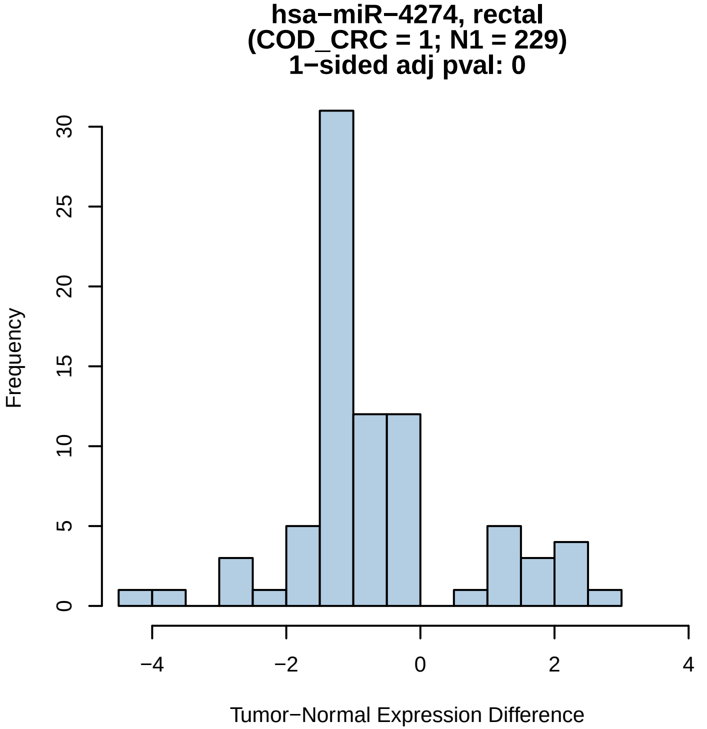
hsa−miR−4274, rectal
(COD_CRC = 1; N1 = 229)
1−sided adj pval: 0
Frequency
0
5
10
15
20
25
30
−4
−2
0
2
4
Tumor−Normal Expression Difference

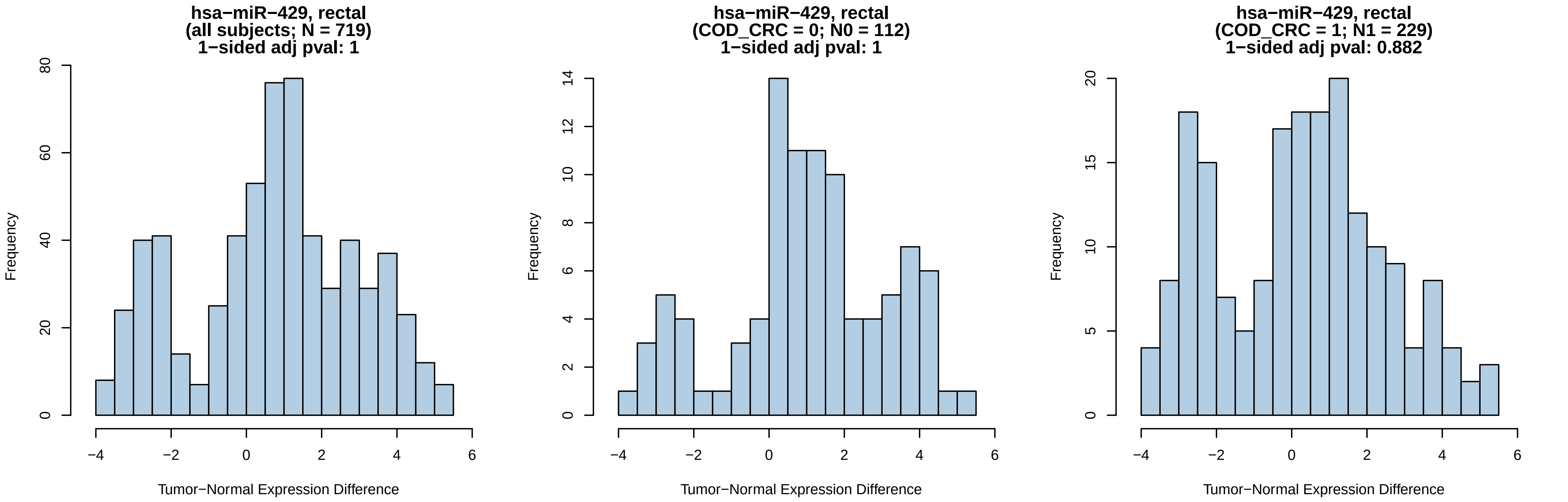
hsa−miR−429, rectal
(all subjects; N = 719)
1−sided adj pval: 1
Frequency
Tumor−Normal Expression Difference
0
20
40
60
80
−4
−2
0
2
4
6
hsa−miR−429, rectal
(COD_CRC = 0; N0 = 112)
1−sided adj pval: 1
Frequency
Tumor−Normal Expression Difference
0
2
4
6
8
10
12
14
−4
−2
0
2
4
6
hsa−miR−429, rectal
(COD_CRC = 1; N1 = 229)
1−sided adj pval: 0.882
Frequency
Tumor−Normal Expression Difference
0
5
10
15
20
−4
−2
0
2
4
6

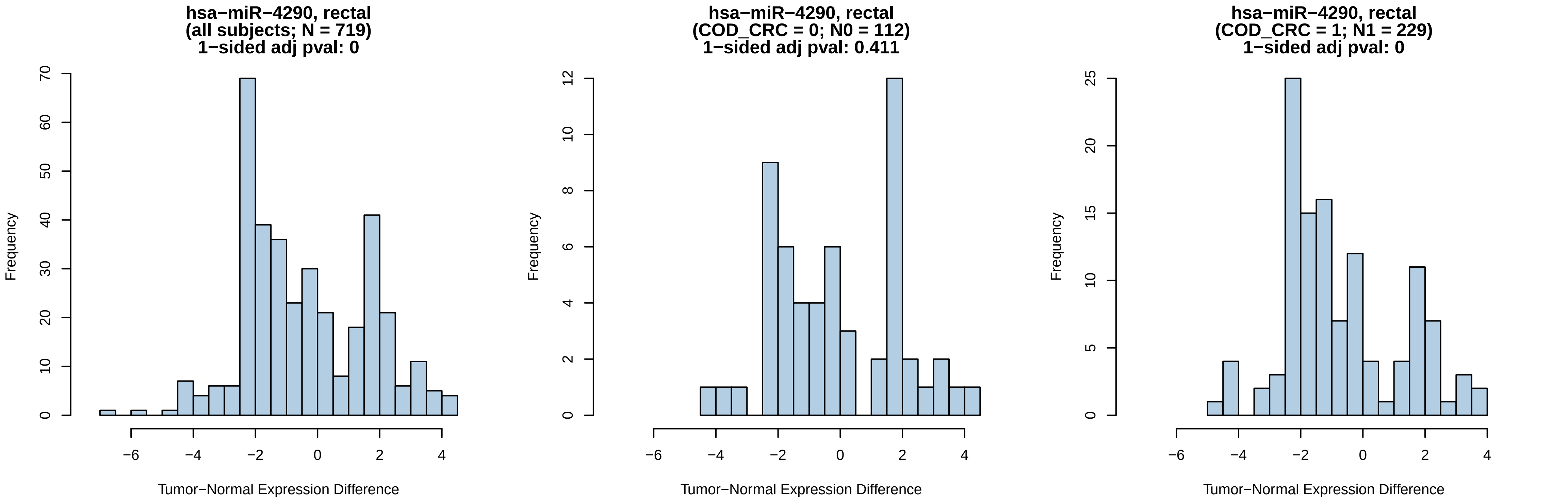
hsa−miR−4290, rectal
(all subjects; N = 719)
1−sided adj pval: 0
Frequency
Tumor−Normal Expression Difference
hsa−miR−4290, rectal
(COD_CRC = 0; N0 = 112)
1−sided adj pval: 0.411
Frequency
Tumor−Normal Expression Difference
hsa−miR−4290, rectal
(COD_CRC = 1; N1 = 229)
1−sided adj pval: 0
Frequency
Tumor−Normal Expression Difference

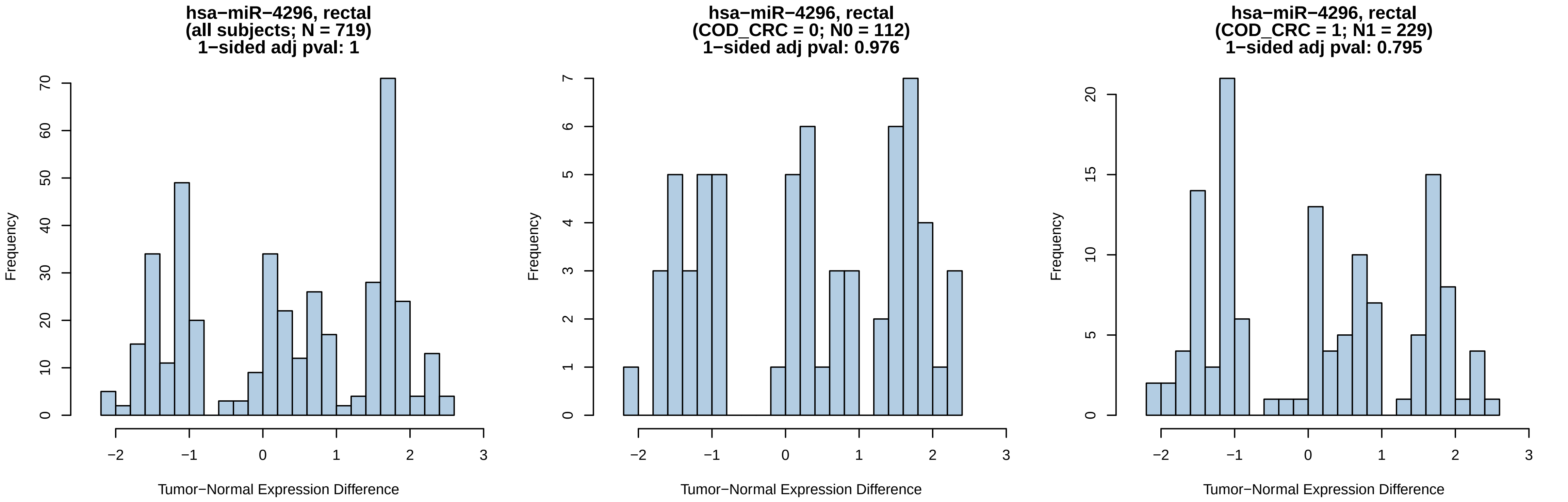
hsa−miR−4296, rectal
(all subjects; N = 719)
1−sided adj pval: 1
Frequency
Tumor−Normal Expression Difference
hsa−miR−4296, rectal
(COD_CRC = 0; N0 = 112)
1−sided adj pval: 0.976
Frequency
Tumor−Normal Expression Difference
hsa−miR−4296, rectal
(COD_CRC = 1; N1 = 229)
1−sided adj pval: 0.795
Frequency
Tumor−Normal Expression Difference

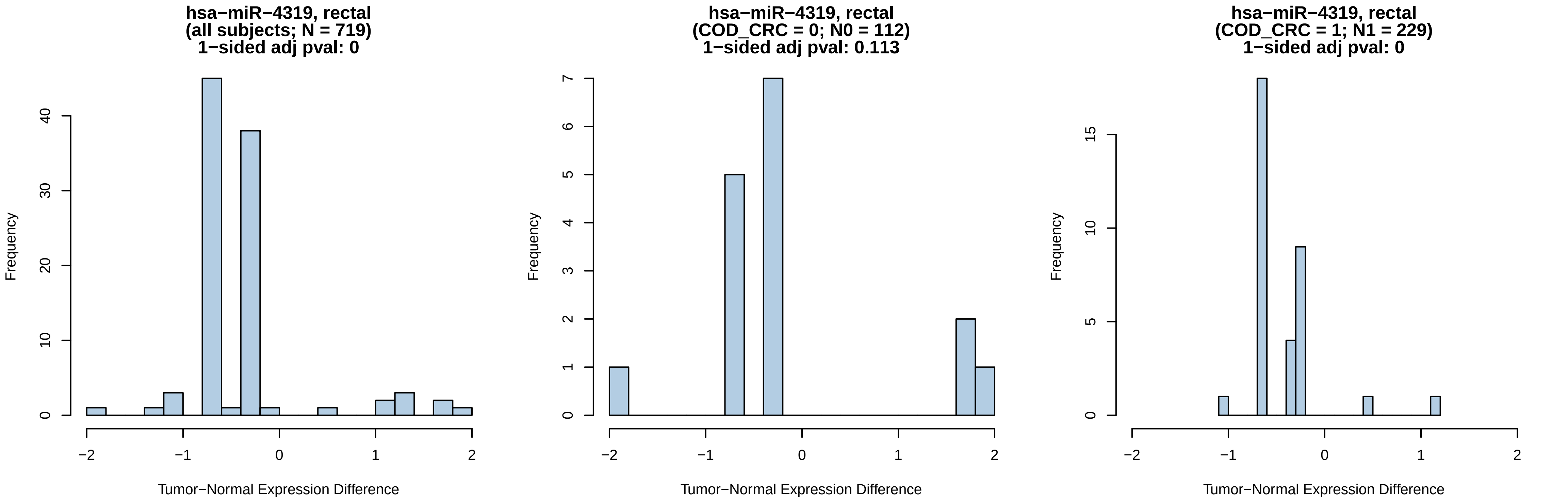
hsa−miR−4319, rectal
(all subjects; N = 719)
1−sided adj pval: 0
Frequency
Tumor−Normal Expression Difference
hsa−miR−4319, rectal
(COD_CRC = 0; N0 = 112)
1−sided adj pval: 0.113
Frequency
Tumor−Normal Expression Difference
hsa−miR−4319, rectal
(COD_CRC = 1; N1 = 229)
1−sided adj pval: 0
Frequency
Tumor−Normal Expression Difference

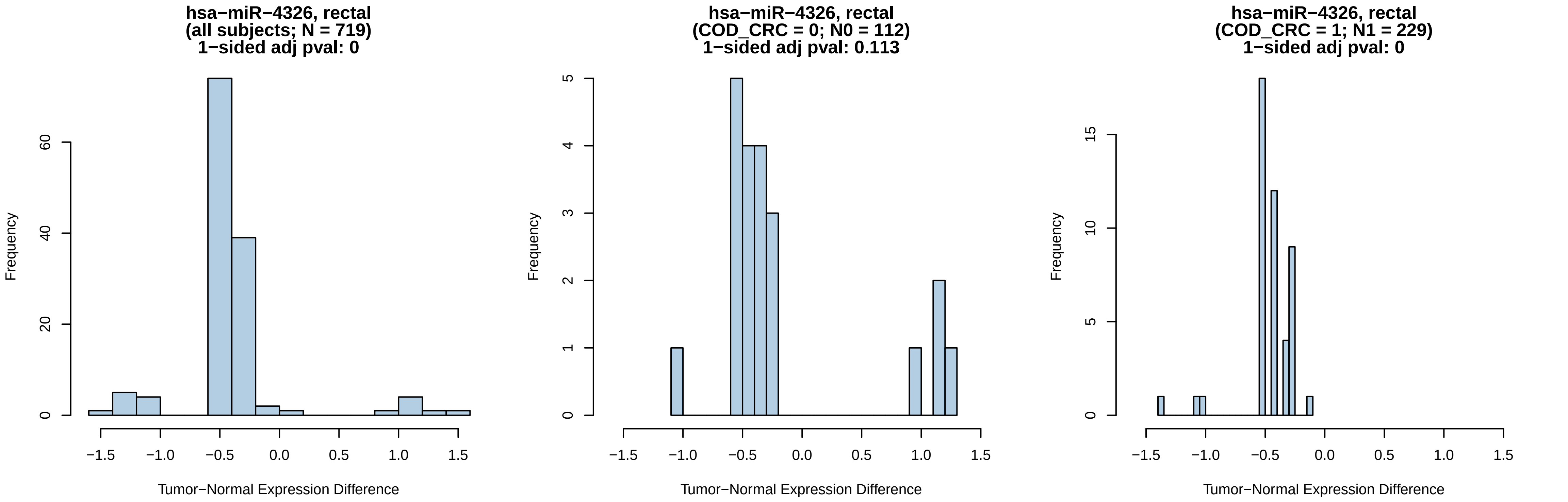

hsa−miR−4326, rectal
(all subjects; N = 719)
1−sided adj pval: 0
Frequency
Tumor−Normal Expression Difference
hsa−miR−4326, rectal
(COD_CRC = 0; N0 = 112)
1−sided adj pval: 0.113
Frequency
Tumor−Normal Expression Difference
hsa−miR−4326, rectal
(COD_CRC = 1; N1 = 229)
1−sided adj pval: 0
Frequency
Tumor−Normal Expression Difference

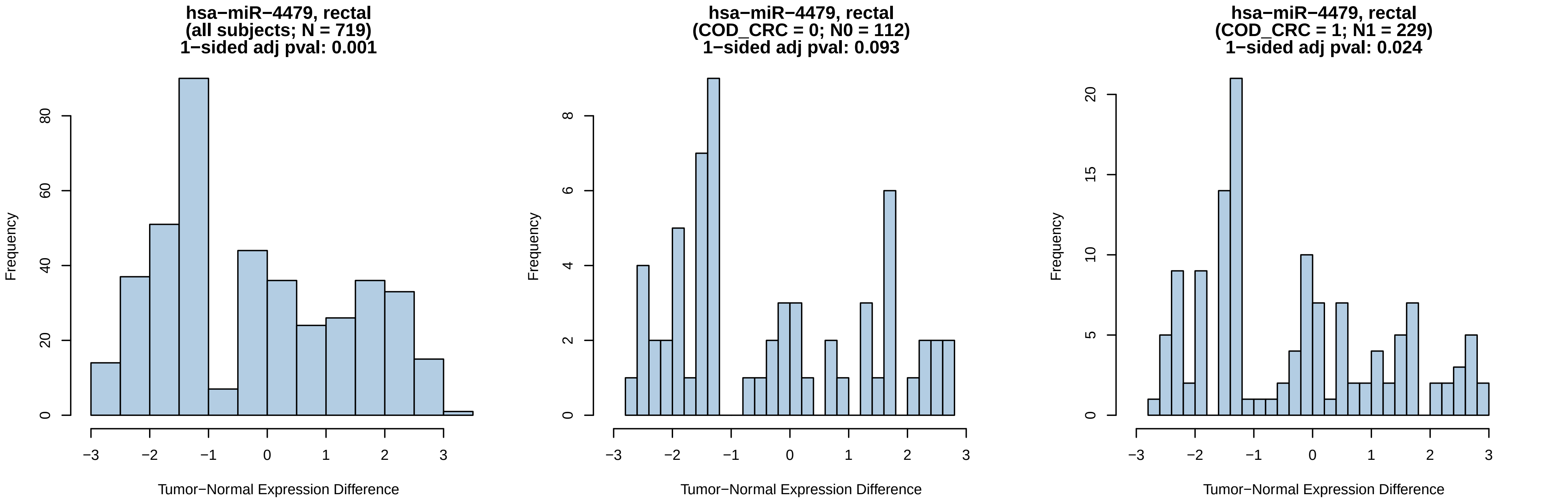
hsa−miR−4479, rectal
(all subjects; N = 719)
1−sided adj pval: 0.001
Frequency
Tumor−Normal Expression Difference
hsa−miR−4479, rectal
(COD_CRC = 0; N0 = 112)
1−sided adj pval: 0.093
Frequency
Tumor−Normal Expression Difference
hsa−miR−4479, rectal
(COD_CRC = 1; N1 = 229)
1−sided adj pval: 0.024
Frequency
Tumor−Normal Expression Difference

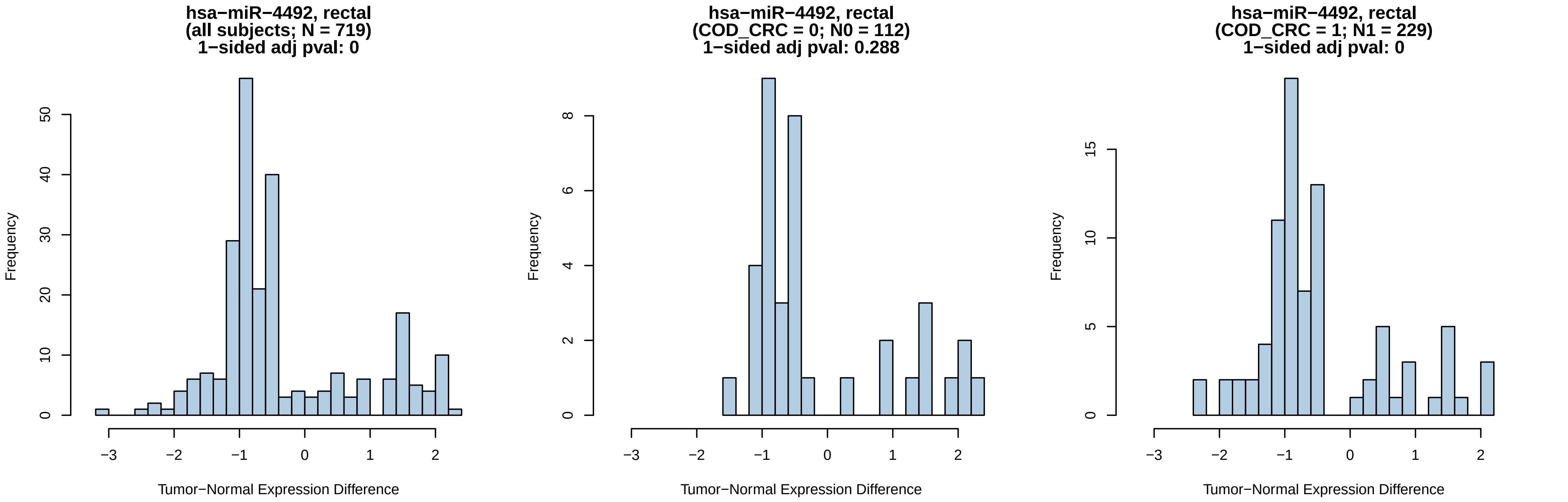
hsa−miR−4492, rectal
(all subjects; N = 719)
1−sided adj pval: 0
Frequency
Tumor−Normal Expression Difference
hsa−miR−4492, rectal
(COD_CRC = 0; N0 = 112)
1−sided adj pval: 0.288
Frequency
Tumor−Normal Expression Difference
hsa−miR−4492, rectal
(COD_CRC = 1; N1 = 229)
1−sided adj pval: 0
Frequency
Tumor−Normal Expression Difference

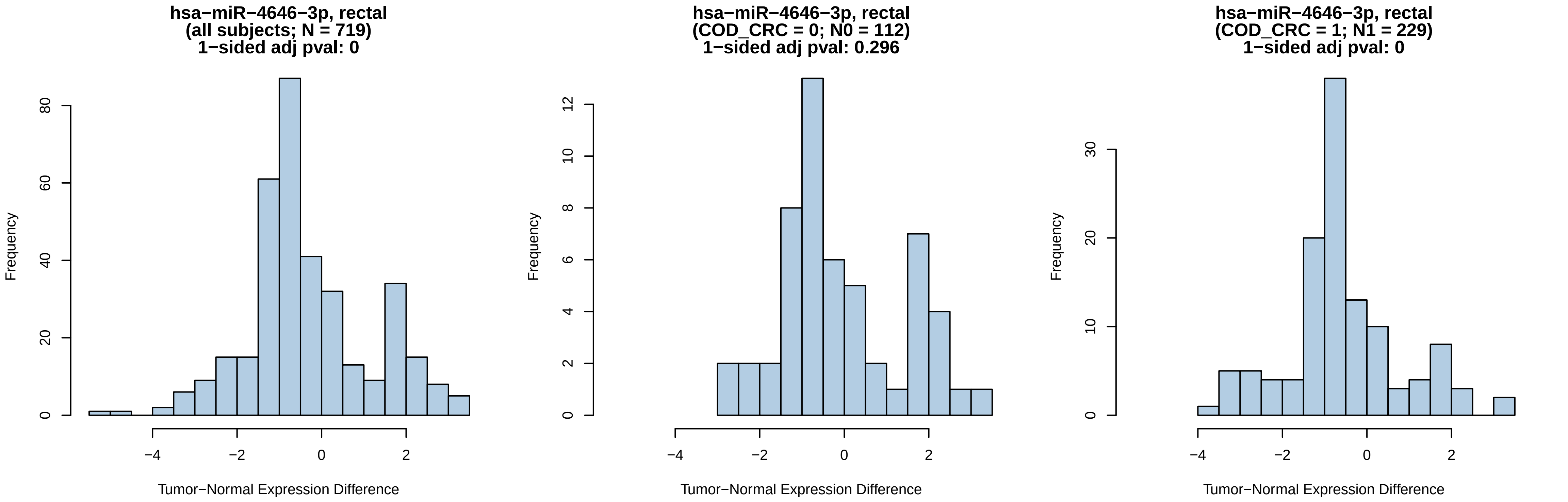
hsa−miR−4646−3p, rectal
(all subjects; N = 719)
1−sided adj pval: 0
hsa−miR−4646−3p, rectal
(COD_CRC = 0; N0 = 112)
1−sided adj pval: 0.296
hsa−miR−4646−3p, rectal
(COD_CRC = 1; N1 = 229)
1−sided adj pval: 0
Frequency
Tumor−Normal Expression Difference

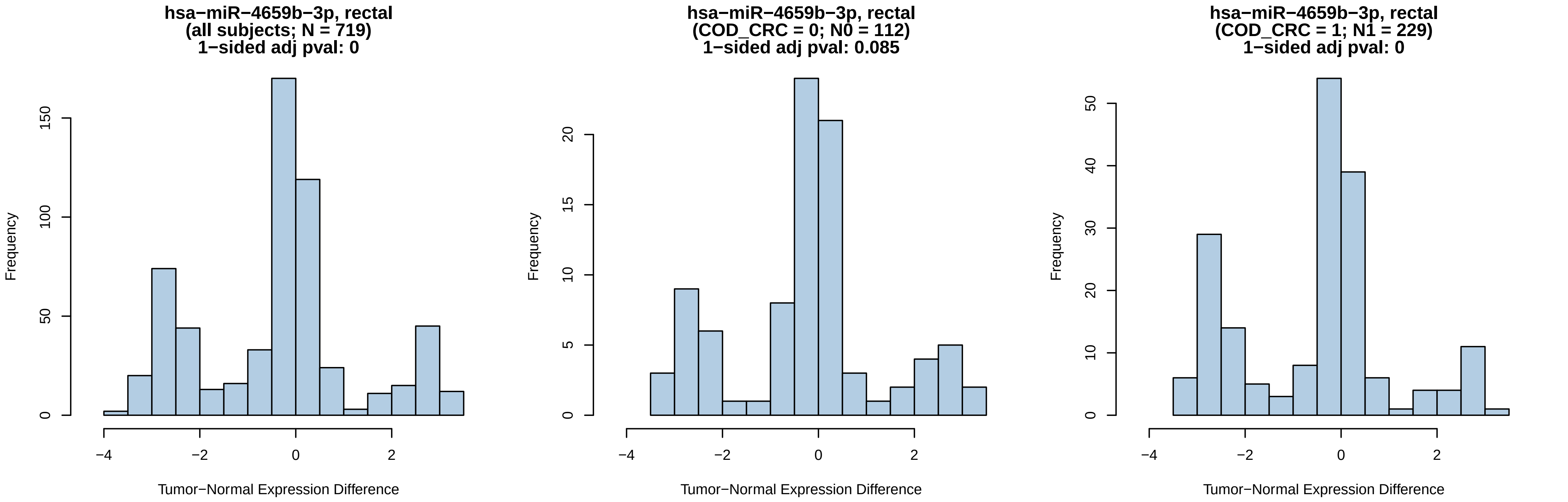

hsa−miR−4659b−3p, rectal
(all subjects; N = 719)
1−sided adj pval: 0
Frequency
0
50
100
150
−4
−2
0
2
Tumor−Normal Expression Difference
hsa−miR−4659b−3p, rectal
(COD_CRC = 0; N0 = 112)
1−sided adj pval: 0.085
Frequency
0
5
10
15
20
−4
−2
0
2
Tumor−Normal Expression Difference
hsa−miR−4659b−3p, rectal
(COD_CRC = 1; N1 = 229)
1−sided adj pval: 0
Frequency
0
10
20
30
40
50
−4
−2
0
2
Tumor−Normal Expression Difference

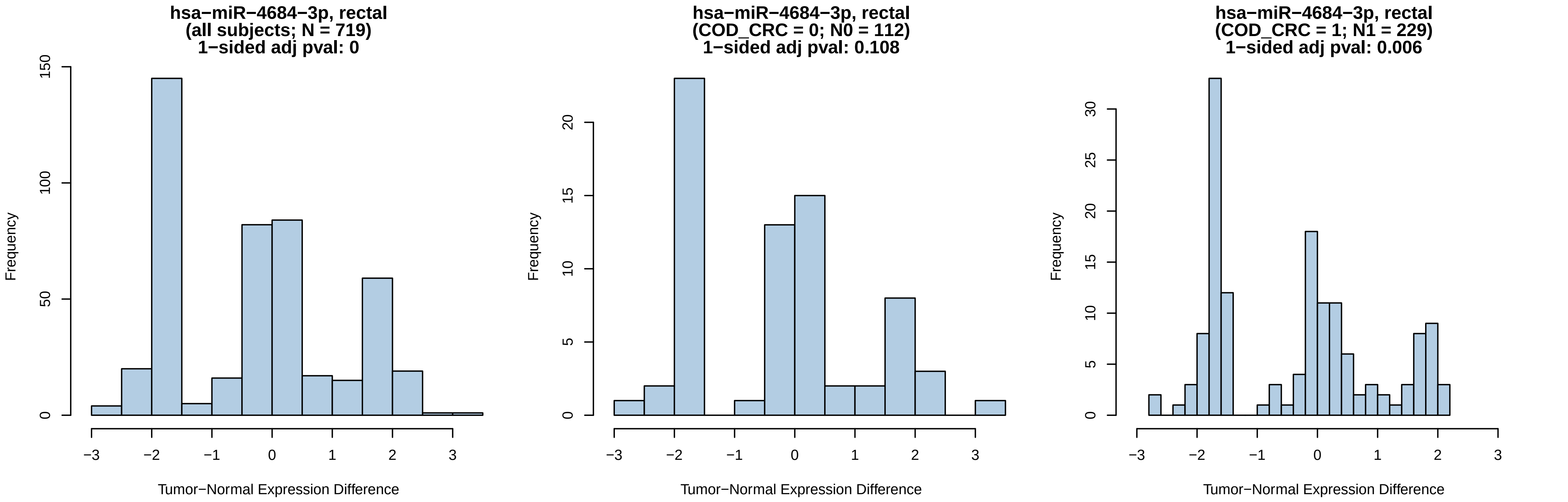
hsa−miR−4684−3p, rectal
(all subjects; N = 719)
1−sided adj pval: 0
Frequency
0
50
100
150
−3
−2
−1
0
1
2
3
Tumor−Normal Expression Difference
hsa−miR−4684−3p, rectal
(COD_CRC = 0; N0 = 112)
1−sided adj pval: 0.108
Frequency
0
5
10
15
20
−3
−2
−1
0
1
2
3
Tumor−Normal Expression Difference
hsa−miR−4684−3p, rectal
(COD_CRC = 1; N1 = 229)
1−sided adj pval: 0.006
Frequency
0
5
10
15
20
25
30
−3
−2
−1
0
1
2
3
Tumor−Normal Expression Difference

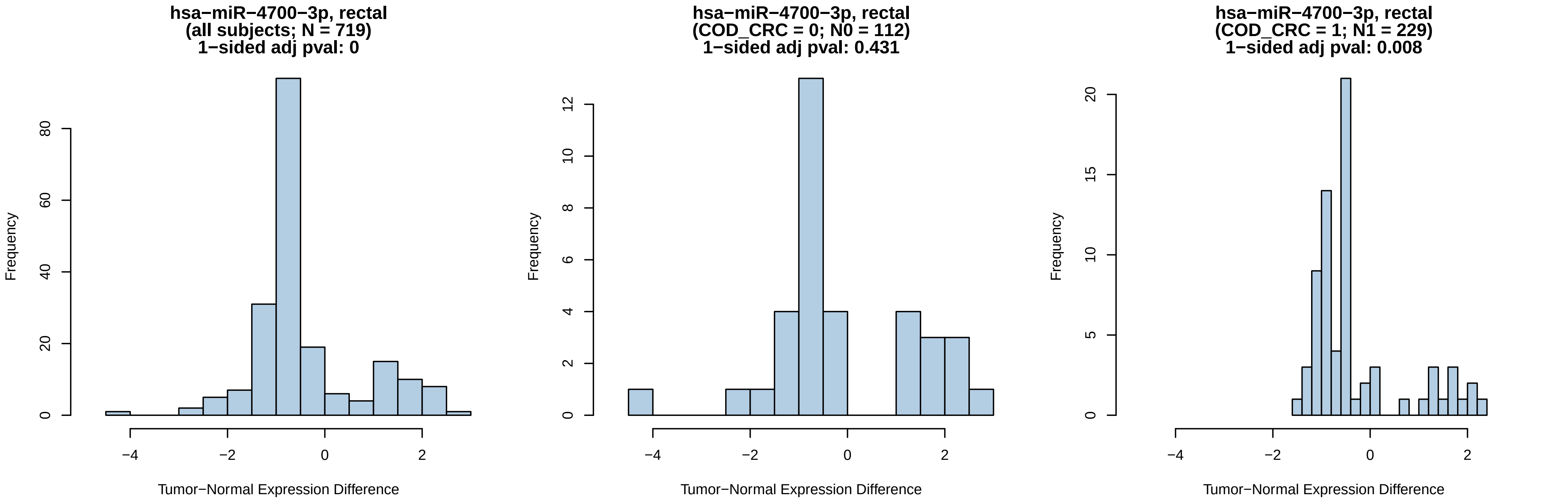
hsa−miR−4700−3p, rectal
(all subjects; N = 719)
1−sided adj pval: 0
Frequency
0
20
40
60
80
−4
−2
0
2
Tumor−Normal Expression Difference
hsa−miR−4700−3p, rectal
(COD_CRC = 0; N0 = 112)
1−sided adj pval: 0.431
Frequency
0
2
4
6
8
10
12
−4
−2
0
2
Tumor−Normal Expression Difference
hsa−miR−4700−3p, rectal
(COD_CRC = 1; N1 = 229)
1−sided adj pval: 0.008
Frequency
0
5
10
15
20
−4
−2
0
2
Tumor−Normal Expression Difference

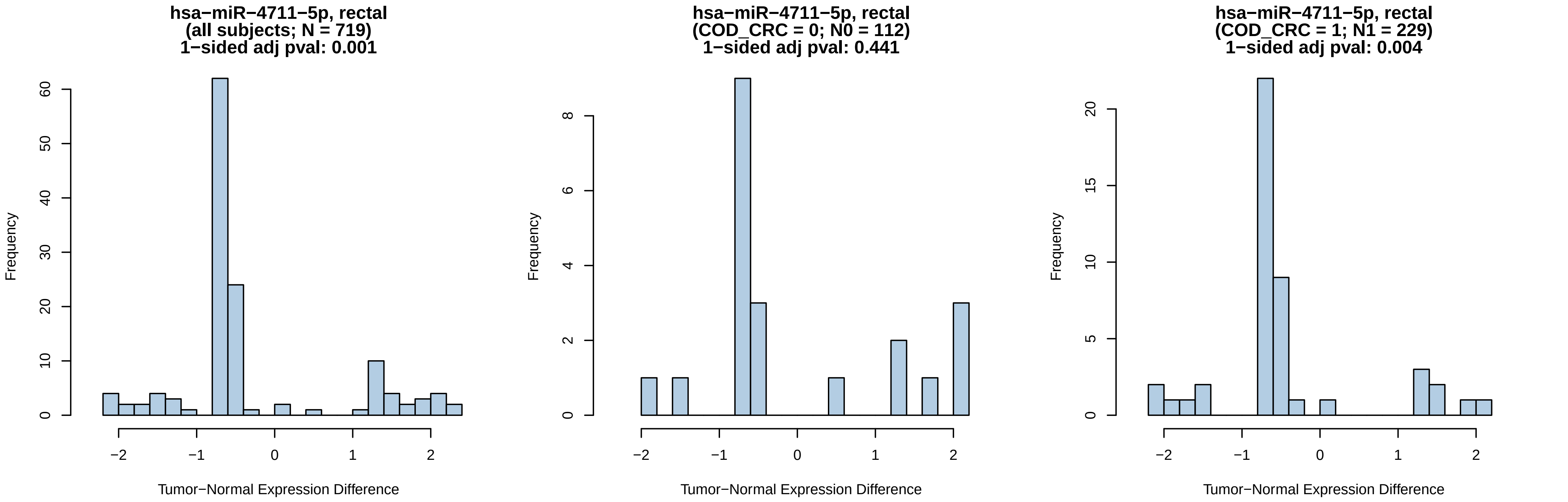
hsa−miR−4711−5p, rectal
(all subjects; N = 719)
1−sided adj pval: 0.001
Frequency
0
10
20
30
40
50
60
−2
−1
0
1
2
Tumor−Normal Expression Difference
hsa−miR−4711−5p, rectal
(COD_CRC = 0; N0 = 112)
1−sided adj pval: 0.441
Frequency
0
2
4
6
8
−2
−1
0
1
2
Tumor−Normal Expression Difference
hsa−miR−4711−5p, rectal
(COD_CRC = 1; N1 = 229)
1−sided adj pval: 0.004
Frequency
0
5
10
15
20
−2
−1
0
1
2
Tumor−Normal Expression Difference

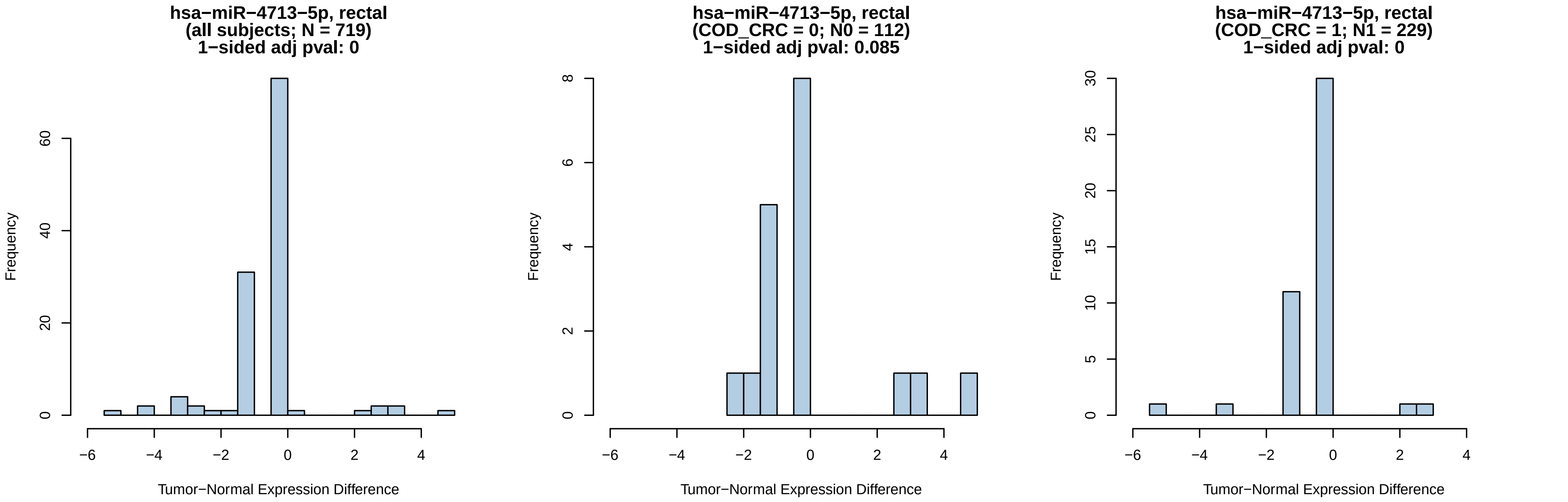
hsa−miR−4713−5p, rectal
(all subjects; N = 719)
1−sided adj pval: 0
hsa−miR−4713−5p, rectal
(COD_CRC = 0; N0 = 112)
1−sided adj pval: 0.085
hsa−miR−4713−5p, rectal
(COD_CRC = 1; N1 = 229)
1−sided adj pval: 0
Frequency
Tumor−Normal Expression Difference

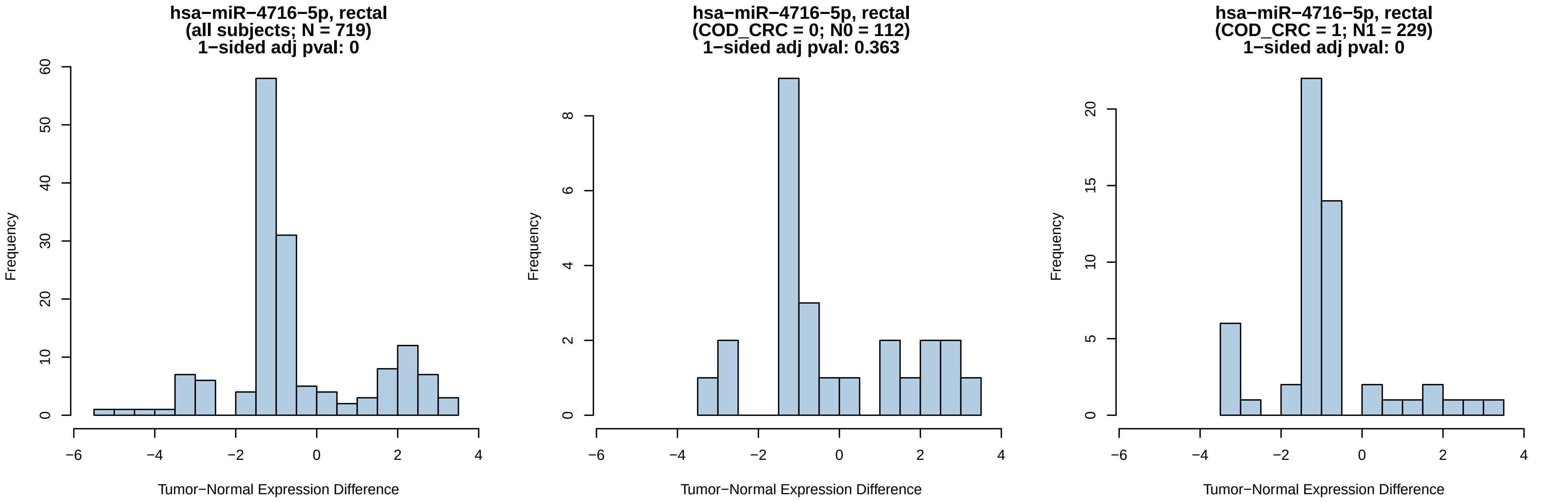
hsa−miR−4716−5p, rectal
(all subjects; N = 719)
1−sided adj pval: 0
Frequency
Tumor−Normal Expression Difference
hsa−miR−4716−5p, rectal
(COD_CRC = 0; N0 = 112)
1−sided adj pval: 0.363
Frequency
Tumor−Normal Expression Difference
hsa−miR−4716−5p, rectal
(COD_CRC = 1; N1 = 229)
1−sided adj pval: 0
Frequency
Tumor−Normal Expression Difference

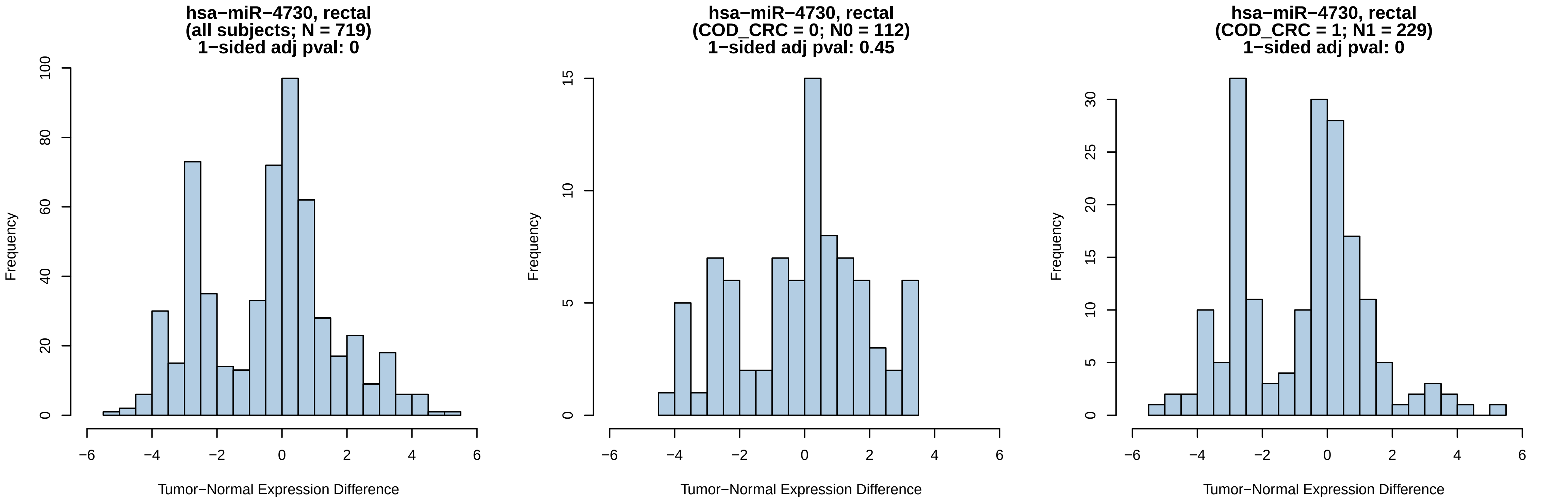

hsa−miR−4730, rectal
(all subjects; N = 719)
1−sided adj pval: 0
Frequency
Tumor−Normal Expression Difference
hsa−miR−4730, rectal
(COD_CRC = 0; N0 = 112)
1−sided adj pval: 0.45
Frequency
Tumor−Normal Expression Difference
hsa−miR−4730, rectal
(COD_CRC = 1; N1 = 229)
1−sided adj pval: 0
Frequency
Tumor−Normal Expression Difference

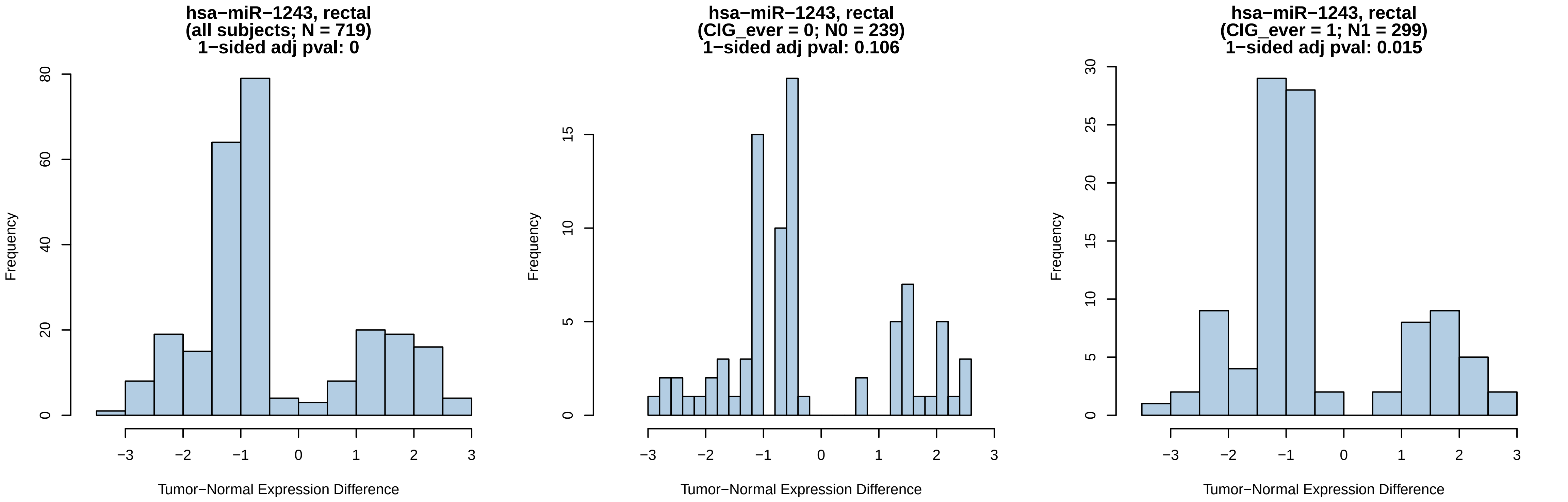
hsa−miR−1243, rectal
(all subjects; N = 719)
1−sided adj pval: 0
Frequency
Tumor−Normal Expression Difference
hsa−miR−1243, rectal
(CIG_ever = 0; N0 = 239)
1−sided adj pval: 0.106
Frequency
Tumor−Normal Expression Difference
hsa−miR−1243, rectal
(CIG_ever = 1; N1 = 299)
1−sided adj pval: 0.015
Frequency
Tumor−Normal Expression Difference

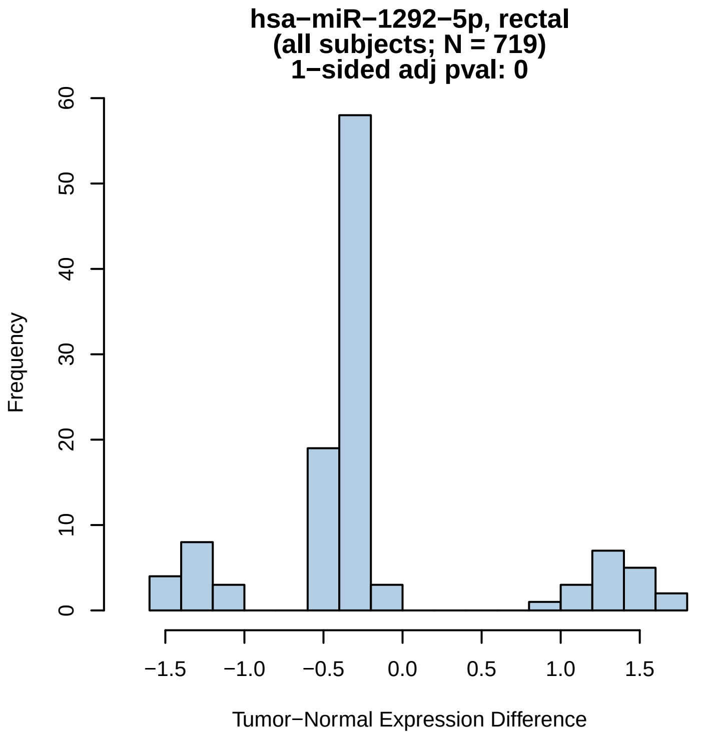
hsa−miR−1292−5p, rectal
(all subjects; N = 719)
1−sided adj pval: 0
Frequency
0
10
20
30
40
50
60
−1.5
−1.0
−0.5
0.0
0.5
1.0
1.5
Tumor−Normal Expression Difference

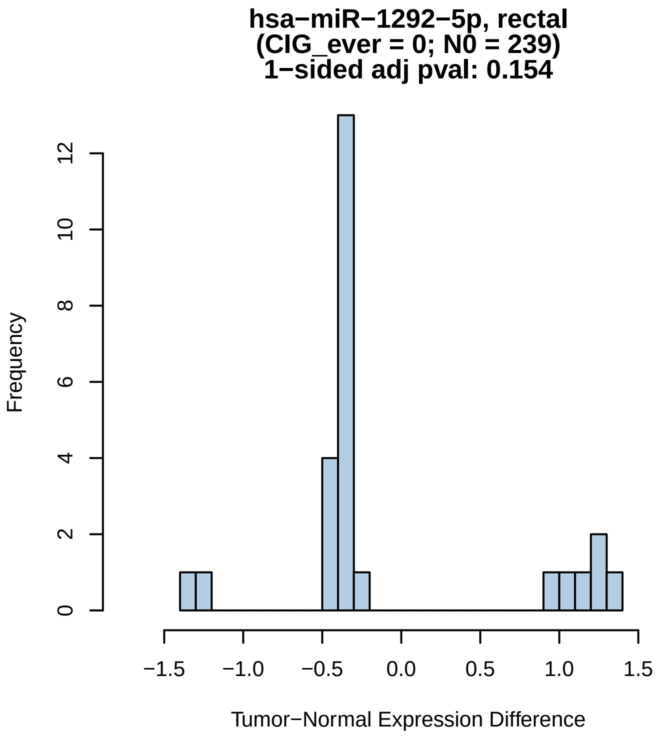
hsa−miR−1292−5p, rectal
(CIG_ever = 0; N0 = 239)
1−sided adj pval: 0.154
Frequency
0
2
4
6
8
10
12
−1.5
−1.0
−0.5
0.0
0.5
1.0
1.5
Tumor−Normal Expression Difference

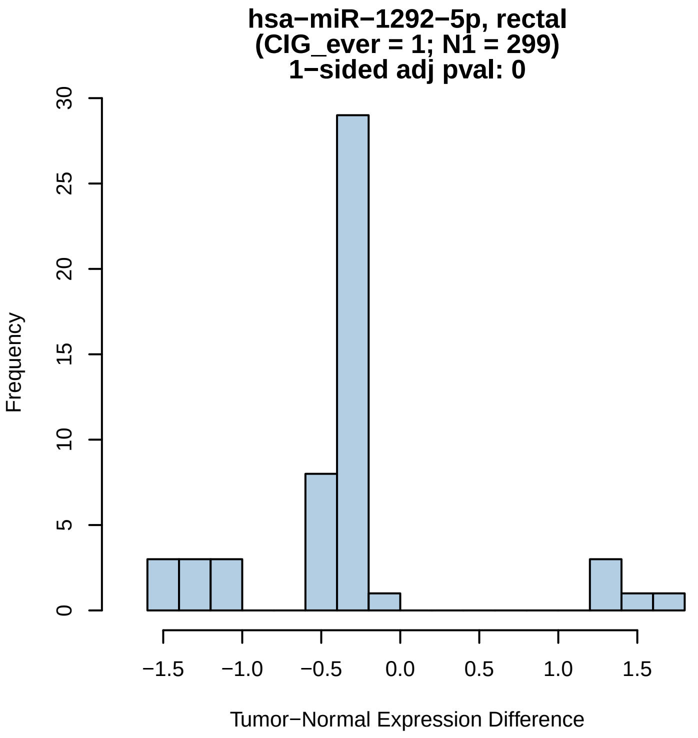
hsa−miR−1292−5p, rectal
(CIG_ever = 1; N1 = 299)
1−sided adj pval: 0
Frequency
0
5
10
15
20
25
30
−1.5
−1.0
−0.5
0.0
0.5
1.0
1.5
Tumor−Normal Expression Difference

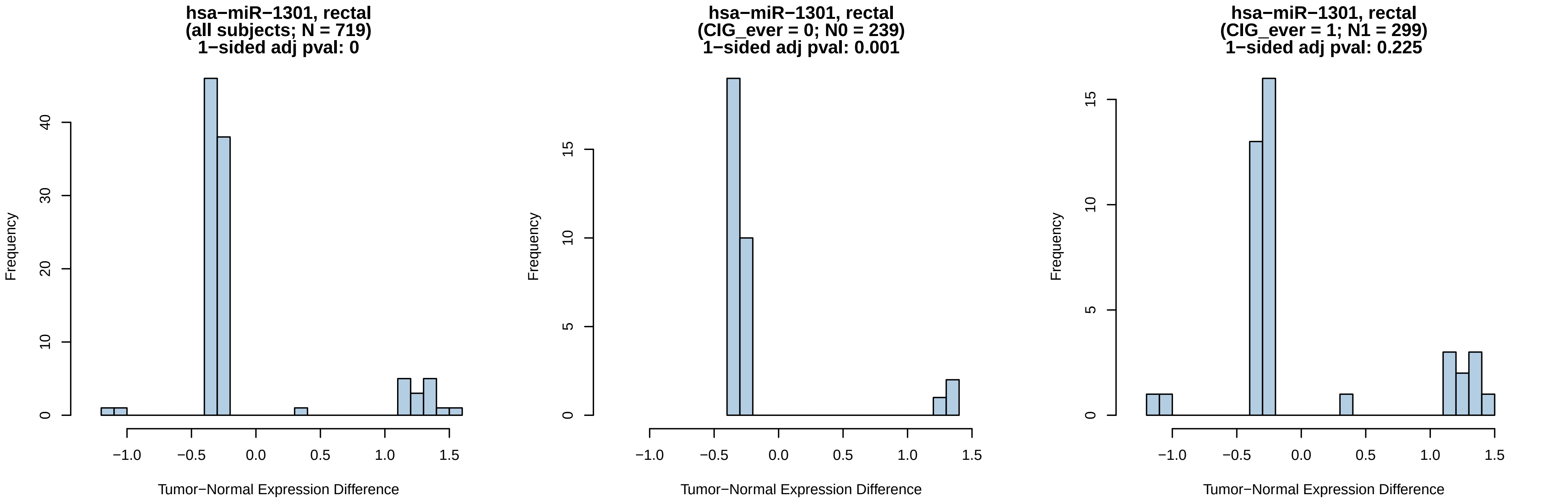
hsa−miR−1301, rectal
(all subjects; N = 719)
1−sided adj pval: 0
hsa−miR−1301, rectal
(CIG_ever = 0; N0 = 239)
1−sided adj pval: 0.001
hsa−miR−1301, rectal
(CIG_ever = 1; N1 = 299)
1−sided adj pval: 0.225
Frequency
Tumor−Normal Expression Difference
0
10
20
30
40
5
15
−1.0
−0.5
0.0
0.5
1.0
1.5

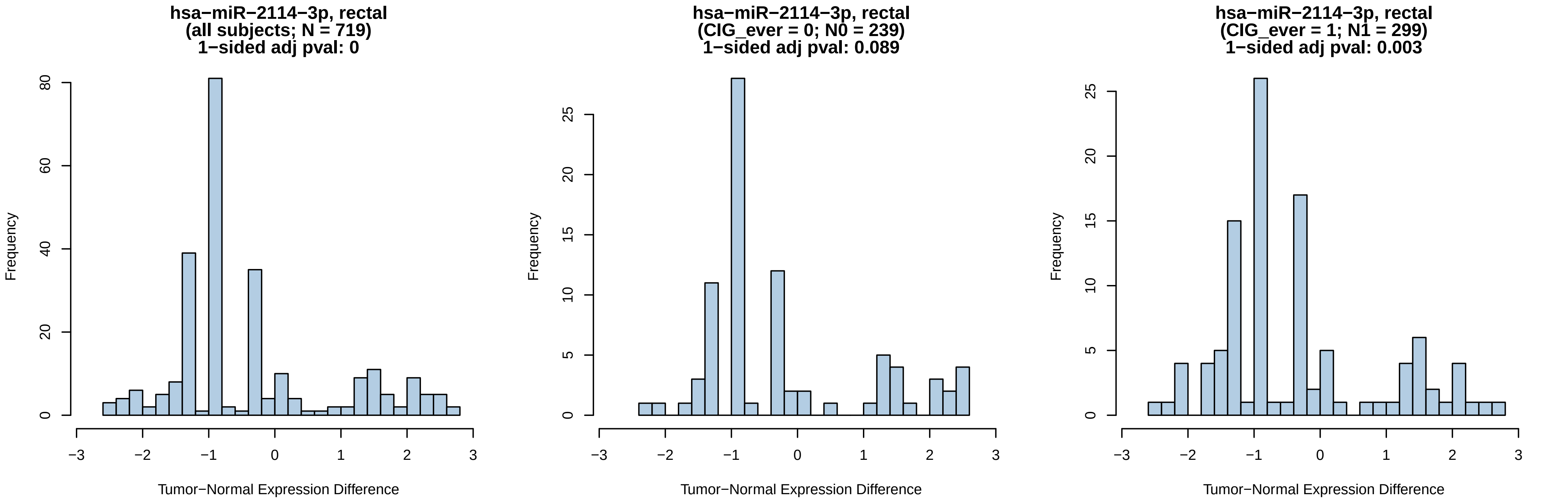

hsa−miR−2114−3p, rectal
(all subjects; N = 719)
1−sided adj pval: 0
Frequency
0
20
40
60
80
−3
−2
−1
0
1
2
3
Tumor−Normal Expression Difference
hsa−miR−2114−3p, rectal
(CIG_ever = 0; N0 = 239)
1−sided adj pval: 0.089
Frequency
0
5
10
15
20
25
−3
−2
−1
0
1
2
3
Tumor−Normal Expression Difference
hsa−miR−2114−3p, rectal
(CIG_ever = 1; N1 = 299)
1−sided adj pval: 0.003
Frequency
0
5
10
15
20
25
−3
−2
−1
0
1
2
3
Tumor−Normal Expression Difference

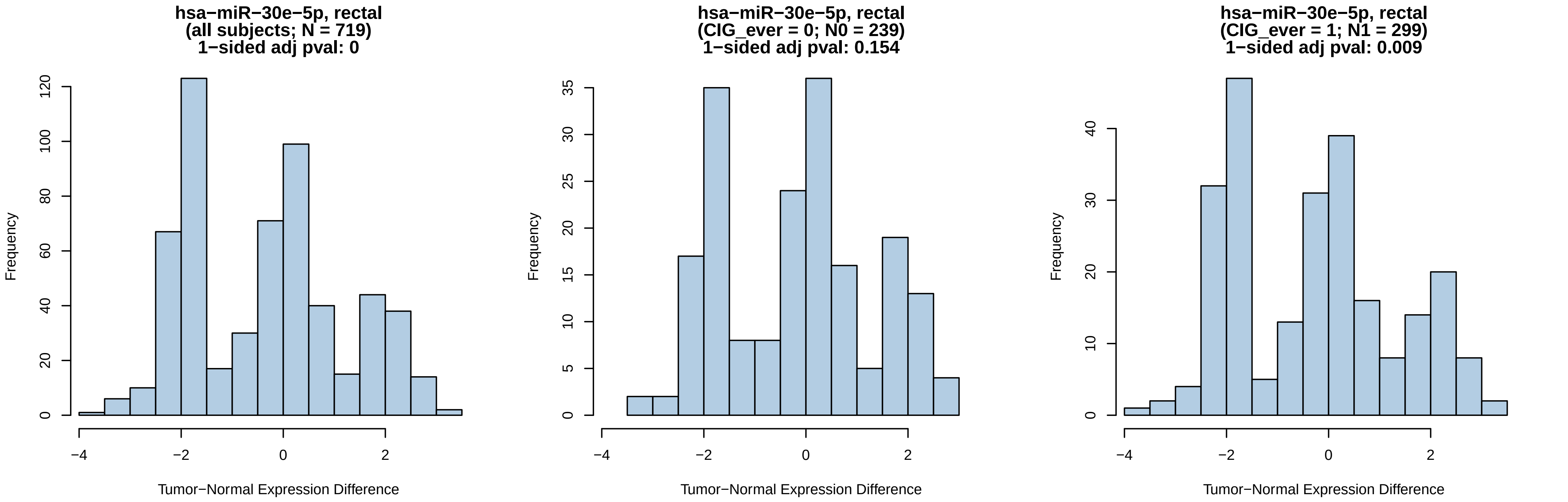
hsa−miR−30e−5p, rectal
(all subjects; N = 719)
1−sided adj pval: 0
Frequency
Tumor−Normal Expression Difference
hsa−miR−30e−5p, rectal
(CIG_ever = 0; N0 = 239)
1−sided adj pval: 0.154
Frequency
Tumor−Normal Expression Difference
hsa−miR−30e−5p, rectal
(CIG_ever = 1; N1 = 299)
1−sided adj pval: 0.009
Frequency
Tumor−Normal Expression Difference

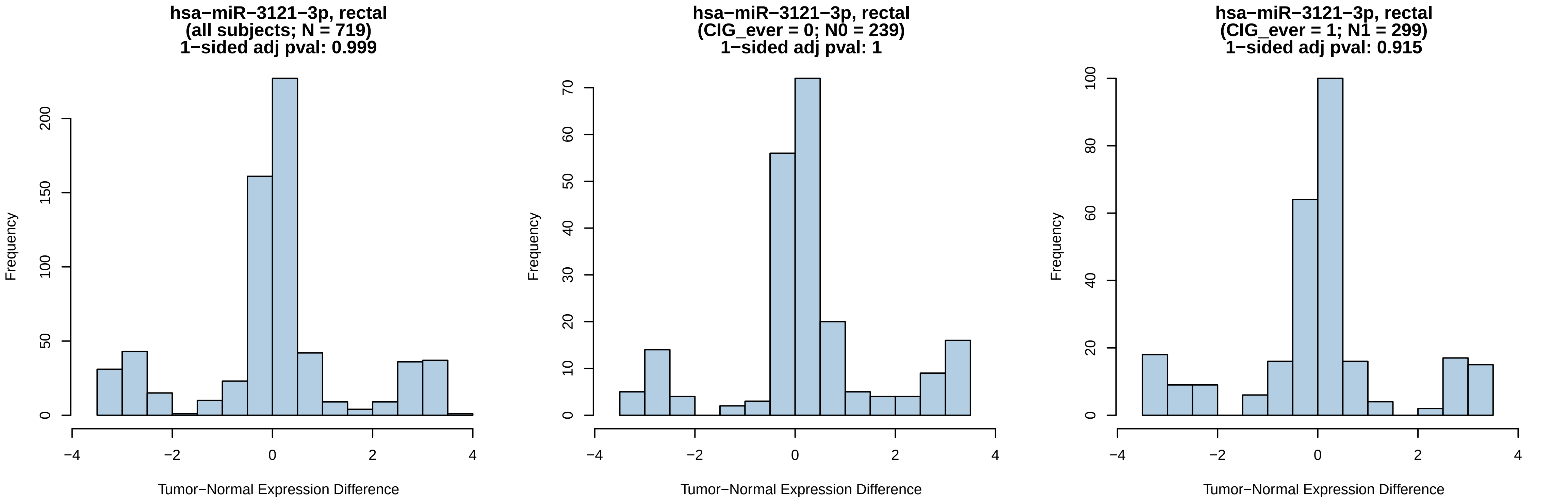

hsa−miR−3121−3p, rectal
(all subjects; N = 719)
1−sided adj pval: 0.999
hsa−miR−3121−3p, rectal
(CIG_ever = 0; N0 = 239)
1−sided adj pval: 1
hsa−miR−3121−3p, rectal
(CIG_ever = 1; N1 = 299)
1−sided adj pval: 0.915
Frequency
Tumor−Normal Expression Difference

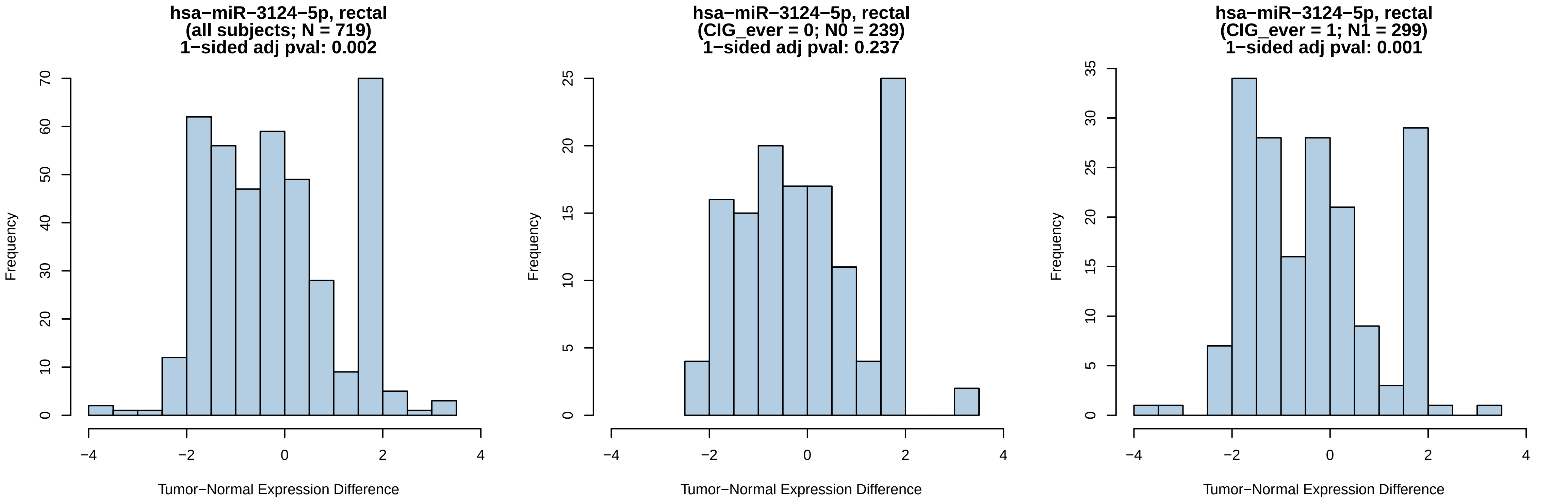

hsa−miR−3124−5p, rectal
(all subjects; N = 719)
1−sided adj pval: 0.002
Frequency
Tumor−Normal Expression Difference
hsa−miR−3124−5p, rectal
(CIG_ever = 0; N0 = 239)
1−sided adj pval: 0.237
Frequency
Tumor−Normal Expression Difference
hsa−miR−3124−5p, rectal
(CIG_ever = 1; N1 = 299)
1−sided adj pval: 0.001
Frequency
Tumor−Normal Expression Difference

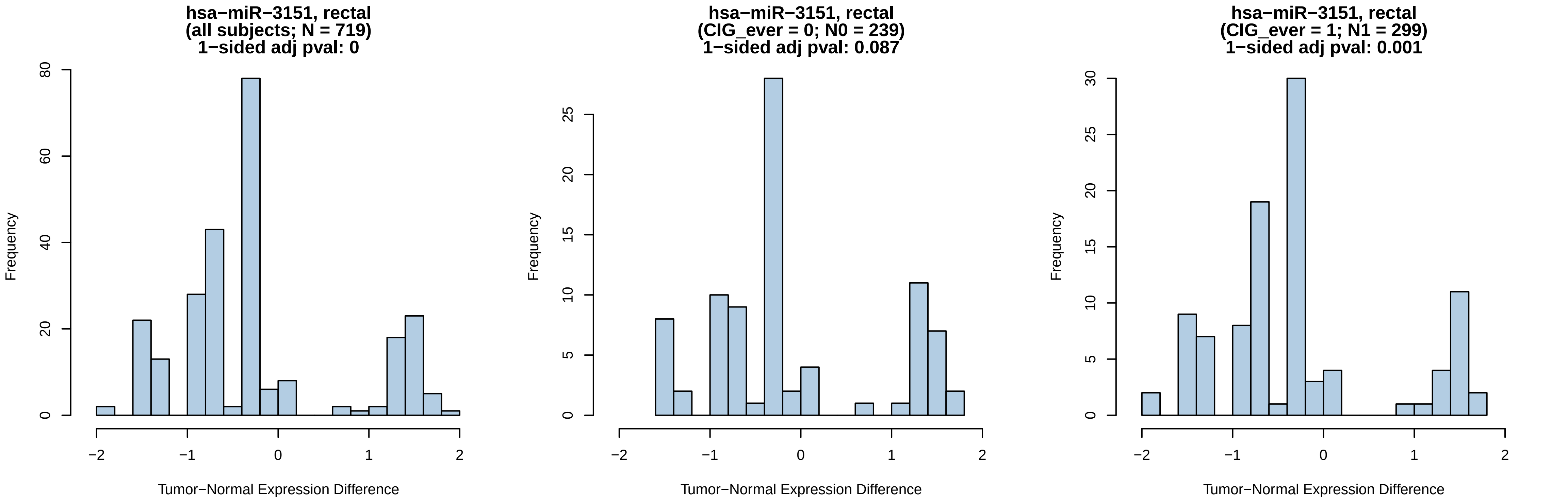
hsa−miR−3151, rectal
(all subjects; N = 719)
1−sided adj pval: 0
Frequency
0
20
40
60
80
−2
−1
0
1
2
Tumor−Normal Expression Difference
hsa−miR−3151, rectal
(CIG_ever = 0; N0 = 239)
1−sided adj pval: 0.087
Frequency
0
5
10
15
20
25
−2
−1
0
1
2
Tumor−Normal Expression Difference
hsa−miR−3151, rectal
(CIG_ever = 1; N1 = 299)
1−sided adj pval: 0.001
Frequency
0
5
10
15
20
25
30
−2
−1
0
1
2
Tumor−Normal Expression Difference

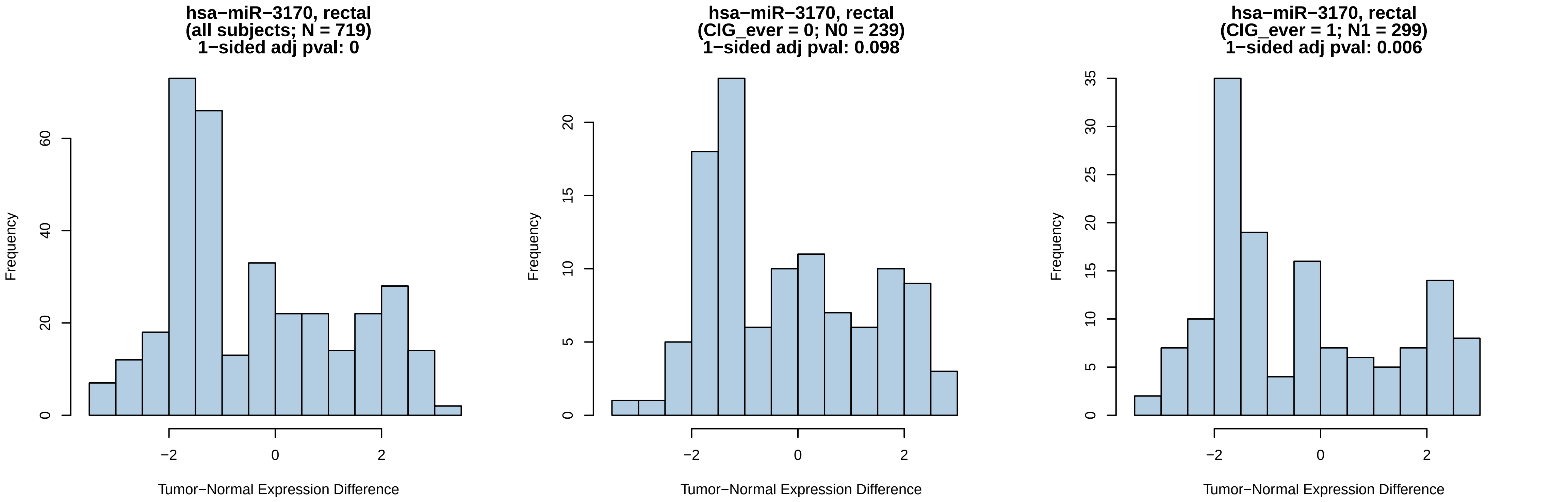

hsa−miR−3170, rectal
(all subjects; N = 719)
1−sided adj pval: 0
Frequency
0
20
40
60
−2
0
2
Tumor−Normal Expression Difference
hsa−miR−3170, rectal
(CIG_ever = 0; N0 = 239)
1−sided adj pval: 0.098
Frequency
0
5
10
15
20
−2
0
2
Tumor−Normal Expression Difference
hsa−miR−3170, rectal
(CIG_ever = 1; N1 = 299)
1−sided adj pval: 0.006
Frequency
0
5
10
15
20
25
30
35
−2
0
2
Tumor−Normal Expression Difference

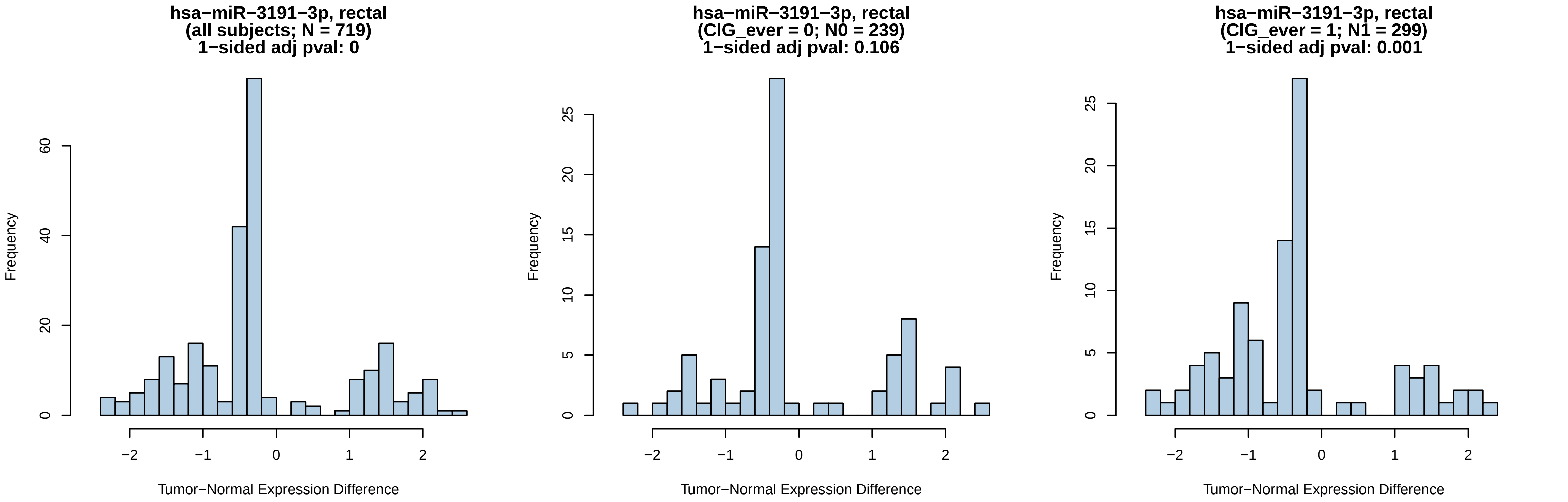

hsa−miR−3191−3p, rectal
(all subjects; N = 719)
1−sided adj pval: 0
Frequency
0
20
40
60
−2
−1
0
1
2
Tumor−Normal Expression Difference
hsa−miR−3191−3p, rectal
(CIG_ever = 0; N0 = 239)
1−sided adj pval: 0.106
Frequency
0
5
10
15
20
25
−2
−1
0
1
2
Tumor−Normal Expression Difference
hsa−miR−3191−3p, rectal
(CIG_ever = 1; N1 = 299)
1−sided adj pval: 0.001
Frequency
0
5
10
15
20
25
−2
−1
0
1
2
Tumor−Normal Expression Difference

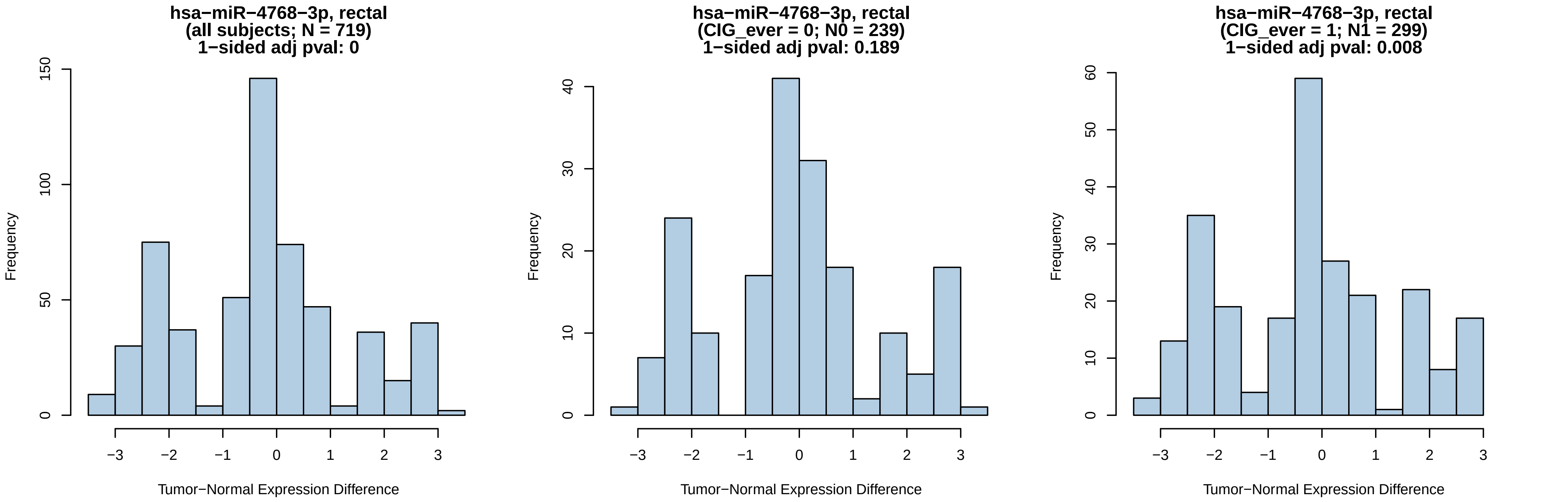

hsa−miR−4768−3p, rectal
(all subjects; N = 719)
1−sided adj pval: 0
Frequency
Tumor−Normal Expression Difference
hsa−miR−4768−3p, rectal
(CIG_ever = 0; N0 = 239)
1−sided adj pval: 0.189
Frequency
Tumor−Normal Expression Difference
hsa−miR−4768−3p, rectal
(CIG_ever = 1; N1 = 299)
1−sided adj pval: 0.008
Frequency
Tumor−Normal Expression Difference

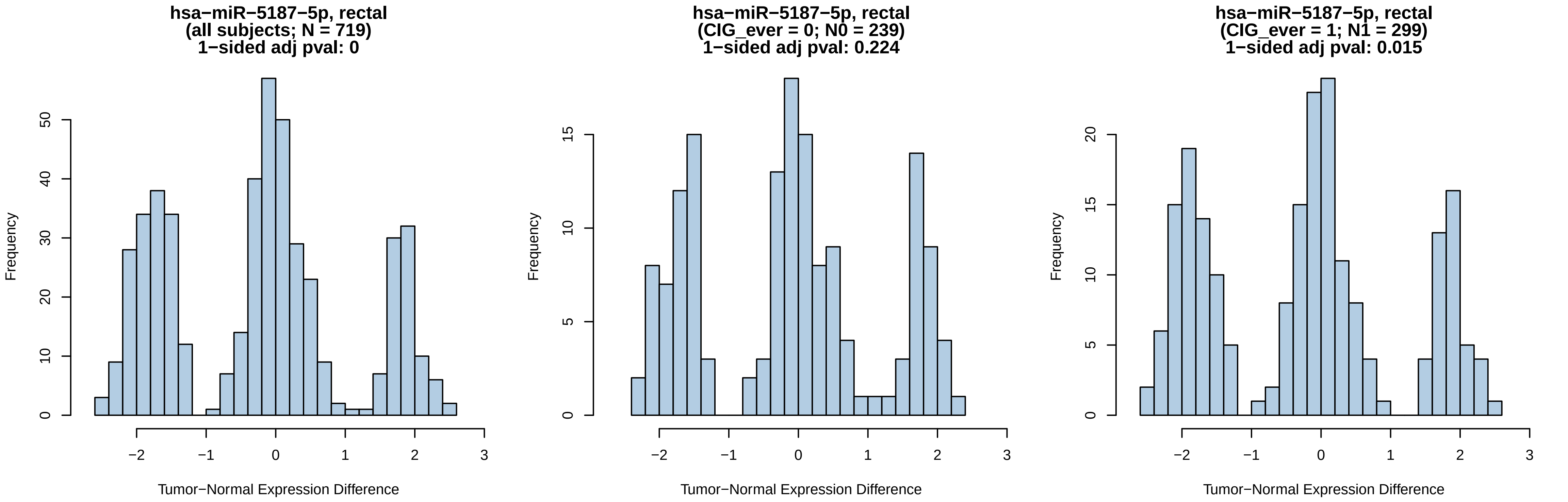
hsa−miR−5187−5p, rectal
(all subjects; N = 719)
1−sided adj pval: 0
Frequency
Tumor−Normal Expression Difference
hsa−miR−5187−5p, rectal
(CIG_ever = 0; N0 = 239)
1−sided adj pval: 0.224
Frequency
Tumor−Normal Expression Difference
hsa−miR−5187−5p, rectal
(CIG_ever = 1; N1 = 299)
1−sided adj pval: 0.015
Frequency
Tumor−Normal Expression Difference

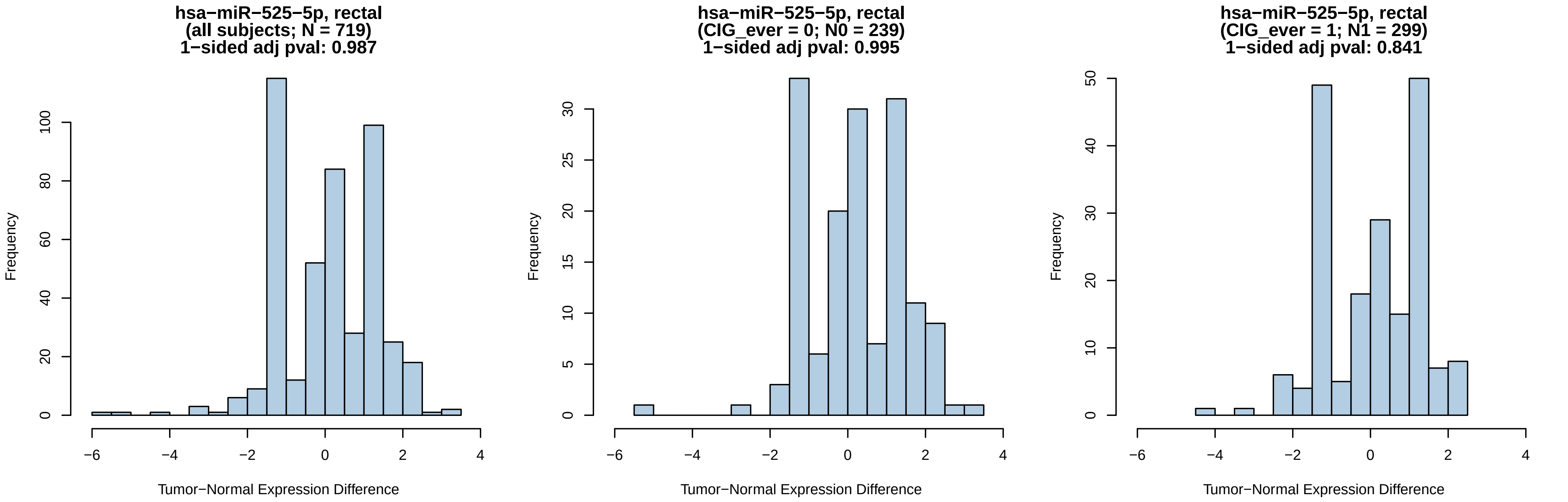
hsa−miR−525−5p, rectal
(all subjects; N = 719)
1−sided adj pval: 0.987
Frequency
Tumor−Normal Expression Difference
hsa−miR−525−5p, rectal
(CIG_ever = 0; N0 = 239)
1−sided adj pval: 0.995
Frequency
Tumor−Normal Expression Difference
hsa−miR−525−5p, rectal
(CIG_ever = 1; N1 = 299)
1−sided adj pval: 0.841
Frequency
Tumor−Normal Expression Difference

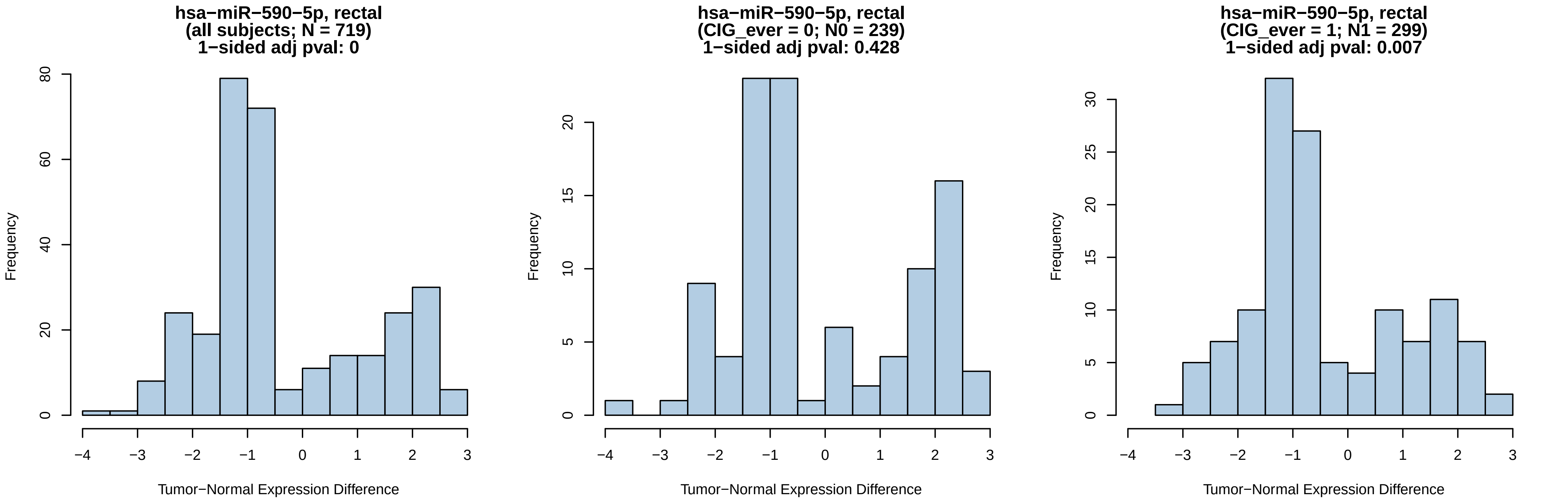
hsa−miR−590−5p, rectal
(all subjects; N = 719)
1−sided adj pval: 0
Frequency
Tumor−Normal Expression Difference
hsa−miR−590−5p, rectal
(CIG_ever = 0; N0 = 239)
1−sided adj pval: 0.428
Frequency
Tumor−Normal Expression Difference
hsa−miR−590−5p, rectal
(CIG_ever = 1; N1 = 299)
1−sided adj pval: 0.007
Frequency
Tumor−Normal Expression Difference

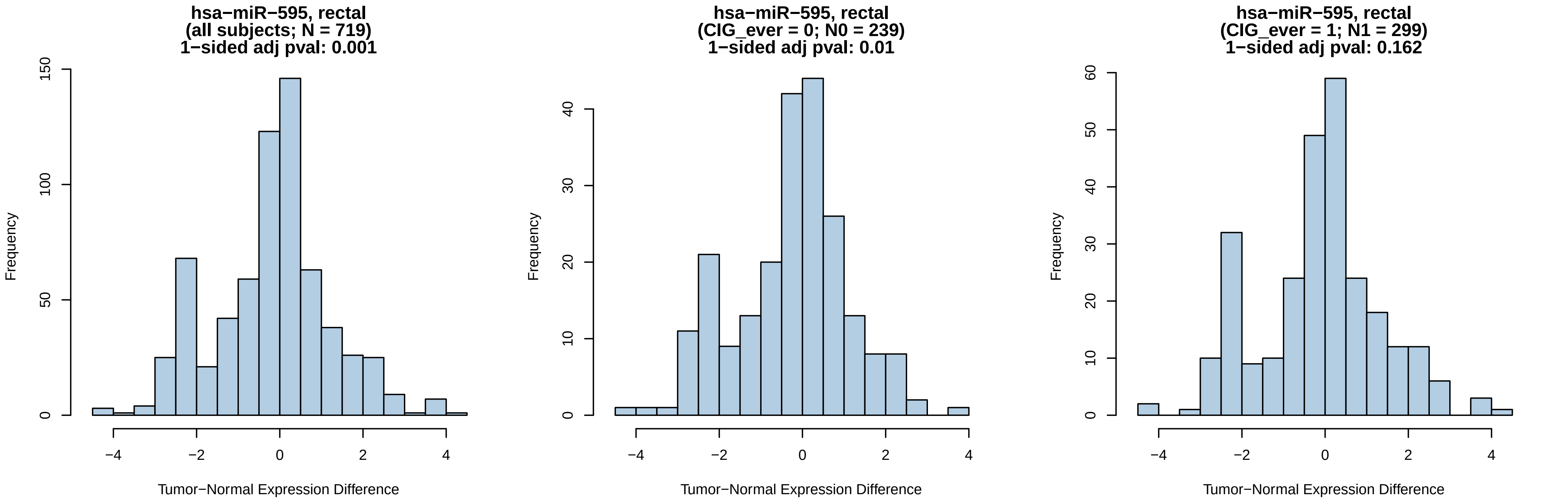
hsa−miR−595, rectal
(all subjects; N = 719)
1−sided adj pval: 0.001
Frequency
Tumor−Normal Expression Difference
hsa−miR−595, rectal
(CIG_ever = 0; N0 = 239)
1−sided adj pval: 0.01
Frequency
Tumor−Normal Expression Difference
hsa−miR−595, rectal
(CIG_ever = 1; N1 = 299)
1−sided adj pval: 0.162
Frequency
Tumor−Normal Expression Difference

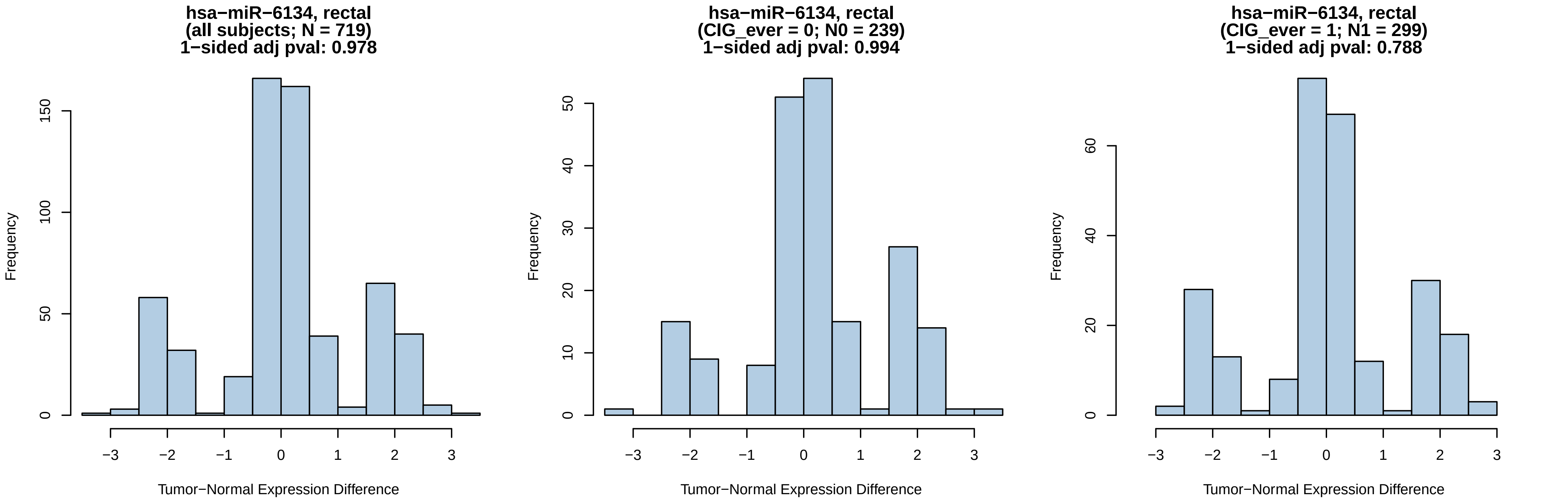

hsa−miR−6134, rectal
(all subjects; N = 719)
1−sided adj pval: 0.978
Frequency
0
50
100
150
−3
−2
−1
0
1
2
3
Tumor−Normal Expression Difference
hsa−miR−6134, rectal
(CIG_ever = 0; N0 = 239)
1−sided adj pval: 0.994
Frequency
0
10
20
30
40
50
−3
−2
−1
0
1
2
3
Tumor−Normal Expression Difference
hsa−miR−6134, rectal
(CIG_ever = 1; N1 = 299)
1−sided adj pval: 0.788
Frequency
0
20
40
60
−3
−2
−1
0
1
2
3
Tumor−Normal Expression Difference

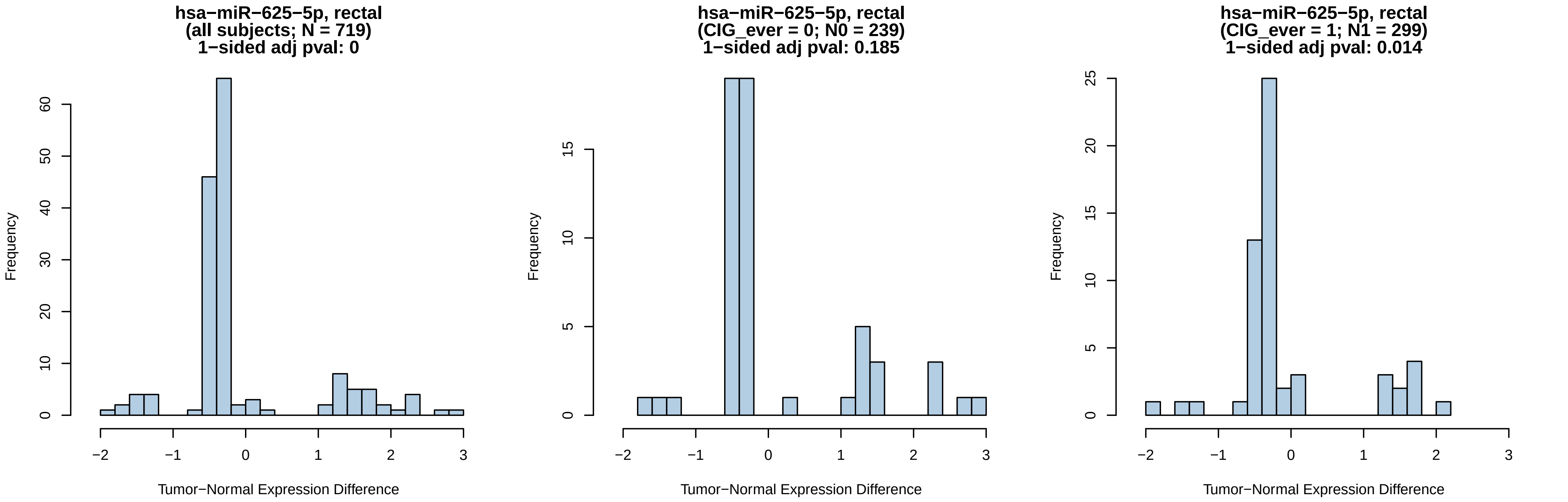
hsa−miR−625−5p, rectal
(all subjects; N = 719)
1−sided adj pval: 0
Frequency
Tumor−Normal Expression Difference
hsa−miR−625−5p, rectal
(CIG_ever = 0; N0 = 239)
1−sided adj pval: 0.185
Frequency
Tumor−Normal Expression Difference
hsa−miR−625−5p, rectal
(CIG_ever = 1; N1 = 299)
1−sided adj pval: 0.014
Frequency
Tumor−Normal Expression Difference

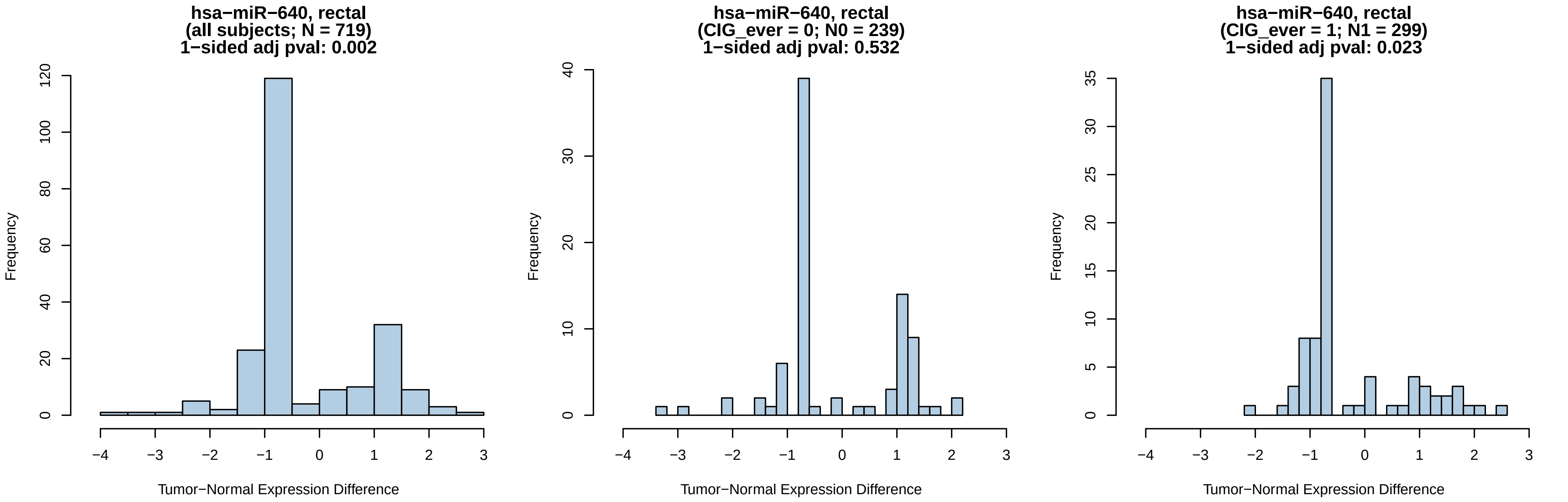
hsa−miR−640, rectal
(all subjects; N = 719)
1−sided adj pval: 0.002
Frequency
Tumor−Normal Expression Difference
hsa−miR−640, rectal
(CIG_ever = 0; N0 = 239)
1−sided adj pval: 0.532
Frequency
Tumor−Normal Expression Difference
hsa−miR−640, rectal
(CIG_ever = 1; N1 = 299)
1−sided adj pval: 0.023
Frequency
Tumor−Normal Expression Difference

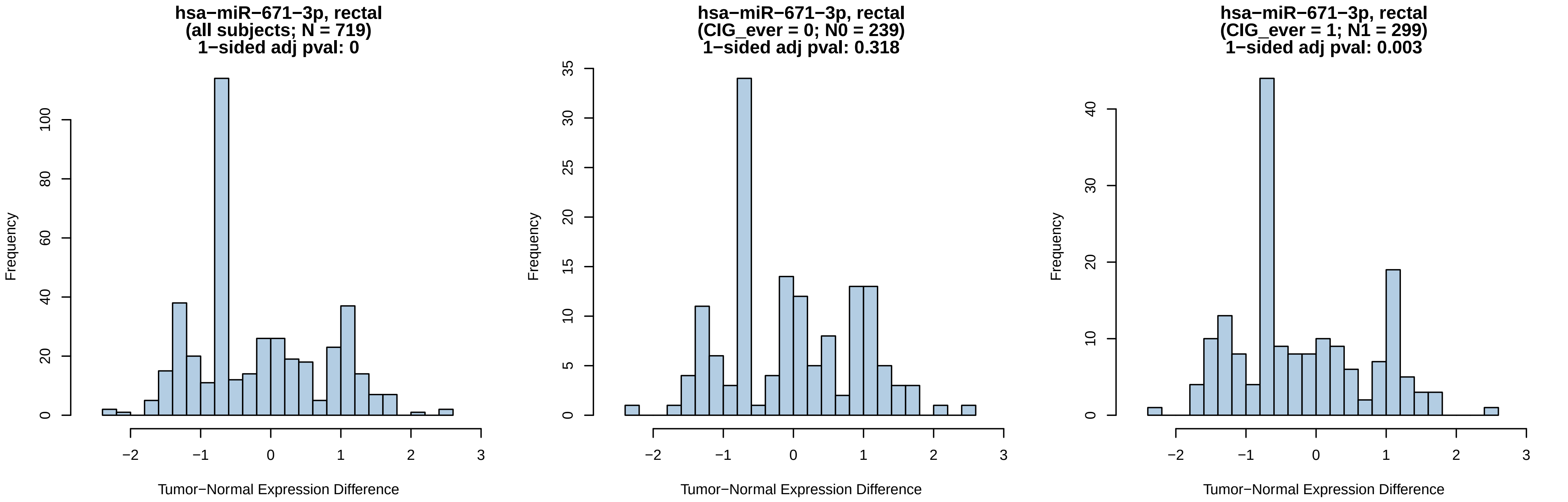
hsa−miR−671−3p, rectal
(all subjects; N = 719)
1−sided adj pval: 0
hsa−miR−671−3p, rectal
(CIG_ever = 0; N0 = 239)
1−sided adj pval: 0.318
hsa−miR−671−3p, rectal
(CIG_ever = 1; N1 = 299)
1−sided adj pval: 0.003
Frequency
Tumor−Normal Expression Difference

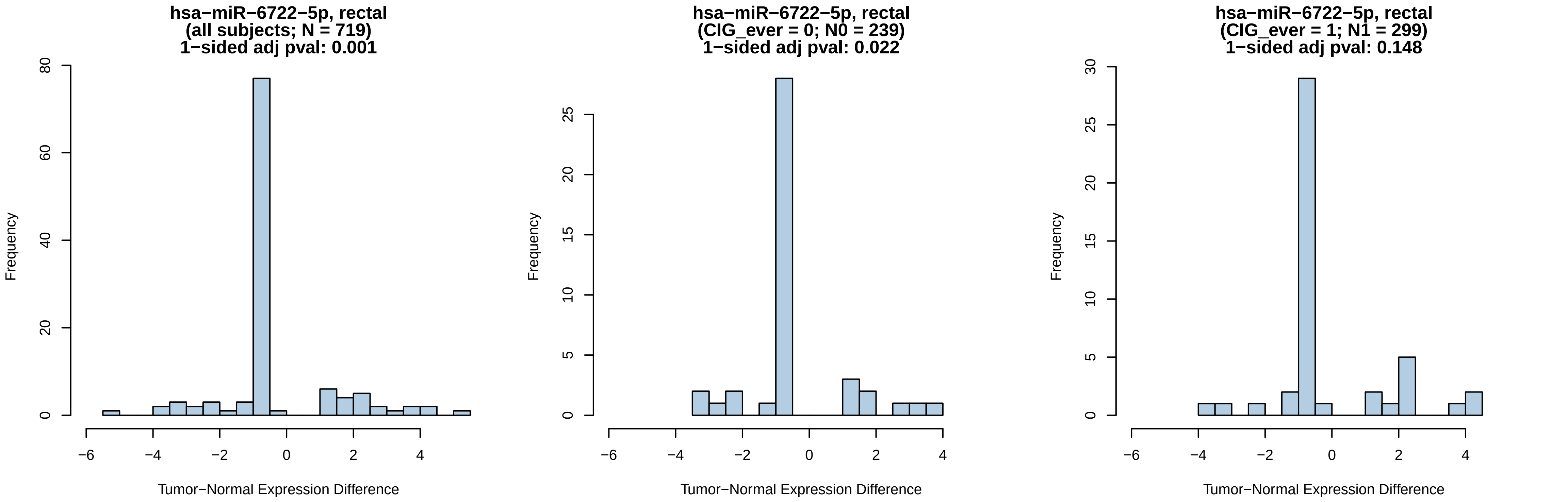
hsa−miR−6722−5p, rectal
(all subjects; N = 719)
1−sided adj pval: 0.001
Frequency
Tumor−Normal Expression Difference
hsa−miR−6722−5p, rectal
(CIG_ever = 0; N0 = 239)
1−sided adj pval: 0.022
Frequency
Tumor−Normal Expression Difference
hsa−miR−6722−5p, rectal
(CIG_ever = 1; N1 = 299)
1−sided adj pval: 0.148
Frequency
Tumor−Normal Expression Difference

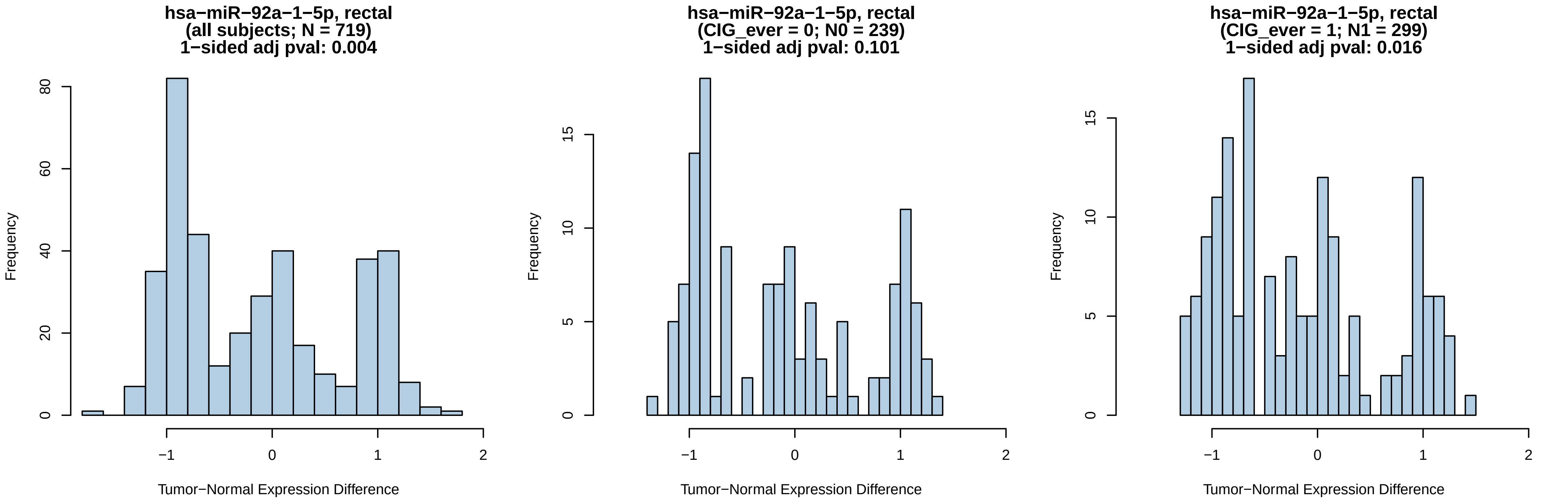
hsa−miR−92a−1−5p, rectal
(all subjects; N = 719)
1−sided adj pval: 0.004
Frequency
Tumor−Normal Expression Difference
hsa−miR−92a−1−5p, rectal
(CIG_ever = 0; N0 = 239)
1−sided adj pval: 0.101
Frequency
Tumor−Normal Expression Difference
hsa−miR−92a−1−5p, rectal
(CIG_ever = 1; N1 = 299)
1−sided adj pval: 0.016
Frequency
Tumor−Normal Expression Difference

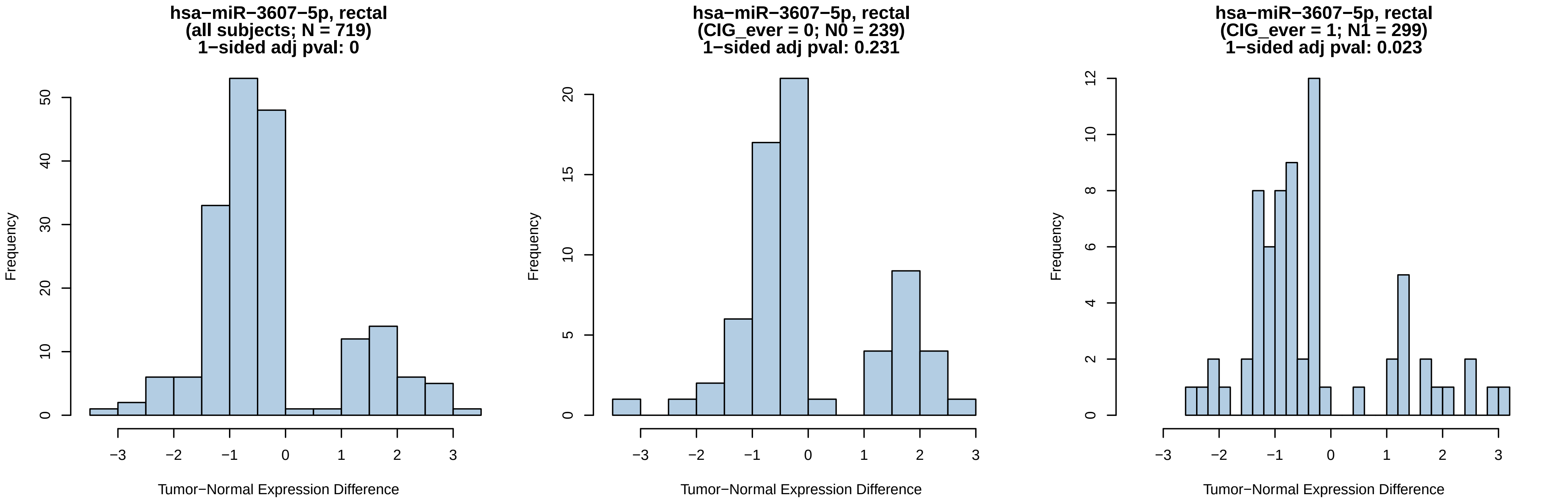
hsa−miR−3607−5p, rectal
(all subjects; N = 719)
1−sided adj pval: 0
Frequency
Tumor−Normal Expression Difference
hsa−miR−3607−5p, rectal
(CIG_ever = 0; N0 = 239)
1−sided adj pval: 0.231
Frequency
Tumor−Normal Expression Difference
hsa−miR−3607−5p, rectal
(CIG_ever = 1; N1 = 299)
1−sided adj pval: 0.023
Frequency
Tumor−Normal Expression Difference

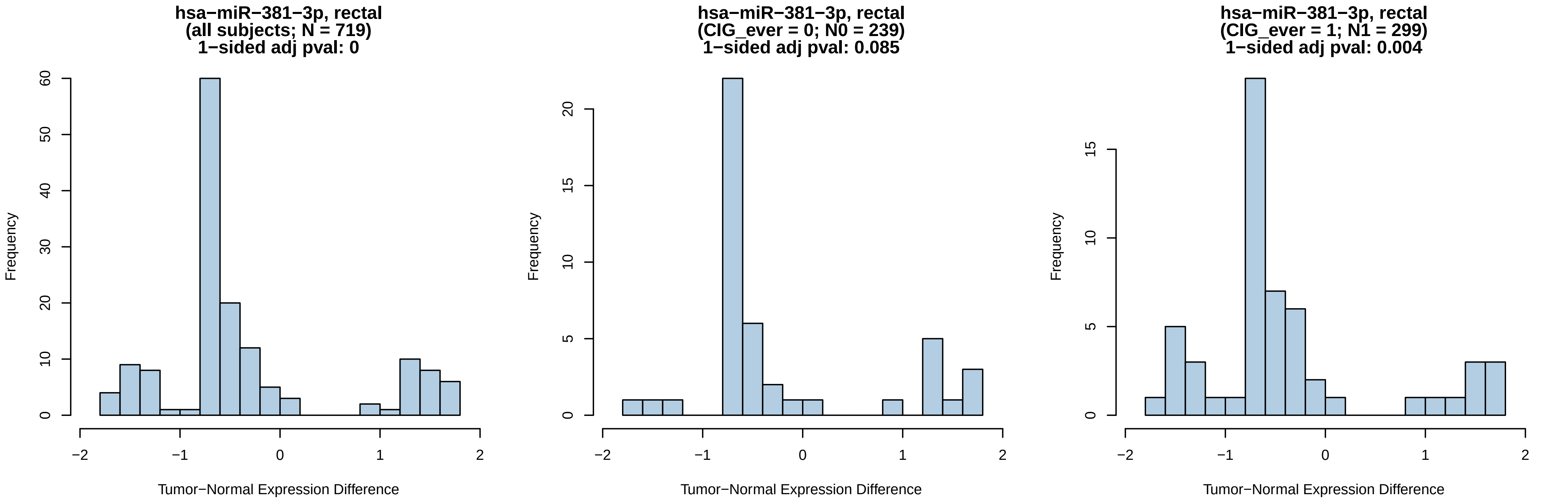
hsa−miR−381−3p, rectal
(all subjects; N = 719)
1−sided adj pval: 0
Frequency
Tumor−Normal Expression Difference
hsa−miR−381−3p, rectal
(CIG_ever = 0; N0 = 239)
1−sided adj pval: 0.085
Frequency
Tumor−Normal Expression Difference
hsa−miR−381−3p, rectal
(CIG_ever = 1; N1 = 299)
1−sided adj pval: 0.004
Frequency
Tumor−Normal Expression Difference

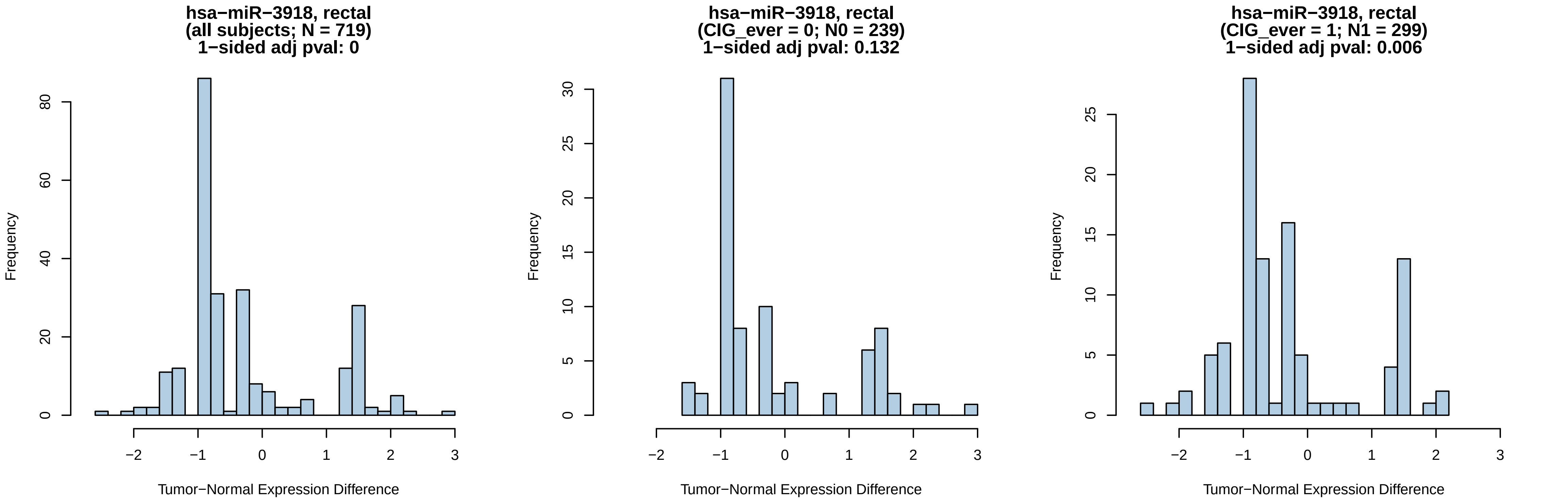

hsa−miR−3918, rectal
(all subjects; N = 719)
1−sided adj pval: 0
Frequency
0
20
40
60
80
−2
−1
0
1
2
3
Tumor−Normal Expression Difference
hsa−miR−3918, rectal
(CIG_ever = 0; N0 = 239)
1−sided adj pval: 0.132
Frequency
0
5
10
15
20
25
30
−2
−1
0
1
2
3
Tumor−Normal Expression Difference
hsa−miR−3918, rectal
(CIG_ever = 1; N1 = 299)
1−sided adj pval: 0.006
Frequency
0
5
10
15
20
25
−2
−1
0
1
2
3
Tumor−Normal Expression Difference

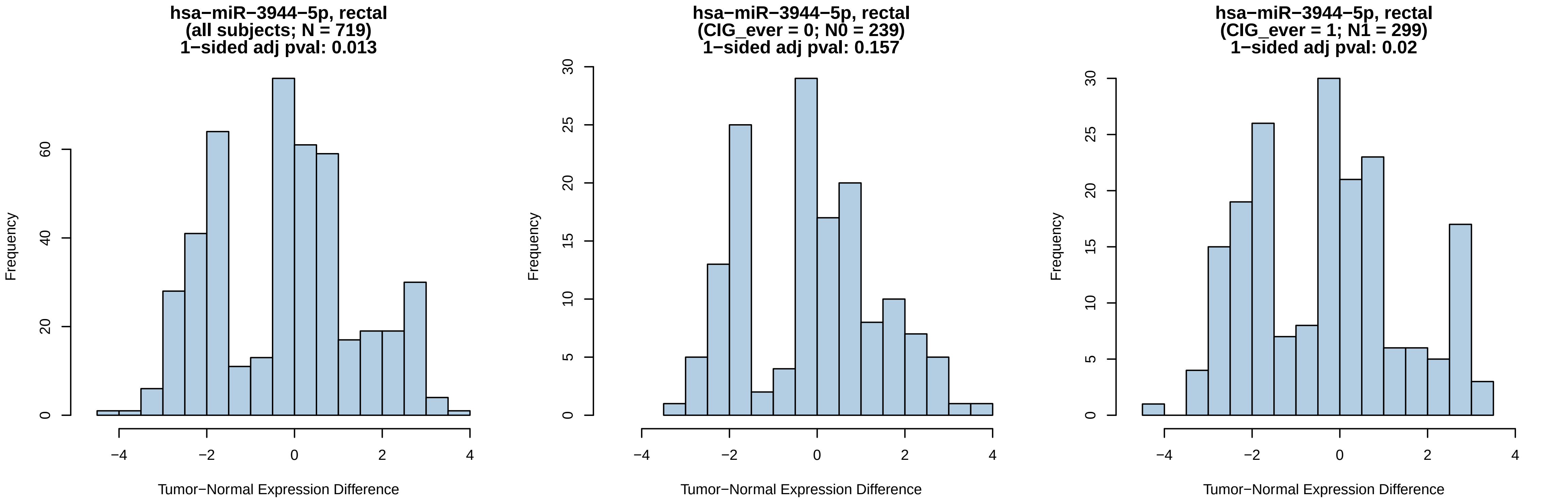
hsa−miR−3944−5p, rectal
(all subjects; N = 719)
1−sided adj pval: 0.013
Frequency
0
20
40
60
−4
−2
0
2
4
Tumor−Normal Expression Difference
hsa−miR−3944−5p, rectal
(CIG_ever = 0; N0 = 239)
1−sided adj pval: 0.157
Frequency
0
5
10
15
20
25
30
−4
−2
0
2
4
Tumor−Normal Expression Difference
hsa−miR−3944−5p, rectal
(CIG_ever = 1; N1 = 299)
1−sided adj pval: 0.02
Frequency
0
5
10
15
20
25
30
−4
−2
0
2
4
Tumor−Normal Expression Difference

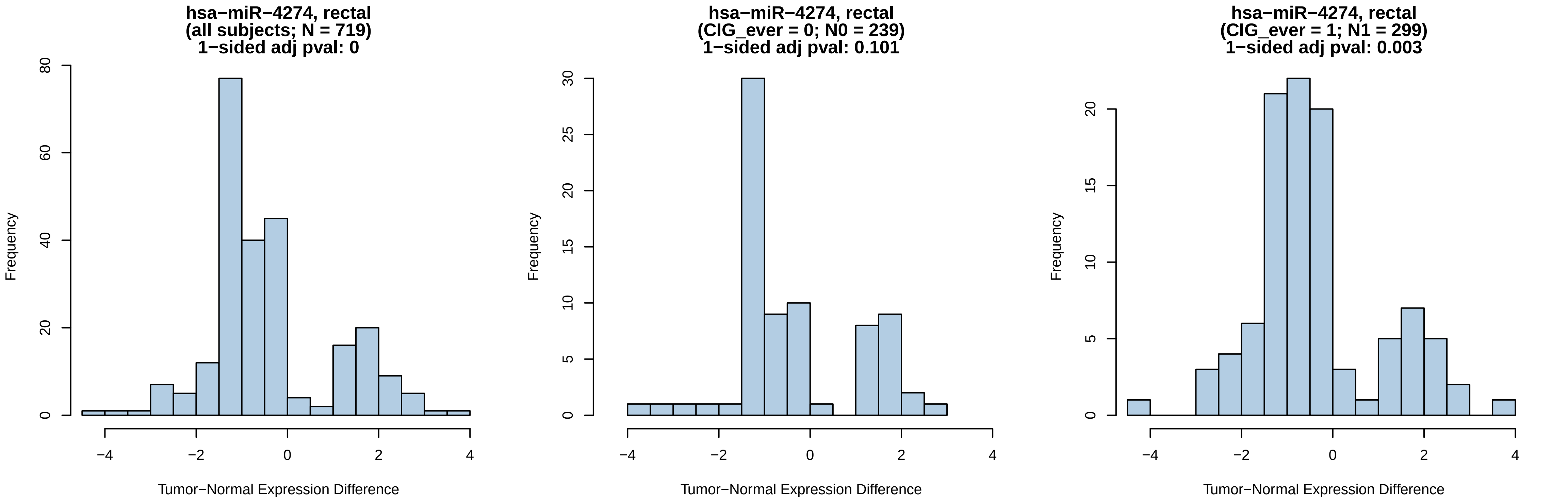

hsa−miR−4274, rectal
(all subjects; N = 719)
1−sided adj pval: 0
hsa−miR−4274, rectal
(CIG_ever = 0; N0 = 239)
1−sided adj pval: 0.101
hsa−miR−4274, rectal
(CIG_ever = 1; N1 = 299)
1−sided adj pval: 0.003
Frequency
Tumor−Normal Expression Difference

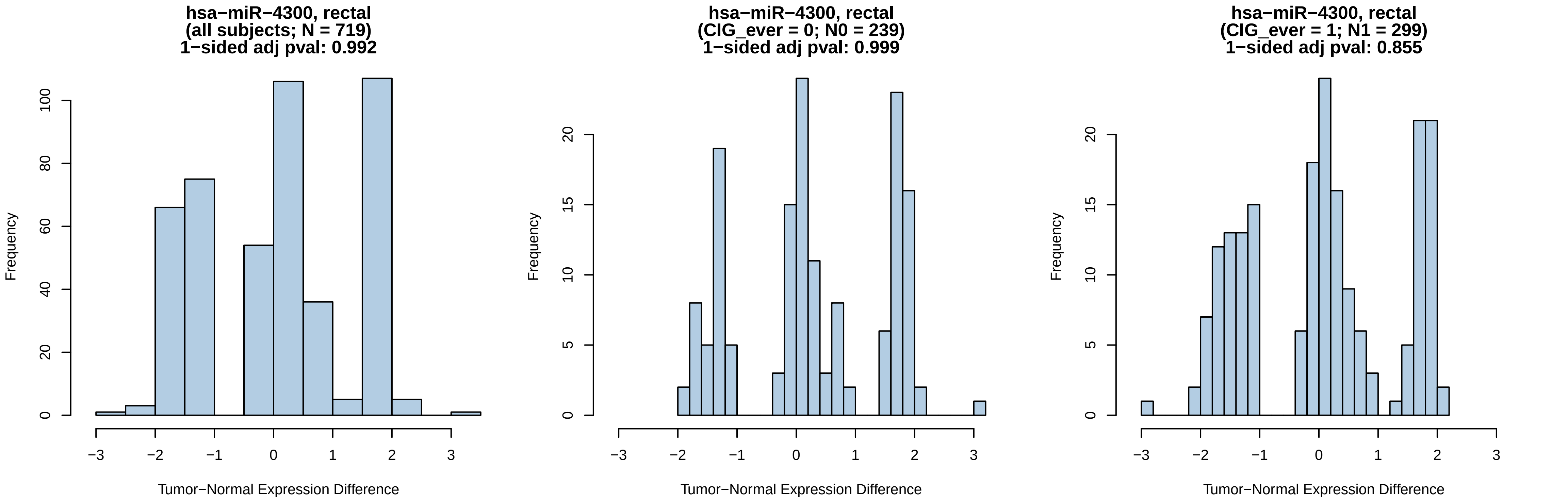
hsa−miR−4300, rectal
(all subjects; N = 719)
1−sided adj pval: 0.992
Frequency
Tumor−Normal Expression Difference
hsa−miR−4300, rectal
(CIG_ever = 0; N0 = 239)
1−sided adj pval: 0.999
Frequency
Tumor−Normal Expression Difference
hsa−miR−4300, rectal
(CIG_ever = 1; N1 = 299)
1−sided adj pval: 0.855
Frequency
Tumor−Normal Expression Difference

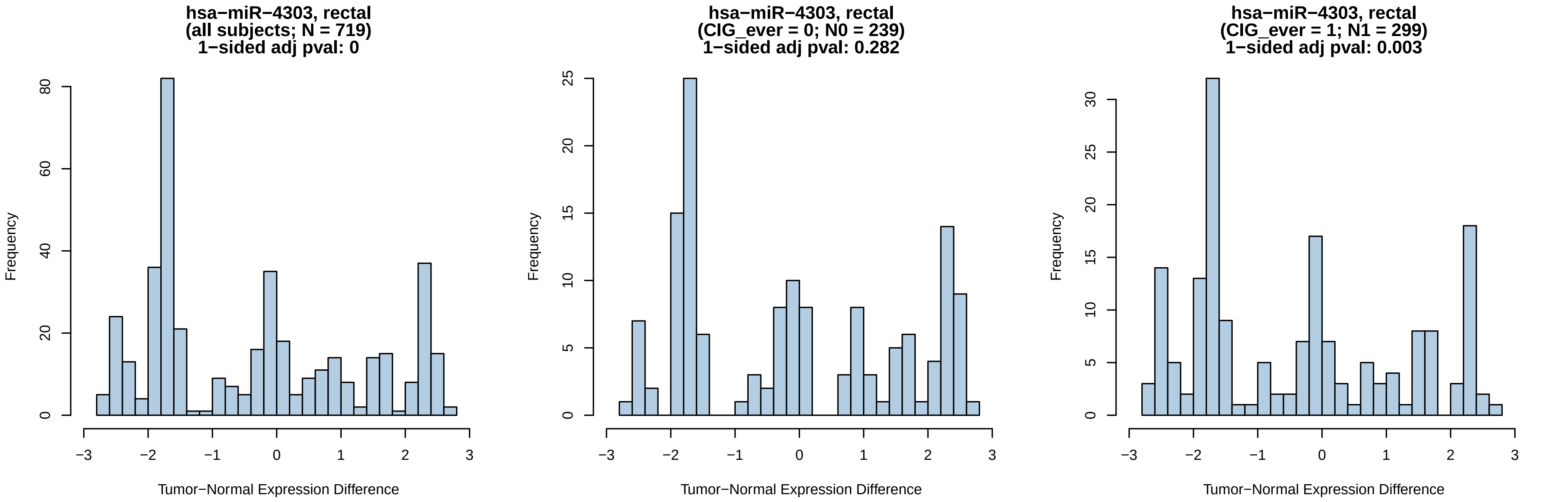

hsa−miR−4303, rectal
(all subjects; N = 719)
1−sided adj pval: 0
hsa−miR−4303, rectal
(CIG_ever = 0; N0 = 239)
1−sided adj pval: 0.282
hsa−miR−4303, rectal
(CIG_ever = 1; N1 = 299)
1−sided adj pval: 0.003
Frequency
Tumor−Normal Expression Difference

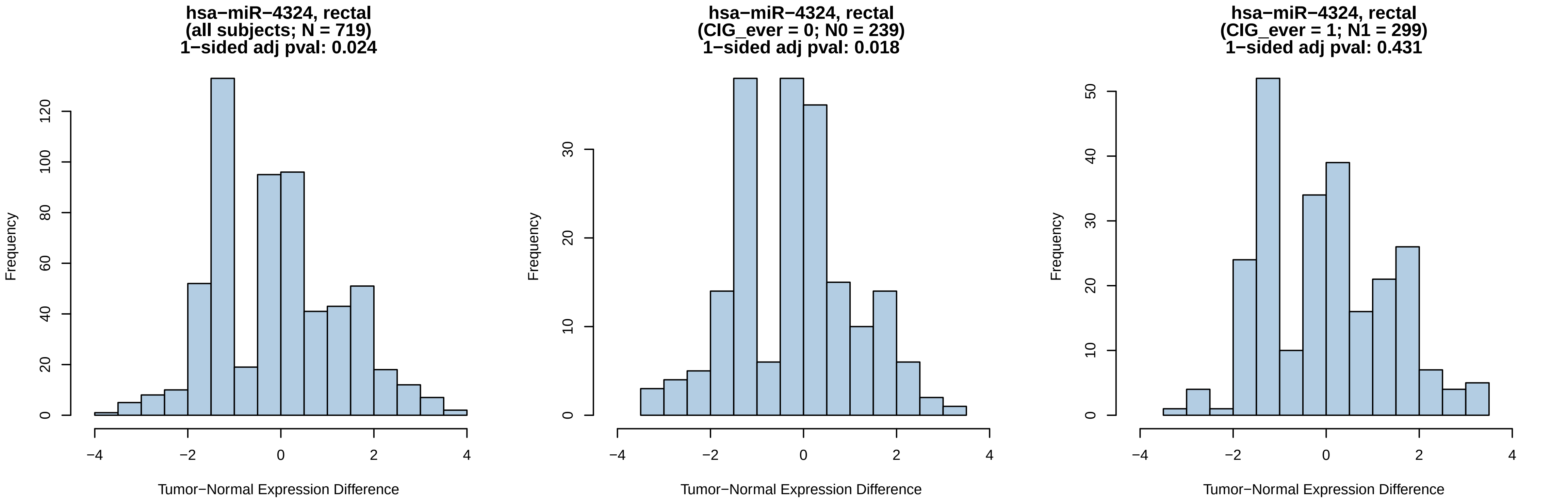

hsa−miR−4324, rectal
(all subjects; N = 719)
1−sided adj pval: 0.024
Frequency
0
20
40
60
80
100
120
−4
−2
0
2
4
Tumor−Normal Expression Difference
hsa−miR−4324, rectal
(CIG_ever = 0; N0 = 239)
1−sided adj pval: 0.018
Frequency
0
10
20
30
−4
−2
0
2
4
Tumor−Normal Expression Difference
hsa−miR−4324, rectal
(CIG_ever = 1; N1 = 299)
1−sided adj pval: 0.431
Frequency
0
10
20
30
40
50
−4
−2
0
2
4
Tumor−Normal Expression Difference

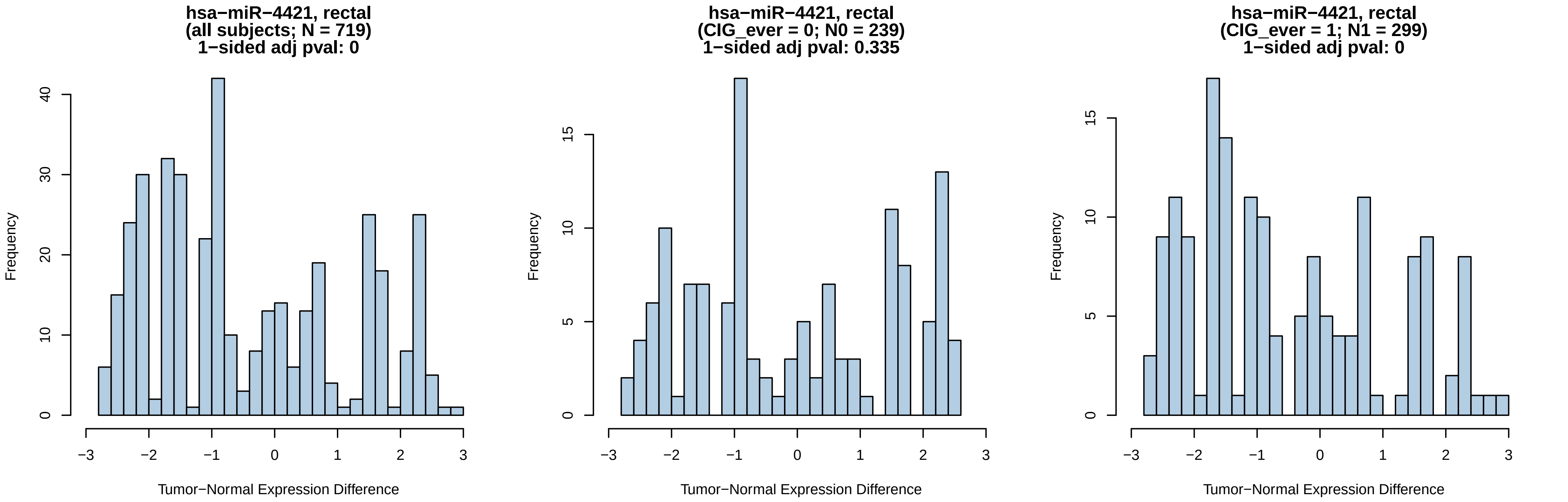

hsa−miR−4421, rectal
(all subjects; N = 719)
1−sided adj pval: 0
hsa−miR−4421, rectal
(CIG_ever = 0; N0 = 239)
1−sided adj pval: 0.335
hsa−miR−4421, rectal
(CIG_ever = 1; N1 = 299)
1−sided adj pval: 0
Frequency
Tumor−Normal Expression Difference

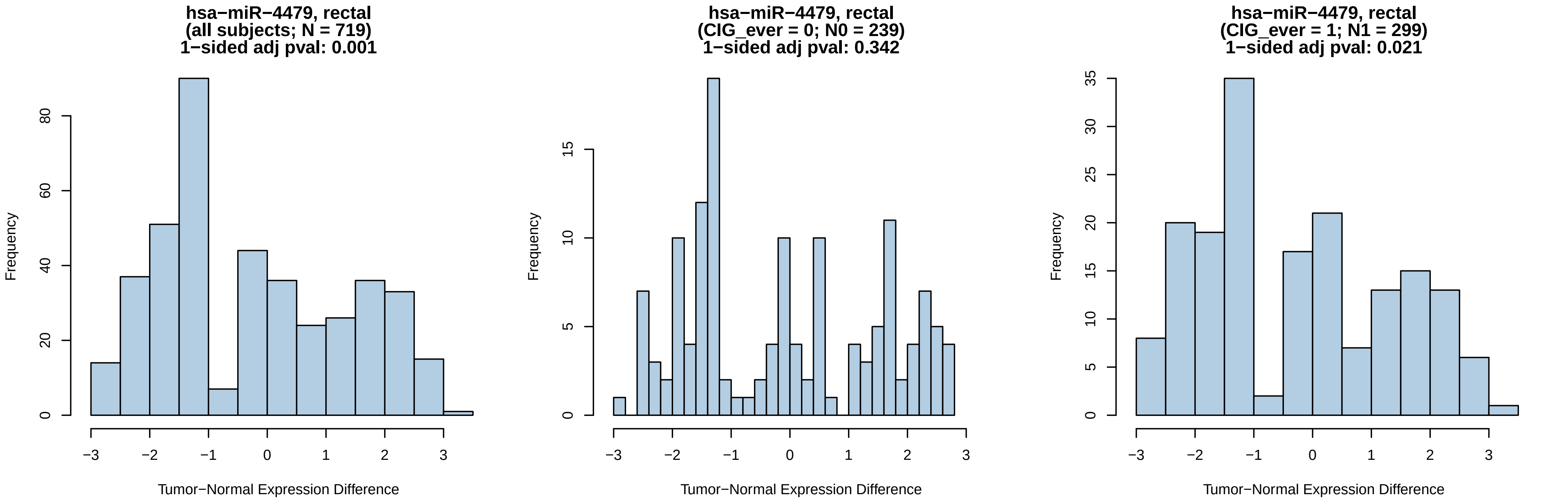

hsa−miR−4479, rectal
(all subjects; N = 719)
1−sided adj pval: 0.001
Frequency
Tumor−Normal Expression Difference
hsa−miR−4479, rectal
(CIG_ever = 0; N0 = 239)
1−sided adj pval: 0.342
Frequency
Tumor−Normal Expression Difference
hsa−miR−4479, rectal
(CIG_ever = 1; N1 = 299)
1−sided adj pval: 0.021
Frequency
Tumor−Normal Expression Difference

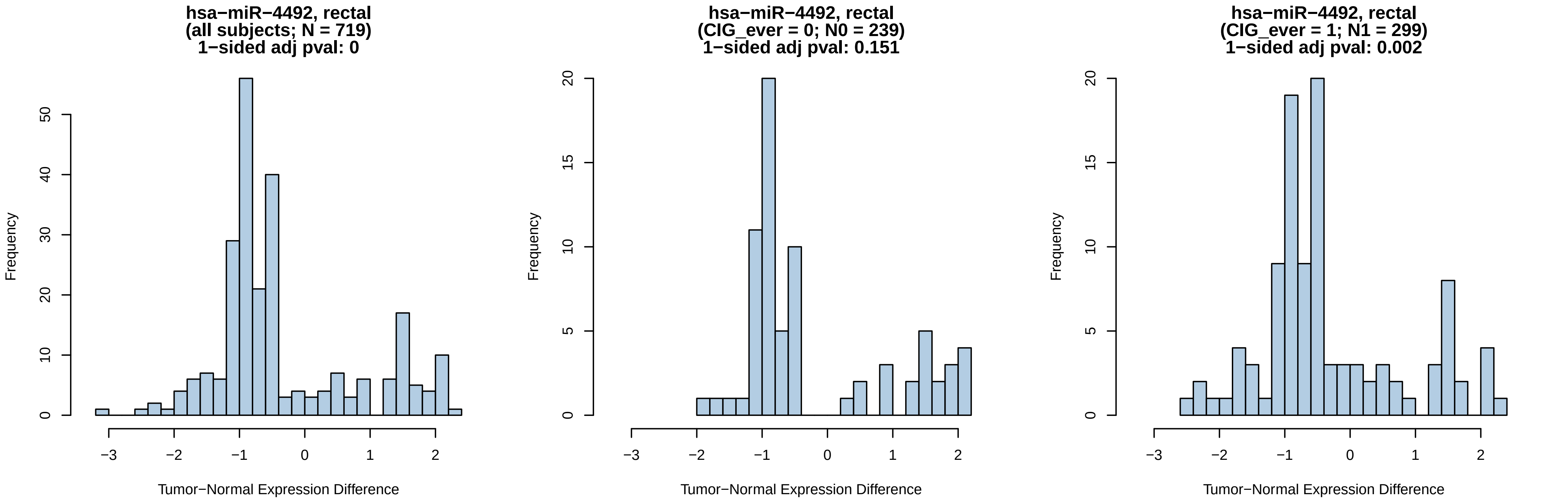
hsa−miR−4492, rectal
(all subjects; N = 719)
1−sided adj pval: 0
Frequency
Tumor−Normal Expression Difference
hsa−miR−4492, rectal
(CIG_ever = 0; N0 = 239)
1−sided adj pval: 0.151
Frequency
Tumor−Normal Expression Difference
hsa−miR−4492, rectal
(CIG_ever = 1; N1 = 299)
1−sided adj pval: 0.002
Frequency
Tumor−Normal Expression Difference

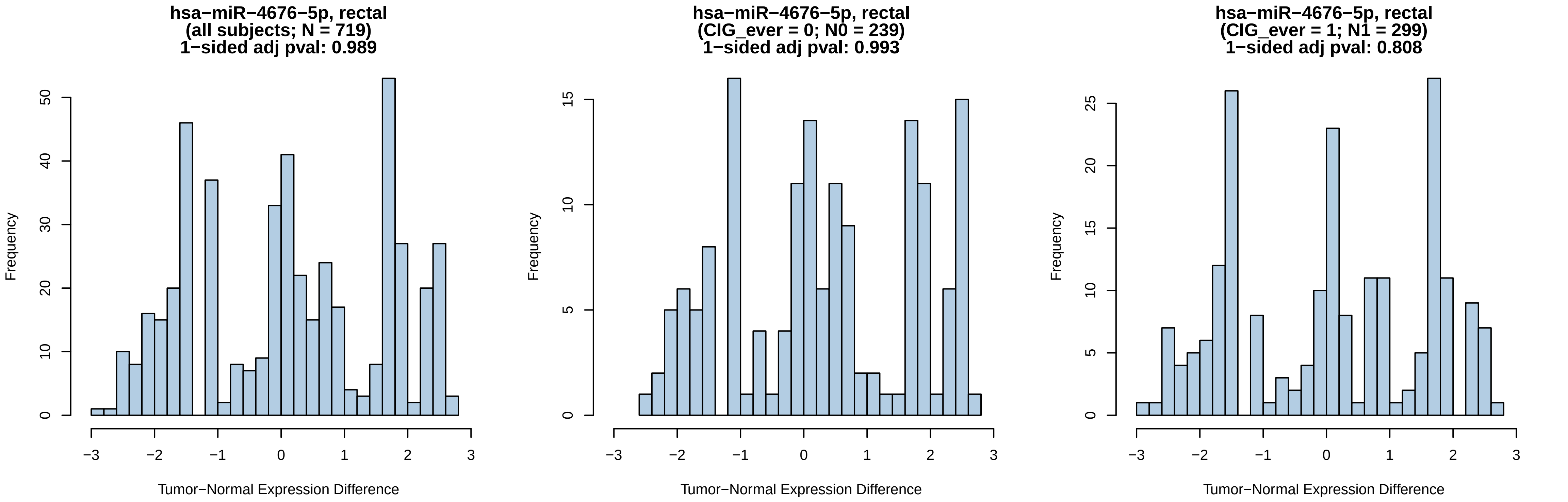

hsa−miR−4676−5p, rectal
(all subjects; N = 719)
1−sided adj pval: 0.989
Frequency
Tumor−Normal Expression Difference
hsa−miR−4676−5p, rectal
(CIG_ever = 0; N0 = 239)
1−sided adj pval: 0.993
Frequency
Tumor−Normal Expression Difference
hsa−miR−4676−5p, rectal
(CIG_ever = 1; N1 = 299)
1−sided adj pval: 0.808
Frequency
Tumor−Normal Expression Difference

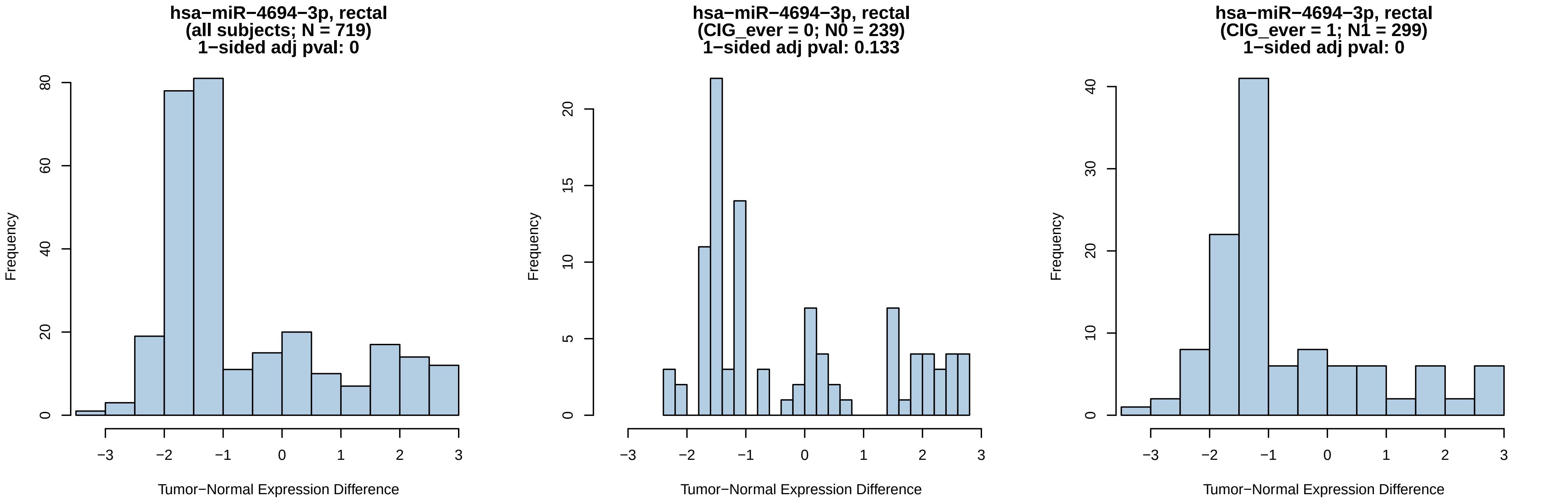

hsa−miR−4694−3p, rectal
(all subjects; N = 719)
1−sided adj pval: 0
Frequency
0
20
40
60
80
−3
−2
−1
0
1
2
3
Tumor−Normal Expression Difference
hsa−miR−4694−3p, rectal
(CIG_ever = 0; N0 = 239)
1−sided adj pval: 0.133
Frequency
0
5
10
15
20
−3
−2
−1
0
1
2
3
Tumor−Normal Expression Difference
hsa−miR−4694−3p, rectal
(CIG_ever = 1; N1 = 299)
1−sided adj pval: 0
Frequency
0
10
20
30
40
−3
−2
−1
0
1
2
3
Tumor−Normal Expression Difference

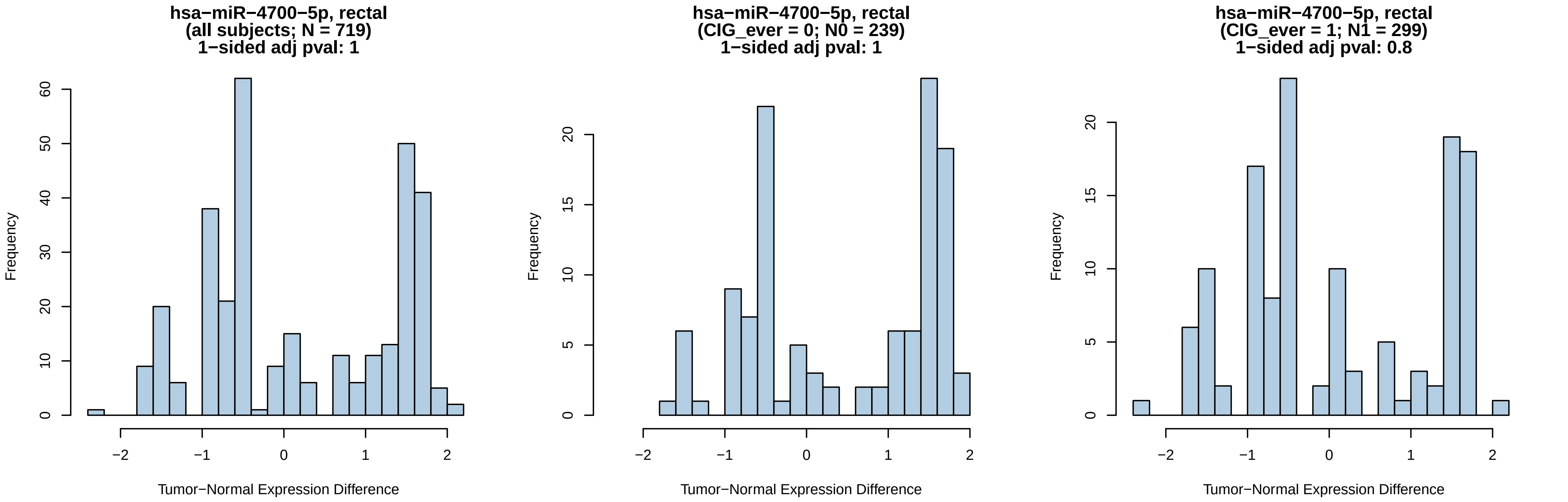

hsa−miR−4700−5p, rectal
(all subjects; N = 719)
1−sided adj pval: 1
hsa−miR−4700−5p, rectal
(CIG_ever = 0; N0 = 239)
1−sided adj pval: 1
hsa−miR−4700−5p, rectal
(CIG_ever = 1; N1 = 299)
1−sided adj pval: 0.8
Frequency
Tumor−Normal Expression Difference

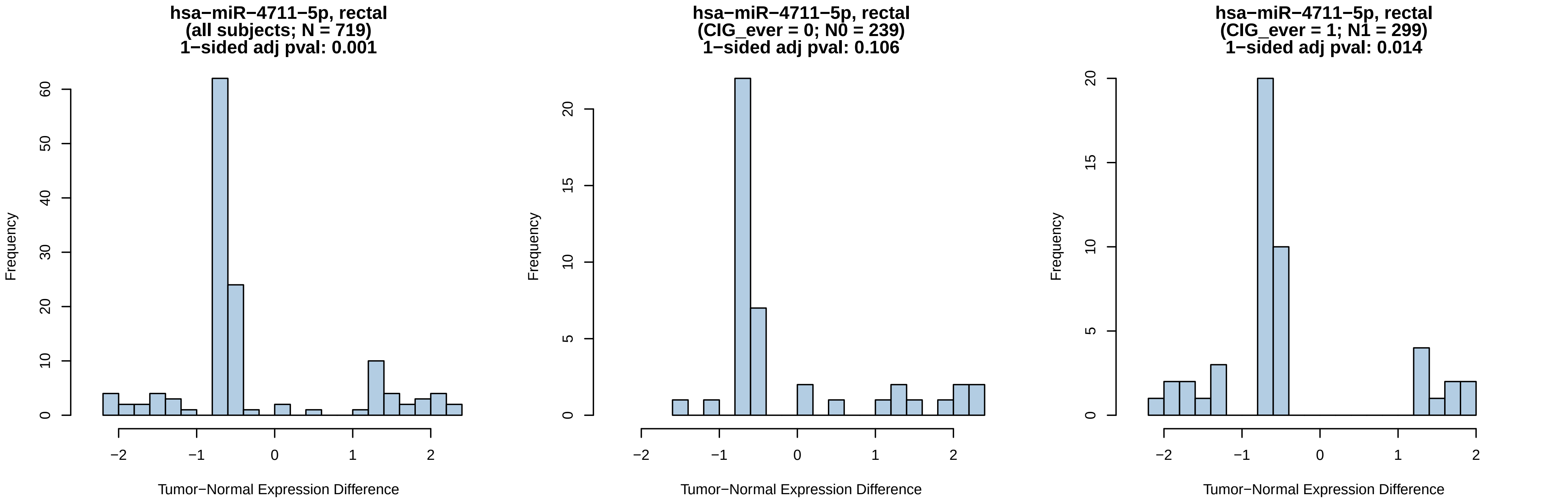

hsa−miR−4711−5p, rectal
(all subjects; N = 719)
1−sided adj pval: 0.001
Frequency
Tumor−Normal Expression Difference
hsa−miR−4711−5p, rectal
(CIG_ever = 0; N0 = 239)
1−sided adj pval: 0.106
Frequency
Tumor−Normal Expression Difference
hsa−miR−4711−5p, rectal
(CIG_ever = 1; N1 = 299)
1−sided adj pval: 0.014
Frequency
Tumor−Normal Expression Difference

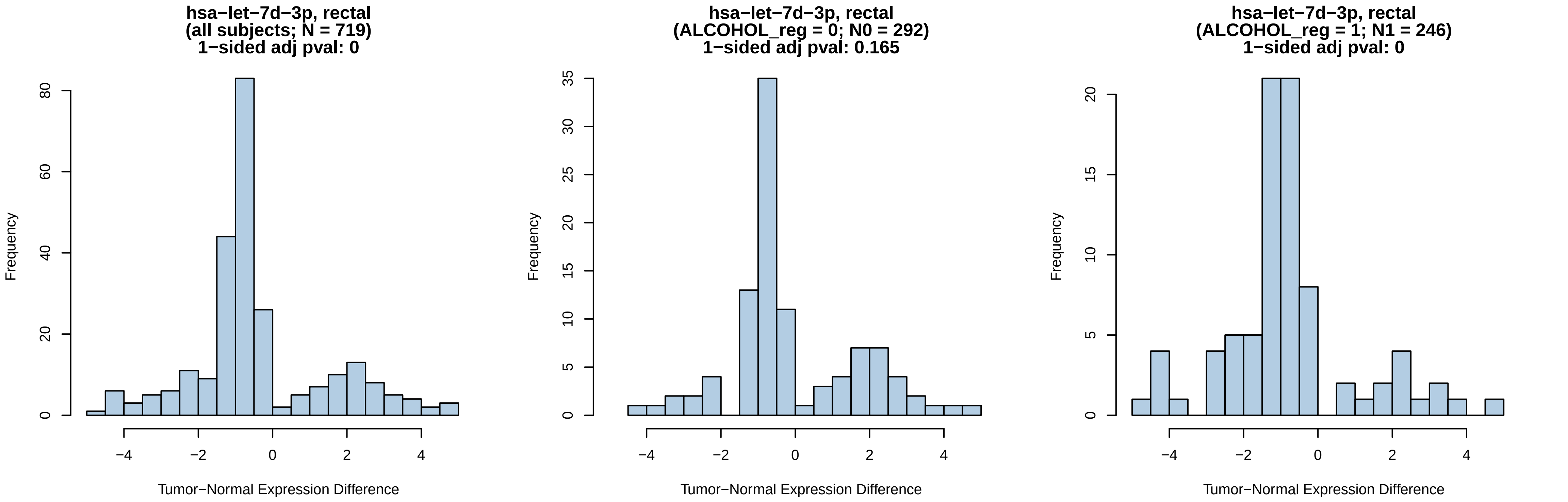
hsa−let−7d−3p, rectal
(all subjects; N = 719)
1−sided adj pval: 0
Frequency
0
20
40
60
80
−4
−2
0
2
4
Tumor−Normal Expression Difference
hsa−let−7d−3p, rectal
(ALCOHOL_reg = 0; N0 = 292)
1−sided adj pval: 0.165
Frequency
0
5
10
15
20
25
30
35
−4
−2
0
2
4
Tumor−Normal Expression Difference
hsa−let−7d−3p, rectal
(ALCOHOL_reg = 1; N1 = 246)
1−sided adj pval: 0
Frequency
0
5
10
15
20
−4
−2
0
2
4
Tumor−Normal Expression Difference

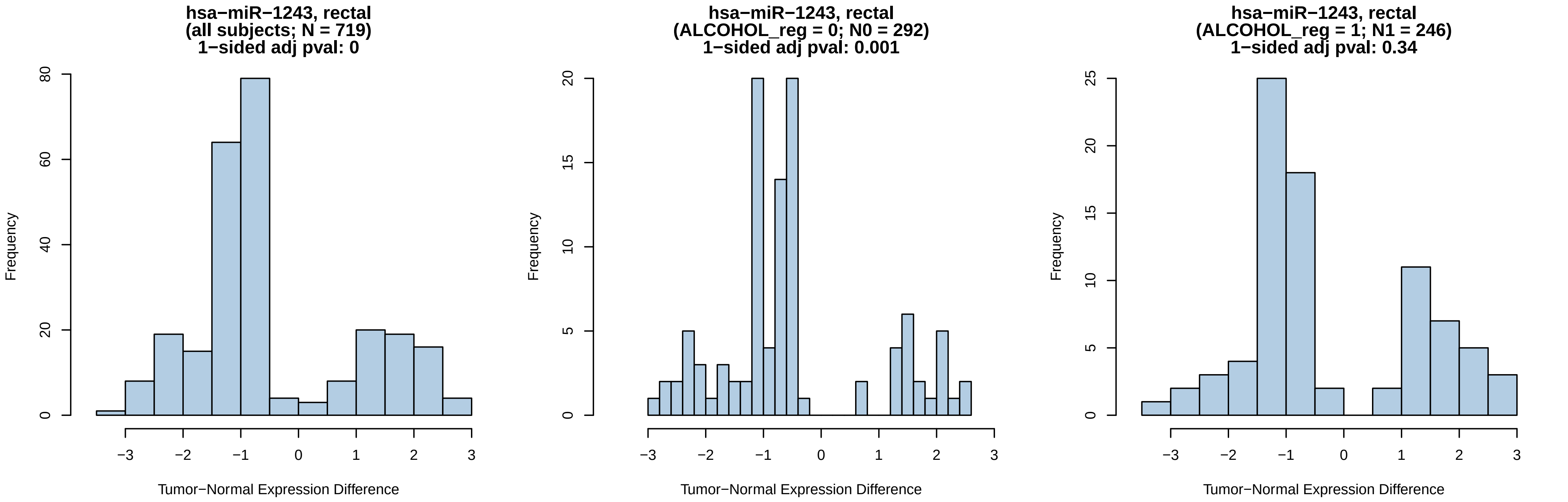

hsa−miR−1243, rectal
(all subjects; N = 719)
1−sided adj pval: 0
Frequency
Tumor−Normal Expression Difference
hsa−miR−1243, rectal
(ALCOHOL_reg = 0; N0 = 292)
1−sided adj pval: 0.001
Frequency
Tumor−Normal Expression Difference
hsa−miR−1243, rectal
(ALCOHOL_reg = 1; N1 = 246)
1−sided adj pval: 0.34
Frequency
Tumor−Normal Expression Difference

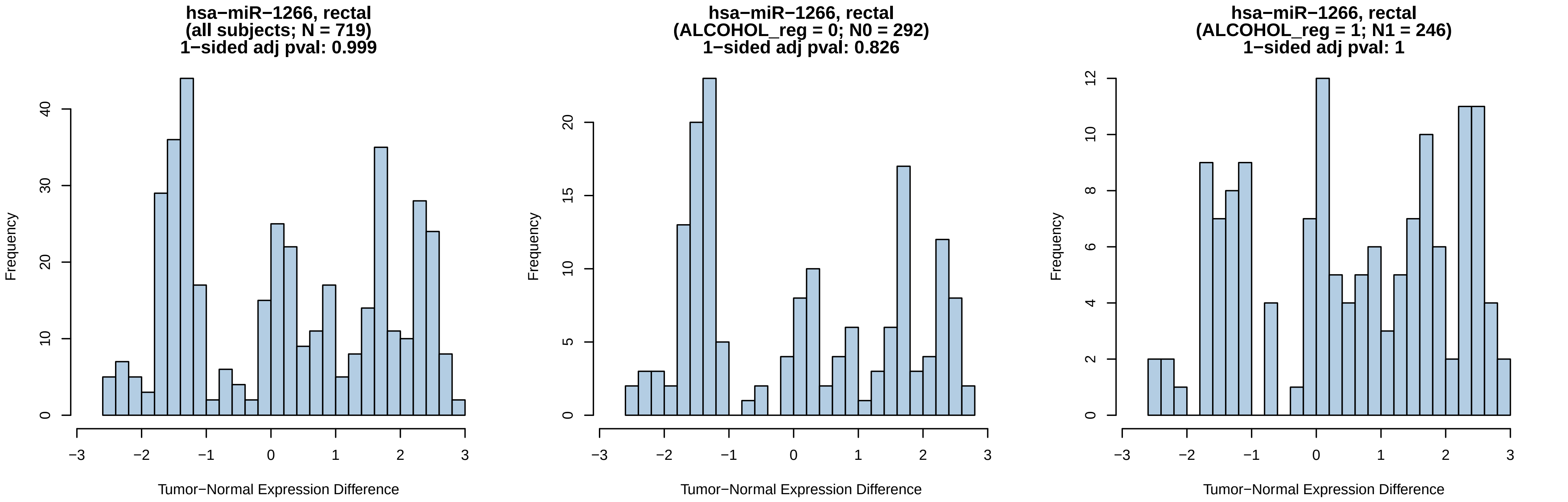
hsa−miR−1266, rectal
(all subjects; N = 719)
1−sided adj pval: 0.999
hsa−miR−1266, rectal
(ALCOHOL_reg = 0; N0 = 292)
1−sided adj pval: 0.826
hsa−miR−1266, rectal
(ALCOHOL_reg = 1; N1 = 246)
1−sided adj pval: 1
Frequency
Tumor−Normal Expression Difference

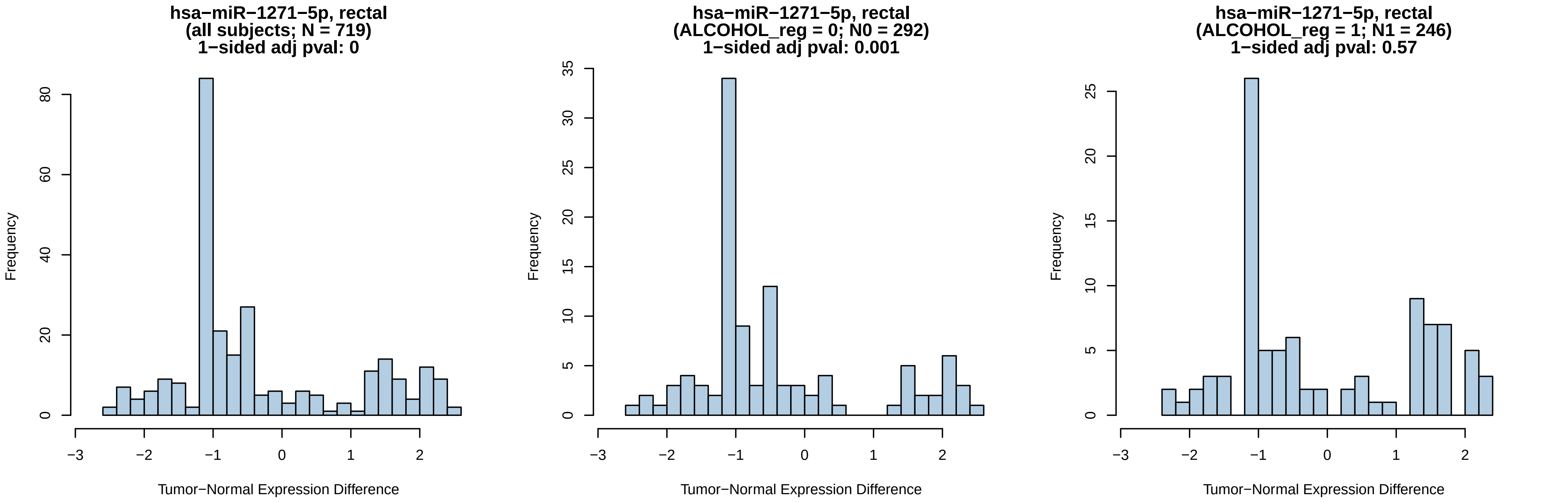
hsa−miR−1271−5p, rectal
(all subjects; N = 719)
1−sided adj pval: 0
hsa−miR−1271−5p, rectal
(ALCOHOL_reg = 0; N0 = 292)
1−sided adj pval: 0.001
hsa−miR−1271−5p, rectal
(ALCOHOL_reg = 1; N1 = 246)
1−sided adj pval: 0.57
Frequency
Tumor−Normal Expression Difference

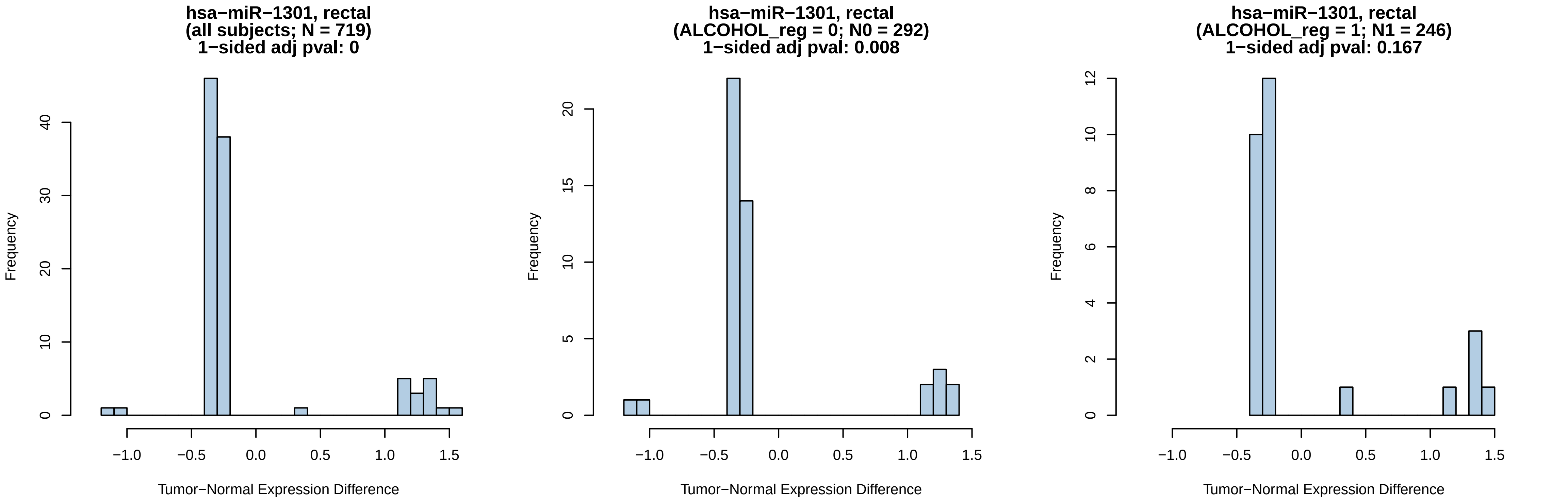
hsa−miR−1301, rectal
(all subjects; N = 719)
1−sided adj pval: 0
Frequency
0
10
20
30
40
−1.0
−0.5
0.0
0.5
1.0
1.5
Tumor−Normal Expression Difference
hsa−miR−1301, rectal
(ALCOHOL_reg = 0; N0 = 292)
1−sided adj pval: 0.008
Frequency
0
5
10
15
20
−1.0
−0.5
0.0
0.5
1.0
1.5
Tumor−Normal Expression Difference
hsa−miR−1301, rectal
(ALCOHOL_reg = 1; N1 = 246)
1−sided adj pval: 0.167
Frequency
0
2
4
6
8
10
12
−1.0
−0.5
0.0
0.5
1.0
1.5
Tumor−Normal Expression Difference

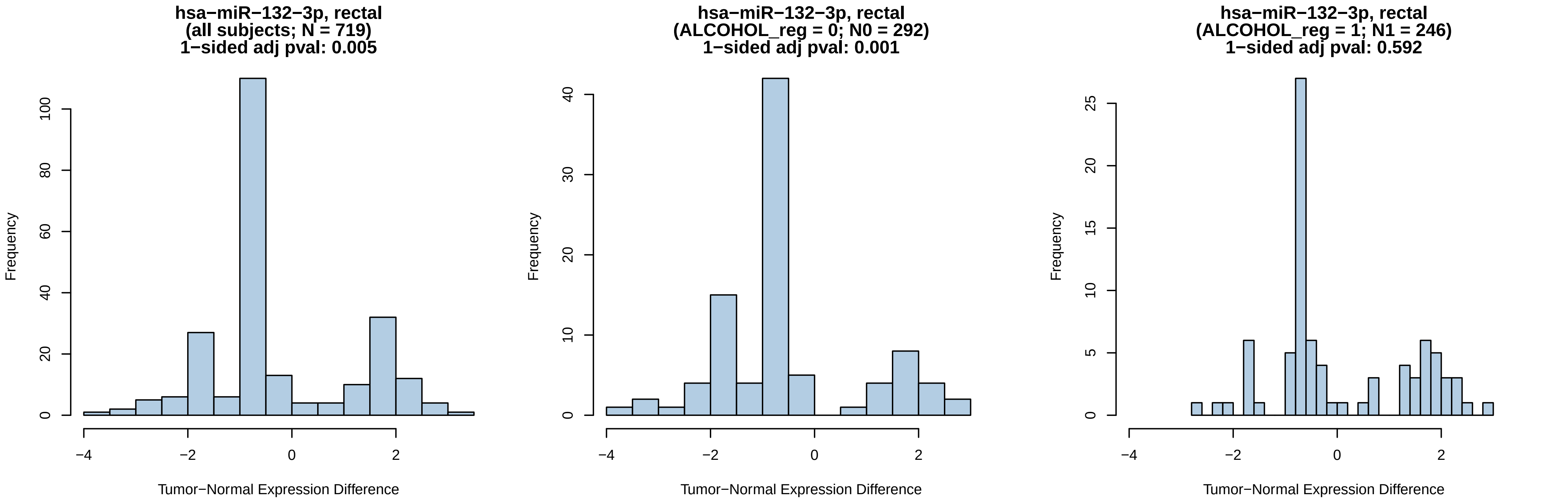
hsa−miR−132−3p, rectal
(all subjects; N = 719)
1−sided adj pval: 0.005
Frequency
Tumor−Normal Expression Difference
hsa−miR−132−3p, rectal
(ALCOHOL_reg = 0; N0 = 292)
1−sided adj pval: 0.001
Frequency
Tumor−Normal Expression Difference
hsa−miR−132−3p, rectal
(ALCOHOL_reg = 1; N1 = 246)
1−sided adj pval: 0.592
Frequency
Tumor−Normal Expression Difference

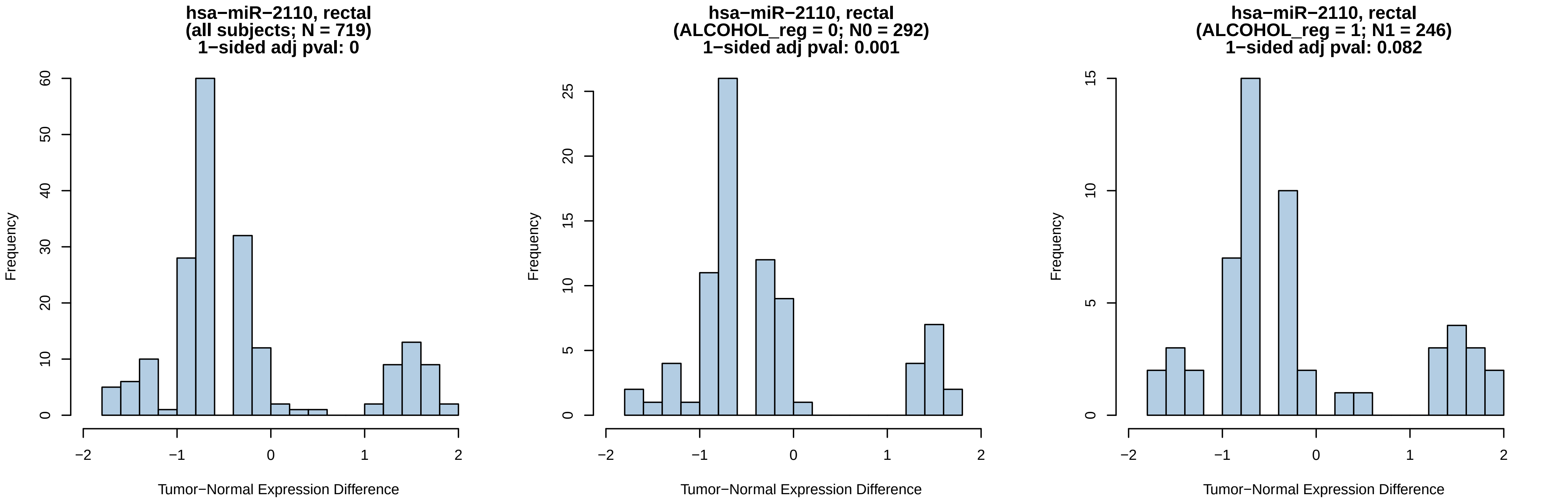
hsa−miR−2110, rectal
(all subjects; N = 719)
1−sided adj pval: 0
Frequency
Tumor−Normal Expression Difference
hsa−miR−2110, rectal
(ALCOHOL_reg = 0; N0 = 292)
1−sided adj pval: 0.001
Frequency
Tumor−Normal Expression Difference
hsa−miR−2110, rectal
(ALCOHOL_reg = 1; N1 = 246)
1−sided adj pval: 0.082
Frequency
Tumor−Normal Expression Difference

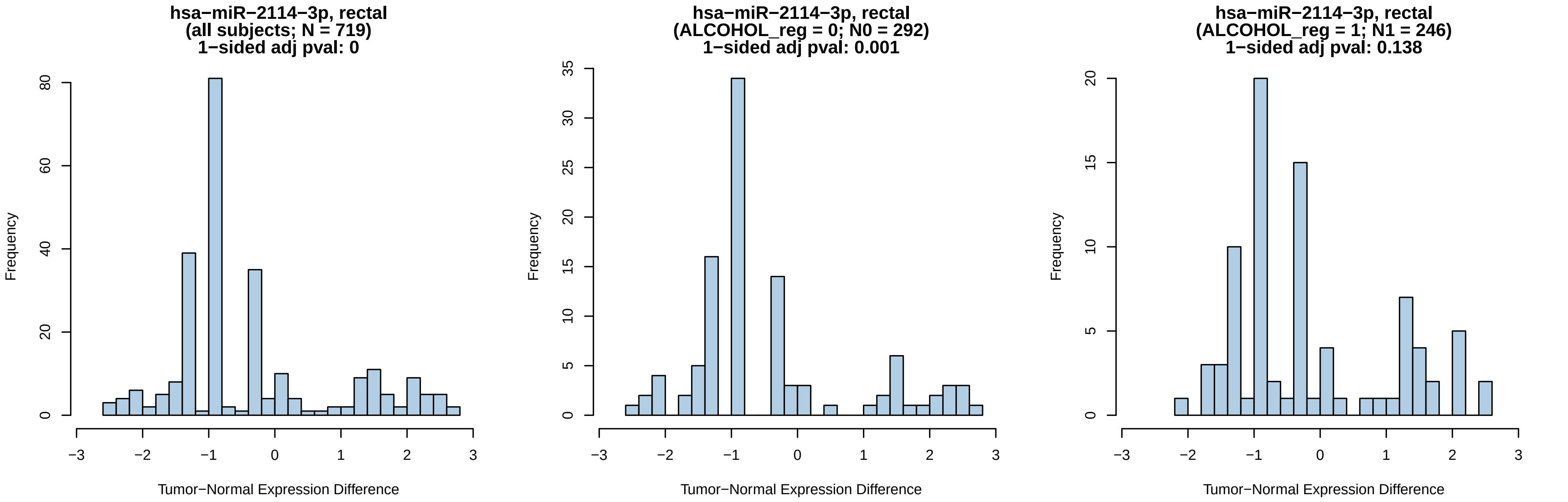

hsa−miR−2114−3p, rectal
(all subjects; N = 719)
1−sided adj pval: 0
Frequency
Tumor−Normal Expression Difference
hsa−miR−2114−3p, rectal
(ALCOHOL_reg = 0; N0 = 292)
1−sided adj pval: 0.001
Frequency
Tumor−Normal Expression Difference
hsa−miR−2114−3p, rectal
(ALCOHOL_reg = 1; N1 = 246)
1−sided adj pval: 0.138
Frequency
Tumor−Normal Expression Difference

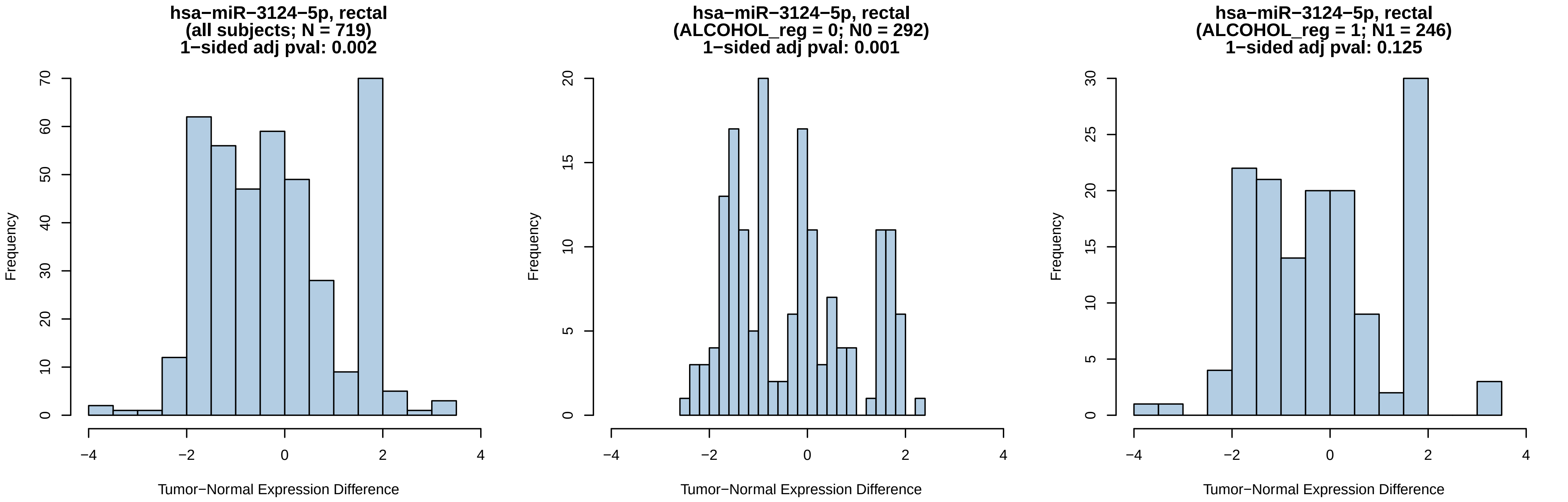
hsa−miR−3124−5p, rectal
(all subjects; N = 719)
1−sided adj pval: 0.002
hsa−miR−3124−5p, rectal
(ALCOHOL_reg = 0; N0 = 292)
1−sided adj pval: 0.001
hsa−miR−3124−5p, rectal
(ALCOHOL_reg = 1; N1 = 246)
1−sided adj pval: 0.125
Frequency
Tumor−Normal Expression Difference

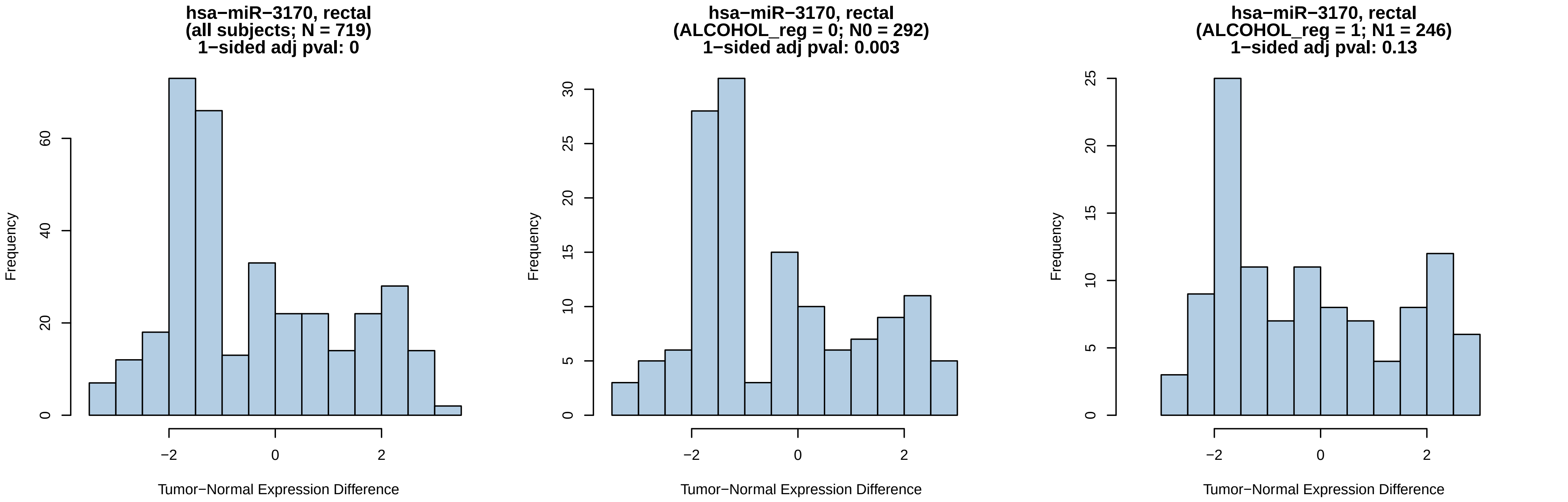
hsa−miR−3170, rectal
(all subjects; N = 719)
1−sided adj pval: 0
hsa−miR−3170, rectal
(ALCOHOL_reg = 0; N0 = 292)
1−sided adj pval: 0.003
hsa−miR−3170, rectal
(ALCOHOL_reg = 1; N1 = 246)
1−sided adj pval: 0.13
Frequency
Tumor−Normal Expression Difference

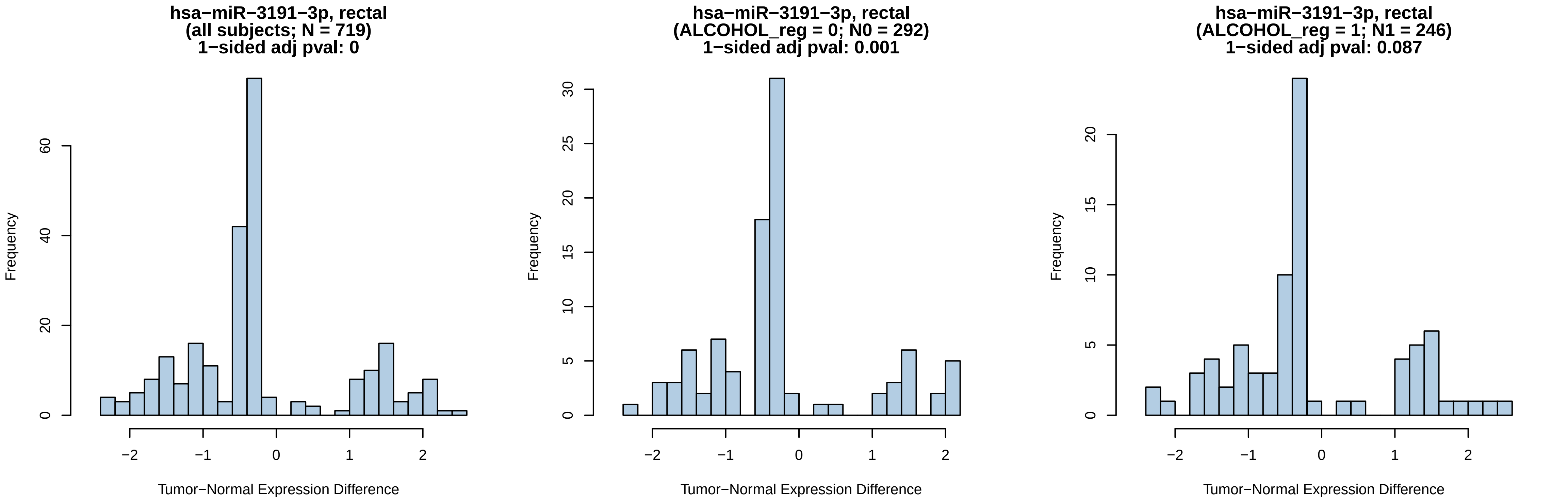

hsa−miR−3191−3p, rectal
(all subjects; N = 719)
1−sided adj pval: 0
Frequency
0
20
40
60
−2
−1
0
1
2
Tumor−Normal Expression Difference
hsa−miR−3191−3p, rectal
(ALCOHOL_reg = 0; N0 = 292)
1−sided adj pval: 0.001
Frequency
0
5
10
15
20
25
30
−2
−1
0
1
2
Tumor−Normal Expression Difference
hsa−miR−3191−3p, rectal
(ALCOHOL_reg = 1; N1 = 246)
1−sided adj pval: 0.087
Frequency
0
5
10
15
20
−2
−1
0
1
2
Tumor−Normal Expression Difference

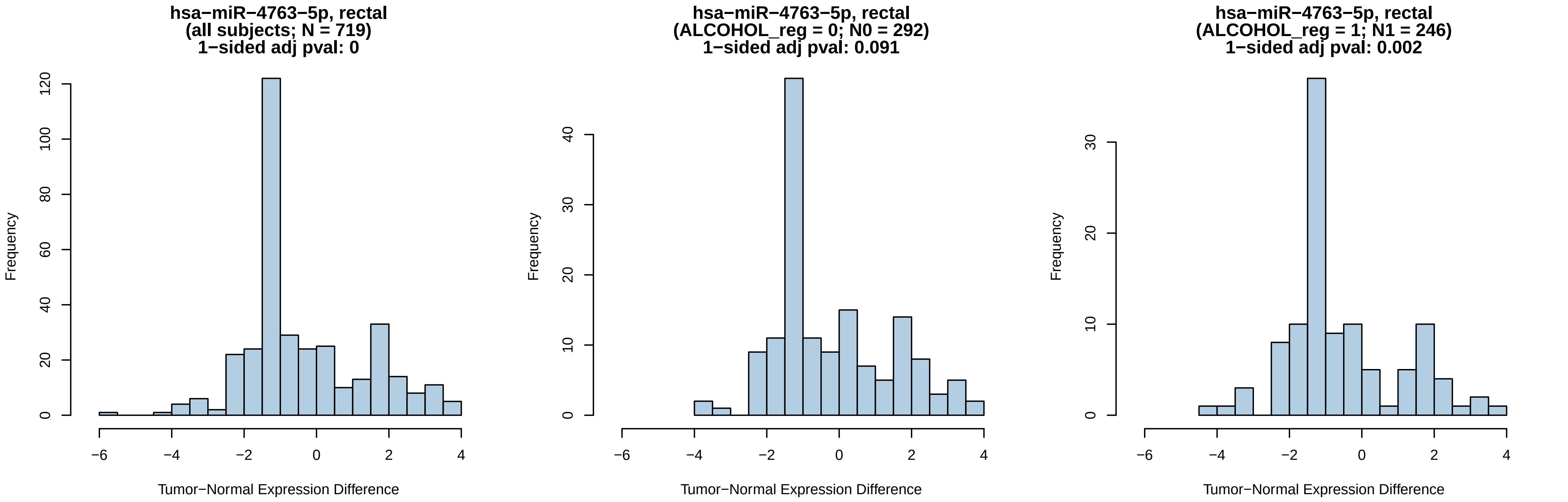
hsa−miR−4763−5p, rectal
(all subjects; N = 719)
1−sided adj pval: 0
Frequency
Tumor−Normal Expression Difference
hsa−miR−4763−5p, rectal
(ALCOHOL_reg = 0; N0 = 292)
1−sided adj pval: 0.091
Frequency
Tumor−Normal Expression Difference
hsa−miR−4763−5p, rectal
(ALCOHOL_reg = 1; N1 = 246)
1−sided adj pval: 0.002
Frequency
Tumor−Normal Expression Difference

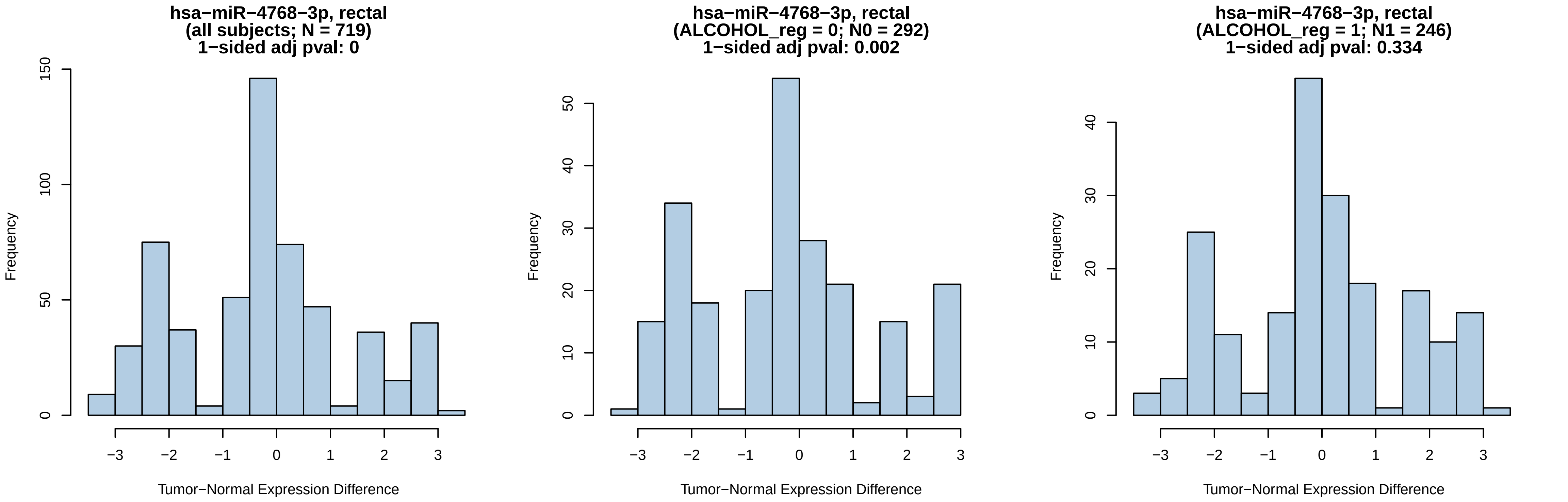
hsa−miR−4768−3p, rectal
(all subjects; N = 719)
1−sided adj pval: 0
Frequency
0
50
100
150
−3
−2
−1
0
1
2
3
Tumor−Normal Expression Difference
hsa−miR−4768−3p, rectal
(ALCOHOL_reg = 0; N0 = 292)
1−sided adj pval: 0.002
Frequency
0
10
20
30
40
50
−3
−2
−1
0
1
2
3
Tumor−Normal Expression Difference
hsa−miR−4768−3p, rectal
(ALCOHOL_reg = 1; N1 = 246)
1−sided adj pval: 0.334
Frequency
0
10
20
30
40
−3
−2
−1
0
1
2
3
Tumor−Normal Expression Difference

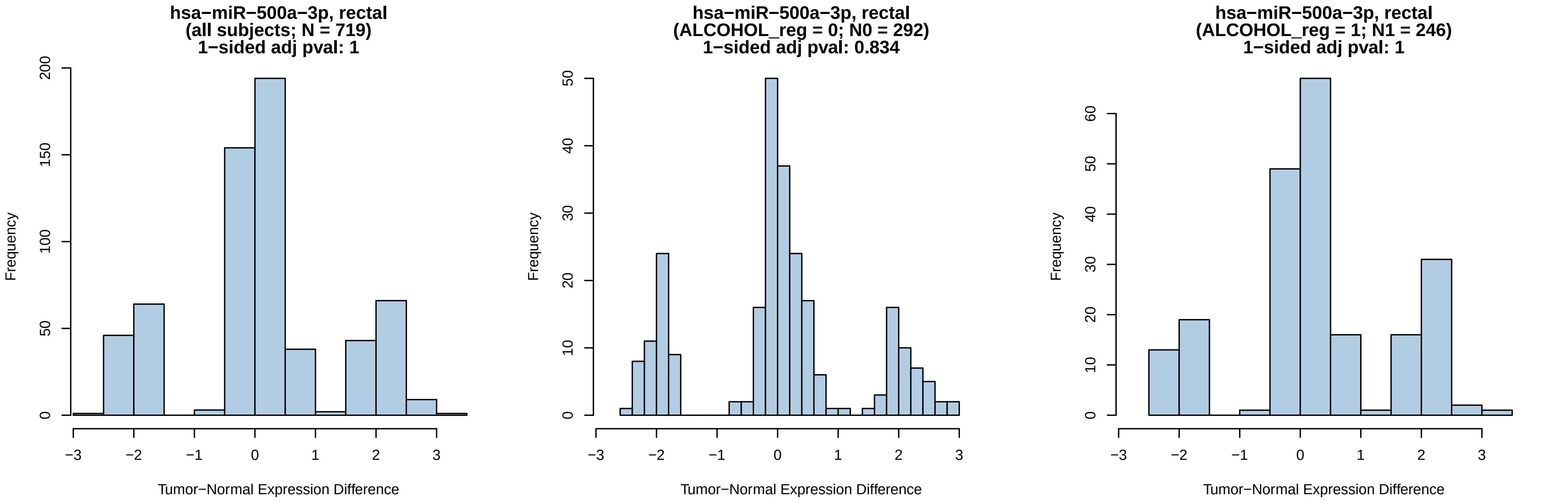

hsa−miR−500a−3p, rectal
(all subjects; N = 719)
1−sided adj pval: 1
Frequency
Tumor−Normal Expression Difference
hsa−miR−500a−3p, rectal
(ALCOHOL_reg = 0; N0 = 292)
1−sided adj pval: 0.834
Frequency
Tumor−Normal Expression Difference
hsa−miR−500a−3p, rectal
(ALCOHOL_reg = 1; N1 = 246)
1−sided adj pval: 1
Frequency
Tumor−Normal Expression Difference

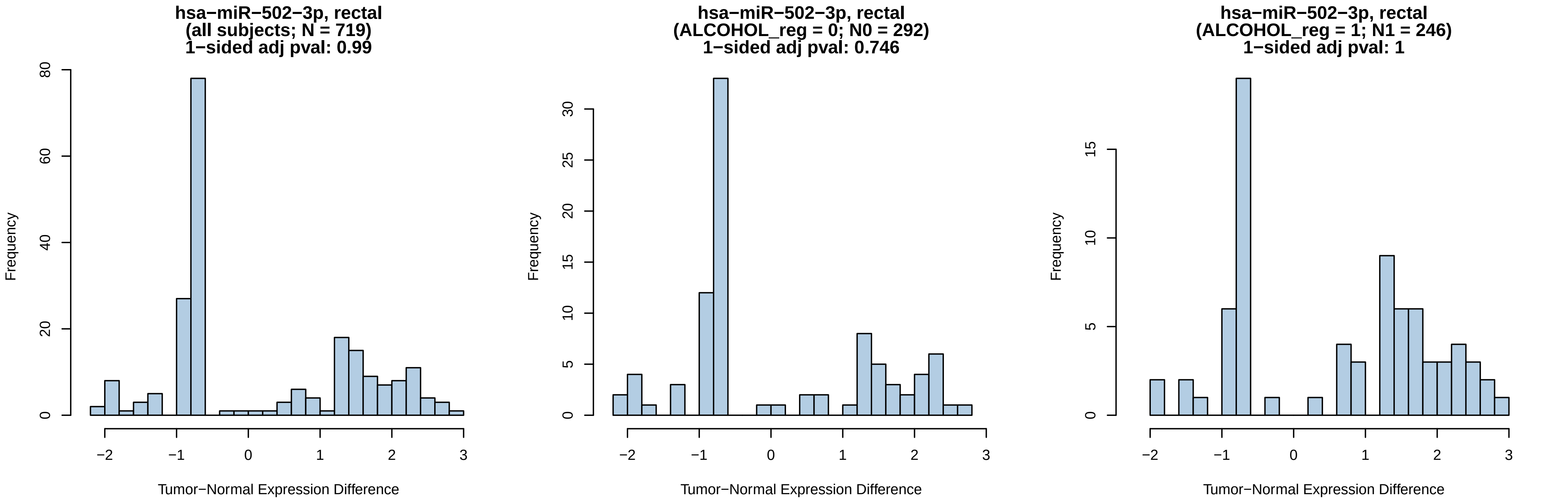
hsa−miR−502−3p, rectal
(all subjects; N = 719)
1−sided adj pval: 0.99
hsa−miR−502−3p, rectal
(ALCOHOL_reg = 0; N0 = 292)
1−sided adj pval: 0.746
hsa−miR−502−3p, rectal
(ALCOHOL_reg = 1; N1 = 246)
1−sided adj pval: 1
Frequency
Tumor−Normal Expression Difference

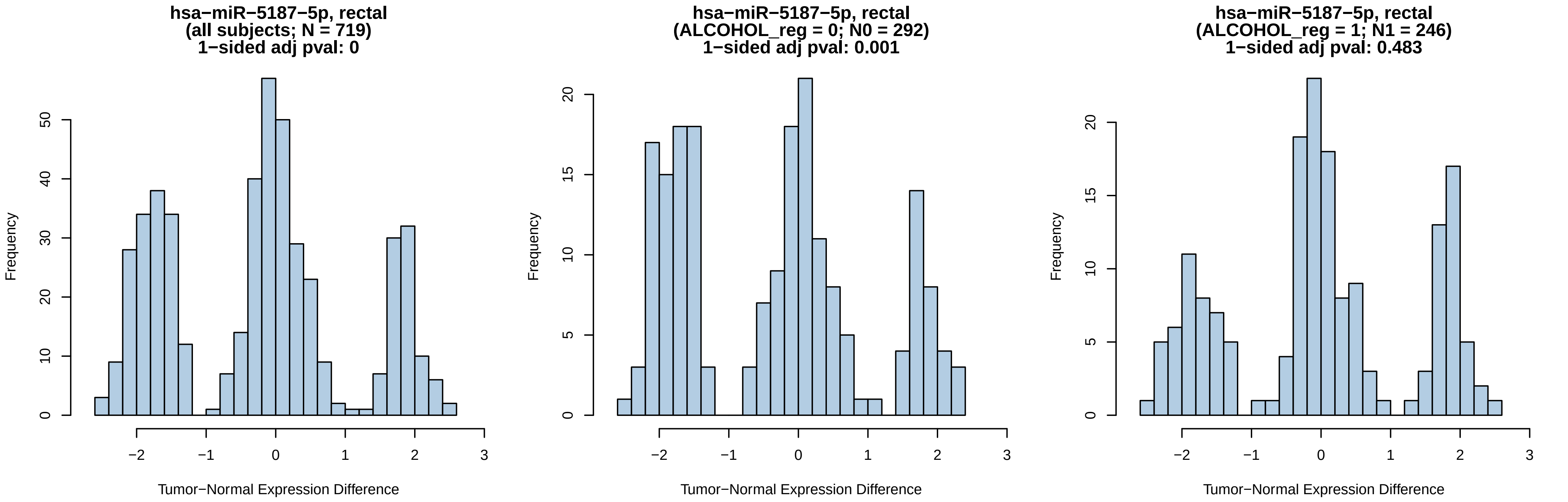
hsa−miR−5187−5p, rectal
(all subjects; N = 719)
1−sided adj pval: 0
Frequency
Tumor−Normal Expression Difference
hsa−miR−5187−5p, rectal
(ALCOHOL_reg = 0; N0 = 292)
1−sided adj pval: 0.001
Frequency
Tumor−Normal Expression Difference
hsa−miR−5187−5p, rectal
(ALCOHOL_reg = 1; N1 = 246)
1−sided adj pval: 0.483
Frequency
Tumor−Normal Expression Difference

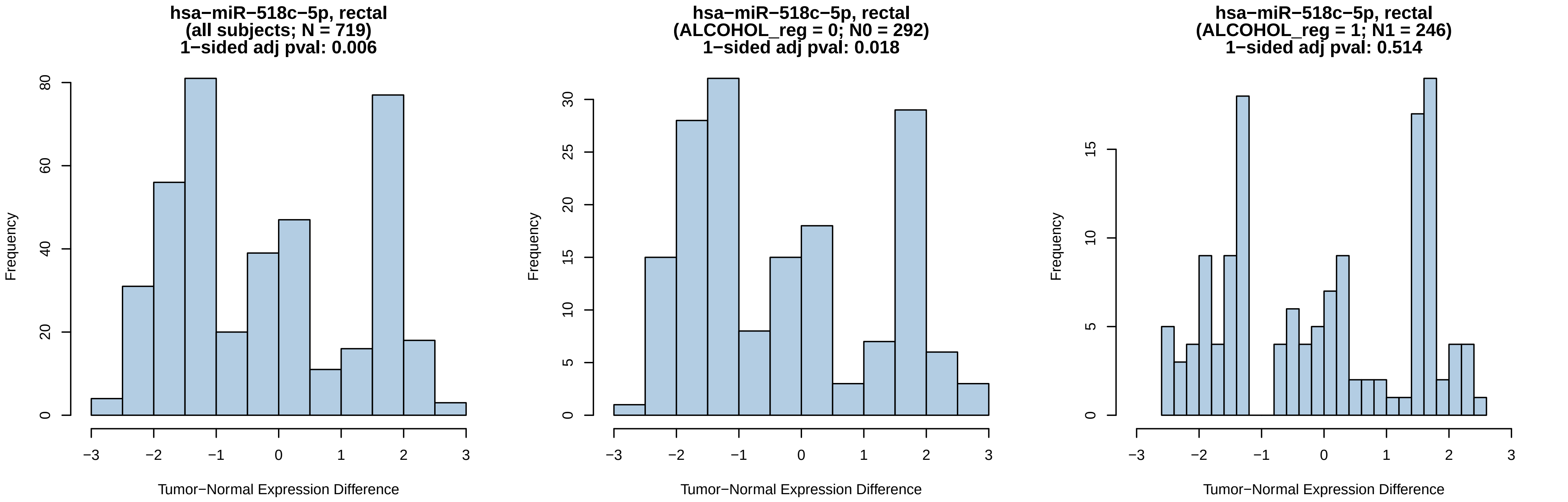

hsa−miR−518c−5p, rectal
(all subjects; N = 719)
1−sided adj pval: 0.006
hsa−miR−518c−5p, rectal
(ALCOHOL_reg = 0; N0 = 292)
1−sided adj pval: 0.018
hsa−miR−518c−5p, rectal
(ALCOHOL_reg = 1; N1 = 246)
1−sided adj pval: 0.514
Frequency
Tumor−Normal Expression Difference

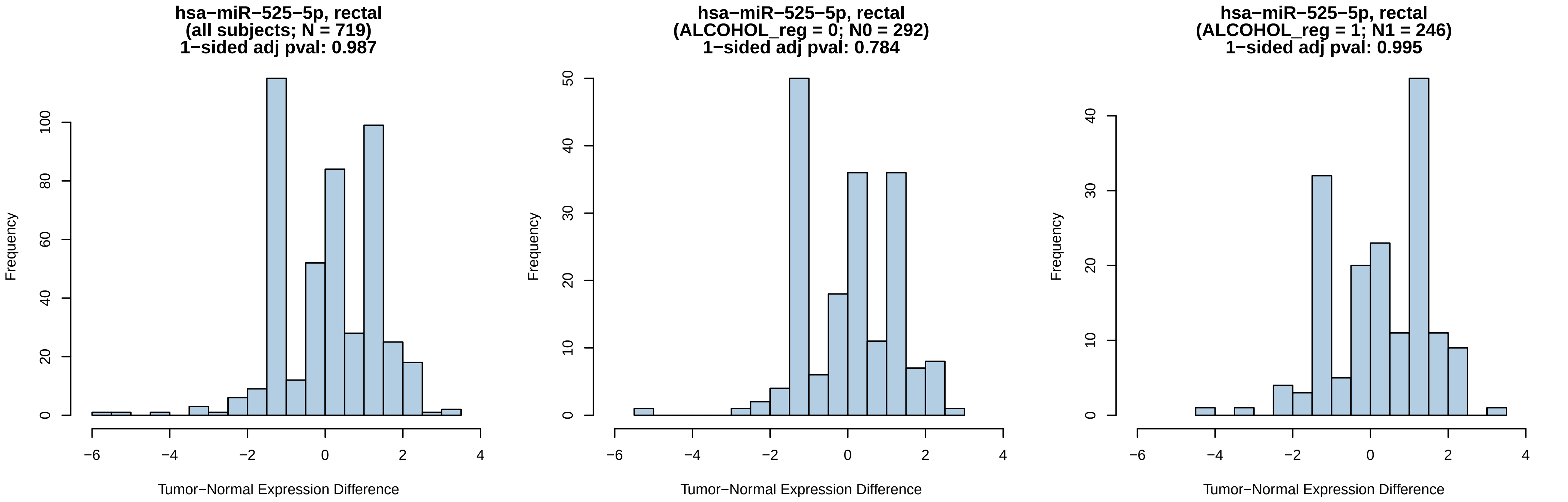

hsa−miR−525−5p, rectal
(all subjects; N = 719)
1−sided adj pval: 0.987
Frequency
Tumor−Normal Expression Difference
hsa−miR−525−5p, rectal
(ALCOHOL_reg = 0; N0 = 292)
1−sided adj pval: 0.784
Frequency
Tumor−Normal Expression Difference
hsa−miR−525−5p, rectal
(ALCOHOL_reg = 1; N1 = 246)
1−sided adj pval: 0.995
Frequency
Tumor−Normal Expression Difference

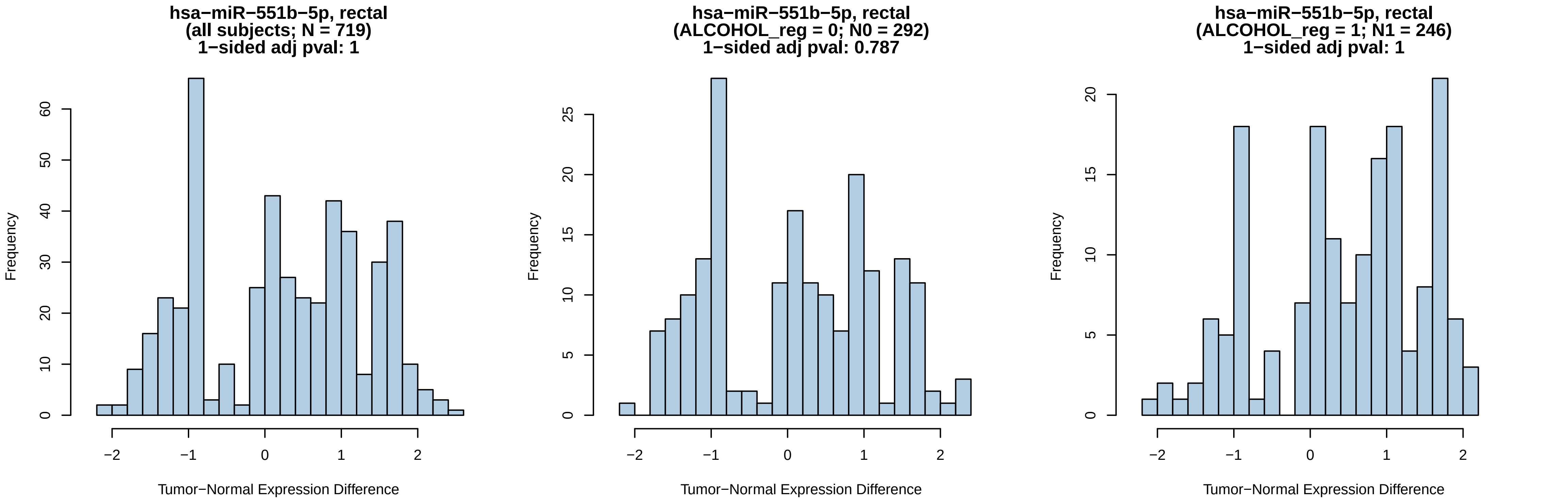

hsa−miR−551b−5p, rectal
(all subjects; N = 719)
1−sided adj pval: 1
Frequency
Tumor−Normal Expression Difference
hsa−miR−551b−5p, rectal
(ALCOHOL_reg = 0; N0 = 292)
1−sided adj pval: 0.787
Frequency
Tumor−Normal Expression Difference
hsa−miR−551b−5p, rectal
(ALCOHOL_reg = 1; N1 = 246)
1−sided adj pval: 1
Frequency
Tumor−Normal Expression Difference

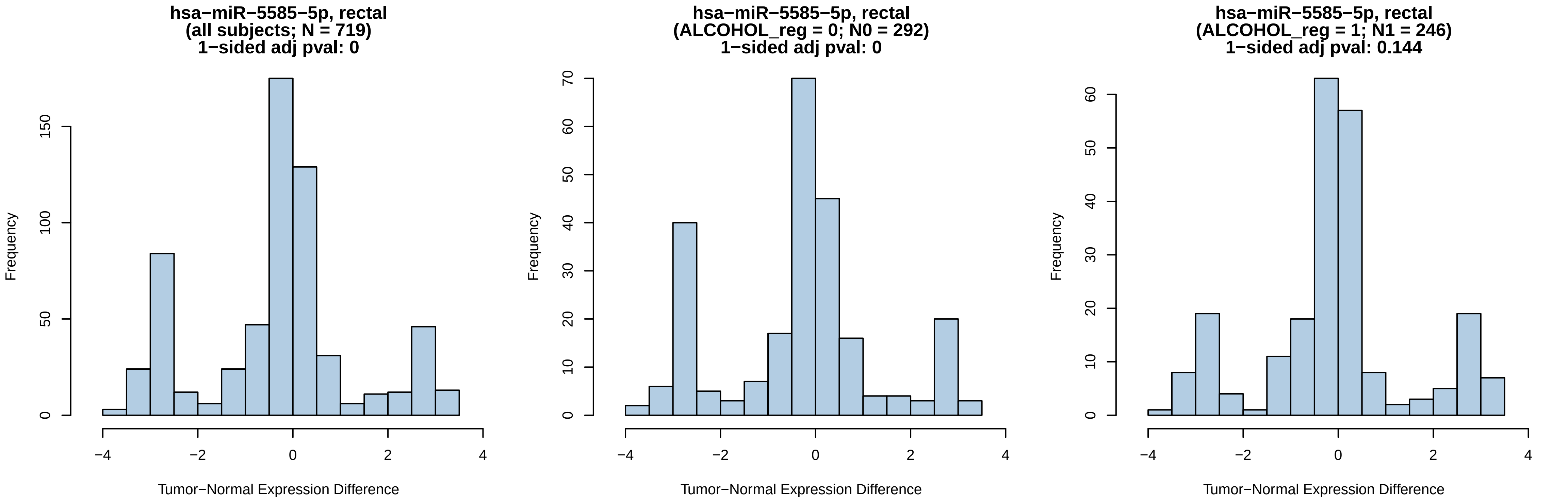
hsa−miR−5585−5p, rectal
(all subjects; N = 719)
1−sided adj pval: 0
hsa−miR−5585−5p, rectal
(ALCOHOL_reg = 0; N0 = 292)
1−sided adj pval: 0
hsa−miR−5585−5p, rectal
(ALCOHOL_reg = 1; N1 = 246)
1−sided adj pval: 0.144
Frequency
Tumor−Normal Expression Difference
0
50
100
150
10
20
30
40
50
60
70
−4
−2
0
2
4

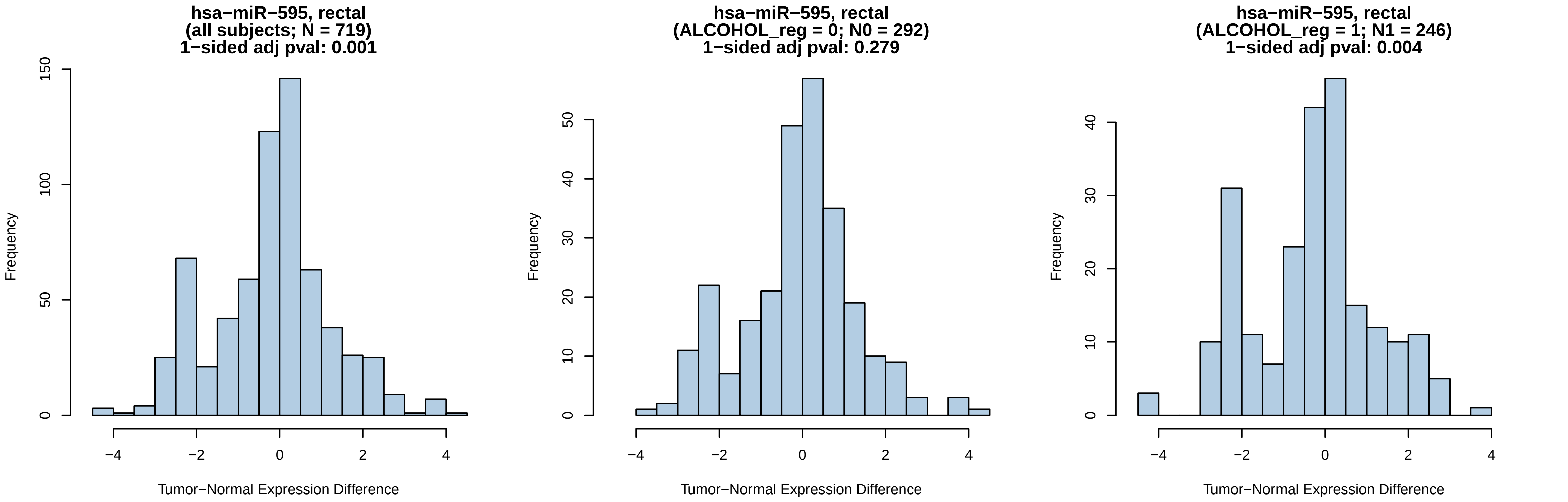

hsa−miR−595, rectal
(all subjects; N = 719)
1−sided adj pval: 0.001
Frequency
Tumor−Normal Expression Difference
hsa−miR−595, rectal
(ALCOHOL_reg = 0; N0 = 292)
1−sided adj pval: 0.279
Frequency
Tumor−Normal Expression Difference
hsa−miR−595, rectal
(ALCOHOL_reg = 1; N1 = 246)
1−sided adj pval: 0.004
Frequency
Tumor−Normal Expression Difference

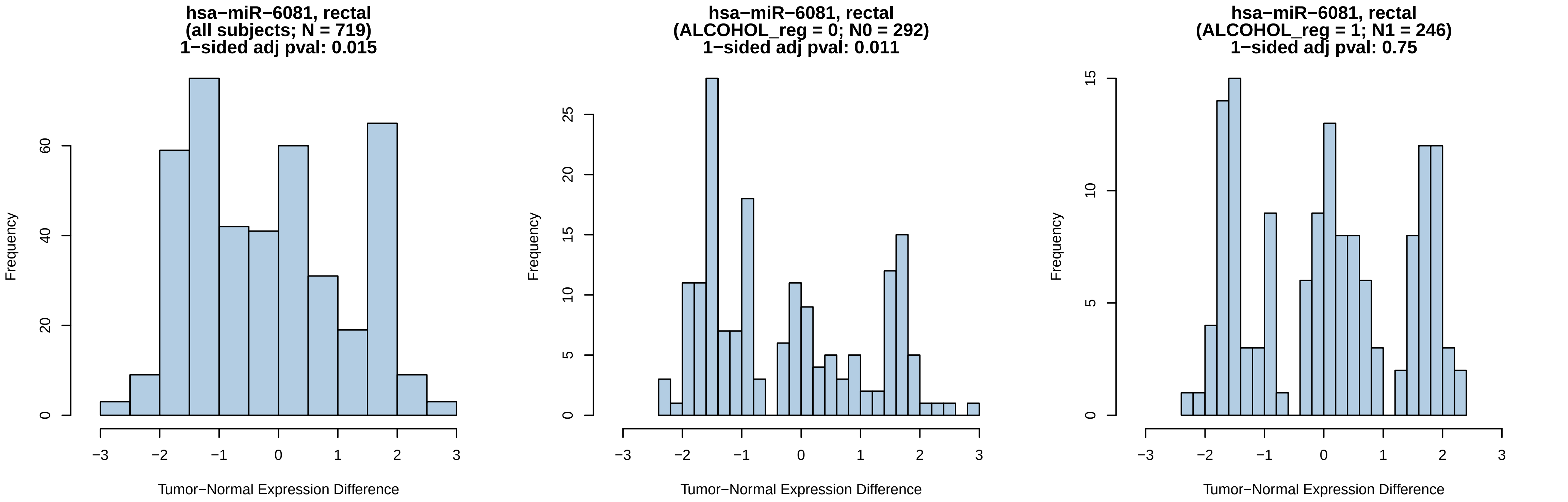
hsa−miR−6081, rectal
(all subjects; N = 719)
1−sided adj pval: 0.015
Frequency
0
20
40
60
−3
−2
−1
0
1
2
3
Tumor−Normal Expression Difference
hsa−miR−6081, rectal
(ALCOHOL_reg = 0; N0 = 292)
1−sided adj pval: 0.011
Frequency
0
5
10
15
20
25
−3
−2
−1
0
1
2
3
Tumor−Normal Expression Difference
hsa−miR−6081, rectal
(ALCOHOL_reg = 1; N1 = 246)
1−sided adj pval: 0.75
Frequency
0
5
10
15
−3
−2
−1
0
1
2
3
Tumor−Normal Expression Difference

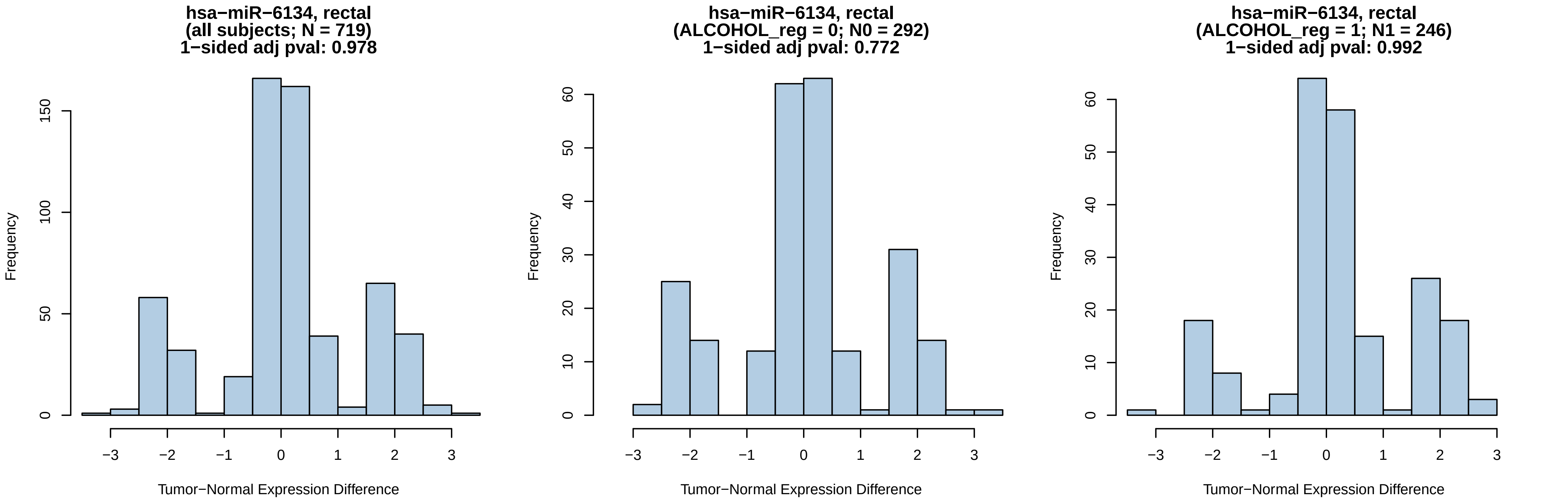
hsa−miR−6134, rectal
(all subjects; N = 719)
1−sided adj pval: 0.978
Frequency
Tumor−Normal Expression Difference
hsa−miR−6134, rectal
(ALCOHOL_reg = 0; N0 = 292)
1−sided adj pval: 0.772
Frequency
Tumor−Normal Expression Difference
hsa−miR−6134, rectal
(ALCOHOL_reg = 1; N1 = 246)
1−sided adj pval: 0.992
Frequency
Tumor−Normal Expression Difference

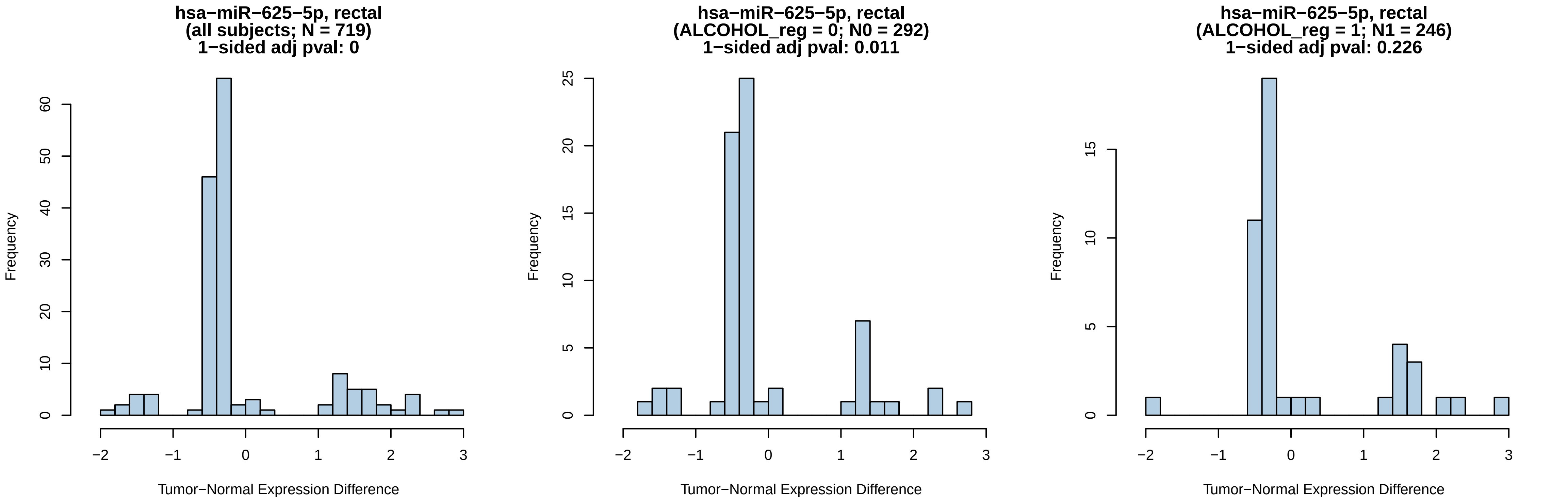

hsa−miR−625−5p, rectal
(all subjects; N = 719)
1−sided adj pval: 0
hsa−miR−625−5p, rectal
(ALCOHOL_reg = 0; N0 = 292)
1−sided adj pval: 0.011
hsa−miR−625−5p, rectal
(ALCOHOL_reg = 1; N1 = 246)
1−sided adj pval: 0.226
Frequency
Tumor−Normal Expression Difference

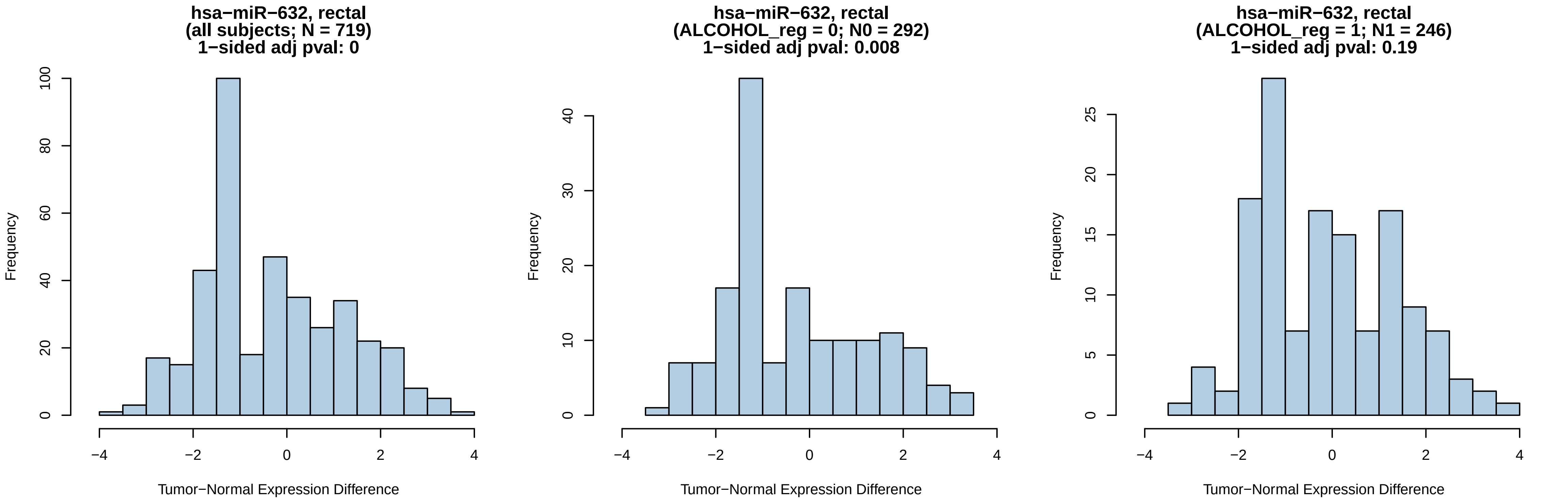

hsa−miR−632, rectal
(all subjects; N = 719)
1−sided adj pval: 0
Frequency
0
20
40
60
80
100
−4
−2
0
2
4
Tumor−Normal Expression Difference
hsa−miR−632, rectal
(ALCOHOL_reg = 0; N0 = 292)
1−sided adj pval: 0.008
Frequency
0
10
20
30
40
−4
−2
0
2
4
Tumor−Normal Expression Difference
hsa−miR−632, rectal
(ALCOHOL_reg = 1; N1 = 246)
1−sided adj pval: 0.19
Frequency
0
5
10
15
20
25
−4
−2
0
2
4
Tumor−Normal Expression Difference

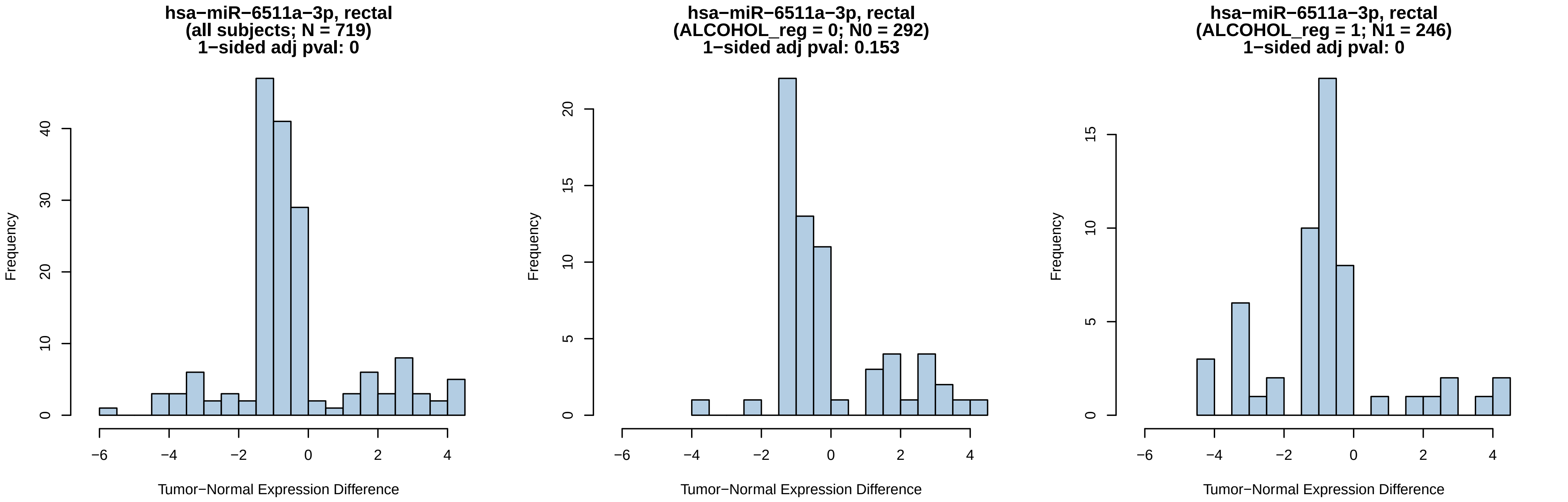
hsa−miR−6511a−3p, rectal
(all subjects; N = 719)
1−sided adj pval: 0
hsa−miR−6511a−3p, rectal
(ALCOHOL_reg = 0; N0 = 292)
1−sided adj pval: 0.153
hsa−miR−6511a−3p, rectal
(ALCOHOL_reg = 1; N1 = 246)
1−sided adj pval: 0
Frequency
Tumor−Normal Expression Difference

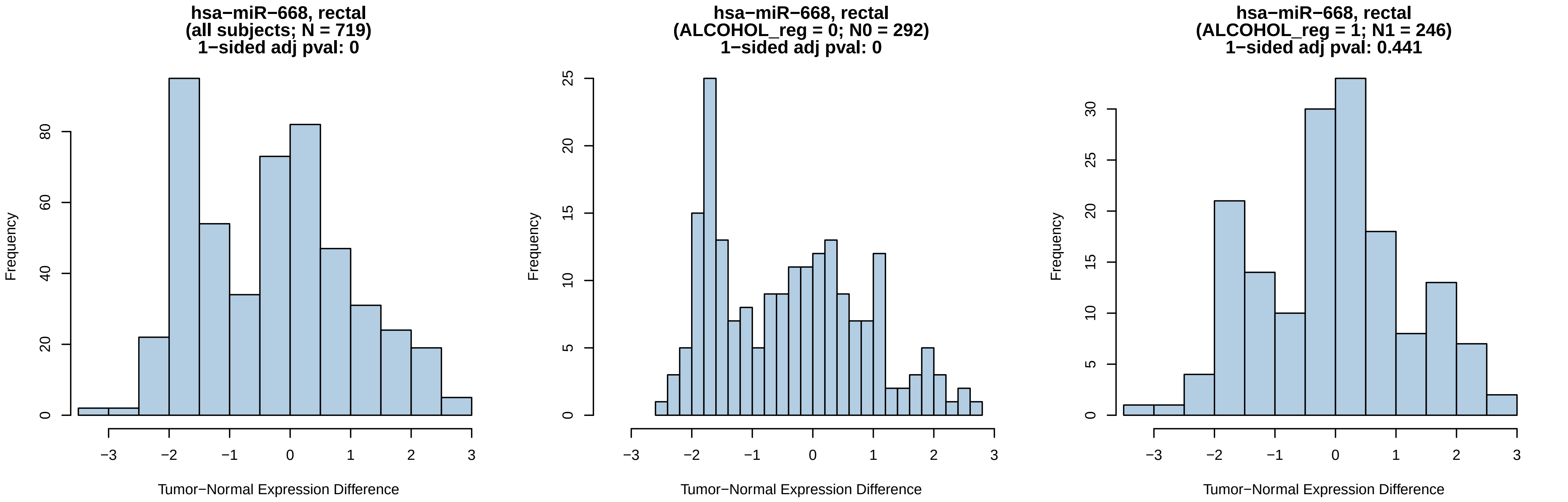
hsa−miR−668, rectal
(all subjects; N = 719)
1−sided adj pval: 0
Frequency
Tumor−Normal Expression Difference
hsa−miR−668, rectal
(ALCOHOL_reg = 0; N0 = 292)
1−sided adj pval: 0
Frequency
Tumor−Normal Expression Difference
hsa−miR−668, rectal
(ALCOHOL_reg = 1; N1 = 246)
1−sided adj pval: 0.441
Frequency
Tumor−Normal Expression Difference

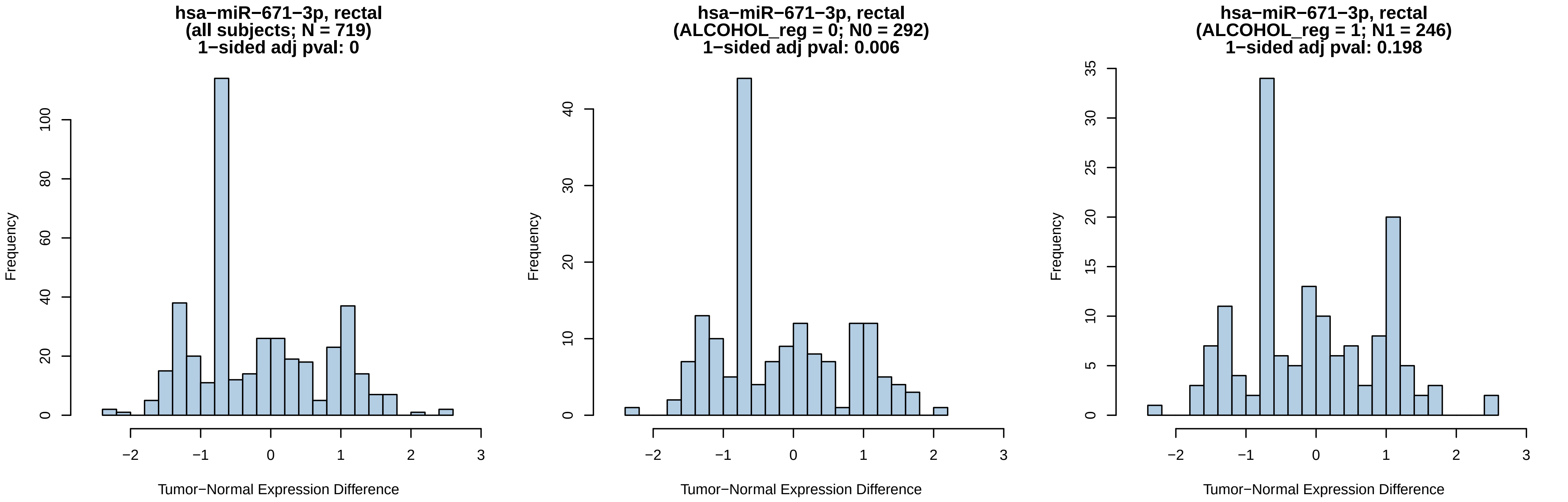

hsa−miR−671−3p, rectal
(all subjects; N = 719)
1−sided adj pval: 0
Frequency
Tumor−Normal Expression Difference
hsa−miR−671−3p, rectal
(ALCOHOL_reg = 0; N0 = 292)
1−sided adj pval: 0.006
Frequency
Tumor−Normal Expression Difference
hsa−miR−671−3p, rectal
(ALCOHOL_reg = 1; N1 = 246)
1−sided adj pval: 0.198
Frequency
Tumor−Normal Expression Difference

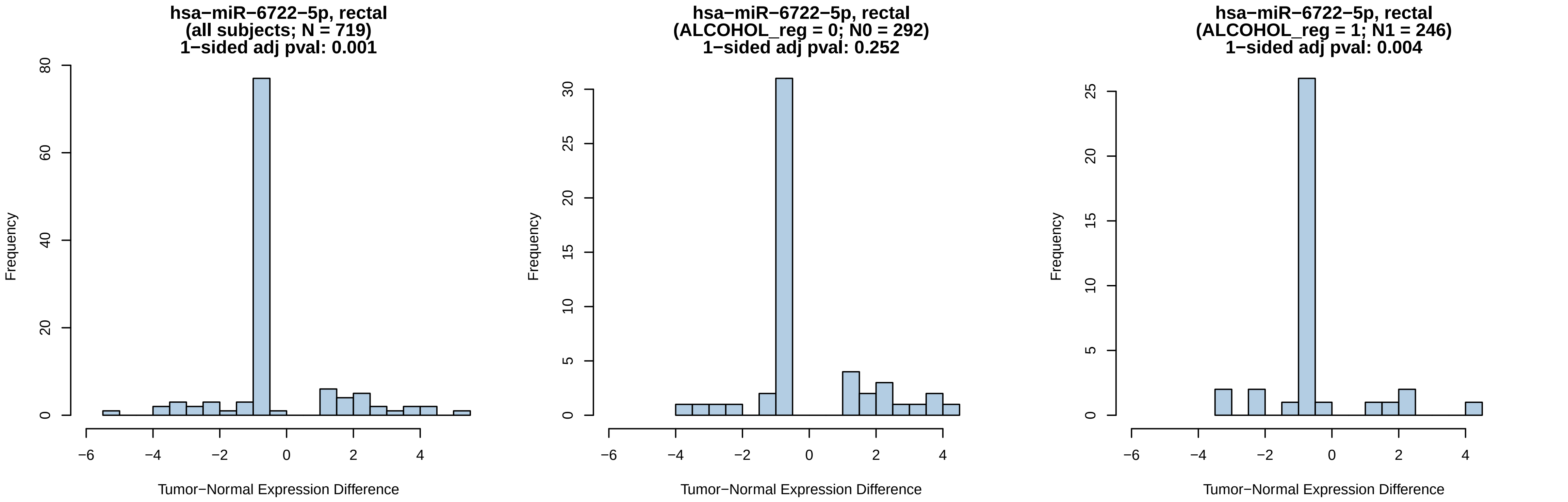
hsa−miR−6722−5p, rectal
(all subjects; N = 719)
1−sided adj pval: 0.001
Frequency
Tumor−Normal Expression Difference
hsa−miR−6722−5p, rectal
(ALCOHOL_reg = 0; N0 = 292)
1−sided adj pval: 0.252
Frequency
Tumor−Normal Expression Difference
hsa−miR−6722−5p, rectal
(ALCOHOL_reg = 1; N1 = 246)
1−sided adj pval: 0.004
Frequency
Tumor−Normal Expression Difference

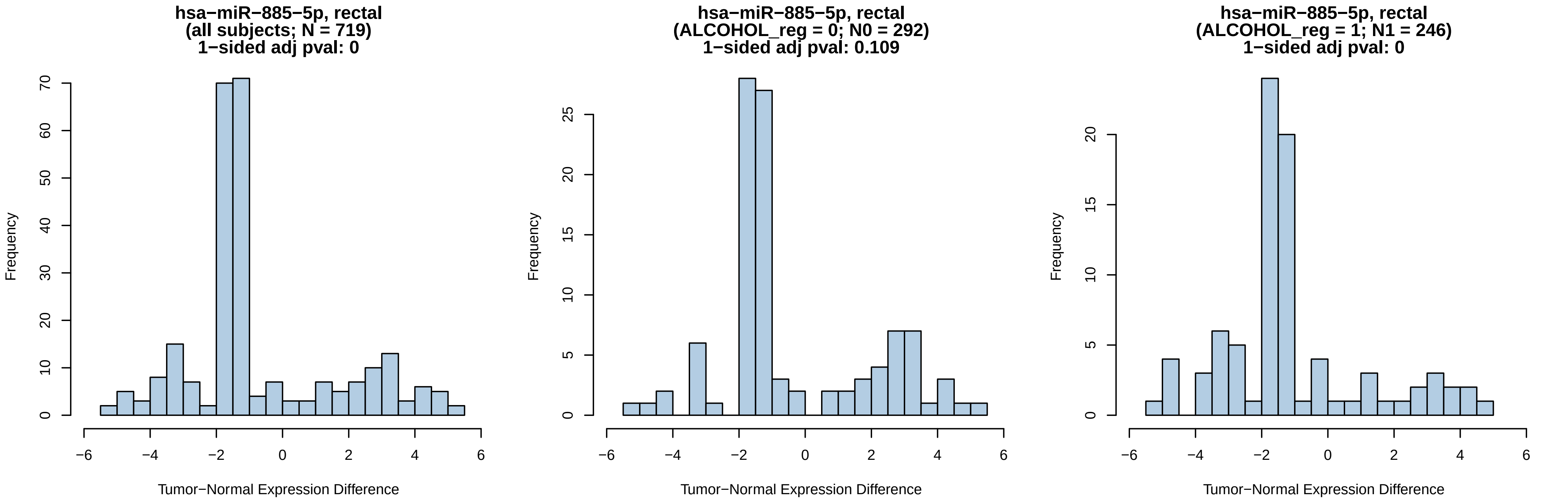
hsa−miR−885−5p, rectal
(all subjects; N = 719)
1−sided adj pval: 0
Frequency
Tumor−Normal Expression Difference
hsa−miR−885−5p, rectal
(ALCOHOL_reg = 0; N0 = 292)
1−sided adj pval: 0.109
Frequency
Tumor−Normal Expression Difference
hsa−miR−885−5p, rectal
(ALCOHOL_reg = 1; N1 = 246)
1−sided adj pval: 0
Frequency
Tumor−Normal Expression Difference

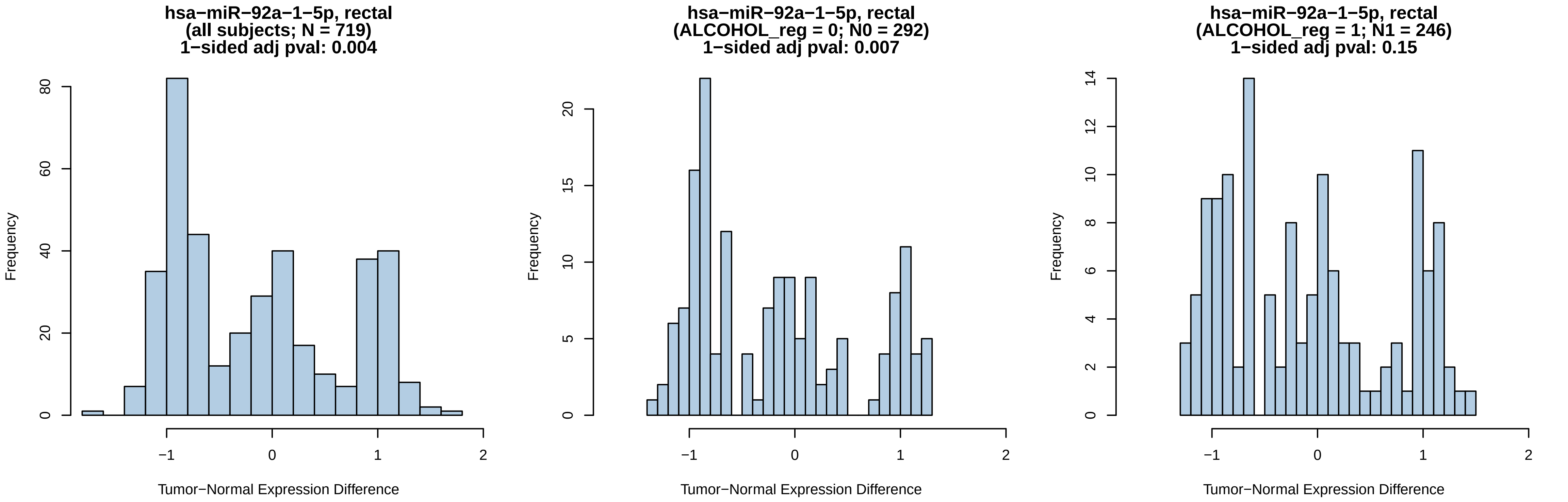
hsa−miR−92a−1−5p, rectal
(all subjects; N = 719)
1−sided adj pval: 0.004
hsa−miR−92a−1−5p, rectal
(ALCOHOL_reg = 0; N0 = 292)
1−sided adj pval: 0.007
hsa−miR−92a−1−5p, rectal
(ALCOHOL_reg = 1; N1 = 246)
1−sided adj pval: 0.15
Frequency
Tumor−Normal Expression Difference

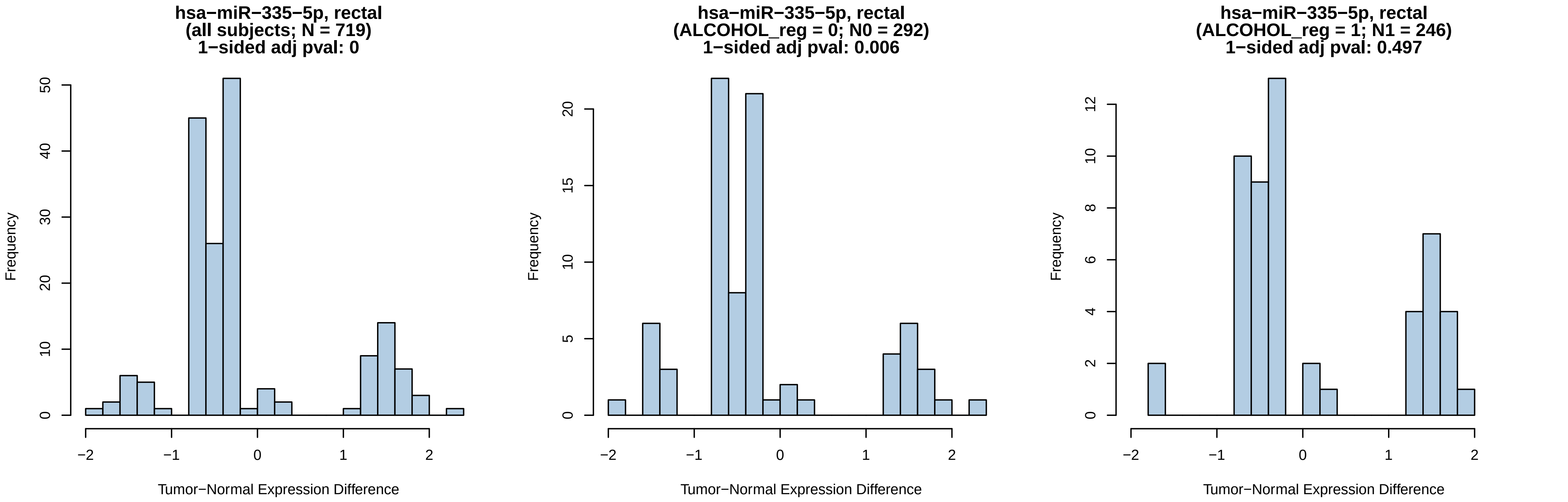
hsa−miR−335−5p, rectal
(all subjects; N = 719)
1−sided adj pval: 0
Frequency
Tumor−Normal Expression Difference
hsa−miR−335−5p, rectal
(ALCOHOL_reg = 0; N0 = 292)
1−sided adj pval: 0.006
Frequency
Tumor−Normal Expression Difference
hsa−miR−335−5p, rectal
(ALCOHOL_reg = 1; N1 = 246)
1−sided adj pval: 0.497
Frequency
Tumor−Normal Expression Difference

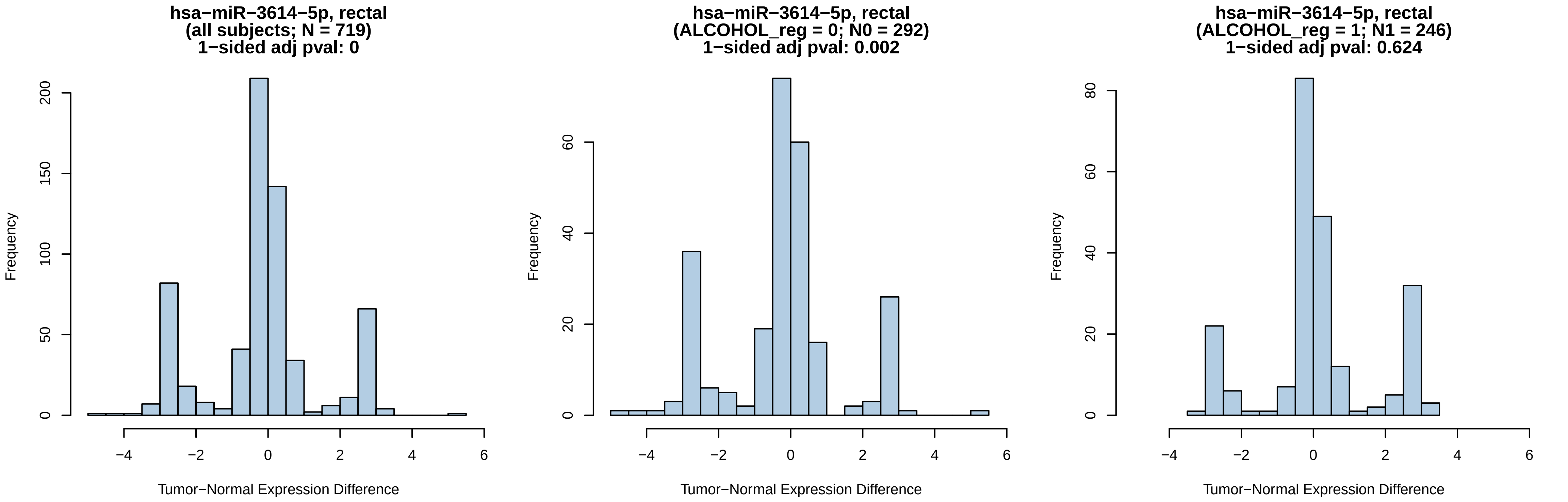
hsa−miR−3614−5p, rectal
(all subjects; N = 719)
1−sided adj pval: 0
Frequency
Tumor−Normal Expression Difference
hsa−miR−3614−5p, rectal
(ALCOHOL_reg = 0; N0 = 292)
1−sided adj pval: 0.002
Frequency
Tumor−Normal Expression Difference
hsa−miR−3614−5p, rectal
(ALCOHOL_reg = 1; N1 = 246)
1−sided adj pval: 0.624
Frequency
Tumor−Normal Expression Difference

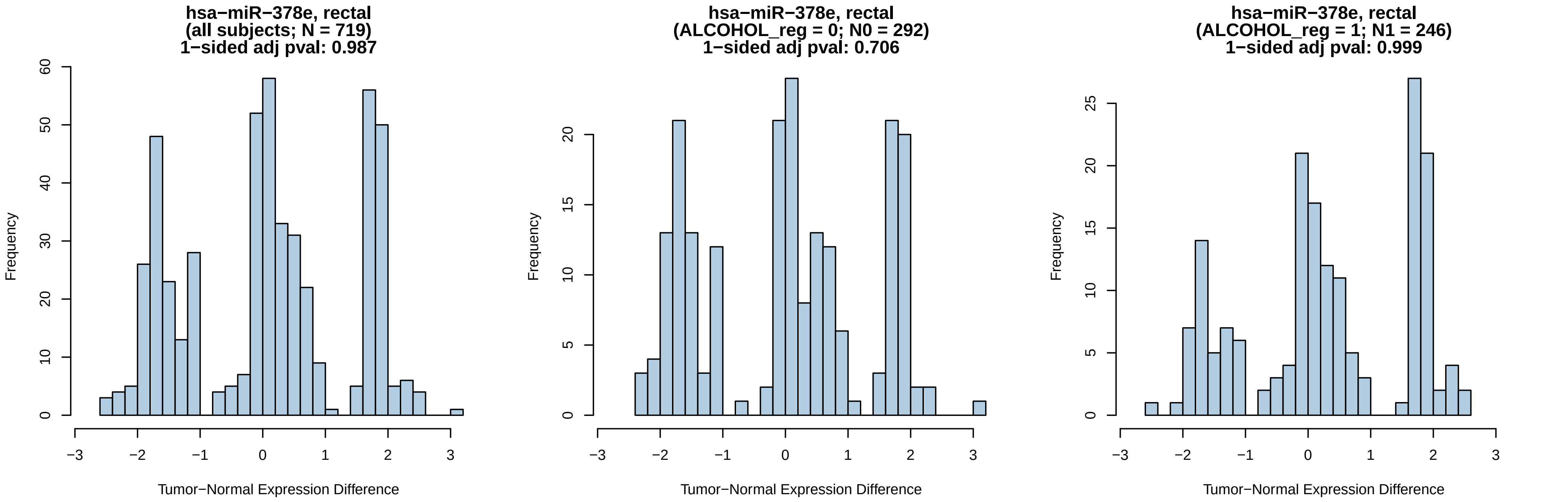

hsa−miR−378e, rectal
(all subjects; N = 719)
1−sided adj pval: 0.987
Frequency
Tumor−Normal Expression Difference
hsa−miR−378e, rectal
(ALCOHOL_reg = 0; N0 = 292)
1−sided adj pval: 0.706
Frequency
Tumor−Normal Expression Difference
hsa−miR−378e, rectal
(ALCOHOL_reg = 1; N1 = 246)
1−sided adj pval: 0.999
Frequency
Tumor−Normal Expression Difference

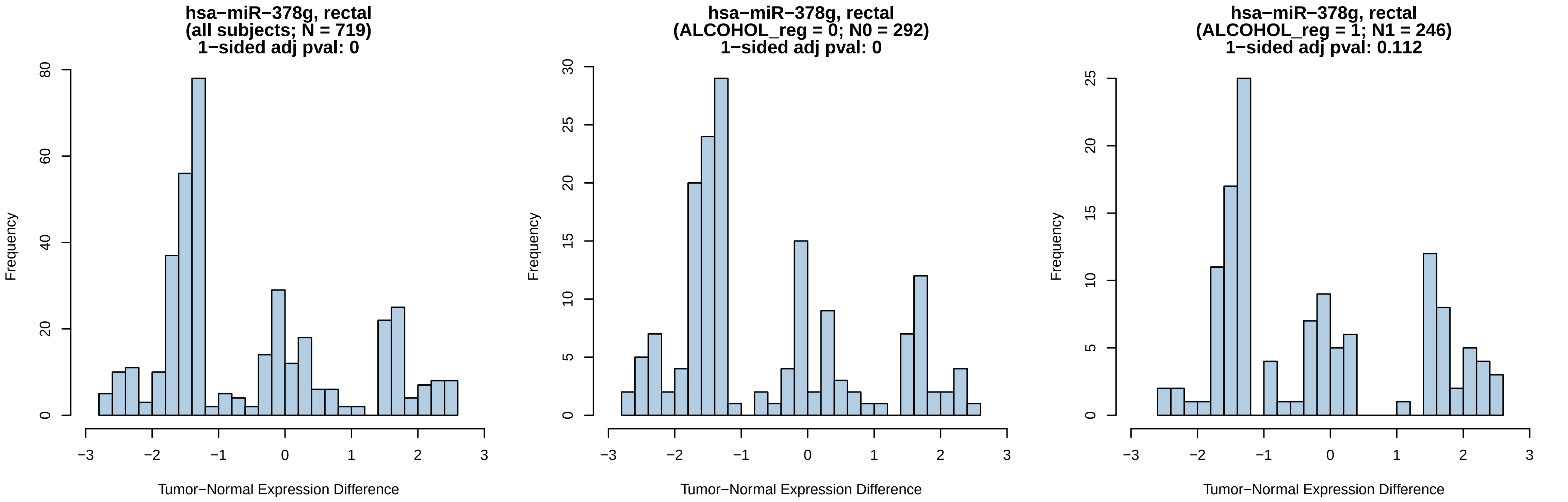
hsa−miR−378g, rectal
(all subjects; N = 719)
1−sided adj pval: 0
Frequency
Tumor−Normal Expression Difference
hsa−miR−378g, rectal
(ALCOHOL_reg = 0; N0 = 292)
1−sided adj pval: 0
Frequency
Tumor−Normal Expression Difference
hsa−miR−378g, rectal
(ALCOHOL_reg = 1; N1 = 246)
1−sided adj pval: 0.112
Frequency
Tumor−Normal Expression Difference

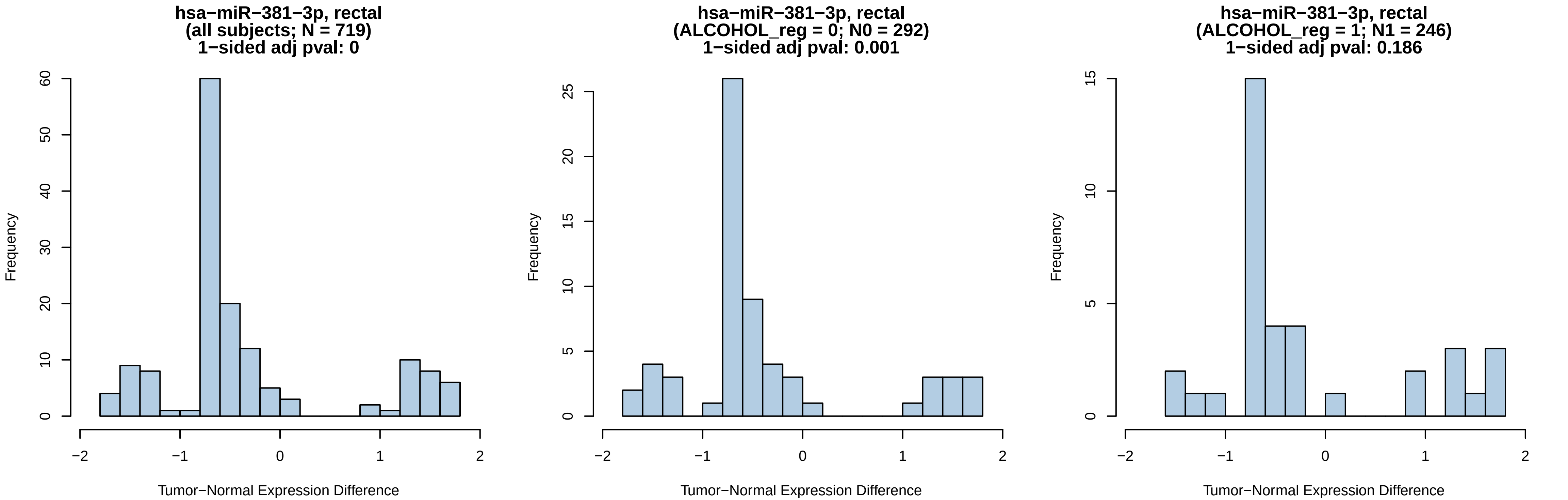
hsa−miR−381−3p, rectal
(all subjects; N = 719)
1−sided adj pval: 0
Frequency
Tumor−Normal Expression Difference
hsa−miR−381−3p, rectal
(ALCOHOL_reg = 0; N0 = 292)
1−sided adj pval: 0.001
Frequency
Tumor−Normal Expression Difference
hsa−miR−381−3p, rectal
(ALCOHOL_reg = 1; N1 = 246)
1−sided adj pval: 0.186
Frequency
Tumor−Normal Expression Difference

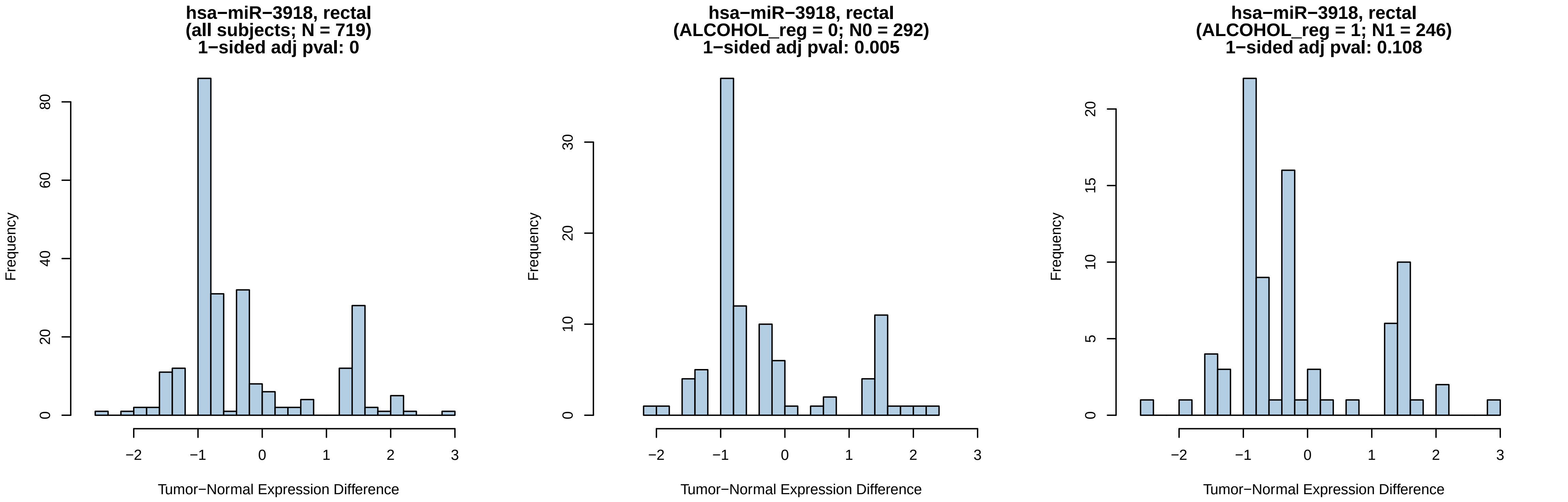

hsa−miR−3918, rectal
(all subjects; N = 719)
1−sided adj pval: 0
Frequency
Tumor−Normal Expression Difference
hsa−miR−3918, rectal
(ALCOHOL_reg = 0; N0 = 292)
1−sided adj pval: 0.005
Frequency
Tumor−Normal Expression Difference
hsa−miR−3918, rectal
(ALCOHOL_reg = 1; N1 = 246)
1−sided adj pval: 0.108
Frequency
Tumor−Normal Expression Difference

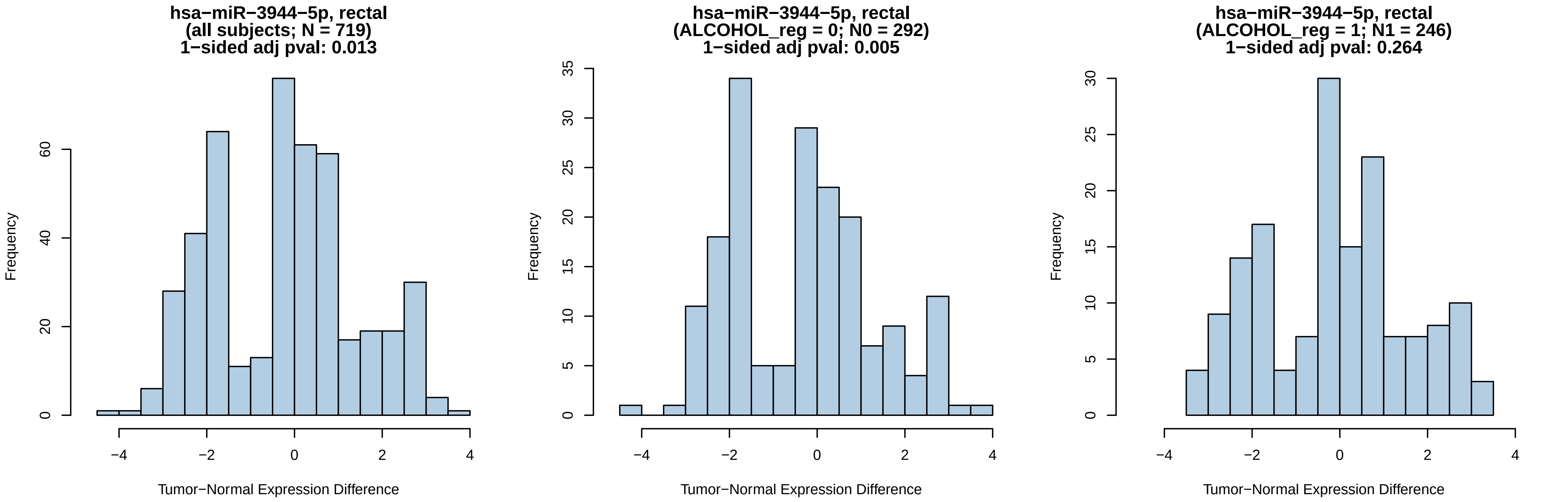

hsa−miR−3944−5p, rectal
(all subjects; N = 719)
1−sided adj pval: 0.013
Frequency
0
20
40
60
−4
−2
0
2
4
Tumor−Normal Expression Difference
hsa−miR−3944−5p, rectal
(ALCOHOL_reg = 0; N0 = 292)
1−sided adj pval: 0.005
Frequency
0
5
10
15
20
25
30
35
−4
−2
0
2
4
Tumor−Normal Expression Difference
hsa−miR−3944−5p, rectal
(ALCOHOL_reg = 1; N1 = 246)
1−sided adj pval: 0.264
Frequency
0
5
10
15
20
25
30
−4
−2
0
2
4
Tumor−Normal Expression Difference

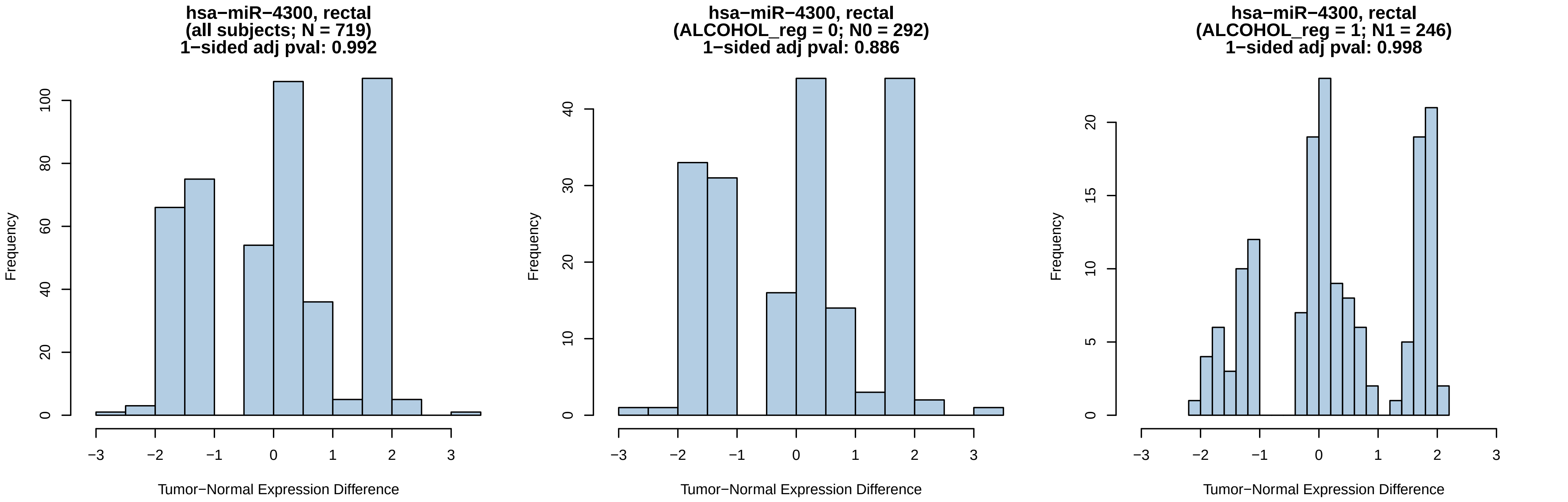

hsa−miR−4300, rectal
(all subjects; N = 719)
1−sided adj pval: 0.992
Frequency
0
20
40
60
80
100
−3
−2
−1
0
1
2
3
Tumor−Normal Expression Difference
hsa−miR−4300, rectal
(ALCOHOL_reg = 0; N0 = 292)
1−sided adj pval: 0.886
Frequency
0
10
20
30
40
−3
−2
−1
0
1
2
3
Tumor−Normal Expression Difference
hsa−miR−4300, rectal
(ALCOHOL_reg = 1; N1 = 246)
1−sided adj pval: 0.998
Frequency
0
5
10
15
20
−3
−2
−1
0
1
2
3
Tumor−Normal Expression Difference

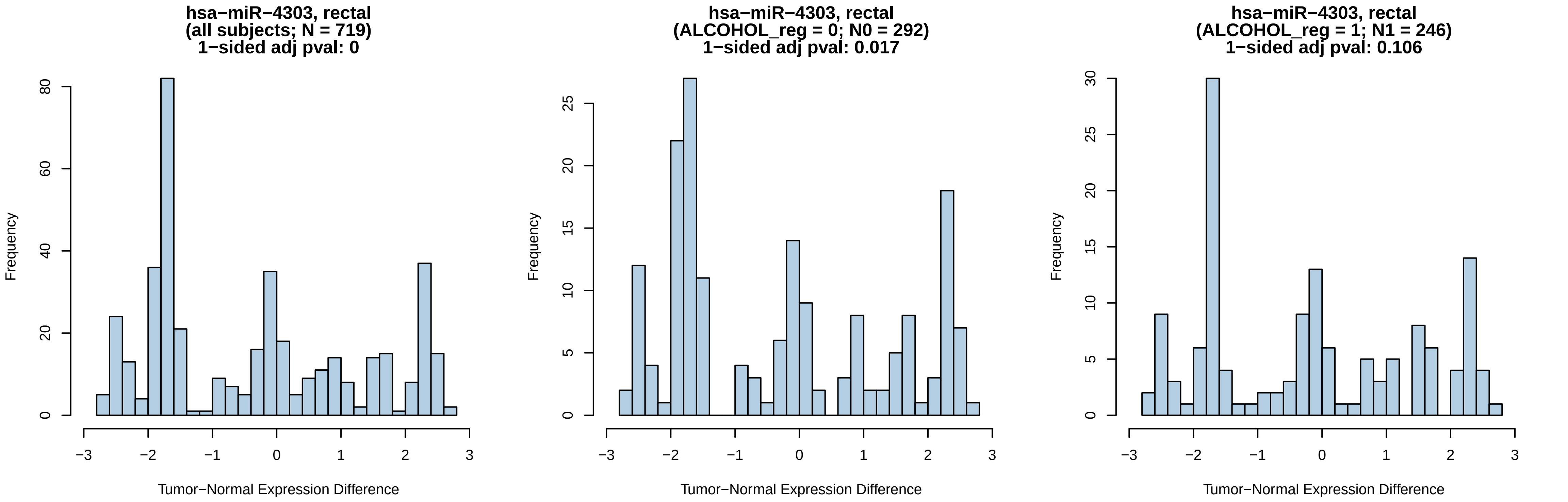
hsa−miR−4303, rectal
(all subjects; N = 719)
1−sided adj pval: 0
hsa−miR−4303, rectal
(ALCOHOL_reg = 0; N0 = 292)
1−sided adj pval: 0.017
hsa−miR−4303, rectal
(ALCOHOL_reg = 1; N1 = 246)
1−sided adj pval: 0.106
Frequency
Tumor−Normal Expression Difference

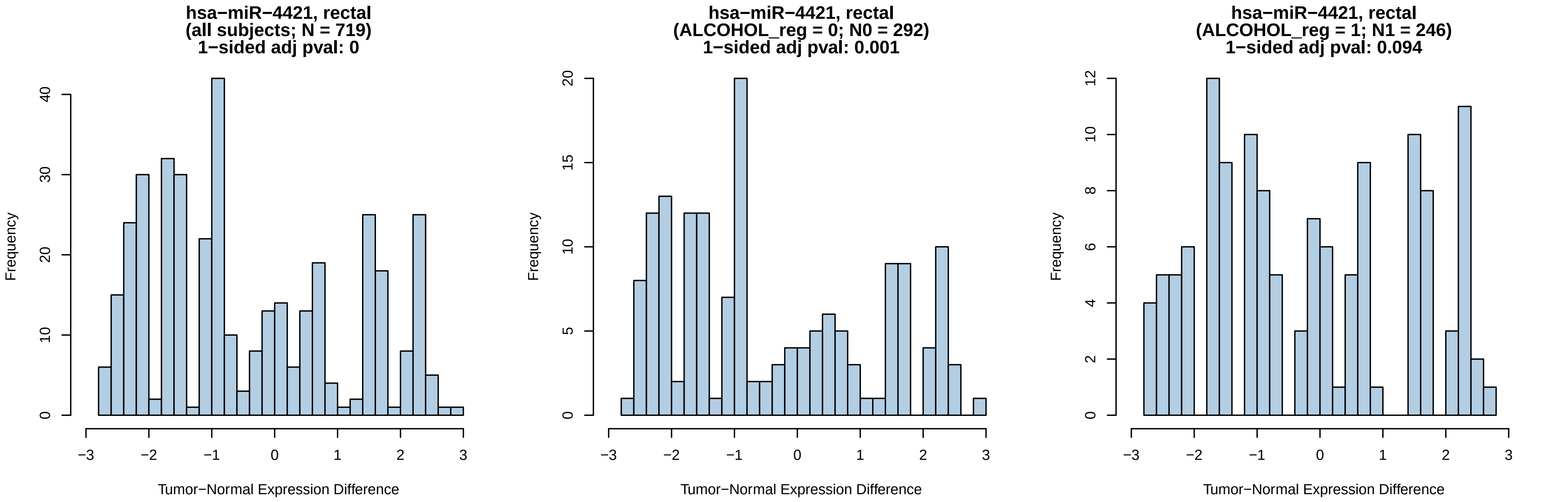
hsa−miR−4421, rectal
(all subjects; N = 719)
1−sided adj pval: 0
Frequency
Tumor−Normal Expression Difference
hsa−miR−4421, rectal
(ALCOHOL_reg = 0; N0 = 292)
1−sided adj pval: 0.001
Frequency
Tumor−Normal Expression Difference
hsa−miR−4421, rectal
(ALCOHOL_reg = 1; N1 = 246)
1−sided adj pval: 0.094
Frequency
Tumor−Normal Expression Difference

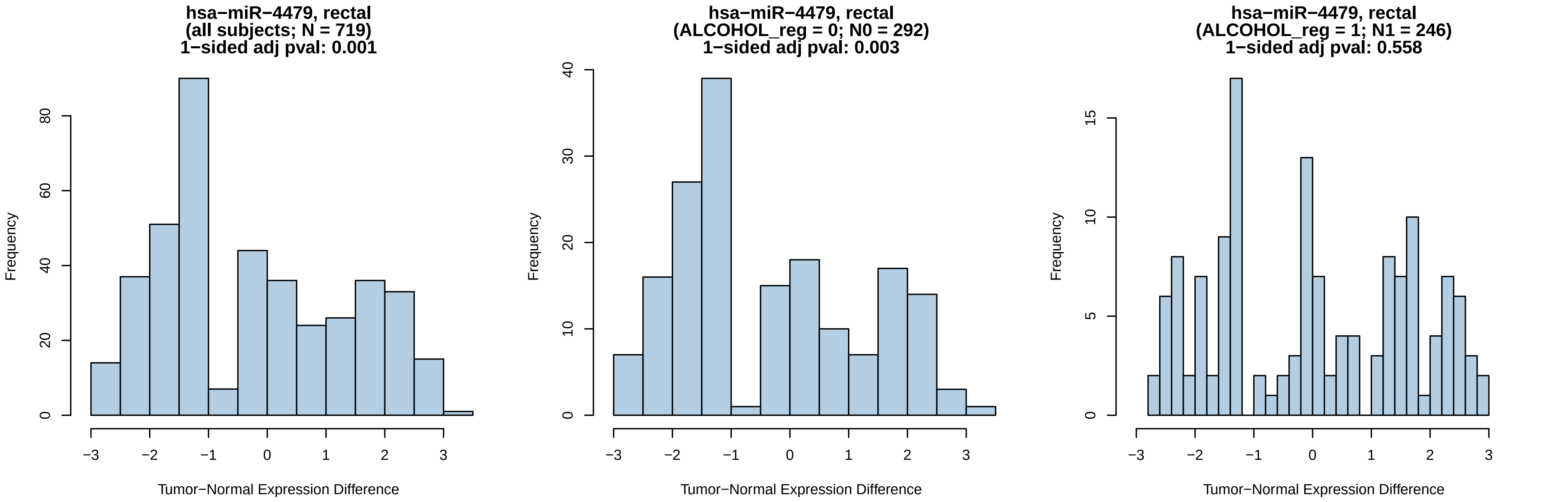
hsa−miR−4479, rectal
(all subjects; N = 719)
1−sided adj pval: 0.001
hsa−miR−4479, rectal
(ALCOHOL_reg = 0; N0 = 292)
1−sided adj pval: 0.003
hsa−miR−4479, rectal
(ALCOHOL_reg = 1; N1 = 246)
1−sided adj pval: 0.558
Frequency
Tumor−Normal Expression Difference

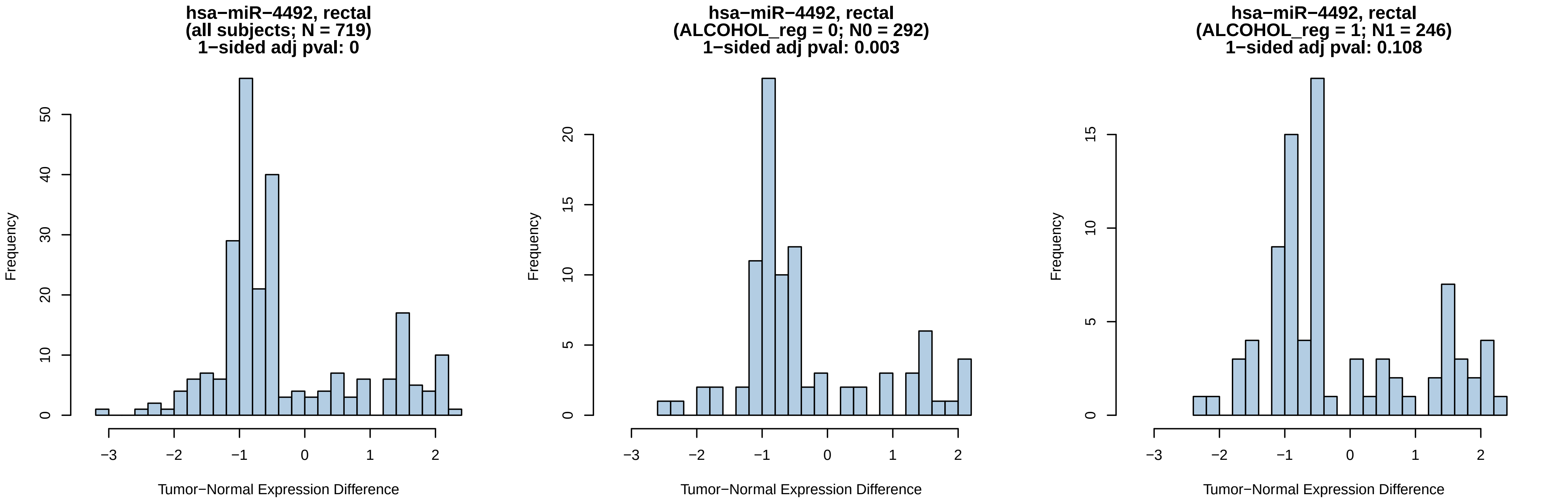

hsa−miR−4492, rectal
(all subjects; N = 719)
1−sided adj pval: 0
Frequency
Tumor−Normal Expression Difference
hsa−miR−4492, rectal
(ALCOHOL_reg = 0; N0 = 292)
1−sided adj pval: 0.003
Frequency
Tumor−Normal Expression Difference
hsa−miR−4492, rectal
(ALCOHOL_reg = 1; N1 = 246)
1−sided adj pval: 0.108
Frequency
Tumor−Normal Expression Difference

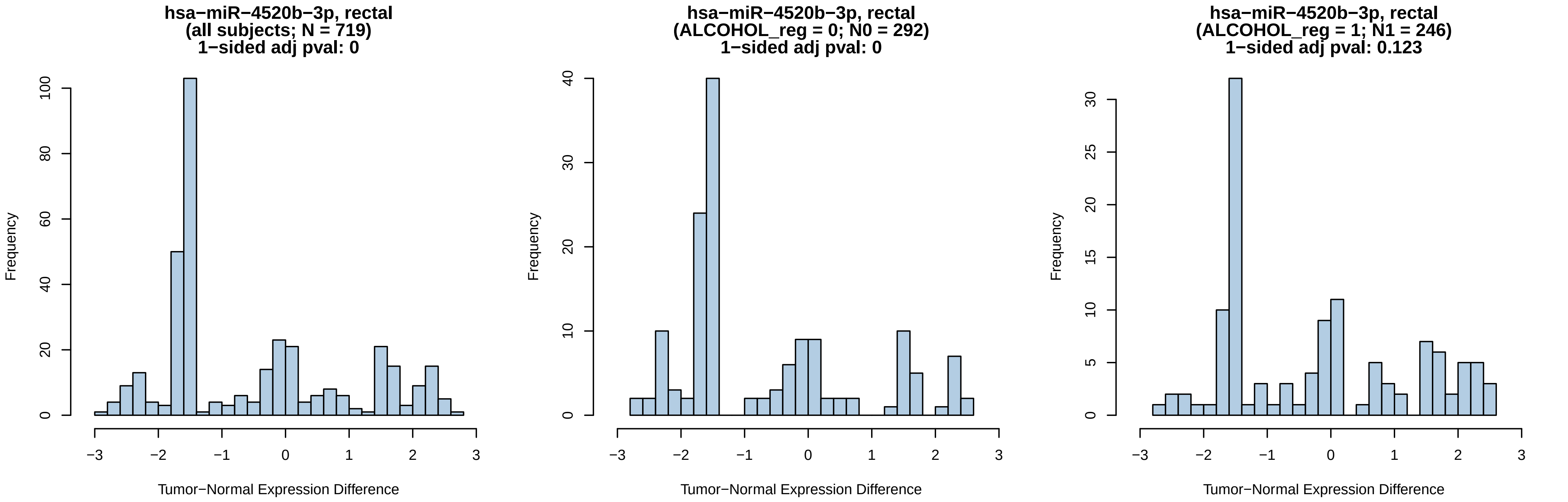
hsa−miR−4520b−3p, rectal
(all subjects; N = 719)
1−sided adj pval: 0
hsa−miR−4520b−3p, rectal
(ALCOHOL_reg = 0; N0 = 292)
1−sided adj pval: 0
hsa−miR−4520b−3p, rectal
(ALCOHOL_reg = 1; N1 = 246)
1−sided adj pval: 0.123
Frequency
Tumor−Normal Expression Difference

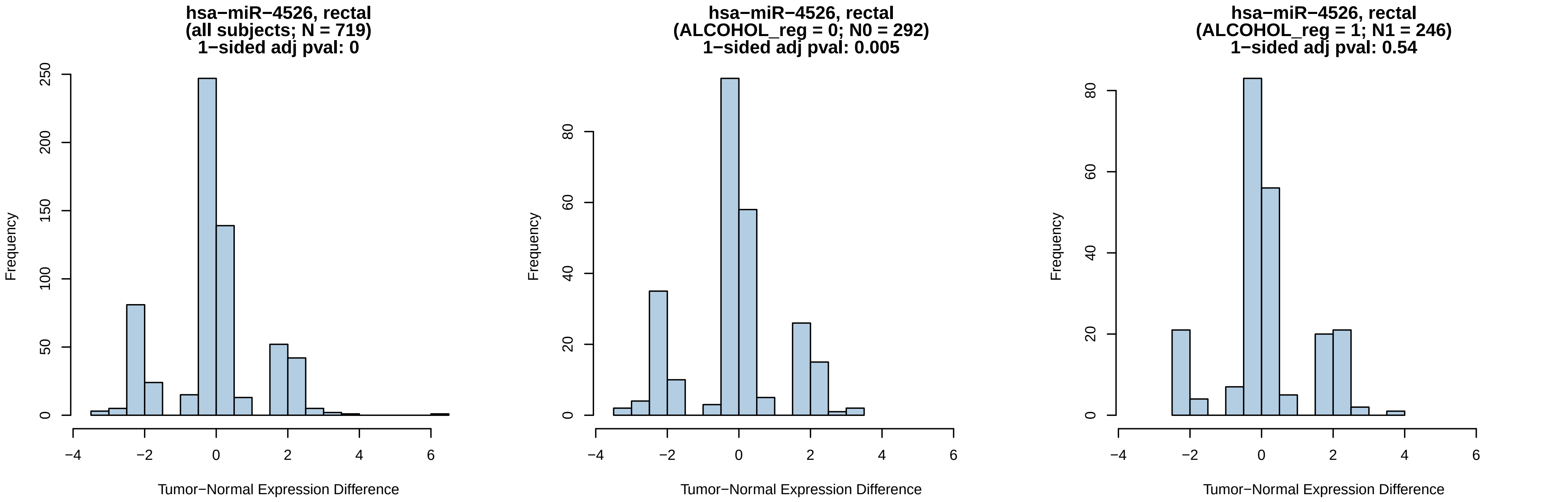
hsa−miR−4526, rectal
(all subjects; N = 719)
1−sided adj pval: 0
Frequency
0
50
100
150
200
250
−4
−2
0
2
4
6
Tumor−Normal Expression Difference
hsa−miR−4526, rectal
(ALCOHOL_reg = 0; N0 = 292)
1−sided adj pval: 0.005
Frequency
0
20
40
60
80
−4
−2
0
2
4
6
Tumor−Normal Expression Difference
hsa−miR−4526, rectal
(ALCOHOL_reg = 1; N1 = 246)
1−sided adj pval: 0.54
Frequency
0
20
40
60
80
−4
−2
0
2
4
6
Tumor−Normal Expression Difference

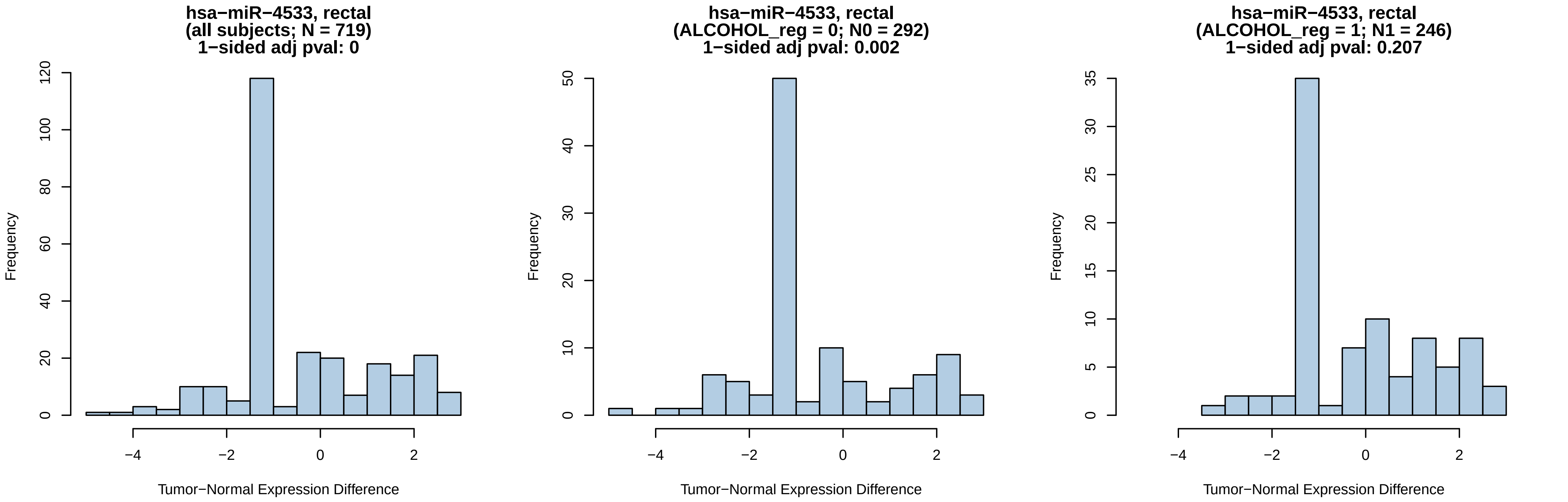
hsa−miR−4533, rectal
(all subjects; N = 719)
1−sided adj pval: 0
Frequency
0
20
40
60
80
100
120
−4
−2
0
2
Tumor−Normal Expression Difference
hsa−miR−4533, rectal
(ALCOHOL_reg = 0; N0 = 292)
1−sided adj pval: 0.002
Frequency
0
10
20
30
40
50
−4
−2
0
2
Tumor−Normal Expression Difference
hsa−miR−4533, rectal
(ALCOHOL_reg = 1; N1 = 246)
1−sided adj pval: 0.207
Frequency
0
5
10
15
20
25
30
35
−4
−2
0
2
Tumor−Normal Expression Difference

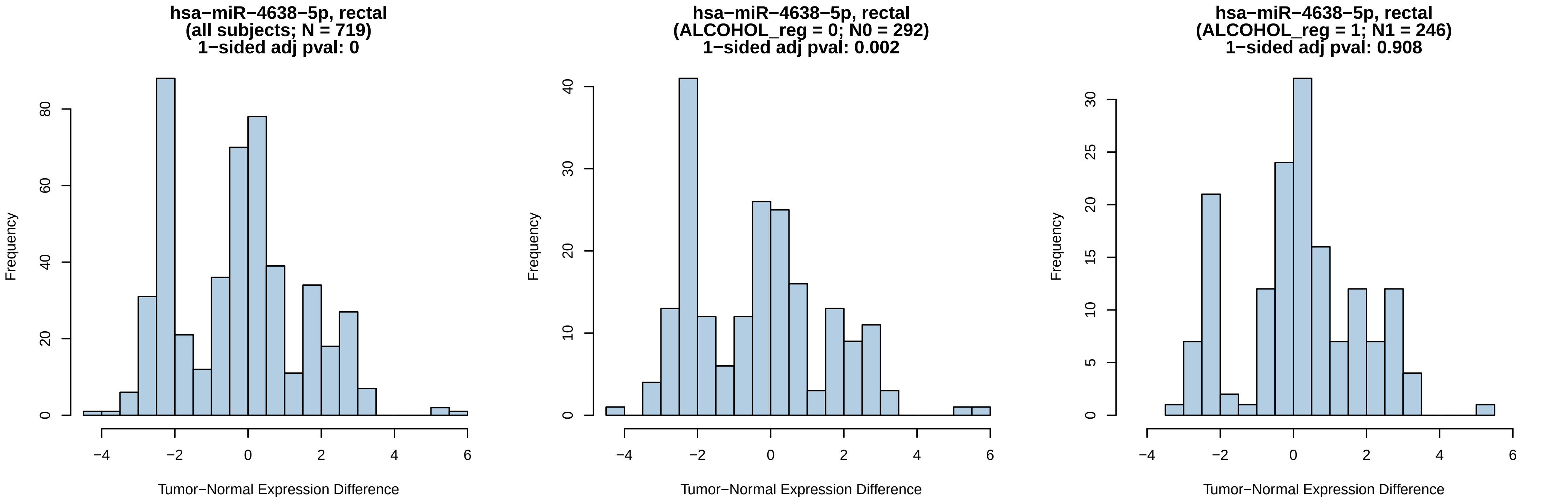

hsa−miR−4638−5p, rectal
(all subjects; N = 719)
1−sided adj pval: 0
hsa−miR−4638−5p, rectal
(ALCOHOL_reg = 0; N0 = 292)
1−sided adj pval: 0.002
hsa−miR−4638−5p, rectal
(ALCOHOL_reg = 1; N1 = 246)
1−sided adj pval: 0.908
Frequency
Tumor−Normal Expression Difference

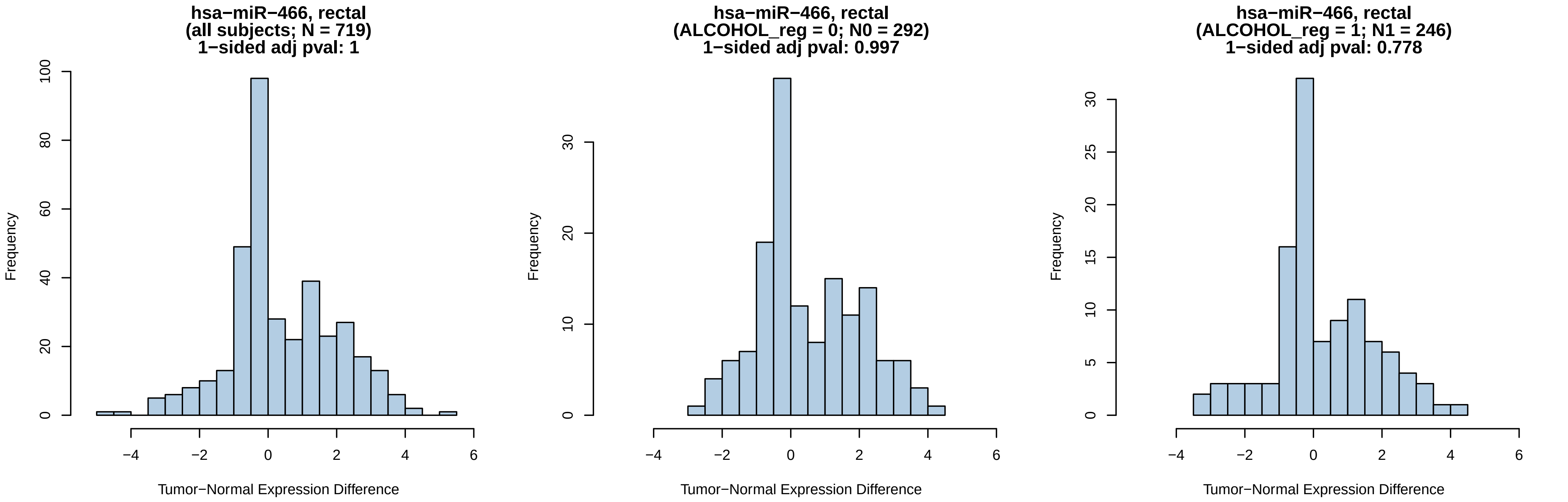
hsa−miR−466, rectal
(all subjects; N = 719)
1−sided adj pval: 1
Frequency
Tumor−Normal Expression Difference
hsa−miR−466, rectal
(ALCOHOL_reg = 0; N0 = 292)
1−sided adj pval: 0.997
Frequency
Tumor−Normal Expression Difference
hsa−miR−466, rectal
(ALCOHOL_reg = 1; N1 = 246)
1−sided adj pval: 0.778
Frequency
Tumor−Normal Expression Difference

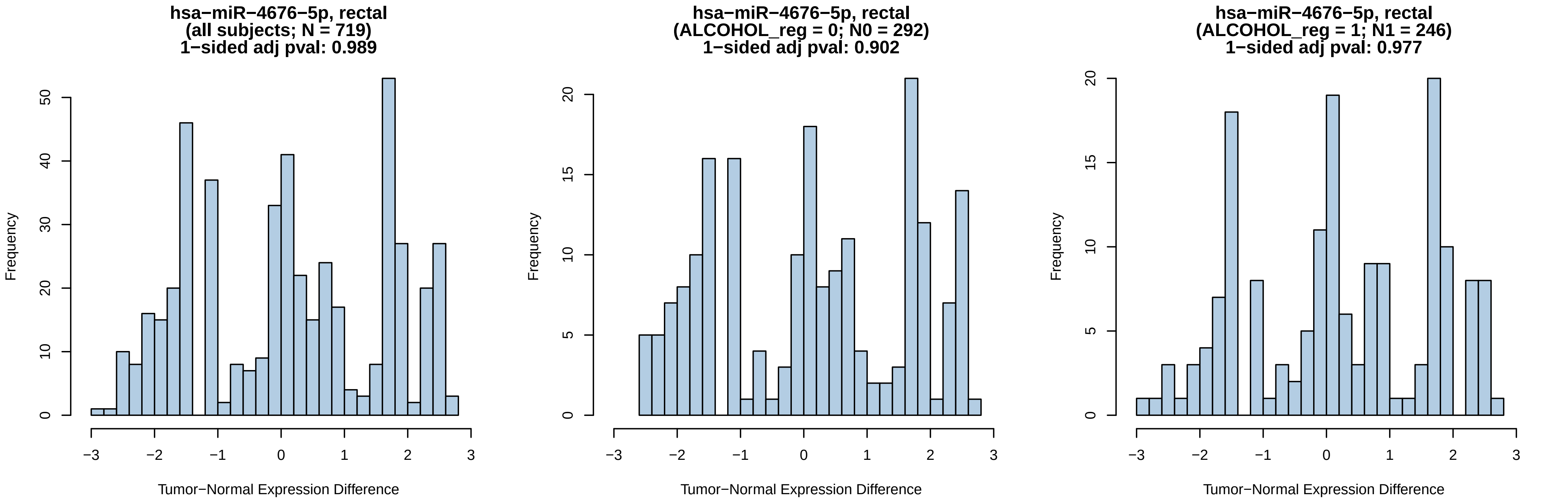
hsa−miR−4676−5p, rectal
(all subjects; N = 719)
1−sided adj pval: 0.989
hsa−miR−4676−5p, rectal
(ALCOHOL_reg = 0; N0 = 292)
1−sided adj pval: 0.902
hsa−miR−4676−5p, rectal
(ALCOHOL_reg = 1; N1 = 246)
1−sided adj pval: 0.977
Frequency
Tumor−Normal Expression Difference

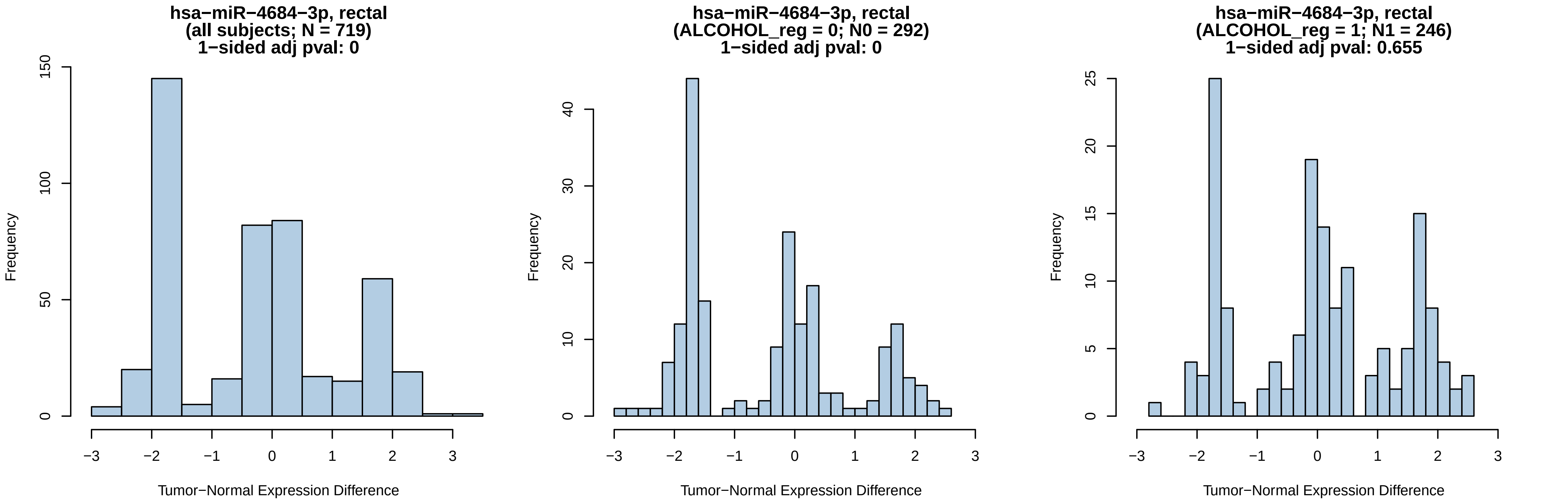

hsa−miR−4684−3p, rectal
(all subjects; N = 719)
1−sided adj pval: 0
Frequency
Tumor−Normal Expression Difference
hsa−miR−4684−3p, rectal
(ALCOHOL_reg = 0; N0 = 292)
1−sided adj pval: 0
Frequency
Tumor−Normal Expression Difference
hsa−miR−4684−3p, rectal
(ALCOHOL_reg = 1; N1 = 246)
1−sided adj pval: 0.655
Frequency
Tumor−Normal Expression Difference

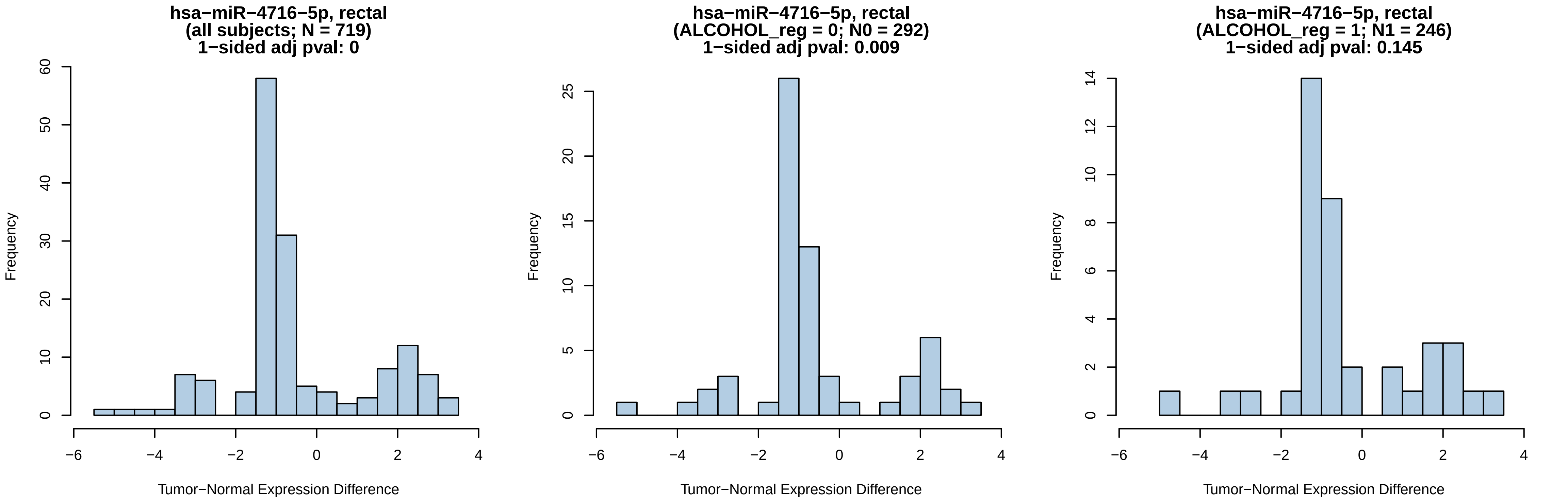
hsa−miR−4716−5p, rectal
(all subjects; N = 719)
1−sided adj pval: 0
Frequency
Tumor−Normal Expression Difference
hsa−miR−4716−5p, rectal
(ALCOHOL_reg = 0; N0 = 292)
1−sided adj pval: 0.009
Frequency
Tumor−Normal Expression Difference
hsa−miR−4716−5p, rectal
(ALCOHOL_reg = 1; N1 = 246)
1−sided adj pval: 0.145
Frequency
Tumor−Normal Expression Difference

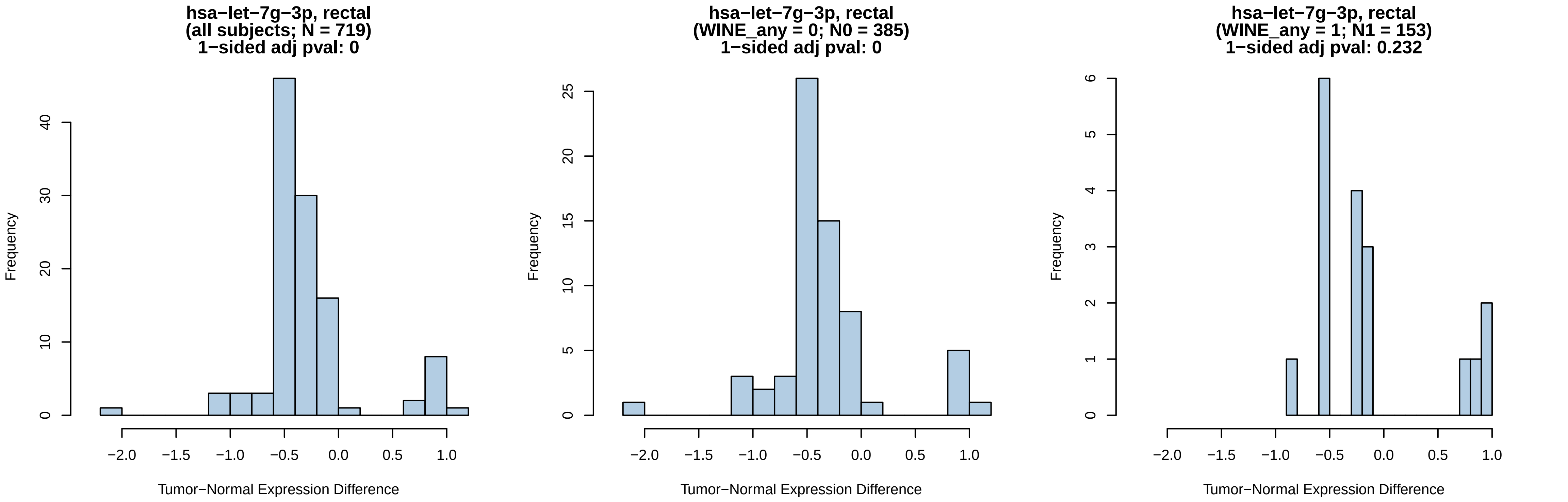

hsa−let−7g−3p, rectal
(all subjects; N = 719)
1−sided adj pval: 0
Frequency
Tumor−Normal Expression Difference
hsa−let−7g−3p, rectal
(WINE_any = 0; N0 = 385)
1−sided adj pval: 0
Frequency
Tumor−Normal Expression Difference
hsa−let−7g−3p, rectal
(WINE_any = 1; N1 = 153)
1−sided adj pval: 0.232
Frequency
Tumor−Normal Expression Difference

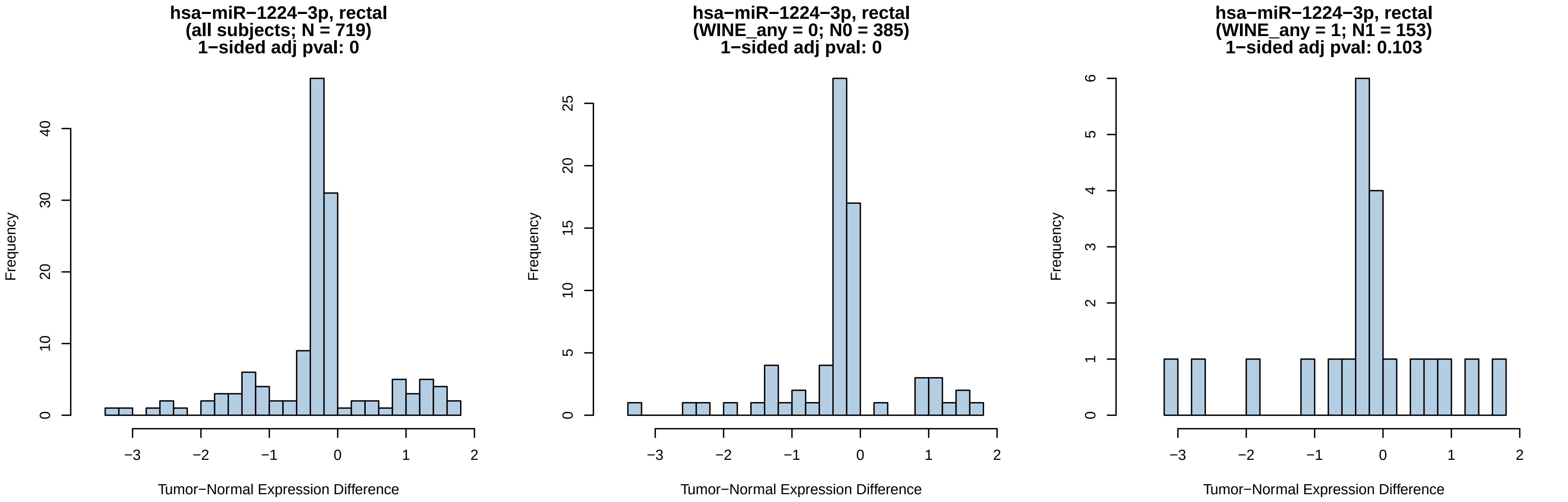

hsa−miR−1224−3p, rectal
(all subjects; N = 719)
1−sided adj pval: 0
Frequency
0
10
20
30
40
−3
−2
−1
0
1
2
Tumor−Normal Expression Difference
hsa−miR−1224−3p, rectal
(WINE_any = 0; N0 = 385)
1−sided adj pval: 0
Frequency
0
5
10
15
20
25
−3
−2
−1
0
1
2
Tumor−Normal Expression Difference
hsa−miR−1224−3p, rectal
(WINE_any = 1; N1 = 153)
1−sided adj pval: 0.103
Frequency
0
1
2
3
4
5
6
−3
−2
−1
0
1
2
Tumor−Normal Expression Difference

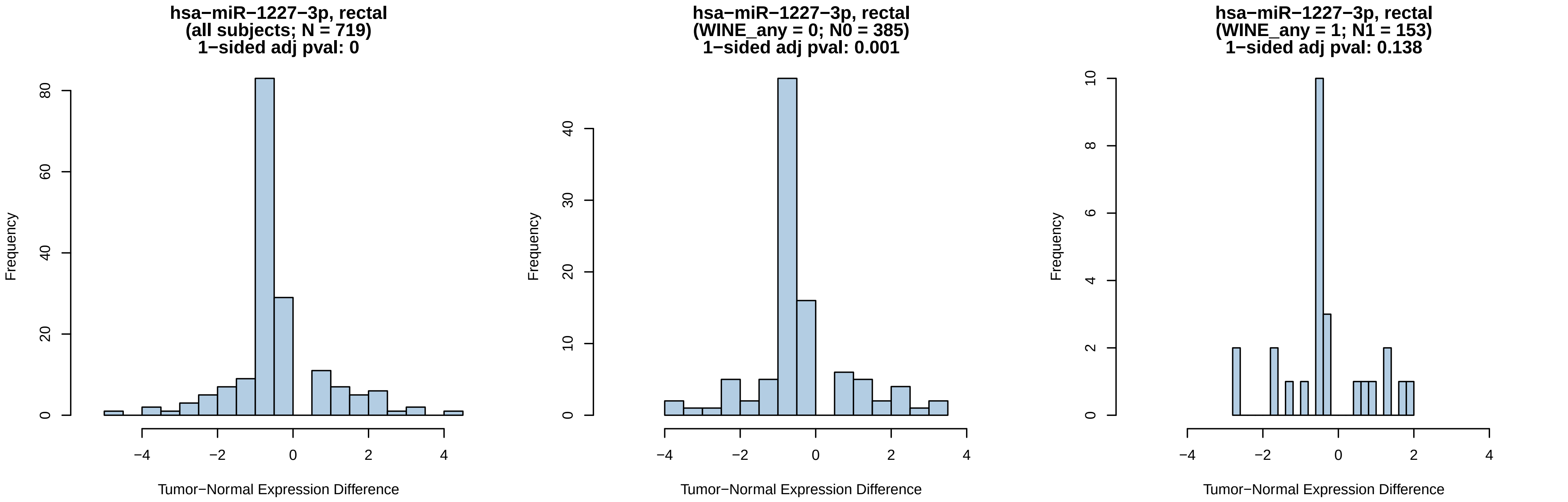
hsa−miR−1227−3p, rectal
(all subjects; N = 719)
1−sided adj pval: 0
Frequency
Tumor−Normal Expression Difference
hsa−miR−1227−3p, rectal
(WINE_any = 0; N0 = 385)
1−sided adj pval: 0.001
Frequency
Tumor−Normal Expression Difference
hsa−miR−1227−3p, rectal
(WINE_any = 1; N1 = 153)
1−sided adj pval: 0.138
Frequency
Tumor−Normal Expression Difference

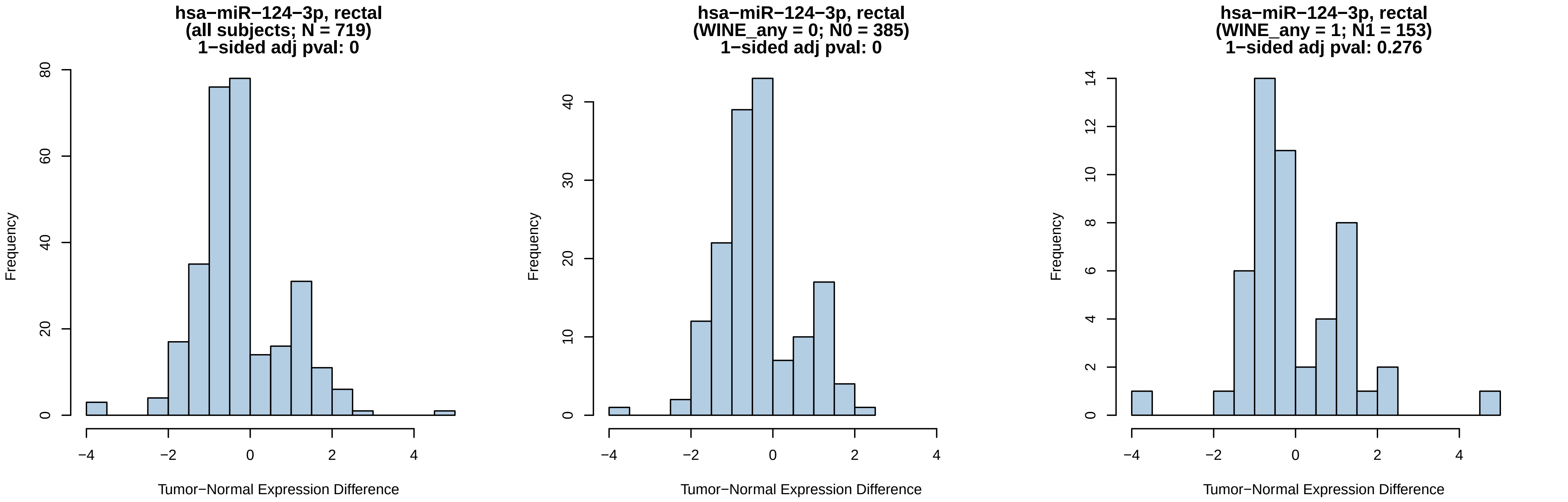
hsa−miR−124−3p, rectal
(all subjects; N = 719)
1−sided adj pval: 0
Frequency
Tumor−Normal Expression Difference
hsa−miR−124−3p, rectal
(WINE_any = 0; N0 = 385)
1−sided adj pval: 0
Frequency
Tumor−Normal Expression Difference
hsa−miR−124−3p, rectal
(WINE_any = 1; N1 = 153)
1−sided adj pval: 0.276
Frequency
Tumor−Normal Expression Difference

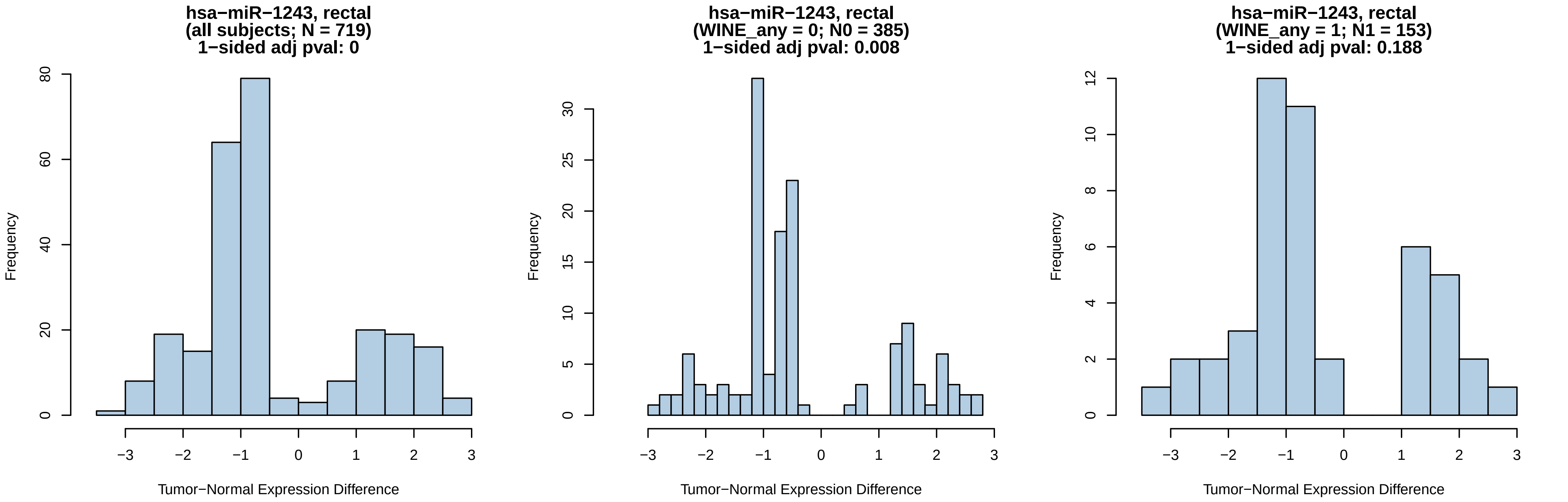
hsa−miR−1243, rectal
(all subjects; N = 719)
1−sided adj pval: 0
hsa−miR−1243, rectal
(WINE_any = 0; N0 = 385)
1−sided adj pval: 0.008
hsa−miR−1243, rectal
(WINE_any = 1; N1 = 153)
1−sided adj pval: 0.188
Frequency
Tumor−Normal Expression Difference

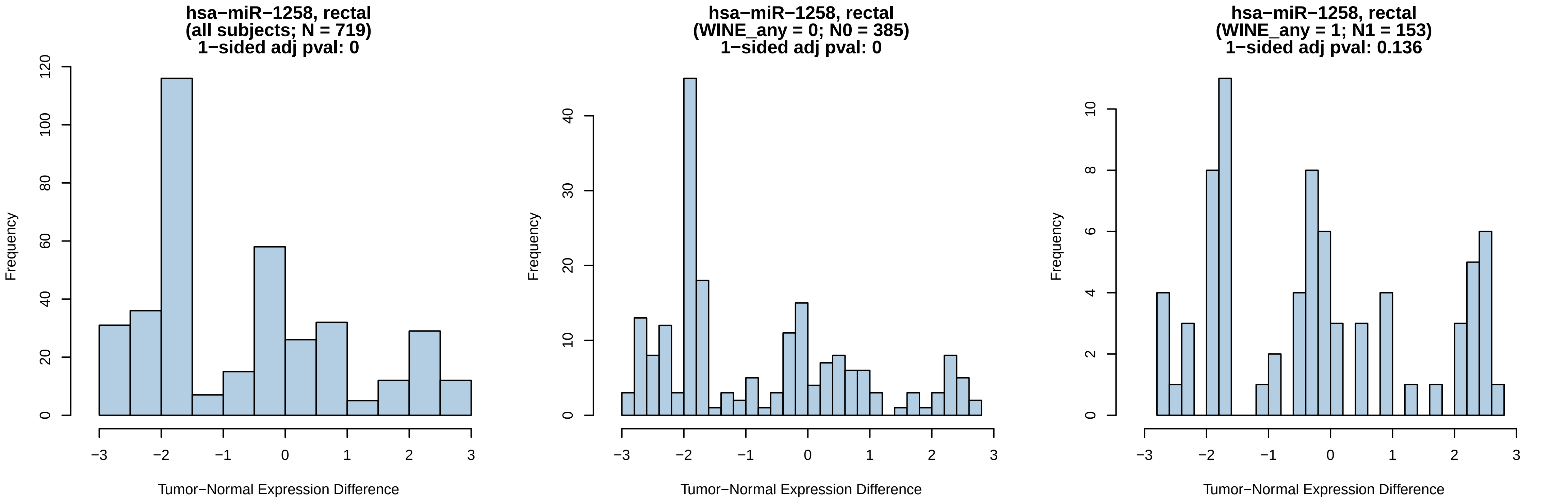

hsa−miR−1258, rectal
(all subjects; N = 719)
1−sided adj pval: 0
Frequency
Tumor−Normal Expression Difference
hsa−miR−1258, rectal
(WINE_any = 0; N0 = 385)
1−sided adj pval: 0
Frequency
Tumor−Normal Expression Difference
hsa−miR−1258, rectal
(WINE_any = 1; N1 = 153)
1−sided adj pval: 0.136
Frequency
Tumor−Normal Expression Difference

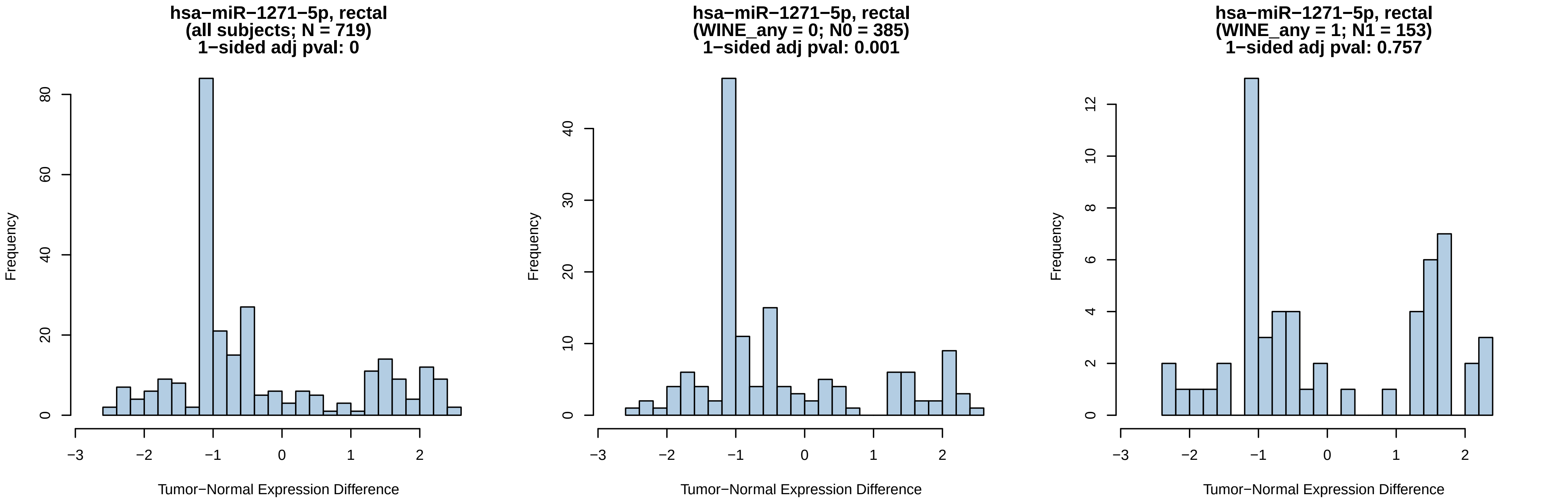

hsa−miR−1271−5p, rectal
(all subjects; N = 719)
1−sided adj pval: 0
hsa−miR−1271−5p, rectal
(WINE_any = 0; N0 = 385)
1−sided adj pval: 0.001
hsa−miR−1271−5p, rectal
(WINE_any = 1; N1 = 153)
1−sided adj pval: 0.757
Frequency
Tumor−Normal Expression Difference

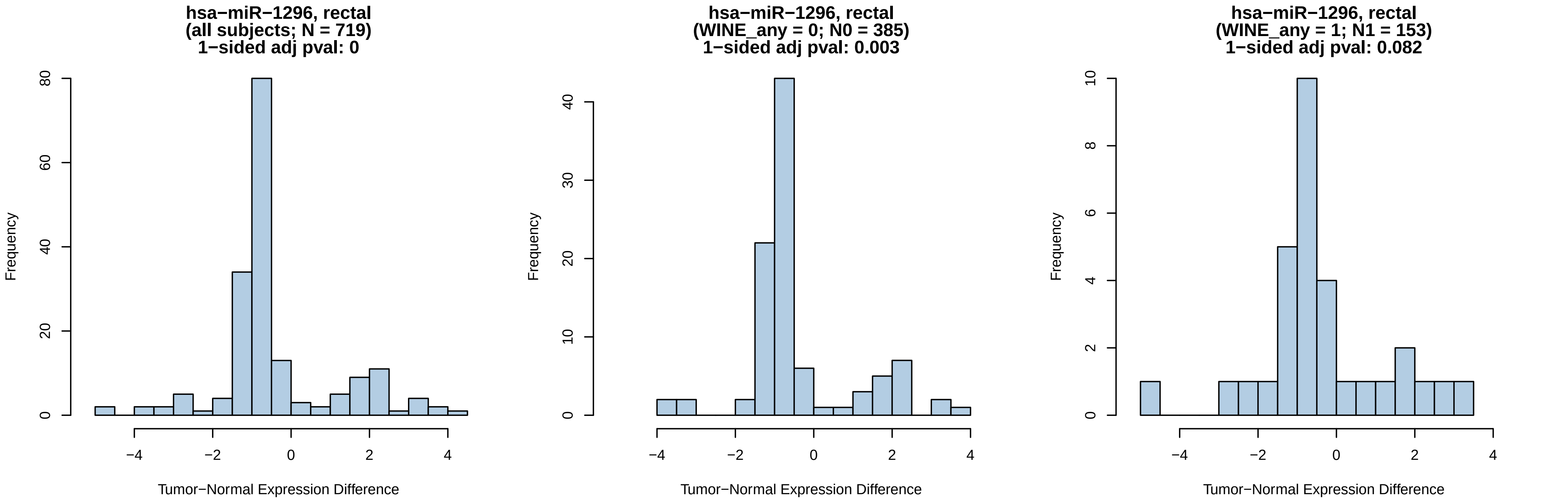
hsa−miR−1296, rectal
(all subjects; N = 719)
1−sided adj pval: 0
hsa−miR−1296, rectal
(WINE_any = 0; N0 = 385)
1−sided adj pval: 0.003
hsa−miR−1296, rectal
(WINE_any = 1; N1 = 153)
1−sided adj pval: 0.082
Frequency
Tumor−Normal Expression Difference

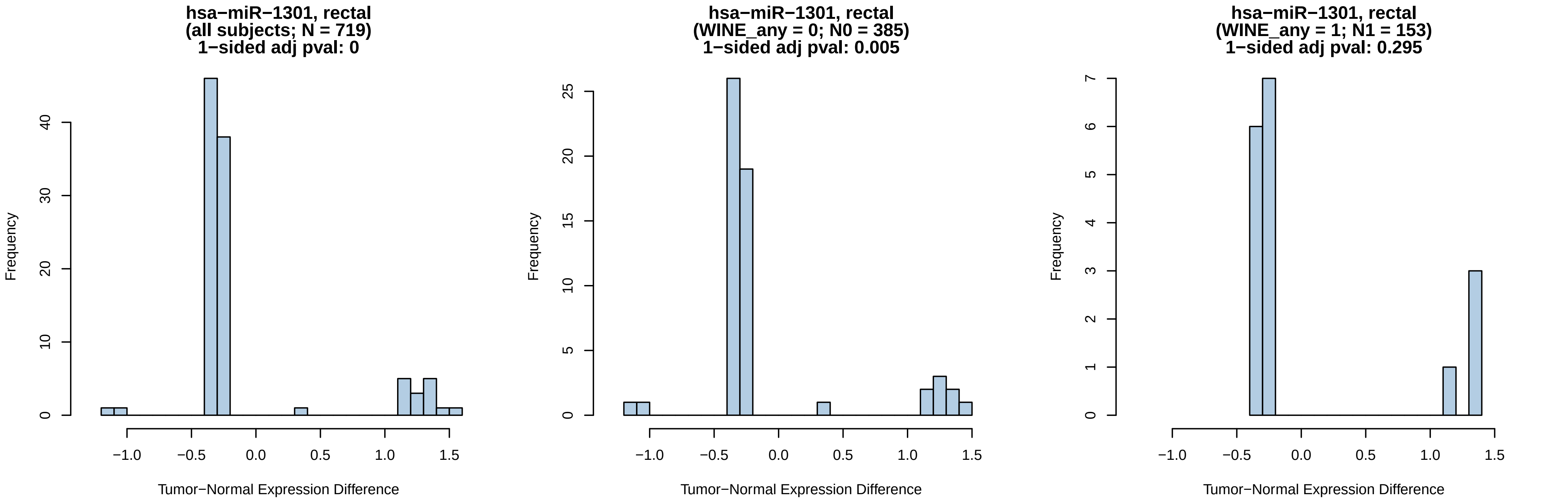

hsa−miR−1301, rectal
(all subjects; N = 719)
1−sided adj pval: 0
Frequency
0
10
20
30
40
−1.0
−0.5
0.0
0.5
1.0
1.5
Tumor−Normal Expression Difference
hsa−miR−1301, rectal
(WINE_any = 0; N0 = 385)
1−sided adj pval: 0.005
Frequency
0
5
10
15
20
25
−1.0
−0.5
0.0
0.5
1.0
1.5
Tumor−Normal Expression Difference
hsa−miR−1301, rectal
(WINE_any = 1; N1 = 153)
1−sided adj pval: 0.295
Frequency
0
1
2
3
4
5
6
7
−1.0
−0.5
0.0
0.5
1.0
1.5
Tumor−Normal Expression Difference

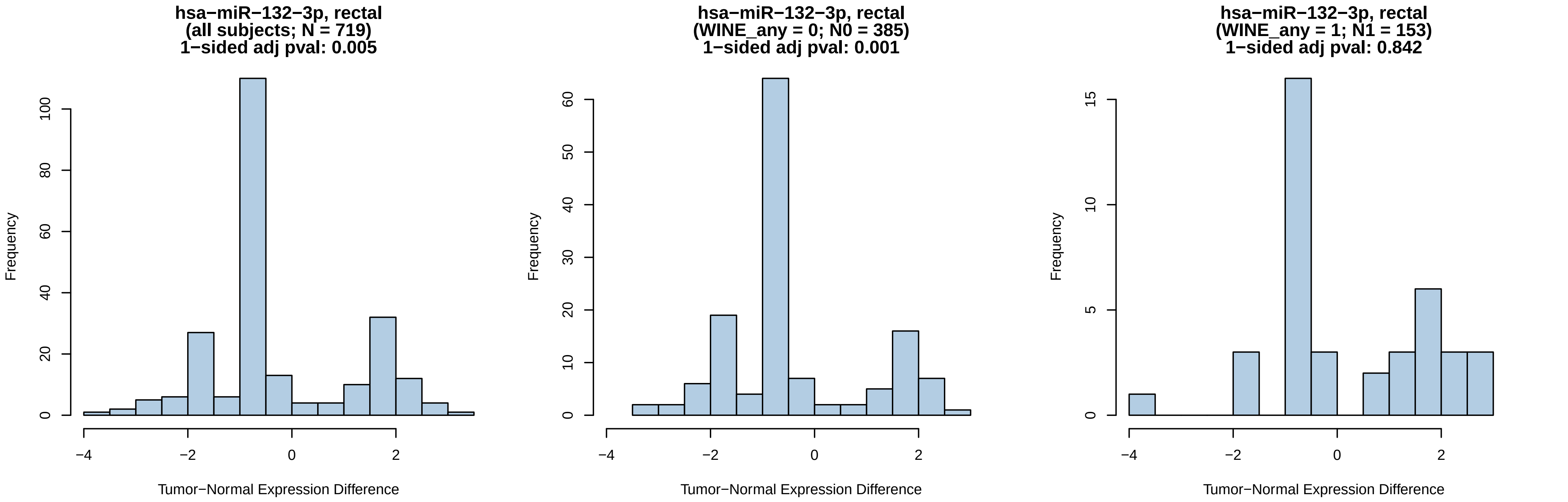

hsa−miR−132−3p, rectal
(all subjects; N = 719)
1−sided adj pval: 0.005
Frequency
Tumor−Normal Expression Difference
hsa−miR−132−3p, rectal
(WINE_any = 0; N0 = 385)
1−sided adj pval: 0.001
Frequency
Tumor−Normal Expression Difference
hsa−miR−132−3p, rectal
(WINE_any = 1; N1 = 153)
1−sided adj pval: 0.842
Frequency
Tumor−Normal Expression Difference

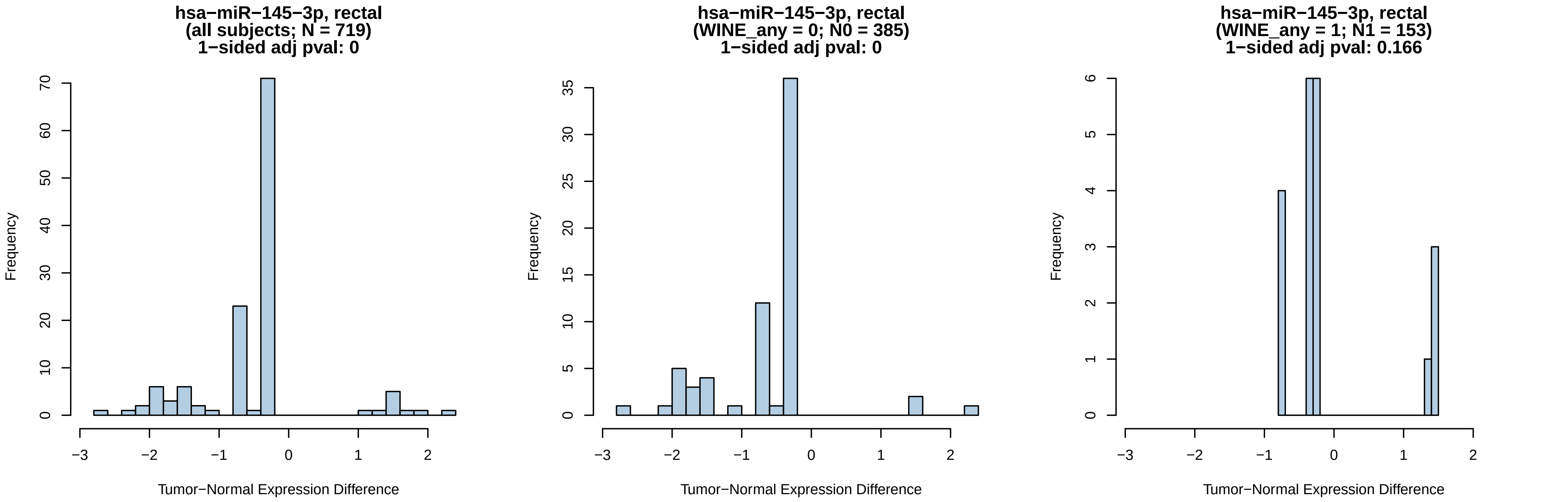
hsa−miR−145−3p, rectal
(all subjects; N = 719)
1−sided adj pval: 0
Frequency
Tumor−Normal Expression Difference
hsa−miR−145−3p, rectal
(WINE_any = 0; N0 = 385)
1−sided adj pval: 0
Frequency
Tumor−Normal Expression Difference
hsa−miR−145−3p, rectal
(WINE_any = 1; N1 = 153)
1−sided adj pval: 0.166
Frequency
Tumor−Normal Expression Difference

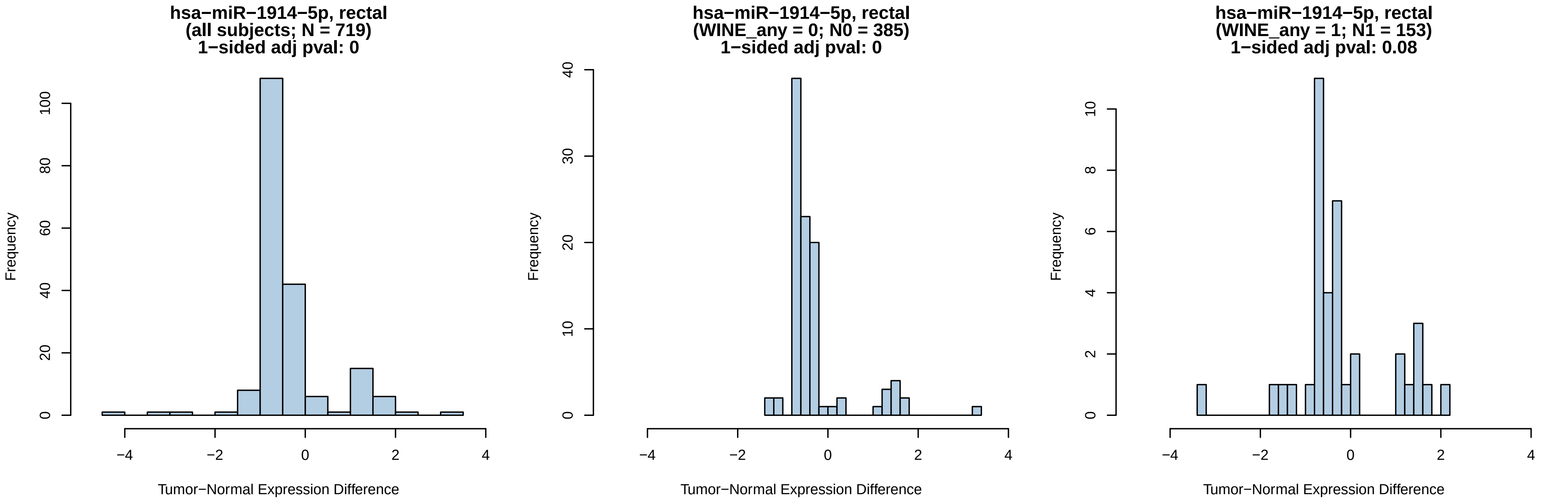
hsa−miR−1914−5p, rectal
(all subjects; N = 719)
1−sided adj pval: 0
hsa−miR−1914−5p, rectal
(WINE_any = 0; N0 = 385)
1−sided adj pval: 0
hsa−miR−1914−5p, rectal
(WINE_any = 1; N1 = 153)
1−sided adj pval: 0.08
Frequency
Tumor−Normal Expression Difference

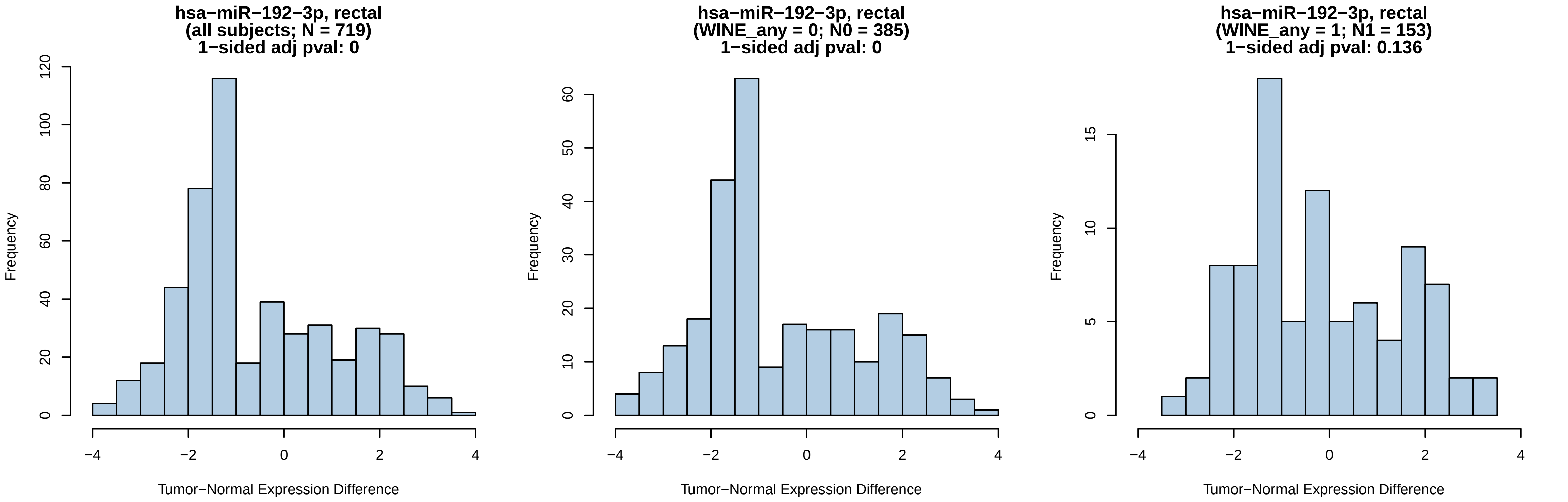
hsa−miR−192−3p, rectal
(all subjects; N = 719)
1−sided adj pval: 0
hsa−miR−192−3p, rectal
(WINE_any = 0; N0 = 385)
1−sided adj pval: 0
hsa−miR−192−3p, rectal
(WINE_any = 1; N1 = 153)
1−sided adj pval: 0.136
Frequency
Tumor−Normal Expression Difference
0
20
40
60
80
100
120
10
30
50
5
15
−4
−2
2
4

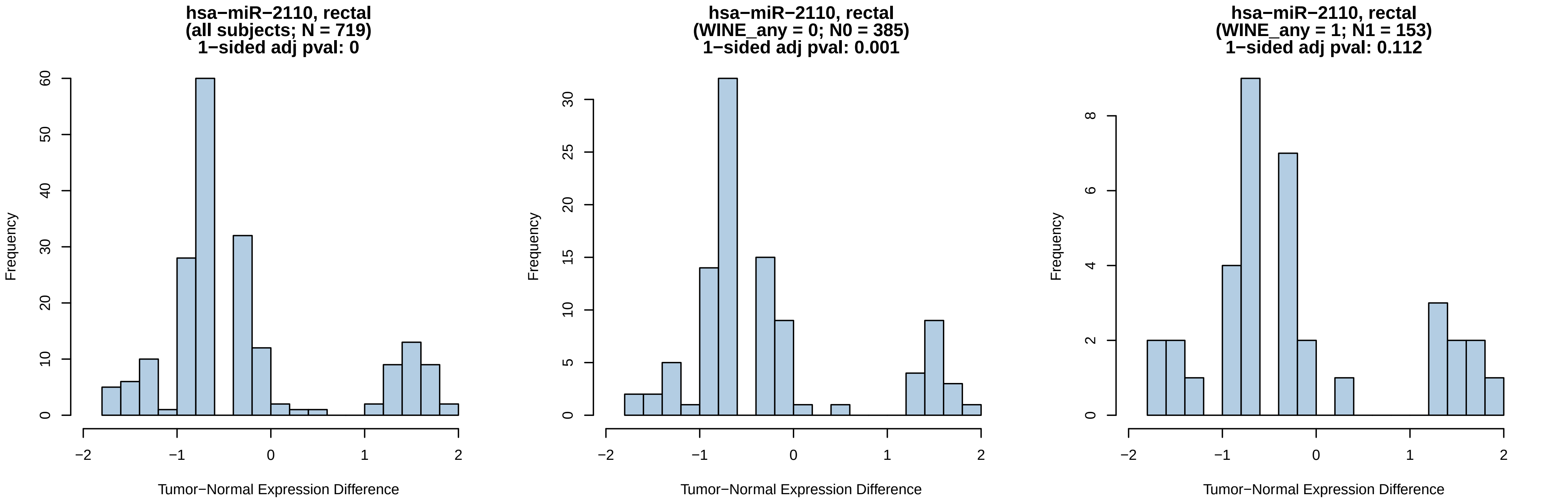

hsa−miR−2110, rectal
(all subjects; N = 719)
1−sided adj pval: 0
Frequency
0
10
20
30
40
50
60
−2
−1
0
1
2
Tumor−Normal Expression Difference
hsa−miR−2110, rectal
(WINE_any = 0; N0 = 385)
1−sided adj pval: 0.001
Frequency
0
5
10
15
20
25
30
−2
−1
0
1
2
Tumor−Normal Expression Difference
hsa−miR−2110, rectal
(WINE_any = 1; N1 = 153)
1−sided adj pval: 0.112
Frequency
0
2
4
6
8
−2
−1
0
1
2
Tumor−Normal Expression Difference

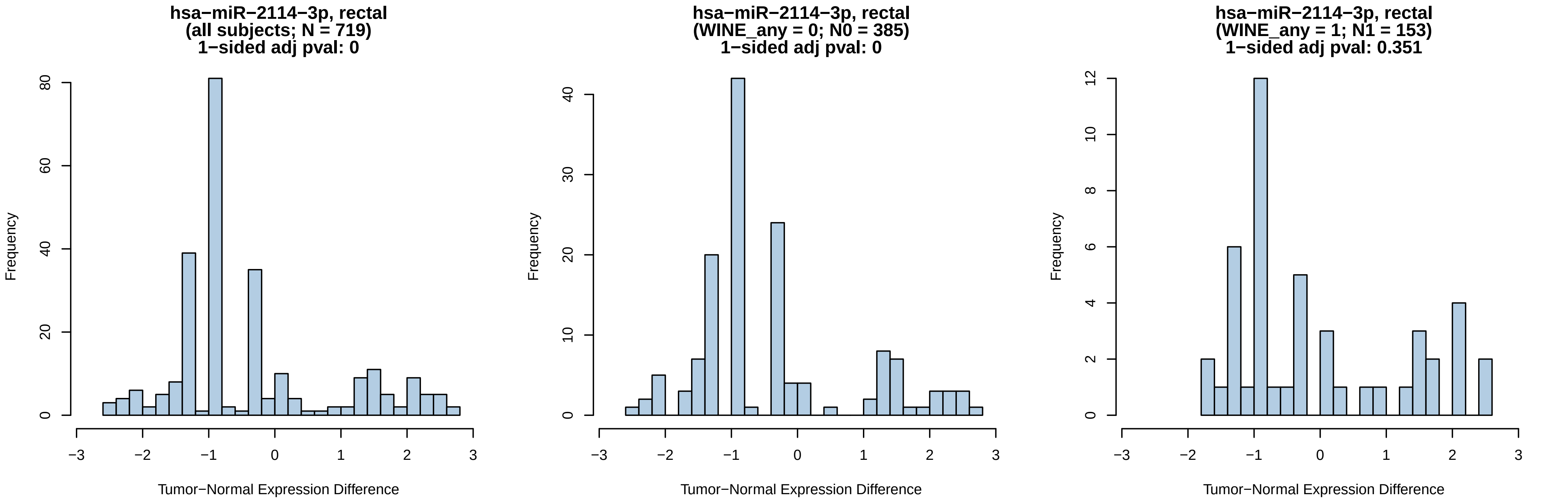
hsa−miR−2114−3p, rectal
(all subjects; N = 719)
1−sided adj pval: 0
Frequency
Tumor−Normal Expression Difference
hsa−miR−2114−3p, rectal
(WINE_any = 0; N0 = 385)
1−sided adj pval: 0
Frequency
Tumor−Normal Expression Difference
hsa−miR−2114−3p, rectal
(WINE_any = 1; N1 = 153)
1−sided adj pval: 0.351
Frequency
Tumor−Normal Expression Difference

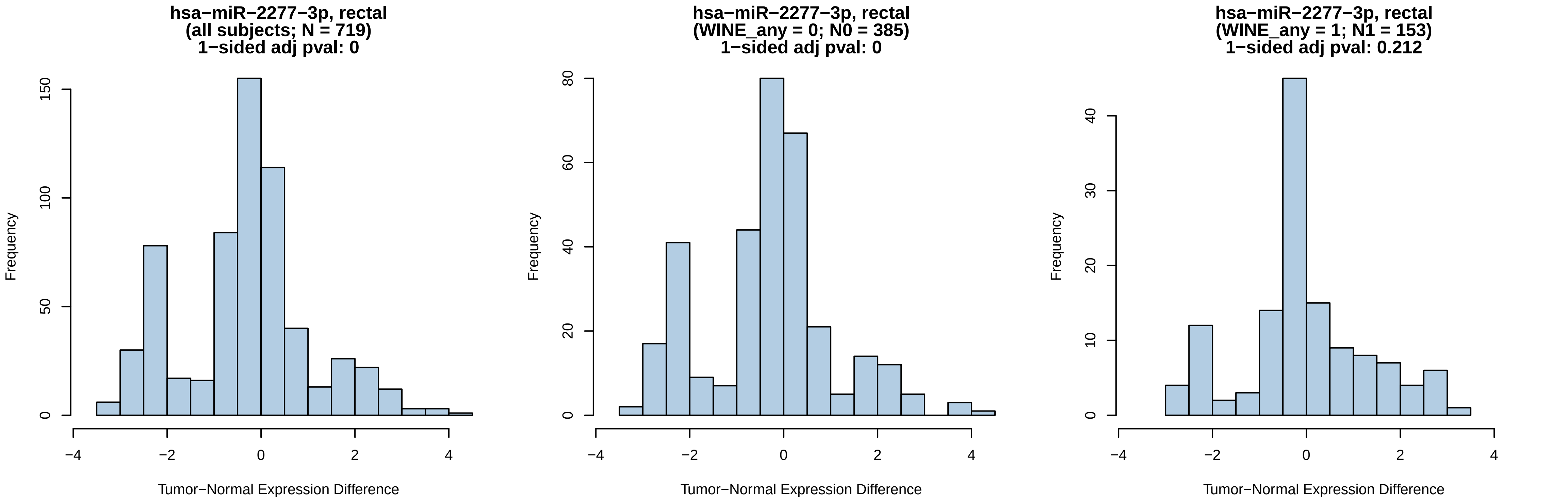

hsa−miR−2277−3p, rectal
(all subjects; N = 719)
1−sided adj pval: 0
Frequency
Tumor−Normal Expression Difference
hsa−miR−2277−3p, rectal
(WINE_any = 0; N0 = 385)
1−sided adj pval: 0
Frequency
Tumor−Normal Expression Difference
hsa−miR−2277−3p, rectal
(WINE_any = 1; N1 = 153)
1−sided adj pval: 0.212
Frequency
Tumor−Normal Expression Difference

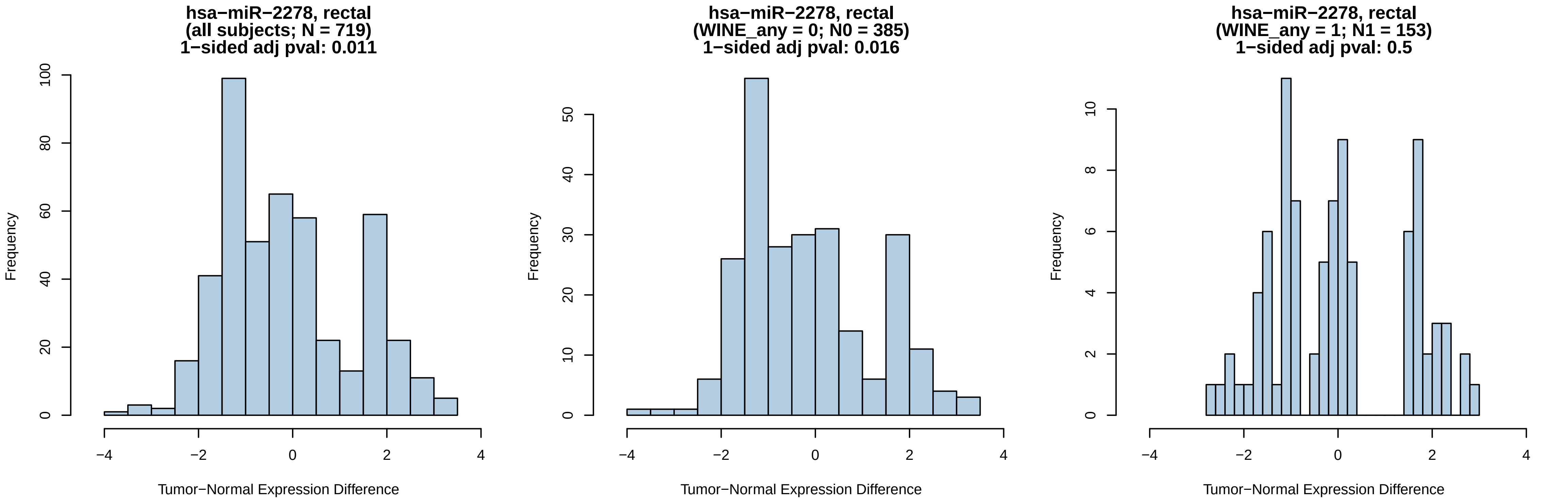
hsa−miR−2278, rectal
(all subjects; N = 719)
1−sided adj pval: 0.011
Frequency
Tumor−Normal Expression Difference
hsa−miR−2278, rectal
(WINE_any = 0; N0 = 385)
1−sided adj pval: 0.016
Frequency
Tumor−Normal Expression Difference
hsa−miR−2278, rectal
(WINE_any = 1; N1 = 153)
1−sided adj pval: 0.5
Frequency
Tumor−Normal Expression Difference

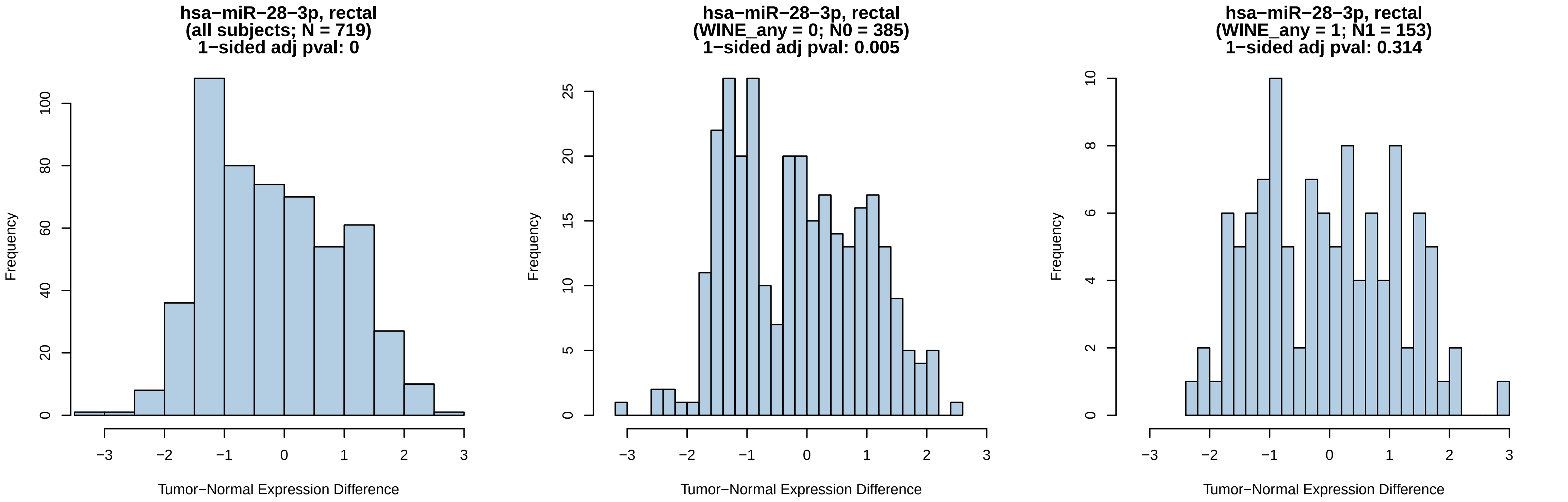

hsa−miR−28−3p, rectal
(all subjects; N = 719)
1−sided adj pval: 0
Frequency
Tumor−Normal Expression Difference
hsa−miR−28−3p, rectal
(WINE_any = 0; N0 = 385)
1−sided adj pval: 0.005
Frequency
Tumor−Normal Expression Difference
hsa−miR−28−3p, rectal
(WINE_any = 1; N1 = 153)
1−sided adj pval: 0.314
Frequency
Tumor−Normal Expression Difference

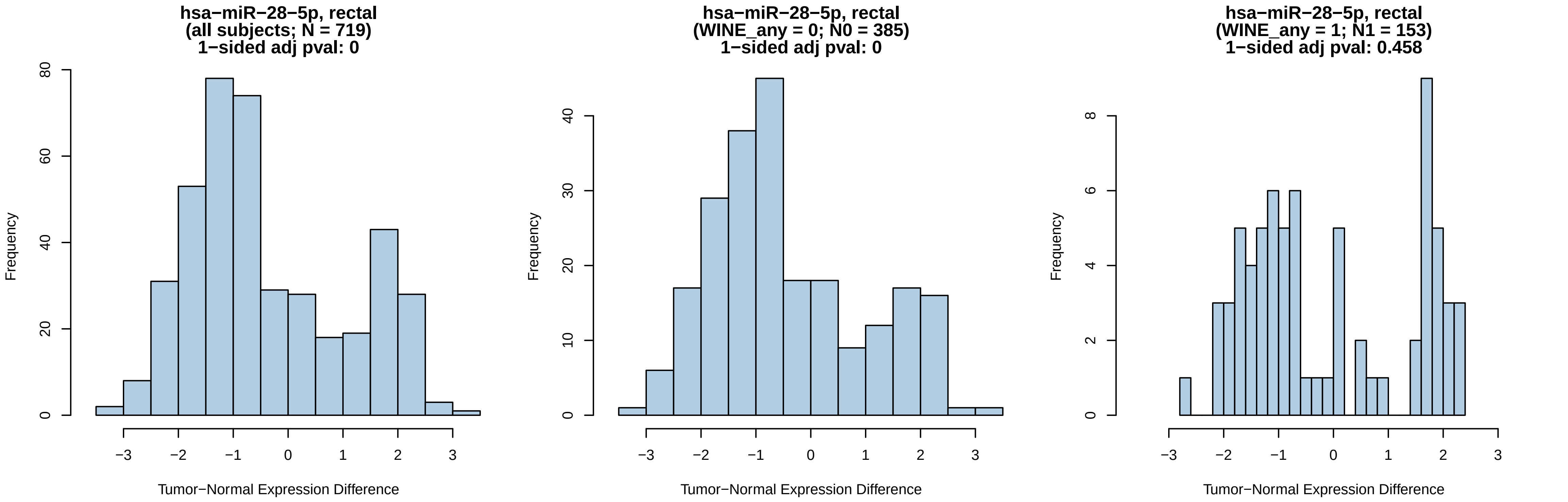
hsa−miR−28−5p, rectal
(all subjects; N = 719)
1−sided adj pval: 0
Frequency
0
20
40
60
80
−3
−2
−1
0
1
2
3
Tumor−Normal Expression Difference
hsa−miR−28−5p, rectal
(WINE_any = 0; N0 = 385)
1−sided adj pval: 0
Frequency
0
10
20
30
40
−3
−2
−1
0
1
2
3
Tumor−Normal Expression Difference
hsa−miR−28−5p, rectal
(WINE_any = 1; N1 = 153)
1−sided adj pval: 0.458
Frequency
0
2
4
6
8
−3
−2
−1
0
1
2
3
Tumor−Normal Expression Difference

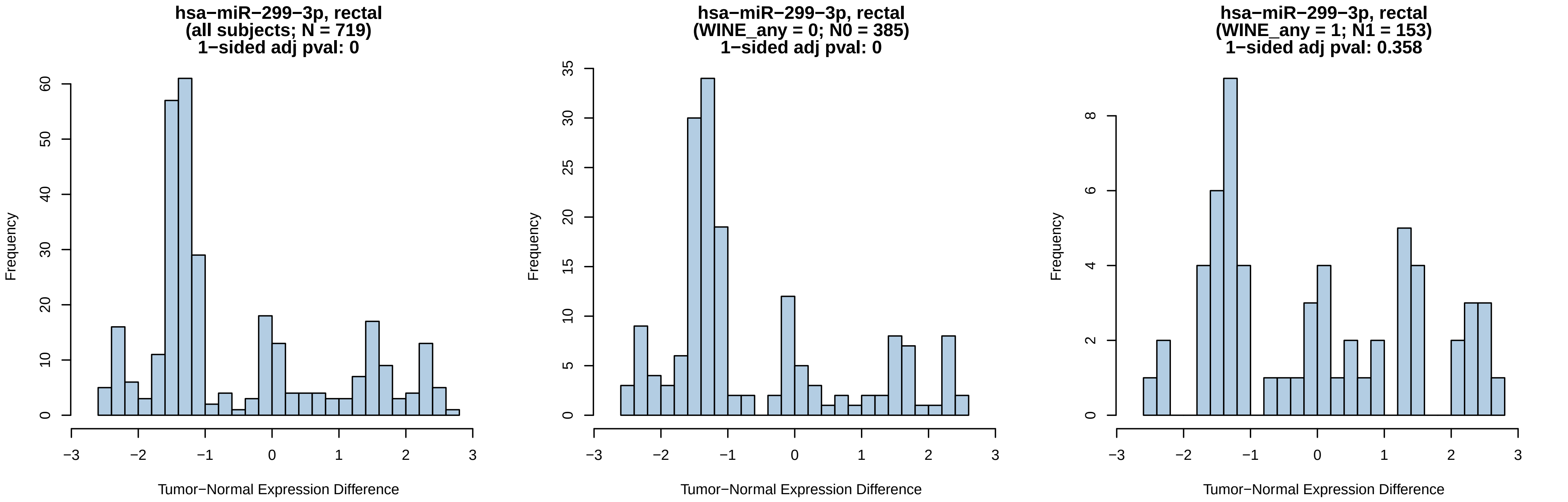
hsa−miR−299−3p, rectal
(all subjects; N = 719)
1−sided adj pval: 0
Frequency
Tumor−Normal Expression Difference
hsa−miR−299−3p, rectal
(WINE_any = 0; N0 = 385)
1−sided adj pval: 0
Frequency
Tumor−Normal Expression Difference
hsa−miR−299−3p, rectal
(WINE_any = 1; N1 = 153)
1−sided adj pval: 0.358
Frequency
Tumor−Normal Expression Difference

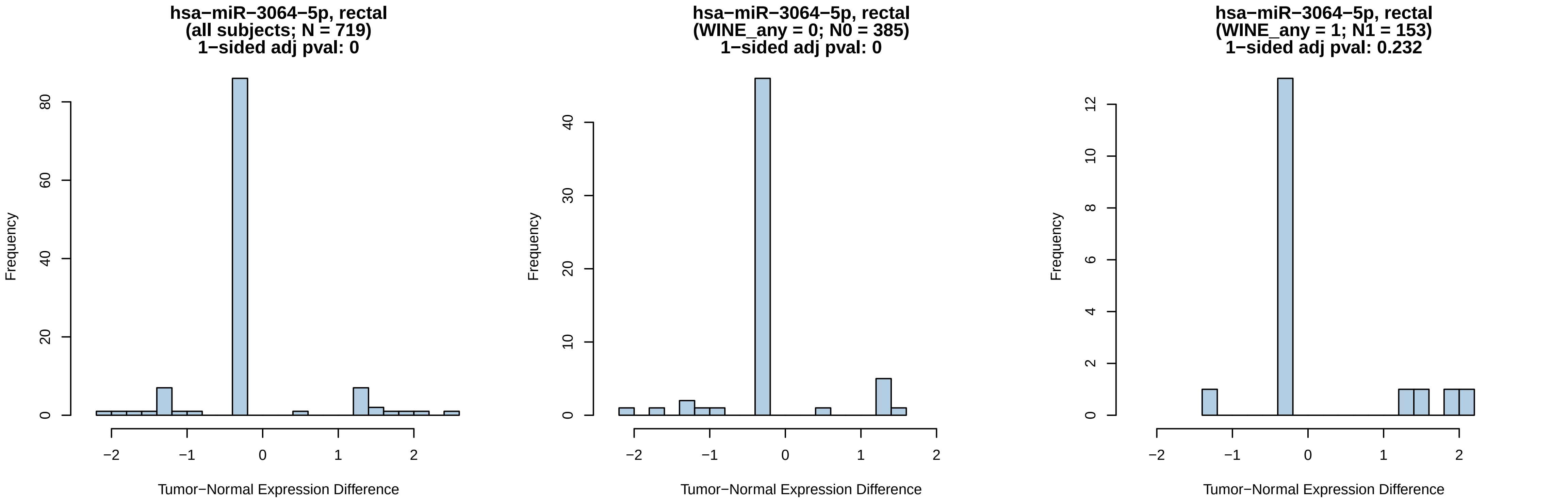

hsa−miR−3064−5p, rectal
(all subjects; N = 719)
1−sided adj pval: 0
hsa−miR−3064−5p, rectal
(WINE_any = 0; N0 = 385)
1−sided adj pval: 0
hsa−miR−3064−5p, rectal
(WINE_any = 1; N1 = 153)
1−sided adj pval: 0.232
Frequency
Tumor−Normal Expression Difference
0
20
40
60
80
10
30
2
4
6
8
12
−2
−1
1

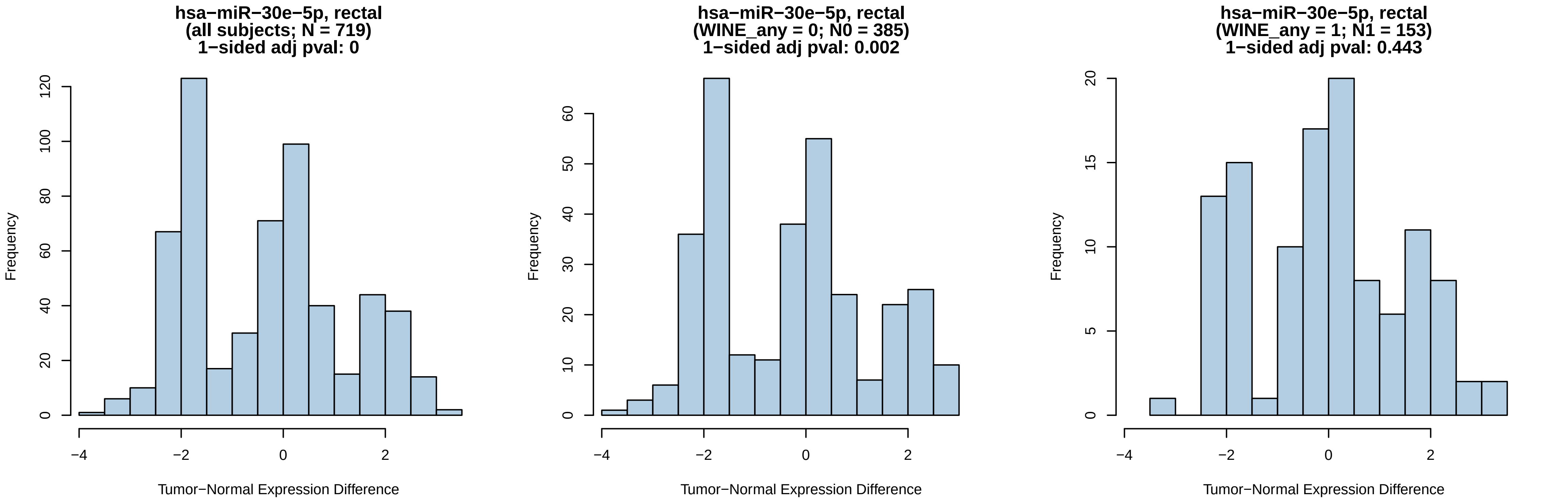
hsa−miR−30e−5p, rectal
(all subjects; N = 719)
1−sided adj pval: 0
Frequency
Tumor−Normal Expression Difference
hsa−miR−30e−5p, rectal
(WINE_any = 0; N0 = 385)
1−sided adj pval: 0.002
Frequency
Tumor−Normal Expression Difference
hsa−miR−30e−5p, rectal
(WINE_any = 1; N1 = 153)
1−sided adj pval: 0.443
Frequency
Tumor−Normal Expression Difference

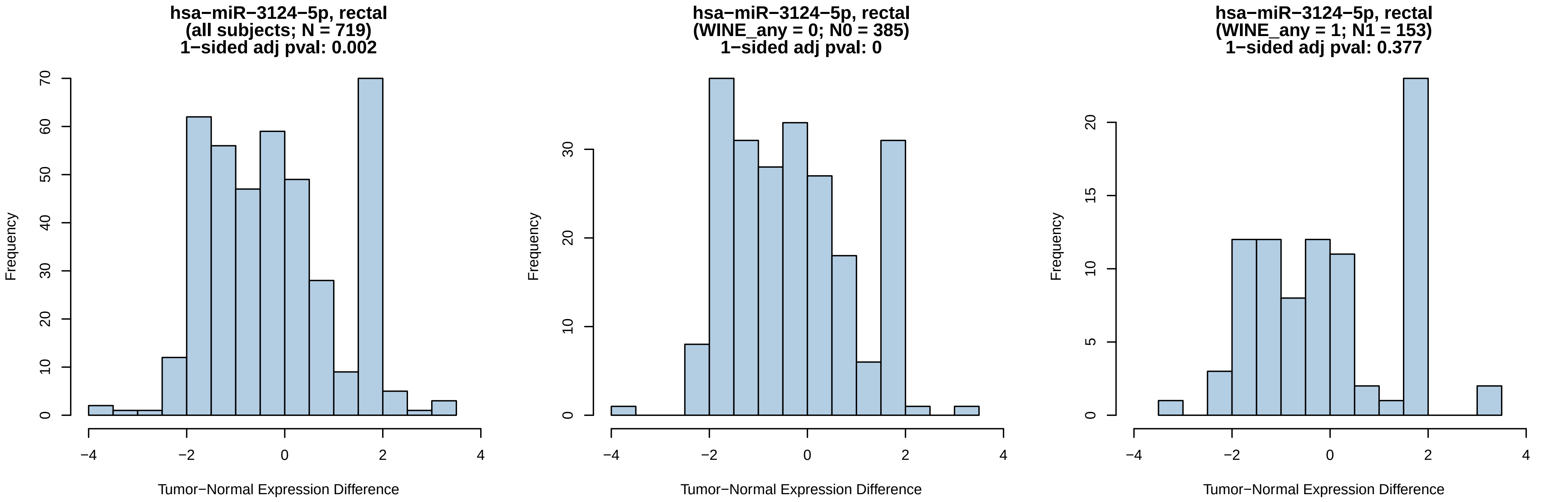
hsa−miR−3124−5p, rectal
(all subjects; N = 719)
1−sided adj pval: 0.002
Frequency
Tumor−Normal Expression Difference
hsa−miR−3124−5p, rectal
(WINE_any = 0; N0 = 385)
1−sided adj pval: 0
Frequency
Tumor−Normal Expression Difference
hsa−miR−3124−5p, rectal
(WINE_any = 1; N1 = 153)
1−sided adj pval: 0.377
Frequency
Tumor−Normal Expression Difference

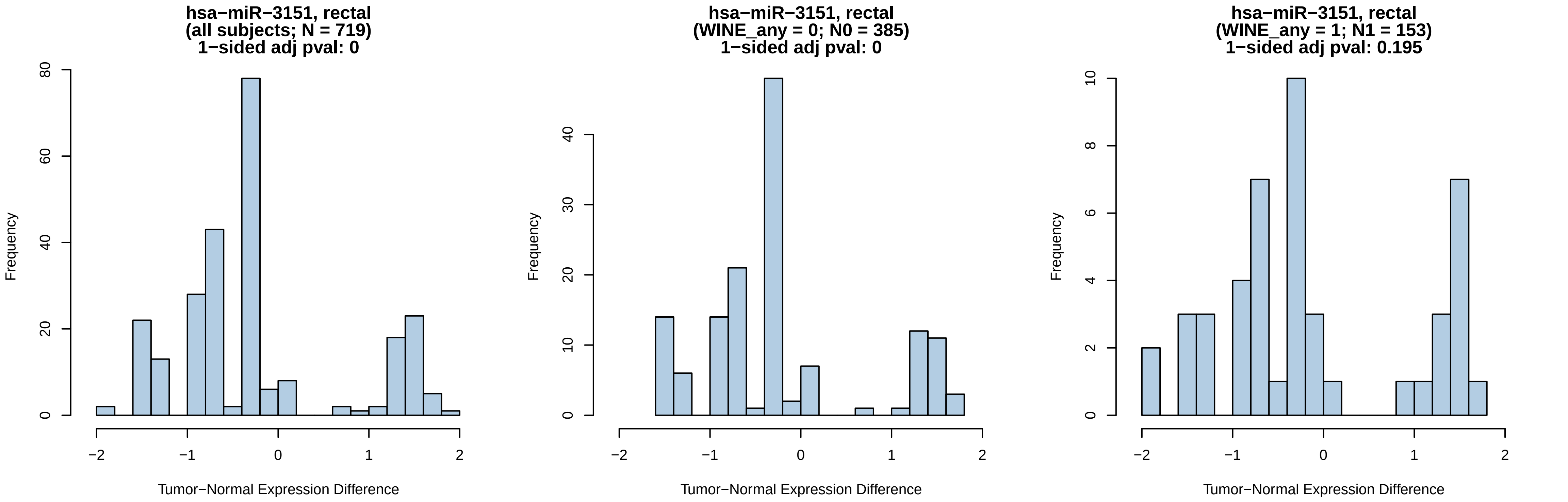
hsa−miR−3151, rectal
(all subjects; N = 719)
1−sided adj pval: 0
Frequency
Tumor−Normal Expression Difference
hsa−miR−3151, rectal
(WINE_any = 0; N0 = 385)
1−sided adj pval: 0
Frequency
Tumor−Normal Expression Difference
hsa−miR−3151, rectal
(WINE_any = 1; N1 = 153)
1−sided adj pval: 0.195
Frequency
Tumor−Normal Expression Difference

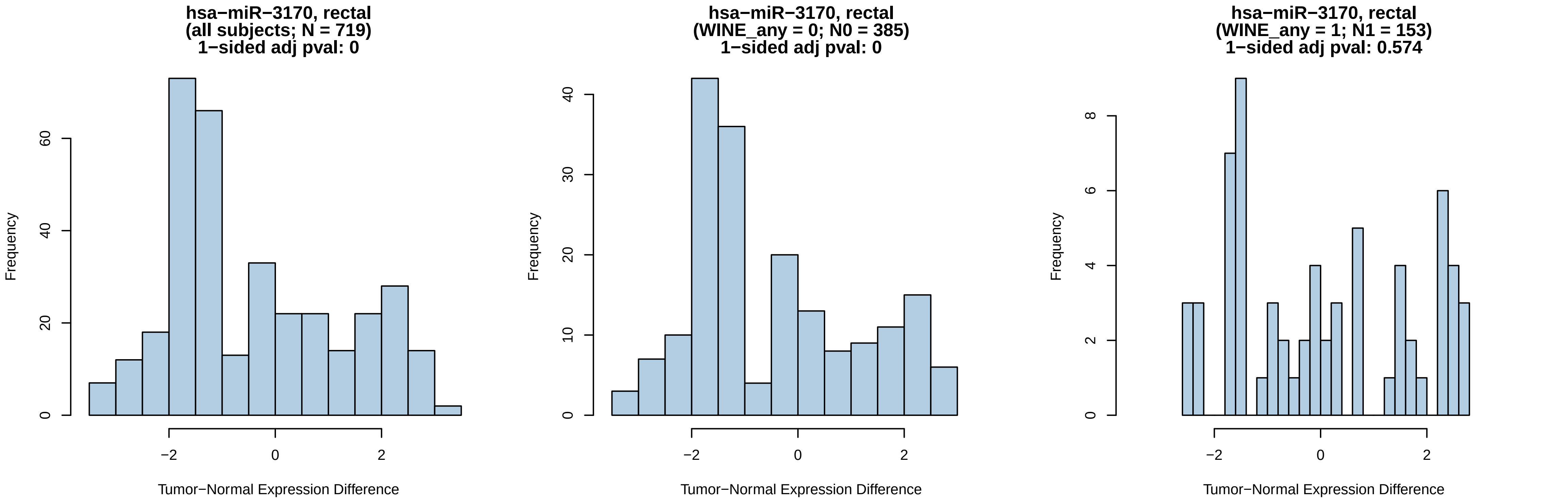

hsa−miR−3170, rectal
(all subjects; N = 719)
1−sided adj pval: 0
Frequency
Tumor−Normal Expression Difference
hsa−miR−3170, rectal
(WINE_any = 0; N0 = 385)
1−sided adj pval: 0
Frequency
Tumor−Normal Expression Difference
hsa−miR−3170, rectal
(WINE_any = 1; N1 = 153)
1−sided adj pval: 0.574
Frequency
Tumor−Normal Expression Difference

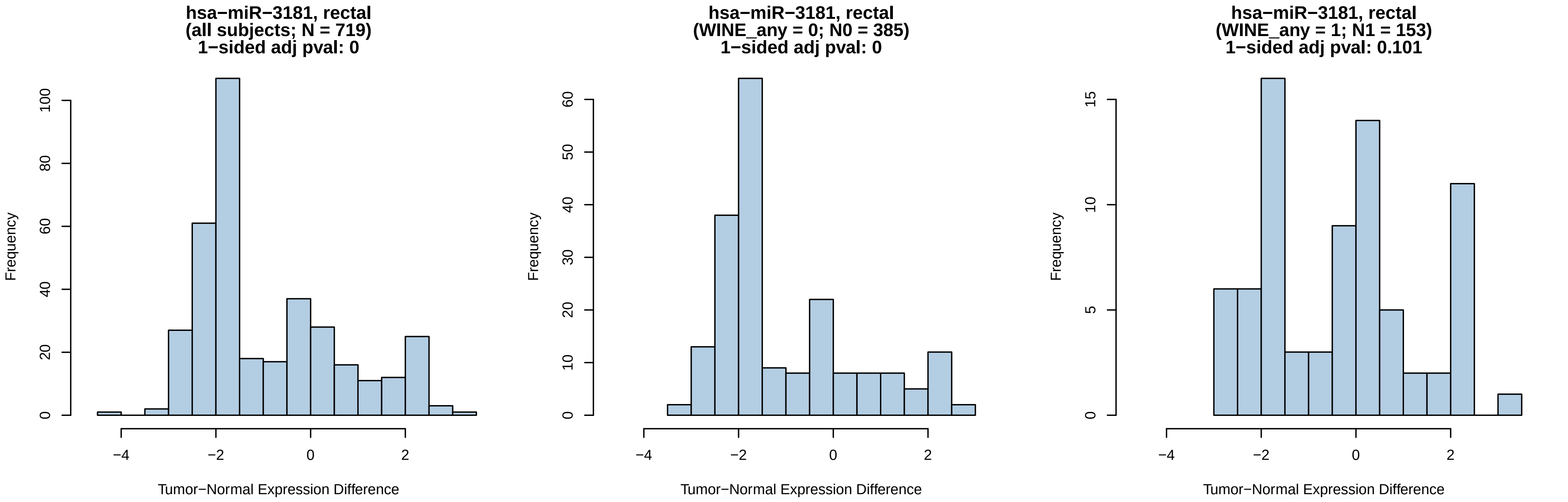
hsa−miR−3181, rectal
(all subjects; N = 719)
1−sided adj pval: 0
Frequency
Tumor−Normal Expression Difference
hsa−miR−3181, rectal
(WINE_any = 0; N0 = 385)
1−sided adj pval: 0
Frequency
Tumor−Normal Expression Difference
hsa−miR−3181, rectal
(WINE_any = 1; N1 = 153)
1−sided adj pval: 0.101
Frequency
Tumor−Normal Expression Difference

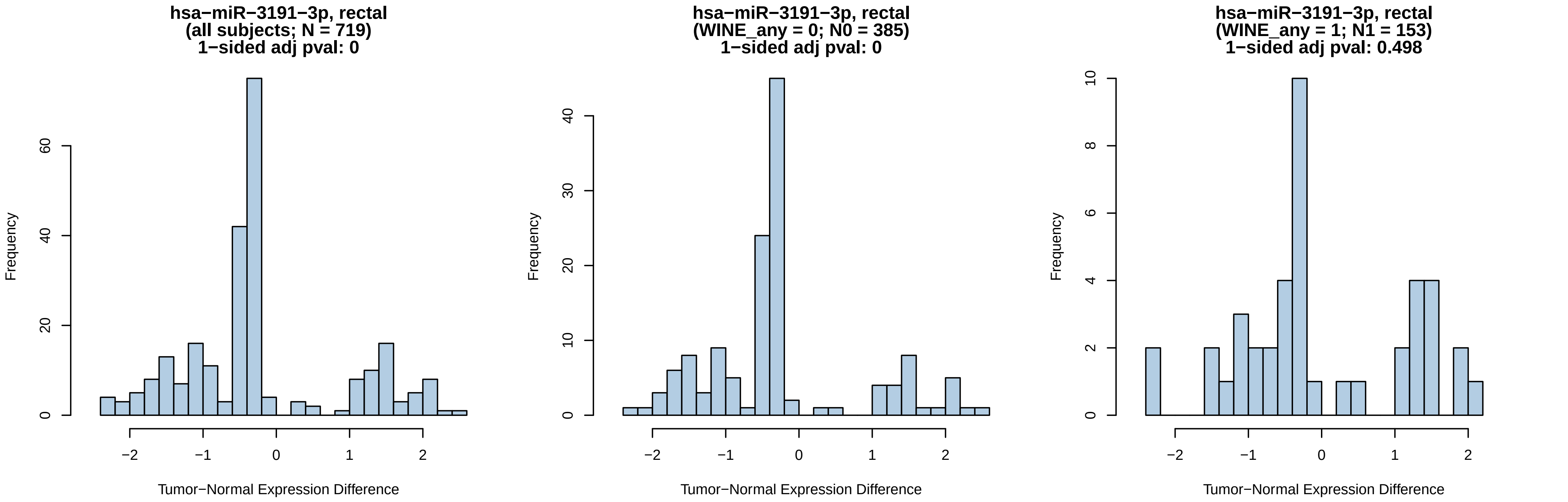

hsa−miR−3191−3p, rectal
(all subjects; N = 719)
1−sided adj pval: 0
Frequency
Tumor−Normal Expression Difference
hsa−miR−3191−3p, rectal
(WINE_any = 0; N0 = 385)
1−sided adj pval: 0
Frequency
Tumor−Normal Expression Difference
hsa−miR−3191−3p, rectal
(WINE_any = 1; N1 = 153)
1−sided adj pval: 0.498
Frequency
Tumor−Normal Expression Difference

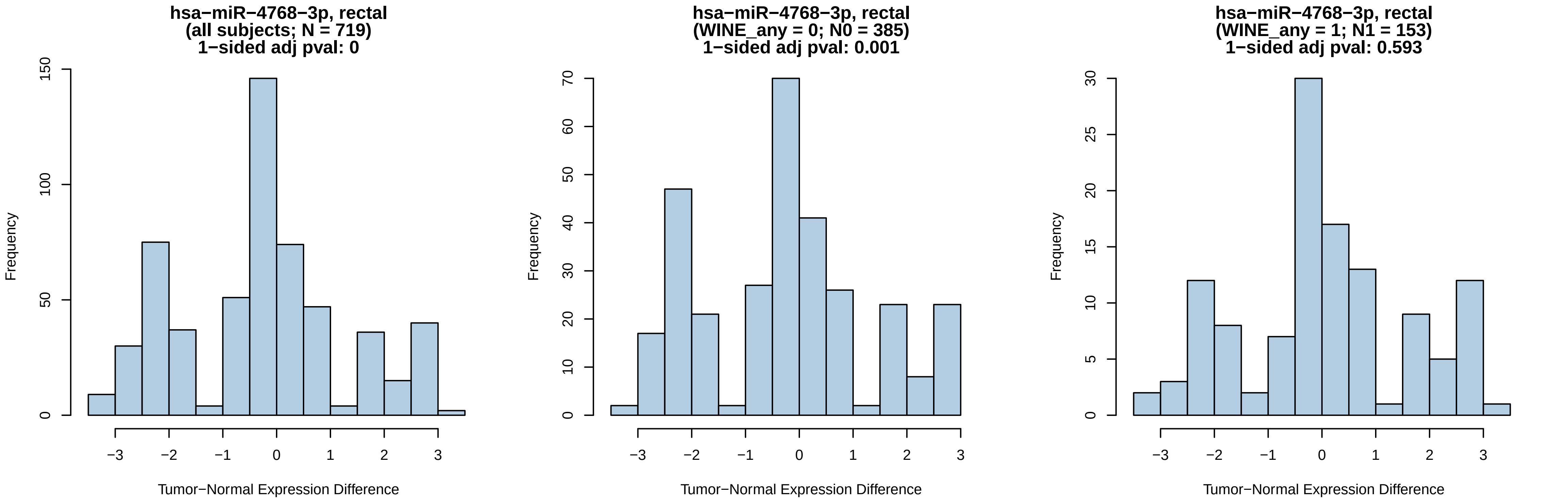

hsa−miR−4768−3p, rectal
(all subjects; N = 719)
1−sided adj pval: 0
hsa−miR−4768−3p, rectal
(WINE_any = 0; N0 = 385)
1−sided adj pval: 0.001
hsa−miR−4768−3p, rectal
(WINE_any = 1; N1 = 153)
1−sided adj pval: 0.593
Frequency
Tumor−Normal Expression Difference

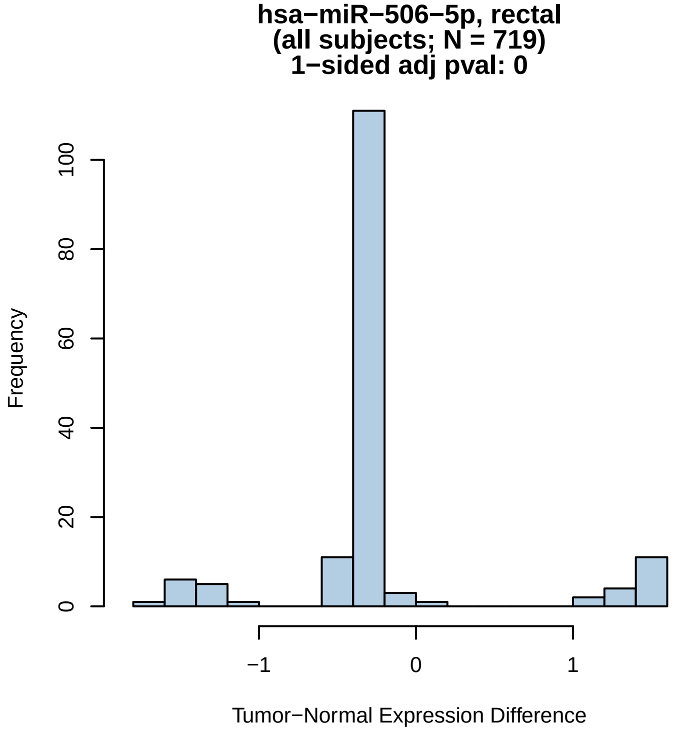

hsa−miR−506−5p, rectal
(all subjects; N = 719)
1−sided adj pval: 0
Frequency
0
20
40
60
80
100
−1
0
1
Tumor−Normal Expression Difference


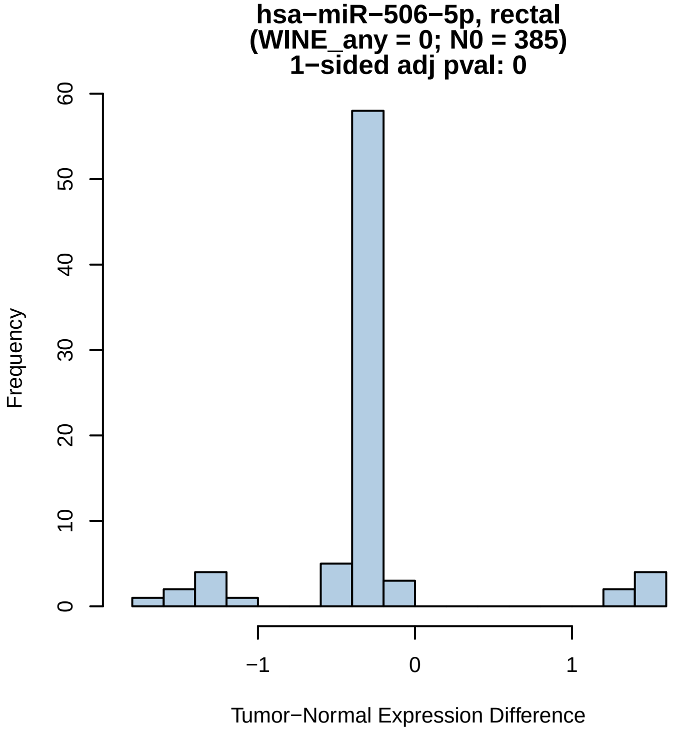

hsa−miR−506−5p, rectal
(WINE_any = 0; N0 = 385)
1−sided adj pval: 0
Frequency
0
10
20
30
40
50
60
−1
0
1
Tumor−Normal Expression Difference


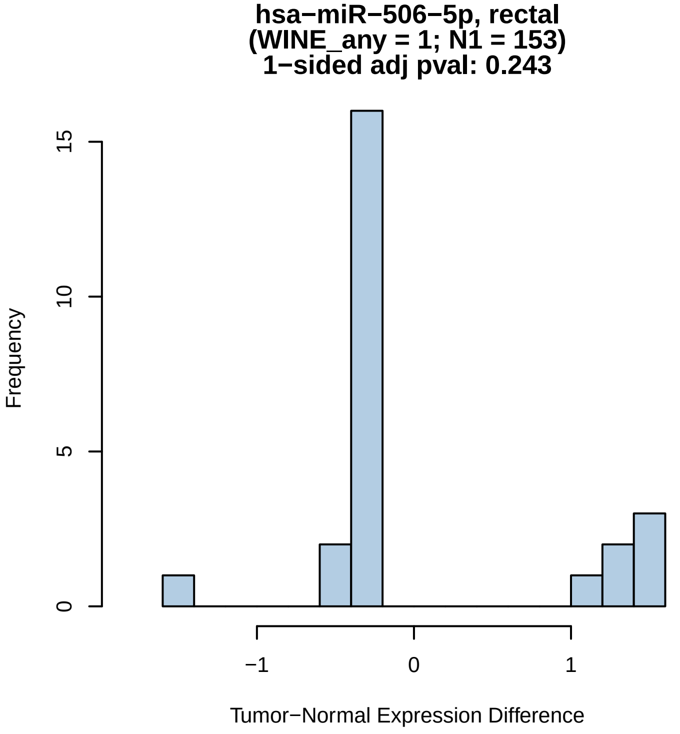

hsa−miR−506−5p, rectal
(WINE_any = 1; N1 = 153)
1−sided adj pval: 0.243
Frequency
0
5
10
15
−1
0
1
Tumor−Normal Expression Difference

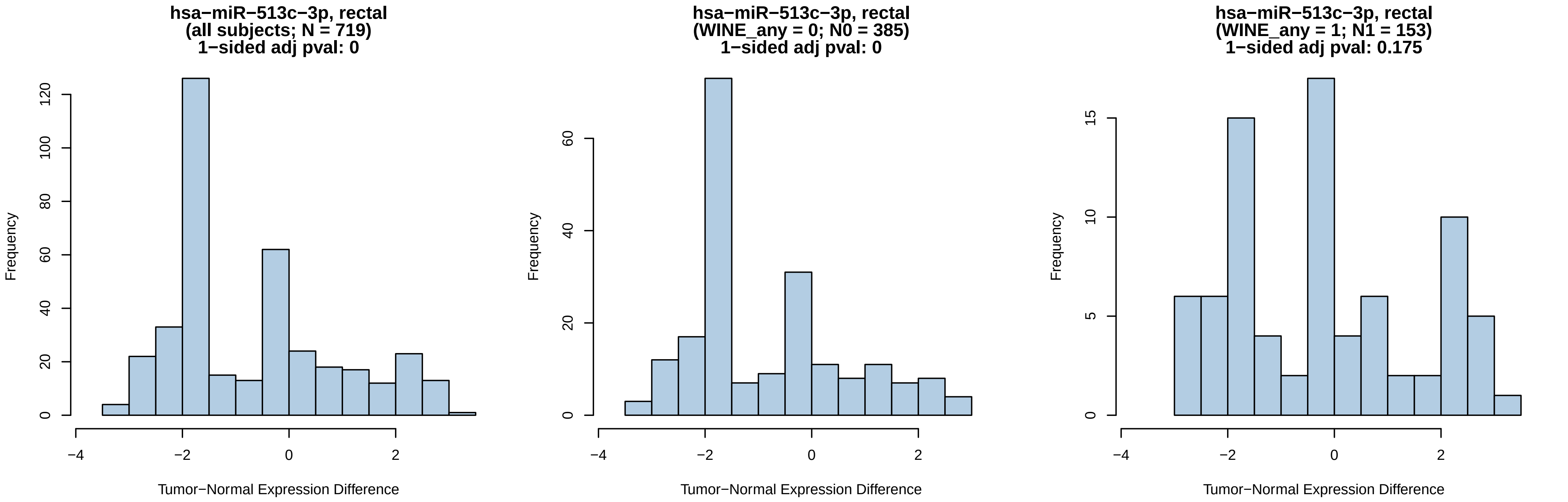

hsa−miR−513c−3p, rectal
(all subjects; N = 719)
1−sided adj pval: 0
Frequency
Tumor−Normal Expression Difference
hsa−miR−513c−3p, rectal
(WINE_any = 0; N0 = 385)
1−sided adj pval: 0
Frequency
Tumor−Normal Expression Difference
hsa−miR−513c−3p, rectal
(WINE_any = 1; N1 = 153)
1−sided adj pval: 0.175
Frequency
Tumor−Normal Expression Difference

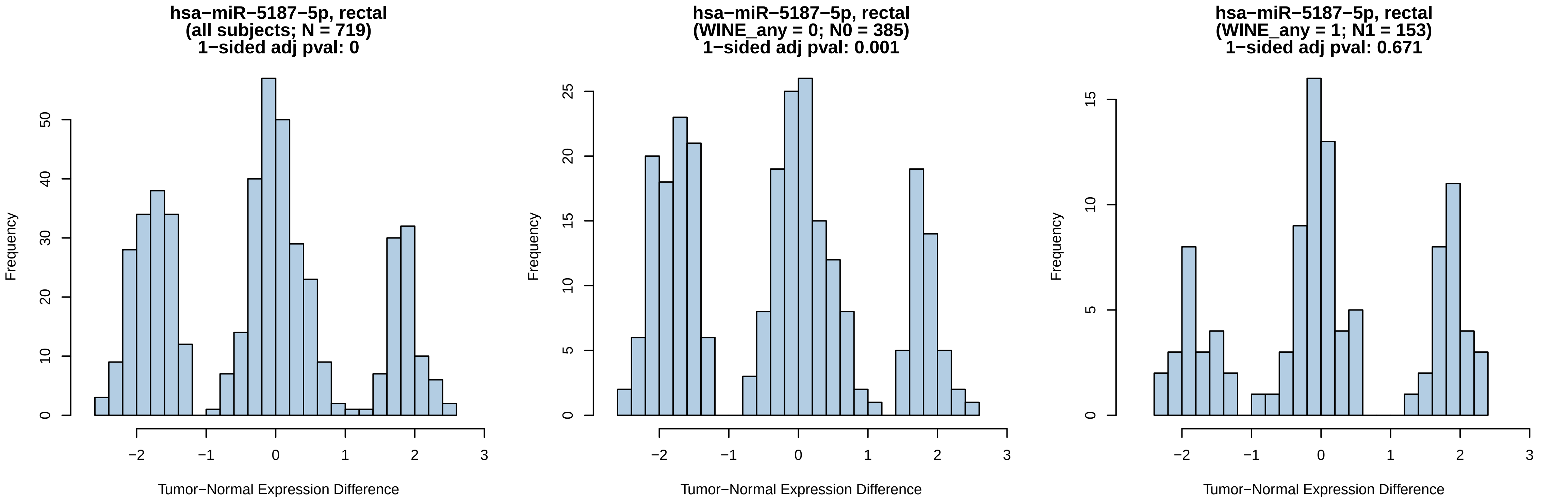
hsa−miR−5187−5p, rectal
(all subjects; N = 719)
1−sided adj pval: 0
Frequency
Tumor−Normal Expression Difference
hsa−miR−5187−5p, rectal
(WINE_any = 0; N0 = 385)
1−sided adj pval: 0.001
Frequency
Tumor−Normal Expression Difference
hsa−miR−5187−5p, rectal
(WINE_any = 1; N1 = 153)
1−sided adj pval: 0.671
Frequency
Tumor−Normal Expression Difference

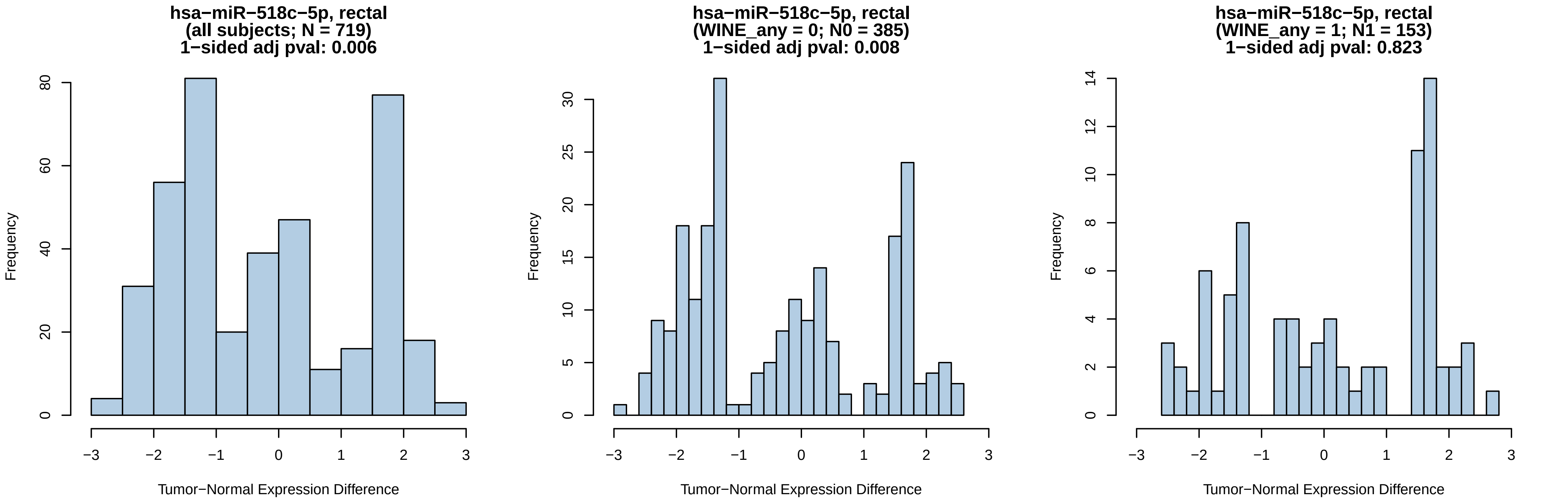
hsa−miR−518c−5p, rectal
(all subjects; N = 719)
1−sided adj pval: 0.006
Frequency
Tumor−Normal Expression Difference
hsa−miR−518c−5p, rectal
(WINE_any = 0; N0 = 385)
1−sided adj pval: 0.008
Frequency
Tumor−Normal Expression Difference
hsa−miR−518c−5p, rectal
(WINE_any = 1; N1 = 153)
1−sided adj pval: 0.823
Frequency
Tumor−Normal Expression Difference

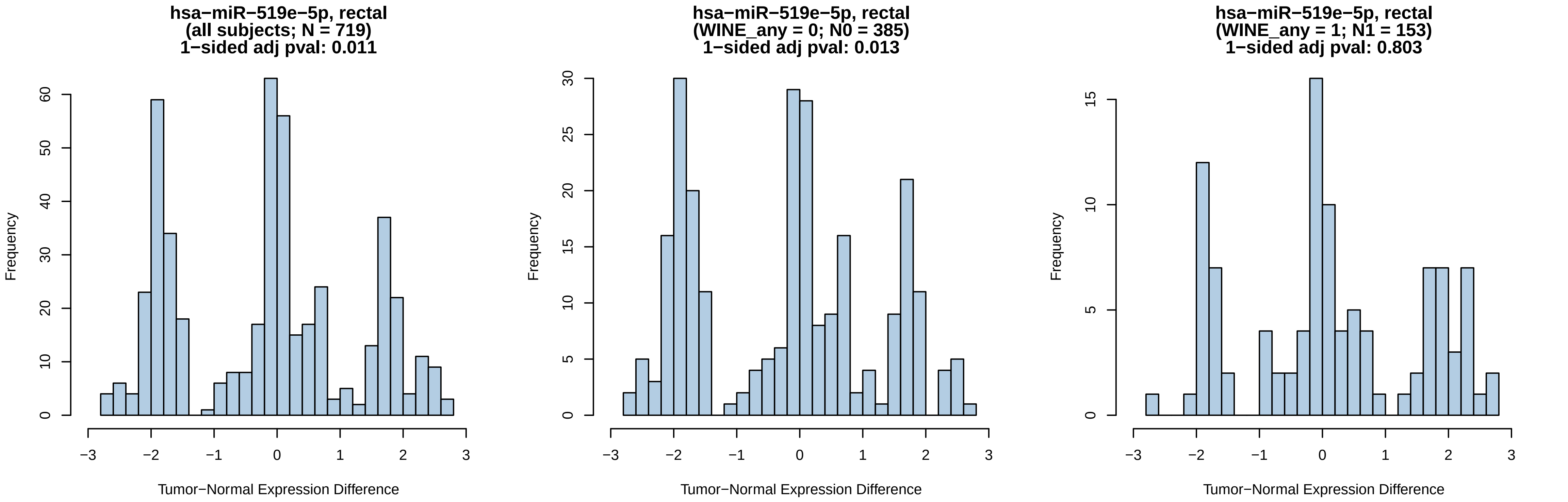

hsa−miR−519e−5p, rectal
(all subjects; N = 719)
1−sided adj pval: 0.011
hsa−miR−519e−5p, rectal
(WINE_any = 0; N0 = 385)
1−sided adj pval: 0.013
hsa−miR−519e−5p, rectal
(WINE_any = 1; N1 = 153)
1−sided adj pval: 0.803
Frequency
Tumor−Normal Expression Difference

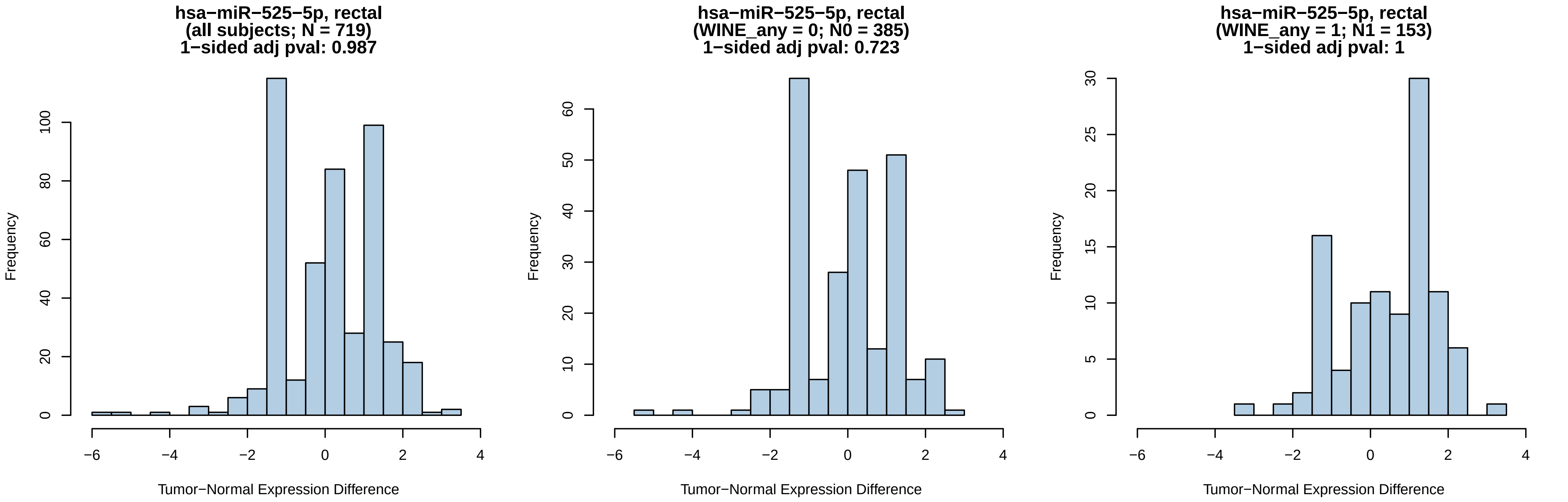

hsa−miR−525−5p, rectal
(all subjects; N = 719)
1−sided adj pval: 0.987
hsa−miR−525−5p, rectal
(WINE_any = 0; N0 = 385)
1−sided adj pval: 0.723
hsa−miR−525−5p, rectal
(WINE_any = 1; N1 = 153)
1−sided adj pval: 1
Frequency
Tumor−Normal Expression Difference

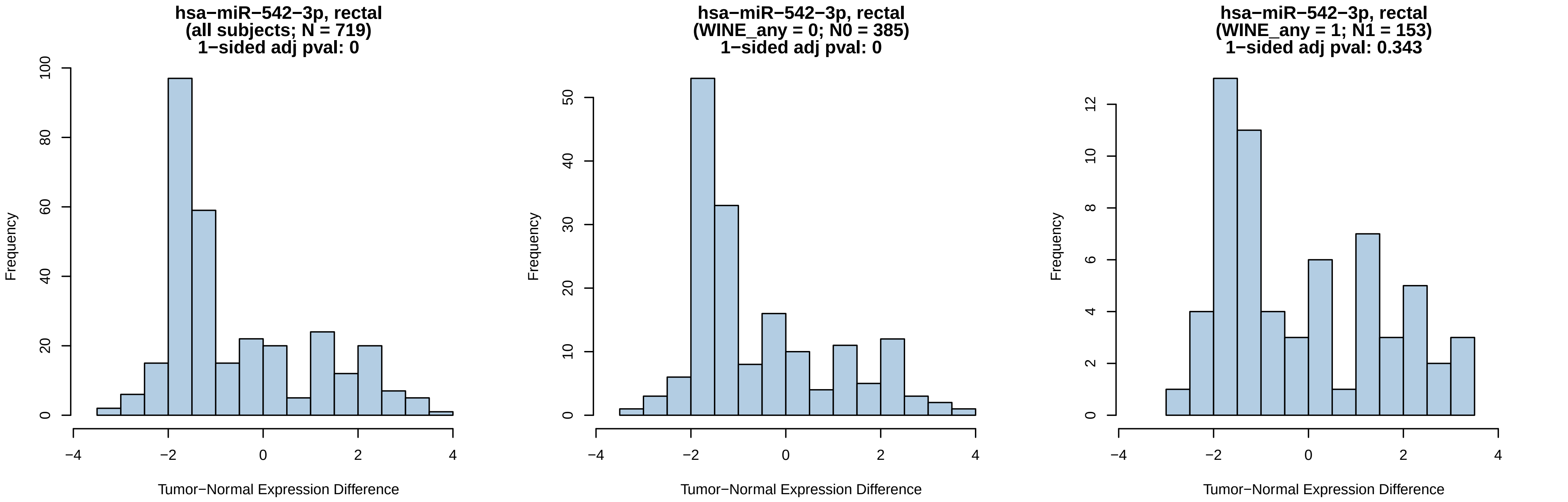
hsa−miR−542−3p, rectal
(all subjects; N = 719)
1−sided adj pval: 0
hsa−miR−542−3p, rectal
(WINE_any = 0; N0 = 385)
1−sided adj pval: 0
hsa−miR−542−3p, rectal
(WINE_any = 1; N1 = 153)
1−sided adj pval: 0.343
Frequency
Tumor−Normal Expression Difference

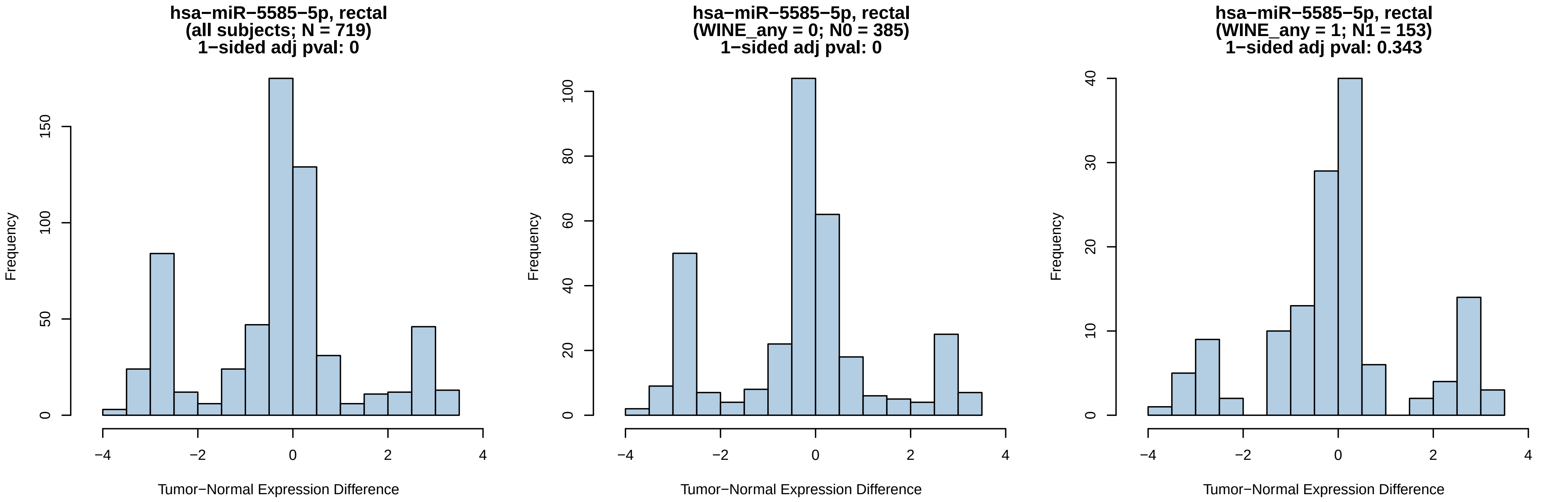

hsa−miR−5585−5p, rectal
(all subjects; N = 719)
1−sided adj pval: 0
Frequency
0
50
100
150
−4
−2
0
2
4
Tumor−Normal Expression Difference
hsa−miR−5585−5p, rectal
(WINE_any = 0; N0 = 385)
1−sided adj pval: 0
Frequency
0
20
40
60
80
100
−4
−2
0
2
4
Tumor−Normal Expression Difference
hsa−miR−5585−5p, rectal
(WINE_any = 1; N1 = 153)
1−sided adj pval: 0.343
Frequency
0
10
20
30
40
−4
−2
0
2
4
Tumor−Normal Expression Difference

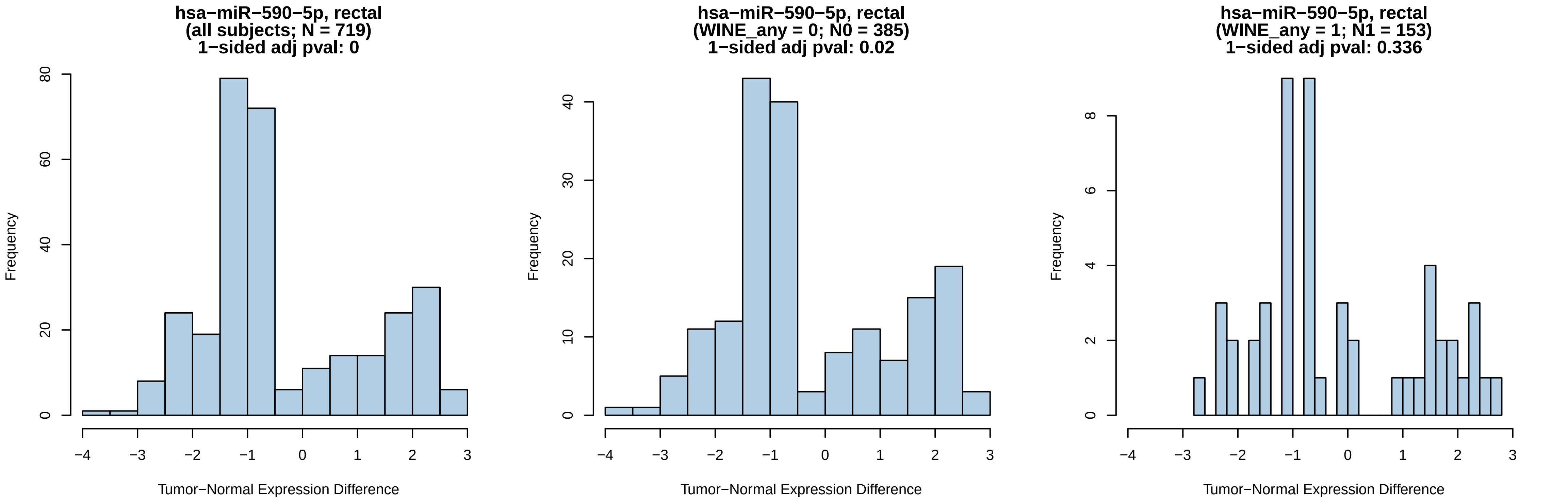

hsa−miR−590−5p, rectal
(all subjects; N = 719)
1−sided adj pval: 0
Frequency
Tumor−Normal Expression Difference
hsa−miR−590−5p, rectal
(WINE_any = 0; N0 = 385)
1−sided adj pval: 0.02
Frequency
Tumor−Normal Expression Difference
hsa−miR−590−5p, rectal
(WINE_any = 1; N1 = 153)
1−sided adj pval: 0.336
Frequency
Tumor−Normal Expression Difference

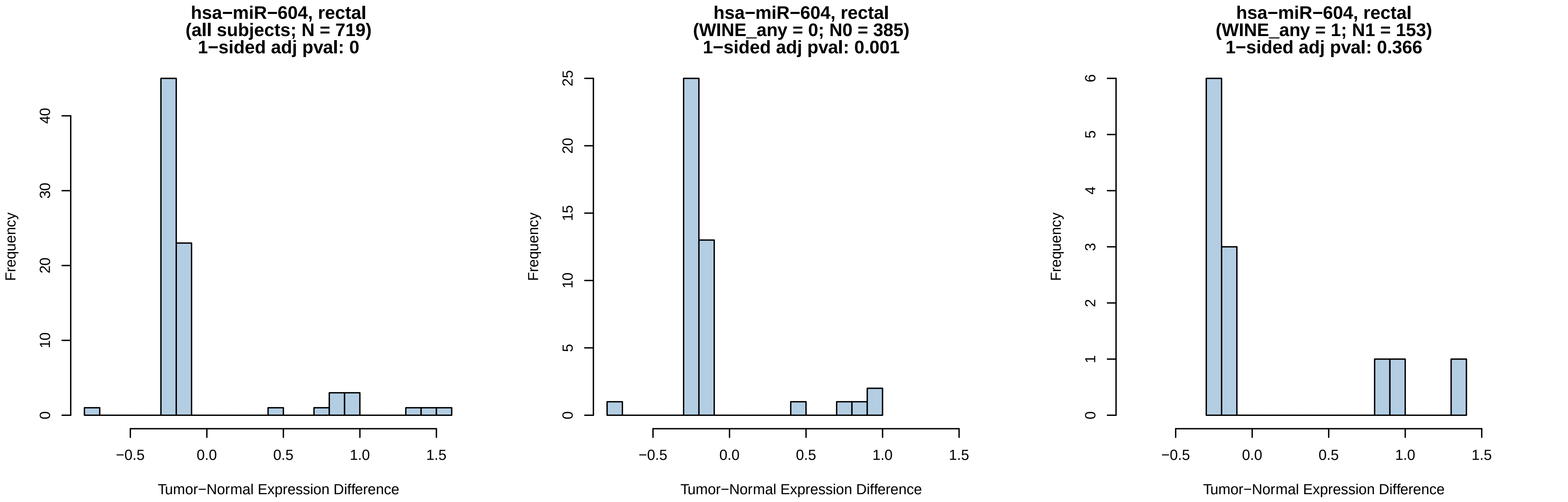
hsa−miR−604, rectal
(all subjects; N = 719)
1−sided adj pval: 0
Frequency
Tumor−Normal Expression Difference
hsa−miR−604, rectal
(WINE_any = 0; N0 = 385)
1−sided adj pval: 0.001
Frequency
Tumor−Normal Expression Difference
hsa−miR−604, rectal
(WINE_any = 1; N1 = 153)
1−sided adj pval: 0.366
Frequency
Tumor−Normal Expression Difference

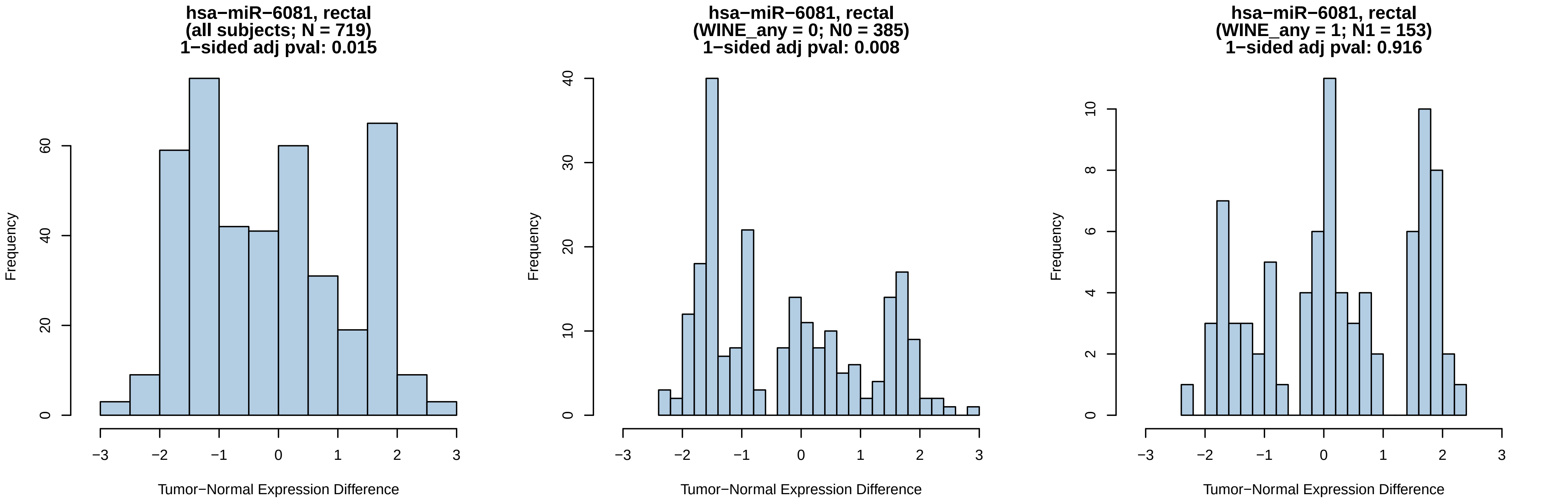
hsa−miR−6081, rectal
(all subjects; N = 719)
1−sided adj pval: 0.015
Frequency
0
20
40
60
−3
−2
−1
0
1
2
3
Tumor−Normal Expression Difference
hsa−miR−6081, rectal
(WINE_any = 0; N0 = 385)
1−sided adj pval: 0.008
Frequency
0
10
20
30
40
−3
−2
−1
0
1
2
3
Tumor−Normal Expression Difference
hsa−miR−6081, rectal
(WINE_any = 1; N1 = 153)
1−sided adj pval: 0.916
Frequency
0
2
4
6
8
10
−3
−2
−1
0
1
2
3
Tumor−Normal Expression Difference

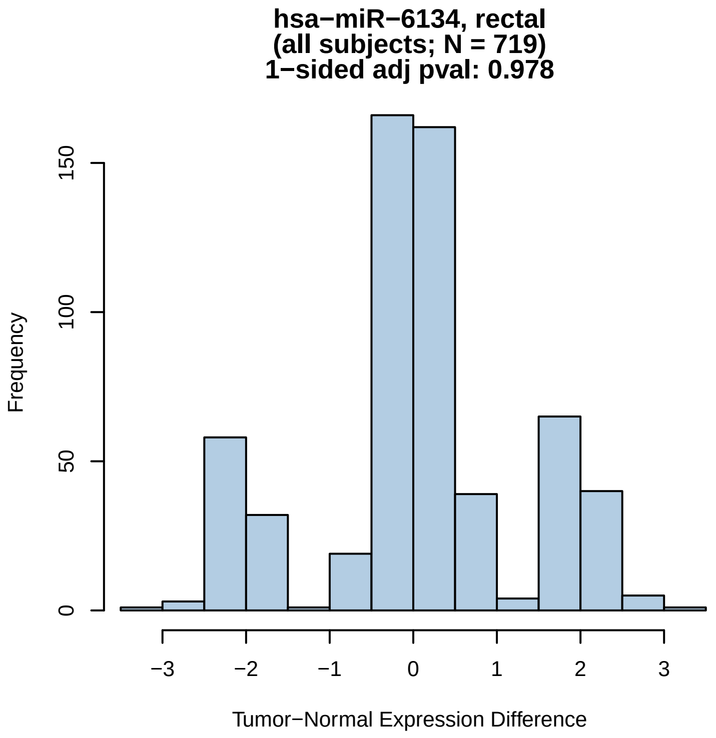
hsa−miR−6134, rectal
(all subjects; N = 719)
1−sided adj pval: 0.978
Frequency
0
50
100
150
−3
−2
−1
0
1
2
3
Tumor−Normal Expression Difference

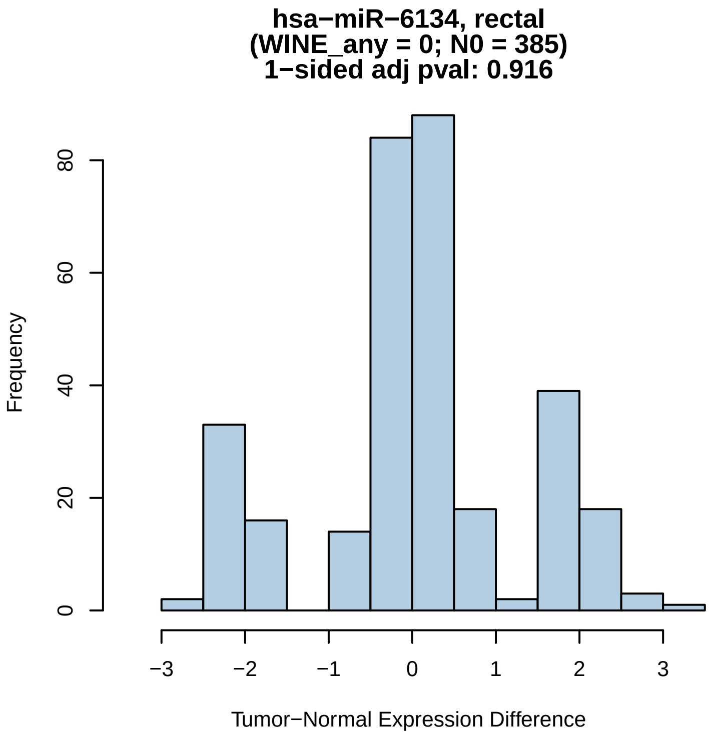
hsa−miR−6134, rectal
(WINE_any = 0; N0 = 385)
1−sided adj pval: 0.916
Frequency
0
20
40
60
80
−3
−2
−1
0
1
2
3
Tumor−Normal Expression Difference

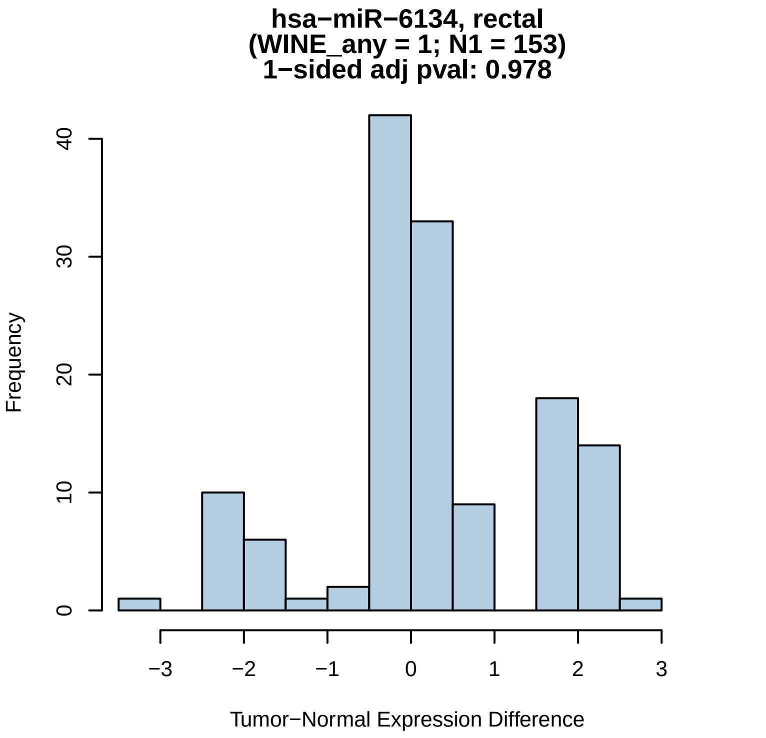
hsa−miR−6134, rectal
(WINE_any = 1; N1 = 153)
1−sided adj pval: 0.978
Frequency
0
10
20
30
40
−3
−2
−1
0
1
2
3
Tumor−Normal Expression Difference

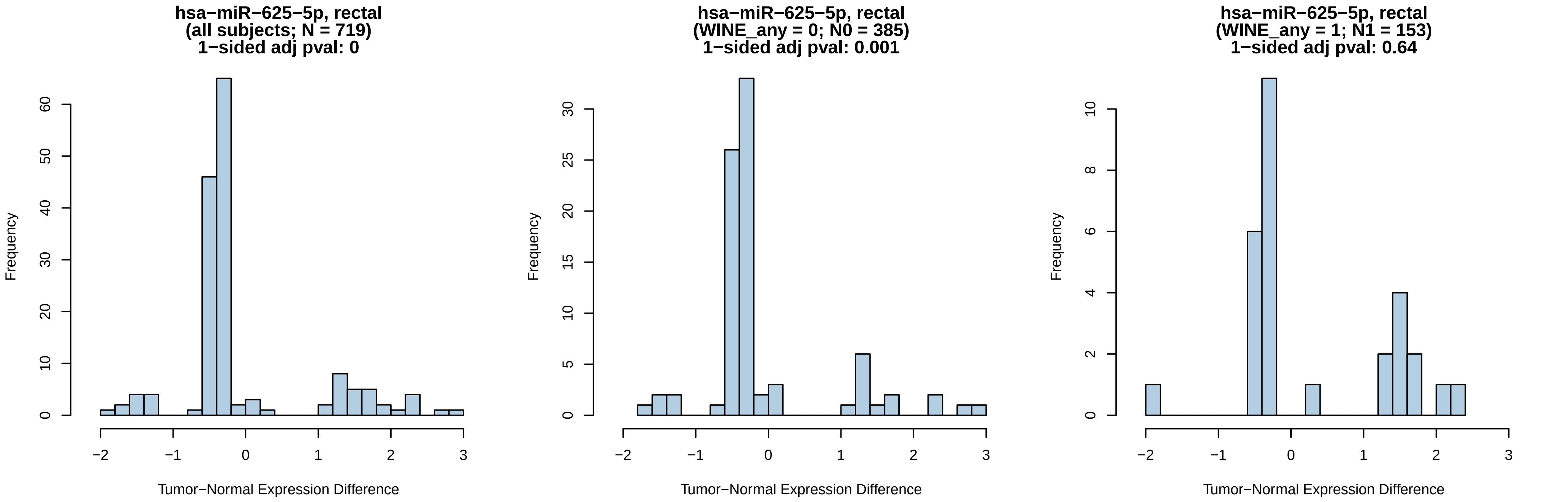
hsa−miR−625−5p, rectal
(all subjects; N = 719)
1−sided adj pval: 0
Frequency
Tumor−Normal Expression Difference
hsa−miR−625−5p, rectal
(WINE_any = 0; N0 = 385)
1−sided adj pval: 0.001
Frequency
Tumor−Normal Expression Difference
hsa−miR−625−5p, rectal
(WINE_any = 1; N1 = 153)
1−sided adj pval: 0.64
Frequency
Tumor−Normal Expression Difference

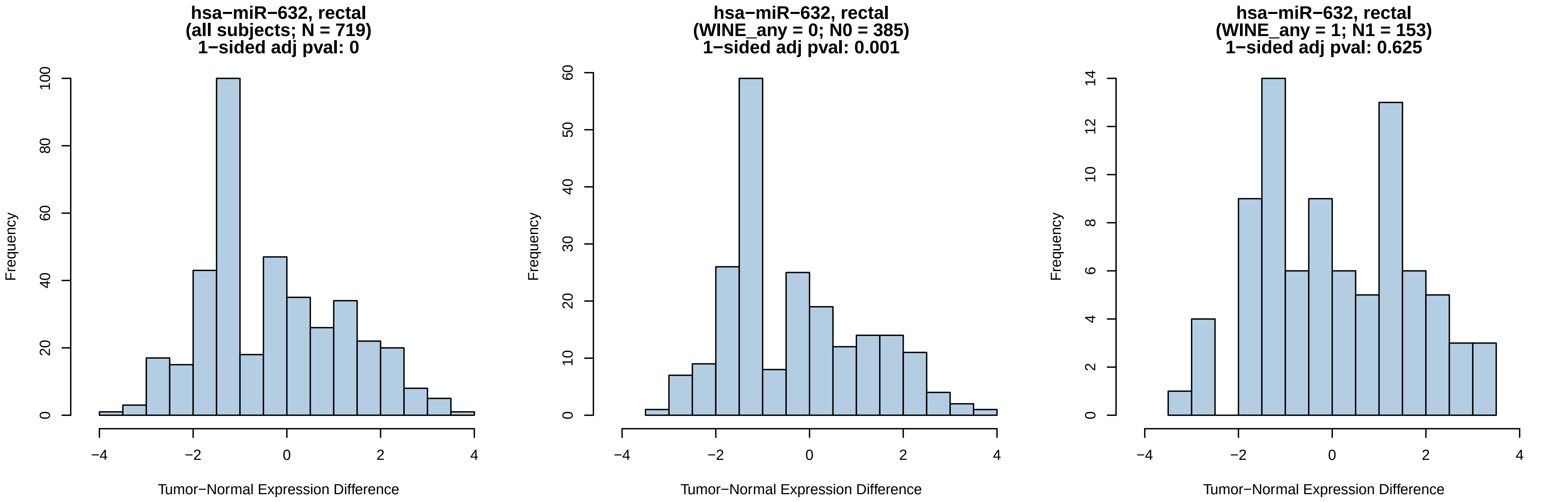
hsa−miR−632, rectal
(all subjects; N = 719)
1−sided adj pval: 0
Frequency
Tumor−Normal Expression Difference
hsa−miR−632, rectal
(WINE_any = 0; N0 = 385)
1−sided adj pval: 0.001
Frequency
Tumor−Normal Expression Difference
hsa−miR−632, rectal
(WINE_any = 1; N1 = 153)
1−sided adj pval: 0.625
Frequency
Tumor−Normal Expression Difference

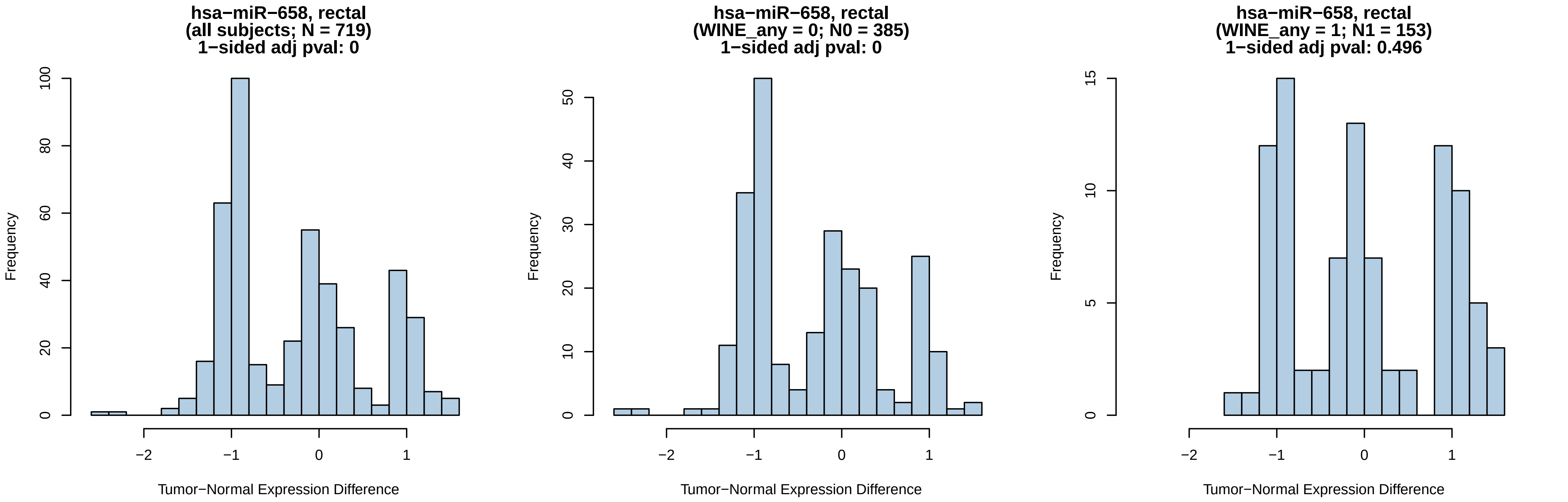
hsa−miR−658, rectal
(all subjects; N = 719)
1−sided adj pval: 0
Frequency
Tumor−Normal Expression Difference
hsa−miR−658, rectal
(WINE_any = 0; N0 = 385)
1−sided adj pval: 0
Frequency
Tumor−Normal Expression Difference
hsa−miR−658, rectal
(WINE_any = 1; N1 = 153)
1−sided adj pval: 0.496
Frequency
Tumor−Normal Expression Difference

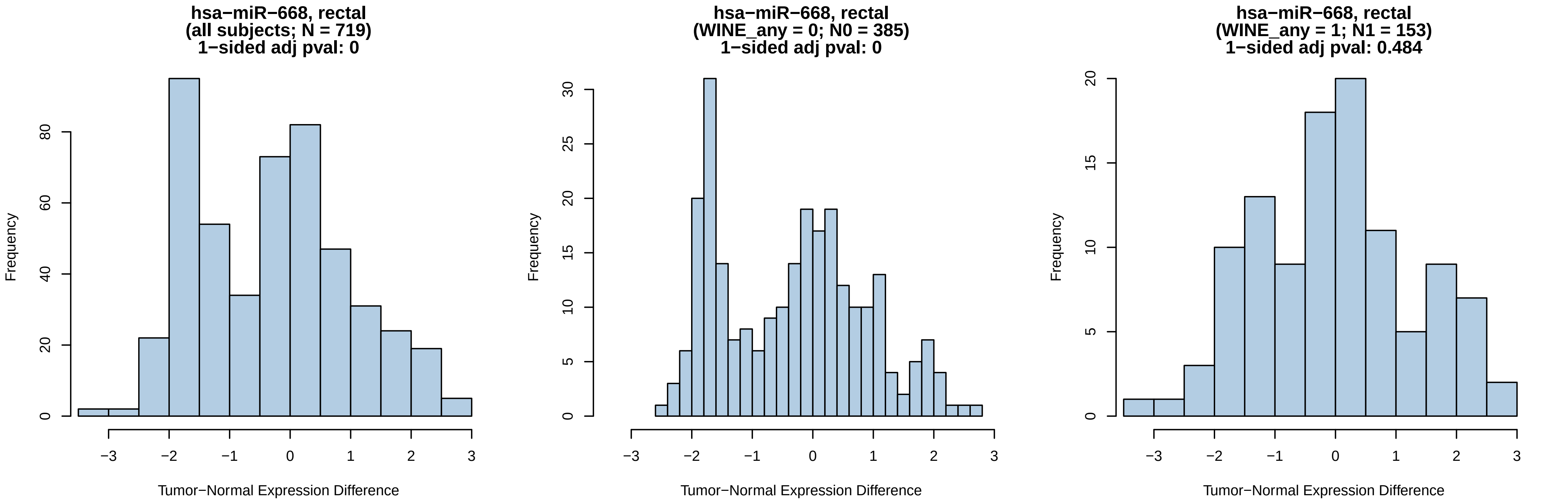

hsa−miR−668, rectal
(all subjects; N = 719)
1−sided adj pval: 0
Frequency
Tumor−Normal Expression Difference
hsa−miR−668, rectal
(WINE_any = 0; N0 = 385)
1−sided adj pval: 0
Frequency
Tumor−Normal Expression Difference
hsa−miR−668, rectal
(WINE_any = 1; N1 = 153)
1−sided adj pval: 0.484
Frequency
Tumor−Normal Expression Difference

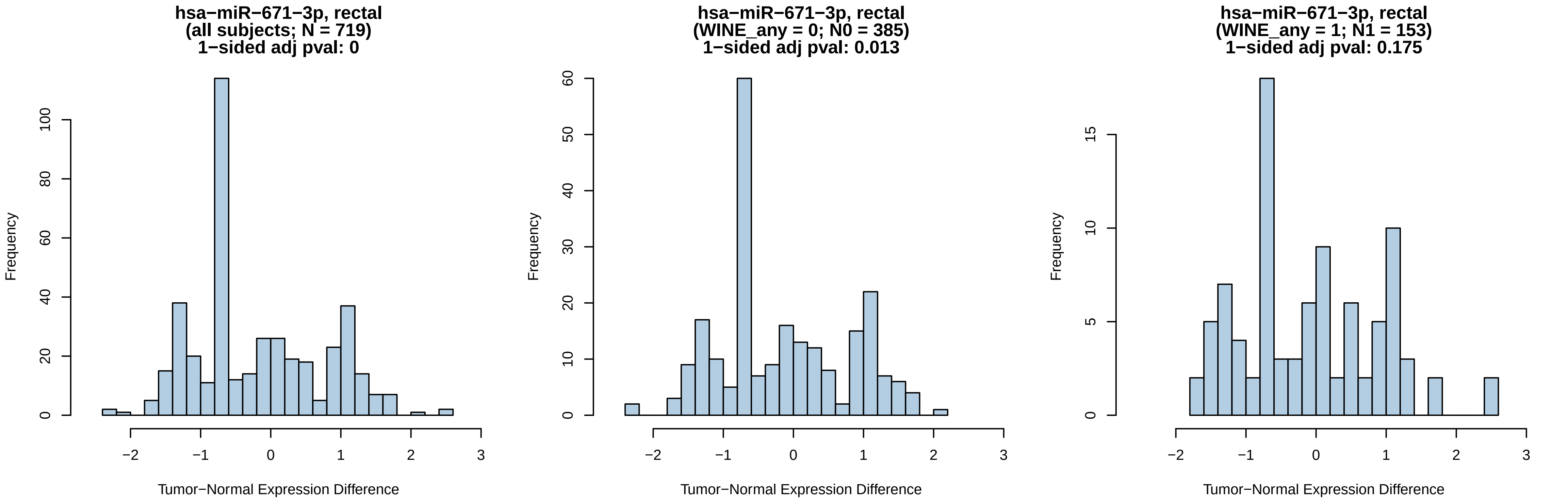
hsa−miR−671−3p, rectal
(all subjects; N = 719)
1−sided adj pval: 0
Frequency
Tumor−Normal Expression Difference
hsa−miR−671−3p, rectal
(WINE_any = 0; N0 = 385)
1−sided adj pval: 0.013
Frequency
Tumor−Normal Expression Difference
hsa−miR−671−3p, rectal
(WINE_any = 1; N1 = 153)
1−sided adj pval: 0.175
Frequency
Tumor−Normal Expression Difference

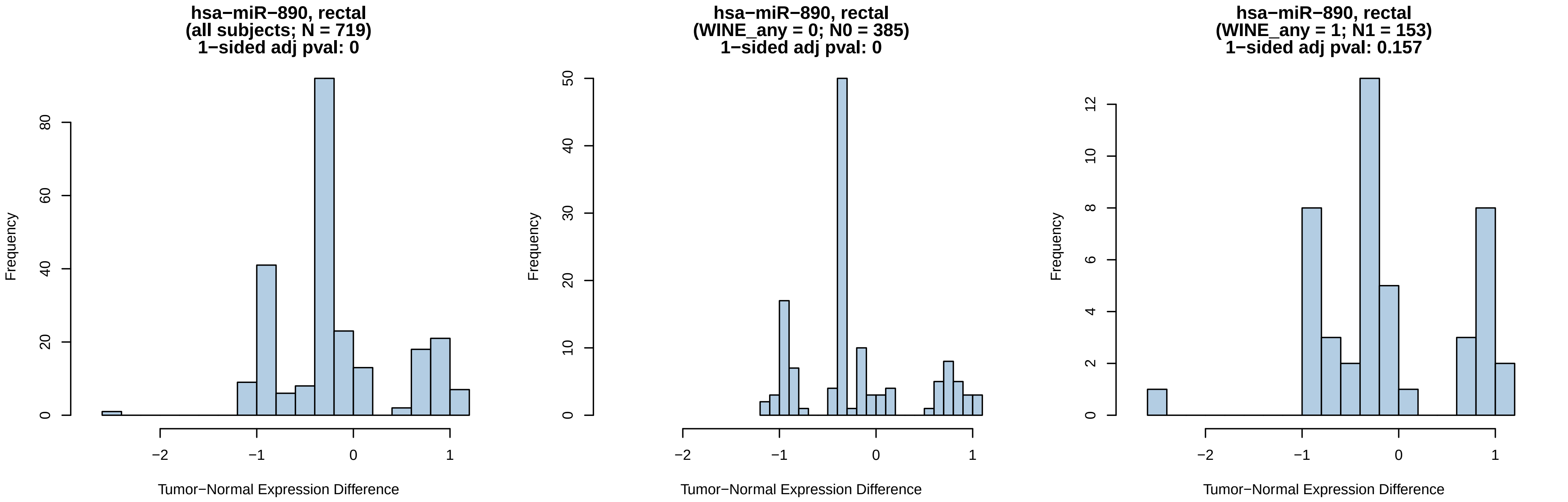

hsa−miR−890, rectal
(all subjects; N = 719)
1−sided adj pval: 0
Frequency
Tumor−Normal Expression Difference
hsa−miR−890, rectal
(WINE_any = 0; N0 = 385)
1−sided adj pval: 0
Frequency
Tumor−Normal Expression Difference
hsa−miR−890, rectal
(WINE_any = 1; N1 = 153)
1−sided adj pval: 0.157
Frequency
Tumor−Normal Expression Difference

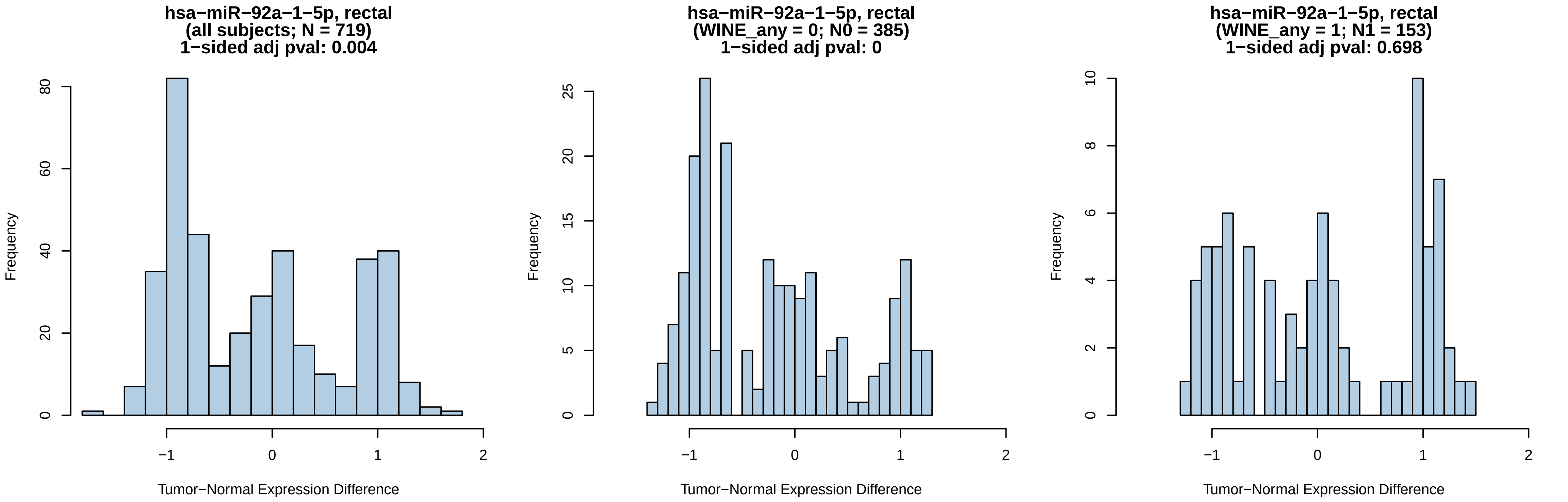
hsa−miR−92a−1−5p, rectal
(all subjects; N = 719)
1−sided adj pval: 0.004
Frequency
Tumor−Normal Expression Difference
hsa−miR−92a−1−5p, rectal
(WINE_any = 0; N0 = 385)
1−sided adj pval: 0
Frequency
Tumor−Normal Expression Difference
hsa−miR−92a−1−5p, rectal
(WINE_any = 1; N1 = 153)
1−sided adj pval: 0.698
Frequency
Tumor−Normal Expression Difference

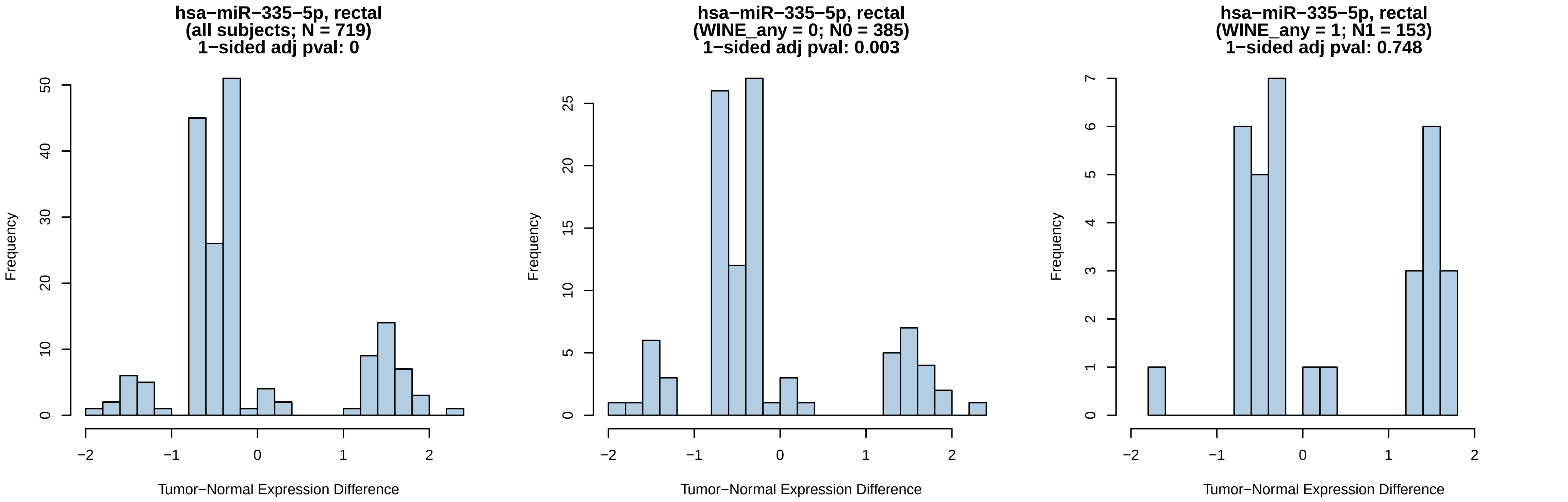
hsa−miR−335−5p, rectal
(all subjects; N = 719)
1−sided adj pval: 0
Frequency
Tumor−Normal Expression Difference
hsa−miR−335−5p, rectal
(WINE_any = 0; N0 = 385)
1−sided adj pval: 0.003
Frequency
Tumor−Normal Expression Difference
hsa−miR−335−5p, rectal
(WINE_any = 1; N1 = 153)
1−sided adj pval: 0.748
Frequency
Tumor−Normal Expression Difference

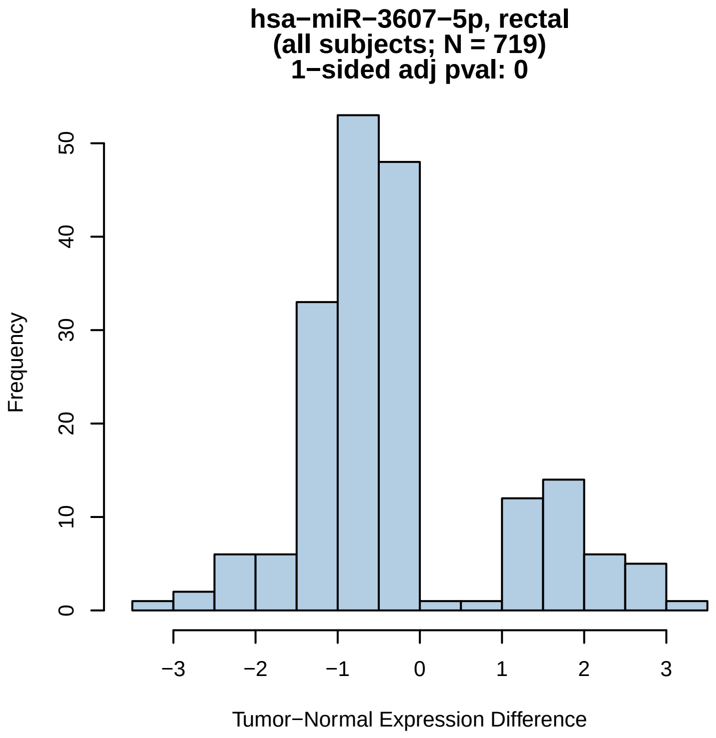
hsa−miR−3607−5p, rectal
(all subjects; N = 719)
1−sided adj pval: 0
Frequency
0
10
20
30
40
50
−3
−2
−1
0
1
2
3
Tumor−Normal Expression Difference

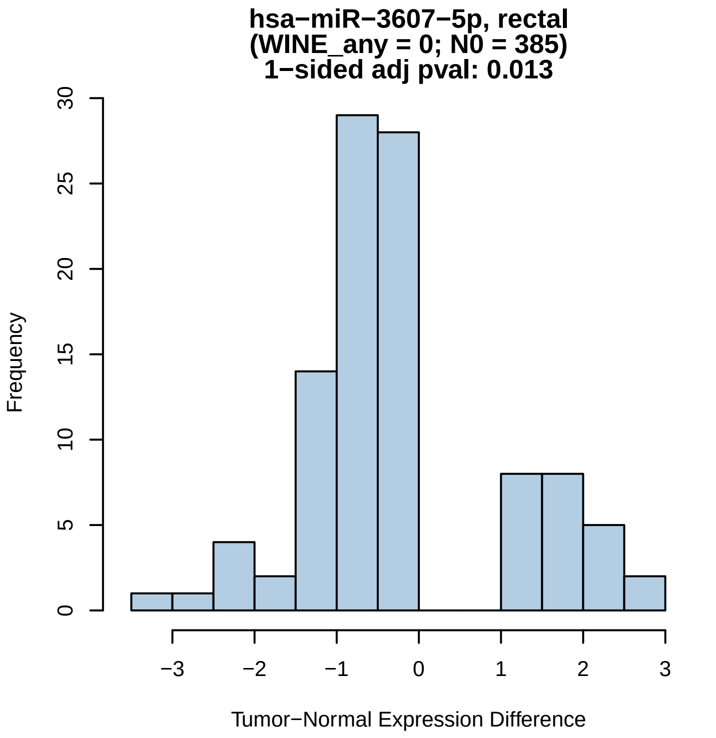
hsa−miR−3607−5p, rectal
(WINE_any = 0; N0 = 385)
1−sided adj pval: 0.013
Frequency
0
5
10
15
20
25
30
−3
−2
−1
0
1
2
3
Tumor−Normal Expression Difference

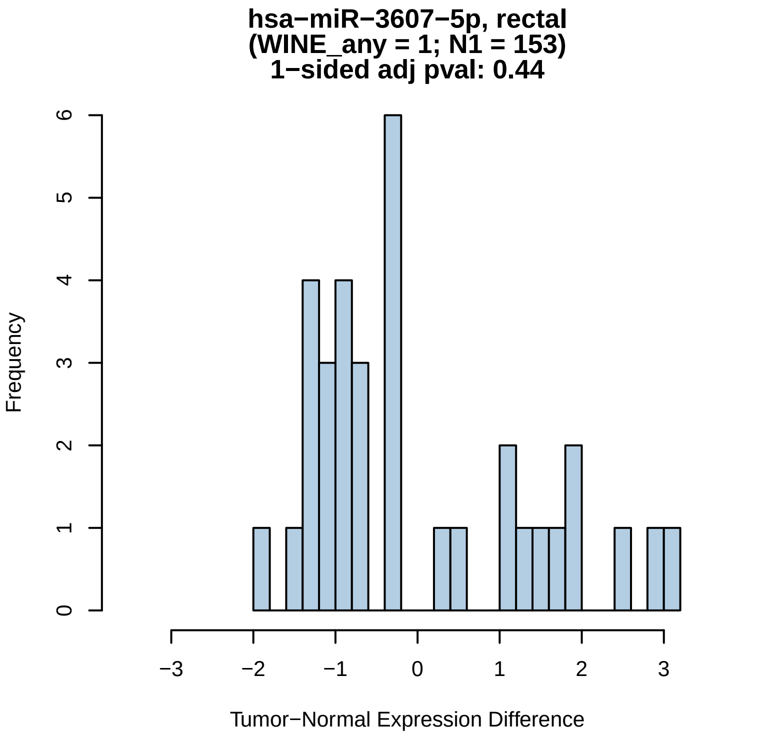
hsa−miR−3607−5p, rectal
(WINE_any = 1; N1 = 153)
1−sided adj pval: 0.44
Frequency
0
1
2
3
4
5
6
−3
−2
−1
0
1
2
3
Tumor−Normal Expression Difference

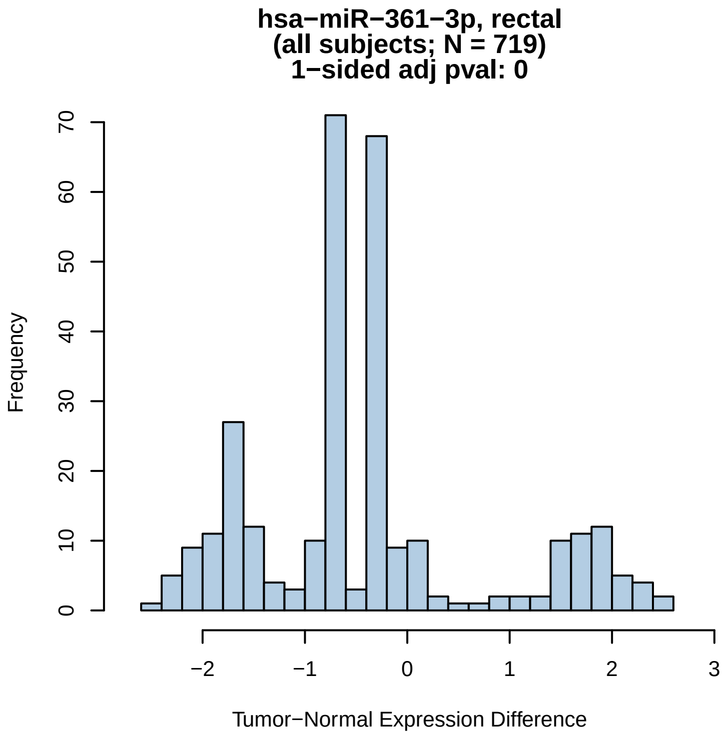

hsa−miR−361−3p, rectal
(all subjects; N = 719)
1−sided adj pval: 0
Frequency
Tumor−Normal Expression Difference


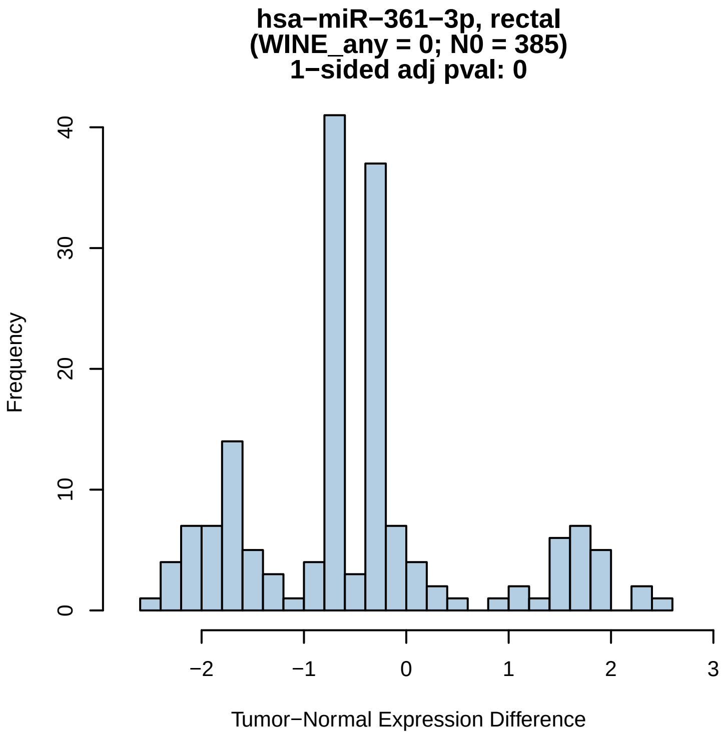

hsa−miR−361−3p, rectal
(WINE_any = 0; N0 = 385)
1−sided adj pval: 0
Frequency
Tumor−Normal Expression Difference


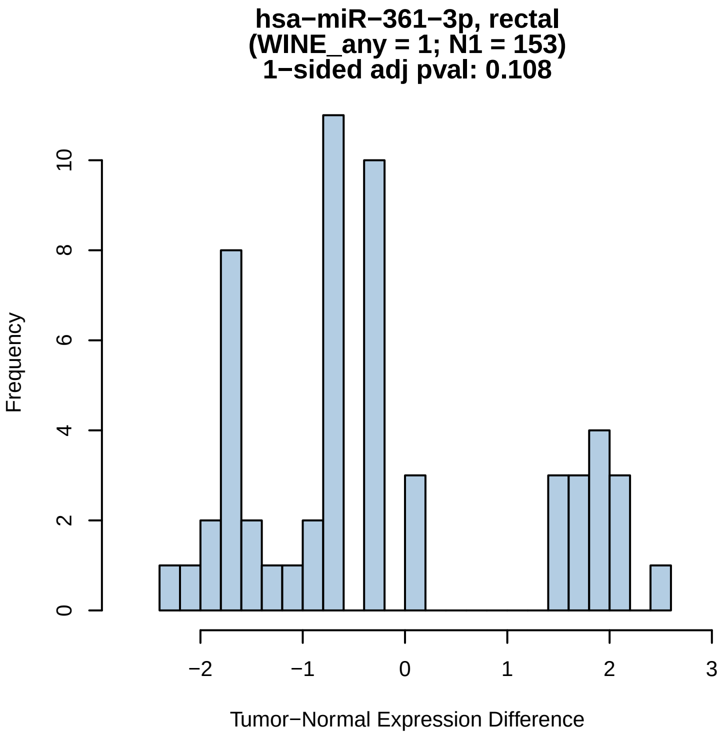

hsa−miR−361−3p, rectal
(WINE_any = 1; N1 = 153)
1−sided adj pval: 0.108
Frequency
Tumor−Normal Expression Difference

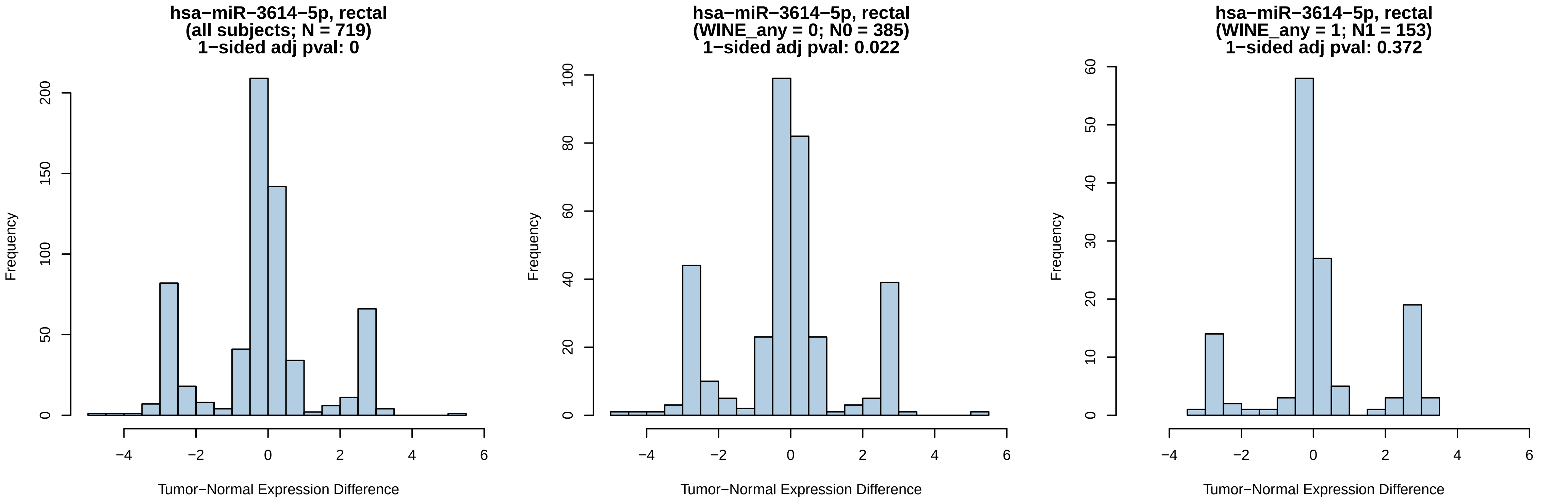

hsa−miR−3614−5p, rectal
(all subjects; N = 719)
1−sided adj pval: 0
Frequency
Tumor−Normal Expression Difference
hsa−miR−3614−5p, rectal
(WINE_any = 0; N0 = 385)
1−sided adj pval: 0.022
Frequency
Tumor−Normal Expression Difference
hsa−miR−3614−5p, rectal
(WINE_any = 1; N1 = 153)
1−sided adj pval: 0.372
Frequency
Tumor−Normal Expression Difference

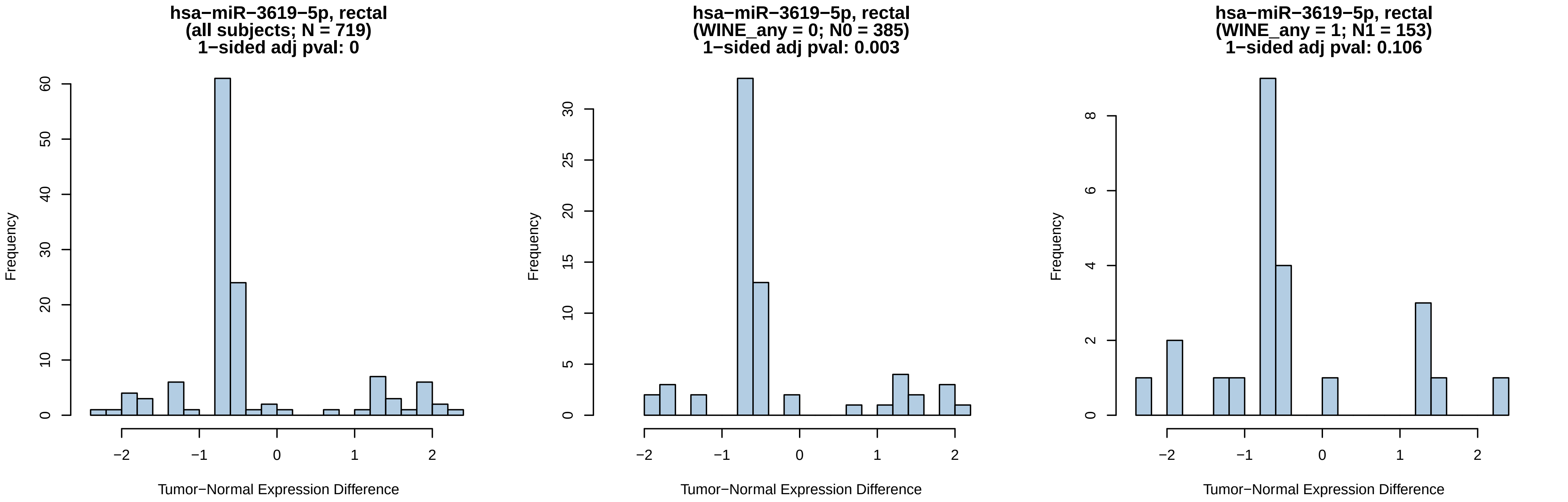
hsa−miR−3619−5p, rectal
(all subjects; N = 719)
1−sided adj pval: 0
Frequency
Tumor−Normal Expression Difference
hsa−miR−3619−5p, rectal
(WINE_any = 0; N0 = 385)
1−sided adj pval: 0.003
Frequency
Tumor−Normal Expression Difference
hsa−miR−3619−5p, rectal
(WINE_any = 1; N1 = 153)
1−sided adj pval: 0.106
Frequency
Tumor−Normal Expression Difference

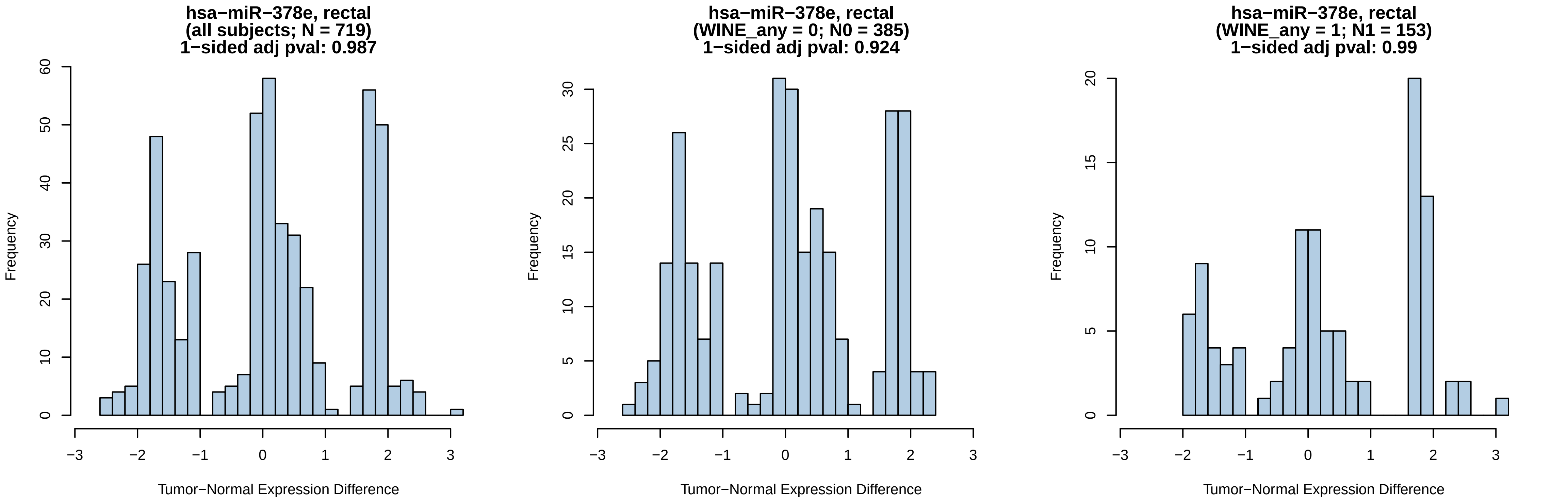
hsa−miR−378e, rectal
(all subjects; N = 719)
1−sided adj pval: 0.987
Frequency
0
10
20
30
40
50
60
−3
−2
−1
0
1
2
3
Tumor−Normal Expression Difference
hsa−miR−378e, rectal
(WINE_any = 0; N0 = 385)
1−sided adj pval: 0.924
Frequency
0
5
10
15
20
25
30
−3
−2
−1
0
1
2
3
Tumor−Normal Expression Difference
hsa−miR−378e, rectal
(WINE_any = 1; N1 = 153)
1−sided adj pval: 0.99
Frequency
0
5
10
15
20
−3
−2
−1
0
1
2
3
Tumor−Normal Expression Difference

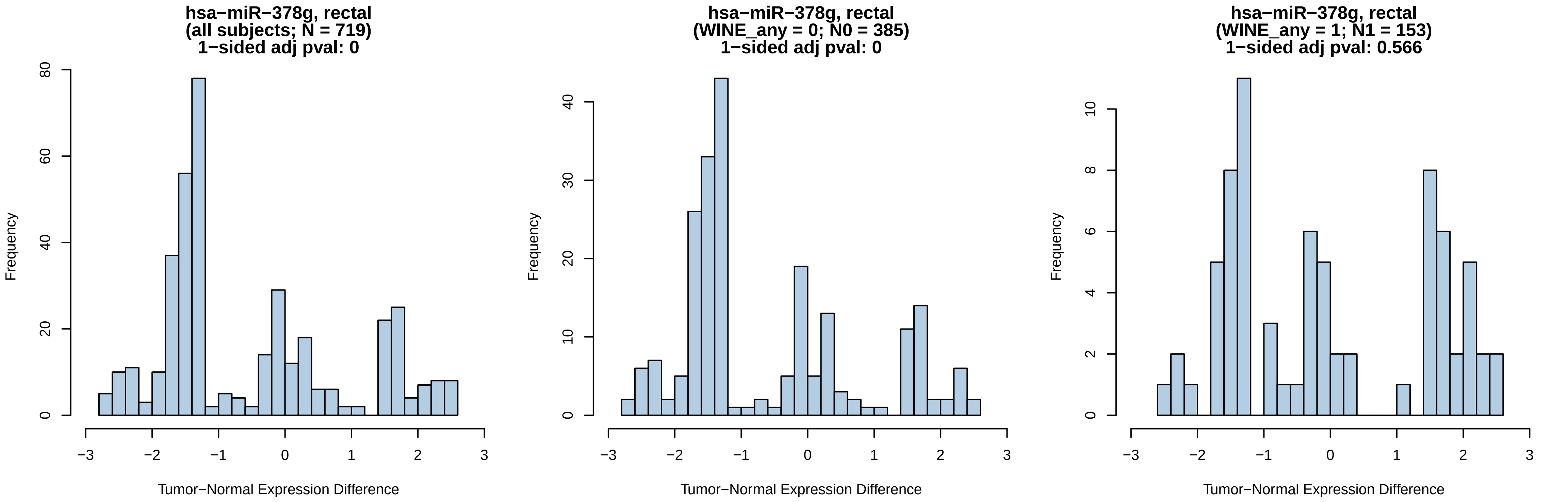
hsa−miR−378g, rectal
(all subjects; N = 719)
1−sided adj pval: 0
hsa−miR−378g, rectal
(WINE_any = 0; N0 = 385)
1−sided adj pval: 0
hsa−miR−378g, rectal
(WINE_any = 1; N1 = 153)
1−sided adj pval: 0.566
Frequency
Tumor−Normal Expression Difference

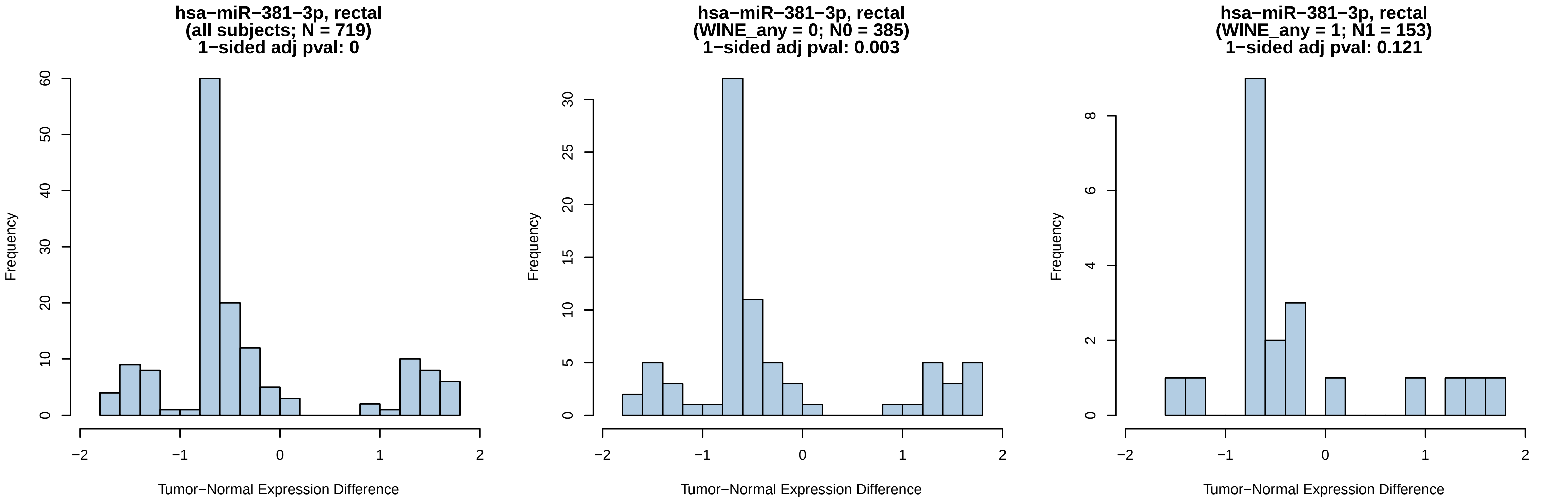
hsa−miR−381−3p, rectal
(all subjects; N = 719)
1−sided adj pval: 0
Frequency
Tumor−Normal Expression Difference
hsa−miR−381−3p, rectal
(WINE_any = 0; N0 = 385)
1−sided adj pval: 0.003
Frequency
Tumor−Normal Expression Difference
hsa−miR−381−3p, rectal
(WINE_any = 1; N1 = 153)
1−sided adj pval: 0.121
Frequency
Tumor−Normal Expression Difference

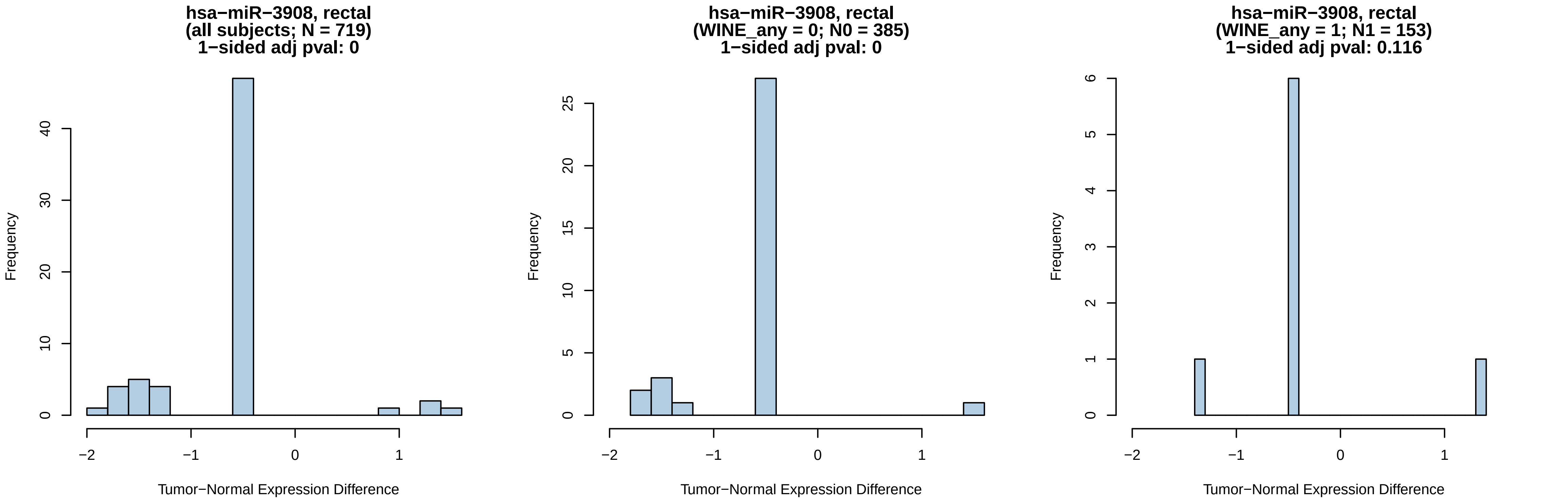

hsa−miR−3908, rectal
(all subjects; N = 719)
1−sided adj pval: 0
Frequency
Tumor−Normal Expression Difference
hsa−miR−3908, rectal
(WINE_any = 0; N0 = 385)
1−sided adj pval: 0
Frequency
Tumor−Normal Expression Difference
hsa−miR−3908, rectal
(WINE_any = 1; N1 = 153)
1−sided adj pval: 0.116
Frequency
Tumor−Normal Expression Difference

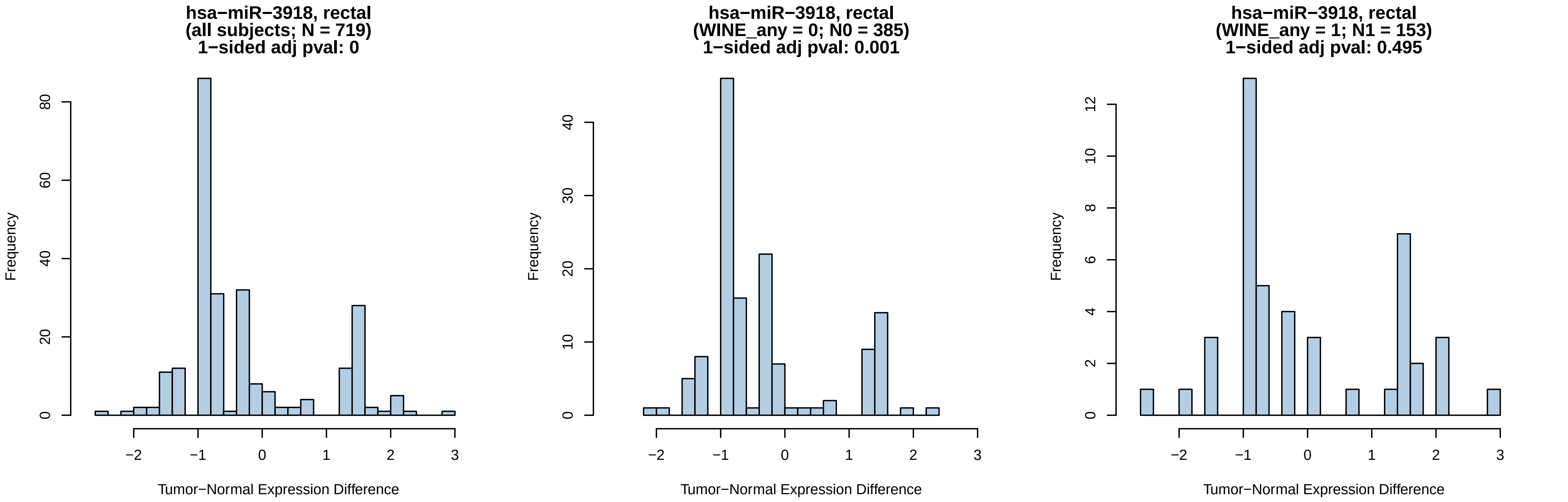

hsa−miR−3918, rectal
(all subjects; N = 719)
1−sided adj pval: 0
Frequency
Tumor−Normal Expression Difference
hsa−miR−3918, rectal
(WINE_any = 0; N0 = 385)
1−sided adj pval: 0.001
Frequency
Tumor−Normal Expression Difference
hsa−miR−3918, rectal
(WINE_any = 1; N1 = 153)
1−sided adj pval: 0.495
Frequency
Tumor−Normal Expression Difference

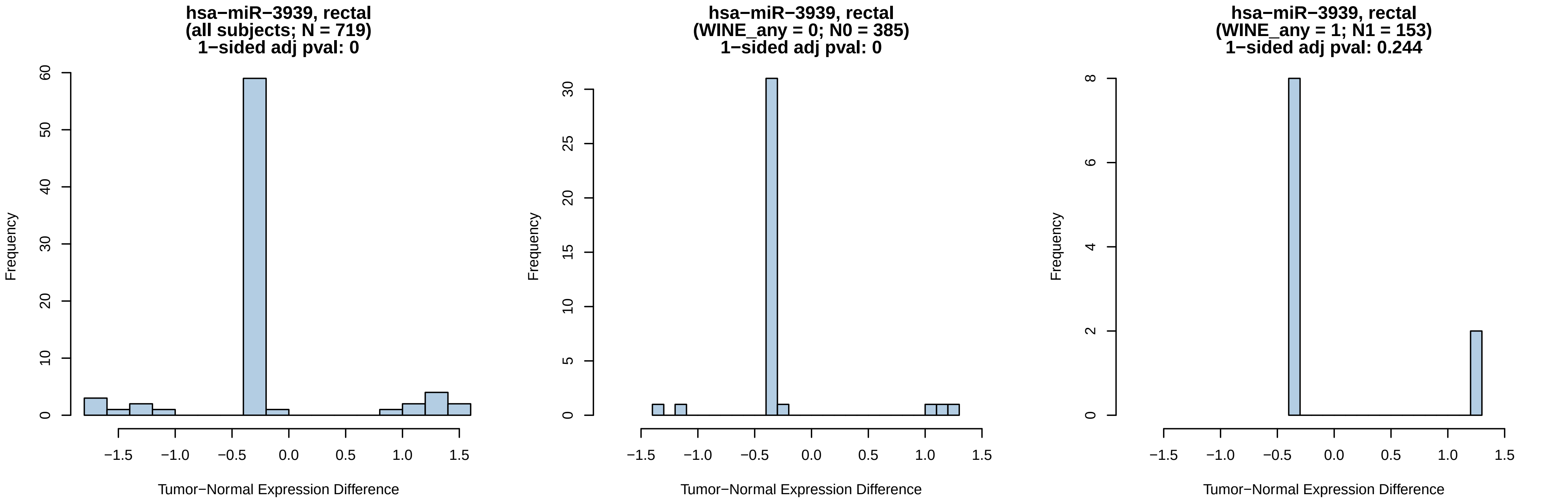
hsa−miR−3939, rectal
(all subjects; N = 719)
1−sided adj pval: 0
Frequency
Tumor−Normal Expression Difference
0
10
20
30
40
50
60
−1.5
−1.0
−0.5
0.0
0.5
1.0
1.5
hsa−miR−3939, rectal
(WINE_any = 0; N0 = 385)
1−sided adj pval: 0
Frequency
Tumor−Normal Expression Difference
0
5
10
15
20
25
30
hsa−miR−3939, rectal
(WINE_any = 1; N1 = 153)
1−sided adj pval: 0.244
Frequency
Tumor−Normal Expression Difference
0
2
4
6
8

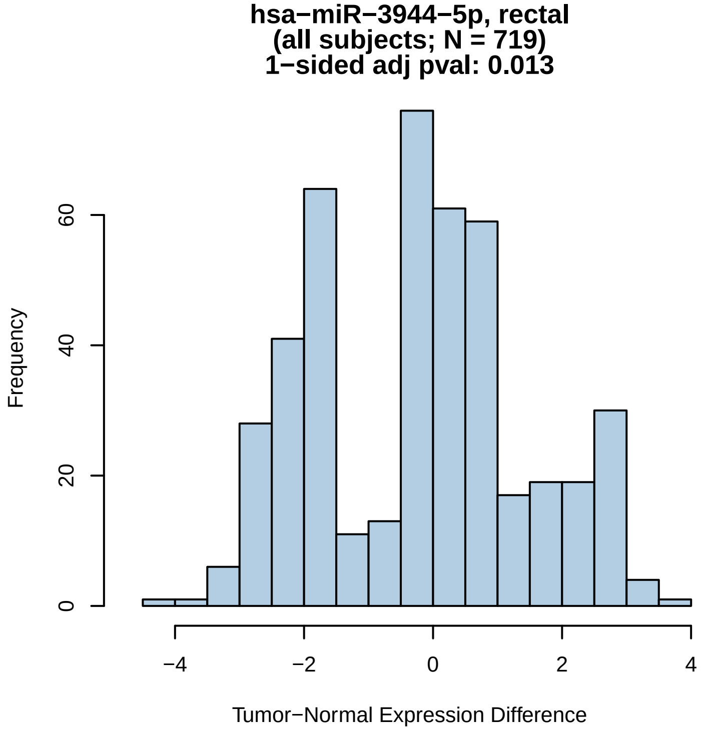
hsa−miR−3944−5p, rectal
(all subjects; N = 719)
1−sided adj pval: 0.013
Frequency
0
20
40
60
−4
−2
0
2
4
Tumor−Normal Expression Difference

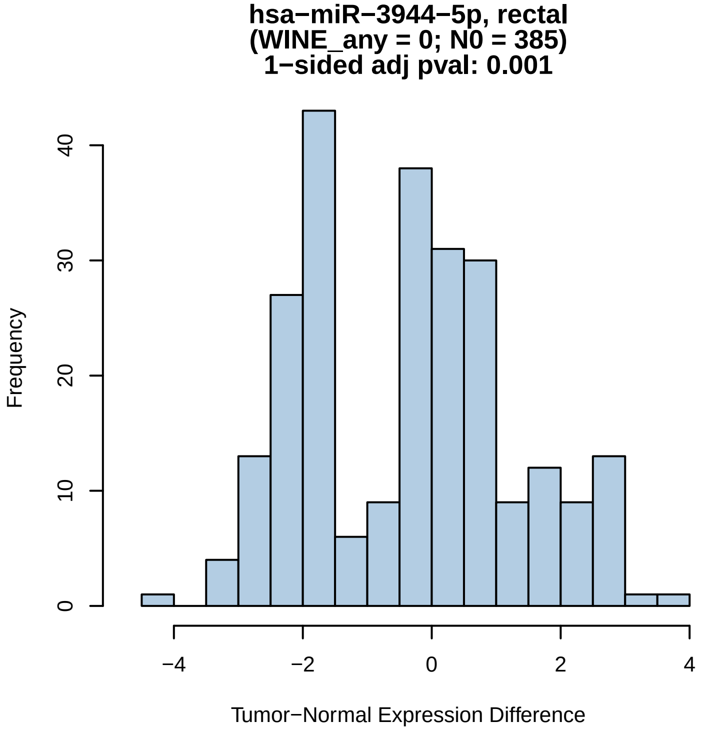
hsa−miR−3944−5p, rectal
(WINE_any = 0; N0 = 385)
1−sided adj pval: 0.001
Frequency
0
10
20
30
40
−4
−2
0
2
4
Tumor−Normal Expression Difference

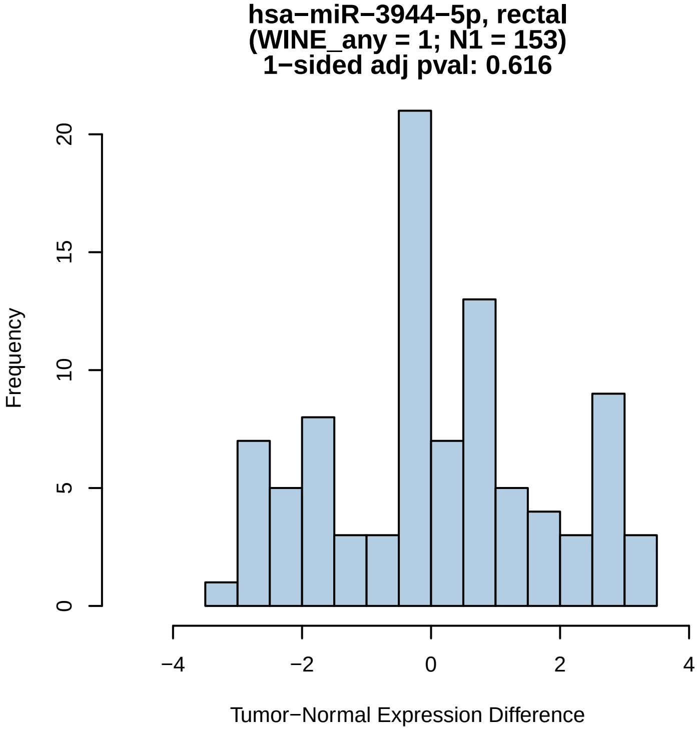
hsa−miR−3944−5p, rectal
(WINE_any = 1; N1 = 153)
1−sided adj pval: 0.616
Frequency
0
5
10
15
20
−4
−2
0
2
4
Tumor−Normal Expression Difference

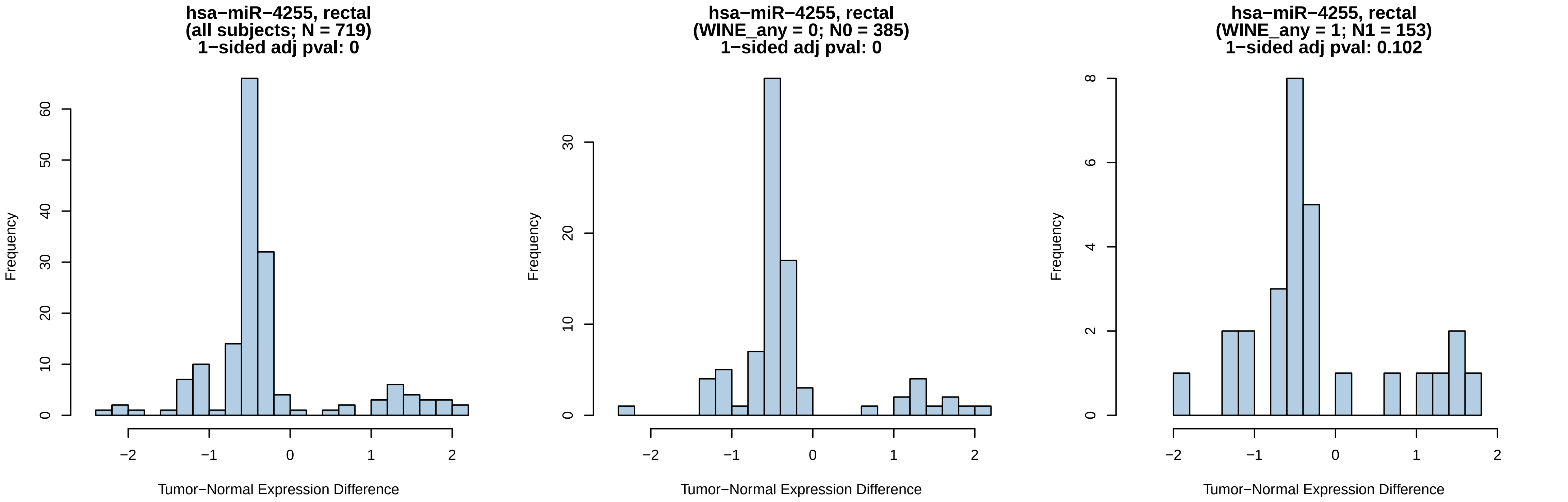

hsa−miR−4255, rectal
(all subjects; N = 719)
1−sided adj pval: 0
Frequency
Tumor−Normal Expression Difference
hsa−miR−4255, rectal
(WINE_any = 0; N0 = 385)
1−sided adj pval: 0
Frequency
Tumor−Normal Expression Difference
hsa−miR−4255, rectal
(WINE_any = 1; N1 = 153)
1−sided adj pval: 0.102
Frequency
Tumor−Normal Expression Difference

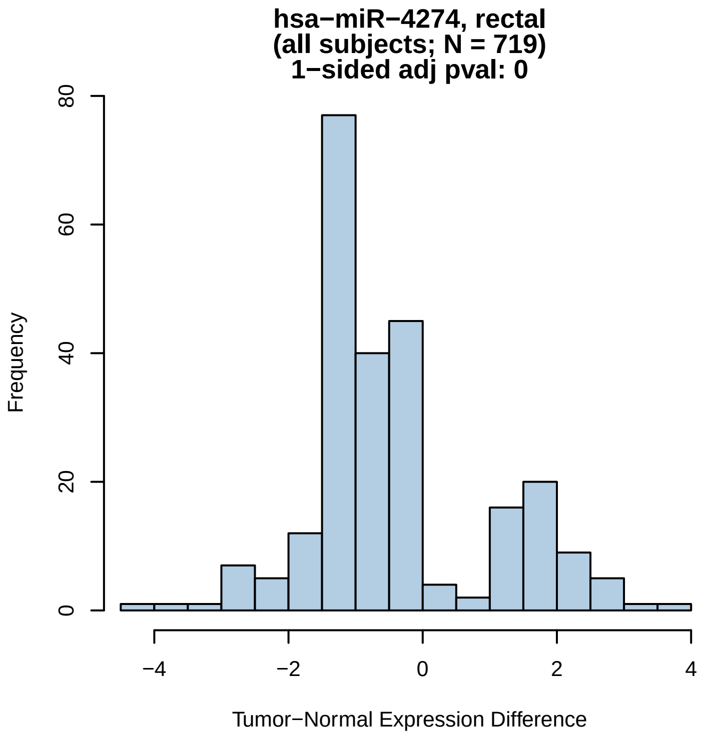

hsa−miR−4274, rectal
(all subjects; N = 719)
1−sided adj pval: 0
Frequency
0
20
40
60
80
−4
−2
0
2
4
Tumor−Normal Expression Difference


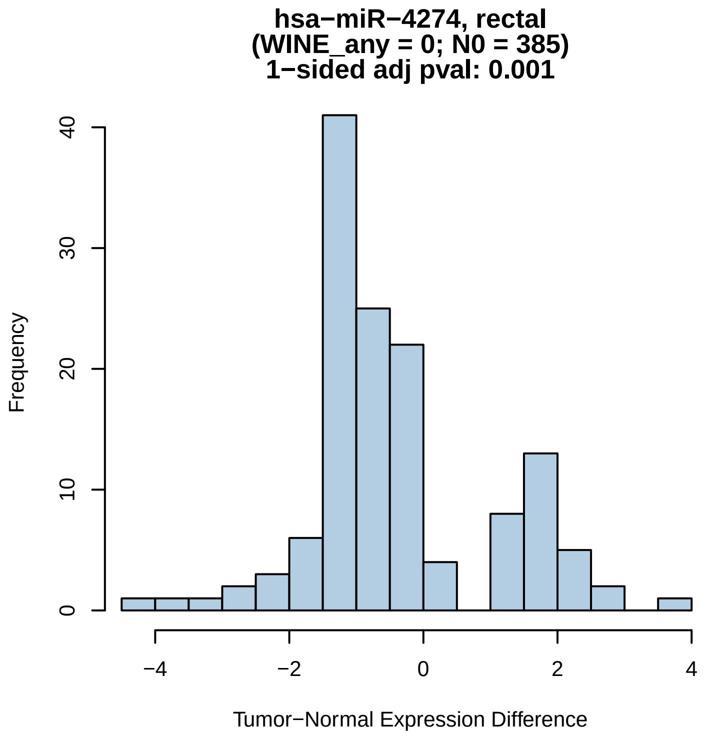

hsa−miR−4274, rectal
(WINE_any = 0; N0 = 385)
1−sided adj pval: 0.001
Frequency
0
10
20
30
40
−4
−2
0
2
4
Tumor−Normal Expression Difference


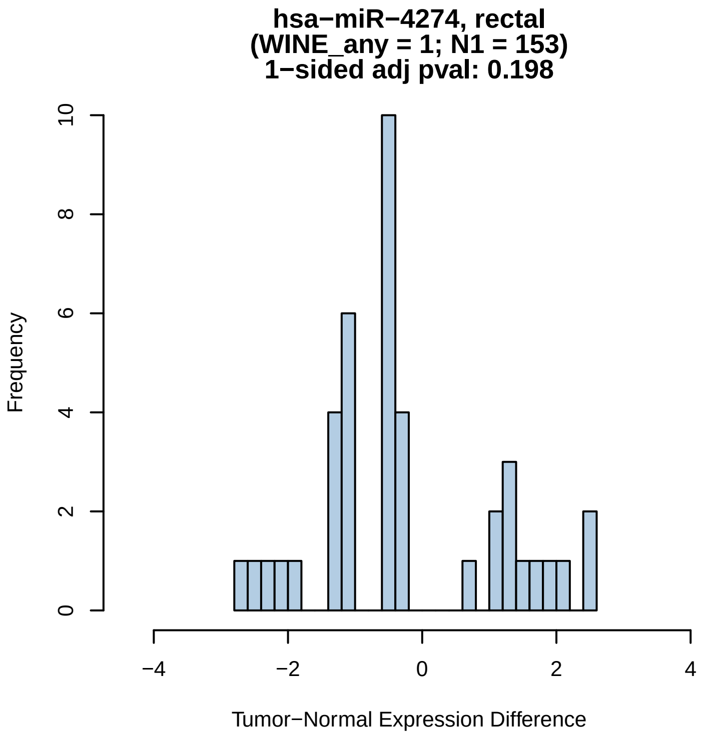

hsa−miR−4274, rectal
(WINE_any = 1; N1 = 153)
1−sided adj pval: 0.198
Frequency
0
2
4
6
8
10
−4
−2
0
2
4
Tumor−Normal Expression Difference

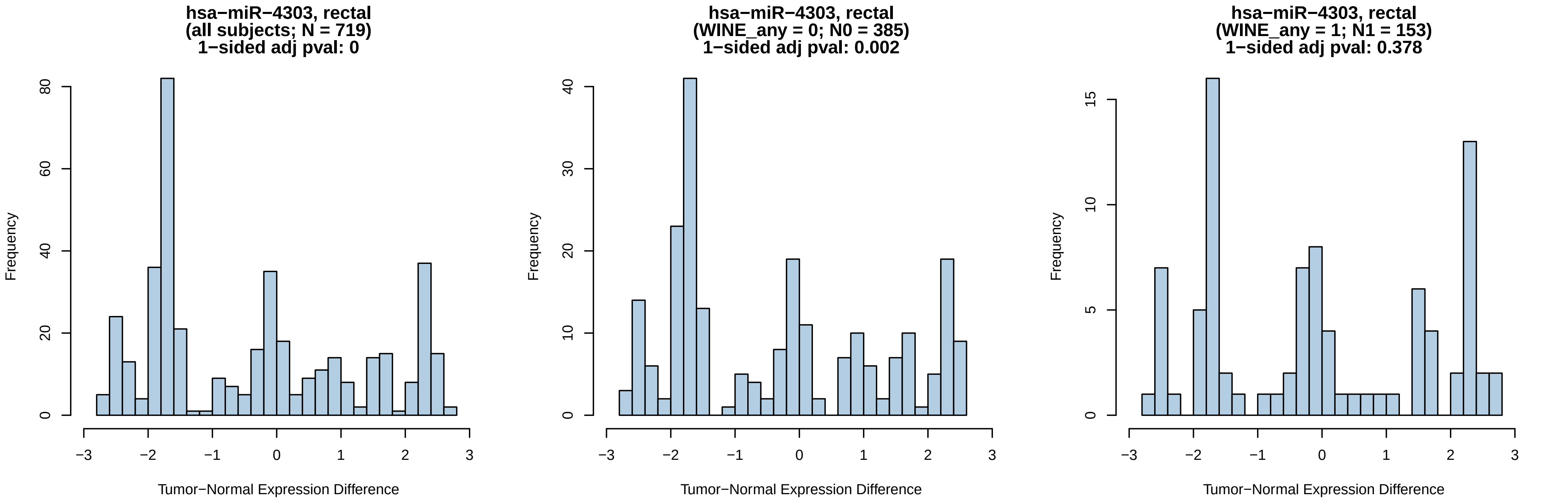
hsa−miR−4303, rectal
(all subjects; N = 719)
1−sided adj pval: 0
Frequency
Tumor−Normal Expression Difference
hsa−miR−4303, rectal
(WINE_any = 0; N0 = 385)
1−sided adj pval: 0.002
Frequency
Tumor−Normal Expression Difference
hsa−miR−4303, rectal
(WINE_any = 1; N1 = 153)
1−sided adj pval: 0.378
Frequency
Tumor−Normal Expression Difference

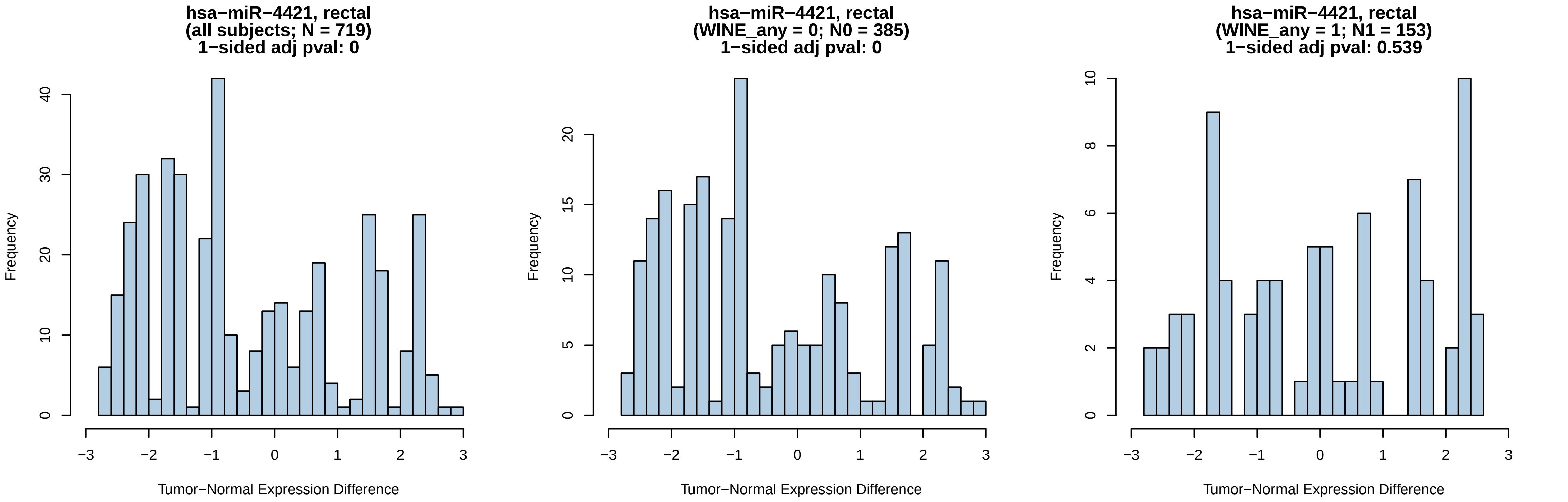
hsa−miR−4421, rectal
(all subjects; N = 719)
1−sided adj pval: 0
hsa−miR−4421, rectal
(WINE_any = 0; N0 = 385)
1−sided adj pval: 0
hsa−miR−4421, rectal
(WINE_any = 1; N1 = 153)
1−sided adj pval: 0.539
Frequency
Tumor−Normal Expression Difference

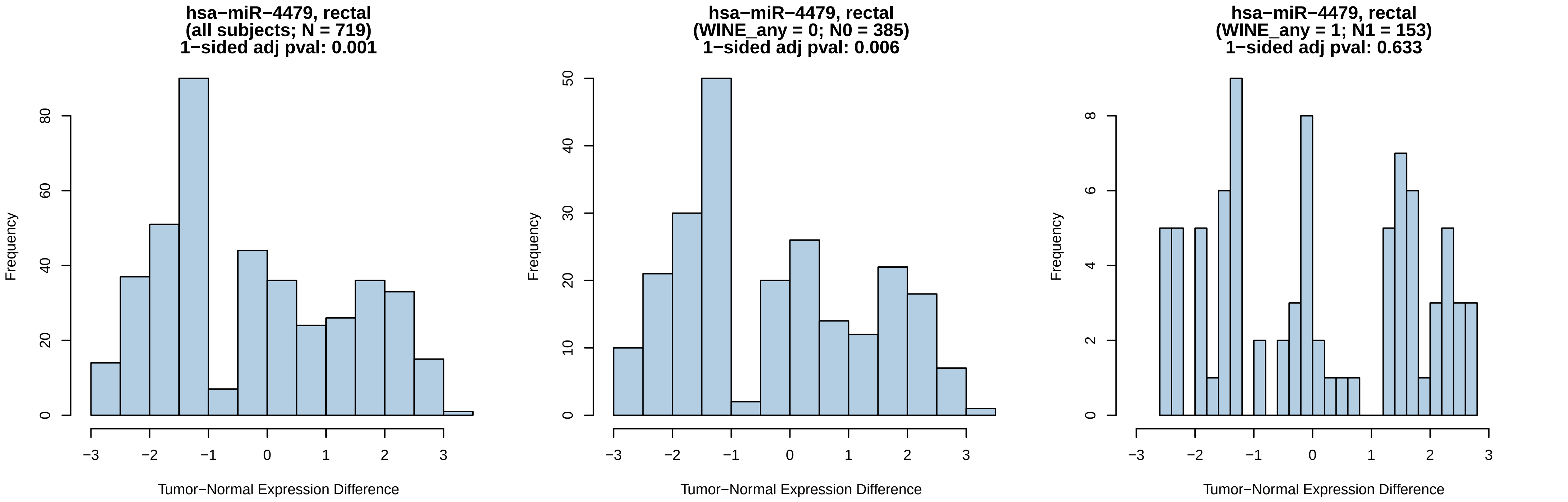

hsa−miR−4479, rectal
(all subjects; N = 719)
1−sided adj pval: 0.001
Frequency
Tumor−Normal Expression Difference
hsa−miR−4479, rectal
(WINE_any = 0; N0 = 385)
1−sided adj pval: 0.006
Frequency
Tumor−Normal Expression Difference
hsa−miR−4479, rectal
(WINE_any = 1; N1 = 153)
1−sided adj pval: 0.633
Frequency
Tumor−Normal Expression Difference

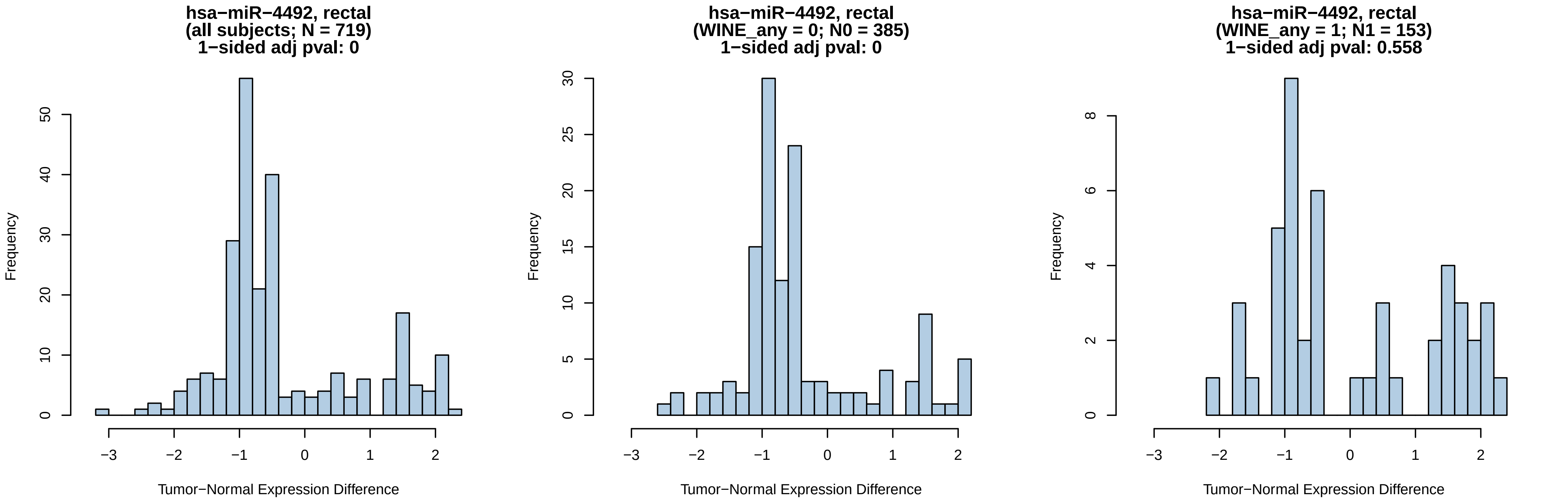

hsa−miR−4492, rectal
(all subjects; N = 719)
1−sided adj pval: 0
Frequency
Tumor−Normal Expression Difference
hsa−miR−4492, rectal
(WINE_any = 0; N0 = 385)
1−sided adj pval: 0
Frequency
Tumor−Normal Expression Difference
hsa−miR−4492, rectal
(WINE_any = 1; N1 = 153)
1−sided adj pval: 0.558
Frequency
Tumor−Normal Expression Difference

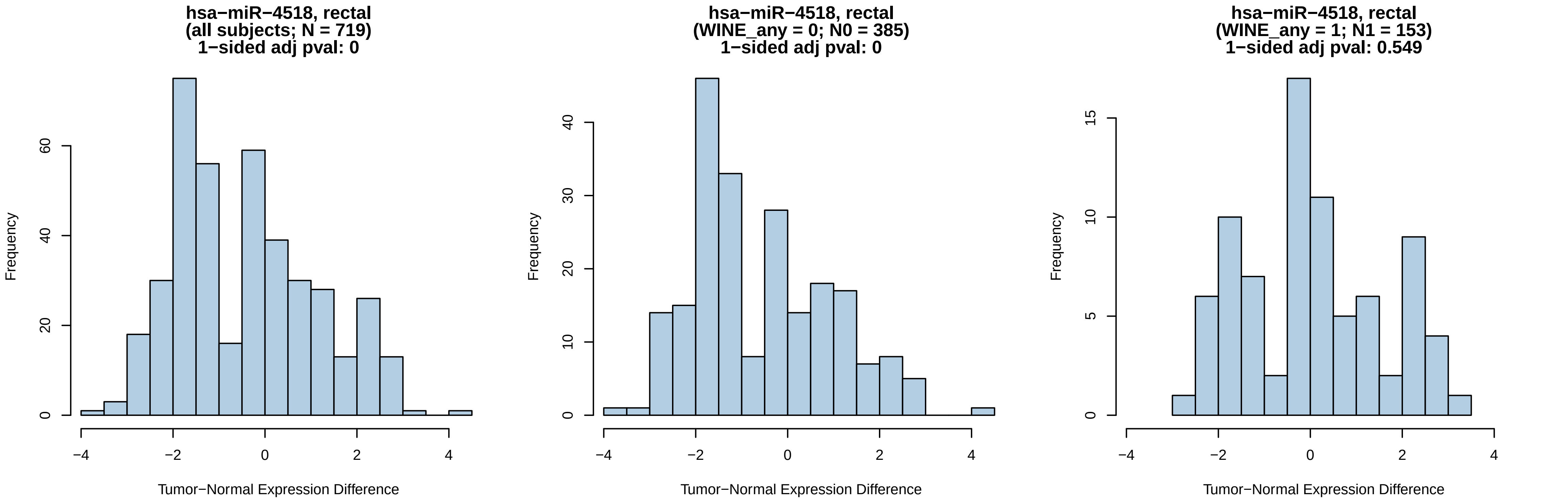
hsa−miR−4518, rectal
(all subjects; N = 719)
1−sided adj pval: 0
Frequency
Tumor−Normal Expression Difference
hsa−miR−4518, rectal
(WINE_any = 0; N0 = 385)
1−sided adj pval: 0
Frequency
Tumor−Normal Expression Difference
hsa−miR−4518, rectal
(WINE_any = 1; N1 = 153)
1−sided adj pval: 0.549
Frequency
Tumor−Normal Expression Difference

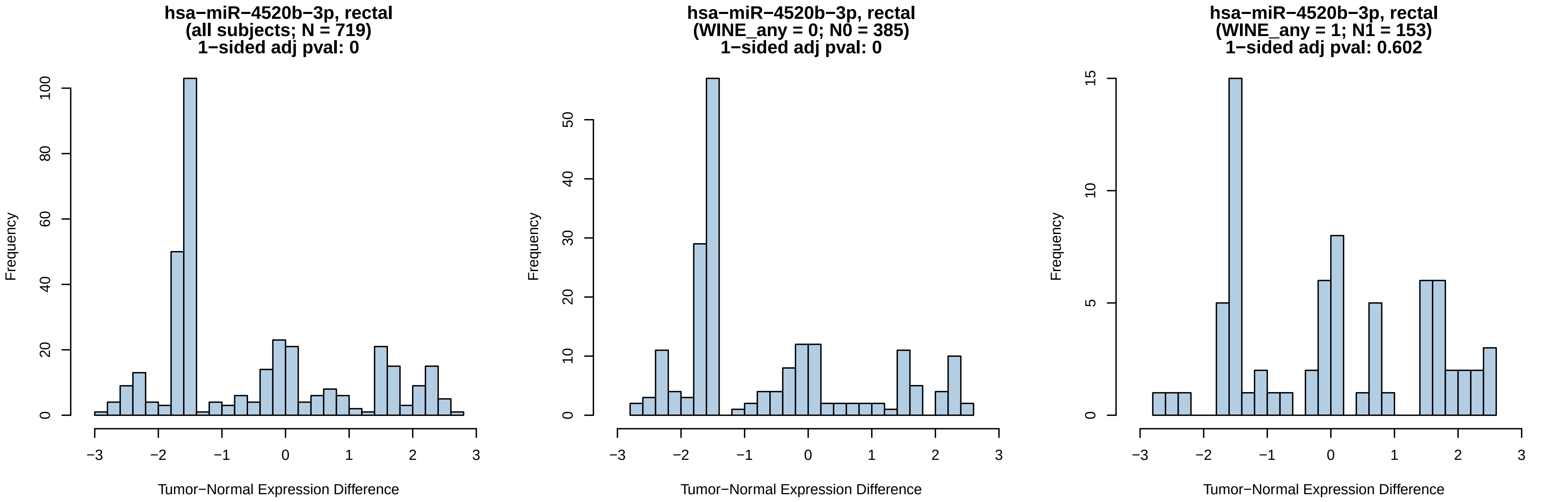
hsa−miR−4520b−3p, rectal
(all subjects; N = 719)
1−sided adj pval: 0
Frequency
Tumor−Normal Expression Difference
hsa−miR−4520b−3p, rectal
(WINE_any = 0; N0 = 385)
1−sided adj pval: 0
Frequency
Tumor−Normal Expression Difference
hsa−miR−4520b−3p, rectal
(WINE_any = 1; N1 = 153)
1−sided adj pval: 0.602
Frequency
Tumor−Normal Expression Difference

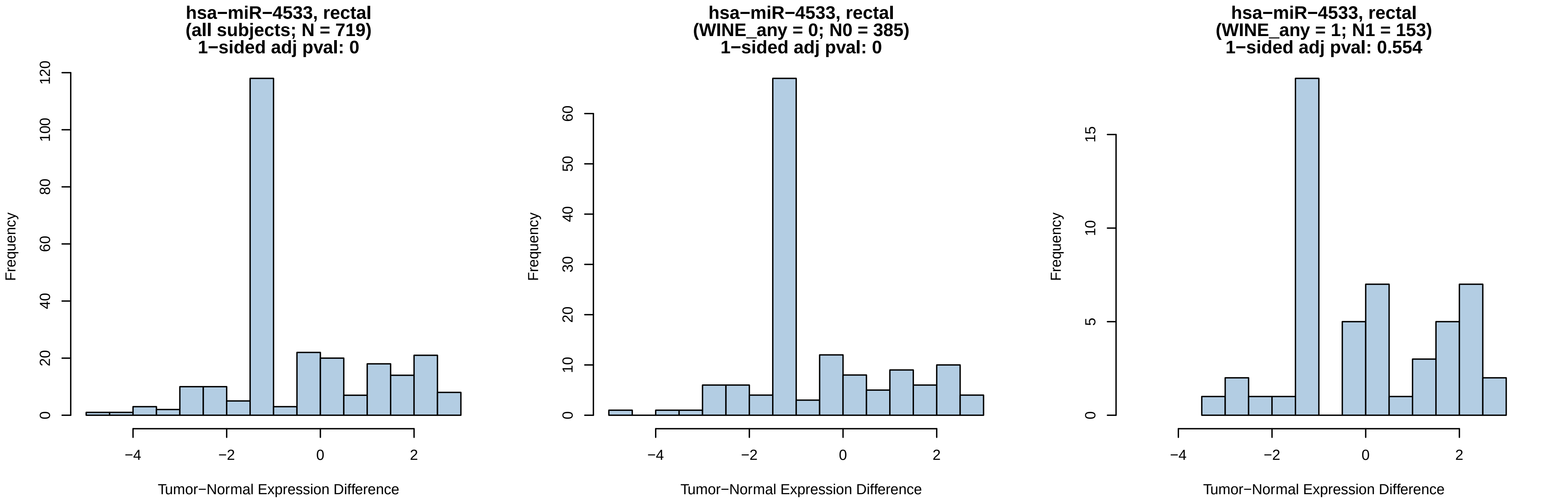

hsa−miR−4533, rectal
(all subjects; N = 719)
1−sided adj pval: 0
Frequency
0
20
40
60
80
100
120
−4
−2
0
2
Tumor−Normal Expression Difference
hsa−miR−4533, rectal
(WINE_any = 0; N0 = 385)
1−sided adj pval: 0
Frequency
0
10
20
30
40
50
60
−4
−2
0
2
Tumor−Normal Expression Difference
hsa−miR−4533, rectal
(WINE_any = 1; N1 = 153)
1−sided adj pval: 0.554
Frequency
0
5
10
15
−4
−2
0
2
Tumor−Normal Expression Difference

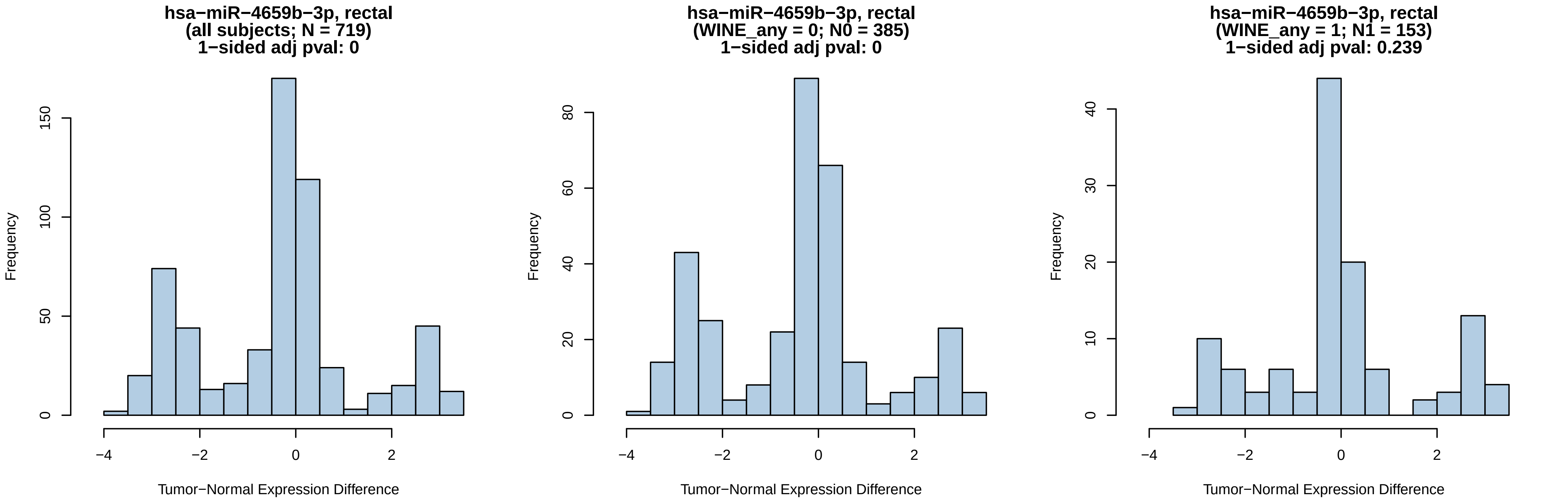
hsa−miR−4659b−3p, rectal
(all subjects; N = 719)
1−sided adj pval: 0
Frequency
Tumor−Normal Expression Difference
hsa−miR−4659b−3p, rectal
(WINE_any = 0; N0 = 385)
1−sided adj pval: 0
Frequency
Tumor−Normal Expression Difference
hsa−miR−4659b−3p, rectal
(WINE_any = 1; N1 = 153)
1−sided adj pval: 0.239
Frequency
Tumor−Normal Expression Difference

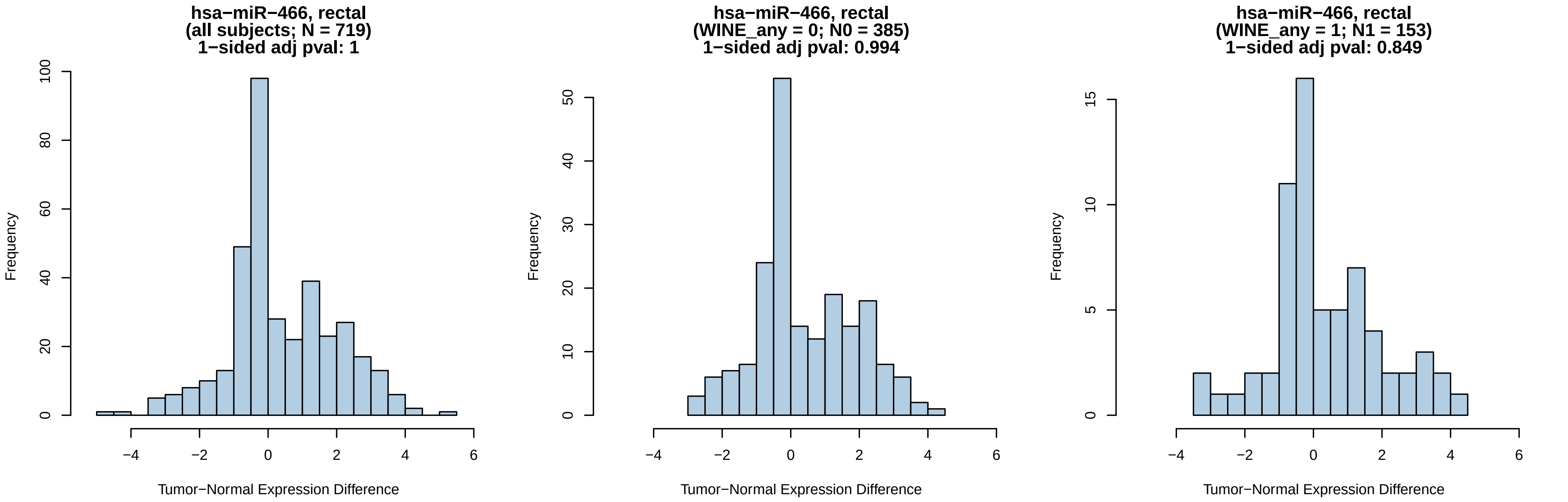
hsa−miR−466, rectal
(all subjects; N = 719)
1−sided adj pval: 1
Frequency
0
20
40
60
80
100
−4
−2
0
2
4
6
Tumor−Normal Expression Difference
hsa−miR−466, rectal
(WINE_any = 0; N0 = 385)
1−sided adj pval: 0.994
Frequency
0
10
20
30
40
50
−4
−2
0
2
4
6
Tumor−Normal Expression Difference
hsa−miR−466, rectal
(WINE_any = 1; N1 = 153)
1−sided adj pval: 0.849
Frequency
0
5
10
15
−4
−2
0
2
4
6
Tumor−Normal Expression Difference

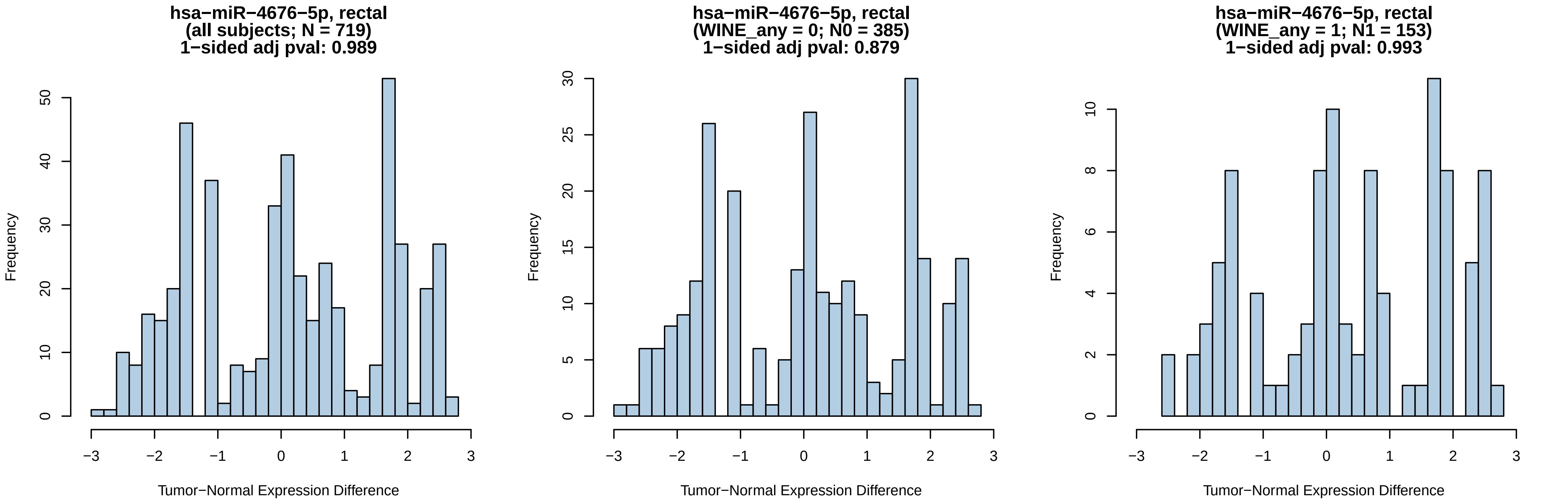

hsa−miR−4676−5p, rectal
(all subjects; N = 719)
1−sided adj pval: 0.989
Frequency
Tumor−Normal Expression Difference
hsa−miR−4676−5p, rectal
(WINE_any = 0; N0 = 385)
1−sided adj pval: 0.879
Frequency
Tumor−Normal Expression Difference
hsa−miR−4676−5p, rectal
(WINE_any = 1; N1 = 153)
1−sided adj pval: 0.993
Frequency
Tumor−Normal Expression Difference

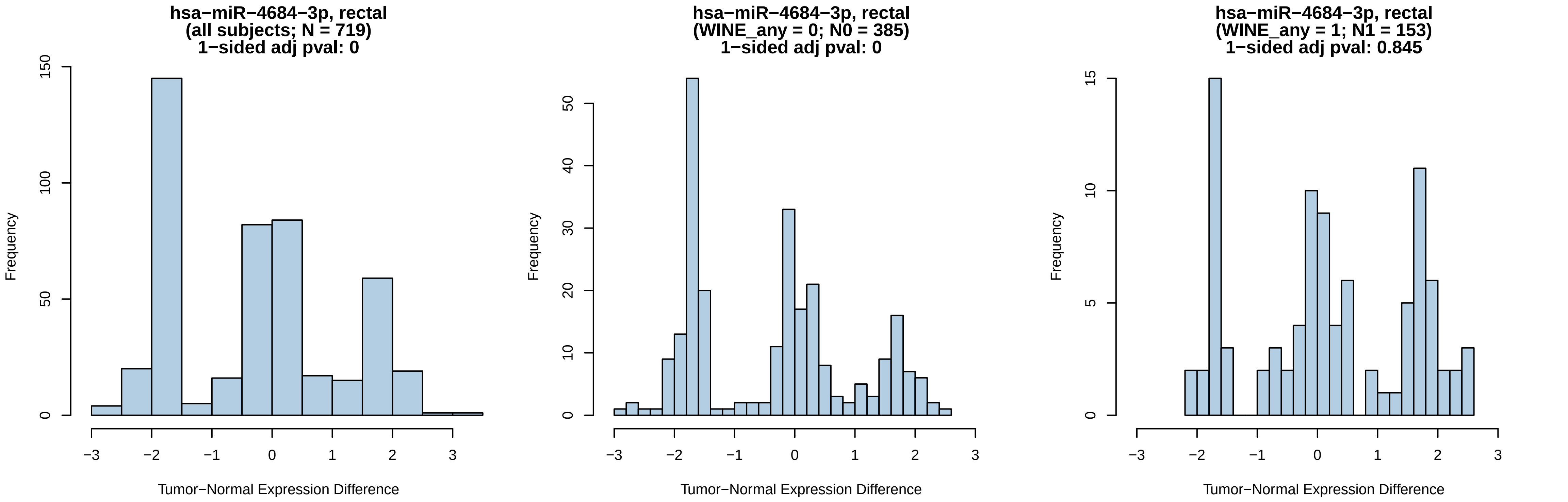
hsa−miR−4684−3p, rectal
(all subjects; N = 719)
1−sided adj pval: 0
hsa−miR−4684−3p, rectal
(WINE_any = 0; N0 = 385)
1−sided adj pval: 0
hsa−miR−4684−3p, rectal
(WINE_any = 1; N1 = 153)
1−sided adj pval: 0.845
Frequency
Tumor−Normal Expression Difference

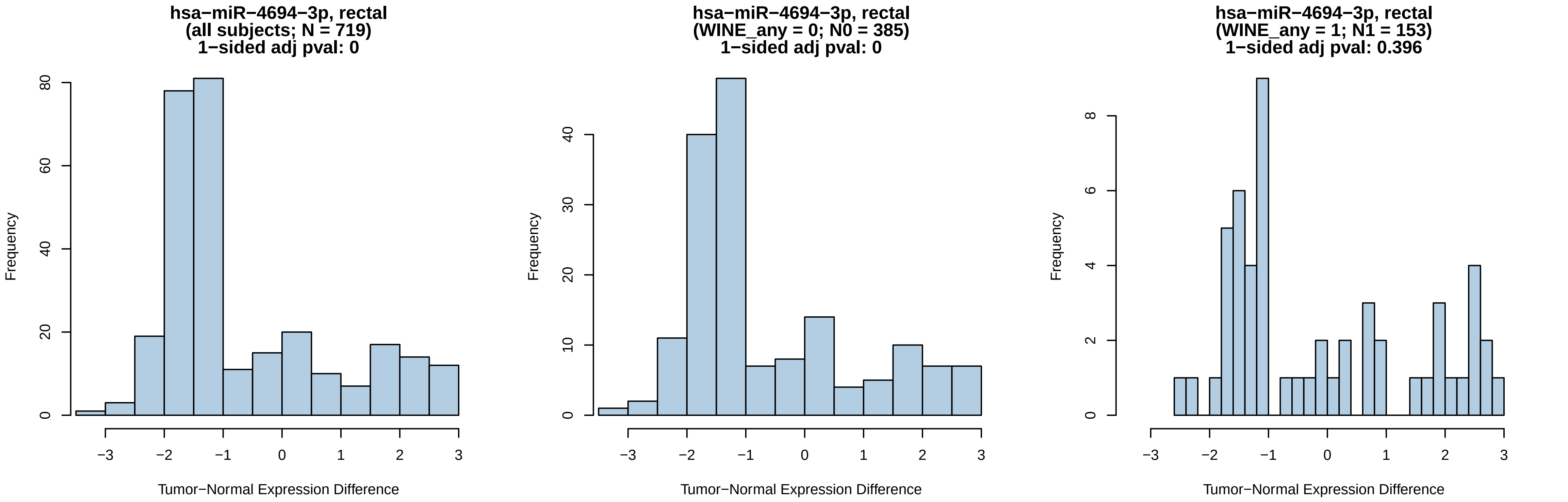
hsa−miR−4694−3p, rectal
(all subjects; N = 719)
1−sided adj pval: 0
hsa−miR−4694−3p, rectal
(WINE_any = 0; N0 = 385)
1−sided adj pval: 0
hsa−miR−4694−3p, rectal
(WINE_any = 1; N1 = 153)
1−sided adj pval: 0.396
Frequency
Tumor−Normal Expression Difference

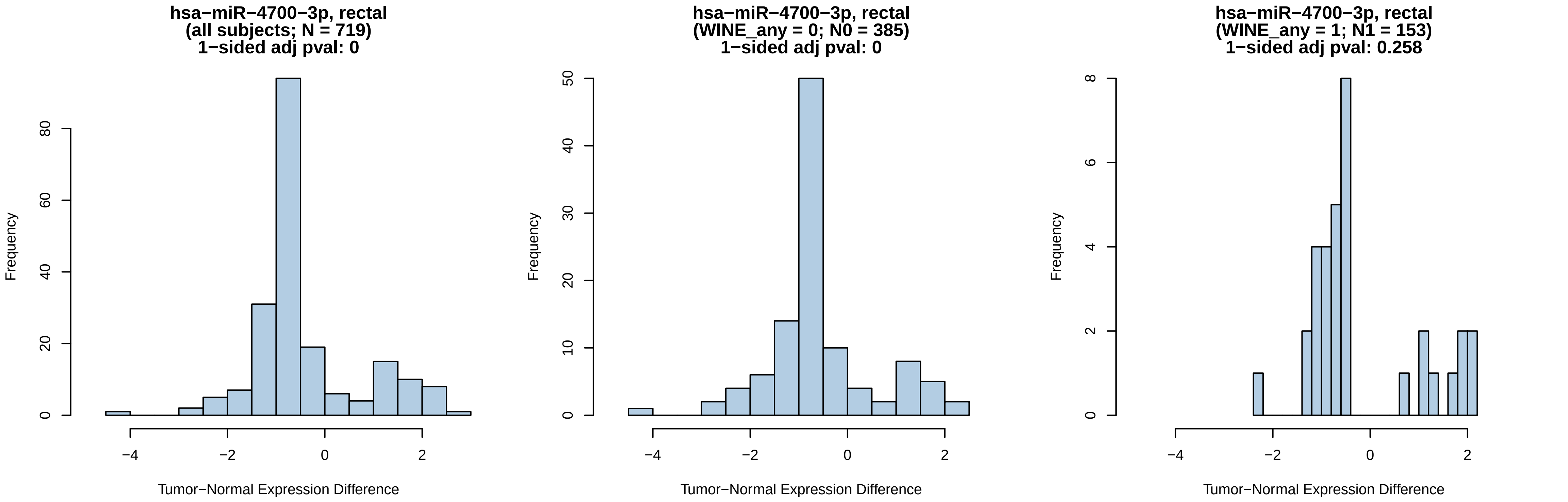
hsa−miR−4700−3p, rectal
(all subjects; N = 719)
1−sided adj pval: 0
Frequency
Tumor−Normal Expression Difference
hsa−miR−4700−3p, rectal
(WINE_any = 0; N0 = 385)
1−sided adj pval: 0
Frequency
Tumor−Normal Expression Difference
hsa−miR−4700−3p, rectal
(WINE_any = 1; N1 = 153)
1−sided adj pval: 0.258
Frequency
Tumor−Normal Expression Difference

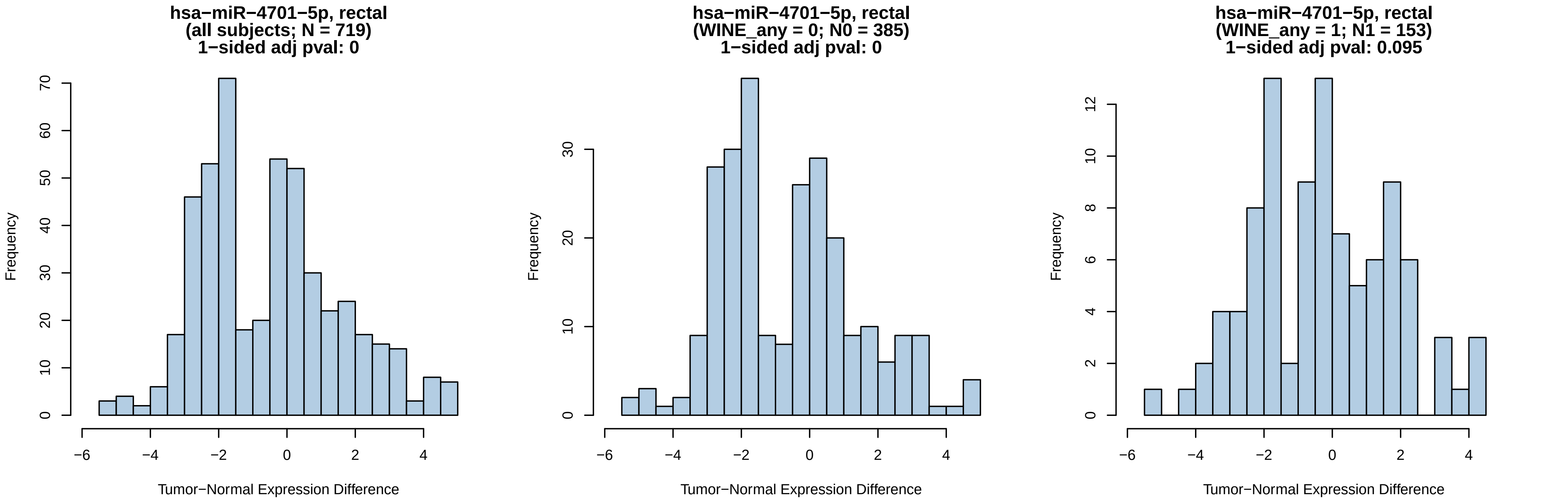
hsa−miR−4701−5p, rectal
(all subjects; N = 719)
1−sided adj pval: 0
hsa−miR−4701−5p, rectal
(WINE_any = 0; N0 = 385)
1−sided adj pval: 0
hsa−miR−4701−5p, rectal
(WINE_any = 1; N1 = 153)
1−sided adj pval: 0.095
Frequency
Tumor−Normal Expression Difference

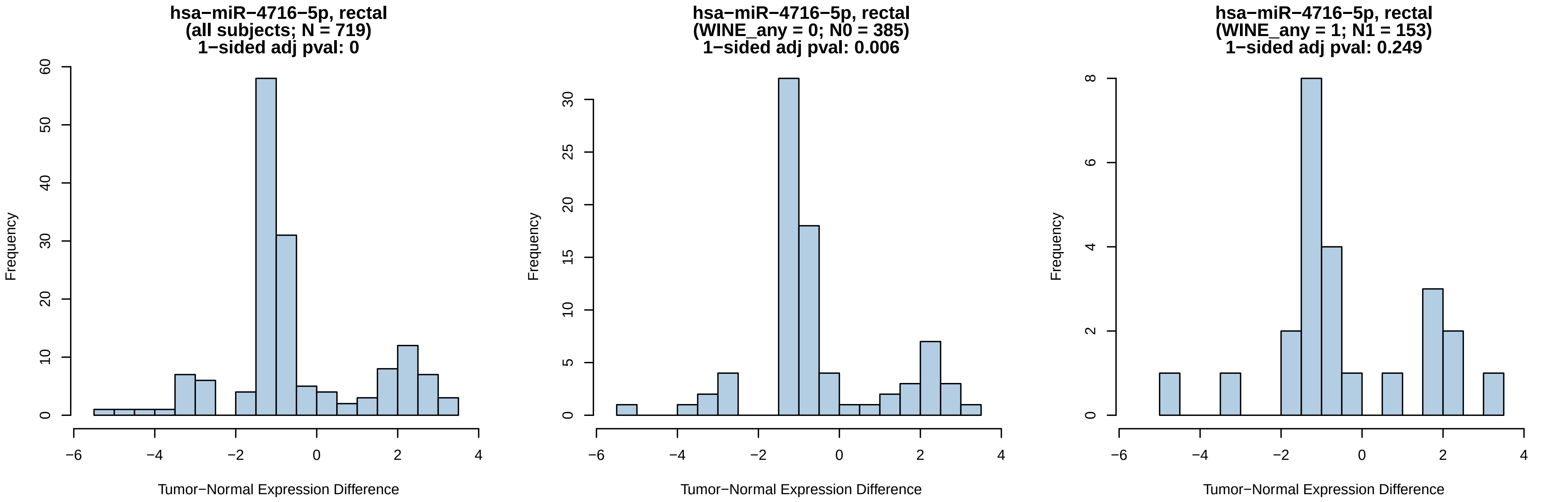
hsa−miR−4716−5p, rectal
(all subjects; N = 719)
1−sided adj pval: 0
Frequency
Tumor−Normal Expression Difference
hsa−miR−4716−5p, rectal
(WINE_any = 0; N0 = 385)
1−sided adj pval: 0.006
Frequency
Tumor−Normal Expression Difference
hsa−miR−4716−5p, rectal
(WINE_any = 1; N1 = 153)
1−sided adj pval: 0.249
Frequency
Tumor−Normal Expression Difference

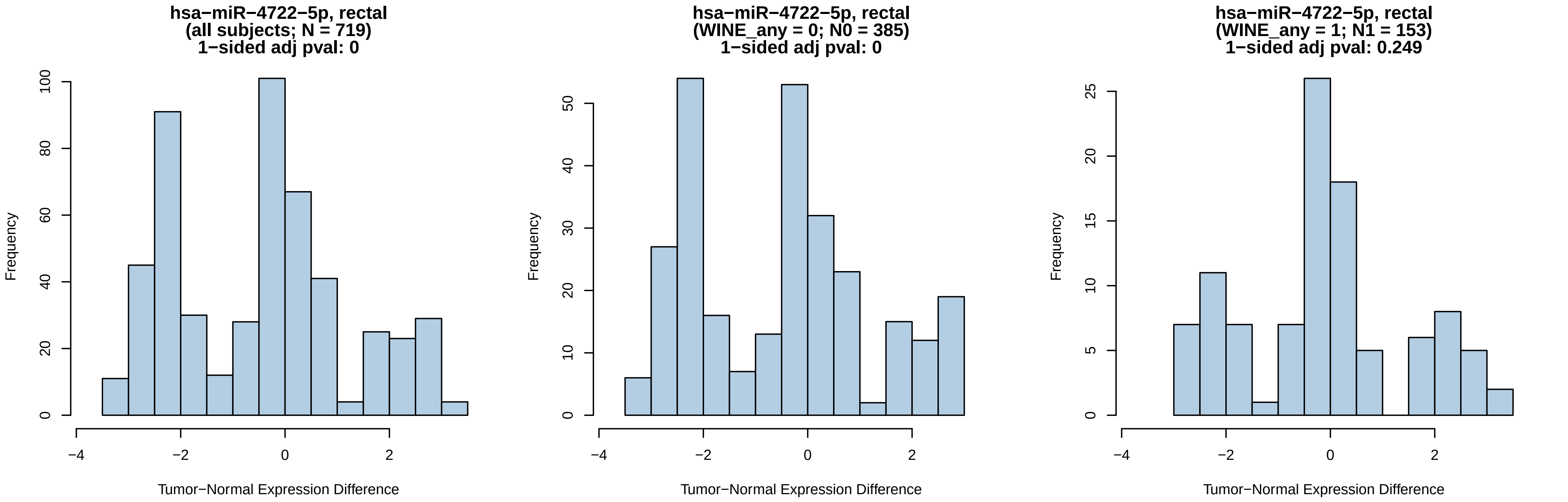

hsa−miR−4722−5p, rectal
(all subjects; N = 719)
1−sided adj pval: 0
hsa−miR−4722−5p, rectal
(WINE_any = 0; N0 = 385)
1−sided adj pval: 0
hsa−miR−4722−5p, rectal
(WINE_any = 1; N1 = 153)
1−sided adj pval: 0.249
Frequency
Tumor−Normal Expression Difference

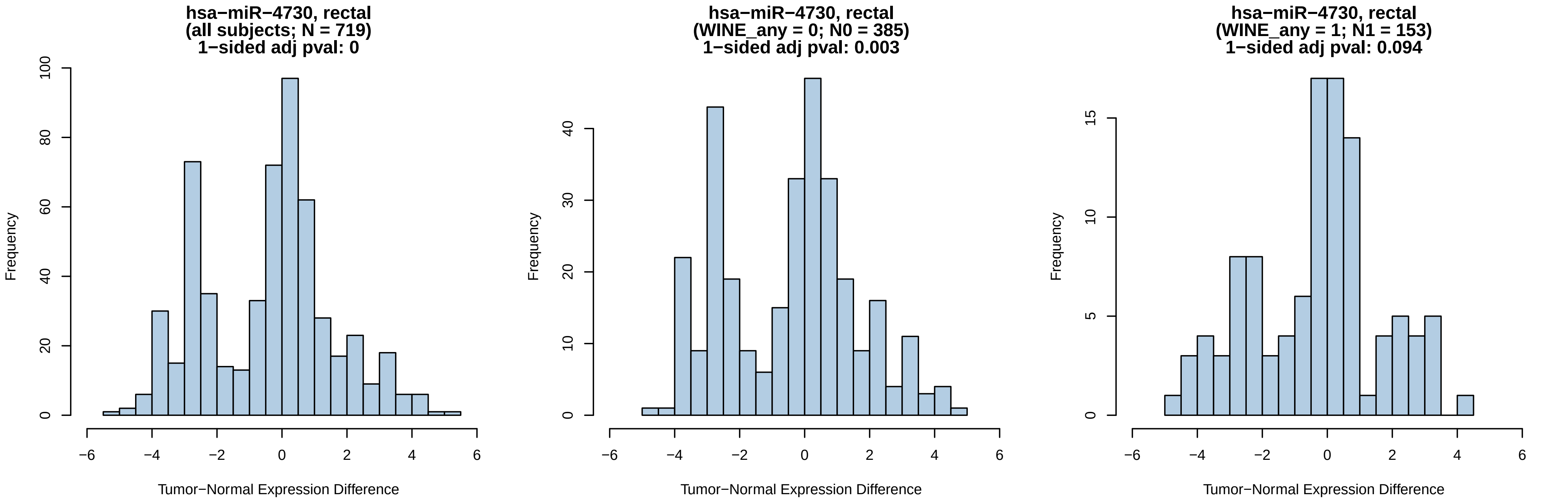
hsa−miR−4730, rectal
(all subjects; N = 719)
1−sided adj pval: 0
hsa−miR−4730, rectal
(WINE_any = 0; N0 = 385)
1−sided adj pval: 0.003
hsa−miR−4730, rectal
(WINE_any = 1; N1 = 153)
1−sided adj pval: 0.094
Frequency
Tumor−Normal Expression Difference
0
20
40
60
80
100
10
30
5
15
−6
−4
−2
0
2
4
6

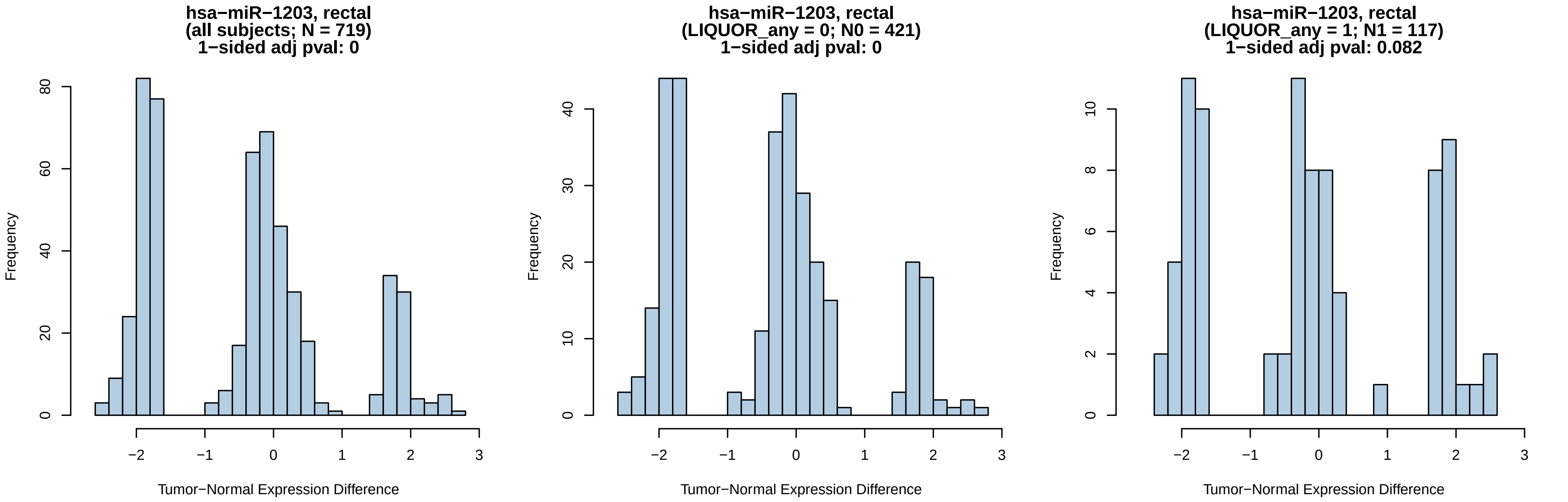
hsa−miR−1203, rectal
(all subjects; N = 719)
1−sided adj pval: 0
hsa−miR−1203, rectal
(LIQUOR_any = 0; N0 = 421)
1−sided adj pval: 0
hsa−miR−1203, rectal
(LIQUOR_any = 1; N1 = 117)
1−sided adj pval: 0.082
Frequency
Tumor−Normal Expression Difference
0
20
40
60
80
10
30
2
4
6
8
−2
−1
1
3

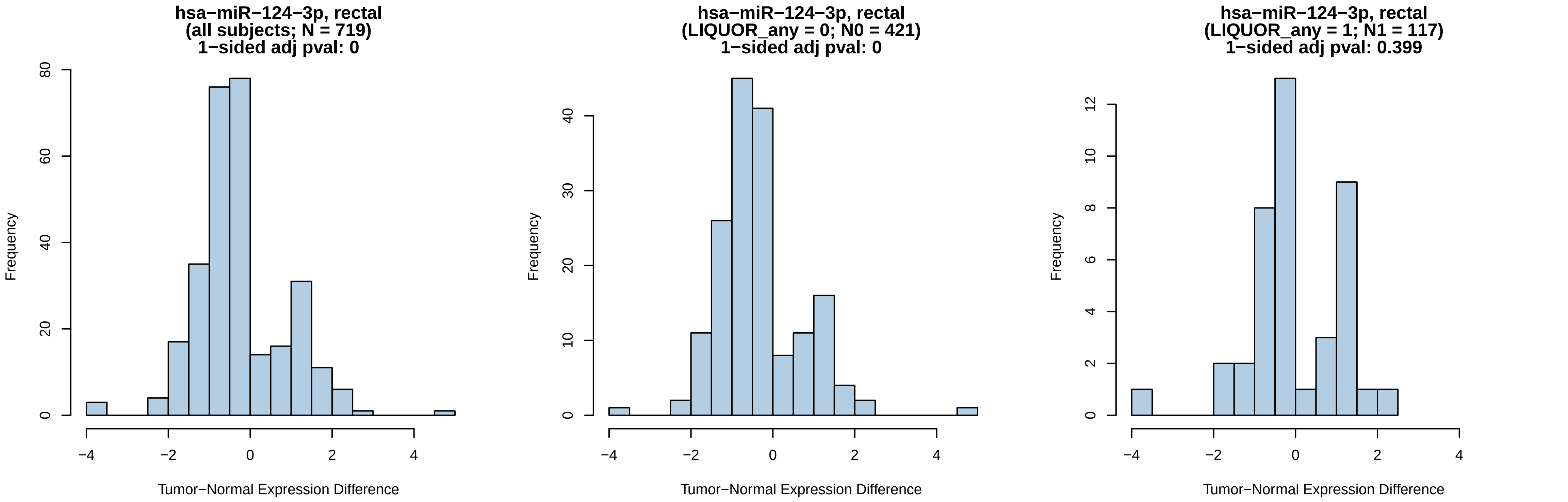

hsa−miR−124−3p, rectal
(all subjects; N = 719)
1−sided adj pval: 0
Frequency
0
20
40
60
80
−4
−2
0
2
4
Tumor−Normal Expression Difference
hsa−miR−124−3p, rectal
(LIQUOR_any = 0; N0 = 421)
1−sided adj pval: 0
Frequency
0
10
20
30
40
−4
−2
0
2
4
Tumor−Normal Expression Difference
hsa−miR−124−3p, rectal
(LIQUOR_any = 1; N1 = 117)
1−sided adj pval: 0.399
Frequency
0
2
4
6
8
10
12
−4
−2
0
2
4
Tumor−Normal Expression Difference

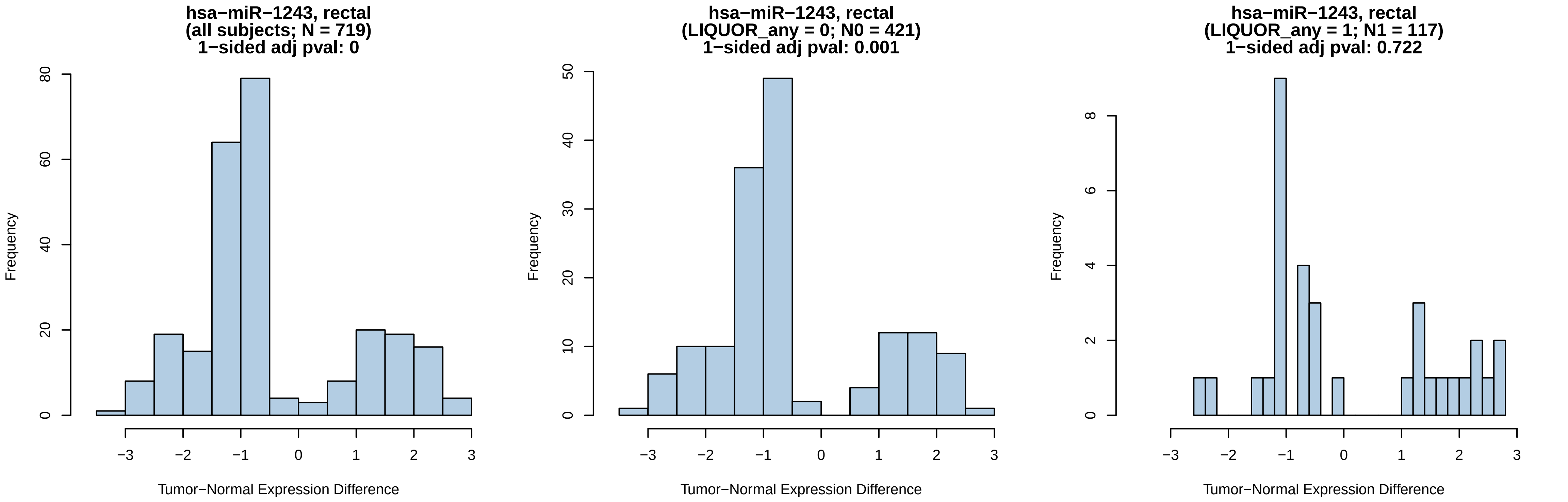

hsa−miR−1243, rectal
(all subjects; N = 719)
1−sided adj pval: 0
hsa−miR−1243, rectal
(LIQUOR_any = 0; N0 = 421)
1−sided adj pval: 0.001
hsa−miR−1243, rectal
(LIQUOR_any = 1; N1 = 117)
1−sided adj pval: 0.722
Frequency
Tumor−Normal Expression Difference

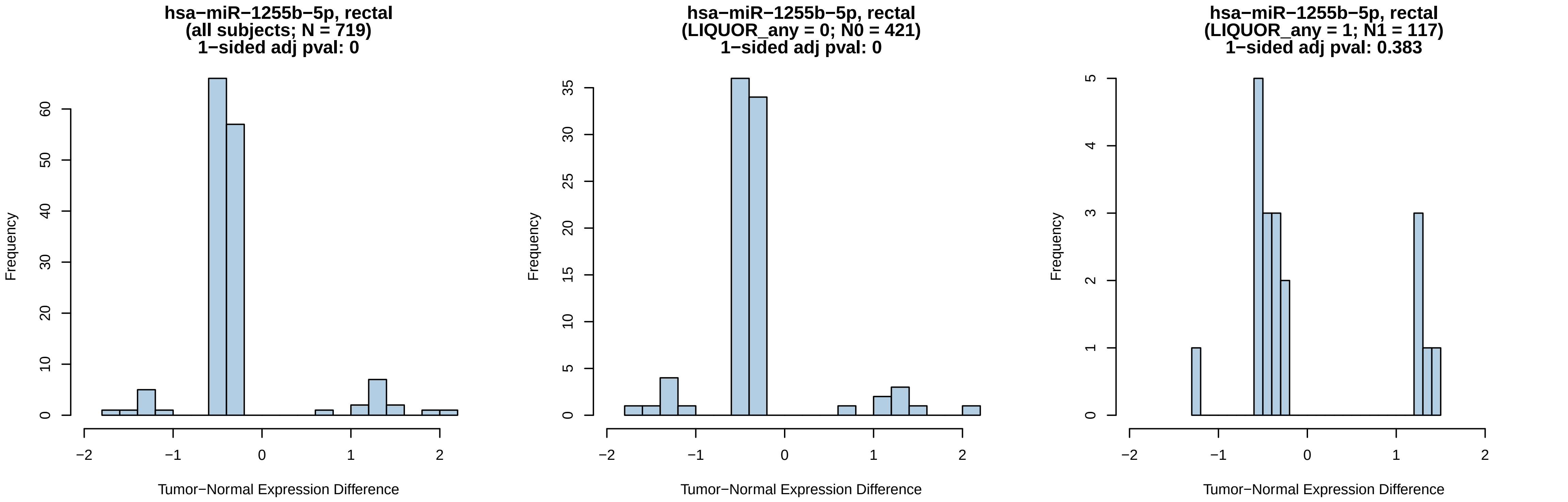

hsa−miR−1255b−5p, rectal
(all subjects; N = 719)
1−sided adj pval: 0
Frequency
Tumor−Normal Expression Difference
hsa−miR−1255b−5p, rectal
(LIQUOR_any = 0; N0 = 421)
1−sided adj pval: 0
Frequency
Tumor−Normal Expression Difference
hsa−miR−1255b−5p, rectal
(LIQUOR_any = 1; N1 = 117)
1−sided adj pval: 0.383
Frequency
Tumor−Normal Expression Difference

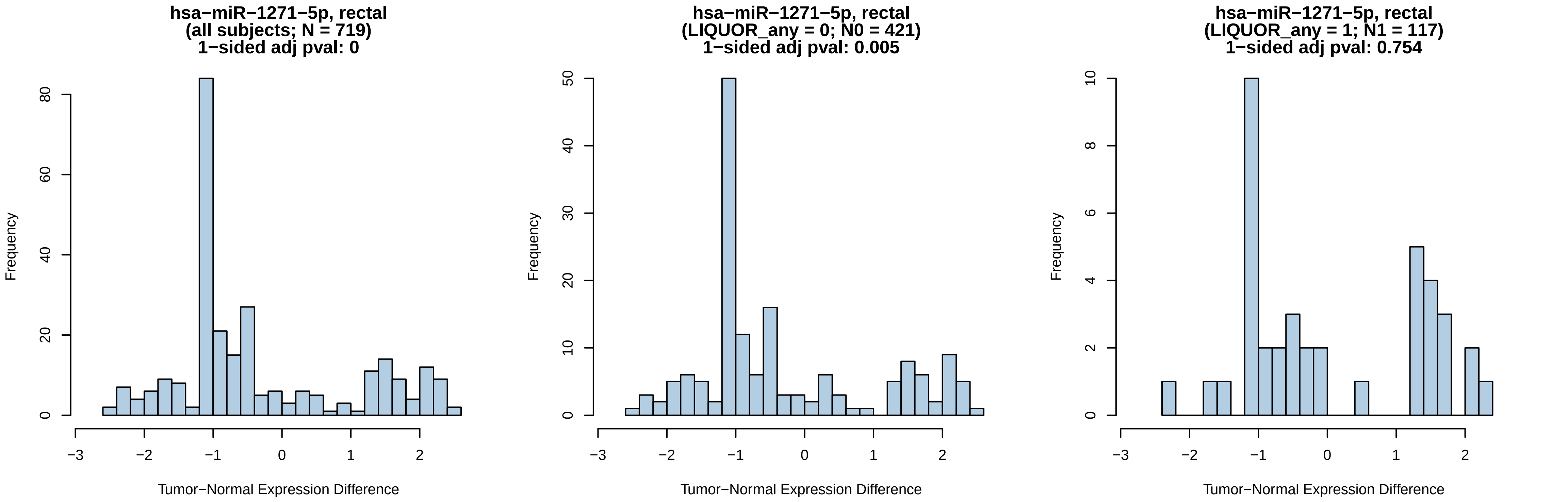
hsa−miR−1271−5p, rectal
(all subjects; N = 719)
1−sided adj pval: 0
hsa−miR−1271−5p, rectal
(LIQUOR_any = 0; N0 = 421)
1−sided adj pval: 0.005
hsa−miR−1271−5p, rectal
(LIQUOR_any = 1; N1 = 117)
1−sided adj pval: 0.754
Frequency
Tumor−Normal Expression Difference

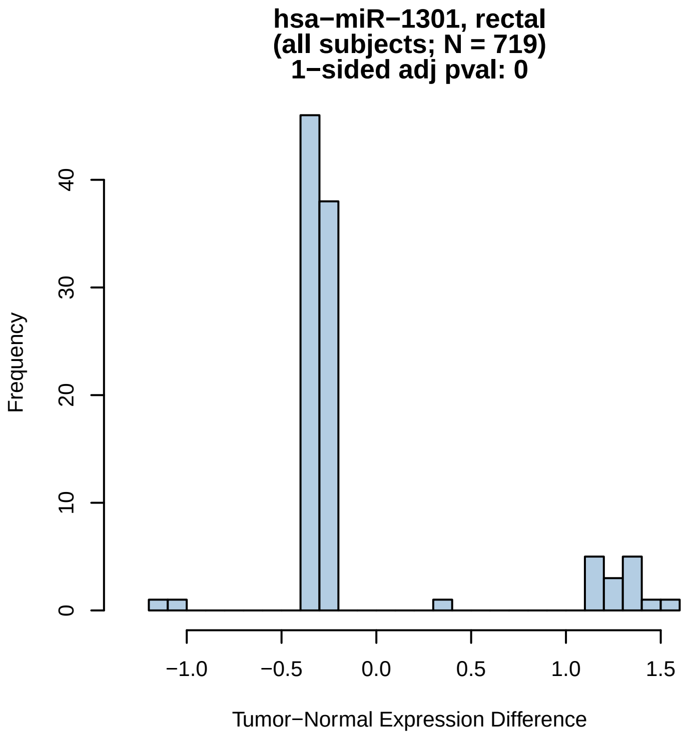
hsa−miR−1301, rectal
(all subjects; N = 719)
1−sided adj pval: 0
Frequency
0
10
20
30
40
−1.0
−0.5
0.0
0.5
1.0
1.5
Tumor−Normal Expression Difference

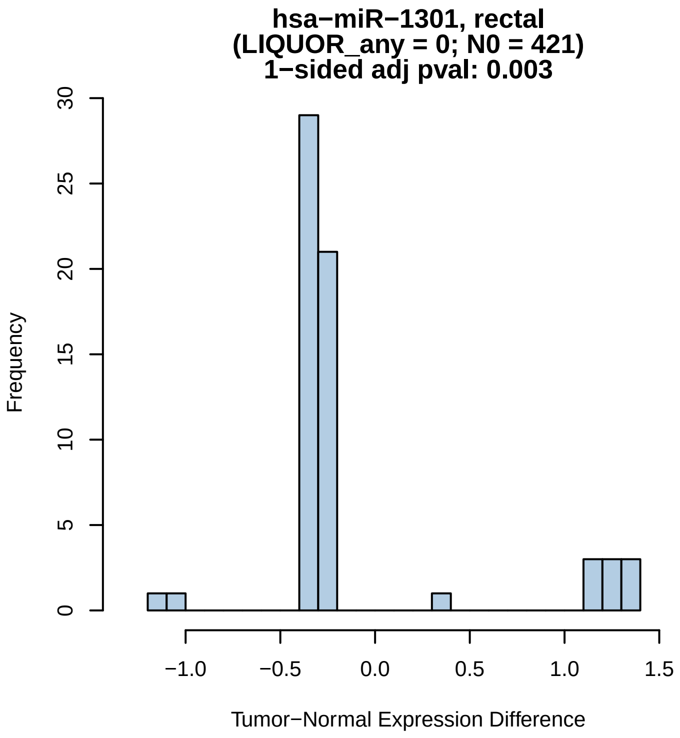
hsa−miR−1301, rectal
(LIQUOR_any = 0; N0 = 421)
1−sided adj pval: 0.003
Frequency
0
5
10
15
20
25
30
−1.0
−0.5
0.0
0.5
1.0
1.5
Tumor−Normal Expression Difference

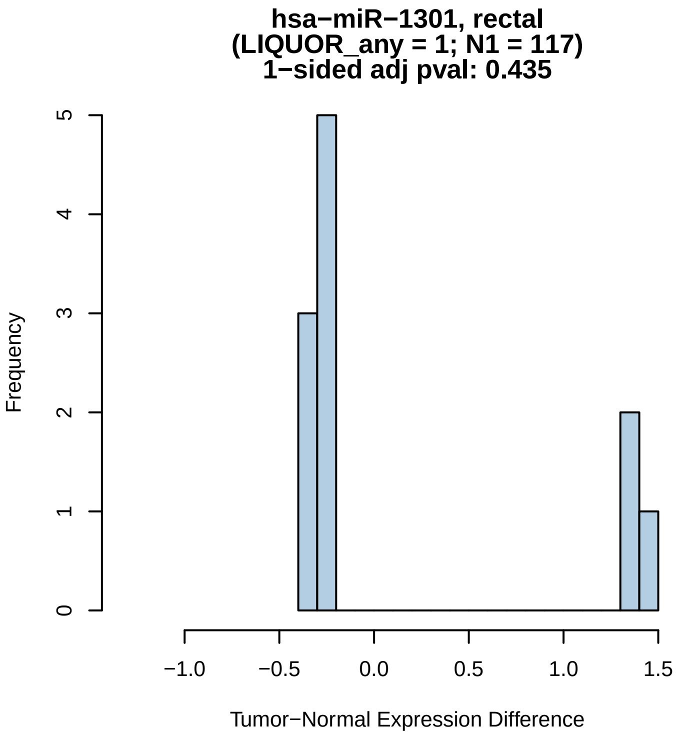
hsa−miR−1301, rectal
(LIQUOR_any = 1; N1 = 117)
1−sided adj pval: 0.435
Frequency
0
1
2
3
4
5
−1.0
−0.5
0.0
0.5
1.0
1.5
Tumor−Normal Expression Difference

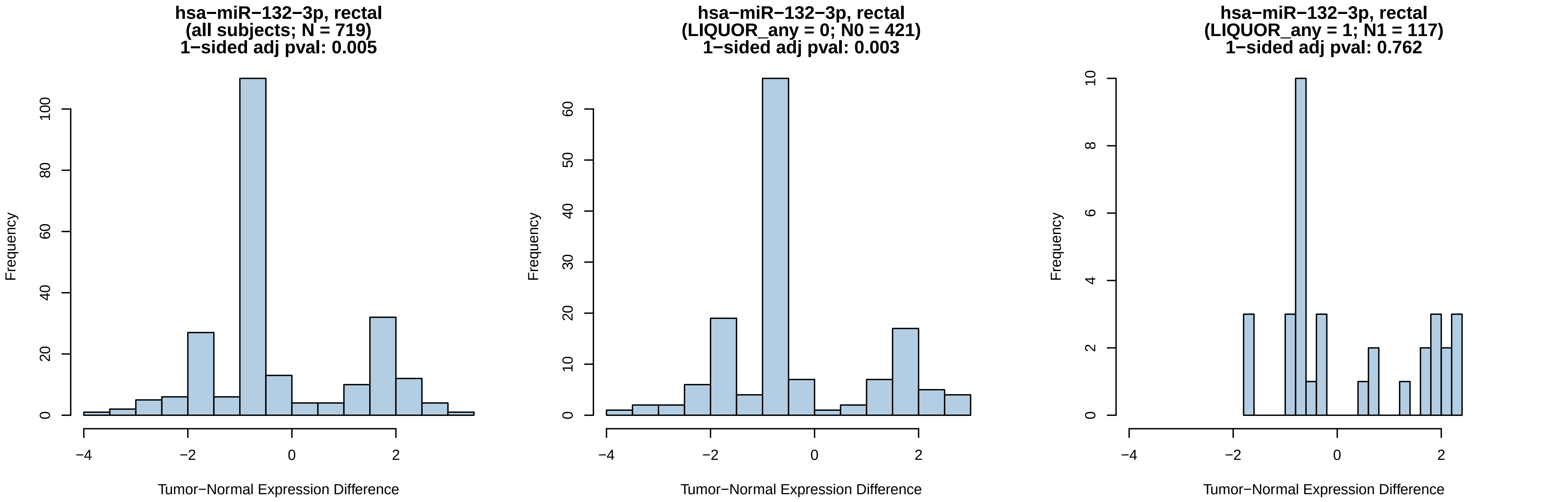

hsa−miR−132−3p, rectal
(all subjects; N = 719)
1−sided adj pval: 0.005
Frequency
Tumor−Normal Expression Difference
hsa−miR−132−3p, rectal
(LIQUOR_any = 0; N0 = 421)
1−sided adj pval: 0.003
Frequency
Tumor−Normal Expression Difference
hsa−miR−132−3p, rectal
(LIQUOR_any = 1; N1 = 117)
1−sided adj pval: 0.762
Frequency
Tumor−Normal Expression Difference

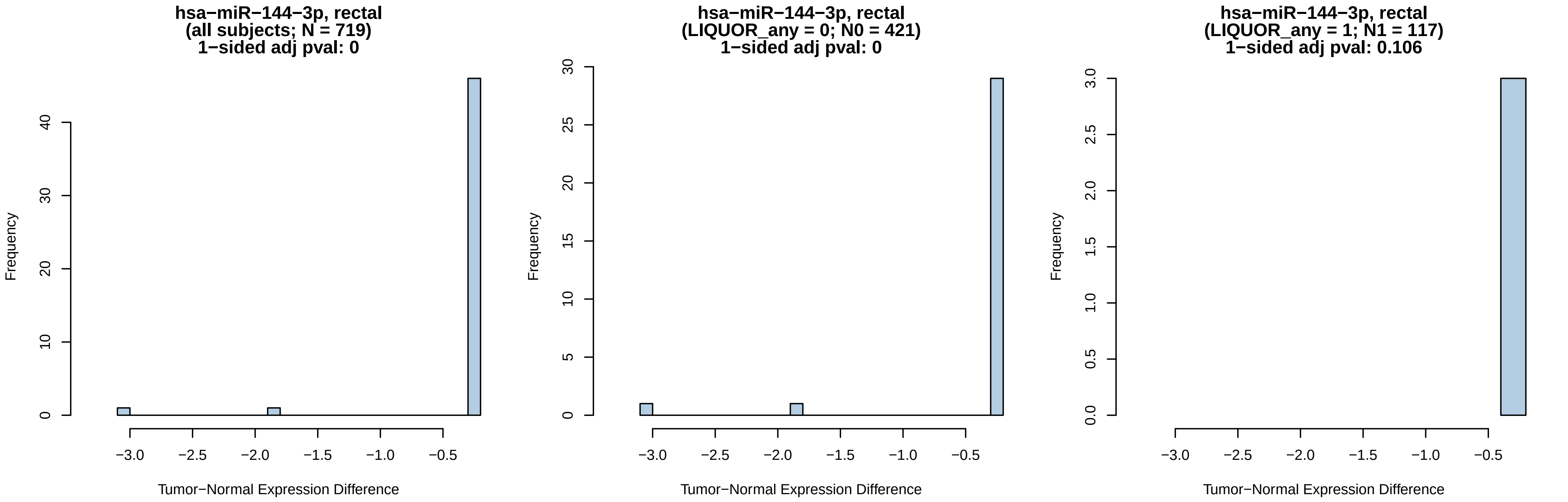

hsa−miR−144−3p, rectal
(all subjects; N = 719)
1−sided adj pval: 0
Frequency
0
10
20
30
40
−3.0
−2.5
−2.0
−1.5
−1.0
−0.5
Tumor−Normal Expression Difference
hsa−miR−144−3p, rectal
(LIQUOR_any = 0; N0 = 421)
1−sided adj pval: 0
Frequency
0
5
10
15
20
25
30
−3.0
−2.5
−2.0
−1.5
−1.0
−0.5
Tumor−Normal Expression Difference
hsa−miR−144−3p, rectal
(LIQUOR_any = 1; N1 = 117)
1−sided adj pval: 0.106
Frequency
0.0
0.5
1.0
1.5
2.0
2.5
3.0
−3.0
−2.5
−2.0
−1.5
−1.0
−0.5
Tumor−Normal Expression Difference

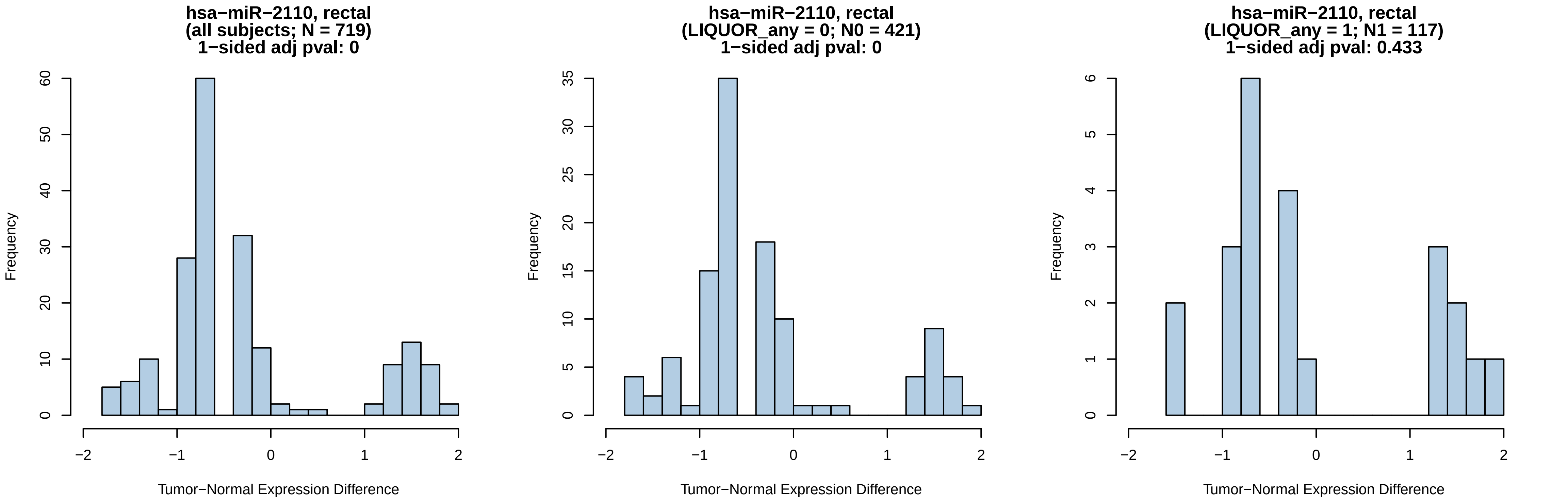
hsa−miR−2110, rectal
(all subjects; N = 719)
1−sided adj pval: 0
Frequency
0
10
20
30
40
50
60
−2
−1
0
1
2
Tumor−Normal Expression Difference
hsa−miR−2110, rectal
(LIQUOR_any = 0; N0 = 421)
1−sided adj pval: 0
Frequency
0
5
10
15
20
25
30
35
−2
−1
0
1
2
Tumor−Normal Expression Difference
hsa−miR−2110, rectal
(LIQUOR_any = 1; N1 = 117)
1−sided adj pval: 0.433
Frequency
0
1
2
3
4
5
6
−2
−1
0
1
2
Tumor−Normal Expression Difference

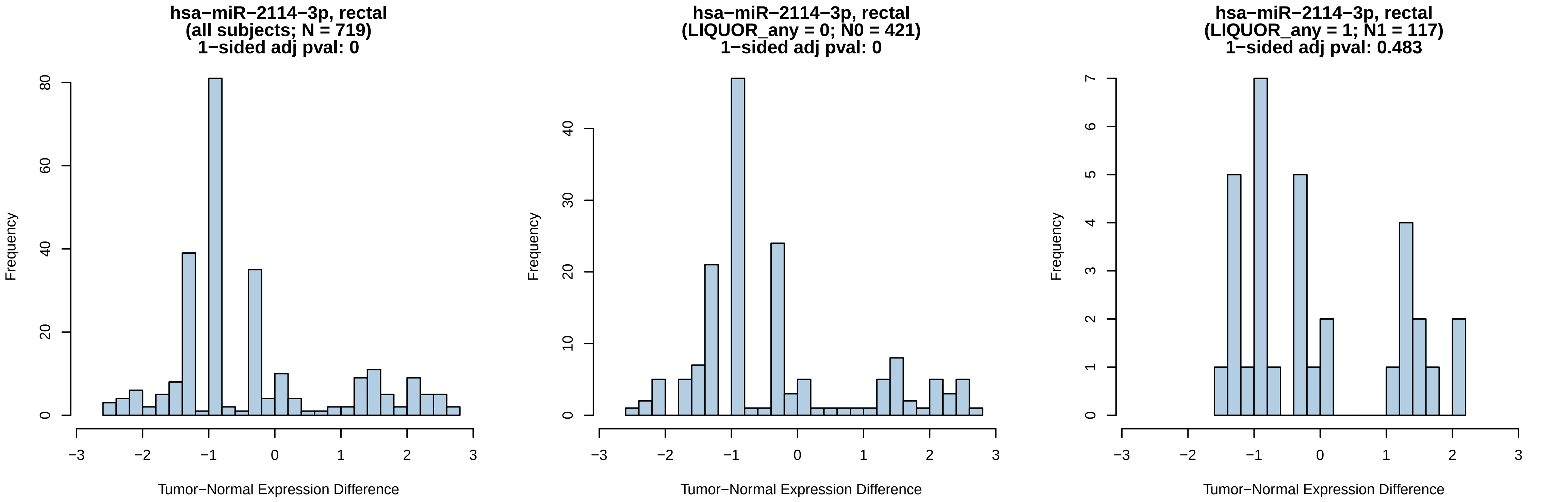
hsa−miR−2114−3p, rectal
(all subjects; N = 719)
1−sided adj pval: 0
hsa−miR−2114−3p, rectal
(LIQUOR_any = 0; N0 = 421)
1−sided adj pval: 0
hsa−miR−2114−3p, rectal
(LIQUOR_any = 1; N1 = 117)
1−sided adj pval: 0.483
Frequency
Tumor−Normal Expression Difference

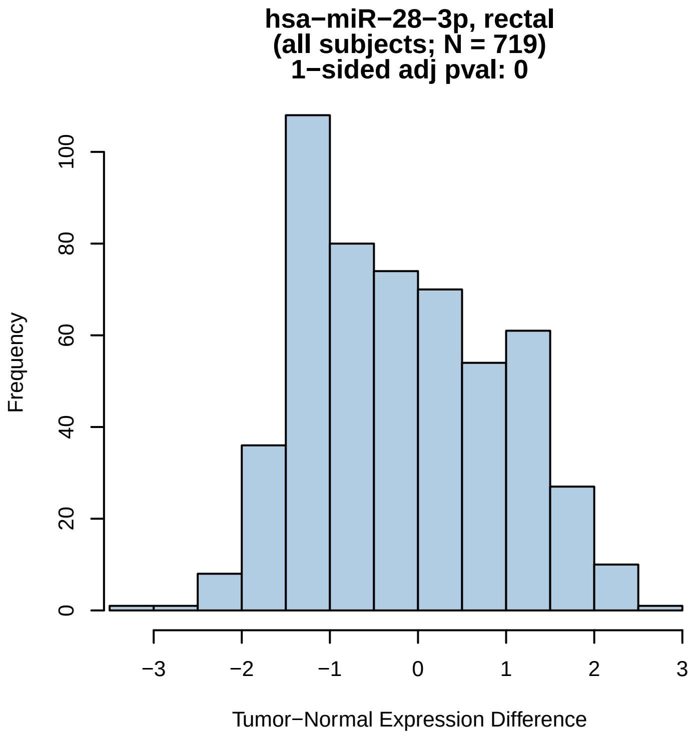

hsa−miR−28−3p, rectal
(all subjects; N = 719)
1−sided adj pval: 0
Frequency
Tumor−Normal Expression Difference


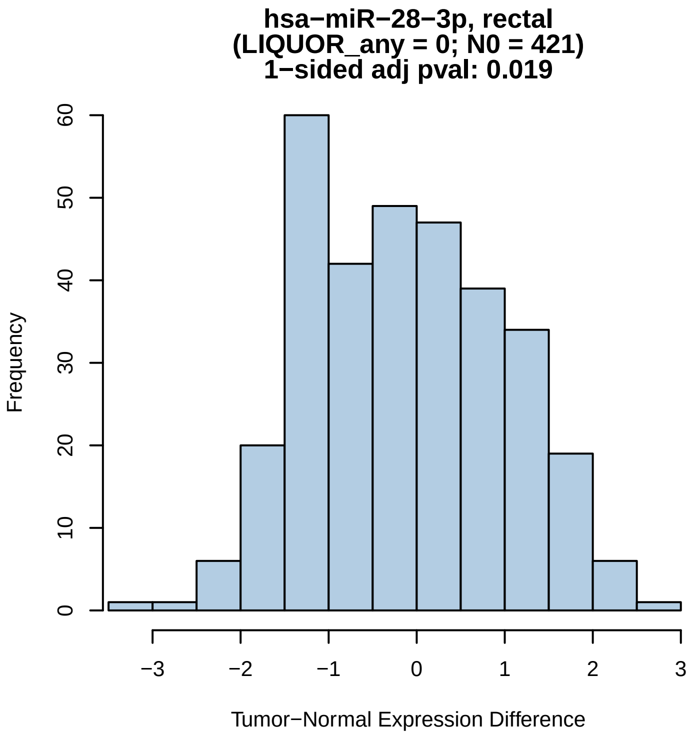

hsa−miR−28−3p, rectal
(LIQUOR_any = 0; N0 = 421)
1−sided adj pval: 0.019
Frequency
Tumor−Normal Expression Difference


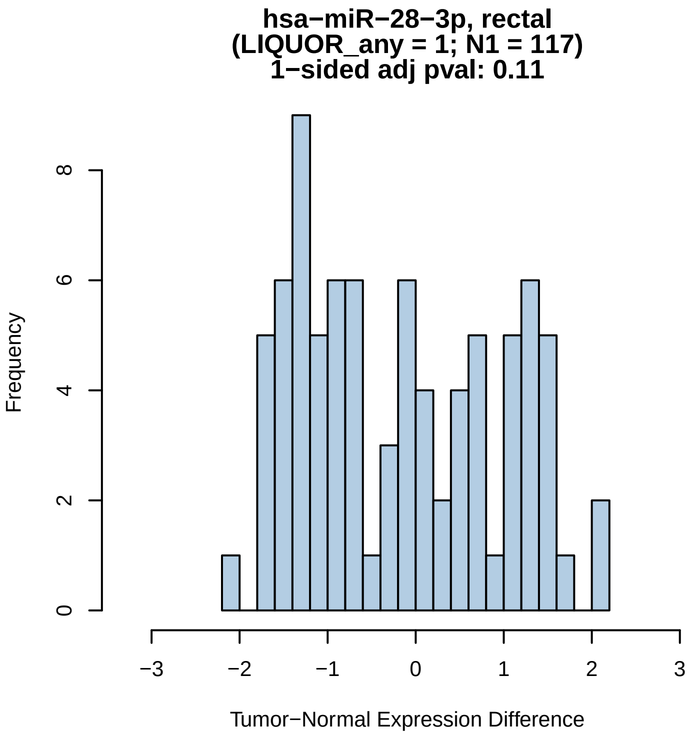

hsa−miR−28−3p, rectal
(LIQUOR_any = 1; N1 = 117)
1−sided adj pval: 0.11
Frequency
Tumor−Normal Expression Difference

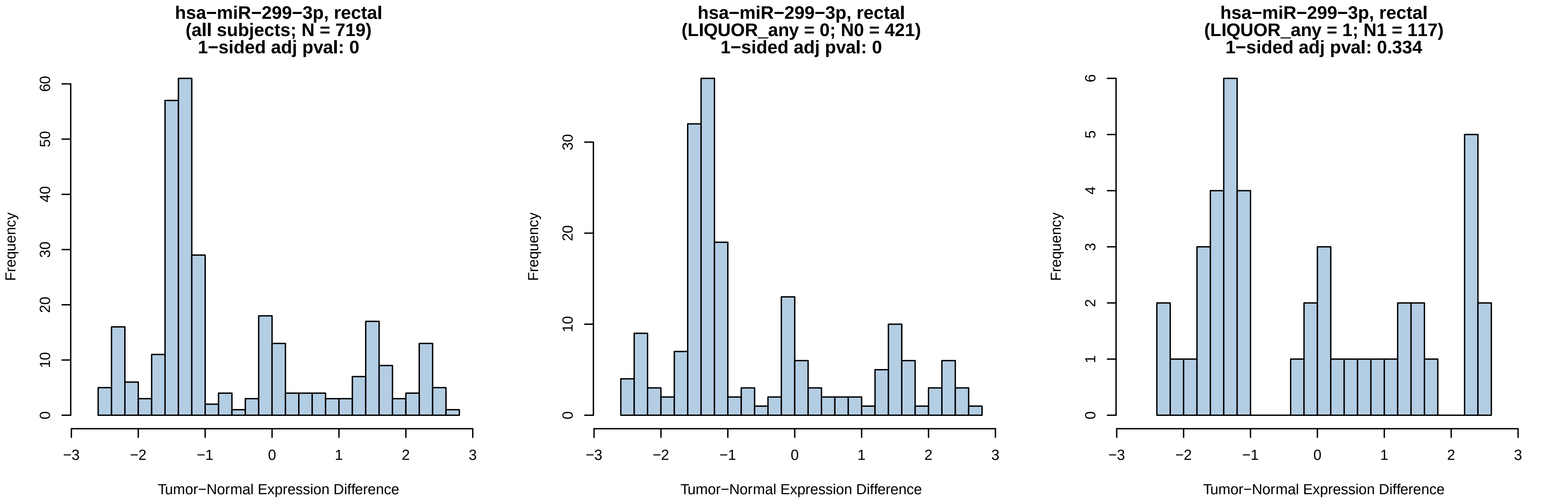
hsa−miR−299−3p, rectal
(all subjects; N = 719)
1−sided adj pval: 0
Frequency
0
10
20
30
40
50
60
−3
−2
−1
0
1
2
3
Tumor−Normal Expression Difference
hsa−miR−299−3p, rectal
(LIQUOR_any = 0; N0 = 421)
1−sided adj pval: 0
Frequency
0
10
20
30
−3
−2
−1
0
1
2
3
Tumor−Normal Expression Difference
hsa−miR−299−3p, rectal
(LIQUOR_any = 1; N1 = 117)
1−sided adj pval: 0.334
Frequency
0
1
2
3
4
5
6
−3
−2
−1
0
1
2
3
Tumor−Normal Expression Difference

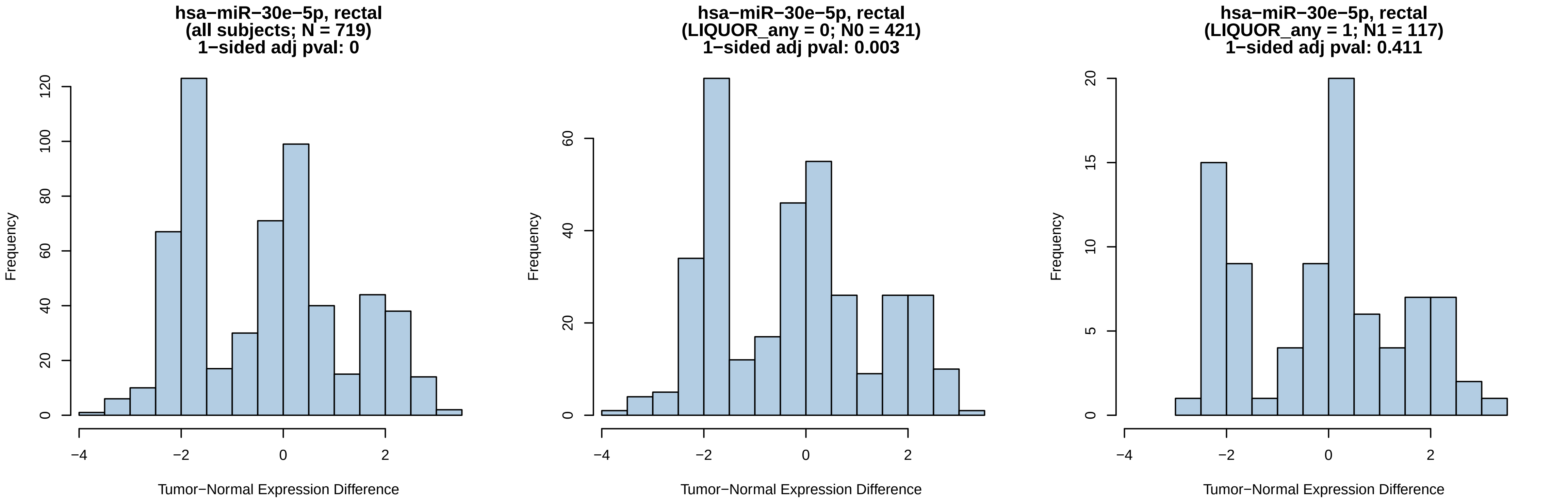
hsa−miR−30e−5p, rectal
(all subjects; N = 719)
1−sided adj pval: 0
Frequency
Tumor−Normal Expression Difference
hsa−miR−30e−5p, rectal
(LIQUOR_any = 0; N0 = 421)
1−sided adj pval: 0.003
Frequency
Tumor−Normal Expression Difference
hsa−miR−30e−5p, rectal
(LIQUOR_any = 1; N1 = 117)
1−sided adj pval: 0.411
Frequency
Tumor−Normal Expression Difference

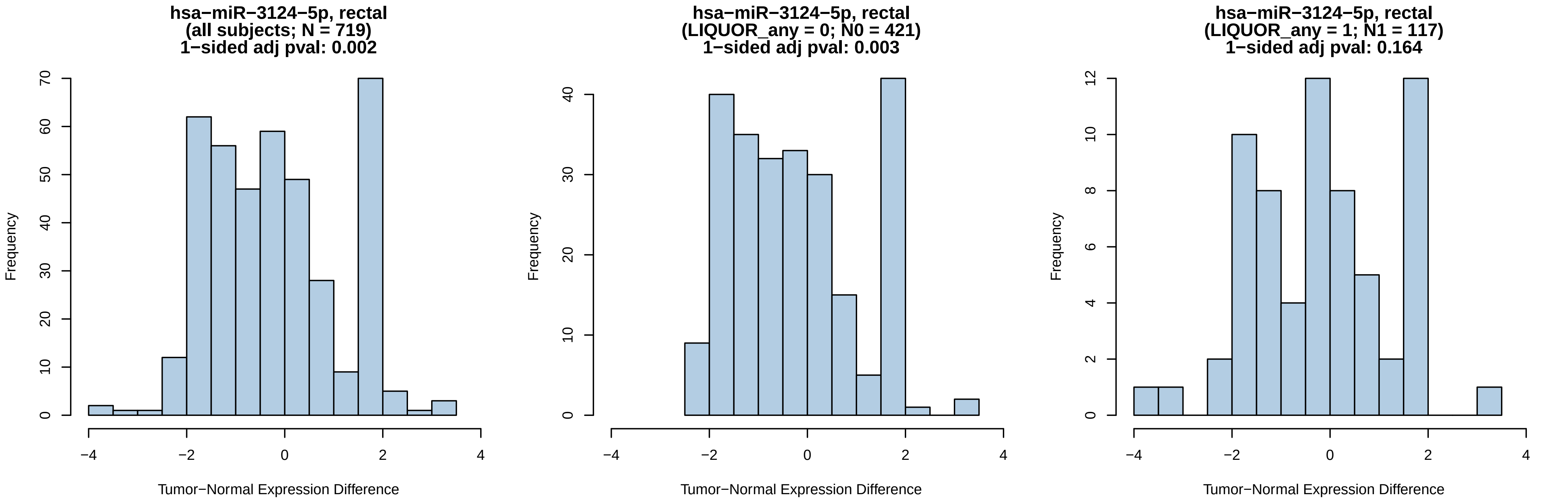

hsa−miR−3124−5p, rectal
(all subjects; N = 719)
1−sided adj pval: 0.002
hsa−miR−3124−5p, rectal
(LIQUOR_any = 0; N0 = 421)
1−sided adj pval: 0.003
hsa−miR−3124−5p, rectal
(LIQUOR_any = 1; N1 = 117)
1−sided adj pval: 0.164
Frequency
Tumor−Normal Expression Difference

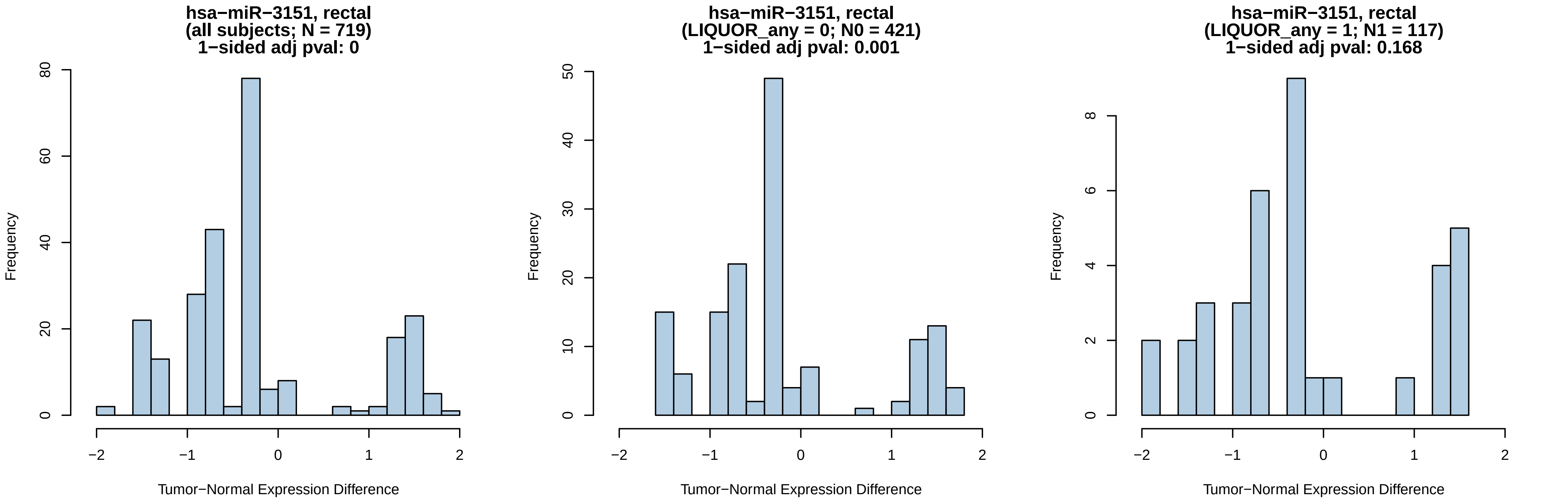
hsa−miR−3151, rectal
(all subjects; N = 719)
1−sided adj pval: 0
Frequency
Tumor−Normal Expression Difference
hsa−miR−3151, rectal
(LIQUOR_any = 0; N0 = 421)
1−sided adj pval: 0.001
Frequency
Tumor−Normal Expression Difference
hsa−miR−3151, rectal
(LIQUOR_any = 1; N1 = 117)
1−sided adj pval: 0.168
Frequency
Tumor−Normal Expression Difference

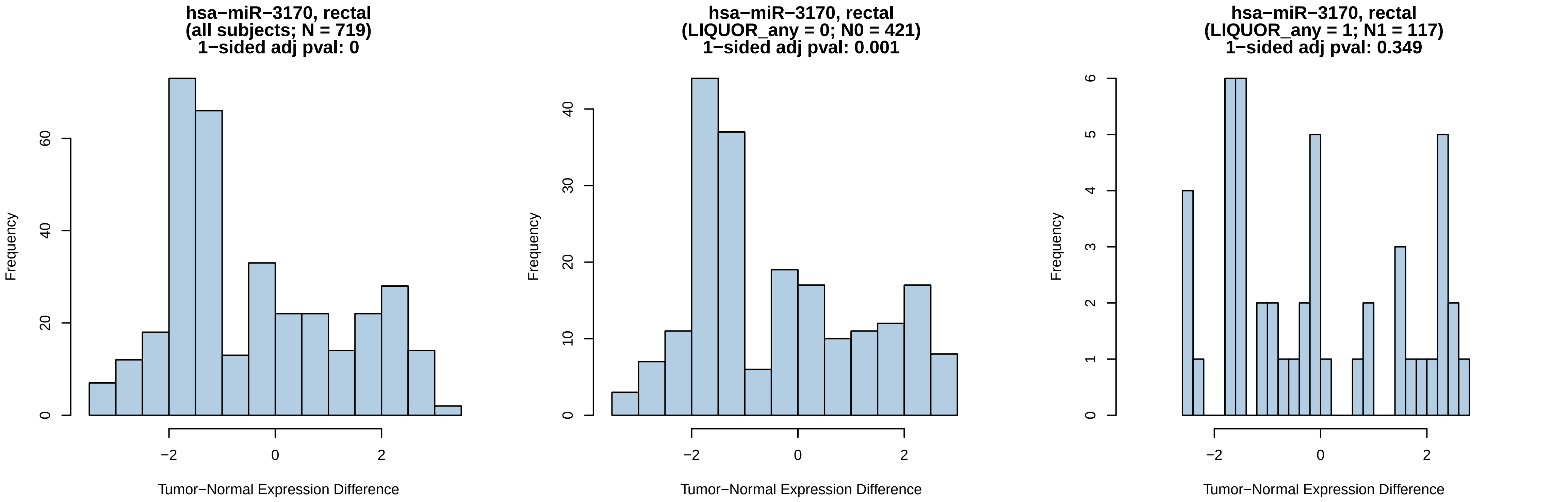

hsa−miR−3170, rectal
(all subjects; N = 719)
1−sided adj pval: 0
Frequency
Tumor−Normal Expression Difference
hsa−miR−3170, rectal
(LIQUOR_any = 0; N0 = 421)
1−sided adj pval: 0.001
Frequency
Tumor−Normal Expression Difference
hsa−miR−3170, rectal
(LIQUOR_any = 1; N1 = 117)
1−sided adj pval: 0.349
Frequency
Tumor−Normal Expression Difference

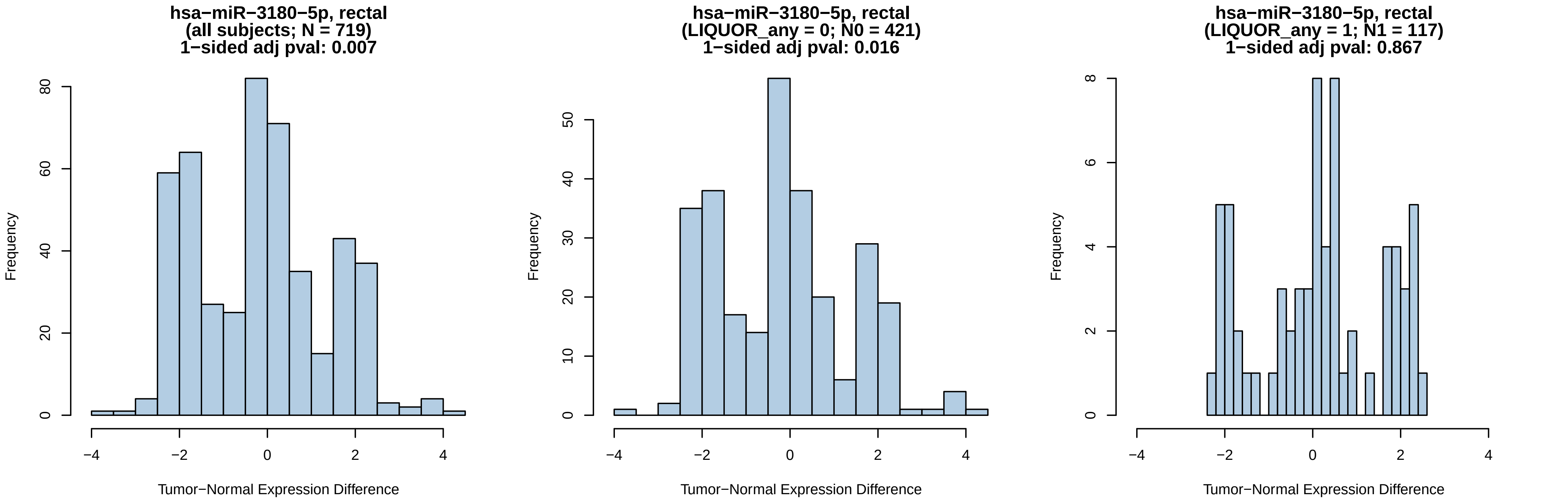

hsa−miR−3180−5p, rectal
(all subjects; N = 719)
1−sided adj pval: 0.007
Frequency
0
20
40
60
80
−4
−2
0
2
4
Tumor−Normal Expression Difference
hsa−miR−3180−5p, rectal
(LIQUOR_any = 0; N0 = 421)
1−sided adj pval: 0.016
Frequency
0
10
20
30
40
50
−4
−2
0
2
4
Tumor−Normal Expression Difference
hsa−miR−3180−5p, rectal
(LIQUOR_any = 1; N1 = 117)
1−sided adj pval: 0.867
Frequency
0
2
4
6
8
−4
−2
0
2
4
Tumor−Normal Expression Difference

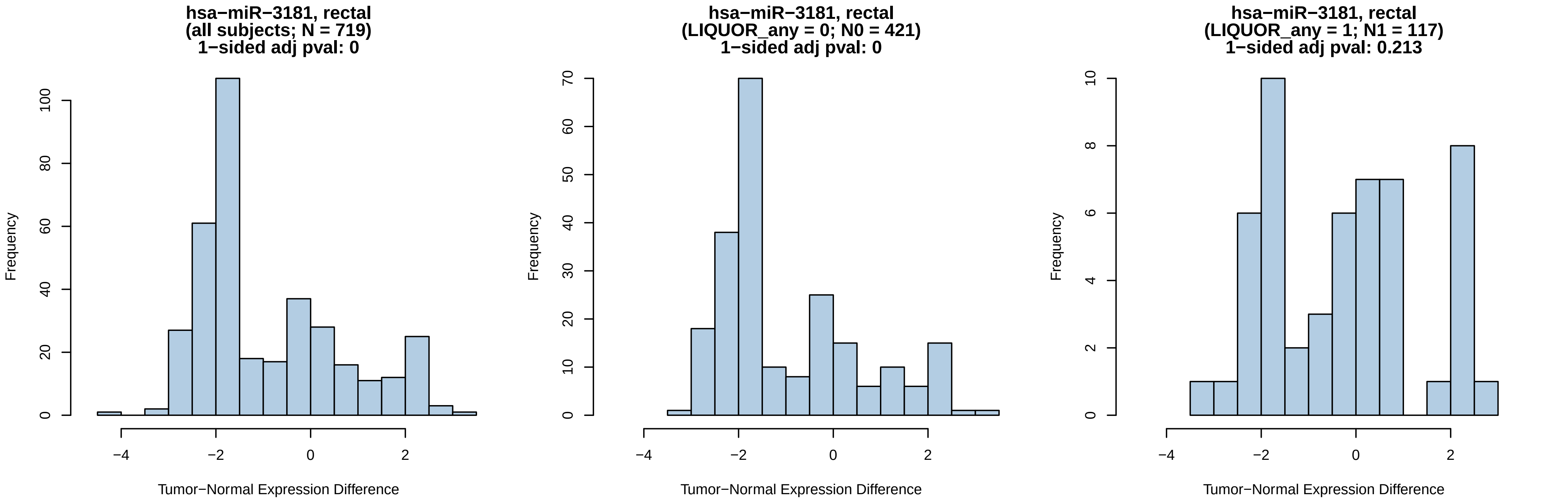

hsa−miR−3181, rectal
(all subjects; N = 719)
1−sided adj pval: 0
Frequency
0
20
40
60
80
100
−4
−2
0
2
Tumor−Normal Expression Difference
hsa−miR−3181, rectal
(LIQUOR_any = 0; N0 = 421)
1−sided adj pval: 0
Frequency
0
10
20
30
40
50
60
70
−4
−2
0
2
Tumor−Normal Expression Difference
hsa−miR−3181, rectal
(LIQUOR_any = 1; N1 = 117)
1−sided adj pval: 0.213
Frequency
0
2
4
6
8
10
−4
−2
0
2
Tumor−Normal Expression Difference

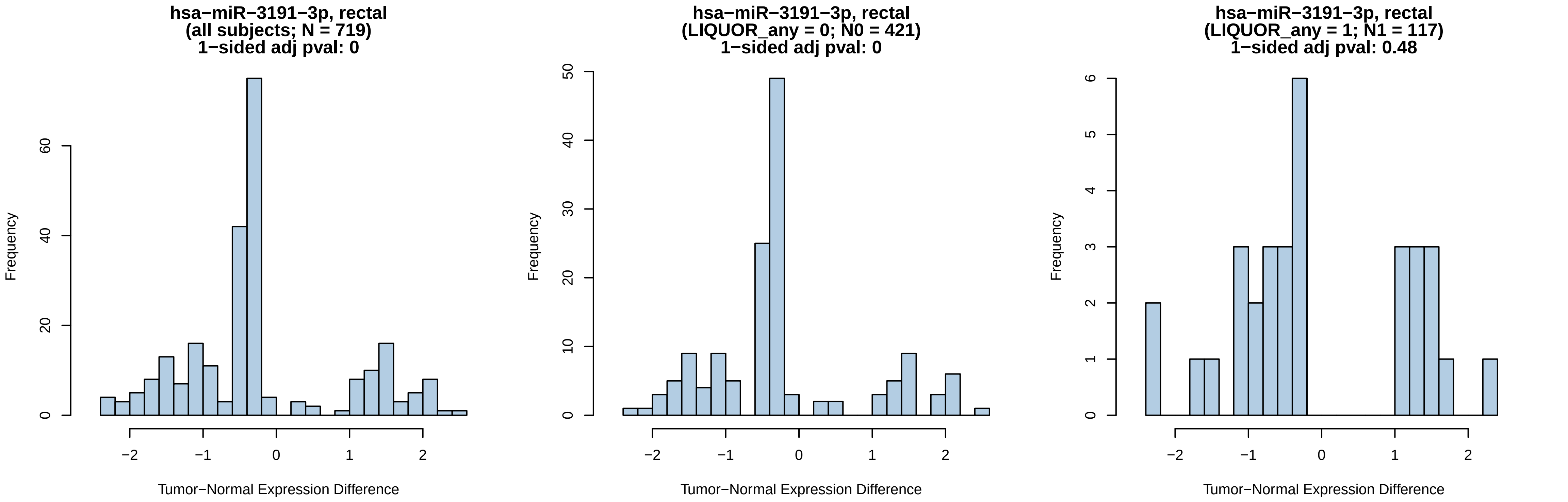

hsa−miR−3191−3p, rectal
(all subjects; N = 719)
1−sided adj pval: 0
hsa−miR−3191−3p, rectal
(LIQUOR_any = 0; N0 = 421)
1−sided adj pval: 0
hsa−miR−3191−3p, rectal
(LIQUOR_any = 1; N1 = 117)
1−sided adj pval: 0.48
Frequency
Tumor−Normal Expression Difference

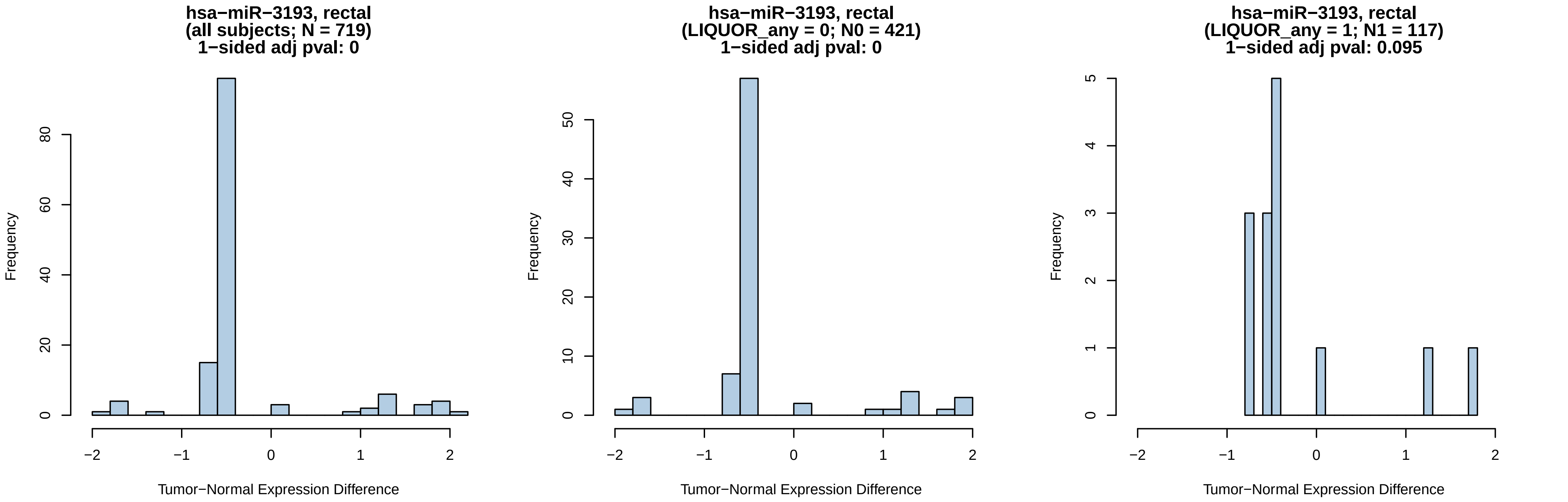

hsa−miR−3193, rectal
(all subjects; N = 719)
1−sided adj pval: 0
Frequency
Tumor−Normal Expression Difference
hsa−miR−3193, rectal
(LIQUOR_any = 0; N0 = 421)
1−sided adj pval: 0
Frequency
Tumor−Normal Expression Difference
hsa−miR−3193, rectal
(LIQUOR_any = 1; N1 = 117)
1−sided adj pval: 0.095
Frequency
Tumor−Normal Expression Difference

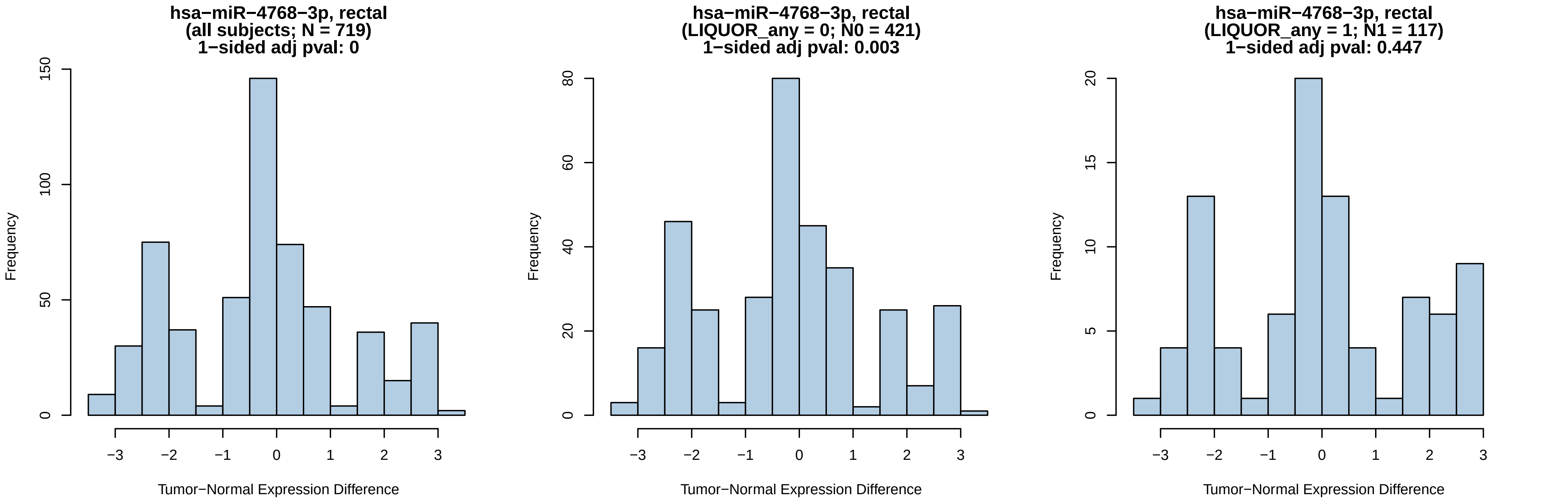

hsa−miR−4768−3p, rectal
(all subjects; N = 719)
1−sided adj pval: 0
Frequency
Tumor−Normal Expression Difference
hsa−miR−4768−3p, rectal
(LIQUOR_any = 0; N0 = 421)
1−sided adj pval: 0.003
Frequency
Tumor−Normal Expression Difference
hsa−miR−4768−3p, rectal
(LIQUOR_any = 1; N1 = 117)
1−sided adj pval: 0.447
Frequency
Tumor−Normal Expression Difference

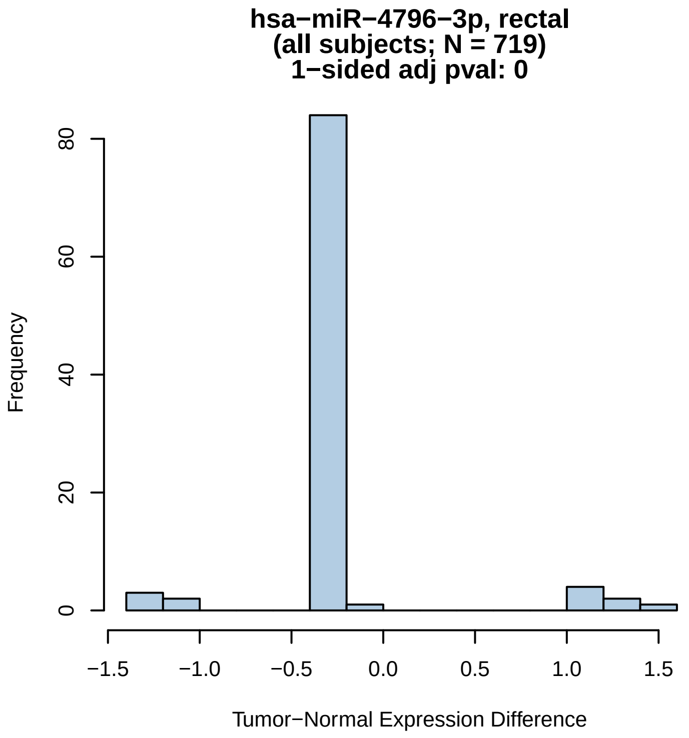

hsa−miR−4796−3p, rectal
(all subjects; N = 719)
1−sided adj pval: 0
Frequency
Tumor−Normal Expression Difference


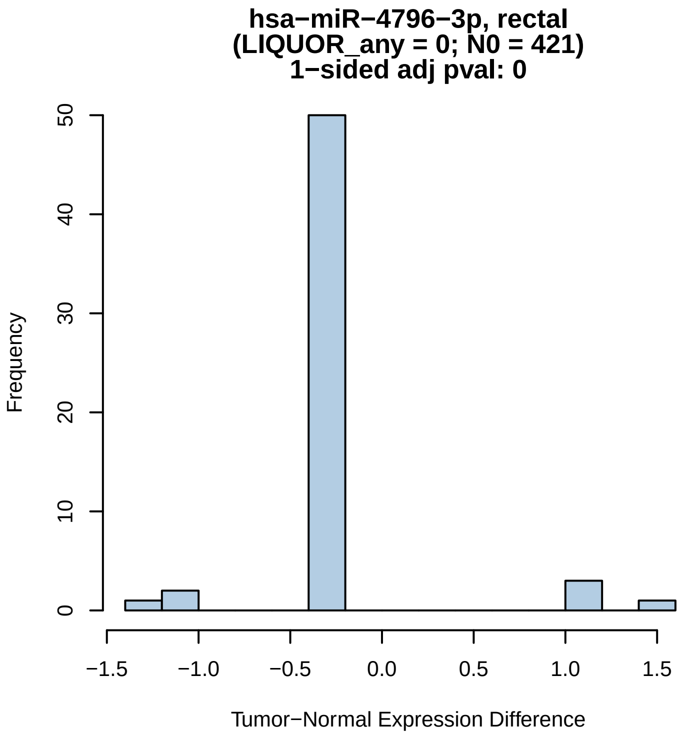

hsa−miR−4796−3p, rectal
(LIQUOR_any = 0; N0 = 421)
1−sided adj pval: 0
Frequency
Tumor−Normal Expression Difference


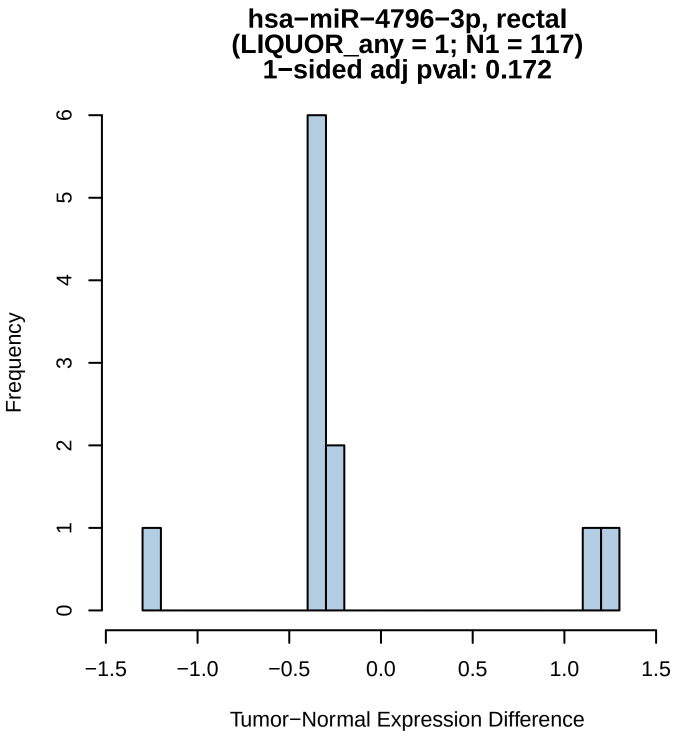

hsa−miR−4796−3p, rectal
(LIQUOR_any = 1; N1 = 117)
1−sided adj pval: 0.172
Frequency
Tumor−Normal Expression Difference

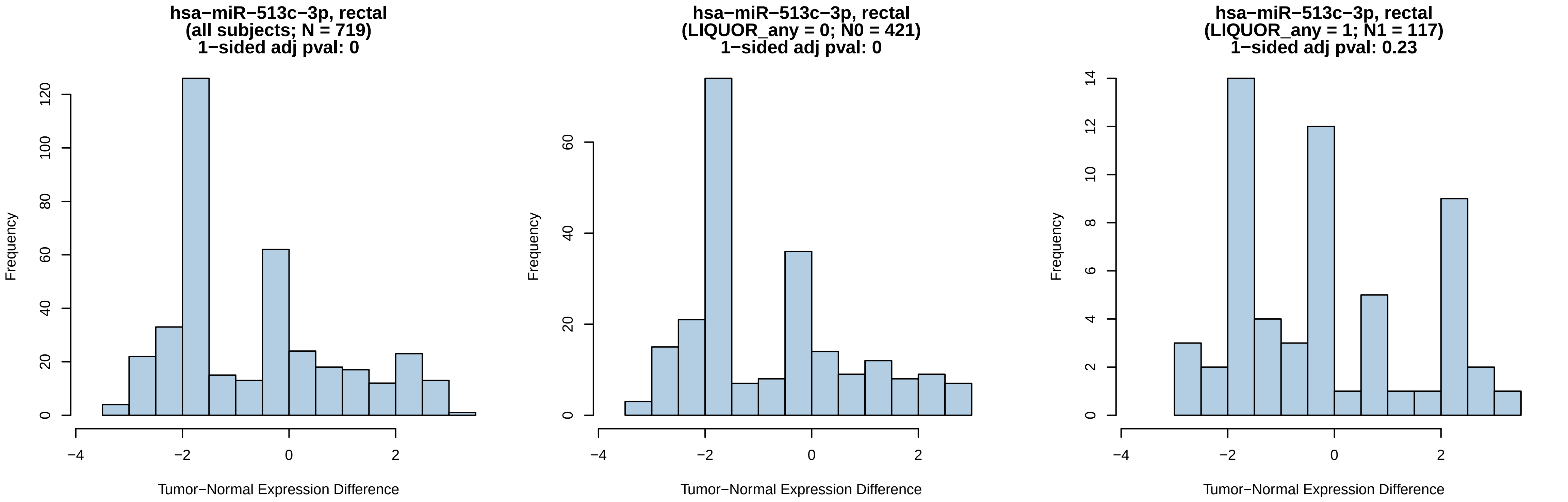
hsa−miR−513c−3p, rectal
(all subjects; N = 719)
1−sided adj pval: 0
hsa−miR−513c−3p, rectal
(LIQUOR_any = 0; N0 = 421)
1−sided adj pval: 0
hsa−miR−513c−3p, rectal
(LIQUOR_any = 1; N1 = 117)
1−sided adj pval: 0.23
Frequency
Tumor−Normal Expression Difference

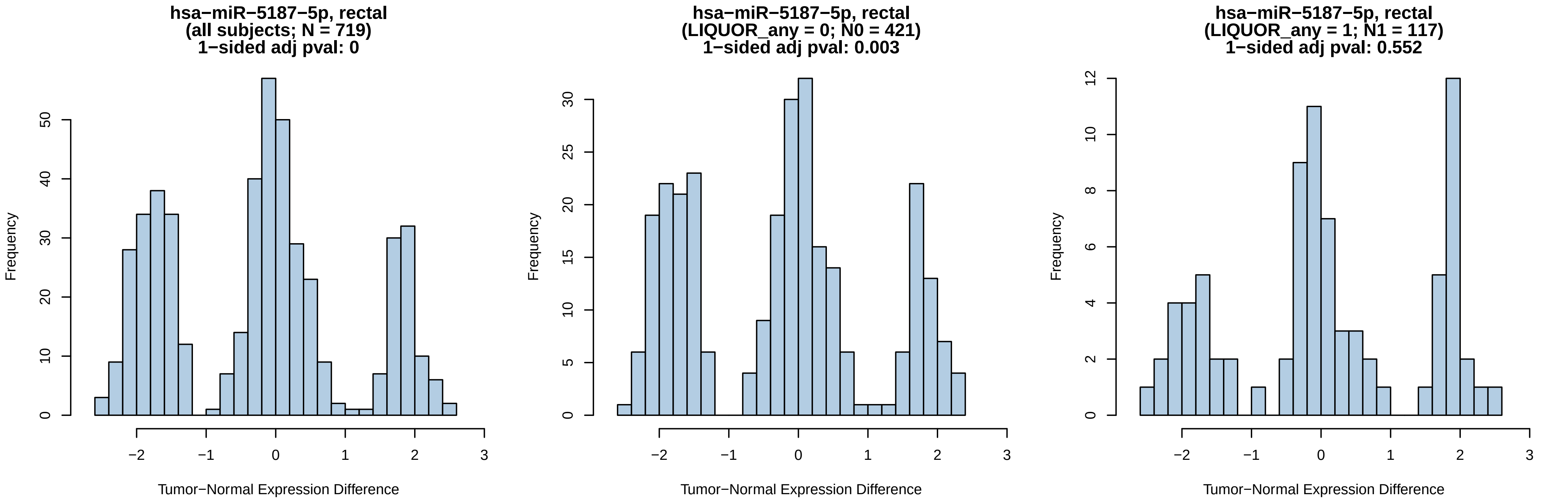
hsa−miR−5187−5p, rectal
(all subjects; N = 719)
1−sided adj pval: 0
Frequency
Tumor−Normal Expression Difference
hsa−miR−5187−5p, rectal
(LIQUOR_any = 0; N0 = 421)
1−sided adj pval: 0.003
Frequency
Tumor−Normal Expression Difference
hsa−miR−5187−5p, rectal
(LIQUOR_any = 1; N1 = 117)
1−sided adj pval: 0.552
Frequency
Tumor−Normal Expression Difference

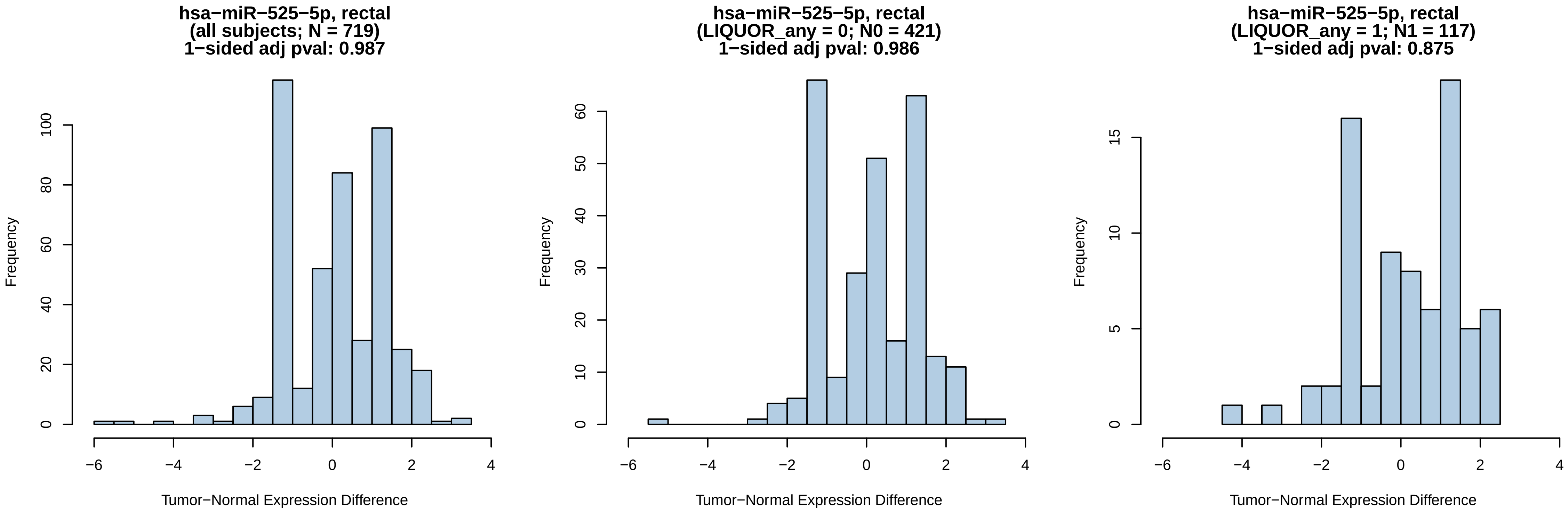
hsa−miR−525−5p, rectal
(all subjects; N = 719)
1−sided adj pval: 0.987
Frequency
Tumor−Normal Expression Difference
hsa−miR−525−5p, rectal
(LIQUOR_any = 0; N0 = 421)
1−sided adj pval: 0.986
Frequency
Tumor−Normal Expression Difference
hsa−miR−525−5p, rectal
(LIQUOR_any = 1; N1 = 117)
1−sided adj pval: 0.875
Frequency
Tumor−Normal Expression Difference

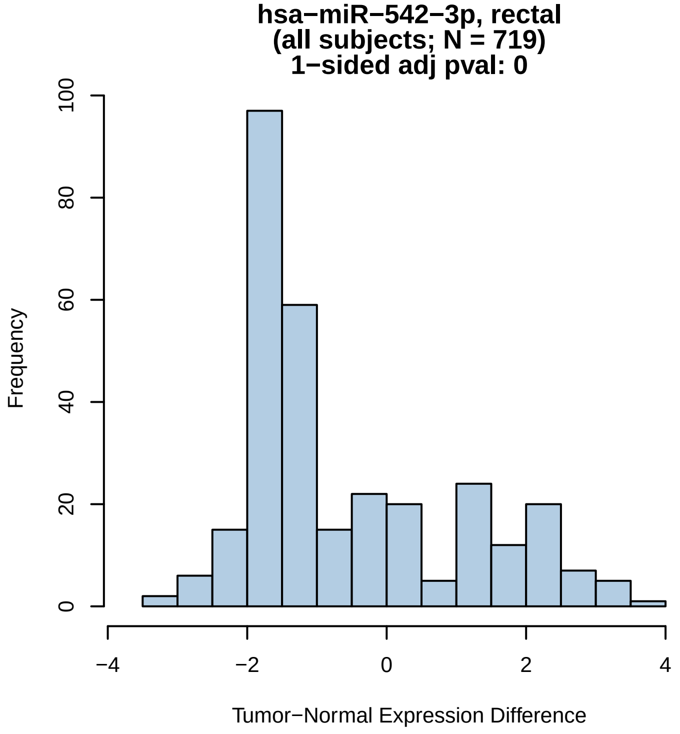

hsa−miR−542−3p, rectal
(all subjects; N = 719)
1−sided adj pval: 0
Frequency
Tumor−Normal Expression Difference


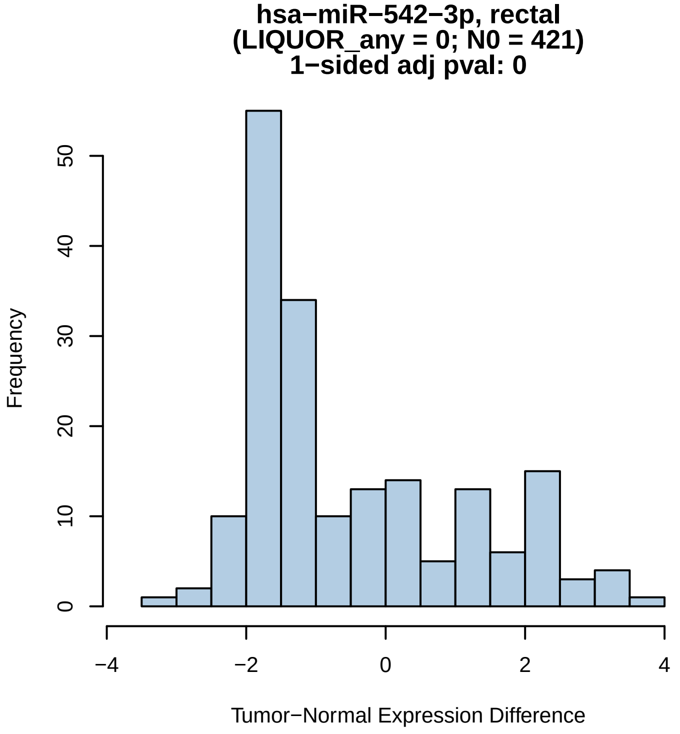

hsa−miR−542−3p, rectal
(LIQUOR_any = 0; N0 = 421)
1−sided adj pval: 0
Frequency
Tumor−Normal Expression Difference


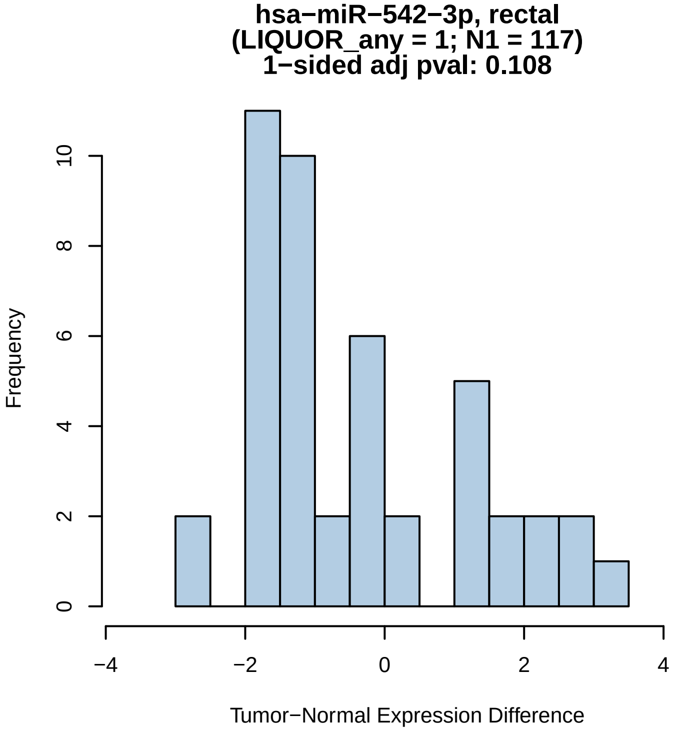

hsa−miR−542−3p, rectal
(LIQUOR_any = 1; N1 = 117)
1−sided adj pval: 0.108
Frequency
Tumor−Normal Expression Difference

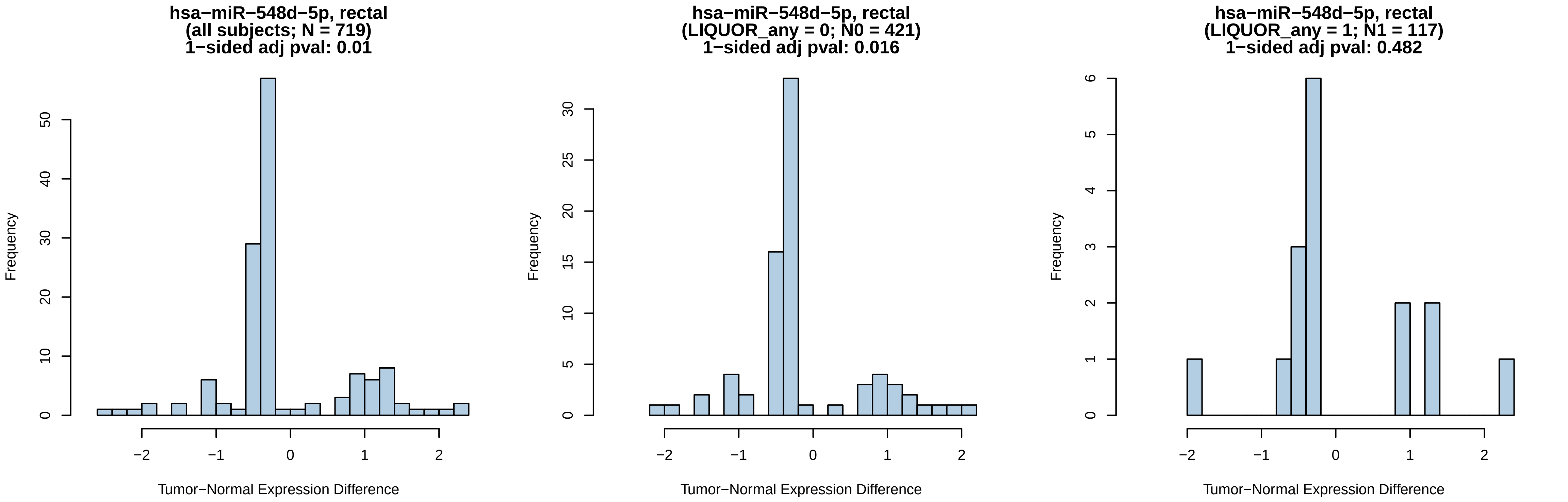
hsa−miR−548d−5p, rectal
(all subjects; N = 719)
1−sided adj pval: 0.01
Frequency
0
10
20
30
40
50
−2
−1
0
1
2
Tumor−Normal Expression Difference
hsa−miR−548d−5p, rectal
(LIQUOR_any = 0; N0 = 421)
1−sided adj pval: 0.016
Frequency
0
5
10
15
20
25
30
−2
−1
0
1
2
Tumor−Normal Expression Difference
hsa−miR−548d−5p, rectal
(LIQUOR_any = 1; N1 = 117)
1−sided adj pval: 0.482
Frequency
0
1
2
3
4
5
6
−2
−1
0
1
2
Tumor−Normal Expression Difference

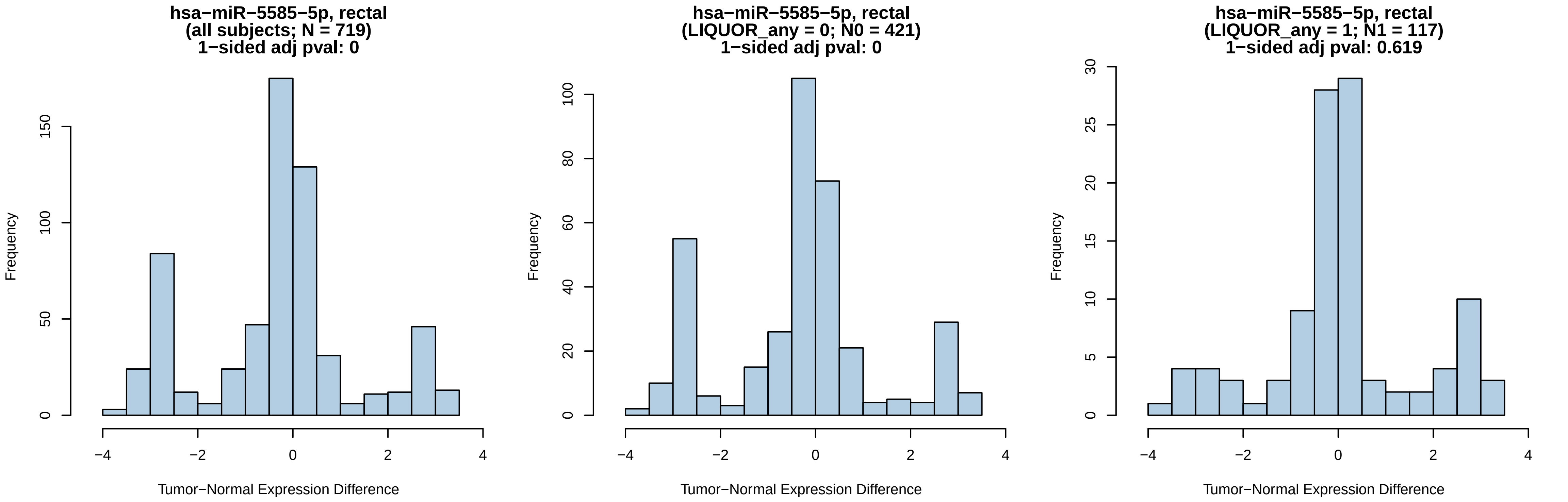

hsa−miR−5585−5p, rectal
(all subjects; N = 719)
1−sided adj pval: 0
Frequency
Tumor−Normal Expression Difference
hsa−miR−5585−5p, rectal
(LIQUOR_any = 0; N0 = 421)
1−sided adj pval: 0
Frequency
Tumor−Normal Expression Difference
hsa−miR−5585−5p, rectal
(LIQUOR_any = 1; N1 = 117)
1−sided adj pval: 0.619
Frequency
Tumor−Normal Expression Difference

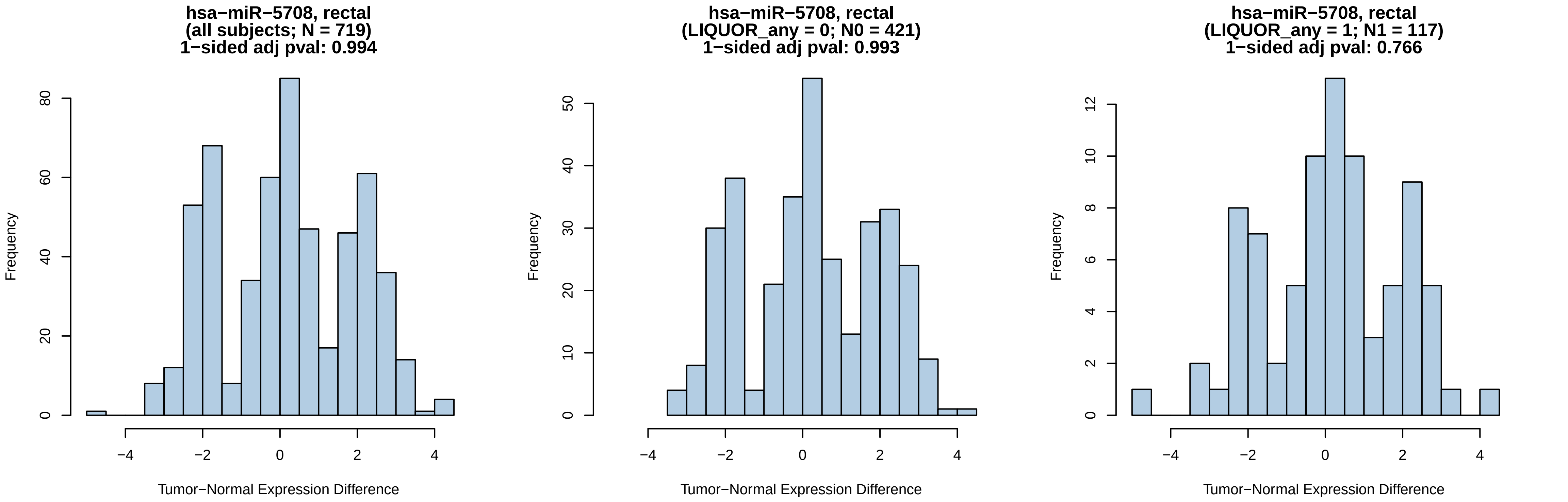

hsa−miR−5708, rectal
(all subjects; N = 719)
1−sided adj pval: 0.994
Frequency
Tumor−Normal Expression Difference
hsa−miR−5708, rectal
(LIQUOR_any = 0; N0 = 421)
1−sided adj pval: 0.993
Frequency
Tumor−Normal Expression Difference
hsa−miR−5708, rectal
(LIQUOR_any = 1; N1 = 117)
1−sided adj pval: 0.766
Frequency
Tumor−Normal Expression Difference

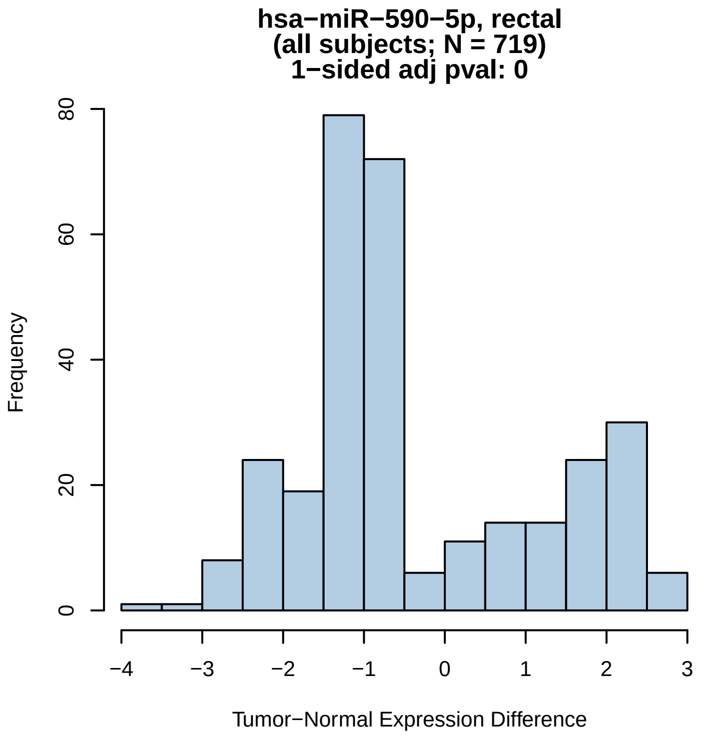

hsa−miR−590−5p, rectal
(all subjects; N = 719)
1−sided adj pval: 0
Frequency
Tumor−Normal Expression Difference


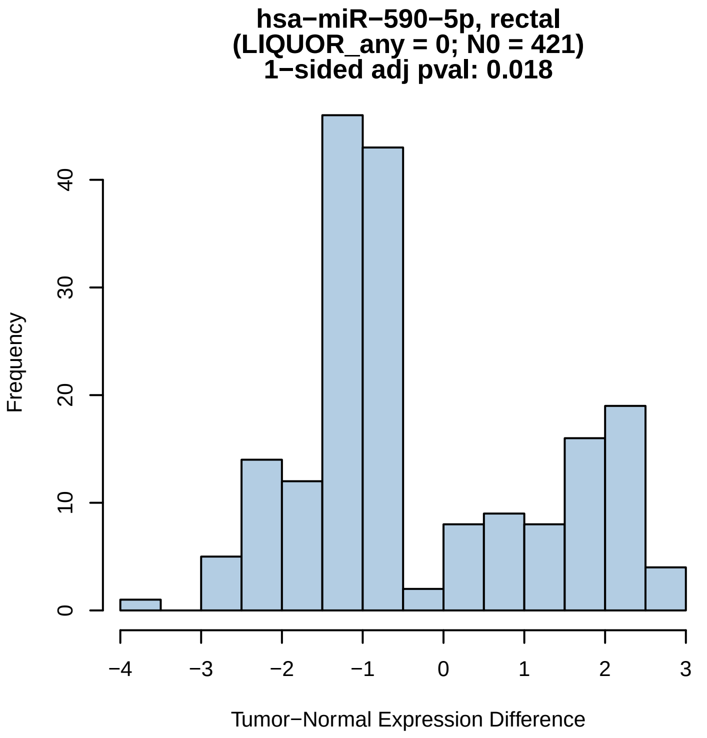

hsa−miR−590−5p, rectal
(LIQUOR_any = 0; N0 = 421)
1−sided adj pval: 0.018
Frequency
Tumor−Normal Expression Difference


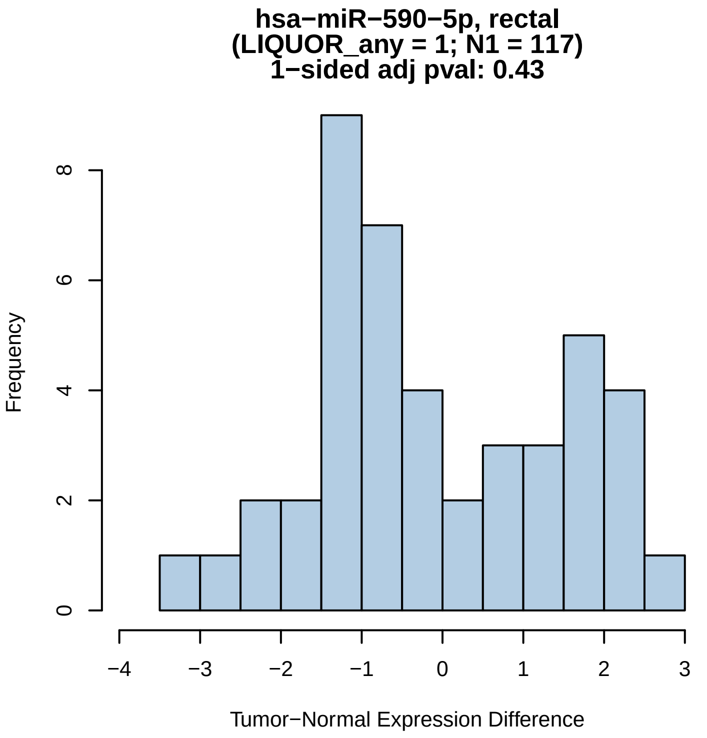

hsa−miR−590−5p, rectal
(LIQUOR_any = 1; N1 = 117)
1−sided adj pval: 0.43
Frequency
Tumor−Normal Expression Difference

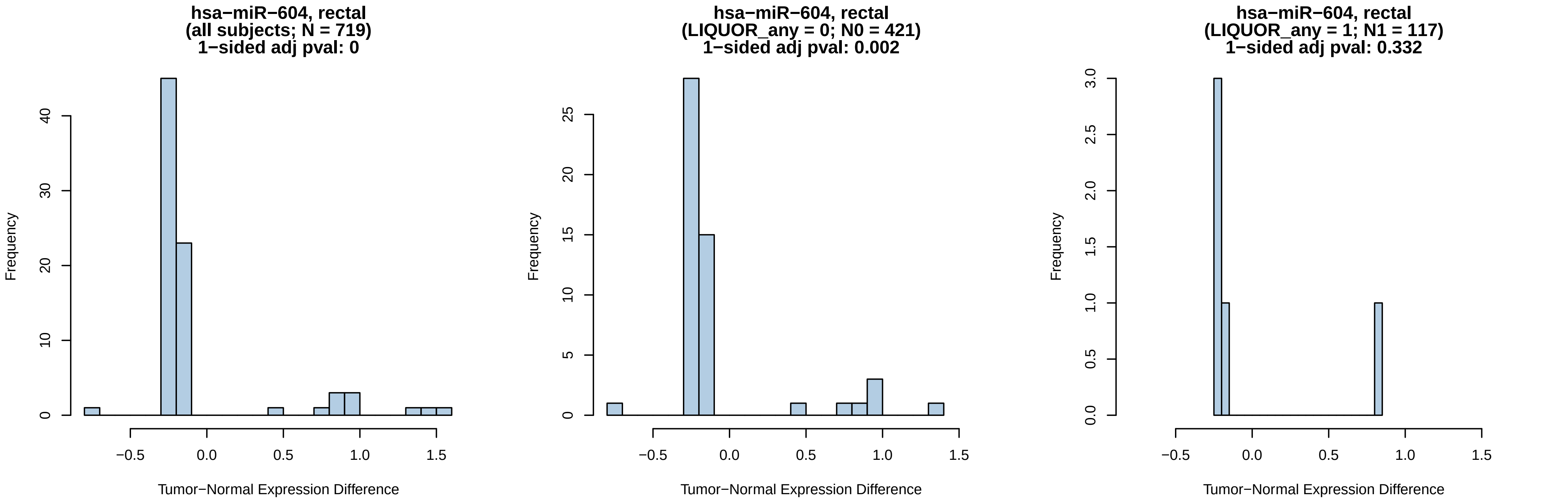
hsa−miR−604, rectal
(all subjects; N = 719)
1−sided adj pval: 0
Frequency
Tumor−Normal Expression Difference
hsa−miR−604, rectal
(LIQUOR_any = 0; N0 = 421)
1−sided adj pval: 0.002
Frequency
Tumor−Normal Expression Difference
hsa−miR−604, rectal
(LIQUOR_any = 1; N1 = 117)
1−sided adj pval: 0.332
Frequency
Tumor−Normal Expression Difference

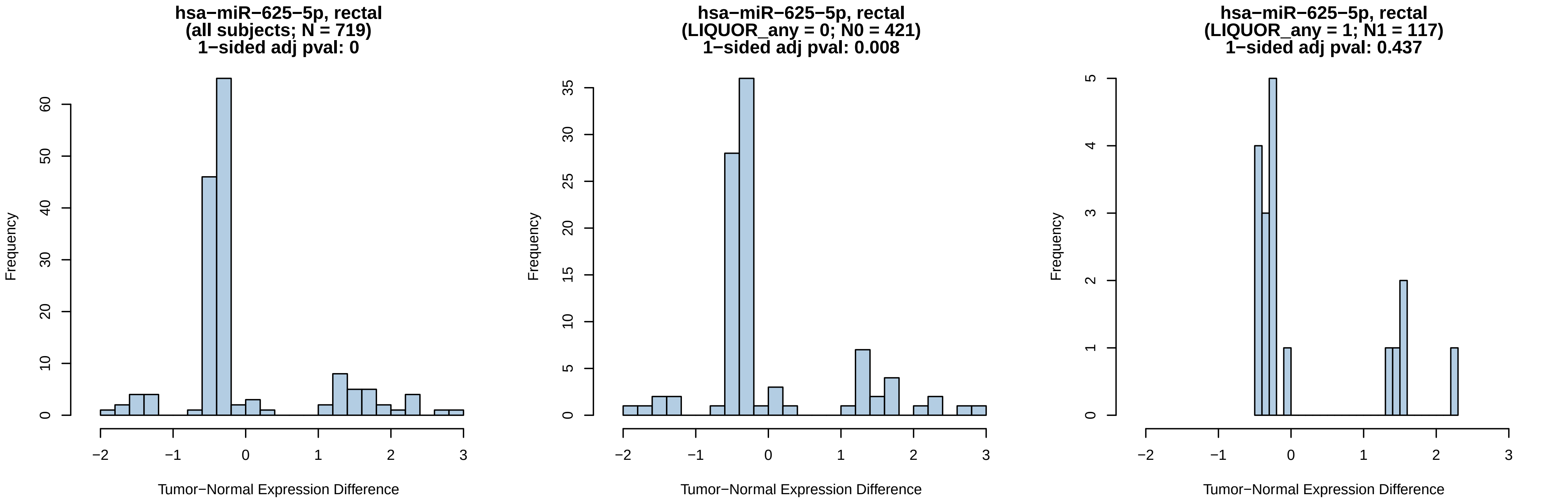
hsa−miR−625−5p, rectal
(all subjects; N = 719)
1−sided adj pval: 0
hsa−miR−625−5p, rectal
(LIQUOR_any = 0; N0 = 421)
1−sided adj pval: 0.008
hsa−miR−625−5p, rectal
(LIQUOR_any = 1; N1 = 117)
1−sided adj pval: 0.437
Frequency
Tumor−Normal Expression Difference

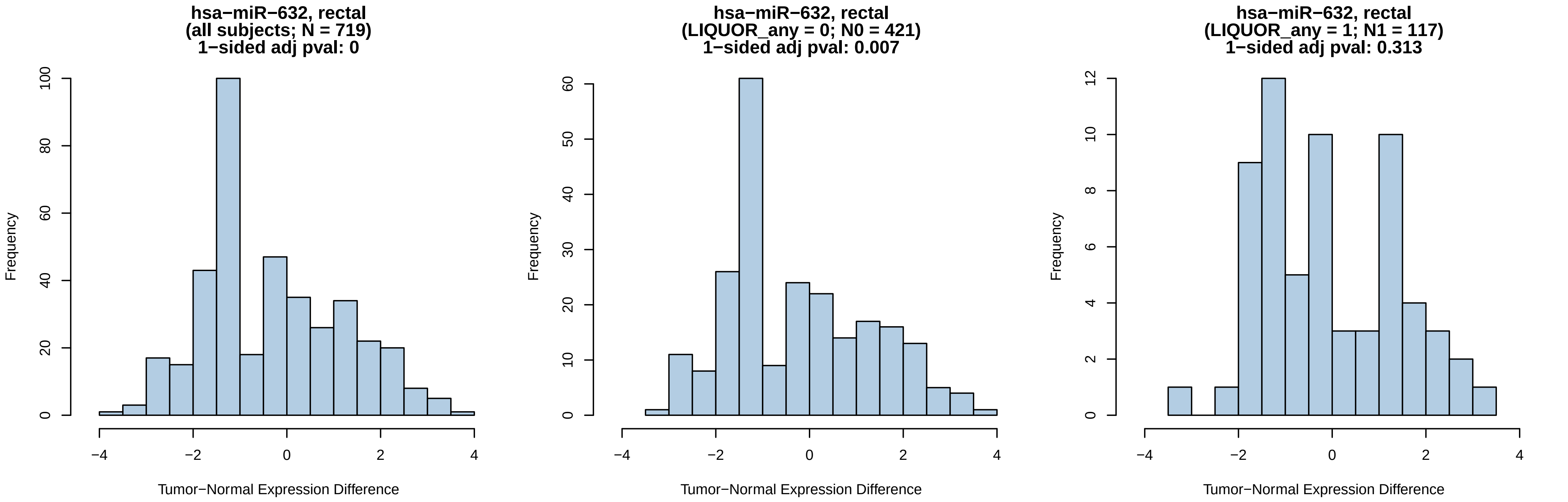

hsa−miR−632, rectal
(all subjects; N = 719)
1−sided adj pval: 0
Frequency
Tumor−Normal Expression Difference
hsa−miR−632, rectal
(LIQUOR_any = 0; N0 = 421)
1−sided adj pval: 0.007
Frequency
Tumor−Normal Expression Difference
hsa−miR−632, rectal
(LIQUOR_any = 1; N1 = 117)
1−sided adj pval: 0.313
Frequency
Tumor−Normal Expression Difference

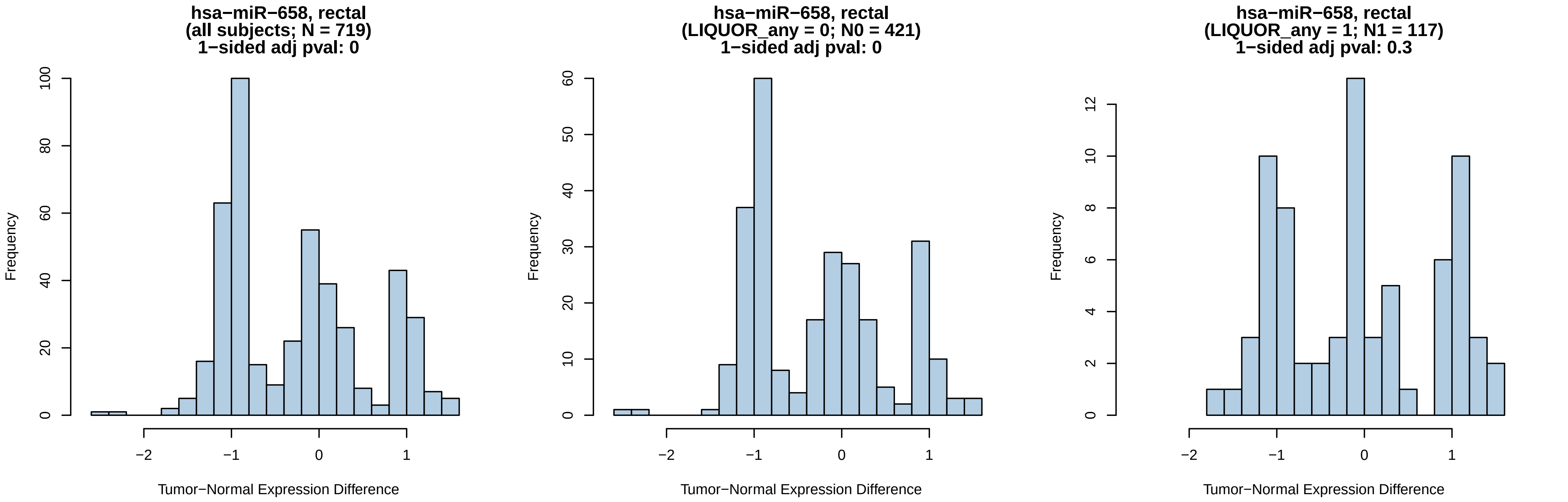
hsa−miR−658, rectal
(all subjects; N = 719)
1−sided adj pval: 0
Frequency
Tumor−Normal Expression Difference
hsa−miR−658, rectal
(LIQUOR_any = 0; N0 = 421)
1−sided adj pval: 0
Frequency
Tumor−Normal Expression Difference
hsa−miR−658, rectal
(LIQUOR_any = 1; N1 = 117)
1−sided adj pval: 0.3
Frequency
Tumor−Normal Expression Difference

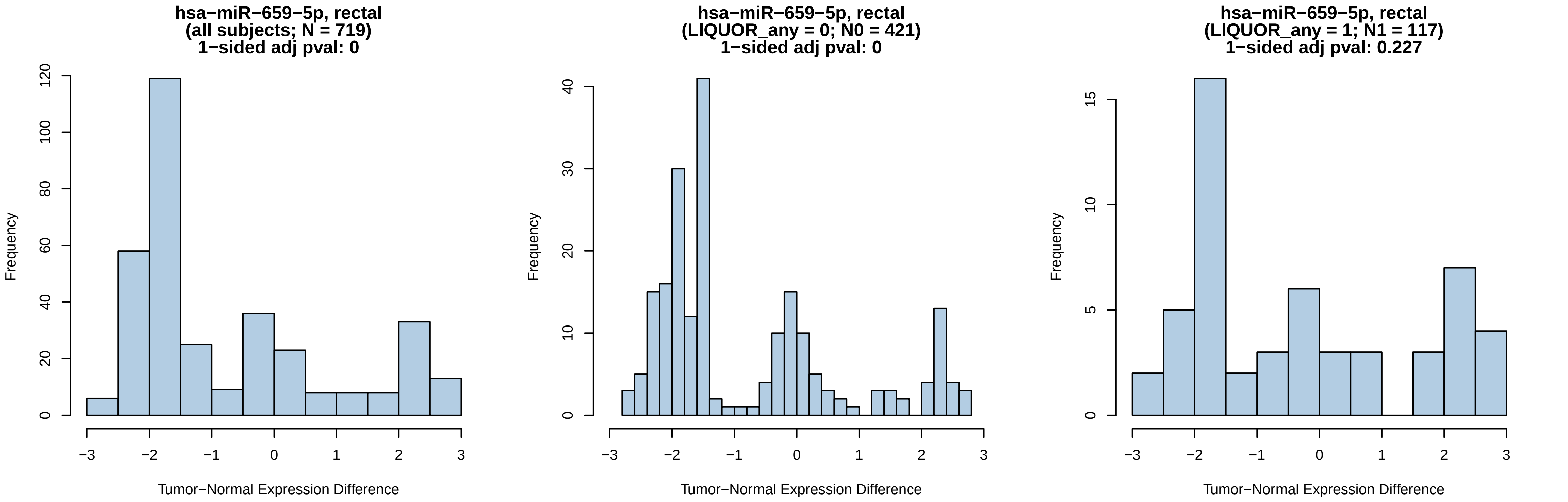
hsa−miR−659−5p, rectal
(all subjects; N = 719)
1−sided adj pval: 0
Frequency
Tumor−Normal Expression Difference
hsa−miR−659−5p, rectal
(LIQUOR_any = 0; N0 = 421)
1−sided adj pval: 0
Frequency
Tumor−Normal Expression Difference
hsa−miR−659−5p, rectal
(LIQUOR_any = 1; N1 = 117)
1−sided adj pval: 0.227
Frequency
Tumor−Normal Expression Difference

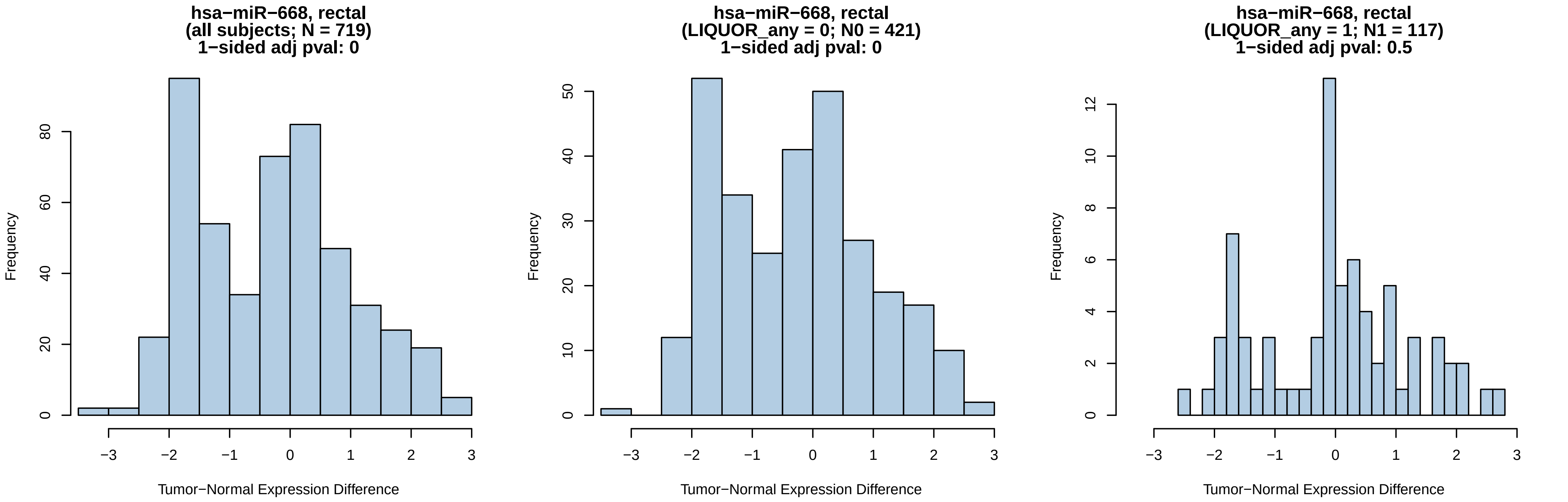
hsa−miR−668, rectal
(all subjects; N = 719)
1−sided adj pval: 0
hsa−miR−668, rectal
(LIQUOR_any = 0; N0 = 421)
1−sided adj pval: 0
hsa−miR−668, rectal
(LIQUOR_any = 1; N1 = 117)
1−sided adj pval: 0.5
Frequency
Tumor−Normal Expression Difference
−3
−2
−1
0
1
2
3
0
20
40
60
80
10
30
50
2
4
6
8
12

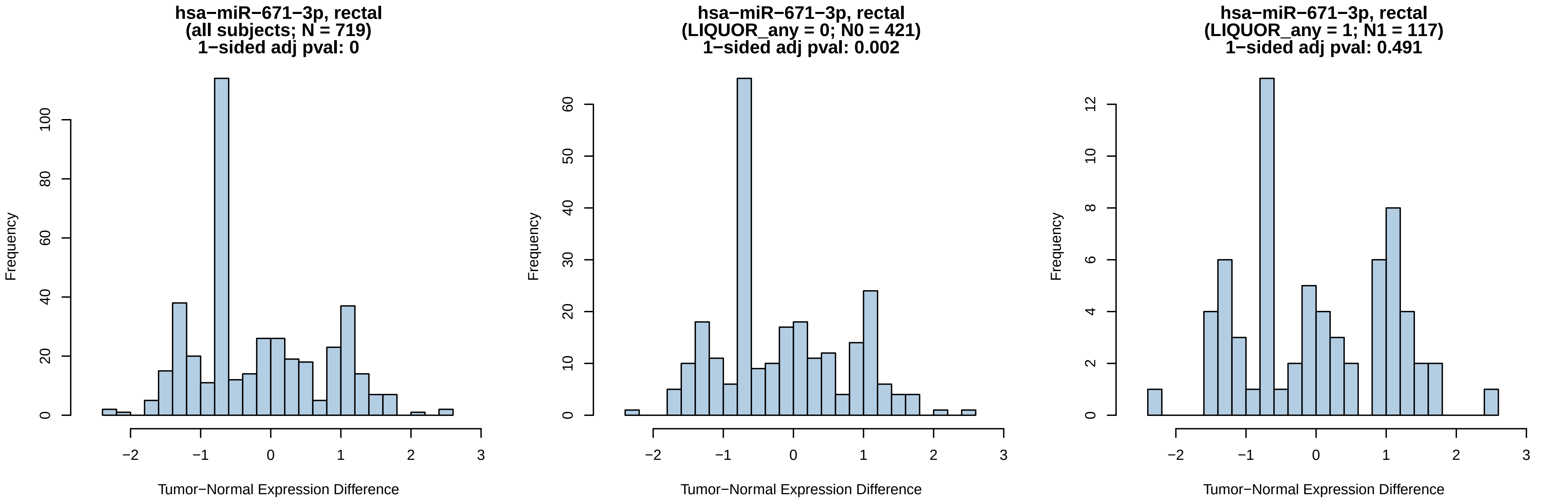
hsa−miR−671−3p, rectal
(all subjects; N = 719)
1−sided adj pval: 0
hsa−miR−671−3p, rectal
(LIQUOR_any = 0; N0 = 421)
1−sided adj pval: 0.002
hsa−miR−671−3p, rectal
(LIQUOR_any = 1; N1 = 117)
1−sided adj pval: 0.491
Frequency
Tumor−Normal Expression Difference

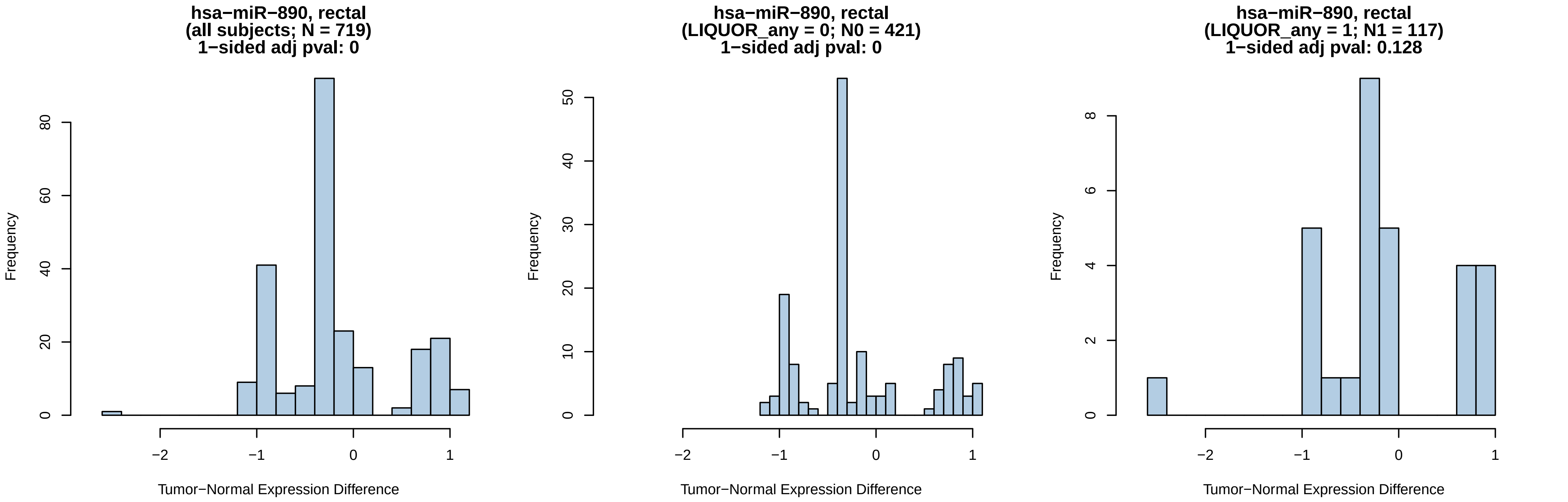
hsa−miR−890, rectal
(all subjects; N = 719)
1−sided adj pval: 0
Frequency
Tumor−Normal Expression Difference
hsa−miR−890, rectal
(LIQUOR_any = 0; N0 = 421)
1−sided adj pval: 0
Frequency
Tumor−Normal Expression Difference
hsa−miR−890, rectal
(LIQUOR_any = 1; N1 = 117)
1−sided adj pval: 0.128
Frequency
Tumor−Normal Expression Difference

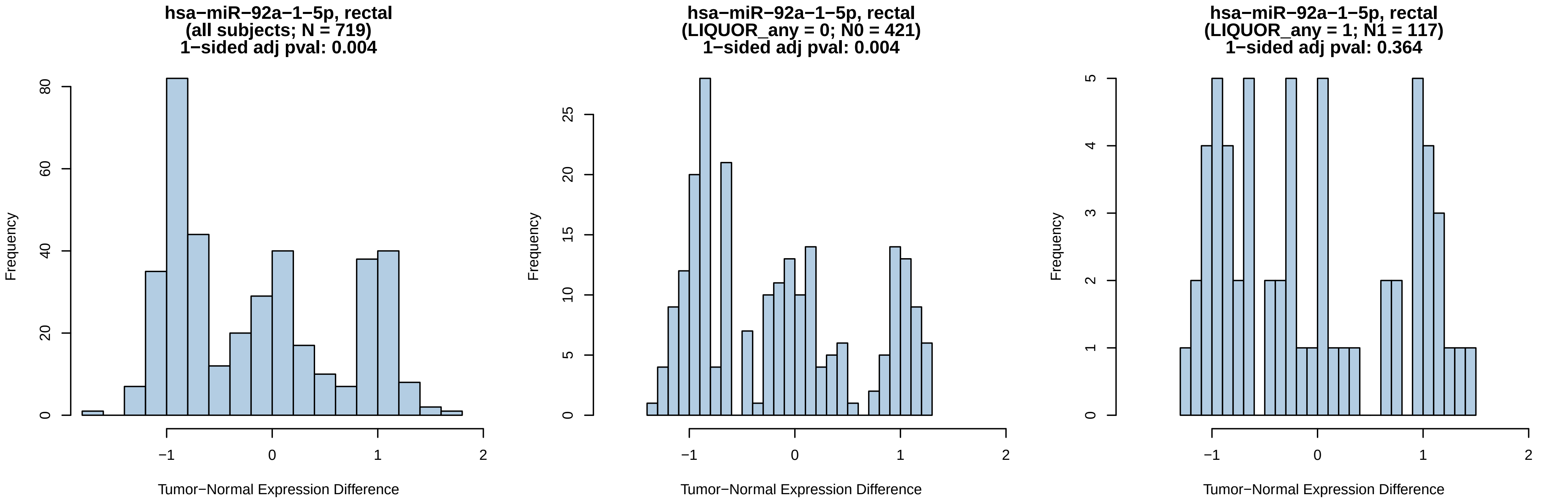

hsa−miR−92a−1−5p, rectal
(all subjects; N = 719)
1−sided adj pval: 0.004
Frequency
0
20
40
60
80
−1
0
1
2
Tumor−Normal Expression Difference
hsa−miR−92a−1−5p, rectal
(LIQUOR_any = 0; N0 = 421)
1−sided adj pval: 0.004
Frequency
0
5
10
15
20
25
−1
0
1
2
Tumor−Normal Expression Difference
hsa−miR−92a−1−5p, rectal
(LIQUOR_any = 1; N1 = 117)
1−sided adj pval: 0.364
Frequency
0
1
2
3
4
5
−1
0
1
2
Tumor−Normal Expression Difference

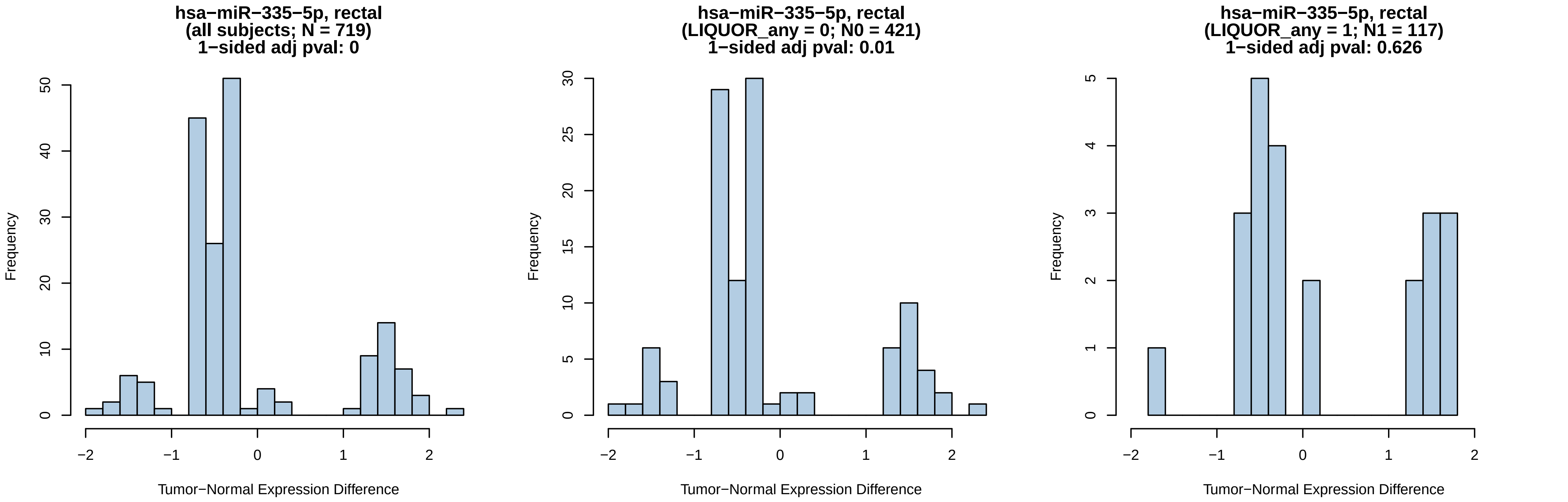

hsa−miR−335−5p, rectal
(all subjects; N = 719)
1−sided adj pval: 0
Frequency
Tumor−Normal Expression Difference
hsa−miR−335−5p, rectal
(LIQUOR_any = 0; N0 = 421)
1−sided adj pval: 0.01
Frequency
Tumor−Normal Expression Difference
hsa−miR−335−5p, rectal
(LIQUOR_any = 1; N1 = 117)
1−sided adj pval: 0.626
Frequency
Tumor−Normal Expression Difference

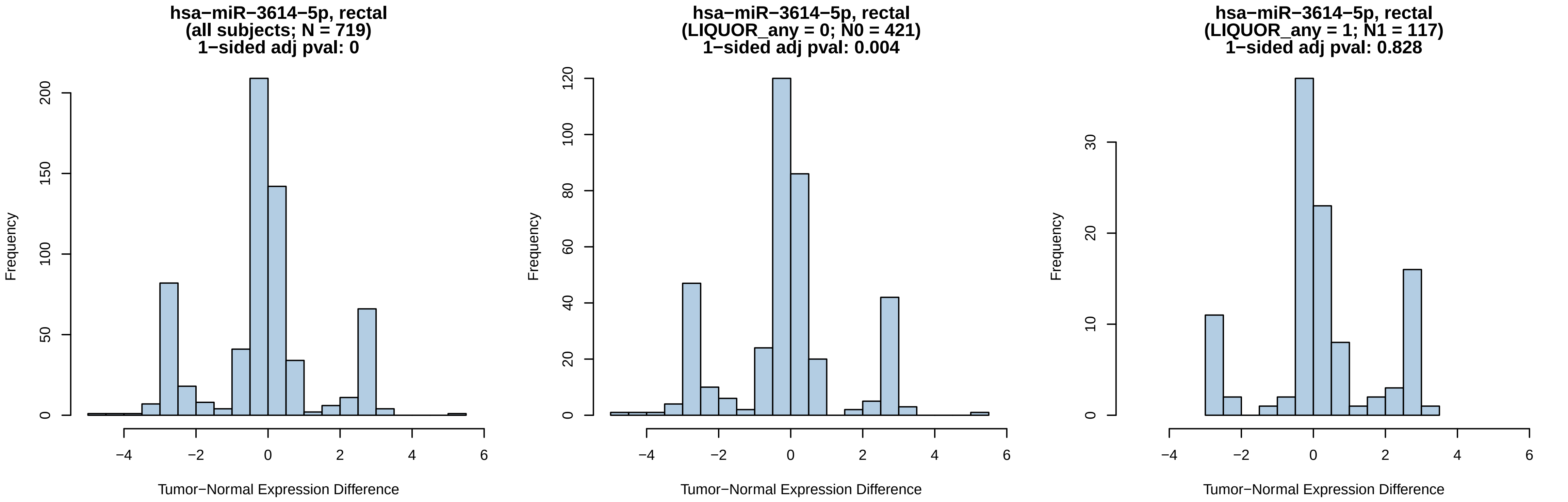
hsa−miR−3614−5p, rectal
(all subjects; N = 719)
1−sided adj pval: 0
Frequency
0
50
100
150
200
−4
−2
0
2
4
6
Tumor−Normal Expression Difference
hsa−miR−3614−5p, rectal
(LIQUOR_any = 0; N0 = 421)
1−sided adj pval: 0.004
Frequency
0
20
40
60
80
100
120
−4
−2
0
2
4
6
Tumor−Normal Expression Difference
hsa−miR−3614−5p, rectal
(LIQUOR_any = 1; N1 = 117)
1−sided adj pval: 0.828
Frequency
0
10
20
30
−4
−2
0
2
4
6
Tumor−Normal Expression Difference

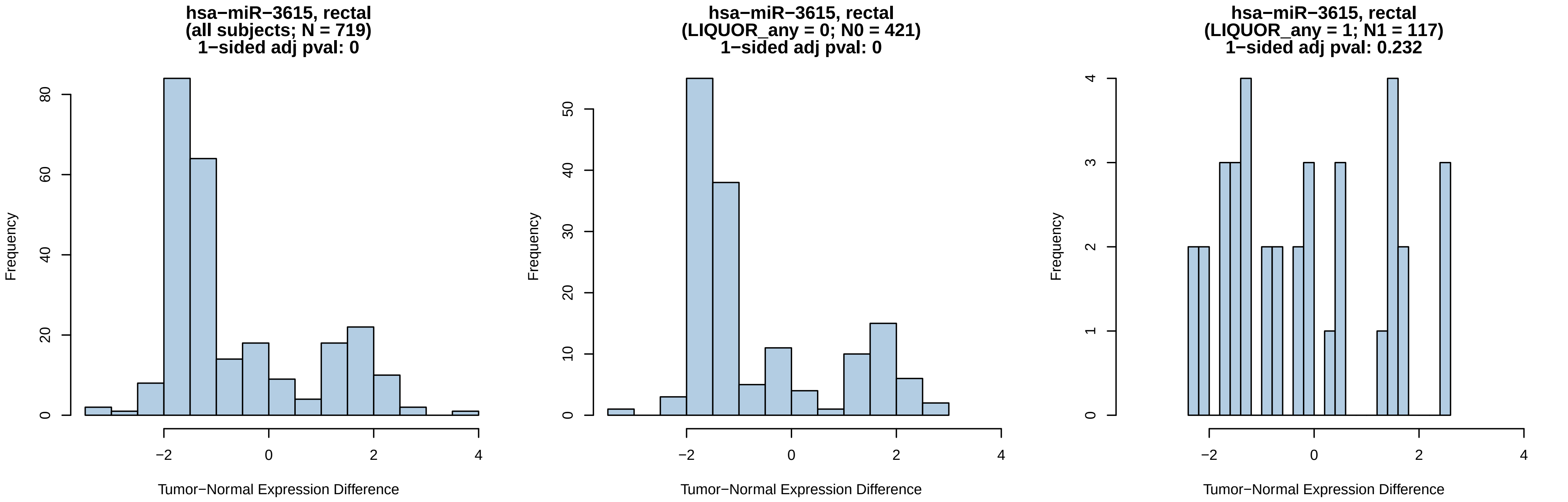
hsa−miR−3615, rectal
(all subjects; N = 719)
1−sided adj pval: 0
Frequency
Tumor−Normal Expression Difference
hsa−miR−3615, rectal
(LIQUOR_any = 0; N0 = 421)
1−sided adj pval: 0
Frequency
Tumor−Normal Expression Difference
hsa−miR−3615, rectal
(LIQUOR_any = 1; N1 = 117)
1−sided adj pval: 0.232
Frequency
Tumor−Normal Expression Difference

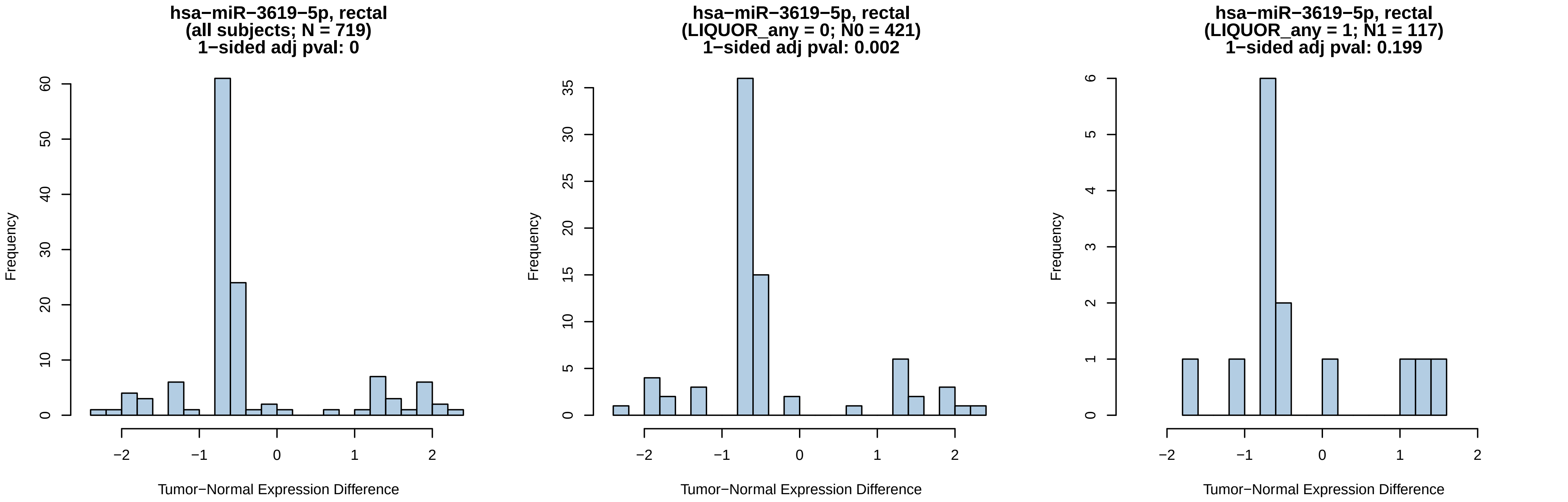

hsa−miR−3619−5p, rectal
(all subjects; N = 719)
1−sided adj pval: 0
Frequency
0
10
20
30
40
50
60
−2
−1
0
1
2
Tumor−Normal Expression Difference
hsa−miR−3619−5p, rectal
(LIQUOR_any = 0; N0 = 421)
1−sided adj pval: 0.002
Frequency
0
5
10
15
20
25
30
35
−2
−1
0
1
2
Tumor−Normal Expression Difference
hsa−miR−3619−5p, rectal
(LIQUOR_any = 1; N1 = 117)
1−sided adj pval: 0.199
Frequency
0
1
2
3
4
5
6
−2
−1
0
1
2
Tumor−Normal Expression Difference

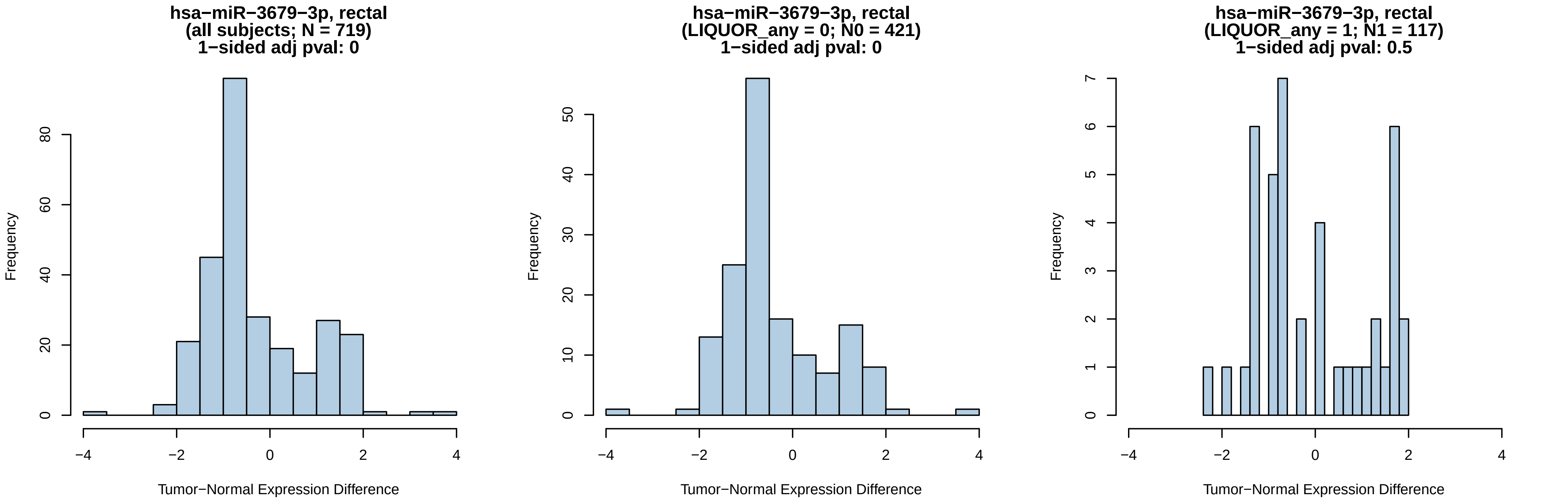
hsa−miR−3679−3p, rectal
(all subjects; N = 719)
1−sided adj pval: 0
Frequency
0
20
40
60
80
−4
−2
0
2
4
Tumor−Normal Expression Difference
hsa−miR−3679−3p, rectal
(LIQUOR_any = 0; N0 = 421)
1−sided adj pval: 0
Frequency
0
10
20
30
40
50
−4
−2
0
2
4
Tumor−Normal Expression Difference
hsa−miR−3679−3p, rectal
(LIQUOR_any = 1; N1 = 117)
1−sided adj pval: 0.5
Frequency
0
1
2
3
4
5
6
7
−4
−2
0
2
4
Tumor−Normal Expression Difference

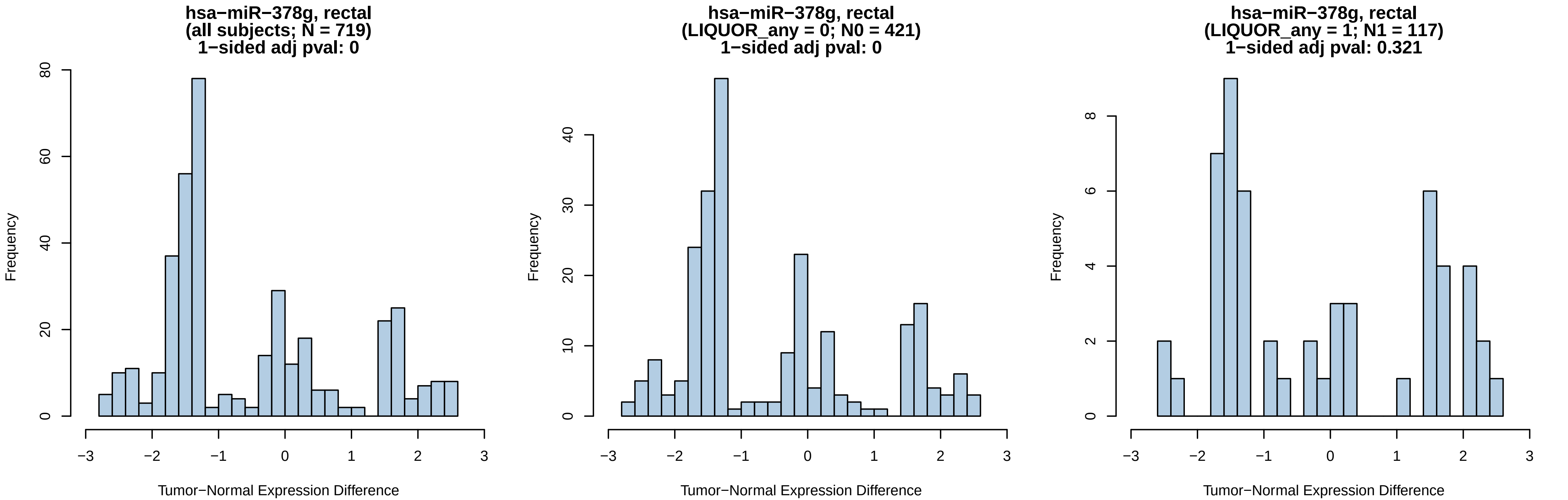
hsa−miR−378g, rectal
(all subjects; N = 719)
1−sided adj pval: 0
Frequency
Tumor−Normal Expression Difference
hsa−miR−378g, rectal
(LIQUOR_any = 0; N0 = 421)
1−sided adj pval: 0
Frequency
Tumor−Normal Expression Difference
hsa−miR−378g, rectal
(LIQUOR_any = 1; N1 = 117)
1−sided adj pval: 0.321
Frequency
Tumor−Normal Expression Difference

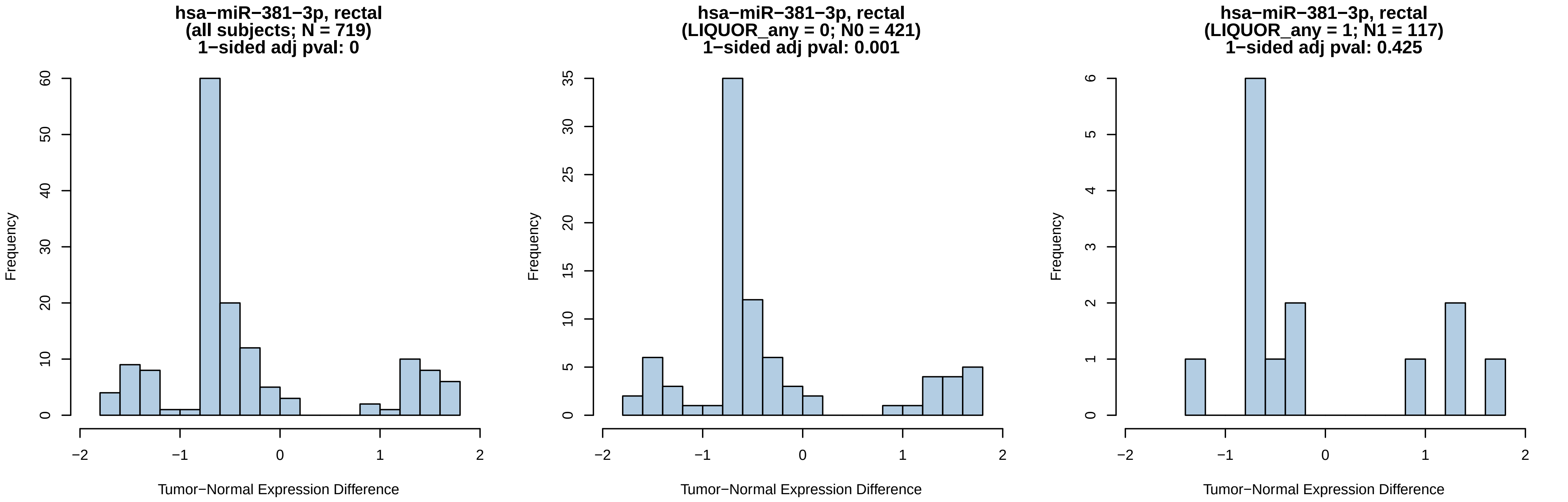
hsa−miR−381−3p, rectal
(all subjects; N = 719)
1−sided adj pval: 0
Frequency
Tumor−Normal Expression Difference
hsa−miR−381−3p, rectal
(LIQUOR_any = 0; N0 = 421)
1−sided adj pval: 0.001
Frequency
Tumor−Normal Expression Difference
hsa−miR−381−3p, rectal
(LIQUOR_any = 1; N1 = 117)
1−sided adj pval: 0.425
Frequency
Tumor−Normal Expression Difference

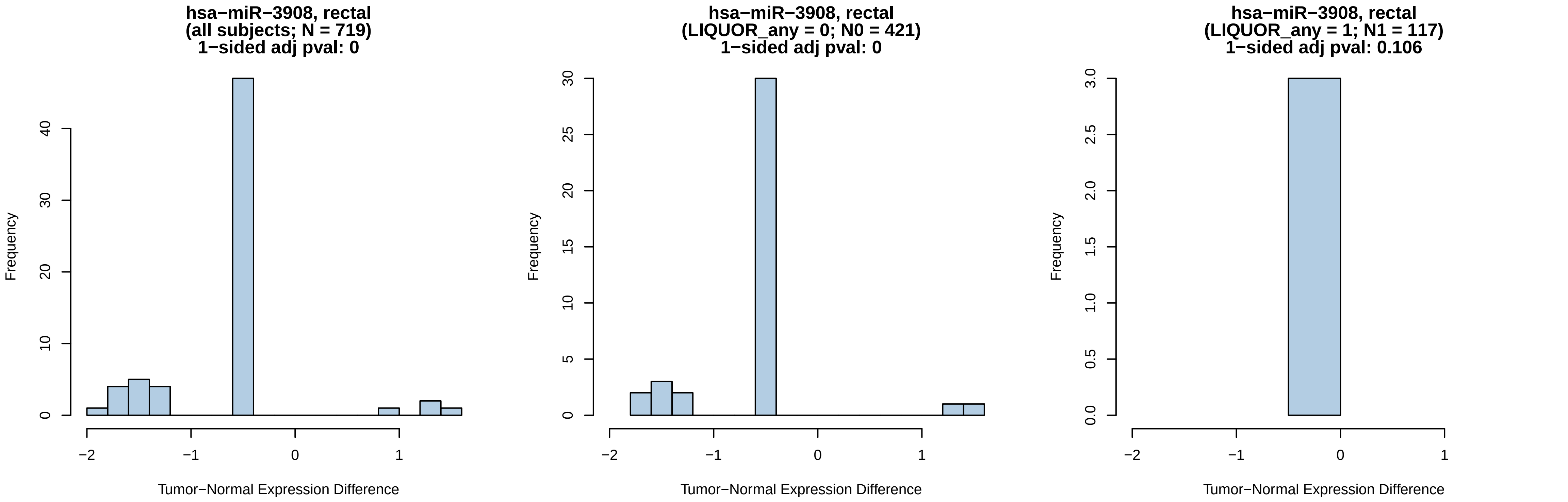

hsa−miR−3908, rectal
(all subjects; N = 719)
1−sided adj pval: 0
hsa−miR−3908, rectal
(LIQUOR_any = 0; N0 = 421)
1−sided adj pval: 0
hsa−miR−3908, rectal
(LIQUOR_any = 1; N1 = 117)
1−sided adj pval: 0.106
Frequency
Tumor−Normal Expression Difference

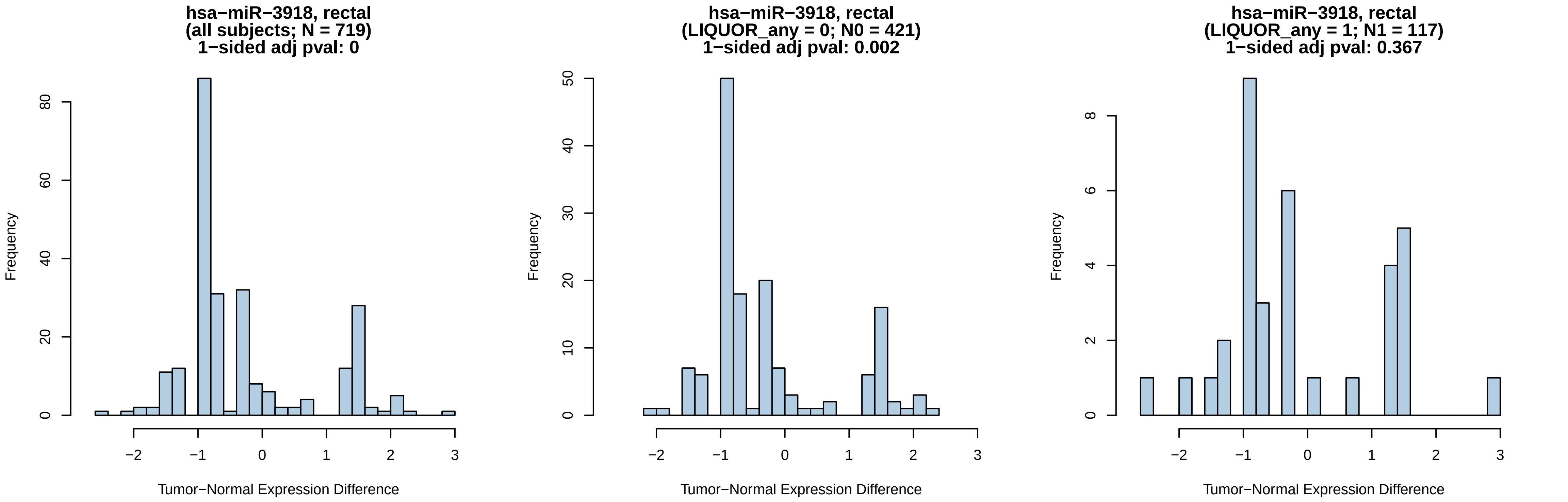
hsa−miR−3918, rectal
(all subjects; N = 719)
1−sided adj pval: 0
Frequency
0
20
40
60
80
−2
−1
0
1
2
3
Tumor−Normal Expression Difference
hsa−miR−3918, rectal
(LIQUOR_any = 0; N0 = 421)
1−sided adj pval: 0.002
Frequency
0
10
20
30
40
50
−2
−1
0
1
2
3
Tumor−Normal Expression Difference
hsa−miR−3918, rectal
(LIQUOR_any = 1; N1 = 117)
1−sided adj pval: 0.367
Frequency
0
2
4
6
8
−2
−1
0
1
2
3
Tumor−Normal Expression Difference

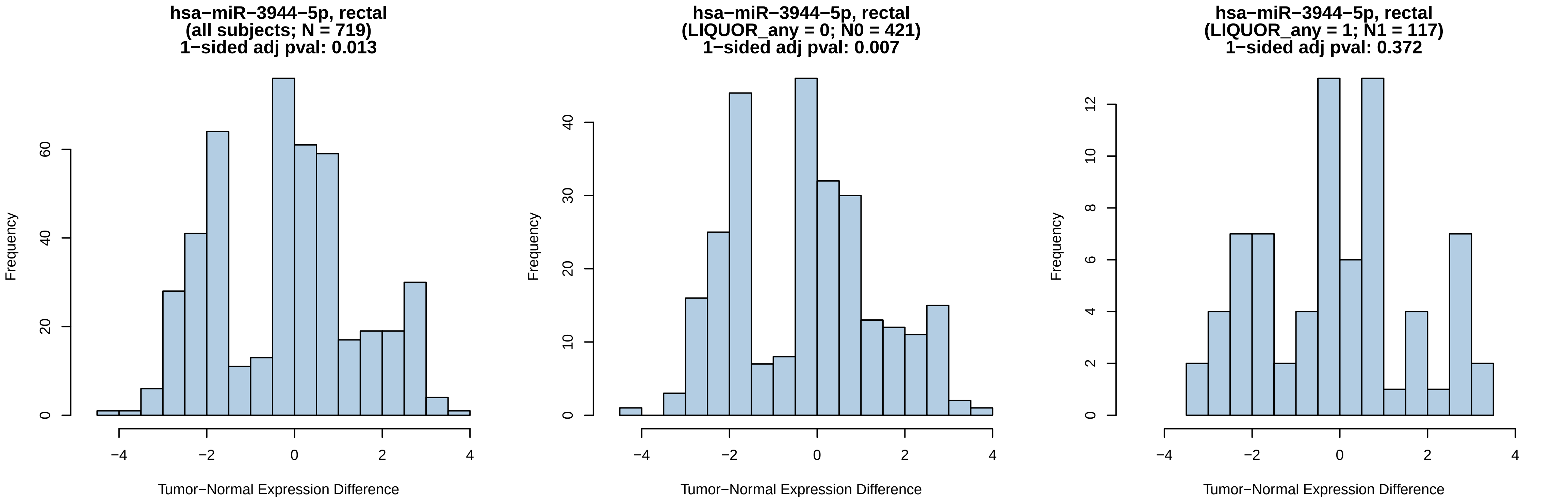
hsa−miR−3944−5p, rectal
(all subjects; N = 719)
1−sided adj pval: 0.013
Frequency
0
20
40
60
−4
−2
0
2
4
Tumor−Normal Expression Difference
hsa−miR−3944−5p, rectal
(LIQUOR_any = 0; N0 = 421)
1−sided adj pval: 0.007
Frequency
0
10
20
30
40
−4
−2
0
2
4
Tumor−Normal Expression Difference
hsa−miR−3944−5p, rectal
(LIQUOR_any = 1; N1 = 117)
1−sided adj pval: 0.372
Frequency
0
2
4
6
8
10
12
−4
−2
0
2
4
Tumor−Normal Expression Difference

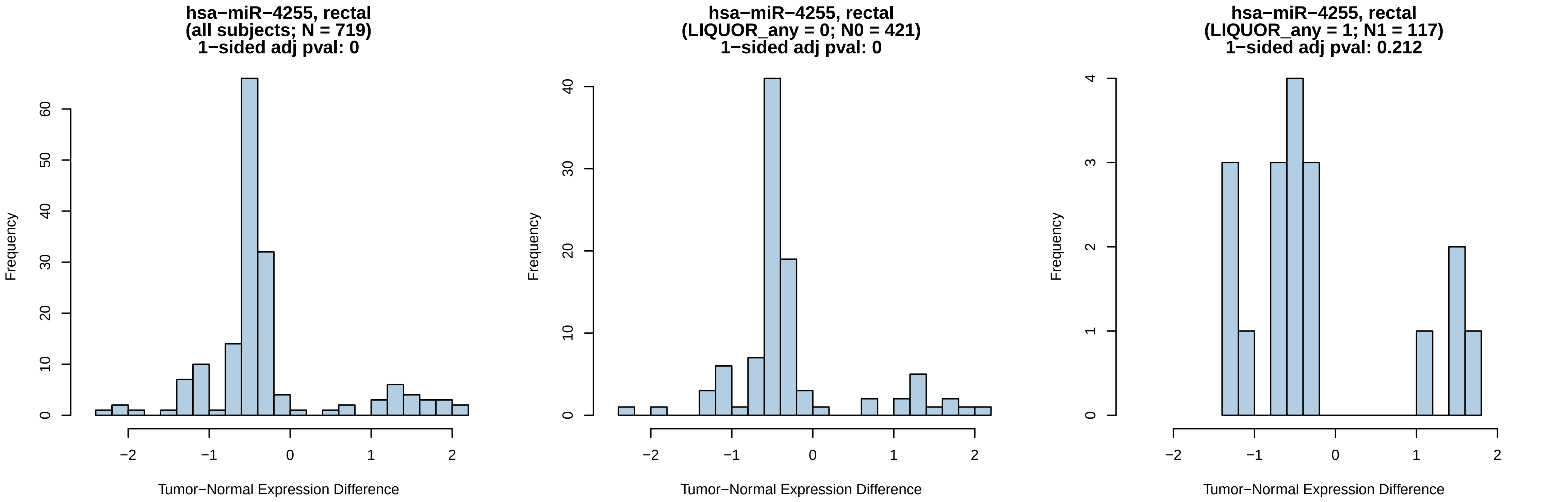
hsa−miR−4255, rectal
(all subjects; N = 719)
1−sided adj pval: 0
Frequency
0
10
20
30
40
50
60
−2
−1
0
1
2
Tumor−Normal Expression Difference
hsa−miR−4255, rectal
(LIQUOR_any = 0; N0 = 421)
1−sided adj pval: 0
Frequency
0
10
20
30
40
−2
−1
0
1
2
Tumor−Normal Expression Difference
hsa−miR−4255, rectal
(LIQUOR_any = 1; N1 = 117)
1−sided adj pval: 0.212
Frequency
0
1
2
3
4
−2
−1
0
1
2
Tumor−Normal Expression Difference

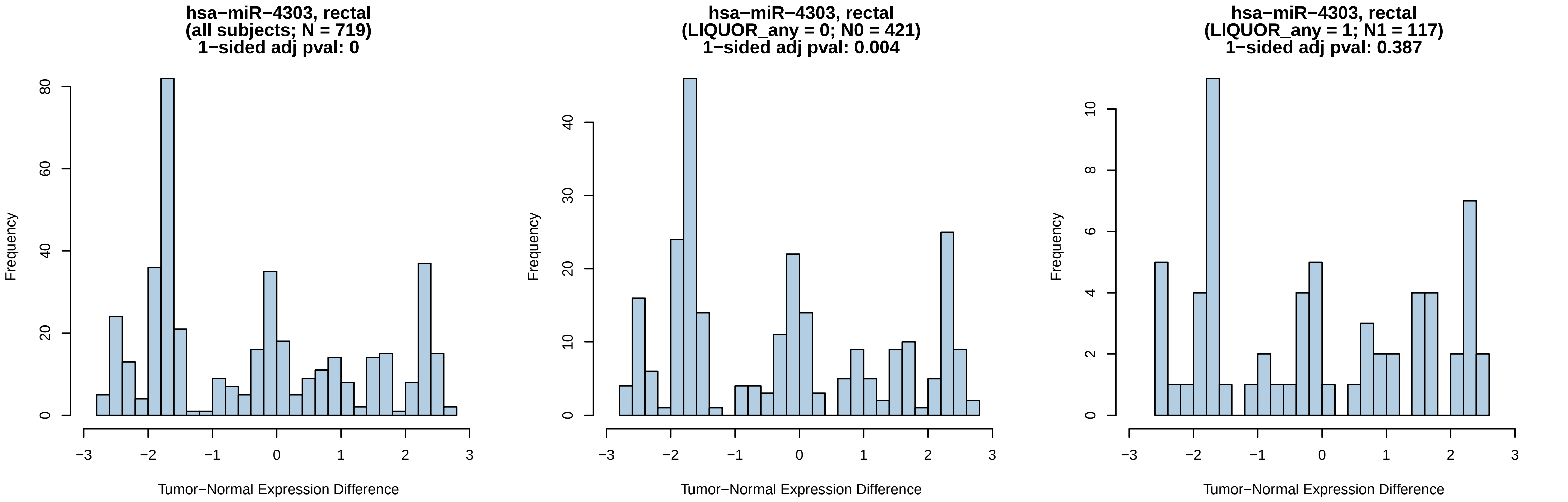
hsa−miR−4303, rectal
(all subjects; N = 719)
1−sided adj pval: 0
Frequency
0
20
40
60
80
−3
−2
−1
0
1
2
3
Tumor−Normal Expression Difference
hsa−miR−4303, rectal
(LIQUOR_any = 0; N0 = 421)
1−sided adj pval: 0.004
Frequency
0
10
20
30
40
−3
−2
−1
0
1
2
3
Tumor−Normal Expression Difference
hsa−miR−4303, rectal
(LIQUOR_any = 1; N1 = 117)
1−sided adj pval: 0.387
Frequency
0
2
4
6
8
10
−3
−2
−1
0
1
2
3
Tumor−Normal Expression Difference

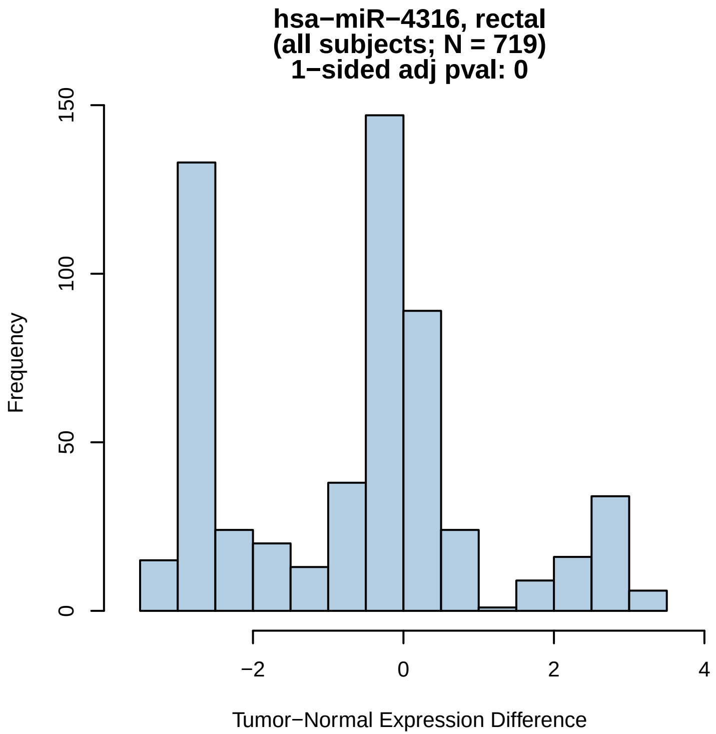
hsa−miR−4316, rectal
(all subjects; N = 719)
1−sided adj pval: 0
Frequency
Tumor−Normal Expression Difference
0
50
100
150
−2
0
2
4

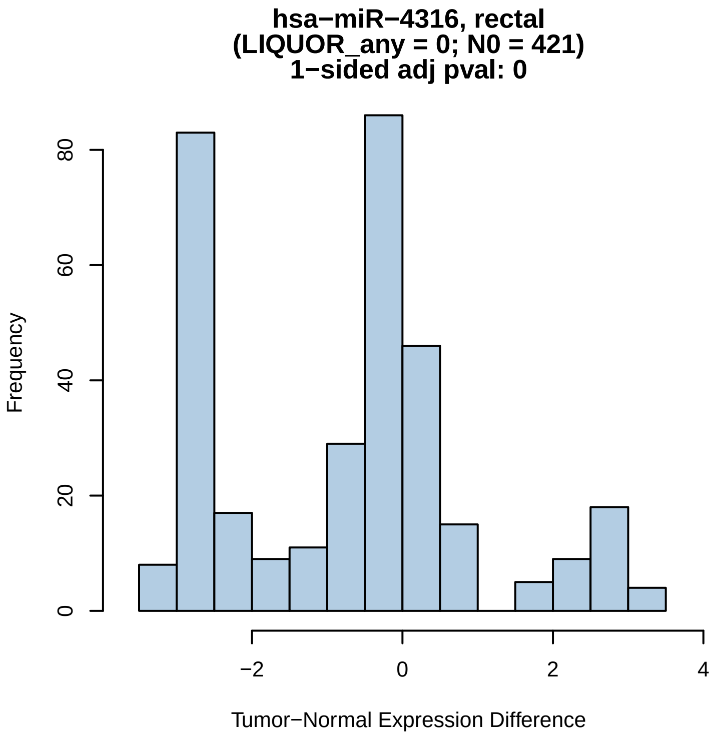
hsa−miR−4316, rectal
(LIQUOR_any = 0; N0 = 421)
1−sided adj pval: 0
Frequency
Tumor−Normal Expression Difference
0
20
40
60
80
−2
0
2
4

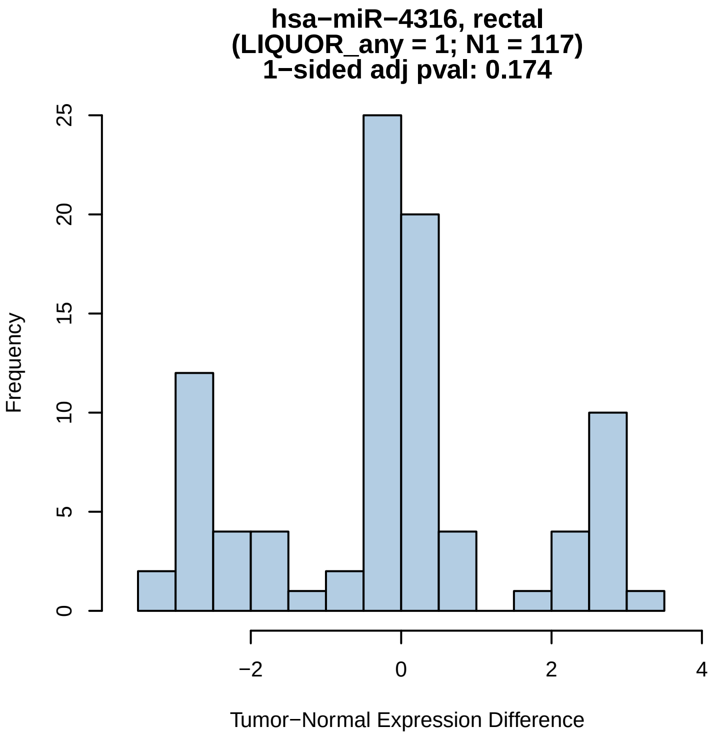
hsa−miR−4316, rectal
(LIQUOR_any = 1; N1 = 117)
1−sided adj pval: 0.174
Frequency
Tumor−Normal Expression Difference
0
5
10
15
20
25
−2
0
2
4

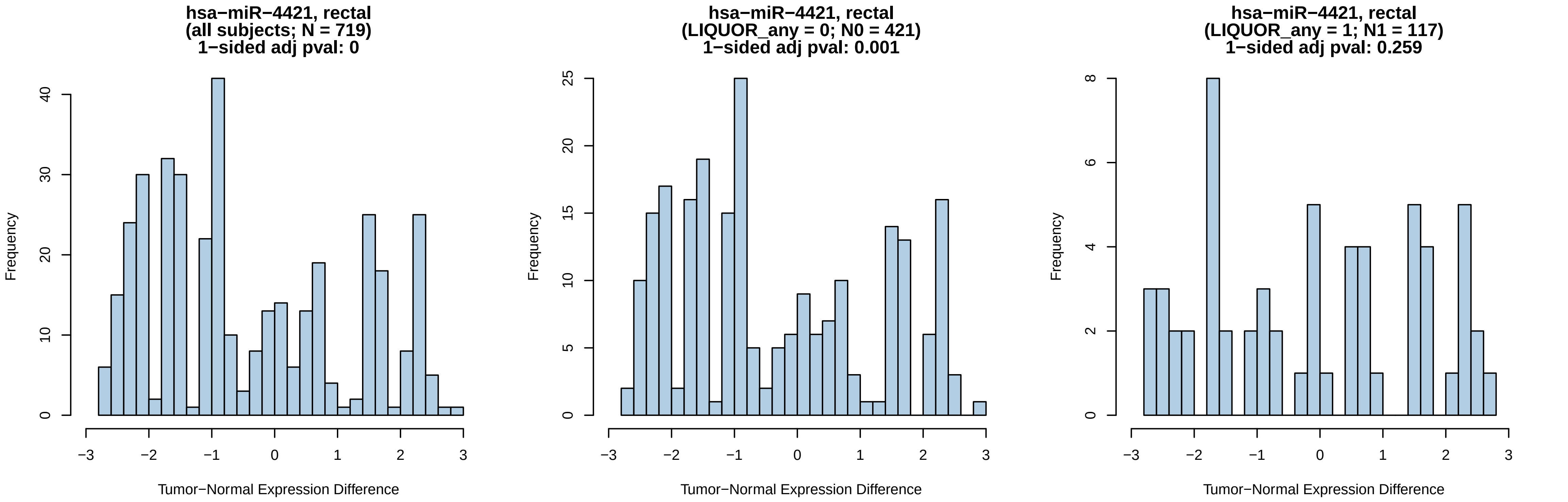

hsa−miR−4421, rectal
(all subjects; N = 719)
1−sided adj pval: 0
Frequency
Tumor−Normal Expression Difference
hsa−miR−4421, rectal
(LIQUOR_any = 0; N0 = 421)
1−sided adj pval: 0.001
Frequency
Tumor−Normal Expression Difference
hsa−miR−4421, rectal
(LIQUOR_any = 1; N1 = 117)
1−sided adj pval: 0.259
Frequency
Tumor−Normal Expression Difference

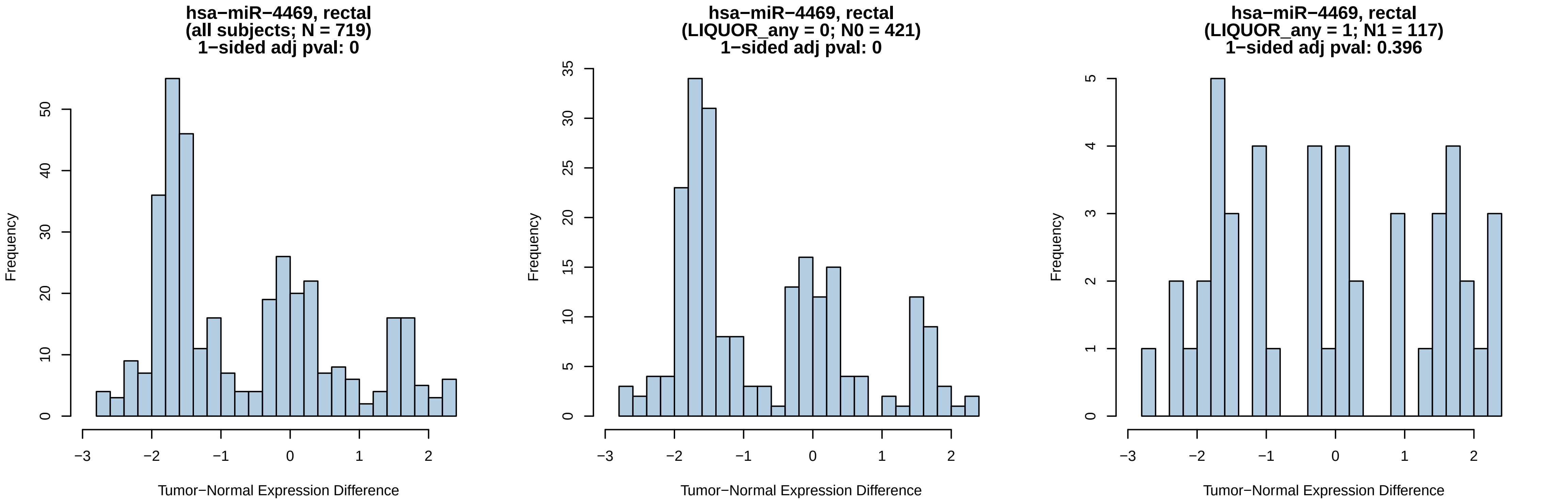
hsa−miR−4469, rectal
(all subjects; N = 719)
1−sided adj pval: 0
hsa−miR−4469, rectal
(LIQUOR_any = 0; N0 = 421)
1−sided adj pval: 0
hsa−miR−4469, rectal
(LIQUOR_any = 1; N1 = 117)
1−sided adj pval: 0.396
Frequency
Tumor−Normal Expression Difference

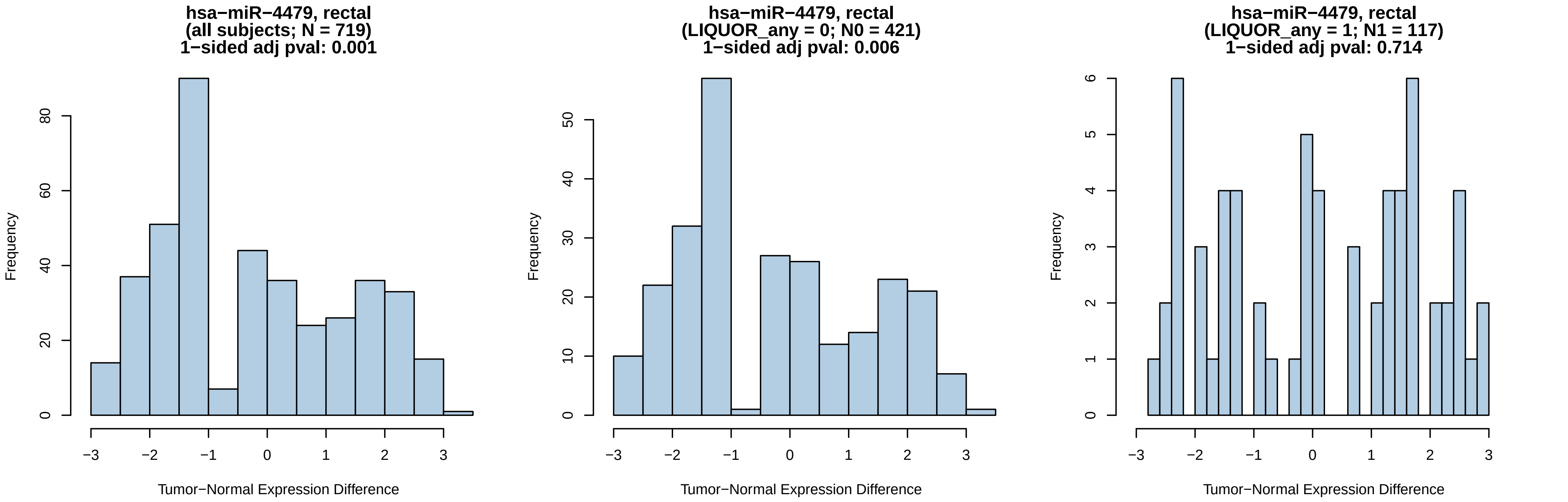
hsa−miR−4479, rectal
(all subjects; N = 719)
1−sided adj pval: 0.001
hsa−miR−4479, rectal
(LIQUOR_any = 0; N0 = 421)
1−sided adj pval: 0.006
hsa−miR−4479, rectal
(LIQUOR_any = 1; N1 = 117)
1−sided adj pval: 0.714
Frequency
Tumor−Normal Expression Difference

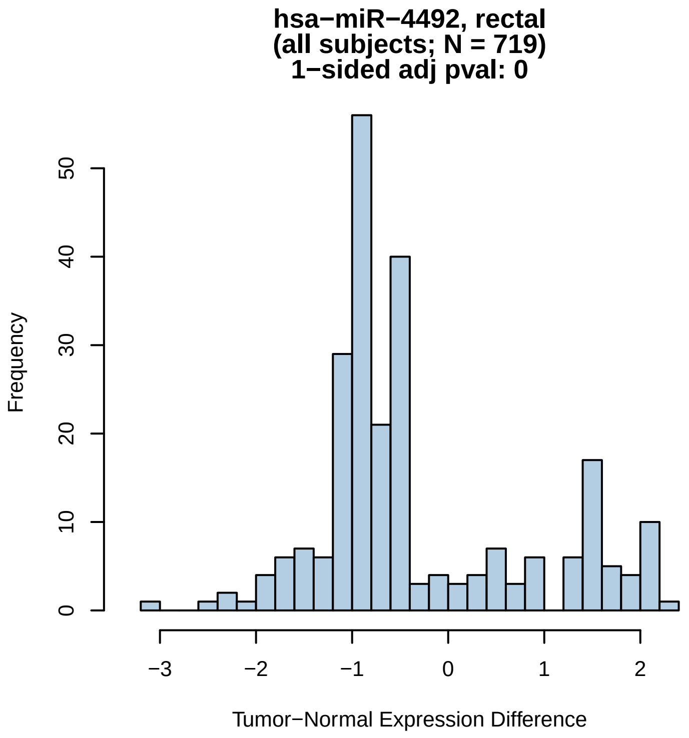
hsa−miR−4492, rectal
(all subjects; N = 719)
1−sided adj pval: 0
Frequency
Tumor−Normal Expression Difference
0
10
20
30
40
50
−3
−2
−1
0
1
2

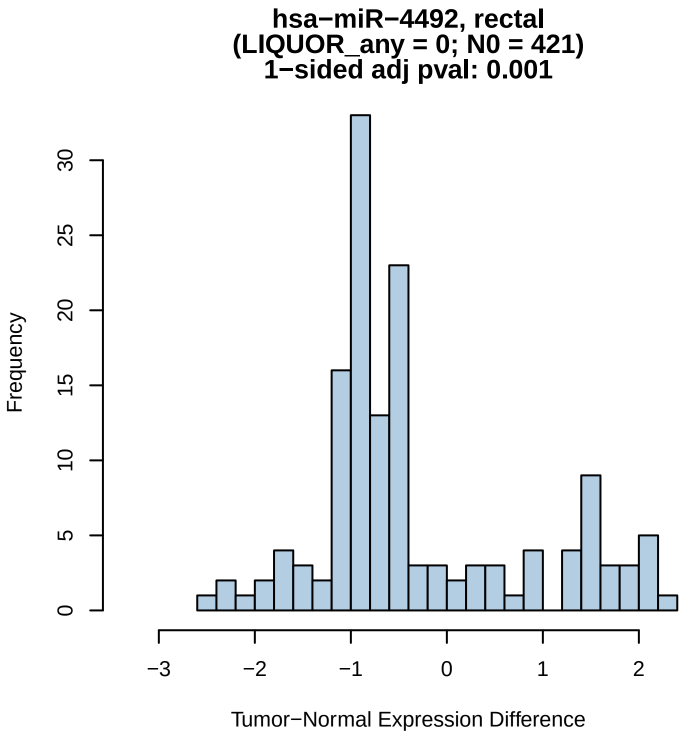
hsa−miR−4492, rectal
(LIQUOR_any = 0; N0 = 421)
1−sided adj pval: 0.001
Frequency
Tumor−Normal Expression Difference
0
5
10
15
20
25
30
−3
−2
−1
0
1
2

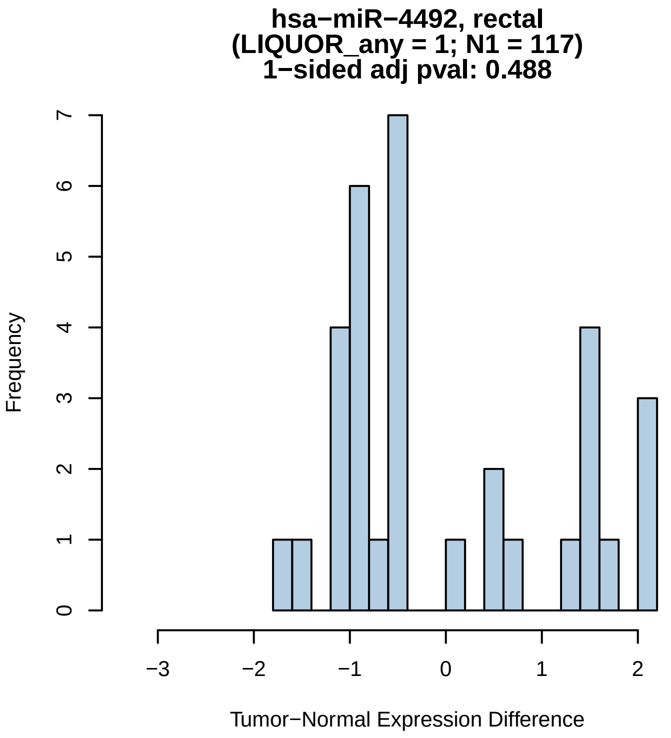
hsa−miR−4492, rectal
(LIQUOR_any = 1; N1 = 117)
1−sided adj pval: 0.488
Frequency
Tumor−Normal Expression Difference
0
1
2
3
4
5
6
7
−3
−2
−1
0
1
2

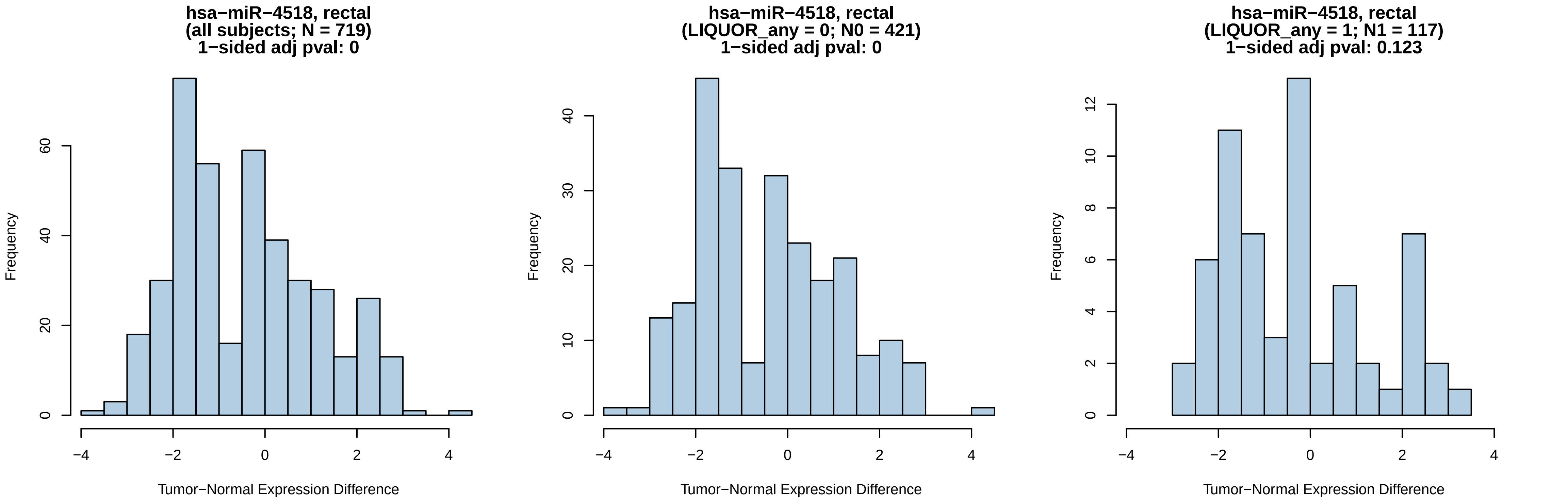
hsa−miR−4518, rectal
(all subjects; N = 719)
1−sided adj pval: 0
Frequency
Tumor−Normal Expression Difference
hsa−miR−4518, rectal
(LIQUOR_any = 0; N0 = 421)
1−sided adj pval: 0
Frequency
Tumor−Normal Expression Difference
hsa−miR−4518, rectal
(LIQUOR_any = 1; N1 = 117)
1−sided adj pval: 0.123
Frequency
Tumor−Normal Expression Difference

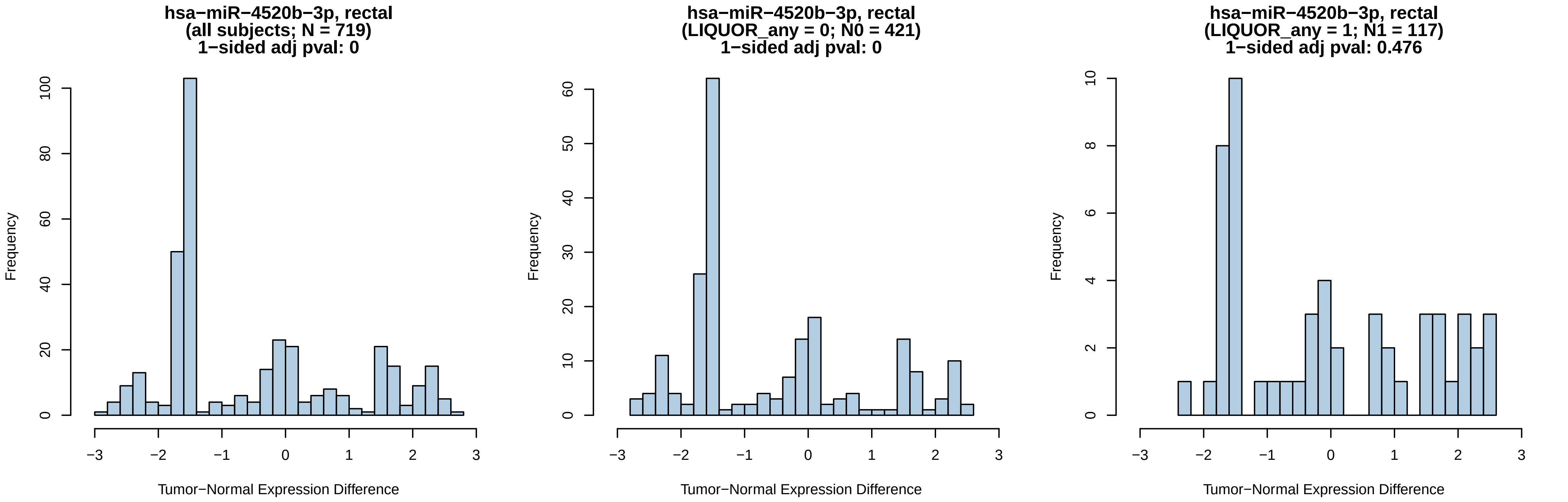
hsa−miR−4520b−3p, rectal
(all subjects; N = 719)
1−sided adj pval: 0
hsa−miR−4520b−3p, rectal
(LIQUOR_any = 0; N0 = 421)
1−sided adj pval: 0
hsa−miR−4520b−3p, rectal
(LIQUOR_any = 1; N1 = 117)
1−sided adj pval: 0.476
Frequency
Tumor−Normal Expression Difference

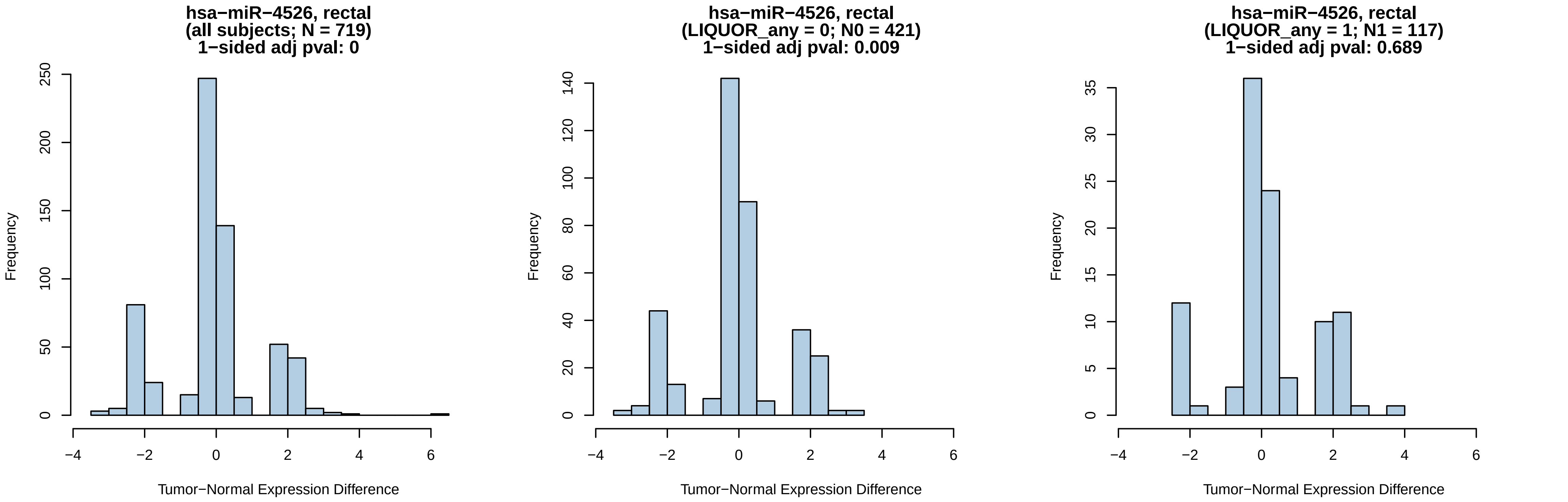
hsa−miR−4526, rectal
(all subjects; N = 719)
1−sided adj pval: 0
hsa−miR−4526, rectal
(LIQUOR_any = 0; N0 = 421)
1−sided adj pval: 0.009
hsa−miR−4526, rectal
(LIQUOR_any = 1; N1 = 117)
1−sided adj pval: 0.689
Frequency
Tumor−Normal Expression Difference

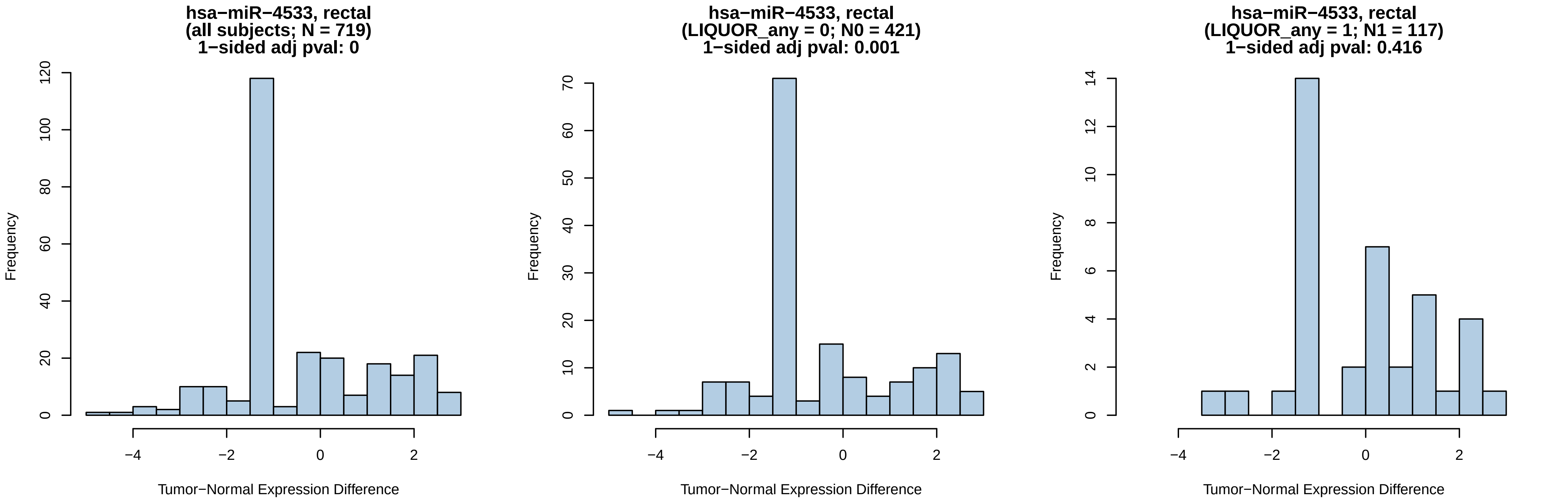

hsa−miR−4533, rectal
(all subjects; N = 719)
1−sided adj pval: 0
Frequency
Tumor−Normal Expression Difference
hsa−miR−4533, rectal
(LIQUOR_any = 0; N0 = 421)
1−sided adj pval: 0.001
Frequency
Tumor−Normal Expression Difference
hsa−miR−4533, rectal
(LIQUOR_any = 1; N1 = 117)
1−sided adj pval: 0.416
Frequency
Tumor−Normal Expression Difference

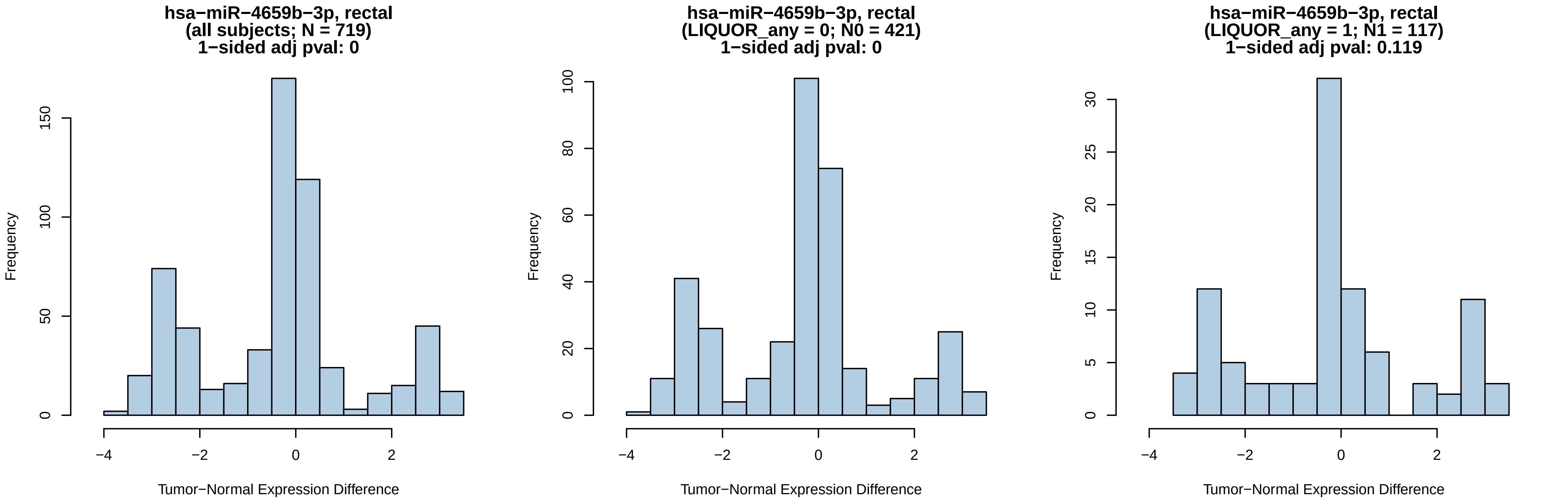
hsa−miR−4659b−3p, rectal
(all subjects; N = 719)
1−sided adj pval: 0
hsa−miR−4659b−3p, rectal
(LIQUOR_any = 0; N0 = 421)
1−sided adj pval: 0
hsa−miR−4659b−3p, rectal
(LIQUOR_any = 1; N1 = 117)
1−sided adj pval: 0.119
Frequency
Tumor−Normal Expression Difference
0
50
100
150
0
20
40
60
80
100
0
5
10
15
20
25
30
−4
−2
0
2

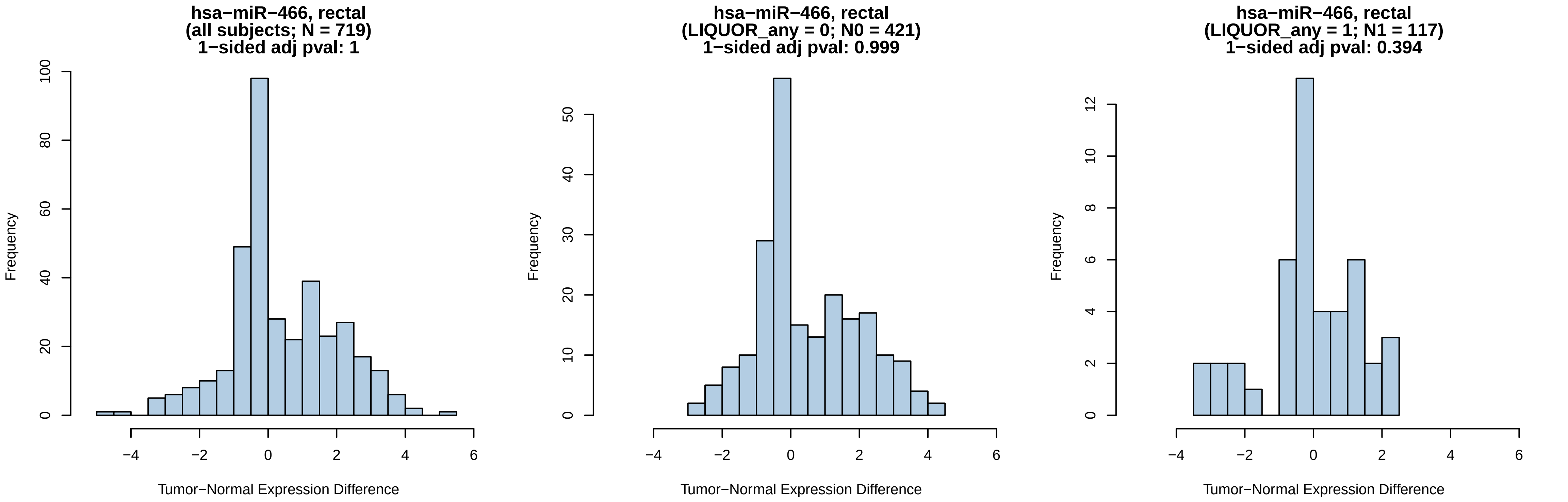

hsa−miR−466, rectal
(all subjects; N = 719)
1−sided adj pval: 1
Frequency
Tumor−Normal Expression Difference
hsa−miR−466, rectal
(LIQUOR_any = 0; N0 = 421)
1−sided adj pval: 0.999
Frequency
Tumor−Normal Expression Difference
hsa−miR−466, rectal
(LIQUOR_any = 1; N1 = 117)
1−sided adj pval: 0.394
Frequency
Tumor−Normal Expression Difference

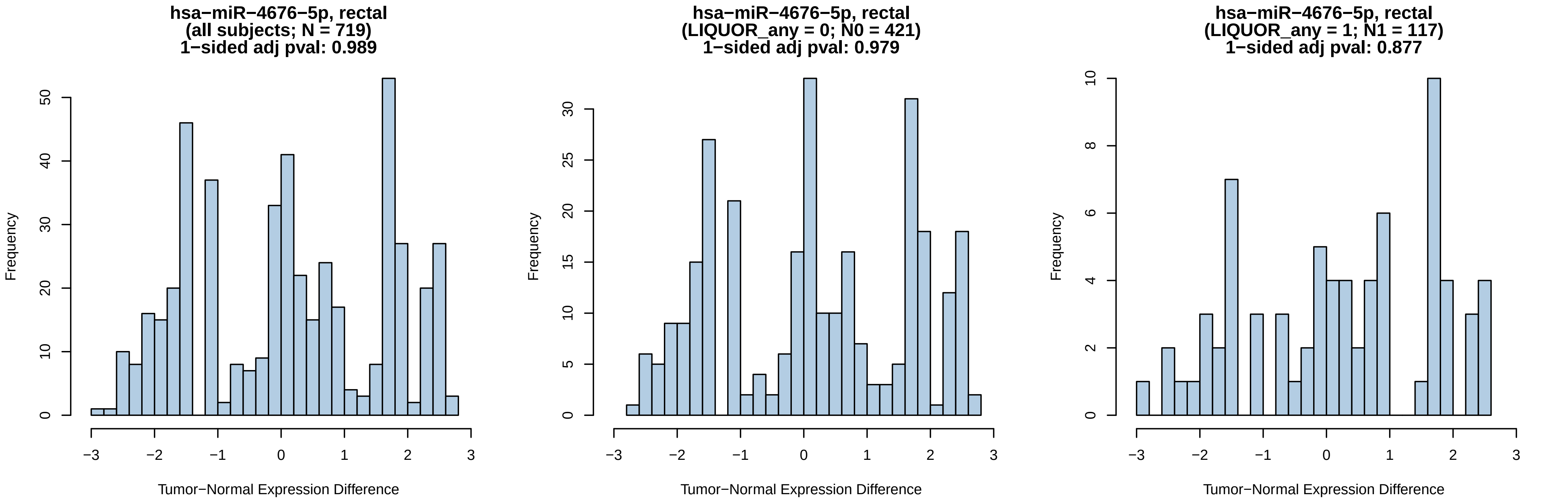
hsa−miR−4676−5p, rectal
(all subjects; N = 719)
1−sided adj pval: 0.989
Frequency
Tumor−Normal Expression Difference
hsa−miR−4676−5p, rectal
(LIQUOR_any = 0; N0 = 421)
1−sided adj pval: 0.979
Frequency
Tumor−Normal Expression Difference
hsa−miR−4676−5p, rectal
(LIQUOR_any = 1; N1 = 117)
1−sided adj pval: 0.877
Frequency
Tumor−Normal Expression Difference

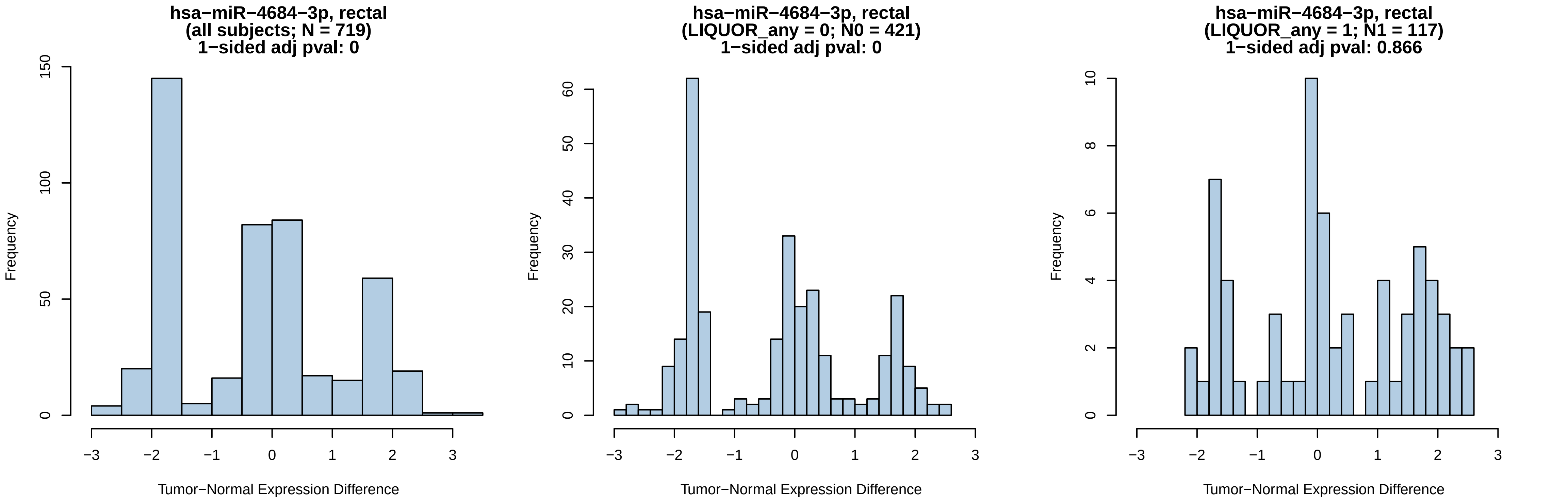
hsa−miR−4684−3p, rectal
(all subjects; N = 719)
1−sided adj pval: 0
hsa−miR−4684−3p, rectal
(LIQUOR_any = 0; N0 = 421)
1−sided adj pval: 0
hsa−miR−4684−3p, rectal
(LIQUOR_any = 1; N1 = 117)
1−sided adj pval: 0.866
Frequency
Tumor−Normal Expression Difference

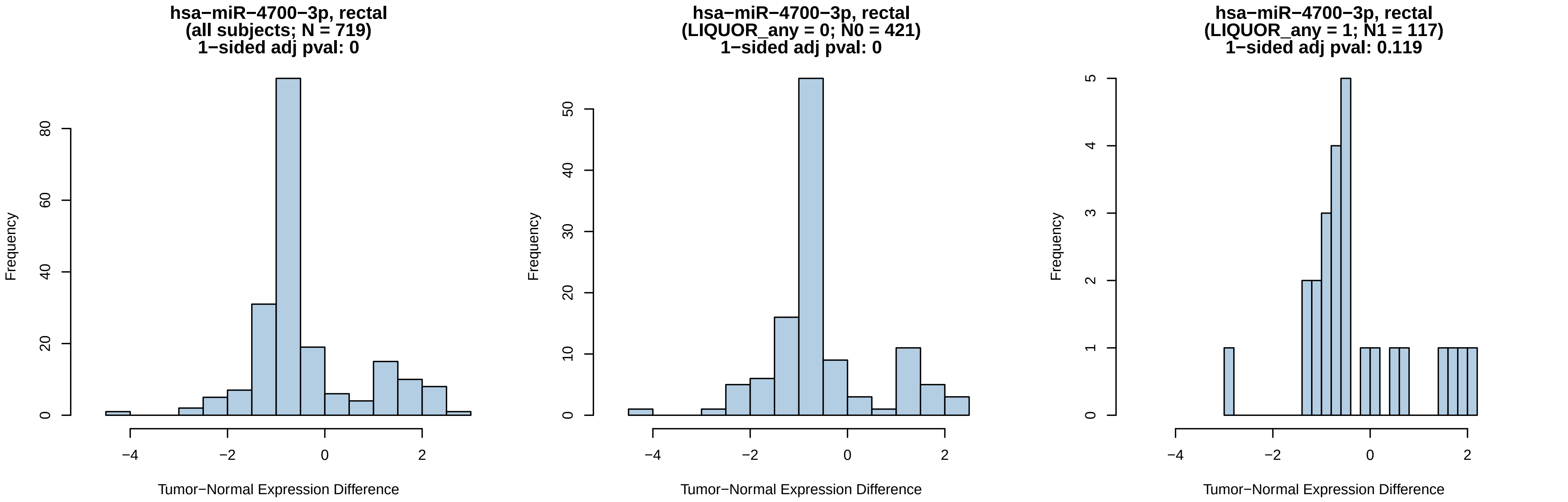

hsa−miR−4700−3p, rectal
(all subjects; N = 719)
1−sided adj pval: 0
Frequency
0
20
40
60
80
−4
−2
0
2
Tumor−Normal Expression Difference
hsa−miR−4700−3p, rectal
(LIQUOR_any = 0; N0 = 421)
1−sided adj pval: 0
Frequency
0
10
20
30
40
50
−4
−2
0
2
Tumor−Normal Expression Difference
hsa−miR−4700−3p, rectal
(LIQUOR_any = 1; N1 = 117)
1−sided adj pval: 0.119
Frequency
0
1
2
3
4
5
−4
−2
0
2
Tumor−Normal Expression Difference

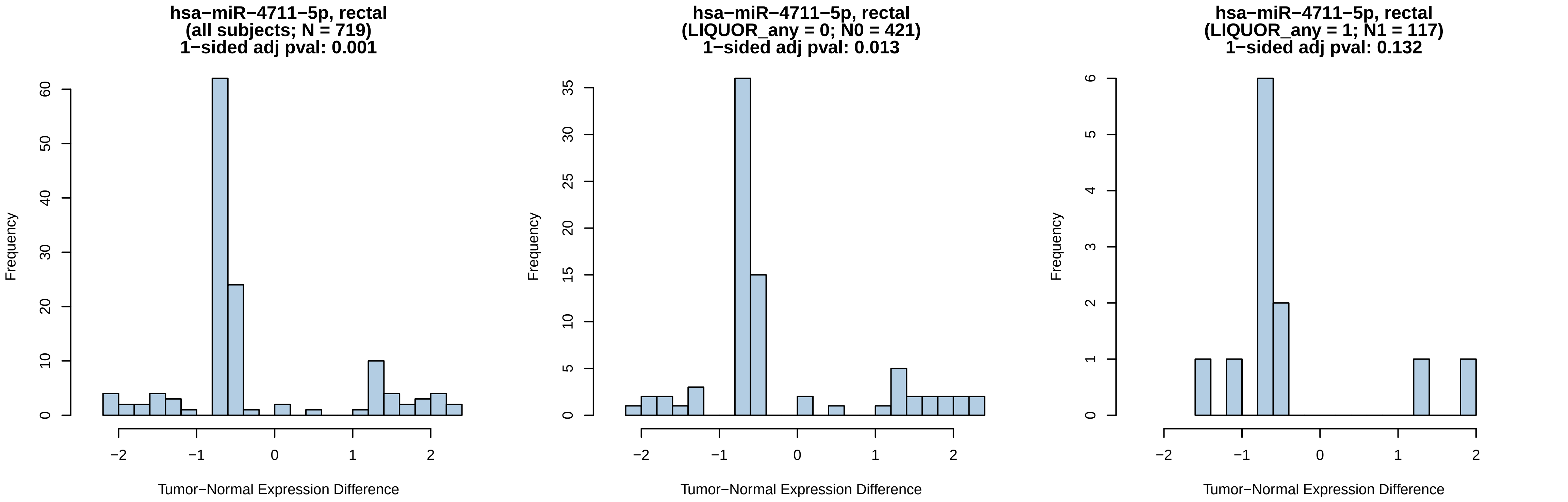

hsa−miR−4711−5p, rectal
(all subjects; N = 719)
1−sided adj pval: 0.001
Frequency
Tumor−Normal Expression Difference
hsa−miR−4711−5p, rectal
(LIQUOR_any = 0; N0 = 421)
1−sided adj pval: 0.013
Frequency
Tumor−Normal Expression Difference
hsa−miR−4711−5p, rectal
(LIQUOR_any = 1; N1 = 117)
1−sided adj pval: 0.132
Frequency
Tumor−Normal Expression Difference

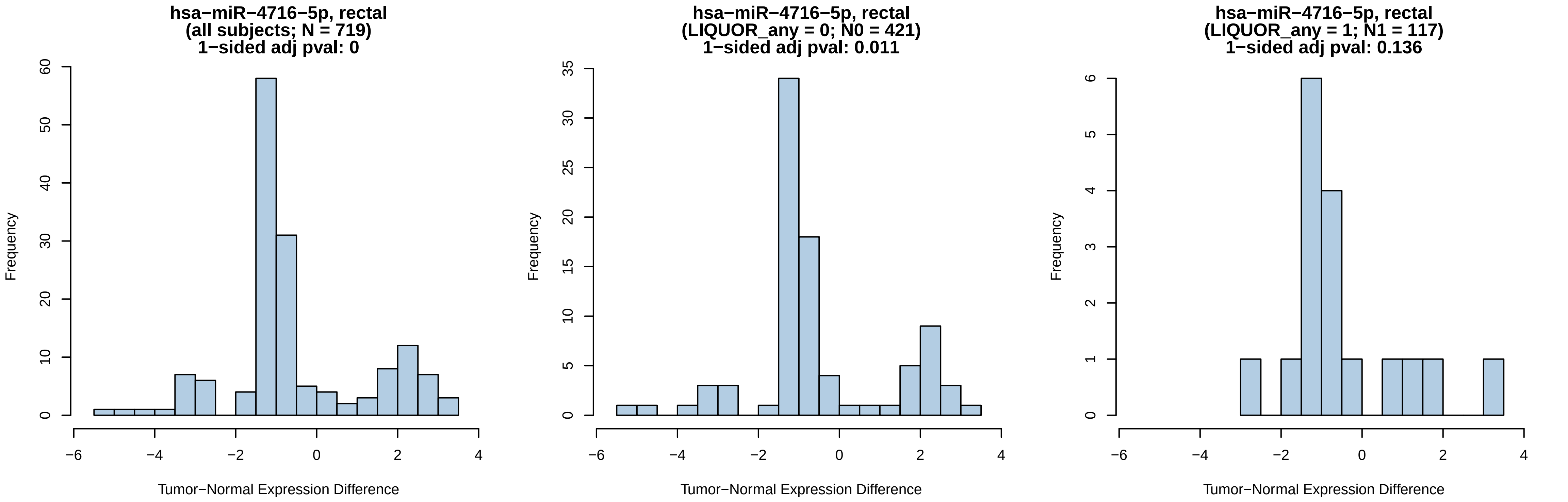

hsa−miR−4716−5p, rectal
(all subjects; N = 719)
1−sided adj pval: 0
Frequency
Tumor−Normal Expression Difference
hsa−miR−4716−5p, rectal
(LIQUOR_any = 0; N0 = 421)
1−sided adj pval: 0.011
Frequency
Tumor−Normal Expression Difference
hsa−miR−4716−5p, rectal
(LIQUOR_any = 1; N1 = 117)
1−sided adj pval: 0.136
Frequency
Tumor−Normal Expression Difference

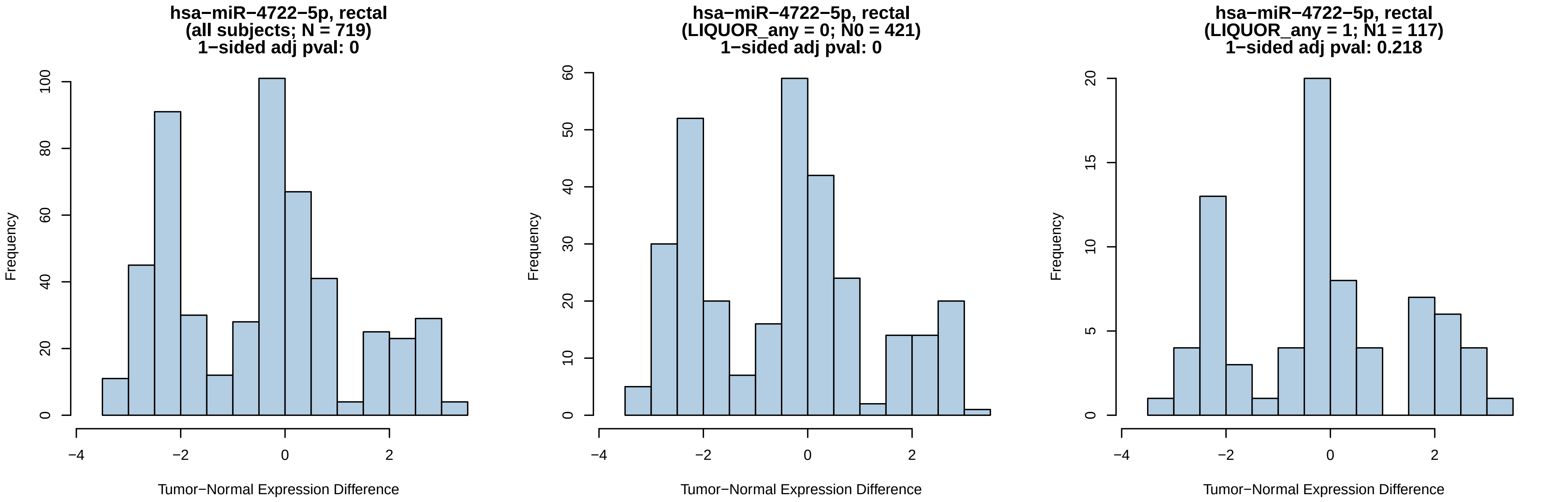

hsa−miR−4722−5p, rectal
(all subjects; N = 719)
1−sided adj pval: 0
hsa−miR−4722−5p, rectal
(LIQUOR_any = 0; N0 = 421)
1−sided adj pval: 0
hsa−miR−4722−5p, rectal
(LIQUOR_any = 1; N1 = 117)
1−sided adj pval: 0.218
Frequency
Tumor−Normal Expression Difference

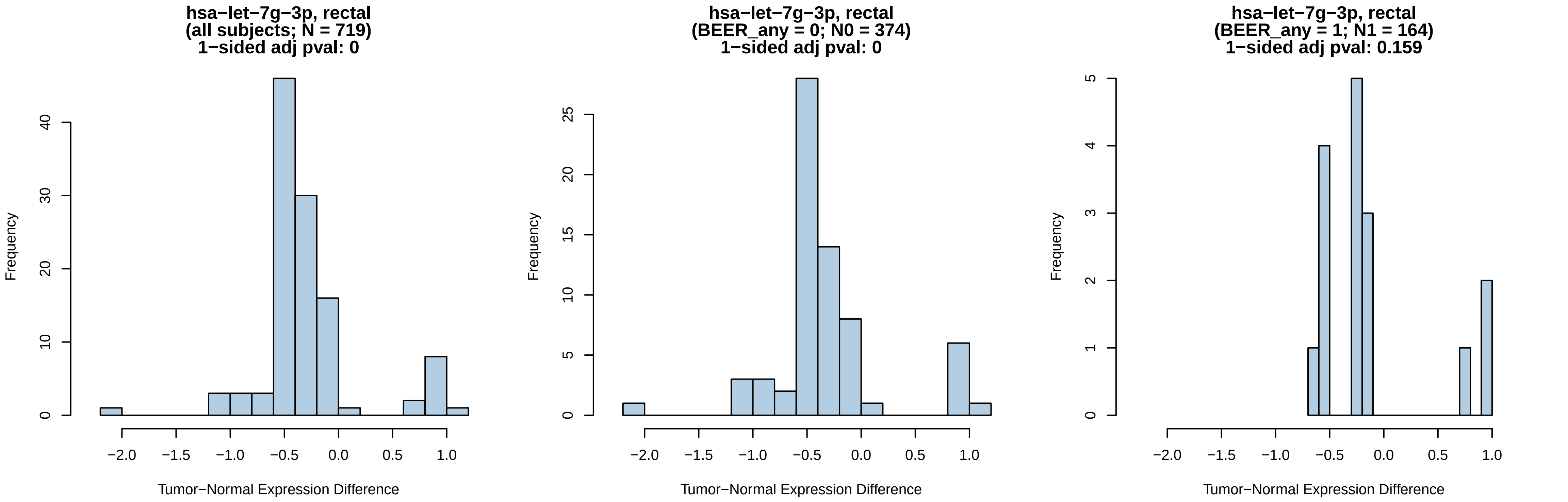

hsa−let−7g−3p, rectal
(all subjects; N = 719)
1−sided adj pval: 0
Frequency
Tumor−Normal Expression Difference
hsa−let−7g−3p, rectal
(BEER_any = 0; N0 = 374)
1−sided adj pval: 0
Frequency
Tumor−Normal Expression Difference
hsa−let−7g−3p, rectal
(BEER_any = 1; N1 = 164)
1−sided adj pval: 0.159
Frequency
Tumor−Normal Expression Difference

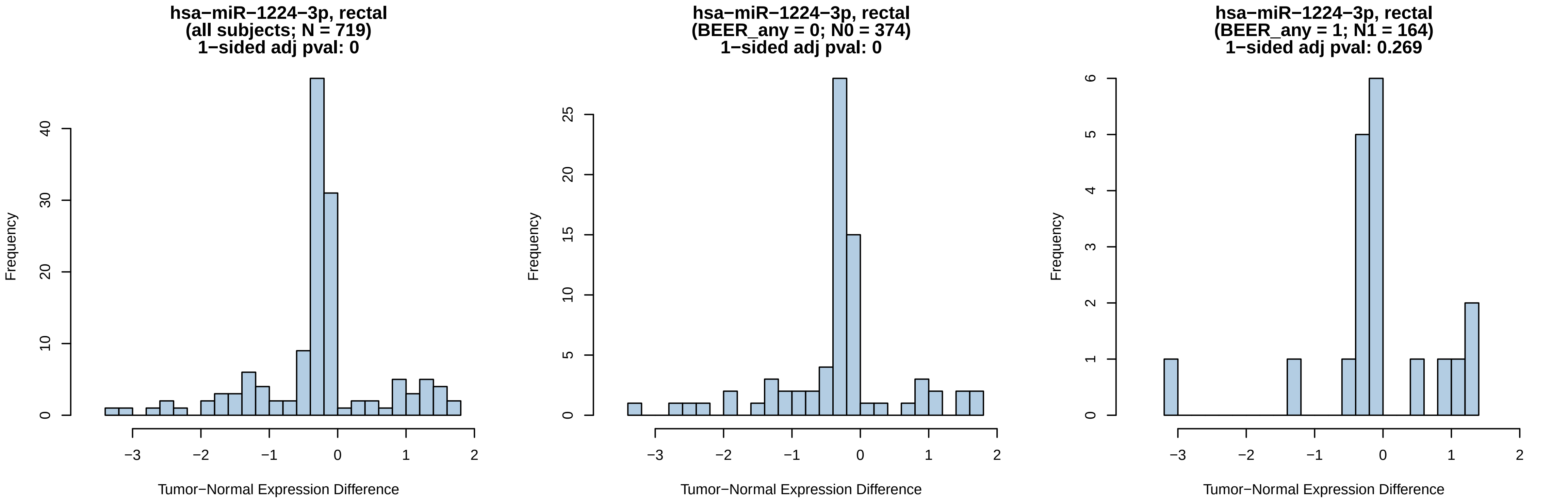
hsa−miR−1224−3p, rectal
(all subjects; N = 719)
1−sided adj pval: 0
Frequency
Tumor−Normal Expression Difference
hsa−miR−1224−3p, rectal
(BEER_any = 0; N0 = 374)
1−sided adj pval: 0
Frequency
Tumor−Normal Expression Difference
hsa−miR−1224−3p, rectal
(BEER_any = 1; N1 = 164)
1−sided adj pval: 0.269
Frequency
Tumor−Normal Expression Difference

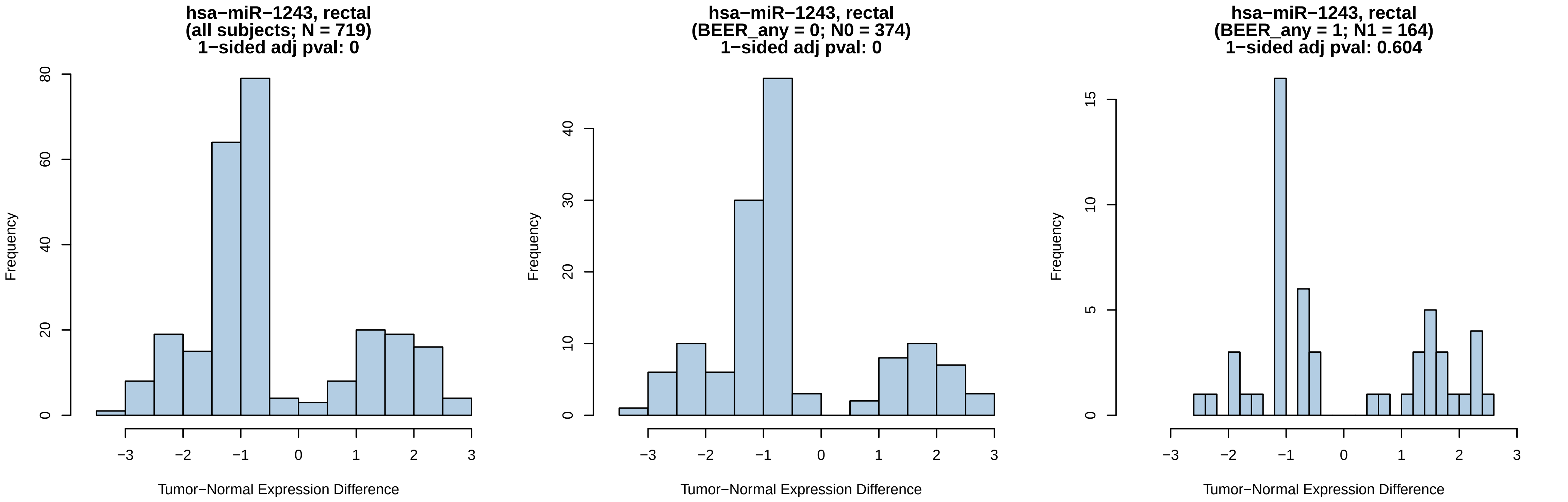

hsa−miR−1243, rectal
(all subjects; N = 719)
1−sided adj pval: 0
Frequency
Tumor−Normal Expression Difference
hsa−miR−1243, rectal
(BEER_any = 0; N0 = 374)
1−sided adj pval: 0
Frequency
Tumor−Normal Expression Difference
hsa−miR−1243, rectal
(BEER_any = 1; N1 = 164)
1−sided adj pval: 0.604
Frequency
Tumor−Normal Expression Difference

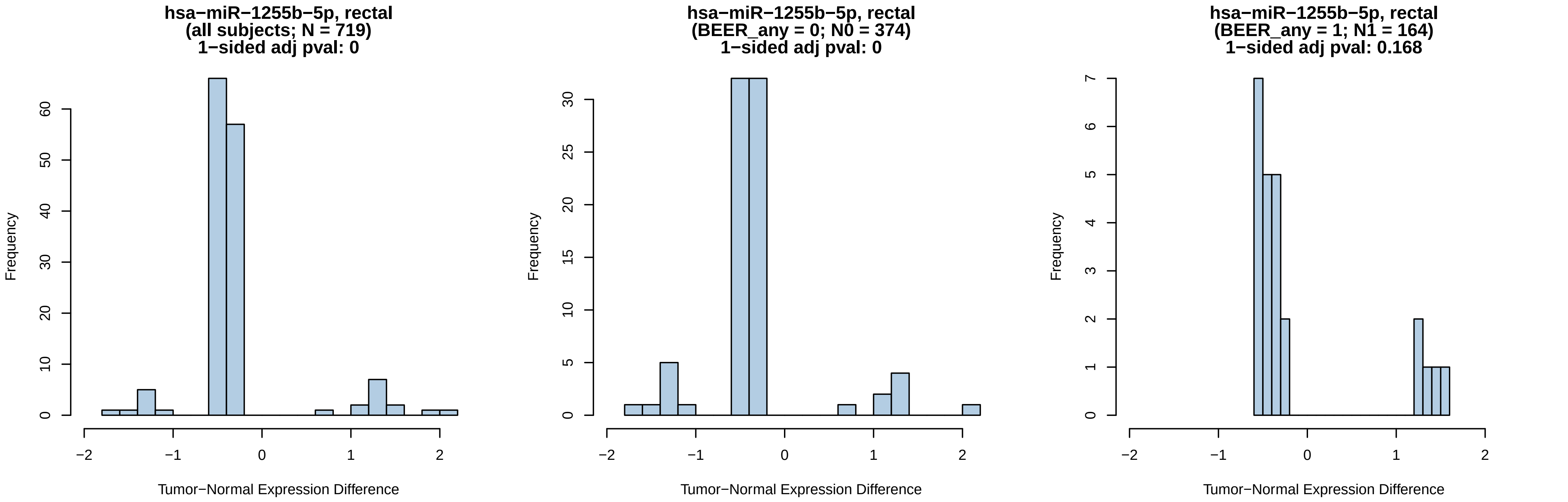
hsa−miR−1255b−5p, rectal
(all subjects; N = 719)
1−sided adj pval: 0
Frequency
0
10
20
30
40
50
60
−2
−1
0
1
2
Tumor−Normal Expression Difference
hsa−miR−1255b−5p, rectal
(BEER_any = 0; N0 = 374)
1−sided adj pval: 0
Frequency
0
5
10
15
20
25
30
−2
−1
0
1
2
Tumor−Normal Expression Difference
hsa−miR−1255b−5p, rectal
(BEER_any = 1; N1 = 164)
1−sided adj pval: 0.168
Frequency
0
1
2
3
4
5
6
7
−2
−1
0
1
2
Tumor−Normal Expression Difference

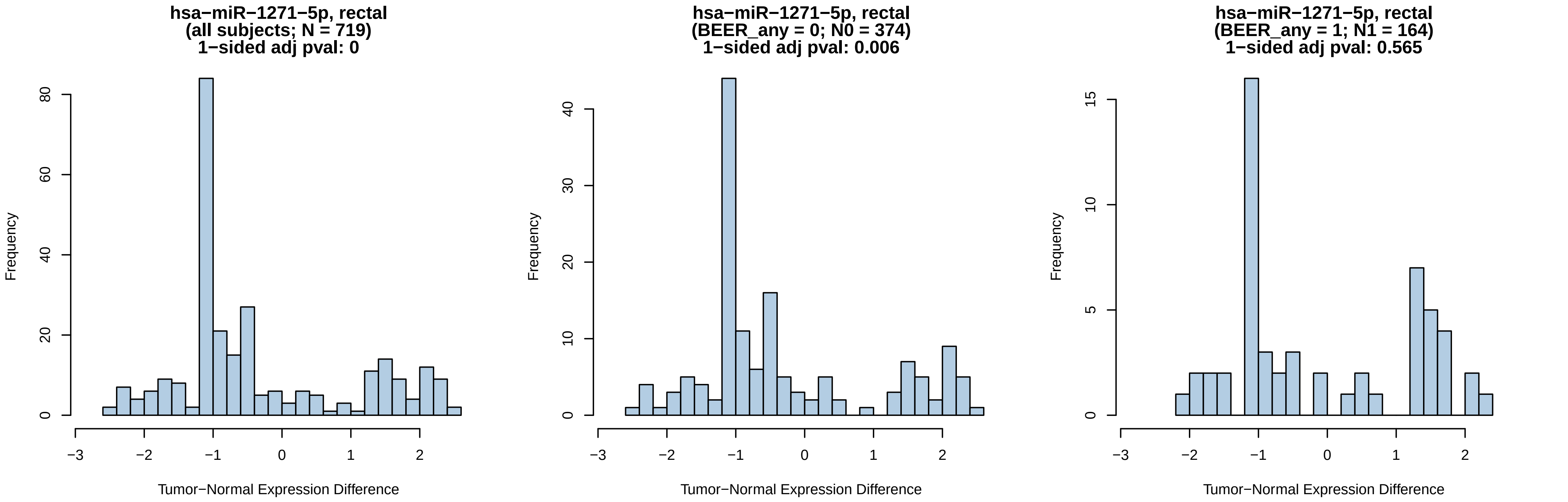

hsa−miR−1271−5p, rectal
(all subjects; N = 719)
1−sided adj pval: 0
Frequency
Tumor−Normal Expression Difference
hsa−miR−1271−5p, rectal
(BEER_any = 0; N0 = 374)
1−sided adj pval: 0.006
Frequency
Tumor−Normal Expression Difference
hsa−miR−1271−5p, rectal
(BEER_any = 1; N1 = 164)
1−sided adj pval: 0.565
Frequency
Tumor−Normal Expression Difference

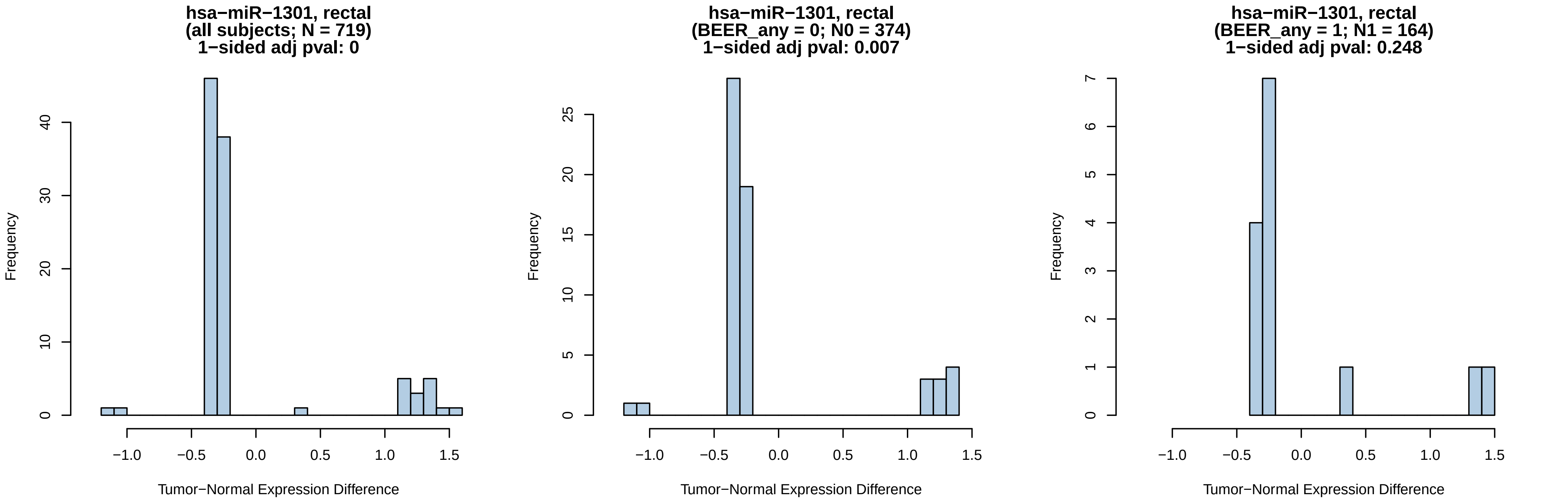
hsa−miR−1301, rectal
(all subjects; N = 719)
1−sided adj pval: 0
hsa−miR−1301, rectal
(BEER_any = 0; N0 = 374)
1−sided adj pval: 0.007
hsa−miR−1301, rectal
(BEER_any = 1; N1 = 164)
1−sided adj pval: 0.248
Frequency
Tumor−Normal Expression Difference

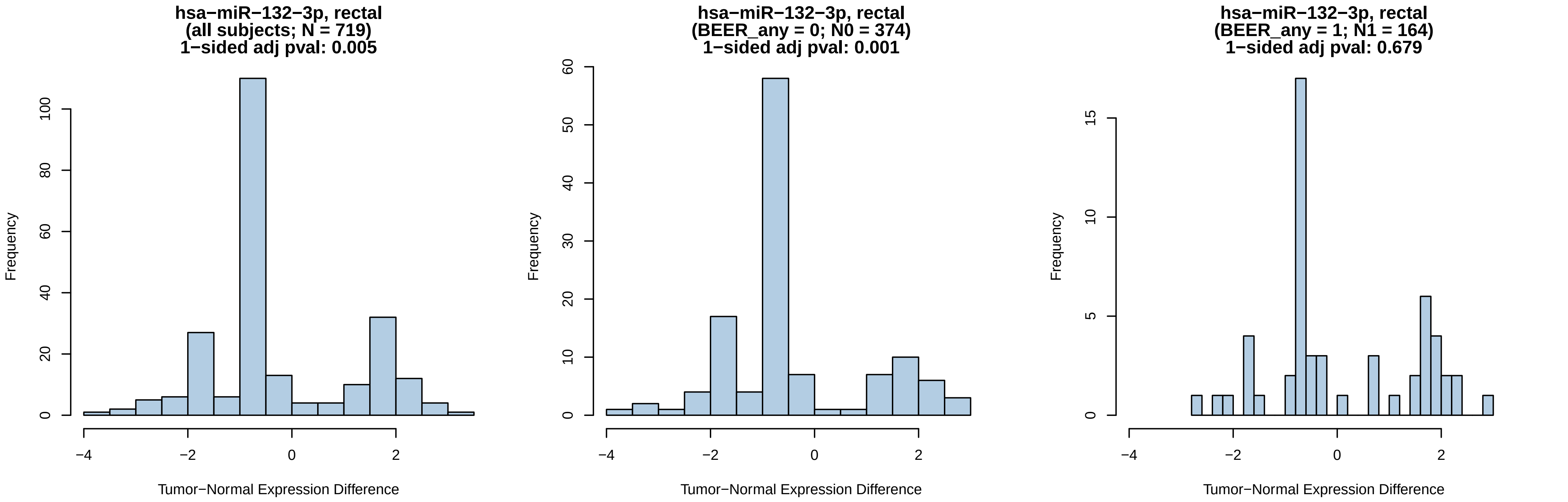
hsa−miR−132−3p, rectal
(all subjects; N = 719)
1−sided adj pval: 0.005
Frequency
Tumor−Normal Expression Difference
hsa−miR−132−3p, rectal
(BEER_any = 0; N0 = 374)
1−sided adj pval: 0.001
Frequency
Tumor−Normal Expression Difference
hsa−miR−132−3p, rectal
(BEER_any = 1; N1 = 164)
1−sided adj pval: 0.679
Frequency
Tumor−Normal Expression Difference

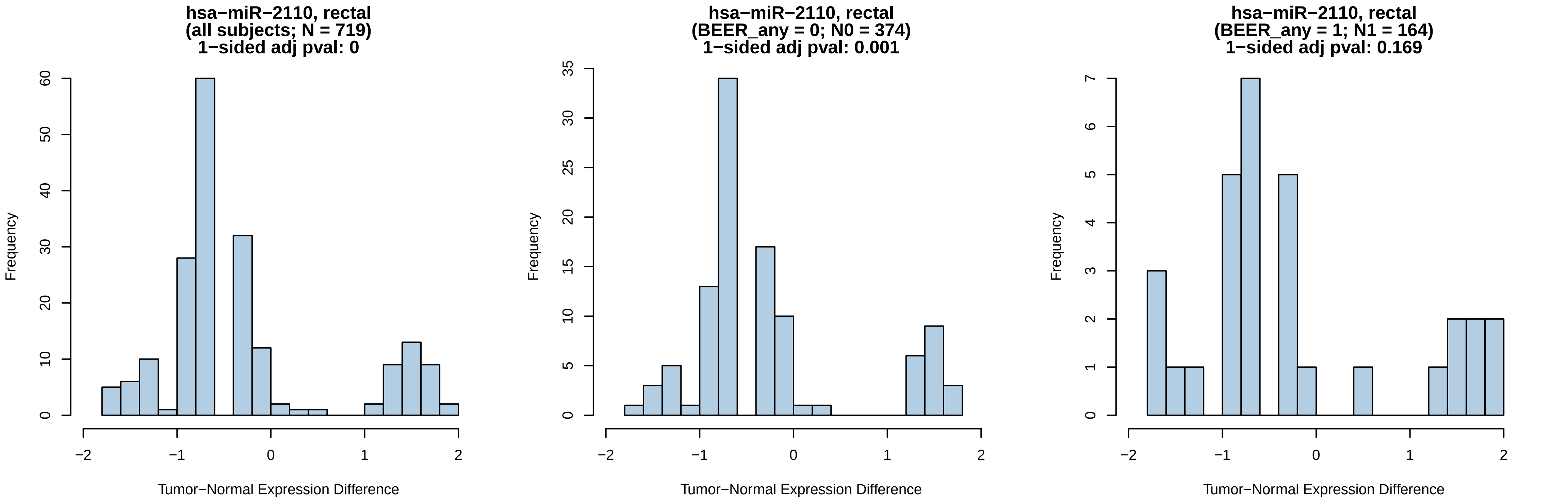

hsa−miR−2110, rectal
(all subjects; N = 719)
1−sided adj pval: 0
Frequency
Tumor−Normal Expression Difference
hsa−miR−2110, rectal
(BEER_any = 0; N0 = 374)
1−sided adj pval: 0.001
Frequency
Tumor−Normal Expression Difference
hsa−miR−2110, rectal
(BEER_any = 1; N1 = 164)
1−sided adj pval: 0.169
Frequency
Tumor−Normal Expression Difference

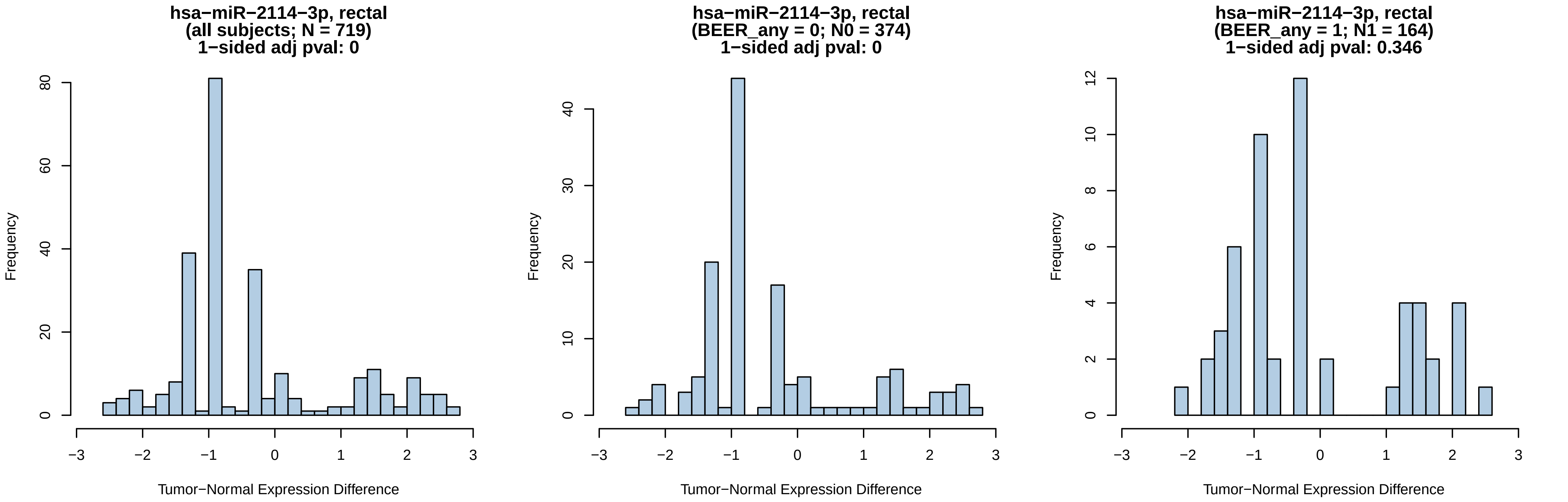

hsa−miR−2114−3p, rectal
(all subjects; N = 719)
1−sided adj pval: 0
Frequency
Tumor−Normal Expression Difference
hsa−miR−2114−3p, rectal
(BEER_any = 0; N0 = 374)
1−sided adj pval: 0
Frequency
Tumor−Normal Expression Difference
hsa−miR−2114−3p, rectal
(BEER_any = 1; N1 = 164)
1−sided adj pval: 0.346
Frequency
Tumor−Normal Expression Difference

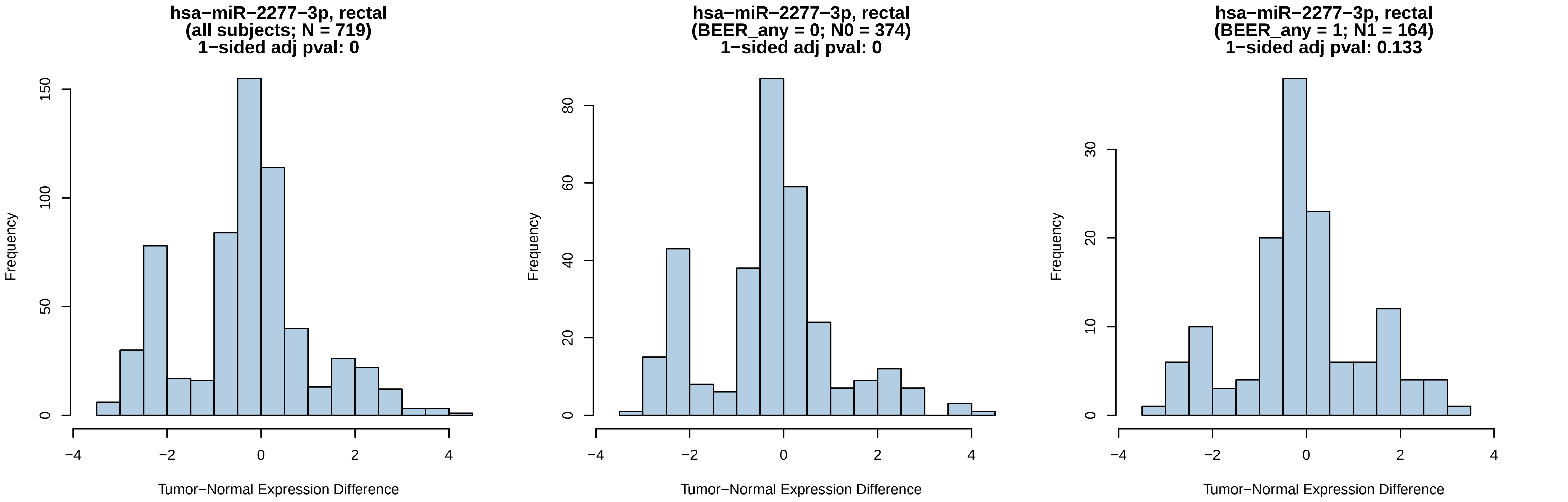
hsa−miR−2277−3p, rectal
(all subjects; N = 719)
1−sided adj pval: 0
hsa−miR−2277−3p, rectal
(BEER_any = 0; N0 = 374)
1−sided adj pval: 0
hsa−miR−2277−3p, rectal
(BEER_any = 1; N1 = 164)
1−sided adj pval: 0.133
Frequency
Tumor−Normal Expression Difference

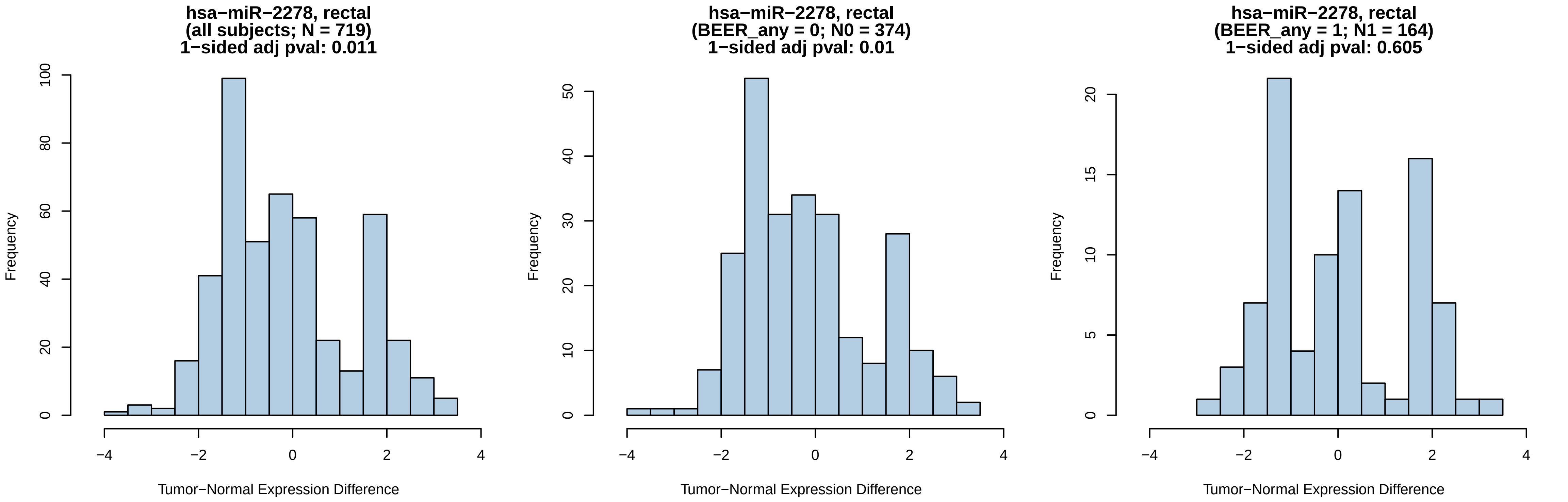
hsa−miR−2278, rectal
(all subjects; N = 719)
1−sided adj pval: 0.011
hsa−miR−2278, rectal
(BEER_any = 0; N0 = 374)
1−sided adj pval: 0.01
hsa−miR−2278, rectal
(BEER_any = 1; N1 = 164)
1−sided adj pval: 0.605
Frequency
Tumor−Normal Expression Difference
0
20
40
60
80
100
10
30
50
5
15
−4
−2
0
2
4

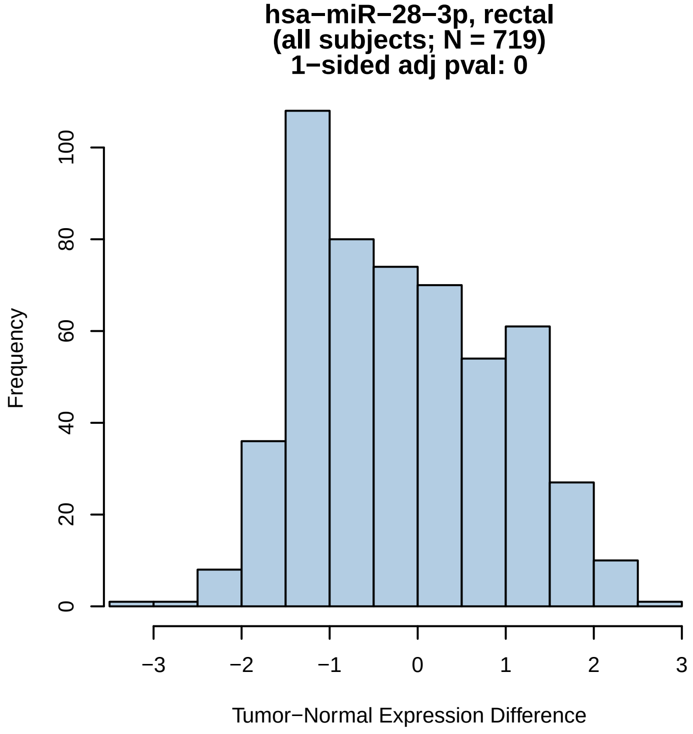
hsa−miR−28−3p, rectal
(all subjects; N = 719)
1−sided adj pval: 0
Frequency
Tumor−Normal Expression Difference

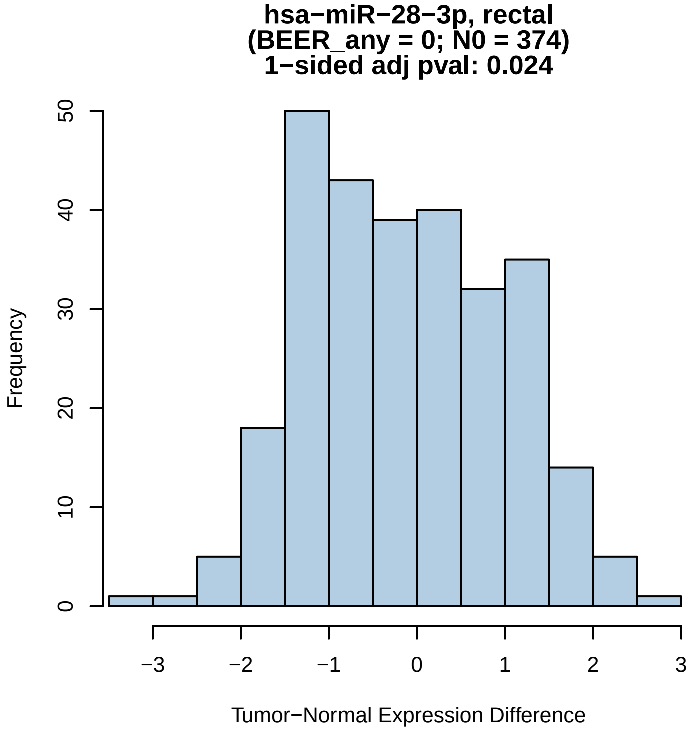
hsa−miR−28−3p, rectal
(BEER_any = 0; N0 = 374)
1−sided adj pval: 0.024
Frequency
Tumor−Normal Expression Difference

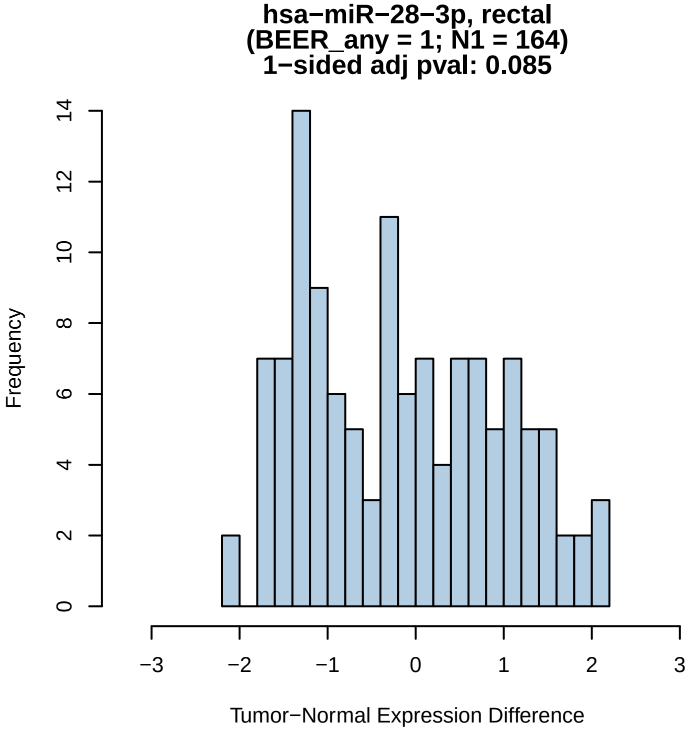
hsa−miR−28−3p, rectal
(BEER_any = 1; N1 = 164)
1−sided adj pval: 0.085
Frequency
Tumor−Normal Expression Difference

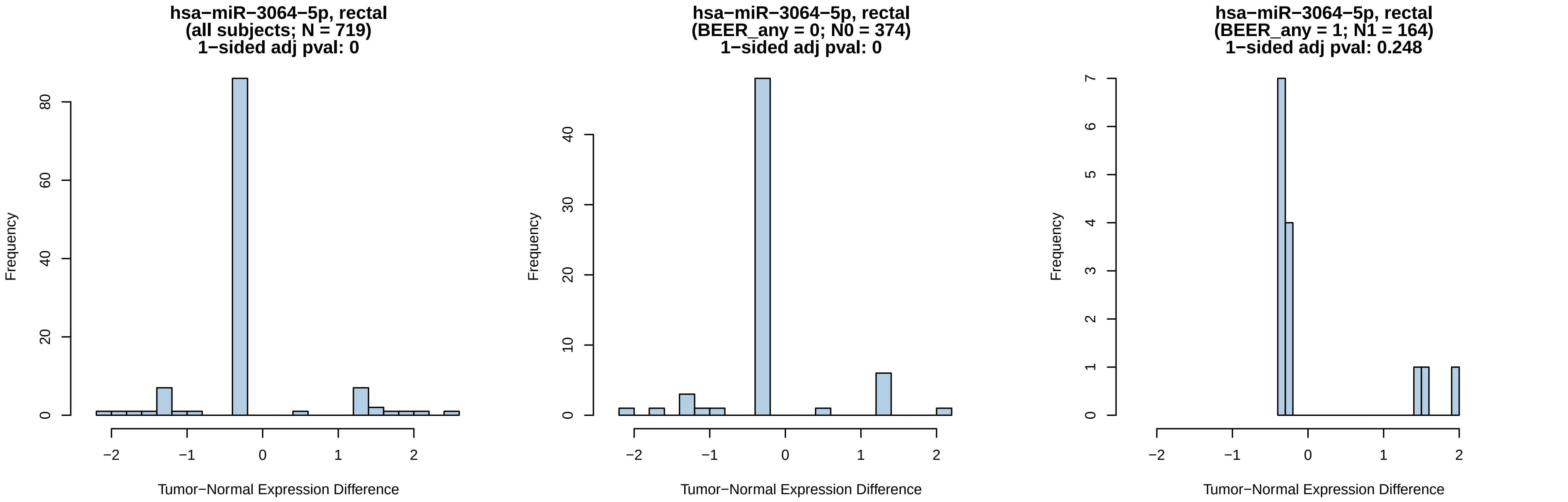

hsa−miR−3064−5p, rectal
(all subjects; N = 719)
1−sided adj pval: 0
Frequency
Tumor−Normal Expression Difference
hsa−miR−3064−5p, rectal
(BEER_any = 0; N0 = 374)
1−sided adj pval: 0
Frequency
Tumor−Normal Expression Difference
hsa−miR−3064−5p, rectal
(BEER_any = 1; N1 = 164)
1−sided adj pval: 0.248
Frequency
Tumor−Normal Expression Difference

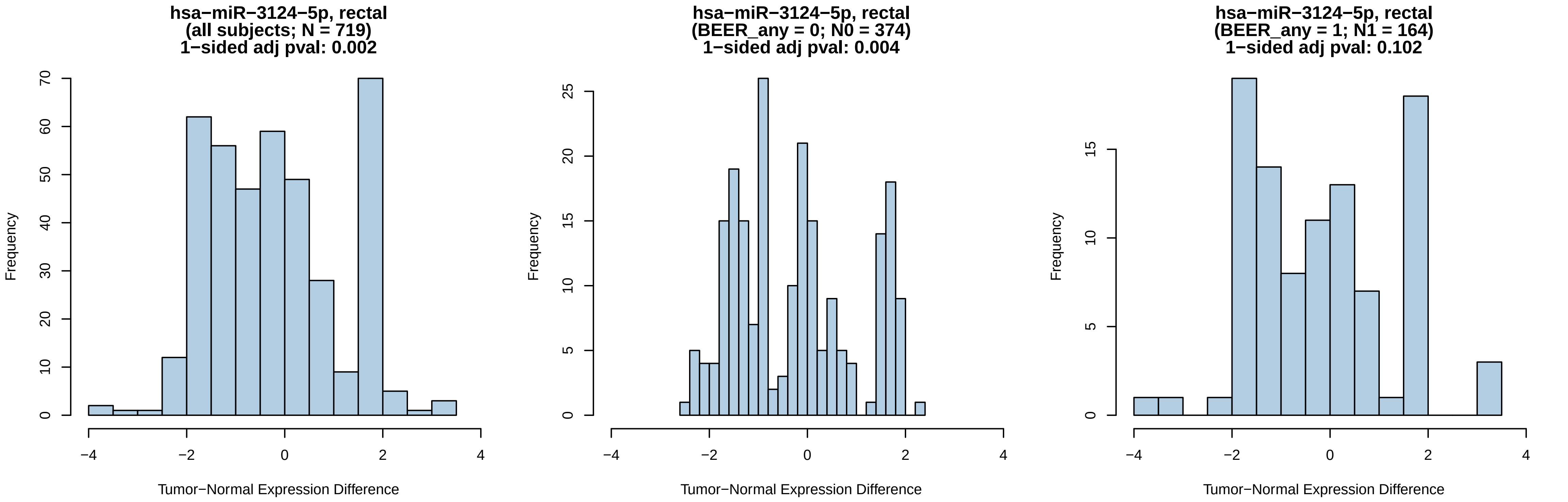
hsa−miR−3124−5p, rectal
(all subjects; N = 719)
1−sided adj pval: 0.002
Frequency
Tumor−Normal Expression Difference
hsa−miR−3124−5p, rectal
(BEER_any = 0; N0 = 374)
1−sided adj pval: 0.004
Frequency
Tumor−Normal Expression Difference
hsa−miR−3124−5p, rectal
(BEER_any = 1; N1 = 164)
1−sided adj pval: 0.102
Frequency
Tumor−Normal Expression Difference

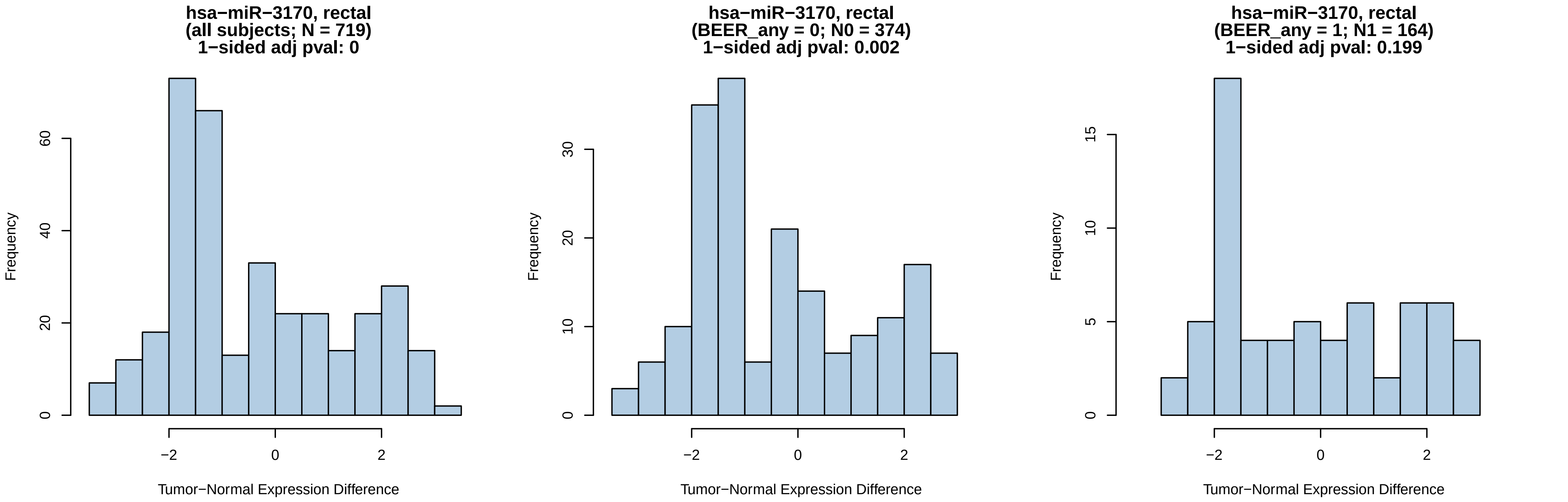

hsa−miR−3170, rectal
(all subjects; N = 719)
1−sided adj pval: 0
Frequency
0
20
40
60
−2
0
2
Tumor−Normal Expression Difference
hsa−miR−3170, rectal
(BEER_any = 0; N0 = 374)
1−sided adj pval: 0.002
Frequency
0
10
20
30
−2
0
2
Tumor−Normal Expression Difference
hsa−miR−3170, rectal
(BEER_any = 1; N1 = 164)
1−sided adj pval: 0.199
Frequency
0
5
10
15
−2
0
2
Tumor−Normal Expression Difference

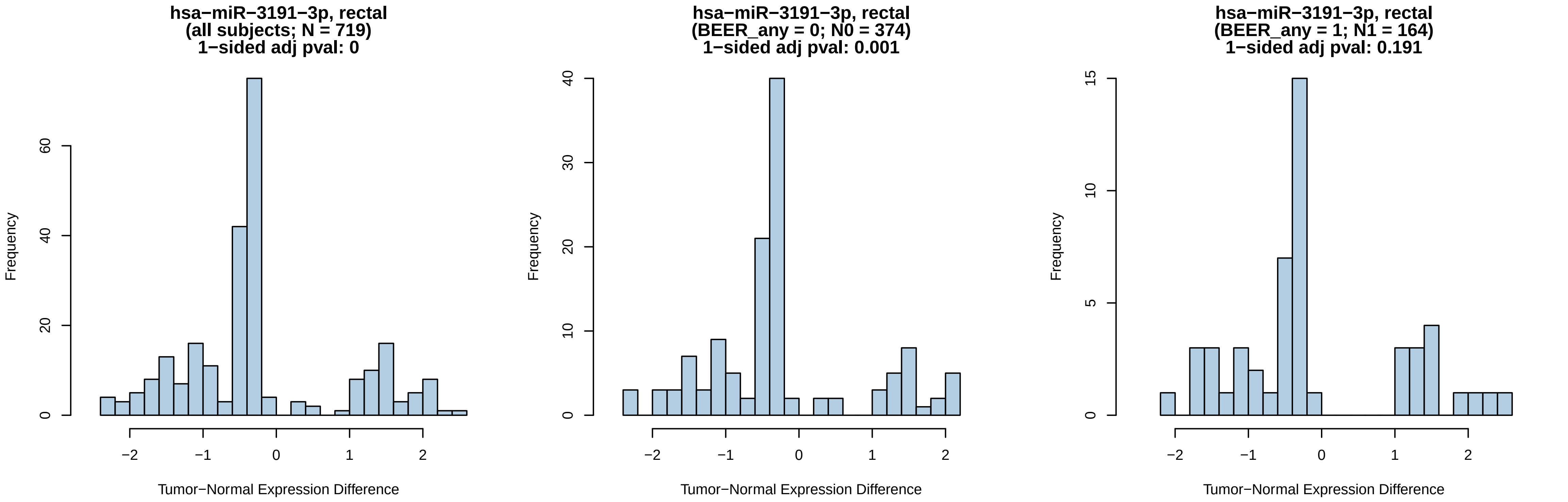
hsa−miR−3191−3p, rectal
(all subjects; N = 719)
1−sided adj pval: 0
Frequency
Tumor−Normal Expression Difference
hsa−miR−3191−3p, rectal
(BEER_any = 0; N0 = 374)
1−sided adj pval: 0.001
Frequency
Tumor−Normal Expression Difference
hsa−miR−3191−3p, rectal
(BEER_any = 1; N1 = 164)
1−sided adj pval: 0.191
Frequency
Tumor−Normal Expression Difference

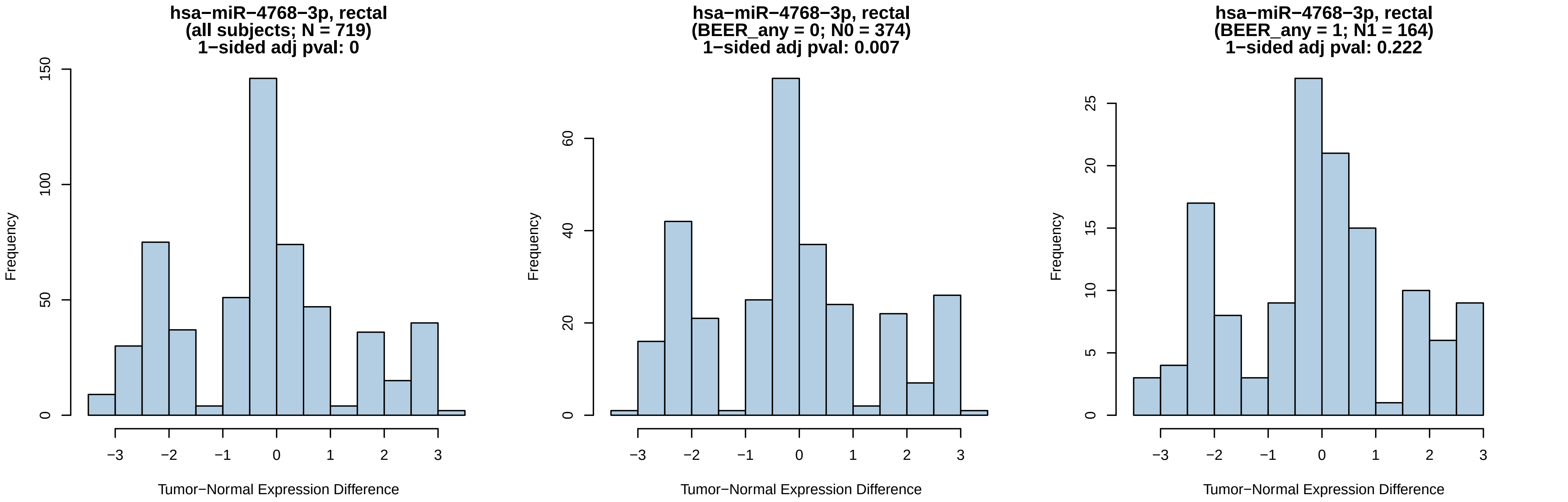

hsa−miR−4768−3p, rectal
(all subjects; N = 719)
1−sided adj pval: 0
Frequency
Tumor−Normal Expression Difference
hsa−miR−4768−3p, rectal
(BEER_any = 0; N0 = 374)
1−sided adj pval: 0.007
Frequency
Tumor−Normal Expression Difference
hsa−miR−4768−3p, rectal
(BEER_any = 1; N1 = 164)
1−sided adj pval: 0.222
Frequency
Tumor−Normal Expression Difference

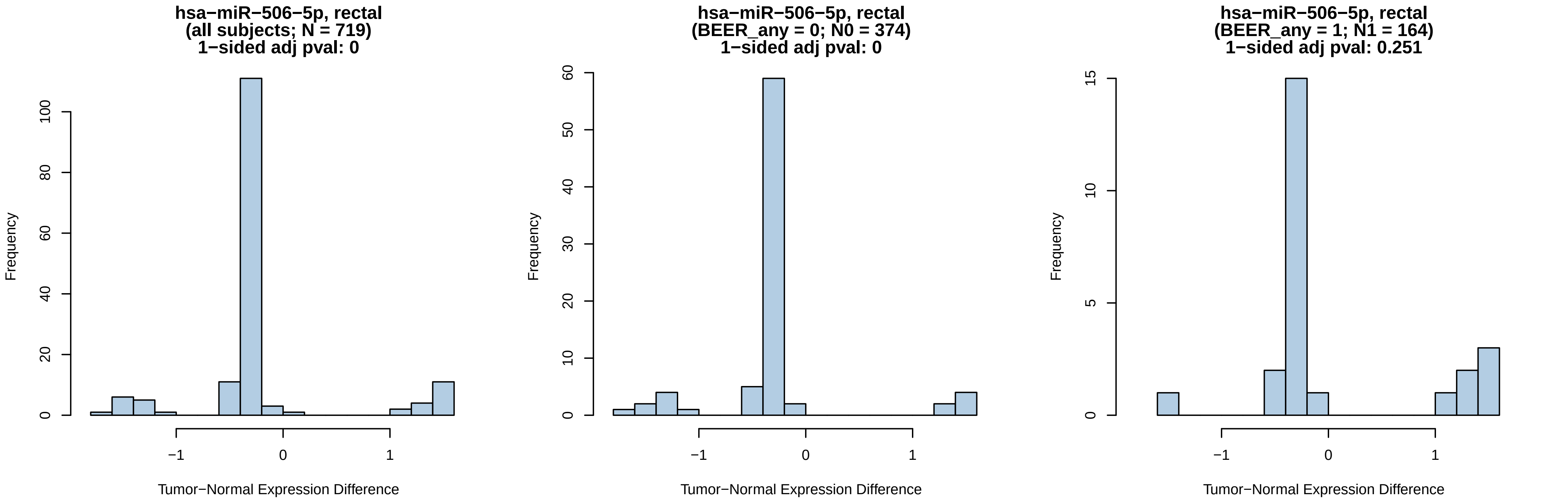

hsa−miR−506−5p, rectal
(all subjects; N = 719)
1−sided adj pval: 0
Frequency
0
20
40
60
80
100
−1
0
1
Tumor−Normal Expression Difference
hsa−miR−506−5p, rectal
(BEER_any = 0; N0 = 374)
1−sided adj pval: 0
Frequency
0
10
20
30
40
50
60
−1
0
1
Tumor−Normal Expression Difference
hsa−miR−506−5p, rectal
(BEER_any = 1; N1 = 164)
1−sided adj pval: 0.251
Frequency
0
5
10
15
−1
0
1
Tumor−Normal Expression Difference

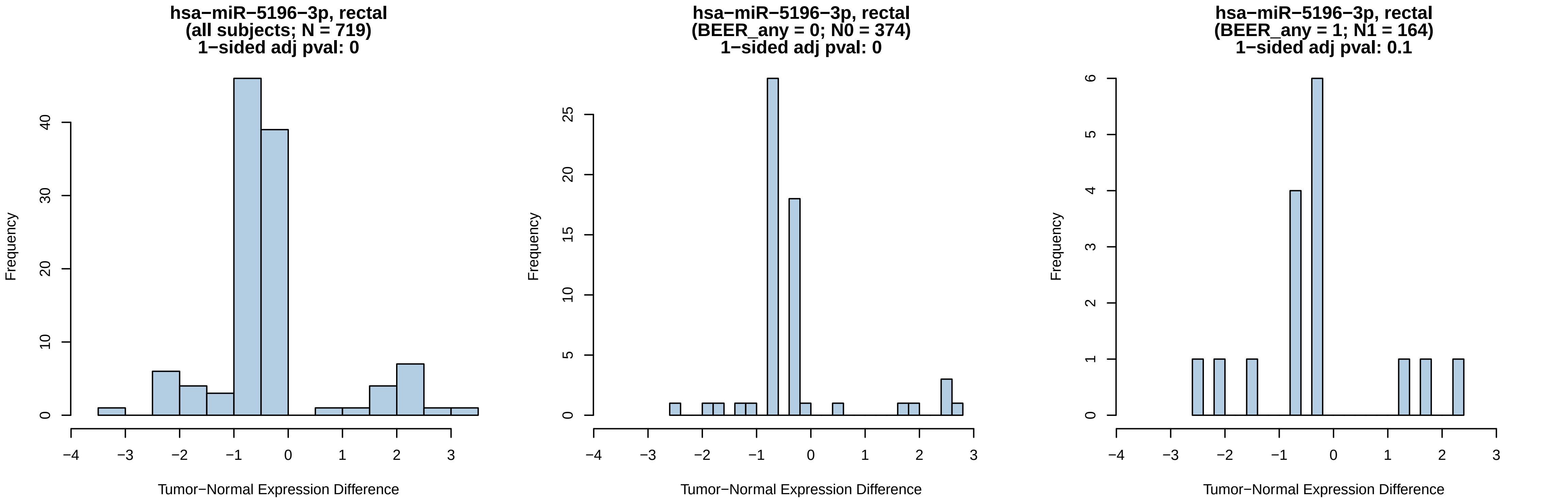
hsa−miR−5196−3p, rectal
(all subjects; N = 719)
1−sided adj pval: 0
Frequency
Tumor−Normal Expression Difference
hsa−miR−5196−3p, rectal
(BEER_any = 0; N0 = 374)
1−sided adj pval: 0
Frequency
Tumor−Normal Expression Difference
hsa−miR−5196−3p, rectal
(BEER_any = 1; N1 = 164)
1−sided adj pval: 0.1
Frequency
Tumor−Normal Expression Difference

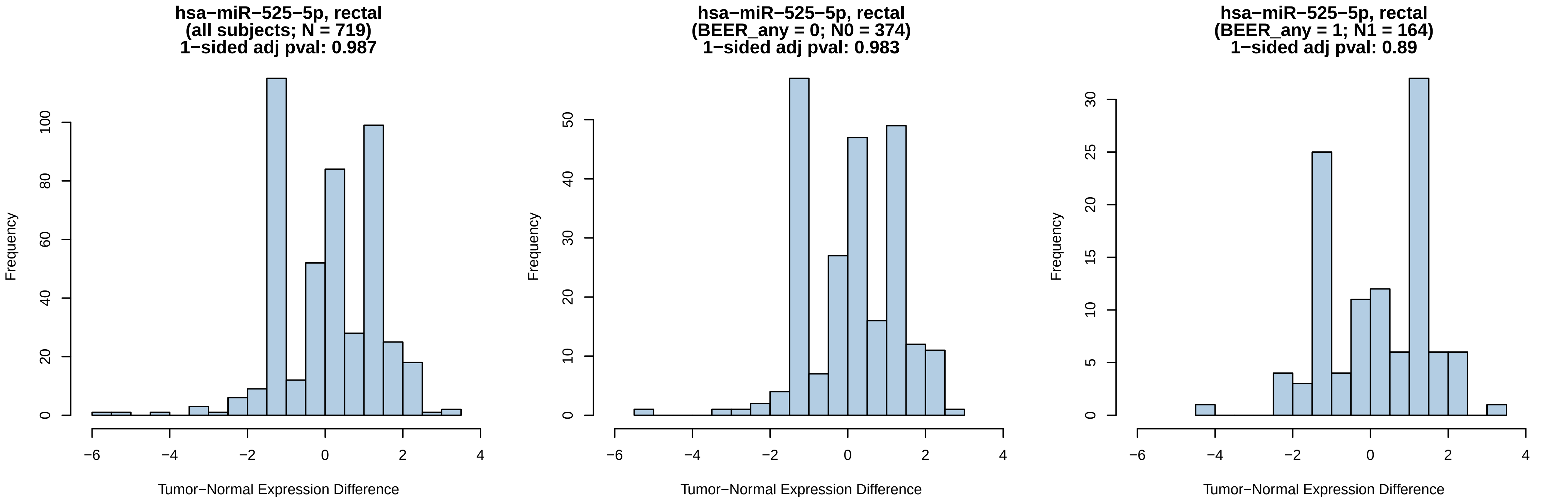
hsa−miR−525−5p, rectal
(all subjects; N = 719)
1−sided adj pval: 0.987
hsa−miR−525−5p, rectal
(BEER_any = 0; N0 = 374)
1−sided adj pval: 0.983
hsa−miR−525−5p, rectal
(BEER_any = 1; N1 = 164)
1−sided adj pval: 0.89
Frequency
Tumor−Normal Expression Difference

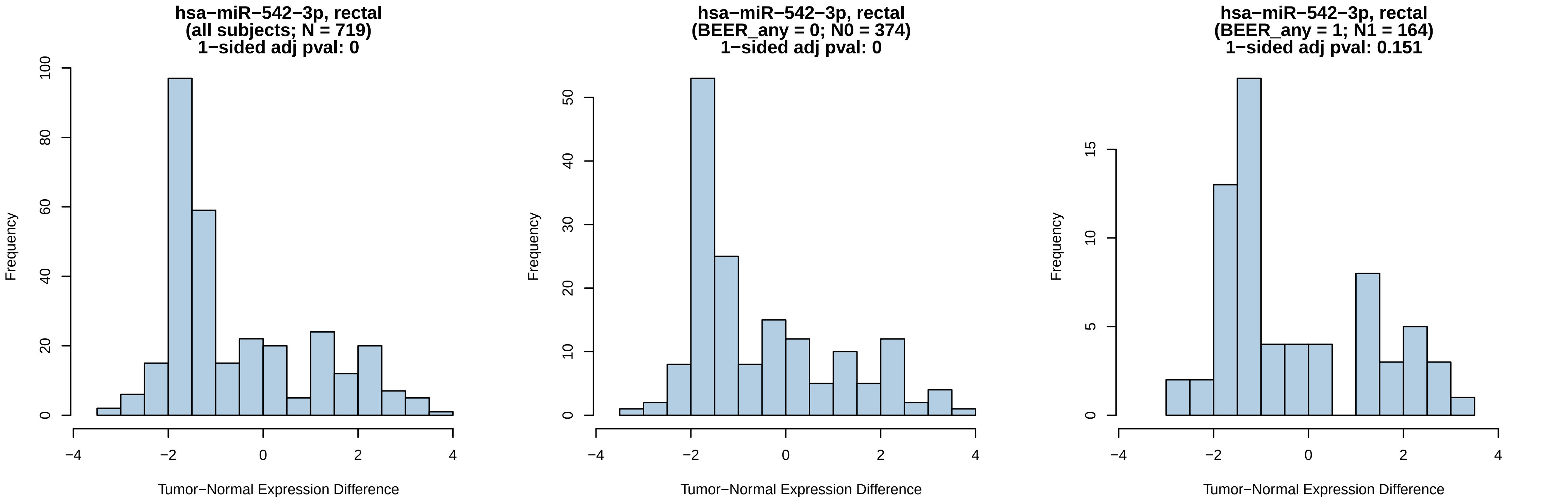
hsa−miR−542−3p, rectal
(all subjects; N = 719)
1−sided adj pval: 0
hsa−miR−542−3p, rectal
(BEER_any = 0; N0 = 374)
1−sided adj pval: 0
hsa−miR−542−3p, rectal
(BEER_any = 1; N1 = 164)
1−sided adj pval: 0.151
Frequency
Tumor−Normal Expression Difference

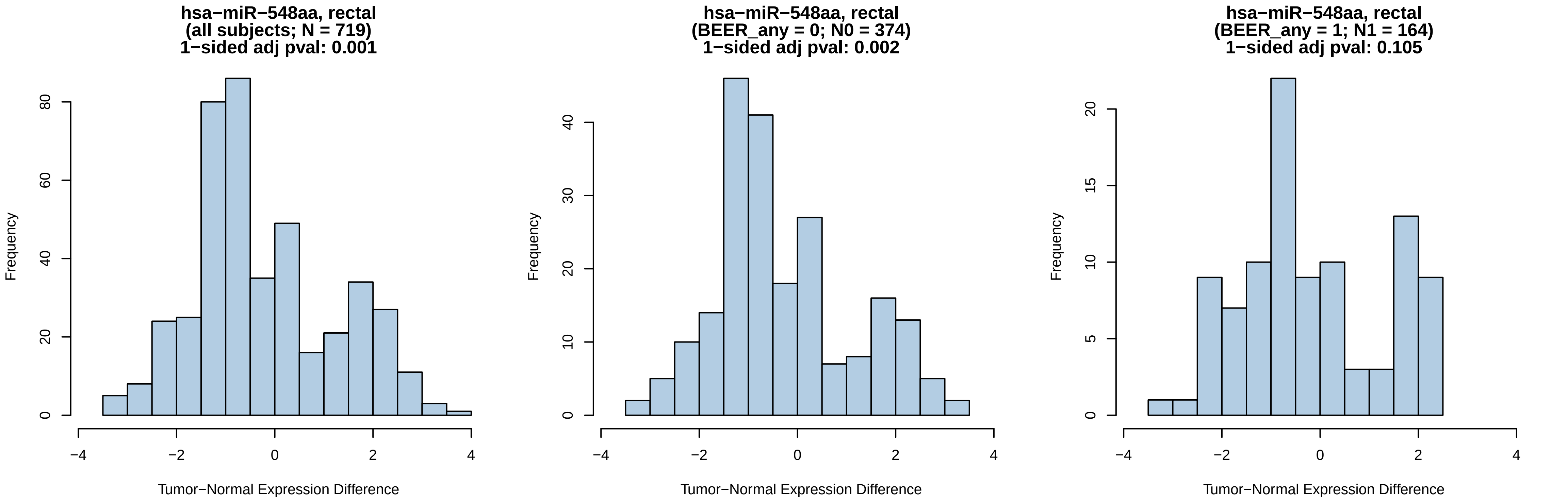
hsa−miR−548aa, rectal
(all subjects; N = 719)
1−sided adj pval: 0.001
hsa−miR−548aa, rectal
(BEER_any = 0; N0 = 374)
1−sided adj pval: 0.002
hsa−miR−548aa, rectal
(BEER_any = 1; N1 = 164)
1−sided adj pval: 0.105
Frequency
Tumor−Normal Expression Difference

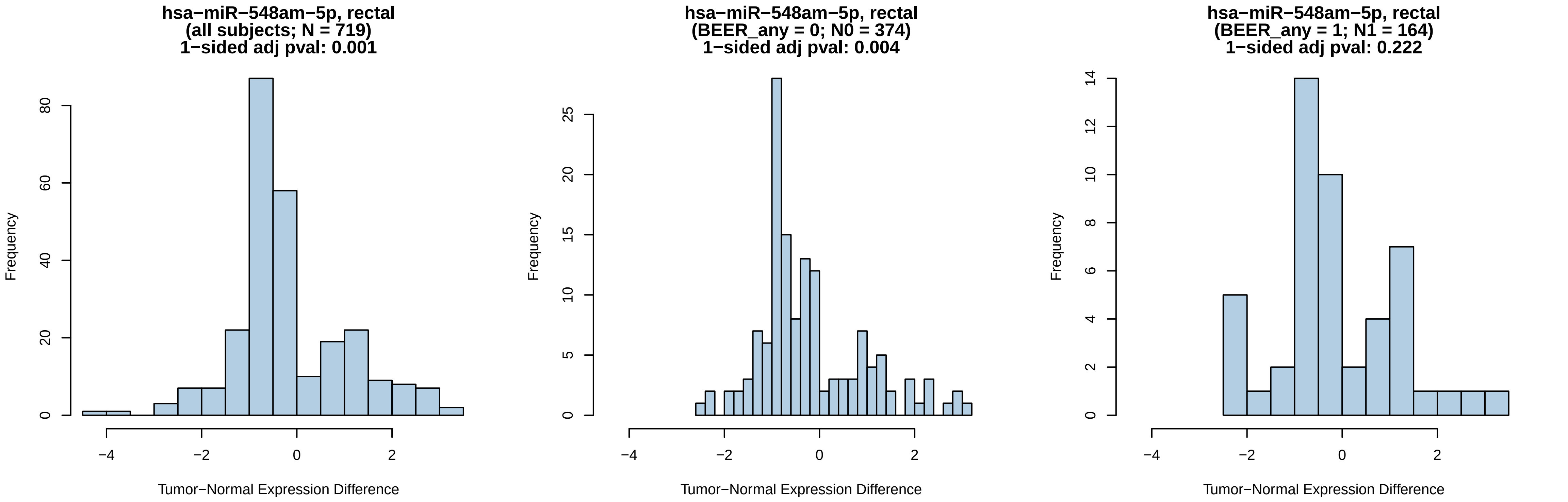
hsa−miR−548am−5p, rectal
(all subjects; N = 719)
1−sided adj pval: 0.001
hsa−miR−548am−5p, rectal
(BEER_any = 0; N0 = 374)
1−sided adj pval: 0.004
hsa−miR−548am−5p, rectal
(BEER_any = 1; N1 = 164)
1−sided adj pval: 0.222
Frequency
Tumor−Normal Expression Difference
0
20
40
60
80
5
10
15
25
2
4
6
8
10
12
14
−4
−2
0
2

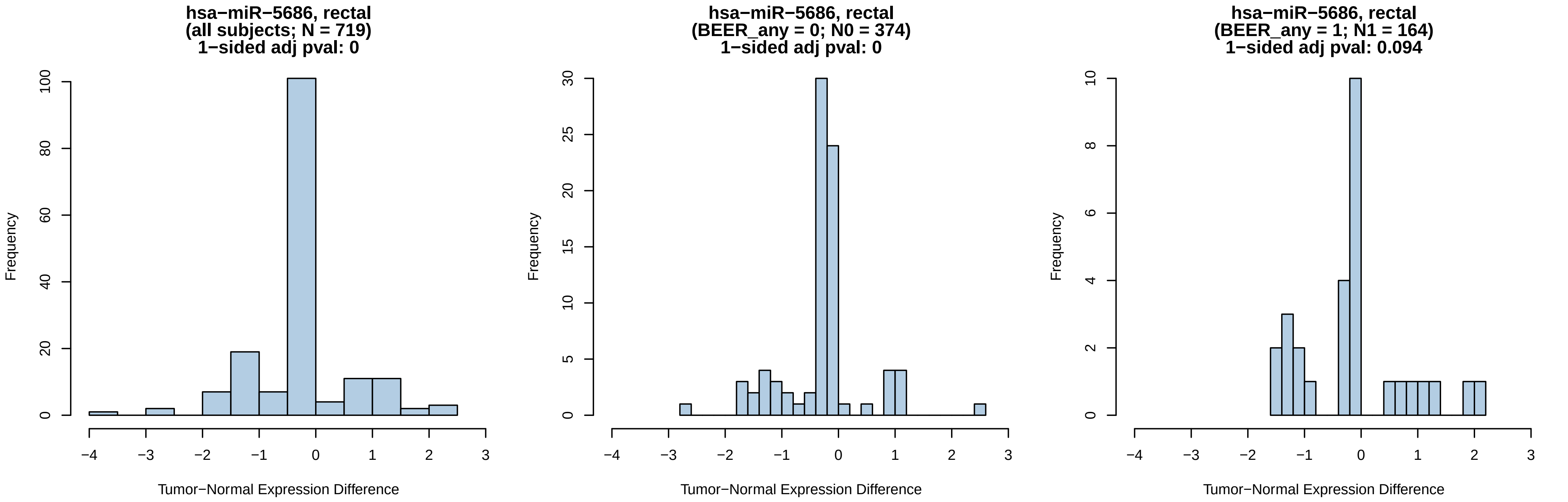

hsa−miR−5686, rectal
(all subjects; N = 719)
1−sided adj pval: 0
hsa−miR−5686, rectal
(BEER_any = 0; N0 = 374)
1−sided adj pval: 0
hsa−miR−5686, rectal
(BEER_any = 1; N1 = 164)
1−sided adj pval: 0.094
Frequency
Tumor−Normal Expression Difference

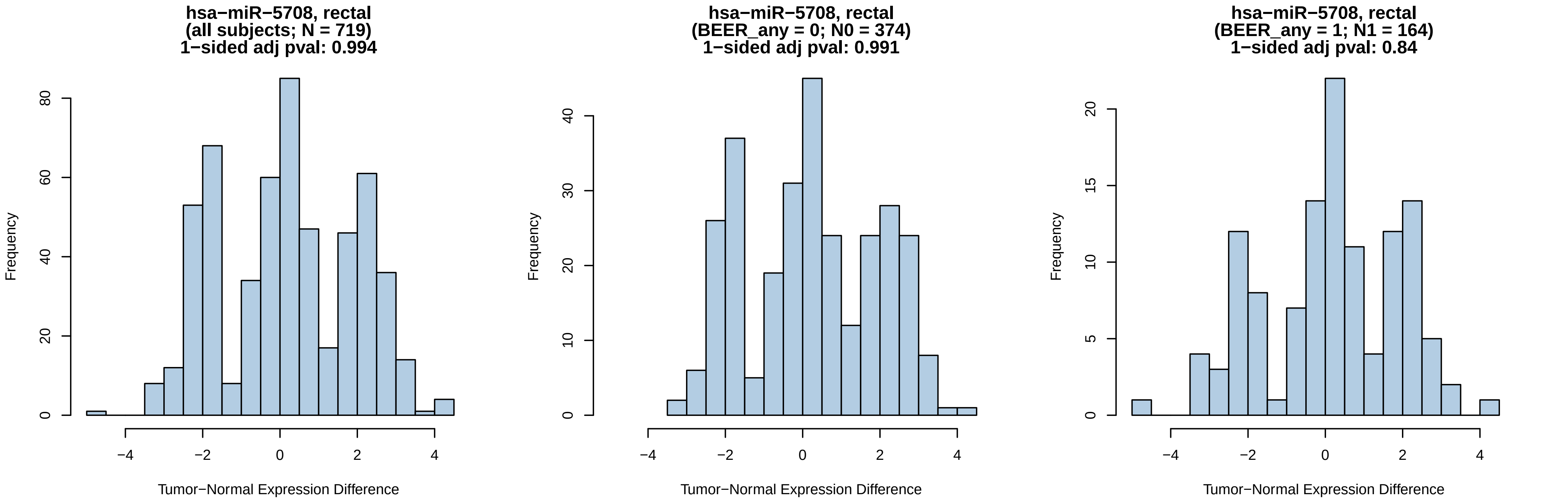

hsa−miR−5708, rectal
(all subjects; N = 719)
1−sided adj pval: 0.994
Frequency
Tumor−Normal Expression Difference
hsa−miR−5708, rectal
(BEER_any = 0; N0 = 374)
1−sided adj pval: 0.991
Frequency
Tumor−Normal Expression Difference
hsa−miR−5708, rectal
(BEER_any = 1; N1 = 164)
1−sided adj pval: 0.84
Frequency
Tumor−Normal Expression Difference

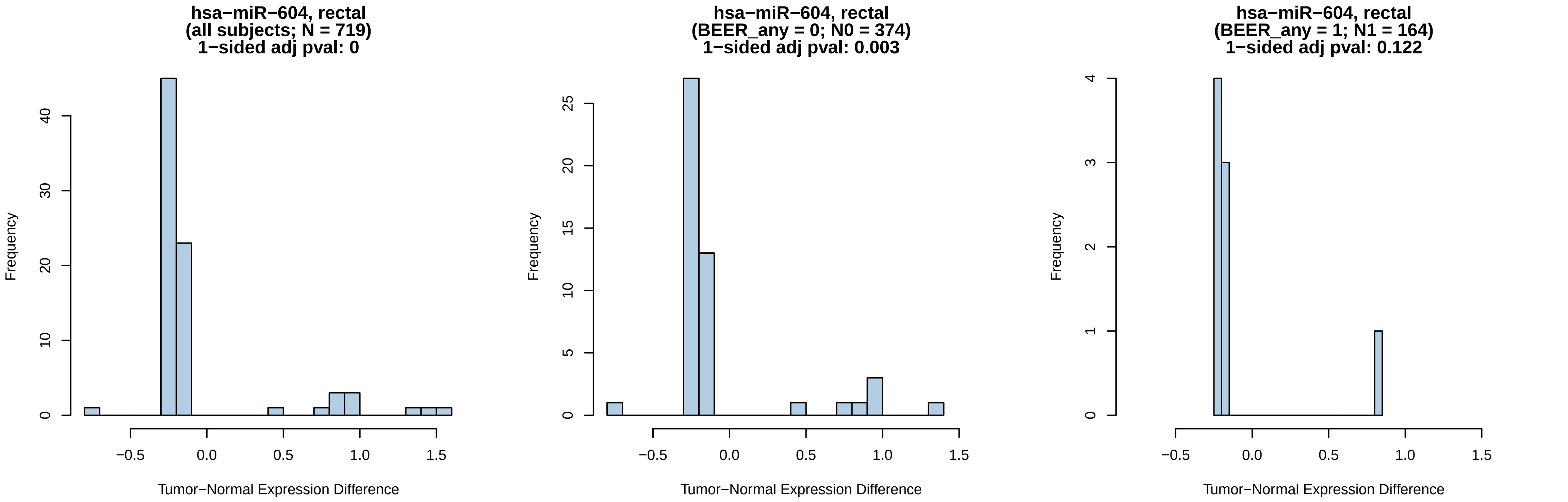

hsa−miR−604, rectal
(all subjects; N = 719)
1−sided adj pval: 0
Frequency
0
10
20
30
40
−0.5
0.0
0.5
1.0
1.5
Tumor−Normal Expression Difference
hsa−miR−604, rectal
(BEER_any = 0; N0 = 374)
1−sided adj pval: 0.003
Frequency
0
5
10
15
20
25
−0.5
0.0
0.5
1.0
1.5
Tumor−Normal Expression Difference
hsa−miR−604, rectal
(BEER_any = 1; N1 = 164)
1−sided adj pval: 0.122
Frequency
0
1
2
3
4
−0.5
0.0
0.5
1.0
1.5
Tumor−Normal Expression Difference

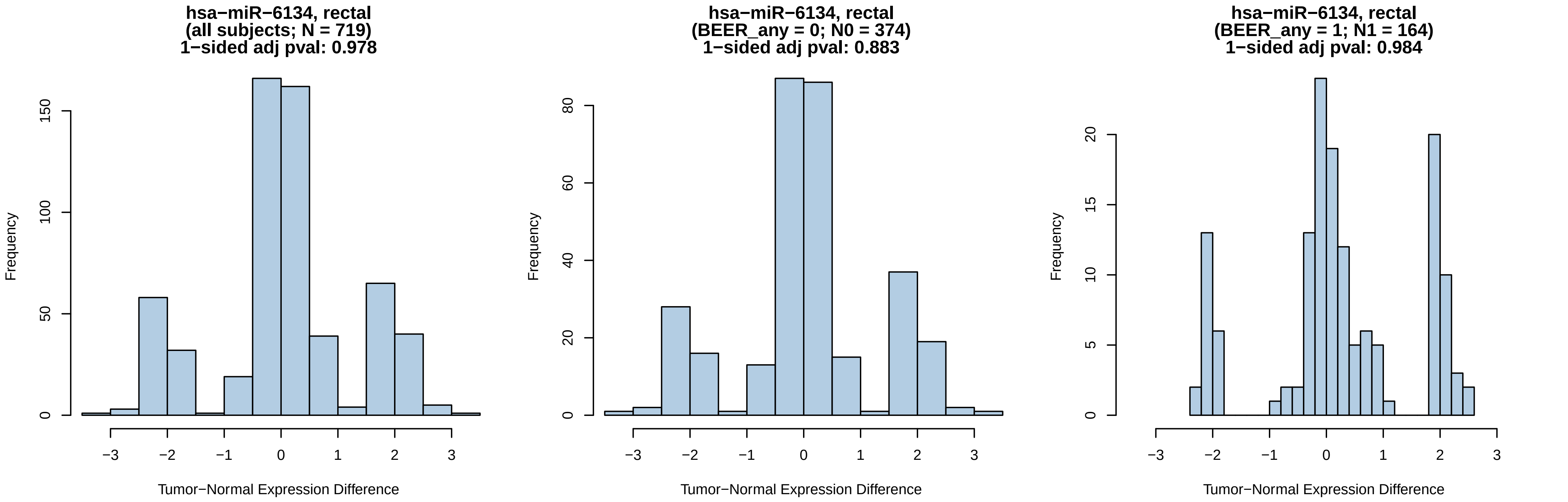
hsa−miR−6134, rectal
(all subjects; N = 719)
1−sided adj pval: 0.978
Frequency
Tumor−Normal Expression Difference
hsa−miR−6134, rectal
(BEER_any = 0; N0 = 374)
1−sided adj pval: 0.883
Frequency
Tumor−Normal Expression Difference
hsa−miR−6134, rectal
(BEER_any = 1; N1 = 164)
1−sided adj pval: 0.984
Frequency
Tumor−Normal Expression Difference

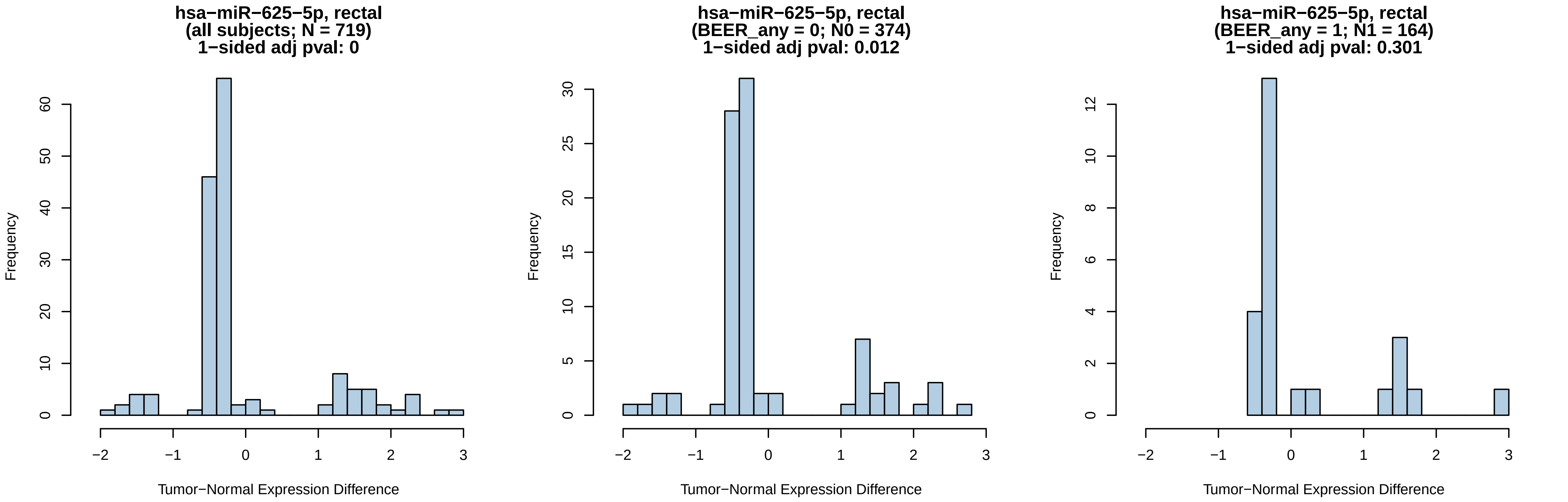
hsa−miR−625−5p, rectal
(all subjects; N = 719)
1−sided adj pval: 0
hsa−miR−625−5p, rectal
(BEER_any = 0; N0 = 374)
1−sided adj pval: 0.012
hsa−miR−625−5p, rectal
(BEER_any = 1; N1 = 164)
1−sided adj pval: 0.301
Frequency
Tumor−Normal Expression Difference

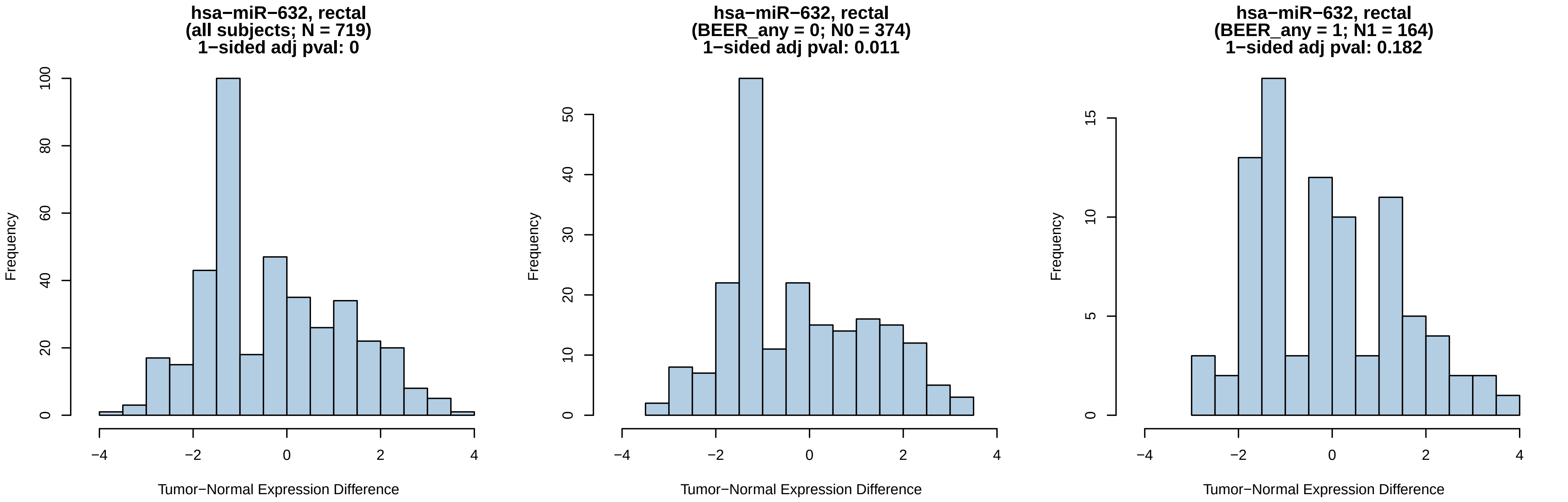
hsa−miR−632, rectal
(all subjects; N = 719)
1−sided adj pval: 0
Frequency
Tumor−Normal Expression Difference
hsa−miR−632, rectal
(BEER_any = 0; N0 = 374)
1−sided adj pval: 0.011
Frequency
Tumor−Normal Expression Difference
hsa−miR−632, rectal
(BEER_any = 1; N1 = 164)
1−sided adj pval: 0.182
Frequency
Tumor−Normal Expression Difference

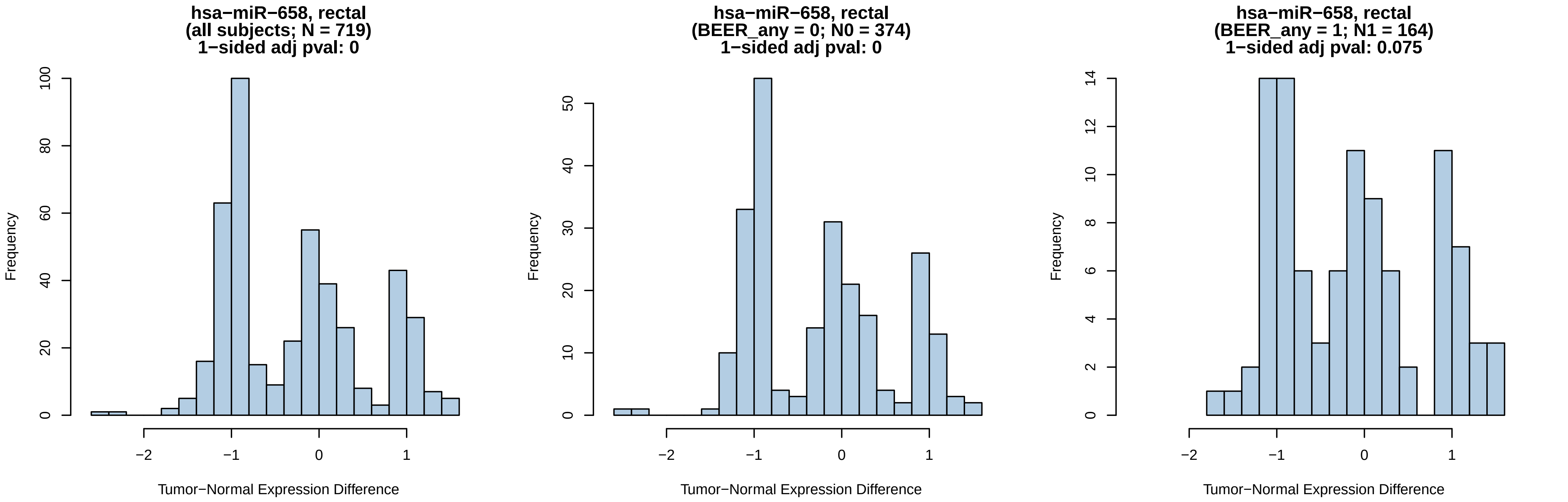
hsa−miR−658, rectal
(all subjects; N = 719)
1−sided adj pval: 0
Frequency
Tumor−Normal Expression Difference
hsa−miR−658, rectal
(BEER_any = 0; N0 = 374)
1−sided adj pval: 0
Frequency
Tumor−Normal Expression Difference
hsa−miR−658, rectal
(BEER_any = 1; N1 = 164)
1−sided adj pval: 0.075
Frequency
Tumor−Normal Expression Difference

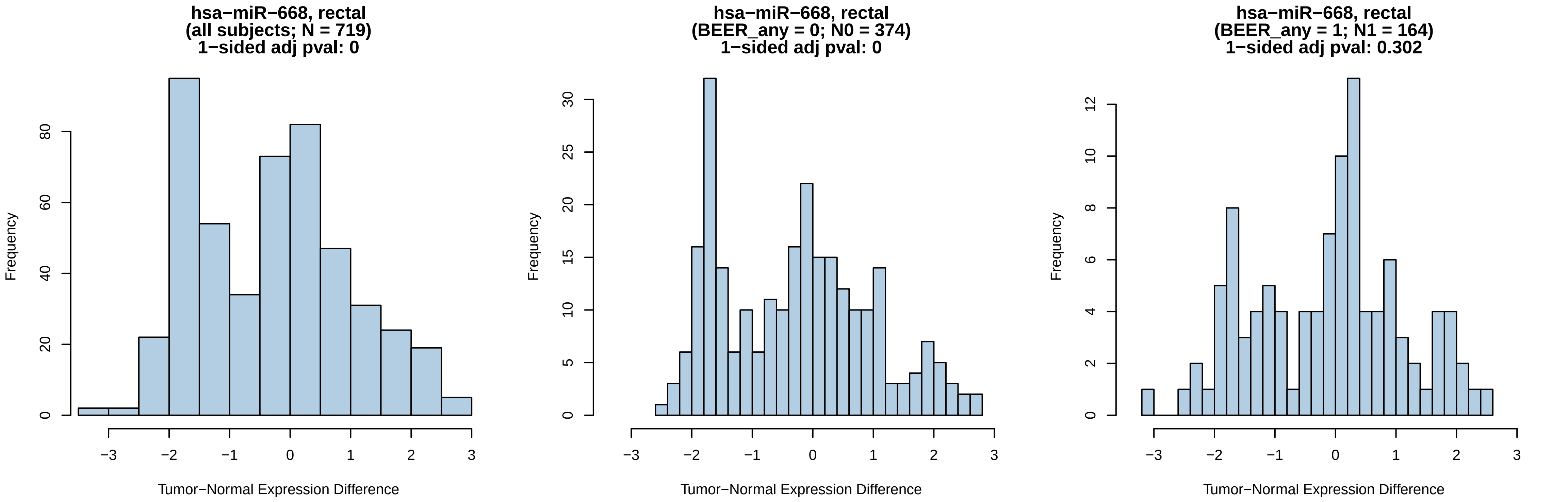
hsa−miR−668, rectal
(all subjects; N = 719)
1−sided adj pval: 0
hsa−miR−668, rectal
(BEER_any = 0; N0 = 374)
1−sided adj pval: 0
hsa−miR−668, rectal
(BEER_any = 1; N1 = 164)
1−sided adj pval: 0.302
Frequency
Tumor−Normal Expression Difference

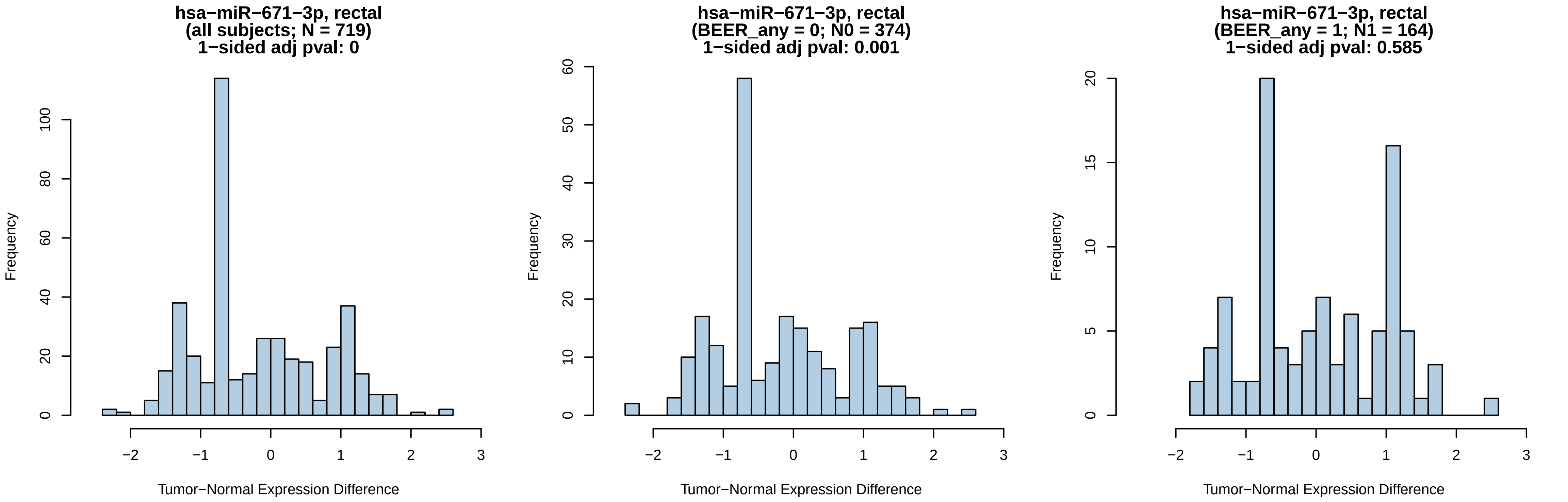
hsa−miR−671−3p, rectal
(all subjects; N = 719)
1−sided adj pval: 0
Frequency
Tumor−Normal Expression Difference
hsa−miR−671−3p, rectal
(BEER_any = 0; N0 = 374)
1−sided adj pval: 0.001
Frequency
Tumor−Normal Expression Difference
hsa−miR−671−3p, rectal
(BEER_any = 1; N1 = 164)
1−sided adj pval: 0.585
Frequency
Tumor−Normal Expression Difference

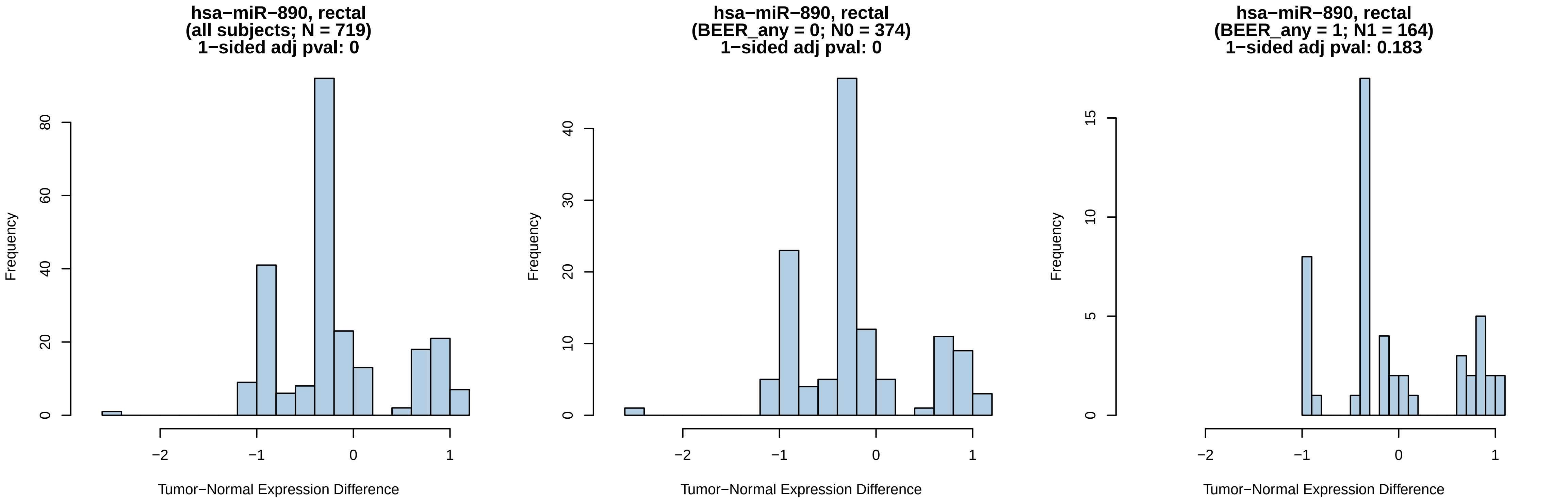
hsa−miR−890, rectal
(all subjects; N = 719)
1−sided adj pval: 0
Frequency
Tumor−Normal Expression Difference
hsa−miR−890, rectal
(BEER_any = 0; N0 = 374)
1−sided adj pval: 0
Frequency
Tumor−Normal Expression Difference
hsa−miR−890, rectal
(BEER_any = 1; N1 = 164)
1−sided adj pval: 0.183
Frequency
Tumor−Normal Expression Difference

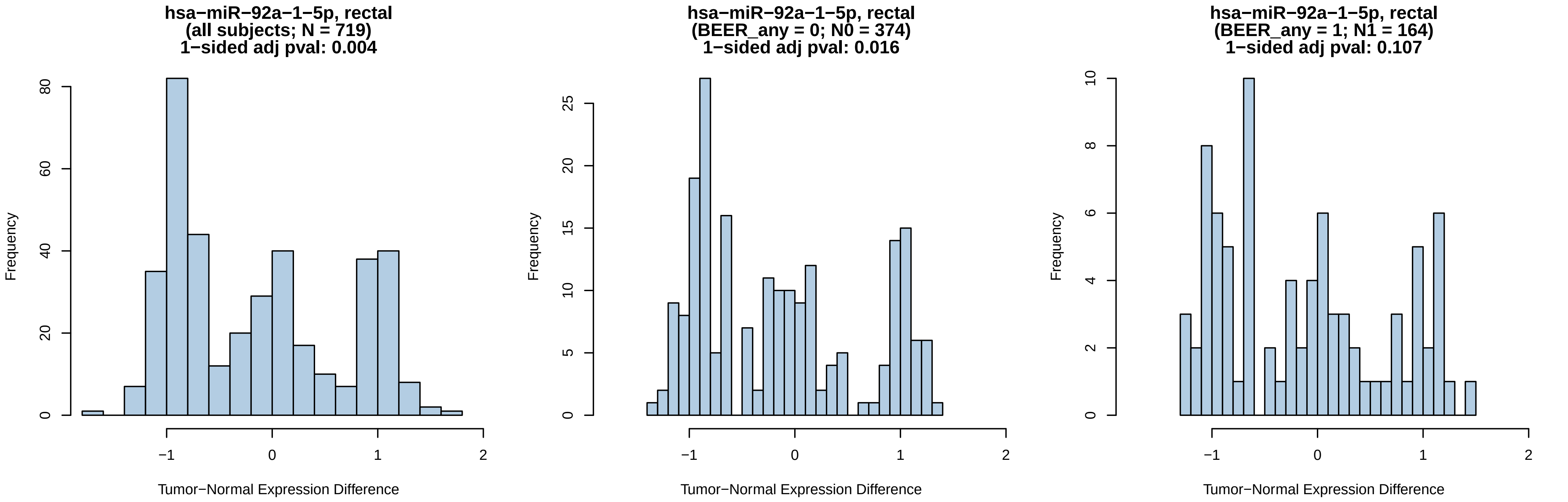
hsa−miR−92a−1−5p, rectal
(all subjects; N = 719)
1−sided adj pval: 0.004
Frequency
Tumor−Normal Expression Difference
hsa−miR−92a−1−5p, rectal
(BEER_any = 0; N0 = 374)
1−sided adj pval: 0.016
Frequency
Tumor−Normal Expression Difference
hsa−miR−92a−1−5p, rectal
(BEER_any = 1; N1 = 164)
1−sided adj pval: 0.107
Frequency
Tumor−Normal Expression Difference

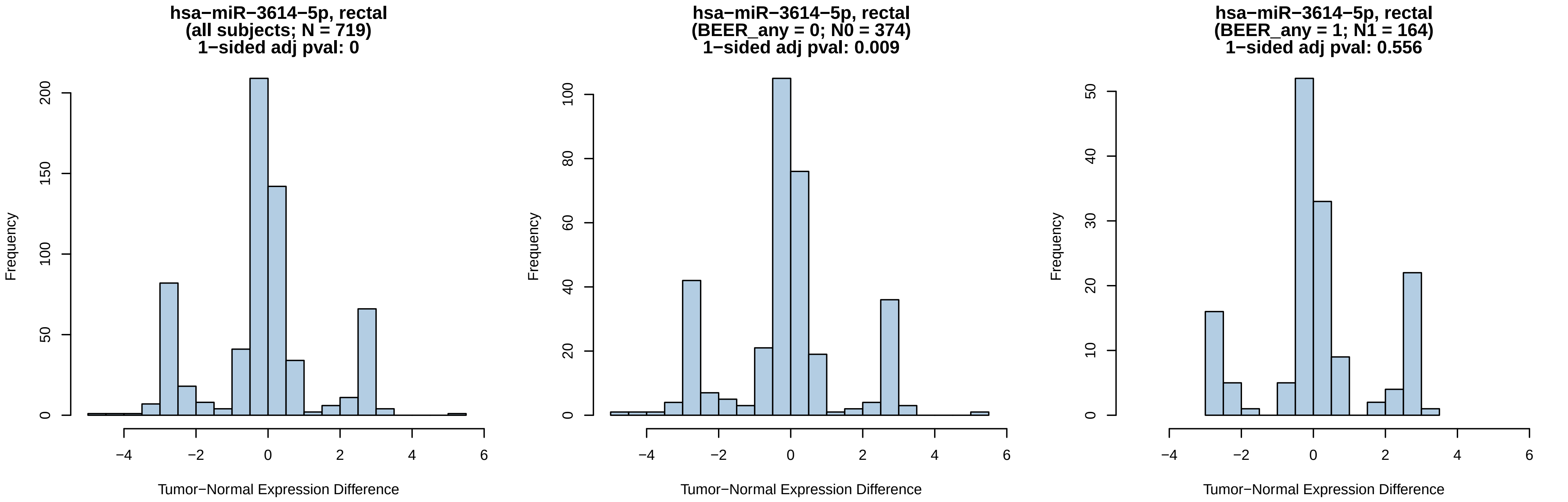
hsa−miR−3614−5p, rectal
(all subjects; N = 719)
1−sided adj pval: 0
Frequency
Tumor−Normal Expression Difference
hsa−miR−3614−5p, rectal
(BEER_any = 0; N0 = 374)
1−sided adj pval: 0.009
Frequency
Tumor−Normal Expression Difference
hsa−miR−3614−5p, rectal
(BEER_any = 1; N1 = 164)
1−sided adj pval: 0.556
Frequency
Tumor−Normal Expression Difference

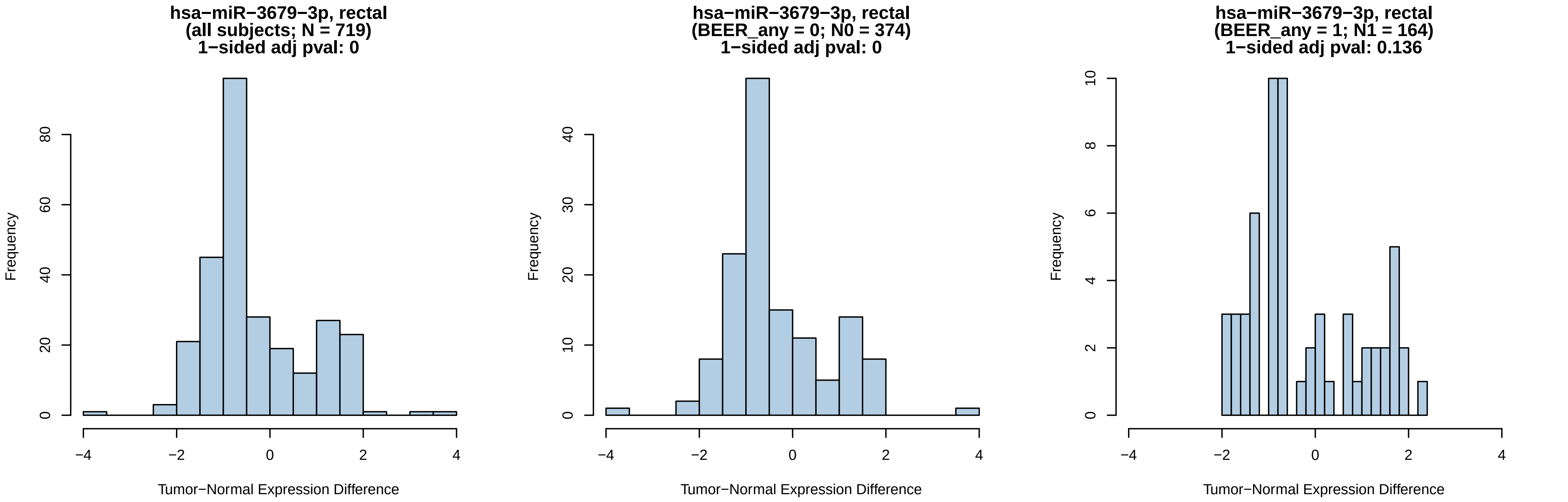
hsa−miR−3679−3p, rectal
(all subjects; N = 719)
1−sided adj pval: 0
Frequency
Tumor−Normal Expression Difference
hsa−miR−3679−3p, rectal
(BEER_any = 0; N0 = 374)
1−sided adj pval: 0
Frequency
Tumor−Normal Expression Difference
hsa−miR−3679−3p, rectal
(BEER_any = 1; N1 = 164)
1−sided adj pval: 0.136
Frequency
Tumor−Normal Expression Difference

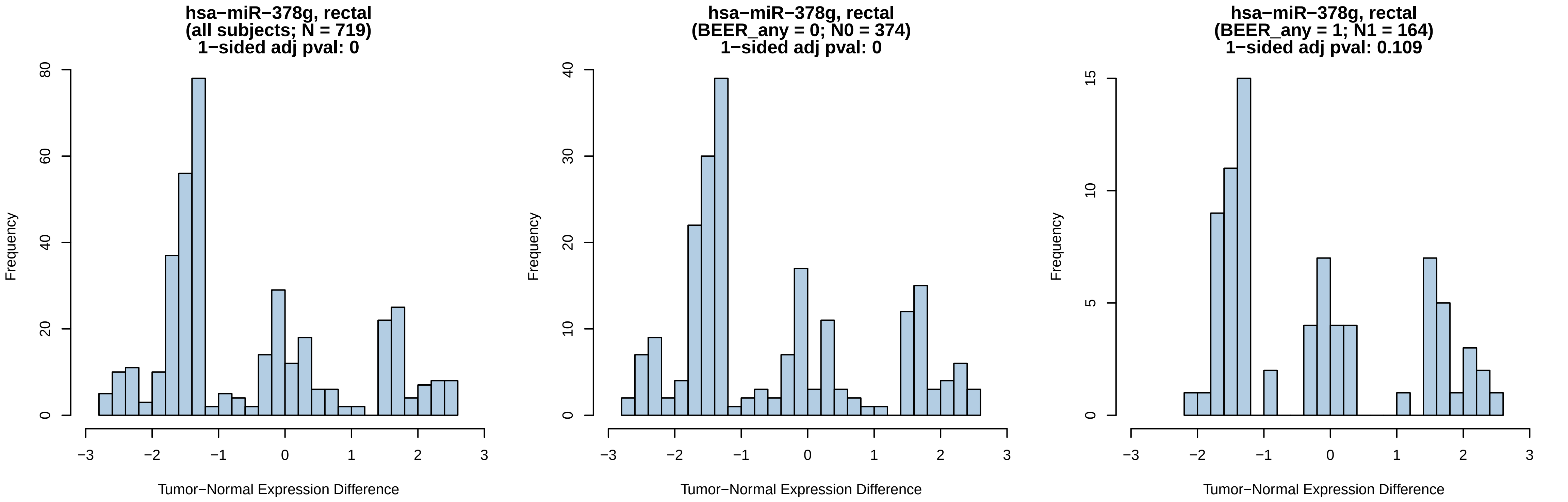

hsa−miR−378g, rectal
(all subjects; N = 719)
1−sided adj pval: 0
Frequency
Tumor−Normal Expression Difference
hsa−miR−378g, rectal
(BEER_any = 0; N0 = 374)
1−sided adj pval: 0
Frequency
Tumor−Normal Expression Difference
hsa−miR−378g, rectal
(BEER_any = 1; N1 = 164)
1−sided adj pval: 0.109
Frequency
Tumor−Normal Expression Difference

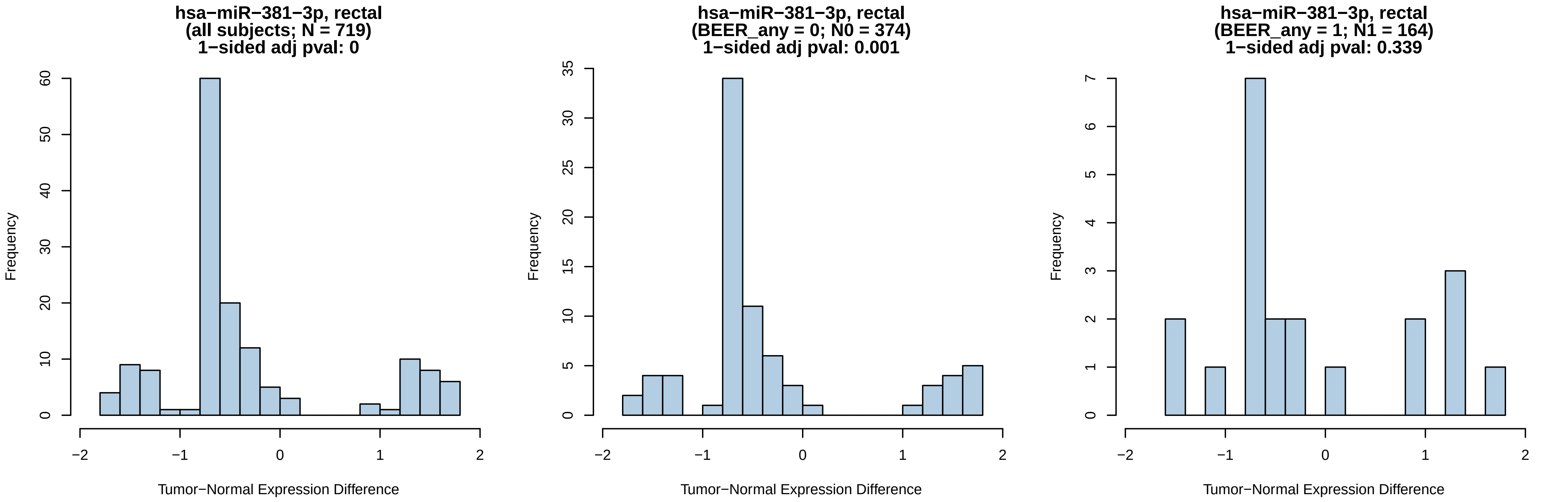

hsa−miR−381−3p, rectal
(all subjects; N = 719)
1−sided adj pval: 0
hsa−miR−381−3p, rectal
(BEER_any = 0; N0 = 374)
1−sided adj pval: 0.001
hsa−miR−381−3p, rectal
(BEER_any = 1; N1 = 164)
1−sided adj pval: 0.339
Frequency
Tumor−Normal Expression Difference

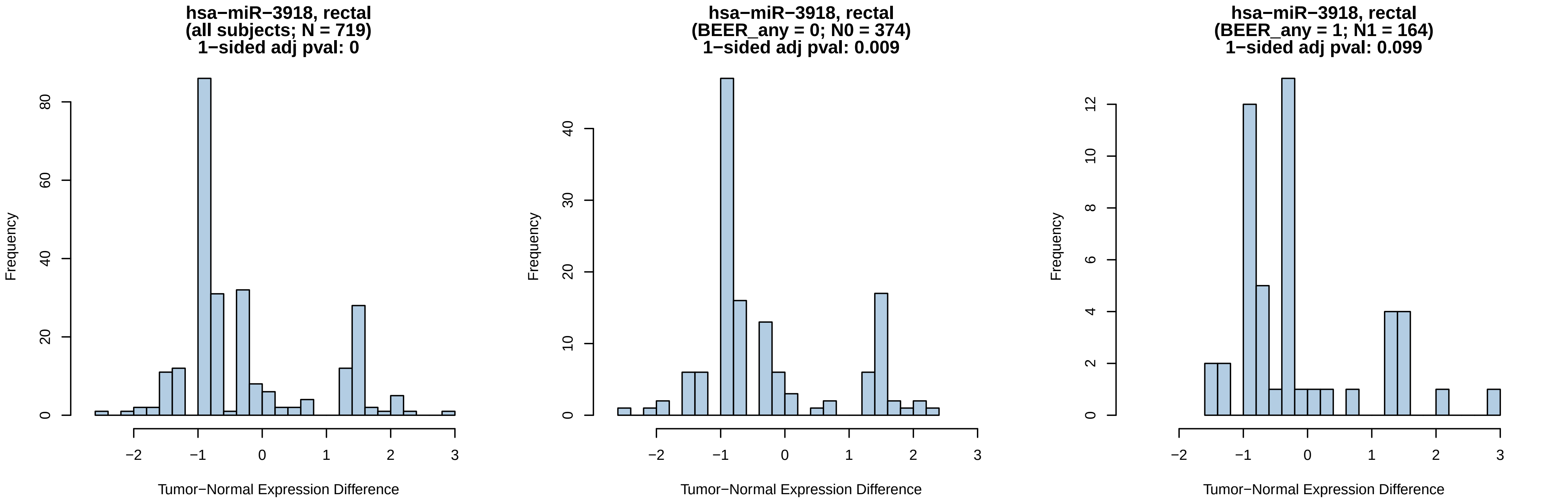

hsa−miR−3918, rectal
(all subjects; N = 719)
1−sided adj pval: 0
hsa−miR−3918, rectal
(BEER_any = 0; N0 = 374)
1−sided adj pval: 0.009
hsa−miR−3918, rectal
(BEER_any = 1; N1 = 164)
1−sided adj pval: 0.099
Frequency
Tumor−Normal Expression Difference

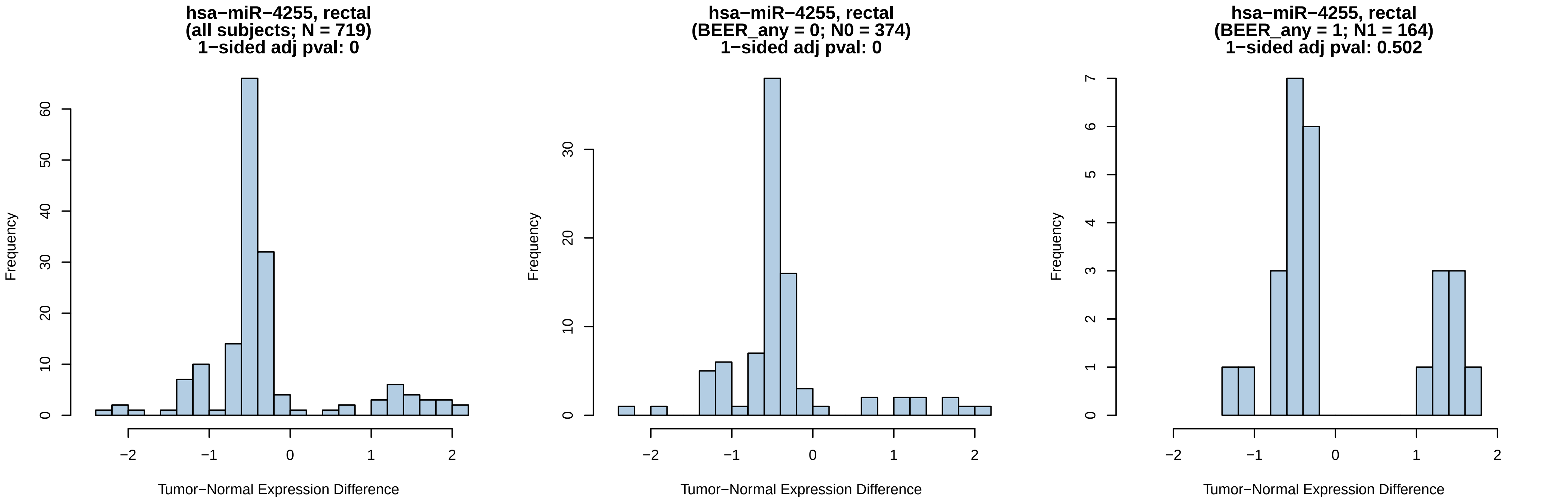
hsa−miR−4255, rectal
(all subjects; N = 719)
1−sided adj pval: 0
Frequency
0
10
20
30
40
50
60
−2
−1
0
1
2
Tumor−Normal Expression Difference
hsa−miR−4255, rectal
(BEER_any = 0; N0 = 374)
1−sided adj pval: 0
Frequency
0
10
20
30
−2
−1
0
1
2
Tumor−Normal Expression Difference
hsa−miR−4255, rectal
(BEER_any = 1; N1 = 164)
1−sided adj pval: 0.502
Frequency
0
1
2
3
4
5
6
7
−2
−1
0
1
2
Tumor−Normal Expression Difference

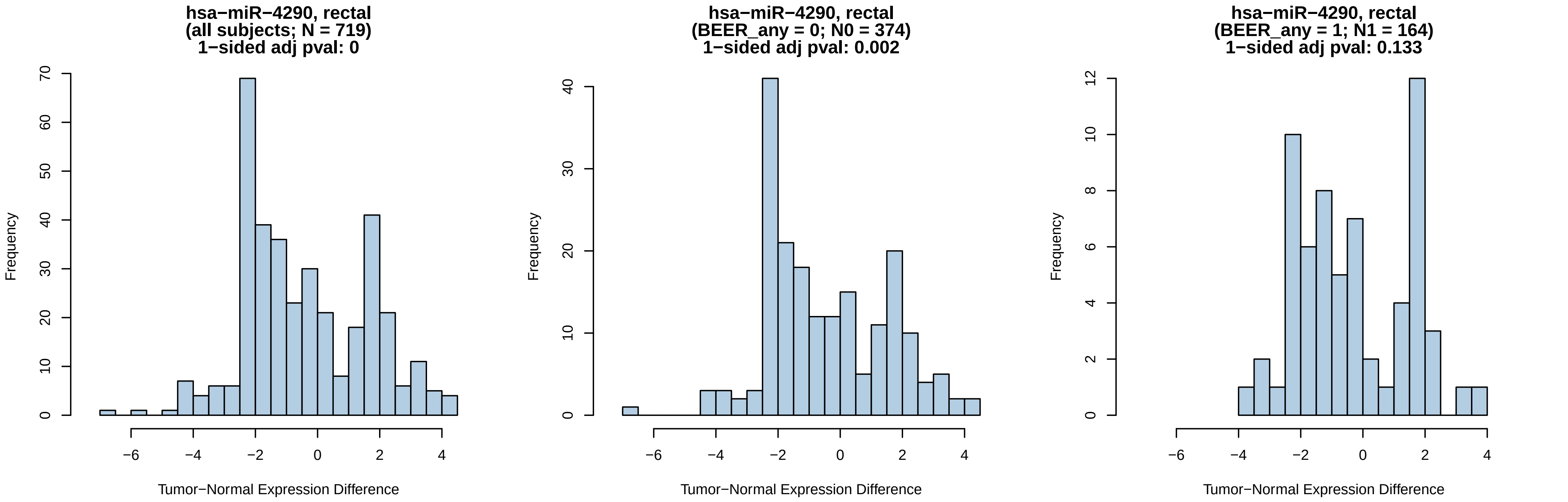
hsa−miR−4290, rectal
(all subjects; N = 719)
1−sided adj pval: 0
Frequency
Tumor−Normal Expression Difference
hsa−miR−4290, rectal
(BEER_any = 0; N0 = 374)
1−sided adj pval: 0.002
Frequency
Tumor−Normal Expression Difference
hsa−miR−4290, rectal
(BEER_any = 1; N1 = 164)
1−sided adj pval: 0.133
Frequency
Tumor−Normal Expression Difference

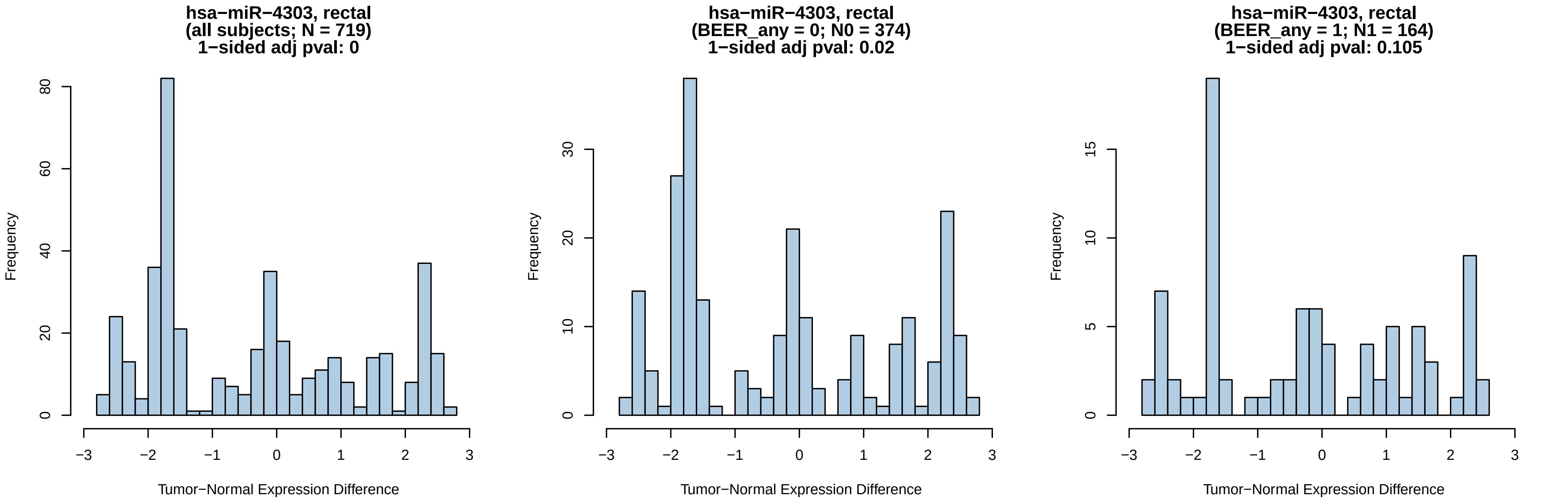
hsa−miR−4303, rectal
(all subjects; N = 719)
1−sided adj pval: 0
Frequency
0
20
40
60
80
−3
−2
−1
0
1
2
3
Tumor−Normal Expression Difference
hsa−miR−4303, rectal
(BEER_any = 0; N0 = 374)
1−sided adj pval: 0.02
Frequency
0
10
20
30
−3
−2
−1
0
1
2
3
Tumor−Normal Expression Difference
hsa−miR−4303, rectal
(BEER_any = 1; N1 = 164)
1−sided adj pval: 0.105
Frequency
0
5
10
15
−3
−2
−1
0
1
2
3
Tumor−Normal Expression Difference

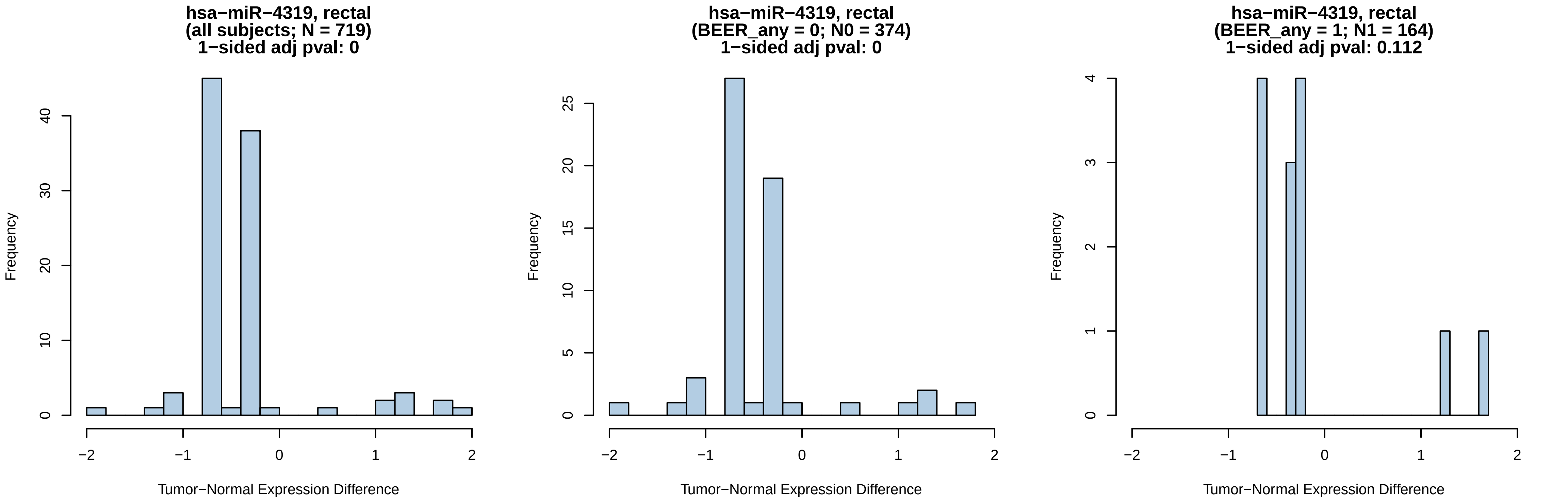

hsa−miR−4319, rectal
(all subjects; N = 719)
1−sided adj pval: 0
Frequency
Tumor−Normal Expression Difference
hsa−miR−4319, rectal
(BEER_any = 0; N0 = 374)
1−sided adj pval: 0
Frequency
Tumor−Normal Expression Difference
hsa−miR−4319, rectal
(BEER_any = 1; N1 = 164)
1−sided adj pval: 0.112
Frequency
Tumor−Normal Expression Difference

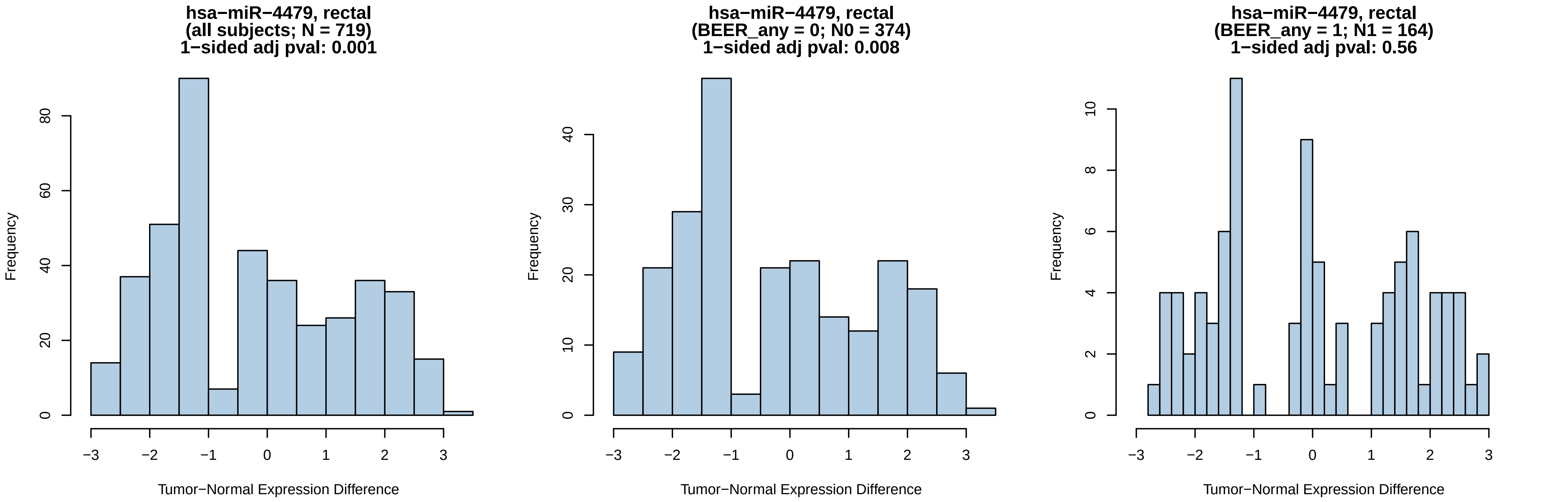

hsa−miR−4479, rectal
(all subjects; N = 719)
1−sided adj pval: 0.001
Frequency
Tumor−Normal Expression Difference
hsa−miR−4479, rectal
(BEER_any = 0; N0 = 374)
1−sided adj pval: 0.008
Frequency
Tumor−Normal Expression Difference
hsa−miR−4479, rectal
(BEER_any = 1; N1 = 164)
1−sided adj pval: 0.56
Frequency
Tumor−Normal Expression Difference

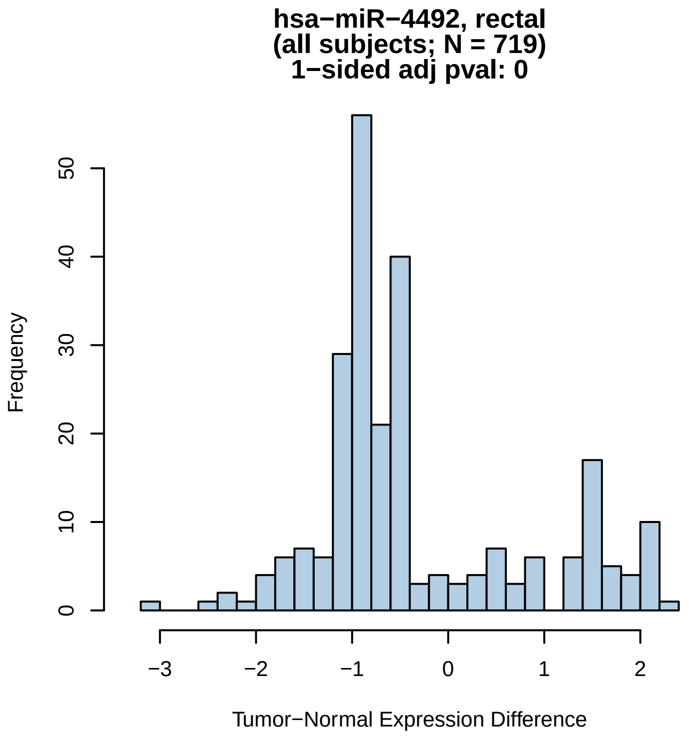
hsa−miR−4492, rectal
(all subjects; N = 719)
1−sided adj pval: 0
Frequency
Tumor−Normal Expression Difference

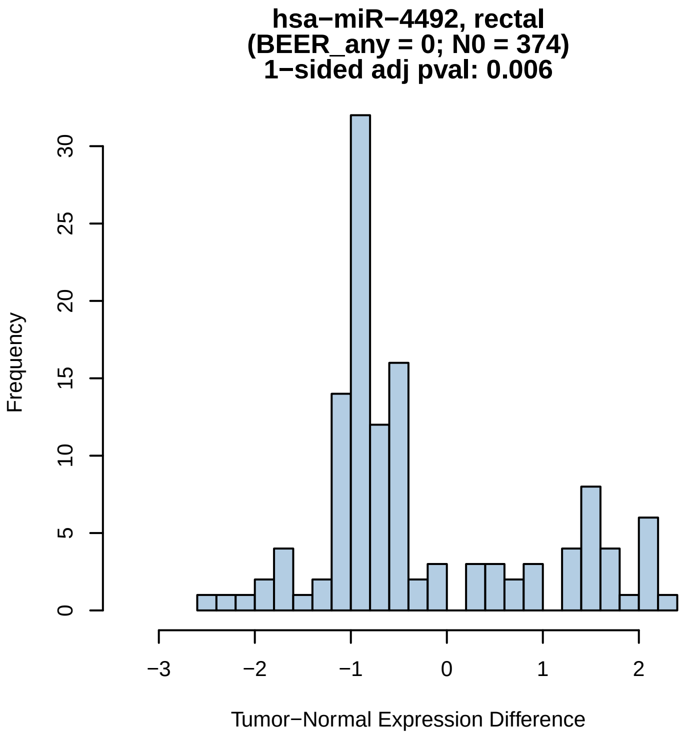
hsa−miR−4492, rectal
(BEER_any = 0; N0 = 374)
1−sided adj pval: 0.006
Frequency
Tumor−Normal Expression Difference

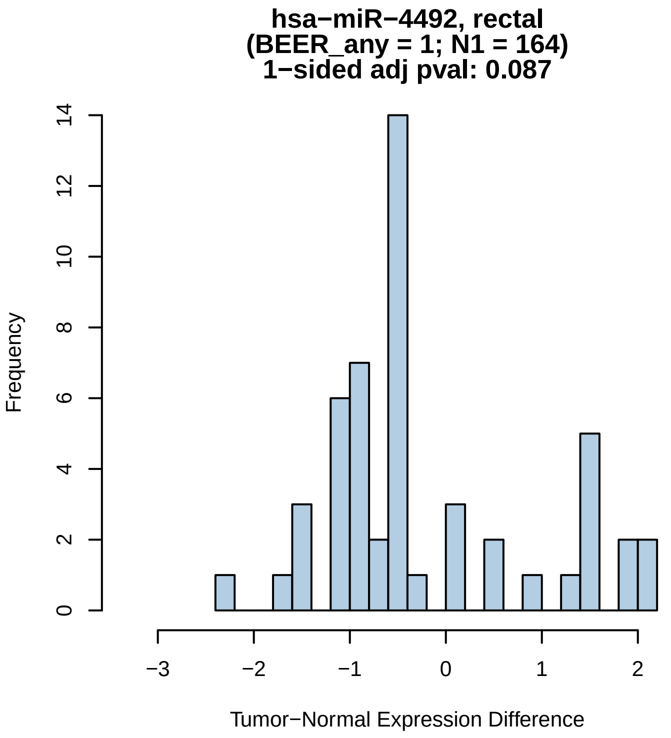
hsa−miR−4492, rectal
(BEER_any = 1; N1 = 164)
1−sided adj pval: 0.087
Frequency
Tumor−Normal Expression Difference

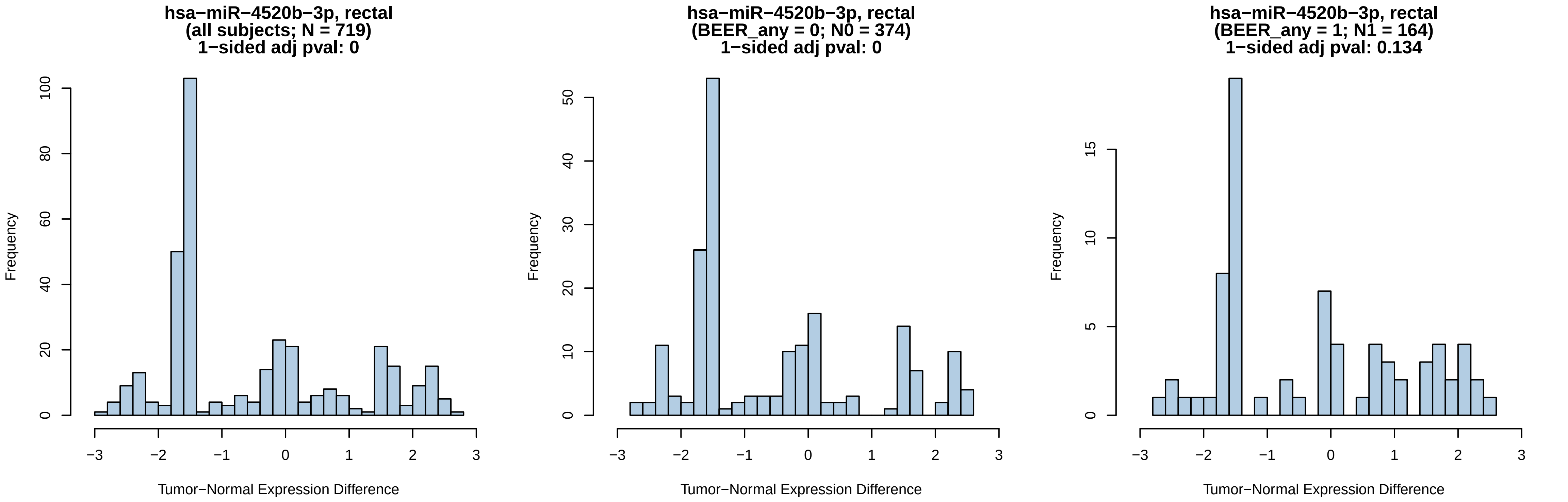
hsa−miR−4520b−3p, rectal
(all subjects; N = 719)
1−sided adj pval: 0
Frequency
0
20
40
60
80
100
−3
−2
−1
0
1
2
3
Tumor−Normal Expression Difference
hsa−miR−4520b−3p, rectal
(BEER_any = 0; N0 = 374)
1−sided adj pval: 0
Frequency
0
10
20
30
40
50
−3
−2
−1
0
1
2
3
Tumor−Normal Expression Difference
hsa−miR−4520b−3p, rectal
(BEER_any = 1; N1 = 164)
1−sided adj pval: 0.134
Frequency
0
5
10
15
−3
−2
−1
0
1
2
3
Tumor−Normal Expression Difference

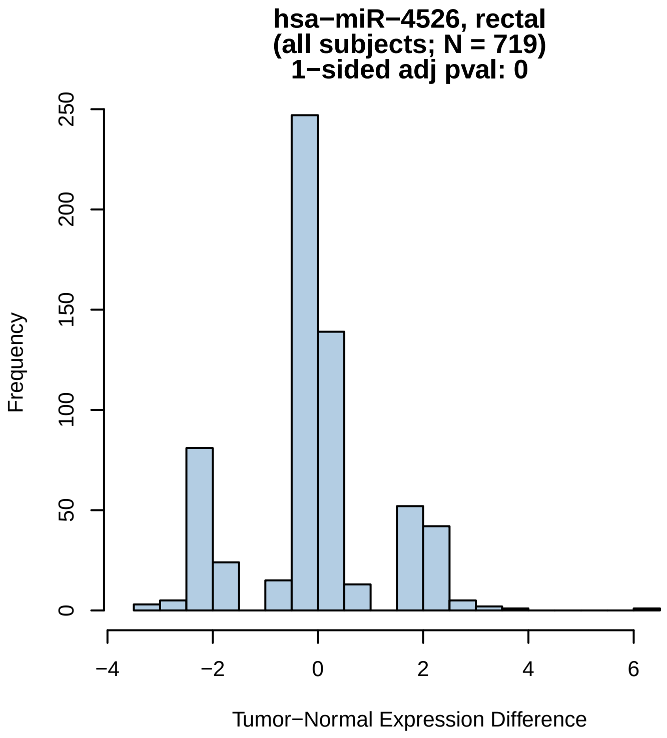

hsa−miR−4526, rectal
(all subjects; N = 719)
1−sided adj pval: 0
Frequency
Tumor−Normal Expression Difference


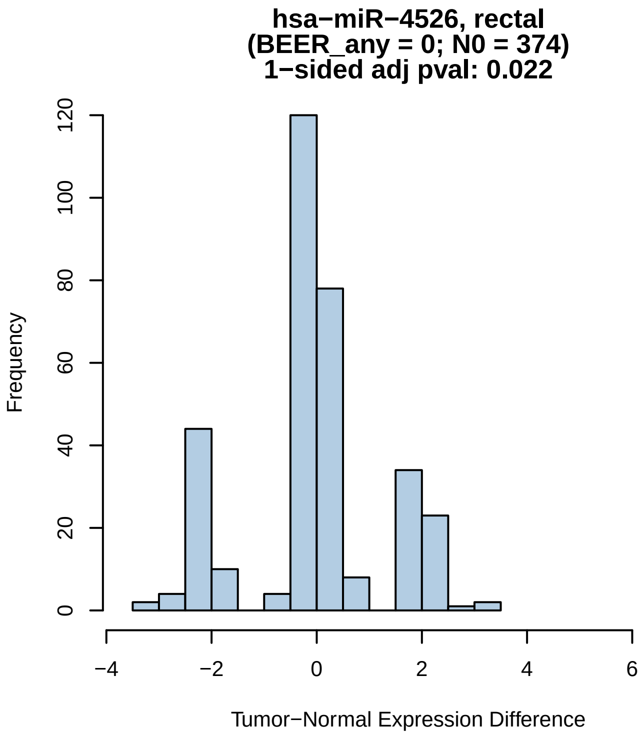

hsa−miR−4526, rectal
(BEER_any = 0; N0 = 374)
1−sided adj pval: 0.022
Frequency
Tumor−Normal Expression Difference


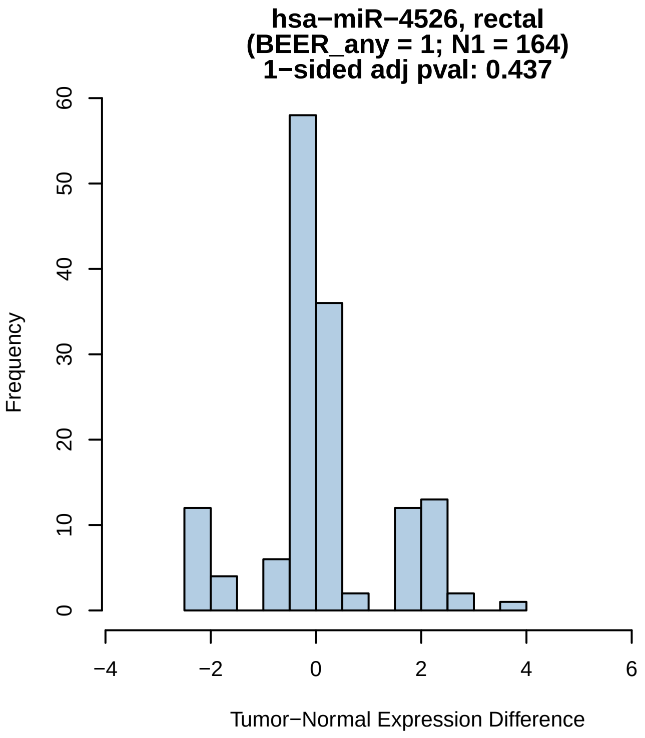

hsa−miR−4526, rectal
(BEER_any = 1; N1 = 164)
1−sided adj pval: 0.437
Frequency
Tumor−Normal Expression Difference

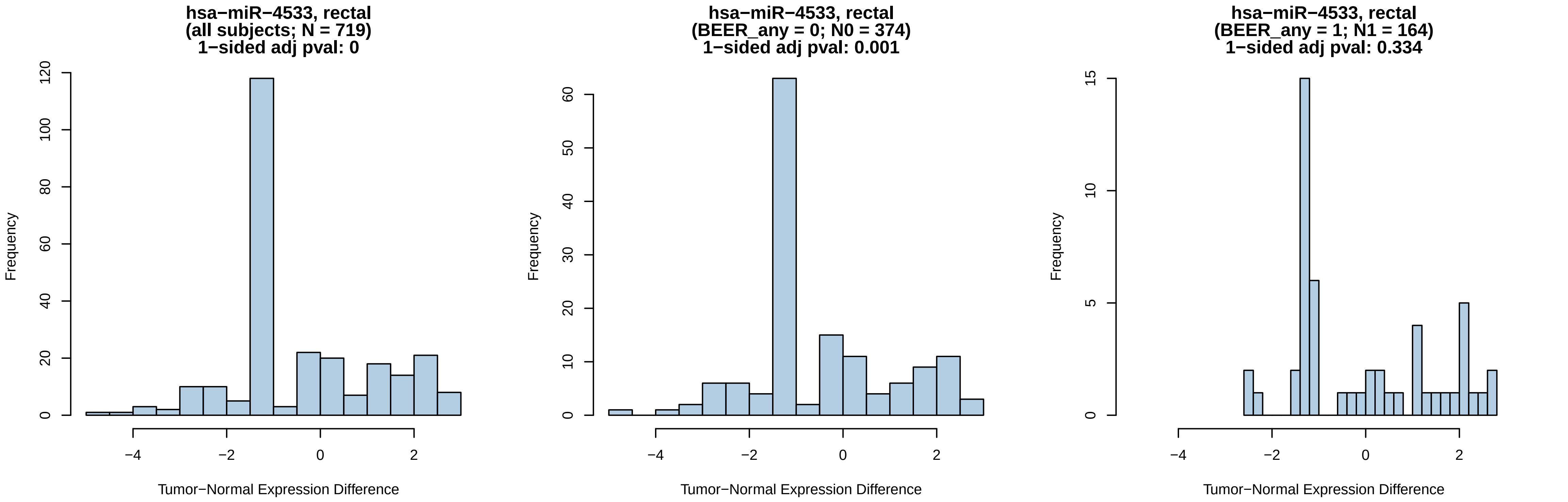

hsa−miR−4533, rectal
(all subjects; N = 719)
1−sided adj pval: 0
Frequency
Tumor−Normal Expression Difference
hsa−miR−4533, rectal
(BEER_any = 0; N0 = 374)
1−sided adj pval: 0.001
Frequency
Tumor−Normal Expression Difference
hsa−miR−4533, rectal
(BEER_any = 1; N1 = 164)
1−sided adj pval: 0.334
Frequency
Tumor−Normal Expression Difference

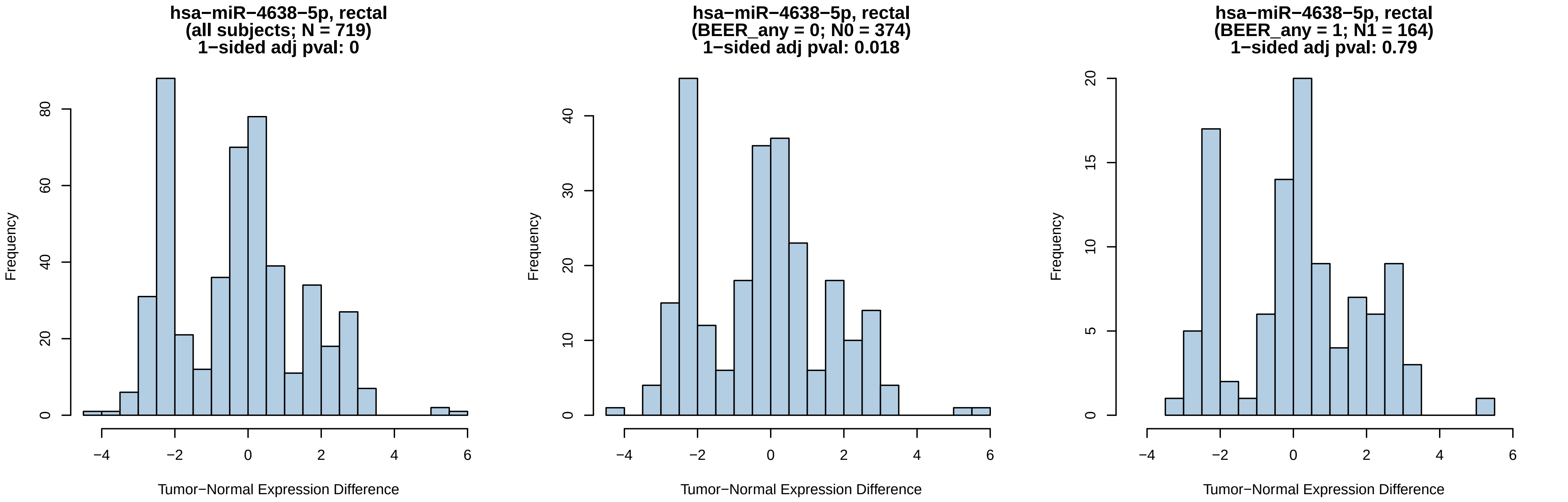
hsa−miR−4638−5p, rectal
(all subjects; N = 719)
1−sided adj pval: 0
Frequency
Tumor−Normal Expression Difference
hsa−miR−4638−5p, rectal
(BEER_any = 0; N0 = 374)
1−sided adj pval: 0.018
Frequency
Tumor−Normal Expression Difference
hsa−miR−4638−5p, rectal
(BEER_any = 1; N1 = 164)
1−sided adj pval: 0.79
Frequency
Tumor−Normal Expression Difference

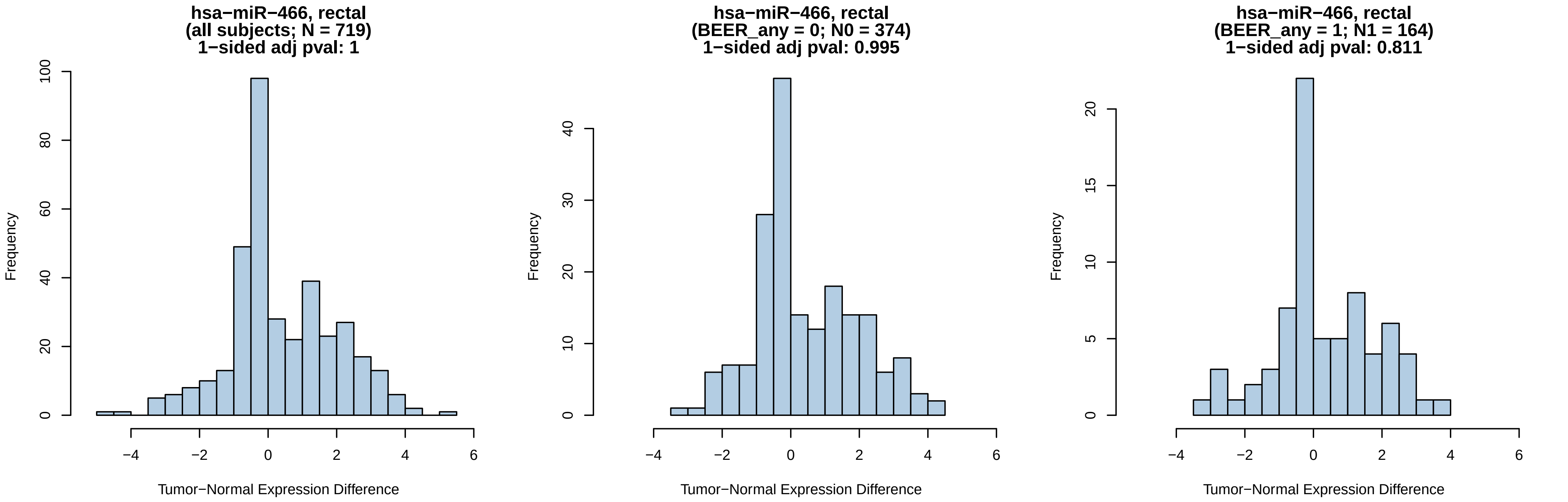

hsa−miR−466, rectal
(all subjects; N = 719)
1−sided adj pval: 1
Frequency
Tumor−Normal Expression Difference
hsa−miR−466, rectal
(BEER_any = 0; N0 = 374)
1−sided adj pval: 0.995
Frequency
Tumor−Normal Expression Difference
hsa−miR−466, rectal
(BEER_any = 1; N1 = 164)
1−sided adj pval: 0.811
Frequency
Tumor−Normal Expression Difference

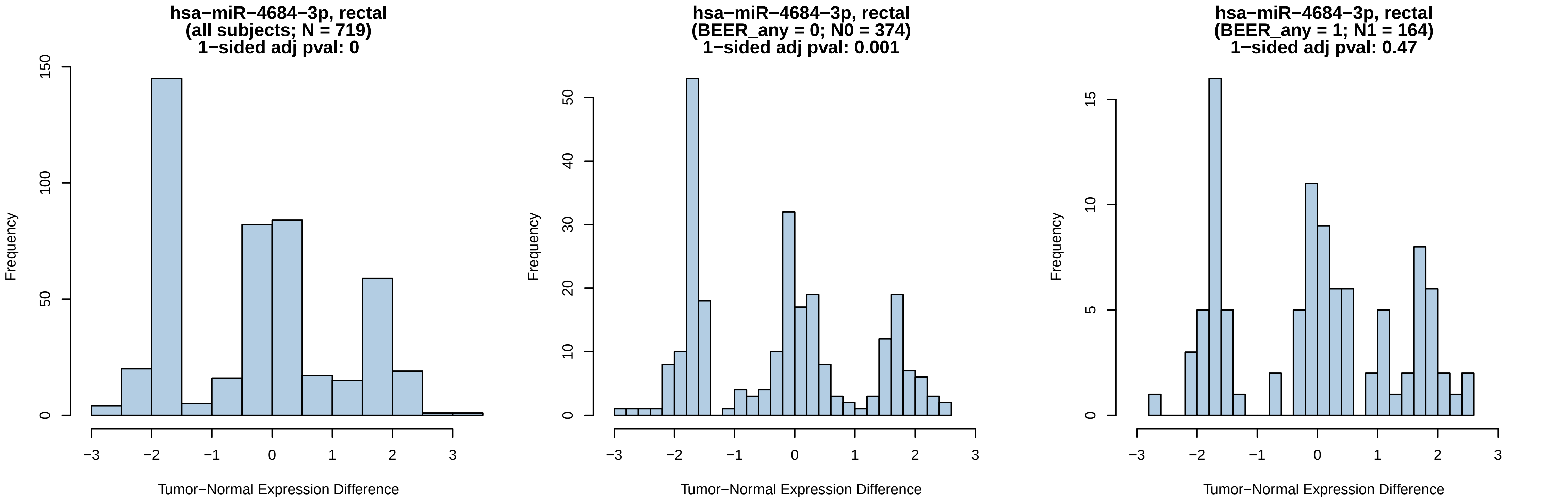
hsa−miR−4684−3p, rectal
(all subjects; N = 719)
1−sided adj pval: 0
Frequency
Tumor−Normal Expression Difference
hsa−miR−4684−3p, rectal
(BEER_any = 0; N0 = 374)
1−sided adj pval: 0.001
Frequency
Tumor−Normal Expression Difference
hsa−miR−4684−3p, rectal
(BEER_any = 1; N1 = 164)
1−sided adj pval: 0.47
Frequency
Tumor−Normal Expression Difference

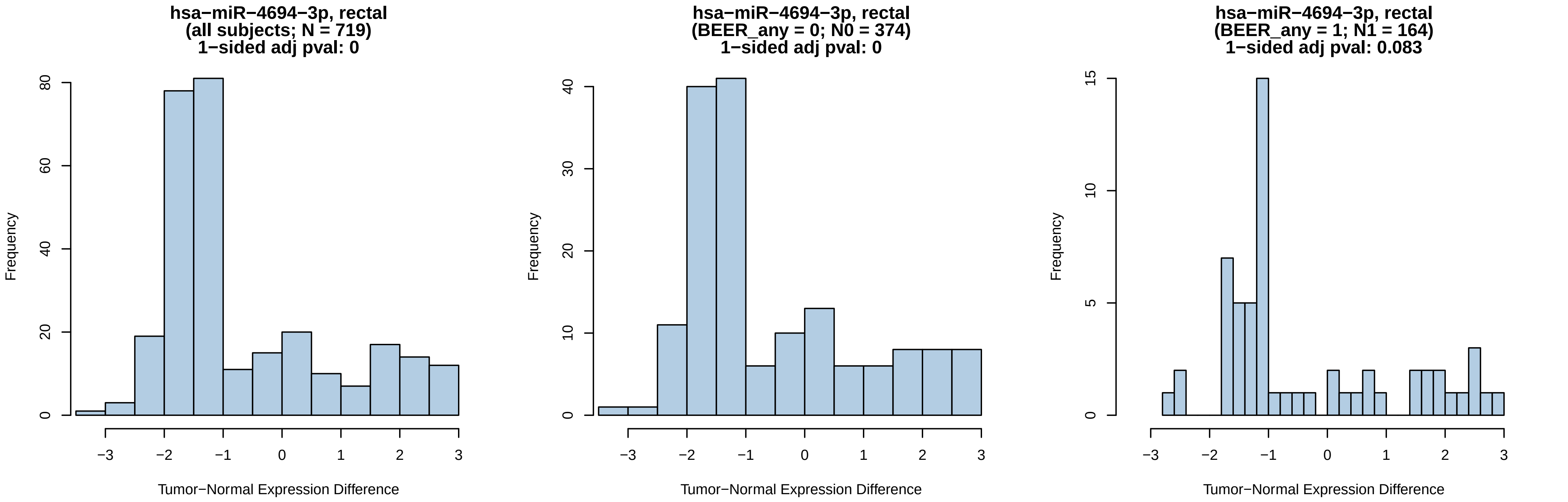

hsa−miR−4694−3p, rectal
(all subjects; N = 719)
1−sided adj pval: 0
Frequency
0
20
40
60
80
−3
−2
−1
0
1
2
3
Tumor−Normal Expression Difference
hsa−miR−4694−3p, rectal
(BEER_any = 0; N0 = 374)
1−sided adj pval: 0
Frequency
0
10
20
30
40
−3
−2
−1
0
1
2
3
Tumor−Normal Expression Difference
hsa−miR−4694−3p, rectal
(BEER_any = 1; N1 = 164)
1−sided adj pval: 0.083
Frequency
0
5
10
15
−3
−2
−1
0
1
2
3
Tumor−Normal Expression Difference

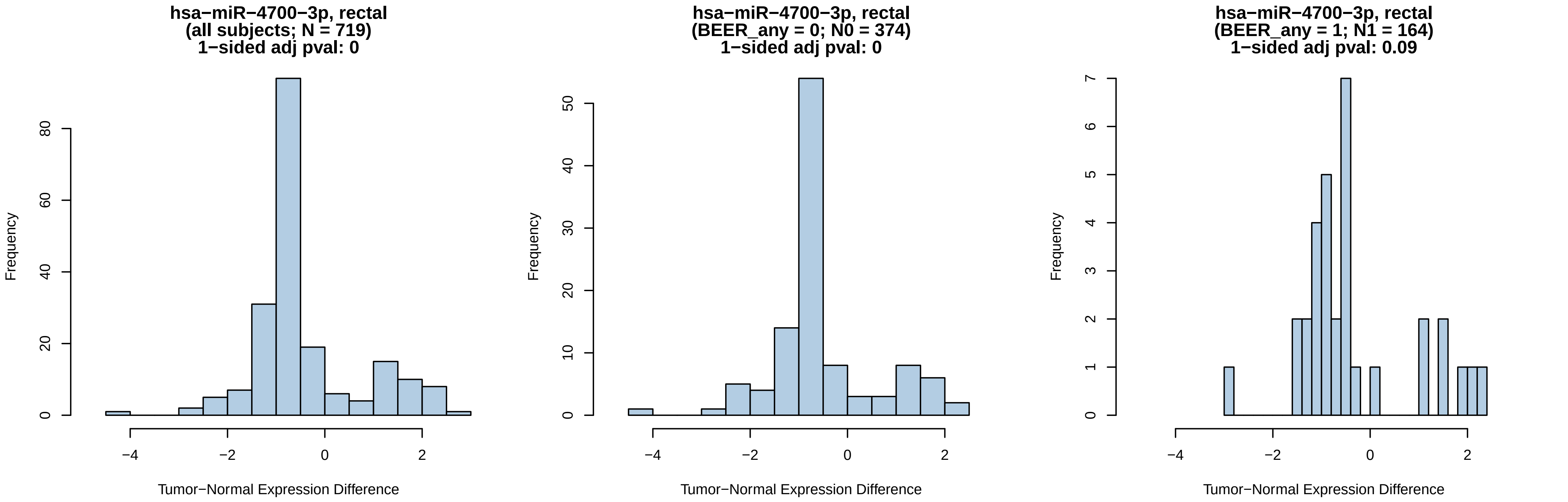
hsa−miR−4700−3p, rectal
(all subjects; N = 719)
1−sided adj pval: 0
Frequency
Tumor−Normal Expression Difference
hsa−miR−4700−3p, rectal
(BEER_any = 0; N0 = 374)
1−sided adj pval: 0
Frequency
Tumor−Normal Expression Difference
hsa−miR−4700−3p, rectal
(BEER_any = 1; N1 = 164)
1−sided adj pval: 0.09
Frequency
Tumor−Normal Expression Difference

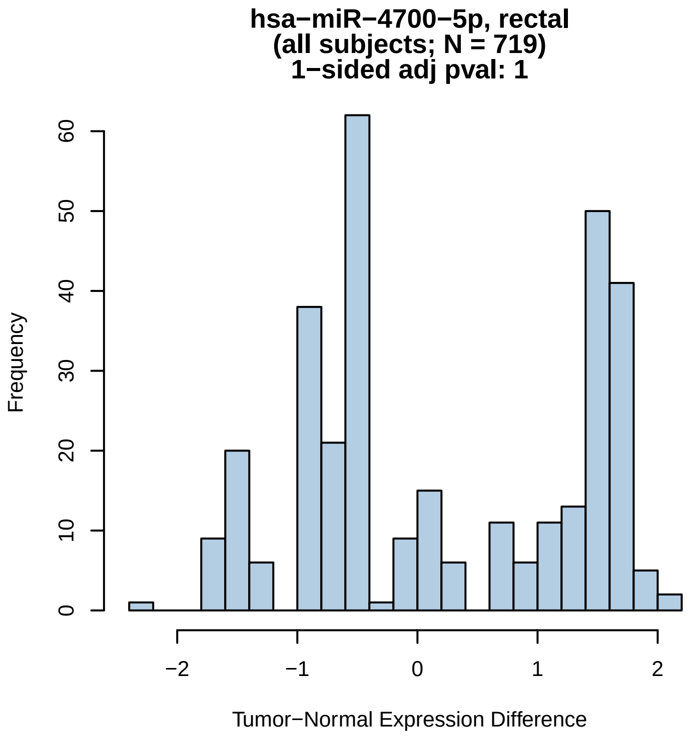

hsa−miR−4700−5p, rectal
(all subjects; N = 719)
1−sided adj pval: 1
Frequency
Tumor−Normal Expression Difference


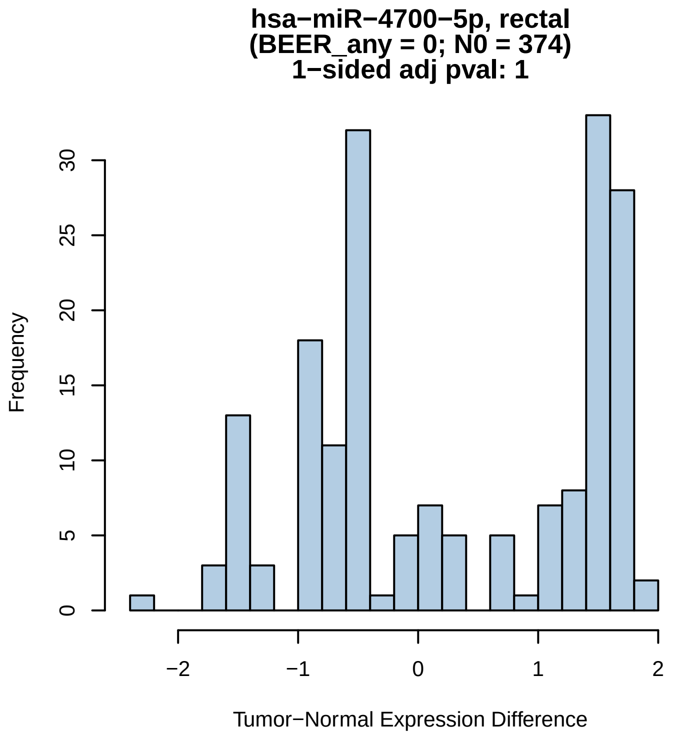

hsa−miR−4700−5p, rectal
(BEER_any = 0; N0 = 374)
1−sided adj pval: 1
Frequency
Tumor−Normal Expression Difference


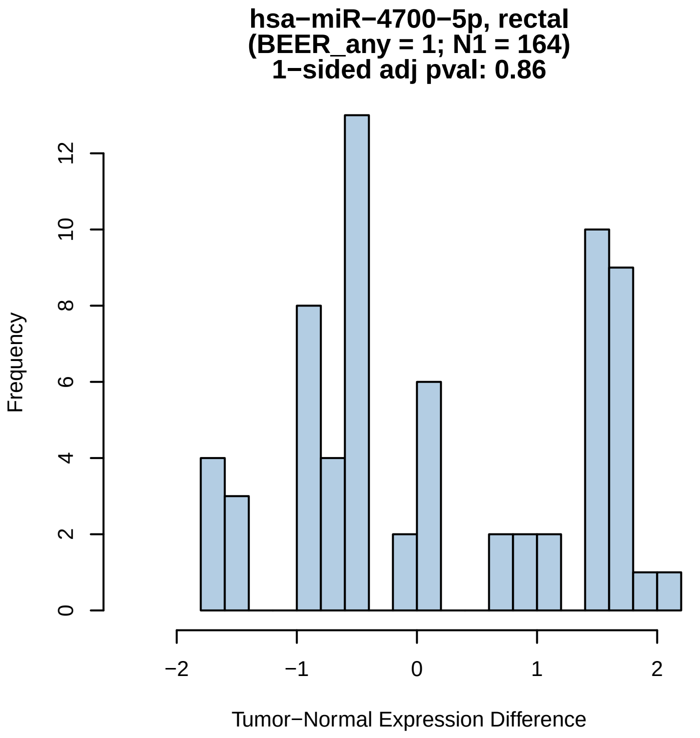

hsa−miR−4700−5p, rectal
(BEER_any = 1; N1 = 164)
1−sided adj pval: 0.86
Frequency
Tumor−Normal Expression Difference

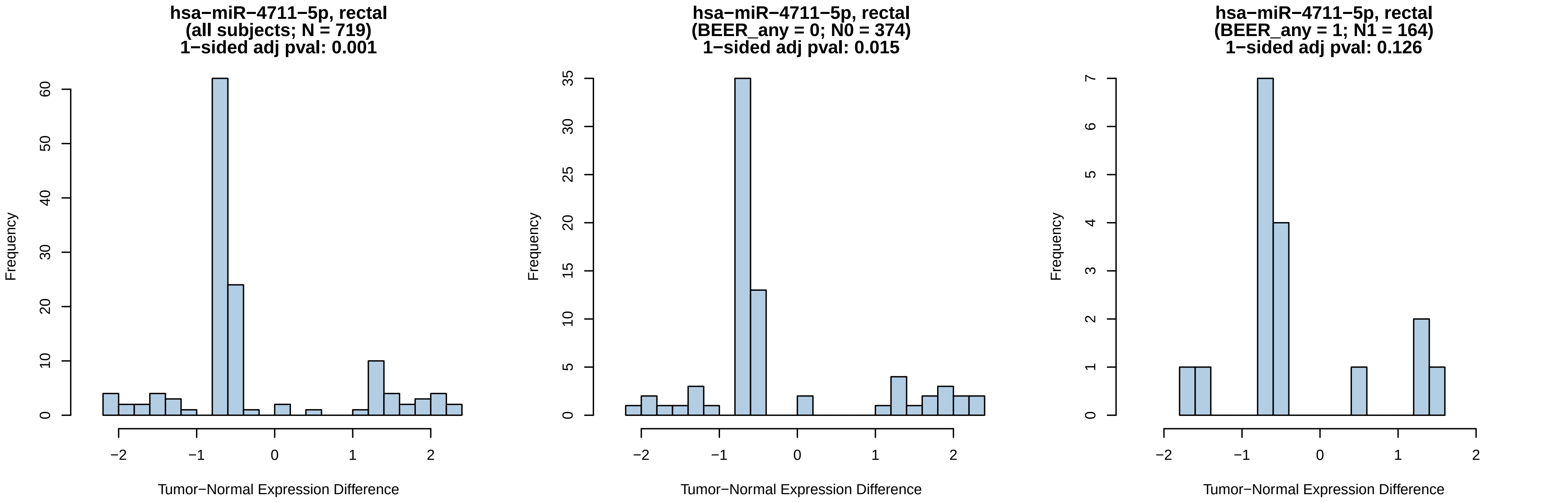

hsa−miR−4711−5p, rectal
(all subjects; N = 719)
1−sided adj pval: 0.001
Frequency
Tumor−Normal Expression Difference
hsa−miR−4711−5p, rectal
(BEER_any = 0; N0 = 374)
1−sided adj pval: 0.015
Frequency
Tumor−Normal Expression Difference
hsa−miR−4711−5p, rectal
(BEER_any = 1; N1 = 164)
1−sided adj pval: 0.126
Frequency
Tumor−Normal Expression Difference

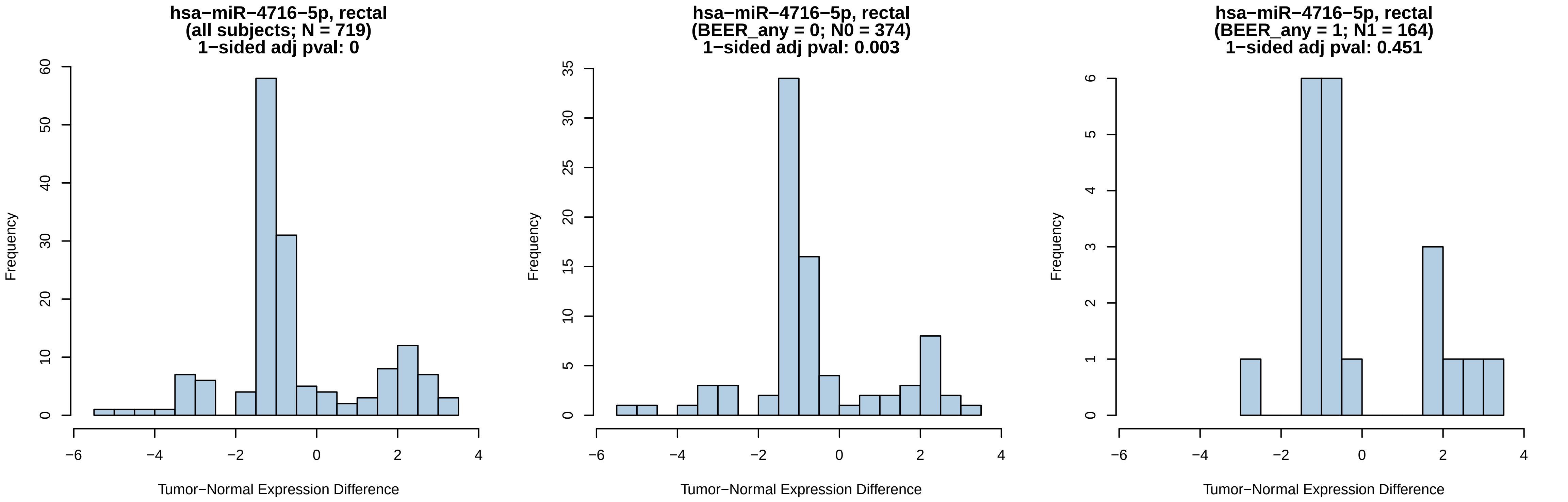
hsa−miR−4716−5p, rectal
(all subjects; N = 719)
1−sided adj pval: 0
Frequency
Tumor−Normal Expression Difference
hsa−miR−4716−5p, rectal
(BEER_any = 0; N0 = 374)
1−sided adj pval: 0.003
Frequency
Tumor−Normal Expression Difference
hsa−miR−4716−5p, rectal
(BEER_any = 1; N1 = 164)
1−sided adj pval: 0.451
Frequency
Tumor−Normal Expression Difference

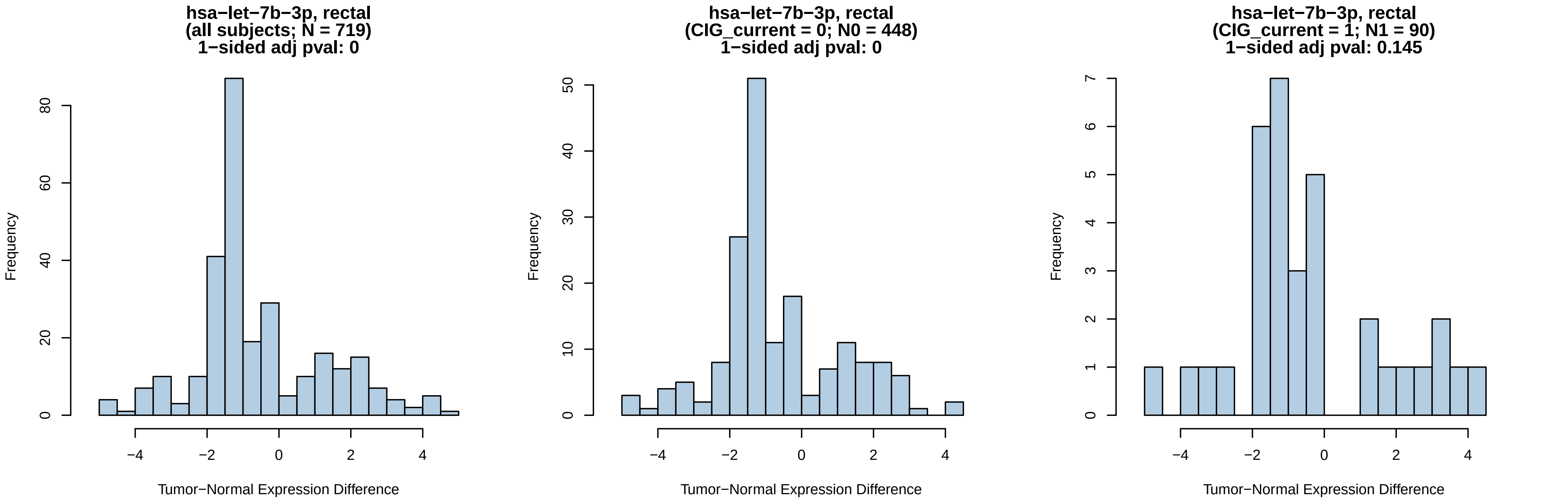

hsa−let−7b−3p, rectal
(all subjects; N = 719)
1−sided adj pval: 0
Frequency
0
20
40
60
80
−4
−2
0
2
4
Tumor−Normal Expression Difference
hsa−let−7b−3p, rectal
(CIG_current = 0; N0 = 448)
1−sided adj pval: 0
Frequency
0
10
20
30
40
50
−4
−2
0
2
4
Tumor−Normal Expression Difference
hsa−let−7b−3p, rectal
(CIG_current = 1; N1 = 90)
1−sided adj pval: 0.145
Frequency
0
1
2
3
4
5
6
7
−4
−2
0
2
4
Tumor−Normal Expression Difference

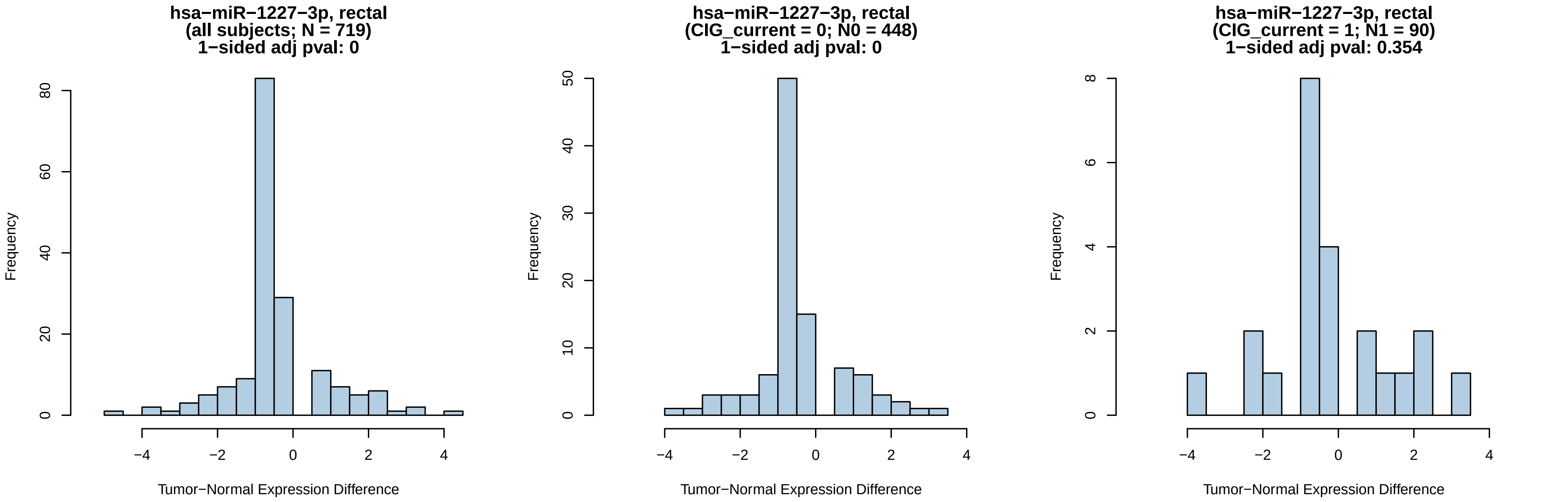

hsa−miR−1227−3p, rectal
(all subjects; N = 719)
1−sided adj pval: 0
Frequency
Tumor−Normal Expression Difference
hsa−miR−1227−3p, rectal
(CIG_current = 0; N0 = 448)
1−sided adj pval: 0
Frequency
Tumor−Normal Expression Difference
hsa−miR−1227−3p, rectal
(CIG_current = 1; N1 = 90)
1−sided adj pval: 0.354
Frequency
Tumor−Normal Expression Difference

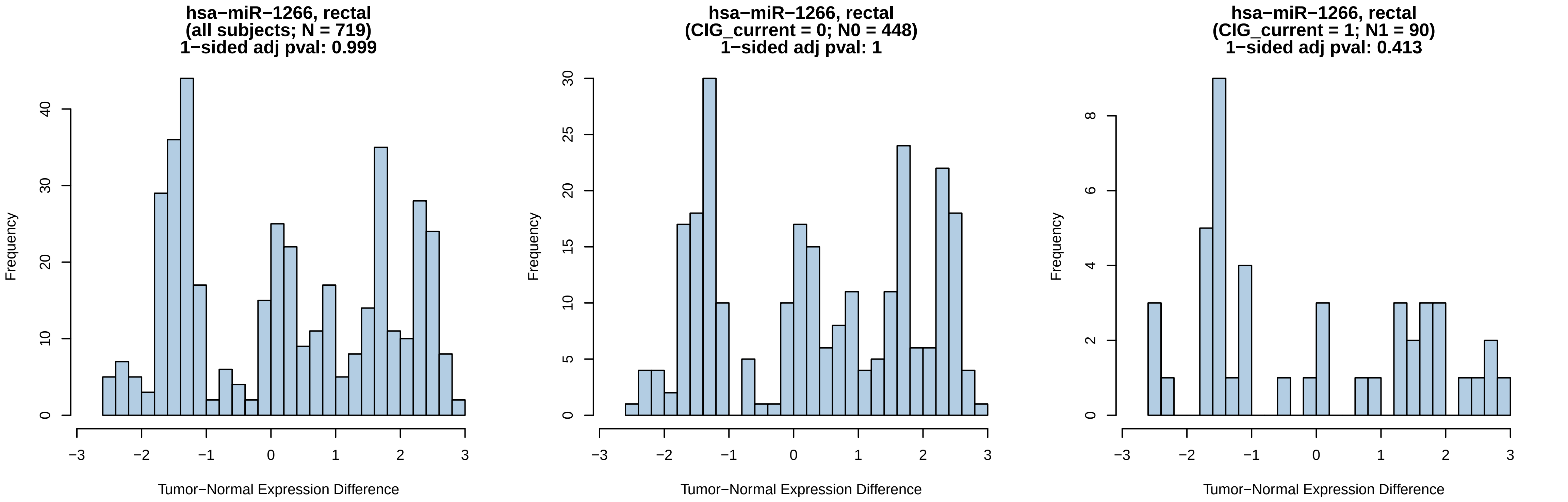
hsa−miR−1266, rectal
(all subjects; N = 719)
1−sided adj pval: 0.999
Frequency
Tumor−Normal Expression Difference
hsa−miR−1266, rectal
(CIG_current = 0; N0 = 448)
1−sided adj pval: 1
Frequency
Tumor−Normal Expression Difference
hsa−miR−1266, rectal
(CIG_current = 1; N1 = 90)
1−sided adj pval: 0.413
Frequency
Tumor−Normal Expression Difference

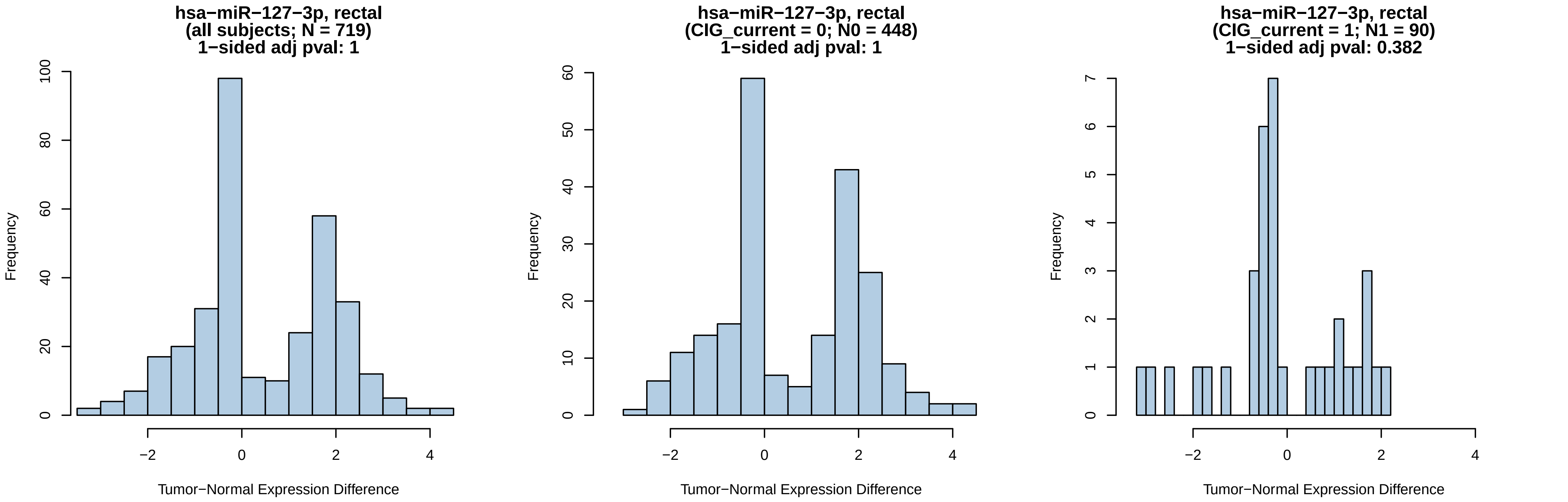

hsa−miR−127−3p, rectal
(all subjects; N = 719)
1−sided adj pval: 1
Frequency
Tumor−Normal Expression Difference
hsa−miR−127−3p, rectal
(CIG_current = 0; N0 = 448)
1−sided adj pval: 1
Frequency
Tumor−Normal Expression Difference
hsa−miR−127−3p, rectal
(CIG_current = 1; N1 = 90)
1−sided adj pval: 0.382
Frequency
Tumor−Normal Expression Difference

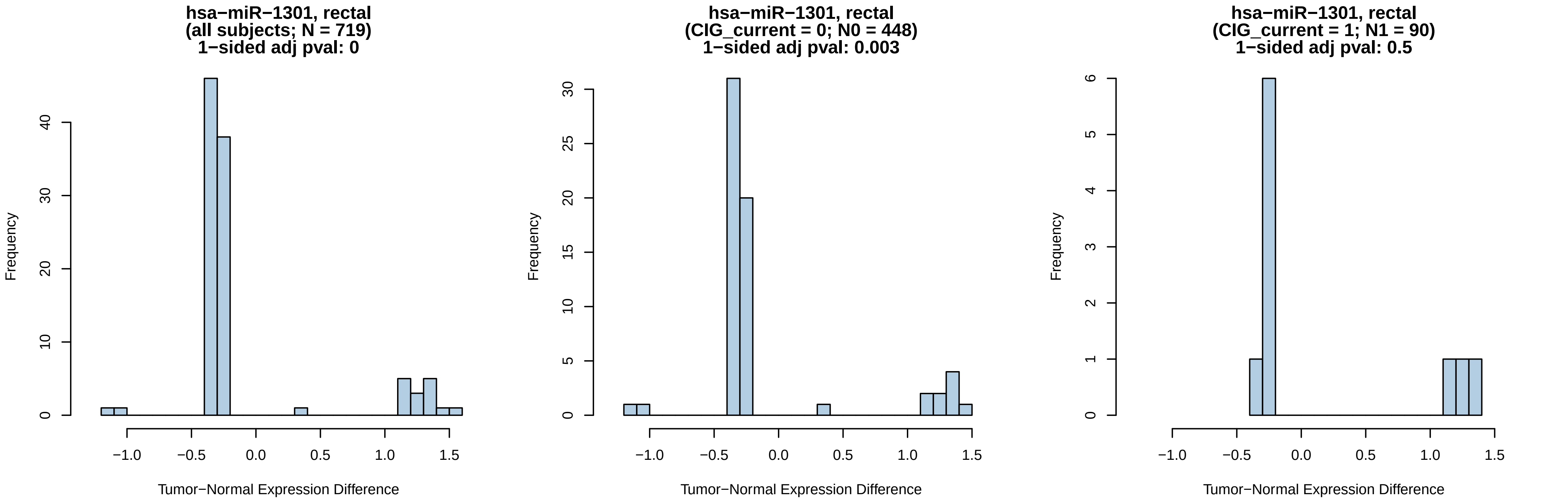

hsa−miR−1301, rectal
(all subjects; N = 719)
1−sided adj pval: 0
hsa−miR−1301, rectal
(CIG_current = 0; N0 = 448)
1−sided adj pval: 0.003
hsa−miR−1301, rectal
(CIG_current = 1; N1 = 90)
1−sided adj pval: 0.5
Frequency
Tumor−Normal Expression Difference

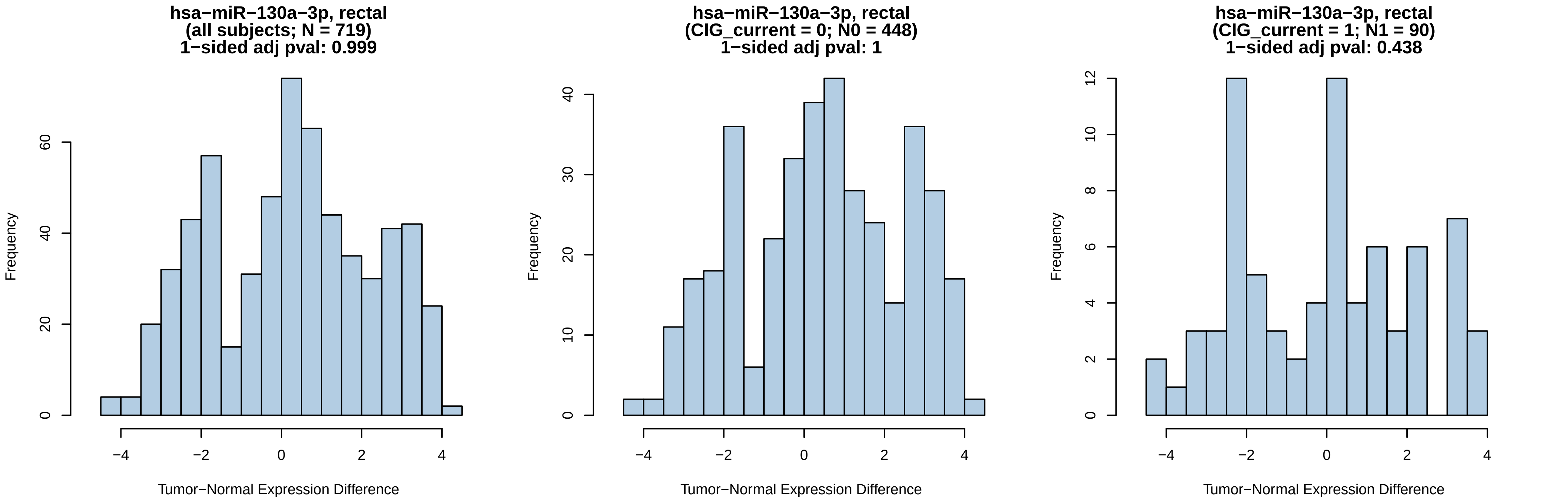
hsa−miR−130a−3p, rectal
(all subjects; N = 719)
1−sided adj pval: 0.999
Frequency
Tumor−Normal Expression Difference
hsa−miR−130a−3p, rectal
(CIG_current = 0; N0 = 448)
1−sided adj pval: 1
Frequency
Tumor−Normal Expression Difference
hsa−miR−130a−3p, rectal
(CIG_current = 1; N1 = 90)
1−sided adj pval: 0.438
Frequency
Tumor−Normal Expression Difference

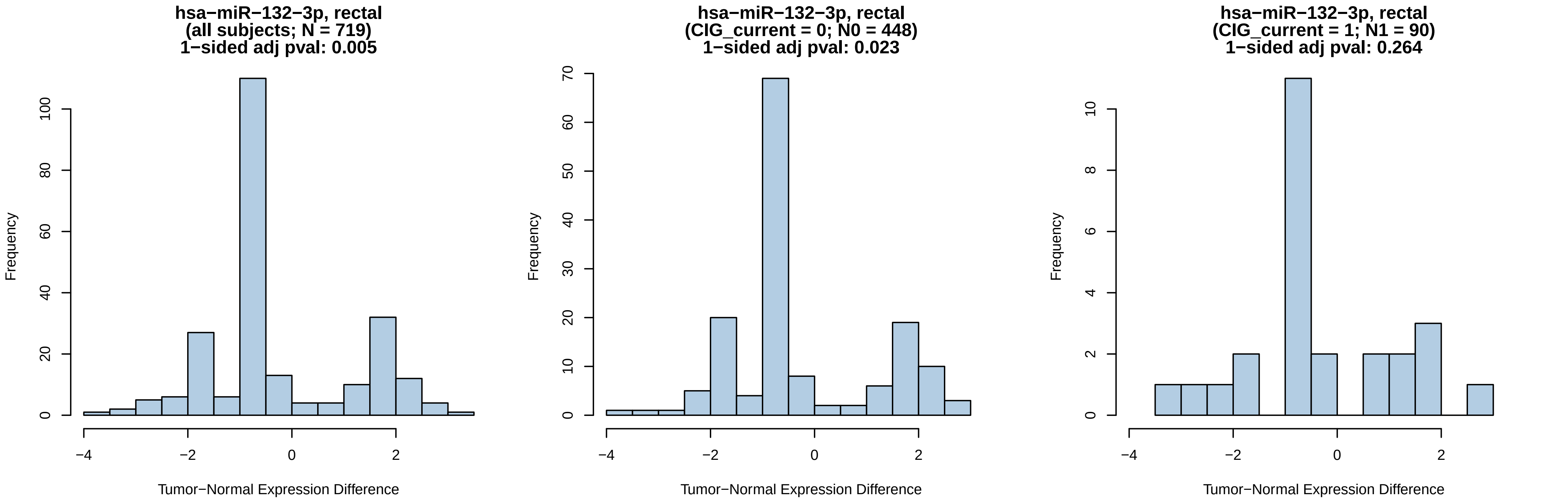
hsa−miR−132−3p, rectal
(all subjects; N = 719)
1−sided adj pval: 0.005
Frequency
Tumor−Normal Expression Difference
hsa−miR−132−3p, rectal
(CIG_current = 0; N0 = 448)
1−sided adj pval: 0.023
Frequency
Tumor−Normal Expression Difference
hsa−miR−132−3p, rectal
(CIG_current = 1; N1 = 90)
1−sided adj pval: 0.264
Frequency
Tumor−Normal Expression Difference

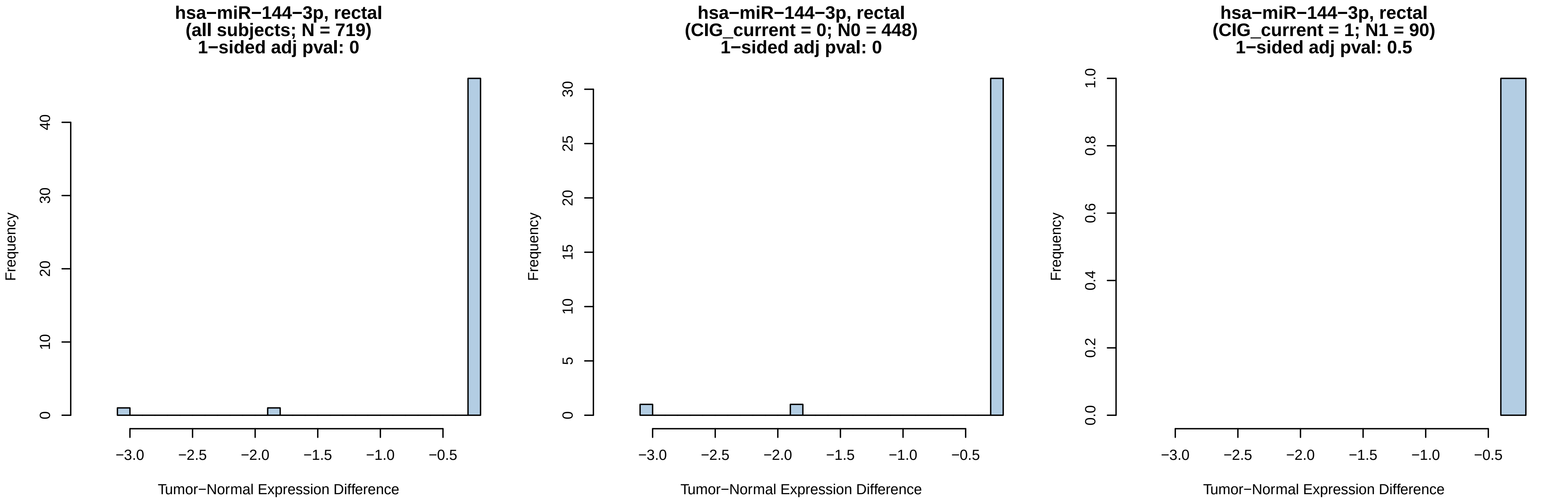
hsa−miR−144−3p, rectal
(all subjects; N = 719)
1−sided adj pval: 0
Frequency
Tumor−Normal Expression Difference
hsa−miR−144−3p, rectal
(CIG_current = 0; N0 = 448)
1−sided adj pval: 0
Frequency
Tumor−Normal Expression Difference
hsa−miR−144−3p, rectal
(CIG_current = 1; N1 = 90)
1−sided adj pval: 0.5
Frequency
Tumor−Normal Expression Difference

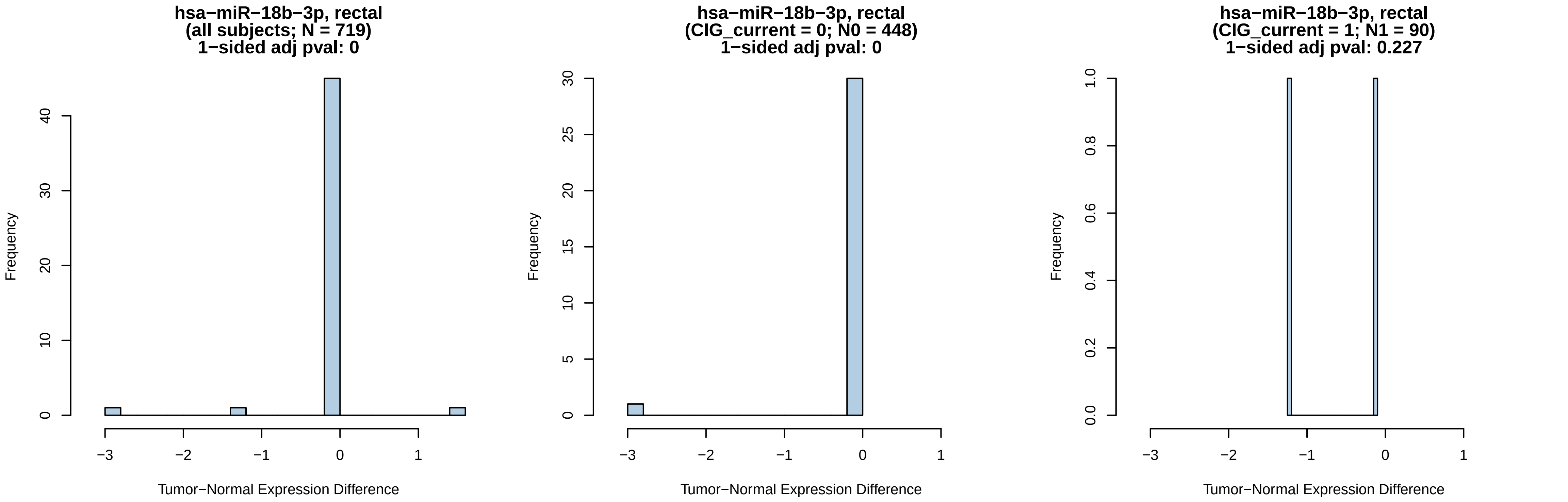
hsa−miR−18b−3p, rectal
(all subjects; N = 719)
1−sided adj pval: 0
Frequency
Tumor−Normal Expression Difference
hsa−miR−18b−3p, rectal
(CIG_current = 0; N0 = 448)
1−sided adj pval: 0
Frequency
Tumor−Normal Expression Difference
hsa−miR−18b−3p, rectal
(CIG_current = 1; N1 = 90)
1−sided adj pval: 0.227
Frequency
Tumor−Normal Expression Difference

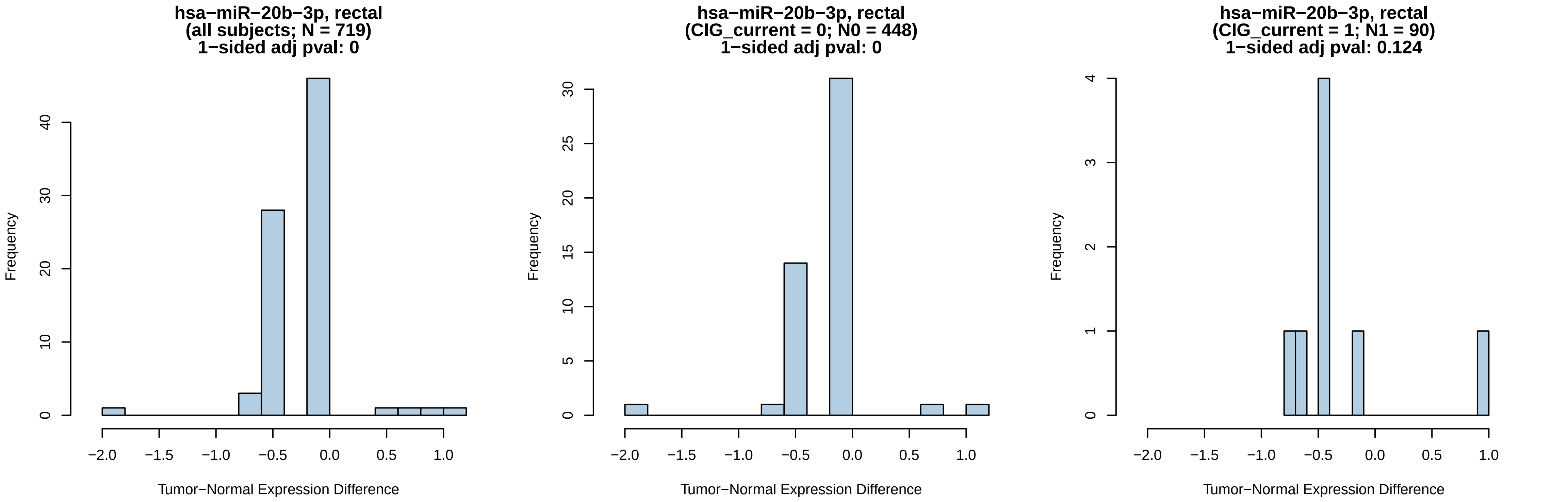

hsa−miR−20b−3p, rectal
(all subjects; N = 719)
1−sided adj pval: 0
Frequency
0
10
20
30
40
−2.0
−1.5
−1.0
−0.5
0.0
0.5
1.0
Tumor−Normal Expression Difference
hsa−miR−20b−3p, rectal
(CIG_current = 0; N0 = 448)
1−sided adj pval: 0
Frequency
0
5
10
15
20
25
30
−2.0
−1.5
−1.0
−0.5
0.0
0.5
1.0
Tumor−Normal Expression Difference
hsa−miR−20b−3p, rectal
(CIG_current = 1; N1 = 90)
1−sided adj pval: 0.124
Frequency
0
1
2
3
4
−2.0
−1.5
−1.0
−0.5
0.0
0.5
1.0
Tumor−Normal Expression Difference

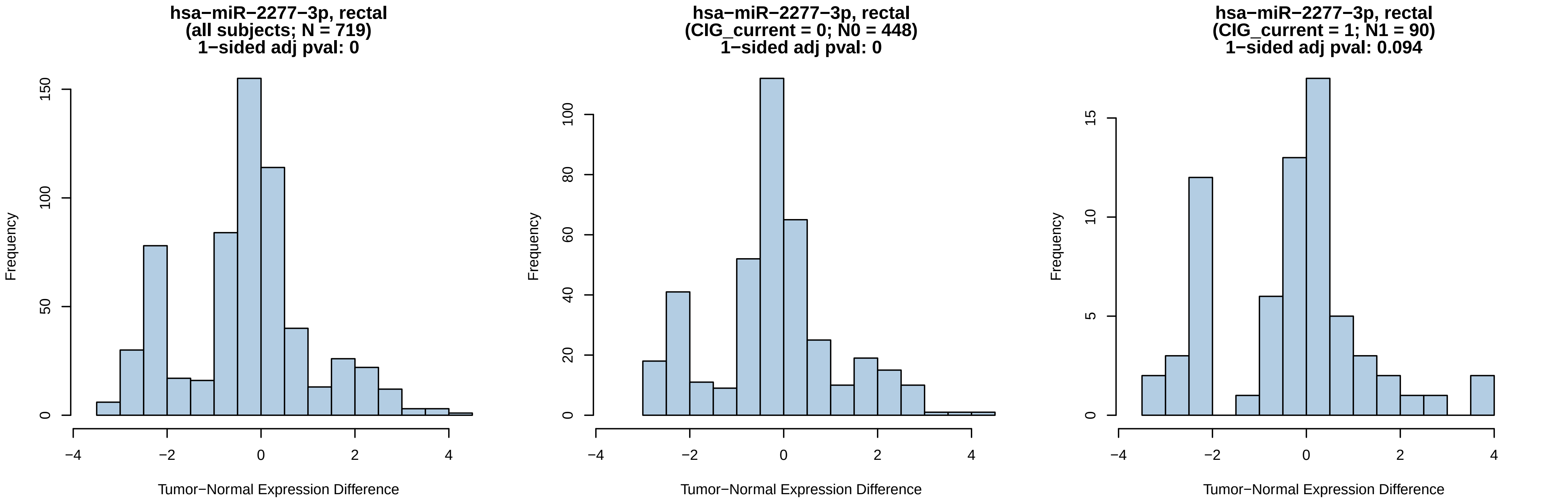
hsa−miR−2277−3p, rectal
(all subjects; N = 719)
1−sided adj pval: 0
Frequency
0
50
100
150
−4
−2
0
2
4
Tumor−Normal Expression Difference
hsa−miR−2277−3p, rectal
(CIG_current = 0; N0 = 448)
1−sided adj pval: 0
Frequency
0
20
40
60
80
100
−4
−2
0
2
4
Tumor−Normal Expression Difference
hsa−miR−2277−3p, rectal
(CIG_current = 1; N1 = 90)
1−sided adj pval: 0.094
Frequency
0
5
10
15
−4
−2
0
2
4
Tumor−Normal Expression Difference

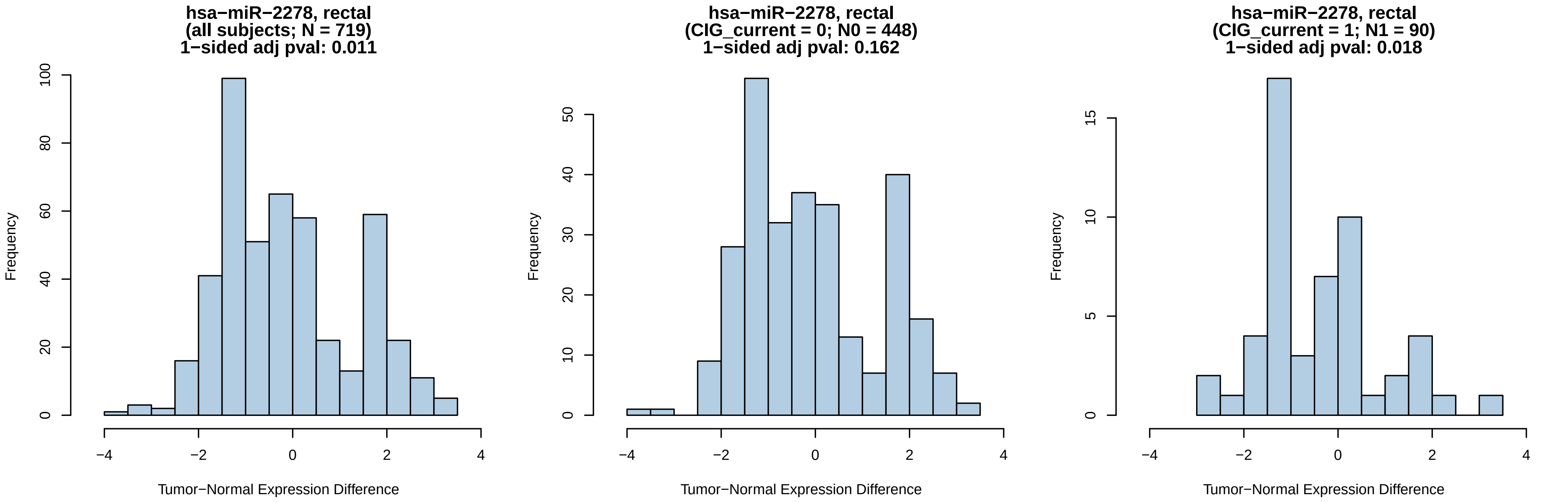

hsa−miR−2278, rectal
(all subjects; N = 719)
1−sided adj pval: 0.011
Frequency
0
20
40
60
80
100
−4
−2
0
2
4
Tumor−Normal Expression Difference
hsa−miR−2278, rectal
(CIG_current = 0; N0 = 448)
1−sided adj pval: 0.162
Frequency
0
10
20
30
40
50
−4
−2
0
2
4
Tumor−Normal Expression Difference
hsa−miR−2278, rectal
(CIG_current = 1; N1 = 90)
1−sided adj pval: 0.018
Frequency
0
5
10
15
−4
−2
0
2
4
Tumor−Normal Expression Difference

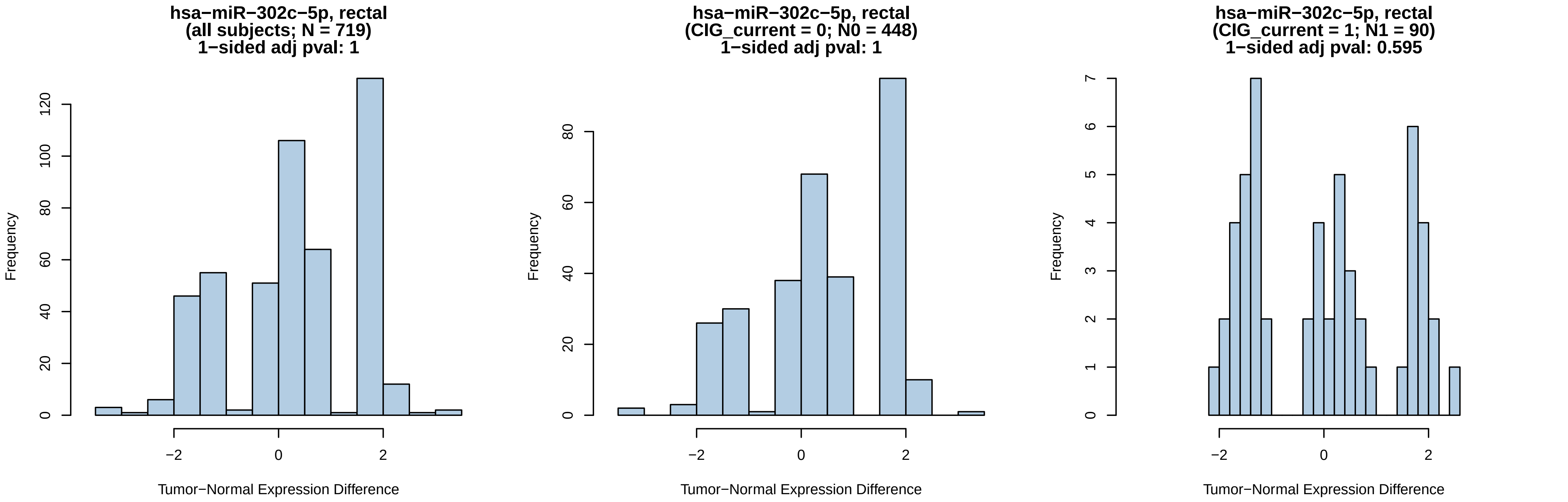

hsa−miR−302c−5p, rectal
(all subjects; N = 719)
1−sided adj pval: 1
Frequency
Tumor−Normal Expression Difference
hsa−miR−302c−5p, rectal
(CIG_current = 0; N0 = 448)
1−sided adj pval: 1
Frequency
Tumor−Normal Expression Difference
hsa−miR−302c−5p, rectal
(CIG_current = 1; N1 = 90)
1−sided adj pval: 0.595
Frequency
Tumor−Normal Expression Difference

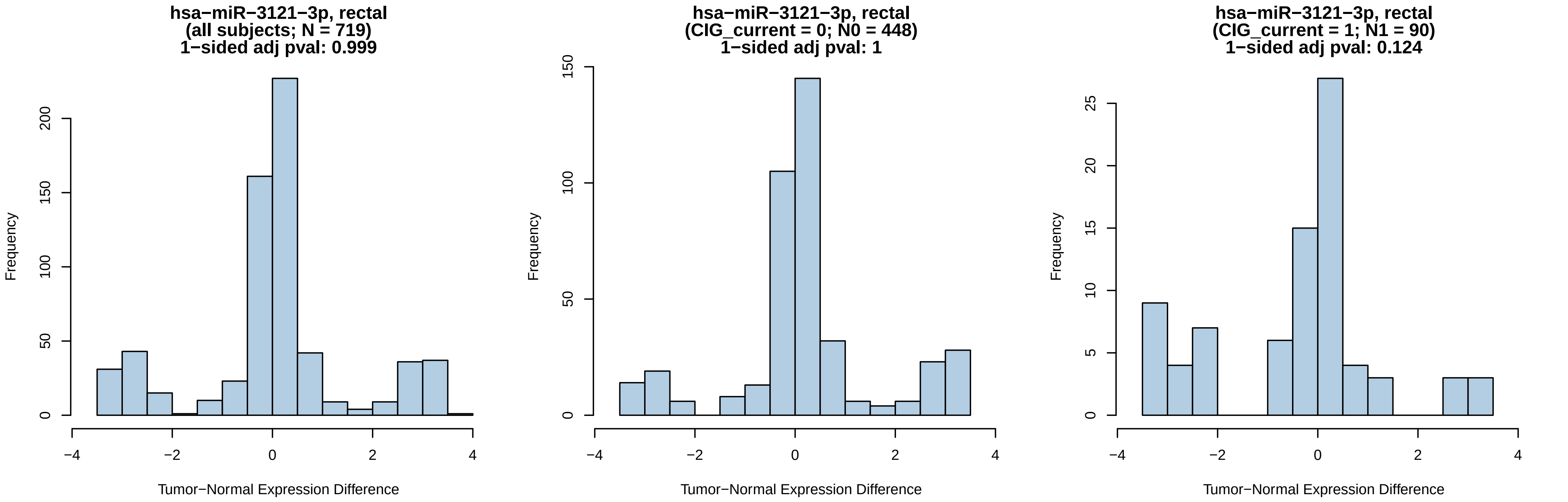

hsa−miR−3121−3p, rectal
(all subjects; N = 719)
1−sided adj pval: 0.999
Frequency
Tumor−Normal Expression Difference
hsa−miR−3121−3p, rectal
(CIG_current = 0; N0 = 448)
1−sided adj pval: 1
Frequency
Tumor−Normal Expression Difference
hsa−miR−3121−3p, rectal
(CIG_current = 1; N1 = 90)
1−sided adj pval: 0.124
Frequency
Tumor−Normal Expression Difference

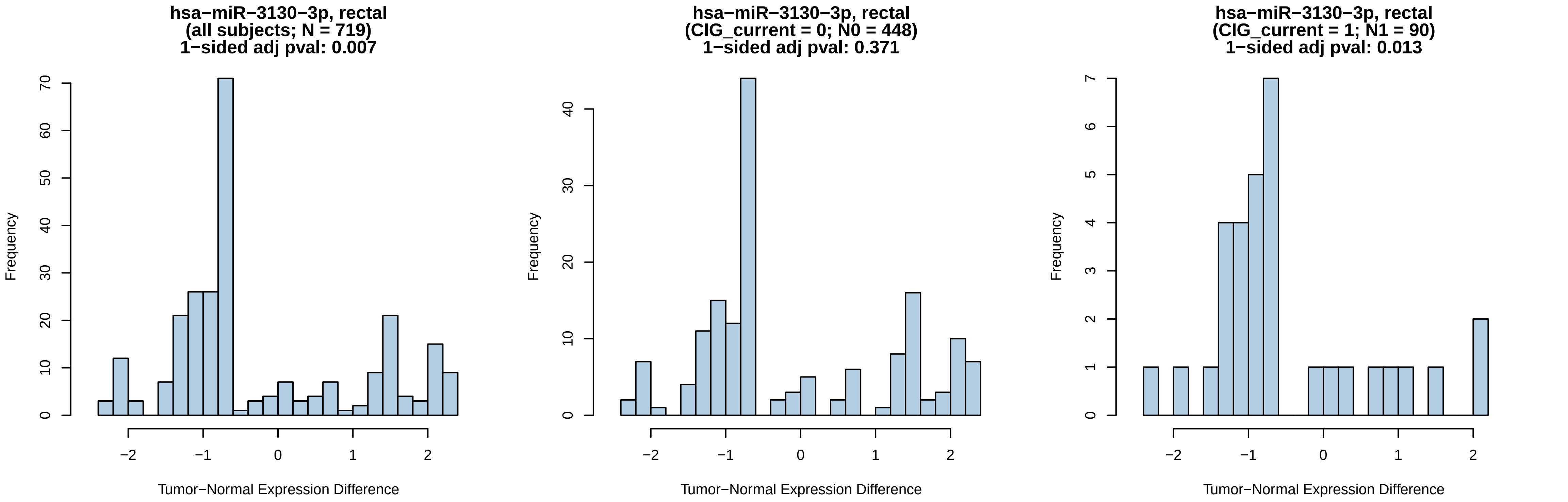

hsa−miR−3130−3p, rectal
(all subjects; N = 719)
1−sided adj pval: 0.007
hsa−miR−3130−3p, rectal
(CIG_current = 0; N0 = 448)
1−sided adj pval: 0.371
hsa−miR−3130−3p, rectal
(CIG_current = 1; N1 = 90)
1−sided adj pval: 0.013
Frequency
Tumor−Normal Expression Difference

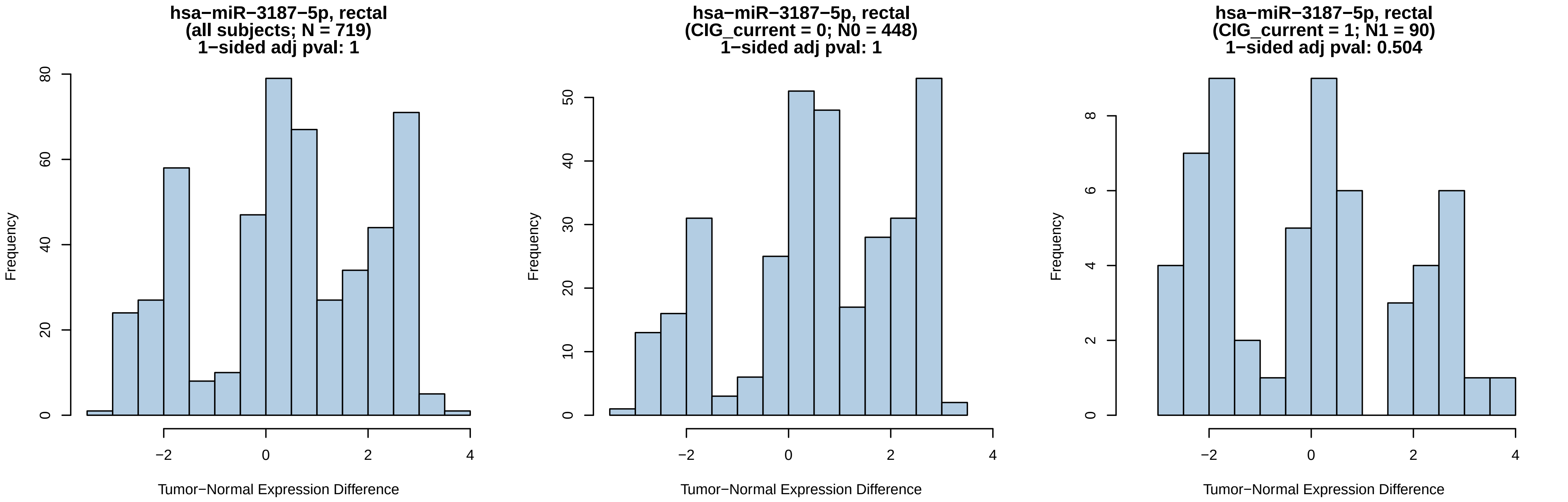

hsa−miR−3187−5p, rectal
(all subjects; N = 719)
1−sided adj pval: 1
Frequency
0
20
40
60
80
−2
0
2
4
Tumor−Normal Expression Difference
hsa−miR−3187−5p, rectal
(CIG_current = 0; N0 = 448)
1−sided adj pval: 1
Frequency
0
10
20
30
40
50
−2
0
2
4
Tumor−Normal Expression Difference
hsa−miR−3187−5p, rectal
(CIG_current = 1; N1 = 90)
1−sided adj pval: 0.504
Frequency
0
2
4
6
8
−2
0
2
4
Tumor−Normal Expression Difference

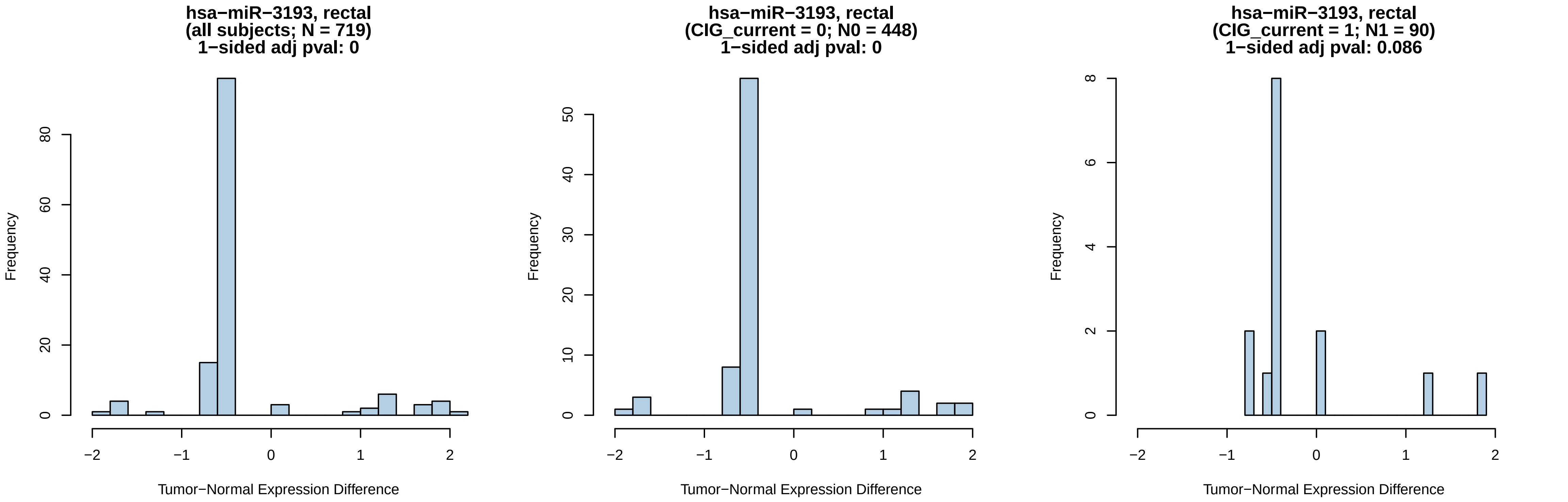
hsa−miR−3193, rectal
(all subjects; N = 719)
1−sided adj pval: 0
Frequency
Tumor−Normal Expression Difference
hsa−miR−3193, rectal
(CIG_current = 0; N0 = 448)
1−sided adj pval: 0
Frequency
Tumor−Normal Expression Difference
hsa−miR−3193, rectal
(CIG_current = 1; N1 = 90)
1−sided adj pval: 0.086
Frequency
Tumor−Normal Expression Difference

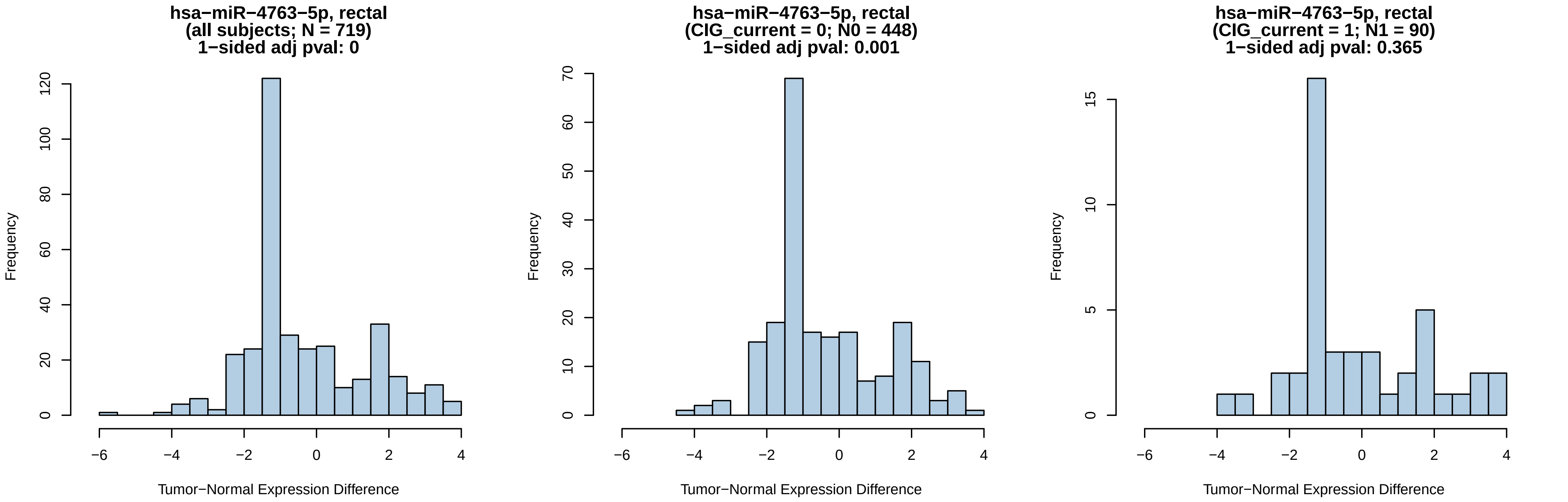
hsa−miR−4763−5p, rectal
(all subjects; N = 719)
1−sided adj pval: 0
Frequency
Tumor−Normal Expression Difference
hsa−miR−4763−5p, rectal
(CIG_current = 0; N0 = 448)
1−sided adj pval: 0.001
Frequency
Tumor−Normal Expression Difference
hsa−miR−4763−5p, rectal
(CIG_current = 1; N1 = 90)
1−sided adj pval: 0.365
Frequency
Tumor−Normal Expression Difference

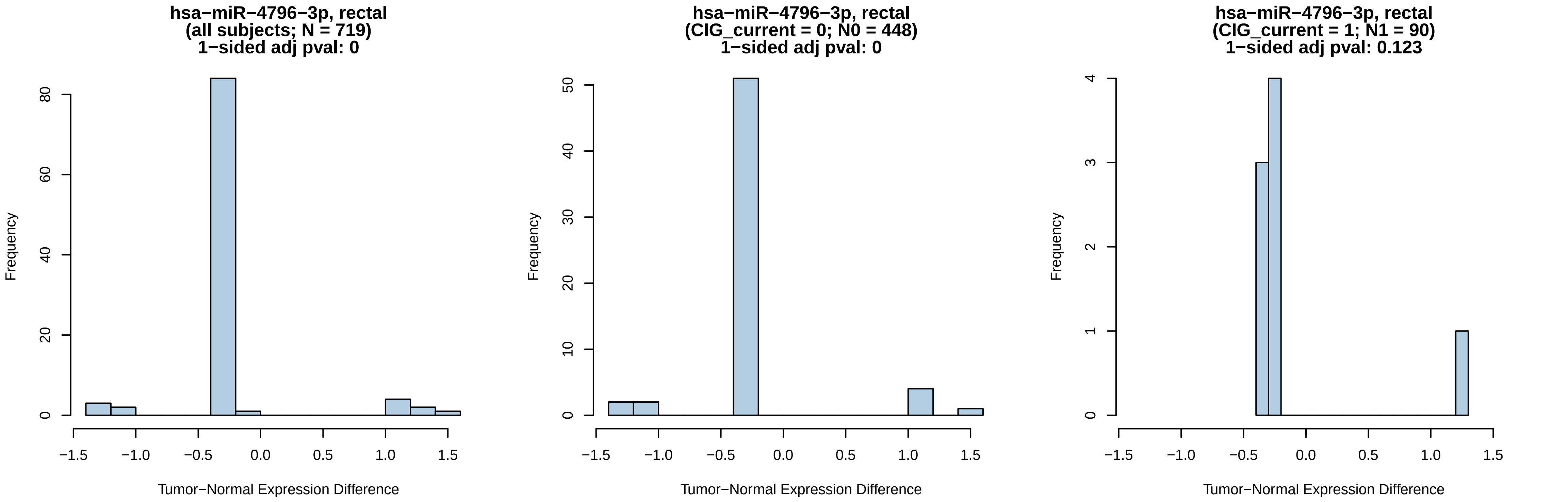
hsa−miR−4796−3p, rectal
(all subjects; N = 719)
1−sided adj pval: 0
Frequency
0
20
40
60
80
−1.5
−1.0
−0.5
0.0
0.5
1.0
1.5
Tumor−Normal Expression Difference
hsa−miR−4796−3p, rectal
(CIG_current = 0; N0 = 448)
1−sided adj pval: 0
Frequency
0
10
20
30
40
50
−1.5
−1.0
−0.5
0.0
0.5
1.0
1.5
Tumor−Normal Expression Difference
hsa−miR−4796−3p, rectal
(CIG_current = 1; N1 = 90)
1−sided adj pval: 0.123
Frequency
0
1
2
3
4
−1.5
−1.0
−0.5
0.0
0.5
1.0
1.5
Tumor−Normal Expression Difference

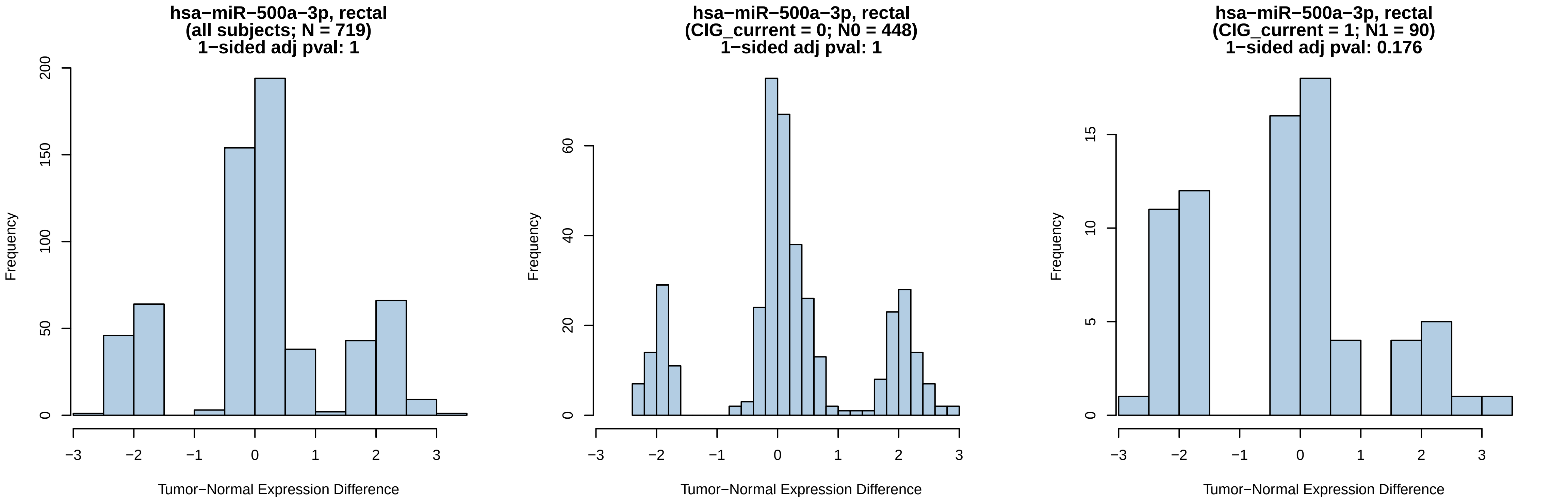
hsa−miR−500a−3p, rectal
(all subjects; N = 719)
1−sided adj pval: 1
Frequency
Tumor−Normal Expression Difference
0
50
100
150
200
−3
−2
−1
0
1
2
3
hsa−miR−500a−3p, rectal
(CIG_current = 0; N0 = 448)
1−sided adj pval: 1
Frequency
Tumor−Normal Expression Difference
0
20
40
60
−3
−2
−1
0
1
2
3
hsa−miR−500a−3p, rectal
(CIG_current = 1; N1 = 90)
1−sided adj pval: 0.176
Frequency
Tumor−Normal Expression Difference
0
5
10
15
−3
−2
−1
0
1
2
3

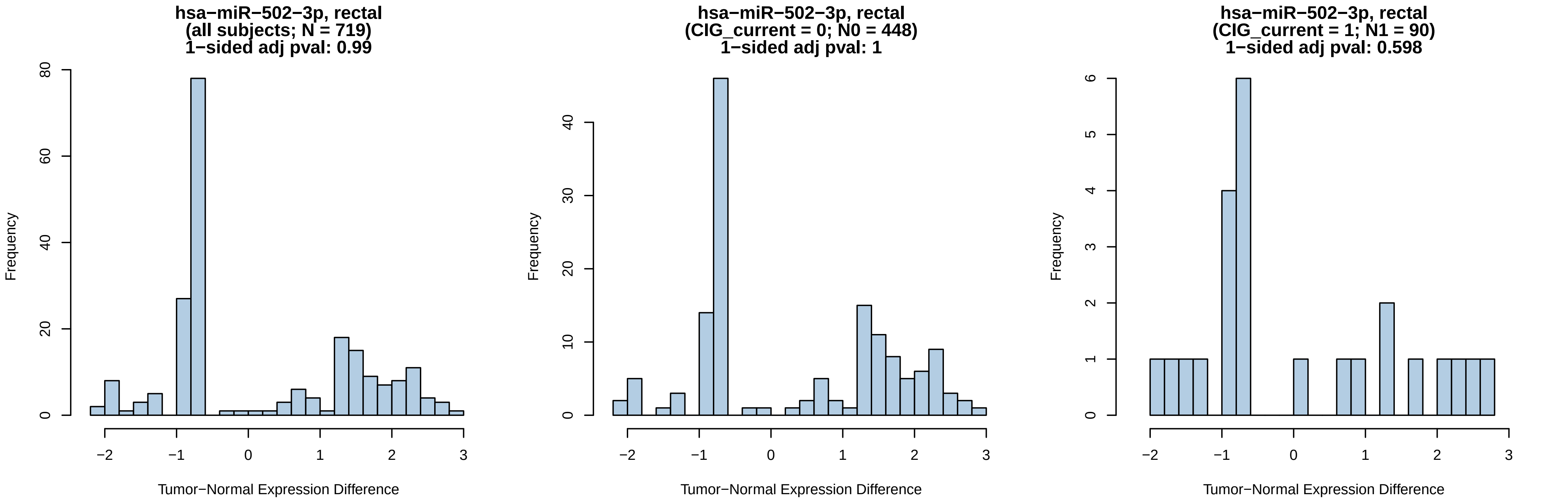

hsa−miR−502−3p, rectal
(all subjects; N = 719)
1−sided adj pval: 0.99
Frequency
0
20
40
60
80
−2
−1
0
1
2
3
Tumor−Normal Expression Difference
hsa−miR−502−3p, rectal
(CIG_current = 0; N0 = 448)
1−sided adj pval: 1
Frequency
0
10
20
30
40
−2
−1
0
1
2
3
Tumor−Normal Expression Difference
hsa−miR−502−3p, rectal
(CIG_current = 1; N1 = 90)
1−sided adj pval: 0.598
Frequency
0
1
2
3
4
5
6
−2
−1
0
1
2
3
Tumor−Normal Expression Difference

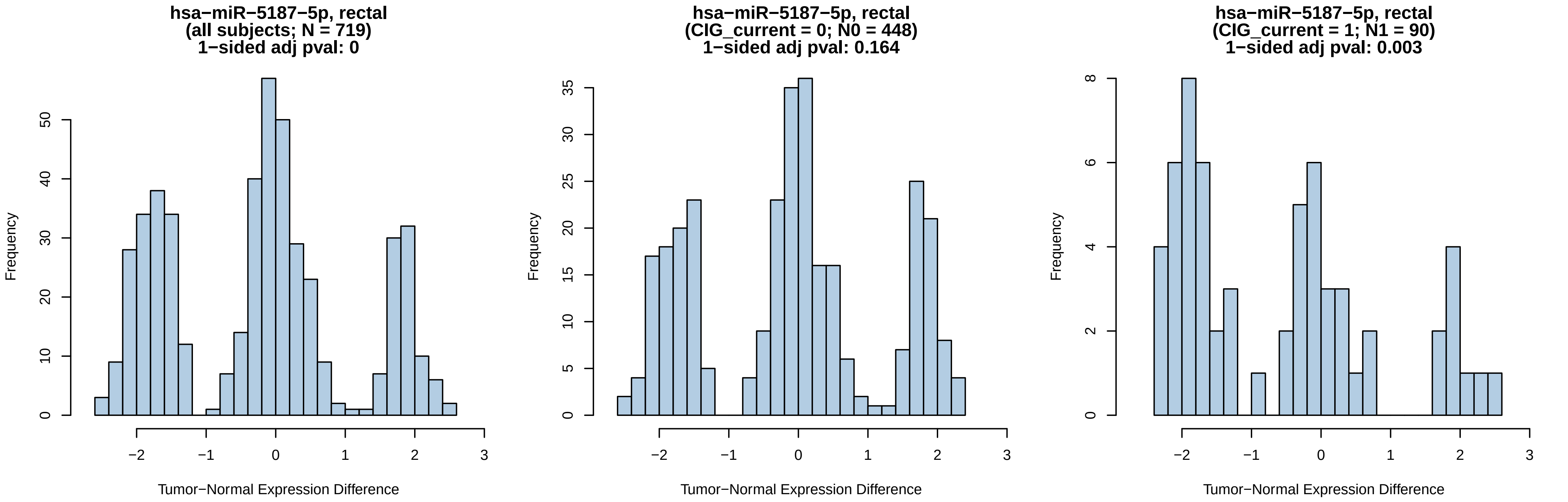
hsa−miR−5187−5p, rectal
(all subjects; N = 719)
1−sided adj pval: 0
Frequency
Tumor−Normal Expression Difference
hsa−miR−5187−5p, rectal
(CIG_current = 0; N0 = 448)
1−sided adj pval: 0.164
Frequency
Tumor−Normal Expression Difference
hsa−miR−5187−5p, rectal
(CIG_current = 1; N1 = 90)
1−sided adj pval: 0.003
Frequency
Tumor−Normal Expression Difference

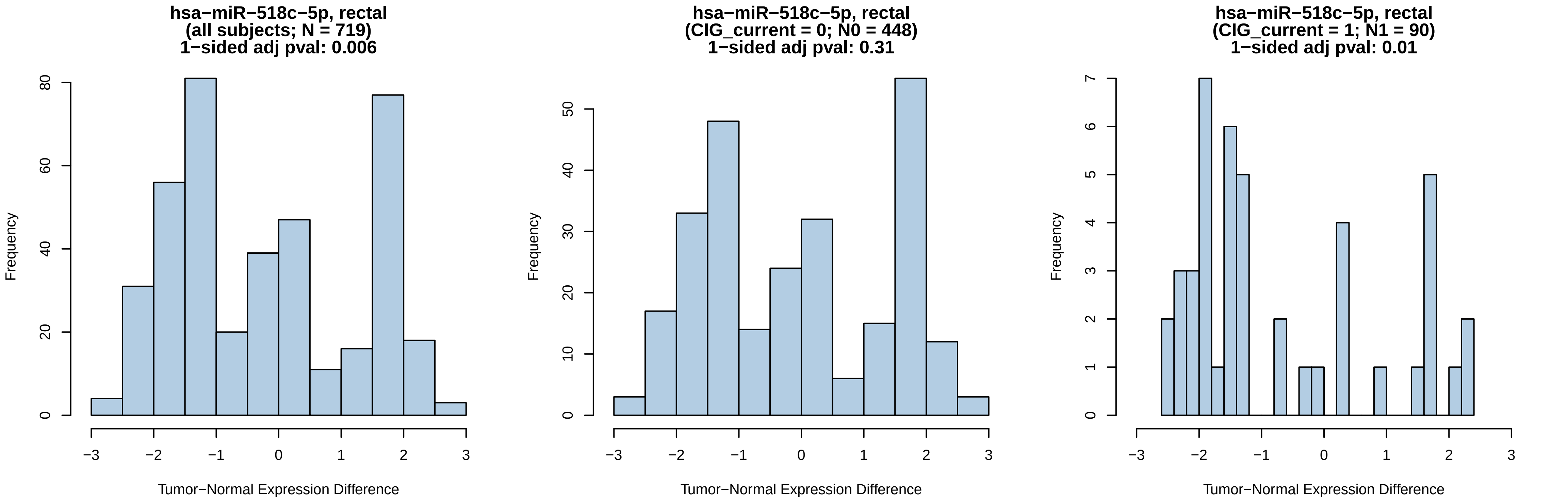
hsa−miR−518c−5p, rectal
(all subjects; N = 719)
1−sided adj pval: 0.006
Frequency
Tumor−Normal Expression Difference
hsa−miR−518c−5p, rectal
(CIG_current = 0; N0 = 448)
1−sided adj pval: 0.31
Frequency
Tumor−Normal Expression Difference
hsa−miR−518c−5p, rectal
(CIG_current = 1; N1 = 90)
1−sided adj pval: 0.01
Frequency
Tumor−Normal Expression Difference

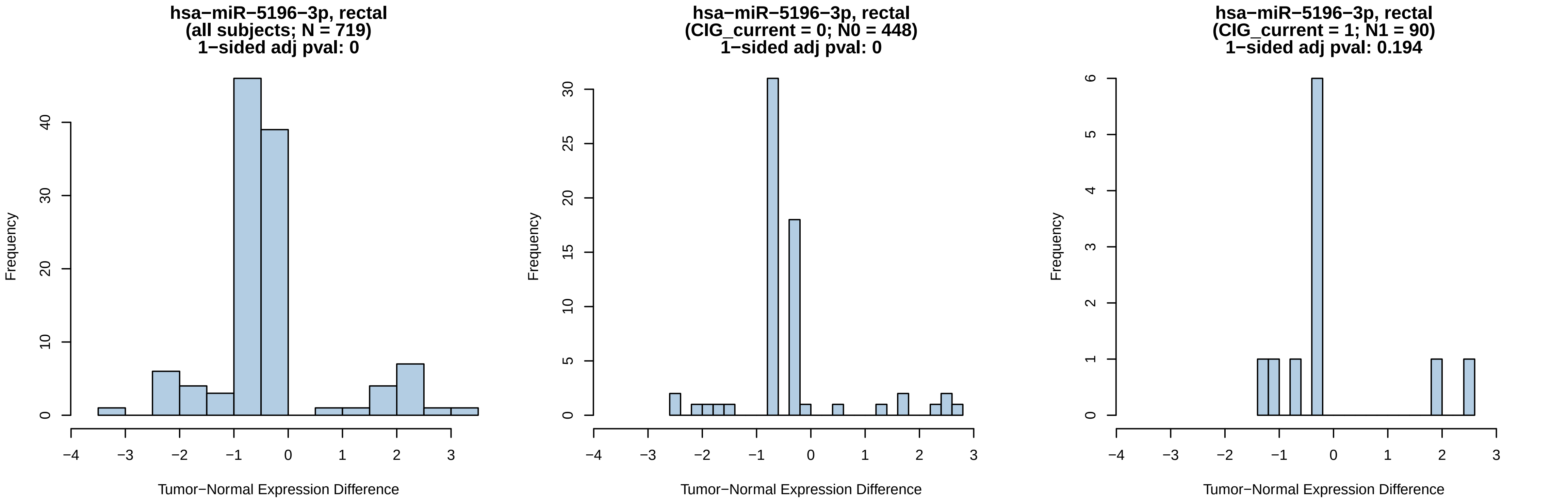
hsa−miR−5196−3p, rectal
(all subjects; N = 719)
1−sided adj pval: 0
Frequency
Tumor−Normal Expression Difference
hsa−miR−5196−3p, rectal
(CIG_current = 0; N0 = 448)
1−sided adj pval: 0
Frequency
Tumor−Normal Expression Difference
hsa−miR−5196−3p, rectal
(CIG_current = 1; N1 = 90)
1−sided adj pval: 0.194
Frequency
Tumor−Normal Expression Difference

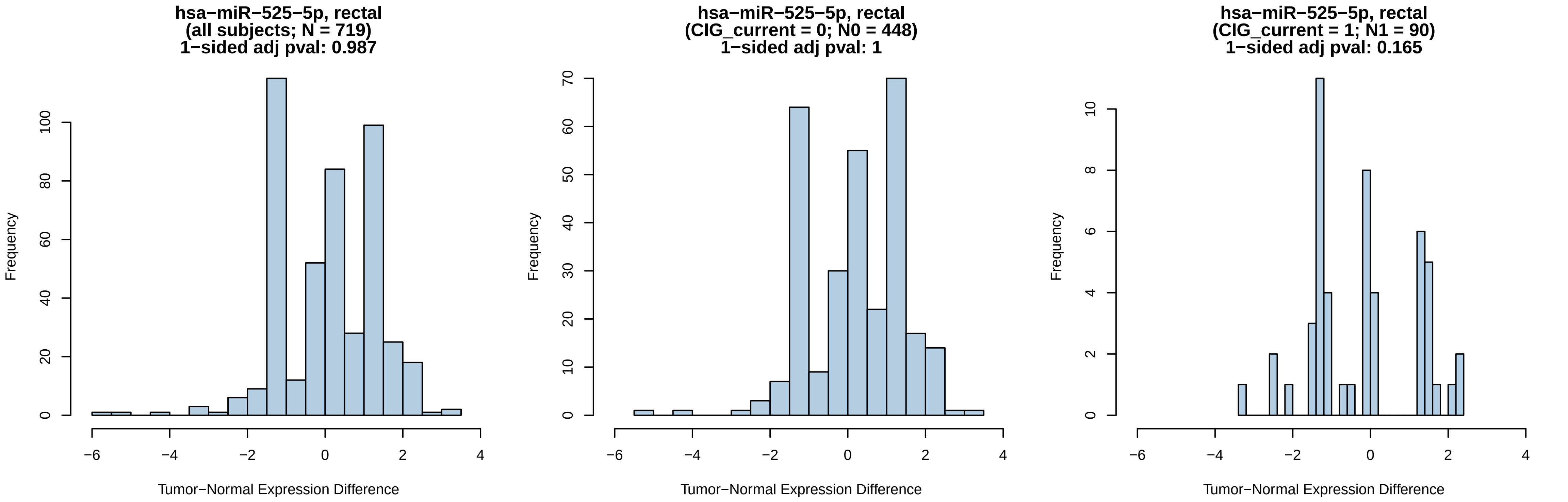

hsa−miR−525−5p, rectal
(all subjects; N = 719)
1−sided adj pval: 0.987
Frequency
Tumor−Normal Expression Difference
hsa−miR−525−5p, rectal
(CIG_current = 0; N0 = 448)
1−sided adj pval: 1
Frequency
Tumor−Normal Expression Difference
hsa−miR−525−5p, rectal
(CIG_current = 1; N1 = 90)
1−sided adj pval: 0.165
Frequency
Tumor−Normal Expression Difference

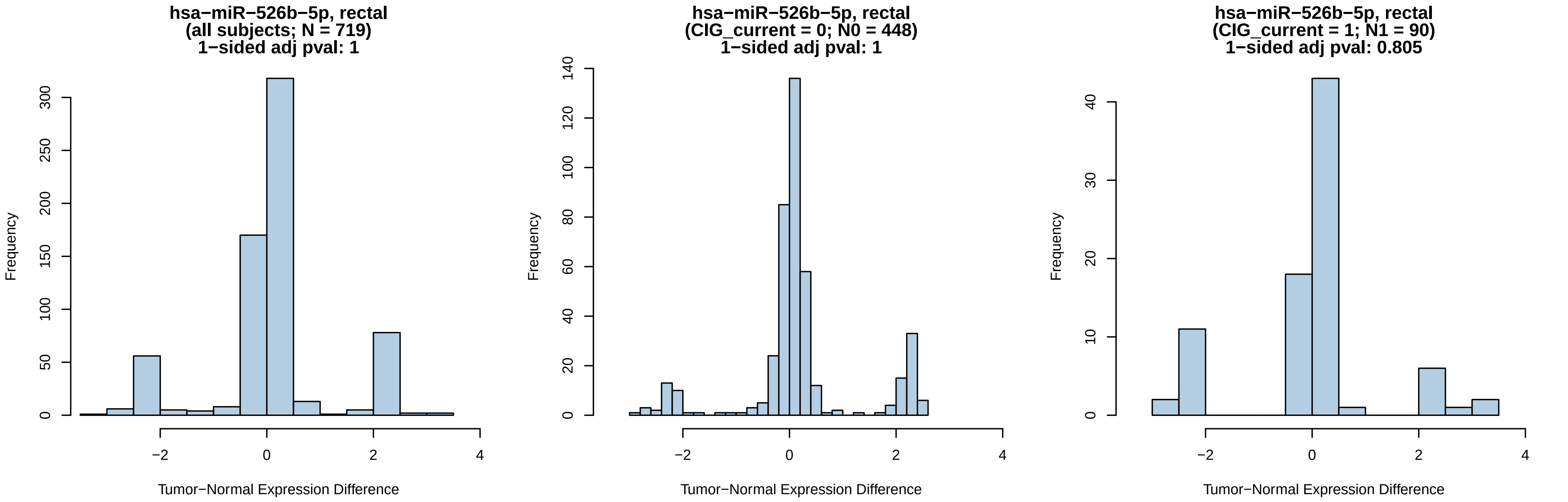

hsa−miR−526b−5p, rectal
(all subjects; N = 719)
1−sided adj pval: 1
Frequency
Tumor−Normal Expression Difference
hsa−miR−526b−5p, rectal
(CIG_current = 0; N0 = 448)
1−sided adj pval: 1
Frequency
Tumor−Normal Expression Difference
hsa−miR−526b−5p, rectal
(CIG_current = 1; N1 = 90)
1−sided adj pval: 0.805
Frequency
Tumor−Normal Expression Difference

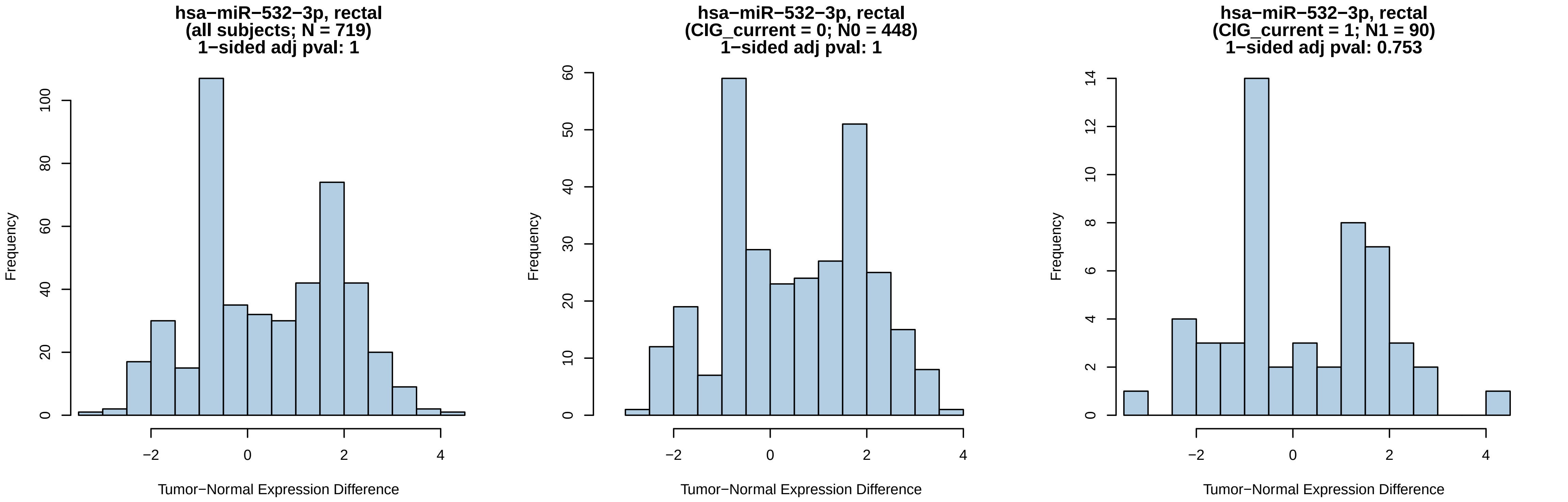

hsa−miR−532−3p, rectal
(all subjects; N = 719)
1−sided adj pval: 1
Frequency
0
20
40
60
80
100
−2
0
2
4
Tumor−Normal Expression Difference
hsa−miR−532−3p, rectal
(CIG_current = 0; N0 = 448)
1−sided adj pval: 1
Frequency
0
10
20
30
40
50
60
−2
0
2
4
Tumor−Normal Expression Difference
hsa−miR−532−3p, rectal
(CIG_current = 1; N1 = 90)
1−sided adj pval: 0.753
Frequency
0
2
4
6
8
10
12
14
−2
0
2
4
Tumor−Normal Expression Difference

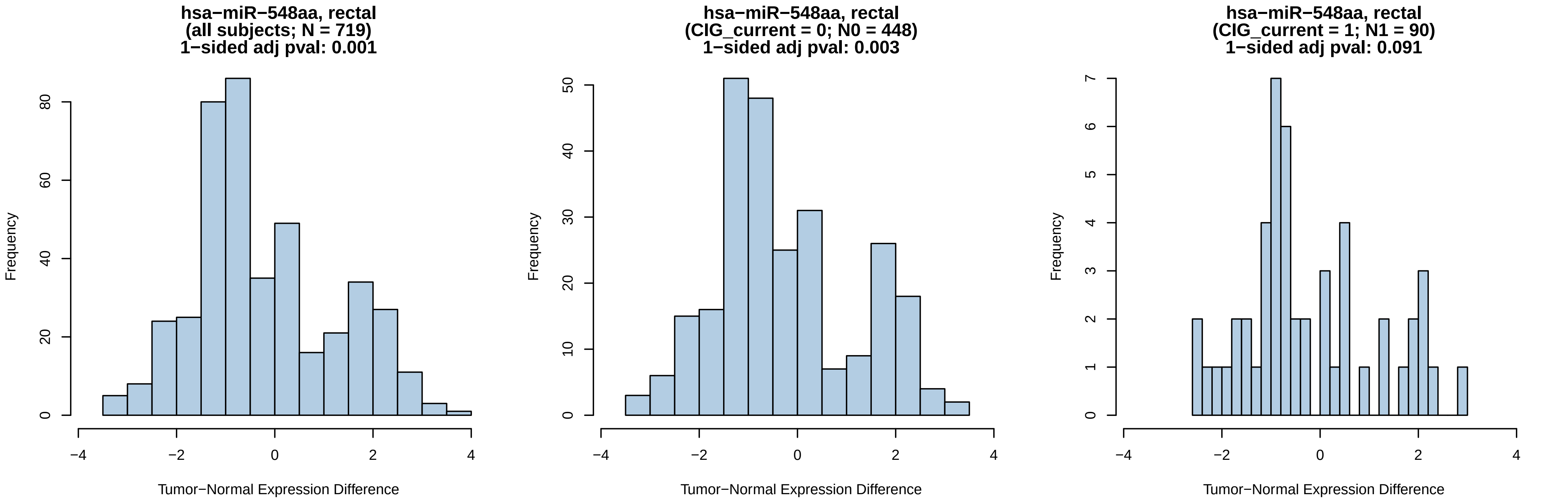

hsa−miR−548aa, rectal
(all subjects; N = 719)
1−sided adj pval: 0.001
Frequency
Tumor−Normal Expression Difference
hsa−miR−548aa, rectal
(CIG_current = 0; N0 = 448)
1−sided adj pval: 0.003
Frequency
Tumor−Normal Expression Difference
hsa−miR−548aa, rectal
(CIG_current = 1; N1 = 90)
1−sided adj pval: 0.091
Frequency
Tumor−Normal Expression Difference

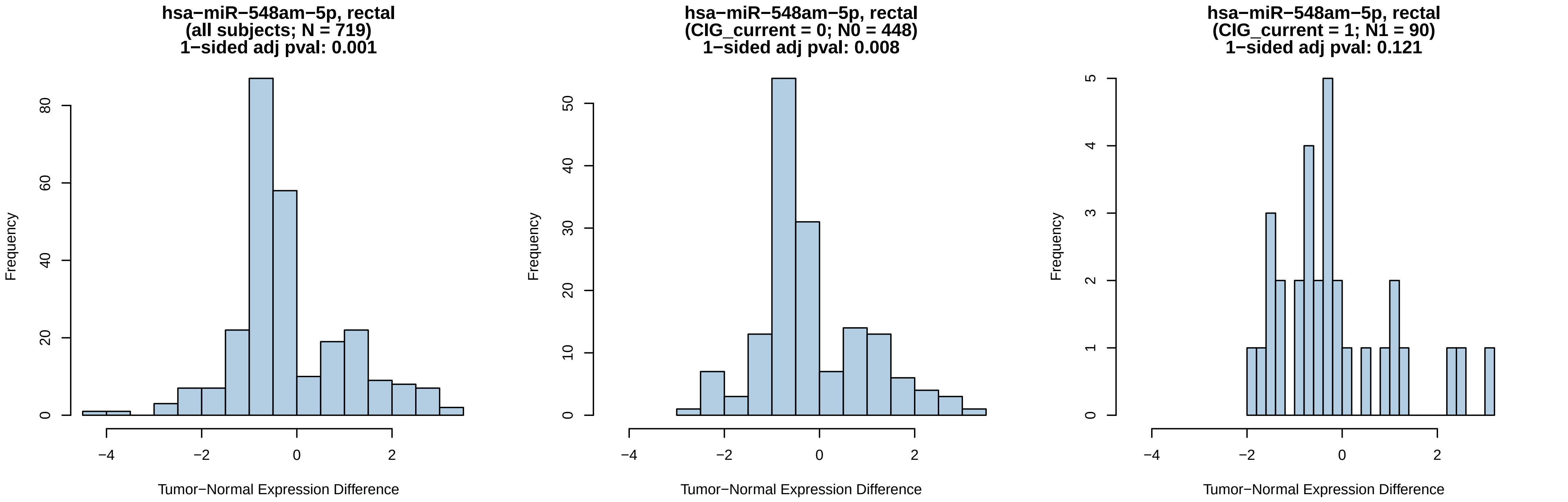

hsa−miR−548am−5p, rectal
(all subjects; N = 719)
1−sided adj pval: 0.001
Frequency
Tumor−Normal Expression Difference
hsa−miR−548am−5p, rectal
(CIG_current = 0; N0 = 448)
1−sided adj pval: 0.008
Frequency
Tumor−Normal Expression Difference
hsa−miR−548am−5p, rectal
(CIG_current = 1; N1 = 90)
1−sided adj pval: 0.121
Frequency
Tumor−Normal Expression Difference

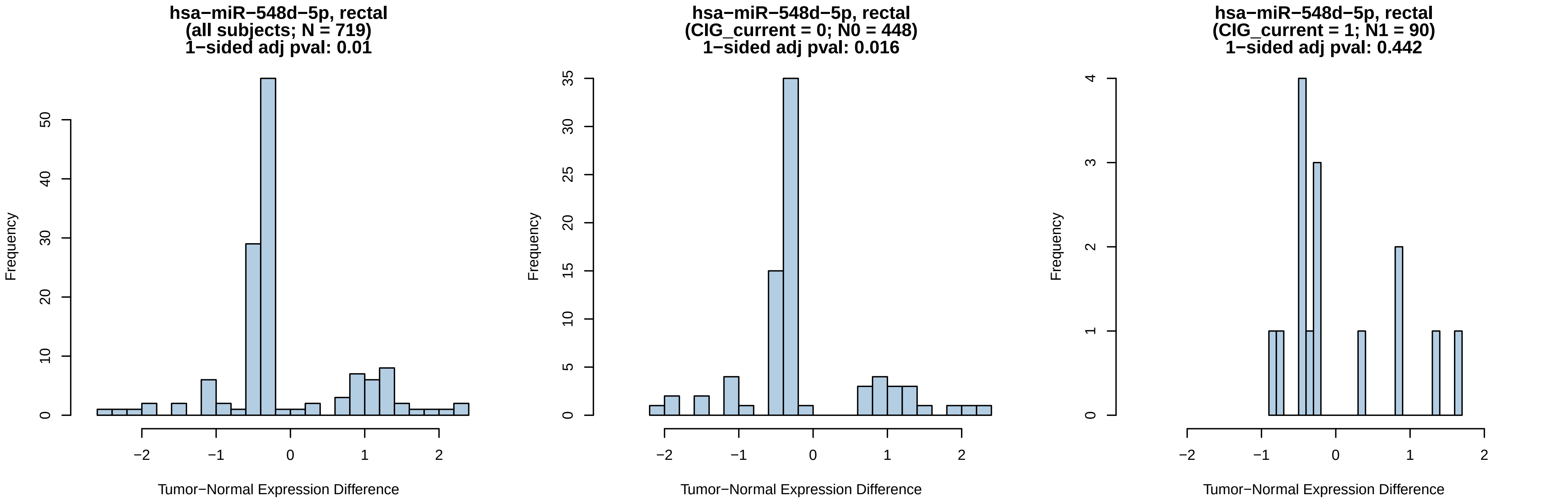
hsa−miR−548d−5p, rectal
(all subjects; N = 719)
1−sided adj pval: 0.01
Frequency
0
10
20
30
40
50
−2
−1
0
1
2
Tumor−Normal Expression Difference
hsa−miR−548d−5p, rectal
(CIG_current = 0; N0 = 448)
1−sided adj pval: 0.016
Frequency
0
5
10
15
20
25
30
35
−2
−1
0
1
2
Tumor−Normal Expression Difference
hsa−miR−548d−5p, rectal
(CIG_current = 1; N1 = 90)
1−sided adj pval: 0.442
Frequency
0
1
2
3
4
−2
−1
0
1
2
Tumor−Normal Expression Difference

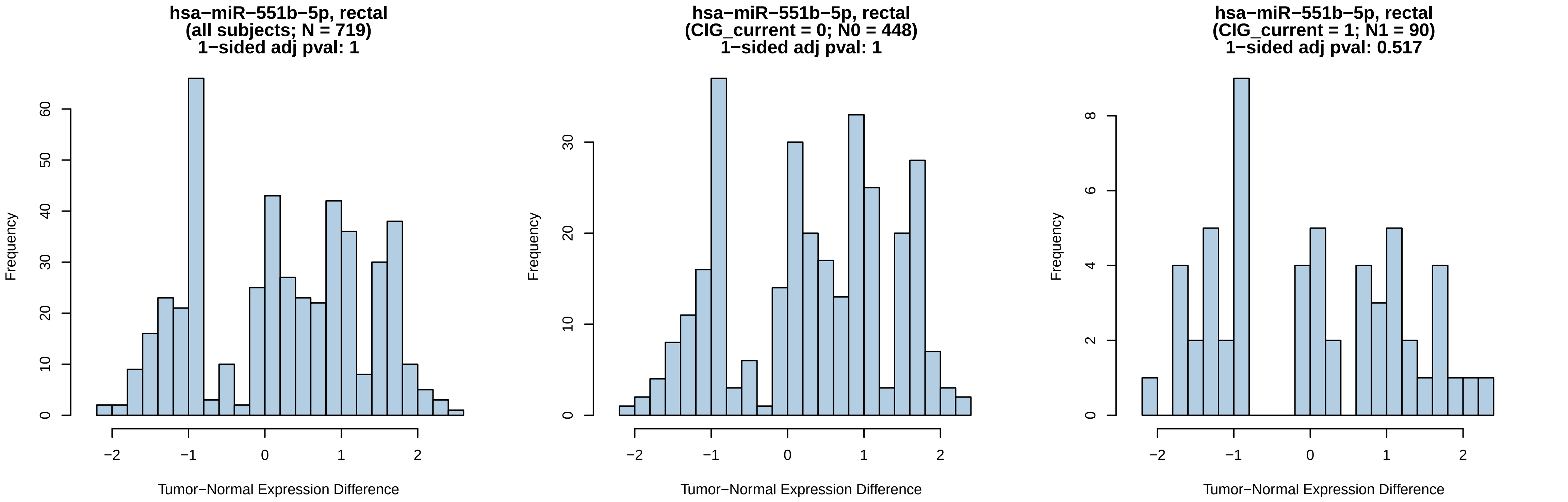
hsa−miR−551b−5p, rectal
(all subjects; N = 719)
1−sided adj pval: 1
Frequency
Tumor−Normal Expression Difference
hsa−miR−551b−5p, rectal
(CIG_current = 0; N0 = 448)
1−sided adj pval: 1
Frequency
Tumor−Normal Expression Difference
hsa−miR−551b−5p, rectal
(CIG_current = 1; N1 = 90)
1−sided adj pval: 0.517
Frequency
Tumor−Normal Expression Difference

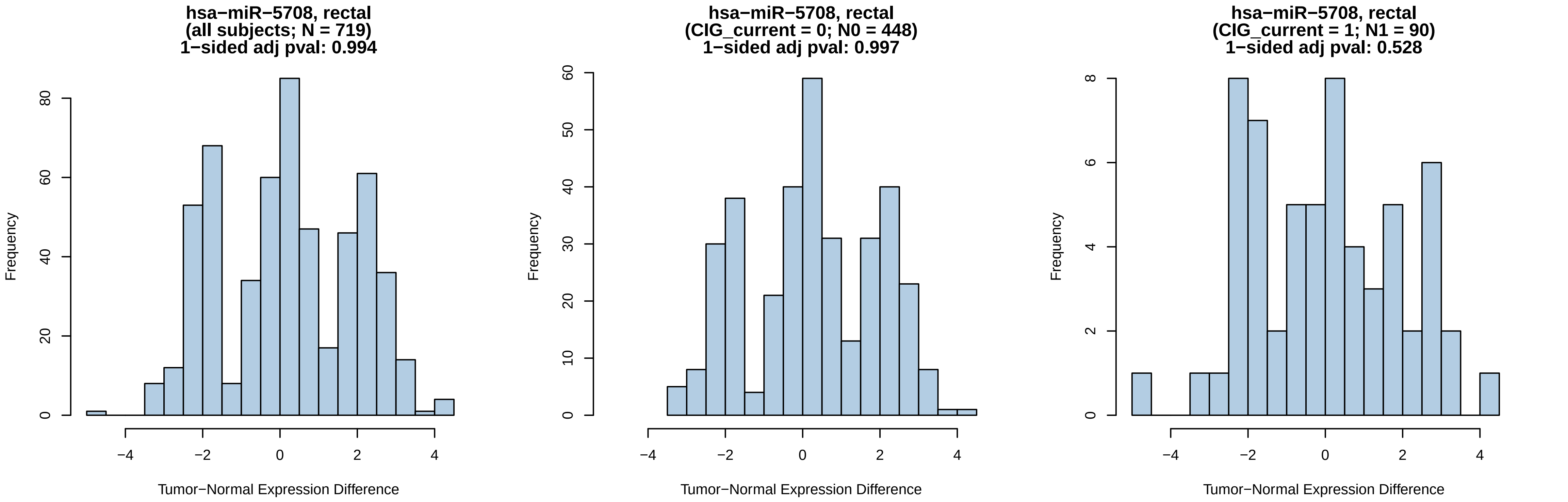

hsa−miR−5708, rectal
(all subjects; N = 719)
1−sided adj pval: 0.994
Frequency
0
20
40
60
80
−4
−2
0
2
4
Tumor−Normal Expression Difference
hsa−miR−5708, rectal
(CIG_current = 0; N0 = 448)
1−sided adj pval: 0.997
Frequency
0
10
20
30
40
50
60
−4
−2
0
2
4
Tumor−Normal Expression Difference
hsa−miR−5708, rectal
(CIG_current = 1; N1 = 90)
1−sided adj pval: 0.528
Frequency
0
2
4
6
8
−4
−2
0
2
4
Tumor−Normal Expression Difference

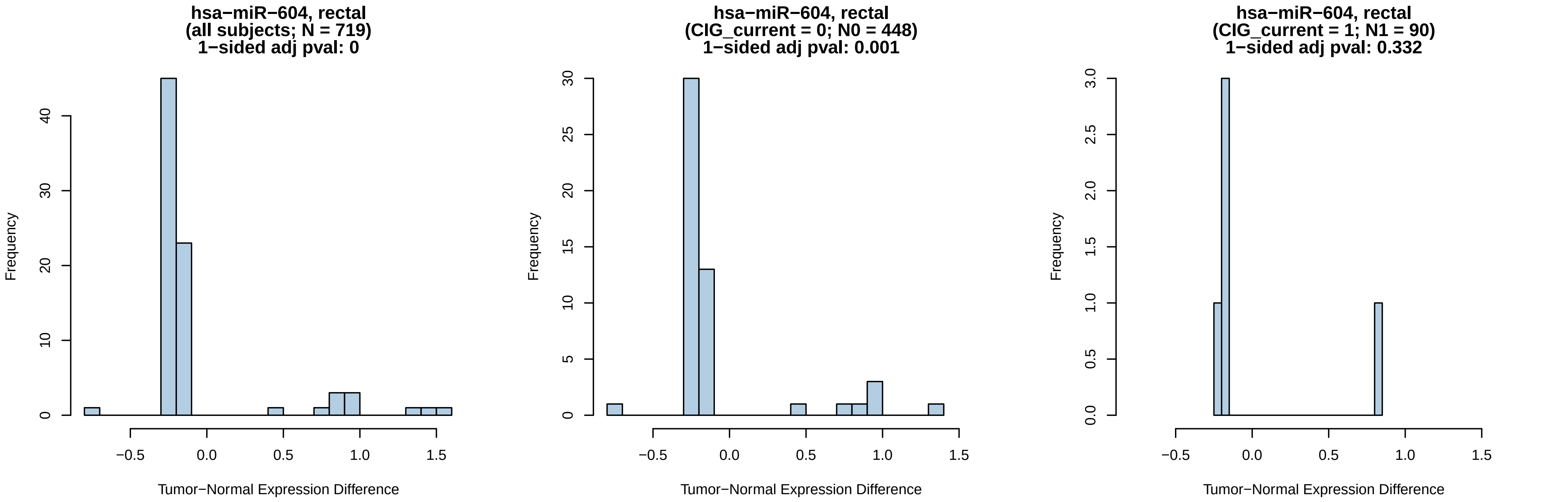
hsa−miR−604, rectal
(all subjects; N = 719)
1−sided adj pval: 0
Frequency
Tumor−Normal Expression Difference
hsa−miR−604, rectal
(CIG_current = 0; N0 = 448)
1−sided adj pval: 0.001
Frequency
Tumor−Normal Expression Difference
hsa−miR−604, rectal
(CIG_current = 1; N1 = 90)
1−sided adj pval: 0.332
Frequency
Tumor−Normal Expression Difference

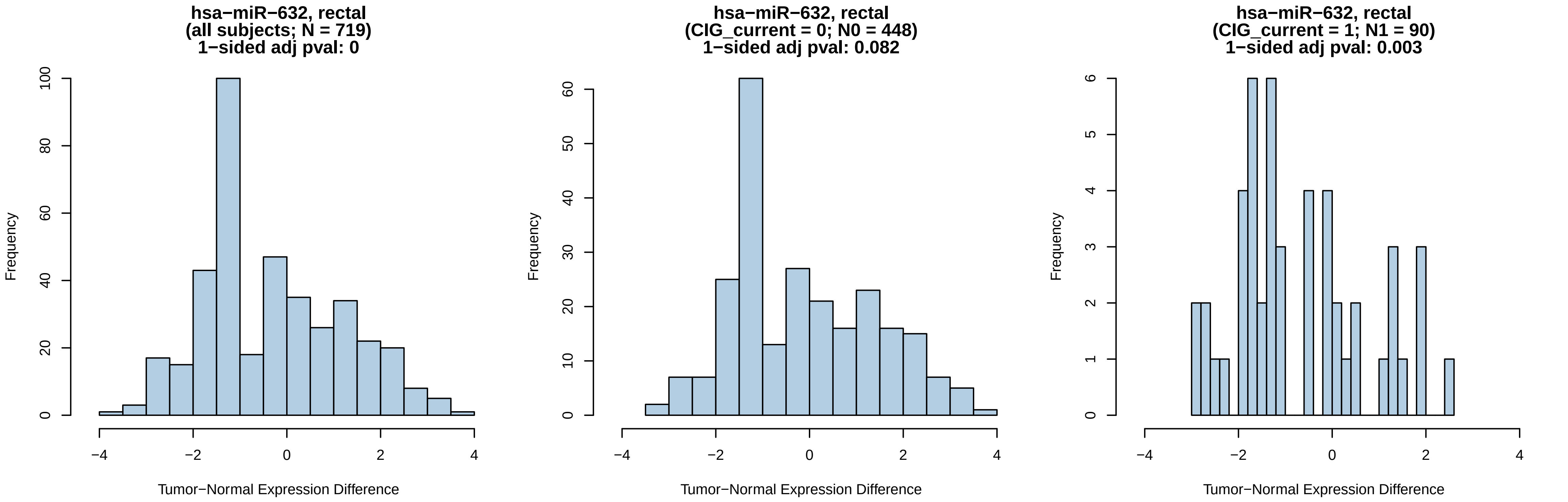
hsa−miR−632, rectal
(all subjects; N = 719)
1−sided adj pval: 0
Frequency
Tumor−Normal Expression Difference
hsa−miR−632, rectal
(CIG_current = 0; N0 = 448)
1−sided adj pval: 0.082
Frequency
Tumor−Normal Expression Difference
hsa−miR−632, rectal
(CIG_current = 1; N1 = 90)
1−sided adj pval: 0.003
Frequency
Tumor−Normal Expression Difference

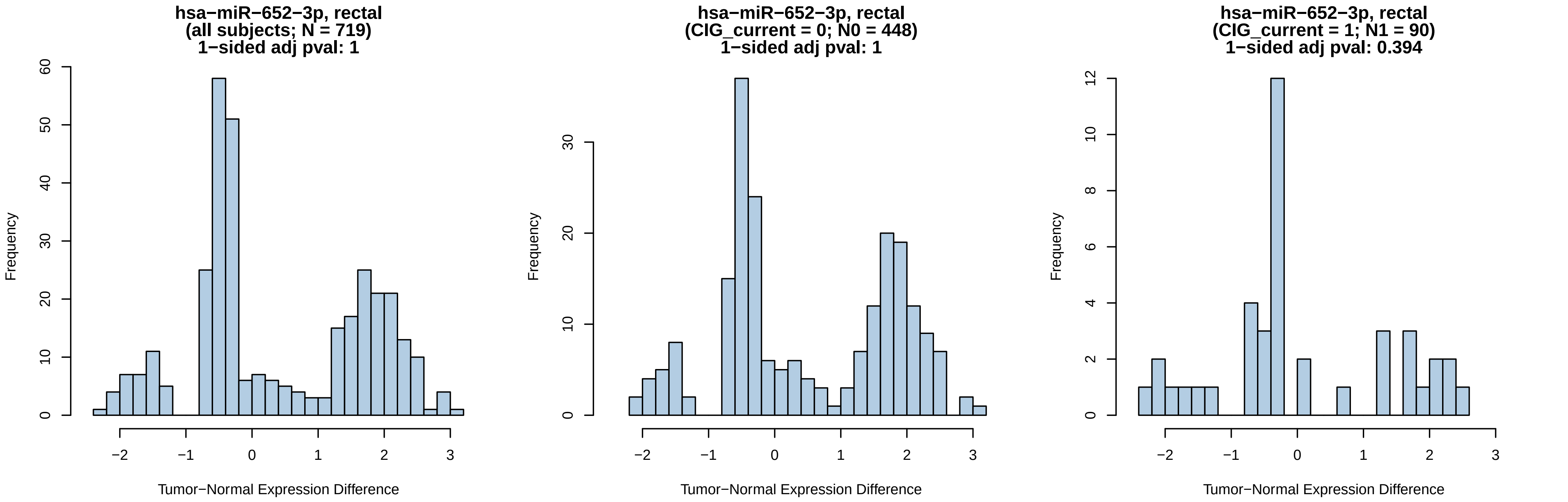
hsa−miR−652−3p, rectal
(all subjects; N = 719)
1−sided adj pval: 1
Frequency
Tumor−Normal Expression Difference
hsa−miR−652−3p, rectal
(CIG_current = 0; N0 = 448)
1−sided adj pval: 1
Frequency
Tumor−Normal Expression Difference
hsa−miR−652−3p, rectal
(CIG_current = 1; N1 = 90)
1−sided adj pval: 0.394
Frequency
Tumor−Normal Expression Difference

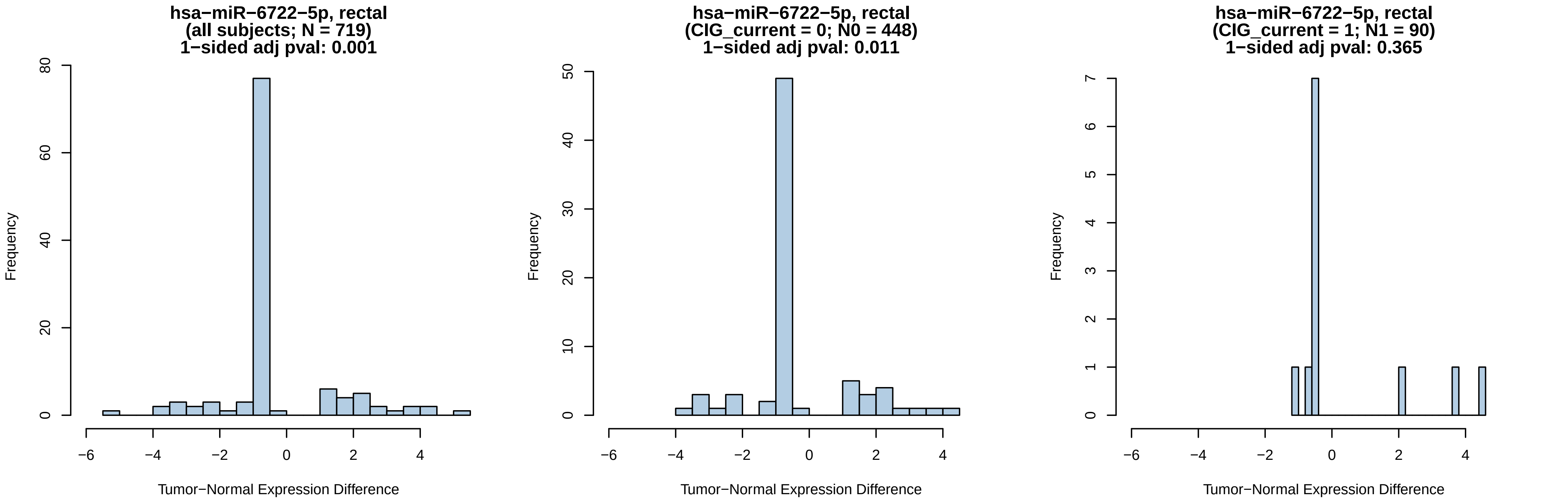
hsa−miR−6722−5p, rectal
(all subjects; N = 719)
1−sided adj pval: 0.001
Frequency
Tumor−Normal Expression Difference
hsa−miR−6722−5p, rectal
(CIG_current = 0; N0 = 448)
1−sided adj pval: 0.011
Frequency
Tumor−Normal Expression Difference
hsa−miR−6722−5p, rectal
(CIG_current = 1; N1 = 90)
1−sided adj pval: 0.365
Frequency
Tumor−Normal Expression Difference

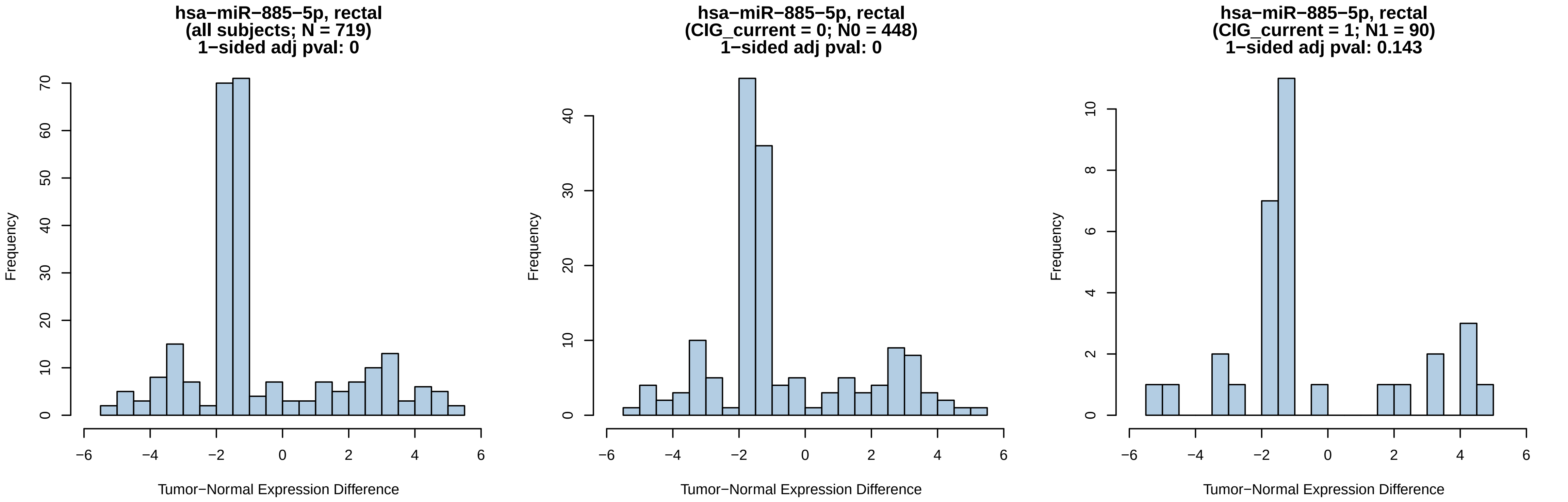

hsa−miR−885−5p, rectal
(all subjects; N = 719)
1−sided adj pval: 0
Frequency
Tumor−Normal Expression Difference
hsa−miR−885−5p, rectal
(CIG_current = 0; N0 = 448)
1−sided adj pval: 0
Frequency
Tumor−Normal Expression Difference
hsa−miR−885−5p, rectal
(CIG_current = 1; N1 = 90)
1−sided adj pval: 0.143
Frequency
Tumor−Normal Expression Difference

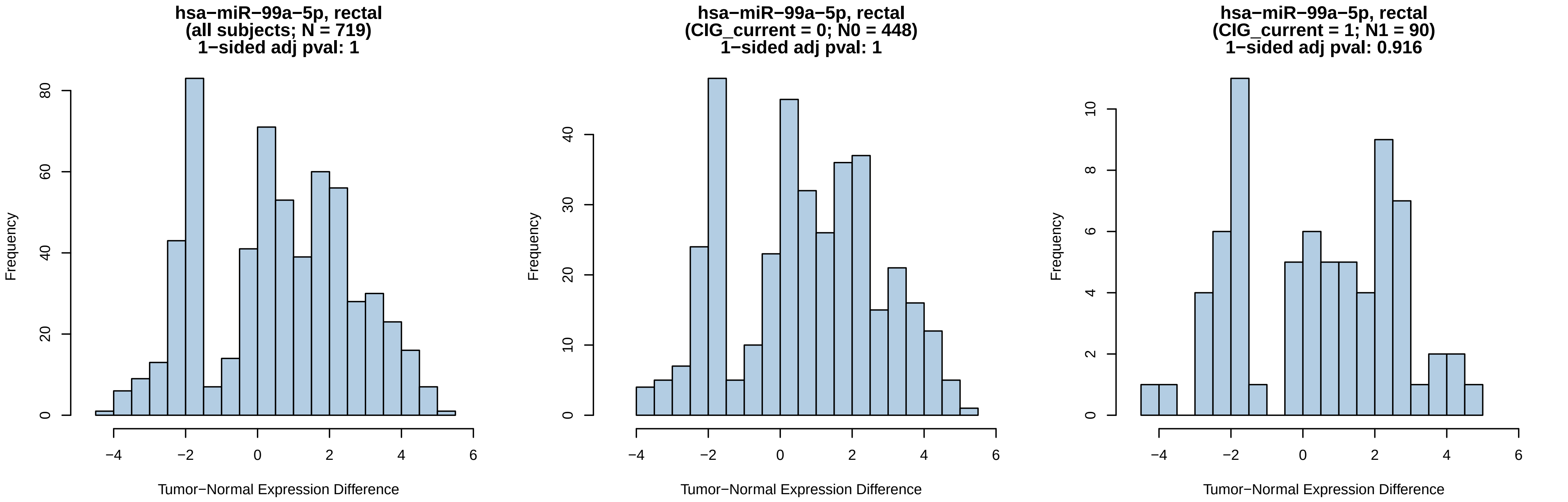

hsa−miR−99a−5p, rectal
(all subjects; N = 719)
1−sided adj pval: 1
hsa−miR−99a−5p, rectal
(CIG_current = 0; N0 = 448)
1−sided adj pval: 1
hsa−miR−99a−5p, rectal
(CIG_current = 1; N1 = 90)
1−sided adj pval: 0.916
Frequency
Tumor−Normal Expression Difference

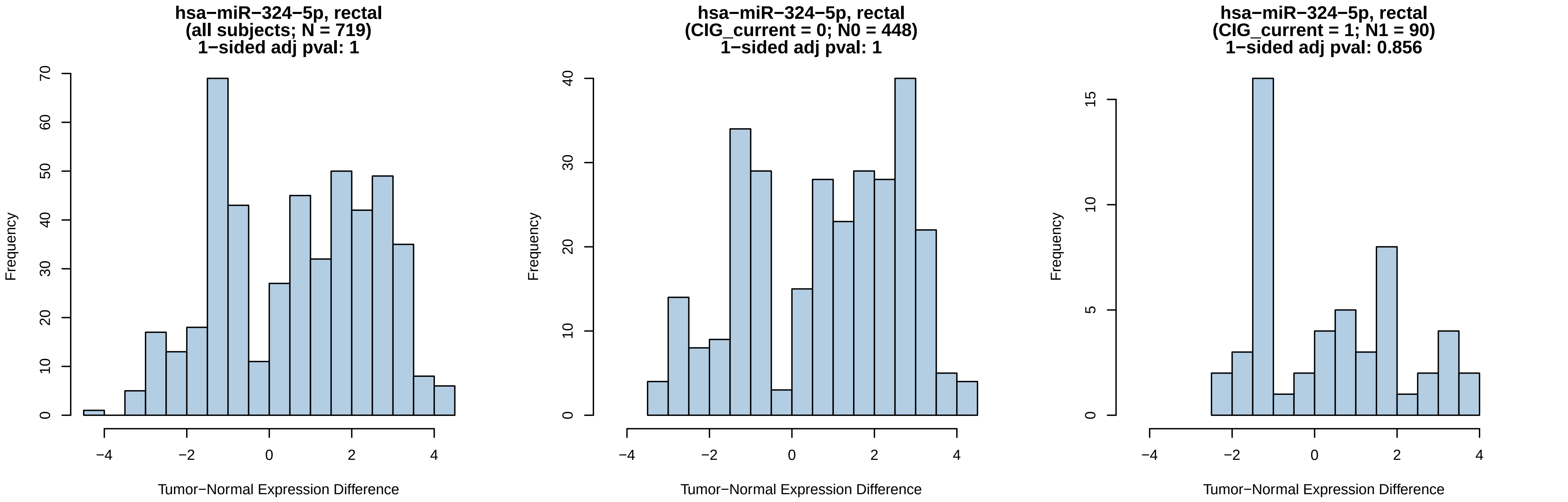
hsa−miR−324−5p, rectal
(all subjects; N = 719)
1−sided adj pval: 1
Frequency
Tumor−Normal Expression Difference
hsa−miR−324−5p, rectal
(CIG_current = 0; N0 = 448)
1−sided adj pval: 1
Frequency
Tumor−Normal Expression Difference
hsa−miR−324−5p, rectal
(CIG_current = 1; N1 = 90)
1−sided adj pval: 0.856
Frequency
Tumor−Normal Expression Difference

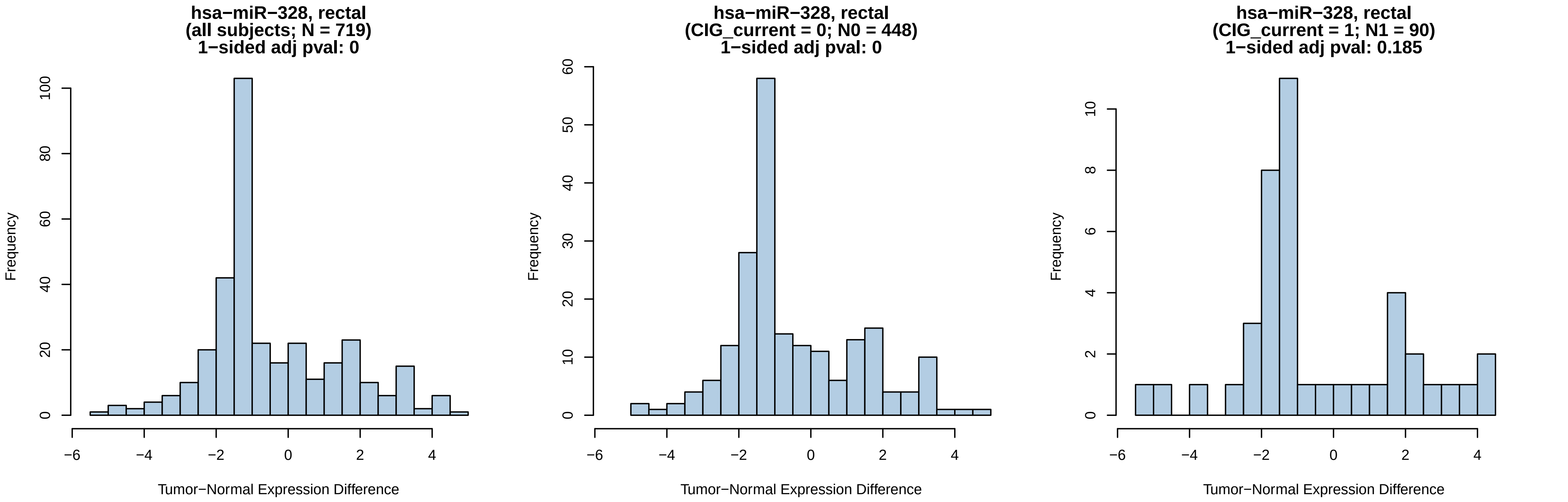
hsa−miR−328, rectal
(all subjects; N = 719)
1−sided adj pval: 0
Frequency
Tumor−Normal Expression Difference
hsa−miR−328, rectal
(CIG_current = 0; N0 = 448)
1−sided adj pval: 0
Frequency
Tumor−Normal Expression Difference
hsa−miR−328, rectal
(CIG_current = 1; N1 = 90)
1−sided adj pval: 0.185
Frequency
Tumor−Normal Expression Difference

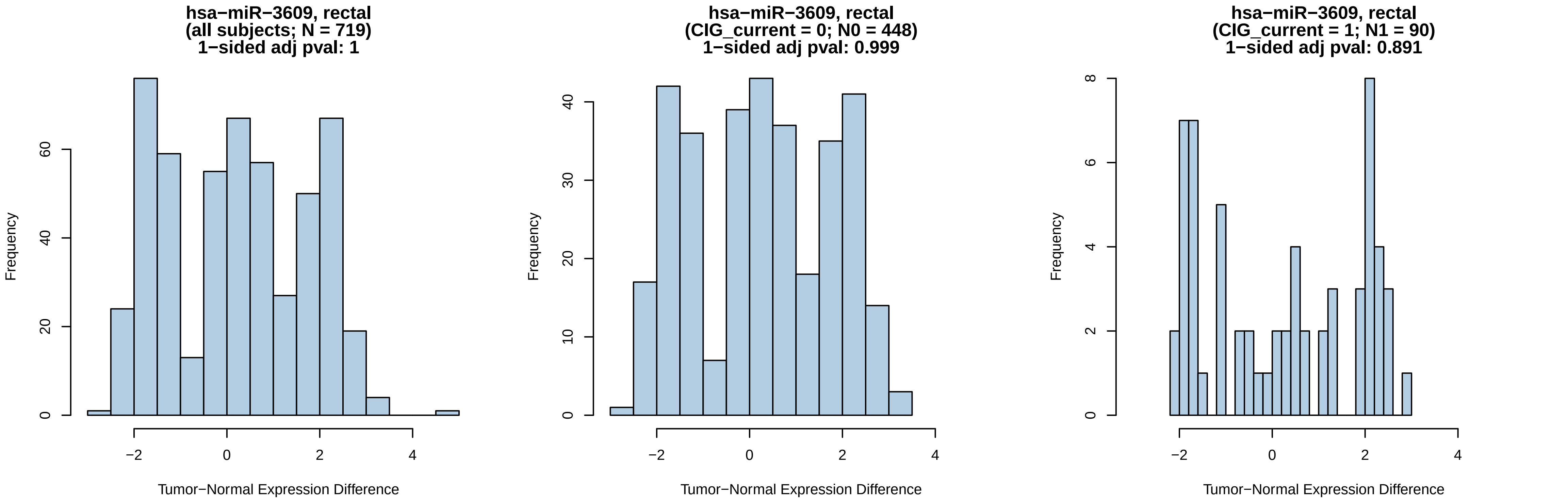
hsa−miR−3609, rectal
(all subjects; N = 719)
1−sided adj pval: 1
Frequency
0
20
40
60
−2
0
2
4
Tumor−Normal Expression Difference
hsa−miR−3609, rectal
(CIG_current = 0; N0 = 448)
1−sided adj pval: 0.999
Frequency
0
10
20
30
40
Tumor−Normal Expression Difference
hsa−miR−3609, rectal
(CIG_current = 1; N1 = 90)
1−sided adj pval: 0.891
Frequency
0
2
4
6
8
Tumor−Normal Expression Difference

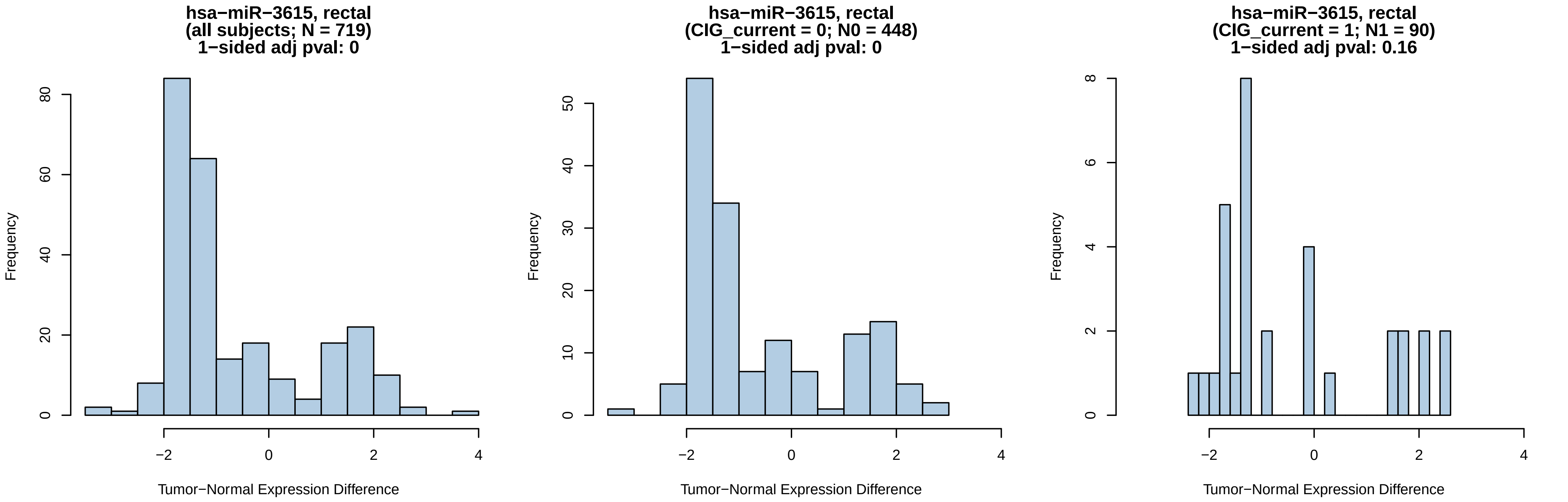
hsa−miR−3615, rectal
(all subjects; N = 719)
1−sided adj pval: 0
Frequency
0
20
40
60
80
−2
0
2
4
Tumor−Normal Expression Difference
hsa−miR−3615, rectal
(CIG_current = 0; N0 = 448)
1−sided adj pval: 0
Frequency
0
10
20
30
40
50
−2
0
2
4
Tumor−Normal Expression Difference
hsa−miR−3615, rectal
(CIG_current = 1; N1 = 90)
1−sided adj pval: 0.16
Frequency
0
2
4
6
8
−2
0
2
4
Tumor−Normal Expression Difference

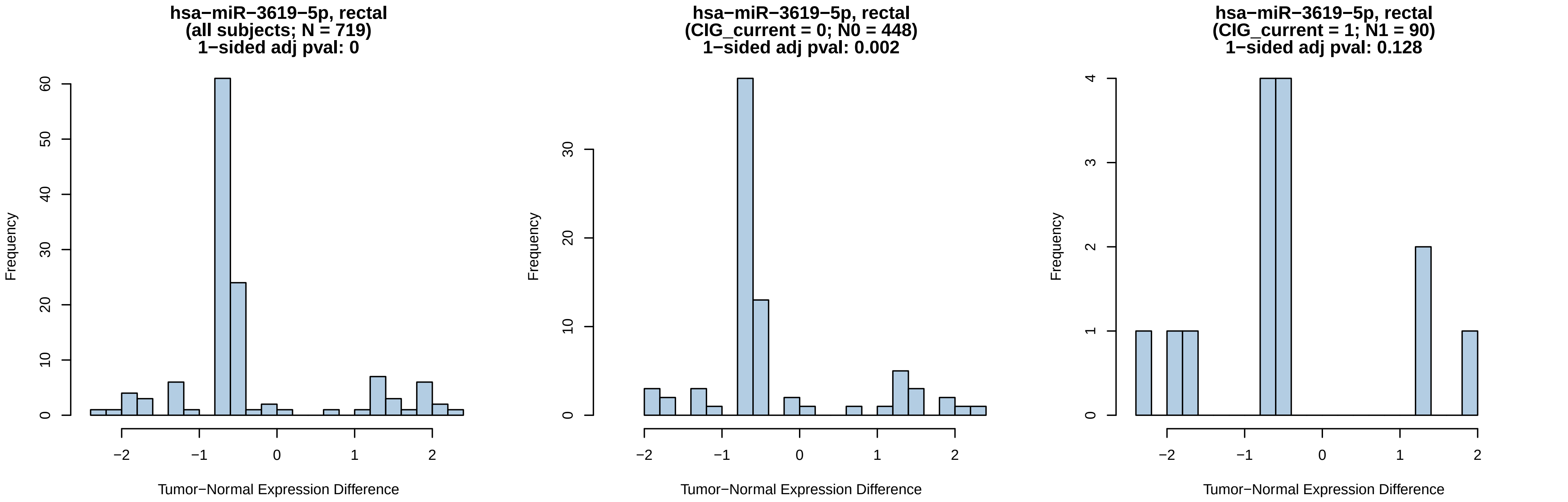

hsa−miR−3619−5p, rectal
(all subjects; N = 719)
1−sided adj pval: 0
Frequency
Tumor−Normal Expression Difference
hsa−miR−3619−5p, rectal
(CIG_current = 0; N0 = 448)
1−sided adj pval: 0.002
Frequency
Tumor−Normal Expression Difference
hsa−miR−3619−5p, rectal
(CIG_current = 1; N1 = 90)
1−sided adj pval: 0.128
Frequency
Tumor−Normal Expression Difference

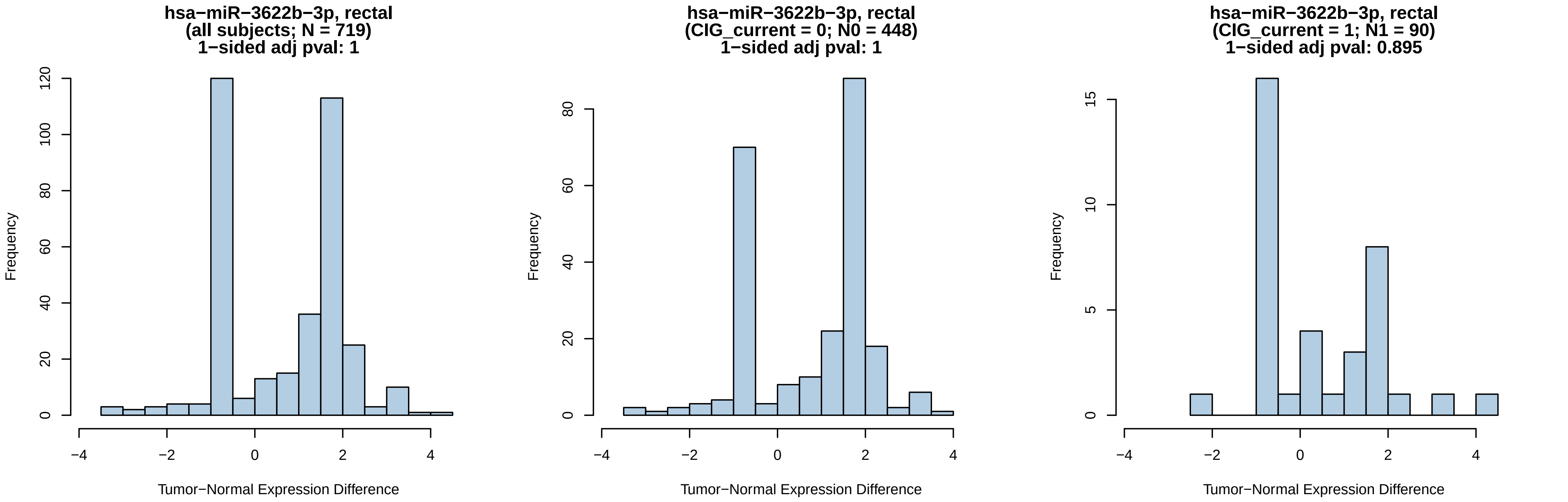
hsa−miR−3622b−3p, rectal
(all subjects; N = 719)
1−sided adj pval: 1
Frequency
0
20
40
60
80
100
120
−4
−2
0
2
4
Tumor−Normal Expression Difference
hsa−miR−3622b−3p, rectal
(CIG_current = 0; N0 = 448)
1−sided adj pval: 1
Frequency
0
20
40
60
80
−4
−2
0
2
4
Tumor−Normal Expression Difference
hsa−miR−3622b−3p, rectal
(CIG_current = 1; N1 = 90)
1−sided adj pval: 0.895
Frequency
0
5
10
15
−4
−2
0
2
4
Tumor−Normal Expression Difference

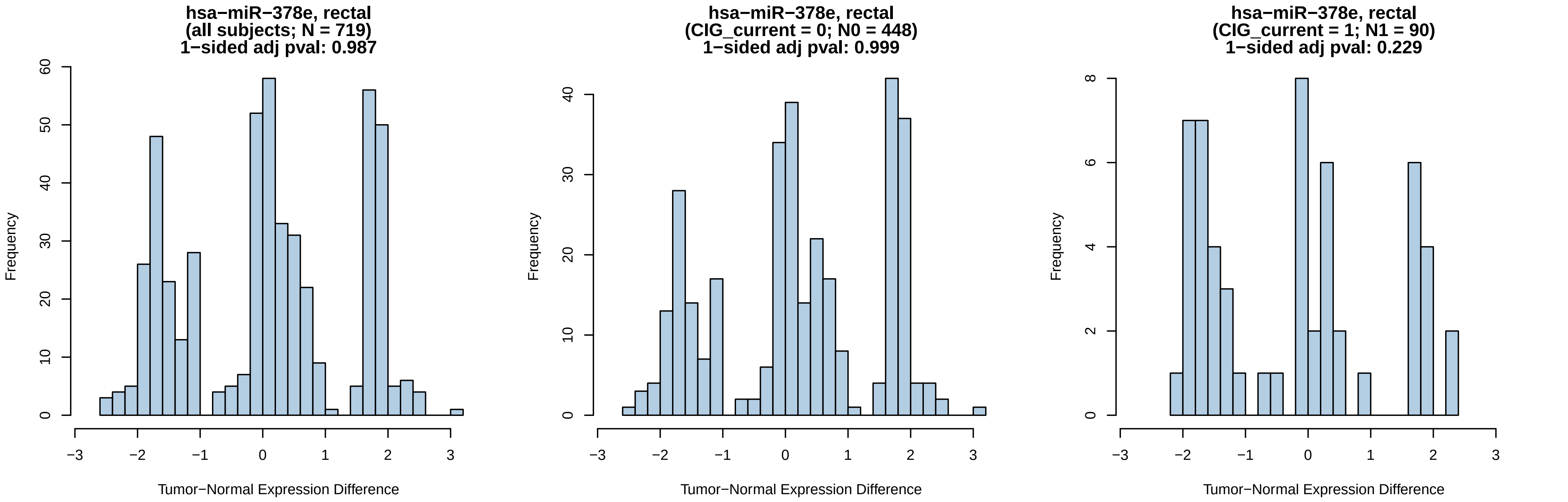

hsa−miR−378e, rectal
(all subjects; N = 719)
1−sided adj pval: 0.987
Frequency
Tumor−Normal Expression Difference
hsa−miR−378e, rectal
(CIG_current = 0; N0 = 448)
1−sided adj pval: 0.999
Frequency
Tumor−Normal Expression Difference
hsa−miR−378e, rectal
(CIG_current = 1; N1 = 90)
1−sided adj pval: 0.229
Frequency
Tumor−Normal Expression Difference

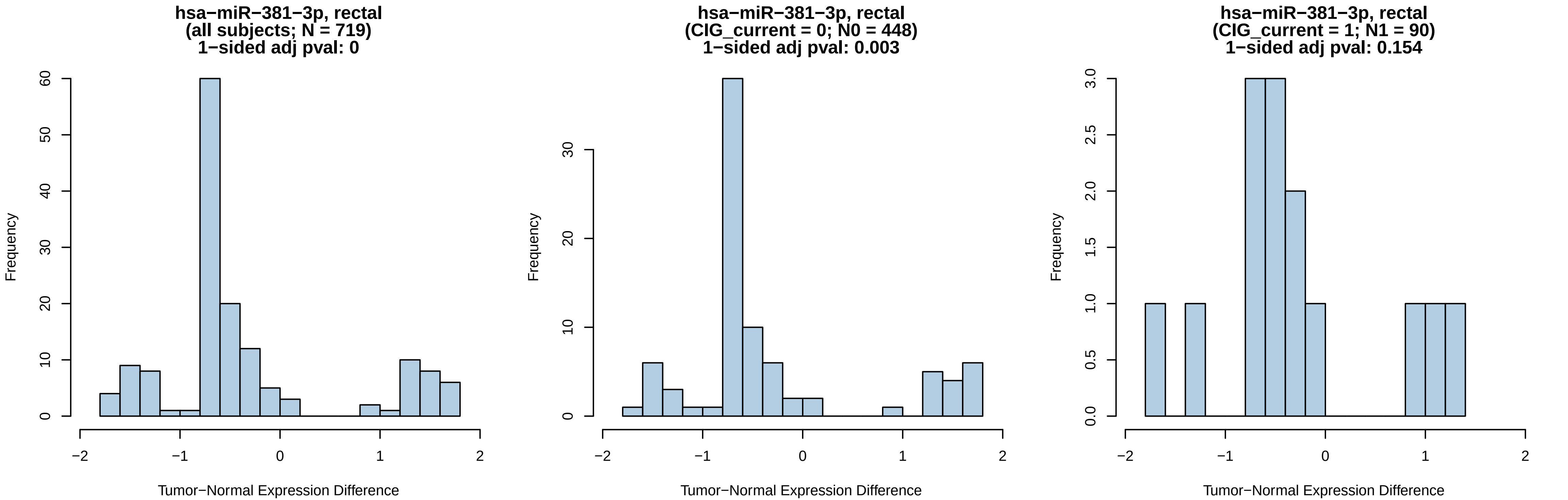
hsa−miR−381−3p, rectal
(all subjects; N = 719)
1−sided adj pval: 0
Frequency
Tumor−Normal Expression Difference
hsa−miR−381−3p, rectal
(CIG_current = 0; N0 = 448)
1−sided adj pval: 0.003
Frequency
Tumor−Normal Expression Difference
hsa−miR−381−3p, rectal
(CIG_current = 1; N1 = 90)
1−sided adj pval: 0.154
Frequency
Tumor−Normal Expression Difference

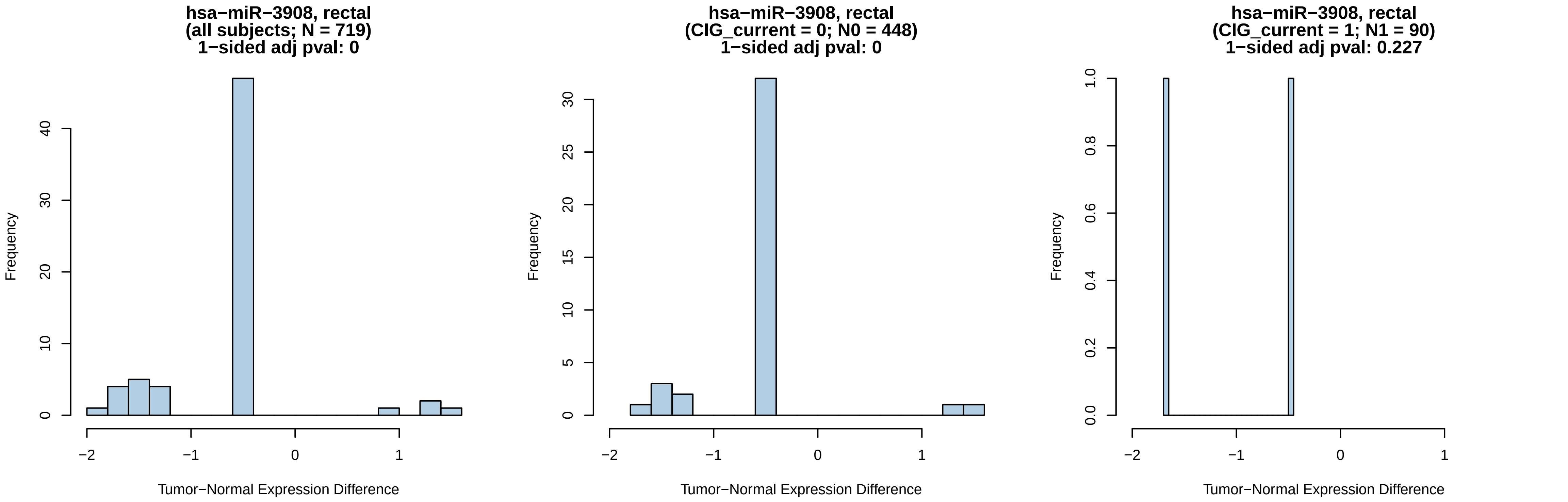

hsa−miR−3908, rectal
(all subjects; N = 719)
1−sided adj pval: 0
Frequency
0
10
20
30
40
−2
−1
0
1
Tumor−Normal Expression Difference
hsa−miR−3908, rectal
(CIG_current = 0; N0 = 448)
1−sided adj pval: 0
Frequency
0
5
10
15
20
25
30
−2
−1
0
1
Tumor−Normal Expression Difference
hsa−miR−3908, rectal
(CIG_current = 1; N1 = 90)
1−sided adj pval: 0.227
Frequency
0.0
0.2
0.4
0.6
0.8
1.0
−2
−1
0
1
Tumor−Normal Expression Difference

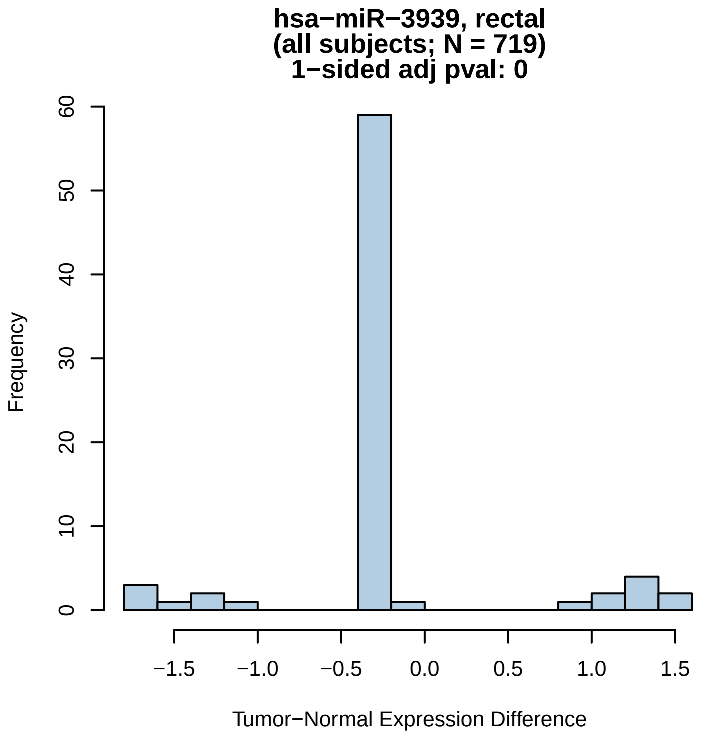
hsa−miR−3939, rectal
(all subjects; N = 719)
1−sided adj pval: 0
Frequency
Tumor−Normal Expression Difference

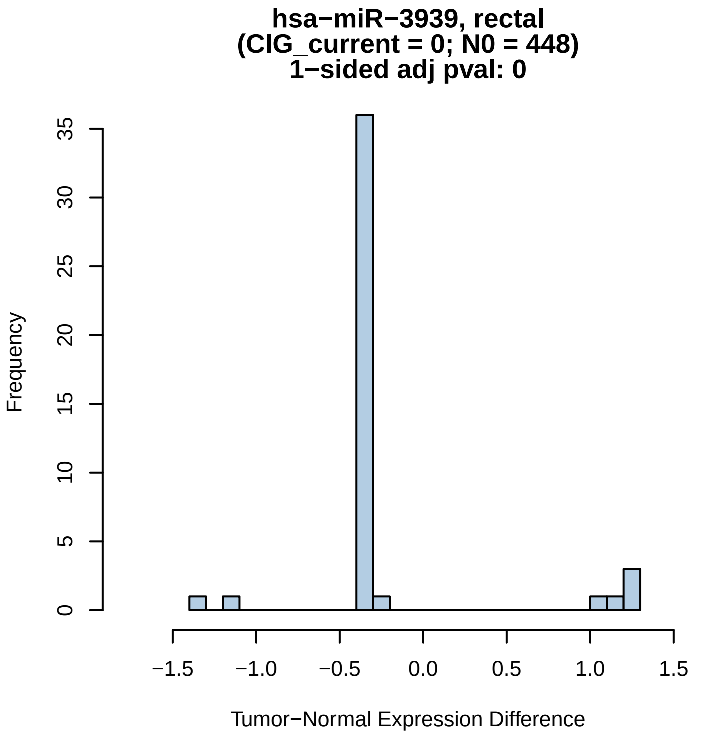
hsa−miR−3939, rectal
(CIG_current = 0; N0 = 448)
1−sided adj pval: 0
Frequency
Tumor−Normal Expression Difference

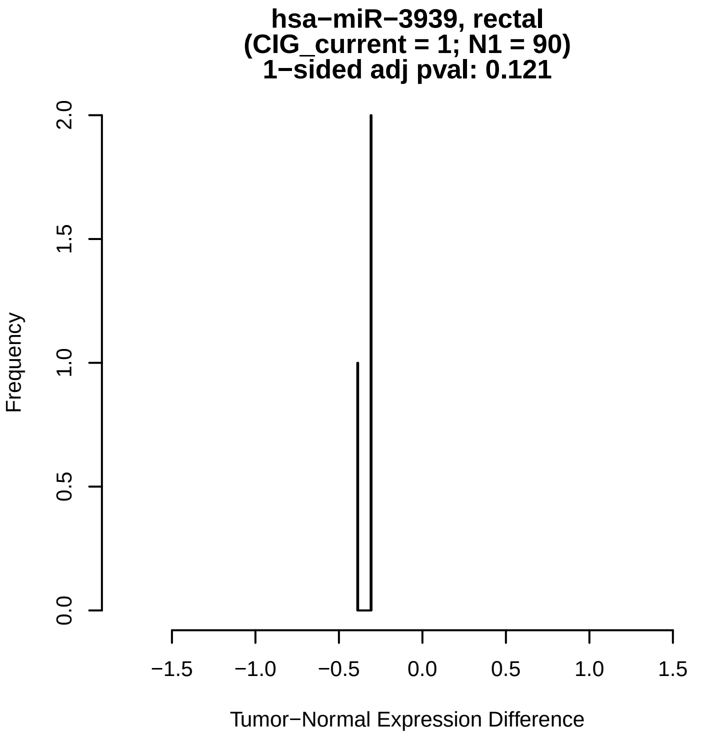
hsa−miR−3939, rectal
(CIG_current = 1; N1 = 90)
1−sided adj pval: 0.121
Frequency
Tumor−Normal Expression Difference

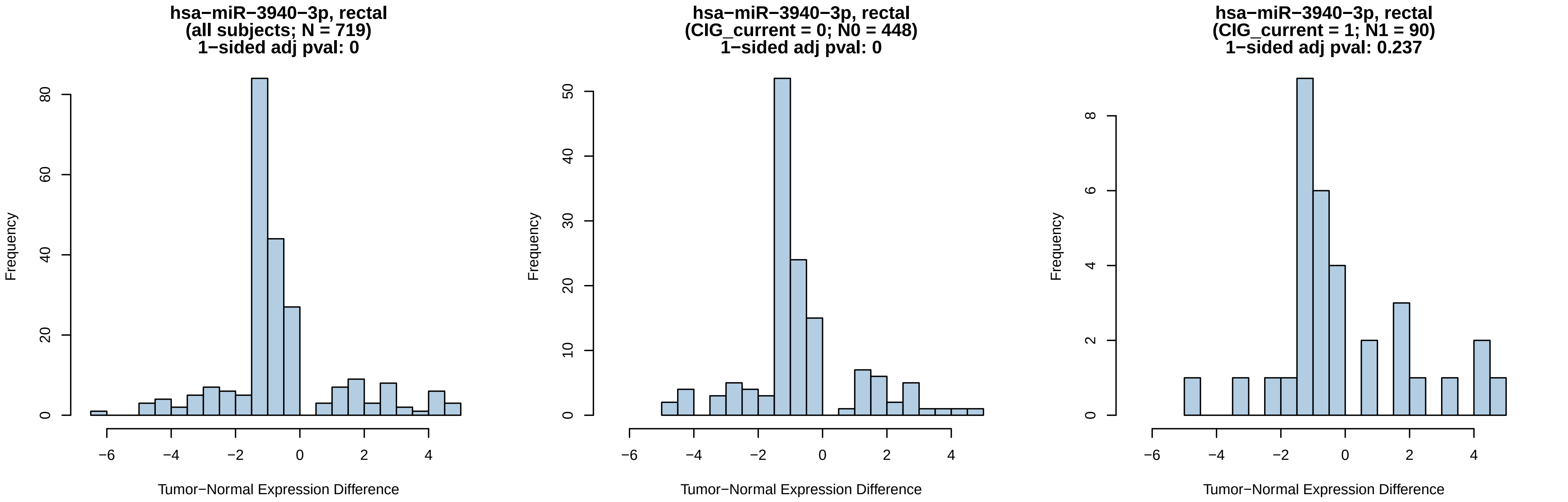
hsa−miR−3940−3p, rectal
(all subjects; N = 719)
1−sided adj pval: 0
Frequency
Tumor−Normal Expression Difference
hsa−miR−3940−3p, rectal
(CIG_current = 0; N0 = 448)
1−sided adj pval: 0
Frequency
Tumor−Normal Expression Difference
hsa−miR−3940−3p, rectal
(CIG_current = 1; N1 = 90)
1−sided adj pval: 0.237
Frequency
Tumor−Normal Expression Difference

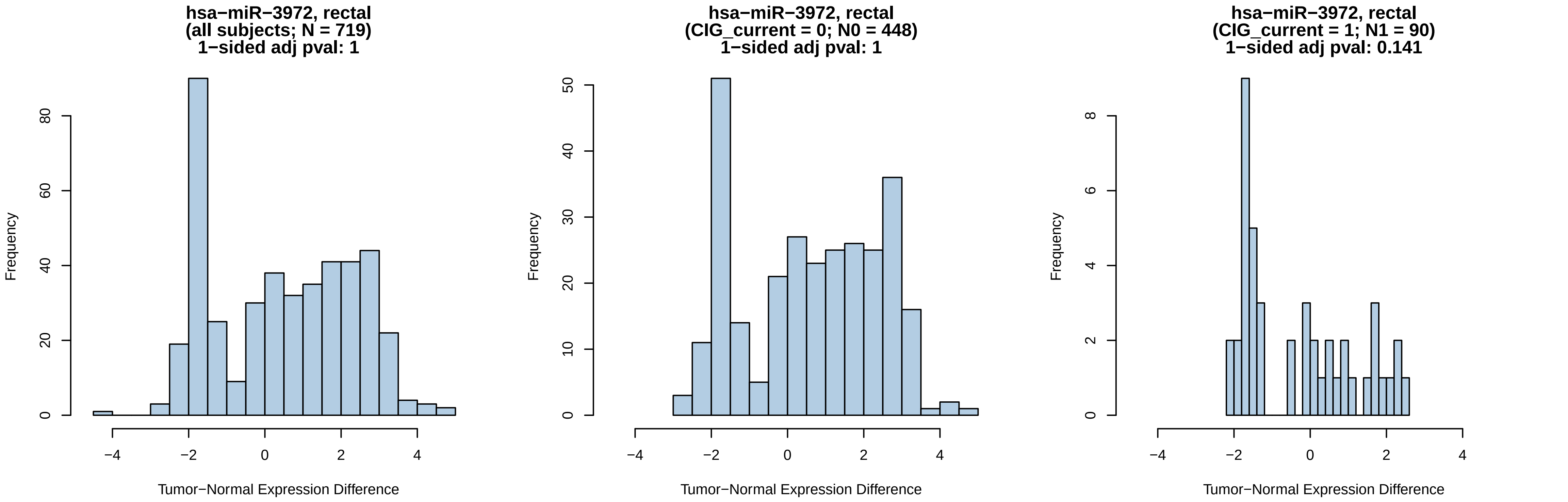

hsa−miR−3972, rectal
(all subjects; N = 719)
1−sided adj pval: 1
Frequency
Tumor−Normal Expression Difference
hsa−miR−3972, rectal
(CIG_current = 0; N0 = 448)
1−sided adj pval: 1
Frequency
Tumor−Normal Expression Difference
hsa−miR−3972, rectal
(CIG_current = 1; N1 = 90)
1−sided adj pval: 0.141
Frequency
Tumor−Normal Expression Difference

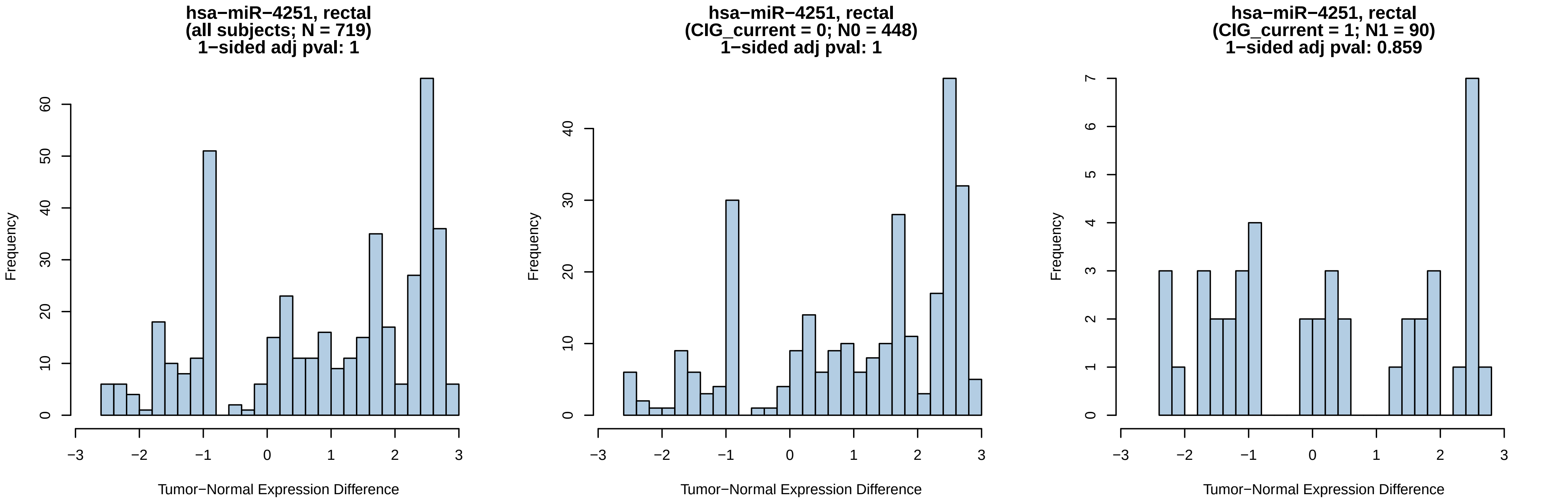
hsa−miR−4251, rectal
(all subjects; N = 719)
1−sided adj pval: 1
Frequency
Tumor−Normal Expression Difference
hsa−miR−4251, rectal
(CIG_current = 0; N0 = 448)
1−sided adj pval: 1
Frequency
Tumor−Normal Expression Difference
hsa−miR−4251, rectal
(CIG_current = 1; N1 = 90)
1−sided adj pval: 0.859
Frequency
Tumor−Normal Expression Difference

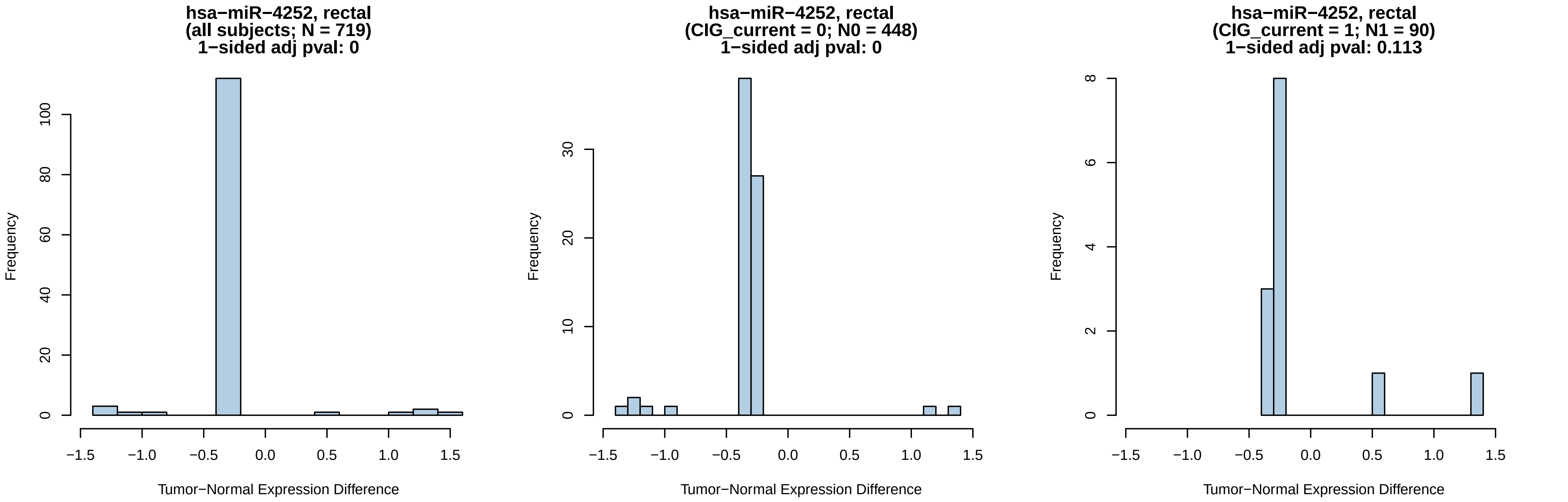
hsa−miR−4252, rectal
(all subjects; N = 719)
1−sided adj pval: 0
hsa−miR−4252, rectal
(CIG_current = 0; N0 = 448)
1−sided adj pval: 0
hsa−miR−4252, rectal
(CIG_current = 1; N1 = 90)
1−sided adj pval: 0.113
Frequency
Tumor−Normal Expression Difference
−1.5
−1.0
−0.5
0.0
0.5
1.0
1.5
0
20
40
60
80
100
10
30
2
4
6
8

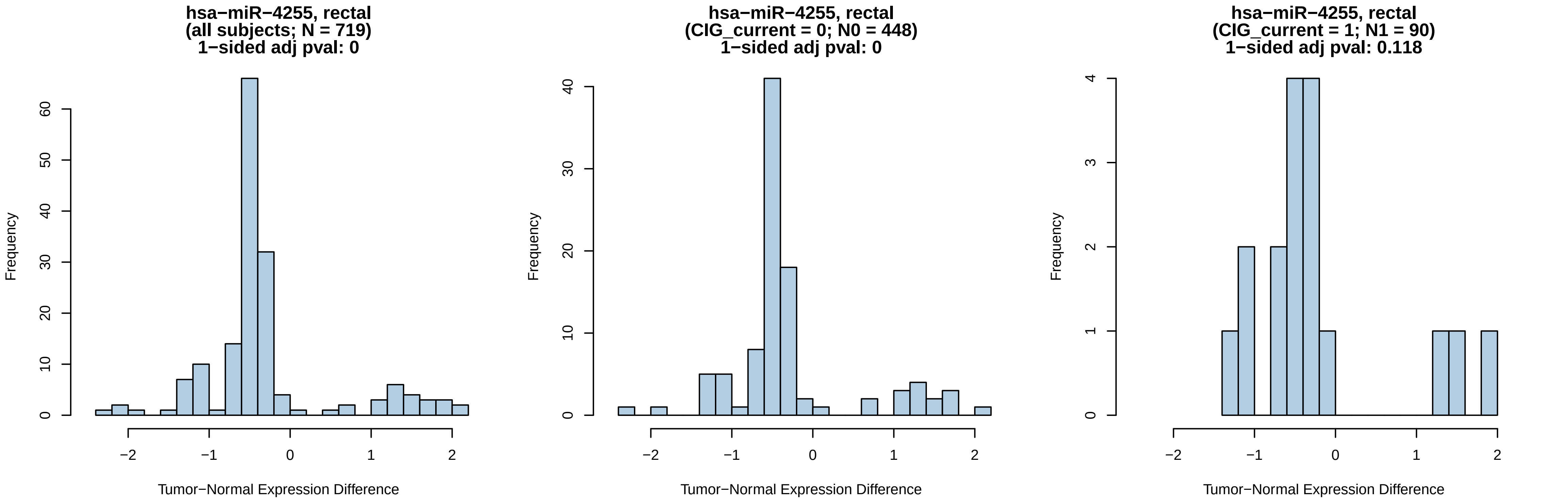
hsa−miR−4255, rectal
(all subjects; N = 719)
1−sided adj pval: 0
Frequency
Tumor−Normal Expression Difference
hsa−miR−4255, rectal
(CIG_current = 0; N0 = 448)
1−sided adj pval: 0
Frequency
Tumor−Normal Expression Difference
hsa−miR−4255, rectal
(CIG_current = 1; N1 = 90)
1−sided adj pval: 0.118
Frequency
Tumor−Normal Expression Difference

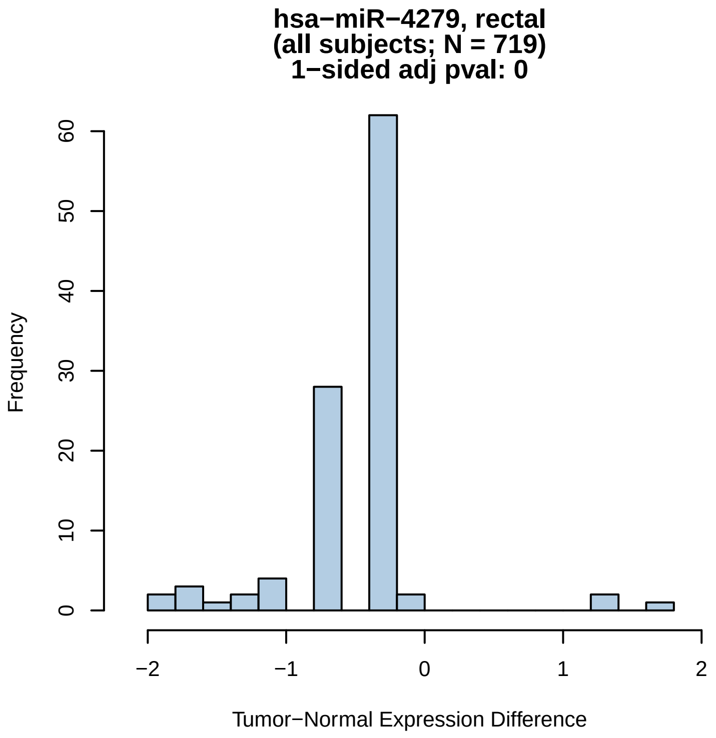
hsa−miR−4279, rectal
(all subjects; N = 719)
1−sided adj pval: 0
Frequency
Tumor−Normal Expression Difference

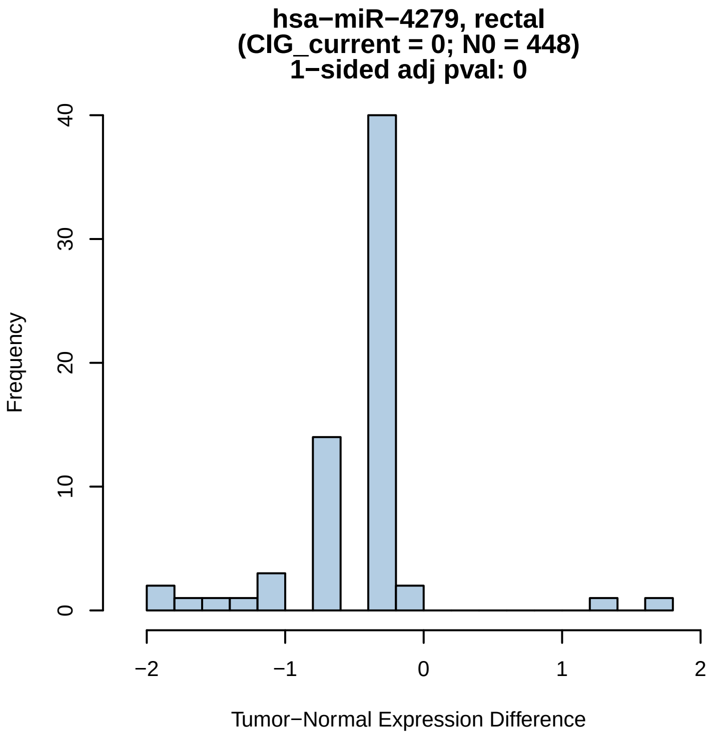
hsa−miR−4279, rectal
(CIG_current = 0; N0 = 448)
1−sided adj pval: 0
Frequency
Tumor−Normal Expression Difference

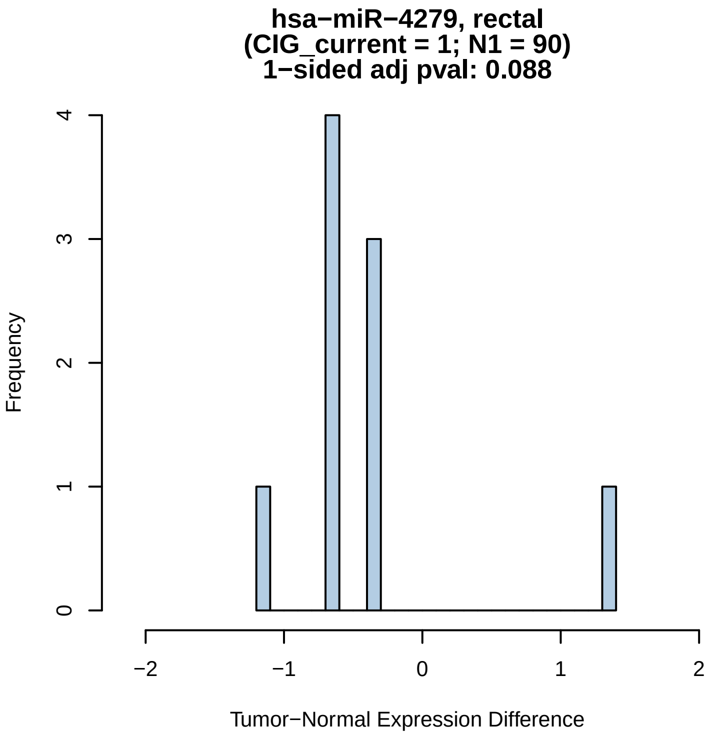
hsa−miR−4279, rectal
(CIG_current = 1; N1 = 90)
1−sided adj pval: 0.088
Frequency
Tumor−Normal Expression Difference

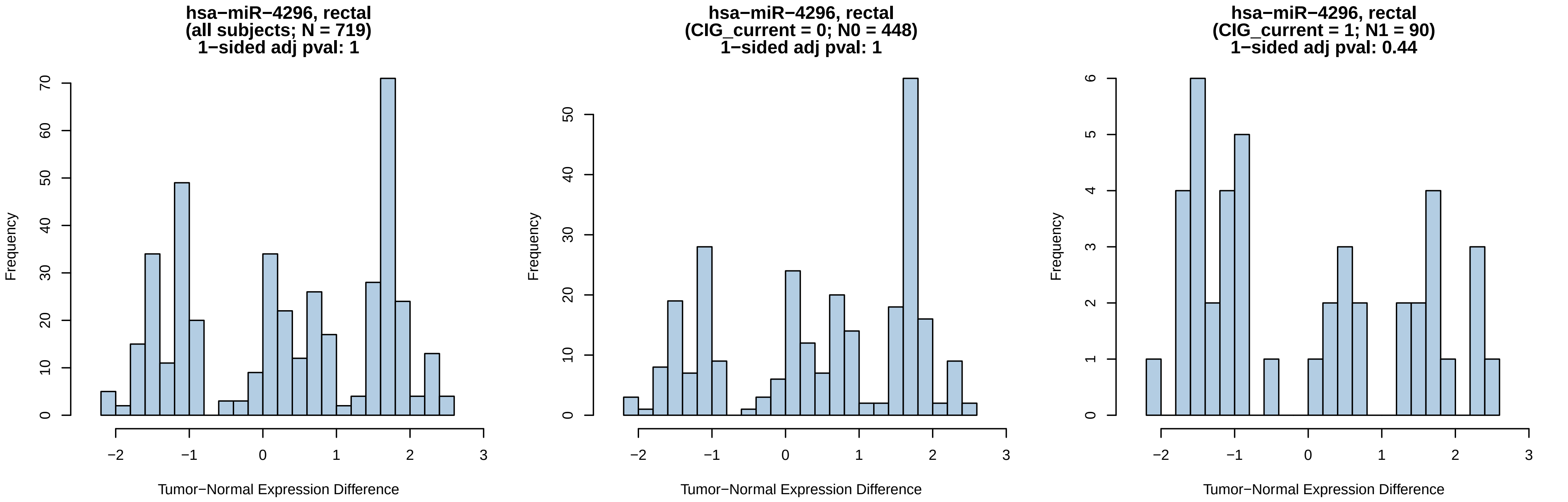
hsa−miR−4296, rectal
(all subjects; N = 719)
1−sided adj pval: 1
hsa−miR−4296, rectal
(CIG_current = 0; N0 = 448)
1−sided adj pval: 1
hsa−miR−4296, rectal
(CIG_current = 1; N1 = 90)
1−sided adj pval: 0.44
Frequency
Tumor−Normal Expression Difference

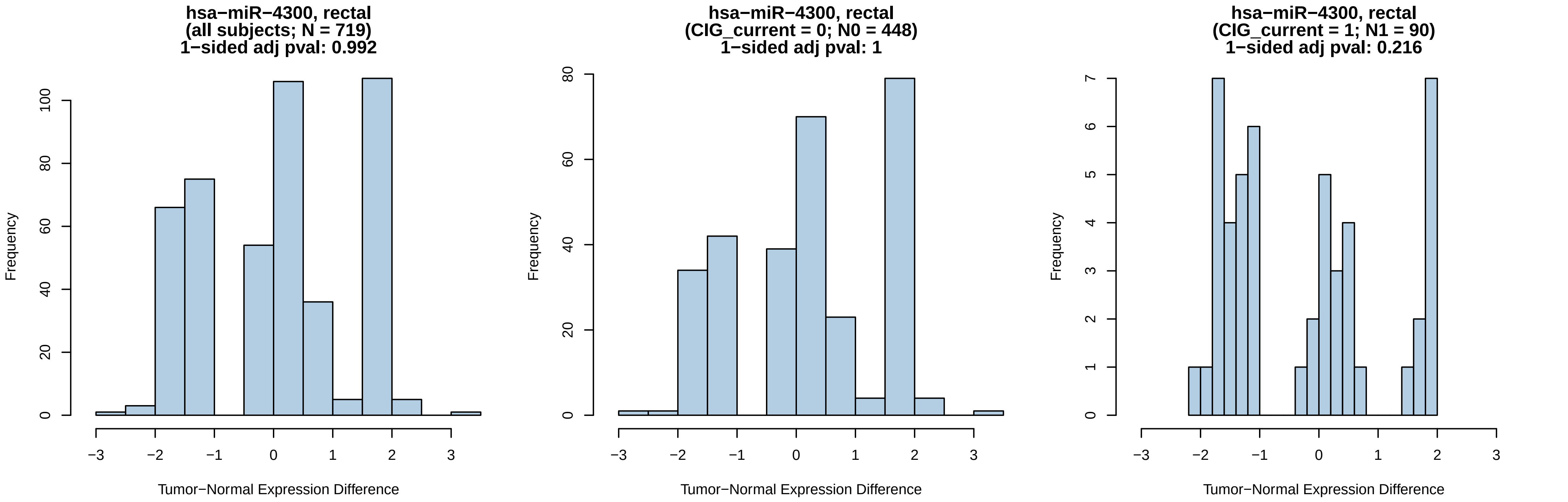

hsa−miR−4300, rectal
(all subjects; N = 719)
1−sided adj pval: 0.992
Frequency
Tumor−Normal Expression Difference
hsa−miR−4300, rectal
(CIG_current = 0; N0 = 448)
1−sided adj pval: 1
Frequency
Tumor−Normal Expression Difference
hsa−miR−4300, rectal
(CIG_current = 1; N1 = 90)
1−sided adj pval: 0.216
Frequency
Tumor−Normal Expression Difference

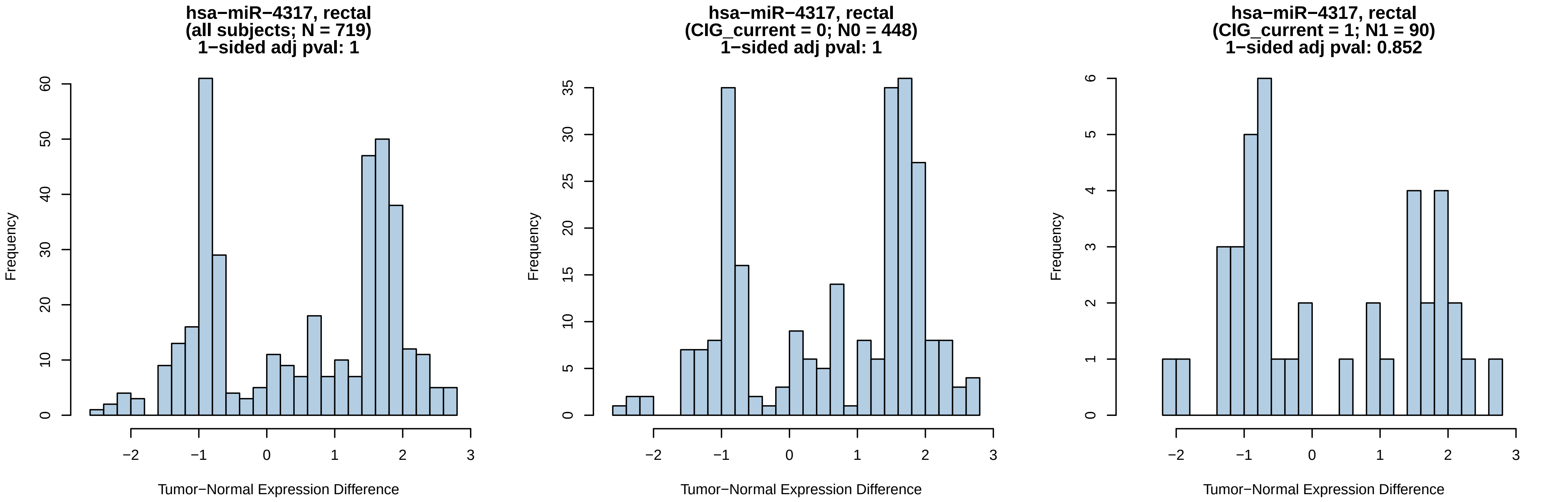

hsa−miR−4317, rectal
(all subjects; N = 719)
1−sided adj pval: 1
hsa−miR−4317, rectal
(CIG_current = 0; N0 = 448)
1−sided adj pval: 1
hsa−miR−4317, rectal
(CIG_current = 1; N1 = 90)
1−sided adj pval: 0.852
Frequency
Tumor−Normal Expression Difference

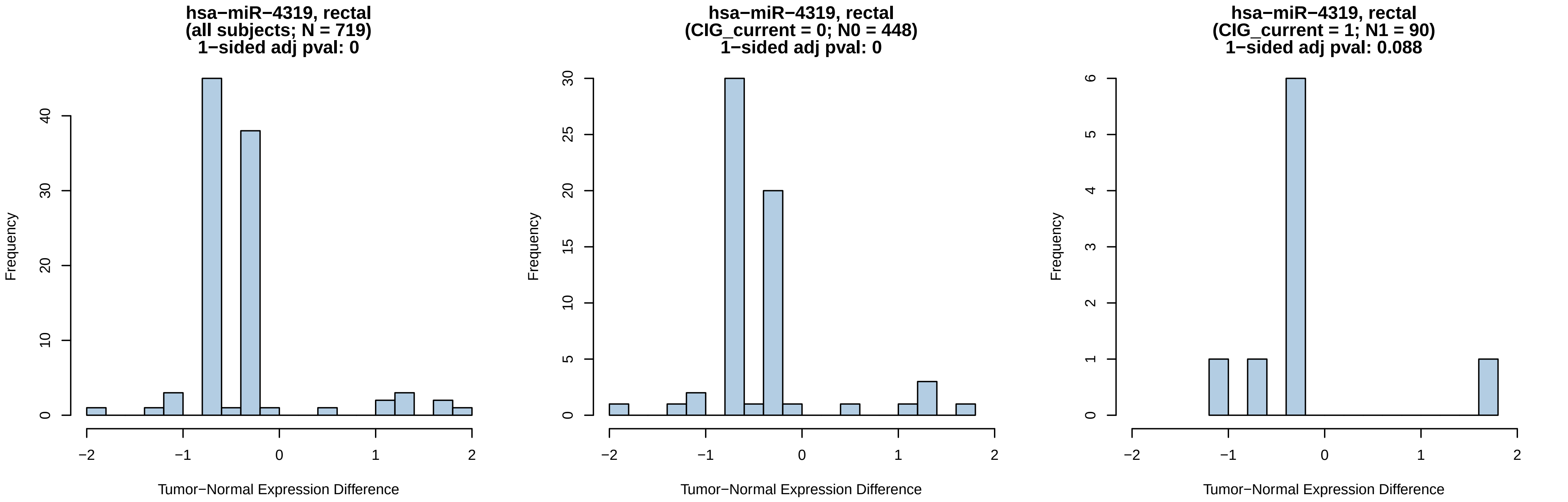

hsa−miR−4319, rectal
(all subjects; N = 719)
1−sided adj pval: 0
Frequency
0
10
20
30
40
−2
−1
0
1
2
Tumor−Normal Expression Difference
hsa−miR−4319, rectal
(CIG_current = 0; N0 = 448)
1−sided adj pval: 0
Frequency
0
5
10
15
20
25
30
−2
−1
0
1
2
Tumor−Normal Expression Difference
hsa−miR−4319, rectal
(CIG_current = 1; N1 = 90)
1−sided adj pval: 0.088
Frequency
0
1
2
3
4
5
6
−2
−1
0
1
2
Tumor−Normal Expression Difference

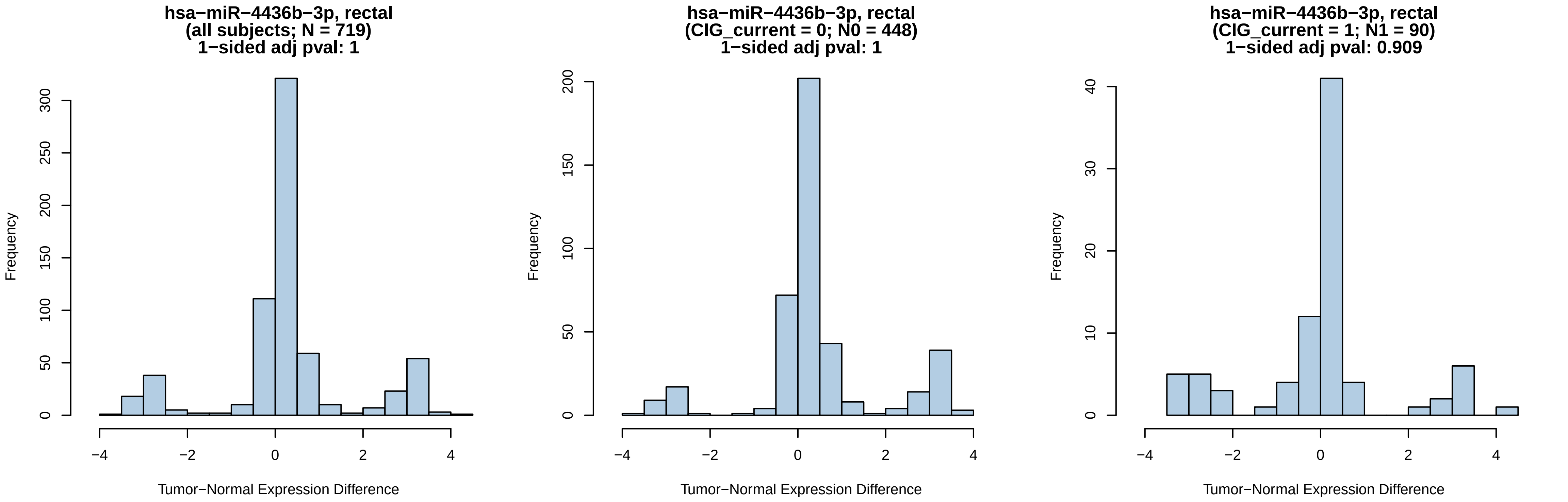
hsa−miR−4436b−3p, rectal
(all subjects; N = 719)
1−sided adj pval: 1
hsa−miR−4436b−3p, rectal
(CIG_current = 0; N0 = 448)
1−sided adj pval: 1
hsa−miR−4436b−3p, rectal
(CIG_current = 1; N1 = 90)
1−sided adj pval: 0.909
Frequency
Tumor−Normal Expression Difference
0
50
100
150
200
250
300
10
20
30
40
−4
−2
2
4

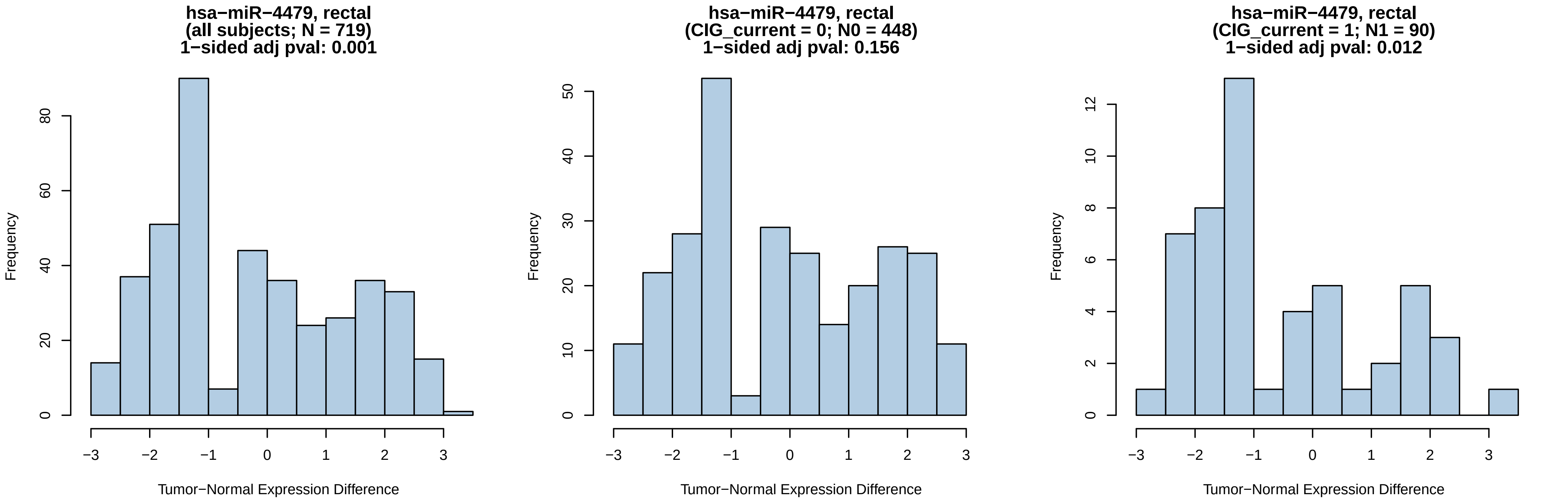
hsa−miR−4479, rectal
(all subjects; N = 719)
1−sided adj pval: 0.001
Frequency
0
20
40
60
80
−3
−2
−1
0
1
2
3
Tumor−Normal Expression Difference
hsa−miR−4479, rectal
(CIG_current = 0; N0 = 448)
1−sided adj pval: 0.156
Frequency
0
10
20
30
40
50
−3
−2
−1
0
1
2
3
Tumor−Normal Expression Difference
hsa−miR−4479, rectal
(CIG_current = 1; N1 = 90)
1−sided adj pval: 0.012
Frequency
0
2
4
6
8
10
12
−3
−2
−1
0
1
2
3
Tumor−Normal Expression Difference

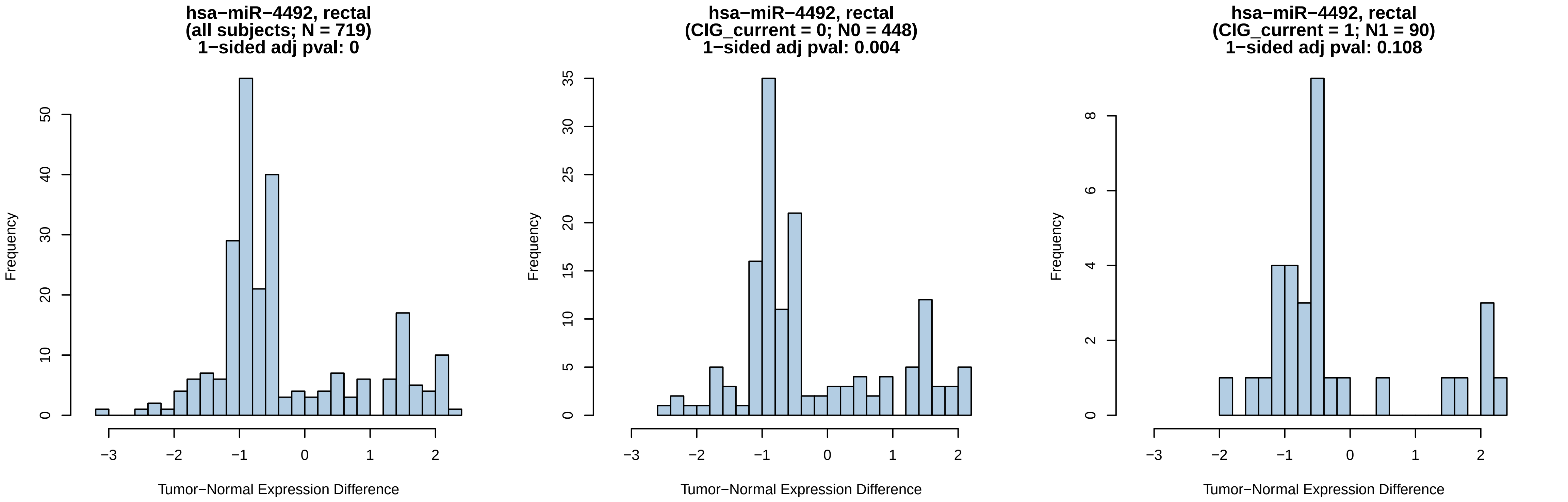

hsa−miR−4492, rectal
(all subjects; N = 719)
1−sided adj pval: 0
Frequency
0
10
20
30
40
50
−3
−2
−1
0
1
2
Tumor−Normal Expression Difference
hsa−miR−4492, rectal
(CIG_current = 0; N0 = 448)
1−sided adj pval: 0.004
Frequency
0
5
10
15
20
25
30
35
−3
−2
−1
0
1
2
Tumor−Normal Expression Difference
hsa−miR−4492, rectal
(CIG_current = 1; N1 = 90)
1−sided adj pval: 0.108
Frequency
0
2
4
6
8
−3
−2
−1
0
1
2
Tumor−Normal Expression Difference

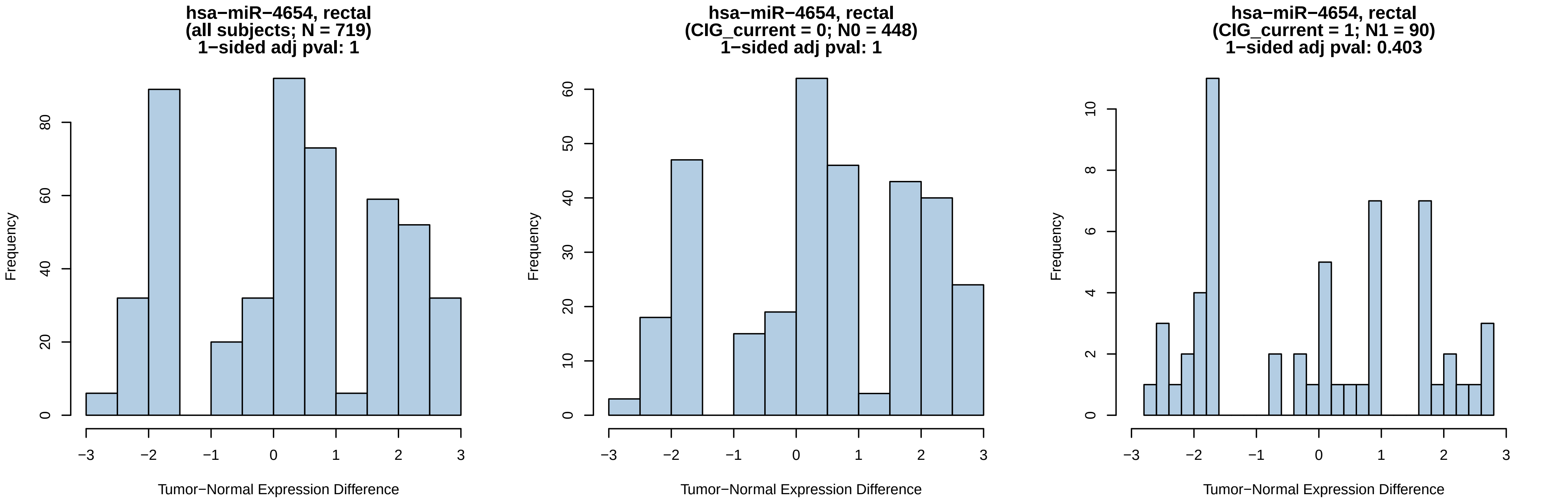

hsa−miR−4654, rectal
(all subjects; N = 719)
1−sided adj pval: 1
Frequency
Tumor−Normal Expression Difference
hsa−miR−4654, rectal
(CIG_current = 0; N0 = 448)
1−sided adj pval: 1
Frequency
Tumor−Normal Expression Difference
hsa−miR−4654, rectal
(CIG_current = 1; N1 = 90)
1−sided adj pval: 0.403
Frequency
Tumor−Normal Expression Difference

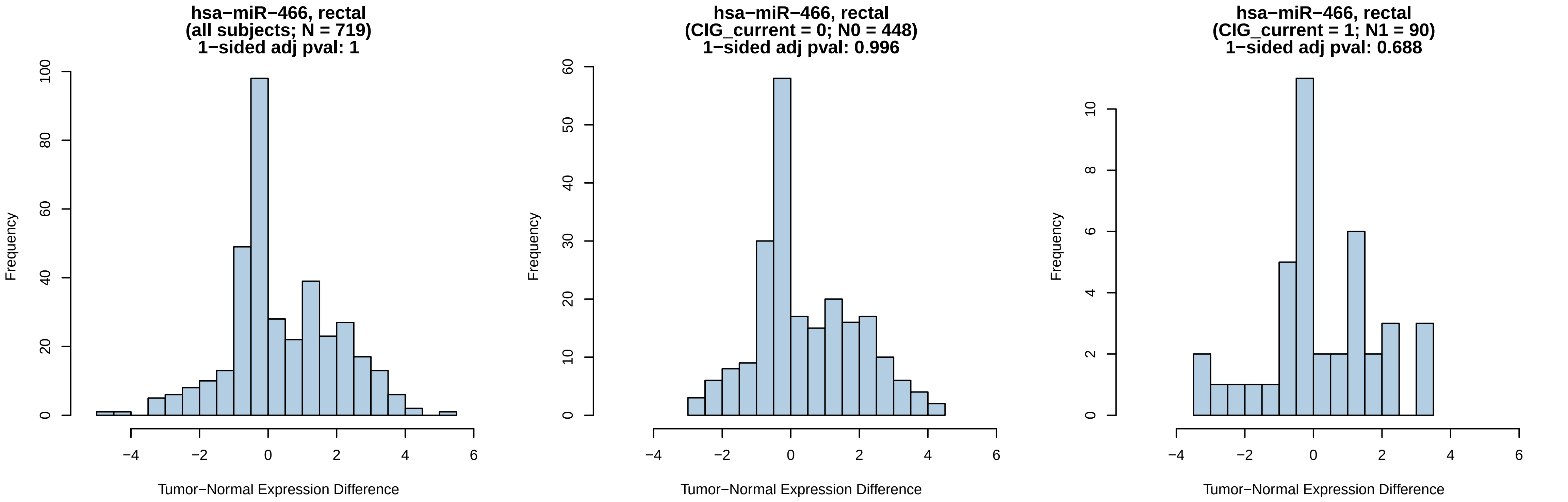
hsa−miR−466, rectal
(all subjects; N = 719)
1−sided adj pval: 1
Frequency
Tumor−Normal Expression Difference
hsa−miR−466, rectal
(CIG_current = 0; N0 = 448)
1−sided adj pval: 0.996
Frequency
Tumor−Normal Expression Difference
hsa−miR−466, rectal
(CIG_current = 1; N1 = 90)
1−sided adj pval: 0.688
Frequency
Tumor−Normal Expression Difference

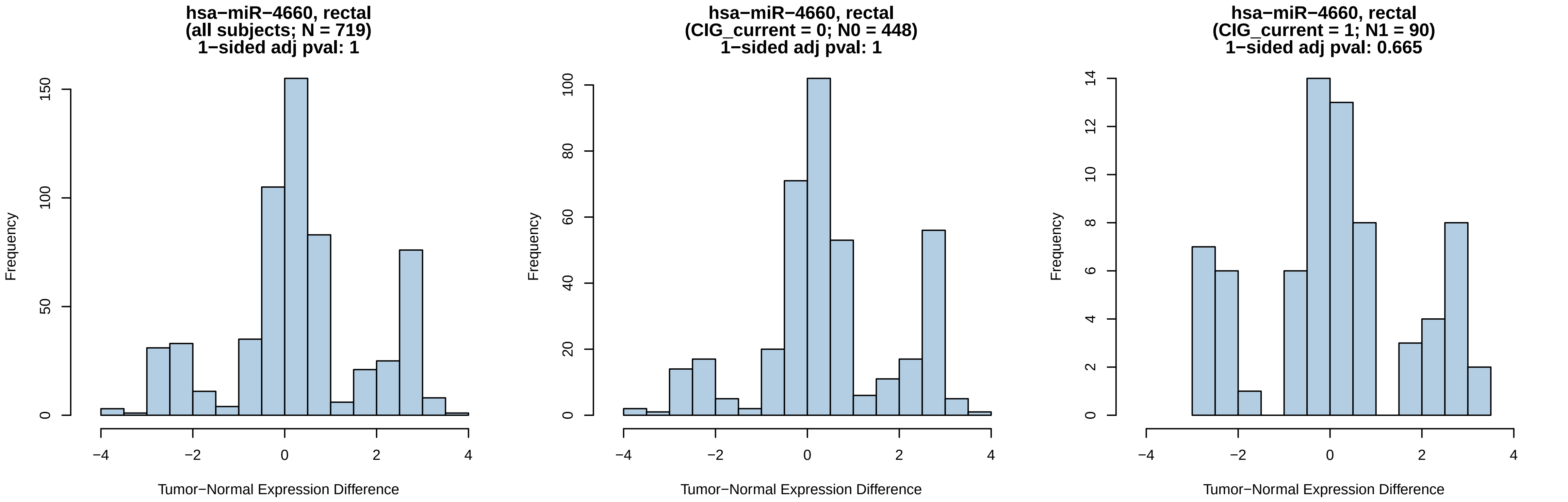
hsa−miR−4660, rectal
(all subjects; N = 719)
1−sided adj pval: 1
Frequency
Tumor−Normal Expression Difference
hsa−miR−4660, rectal
(CIG_current = 0; N0 = 448)
1−sided adj pval: 1
Frequency
Tumor−Normal Expression Difference
hsa−miR−4660, rectal
(CIG_current = 1; N1 = 90)
1−sided adj pval: 0.665
Frequency
Tumor−Normal Expression Difference

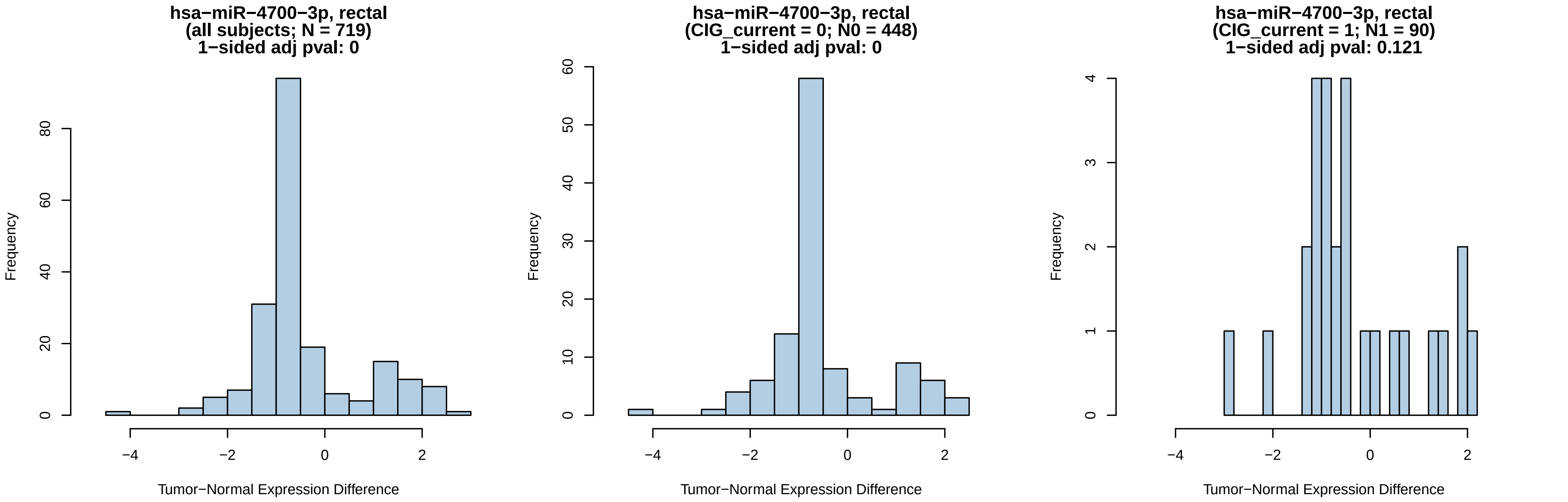
hsa−miR−4700−3p, rectal
(all subjects; N = 719)
1−sided adj pval: 0
Frequency
Tumor−Normal Expression Difference
hsa−miR−4700−3p, rectal
(CIG_current = 0; N0 = 448)
1−sided adj pval: 0
Frequency
Tumor−Normal Expression Difference
hsa−miR−4700−3p, rectal
(CIG_current = 1; N1 = 90)
1−sided adj pval: 0.121
Frequency
Tumor−Normal Expression Difference

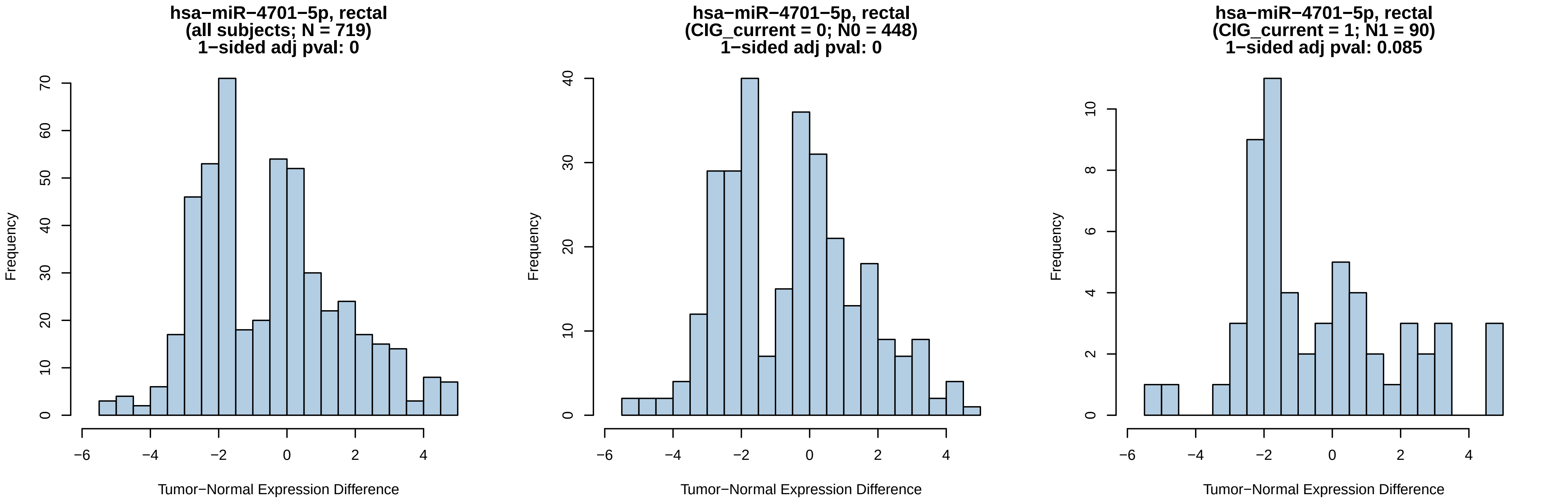
hsa−miR−4701−5p, rectal
(all subjects; N = 719)
1−sided adj pval: 0
Frequency
Tumor−Normal Expression Difference
hsa−miR−4701−5p, rectal
(CIG_current = 0; N0 = 448)
1−sided adj pval: 0
Frequency
Tumor−Normal Expression Difference
hsa−miR−4701−5p, rectal
(CIG_current = 1; N1 = 90)
1−sided adj pval: 0.085
Frequency
Tumor−Normal Expression Difference

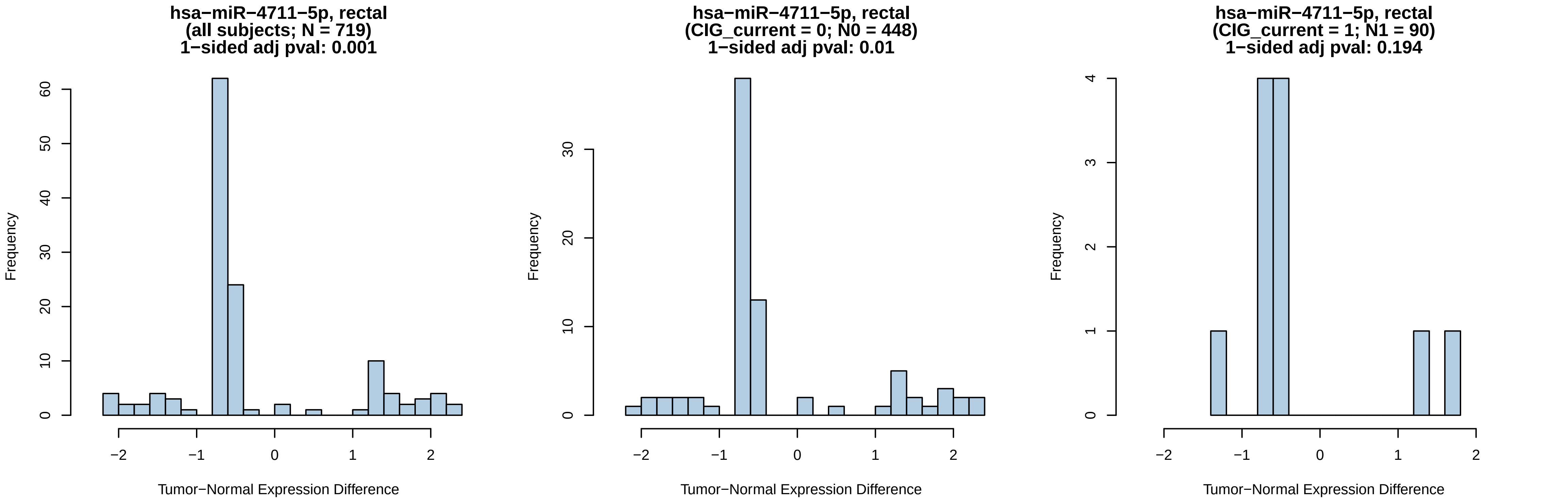
hsa−miR−4711−5p, rectal
(all subjects; N = 719)
1−sided adj pval: 0.001
Frequency
0
10
20
30
40
50
60
−2
−1
0
1
2
Tumor−Normal Expression Difference
hsa−miR−4711−5p, rectal
(CIG_current = 0; N0 = 448)
1−sided adj pval: 0.01
Frequency
0
10
20
30
−2
−1
0
1
2
Tumor−Normal Expression Difference
hsa−miR−4711−5p, rectal
(CIG_current = 1; N1 = 90)
1−sided adj pval: 0.194
Frequency
0
1
2
3
4
−2
−1
0
1
2
Tumor−Normal Expression Difference

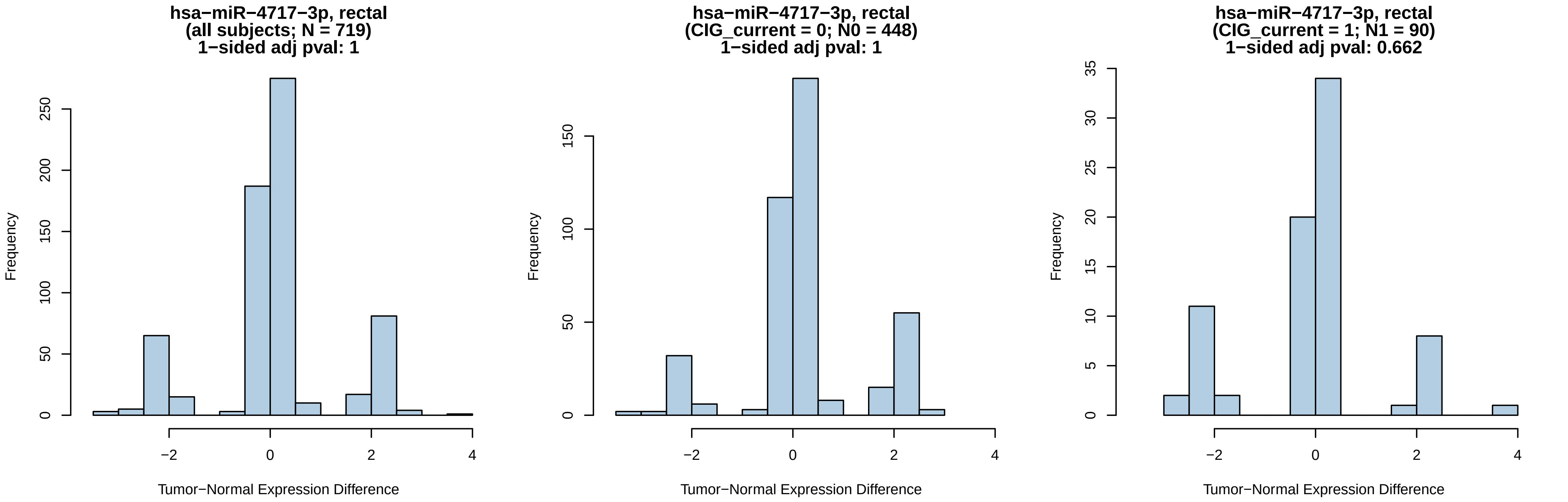
hsa−miR−4717−3p, rectal
(all subjects; N = 719)
1−sided adj pval: 1
Frequency
Tumor−Normal Expression Difference
hsa−miR−4717−3p, rectal
(CIG_current = 0; N0 = 448)
1−sided adj pval: 1
Frequency
Tumor−Normal Expression Difference
hsa−miR−4717−3p, rectal
(CIG_current = 1; N1 = 90)
1−sided adj pval: 0.662
Frequency
Tumor−Normal Expression Difference

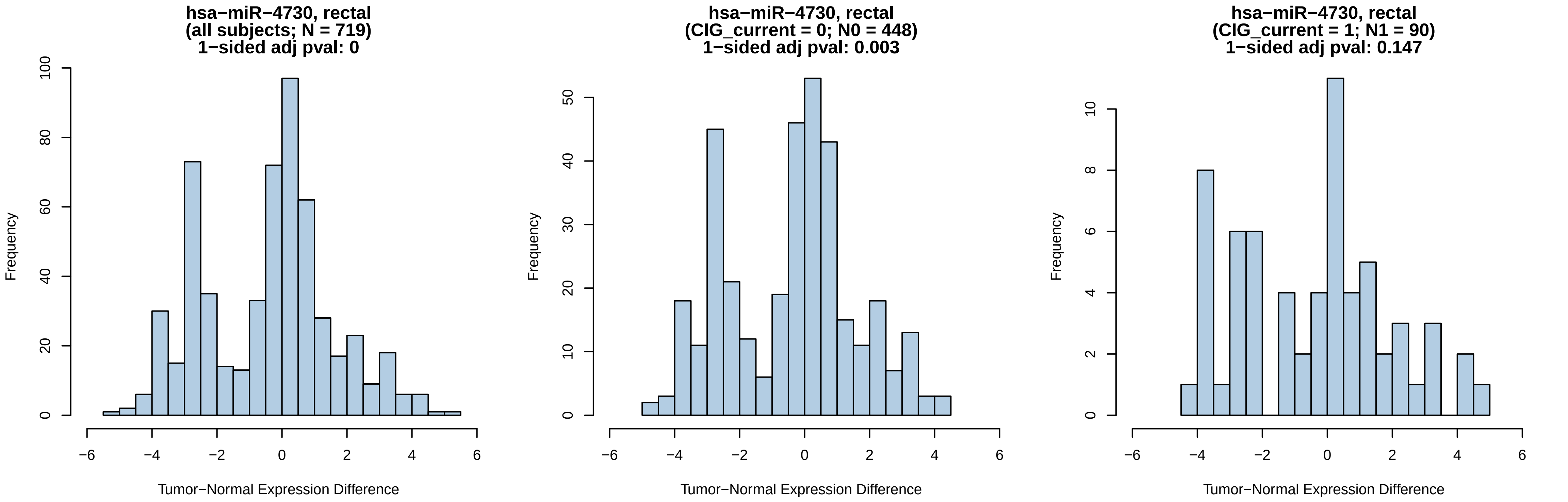
hsa−miR−4730, rectal
(all subjects; N = 719)
1−sided adj pval: 0
hsa−miR−4730, rectal
(CIG_current = 0; N0 = 448)
1−sided adj pval: 0.003
hsa−miR−4730, rectal
(CIG_current = 1; N1 = 90)
1−sided adj pval: 0.147
Frequency
Tumor−Normal Expression Difference

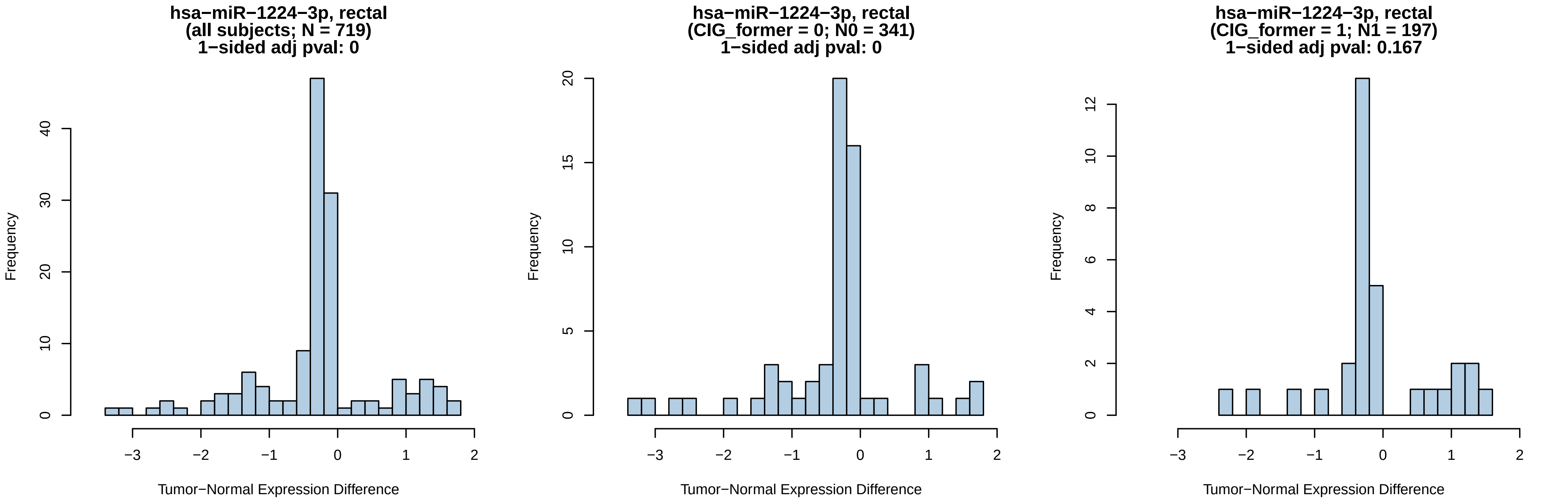
hsa−miR−1224−3p, rectal
(all subjects; N = 719)
1−sided adj pval: 0
Frequency
Tumor−Normal Expression Difference
hsa−miR−1224−3p, rectal
(CIG_former = 0; N0 = 341)
1−sided adj pval: 0
Frequency
Tumor−Normal Expression Difference
hsa−miR−1224−3p, rectal
(CIG_former = 1; N1 = 197)
1−sided adj pval: 0.167
Frequency
Tumor−Normal Expression Difference

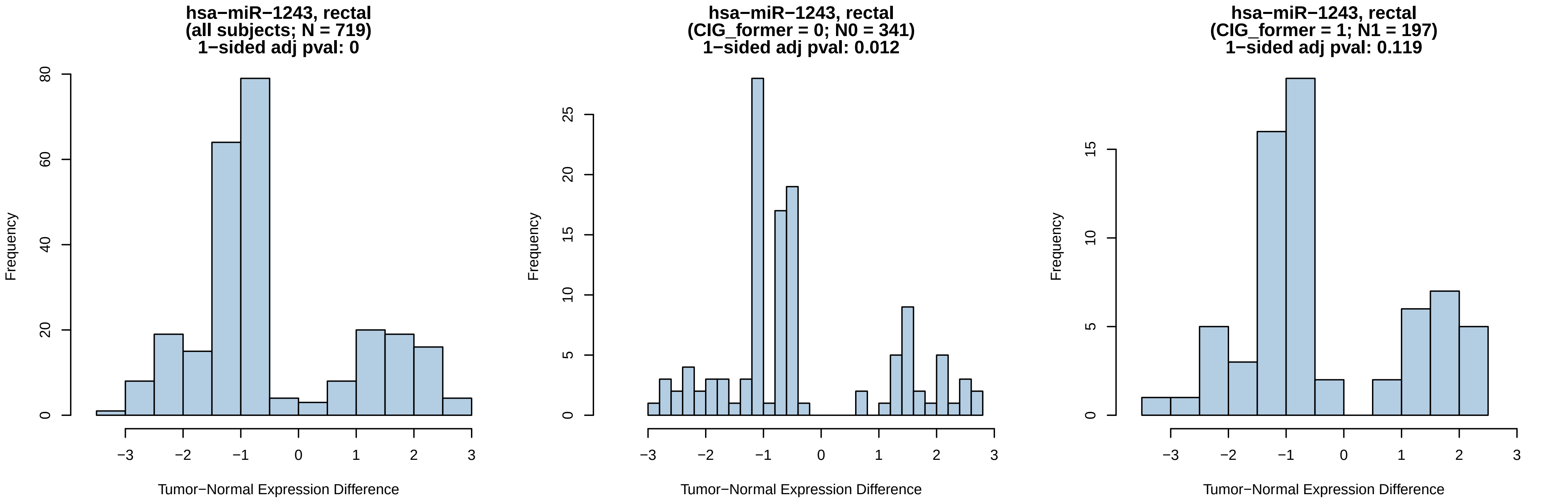
hsa−miR−1243, rectal
(all subjects; N = 719)
1−sided adj pval: 0
Frequency
Tumor−Normal Expression Difference
hsa−miR−1243, rectal
(CIG_former = 0; N0 = 341)
1−sided adj pval: 0.012
Frequency
Tumor−Normal Expression Difference
hsa−miR−1243, rectal
(CIG_former = 1; N1 = 197)
1−sided adj pval: 0.119
Frequency
Tumor−Normal Expression Difference

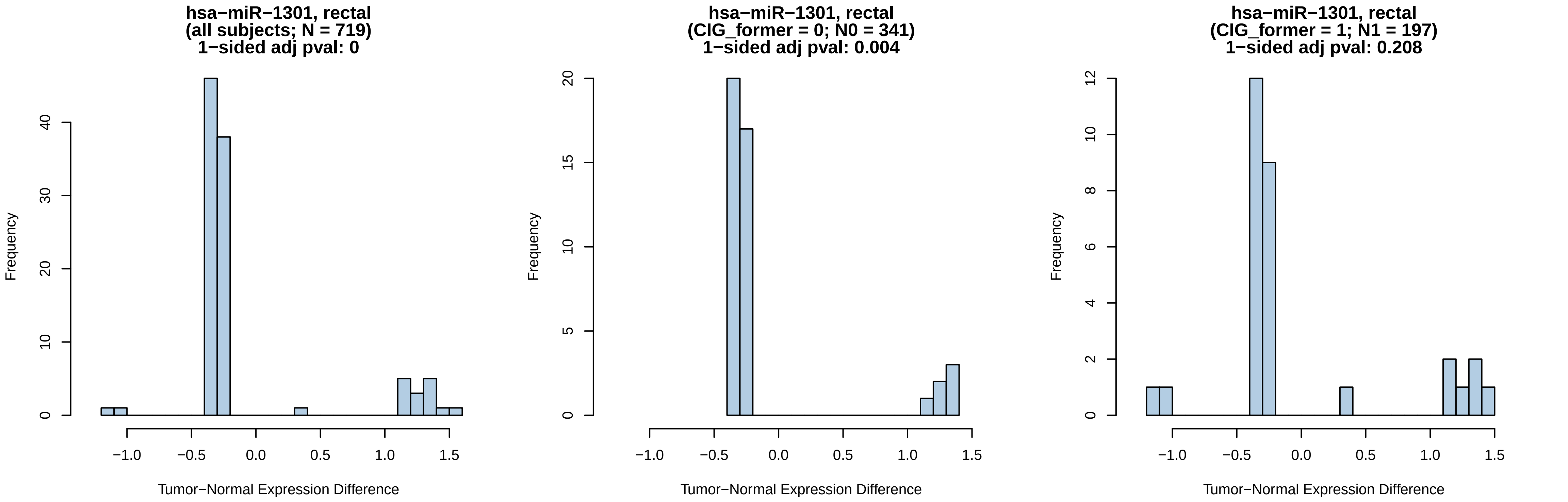

hsa−miR−1301, rectal
(all subjects; N = 719)
1−sided adj pval: 0
Frequency
0
10
20
30
40
−1.0
−0.5
0.0
0.5
1.0
1.5
Tumor−Normal Expression Difference
hsa−miR−1301, rectal
(CIG_former = 0; N0 = 341)
1−sided adj pval: 0.004
Frequency
0
5
10
15
20
−1.0
−0.5
0.0
0.5
1.0
1.5
Tumor−Normal Expression Difference
hsa−miR−1301, rectal
(CIG_former = 1; N1 = 197)
1−sided adj pval: 0.208
Frequency
0
2
4
6
8
10
12
−1.0
−0.5
0.0
0.5
1.0
1.5
Tumor−Normal Expression Difference

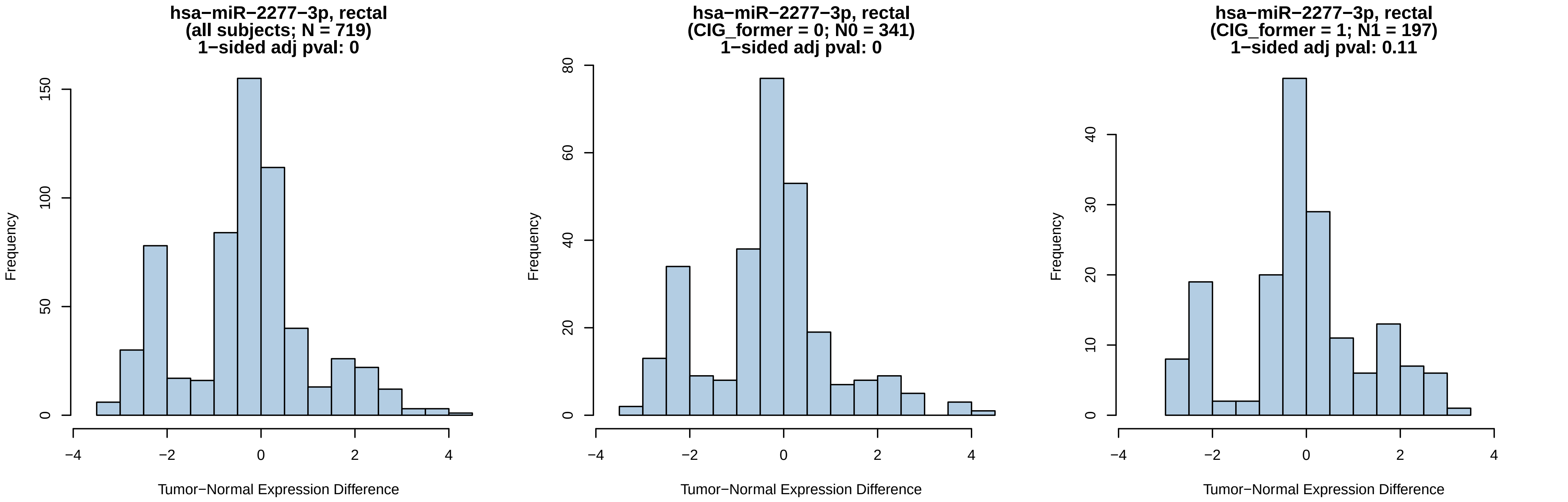

hsa−miR−2277−3p, rectal
(all subjects; N = 719)
1−sided adj pval: 0
Frequency
0
50
100
150
−4
−2
0
2
4
Tumor−Normal Expression Difference
hsa−miR−2277−3p, rectal
(CIG_former = 0; N0 = 341)
1−sided adj pval: 0
Frequency
0
20
40
60
80
−4
−2
0
2
4
Tumor−Normal Expression Difference
hsa−miR−2277−3p, rectal
(CIG_former = 1; N1 = 197)
1−sided adj pval: 0.11
Frequency
0
10
20
30
40
−4
−2
0
2
4
Tumor−Normal Expression Difference

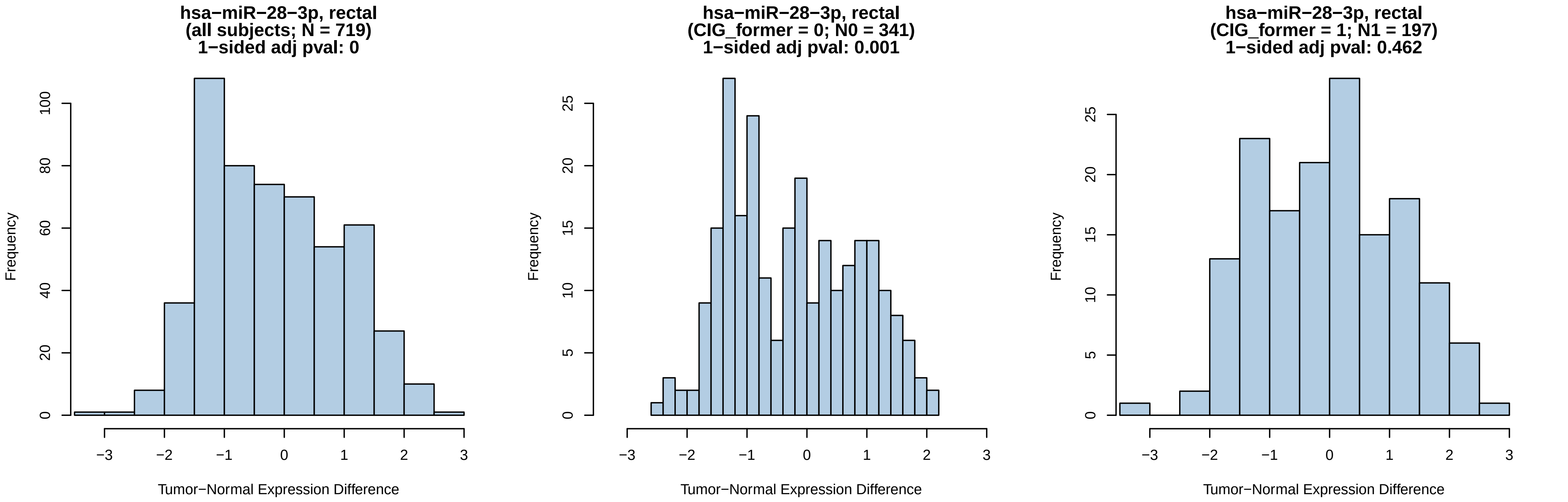
hsa−miR−28−3p, rectal
(all subjects; N = 719)
1−sided adj pval: 0
Frequency
Tumor−Normal Expression Difference
hsa−miR−28−3p, rectal
(CIG_former = 0; N0 = 341)
1−sided adj pval: 0.001
Frequency
Tumor−Normal Expression Difference
hsa−miR−28−3p, rectal
(CIG_former = 1; N1 = 197)
1−sided adj pval: 0.462
Frequency
Tumor−Normal Expression Difference

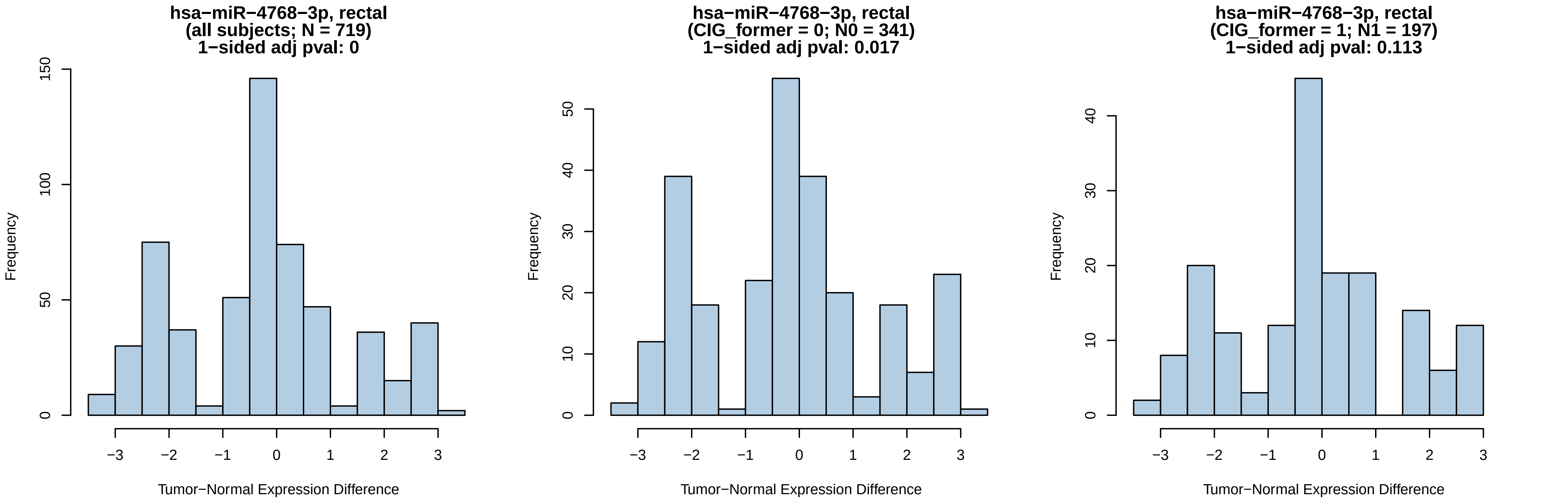

hsa−miR−4768−3p, rectal
(all subjects; N = 719)
1−sided adj pval: 0
Frequency
Tumor−Normal Expression Difference
hsa−miR−4768−3p, rectal
(CIG_former = 0; N0 = 341)
1−sided adj pval: 0.017
Frequency
Tumor−Normal Expression Difference
hsa−miR−4768−3p, rectal
(CIG_former = 1; N1 = 197)
1−sided adj pval: 0.113
Frequency
Tumor−Normal Expression Difference

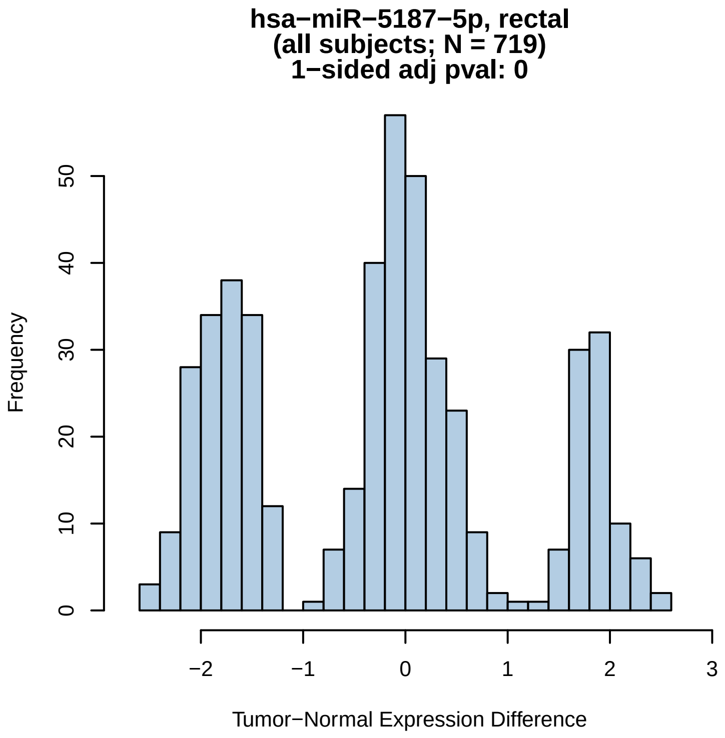

hsa−miR−5187−5p, rectal
(all subjects; N = 719)
1−sided adj pval: 0
Frequency
Tumor−Normal Expression Difference


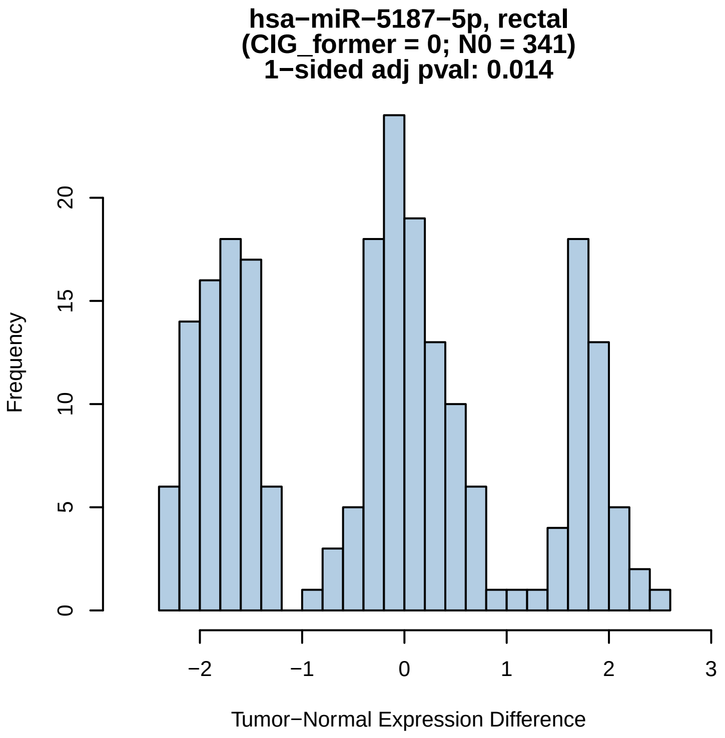

hsa−miR−5187−5p, rectal
(CIG_former = 0; N0 = 341)
1−sided adj pval: 0.014
Frequency
Tumor−Normal Expression Difference


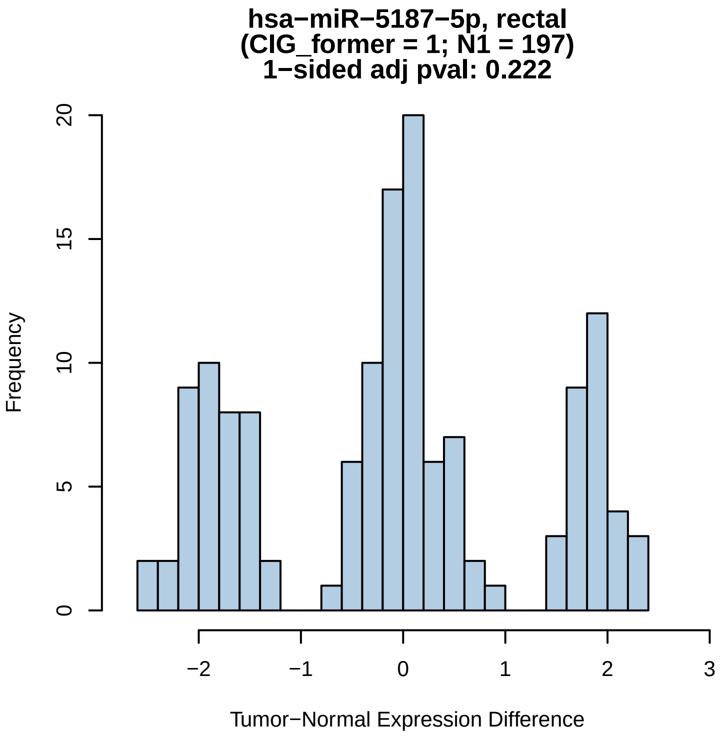

hsa−miR−5187−5p, rectal
(CIG_former = 1; N1 = 197)
1−sided adj pval: 0.222
Frequency
Tumor−Normal Expression Difference

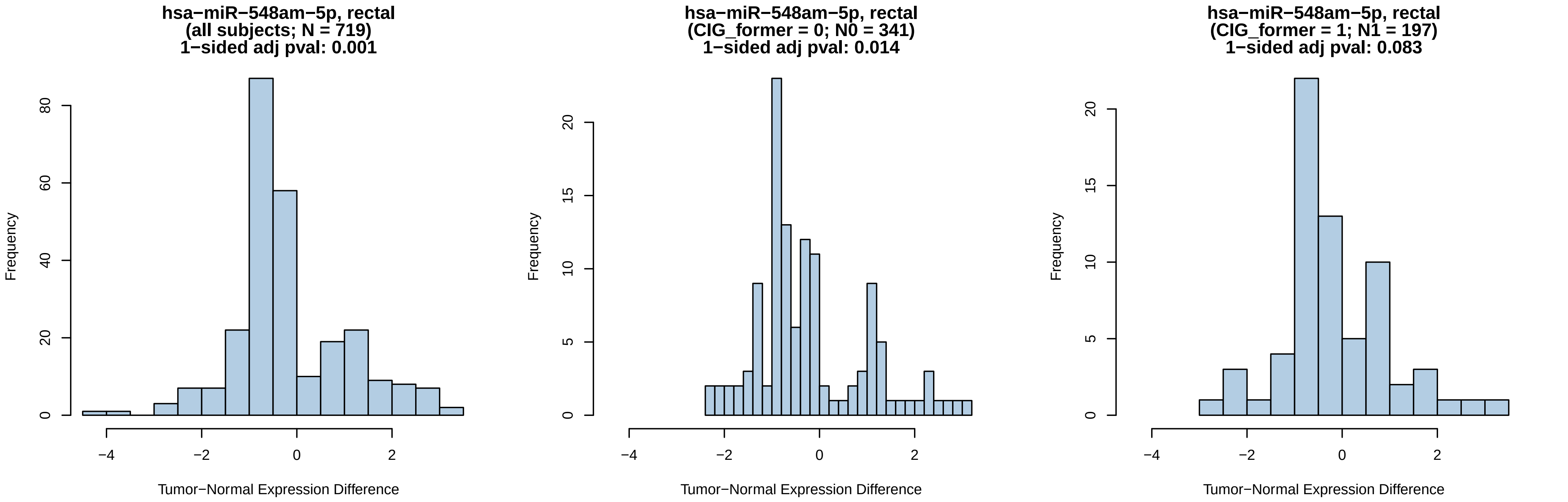

hsa−miR−548am−5p, rectal
(all subjects; N = 719)
1−sided adj pval: 0.001
hsa−miR−548am−5p, rectal
(CIG_former = 0; N0 = 341)
1−sided adj pval: 0.014
hsa−miR−548am−5p, rectal
(CIG_former = 1; N1 = 197)
1−sided adj pval: 0.083
Frequency
Tumor−Normal Expression Difference

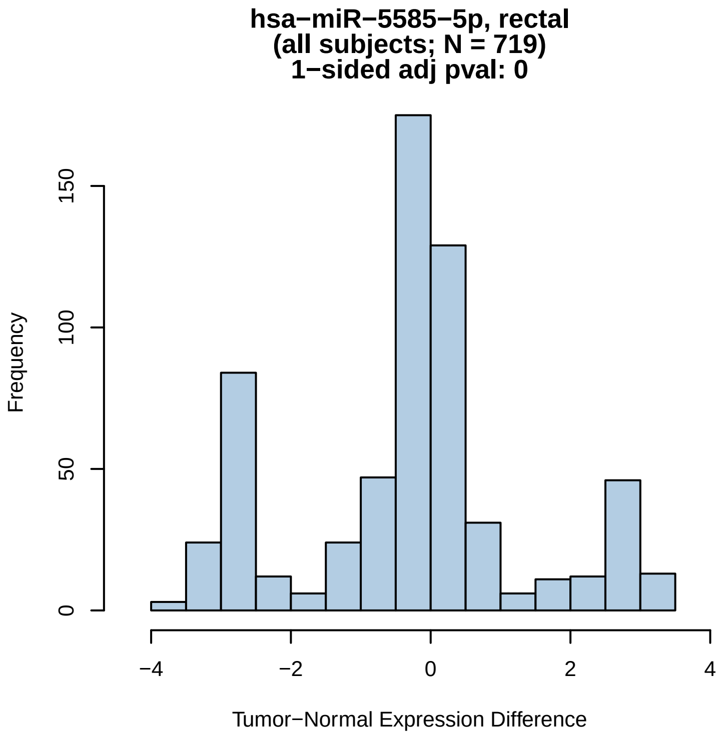
hsa−miR−5585−5p, rectal
(all subjects; N = 719)
1−sided adj pval: 0
Frequency
0
50
100
150
−4
−2
0
2
4
Tumor−Normal Expression Difference

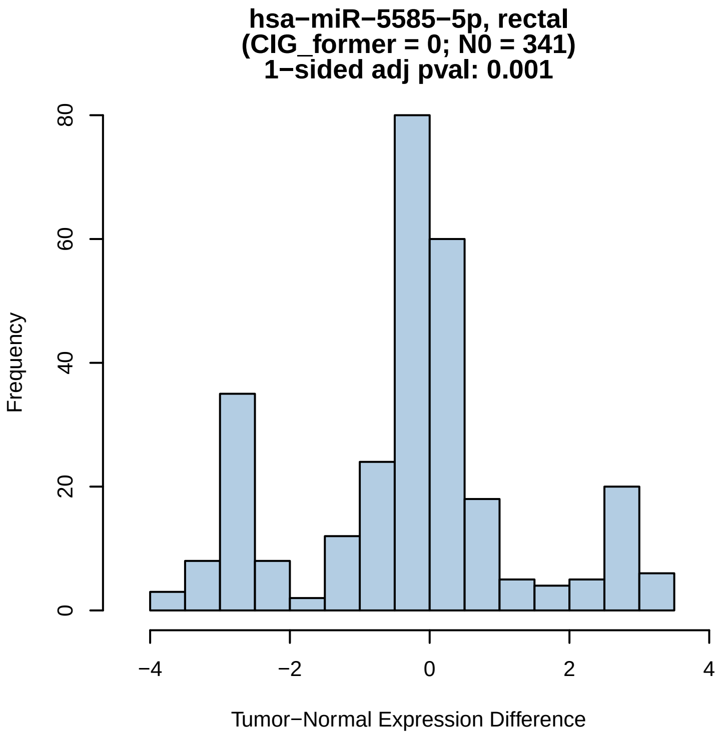
hsa−miR−5585−5p, rectal
(CIG_former = 0; N0 = 341)
1−sided adj pval: 0.001
Frequency
0
20
40
60
80
−4
−2
0
2
4
Tumor−Normal Expression Difference

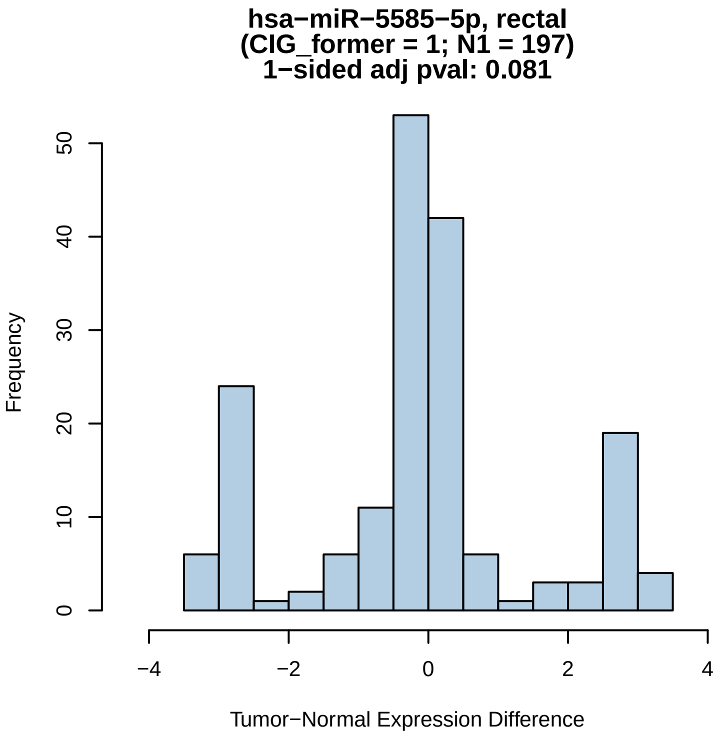
hsa−miR−5585−5p, rectal
(CIG_former = 1; N1 = 197)
1−sided adj pval: 0.081
Frequency
0
10
20
30
40
50
−4
−2
0
2
4
Tumor−Normal Expression Difference

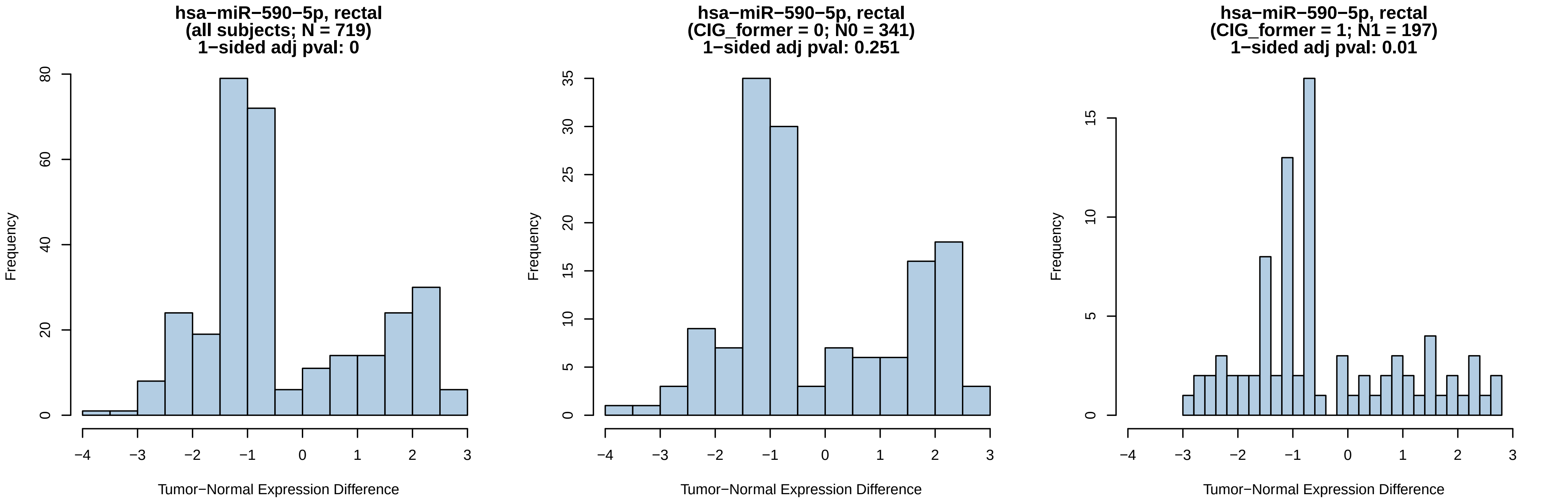
hsa−miR−590−5p, rectal
(all subjects; N = 719)
1−sided adj pval: 0
Frequency
Tumor−Normal Expression Difference
hsa−miR−590−5p, rectal
(CIG_former = 0; N0 = 341)
1−sided adj pval: 0.251
Frequency
Tumor−Normal Expression Difference
hsa−miR−590−5p, rectal
(CIG_former = 1; N1 = 197)
1−sided adj pval: 0.01
Frequency
Tumor−Normal Expression Difference

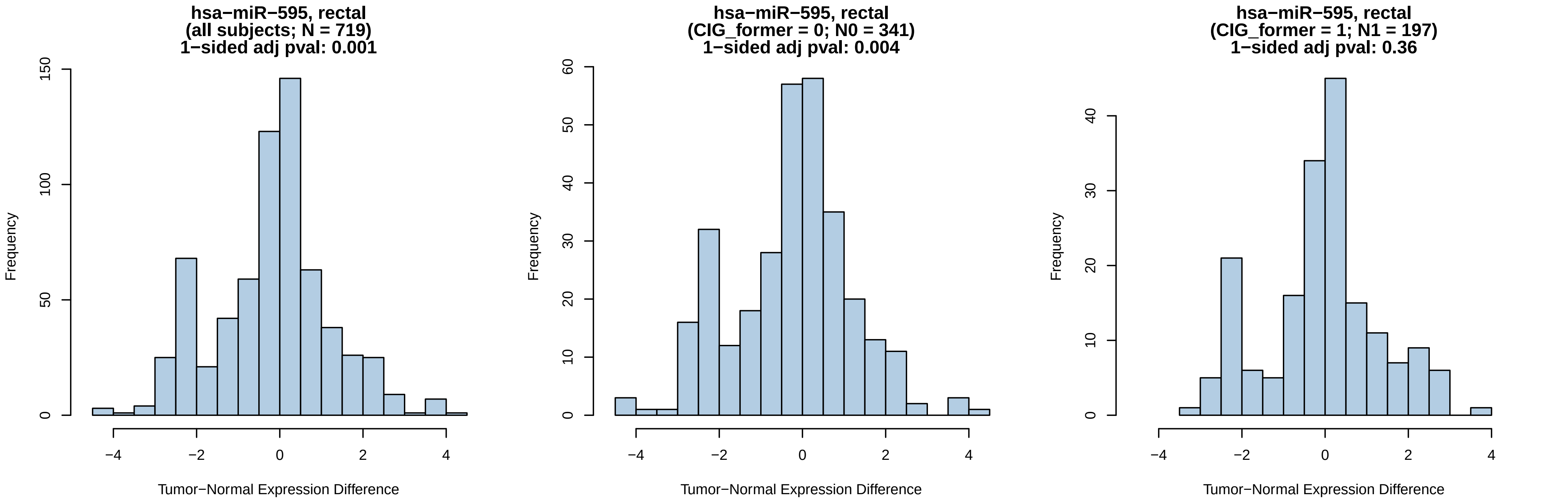
hsa−miR−595, rectal
(all subjects; N = 719)
1−sided adj pval: 0.001
Frequency
0
50
100
150
−4
−2
0
2
4
Tumor−Normal Expression Difference
hsa−miR−595, rectal
(CIG_former = 0; N0 = 341)
1−sided adj pval: 0.004
Frequency
0
10
20
30
40
50
60
−4
−2
0
2
4
Tumor−Normal Expression Difference
hsa−miR−595, rectal
(CIG_former = 1; N1 = 197)
1−sided adj pval: 0.36
Frequency
0
10
20
30
40
−4
−2
0
2
4
Tumor−Normal Expression Difference

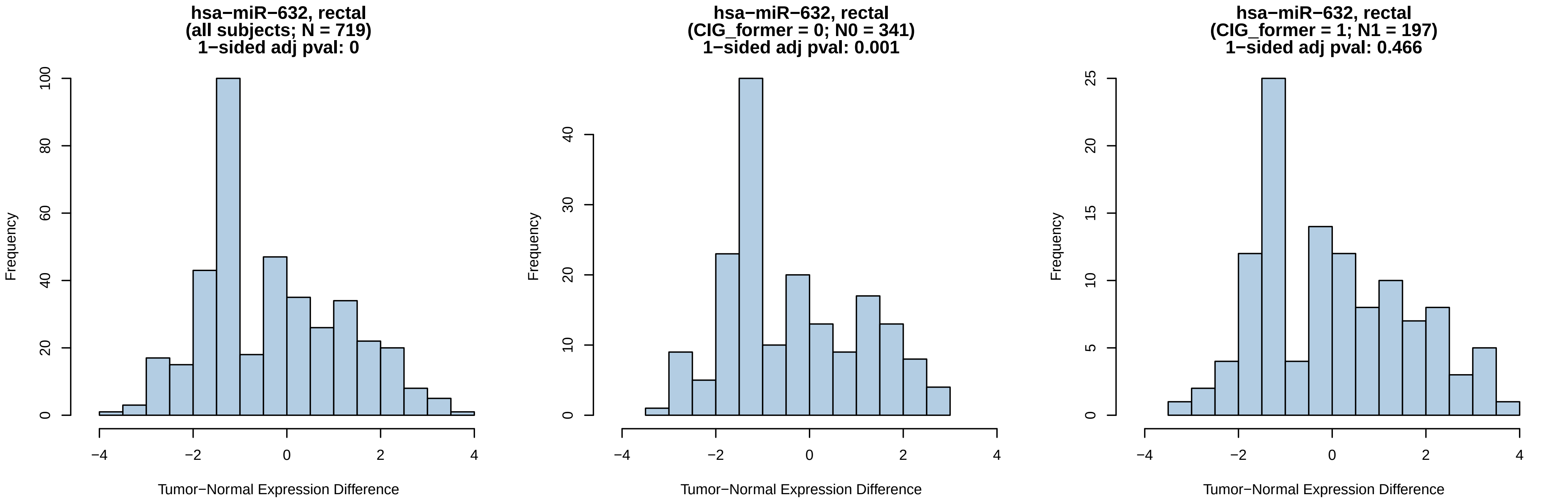
hsa−miR−632, rectal
(all subjects; N = 719)
1−sided adj pval: 0
Frequency
0
20
40
60
80
100
−4
−2
0
2
4
Tumor−Normal Expression Difference
hsa−miR−632, rectal
(CIG_former = 0; N0 = 341)
1−sided adj pval: 0.001
Frequency
0
10
20
30
40
−4
−2
0
2
4
Tumor−Normal Expression Difference
hsa−miR−632, rectal
(CIG_former = 1; N1 = 197)
1−sided adj pval: 0.466
Frequency
0
5
10
15
20
25
−4
−2
0
2
4
Tumor−Normal Expression Difference

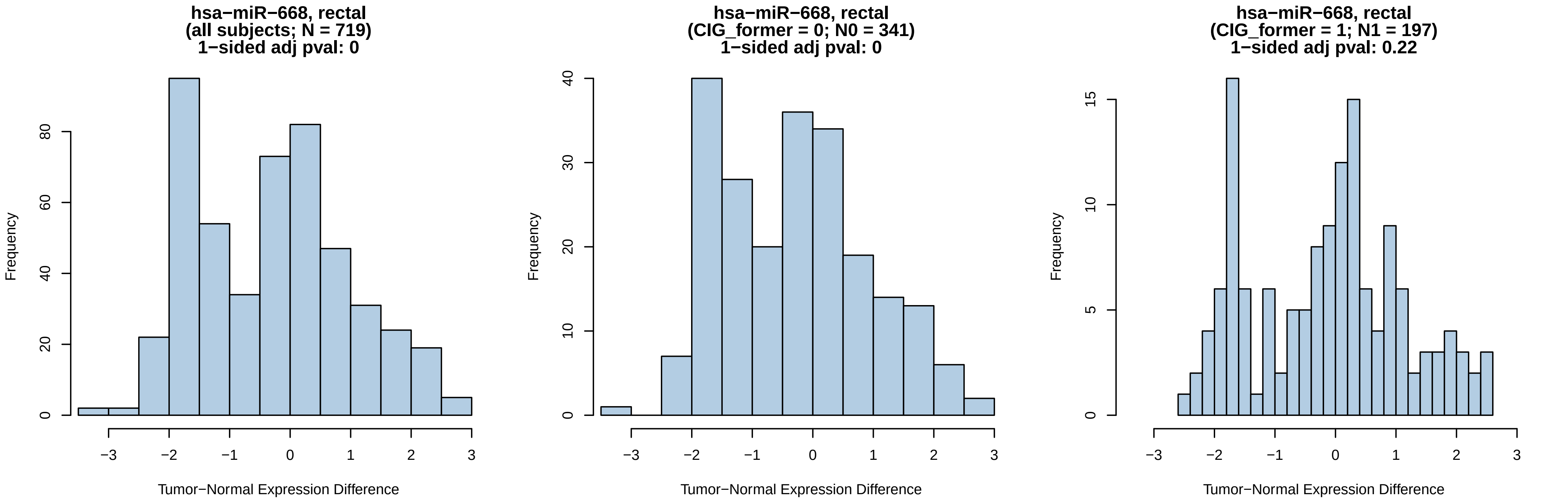

hsa−miR−668, rectal
(all subjects; N = 719)
1−sided adj pval: 0
Frequency
Tumor−Normal Expression Difference
hsa−miR−668, rectal
(CIG_former = 0; N0 = 341)
1−sided adj pval: 0
Frequency
Tumor−Normal Expression Difference
hsa−miR−668, rectal
(CIG_former = 1; N1 = 197)
1−sided adj pval: 0.22
Frequency
Tumor−Normal Expression Difference

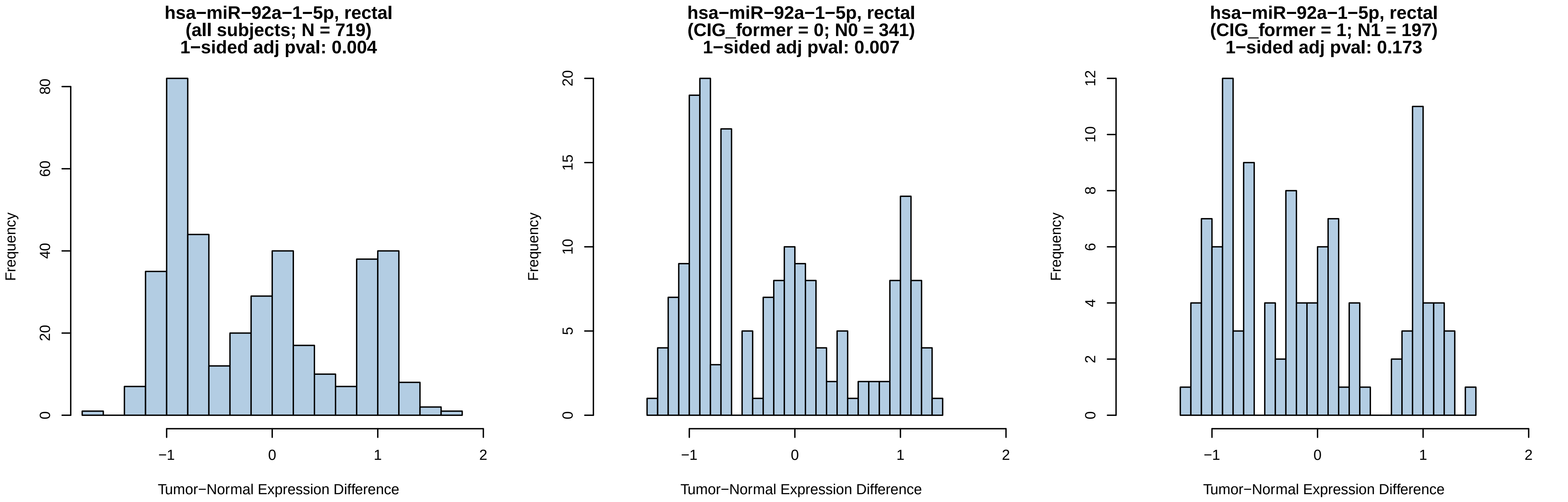

hsa−miR−92a−1−5p, rectal
(all subjects; N = 719)
1−sided adj pval: 0.004
Frequency
Tumor−Normal Expression Difference
hsa−miR−92a−1−5p, rectal
(CIG_former = 0; N0 = 341)
1−sided adj pval: 0.007
Frequency
Tumor−Normal Expression Difference
hsa−miR−92a−1−5p, rectal
(CIG_former = 1; N1 = 197)
1−sided adj pval: 0.173
Frequency
Tumor−Normal Expression Difference

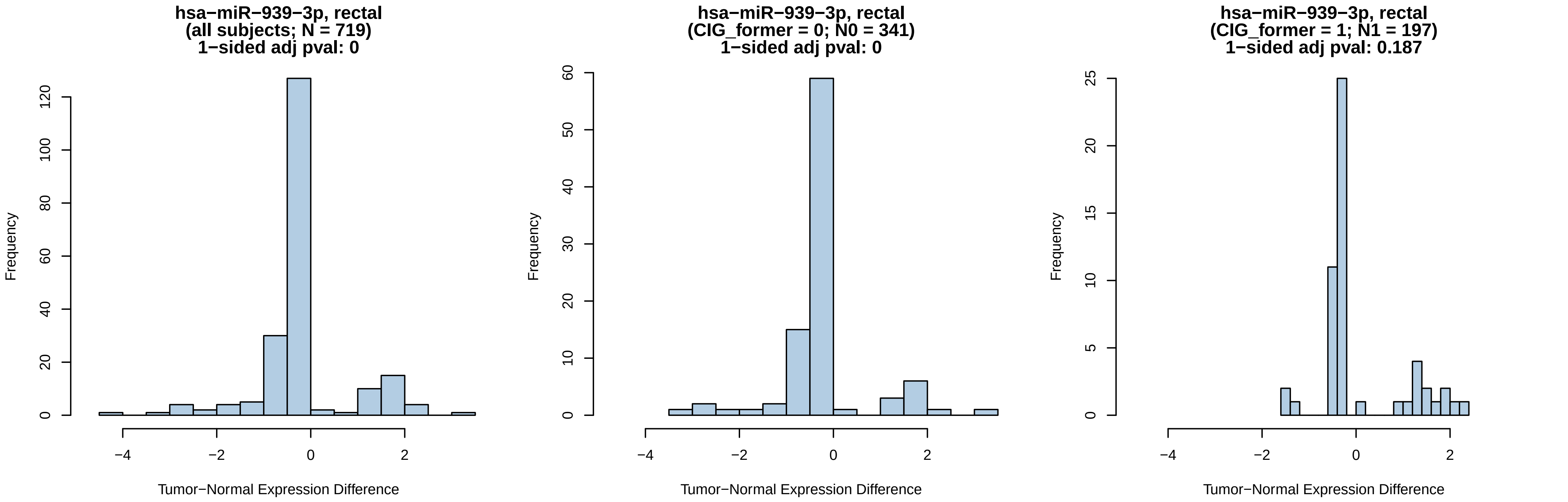

hsa−miR−939−3p, rectal
(all subjects; N = 719)
1−sided adj pval: 0
Frequency
Tumor−Normal Expression Difference
hsa−miR−939−3p, rectal
(CIG_former = 0; N0 = 341)
1−sided adj pval: 0
Frequency
Tumor−Normal Expression Difference
hsa−miR−939−3p, rectal
(CIG_former = 1; N1 = 197)
1−sided adj pval: 0.187
Frequency
Tumor−Normal Expression Difference

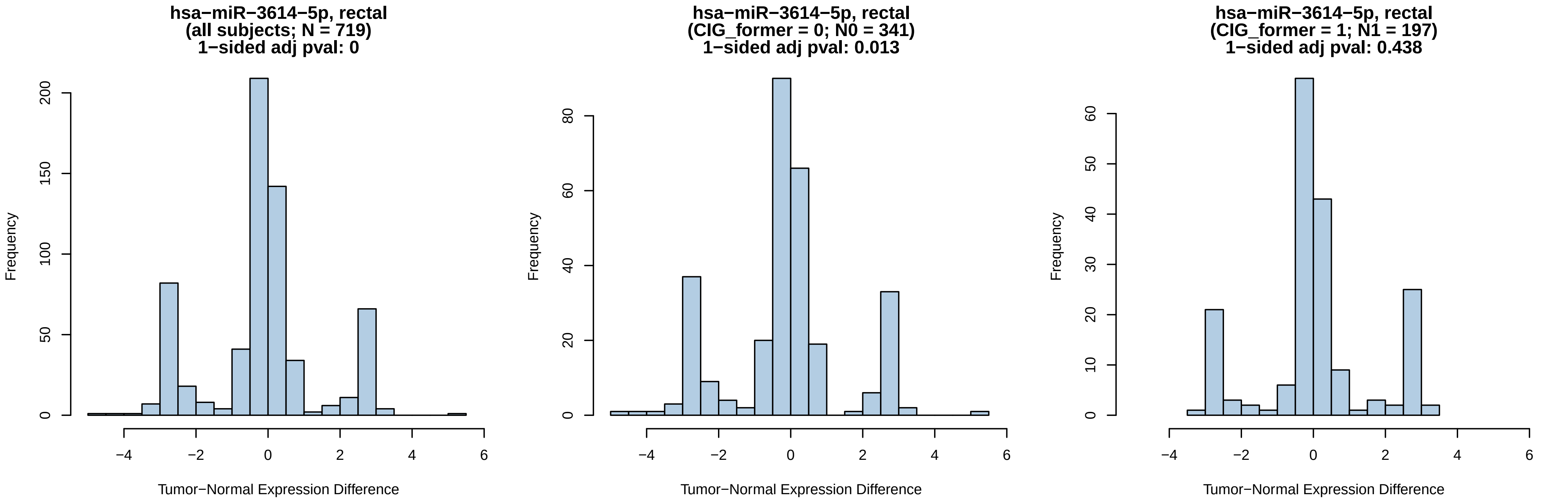
hsa−miR−3614−5p, rectal
(all subjects; N = 719)
1−sided adj pval: 0
Frequency
Tumor−Normal Expression Difference
hsa−miR−3614−5p, rectal
(CIG_former = 0; N0 = 341)
1−sided adj pval: 0.013
Frequency
Tumor−Normal Expression Difference
hsa−miR−3614−5p, rectal
(CIG_former = 1; N1 = 197)
1−sided adj pval: 0.438
Frequency
Tumor−Normal Expression Difference

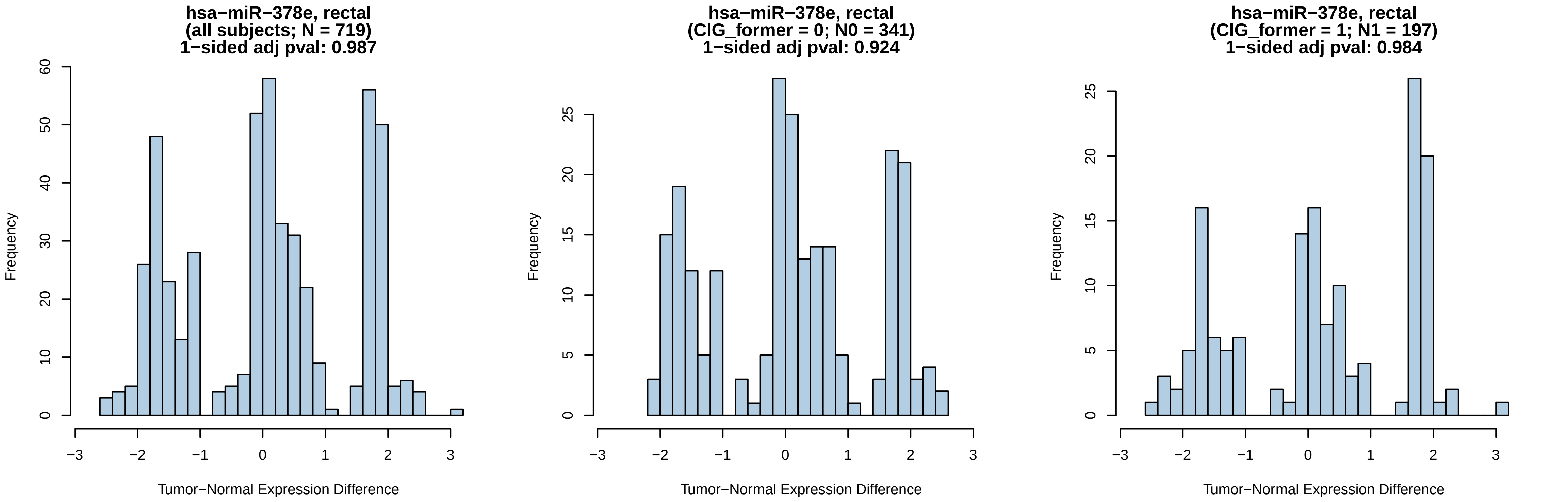

hsa−miR−378e, rectal
(all subjects; N = 719)
1−sided adj pval: 0.987
Frequency
Tumor−Normal Expression Difference
hsa−miR−378e, rectal
(CIG_former = 0; N0 = 341)
1−sided adj pval: 0.924
Frequency
Tumor−Normal Expression Difference
hsa−miR−378e, rectal
(CIG_former = 1; N1 = 197)
1−sided adj pval: 0.984
Frequency
Tumor−Normal Expression Difference

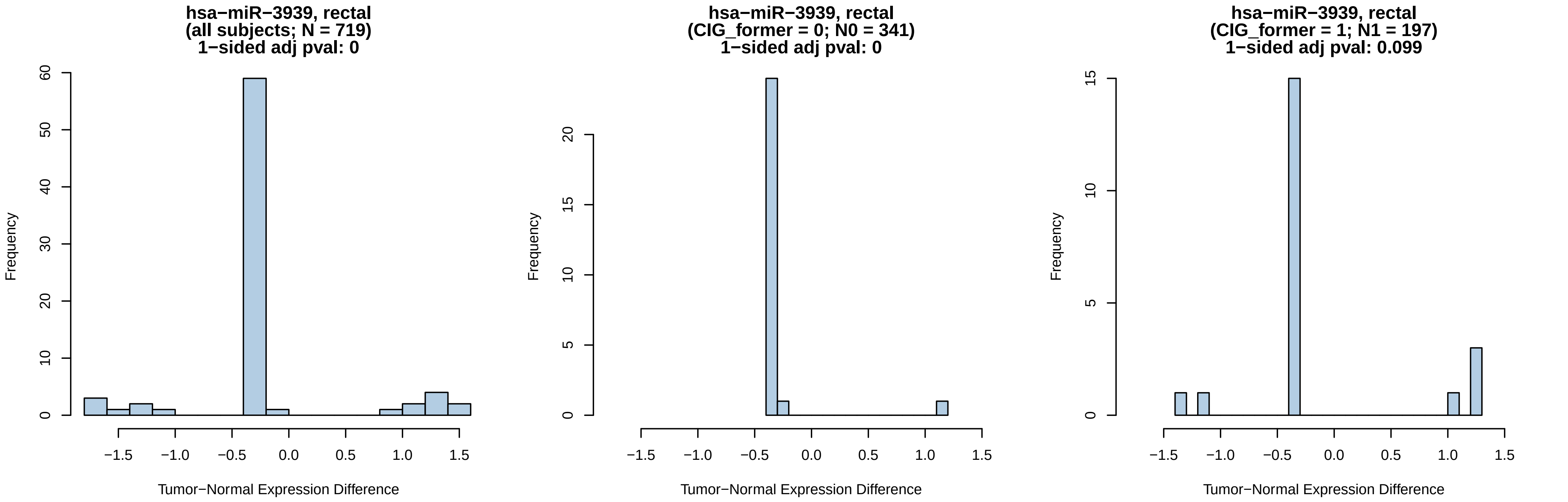

hsa−miR−3939, rectal
(all subjects; N = 719)
1−sided adj pval: 0
Frequency
Tumor−Normal Expression Difference
hsa−miR−3939, rectal
(CIG_former = 0; N0 = 341)
1−sided adj pval: 0
Frequency
Tumor−Normal Expression Difference
hsa−miR−3939, rectal
(CIG_former = 1; N1 = 197)
1−sided adj pval: 0.099
Frequency
Tumor−Normal Expression Difference

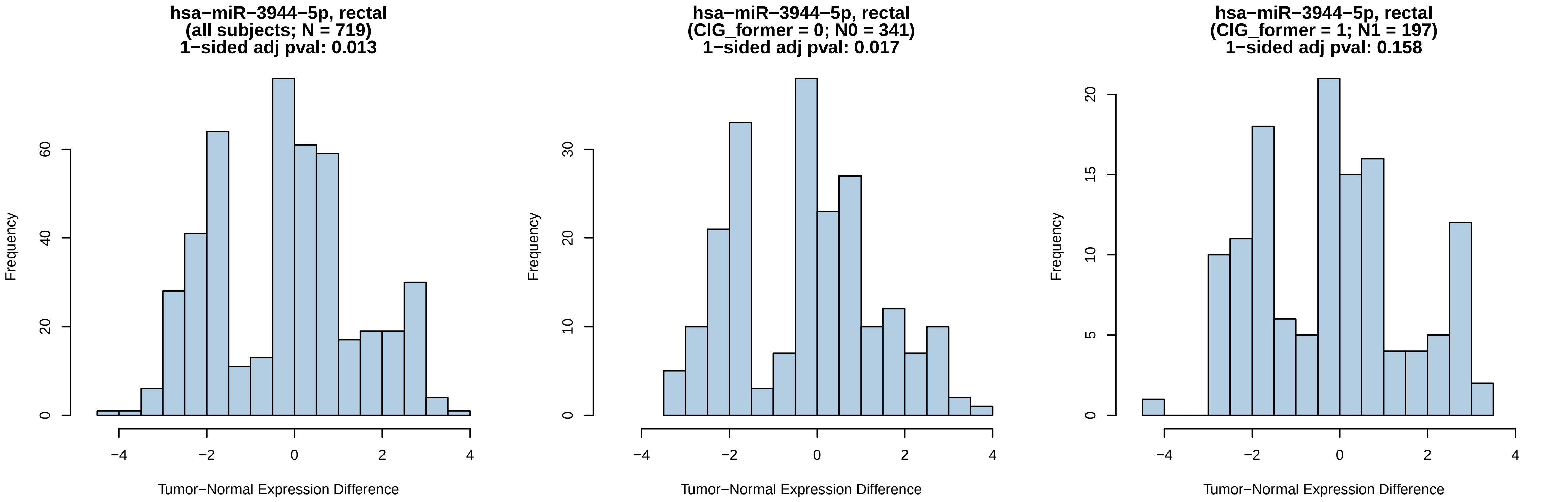
hsa−miR−3944−5p, rectal
(all subjects; N = 719)
1−sided adj pval: 0.013
hsa−miR−3944−5p, rectal
(CIG_former = 0; N0 = 341)
1−sided adj pval: 0.017
hsa−miR−3944−5p, rectal
(CIG_former = 1; N1 = 197)
1−sided adj pval: 0.158
Frequency
Tumor−Normal Expression Difference

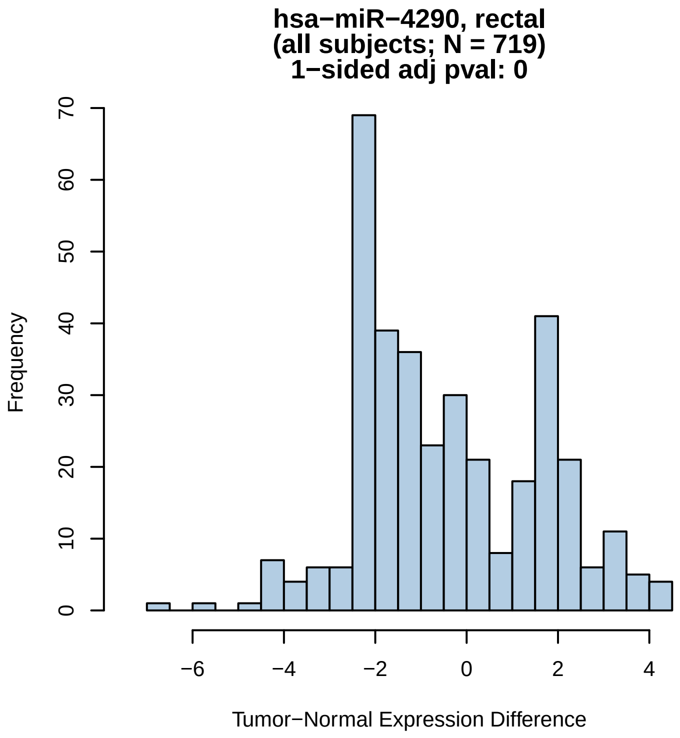
hsa−miR−4290, rectal
(all subjects; N = 719)
1−sided adj pval: 0
Frequency
Tumor−Normal Expression Difference

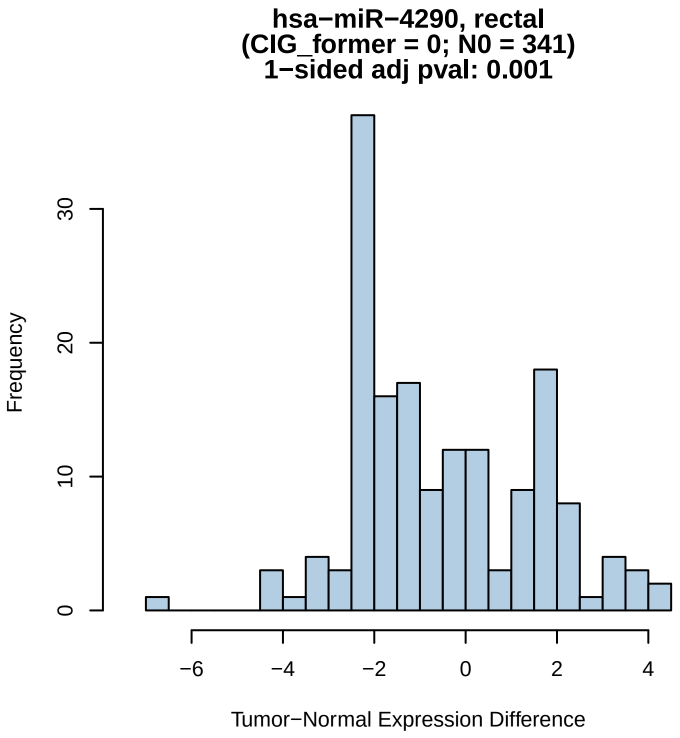
hsa−miR−4290, rectal
(CIG_former = 0; N0 = 341)
1−sided adj pval: 0.001
Frequency
Tumor−Normal Expression Difference

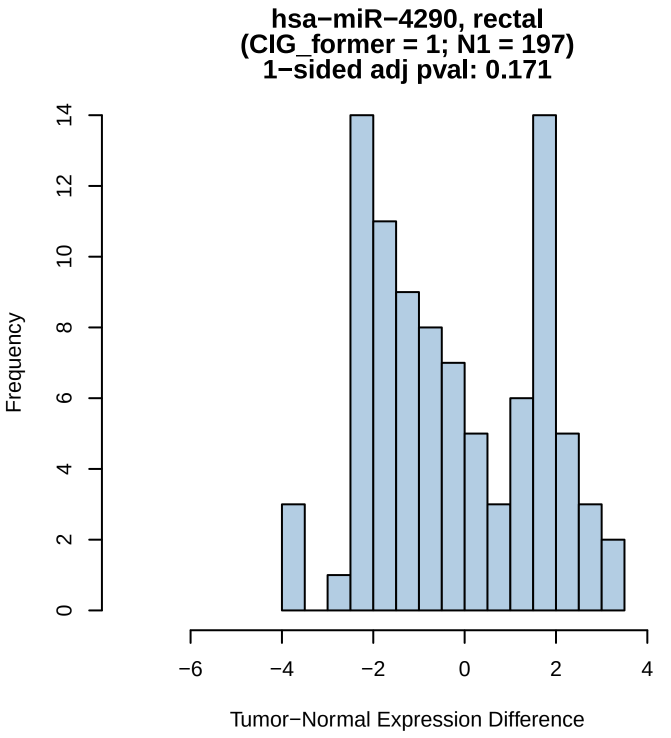
hsa−miR−4290, rectal
(CIG_former = 1; N1 = 197)
1−sided adj pval: 0.171
Frequency
Tumor−Normal Expression Difference

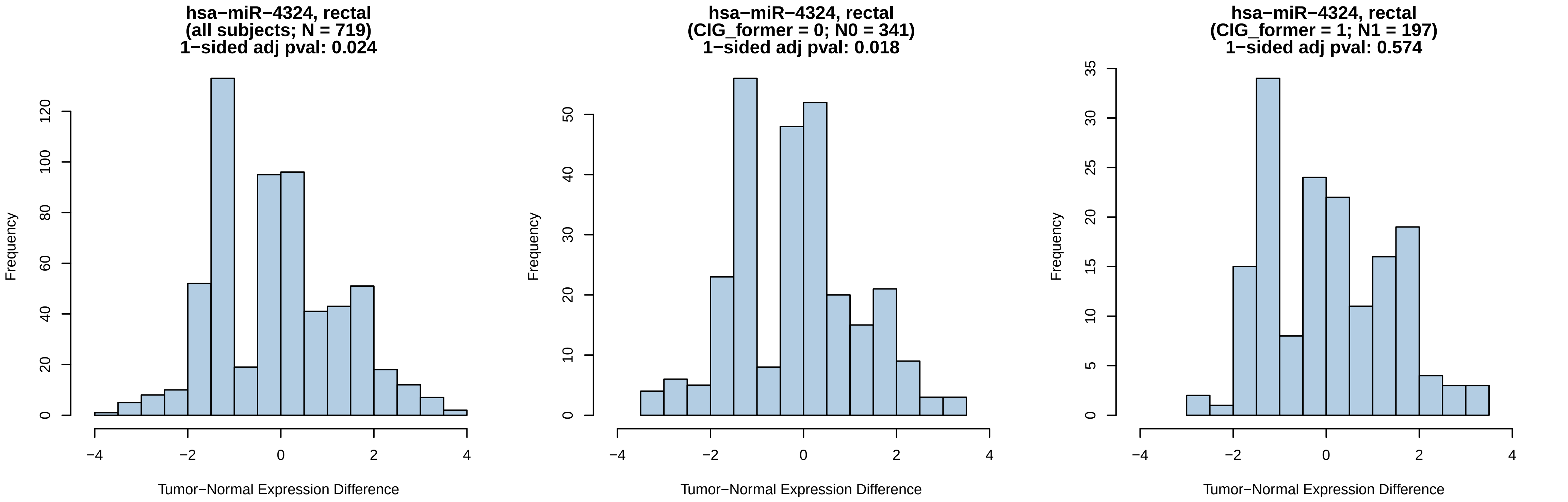

hsa−miR−4324, rectal
(all subjects; N = 719)
1−sided adj pval: 0.024
hsa−miR−4324, rectal
(CIG_former = 0; N0 = 341)
1−sided adj pval: 0.018
hsa−miR−4324, rectal
(CIG_former = 1; N1 = 197)
1−sided adj pval: 0.574
Frequency
0
20
40
60
80
100
120
0
10
20
30
40
50
0
5
10
15
20
25
30
35
−4
−2
0
2
4
Tumor−Normal Expression Difference

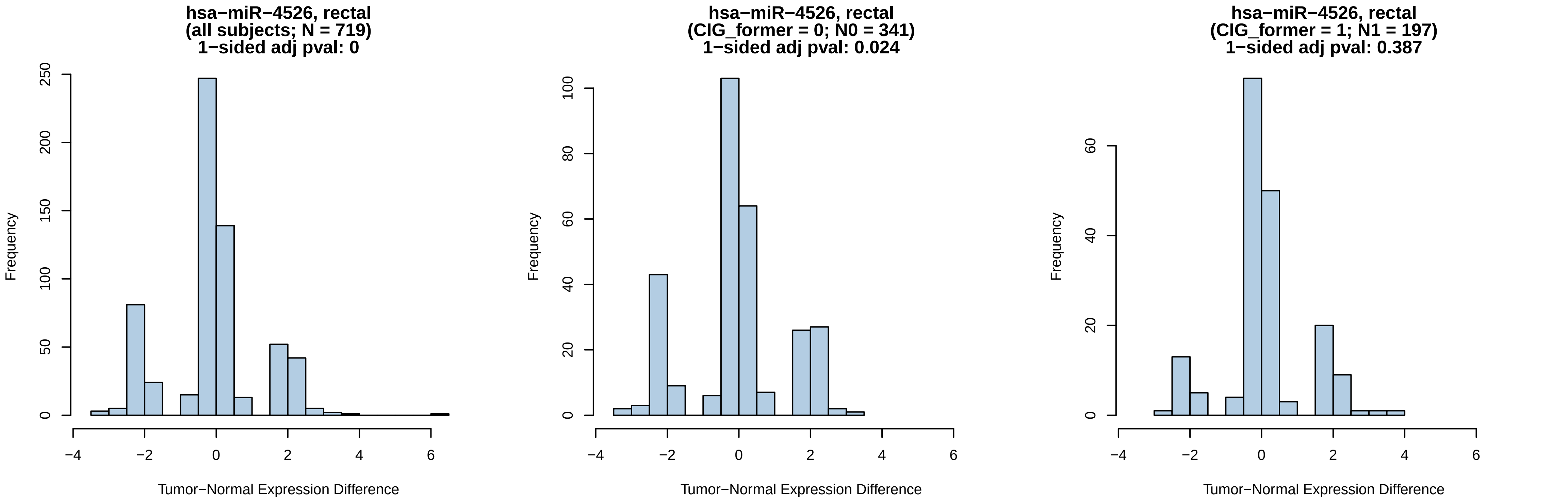
hsa−miR−4526, rectal
(all subjects; N = 719)
1−sided adj pval: 0
Frequency
Tumor−Normal Expression Difference
hsa−miR−4526, rectal
(CIG_former = 0; N0 = 341)
1−sided adj pval: 0.024
Frequency
Tumor−Normal Expression Difference
hsa−miR−4526, rectal
(CIG_former = 1; N1 = 197)
1−sided adj pval: 0.387
Frequency
Tumor−Normal Expression Difference

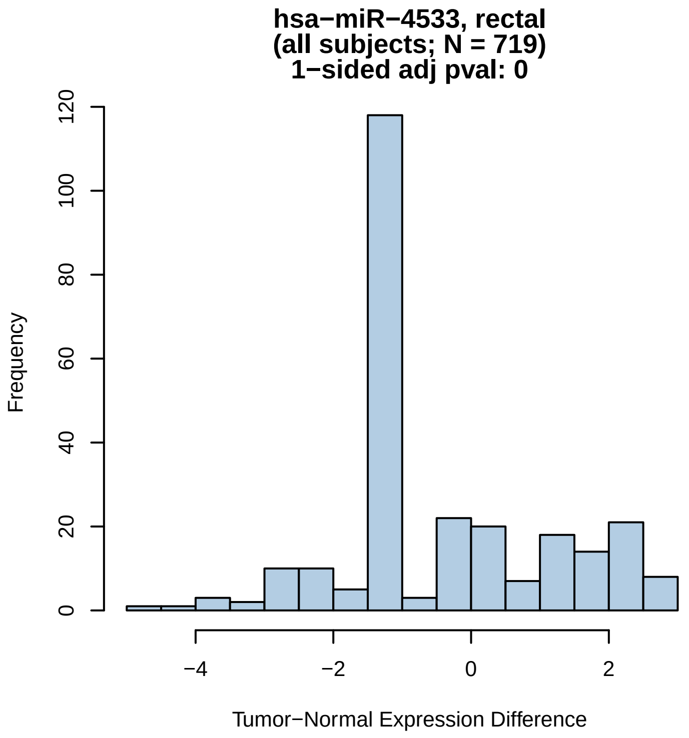
hsa−miR−4533, rectal
(all subjects; N = 719)
1−sided adj pval: 0
Frequency
0
20
40
60
80
100
120
−4
−2
0
2
Tumor−Normal Expression Difference

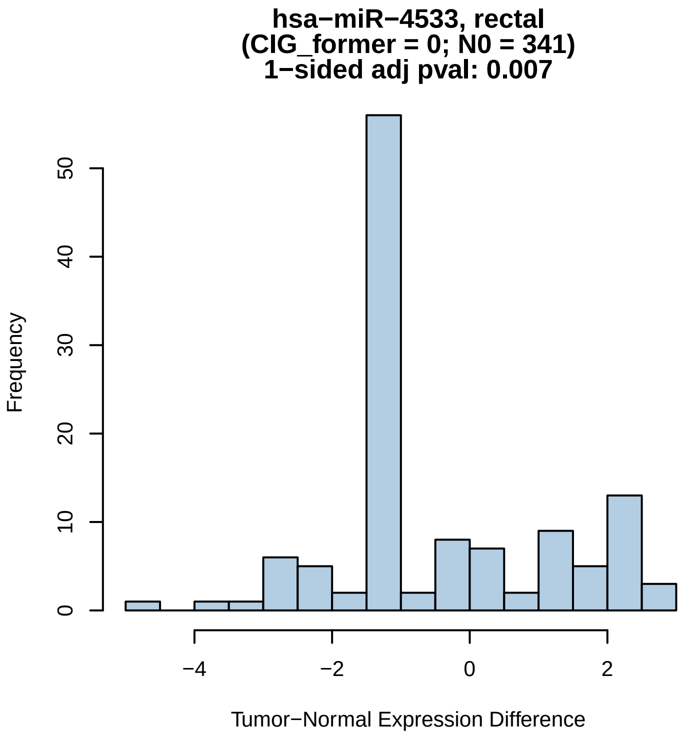
hsa−miR−4533, rectal
(CIG_former = 0; N0 = 341)
1−sided adj pval: 0.007
Frequency
0
10
20
30
40
50
−4
−2
0
2
Tumor−Normal Expression Difference

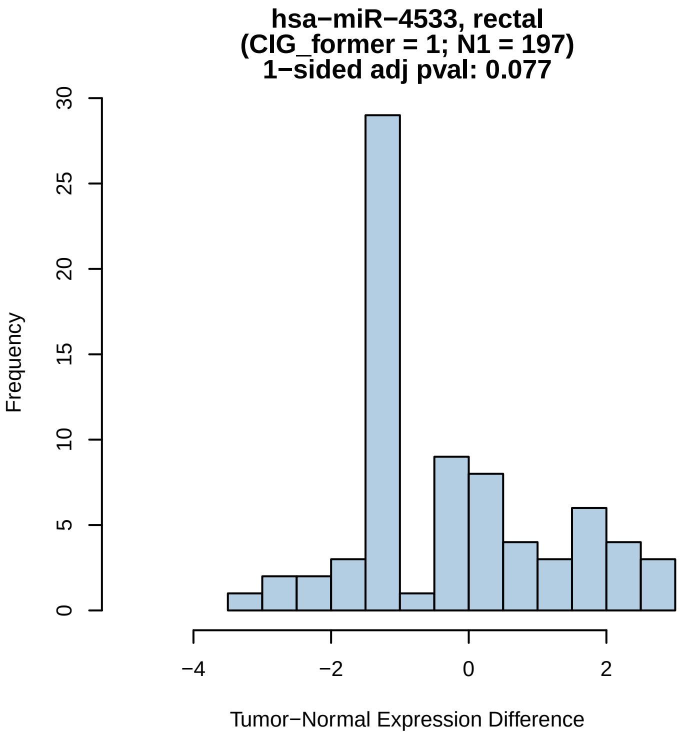
hsa−miR−4533, rectal
(CIG_former = 1; N1 = 197)
1−sided adj pval: 0.077
Frequency
0
5
10
15
20
25
30
−4
−2
0
2
Tumor−Normal Expression Difference

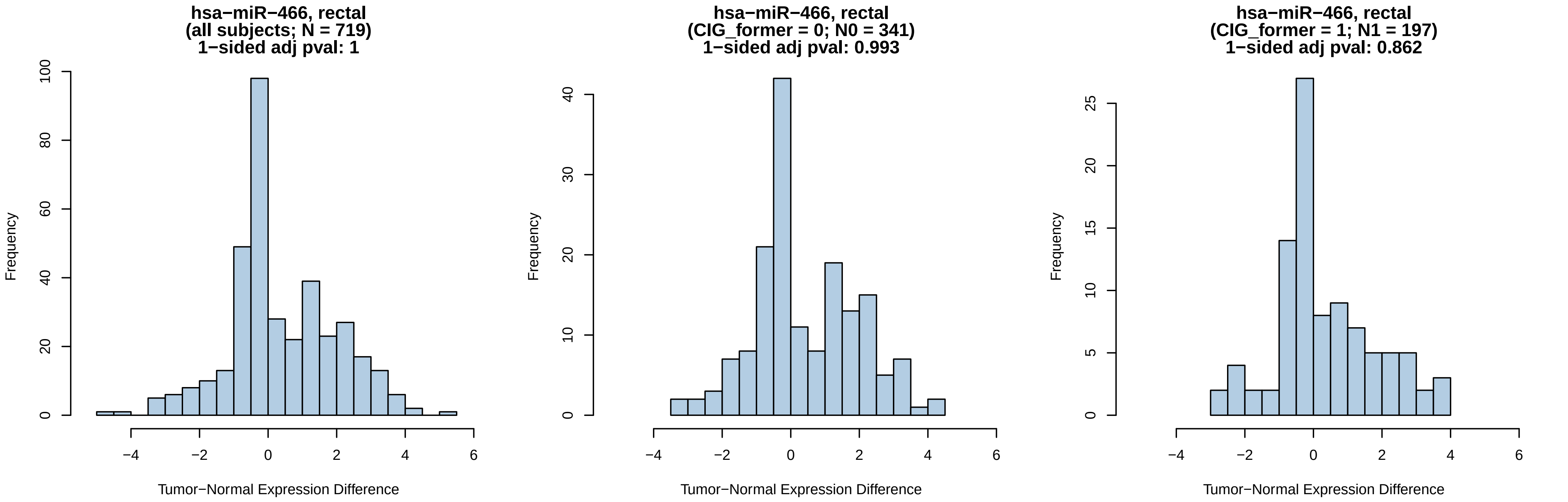
hsa−miR−466, rectal
(all subjects; N = 719)
1−sided adj pval: 1
Frequency
Tumor−Normal Expression Difference
hsa−miR−466, rectal
(CIG_former = 0; N0 = 341)
1−sided adj pval: 0.993
Frequency
Tumor−Normal Expression Difference
hsa−miR−466, rectal
(CIG_former = 1; N1 = 197)
1−sided adj pval: 0.862
Frequency
Tumor−Normal Expression Difference

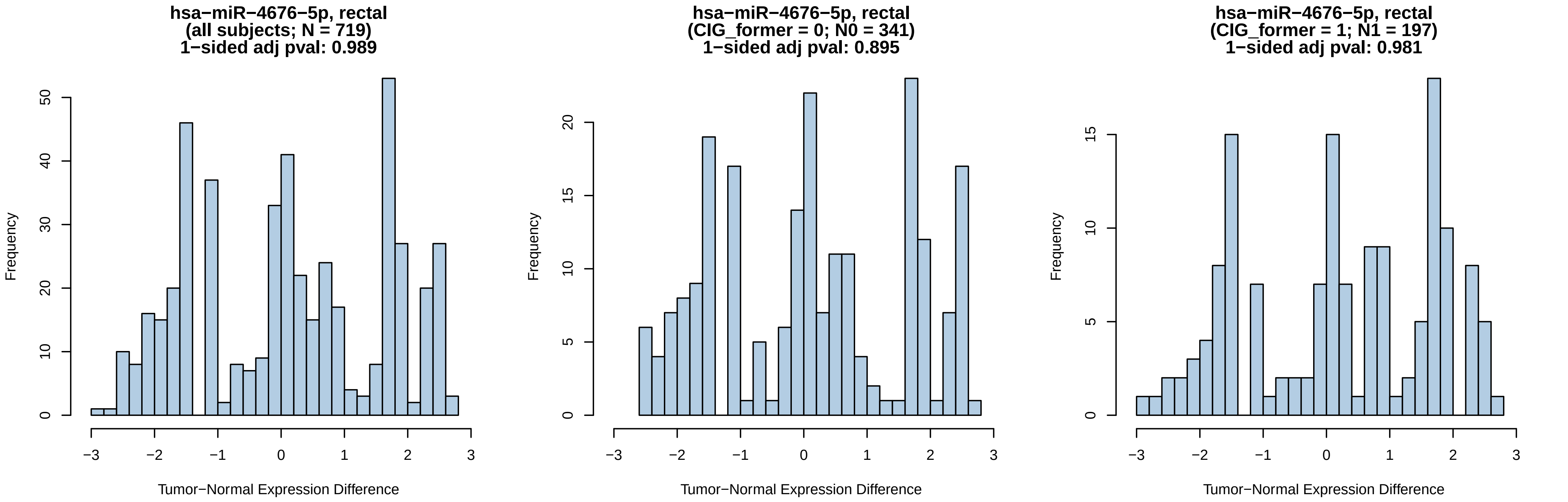

hsa−miR−4676−5p, rectal
(all subjects; N = 719)
1−sided adj pval: 0.989
Frequency
Tumor−Normal Expression Difference
hsa−miR−4676−5p, rectal
(CIG_former = 0; N0 = 341)
1−sided adj pval: 0.895
Frequency
Tumor−Normal Expression Difference
hsa−miR−4676−5p, rectal
(CIG_former = 1; N1 = 197)
1−sided adj pval: 0.981
Frequency
Tumor−Normal Expression Difference

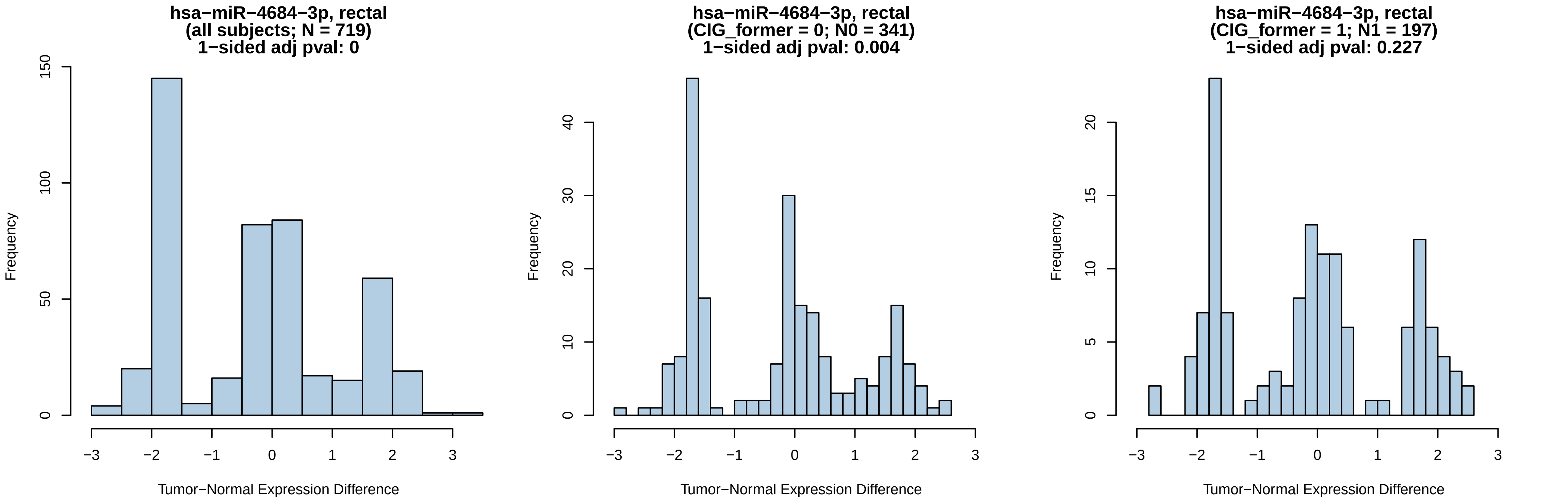
hsa−miR−4684−3p, rectal
(all subjects; N = 719)
1−sided adj pval: 0
hsa−miR−4684−3p, rectal
(CIG_former = 0; N0 = 341)
1−sided adj pval: 0.004
hsa−miR−4684−3p, rectal
(CIG_former = 1; N1 = 197)
1−sided adj pval: 0.227
Frequency
Tumor−Normal Expression Difference

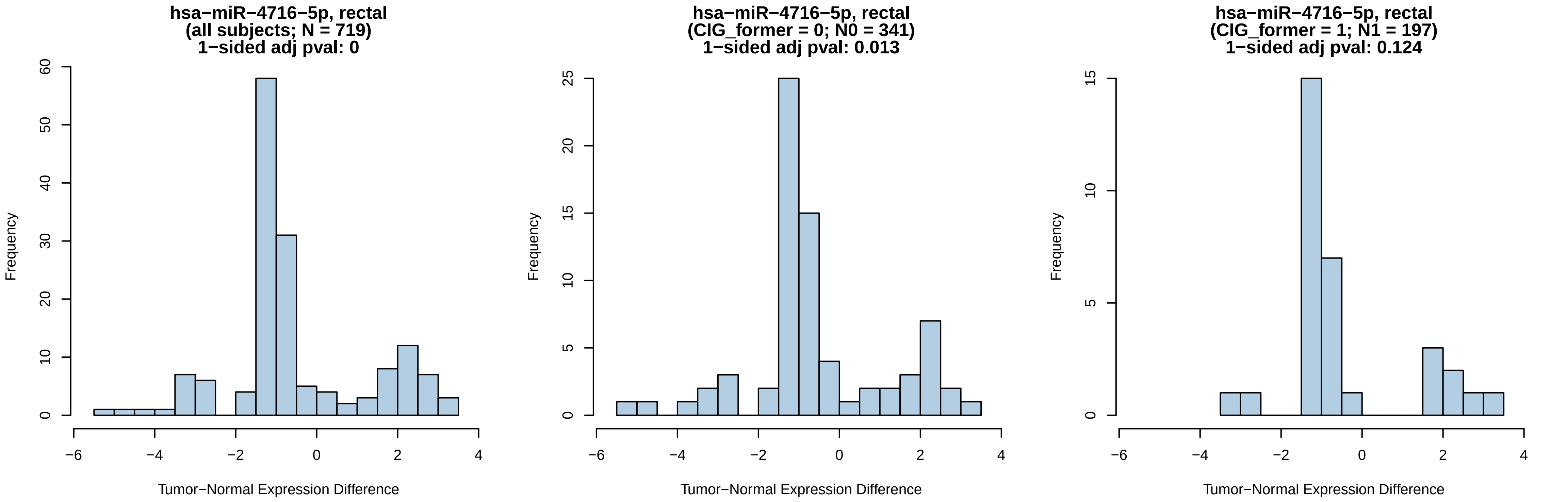

hsa−miR−4716−5p, rectal
(all subjects; N = 719)
1−sided adj pval: 0
Frequency
0
10
20
30
40
50
60
−6
−4
−2
0
2
4
Tumor−Normal Expression Difference
hsa−miR−4716−5p, rectal
(CIG_former = 0; N0 = 341)
1−sided adj pval: 0.013
Frequency
0
5
10
15
20
25
−6
−4
−2
0
2
4
Tumor−Normal Expression Difference
hsa−miR−4716−5p, rectal
(CIG_former = 1; N1 = 197)
1−sided adj pval: 0.124
Frequency
0
5
10
15
−6
−4
−2
0
2
4
Tumor−Normal Expression Difference

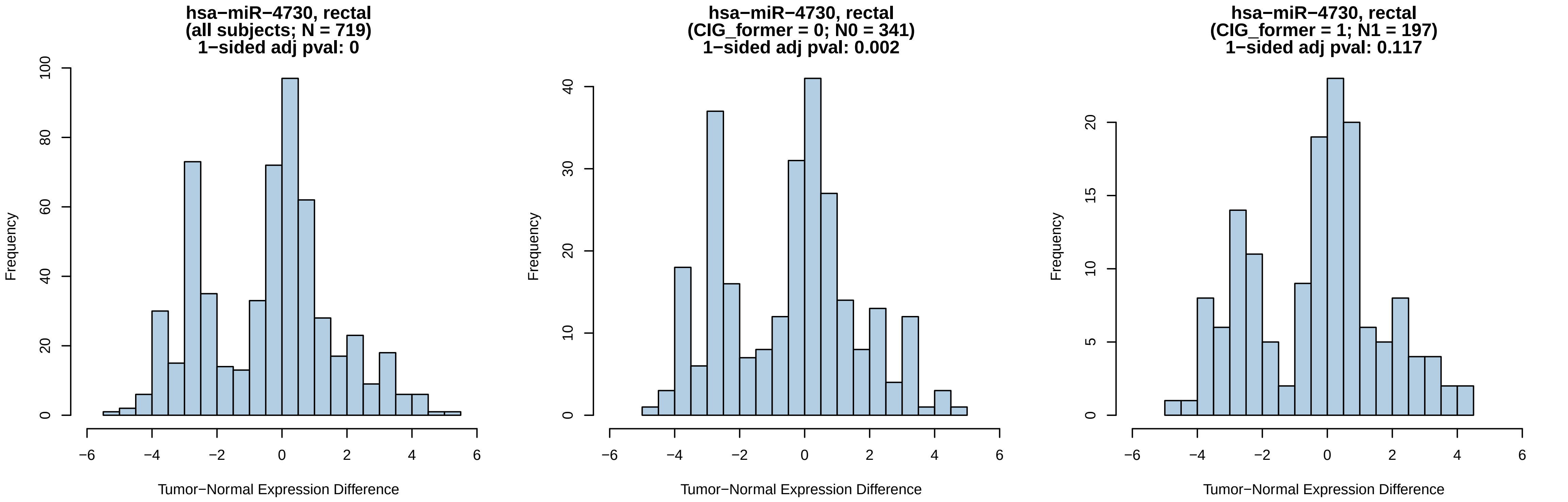
hsa−miR−4730, rectal
(all subjects; N = 719)
1−sided adj pval: 0
hsa−miR−4730, rectal
(CIG_former = 0; N0 = 341)
1−sided adj pval: 0.002
hsa−miR−4730, rectal
(CIG_former = 1; N1 = 197)
1−sided adj pval: 0.117
Frequency
Tumor−Normal Expression Difference

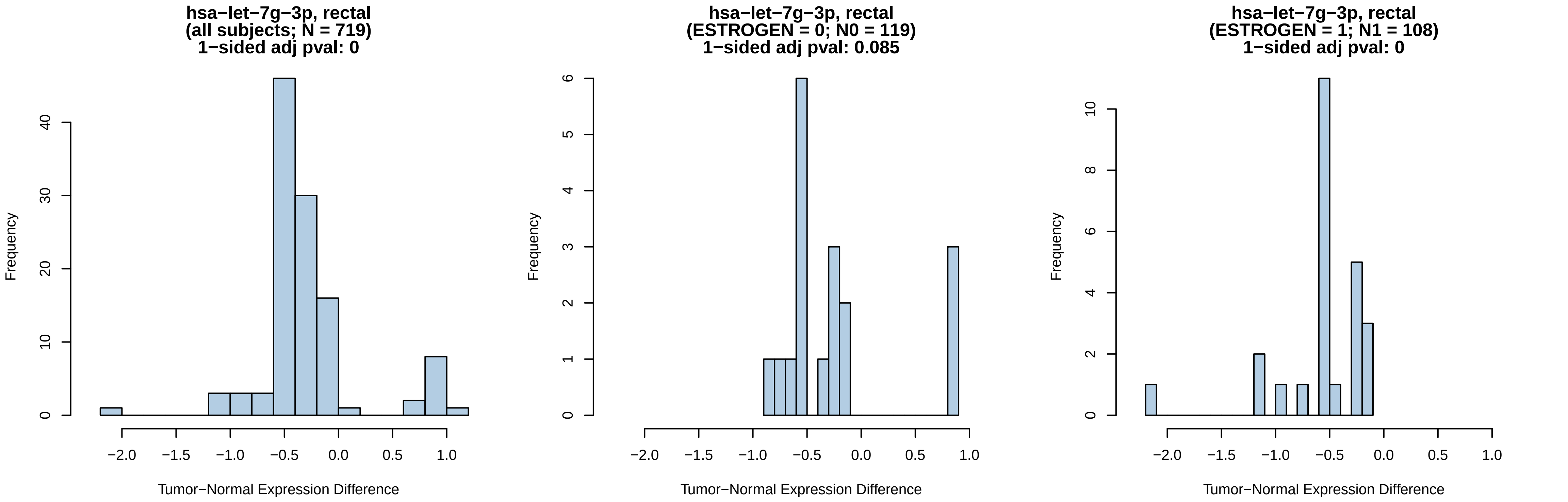

hsa−let−7g−3p, rectal
(all subjects; N = 719)
1−sided adj pval: 0
hsa−let−7g−3p, rectal
(ESTROGEN = 0; N0 = 119)
1−sided adj pval: 0.085
hsa−let−7g−3p, rectal
(ESTROGEN = 1; N1 = 108)
1−sided adj pval: 0
Frequency
Tumor−Normal Expression Difference

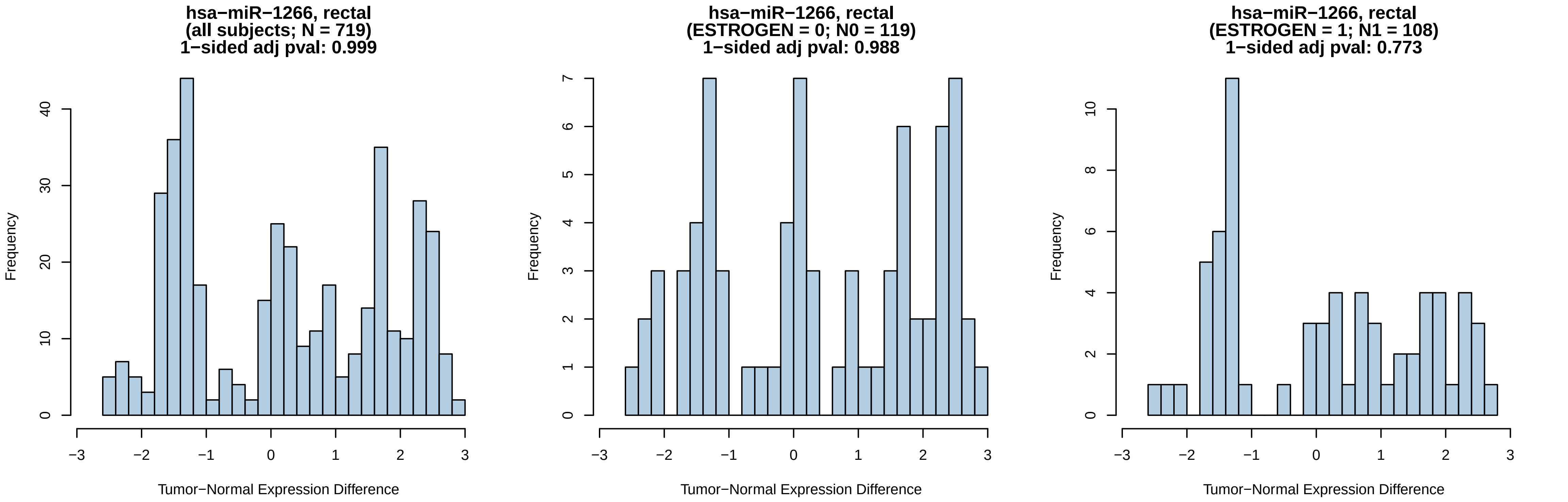
hsa−miR−1266, rectal
(all subjects; N = 719)
1−sided adj pval: 0.999
hsa−miR−1266, rectal
(ESTROGEN = 0; N0 = 119)
1−sided adj pval: 0.988
hsa−miR−1266, rectal
(ESTROGEN = 1; N1 = 108)
1−sided adj pval: 0.773
Frequency
Tumor−Normal Expression Difference
−3
−2
−1
0
1
2
3
0
10
20
30
40
1
2
3
4
5
6
7
8

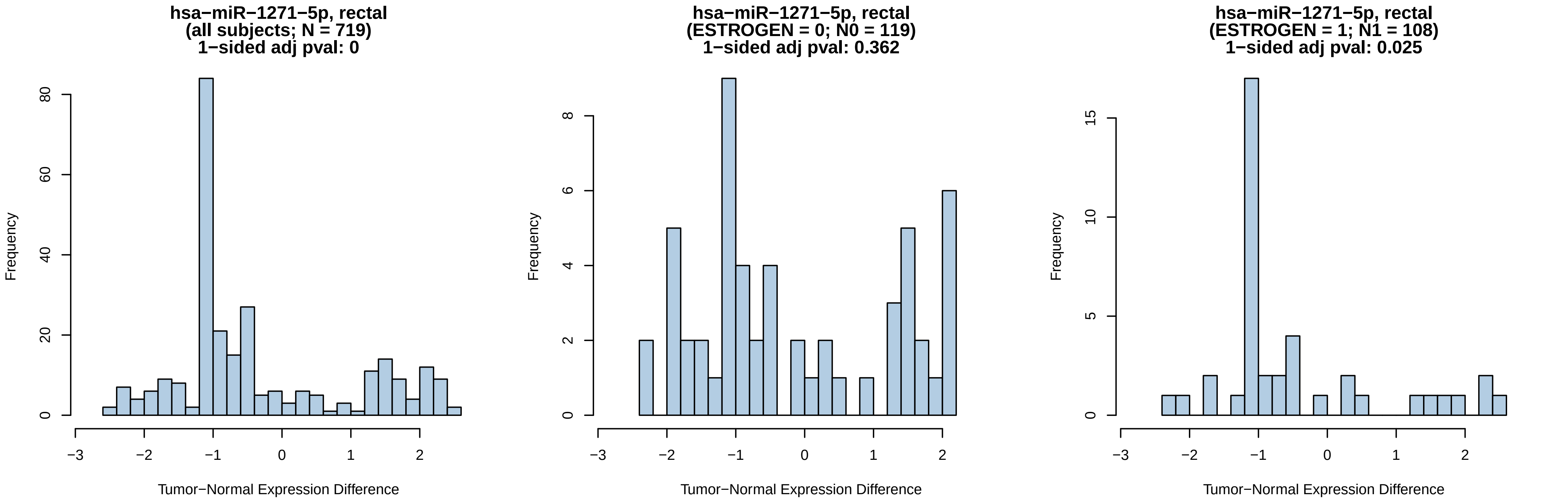

hsa−miR−1271−5p, rectal
(all subjects; N = 719)
1−sided adj pval: 0
Frequency
Tumor−Normal Expression Difference
hsa−miR−1271−5p, rectal
(ESTROGEN = 0; N0 = 119)
1−sided adj pval: 0.362
Frequency
Tumor−Normal Expression Difference
hsa−miR−1271−5p, rectal
(ESTROGEN = 1; N1 = 108)
1−sided adj pval: 0.025
Frequency
Tumor−Normal Expression Difference

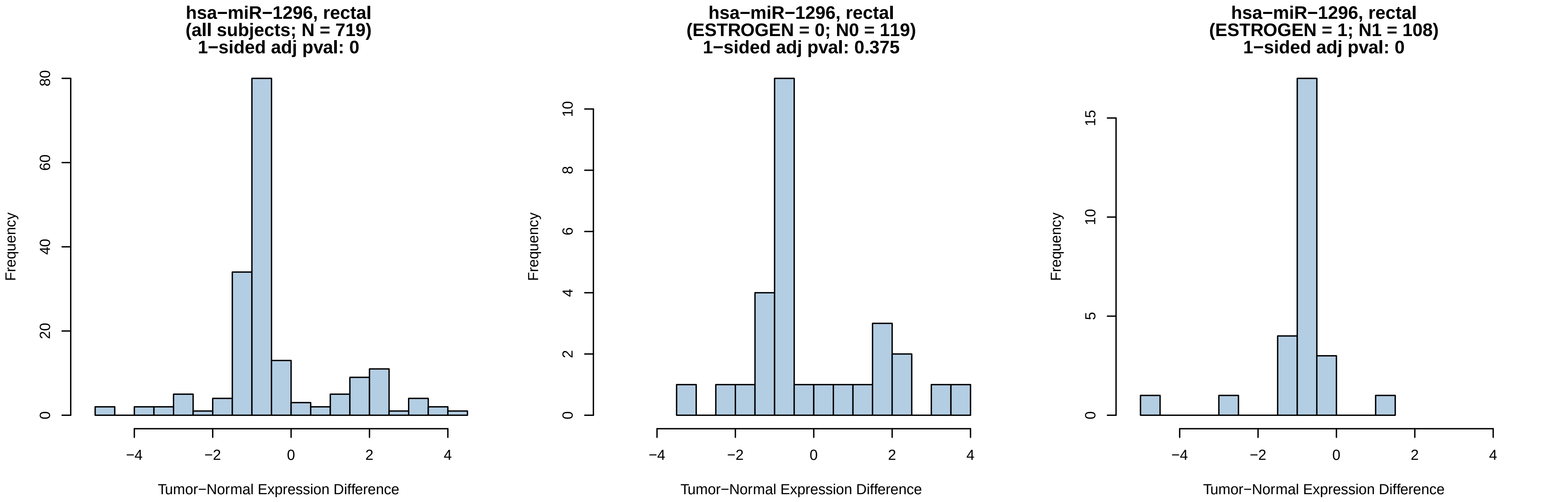
hsa−miR−1296, rectal
(all subjects; N = 719)
1−sided adj pval: 0
Frequency
Tumor−Normal Expression Difference
hsa−miR−1296, rectal
(ESTROGEN = 0; N0 = 119)
1−sided adj pval: 0.375
Frequency
Tumor−Normal Expression Difference
hsa−miR−1296, rectal
(ESTROGEN = 1; N1 = 108)
1−sided adj pval: 0
Frequency
Tumor−Normal Expression Difference

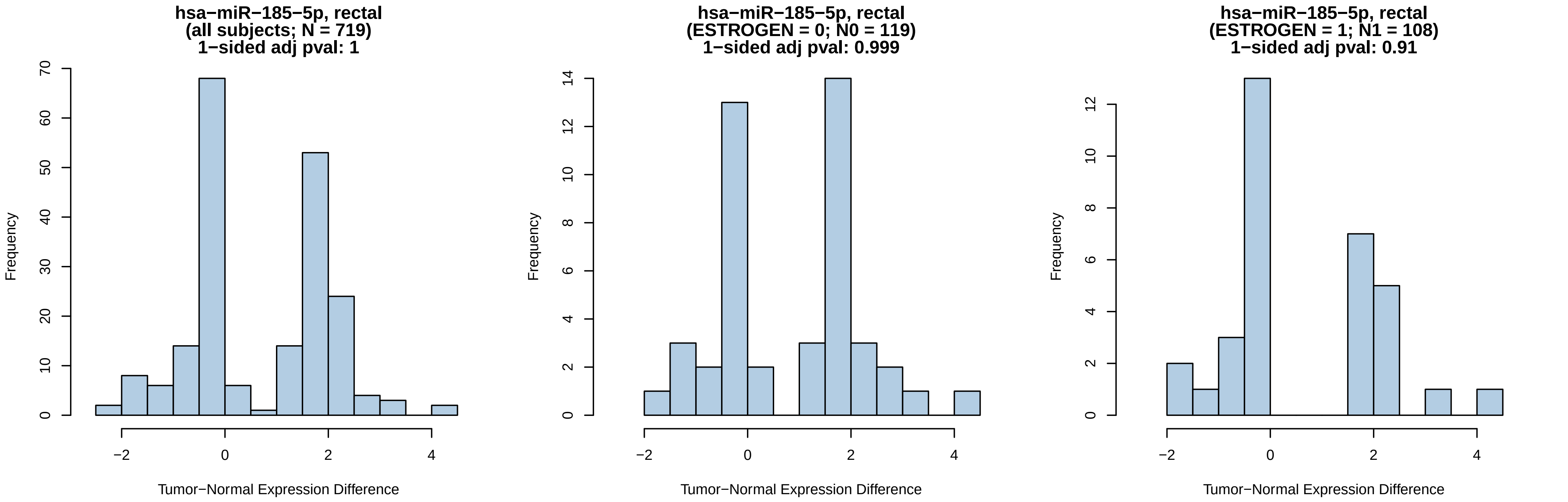
hsa−miR−185−5p, rectal
(all subjects; N = 719)
1−sided adj pval: 1
Frequency
Tumor−Normal Expression Difference
hsa−miR−185−5p, rectal
(ESTROGEN = 0; N0 = 119)
1−sided adj pval: 0.999
Frequency
Tumor−Normal Expression Difference
hsa−miR−185−5p, rectal
(ESTROGEN = 1; N1 = 108)
1−sided adj pval: 0.91
Frequency
Tumor−Normal Expression Difference

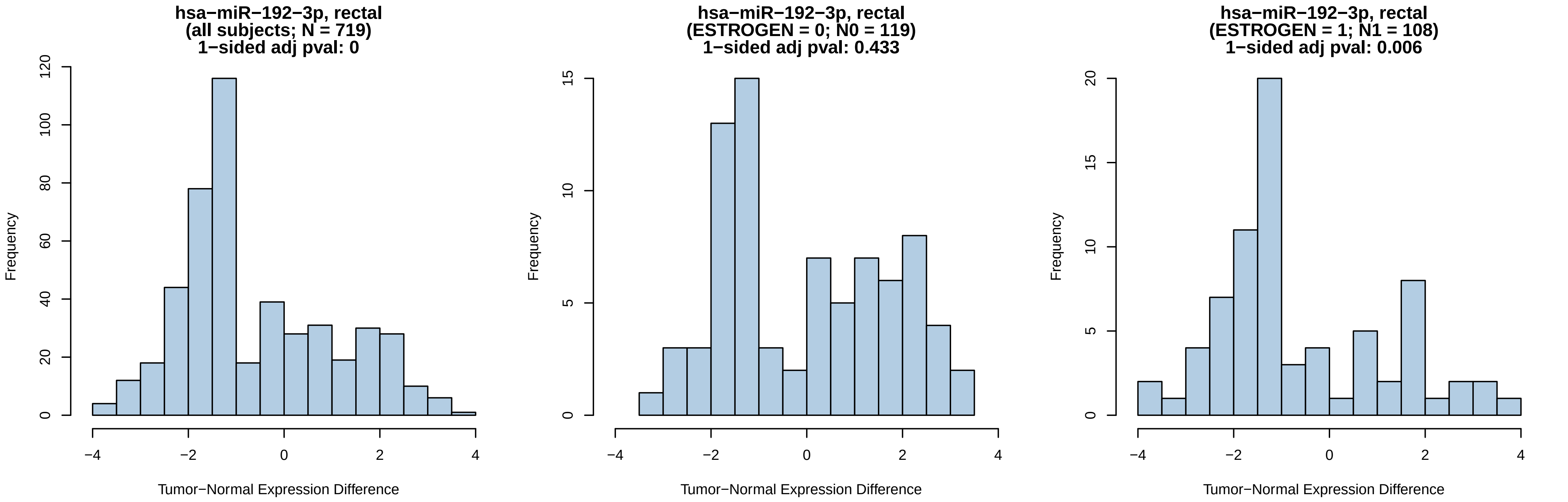

hsa−miR−192−3p, rectal
(all subjects; N = 719)
1−sided adj pval: 0
Frequency
0
20
40
60
80
100
120
−4
−2
0
2
4
Tumor−Normal Expression Difference
hsa−miR−192−3p, rectal
(ESTROGEN = 0; N0 = 119)
1−sided adj pval: 0.433
Frequency
0
5
10
15
−4
−2
0
2
4
Tumor−Normal Expression Difference
hsa−miR−192−3p, rectal
(ESTROGEN = 1; N1 = 108)
1−sided adj pval: 0.006
Frequency
0
5
10
15
20
−4
−2
0
2
4
Tumor−Normal Expression Difference

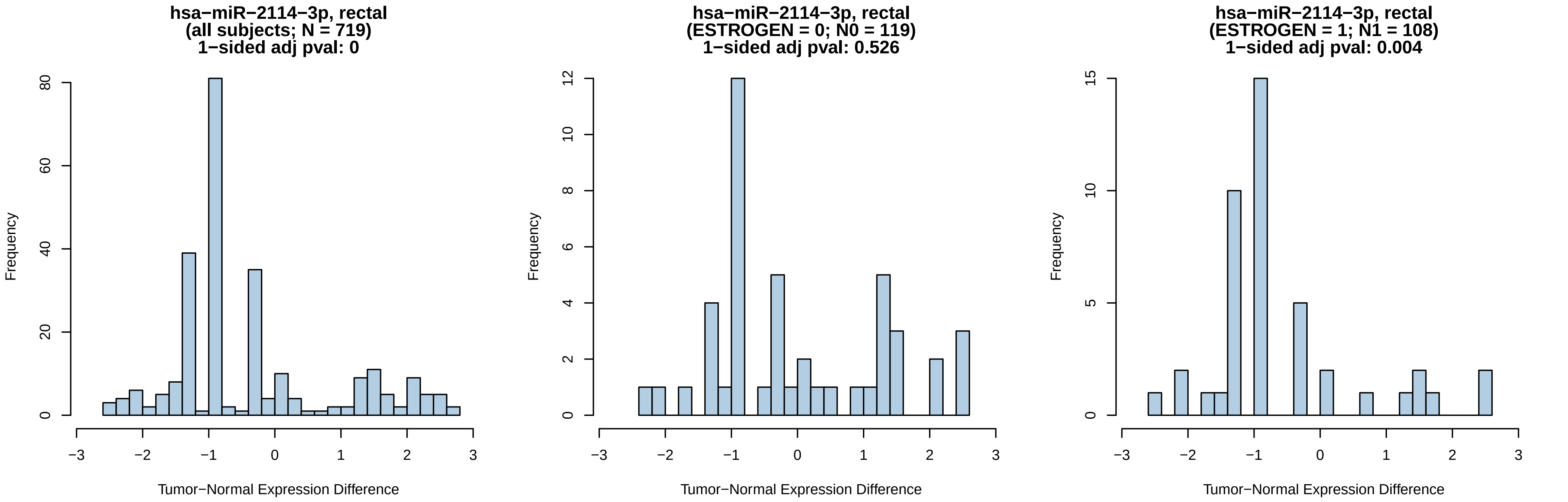

hsa−miR−2114−3p, rectal
(all subjects; N = 719)
1−sided adj pval: 0
hsa−miR−2114−3p, rectal
(ESTROGEN = 0; N0 = 119)
1−sided adj pval: 0.526
hsa−miR−2114−3p, rectal
(ESTROGEN = 1; N1 = 108)
1−sided adj pval: 0.004
Frequency
Tumor−Normal Expression Difference

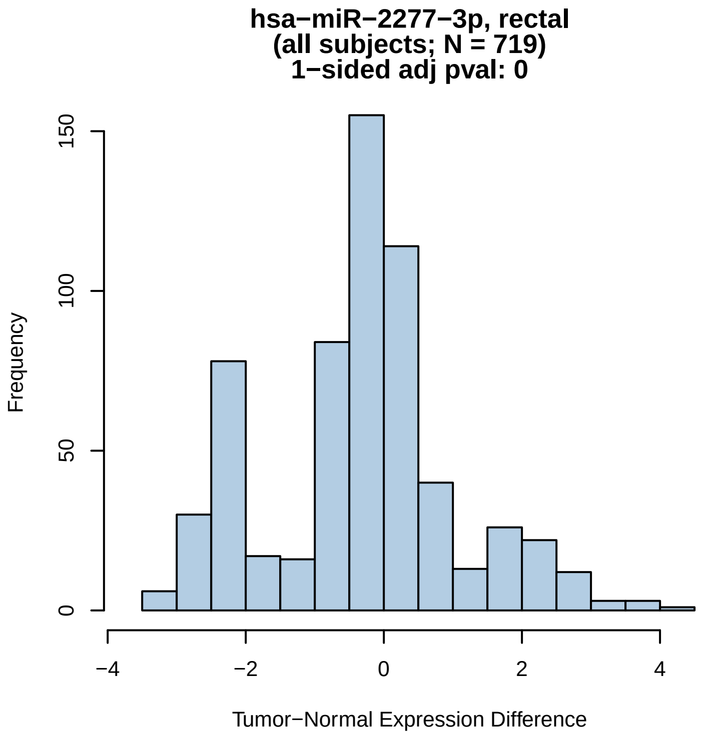

hsa−miR−2277−3p, rectal
(all subjects; N = 719)
1−sided adj pval: 0
Frequency
Tumor−Normal Expression Difference


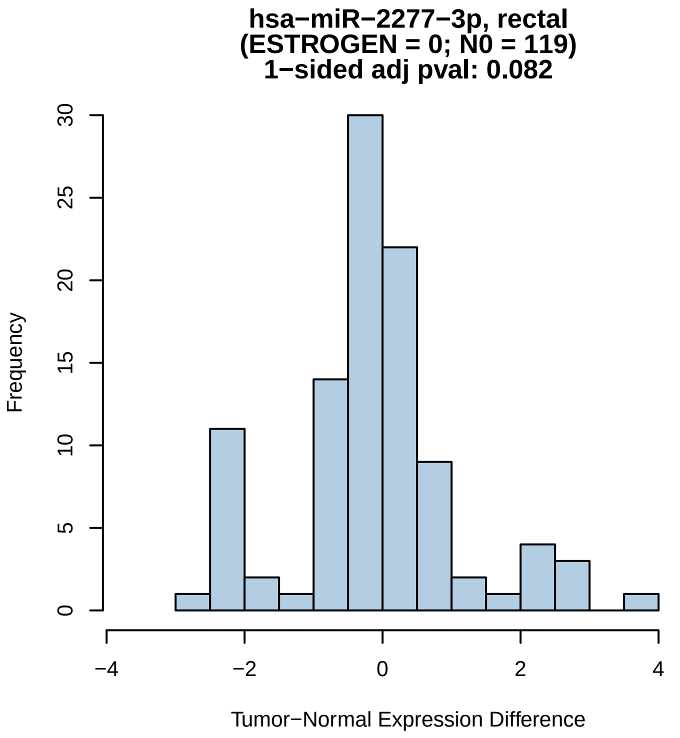

hsa−miR−2277−3p, rectal
(ESTROGEN = 0; N0 = 119)
1−sided adj pval: 0.082
Frequency
Tumor−Normal Expression Difference


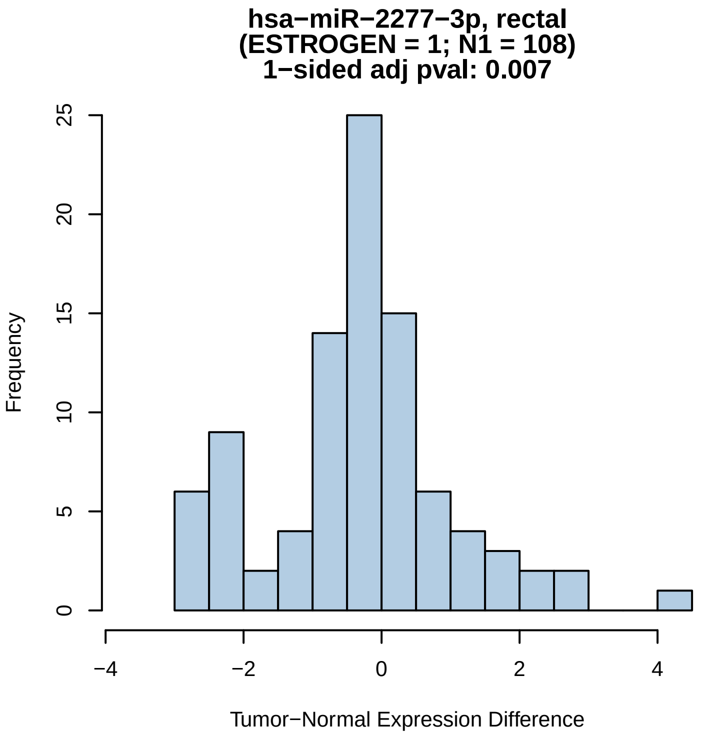

hsa−miR−2277−3p, rectal
(ESTROGEN = 1; N1 = 108)
1−sided adj pval: 0.007
Frequency
Tumor−Normal Expression Difference

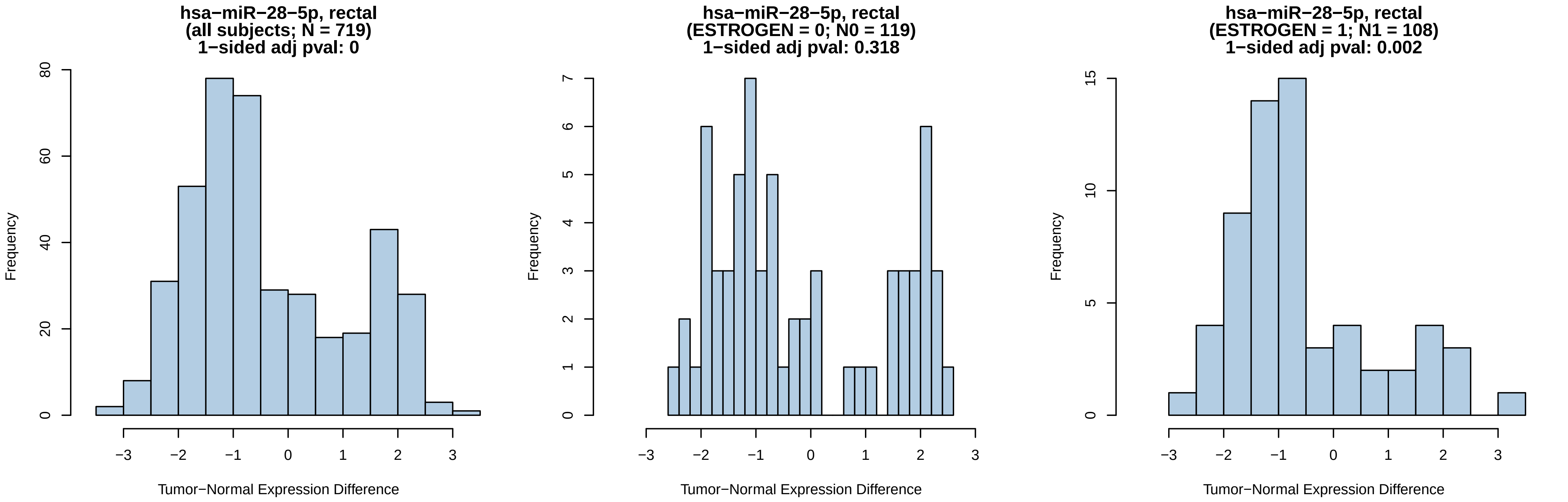

hsa−miR−28−5p, rectal
(all subjects; N = 719)
1−sided adj pval: 0
Frequency
0
20
40
60
80
−3
−2
−1
0
1
2
3
Tumor−Normal Expression Difference
hsa−miR−28−5p, rectal
(ESTROGEN = 0; N0 = 119)
1−sided adj pval: 0.318
Frequency
0
1
2
3
4
5
6
7
−3
−2
−1
0
1
2
3
Tumor−Normal Expression Difference
hsa−miR−28−5p, rectal
(ESTROGEN = 1; N1 = 108)
1−sided adj pval: 0.002
Frequency
0
5
10
15
−3
−2
−1
0
1
2
3
Tumor−Normal Expression Difference

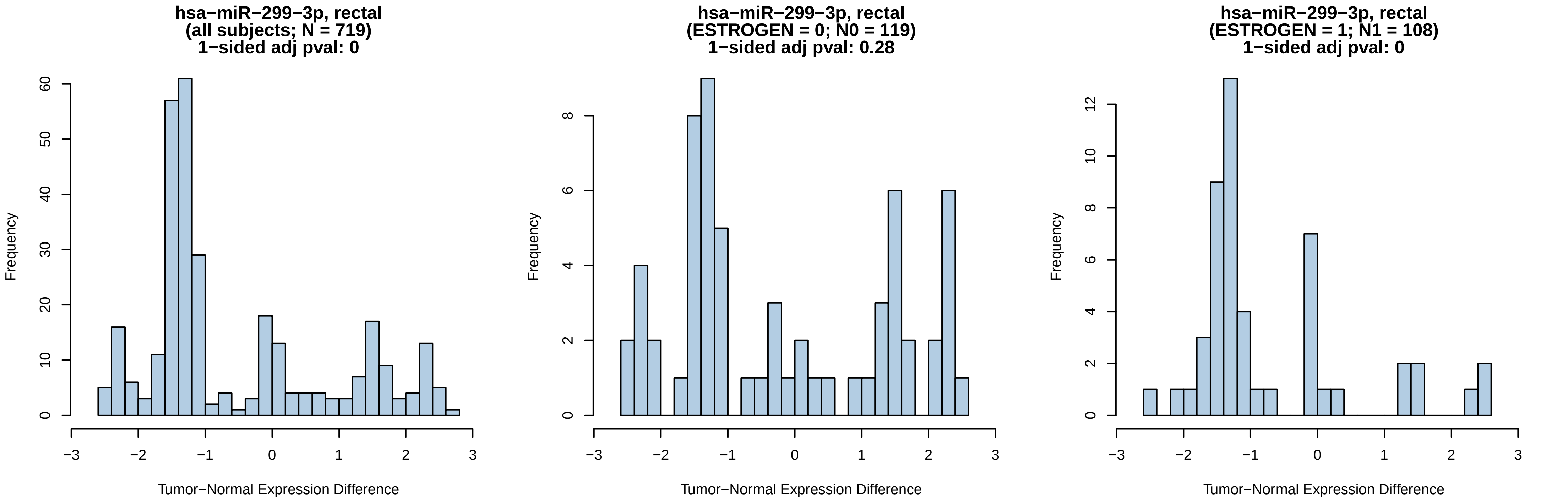
hsa−miR−299−3p, rectal
(all subjects; N = 719)
1−sided adj pval: 0
hsa−miR−299−3p, rectal
(ESTROGEN = 0; N0 = 119)
1−sided adj pval: 0.28
hsa−miR−299−3p, rectal
(ESTROGEN = 1; N1 = 108)
1−sided adj pval: 0
Frequency
Tumor−Normal Expression Difference

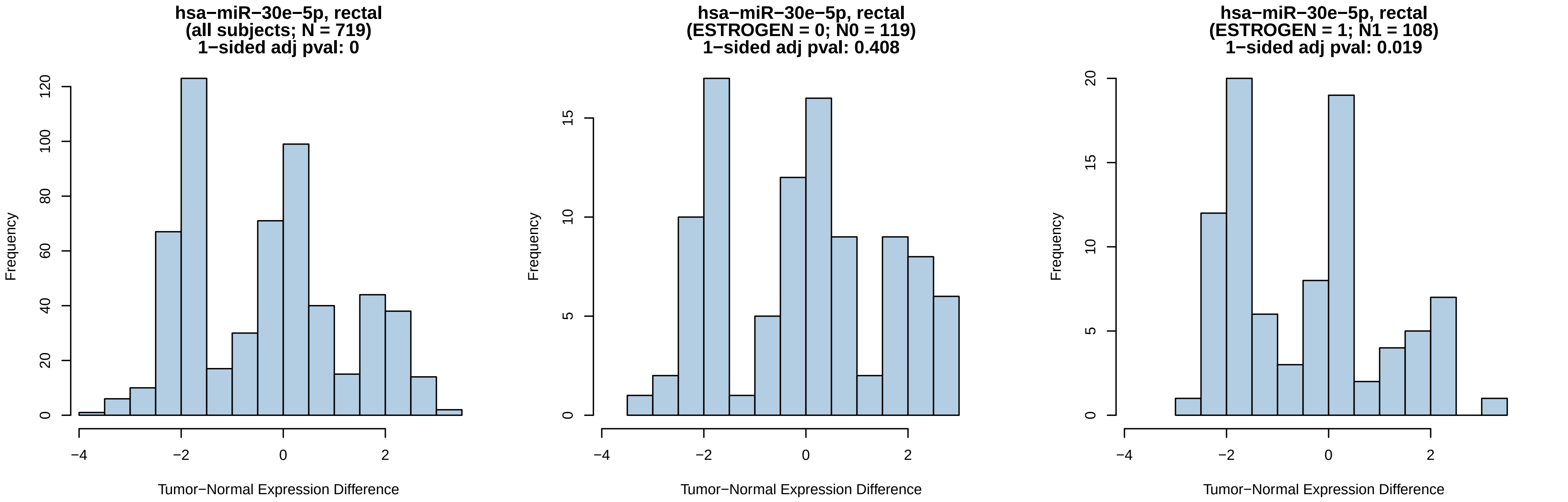

hsa−miR−30e−5p, rectal
(all subjects; N = 719)
1−sided adj pval: 0
hsa−miR−30e−5p, rectal
(ESTROGEN = 0; N0 = 119)
1−sided adj pval: 0.408
hsa−miR−30e−5p, rectal
(ESTROGEN = 1; N1 = 108)
1−sided adj pval: 0.019
Frequency
Tumor−Normal Expression Difference

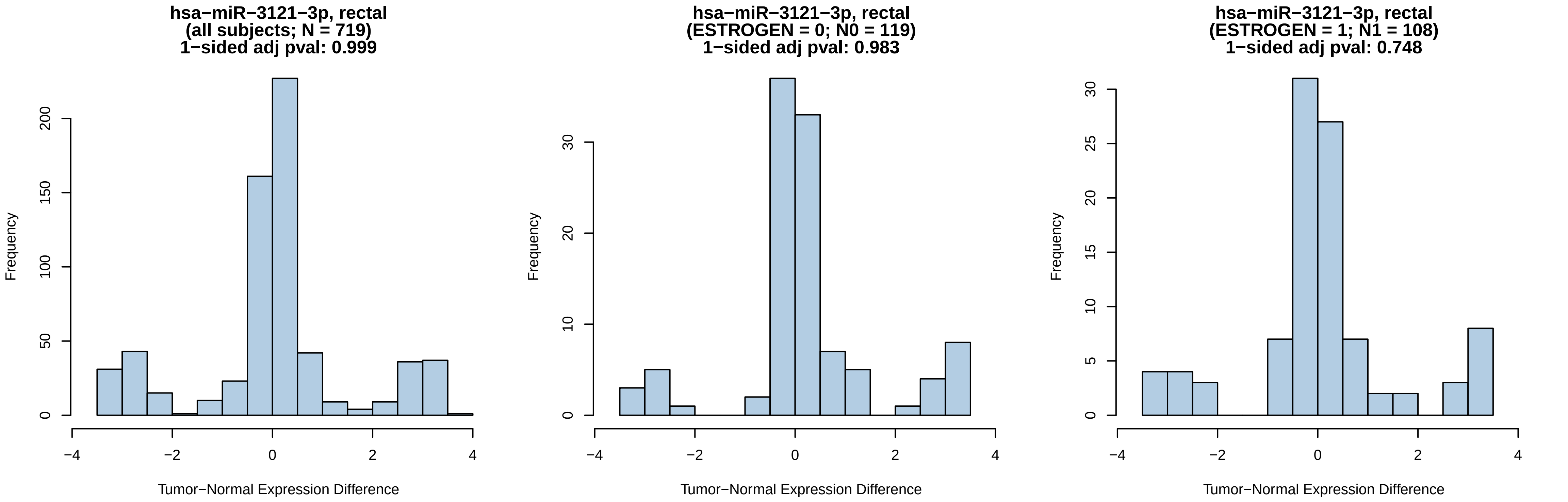

hsa−miR−3121−3p, rectal
(all subjects; N = 719)
1−sided adj pval: 0.999
Frequency
Tumor−Normal Expression Difference
hsa−miR−3121−3p, rectal
(ESTROGEN = 0; N0 = 119)
1−sided adj pval: 0.983
Frequency
Tumor−Normal Expression Difference
hsa−miR−3121−3p, rectal
(ESTROGEN = 1; N1 = 108)
1−sided adj pval: 0.748
Frequency
Tumor−Normal Expression Difference

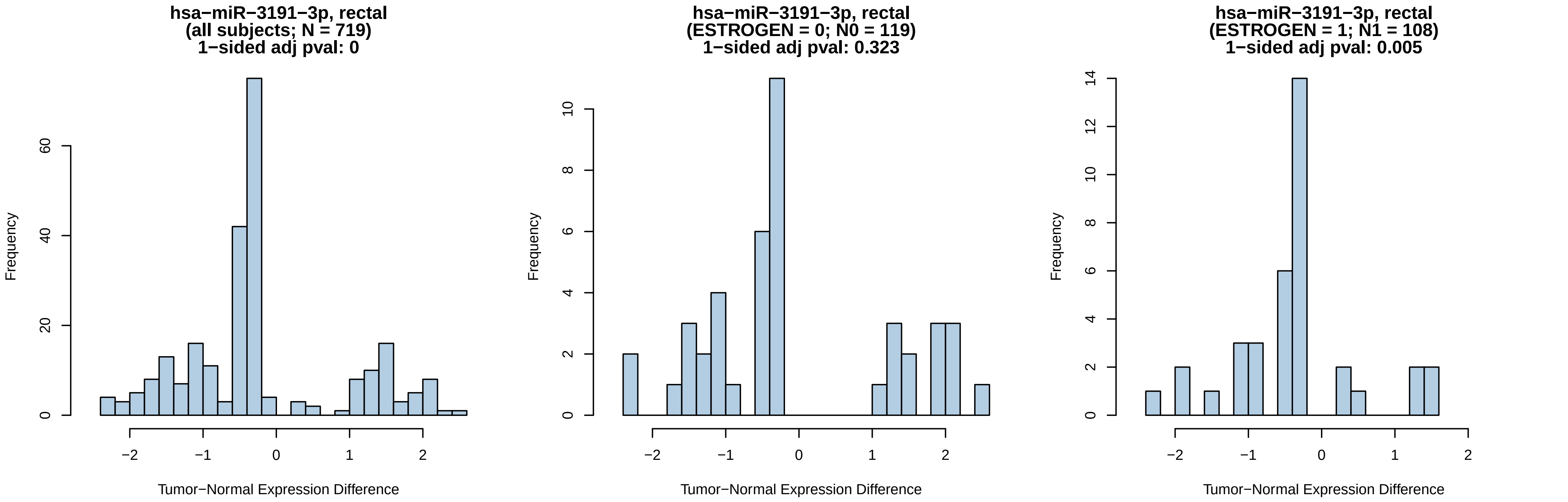
hsa−miR−3191−3p, rectal
(all subjects; N = 719)
1−sided adj pval: 0
Frequency
0
20
40
60
−2
−1
0
1
2
Tumor−Normal Expression Difference
hsa−miR−3191−3p, rectal
(ESTROGEN = 0; N0 = 119)
1−sided adj pval: 0.323
Frequency
0
2
4
6
8
10
−2
−1
0
1
2
Tumor−Normal Expression Difference
hsa−miR−3191−3p, rectal
(ESTROGEN = 1; N1 = 108)
1−sided adj pval: 0.005
Frequency
0
2
4
6
8
10
12
14
−2
−1
0
1
2
Tumor−Normal Expression Difference

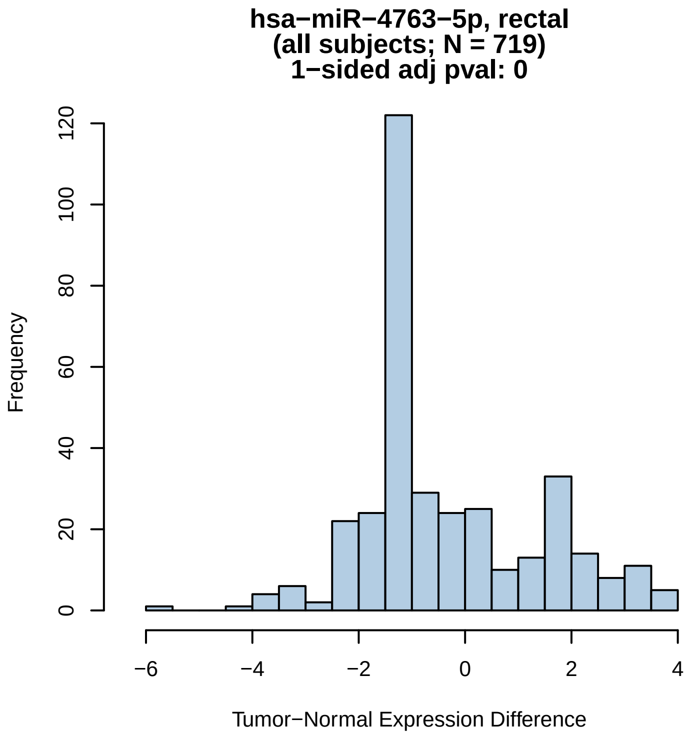

hsa−miR−4763−5p, rectal
(all subjects; N = 719)
1−sided adj pval: 0
Frequency
Tumor−Normal Expression Difference


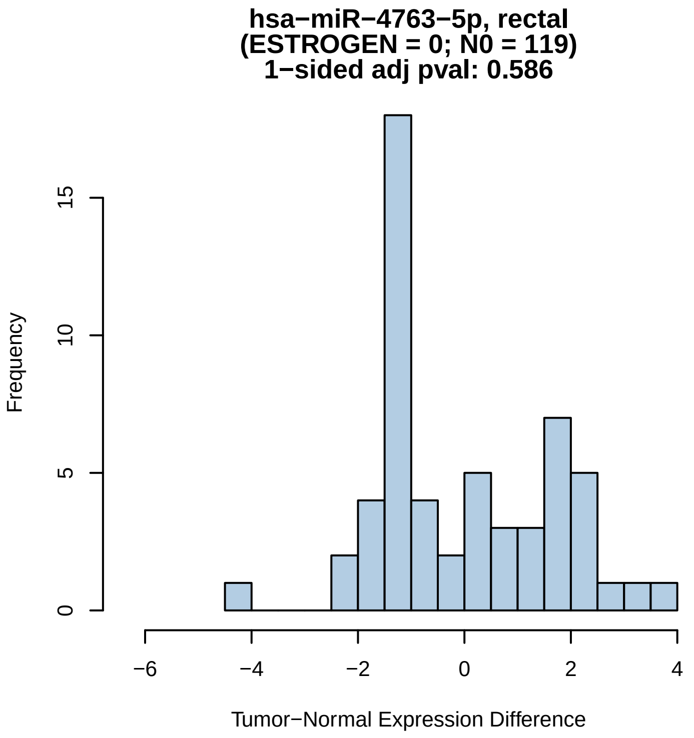

hsa−miR−4763−5p, rectal
(ESTROGEN = 0; N0 = 119)
1−sided adj pval: 0.586
Frequency
Tumor−Normal Expression Difference


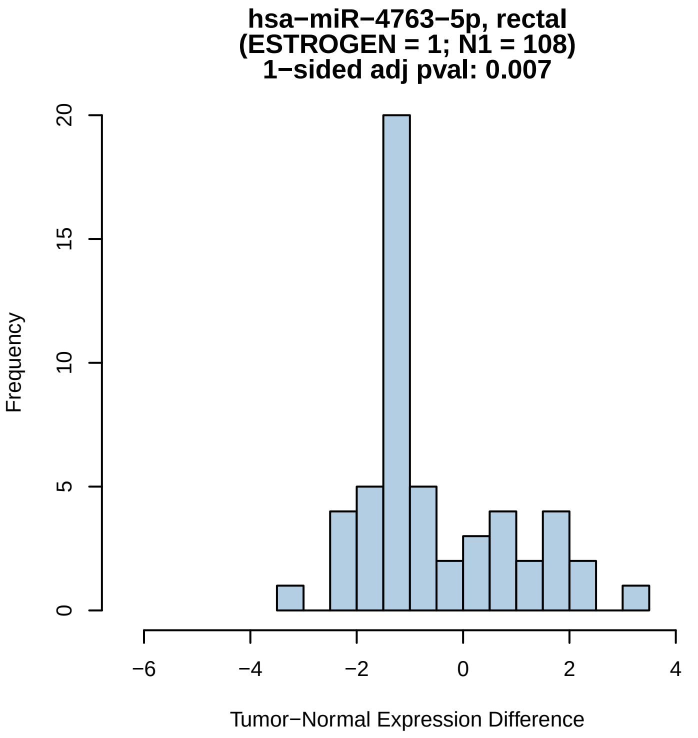

hsa−miR−4763−5p, rectal
(ESTROGEN = 1; N1 = 108)
1−sided adj pval: 0.007
Frequency
Tumor−Normal Expression Difference

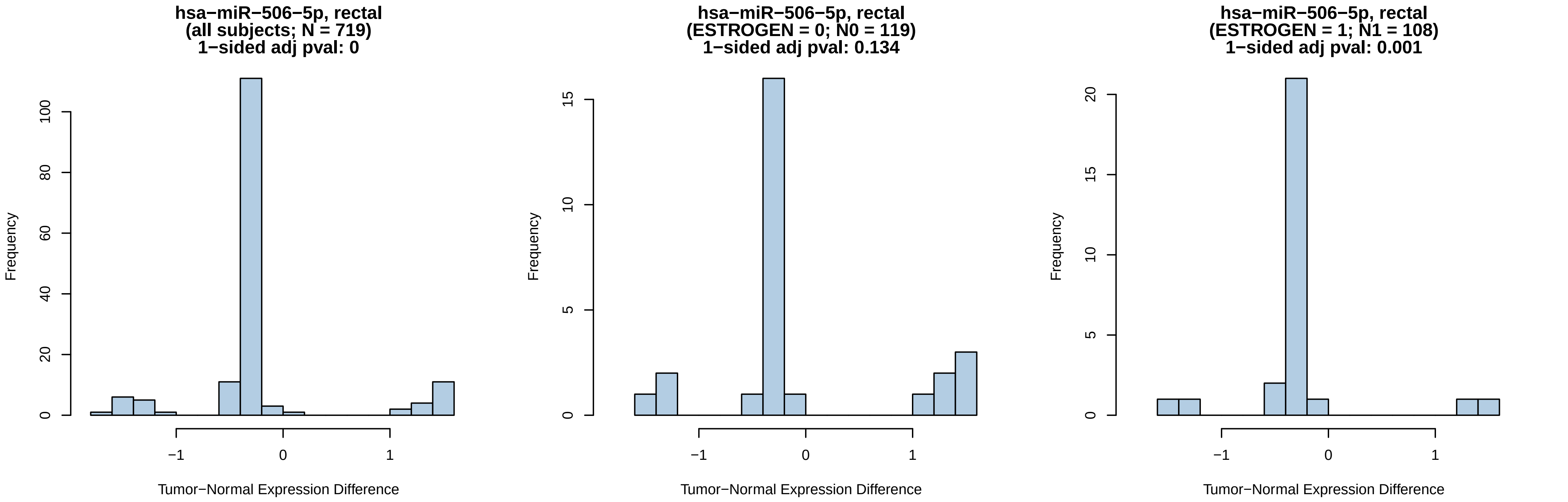
hsa−miR−506−5p, rectal
(all subjects; N = 719)
1−sided adj pval: 0
Frequency
Tumor−Normal Expression Difference
hsa−miR−506−5p, rectal
(ESTROGEN = 0; N0 = 119)
1−sided adj pval: 0.134
Frequency
Tumor−Normal Expression Difference
hsa−miR−506−5p, rectal
(ESTROGEN = 1; N1 = 108)
1−sided adj pval: 0.001
Frequency
Tumor−Normal Expression Difference

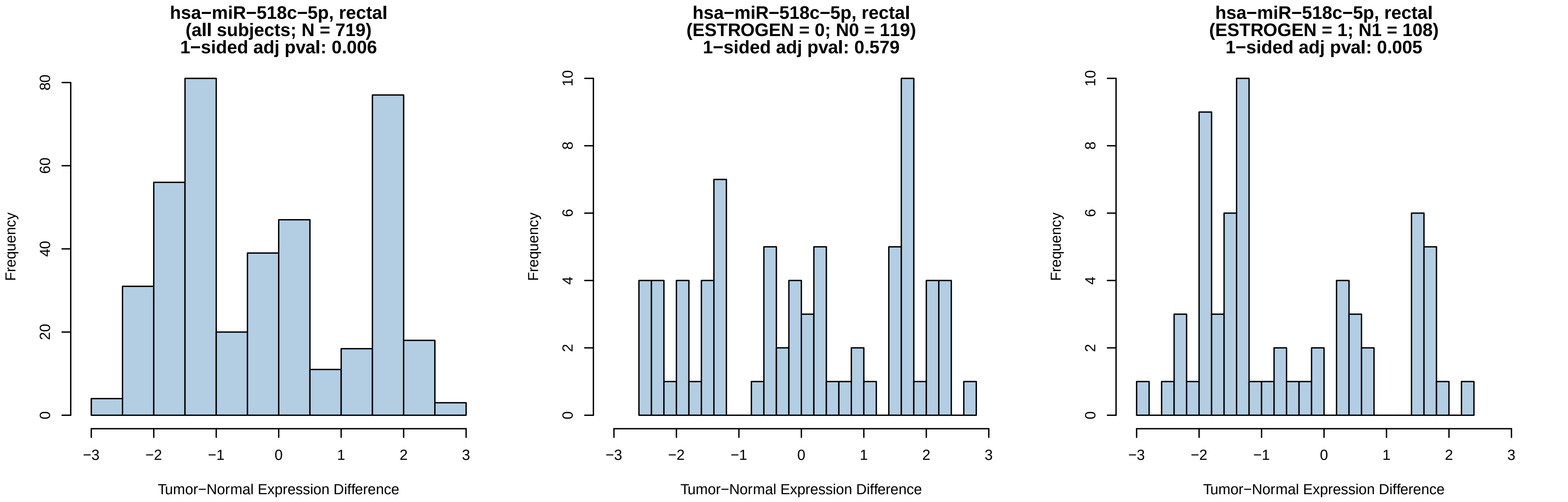
hsa−miR−518c−5p, rectal
(all subjects; N = 719)
1−sided adj pval: 0.006
hsa−miR−518c−5p, rectal
(ESTROGEN = 0; N0 = 119)
1−sided adj pval: 0.579
hsa−miR−518c−5p, rectal
(ESTROGEN = 1; N1 = 108)
1−sided adj pval: 0.005
Frequency
Tumor−Normal Expression Difference

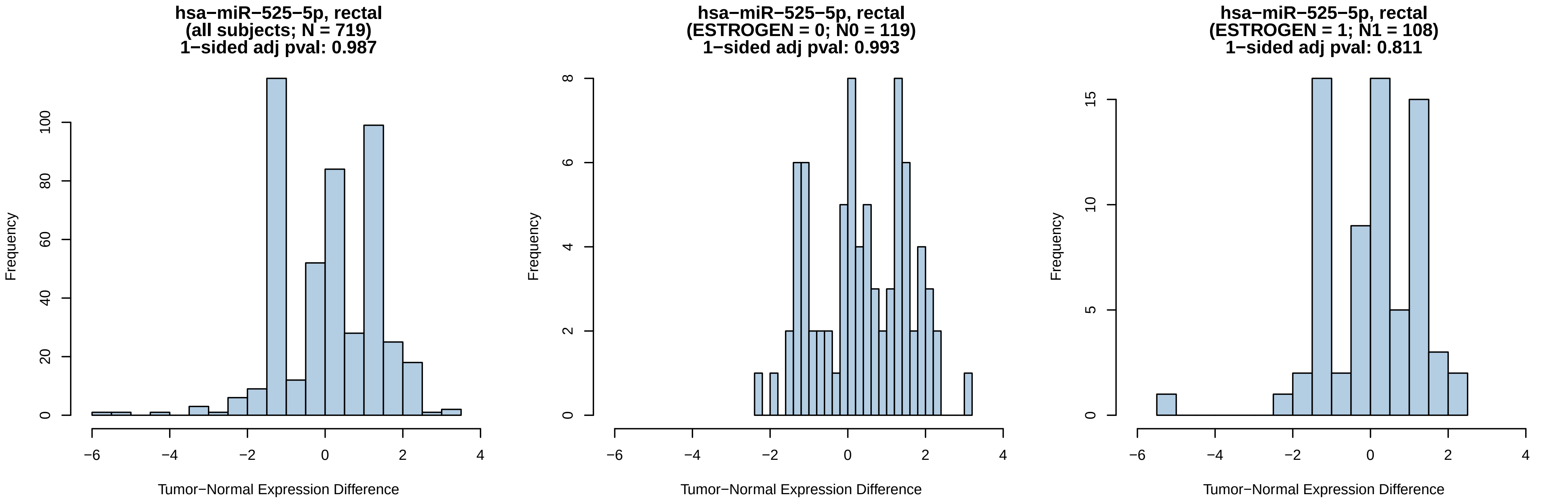
hsa−miR−525−5p, rectal
(all subjects; N = 719)
1−sided adj pval: 0.987
Frequency
0
20
40
60
80
100
−6
−4
−2
0
2
4
Tumor−Normal Expression Difference
hsa−miR−525−5p, rectal
(ESTROGEN = 0; N0 = 119)
1−sided adj pval: 0.993
Frequency
0
2
4
6
8
−6
−4
−2
0
2
4
Tumor−Normal Expression Difference
hsa−miR−525−5p, rectal
(ESTROGEN = 1; N1 = 108)
1−sided adj pval: 0.811
Frequency
0
5
10
15
−6
−4
−2
0
2
4
Tumor−Normal Expression Difference

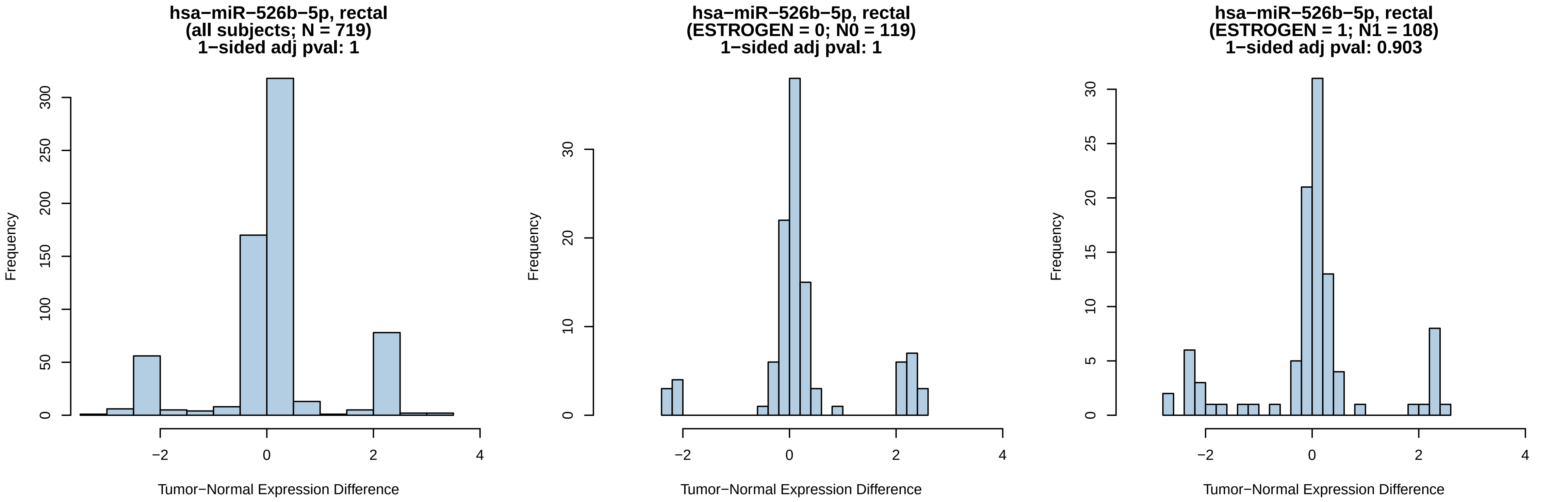
hsa−miR−526b−5p, rectal
(all subjects; N = 719)
1−sided adj pval: 1
hsa−miR−526b−5p, rectal
(ESTROGEN = 0; N0 = 119)
1−sided adj pval: 1
hsa−miR−526b−5p, rectal
(ESTROGEN = 1; N1 = 108)
1−sided adj pval: 0.903
Frequency
Tumor−Normal Expression Difference

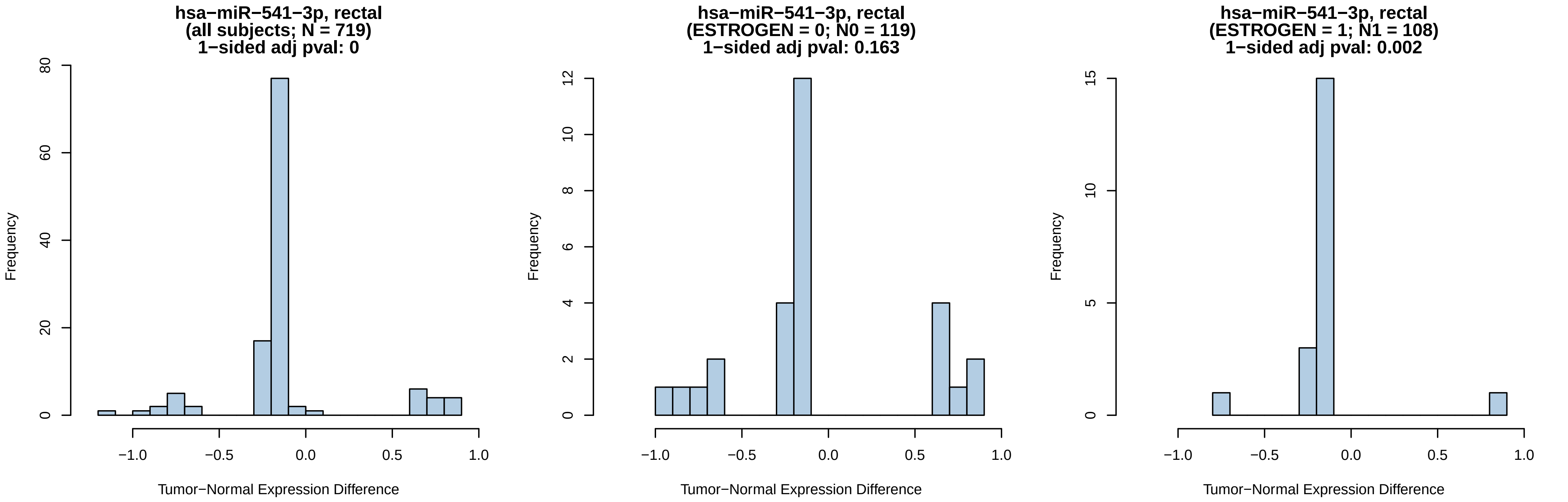
hsa−miR−541−3p, rectal
(all subjects; N = 719)
1−sided adj pval: 0
hsa−miR−541−3p, rectal
(ESTROGEN = 0; N0 = 119)
1−sided adj pval: 0.163
hsa−miR−541−3p, rectal
(ESTROGEN = 1; N1 = 108)
1−sided adj pval: 0.002
Frequency
Tumor−Normal Expression Difference
0
20
40
60
80
2
4
6
8
10
12
5
10
15
−1.0
−0.5
0.0
0.5
1.0

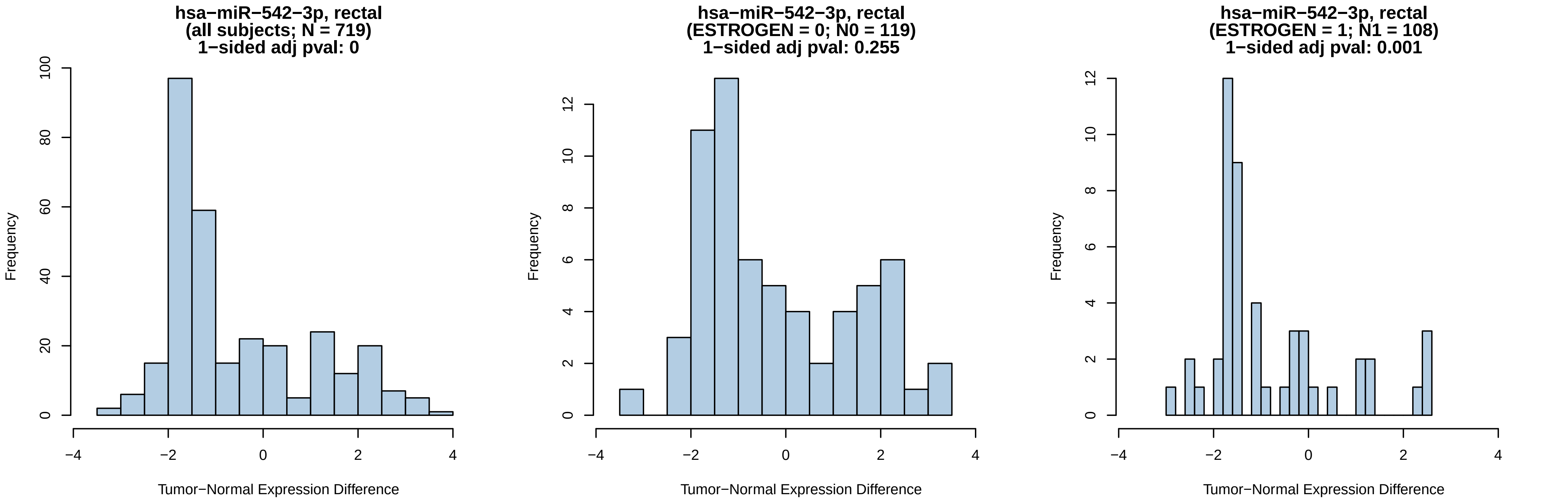

hsa−miR−542−3p, rectal
(all subjects; N = 719)
1−sided adj pval: 0
Frequency
Tumor−Normal Expression Difference
hsa−miR−542−3p, rectal
(ESTROGEN = 0; N0 = 119)
1−sided adj pval: 0.255
Frequency
Tumor−Normal Expression Difference
hsa−miR−542−3p, rectal
(ESTROGEN = 1; N1 = 108)
1−sided adj pval: 0.001
Frequency
Tumor−Normal Expression Difference

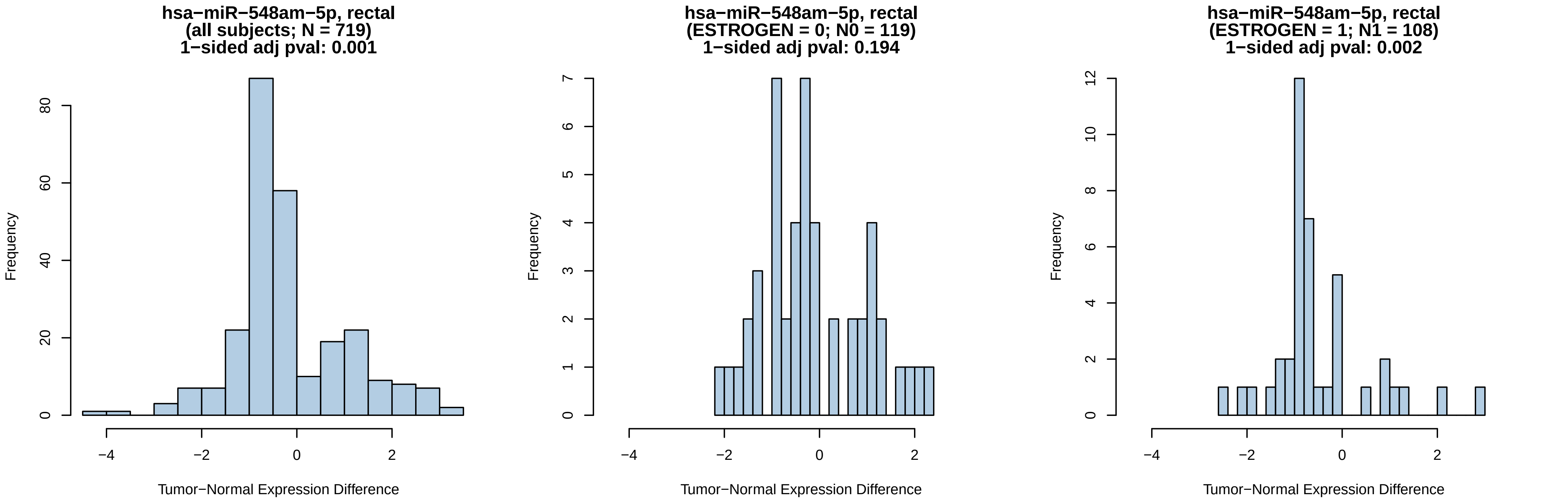
hsa−miR−548am−5p, rectal
(all subjects; N = 719)
1−sided adj pval: 0.001
Frequency
0
20
40
60
80
−4
−2
0
2
Tumor−Normal Expression Difference
hsa−miR−548am−5p, rectal
(ESTROGEN = 0; N0 = 119)
1−sided adj pval: 0.194
Frequency
0
1
2
3
4
5
6
7
−4
−2
0
2
Tumor−Normal Expression Difference
hsa−miR−548am−5p, rectal
(ESTROGEN = 1; N1 = 108)
1−sided adj pval: 0.002
Frequency
0
2
4
6
8
10
12
−4
−2
0
2
Tumor−Normal Expression Difference

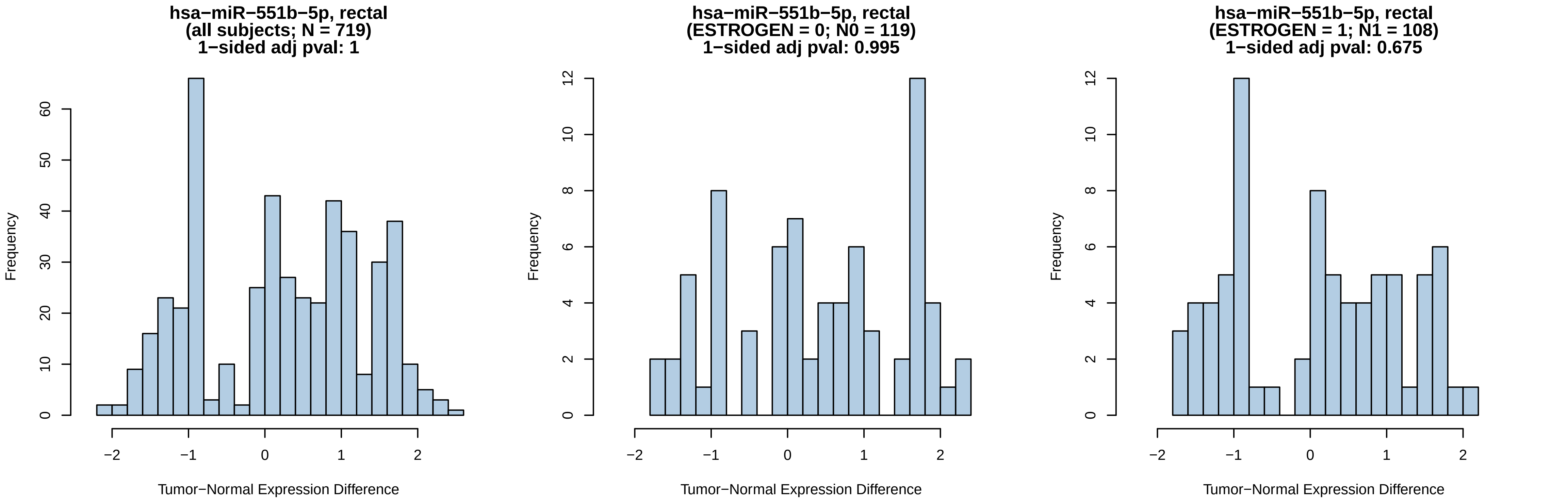
hsa−miR−551b−5p, rectal
(all subjects; N = 719)
1−sided adj pval: 1
Frequency
Tumor−Normal Expression Difference
hsa−miR−551b−5p, rectal
(ESTROGEN = 0; N0 = 119)
1−sided adj pval: 0.995
Frequency
Tumor−Normal Expression Difference
hsa−miR−551b−5p, rectal
(ESTROGEN = 1; N1 = 108)
1−sided adj pval: 0.675
Frequency
Tumor−Normal Expression Difference

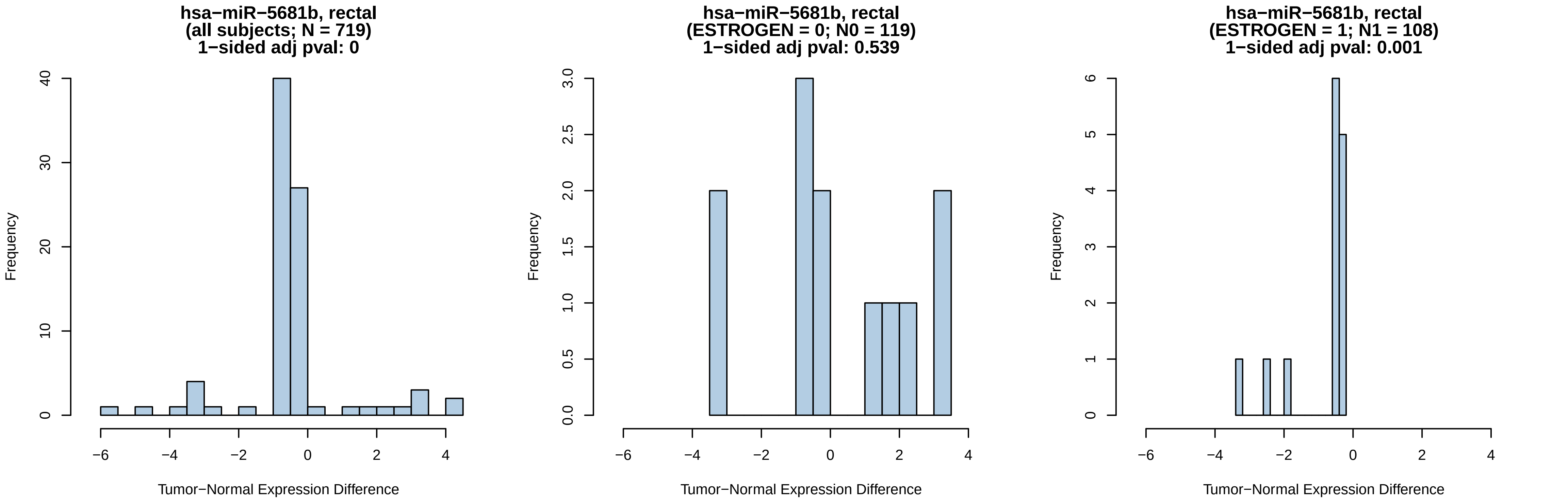

hsa−miR−5681b, rectal
(all subjects; N = 719)
1−sided adj pval: 0
hsa−miR−5681b, rectal
(ESTROGEN = 0; N0 = 119)
1−sided adj pval: 0.539
hsa−miR−5681b, rectal
(ESTROGEN = 1; N1 = 108)
1−sided adj pval: 0.001
Frequency
Tumor−Normal Expression Difference

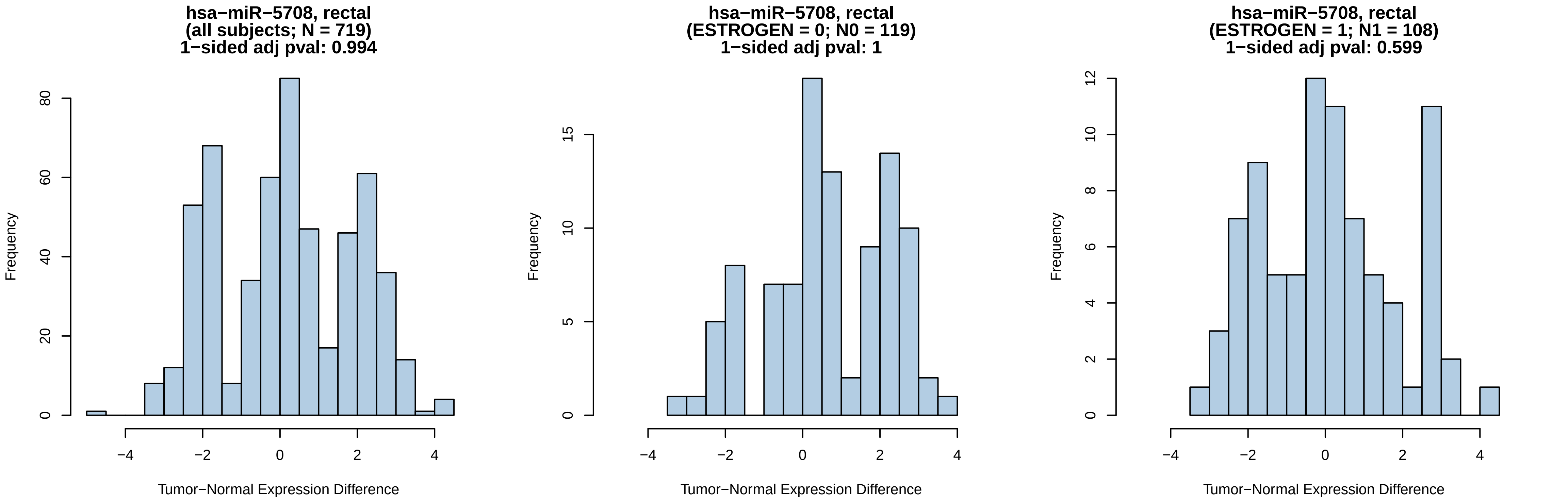
hsa−miR−5708, rectal
(all subjects; N = 719)
1−sided adj pval: 0.994
Frequency
0
20
40
60
80
−4
−2
0
2
4
Tumor−Normal Expression Difference
hsa−miR−5708, rectal
(ESTROGEN = 0; N0 = 119)
1−sided adj pval: 1
Frequency
0
5
10
15
−4
−2
0
2
4
Tumor−Normal Expression Difference
hsa−miR−5708, rectal
(ESTROGEN = 1; N1 = 108)
1−sided adj pval: 0.599
Frequency
0
2
4
6
8
10
12
−4
−2
0
2
4
Tumor−Normal Expression Difference

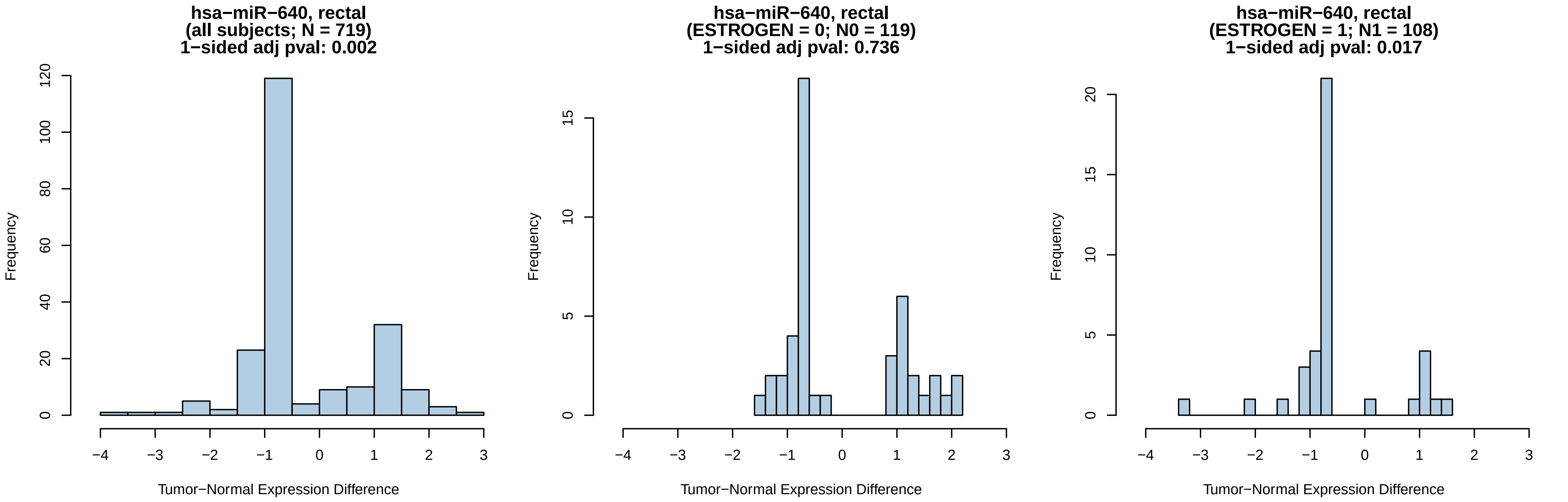
hsa−miR−640, rectal
(all subjects; N = 719)
1−sided adj pval: 0.002
Frequency
Tumor−Normal Expression Difference
hsa−miR−640, rectal
(ESTROGEN = 0; N0 = 119)
1−sided adj pval: 0.736
Frequency
Tumor−Normal Expression Difference
hsa−miR−640, rectal
(ESTROGEN = 1; N1 = 108)
1−sided adj pval: 0.017
Frequency
Tumor−Normal Expression Difference

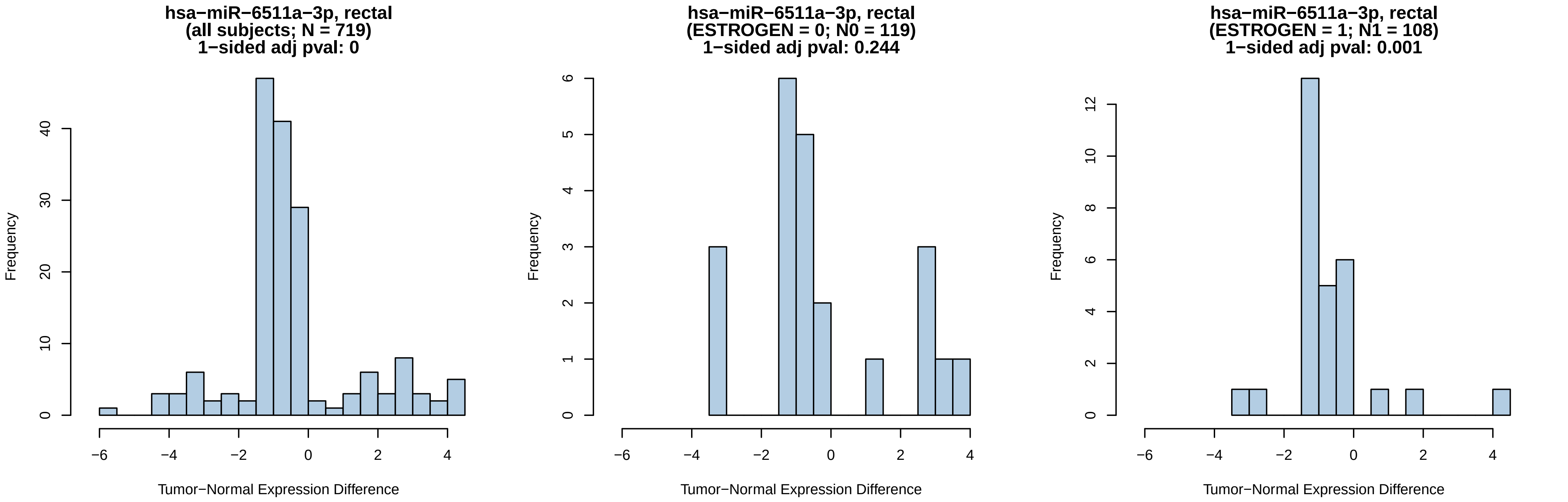
hsa−miR−6511a−3p, rectal
(all subjects; N = 719)
1−sided adj pval: 0
Frequency
0
10
20
30
40
−6
−4
−2
0
2
4
Tumor−Normal Expression Difference
hsa−miR−6511a−3p, rectal
(ESTROGEN = 0; N0 = 119)
1−sided adj pval: 0.244
Frequency
0
1
2
3
4
5
6
−6
−4
−2
0
2
4
Tumor−Normal Expression Difference
hsa−miR−6511a−3p, rectal
(ESTROGEN = 1; N1 = 108)
1−sided adj pval: 0.001
Frequency
0
2
4
6
8
10
12
−6
−4
−2
0
2
4
Tumor−Normal Expression Difference

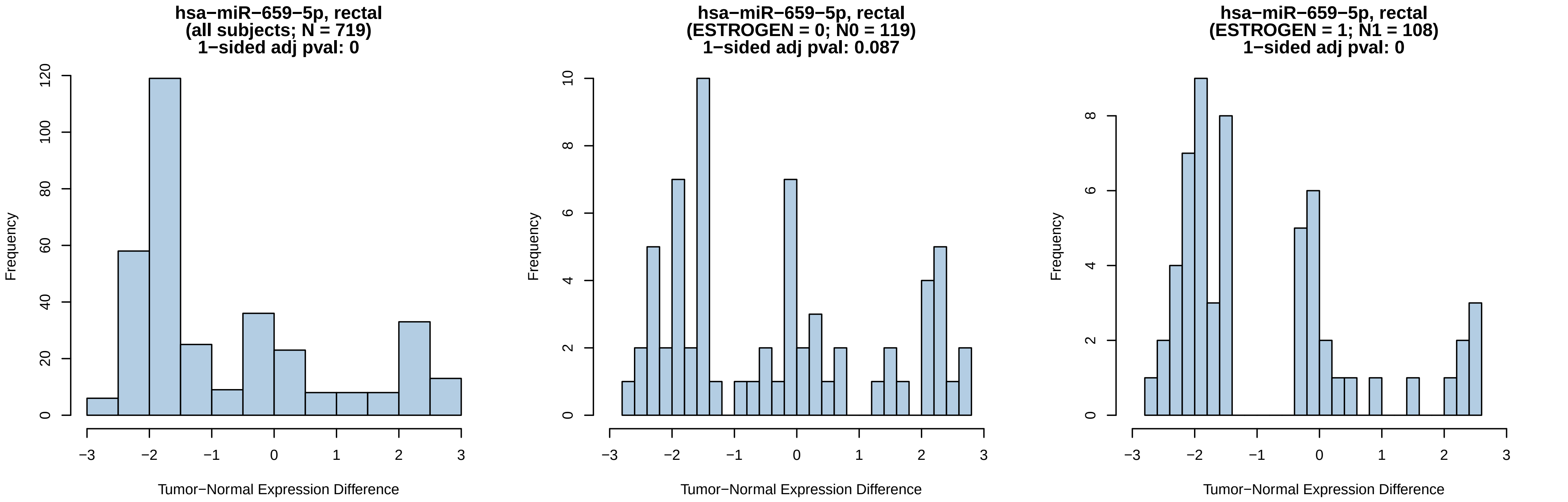

hsa−miR−659−5p, rectal
(all subjects; N = 719)
1−sided adj pval: 0
Frequency
Tumor−Normal Expression Difference
hsa−miR−659−5p, rectal
(ESTROGEN = 0; N0 = 119)
1−sided adj pval: 0.087
Frequency
Tumor−Normal Expression Difference
hsa−miR−659−5p, rectal
(ESTROGEN = 1; N1 = 108)
1−sided adj pval: 0
Frequency
Tumor−Normal Expression Difference

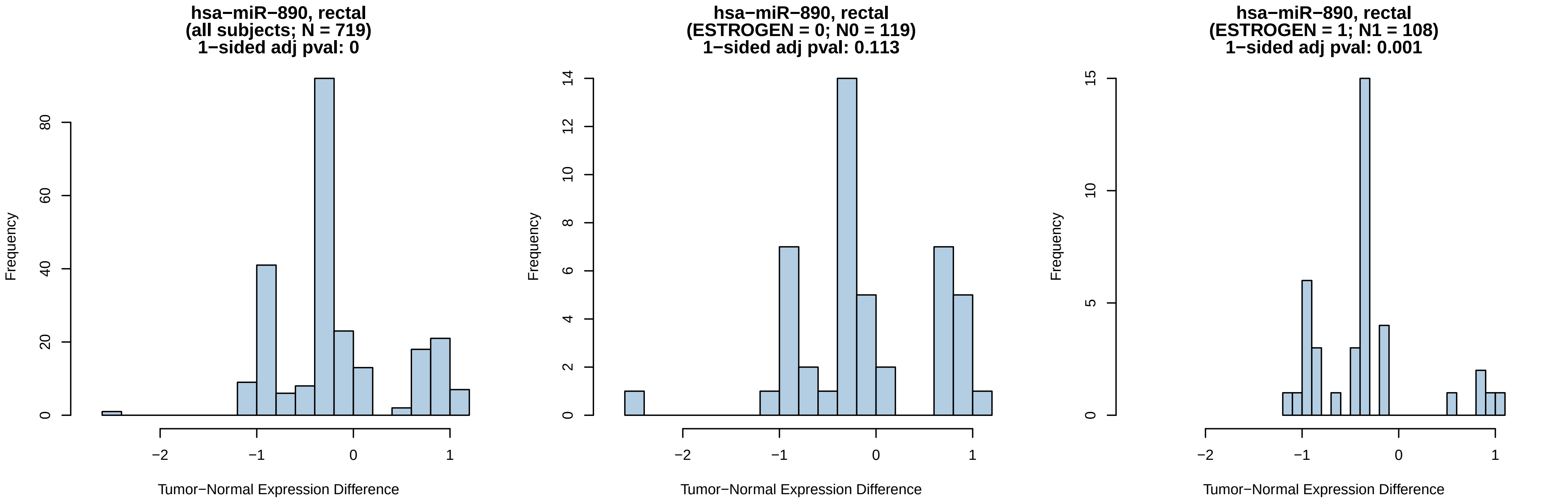
hsa−miR−890, rectal
(all subjects; N = 719)
1−sided adj pval: 0
Frequency
Tumor−Normal Expression Difference
hsa−miR−890, rectal
(ESTROGEN = 0; N0 = 119)
1−sided adj pval: 0.113
Frequency
Tumor−Normal Expression Difference
hsa−miR−890, rectal
(ESTROGEN = 1; N1 = 108)
1−sided adj pval: 0.001
Frequency
Tumor−Normal Expression Difference

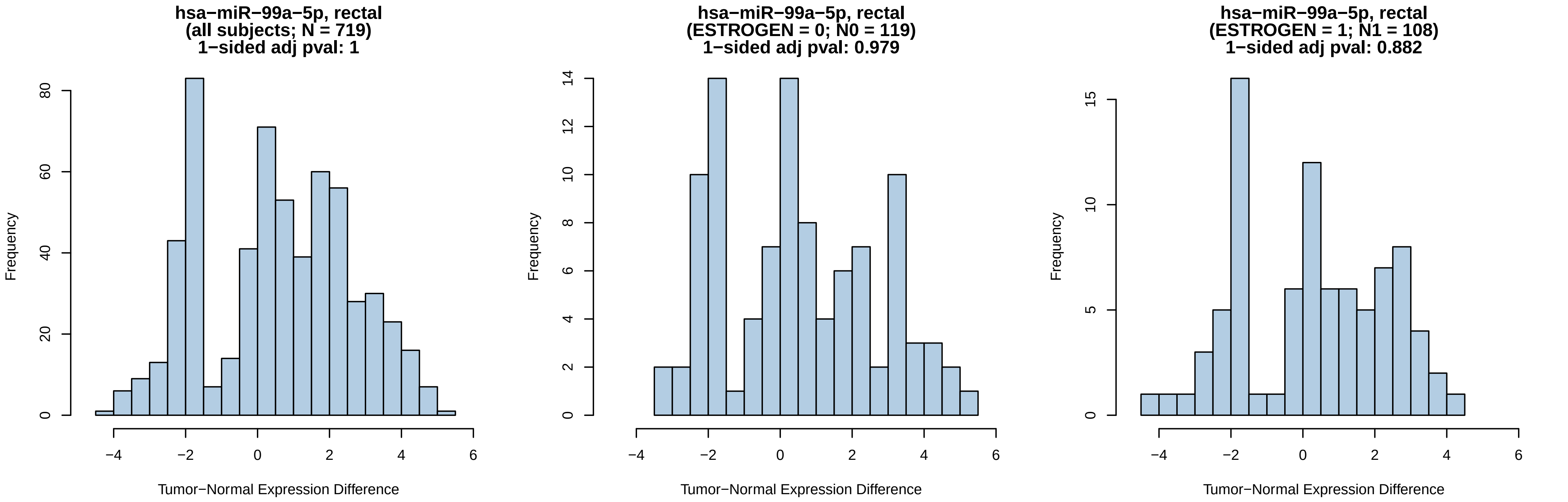

hsa−miR−99a−5p, rectal
(all subjects; N = 719)
1−sided adj pval: 1
Frequency
Tumor−Normal Expression Difference
hsa−miR−99a−5p, rectal
(ESTROGEN = 0; N0 = 119)
1−sided adj pval: 0.979
Frequency
Tumor−Normal Expression Difference
hsa−miR−99a−5p, rectal
(ESTROGEN = 1; N1 = 108)
1−sided adj pval: 0.882
Frequency
Tumor−Normal Expression Difference

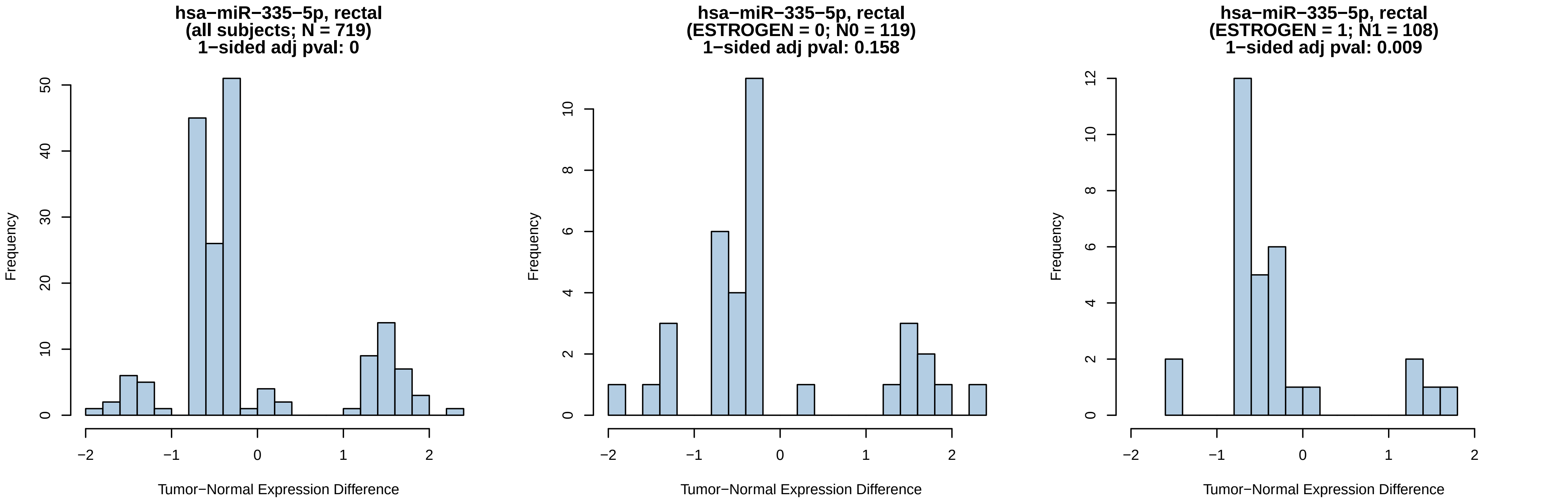
hsa−miR−335−5p, rectal
(all subjects; N = 719)
1−sided adj pval: 0
hsa−miR−335−5p, rectal
(ESTROGEN = 0; N0 = 119)
1−sided adj pval: 0.158
hsa−miR−335−5p, rectal
(ESTROGEN = 1; N1 = 108)
1−sided adj pval: 0.009
Frequency
Tumor−Normal Expression Difference

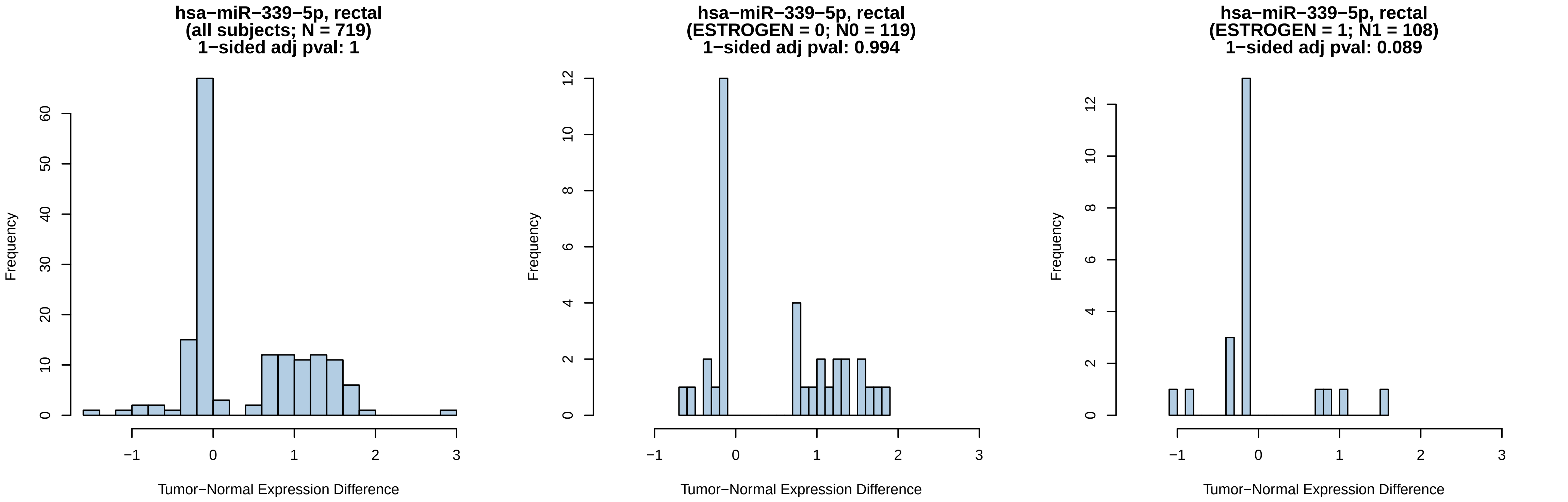
hsa−miR−339−5p, rectal
(all subjects; N = 719)
1−sided adj pval: 1
Frequency
Tumor−Normal Expression Difference
hsa−miR−339−5p, rectal
(ESTROGEN = 0; N0 = 119)
1−sided adj pval: 0.994
Frequency
Tumor−Normal Expression Difference
hsa−miR−339−5p, rectal
(ESTROGEN = 1; N1 = 108)
1−sided adj pval: 0.089
Frequency
Tumor−Normal Expression Difference

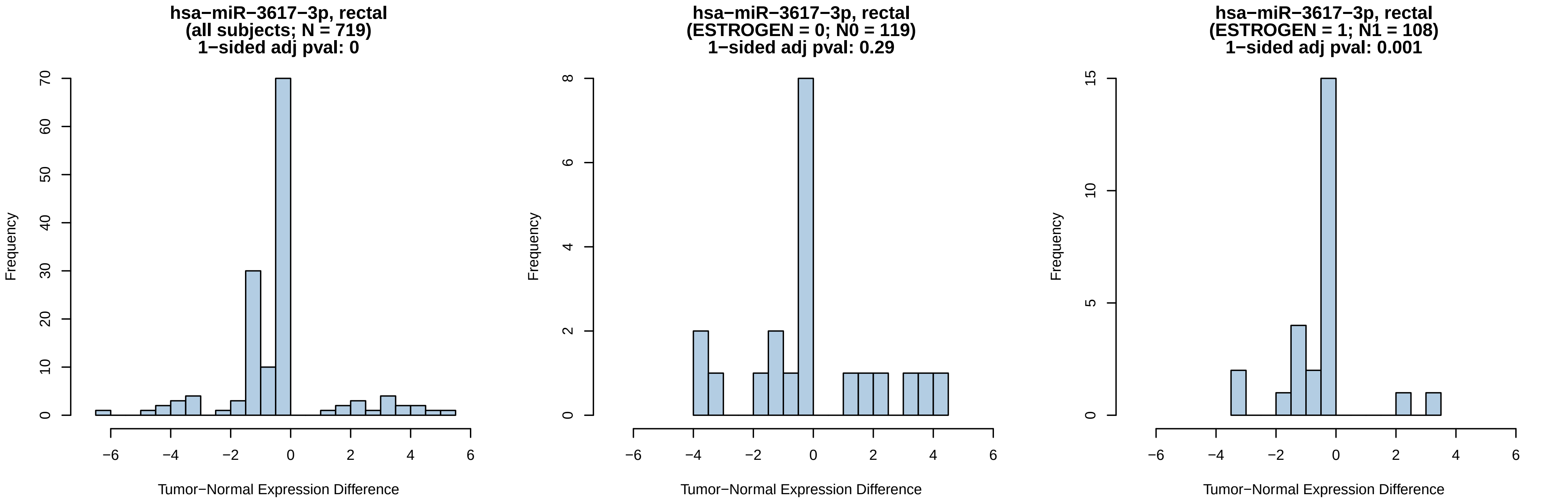

hsa−miR−3617−3p, rectal
(all subjects; N = 719)
1−sided adj pval: 0
hsa−miR−3617−3p, rectal
(ESTROGEN = 0; N0 = 119)
1−sided adj pval: 0.29
hsa−miR−3617−3p, rectal
(ESTROGEN = 1; N1 = 108)
1−sided adj pval: 0.001
Frequency
Tumor−Normal Expression Difference

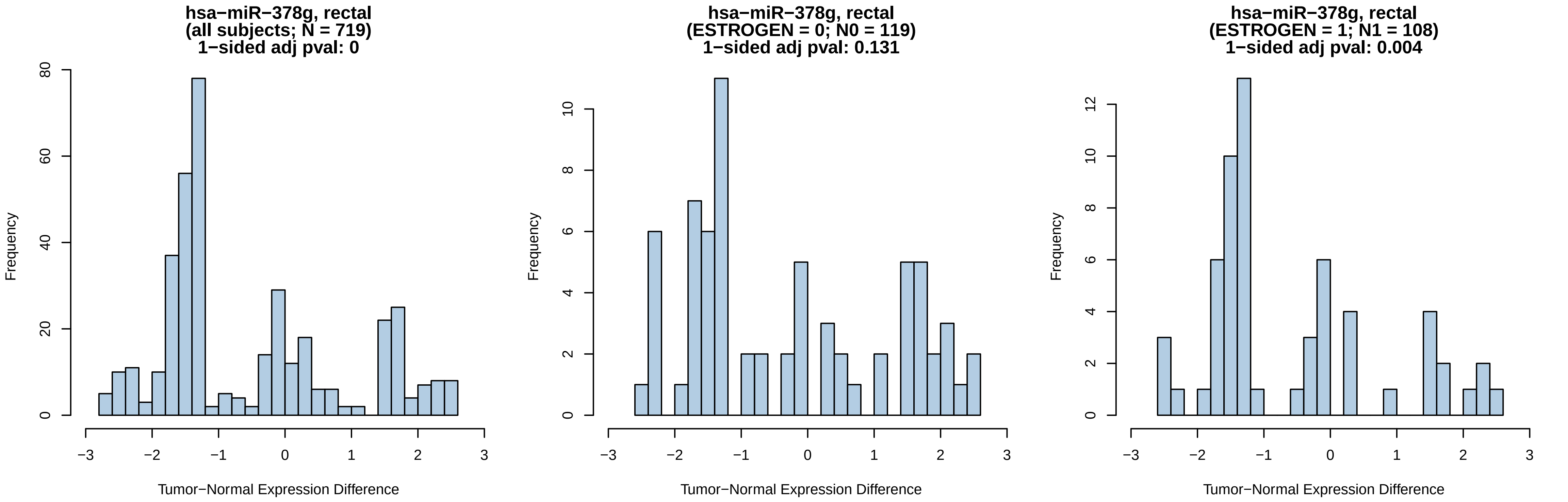

hsa−miR−378g, rectal
(all subjects; N = 719)
1−sided adj pval: 0
hsa−miR−378g, rectal
(ESTROGEN = 0; N0 = 119)
1−sided adj pval: 0.131
hsa−miR−378g, rectal
(ESTROGEN = 1; N1 = 108)
1−sided adj pval: 0.004
Frequency
Tumor−Normal Expression Difference

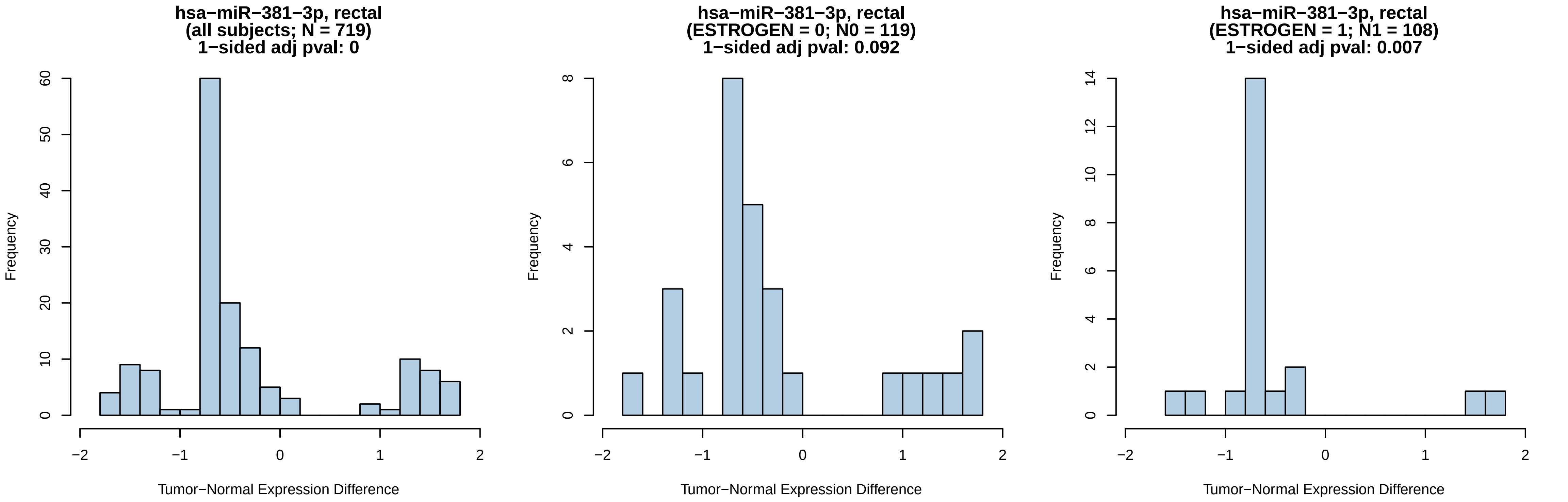

hsa−miR−381−3p, rectal
(all subjects; N = 719)
1−sided adj pval: 0
Frequency
Tumor−Normal Expression Difference
hsa−miR−381−3p, rectal
(ESTROGEN = 0; N0 = 119)
1−sided adj pval: 0.092
Frequency
Tumor−Normal Expression Difference
hsa−miR−381−3p, rectal
(ESTROGEN = 1; N1 = 108)
1−sided adj pval: 0.007
Frequency
Tumor−Normal Expression Difference

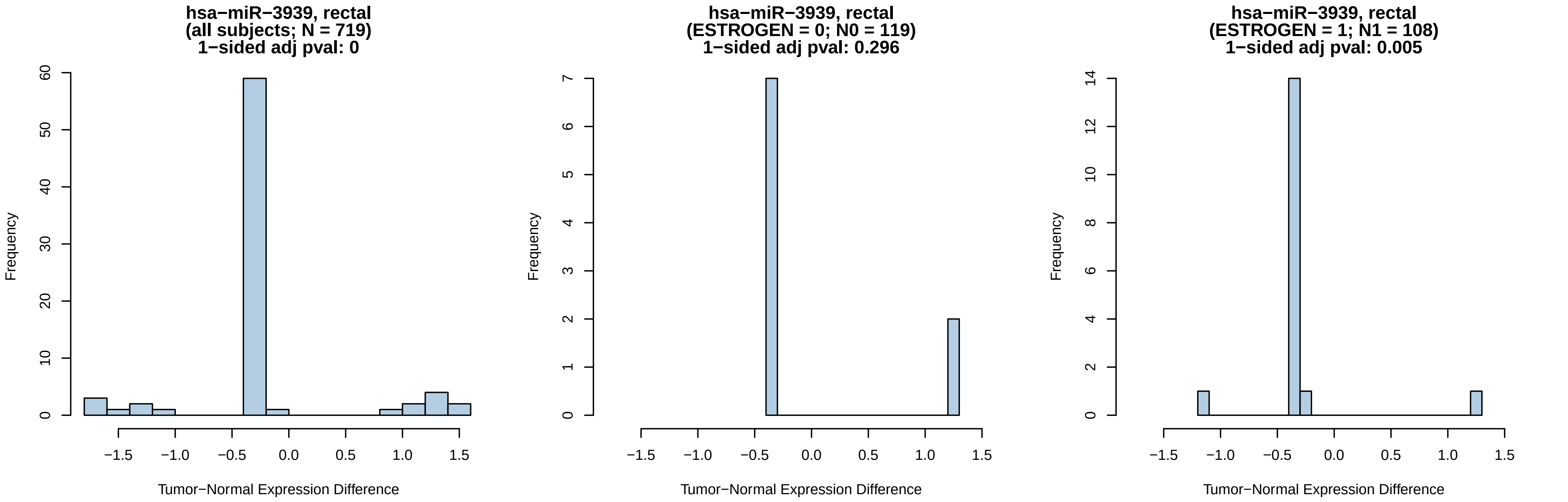

hsa−miR−3939, rectal
(all subjects; N = 719)
1−sided adj pval: 0
Frequency
Tumor−Normal Expression Difference
hsa−miR−3939, rectal
(ESTROGEN = 0; N0 = 119)
1−sided adj pval: 0.296
Frequency
Tumor−Normal Expression Difference
hsa−miR−3939, rectal
(ESTROGEN = 1; N1 = 108)
1−sided adj pval: 0.005
Frequency
Tumor−Normal Expression Difference

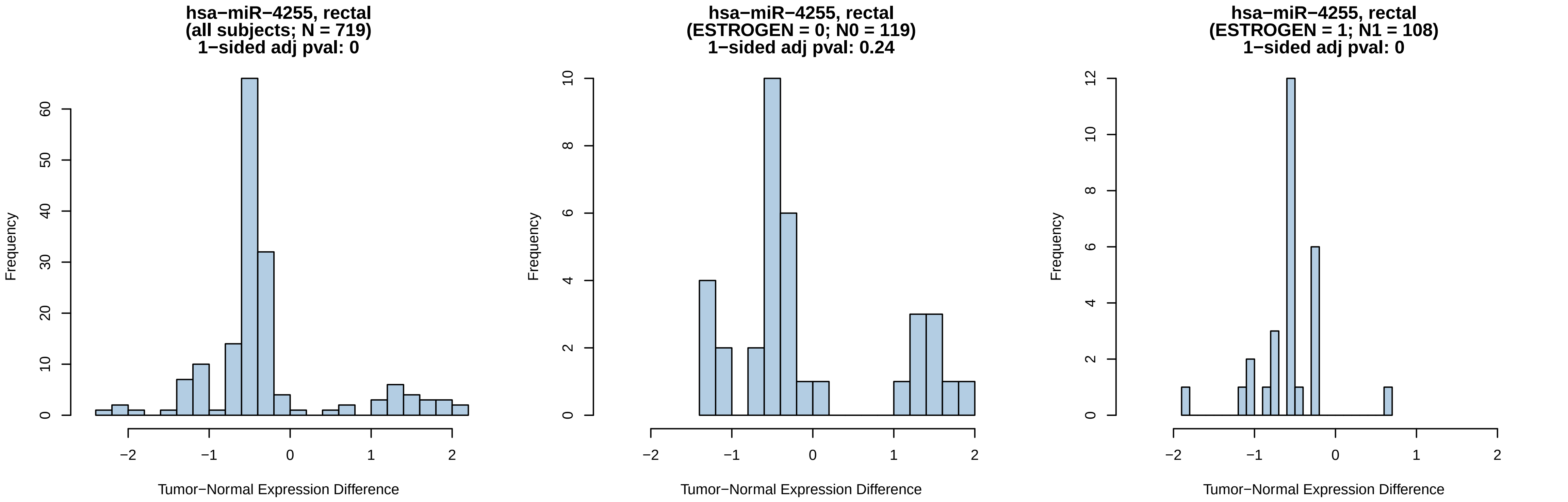
hsa−miR−4255, rectal
(all subjects; N = 719)
1−sided adj pval: 0
Frequency
0
10
20
30
40
50
60
−2
−1
0
1
2
Tumor−Normal Expression Difference
hsa−miR−4255, rectal
(ESTROGEN = 0; N0 = 119)
1−sided adj pval: 0.24
Frequency
0
2
4
6
8
10
−2
−1
0
1
2
Tumor−Normal Expression Difference
hsa−miR−4255, rectal
(ESTROGEN = 1; N1 = 108)
1−sided adj pval: 0
Frequency
0
2
4
6
8
10
12
−2
−1
0
1
2
Tumor−Normal Expression Difference

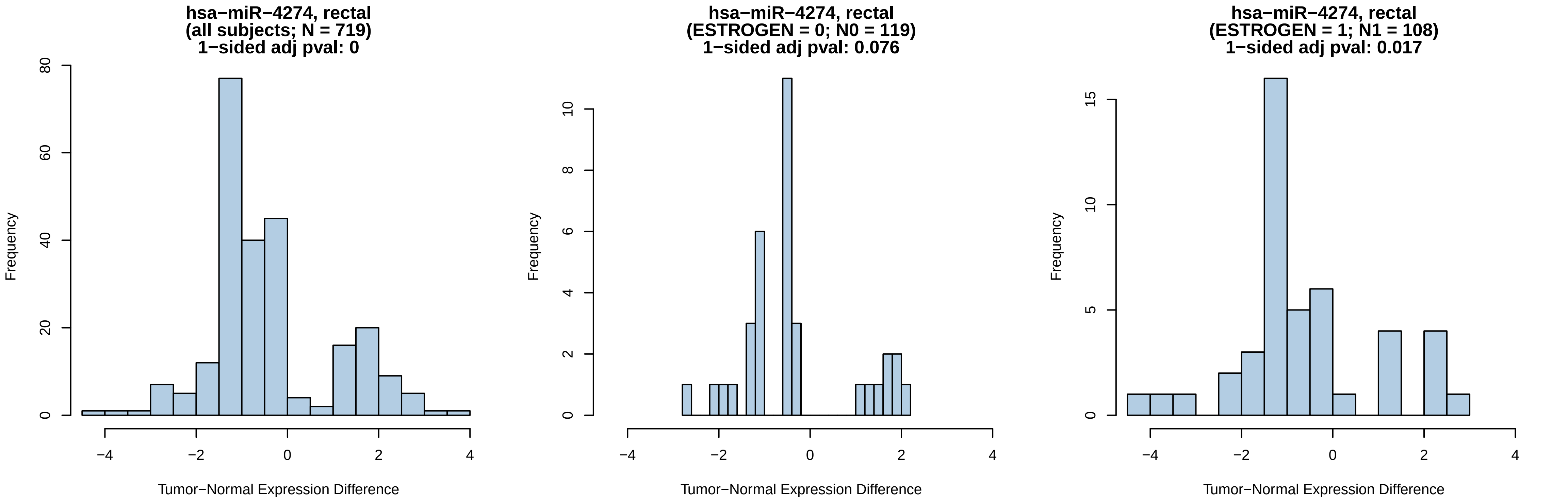

hsa−miR−4274, rectal
(all subjects; N = 719)
1−sided adj pval: 0
Frequency
0
20
40
60
80
−4
−2
0
2
4
Tumor−Normal Expression Difference
hsa−miR−4274, rectal
(ESTROGEN = 0; N0 = 119)
1−sided adj pval: 0.076
Frequency
0
2
4
6
8
10
−4
−2
0
2
4
Tumor−Normal Expression Difference
hsa−miR−4274, rectal
(ESTROGEN = 1; N1 = 108)
1−sided adj pval: 0.017
Frequency
0
5
10
15
−4
−2
0
2
4
Tumor−Normal Expression Difference

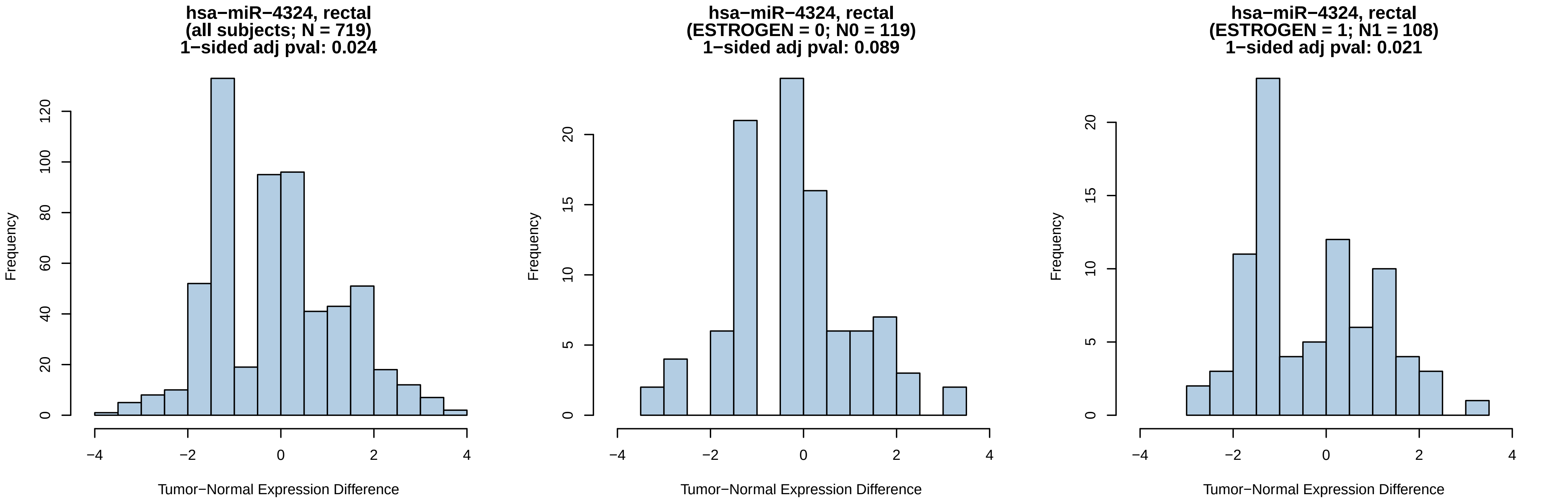

hsa−miR−4324, rectal
(all subjects; N = 719)
1−sided adj pval: 0.024
Frequency
0
20
40
60
80
100
120
−4
−2
0
2
4
Tumor−Normal Expression Difference
hsa−miR−4324, rectal
(ESTROGEN = 0; N0 = 119)
1−sided adj pval: 0.089
Frequency
0
5
10
15
20
−4
−2
0
2
4
Tumor−Normal Expression Difference
hsa−miR−4324, rectal
(ESTROGEN = 1; N1 = 108)
1−sided adj pval: 0.021
Frequency
0
5
10
15
20
−4
−2
0
2
4
Tumor−Normal Expression Difference

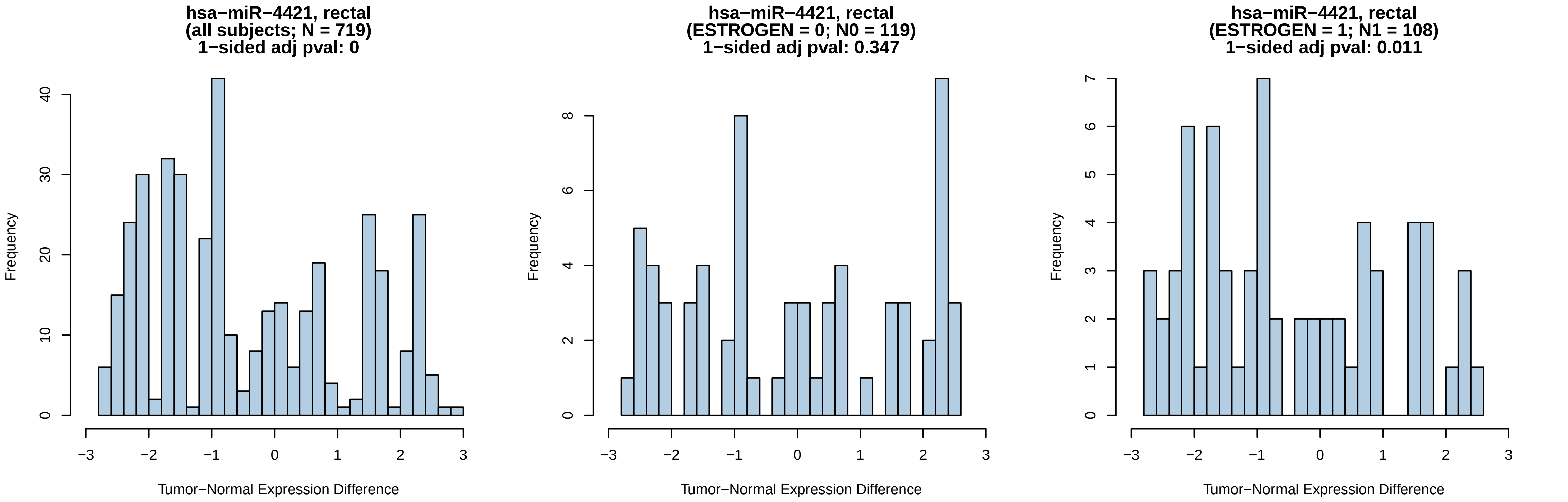
hsa−miR−4421, rectal
(all subjects; N = 719)
1−sided adj pval: 0
Frequency
Tumor−Normal Expression Difference
hsa−miR−4421, rectal
(ESTROGEN = 0; N0 = 119)
1−sided adj pval: 0.347
Frequency
Tumor−Normal Expression Difference
hsa−miR−4421, rectal
(ESTROGEN = 1; N1 = 108)
1−sided adj pval: 0.011
Frequency
Tumor−Normal Expression Difference

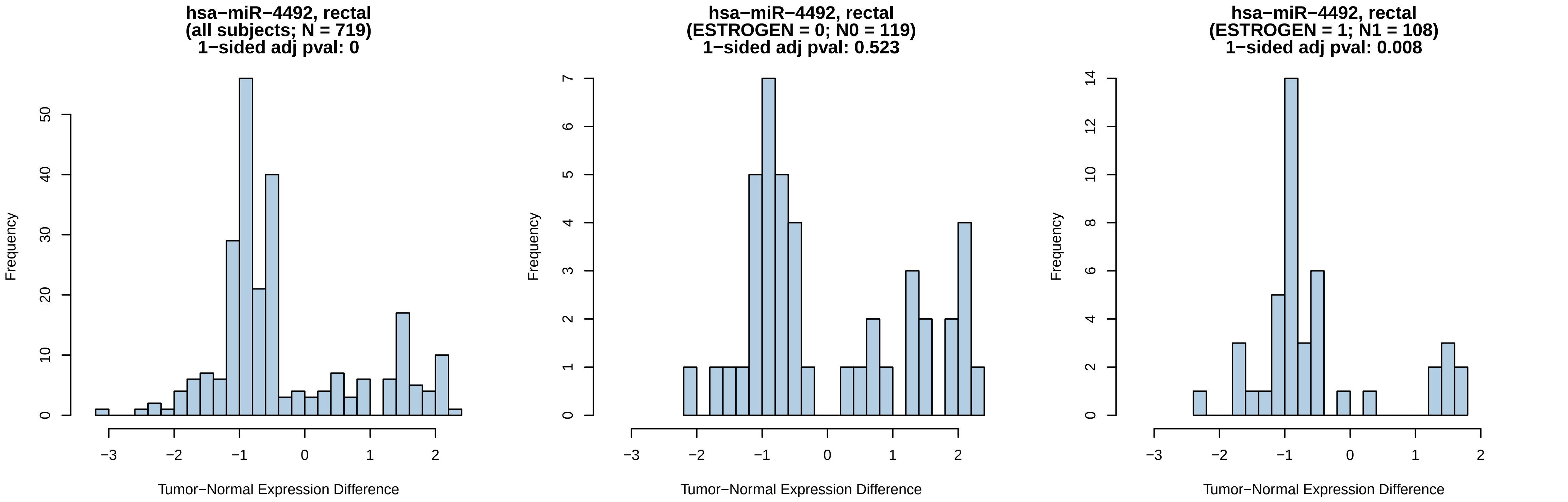

hsa−miR−4492, rectal
(all subjects; N = 719)
1−sided adj pval: 0
hsa−miR−4492, rectal
(ESTROGEN = 0; N0 = 119)
1−sided adj pval: 0.523
hsa−miR−4492, rectal
(ESTROGEN = 1; N1 = 108)
1−sided adj pval: 0.008
Frequency
Tumor−Normal Expression Difference

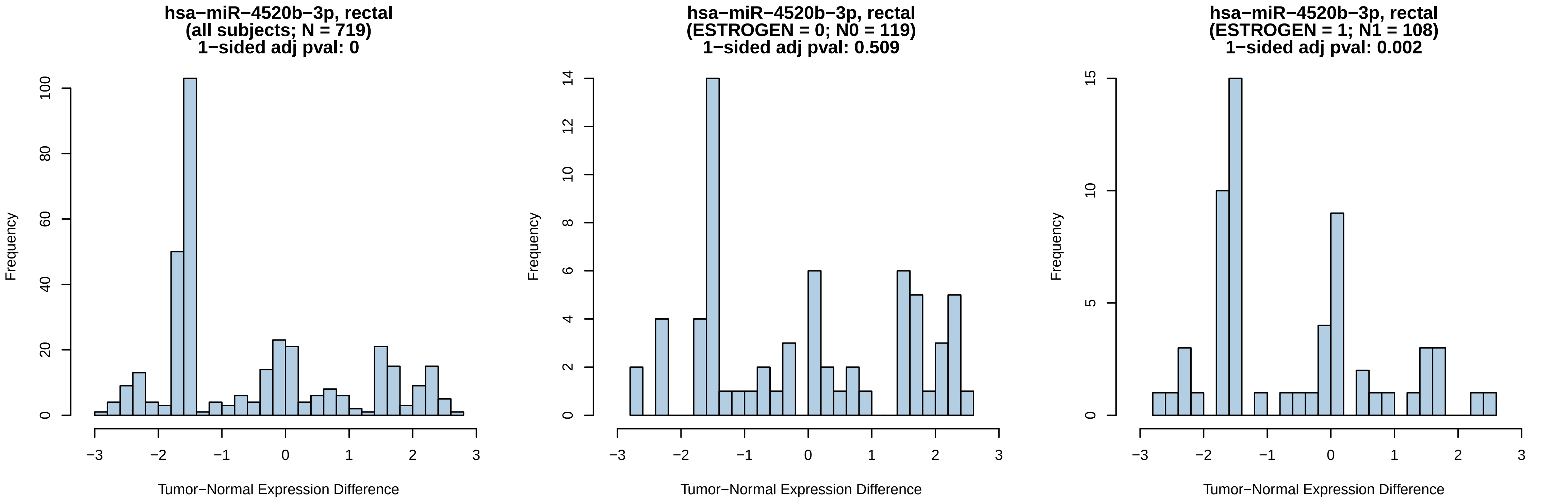

hsa−miR−4520b−3p, rectal
(all subjects; N = 719)
1−sided adj pval: 0
hsa−miR−4520b−3p, rectal
(ESTROGEN = 0; N0 = 119)
1−sided adj pval: 0.509
hsa−miR−4520b−3p, rectal
(ESTROGEN = 1; N1 = 108)
1−sided adj pval: 0.002
Frequency
Tumor−Normal Expression Difference

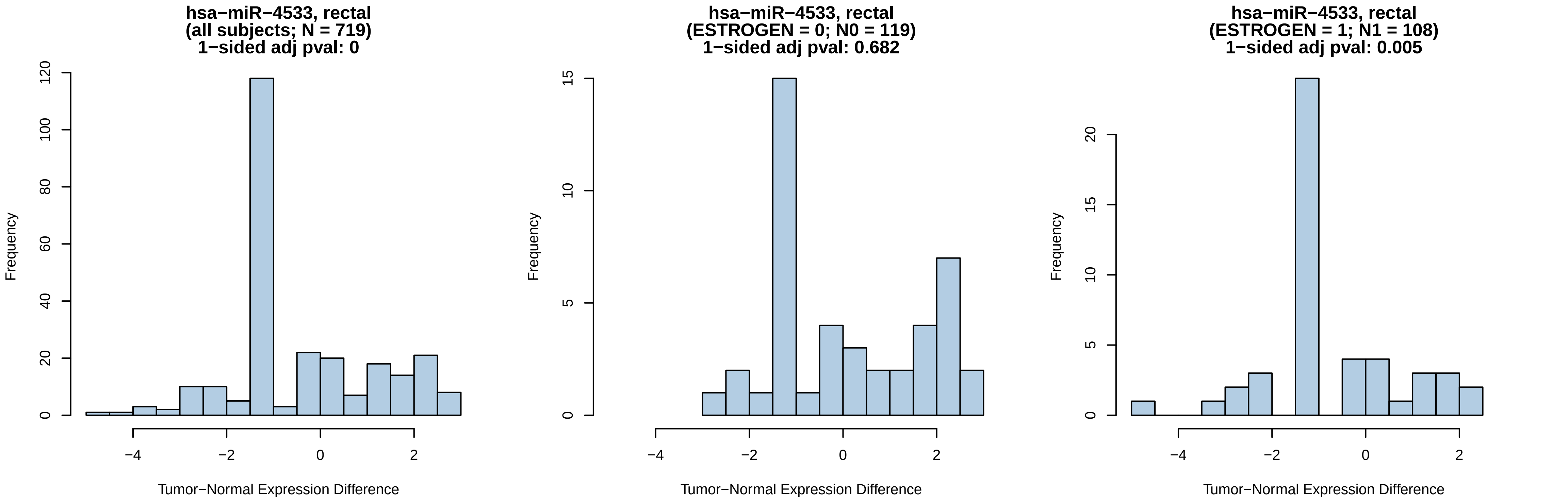

hsa−miR−4533, rectal
(all subjects; N = 719)
1−sided adj pval: 0
hsa−miR−4533, rectal
(ESTROGEN = 0; N0 = 119)
1−sided adj pval: 0.682
hsa−miR−4533, rectal
(ESTROGEN = 1; N1 = 108)
1−sided adj pval: 0.005
Frequency
Tumor−Normal Expression Difference

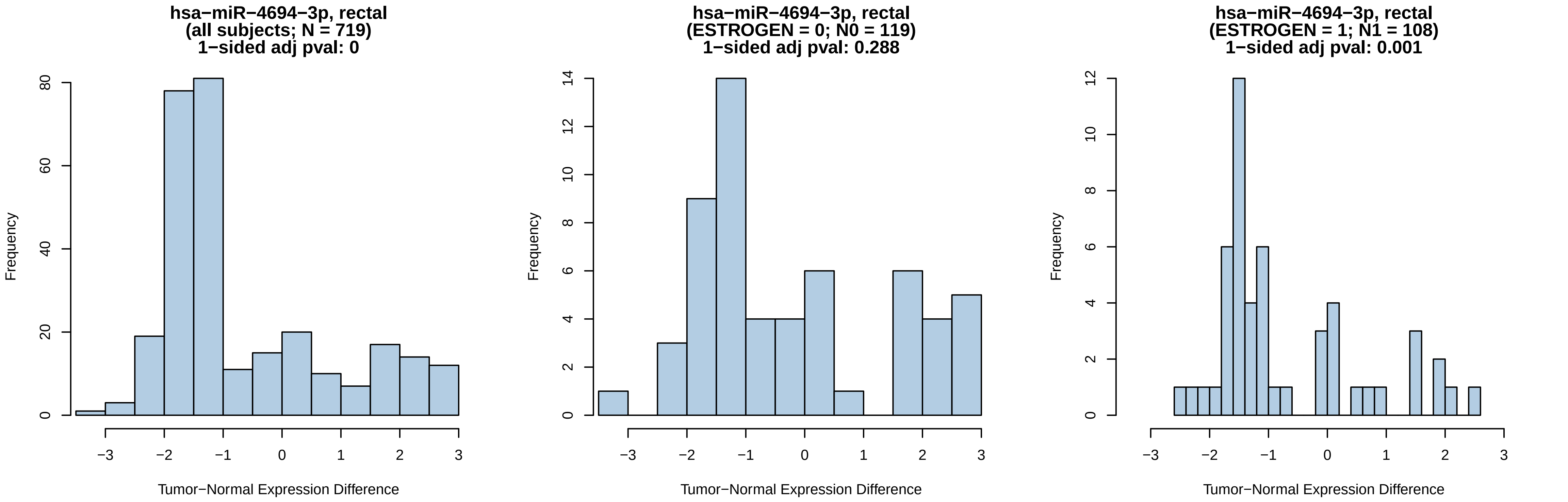

hsa−miR−4694−3p, rectal
(all subjects; N = 719)
1−sided adj pval: 0
Frequency
Tumor−Normal Expression Difference
hsa−miR−4694−3p, rectal
(ESTROGEN = 0; N0 = 119)
1−sided adj pval: 0.288
Frequency
Tumor−Normal Expression Difference
hsa−miR−4694−3p, rectal
(ESTROGEN = 1; N1 = 108)
1−sided adj pval: 0.001
Frequency
Tumor−Normal Expression Difference

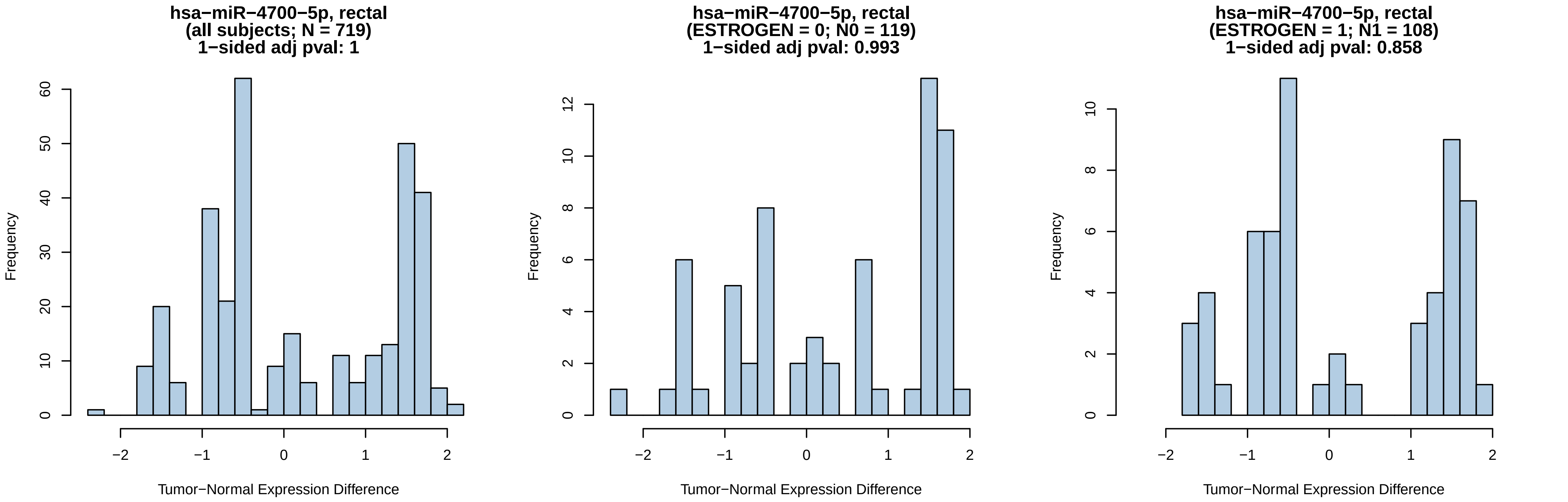

hsa−miR−4700−5p, rectal
(all subjects; N = 719)
1−sided adj pval: 1
hsa−miR−4700−5p, rectal
(ESTROGEN = 0; N0 = 119)
1−sided adj pval: 0.993
hsa−miR−4700−5p, rectal
(ESTROGEN = 1; N1 = 108)
1−sided adj pval: 0.858
Frequency
Tumor−Normal Expression Difference

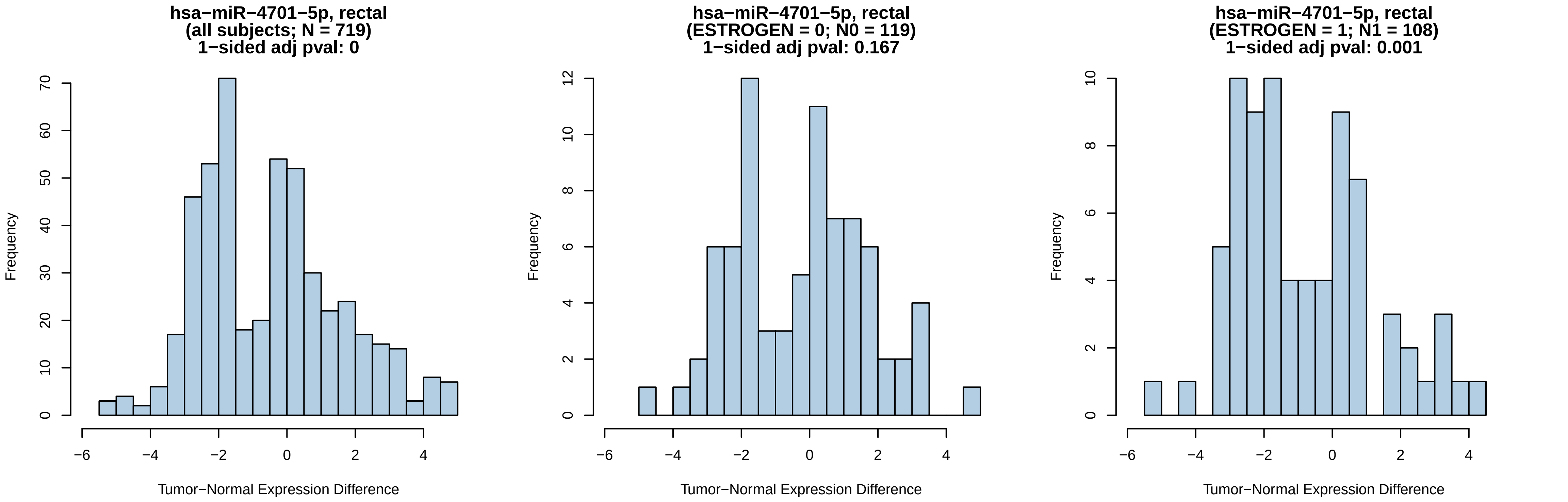

hsa−miR−4701−5p, rectal
(all subjects; N = 719)
1−sided adj pval: 0
Frequency
Tumor−Normal Expression Difference
hsa−miR−4701−5p, rectal
(ESTROGEN = 0; N0 = 119)
1−sided adj pval: 0.167
Frequency
Tumor−Normal Expression Difference
hsa−miR−4701−5p, rectal
(ESTROGEN = 1; N1 = 108)
1−sided adj pval: 0.001
Frequency
Tumor−Normal Expression Difference

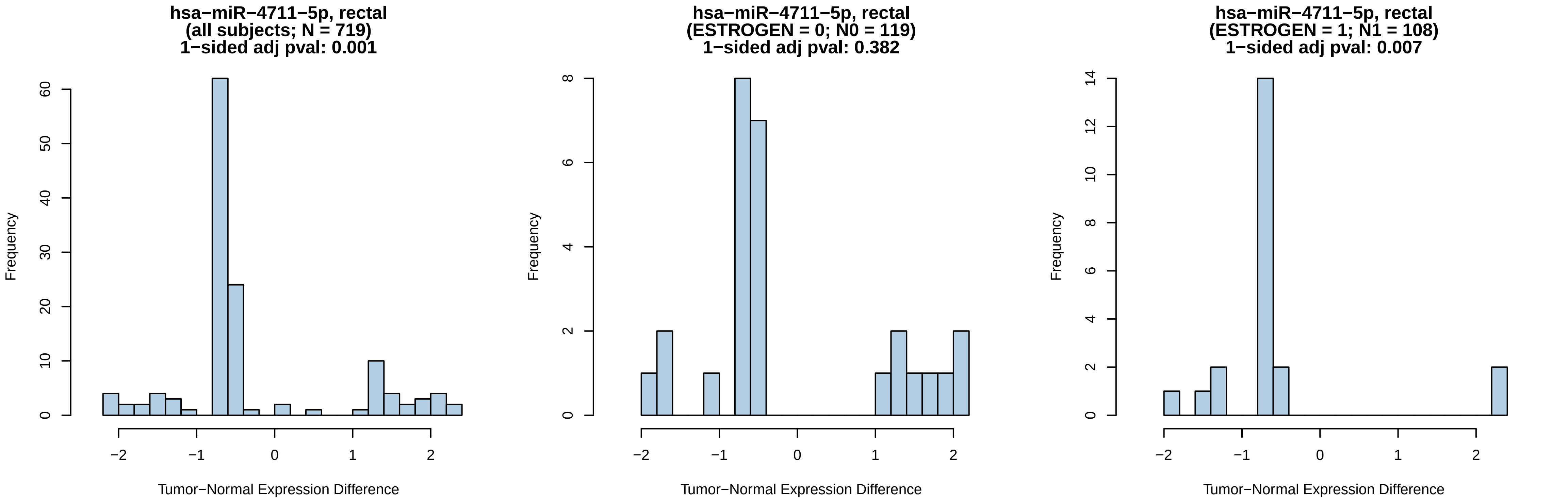
hsa−miR−4711−5p, rectal
(all subjects; N = 719)
1−sided adj pval: 0.001
Frequency
Tumor−Normal Expression Difference
hsa−miR−4711−5p, rectal
(ESTROGEN = 0; N0 = 119)
1−sided adj pval: 0.382
Frequency
Tumor−Normal Expression Difference
hsa−miR−4711−5p, rectal
(ESTROGEN = 1; N1 = 108)
1−sided adj pval: 0.007
Frequency
Tumor−Normal Expression Difference

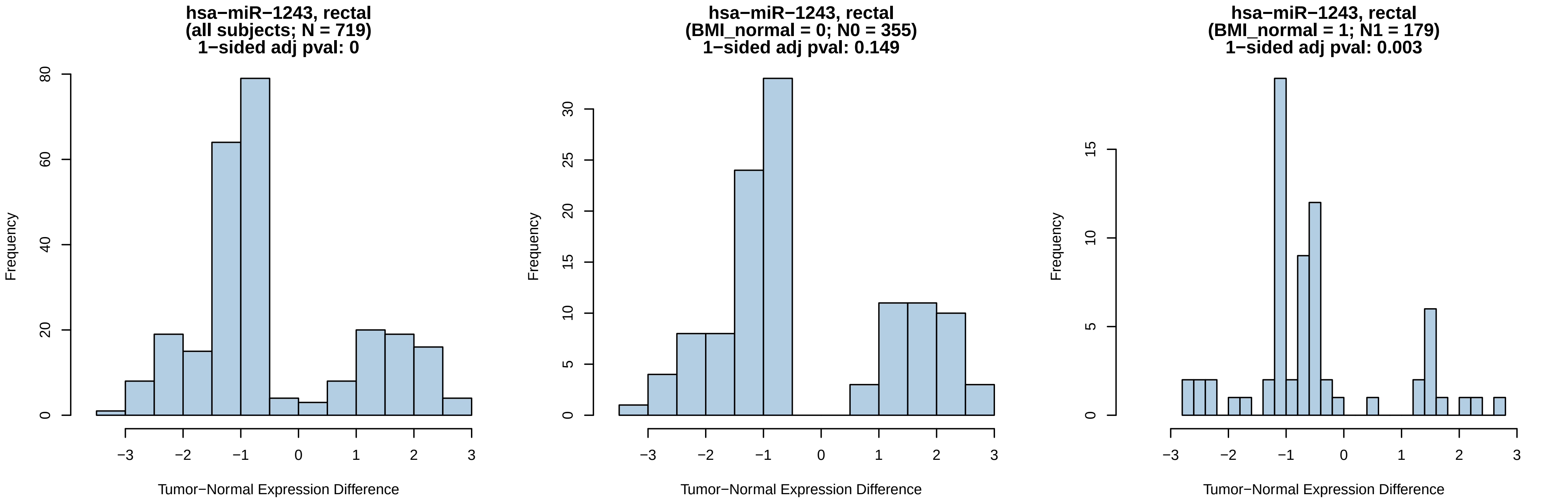
hsa−miR−1243, rectal
(all subjects; N = 719)
1−sided adj pval: 0
Frequency
Tumor−Normal Expression Difference
hsa−miR−1243, rectal
(BMI_normal = 0; N0 = 355)
1−sided adj pval: 0.149
Frequency
Tumor−Normal Expression Difference
hsa−miR−1243, rectal
(BMI_normal = 1; N1 = 179)
1−sided adj pval: 0.003
Frequency
Tumor−Normal Expression Difference

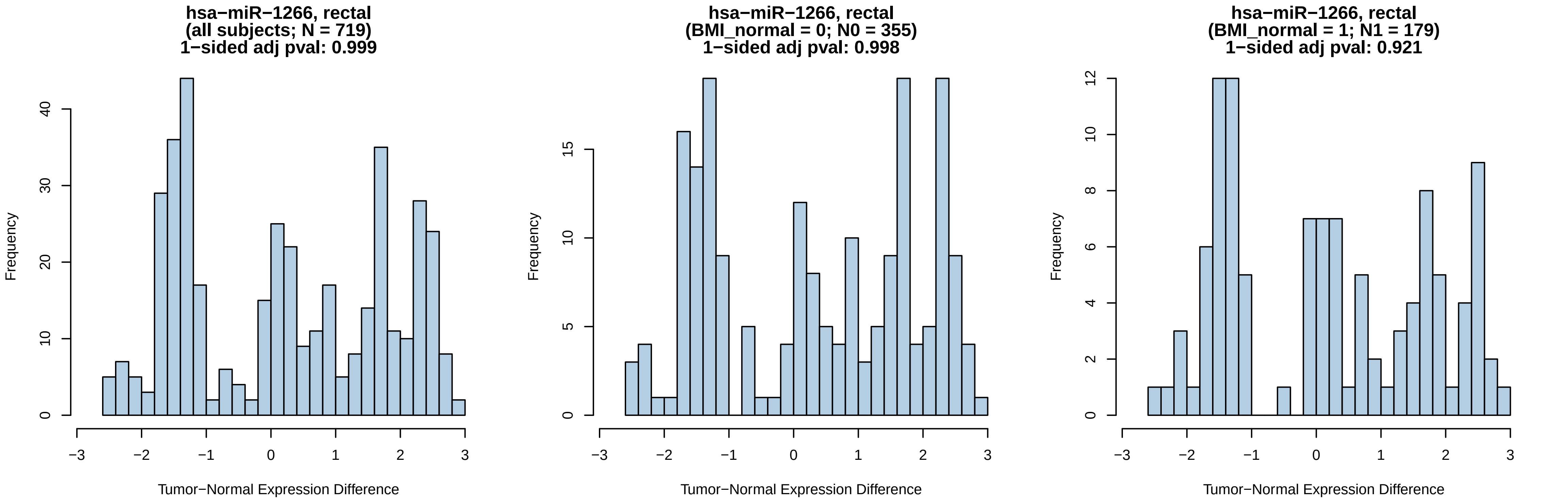
hsa−miR−1266, rectal
(all subjects; N = 719)
1−sided adj pval: 0.999
Frequency
Tumor−Normal Expression Difference
hsa−miR−1266, rectal
(BMI_normal = 0; N0 = 355)
1−sided adj pval: 0.998
Frequency
Tumor−Normal Expression Difference
hsa−miR−1266, rectal
(BMI_normal = 1; N1 = 179)
1−sided adj pval: 0.921
Frequency
Tumor−Normal Expression Difference

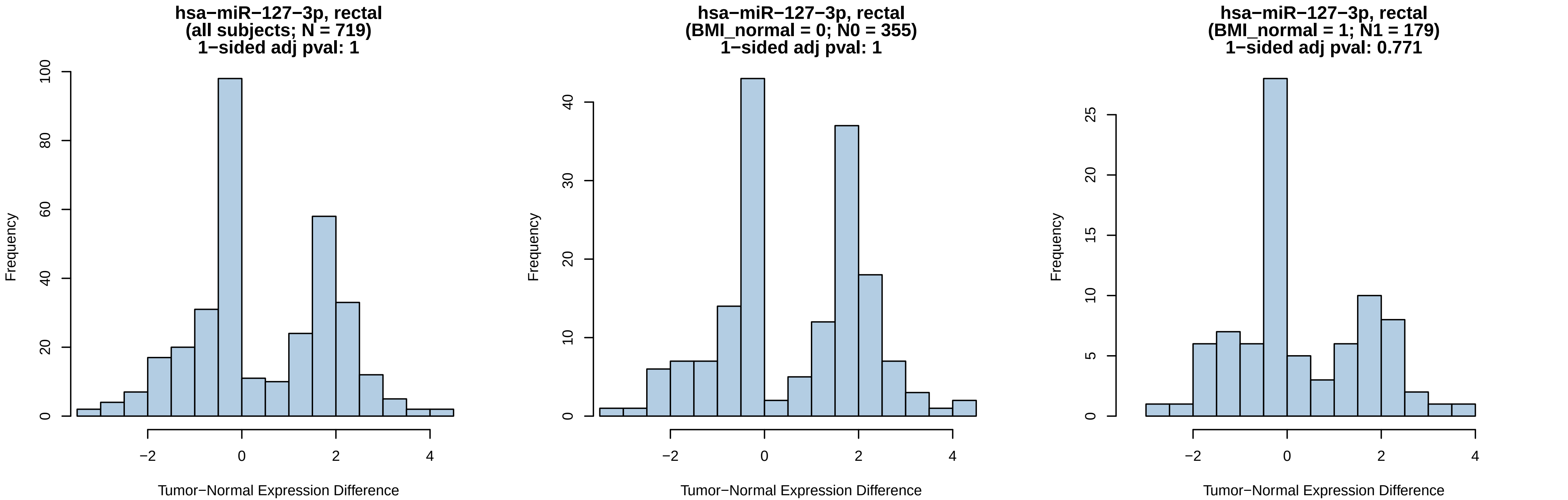

hsa−miR−127−3p, rectal
(all subjects; N = 719)
1−sided adj pval: 1
Frequency
0
20
40
60
80
100
−2
0
2
4
Tumor−Normal Expression Difference
hsa−miR−127−3p, rectal
(BMI_normal = 0; N0 = 355)
1−sided adj pval: 1
Frequency
0
10
20
30
40
−2
0
2
4
Tumor−Normal Expression Difference
hsa−miR−127−3p, rectal
(BMI_normal = 1; N1 = 179)
1−sided adj pval: 0.771
Frequency
0
5
10
15
20
25
−2
0
2
4
Tumor−Normal Expression Difference

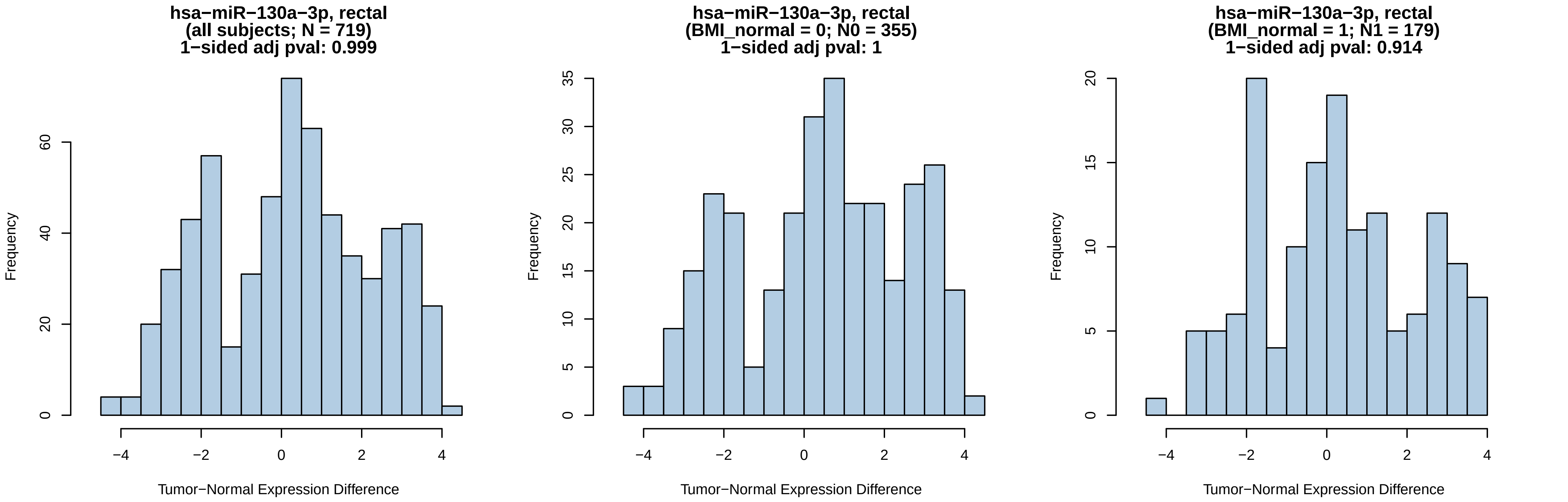
hsa−miR−130a−3p, rectal
(all subjects; N = 719)
1−sided adj pval: 0.999
Frequency
0
20
40
60
−4
−2
0
2
4
Tumor−Normal Expression Difference
hsa−miR−130a−3p, rectal
(BMI_normal = 0; N0 = 355)
1−sided adj pval: 1
Frequency
0
5
10
15
20
25
30
35
−4
−2
0
2
4
Tumor−Normal Expression Difference
hsa−miR−130a−3p, rectal
(BMI_normal = 1; N1 = 179)
1−sided adj pval: 0.914
Frequency
0
5
10
15
20
−4
−2
0
2
4
Tumor−Normal Expression Difference

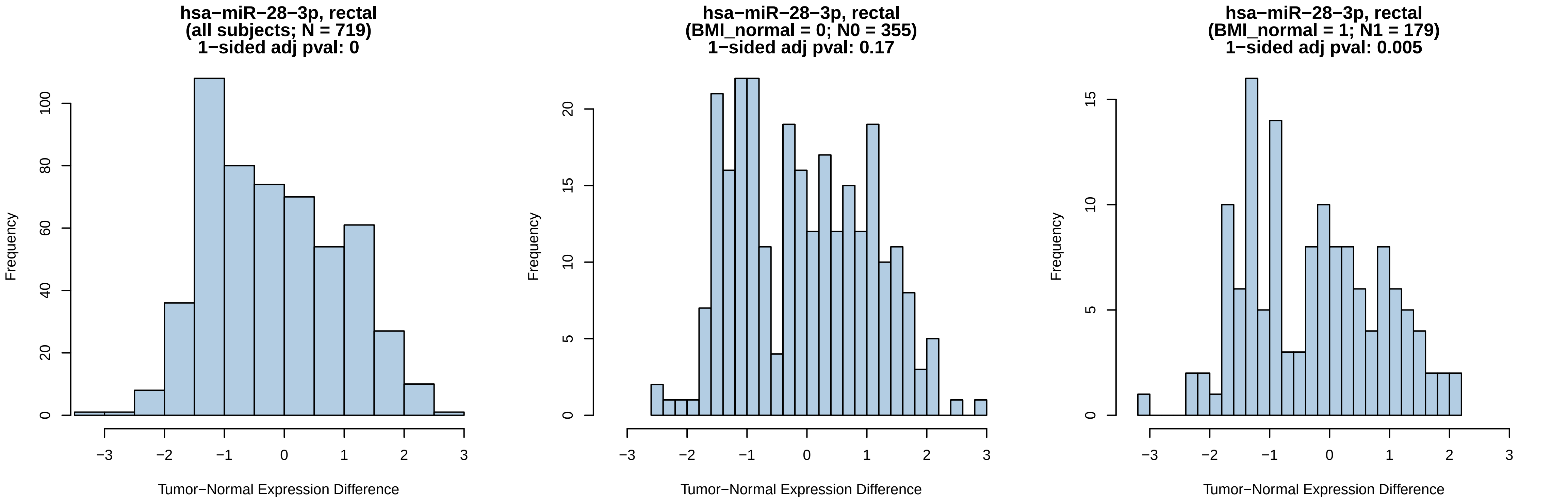

hsa−miR−28−3p, rectal
(all subjects; N = 719)
1−sided adj pval: 0
Frequency
Tumor−Normal Expression Difference
hsa−miR−28−3p, rectal
(BMI_normal = 0; N0 = 355)
1−sided adj pval: 0.17
Frequency
Tumor−Normal Expression Difference
hsa−miR−28−3p, rectal
(BMI_normal = 1; N1 = 179)
1−sided adj pval: 0.005
Frequency
Tumor−Normal Expression Difference

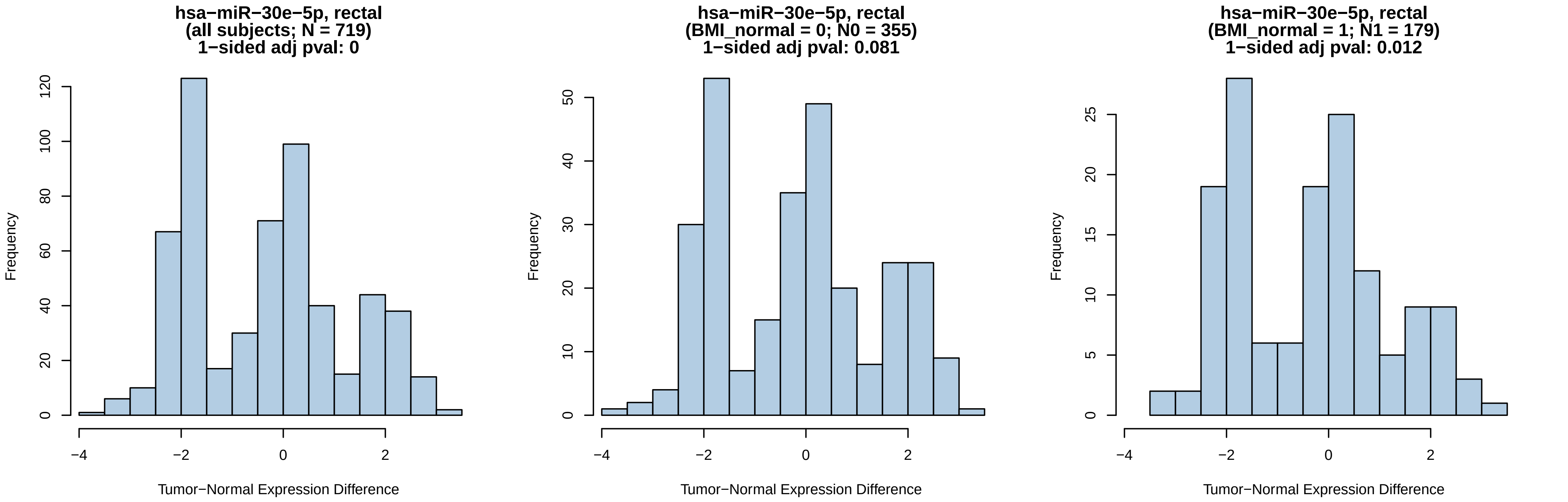
hsa−miR−30e−5p, rectal
(all subjects; N = 719)
1−sided adj pval: 0
Frequency
Tumor−Normal Expression Difference
hsa−miR−30e−5p, rectal
(BMI_normal = 0; N0 = 355)
1−sided adj pval: 0.081
Frequency
Tumor−Normal Expression Difference
hsa−miR−30e−5p, rectal
(BMI_normal = 1; N1 = 179)
1−sided adj pval: 0.012
Frequency
Tumor−Normal Expression Difference

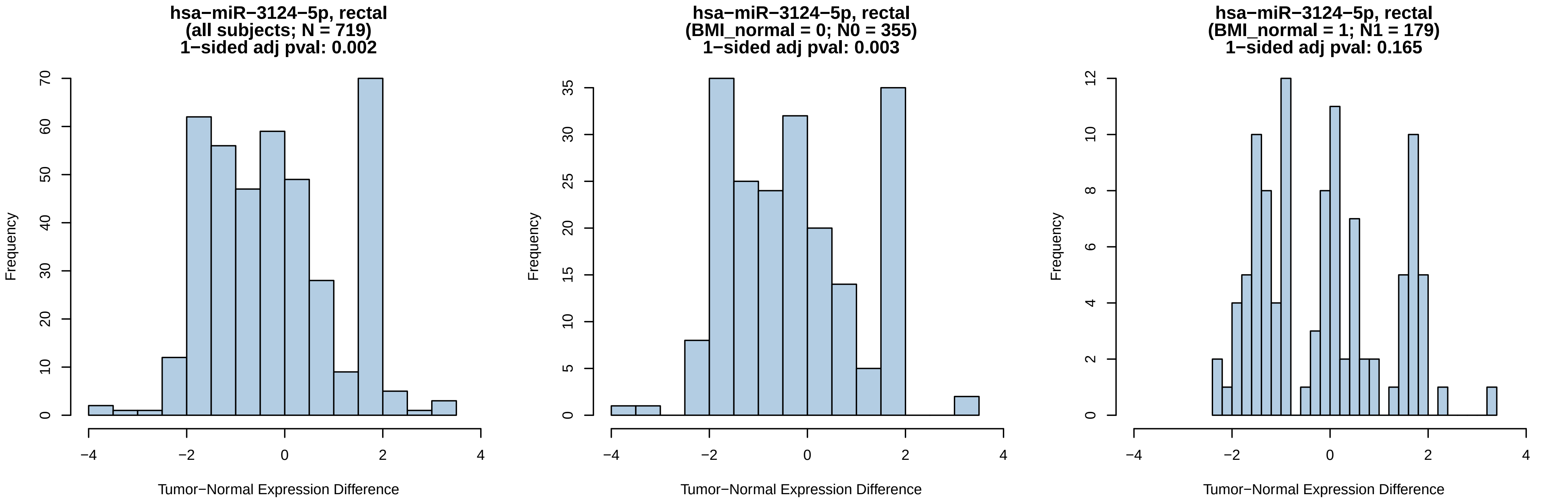

hsa−miR−3124−5p, rectal
(all subjects; N = 719)
1−sided adj pval: 0.002
Frequency
Tumor−Normal Expression Difference
hsa−miR−3124−5p, rectal
(BMI_normal = 0; N0 = 355)
1−sided adj pval: 0.003
Frequency
Tumor−Normal Expression Difference
hsa−miR−3124−5p, rectal
(BMI_normal = 1; N1 = 179)
1−sided adj pval: 0.165
Frequency
Tumor−Normal Expression Difference

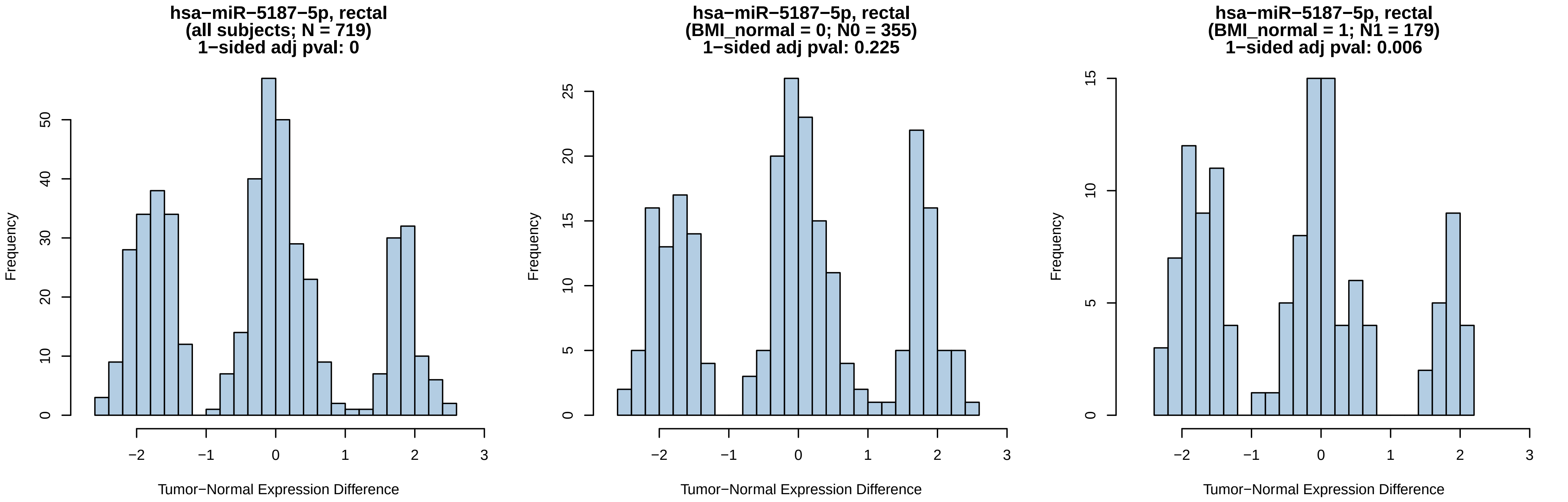
hsa−miR−5187−5p, rectal
(all subjects; N = 719)
1−sided adj pval: 0
Frequency
0
10
20
30
40
50
−2
−1
0
1
2
3
Tumor−Normal Expression Difference
hsa−miR−5187−5p, rectal
(BMI_normal = 0; N0 = 355)
1−sided adj pval: 0.225
Frequency
0
5
10
15
20
25
−2
−1
0
1
2
3
Tumor−Normal Expression Difference
hsa−miR−5187−5p, rectal
(BMI_normal = 1; N1 = 179)
1−sided adj pval: 0.006
Frequency
0
5
10
15
−2
−1
0
1
2
3
Tumor−Normal Expression Difference

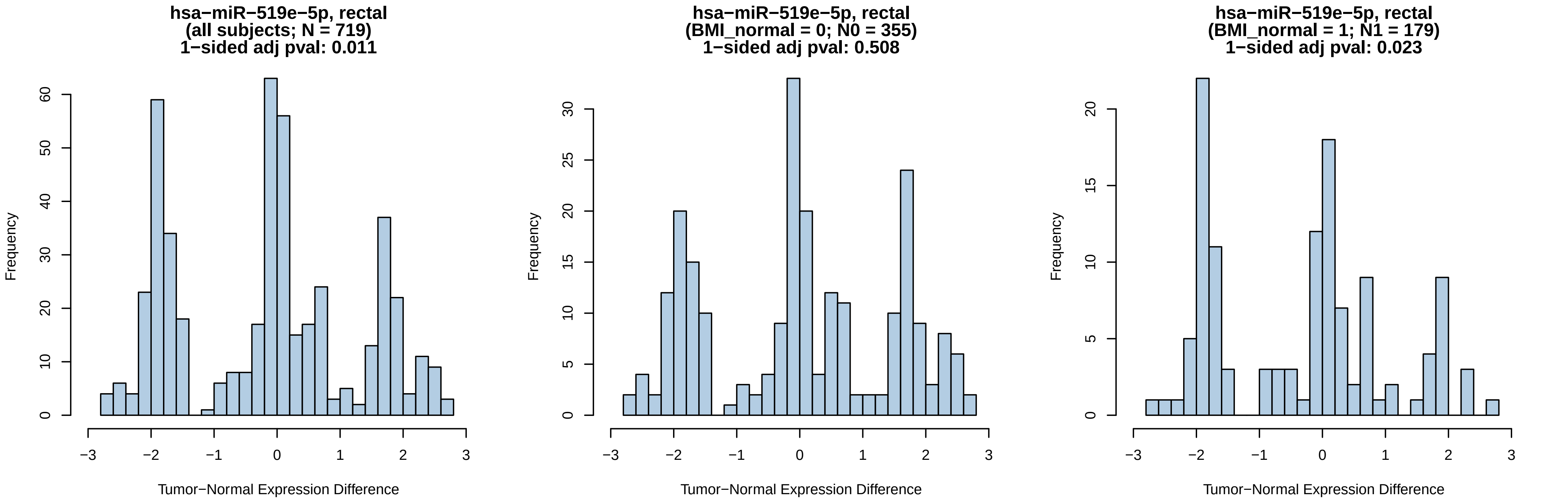
hsa−miR−519e−5p, rectal
(all subjects; N = 719)
1−sided adj pval: 0.011
Frequency
Tumor−Normal Expression Difference
hsa−miR−519e−5p, rectal
(BMI_normal = 0; N0 = 355)
1−sided adj pval: 0.508
Frequency
Tumor−Normal Expression Difference
hsa−miR−519e−5p, rectal
(BMI_normal = 1; N1 = 179)
1−sided adj pval: 0.023
Frequency
Tumor−Normal Expression Difference

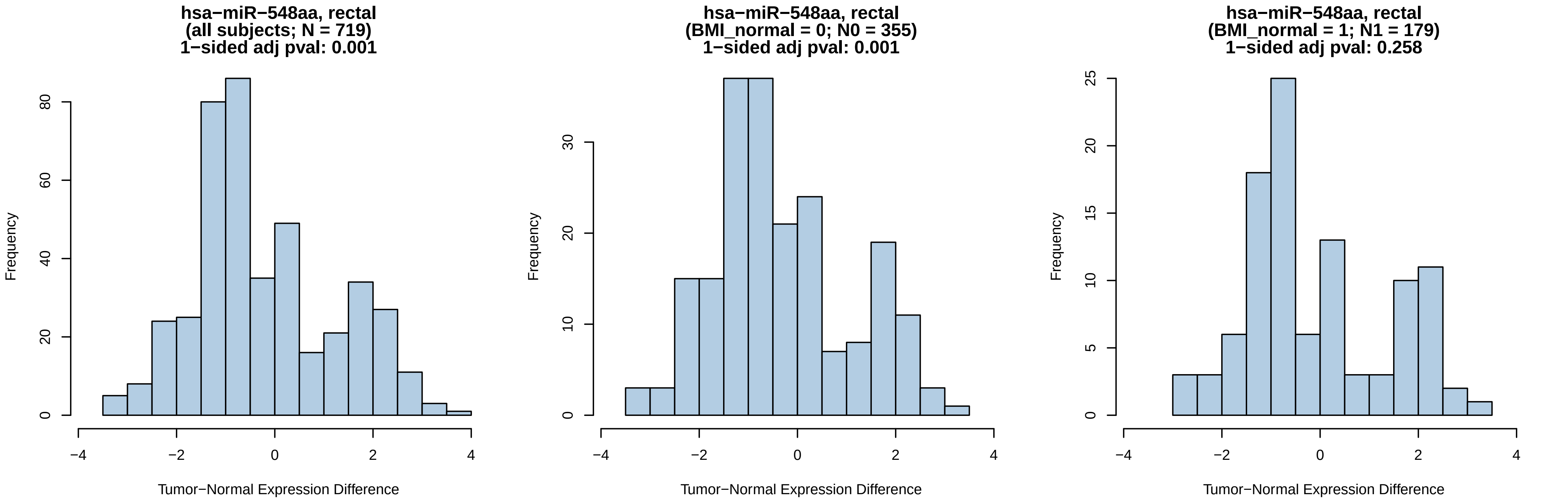
hsa−miR−548aa, rectal
(all subjects; N = 719)
1−sided adj pval: 0.001
Frequency
Tumor−Normal Expression Difference
hsa−miR−548aa, rectal
(BMI_normal = 0; N0 = 355)
1−sided adj pval: 0.001
Frequency
Tumor−Normal Expression Difference
hsa−miR−548aa, rectal
(BMI_normal = 1; N1 = 179)
1−sided adj pval: 0.258
Frequency
Tumor−Normal Expression Difference

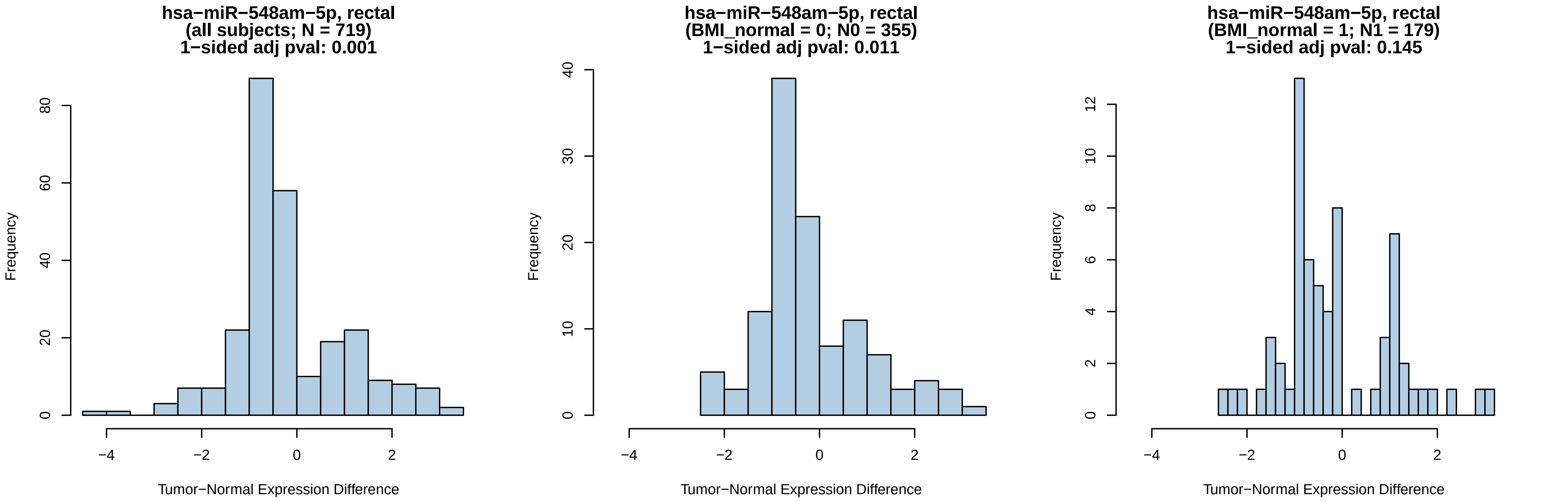

hsa−miR−548am−5p, rectal
(all subjects; N = 719)
1−sided adj pval: 0.001
Frequency
0
20
40
60
80
−4
−2
0
2
Tumor−Normal Expression Difference
hsa−miR−548am−5p, rectal
(BMI_normal = 0; N0 = 355)
1−sided adj pval: 0.011
Frequency
0
10
20
30
40
−4
−2
0
2
Tumor−Normal Expression Difference
hsa−miR−548am−5p, rectal
(BMI_normal = 1; N1 = 179)
1−sided adj pval: 0.145
Frequency
0
2
4
6
8
10
12
−4
−2
0
2
Tumor−Normal Expression Difference

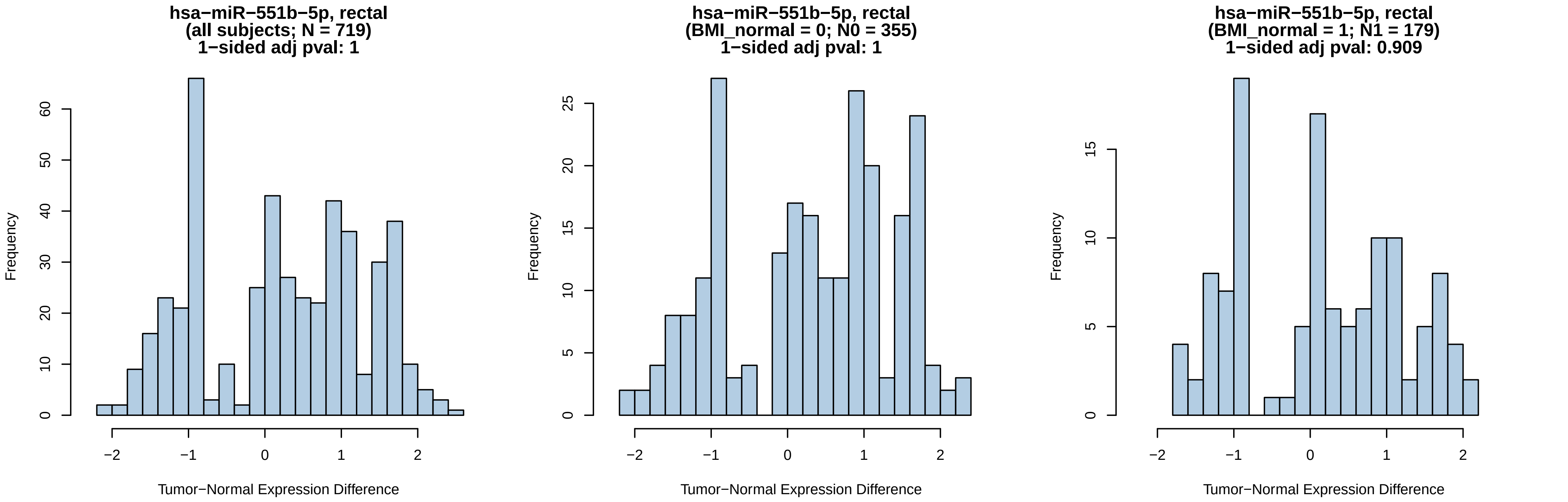

hsa−miR−551b−5p, rectal
(all subjects; N = 719)
1−sided adj pval: 1
Frequency
Tumor−Normal Expression Difference
hsa−miR−551b−5p, rectal
(BMI_normal = 0; N0 = 355)
1−sided adj pval: 1
Frequency
Tumor−Normal Expression Difference
hsa−miR−551b−5p, rectal
(BMI_normal = 1; N1 = 179)
1−sided adj pval: 0.909
Frequency
Tumor−Normal Expression Difference

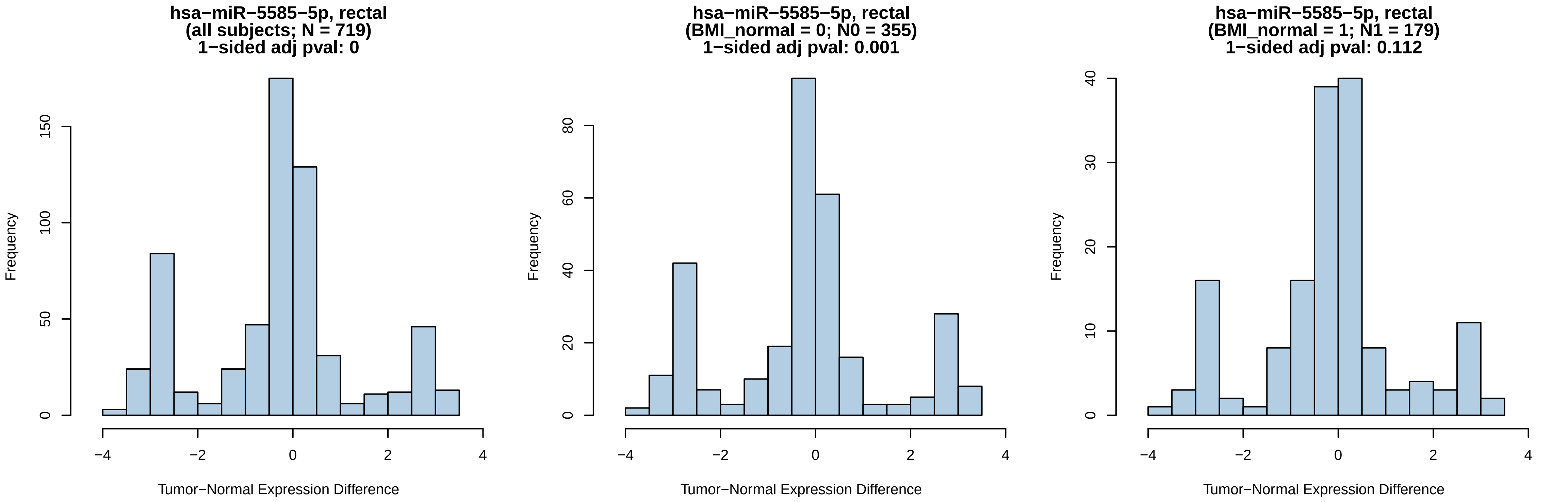
hsa−miR−5585−5p, rectal
(all subjects; N = 719)
1−sided adj pval: 0
Frequency
Tumor−Normal Expression Difference
hsa−miR−5585−5p, rectal
(BMI_normal = 0; N0 = 355)
1−sided adj pval: 0.001
Frequency
Tumor−Normal Expression Difference
hsa−miR−5585−5p, rectal
(BMI_normal = 1; N1 = 179)
1−sided adj pval: 0.112
Frequency
Tumor−Normal Expression Difference

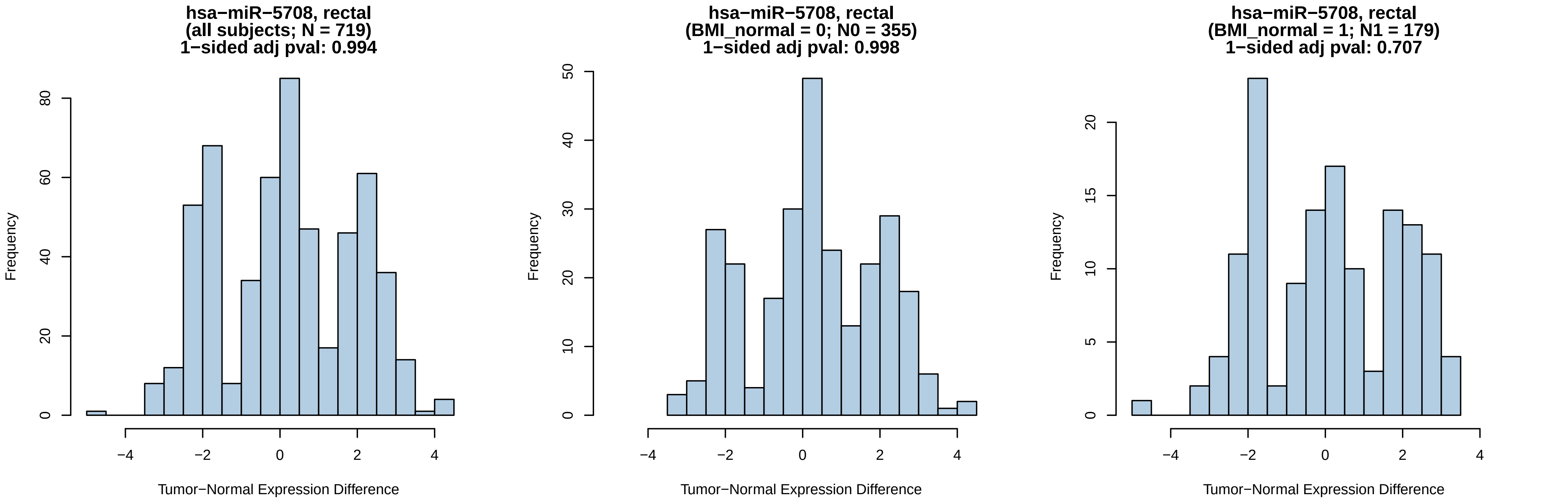
hsa−miR−5708, rectal
(all subjects; N = 719)
1−sided adj pval: 0.994
Frequency
Tumor−Normal Expression Difference
hsa−miR−5708, rectal
(BMI_normal = 0; N0 = 355)
1−sided adj pval: 0.998
Frequency
Tumor−Normal Expression Difference
hsa−miR−5708, rectal
(BMI_normal = 1; N1 = 179)
1−sided adj pval: 0.707
Frequency
Tumor−Normal Expression Difference

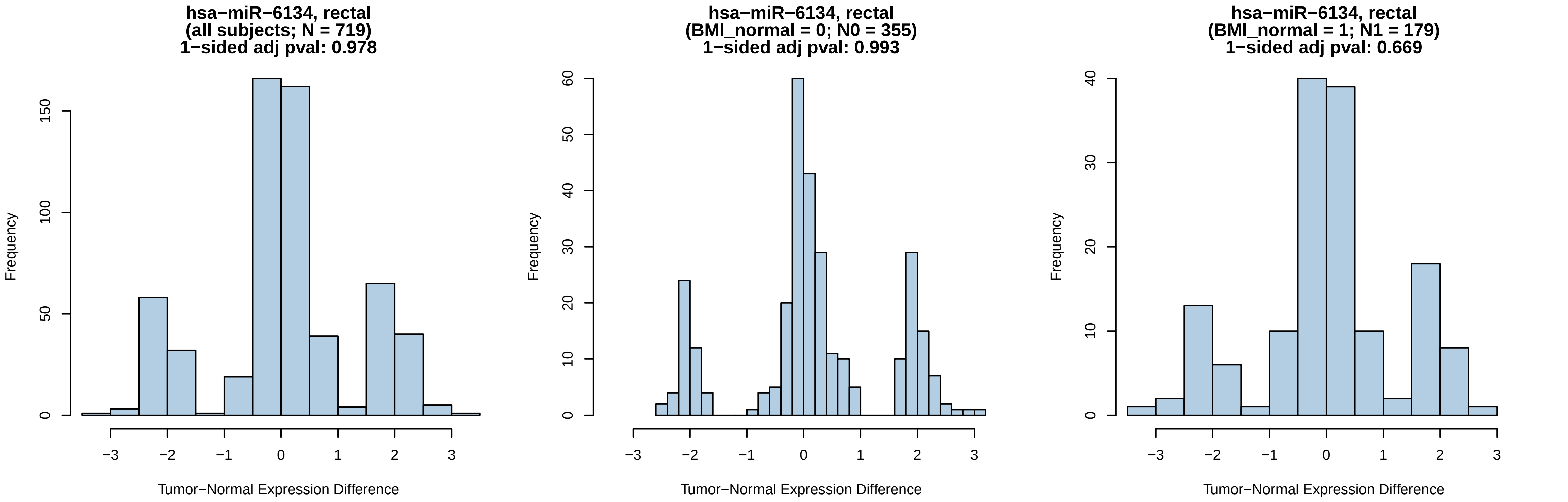
hsa−miR−6134, rectal
(all subjects; N = 719)
1−sided adj pval: 0.978
Frequency
Tumor−Normal Expression Difference
hsa−miR−6134, rectal
(BMI_normal = 0; N0 = 355)
1−sided adj pval: 0.993
Frequency
Tumor−Normal Expression Difference
hsa−miR−6134, rectal
(BMI_normal = 1; N1 = 179)
1−sided adj pval: 0.669
Frequency
Tumor−Normal Expression Difference

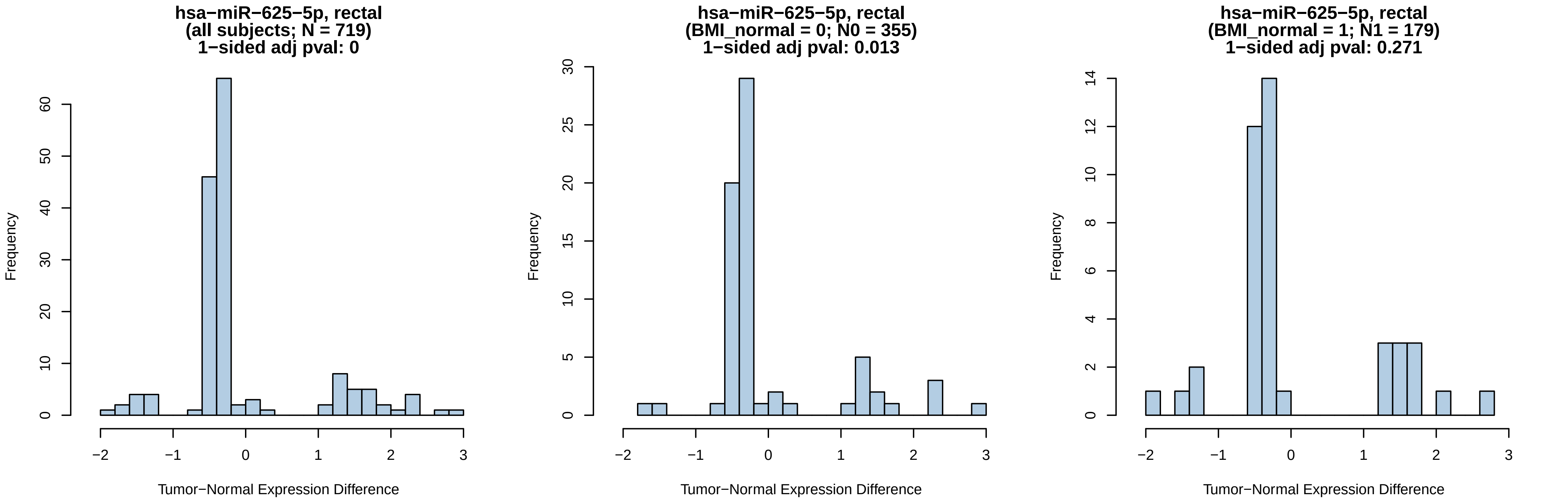

hsa−miR−625−5p, rectal
(all subjects; N = 719)
1−sided adj pval: 0
Frequency
Tumor−Normal Expression Difference
hsa−miR−625−5p, rectal
(BMI_normal = 0; N0 = 355)
1−sided adj pval: 0.013
Frequency
Tumor−Normal Expression Difference
hsa−miR−625−5p, rectal
(BMI_normal = 1; N1 = 179)
1−sided adj pval: 0.271
Frequency
Tumor−Normal Expression Difference

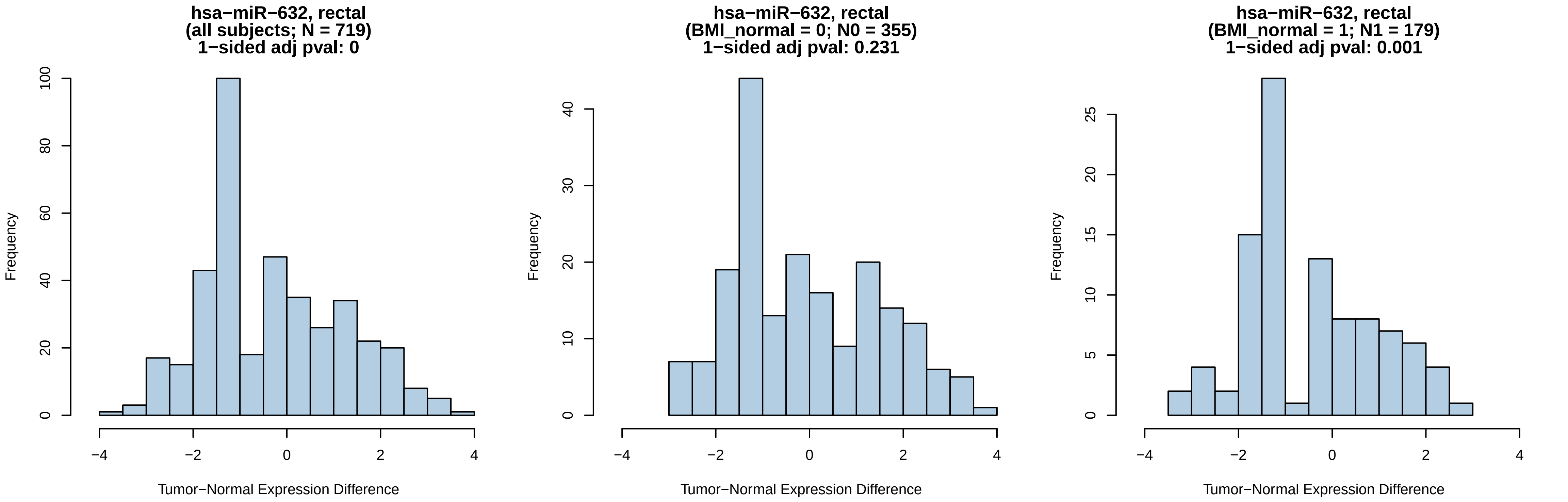
hsa−miR−632, rectal
(all subjects; N = 719)
1−sided adj pval: 0
hsa−miR−632, rectal
(BMI_normal = 0; N0 = 355)
1−sided adj pval: 0.231
hsa−miR−632, rectal
(BMI_normal = 1; N1 = 179)
1−sided adj pval: 0.001
Frequency
Tumor−Normal Expression Difference

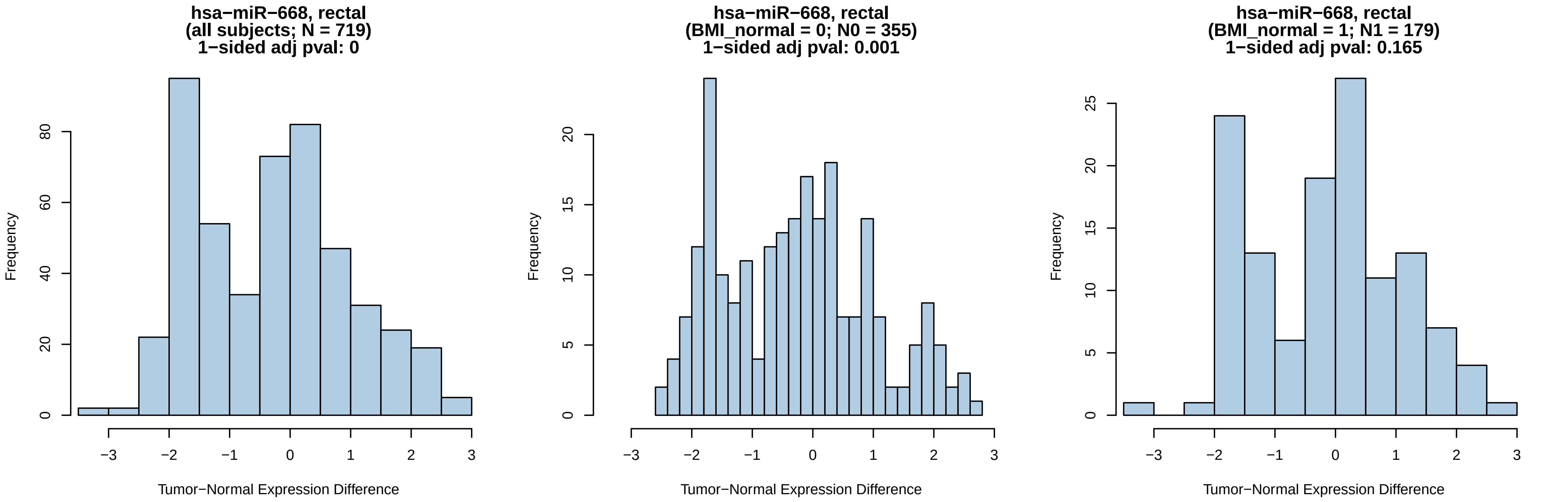
hsa−miR−668, rectal
(all subjects; N = 719)
1−sided adj pval: 0
Frequency
0
20
40
60
80
−3
−2
−1
0
1
2
3
Tumor−Normal Expression Difference
hsa−miR−668, rectal
(BMI_normal = 0; N0 = 355)
1−sided adj pval: 0.001
Frequency
0
5
10
15
20
−3
−2
−1
0
1
2
3
Tumor−Normal Expression Difference
hsa−miR−668, rectal
(BMI_normal = 1; N1 = 179)
1−sided adj pval: 0.165
Frequency
0
5
10
15
20
25
−3
−2
−1
0
1
2
3
Tumor−Normal Expression Difference

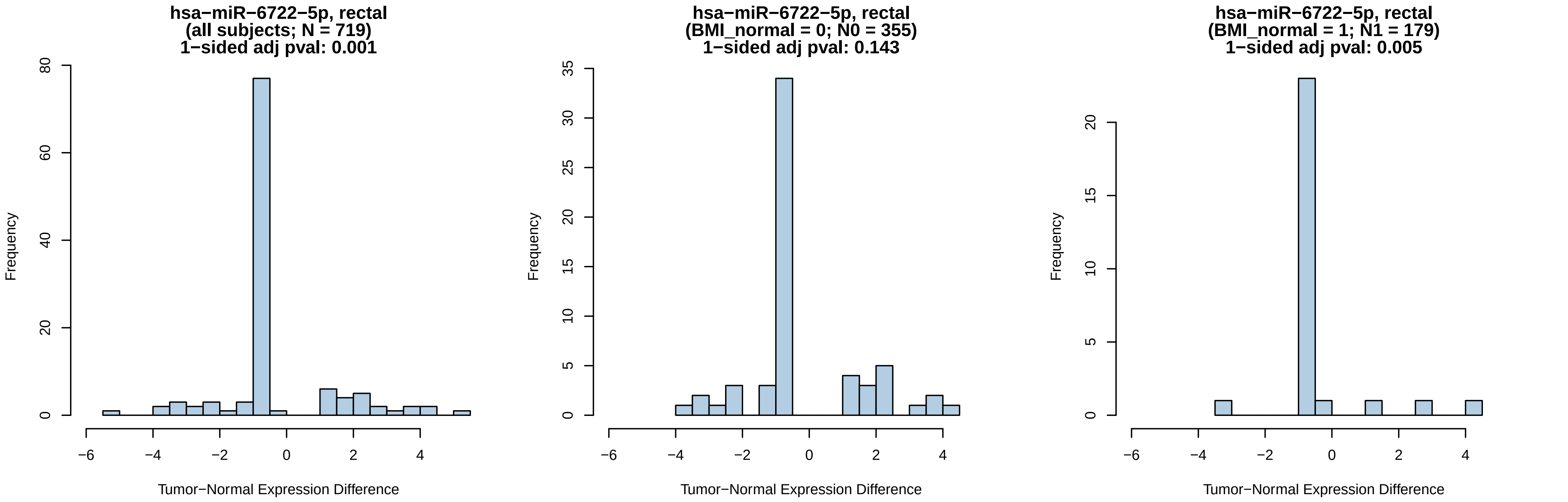
hsa−miR−6722−5p, rectal
(all subjects; N = 719)
1−sided adj pval: 0.001
Frequency
0
20
40
60
80
−6
−4
−2
0
2
4
Tumor−Normal Expression Difference
hsa−miR−6722−5p, rectal
(BMI_normal = 0; N0 = 355)
1−sided adj pval: 0.143
Frequency
0
5
10
15
20
25
30
35
−6
−4
−2
0
2
4
Tumor−Normal Expression Difference
hsa−miR−6722−5p, rectal
(BMI_normal = 1; N1 = 179)
1−sided adj pval: 0.005
Frequency
0
5
10
15
20
−6
−4
−2
0
2
4
Tumor−Normal Expression Difference

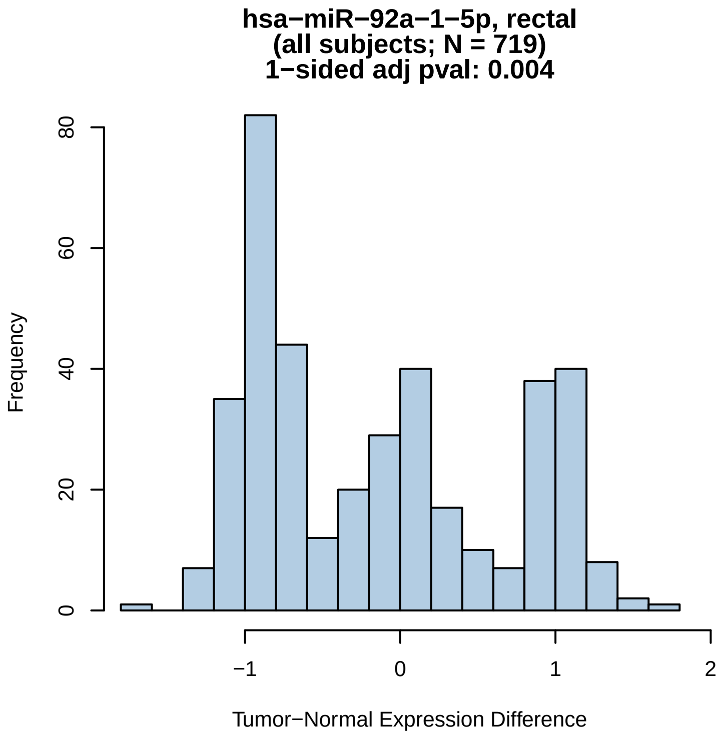

hsa−miR−92a−1−5p, rectal
(all subjects; N = 719)
1−sided adj pval: 0.004
Frequency
Tumor−Normal Expression Difference


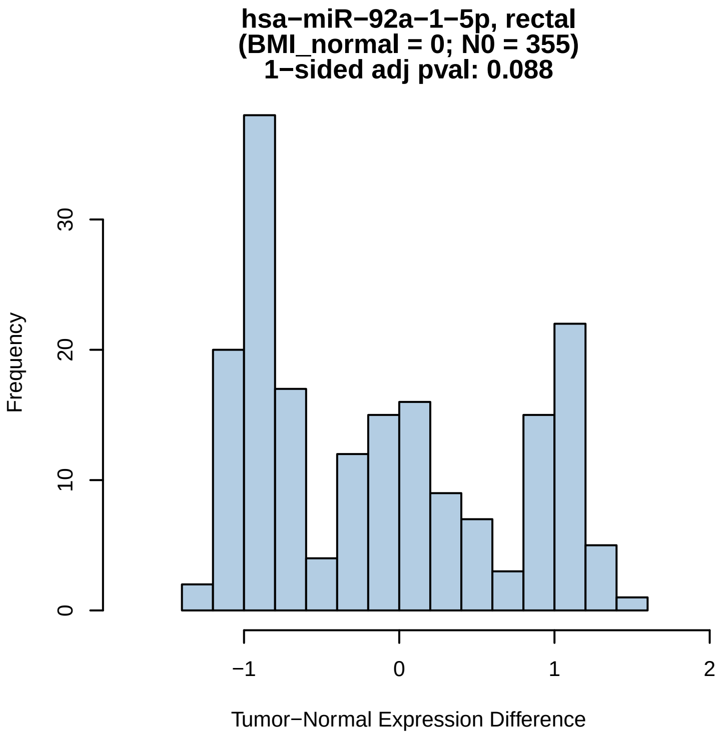

hsa−miR−92a−1−5p, rectal
(BMI_normal = 0; N0 = 355)
1−sided adj pval: 0.088
Frequency
Tumor−Normal Expression Difference


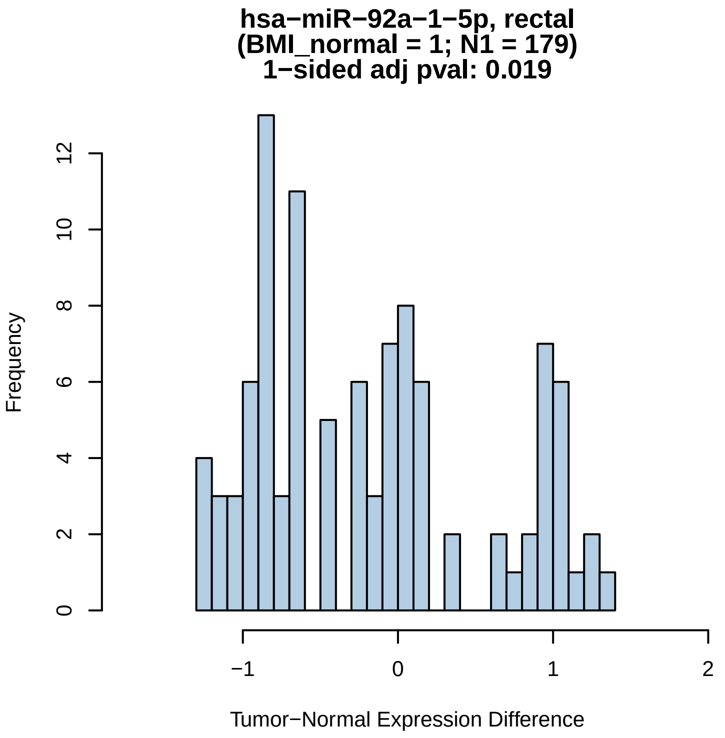

hsa−miR−92a−1−5p, rectal
(BMI_normal = 1; N1 = 179)
1−sided adj pval: 0.019
Frequency
Tumor−Normal Expression Difference

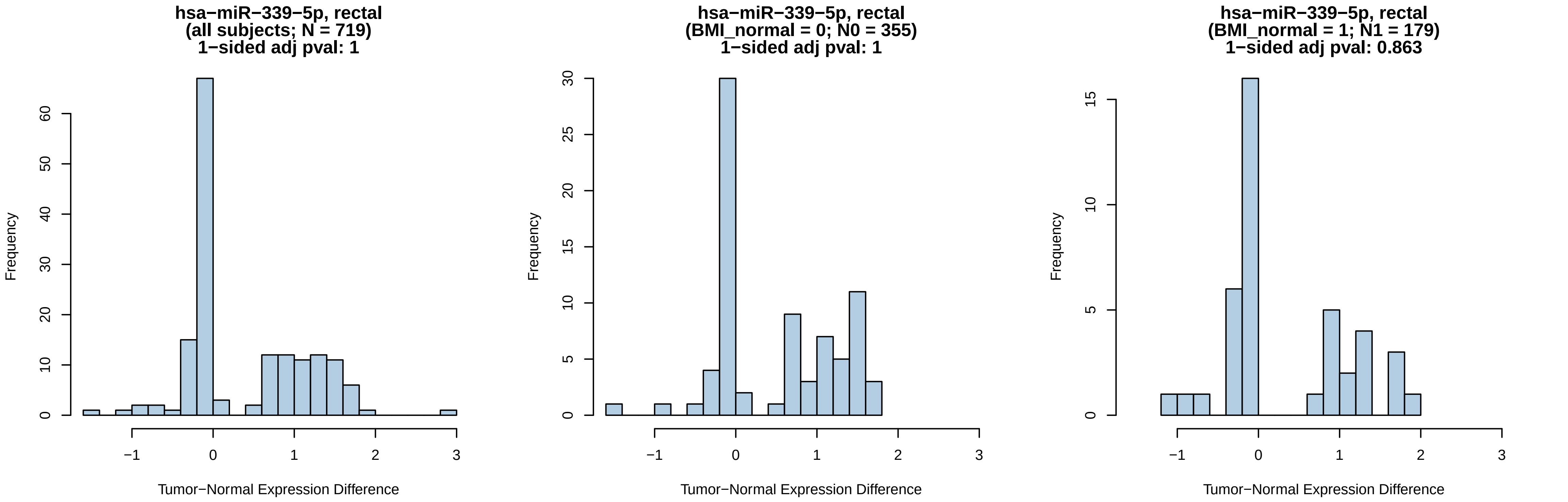
hsa−miR−339−5p, rectal
(all subjects; N = 719)
1−sided adj pval: 1
Frequency
0
10
20
30
40
50
60
−1
0
1
2
3
Tumor−Normal Expression Difference
hsa−miR−339−5p, rectal
(BMI_normal = 0; N0 = 355)
1−sided adj pval: 1
Frequency
0
5
10
15
20
25
30
−1
0
1
2
3
Tumor−Normal Expression Difference
hsa−miR−339−5p, rectal
(BMI_normal = 1; N1 = 179)
1−sided adj pval: 0.863
Frequency
0
5
10
15
−1
0
1
2
3
Tumor−Normal Expression Difference

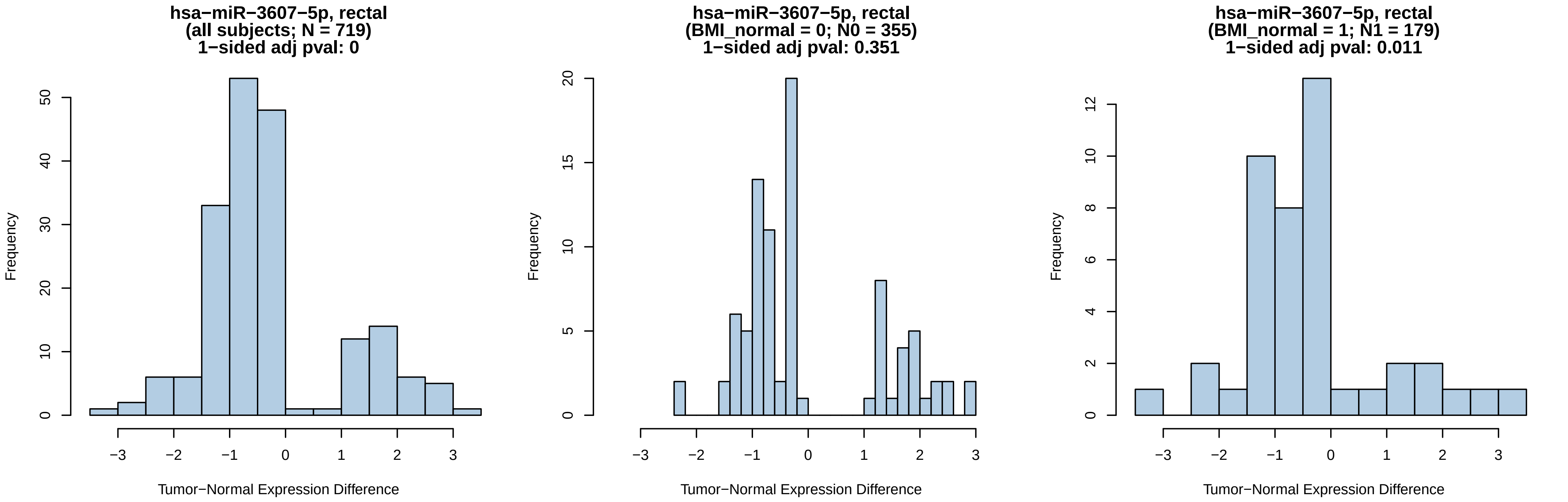
hsa−miR−3607−5p, rectal
(all subjects; N = 719)
1−sided adj pval: 0
Frequency
Tumor−Normal Expression Difference
hsa−miR−3607−5p, rectal
(BMI_normal = 0; N0 = 355)
1−sided adj pval: 0.351
Frequency
Tumor−Normal Expression Difference
hsa−miR−3607−5p, rectal
(BMI_normal = 1; N1 = 179)
1−sided adj pval: 0.011
Frequency
Tumor−Normal Expression Difference

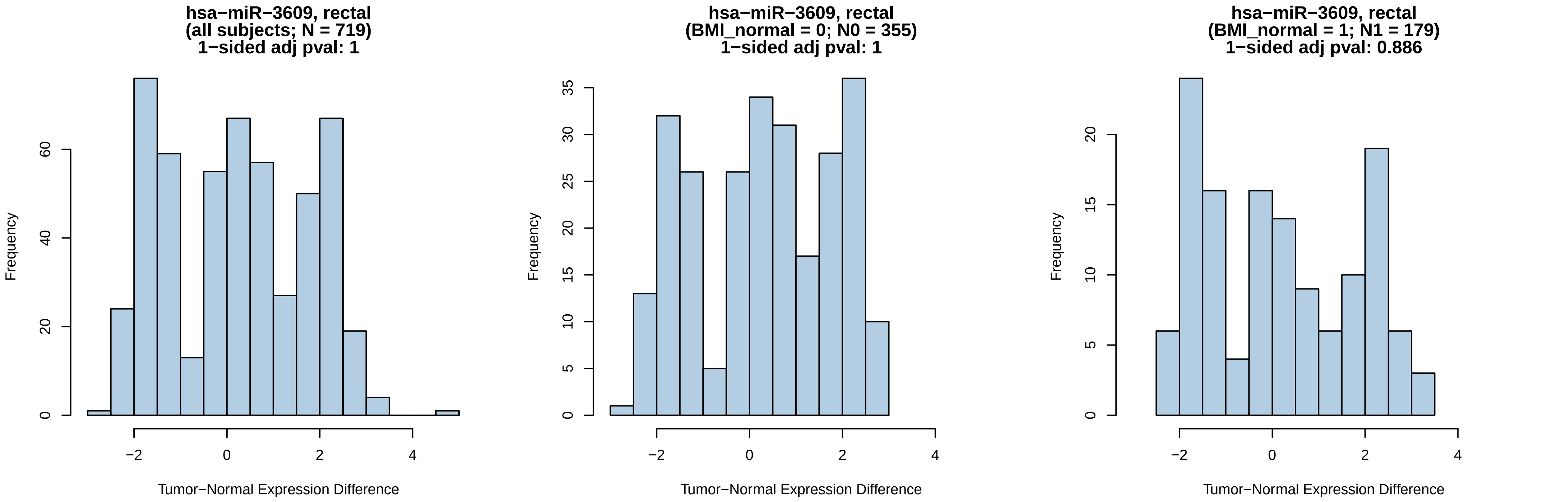
hsa−miR−3609, rectal
(all subjects; N = 719)
1−sided adj pval: 1
Frequency
Tumor−Normal Expression Difference
hsa−miR−3609, rectal
(BMI_normal = 0; N0 = 355)
1−sided adj pval: 1
Frequency
Tumor−Normal Expression Difference
hsa−miR−3609, rectal
(BMI_normal = 1; N1 = 179)
1−sided adj pval: 0.886
Frequency
Tumor−Normal Expression Difference

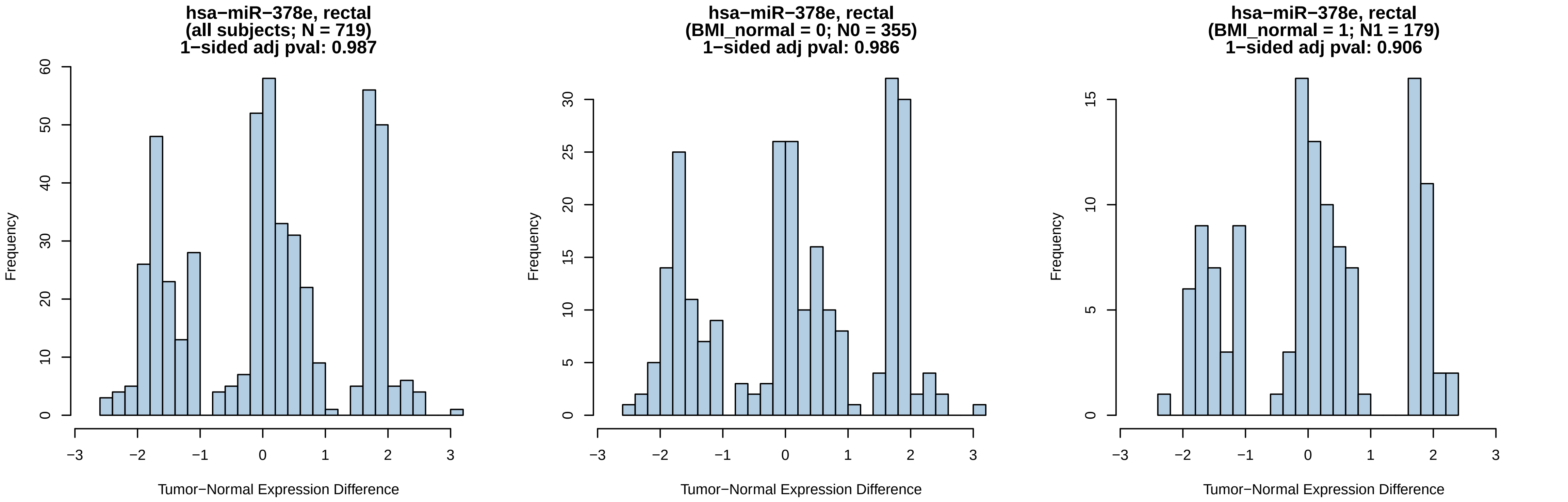
hsa−miR−378e, rectal
(all subjects; N = 719)
1−sided adj pval: 0.987
Frequency
Tumor−Normal Expression Difference
0
10
20
30
40
50
60
−3
−2
−1
0
1
2
3
hsa−miR−378e, rectal
(BMI_normal = 0; N0 = 355)
1−sided adj pval: 0.986
Frequency
Tumor−Normal Expression Difference
0
5
10
15
20
25
30
−3
−2
−1
0
1
2
3
hsa−miR−378e, rectal
(BMI_normal = 1; N1 = 179)
1−sided adj pval: 0.906
Frequency
Tumor−Normal Expression Difference
0
5
10
15
−3
−2
−1
0
1
2
3

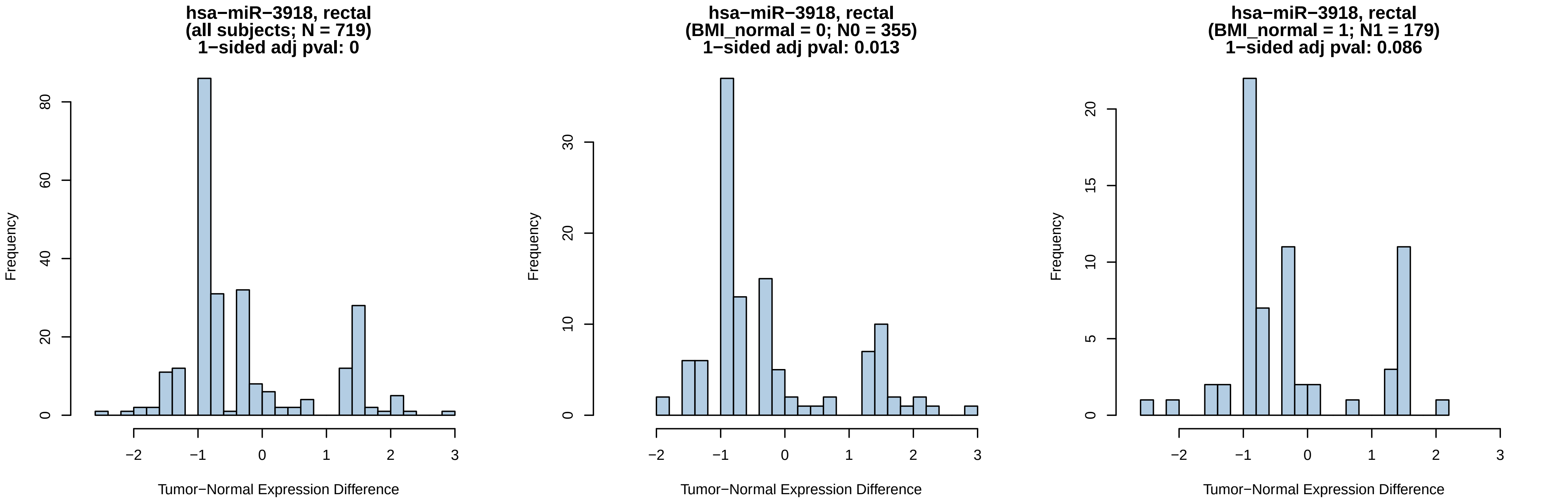
hsa−miR−3918, rectal
(all subjects; N = 719)
1−sided adj pval: 0
Frequency
Tumor−Normal Expression Difference
hsa−miR−3918, rectal
(BMI_normal = 0; N0 = 355)
1−sided adj pval: 0.013
Frequency
Tumor−Normal Expression Difference
hsa−miR−3918, rectal
(BMI_normal = 1; N1 = 179)
1−sided adj pval: 0.086
Frequency
Tumor−Normal Expression Difference

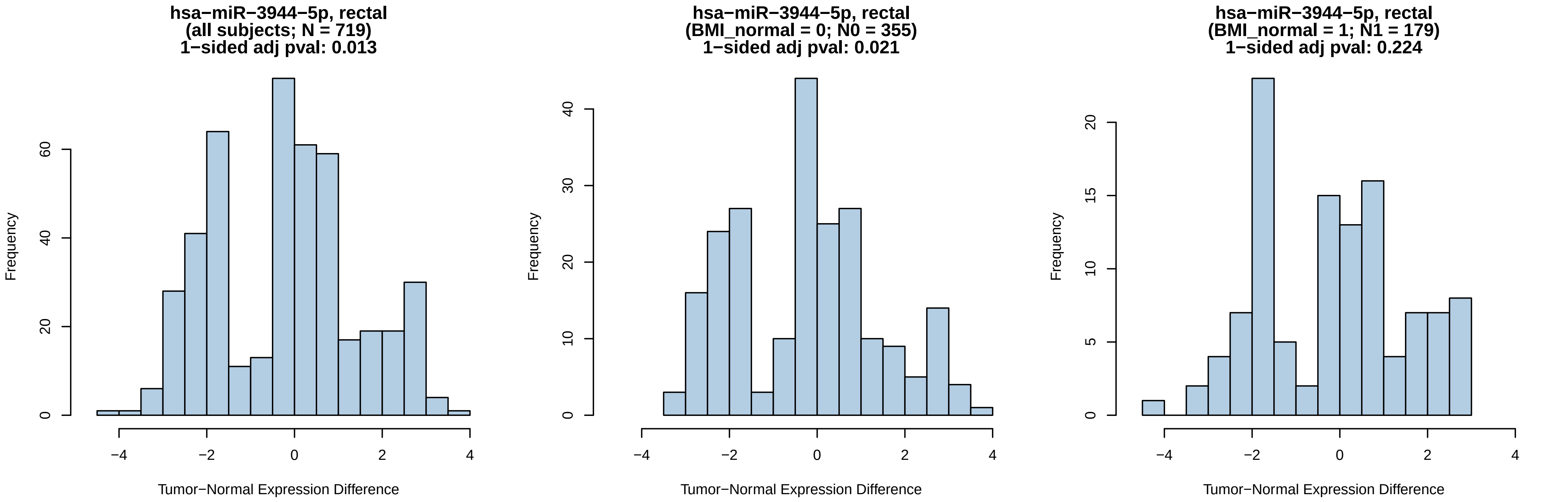
hsa−miR−3944−5p, rectal
(all subjects; N = 719)
1−sided adj pval: 0.013
Frequency
0
20
40
60
−4
−2
0
2
4
Tumor−Normal Expression Difference
hsa−miR−3944−5p, rectal
(BMI_normal = 0; N0 = 355)
1−sided adj pval: 0.021
Frequency
0
10
20
30
40
−4
−2
0
2
4
Tumor−Normal Expression Difference
hsa−miR−3944−5p, rectal
(BMI_normal = 1; N1 = 179)
1−sided adj pval: 0.224
Frequency
0
5
10
15
20
−4
−2
0
2
4
Tumor−Normal Expression Difference

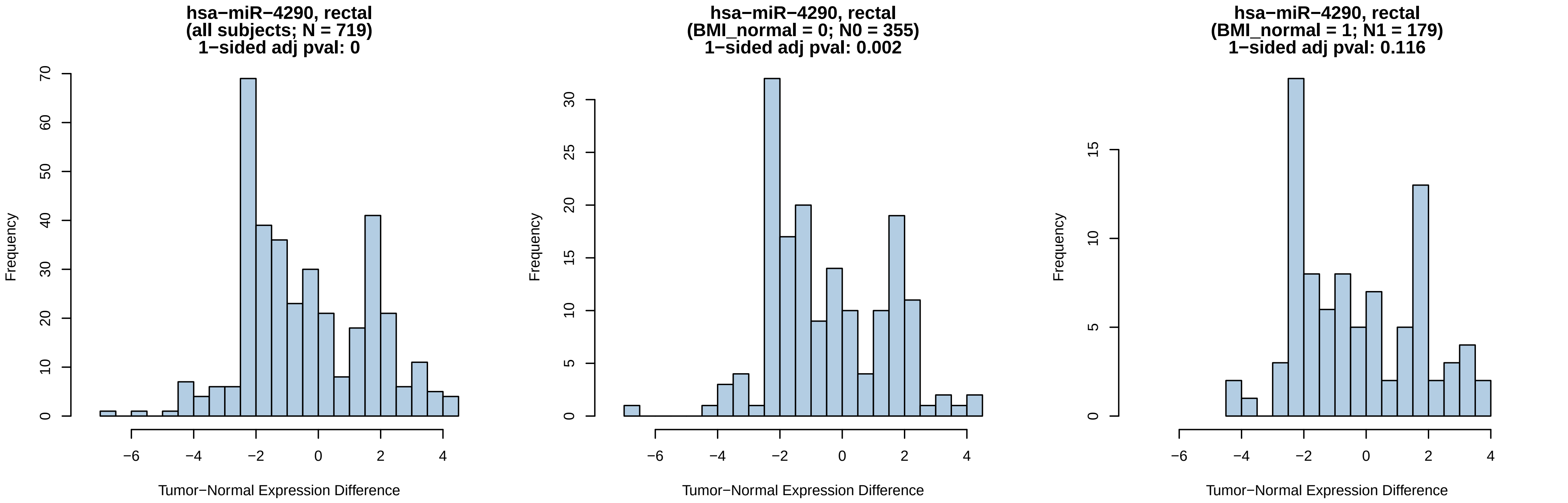
hsa−miR−4290, rectal
(all subjects; N = 719)
1−sided adj pval: 0
Frequency
0
10
20
30
40
50
60
70
−6
−4
−2
0
2
4
Tumor−Normal Expression Difference
hsa−miR−4290, rectal
(BMI_normal = 0; N0 = 355)
1−sided adj pval: 0.002
Frequency
0
5
10
15
20
25
30
−6
−4
−2
0
2
4
Tumor−Normal Expression Difference
hsa−miR−4290, rectal
(BMI_normal = 1; N1 = 179)
1−sided adj pval: 0.116
Frequency
0
5
10
15
−6
−4
−2
0
2
4
Tumor−Normal Expression Difference

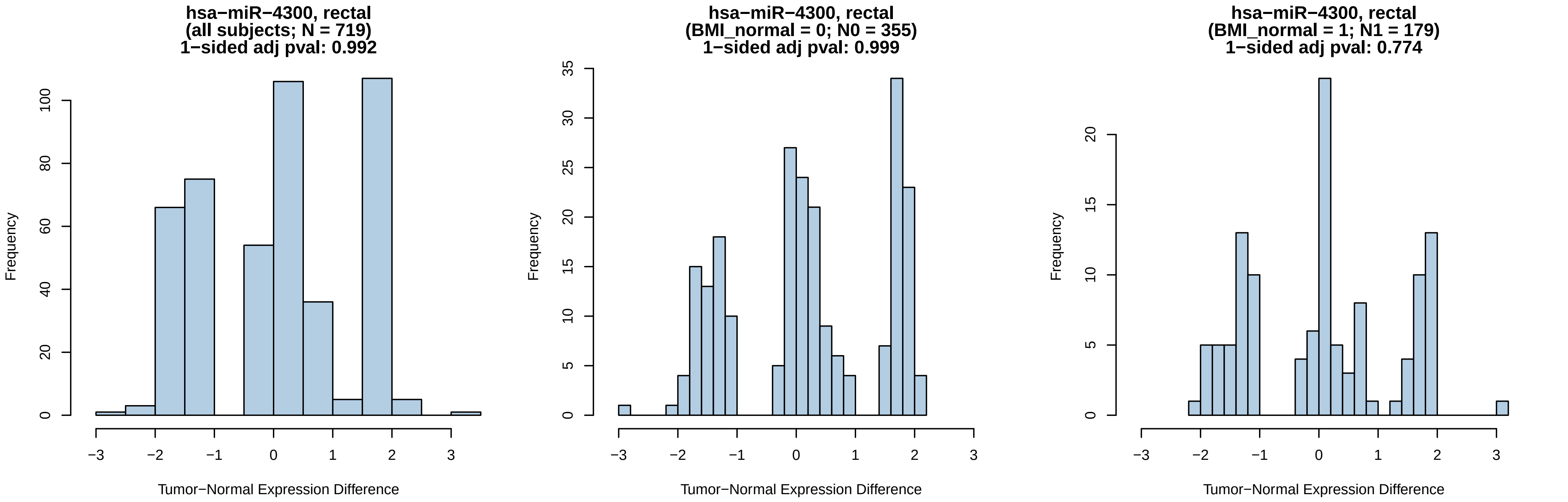
hsa−miR−4300, rectal
(all subjects; N = 719)
1−sided adj pval: 0.992
Frequency
Tumor−Normal Expression Difference
hsa−miR−4300, rectal
(BMI_normal = 0; N0 = 355)
1−sided adj pval: 0.999
Frequency
Tumor−Normal Expression Difference
hsa−miR−4300, rectal
(BMI_normal = 1; N1 = 179)
1−sided adj pval: 0.774
Frequency
Tumor−Normal Expression Difference

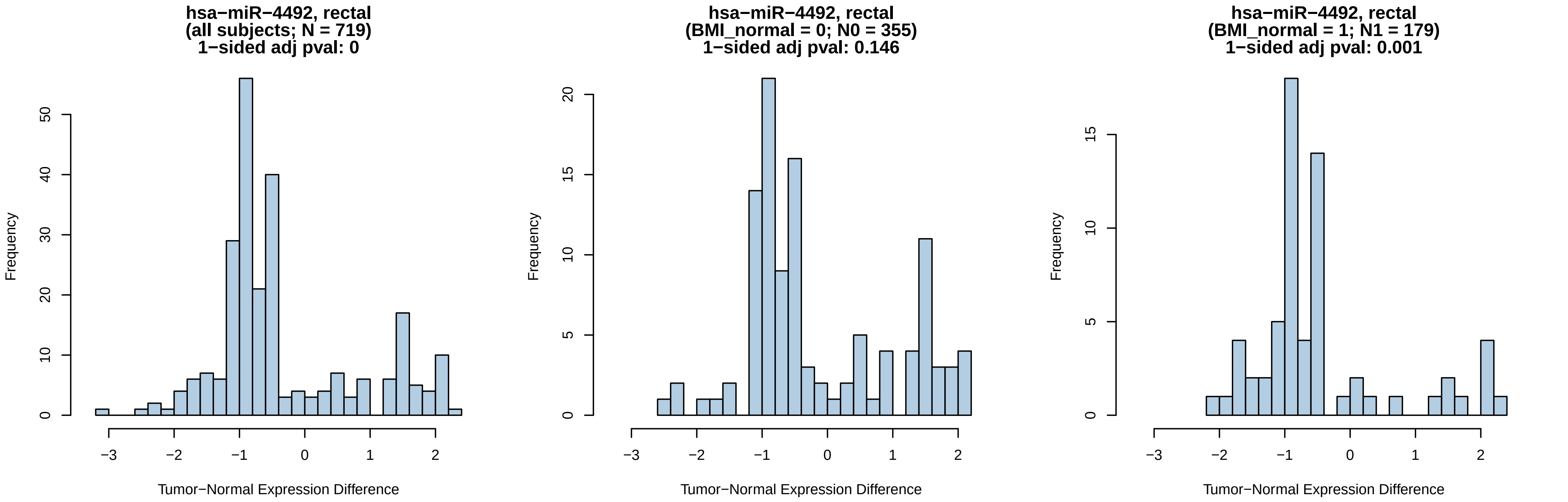
hsa−miR−4492, rectal
(all subjects; N = 719)
1−sided adj pval: 0
Frequency
Tumor−Normal Expression Difference
hsa−miR−4492, rectal
(BMI_normal = 0; N0 = 355)
1−sided adj pval: 0.146
Frequency
Tumor−Normal Expression Difference
hsa−miR−4492, rectal
(BMI_normal = 1; N1 = 179)
1−sided adj pval: 0.001
Frequency
Tumor−Normal Expression Difference

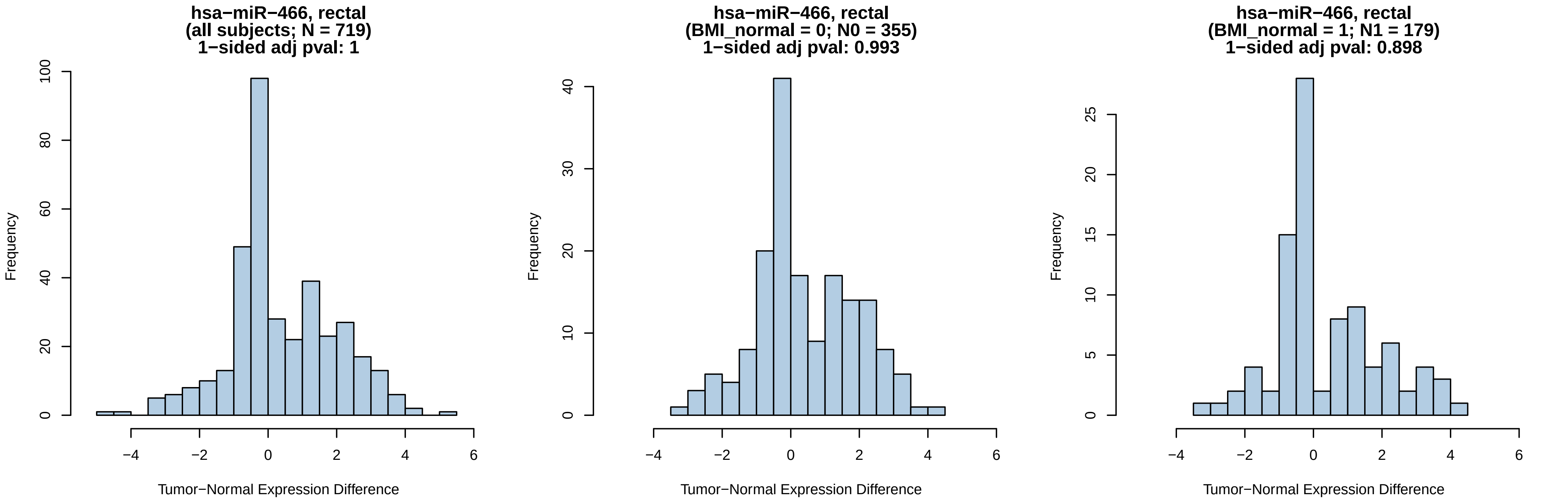
hsa−miR−466, rectal
(all subjects; N = 719)
1−sided adj pval: 1
Frequency
Tumor−Normal Expression Difference
hsa−miR−466, rectal
(BMI_normal = 0; N0 = 355)
1−sided adj pval: 0.993
Frequency
Tumor−Normal Expression Difference
hsa−miR−466, rectal
(BMI_normal = 1; N1 = 179)
1−sided adj pval: 0.898
Frequency
Tumor−Normal Expression Difference

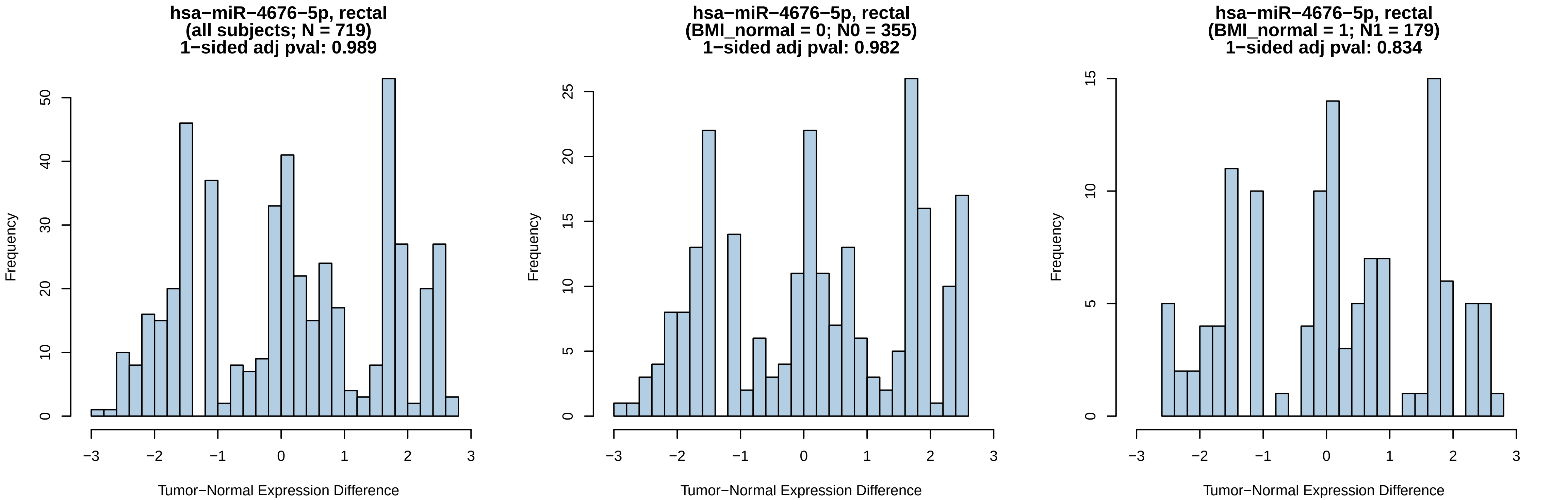

hsa−miR−4676−5p, rectal
(all subjects; N = 719)
1−sided adj pval: 0.989
hsa−miR−4676−5p, rectal
(BMI_normal = 0; N0 = 355)
1−sided adj pval: 0.982
hsa−miR−4676−5p, rectal
(BMI_normal = 1; N1 = 179)
1−sided adj pval: 0.834
Frequency
Tumor−Normal Expression Difference

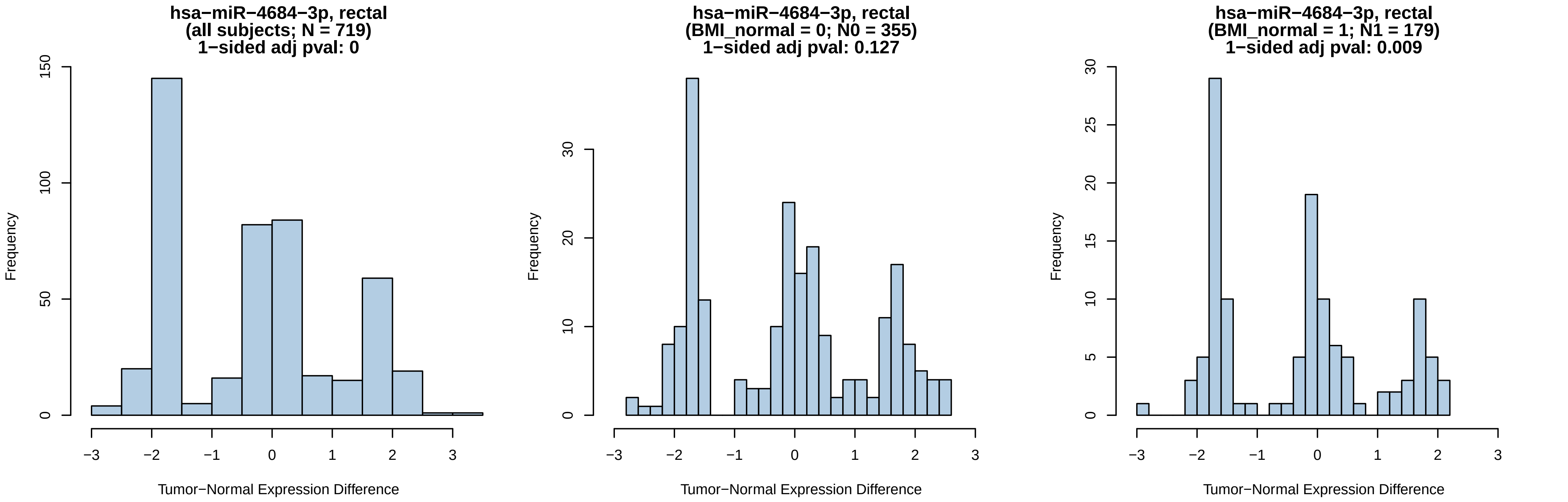
hsa−miR−4684−3p, rectal
(all subjects; N = 719)
1−sided adj pval: 0
Frequency
Tumor−Normal Expression Difference
hsa−miR−4684−3p, rectal
(BMI_normal = 0; N0 = 355)
1−sided adj pval: 0.127
Frequency
Tumor−Normal Expression Difference
hsa−miR−4684−3p, rectal
(BMI_normal = 1; N1 = 179)
1−sided adj pval: 0.009
Frequency
Tumor−Normal Expression Difference

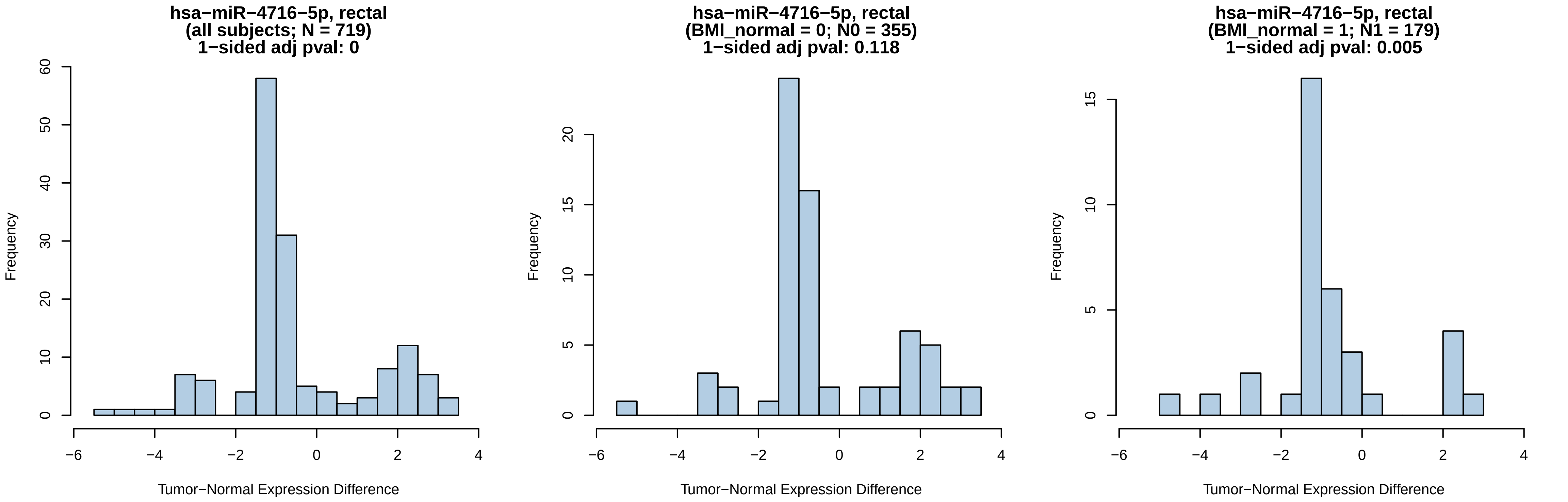
hsa−miR−4716−5p, rectal
(all subjects; N = 719)
1−sided adj pval: 0
Frequency
Tumor−Normal Expression Difference
hsa−miR−4716−5p, rectal
(BMI_normal = 0; N0 = 355)
1−sided adj pval: 0.118
Frequency
Tumor−Normal Expression Difference
hsa−miR−4716−5p, rectal
(BMI_normal = 1; N1 = 179)
1−sided adj pval: 0.005
Frequency
Tumor−Normal Expression Difference

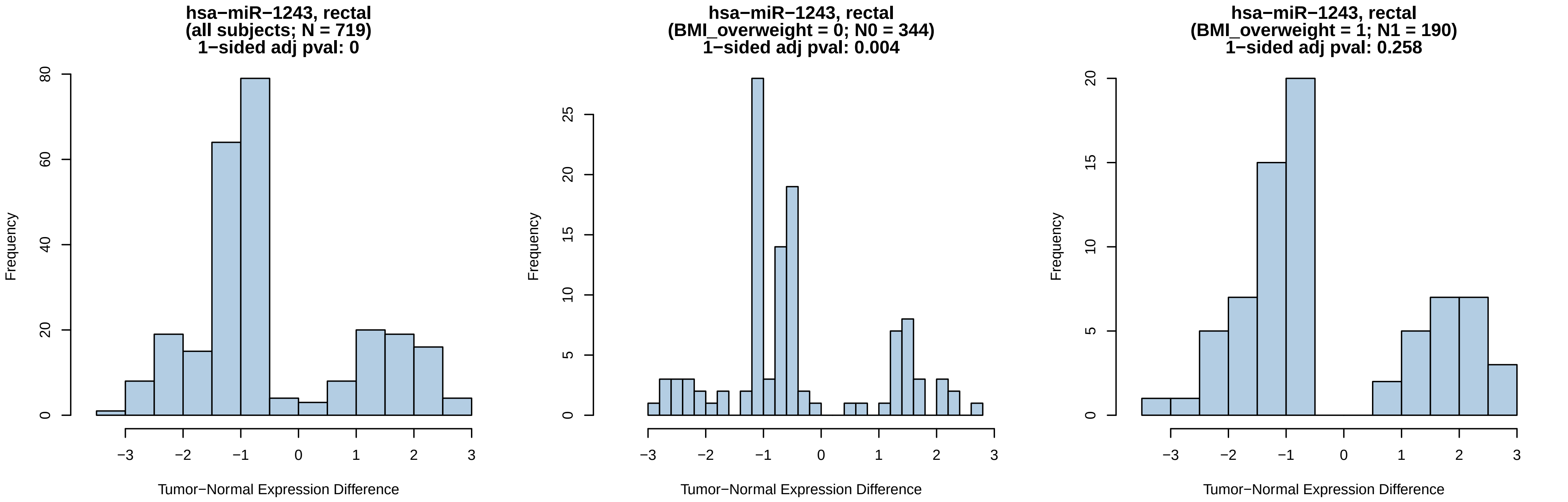
hsa−miR−1243, rectal
(all subjects; N = 719)
1−sided adj pval: 0
Frequency
Tumor−Normal Expression Difference
hsa−miR−1243, rectal
(BMI_overweight = 0; N0 = 344)
1−sided adj pval: 0.004
Frequency
Tumor−Normal Expression Difference
hsa−miR−1243, rectal
(BMI_overweight = 1; N1 = 190)
1−sided adj pval: 0.258
Frequency
Tumor−Normal Expression Difference

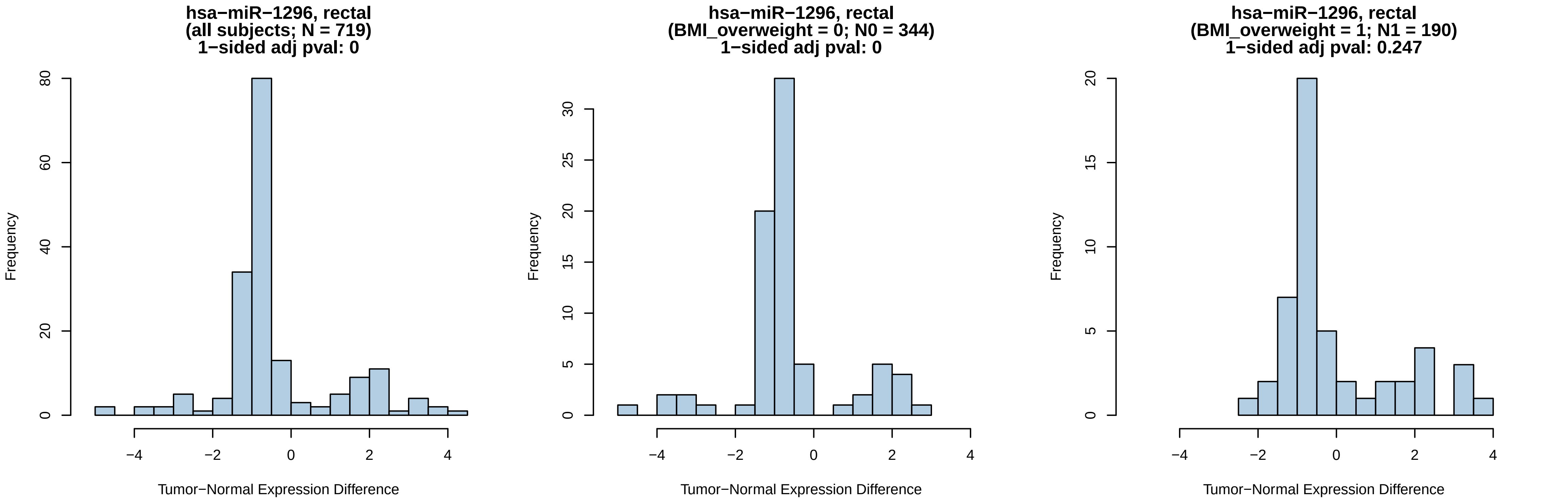
hsa−miR−1296, rectal
(all subjects; N = 719)
1−sided adj pval: 0
Frequency
Tumor−Normal Expression Difference
hsa−miR−1296, rectal
(BMI_overweight = 0; N0 = 344)
1−sided adj pval: 0
Frequency
Tumor−Normal Expression Difference
hsa−miR−1296, rectal
(BMI_overweight = 1; N1 = 190)
1−sided adj pval: 0.247
Frequency
Tumor−Normal Expression Difference

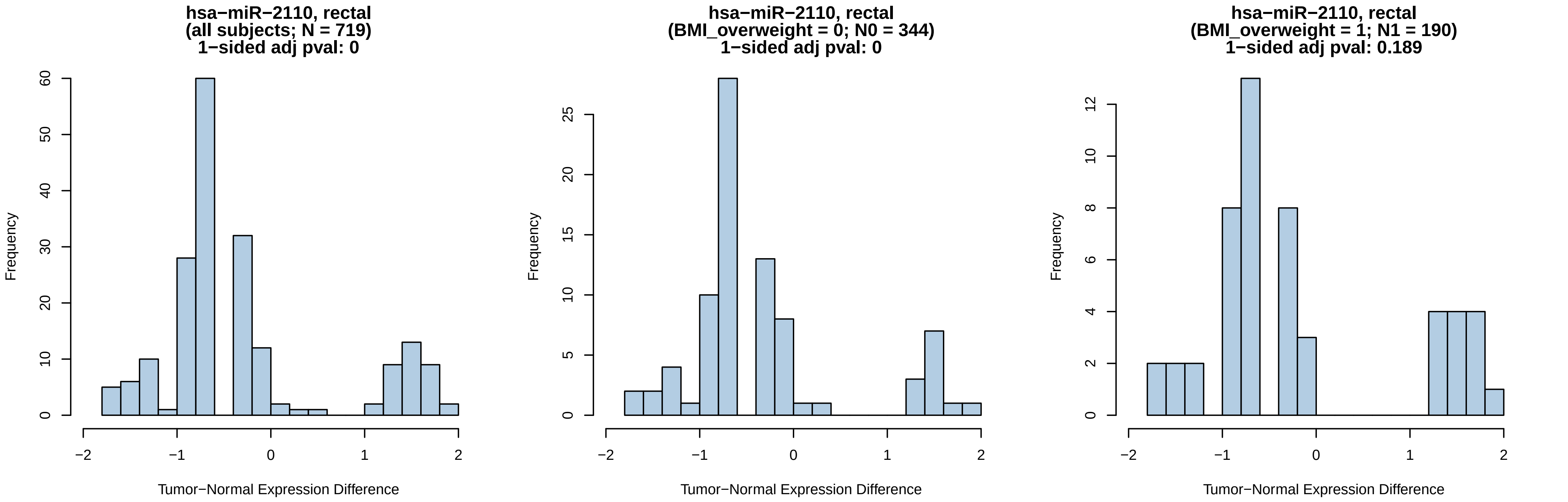

hsa−miR−2110, rectal
(all subjects; N = 719)
1−sided adj pval: 0
Frequency
Tumor−Normal Expression Difference
hsa−miR−2110, rectal
(BMI_overweight = 0; N0 = 344)
1−sided adj pval: 0
Frequency
Tumor−Normal Expression Difference
hsa−miR−2110, rectal
(BMI_overweight = 1; N1 = 190)
1−sided adj pval: 0.189
Frequency
Tumor−Normal Expression Difference

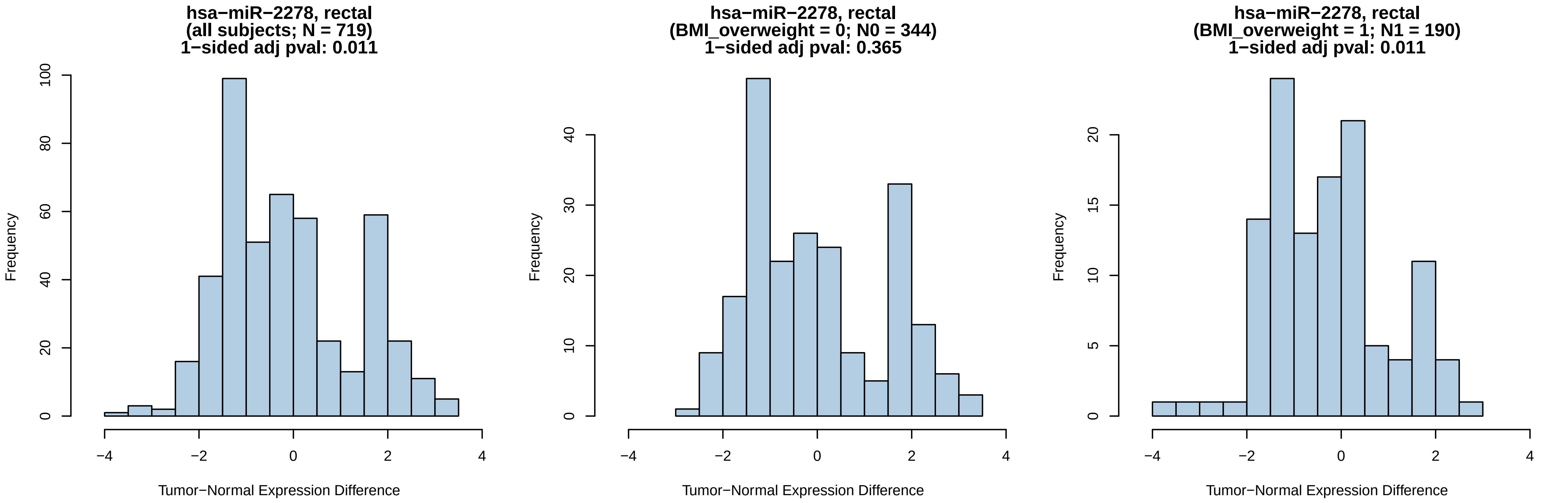
hsa−miR−2278, rectal
(all subjects; N = 719)
1−sided adj pval: 0.011
hsa−miR−2278, rectal
(BMI_overweight = 0; N0 = 344)
1−sided adj pval: 0.365
hsa−miR−2278, rectal
(BMI_overweight = 1; N1 = 190)
1−sided adj pval: 0.011
Frequency
Tumor−Normal Expression Difference

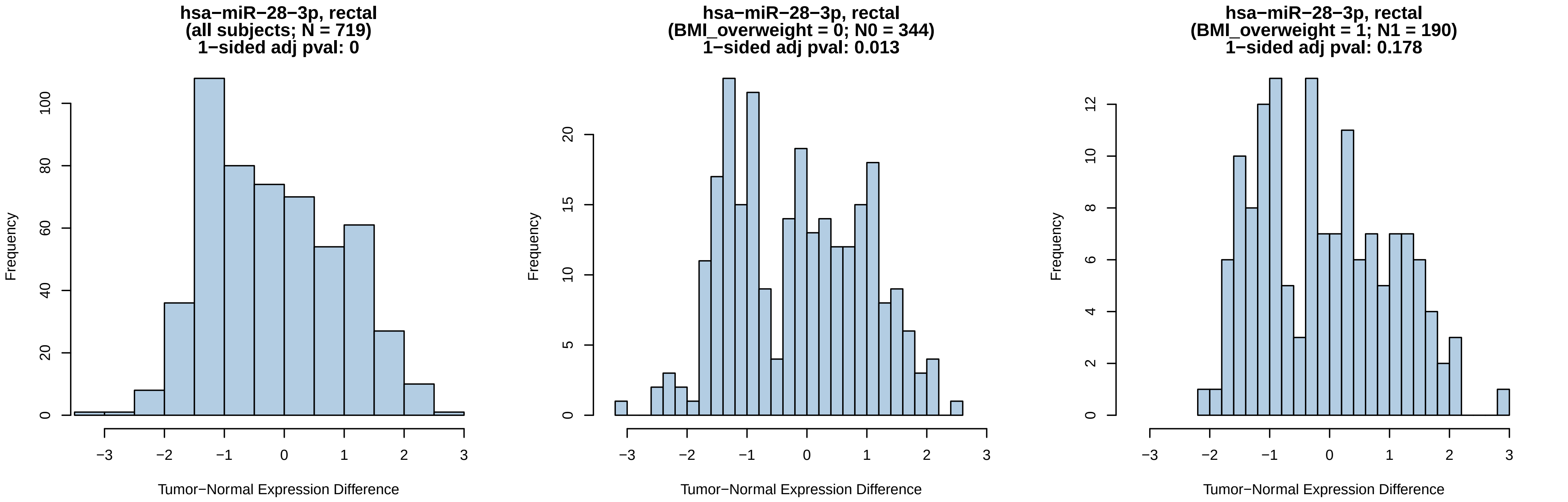
hsa−miR−28−3p, rectal
(all subjects; N = 719)
1−sided adj pval: 0
Frequency
Tumor−Normal Expression Difference
hsa−miR−28−3p, rectal
(BMI_overweight = 0; N0 = 344)
1−sided adj pval: 0.013
Frequency
Tumor−Normal Expression Difference
hsa−miR−28−3p, rectal
(BMI_overweight = 1; N1 = 190)
1−sided adj pval: 0.178
Frequency
Tumor−Normal Expression Difference

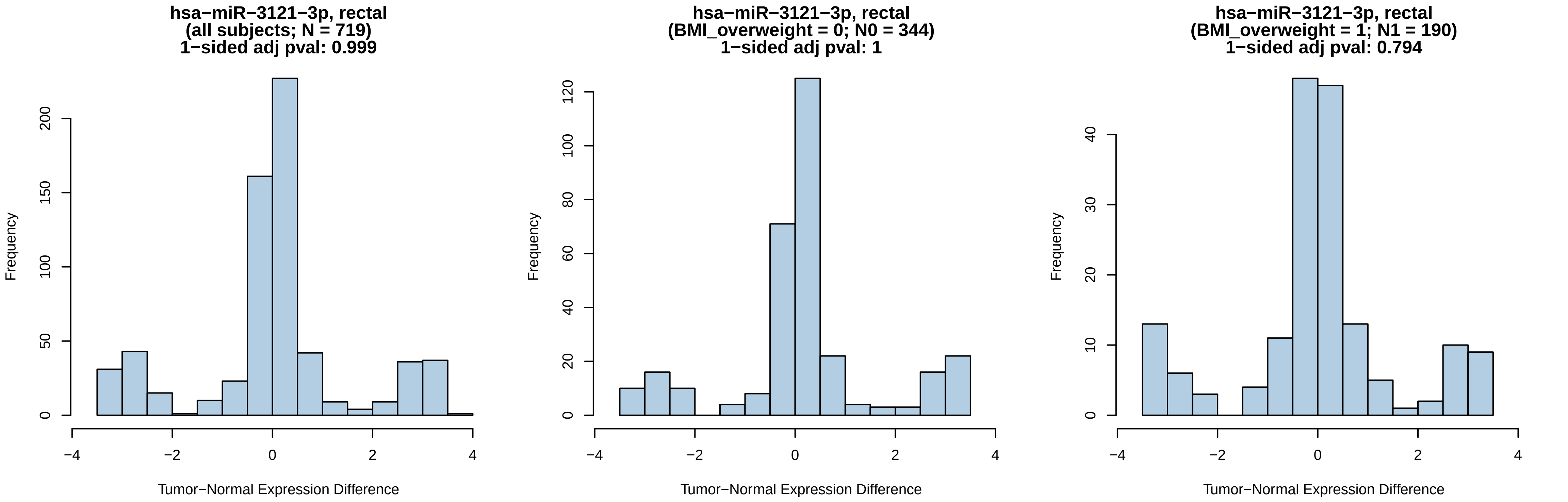

hsa−miR−3121−3p, rectal
(all subjects; N = 719)
1−sided adj pval: 0.999
Frequency
Tumor−Normal Expression Difference
hsa−miR−3121−3p, rectal
(BMI_overweight = 0; N0 = 344)
1−sided adj pval: 1
Frequency
Tumor−Normal Expression Difference
hsa−miR−3121−3p, rectal
(BMI_overweight = 1; N1 = 190)
1−sided adj pval: 0.794
Frequency
Tumor−Normal Expression Difference

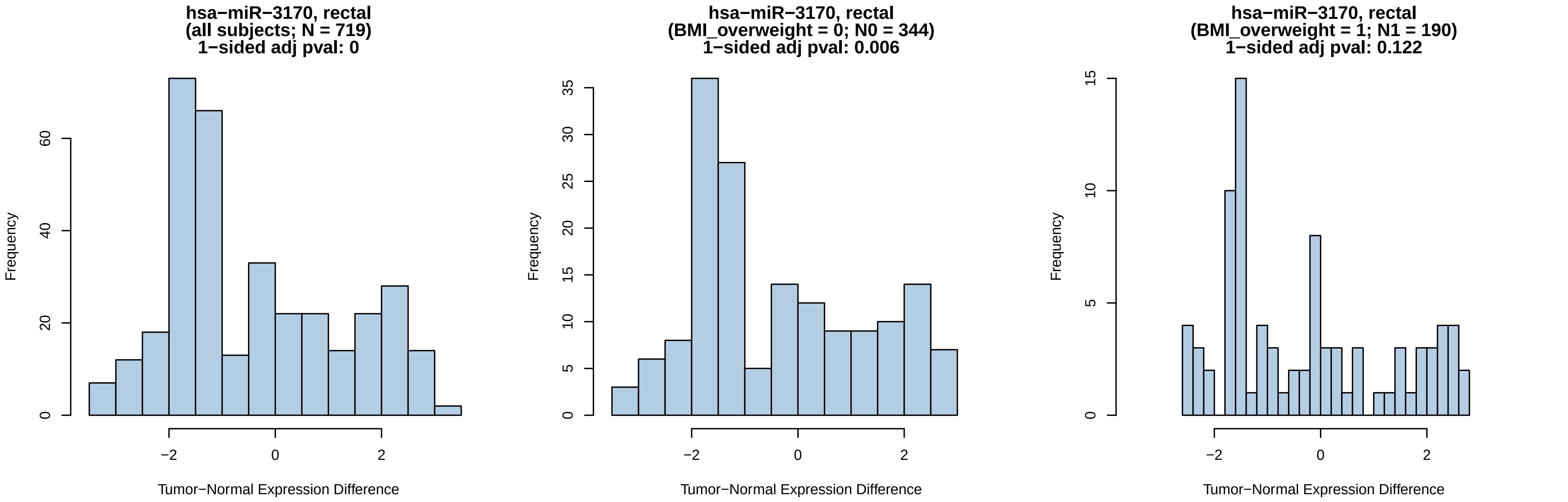
hsa−miR−3170, rectal
(all subjects; N = 719)
1−sided adj pval: 0
Frequency
Tumor−Normal Expression Difference
hsa−miR−3170, rectal
(BMI_overweight = 0; N0 = 344)
1−sided adj pval: 0.006
Frequency
Tumor−Normal Expression Difference
hsa−miR−3170, rectal
(BMI_overweight = 1; N1 = 190)
1−sided adj pval: 0.122
Frequency
Tumor−Normal Expression Difference

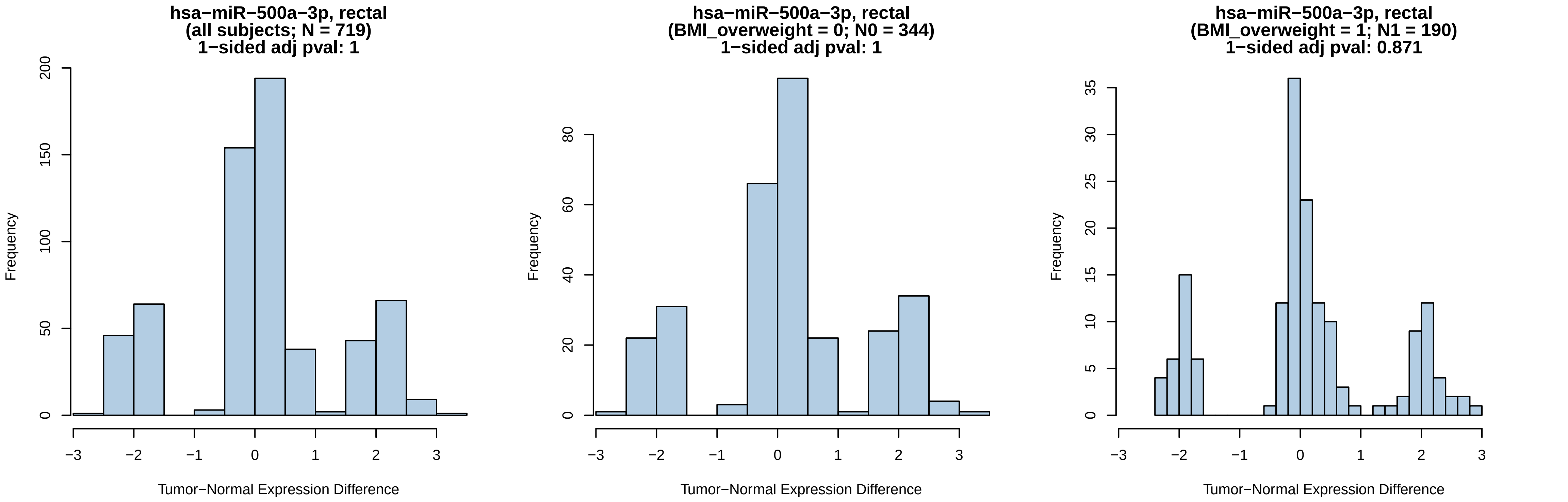

hsa−miR−500a−3p, rectal
(all subjects; N = 719)
1−sided adj pval: 1
Frequency
Tumor−Normal Expression Difference
hsa−miR−500a−3p, rectal
(BMI_overweight = 0; N0 = 344)
1−sided adj pval: 1
Frequency
Tumor−Normal Expression Difference
hsa−miR−500a−3p, rectal
(BMI_overweight = 1; N1 = 190)
1−sided adj pval: 0.871
Frequency
Tumor−Normal Expression Difference

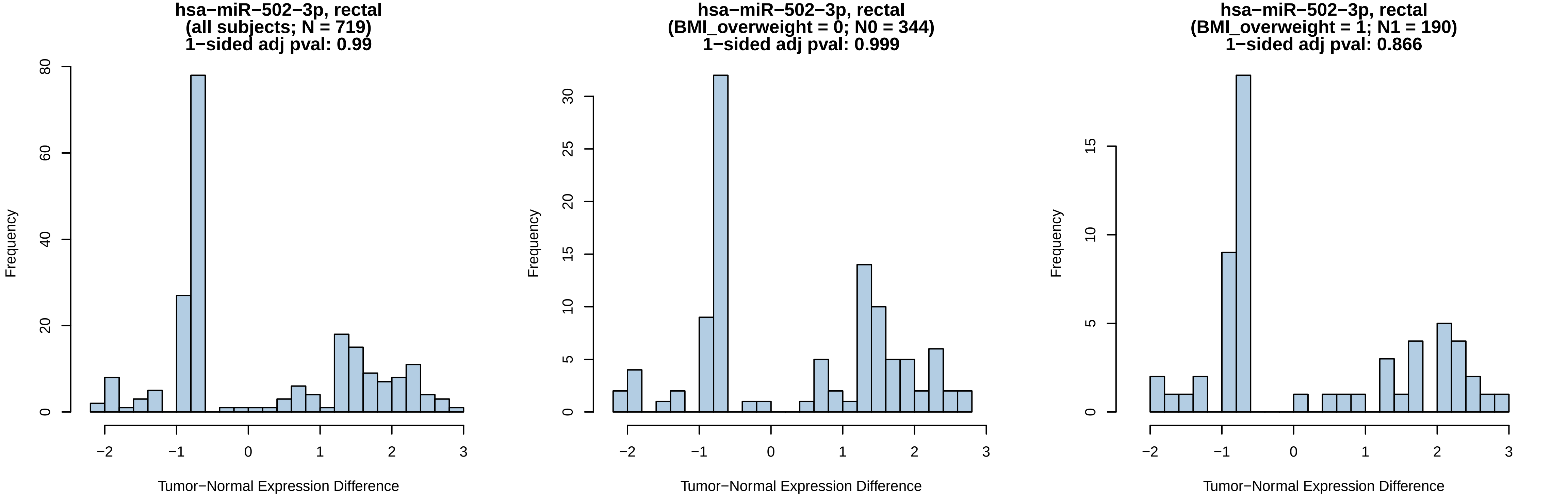
hsa−miR−502−3p, rectal
(all subjects; N = 719)
1−sided adj pval: 0.99
Frequency
Tumor−Normal Expression Difference
hsa−miR−502−3p, rectal
(BMI_overweight = 0; N0 = 344)
1−sided adj pval: 0.999
Frequency
Tumor−Normal Expression Difference
hsa−miR−502−3p, rectal
(BMI_overweight = 1; N1 = 190)
1−sided adj pval: 0.866
Frequency
Tumor−Normal Expression Difference

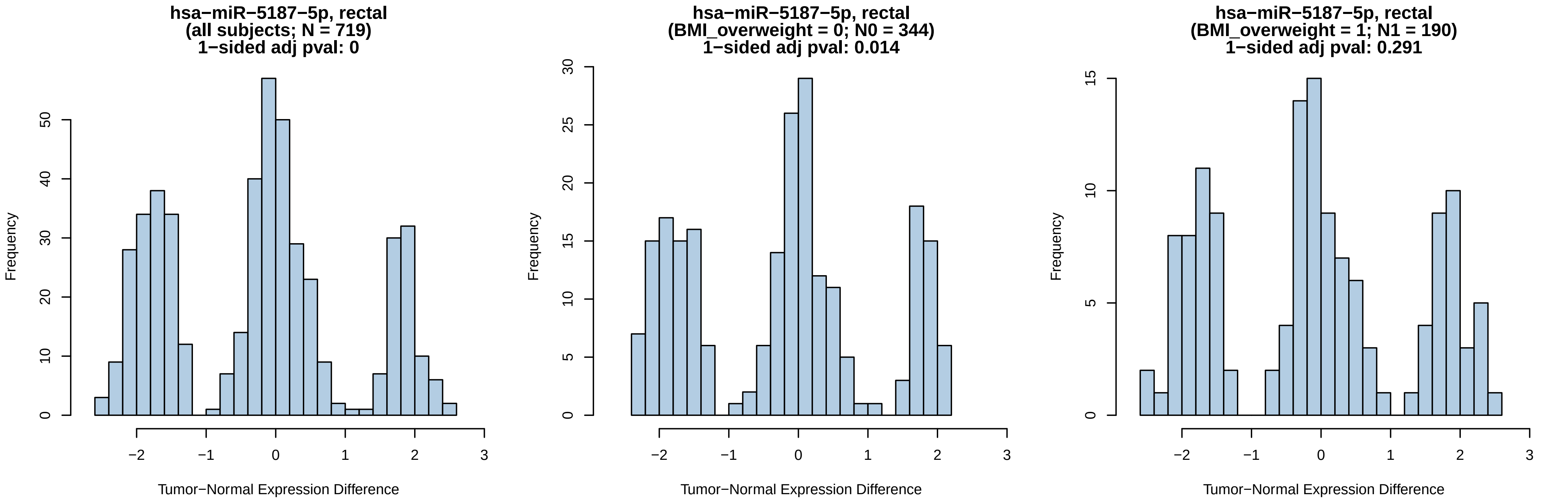
hsa−miR−5187−5p, rectal
(all subjects; N = 719)
1−sided adj pval: 0
Frequency
Tumor−Normal Expression Difference
hsa−miR−5187−5p, rectal
(BMI_overweight = 0; N0 = 344)
1−sided adj pval: 0.014
Frequency
Tumor−Normal Expression Difference
hsa−miR−5187−5p, rectal
(BMI_overweight = 1; N1 = 190)
1−sided adj pval: 0.291
Frequency
Tumor−Normal Expression Difference

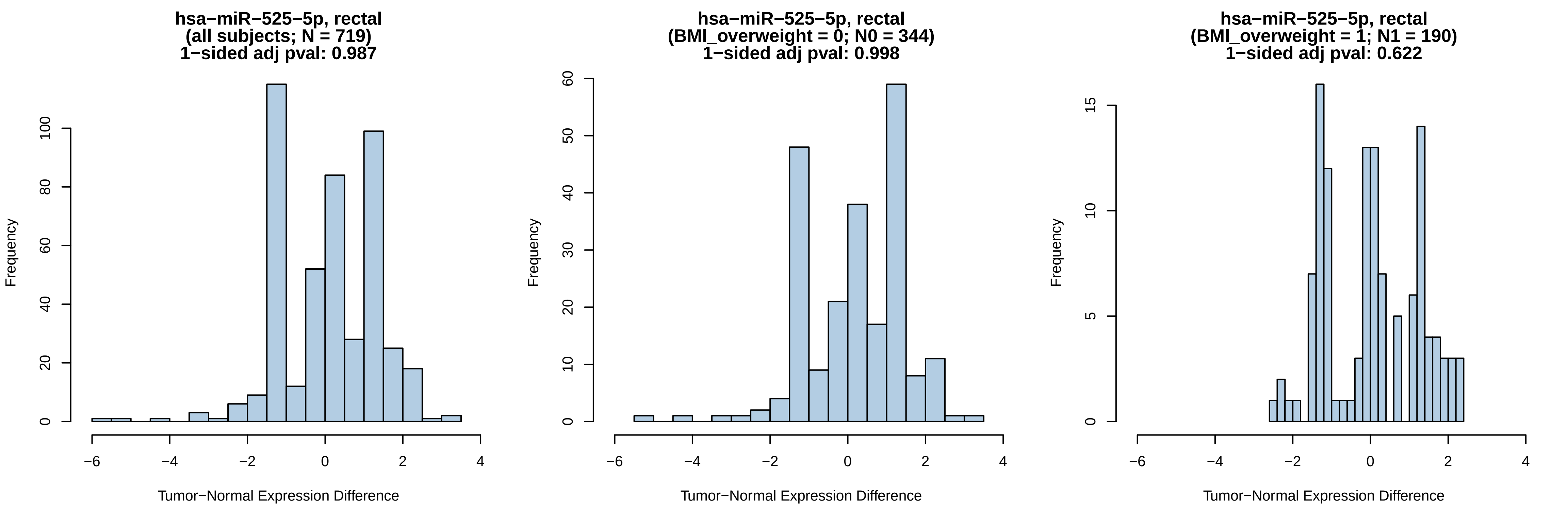
hsa−miR−525−5p, rectal
(all subjects; N = 719)
1−sided adj pval: 0.987
Frequency
Tumor−Normal Expression Difference
hsa−miR−525−5p, rectal
(BMI_overweight = 0; N0 = 344)
1−sided adj pval: 0.998
Frequency
Tumor−Normal Expression Difference
hsa−miR−525−5p, rectal
(BMI_overweight = 1; N1 = 190)
1−sided adj pval: 0.622
Frequency
Tumor−Normal Expression Difference

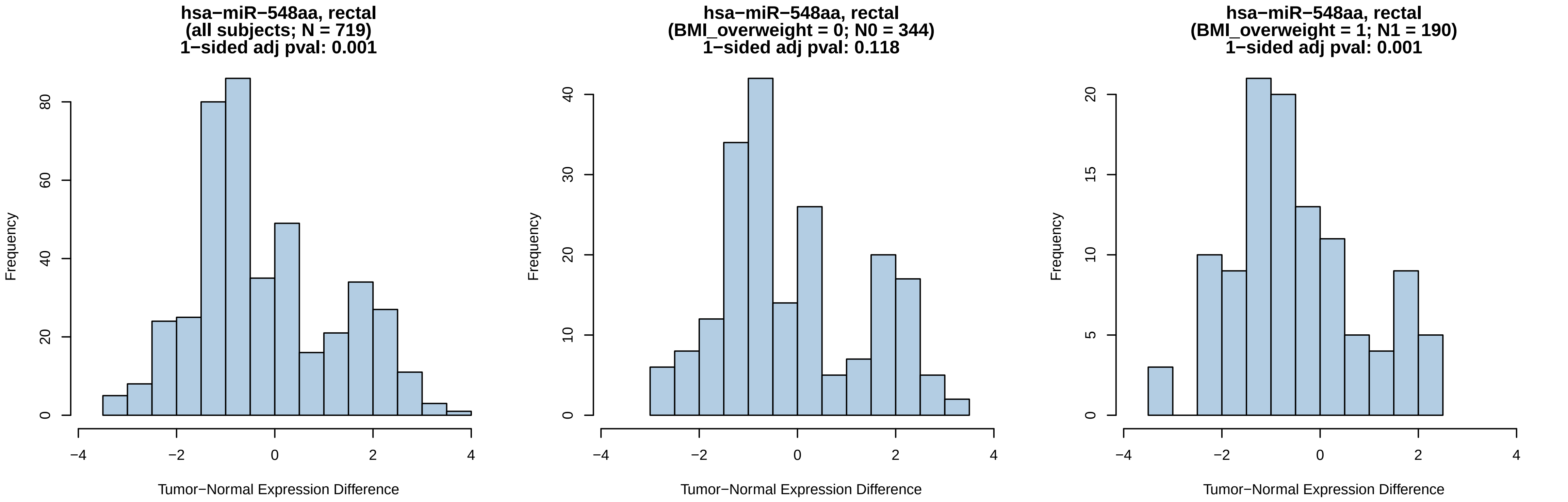
hsa−miR−548aa, rectal
(all subjects; N = 719)
1−sided adj pval: 0.001
Frequency
Tumor−Normal Expression Difference
hsa−miR−548aa, rectal
(BMI_overweight = 0; N0 = 344)
1−sided adj pval: 0.118
Frequency
Tumor−Normal Expression Difference
hsa−miR−548aa, rectal
(BMI_overweight = 1; N1 = 190)
1−sided adj pval: 0.001
Frequency
Tumor−Normal Expression Difference

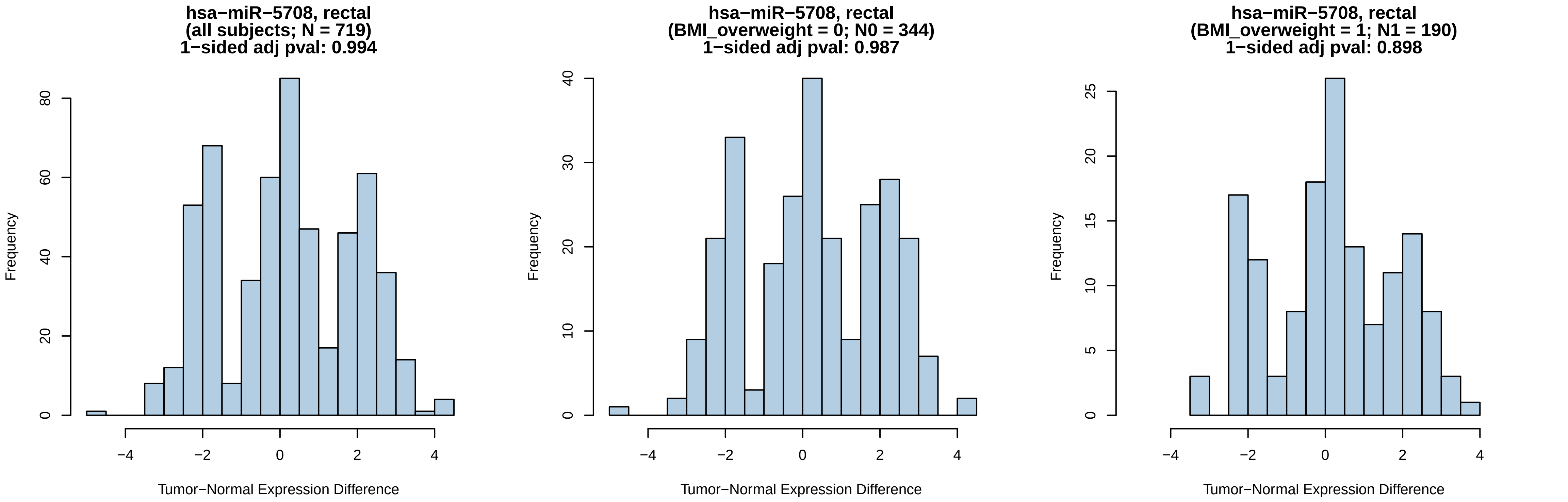

hsa−miR−5708, rectal
(all subjects; N = 719)
1−sided adj pval: 0.994
Frequency
Tumor−Normal Expression Difference
hsa−miR−5708, rectal
(BMI_overweight = 0; N0 = 344)
1−sided adj pval: 0.987
Frequency
Tumor−Normal Expression Difference
hsa−miR−5708, rectal
(BMI_overweight = 1; N1 = 190)
1−sided adj pval: 0.898
Frequency
Tumor−Normal Expression Difference

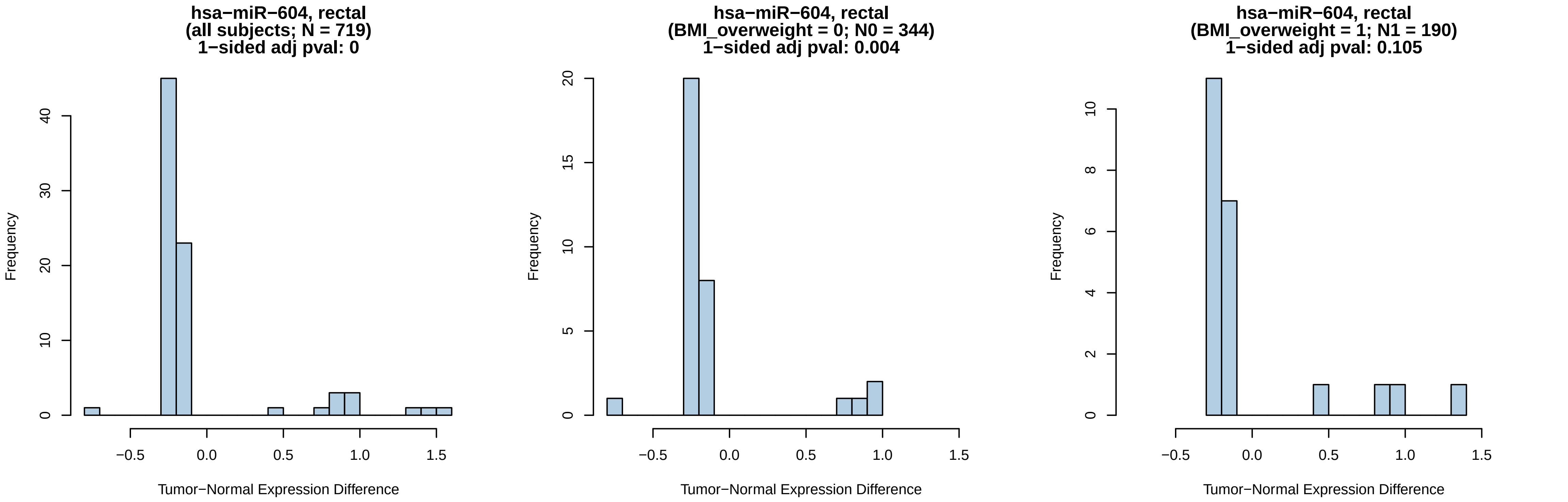
hsa−miR−604, rectal
(all subjects; N = 719)
1−sided adj pval: 0
hsa−miR−604, rectal
(BMI_overweight = 0; N0 = 344)
1−sided adj pval: 0.004
hsa−miR−604, rectal
(BMI_overweight = 1; N1 = 190)
1−sided adj pval: 0.105
Frequency
Tumor−Normal Expression Difference

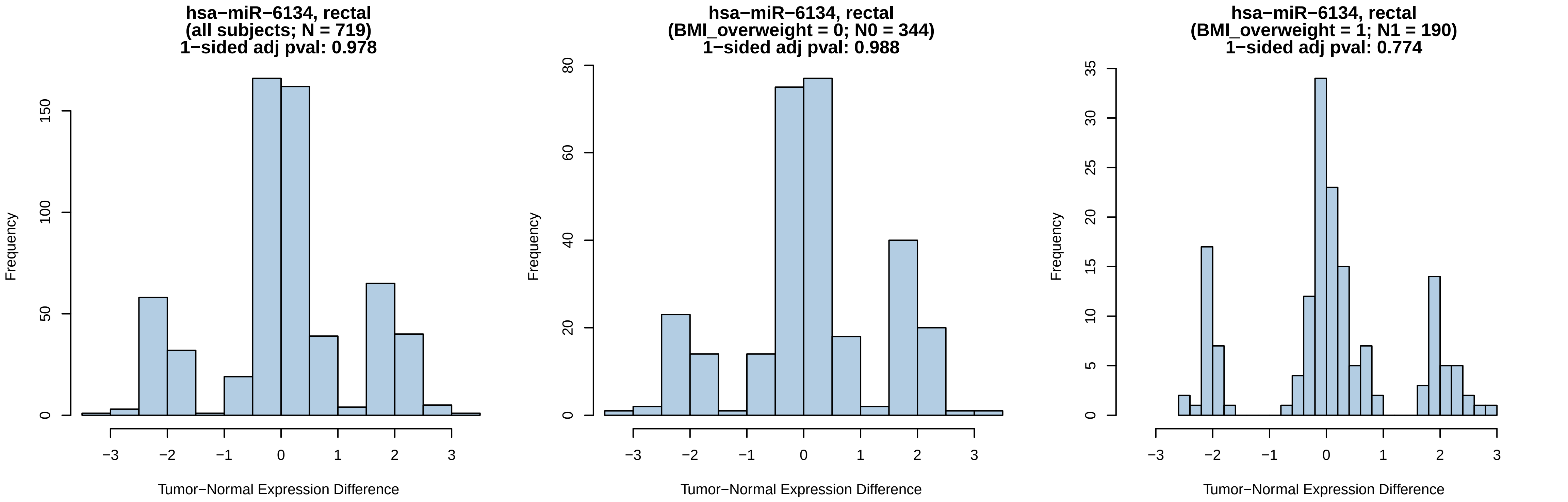

hsa−miR−6134, rectal
(all subjects; N = 719)
1−sided adj pval: 0.978
Frequency
Tumor−Normal Expression Difference
hsa−miR−6134, rectal
(BMI_overweight = 0; N0 = 344)
1−sided adj pval: 0.988
Frequency
Tumor−Normal Expression Difference
hsa−miR−6134, rectal
(BMI_overweight = 1; N1 = 190)
1−sided adj pval: 0.774
Frequency
Tumor−Normal Expression Difference

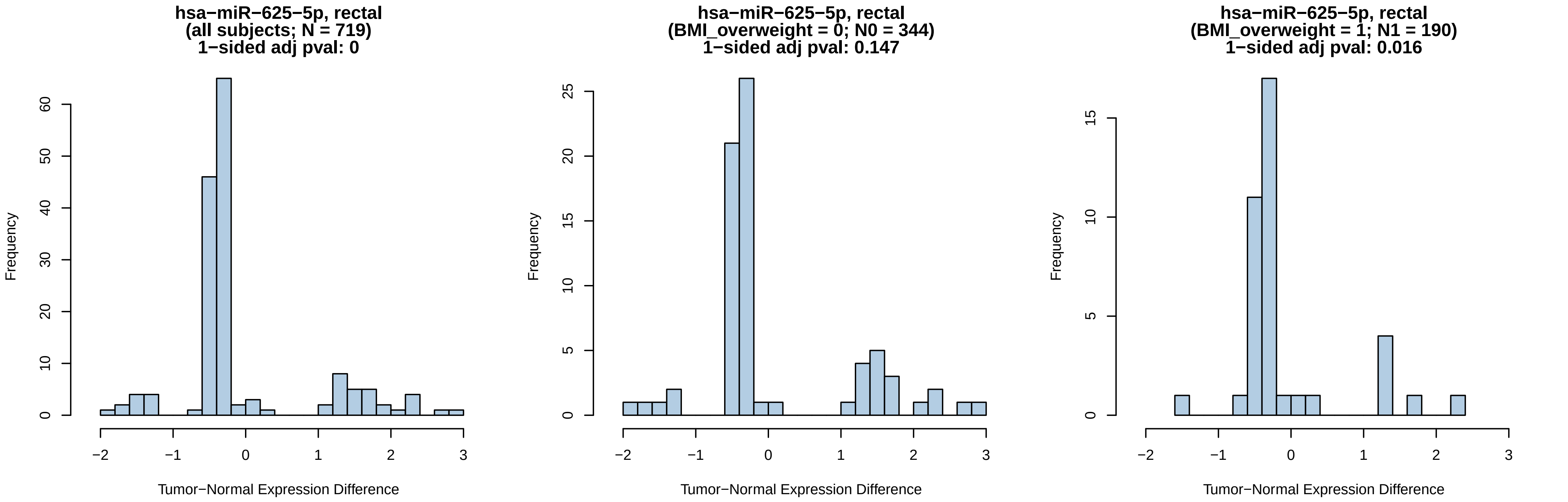
hsa−miR−625−5p, rectal
(all subjects; N = 719)
1−sided adj pval: 0
hsa−miR−625−5p, rectal
(BMI_overweight = 0; N0 = 344)
1−sided adj pval: 0.147
hsa−miR−625−5p, rectal
(BMI_overweight = 1; N1 = 190)
1−sided adj pval: 0.016
Frequency
Tumor−Normal Expression Difference

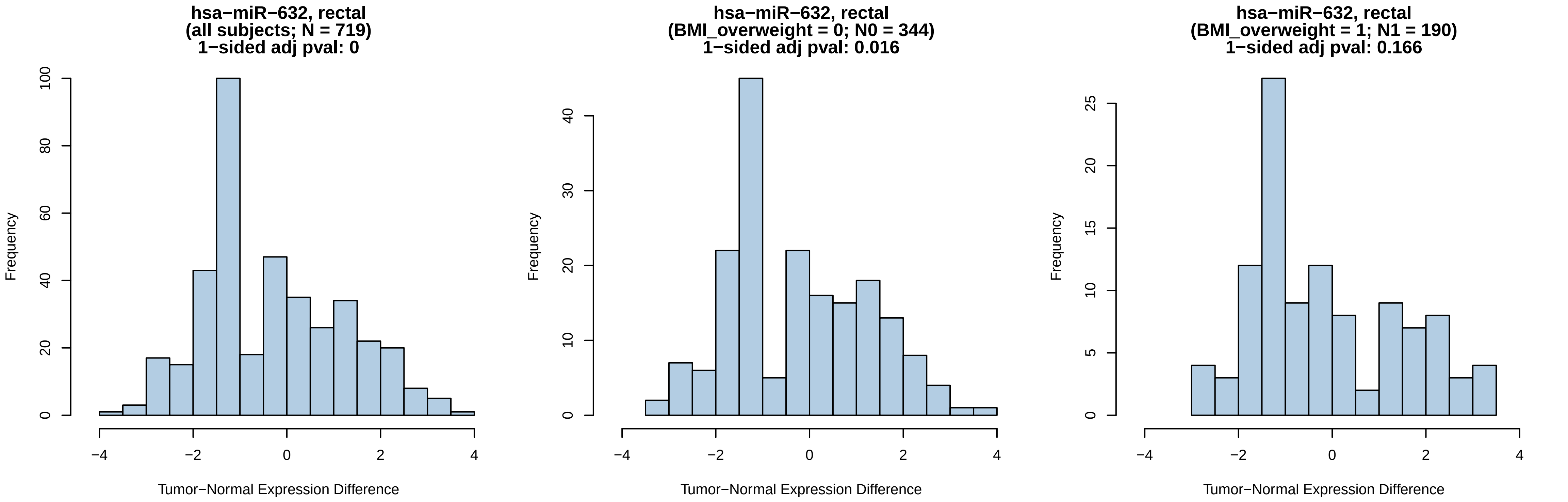
hsa−miR−632, rectal
(all subjects; N = 719)
1−sided adj pval: 0
Frequency
0
20
40
60
80
100
−4
−2
0
2
4
Tumor−Normal Expression Difference
hsa−miR−632, rectal
(BMI_overweight = 0; N0 = 344)
1−sided adj pval: 0.016
Frequency
0
10
20
30
40
−4
−2
0
2
4
Tumor−Normal Expression Difference
hsa−miR−632, rectal
(BMI_overweight = 1; N1 = 190)
1−sided adj pval: 0.166
Frequency
0
5
10
15
20
25
−4
−2
0
2
4
Tumor−Normal Expression Difference

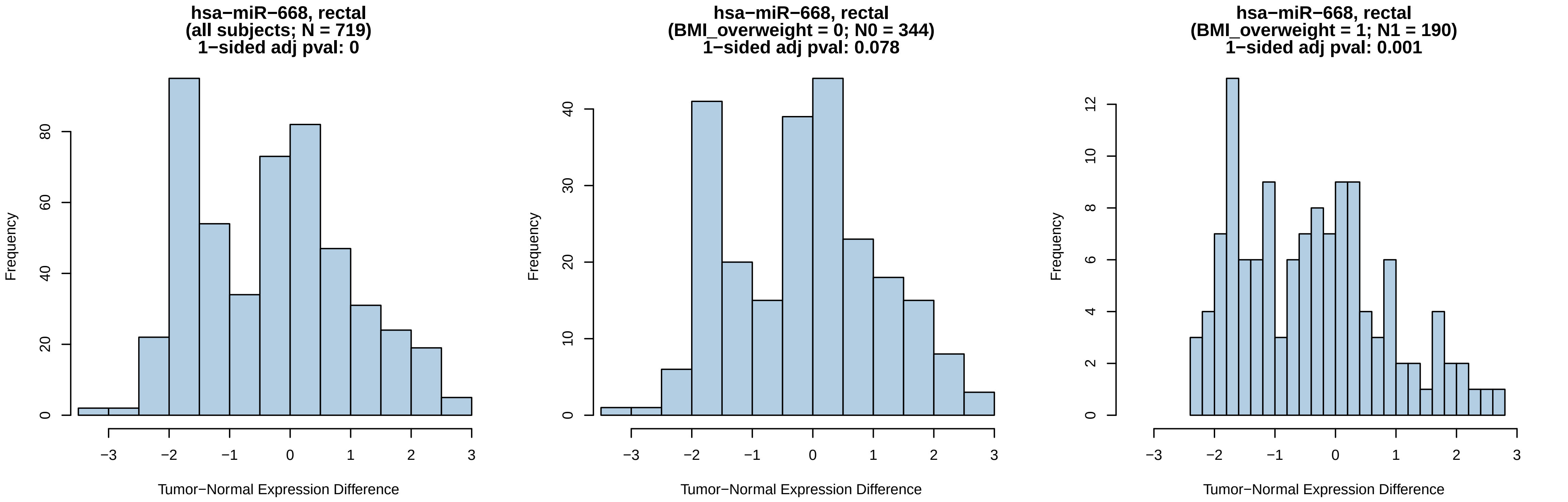
hsa−miR−668, rectal
(all subjects; N = 719)
1−sided adj pval: 0
hsa−miR−668, rectal
(BMI_overweight = 0; N0 = 344)
1−sided adj pval: 0.078
hsa−miR−668, rectal
(BMI_overweight = 1; N1 = 190)
1−sided adj pval: 0.001
Frequency
Tumor−Normal Expression Difference

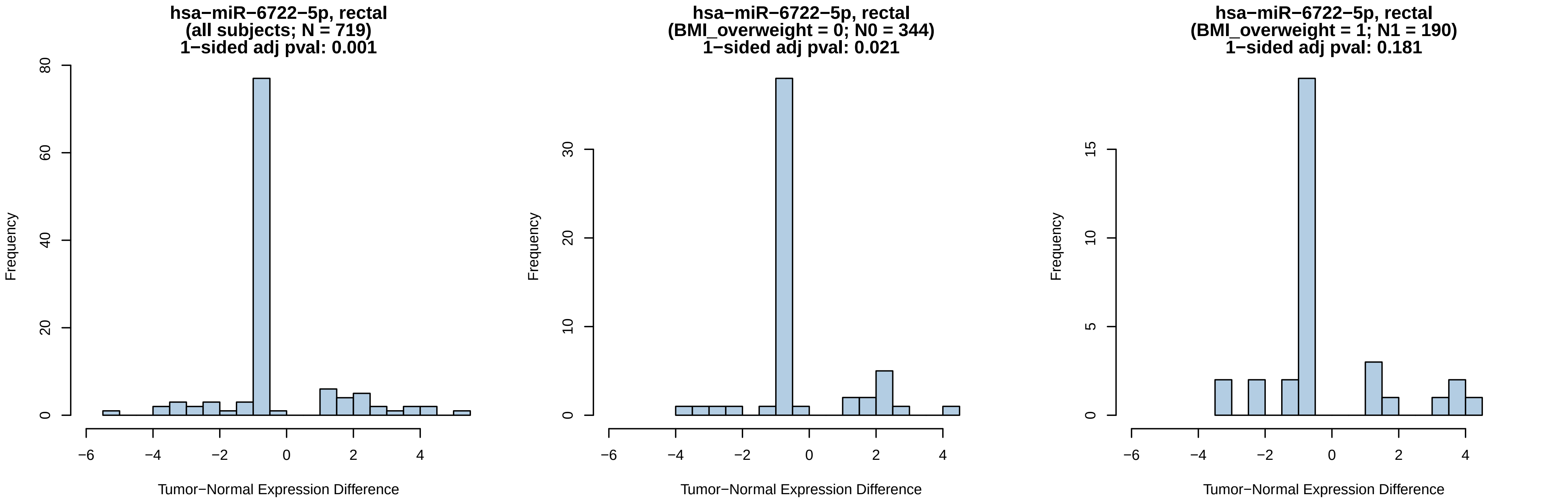
hsa−miR−6722−5p, rectal
(all subjects; N = 719)
1−sided adj pval: 0.001
Frequency
0
20
40
60
80
−6
−4
−2
0
2
4
Tumor−Normal Expression Difference
hsa−miR−6722−5p, rectal
(BMI_overweight = 0; N0 = 344)
1−sided adj pval: 0.021
Frequency
0
10
20
30
−6
−4
−2
0
2
4
Tumor−Normal Expression Difference
hsa−miR−6722−5p, rectal
(BMI_overweight = 1; N1 = 190)
1−sided adj pval: 0.181
Frequency
0
5
10
15
−6
−4
−2
0
2
4
Tumor−Normal Expression Difference

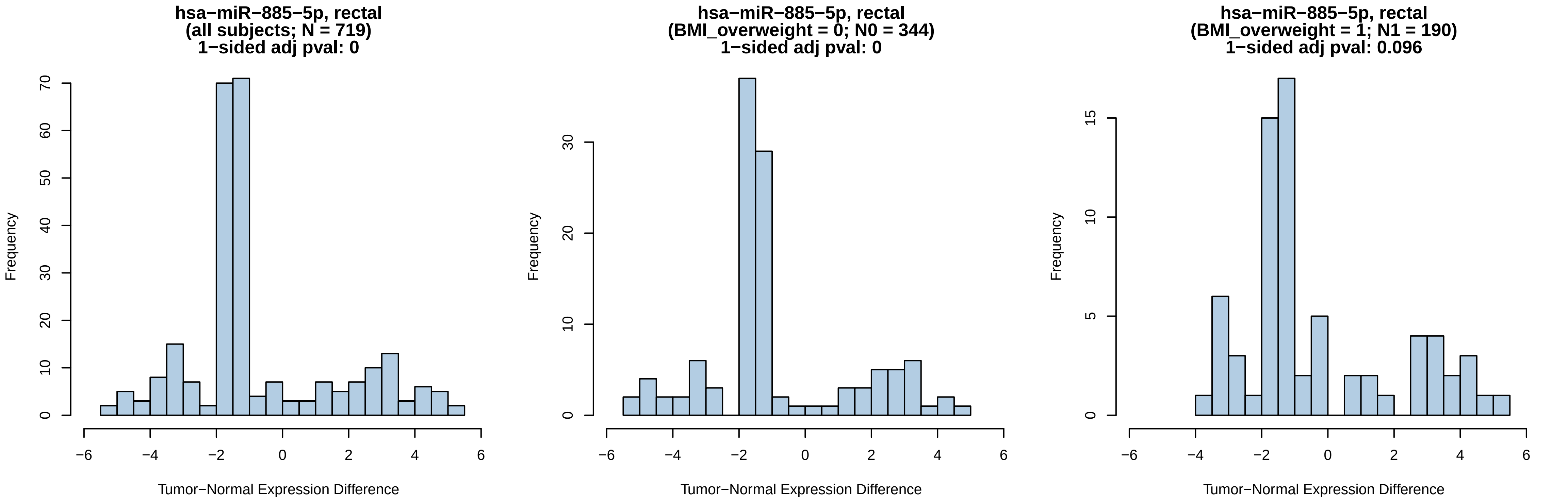

hsa−miR−885−5p, rectal
(all subjects; N = 719)
1−sided adj pval: 0
Frequency
Tumor−Normal Expression Difference
hsa−miR−885−5p, rectal
(BMI_overweight = 0; N0 = 344)
1−sided adj pval: 0
Frequency
Tumor−Normal Expression Difference
hsa−miR−885−5p, rectal
(BMI_overweight = 1; N1 = 190)
1−sided adj pval: 0.096
Frequency
Tumor−Normal Expression Difference

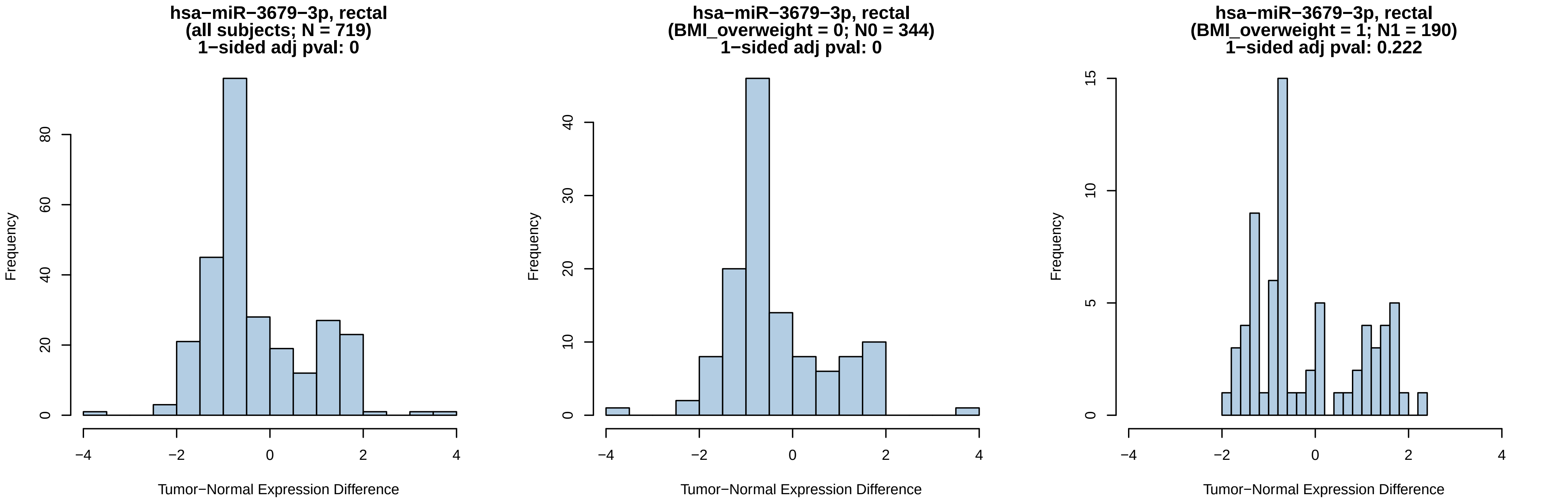

hsa−miR−3679−3p, rectal
(all subjects; N = 719)
1−sided adj pval: 0
Frequency
0
20
40
60
80
−4
−2
0
2
4
Tumor−Normal Expression Difference
hsa−miR−3679−3p, rectal
(BMI_overweight = 0; N0 = 344)
1−sided adj pval: 0
Frequency
0
10
20
30
40
−4
−2
0
2
4
Tumor−Normal Expression Difference
hsa−miR−3679−3p, rectal
(BMI_overweight = 1; N1 = 190)
1−sided adj pval: 0.222
Frequency
0
5
10
15
−4
−2
0
2
4
Tumor−Normal Expression Difference

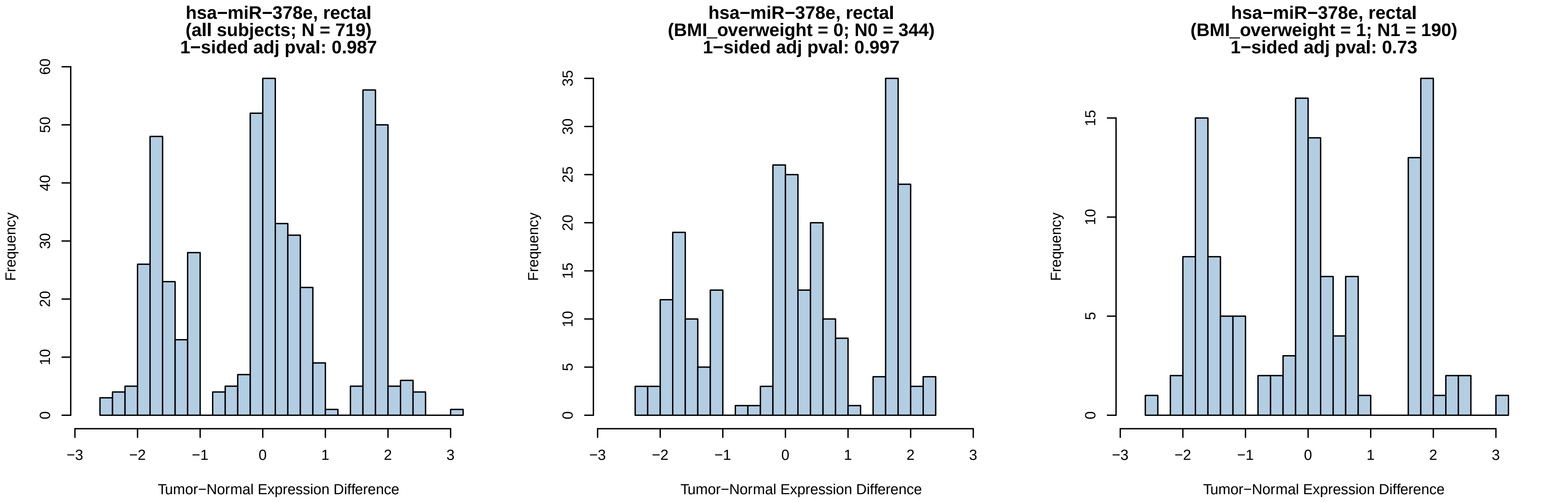
hsa−miR−378e, rectal
(all subjects; N = 719)
1−sided adj pval: 0.987
Frequency
Tumor−Normal Expression Difference
hsa−miR−378e, rectal
(BMI_overweight = 0; N0 = 344)
1−sided adj pval: 0.997
Frequency
Tumor−Normal Expression Difference
hsa−miR−378e, rectal
(BMI_overweight = 1; N1 = 190)
1−sided adj pval: 0.73
Frequency
Tumor−Normal Expression Difference

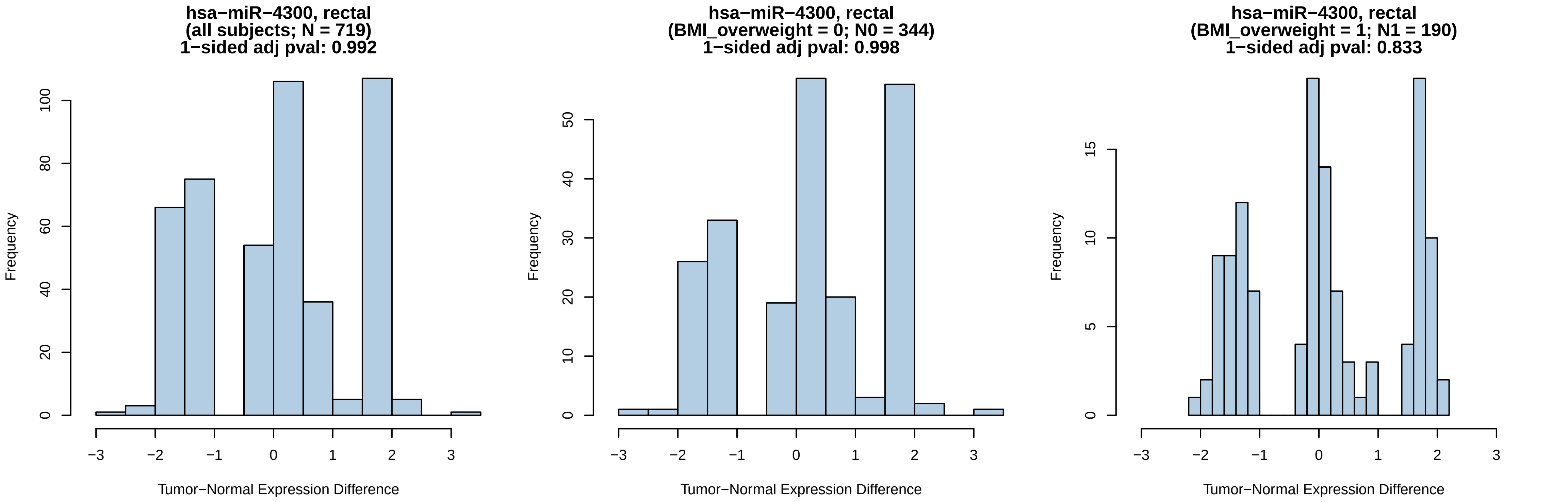
hsa−miR−4300, rectal
(all subjects; N = 719)
1−sided adj pval: 0.992
Frequency
Tumor−Normal Expression Difference
hsa−miR−4300, rectal
(BMI_overweight = 0; N0 = 344)
1−sided adj pval: 0.998
Frequency
Tumor−Normal Expression Difference
hsa−miR−4300, rectal
(BMI_overweight = 1; N1 = 190)
1−sided adj pval: 0.833
Frequency
Tumor−Normal Expression Difference

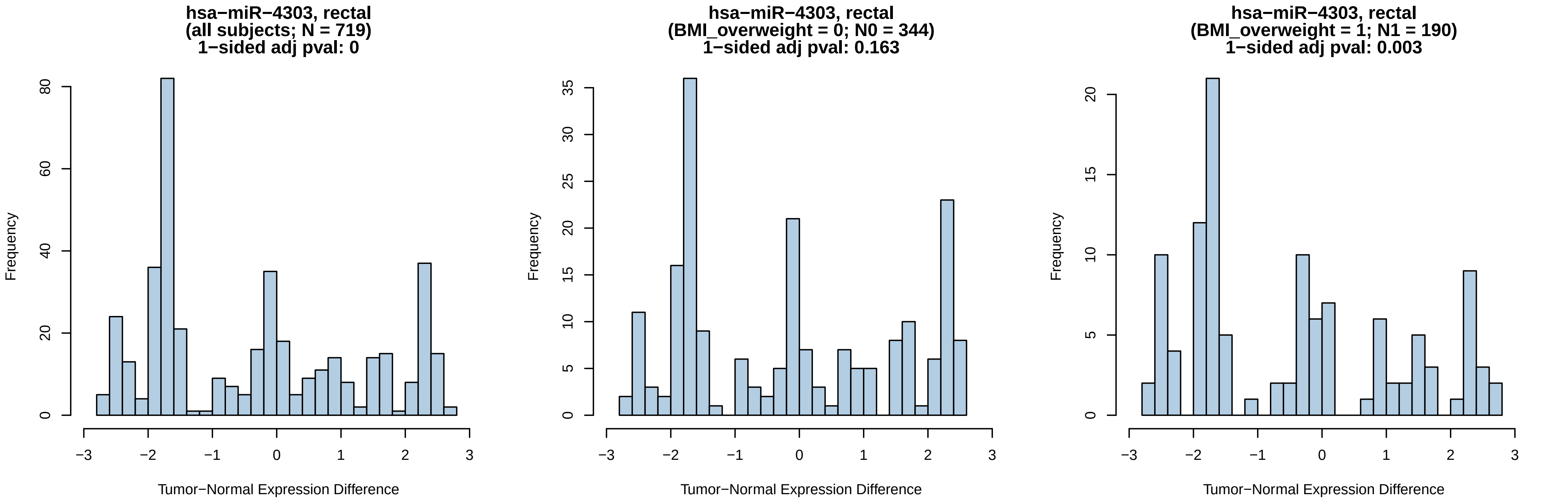
hsa−miR−4303, rectal
(all subjects; N = 719)
1−sided adj pval: 0
Frequency
Tumor−Normal Expression Difference
hsa−miR−4303, rectal
(BMI_overweight = 0; N0 = 344)
1−sided adj pval: 0.163
Frequency
Tumor−Normal Expression Difference
hsa−miR−4303, rectal
(BMI_overweight = 1; N1 = 190)
1−sided adj pval: 0.003
Frequency
Tumor−Normal Expression Difference

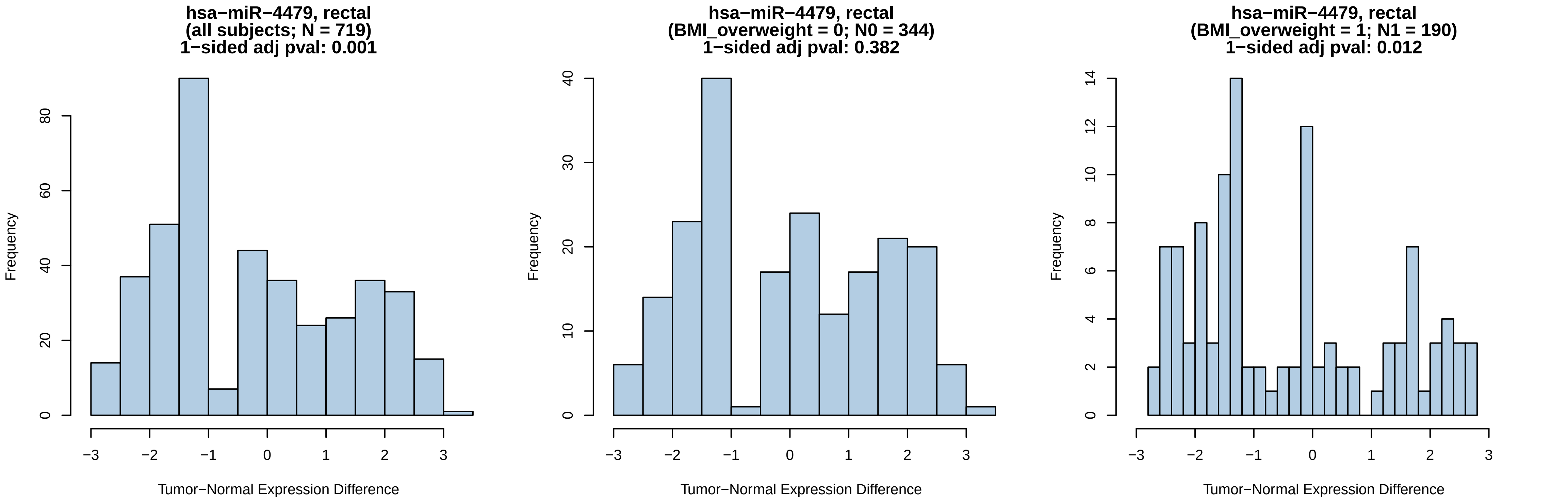

hsa−miR−4479, rectal
(all subjects; N = 719)
1−sided adj pval: 0.001
Frequency
0
20
40
60
80
−3
−2
−1
0
1
2
3
Tumor−Normal Expression Difference
hsa−miR−4479, rectal
(BMI_overweight = 0; N0 = 344)
1−sided adj pval: 0.382
Frequency
0
10
20
30
40
−3
−2
−1
0
1
2
3
Tumor−Normal Expression Difference
hsa−miR−4479, rectal
(BMI_overweight = 1; N1 = 190)
1−sided adj pval: 0.012
Frequency
0
2
4
6
8
10
12
14
−3
−2
−1
0
1
2
3
Tumor−Normal Expression Difference

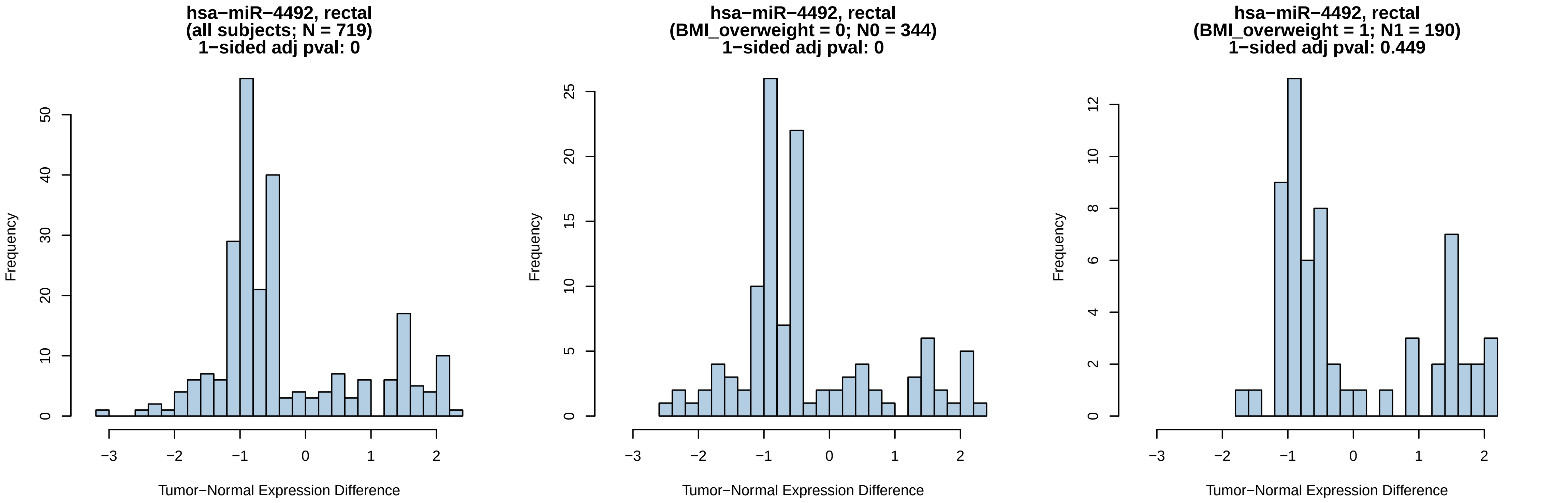
hsa−miR−4492, rectal
(all subjects; N = 719)
1−sided adj pval: 0
Frequency
Tumor−Normal Expression Difference
hsa−miR−4492, rectal
(BMI_overweight = 0; N0 = 344)
1−sided adj pval: 0
Frequency
Tumor−Normal Expression Difference
hsa−miR−4492, rectal
(BMI_overweight = 1; N1 = 190)
1−sided adj pval: 0.449
Frequency
Tumor−Normal Expression Difference

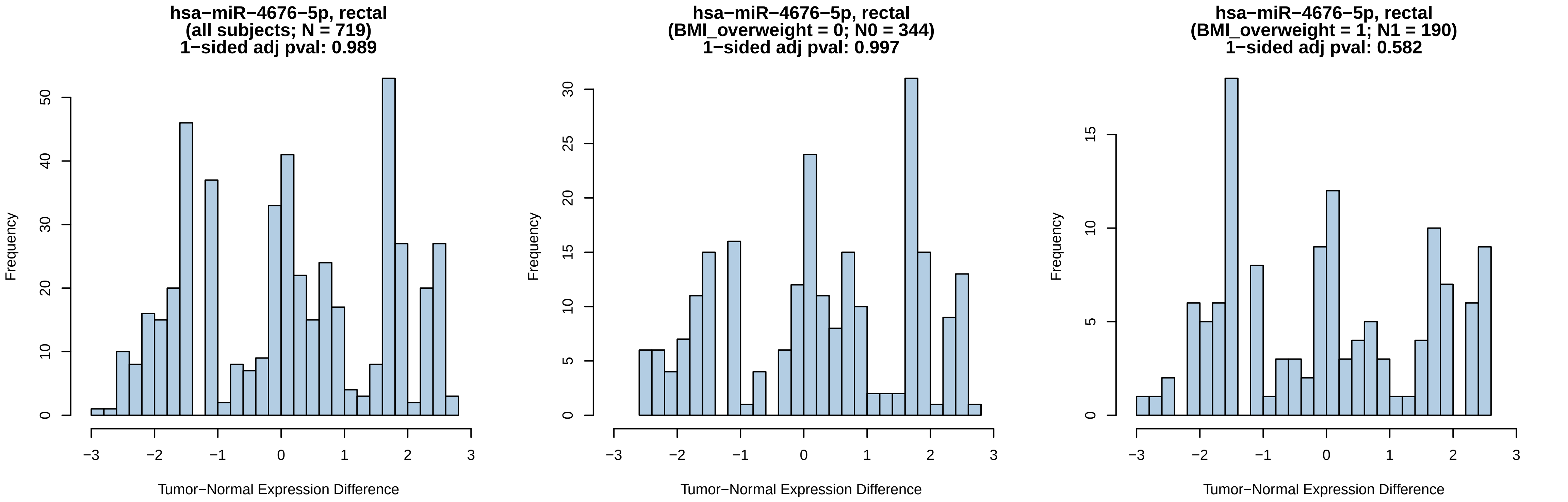

hsa−miR−4676−5p, rectal
(all subjects; N = 719)
1−sided adj pval: 0.989
hsa−miR−4676−5p, rectal
(BMI_overweight = 0; N0 = 344)
1−sided adj pval: 0.997
hsa−miR−4676−5p, rectal
(BMI_overweight = 1; N1 = 190)
1−sided adj pval: 0.582
Frequency
Tumor−Normal Expression Difference

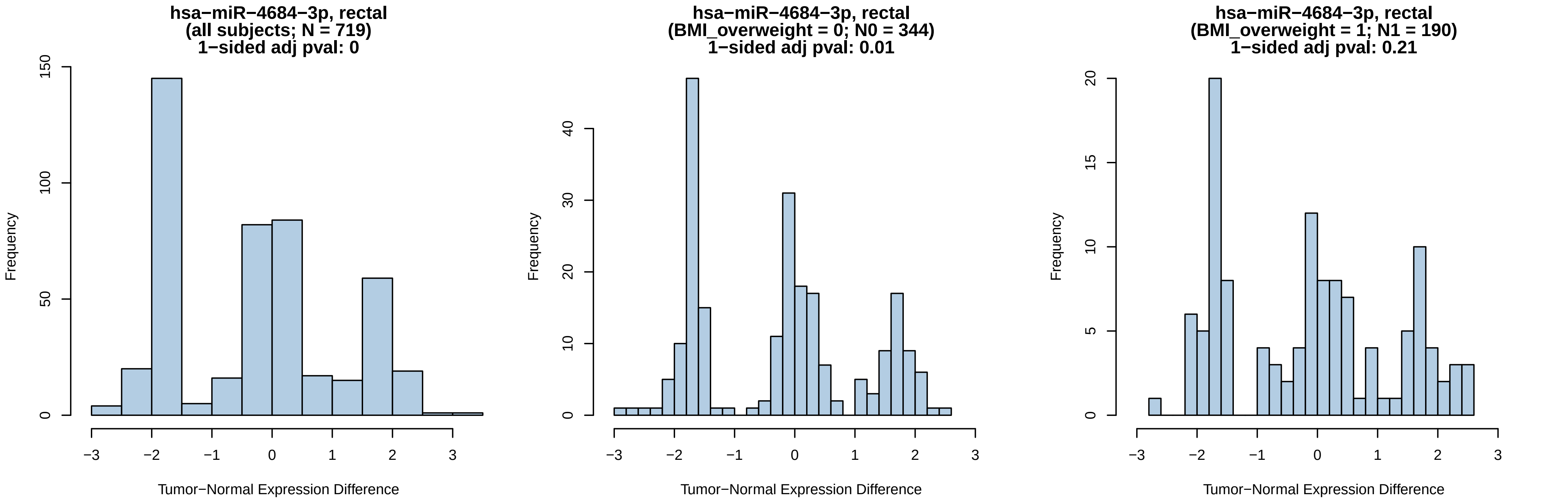
hsa−miR−4684−3p, rectal
(all subjects; N = 719)
1−sided adj pval: 0
Frequency
Tumor−Normal Expression Difference
hsa−miR−4684−3p, rectal
(BMI_overweight = 0; N0 = 344)
1−sided adj pval: 0.01
Frequency
Tumor−Normal Expression Difference
hsa−miR−4684−3p, rectal
(BMI_overweight = 1; N1 = 190)
1−sided adj pval: 0.21
Frequency
Tumor−Normal Expression Difference

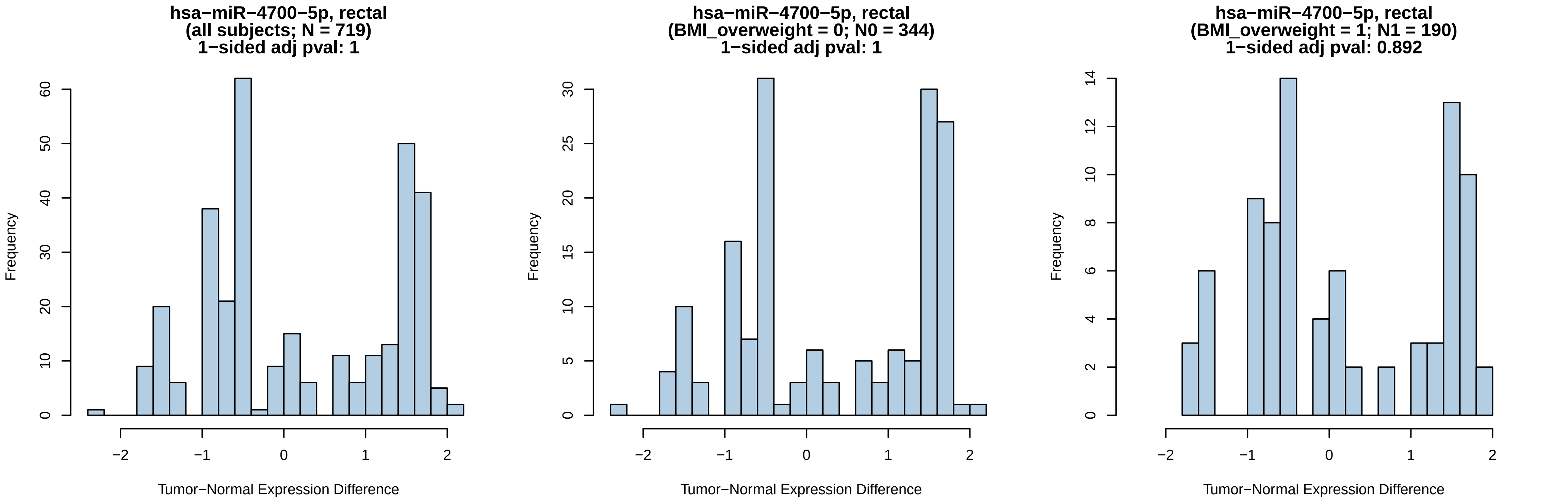
hsa−miR−4700−5p, rectal
(all subjects; N = 719)
1−sided adj pval: 1
Frequency
Tumor−Normal Expression Difference
0
10
20
30
40
50
60
−2
−1
0
1
2
hsa−miR−4700−5p, rectal
(BMI_overweight = 0; N0 = 344)
1−sided adj pval: 1
Frequency
Tumor−Normal Expression Difference
0
5
10
15
20
25
30
hsa−miR−4700−5p, rectal
(BMI_overweight = 1; N1 = 190)
1−sided adj pval: 0.892
Frequency
Tumor−Normal Expression Difference
0
2
4
6
8
10
12
14

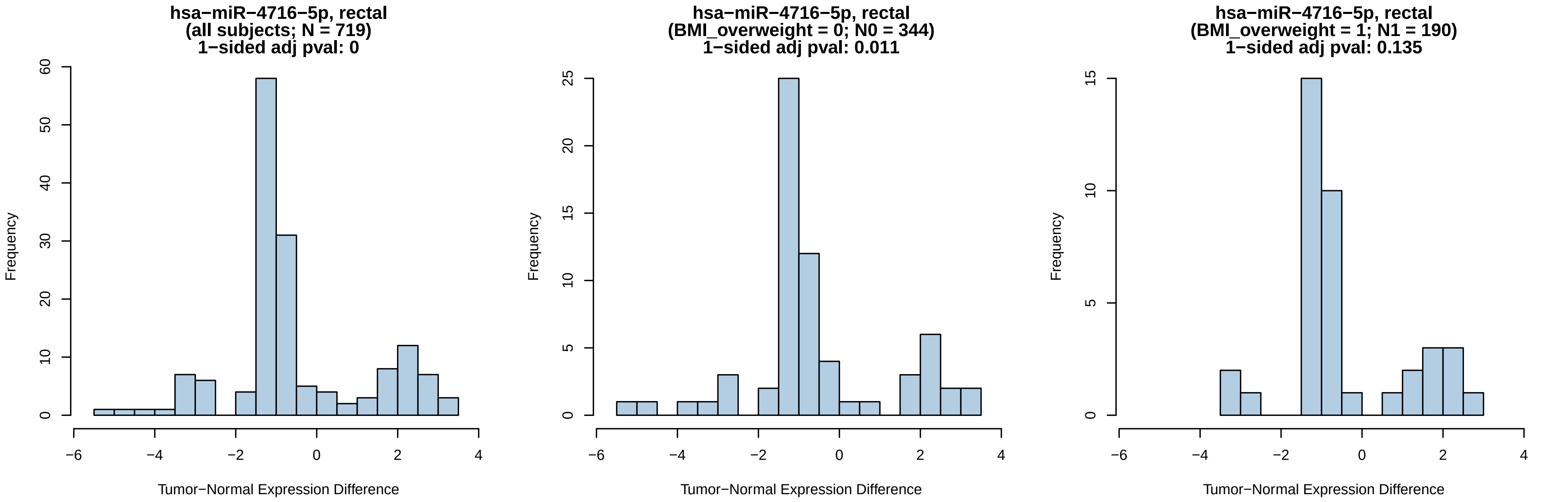
hsa−miR−4716−5p, rectal
(all subjects; N = 719)
1−sided adj pval: 0
Frequency
Tumor−Normal Expression Difference
hsa−miR−4716−5p, rectal
(BMI_overweight = 0; N0 = 344)
1−sided adj pval: 0.011
Frequency
Tumor−Normal Expression Difference
hsa−miR−4716−5p, rectal
(BMI_overweight = 1; N1 = 190)
1−sided adj pval: 0.135
Frequency
Tumor−Normal Expression Difference

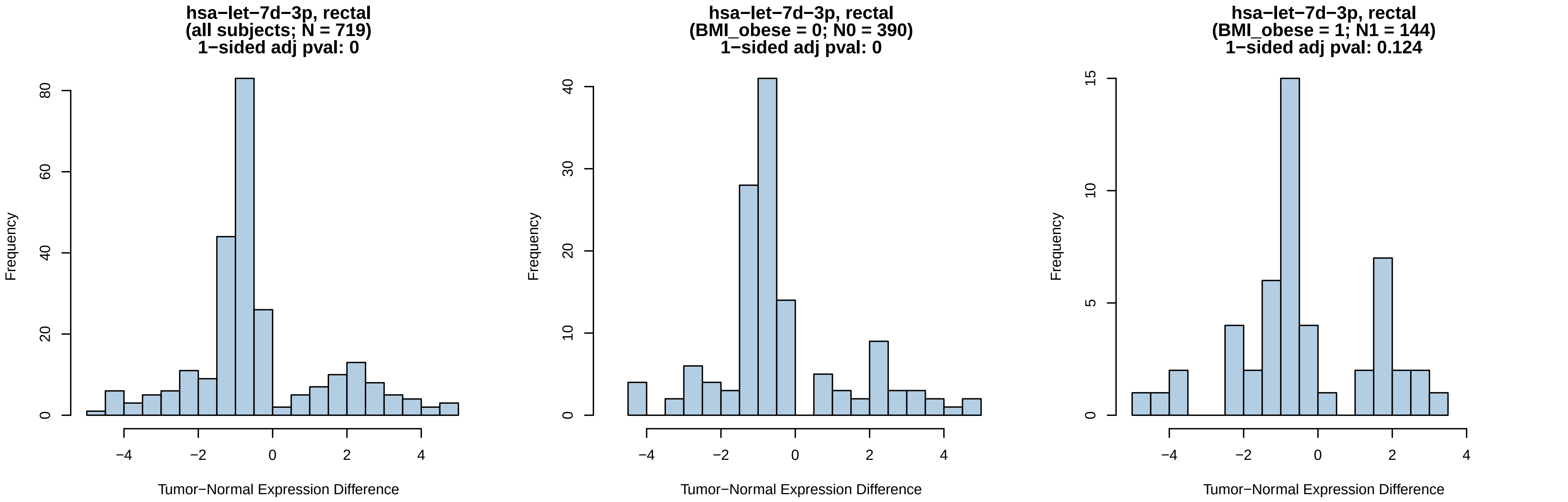
hsa−let−7d−3p, rectal
(all subjects; N = 719)
1−sided adj pval: 0
hsa−let−7d−3p, rectal
(BMI_obese = 0; N0 = 390)
1−sided adj pval: 0
hsa−let−7d−3p, rectal
(BMI_obese = 1; N1 = 144)
1−sided adj pval: 0.124
Frequency
Tumor−Normal Expression Difference

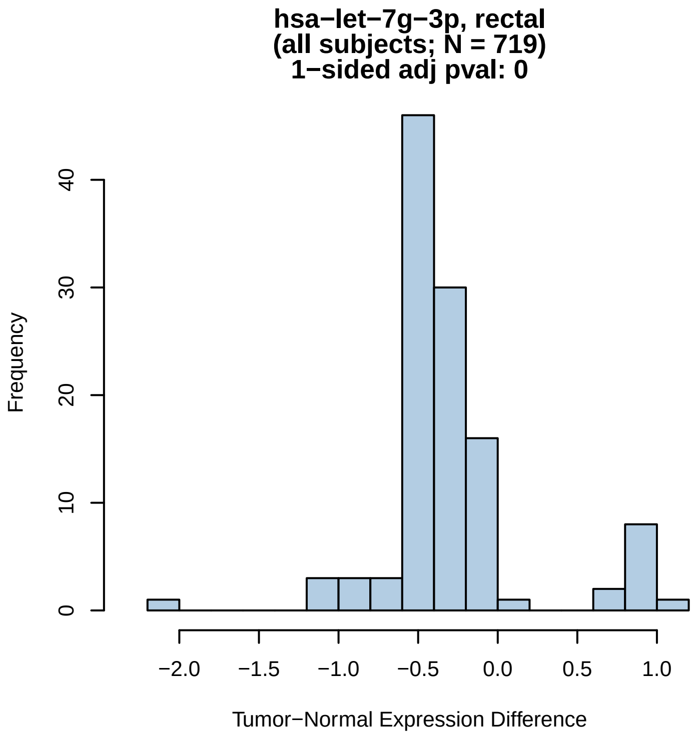

hsa−let−7g−3p, rectal
(all subjects; N = 719)
1−sided adj pval: 0
Frequency
Tumor−Normal Expression Difference


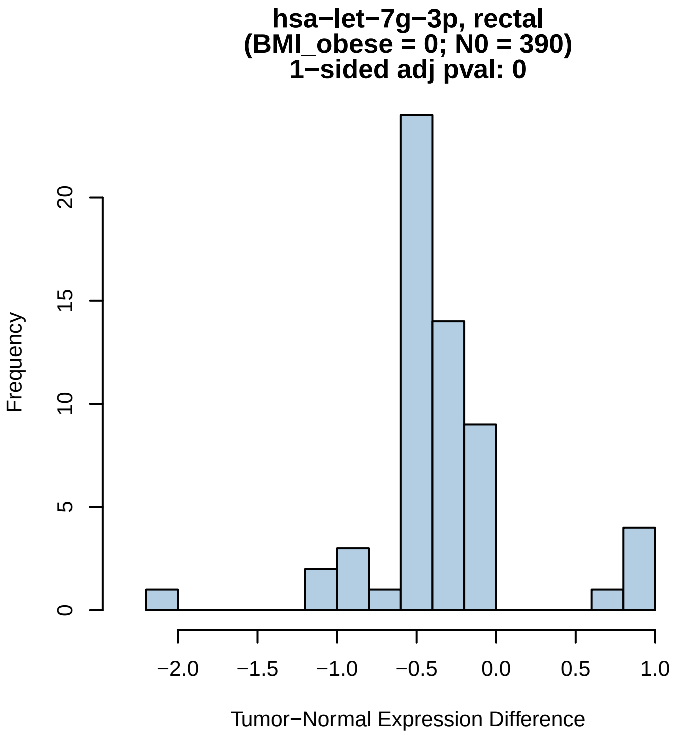

hsa−let−7g−3p, rectal
(BMI_obese = 0; N0 = 390)
1−sided adj pval: 0
Frequency
Tumor−Normal Expression Difference


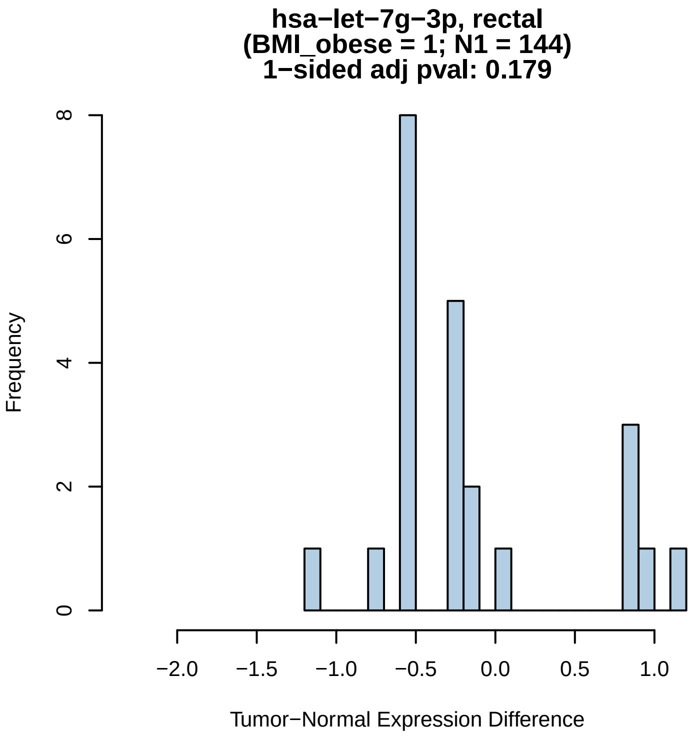

hsa−let−7g−3p, rectal
(BMI_obese = 1; N1 = 144)
1−sided adj pval: 0.179
Frequency
Tumor−Normal Expression Difference

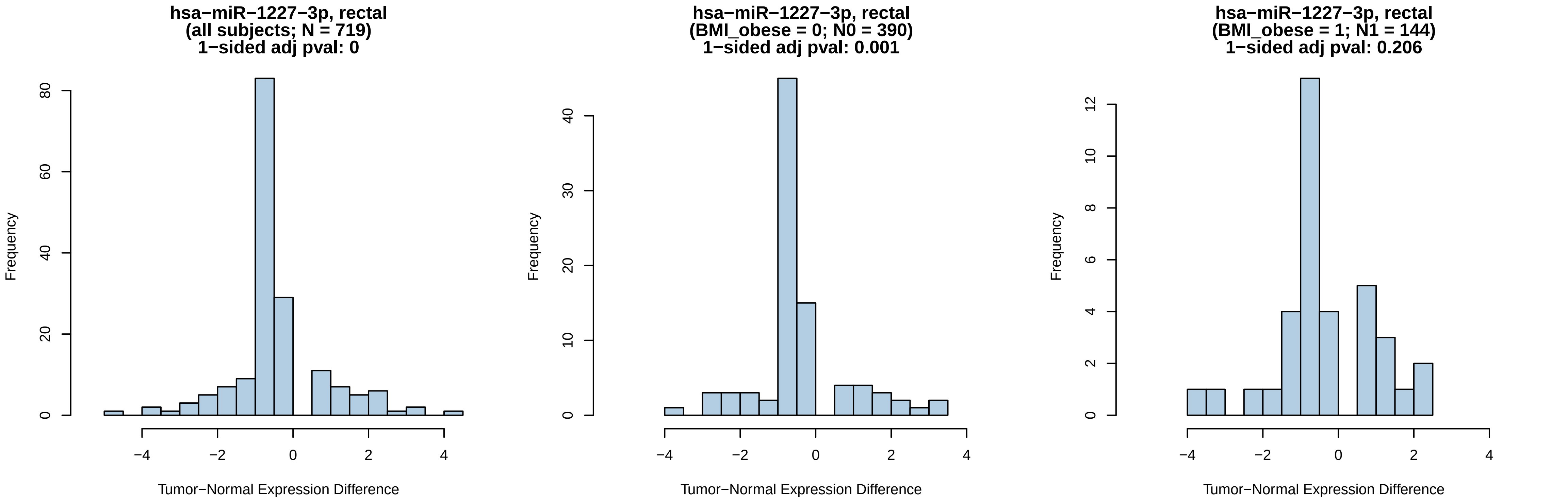
hsa−miR−1227−3p, rectal
(all subjects; N = 719)
1−sided adj pval: 0
Frequency
Tumor−Normal Expression Difference
hsa−miR−1227−3p, rectal
(BMI_obese = 0; N0 = 390)
1−sided adj pval: 0.001
Frequency
Tumor−Normal Expression Difference
hsa−miR−1227−3p, rectal
(BMI_obese = 1; N1 = 144)
1−sided adj pval: 0.206
Frequency
Tumor−Normal Expression Difference

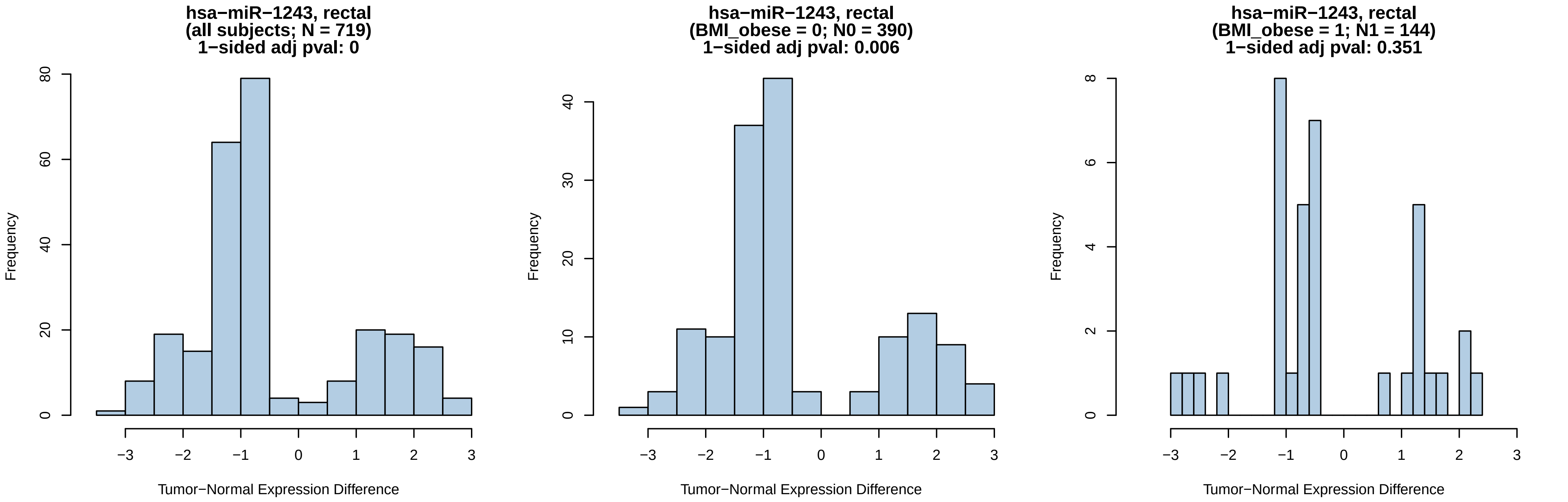
hsa−miR−1243, rectal
(all subjects; N = 719)
1−sided adj pval: 0
Frequency
0
20
40
60
80
−3
−2
−1
0
1
2
3
Tumor−Normal Expression Difference
hsa−miR−1243, rectal
(BMI_obese = 0; N0 = 390)
1−sided adj pval: 0.006
Frequency
0
10
20
30
40
−3
−2
−1
0
1
2
3
Tumor−Normal Expression Difference
hsa−miR−1243, rectal
(BMI_obese = 1; N1 = 144)
1−sided adj pval: 0.351
Frequency
0
2
4
6
8
−3
−2
−1
0
1
2
3
Tumor−Normal Expression Difference

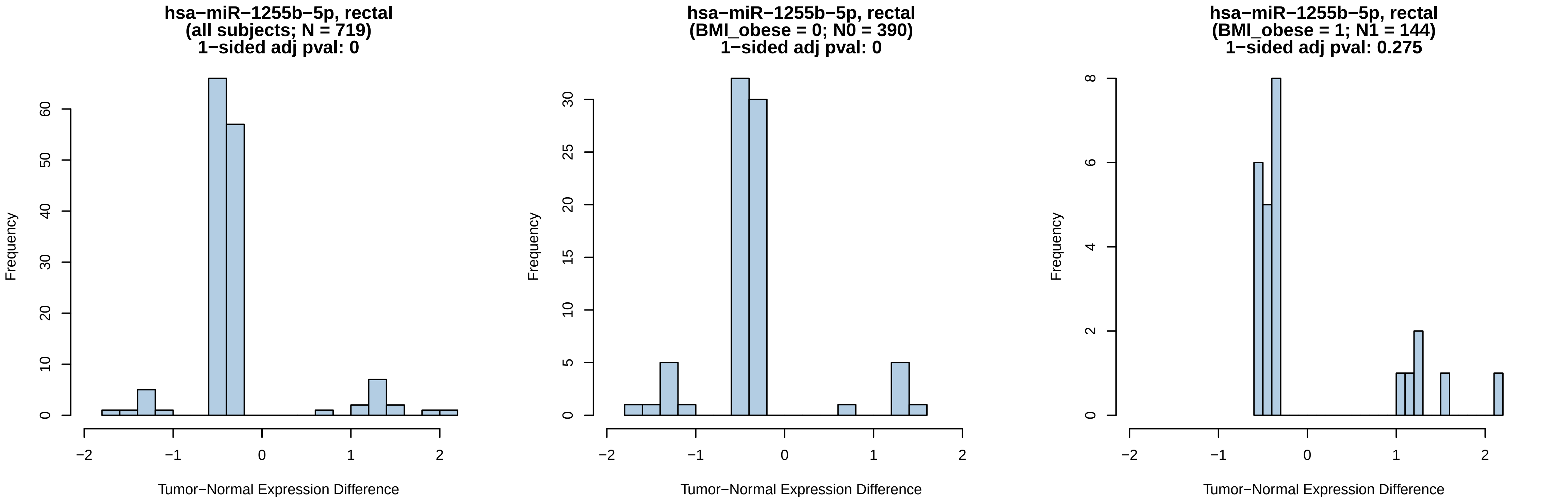
hsa−miR−1255b−5p, rectal
(all subjects; N = 719)
1−sided adj pval: 0
Frequency
Tumor−Normal Expression Difference
hsa−miR−1255b−5p, rectal
(BMI_obese = 0; N0 = 390)
1−sided adj pval: 0
Frequency
Tumor−Normal Expression Difference
hsa−miR−1255b−5p, rectal
(BMI_obese = 1; N1 = 144)
1−sided adj pval: 0.275
Frequency
Tumor−Normal Expression Difference

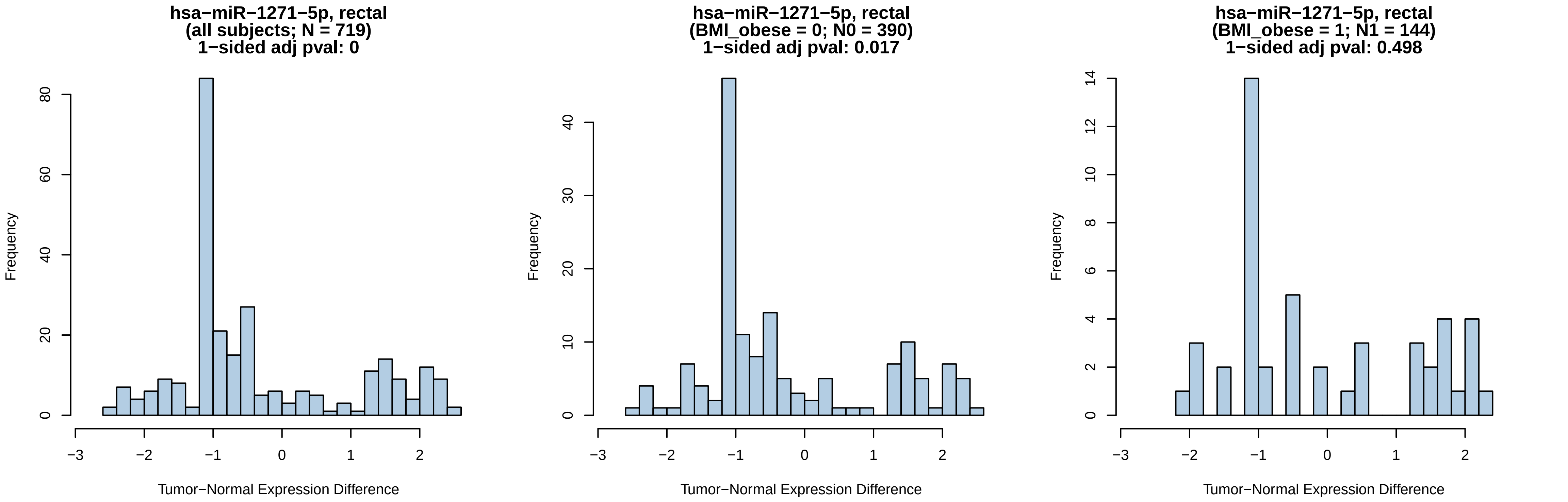
hsa−miR−1271−5p, rectal
(all subjects; N = 719)
1−sided adj pval: 0
Frequency
Tumor−Normal Expression Difference
hsa−miR−1271−5p, rectal
(BMI_obese = 0; N0 = 390)
1−sided adj pval: 0.017
Frequency
Tumor−Normal Expression Difference
hsa−miR−1271−5p, rectal
(BMI_obese = 1; N1 = 144)
1−sided adj pval: 0.498
Frequency
Tumor−Normal Expression Difference

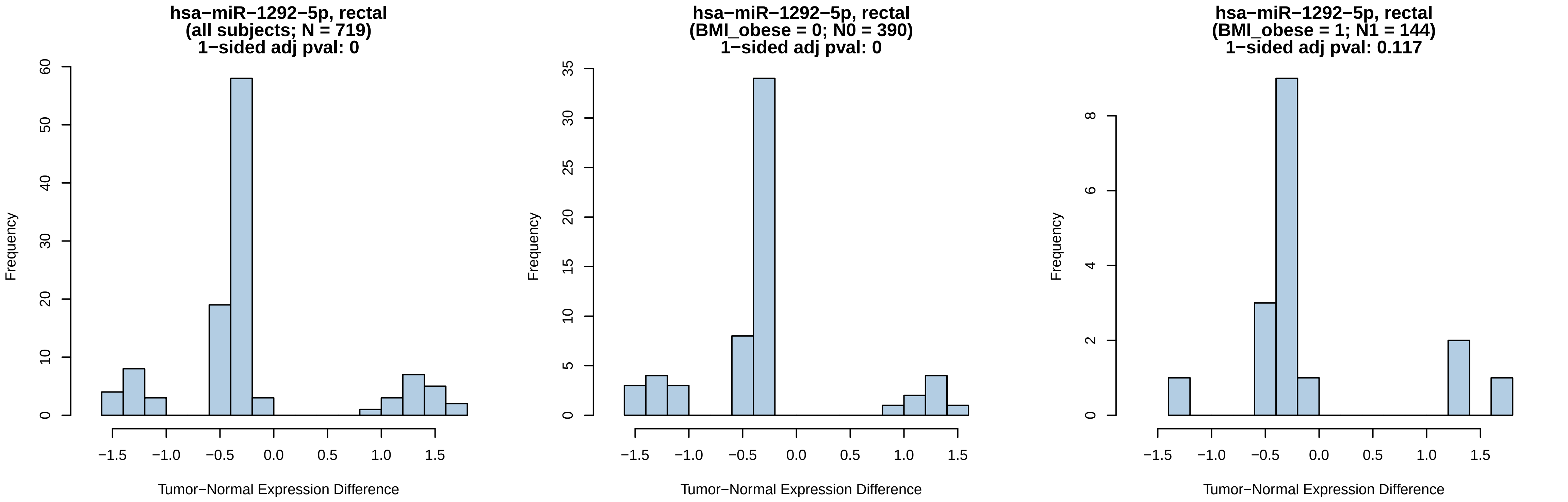
hsa−miR−1292−5p, rectal
(all subjects; N = 719)
1−sided adj pval: 0
Frequency
Tumor−Normal Expression Difference
hsa−miR−1292−5p, rectal
(BMI_obese = 0; N0 = 390)
1−sided adj pval: 0
Frequency
Tumor−Normal Expression Difference
hsa−miR−1292−5p, rectal
(BMI_obese = 1; N1 = 144)
1−sided adj pval: 0.117
Frequency
Tumor−Normal Expression Difference

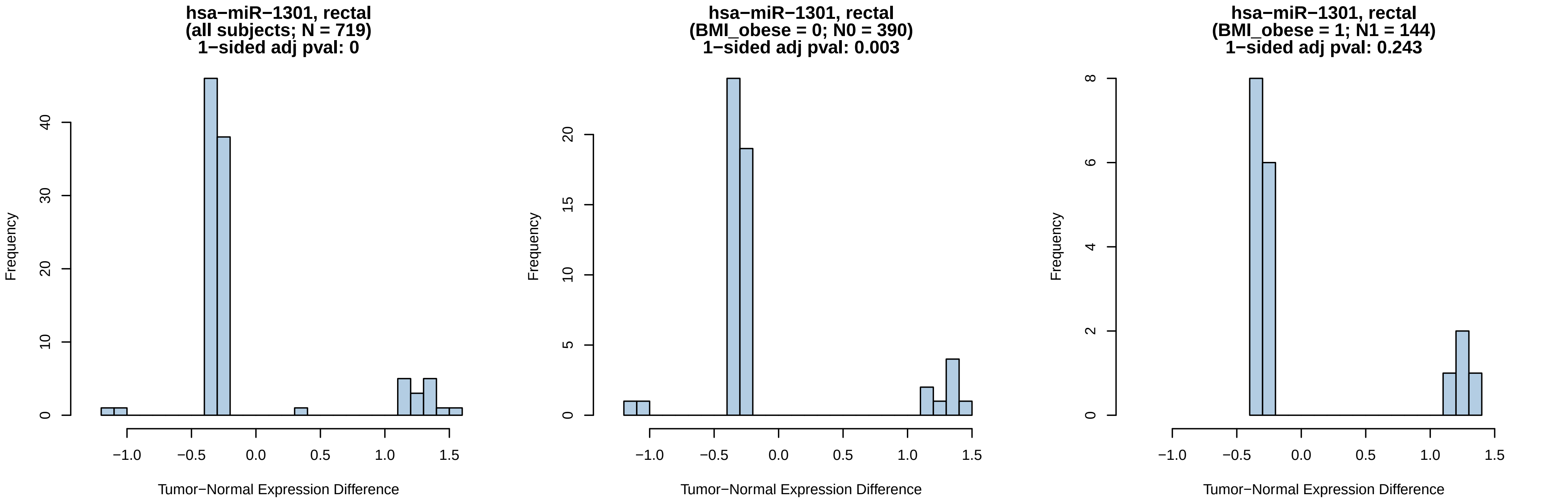
hsa−miR−1301, rectal
(all subjects; N = 719)
1−sided adj pval: 0
Frequency
Tumor−Normal Expression Difference
hsa−miR−1301, rectal
(BMI_obese = 0; N0 = 390)
1−sided adj pval: 0.003
Frequency
Tumor−Normal Expression Difference
hsa−miR−1301, rectal
(BMI_obese = 1; N1 = 144)
1−sided adj pval: 0.243
Frequency
Tumor−Normal Expression Difference

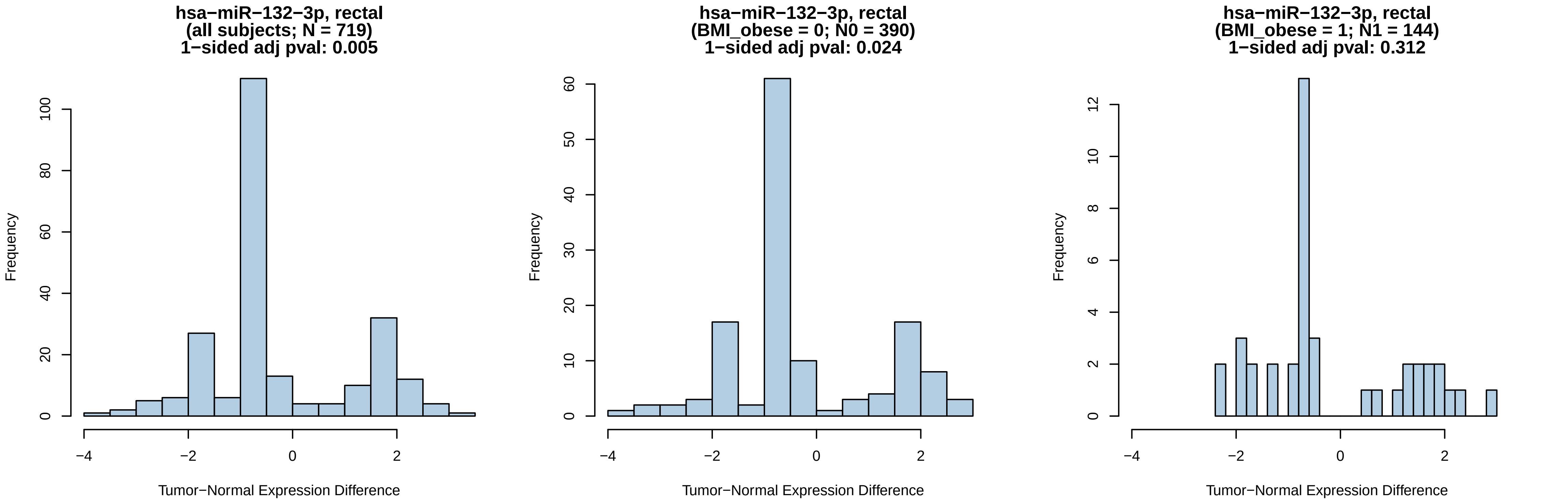
hsa−miR−132−3p, rectal
(all subjects; N = 719)
1−sided adj pval: 0.005
Frequency
Tumor−Normal Expression Difference
hsa−miR−132−3p, rectal
(BMI_obese = 0; N0 = 390)
1−sided adj pval: 0.024
Frequency
Tumor−Normal Expression Difference
hsa−miR−132−3p, rectal
(BMI_obese = 1; N1 = 144)
1−sided adj pval: 0.312
Frequency
Tumor−Normal Expression Difference

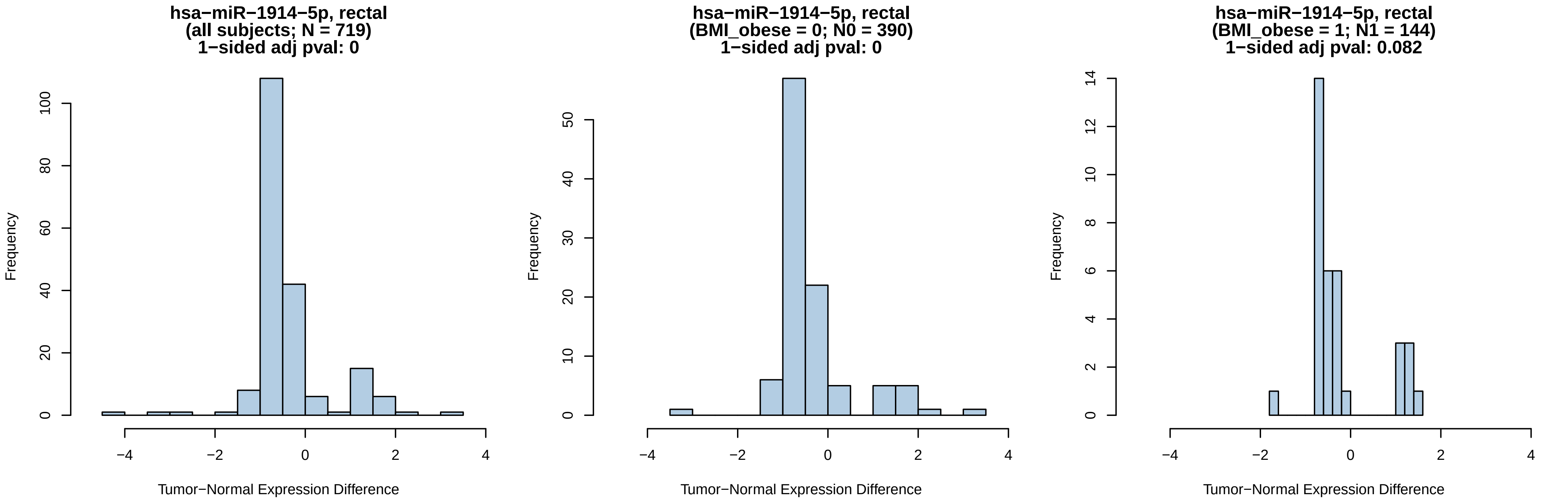

hsa−miR−1914−5p, rectal
(all subjects; N = 719)
1−sided adj pval: 0
Frequency
Tumor−Normal Expression Difference
hsa−miR−1914−5p, rectal
(BMI_obese = 0; N0 = 390)
1−sided adj pval: 0
Frequency
Tumor−Normal Expression Difference
hsa−miR−1914−5p, rectal
(BMI_obese = 1; N1 = 144)
1−sided adj pval: 0.082
Frequency
Tumor−Normal Expression Difference

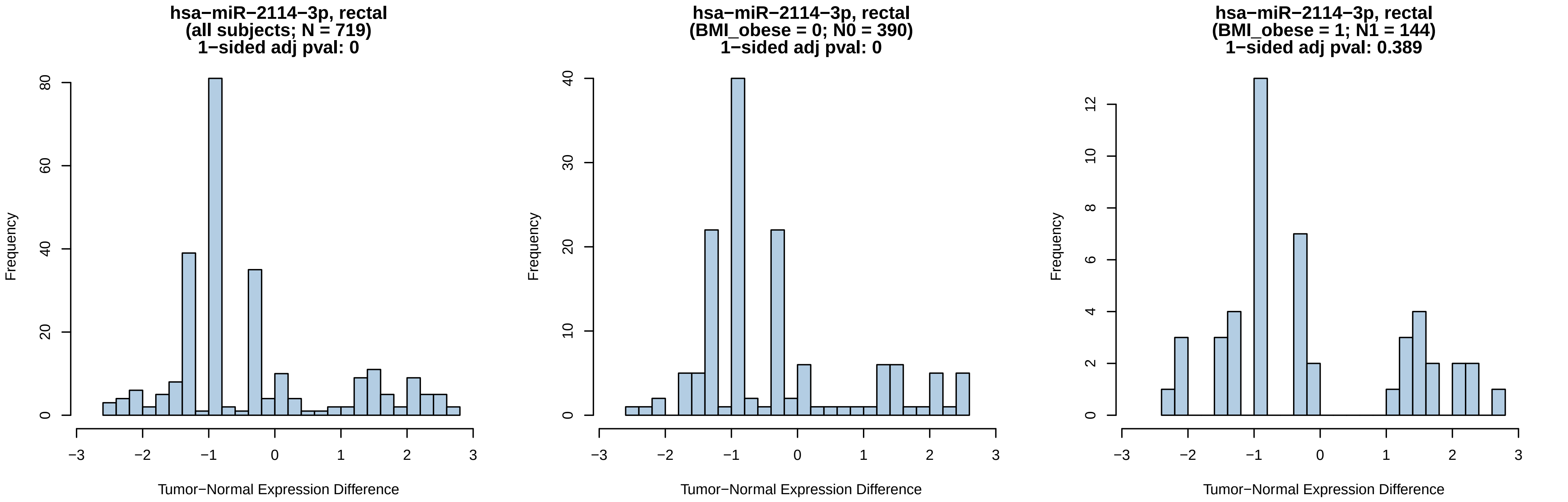

hsa−miR−2114−3p, rectal
(all subjects; N = 719)
1−sided adj pval: 0
Frequency
Tumor−Normal Expression Difference
hsa−miR−2114−3p, rectal
(BMI_obese = 0; N0 = 390)
1−sided adj pval: 0
Frequency
Tumor−Normal Expression Difference
hsa−miR−2114−3p, rectal
(BMI_obese = 1; N1 = 144)
1−sided adj pval: 0.389
Frequency
Tumor−Normal Expression Difference

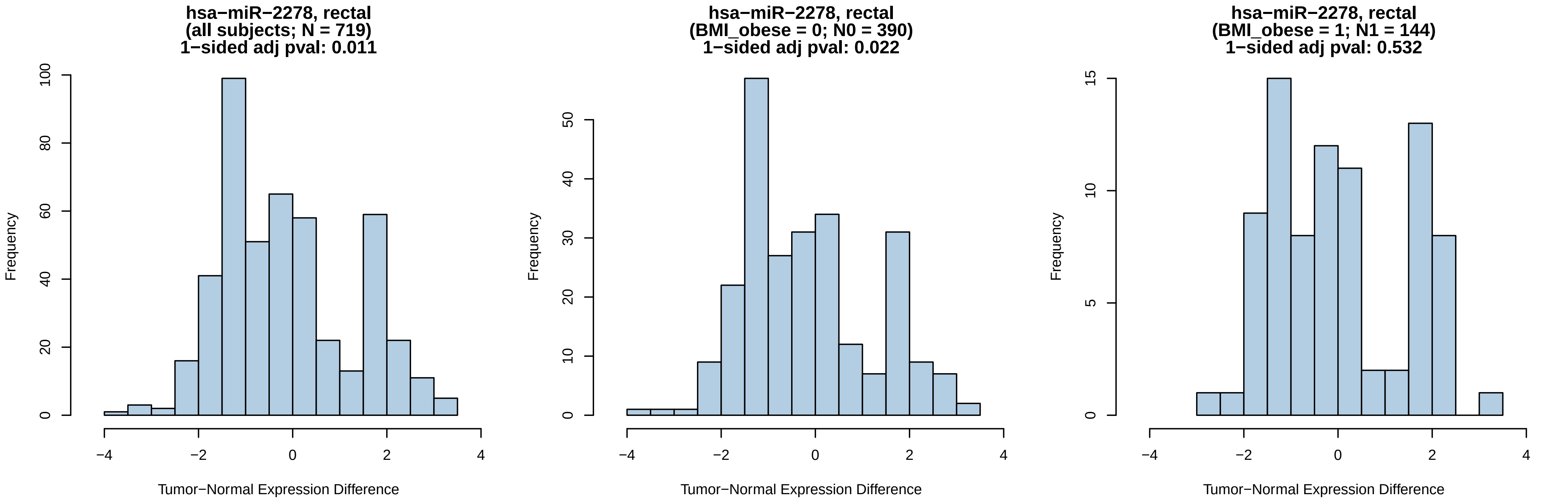
hsa−miR−2278, rectal
(all subjects; N = 719)
1−sided adj pval: 0.011
hsa−miR−2278, rectal
(BMI_obese = 0; N0 = 390)
1−sided adj pval: 0.022
hsa−miR−2278, rectal
(BMI_obese = 1; N1 = 144)
1−sided adj pval: 0.532
Frequency
Tumor−Normal Expression Difference

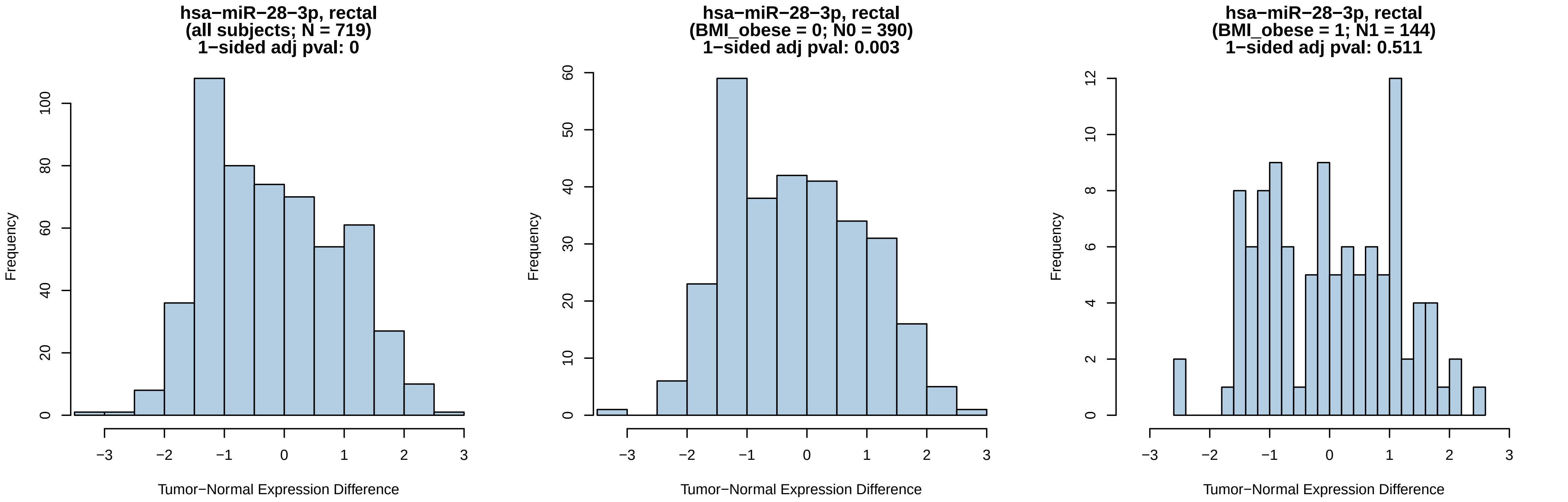

hsa−miR−28−3p, rectal
(all subjects; N = 719)
1−sided adj pval: 0
hsa−miR−28−3p, rectal
(BMI_obese = 0; N0 = 390)
1−sided adj pval: 0.003
hsa−miR−28−3p, rectal
(BMI_obese = 1; N1 = 144)
1−sided adj pval: 0.511
Frequency
Tumor−Normal Expression Difference

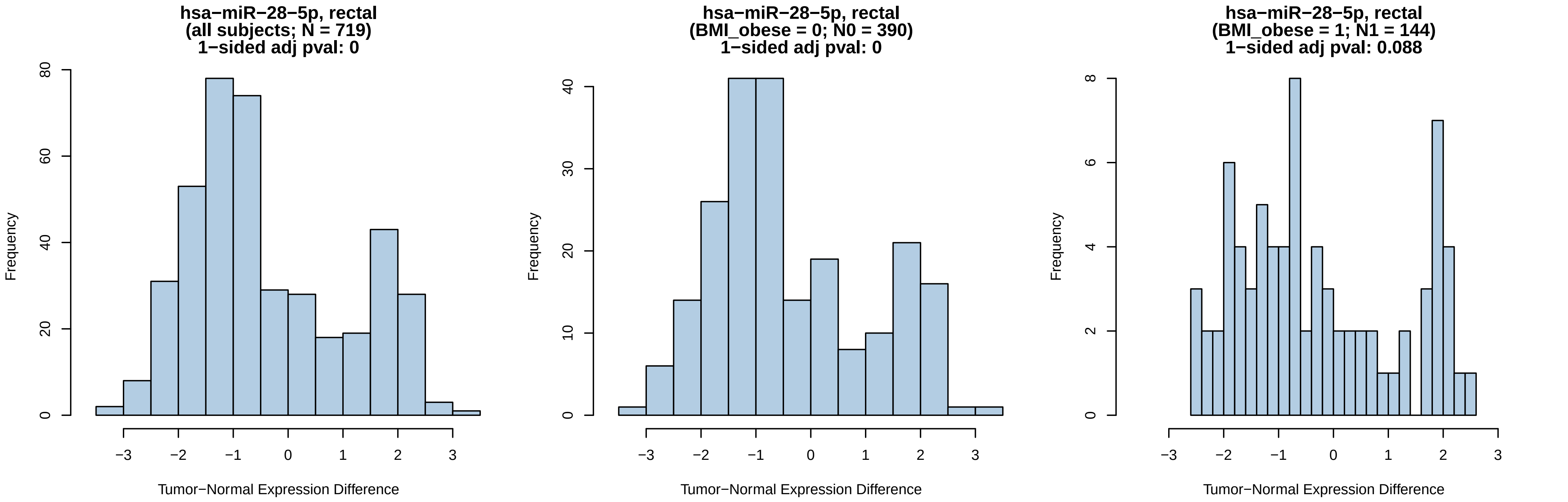

hsa−miR−28−5p, rectal
(all subjects; N = 719)
1−sided adj pval: 0
hsa−miR−28−5p, rectal
(BMI_obese = 0; N0 = 390)
1−sided adj pval: 0
hsa−miR−28−5p, rectal
(BMI_obese = 1; N1 = 144)
1−sided adj pval: 0.088
Frequency
Tumor−Normal Expression Difference

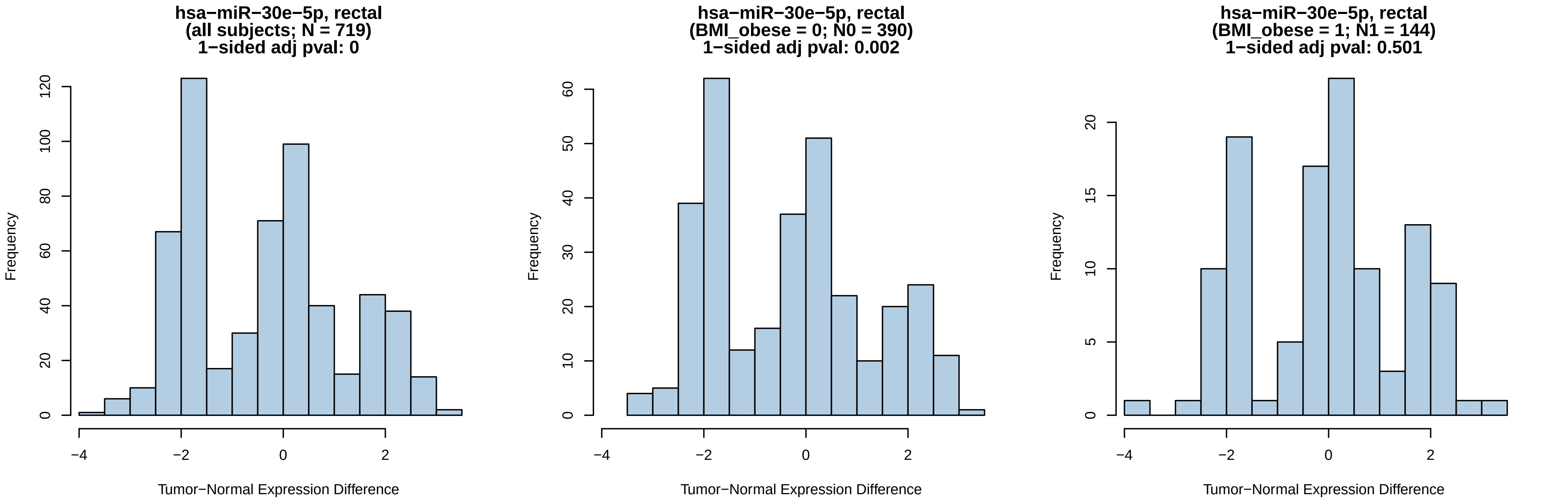
hsa−miR−30e−5p, rectal
(all subjects; N = 719)
1−sided adj pval: 0
Frequency
0
20
40
60
80
100
120
−4
−2
0
2
Tumor−Normal Expression Difference
hsa−miR−30e−5p, rectal
(BMI_obese = 0; N0 = 390)
1−sided adj pval: 0.002
Frequency
0
10
20
30
40
50
60
−4
−2
0
2
Tumor−Normal Expression Difference
hsa−miR−30e−5p, rectal
(BMI_obese = 1; N1 = 144)
1−sided adj pval: 0.501
Frequency
0
5
10
15
20
−4
−2
0
2
Tumor−Normal Expression Difference

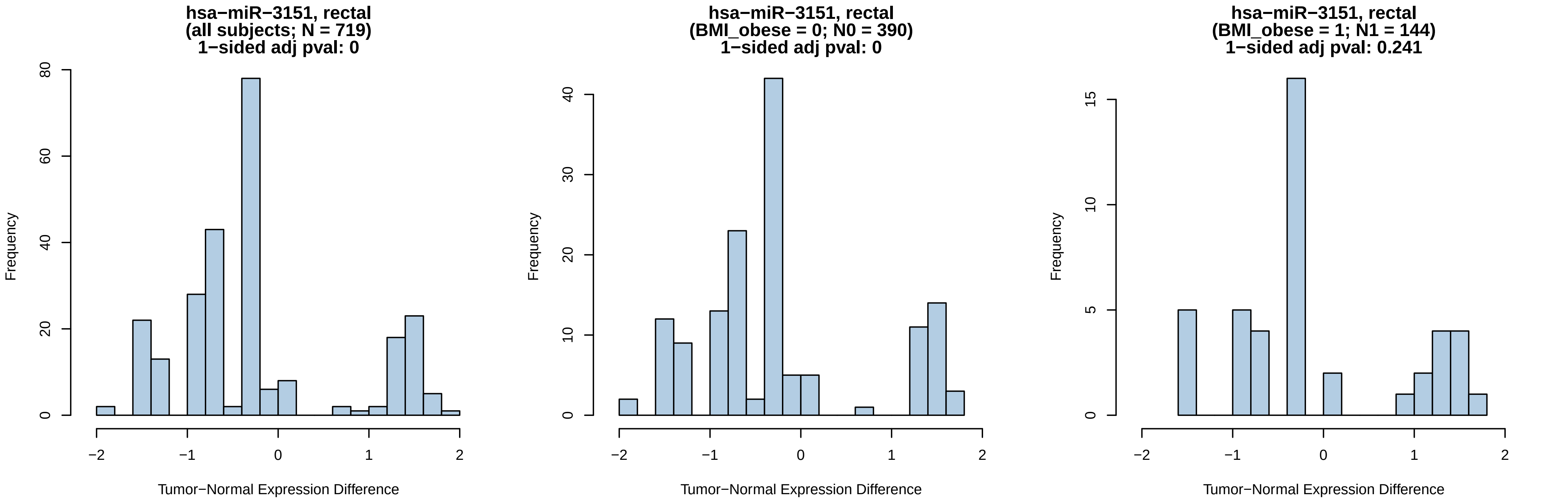

hsa−miR−3151, rectal
(all subjects; N = 719)
1−sided adj pval: 0
Frequency
0
20
40
60
80
−2
−1
0
1
2
Tumor−Normal Expression Difference
hsa−miR−3151, rectal
(BMI_obese = 0; N0 = 390)
1−sided adj pval: 0
Frequency
0
10
20
30
40
−2
−1
0
1
2
Tumor−Normal Expression Difference
hsa−miR−3151, rectal
(BMI_obese = 1; N1 = 144)
1−sided adj pval: 0.241
Frequency
0
5
10
15
−2
−1
0
1
2
Tumor−Normal Expression Difference

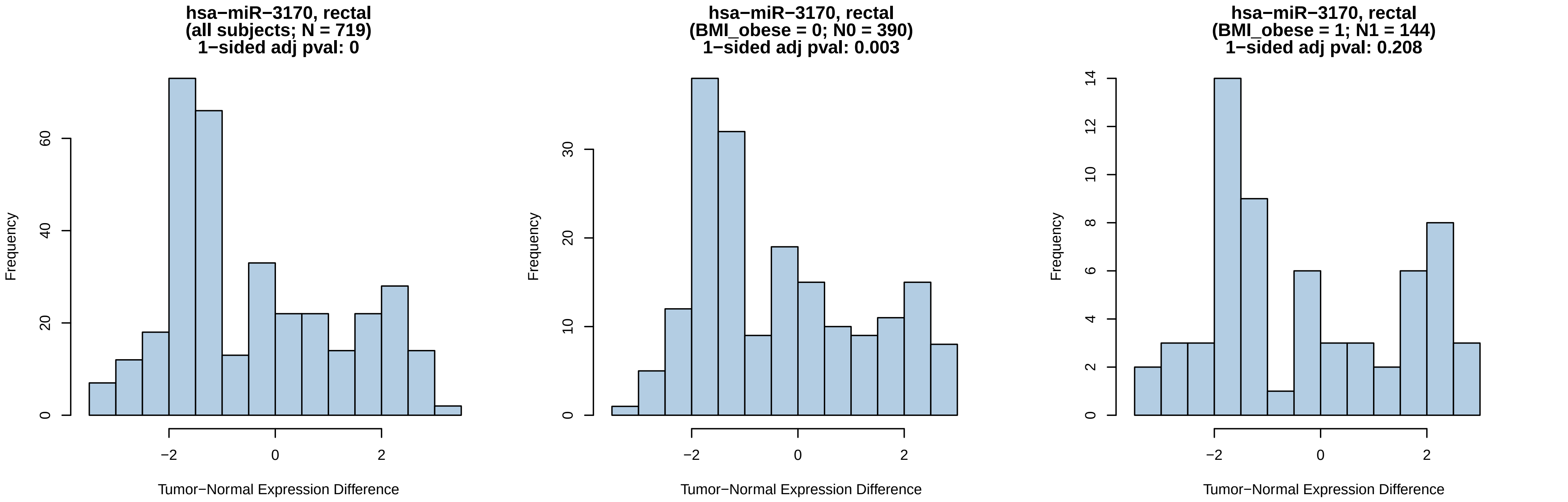

hsa−miR−3170, rectal
(all subjects; N = 719)
1−sided adj pval: 0
Frequency
0
20
40
60
−2
0
2
Tumor−Normal Expression Difference
hsa−miR−3170, rectal
(BMI_obese = 0; N0 = 390)
1−sided adj pval: 0.003
Frequency
0
10
20
30
−2
0
2
Tumor−Normal Expression Difference
hsa−miR−3170, rectal
(BMI_obese = 1; N1 = 144)
1−sided adj pval: 0.208
Frequency
0
2
4
6
8
10
12
14
−2
0
2
Tumor−Normal Expression Difference

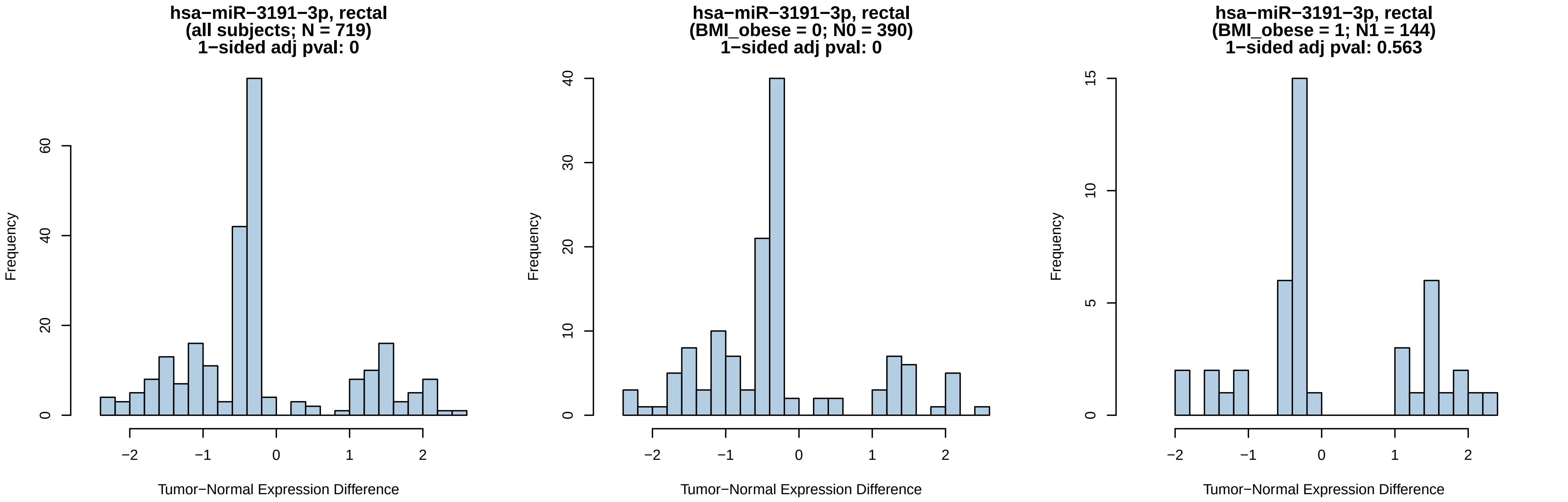

hsa−miR−3191−3p, rectal
(all subjects; N = 719)
1−sided adj pval: 0
Frequency
0
20
40
60
−2
−1
0
1
2
Tumor−Normal Expression Difference
hsa−miR−3191−3p, rectal
(BMI_obese = 0; N0 = 390)
1−sided adj pval: 0
Frequency
0
10
20
30
40
−2
−1
0
1
2
Tumor−Normal Expression Difference
hsa−miR−3191−3p, rectal
(BMI_obese = 1; N1 = 144)
1−sided adj pval: 0.563
Frequency
0
5
10
15
−2
−1
0
1
2
Tumor−Normal Expression Difference

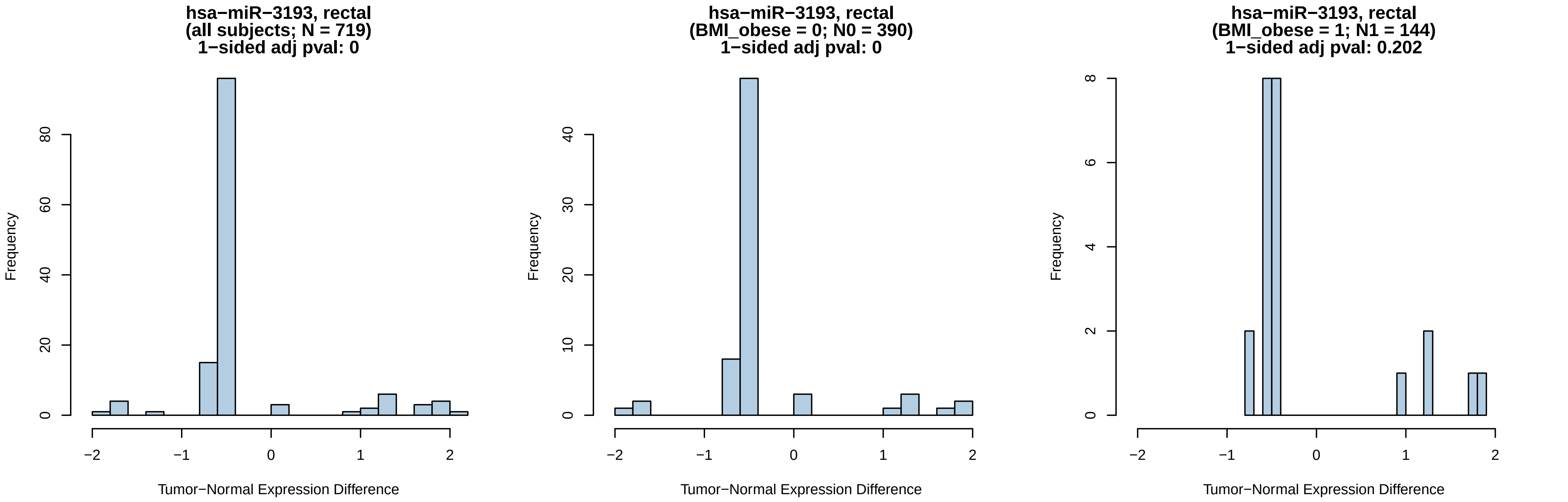
hsa−miR−3193, rectal
(all subjects; N = 719)
1−sided adj pval: 0
Frequency
Tumor−Normal Expression Difference
0
20
40
60
80
−2
−1
0
1
2
hsa−miR−3193, rectal
(BMI_obese = 0; N0 = 390)
1−sided adj pval: 0
Frequency
Tumor−Normal Expression Difference
0
10
20
30
40
−2
−1
0
1
2
hsa−miR−3193, rectal
(BMI_obese = 1; N1 = 144)
1−sided adj pval: 0.202
Frequency
Tumor−Normal Expression Difference
0
2
4
6
8
−2
−1
0
1
2

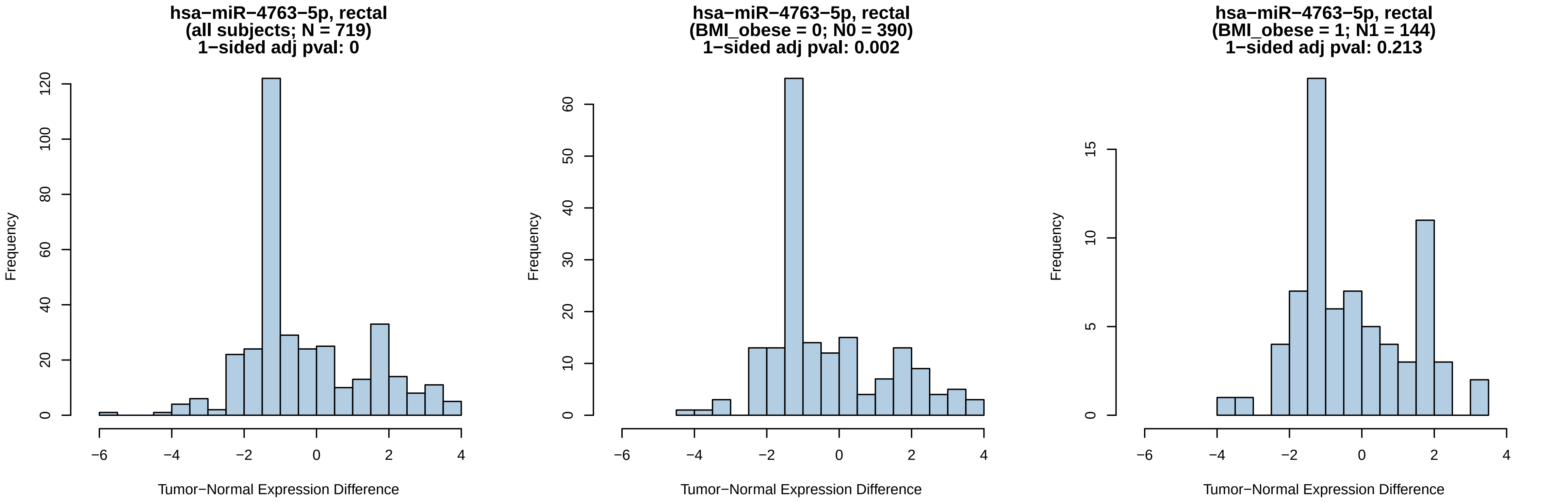
hsa−miR−4763−5p, rectal
(all subjects; N = 719)
1−sided adj pval: 0
Frequency
Tumor−Normal Expression Difference
hsa−miR−4763−5p, rectal
(BMI_obese = 0; N0 = 390)
1−sided adj pval: 0.002
Frequency
Tumor−Normal Expression Difference
hsa−miR−4763−5p, rectal
(BMI_obese = 1; N1 = 144)
1−sided adj pval: 0.213
Frequency
Tumor−Normal Expression Difference

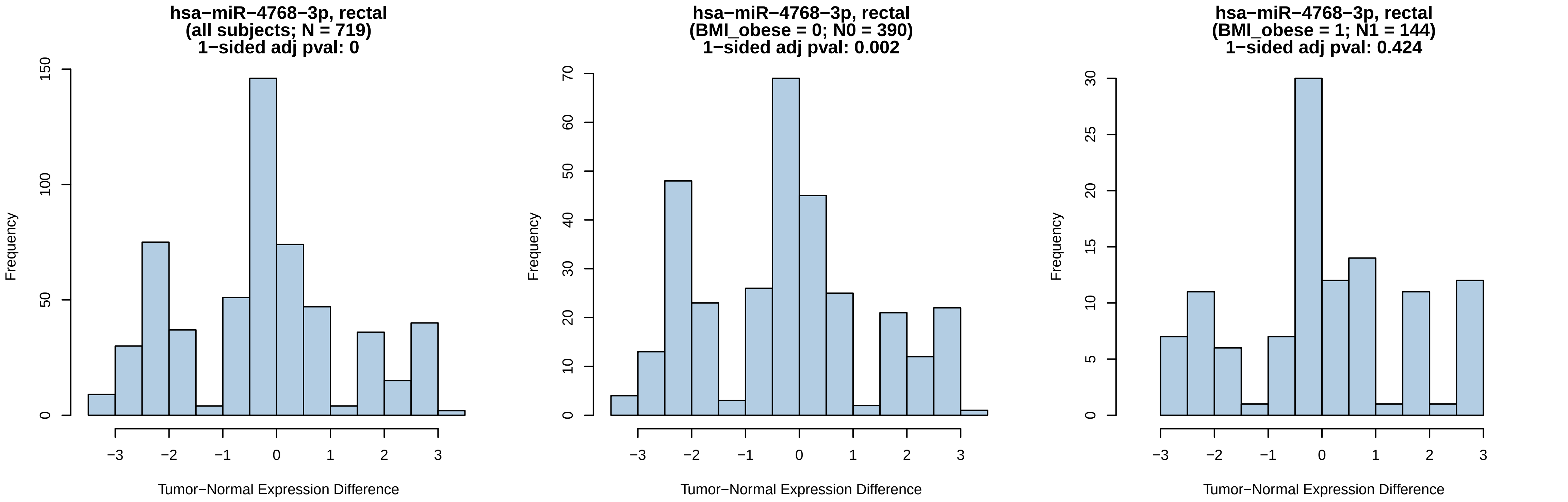

hsa−miR−4768−3p, rectal
(all subjects; N = 719)
1−sided adj pval: 0
Frequency
Tumor−Normal Expression Difference
hsa−miR−4768−3p, rectal
(BMI_obese = 0; N0 = 390)
1−sided adj pval: 0.002
Frequency
Tumor−Normal Expression Difference
hsa−miR−4768−3p, rectal
(BMI_obese = 1; N1 = 144)
1−sided adj pval: 0.424
Frequency
Tumor−Normal Expression Difference

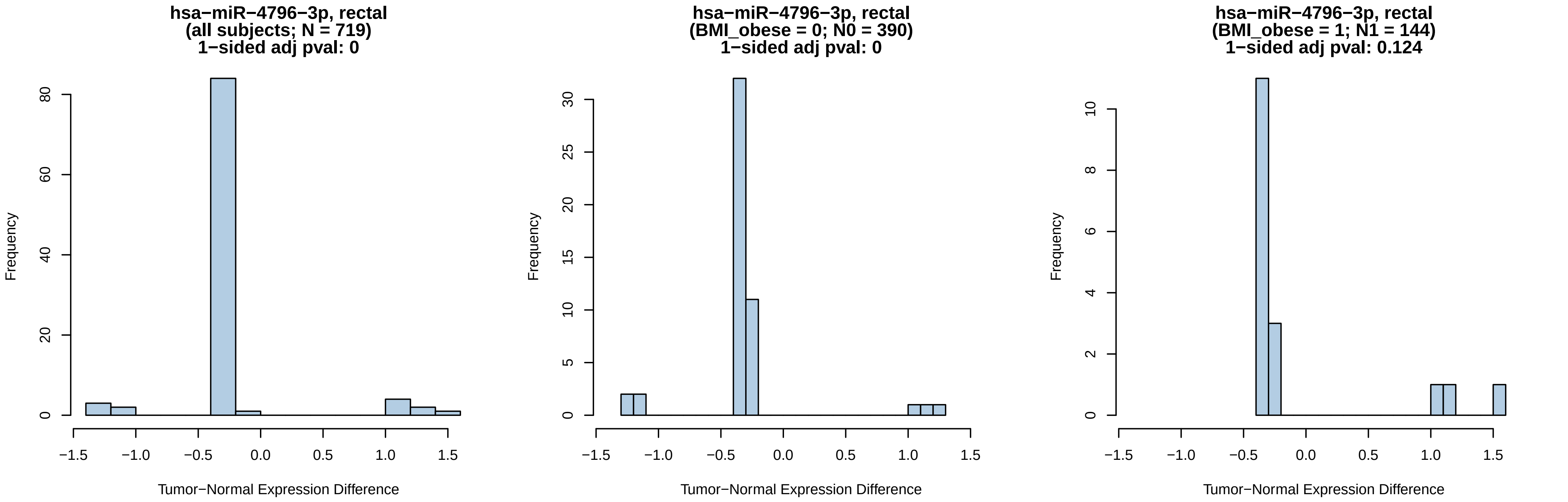

hsa−miR−4796−3p, rectal
(all subjects; N = 719)
1−sided adj pval: 0
Frequency
0
20
40
60
80
−1.5
−1.0
−0.5
0.0
0.5
1.0
1.5
Tumor−Normal Expression Difference
hsa−miR−4796−3p, rectal
(BMI_obese = 0; N0 = 390)
1−sided adj pval: 0
Frequency
0
5
10
15
20
25
30
−1.5
−1.0
−0.5
0.0
0.5
1.0
1.5
Tumor−Normal Expression Difference
hsa−miR−4796−3p, rectal
(BMI_obese = 1; N1 = 144)
1−sided adj pval: 0.124
Frequency
0
2
4
6
8
10
−1.5
−1.0
−0.5
0.0
0.5
1.0
1.5
Tumor−Normal Expression Difference

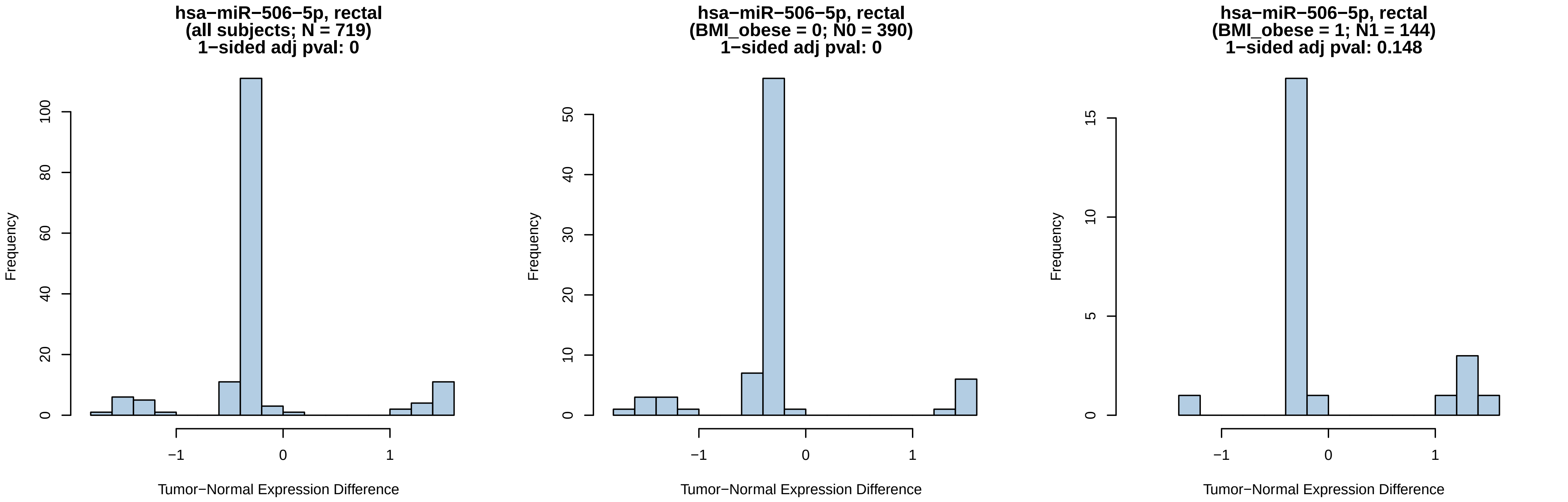

hsa−miR−506−5p, rectal
(all subjects; N = 719)
1−sided adj pval: 0
Frequency
0
20
40
60
80
100
−1
0
1
Tumor−Normal Expression Difference
hsa−miR−506−5p, rectal
(BMI_obese = 0; N0 = 390)
1−sided adj pval: 0
Frequency
0
10
20
30
40
50
−1
0
1
Tumor−Normal Expression Difference
hsa−miR−506−5p, rectal
(BMI_obese = 1; N1 = 144)
1−sided adj pval: 0.148
Frequency
0
5
10
15
−1
0
1
Tumor−Normal Expression Difference

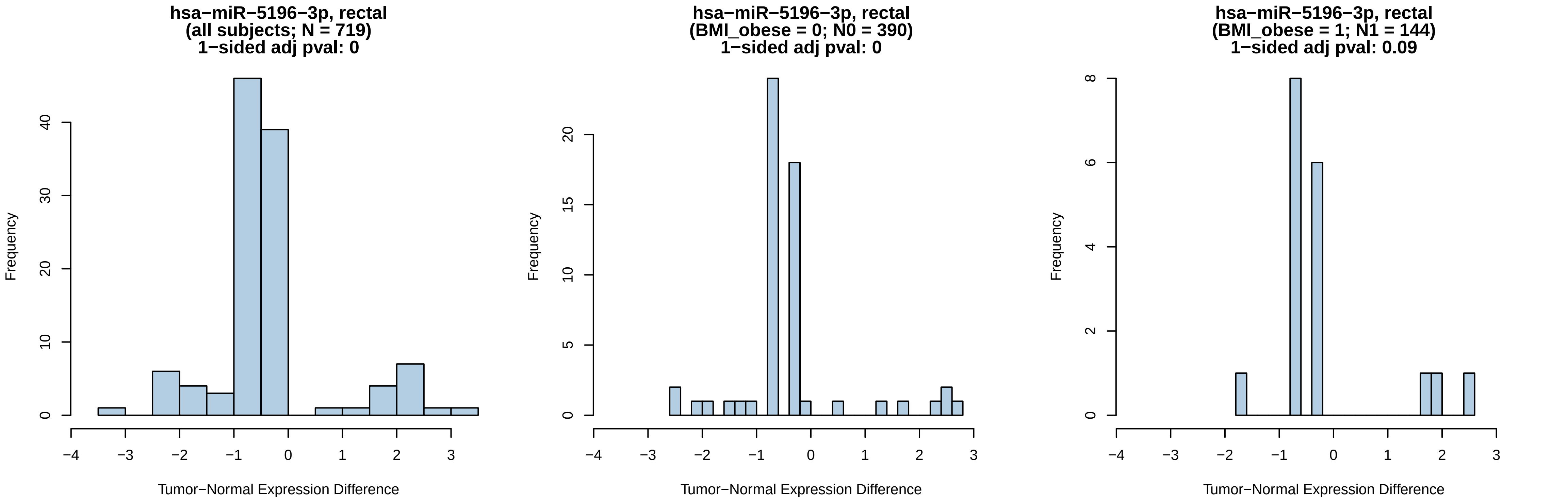

hsa−miR−5196−3p, rectal
(all subjects; N = 719)
1−sided adj pval: 0
Frequency
0
10
20
30
40
−4
−3
−2
−1
0
1
2
3
Tumor−Normal Expression Difference
hsa−miR−5196−3p, rectal
(BMI_obese = 0; N0 = 390)
1−sided adj pval: 0
Frequency
0
5
10
15
20
−4
−3
−2
−1
0
1
2
3
Tumor−Normal Expression Difference
hsa−miR−5196−3p, rectal
(BMI_obese = 1; N1 = 144)
1−sided adj pval: 0.09
Frequency
0
2
4
6
8
−4
−3
−2
−1
0
1
2
3
Tumor−Normal Expression Difference

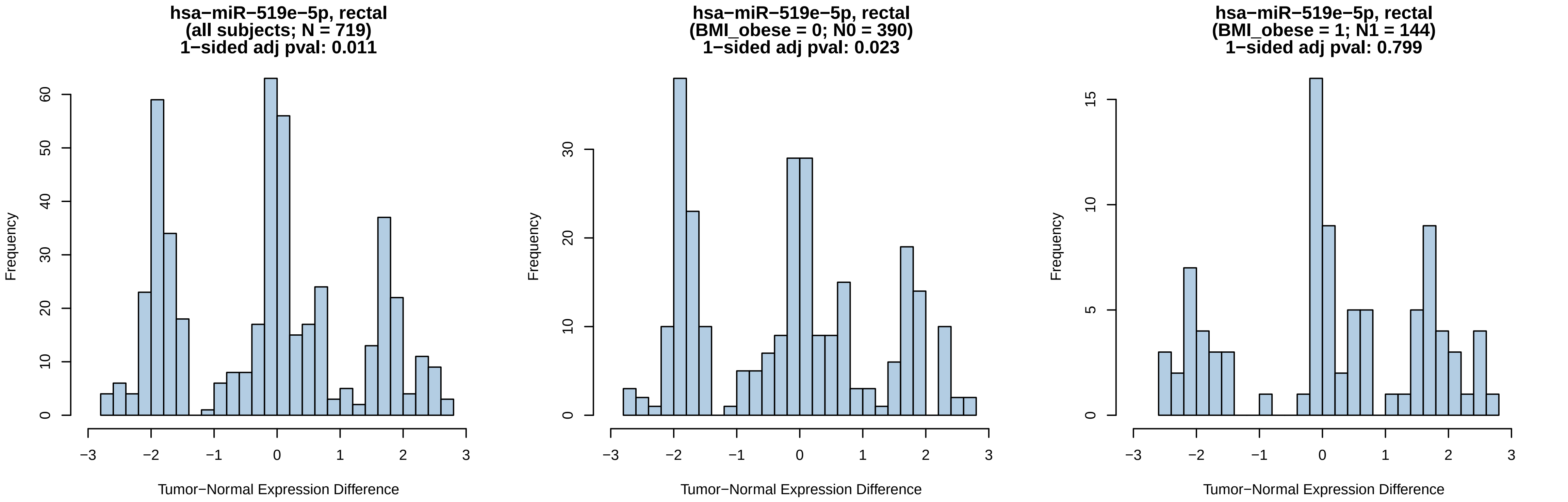

hsa−miR−519e−5p, rectal
(all subjects; N = 719)
1−sided adj pval: 0.011
Frequency
Tumor−Normal Expression Difference
hsa−miR−519e−5p, rectal
(BMI_obese = 0; N0 = 390)
1−sided adj pval: 0.023
Frequency
Tumor−Normal Expression Difference
hsa−miR−519e−5p, rectal
(BMI_obese = 1; N1 = 144)
1−sided adj pval: 0.799
Frequency
Tumor−Normal Expression Difference

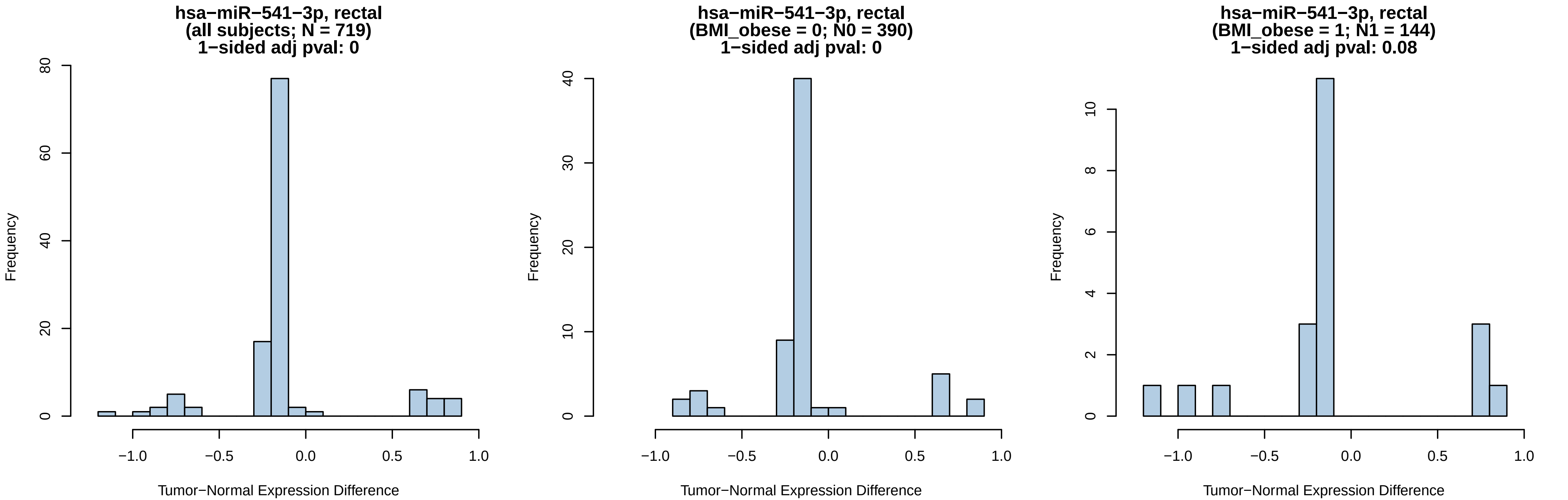
hsa−miR−541−3p, rectal
(all subjects; N = 719)
1−sided adj pval: 0
Frequency
Tumor−Normal Expression Difference
hsa−miR−541−3p, rectal
(BMI_obese = 0; N0 = 390)
1−sided adj pval: 0
Frequency
Tumor−Normal Expression Difference
hsa−miR−541−3p, rectal
(BMI_obese = 1; N1 = 144)
1−sided adj pval: 0.08
Frequency
Tumor−Normal Expression Difference

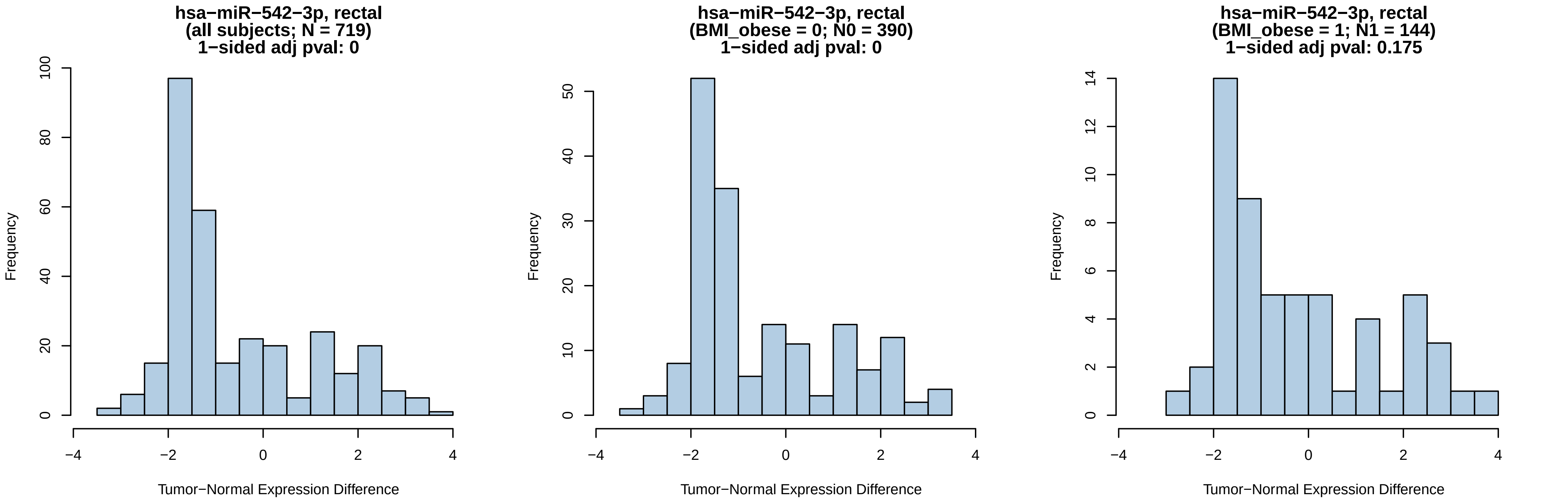

hsa−miR−542−3p, rectal
(all subjects; N = 719)
1−sided adj pval: 0
Frequency
Tumor−Normal Expression Difference
hsa−miR−542−3p, rectal
(BMI_obese = 0; N0 = 390)
1−sided adj pval: 0
Frequency
Tumor−Normal Expression Difference
hsa−miR−542−3p, rectal
(BMI_obese = 1; N1 = 144)
1−sided adj pval: 0.175
Frequency
Tumor−Normal Expression Difference

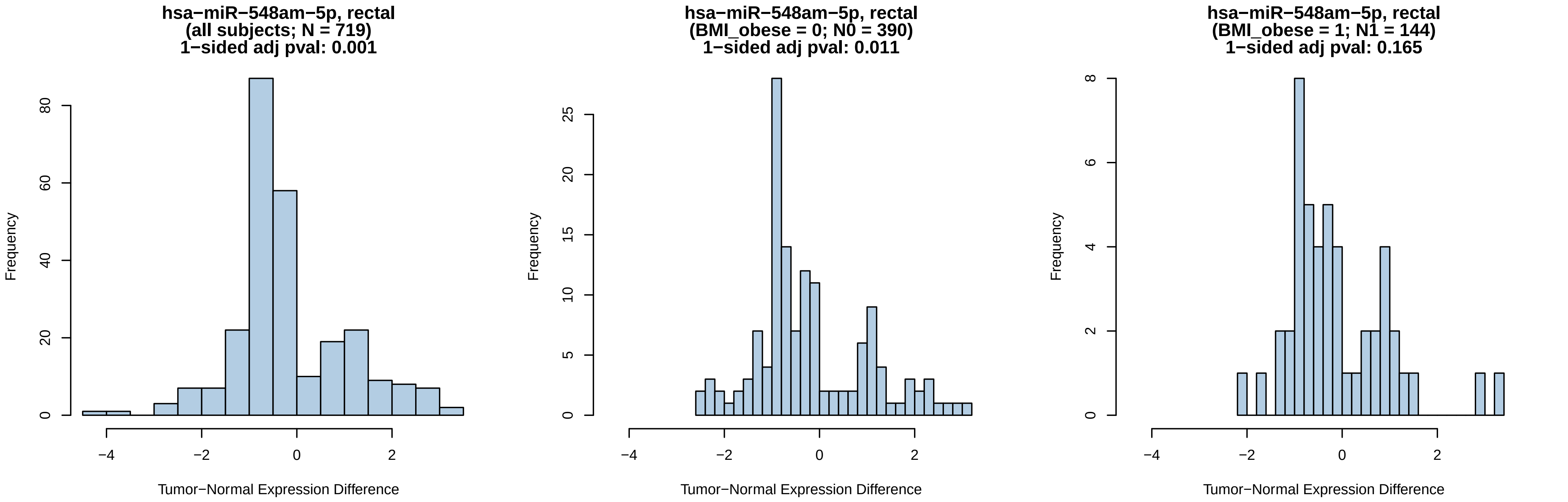

hsa−miR−548am−5p, rectal
(all subjects; N = 719)
1−sided adj pval: 0.001
Frequency
0
20
40
60
80
−4
−2
0
2
Tumor−Normal Expression Difference
hsa−miR−548am−5p, rectal
(BMI_obese = 0; N0 = 390)
1−sided adj pval: 0.011
Frequency
0
5
10
15
20
25
−4
−2
0
2
Tumor−Normal Expression Difference
hsa−miR−548am−5p, rectal
(BMI_obese = 1; N1 = 144)
1−sided adj pval: 0.165
Frequency
0
2
4
6
8
−4
−2
0
2
Tumor−Normal Expression Difference

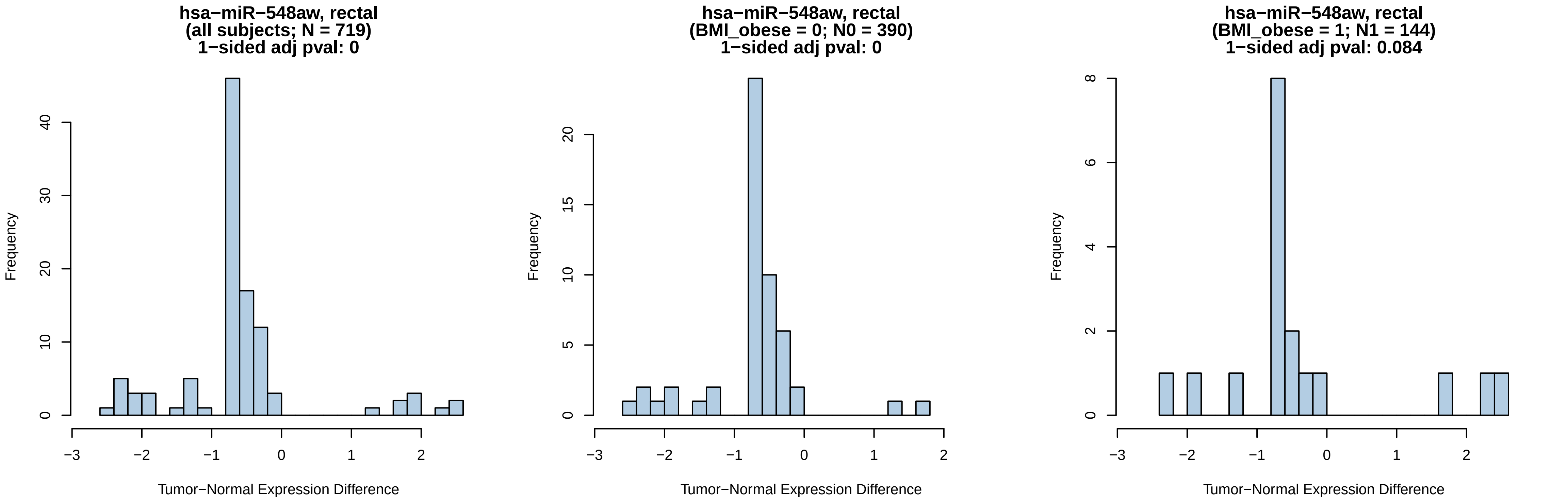
hsa−miR−548aw, rectal
(all subjects; N = 719)
1−sided adj pval: 0
Frequency
Tumor−Normal Expression Difference
hsa−miR−548aw, rectal
(BMI_obese = 0; N0 = 390)
1−sided adj pval: 0
Frequency
Tumor−Normal Expression Difference
hsa−miR−548aw, rectal
(BMI_obese = 1; N1 = 144)
1−sided adj pval: 0.084
Frequency
Tumor−Normal Expression Difference

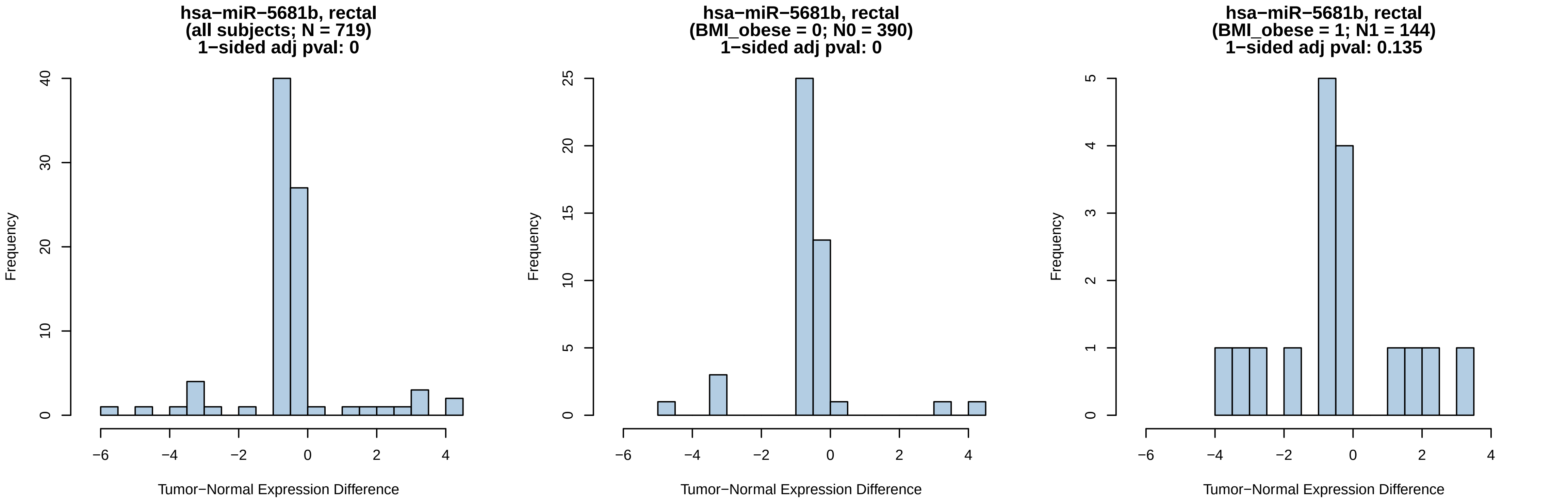

hsa−miR−5681b, rectal
(all subjects; N = 719)
1−sided adj pval: 0
Frequency
0
10
20
30
40
−6
−4
−2
0
2
4
Tumor−Normal Expression Difference
hsa−miR−5681b, rectal
(BMI_obese = 0; N0 = 390)
1−sided adj pval: 0
Frequency
0
5
10
15
20
25
−6
−4
−2
0
2
4
Tumor−Normal Expression Difference
hsa−miR−5681b, rectal
(BMI_obese = 1; N1 = 144)
1−sided adj pval: 0.135
Frequency
0
1
2
3
4
5
−6
−4
−2
0
2
4
Tumor−Normal Expression Difference

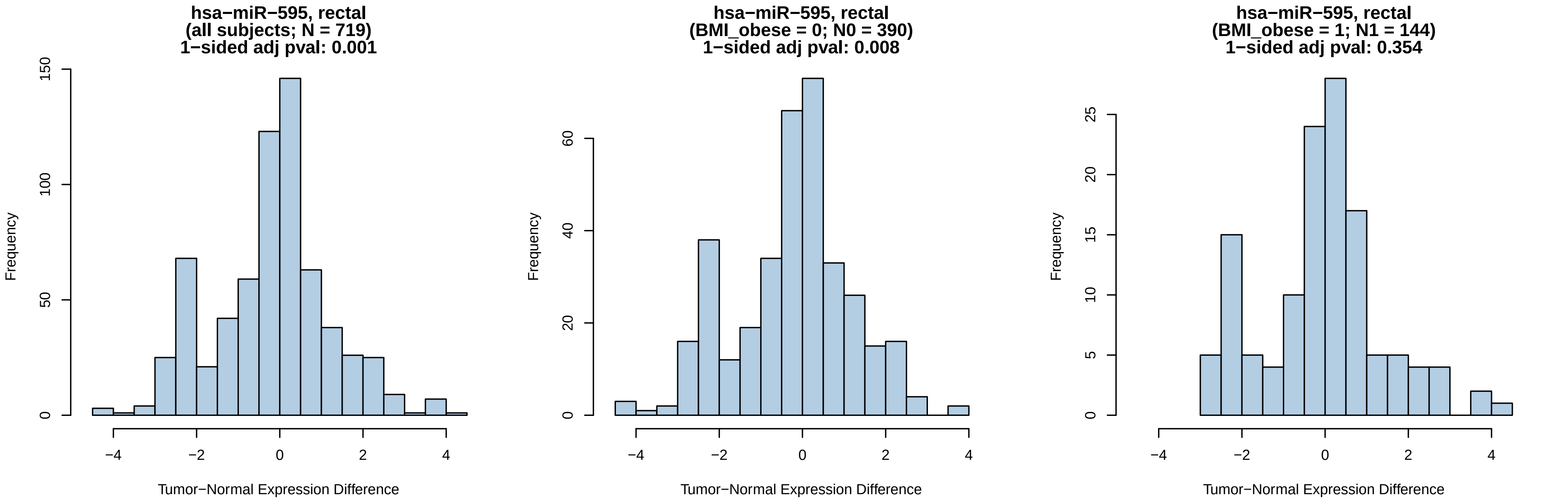
hsa−miR−595, rectal
(all subjects; N = 719)
1−sided adj pval: 0.001
Frequency
Tumor−Normal Expression Difference
hsa−miR−595, rectal
(BMI_obese = 0; N0 = 390)
1−sided adj pval: 0.008
Frequency
Tumor−Normal Expression Difference
hsa−miR−595, rectal
(BMI_obese = 1; N1 = 144)
1−sided adj pval: 0.354
Frequency
Tumor−Normal Expression Difference

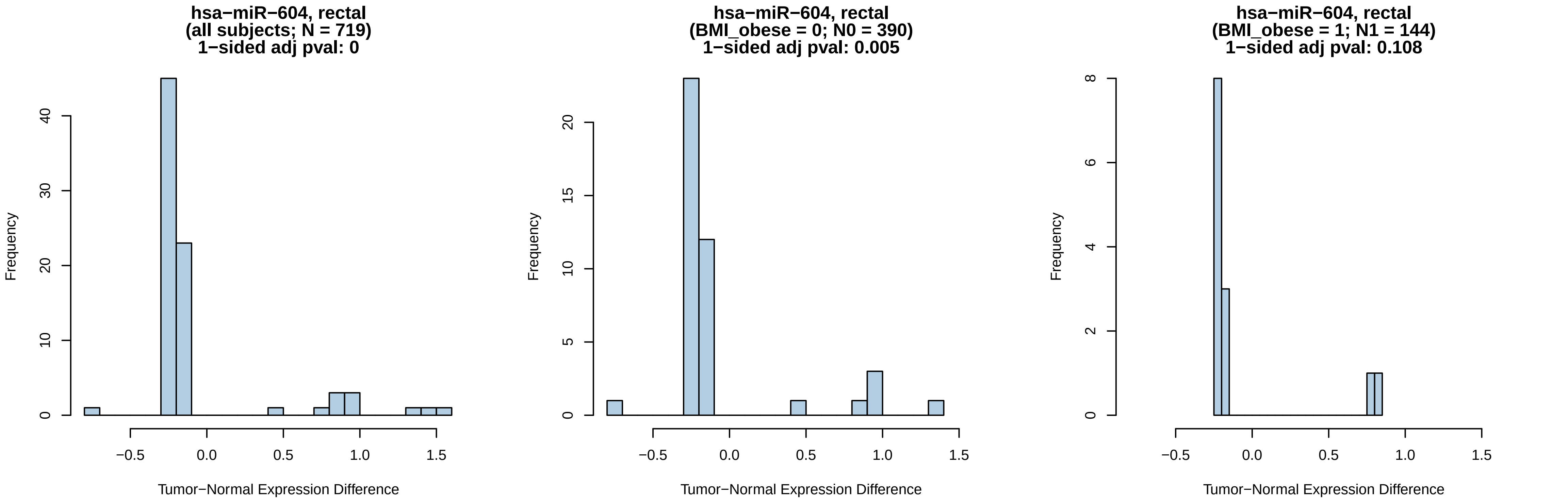

hsa−miR−604, rectal
(all subjects; N = 719)
1−sided adj pval: 0
Frequency
Tumor−Normal Expression Difference
hsa−miR−604, rectal
(BMI_obese = 0; N0 = 390)
1−sided adj pval: 0.005
Frequency
Tumor−Normal Expression Difference
hsa−miR−604, rectal
(BMI_obese = 1; N1 = 144)
1−sided adj pval: 0.108
Frequency
Tumor−Normal Expression Difference

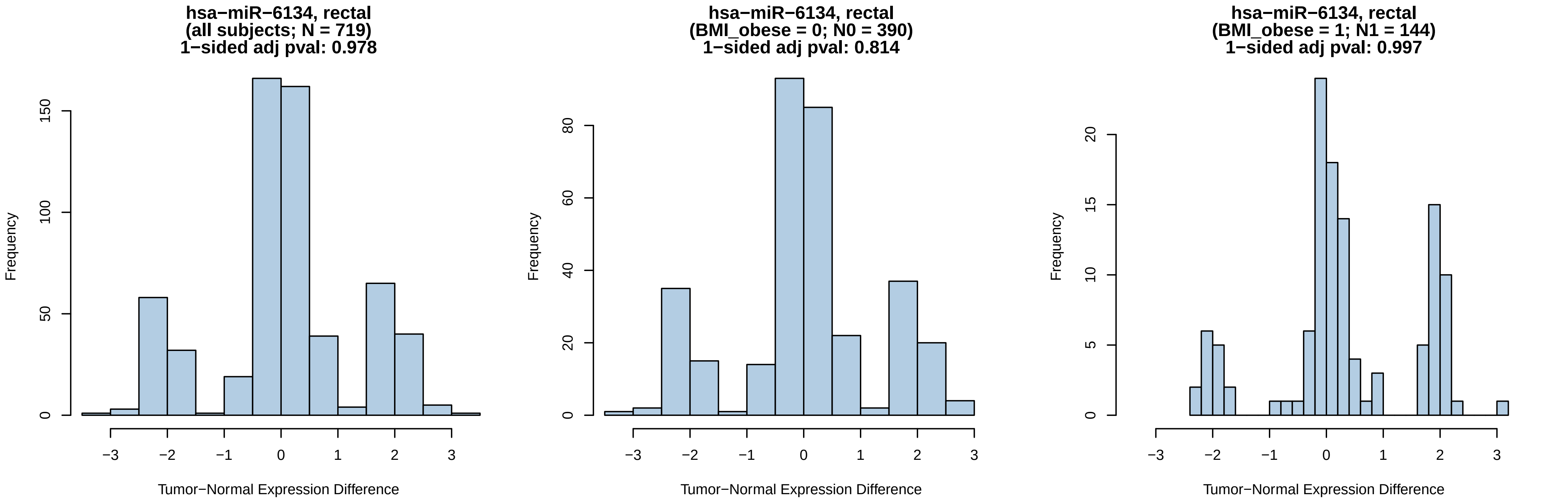

hsa−miR−6134, rectal
(all subjects; N = 719)
1−sided adj pval: 0.978
Frequency
Tumor−Normal Expression Difference
hsa−miR−6134, rectal
(BMI_obese = 0; N0 = 390)
1−sided adj pval: 0.814
Frequency
Tumor−Normal Expression Difference
hsa−miR−6134, rectal
(BMI_obese = 1; N1 = 144)
1−sided adj pval: 0.997
Frequency
Tumor−Normal Expression Difference

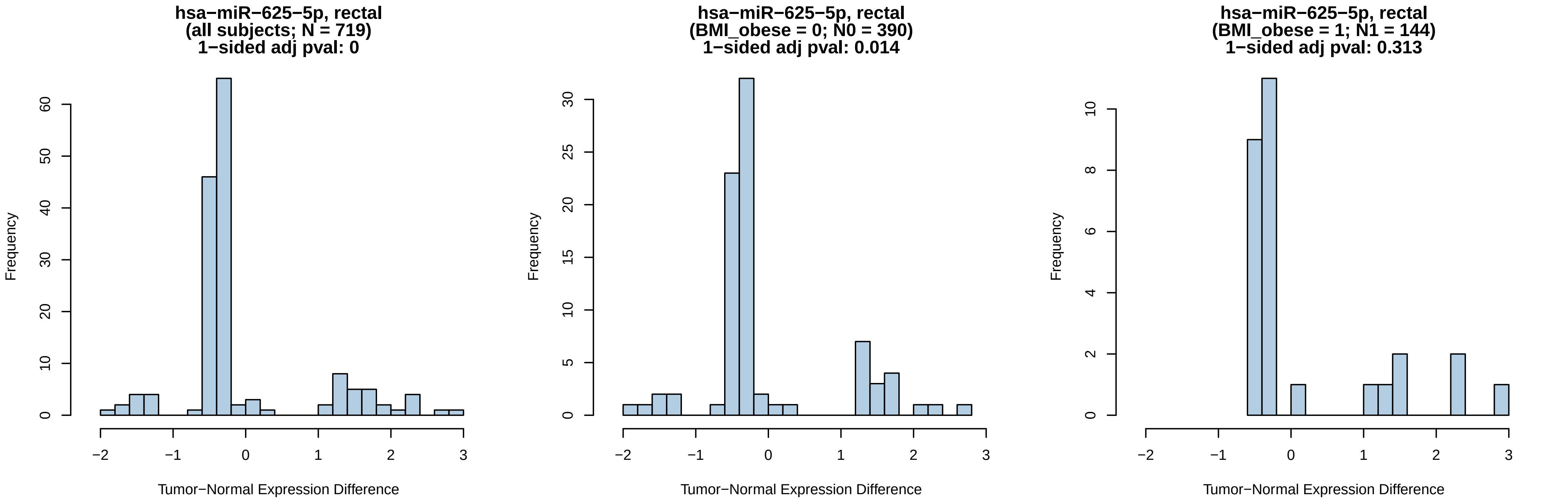
hsa−miR−625−5p, rectal
(all subjects; N = 719)
1−sided adj pval: 0
Frequency
0
10
20
30
40
50
60
−2
−1
0
1
2
3
Tumor−Normal Expression Difference
hsa−miR−625−5p, rectal
(BMI_obese = 0; N0 = 390)
1−sided adj pval: 0.014
Frequency
0
5
10
15
20
25
30
−2
−1
0
1
2
3
Tumor−Normal Expression Difference
hsa−miR−625−5p, rectal
(BMI_obese = 1; N1 = 144)
1−sided adj pval: 0.313
Frequency
0
2
4
6
8
10
−2
−1
0
1
2
3
Tumor−Normal Expression Difference

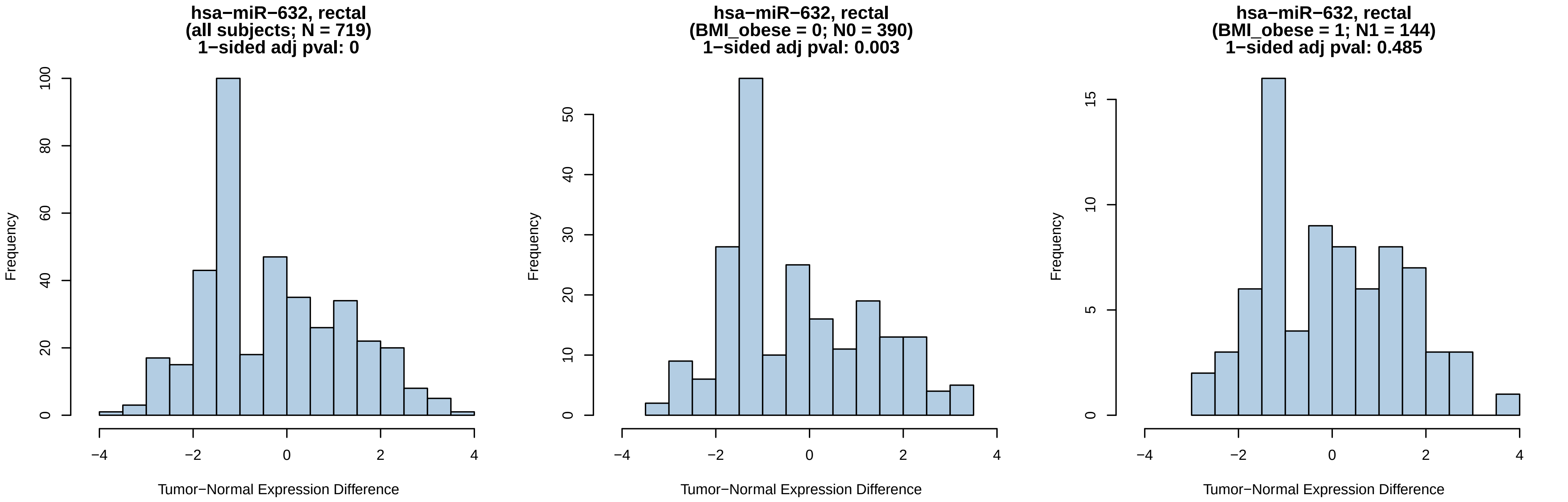
hsa−miR−632, rectal
(all subjects; N = 719)
1−sided adj pval: 0
hsa−miR−632, rectal
(BMI_obese = 0; N0 = 390)
1−sided adj pval: 0.003
hsa−miR−632, rectal
(BMI_obese = 1; N1 = 144)
1−sided adj pval: 0.485
Frequency
Tumor−Normal Expression Difference
0
20
40
60
80
100
10
30
50
5
15
−4
−2
2
4

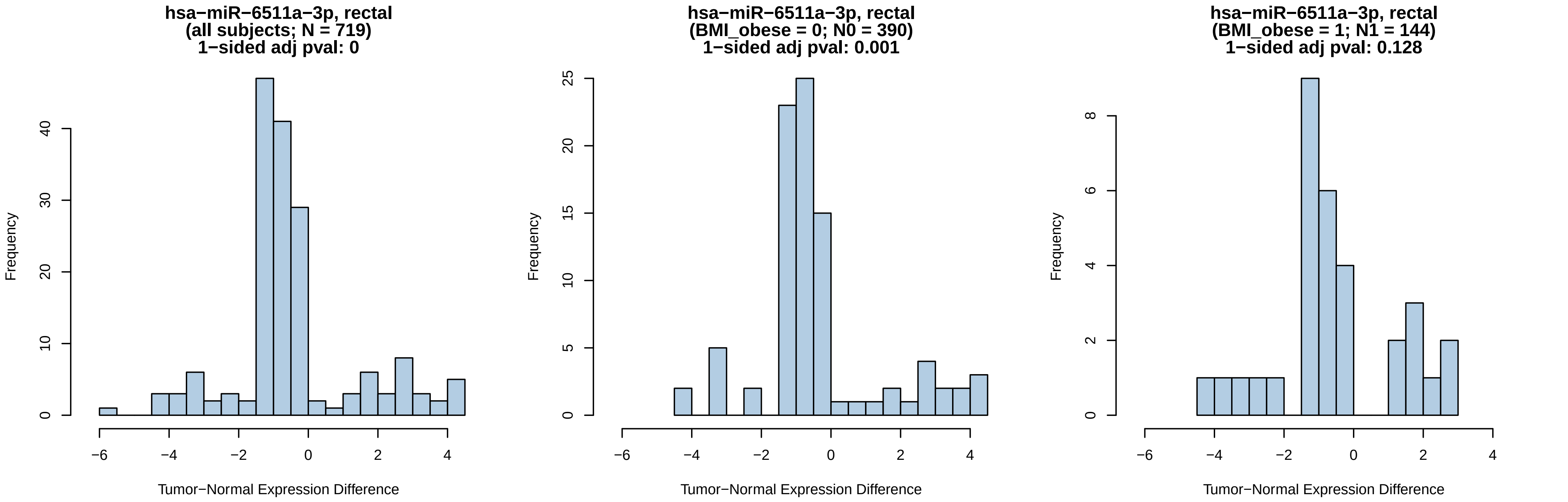
hsa−miR−6511a−3p, rectal
(all subjects; N = 719)
1−sided adj pval: 0
Frequency
Tumor−Normal Expression Difference
hsa−miR−6511a−3p, rectal
(BMI_obese = 0; N0 = 390)
1−sided adj pval: 0.001
Frequency
Tumor−Normal Expression Difference
hsa−miR−6511a−3p, rectal
(BMI_obese = 1; N1 = 144)
1−sided adj pval: 0.128
Frequency
Tumor−Normal Expression Difference

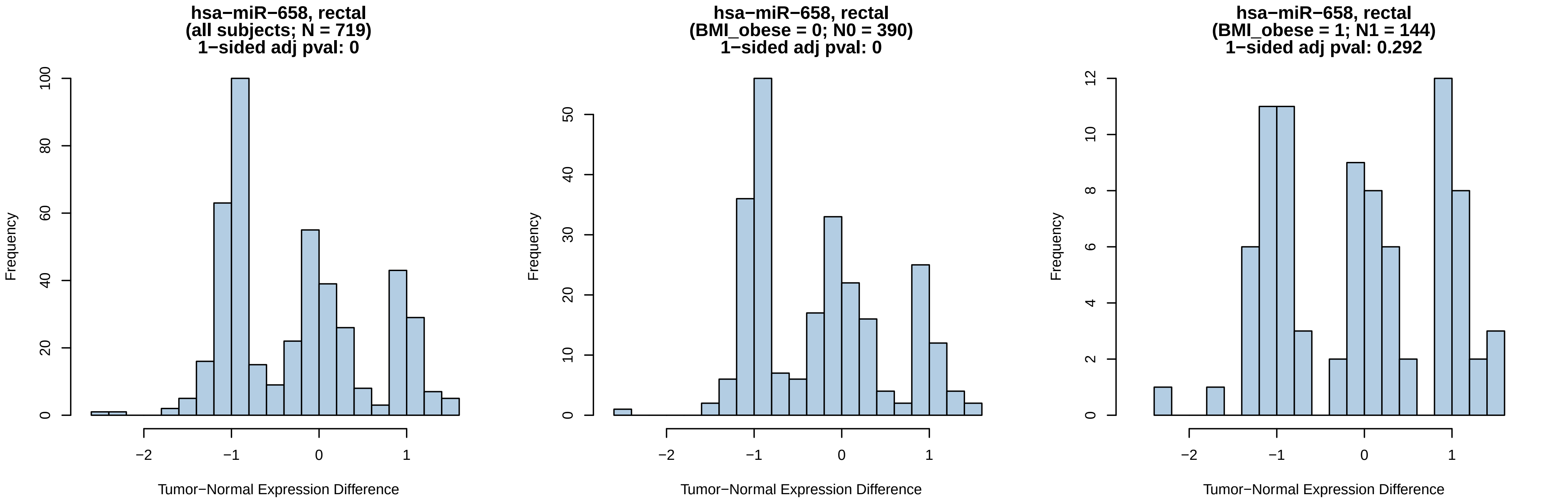
hsa−miR−658, rectal
(all subjects; N = 719)
1−sided adj pval: 0
Frequency
Tumor−Normal Expression Difference
hsa−miR−658, rectal
(BMI_obese = 0; N0 = 390)
1−sided adj pval: 0
Frequency
Tumor−Normal Expression Difference
hsa−miR−658, rectal
(BMI_obese = 1; N1 = 144)
1−sided adj pval: 0.292
Frequency
Tumor−Normal Expression Difference

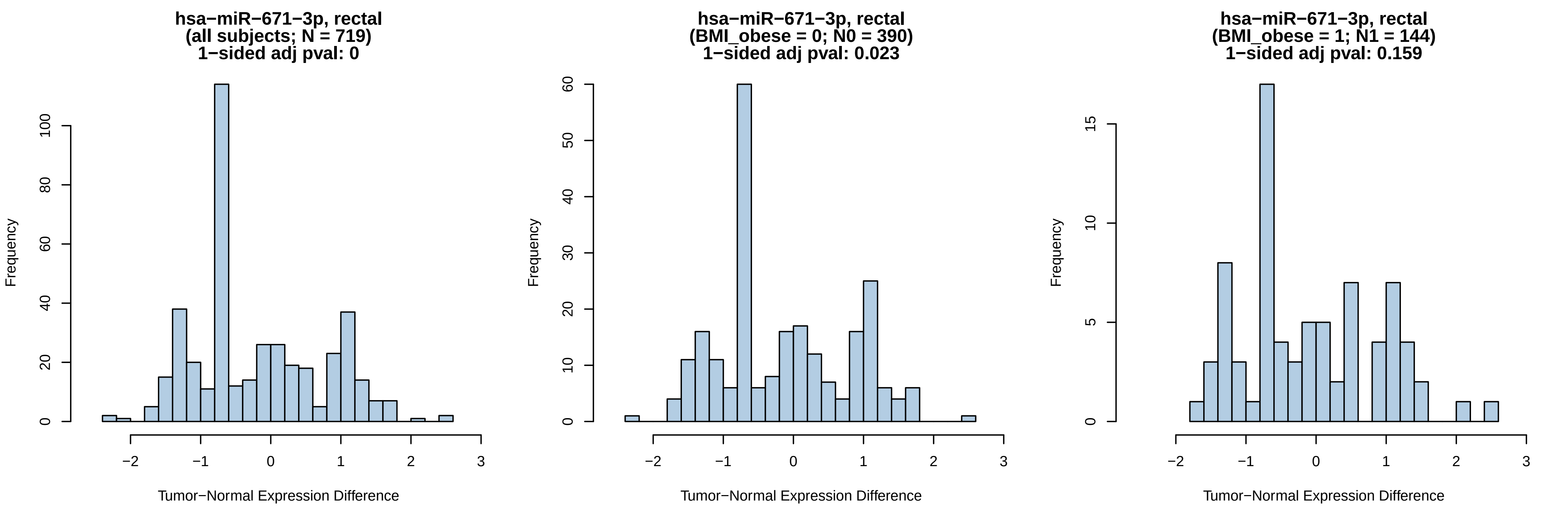

hsa−miR−671−3p, rectal
(all subjects; N = 719)
1−sided adj pval: 0
hsa−miR−671−3p, rectal
(BMI_obese = 0; N0 = 390)
1−sided adj pval: 0.023
hsa−miR−671−3p, rectal
(BMI_obese = 1; N1 = 144)
1−sided adj pval: 0.159
Frequency
Tumor−Normal Expression Difference

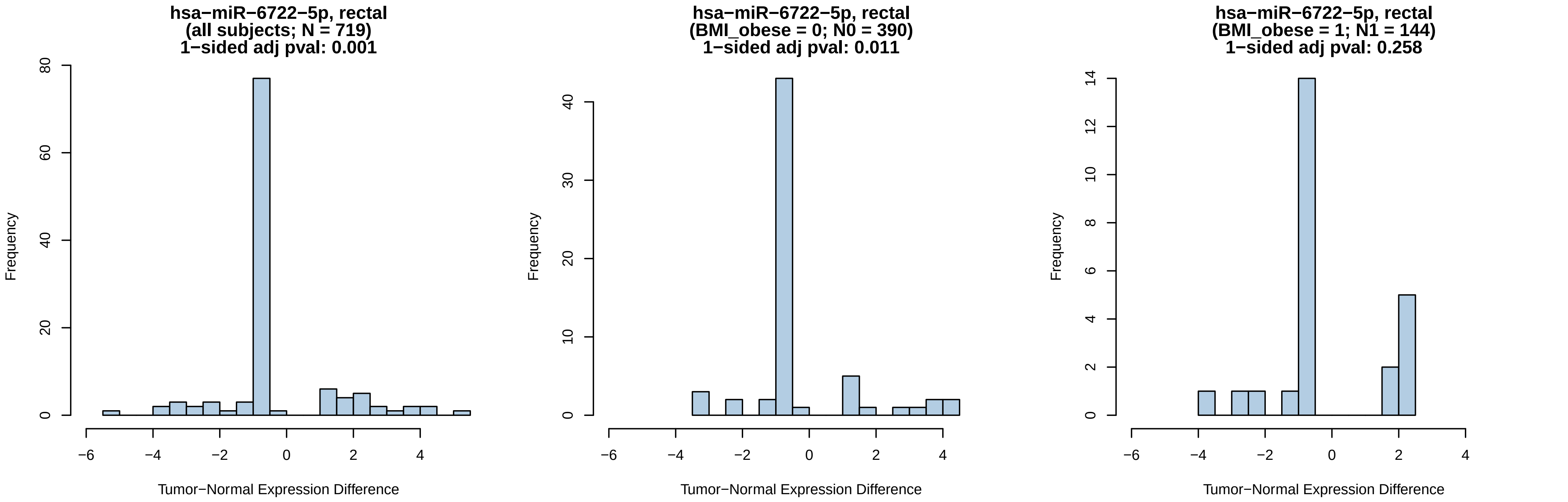

hsa−miR−6722−5p, rectal
(all subjects; N = 719)
1−sided adj pval: 0.001
Frequency
Tumor−Normal Expression Difference
hsa−miR−6722−5p, rectal
(BMI_obese = 0; N0 = 390)
1−sided adj pval: 0.011
Frequency
Tumor−Normal Expression Difference
hsa−miR−6722−5p, rectal
(BMI_obese = 1; N1 = 144)
1−sided adj pval: 0.258
Frequency
Tumor−Normal Expression Difference

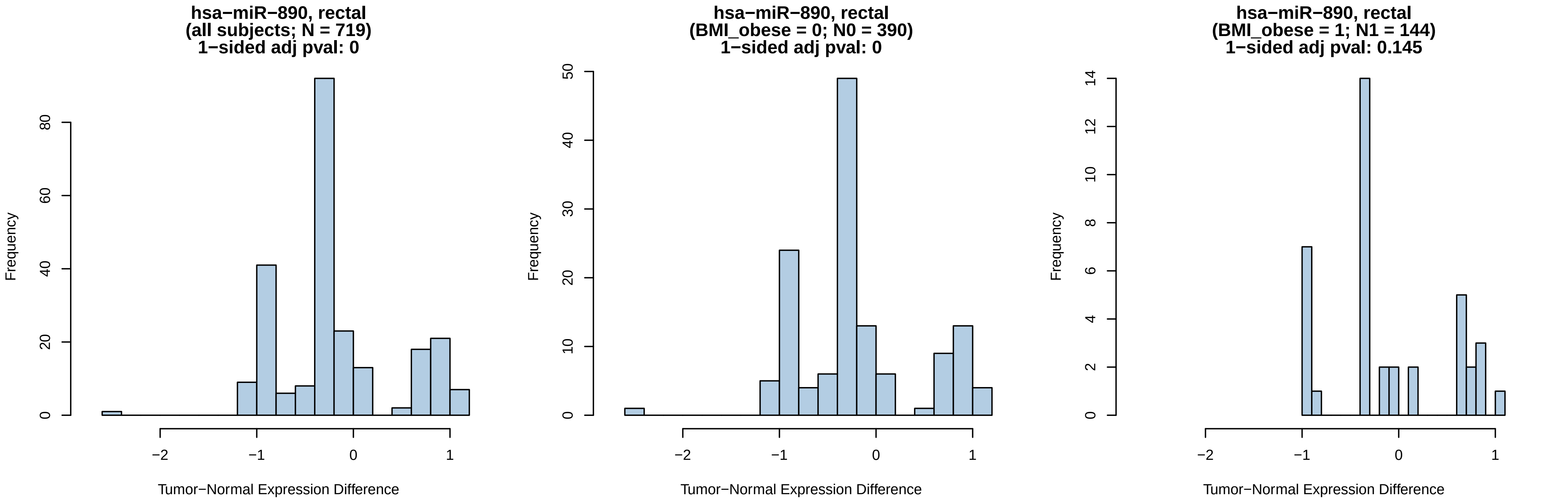

hsa−miR−890, rectal
(all subjects; N = 719)
1−sided adj pval: 0
Frequency
0
20
40
60
80
−2
−1
0
1
Tumor−Normal Expression Difference
hsa−miR−890, rectal
(BMI_obese = 0; N0 = 390)
1−sided adj pval: 0
Frequency
0
10
20
30
40
50
−2
−1
0
1
Tumor−Normal Expression Difference
hsa−miR−890, rectal
(BMI_obese = 1; N1 = 144)
1−sided adj pval: 0.145
Frequency
0
2
4
6
8
10
12
14
−2
−1
0
1
Tumor−Normal Expression Difference

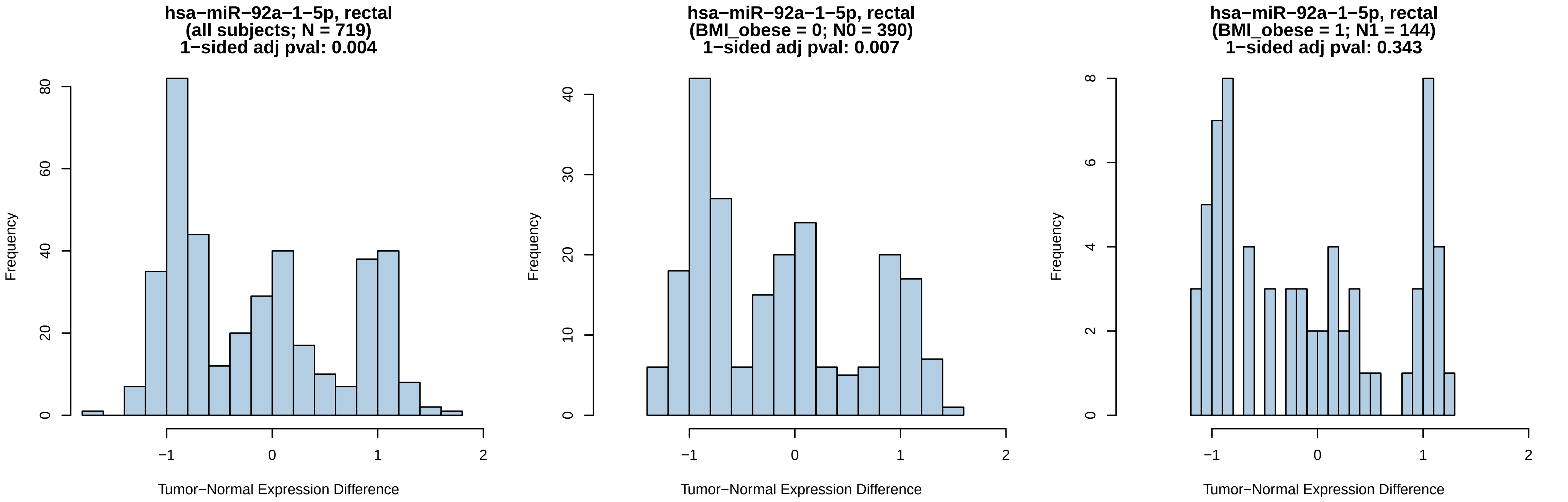

hsa−miR−92a−1−5p, rectal
(all subjects; N = 719)
1−sided adj pval: 0.004
hsa−miR−92a−1−5p, rectal
(BMI_obese = 0; N0 = 390)
1−sided adj pval: 0.007
hsa−miR−92a−1−5p, rectal
(BMI_obese = 1; N1 = 144)
1−sided adj pval: 0.343
Frequency
Tumor−Normal Expression Difference

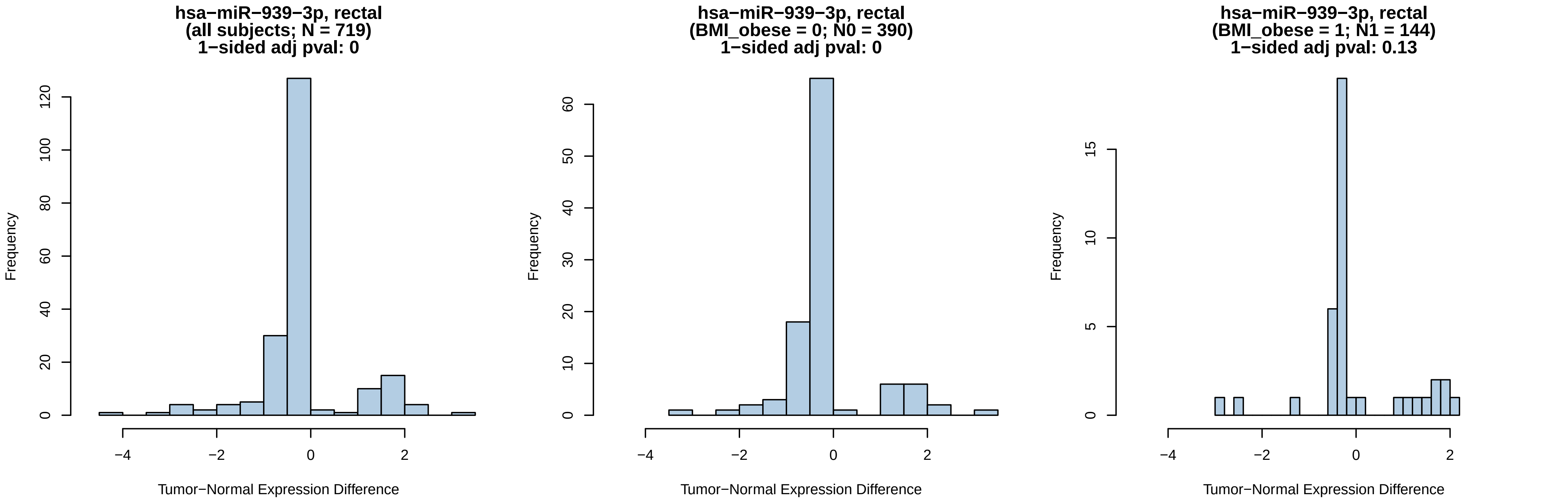
hsa−miR−939−3p, rectal
(all subjects; N = 719)
1−sided adj pval: 0
Frequency
0
20
40
60
80
100
120
−4
−2
0
2
Tumor−Normal Expression Difference
hsa−miR−939−3p, rectal
(BMI_obese = 0; N0 = 390)
1−sided adj pval: 0
Frequency
0
10
20
30
40
50
60
−4
−2
0
2
Tumor−Normal Expression Difference
hsa−miR−939−3p, rectal
(BMI_obese = 1; N1 = 144)
1−sided adj pval: 0.13
Frequency
0
5
10
15
−4
−2
0
2
Tumor−Normal Expression Difference

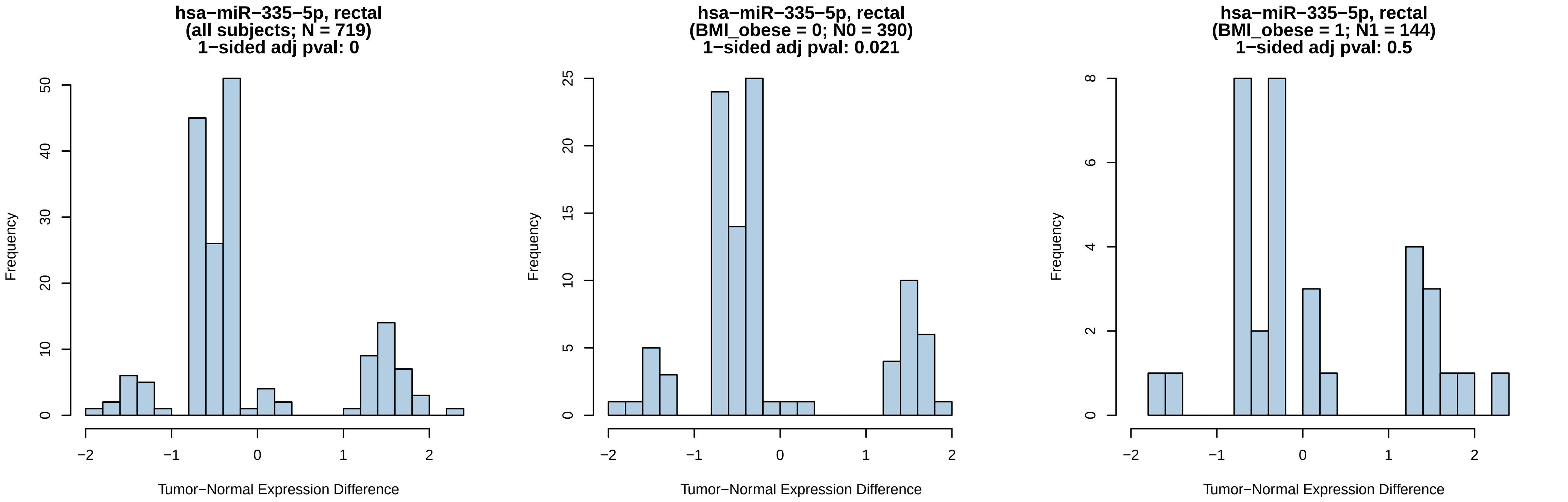
hsa−miR−335−5p, rectal
(all subjects; N = 719)
1−sided adj pval: 0
Frequency
Tumor−Normal Expression Difference
hsa−miR−335−5p, rectal
(BMI_obese = 0; N0 = 390)
1−sided adj pval: 0.021
Frequency
Tumor−Normal Expression Difference
hsa−miR−335−5p, rectal
(BMI_obese = 1; N1 = 144)
1−sided adj pval: 0.5
Frequency
Tumor−Normal Expression Difference

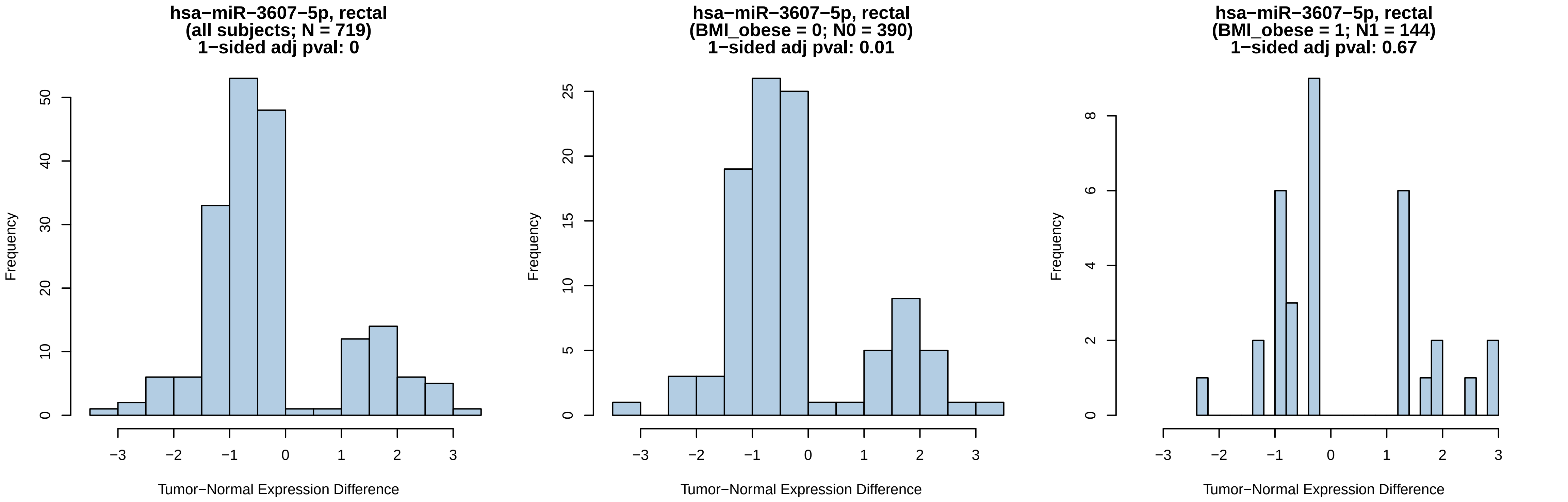

hsa−miR−3607−5p, rectal
(all subjects; N = 719)
1−sided adj pval: 0
Frequency
Tumor−Normal Expression Difference
hsa−miR−3607−5p, rectal
(BMI_obese = 0; N0 = 390)
1−sided adj pval: 0.01
Frequency
Tumor−Normal Expression Difference
hsa−miR−3607−5p, rectal
(BMI_obese = 1; N1 = 144)
1−sided adj pval: 0.67
Frequency
Tumor−Normal Expression Difference

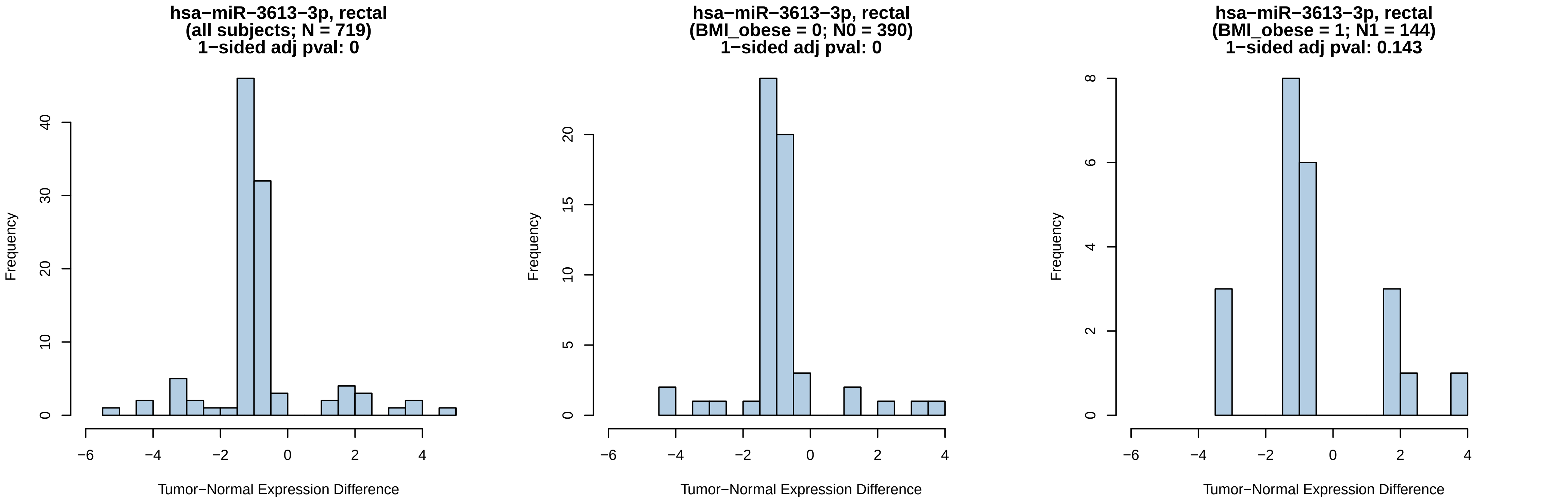

hsa−miR−3613−3p, rectal
(all subjects; N = 719)
1−sided adj pval: 0
Frequency
Tumor−Normal Expression Difference
hsa−miR−3613−3p, rectal
(BMI_obese = 0; N0 = 390)
1−sided adj pval: 0
Frequency
Tumor−Normal Expression Difference
hsa−miR−3613−3p, rectal
(BMI_obese = 1; N1 = 144)
1−sided adj pval: 0.143
Frequency
Tumor−Normal Expression Difference

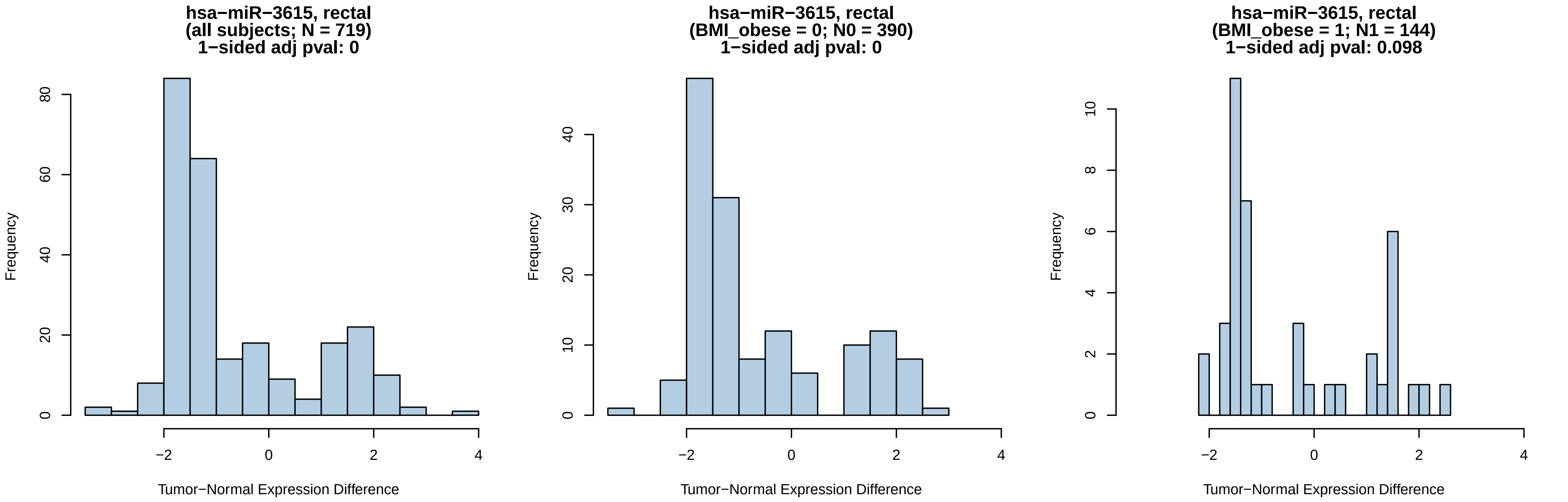
hsa−miR−3615, rectal
(all subjects; N = 719)
1−sided adj pval: 0
Frequency
Tumor−Normal Expression Difference
hsa−miR−3615, rectal
(BMI_obese = 0; N0 = 390)
1−sided adj pval: 0
Frequency
Tumor−Normal Expression Difference
hsa−miR−3615, rectal
(BMI_obese = 1; N1 = 144)
1−sided adj pval: 0.098
Frequency
Tumor−Normal Expression Difference

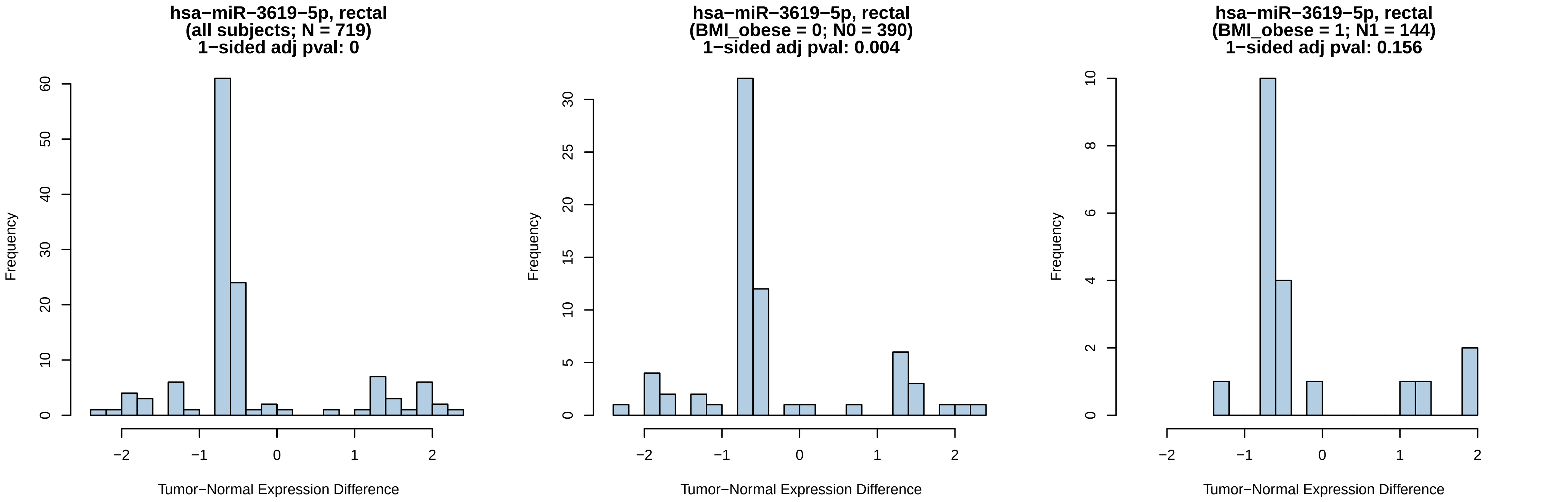

hsa−miR−3619−5p, rectal
(all subjects; N = 719)
1−sided adj pval: 0
Frequency
0
10
20
30
40
50
60
−2
−1
0
1
2
Tumor−Normal Expression Difference
hsa−miR−3619−5p, rectal
(BMI_obese = 0; N0 = 390)
1−sided adj pval: 0.004
Frequency
0
5
10
15
20
25
30
−2
−1
0
1
2
Tumor−Normal Expression Difference
hsa−miR−3619−5p, rectal
(BMI_obese = 1; N1 = 144)
1−sided adj pval: 0.156
Frequency
0
2
4
6
8
10
−2
−1
0
1
2
Tumor−Normal Expression Difference

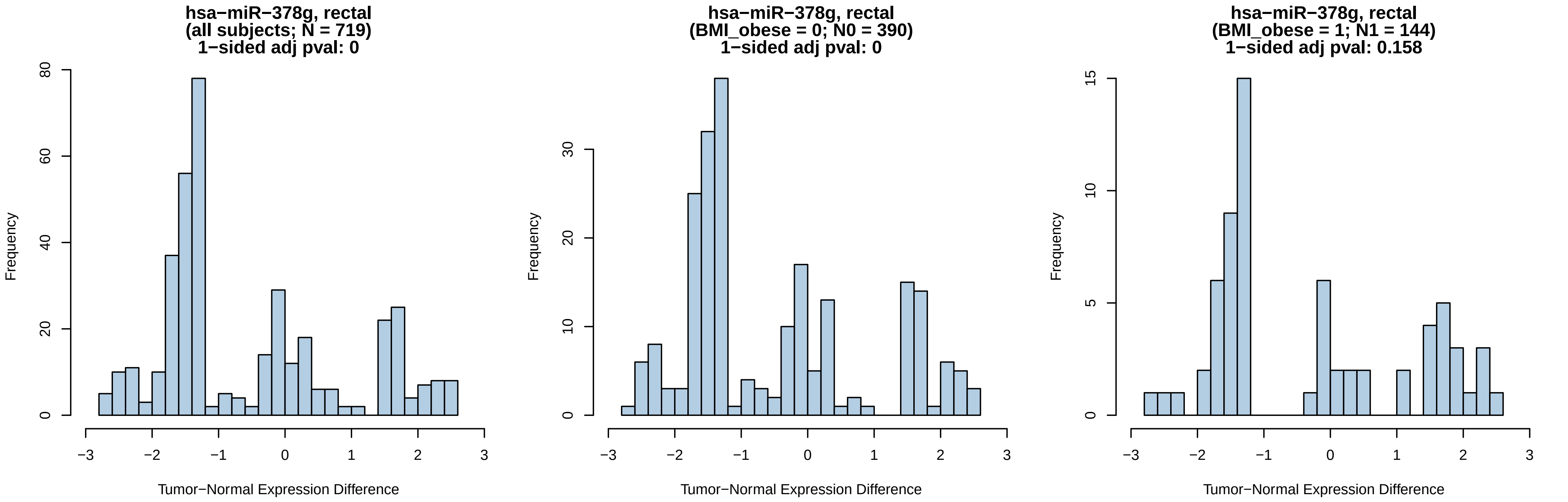

hsa−miR−378g, rectal
(all subjects; N = 719)
1−sided adj pval: 0
hsa−miR−378g, rectal
(BMI_obese = 0; N0 = 390)
1−sided adj pval: 0
hsa−miR−378g, rectal
(BMI_obese = 1; N1 = 144)
1−sided adj pval: 0.158
Frequency
Tumor−Normal Expression Difference

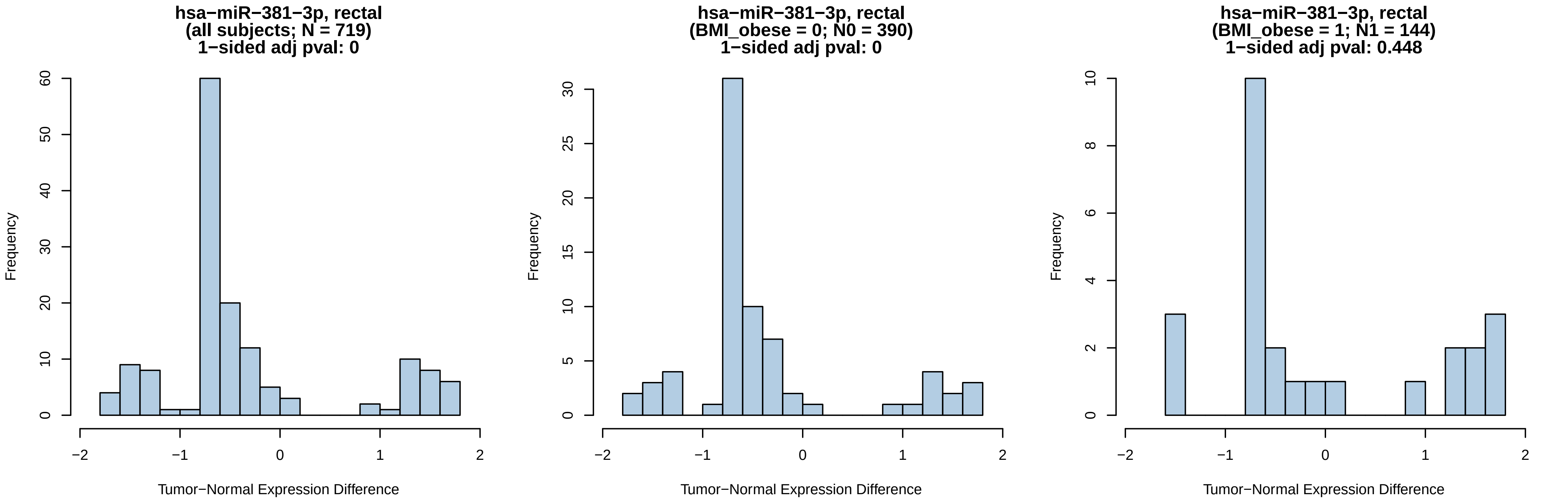
hsa−miR−381−3p, rectal
(all subjects; N = 719)
1−sided adj pval: 0
Frequency
Tumor−Normal Expression Difference
hsa−miR−381−3p, rectal
(BMI_obese = 0; N0 = 390)
1−sided adj pval: 0
Frequency
Tumor−Normal Expression Difference
hsa−miR−381−3p, rectal
(BMI_obese = 1; N1 = 144)
1−sided adj pval: 0.448
Frequency
Tumor−Normal Expression Difference

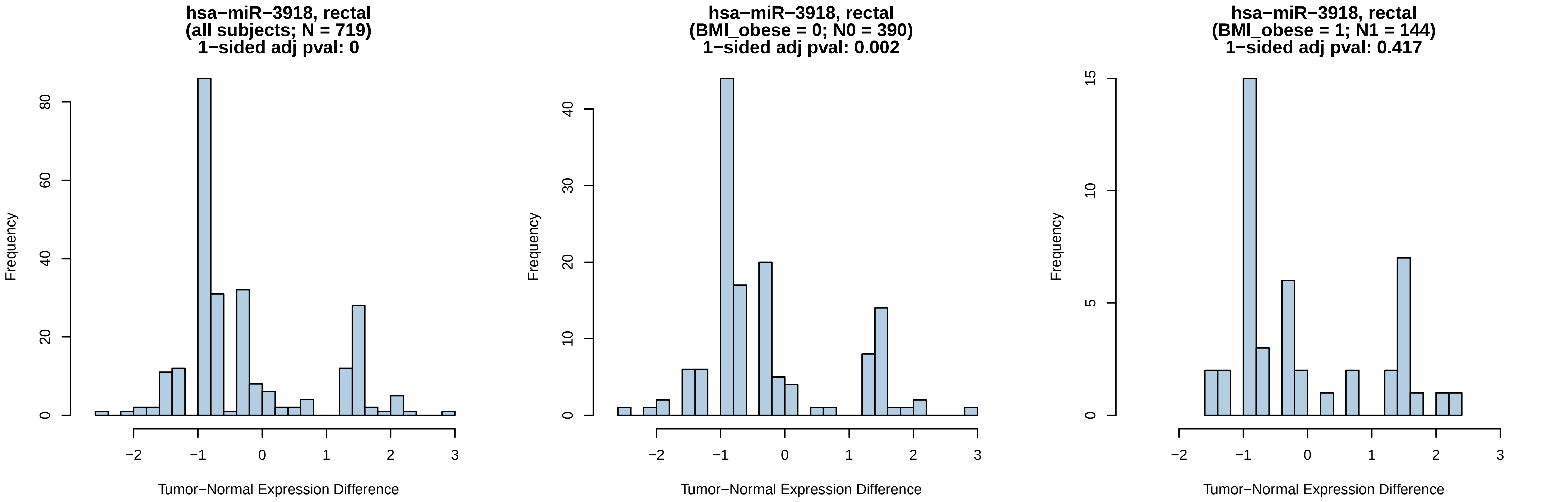

hsa−miR−3918, rectal
(all subjects; N = 719)
1−sided adj pval: 0
hsa−miR−3918, rectal
(BMI_obese = 0; N0 = 390)
1−sided adj pval: 0.002
hsa−miR−3918, rectal
(BMI_obese = 1; N1 = 144)
1−sided adj pval: 0.417
Frequency
Tumor−Normal Expression Difference

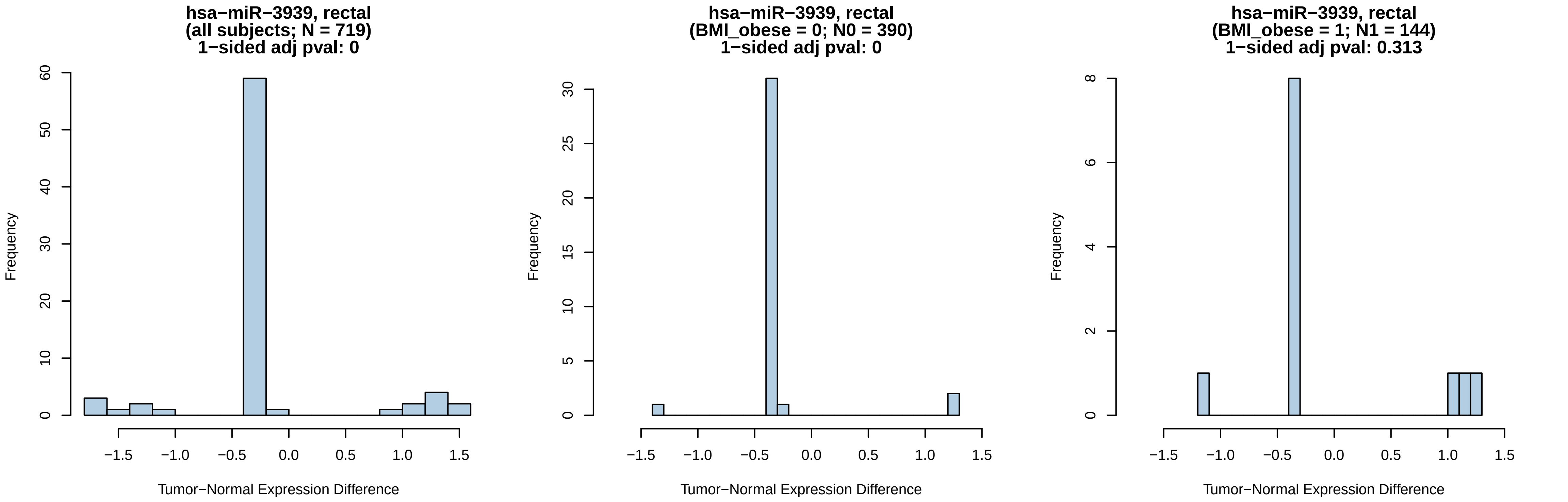

hsa−miR−3939, rectal
(all subjects; N = 719)
1−sided adj pval: 0
Frequency
Tumor−Normal Expression Difference
hsa−miR−3939, rectal
(BMI_obese = 0; N0 = 390)
1−sided adj pval: 0
Frequency
Tumor−Normal Expression Difference
hsa−miR−3939, rectal
(BMI_obese = 1; N1 = 144)
1−sided adj pval: 0.313
Frequency
Tumor−Normal Expression Difference

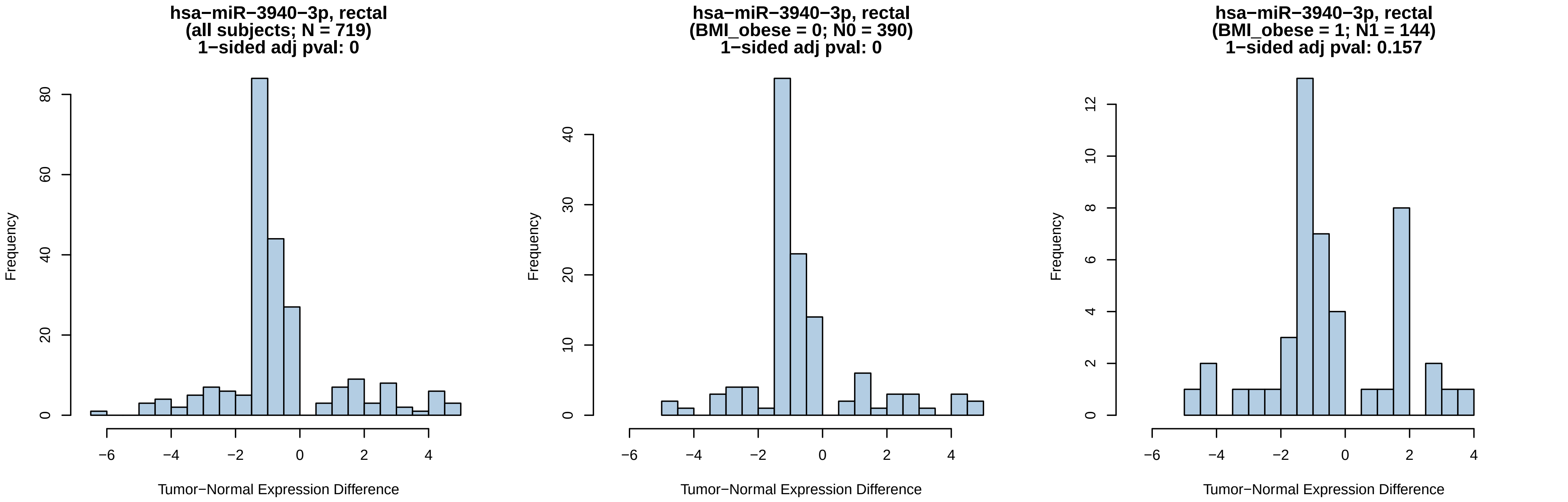
hsa−miR−3940−3p, rectal
(all subjects; N = 719)
1−sided adj pval: 0
Frequency
Tumor−Normal Expression Difference
hsa−miR−3940−3p, rectal
(BMI_obese = 0; N0 = 390)
1−sided adj pval: 0
Frequency
Tumor−Normal Expression Difference
hsa−miR−3940−3p, rectal
(BMI_obese = 1; N1 = 144)
1−sided adj pval: 0.157
Frequency
Tumor−Normal Expression Difference

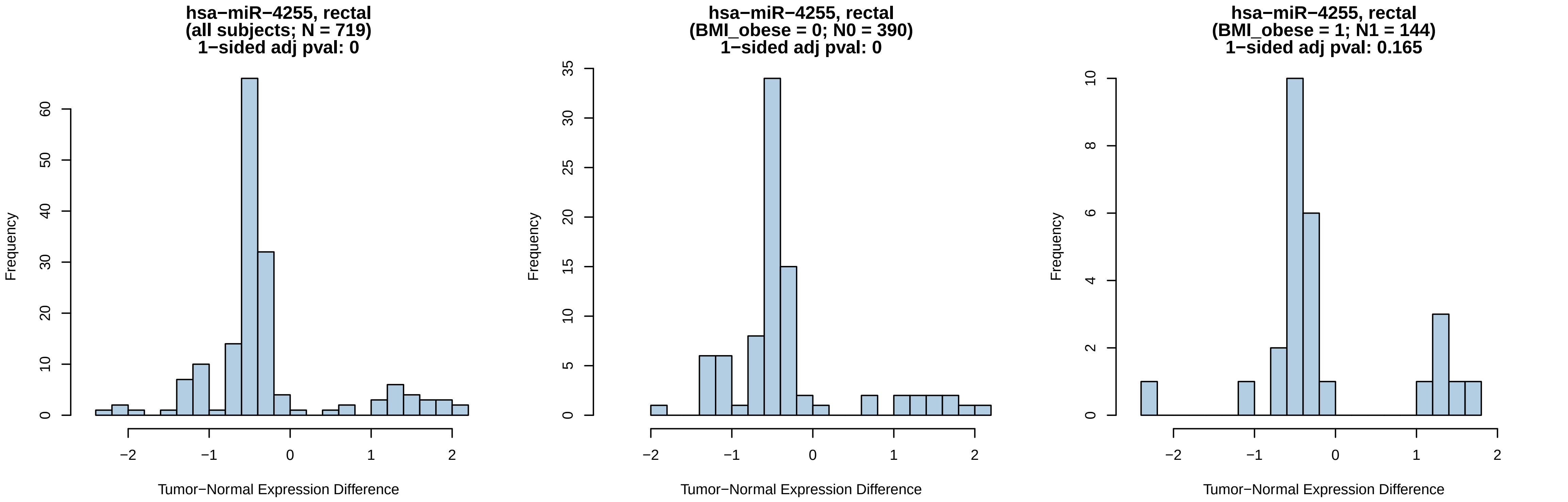
hsa−miR−4255, rectal
(all subjects; N = 719)
1−sided adj pval: 0
Frequency
0
10
20
30
40
50
60
−2
−1
0
1
2
Tumor−Normal Expression Difference
hsa−miR−4255, rectal
(BMI_obese = 0; N0 = 390)
1−sided adj pval: 0
Frequency
0
5
10
15
20
25
30
35
−2
−1
0
1
2
Tumor−Normal Expression Difference
hsa−miR−4255, rectal
(BMI_obese = 1; N1 = 144)
1−sided adj pval: 0.165
Frequency
0
2
4
6
8
10
−2
−1
0
1
2
Tumor−Normal Expression Difference

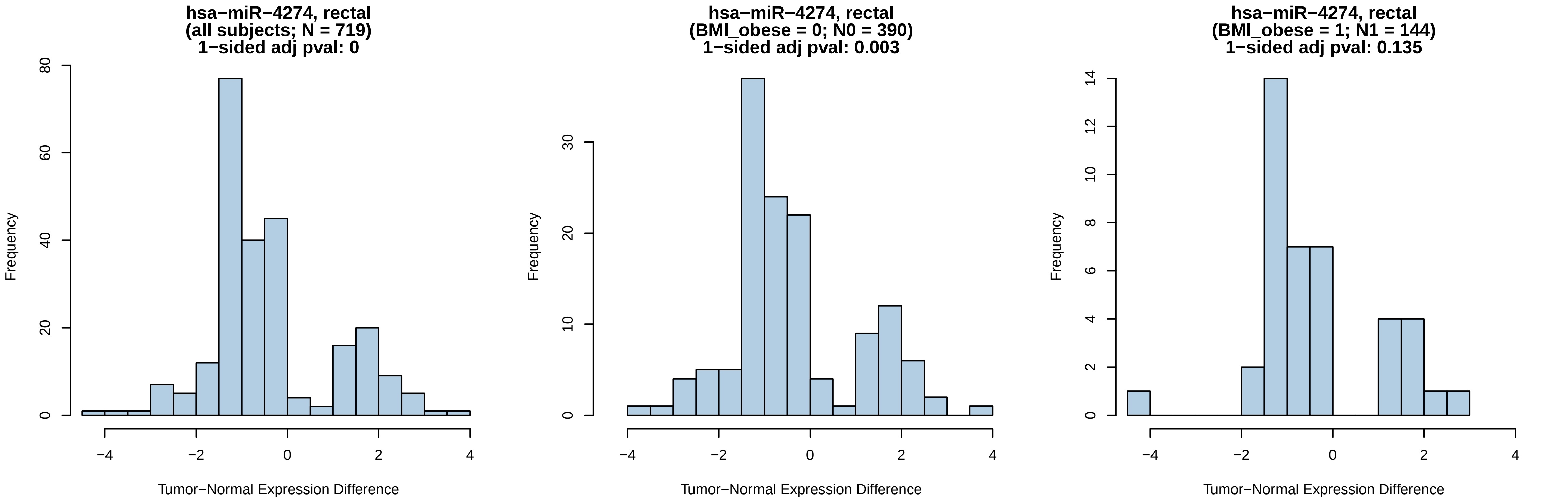

hsa−miR−4274, rectal
(all subjects; N = 719)
1−sided adj pval: 0
Frequency
Tumor−Normal Expression Difference
hsa−miR−4274, rectal
(BMI_obese = 0; N0 = 390)
1−sided adj pval: 0.003
Frequency
Tumor−Normal Expression Difference
hsa−miR−4274, rectal
(BMI_obese = 1; N1 = 144)
1−sided adj pval: 0.135
Frequency
Tumor−Normal Expression Difference

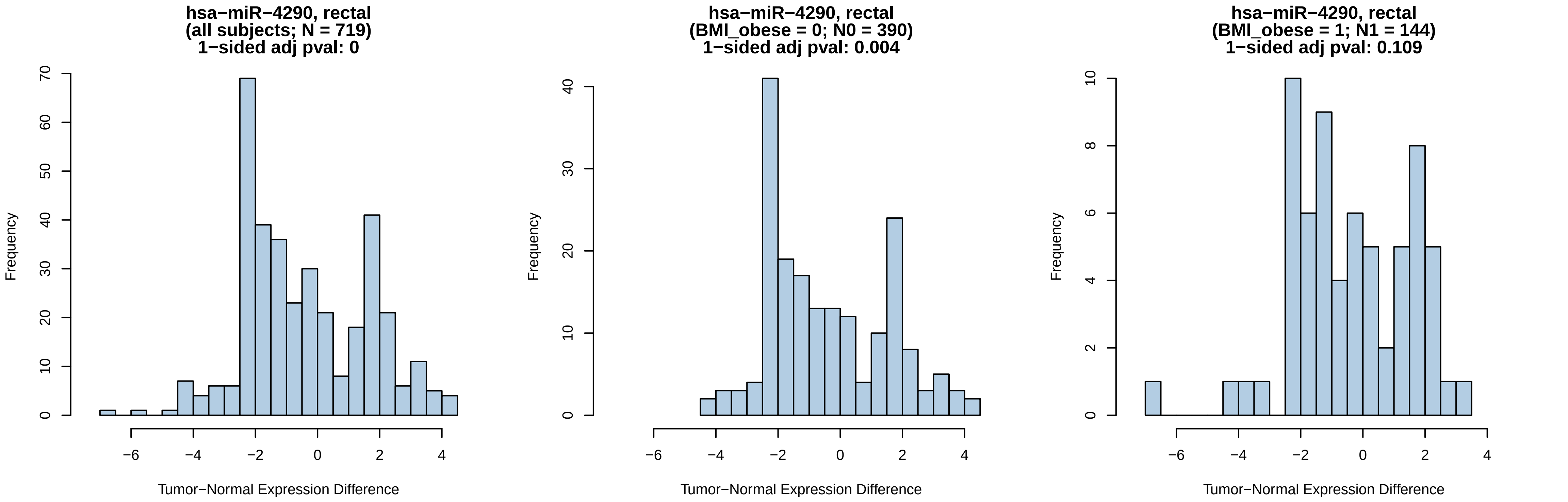
hsa−miR−4290, rectal
(all subjects; N = 719)
1−sided adj pval: 0
Frequency
Tumor−Normal Expression Difference
hsa−miR−4290, rectal
(BMI_obese = 0; N0 = 390)
1−sided adj pval: 0.004
Frequency
Tumor−Normal Expression Difference
hsa−miR−4290, rectal
(BMI_obese = 1; N1 = 144)
1−sided adj pval: 0.109
Frequency
Tumor−Normal Expression Difference

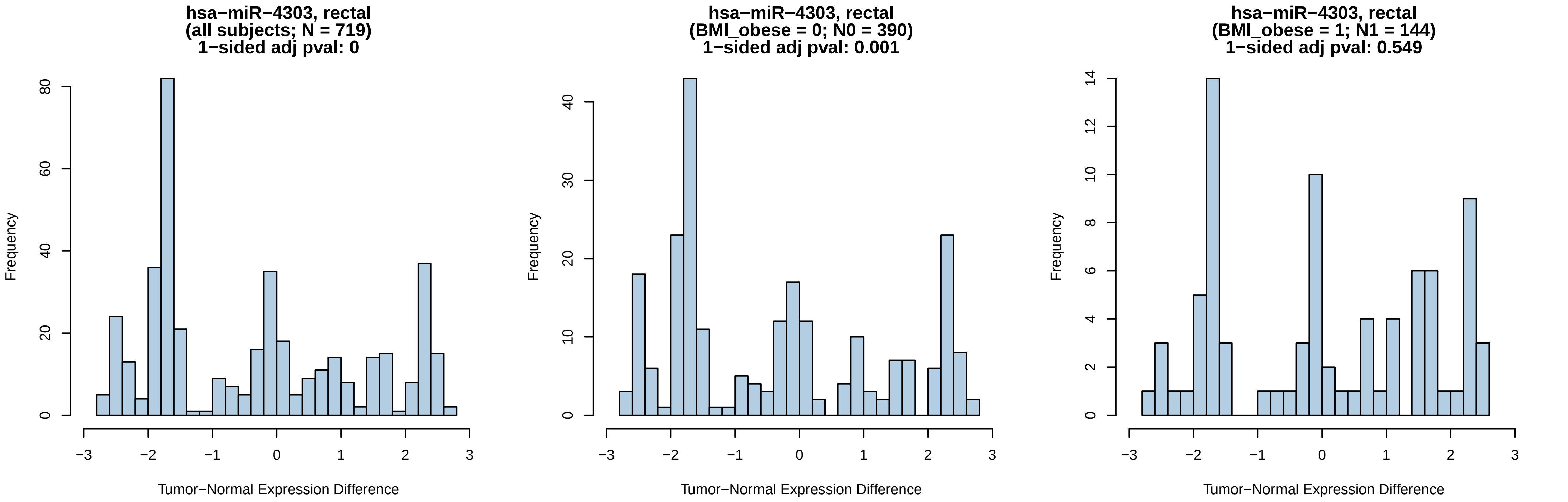

hsa−miR−4303, rectal
(all subjects; N = 719)
1−sided adj pval: 0
hsa−miR−4303, rectal
(BMI_obese = 0; N0 = 390)
1−sided adj pval: 0.001
hsa−miR−4303, rectal
(BMI_obese = 1; N1 = 144)
1−sided adj pval: 0.549
Frequency
Tumor−Normal Expression Difference

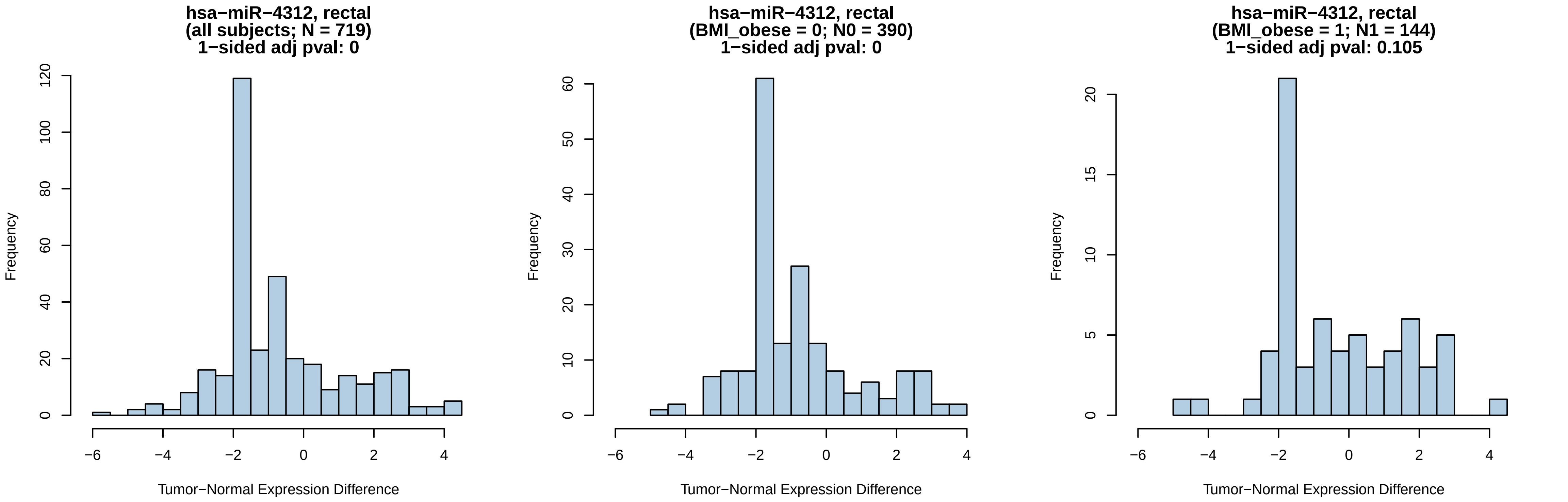

hsa−miR−4312, rectal
(all subjects; N = 719)
1−sided adj pval: 0
Frequency
Tumor−Normal Expression Difference
hsa−miR−4312, rectal
(BMI_obese = 0; N0 = 390)
1−sided adj pval: 0
Frequency
Tumor−Normal Expression Difference
hsa−miR−4312, rectal
(BMI_obese = 1; N1 = 144)
1−sided adj pval: 0.105
Frequency
Tumor−Normal Expression Difference

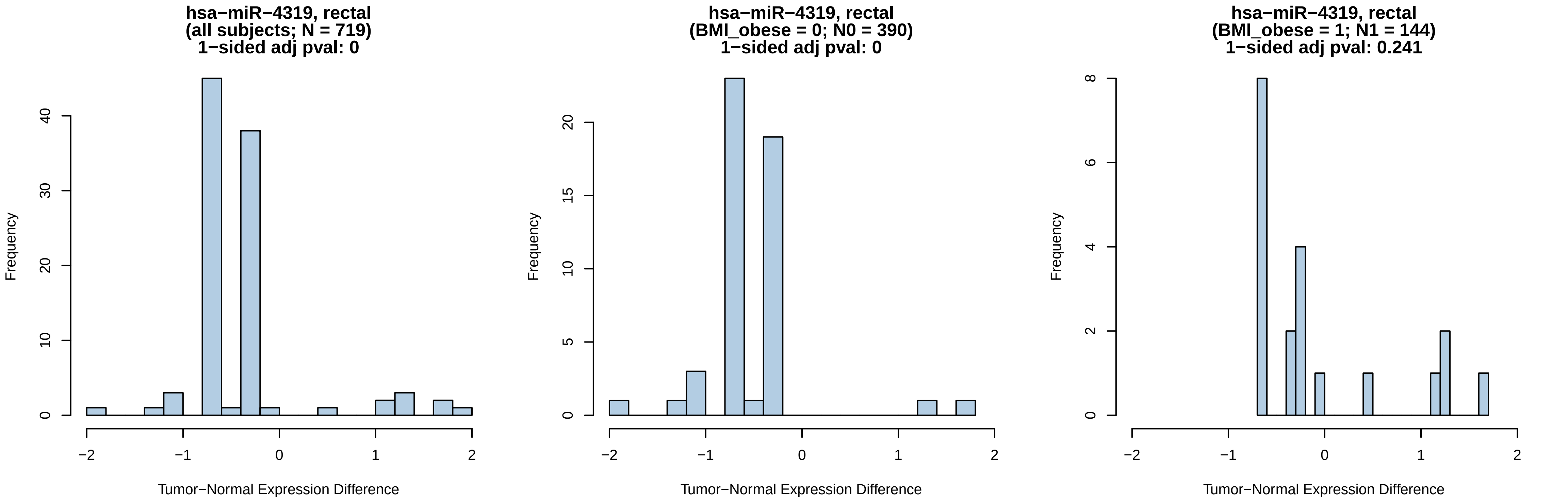
hsa−miR−4319, rectal
(all subjects; N = 719)
1−sided adj pval: 0
Frequency
Tumor−Normal Expression Difference
hsa−miR−4319, rectal
(BMI_obese = 0; N0 = 390)
1−sided adj pval: 0
Frequency
Tumor−Normal Expression Difference
hsa−miR−4319, rectal
(BMI_obese = 1; N1 = 144)
1−sided adj pval: 0.241
Frequency
Tumor−Normal Expression Difference

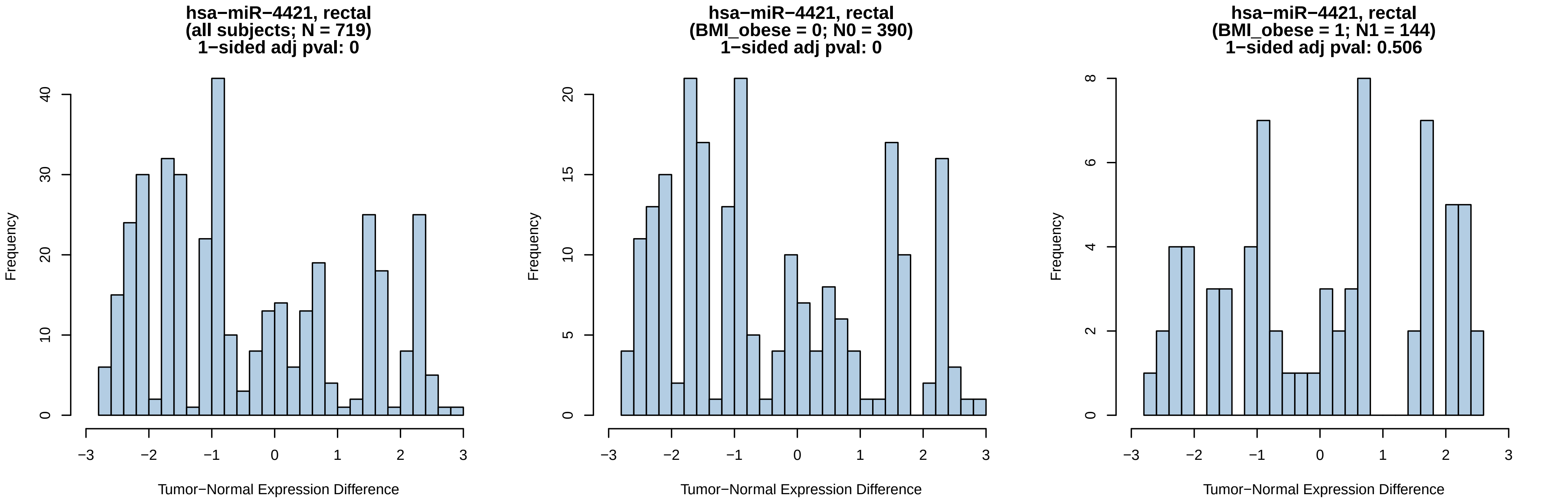
hsa−miR−4421, rectal
(all subjects; N = 719)
1−sided adj pval: 0
Frequency
Tumor−Normal Expression Difference
hsa−miR−4421, rectal
(BMI_obese = 0; N0 = 390)
1−sided adj pval: 0
Frequency
Tumor−Normal Expression Difference
hsa−miR−4421, rectal
(BMI_obese = 1; N1 = 144)
1−sided adj pval: 0.506
Frequency
Tumor−Normal Expression Difference

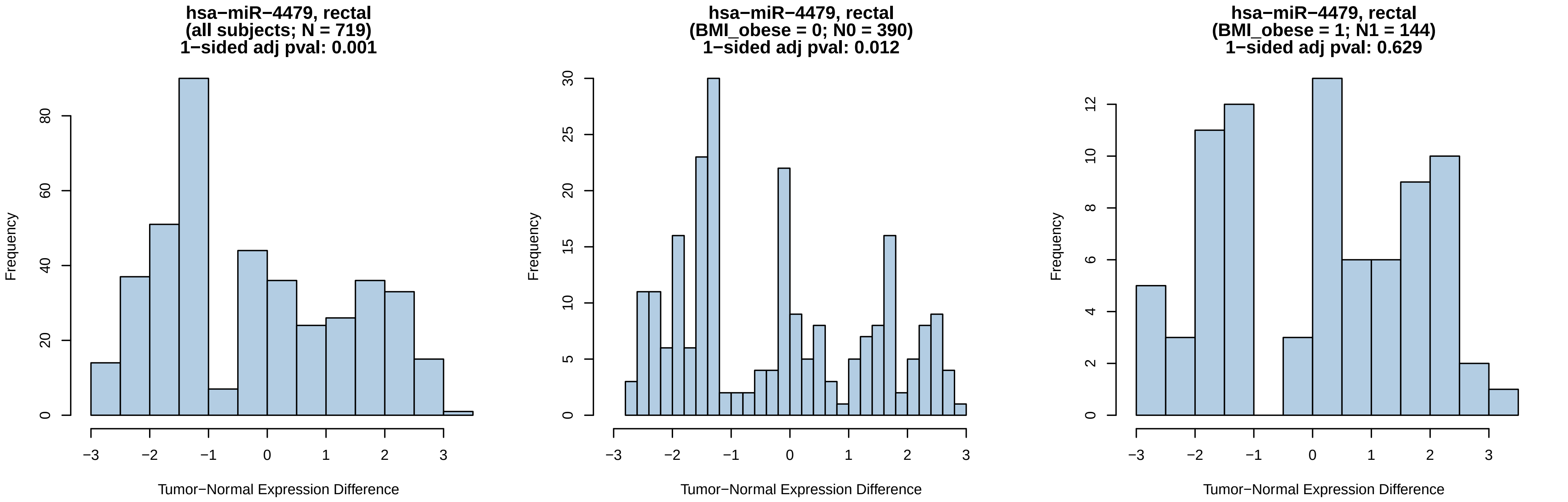
hsa−miR−4479, rectal
(all subjects; N = 719)
1−sided adj pval: 0.001
hsa−miR−4479, rectal
(BMI_obese = 0; N0 = 390)
1−sided adj pval: 0.012
hsa−miR−4479, rectal
(BMI_obese = 1; N1 = 144)
1−sided adj pval: 0.629
Frequency
Tumor−Normal Expression Difference

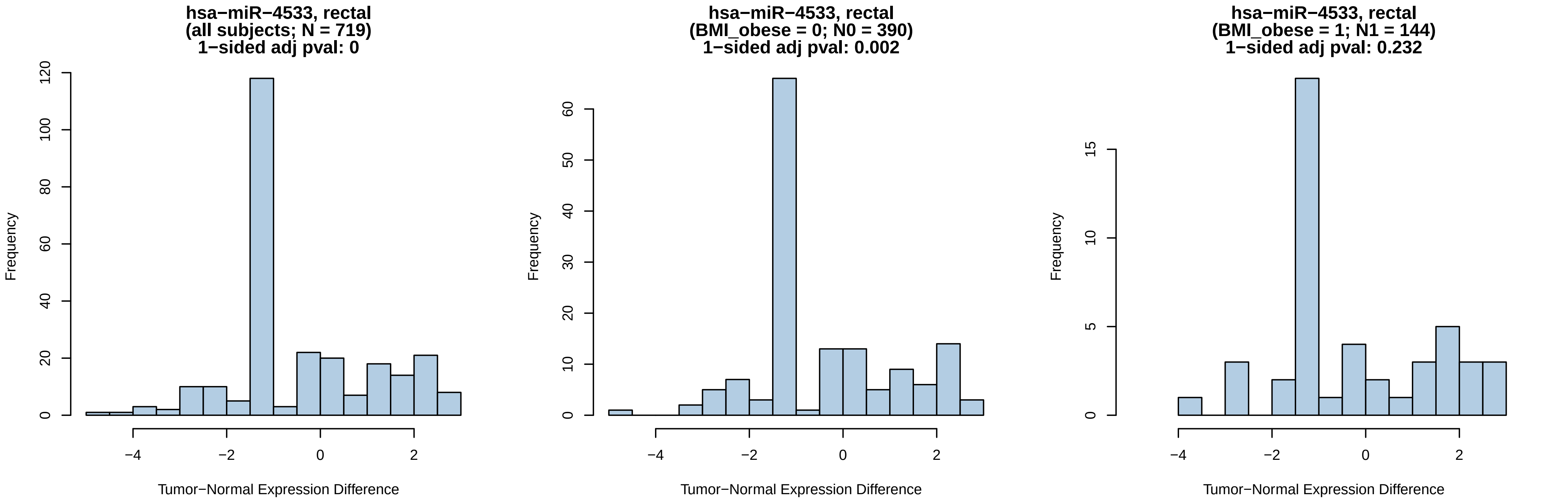
hsa−miR−4533, rectal
(all subjects; N = 719)
1−sided adj pval: 0
Frequency
0
20
40
60
80
100
120
−4
−2
0
2
Tumor−Normal Expression Difference
hsa−miR−4533, rectal
(BMI_obese = 0; N0 = 390)
1−sided adj pval: 0.002
Frequency
0
10
20
30
40
50
60
−4
−2
0
2
Tumor−Normal Expression Difference
hsa−miR−4533, rectal
(BMI_obese = 1; N1 = 144)
1−sided adj pval: 0.232
Frequency
0
5
10
15
−4
−2
0
2
Tumor−Normal Expression Difference

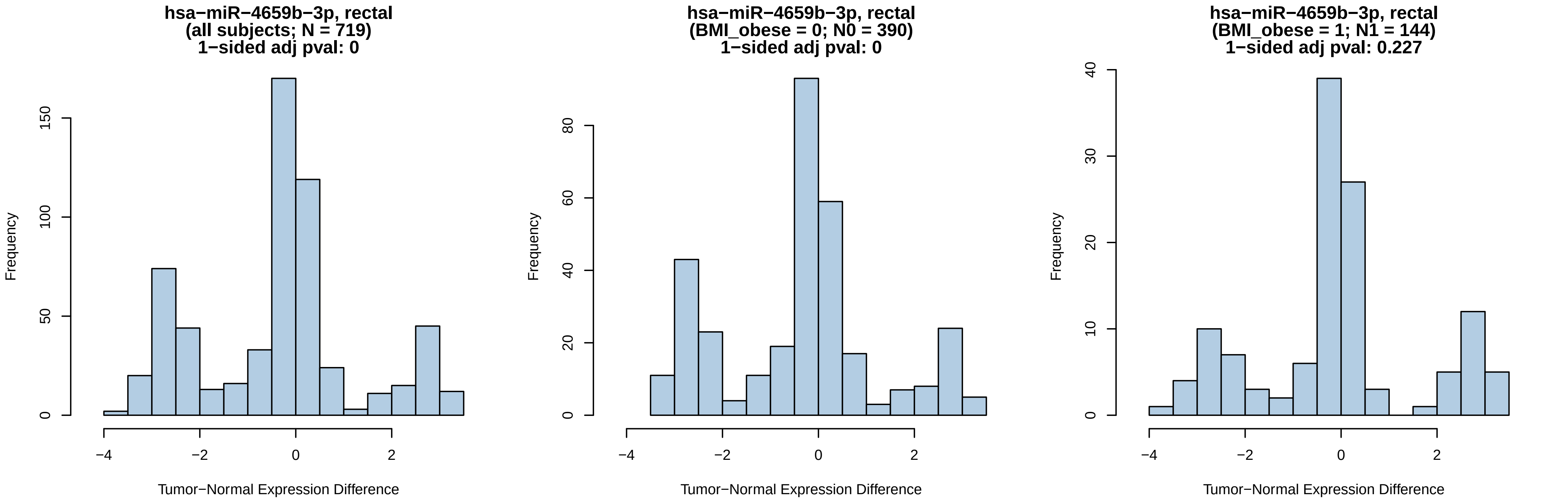

hsa−miR−4659b−3p, rectal
(all subjects; N = 719)
1−sided adj pval: 0
Frequency
Tumor−Normal Expression Difference
hsa−miR−4659b−3p, rectal
(BMI_obese = 0; N0 = 390)
1−sided adj pval: 0
Frequency
Tumor−Normal Expression Difference
hsa−miR−4659b−3p, rectal
(BMI_obese = 1; N1 = 144)
1−sided adj pval: 0.227
Frequency
Tumor−Normal Expression Difference

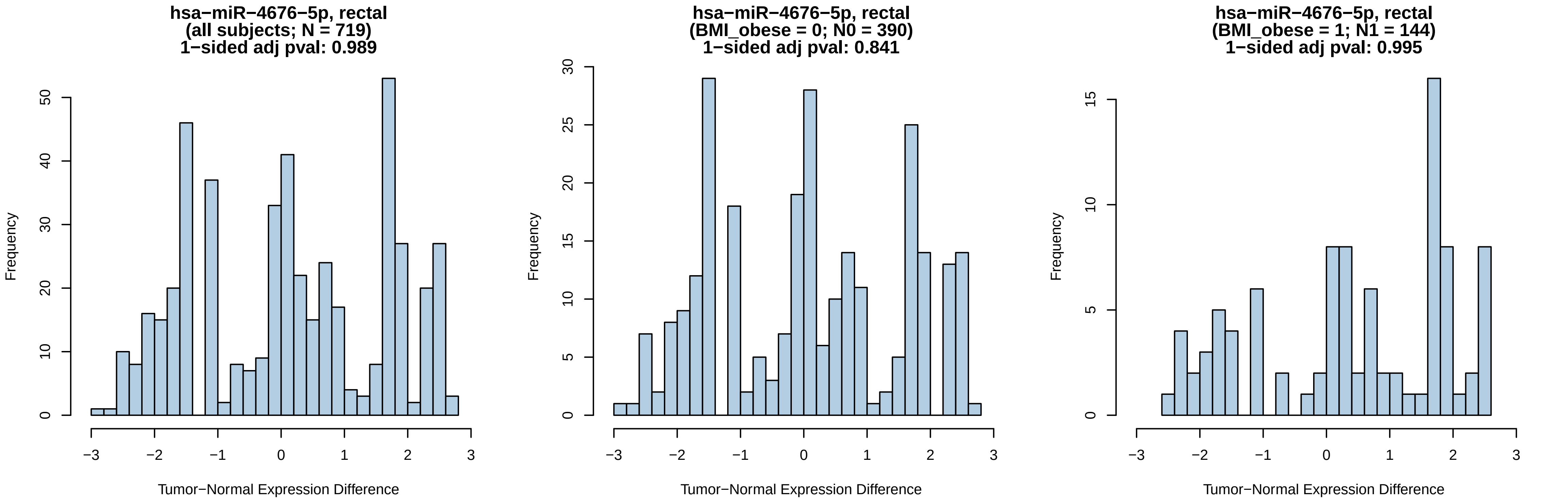

hsa−miR−4676−5p, rectal
(all subjects; N = 719)
1−sided adj pval: 0.989
Frequency
Tumor−Normal Expression Difference
hsa−miR−4676−5p, rectal
(BMI_obese = 0; N0 = 390)
1−sided adj pval: 0.841
Frequency
Tumor−Normal Expression Difference
hsa−miR−4676−5p, rectal
(BMI_obese = 1; N1 = 144)
1−sided adj pval: 0.995
Frequency
Tumor−Normal Expression Difference

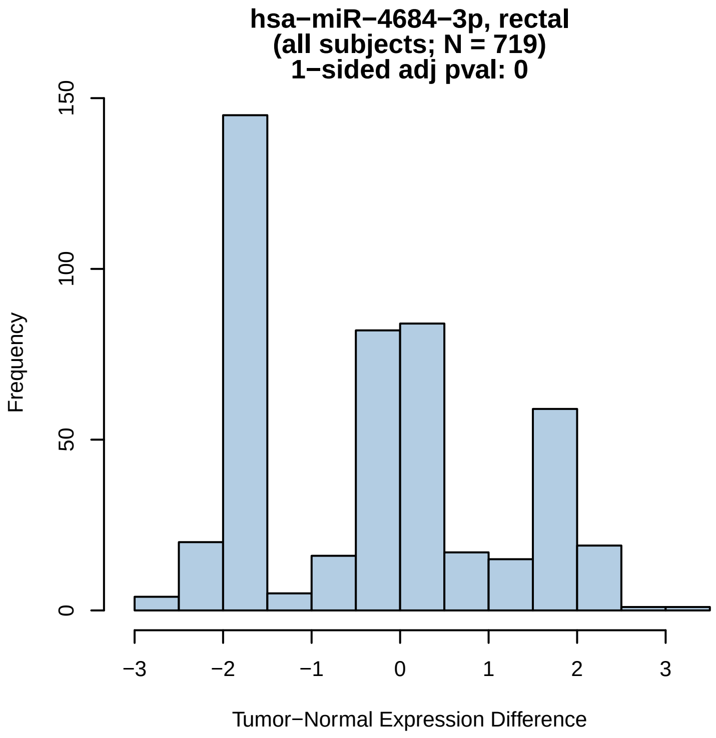
hsa−miR−4684−3p, rectal
(all subjects; N = 719)
1−sided adj pval: 0
Frequency
Tumor−Normal Expression Difference
0
50
100
150
−3
−2
−1
0
1
2
3

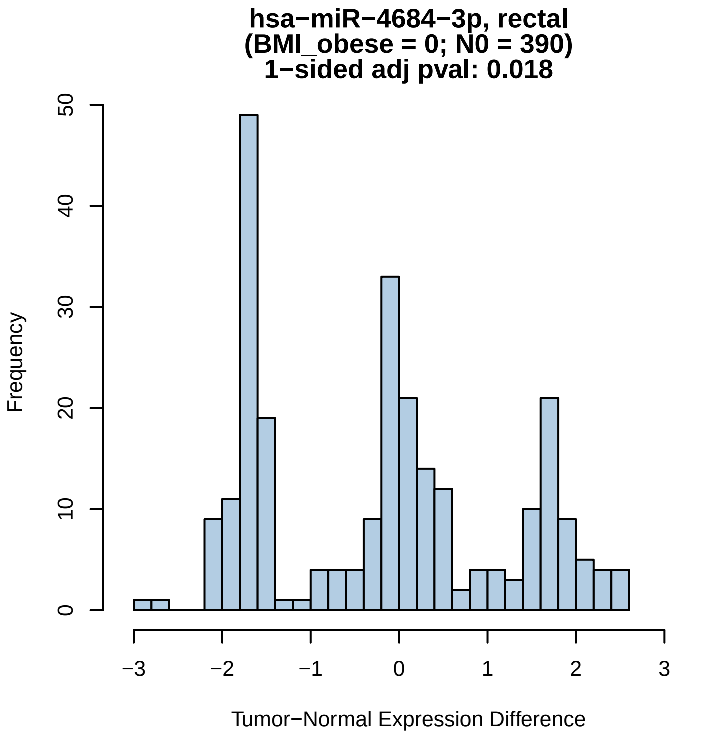
hsa−miR−4684−3p, rectal
(BMI_obese = 0; N0 = 390)
1−sided adj pval: 0.018
Frequency
Tumor−Normal Expression Difference
0
10
20
30
40
50
−3
−2
−1
0
1
2
3

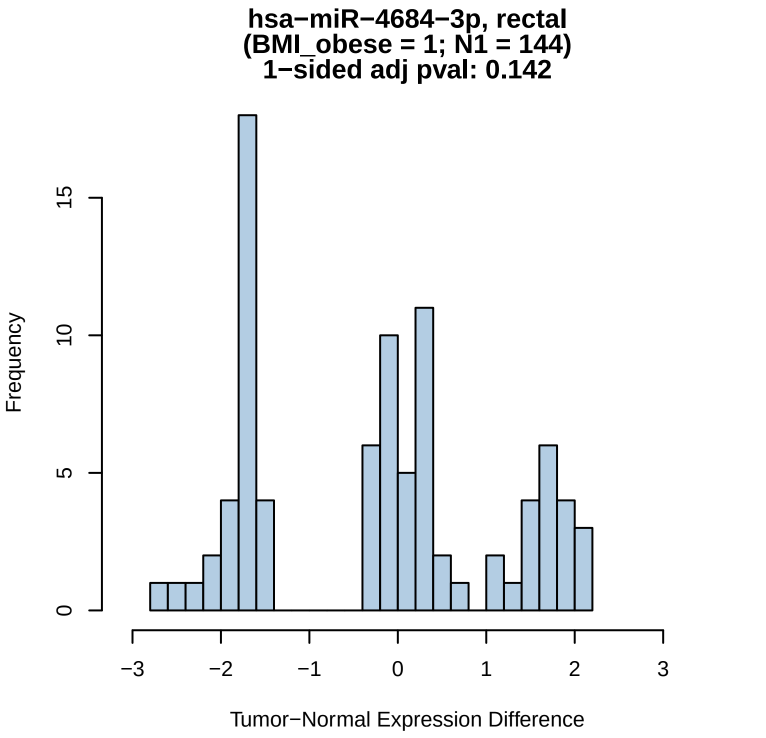
hsa−miR−4684−3p, rectal
(BMI_obese = 1; N1 = 144)
1−sided adj pval: 0.142
Frequency
Tumor−Normal Expression Difference
0
5
10
15
−3
−2
−1
0
1
2
3

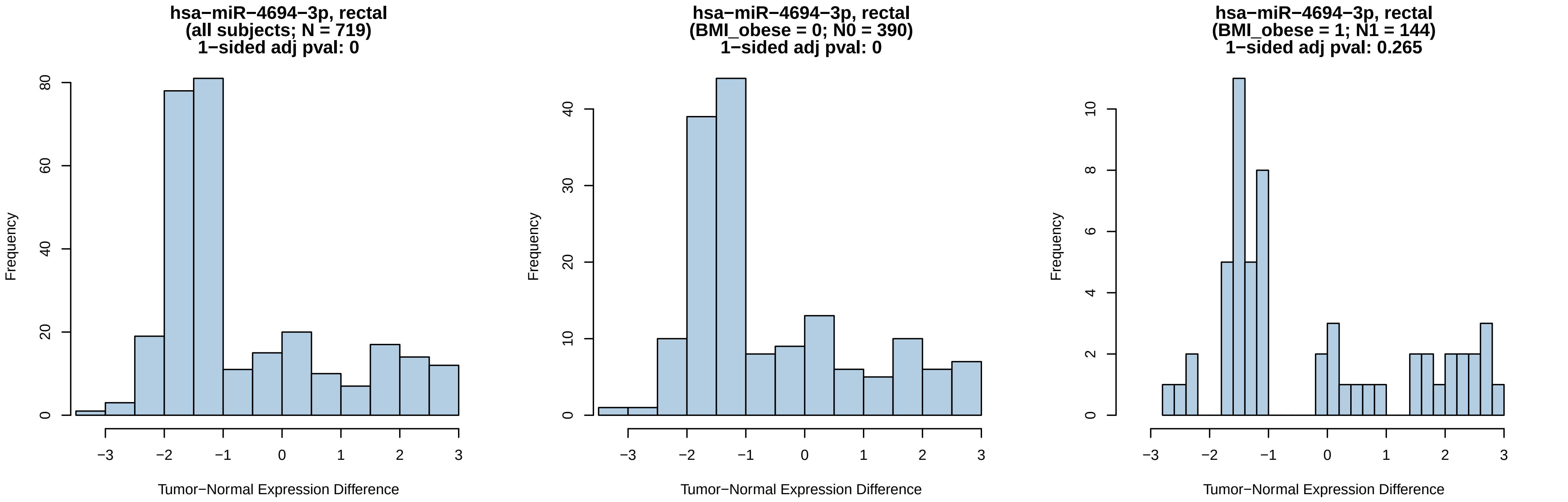

hsa−miR−4694−3p, rectal
(all subjects; N = 719)
1−sided adj pval: 0
Frequency
Tumor−Normal Expression Difference
hsa−miR−4694−3p, rectal
(BMI_obese = 0; N0 = 390)
1−sided adj pval: 0
Frequency
Tumor−Normal Expression Difference
hsa−miR−4694−3p, rectal
(BMI_obese = 1; N1 = 144)
1−sided adj pval: 0.265
Frequency
Tumor−Normal Expression Difference

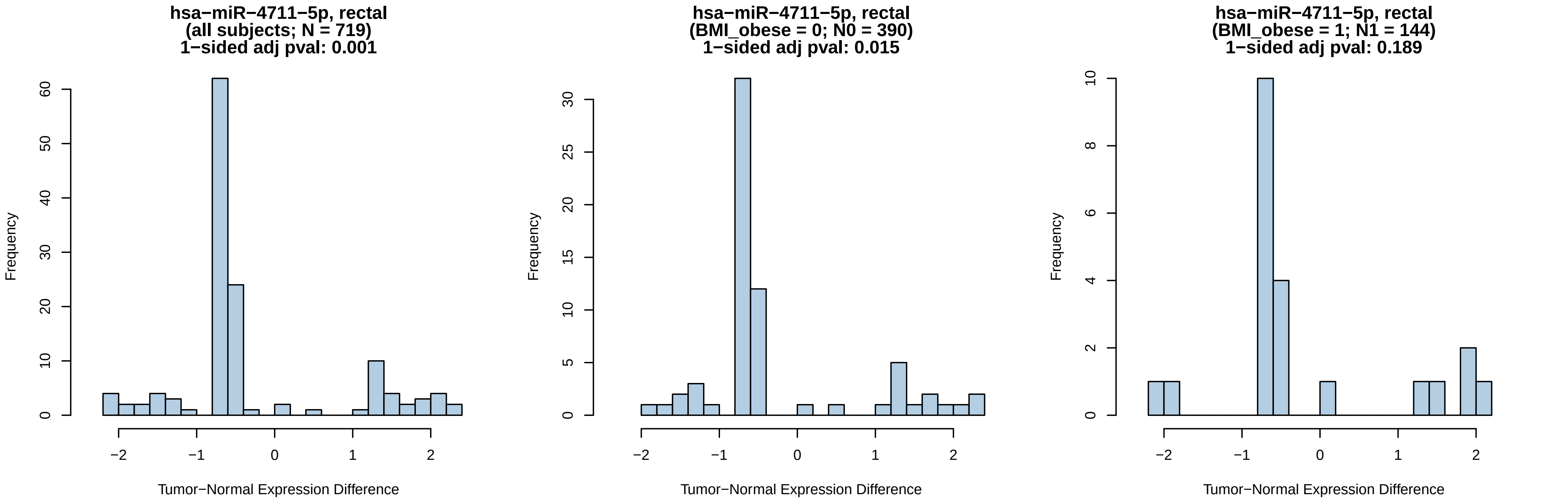
hsa−miR−4711−5p, rectal
(all subjects; N = 719)
1−sided adj pval: 0.001
Frequency
Tumor−Normal Expression Difference
hsa−miR−4711−5p, rectal
(BMI_obese = 0; N0 = 390)
1−sided adj pval: 0.015
Frequency
Tumor−Normal Expression Difference
hsa−miR−4711−5p, rectal
(BMI_obese = 1; N1 = 144)
1−sided adj pval: 0.189
Frequency
Tumor−Normal Expression Difference

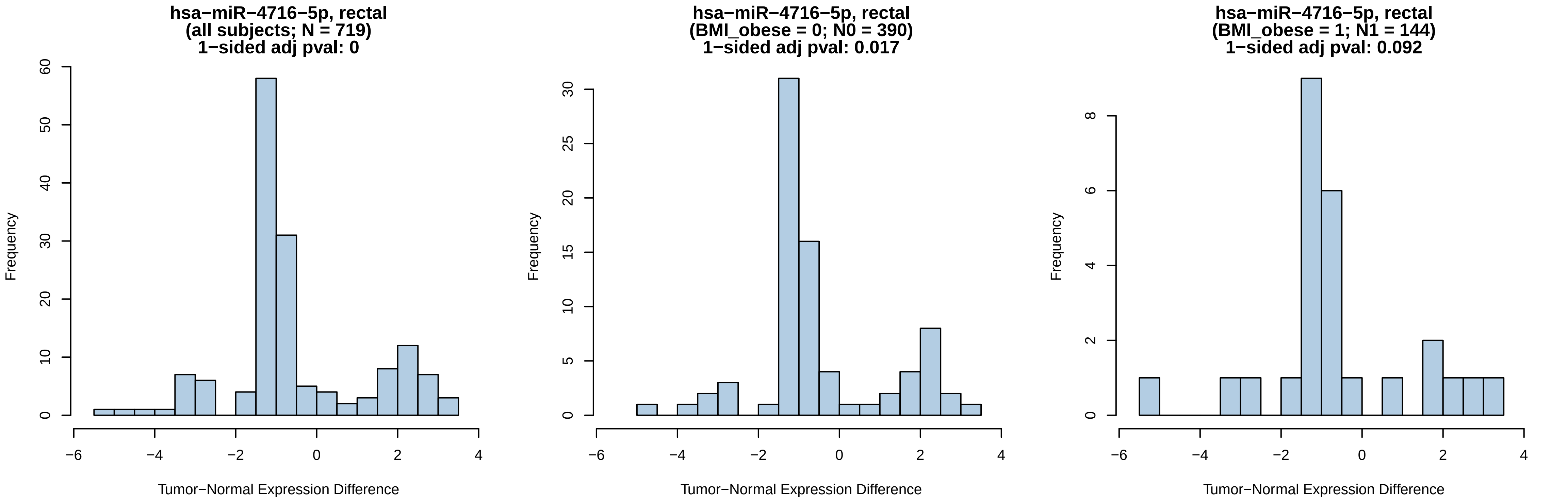

hsa−miR−4716−5p, rectal
(all subjects; N = 719)
1−sided adj pval: 0
Frequency
Tumor−Normal Expression Difference
hsa−miR−4716−5p, rectal
(BMI_obese = 0; N0 = 390)
1−sided adj pval: 0.017
Frequency
Tumor−Normal Expression Difference
hsa−miR−4716−5p, rectal
(BMI_obese = 1; N1 = 144)
1−sided adj pval: 0.092
Frequency
Tumor−Normal Expression Difference

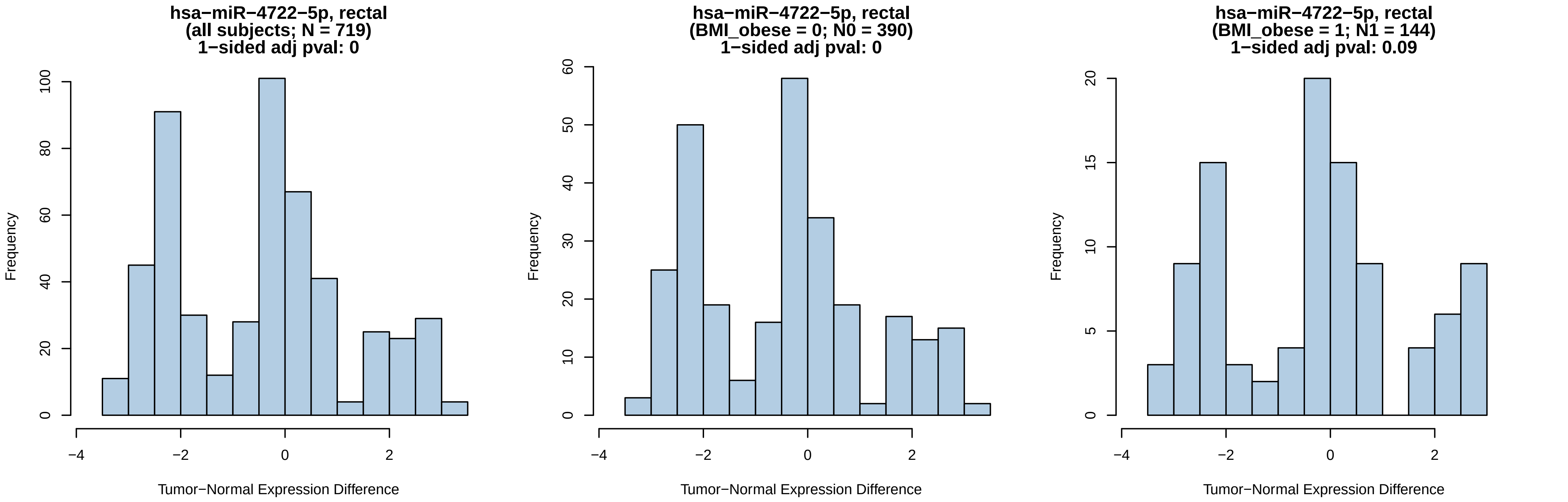

hsa−miR−4722−5p, rectal
(all subjects; N = 719)
1−sided adj pval: 0
Frequency
Tumor−Normal Expression Difference
hsa−miR−4722−5p, rectal
(BMI_obese = 0; N0 = 390)
1−sided adj pval: 0
Frequency
Tumor−Normal Expression Difference
hsa−miR−4722−5p, rectal
(BMI_obese = 1; N1 = 144)
1−sided adj pval: 0.09
Frequency
Tumor−Normal Expression Difference

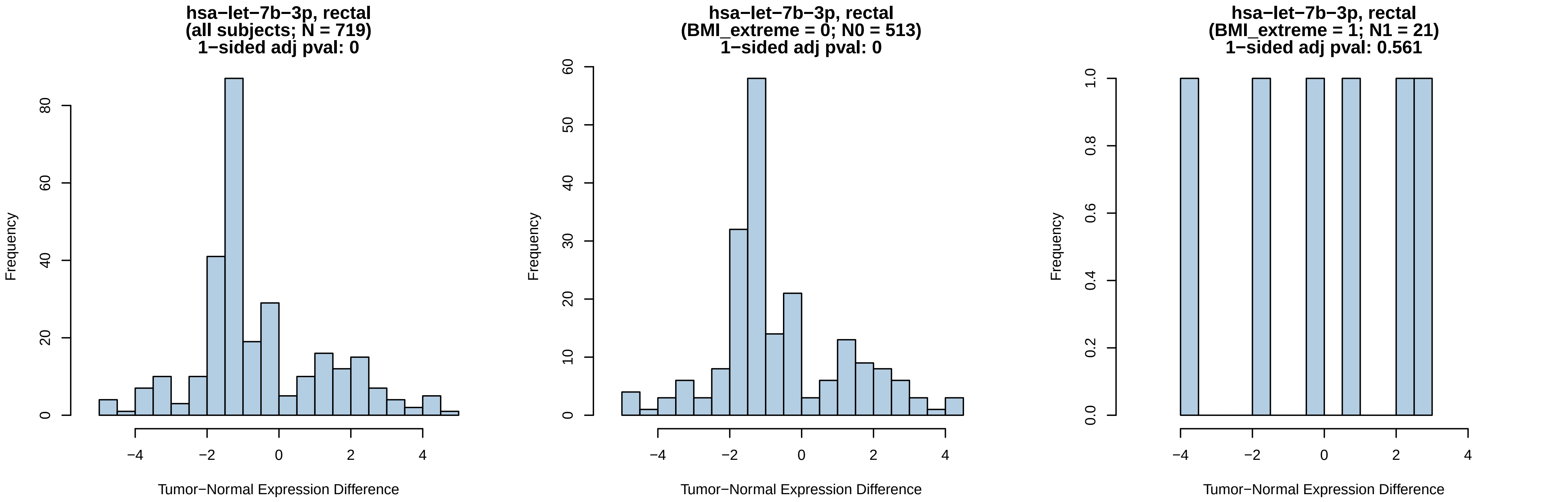
hsa−let−7b−3p, rectal
(all subjects; N = 719)
1−sided adj pval: 0
Frequency
Tumor−Normal Expression Difference
hsa−let−7b−3p, rectal
(BMI_extreme = 0; N0 = 513)
1−sided adj pval: 0
Frequency
Tumor−Normal Expression Difference
hsa−let−7b−3p, rectal
(BMI_extreme = 1; N1 = 21)
1−sided adj pval: 0.561
Frequency
Tumor−Normal Expression Difference

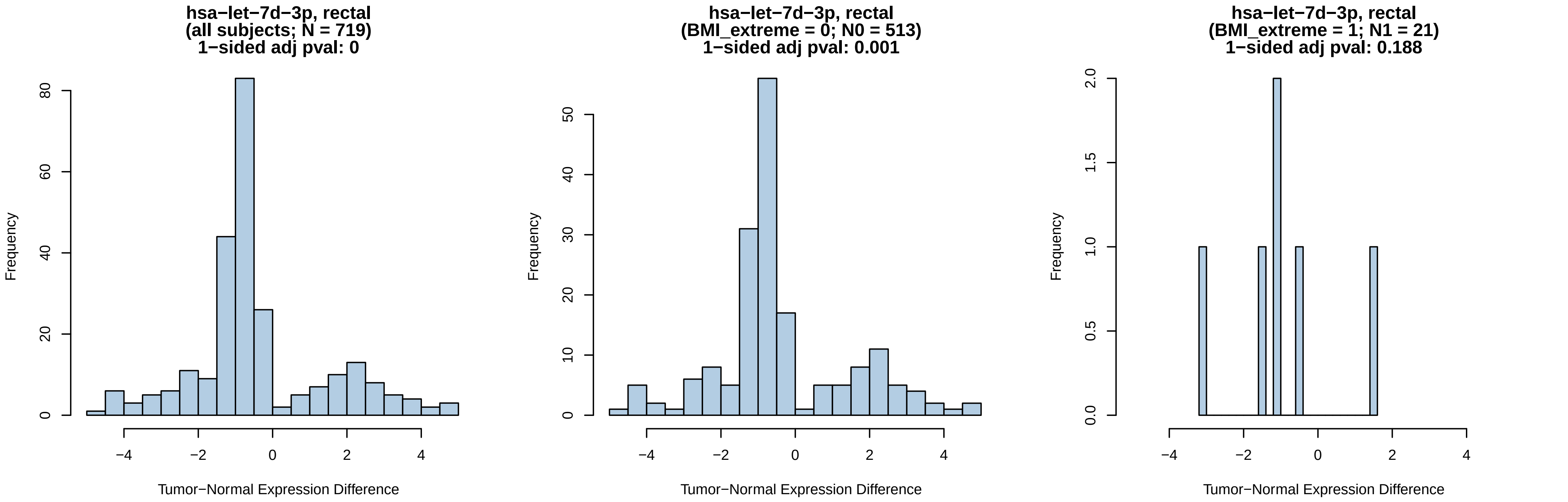
hsa−let−7d−3p, rectal
(all subjects; N = 719)
1−sided adj pval: 0
Frequency
Tumor−Normal Expression Difference
hsa−let−7d−3p, rectal
(BMI_extreme = 0; N0 = 513)
1−sided adj pval: 0.001
Frequency
Tumor−Normal Expression Difference
hsa−let−7d−3p, rectal
(BMI_extreme = 1; N1 = 21)
1−sided adj pval: 0.188
Frequency
Tumor−Normal Expression Difference

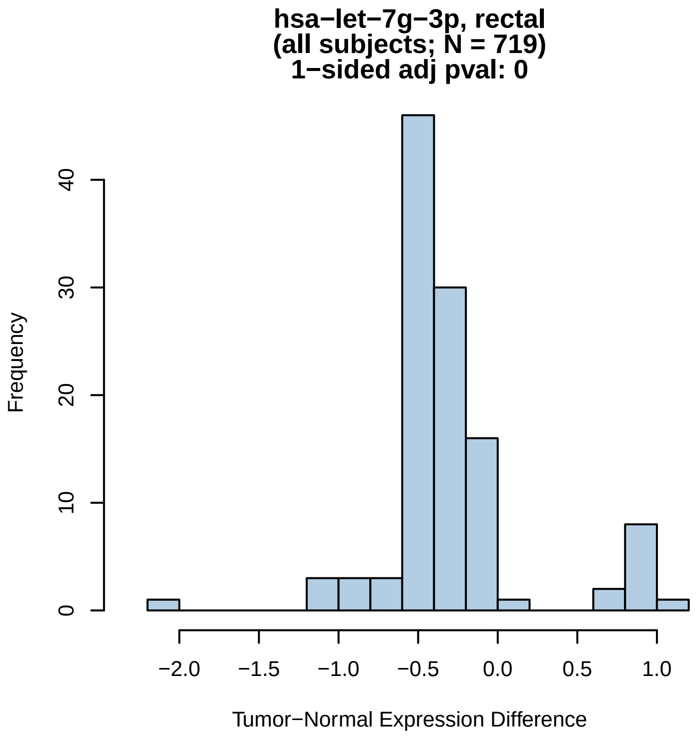

hsa−let−7g−3p, rectal
(all subjects; N = 719)
1−sided adj pval: 0
Frequency
0
10
20
30
40
−2.0
−1.5
−1.0
−0.5
0.0
0.5
1.0
Tumor−Normal Expression Difference


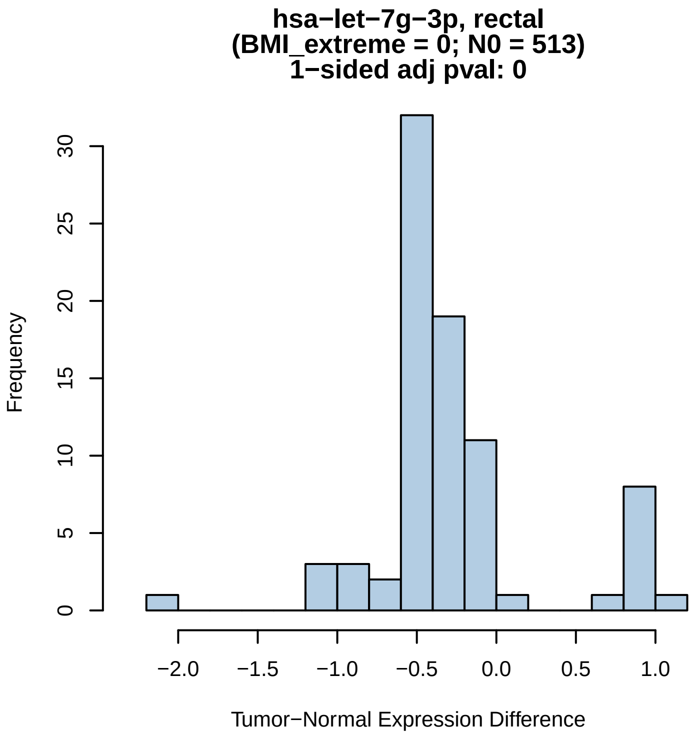

hsa−let−7g−3p, rectal
(BMI_extreme = 0; N0 = 513)
1−sided adj pval: 0
Frequency
0
5
10
15
20
25
30
−2.0
−1.5
−1.0
−0.5
0.0
0.5
1.0
Tumor−Normal Expression Difference

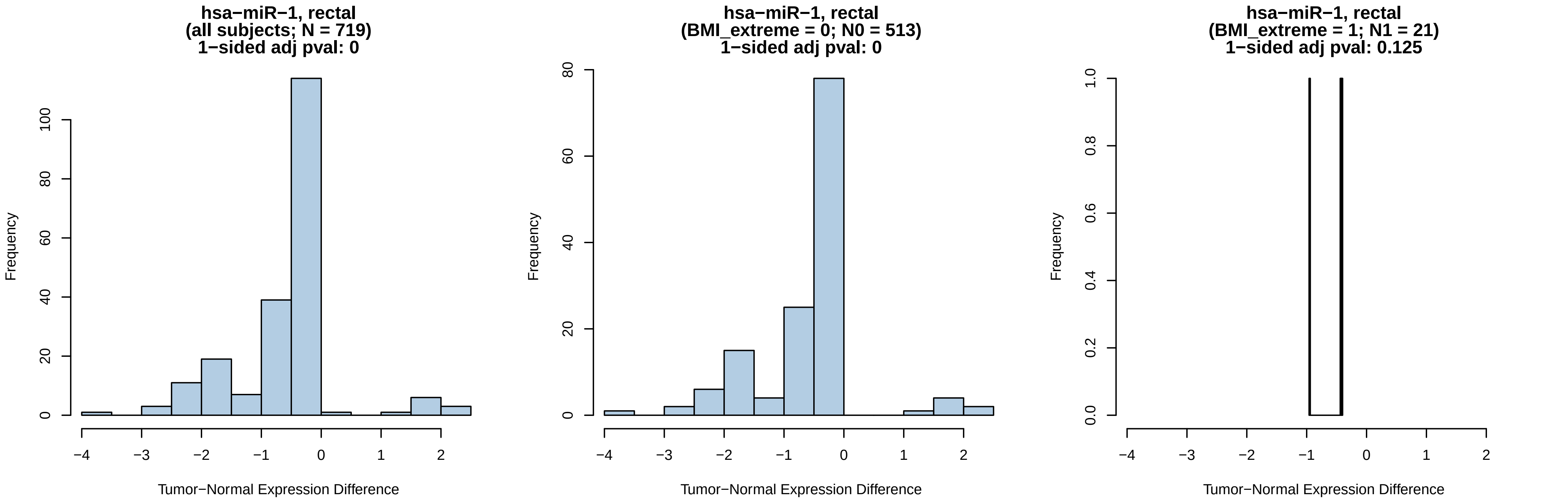
hsa−miR−1, rectal
(all subjects; N = 719)
1−sided adj pval: 0
Frequency
Tumor−Normal Expression Difference
hsa−miR−1, rectal
(BMI_extreme = 0; N0 = 513)
1−sided adj pval: 0
Frequency
Tumor−Normal Expression Difference
hsa−miR−1, rectal
(BMI_extreme = 1; N1 = 21)
1−sided adj pval: 0.125
Frequency
Tumor−Normal Expression Difference

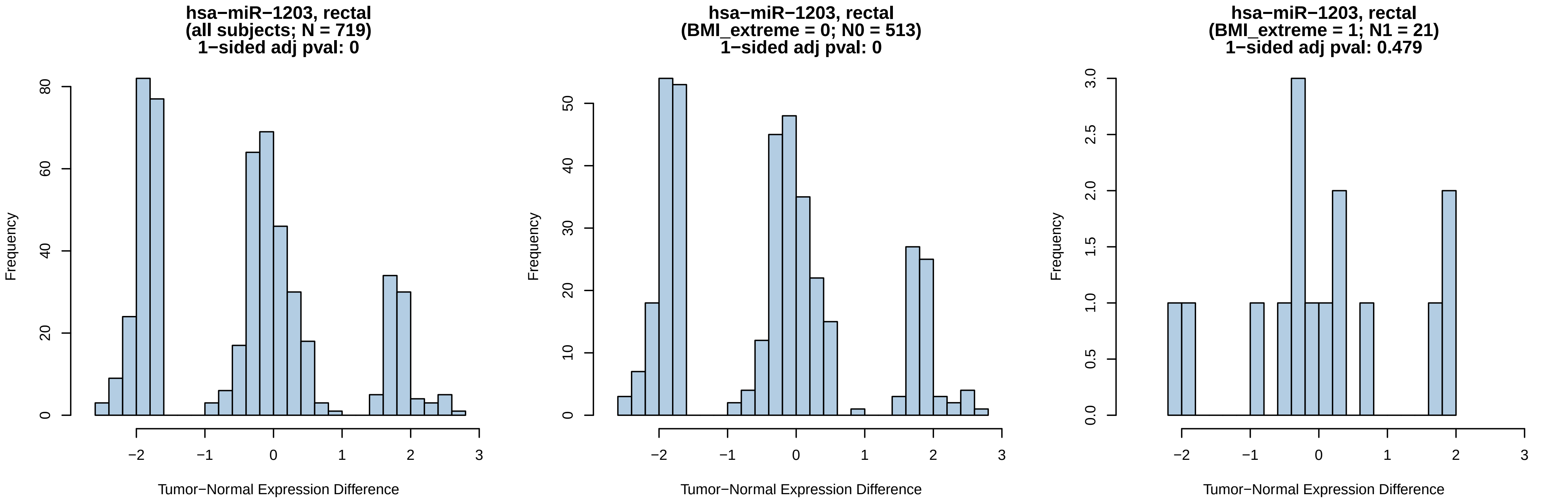

hsa−miR−1203, rectal
(all subjects; N = 719)
1−sided adj pval: 0
Frequency
Tumor−Normal Expression Difference
hsa−miR−1203, rectal
(BMI_extreme = 0; N0 = 513)
1−sided adj pval: 0
Frequency
Tumor−Normal Expression Difference
hsa−miR−1203, rectal
(BMI_extreme = 1; N1 = 21)
1−sided adj pval: 0.479
Frequency
Tumor−Normal Expression Difference

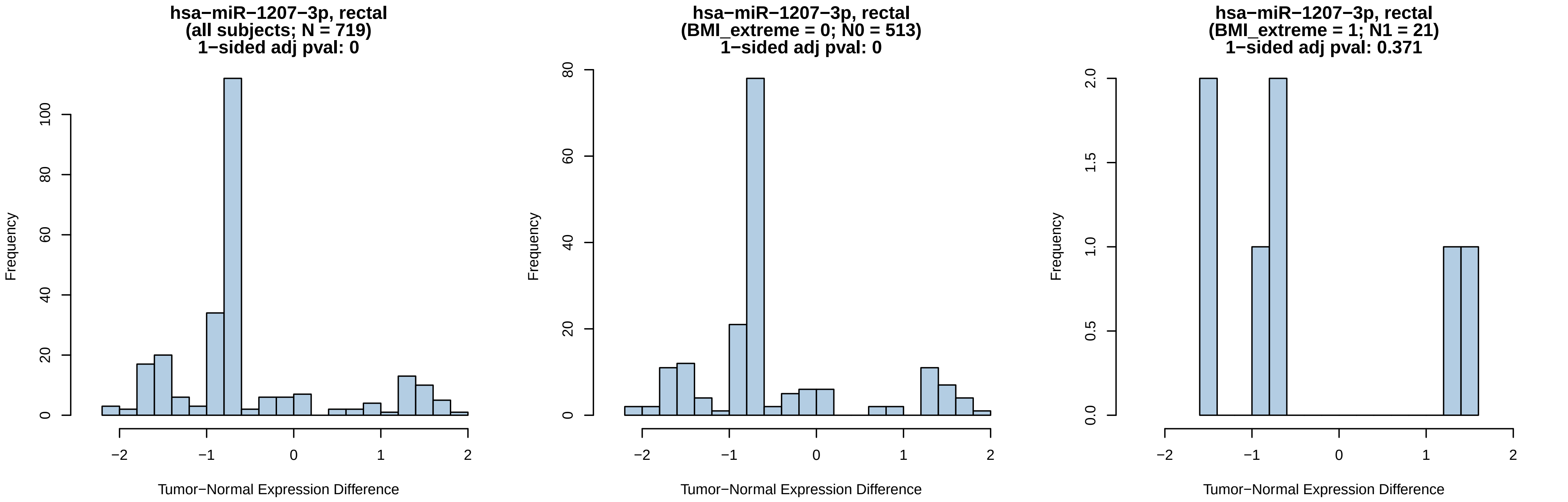
hsa−miR−1207−3p, rectal
(all subjects; N = 719)
1−sided adj pval: 0
Frequency
Tumor−Normal Expression Difference
hsa−miR−1207−3p, rectal
(BMI_extreme = 0; N0 = 513)
1−sided adj pval: 0
Frequency
Tumor−Normal Expression Difference
hsa−miR−1207−3p, rectal
(BMI_extreme = 1; N1 = 21)
1−sided adj pval: 0.371
Frequency
Tumor−Normal Expression Difference

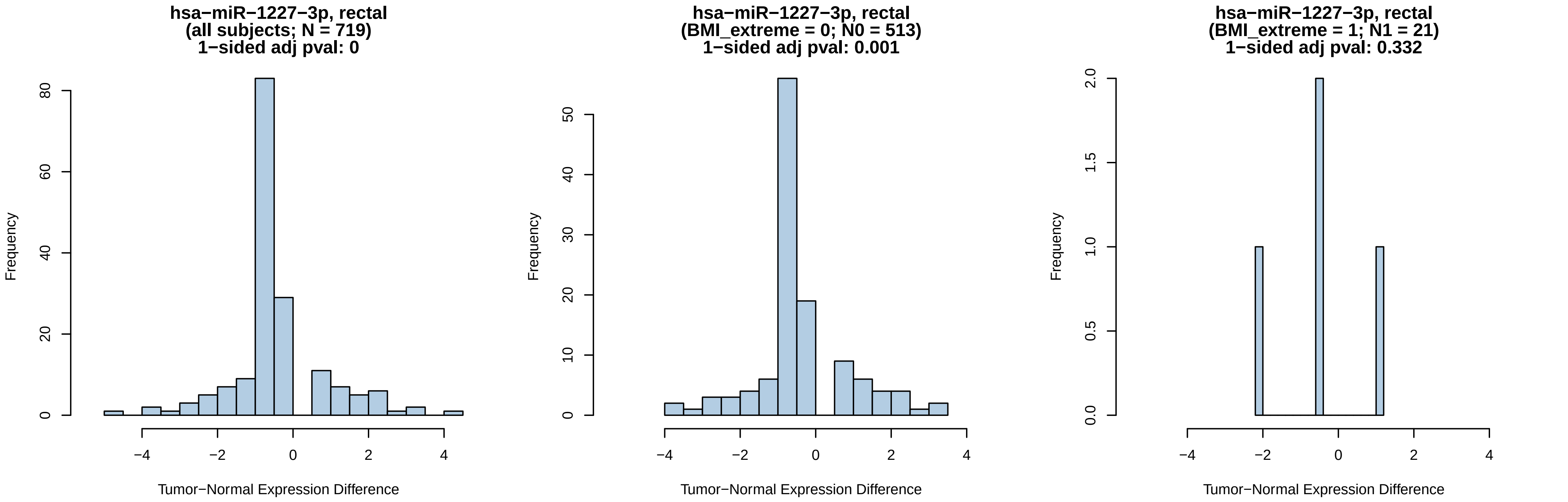
hsa−miR−1227−3p, rectal
(all subjects; N = 719)
1−sided adj pval: 0
Frequency
Tumor−Normal Expression Difference
hsa−miR−1227−3p, rectal
(BMI_extreme = 0; N0 = 513)
1−sided adj pval: 0.001
Frequency
Tumor−Normal Expression Difference
hsa−miR−1227−3p, rectal
(BMI_extreme = 1; N1 = 21)
1−sided adj pval: 0.332
Frequency
Tumor−Normal Expression Difference

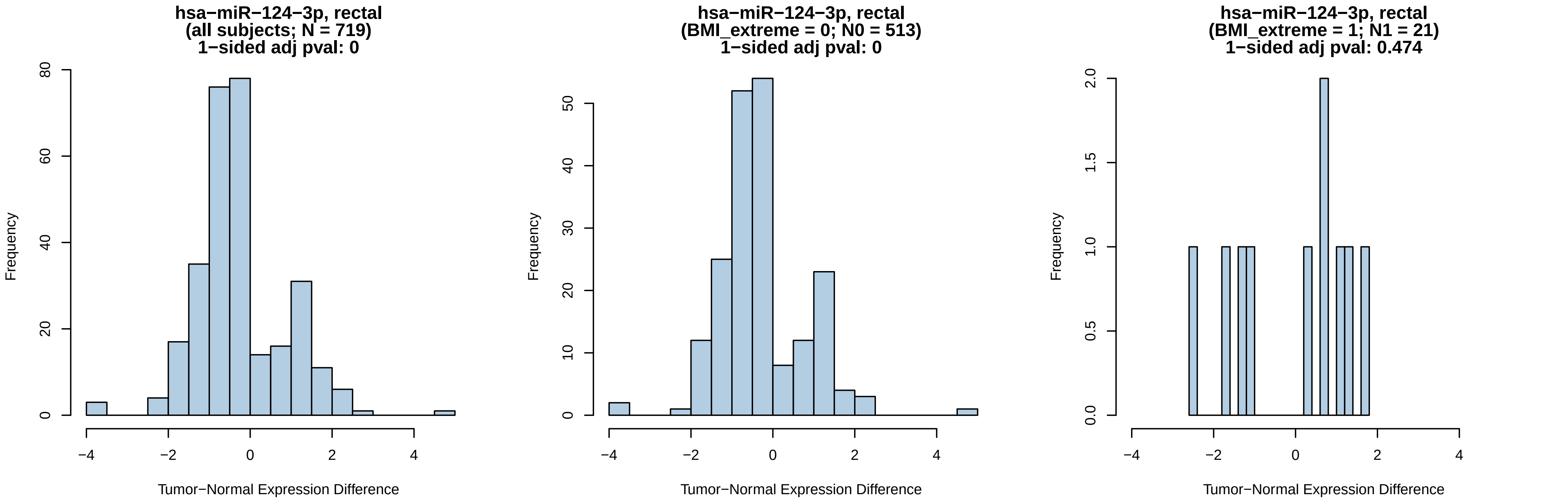
hsa−miR−124−3p, rectal
(all subjects; N = 719)
1−sided adj pval: 0
hsa−miR−124−3p, rectal
(BMI_extreme = 0; N0 = 513)
1−sided adj pval: 0
hsa−miR−124−3p, rectal
(BMI_extreme = 1; N1 = 21)
1−sided adj pval: 0.474
Frequency
Tumor−Normal Expression Difference

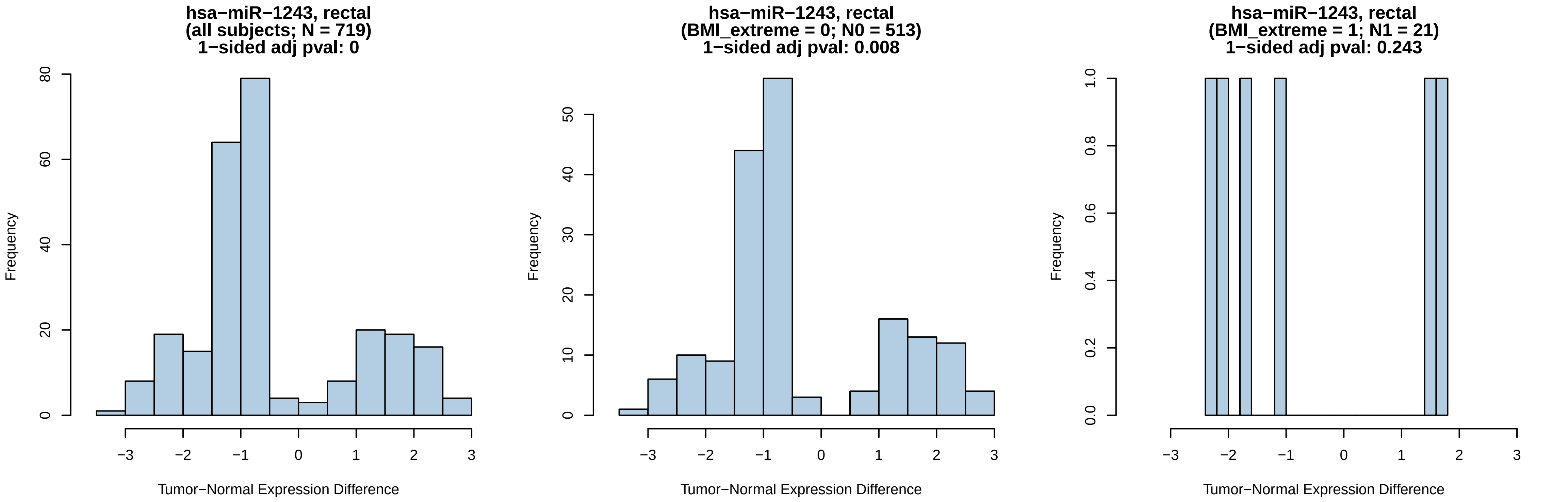

hsa−miR−1243, rectal
(all subjects; N = 719)
1−sided adj pval: 0
Frequency
Tumor−Normal Expression Difference
hsa−miR−1243, rectal
(BMI_extreme = 0; N0 = 513)
1−sided adj pval: 0.008
Frequency
Tumor−Normal Expression Difference
hsa−miR−1243, rectal
(BMI_extreme = 1; N1 = 21)
1−sided adj pval: 0.243
Frequency
Tumor−Normal Expression Difference

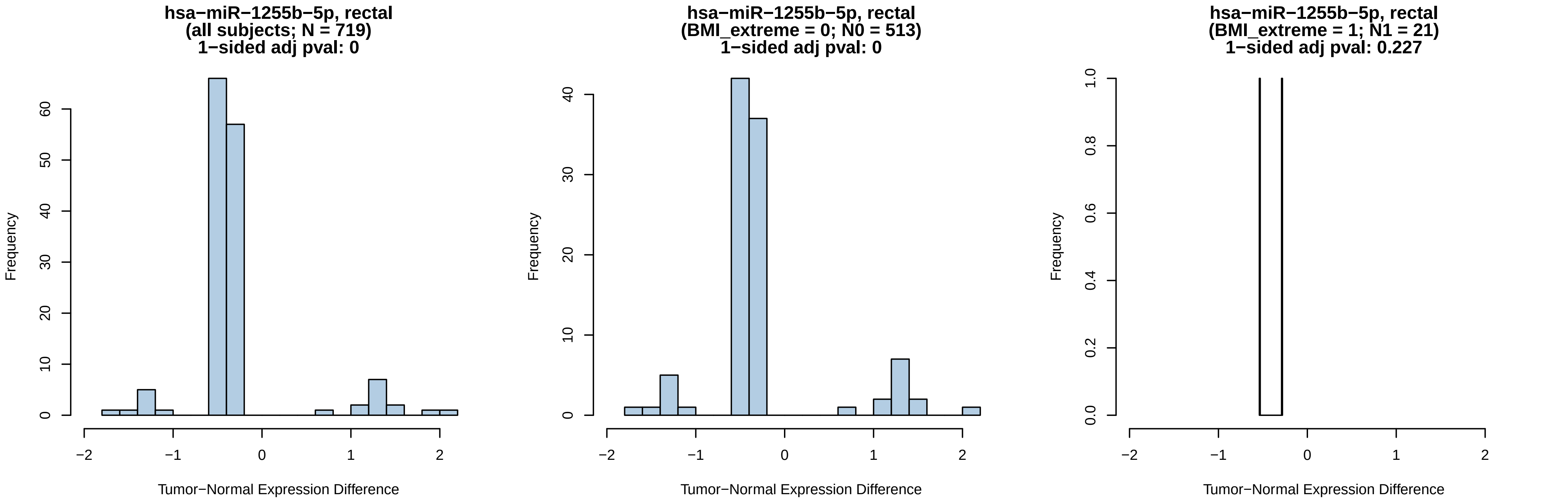

hsa−miR−1255b−5p, rectal
(all subjects; N = 719)
1−sided adj pval: 0
hsa−miR−1255b−5p, rectal
(BMI_extreme = 0; N0 = 513)
1−sided adj pval: 0
hsa−miR−1255b−5p, rectal
(BMI_extreme = 1; N1 = 21)
1−sided adj pval: 0.227
Frequency
Tumor−Normal Expression Difference

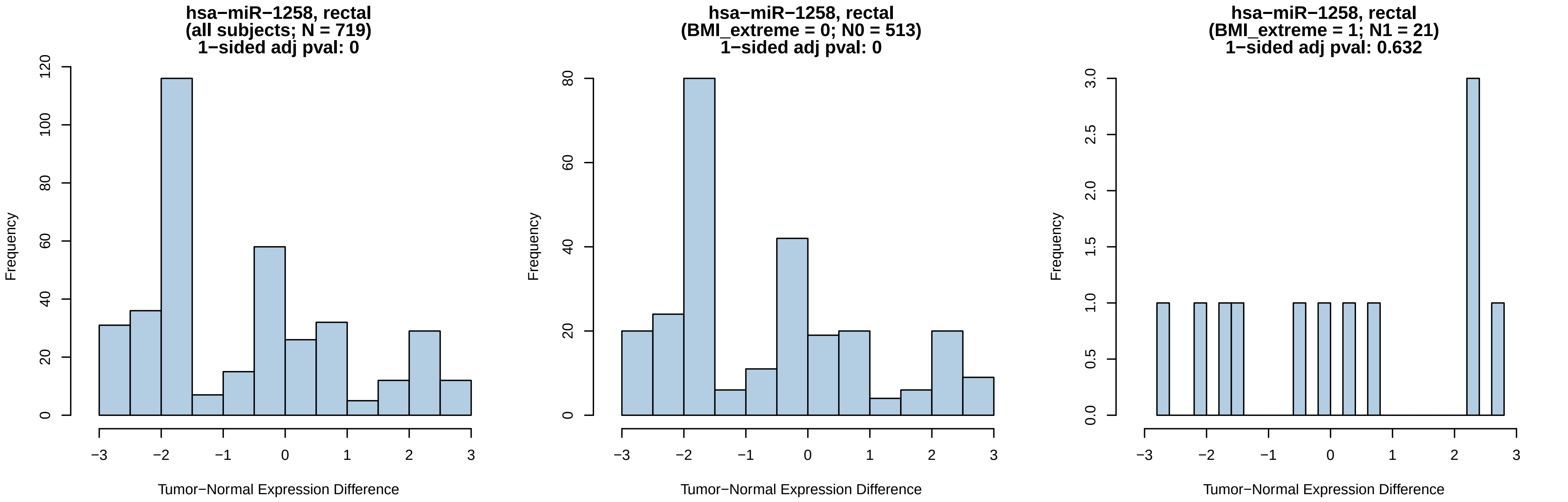
hsa−miR−1258, rectal
(all subjects; N = 719)
1−sided adj pval: 0
Frequency
Tumor−Normal Expression Difference
hsa−miR−1258, rectal
(BMI_extreme = 0; N0 = 513)
1−sided adj pval: 0
Frequency
Tumor−Normal Expression Difference
hsa−miR−1258, rectal
(BMI_extreme = 1; N1 = 21)
1−sided adj pval: 0.632
Frequency
Tumor−Normal Expression Difference

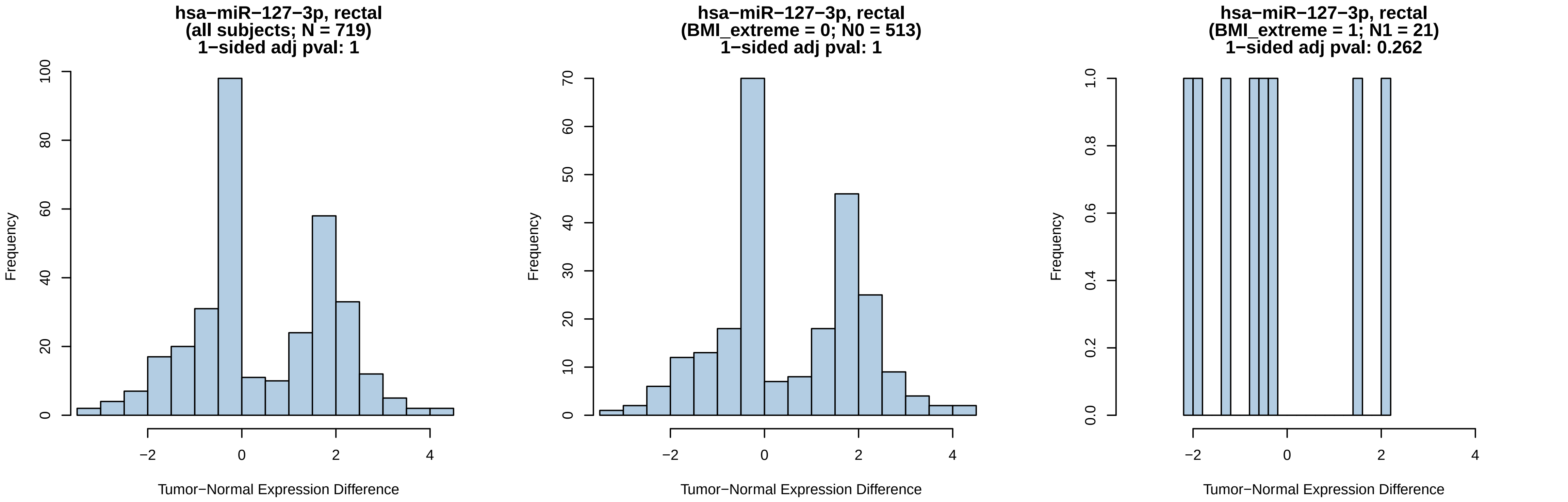

hsa−miR−127−3p, rectal
(all subjects; N = 719)
1−sided adj pval: 1
Frequency
0
20
40
60
80
100
−2
0
2
4
Tumor−Normal Expression Difference
hsa−miR−127−3p, rectal
(BMI_extreme = 0; N0 = 513)
1−sided adj pval: 1
Frequency
0
10
20
30
40
50
60
70
−2
0
2
4
Tumor−Normal Expression Difference
hsa−miR−127−3p, rectal
(BMI_extreme = 1; N1 = 21)
1−sided adj pval: 0.262
Frequency
0.0
0.2
0.4
0.6
0.8
1.0
−2
0
2
4
Tumor−Normal Expression Difference

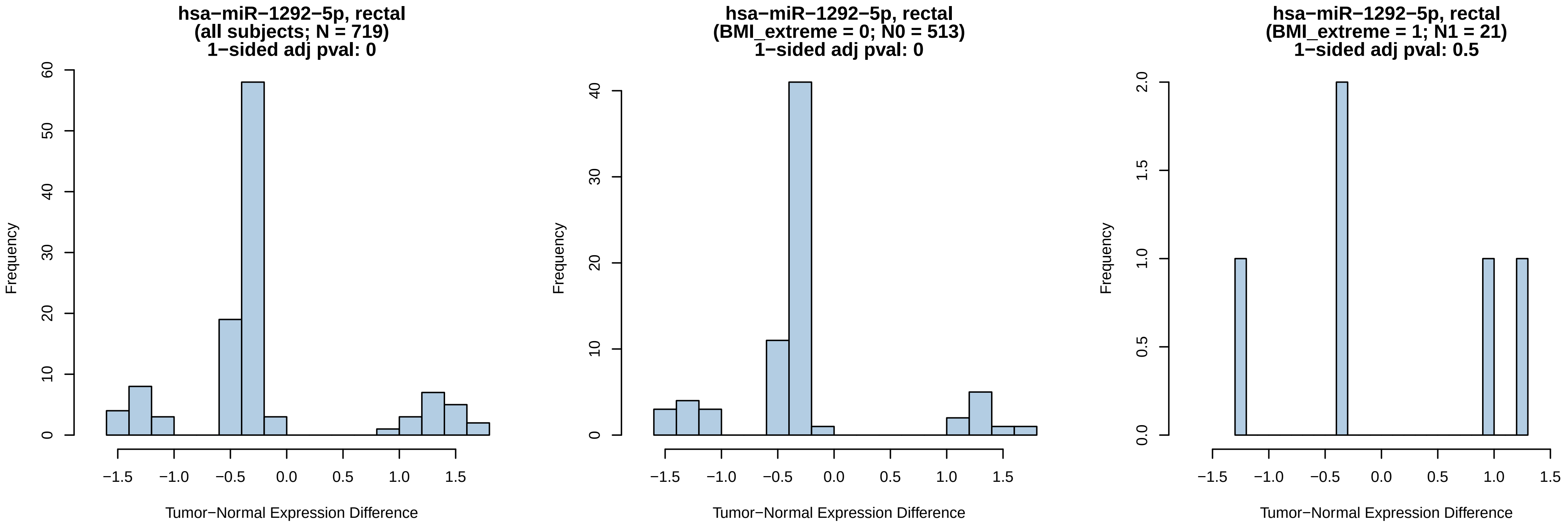
hsa−miR−1292−5p, rectal
(all subjects; N = 719)
1−sided adj pval: 0
Frequency
Tumor−Normal Expression Difference
hsa−miR−1292−5p, rectal
(BMI_extreme = 0; N0 = 513)
1−sided adj pval: 0
Frequency
Tumor−Normal Expression Difference
hsa−miR−1292−5p, rectal
(BMI_extreme = 1; N1 = 21)
1−sided adj pval: 0.5
Frequency
Tumor−Normal Expression Difference

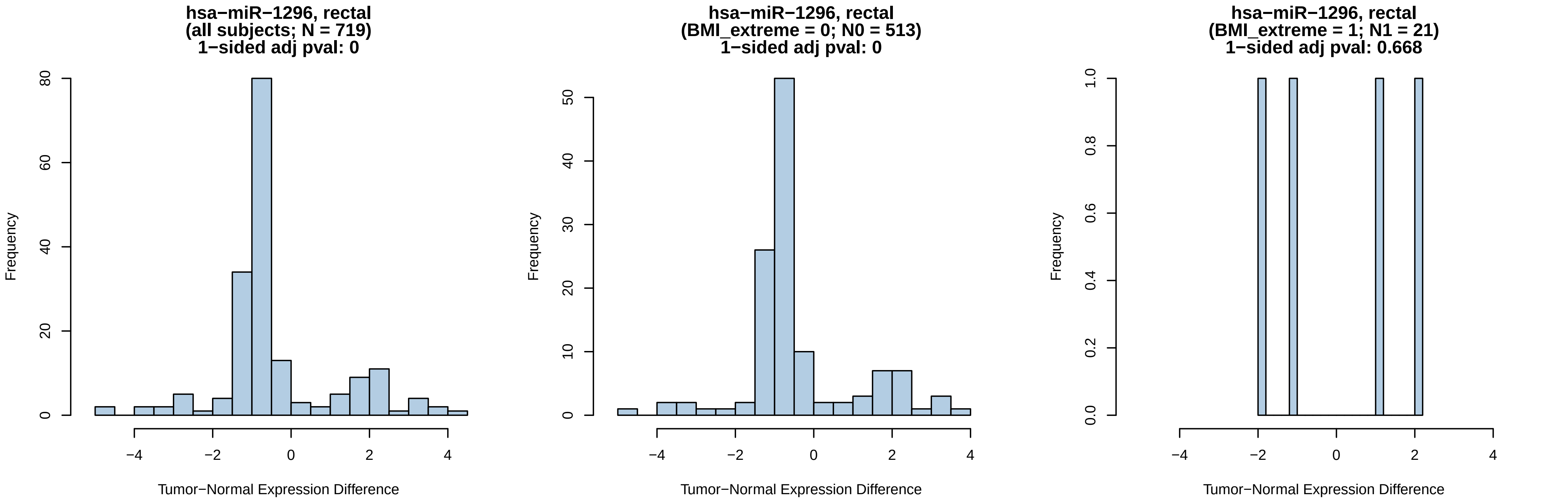
hsa−miR−1296, rectal
(all subjects; N = 719)
1−sided adj pval: 0
Frequency
Tumor−Normal Expression Difference
hsa−miR−1296, rectal
(BMI_extreme = 0; N0 = 513)
1−sided adj pval: 0
Frequency
Tumor−Normal Expression Difference
hsa−miR−1296, rectal
(BMI_extreme = 1; N1 = 21)
1−sided adj pval: 0.668
Frequency
Tumor−Normal Expression Difference

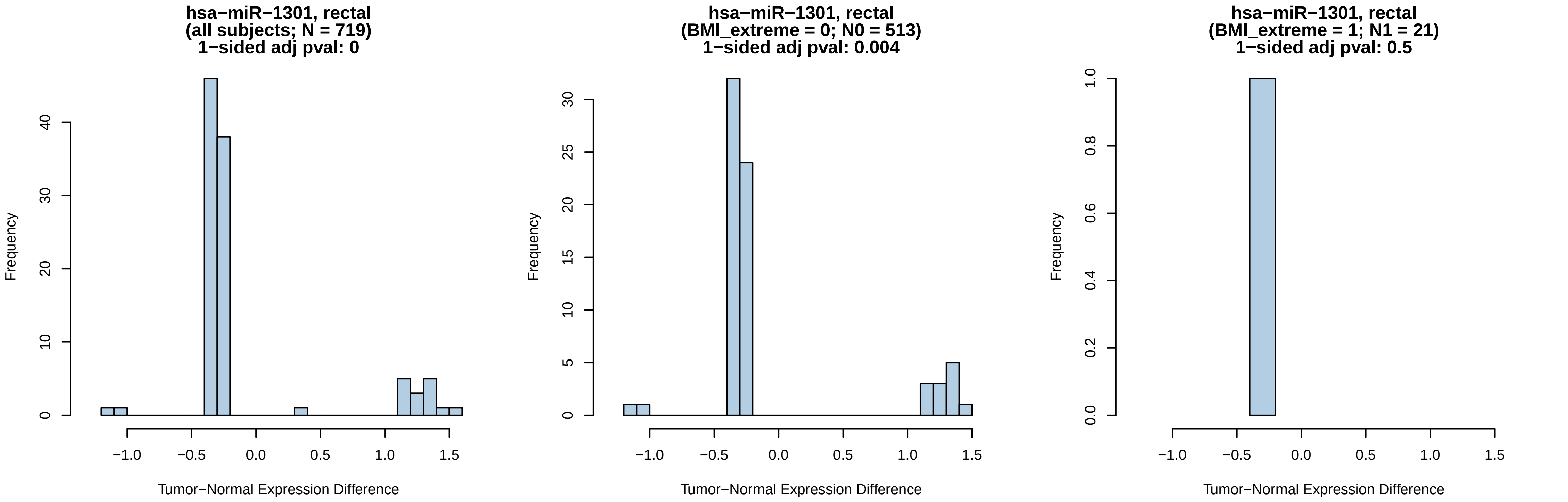
hsa−miR−1301, rectal
(all subjects; N = 719)
1−sided adj pval: 0
Frequency
0
10
20
30
40
−1.0
−0.5
0.0
0.5
1.0
1.5
Tumor−Normal Expression Difference
hsa−miR−1301, rectal
(BMI_extreme = 0; N0 = 513)
1−sided adj pval: 0.004
Frequency
0
5
10
15
20
25
30
−1.0
−0.5
0.0
0.5
1.0
1.5
Tumor−Normal Expression Difference
hsa−miR−1301, rectal
(BMI_extreme = 1; N1 = 21)
1−sided adj pval: 0.5
Frequency
0.0
0.2
0.4
0.6
0.8
1.0
−1.0
−0.5
0.0
0.5
1.0
1.5
Tumor−Normal Expression Difference

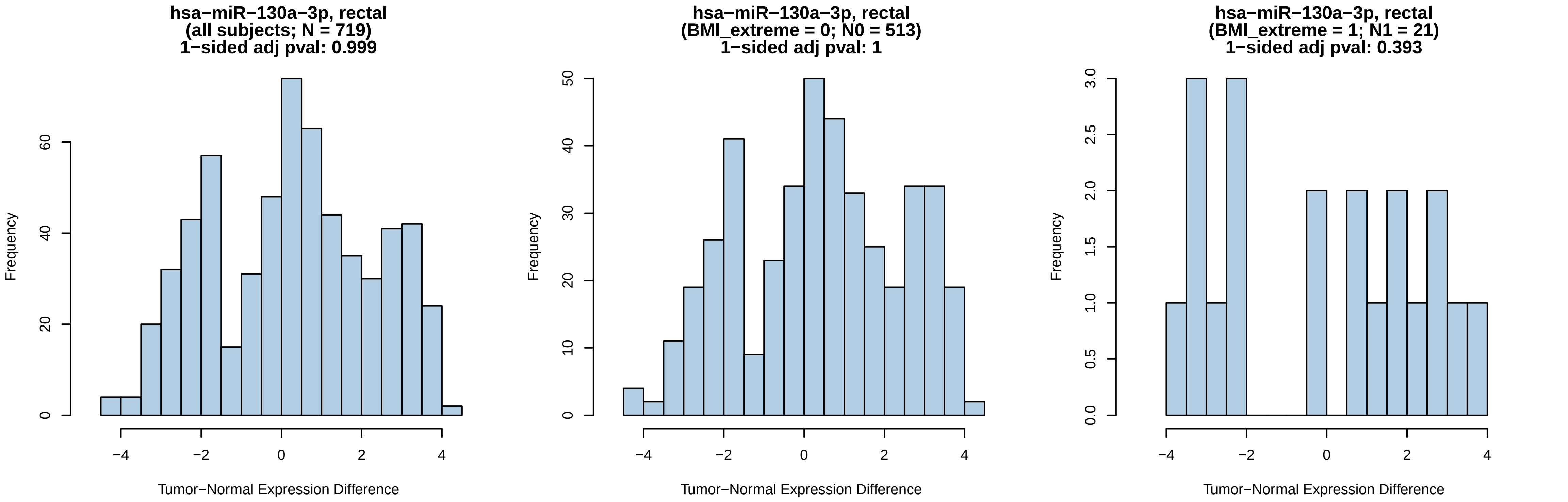
hsa−miR−130a−3p, rectal
(all subjects; N = 719)
1−sided adj pval: 0.999
Frequency
Tumor−Normal Expression Difference
hsa−miR−130a−3p, rectal
(BMI_extreme = 0; N0 = 513)
1−sided adj pval: 1
Frequency
Tumor−Normal Expression Difference
hsa−miR−130a−3p, rectal
(BMI_extreme = 1; N1 = 21)
1−sided adj pval: 0.393
Frequency
Tumor−Normal Expression Difference

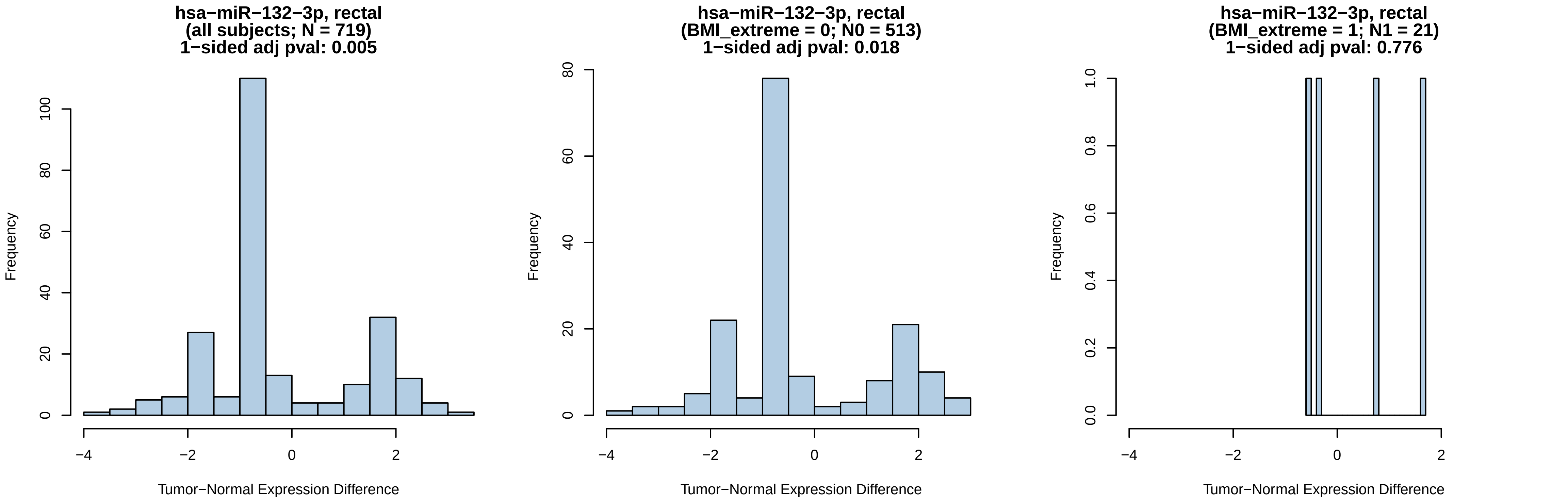

hsa−miR−132−3p, rectal
(all subjects; N = 719)
1−sided adj pval: 0.005
Frequency
Tumor−Normal Expression Difference
hsa−miR−132−3p, rectal
(BMI_extreme = 0; N0 = 513)
1−sided adj pval: 0.018
Frequency
Tumor−Normal Expression Difference
hsa−miR−132−3p, rectal
(BMI_extreme = 1; N1 = 21)
1−sided adj pval: 0.776
Frequency
Tumor−Normal Expression Difference

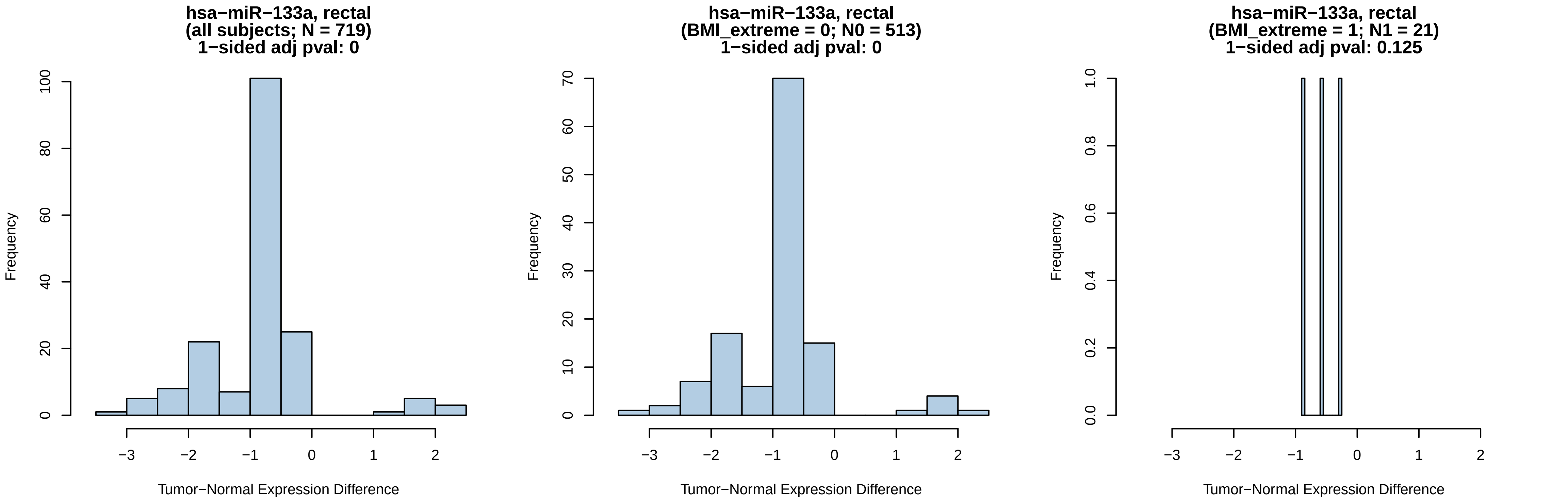

hsa−miR−133a, rectal
(all subjects; N = 719)
1−sided adj pval: 0
Frequency
0
20
40
60
80
100
−3
−2
−1
0
1
2
Tumor−Normal Expression Difference
hsa−miR−133a, rectal
(BMI_extreme = 0; N0 = 513)
1−sided adj pval: 0
Frequency
0
10
20
30
40
50
60
70
−3
−2
−1
0
1
2
Tumor−Normal Expression Difference
hsa−miR−133a, rectal
(BMI_extreme = 1; N1 = 21)
1−sided adj pval: 0.125
Frequency
0.0
0.2
0.4
0.6
0.8
1.0
−3
−2
−1
0
1
2
Tumor−Normal Expression Difference

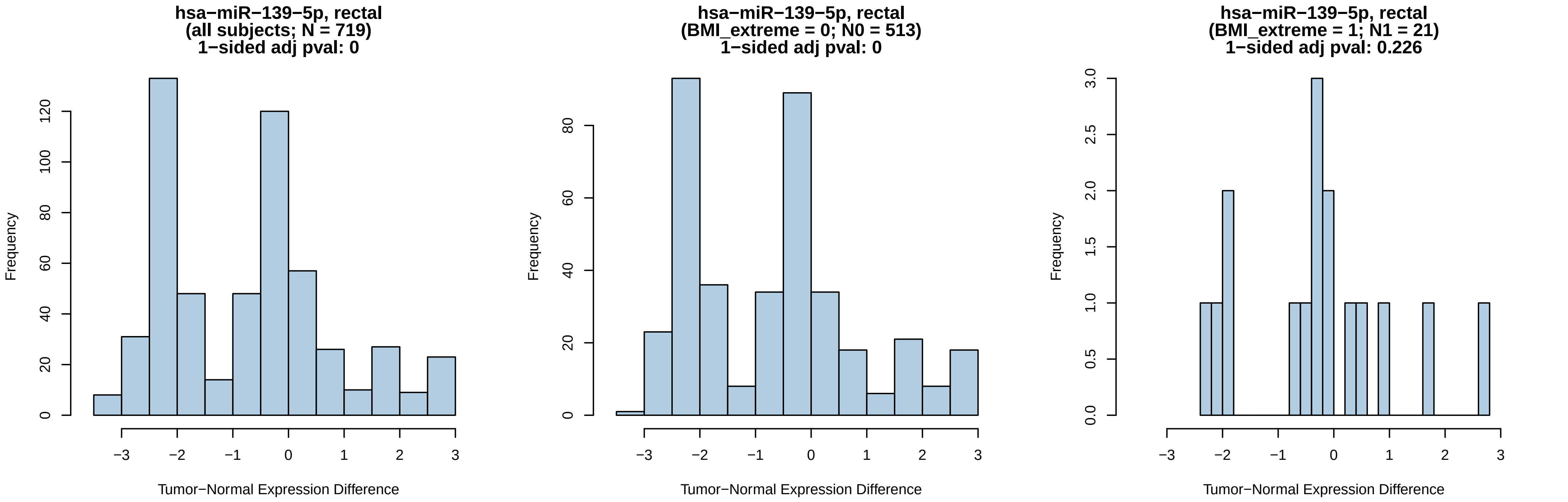
hsa−miR−139−5p, rectal
(all subjects; N = 719)
1−sided adj pval: 0
Frequency
0
20
40
60
80
100
120
−3
−2
−1
0
1
2
3
Tumor−Normal Expression Difference
hsa−miR−139−5p, rectal
(BMI_extreme = 0; N0 = 513)
1−sided adj pval: 0
Frequency
0
20
40
60
80
−3
−2
−1
0
1
2
3
Tumor−Normal Expression Difference
hsa−miR−139−5p, rectal
(BMI_extreme = 1; N1 = 21)
1−sided adj pval: 0.226
Frequency
0.0
0.5
1.0
1.5
2.0
2.5
3.0
−3
−2
−1
0
1
2
3
Tumor−Normal Expression Difference

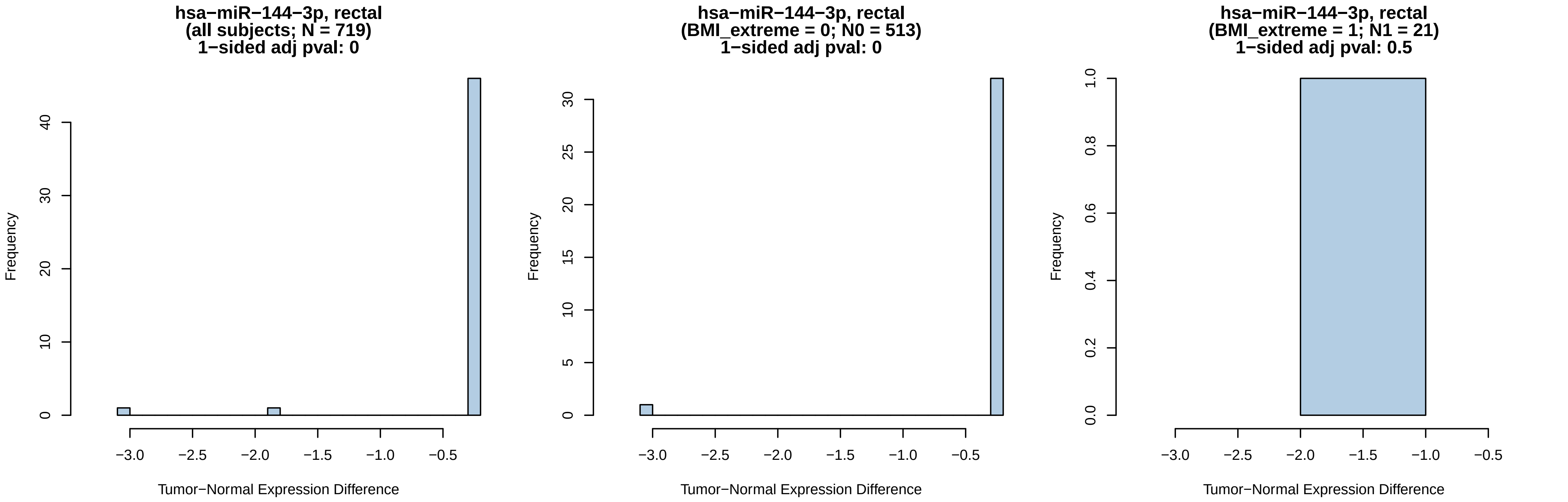
hsa−miR−144−3p, rectal
(all subjects; N = 719)
1−sided adj pval: 0
Frequency
0
10
20
30
40
−3.0
−2.5
−2.0
−1.5
−1.0
−0.5
Tumor−Normal Expression Difference
hsa−miR−144−3p, rectal
(BMI_extreme = 0; N0 = 513)
1−sided adj pval: 0
Frequency
0
5
10
15
20
25
30
−3.0
−2.5
−2.0
−1.5
−1.0
−0.5
Tumor−Normal Expression Difference
hsa−miR−144−3p, rectal
(BMI_extreme = 1; N1 = 21)
1−sided adj pval: 0.5
Frequency
0.0
0.2
0.4
0.6
0.8
1.0
−3.0
−2.5
−2.0
−1.5
−1.0
−0.5
Tumor−Normal Expression Difference

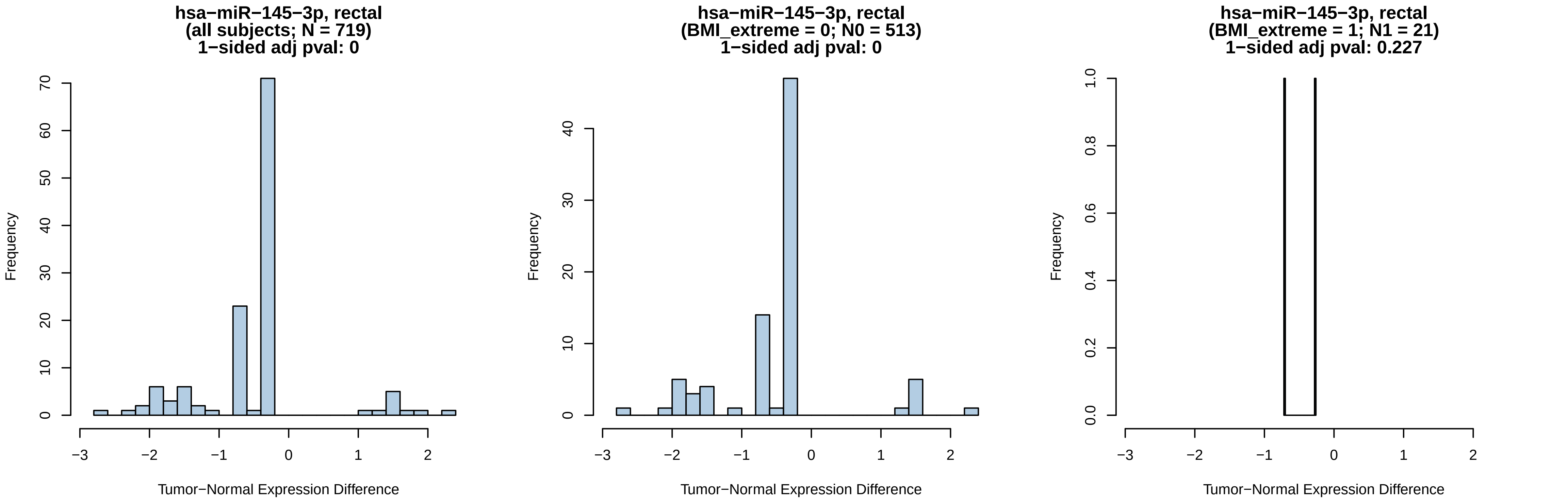

hsa−miR−145−3p, rectal
(all subjects; N = 719)
1−sided adj pval: 0
Frequency
Tumor−Normal Expression Difference
hsa−miR−145−3p, rectal
(BMI_extreme = 0; N0 = 513)
1−sided adj pval: 0
Frequency
Tumor−Normal Expression Difference
hsa−miR−145−3p, rectal
(BMI_extreme = 1; N1 = 21)
1−sided adj pval: 0.227
Frequency
Tumor−Normal Expression Difference

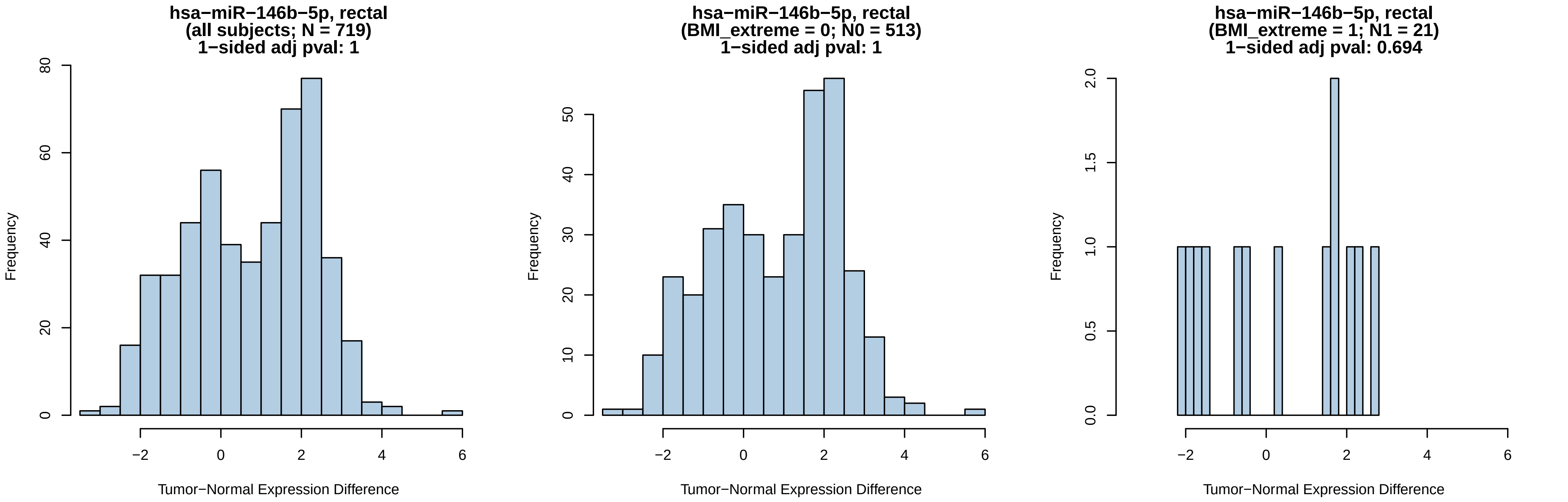
hsa−miR−146b−5p, rectal
(all subjects; N = 719)
1−sided adj pval: 1
Frequency
Tumor−Normal Expression Difference
hsa−miR−146b−5p, rectal
(BMI_extreme = 0; N0 = 513)
1−sided adj pval: 1
Frequency
Tumor−Normal Expression Difference
hsa−miR−146b−5p, rectal
(BMI_extreme = 1; N1 = 21)
1−sided adj pval: 0.694
Frequency
Tumor−Normal Expression Difference

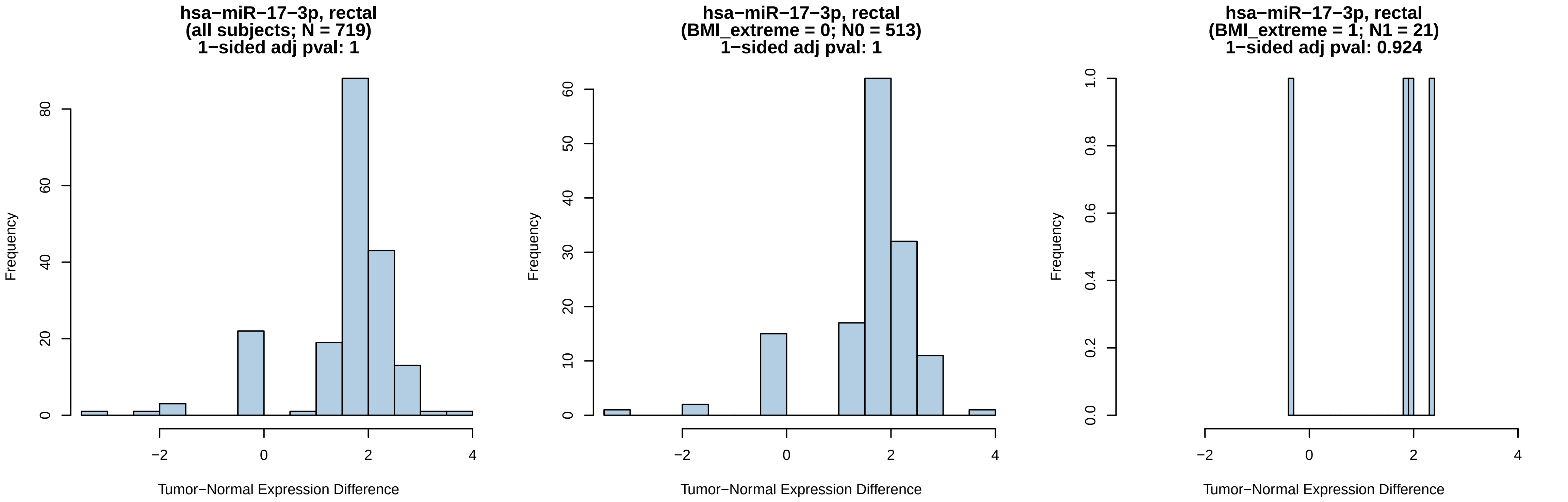

hsa−miR−17−3p, rectal
(all subjects; N = 719)
1−sided adj pval: 1
Frequency
0
20
40
60
80
−2
0
2
4
Tumor−Normal Expression Difference
hsa−miR−17−3p, rectal
(BMI_extreme = 0; N0 = 513)
1−sided adj pval: 1
Frequency
0
10
20
30
40
50
60
−2
0
2
4
Tumor−Normal Expression Difference
hsa−miR−17−3p, rectal
(BMI_extreme = 1; N1 = 21)
1−sided adj pval: 0.924
Frequency
0.0
0.2
0.4
0.6
0.8
1.0
−2
0
2
4
Tumor−Normal Expression Difference

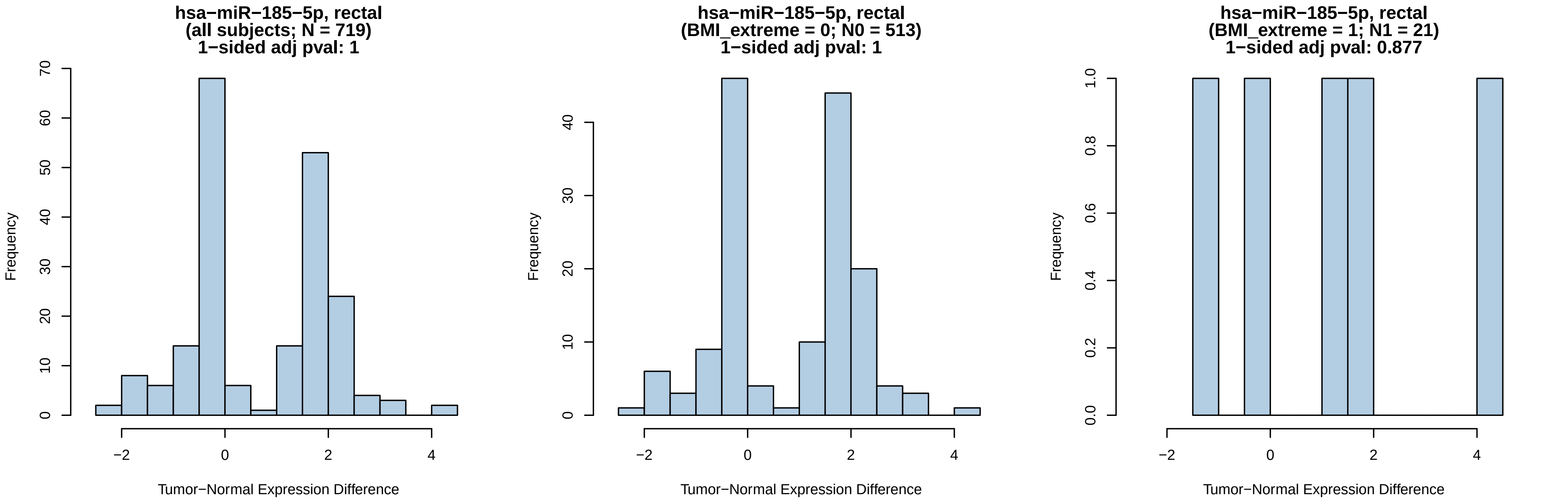
hsa−miR−185−5p, rectal
(all subjects; N = 719)
1−sided adj pval: 1
Frequency
Tumor−Normal Expression Difference
hsa−miR−185−5p, rectal
(BMI_extreme = 0; N0 = 513)
1−sided adj pval: 1
Frequency
Tumor−Normal Expression Difference
hsa−miR−185−5p, rectal
(BMI_extreme = 1; N1 = 21)
1−sided adj pval: 0.877
Frequency
Tumor−Normal Expression Difference

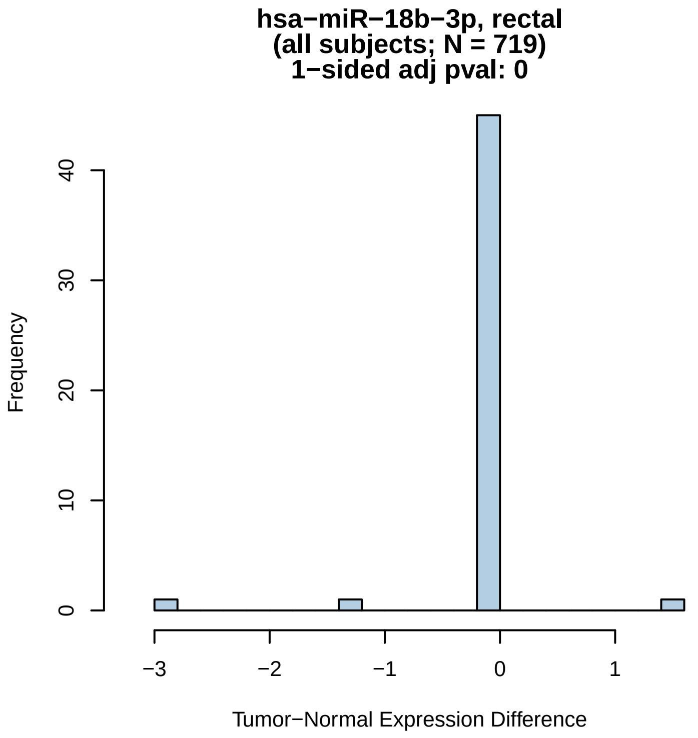
hsa−miR−18b−3p, rectal
(all subjects; N = 719)
1−sided adj pval: 0
Frequency
0
10
20
30
40
−3
−2
−1
0
1
Tumor−Normal Expression Difference

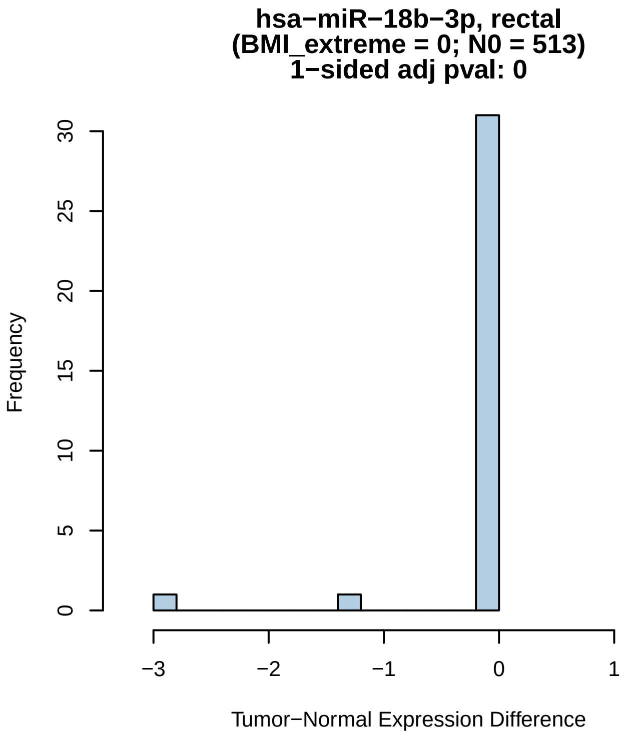
hsa−miR−18b−3p, rectal
(BMI_extreme = 0; N0 = 513)
1−sided adj pval: 0
Frequency
0
5
10
15
20
25
30
−3
−2
−1
0
1
Tumor−Normal Expression Difference

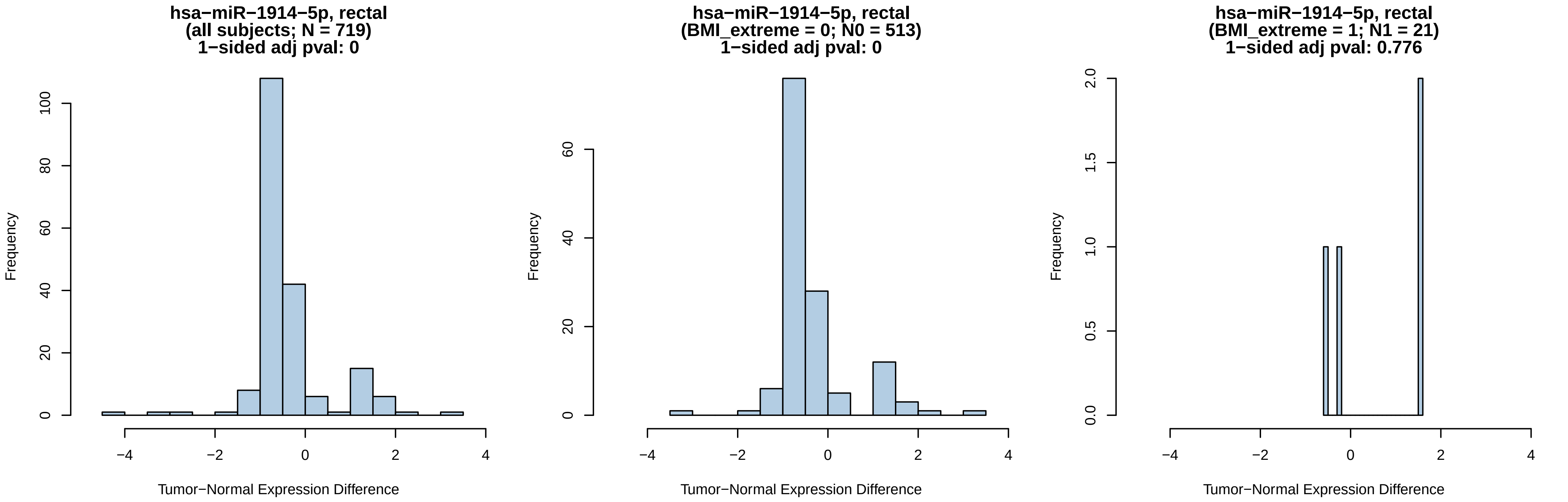
hsa−miR−1914−5p, rectal
(all subjects; N = 719)
1−sided adj pval: 0
Frequency
Tumor−Normal Expression Difference
hsa−miR−1914−5p, rectal
(BMI_extreme = 0; N0 = 513)
1−sided adj pval: 0
Frequency
Tumor−Normal Expression Difference
hsa−miR−1914−5p, rectal
(BMI_extreme = 1; N1 = 21)
1−sided adj pval: 0.776
Frequency
Tumor−Normal Expression Difference

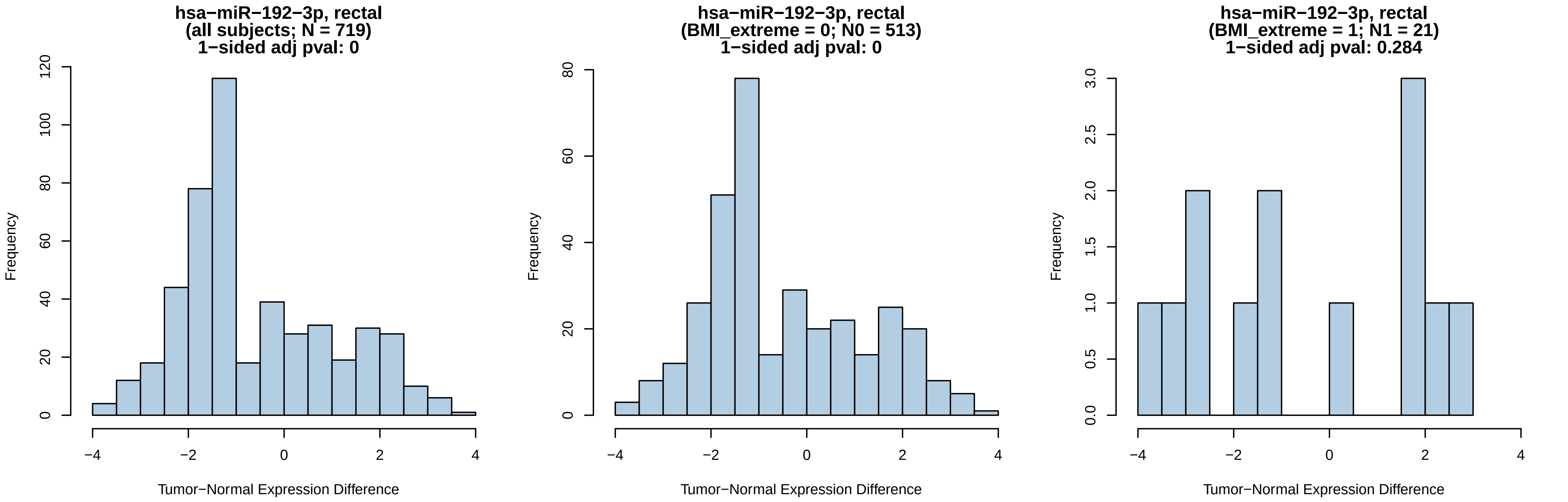

hsa−miR−192−3p, rectal
(all subjects; N = 719)
1−sided adj pval: 0
Frequency
Tumor−Normal Expression Difference
hsa−miR−192−3p, rectal
(BMI_extreme = 0; N0 = 513)
1−sided adj pval: 0
Frequency
Tumor−Normal Expression Difference
hsa−miR−192−3p, rectal
(BMI_extreme = 1; N1 = 21)
1−sided adj pval: 0.284
Frequency
Tumor−Normal Expression Difference

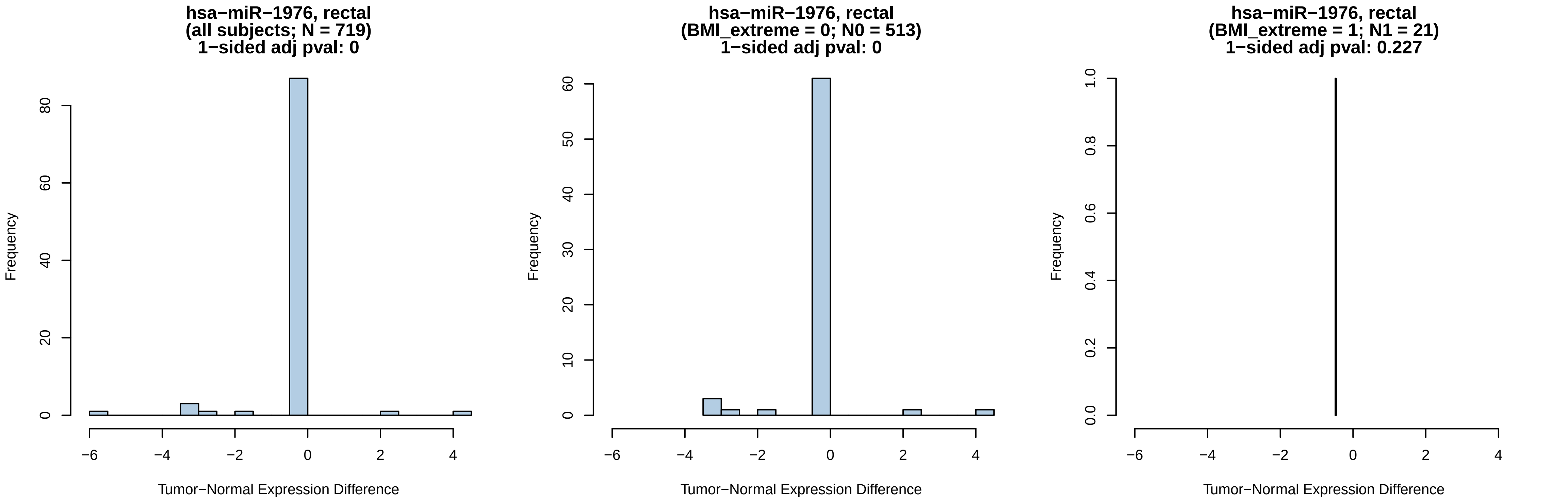
hsa−miR−1976, rectal
(all subjects; N = 719)
1−sided adj pval: 0
Frequency
Tumor−Normal Expression Difference
hsa−miR−1976, rectal
(BMI_extreme = 0; N0 = 513)
1−sided adj pval: 0
Frequency
Tumor−Normal Expression Difference
hsa−miR−1976, rectal
(BMI_extreme = 1; N1 = 21)
1−sided adj pval: 0.227
Frequency
Tumor−Normal Expression Difference

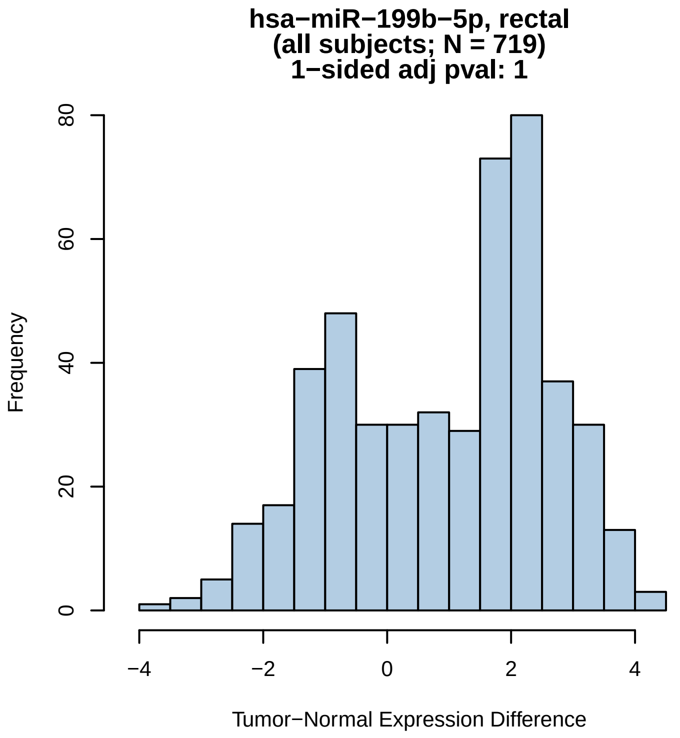
hsa−miR−199b−5p, rectal
(all subjects; N = 719)
1−sided adj pval: 1
Frequency
Tumor−Normal Expression Difference
0
20
40
60
80
−4
−2
0
2
4

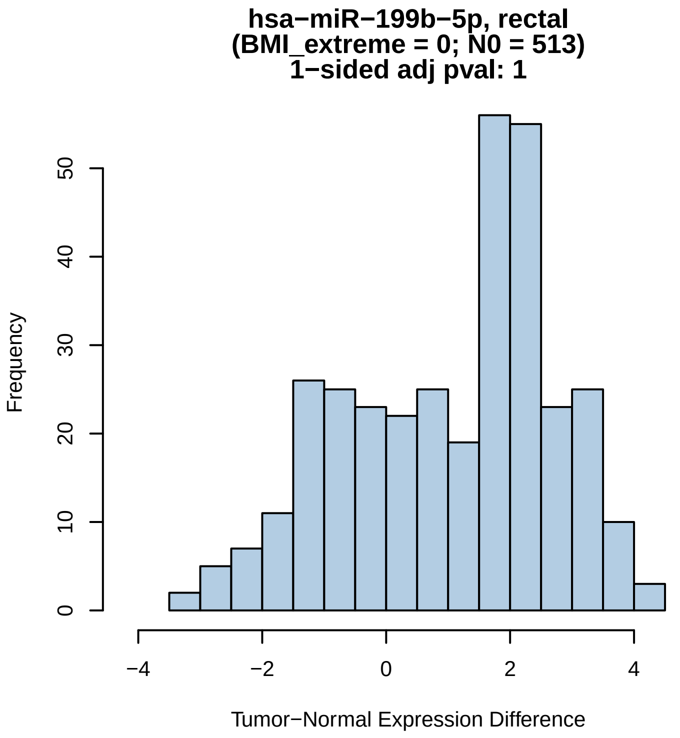
hsa−miR−199b−5p, rectal
(BMI_extreme = 0; N0 = 513)
1−sided adj pval: 1
Frequency
Tumor−Normal Expression Difference
0
10
20
30
40
50
−4
−2
0
2
4

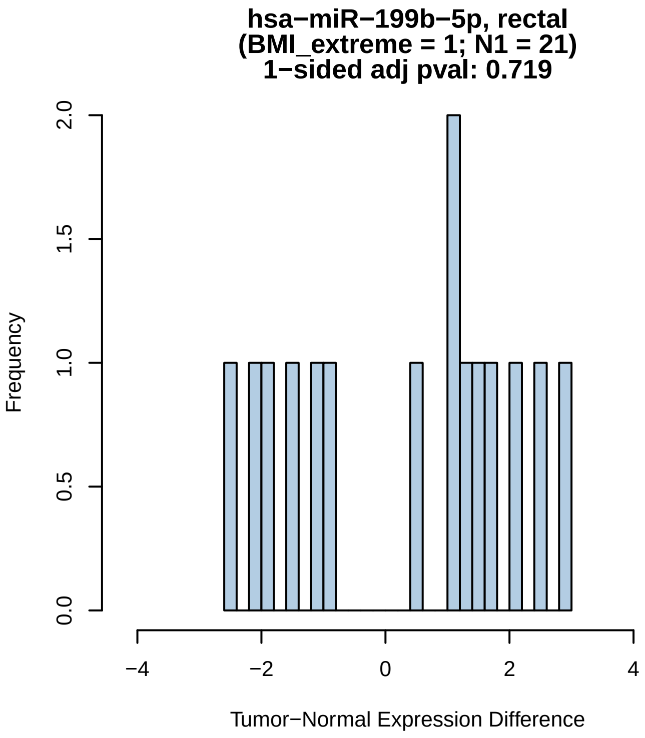
hsa−miR−199b−5p, rectal
(BMI_extreme = 1; N1 = 21)
1−sided adj pval: 0.719
Frequency
Tumor−Normal Expression Difference
0.0
0.5
1.0
1.5
2.0
−4
−2
0
2
4

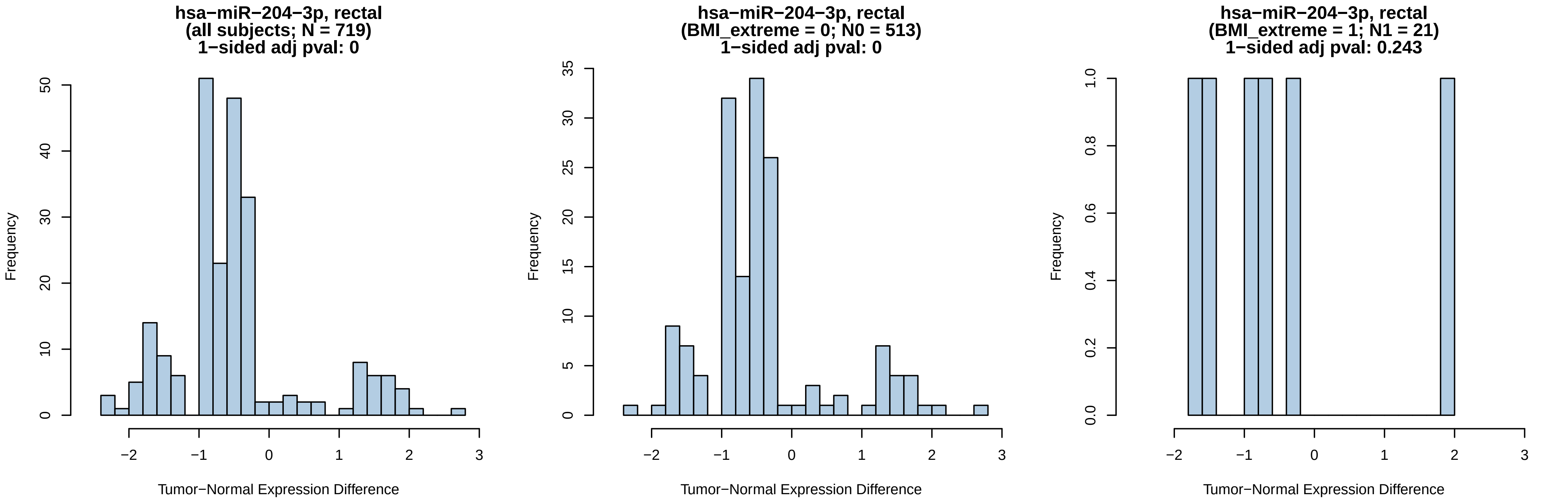
hsa−miR−204−3p, rectal
(all subjects; N = 719)
1−sided adj pval: 0
Frequency
Tumor−Normal Expression Difference
hsa−miR−204−3p, rectal
(BMI_extreme = 0; N0 = 513)
1−sided adj pval: 0
Frequency
Tumor−Normal Expression Difference
hsa−miR−204−3p, rectal
(BMI_extreme = 1; N1 = 21)
1−sided adj pval: 0.243
Frequency
Tumor−Normal Expression Difference

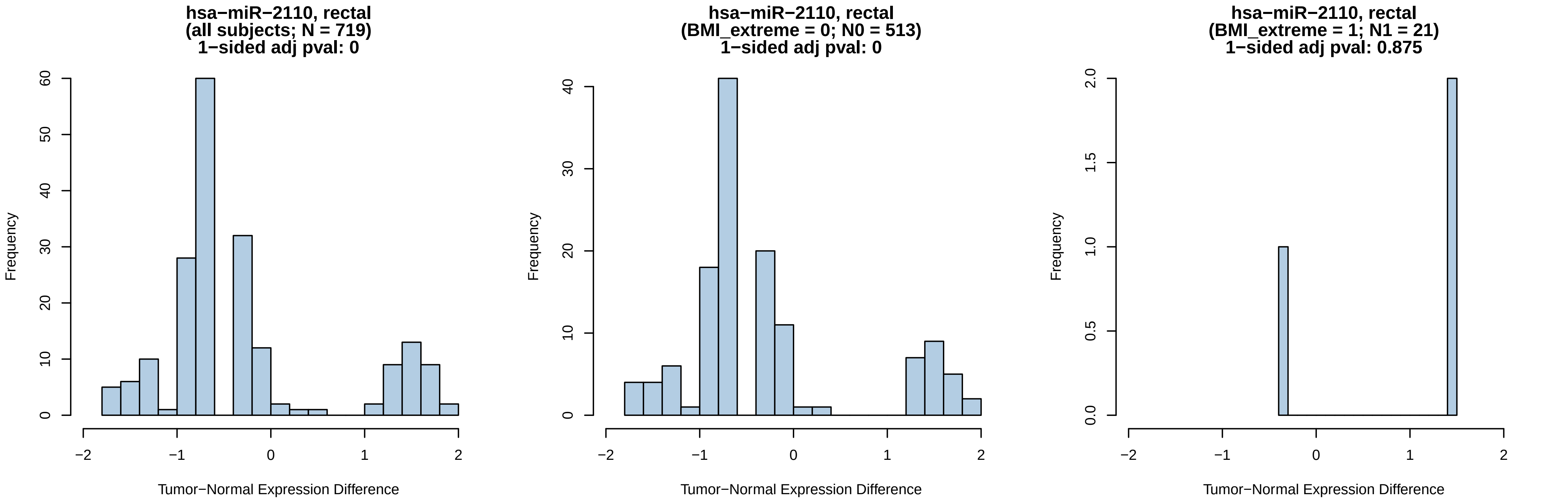

hsa−miR−2110, rectal
(all subjects; N = 719)
1−sided adj pval: 0
Frequency
Tumor−Normal Expression Difference
hsa−miR−2110, rectal
(BMI_extreme = 0; N0 = 513)
1−sided adj pval: 0
Frequency
Tumor−Normal Expression Difference
hsa−miR−2110, rectal
(BMI_extreme = 1; N1 = 21)
1−sided adj pval: 0.875
Frequency
Tumor−Normal Expression Difference

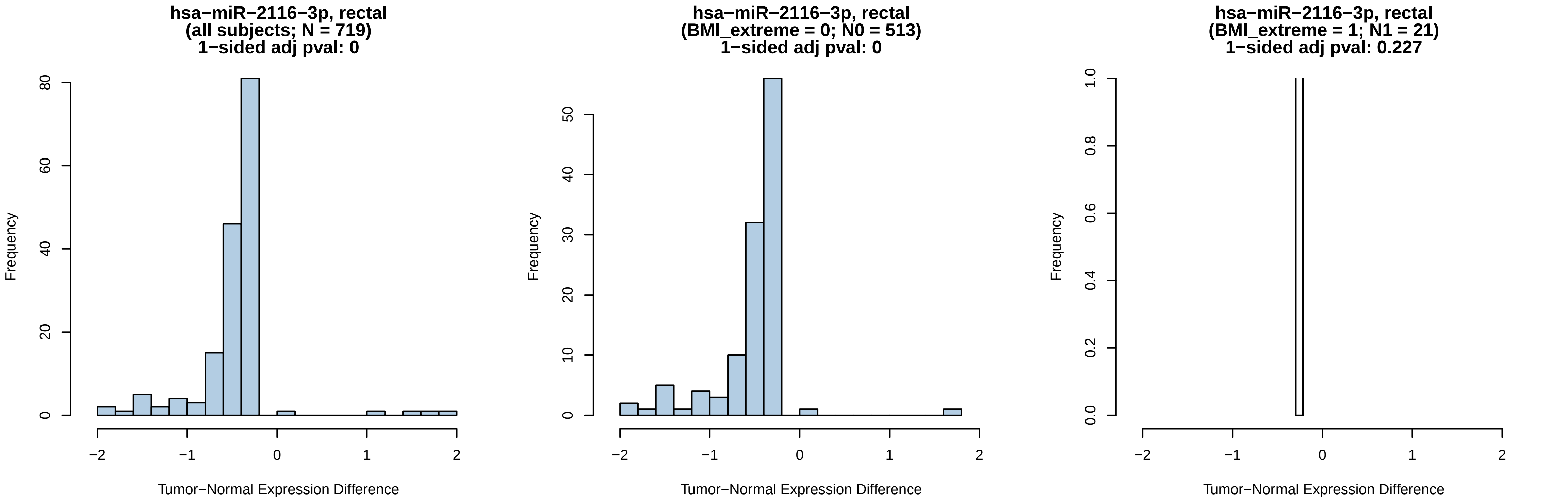
hsa−miR−2116−3p, rectal
(all subjects; N = 719)
1−sided adj pval: 0
Frequency
Tumor−Normal Expression Difference
hsa−miR−2116−3p, rectal
(BMI_extreme = 0; N0 = 513)
1−sided adj pval: 0
Frequency
Tumor−Normal Expression Difference
hsa−miR−2116−3p, rectal
(BMI_extreme = 1; N1 = 21)
1−sided adj pval: 0.227
Frequency
Tumor−Normal Expression Difference

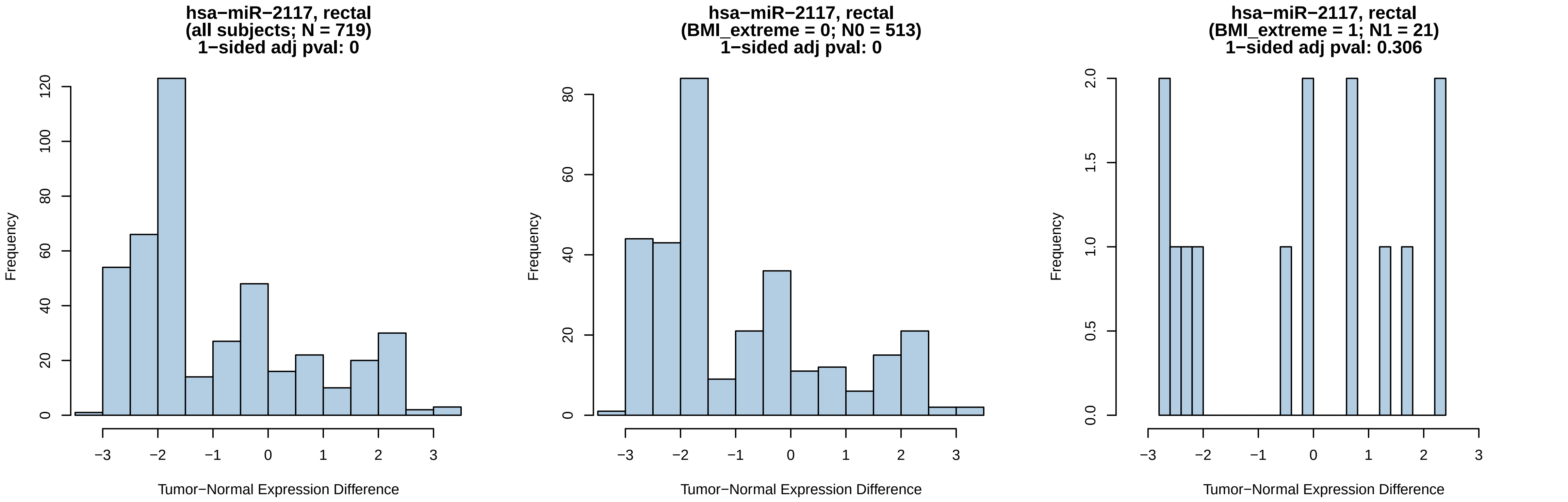

hsa−miR−2117, rectal
(all subjects; N = 719)
1−sided adj pval: 0
Frequency
0
20
40
60
80
100
120
−3
−2
−1
0
1
2
3
Tumor−Normal Expression Difference
hsa−miR−2117, rectal
(BMI_extreme = 0; N0 = 513)
1−sided adj pval: 0
Frequency
0
20
40
60
80
−3
−2
−1
0
1
2
3
Tumor−Normal Expression Difference
hsa−miR−2117, rectal
(BMI_extreme = 1; N1 = 21)
1−sided adj pval: 0.306
Frequency
0.0
0.5
1.0
1.5
2.0
−3
−2
−1
0
1
2
3
Tumor−Normal Expression Difference

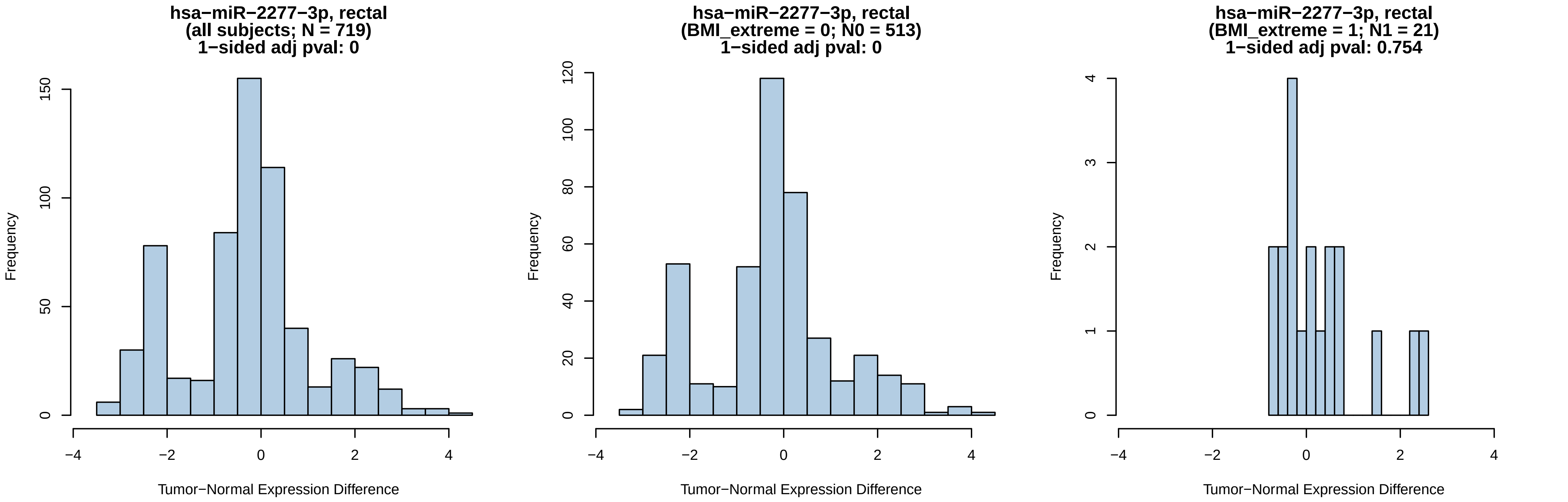

hsa−miR−2277−3p, rectal
(all subjects; N = 719)
1−sided adj pval: 0
Frequency
Tumor−Normal Expression Difference
hsa−miR−2277−3p, rectal
(BMI_extreme = 0; N0 = 513)
1−sided adj pval: 0
Frequency
Tumor−Normal Expression Difference
hsa−miR−2277−3p, rectal
(BMI_extreme = 1; N1 = 21)
1−sided adj pval: 0.754
Frequency
Tumor−Normal Expression Difference

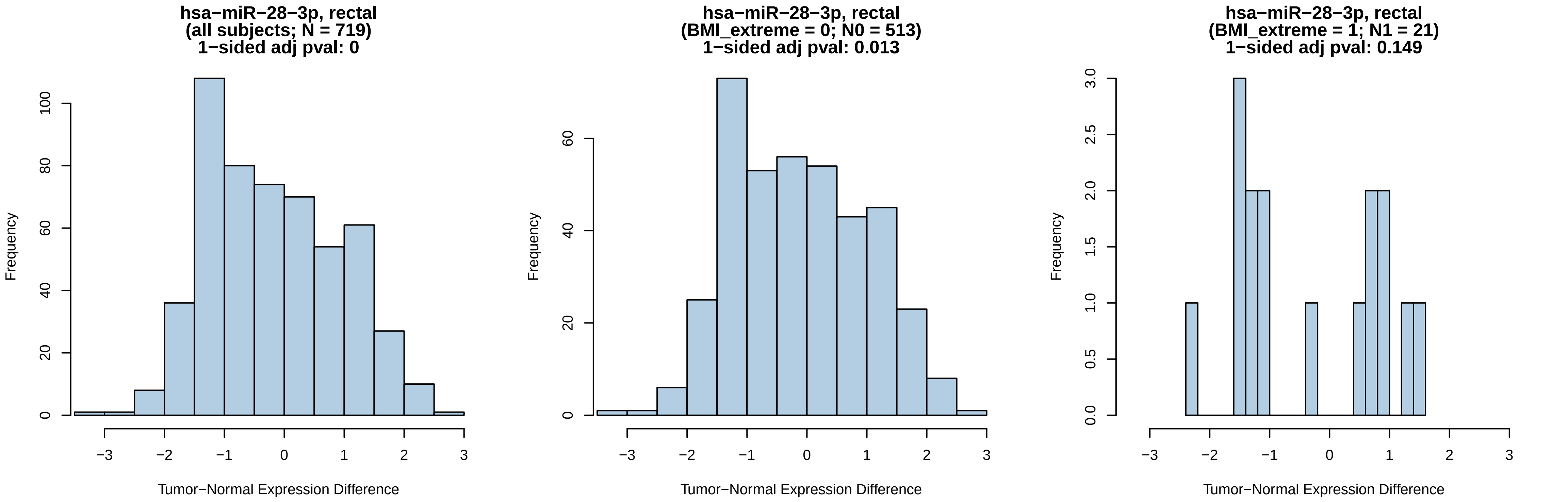

hsa−miR−28−3p, rectal
(all subjects; N = 719)
1−sided adj pval: 0
hsa−miR−28−3p, rectal
(BMI_extreme = 0; N0 = 513)
1−sided adj pval: 0.013
hsa−miR−28−3p, rectal
(BMI_extreme = 1; N1 = 21)
1−sided adj pval: 0.149
Frequency
Tumor−Normal Expression Difference

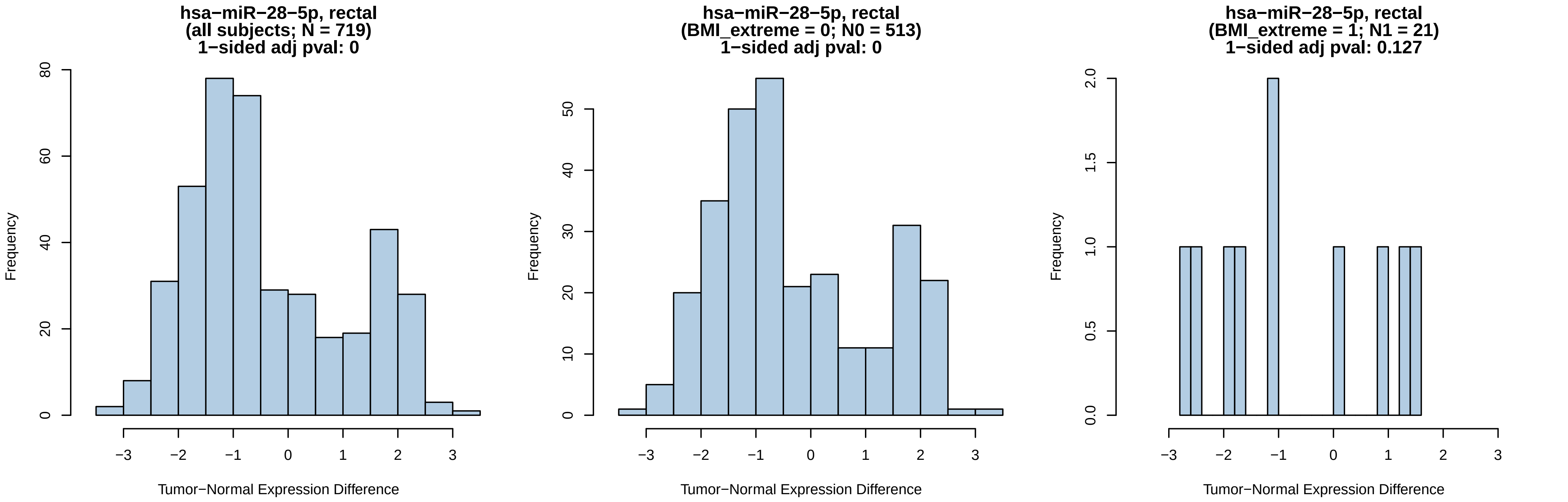

hsa−miR−28−5p, rectal
(all subjects; N = 719)
1−sided adj pval: 0
hsa−miR−28−5p, rectal
(BMI_extreme = 0; N0 = 513)
1−sided adj pval: 0
hsa−miR−28−5p, rectal
(BMI_extreme = 1; N1 = 21)
1−sided adj pval: 0.127
Frequency
Tumor−Normal Expression Difference

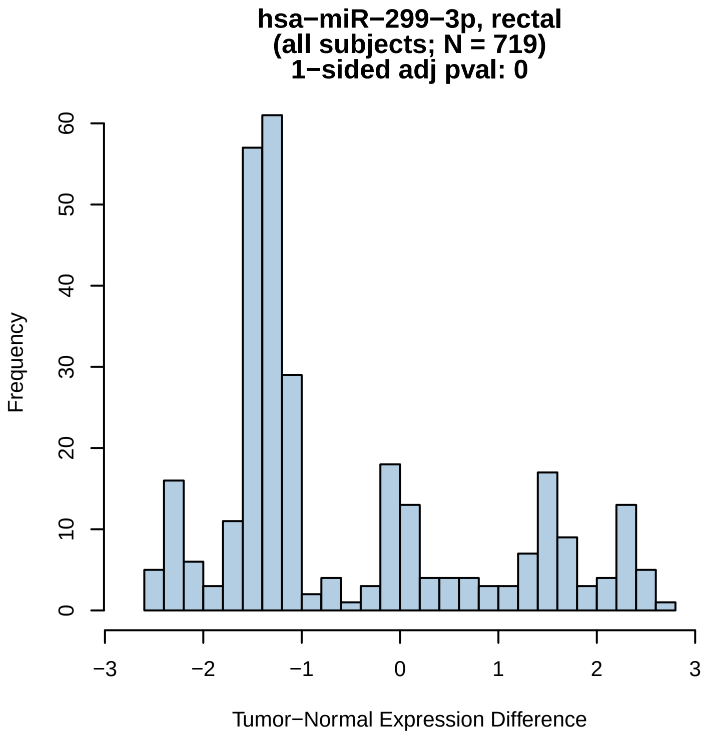
hsa−miR−299−3p, rectal
(all subjects; N = 719)
1−sided adj pval: 0
Frequency
0
10
20
30
40
50
60
−3
−2
−1
0
1
2
3
Tumor−Normal Expression Difference

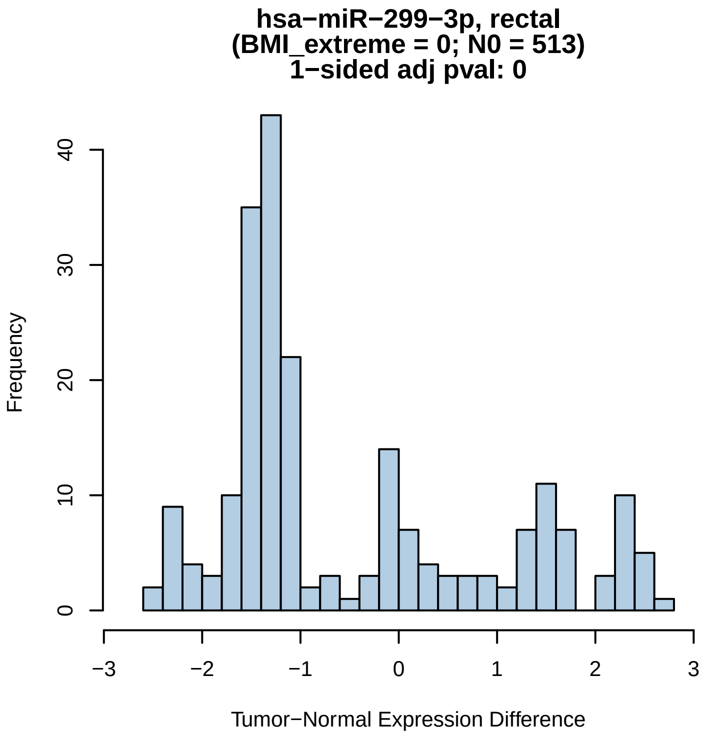
hsa−miR−299−3p, rectal
(BMI_extreme = 0; N0 = 513)
1−sided adj pval: 0
Frequency
0
10
20
30
40
−3
−2
−1
0
1
2
3
Tumor−Normal Expression Difference

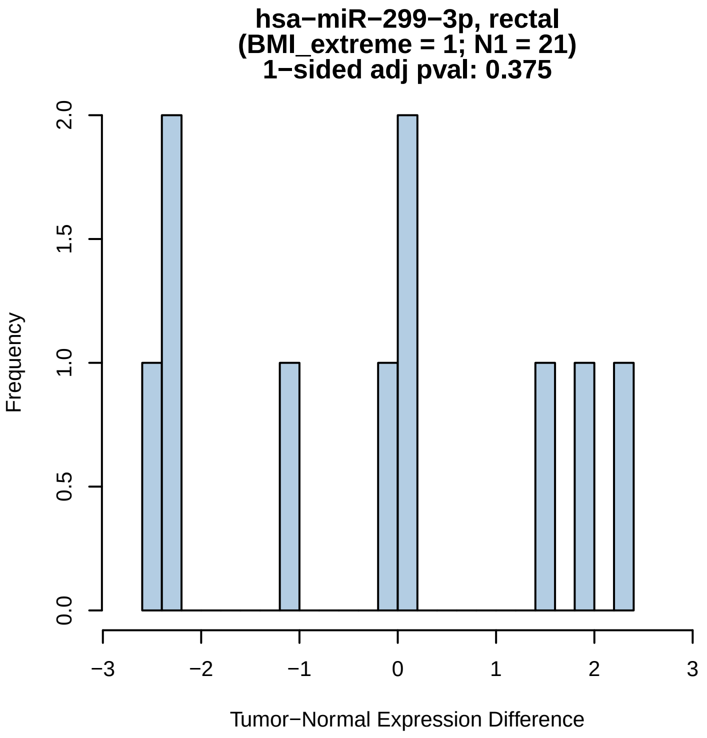
hsa−miR−299−3p, rectal
(BMI_extreme = 1; N1 = 21)
1−sided adj pval: 0.375
Frequency
0.0
0.5
1.0
1.5
2.0
−3
−2
−1
0
1
2
3
Tumor−Normal Expression Difference

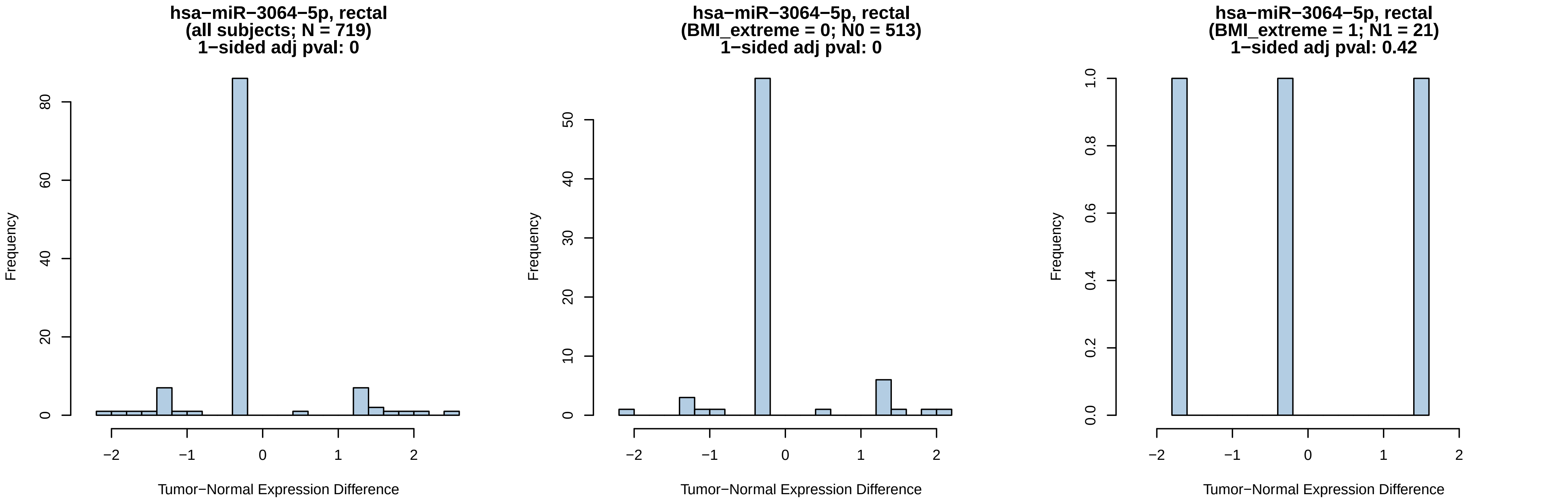
hsa−miR−3064−5p, rectal
(all subjects; N = 719)
1−sided adj pval: 0
Frequency
0
20
40
60
80
−2
−1
0
1
2
Tumor−Normal Expression Difference
hsa−miR−3064−5p, rectal
(BMI_extreme = 0; N0 = 513)
1−sided adj pval: 0
Frequency
0
10
20
30
40
50
−2
−1
0
1
2
Tumor−Normal Expression Difference
hsa−miR−3064−5p, rectal
(BMI_extreme = 1; N1 = 21)
1−sided adj pval: 0.42
Frequency
0.0
0.2
0.4
0.6
0.8
1.0
−2
−1
0
1
2
Tumor−Normal Expression Difference

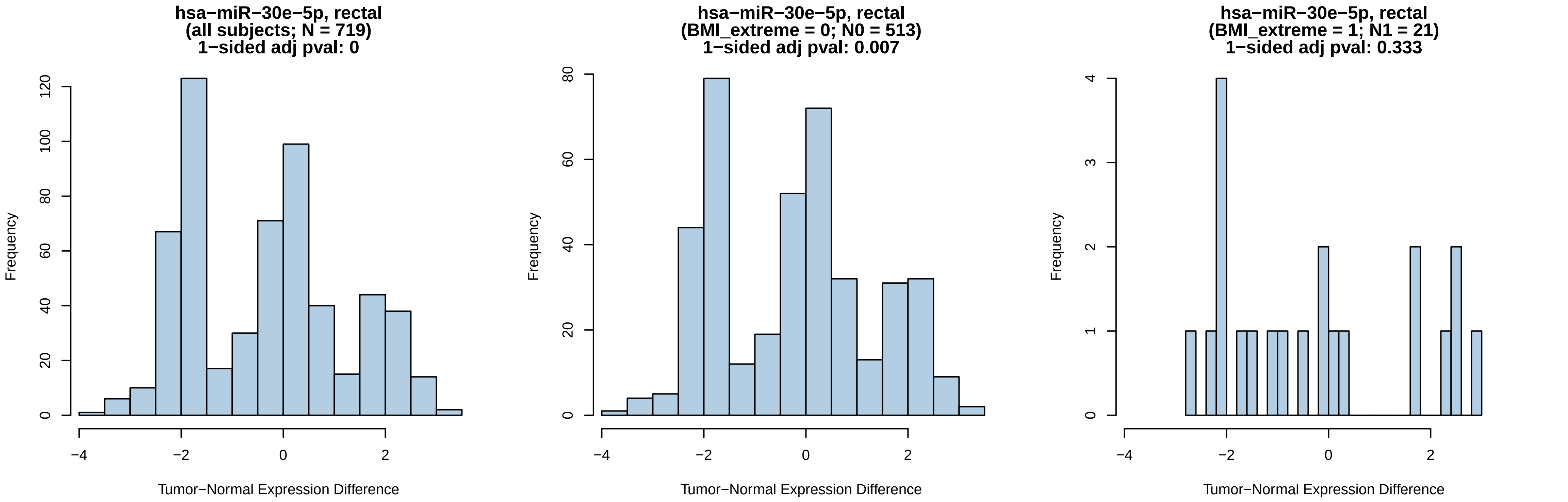
hsa−miR−30e−5p, rectal
(all subjects; N = 719)
1−sided adj pval: 0
hsa−miR−30e−5p, rectal
(BMI_extreme = 0; N0 = 513)
1−sided adj pval: 0.007
hsa−miR−30e−5p, rectal
(BMI_extreme = 1; N1 = 21)
1−sided adj pval: 0.333
Frequency
Tumor−Normal Expression Difference

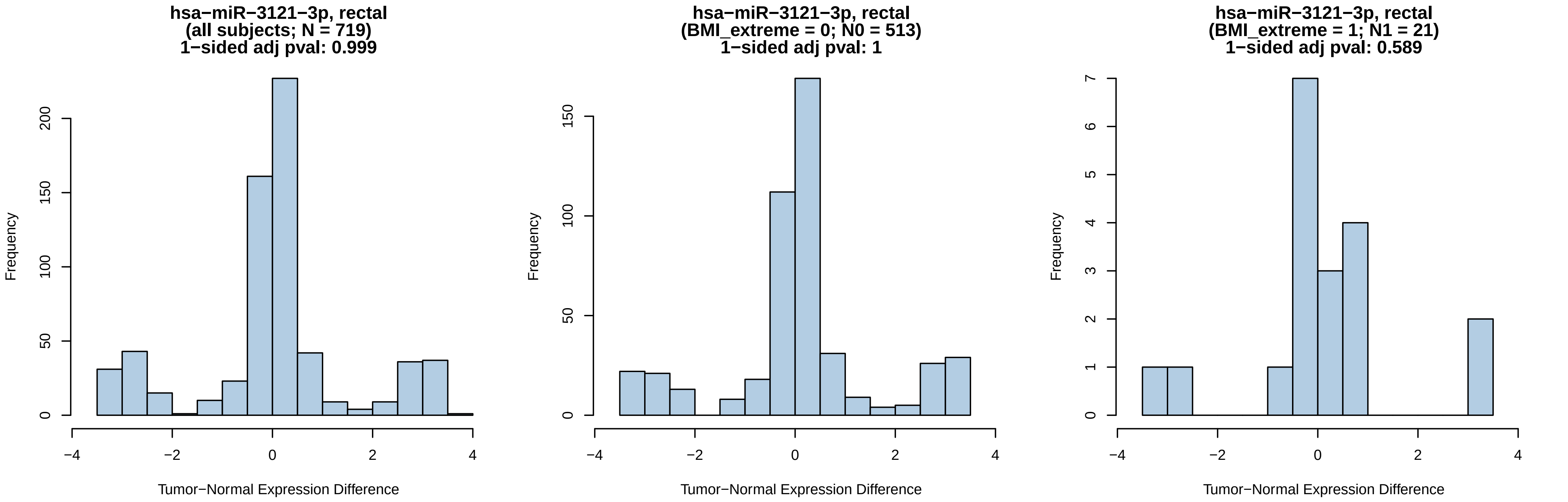
hsa−miR−3121−3p, rectal
(all subjects; N = 719)
1−sided adj pval: 0.999
Frequency
Tumor−Normal Expression Difference
hsa−miR−3121−3p, rectal
(BMI_extreme = 0; N0 = 513)
1−sided adj pval: 1
Frequency
Tumor−Normal Expression Difference
hsa−miR−3121−3p, rectal
(BMI_extreme = 1; N1 = 21)
1−sided adj pval: 0.589
Frequency
Tumor−Normal Expression Difference

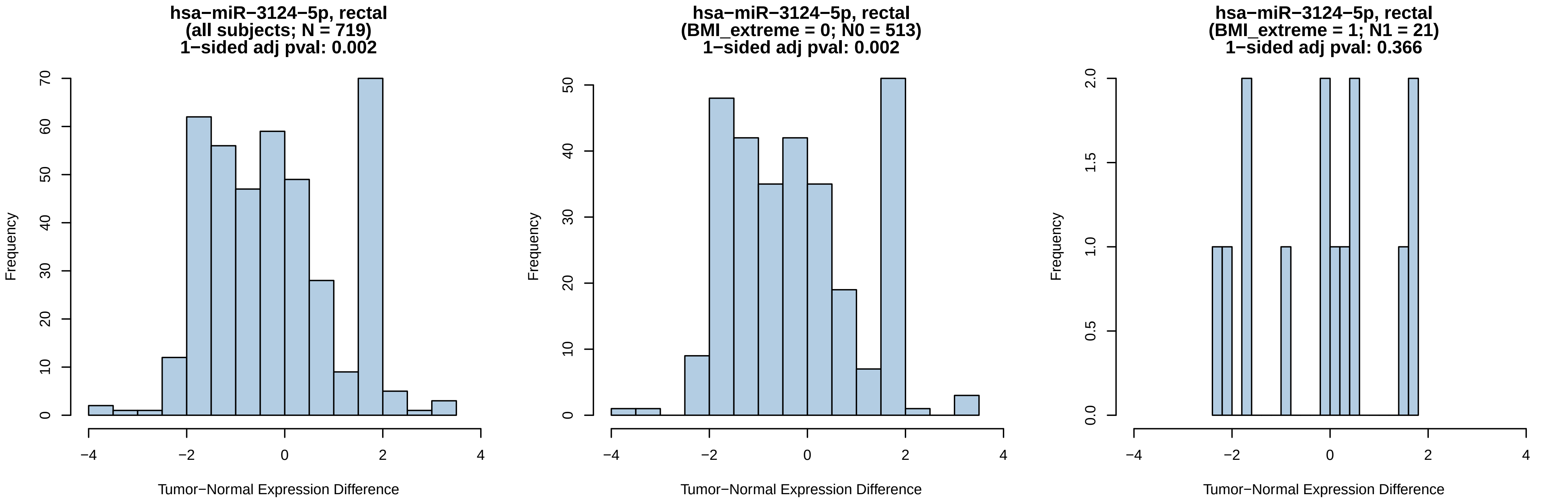
hsa−miR−3124−5p, rectal
(all subjects; N = 719)
1−sided adj pval: 0.002
hsa−miR−3124−5p, rectal
(BMI_extreme = 0; N0 = 513)
1−sided adj pval: 0.002
hsa−miR−3124−5p, rectal
(BMI_extreme = 1; N1 = 21)
1−sided adj pval: 0.366
Frequency
Tumor−Normal Expression Difference

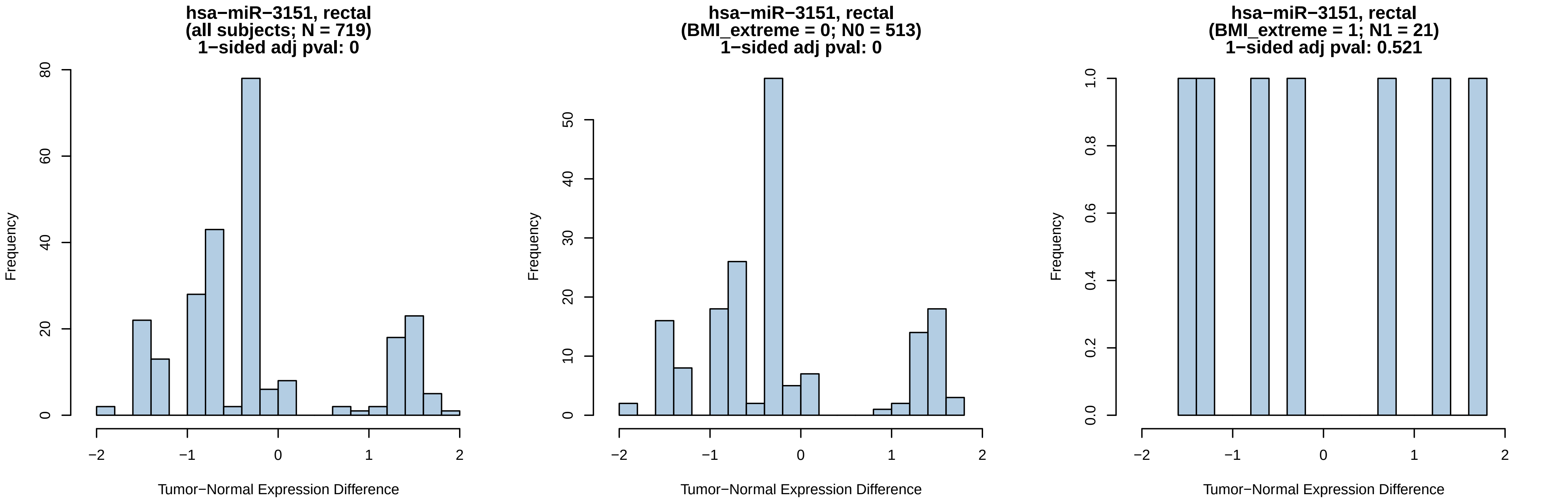
hsa−miR−3151, rectal
(all subjects; N = 719)
1−sided adj pval: 0
Frequency
Tumor−Normal Expression Difference
hsa−miR−3151, rectal
(BMI_extreme = 0; N0 = 513)
1−sided adj pval: 0
Frequency
Tumor−Normal Expression Difference
hsa−miR−3151, rectal
(BMI_extreme = 1; N1 = 21)
1−sided adj pval: 0.521
Frequency
Tumor−Normal Expression Difference

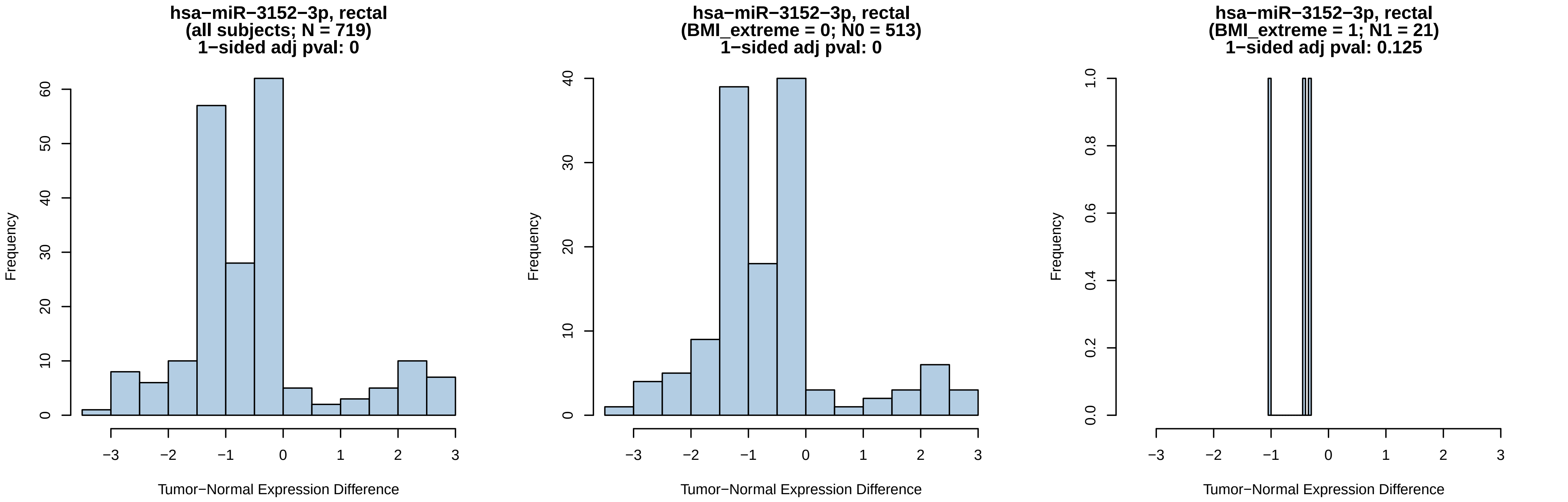

hsa−miR−3152−3p, rectal
(all subjects; N = 719)
1−sided adj pval: 0
Frequency
Tumor−Normal Expression Difference
hsa−miR−3152−3p, rectal
(BMI_extreme = 0; N0 = 513)
1−sided adj pval: 0
Frequency
Tumor−Normal Expression Difference
hsa−miR−3152−3p, rectal
(BMI_extreme = 1; N1 = 21)
1−sided adj pval: 0.125
Frequency
Tumor−Normal Expression Difference

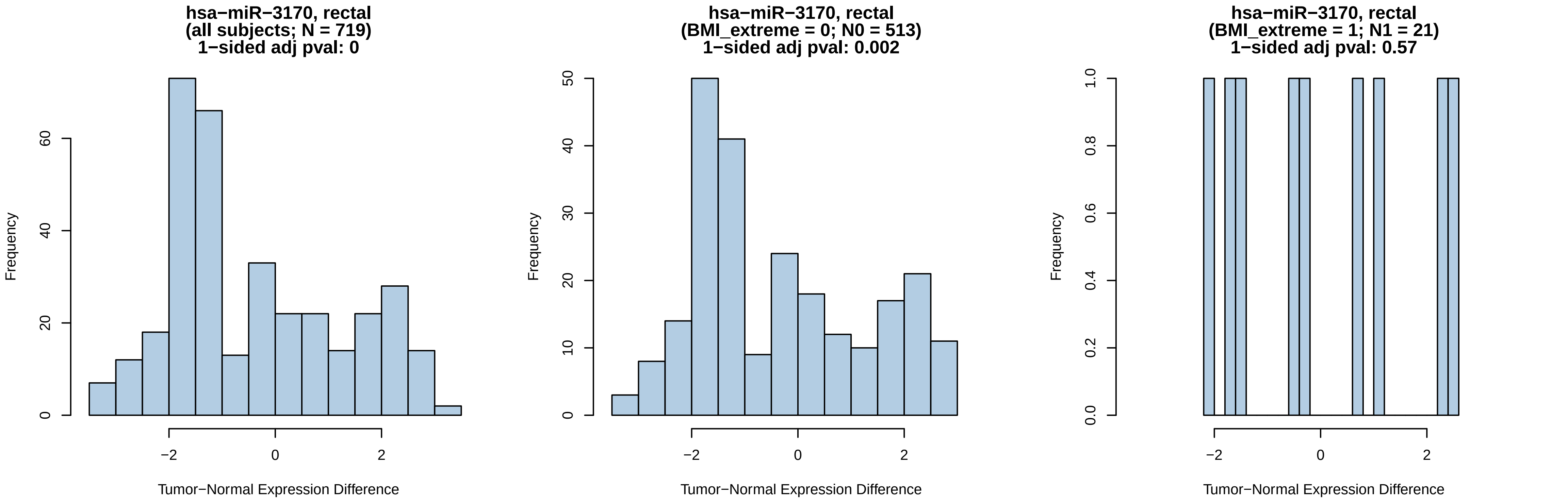
hsa−miR−3170, rectal
(all subjects; N = 719)
1−sided adj pval: 0
Frequency
0
20
40
60
−2
0
2
Tumor−Normal Expression Difference
hsa−miR−3170, rectal
(BMI_extreme = 0; N0 = 513)
1−sided adj pval: 0.002
Frequency
0
10
20
30
40
50
−2
0
2
Tumor−Normal Expression Difference
hsa−miR−3170, rectal
(BMI_extreme = 1; N1 = 21)
1−sided adj pval: 0.57
Frequency
0.0
0.2
0.4
0.6
0.8
1.0
−2
0
2
Tumor−Normal Expression Difference

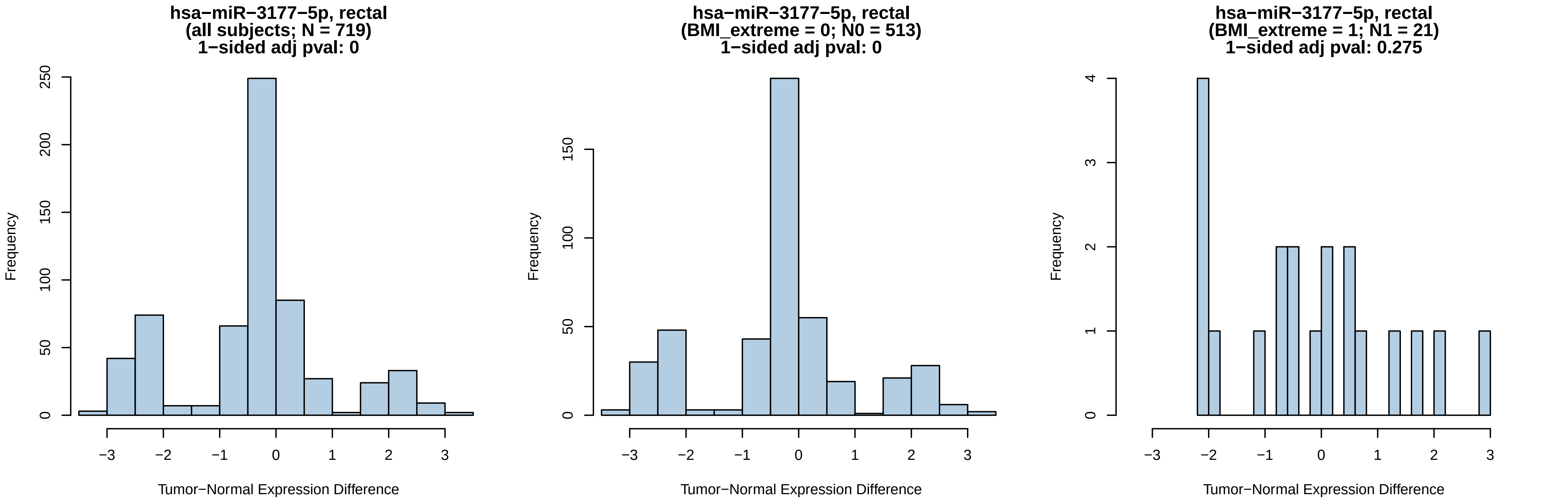
hsa−miR−3177−5p, rectal
(all subjects; N = 719)
1−sided adj pval: 0
Frequency
Tumor−Normal Expression Difference
hsa−miR−3177−5p, rectal
(BMI_extreme = 0; N0 = 513)
1−sided adj pval: 0
Frequency
Tumor−Normal Expression Difference
hsa−miR−3177−5p, rectal
(BMI_extreme = 1; N1 = 21)
1−sided adj pval: 0.275
Frequency
Tumor−Normal Expression Difference

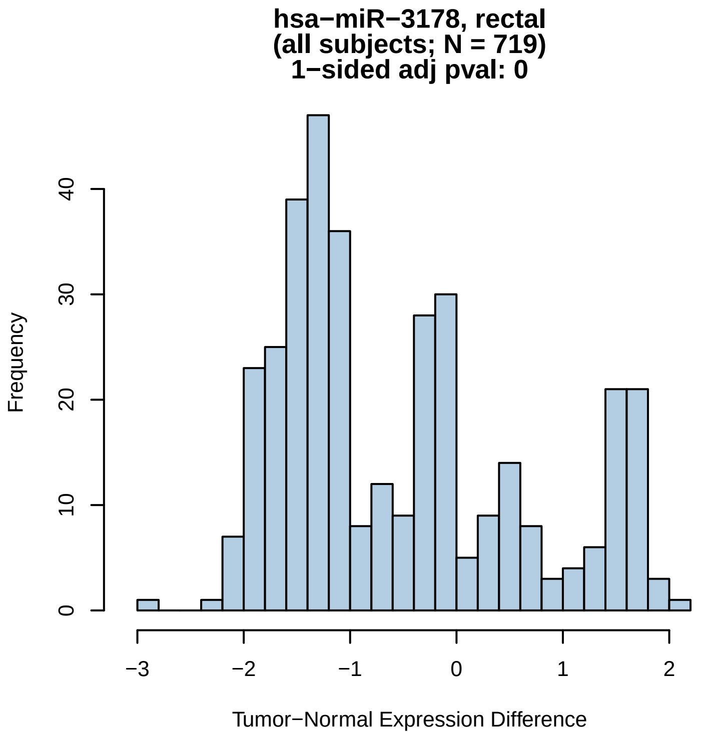
hsa−miR−3178, rectal
(all subjects; N = 719)
1−sided adj pval: 0
Frequency
0
10
20
30
40
−3
−2
−1
0
1
2
Tumor−Normal Expression Difference
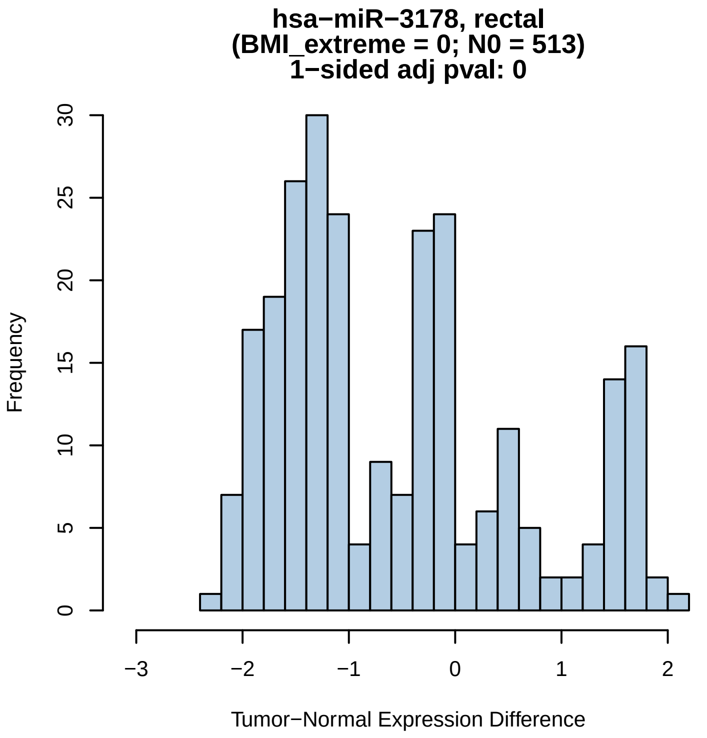
hsa−miR−3178, rectal
(BMI_extreme = 0; N0 = 513)
1−sided adj pval: 0
Frequency
0
5
10
15
20
25
30
−3
−2
−1
0
1
2
Tumor−Normal Expression Difference
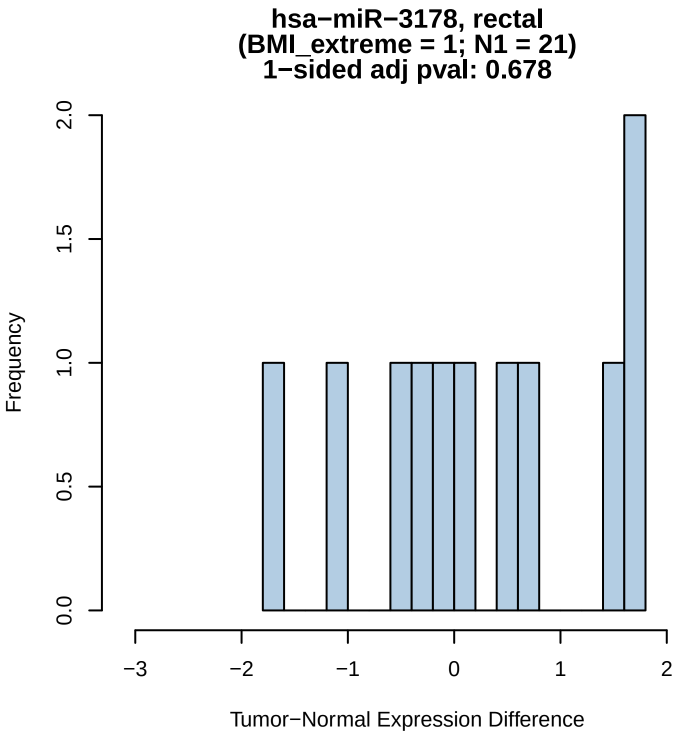
hsa−miR−3178, rectal
(BMI_extreme = 1; N1 = 21)
1−sided adj pval: 0.678
Frequency
0.0
0.5
1.0
1.5
2.0
−3
−2
−1
0
1
2
Tumor−Normal Expression Difference

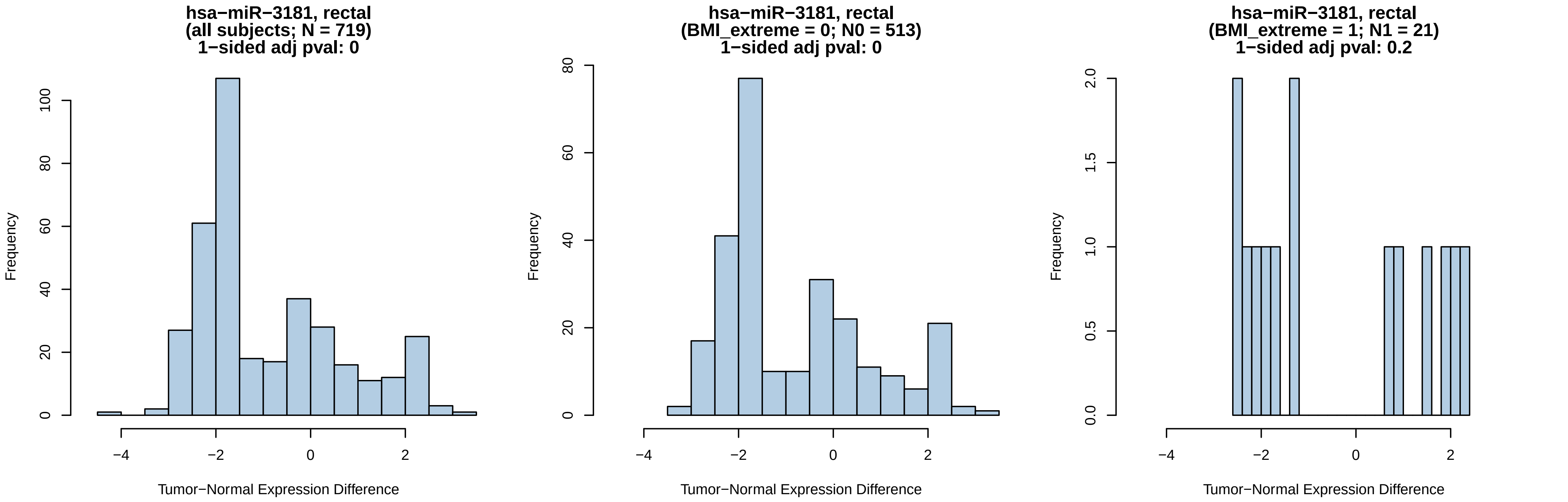

hsa−miR−3181, rectal
(all subjects; N = 719)
1−sided adj pval: 0
Frequency
0
20
40
60
80
100
−4
−2
0
2
Tumor−Normal Expression Difference
hsa−miR−3181, rectal
(BMI_extreme = 0; N0 = 513)
1−sided adj pval: 0
Frequency
0
20
40
60
80
−4
−2
0
2
Tumor−Normal Expression Difference
hsa−miR−3181, rectal
(BMI_extreme = 1; N1 = 21)
1−sided adj pval: 0.2
Frequency
0.0
0.5
1.0
1.5
2.0
−4
−2
0
2
Tumor−Normal Expression Difference

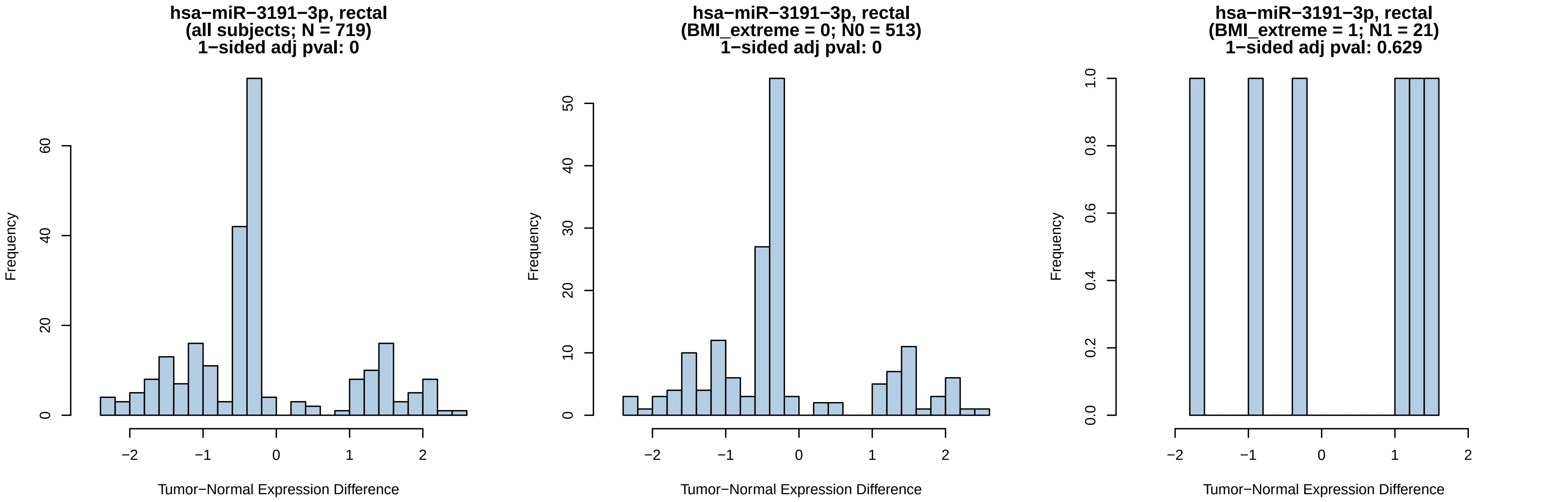

hsa−miR−3191−3p, rectal
(all subjects; N = 719)
1−sided adj pval: 0
Frequency
Tumor−Normal Expression Difference
hsa−miR−3191−3p, rectal
(BMI_extreme = 0; N0 = 513)
1−sided adj pval: 0
Frequency
Tumor−Normal Expression Difference
hsa−miR−3191−3p, rectal
(BMI_extreme = 1; N1 = 21)
1−sided adj pval: 0.629
Frequency
Tumor−Normal Expression Difference

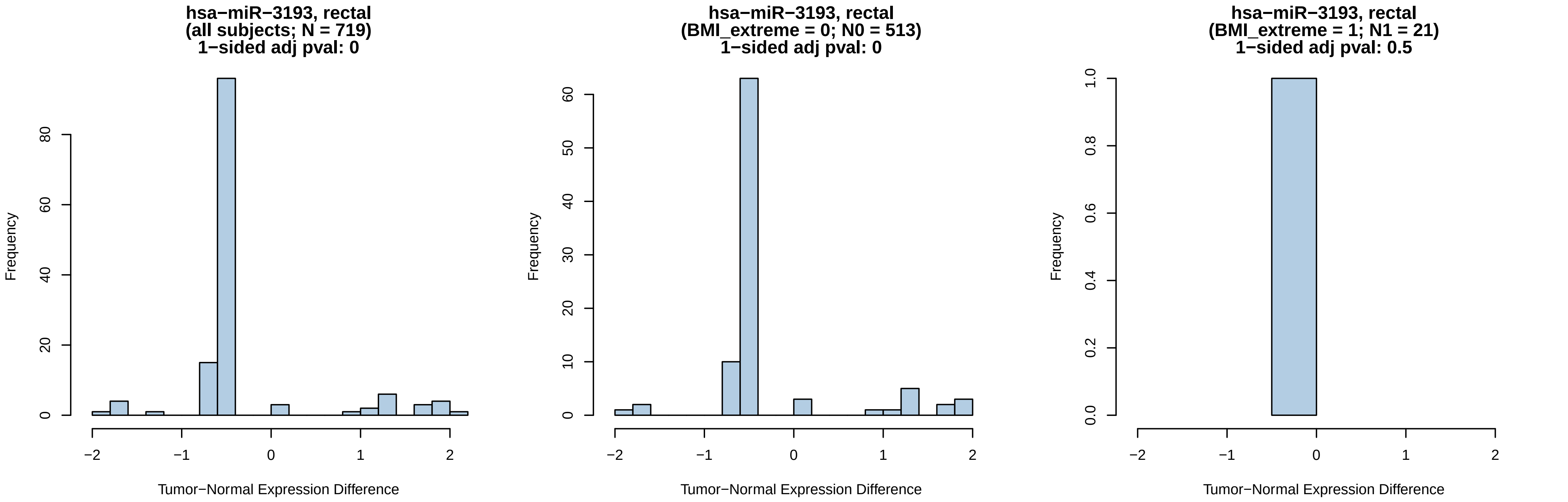
hsa−miR−3193, rectal
(all subjects; N = 719)
1−sided adj pval: 0
Frequency
0
20
40
60
80
−2
−1
0
1
2
Tumor−Normal Expression Difference
hsa−miR−3193, rectal
(BMI_extreme = 0; N0 = 513)
1−sided adj pval: 0
Frequency
0
10
20
30
40
50
60
−2
−1
0
1
2
Tumor−Normal Expression Difference
hsa−miR−3193, rectal
(BMI_extreme = 1; N1 = 21)
1−sided adj pval: 0.5
Frequency
0.0
0.2
0.4
0.6
0.8
1.0
−2
−1
0
1
2
Tumor−Normal Expression Difference

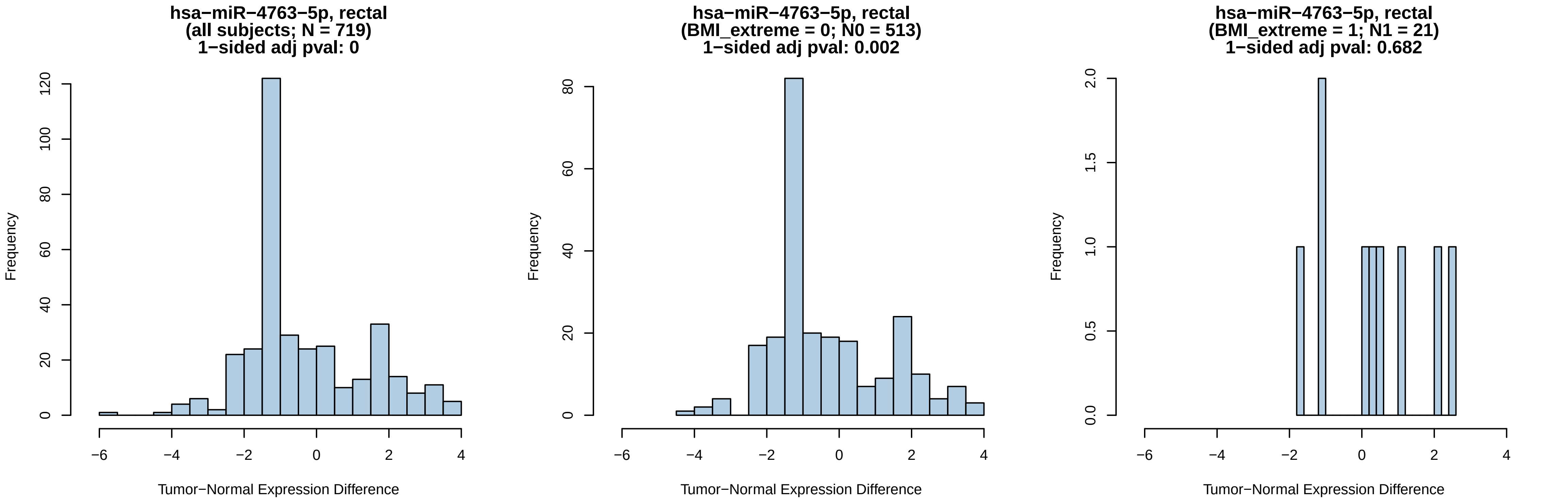
hsa−miR−4763−5p, rectal
(all subjects; N = 719)
1−sided adj pval: 0
hsa−miR−4763−5p, rectal
(BMI_extreme = 0; N0 = 513)
1−sided adj pval: 0.002
hsa−miR−4763−5p, rectal
(BMI_extreme = 1; N1 = 21)
1−sided adj pval: 0.682
Frequency
Tumor−Normal Expression Difference

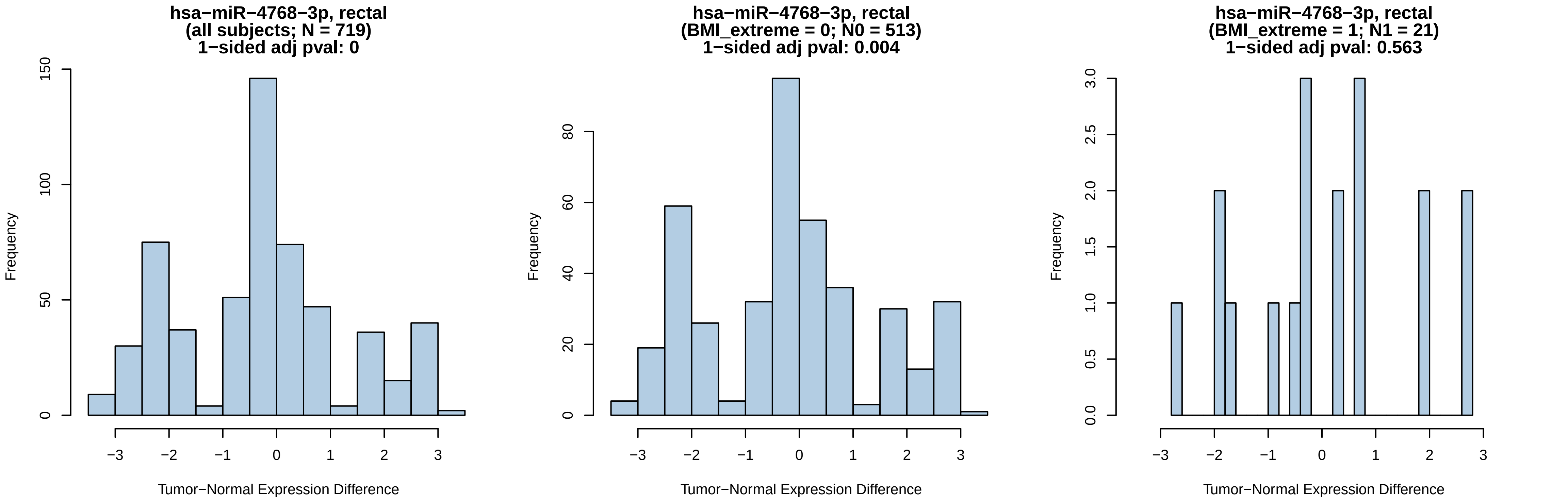
hsa−miR−4768−3p, rectal
(all subjects; N = 719)
1−sided adj pval: 0
Frequency
0
50
100
150
−3
−2
−1
0
1
2
3
Tumor−Normal Expression Difference
hsa−miR−4768−3p, rectal
(BMI_extreme = 0; N0 = 513)
1−sided adj pval: 0.004
Frequency
0
20
40
60
80
−3
−2
−1
0
1
2
3
Tumor−Normal Expression Difference
hsa−miR−4768−3p, rectal
(BMI_extreme = 1; N1 = 21)
1−sided adj pval: 0.563
Frequency
0.0
0.5
1.0
1.5
2.0
2.5
3.0
−3
−2
−1
0
1
2
3
Tumor−Normal Expression Difference

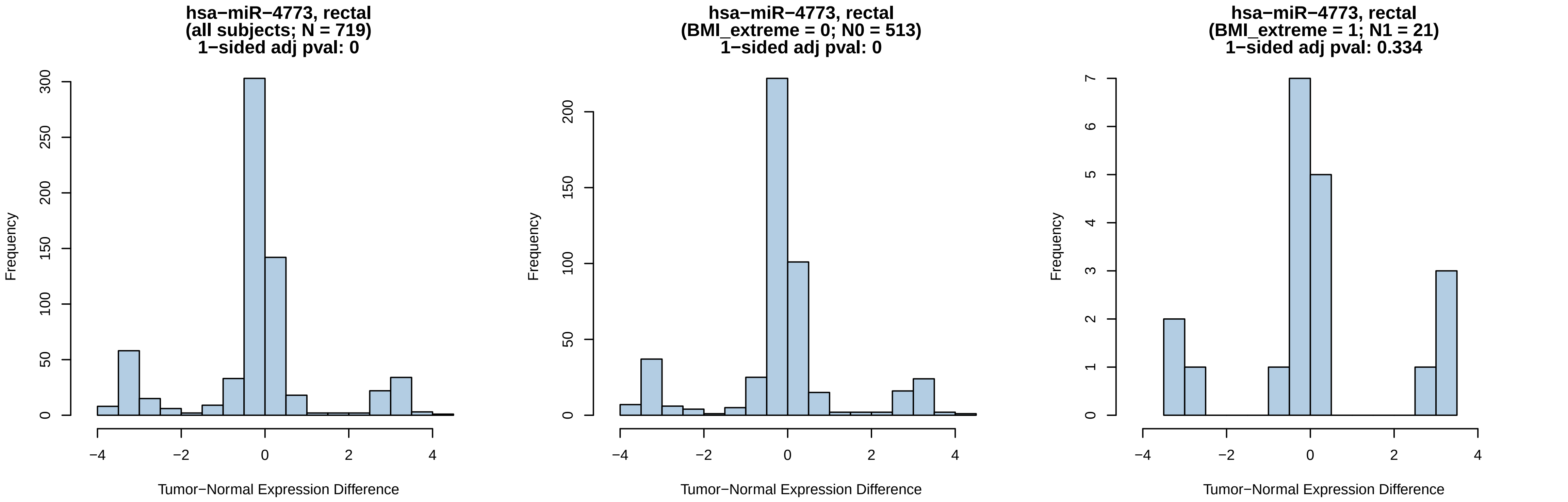
hsa−miR−4773, rectal
(all subjects; N = 719)
1−sided adj pval: 0
Frequency
Tumor−Normal Expression Difference
hsa−miR−4773, rectal
(BMI_extreme = 0; N0 = 513)
1−sided adj pval: 0
Frequency
Tumor−Normal Expression Difference
hsa−miR−4773, rectal
(BMI_extreme = 1; N1 = 21)
1−sided adj pval: 0.334
Frequency
Tumor−Normal Expression Difference

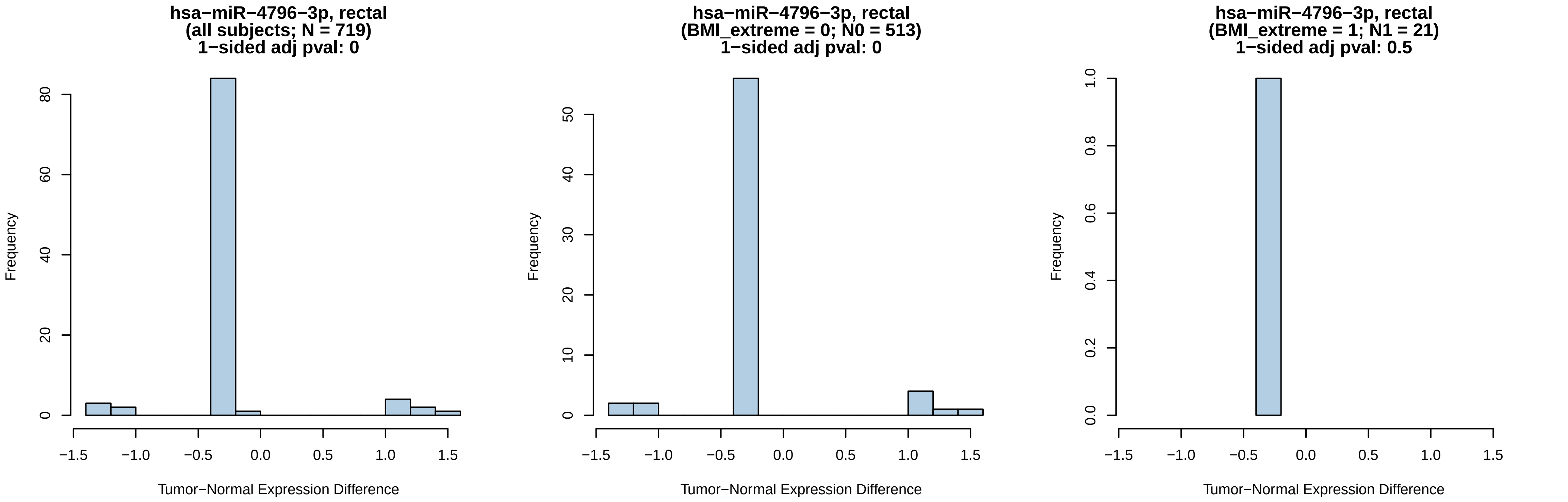
hsa−miR−4796−3p, rectal
(all subjects; N = 719)
1−sided adj pval: 0
Frequency
0
20
40
60
80
−1.5
−1.0
−0.5
0.0
0.5
1.0
1.5
Tumor−Normal Expression Difference
hsa−miR−4796−3p, rectal
(BMI_extreme = 0; N0 = 513)
1−sided adj pval: 0
Frequency
0
10
20
30
40
50
−1.5
−1.0
−0.5
0.0
0.5
1.0
1.5
Tumor−Normal Expression Difference
hsa−miR−4796−3p, rectal
(BMI_extreme = 1; N1 = 21)
1−sided adj pval: 0.5
Frequency
0.0
0.2
0.4
0.6
0.8
1.0
−1.5
−1.0
−0.5
0.0
0.5
1.0
1.5
Tumor−Normal Expression Difference

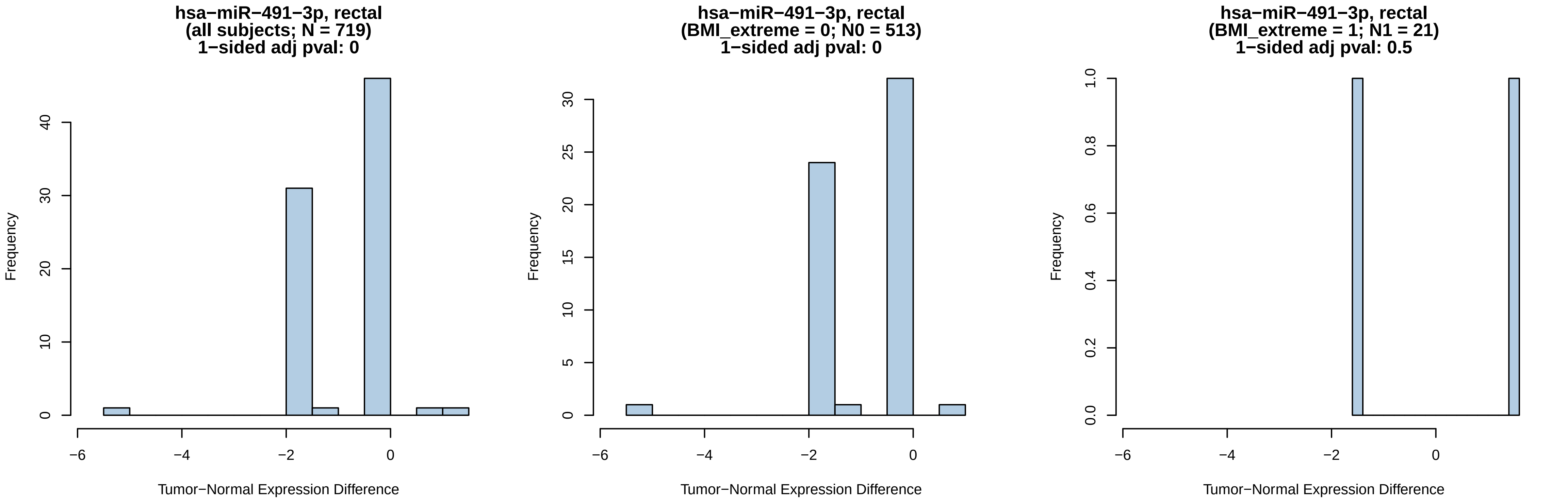

hsa−miR−491−3p, rectal
(all subjects; N = 719)
1−sided adj pval: 0
Frequency
Tumor−Normal Expression Difference
hsa−miR−491−3p, rectal
(BMI_extreme = 0; N0 = 513)
1−sided adj pval: 0
Frequency
Tumor−Normal Expression Difference
hsa−miR−491−3p, rectal
(BMI_extreme = 1; N1 = 21)
1−sided adj pval: 0.5
Frequency
Tumor−Normal Expression Difference

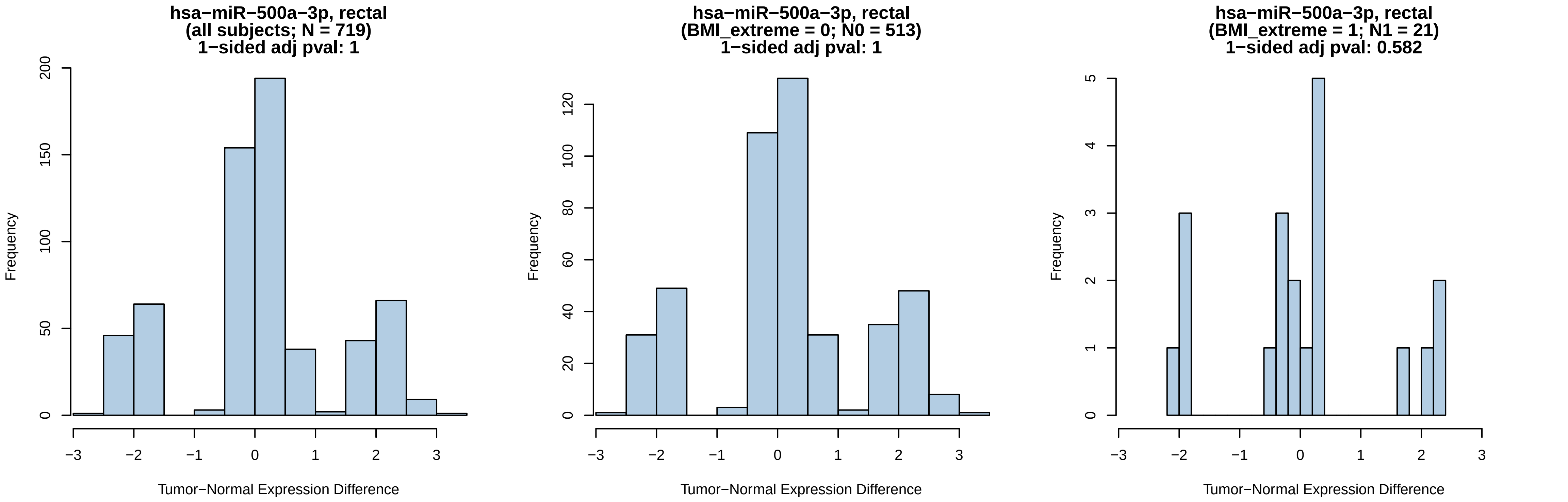

hsa−miR−500a−3p, rectal
(all subjects; N = 719)
1−sided adj pval: 1
Frequency
Tumor−Normal Expression Difference
hsa−miR−500a−3p, rectal
(BMI_extreme = 0; N0 = 513)
1−sided adj pval: 1
Frequency
Tumor−Normal Expression Difference
hsa−miR−500a−3p, rectal
(BMI_extreme = 1; N1 = 21)
1−sided adj pval: 0.582
Frequency
Tumor−Normal Expression Difference

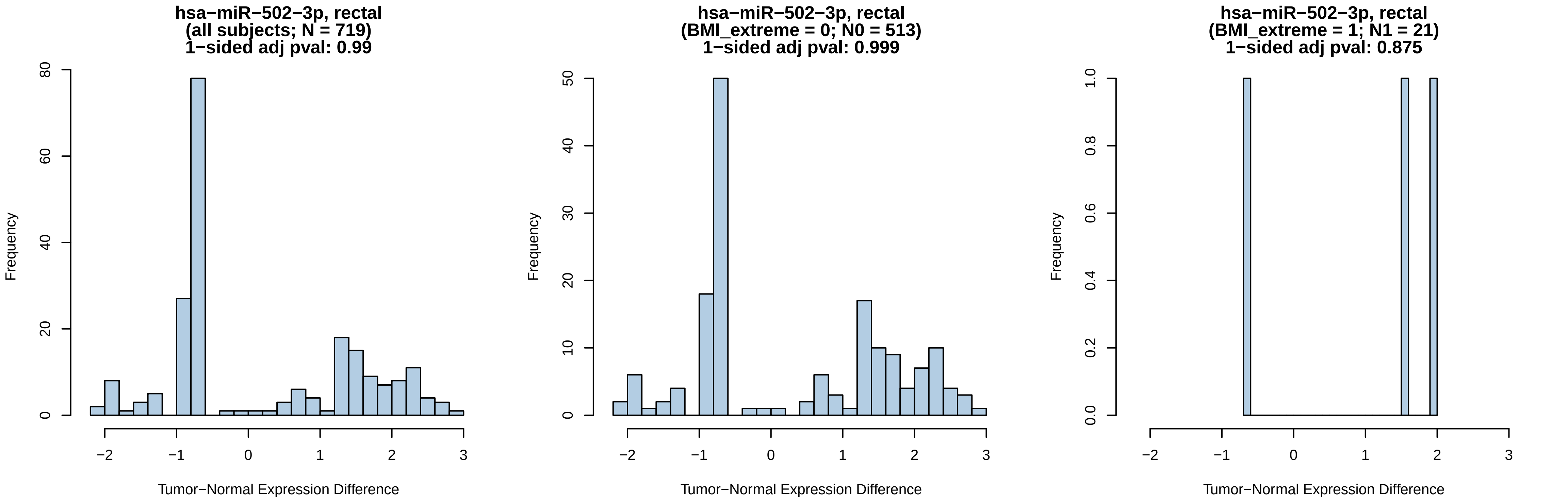

hsa−miR−502−3p, rectal
(all subjects; N = 719)
1−sided adj pval: 0.99
Frequency
Tumor−Normal Expression Difference
hsa−miR−502−3p, rectal
(BMI_extreme = 0; N0 = 513)
1−sided adj pval: 0.999
Frequency
Tumor−Normal Expression Difference
hsa−miR−502−3p, rectal
(BMI_extreme = 1; N1 = 21)
1−sided adj pval: 0.875
Frequency
Tumor−Normal Expression Difference

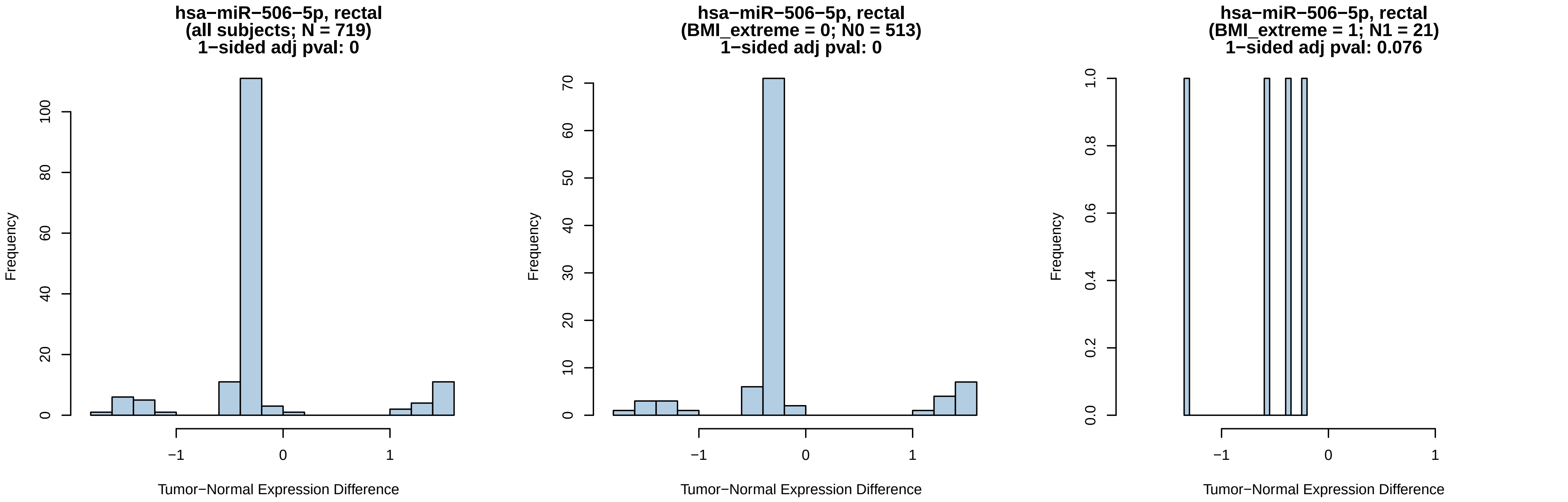

hsa−miR−506−5p, rectal
(all subjects; N = 719)
1−sided adj pval: 0
Frequency
0
20
40
60
80
100
−1
0
1
Tumor−Normal Expression Difference
hsa−miR−506−5p, rectal
(BMI_extreme = 0; N0 = 513)
1−sided adj pval: 0
Frequency
0
10
20
30
40
50
60
70
−1
0
1
Tumor−Normal Expression Difference
hsa−miR−506−5p, rectal
(BMI_extreme = 1; N1 = 21)
1−sided adj pval: 0.076
Frequency
0.0
0.2
0.4
0.6
0.8
1.0
−1
0
1
Tumor−Normal Expression Difference

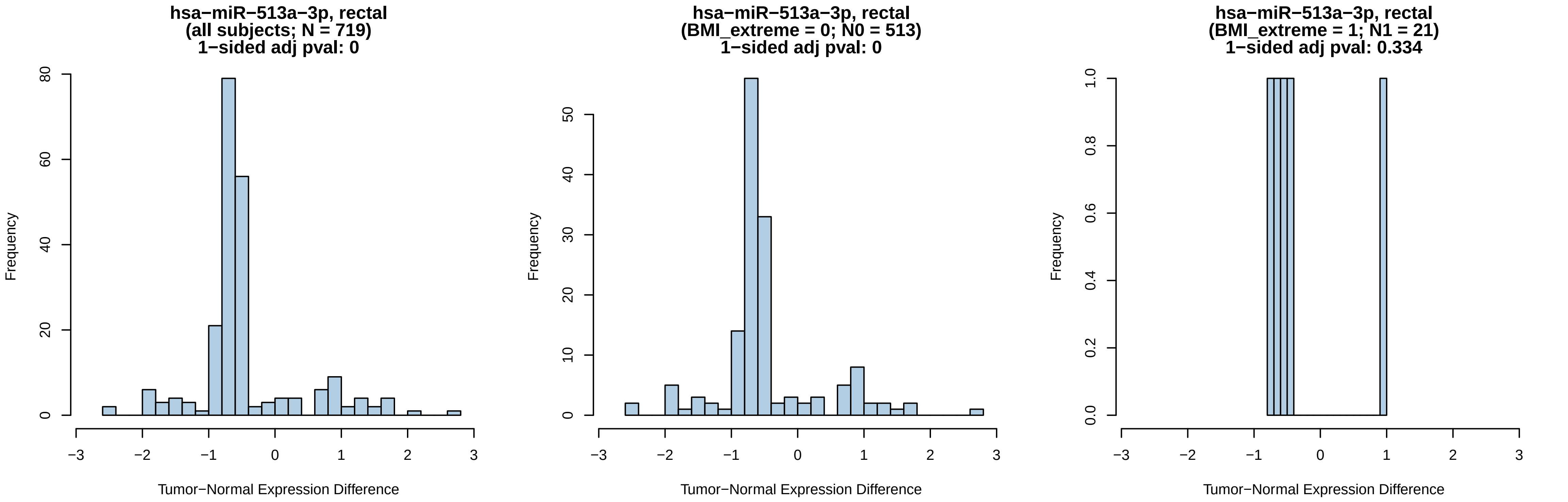
hsa−miR−513a−3p, rectal
(all subjects; N = 719)
1−sided adj pval: 0
hsa−miR−513a−3p, rectal
(BMI_extreme = 0; N0 = 513)
1−sided adj pval: 0
hsa−miR−513a−3p, rectal
(BMI_extreme = 1; N1 = 21)
1−sided adj pval: 0.334
Frequency
Tumor−Normal Expression Difference

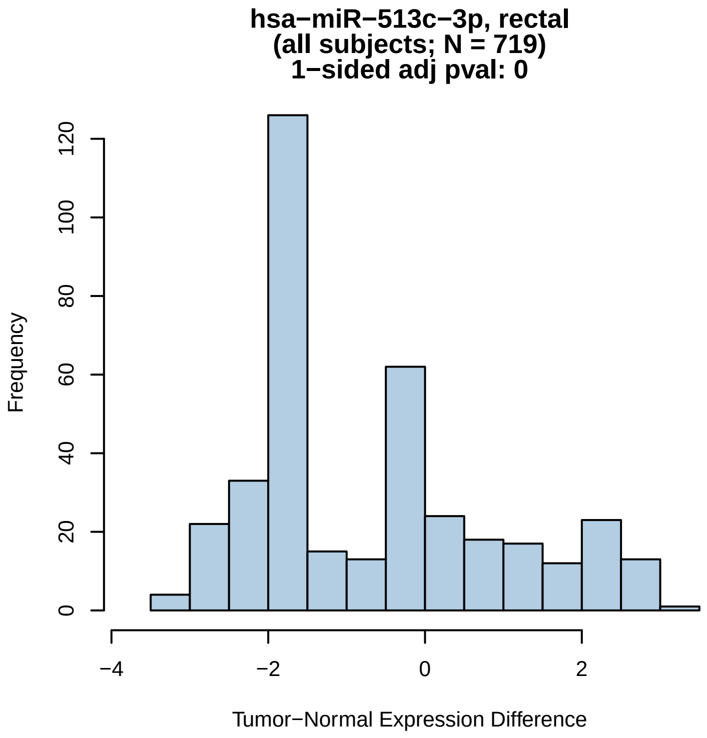
hsa−miR−513c−3p, rectal
(all subjects; N = 719)
1−sided adj pval: 0
Frequency
Tumor−Normal Expression Difference

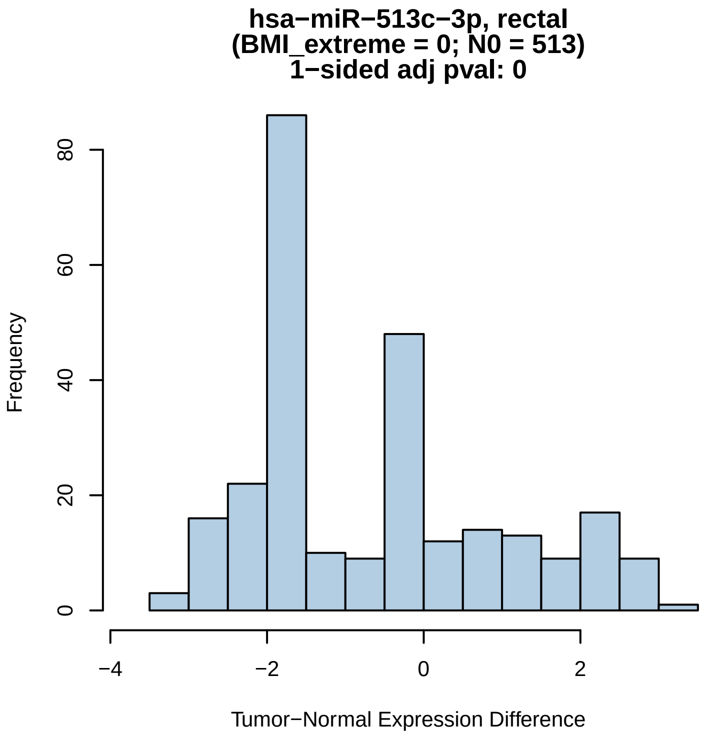
hsa−miR−513c−3p, rectal
(BMI_extreme = 0; N0 = 513)
1−sided adj pval: 0
Frequency
Tumor−Normal Expression Difference

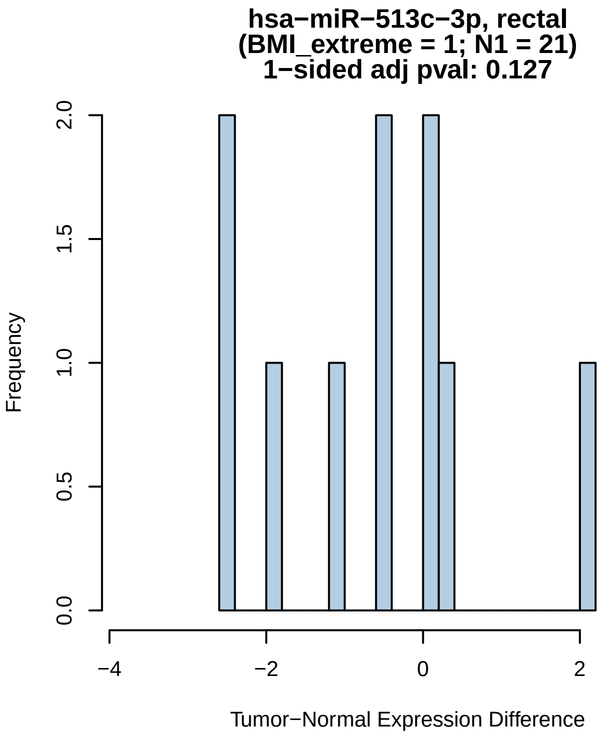
hsa−miR−513c−3p, rectal
(BMI_extreme = 1; N1 = 21)
1−sided adj pval: 0.127
Frequency
Tumor−Normal Expression Difference

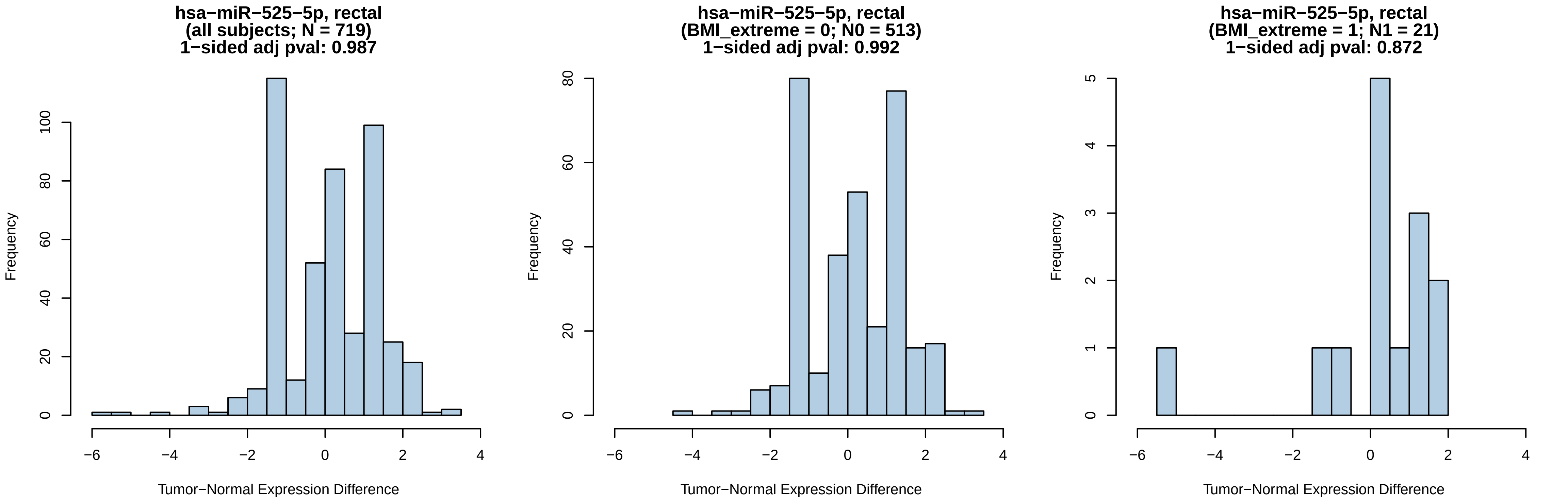
hsa−miR−525−5p, rectal
(all subjects; N = 719)
1−sided adj pval: 0.987
Frequency
Tumor−Normal Expression Difference
hsa−miR−525−5p, rectal
(BMI_extreme = 0; N0 = 513)
1−sided adj pval: 0.992
Frequency
Tumor−Normal Expression Difference
hsa−miR−525−5p, rectal
(BMI_extreme = 1; N1 = 21)
1−sided adj pval: 0.872
Frequency
Tumor−Normal Expression Difference

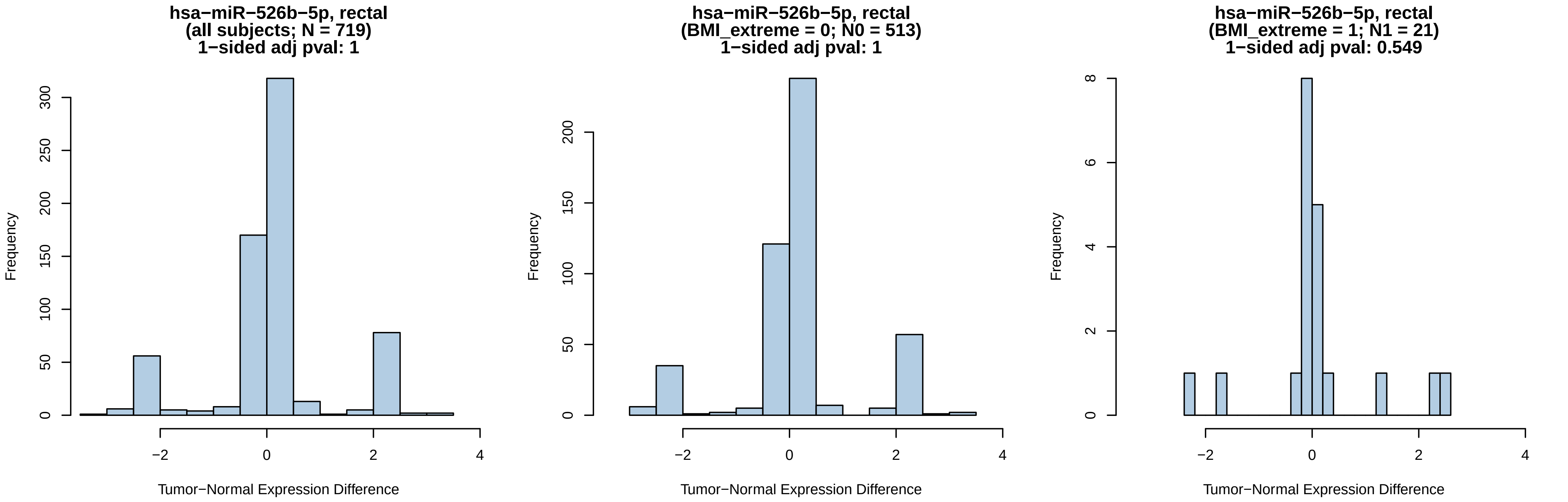
hsa−miR−526b−5p, rectal
(all subjects; N = 719)
1−sided adj pval: 1
Frequency
Tumor−Normal Expression Difference
hsa−miR−526b−5p, rectal
(BMI_extreme = 0; N0 = 513)
1−sided adj pval: 1
Frequency
Tumor−Normal Expression Difference
hsa−miR−526b−5p, rectal
(BMI_extreme = 1; N1 = 21)
1−sided adj pval: 0.549
Frequency
Tumor−Normal Expression Difference

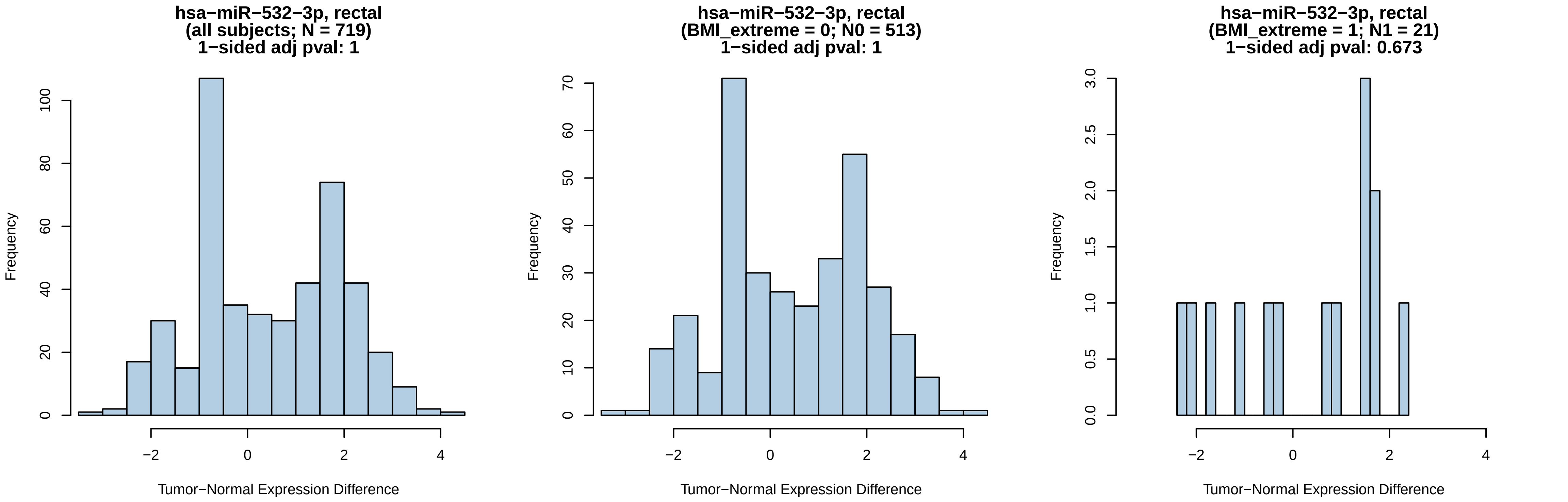
hsa−miR−532−3p, rectal
(all subjects; N = 719)
1−sided adj pval: 1
Frequency
Tumor−Normal Expression Difference
hsa−miR−532−3p, rectal
(BMI_extreme = 0; N0 = 513)
1−sided adj pval: 1
Frequency
Tumor−Normal Expression Difference
hsa−miR−532−3p, rectal
(BMI_extreme = 1; N1 = 21)
1−sided adj pval: 0.673
Frequency
Tumor−Normal Expression Difference

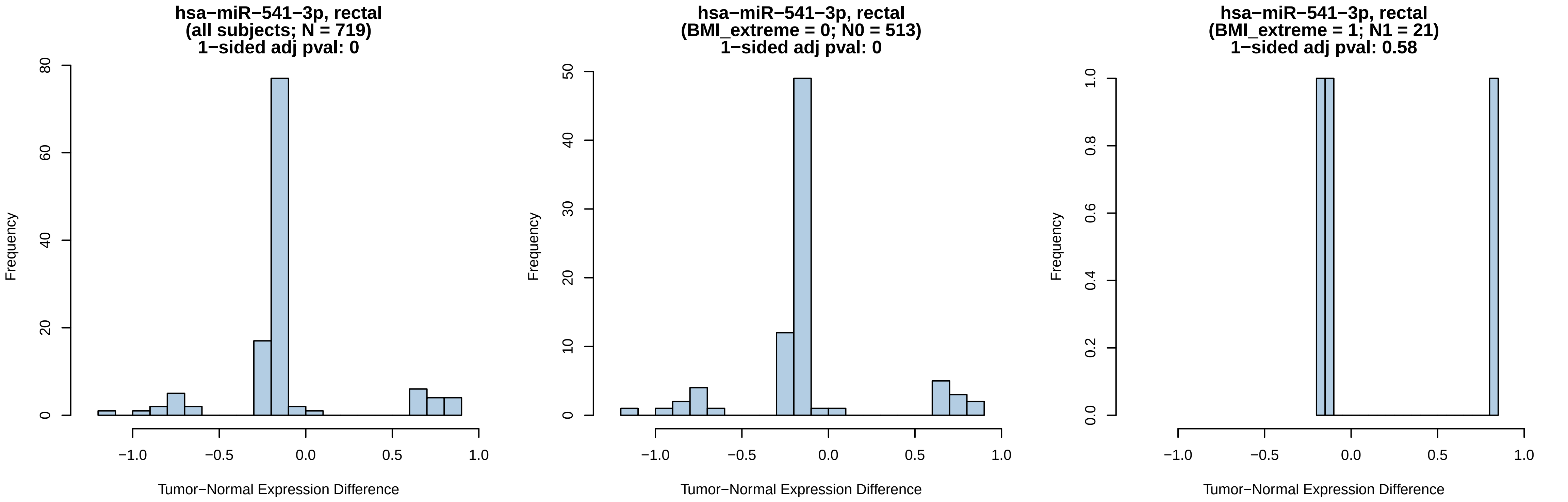

hsa−miR−541−3p, rectal
(all subjects; N = 719)
1−sided adj pval: 0
Frequency
0
20
40
60
80
−1.0
−0.5
0.0
0.5
1.0
Tumor−Normal Expression Difference
hsa−miR−541−3p, rectal
(BMI_extreme = 0; N0 = 513)
1−sided adj pval: 0
Frequency
0
10
20
30
40
50
−1.0
−0.5
0.0
0.5
1.0
Tumor−Normal Expression Difference
hsa−miR−541−3p, rectal
(BMI_extreme = 1; N1 = 21)
1−sided adj pval: 0.58
Frequency
0.0
0.2
0.4
0.6
0.8
1.0
−1.0
−0.5
0.0
0.5
1.0
Tumor−Normal Expression Difference

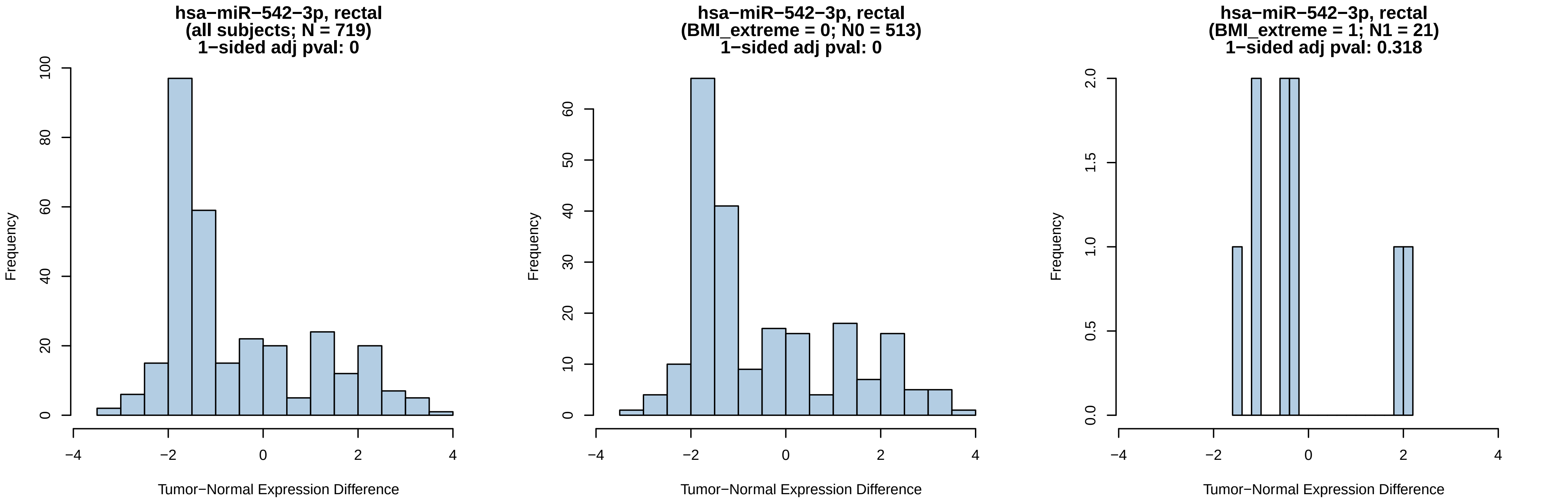
hsa−miR−542−3p, rectal
(all subjects; N = 719)
1−sided adj pval: 0
Frequency
Tumor−Normal Expression Difference
hsa−miR−542−3p, rectal
(BMI_extreme = 0; N0 = 513)
1−sided adj pval: 0
Frequency
Tumor−Normal Expression Difference
hsa−miR−542−3p, rectal
(BMI_extreme = 1; N1 = 21)
1−sided adj pval: 0.318
Frequency
Tumor−Normal Expression Difference

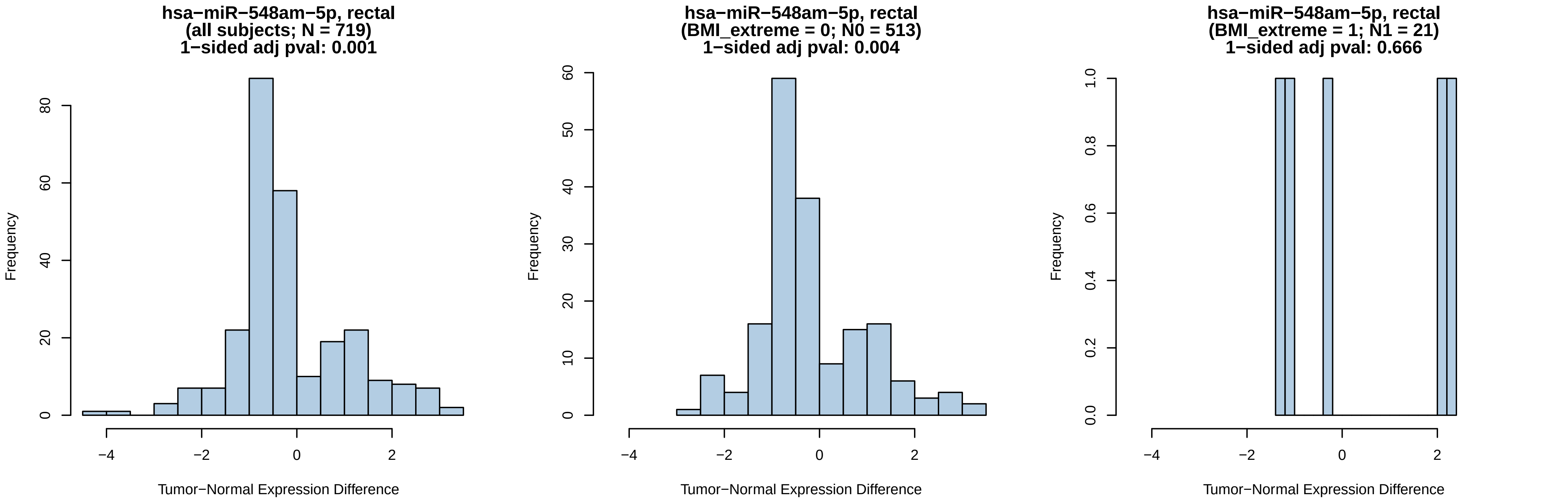

hsa−miR−548am−5p, rectal
(all subjects; N = 719)
1−sided adj pval: 0.001
Frequency
0
20
40
60
80
−4
−2
0
2
Tumor−Normal Expression Difference
hsa−miR−548am−5p, rectal
(BMI_extreme = 0; N0 = 513)
1−sided adj pval: 0.004
Frequency
0
10
20
30
40
50
60
−4
−2
0
2
Tumor−Normal Expression Difference
hsa−miR−548am−5p, rectal
(BMI_extreme = 1; N1 = 21)
1−sided adj pval: 0.666
Frequency
0.0
0.2
0.4
0.6
0.8
1.0
−4
−2
0
2
Tumor−Normal Expression Difference

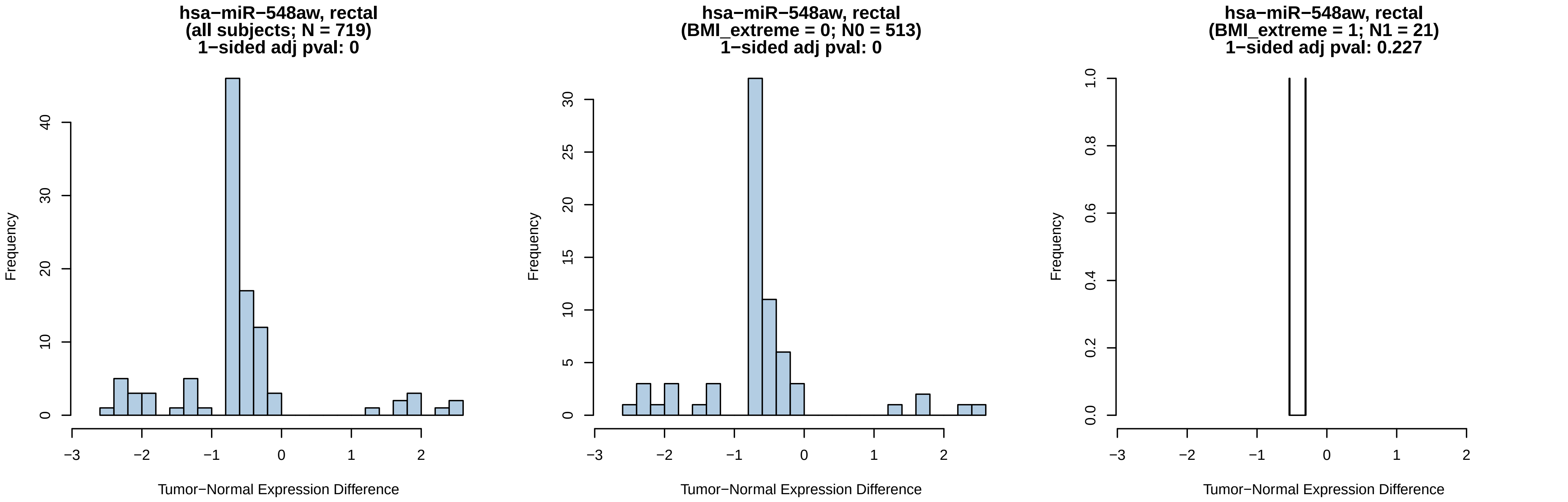
hsa−miR−548aw, rectal
(all subjects; N = 719)
1−sided adj pval: 0
Frequency
Tumor−Normal Expression Difference
hsa−miR−548aw, rectal
(BMI_extreme = 0; N0 = 513)
1−sided adj pval: 0
Frequency
Tumor−Normal Expression Difference
hsa−miR−548aw, rectal
(BMI_extreme = 1; N1 = 21)
1−sided adj pval: 0.227
Frequency
Tumor−Normal Expression Difference

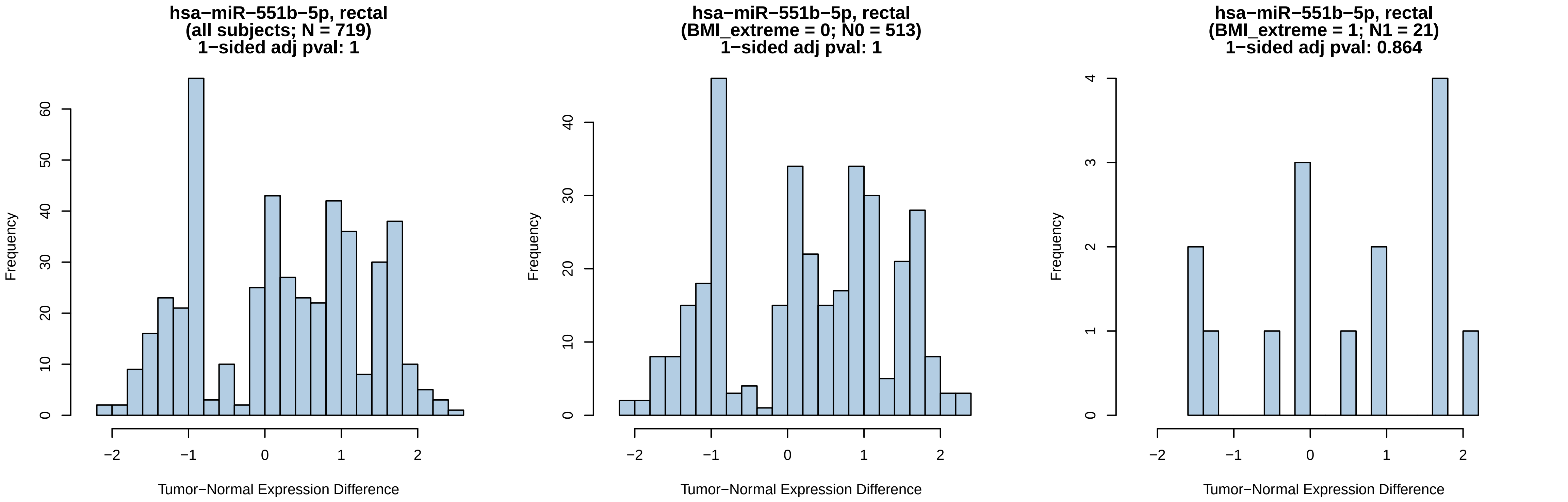

hsa−miR−551b−5p, rectal
(all subjects; N = 719)
1−sided adj pval: 1
Frequency
0
10
20
30
40
50
60
−2
−1
0
1
2
Tumor−Normal Expression Difference
hsa−miR−551b−5p, rectal
(BMI_extreme = 0; N0 = 513)
1−sided adj pval: 1
Frequency
0
10
20
30
40
−2
−1
0
1
2
Tumor−Normal Expression Difference
hsa−miR−551b−5p, rectal
(BMI_extreme = 1; N1 = 21)
1−sided adj pval: 0.864
Frequency
0
1
2
3
4
−2
−1
0
1
2
Tumor−Normal Expression Difference

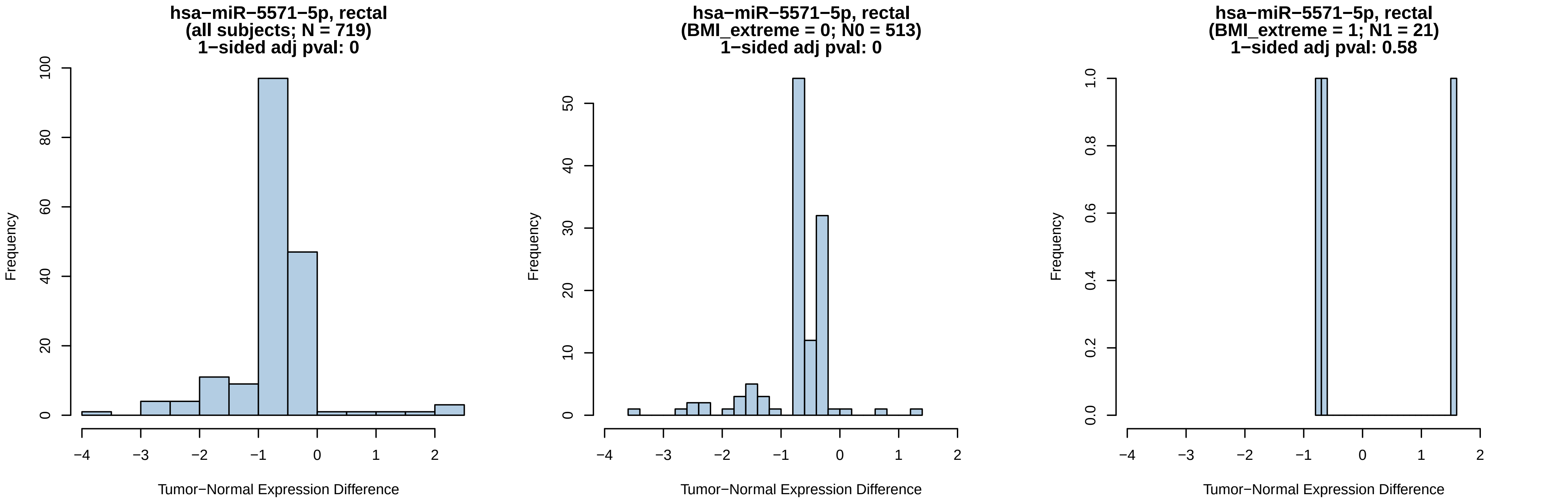

hsa−miR−5571−5p, rectal
(all subjects; N = 719)
1−sided adj pval: 0
Frequency
Tumor−Normal Expression Difference
hsa−miR−5571−5p, rectal
(BMI_extreme = 0; N0 = 513)
1−sided adj pval: 0
Frequency
Tumor−Normal Expression Difference
hsa−miR−5571−5p, rectal
(BMI_extreme = 1; N1 = 21)
1−sided adj pval: 0.58
Frequency
Tumor−Normal Expression Difference

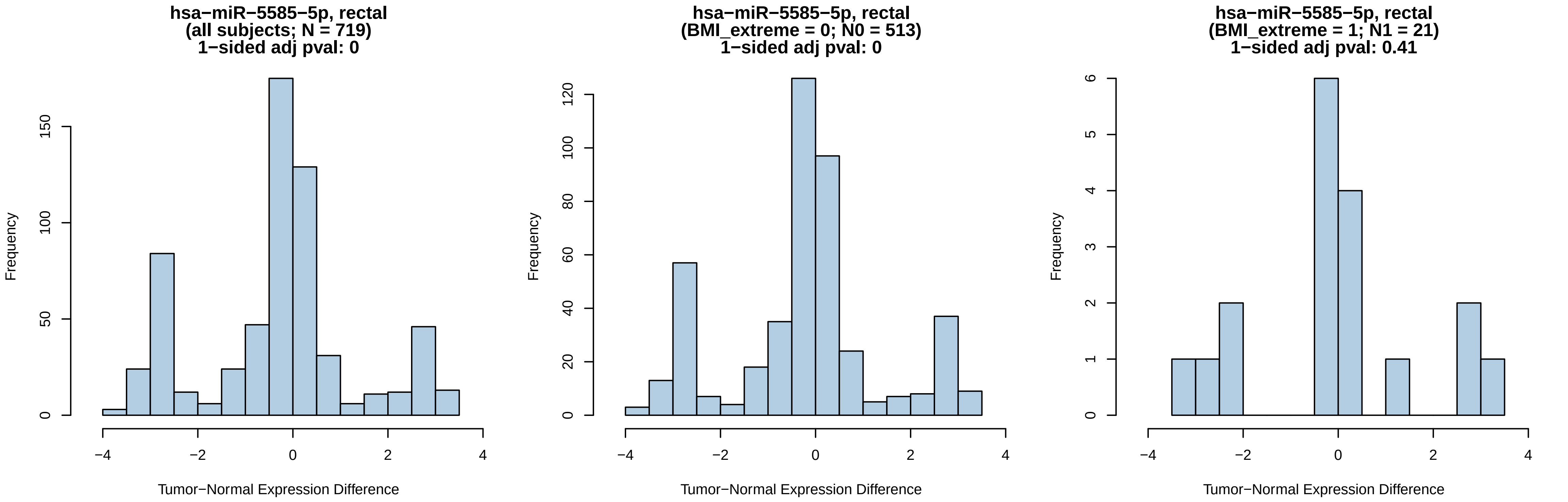
hsa−miR−5585−5p, rectal
(all subjects; N = 719)
1−sided adj pval: 0
Frequency
0
50
100
150
−4
−2
0
2
4
Tumor−Normal Expression Difference
hsa−miR−5585−5p, rectal
(BMI_extreme = 0; N0 = 513)
1−sided adj pval: 0
Frequency
0
20
40
60
80
100
120
−4
−2
0
2
4
Tumor−Normal Expression Difference
hsa−miR−5585−5p, rectal
(BMI_extreme = 1; N1 = 21)
1−sided adj pval: 0.41
Frequency
0
1
2
3
4
5
6
−4
−2
0
2
4
Tumor−Normal Expression Difference

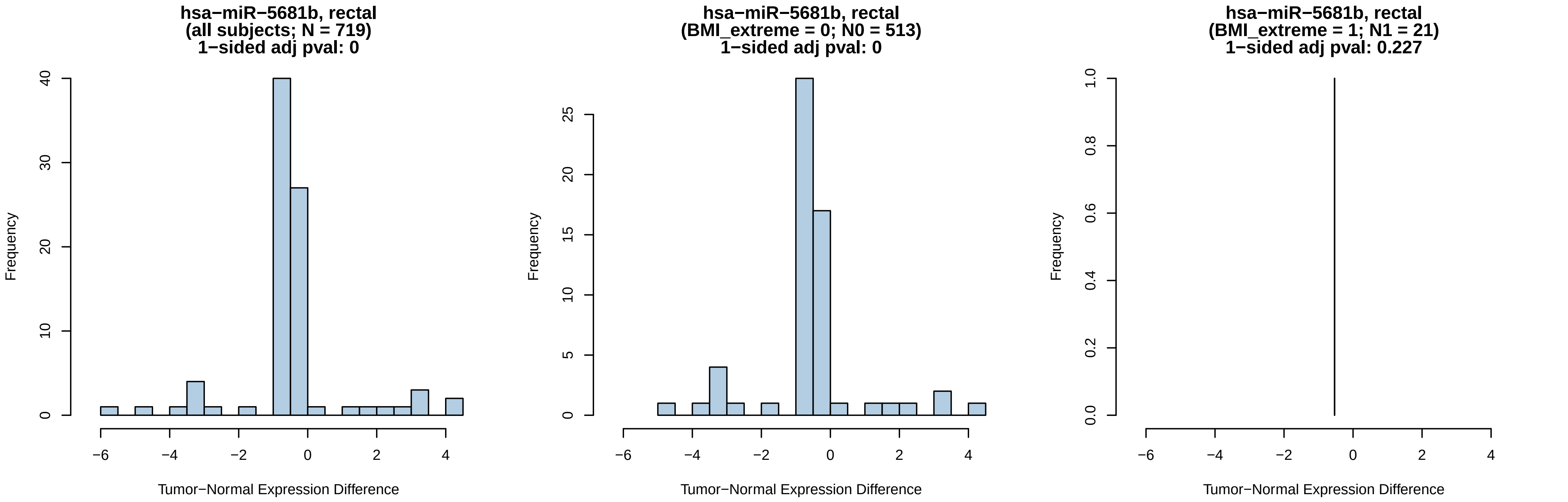

hsa−miR−5681b, rectal
(all subjects; N = 719)
1−sided adj pval: 0
Frequency
Tumor−Normal Expression Difference
hsa−miR−5681b, rectal
(BMI_extreme = 0; N0 = 513)
1−sided adj pval: 0
Frequency
Tumor−Normal Expression Difference
hsa−miR−5681b, rectal
(BMI_extreme = 1; N1 = 21)
1−sided adj pval: 0.227
Frequency
Tumor−Normal Expression Difference

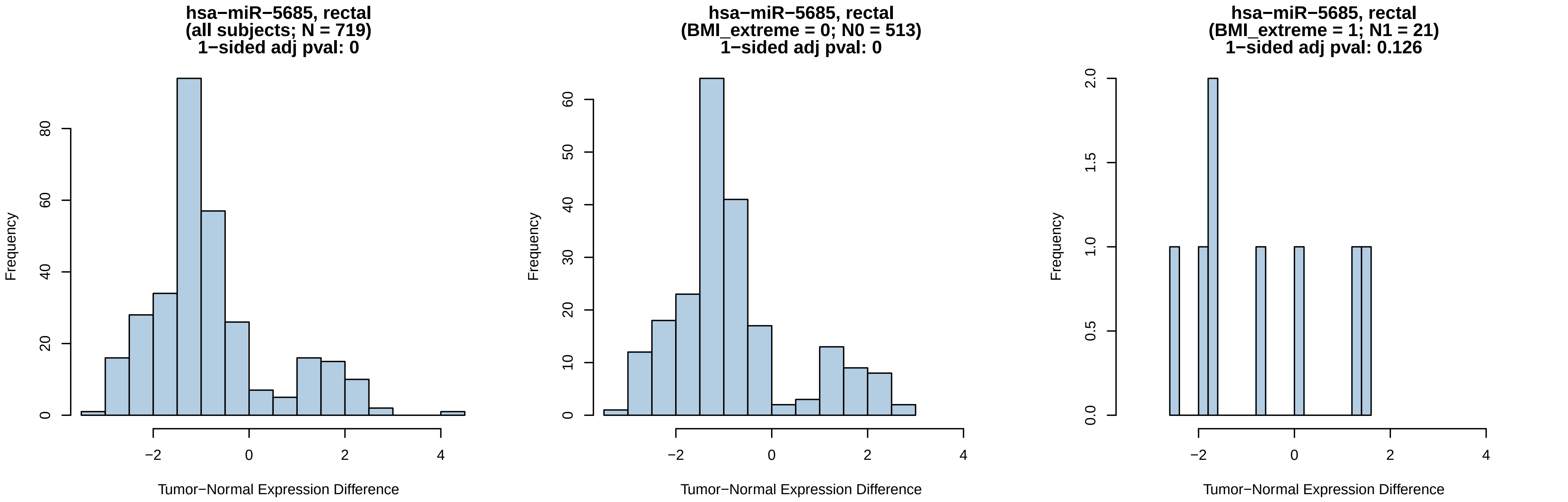
hsa−miR−5685, rectal
(all subjects; N = 719)
1−sided adj pval: 0
hsa−miR−5685, rectal
(BMI_extreme = 0; N0 = 513)
1−sided adj pval: 0
hsa−miR−5685, rectal
(BMI_extreme = 1; N1 = 21)
1−sided adj pval: 0.126
Frequency
Tumor−Normal Expression Difference

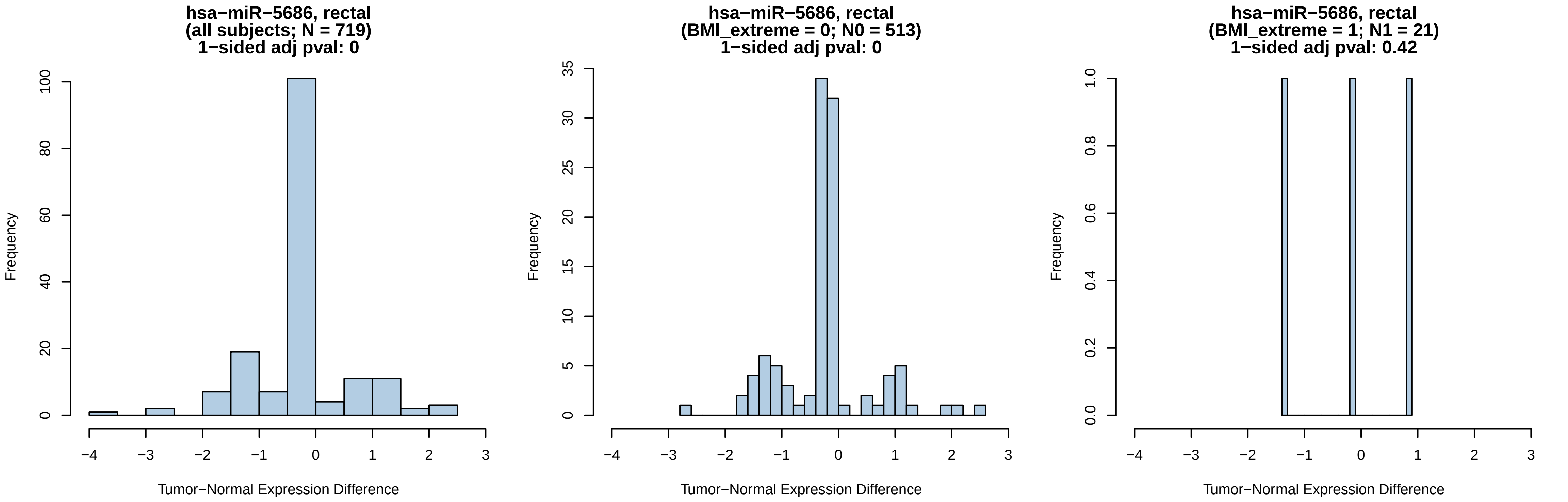

hsa−miR−5686, rectal
(all subjects; N = 719)
1−sided adj pval: 0
Frequency
Tumor−Normal Expression Difference
hsa−miR−5686, rectal
(BMI_extreme = 0; N0 = 513)
1−sided adj pval: 0
Frequency
Tumor−Normal Expression Difference
hsa−miR−5686, rectal
(BMI_extreme = 1; N1 = 21)
1−sided adj pval: 0.42
Frequency
Tumor−Normal Expression Difference

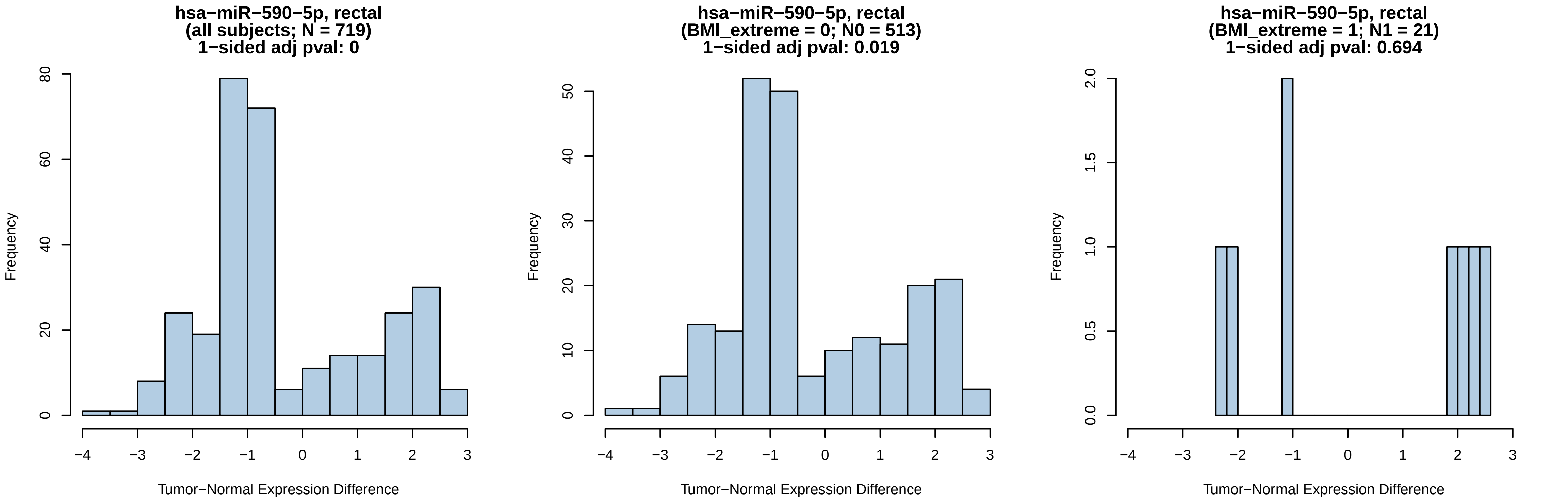

hsa−miR−590−5p, rectal
(all subjects; N = 719)
1−sided adj pval: 0
hsa−miR−590−5p, rectal
(BMI_extreme = 0; N0 = 513)
1−sided adj pval: 0.019
hsa−miR−590−5p, rectal
(BMI_extreme = 1; N1 = 21)
1−sided adj pval: 0.694
Frequency
Tumor−Normal Expression Difference

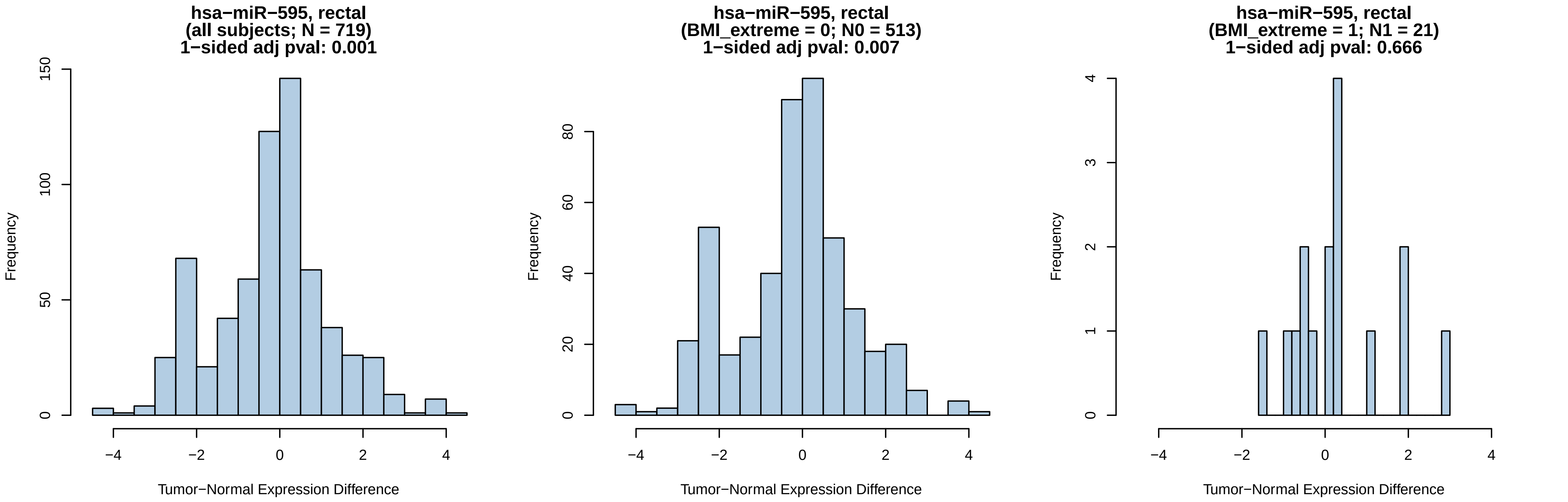

hsa−miR−595, rectal
(all subjects; N = 719)
1−sided adj pval: 0.001
Frequency
Tumor−Normal Expression Difference
hsa−miR−595, rectal
(BMI_extreme = 0; N0 = 513)
1−sided adj pval: 0.007
Frequency
Tumor−Normal Expression Difference
hsa−miR−595, rectal
(BMI_extreme = 1; N1 = 21)
1−sided adj pval: 0.666
Frequency
Tumor−Normal Expression Difference

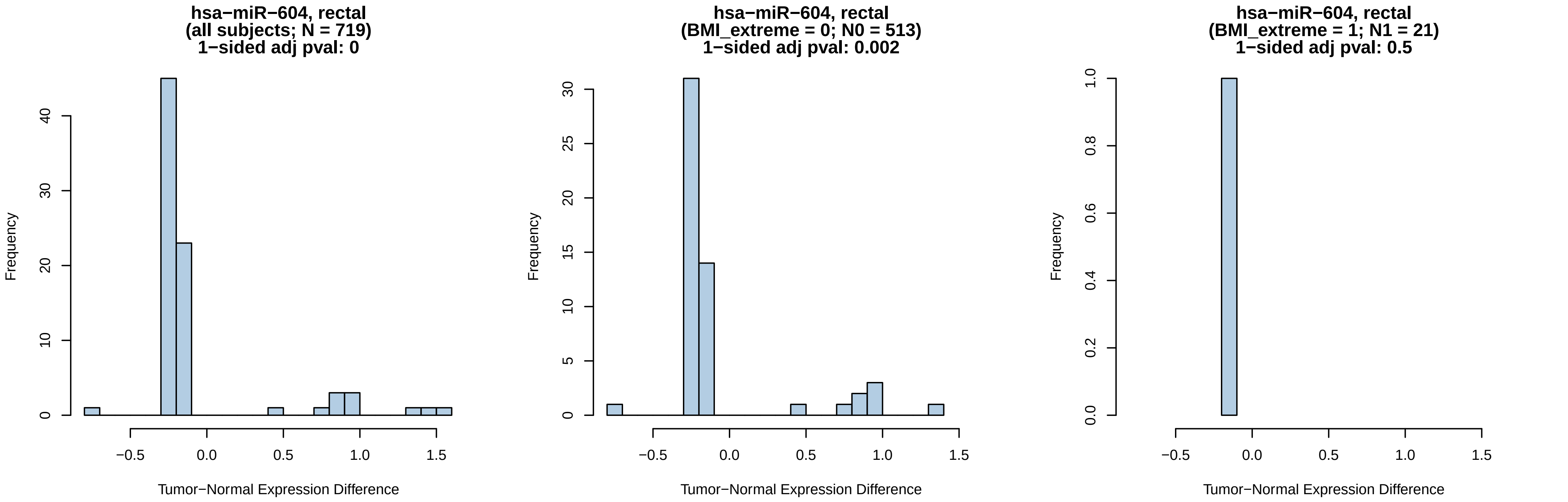
hsa−miR−604, rectal
(all subjects; N = 719)
1−sided adj pval: 0
Frequency
Tumor−Normal Expression Difference
hsa−miR−604, rectal
(BMI_extreme = 0; N0 = 513)
1−sided adj pval: 0.002
Frequency
Tumor−Normal Expression Difference
hsa−miR−604, rectal
(BMI_extreme = 1; N1 = 21)
1−sided adj pval: 0.5
Frequency
Tumor−Normal Expression Difference

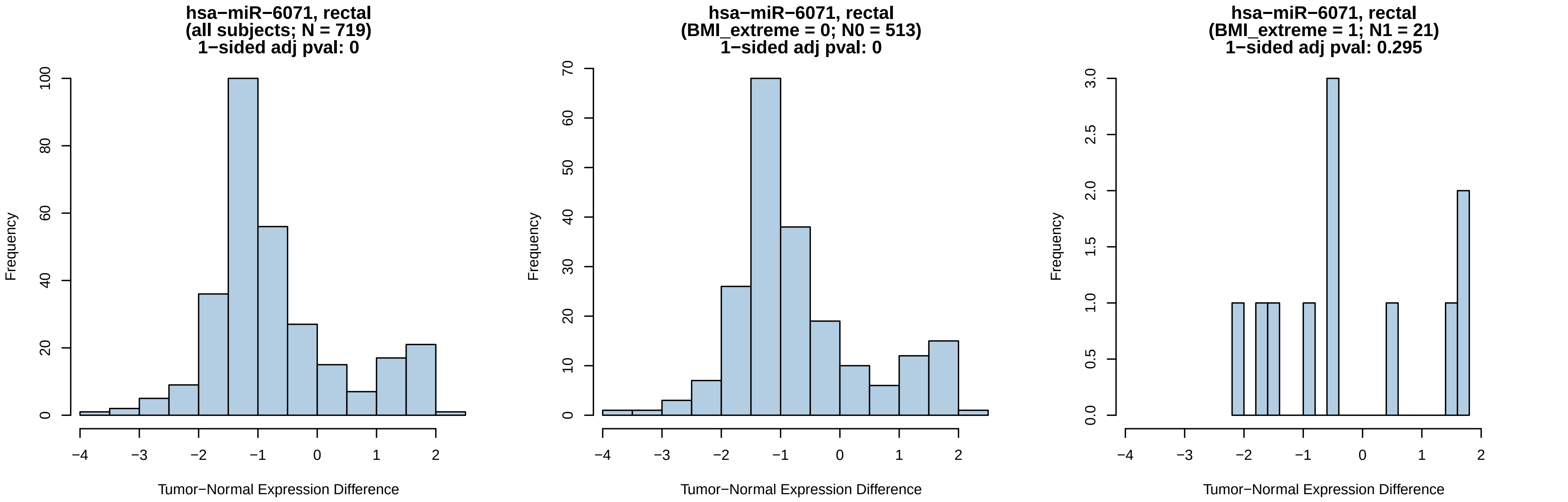
hsa−miR−6071, rectal
(all subjects; N = 719)
1−sided adj pval: 0
Frequency
Tumor−Normal Expression Difference
hsa−miR−6071, rectal
(BMI_extreme = 0; N0 = 513)
1−sided adj pval: 0
Frequency
Tumor−Normal Expression Difference
hsa−miR−6071, rectal
(BMI_extreme = 1; N1 = 21)
1−sided adj pval: 0.295
Frequency
Tumor−Normal Expression Difference

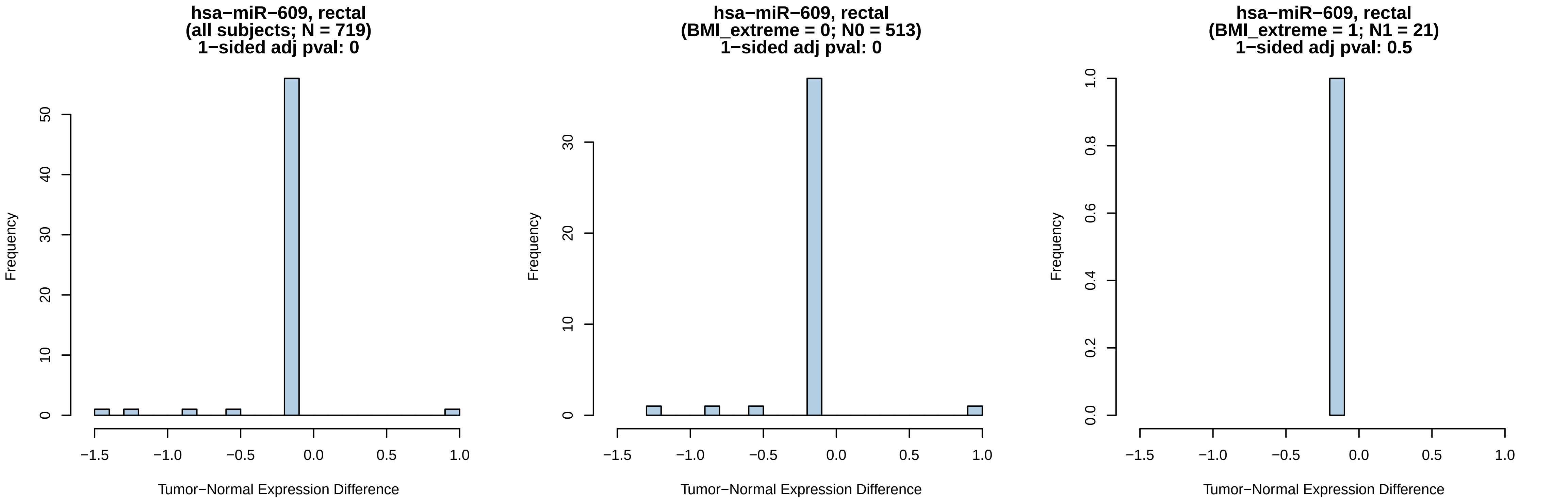
hsa−miR−609, rectal
(all subjects; N = 719)
1−sided adj pval: 0
Frequency
0
10
20
30
40
50
−1.5
−1.0
−0.5
0.0
0.5
1.0
Tumor−Normal Expression Difference
hsa−miR−609, rectal
(BMI_extreme = 0; N0 = 513)
1−sided adj pval: 0
Frequency
0
10
20
30
−1.5
−1.0
−0.5
0.0
0.5
1.0
Tumor−Normal Expression Difference
hsa−miR−609, rectal
(BMI_extreme = 1; N1 = 21)
1−sided adj pval: 0.5
Frequency
0.0
0.2
0.4
0.6
0.8
1.0
−1.5
−1.0
−0.5
0.0
0.5
1.0
Tumor−Normal Expression Difference

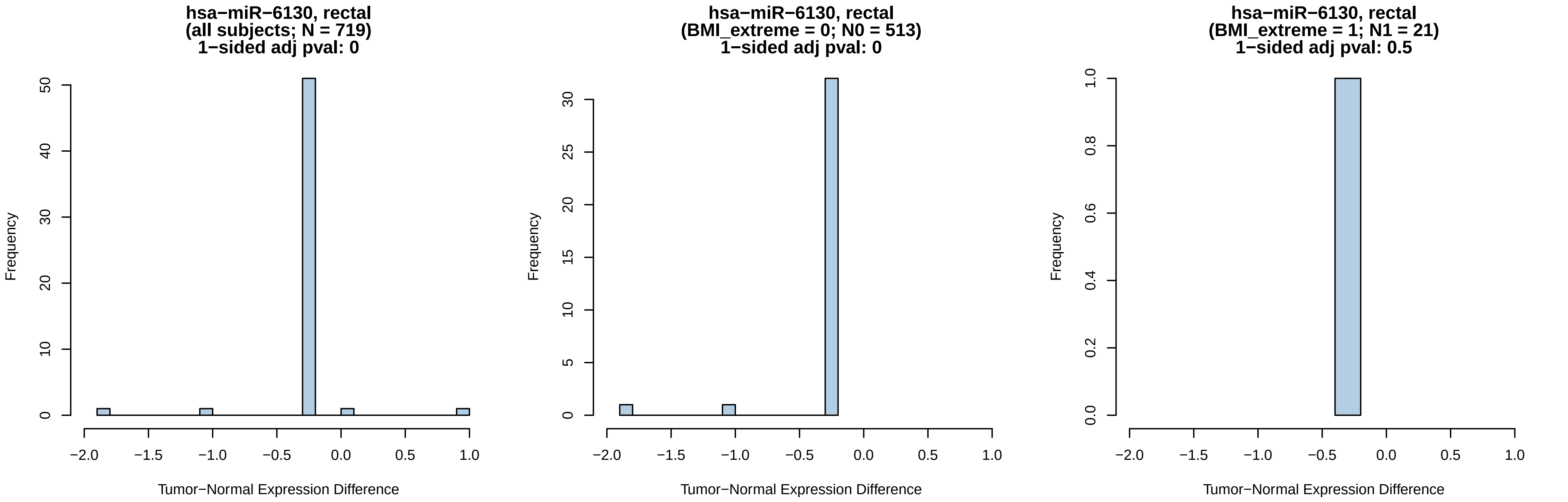

hsa−miR−6130, rectal
(all subjects; N = 719)
1−sided adj pval: 0
Frequency
0
10
20
30
40
50
−2.0
−1.5
−1.0
−0.5
0.0
0.5
1.0
Tumor−Normal Expression Difference
hsa−miR−6130, rectal
(BMI_extreme = 0; N0 = 513)
1−sided adj pval: 0
Frequency
0
5
10
15
20
25
30
−2.0
−1.5
−1.0
−0.5
0.0
0.5
1.0
Tumor−Normal Expression Difference
hsa−miR−6130, rectal
(BMI_extreme = 1; N1 = 21)
1−sided adj pval: 0.5
Frequency
0.0
0.2
0.4
0.6
0.8
1.0
−2.0
−1.5
−1.0
−0.5
0.0
0.5
1.0
Tumor−Normal Expression Difference

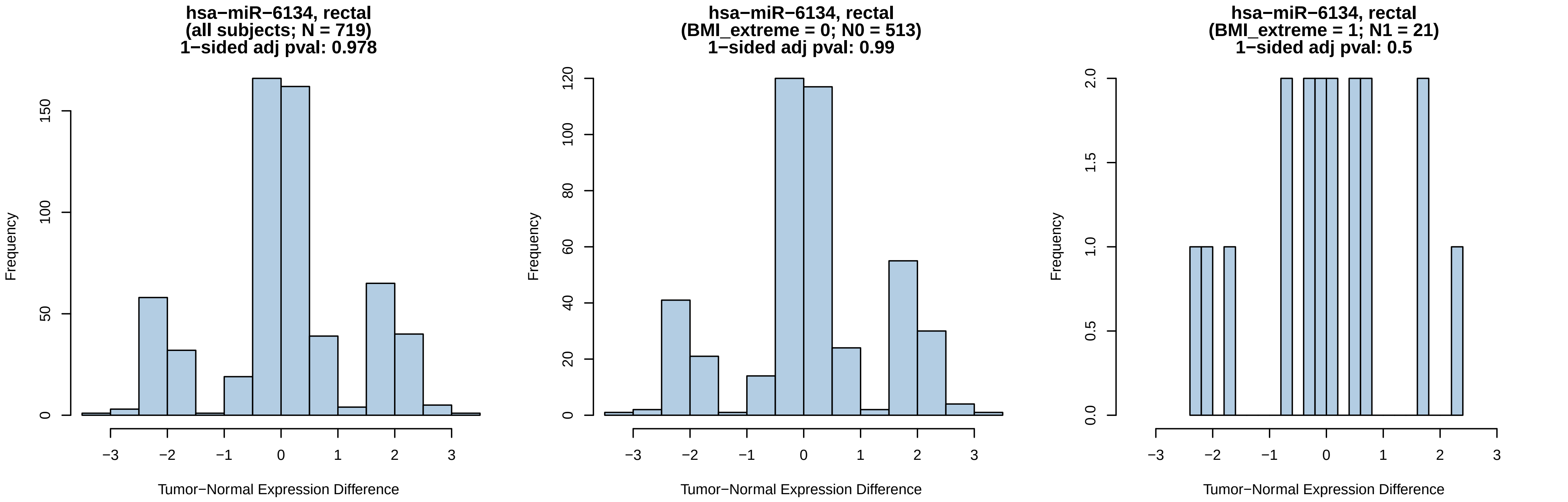
hsa−miR−6134, rectal
(all subjects; N = 719)
1−sided adj pval: 0.978
Frequency
0
50
100
150
−3
−2
−1
0
1
2
3
Tumor−Normal Expression Difference
hsa−miR−6134, rectal
(BMI_extreme = 0; N0 = 513)
1−sided adj pval: 0.99
Frequency
0
20
40
60
80
100
120
−3
−2
−1
0
1
2
3
Tumor−Normal Expression Difference
hsa−miR−6134, rectal
(BMI_extreme = 1; N1 = 21)
1−sided adj pval: 0.5
Frequency
0.0
0.5
1.0
1.5
2.0
−3
−2
−1
0
1
2
3
Tumor−Normal Expression Difference

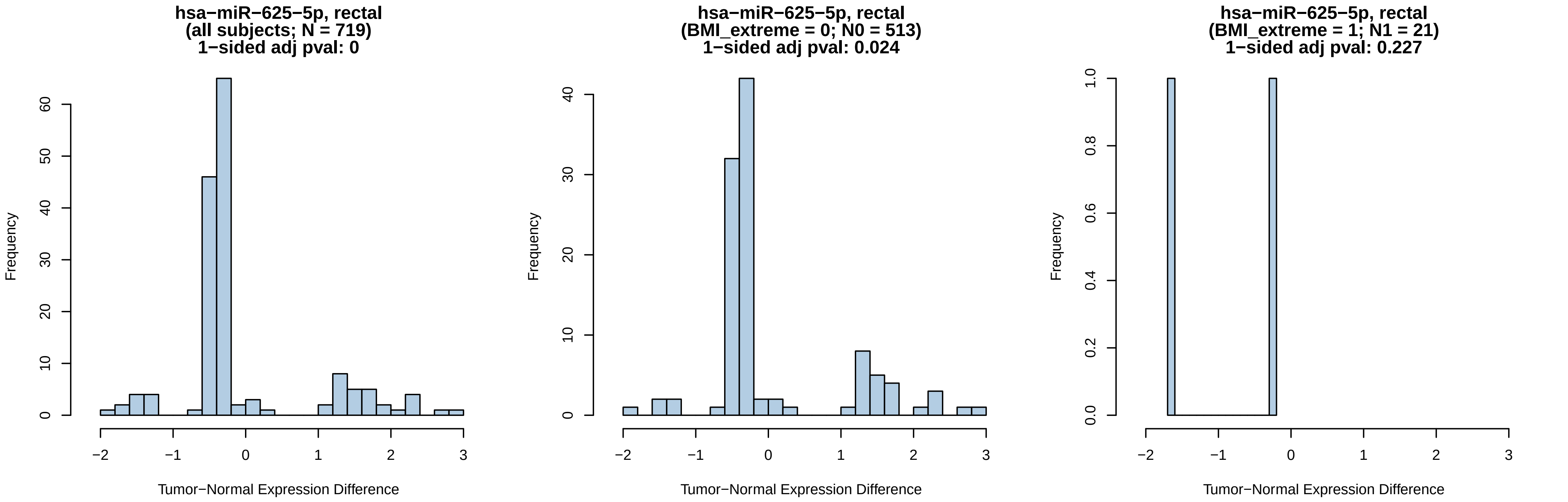
hsa−miR−625−5p, rectal
(all subjects; N = 719)
1−sided adj pval: 0
hsa−miR−625−5p, rectal
(BMI_extreme = 0; N0 = 513)
1−sided adj pval: 0.024
hsa−miR−625−5p, rectal
(BMI_extreme = 1; N1 = 21)
1−sided adj pval: 0.227
Frequency
Tumor−Normal Expression Difference

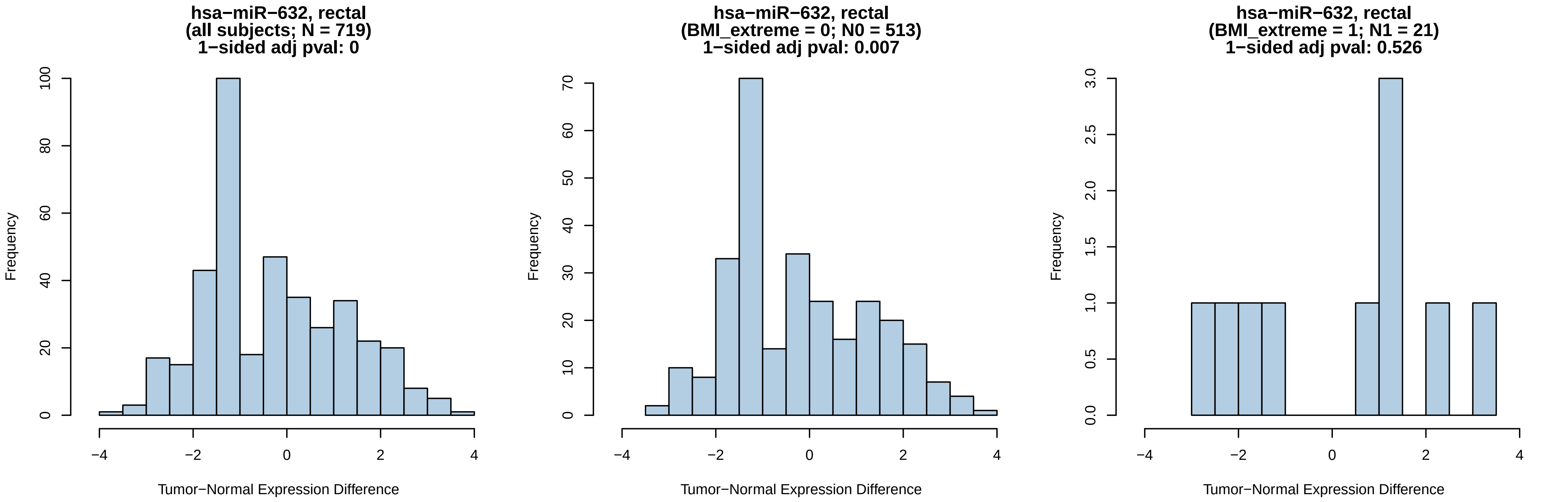
hsa−miR−632, rectal
(all subjects; N = 719)
1−sided adj pval: 0
hsa−miR−632, rectal
(BMI_extreme = 0; N0 = 513)
1−sided adj pval: 0.007
hsa−miR−632, rectal
(BMI_extreme = 1; N1 = 21)
1−sided adj pval: 0.526
Frequency
Tumor−Normal Expression Difference
0
20
40
60
80
100
10
30
50
70
0.0
0.5
1.0
1.5
2.0
2.5
3.0
−4
−2
0
2
4

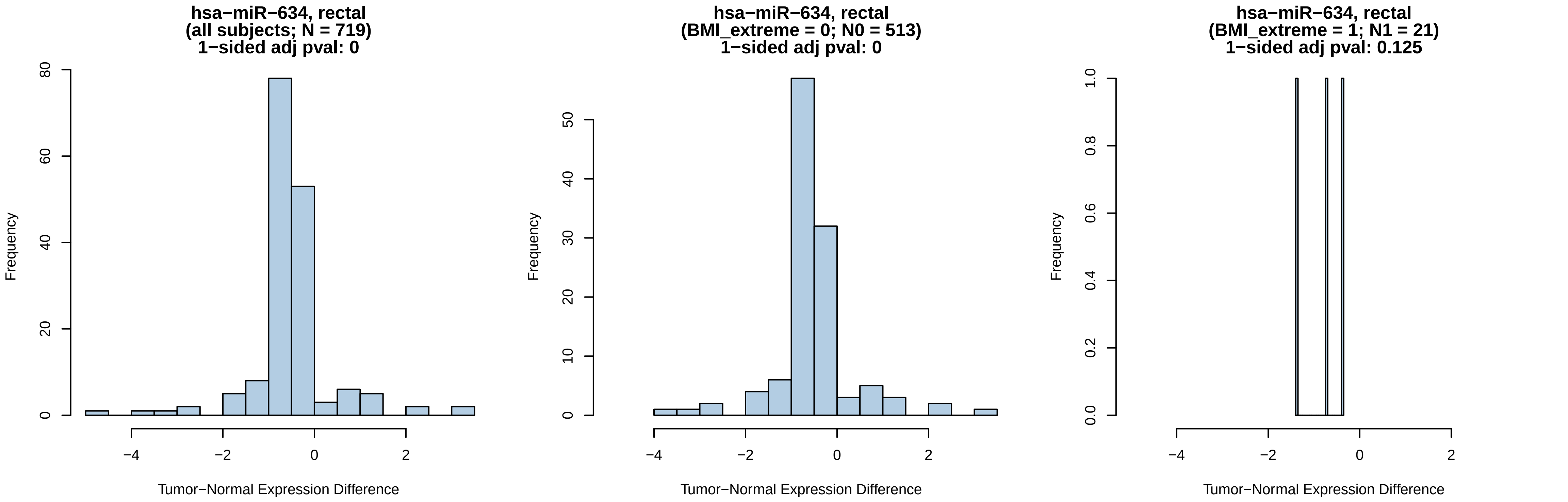

hsa−miR−634, rectal
(all subjects; N = 719)
1−sided adj pval: 0
hsa−miR−634, rectal
(BMI_extreme = 0; N0 = 513)
1−sided adj pval: 0
hsa−miR−634, rectal
(BMI_extreme = 1; N1 = 21)
1−sided adj pval: 0.125
Frequency
Tumor−Normal Expression Difference

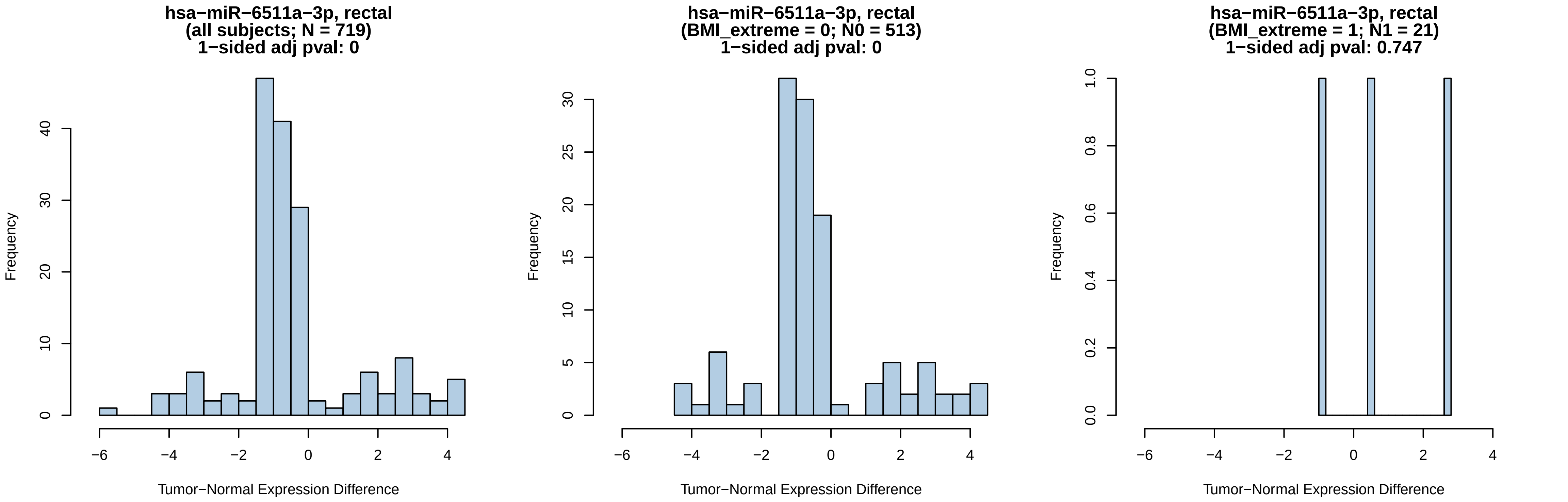
hsa−miR−6511a−3p, rectal
(all subjects; N = 719)
1−sided adj pval: 0
Frequency
Tumor−Normal Expression Difference
hsa−miR−6511a−3p, rectal
(BMI_extreme = 0; N0 = 513)
1−sided adj pval: 0
Frequency
Tumor−Normal Expression Difference
hsa−miR−6511a−3p, rectal
(BMI_extreme = 1; N1 = 21)
1−sided adj pval: 0.747
Frequency
Tumor−Normal Expression Difference

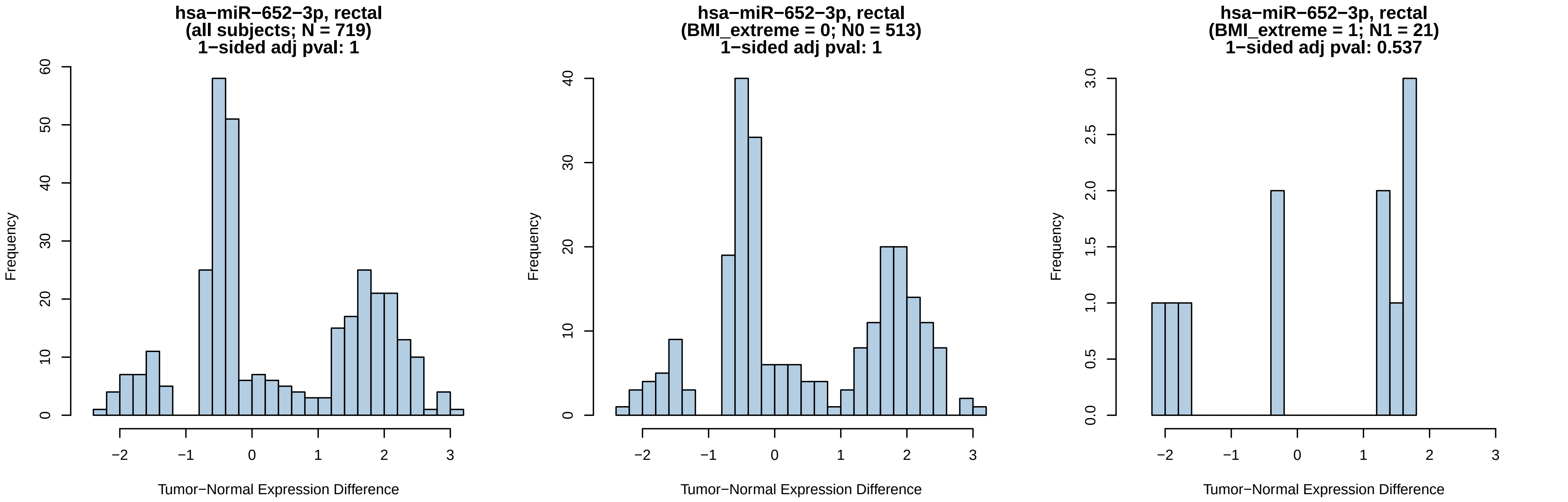

hsa−miR−652−3p, rectal
(all subjects; N = 719)
1−sided adj pval: 1
Frequency
Tumor−Normal Expression Difference
0
10
20
30
40
50
60
−2
−1
0
1
2
3
hsa−miR−652−3p, rectal
(BMI_extreme = 0; N0 = 513)
1−sided adj pval: 1
Frequency
Tumor−Normal Expression Difference
0
10
20
30
40
−2
−1
0
1
2
3
hsa−miR−652−3p, rectal
(BMI_extreme = 1; N1 = 21)
1−sided adj pval: 0.537
Frequency
Tumor−Normal Expression Difference
0.0
0.5
1.0
1.5
2.0
2.5
3.0
−2
−1
0
1
2
3

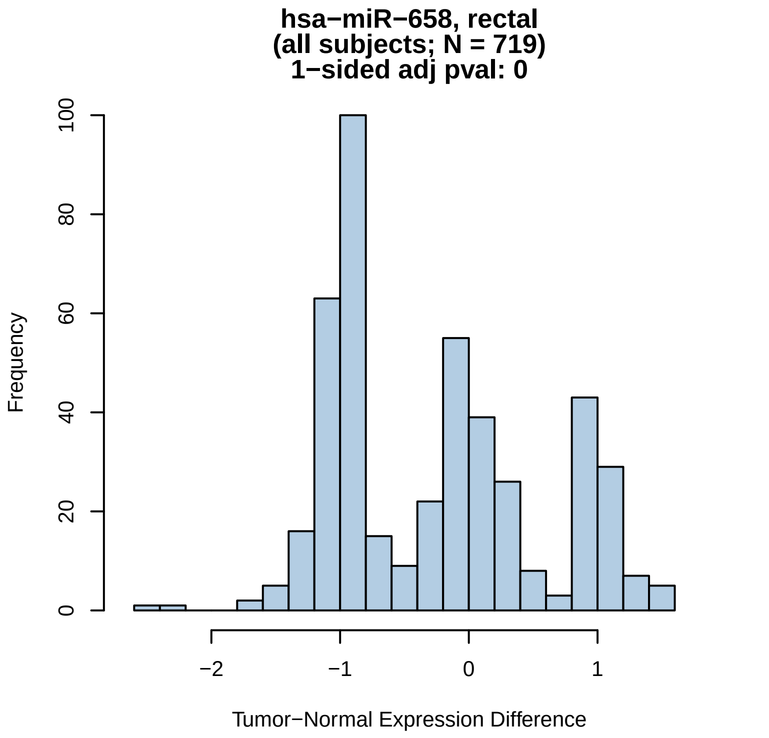
hsa−miR−658, rectal
(all subjects; N = 719)
1−sided adj pval: 0
Frequency
Tumor−Normal Expression Difference
0
20
40
60
80
100
−2
−1
0
1

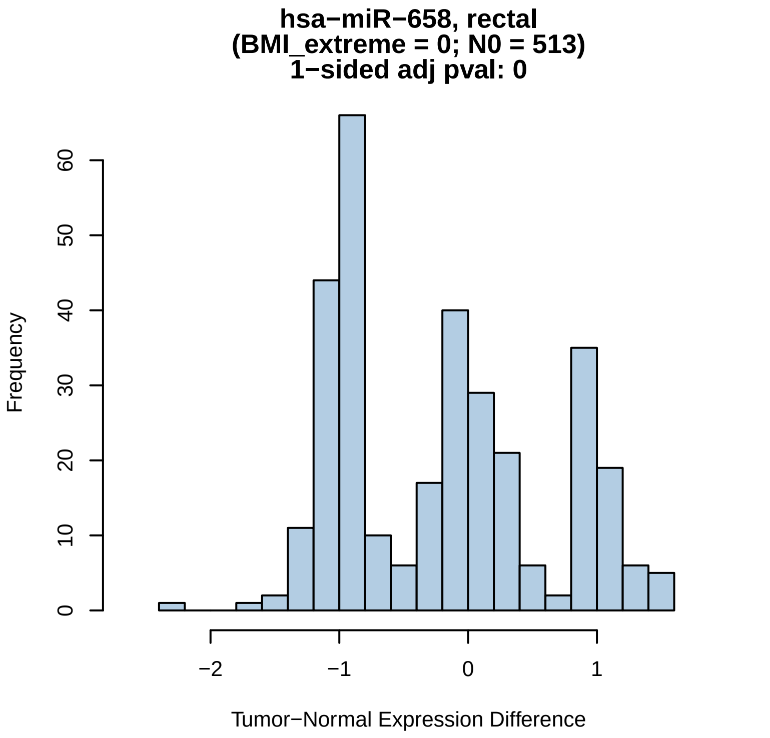
hsa−miR−658, rectal
(BMI_extreme = 0; N0 = 513)
1−sided adj pval: 0
Frequency
Tumor−Normal Expression Difference
0
10
20
30
40
50
60
−2
−1
0
1

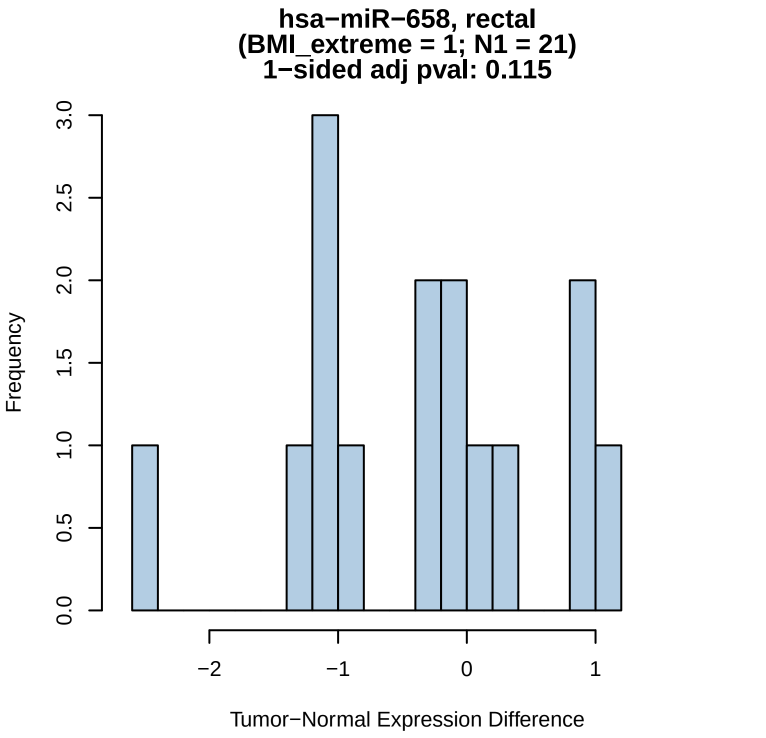
hsa−miR−658, rectal
(BMI_extreme = 1; N1 = 21)
1−sided adj pval: 0.115
Frequency
Tumor−Normal Expression Difference
0.0
0.5
1.0
1.5
2.0
2.5
3.0
−2
−1
0
1

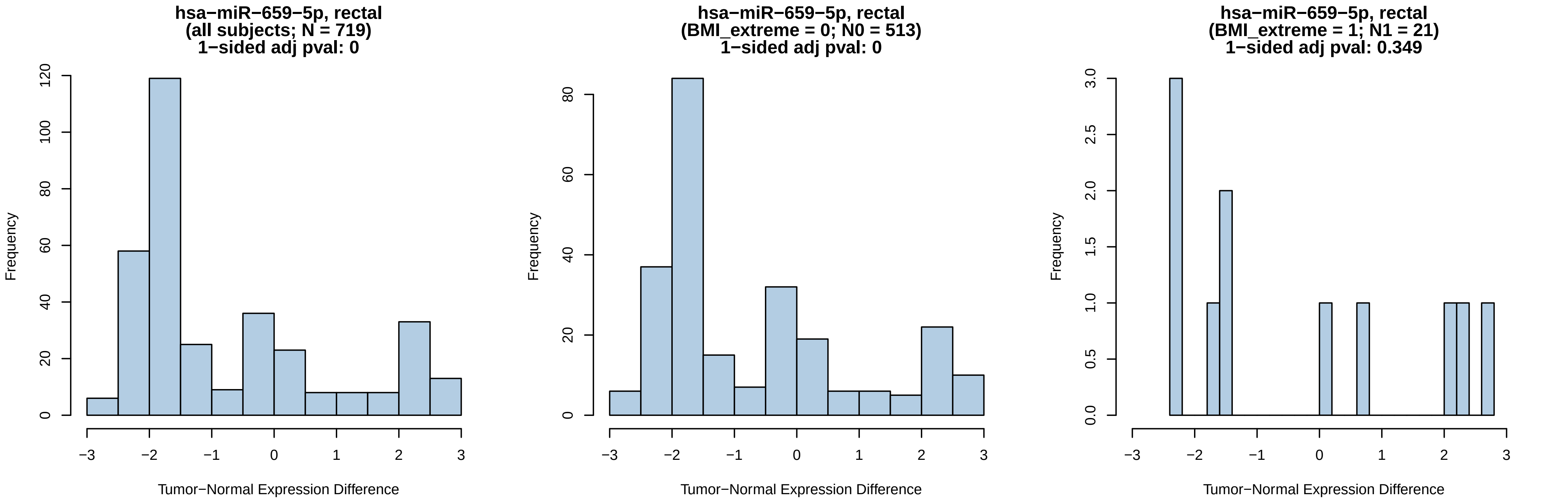

hsa−miR−659−5p, rectal
(all subjects; N = 719)
1−sided adj pval: 0
Frequency
0
20
40
60
80
100
120
−3
−2
−1
0
1
2
3
Tumor−Normal Expression Difference
hsa−miR−659−5p, rectal
(BMI_extreme = 0; N0 = 513)
1−sided adj pval: 0
Frequency
0
20
40
60
80
−3
−2
−1
0
1
2
3
Tumor−Normal Expression Difference
hsa−miR−659−5p, rectal
(BMI_extreme = 1; N1 = 21)
1−sided adj pval: 0.349
Frequency
0.0
0.5
1.0
1.5
2.0
2.5
3.0
−3
−2
−1
0
1
2
3
Tumor−Normal Expression Difference

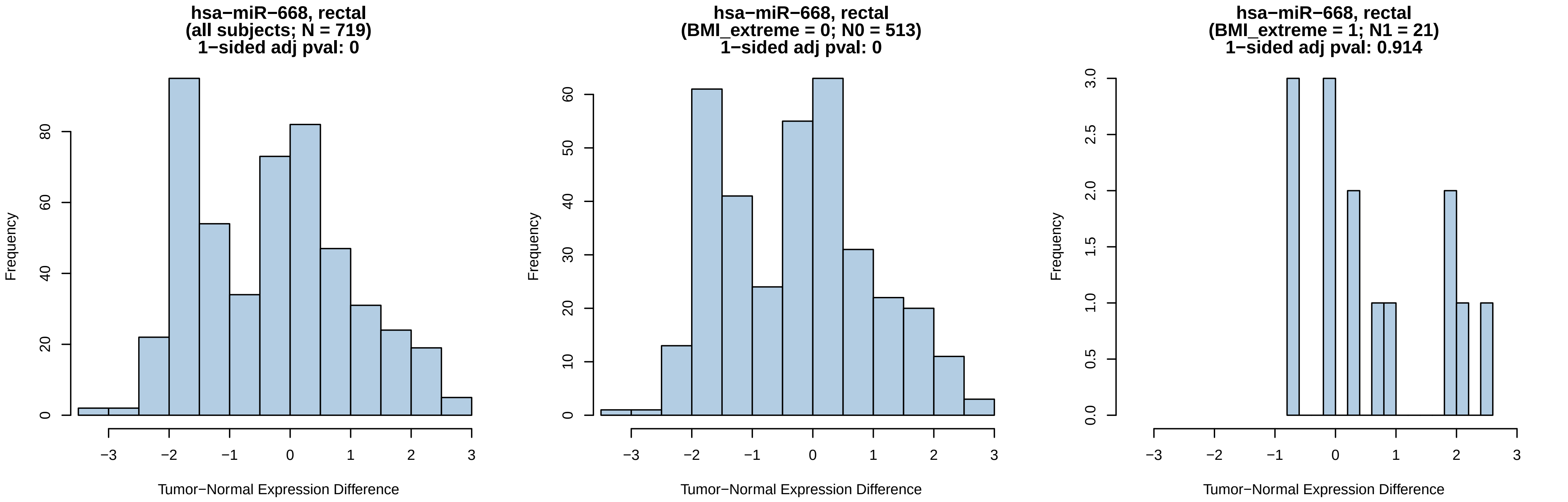
hsa−miR−668, rectal
(all subjects; N = 719)
1−sided adj pval: 0
Frequency
Tumor−Normal Expression Difference
hsa−miR−668, rectal
(BMI_extreme = 0; N0 = 513)
1−sided adj pval: 0
Frequency
Tumor−Normal Expression Difference
hsa−miR−668, rectal
(BMI_extreme = 1; N1 = 21)
1−sided adj pval: 0.914
Frequency
Tumor−Normal Expression Difference

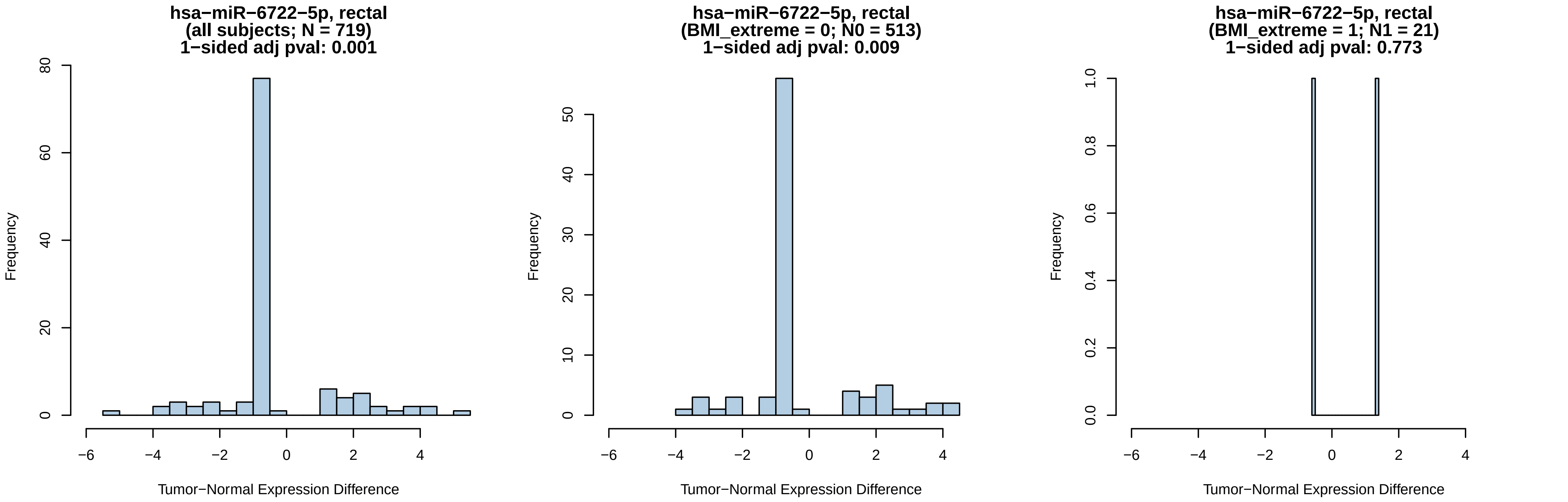
hsa−miR−6722−5p, rectal
(all subjects; N = 719)
1−sided adj pval: 0.001
Frequency
Tumor−Normal Expression Difference
hsa−miR−6722−5p, rectal
(BMI_extreme = 0; N0 = 513)
1−sided adj pval: 0.009
Frequency
Tumor−Normal Expression Difference
hsa−miR−6722−5p, rectal
(BMI_extreme = 1; N1 = 21)
1−sided adj pval: 0.773
Frequency
Tumor−Normal Expression Difference

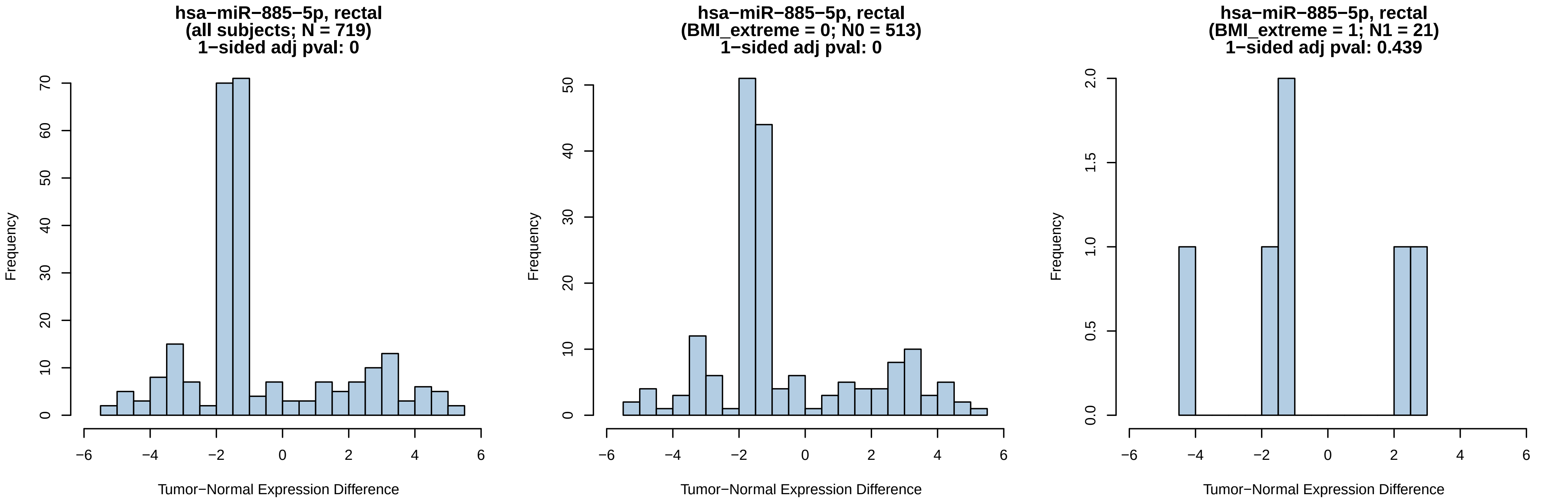
hsa−miR−885−5p, rectal
(all subjects; N = 719)
1−sided adj pval: 0
Frequency
Tumor−Normal Expression Difference
hsa−miR−885−5p, rectal
(BMI_extreme = 0; N0 = 513)
1−sided adj pval: 0
Frequency
Tumor−Normal Expression Difference
hsa−miR−885−5p, rectal
(BMI_extreme = 1; N1 = 21)
1−sided adj pval: 0.439
Frequency
Tumor−Normal Expression Difference

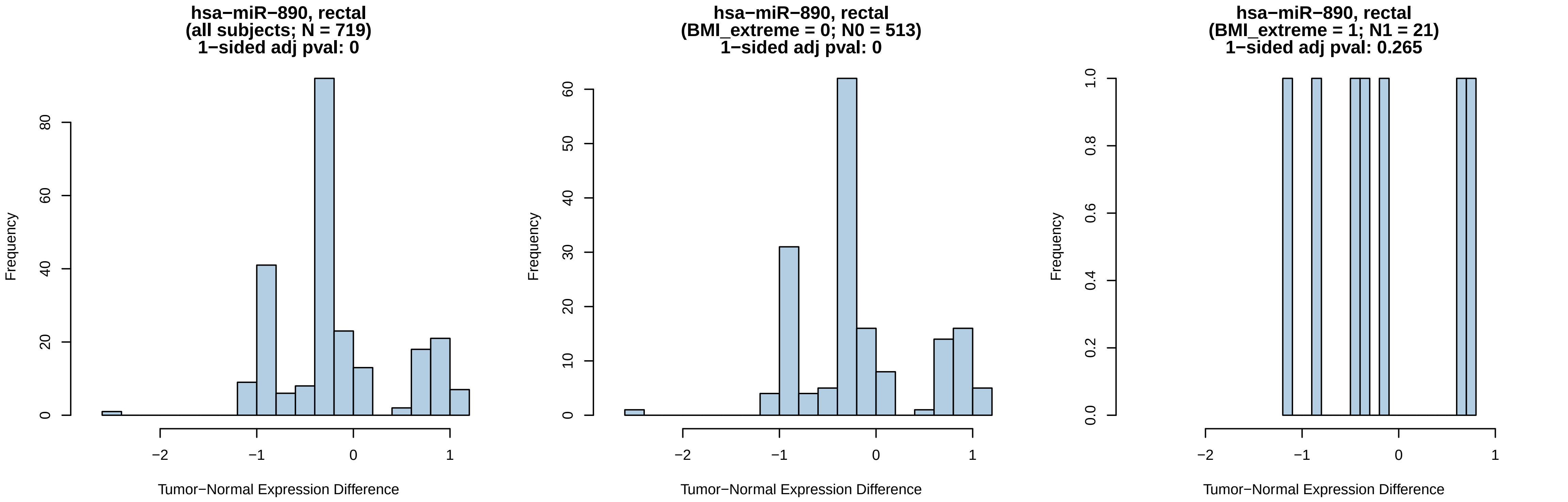

hsa−miR−890, rectal
(all subjects; N = 719)
1−sided adj pval: 0
Frequency
Tumor−Normal Expression Difference
hsa−miR−890, rectal
(BMI_extreme = 0; N0 = 513)
1−sided adj pval: 0
Frequency
Tumor−Normal Expression Difference
hsa−miR−890, rectal
(BMI_extreme = 1; N1 = 21)
1−sided adj pval: 0.265
Frequency
Tumor−Normal Expression Difference

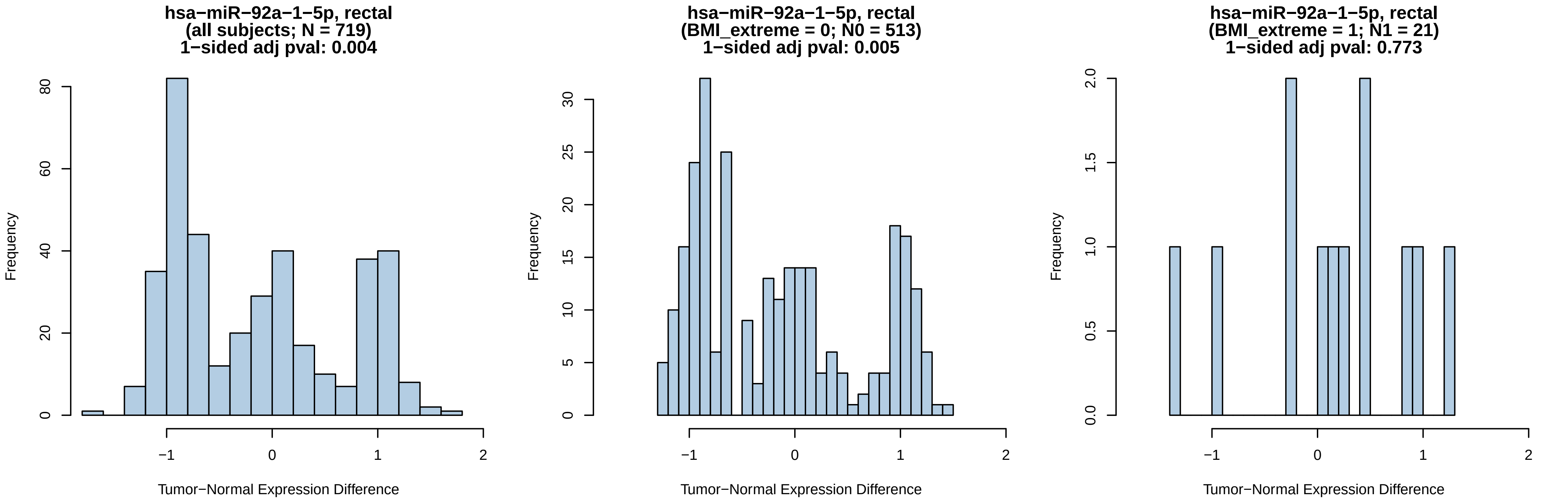
hsa−miR−92a−1−5p, rectal
(all subjects; N = 719)
1−sided adj pval: 0.004
Frequency
Tumor−Normal Expression Difference
hsa−miR−92a−1−5p, rectal
(BMI_extreme = 0; N0 = 513)
1−sided adj pval: 0.005
Frequency
Tumor−Normal Expression Difference
hsa−miR−92a−1−5p, rectal
(BMI_extreme = 1; N1 = 21)
1−sided adj pval: 0.773
Frequency
Tumor−Normal Expression Difference

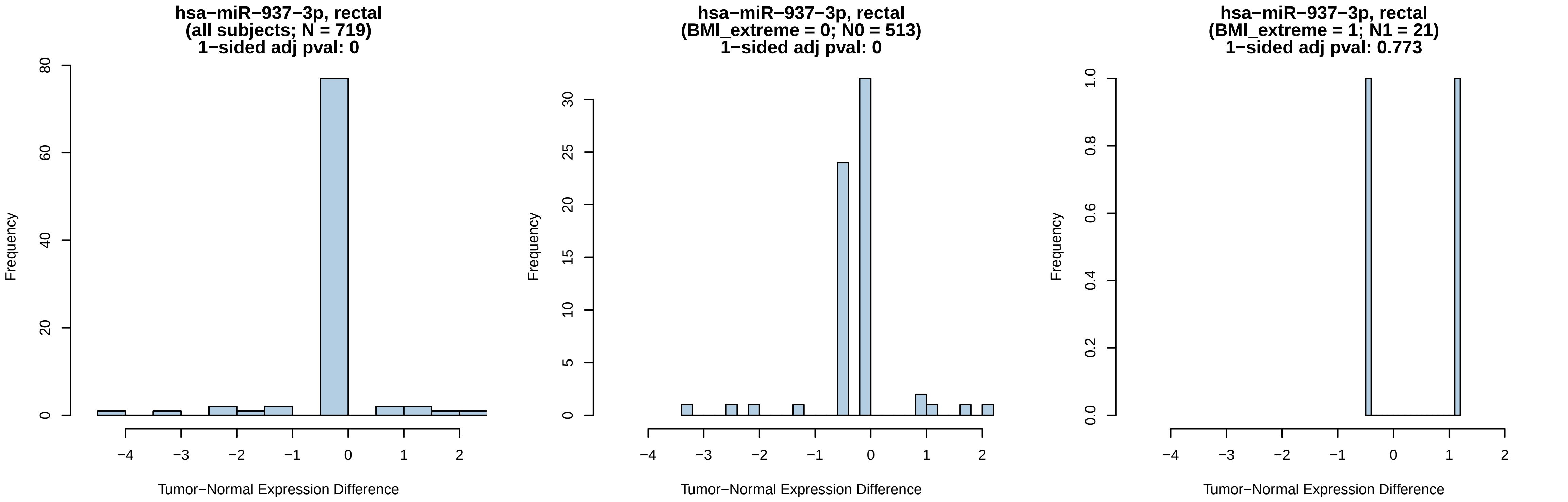

hsa−miR−937−3p, rectal
(all subjects; N = 719)
1−sided adj pval: 0
Frequency
Tumor−Normal Expression Difference
hsa−miR−937−3p, rectal
(BMI_extreme = 0; N0 = 513)
1−sided adj pval: 0
Frequency
Tumor−Normal Expression Difference
hsa−miR−937−3p, rectal
(BMI_extreme = 1; N1 = 21)
1−sided adj pval: 0.773
Frequency
Tumor−Normal Expression Difference

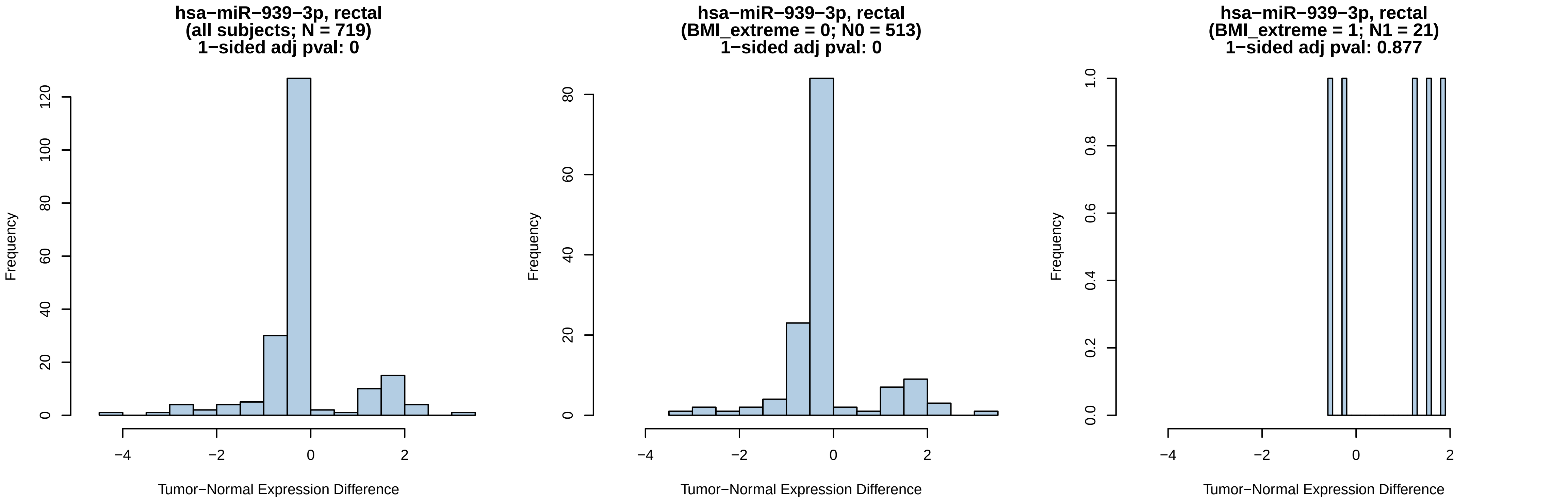
hsa−miR−939−3p, rectal
(all subjects; N = 719)
1−sided adj pval: 0
hsa−miR−939−3p, rectal
(BMI_extreme = 0; N0 = 513)
1−sided adj pval: 0
hsa−miR−939−3p, rectal
(BMI_extreme = 1; N1 = 21)
1−sided adj pval: 0.877
Frequency
Tumor−Normal Expression Difference

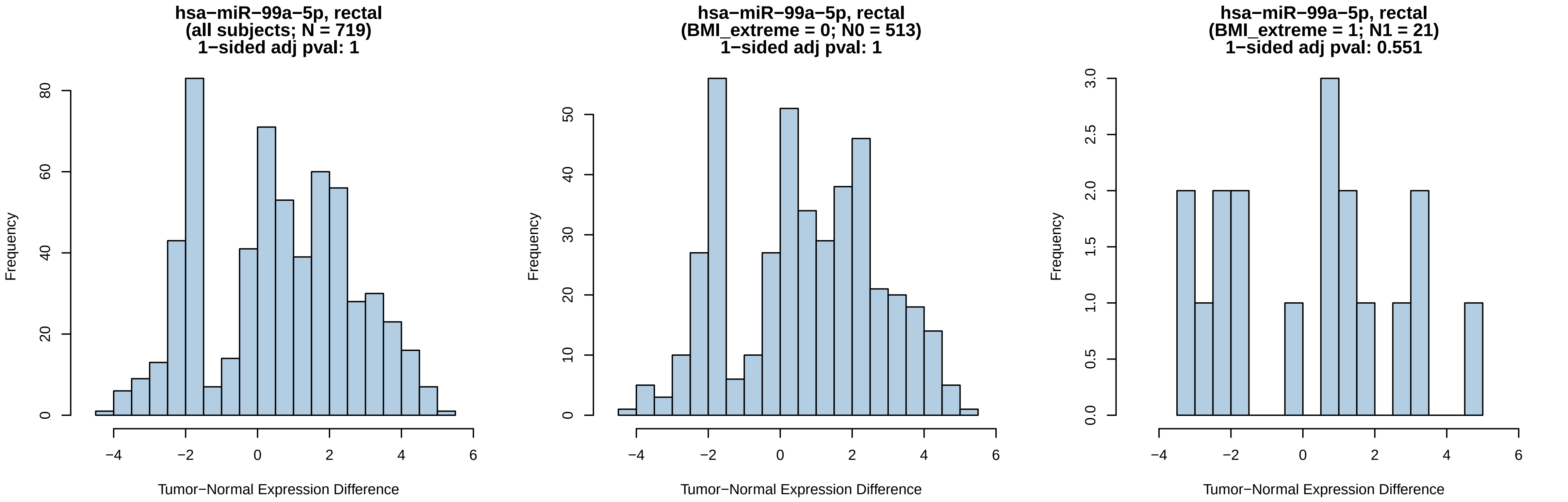

hsa−miR−99a−5p, rectal
(all subjects; N = 719)
1−sided adj pval: 1
Frequency
0
20
40
60
80
−4
−2
0
2
4
6
Tumor−Normal Expression Difference
hsa−miR−99a−5p, rectal
(BMI_extreme = 0; N0 = 513)
1−sided adj pval: 1
Frequency
0
10
20
30
40
50
−4
−2
0
2
4
6
Tumor−Normal Expression Difference
hsa−miR−99a−5p, rectal
(BMI_extreme = 1; N1 = 21)
1−sided adj pval: 0.551
Frequency
0.0
0.5
1.0
1.5
2.0
2.5
3.0
−4
−2
0
2
4
6
Tumor−Normal Expression Difference

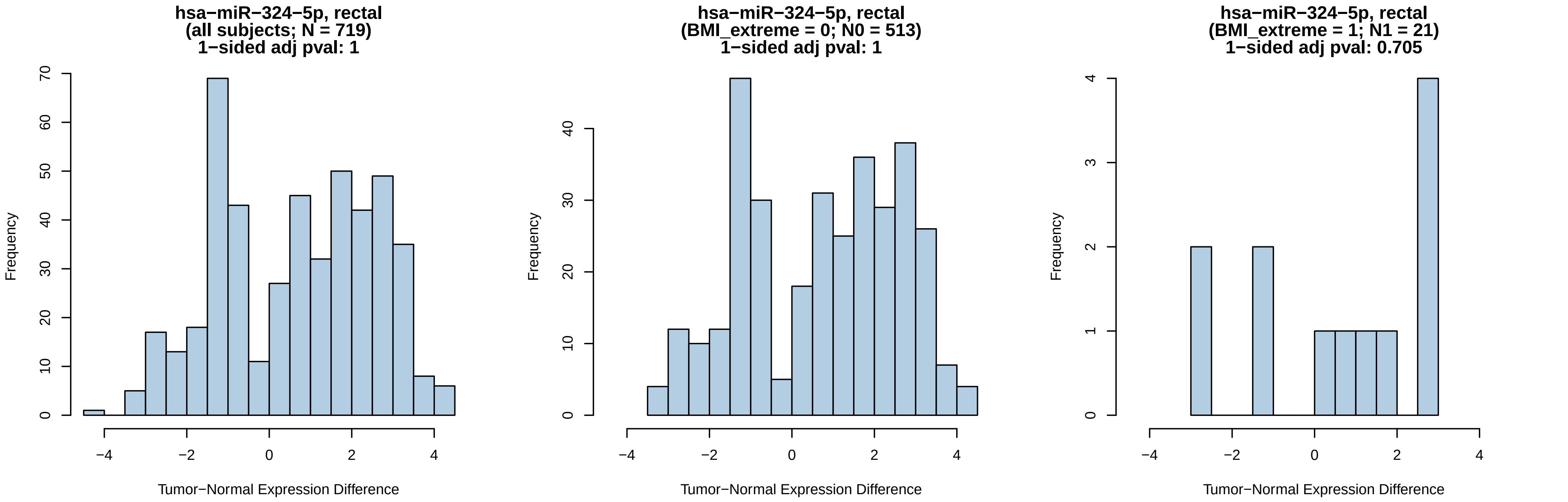

hsa−miR−324−5p, rectal
(all subjects; N = 719)
1−sided adj pval: 1
Frequency
Tumor−Normal Expression Difference
hsa−miR−324−5p, rectal
(BMI_extreme = 0; N0 = 513)
1−sided adj pval: 1
Frequency
Tumor−Normal Expression Difference
hsa−miR−324−5p, rectal
(BMI_extreme = 1; N1 = 21)
1−sided adj pval: 0.705
Frequency
Tumor−Normal Expression Difference

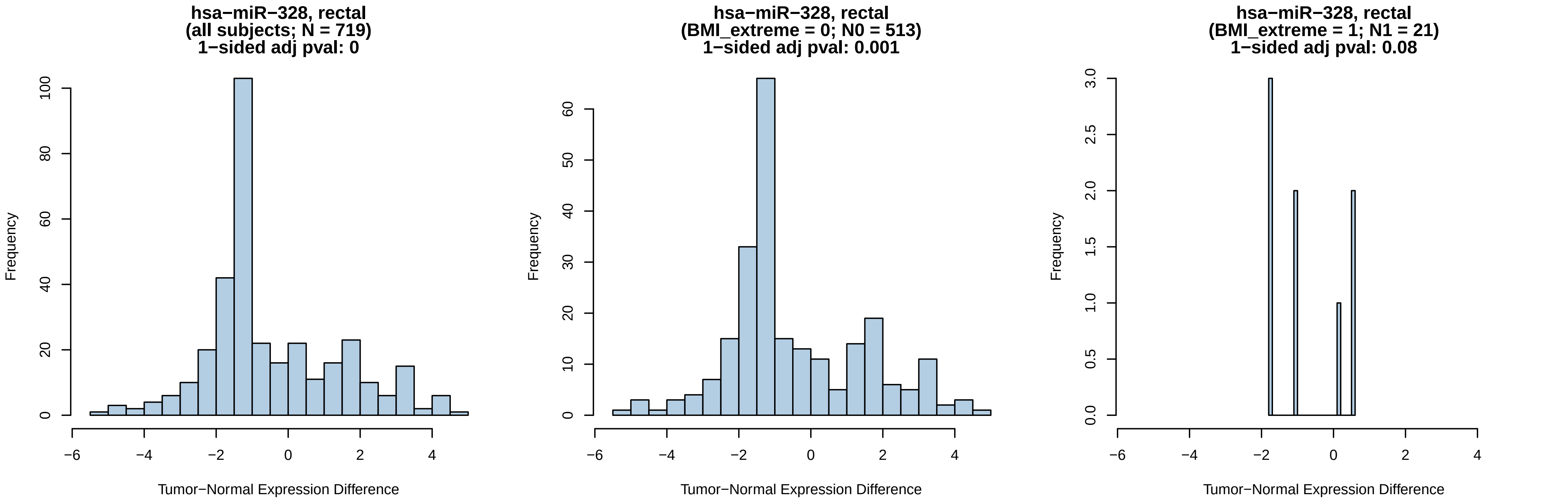
hsa−miR−328, rectal
(all subjects; N = 719)
1−sided adj pval: 0
Frequency
Tumor−Normal Expression Difference
hsa−miR−328, rectal
(BMI_extreme = 0; N0 = 513)
1−sided adj pval: 0.001
Frequency
Tumor−Normal Expression Difference
hsa−miR−328, rectal
(BMI_extreme = 1; N1 = 21)
1−sided adj pval: 0.08
Frequency
Tumor−Normal Expression Difference

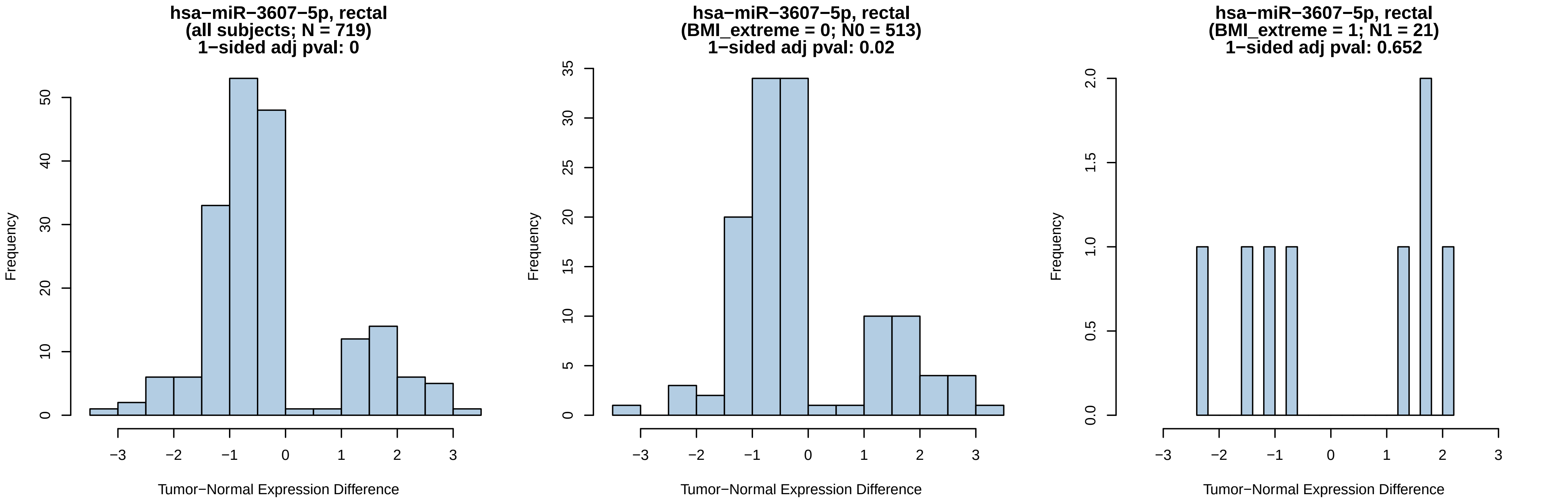
hsa−miR−3607−5p, rectal
(all subjects; N = 719)
1−sided adj pval: 0
Frequency
Tumor−Normal Expression Difference
hsa−miR−3607−5p, rectal
(BMI_extreme = 0; N0 = 513)
1−sided adj pval: 0.02
Frequency
Tumor−Normal Expression Difference
hsa−miR−3607−5p, rectal
(BMI_extreme = 1; N1 = 21)
1−sided adj pval: 0.652
Frequency
Tumor−Normal Expression Difference

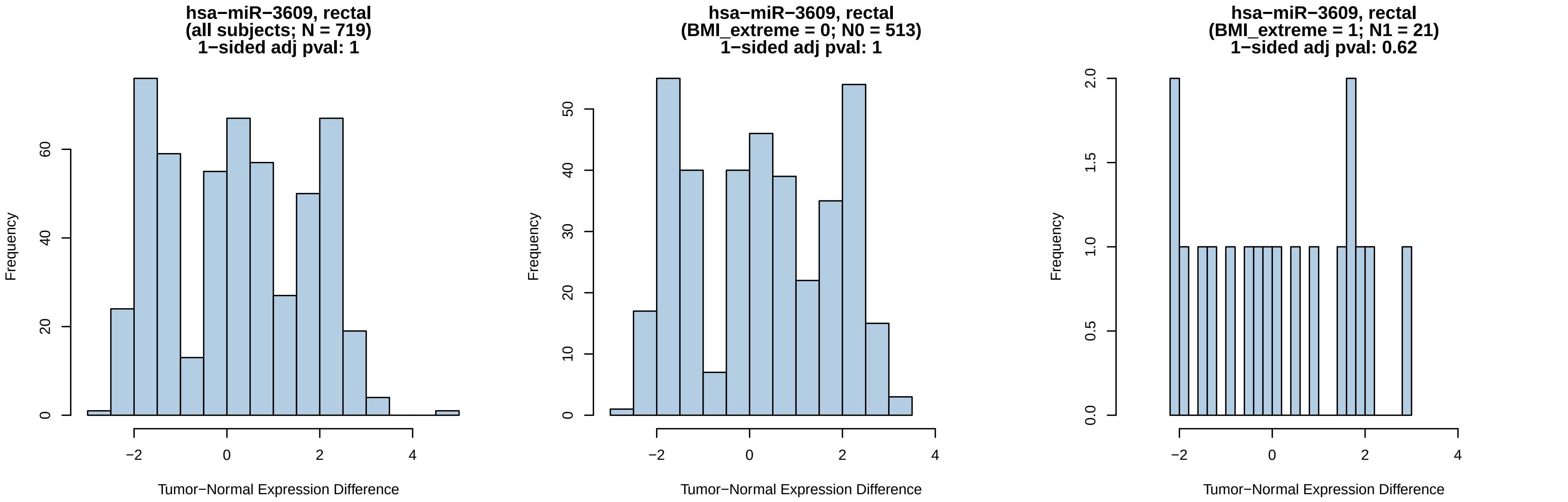

hsa−miR−3609, rectal
(all subjects; N = 719)
1−sided adj pval: 1
Frequency
Tumor−Normal Expression Difference
hsa−miR−3609, rectal
(BMI_extreme = 0; N0 = 513)
1−sided adj pval: 1
Frequency
Tumor−Normal Expression Difference
hsa−miR−3609, rectal
(BMI_extreme = 1; N1 = 21)
1−sided adj pval: 0.62
Frequency
Tumor−Normal Expression Difference

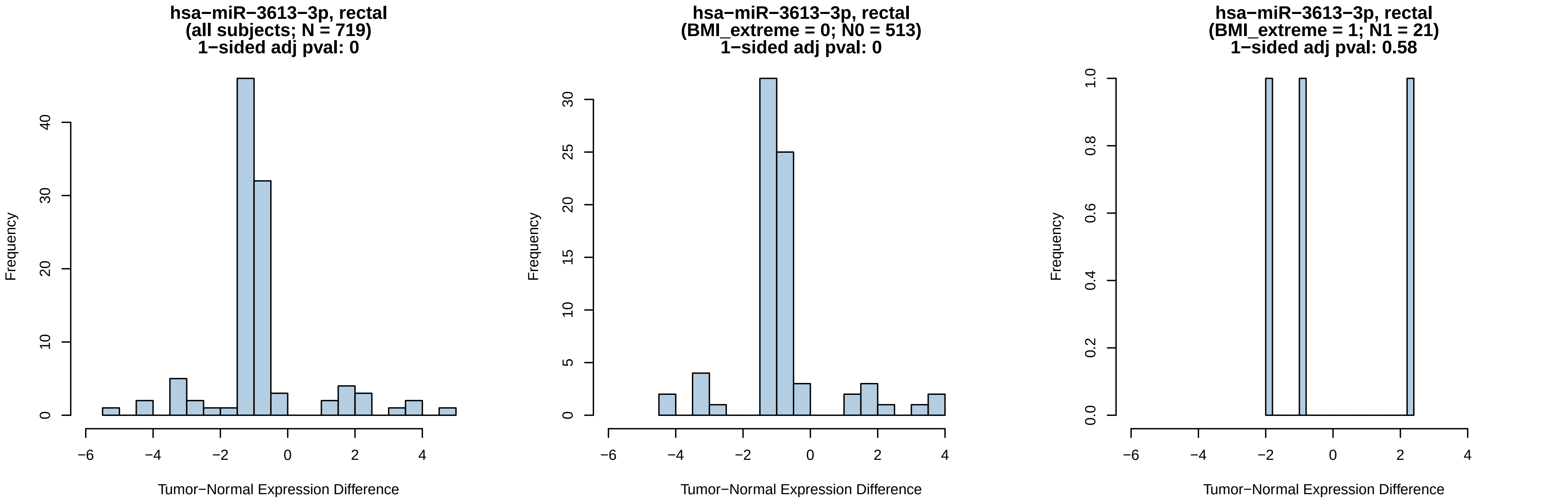
hsa−miR−3613−3p, rectal
(all subjects; N = 719)
1−sided adj pval: 0
Frequency
Tumor−Normal Expression Difference
hsa−miR−3613−3p, rectal
(BMI_extreme = 0; N0 = 513)
1−sided adj pval: 0
Frequency
Tumor−Normal Expression Difference
hsa−miR−3613−3p, rectal
(BMI_extreme = 1; N1 = 21)
1−sided adj pval: 0.58
Frequency
Tumor−Normal Expression Difference

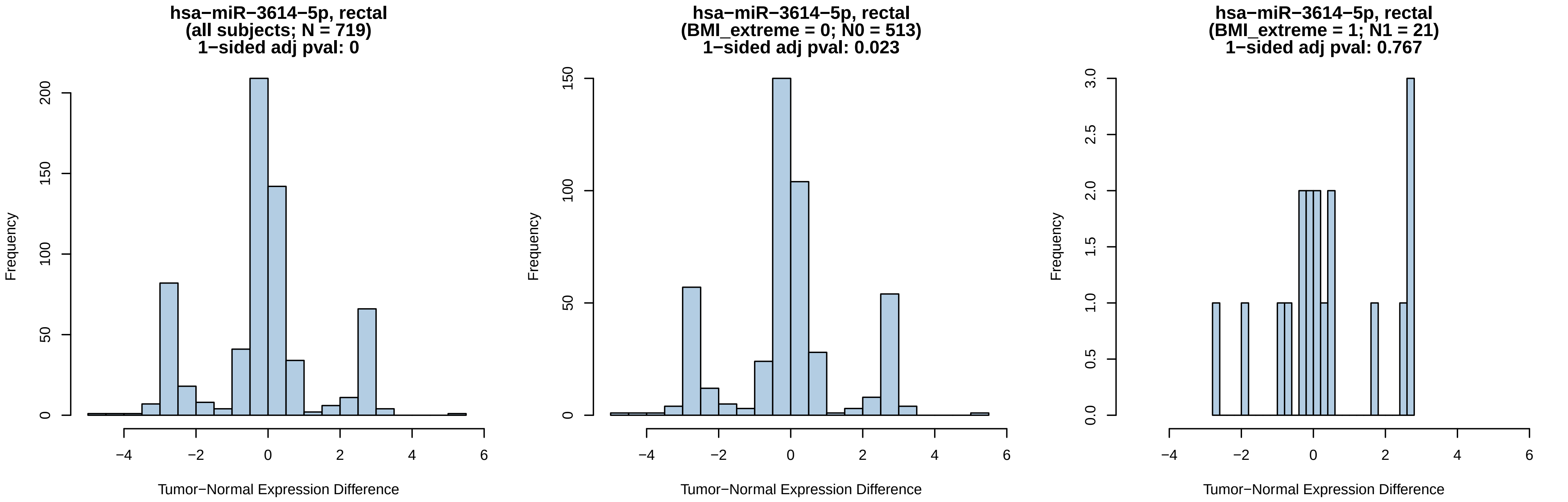

hsa−miR−3614−5p, rectal
(all subjects; N = 719)
1−sided adj pval: 0
Frequency
Tumor−Normal Expression Difference
hsa−miR−3614−5p, rectal
(BMI_extreme = 0; N0 = 513)
1−sided adj pval: 0.023
Frequency
Tumor−Normal Expression Difference
hsa−miR−3614−5p, rectal
(BMI_extreme = 1; N1 = 21)
1−sided adj pval: 0.767
Frequency
Tumor−Normal Expression Difference

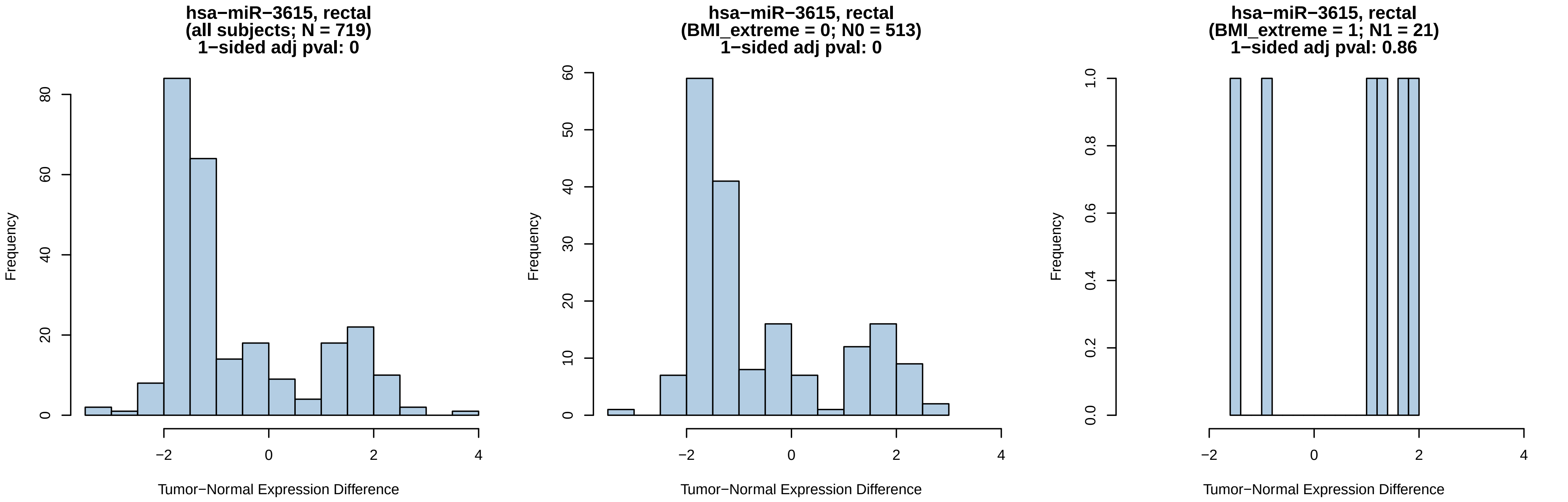
hsa−miR−3615, rectal
(all subjects; N = 719)
1−sided adj pval: 0
Frequency
Tumor−Normal Expression Difference
hsa−miR−3615, rectal
(BMI_extreme = 0; N0 = 513)
1−sided adj pval: 0
Frequency
Tumor−Normal Expression Difference
hsa−miR−3615, rectal
(BMI_extreme = 1; N1 = 21)
1−sided adj pval: 0.86
Frequency
Tumor−Normal Expression Difference

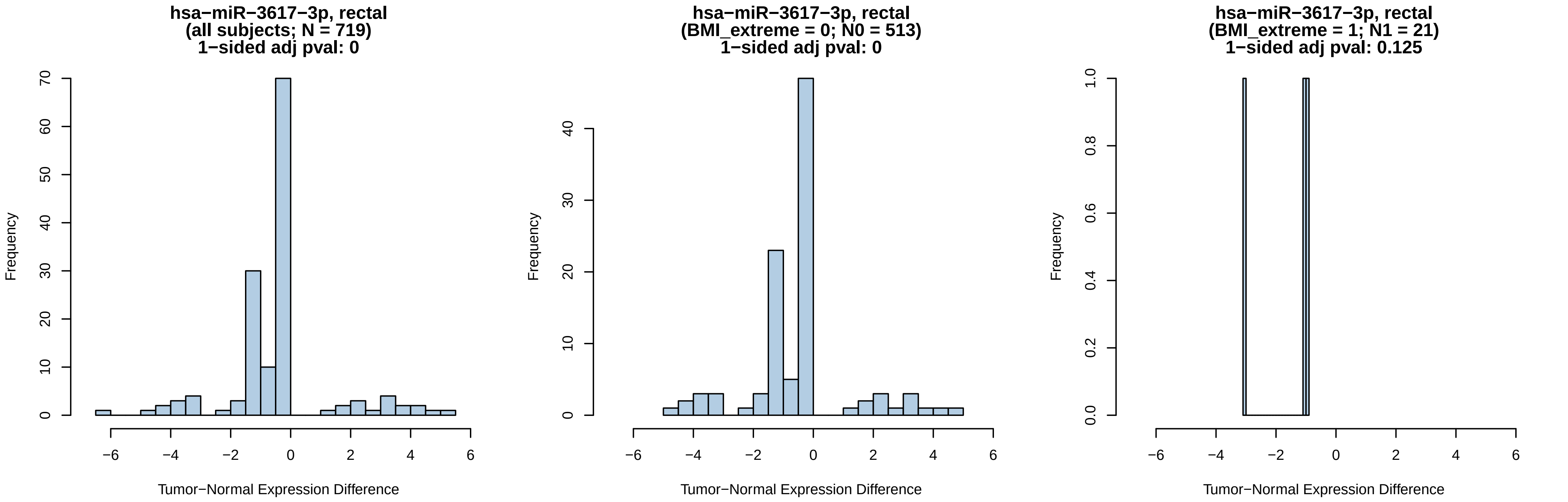
hsa−miR−3617−3p, rectal
(all subjects; N = 719)
1−sided adj pval: 0
Frequency
Tumor−Normal Expression Difference
hsa−miR−3617−3p, rectal
(BMI_extreme = 0; N0 = 513)
1−sided adj pval: 0
Frequency
Tumor−Normal Expression Difference
hsa−miR−3617−3p, rectal
(BMI_extreme = 1; N1 = 21)
1−sided adj pval: 0.125
Frequency
Tumor−Normal Expression Difference

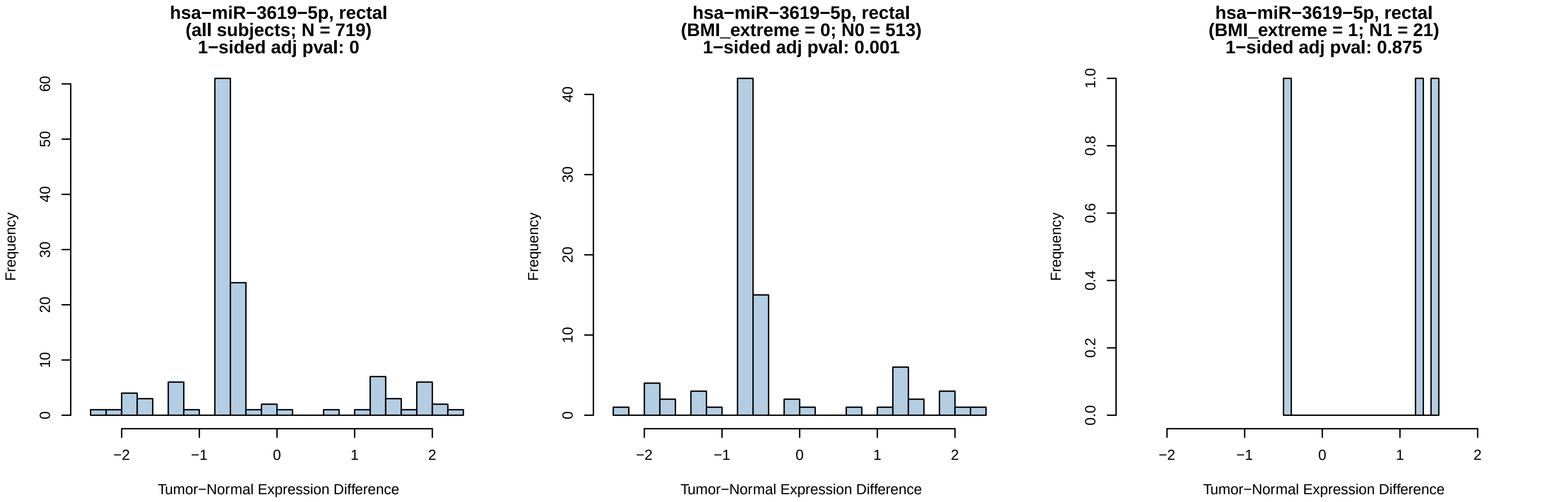

hsa−miR−3619−5p, rectal
(all subjects; N = 719)
1−sided adj pval: 0
Frequency
Tumor−Normal Expression Difference
hsa−miR−3619−5p, rectal
(BMI_extreme = 0; N0 = 513)
1−sided adj pval: 0.001
Frequency
Tumor−Normal Expression Difference
hsa−miR−3619−5p, rectal
(BMI_extreme = 1; N1 = 21)
1−sided adj pval: 0.875
Frequency
Tumor−Normal Expression Difference

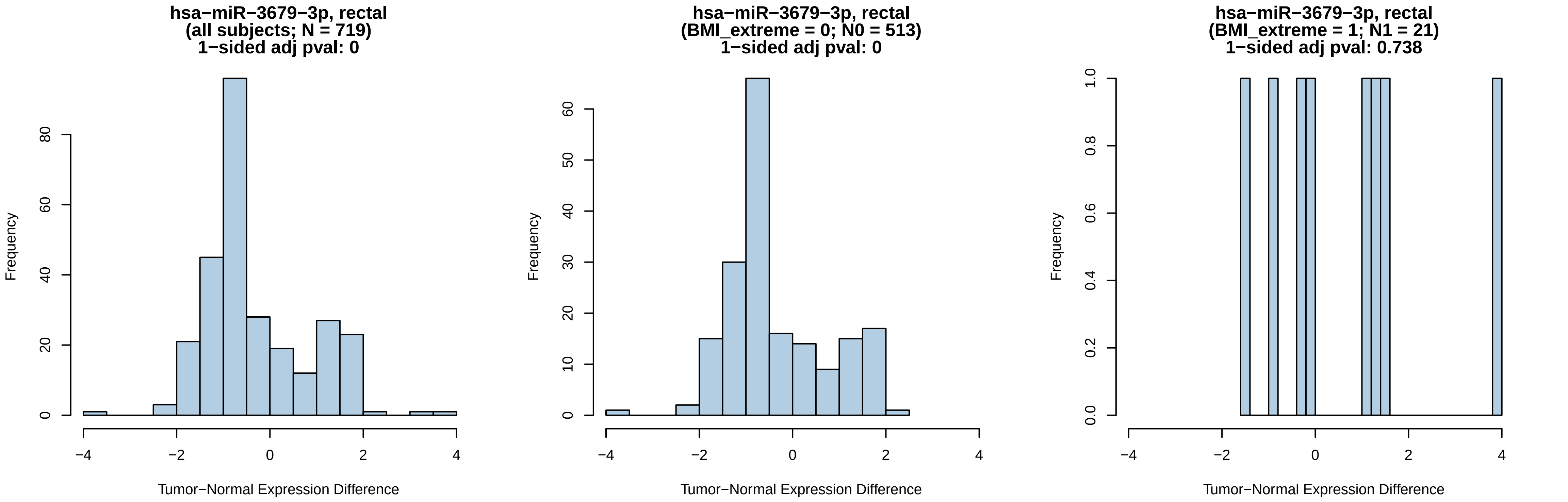
hsa−miR−3679−3p, rectal
(all subjects; N = 719)
1−sided adj pval: 0
Frequency
0
20
40
60
80
−4
−2
0
2
4
Tumor−Normal Expression Difference
hsa−miR−3679−3p, rectal
(BMI_extreme = 0; N0 = 513)
1−sided adj pval: 0
Frequency
0
10
20
30
40
50
60
−4
−2
0
2
4
Tumor−Normal Expression Difference
hsa−miR−3679−3p, rectal
(BMI_extreme = 1; N1 = 21)
1−sided adj pval: 0.738
Frequency
0.0
0.2
0.4
0.6
0.8
1.0
−4
−2
0
2
4
Tumor−Normal Expression Difference

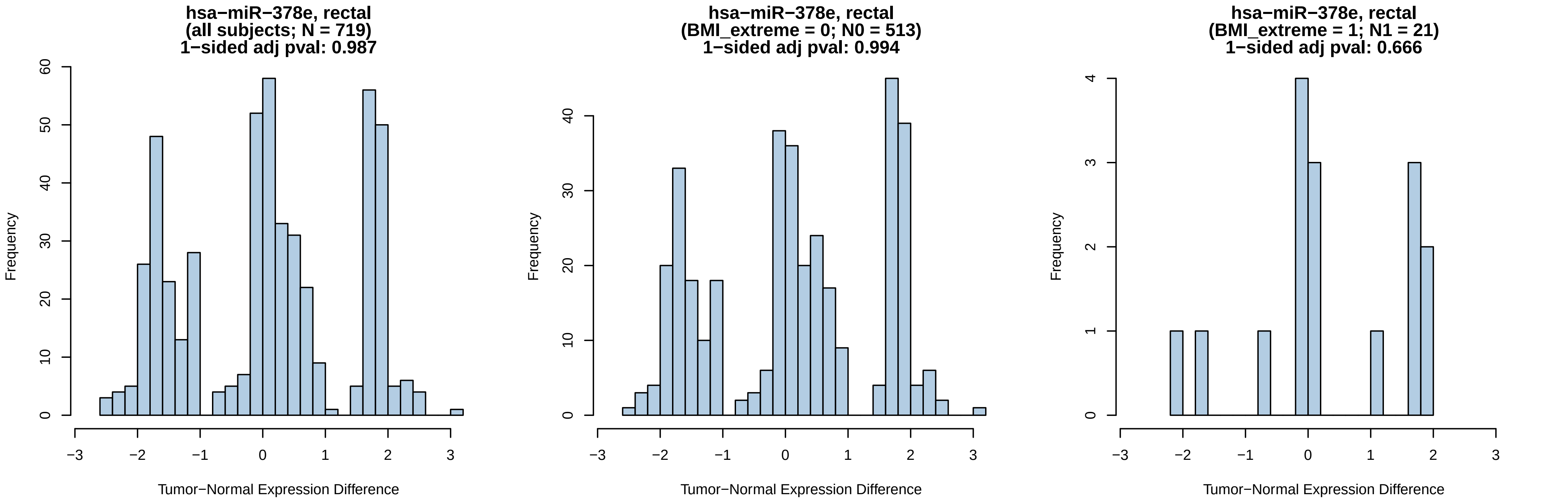
hsa−miR−378e, rectal
(all subjects; N = 719)
1−sided adj pval: 0.987
Frequency
Tumor−Normal Expression Difference
hsa−miR−378e, rectal
(BMI_extreme = 0; N0 = 513)
1−sided adj pval: 0.994
Frequency
Tumor−Normal Expression Difference
hsa−miR−378e, rectal
(BMI_extreme = 1; N1 = 21)
1−sided adj pval: 0.666
Frequency
Tumor−Normal Expression Difference

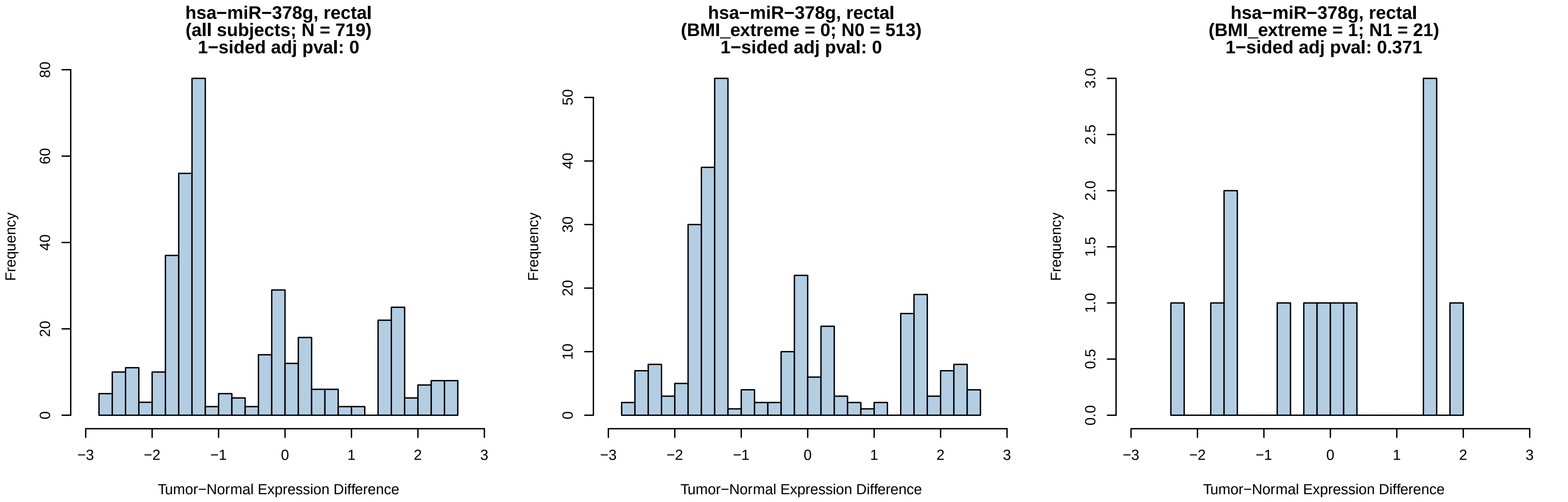
hsa−miR−378g, rectal
(all subjects; N = 719)
1−sided adj pval: 0
Frequency
Tumor−Normal Expression Difference
hsa−miR−378g, rectal
(BMI_extreme = 0; N0 = 513)
1−sided adj pval: 0
Frequency
Tumor−Normal Expression Difference
hsa−miR−378g, rectal
(BMI_extreme = 1; N1 = 21)
1−sided adj pval: 0.371
Frequency
Tumor−Normal Expression Difference

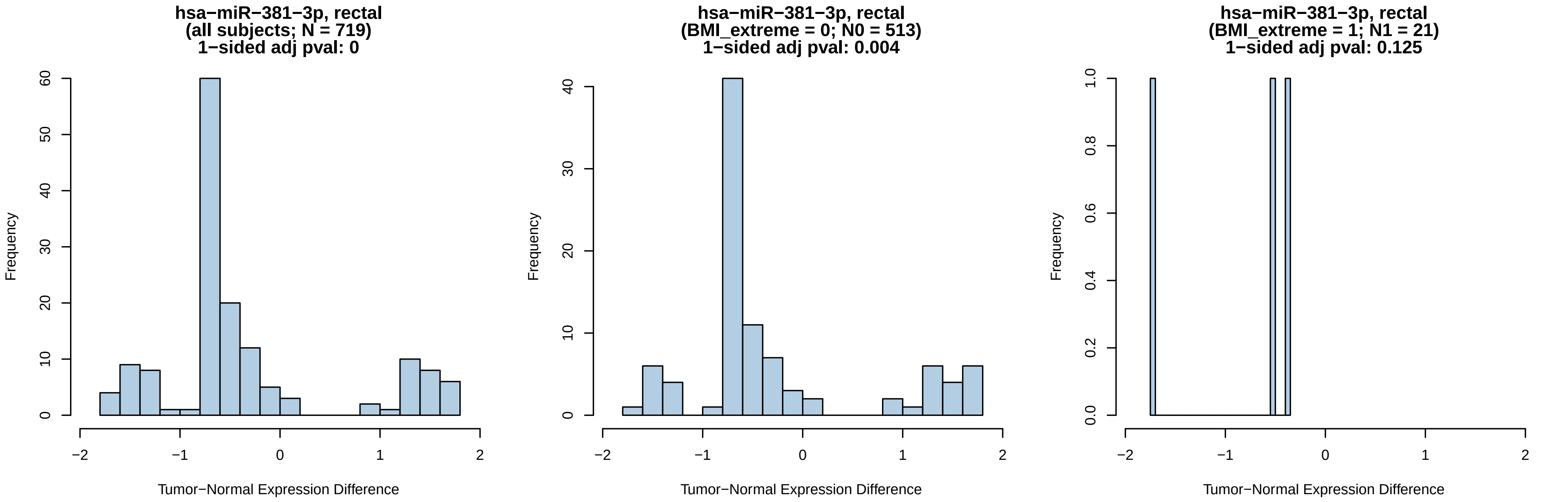
hsa−miR−381−3p, rectal
(all subjects; N = 719)
1−sided adj pval: 0
Frequency
Tumor−Normal Expression Difference
hsa−miR−381−3p, rectal
(BMI_extreme = 0; N0 = 513)
1−sided adj pval: 0.004
Frequency
Tumor−Normal Expression Difference
hsa−miR−381−3p, rectal
(BMI_extreme = 1; N1 = 21)
1−sided adj pval: 0.125
Frequency
Tumor−Normal Expression Difference

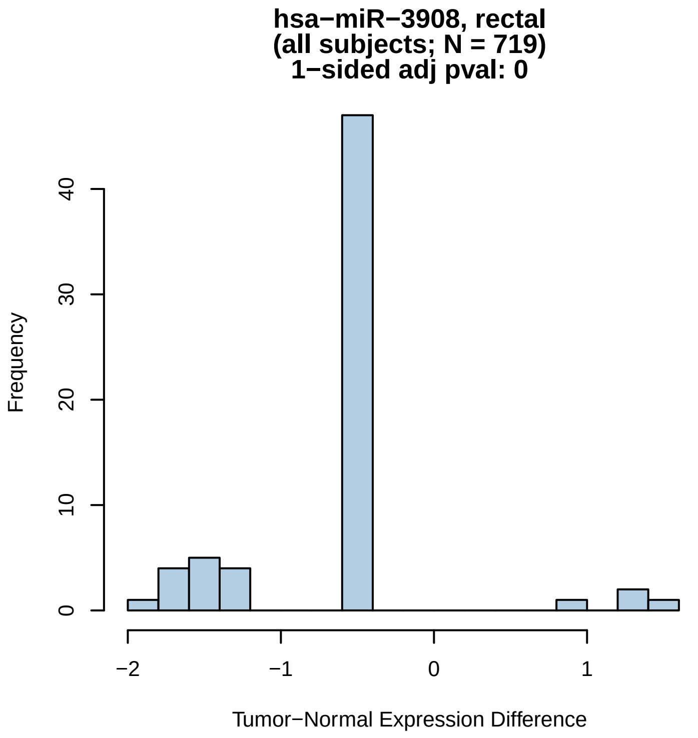
hsa−miR−3908, rectal
(all subjects; N = 719)
1−sided adj pval: 0
Frequency
Tumor−Normal Expression Difference
0
10
20
30
40
−2
−1
0
1

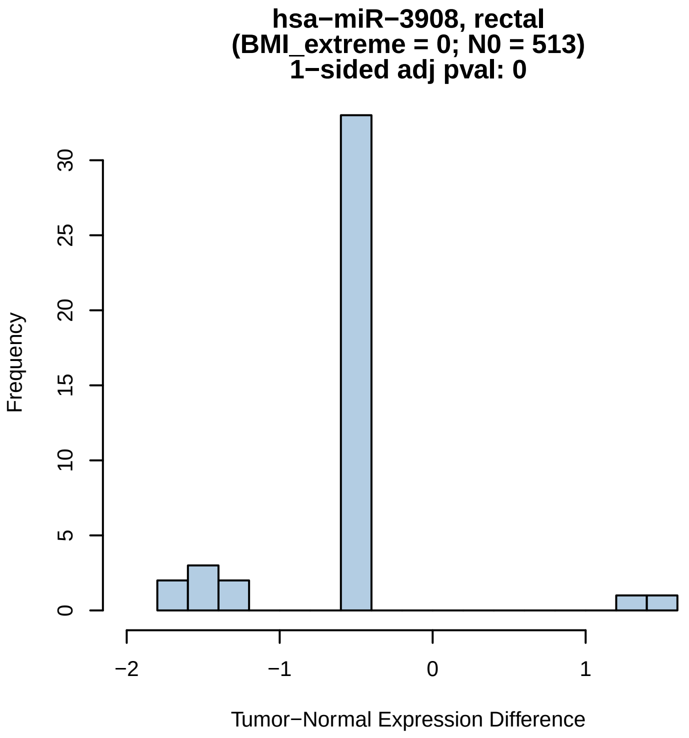
hsa−miR−3908, rectal
(BMI_extreme = 0; N0 = 513)
1−sided adj pval: 0
Frequency
Tumor−Normal Expression Difference
0
5
10
15
20
25
30
−2
−1
0
1

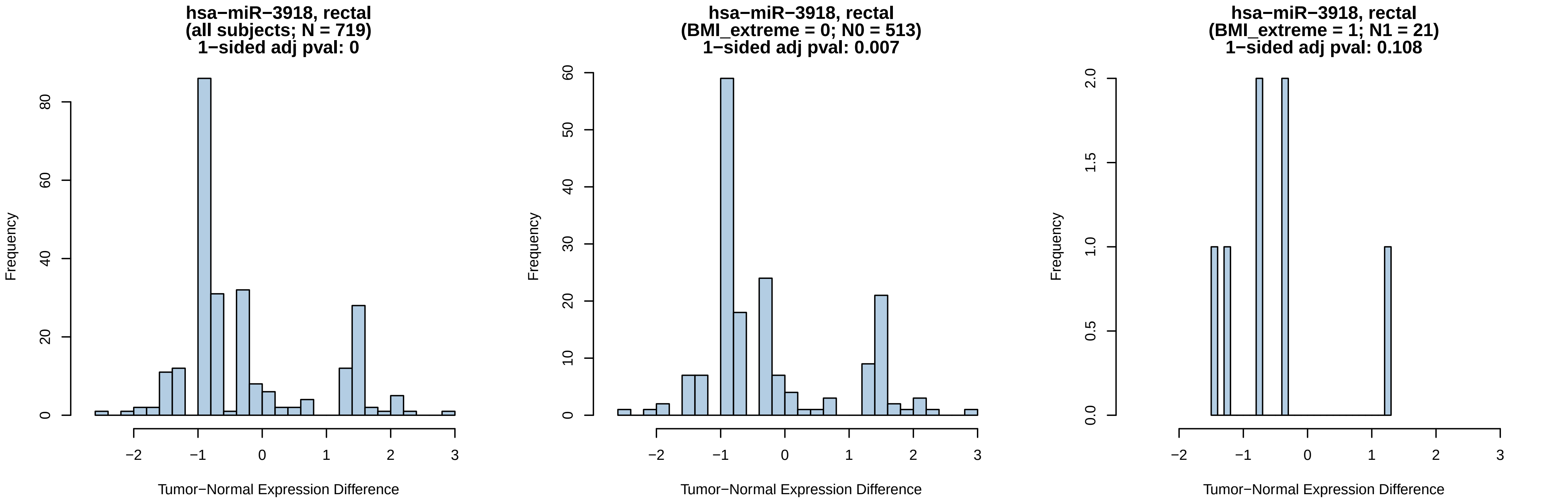

hsa−miR−3918, rectal
(all subjects; N = 719)
1−sided adj pval: 0
hsa−miR−3918, rectal
(BMI_extreme = 0; N0 = 513)
1−sided adj pval: 0.007
hsa−miR−3918, rectal
(BMI_extreme = 1; N1 = 21)
1−sided adj pval: 0.108
Frequency
Tumor−Normal Expression Difference
0
20
40
60
80
10
30
50
0.0
0.5
1.0
1.5
2.0
−2
−1
1
2
3

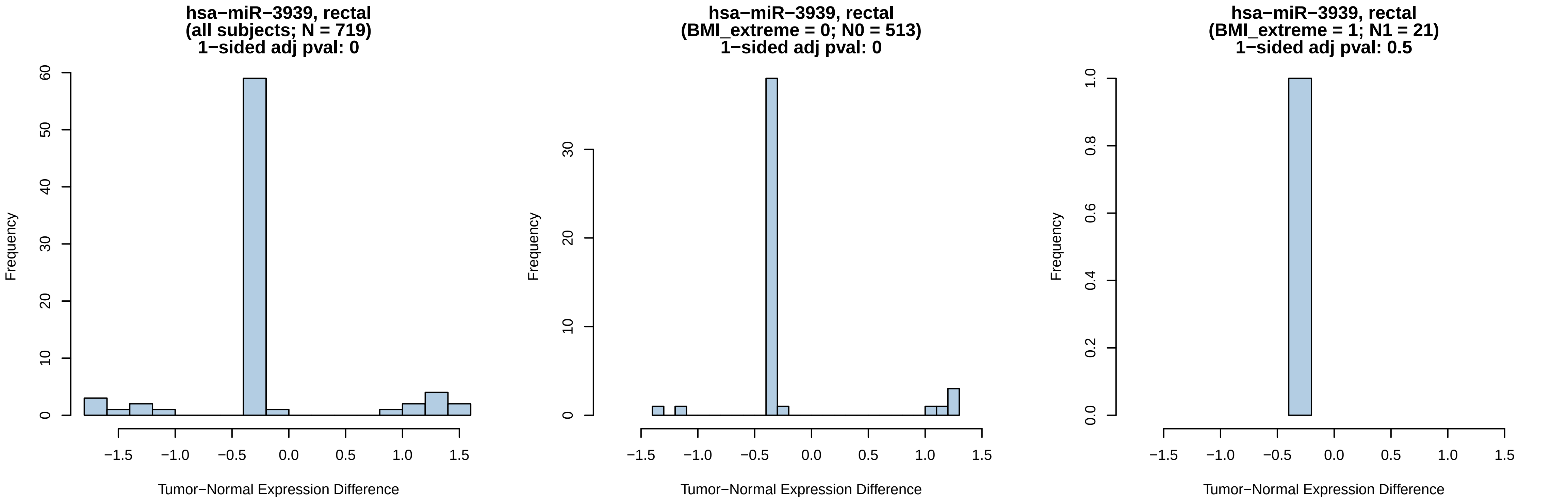

hsa−miR−3939, rectal
(all subjects; N = 719)
1−sided adj pval: 0
hsa−miR−3939, rectal
(BMI_extreme = 0; N0 = 513)
1−sided adj pval: 0
hsa−miR−3939, rectal
(BMI_extreme = 1; N1 = 21)
1−sided adj pval: 0.5
Frequency
Tumor−Normal Expression Difference

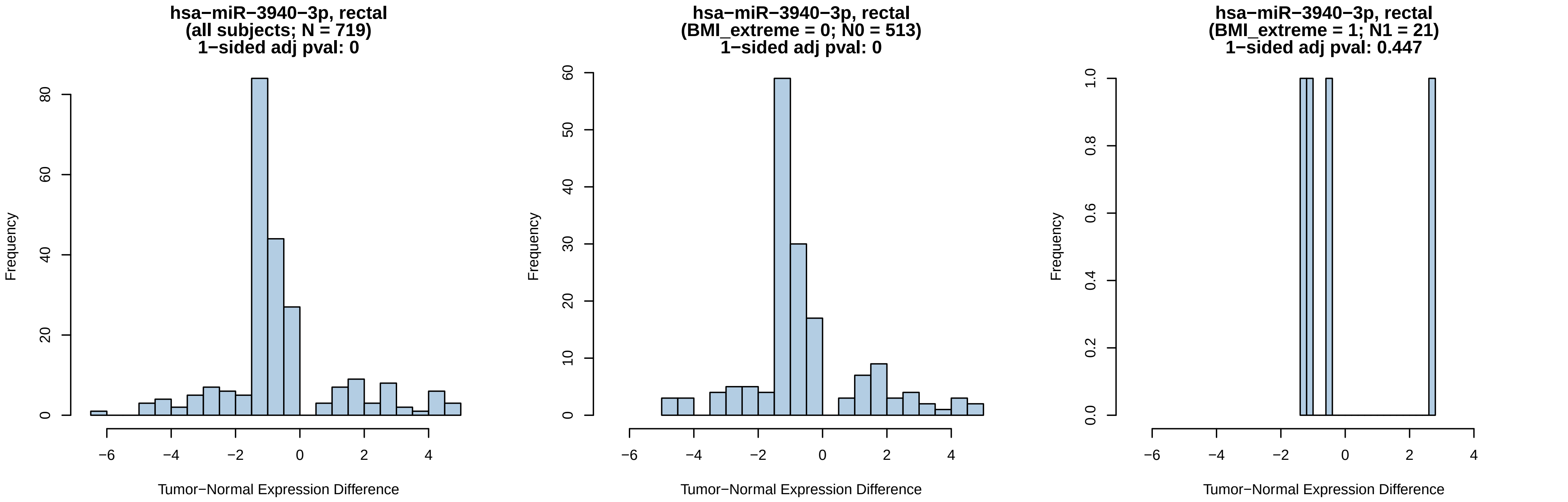

hsa−miR−3940−3p, rectal
(all subjects; N = 719)
1−sided adj pval: 0
Frequency
0
20
40
60
80
−6
−4
−2
0
2
4
Tumor−Normal Expression Difference
hsa−miR−3940−3p, rectal
(BMI_extreme = 0; N0 = 513)
1−sided adj pval: 0
Frequency
0
10
20
30
40
50
60
−6
−4
−2
0
2
4
Tumor−Normal Expression Difference
hsa−miR−3940−3p, rectal
(BMI_extreme = 1; N1 = 21)
1−sided adj pval: 0.447
Frequency
0.0
0.2
0.4
0.6
0.8
1.0
−6
−4
−2
0
2
4
Tumor−Normal Expression Difference

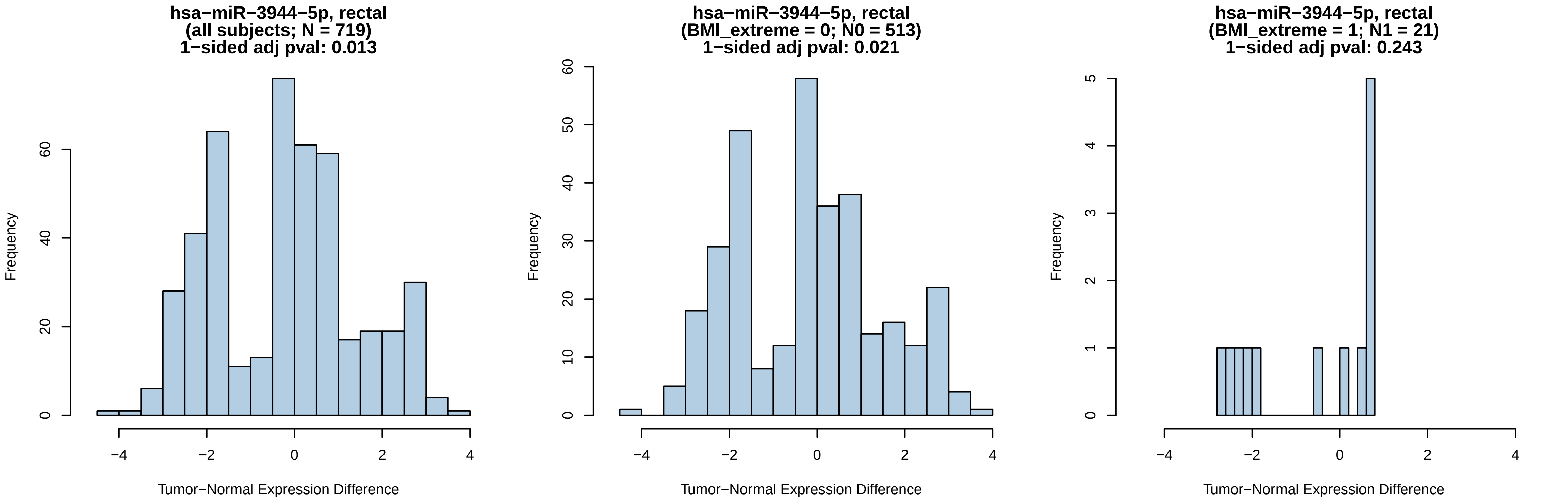
hsa−miR−3944−5p, rectal
(all subjects; N = 719)
1−sided adj pval: 0.013
Frequency
0
20
40
60
−4
−2
0
2
4
Tumor−Normal Expression Difference
hsa−miR−3944−5p, rectal
(BMI_extreme = 0; N0 = 513)
1−sided adj pval: 0.021
Frequency
0
10
20
30
40
50
60
−4
−2
0
2
4
Tumor−Normal Expression Difference
hsa−miR−3944−5p, rectal
(BMI_extreme = 1; N1 = 21)
1−sided adj pval: 0.243
Frequency
0
1
2
3
4
5
−4
−2
0
2
4
Tumor−Normal Expression Difference

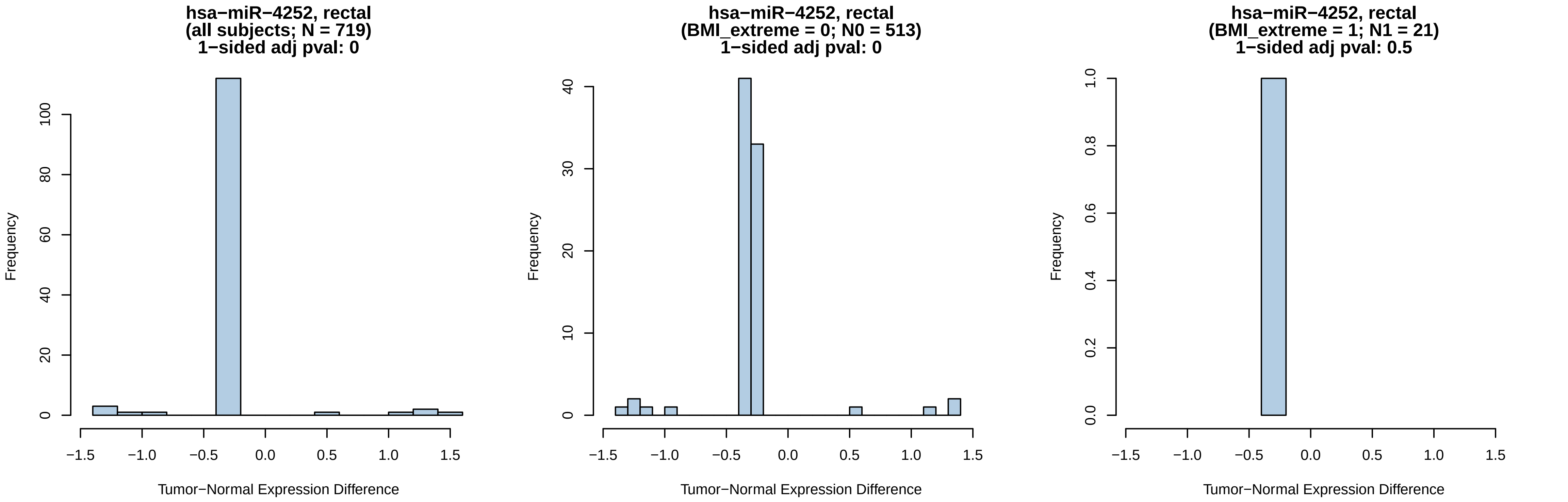

hsa−miR−4252, rectal
(all subjects; N = 719)
1−sided adj pval: 0
hsa−miR−4252, rectal
(BMI_extreme = 0; N0 = 513)
1−sided adj pval: 0
hsa−miR−4252, rectal
(BMI_extreme = 1; N1 = 21)
1−sided adj pval: 0.5
Frequency
Tumor−Normal Expression Difference

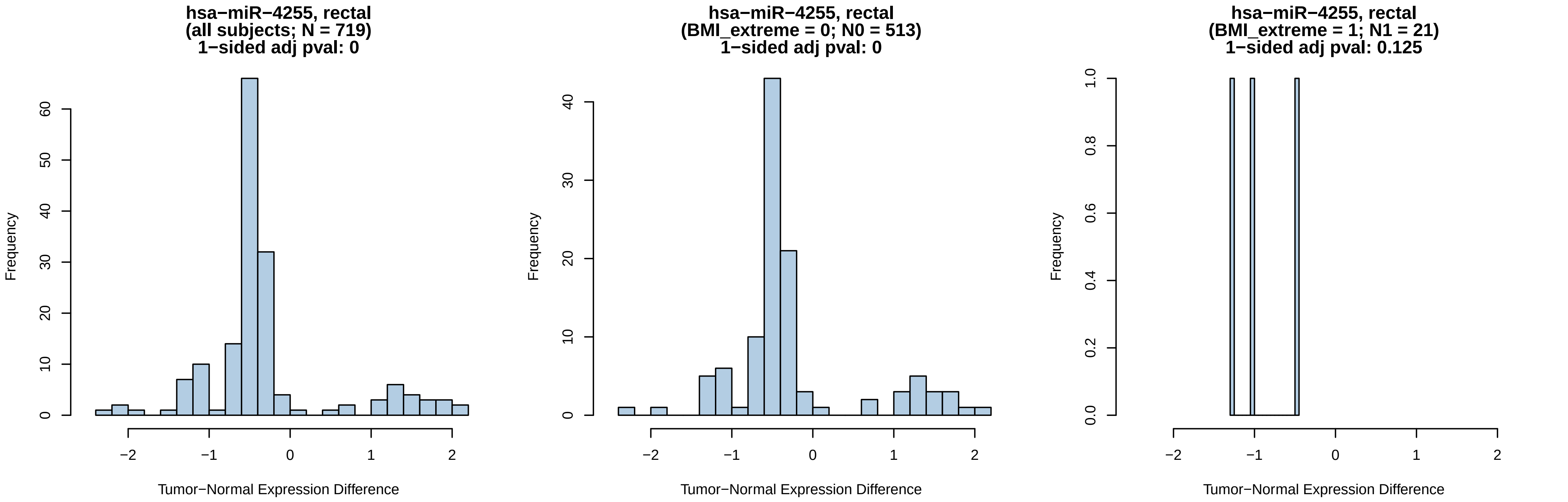
hsa−miR−4255, rectal
(all subjects; N = 719)
1−sided adj pval: 0
Frequency
Tumor−Normal Expression Difference
hsa−miR−4255, rectal
(BMI_extreme = 0; N0 = 513)
1−sided adj pval: 0
Frequency
Tumor−Normal Expression Difference
hsa−miR−4255, rectal
(BMI_extreme = 1; N1 = 21)
1−sided adj pval: 0.125
Frequency
Tumor−Normal Expression Difference

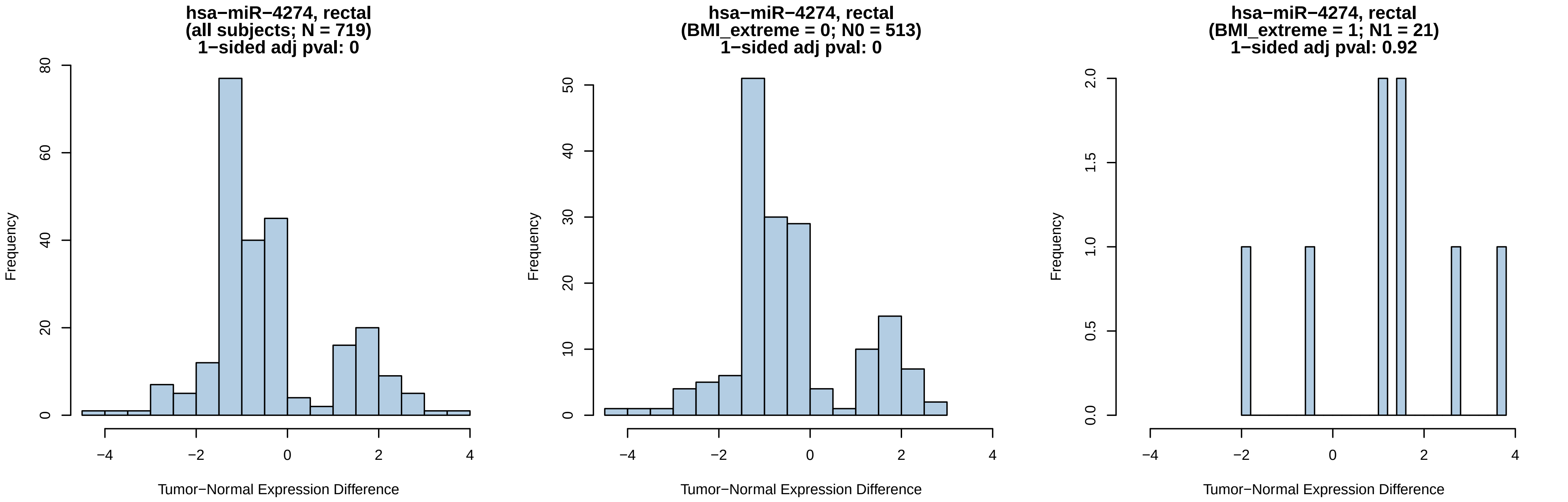
hsa−miR−4274, rectal
(all subjects; N = 719)
1−sided adj pval: 0
Frequency
Tumor−Normal Expression Difference
hsa−miR−4274, rectal
(BMI_extreme = 0; N0 = 513)
1−sided adj pval: 0
Frequency
Tumor−Normal Expression Difference
hsa−miR−4274, rectal
(BMI_extreme = 1; N1 = 21)
1−sided adj pval: 0.92
Frequency
Tumor−Normal Expression Difference

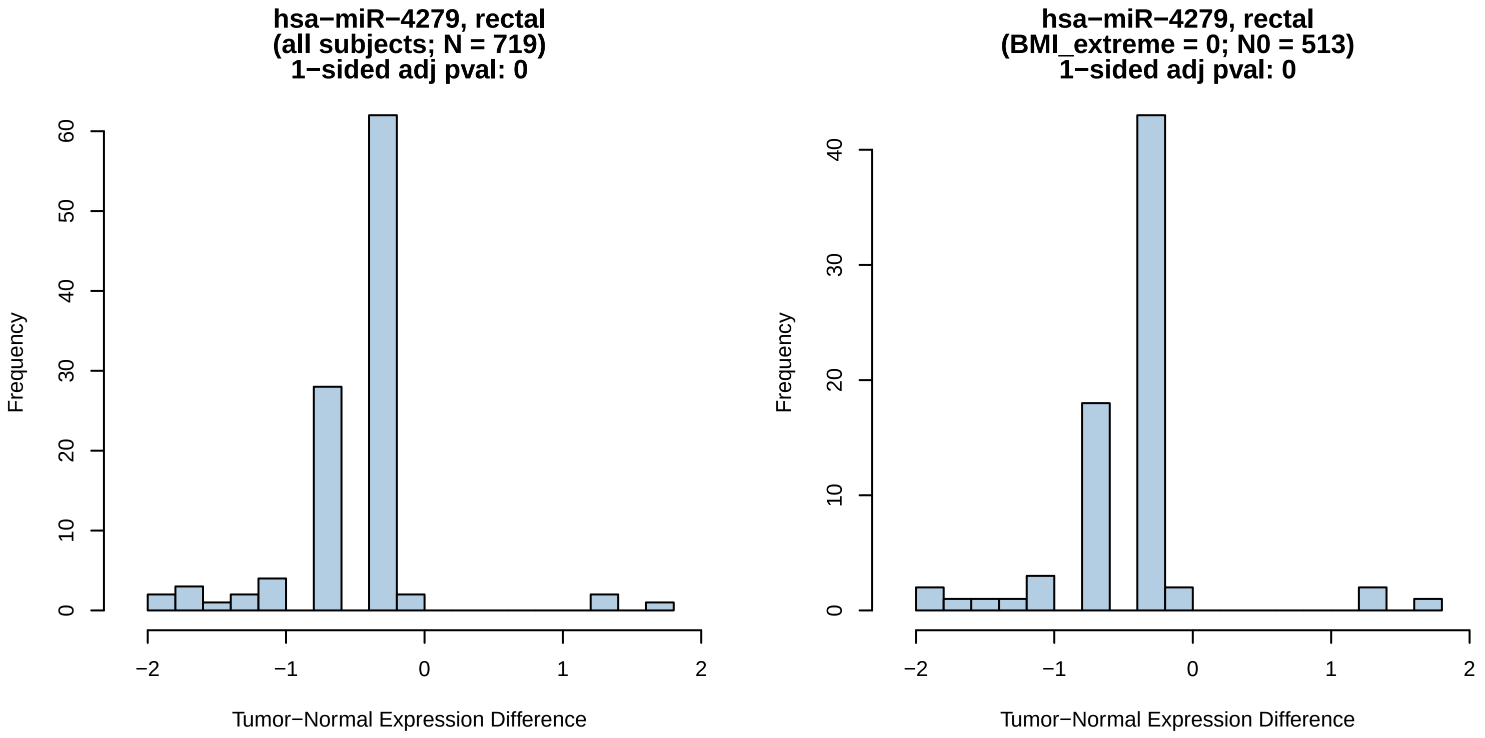
hsa−miR−4279, rectal
(all subjects; N = 719)
1−sided adj pval: 0
Frequency
Tumor−Normal Expression Difference
hsa−miR−4279, rectal
(BMI_extreme = 0; N0 = 513)
1−sided adj pval: 0
Frequency
Tumor−Normal Expression Difference

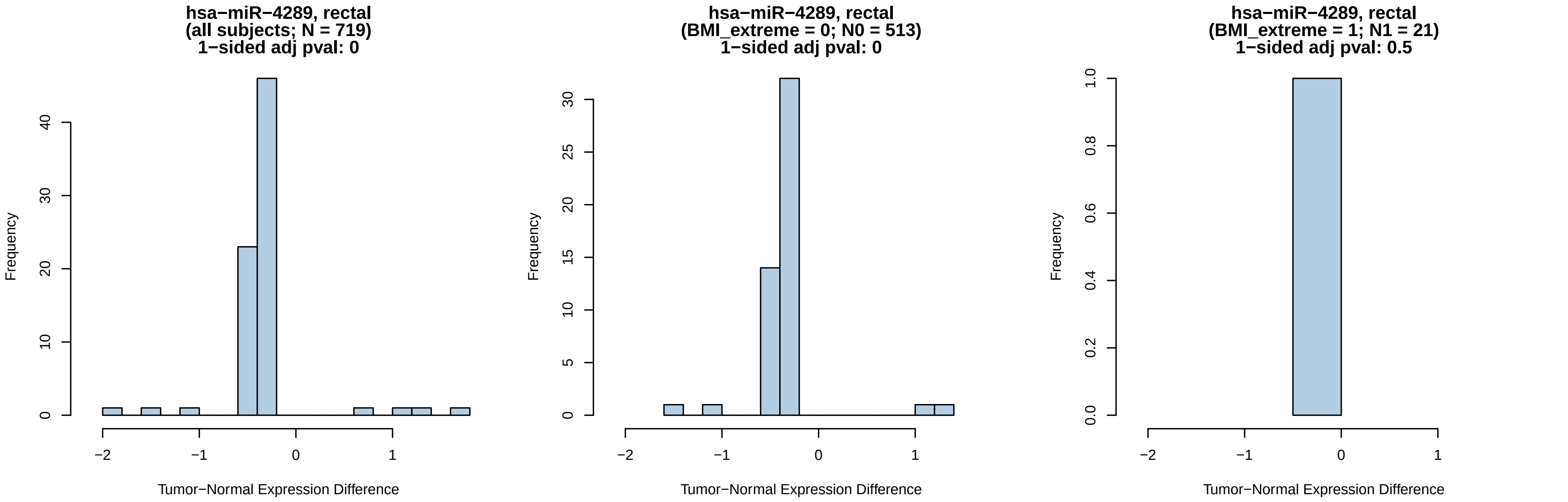
hsa−miR−4289, rectal
(all subjects; N = 719)
1−sided adj pval: 0
Frequency
Tumor−Normal Expression Difference
hsa−miR−4289, rectal
(BMI_extreme = 0; N0 = 513)
1−sided adj pval: 0
Frequency
Tumor−Normal Expression Difference
hsa−miR−4289, rectal
(BMI_extreme = 1; N1 = 21)
1−sided adj pval: 0.5
Frequency
Tumor−Normal Expression Difference

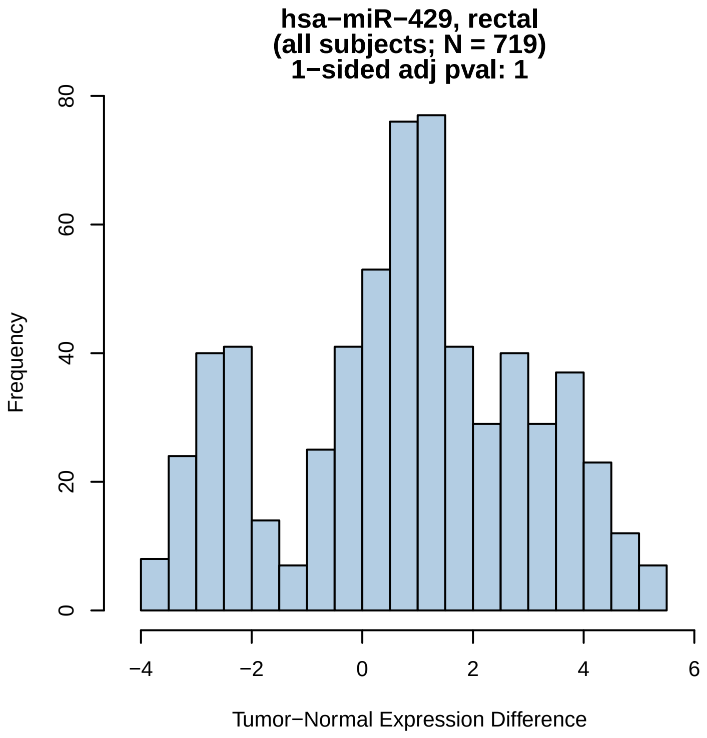
hsa−miR−429, rectal
(all subjects; N = 719)
1−sided adj pval: 1
Frequency
Tumor−Normal Expression Difference

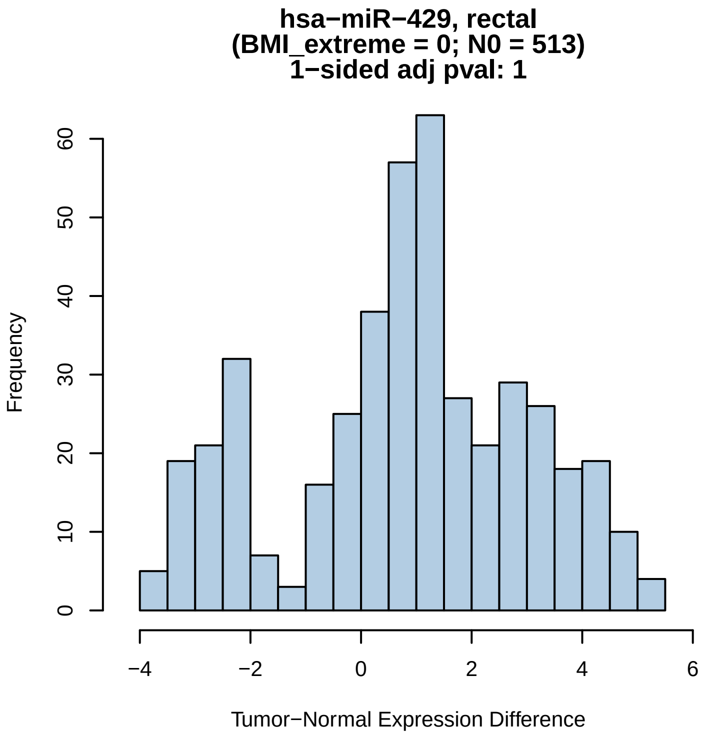
hsa−miR−429, rectal
(BMI_extreme = 0; N0 = 513)
1−sided adj pval: 1
Frequency
Tumor−Normal Expression Difference

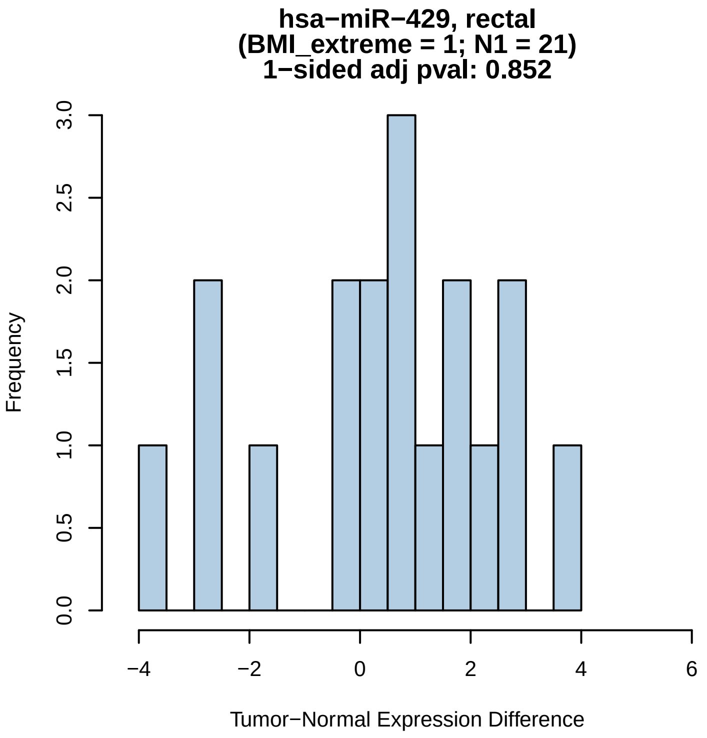
hsa−miR−429, rectal
(BMI_extreme = 1; N1 = 21)
1−sided adj pval: 0.852
Frequency
Tumor−Normal Expression Difference

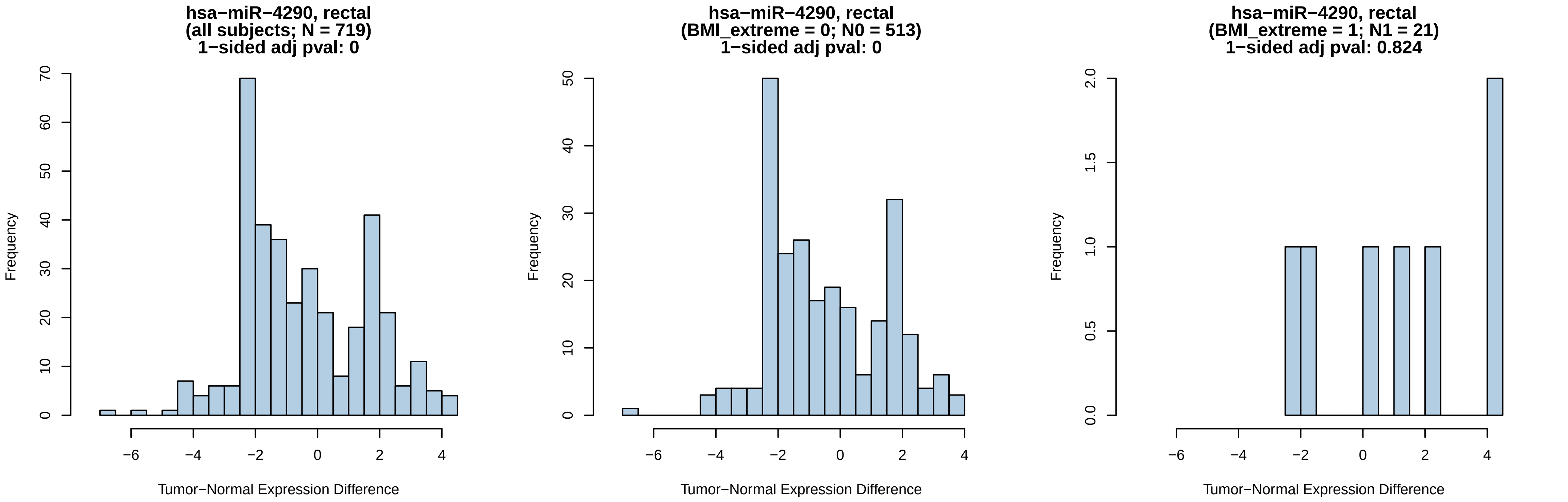

hsa−miR−4290, rectal
(all subjects; N = 719)
1−sided adj pval: 0
hsa−miR−4290, rectal
(BMI_extreme = 0; N0 = 513)
1−sided adj pval: 0
hsa−miR−4290, rectal
(BMI_extreme = 1; N1 = 21)
1−sided adj pval: 0.824
Frequency
Tumor−Normal Expression Difference

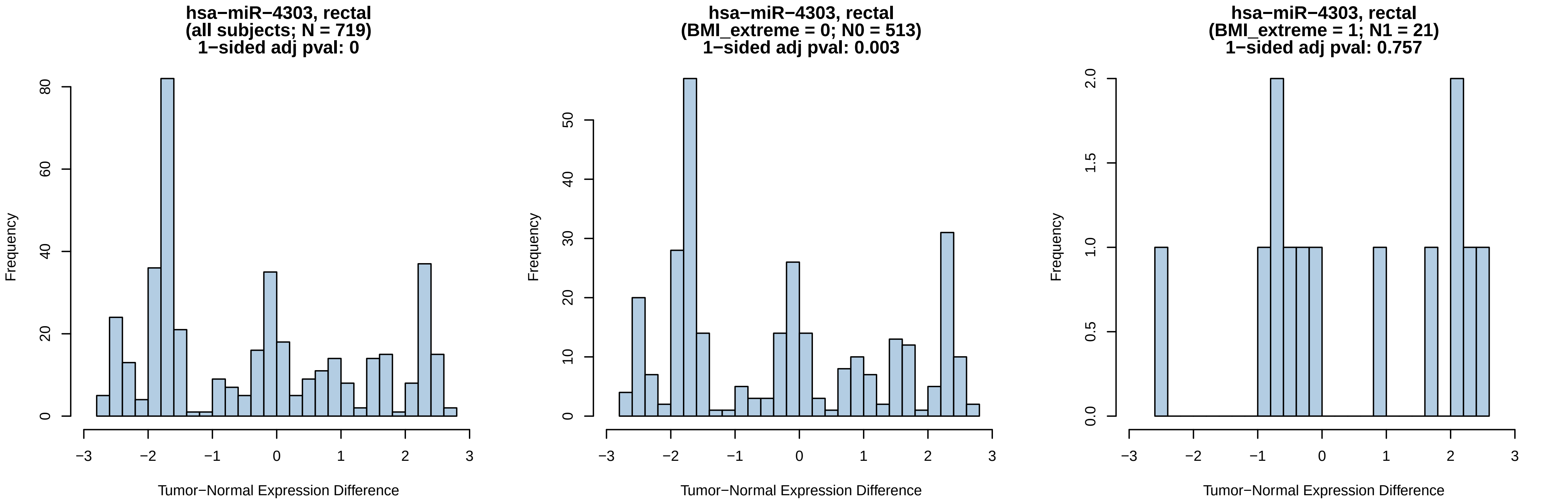

hsa−miR−4303, rectal
(all subjects; N = 719)
1−sided adj pval: 0
Frequency
Tumor−Normal Expression Difference
hsa−miR−4303, rectal
(BMI_extreme = 0; N0 = 513)
1−sided adj pval: 0.003
Frequency
Tumor−Normal Expression Difference
hsa−miR−4303, rectal
(BMI_extreme = 1; N1 = 21)
1−sided adj pval: 0.757
Frequency
Tumor−Normal Expression Difference

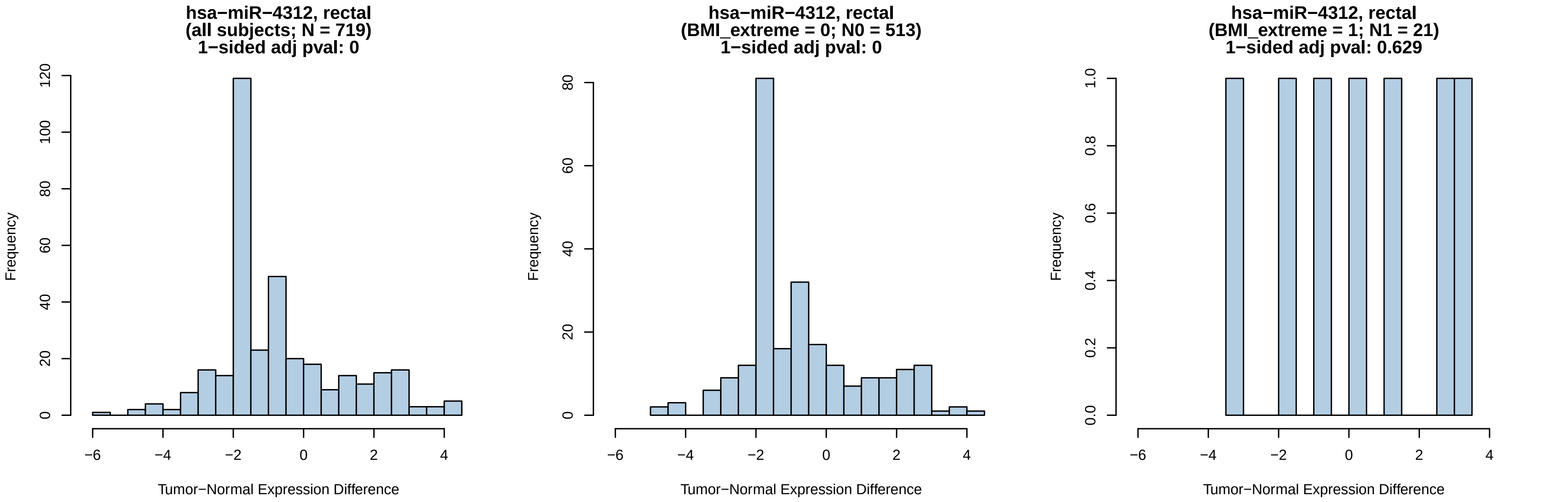

hsa−miR−4312, rectal
(all subjects; N = 719)
1−sided adj pval: 0
Frequency
Tumor−Normal Expression Difference
0
20
40
60
80
100
120
−6
−4
−2
0
2
4
hsa−miR−4312, rectal
(BMI_extreme = 0; N0 = 513)
1−sided adj pval: 0
Frequency
Tumor−Normal Expression Difference
0
20
40
60
80
−6
−4
−2
0
2
4
hsa−miR−4312, rectal
(BMI_extreme = 1; N1 = 21)
1−sided adj pval: 0.629
Frequency
Tumor−Normal Expression Difference
0.0
0.2
0.4
0.6
0.8
1.0
−6
−4
−2
0
2
4

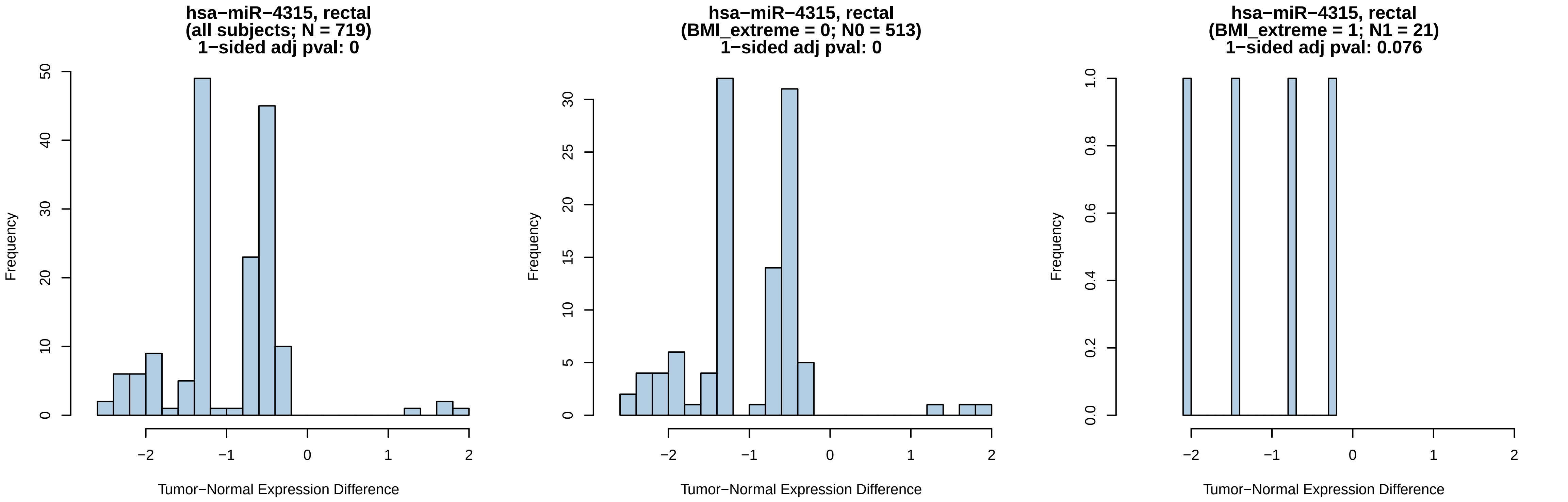

hsa−miR−4315, rectal
(all subjects; N = 719)
1−sided adj pval: 0
Frequency
Tumor−Normal Expression Difference
hsa−miR−4315, rectal
(BMI_extreme = 0; N0 = 513)
1−sided adj pval: 0
Frequency
Tumor−Normal Expression Difference
hsa−miR−4315, rectal
(BMI_extreme = 1; N1 = 21)
1−sided adj pval: 0.076
Frequency
Tumor−Normal Expression Difference

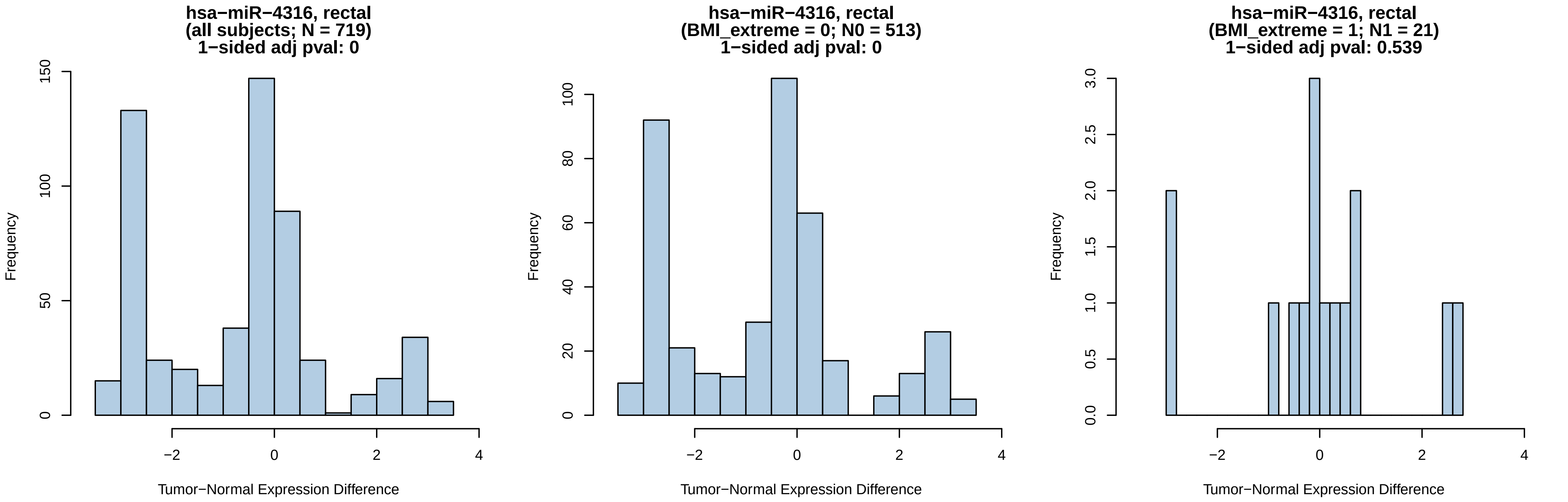
hsa−miR−4316, rectal
(all subjects; N = 719)
1−sided adj pval: 0
Frequency
Tumor−Normal Expression Difference
hsa−miR−4316, rectal
(BMI_extreme = 0; N0 = 513)
1−sided adj pval: 0
Frequency
Tumor−Normal Expression Difference
hsa−miR−4316, rectal
(BMI_extreme = 1; N1 = 21)
1−sided adj pval: 0.539
Frequency
Tumor−Normal Expression Difference

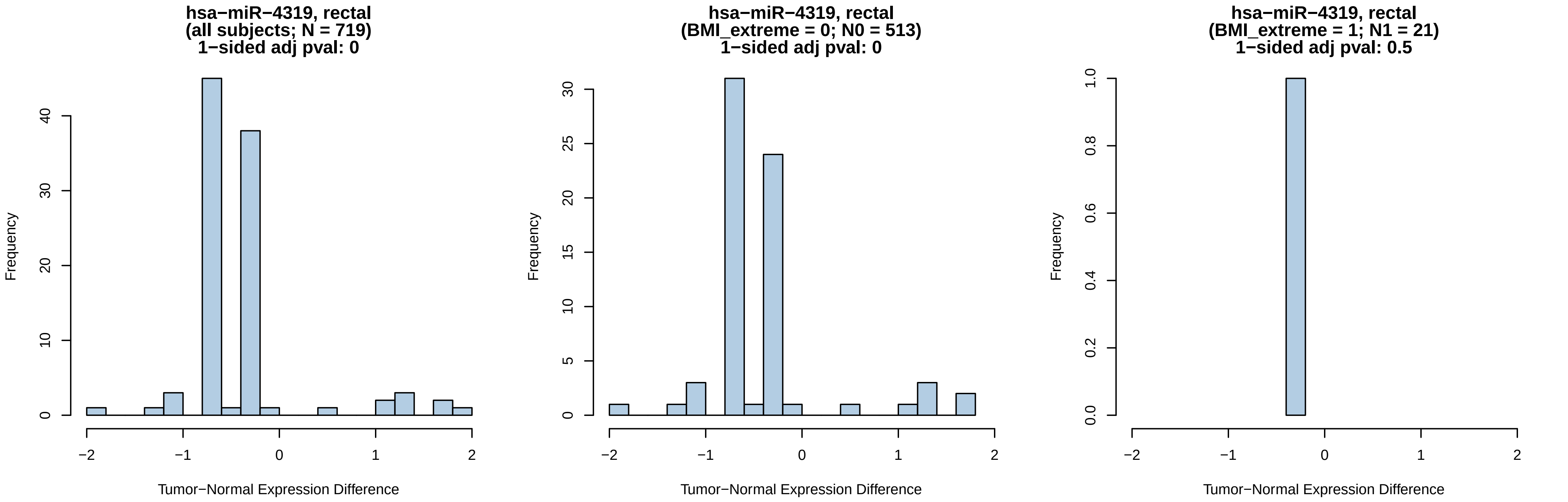

hsa−miR−4319, rectal
(all subjects; N = 719)
1−sided adj pval: 0
hsa−miR−4319, rectal
(BMI_extreme = 0; N0 = 513)
1−sided adj pval: 0
hsa−miR−4319, rectal
(BMI_extreme = 1; N1 = 21)
1−sided adj pval: 0.5
Frequency
Tumor−Normal Expression Difference

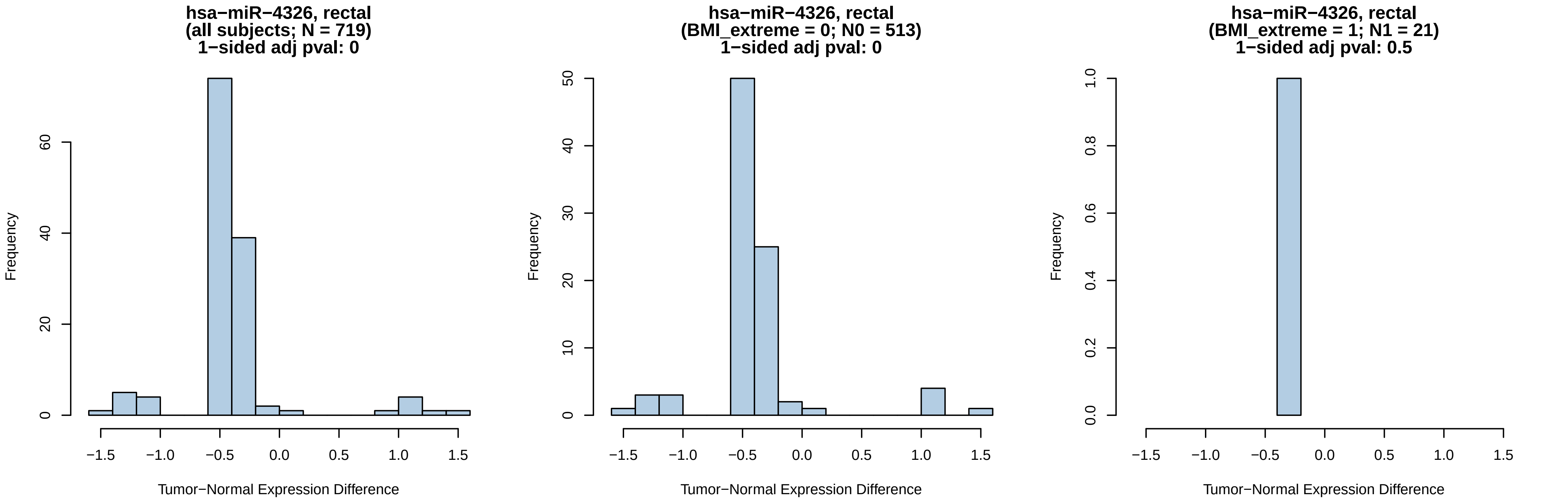

hsa−miR−4326, rectal
(all subjects; N = 719)
1−sided adj pval: 0
Frequency
0
20
40
60
−1.5
−1.0
−0.5
0.0
0.5
1.0
1.5
Tumor−Normal Expression Difference
hsa−miR−4326, rectal
(BMI_extreme = 0; N0 = 513)
1−sided adj pval: 0
Frequency
0
10
20
30
40
50
−1.5
−1.0
−0.5
0.0
0.5
1.0
1.5
Tumor−Normal Expression Difference
hsa−miR−4326, rectal
(BMI_extreme = 1; N1 = 21)
1−sided adj pval: 0.5
Frequency
0.0
0.2
0.4
0.6
0.8
1.0
−1.5
−1.0
−0.5
0.0
0.5
1.0
1.5
Tumor−Normal Expression Difference

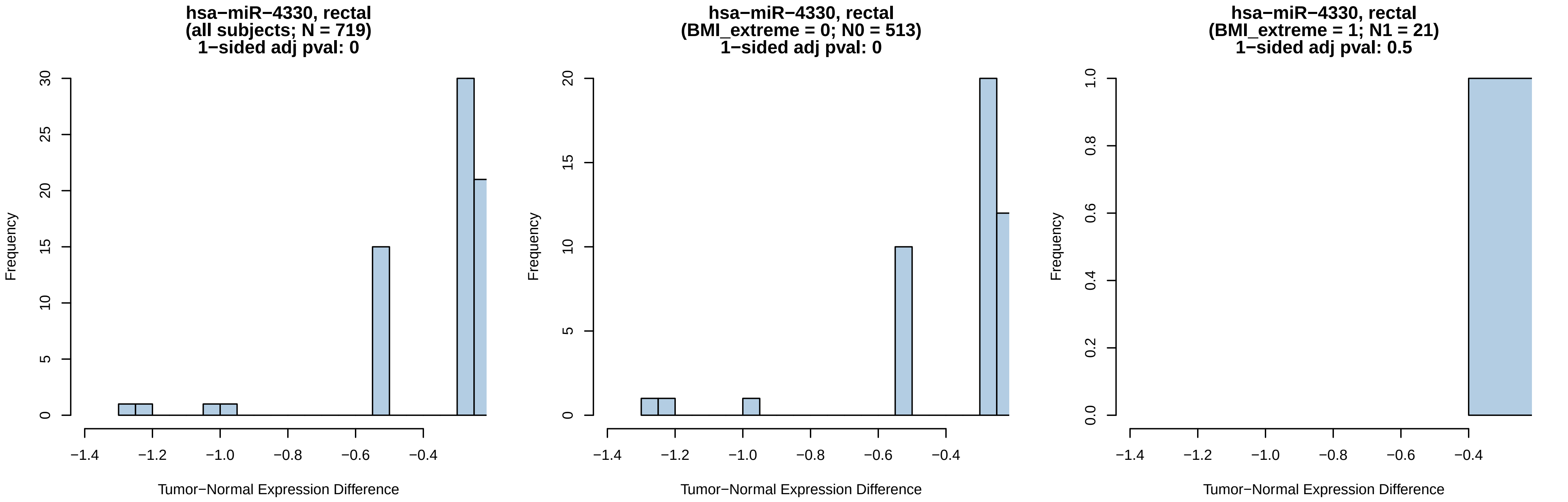

hsa−miR−4330, rectal
(all subjects; N = 719)
1−sided adj pval: 0
Frequency
0
5
10
15
20
25
30
−1.4
−1.2
−1.0
−0.8
−0.6
−0.4
Tumor−Normal Expression Difference
hsa−miR−4330, rectal
(BMI_extreme = 0; N0 = 513)
1−sided adj pval: 0
Frequency
0
5
10
15
20
−1.4
−1.2
−1.0
−0.8
−0.6
−0.4
Tumor−Normal Expression Difference
hsa−miR−4330, rectal
(BMI_extreme = 1; N1 = 21)
1−sided adj pval: 0.5
Frequency
0.0
0.2
0.4
0.6
0.8
1.0
−1.4
−1.2
−1.0
−0.8
−0.6
−0.4
Tumor−Normal Expression Difference

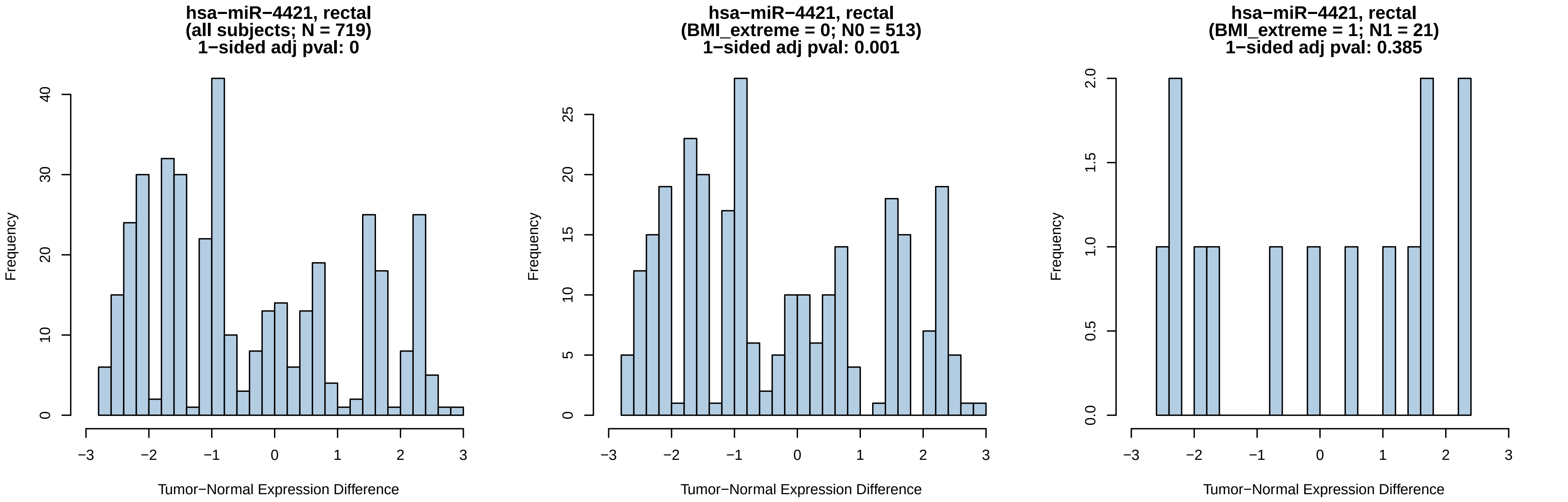
hsa−miR−4421, rectal
(all subjects; N = 719)
1−sided adj pval: 0
Frequency
Tumor−Normal Expression Difference
hsa−miR−4421, rectal
(BMI_extreme = 0; N0 = 513)
1−sided adj pval: 0.001
Frequency
Tumor−Normal Expression Difference
hsa−miR−4421, rectal
(BMI_extreme = 1; N1 = 21)
1−sided adj pval: 0.385
Frequency
Tumor−Normal Expression Difference

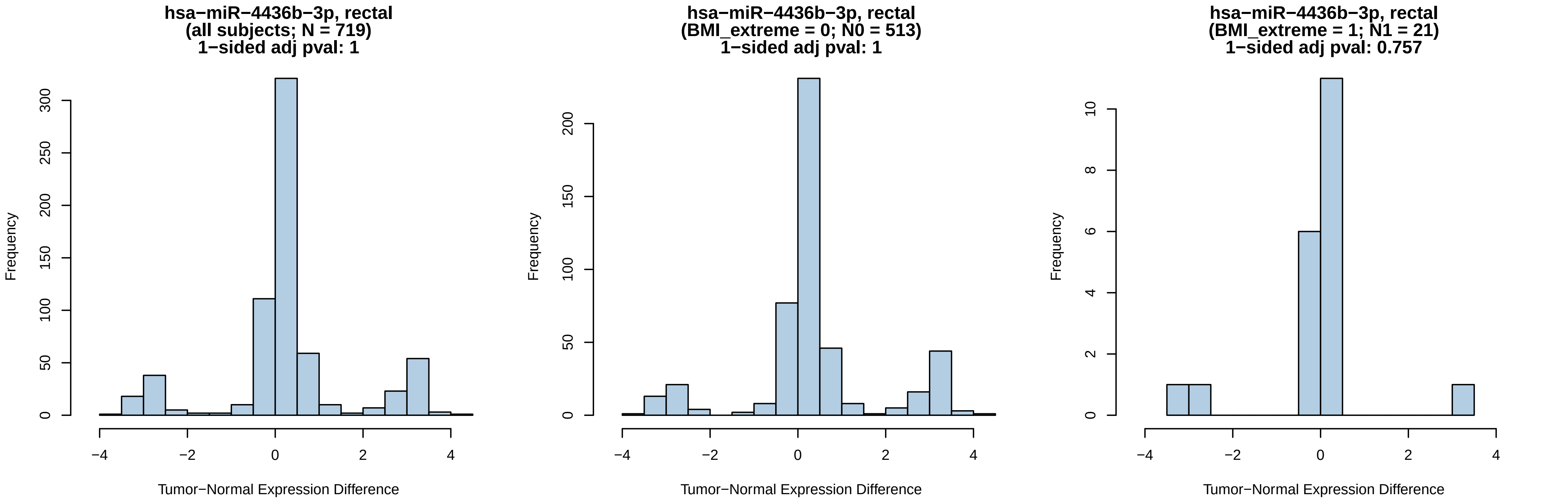

hsa−miR−4436b−3p, rectal
(all subjects; N = 719)
1−sided adj pval: 1
Frequency
Tumor−Normal Expression Difference
hsa−miR−4436b−3p, rectal
(BMI_extreme = 0; N0 = 513)
1−sided adj pval: 1
Frequency
Tumor−Normal Expression Difference
hsa−miR−4436b−3p, rectal
(BMI_extreme = 1; N1 = 21)
1−sided adj pval: 0.757
Frequency
Tumor−Normal Expression Difference

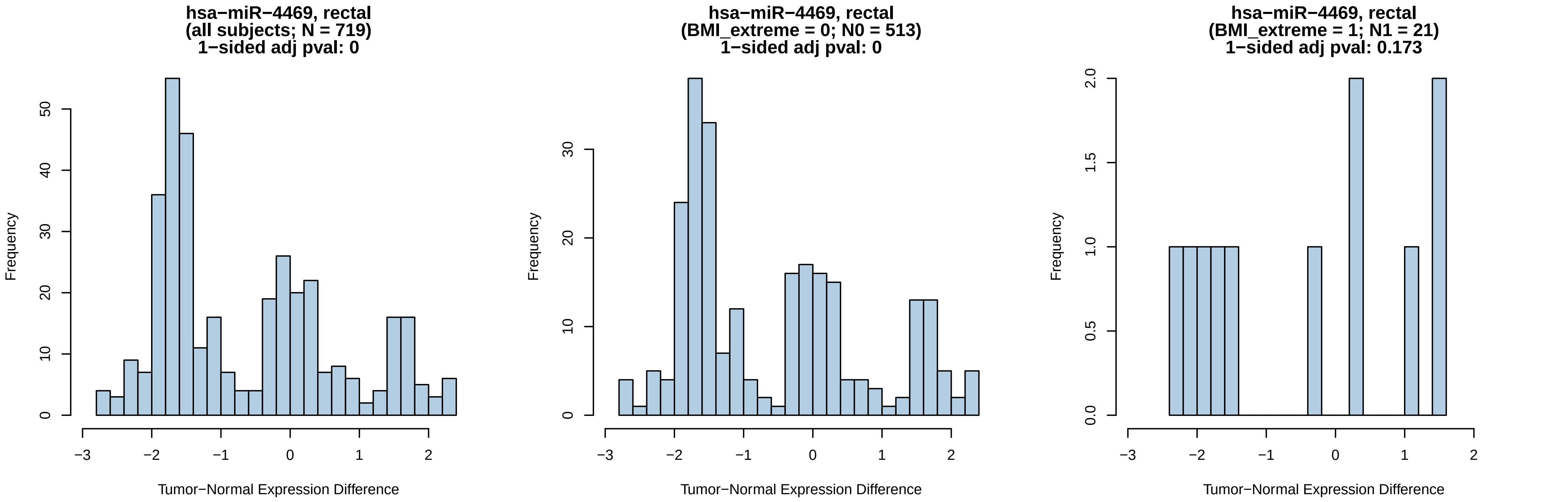

hsa−miR−4469, rectal
(all subjects; N = 719)
1−sided adj pval: 0
Frequency
0
10
20
30
40
50
−3
−2
−1
0
1
2
Tumor−Normal Expression Difference
hsa−miR−4469, rectal
(BMI_extreme = 0; N0 = 513)
1−sided adj pval: 0
Frequency
0
10
20
30
−3
−2
−1
0
1
2
Tumor−Normal Expression Difference
hsa−miR−4469, rectal
(BMI_extreme = 1; N1 = 21)
1−sided adj pval: 0.173
Frequency
0.0
0.5
1.0
1.5
2.0
−3
−2
−1
0
1
2
Tumor−Normal Expression Difference

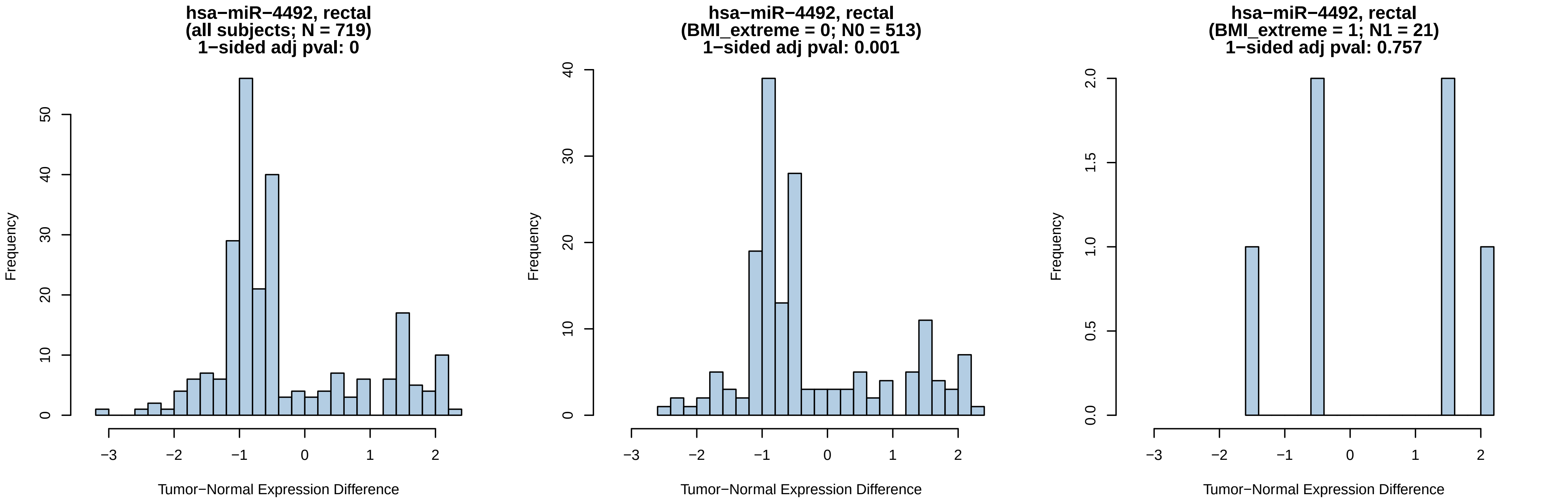

hsa−miR−4492, rectal
(all subjects; N = 719)
1−sided adj pval: 0
hsa−miR−4492, rectal
(BMI_extreme = 0; N0 = 513)
1−sided adj pval: 0.001
hsa−miR−4492, rectal
(BMI_extreme = 1; N1 = 21)
1−sided adj pval: 0.757
Frequency
Tumor−Normal Expression Difference

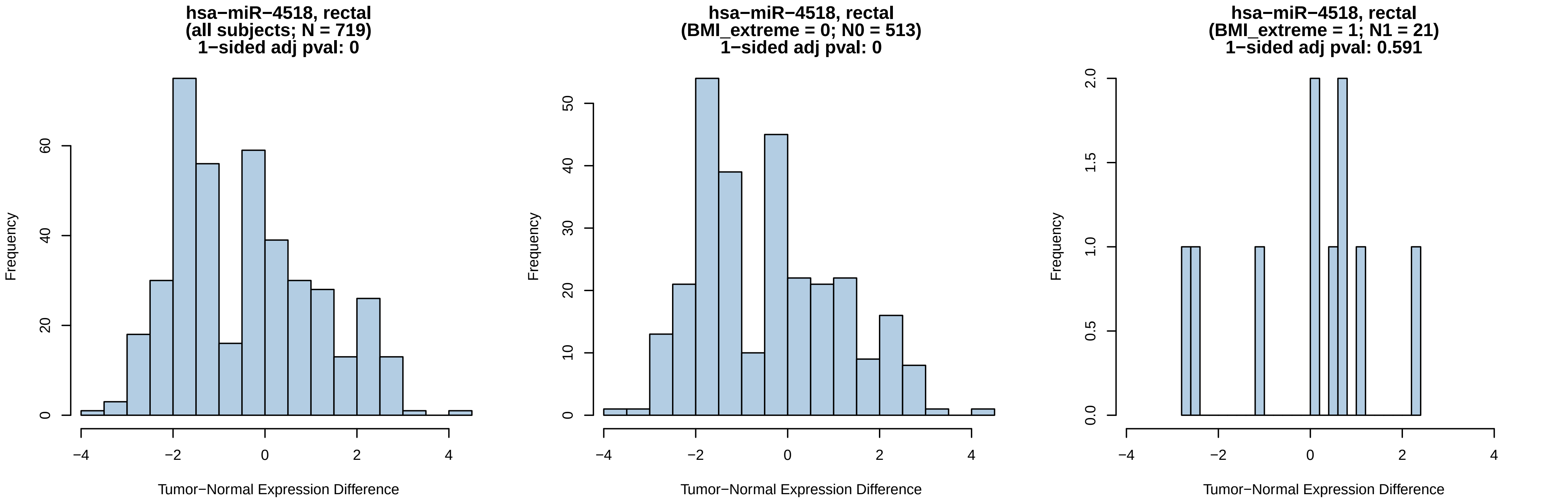
hsa−miR−4518, rectal
(all subjects; N = 719)
1−sided adj pval: 0
Frequency
0
20
40
60
−4
−2
0
2
4
Tumor−Normal Expression Difference
hsa−miR−4518, rectal
(BMI_extreme = 0; N0 = 513)
1−sided adj pval: 0
Frequency
0
10
20
30
40
50
−4
−2
0
2
4
Tumor−Normal Expression Difference
hsa−miR−4518, rectal
(BMI_extreme = 1; N1 = 21)
1−sided adj pval: 0.591
Frequency
0.0
0.5
1.0
1.5
2.0
−4
−2
0
2
4
Tumor−Normal Expression Difference

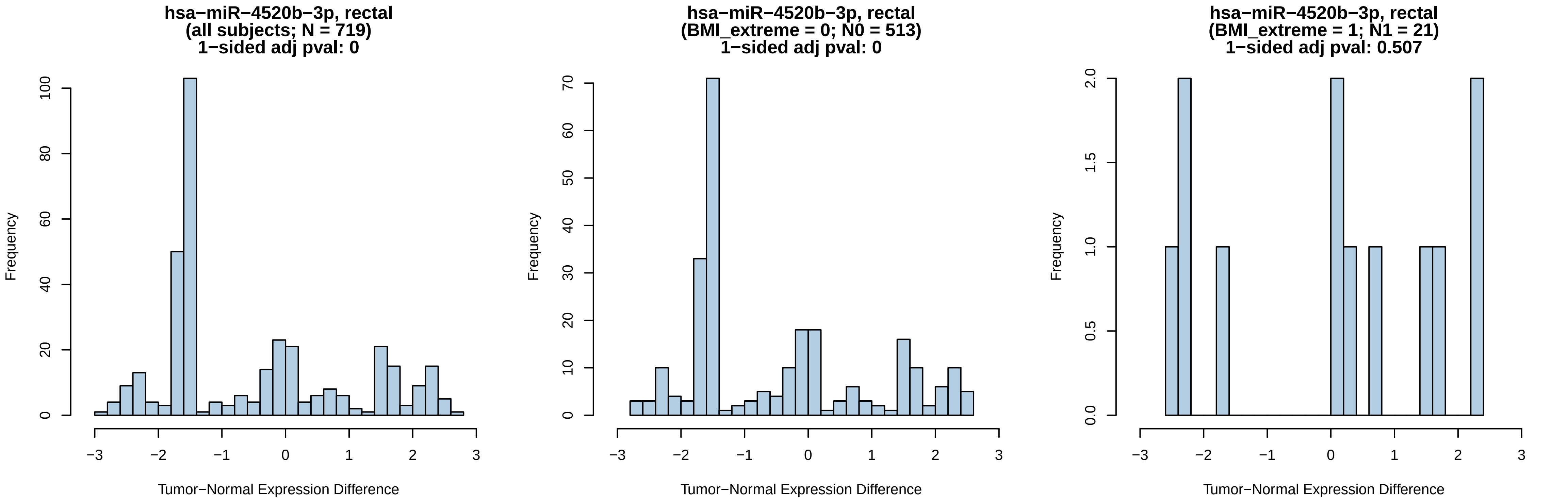
hsa−miR−4520b−3p, rectal
(all subjects; N = 719)
1−sided adj pval: 0
Frequency
Tumor−Normal Expression Difference
hsa−miR−4520b−3p, rectal
(BMI_extreme = 0; N0 = 513)
1−sided adj pval: 0
Frequency
Tumor−Normal Expression Difference
hsa−miR−4520b−3p, rectal
(BMI_extreme = 1; N1 = 21)
1−sided adj pval: 0.507
Frequency
Tumor−Normal Expression Difference

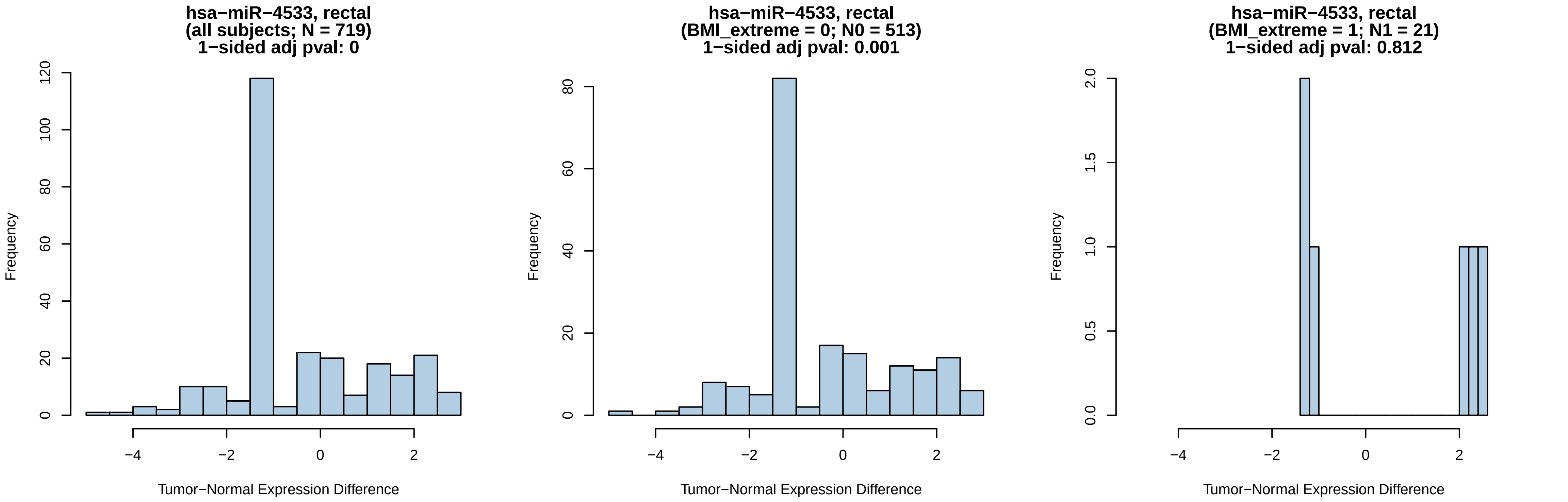

hsa−miR−4533, rectal
(all subjects; N = 719)
1−sided adj pval: 0
Frequency
Tumor−Normal Expression Difference
hsa−miR−4533, rectal
(BMI_extreme = 0; N0 = 513)
1−sided adj pval: 0.001
Frequency
Tumor−Normal Expression Difference
hsa−miR−4533, rectal
(BMI_extreme = 1; N1 = 21)
1−sided adj pval: 0.812
Frequency
Tumor−Normal Expression Difference

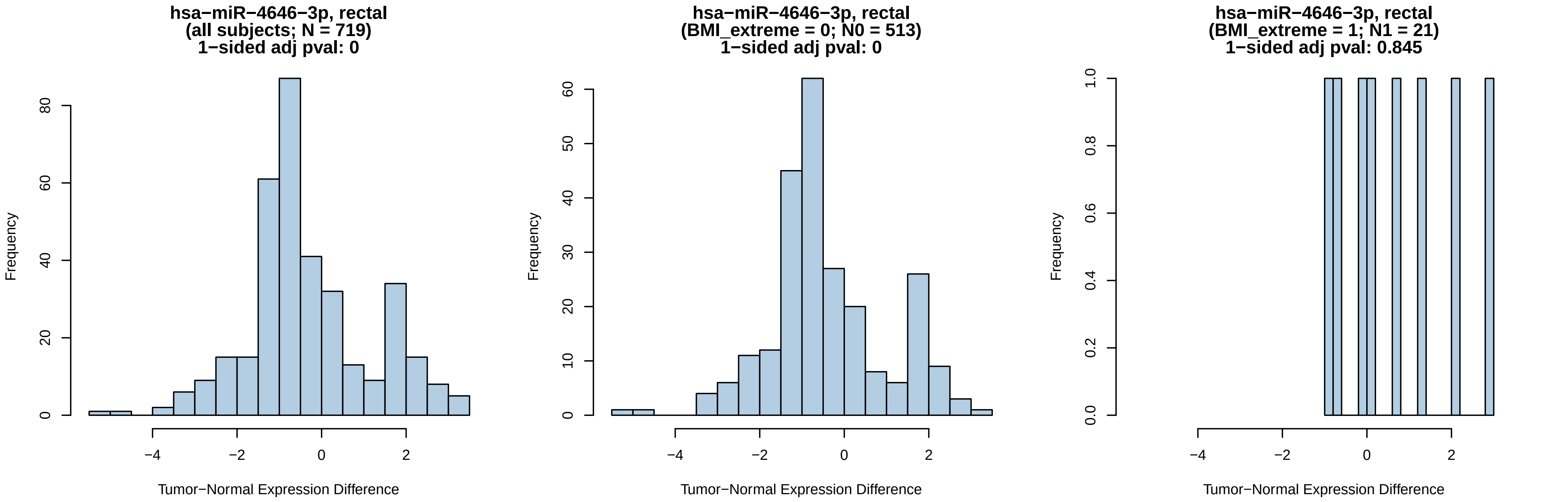
hsa−miR−4646−3p, rectal
(all subjects; N = 719)
1−sided adj pval: 0
Frequency
Tumor−Normal Expression Difference
hsa−miR−4646−3p, rectal
(BMI_extreme = 0; N0 = 513)
1−sided adj pval: 0
Frequency
Tumor−Normal Expression Difference
hsa−miR−4646−3p, rectal
(BMI_extreme = 1; N1 = 21)
1−sided adj pval: 0.845
Frequency
Tumor−Normal Expression Difference

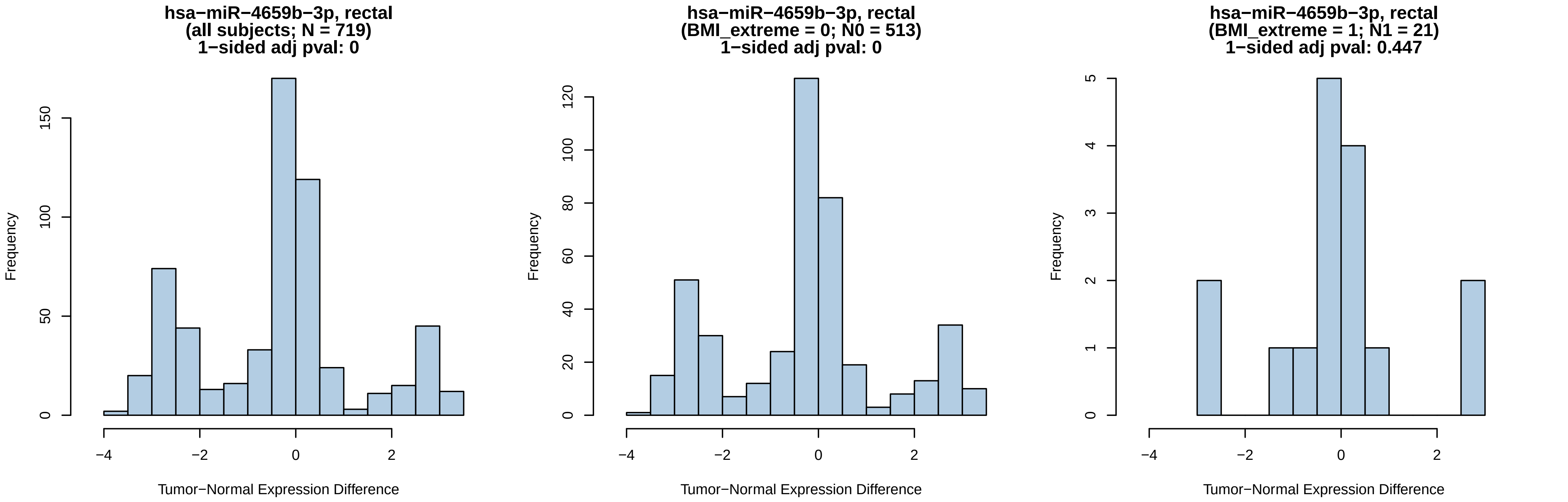
hsa−miR−4659b−3p, rectal
(all subjects; N = 719)
1−sided adj pval: 0
hsa−miR−4659b−3p, rectal
(BMI_extreme = 0; N0 = 513)
1−sided adj pval: 0
hsa−miR−4659b−3p, rectal
(BMI_extreme = 1; N1 = 21)
1−sided adj pval: 0.447
Frequency
Tumor−Normal Expression Difference
0
50
100
150
0
20
40
60
80
100
120
0
1
2
3
4
5
−4
−2
0
2

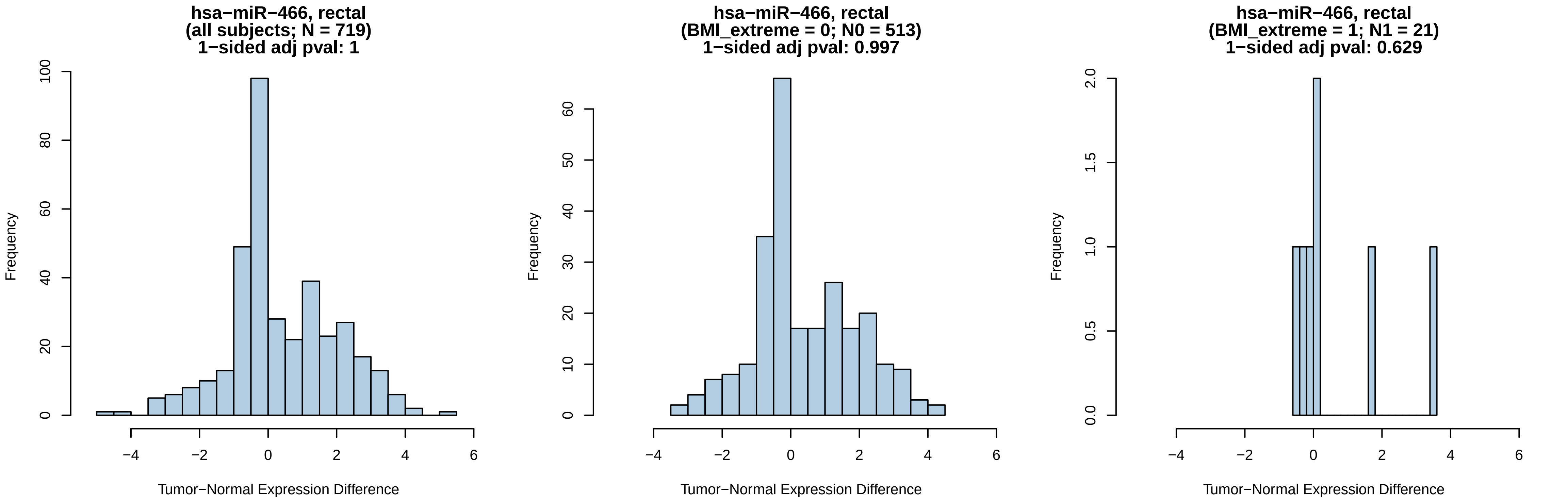

hsa−miR−466, rectal
(all subjects; N = 719)
1−sided adj pval: 1
Frequency
Tumor−Normal Expression Difference
hsa−miR−466, rectal
(BMI_extreme = 0; N0 = 513)
1−sided adj pval: 0.997
Frequency
Tumor−Normal Expression Difference
hsa−miR−466, rectal
(BMI_extreme = 1; N1 = 21)
1−sided adj pval: 0.629
Frequency
Tumor−Normal Expression Difference

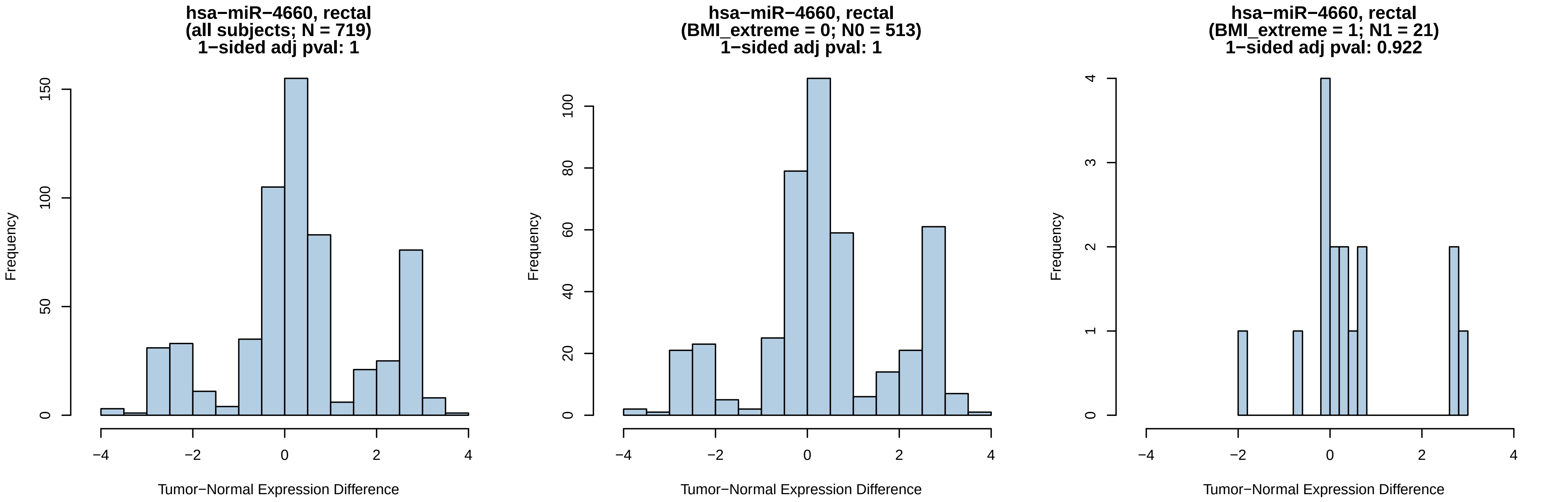

hsa−miR−4660, rectal
(all subjects; N = 719)
1−sided adj pval: 1
Frequency
0
50
100
150
−4
−2
0
2
4
Tumor−Normal Expression Difference
hsa−miR−4660, rectal
(BMI_extreme = 0; N0 = 513)
1−sided adj pval: 1
Frequency
0
20
40
60
80
100
−4
−2
0
2
4
Tumor−Normal Expression Difference
hsa−miR−4660, rectal
(BMI_extreme = 1; N1 = 21)
1−sided adj pval: 0.922
Frequency
0
1
2
3
4
−4
−2
0
2
4
Tumor−Normal Expression Difference

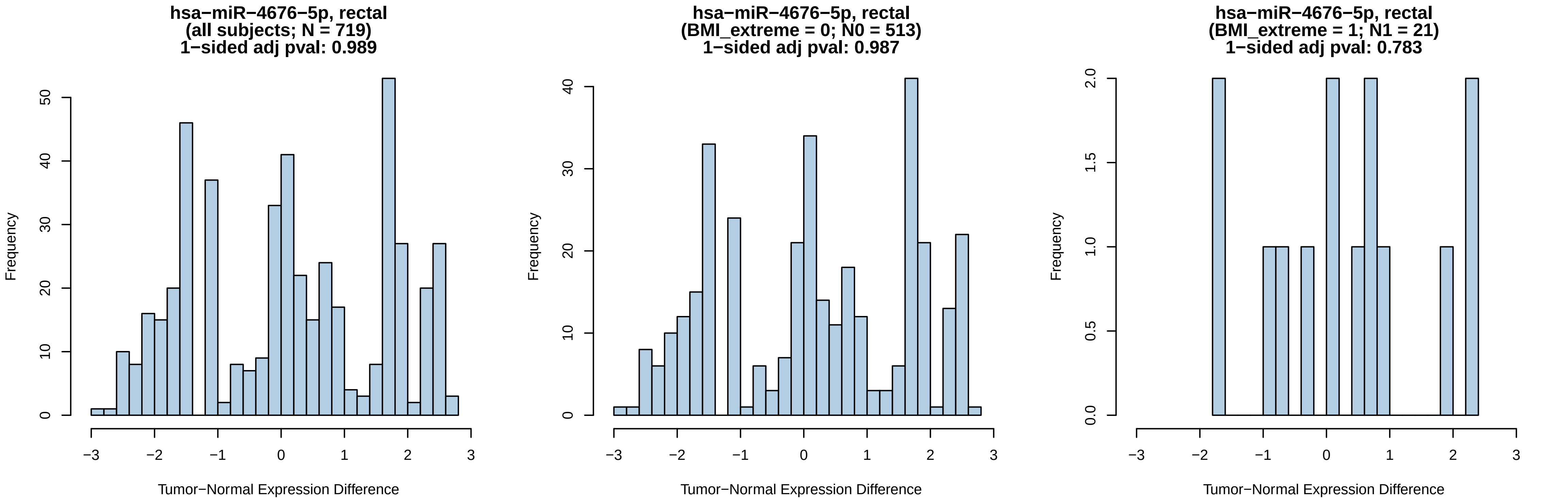
hsa−miR−4676−5p, rectal
(all subjects; N = 719)
1−sided adj pval: 0.989
Frequency
Tumor−Normal Expression Difference
hsa−miR−4676−5p, rectal
(BMI_extreme = 0; N0 = 513)
1−sided adj pval: 0.987
Frequency
Tumor−Normal Expression Difference
hsa−miR−4676−5p, rectal
(BMI_extreme = 1; N1 = 21)
1−sided adj pval: 0.783
Frequency
Tumor−Normal Expression Difference

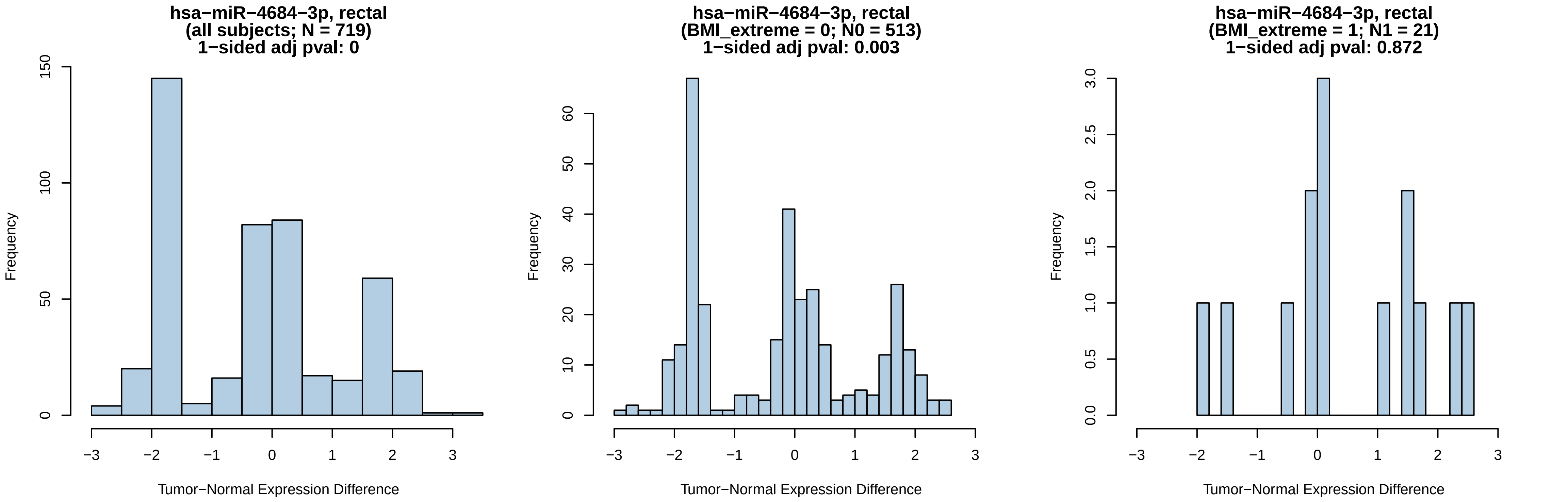
hsa−miR−4684−3p, rectal
(all subjects; N = 719)
1−sided adj pval: 0
Frequency
0
50
100
150
−3
−2
−1
0
1
2
3
Tumor−Normal Expression Difference
hsa−miR−4684−3p, rectal
(BMI_extreme = 0; N0 = 513)
1−sided adj pval: 0.003
Frequency
0
10
20
30
40
50
60
−3
−2
−1
0
1
2
3
Tumor−Normal Expression Difference
hsa−miR−4684−3p, rectal
(BMI_extreme = 1; N1 = 21)
1−sided adj pval: 0.872
Frequency
0.0
0.5
1.0
1.5
2.0
2.5
3.0
−3
−2
−1
0
1
2
3
Tumor−Normal Expression Difference

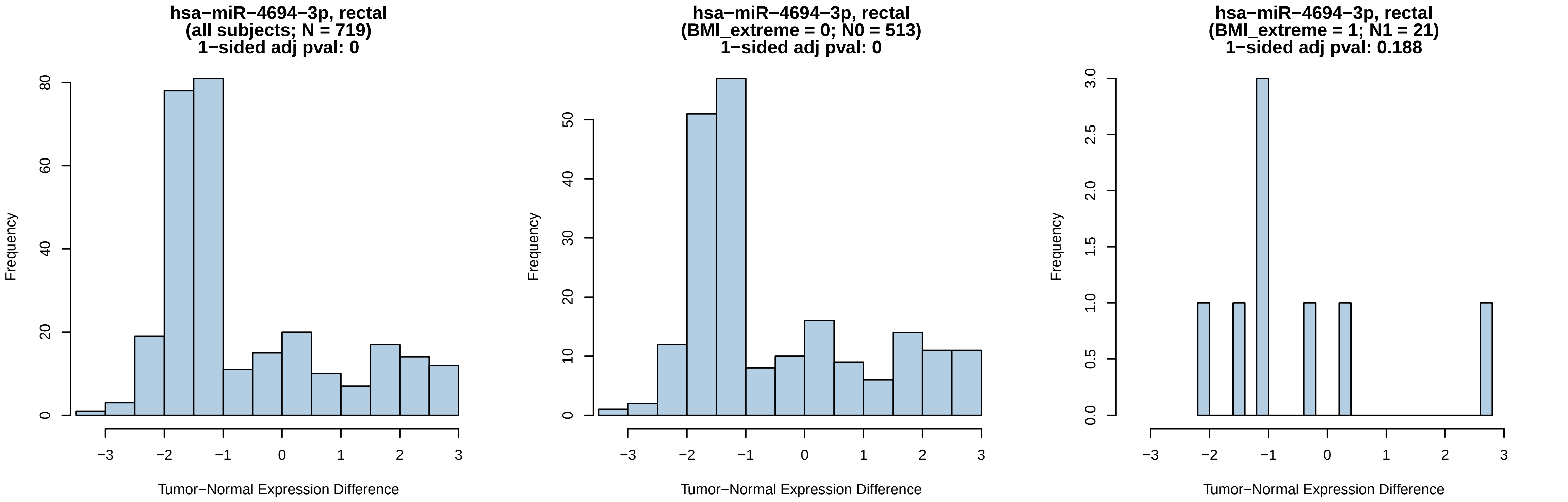
hsa−miR−4694−3p, rectal
(all subjects; N = 719)
1−sided adj pval: 0
Frequency
Tumor−Normal Expression Difference
hsa−miR−4694−3p, rectal
(BMI_extreme = 0; N0 = 513)
1−sided adj pval: 0
Frequency
Tumor−Normal Expression Difference
hsa−miR−4694−3p, rectal
(BMI_extreme = 1; N1 = 21)
1−sided adj pval: 0.188
Frequency
Tumor−Normal Expression Difference

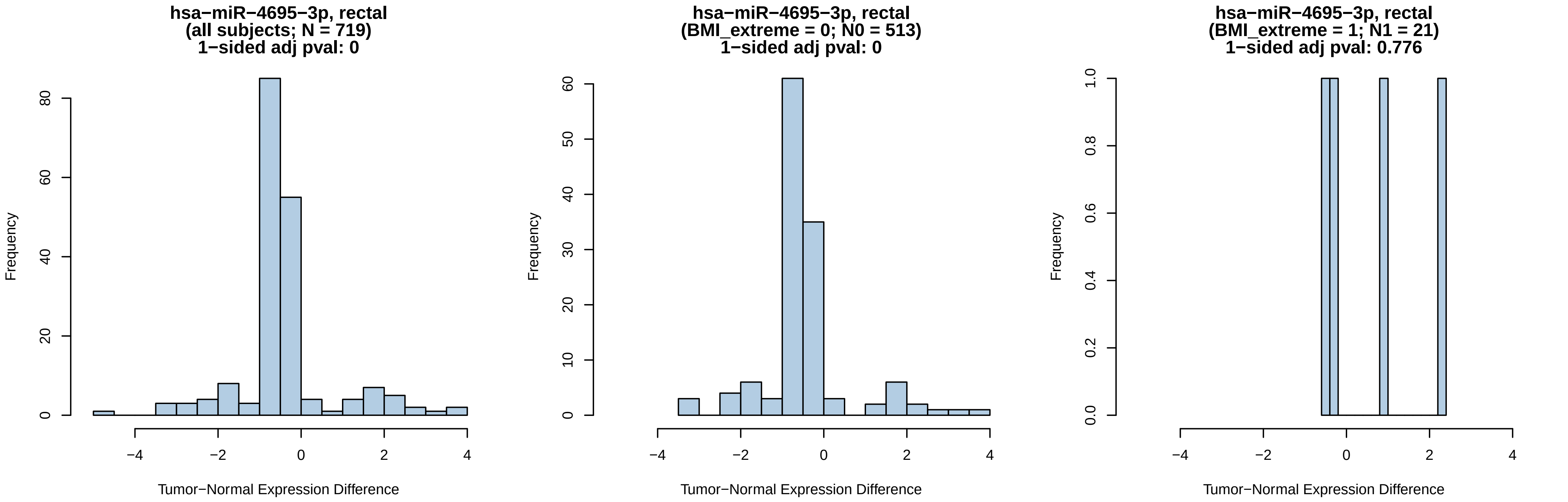

hsa−miR−4695−3p, rectal
(all subjects; N = 719)
1−sided adj pval: 0
Frequency
Tumor−Normal Expression Difference
hsa−miR−4695−3p, rectal
(BMI_extreme = 0; N0 = 513)
1−sided adj pval: 0
Frequency
Tumor−Normal Expression Difference
hsa−miR−4695−3p, rectal
(BMI_extreme = 1; N1 = 21)
1−sided adj pval: 0.776
Frequency
Tumor−Normal Expression Difference

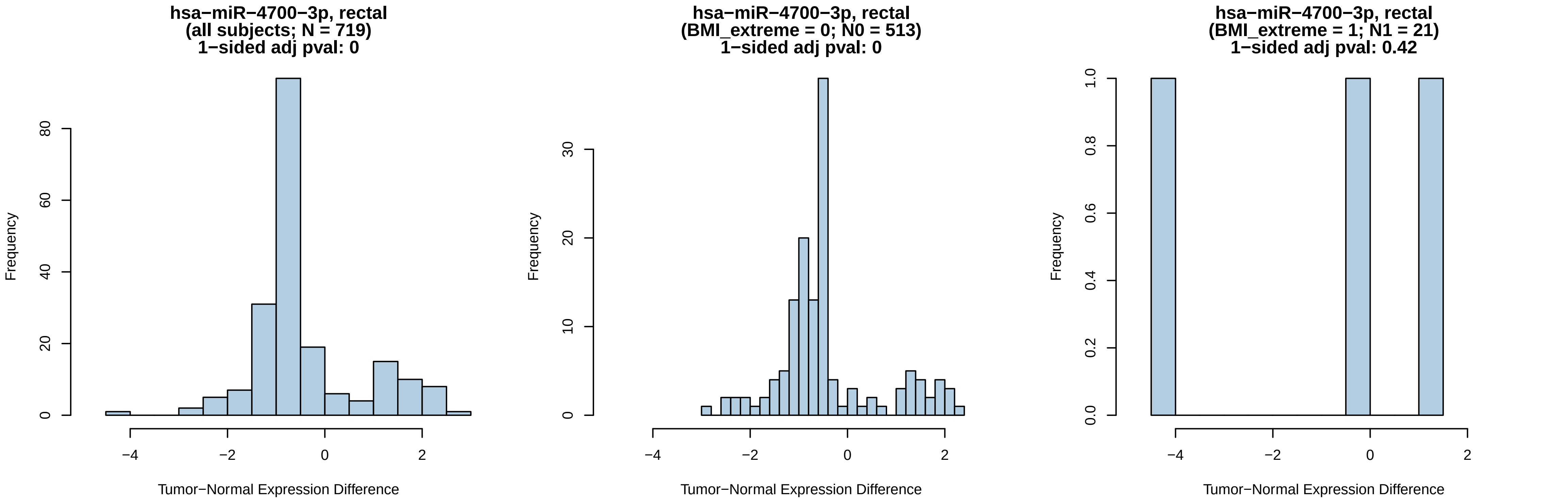
hsa−miR−4700−3p, rectal
(all subjects; N = 719)
1−sided adj pval: 0
Frequency
0
20
40
60
80
−4
−2
0
2
Tumor−Normal Expression Difference
hsa−miR−4700−3p, rectal
(BMI_extreme = 0; N0 = 513)
1−sided adj pval: 0
Frequency
0
10
20
30
−4
−2
0
2
Tumor−Normal Expression Difference
hsa−miR−4700−3p, rectal
(BMI_extreme = 1; N1 = 21)
1−sided adj pval: 0.42
Frequency
0.0
0.2
0.4
0.6
0.8
1.0
−4
−2
0
2
Tumor−Normal Expression Difference

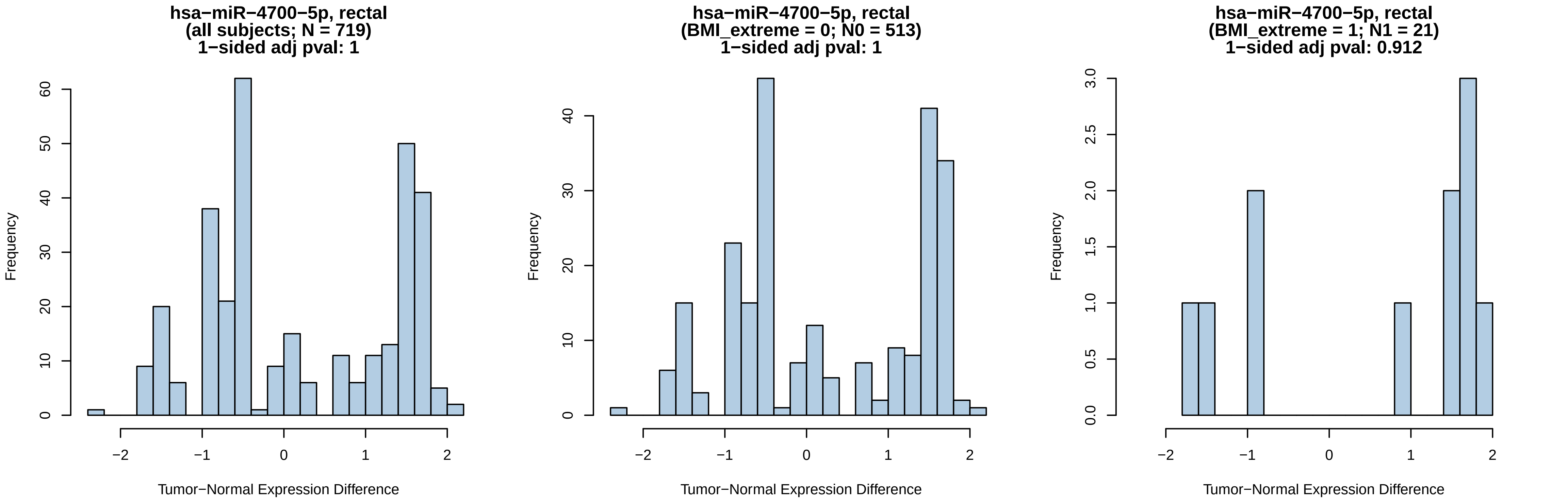
hsa−miR−4700−5p, rectal
(all subjects; N = 719)
1−sided adj pval: 1
Frequency
Tumor−Normal Expression Difference
hsa−miR−4700−5p, rectal
(BMI_extreme = 0; N0 = 513)
1−sided adj pval: 1
Frequency
Tumor−Normal Expression Difference
hsa−miR−4700−5p, rectal
(BMI_extreme = 1; N1 = 21)
1−sided adj pval: 0.912
Frequency
Tumor−Normal Expression Difference

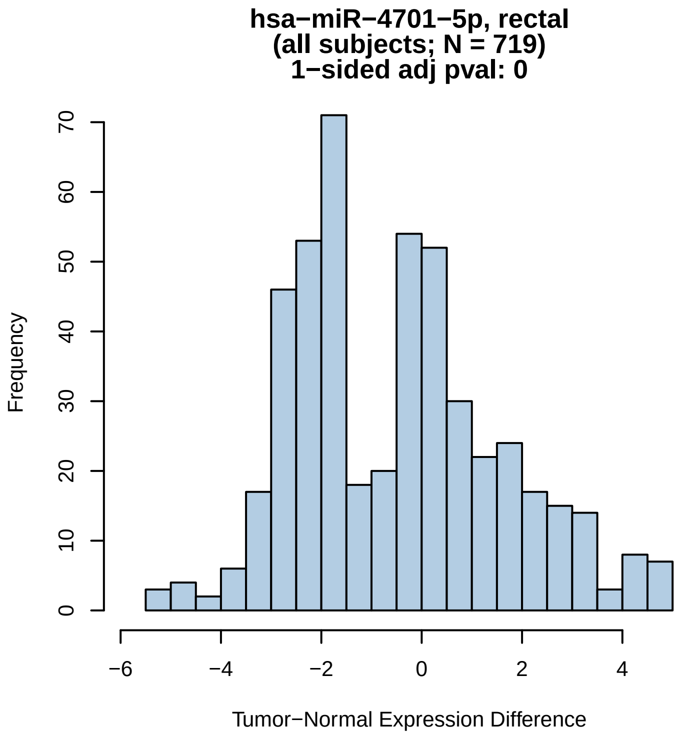
hsa−miR−4701−5p, rectal
(all subjects; N = 719)
1−sided adj pval: 0
Frequency
Tumor−Normal Expression Difference

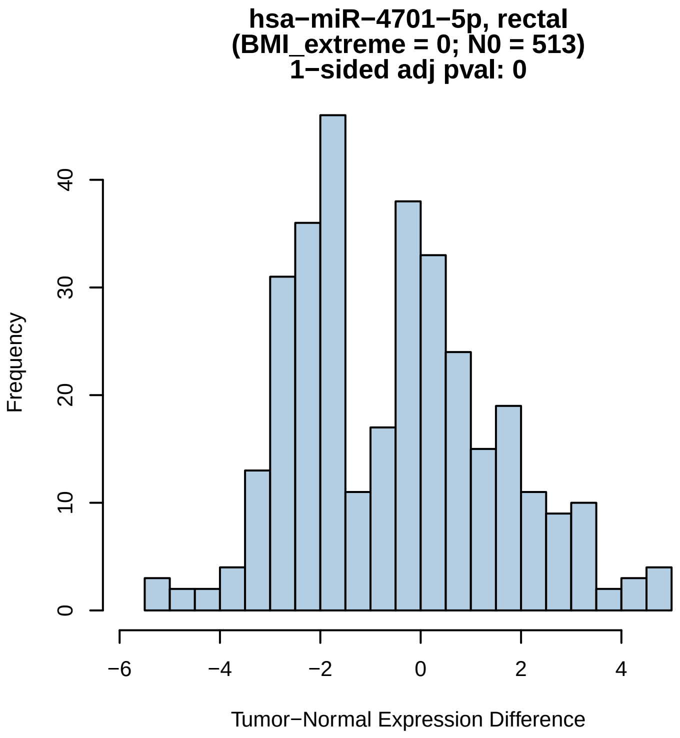
hsa−miR−4701−5p, rectal
(BMI_extreme = 0; N0 = 513)
1−sided adj pval: 0
Frequency
Tumor−Normal Expression Difference

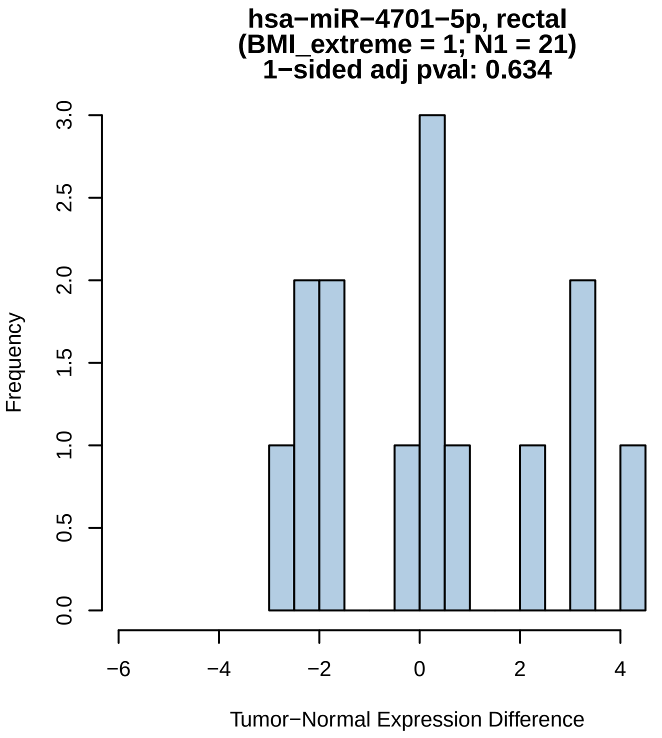
hsa−miR−4701−5p, rectal
(BMI_extreme = 1; N1 = 21)
1−sided adj pval: 0.634
Frequency
Tumor−Normal Expression Difference

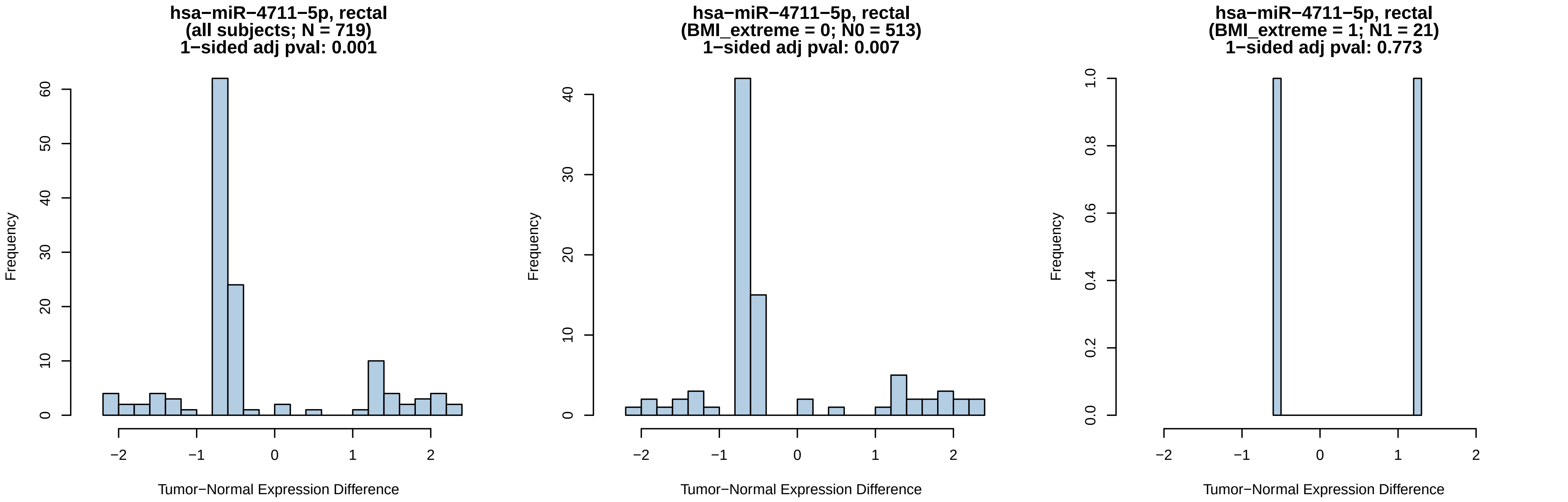
hsa−miR−4711−5p, rectal
(all subjects; N = 719)
1−sided adj pval: 0.001
Frequency
0
10
20
30
40
50
60
−2
−1
0
1
2
Tumor−Normal Expression Difference
hsa−miR−4711−5p, rectal
(BMI_extreme = 0; N0 = 513)
1−sided adj pval: 0.007
Frequency
0
10
20
30
40
−2
−1
0
1
2
Tumor−Normal Expression Difference
hsa−miR−4711−5p, rectal
(BMI_extreme = 1; N1 = 21)
1−sided adj pval: 0.773
Frequency
0.0
0.2
0.4
0.6
0.8
1.0
−2
−1
0
1
2
Tumor−Normal Expression Difference

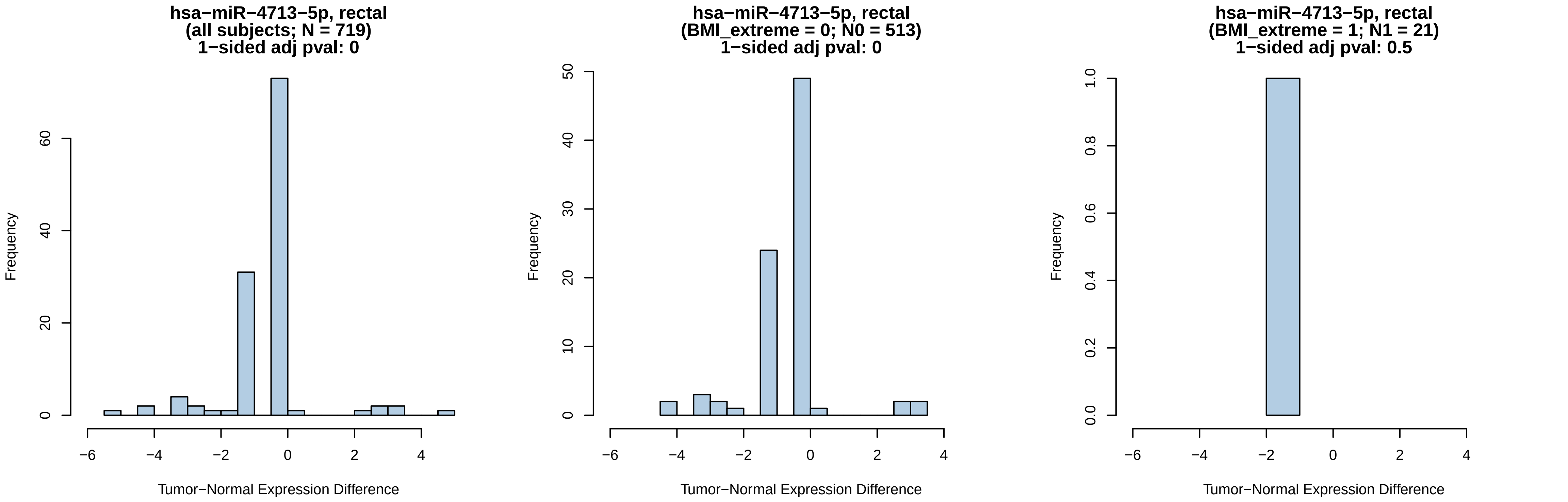
hsa−miR−4713−5p, rectal
(all subjects; N = 719)
1−sided adj pval: 0
Frequency
Tumor−Normal Expression Difference
hsa−miR−4713−5p, rectal
(BMI_extreme = 0; N0 = 513)
1−sided adj pval: 0
Frequency
Tumor−Normal Expression Difference
hsa−miR−4713−5p, rectal
(BMI_extreme = 1; N1 = 21)
1−sided adj pval: 0.5
Frequency
Tumor−Normal Expression Difference

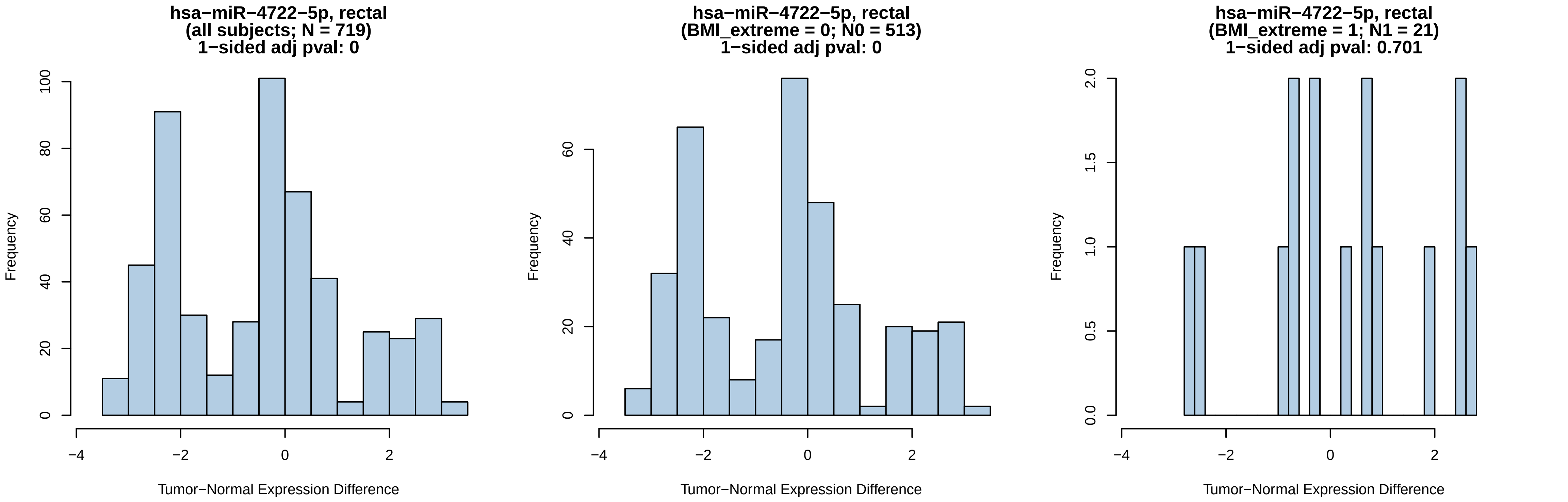
hsa−miR−4722−5p, rectal
(all subjects; N = 719)
1−sided adj pval: 0
Frequency
0
20
40
60
80
100
−4
−2
0
2
Tumor−Normal Expression Difference
hsa−miR−4722−5p, rectal
(BMI_extreme = 0; N0 = 513)
1−sided adj pval: 0
Frequency
0
20
40
60
−4
−2
0
2
Tumor−Normal Expression Difference
hsa−miR−4722−5p, rectal
(BMI_extreme = 1; N1 = 21)
1−sided adj pval: 0.701
Frequency
0.0
0.5
1.0
1.5
2.0
−4
−2
0
2
Tumor−Normal Expression Difference

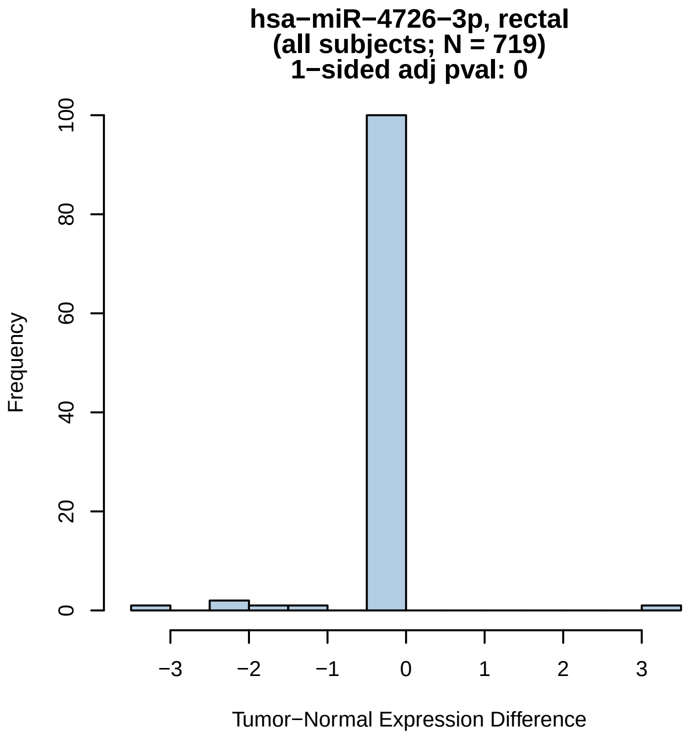
hsa−miR−4726−3p, rectal
(all subjects; N = 719)
1−sided adj pval: 0
Frequency
0
20
40
60
80
100
−3
−2
−1
0
1
2
3
Tumor−Normal Expression Difference

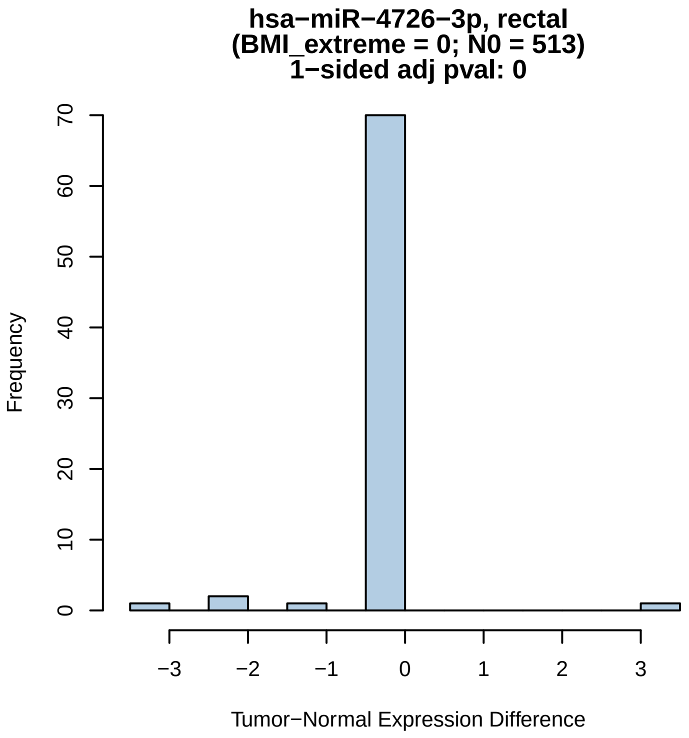
hsa−miR−4726−3p, rectal
(BMI_extreme = 0; N0 = 513)
1−sided adj pval: 0
Frequency
0
10
20
30
40
50
60
70
−3
−2
−1
0
1
2
3
Tumor−Normal Expression Difference

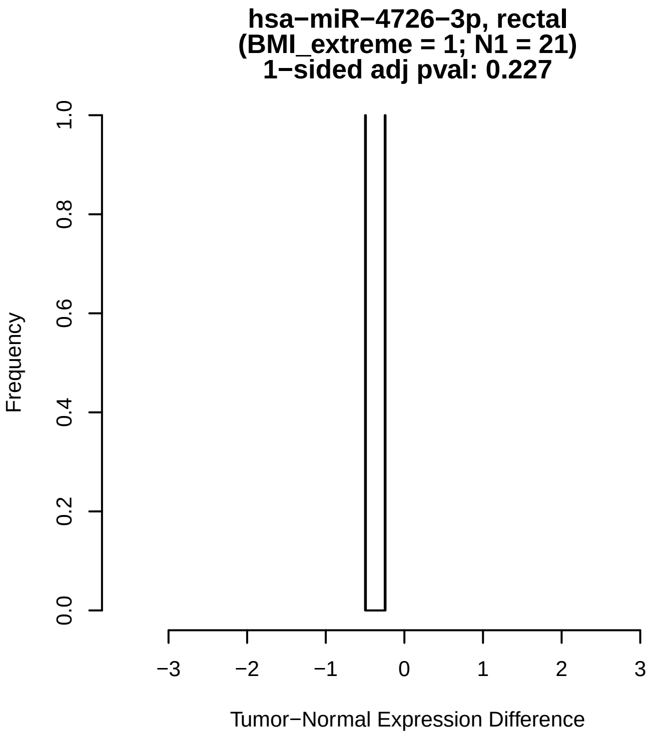
hsa−miR−4726−3p, rectal
(BMI_extreme = 1; N1 = 21)
1−sided adj pval: 0.227
Frequency
0.0
0.2
0.4
0.6
0.8
1.0
−3
−2
−1
0
1
2
3
Tumor−Normal Expression Difference

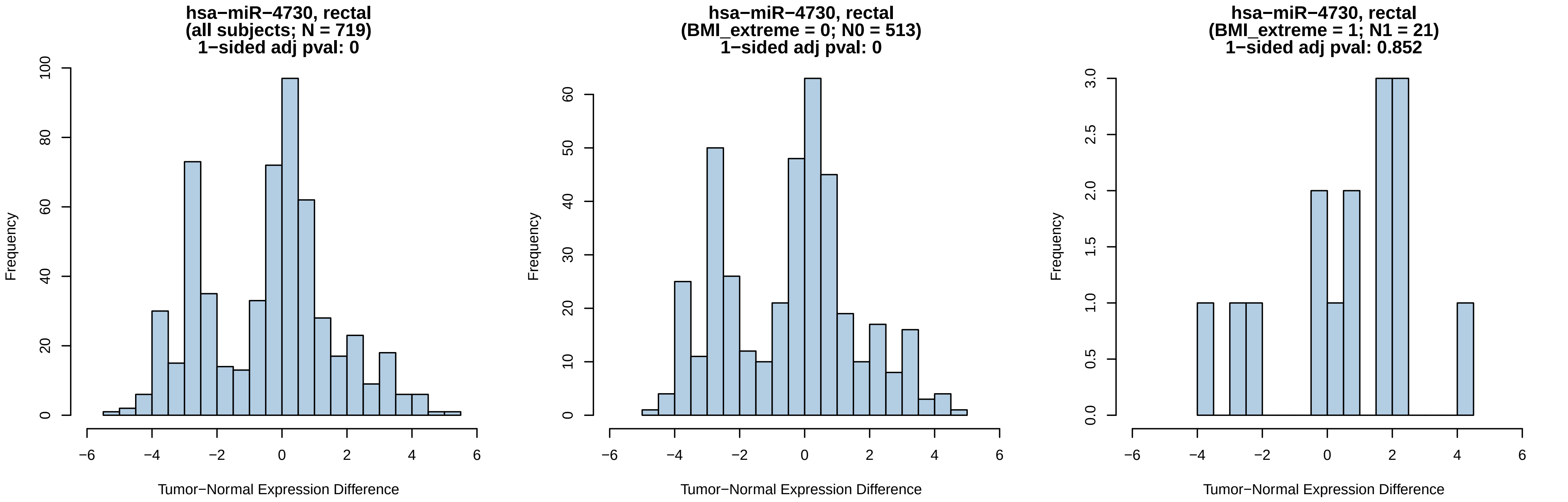
hsa−miR−4730, rectal
(all subjects; N = 719)
1−sided adj pval: 0
Frequency
Tumor−Normal Expression Difference
hsa−miR−4730, rectal
(BMI_extreme = 0; N0 = 513)
1−sided adj pval: 0
Frequency
Tumor−Normal Expression Difference
hsa−miR−4730, rectal
(BMI_extreme = 1; N1 = 21)
1−sided adj pval: 0.852
Frequency
Tumor−Normal Expression Difference

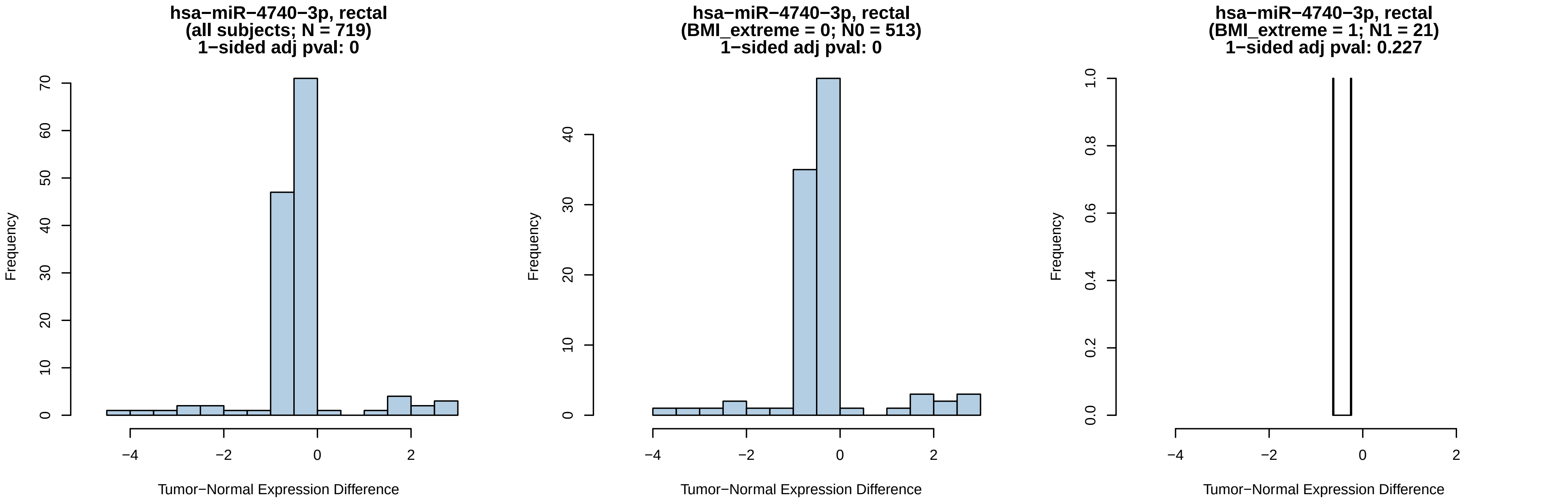
hsa−miR−4740−3p, rectal
(all subjects; N = 719)
1−sided adj pval: 0
Frequency
0
10
20
30
40
50
60
70
−4
−2
0
2
Tumor−Normal Expression Difference
hsa−miR−4740−3p, rectal
(BMI_extreme = 0; N0 = 513)
1−sided adj pval: 0
Frequency
0
10
20
30
40
−4
−2
0
2
Tumor−Normal Expression Difference
hsa−miR−4740−3p, rectal
(BMI_extreme = 1; N1 = 21)
1−sided adj pval: 0.227
Frequency
0.0
0.2
0.4
0.6
0.8
1.0
−4
−2
0
2
Tumor−Normal Expression Difference
